# Targeting pro-inflammatory T cells as a novel therapeutic approach to potentially resolve atherosclerosis in humans

**DOI:** 10.1038/s41422-024-00945-0

**Published:** 2024-03-15

**Authors:** Lin Fan, Junwei Liu, Wei Hu, Zexin Chen, Jie Lan, Tongtong Zhang, Yang Zhang, Xianpeng Wu, Zhiwei Zhong, Danyang Zhang, Jinlong Zhang, Rui Qin, Hui Chen, Yunfeng Zong, Jianmin Zhang, Bing Chen, Jun Jiang, Jifang Cheng, Jingyi Zhou, Zhiwei Gao, Zhenjie Liu, Ying Chai, Junqiang Fan, Pin Wu, Yinxuan Chen, Yuefeng Zhu, Kai Wang, Ying Yuan, Pintong Huang, Ying Zhang, Huiqin Feng, Kaichen Song, Xun Zeng, Wei Zhu, Xinyang Hu, Weiwei Yin, Wei Chen, Jian’an Wang

**Affiliations:** 1 Department of Cardiology, The Second Affiliated Hospital, Zhejiang University School of Medicine, Hangzhou, Zhejiang China; 2 Cardiovascular Key Laboratory of Zhejiang Province, Hangzhou, Zhejiang China; 3 Research Center for Life Science and Human Health, Binjiang Institute of Zhejiang University, Hangzhou, Zhejiang China; 4 Department of Cell Biology, Zhejiang University School of Medicine, and Liangzhu Laboratory, Zhejiang University, Hangzhou, Zhejiang China; 5 Key Laboratory for Biomedical Engineering of the Ministry of Education, College of Biomedical Engineering and Instrument Science, Zhejiang University, Hangzhou, Zhejiang China; 6 Guangzhou National Laboratory, Guangzhou, Guangdong China; 7 Kidney Disease Center, The First Affiliated Hospital, Zhejiang University School of Medicine, Hangzhou, Zhejiang China; 8 Center of Clinical Epidemiology and Biostatistics and Department of Scientific Research, The Second Affiliated Hospital, Zhejiang University School of Medicine, Hangzhou, Zhejiang China; 9 National Laboratory of Biomacromolecules, Institute of Biophysics, University of Chinese Academy of Sciences, Beijing, China; 10 Department of Bioinformatics, The Basic Medical School of Chongqing Medical University, Chongqing, China; 11 Department of Hepatobiliary and Pancreatic Surgery, The Center for Integrated Oncology and Precision Medicine, Affiliated Hangzhou First People’s Hospital, Zhejiang University School of Medicine, Hangzhou, Zhejiang China; 12 The MOE Frontier Science Center for Brain Science & Brain-machine Integration, Zhejiang University, Hangzhou, Zhejiang China; 13 National Clinical Research Center for Infectious Diseases, The First Affiliated Hospital, Zhejiang University School of Medicine, Hangzhou, Zhejiang China; 14 Department of Neurosurgery, The Second Affiliated Hospital, Zhejiang University School of Medicine, Hangzhou, Zhejiang China; 15 Department of Vascular Surgery, The Second Affiliated Hospital, Zhejiang University School of Medicine, Hangzhou, Zhejiang China; 16 Department of Thoracic Surgery, The Second Affiliated Hospital, Zhejiang University School of Medicine, Hangzhou, Zhejiang China; 17 Department of Vascular Surgery, Sir Run Run Shaw Hospital, Zhejiang University School of Medicine, Hangzhou, Zhejiang China; 18 Department of Respiratory, The Second Affiliated Hospital, Zhejiang University School of Medicine, Hangzhou, Zhejiang China; 19 Department of Oncology, The Second Affiliated Hospital, Zhejiang University School of Medicine, Hangzhou, Zhejiang China; 20 Department of Ultrasound in Medicine, The Second Affiliated Hospital, Zhejiang University School of Medicine, Hangzhou, Zhejiang China; 21 Department of Clinical Research Center, The Second Affiliated Hospital, Zhejiang University School of Medicine, Hangzhou, Zhejiang China; 22 Zhejiang Provincial Key Laboratory of Cardio-Cerebral Vascular Detection Technology and Medicinal Effectiveness Appraisal, Zhejiang University, Hangzhou, Zhejiang China

**Keywords:** Mechanisms of disease, Autoimmunity

## Abstract

Atherosclerosis (AS), a leading cause of cardio-cerebrovascular disease worldwide, is driven by the accumulation of lipid contents and chronic inflammation. Traditional strategies primarily focus on lipid reduction to control AS progression, leaving residual inflammatory risks for major adverse cardiovascular events (MACEs). While anti-inflammatory therapies targeting innate immunity have reduced MACEs, many patients continue to face significant risks. Another key component in AS progression is adaptive immunity, but its potential role in preventing AS remains unclear. To investigate this, we conducted a retrospective cohort study on tumor patients with AS plaques. We found that anti-programmed cell death protein 1 (PD-1) monoclonal antibody (mAb) significantly reduces AS plaque size. With multi-omics single-cell analyses, we comprehensively characterized AS plaque-specific PD-1^+^ T cells, which are activated and pro-inflammatory. We demonstrated that anti-PD-1 mAb, when captured by myeloid-expressed Fc gamma receptors (FcγRs), interacts with PD-1 expressed on T cells. This interaction turns the anti-PD-1 mAb into a substitute PD-1 ligand, suppressing T-cell functions in the PD-1 ligands-deficient context of AS plaques. Further, we conducted a prospective cohort study on tumor patients treated with anti-PD-1 mAb with or without FcγR-binding capability. Our analysis shows that anti-PD-1 mAb with FcγR-binding capability effectively reduces AS plaque size, while anti-PD-1 mAb without FcγR-binding capability does not. Our work suggests that T cell-targeting immunotherapy can be an effective strategy to resolve AS in humans.

## Introduction

Atherosclerosis (AS) is a chronic disease that leads to clinical complications known as major adverse cardiovascular events (MACEs) and is the leading cause of death worldwide.^1–4^ It is characterized by excessive lipid deposition in the intimal space of larger arteries where low-density lipoprotein cholesterol (LDL-C) retention elicits arterial inflammation. To date, the most common clinical treatment is to use lipid-lowering agents (e.g., statins) to effectively reduce blood lipid levels.^5^ Intensive lipid-lowering agents can effectively reduce the incidence of MACEs by 30%, but many patients still develop MACEs.^6^ Nearly 40% of patients who suffered from MACEs had already reduced their total cholesterol (TC) levels to normal,^7^ indicating that only lowering lipids is far from enough to eliminate the occurrence of MACEs for AS patients. A recent multinational trial study analyzed statin-treated patients and reported that the occurrence of MACEs was predominantly associated with the residual inflammatory risk (high C-reactive protein (CRP)) rather than the residual cholesterol risk (high LDL-C),^8^ which suggests that statin alone is not sufficient to prevent MACEs.

Chronic and low-grade inflammation is present at all stages of atherosclerosis, with both innate and adaptive immunity involved in the modulation of AS inflammation.^9,10^ Recruitment and differentiation of inflammatory macrophages and lipid-load foam cells are essential players in the formation and maturation of AS plaques. To target innate immunity, further intervention in human atherosclerosis might require the cooperation of anti-inflammatory and lipid-lowering strategies to improve clinical efficacy. Indeed, recent clinical trials have shown some efficacy in reducing MACEs by targeting innate immunity.^11–13^ For example, the Canakinumab Anti-inflammatory Thrombosis Outcome Study (CANTOS) exploited a combination of lipid-lowering and anti-inflammatory agents, such as anti-interleukin (IL)-1β monoclonal antibody (mAb), which neutralizes the NLRP3/IL-1β pathway and synthesis of pro-inflammatory cytokines.^11^ Subsequent clinical trials, such as the Colchicine Cardiovascular Outcomes Trial (COLCOT) and Low-Dose Colchicine 2 Trial (LoDoCo2), both have shown that the simultaneous use of low-dose colchicine (which inhibits microtubule polymerization to prevent IL-1β production) with statins can further reduce the incidence of MACEs by approximately 15%–30%.^12,13^ However, it still leaves much space for improvement in resolving residual inflammatory risk, which requires exploring and identifying more efficient immune targets.

Adaptive immunity is another important branch of the immune system that regulates inflammation in atherosclerosis.^14^ However, harnessing adaptive immunity to regulate human atherosclerosis remains challenging. Increasing evidence supports the causal role of adaptive immunity as an essential modulator of human atherosclerosis.^15^ By single-cell techniques, previous studies identified T cells as a substantial immune cell type with diverse phenotypes that infiltrate human AS plaques.^16^ In response to local antigens presented in human AS plaques, T cells contribute to atherogenesis by interacting with various antigen-presenting cells, such as foam cells, macrophages, and dendritic cells. These interactions activate antigen-specific T cells and clonally expand their T-cell receptor (TCR) repertoire, leading to differentiation into diverse subsets and the production of inflammatory cytokines that promote AS progression.^17–20^ On the other hand, recent single-cell analyses have reported that autoreactive CD4^+^ T cells are an autoimmune component that drives human atherosclerosis.^21^ Additionally, AS plaque-resident T cells have a PD-1^+^ subset exhibiting an “early-exhaustion” phenotype,^16^ which might suggest that these T cells are unlike fully exhausted ones in tumors and could be tunable to reshape T-cell responses. Therefore, we propose that these T cells could be valuable targets to inhibit T cell-mediated inflammatory response in human AS plaques, but further investigation is needed.

In this study, we investigated the clinical effects of anti-PD-1 treatment on AS plaques in a retrospective cohort of tumor patients who were treated with anti-PD-1 mAb or not. Surprisingly, we found that anti-PD-1 treatment played a significant role in reducing AS plaque areas in humans. Additionally, we discovered that AS plaque-specific PD-1^+^ T cells are activated and pro-inflammatory. Targeting these cells could inhibit T-cell responses ex vivo. We then dissected one of its working mechanisms by which FcγR-binding anti-PD-1 mAb can be captured by FcγRs on myeloid cells and serve as a “proxy PD-1 ligand” to suppress the activation of these PD-1^+^ T cells. To support this mechanism, we further examined a prospective cohort and found that only anti-PD-1 mAb with FcγR-binding capability was effective in reducing AS plaque size, while anti-PD-1 mAb without FcγR-binding capability was not.

## Results

### Retrospective cohort reveals clinical benefits of anti-PD-1 treatment in resolving AS plaques

**Fig. 1:**
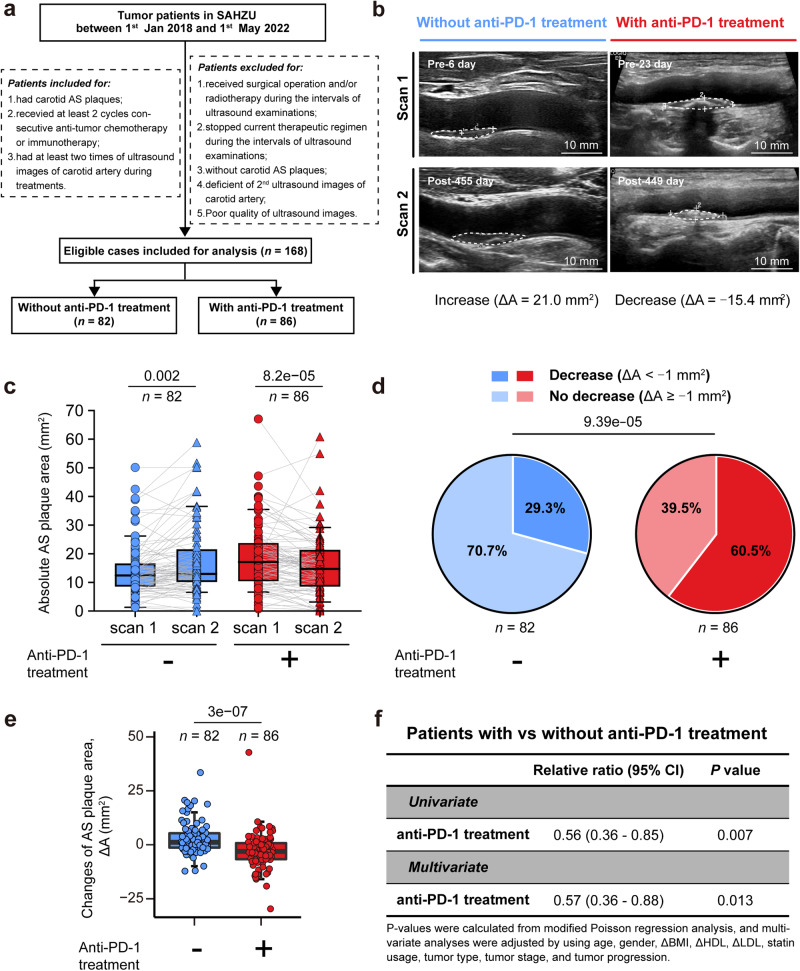
Retrospective cohort analyses reveal that anti-PD-1 therapy impedes and reverses AS plaque progression in vivo. **a** Flowchart showing the identification of eligible patients in the retrospective cohort study. **b**, **c** Representative ultrasound images of carotid plaques (**b**) and comparisons of AS plaque areas (**c**) in patients treated with or without anti-PD-1 mAb at two scanning time points (Scan 1 and Scan 2). Scar bars, 10 mm. **d** Comparison of the compositions of AS plaque progression (decrease: ΔA < –1 mm^2^; no decrease: ΔA ≥ –1 mm^2^) in groups treated without (*n* = 82) or with (*n* = 86) anti-PD-1 treatment. **e** Comparison of the changes of AS plaque areas (ΔA) between patients with or without anti-PD-1 treatment. **f** Univariate and multivariate (modified Poisson) regression analysis of the relative ratio (RR) of anti-PD-1 treatment to AS plaque progression in tumor patients (*n* = 168). Multivariate analyses were adjusted using age, gender, ΔBMI, ΔHDL, ΔLDL, statin usage, tumor types, tumor stage, and tumor progression. Data are represented as median with interquartile range (IQR) in **c** and **e**. Paired Mann–Whitney test was used in **c** and unpaired Mann–Whitney test was in **e**, and the χ2 test was in **d**.

To explore the potential impact of anti-PD-1 treatment on the progression of human AS plaques, we conducted a retrospective cohort study. Our study enrolled the tumor patients who were diagnosed and treated at The Second Affiliated Hospital of Zhejiang University (SAHZU) from 1st Jan 2018 to 1st May 2022 (Fig. 1a) and had at least two eligible ultrasound imaging records of carotid plaques before and/or during anti-tumor therapy (Supplementary information, Fig. S1). A total of 168 patients were enrolled. Among them, 86 patients received chemotherapy combined with anti-PD-1 treatment. And 82 patients received only chemotherapy without any anti-PD-1 treatment.

By double-blinded analysis of the ultrasound images, we measured the changes in the area of carotid plaque between consecutive images (Scan 1 and Scan 2) for individual patient (Fig. 1b). We discovered a significant reduction in plaque areas (*P* = 8.2e–05) in patients who received anti-PD-1 treatment (Fig. 1c), among which 60.5% (52 out of 86) had decreased plaque areas (Fig. 1d). In contrast, patients who did not receive anti-PD-1 treatment experienced an increase in AS plaque areas (*P* = 0.002) (Fig. 1c). Although 29.3% (24 out of 82) of these patients also had decreased areas of AS plaques, this was still significantly lower than the group who received anti-PD-1 treatment (Fig. 1d). The median change of AS plaque areas (ΔA: the change of AS plaque area) was reduced in patients who received anti-PD-1 treatment (ΔA = −3.0 (−7.0, 1.0) mm^2^), in contrast to the increased median areas in those who did not receive anti-PD-1 treatment (ΔA = 1.0 (−1.0, 5.0) mm^2^) (Fig. 1e). This decrease in carotid plaque area was observed in individuals with relatively older ages and slightly reduced high-density lipoprotein (HDL) levels. However, no significant differences were observed for other clinical variables such as Body Mass Index (BMI), gender, LDL, statin usage, tumor progression, tumor stage, and tumor type in the anti-PD-1-treated group (Supplementary information, Fig. S1). We further conducted univariate and multivariate (Modified Poisson)^22^ regression analyses and found that anti-PD-1 treatment was an independent and significant protective factor for the reduction of carotid plaques when comparing the patients who received or did not receive anti-PD-1 treatment (relative ratio (RR) = 0.56 (0.36–0.85), *P* = 0.007; RR = 0.57 (0.36–0.88), *P* = 0.013) (Fig. 1f). Collectively, our clinical evidence suggests that anti-PD-1 treatment potentially reduces AS plaques in tumors of patients.

### T-cell atlas of human atherosclerosis

**Fig. 2:**
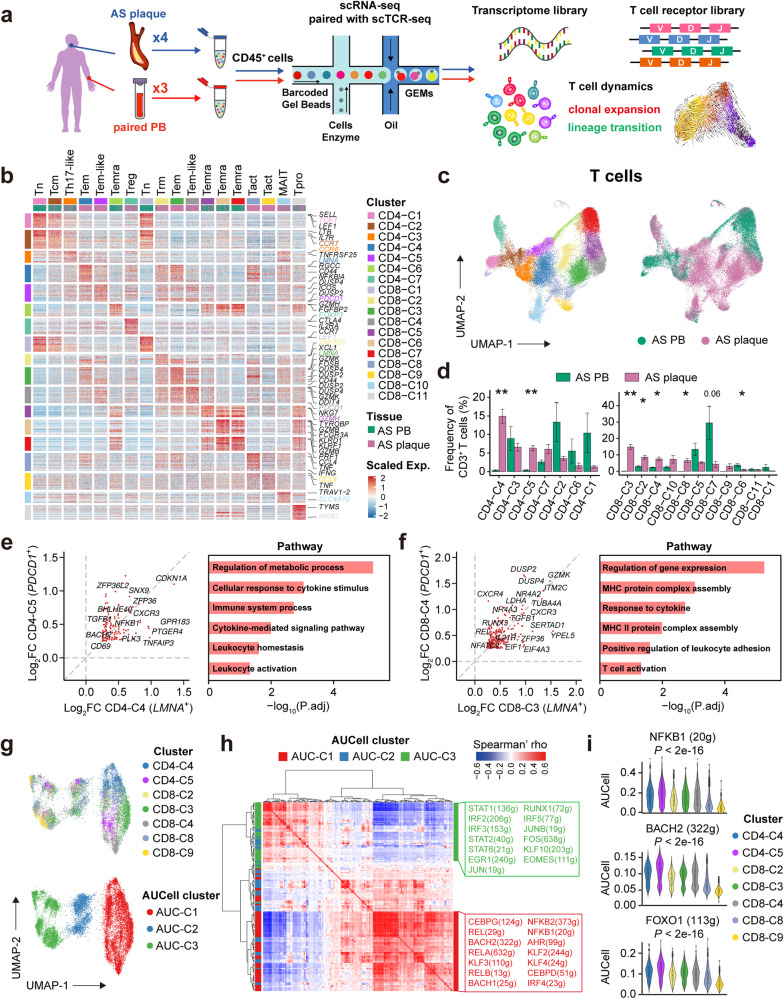
scRNA-seq profiling reveals the T-cell atlas of human AS plaques. **a** Experimental design for paired scRNA-seq and αβTCR-seq analyses. **b** DEGs of T cell clusters, the phenotypical definition of each cluster was labeled on the top, and the clusters are colored by both clusters (left and top) and tissue sources of individual cells (top). Typical genes of each cluster are labeled on the right. **c** Uniform Manifold Approximation and Projection (UMAP) plots of 40,985 T cells from scRNA-seq data, colored by clusters (left) and tissue sources (right). **d** Composition of CD4^+^ (left) and CD8^+^ (right) T cell clusters, colored by sample sources, and clusters were ranked by mean frequencies in AS plaques. Data are represented as means ± SEM. **e**, **f** Scatter plots showing log_2_(fold change) of overlapped DEGs (left) and enriched pathways (right) between CD4-C4 and CD4-C5 clusters (**e**) and between CD8-C3 and CD8-C4 clusters (**f**). **g** UMAP plots of T cells in plaque-specific T cell clusters (mapping based on cell–regulon expression matrix), colored by T cell clusters (top) and AUCell clusters (bottom). **h** Heatmap showing pairwise TF–regulon correlations, the left bar is colored with the most expressed AUCell cluster, and the right bar is labeled with the dominant AUCell cluster and typical TF–regulons. **i** Violin plots showing AUCell scores of regulons on identified plaque-specific T cell clusters. Data are represented as means ± SEM in **d**. A two-sided Student’s *t*-test with Benjamini–Hochberg adjustment was used in **d**, and the one-way ANOVA test was used in **i**.

To investigate the mechanism by which anti-PD-1 mAb reduces human AS plaques, we used single-cell RNA sequencing (scRNA-seq) and paired single-cell TCR sequencing (scTCR-seq) to characterize CD45^+^ cells from 4 human AS plaques and 3 paired peripheral blood mononuclear cells (PBMCs) (Fig. 2a). In total, we obtained 62,522 CD45^+^ cells with an average of 1641 genes per cell and partitioned them into 26 clusters. Based on lineage-specific genes, we annotated 17 T cell clusters (42,921 cells), 2 B cell clusters (2,099 cells), 5 natural killer (NK) cell clusters (15,289 cells), and 2 myeloid cell clusters (2,213 cells) (Fig. 2b; Supplementary information, Fig. S2a–e). Among the cells extracted from human AS plaques through single-cell processing, T cells were the most abundant immune cell type (Supplementary information, Fig. S2f), consistent with the previously published findings.^16^ We further re-clustered T cells into 7 CD4^+^ and 11 CD8^+^ T cell clusters. These T cell clusters had distinct tissue distributions (Fig. 2c, d) and were annotated as different functional phenotypes based on their differentially expressed genes (DEGs) (Fig. 2b, c; Supplementary information, Table S1), including naïve/central memory T (Tn/Tcm) cells, effector memory cells re-expressing CD45RA (Temra) T cells, mucosal-associated invariant T (MAIT) cells, and regulatory T (Treg) cells. We also identified T helper (Th)17-like cells that expressed *CCR6* (CD4-C3), tissue-resident memory T (Trm) cells (CD8-C2) that expressed *ZNF683* and *RUNX3*,^23^ and proliferating T cells (Tpro; CD8-C11) that expressed a series of proliferation-related genes (*STMN1*, *MKI67*, *HMGB2*, and *TPX2*). Furthermore, we identified two activated CD8^+^ T cell clusters (Tact; CD8-C8, -C9) that highly expressed genes encoding inflammatory cytokines (*TNF* and *IFNG*), T-cell activation (*CD69*, *JUN*, and *FOS*), and mitochondrial-related metabolic programming (*MT-ND2*, *MT-CO1*, and *MT-ND6*)^24^ rather than cytotoxic-related genes (*NKG7*, *GZMB*, and *GNLY*) (Fig. 2b), indicating their pro-inflammatory rather than cytotoxic phenotypes in AS plaques.

After analyzing the tissue distributions of T cells (Fig. 2d), we identified two CD4^+^ (CD4-C4, -C5) and four CD8^+^ (CD8-C2, -C3, -C4, and -C8) T cell clusters that were predominantly or even exclusively distributed in AS plaques (Fig. 2c, d). Among them, CD4-C4 and CD8-C3 clusters were the most abundant CD4^+^ (14.9%) and CD8^+^ (14.8%) T cell clusters in AS plaques, respectively. Both clusters expressed higher levels of *LMNA, MCL1, CXCR3*, and activation genes (*CD44, FOS*, and *KLF6*) than other T cells (Fig. 2b, c; Supplementary information, Fig. S2g), indicating their effector memory-like phenotype. Therefore, we defined them as *LMNA*^+^ effector memory T (Tem) cells. Besides, CD4-C5 and CD8-C4 clusters were two plaque-specific Tem-like clusters that not only shared a part of DEGs with *LMNA*^+^ Tem cells but also highly expressed *PDCD1* (defined as *PDCD1*^*+*^ Tem cells) (Fig. 2b, c; Supplementary information, Fig. S2g). We further found that in CD4^+^ and CD8^+^ T cells, both *LMNA*^+^ and *PDCD1*^*+*^ Tem cells highly expressed genes of chemokine receptors (*CXCR3* and *CXCR4*), *ZFP36* and *TNFAIP3*; and *IL21R*, *DUSP2*, and *DUSP4* were expressed exclusively in CD8^+^ T cells. These data suggest that all of these gene expressions may contribute to restraining the effector functions of T cells, forming the long-lived Tem cells. More importantly, pathway analysis further confirms that plaque-specific *LMNA*^+^ and *PDCD1*^+^ Tem cells were both enriched in the signaling pathways of “leukocyte activation”, “leukocyte homeostasis”, and “cytokine response” (Fig. 2e, f). Our results imply the potential inflammatory role of these T cells, which might contribute to sustaining the chronic inflammatory homeostasis of atherosclerotic plaques.^25–28^

We next applied the single-cell regulatory network inference and clustering (SCENIC) pipeline^29^ to dissect the key regulons that included essential transcription factors (TFs) and their target genes in plaque-specific T cell clusters. As a result, we identified three regulon-based AUCell clusters (AUC-C1, -C2, and -C3) and found uneven distributions of T cell clusters (Fig. 2g). Tem cells expressing *LMNA*^+^ (CD4-C4 and CD8-C3) and *PDCD1*^+^ (CD4-C5 and CD8-C4) were mainly distributed in the AUC-C1 cluster, indicating that these cells shared similar transcriptional regulatory pathways (Supplementary information, Fig. S2h). Correlated regulation analysis revealed that T cells in the AUC-C1 cluster highly expressed regulons like REL (29 g), RELA (632 g), RELB (13 g), NFKB1 (20 g), and NFKB2 (25 g) (bottom block in Fig. 2h), suggesting that the activation of nuclear factor-κB (NF-κB) signaling was involved in programming the inflammatory states of *LMNA*^+^ and *PDCD1*^+^ Tem cells (Fig. 2h, i; Supplementary information, Fig. S2i). Meanwhile, these cells were also enriched in BACH2 (332 g) and FOXO1 (113 g) regulons^30,31^ that were related to the differentiation of Tem cells (Fig. 2h, i). In contrast, the regulons for IRFs and JAK-STAT signaling pathways (top block in Fig. 2h, i; Supplementary information, Fig. S2i) were highly expressed in the AUC-C3 cluster (mainly CD8-C8, -C9), indicating their distinct activation pathways. Collectively, we identified two distinct pro-inflammatory signaling pathways that independently remodeled the functional states of T cells in AS plaques, and that the activation of the NF-κB signaling pathway was dominated in plaque-specific *LMNA*^+^ and *PDCD1*^+^ Tem cells, supporting the transcriptional regulation of these T cells in sustaining the chronic inflammation of AS plaques.^32,33^

### *LMNA*^*+*^ and *PDCD1*^*+*^ Tem cells are exclusively enriched in human AS plaques

To distinguish AS-specific T cells from those found in other tissues and diseases, we integrated our T cell scRNA-seq data with those of normal colon tissue,^34^ immunotherapy-induced colitis tissue,^34^ immunotherapy-induced inflammatory arthritis synovial fluid,^35^ and lung tumor tissue.^36^ We obtained 12 CD4^+^, 7 CD8^+^, and 1 γδ T cell clusters (Supplementary information, Fig. S3a–d and Table S2), which were annotated as resting T cells (Meta_CD4_C1, _C2, and Meta_CD8_C1), Treg cells (Meta_CD4_C10, _C11), Th17 cells (Meta_CD4_C5), *CXCR5*^+^ T follicular helper (Tfh) cells (Meta_CD4 _C7), Trm cells (Meta_CD8_C6), and Temra cells (Meta_CD8_C4, _C5). To compare the similarities of T cell clusters from the other disease datasets, we calculated the scaled expressions of (AS plaque) T cell cluster gene signature (top 30 DEGs) in individual Meta-T cell clusters (Supplementary information, Fig. S3e). We found that Meta_CD8_C2 and _C3 most resembled the *LMNA*^*+*^ and *PDCD1*^*+*^CD8^+^ T cell clusters (CD8-C3 and CD8-C4) in AS plaques, respectively. Meta_CD4_C6 was similar to the *PDCD1*^*+*^CD4^+^ T cell cluster (CD4-C5) in AS plaques, and Meta_CD4_C12 highly expressed the gene signature of both *LMNA*^*+*^ T cell clusters (CD4-C4 and CD8-C3) in AS plaques. These T cell clusters, especially CD4^+^ T cell clusters, had the highest median cell frequencies in AS plaques (Supplementary information, Fig. S3f). We also identified T cell clusters that were mostly enriched in different tissues, including Th17 cells (Meta_CD4_C5) in normal colon tissues, γδT cells (Meta_γδT) in colitis tissues, *CXCR5*^+^ Tfh cells (Meta_CD4_C7) in lung tumor tissues, and interferon-responsive T cells (Meta_CD8_C7) in arthritis synovial fluid (Supplementary information, Fig. S3f). Altogether, these results support that the enrichments of *LMNA*^*+*^ and *PDCD1*^*+*^ T cells with pro-inflammatory phenotypes are human AS-specific.

### Cytometry by Time-Of-Flight (CyTOF) analysis reveals that PD-1^+^ T cells in AS plaques are still in the activated state

To characterize the T-cell atlas and functional phenotypes of PD-1^+^ T cells in human AS plaques, we performed single-cell CyTOF analysis of CD45^+^ cells from 64 human samples, including 44 AS peripheral blood (PB) and 20 AS plaques. We designed two independent antibody-staining panels (T- and myeloid cell panel; Supplementary information, Table S3) to profile T cells and myeloid cells in depth. After removing Gadolinium (Gd) contamination in AS plaque samples^37^ and pre-processing CyTOF raw data (Supplementary information, Fig. S4a, b), we performed single-cell clustering analyses followed by frequency correlation analysis between major immune cell types from the two antibody-staining panels, confirming the high consistency of major immune cell type distributions in our parallel experiments (Supplementary information, Fig. S4c–e). We confirmed that T and myeloid cells were the two predominant immune subtypes in AS plaques (62% and 18%, respectively) (Supplementary information, Fig. S4f).

**Fig. 3:**
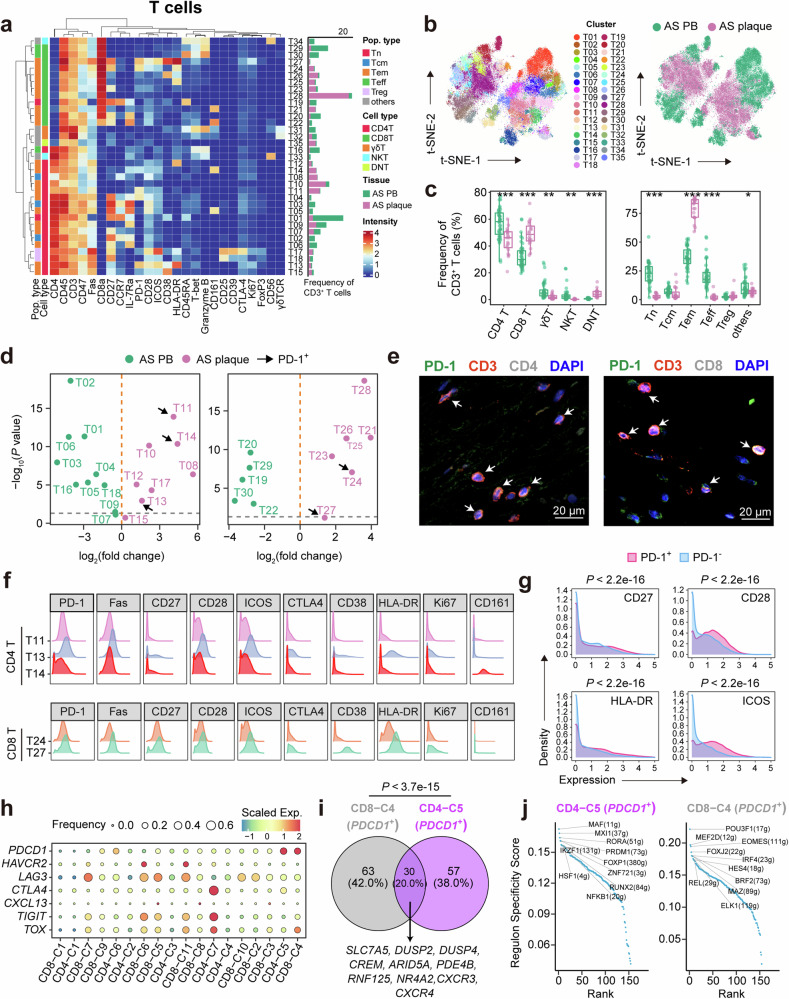
T-cell atlas of human AS plaques and AS PB revealed by single-cell CyTOF analysis. **a** Heatmap displaying the median expression of 35 T cell clusters (T-cell panel), labeled with major or functional subsets (left) and cluster frequency (right). **b** t-SNE plots of T cells, colored by clusters or sample groups. **c** Compositions of major (left) and functional (right) T cell subsets in AS PB and AS plaques. **d** Volcano plots showing different frequencies of CD4^+^ (left) and CD8^+^ (right) T cell clusters in AS plaques compared to those in AS PB, colored by dominating tissue types, and arrows indicate PD-1^+^ T cell clusters. **e** Multicolor IFC staining confirming PD-1^+^CD4^+^ and PD-1^+^CD8^+^ T cells in a representative human AS plaque. Scale bars, 20 μm. **f** Histograms showing selected functional marker expressions on PD-1^+^CD4^+^ (top) and PD-1^+^CD8^+^ (bottom) T cell clusters. **g** Histograms showing ICOS, HLA-DR, CD27, and CD28 expressions on plaque-derived PD-1^+^ and PD-1^–^ T cells. **h** Dot plots showing expressions of exhaustion-related co-inhibitory and regulatory genes in T cell clusters, colored by scaled mean expression and sized by fraction of cells expressing specified genes. T cell clusters were ranked by their AUCell scores of *PDCD1* gene signature as displayed in Supplementary information, Fig. S4l. **i** Venn plot of shared DEGs between *PDCD1*^*+*^ T cell clusters (CD4-C5 and CD8-C4), with the numbers of intersected or exclusive genes labeled. **j** Dot plots showing the calculated regulon specificity scores of *PDCD1*^*+*^ T cell clusters (CD4-C5 and CD8-C4), with the top-10 regulons labeled. Data are presented as median with IQR in **c**. A two-sided Student’s *t*-test with Benjamini–Hochberg adjustment was used for statistical analyses in **c** and **d**. The Kolmogorov–Smirnov test was used in **g** and the hypergeometric test in **i**.

We further analyzed T-cell compositions and identified 35 T cell clusters (Fig. 3a, b; Supplementary information, Fig. S4g), including 18 CD4^+^ (T01-T18), 12 CD8^+^ (T19-T30), 2 γδT (T31 and T32), 2 NKT (natural killer T; T33 and T34), and 1 DNT (double negative T; T35) cell clusters. T cell compositions dramatically altered across tissues, with increased fractions of CD8^+^ T cells and decreased fractions of CD4^+^ T, γδT, and NKT cells in AS plaques compared to those in AS PB (Fig. 3c; Supplementary information, Fig. S4h). Moreover, plaque-specific T cells mostly consisted of non-cytotoxic Tem cells (CD45RA^–^CCR7^–^) with reduced fractions of Tn (CD45RA^+^CCR7^+^) and effector T (Teff; Granzyme B^+^CD45RA^+^T-bet^+^) cells.

We compared the fold changes in cell frequencies of CD4^+^ and CD8^+^ T cell clusters (Fig. 3d) and identified that 5 PD-1^+^ (T11, T13, and T14 for CD4^+^; T24 and T27 for CD8^+^) T cell clusters were exclusively enriched in AS plaques, and the existence of CD4^+^PD-1^+^ and CD8^+^PD-1^+^ T cells was further supported by immunofluorescence staining of human AS plaques (Fig. 3e; Supplementary information, Fig. S4i). Subsequently, we found that co-stimulatory molecules (CD28 and ICOS) were co-expressed on PD-1^+^ T cells, activating molecules (HLA-DR and CD27) were particularly highly expressed on PD-1^+^CD8^+^ T cells (Fig. 3f). Furthermore, these 4 functional markers were significantly higher expressed on PD-1^+^ T cells than PD-1^–^ T cells in AS plaques (Fig. 3g), and their expression levels were also highly correlated with PD-1 expression (Supplementary information, Fig. S4j). Besides, activating molecule CD38^38^ was also highly expressed on PD-1^+^ T cells (T13 and T27) (Fig. 3f). Altogether, these results indicate that PD-1^+^ T cells are mainly located in human AS plaques and suggest that they do not resemble terminally-differentiated exhausted T (Tex) cells in cancers.^39^

### scRNA-seq confirms the functionally activated state of *PDCD1*^*+*^ T cells in human AS plaques

PD-1 expression is theoretically induced by T-cell activation and contributes to T-cell inhibition, memory, and homeostasis, as well as immune tolerance.^40^ The sustained co-expression of PD-1 with other co-inhibitory immune checkpoint receptors, such as lymphocyte-activation gene-3 (Lag-3), T cell immunoglobulin and mucin-domain containing-3 (Tim-3), and T cell immunoglobulin and ITIM (immunoreceptor tyrosine-based inhibitory motif) domain (TIGIT), has been validated as the hallmark of T-cell exhaustion in tumor diseases.^41,42^ We examined the functionalities of PD-1^+^ T cells in AS plaques by deeply analyzing the single-cell transcriptomes of plaque-specific T cells. We identified the top 30 genes that were highly correlated with *PDCD1* expression in T cells, including *DUSP2*, *DUSP4*, *CXCR3*, *CXCR4*, *ICOS*, etc., and defined them as the plaque-specific *PDCD1* gene signature (Supplementary information, Fig. S4k). We calculated and ranked the AUCell score^29^ for each T cell cluster and found that CD4-C5 and CD8-C4 clusters, which expressed the highest level of *PDCD1*, were ranked at the top and defined as *PDCD1*^+^ T cell clusters in AS plaques (Supplementary information, Fig. S4l). Consistent with the aforementioned CyTOF results (Fig. 3f, g), *ICOS* was co-expressed with *PDCD1* at the transcriptome level (Supplementary information, Fig. S4k). However, the typical co-inhibitory molecules or regulators related to T-cell exhaustion, such as *HAVCR2*, *LAG3*, *TIGIT*, and *TOX*,^41^ were not co-expressed on these cells, thereby not being ranked within the genes correlated with *PDCD1* (Fig. 3h; Supplementary information, Fig. S4k). Furthermore, we did not observe significant expressions of dysfunctional T cell-associated genes^43^ in our *PDCD1*^+^ T cell clusters (Supplementary information, Fig. S4m). Collectively, these findings indicate that *PDCD1*^+^ T cells in human AS plaques are functionally distinct from those exhausted *PDCD1*^+^ tumor-infiltrating lymphocytes (TILs).^43–45^

The PD-1 signaling pathway, accompanied by the activation of TCR signals,^46,47^ also contributes to the maintenance of T cell memory. By analyzing the DEGs of *PDCD1*^+^ T cells (CD4-C5 and CD8-C4), we identified 30 genes that were shared by these two clusters (Fig. 3i), including genes associated with amino acid transport (*SLC7A5*), T-cell activation (*DUSP2*, *DUSP4*, *CREM*, *ARID5A*, *PDE4B*, *RNF125*, and *NR4A2*), and chemotaxis (*CXCR3* and *CXCR4*) in response to inflammation.^48–52^ We further calculated the regulon specificity scores^29^ of these two *PDCD1*^+^ T cell clusters to identify their transcriptomic regulations (Fig. 3j) and found key regulators in the CD4-C5 cluster, such as transcription factors *RORA* related to colitis and inflammation,^53,54^ and *PRDM1* related to effector functions of T cells.^55^ Meanwhile, we also found key regulators in the CD8-C4 cluster, such as the transcription factors *IRF4*, *EOMES*, and *REL*, which all play important roles in regulating the differentiation and effector function of T cells.^56–58^ Herein, these analyses reveal that the transcriptomic regulations of *PDCD1*^+^ T cells in AS plaques are different from those of terminally-differentiated exhausted TILs, but rather are more activated.

Besides, we compared our transcriptomic dataset of T cells in AS plaques with that of the previous study^16^ and identified 8 T cell clusters. Among them, F_C0 and F_C2 highly expressed genes, such as *NFKBIA, FOS, DUSP1, DUSP2*, and *LMNA*, which shared a similar phenotype with *LMNA*^*+*^ Tem cells (CD4-C4 and CD8-C3) in our dataset (Fig. 2b; Supplementary information, S5a–c and Table S4). We then calculated the pairwise AUCell scores of T cell clusters in their dataset by using the top 30 DEGs of T cell clusters identified in our dataset and found that F_C2 represented the mixture of *LMNA*^*+*^ Tem cells (CD4-C4 and CD8-C3), *PDCD1*^*+*^ Tem cells (CD4-C5 and CD8-C4), and activated T cells (CD8-C8 and CD8-C9) in our dataset, which also had the highest expression level of *PDCD1* (Supplementary information, Fig. S5d). Meanwhile, F_C2 did not express the other T cell exhaustion-related genes, such as *HAVCR2*, *LAG3*, *CTLA4*, *TIGIT*, and *TOX* (Supplementary information, Fig. S5e), and was not enriched in the dysfunctional gene signature,^43^ indicating the existence of unexhausted *PDCD1*^*+*^ T cells also in their datasets (Supplementary information, Fig. S5f). Altogether, this independent study also supports our conclusions about the existence of pro-inflammatory and non-exhausted *LMNA*^*+*^ and *PDCD1*^*+*^ Tem cells in human AS plaques.

### Epigenetic footprints and regulations of AS plaque-specific *PDCD1*^*+*^ T cells

Single-cell chromatin landscape can reveal both the chromatin accessibility states of cell types and the critical gene regulators that program cellular functions. To investigate the chromatin accessibility states of T cell activation- or exhaustion-related genes in AS plaque-specific T cells, CD3^+^ T cells were sorted from four AS plaque samples (Supplementary information, Fig. S6a), and a single-nucleus assay for transposase-accessible chromatin using sequencing (snATAC-seq) analyses was performed. After data processing and quality control, 5,598 single nuclei were obtained and segregated into 12 T cell clusters (Supplementary information, Fig. S6b and Table S5). Comparing the inferred gene activity scores of each cluster (Supplementary information, Fig. S6c, d), we annotated these clusters as the resting CD4^+^ (C8) and CD8^+^ (C1) T cells (Tres), CD8^+^ Teff cells (C4), Treg cells (C11), Th17-like cells (C10), CD8^+^ activated T cells (C6 and C7), and γδT cells (C12). Consistent with the gene signature of *LMNA*^*+*^ and *PDCD1*^*+*^ T cells in AS plaques from scRNA-seq data (Fig. 2b), we found CD4^+^ Tem cells (C9) and CD8^+^ Tem cells (C3 and C5) with high gene activity scores of *CD44*, *CD69*, *DUSP4*, *LMNA*, and *PDCD1* (Supplementary information, Fig. S6d). C2 cluster was annotated as Tem-like cells because of their lower gene activities of cytotoxic genes (e.g., *PRF1*, *GZMB*, and *NKG7*) but higher gene activity of *LMNA* compared to CD8^+^ Teff cells (C4) (Supplementary information, Fig. S6d). We then used chromVAR^59^ to identify cluster-specific TF regulatory elements and found LEF1 and TCF7 particularly enriched in the resting T cells (C1 and C8), RORA in Th17-like T cells (C10), and YY1 in Treg cells (C11) (Supplementary information, Fig. S6e–g). Consistent with SCENIC analyses (Fig. 2g–i), the plaque-specific Tem and Tem-like cells (C2, C3, C5, and C9) were enriched in the motifs of activator protein-1 (AP-1) TFs (*FOS* and *JUNB*), BATF, and BACH2, whereas the activated CD8^+^ T cells (C6 and C7) were enriched in motifs of IRF3, STAT1, and STAT2 (Supplementary information, Fig. S6e–g).

**Fig. 4:**
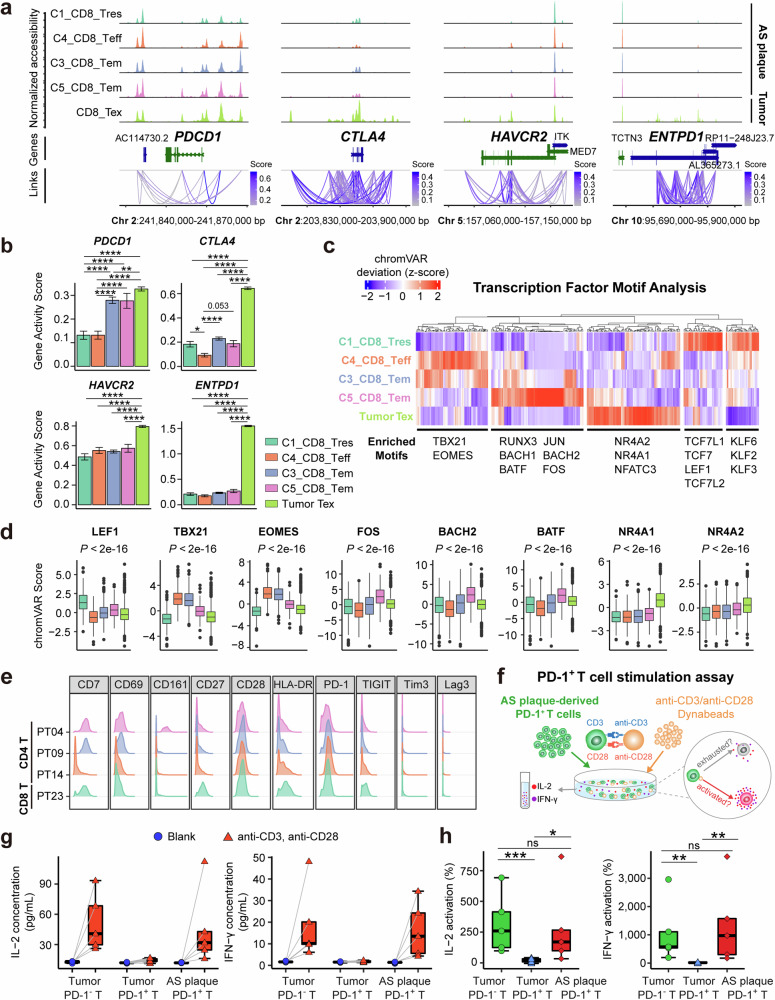
Ex vivo validations of the activated phenotype of AS plaque-specific PD-1^+^ T cells. **a** Coverage plots of the chromatin accessibilities of identified gene loci, colored by T cell clusters. **b** Bar plots showing the inferred gene activity scores for specified genes, colored by T cell clusters. **c** Heatmap plot showing the scaled chromVAR scores of TF motifs in T cell clusters, and motifs were clustered by hierarchy clustering. **d** Boxplots of chromVAR scores of selected TF motifs across T cell clusters. A one-way ANOVA test was used to compare chromVAR scores between AS plaque-specific T cells and tumor-specific Tex cells. **e** Histograms of selected T-cell functional marker expressions on PD-1^+^CD4^+^ (PT04, PT09, and PT14) and PD-1^+^CD8^+^ (PT23) T cell clusters. **f **Schematic diagram showing ex vivo T-cell stimulation assay for activating PD-1^+^ T cells derived from lung tumors (*n* = 11) or AS plaques (*n* = 5) to secret cytokines, in comparison to PD-1^–^ T cells derived from lung tumors (*n* = 5). **g**, **h** Concentrations of IL-2 and IFN-γ in the supernatant were displayed (**g**) and their relative activation levels (**h**) were compared. Data are presented as means ± SEM in **b** and as the median with IQR in **g** and **h**. A two-sided Student’s *t*-test with Benjamini–Hochberg adjustment was used in **b** and **h**.

To investigate the differential chromatin accessibility of AS plaque-specific CD8^+^PD-1^+^ Tem cells (C3 and C5), we integrated our snATAC-seq dataset with the exhausted CD8^+^ T cells from basal cell carcinoma.^60^ Compared with CD8^+^ Tres and Teff cells (C1 and C4), both CD8^+^ Tex (tumor-specific) and Tem (C3, C5; AS plaque-specific) cells exhibited higher accessibility of +5Kb and –5Kb cis-elements of *PDCD1* locus,^60,61^ and also higher inferred gene activity of *PDCD1* (Fig. 4a, b). However, the chromatin accessibilities of other T cell exhaustion-related genes (e.g., *CTLA4*, *HAVCR2*, and *ENTPD1*)^60^ were not enriched in plaque-specific Tem cells but were exclusively enriched in tumor-specific Tex cells (Fig. 4a, b). This indicates that the chromatin regulatory program in AS plaque-specific PD-1^+^ Tem cells was distinct from those PD-1^+^ Tex in tumors, despite both expressing PD-1.

Subsequently, we applied chromVAR^59^ to compare TF motif enrichments with the integrated snATAC-seq datasets (Fig. 4c). Using hierarchy clustering, we revealed the dominant subgroups of TF-binding motifs within T cell clusters, including the accessibility of TBX21 and EOMES motifs enriched in C3_CD8_Tem and C4_CD8_Teff clusters, and the accessibility of AP-1 family (FOS, JUN, BATF, and BATF3) and memory-associated motifs (RUNX3, BACH1, and BACH2) enriched in C3_CD8_ and C5_CD8_Tem clusters (Fig. 4c, d; Supplementary information, Fig. S6h). In contrast, the TF motifs (e.g., NR4A1, NR4A2, and NR4A3) orchestrating T-cell exhaustion were exclusively enriched in tumor-specific Tex cells but not in plaque-specific Tem cells (Fig. 4c, d; Supplementary information, Fig. S6h). Together, these data further support that *PDCD1*^*+*^ Tem cells (C3_CD8 and C5_CD8) in AS plaques remain activated and pro-inflammatory at the single-cell epigenetic level.

### Human AS plaque-specific PD-1^+^ T cells can be re-activated ex vivo

We next performed a series of ex vivo functional assays to validate whether human AS plaque-specific PD-1^+^ T cells could be re-activated. An additional CyTOF analysis of immune cells from 4 human AS plaques with the third antibody-staining panel was performed to evaluate the activation and exhaustion states of local T cells (Supplementary information, Fig. S7a and Table S3). AS plaque-specific PD-1^+^ T cells (PT04, PT09, PT14, and PT23) did not express the other T-cell exhaustion markers (e.g., Lag-3, Tim-3, and TIGIT) (Fig. 4e; Supplementary information, Fig. S7b), but highly expressed co-stimulatory molecules associated with T-cell activation (CD28, CD69, and HLA-DR) (Fig. 4e). These data confirm that PD-1^+^ T cells in human AS plaques are not functionally exhausted, but rather maintain a biologically activated state.

We next ex vivo stimulated PD-1^+^ T cells that were sorted from human AS plaques and lung tumor samples (Fig. 4f; Supplementary information, Fig. S7c). Similar to lung tumor-derived PD-1^–^ T cells, AS plaque-specific PD-1^+^ T cells were still capable of secreting pro-inflammatory cytokines (IL-2 and interferon (IFN)-γ), in contrast to lung tumor-derived PD-1^+^ T cells (Fig. 4g, h). We further sorted plaque-specific CD4^+^PD-1^+^ and CD8^+^PD-1^+^ T cells to analyze their cytokine-releasing capabilities. IFN-γ, IL-2, and tumor necrosis factor (TNF)-α were highly secreted from both CD4^+^ and CD8^+^ T cells, with IFN-γ being the highest; whereas IL-1β and IL-6 were seldomly released even after strong stimulation (Supplementary information, Fig. S7d–g). Besides, we observed a higher abundance of cytokines released from AS plaque-specific CD4^+^PD-1^+^ T cells than CD8^+^PD-1^+^ T cells, suggesting a stronger pro-inflammatory role of CD4^+^PD-1^+^ T cells in human AS plaques.

### scTCR-seq analysis reveals T-cell lineage dynamics in human AS plaques

We next aimed to explore the lineage relationship among different T cell clusters in AS plaques, particularly in regards to *LMNA*^+^ and *PDCD1*^+^ T cells, and to reveal how they are dynamically remodeled by the AS microenvironment. To accomplish this, we constructed human AS-specific TCR repertoires using the paired scTCR-seq and scRNA-seq datasets. Using these data, we identified 24,407 T cells (~56.9% of total T cells) with paired TCR-α and -β chains, and 12,259 different TCR clonotypes for downstream lineage tracing (Supplementary information, Table S6). Among these, 1,005 TCR clonotypes detected in three or more T cells were annotated as clonally expanded TCRs. Temra cells (CD4^+^: CD4-C6; CD8^+^: CD8-C5 and CD8-C7) demonstrated the highest clonally expansions both in AS plaques and AS PB. CD8^+^ Trm cells (CD8-C2), CD8^+^*LMNA*^*+*^ Tem (CD8-C3), CD8^+^*PDCD1*^*+*^ Tem (CD8-C4), and MAIT cells (CD8-C10) were also clonally expanded, but to a lesser extent than Temra cells and only in AS plaques^62^ (Supplementary information, Fig. S8a, b).

**Fig. 5:**
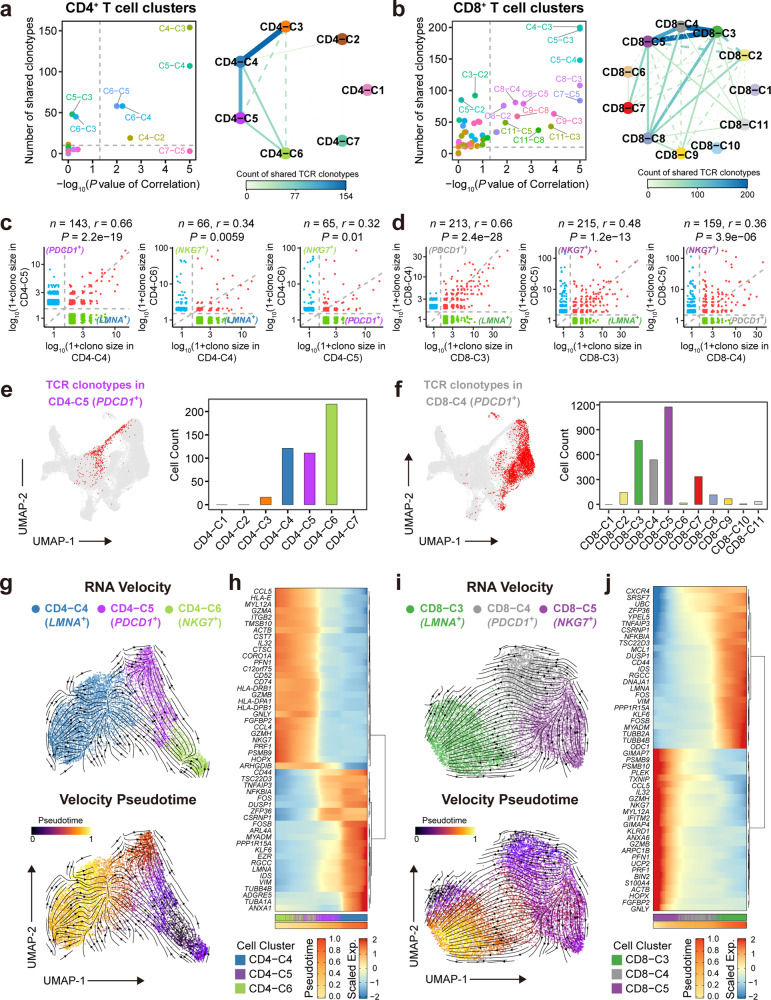
Paired scRNA-seq and αβTCR-seq reveal T-cell dynamics and differentiation trajectory of T cells in AS plaque. **a**, **b** Scatter plots (left) showing the counts and correlation tests of shared TCR clonal size in T cell cluster pairs, and gray dashed lines indicate counts equal to 10 (horizontal line) and *P* value equal to 0.05 (vertical line); graph plots (right) showing T cell cluster connections, with color and line width corresponding to counts of shared TCR clonotypes, and solid lines indicate T cell cluster pairs with significant (*P* < 0.05) correlation of the shared TCR clonal size in CD4^+^ (**a**) and CD8^+^ (**b**) T cell clusters. **c**, **d** Scatter plots showing the clone size of TCR clonotypes across the selected CD4^+^ (**c**) and CD8^+^ (**d**) T cell cluster-pairs, colored by shared (red) or non-shared (blue and green) TCR clonotypes. The diagonal line indicates equal TCR clone sizes, and other dashed lines separate non-shared clonotypes, *n* represents the number of shared clonotypes, and the correlation coefficient (*r*) and *P* value were labeled. **e**, **f** UMAP plots of T cells with identified TCR clonotypes in CD4-C5 (**e**) and CD8-C4 (**f**) clusters (left), and the related cell counts in individual T cell clusters (right). **g**, **i** UMAP plots showing RNA velocities for CD4-C4, CD4-C5, and CD4-C6 (**g**) and CD8-C3, CD8-C4, and CD8-C5 (**i**), colored by clusters (top) and cellular pseudotime (bottom). Arrows indicate the directions of T-cell differentiation. **h**, **j** Heatmaps showing the top 50 DEGs in analyzed CD4^+^ (**h**) or CD8^+^ (**j**) T cells arranged by pseudotime, and rows indicate genes and columns denote cells. Pseudotime and clusters of analyzed T cells were labeled on the bottom, and genes were labeled on the left. Pearson’s correlation test was used in **a**–**d**.

Rather than the local expansion, T cell differentiation and transition among different phenotypic clusters are also crucial for T cell responses in inflamed tissues.^34^ We found evident cross-sharing of TCR clonotypes among *LMNA*^*+*^ (CD4-C4), *PDCD1*^*+*^ (CD4-C5), and *NKG7*^*+*^ (CD4-C6) T cell clusters in CD4^+^ lineages, with 33 unique TCR clonotypes (464 cells) shared among the three clusters (Supplementary information, Fig. S8c, e). In CD8^+^ lineages, 126 unique TCR clonotypes (3,203 T cells), accounting for 40%–50% of TCR clonotypes in individual clusters, were shared among *LMNA*^+^ (CD8-C3), *PDCD1*^+^ (CD8-C4), and *NKG7*^+^ (CD8-C5) T cell clusters (Supplementary information, Fig. S8d, f). Clone size correlation analysis^63^ of the shared TCRs for every pair of CD4^+^ or CD8^+^ T cell clusters further confirmed that *LMNA*^*+*^, *PDCD1*^*+*^, and *NKG7*^*+*^ T cell clusters all had the significant and abundant sharing of TCR clonotypes (Fig. 5a–f). The cross-sharing of TCR clonotypes also existed between other T cell cluster pairs, including Th17-like (CD4-C3), *LMNA*^*+*^CD4^+^ T cells (CD4-C4), and across Temra cell clusters (CD8-C5, -C6, and -C7), suggesting dynamic transitions among these clusters in AS plaques (Fig. 5a, b).

To determine whether T cells with the same TCR clonotypes had different functional phenotypes, we analyzed the transcriptomes of T cells bearing the common TCR clonotypes among CD4-C4, -C5, and -C6 clusters or among CD8-C3, -C4, and -C5 clusters. Their gene expressions were strongly accordant with their designated T cell clusters but independent of their TCR clonotypes, providing additional evidence for inter-cluster lineage differentiation of T cells in AS plaques (Supplementary information, Fig. S8g, h). These results suggest the dominant clonal expansion and local differentiation of T cells among *LMNA*^*+*^, *PDCD1*^*+*^, and *NKG7*^*+*^ T cells in AS plaques.

### *PDCD1*^+^ Tem cells are not terminally differentiated but in an intermediate state in AS plaques

We inferred the directional differentiation trajectories among *PDCD1*^+^, *LMNA*^+^, and *NKG7*^+^ T cells in CD4^+^ and CD8^+^ T cell clusters. Using RNA velocity analysis,^64,65^ we found that *NKG7*^+^ Temra cells (CD4-C6 and CD8-C5) had two distinct differentiation directions. One direction pointed to itself, indicating that some of these cells could self-renew locally, while the other pointed to *PDCD1*^+^ T cells, moving towards and finally terminating in *LMNA*^+^ T cells. This suggested the transition trajectory from *NKG7*^+^ Temra cells toward *PDCD1*^+^ Tem cells (CD4-C5 and CD8-C4) and subsequently towards the differentiation terminals of *LMNA*^+^ Tem cells (CD4-C4 and CD8-C3) (Fig. 5g, i). Furthermore, we identified the top 50 genes that significantly changed along with cellular pseudotime. We found that the cytotoxic genes (*NKG7* and *PRF1*) were gradually downregulated, whereas the genes related to T-cell activation (*CD44*, *FOS*, *NFKBIA*, and *LMNA*) and cytoskeleton (*TUBB4B*, *TUBB2A*, and *VIM*) were continuously upregulated along with the differentiation trajectory from *NKG7*^+^ Temra to *LMNA*^+^ Tem via *PDCD1*^+^ Tem cells (Fig. 5h, j). This result suggests that these T cells may gradually lose cytotoxicity as they differentiate and possibly reach a persistently activated memory state.

We also utilized additional algorithms (CytoTRACE and Monocle 3)^66,67^ and obtained consistent results (Supplementary information, Fig. S9a–f), confirming that *PDCD1*-expressing CD4^+^ and CD8^+^ Tem cells served as an intermediate differentiation state from *NKG7*^+^ Temra transiting into long-lived *LMNA*^+^ Tem cells. Furthermore, we confirmed the terminally differentiated state of CD8^+^*LMNA*^*+*^ T cells (F_C2) in human AS plaques from the previously published scRNA-seq dataset^16^ by using CytoTRACE (Supplementary information, Fig. S9g). Interestingly, the decrease in the inferred CytoTRACE score was well accordant with the declined expressions of cytotoxic genes,^43^ confirming that T cells may gradually lose cytotoxicity as they differentiated into long-lived *LMNA*^*+*^ Tem cells in AS plaques (Supplementary information, Fig. S9h). Altogether, these analyses suggest that the *PDCD1*^+^ Tem cells should be the essential intermediate for *NKG7*^+^ Temra cells to transition into the long-lived *LMNA*^+^ Tem cells, potentially serving as the essential cell source for pro-inflammatory *LMNA*^+^ Tem cells in human AS plaques.

### Deficient expression of PD-1’s ligands in human AS plaques

**Fig. 6:**
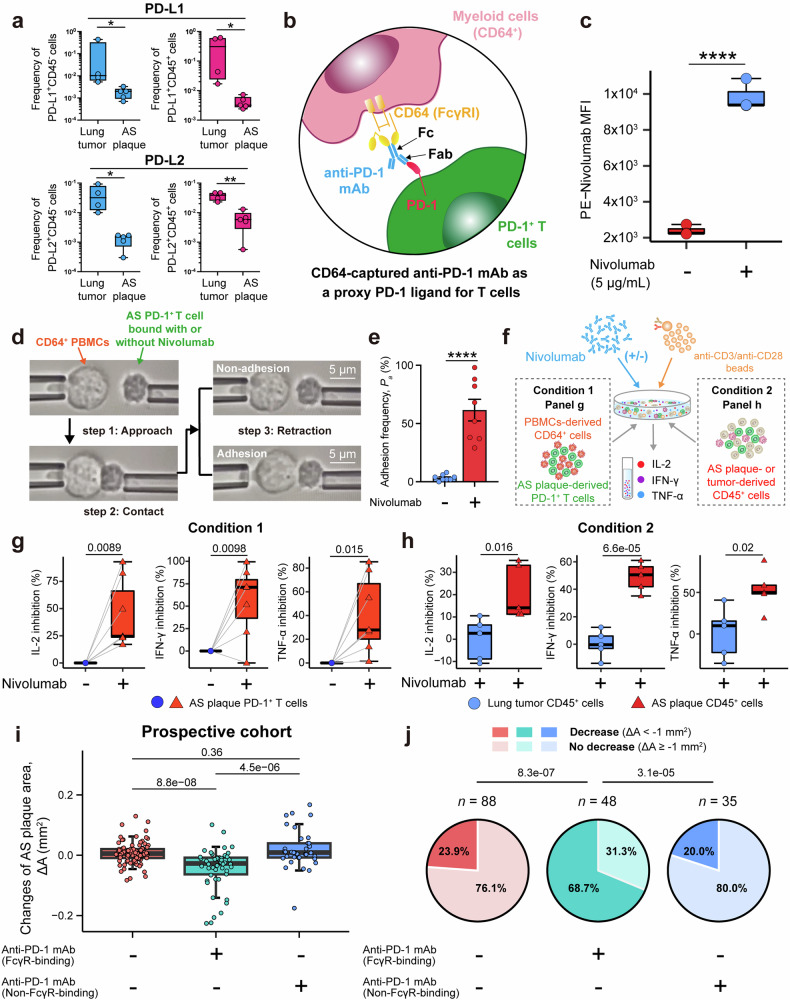
FcγRI-captured anti-PD-1 mAb serves as a proxy PD-1 ligand to suppress AS-derived PD-1^+^ T cells in vitro and reduces AS plaque areas in humans. **a** Frequency comparison of PD-L1^+^ and PD-L2^+^ cells in CD45^–^ and CD45^+^ cells in lung tumors (*n* = 4) and AS plaques (*n* = 5). **b** Diagram showing that CD64-captured anti-PD-1 mAb served as the proxy PD-1 ligand. **c** Mean fluorescence intensity (MFI) of PE-Nivolumab on PBMC-derived CD64^+^ cells treated with Nivolumab (5 μg/mL) or not. **d** Diagram showing micro-pipette adhesion assay for examining the cell–cell adhesion frequency (*Pa%*) between CD64^+^ PBMCs and primary PD-1^+^ T cells bound with Nivolumab or not. **e** Quantification of the cell–cell adhesion frequency (*P*_*a*_*%*) between CD64^+^ PBMCs and PD-1^+^ T cells bound with Nivolumab or not. **f–h** Schematic diagram (**f**) showing ex vivo stimulation assays of primary CD64^+^ myeloid cells sorted from PBMC (*n* = 7) and AS plaque-derived PD-1^+^ T cells (*n* = 7) co-cultured with anti-PD-1 mAb (Condition 1; **g**), or CD45^+^ cells sorted from either lung tumors (*n* = 5) or AS plaques (*n* = 5) (Condition 2; **h**) co-cultured with Nivolumab. Concentrations of IL-2, IFN-γ, and TNF-α in the supernatant of Condition 1 (**g**) or Condition 2 (**h**) were measured, and the relative inhibition levels (%) of cytokines in different CD45^+^ cells treated with Nivolumab were compared. **i**, **j** Comparing the changes of AS plaque areas (ΔA) (**i**) and the compositions of AS plaque progression (**j**) among three groups treated without (*n* = 88) or with anti-PD-1 mAb of FcγR-binding (*n* = 48) or non-FcγR-binding capability (*n* = 35) (decrease: ΔA < –1 mm^2^; no decrease: ΔA ≥ –1 mm^2^). Data are presented as means ± SEM in **e** and as median with IQR in **a**, **c**, **g**, **h**, **i**. Unpaired Student’s *t*-test was used in **a** and **c**, the paired *t*-tests in **e**, **g**, and **h**, the Mann–Whitney test in **i**, and the χ2 test in **j**.

We next investigated why human AS plaque-specific PD-1^+^ T cells still had pro-inflammatory functions and cytokine-releasing capability and did not differentiate into exhausted states as TILs in tumor microenvironments. Activating PD-1’s inhibitory function requires the presence and engagement of its natural ligands, e.g., programmed cell death 1 ligand-1 (PD-L1; CD274) and programmed cell death 1 ligand-2 (PD-L2 ; CD273).^68,69^ We thus examined the expressions of PD-1 ligands in human AS plaques. Almost no CD45^+^ immune cells and CD45^–^ non-immune cells expressed either PD-L1 or PD-L2 as revealed by immunohistochemistry (IHC) staining (Supplementary information, Fig. S10a) and flow cytometric analyses (Fig. 6a; Supplementary information, Fig. S10b). This is in contrast to the counterparts in *Ldlr*^*−/−*^ mice AS plaques,^70^ lung cancer, or other tumors.^71^ These results suggest that the deficient expression of PD-1 ligands in human AS plaques might contribute to forming the pro-inflammatory phenotype of PD-1^+^ T cells and their differentiation routines into the long-lived *LMNA*^+^ Tem cells.

### Surface-captured anti-PD-1 mAb serves as a proxy PD-1 ligand to suppress T cell activation

The engagement with PD-1 ligands (PD-L1 and/or PD-L2) can trigger the inhibitory function of PD-1, thereby suppressing T-cell activation. This mechanism has been used to treat cancers via immune checkpoint blockade (ICB)^45^ and to preliminarily relieve autoimmune disease with PD-1 agonists.^72,73^ We observed high expressions of CD64 (FcγRI) and CD32 (FcγRII) on myeloid cells in human AS plaques (Supplementary information, Fig. S10c–e). These receptors can bind the Fc domain of various immunoglobulin G (IgG) well.^74^ In particular, CD64 can bind anti-human IgG4 mAb (e.g., Nivolumab) with high affinity.^75,76^ Besides, we also found a close spatial vicinity of PD-1^+^ T cells and CD64^+^ cells, as revealed by IHC co-staining of PD-1 and CD64 in human AS plaques, greatly increasing the feasibility and availability for CD64-captured-Nivolumab to bind PD-1 (Supplementary information, Fig. S10f). Therefore, we hypothesized that surface-captured anti-PD-1 mAb via FcγRs (e.g., FcγRI/CD64) on human AS plaque-specific myeloid cells might serve as the proxy PD-1 ligand to suppress T-cell activation (Fig. 6b).

We first verified that Nivolumab (one kind of anti-human-PD-1 IgG4 mAb) could be captured by CD64^+^ human PBMCs (Fig. 6c; Supplementary information, Fig. S10g) and CD64^+^ cell lines (Supplementary information, Fig. S10h). The in vitro cell–cell conjugation assay confirmed the direct binding between CD64^+ ^HEK293 cells and PD-1^+^ Jurkat T cells in the presence of Nivolumab (Supplementary information, Fig. S10i). We further validated this by using a single-cell biomechanical adhesion assay,^77^ which detected a significantly increased adhesion frequency (*P*_a_*%*) between the Nivolumab-bound primary PD-1^+^ T cells from human AS plaques and PBMC-derived CD64^+^ cells (61.5%) as compared to the non-Nivolumab-bound condition (3.5%) (Fig. 6d, e).

We then conducted experiments to determine whether the interaction between CD64-captured anti-PD-1 mAb (e.g., Nivolumab) and PD-1 could activate the PD-1 downstream signaling pathway to suppress T-cell activation. By conjugating PD-1^+^ Jurkat T cells with beads coated with both Nivolumab and anti-CD3/anti-CD28 mAb, we found a significantly impaired cytokine release (IL-2) from PD-1^+^ Jurkat T cells (Supplementary information, Fig. S11a, b) and weakened expression of T-cell activation marker (NFAT, nuclear factor of activated T cells) (Supplementary information, Fig. S11c, d). Subsequently, we further confirmed this in primary PD-1^+^ T cells from human AS plaques by coculturing them with CD64^+^ cells from human PBMCs ex vivo in the presence or absence of Nivolumab (Condition 1 in Fig. 6f). Nivolumab was able to significantly inhibit the secretion of inflammatory cytokines (IL-2, TNF-α, and IFN-γ) by 25%–70% from AS plaque-specific PD-1^+^ T cells (Fig. 6g; Supplementary information, Fig. S11e). Furthermore, we performed an ex vivo coculture stimulation assay by coculturing human AS plaque- or lung-tumor-derived CD45^+^ cells with anti-CD3/anti-CD28-mAb-coated beads in the presence or absence of Nivolumab (Condition 2 in Fig. 6f). We found that the presence of Nivolumab significantly reduced the secretion of inflammatory cytokines (IL-2, IFN-γ, and TNF-α) by 15%–50% from AS plaque-derived CD45^+^ cells, but not from lung tumor-derived immune cells (Fig. 6h; Supplementary information, Fig. S11f).

### A prospective cohort validates the effectiveness of anti-PD-1 mAb on AS plaques

To further support this functional mechanism of the “proxy PD-1 ligand” in human atherosclerosis and confirm our findings from the retrospective cohort, we conducted a prospective cohort study. We prospectively recruited 171 tumor patients who received anti-tumor chemotherapy combined with (*n* = 83) or without (*n* = 88) anti-PD-1 treatment at SAHZU between 16th Sep 2022 and 24th Feb 2023 (Supplementary information, Fig. S11g). Of the 83 patients, 48 were treated with anti-PD-1 mAb of FcγR-binding capability, and 35 were treated with anti-PD-1 mAb of non-FcγR-binding capability. The recruited patients from 3 groups underwent a second ultrasound examination with an average follow-up of 3 months. The median treatment cycle was not significantly different across the three groups (Supplementary information, Fig. S12). Consistent with the findings from the retrospective cohort (Fig. 1e), patients with anti-PD-1 treatment (with or without FcγR-binding capability) showed significant (*P* = 0.0018) reductions of AS plaque areas (ΔA) as compared to those without anti-PD-1 treatment (Supplementary information, Fig. S11h). We found a significant reduction of ΔA only in patients treated with anti-PD-1 mAb of FcγR-binding capability (ΔA = −2.72 (−6.36, −0.80) mm^2^), compared to those either without anti-PD-1 treatment (ΔA = 0.55 (−0.92, 2.05) mm^2^; *P* = 8.8e–08) or with anti-PD-1 mAb of non-FcγR-binding capability (ΔA = 0.88 (−0.78, 3.98) mm^2^; *P* = 4.5e–06) (Fig. 6i).

In the group treated with anti-PD-1 mAb with FcγR-binding capability, a majority of patients (33 out of 48; 68.7%) experienced AS plaque reductions, which was significantly higher than the other two groups. The group treated with anti-PD-1 mAb with non-FcγR-binding capability had a plaque reduction frequency of 20.0%, while the group without anti-PD-1 treatment had a frequency of 23.9% (Fig. 6j). We compared the clinical characteristics and found significant differences that existed in multiple factors (Supplementary information, Fig. S12), including age, gender, the change in BMI, the change in triglyceride (ΔTG), the change in LDL (ΔLDL), tumor stage, tumor type, etc. across different treatment groups. By taking these confounding factors into account, we conducted both univariate and multivariate regression analyses, and these results confirmed that anti-PD-1 mAb with FcγR-binding capability played a protective role in human atherosclerosis, compared to the group without anti-PD-1 treatment (RR = 0.41 (0.23–0.70), *P* = 0.00182; RR = 0.42 (0.21–0.77), *P* = 0.0067) and the group treated with anti-PD-1 mAb of non-FcγR-binding capability (RR = 0.39 (0.20–0.72), *P* = 0.0033; RR = 0.31 (0.13–0.69), *P* = 0.0056) (Supplementary information, Fig. S11i, j).

Altogether, our findings suggest that anti-PD-1 mAb with FcγR-binding capability can serve as a proxy PD-1 ligand, rather than the traditional blockade method, to suppress T-cell activation in human AS plaques.

## Discussion

In this study, we discovered that the treatment with FcγR-binding anti-PD-1 mAb (e.g., Nivolumab) could reduce the sizes of AS plaques and potentially resolve human atherosclerosis, based on a retrospective cohort analysis. Furthermore, we revealed one of the possible working mechanisms: the FcγR-binding capability of anti-PD-1 IgG4 mAb allows it to be captured by FcγRs, acting as a proxy PD-1 ligand to suppress activated and pro-inflammatory PD-1^+^ T-cell functions in AS plaques. This finding is crucial for the field of human AS as it suggests a potential therapeutic approach to resolve local inflammation via suppressing pro-inflammatory T-cell functions in human AS plaques.

Our analysis of scRNA-seq reveals that PD-1^+^ T cells infiltrating AS plaques remain functionally activated and pro-inflammatory, whereas they are not terminally differentiated (Figs. 2e, f and 5g, i). These PD-1^+^ Tem cells do not co-express other typical immune checkpoints (Lag-3, Tim-3, and TIGIT) at both the transcriptomic and protein levels (Figs. 3h, 4e). Beyond *PDCD1*, PD-1^+^ Tem cells infiltrating human AS plaques do not show epigenetic footprints of key regulators modulating T-cell exhaustion, such as *CTLA4*, *HAVCR2*, and *ENTPD1*. More remarkably, the cytokine-secretion ability of these PD-1^+^ Tem cells is not impaired ex vivo (Fig. 4g, h; Supplementary information, Fig. S7d–g), resembling the non-exhausted PD-1^+^ T cells that were previously reported in other non-cancer diseases (e.g., SARS-CoV-2, juvenile idiopathic arthritis, and chronic pancreatitis).^78–80^ Instead, the trajectory analyses proposed that they may adopt a distinctive differentiation route by directionally switching to the long-lived and plaque-resident inflammatory *LMNA*^+^ Tem cells (Fig. 5g, i). These findings suggest that *PDCD1*^+^ Tem cells may undergo functional adaptations in human AS plaques. We speculate that it may be potentially due to distinct antigen stimulation, cytokine signals, and transcriptomic reprogramming in AS context.

The distinct functional phenotype of AS plaque-specific PD-1^+^ T cells suggests their distinct local microenvironments. Interestingly, we observed extremely low or almost no expression of PD-L1/L2 in human AS plaques, which was validated in multiple ways (Fig. 6a; Supplementary information, Fig. S10a, b). This significantly differs from the tumor microenvironment or atherosclerotic murine models.^70,81^ This lack of expression may be a possible reason for preventing *PDCD1*^+^ Tem cells from differentiating into a terminal exhausted state but instead transforming into pro-inflammatory long-lived *LMNA*^+^ Tem cells. Additionally, PD-1^+^ Tem cells in AS plaques express TFs related to the activation of NF-κB signaling, such as REL, RELA, RELB, NFKB1, and NFKB2. These TFs may contribute to reshaping these T cells into pro-inflammatory phenotypes, thereby driving the development of AS plaque.^82–84^ Based on these findings, we speculate that these PD-1^+^ Tem cells may play a pivotal role in maintaining chronic and non-resolved inflammation in human AS plaques.

In this scenario, it is essential to answer fundamental questions about how to regulate PD-1^+^ T cells and whether suppressing these T cells could resolve local inflammation or inhibit human AS progression. Our retrospective cohort study suggests that the anti-PD-1 treatment benefits the shrinkages of human AS plaques, which is consistent with the case report that demonstrated a similar reduction of AS plaques in patients after 8 weeks of Nivolumab treatment.^85^ However, this result is inconsistent with previous studies that reported immune checkpoint blockade therapies with anti-cytotoxic T lymphocyte-associated antigen-4 (CTLA-4) and/or anti-PD-1 mAb might induce T cell-mediated inflammation and promote AS plaque progression in tumor patients^86–89^ and *Ldlr*^*−/−*^ mice.^81,87^ Three possible explanations for these discrepancies include: (1) patients in our cohorts were only treated with one kind of immunotherapy (i.e., anti-PD-1 mAb), whereas patients in the previous studies received a mix of anti-CTLA-4 and/or anti-PD-1 mAb. The role of anti-CTLA-4 mAb on T cells in human AS plaques is not clear, and this may affect the overall outcome of mixed treatment; (2) the immune systems of mice and humans are intrinsically different, leading to different immune mechanisms; (3) the expression of PD-1 ligands in AS plaques differs between humans and genetically modified mice, likely inducing differential functional states of PD-1^+^ T cells in human AS plaques compared to those in mouse models.

The activation of PD-1 signaling to treat autoimmune diseases^72,73^ has inspired us to target these pro-inflammatory PD-1^+^ T cells to resolve AS plaque inflammations. The absence of PD-1 ligands in AS plaques creates a specific microenvironment for targeting PD-1 expressed in these T cells. A previous study reported cell-targeted PD-1 agonist immune-modulating monoclonal TCR against autoimmunity (ImmTAAI) molecule, which mimics PD-L1, to activate PD-1 signaling on T cells and suppress T-cell functions.^73^ Another study reported an immunotoxin (consisting of an anti-PD-1 single-chain variable fragment, an albumin-binding domain, and pseudomonas exotoxin) to specifically deplete PD-1^+^ T cells, thereby assuaging inflammatory responses in the context of autoimmune diseases.^90^ Additionally, the anti-CTLA-4 antibodies with the Fc domain were reported to induce colitis by targeting Treg cells.^91^ These findings support our approach of targeting PD-1^+^ T cells via the FcγRs-captured anti-PD-1 mAb. Our in-vitro and ex-vivo functional analysis, and biophysical analysis all support the downstream suppression of T-cell functions. Tislelizumab, also known as BGB-A317, is specifically designed to abolish the binding of IgG4 and FcγRI such that it cannot behave as a proxy ligand to suppress T-cell activation.^92^ Thus, within the anti-PD-1-treated patients in our prospective cohort, the Tislelizumab-treated patients served as a negative control, and the corresponding cohort analysis results support that the binding of anti-PD-1 mAb and FcγRI is crucial to activating the inhibitory function of PD-1 in AS plaque-specific T-cell activation, further supporting our idea that only the anti-PD-1 mAb with FcγR-binding capability could potentially lead to AS plaque shrinkages in humans.

This study still has several limitations. First, while multi-omics single-cell techniques have been used to give in-detail profiling of the immune microenvironment of human AS plaques, the dynamic changes in the local microenvironment along with disease progression remain unknown. Additionally, the single-cell isolation process, due to its technical defects, may potentially lead to biased and different identification of myeloid cell compositions compared to other pathology studies.^91^ Second, we acknowledge that there might be other working mechanisms of anti-PD-1 mAb in human AS plaques in vivo, such as activating the other FcRs signaling to delay T-cell activation or induce T-cell death.^93^ However, due to ethical limitations, it has been impossible to obtain AS plaque samples to investigate AS plaque progression from non-cancer patients after anti-PD-1 therapy, as this therapy is currently only approved for anti-cancer treatment worldwide. Third, the anti-PD-1 mAb used in our clinical cohort study was not specifically designed to treat human AS. Therefore, a more desirable and meaningful approach would be a multi-center, prospective cohort design without other disease interventions. Finally, as several ICB-associated immune-related adverse events (irAEs) have been reported,^94^ there is a need for PD-1 agonists tailored to inhibit T-cell activation in the human AS plaques, which requires further investigation in the future.

In conclusion, we have discovered activated and pro-inflammatory PD-1^+^ T cells in human AS. We have proposed a potential therapeutic approach to target these cells to resolve human AS. Our finding was driven by retrospective clinical investigations, and its working mechanism was revealed and systematically validated by multi-omic single-cell analysis, basic immunological functional and biophysical analyses, as well as a prospective cohort investigation. However, to further evaluate the safety and efficacy of anti-PD-1 treatment for anti-atherosclerosis in humans, a long-term systematic and prospective human cohort study will be required. Our work also enhances the understanding of the pathogenesis of AS from the perspective of adaptive immune remodeling. It opens up the possibility of T cell-based immunotherapy for combating human AS.

## Materials and methods

### Study subjects

Samples of peripheral blood (PB), atherosclerotic plaque (AS plaque), and lung tumors were collected from The Second Affiliated Hospital of Zhejiang University School of Medicine (China). The study was performed in accordance with the declaration of Helsinki and the study protocol was approved by the Ethics Committee of The Second Affiliated Hospital of Zhejiang University School of Medicine (SAHZU) (ID: #2017-102). Participants or their legal guardians provided written informed consent before obtaining peripheral blood or tissue samples. Participants in the retrospective cohort (ID: #2021-0533) and prospective cohort (ID: #2022-0803) were recruited from The Second Affiliated Hospital of Zhejiang University School of Medicine, and all protocols for processing human medical information were performed in accordance with the declaration of Helsinki and approved by the hospital. Participants or their legal guardians provided written informed consent before the recruitment in cohort study.

Samples of atherosclerotic blood (AS PB) were obtained from diagnosed atherosclerotic patients with definite imaging signs of atherosclerotic lesions in coronary arteries (artery stenosis > 50%) and/or peripheral arteries. AS plaque samples were obtained from the patients undergoing endarterectomy from the Department of Surgery, and the patients with a known history of tumor diseases, infectious diseases, abnormal liver or renal tests, vascular diseases, or those who had received systematic chemotherapy or radiotherapy were excluded. For functional assays, we additionally enrolled and collected the treatment-naïve lung tumor tissues from patients at the Department of Surgery who had not received anti-tumor therapy, chemotherapy, or radiotherapy before tumor resection.

### Retrospective cohort study of anti-PD-1 therapy’s effects on carotid plaque progression

To investigate the clinical effects of anti-PD-1 treatment on AS plaque progression in vivo, we retrospectively analyzed a cohort of tumor patients who received chemotherapy either combined with anti-PD-1 immunotherapy (anti-PD-1-treated group) or not (non-anti-PD-1 treated group) at The Second Affiliated Hospital of Zhejiang University School of Medicine (SAHZU, China) between 1st Jan 2018 and 1st May 2022 (ID: #2021-0533). The anti-PD-1-treated group included tumor patients who received Nivolumab (Bristol-Myers Squibb), Pembrolizumab (Merck Sharp & Dohme), Sintilimab (Innovent Biologics), Serplulimab (Henlius), Toripalimab (TopAlliance), Camrelizumab (Hengrui). Whether or not a patient received anti-PD-1 therapy and which anti-PD-1 treatment to receive was jointly decided by the tumor physicians and the patients themselves, without any of our intervention. We measured the changes in the carotid plaque area using ultrasound images by assessing the cross-sectional area of longitudinal views of all visible plaques as previously described.^95,96^ Ultrasound-image-based AS plaque areas were analyzed by two independent ultrasound professionals with ImageJ software in a double-blinded manner. Due to intrinsic and systematic errors of ultrasound instruments and measurements of area changes (ΔA) of AS plaques, the decrease (ΔA < –1 mm^2^) and no decrease (ΔA ≥ –1 mm^2^) were defined for downstream analyses to enhance the accuracy of evaluation.

To be eligible for the study, patients had to meet the following criteria: (1) aged over 18; (2) have carotid plaques; (3) have received ≥ 2 cycles of immuno- or chemotherapy; (4) have had at least two ultrasound imaging records of AS plaques during immunotherapy or chemotherapy period (when > 1 ultrasound images were available for the baseline study (Scan 1), the oldest ultrasound images were analyzed, and when > 1 ultrasound images were available for the follow-up study (Scan 2) then the most recent study was analyzed); and (5) have received regular immuno- or chemotherapy between the baseline study (Scan 1) and follow-up study (Scan 2). Exclusive criteria are for those patients who (1) had received surgical operations after Scan 1; (2) had received radiotherapy after Scan 1; or (3) were deficient in follow-up (Scan 2). The clinical information of included patients is shown in the Supplementary information, Fig. S1.

We used both univariate and multivariate (Modified Poisson)^22^ regression models to calculate the relative ratio (RR) and 95% confidence interval (CI) to estimate the RR of AS plaque progression by chemotherapy with or without anti-PD-1 treatment. The estimate of RR refers to the increase of AS plaque area after chemotherapy with or without anti-PD-1 treatment. RR < 1 represents a risk factor that negatively correlates with the increase of AS plaque area, whereas RR > 1 represents a risk factor that positively correlates with the increase of AS plaque area. *P* values were calculated from Modified Poisson regression analysis, and multivariate analyses were adjusted by using age, gender, the changes in BMI (ΔBMI), the changes in HDL (ΔHDL), ΔLDL, statin usage, tumor type, tumor stage, and tumor progression.

### A prospective cohort study of anti-PD-1 therapy’s effects on carotid plaque progression

To further validate our clinical finding that anti-PD-1 mAb could reduce carotid plaques, we established a prospective cohort of tumor patients who were about to receive chemotherapy, either combined with anti-PD-1 therapy or not. These patients were prospectively recruited from the Department of Oncology and Department of Respiratory of The Second Affiliated Hospital of Zhejiang University School of Medicine (SAHZU, China) between 16th Sep 2022 and 24th Feb 2023 (ID: #2022-0803; NCT05549336). Whether or not a patient received anti-PD-1 therapy and which anti-PD-1 treatment to receive was jointly decided by the tumor physicians and the patients themselves, without any of our intervention. Ultrasound examinations of carotid arteries were performed at baseline when the patients received the first dose of either immunotherapy or chemotherapy.

Inclusive criteria for patients: (1) were aged over 18, (2) were never treated with anti-tumor therapy before enrollment, (3) ultrasound-diagnosed carotid plaques, and (4) have not received radiotherapy or surgical operation after the baseline ultrasound examination. Patients satisfying all of the inclusive criteria were subsequently seen for a second ultrasound examination with an average follow-up of 3 months. Among eligible patients (*n* = 196), 25 were excluded from the final analysis due to withdrawal of informed consent (*n* = 17), missed follow-up (*n* = 6), or poor image quality (*n* = 2). The anti-PD-1-treated patients were divided into two subgroups: one subgroup of patients receiving anti-PD-1 mAb with FcγR-binding capability (the group treated with anti-PD-1 mAb with FcγR-binding capability), including Nivolumab, Keytruda, Sintilimab, Serplulimab, Toripalimab, and Camrelizumab; and the other subgroup of patients receiving Tislelizumab, a kind of anti-PD-1 mAb without FcγR-binding capability (the group treated with anti-PD-1 mAb with non-FcγR-binding capability). In total, we enrolled 171 eligible patients for prospective cohort analyses, of which 88 were in the group without anti-PD-1 mAb, 48 were in the group with anti-PD-1 mAb with FcγR-binding capability, and 35 were in the group with anti-PD-1 mAb with non-FcγR-binding capability. The modified Poisson regression analysis for the prospective cohort was performed as previously described. Multivariate analysis was adjusted by using age, gender, ΔBMI, ΔHDL, ΔLDL, statin usage, tumor type, tumor stage, and tumor progression when comparing FcγR-binding anti-PD-1 mAb and non-anti-PD-1 mAb groups. It was adjusted by using age, gender, ΔBMI, TG-1, ΔTG, ΔLDL, statin usage, tumor type, tumor stage, and tumor progression when comparing FcγR-binding anti-PD-1 mAb and non-FcγR-binding anti-PD-1 mAb groups.

### Cell lines

CD64^+^ HEK293 and PD-1^+^ Jurkat T cells were respectively provided by Dr. Tong Zhang’ Lab from BeiGene (Beijing, China) and Dr. Jizhong Lou’s Lab at the Institute of Biophysics, University of Chinese Academy of Sciences (Beijing, China). They were cultured in Dulbecco’s Modified Eagle Medium (DMEM) and Roswell Park Memorial Institute (RPMI)-1640 medium (Basal Media) supplemented with 100 μg/mL Penicillin, 100 U/mL Streptomycin (Gibco), and 10% Fetal Bovine Serum (FBS) at 37 °C in 5% CO_2_. Mycoplasma tests were negative in both cell lines.

### Single-cell processing of human samples

Fresh PB samples were collected (10 mL/sample) into Ethylene Diamine Tetraacetic Acid (EDTA) anti-coagulation tubes (BD Biosciences) and treated with Ammonium-Chloride-Potassium (ACK) lysing buffer (Solarbio Life Sciences) to remove red blood cells. The cells were washed, counted, and resuspended in the fluorescence-activated cell sorting (FACS) buffer. For PB samples used in the scRNA-seq analyses, fresh PB samples were processed into PBMCs with Ficoll-Paque PLUS (GE Healthcare) following the manufacturer’s instructions. PBMCs were then treated with ACK lysis buffer as described above, counted, assessed for cell viability, and finally resuspended in FACS buffer.

Fresh AS plaque tissues were stored in MACS Tissue Storage Solution (Miltenyi Biotech) immediately after resection. The tissues were minced and transferred into 5 mL of enzymatic digestion mix consisting of RPMI-1640 medium supplemented with Collagenase IV (4 mg/mL), Hyaluronidase (250 μg/mL), and DNase I (20 μg/mL). Tissue digestions were carried out at room temperature, 60 rpm for 4 h and terminated by adding 5 mL Hank’s Balanced Salt Solution (HBSS) buffer mix (Ca^2+^/Mg^2+^-free, Thermo Fisher) containing 2 mM EDTA (Sangon Biotech) and 0.5% Bovine Serum Albumin (BSA; Sigma-Aldrich). The cells were then filtered through a 70-μm nylon cell strainer (Corning), and treated with ACK lysis buffer for 2–3 min. Subsequently, the cells were pelleted, washed, and resuspended in the FACS buffer.

Fresh lung tumors were manually cut up and then transferred into a 5 mL enzymatic digestion mix consisting of RPMI-1640 medium supplemented with Collagenase IV (2 mg/mL), 250 μg/mL Hyaluronidase (Sigma-Aldrich), and DNase I (20 μg/mL). Tissue digestions were processed at 37 °C, 145 rpm for 1 h and terminated by adding 5 mL HBSS buffer mix. The cells were then filtered through a 70-μm nylon cell strainer (Corning), and treated with ACK lysis buffer as described before. Finally, the cells were pelleted, washed, and resuspended in the FACS buffer.

### Antibody labeling, staining, and barcoding for CyTOF assay

Antibodies were conjugated with the indicated metal tags using the Maxpar Antibody Labeling Kit (Fluidigm), following the manufacturer’s standard instructions. After metal-tag conjugation, the concentration of each antibody was determined using Nano-100 (Allsheng Instrument), and then the concentration was validated and optimized for the final staining concentration for CyTOF analyses. A maximum of 3 × 10^6^ cells from PB and AS plaque samples were labeled with ^194^Pt (Platinum) (Cisplatin, Fluidigm) for 5 min, and incubated with Fc receptors (FcRs) blocking reagent mix (human, mouse, rat, and hamster IgG, Equitech-bio) for 20 min. These cells were then resuspended with 100 μL Surface Marker Staining Mix in FACS buffer for 30 min. After washing, the cells were incubated with Fix-and-Perm Buffer (Fluidigm) containing 250 μM DNA Intercalator iridium (^191^Ir and ^193^Ir, Fluidigm) overnight at 4 °C.

For intracellular staining, the cells were stained with an Intracellular Marker Staining Mix in Permeabilization Buffer (eBioscience) for 30 min. Mass-tag cellular barcoding of cells was performed before introducing cells into the CyTOF system. Briefly, five palladium isotopes (^104^Pd, ^105^Pd, ^106^Pd, ^108^Pd, and ^110^Pd, TRACE) were conjugated to bromoacetamidobenzyl-EDTA (BABE, Dojindo), following the manufacturer’s instruction.^97^ Cells from AS PB and AS plaques were then resuspended and barcoded in 100 μL Phosphate Buffered Solution (PBS) buffer containing two palladium isotopes for 30 min. After removal of the supernatant, the cells were washed with FACS buffer and ddH_2_O respectively before being pooled into a FACS tube (BD Biosciences) for CyTOF analyses.

### CyTOF data acquisition

Before introducing the cells into the CyTOF system, we performed a tuning and quality control procedure to calibrate the CyTOF system with the Tuning Solution (Fluidigm) and EQ^TM^ Four Element Calibration Beads (Fluidigm). We diluted the cells to a concentration of 1 × 10^6^ cells/mL in ddH_2_O with 20% EQ Beads and then flowed into a new 35-μm filter-cap FACS tube. We acquired data with the CyTOF system at an average rate of 300 to 500 events/s and recorded raw data for over 1 million events for each sample.

### Immunofluorescence (IFC) staining

Frozen tissue sections, 7-μm thick, were air-dried on glass slides and fixed in 4% formaldehyde for 10 min at room temperature. The cells were then permeabilized with 0.5% Triton X-100 (Thermo Fisher) for 10 min and non-specific antibody binding was blocked by incubation with PBS buffer containing 3% BSA for 1 h. The slides were then incubated with primary antibodies, including rat anti-human CD3 antibody (1:200, CD3-12, Abcam), mouse anti-human PD-1 antibody (Ready-to-use, MX033, MXB Biotech), rabbit anti-human CD4 antibody (1:200, EP204, ZSGB-BIO), and rabbit anti-human CD8 antibody (1:100, SP16, ZSGB-BIO). After washing, slides were incubated with fluorescent secondary antibodies (1:500, Abcam) for 1 h and covered with DAPI-containing Mounting Medium (Vector Lab). Images were acquired using laser confocal scanning microscopy (Leica Microsystems).

### IHC staining

For PD-L1 staining, 3-μm thick formalin-fixed paraffin-embedded (FFPE) tissue sections on glass slides were heated at 68 °C overnight. The sections were then deparaffinized in xylene and rehydrated in graded ethanol. Heat-mediated antigen retrieval was performed in retrieval solution (supplement with sodium citrate, pH = 6) in the pressure cooker for 3 min. Tissue sections were blocked by PBS buffer with 3% BSA for 1 h and treated with 0.3% H_2_O_2_ for 10 min. The sections were incubated with primary antibody at room temperature for 2 h. The samples were then incubated with either an anti-human PD-L1 antibody (Ready-to-use, 22C3, Agilent) or an anti-human PD-L1 antibody (Ready-to-use, SP263, Roche). After being washed with PBS buffer, the slides were incubated with Horseradish Peroxidase (HRP)-conjugated secondary antibodies for 1 h, and then with 3,3′-diaminobenzidine (DAB, ZSGB-BIO) for 10 min. The sections were then processed with hematoxylin counterstain before graded ethanol dehydration and xylene penetration. For co-localization staining, 7-μm thick frozen and serial tissue sections were respectively incubated with primary antibodies of anti-human PD-1 antibody (Ready-to-use, MX033, MAB Biotech), anti-human PD-L1 antibody (1:100, 29E.2A3, BioLegend), and anti-human CD64 antibody (1:100, 10.1, BioLegend) according to the abovementioned procedure. The images were acquired by light microscopy (Leica Microsystems).

### Flow cytometric analysis of PD-L1 and PD-L2 expression

To analyze the expression of PD-L1 and PD-L2 on human AS plaque-derived cells, single-cell suspensions were made after tissue digestion. The suspensions were then resuspended with ACK lysis buffer to remove RBCs, as described previously. No further cell purification procedure was performed. The single-cell suspensions were then resuspended with FcRs Blocking Reagent for 30 min and stained with APC anti-human CD45 (HI30, BioLegend) and PE anti-human PD-L1 (1:50, MIH1, BioLegend) or PE anti-human PD-L2 (24 F.10C12, BioLegend) in FACS buffer. After washing, flow cytometric analysis of PD-L1 and PD-L2 expressions on immune (CD45^+^) and non-immune (CD45^–^) cells either derived from AS plaques or lung tumors were performed on a BD FACS Aria II (BD Biosciences), and the results were analyzed with FlowJo (Tree Star).

### scRNA-seq library preparation for paired messenger (m)RNA and αβTCR V(D)J sequencing

Single-cell suspensions of AS PB and AS plaque samples were prepared as previously described. For antibody staining, PBMCs were incubated with FcRs Blocking Reagent Mix for 30 min on ice and then stained with PE anti-human CD45 (HI30, BioLegend) and PE/Cy7 anti-human CD66b (G10F5, BioLegend) in FACS buffer. Cells from AS plaque samples were incubated with FcRs Blocking Reagent Mix for 30 min and then stained with PE anti-human CD45 (HI30, BioLegend). Subsequently, the targeted CD45^+^CD66b^–^ cells from PB samples and CD45^+^ cells from AS plaque samples were sorted on BD FACS Aria II (BD Biosciences). FACS-sorted target cells were assessed for quantity and cell viability by Trypan Blue Staining and Hemocytometer (Bio-Rad TC20). Cells with over 90% viability were then resuspended in FACS buffer at a concentration of 1000 cells/μL.

Target cells were introduced to the GemCode Single Cell Platform by adopting Chromium Single Cell 5′ Library and Gel Bead Kit (10X Genomics, PN-1000006), and Chromium Single Cell A Chip Kit (10X Genomics, PN-120236). Simultaneously, Chromium Single Cell V(D)J Enrichment Kits (10X Genomics, PN-1000005) was used to enrich full-length V(D)J segments of T cells. Approximately 16,000 cells per sample from single-cell suspensions were transferred to A chips, and an average of ~8000 to 10,000 cells per sample were recovered. Single cells were packed as barcoded Gel Beads in Emulsion (GEMs) in the Chromium Single Cell Controller system. The cells in GEMs were lysed and produced barcoded full-length cDNA from mRNA. Afterward, full-length cDNA was amplified, fragmentized, and added with unique sample indexes using Chromium i7 Multiplex Kit (10X Genomics, PN-120262). The constructed libraries for single-cell 3′ mRNA and 5′ V(D)J were separately sequenced on the NovaSeq 6000 platform (Illumina).

### snATAC-seq library preparation for CD3^+^ T cells in human AS plaques

Single-cell suspensions of AS plaque samples were prepared as previously described. For antibody staining, single cells were incubated with FcRs blocking reagent mix for 30 min and then stained with Live/Dead Aqua (Thermo Fisher), APC anti-human CD45 (HI30, BioLegend), APC/Cy7 anti-human CD3 (UCHT1, BioLegend), and PE anti-human PD-1 (EH12.2H7, Bristol-Myers Squibb) antibodies. Subsequently, CD3^+^ T cells from AS plaque samples (*n* = 4) were then sorted on a BD FACS Aria II (BD Biosciences). FACS-sorted target cells were assessed for quantity and cell viability by Trypan Blue and Hemocytometer (Bio-Rad TC20). 1.1 × 10^5^ target cells were used for single nuclei isolation by adding lysis buffer (1% BSA, 3 mM MgCl_2_, 10 mM Tris–HCl, 10 mM NaCl, 0.1% Tween-20, and 0.1% Nonidet P40 Substitute) for 5 min. Nuclei were then washed and diluted to a concentration of 1000 nuclei/μL.

The nuclei suspension was incubated with the transposition mix (ATAC Buffer B and ATAC Enzyme) at 37 °C for 1 h. Then it was mixed with the Master mix (Barcoding Reagent B, Reducing Agent B, and Barcoding Enzyme). Finally, the transposed nuclei suspension, barcoded Gel beads, and partitioning oil were introduced to the chromium next to GEM chip E for constructing a single-nuclei library. The generated single nuclei GEMs were collected and further linearly amplified according to the manufacturer’s protocol as previously described.^60^ The constructed library was then sequenced on a NovaSeq 6000 platform (Illumina).

### PE/Alexa Fluor 405 labeling of anti-PD-1 mAb

Freshly opened anti-PD-1 mAb (Nivolumab, Bristol-Myers Squibb) was diluted to 5 mg/mL and treated with 20 mM DL-Dithiothreitol (DTT) in 200 μL MES buffer (50 mM 2-(N-Morpholino) Ethanesulfonic Acid, 2 mM EDTA, pH = 6.0) for 30 min at room temperature. Meanwhile, 1.6 mg PE-dyes (AAT Bioquest) were calculated and incubated with 0.5 mM Succinimidyl 4-(N-maleimidomethyl) cyclohexane-1-carboxylate (SMCC, Thermo Fisher) in 200 μL PBS (2 mM KH_2_PO_4_, 8 mM Na_2_HPO_4_, 136 mM NaCl, 2.6 mM KCl, 2.5 mM EDTA, pH = 7.2) for 30 min at room temperature. Then, the two mixtures were independently flown through the HiTrap Desalting Column with Sephadex G-25 resin (GE Healthcare) with MES buffer to remove residual DTT and SMCC. The reduced anti-PD-1 mAb was then conjugated with PE-SMCC at room temperature for 1 h in a dark place. This reaction was terminated with 34 μL 1 mg/mL NEM (Sigma) for 20 min. For Alexa Fluor-405 labeling, 10 μM anti-PD-1 mAb was treated with 50 μM Alexa Fluor 405 NHS ester (Thermo Fisher) in PBS at room temperature for 1 h. Then, the mixture was flown through a HiTrap Desalting Column with Sephadex G-25 resin (GE Healthcare) with PBS buffer to remove free Alexa Fluor 405 dye. PE- and Alexa Fluor-405-labeled anti-PD-1 mAb were further validated with the staining of PD-1^+^ Jurkat T cells by flow cytometry and kept in PBS buffer (with 0.01% NaN_3_) at the concentration of 1 mg/mL for preservation at 4 °C.

### Cytometric Bead Array (CBA)

The concentrations of IL-2, IFN-γ, TNF-α, IL-1β, or IL-6 in the supernatant were assessed using a Cytometric Bead Array (CBA, BD Biosciences). The supernatant containing cytokines was diluted in RPMI-1640 medium followed by the manufacturer’s standard protocol. In brief, 50 μL cytokine containing RPMI-1640 medium was diluted with an equal volume of FACS buffer containing 0.2 μL capture beads to enrich and capture specific cytokine at room temperature for 1 h. Capture beads were then incubated with 50 μL FACS buffer containing 0.2 μL relevant PE Detection Reagent at room temperature for another 1 h. The MFI of these beads was detected and quantified by flow cytometry (Beckman Coulter). The concentrations of IL-2, IFN-γ, TNF-α, IL-1β, or IL-6 in the supernatant were calculated by comparing with a series of known-concentration standard samples, which were also performed as described above. The percentage changes of activation or inhibition of cytokine releasing were calculated by the ratios of increased or decreased volume of the detected cytokines across different experimental conditions.

### In vitro stimulation of primary PD-1^+^ T cells and Jurkat T cells

For PD-1^+^ T cells from AS plaques and lung tumors, single-cell suspension was stained with Live/Dead Aqua (Thermo Fisher), APC anti-human CD45 (1:20, HI30, BioLegend), PE/Cy7 anti-human CD4 (1:20, RPA-T4, BioLegend), FITC anti-human CD8 (1:20, RPA-T8, BioLegend), and PE anti-human PD-1 (1:50, Bristol-Myers Squibb). CD4^+^PD-1^+^ and CD8^+^PD-1^+^ T cells from AS plaques and lung tumors, as well as CD4^+^PD-1^+^ and CD8^+^PD-1^+^ T cells from AS plaques, were sorted by BD FACS Aria II (BD Biosciences). Next, target cells were rested in complete RPMI medium (RPMI-1640 medium supplemented with 10% FBS, 1 mM sodium pyruvate, 1× MEM non-essential amino acids, 100 ng/mL penicillin/streptomycin, 2 mM _L_-glutamine, 1 mg/mL ciproxin) at 37 °C in 5% CO_2_ for 2 h. Afterward, 2 × 10^4^ PD-1^+^ T cells from each sample were stimulated with Human T-Activator CD3/CD28 Dynabeads (Thermo Fisher) at a ratio of 1:3 in 200 μL RPMI-1640 medium at 37 °C in 5% CO_2_ for 48 h. The supernatants were collected and analyzed by Cytometric Bead Array to detect IL-2, IFN-γ, TNF-α, IL-1β, or IL-6 as described before.

For Jurkat T cells, biotinylated PD-L1 or anti-PD-1 mAb was co-conjugated with anti-CD3/anti-CD28 biotinylated antibodies (BioLegend) onto Streptavidin Coated Polystyrene Particles (Spherotech) at a 1:1:1 ratio for a total of 12 μg/mL protein per 2 × 10^6^ particles at 37 °C for 30 min. For the positive and negative control, streptavidin-coated polystyrene particles were linked with or without anti-CD3/anti-CD28 biotinylated antibodies. Then, 2 × 10^5^ Jurkat T cells were respectively co-cultured with the above polystyrene particles in 200 μL complete RPMI-1640 medium at 37 °C in 5% CO_2_ for 24 h. The supernatant was collected and analyzed by Cytometric Bead Array assay to detect IL-2 as described before.

### Determining the binding ability of CD64^+^ HEK293 cells to anti-PD-1 mAb in vitro

2 × 10^5^ CD64^+^ HEK293 cells or CD64^–^ HEK293 cells were respectively incubated with different concentrations (0.00005, 0.0005, 0.005, 0.05, 0.5, and 5 μg/mL) of anti-PD-1 mAb (Nivolumab, Bristol-Myers Squibb) in FACS buffer for 30 min on ice. The cells were then stained with PE-conjugated goat F(ab’)2 anti-human IgG (F(ab’)2) fragment (Jackson ImmunoResearch) for flow cytometric analyses. The binding abilities of CD64^+^ HEK293 or CD64^–^ HEK293 cells to anti-PD-1 mAb were quantified by MFI.

### In vitro cell–cell conjugation assay of PD-1^+^ and CD64^+^ cells

1 × 10^6^ GFP-fusing PD-1^+^ Jurkat T cells were pre-incubated with 1 μg/mL Alexa Fluor 405-labeled anti-PD-1 mAb on ice for 30 min. The free mAb was then washed off with PBS buffer. Meanwhile, 1 × 10^6^ CD64^+^ HEK293 cells or CD64^–^ HEK293 cells were separately prepared and mixed with the above PD-1^+^ Jurkat T cells in a 1.5 mL EP tube on ice. The mixed cells were centrifuged at 300× *g* for 5 min at 4 °C to initiate cell–cell conjugation and then were gently resuspended and transferred to a confocal imaging dish. Images were acquired with an A1RSi confocal microscope (Nikon). Cell–cell conjugation was confirmed by observing PD-1 and anti-PD-1 mAb (Nivolumab) enrichment at the interface in the bright field.

### In-situ detection of CD64/anti-PD-1-mAb/PD-1 interaction on living primary cells

To detect the interaction between CD64/anti-PD-1-mAb/PD-1 on living primary cells, we stained the single-cell suspension from human AS plaques with Live/Dead Aqua (Thermo Fisher) and PE anti-human PD-1 (1:50, Nivolumab, Bristol-Myers Squibb), after treating FcRs Blocking Reagent Mix (anti-human, anti-mouse, anti-rat, and anti-hamster IgG, Equitech-Bio). PBMCs from fresh human PB samples were stained with Live/Dead Aqua (Thermo Fisher) and Alexa Fluor-647 anti-human CD64 antibody (1:100, 10.1, BioLegend), without FcRs blocking. Primary PD-1^+^ cells from human AS plaques and CD64^+^ cells from human PBMCs were sorted by BD FACS Aria II (BD Biosciences). Then, we replaced PE anti-human PD-1 antibodies on PD-1^+^ cells with unlabeled anti-PD-1 mAb (1 μg/μL, Bristol-Myers Squibb) for 1 h at 37 °C.

We performed a micro-pipette adhesion assay^77^ to qualitatively determine the in-situ interaction of primary CD64^+^ cells and primary PD-1^+^ T cells treated with Nivolumab or not. Briefly, PD-1^+^ T cell-bound with anti-PD-1 mAb (Nivolumab) were brought into contact with CD64^+^ cells for 0.1 s and retracted to mechanically detect adhesion event occurrence in RPMI medium supplemented with 0.5% BSA. The cell–cell adhesion frequency (*P*_a_%) was used to characterize CD64/anti-PD-1-mAb/PD-1 interaction and was obtained after 50 cycles. Images of primary cell–cell adhesion were acquired using high-definition cameras (Allied Vision, Model: GC1290).

### Ex vivo stimulation of primary PD-1^+^ T cells and CD64^+^ cells co-culture

Primary CD4^+^PD-1^+^ or CD8^+^PD-1^+^ T cells from human AS plaques were sorted by staining with Live/Dead Aqua (Thermo Fisher), APC anti-human CD45 (1:20, HI30, BioLegend), FITC anti-human CD4 (1:20, RPA-T4, BioLegend), FITC anti-human CD8 (1:20, RPA-T8, BioLegend) and PE anti-human PD-1 (1:50, Bristol-Myers Squibb) antibodies. PE anti-human PD-1 on PD-1^+^ cells was replaced with unlabeled anti-PD-1 mAb (1 μg/μL, Bristol-Myers Squibb). CD64^+^ cells from human PBMCs were sorted by staining with Live/Dead Aqua (Thermo Fisher), PE anti-human CD45 (1:20, HI30, BioLegend), and Alexa Fluor-647 anti-human CD64 antibody (1:100, 10.1, BioLegend) without FcRs blocking. About 2 × 10^4^ PD-1^+^ T cells bound with anti-PD-1 mAb were co-cultured with 1 × 10^5^ CD64^+^ cells and Human T-Activator CD3/CD28 Dynabeads (Thermo Fisher) in complete RPMI-1640 medium at 37 °C in 5% CO_2_ for 48 h. The supernatant was collected and analyzed by Cytometric Bead Array assay to detect IL-2, IFN-γ, and TNF-α as described before.

### Ex vivo stimulation of primary CD45^+^ cells

Viable CD45^+^ cells were isolated from single-cell suspensions of AS plaques and lung tumors by staining with Live/Dead Aqua (Thermo Fisher) and APC anti-human CD45 (1:20, HI30, BioLegend), and then sorted on BD FACS Aria II (BD Biosciences). CD45^+^ cells from each sample were rested in a complete RPMI-1640 medium at 37 °C in 5% CO_2_ for 2 h. Next, 5 × 10^4^ CD45^+^ cells from each sample were used as the negative control, while another 5 × 10^4^ CD45^+^ cells were incubated with 10 μg/mL anti-PD-1 mAb (Nivolumab, Bristol-Myers Squibb) in complete RPMI-1640 medium at 37 °C in 5% CO_2_ for 1 h. After washing twice to remove free anti-PD-1 mAb, the CD45^+^ cells were stimulated with Human T-Activator CD3/CD28 Dynabeads (Thermo Fisher) for 48 h. The concentrations of IL-2, IFN-γ, and TNF-α in the supernatants were collected and analyzed by Cytometric Bead Array assay, as described before.

### Pre-processing and Gadolinium contamination cleaning of CyTOF data

The raw data from CyTOF were collected and normalized using an automated normalizer based on bead standards.^98^ Samples were de-barcoded with a deconvolution algorithm using mass-tagged barcodes.^99^ GdClean R package^37^ was used to compensate for potential Gd contamination in CyTOF raw data of AS plaques within selected Gd isotope channels (Supplementary information, Fig. S4a). All CyTOF raw data were manually gated in FlowJo (v10.0.7) to exclude dead cells, debris, doublets, non-immune cells, and beads. Granulocytes (CD66b^+^CD45^low^) and other immune cells (CD66^–^CD45^+^) were separately gated and then concatenated together (Supplementary information, Fig. S4b). The obtained CyTOF data for single, alive, and intact immune cells were arcsinh-transformed with a cofactor 5 before applying downstream analyses.

### Clustering and visualization of CyTOF data

To identify the immune cell subsets, we applied a Self-Organizing Map (SOM)-based clustering approach.^100^ Firstly, we initialized a SOM network with suitable dimensions (e.g., 10 × 10) and trained this SOM network using sub-sampled cells of interest. Each sample file was randomly selected with no more than 10,000 cells. We used the MATLAB neural network toolbox with a training step set to 200 for this purpose. Next, we used the obtained SOM network to assign immune cells into identified SOM sub-clusters. Obtaining SOM sub-clusters was designed for over-clustering and required subsequent meta-clustering to get biologically and immunologically meaningful immune cell subsets. We applied hierarchical clustering with cosine distance and average linkage to evaluate similarities between SOM sub-clusters and constructed merging strategies based on expert experience and priority levels of lineage markers. We repeatedly used this procedure for clustering cell populations, including CD45^+^ cells, T cells, myeloid cells, etc. For visualization of CyTOF datasets, we used FIt-tSNE (fast Fourier transform-accelerated interpolation-based t-SNE), a modified t-distributed stochastic neighbor embedding (t-SNE) that has better performance and allows visualization of more cells. We set the parameters to iteration = 1000 and perplexity = 100.^101,102^

### Pre-processing of paired scRNA-seq and αβTCR-seq data

The pre-processing of paired scRNA and αβTCR-seq data, including gene alignment, cell barcode matching, and unique molecular identifier (UMI) counting, was implemented using the Cell Ranger (v3.0.2) single-cell software suite provided by 10X Genomics. The GRCh38 human reference genome was used as the alignment reference. The resulting filtered gene-cell matrices (called count matrices below) from Cell Ranger recorded the raw unique counts of UMIs for each gene feature associated with each cell barcode and were used for downstream scRNA-seq analyses.

For each sample, the paired TCR library was sequenced separately, and the contig information of each sequence was annotated using the ‘cellranger vdj’ function in Cell Ranger (v3.0.2). Based on cell barcodes provided by 10X Genomics, the productive TCR-α or -β chains for each T cell were identified. Cells with only a single α or β chain were discarded and not included in the downstream analyses. Cells with two or more different TCR-α or -β chains were annotated by the most abundant and productive TCR-αβ pair. Unique pairs of TCR-αβ chains were identified as TCR clonotypes, and those TCR clonotypes expressed in three or more cells were annotated as clonal TCR clonotypes. To integrate with the paired single-cell transcriptomic profiles, TCR-based analyses were performed on cells passing quality control in the scRNA-seq processing pipeline. A total of 24,407 T cells with effective TCR information were used in the downstream analyses.

### Quality control, batch correction, and clustering of scRNA-seq data with Seurat

We used Seurat^103^ (v4.0.5) for the scRNA-seq data analysis. To ensure high-quality data, we discarded cells with less than 200 unique genes, more than 3500 unique genes, or more than 10% mitochondrial genes. Additionally, we discarded genes detected in less than 0.1% of cells. The filtered count matrices were normalized, and high-variable genes were identified using the default setting. We identified ‘Anchors’ genes across different samples and integrated all samples into one dataset for batch effect correction. To obtain biologically meaningful immune cell clusters, we temporarily excluded genes describing TCR, B cell receptor (BCR), cell cycle, ribosome, and mitochondria before dimension reduction and data clustering. We conducted the principal component analysis (PCA) with filtered genes and selected the top 30 principal components (pcs) for t-SNE and Uniform Manifold Approximation and Projection (UMAP) visualization. The cells were clustered with the Louvain graph clustering algorithm,^104^ with a resolution = 1. We annotated the identified clusters with typical lineage marker genes of immune cells. For further clustering analysis of immune cell subsets, we repeated the aforementioned procedures, including normalization, integration, and clustering.

### Integrating and characterizing the T-cell atlas across tissues and diseases

We accessed the raw scRNA-seq datasets of normal colon tissues,^34^ immunotherapy-induced colitis tissues,^34^ immunotherapy-induced inflammatory arthritis synovial fluid,^35^ and lung tumor tissues^36^ via GSE144469, GSE173303, and Lambrechts Lab website (https://lambrechtslab.sites.vib.be/). We obtained T-cell data from individual datasets based on pre-annotated cell labels or specific expressions of T-cell lineage genes such as *CD3D*, *CD3E*, *CD8A*, and *CD4*. After preprocessing the T-cell datasets as described previously, we merged them and performed principal component analysis. We used the Harmony method^105^ to integrate T cells from different datasets for downstream dimension reduction and cell clustering analyses.

### Pre-processing of snATAC-seq data

The sequence data for the snATAC-seq experiment was preprocessed with the “cellranger-atac count” function in the “cellranger-atac” software suite (v2.0.0) provided by 10X Genomics. The transposase cut sites and peak regions were identified, and the chromatin accessibilities of single cells were accessed by peak alignment, cell barcode matching, and UMI counting. Finally, the filtered cell-peaks matrix and fragment information of peaks were used for downstream analyses. The preprocessing procedure was independently performed for the two parallel snATAC-seq experiments.

### Quality control, integration, and clustering analyses of snATAC-seq data

The bioinformatics analysis of snATAC-seq was performed using Signac.^106^ The single-cell datasets of two parallel experiments were loaded with the “Create Chromatin Assay” function. Single-cell data quality control was performed by filtering out single cells based on the number of unique peaks detected, the percentage of reads in peaks, and the enrichment score of the Transcription Start Site (TSS). The peaks were recalled using MACS2,^107^ and the single-cell datasets of two parallel experiments were integrated with Harmony.^105^ The integrated single-cell dataset was further clustered with the Louvain Graph Clustering Algorithm^104^ using a resolution of 0.8 and visualized by UMAP. Differently expressed peaks of cell clusters were identified by model-based analysis of single-cell transcriptomics (MAST)^108^ and annotated by the “ClosestFeature” function. Analysis of TF-binding motifs was performed using the “Add Motifs” function. The peak enrichments of cell clusters were visualized by the “Coverage Plot” function, and the co-accessible networks were calculated using Cicero (v1.3.4).^109^ To integrate the snATAC-seq dataset of CD8^+^ Tex cells, the raw cell-peaks matrix was assessed via GSE129785,^60^ and the peak sites were lifted over from Hg19 to Hg38 human gene reference using the “liftover” function. The single-cell dataset of exhausted T cells was then retrieved based on the provided cell-cluster information. The retrieved dataset was further merged with the CD8^+^ T cell dataset of AS plaques for downstream analysis.

### Gene signatures and AUCell scoring

To identify the co-expression pattern of selected genes in the scRNA-seq data, we calculated the Spearman correlation of normalized gene expressions between the selected and other genes. We selected the top 30 highly correlated genes as the relevant gene signatures. To quantify the expression of the generated gene signature, we applied the “AUCell” algorithm^29^ to assess the enrichment of the identified gene set on single cells for particular clusters.

### Pathway analysis

DEGs between different T cell clusters were calculated using the “MAST” algorithm,^108^ with logFC > 0.25 and min. pct > 0.1 in the Seurat framework. The generated DEGs were then imported into “g:GOSt”, a function in g: Profiler for functional enrichment analysis.^110^ We used the unordered gene query and the reference Gene Ontology (GO) biological process pathway set for functional profiling.

### Transcriptional regulation analysis

To analyze the gene regulatory network from scRNA-seq data, we used the “pySCENIC” algorithm^29^ with normalized transcriptomic data from Seurat. This helped us identify potential target genes that were highly correlated with TFs. We then pruned these target genes using the *cis*-regulatory database to generate the most precise TF-gene regulons. We calculated the AUCell scores^29^ of identified regulons on single cells, generated a cell-regulon matrix, and used it for UMAP visualization and cell clustering. To infer the potential common TF-regulon regulations across plaque-specific T cells, we calculated Spearman correlations between expressions of TF-regulons and clustered them hierarchically. The Regulon Specificity Score (RSS) of identified T cell clusters was calculated with the ‘calcRSS’ function and the top 10 highest cluster-specific regulons were labeled.

### TCR repertoire analysis

To quantify the clonality of T cell clusters, we examined the index of “1 – normalized Shannon entropy” based on the compositions of TCR clonotypes within T cells across tissues or clusters.^62^ To quantify the transition between T cell clusters, we counted the number of shared clonal TCR clonotypes between T cell clusters. The significant shared T cell cluster pairs were tested using the one-sided Fisher’s exact test, followed by Benjamini–Hochberg correction.^34^ Additionally, to consider the clonal status of shared TCR clonotypes between T cell clusters in T cell transition, we applied Pearson’s correlation test to identify T cell cluster pairs with significantly correlated clone size of identity TCR clonotypes in both T cell clusters, as described before. ^63^

### Trajectory inference analysis of T cells

We applied multiple trajectory inference methods, including RNA velocity,^64^ CytoTRACE,^66^ and Monocle 3,^67^ to infer and validate the lineage trajectories of T cells in our data. To perform RNA velocity analyses in the targeted T cells, we imported the bam files generated from the Cell Ranger workflow into the “run10x” function in the “veloctyto” python package to recount the spliced and un-spliced mRNA of single cells.^64^ We then used “scVelo” python pipeline^65^ to uncover the cell differential dynamics with calculated splicing information of single cells. The “Dynamical” model was used with the assumption that the splicing kinetics of each gene were different, and the transcriptional dynamic of each gene was estimated before calculating the cell transition probability. We uncovered the pseudotime of single cells using the “tl.velocity_pseduotime” function, with the identified root cell and cell transition matrix. The DEGs along with pseudotime were evaluated with the Generalized Additive Model (GAM) by the “gam” package in R. We used the pseudotime and directional arrows to infer the differentiation pathways of T cells and to identify pseudotime-based gene variation and cluster distribution.

For CytoTRACE analysis, the raw UMI counts of selected T cells were imported into the “CytoTRACE” function in the “CytoTRACE” R package with default settings. The resulting inferred gene counts, gene counts signature (GCS), and CytoTRACE indexes were then used to order T cells. The CytoTRACE indexes were then projected on the UMAP projection of T cells from Seurat data. For Monocle 3 analysis, the distance graph of selected T cells was recalculated using the “learn_graph” function. The cells were then ordered using the “order_cells” function in the “monocle 3” R package. The root cell was manually selected based on the result of RNA velocity and CytoTRACE. The inferred pseudotime was then projected onto the UMAP projection of T cells from Seurat data. To compare the inferred cell trajectories across different methods, we performed Spearman correlation analyses on the inferred pseudotime from RNA velocity with the inferred CytoTRACE index or inferred pseudotime by Monocle 3 individually.

### External analysis for independent scRNA-seq data of T cells in AS plaques

We analyzed the scRNA-seq dataset of T cells in human atherosclerosis.^16^ The normalized and merged UMI data, including 4162 T cells with the top 10,000 selected genes as described in the paper, were downloaded from the provided link (https://figshare.com/s/c00d88b1b25ef0c5c788). We imported the dataset into the Seurat analysis platform and divided it into sample-based sub-datasets, which were integrated with Harmony.^105^ We then performed downstream analyses, including cell clustering, DEG analysis, AUCell gene signature expression, and cellular trajectory inference by CytoTRACE, using the same methods as previously described.

## Supplementary information

Supplementary information, Fig. S1

Supplementary information, Fig. S2

Supplementary information, Fig. S3

Supplementary information, Fig. S4

Supplementary information, Fig. S5

Supplementary information, Fig. S6

Supplementary information, Fig. S7

Supplementary information, Fig. S8

Supplementary information, Fig. S9

Supplementary information, Fig. S10

Supplementary information, Fig. S11

Supplementary information, Fig. S12

Supplementary information, Table S1

Supplementary information, Table S2

Supplementary information, Table S3

Supplementary information, Table S4

Supplementary information, Table S5

Supplementary information, Table S6
