# Author Correction: Targeting pro-inflammatory T cells as a novel therapeutic approach to potentially resolve atherosclerosis in humans

**DOI:** 10.1038/s41422-024-01003-5

**Published:** 2024-09-18

**Authors:** Lin Fan, Junwei Liu, Wei Hu, Zexin Chen, Jie Lan, Tongtong Zhang, Yang Zhang, Xianpeng Wu, Zhiwei Zhong, Danyang Zhang, Jinlong Zhang, Rui Qin, Hui Chen, Yunfeng Zong, Jianmin Zhang, Bing Chen, Jun Jiang, Jifang Cheng, Jingyi Zhou, Zhiwei Gao, Zhenjie Liu, Ying Chai, Junqiang Fan, Pin Wu, Yinxuan Chen, Yuefeng Zhu, Kai Wang, Ying Yuan, Pintong Huang, Ying Zhang, Huiqin Feng, Kaichen Song, Xun Zeng, Wei Zhu, Xinyang Hu, Weiwei Yin, Wei Chen, Jian’an Wang

**Affiliations:** 1 Department of Cardiology, The Second Affiliated Hospital, Zhejiang University School of Medicine, Hangzhou, Zhejiang China; 2 Cardiovascular Key Laboratory of Zhejiang Province, Hangzhou, Zhejiang China; 3 Research Center for Life Science and Human Health, Binjiang Institute of Zhejiang University, Hangzhou, Zhejiang China; 4 Department of Cell Biology, Zhejiang University School of Medicine, and Liangzhu Laboratory, Zhejiang University, Hangzhou, Zhejiang China; 5 Key Laboratory for Biomedical Engineering of the Ministry of Education, College of Biomedical Engineering and Instrument Science, Zhejiang University, Hangzhou, Zhejiang China; 6 Guangzhou National Laboratory, Guangzhou, Guangdong China; 7 Kidney Disease Center, The First Affiliated Hospital, Zhejiang University School of Medicine, Hangzhou, Zhejiang China; 8 Center of Clinical Epidemiology and Biostatistics and Department of Scientific Research, The Second Affiliated Hospital, Zhejiang University School of Medicine, Hangzhou, Zhejiang China; 9 National Laboratory of Biomacromolecules, Institute of Biophysics, University of Chinese Academy of Sciences, Beijing, China; 10 Department of Bioinformatics, The Basic Medical School of Chongqing Medical University, Chongqing, China; 11 Department of Hepatobiliary and Pancreatic Surgery, The Center for Integrated Oncology and Precision Medicine, Affiliated Hangzhou First People’s Hospital, Zhejiang University School of Medicine, Hangzhou, Zhejiang China; 12 The MOE Frontier Science Center for Brain Science & Brain-machine Integration, Zhejiang University, Hangzhou, Zhejiang China; 13 National Clinical Research Center for Infectious Diseases, The First Affiliated Hospital, Zhejiang University School of Medicine, Hangzhou, Zhejiang China; 14 Department of Neurosurgery, The Second Affiliated Hospital, Zhejiang University School of Medicine, Hangzhou, Zhejiang China; 15 Department of Vascular Surgery, The Second Affiliated Hospital, Zhejiang University School of Medicine, Hangzhou, Zhejiang China; 16 Department of Thoracic Surgery, The Second Affiliated Hospital, Zhejiang University School of Medicine, Hangzhou, Zhejiang China; 17 Department of Vascular Surgery, Sir Run Run Shaw Hospital, Zhejiang University School of Medicine, Hangzhou, Zhejiang China; 18 Department of Respiratory, The Second Affiliated Hospital, Zhejiang University School of Medicine, Hangzhou, Zhejiang China; 19 Department of Oncology, The Second Affiliated Hospital, Zhejiang University School of Medicine, Hangzhou, Zhejiang China; 20 Department of Ultrasound in Medicine, The Second Affiliated Hospital, Zhejiang University School of Medicine, Hangzhou, Zhejiang China; 21 Department of Clinical Research Center, The Second Affiliated Hospital, Zhejiang University School of Medicine, Hangzhou, Zhejiang China; 22 Zhejiang Provincial Key Laboratory of Cardio-Cerebral Vascular Detection Technology and Medicinal Effectiveness Appraisal, Zhejiang University, Hangzhou, Zhejiang China

**Keywords:** Mechanisms of disease, Autoimmunity

Correction to: *Cell Research* 10.1038/s41422-024-00945-0, published online 15 March 2024

In the original publication of the paper, there were several mistakes in the text and figures.

First, the equal contribution statement was wrongly assigned number 24 instead of number 23. The correct statement should be “23 These authors contributed equally: Lin Fan, Junwei Liu, Wei Hu.”

In Fig. 3a, the cluster frequency annotation was incorrect and has been re-ordered to match the rows of the heatmap.

In Fig. 4g, a typo was corrected in the two y-axis: they should read ‘IL-2 concentration (pg/mL)’ and ‘IFN-γ concentration (pg/mL) respectively.

In Fig. 6a, the Y-Axis of the purple bar graph was corrected from “Frequency of PD-L1^+^CD45^–^ cells” to ““Frequency of PD-L1^+^CD45^+^ cells”.

In the caption of Fig. 6, the description “d Diagram showing micro-peptide adhesion assay for examining the cell-cell adhesion frequency (Pa%) between CD64^+^ PBMCs and primary PD-1^+^ T cells bound with Nivolumab or not” was corrected to “d Diagram showing micro-pipette adhesion assay for examining the cell-cell adhesion frequency (Pa%) between CD64^+^ PBMCs and primary PD-1^+^ T cells bound with Nivolumab or not.”

Similarly, in the section “In-situ detection of CD64/anti-PD-1-mAb/PD-1 interaction on living primary cells”, the following sentence “We performed a micro-peptide adhesion assay to qualitatively determine the in-situ interaction of primary CD64^+^ cells and primary PD-1^+^ T cells treated with Nivolumab or not” was corrected to “We performed a micro-pipette adhesion assay to qualitatively determine the in-situ interaction of primary CD64^+^ cells and primary PD-1^+^ T cells treated with Nivolumab or not”.

Furthermore, the terms ‘Fc-binding’ and ‘non-Fc-binding’ have been corrected to ‘FcγR-binding’ and ‘non-FcγR-binding’, throughout the text, in Fig. 6 as well as in the Supplementary information, Figs. S11 and S12. Finally, the reference 53 was corrected from “Liora, V. et al. Rora regulates activated T helper cells during inflammation. bioRxiv. 10.1101/709998 (2020)” to “Haim-Vilmovsky, L. et al. Mapping Rora expression in resting and activated CD4^+^ T cells. *PLoS ONE* **16**, e0251233 (2021)”.

We are sorry for these mistakes, and confirm that these corrections do not affect the results or the conclusion of this work. The original article has been corrected.

## Supplementary information

Supplementary information, Fig. S11

Supplementary information, Fig. S12

