# Supplementary material for: Targeting pro-inflammatory T cells as a novel therapeutic approach to potentially resolve atherosclerosis in humans
Source: Cell Res. 2024 Mar 15;34(6):407–27. doi: 10.1038/s41422-024-00945-0 (PMC11143203; doi:10.1038/s41422-024-00945-0)
Supplement: Supplementary file 1 — Supplementary information, Fig. S1 [file 41422_2024_945_MOESM1_ESM.pdf]

Parameter		Overall (*n* = 168)	Without anti-PD-1 treatment (*n* = 82)	With anti-PD-1 treatment (*n* = 86)	*P* value
**Age, years**		67.78 (7.69)	66.35 (7.87)	69.14 (7.30)	0.018
**Gender, n (%)**	F	26 (15.48)	14 (17.07)	12 (13.95)	0.730
	M	142 (84.52)	68 (82.93)	74 (86.05)	
**BMI, kg/m^2**					
- Baseline		22.77 [20.20, 24.85]	23.00 [20.43, 25.68]	22.17 [19.88, 24.16]	0.167
- After treatment		22.50 [20.32, 24.45]	22.94 [21.05, 25.02]	22.09 [19.94, 24.20]	0.134
- Changes		-0.23 [-1.09, 1.26]	-0.10 [-1.19, 1.33]	-0.25 [-1.02, 1.05]	0.954
**Systolic BP, mmHg**					
- Baseline		129.00 [117.75, 140.25]	129.50 [117.00, 140.75]	128.50 [118.00, 139.50]	0.858
- After treatment		126.00 [116.00, 139.25]	126.00 [116.25, 141.50]	127.00 [116.00, 138.75]	0.962
- Changes		-3.50 [-13.25, 9.00]	-4.00 [-13.00, 8.00]	-3.00 [-14.00, 9.75]	0.954
**Diastolic BP, mmHg**					
- Baseline		74.50 [68.00, 83.00]	77.00 [68.25, 84.00]	73.00 [68.00, 82.00]	0.197
- After treatment		74.00 [67.00, 84.00]	77.00 [68.25, 86.00]	71.00 [65.25, 82.00]	0.042
- Changes		-0.50 [-9.00, 7.25]	-1.50 [-9.00, 8.00]	0.00 [-9.00, 6.00]	0.706
**Total cholesterol, mmol/L**					
- Baseline		4.70 [3.68, 5.45]	4.72 [3.72, 5.44]	4.67 [3.69, 5.43]	0.962
- After treatment		4.62 [3.94, 5.39]	4.80 [4.16, 5.40]	4.45 [3.91, 5.34]	0.200
- Changes		0.02 [-0.43, 0.63]	0.07 [-0.36, 0.69]	-0.08 [-0.48, 0.49]	0.176
**Triglyceride, mmol/L**					
- Baseline		1.21 [0.92, 1.69]	1.33 [0.94, 1.70]	1.10 [0.91, 1.64]	0.117
- After treatment		1.38 [0.99, 1.80]	1.48 [1.08, 1.77]	1.35 [0.88, 1.84]	0.315
- Changes		0.14 [-0.21, 0.50]	0.17 [-0.25, 0.50]	0.06 [-0.18, 0.48]	0.956
**HDL, mmol/L**					
- Baseline		1.17 [1.00, 1.40]	1.21 [0.96, 1.40]	1.17 [1.01, 1.41]	0.856
- After treatment		1.17 [0.98, 1.36]	1.23 [1.03, 1.44]	1.13 [0.95, 1.27]	0.024
- Changes		-0.02 [-0.16, 0.13]	0.04 [-0.09, 0.17]	-0.07 [-0.20, 0.06]	0.011
**LDL, mmol/L**					
- Baseline		2.67 [1.91, 3.09]	2.50 [1.87, 2.99]	2.74 [1.96, 3.10]	0.351
- After treatment		2.56 [2.10, 3.12]	2.62 [2.16, 3.10]	2.51 [2.02, 3.15]	0.608
- Changes		0.10 [-0.22, 0.49]	0.18 [-0.18, 0.64]	0.02 [-0.24, 0.42]	0.119
**Fasting blood glucose, mmol/L**					
- Baseline		5.44 [4.86, 6.06]	5.47 [4.96, 6.32]	5.31 [4.80, 5.88]	0.183
- After treatment		5.31 [4.86, 6.26]	5.50 [4.88, 6.46]	5.28 [4.85, 6.12]	0.464
- Changes		0.01 [-0.59, 0.64]	-0.09 [-0.62, 0.53]	0.05 [-0.50, 0.69]	0.591
**Statin, n (%)**	N	103 (61.31)	50 (60.98)	53 (61.63)	1.000
	Y	65 (38.69)	32 (39.02)	33 (38.37)	
**Tumor type, n (%)**					
- Digestive system		81 (48.2)	44 (53.7)	36 (41.9)	0.178
- Respiratory system		72 (42.9)	33 (40.2)	39 (45.3)	
- Others		15 (8.9)	5 (6.1)	11 (12.8)	
**Treatment cycles, months**		3.50 [2.50, 7.45]	4.20 [2.68, 7.83]	3.50 [2.30, 6.58]	0.249
**Changes of plaque areas, (ΔA)**		0.0 [-4.0, 3.0]	1.0 [-1.0, 5.0]	-3.0 [-7.0, 1.0]	< 0.001

Supplementary information, Fig. S1. Clinical characteristics of the retrospective cohort. Categorical variables are presented as numbers (*n*) and percentages (%). Continuous variables are presented as median and interquartile range (IQR). Chi-squared test and Mann-Whitney test are used.
