# Supplementary material for: Targeting pro-inflammatory T cells as a novel therapeutic approach to potentially resolve atherosclerosis in humans
Source: Cell Res. 2024 Mar 15;34(6):407–27. doi: 10.1038/s41422-024-00945-0 (PMC11143203; doi:10.1038/s41422-024-00945-0)
Supplement: Supplementary file 2 — Supplementary information, Fig. S2 [file 41422_2024_945_MOESM2_ESM.pdf]

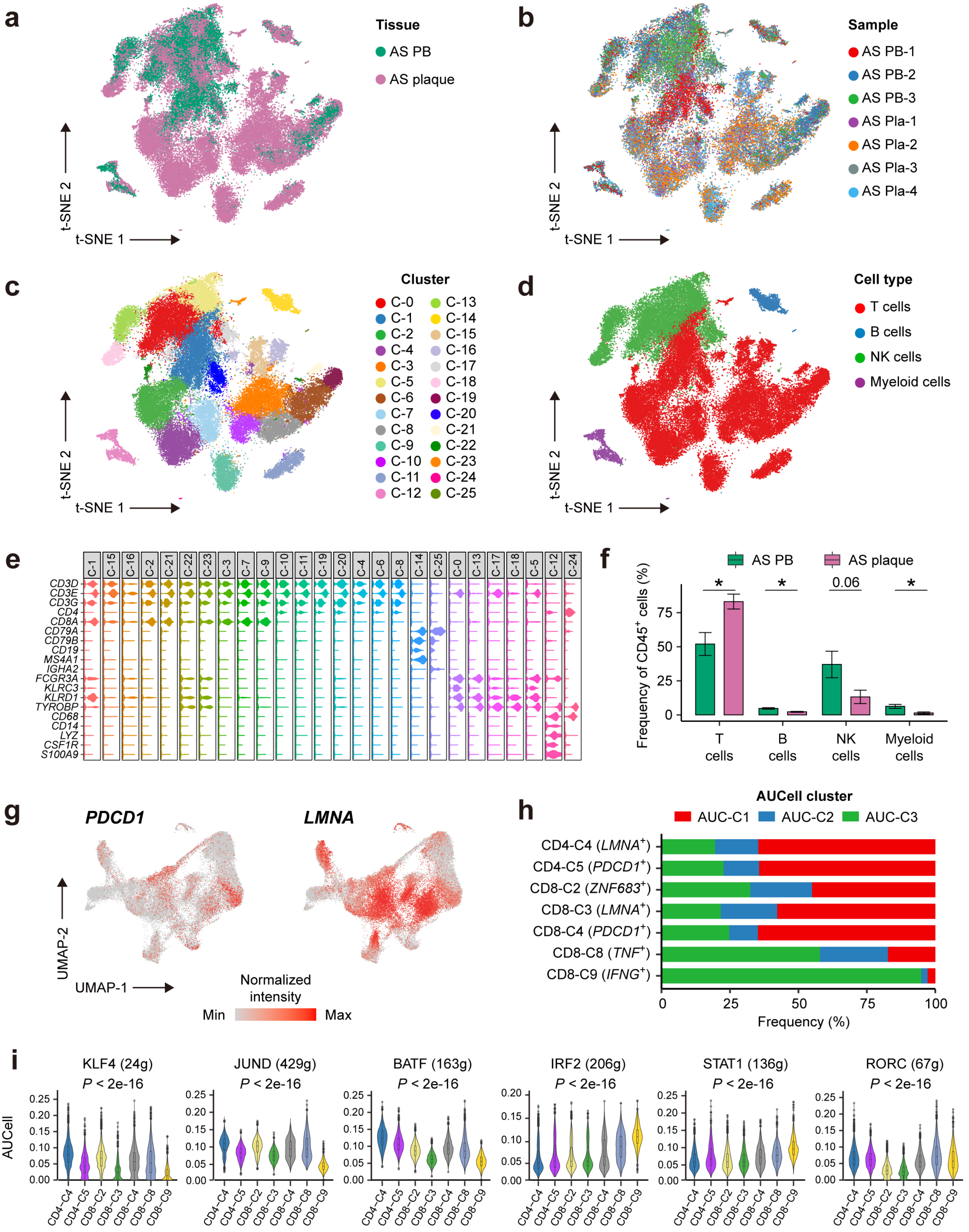
a
Tissue
AS PB
AS plaque
t-SNE 2
t-SNE 1
b
Sample
AS PB-1
AS PB-2
AS PB-3
AS Pla-1
AS Pla-2
AS Pla-3
AS Pla-4
t-SNE 2
t-SNE 1
c
Cluster
C-0
C-1
C-2
C-4
C-3
C-5
C-6
C-7
C-8
C-9
C-10
C-11
C-12
C-13
C-14
C-15
C-16
C-17
C-18
C-19
C-20
C-21
C-22
C-23
C-24
C-25
t-SNE 2
t-SNE 1
d
Cell type
T cells
B cells
NK cells
Myeloid cells
t-SNE 2
t-SNE 1
e
C-1 C-15 C-16 C-2 C-21 C-22 C-23 C-3 C-7 C-9 C-10 C-11 C-19 C-20 C-4 C-6 C-8 C-14 C-25 C-0 C-13 C-17 C-18 C-5 C-12 C-24
CD3D
CD3E
CD3G
CD4
CD8A
CD79A
CD79B
CD19
MS4A1
IGHA2
FCGR3A
KLRC3
KLRD1
TYROBP
CD68
CD14
LYZ
CSF1R
S100A9
f
AS PB
AS plaque
Frequency of CD45+ cells (%)
*
*
0.06
*
0
25
50
75
T cells
B cells
NK cells
Myeloid cells
g
PDCD1
LMNA
UMAP-2
UMAP-1
Normalized intensity
Min
Max
h
AUCell cluster
AUC-C1
AUC-C2
AUC-C3
CD4-C4 (LMNA+)
CD4-C5 (PDCD1+)
CD8-C2 (ZNF683+)
CD8-C3 (LMNA+)
CD8-C4 (PDCD1+)
CD8-C8 (TNF+)
CD8-C9 (IFNG+)
0
25
50
75
100
Frequency (%)
i
AUCell
KLF4 (24g)
JUND (429g)
BATF (163g)
IRF2 (206g)
STAT1 (136g)
RORC (67g)
P < 2e-16
CD4-C4 CD4-C5 CD8-C2 CD8-C3 CD8-C4 CD8-C8 CD8-C9

Supplementary information, Fig. S2. scRNA-seq profiling of CD45$^+$ cells from AS plaques and AS PB.

a-d t-SNE plots of CD45$^+$ cells from AS PB (n = 3) and AS plaques (n = 4), colored by tissue sources (**a**), sample sources (**b**), cell clusters (**c**), and immune cell subtypes (**d**).

e Violin plots showing the expressions of selected genes on identified clusters as in (**c**).

f Frequency comparisons of immune cell subtypes as in (**d**) between AS PB and AS plaques. Data are represented as mean ± SEM.

g UMAP plots of T cells from AS plaques and AS PB, colored by normalized expressions of *PDCD1* and *LMNA*.

h Bar plots showing cell compositions in terms of AUCell clusters in T cell clusters.

i Violin plots showing AUCell scores of plaque-specific T cell clusters for selected regulons. Data are represented as the mean and standard error of mean (SEM) in (**f**).

A two-sided Student's t-test with Benjamini-Hochberg adjustment was used in (**f**) and a one-way ANOVA test in (**i**).
