# Supplementary material for: Targeting pro-inflammatory T cells as a novel therapeutic approach to potentially resolve atherosclerosis in humans
Source: Cell Res. 2024 Mar 15;34(6):407–27. doi: 10.1038/s41422-024-00945-0 (PMC11143203; doi:10.1038/s41422-024-00945-0)
Supplement: Supplementary file 3 — Supplementary information, Fig. S3 [file 41422_2024_945_MOESM3_ESM.pdf]

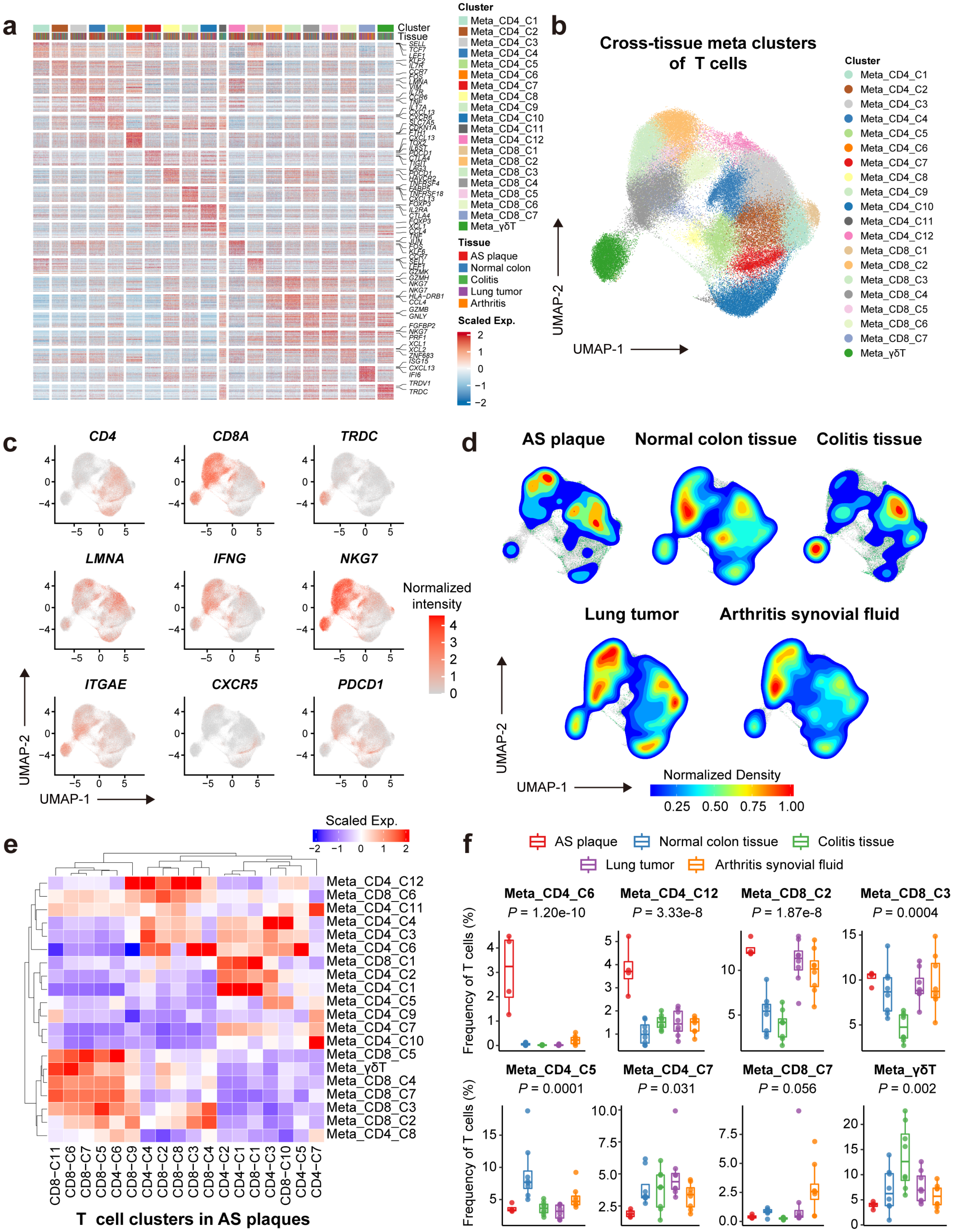

a
Cluster
Tissue
SELL
TCF7
LEF1
IL7R
CCR7
LMNA
ITGAE
CXCL13
PDCD1
CTLA4
TIGIT
FOXP3
IL2RA
NKG7
GZMK
GZMH
PRF1
HLA−DRB1
CCL4
GZMB
GNLY
FGFBP2
XCL1
XCL2
ZNF683
ISG15
IFI6
TRDV1
TRDC
Cluster
Meta_CD4_C1
Meta_CD4_C2
Meta_CD4_C3
Meta_CD4_C4
Meta_CD4_C5
Meta_CD4_C6
Meta_CD4_C7
Meta_CD4_C8
Meta_CD4_C9
Meta_CD4_C10
Meta_CD4_C11
Meta_CD4_C12
Meta_CD8_C1
Meta_CD8_C2
Meta_CD8_C3
Meta_CD8_C4
Meta_CD8_C5
Meta_CD8_C6
Meta_CD8_C7
Meta_γδT
Tissue
AS plaque
Normal colon
Colitis
Lung tumor
Arthritis
Scaled Exp.
2
1
0
−1
−2
b
Cross-tissue meta clusters of T cells
UMAP-2
UMAP-1
Cluster
Meta_CD4_C1
Meta_CD4_C2
Meta_CD4_C3
Meta_CD4_C4
Meta_CD4_C5
Meta_CD4_C6
Meta_CD4_C7
Meta_CD4_C8
Meta_CD4_C9
Meta_CD4_C10
Meta_CD4_C11
Meta_CD4_C12
Meta_CD8_C1
Meta_CD8_C2
Meta_CD8_C3
Meta_CD8_C4
Meta_CD8_C5
Meta_CD8_C6
Meta_CD8_C7
Meta_γδT
c
CD4
CD8A
TRDC
LMNA
IFNG
NKG7
ITGAE
CXCR5
PDCD1
Normalized intensity
4
3
2
1
0
UMAP-2
UMAP-1
d
AS plaque
Normal colon tissue
Colitis tissue
Lung tumor
Arthritis synovial fluid
UMAP-2
UMAP-1
Normalized Density
0.25
0.50
0.75
1.00
e
Scaled Exp.
−2
−1
0
1
2
Meta_CD4_C12
Meta_CD8_C6
Meta_CD4_C11
Meta_CD4_C4
Meta_CD4_C3
Meta_CD4_C6
Meta_CD8_C1
Meta_CD4_C2
Meta_CD4_C1
Meta_CD4_C5
Meta_CD4_C9
Meta_CD4_C7
Meta_CD4_C10
Meta_CD8_C5
Meta_γδT
Meta_CD8_C4
Meta_CD8_C7
Meta_CD8_C3
Meta_CD8_C2
Meta_CD4_C8
CD8−C11
CD8−C6
CD8−C7
CD8−C5
CD4−C6
CD8−C9
CD4−C4
CD8−C2
CD8−C8
CD8−C3
CD8−C4
CD4−C2
CD4−C1
CD8−C1
CD4−C3
CD8−C10
CD4−C5
CD4−C7
T cell clusters in AS plaques
f
AS plaque
Normal colon tissue
Colitis tissue
Lung tumor
Arthritis synovial fluid
Meta_CD4_C6
P = 1.20e-10
Meta_CD4_C12
P = 3.33e-8
Meta_CD8_C2
P = 1.87e-8
Meta_CD8_C3
P = 0.0004
Meta_CD4_C5
P = 0.0001
Meta_CD4_C7
P = 0.031
Meta_CD8_C7
P = 0.056
Meta_γδT
P = 0.002
Frequency of T cells (%)

Supplementary information, Fig. S3. scRNA-seq profiling of T cell atlas in AS plaques and other tissues.

a DEGs of Meta-T cell clusters identified in the integrated T cell datasets, colored by clusters (top), tissue sources (top), and typical genes were labeled on the right.

b, c UMAP plots of Meta-T cells, colored by clusters (**b**) and normalized expressions of selected genes (**c**).

d UMAP plots showing T cell density distribution across different tissues.

e Heatmap showing the scaled mean expression of the top-30 DEGs of our defined T cell clusters as in Fig. 2b in Meta-T cell clusters.

f Frequency compositions of selected Meta-T cell clusters across different tissue sources. Data are represented as median with interquartile range (IQR) in (**f**).

A one-way ANOVA test was used in (**f**).
