# Supplementary material for: Targeting pro-inflammatory T cells as a novel therapeutic approach to potentially resolve atherosclerosis in humans
Source: Cell Res. 2024 Mar 15;34(6):407–27. doi: 10.1038/s41422-024-00945-0 (PMC11143203; doi:10.1038/s41422-024-00945-0)
Supplement: Supplementary file 4 — Supplementary information, Fig. S4 [file 41422_2024_945_MOESM4_ESM.pdf]

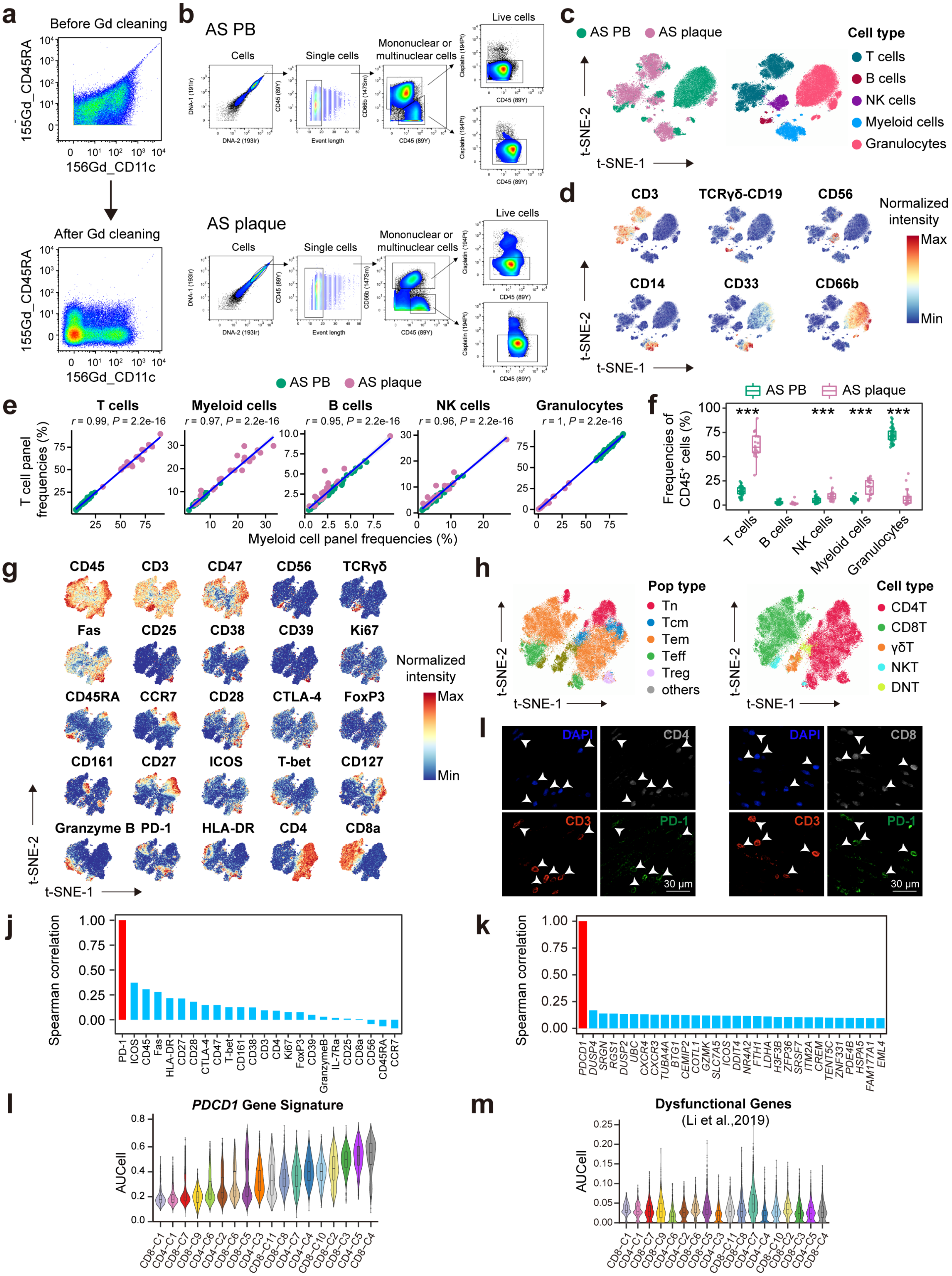
a
Before Gd cleaning
155Gd_CD45RA
156Gd_CD11c
After Gd cleaning
155Gd_CD45RA
156Gd_CD11c
b
AS PB
Cells
Single cells
Mononuclear or multinuclear cells
Live cells
AS plaque
Cells
Single cells
Mononuclear or multinuclear cells
Live cells
c
AS PB
AS plaque
Cell type
T cells
B cells
NK cells
Myeloid cells
Granulocytes
t-SNE-2
t-SNE-1
d
CD3
TCRγδ-CD19
CD56
CD14
CD33
CD66b
Normalized intensity
Max
Min
t-SNE-2
t-SNE-1
AS PB
AS plaque
e
T cells
r = 0.99, P = 2.2e-16
Myeloid cells
r = 0.97, P = 2.2e-16
B cells
r = 0.95, P = 2.2e-16
NK cells
r = 0.96, P = 2.2e-16
Granulocytes
r = 1, P = 2.2e-16
T cell panel frequencies (%)
Myeloid cell panel frequencies (%)
f
AS PB
AS plaque
Frequencies of CD45⁺ cells (%)
***
***
***
***
T cells
B cells
NK cells
Myeloid cells
Granulocytes
g
CD45
CD3
CD47
CD56
TCRγδ
Fas
CD25
CD38
CD39
Ki67
CD45RA
CCR7
CD28
CTLA-4
FoxP3
CD161
CD27
ICOS
T-bet
CD127
Granzyme B
PD-1
HLA-DR
CD4
CD8a
Normalized intensity
Max
Min
t-SNE-2
t-SNE-1
h
Pop type
Tn
Tcm
Tem
Teff
Treg
others
Cell type
CD4T
CD8T
γδT
NKT
DNT
t-SNE-2
t-SNE-1
i
DAPI
CD4
CD3
PD-1
30 μm
DAPI
CD8
CD3
PD-1
30 μm
j
Spearman correlation
PD-1
ICOS
CD45
Fas
HLA-DR
CD27
CD28
CTLA-4
CD47
T-bet
CD161
CD38
CD3
CD4
Ki67
FoxP3
CD39
GranzymeB
IL-7Ra
CD25
CD8a
CD56
CD45RA
CCR7
k
Spearman correlation
PDCD1
DUSP4
SRGN
RGS1
DUSP2
UBC
CXCR4
CXCR3
TUBA4A
BTG1
CEMIP2
COTL1
GZMK
SLC7A5
ICOS
DDIT4
NR4A2
FTH1
LDHA
H3F3B
ZFP36
SRSF7
ITM2A
CREM
TENT5C
ZNF331
PDE4B
HSPA5
FAM177A1
EML4
l
PDCD1 Gene Signature
AUCell
CD8−C1
CD4−C1
CD8−C7
CD8−C9
CD4−C6
CD4−C2
CD8−C6
CD8−C5
CD4−C3
CD8−C11
CD8−C8
CD4−C7
CD4−C4
CD8−C10
CD8−C2
CD8−C3
CD4−C5
CD8−C4
m
Dysfunctional Genes
(Li et al.,2019)
AUCell
CD8−C1
CD4−C1
CD8−C7
CD8−C9
CD4−C6
CD4−C2
CD8−C6
CD8−C5
CD4−C3
CD8−C11
CD8−C8
CD4−C7
CD4−C4
CD8−C10
CD8−C2
CD8−C3
CD4−C5
CD8−C4

Supplementary information, Fig. S4. Single-cell CyTOF profiling of CD45$^+$ cells in human atherosclerosis.

a Representative CyTOF data for Gd-contaminated AS plaque samples before (top) and after (bottom) Gd-cleaning procedure.

b Gating strategy for CyTOF data to obtain valid single immune cells from AS PB (top) and AS plaques (bottom).

c, d t-SNE plots of CD45$^+$ cells, colored either by sample groups (left in **c**), immune cell subsets (right in **c**), or selected lineage markers (**d**).

e Frequency correlation analysis of major immune cell subset identified in two independent staining panels, colored by tissue sources.

f Frequency comparisons of major immune cell subsets in AS PB and AS plaques.

g, h t-SNE plots of T cells as in Fig. 3b, colored either by normalized expression of selected markers from T cell panel (**g**), functional T cell subsets (left in **h**), or major T cell subsets (right in **h**).

i Multi-color IFC staining of PD-1$^+$CD4$^+$ (left) and PD-1$^+$CD8$^+$ (right) T cells in a representative human AS plaque as in Fig. 3e. Scar bar: 30 μm.

j Bar plots showing Spearman correlations of PD-1 with all markers from the T cell panel.

k Bar plots showing Spearman correlations of *PDCD1* with other genes in the scRNA-seq dataset, and the top 30 highly correlated genes are shown.

l, m Violin plots showing AUCell scores of *PDCD1* gene signature (**l**) and dysfunctional gene signature[43] (**m**) in T cell clusters, ordered by mean scores of *PDCD1* gene signature.

Data are represented as median with interquartile range (IQR) in (**f**). A two-sided student's t-test with Benjamini-Hochberg adjustment was used for statistical analyses in (**f**). Spearman correlation test was used in (**e**), (**j**), and (**k**), with correlation coefficient (*r*-value) and *P* value labeled in (**e**).
