# Supplementary material for: Targeting pro-inflammatory T cells as a novel therapeutic approach to potentially resolve atherosclerosis in humans
Source: Cell Res. 2024 Mar 15;34(6):407–27. doi: 10.1038/s41422-024-00945-0 (PMC11143203; doi:10.1038/s41422-024-00945-0)
Supplement: Supplementary file 5 — Supplementary information, Fig. S5 [file 41422_2024_945_MOESM5_ESM.pdf]

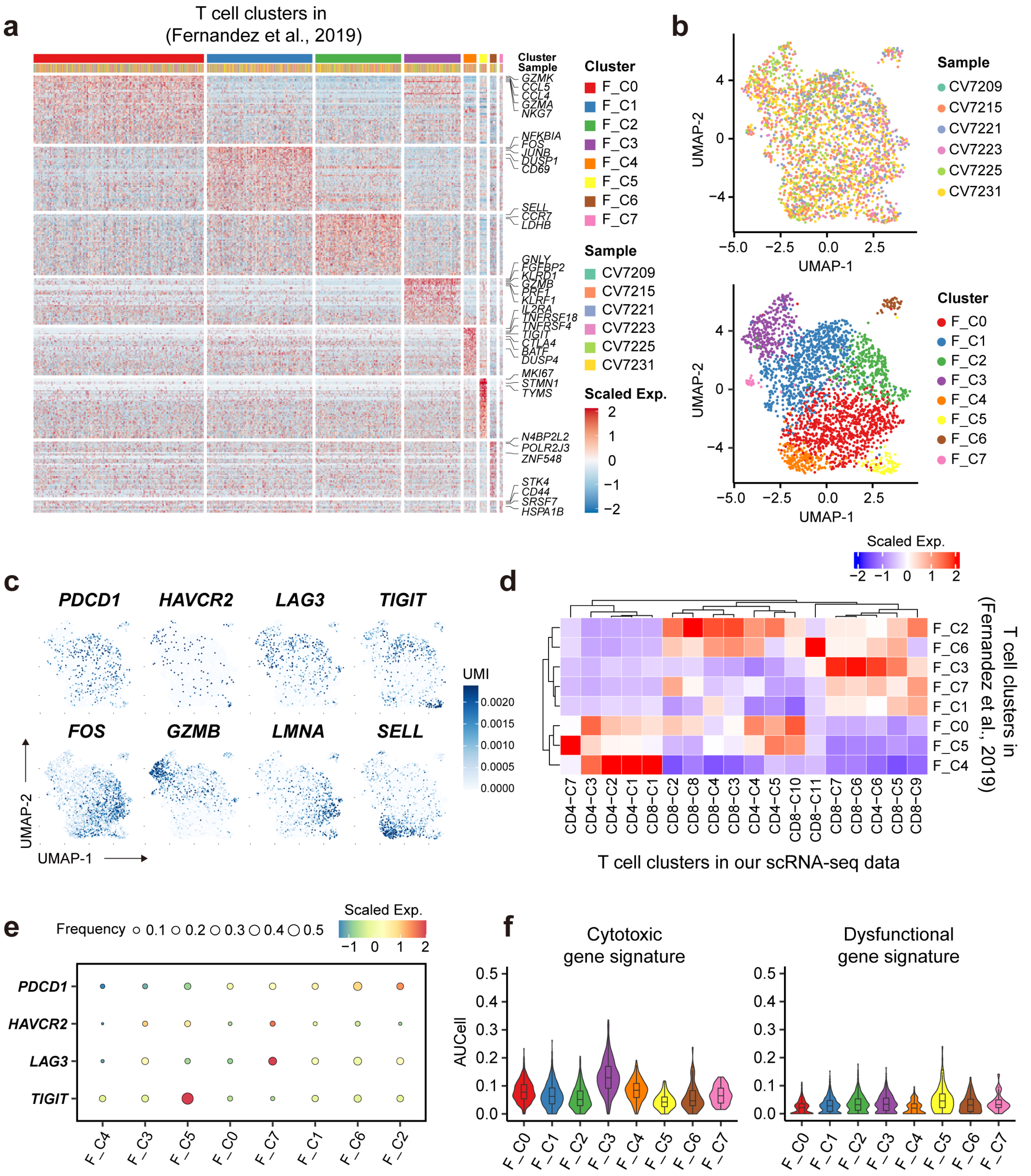

a
T cell clusters in
(Fernandez et al., 2019)
Cluster
Sample
GZMK
CCL5
CCL4
GZMA
NKG7
NFKBIA
FOS
JUNB
DUSP1
CD69
SELL
CCR7
LDHB
GNLY
FGFBP2
KLRD1
GZMB
PRF1
KLRF1
IL2RA
TNFRSF18
TNFRSF4
TIGIT
CTLA4
BATF
DUSP4
MKI67
STMN1
TYMS
N4BP2L2
POLR2J3
ZNF548
STK4
CD44
SRSF7
HSPA1B
Cluster
F_C0
F_C1
F_C2
F_C3
F_C4
F_C5
F_C6
F_C7
Sample
CV7209
CV7215
CV7221
CV7223
CV7225
CV7231
Scaled Exp.
2
1
0
−1
−2
b
Sample
CV7209
CV7215
CV7221
CV7223
CV7225
CV7231
UMAP-2
UMAP-1
Cluster
F_C0
F_C1
F_C2
F_C3
F_C4
F_C5
F_C6
F_C7
c
PDCD1
HAVCR2
LAG3
TIGIT
FOS
GZMB
LMNA
SELL
UMI
0.0020
0.0015
0.0010
0.0005
0.0000
UMAP-2
UMAP-1
d
Scaled Exp.
−2 −1 0 1 2
F_C2
F_C6
F_C3
F_C7
F_C1
F_C0
F_C5
F_C4
T cell clusters in (Fernandez et al., 2019)
CD4−C7
CD4−C3
CD4−C2
CD4−C1
CD8−C1
CD8−C2
CD8−C8
CD8−C4
CD8−C3
CD4−C4
CD4−C5
CD8−C10
CD8−C11
CD8−C7
CD8−C6
CD4−C6
CD8−C5
CD8−C9
T cell clusters in our scRNA-seq data
e
Frequency 0.1 0.2 0.3 0.4 0.5
Scaled Exp.
−1 0 1 2
PDCD1
HAVCR2
LAG3
TIGIT
F_C4
F_C3
F_C5
F_C0
F_C7
F_C1
F_C6
F_C2
f
Cytotoxic
gene signature
Dysfunctional
gene signature
AUCell
0.5
0.4
0.3
0.2
0.1
0.0
F_C0
F_C1
F_C2
F_C3
F_C4
F_C5
F_C6
F_C7

Supplementary information, Fig. S5. External validation using published scRNA-seq data for T cells in human atherosclerosis.

a Heatmap showing differentially expressed genes (DEGs) in T cell clusters identified in published scRNA-seq data from Fernandez et al.[16] Cluster IDs and sample IDs are labeled on the top, and typical genes are labeled on the right.

b UMAP plots of T cells in Fernandez et al.,[16] colored by sample ID (top) or clusters (bottom).

c UMAP plots colored by normalized expressions of selected genes.

d Heatmap showing the scaled AUCell scores of T cell clusters (in Fernandez et al.) on the top-30 DEGs of T cell clusters as in Fig. 2b.

e Dot plots showing the expressions of typical exhaustion-related genes in T cell clusters, colored by scaled mean expression, and sized by cell fraction. T-cell clusters were ordered by the expression level of *PDCD1*.

f Violin plots showing AUCell scores of Cytotoxic (left) and Dysfunctional (right) gene signatures[43] in T cell clusters.
