# Supplementary material for: Targeting pro-inflammatory T cells as a novel therapeutic approach to potentially resolve atherosclerosis in humans
Source: Cell Res. 2024 Mar 15;34(6):407–27. doi: 10.1038/s41422-024-00945-0 (PMC11143203; doi:10.1038/s41422-024-00945-0)
Supplement: Supplementary file 6 — Supplementary information, Fig. S6 [file 41422_2024_945_MOESM6_ESM.pdf]

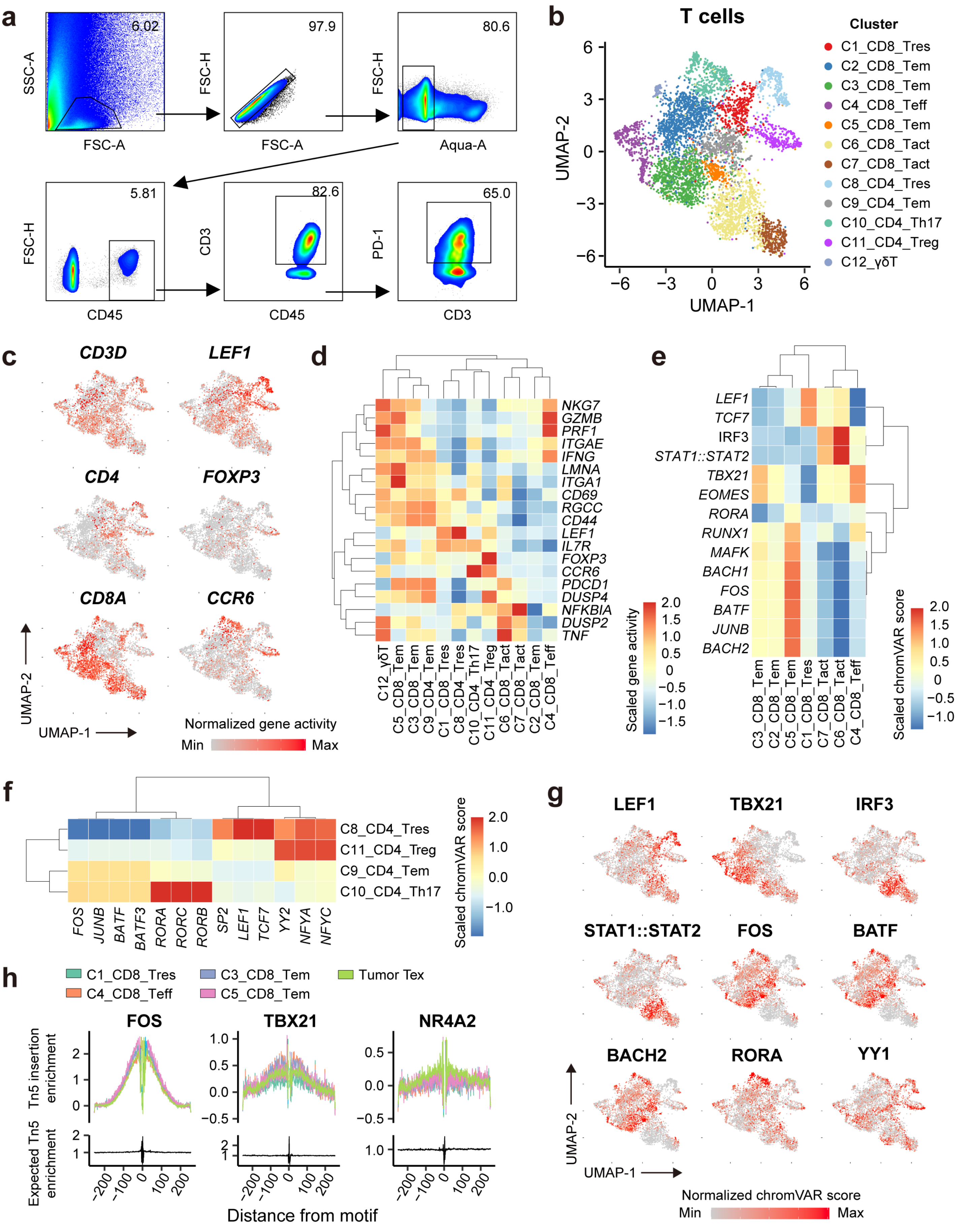
a
SSC-A
FSC-A
6.02
FSC-H
FSC-A
97.9
FSC-H
Aqua-A
80.6
FSC-H
CD45
5.81
CD3
CD45
82.6
PD-1
CD3
65.0
b
T cells
Cluster
C1_CD8_Tres
C2_CD8_Tem
C3_CD8_Tem
C4_CD8_Teff
C5_CD8_Tem
C6_CD8_Tact
C7_CD8_Tact
C8_CD4_Tres
C9_CD4_Tem
C10_CD4_Th17
C11_CD4_Treg
C12_γδT
UMAP-1
UMAP-2
c
CD3D
LEF1
CD4
FOXP3
CD8A
CCR6
UMAP-1
UMAP-2
Normalized gene activity
Min
Max
d
NKG7
GZMB
PRF1
ITGAE
IFNG
LMNA
ITGA1
CD69
RGCC
CD44
LEF1
IL7R
FOXP3
CCR6
PDCD1
DUSP4
NFKBIA
DUSP2
TNF
C12_γδT
C5_CD8_Tem
C3_CD8_Tem
C9_CD4_Tem
C1_CD8_Tres
C8_CD4_Tres
C10_CD4_Th17
C11_CD4_Treg
C6_CD8_Tact
C7_CD8_Tact
C2_CD8_Tem
C4_CD8_Teff
Scaled gene activity
e
LEF1
TCF7
IRF3
STAT1::STAT2
TBX21
EOMES
RORA
RUNX1
MAFK
BACH1
FOS
BATF
JUNB
BACH2
C3_CD8_Tem
C2_CD8_Tem
C5_CD8_Tem
C1_CD8_Tres
C7_CD8_Tact
C6_CD8_Tact
C4_CD8_Teff
Scaled chromVAR score
f
C8_CD4_Tres
C11_CD4_Treg
C9_CD4_Tem
C10_CD4_Th17
FOS
JUNB
BATF
BATF3
RORA
RORC
RORB
SP2
LEF1
TCF7
YY2
NFYA
NFYC
Scaled chromVAR score
g
LEF1
TBX21
IRF3
STAT1::STAT2
FOS
BATF
BACH2
RORA
YY1
UMAP-1
UMAP-2
Normalized chromVAR score
Min
Max
h
C1_CD8_Tres
C4_CD8_Teff
C3_CD8_Tem
C5_CD8_Tem
Tumor Tex
FOS
TBX21
NR4A2
Tn5 insertion enrichment
Expected Tn5 enrichment
Distance from motif

Supplementary information, Fig. S6. Single-cell chromatin accessibility landscape of $CD3^+$ T cells in human AS plaques.

a Gating strategy of $CD3^+$ T cells sorted from AS plaques (n = 4) for snATAC-seq.

b, c UMAP plots of T cells in snATAC-seq datasets, colored by clusters (**b**) and normalized gene activity score of selected genes (**c**).

d Heatmap showing the scaled mean activities of selected genes in corresponding clusters, and the genes and clusters were organized by hierarchy clustering.

e, f Heatmap showing the scaled mean chromVAR scores of selected TF motifs in $CD8^+$ (**e**) and $CD4^+$ (**f**) T cell clusters, and the motifs and clusters were organized by hierarchy clustering.

g UMAP showing $CD3^+$ T cells as in (**c**), colored by the chromVAR scores of selected TF motifs.

h TF footprints of FOS, TBX21, and NR4A2 in (colored by) T cell clusters.
