# Supplementary material for: Targeting pro-inflammatory T cells as a novel therapeutic approach to potentially resolve atherosclerosis in humans
Source: Cell Res. 2024 Mar 15;34(6):407–27. doi: 10.1038/s41422-024-00945-0 (PMC11143203; doi:10.1038/s41422-024-00945-0)
Supplement: Supplementary file 7 — Supplementary information, Fig. S7 [file 41422_2024_945_MOESM7_ESM.pdf]

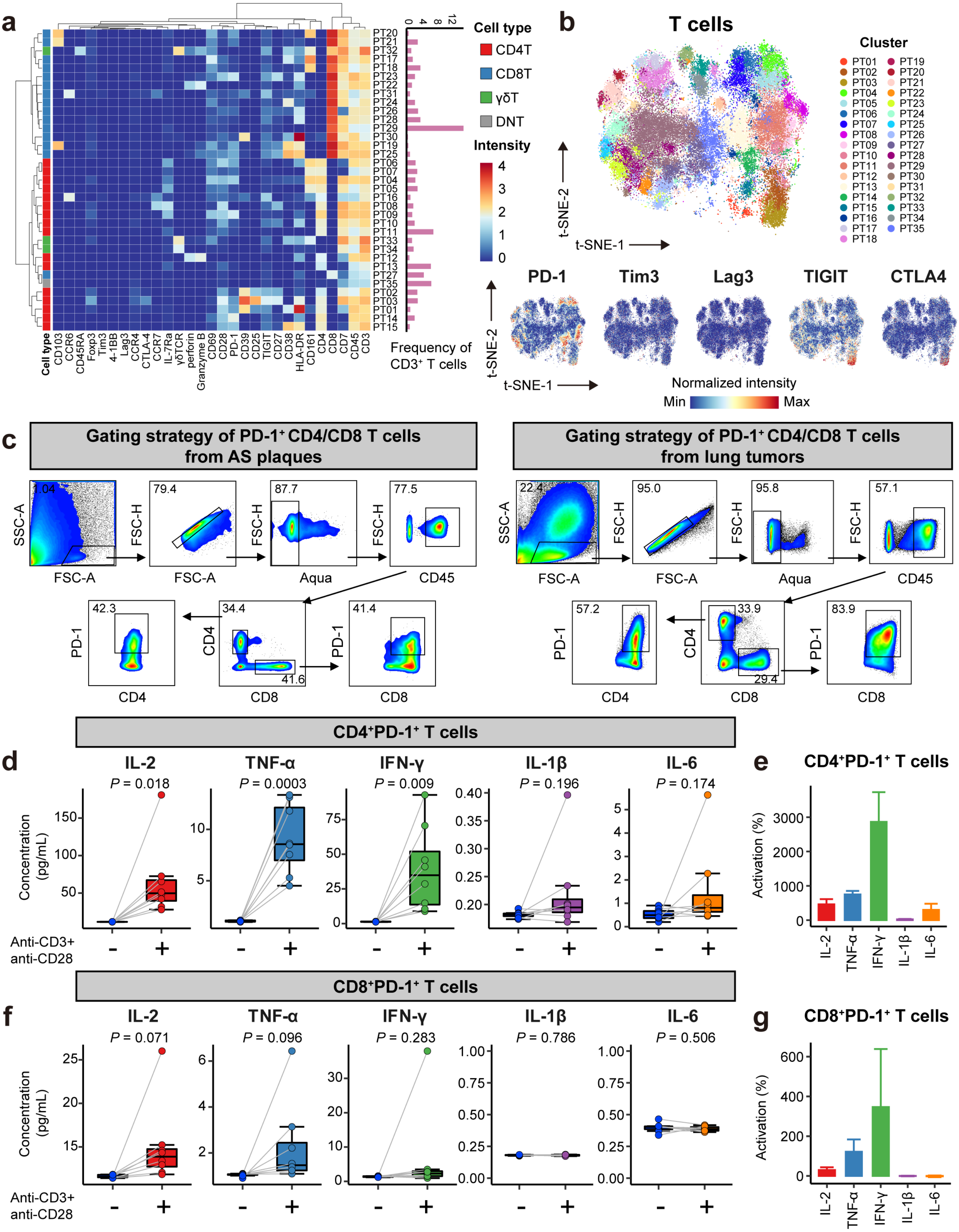

a
Cell type
CD4T
CD8T
γδT
DNT
Intensity
4
3
2
1
0
0
4
8
12
PT20
PT21
PT32
PT17
PT18
PT23
PT22
PT31
PT24
PT26
PT28
PT29
PT30
PT19
PT25
PT06
PT07
PT04
PT05
PT16
PT08
PT09
PT10
PT11
PT33
PT34
PT12
PT13
PT27
PT35
PT02
PT03
PT01
PT14
PT15
Cell type
CD103
CCR6
CD45RA
Foxp3
Tim3
4-1BB
Lag3
CCR4
CTLA-4
CCR7
IL-7Ra
γδTCR
perforin
Granzyme B
CD69
CD28
PD-1
CD39
CD25
TIGIT
CD27
CD38
HLA-DR
CD161
CD4
CD8
CD7
CD45
CD3
Frequency of CD3+ T cells
b
T cells
Cluster
PT01
PT02
PT03
PT04
PT05
PT06
PT07
PT08
PT09
PT10
PT11
PT12
PT13
PT14
PT15
PT16
PT17
PT18
PT19
PT20
PT21
PT22
PT23
PT24
PT25
PT26
PT27
PT28
PT29
PT30
PT31
PT32
PT33
PT34
PT35
t-SNE-2
t-SNE-1
PD-1
Tim3
Lag3
TIGIT
CTLA4
Normalized intensity
Min
Max
c
Gating strategy of PD-1+ CD4/CD8 T cells from AS plaques
Gating strategy of PD-1+ CD4/CD8 T cells from lung tumors
1.04
79.4
87.7
77.5
SSC-A
FSC-H
FSC-A
Aqua
CD45
42.3
34.4
41.4
41.6
PD-1
CD4
CD8
22.4
95.0
95.8
57.1
57.2
33.9
29.4
83.9
CD4+PD-1+ T cells
d
IL-2
TNF-α
IFN-γ
IL-1β
IL-6
P = 0.018
P = 0.0003
P = 0.009
P = 0.196
P = 0.174
Concentration (pg/mL)
Anti-CD3+ anti-CD28
-
+
e
CD4+PD-1+ T cells
Activation (%)
3000
2000
1000
0
CD8+PD-1+ T cells
f
P = 0.071
P = 0.096
P = 0.283
P = 0.786
P = 0.506
g
CD8+PD-1+ T cells
600
400
200

Supplementary information, Fig. S7. PD-1^{+} T cells remained activated but not exhausted in human AS plaques.

a Heatmap showing the median expression of T-cell activation and exhaustion panel markers in 35 identified T cell clusters (PT) labeled with major subsets (left) and cluster frequency (right).

b t-SNE plots of T cells from additional AS plaque samples (n = 4), colored by clusters (top) and normalized expressions of selected markers (bottom).

c Gating strategy of PD-1^{+}CD4^{+} and PD-1^{+}CD8^{+} T cells from AS plaques and lung tumors for T-cell stimulation assays.

d, f Comparisons of cytokine secretion capabilities (pg/mL) including IL-2, TNF-α, IFN-γ, IL-1β, and IL-6 from CD4^{+}PD-1^{+} (**d**) and CD8^{+}PD-1^{+} (**f**) T cells derived from human AS plaques (n = 8) before or after stimulation. Paired student's t-test was used, and P values were labeled.

e, g Comparisons of the activation levels (%) of IL-2, TNF-α, IFN-γ, IL-1β, and IL-6 from CD4^{+} (**e**) and CD8^{+} (**g**) PD-1^{+} T cells upon stimulation as in (**d**) and (**f**).

Data are represented as the mean and standard error of mean (SEM) in (**e**) and (**g**) and as median with interquartile range (IQR) in (**d**) and (**f**). Paired student's t-test was used in (**d**) and (**f**), and P values were labeled.
