# Supplementary material for: Targeting pro-inflammatory T cells as a novel therapeutic approach to potentially resolve atherosclerosis in humans
Source: Cell Res. 2024 Mar 15;34(6):407–27. doi: 10.1038/s41422-024-00945-0 (PMC11143203; doi:10.1038/s41422-024-00945-0)
Supplement: Supplementary file 8 — Supplementary information, Fig. S8 [file 41422_2024_945_MOESM8_ESM.pdf]

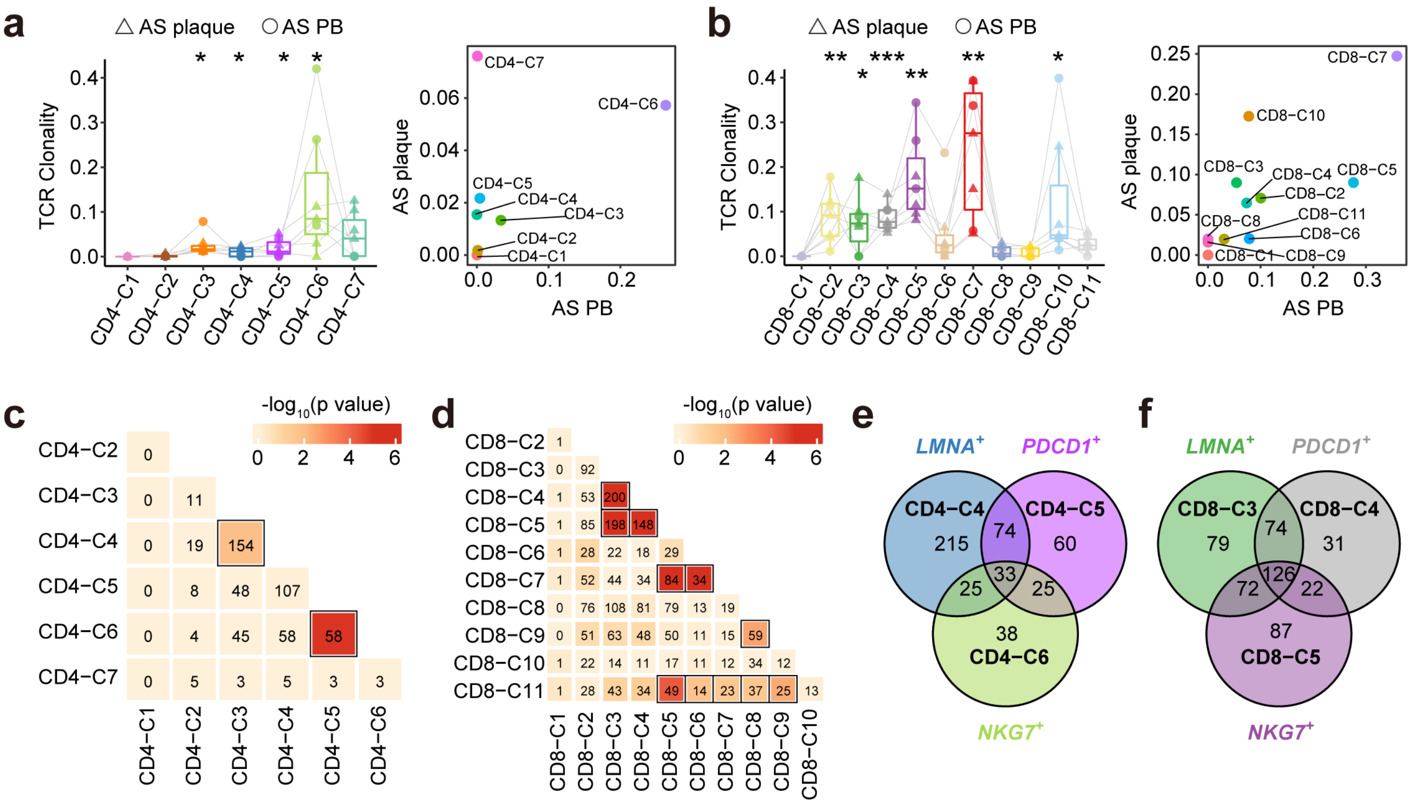

g

CD4⁺ T

Clonotype Names	CDR3 amino acid sequence: CDR3-α Sequence	CDR3-β Sequence	CD4-C4 (n) AS PB	CD4-C4 (n) AS pla.	CD4-C5 (n) AS PB	CD4-C5 (n) AS pla.	CD4-C6 (n) AS PB	CD4-C6 (n) AS pla.
P1_48	CAEVSGYSTLTF	CASSFPTGGRTGRTEAFF	1	2	0	3	6	3
P2_18	CAATSSSNFGNEKLTF	CAWSRAALTEAFF	0	9	0	7	7	1
P2_32	CAMREANVGADGLTF	CASSTRIAGGPFPETQYF	0	8	0	5	0	3
P4_31	CAARKGYQKVTF	CASSFGGTNTGELFF	NA	5	NA	5	NA	4
P4_11	CAVGAKQGGSEKLVF	CASSTRPGGSEKLFF	NA	12	NA	13	NA	6
P4_6	CVVSSSGTYKYIF	CASSTRPGGSEKLFF	NA	14	NA	17	NA	11
P4_44	CAVGSSGSARQLTF	CSALAGGGTGELFF	NA	5	NA	6	NA	3

CD8⁺ T

Clonotype Names	CDR3 amino acid sequence: CDR3-α Sequence	CDR3-β Sequence	CD4-C4 (n) AS PB	CD4-C4 (n) AS pla.	CD4-C5 (n) AS PB	CD4-C5 (n) AS pla.	CD4-C6 (n) AS PB	CD4-C6 (n) AS pla.
P1_20	CVVVGGRALTF	CASSLATNPHETQYF	0	31	4	11	0	9
P1_23	CALRAITQGGSEKLVF	CASSLTGAVYGYTF	0	24	8	11	5	11
P1_37	CAVNIVGNQFYF	CASSPLGTEAFF	0	7	10	11	2	4
P2_2	CVVPNTNAGKSTF	CASSLQGSTYNEQFF	4	68	8	33	50	23
P2_3	CAVNPLESSYKLIF	CASSPGTANTGELFF	0	23	0	5	41	37
P2_8	CAALEGDNYGQNFVF	CASSPTWGGVRDTQYF	0	16	7	5	13	4
P3_3	CVVPWMDSSYKLIF	CASSQRAGDGNYGYTF	0	14	0	6	7	10
P3_5	CALRNSNYQLIW	CASSLGDRAYNEQFF	0	10	0	5	6	12
P4_5	CATYSGNTPLVF	CASSWWGEGTEAFF	NA	12	NA	11	NA	16
P4_13	CVVSDGYGGATNKLIF	CASSAISKEGPYGYTF	NA	15	NA	3	NA	9

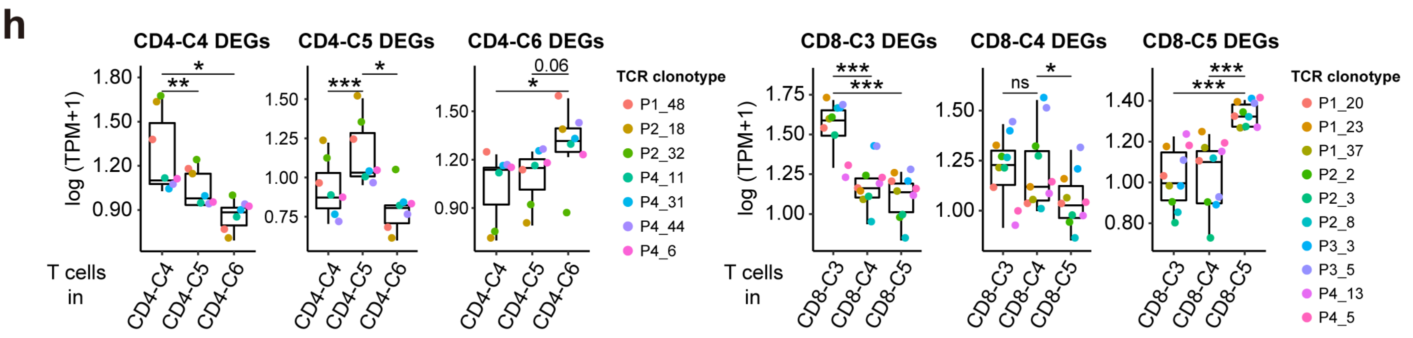

Supplementary information, Fig. S8. TCR repertoire analyses reveal lineage differentiation of T cells in AS plaques.

a, b Boxplots (left) showing the TCR clonality index of $CD4^+$ (**a**) and $CD8^+$ (**b**) T cell clusters. Points are colored by clusters and shaped by tissue sources. Scatter plot (right) showing the TCR clonality index in different tissues.

c, d Numbers of shared TCR clonotypes between $CD4^+$ (**c**) or $CD8^+$ (**d**) T cell clusters, colored by the calculated *P* values, and the black box marks $P < 0.05$. One-sided Fisher's exact test was used with Benjamini-Hochberg adjustment.

e, f Venn plots showing the shared TCR clonotypes among $PDCD1^+$, $LMNA^+$, and $NKG7^+$ T cell clusters of $CD4^+$ (**e**) and $CD8^+$ (**f**) lineages.

g Detailed information of shared TCR clonotypes among $PDCD1^+$, $LMNA^+$, and $NKG7^+$ T cell clusters of $CD4^+$ (**e**) and $CD8^+$ (**f**) lineages.

h Paired comparisons of cluster-specific DEGs on cells with shared TCR clonotypes as identified in (**g**) among $PDCD1^+$, $LMNA^+$, and $NKG7^+$ T cell clusters of $CD4^+$ (**e**) and $CD8^+$ (**f**) lineages.

Data are represented as median with interquartile range (IQR) in (**a**), (**b**), and (**h**). Paired student's t-test was used in (**a**) and (**b**), and the two-sided student's t-test in (**h**).
