# Supplementary material for: Targeting pro-inflammatory T cells as a novel therapeutic approach to potentially resolve atherosclerosis in humans
Source: Cell Res. 2024 Mar 15;34(6):407–27. doi: 10.1038/s41422-024-00945-0 (PMC11143203; doi:10.1038/s41422-024-00945-0)
Supplement: Supplementary file 9 — Supplementary information, Fig. S9 [file 41422_2024_945_MOESM9_ESM.pdf]

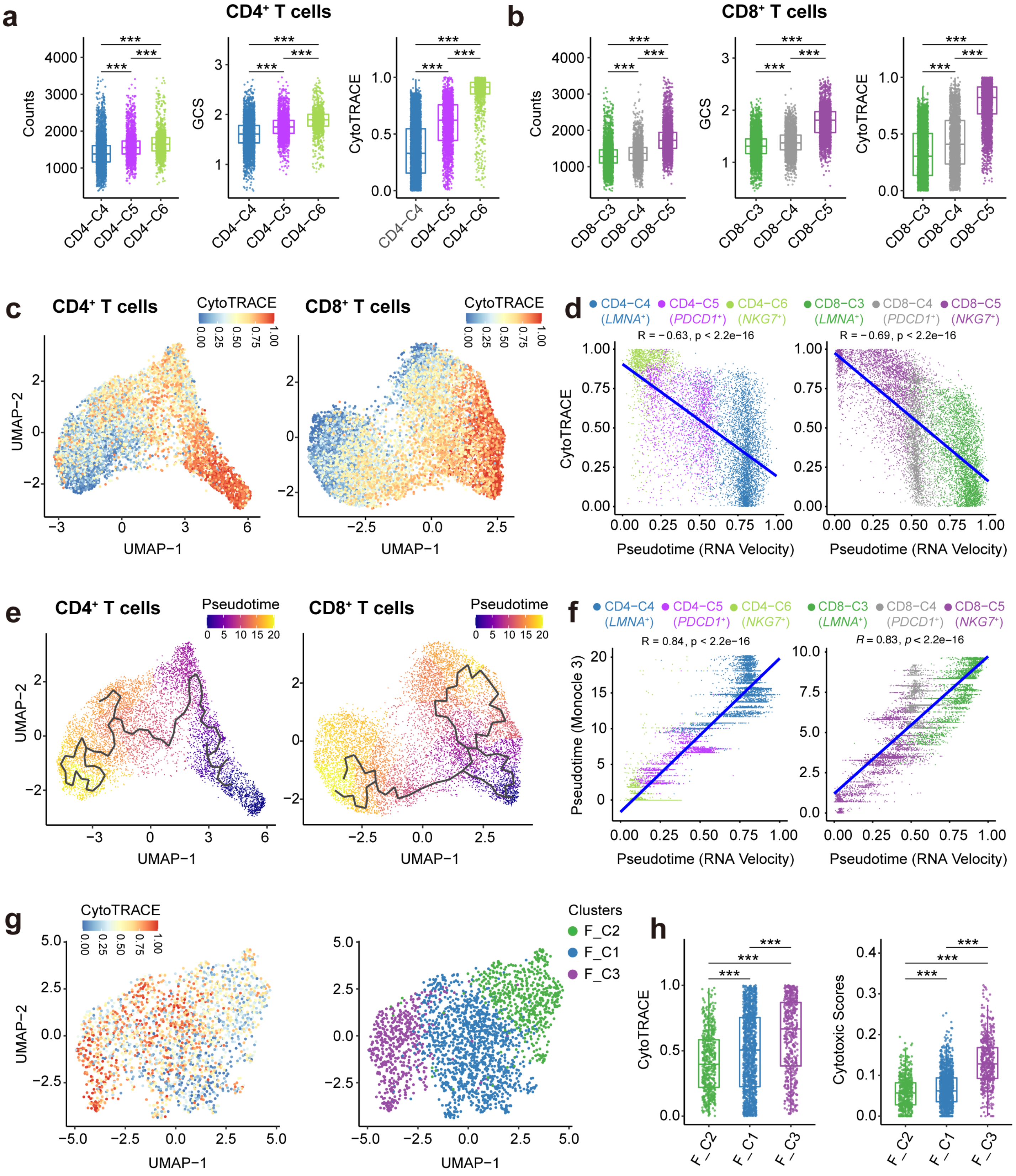
a
CD4⁺ T cells
Counts
GCS
CytoTRACE
CD4−C4
CD4−C5
CD4−C6
b
CD8⁺ T cells
CD8−C3
CD8−C4
CD8−C5
c
CD4⁺ T cells
CytoTRACE
CD8⁺ T cells
UMAP−1
UMAP−2
d
CD4−C4 (LMNA⁺) CD4−C5 (PDCD1⁺) CD4−C6 (NKG7⁺)
CD8−C3 (LMNA⁺) CD8−C4 (PDCD1⁺) CD8−C5 (NKG7⁺)
R = −0.63, p < 2.2e−16
R = −0.69, p < 2.2e−16
Pseudotime (RNA Velocity)
e
Pseudotime
f
R = 0.84, p < 2.2e−16
R = 0.83, p < 2.2e−16
Pseudotime (Monocle 3)
g
Clusters
F_C2
F_C1
F_C3
h
Cytotoxic Scores

Supplementary information, Fig. S9. Directional differentiation of *PDCD1*$^+$ T cells in AS plaques.

a, b Comparisons of unique gene counts (left), GCS (middle), and CytoTRACE (right) index calculated by CytoTRACE between CD4-C4, CD4-C5, and CD4-C6 cluster (**a**) and CD8-C3, CD8-C4, and CD8-C5 cluster (**b**).

c, e UMAP plots of T cells in CD4-C4, CD4-C5, and CD4-C6 clusters (left) and CD8-C3, CD8-C4, and CD8-C5 clusters (right), colored by CytoTRACE index (**c**) and pseudotime from Monocle 3 and labeled with inferred routes (**e**).

d, f Correlation analysis of pseudotime from RNA velocity with CytoTRACE index (**d**) or with pseudotime from Monocle 3 (**f**) for T cells in CD4-C4, CD4-C5, and CD4-C6 clusters (left) and CD8-C3, CD8-C4, and CD8-C5 clusters (right), Spearman correlation was used. Their correlation coefficients (*r*-value) and *P* values were labeled.

g UMAP plots of T cells in F_C1, F_C2, and F_C3 clusters from scRNA-seq data of Fernandez et al. as in supplementary information, Fig. S5a, colored by CytoTRACE index (left) and clusters (right).

h Comparisons of CytoTRACE index (left) and cytotoxic scores (right) among CD8$^+$ T cell clusters in (**g**).

Data are represented as median with interquartile range (IQR) in (**a**), (**b**), and (**h**). Unpaired student's t-test with Benjamini-Hochberg adjustment was used in (**a**), (**b**), and (**h**).
