# Supplementary material for: Targeting pro-inflammatory T cells as a novel therapeutic approach to potentially resolve atherosclerosis in humans
Source: Cell Res. 2024 Mar 15;34(6):407–27. doi: 10.1038/s41422-024-00945-0 (PMC11143203; doi:10.1038/s41422-024-00945-0)
Supplement: Supplementary file 10 — Supplementary information, Fig. S10 [file 41422_2024_945_MOESM10_ESM.pdf]

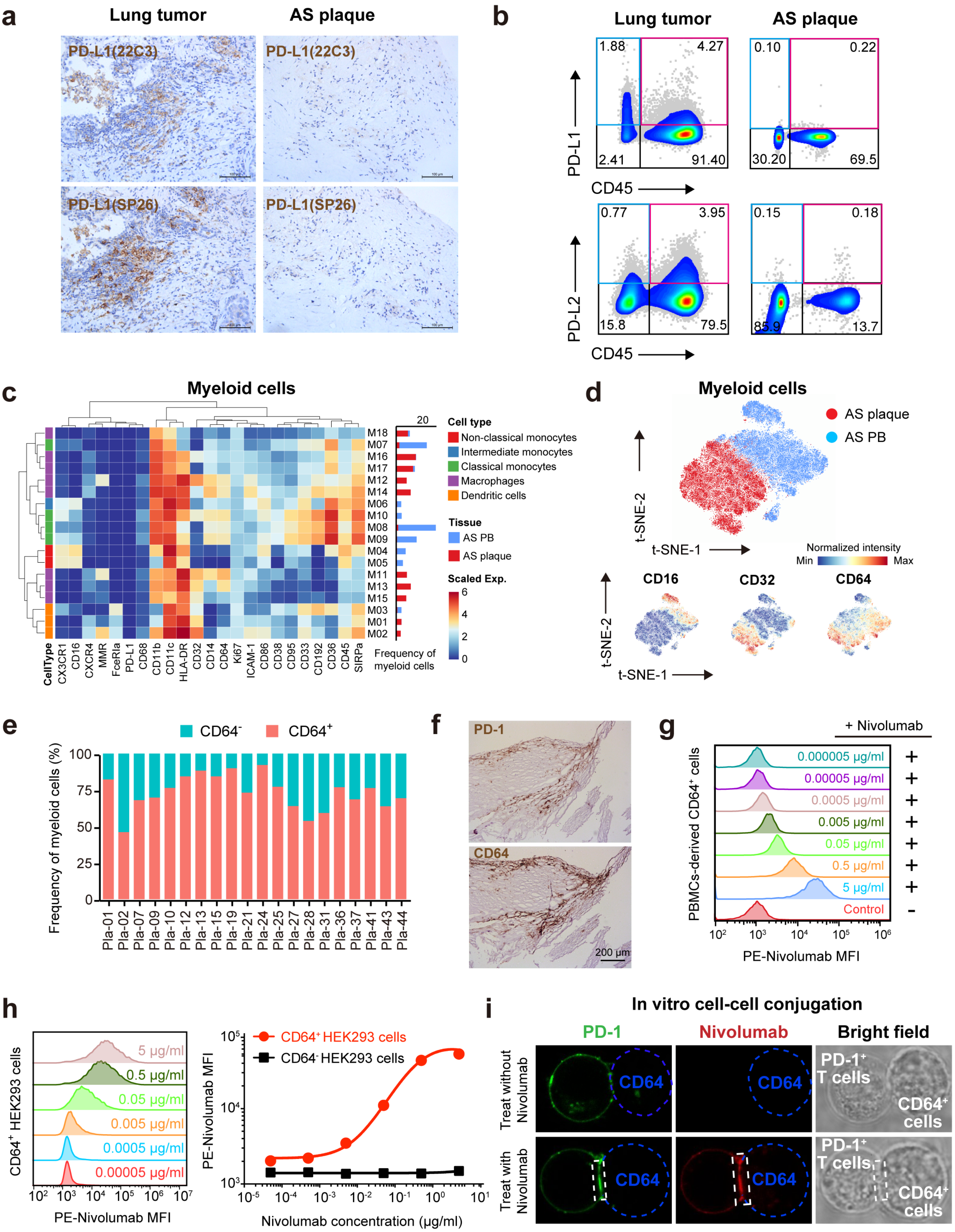
a
Lung tumor
AS plaque
PD-L1(22C3)
PD-L1(SP26)
b
Lung tumor
AS plaque
1.88
4.27
2.41
91.40
0.10
0.22
30.20
69.5
PD-L1
CD45
0.77
3.95
15.8
79.5
0.15
0.18
85.9
13.7
PD-L2
CD45
c
Myeloid cells
Cell type
Non-classical monocytes
Intermediate monocytes
Classical monocytes
Macrophages
Dendritic cells
Tissue
AS PB
AS plaque
Scaled Exp.
Frequency of myeloid cells
CellType
d
Myeloid cells
AS plaque
AS PB
t-SNE-1
t-SNE-2
Normalized intensity
Min
Max
CD16
CD32
CD64
e
CD64-
CD64+
Frequency of myeloid cells (%)
f
PD-1
CD64
200 μm
g
+ Nivolumab
PBMCs-derived CD64+ cells
0.000005 μg/ml
0.00005 μg/ml
0.0005 μg/ml
0.005 μg/ml
0.05 μg/ml
0.5 μg/ml
5 μg/ml
Control
PE-Nivolumab MFI
h
CD64+ HEK293 cells
5 μg/ml
0.5 μg/ml
0.05 μg/ml
0.005 μg/ml
0.0005 μg/ml
0.00005 μg/ml
PE-Nivolumab MFI
CD64+ HEK293 cells
CD64- HEK293 cells
PE-Nivolumab MFI
Nivolumab concentration (μg/ml)
i
In vitro cell-cell conjugation
PD-1
Nivolumab
Bright field
Treat without Nivolumab
Treat with Nivolumab
CD64
PD-1+ T cells
CD64+ cells

Supplementary information, Fig. S10. Single-cell CyTOF atlas of myeloid cells in AS plaques.

a Representative sample of lung tumors (*n* = 3; left) and AS plaques (*n* = 3, right) with staining of PD-L1 (with clone 22C3 and SP26). Scar bar: 100 μm.

b Flow cytometric analysis of PD-L1$^+$ and PD-L2$^+$ cells in CD45$^-$ and CD45$^+$ cells in lung tumors (*n* = 4) and AS plaques (*n* = 5).

c Heatmap showing the median marker expressions of the myeloid cell panel for 18 myeloid cell clusters, labeled with major subsets (left) and cluster frequency (right).

d t-SNE plots of myeloid cells, colored either by sample groups or normalized expressions of CD16, CD32, and CD64.

e Compositions of CD64$^+$ and CD64$^-$ cells for AS plaque samples (*n* = 20).

f Representative IHC staining showing physical co-localization of PD-1 and CD64 on serial sections from a carotid plaque. Scale bar: 200 μm.

g Flow cytometric analysis of binding ability between anti-PD-1 mAb (Nivolumab) and PBMC-derived CD64$^+$ cells.

h Flow cytometric analysis of binding ability between anti-PD-1 mAb (Nivolumab) and CD64$^+$ HEK293 cells (left), and MFI of PE-Nivolumab on CD64$^+$HEK293 cells (*n* = 4) (right).

i Cell-cell conjugation assay showing PD-1$^+$ Jurkat T cells (green) were conjugated with CD64$^+$ HEK293 cells in the presence of Nivolumab (red) as displayed by confocal images and corresponding bright-field.
