# Supplementary material for: Targeting pro-inflammatory T cells as a novel therapeutic approach to potentially resolve atherosclerosis in humans
Source: Cell Res. 2024 Mar 15;34(6):407–27. doi: 10.1038/s41422-024-00945-0 (PMC11143203; doi:10.1038/s41422-024-00945-0)
Supplement: Supplementary file 11 — Supplementary information, Fig. S11 [file 41422_2024_945_MOESM11_ESM.pdf]

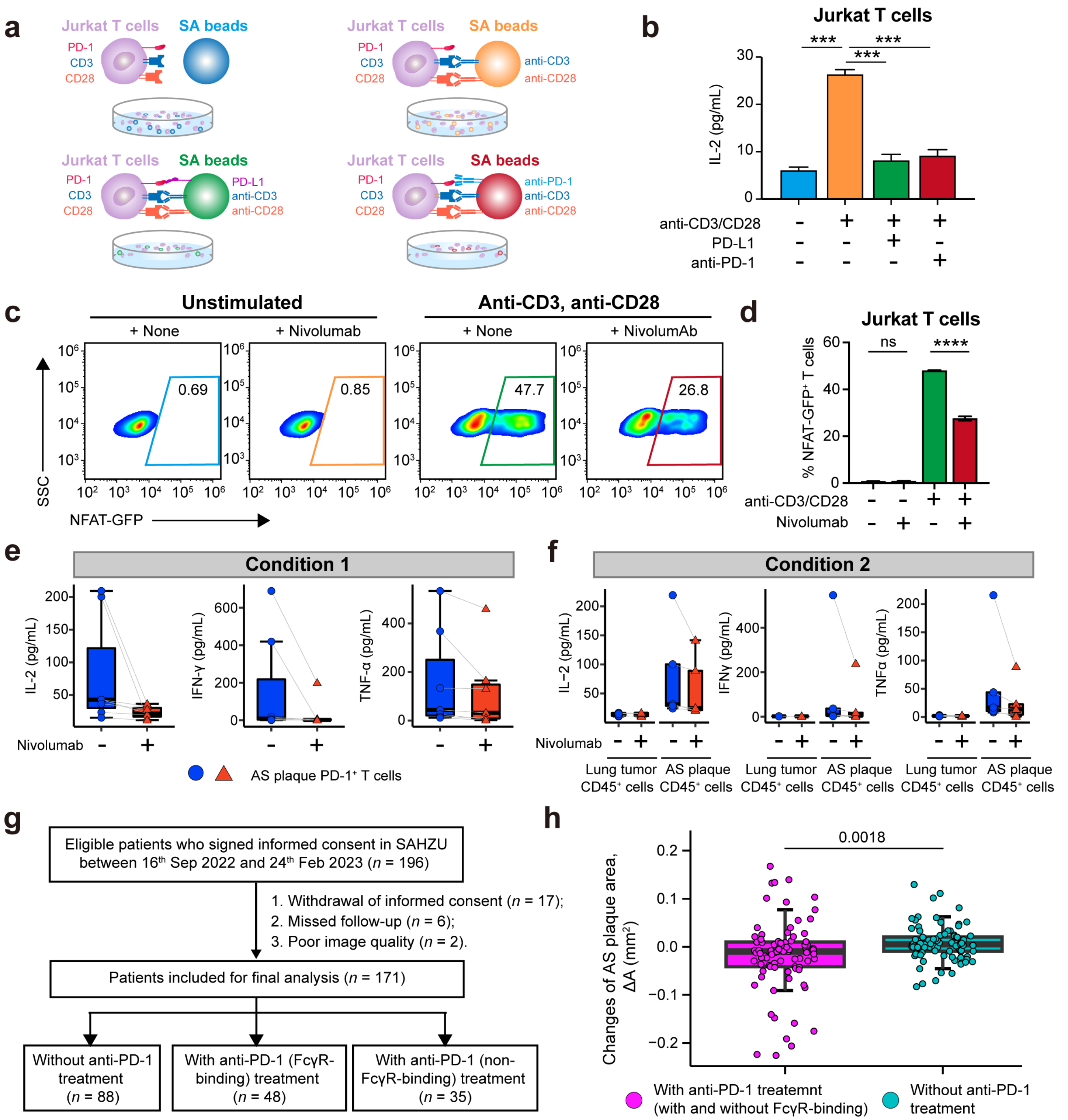

i **Patients with anti-PD-1 (FcγR-binding) vs non-anti-PD-1 treatment**

	Relative ratio (95% CI)	*P* value
Univariate		
**Anti-PD-1 mAb (FcγR-binding)**	0.41 (0.23 - 0.70)	0.00182
Multivariate		
**Anti-PD-1 mAb (FcγR-binding)**	0.42 (0.21 - 0.77)	0.0067

P-values were calculated from modified Poisson regression analysis, and multivariate analyses were adjusted by using age, gender, ΔBMI, ΔHDL, ΔLDL, statin usage, tumor type, tumor stage, and tumor progression.

j **Patients with anti-PD-1 (FcγR-binding) vs anti-PD-1 (non-FcγR-binding) treatment**

	Relative ratio (95% CI)	*P* value
Univariate		
**Anti-PD-1 mAb (FcγR-binding)**	0.39 (0.20 - 0.72)	0.0033
Multivariate		
**Anti-PD-1 mAb (FcγR-binding)**	0.31 (0.13 - 0.69)	0.0056

P-values were calculated from modified Poisson regression analysis, and multivariate analyses were adjusted by using age, gender, ΔBMI, TG-1, ΔTG, ΔLDL, statin usage, tumor type, tumor stage, and tumor progression.

Supplementary information, Fig. S11. CD64-captured anti-PD-1 mAb suppresses T cell activation in AS plaque.

a, b Schematic (**a**) of an *in-vitro* stimulation assay for PD-1$^+$ Jurkat T cells (n = 4) with different stimulations, and the calculation of IL-2 concentration in the supernatant (**b**).

c, d Flow cytometric analysis of NFAT-GFP intensity in PD-1$^+$ Jurkat T cells upon different stimulations (**c**), and frequency comparison (**d**) of NFAT-GFP expressing T cells (n = 4).

e, f Concentrations (pg/mL) of IL-2, IFN-γ, and TNF-α in the supernatant of Condition 1 (**e**) and Condition 2 (**f**) in Fig. 6f.

g Flowchart of identifying eligible patients in the prospective cohort study.

h Comparison of AS plaque areas (ΔA) between two scanning time points in the patients without anti-PD-1 mAb (n = 88) and with anti-PD-1 mAb (with or without FcγR-binding ability; n = 83).

i, j Univariate and multivariate (Modified Poisson) regression analysis of the relative ratio (RR) of anti-PD-1 (FcγR-binding) treatment to AS plaque progression in comparisons to without anti-PD-1 treatment (**i**) and anti-PD-1 (non-FcγR-binding) treatment (**j**). Multivariate analysis was adjusted by using age, gender, ΔBMI, ΔHDL, ΔLDL, statin usage, tumor type, tumor stage, and tumor progression in (**i**). It was adjusted by using age, gender, ΔBMI, TG-1, ΔTG, ΔLDL, statin usage, tumor type, tumor stage, and tumor progression in (**j**).

Data are represented as the mean and standard error of mean (SEM) in (**b**) and (**d**) and as median with interquartile range (IQR) in (**e**), (**f**), and (**h**). Paired Student's t-test was used in (**b**), (**d**), (**e**), and (**f**), and the Mann-Whitney test was in (**h**).
