# Supplementary material for: Targeting pro-inflammatory T cells as a novel therapeutic approach to potentially resolve atherosclerosis in humans
Source: Cell Res. 2024 Mar 15;34(6):407–27. doi: 10.1038/s41422-024-00945-0 (PMC11143203; doi:10.1038/s41422-024-00945-0)
Supplement: Supplementary file 12 — Supplementary information, Fig. S12 [file 41422_2024_945_MOESM12_ESM.pdf]

Parameter		Overall (n = 171)	Non-anti-PD-1 (n = 88)	Anti-PD-1 (FcγR-binding) (n = 48)	Anti-PD-1 (non-FcγR-binding) (n = 35)	P value
**Age, years**		65.32 (7.53)	63.99 (7.66)	66.19 (7.09)	67.46 (7.33) #	0.044
**Gender, n (%)**	F	31 (18.13)	24 (27.27)	6 (12.50)	1 (2.86)	0.002
	M	140 (81.87)	64 (72.73)	42 (87.50)	34 (97.14)	
**BMI, kg/m^2**						
- Baseline		22.15 [20.83, 24.43]	22.68 [20.94, 25.39]	22.85 [21.25, 24.26]	21.45 [19.88, 22.28] #	0.018
- After treatment		22.49 [20.44, 24.85]	23.47 [21.21, 25.06]	22.66 [19.82, 25.41]	21.30 [19.37, 22.76] #	0.009
- Changes		0.00 [-0.53, 0.82]	0.00 [-0.41, 0.71]	0.08 [-0.69, 0.80]	0.00 [-0.73, 1.05]	0.955
**Systolic BP, mmHg**						
- Baseline		124.00 [117.00, 131.00]	124.00 [117.00, 130.25]	124.50 [116.00, 131.25]	123.00 [116.00, 129.00]	0.710
- After treatment		124.00 [117.00, 131.00]	125.00 [117.00, 131.25]	123.50 [120.00, 131.00]	122.00 [114.50, 127.50]	0.305
- Changes		0.00 [-6.00, 6.00]	1.00 [-6.50, 8.25]	1.50 [-4.25, 5.25]	-1.00 [-6.50, 4.00]	0.412
**Diastolic BP, mmHg**						
- Baseline		74.00 [69.00, 79.00]	74.00 [68.00, 80.00]	74.50 [70.00, 79.00]	75.00 [69.50, 78.50]	0.805
- After treatment		73.00 [67.00, 80.00]	74.50 [67.00, 80.00]	72.50 [67.75, 80.25]	69.00 [66.00, 78.00]	0.611
- Changes		-1.00 [-6.00, 4.00]	-1.00 [-5.25, 5.00]	-1.00 [-4.25, 1.25]	-2.00 [-10.00, 8.00]	0.628
**Fasting blood glucose, mmol/L**						
- Baseline		5.53 [4.95, 6.40]	5.60 [5.04, 6.48]	5.52 [4.90, 6.83]	5.32 [4.90, 5.86]	0.367
- After treatment		5.60 [5.04, 6.31]	5.54 [5.10, 6.36]	5.65 [5.05, 6.21]	5.72 [4.91, 6.24]	0.938
- Changes		0.02 [-0.64, 0.66]	0.09 [-0.64, 0.55]	-0.19 [-0.95, 0.72]	0.07 [-0.36, 0.98]	0.381
**Total cholesterol, mmol/L**						
- Baseline		4.72 [3.89, 5.39]	4.97 [4.00, 5.55]	4.56 [4.00, 5.22]	4.56 [3.40, 4.88] #	0.040
- After treatment		4.97 [4.29, 5.65]	5.11 [4.34, 5.66]	4.78 [4.19, 5.55]	4.77 [4.39, 5.80]	0.557
- Changes		0.26 [-0.21, 0.84]	0.20 [-0.13, 0.74]	0.21 [-0.47, 0.91]	0.67 [-0.04, 1.28]	0.056
**Triglyceride, mmol/L**						
- Baseline		1.28 [0.88, 1.70]	1.38 [1.01, 1.95]	1.27 [0.86, 1.69]	1.03 [0.81, 1.30] # $	0.001
- After treatment		1.56 [1.17, 2.12]	1.64 [1.32, 2.20]	1.33 [1.03, 2.01]	1.43 [1.14, 2.00]	0.041
- Changes		0.23 [-0.11, 0.63]	0.23 [-0.15, 0.62]	0.04 [-0.15, 0.46]	0.44 [0.06, 0.85] $	0.043
**HDL, mmol/L**						
- Baseline		1.07 [0.95, 1.25]	1.10 [0.99, 1.25]	1.04 [0.92, 1.33]	1.05 [0.90, 1.19]	0.300
- After treatment		1.22 [1.02, 1.40]	1.26 [1.07, 1.47]	1.17 [1.00, 1.36]	1.18 [0.98, 1.38]	0.369
- Changes		0.12 [-0.07, 0.28]	0.13 [0.00, 0.28]	0.11 [-0.10, 0.26]	0.14 [-0.13, 0.38]	0.791
**LDL, mmol/L**						
- Baseline		2.74 [2.11, 3.26]	2.92 [2.11, 3.36]	2.58 [2.28, 3.27]	2.65 [1.97, 2.82]	0.112
- After treatment		2.83 [2.27, 3.47]	2.94 [2.25, 3.46]	2.73 [2.20, 3.46]	2.83 [2.44, 3.45]	0.776
- Changes		0.12 [-0.25, 0.48]	0.07 [-0.30, 0.43]	-0.07 [-0.43, 0.38]	0.28 [0.02, 0.79] # $	0.007
**Statin, n (%)**	N	125 (73.10)	61 (69.32)	38 (79.17)	26 (74.29)	0.458
	Y	46 (26.90)	27 (30.68)	10 (20.83)	9 (25.71)	
**Tumor type, n (%)**						
- Digestive system		46 (26.90)	32 (36.36)	11 (22.92)	3 (8.57)	
- Respiratory system		104 (60.82)	41 (46.59)	34 (70.83)	29 (82.86)	0.0015
- Others		21 (12.28)	15 (17.05)	3 (6.25)	3 (8.57)	
**Treatment cycles, months**		3.3667 [3.0317, 3.7300]	3.3517 [3.0325, 3.7400]	3.3150 [3.0000, 3.7300]	3.4000 [3.1000, 3.7300]	0.890
**Changes of plaque areas (ΔA)**		-0.05 [-2.48, 1.62]	0.55 [-0.92, 2.05]	-2.72 [-6.36, -0.80]	0.88 [-0.78, 3.98]	<0.0001

Supplementary information, Fig. S12. Clinical characteristics of the prospective cohort. Categorical variables are presented as numbers (n) and percentages (%). Continuous variables are presented as the median and interquartile range (IQR). The Chi-squared test and Mann-Whitney test are used. The symbol # represents adjusted $P < 0.05$ by comparing the patients of anti-PD-1 (non-FcγR-binding) treatment with those without anti-PD-1 treatment, and the symbol $ represents adjusted $P < 0.05$ by comparing the patients of anti-PD-1 (non-FcγR-binding) treatment with those of anti-PD-1 (FcγR-binding) treatment in pairwise comparison.
