# Supplementary material for: Targeting pro-inflammatory T cells as a novel therapeutic approach to potentially resolve atherosclerosis in humans
Source: Cell Res. 2024 Mar 15;34(6):407–27. doi: 10.1038/s41422-024-00945-0 (PMC11143203; doi:10.1038/s41422-024-00945-0)
Supplement: Supplementary file 13 — Supplementary information, Table S1 [file 41422_2024_945_MOESM13_ESM.pdf]

Supplementary information, Table S1. Differentially expressed genes (DEGs) of identified T cell clusters.

Differentially expressed genes (DEGs) of identified T cell clusters.

Gene	*P* value	avg_logFC	pct.1	pct.2	adjusted *P* value	cluster
RPS13	1.02E-312	0.743855685	1.000	0.995	0.00E+00	CD4-C1
RPL32	7.12E-300	0.637690459	0.999	0.998	1.18E-295	CD4-C1
CCR7	8.75E-280	1.375292071	0.844	0.119	1.46E-275	CD4-C1
RPL11	6.99E-272	0.54815563	0.999	0.998	1.16E-267	CD4-C1
LEF1	9.57E-265	1.275153431	0.822	0.118	1.59E-260	CD4-C1
RPS8	6.11E-263	0.62106959	0.999	0.997	1.02E-258	CD4-C1
TCF7	1.16E-257	1.345776459	0.872	0.201	1.94E-253	CD4-C1
RPL22	4.41E-257	0.721114161	0.999	0.976	7.33E-253	CD4-C1
SELL	9.35E-251	1.371947062	0.876	0.180	1.56E-246	CD4-C1
LDHB	3.32E-250	0.996143416	0.980	0.663	5.52E-246	CD4-C1
RPS23	8.34E-240	0.541587829	1.000	0.996	1.39E-235	CD4-C1
RPS5	1.11E-233	0.699392076	0.999	0.980	1.85E-229	CD4-C1
RPL19	2.24E-228	0.521630607	1.000	0.998	3.72E-224	CD4-C1
RPS12	2.07E-224	0.532152643	0.999	0.998	3.45E-220	CD4-C1
RPL30	3.40E-223	0.473942684	1.000	0.998	5.65E-219	CD4-C1
RPL5	3.70E-217	0.613833799	0.999	0.986	6.14E-213	CD4-C1
RPL34	1.67E-212	0.49175112	0.999	0.998	2.77E-208	CD4-C1
RPL18	1.62E-196	0.493543331	0.999	0.995	2.70E-192	CD4-C1
EEF1B2	5.53E-195	0.645594102	0.998	0.964	9.20E-191	CD4-C1
RPL29	5.43E-194	0.492275959	0.999	0.993	9.03E-190	CD4-C1
LTB	2.84E-193	0.974479665	0.963	0.486	4.71E-189	CD4-C1
RPL13	1.97E-191	0.472224557	1.000	0.999	3.27E-187	CD4-C1
MAL	3.88E-191	1.088140037	0.732	0.107	6.45E-187	CD4-C1
PABPC1	3.47E-185	0.661804261	0.999	0.948	5.77E-181	CD4-C1
EEF1A1	5.76E-183	0.429702301	1.000	1.000	9.59E-179	CD4-C1
RPS14	1.28E-182	0.445546772	0.999	0.997	2.14E-178	CD4-C1
RPS28	1.12E-181	0.445802121	0.998	0.997	1.87E-177	CD4-C1
NOSIP	1.02E-178	1.090703051	0.861	0.336	1.69E-174	CD4-C1
RPS6	2.63E-173	0.51616554	0.998	0.992	4.37E-169	CD4-C1
EEF1G	1.28E-172	0.677669642	0.995	0.890	2.14E-168	CD4-C1
RPL35A	3.39E-166	0.426123479	0.999	0.995	5.65E-162	CD4-C1
RPL39	8.19E-165	0.409414645	1.000	0.997	1.36E-160	CD4-C1
RPS27A	4.48E-158	0.400905374	1.000	0.998	7.46E-154	CD4-C1
RPL37	2.98E-157	0.415566966	0.999	0.997	4.96E-153	CD4-C1
RPL18A	1.87E-154	0.435024001	1.000	0.995	3.11E-150	CD4-C1
RACK1	4.50E-152	0.476402293	0.998	0.980	7.48E-148	CD4-C1
RPS4X	1.64E-150	0.41637708	1.000	0.994	2.73E-146	CD4-C1
RPL10	3.16E-150	0.397910122	1.000	0.999	5.25E-146	CD4-C1
NACA	2.74E-146	0.45785451	0.999	0.984	4.56E-142	CD4-C1
RPL10A	9.93E-141	0.477848711	0.997	0.981	1.65E-136	CD4-C1
RPL14	1.22E-138	0.38055397	0.999	0.995	2.04E-134	CD4-C1
RPL12	4.08E-135	0.401401126	1.000	0.995	6.79E-131	CD4-C1
RPL9	3.29E-133	0.431442374	0.998	0.989	5.47E-129	CD4-C1
IL7R	6.60E-128	0.633992504	0.912	0.485	1.10E-123	CD4-C1
ACTN1	2.41E-126	0.751118317	0.463	0.036	4.01E-122	CD4-C1
RPL8	2.84E-120	0.379136269	0.999	0.992	4.73E-116	CD4-C1
RPS3A	1.53E-119	0.479634206	1.000	0.993	2.54E-115	CD4-C1

EIF3E	9.93E-119	0.703802758	0.922	0.602	1.65E-114	CD4-C1
RPL21	1.13E-118	0.435943627	0.997	0.984	1.87E-114	CD4-C1
TSHZ2	2.57E-114	0.76223332	0.433	0.026	4.27E-110	CD4-C1
RPSA	5.56E-114	0.395228691	0.999	0.983	9.24E-110	CD4-C1
RPL7	2.06E-112	0.490437669	0.995	0.935	3.43E-108	CD4-C1
RPS21	9.07E-112	0.346935241	1.000	0.995	1.51E-107	CD4-C1
RPS18	2.92E-110	0.392417882	0.999	0.995	4.85E-106	CD4-C1
LINC00861	5.04E-109	0.79963439	0.725	0.272	8.38E-105	CD4-C1
TXNIP	2.90E-108	0.596983657	0.972	0.746	4.83E-104	CD4-C1
RPL36	1.29E-107	0.356426168	0.999	0.992	2.15E-103	CD4-C1
RPS25	9.49E-102	0.349970302	0.999	0.996	1.58E-97	CD4-C1
TRABD2A	4.67E-99	0.675393259	0.449	0.064	7.77E-95	CD4-C1
RPL4	7.60E-98	0.475569481	0.993	0.944	1.26E-93	CD4-C1
RPL3	2.55E-97	0.379188636	0.999	0.988	4.24E-93	CD4-C1
FHIT	1.99E-95	0.664740322	0.368	0.022	3.30E-91	CD4-C1
FCMR	3.41E-95	0.684862454	0.701	0.279	5.67E-91	CD4-C1
RPS9	1.38E-94	0.403535786	0.997	0.976	2.30E-90	CD4-C1
EEF2	2.55E-94	0.397294917	0.994	0.943	4.25E-90	CD4-C1
C1orf162	3.29E-92	0.653560009	0.509	0.116	5.46E-88	CD4-C1
ADTRP	3.94E-92	0.628420584	0.323	0.012	6.55E-88	CD4-C1
BEX3	7.52E-91	0.556360345	0.356	0.037	1.25E-86	CD4-C1
CHRM3-AS2	3.52E-89	0.600256277	0.362	0.029	5.85E-85	CD4-C1
AIF1	4.90E-87	0.703703984	0.408	0.045	8.15E-83	CD4-C1
SATB1	2.53E-86	0.631675412	0.552	0.163	4.21E-82	CD4-C1
PIK3IP1	2.68E-86	0.635677507	0.876	0.516	4.45E-82	CD4-C1
TMEM123	6.02E-85	0.598635537	0.827	0.438	1.00E-80	CD4-C1
ST13	7.10E-81	0.576740582	0.814	0.471	1.18E-76	CD4-C1
EPHX2	1.89E-79	0.617500778	0.393	0.050	3.15E-75	CD4-C1
LIMD2	2.83E-79	0.53591531	0.939	0.681	4.70E-75	CD4-C1
GIMAP7	4.16E-78	0.62982037	0.843	0.446	6.92E-74	CD4-C1
NOP53	1.49E-76	0.461885542	0.975	0.851	2.49E-72	CD4-C1
TCEA3	6.88E-72	0.473396143	0.284	0.019	1.14E-67	CD4-C1
ADD3	8.64E-70	0.532449335	0.705	0.349	1.44E-65	CD4-C1
RPL31	1.28E-69	0.352518349	0.992	0.948	2.13E-65	CD4-C1
MYC	8.83E-69	0.664639299	0.518	0.158	1.47E-64	CD4-C1
CAMK4	1.96E-68	0.566357926	0.741	0.361	3.26E-64	CD4-C1
RIPOR2	2.97E-65	0.57554999	0.695	0.347	4.94E-61	CD4-C1
EIF2S3	5.83E-64	0.566530238	0.732	0.368	9.69E-60	CD4-C1
NDFIP1	1.93E-63	0.541140816	0.858	0.557	3.21E-59	CD4-C1
RPS10	6.04E-63	0.389874397	0.994	0.976	1.00E-58	CD4-C1
AES	1.45E-62	0.488927926	0.908	0.629	2.41E-58	CD4-C1
CD27	2.14E-61	0.40161111	0.628	0.278	3.56E-57	CD4-C1
EIF3L	6.86E-60	0.474079727	0.890	0.637	1.14E-55	CD4-C1
GSTM2	8.57E-60	0.390093148	0.241	0.032	1.42E-55	CD4-C1
EIF4B	3.75E-59	0.445450984	0.885	0.630	6.24E-55	CD4-C1
COX7C	6.13E-57	0.349361383	0.979	0.880	1.02E-52	CD4-C1
FOXP1	3.46E-55	0.466830934	0.768	0.433	5.75E-51	CD4-C1
TXK	1.17E-54	0.430700612	0.317	0.060	1.94E-50	CD4-C1
FYB1	5.46E-54	0.50215458	0.885	0.630	9.08E-50	CD4-C1
MDS2	9.01E-54	0.368389704	0.195	0.014	1.50E-49	CD4-C1
EIF3H	2.80E-53	0.398680126	0.912	0.705	4.65E-49	CD4-C1
IL6ST	1.19E-52	0.509452639	0.575	0.244	1.98E-48	CD4-C1

LINC01550	1.72E-50	0.4578589	0.335	0.073	2.85E-46	CD4-C1
TRAF3IP3	9.78E-50	0.451613975	0.752	0.445	1.63E-45	CD4-C1
DGKA	1.28E-49	0.493672164	0.522	0.194	2.13E-45	CD4-C1
FLT3LG	3.00E-49	0.473944002	0.761	0.436	4.99E-45	CD4-C1
GPSM3	3.39E-48	0.401793586	0.908	0.675	5.64E-44	CD4-C1
RCAN3	1.57E-47	0.469565047	0.566	0.246	2.61E-43	CD4-C1
LDLRAP1	2.90E-47	0.470487611	0.469	0.168	4.82E-43	CD4-C1
TESPA1	7.37E-45	0.423388145	0.392	0.129	1.23E-40	CD4-C1
AC243960.1	1.24E-43	0.48226776	0.533	0.234	2.06E-39	CD4-C1
TRAT1	1.60E-43	0.3916651	0.420	0.169	2.65E-39	CD4-C1
RASGRP2	2.66E-43	0.456951743	0.564	0.257	4.43E-39	CD4-C1
RNASET2	7.81E-43	0.365728069	0.745	0.468	1.30E-38	CD4-C1
NELL2	1.41E-42	0.458149934	0.381	0.122	2.34E-38	CD4-C1
SPINT2	7.88E-41	0.359815455	0.344	0.098	1.31E-36	CD4-C1
PCED1B	5.17E-40	0.483395079	0.463	0.196	8.60E-36	CD4-C1
AC119396.1	2.82E-39	0.402255573	0.337	0.108	4.70E-35	CD4-C1
SELENOH	1.53E-38	0.371417494	0.687	0.398	2.54E-34	CD4-C1
ANP32B	1.49E-37	0.387483738	0.840	0.604	2.49E-33	CD4-C1
PDE3B	6.57E-37	0.393117113	0.437	0.171	1.09E-32	CD4-C1
RGS10	8.66E-37	0.407863525	0.807	0.534	1.44E-32	CD4-C1
PRMT2	1.29E-36	0.398536297	0.731	0.449	2.14E-32	CD4-C1
SH3YL1	9.52E-36	0.386832683	0.325	0.090	1.58E-31	CD4-C1
UCP2	4.95E-35	0.382035601	0.705	0.440	8.24E-31	CD4-C1
ARMH1	8.06E-35	0.363737089	0.254	0.055	1.34E-30	CD4-C1
MHENCR	9.82E-35	0.373117298	0.319	0.101	1.63E-30	CD4-C1
GIMAP5	1.27E-34	0.365954847	0.323	0.103	2.12E-30	CD4-C1
TMIGD2	3.41E-34	0.35240729	0.284	0.058	5.67E-30	CD4-C1
SAMHD1	4.57E-34	0.352820445	0.526	0.275	7.61E-30	CD4-C1
INPP4B	5.66E-34	0.403420417	0.456	0.194	9.41E-30	CD4-C1
HSPB1	7.97E-34	0.375555216	0.599	0.330	1.32E-29	CD4-C1
MAML2	2.06E-33	0.348600258	0.319	0.106	3.42E-29	CD4-C1
PLAC8	3.31E-33	0.347834434	0.431	0.180	5.51E-29	CD4-C1
APEX1	6.17E-33	0.368246665	0.539	0.277	1.03E-28	CD4-C1
GIMAP1	6.68E-33	0.392750652	0.490	0.239	1.11E-28	CD4-C1
SESN3	1.04E-32	0.392711041	0.293	0.078	1.73E-28	CD4-C1
TMEM204	1.12E-32	0.352977667	0.275	0.074	1.86E-28	CD4-C1
STMN3	2.17E-32	0.355505255	0.405	0.177	3.60E-28	CD4-C1
CD55	1.70E-31	0.395376825	0.525	0.273	2.82E-27	CD4-C1
NUCB2	2.59E-31	0.395570106	0.501	0.228	4.31E-27	CD4-C1
OXNAD1	5.02E-29	0.346873792	0.501	0.253	8.35E-25	CD4-C1
SMDT1	1.19E-24	0.349994638	0.779	0.549	1.99E-20	CD4-C1
STAT1	2.93E-24	0.429418844	0.422	0.227	4.87E-20	CD4-C1
RPS8	9.40E-171	0.50403364	0.999	0.997	1.56E-166	CD4-C2
LTB	7.41E-144	0.951140237	0.918	0.478	1.23E-139	CD4-C2
LDHB	6.25E-143	0.839401968	0.942	0.658	1.04E-138	CD4-C2
RPL32	9.66E-141	0.414230699	0.999	0.998	1.61E-136	CD4-C2
RPL11	5.49E-136	0.405699376	1.000	0.998	9.12E-132	CD4-C2
RPS13	7.08E-127	0.435609869	0.998	0.995	1.18E-122	CD4-C2
SELL	3.21E-123	1.033638651	0.666	0.178	5.33E-119	CD4-C2
RPL22	5.78E-119	0.469156283	0.994	0.975	9.61E-115	CD4-C2
EEF1A1	6.85E-113	0.351961232	1.000	1.000	1.14E-108	CD4-C2
RPL29	6.80E-110	0.360768563	0.997	0.993	1.13E-105	CD4-C2

MAL	2.16E-107	0.77352298	0.551	0.105	3.59E-103	CD4-C2
LEF1	1.33E-104	0.708523132	0.542	0.120	2.22E-100	CD4-C2
RPL5	1.75E-103	0.420693815	0.998	0.986	2.91E-99	CD4-C2
EEF1G	2.64E-102	0.47339301	0.981	0.888	4.38E-98	CD4-C2
EEF2	9.31E-100	0.403396512	0.988	0.943	1.55E-95	CD4-C2
IL7R	2.47E-98	0.741271032	0.870	0.477	4.12E-94	CD4-C2
RPS12	1.06E-95	0.364209709	0.998	0.998	1.76E-91	CD4-C2
PABPC1	2.96E-92	0.495068154	0.996	0.947	4.93E-88	CD4-C2
RPS6	2.30E-90	0.386490542	0.998	0.992	3.83E-86	CD4-C2
RPS5	2.81E-88	0.39521309	0.993	0.980	4.67E-84	CD4-C2
FXYD5	4.56E-87	0.483295639	0.989	0.896	7.59E-83	CD4-C2
RPL4	3.73E-85	0.42617619	0.986	0.943	6.20E-81	CD4-C2
CCR7	4.34E-85	0.642308106	0.491	0.126	7.22E-81	CD4-C2
RACK1	4.70E-84	0.361314723	0.995	0.979	7.81E-80	CD4-C2
TCF7	4.23E-80	0.793523893	0.620	0.203	7.04E-76	CD4-C2
EEF1B2	9.60E-77	0.377459317	0.990	0.963	1.60E-72	CD4-C2
RPS18	5.64E-73	0.352940458	0.997	0.995	9.38E-69	CD4-C2
TMEM123	1.18E-72	0.565642937	0.810	0.430	1.97E-68	CD4-C2
C1orf162	4.71E-65	0.554493085	0.421	0.112	7.83E-61	CD4-C2
NOSIP	4.64E-63	0.73079251	0.679	0.336	7.72E-59	CD4-C2
TXNIP	4.16E-62	0.51888554	0.949	0.743	6.92E-58	CD4-C2
RPL7	2.55E-61	0.354940906	0.984	0.934	4.23E-57	CD4-C2
IL6R	6.50E-53	0.386974474	0.260	0.041	1.08E-48	CD4-C2
EIF3E	8.92E-52	0.44968333	0.855	0.599	1.48E-47	CD4-C2
RNASET2	1.93E-49	0.479235602	0.772	0.459	3.21E-45	CD4-C2
RCAN3	3.70E-47	0.390378749	0.544	0.240	6.16E-43	CD4-C2
TSHZ2	7.82E-44	0.371742777	0.219	0.031	1.30E-39	CD4-C2
SESN3	5.37E-43	0.464963429	0.298	0.073	8.93E-39	CD4-C2
INPP4B	7.40E-43	0.42643486	0.462	0.188	1.23E-38	CD4-C2
FLT3LG	1.89E-42	0.375612906	0.717	0.431	3.14E-38	CD4-C2
CRIP2	3.99E-41	0.428158024	0.274	0.059	6.63E-37	CD4-C2
MYC	5.01E-40	0.414879623	0.415	0.156	8.33E-36	CD4-C2
TESPA1	1.81E-39	0.378981903	0.371	0.124	3.02E-35	CD4-C2
TRAT1	1.83E-39	0.362754074	0.407	0.164	3.04E-35	CD4-C2
CAMK4	5.09E-39	0.350136704	0.645	0.359	8.47E-35	CD4-C2
DGKA	6.19E-36	0.406764785	0.464	0.190	1.03E-31	CD4-C2
ADD3	3.91E-35	0.409268554	0.644	0.345	6.50E-31	CD4-C2
ARHGAP15	4.55E-34	0.356437539	0.645	0.385	7.57E-30	CD4-C2
GIMAP7	5.78E-30	0.421296137	0.709	0.446	9.62E-26	CD4-C2
TRADD	4.86E-28	0.364548513	0.437	0.208	8.08E-24	CD4-C2
AES	1.46E-25	0.351967656	0.834	0.627	2.44E-21	CD4-C2
RIPOR2	2.69E-25	0.364151228	0.583	0.346	4.47E-21	CD4-C2
STAT1	3.25E-25	0.566752483	0.417	0.223	5.40E-21	CD4-C2
PASK	1.46E-23	0.347619031	0.222	0.057	2.42E-19	CD4-C2
FYB1	6.72E-23	0.364586911	0.792	0.630	1.12E-18	CD4-C2
IL7R	4.89E-112	1.09428028	0.878	0.474	8.14E-108	CD4-C3
RPLP0	6.59E-106	0.464746698	0.999	0.989	1.10E-101	CD4-C3
CCR6	4.56E-103	0.771549096	0.486	0.073	7.58E-99	CD4-C3
LTB	2.05E-95	0.849266647	0.860	0.479	3.41E-91	CD4-C3
TNFRSF25	1.45E-85	0.633252474	0.609	0.207	2.41E-81	CD4-C3
AQP3	1.89E-78	0.650551416	0.733	0.318	3.15E-74	CD4-C3
FLT3LG	1.46E-62	0.442727483	0.753	0.426	2.43E-58	CD4-C3

EEF1G	1.98E-56	0.35098109	0.978	0.888	3.29E-52	CD4-C3
KDSR	1.26E-52	0.432735544	0.433	0.154	2.09E-48	CD4-C3
CD4	6.78E-51	0.37118592	0.472	0.174	1.13E-46	CD4-C3
CTSH	3.72E-47	0.39515272	0.303	0.064	6.18E-43	CD4-C3
PDE4D	2.46E-41	0.485870119	0.614	0.367	4.10E-37	CD4-C3
LDHB	4.21E-41	0.379079258	0.864	0.662	7.01E-37	CD4-C3
ERN1	6.61E-40	0.388915358	0.471	0.201	1.10E-35	CD4-C3
LGALS3	7.23E-39	0.458036604	0.508	0.227	1.20E-34	CD4-C3
KLRB1	1.19E-37	0.387872884	0.600	0.336	1.98E-33	CD4-C3
DPP4	1.77E-37	0.363331057	0.326	0.093	2.95E-33	CD4-C3
GNA15	1.47E-36	0.388223995	0.262	0.073	2.44E-32	CD4-C3
TNFAIP8	1.49E-32	0.371183878	0.567	0.313	2.47E-28	CD4-C3
FURIN	3.11E-25	0.449806479	0.254	0.060	5.17E-21	CD4-C3
LMNA	3.38E-170	1.21787792	0.919	0.440	5.62E-166	CD4-C4
RGCC	3.82E-145	1.019199639	0.907	0.492	6.36E-141	CD4-C4
VIM	4.06E-145	0.861977212	0.998	0.975	6.75E-141	CD4-C4
PPP1R15A	7.30E-123	0.742058246	0.966	0.695	1.21E-118	CD4-C4
ANXA1	1.42E-114	1.048478005	0.933	0.732	2.36E-110	CD4-C4
TNFAIP3	1.08E-111	0.676372126	0.985	0.788	1.80E-107	CD4-C4
KLF6	3.25E-110	0.735286634	0.988	0.883	5.40E-106	CD4-C4
CD44	2.23E-99	0.681992814	0.957	0.798	3.72E-95	CD4-C4
CSRNP1	8.56E-93	0.578183877	0.906	0.583	1.42E-88	CD4-C4
FOS	1.11E-85	0.664249696	0.923	0.612	1.84E-81	CD4-C4
MYADM	3.35E-85	0.901734164	0.757	0.355	5.57E-81	CD4-C4
TSC22D3	6.26E-85	0.505601525	0.988	0.935	1.04E-80	CD4-C4
CDKN1A	6.31E-81	0.937745619	0.619	0.217	1.05E-76	CD4-C4
FOSB	1.25E-76	0.595780347	0.763	0.352	2.08E-72	CD4-C4
IDS	1.10E-67	0.683654296	0.878	0.666	1.83E-63	CD4-C4
ZFP36	2.30E-62	0.498830837	0.974	0.887	3.82E-58	CD4-C4
MCL1	3.19E-62	0.548858179	0.850	0.650	5.30E-58	CD4-C4
PER1	9.13E-61	0.668548145	0.691	0.364	1.52E-56	CD4-C4
PTGER4	3.61E-58	0.661713913	0.759	0.464	6.00E-54	CD4-C4
NFKBIA	1.09E-57	0.455398886	0.955	0.797	1.81E-53	CD4-C4
RPS16	4.91E-55	0.347913464	0.998	0.992	8.16E-51	CD4-C4
EZR	1.18E-53	0.683736286	0.831	0.678	1.96E-49	CD4-C4
FTH1	2.84E-53	0.42057128	0.998	0.989	4.72E-49	CD4-C4
S100A10	5.13E-53	0.501775551	0.939	0.864	8.53E-49	CD4-C4
GPR183	3.14E-47	0.57811055	0.573	0.306	5.23E-43	CD4-C4
TAGLN2	1.63E-45	0.466448956	0.906	0.810	2.72E-41	CD4-C4
ARL4A	5.05E-43	0.587748333	0.578	0.272	8.40E-39	CD4-C4
YPEL5	9.90E-43	0.450128067	0.855	0.622	1.65E-38	CD4-C4
ATP2B1	3.83E-42	0.630614867	0.509	0.249	6.36E-38	CD4-C4
AHR	6.84E-42	0.598183262	0.509	0.214	1.14E-37	CD4-C4
PNP	7.43E-42	0.595989637	0.497	0.271	1.23E-37	CD4-C4
GATA3	3.90E-41	0.631941018	0.708	0.455	6.48E-37	CD4-C4
EMP1	1.01E-40	0.412156793	0.180	0.019	1.67E-36	CD4-C4
TUBB4B	1.98E-40	0.46142787	0.754	0.524	3.29E-36	CD4-C4
LRRFIP1	5.82E-40	0.454515713	0.776	0.658	9.68E-36	CD4-C4
KDM6B	2.45E-39	0.483121193	0.526	0.283	4.08E-35	CD4-C4
AHNAK	3.80E-39	0.506691319	0.855	0.703	6.32E-35	CD4-C4
PBX4	3.43E-37	0.562621126	0.597	0.322	5.70E-33	CD4-C4
PLK3	4.06E-36	0.531691913	0.538	0.280	6.76E-32	CD4-C4

MBNL1	4.32E-36	0.407804811	0.876	0.791	7.19E-32	CD4-C4
ADGRE5	3.03E-35	0.532390545	0.823	0.646	5.04E-31	CD4-C4
ANKRD28	4.71E-35	0.54153712	0.321	0.099	7.83E-31	CD4-C4
S100A11	9.93E-33	0.560236085	0.670	0.500	1.65E-28	CD4-C4
CRIP1	1.50E-31	0.382354483	0.931	0.854	2.49E-27	CD4-C4
CXCR3	1.10E-30	0.462572359	0.658	0.437	1.83E-26	CD4-C4
RFX2	1.27E-29	0.483841375	0.229	0.044	2.12E-25	CD4-C4
TUBA1A	1.22E-28	0.527639278	0.750	0.595	2.03E-24	CD4-C4
NINJ1	6.48E-28	0.476653932	0.544	0.332	1.08E-23	CD4-C4
TIPARP	9.47E-28	0.472293188	0.504	0.276	1.57E-23	CD4-C4
IRF2BP2	1.39E-27	0.40159476	0.643	0.443	2.31E-23	CD4-C4
PIM1	1.89E-27	0.440421442	0.613	0.478	3.14E-23	CD4-C4
CFAP20	2.91E-26	0.434131714	0.361	0.167	4.84E-22	CD4-C4
CDK17	5.14E-26	0.404279795	0.547	0.357	8.54E-22	CD4-C4
SLC2A3	5.14E-26	0.393663939	0.761	0.608	8.55E-22	CD4-C4
IVNS1ABP	1.16E-25	0.511900984	0.508	0.290	1.93E-21	CD4-C4
JUND	2.73E-25	0.496525873	0.612	0.367	4.55E-21	CD4-C4
AKAP13	4.62E-25	0.380383134	0.698	0.579	7.68E-21	CD4-C4
SNX9	5.54E-25	0.46252662	0.477	0.225	9.21E-21	CD4-C4
KMT5C	1.31E-24	0.393535578	0.287	0.110	2.18E-20	CD4-C4
ATF3	5.75E-23	0.361936539	0.351	0.160	9.55E-19	CD4-C4
TPPP	7.48E-23	0.375756309	0.181	0.040	1.24E-18	CD4-C4
FAM107B	1.08E-22	0.471297467	0.535	0.388	1.79E-18	CD4-C4
PERP	4.46E-22	0.419673028	0.381	0.196	7.41E-18	CD4-C4
SRGN	5.50E-22	0.349557096	0.948	0.895	9.14E-18	CD4-C4
TXN	1.41E-21	0.42575217	0.611	0.524	2.35E-17	CD4-C4
ANXA2	1.64E-21	0.389510142	0.604	0.464	2.73E-17	CD4-C4
ARL4C	2.61E-21	0.361433008	0.764	0.649	4.34E-17	CD4-C4
AC016831.7	3.22E-21	0.351044186	0.666	0.490	5.36E-17	CD4-C4
AAED1	3.29E-20	0.398268094	0.324	0.154	5.47E-16	CD4-C4
TOB1	5.04E-20	0.453306605	0.630	0.446	8.38E-16	CD4-C4
IRS2	5.55E-20	0.379697252	0.234	0.077	9.23E-16	CD4-C4
TUBA1C	1.27E-19	0.373945511	0.463	0.319	2.12E-15	CD4-C4
JMJD1C	2.74E-19	0.44849265	0.620	0.431	4.56E-15	CD4-C4
ZC3H12A	4.36E-19	0.363860488	0.512	0.331	7.26E-15	CD4-C4
RELB	1.60E-18	0.422893059	0.574	0.355	2.66E-14	CD4-C4
ANKRD12	3.39E-18	0.388124823	0.728	0.635	5.64E-14	CD4-C4
ODC1	8.51E-18	0.382560326	0.621	0.418	1.42E-13	CD4-C4
MAP3K2	5.28E-17	0.37677232	0.380	0.207	8.78E-13	CD4-C4
RARA	9.57E-17	0.367286428	0.388	0.227	1.59E-12	CD4-C4
NFKB1	2.15E-16	0.347947396	0.434	0.268	3.57E-12	CD4-C4
TRAF4	3.20E-15	0.418268544	0.395	0.209	5.32E-11	CD4-C4
RORA	3.00E-11	0.34764304	0.558	0.426	4.99E-07	CD4-C4
FTH1	5.85E-116	0.850093211	1.000	0.989	9.73E-112	CD4-C5
DDIT4	1.33E-94	0.889377006	0.922	0.650	2.21E-90	CD4-C5
ZFP36L2	5.86E-88	0.686586419	0.996	0.968	9.75E-84	CD4-C5
DUSP4	8.45E-86	0.736293243	0.745	0.332	1.41E-81	CD4-C5
BTG1	1.09E-80	0.534432901	0.998	0.983	1.81E-76	CD4-C5
SPOCK2	2.78E-72	0.570500013	0.926	0.719	4.63E-68	CD4-C5
PDE4B	1.67E-71	0.659182928	0.724	0.370	2.77E-67	CD4-C5
CDKN1A	1.50E-69	0.762534697	0.626	0.241	2.49E-65	CD4-C5
CSRNP1	1.24E-68	0.388899005	0.915	0.603	2.06E-64	CD4-C5

SRGN	7.67E-68	0.547361133	0.988	0.896	1.28E-63	CD4-C5
LAPTM5	7.96E-63	0.391213062	0.989	0.929	1.32E-58	CD4-C5
SLC2A3	1.33E-62	0.773706451	0.893	0.611	2.21E-58	CD4-C5
ZFP36	2.92E-61	0.527782834	0.981	0.892	4.86E-57	CD4-C5
PBX4	3.05E-60	0.559302284	0.647	0.336	5.08E-56	CD4-C5
SAMSN1	1.38E-59	0.636314616	0.678	0.347	2.30E-55	CD4-C5
ZNF331	1.80E-58	0.667515193	0.655	0.335	2.99E-54	CD4-C5
SLC7A5	2.96E-56	0.511511609	0.747	0.438	4.92E-52	CD4-C5
SNX9	6.33E-55	0.635253291	0.540	0.237	1.05E-50	CD4-C5
NR4A2	6.76E-54	0.524348884	0.794	0.481	1.12E-49	CD4-C5
FXYD5	1.34E-53	0.407722463	0.980	0.898	2.23E-49	CD4-C5
LEPROTL1	8.16E-53	0.614432969	0.813	0.586	1.36E-48	CD4-C5
TENT5C	3.90E-52	0.50171348	0.742	0.407	6.49E-48	CD4-C5
FAM177A1	6.11E-51	0.488303481	0.732	0.460	1.02E-46	CD4-C5
CXCR3	9.28E-51	0.499511423	0.766	0.445	1.54E-46	CD4-C5
CEMIP2	1.24E-50	0.513123268	0.722	0.407	2.06E-46	CD4-C5
ICOS	1.86E-50	0.59778531	0.586	0.264	3.10E-46	CD4-C5
SLBP	2.54E-50	0.751482783	0.604	0.357	4.22E-46	CD4-C5
CXCR4	3.29E-50	0.431469895	0.925	0.710	5.47E-46	CD4-C5
RGS1	4.16E-45	0.478220766	0.583	0.296	6.91E-41	CD4-C5
CD4	1.98E-44	0.50031546	0.491	0.181	3.30E-40	CD4-C5
GNA15	1.43E-43	0.549968254	0.309	0.076	2.39E-39	CD4-C5
PDCL3	1.53E-43	0.551947608	0.558	0.291	2.55E-39	CD4-C5
TRAF4	2.41E-42	0.482212296	0.469	0.217	4.00E-38	CD4-C5
EML4	8.75E-42	0.495561321	0.809	0.563	1.46E-37	CD4-C5
ELL2	8.81E-42	0.52269475	0.522	0.230	1.46E-37	CD4-C5
ABCG1	1.24E-41	0.50964595	0.482	0.212	2.07E-37	CD4-C5
GRAMD1B	1.71E-41	0.458446135	0.352	0.119	2.84E-37	CD4-C5
DUSP2	3.01E-41	0.4132555	0.907	0.674	5.01E-37	CD4-C5
TIPARP	4.13E-41	0.468212634	0.569	0.287	6.86E-37	CD4-C5
TMEM173	1.12E-40	0.564322127	0.590	0.306	1.87E-36	CD4-C5
UBE2D3	3.50E-40	0.383682288	0.921	0.805	5.82E-36	CD4-C5
DDX24	2.05E-39	0.362448698	0.880	0.722	3.40E-35	CD4-C5
BHLHE40	8.91E-38	0.441453747	0.754	0.489	1.48E-33	CD4-C5
CACYBP	1.02E-36	0.455149896	0.709	0.474	1.70E-32	CD4-C5
ATP1B1	3.66E-35	0.484077967	0.308	0.089	6.08E-31	CD4-C5
PFKFB3	1.07E-34	0.476087386	0.396	0.150	1.78E-30	CD4-C5
FAM102A	6.62E-34	0.413325735	0.573	0.325	1.10E-29	CD4-C5
COTL1	3.39E-33	0.414639038	0.879	0.640	5.65E-29	CD4-C5
CREM	4.67E-33	0.482308752	0.537	0.288	7.77E-29	CD4-C5
IDI1	1.81E-32	0.403561376	0.630	0.392	3.00E-28	CD4-C5
RGS16	2.20E-32	0.584368207	0.299	0.092	3.66E-28	CD4-C5
SMIM3	9.28E-32	0.473410669	0.316	0.104	1.54E-27	CD4-C5
PRDM1	2.57E-31	0.426639735	0.596	0.363	4.27E-27	CD4-C5
PIK3R1	5.64E-31	0.44190181	0.658	0.448	9.37E-27	CD4-C5
RNF125	3.51E-30	0.445945912	0.700	0.459	5.84E-26	CD4-C5
ATP1B3	4.88E-30	0.490843407	0.429	0.228	8.11E-26	CD4-C5
CTSL	9.10E-30	0.376966728	0.163	0.023	1.51E-25	CD4-C5
STK17A	8.24E-29	0.386509179	0.810	0.628	1.37E-24	CD4-C5
ARHGEF7	8.32E-29	0.456518471	0.457	0.239	1.38E-24	CD4-C5
DNAJB6	4.73E-28	0.362892208	0.803	0.599	7.86E-24	CD4-C5
FOXP1	8.66E-28	0.462509234	0.675	0.436	1.44E-23	CD4-C5

DUSP5	1.54E-27	0.348046994	0.292	0.100	2.56E-23	CD4-C5
CD6	3.03E-27	0.37719064	0.832	0.669	5.04E-23	CD4-C5
ARID5A	4.50E-27	0.418566749	0.696	0.477	7.48E-23	CD4-C5
JUNB	3.26E-26	0.504152382	0.954	0.877	5.41E-22	CD4-C5
PELI1	8.90E-26	0.427447985	0.435	0.207	1.48E-21	CD4-C5
SYNJ2	9.12E-26	0.378153887	0.326	0.130	1.52E-21	CD4-C5
TGFB1	1.06E-25	0.358688569	0.796	0.598	1.77E-21	CD4-C5
NINJ1	1.47E-25	0.466659074	0.563	0.344	2.45E-21	CD4-C5
PLIN2	3.16E-24	0.449300557	0.461	0.242	5.25E-20	CD4-C5
PTP4A1	1.14E-23	0.398993896	0.642	0.422	1.90E-19	CD4-C5
ARID5B	1.14E-23	0.390317878	0.547	0.324	1.90E-19	CD4-C5
KDM6B	1.52E-23	0.361930762	0.513	0.298	2.53E-19	CD4-C5
RASSF5	5.41E-23	0.35780976	0.644	0.460	9.00E-19	CD4-C5
YIPF5	1.92E-22	0.406108082	0.515	0.327	3.19E-18	CD4-C5
ZFP36L1	3.52E-22	0.398234557	0.773	0.640	5.86E-18	CD4-C5
ID2	4.58E-22	0.375587082	0.733	0.591	7.62E-18	CD4-C5
MAFF	2.88E-21	0.370081914	0.338	0.167	4.79E-17	CD4-C5
PDCD1	3.20E-21	0.411842299	0.278	0.088	5.32E-17	CD4-C5
LAPTM4A	3.46E-21	0.385056582	0.626	0.434	5.75E-17	CD4-C5
CD5	3.79E-21	0.439376841	0.672	0.471	6.30E-17	CD4-C5
NCF1	3.86E-21	0.356713768	0.470	0.255	6.42E-17	CD4-C5
P2RY8	6.88E-20	0.363019892	0.631	0.464	1.14E-15	CD4-C5
HAUS3	3.03E-17	0.35378937	0.502	0.288	5.04E-13	CD4-C5
GPR183	9.70E-15	0.366941443	0.530	0.324	1.61E-10	CD4-C5
CYTOR	6.94E-11	0.350019612	0.596	0.445	1.15E-06	CD4-C5
MT2A	1.98E-08	0.402806424	0.570	0.482	0.000328945	CD4-C5
GZMH	4.05E-163	1.025777449	0.941	0.434	6.74E-159	CD4-C6
B2M	8.77E-129	0.390008023	1.000	0.999	1.46E-124	CD4-C6
FGFBP2	4.11E-125	0.90057646	0.745	0.213	6.83E-121	CD4-C6
S100A4	7.59E-112	0.580120453	0.998	0.885	1.26E-107	CD4-C6
GZMA	1.50E-95	0.532766305	0.964	0.655	2.50E-91	CD4-C6
SH3BGRL3	3.67E-90	0.461981633	1.000	0.979	6.11E-86	CD4-C6
ITGB2	3.09E-88	0.574395682	0.954	0.748	5.14E-84	CD4-C6
HLA-C	2.26E-87	0.392158956	1.000	0.996	3.75E-83	CD4-C6
NKG7	7.41E-87	0.694603399	0.912	0.614	1.23E-82	CD4-C6
HOPX	3.35E-83	0.735890782	0.750	0.325	5.57E-79	CD4-C6
CD52	2.09E-82	0.483917155	0.994	0.934	3.47E-78	CD4-C6
CCL5	4.67E-77	0.391519314	0.990	0.783	7.76E-73	CD4-C6
PFN1	5.63E-76	0.495520228	0.997	0.963	9.36E-72	CD4-C6
LAIR2	2.53E-70	0.740826206	0.482	0.126	4.21E-66	CD4-C6
ITGB1	3.73E-69	0.677159181	0.748	0.388	6.20E-65	CD4-C6
MYL12A	8.07E-66	0.533146931	0.962	0.782	1.34E-61	CD4-C6
GZMB	1.00E-65	0.526471492	0.635	0.262	1.67E-61	CD4-C6
CD99	1.56E-65	0.371916539	0.990	0.919	2.59E-61	CD4-C6
PRF1	2.27E-62	0.668055063	0.685	0.288	3.77E-58	CD4-C6
CD4	2.20E-61	0.558188034	0.510	0.186	3.66E-57	CD4-C6
CX3CR1	2.45E-61	0.625654999	0.430	0.114	4.08E-57	CD4-C6
C12orf75	1.01E-59	0.497301962	0.782	0.459	1.67E-55	CD4-C6
IL32	1.21E-59	0.423833728	0.985	0.909	2.02E-55	CD4-C6
ADGRG1	8.01E-59	0.576417353	0.513	0.161	1.33E-54	CD4-C6
PLEK	2.42E-53	0.525672835	0.570	0.229	4.02E-49	CD4-C6
MT-CO1	3.00E-48	0.352491525	0.995	0.991	4.99E-44	CD4-C6

GIMAP7	7.77E-48	0.532931732	0.749	0.454	1.29E-43	CD4-C6
CTSC	2.59E-47	0.470141485	0.738	0.451	4.31E-43	CD4-C6
SLC9A3R1	5.33E-45	0.488502425	0.819	0.597	8.86E-41	CD4-C6
IFITM2	8.32E-45	0.401119088	0.969	0.835	1.38E-40	CD4-C6
ACTB	1.38E-44	0.42185111	1.000	0.997	2.30E-40	CD4-C6
ARPC1B	1.89E-43	0.408903076	0.838	0.598	3.14E-39	CD4-C6
GNLY	8.48E-43	0.733559423	0.549	0.237	1.41E-38	CD4-C6
TRAV6	8.92E-43	0.760267065	0.199	0.018	1.48E-38	CD4-C6
ANXA6	1.19E-42	0.47149091	0.720	0.470	1.97E-38	CD4-C6
MYOM2	1.02E-41	0.556515148	0.276	0.051	1.70E-37	CD4-C6
S1PR5	1.34E-41	0.431892207	0.432	0.161	2.22E-37	CD4-C6
KLRG1	5.11E-41	0.48393655	0.527	0.243	8.49E-37	CD4-C6
CD40LG	3.18E-39	0.437893682	0.333	0.095	5.30E-35	CD4-C6
FCRL6	3.49E-39	0.43250889	0.458	0.171	5.80E-35	CD4-C6
RAP1B	1.32E-38	0.412485209	0.838	0.651	2.20E-34	CD4-C6
OAZ1	4.87E-38	0.354778232	0.933	0.771	8.10E-34	CD4-C6
SPON2	5.97E-38	0.559867737	0.414	0.168	9.92E-34	CD4-C6
TSPO	7.87E-37	0.374476315	0.699	0.487	1.31E-32	CD4-C6
SUN2	1.00E-36	0.47565667	0.728	0.476	1.67E-32	CD4-C6
IL10RA	2.38E-36	0.420143179	0.733	0.517	3.95E-32	CD4-C6
RARRES3	4.07E-36	0.354156647	0.837	0.631	6.76E-32	CD4-C6
CORO1A	4.74E-34	0.364118317	0.942	0.829	7.87E-30	CD4-C6
PPP1CA	2.30E-33	0.39527104	0.805	0.568	3.83E-29	CD4-C6
PSMB9	5.73E-33	0.376375199	0.755	0.520	9.54E-29	CD4-C6
PRSS23	5.74E-33	0.425999747	0.350	0.112	9.55E-29	CD4-C6
CAP1	3.40E-32	0.428019947	0.755	0.536	5.65E-28	CD4-C6
CLEC2D	4.34E-32	0.432586383	0.592	0.334	7.22E-28	CD4-C6
CD320	5.98E-32	0.517622236	0.436	0.186	9.95E-28	CD4-C6
SAMD3	6.44E-32	0.413954832	0.539	0.272	1.07E-27	CD4-C6
EMP3	4.54E-31	0.358932476	0.918	0.778	7.55E-27	CD4-C6
SELPLG	5.29E-31	0.444492333	0.636	0.384	8.80E-27	CD4-C6
LYAR	3.81E-30	0.391303428	0.600	0.363	6.33E-26	CD4-C6
LGALS1	5.01E-30	0.352811066	0.734	0.497	8.33E-26	CD4-C6
PPP1R18	1.12E-29	0.399540168	0.680	0.416	1.87E-25	CD4-C6
FLNA	6.51E-29	0.373339028	0.787	0.578	1.08E-24	CD4-C6
CD300A	1.44E-28	0.414943804	0.316	0.103	2.40E-24	CD4-C6
ITGAM	4.12E-28	0.363562447	0.229	0.061	6.85E-24	CD4-C6
AES	1.63E-27	0.365837289	0.816	0.635	2.72E-23	CD4-C6
ARPC5	7.36E-27	0.413521978	0.635	0.379	1.22E-22	CD4-C6
TRBV7-3	3.13E-26	1.244142796	0.110	0.035	5.21E-22	CD4-C6
CD3G	2.10E-25	0.350468102	0.847	0.661	3.48E-21	CD4-C6
MYO1F	2.46E-25	0.391760903	0.548	0.303	4.09E-21	CD4-C6
ADAM8	2.05E-23	0.354282491	0.512	0.282	3.42E-19	CD4-C6
BIN2	2.25E-23	0.398365519	0.690	0.468	3.74E-19	CD4-C6
CTSB	1.16E-22	0.350318831	0.391	0.186	1.94E-18	CD4-C6
BIN1	1.50E-22	0.35093752	0.590	0.384	2.49E-18	CD4-C6
CD5	3.33E-22	0.386345132	0.688	0.474	5.54E-18	CD4-C6
CDK2AP2	1.62E-21	0.362527133	0.440	0.242	2.70E-17	CD4-C6
TRGV10	6.61E-21	0.574008241	0.232	0.094	1.10E-16	CD4-C6
MYO1G	1.02E-20	0.354933396	0.616	0.400	1.70E-16	CD4-C6
TRGV8	1.04E-18	0.4554404	0.173	0.042	1.73E-14	CD4-C6
TRBV5-1	3.84E-18	1.023231578	0.149	0.045	6.39E-14	CD4-C6

TRAV12-1	1.36E-12	0.465081292	0.130	0.043	2.26E-08	CD4-C6
TRBV28	1.54E-09	0.411051817	0.147	0.069	2.57E-05	CD4-C6
FOXP3	3.33E-258	1.534972422	0.645	0.008	5.54E-254	CD4-C7
CTLA4	1.22E-191	1.444539638	0.630	0.057	2.03E-187	CD4-C7
BATF	1.62E-165	1.653794931	0.765	0.280	2.70E-161	CD4-C7
STAM	1.70E-144	1.156588131	0.637	0.124	2.83E-140	CD4-C7
TNFRSF18	3.58E-136	1.525201775	0.545	0.056	5.96E-132	CD4-C7
IL2RA	2.01E-120	0.940676059	0.389	0.018	3.35E-116	CD4-C7
TNFRSF4	8.52E-116	1.637582275	0.490	0.063	1.42E-111	CD4-C7
PELI1	1.30E-109	1.151343094	0.659	0.194	2.16E-105	CD4-C7
LAYN	6.88E-99	0.7461915	0.321	0.003	1.14E-94	CD4-C7
TBC1D4	3.18E-96	0.735199725	0.369	0.032	5.30E-92	CD4-C7
SAT1	2.78E-94	1.032823217	0.848	0.594	4.62E-90	CD4-C7
LINC01943	9.07E-94	0.93435366	0.425	0.076	1.51E-89	CD4-C7
RHBDD2	7.83E-91	0.99220801	0.718	0.397	1.30E-86	CD4-C7
CCR8	5.08E-89	0.73238897	0.282	0.002	8.44E-85	CD4-C7
IL32	3.74E-81	0.8890818	0.976	0.907	6.21E-77	CD4-C7
FAS	1.94E-80	0.921977192	0.632	0.248	3.23E-76	CD4-C7
LTB	6.37E-77	0.825977829	0.845	0.488	1.06E-72	CD4-C7
ARID5B	1.47E-75	0.831982382	0.698	0.314	2.44E-71	CD4-C7
TIGIT	1.46E-74	0.830483018	0.478	0.117	2.42E-70	CD4-C7
ICOS	3.01E-72	0.904251793	0.637	0.260	5.00E-68	CD4-C7
PBXIP1	2.52E-71	0.773792176	0.789	0.464	4.19E-67	CD4-C7
PMAIP1	1.06E-67	0.855738715	0.658	0.321	1.76E-63	CD4-C7
TNFRSF9	1.17E-65	0.618281413	0.267	0.017	1.95E-61	CD4-C7
IKZF2	4.96E-60	0.518692789	0.265	0.022	8.25E-56	CD4-C7
SNX9	1.75E-59	0.771680998	0.561	0.234	2.91E-55	CD4-C7
GLRX	6.03E-59	0.810841106	0.518	0.217	1.00E-54	CD4-C7
CD177	2.79E-57	0.854249418	0.170	0.000	4.64E-53	CD4-C7
ID3	5.65E-56	0.9896957	0.322	0.056	9.40E-52	CD4-C7
SETD7	1.01E-54	0.530059461	0.277	0.051	1.68E-50	CD4-C7
AC017002.3	9.09E-54	0.46939959	0.205	0.015	1.51E-49	CD4-C7
TTN	1.01E-53	0.516119861	0.270	0.045	1.68E-49	CD4-C7
DNPH1	3.37E-52	0.607663138	0.421	0.136	5.61E-48	CD4-C7
LINC02195	8.47E-51	0.42647318	0.181	0.006	1.41E-46	CD4-C7
SQSTM1	1.99E-50	0.586040136	0.746	0.545	3.30E-46	CD4-C7
PRDM1	2.29E-50	0.572075956	0.638	0.359	3.81E-46	CD4-C7
MIR4435-2HG	7.72E-50	0.74055071	0.499	0.195	1.28E-45	CD4-C7
CD27	3.63E-49	0.634661061	0.586	0.276	6.03E-45	CD4-C7
MAGEH1	8.56E-49	0.838394901	0.297	0.058	1.42E-44	CD4-C7
NCF4	9.76E-49	0.479505877	0.327	0.078	1.62E-44	CD4-C7
RHOG	2.27E-48	0.537923856	0.683	0.427	3.78E-44	CD4-C7
PVT1	3.08E-48	0.528374954	0.411	0.130	5.12E-44	CD4-C7
UBC	6.67E-48	0.489129857	0.996	0.991	1.11E-43	CD4-C7
IL10RA	7.32E-48	0.539225708	0.757	0.510	1.22E-43	CD4-C7
RTKN2	8.91E-48	0.454727814	0.177	0.007	1.48E-43	CD4-C7
CORO1B	1.54E-47	0.760713264	0.584	0.307	2.57E-43	CD4-C7
SELL	5.33E-47	0.567241072	0.487	0.194	8.86E-43	CD4-C7
SGMS1	5.67E-47	0.561044587	0.371	0.098	9.43E-43	CD4-C7
PHTF2	7.60E-47	0.504749218	0.395	0.143	1.26E-42	CD4-C7
SRGN	1.36E-46	0.581131635	0.942	0.898	2.26E-42	CD4-C7
LAIR2	1.71E-46	0.595867781	0.405	0.121	2.84E-42	CD4-C7

NAMPT	5.24E-46	0.580680353	0.473	0.209	8.71E-42	CD4-C7
CD4	7.05E-46	0.519531072	0.478	0.180	1.17E-41	CD4-C7
ARHGDIB	1.09E-45	0.386073789	0.974	0.946	1.81E-41	CD4-C7
ACP5	3.33E-45	0.585626757	0.359	0.106	5.54E-41	CD4-C7
AKIRIN2	6.97E-45	0.544655639	0.570	0.350	1.16E-40	CD4-C7
NDUFV2	1.58E-44	0.614424453	0.661	0.399	2.63E-40	CD4-C7
BACH1	4.53E-44	0.570873626	0.358	0.096	7.54E-40	CD4-C7
CIRBP	1.27E-43	0.419166186	0.968	0.917	2.11E-39	CD4-C7
PHLDA1	2.39E-43	0.542996817	0.322	0.105	3.98E-39	CD4-C7
FXYD5	4.60E-43	0.36490573	0.966	0.898	7.65E-39	CD4-C7
S100A4	1.68E-42	0.503001079	0.974	0.883	2.79E-38	CD4-C7
LAPTM4A	4.34E-42	0.665356823	0.648	0.432	7.22E-38	CD4-C7
MAST4	6.40E-42	0.455462377	0.249	0.045	1.06E-37	CD4-C7
TRIB1	1.41E-40	0.366419357	0.185	0.019	2.34E-36	CD4-C7
IL2RB	5.79E-40	0.593864026	0.623	0.374	9.62E-36	CD4-C7
NOP58	6.54E-40	0.57201751	0.675	0.449	1.09E-35	CD4-C7
HPGD	1.40E-39	0.575470161	0.268	0.057	2.33E-35	CD4-C7
RGS1	3.47E-39	0.509764193	0.569	0.295	5.77E-35	CD4-C7
SAMSN1	7.41E-39	0.592555476	0.622	0.348	1.23E-34	CD4-C7
TNFRSF1B	8.21E-39	0.580831482	0.555	0.334	1.36E-34	CD4-C7
GADD45A	1.18E-38	0.729382227	0.420	0.166	1.96E-34	CD4-C7
CACYBP	3.39E-37	0.660816737	0.676	0.474	5.65E-33	CD4-C7
BIRC3	4.88E-37	0.529278248	0.359	0.138	8.12E-33	CD4-C7
LGALS3	6.06E-37	0.561299274	0.480	0.235	1.01E-32	CD4-C7
PIM2	1.14E-36	0.592516366	0.624	0.358	1.89E-32	CD4-C7
BCL2L11	1.17E-36	0.501216676	0.297	0.088	1.95E-32	CD4-C7
FOXP1	2.08E-36	0.579651073	0.633	0.437	3.46E-32	CD4-C7
ZC3H12D	2.39E-35	0.374888759	0.236	0.059	3.97E-31	CD4-C7
TENT5C	8.30E-35	0.502985366	0.651	0.410	1.38E-30	CD4-C7
HLA-DRB1	9.52E-35	0.546992999	0.723	0.508	1.58E-30	CD4-C7
SIRPG	4.14E-34	0.524123697	0.408	0.158	6.88E-30	CD4-C7
DUSP4	4.37E-34	0.570112096	0.619	0.336	7.27E-30	CD4-C7
SOX4	5.10E-34	0.524070341	0.134	0.007	8.49E-30	CD4-C7
SPOCK2	6.11E-34	0.40710516	0.887	0.720	1.02E-29	CD4-C7
FKBP1A	1.22E-33	0.435003112	0.588	0.413	2.03E-29	CD4-C7
CRADD	1.66E-33	0.407147413	0.193	0.038	2.77E-29	CD4-C7
ZEB1	3.65E-33	0.465093455	0.337	0.128	6.07E-29	CD4-C7
RAB33A	3.74E-33	0.36611248	0.211	0.056	6.21E-29	CD4-C7
GBP2	4.60E-33	0.466081551	0.435	0.222	7.65E-29	CD4-C7
CD247	6.47E-33	0.457437696	0.816	0.638	1.08E-28	CD4-C7
CREM	7.15E-33	0.561544785	0.516	0.288	1.19E-28	CD4-C7
PRNP	7.84E-33	0.442944343	0.580	0.372	1.30E-28	CD4-C7
SLAMF1	1.65E-32	0.484288874	0.321	0.114	2.74E-28	CD4-C7
RPS27L	4.43E-32	0.454373133	0.681	0.488	7.37E-28	CD4-C7
CNST	6.41E-32	0.456337207	0.465	0.225	1.07E-27	CD4-C7
SARAF	9.86E-32	0.401304559	0.975	0.953	1.64E-27	CD4-C7
BTG3	1.72E-31	0.439144749	0.459	0.231	2.87E-27	CD4-C7
DUSP16	2.55E-31	0.416693241	0.263	0.062	4.24E-27	CD4-C7
MAPK1IP1L	5.08E-31	0.421180511	0.693	0.498	8.45E-27	CD4-C7
ISG20	1.19E-30	0.456270867	0.625	0.470	1.98E-26	CD4-C7
PPP1R2	1.38E-30	0.469623967	0.754	0.570	2.30E-26	CD4-C7
CTSA	1.40E-30	0.418167114	0.474	0.274	2.34E-26	CD4-C7

NR3C1	1.50E-30	0.539941554	0.680	0.488	2.49E-26	CD4-C7
SEC14L1	1.96E-30	0.46904546	0.377	0.171	3.25E-26	CD4-C7
PTTG1	2.11E-30	0.358035437	0.282	0.097	3.51E-26	CD4-C7
ZNF292	5.60E-30	0.4402164	0.513	0.297	9.30E-26	CD4-C7
ICAM3	9.36E-30	0.347929898	0.788	0.649	1.56E-25	CD4-C7
GBP5	1.63E-29	0.435124391	0.530	0.330	2.71E-25	CD4-C7
SOD1	4.01E-29	0.405929646	0.845	0.707	6.66E-25	CD4-C7
PKM	5.21E-29	0.589658559	0.731	0.591	8.66E-25	CD4-C7
SAMHD1	7.04E-29	0.431433265	0.461	0.276	1.17E-24	CD4-C7
HAPLN3	1.16E-28	0.390914125	0.228	0.049	1.94E-24	CD4-C7
CD79B	1.30E-27	0.354929615	0.244	0.065	2.16E-23	CD4-C7
CARD16	2.49E-27	0.496577176	0.480	0.288	4.14E-23	CD4-C7
TAGLN2	1.91E-26	0.419094235	0.893	0.816	3.18E-22	CD4-C7
P2RY10	3.26E-26	0.366507385	0.307	0.122	5.42E-22	CD4-C7
PIM3	5.72E-26	0.468283375	0.521	0.317	9.51E-22	CD4-C7
RCAN3	6.41E-26	0.399772223	0.466	0.249	1.07E-21	CD4-C7
DUSP10	9.86E-26	0.363369143	0.383	0.198	1.64E-21	CD4-C7
PAIP2	1.63E-25	0.379665937	0.719	0.565	2.72E-21	CD4-C7
UBE2B	2.93E-25	0.395895226	0.688	0.508	4.88E-21	CD4-C7
CTSC	8.60E-25	0.610645908	0.596	0.452	1.43E-20	CD4-C7
RAP1A	9.87E-25	0.368645352	0.627	0.425	1.64E-20	CD4-C7
STK17B	1.76E-24	0.406916554	0.823	0.700	2.92E-20	CD4-C7
ELF1	1.83E-24	0.443204163	0.708	0.512	3.03E-20	CD4-C7
SLCO4A1	1.84E-24	0.36795781	0.176	0.036	3.06E-20	CD4-C7
PDE4A	2.20E-24	0.353845498	0.204	0.055	3.66E-20	CD4-C7
IGFLR1	3.36E-24	0.369498314	0.315	0.143	5.58E-20	CD4-C7
EZR	3.93E-24	0.414231878	0.844	0.686	6.54E-20	CD4-C7
ANKRD12	6.13E-24	0.419665373	0.789	0.636	1.02E-19	CD4-C7
STAT3	1.27E-23	0.413736663	0.641	0.455	2.11E-19	CD4-C7
INSIG1	2.23E-23	0.444523691	0.374	0.195	3.71E-19	CD4-C7
FAM129A	2.57E-23	0.409402756	0.425	0.210	4.28E-19	CD4-C7
FCMR	3.69E-23	0.440440038	0.449	0.288	6.13E-19	CD4-C7
PIK3IP1	1.71E-22	0.362034642	0.707	0.521	2.84E-18	CD4-C7
CD28	8.09E-22	0.373603545	0.301	0.117	1.35E-17	CD4-C7
TMEM173	9.50E-22	0.400012545	0.488	0.309	1.58E-17	CD4-C7
SNU13	1.16E-21	0.365314287	0.730	0.558	1.94E-17	CD4-C7
SURF4	1.86E-21	0.360709831	0.547	0.388	3.10E-17	CD4-C7
SDF4	2.54E-21	0.359537265	0.548	0.373	4.23E-17	CD4-C7
TSC22D3	6.00E-21	0.371650457	0.968	0.939	9.98E-17	CD4-C7
TLK1	1.19E-20	0.347361463	0.413	0.228	1.98E-16	CD4-C7
SLC3A2	1.44E-20	0.381985338	0.719	0.546	2.39E-16	CD4-C7
THADA	1.45E-20	0.378808187	0.185	0.052	2.41E-16	CD4-C7
IFI6	1.99E-20	0.490998441	0.418	0.244	3.30E-16	CD4-C7
USP15	2.03E-20	0.391741269	0.612	0.425	3.38E-16	CD4-C7
TYMP	3.52E-20	0.456214732	0.292	0.125	5.86E-16	CD4-C7
CXCR6	6.80E-20	0.379559883	0.203	0.066	1.13E-15	CD4-C7
IL6ST	9.92E-20	0.408649852	0.454	0.247	1.65E-15	CD4-C7
SYNGR2	1.35E-19	0.373364227	0.520	0.368	2.25E-15	CD4-C7
NFKBIZ	3.24E-19	0.449259537	0.482	0.292	5.40E-15	CD4-C7
ARPC1B	5.66E-19	0.407711066	0.699	0.600	9.40E-15	CD4-C7
CALM3	1.80E-17	0.350498195	0.640	0.499	3.00E-13	CD4-C7
CLK1	2.33E-17	0.380391321	0.676	0.501	3.87E-13	CD4-C7

GPR183	3.12E-17	0.45455417	0.509	0.324	5.18E-13	CD4-C7
LEPROTL1	5.21E-17	0.372761387	0.763	0.587	8.66E-13	CD4-C7
C4orf48	5.26E-17	0.356918332	0.453	0.279	8.75E-13	CD4-C7
ABI1	9.02E-17	0.353218603	0.547	0.361	1.50E-12	CD4-C7
RHOH	1.70E-16	0.350008887	0.624	0.453	2.82E-12	CD4-C7
ENO1	3.25E-16	0.350228267	0.632	0.566	5.40E-12	CD4-C7
PLIN2	3.85E-16	0.435050089	0.386	0.245	6.41E-12	CD4-C7
CAMK4	2.36E-15	0.348139191	0.544	0.368	3.92E-11	CD4-C7
CFAP20	5.56E-15	0.402284251	0.328	0.180	9.25E-11	CD4-C7
WDR74	6.44E-15	0.410448592	0.465	0.299	1.07E-10	CD4-C7
NUP58	2.07E-14	0.357644828	0.447	0.277	3.45E-10	CD4-C7
CD7	1.01E-13	0.390300902	0.714	0.624	1.68E-09	CD4-C7
GABARAPL1	1.31E-13	0.426204811	0.568	0.427	2.18E-09	CD4-C7
GATA3	3.11E-11	0.437793651	0.636	0.473	5.17E-07	CD4-C7
UCP2	5.51E-11	0.380222138	0.563	0.445	9.17E-07	CD4-C7
JUNB	5.55E-10	0.357225651	0.919	0.878	9.24E-06	CD4-C7
HLA-DQA1	2.19E-08	0.417027873	0.307	0.201	0.000364497	CD4-C7
TRBV20-1	3.81E-07	0.865173809	0.142	0.094	0.00634027	CD4-C7
LGALS1	0.000479771	0.352023283	0.544	0.502	1	CD4-C7
KLRB1	0	1.769429586	0.976	0.315	0	CD8-C10
TRAV1-2	2.22E-180	1.264425115	0.558	0.029	3.68E-176	CD8-C10
NCR3	2.67E-141	1.094048291	0.584	0.095	4.44E-137	CD8-C10
TMIGD2	4.64E-122	0.867578204	0.461	0.041	7.72E-118	CD8-C10
AQP3	9.65E-102	1.059078916	0.740	0.322	1.60E-97	CD8-C10
SLC4A10	1.62E-94	0.617079853	0.309	0.015	2.70E-90	CD8-C10
ZBTB16	2.23E-89	0.615682681	0.333	0.023	3.71E-85	CD8-C10
CEBPD	2.38E-76	0.722922795	0.377	0.048	3.96E-72	CD8-C10
LST1	6.39E-72	0.689417585	0.327	0.046	1.06E-67	CD8-C10
DUSP1	3.78E-64	0.690373072	0.952	0.794	6.29E-60	CD8-C10
CTSW	6.81E-64	0.436507731	0.867	0.561	1.13E-59	CD8-C10
SPOCK2	5.71E-61	0.55928812	0.911	0.716	9.49E-57	CD8-C10
GYG1	1.85E-58	0.601003968	0.615	0.297	3.07E-54	CD8-C10
RORC	1.53E-57	0.471434352	0.235	0.022	2.54E-53	CD8-C10
NFKBIA	2.51E-57	0.649304629	0.949	0.805	4.18E-53	CD8-C10
LTB	6.95E-56	0.691968666	0.798	0.487	1.16E-51	CD8-C10
TLE1	2.83E-55	0.527683778	0.263	0.020	4.70E-51	CD8-C10
CCR6	3.14E-54	0.59749169	0.368	0.086	5.22E-50	CD8-C10
ME1	3.33E-54	0.383558327	0.194	0.009	5.53E-50	CD8-C10
CA2	1.16E-53	0.490711494	0.207	0.011	1.93E-49	CD8-C10
ID2	1.47E-53	0.573325877	0.814	0.583	2.44E-49	CD8-C10
TRBV6-4	8.74E-53	0.78945432	0.179	0.008	1.45E-48	CD8-C10
SPRY1	1.93E-52	0.490363761	0.245	0.028	3.20E-48	CD8-C10
TNFAIP3	5.72E-49	0.435908996	0.960	0.799	9.51E-45	CD8-C10
JAML	8.47E-44	0.5151245	0.373	0.109	1.41E-39	CD8-C10
IL4I1	1.61E-41	0.408169643	0.198	0.025	2.68E-37	CD8-C10
CTSH	4.28E-40	0.373676205	0.269	0.069	7.12E-36	CD8-C10
BHLHE40	3.51E-36	0.513113872	0.748	0.485	5.84E-32	CD8-C10
DPP4	6.40E-36	0.364248887	0.290	0.098	1.06E-31	CD8-C10
HSPA5	1.06E-35	0.357395367	0.923	0.800	1.76E-31	CD8-C10
ZFP36L2	1.17E-35	0.364577054	0.989	0.968	1.95E-31	CD8-C10
ZFP36	1.49E-35	0.404775996	0.967	0.892	2.48E-31	CD8-C10
FKBP11	1.71E-35	0.402371235	0.564	0.325	2.85E-31	CD8-C10

TRBV6-1	1.29E-34	0.958676486	0.211	0.026	2.15E-30	CD8-C10
ERN1	3.17E-33	0.454533457	0.455	0.205	5.27E-29	CD8-C10
DDIT4	3.25E-32	0.470085538	0.827	0.651	5.40E-28	CD8-C10
ABCA1	2.44E-31	0.485212962	0.235	0.051	4.07E-27	CD8-C10
FOS	1.09E-29	0.455356377	0.836	0.632	1.81E-25	CD8-C10
LINC01871	4.39E-29	0.471809819	0.373	0.158	7.31E-25	CD8-C10
GTF3C1	7.29E-28	0.469176891	0.357	0.144	1.21E-23	CD8-C10
KLRG1	1.19E-26	0.410567428	0.487	0.236	1.98E-22	CD8-C10
Z93241.1	5.58E-25	0.427825086	0.270	0.099	9.28E-21	CD8-C10
TNFRSF25	1.79E-24	0.388897815	0.450	0.222	2.97E-20	CD8-C10
PERP	4.46E-22	0.394901247	0.414	0.203	7.42E-18	CD8-C10
MT2A	1.27E-20	0.738393121	0.618	0.477	2.12E-16	CD8-C10
PDE4D	9.80E-20	0.400309953	0.576	0.372	1.63E-15	CD8-C10
TRGV9	1.73E-17	0.50016456	0.158	0.046	2.87E-13	CD8-C10
CITED2	6.55E-15	0.400778864	0.642	0.509	1.09E-10	CD8-C10
PER1	3.06E-14	0.363213908	0.564	0.387	5.09E-10	CD8-C10
NINJ1	5.62E-14	0.479341548	0.515	0.343	9.35E-10	CD8-C10
SLC7A5	6.55E-14	0.425824782	0.608	0.442	1.09E-09	CD8-C10
TRBV20-1	9.27E-13	0.501432535	0.205	0.089	1.54E-08	CD8-C10
STMN1	5.02E-275	2.583139196	0.977	0.119	8.35E-271	CD8-C11
TYMS	1.28E-187	1.703623093	0.698	0.004	2.13E-183	CD8-C11
TUBA1B	1.45E-182	1.873378212	0.984	0.681	2.40E-178	CD8-C11
HMGN2	1.06E-175	1.364424277	0.987	0.758	1.77E-171	CD8-C11
TUBB	2.25E-151	1.774536865	0.940	0.462	3.75E-147	CD8-C11
PCLAF	8.79E-147	1.041554503	0.583	0.004	1.46E-142	CD8-C11
MCM7	2.28E-131	1.30396078	0.755	0.104	3.79E-127	CD8-C11
MCM5	2.22E-120	1.128278405	0.719	0.084	3.70E-116	CD8-C11
CFL1	1.92E-118	0.779208774	0.997	0.952	3.19E-114	CD8-C11
CDT1	3.85E-116	0.769909863	0.500	0.004	6.41E-112	CD8-C11
UHRF1	6.55E-115	0.832922173	0.456	0.001	1.09E-110	CD8-C11
DHFR	1.50E-114	0.896388975	0.531	0.012	2.49E-110	CD8-C11
DUT	2.68E-114	1.307410753	0.865	0.316	4.45E-110	CD8-C11
TK1	4.92E-112	0.998645604	0.490	0.005	8.18E-108	CD8-C11
CLSPN	2.40E-108	0.823970185	0.500	0.013	3.99E-104	CD8-C11
MCM2	4.44E-106	0.771776504	0.521	0.022	7.39E-102	CD8-C11
PPIA	6.70E-106	0.762283441	0.997	0.928	1.11E-101	CD8-C11
GAPDH	2.31E-104	0.759521681	1.000	0.982	3.83E-100	CD8-C11
GINS2	1.32E-102	0.718873944	0.464	0.007	2.19E-98	CD8-C11
DEK	2.30E-101	1.034416265	0.891	0.404	3.83E-97	CD8-C11
HMGB2	1.29E-100	1.303026676	0.893	0.390	2.15E-96	CD8-C11
ZWINT	1.45E-97	0.708408128	0.445	0.007	2.40E-93	CD8-C11
ACTB	5.38E-96	0.872191011	1.000	0.997	8.94E-92	CD8-C11
PFN1	5.52E-96	0.828270305	1.000	0.964	9.17E-92	CD8-C11
PKMYT1	6.69E-95	0.719453139	0.385	0.001	1.11E-90	CD8-C11
RRM2	1.56E-94	1.028284964	0.404	0.005	2.59E-90	CD8-C11
CENPM	2.88E-94	0.744088527	0.477	0.016	4.79E-90	CD8-C11
CENPU	7.79E-93	0.767650648	0.471	0.022	1.29E-88	CD8-C11
MKI67	7.53E-91	1.008008928	0.438	0.008	1.25E-86	CD8-C11
LIG1	1.07E-89	0.7251003	0.583	0.077	1.77E-85	CD8-C11
TPI1	2.51E-88	0.930441537	0.930	0.567	4.17E-84	CD8-C11
MCM3	1.71E-87	0.877852925	0.552	0.073	2.84E-83	CD8-C11
H2AFZ	2.47E-85	0.978591956	0.951	0.563	4.11E-81	CD8-C11

PCNA	3.87E-85	0.976969942	0.622	0.114	6.44E-81	CD8-C11
CDC45	2.55E-84	0.539553211	0.372	0.002	4.24E-80	CD8-C11
COX8A	4.39E-84	0.726522236	0.977	0.674	7.30E-80	CD8-C11
WDR34	4.11E-83	0.622188723	0.432	0.016	6.83E-79	CD8-C11
GGH	2.97E-82	0.554019443	0.406	0.013	4.94E-78	CD8-C11
PTMA	5.29E-81	0.543077973	1.000	0.996	8.80E-77	CD8-C11
IDH2	6.09E-81	0.876149806	0.862	0.336	1.01E-76	CD8-C11
HELLS	1.38E-80	0.709368356	0.469	0.030	2.30E-76	CD8-C11
TMEM106C	3.68E-80	0.871349744	0.654	0.111	6.12E-76	CD8-C11
SMC2	3.21E-79	0.591658601	0.448	0.033	5.34E-75	CD8-C11
CENPX	3.69E-79	0.752242211	0.742	0.211	6.14E-75	CD8-C11
MCM4	5.10E-78	0.624899062	0.492	0.038	8.49E-74	CD8-C11
H2AFY	1.20E-77	0.780809638	0.740	0.216	1.99E-73	CD8-C11
CRIP1	2.70E-77	0.764699355	0.995	0.861	4.48E-73	CD8-C11
GSTP1	2.78E-77	0.873002246	0.927	0.519	4.63E-73	CD8-C11
HNRNPA2B1	3.98E-76	0.746443106	0.979	0.791	6.61E-72	CD8-C11
NUSAP1	9.90E-75	0.735188848	0.378	0.014	1.65E-70	CD8-C11
TCF19	8.98E-74	0.70352891	0.464	0.027	1.49E-69	CD8-C11
DNMT1	1.55E-73	0.742675187	0.753	0.219	2.58E-69	CD8-C11
CKS1B	7.74E-73	0.557358897	0.406	0.031	1.29E-68	CD8-C11
CENPH	2.17E-72	0.542955923	0.458	0.041	3.62E-68	CD8-C11
GZMA	1.56E-70	0.780537927	0.971	0.661	2.59E-66	CD8-C11
FANCI	1.71E-69	0.493746044	0.362	0.014	2.85E-65	CD8-C11
NRM	2.48E-69	0.573306365	0.471	0.056	4.12E-65	CD8-C11
BIRC5	9.73E-69	0.653535152	0.310	0.000	1.62E-64	CD8-C11
RPA3	1.86E-68	0.612357363	0.641	0.171	3.09E-64	CD8-C11
NUDT1	3.94E-68	0.718325232	0.661	0.147	6.56E-64	CD8-C11
TIMELESS	5.28E-67	0.480796212	0.349	0.015	8.78E-63	CD8-C11
CLIC1	9.66E-67	0.64232996	0.974	0.726	1.61E-62	CD8-C11
ANP32B	1.90E-66	0.682695012	0.943	0.611	3.16E-62	CD8-C11
PPDPF	4.81E-66	0.627044124	0.979	0.857	7.99E-62	CD8-C11
PSME2	6.44E-66	0.740030345	0.932	0.573	1.07E-61	CD8-C11
DDX11	1.39E-65	0.455694641	0.349	0.023	2.31E-61	CD8-C11
CARHSP1	4.66E-65	0.70761232	0.706	0.188	7.75E-61	CD8-C11
ACOT7	4.98E-65	0.473669558	0.422	0.052	8.27E-61	CD8-C11
ENO1	5.98E-65	0.743920606	0.943	0.566	9.95E-61	CD8-C11
CKS2	6.32E-65	0.634413934	0.495	0.073	1.05E-60	CD8-C11
H2AFV	7.05E-65	0.743353888	0.857	0.443	1.17E-60	CD8-C11
HMGB1	1.81E-64	0.720251124	0.995	0.893	3.01E-60	CD8-C11
NUCKS1	3.75E-64	0.778143141	0.844	0.383	6.23E-60	CD8-C11
ASF1B	1.80E-63	0.559806883	0.341	0.011	2.99E-59	CD8-C11
MCM6	4.64E-63	0.670131584	0.552	0.084	7.72E-59	CD8-C11
SMC4	2.64E-61	0.729388766	0.586	0.119	4.40E-57	CD8-C11
MT2A	4.23E-61	0.852260513	0.914	0.482	7.03E-57	CD8-C11
RAD51AP1	7.62E-61	0.402541962	0.276	0.005	1.27E-56	CD8-C11
POLD1	2.26E-60	0.501384601	0.391	0.040	3.76E-56	CD8-C11
SNRPB	4.33E-60	0.560403819	0.938	0.610	7.20E-56	CD8-C11
BANF1	8.70E-60	0.613158724	0.732	0.265	1.45E-55	CD8-C11
RALY	1.18E-59	0.645326458	0.878	0.524	1.96E-55	CD8-C11
IFI27L1	8.20E-59	0.473831883	0.388	0.037	1.36E-54	CD8-C11
NUCB2	9.58E-59	0.688427045	0.701	0.235	1.59E-54	CD8-C11
FEN1	8.16E-58	0.591091318	0.406	0.049	1.36E-53	CD8-C11

RNASEH2A	3.09E-57	0.501766951	0.391	0.043	5.14E-53	CD8-C11
CHEK1	3.86E-57	0.436863366	0.331	0.018	6.42E-53	CD8-C11
PGAM1	5.64E-57	0.631305023	0.896	0.515	9.38E-53	CD8-C11
TPM3	9.97E-57	0.515213727	0.979	0.830	1.66E-52	CD8-C11
DTYMK	4.47E-56	0.51544536	0.461	0.071	7.44E-52	CD8-C11
MYL6	5.04E-56	0.487925063	1.000	0.929	8.38E-52	CD8-C11
CENPW	6.05E-56	0.438419194	0.326	0.015	1.01E-51	CD8-C11
DTL	1.11E-55	0.470833865	0.302	0.008	1.85E-51	CD8-C11
CDC6	1.20E-55	0.381227816	0.273	0.005	1.99E-51	CD8-C11
HNRNPA3	1.66E-55	0.621536621	0.956	0.629	2.76E-51	CD8-C11
UBE2C	5.64E-55	0.535907955	0.253	0.001	9.38E-51	CD8-C11
E2F1	1.01E-54	0.399667284	0.263	0.004	1.69E-50	CD8-C11
SAE1	2.86E-54	0.505301402	0.534	0.129	4.76E-50	CD8-C11
RHOA	3.31E-54	0.474316814	0.977	0.857	5.51E-50	CD8-C11
PPP1CA	4.04E-54	0.583346651	0.935	0.571	6.71E-50	CD8-C11
TPX2	5.17E-54	0.591481508	0.302	0.012	8.59E-50	CD8-C11
MTHFD1	6.27E-54	0.427170798	0.375	0.047	1.04E-49	CD8-C11
KIFC1	6.34E-54	0.420205186	0.263	0.002	1.05E-49	CD8-C11
HIRIP3	1.66E-53	0.445919861	0.404	0.056	2.76E-49	CD8-C11
HPRT1	4.07E-53	0.604004537	0.602	0.166	6.77E-49	CD8-C11
LCP1	7.86E-53	0.595954581	0.971	0.749	1.31E-48	CD8-C11
MAD2L1	3.48E-52	0.437825353	0.375	0.040	5.78E-48	CD8-C11
TALDO1	4.61E-52	0.588866232	0.703	0.269	7.67E-48	CD8-C11
LMNB1	5.16E-52	0.502661133	0.544	0.155	8.58E-48	CD8-C11
CCDC167	5.48E-52	0.476838866	0.508	0.116	9.12E-48	CD8-C11
ANXA2	7.27E-52	0.589515422	0.883	0.475	1.21E-47	CD8-C11
AP2S1	7.54E-52	0.508450317	0.740	0.285	1.25E-47	CD8-C11
EZH2	1.46E-51	0.467280585	0.396	0.067	2.43E-47	CD8-C11
PSMB9	3.97E-51	0.677215014	0.901	0.524	6.60E-47	CD8-C11
LSM4	5.64E-51	0.591604673	0.698	0.263	9.38E-47	CD8-C11
ARPC2	1.18E-50	0.507655388	0.990	0.877	1.96E-46	CD8-C11
SERF2	3.20E-50	0.422662122	0.992	0.923	5.31E-46	CD8-C11
SLC25A5	5.39E-50	0.597064041	0.935	0.654	8.96E-46	CD8-C11
ITGB3BP	5.48E-50	0.44145689	0.365	0.043	9.12E-46	CD8-C11
NKG7	5.72E-50	0.71856045	0.948	0.620	9.51E-46	CD8-C11
DBI	7.15E-50	0.612123709	0.891	0.528	1.19E-45	CD8-C11
NASP	1.46E-49	0.577519354	0.776	0.357	2.43E-45	CD8-C11
RPL39L	1.79E-49	0.388857768	0.354	0.044	2.98E-45	CD8-C11
CDCA8	1.87E-49	0.397986234	0.276	0.012	3.10E-45	CD8-C11
MAD2L2	2.20E-49	0.491871417	0.615	0.202	3.66E-45	CD8-C11
YBX1	3.28E-49	0.447950003	0.987	0.882	5.45E-45	CD8-C11
PSMB10	4.13E-49	0.568753922	0.805	0.400	6.86E-45	CD8-C11
PRDX3	5.42E-49	0.440347813	0.471	0.099	9.01E-45	CD8-C11
PSMB2	8.65E-49	0.450830351	0.690	0.274	1.44E-44	CD8-C11
PKM	8.78E-49	0.614664	0.911	0.595	1.46E-44	CD8-C11
CTSW	9.89E-49	0.845818063	0.883	0.577	1.65E-44	CD8-C11
CENPK	9.99E-49	0.513608539	0.466	0.094	1.66E-44	CD8-C11
DNAJC9	1.15E-48	0.678179941	0.706	0.267	1.92E-44	CD8-C11
ANAPC11	1.44E-48	0.564568193	0.831	0.403	2.40E-44	CD8-C11
ORC6	1.50E-48	0.352801499	0.240	0.011	2.49E-44	CD8-C11
CDCA5	1.83E-48	0.366660227	0.234	0.003	3.04E-44	CD8-C11
HMGN1	2.06E-48	0.537125089	0.940	0.657	3.42E-44	CD8-C11

CDKN2A	2.67E-48	0.489862673	0.404	0.061	4.43E-44	CD8-C11
CDKN3	5.83E-48	0.419993081	0.245	0.009	9.70E-44	CD8-C11
BRCA1	6.26E-48	0.360386523	0.247	0.007	1.04E-43	CD8-C11
SSRP1	7.19E-48	0.4415486	0.573	0.187	1.20E-43	CD8-C11
ATAD2	7.23E-48	0.569935434	0.427	0.072	1.20E-43	CD8-C11
TOP2A	9.54E-48	0.488385621	0.297	0.021	1.59E-43	CD8-C11
COMMD4	1.73E-47	0.34881267	0.424	0.098	2.88E-43	CD8-C11
TMEM109	2.16E-47	0.623392797	0.750	0.334	3.58E-43	CD8-C11
CENPF	2.67E-47	0.890446958	0.370	0.048	4.44E-43	CD8-C11
FAM111B	3.43E-47	0.382180197	0.219	0.001	5.71E-43	CD8-C11
ACTG1	4.82E-47	0.661728532	0.997	0.939	8.01E-43	CD8-C11
CDK4	9.71E-47	0.44559094	0.448	0.096	1.61E-42	CD8-C11
FABP5	1.47E-46	0.485310497	0.536	0.154	2.44E-42	CD8-C11
BLOC1S1	2.33E-46	0.531566196	0.643	0.216	3.87E-42	CD8-C11
COX5A	2.72E-46	0.551441555	0.880	0.499	4.53E-42	CD8-C11
SMC1A	3.25E-46	0.495607037	0.487	0.112	5.41E-42	CD8-C11
HLA-DRA	3.41E-46	0.885253349	0.703	0.266	5.67E-42	CD8-C11
COX6C	3.46E-46	0.456276128	0.966	0.789	5.75E-42	CD8-C11
CHTF18	6.55E-46	0.359749224	0.310	0.030	1.09E-41	CD8-C11
TRDC	6.58E-46	0.85834441	0.357	0.039	1.09E-41	CD8-C11
GSTO1	7.49E-46	0.374174764	0.521	0.164	1.25E-41	CD8-C11
WDR76	8.45E-46	0.375724141	0.315	0.035	1.40E-41	CD8-C11
RANBP1	1.73E-45	0.692300606	0.732	0.308	2.88E-41	CD8-C11
NDUFB2	1.95E-45	0.574332851	0.891	0.543	3.24E-41	CD8-C11
CENPN	2.30E-45	0.445788804	0.453	0.106	3.82E-41	CD8-C11
GUSB	5.46E-45	0.433413667	0.602	0.200	9.09E-41	CD8-C11
TROAP	1.07E-44	0.380925748	0.221	0.002	1.78E-40	CD8-C11
ANXA5	2.29E-44	0.626368129	0.784	0.352	3.81E-40	CD8-C11
HNRNPAB	2.43E-44	0.419988439	0.497	0.148	4.03E-40	CD8-C11
CBX3	6.13E-44	0.459063981	0.766	0.365	1.02E-39	CD8-C11
MIR4435-2HG	9.89E-44	0.422528534	0.607	0.207	1.64E-39	CD8-C11
MRPL11	1.26E-43	0.388187332	0.573	0.205	2.09E-39	CD8-C11
CALM3	1.43E-43	0.621928134	0.872	0.503	2.38E-39	CD8-C11
PDIA6	1.48E-43	0.530427204	0.763	0.340	2.46E-39	CD8-C11
PSMB3	2.13E-43	0.501162092	0.852	0.464	3.54E-39	CD8-C11
H3F3A	2.29E-43	0.461923056	0.992	0.917	3.80E-39	CD8-C11
ATP5PF	6.81E-43	0.409118163	0.831	0.457	1.13E-38	CD8-C11
ARPC5	7.29E-43	0.564857341	0.799	0.383	1.21E-38	CD8-C11
MRPL51	8.05E-43	0.409382305	0.586	0.214	1.34E-38	CD8-C11
JPT1	9.12E-43	0.509510208	0.685	0.297	1.52E-38	CD8-C11
UBE2T	1.50E-42	0.377684265	0.279	0.015	2.49E-38	CD8-C11
BRCA2	1.51E-42	0.355281198	0.273	0.022	2.51E-38	CD8-C11
CDK2	2.14E-42	0.350666711	0.294	0.026	3.55E-38	CD8-C11
C12orf75	2.17E-42	0.560409604	0.857	0.465	3.61E-38	CD8-C11
TYROBP	3.89E-42	1.097342175	0.419	0.087	6.47E-38	CD8-C11
NAA38	4.15E-42	0.436523529	0.703	0.302	6.90E-38	CD8-C11
CORO1A	8.29E-42	0.536951053	0.984	0.831	1.38E-37	CD8-C11
CBX5	9.63E-42	0.373779326	0.388	0.081	1.60E-37	CD8-C11
CKAP2L	1.73E-41	0.36603612	0.203	0.004	2.88E-37	CD8-C11
EBP	4.13E-41	0.384856579	0.586	0.206	6.87E-37	CD8-C11
PSMC3	6.04E-41	0.446950935	0.682	0.283	1.00E-36	CD8-C11
LSM3	6.90E-41	0.470035581	0.693	0.282	1.15E-36	CD8-C11

HSPB11	7.26E-41	0.516657255	0.599	0.198	1.21E-36	CD8-C11
GLIPR2	9.90E-41	0.428738452	0.664	0.274	1.65E-36	CD8-C11
ATP5MC3	9.92E-41	0.548223345	0.896	0.558	1.65E-36	CD8-C11
HLA-DRB1	1.06E-40	0.801204718	0.883	0.515	1.76E-36	CD8-C11
ATP5MF	1.21E-40	0.503154986	0.854	0.457	2.02E-36	CD8-C11
SKA2	1.34E-40	0.351023718	0.398	0.089	2.23E-36	CD8-C11
RFC4	1.68E-40	0.397438723	0.349	0.057	2.80E-36	CD8-C11
POLD2	2.40E-40	0.406200674	0.419	0.097	3.99E-36	CD8-C11
RECQL	2.62E-40	0.390484346	0.518	0.177	4.36E-36	CD8-C11
ACAA2	3.28E-40	0.404609109	0.586	0.217	5.46E-36	CD8-C11
PRKDC	1.13E-39	0.459117555	0.521	0.176	1.88E-35	CD8-C11
HNRNPF	1.39E-39	0.589803438	0.794	0.433	2.31E-35	CD8-C11
NDUFA2	1.50E-39	0.408319861	0.745	0.366	2.50E-35	CD8-C11
UNG	2.43E-39	0.542754197	0.346	0.066	4.04E-35	CD8-C11
GZMH	2.57E-39	0.462057612	0.823	0.445	4.28E-35	CD8-C11
BRK1	3.16E-39	0.36677894	0.844	0.481	5.26E-35	CD8-C11
IFI27L2	3.54E-39	0.43361174	0.667	0.274	5.89E-35	CD8-C11
CKLF	3.67E-39	0.435623076	0.727	0.351	6.10E-35	CD8-C11
SNRPD3	3.74E-39	0.469114944	0.737	0.333	6.22E-35	CD8-C11
PSMA6	5.63E-39	0.36817485	0.797	0.426	9.36E-35	CD8-C11
SHMT2	1.06E-38	0.402229947	0.461	0.125	1.76E-34	CD8-C11
RFC2	1.23E-38	0.406191111	0.359	0.058	2.05E-34	CD8-C11
NDUFS6	1.74E-38	0.465453854	0.711	0.318	2.90E-34	CD8-C11
LAT2	1.85E-38	0.361976659	0.242	0.025	3.08E-34	CD8-C11
XRCC5	2.10E-38	0.450392702	0.763	0.373	3.48E-34	CD8-C11
WDR1	3.00E-38	0.5190227	0.859	0.501	4.98E-34	CD8-C11
IGFBP7	3.18E-38	0.615952938	0.271	0.026	5.29E-34	CD8-C11
TMPO	3.47E-38	0.520386834	0.536	0.162	5.77E-34	CD8-C11
RNASEH2C	5.40E-38	0.470190709	0.555	0.197	8.98E-34	CD8-C11
HINT2	6.60E-38	0.39032613	0.482	0.139	1.10E-33	CD8-C11
ARPC1B	7.84E-38	0.52533037	0.911	0.602	1.30E-33	CD8-C11
NDUFS8	1.15E-37	0.400802851	0.695	0.308	1.92E-33	CD8-C11
GNG5	1.20E-37	0.517751381	0.849	0.494	2.00E-33	CD8-C11
COX6A1	1.40E-37	0.37696088	0.971	0.817	2.32E-33	CD8-C11
NDUFB3	1.64E-37	0.384002152	0.568	0.211	2.72E-33	CD8-C11
SNRPF	1.66E-37	0.360577608	0.807	0.429	2.77E-33	CD8-C11
NAP1L1	2.11E-37	0.422298743	0.846	0.515	3.50E-33	CD8-C11
RBBP7	4.67E-37	0.464708211	0.695	0.314	7.76E-33	CD8-C11
CAPZB	6.09E-37	0.460017929	0.901	0.627	1.01E-32	CD8-C11
COPS3	9.04E-37	0.362195999	0.641	0.290	1.50E-32	CD8-C11
CD74	9.27E-37	0.636036983	0.987	0.894	1.54E-32	CD8-C11
SLBP	1.54E-36	0.46383506	0.740	0.365	2.56E-32	CD8-C11
HDAC1	1.82E-36	0.38510366	0.651	0.283	3.02E-32	CD8-C11
LSM2	2.00E-36	0.383714715	0.549	0.218	3.33E-32	CD8-C11
ASPM	2.51E-36	0.436845136	0.174	0.003	4.17E-32	CD8-C11
NSD2	3.06E-36	0.370993128	0.299	0.042	5.08E-32	CD8-C11
MRPL52	4.06E-36	0.35144624	0.609	0.243	6.74E-32	CD8-C11
MEA1	5.69E-36	0.402275291	0.594	0.219	9.47E-32	CD8-C11
PHF19	5.88E-36	0.381327295	0.333	0.052	9.78E-32	CD8-C11
FCGR3A	5.90E-36	1.0257561	0.453	0.119	9.81E-32	CD8-C11
SUPT16H	6.89E-36	0.400972107	0.508	0.181	1.15E-31	CD8-C11
ANP32E	1.57E-35	0.479208009	0.599	0.239	2.61E-31	CD8-C11

PARK7	1.97E-35	0.403703552	0.844	0.495	3.27E-31	CD8-C11
PDCD5	2.18E-35	0.369241537	0.625	0.274	3.63E-31	CD8-C11
DCTN3	2.59E-35	0.403935703	0.753	0.386	4.30E-31	CD8-C11
C19orf48	3.49E-35	0.354280974	0.391	0.094	5.80E-31	CD8-C11
UQCRQ	3.82E-35	0.438555345	0.833	0.486	6.35E-31	CD8-C11
DUSP23	7.01E-35	0.358634031	0.469	0.143	1.17E-30	CD8-C11
USP1	7.74E-35	0.470305569	0.570	0.208	1.29E-30	CD8-C11
CHCHD2	1.03E-34	0.40165084	0.979	0.852	1.71E-30	CD8-C11
PRELID1	1.11E-34	0.357638594	0.794	0.452	1.85E-30	CD8-C11
RPA2	1.15E-34	0.376149356	0.508	0.187	1.91E-30	CD8-C11
NDUFB7	1.75E-34	0.429189392	0.781	0.400	2.91E-30	CD8-C11
ANXA6	2.19E-34	0.486526174	0.826	0.474	3.64E-30	CD8-C11
RPA1	3.13E-34	0.378340997	0.435	0.121	5.20E-30	CD8-C11
SMC3	3.13E-34	0.447271691	0.557	0.194	5.21E-30	CD8-C11
SIVA1	3.94E-34	0.494504138	0.792	0.460	6.54E-30	CD8-C11
RAC2	5.17E-34	0.478894055	0.964	0.787	8.60E-30	CD8-C11
P4HB	5.90E-34	0.429759033	0.883	0.557	9.81E-30	CD8-C11
CDCA7	7.23E-34	0.402531141	0.255	0.023	1.20E-29	CD8-C11
GZMB	7.43E-34	1.091032341	0.615	0.269	1.24E-29	CD8-C11
POMP	8.75E-34	0.355109322	0.844	0.505	1.46E-29	CD8-C11
RBBP4	8.82E-34	0.441657624	0.669	0.296	1.47E-29	CD8-C11
GMPS	9.13E-34	0.346674716	0.466	0.142	1.52E-29	CD8-C11
KIF22	1.31E-33	0.451124471	0.547	0.193	2.18E-29	CD8-C11
CAP1	1.92E-33	0.509083899	0.867	0.540	3.20E-29	CD8-C11
NDUFB6	3.74E-33	0.350812617	0.612	0.258	6.23E-29	CD8-C11
CISD3	5.41E-33	0.401830942	0.492	0.167	9.00E-29	CD8-C11
SNRPE	1.57E-32	0.374545687	0.646	0.291	2.61E-28	CD8-C11
UBE2L6	2.13E-32	0.400136706	0.682	0.322	3.54E-28	CD8-C11
SNRPG	2.40E-32	0.38219649	0.805	0.455	3.99E-28	CD8-C11
LYN	4.22E-32	0.37776962	0.289	0.049	7.02E-28	CD8-C11
CTSC	4.40E-32	0.400351402	0.802	0.456	7.31E-28	CD8-C11
PSME1	5.25E-32	0.392716555	0.974	0.827	8.74E-28	CD8-C11
RRM1	7.07E-32	0.399286653	0.391	0.098	1.18E-27	CD8-C11
ARPC3	8.21E-32	0.438355792	0.964	0.783	1.37E-27	CD8-C11
PSMB8	8.89E-32	0.419377911	0.841	0.522	1.48E-27	CD8-C11
SPN	9.07E-32	0.375532038	0.630	0.291	1.51E-27	CD8-C11
OAZ1	9.23E-32	0.391522655	0.969	0.774	1.54E-27	CD8-C11
RBX1	1.43E-31	0.3722634	0.654	0.298	2.37E-27	CD8-C11
TPM4	1.88E-31	0.408161902	0.630	0.282	3.13E-27	CD8-C11
RAD21	2.92E-31	0.447116291	0.781	0.438	4.85E-27	CD8-C11
CDK2AP2	3.16E-31	0.535869666	0.591	0.245	5.26E-27	CD8-C11
PA2G4	3.47E-31	0.470278338	0.771	0.428	5.76E-27	CD8-C11
TAF15	3.63E-31	0.378233785	0.646	0.295	6.04E-27	CD8-C11
TRAPPC1	3.75E-31	0.408201369	0.776	0.428	6.23E-27	CD8-C11
FDPS	4.05E-31	0.448621323	0.555	0.203	6.73E-27	CD8-C11
PDIA3	4.08E-31	0.474565907	0.927	0.705	6.78E-27	CD8-C11
UQCRC1	4.15E-31	0.351211063	0.651	0.310	6.89E-27	CD8-C11
CDC20	6.96E-31	0.399229261	0.151	0.003	1.16E-26	CD8-C11
MT1E	9.32E-31	0.447699424	0.424	0.126	1.55E-26	CD8-C11
HLA-DPA1	1.67E-30	0.482897283	0.880	0.555	2.78E-26	CD8-C11
SET	1.89E-30	0.440635471	0.755	0.385	3.15E-26	CD8-C11
DDX39A	2.26E-30	0.469396176	0.841	0.499	3.76E-26	CD8-C11

BSG	2.68E-30	0.35726069	0.836	0.503	4.46E-26	CD8-C11
PSIP1	3.31E-30	0.444568792	0.714	0.360	5.51E-26	CD8-C11
HSP90AA1	3.52E-30	0.411439314	0.982	0.857	5.85E-26	CD8-C11
KLRF1	6.90E-30	0.621656888	0.341	0.063	1.15E-25	CD8-C11
RAB1B	7.97E-30	0.366787023	0.703	0.340	1.33E-25	CD8-C11
DCXR	1.65E-29	0.368180249	0.599	0.266	2.74E-25	CD8-C11
AKR7A2	2.05E-29	0.351423605	0.424	0.136	3.41E-25	CD8-C11
IFI16	2.81E-29	0.430472137	0.578	0.236	4.66E-25	CD8-C11
NCR3	4.41E-29	0.370495119	0.396	0.123	7.33E-25	CD8-C11
CD38	4.68E-29	0.416865062	0.247	0.034	7.78E-25	CD8-C11
HNRNPR	1.37E-28	0.38809698	0.753	0.407	2.27E-24	CD8-C11
ITGB2	3.55E-28	0.452848179	0.956	0.752	5.91E-24	CD8-C11
DCTPP1	7.83E-28	0.367581439	0.362	0.097	1.30E-23	CD8-C11
POLR2L	1.10E-27	0.372836079	0.880	0.609	1.84E-23	CD8-C11
KIR3DL2	1.96E-27	0.495711131	0.281	0.050	3.25E-23	CD8-C11
KLRC2	2.01E-27	0.403390428	0.260	0.042	3.34E-23	CD8-C11
LGALS1	3.42E-27	1.019964726	0.784	0.501	5.69E-23	CD8-C11
MSN	9.09E-27	0.362824378	0.849	0.549	1.51E-22	CD8-C11
ATP5F1C	2.33E-26	0.356196425	0.773	0.425	3.88E-22	CD8-C11
PLEK	2.40E-26	0.486560279	0.536	0.236	3.99E-22	CD8-C11
ABI3	2.45E-26	0.368537074	0.518	0.214	4.08E-22	CD8-C11
NDUFB1	3.28E-26	0.38573094	0.622	0.295	5.45E-22	CD8-C11
ATP5F1E	3.90E-26	0.349806398	0.990	0.927	6.48E-22	CD8-C11
7-Sep	4.59E-26	0.382448836	0.938	0.696	7.64E-22	CD8-C11
TTC38	5.15E-26	0.462315218	0.404	0.124	8.57E-22	CD8-C11
SH2D1B	5.28E-26	0.420381565	0.180	0.009	8.77E-22	CD8-C11
PARP1	6.09E-26	0.372505089	0.508	0.204	1.01E-21	CD8-C11
KLRC3	6.28E-26	0.408064875	0.273	0.062	1.05E-21	CD8-C11
ITGB7	9.85E-26	0.365839202	0.716	0.399	1.64E-21	CD8-C11
PTTG1	2.38E-25	0.543163549	0.385	0.103	3.96E-21	CD8-C11
PSMA5	3.98E-25	0.363487084	0.591	0.283	6.62E-21	CD8-C11
HIST1H1B	4.79E-25	0.426487541	0.159	0.009	7.97E-21	CD8-C11
SUMO2	5.03E-25	0.36996121	0.958	0.792	8.37E-21	CD8-C11
CD63	5.63E-25	0.441985931	0.685	0.385	9.36E-21	CD8-C11
HLA-DPB1	8.35E-25	0.358096233	0.846	0.544	1.39E-20	CD8-C11
ARL6IP1	9.92E-25	0.412356381	0.771	0.472	1.65E-20	CD8-C11
KLRD1	1.57E-24	0.490890196	0.523	0.223	2.62E-20	CD8-C11
CYBA	2.81E-24	0.411682989	0.948	0.772	4.67E-20	CD8-C11
KIR2DL3	4.06E-24	0.458537274	0.224	0.028	6.75E-20	CD8-C11
H2AFX	4.79E-24	0.420501058	0.534	0.253	7.97E-20	CD8-C11
CST7	1.20E-23	0.348385351	0.927	0.695	1.99E-19	CD8-C11
MIF	1.62E-23	0.358153305	0.951	0.841	2.69E-19	CD8-C11
HLA-DQA1	2.72E-23	0.415307832	0.466	0.204	4.52E-19	CD8-C11
PTMS	2.78E-23	0.438050808	0.380	0.148	4.62E-19	CD8-C11
EMP3	3.55E-23	0.347747834	0.964	0.781	5.90E-19	CD8-C11
UCP2	3.91E-23	0.359884122	0.758	0.448	6.51E-19	CD8-C11
PRF1	1.08E-22	0.764953677	0.604	0.297	1.80E-18	CD8-C11
PSMA4	1.79E-22	0.364180663	0.557	0.243	2.97E-18	CD8-C11
CALR	4.90E-22	0.466252536	0.904	0.696	8.15E-18	CD8-C11
HIST1H4C	1.13E-21	1.108790389	0.844	0.643	1.88E-17	CD8-C11
ACTR3	4.42E-21	0.350913968	0.768	0.465	7.36E-17	CD8-C11
SRP9	6.34E-21	0.372194668	0.711	0.400	1.05E-16	CD8-C11

HIST1H1D	6.56E-21	0.349654329	0.456	0.202	1.09E-16	CD8-C11
FGFBP2	1.64E-20	0.491913437	0.497	0.226	2.73E-16	CD8-C11
FCER1G	7.64E-20	0.602777225	0.148	0.016	1.27E-15	CD8-C11
HOPX	1.10E-19	0.52840294	0.612	0.335	1.83E-15	CD8-C11
XCL1	1.81E-19	0.418728366	0.349	0.128	3.01E-15	CD8-C11
IFITM3	1.82E-19	0.367382314	0.333	0.129	3.03E-15	CD8-C11
XCL2	6.57E-19	0.466462073	0.388	0.151	1.09E-14	CD8-C11
HLA-DRB5	8.12E-19	0.4747613	0.409	0.150	1.35E-14	CD8-C11
ADGRG1	2.51E-18	0.402651349	0.417	0.169	4.17E-14	CD8-C11
TRBC1	3.01E-18	0.50817238	0.596	0.322	5.00E-14	CD8-C11
IFNG	4.17E-18	0.358576276	0.346	0.135	6.94E-14	CD8-C11
CD247	4.75E-18	0.417922217	0.846	0.646	7.90E-14	CD8-C11
GNLY	8.68E-18	0.967433621	0.490	0.244	1.44E-13	CD8-C11
Z93241.1	1.91E-17	0.474514328	0.310	0.107	3.18E-13	CD8-C11
CX3CR1	6.56E-16	0.35708103	0.312	0.121	1.09E-11	CD8-C11
LY6E	1.40E-13	0.385343229	0.875	0.717	2.34E-09	CD8-C11
CCL3	1.01E-10	0.379529409	0.240	0.081	1.68E-06	CD8-C11
IFITM2	4.53E-08	0.383496273	0.924	0.838	0.000752804	CD8-C11
SPON2	8.75E-08	0.536516948	0.312	0.173	0.001455405	CD8-C11
COTL1	5.20E-07	0.392342728	0.688	0.650	0.008654705	CD8-C11
RPS13	4.87E-164	0.71872714	1.000	0.995	8.10E-160	CD8-C1
RPS5	2.98E-148	0.746027894	1.000	0.981	4.96E-144	CD8-C1
RPL32	8.13E-145	0.621923645	1.000	0.998	1.35E-140	CD8-C1
RPS8	7.61E-142	0.623999704	1.000	0.997	1.26E-137	CD8-C1
CCR7	7.06E-140	1.19912854	0.835	0.143	1.17E-135	CD8-C1
RPS12	3.91E-131	0.576927738	1.000	0.998	6.51E-127	CD8-C1
CD8B	2.14E-128	1.117863494	0.970	0.366	3.56E-124	CD8-C1
RPL22	3.69E-128	0.693497926	0.998	0.976	6.13E-124	CD8-C1
RPS23	1.86E-126	0.556210033	1.000	0.996	3.10E-122	CD8-C1
LEF1	3.89E-125	1.123087539	0.810	0.141	6.47E-121	CD8-C1
RPL19	4.55E-125	0.513651871	1.000	0.998	7.56E-121	CD8-C1
AIF1	2.72E-120	0.985125833	0.643	0.054	4.52E-116	CD8-C1
RPL34	2.68E-119	0.481562959	1.000	0.998	4.45E-115	CD8-C1
TCF7	4.93E-118	1.096949675	0.870	0.223	8.20E-114	CD8-C1
LDHB	1.19E-116	0.937831731	0.983	0.673	1.97E-112	CD8-C1
RPL18	7.79E-113	0.491670797	0.998	0.995	1.29E-108	CD8-C1
EEF1B2	9.21E-112	0.660291621	1.000	0.965	1.53E-107	CD8-C1
RPL13	1.51E-111	0.499718017	1.000	0.999	2.51E-107	CD8-C1
RPL5	6.49E-110	0.617733224	1.000	0.986	1.08E-105	CD8-C1
SELL	5.21E-105	1.042587819	0.850	0.203	8.66E-101	CD8-C1
RPS28	3.22E-104	0.466677981	1.000	0.997	5.36E-100	CD8-C1
RPS6	4.57E-103	0.545783721	1.000	0.992	7.59E-99	CD8-C1
EEF1A1	4.11E-101	0.45126352	1.000	1.000	6.83E-97	CD8-C1
RPS14	4.18E-100	0.432416357	1.000	0.997	6.95E-96	CD8-C1
RPL11	2.86E-98	0.463534767	1.000	0.998	4.75E-94	CD8-C1
RPL12	6.36E-98	0.484303246	1.000	0.995	1.06E-93	CD8-C1
RPL29	9.12E-96	0.475103324	1.000	0.993	1.52E-91	CD8-C1
EEF1G	6.33E-95	0.645814889	0.998	0.893	1.05E-90	CD8-C1
NELL2	2.20E-94	0.918425643	0.678	0.127	3.66E-90	CD8-C1
PABPC1	5.95E-92	0.606508884	1.000	0.950	9.90E-88	CD8-C1
LINC02446	2.36E-90	1.2344865	0.539	0.044	3.92E-86	CD8-C1
ACTN1	2.59E-87	0.813205443	0.546	0.049	4.31E-83	CD8-C1

RPS4X	3.32E-87	0.45843073	1.000	0.994	5.52E-83	CD8-C1
RPL37	4.64E-87	0.412543812	1.000	0.997	7.71E-83	CD8-C1
RPL30	1.01E-83	0.403972157	1.000	0.998	1.67E-79	CD8-C1
RPS27A	1.74E-83	0.383065986	1.000	0.998	2.89E-79	CD8-C1
NOSIP	3.27E-83	0.866472477	0.870	0.353	5.43E-79	CD8-C1
RPL18A	8.09E-83	0.415752686	1.000	0.995	1.35E-78	CD8-C1
RPL10A	1.10E-82	0.501221448	1.000	0.981	1.83E-78	CD8-C1
RPSA	6.72E-82	0.473552723	1.000	0.984	1.12E-77	CD8-C1
RACK1	1.56E-81	0.485237652	1.000	0.980	2.59E-77	CD8-C1
RPL10	2.35E-81	0.393991897	1.000	0.999	3.91E-77	CD8-C1
RPL35A	8.48E-81	0.400350749	1.000	0.995	1.41E-76	CD8-C1
RPL14	1.40E-79	0.417155914	1.000	0.995	2.33E-75	CD8-C1
RPL7	1.01E-77	0.494794847	0.995	0.937	1.67E-73	CD8-C1
NACA	1.66E-75	0.456248842	1.000	0.984	2.76E-71	CD8-C1
RPS21	2.68E-74	0.399839014	1.000	0.995	4.45E-70	CD8-C1
RPS15	2.63E-73	0.346743364	1.000	0.997	4.37E-69	CD8-C1
MAL	1.78E-71	0.739549191	0.611	0.129	2.96E-67	CD8-C1
RPL21	6.19E-70	0.45311957	1.000	0.985	1.03E-65	CD8-C1
TRABD2A	4.65E-66	0.631063287	0.481	0.076	7.73E-62	CD8-C1
EIF3E	2.88E-65	0.654789447	0.943	0.612	4.78E-61	CD8-C1
RPL4	3.59E-62	0.47717138	1.000	0.945	5.97E-58	CD8-C1
NT5E	1.22E-61	0.49603886	0.317	0.012	2.03E-57	CD8-C1
RPS18	3.92E-61	0.40705177	1.000	0.995	6.51E-57	CD8-C1
RPL8	1.06E-60	0.377866922	1.000	0.992	1.77E-56	CD8-C1
RPL9	2.81E-58	0.379950131	1.000	0.989	4.67E-54	CD8-C1
RPS3A	5.30E-58	0.401991179	1.000	0.994	8.81E-54	CD8-C1
RPS10	5.62E-58	0.45279671	1.000	0.976	9.34E-54	CD8-C1
CLEC11A	3.91E-56	0.415520683	0.262	0.008	6.50E-52	CD8-C1
LTB	1.39E-54	0.418420859	0.923	0.502	2.31E-50	CD8-C1
RPS4Y1	1.53E-53	0.459510147	0.978	0.912	2.54E-49	CD8-C1
PDE3B	6.25E-53	0.63866401	0.623	0.178	1.04E-48	CD8-C1
RPL3	3.89E-52	0.361331874	1.000	0.988	6.47E-48	CD8-C1
RPS9	8.59E-51	0.39402068	0.995	0.977	1.43E-46	CD8-C1
PIK3IP1	1.02E-50	0.587492158	0.895	0.527	1.70E-46	CD8-C1
IL7R	1.42E-50	0.456091661	0.908	0.499	2.36E-46	CD8-C1
NUCB2	2.61E-50	0.602298001	0.663	0.235	4.35E-46	CD8-C1
PCSK1N	1.51E-48	0.523091376	0.299	0.021	2.51E-44	CD8-C1
SPINT2	4.37E-48	0.4886071	0.471	0.104	7.27E-44	CD8-C1
LIMD2	4.08E-46	0.545864143	0.950	0.690	6.78E-42	CD8-C1
COX4I1	1.08E-44	0.372894891	0.995	0.933	1.80E-40	CD8-C1
TXNIP	1.53E-44	0.564911284	0.978	0.754	2.55E-40	CD8-C1
ST13	1.56E-44	0.569375246	0.840	0.482	2.60E-40	CD8-C1
LRRN3	2.43E-43	0.418780557	0.229	0.010	4.04E-39	CD8-C1
CD248	7.22E-43	0.347856941	0.209	0.004	1.20E-38	CD8-C1
NDFIP1	7.28E-43	0.543119219	0.900	0.567	1.21E-38	CD8-C1
SATB1	1.24E-42	0.5558312	0.554	0.176	2.06E-38	CD8-C1
CARS	1.80E-42	0.488044132	0.404	0.099	2.99E-38	CD8-C1
CD55	3.68E-42	0.48599405	0.666	0.280	6.13E-38	CD8-C1
RPL31	9.81E-42	0.37071038	0.998	0.949	1.63E-37	CD8-C1
CD27	1.41E-41	0.441784835	0.716	0.288	2.34E-37	CD8-C1
PRMT2	1.28E-40	0.574130874	0.835	0.457	2.13E-36	CD8-C1
CD7	4.16E-40	0.480912541	0.938	0.626	6.91E-36	CD8-C1

RGS10	6.67E-40	0.504621786	0.873	0.542	1.11E-35	CD8-C1
EEF2	2.49E-38	0.351082789	0.998	0.945	4.15E-34	CD8-C1
EPHX2	1.25E-36	0.439561038	0.329	0.062	2.08E-32	CD8-C1
SELENOH	4.43E-36	0.487465244	0.781	0.406	7.37E-32	CD8-C1
EIF2S3	1.46E-35	0.52021301	0.751	0.379	2.43E-31	CD8-C1
LDLRAP1	3.74E-35	0.520171252	0.541	0.177	6.21E-31	CD8-C1
PCED1B	2.07E-33	0.534569647	0.554	0.204	3.44E-29	CD8-C1
AES	4.16E-33	0.384234684	0.930	0.638	6.92E-29	CD8-C1
TMEM123	2.79E-32	0.451904821	0.800	0.451	4.64E-28	CD8-C1
C12orf57	8.65E-32	0.350860984	0.970	0.835	1.44E-27	CD8-C1
ARMH1	1.60E-31	0.417822958	0.317	0.061	2.67E-27	CD8-C1
FCMR	2.93E-31	0.483725156	0.656	0.293	4.87E-27	CD8-C1
EIF4B	7.66E-31	0.414151149	0.905	0.638	1.27E-26	CD8-C1
EIF3H	2.97E-30	0.408325133	0.930	0.711	4.94E-26	CD8-C1
TXK	9.29E-30	0.396594684	0.329	0.068	1.55E-25	CD8-C1
EIF3L	1.27E-29	0.428291893	0.898	0.645	2.12E-25	CD8-C1
FLT3LG	1.77E-29	0.44021647	0.781	0.446	2.94E-25	CD8-C1
LINC00861	3.24E-29	0.400010149	0.626	0.287	5.39E-25	CD8-C1
FBL	4.13E-29	0.430635666	0.751	0.415	6.87E-25	CD8-C1
RCAN3	8.51E-29	0.424360195	0.576	0.257	1.42E-24	CD8-C1
COX7C	1.19E-28	0.358457997	0.978	0.883	1.98E-24	CD8-C1
NOP53	2.07E-27	0.354946147	0.983	0.855	3.44E-23	CD8-C1
MYC	5.20E-27	0.528711013	0.481	0.170	8.65E-23	CD8-C1
RASGRP2	1.78E-26	0.37852565	0.591	0.267	2.95E-22	CD8-C1
IMPDH2	2.39E-26	0.403086809	0.444	0.166	3.97E-22	CD8-C1
GIMAP7	3.06E-26	0.35086305	0.793	0.459	5.08E-22	CD8-C1
ALKBH7	5.03E-26	0.426091416	0.621	0.319	8.36E-22	CD8-C1
C1orf162	6.04E-26	0.392843331	0.416	0.130	1.00E-21	CD8-C1
APEX1	7.24E-26	0.408693458	0.628	0.285	1.20E-21	CD8-C1
RIPOR2	2.10E-25	0.407010079	0.703	0.358	3.49E-21	CD8-C1
ATM	2.27E-25	0.364403829	0.539	0.250	3.78E-21	CD8-C1
DGKA	1.12E-24	0.40231993	0.511	0.205	1.85E-20	CD8-C1
OXNAD1	1.43E-24	0.38794068	0.561	0.260	2.38E-20	CD8-C1
BEX3	1.83E-24	0.37603394	0.289	0.048	3.05E-20	CD8-C1
CAMK4	1.02E-23	0.376270296	0.678	0.374	1.69E-19	CD8-C1
STMN3	1.39E-23	0.38804861	0.474	0.184	2.31E-19	CD8-C1
CHCHD10	7.91E-23	0.358571348	0.623	0.318	1.32E-18	CD8-C1
SNHG8	1.18E-22	0.351244459	0.838	0.551	1.97E-18	CD8-C1
UXT	3.40E-22	0.376167242	0.848	0.594	5.65E-18	CD8-C1
SH3YL1	1.11E-21	0.4227899	0.352	0.098	1.84E-17	CD8-C1
TGFBR2	2.47E-21	0.365254467	0.489	0.218	4.11E-17	CD8-C1
TSPAN32	3.70E-21	0.348832983	0.419	0.160	6.16E-17	CD8-C1
HSPB1	8.88E-21	0.395999029	0.661	0.338	1.48E-16	CD8-C1
SNRPN	9.29E-18	0.368185325	0.678	0.414	1.54E-13	CD8-C1
AC243960.1	4.62E-17	0.361932819	0.516	0.244	7.68E-13	CD8-C1
STAT1	1.19E-09	0.4133987	0.431	0.234	1.97E-05	CD8-C1
TRBV9	9.69E-05	0.380641881	0.110	0.038	1	CD8-C1
CD8A	5.05E-107	0.569528863	0.907	0.439	8.40E-103	CD8-C2
CD8B	5.13E-89	0.520540422	0.784	0.343	8.53E-85	CD8-C2
ZFP36L2	3.40E-69	0.579336417	0.997	0.968	5.65E-65	CD8-C2
TOB1	2.91E-62	0.850947315	0.763	0.444	4.84E-58	CD8-C2
CD7	1.84E-50	0.533038406	0.855	0.613	3.05E-46	CD8-C2

ZNF683	1.65E-48	0.752868051	0.370	0.085	2.74E-44	CD8-C2
FXYD2	1.94E-38	0.43077558	0.185	0.022	3.23E-34	CD8-C2
SMIM3	6.17E-38	0.5371433	0.334	0.098	1.03E-33	CD8-C2
CXCR3	1.62E-36	0.477903822	0.715	0.442	2.69E-32	CD8-C2
ZFP36	1.22E-34	0.484377703	0.968	0.891	2.04E-30	CD8-C2
VIM	1.77E-34	0.486832609	0.997	0.976	2.94E-30	CD8-C2
NR4A2	2.71E-31	0.446858873	0.719	0.479	4.50E-27	CD8-C2
TNFAIP3	1.64E-30	0.397023084	0.945	0.799	2.72E-26	CD8-C2
WHRN	2.88E-29	0.416006019	0.289	0.096	4.79E-25	CD8-C2
LMNA	1.23E-28	0.489839216	0.707	0.475	2.05E-24	CD8-C2
CSRNP1	1.07E-27	0.4057283	0.815	0.603	1.78E-23	CD8-C2
XCL1	1.08E-27	0.73748667	0.311	0.118	1.80E-23	CD8-C2
FOS	1.72E-27	0.435293623	0.840	0.631	2.86E-23	CD8-C2
KLRC1	4.40E-23	0.359788154	0.139	0.017	7.31E-19	CD8-C2
FOSB	3.06E-21	0.352298199	0.609	0.379	5.09E-17	CD8-C2
ATP1B1	3.24E-20	0.379931455	0.258	0.088	5.39E-16	CD8-C2
RUNX3	2.53E-19	0.37425123	0.681	0.485	4.21E-15	CD8-C2
GADD45B	6.02E-19	0.351435794	0.791	0.617	1.00E-14	CD8-C2
ID2	4.39E-17	0.3594146	0.763	0.586	7.30E-13	CD8-C2
BHLHE40	7.17E-17	0.41100299	0.700	0.487	1.19E-12	CD8-C2
RGS16	2.54E-11	0.358020194	0.225	0.092	4.22E-07	CD8-C2
RGCC	4.86E-151	1.120962226	0.917	0.492	8.09E-147	CD8-C3
LMNA	4.22E-136	1.102153352	0.888	0.445	7.01E-132	CD8-C3
YPEL5	1.56E-137	1.069481925	0.887	0.619	2.60E-133	CD8-C3
GZMK	3.07E-155	1.025744998	0.871	0.375	5.10E-151	CD8-C3
MYADM	3.44E-78	0.950550935	0.729	0.359	5.72E-74	CD8-C3
ITM2C	2.38E-95	0.93423971	0.699	0.304	3.96E-91	CD8-C3
TUBA4A	5.25E-114	0.914995009	0.911	0.669	8.73E-110	CD8-C3
ODC1	7.91E-83	0.907311078	0.714	0.408	1.32E-78	CD8-C3
TUBB2A	2.53E-55	0.84931117	0.478	0.180	4.20E-51	CD8-C3
SERTAD1	2.23E-90	0.789040939	0.695	0.366	3.71E-86	CD8-C3
SRRT	5.87E-48	0.76570804	0.575	0.384	9.75E-44	CD8-C3
TUBB4B	3.30E-76	0.758137062	0.793	0.520	5.49E-72	CD8-C3
IDS	1.28E-78	0.723442221	0.845	0.670	2.13E-74	CD8-C3
FOSB	1.56E-86	0.713347214	0.747	0.354	2.59E-82	CD8-C3
DNAJA1	9.75E-81	0.71126032	0.805	0.564	1.62E-76	CD8-C3
MCL1	1.98E-73	0.700817963	0.848	0.651	3.29E-69	CD8-C3
EZR	7.97E-54	0.685811031	0.817	0.680	1.32E-49	CD8-C3
DUSP4	5.68E-57	0.684625139	0.675	0.314	9.44E-53	CD8-C3
EIF4A3	2.72E-58	0.674334776	0.635	0.378	4.52E-54	CD8-C3
CXCR3	1.46E-71	0.670703541	0.745	0.427	2.43E-67	CD8-C3
DUSP2	1.89E-80	0.669908409	0.903	0.660	3.14E-76	CD8-C3
CSRNP1	7.35E-86	0.663818273	0.906	0.584	1.22E-81	CD8-C3
MARCKSL1	1.59E-48	0.655463748	0.423	0.201	2.65E-44	CD8-C3
NR4A2	1.43E-62	0.652955993	0.787	0.462	2.37E-58	CD8-C3
PPP1R15A	9.60E-96	0.652637031	0.930	0.700	1.60E-91	CD8-C3
HSPA5	3.15E-81	0.649873898	0.940	0.793	5.24E-77	CD8-C3
TSPYL2	8.43E-44	0.645765734	0.708	0.482	1.40E-39	CD8-C3
SLC1A5	9.29E-54	0.643621239	0.497	0.239	1.55E-49	CD8-C3
LDHA	2.45E-70	0.642898779	0.916	0.816	4.07E-66	CD8-C3
ADGRE5	2.77E-52	0.640154714	0.808	0.649	4.60E-48	CD8-C3
ZFP36	1.30E-97	0.627192183	0.985	0.886	2.16E-93	CD8-C3

VIM	1.03E-69	0.620723527	0.995	0.975	1.71E-65	CD8-C3
SRSF7	3.03E-87	0.600890081	0.944	0.858	5.04E-83	CD8-C3
HSPH1	2.14E-46	0.5988188	0.471	0.255	3.56E-42	CD8-C3
SRSF2	2.36E-72	0.588965078	0.833	0.692	3.93E-68	CD8-C3
CD44	1.99E-79	0.587475751	0.935	0.801	3.30E-75	CD8-C3
H2AFX	2.54E-41	0.584045188	0.451	0.234	4.23E-37	CD8-C3
OASL	6.65E-31	0.573483811	0.472	0.261	1.11E-26	CD8-C3
AMD1	2.48E-44	0.567694937	0.622	0.416	4.12E-40	CD8-C3
UBC	1.44E-100	0.555247804	0.999	0.991	2.40E-96	CD8-C3
FOS	5.61E-69	0.550271344	0.878	0.618	9.34E-65	CD8-C3
DNAJB6	1.59E-40	0.548878053	0.749	0.593	2.64E-36	CD8-C3
EIF1	7.32E-157	0.54819058	1.000	0.997	1.22E-152	CD8-C3
CD74	2.02E-53	0.542137557	0.948	0.889	3.36E-49	CD8-C3
TGFB1	1.33E-35	0.541976546	0.726	0.594	2.21E-31	CD8-C3
CXCR4	1.09E-56	0.537243998	0.901	0.699	1.81E-52	CD8-C3
DUSP1	1.87E-51	0.522459912	0.905	0.792	3.11E-47	CD8-C3
CMC2	3.02E-28	0.520388869	0.542	0.373	5.03E-24	CD8-C3
PLK3	1.65E-26	0.518206144	0.476	0.288	2.74E-22	CD8-C3
ENC1	5.08E-31	0.513504556	0.307	0.106	8.44E-27	CD8-C3
ZC3H12A	6.83E-32	0.510004798	0.532	0.329	1.14E-27	CD8-C3
SLC7A5	2.57E-40	0.506240229	0.696	0.425	4.28E-36	CD8-C3
DNAJB1	8.46E-45	0.502662589	0.804	0.678	1.41E-40	CD8-C3
HLA-DRB1	1.11E-34	0.501852473	0.731	0.495	1.84E-30	CD8-C3
IFRD1	8.07E-32	0.496223324	0.561	0.395	1.34E-27	CD8-C3
TUBA1A	1.16E-30	0.49594152	0.729	0.597	1.93E-26	CD8-C3
GLUL	8.86E-19	0.49279297	0.319	0.263	1.47E-14	CD8-C3
GNG2	4.23E-38	0.49055758	0.730	0.602	7.04E-34	CD8-C3
COTL1	7.00E-31	0.480134737	0.761	0.638	1.16E-26	CD8-C3
TNFAIP3	3.28E-74	0.475913057	0.971	0.790	5.46E-70	CD8-C3
NEU1	8.91E-25	0.472810452	0.362	0.182	1.48E-20	CD8-C3
GGA2	3.92E-30	0.472631716	0.432	0.254	6.52E-26	CD8-C3
JUND	3.92E-29	0.469300327	0.582	0.371	6.51E-25	CD8-C3
FABP5	1.34E-25	0.467347538	0.296	0.142	2.22E-21	CD8-C3
YBX3	2.00E-22	0.463185644	0.200	0.056	3.33E-18	CD8-C3
TSC22D3	2.38E-72	0.457777167	0.982	0.935	3.95E-68	CD8-C3
H3F3B	3.23E-97	0.454730445	0.999	0.988	5.37E-93	CD8-C3
BRD2	1.68E-34	0.454210957	0.620	0.489	2.80E-30	CD8-C3
TRMT112	2.37E-42	0.453773178	0.685	0.581	3.94E-38	CD8-C3
NASP	6.79E-26	0.450744205	0.485	0.346	1.13E-21	CD8-C3
PLP2	8.59E-34	0.449105394	0.775	0.640	1.43E-29	CD8-C3
HSP90AB1	3.87E-42	0.434473191	0.941	0.886	6.44E-38	CD8-C3
SERTAD3	9.98E-23	0.434252517	0.284	0.125	1.66E-18	CD8-C3
APOBEC3G	1.09E-37	0.430378929	0.584	0.421	1.81E-33	CD8-C3
KMT5C	1.66E-20	0.428460065	0.264	0.113	2.76E-16	CD8-C3
IER2	5.61E-33	0.426385336	0.842	0.790	9.33E-29	CD8-C3
UBB	1.02E-38	0.425305706	0.896	0.869	1.70E-34	CD8-C3
MLF1	2.85E-22	0.422936442	0.179	0.064	4.74E-18	CD8-C3
KDM6B	2.97E-18	0.421394952	0.466	0.290	4.94E-14	CD8-C3
RBM8A	4.55E-37	0.420354244	0.763	0.678	7.57E-33	CD8-C3
JMJD6	7.45E-26	0.420076165	0.454	0.291	1.24E-21	CD8-C3
NR4A3	1.62E-24	0.418581122	0.336	0.180	2.69E-20	CD8-C3
CITED2	5.16E-21	0.418330058	0.591	0.509	8.58E-17	CD8-C3

ANXA1	1.42E-10	0.417485942	0.763	0.751	2.36E-06	CD8-C3
AC016831.7	3.95E-30	0.415405732	0.637	0.494	6.57E-26	CD8-C3
RGS1	1.49E-22	0.408351231	0.471	0.290	2.47E-18	CD8-C3
TNFSF9	9.17E-24	0.406706104	0.196	0.057	1.52E-19	CD8-C3
KDM2A	9.38E-21	0.405828521	0.445	0.313	1.56E-16	CD8-C3
SRGN	8.70E-41	0.404097868	0.961	0.894	1.45E-36	CD8-C3
CEMIP2	1.81E-18	0.403940674	0.564	0.405	3.01E-14	CD8-C3
BUD31	8.97E-31	0.403379835	0.584	0.462	1.49E-26	CD8-C3
MIDN	2.13E-16	0.400553666	0.339	0.195	3.54E-12	CD8-C3
HLA-DRA	1.14E-16	0.398092327	0.417	0.254	1.89E-12	CD8-C3
KPNA2	2.35E-14	0.397126858	0.356	0.211	3.91E-10	CD8-C3
CALM1	2.66E-40	0.396086939	0.974	0.974	4.42E-36	CD8-C3
RBM39	2.62E-41	0.394577614	0.900	0.839	4.36E-37	CD8-C3
CFAP20	3.86E-18	0.393985221	0.310	0.173	6.41E-14	CD8-C3
HNRNPA0	3.57E-52	0.39310202	0.890	0.825	5.94E-48	CD8-C3
DDX3X	9.52E-21	0.39185573	0.540	0.427	1.58E-16	CD8-C3
SAP18	2.19E-48	0.379060201	0.851	0.784	3.64E-44	CD8-C3
TUBA1C	2.33E-14	0.376694712	0.428	0.323	3.87E-10	CD8-C3
GABARAPL1	2.57E-10	0.376421704	0.541	0.422	4.28E-06	CD8-C3
HLA-DQA1	6.44E-11	0.376296018	0.343	0.192	1.07E-06	CD8-C3
HERPUD1	3.15E-28	0.375104213	0.688	0.534	5.24E-24	CD8-C3
RUNX3	2.35E-26	0.373229889	0.617	0.484	3.91E-22	CD8-C3
DKK3	3.51E-16	0.372951325	0.129	0.027	5.84E-12	CD8-C3
UBE2A	6.37E-20	0.372328413	0.393	0.290	1.06E-15	CD8-C3
HLA-DQB1	2.34E-13	0.371675784	0.311	0.177	3.89E-09	CD8-C3
ARL4A	1.37E-18	0.371372154	0.427	0.290	2.27E-14	CD8-C3
KLF6	6.83E-50	0.36662701	0.960	0.886	1.14E-45	CD8-C3
ITM2A	2.97E-21	0.36492213	0.623	0.535	4.94E-17	CD8-C3
EIF4G2	4.33E-36	0.36230829	0.775	0.716	7.20E-32	CD8-C3
UBE2S	1.59E-19	0.361837366	0.232	0.097	2.65E-15	CD8-C3
SYAP1	7.37E-22	0.356552808	0.415	0.310	1.23E-17	CD8-C3
HLA-DMA	9.38E-20	0.356202533	0.337	0.196	1.56E-15	CD8-C3
H2AFZ	3.23E-22	0.355759599	0.606	0.562	5.37E-18	CD8-C3
GAPDH	1.87E-34	0.354701785	0.990	0.981	3.12E-30	CD8-C3
DDX24	5.22E-30	0.354689077	0.797	0.722	8.68E-26	CD8-C3
PNP	1.90E-18	0.354680851	0.395	0.283	3.15E-14	CD8-C3
B4GALT1	1.09E-19	0.352461988	0.373	0.260	1.81E-15	CD8-C3
MAPRE2	6.89E-14	0.349354622	0.513	0.425	1.15E-09	CD8-C3
RPS26	2.55E-38	0.348875507	0.984	0.969	4.24E-34	CD8-C3
DUSP2	5.34E-178	1.151042183	0.959	0.667	8.89E-174	CD8-C4
GZMK	2.79E-166	1.073379162	0.910	0.394	4.64E-162	CD8-C4
DUSP4	6.08E-93	1.130688396	0.696	0.329	1.01E-88	CD8-C4
TUBA4A	1.20E-82	0.834356457	0.882	0.682	1.99E-78	CD8-C4
ITM2C	8.17E-79	0.944803043	0.681	0.323	1.36E-74	CD8-C4
DDIT4	1.17E-77	0.805512428	0.834	0.651	1.95E-73	CD8-C4
BTG1	5.32E-76	0.529150694	0.995	0.983	8.84E-72	CD8-C4
SRRT	1.27E-74	1.05251316	0.652	0.387	2.11E-70	CD8-C4
CD74	4.40E-72	0.582773786	0.966	0.890	7.32E-68	CD8-C4
CXCR4	6.48E-72	0.6659946	0.903	0.708	1.08E-67	CD8-C4
LDHA	2.60E-71	0.69571105	0.899	0.822	4.32E-67	CD8-C4
CEMIP2	1.16E-62	0.720453866	0.646	0.407	1.92E-58	CD8-C4
COTL1	3.08E-61	0.583016376	0.819	0.640	5.13E-57	CD8-C4

ENC1	4.48E-59	0.748997926	0.396	0.110	7.45E-55	CD8-C4
NR4A2	2.34E-57	0.745488875	0.769	0.478	3.89E-53	CD8-C4
SLC7A5	1.64E-55	0.786513051	0.693	0.437	2.72E-51	CD8-C4
EIF1	7.79E-54	0.360545985	0.999	0.997	1.30E-49	CD8-C4
ZNF331	3.36E-53	0.712738002	0.592	0.334	5.58E-49	CD8-C4
GGA2	2.61E-52	0.655083892	0.487	0.258	4.34E-48	CD8-C4
H3F3B	3.04E-52	0.435009463	0.992	0.989	5.06E-48	CD8-C4
RUNX3	1.54E-49	0.547917835	0.678	0.487	2.55E-45	CD8-C4
CXCR3	1.91E-48	0.644156553	0.726	0.443	3.18E-44	CD8-C4
CD8A	4.65E-47	0.535147157	0.786	0.450	7.74E-43	CD8-C4
UBC	1.92E-46	0.372556828	0.997	0.991	3.20E-42	CD8-C4
SRSF2	8.78E-46	0.505815416	0.809	0.700	1.46E-41	CD8-C4
PIK3R1	1.69E-45	0.575009666	0.591	0.449	2.81E-41	CD8-C4
DDX24	2.70E-44	0.417478394	0.813	0.724	4.49E-40	CD8-C4
HMGB1	9.73E-42	0.40354179	0.928	0.892	1.62E-37	CD8-C4
TSPYL2	1.19E-41	0.625299808	0.706	0.493	1.98E-37	CD8-C4
SRSF5	1.40E-40	0.392415536	0.931	0.880	2.33E-36	CD8-C4
JMJD6	6.46E-40	0.518822731	0.499	0.296	1.07E-35	CD8-C4
PDE4B	7.54E-39	0.579813539	0.579	0.374	1.25E-34	CD8-C4
IDI1	7.85E-38	0.50694351	0.558	0.393	1.31E-33	CD8-C4
RGS1	1.30E-37	0.627620724	0.551	0.293	2.16E-33	CD8-C4
FAM177A1	2.77E-37	0.531325856	0.622	0.463	4.61E-33	CD8-C4
PPP1R14B	3.16E-37	0.649921041	0.327	0.114	5.26E-33	CD8-C4
PSMA7	1.35E-36	0.388452607	0.772	0.710	2.25E-32	CD8-C4
HERPUD1	1.71E-36	0.517396448	0.723	0.539	2.84E-32	CD8-C4
SRSF3	3.45E-35	0.3978787	0.777	0.685	5.73E-31	CD8-C4
HSPA5	5.14E-34	0.473319269	0.894	0.802	8.54E-30	CD8-C4
NR4A3	5.80E-34	0.613346675	0.404	0.183	9.64E-30	CD8-C4
HERPUD2	1.54E-32	0.397127083	0.551	0.441	2.56E-28	CD8-C4
BTG2	3.67E-32	0.504629067	0.636	0.474	6.10E-28	CD8-C4
TGFB1	1.36E-30	0.477535722	0.706	0.601	2.26E-26	CD8-C4
AP3S1	5.00E-30	0.386428561	0.445	0.326	8.31E-26	CD8-C4
LITAF	5.17E-30	0.501981079	0.786	0.670	8.60E-26	CD8-C4
YPEL5	5.69E-30	0.404700578	0.793	0.637	9.47E-26	CD8-C4
ZDHHC7	6.03E-30	0.478038837	0.373	0.201	1.00E-25	CD8-C4
NFATC2	7.75E-30	0.408362104	0.518	0.390	1.29E-25	CD8-C4
CSNK1D	1.76E-29	0.461120969	0.515	0.372	2.93E-25	CD8-C4
SLBP	3.88E-29	0.372363027	0.444	0.364	6.46E-25	CD8-C4
ACHE	4.14E-29	0.529567284	0.157	0.024	6.88E-25	CD8-C4
HNRNPH3	1.00E-28	0.38096233	0.648	0.564	1.67E-24	CD8-C4
SRRM1	1.06E-28	0.365530254	0.809	0.744	1.75E-24	CD8-C4
DNAJB6	7.80E-28	0.360485853	0.707	0.602	1.30E-23	CD8-C4
TOM1	2.08E-27	0.36483343	0.345	0.235	3.46E-23	CD8-C4
CCL3L1	7.98E-27	0.461173414	0.197	0.062	1.33E-22	CD8-C4
KPNA2	1.08E-26	0.397423867	0.370	0.217	1.79E-22	CD8-C4
HAUS3	1.23E-26	0.448821829	0.439	0.289	2.04E-22	CD8-C4
CRTAM	1.93E-26	0.420862721	0.250	0.089	3.21E-22	CD8-C4
CD5	2.34E-26	0.347105272	0.550	0.476	3.89E-22	CD8-C4
HLA-DRB1	3.41E-25	0.366088046	0.733	0.505	5.68E-21	CD8-C4
DKK3	5.48E-25	0.453582509	0.148	0.031	9.11E-21	CD8-C4
ZFP36	6.88E-25	0.361137403	0.946	0.893	1.14E-20	CD8-C4
CMC2	1.31E-24	0.437125407	0.523	0.382	2.18E-20	CD8-C4

IFRD1	4.11E-24	0.430488291	0.527	0.404	6.84E-20	CD8-C4
B3GNT2	5.39E-24	0.445330932	0.385	0.227	8.96E-20	CD8-C4
PDCD1	9.82E-24	0.445632607	0.242	0.087	1.63E-19	CD8-C4
SKI	2.91E-23	0.403209425	0.326	0.186	4.84E-19	CD8-C4
ITM2A	1.10E-22	0.391910103	0.653	0.537	1.82E-18	CD8-C4
CENPN	1.10E-22	0.447438835	0.253	0.101	1.84E-18	CD8-C4
REL	1.24E-22	0.434577525	0.588	0.454	2.06E-18	CD8-C4
SLC1A5	1.88E-22	0.471378009	0.442	0.254	3.13E-18	CD8-C4
CMC1	3.10E-22	0.480584926	0.417	0.241	5.15E-18	CD8-C4
ARID5A	5.69E-22	0.397747007	0.615	0.479	9.46E-18	CD8-C4
SYAP1	7.01E-22	0.386049296	0.423	0.314	1.17E-17	CD8-C4
CARHSP1	4.57E-21	0.385247933	0.290	0.186	7.59E-17	CD8-C4
ELL2	1.09E-20	0.428438392	0.393	0.234	1.81E-16	CD8-C4
TNFSF9	3.40E-20	0.405248336	0.205	0.063	5.65E-16	CD8-C4
PPP1R16B	4.54E-20	0.369738934	0.323	0.193	7.55E-16	CD8-C4
LDLRAD4	4.90E-20	0.388167441	0.336	0.203	8.15E-16	CD8-C4
IL21R	1.57E-19	0.407754042	0.387	0.235	2.61E-15	CD8-C4
SERTAD1	8.37E-18	0.401496109	0.541	0.391	1.39E-13	CD8-C4
CREM	1.72E-17	0.385806702	0.417	0.292	2.85E-13	CD8-C4
METRNL	4.01E-17	0.355821658	0.223	0.088	6.67E-13	CD8-C4
MAP3K8	4.93E-17	0.350143939	0.421	0.321	8.21E-13	CD8-C4
MAFF	6.35E-17	0.413445604	0.305	0.167	1.06E-12	CD8-C4
RNF125	1.71E-16	0.350151017	0.555	0.464	2.84E-12	CD8-C4
GABARAPL1	2.86E-16	0.428078065	0.550	0.427	4.76E-12	CD8-C4
SKIL	6.50E-16	0.3601225	0.391	0.252	1.08E-11	CD8-C4
RBM38	1.67E-15	0.370979836	0.396	0.264	2.77E-11	CD8-C4
JUNB	1.46E-13	0.417638601	0.910	0.878	2.42E-09	CD8-C4
CH25H	1.14E-09	0.474840539	0.131	0.062	1.89E-05	CD8-C4
NKG7	3.21E-199	0.98338727	0.995	0.591	5.34E-195	CD8-C5
CCL5	1.42E-175	0.802021127	1.000	0.771	2.36E-171	CD8-C5
GZMH	1.30E-166	0.932670017	0.939	0.406	2.17E-162	CD8-C5
HLA-DRB1	1.74E-146	0.991699939	0.936	0.482	2.89E-142	CD8-C5
CST7	1.51E-138	0.703672695	0.980	0.673	2.52E-134	CD8-C5
CD8B	3.42E-125	1.010995436	0.832	0.332	5.69E-121	CD8-C5
CD74	3.96E-123	0.832175038	0.993	0.886	6.59E-119	CD8-C5
GZMA	7.50E-121	0.721128715	0.972	0.637	1.25E-116	CD8-C5
HLA-DPB1	3.08E-120	0.765387051	0.918	0.515	5.11E-116	CD8-C5
CD8A	6.19E-119	0.872561406	0.899	0.432	1.03E-114	CD8-C5
HLA-DPA1	2.45E-117	0.781776699	0.913	0.527	4.08E-113	CD8-C5
HLA-DRA	4.87E-91	1.020526816	0.669	0.236	8.09E-87	CD8-C5
HLA-DQA1	3.34E-82	0.825030135	0.589	0.174	5.56E-78	CD8-C5
CMC1	1.85E-80	0.946358953	0.608	0.221	3.07E-76	CD8-C5
KLRD1	9.81E-76	0.80682971	0.601	0.193	1.63E-71	CD8-C5
GZMB	1.33E-67	0.611361989	0.619	0.242	2.21E-63	CD8-C5
PLEK	2.12E-65	0.72326709	0.589	0.209	3.53E-61	CD8-C5
GZMM	1.44E-64	0.534168447	0.863	0.602	2.40E-60	CD8-C5
PFN1	1.51E-64	0.470262811	0.994	0.961	2.51E-60	CD8-C5
FGFBP2	2.02E-63	0.425158051	0.510	0.204	3.36E-59	CD8-C5
ITGB2	3.35E-63	0.494575593	0.919	0.740	5.57E-59	CD8-C5
PTPRCAP	2.04E-58	0.432538564	0.978	0.891	3.39E-54	CD8-C5
ACTB	4.38E-58	0.455489397	1.000	0.996	7.28E-54	CD8-C5
C12orf75	6.52E-58	0.489190592	0.766	0.442	1.08E-53	CD8-C5

FCRL6	2.24E-56	0.570718708	0.477	0.153	3.73E-52	CD8-C5
PRF1	1.33E-55	0.618509948	0.606	0.273	2.21E-51	CD8-C5
HCST	1.52E-53	0.379354012	0.968	0.849	2.53E-49	CD8-C5
CTSW	5.79E-52	0.521016903	0.853	0.556	9.63E-48	CD8-C5
PTPRC	2.52E-50	0.430297098	0.953	0.847	4.18E-46	CD8-C5
LITAF	9.77E-50	0.449540022	0.899	0.658	1.63E-45	CD8-C5
HLA-DQB1	5.58E-48	0.544214376	0.475	0.166	9.27E-44	CD8-C5
ADGRG1	1.71E-45	0.45155003	0.399	0.152	2.85E-41	CD8-C5
ZEB2	2.73E-44	0.359600347	0.535	0.264	4.54E-40	CD8-C5
XCL2	9.01E-44	0.393529655	0.384	0.133	1.50E-39	CD8-C5
FGR	6.59E-42	0.447612619	0.345	0.106	1.10E-37	CD8-C5
AOAH	4.44E-41	0.426308989	0.376	0.127	7.38E-37	CD8-C5
LCP1	1.10E-40	0.37808753	0.908	0.738	1.82E-36	CD8-C5
PPP1CA	3.45E-40	0.405351572	0.776	0.557	5.74E-36	CD8-C5
CLIC1	4.25E-40	0.389918076	0.887	0.714	7.08E-36	CD8-C5
S1PR5	5.08E-40	0.444588754	0.399	0.149	8.45E-36	CD8-C5
RARRES3	2.31E-38	0.435397373	0.817	0.622	3.84E-34	CD8-C5
IL32	3.48E-38	0.386081341	0.973	0.906	5.78E-34	CD8-C5
PSMB9	2.25E-37	0.481151339	0.757	0.507	3.75E-33	CD8-C5
APMAP	2.27E-37	0.425417859	0.681	0.417	3.78E-33	CD8-C5
ANXA6	2.84E-37	0.435608464	0.694	0.459	4.73E-33	CD8-C5
KLRG1	4.03E-37	0.540524335	0.499	0.230	6.70E-33	CD8-C5
CX3CR1	1.03E-36	0.443148536	0.312	0.106	1.71E-32	CD8-C5
APOBEC3G	1.59E-36	0.431834085	0.698	0.415	2.64E-32	CD8-C5
SAMD3	2.71E-36	0.424321917	0.525	0.258	4.50E-32	CD8-C5
CORO1A	2.60E-34	0.394601857	0.934	0.824	4.32E-30	CD8-C5
MATK	3.63E-34	0.39033159	0.583	0.327	6.04E-30	CD8-C5
TPST2	5.47E-34	0.418649099	0.497	0.242	9.10E-30	CD8-C5
SH2D1A	7.02E-34	0.428425043	0.481	0.227	1.17E-29	CD8-C5
HLA-DMA	1.90E-33	0.466338448	0.458	0.189	3.16E-29	CD8-C5
GSTP1	6.10E-33	0.372997549	0.732	0.504	1.01E-28	CD8-C5
ABI3	1.04E-31	0.401274887	0.439	0.198	1.73E-27	CD8-C5
CTSC	3.45E-31	0.417841534	0.699	0.439	5.74E-27	CD8-C5
CAP1	2.48E-30	0.410590099	0.747	0.525	4.13E-26	CD8-C5
PPP1R18	2.62E-29	0.392789081	0.633	0.406	4.36E-25	CD8-C5
MYL12A	3.73E-28	0.372033842	0.884	0.779	6.21E-24	CD8-C5
ACTG1	4.98E-28	0.387420982	0.983	0.936	8.29E-24	CD8-C5
WDR1	7.04E-28	0.357259451	0.683	0.489	1.17E-23	CD8-C5
SLC9A3R1	4.51E-27	0.375894541	0.777	0.588	7.50E-23	CD8-C5
LAG3	1.73E-26	0.348794211	0.470	0.250	2.87E-22	CD8-C5
PYHIN1	1.32E-25	0.349854827	0.422	0.217	2.19E-21	CD8-C5
PSMB10	1.44E-23	0.37825842	0.612	0.386	2.40E-19	CD8-C5
FCGR3A	3.69E-23	0.386054622	0.283	0.108	6.14E-19	CD8-C5
SASH3	6.19E-23	0.369493603	0.555	0.325	1.03E-18	CD8-C5
GIMAP4	2.32E-22	0.446353585	0.525	0.297	3.86E-18	CD8-C5
HLA-DRB5	1.60E-21	0.473257978	0.306	0.139	2.66E-17	CD8-C5
TRBV13	2.09E-20	1.048279819	0.126	0.022	3.47E-16	CD8-C5
TRBC2	1.01E-18	0.368883151	0.733	0.577	1.68E-14	CD8-C5
GZMK	3.14E-15	0.460978586	0.608	0.409	5.22E-11	CD8-C5
TRAV14DV4	1.00E-13	0.673648138	0.126	0.041	1.66E-09	CD8-C5
COTL1	3.66E-13	0.404216052	0.711	0.645	6.08E-09	CD8-C5
TRGV2	9.85E-13	0.42379573	0.176	0.059	1.64E-08	CD8-C5

TRBV28	3.43E-11	0.529425038	0.118	0.067	5.70E-07	CD8-C5
TYROBP	2.69E-250	1.724994515	0.827	0.076	4.48E-246	CD8-C6
GNLY	6.64E-200	1.940406744	0.899	0.234	1.10E-195	CD8-C6
KLRC3	4.52E-187	1.103004665	0.676	0.052	7.51E-183	CD8-C6
NKG7	1.64E-176	1.035951311	0.996	0.616	2.73E-172	CD8-C6
KLRD1	1.32E-174	0.924381109	0.861	0.214	2.19E-170	CD8-C6
GZMB	6.87E-166	1.226988443	0.891	0.261	1.14E-161	CD8-C6
KLRC2	1.43E-160	0.989610852	0.575	0.034	2.38E-156	CD8-C6
CTSW	5.11E-146	0.918215932	0.983	0.572	8.49E-142	CD8-C6
FCGR3A	3.67E-144	1.163794884	0.688	0.111	6.10E-140	CD8-C6
FGFBP2	4.45E-135	0.982396155	0.803	0.218	7.39E-131	CD8-C6
KIR2DL3	1.26E-131	0.823155377	0.475	0.021	2.09E-127	CD8-C6
CST7	6.32E-112	0.754086651	0.979	0.692	1.05E-107	CD8-C6
GZMH	1.49E-107	0.709472853	0.925	0.440	2.47E-103	CD8-C6
AC068775.1	1.80E-106	0.634821676	0.388	0.020	2.99E-102	CD8-C6
TRDC	1.49E-105	0.918129588	0.441	0.034	2.47E-101	CD8-C6
KIR3DL2	3.03E-104	0.686028306	0.462	0.044	5.03E-100	CD8-C6
PRF1	5.43E-103	1.055288884	0.793	0.290	9.04E-99	CD8-C6
KLRF1	2.43E-94	0.706099414	0.470	0.058	4.05E-90	CD8-C6
ADGRG1	7.23E-94	0.712104528	0.631	0.163	1.20E-89	CD8-C6
S1PR5	1.45E-86	0.611071813	0.614	0.161	2.42E-82	CD8-C6
CCL5	2.98E-86	0.596595753	0.991	0.785	4.96E-82	CD8-C6
HOPX	7.31E-86	0.706783884	0.787	0.329	1.22E-81	CD8-C6
IFITM2	8.04E-85	0.649212858	0.988	0.836	1.34E-80	CD8-C6
TRBC1	2.35E-81	0.812339238	0.749	0.317	3.90E-77	CD8-C6
FCRL6	2.59E-79	0.594474336	0.604	0.171	4.31E-75	CD8-C6
LITAF	8.02E-77	0.536895417	0.951	0.672	1.33E-72	CD8-C6
PRSS23	1.22E-73	0.613635468	0.504	0.112	2.03E-69	CD8-C6
SYNGR1	2.52E-72	0.439781696	0.444	0.103	4.19E-68	CD8-C6
TTC38	6.35E-72	0.576053389	0.505	0.119	1.06E-67	CD8-C6
CD300A	1.95E-71	0.482688554	0.457	0.103	3.25E-67	CD8-C6
CD247	3.04E-71	0.589590887	0.920	0.642	5.05E-67	CD8-C6
CD7	9.14E-71	0.574071821	0.925	0.623	1.52E-66	CD8-C6
PLEK	1.38E-70	0.633825643	0.664	0.231	2.29E-66	CD8-C6
APMAP	2.57E-67	0.441677656	0.802	0.432	4.28E-63	CD8-C6
FGR	3.42E-67	0.557266789	0.472	0.119	5.69E-63	CD8-C6
AOAH	6.60E-66	0.547007548	0.528	0.140	1.10E-61	CD8-C6
ZEB2	8.19E-65	0.429642927	0.673	0.278	1.36E-60	CD8-C6
IFITM1	2.75E-63	0.457839975	1.000	0.942	4.57E-59	CD8-C6
NCR3	4.03E-63	0.483628339	0.457	0.119	6.70E-59	CD8-C6
SH2D1B	1.32E-59	0.447740741	0.228	0.006	2.20E-55	CD8-C6
CD63	2.25E-57	0.490993736	0.739	0.381	3.73E-53	CD8-C6
IGFBP7	3.48E-57	0.447628739	0.244	0.024	5.79E-53	CD8-C6
CEP78	1.38E-56	0.40149968	0.394	0.101	2.30E-52	CD8-C6
LYN	1.47E-55	0.425416094	0.316	0.046	2.45E-51	CD8-C6
C1orf21	8.99E-55	0.424288944	0.454	0.125	1.50E-50	CD8-C6
CHST12	2.54E-52	0.348980698	0.576	0.258	4.23E-48	CD8-C6
HLA-DPB1	3.90E-51	0.352143321	0.873	0.541	6.49E-47	CD8-C6
TXK	6.53E-51	0.404817895	0.335	0.066	1.09E-46	CD8-C6
ADAM8	1.25E-49	0.462511093	0.635	0.282	2.08E-45	CD8-C6
MATK	8.06E-48	0.361640739	0.690	0.341	1.34E-43	CD8-C6
HSH2D	3.84E-46	0.416857539	0.398	0.114	6.38E-42	CD8-C6

ITGB7	1.19E-45	0.408711504	0.728	0.395	1.97E-41	CD8-C6
CX3CR1	1.35E-45	0.512980774	0.413	0.117	2.25E-41	CD8-C6
IRF1	2.07E-45	0.498252086	0.807	0.501	3.45E-41	CD8-C6
LGALS1	8.59E-45	0.481958274	0.819	0.498	1.43E-40	CD8-C6
CYBA	2.28E-44	0.473886937	0.942	0.771	3.80E-40	CD8-C6
TPST2	3.37E-44	0.399323473	0.583	0.256	5.61E-40	CD8-C6
ITGB2	3.88E-44	0.421828743	0.938	0.751	6.45E-40	CD8-C6
GSTP1	6.24E-42	0.404344435	0.844	0.516	1.04E-37	CD8-C6
CMC1	1.78E-40	0.395819573	0.546	0.246	2.96E-36	CD8-C6
RASSF1	6.68E-39	0.412861479	0.643	0.326	1.11E-34	CD8-C6
BIN2	1.02E-37	0.384048194	0.760	0.469	1.70E-33	CD8-C6
PYHIN1	1.33E-37	0.379565094	0.526	0.228	2.21E-33	CD8-C6
CLIC3	6.18E-37	0.419260325	0.457	0.175	1.03E-32	CD8-C6
SH2D2A	3.87E-36	0.385612122	0.629	0.321	6.43E-32	CD8-C6
TRG-AS1	1.05E-35	0.419595193	0.394	0.134	1.75E-31	CD8-C6
HLA-DRA	2.25E-34	0.360360531	0.524	0.266	3.74E-30	CD8-C6
FLNA	6.20E-33	0.352501199	0.833	0.580	1.03E-28	CD8-C6
IFITM3	5.18E-31	0.356125509	0.365	0.127	8.62E-27	CD8-C6
SELPLG	1.12E-30	0.359625282	0.665	0.387	1.86E-26	CD8-C6
LINC02446	4.48E-30	0.556773018	0.248	0.045	7.45E-26	CD8-C6
TXNIP	1.76E-29	0.415663983	0.928	0.753	2.92E-25	CD8-C6
FCER1G	1.70E-28	0.48544883	0.157	0.014	2.83E-24	CD8-C6
PLAC8	4.79E-27	0.384844797	0.446	0.186	7.97E-23	CD8-C6
SPON2	7.47E-27	0.440568836	0.399	0.170	1.24E-22	CD8-C6
TRDV1	7.55E-27	1.001870176	0.123	0.016	1.26E-22	CD8-C6
UCP2	3.96E-25	0.371974727	0.701	0.446	6.58E-21	CD8-C6
ZNF683	1.62E-21	0.408899526	0.277	0.101	2.70E-17	CD8-C6
PTGDS	5.19E-20	0.659357092	0.156	0.032	8.63E-16	CD8-C6
MYOM2	1.07E-14	0.410039879	0.161	0.055	1.79E-10	CD8-C6
TRGV3	1.87E-12	0.357785954	0.165	0.062	3.12E-08	CD8-C6
FGFBP2	0	2.005847494	0.967	0.127	0	CD8-C7
NKG7	0	1.609154637	1.000	0.572	0	CD8-C7
GZMH	0	1.429495632	0.993	0.375	0	CD8-C7
GZMB	6573869919905e-	1.687726844	0.936	0.182	3.44E-308	CD8-C7
GNLY	2.83E-292	2.098026478	0.887	0.159	4.71E-288	CD8-C7
KLRD1	1.21E-252	1.530442587	0.854	0.140	2.01E-248	CD8-C7
ADGRG1	3.38E-229	1.345265467	0.762	0.091	5.63E-225	CD8-C7
PRF1	1.44E-221	1.679544909	0.842	0.226	2.40E-217	CD8-C7
CST7	6.26E-212	0.998493927	0.992	0.657	1.04E-207	CD8-C7
CCL5	6.19E-198	0.845865112	1.000	0.760	1.03E-193	CD8-C7
PRSS23	5.37E-173	1.141776462	0.626	0.050	8.92E-169	CD8-C7
PLEK	8.80E-172	1.174115976	0.759	0.168	1.46E-167	CD8-C7
B2M	7.66E-154	0.407941091	1.000	0.999	1.27E-149	CD8-C7
FCRL6	1.21E-150	1.001909972	0.664	0.113	2.01E-146	CD8-C7
FCGR3A	1.71E-146	1.439453281	0.574	0.060	2.84E-142	CD8-C7
CTSW	3.69E-142	0.882517556	0.931	0.531	6.13E-138	CD8-C7
HOPX	2.03E-140	0.93612156	0.786	0.276	3.38E-136	CD8-C7
ITGB2	9.07E-138	0.795989578	0.967	0.725	1.51E-133	CD8-C7
FGR	1.45E-137	0.934944649	0.547	0.068	2.40E-133	CD8-C7
CX3CR1	1.28E-134	1.041008867	0.553	0.064	2.13E-130	CD8-C7
S1PR5	4.56E-129	0.991391452	0.625	0.107	7.59E-125	CD8-C7
IFITM2	6.65E-128	0.812446387	0.977	0.820	1.11E-123	CD8-C7

MYL12A	2.62E-117	0.688446548	0.969	0.762	4.35E-113	CD8-C7
SLC9A3R1	3.18E-117	0.736897113	0.904	0.562	5.29E-113	CD8-C7
HLA-E	2.98E-113	0.517251995	0.997	0.954	4.95E-109	CD8-C7
TTC38	5.56E-113	0.862350149	0.511	0.074	9.25E-109	CD8-C7
LITAF	1.07E-107	0.625563638	0.940	0.641	1.77E-103	CD8-C7
HLA-C	5.78E-107	0.395395342	1.000	0.996	9.62E-103	CD8-C7
HLA-DPB1	5.92E-105	0.570182733	0.873	0.503	9.84E-101	CD8-C7
PFN1	8.91E-104	0.469171934	0.997	0.960	1.48E-99	CD8-C7
TPST2	1.31E-103	0.828628797	0.658	0.208	2.17E-99	CD8-C7
S100A4	1.59E-102	0.5110669	0.994	0.874	2.65E-98	CD8-C7
SPON2	2.75E-101	0.992176554	0.551	0.123	4.57E-97	CD8-C7
RAP1B	1.14E-96	0.694343914	0.885	0.626	1.90E-92	CD8-C7
ASCL2	1.49E-95	0.690771579	0.363	0.025	2.48E-91	CD8-C7
APMAP	1.85E-95	0.671823136	0.778	0.392	3.07E-91	CD8-C7
GZMA	9.14E-95	0.503944817	0.957	0.624	1.52E-90	CD8-C7
C12orf75	3.31E-94	0.640954701	0.805	0.422	5.50E-90	CD8-C7
IFITM1	1.24E-91	0.476894778	0.997	0.935	2.06E-87	CD8-C7
BIN2	9.76E-89	0.70394633	0.807	0.429	1.62E-84	CD8-C7
SH3BGRL3	1.99E-88	0.411154862	0.998	0.977	3.32E-84	CD8-C7
ZEB2	1.81E-87	0.727770382	0.663	0.234	3.01E-83	CD8-C7
MT-CO1	5.68E-87	0.424779247	1.000	0.990	9.44E-83	CD8-C7
C1orf21	1.11E-86	0.702655539	0.476	0.084	1.84E-82	CD8-C7
FLNA	4.61E-86	0.589434518	0.845	0.549	7.66E-82	CD8-C7
CYBA	1.87E-83	0.571514912	0.945	0.751	3.11E-79	CD8-C7
PTPRC	5.83E-81	0.595579304	0.969	0.840	9.70E-77	CD8-C7
ITGB1	1.74E-80	0.665838497	0.720	0.354	2.90E-76	CD8-C7
MYOM2	2.92E-78	0.841738711	0.295	0.025	4.85E-74	CD8-C7
S100A6	2.83E-77	0.38349682	0.992	0.909	4.71E-73	CD8-C7
RASSF1	3.09E-77	0.635175555	0.658	0.287	5.13E-73	CD8-C7
LINC02384	1.79E-76	0.689890719	0.381	0.040	2.98E-72	CD8-C7
CLIC3	1.06E-75	0.75274015	0.492	0.137	1.76E-71	CD8-C7
ANXA6	6.99E-74	0.589728209	0.769	0.438	1.16E-69	CD8-C7
LAIR2	1.23E-73	0.765268852	0.446	0.094	2.04E-69	CD8-C7
ZNF683	3.17E-72	0.835145254	0.366	0.068	5.28E-68	CD8-C7
LILRB1	1.04E-70	0.631568467	0.364	0.035	1.73E-66	CD8-C7
CD8A	9.52E-70	0.669178375	0.729	0.434	1.58E-65	CD8-C7
MATK	1.02E-69	0.59591219	0.677	0.303	1.70E-65	CD8-C7
ITGAM	4.14E-69	0.547856469	0.309	0.033	6.89E-65	CD8-C7
LGALS1	2.18E-67	0.577102918	0.808	0.463	3.62E-63	CD8-C7
ARPC2	2.72E-67	0.426419073	0.976	0.865	4.52E-63	CD8-C7
PSMB9	1.45E-66	0.598413779	0.819	0.487	2.42E-62	CD8-C7
SUN2	7.88E-66	0.618595048	0.778	0.443	1.31E-61	CD8-C7
RARRES3	1.27E-64	0.597432444	0.864	0.606	2.11E-60	CD8-C7
LSP1	2.13E-64	0.430563954	0.964	0.825	3.55E-60	CD8-C7
HMGN3	6.21E-64	0.564183666	0.633	0.303	1.03E-59	CD8-C7
GZMM	7.34E-63	0.454309442	0.865	0.589	1.22E-58	CD8-C7
IL32	5.72E-62	0.386472434	0.980	0.902	9.51E-58	CD8-C7
GSTP1	2.45E-61	0.546881749	0.801	0.484	4.08E-57	CD8-C7
PYHIN1	2.82E-61	0.636514974	0.540	0.191	4.69E-57	CD8-C7
SELPLG	1.74E-60	0.609252466	0.685	0.352	2.89E-56	CD8-C7
PPIA	2.12E-60	0.360163292	0.984	0.921	3.52E-56	CD8-C7
TRG-AS1	1.58E-59	0.748497202	0.424	0.100	2.63E-55	CD8-C7

ACTB	1.89E-59	0.351091167	1.000	0.996	3.13E-55	CD8-C7
UCP2	1.22E-58	0.671490923	0.752	0.410	2.03E-54	CD8-C7
EMP3	4.45E-58	0.434415353	0.935	0.762	7.40E-54	CD8-C7
MYO1F	1.82E-57	0.551521034	0.601	0.270	3.03E-53	CD8-C7
OAZ1	3.30E-57	0.436588592	0.937	0.754	5.49E-53	CD8-C7
PPP1R18	4.37E-57	0.526634419	0.709	0.385	7.27E-53	CD8-C7
PXN	4.99E-57	0.564774002	0.533	0.213	8.29E-53	CD8-C7
CHST12	5.92E-57	0.52412778	0.547	0.225	9.84E-53	CD8-C7
CDK2AP2	3.30E-56	0.562238942	0.521	0.211	5.49E-52	CD8-C7
CD8B	6.38E-56	0.573591317	0.648	0.334	1.06E-51	CD8-C7
PTPRCAP	6.39E-56	0.391684784	0.979	0.887	1.06E-51	CD8-C7
CTSC	6.95E-56	0.538525479	0.757	0.419	1.16E-51	CD8-C7
MYO1G	1.21E-55	0.571419827	0.693	0.367	2.01E-51	CD8-C7
HLA-DPA1	5.18E-54	0.38679587	0.822	0.522	8.62E-50	CD8-C7
TXNIP	1.04E-53	0.483552535	0.941	0.731	1.73E-49	CD8-C7
CLEC2D	1.33E-53	0.594303008	0.621	0.303	2.22E-49	CD8-C7
PPP1CA	3.43E-53	0.477643324	0.826	0.540	5.70E-49	CD8-C7
CD244	1.22E-52	0.484785737	0.316	0.065	2.02E-48	CD8-C7
SYNGR1	2.46E-52	0.5712457	0.352	0.076	4.09E-48	CD8-C7
GPR141	4.32E-52	0.385128815	0.205	0.014	7.19E-48	CD8-C7
CLIC1	2.04E-51	0.411589951	0.917	0.702	3.39E-47	CD8-C7
ZAP70	6.84E-51	0.456505244	0.833	0.610	1.14E-46	CD8-C7
KLRF1	1.05E-50	0.711425969	0.283	0.036	1.75E-46	CD8-C7
HCST	4.65E-50	0.349814396	0.958	0.845	7.73E-46	CD8-C7
RAB37	1.70E-49	0.518864999	0.390	0.117	2.82E-45	CD8-C7
PATL2	1.78E-49	0.519942102	0.387	0.119	2.97E-45	CD8-C7
PSMB10	1.89E-49	0.473189277	0.640	0.372	3.15E-45	CD8-C7
GIMAP7	8.56E-49	0.595201796	0.756	0.423	1.42E-44	CD8-C7
PLAC8	1.01E-48	0.574976497	0.436	0.157	1.68E-44	CD8-C7
ADAM8	2.60E-48	0.526989546	0.546	0.254	4.33E-44	CD8-C7
ABI3	2.62E-48	0.538706466	0.480	0.181	4.36E-44	CD8-C7
SPN	3.59E-48	0.585734171	0.563	0.257	5.98E-44	CD8-C7
ATP6V0E1	4.28E-48	0.398929002	0.724	0.471	7.11E-44	CD8-C7
LCK	5.61E-48	0.421426799	0.867	0.647	9.33E-44	CD8-C7
IL10RA	7.42E-48	0.452456259	0.759	0.491	1.23E-43	CD8-C7
CDC25B	1.12E-47	0.489099888	0.568	0.274	1.86E-43	CD8-C7
TGFBR3	1.20E-47	0.55789007	0.486	0.182	1.99E-43	CD8-C7
RPS6KA1	2.23E-47	0.5049839	0.440	0.167	3.71E-43	CD8-C7
ARPC5	1.42E-46	0.551820441	0.664	0.349	2.36E-42	CD8-C7
SASH3	1.84E-46	0.524517681	0.614	0.306	3.06E-42	CD8-C7
ITGB7	1.91E-46	0.53761709	0.661	0.366	3.18E-42	CD8-C7
TSPAN32	4.06E-46	0.476517189	0.394	0.131	6.74E-42	CD8-C7
CCND3	1.72E-45	0.482667881	0.815	0.545	2.86E-41	CD8-C7
SAMD3	3.01E-45	0.487982081	0.546	0.243	5.01E-41	CD8-C7
STK38	3.33E-45	0.550377988	0.497	0.199	5.54E-41	CD8-C7
PRKCB	8.09E-45	0.447541543	0.379	0.137	1.34E-40	CD8-C7
PLEKHF1	1.16E-44	0.477084233	0.485	0.200	1.93E-40	CD8-C7
PSME1	1.68E-44	0.356815735	0.952	0.812	2.80E-40	CD8-C7
GABARAP	1.81E-44	0.374065932	0.856	0.649	3.01E-40	CD8-C7
TBX21	1.96E-44	0.498179217	0.458	0.162	3.26E-40	CD8-C7
GIMAP4	2.23E-44	0.602925427	0.612	0.275	3.71E-40	CD8-C7
LPCAT1	2.30E-44	0.485275524	0.357	0.096	3.82E-40	CD8-C7

MBP	2.93E-44	0.543849816	0.746	0.468	4.87E-40	CD8-C7
CEP78	6.61E-44	0.518756442	0.321	0.078	1.10E-39	CD8-C7
BTN3A2	1.96E-43	0.507714765	0.529	0.249	3.26E-39	CD8-C7
RAB29	2.23E-43	0.487755018	0.455	0.180	3.70E-39	CD8-C7
ARL6IP5	2.34E-42	0.482460826	0.801	0.551	3.89E-38	CD8-C7
KLRG1	4.08E-42	0.52969748	0.471	0.221	6.79E-38	CD8-C7
AES	1.54E-41	0.402096199	0.846	0.612	2.56E-37	CD8-C7
MT-ND4	2.08E-41	0.404050127	0.952	0.813	3.45E-37	CD8-C7
VCL	2.67E-41	0.493493458	0.321	0.075	4.44E-37	CD8-C7
PAXX	5.76E-41	0.35747627	0.847	0.644	9.57E-37	CD8-C7
ARHGAP25	2.18E-40	0.475270501	0.422	0.163	3.63E-36	CD8-C7
LYN	2.90E-40	0.393225212	0.208	0.030	4.82E-36	CD8-C7
TTC16	2.92E-40	0.406016912	0.238	0.039	4.86E-36	CD8-C7
OSBPL5	4.24E-40	0.393647084	0.220	0.028	7.05E-36	CD8-C7
SYNE1	6.10E-40	0.510676942	0.584	0.293	1.01E-35	CD8-C7
PTGDS	1.95E-39	0.639901932	0.164	0.017	3.24E-35	CD8-C7
MTSS1	2.91E-39	0.448531631	0.284	0.060	4.83E-35	CD8-C7
TRAF3IP3	4.98E-39	0.467028593	0.718	0.422	8.28E-35	CD8-C7
PLEKHG3	7.42E-39	0.377043391	0.241	0.051	1.23E-34	CD8-C7
TYROBP	9.35E-39	1.195934779	0.286	0.063	1.56E-34	CD8-C7
RAC2	9.92E-39	0.415615553	0.935	0.768	1.65E-34	CD8-C7
SH2D2A	1.18E-38	0.471867322	0.564	0.294	1.97E-34	CD8-C7
ANXA4	4.74E-38	0.374866532	0.287	0.088	7.88E-34	CD8-C7
GNPTAB	5.74E-38	0.461206278	0.395	0.156	9.55E-34	CD8-C7
ARRB2	1.03E-37	0.399194846	0.684	0.441	1.71E-33	CD8-C7
PSMB8	2.22E-37	0.383184879	0.724	0.498	3.70E-33	CD8-C7
DHRS7	1.38E-36	0.444943743	0.669	0.403	2.29E-32	CD8-C7
CAP1	3.12E-36	0.43220076	0.753	0.514	5.18E-32	CD8-C7
AC116366.3	3.57E-36	0.511355625	0.472	0.195	5.94E-32	CD8-C7
RAP1GAP2	1.84E-35	0.352264351	0.212	0.039	3.06E-31	CD8-C7
VAMP8	2.03E-35	0.42376061	0.660	0.414	3.37E-31	CD8-C7
PRMT2	3.56E-35	0.459676872	0.711	0.427	5.92E-31	CD8-C7
PYCARD	3.93E-35	0.40140918	0.405	0.178	6.54E-31	CD8-C7
PTMS	4.23E-35	0.419052431	0.356	0.123	7.04E-31	CD8-C7
DBI	1.40E-34	0.35293799	0.717	0.506	2.32E-30	CD8-C7
ACTR3	1.71E-34	0.418481735	0.669	0.440	2.84E-30	CD8-C7
CALHM2	3.33E-34	0.363405903	0.281	0.087	5.54E-30	CD8-C7
HNRNPA2B1	3.50E-34	0.387235958	0.930	0.774	5.82E-30	CD8-C7
APBB1IP	3.81E-34	0.45507491	0.632	0.358	6.33E-30	CD8-C7
AOAH	4.51E-34	0.478150433	0.357	0.118	7.50E-30	CD8-C7
KLRC3	6.22E-34	0.616956503	0.239	0.040	1.03E-29	CD8-C7
SCP2	1.85E-33	0.449617542	0.565	0.309	3.07E-29	CD8-C7
CD53	1.89E-33	0.449662538	0.740	0.502	3.14E-29	CD8-C7
DSTN	2.08E-33	0.372500536	0.625	0.400	3.46E-29	CD8-C7
ITGAL	2.78E-33	0.412881926	0.586	0.324	4.62E-29	CD8-C7
C11orf21	4.62E-33	0.411863265	0.287	0.079	7.69E-29	CD8-C7
GBP5	4.77E-33	0.448545744	0.573	0.309	7.93E-29	CD8-C7
SYTL1	9.58E-33	0.428514023	0.617	0.371	1.59E-28	CD8-C7
PTGER2	1.35E-32	0.390240495	0.390	0.168	2.24E-28	CD8-C7
CD63	1.62E-32	0.416764872	0.601	0.359	2.69E-28	CD8-C7
NDUFB7	1.90E-32	0.389180454	0.601	0.377	3.16E-28	CD8-C7
PTPN4	2.39E-32	0.459337893	0.481	0.230	3.98E-28	CD8-C7

HCLS1	2.95E-32	0.353997807	0.815	0.623	4.90E-28	CD8-C7
SLAMF7	5.01E-32	0.353722612	0.279	0.090	8.33E-28	CD8-C7
USP28	7.53E-32	0.353979994	0.249	0.061	1.25E-27	CD8-C7
CD247	1.12E-31	0.505758095	0.811	0.625	1.85E-27	CD8-C7
RIPOR2	1.96E-31	0.407504001	0.600	0.329	3.27E-27	CD8-C7
CARD16	1.98E-31	0.353003222	0.495	0.271	3.30E-27	CD8-C7
TRBV3-1	2.38E-31	1.200625058	0.176	0.038	3.96E-27	CD8-C7
PRR5L	3.90E-31	0.379802026	0.249	0.052	6.49E-27	CD8-C7
TSPAN2	4.04E-31	0.399668643	0.244	0.045	6.72E-27	CD8-C7
HNRNPF	4.90E-31	0.381150338	0.631	0.410	8.15E-27	CD8-C7
LAG3	7.46E-31	0.388141562	0.499	0.235	1.24E-26	CD8-C7
KIR3DL2	8.95E-31	0.407556505	0.195	0.033	1.49E-26	CD8-C7
TRGV7	1.05E-30	0.471780147	0.156	0.026	1.75E-26	CD8-C7
LINC00861	1.49E-30	0.42298881	0.518	0.259	2.48E-26	CD8-C7
TRAPPC1	2.47E-30	0.383663585	0.642	0.403	4.11E-26	CD8-C7
LYAR	3.02E-30	0.371961555	0.579	0.341	5.02E-26	CD8-C7
MFSD10	7.11E-30	0.361489725	0.552	0.330	1.18E-25	CD8-C7
KLRC4	8.47E-30	0.385758393	0.241	0.056	1.41E-25	CD8-C7
MAT2B	4.52E-29	0.366154838	0.538	0.323	7.52E-25	CD8-C7
ARHGAP30	2.66E-28	0.397202341	0.555	0.317	4.42E-24	CD8-C7
EFHD2	2.71E-28	0.385919629	0.603	0.343	4.51E-24	CD8-C7
LINC01871	5.93E-28	0.370718666	0.363	0.145	9.86E-24	CD8-C7
CYTH4	6.34E-28	0.381270459	0.413	0.192	1.05E-23	CD8-C7
CASP1	9.53E-28	0.416061156	0.439	0.198	1.58E-23	CD8-C7
ADRB2	1.61E-27	0.350314126	0.379	0.161	2.69E-23	CD8-C7
TAP1	1.99E-27	0.378269566	0.579	0.349	3.30E-23	CD8-C7
TRBV4-2	7.30E-27	0.916854246	0.129	0.025	1.21E-22	CD8-C7
PTGDR	7.96E-27	0.433631543	0.371	0.125	1.32E-22	CD8-C7
S1PR1	1.58E-26	0.391551974	0.455	0.238	2.63E-22	CD8-C7
IGFBP7	2.21E-26	0.440355472	0.145	0.012	3.68E-22	CD8-C7
CD300A	5.38E-26	0.452273117	0.279	0.086	8.94E-22	CD8-C7
AC004687.1	3.04E-25	0.36345414	0.431	0.210	5.05E-21	CD8-C7
PLEKHA1	4.95E-25	0.354161861	0.301	0.113	8.23E-21	CD8-C7
BST2	5.46E-25	0.347352264	0.542	0.325	9.07E-21	CD8-C7
TRGV2	1.84E-24	0.530258471	0.205	0.050	3.06E-20	CD8-C7
LAIR1	1.98E-24	0.355638633	0.307	0.115	3.30E-20	CD8-C7
CDC42SE1	3.27E-24	0.349891214	0.499	0.292	5.43E-20	CD8-C7
KLRC2	3.91E-24	0.395154561	0.173	0.026	6.50E-20	CD8-C7
TRAV12-3	4.12E-24	0.835969151	0.152	0.030	6.85E-20	CD8-C7
CAST	7.55E-24	0.386273258	0.670	0.458	1.26E-19	CD8-C7
SRPK2	1.08E-23	0.365956178	0.399	0.193	1.79E-19	CD8-C7
DOK2	1.17E-23	0.349805086	0.648	0.416	1.94E-19	CD8-C7
CAPN2	1.38E-23	0.358303343	0.643	0.436	2.30E-19	CD8-C7
SH3BP5	1.57E-23	0.351279634	0.423	0.217	2.61E-19	CD8-C7
CD320	2.14E-23	0.35291943	0.358	0.171	3.55E-19	CD8-C7
ZBTB38	2.36E-23	0.377859721	0.420	0.207	3.93E-19	CD8-C7
G6PD	2.90E-23	0.37193047	0.334	0.137	4.83E-19	CD8-C7
FUT11	3.02E-23	0.353289746	0.246	0.068	5.03E-19	CD8-C7
WDR1	4.52E-23	0.393566325	0.699	0.478	7.52E-19	CD8-C7
OSTF1	2.17E-22	0.389267435	0.620	0.383	3.60E-18	CD8-C7
TLN1	5.05E-22	0.347900987	0.439	0.242	8.40E-18	CD8-C7
TRGV8	7.49E-22	0.528743939	0.163	0.030	1.25E-17	CD8-C7

ADD3	2.57E-21	0.352586973	0.551	0.339	4.27E-17	CD8-C7
DENND2D	3.98E-21	0.401784186	0.470	0.244	6.62E-17	CD8-C7
TRBC1	5.58E-21	0.657405274	0.442	0.309	9.28E-17	CD8-C7
PTPN6	2.43E-19	0.35655516	0.474	0.283	4.03E-15	CD8-C7
RASA3	2.85E-18	0.350824101	0.559	0.363	4.74E-14	CD8-C7
ITGA4	3.06E-18	0.359969674	0.585	0.360	5.09E-14	CD8-C7
LINC02446	3.43E-15	0.388452849	0.141	0.037	5.70E-11	CD8-C7
TRAV12-1	6.39E-14	0.413282246	0.105	0.038	1.06E-09	CD8-C7
MT1E	1.86E-07	0.372468904	0.203	0.118	0.003088157	CD8-C7
FOS	4.52E-235	1.820046951	0.980	0.629	7.51E-231	CD8-C8
FOSB	5.53E-230	1.713199313	0.903	0.371	9.20E-226	CD8-C8
KLF6	1.16E-218	1.433209658	0.988	0.890	1.93E-214	CD8-C8
JUN	9.56E-193	1.518463134	0.980	0.855	1.59E-188	CD8-C8
CD69	6.27E-182	1.35736103	0.913	0.733	1.04E-177	CD8-C8
DUSP1	1.91E-177	1.257025216	0.967	0.796	3.17E-173	CD8-C8
PPP1R15A	5.72E-131	1.061972541	0.909	0.715	9.51E-127	CD8-C8
TAGAP	1.27E-106	1.125406815	0.709	0.455	2.11E-102	CD8-C8
SRSF7	3.34E-104	0.857111984	0.928	0.864	5.55E-100	CD8-C8
NFKBIA	8.71E-101	0.842084718	0.951	0.807	1.45E-96	CD8-C8
GADD45B	8.42E-96	1.280990161	0.816	0.620	1.40E-91	CD8-C8
IER2	4.40E-93	1.053284895	0.900	0.791	7.32E-89	CD8-C8
NEU1	6.07E-78	1.104163599	0.504	0.186	1.01E-73	CD8-C8
CITED2	6.09E-76	1.061183295	0.718	0.508	1.01E-71	CD8-C8
ATF3	2.30E-74	1.26364538	0.475	0.166	3.82E-70	CD8-C8
EGR1	2.53E-73	1.350862764	0.345	0.062	4.22E-69	CD8-C8
BRD2	1.63E-72	0.872494624	0.662	0.495	2.70E-68	CD8-C8
TSC22D3	1.52E-71	0.655277711	0.947	0.940	2.53E-67	CD8-C8
KLF2	4.27E-70	1.001893978	0.543	0.488	7.11E-66	CD8-C8
NEAT1	1.40E-69	0.838674308	0.646	0.470	2.32E-65	CD8-C8
ATP2B1-AS1	1.55E-66	0.970771768	0.417	0.152	2.58E-62	CD8-C8
JUND	6.53E-66	1.130450596	0.639	0.381	1.09E-61	CD8-C8
CCL4	9.37E-66	2.030675144	0.483	0.381	1.56E-61	CD8-C8
TNFAIP3	2.29E-60	0.712939432	0.924	0.803	3.82E-56	CD8-C8
TNF	6.86E-59	1.436036262	0.431	0.150	1.14E-54	CD8-C8
RSRP1	8.11E-59	0.546488658	0.694	0.669	1.35E-54	CD8-C8
DNAJA1	5.81E-57	0.714162102	0.696	0.584	9.66E-53	CD8-C8
MCL1	1.01E-56	0.649956876	0.781	0.666	1.68E-52	CD8-C8
CCNL1	7.28E-55	0.606762429	0.719	0.651	1.21E-50	CD8-C8
SLC38A2	9.54E-53	0.73746907	0.581	0.391	1.59E-48	CD8-C8
MT-ND2	2.58E-52	0.516831554	0.903	0.914	4.28E-48	CD8-C8
TUBA1A	1.58E-51	0.679886046	0.654	0.609	2.62E-47	CD8-C8
CSKMT	2.46E-50	0.993633487	0.383	0.143	4.09E-46	CD8-C8
KMT2E-AS1	3.26E-50	0.815953748	0.400	0.202	5.41E-46	CD8-C8
DUSP6	7.06E-50	1.013422967	0.329	0.096	1.17E-45	CD8-C8
RHOB	5.05E-49	0.983190206	0.328	0.137	8.40E-45	CD8-C8
PTGER4	1.15E-48	0.668120878	0.638	0.488	1.92E-44	CD8-C8
WSB1	1.28E-47	0.52189561	0.516	0.447	2.12E-43	CD8-C8
MAPRE2	2.45E-47	0.615035943	0.524	0.430	4.07E-43	CD8-C8
TBCC	9.75E-46	0.576725695	0.474	0.361	1.62E-41	CD8-C8
EIF4A2	1.48E-44	0.386303869	0.664	0.680	2.46E-40	CD8-C8
ARRDC3	1.57E-44	0.599463165	0.272	0.109	2.61E-40	CD8-C8
TUBB4B	2.91E-44	0.84340889	0.683	0.542	4.84E-40	CD8-C8

RPL22L1	1.25E-43	0.385746318	0.511	0.518	2.07E-39	CD8-C8
Z93241.1	1.46E-43	1.064622389	0.311	0.100	2.43E-39	CD8-C8
PHLDA1	1.95E-43	0.806929809	0.310	0.107	3.25E-39	CD8-C8
SLC2A3	1.27E-42	0.567988342	0.672	0.622	2.12E-38	CD8-C8
RGCC	2.71E-42	0.798624688	0.713	0.527	4.50E-38	CD8-C8
IFRD1	5.81E-42	0.642465475	0.534	0.406	9.66E-38	CD8-C8
CD44	7.28E-42	0.416191662	0.836	0.814	1.21E-37	CD8-C8
STK17B	6.41E-41	0.393186027	0.718	0.706	1.07E-36	CD8-C8
ID2	2.37E-40	0.615341397	0.706	0.592	3.95E-36	CD8-C8
PNRC1	3.22E-40	0.373528381	0.775	0.815	5.35E-36	CD8-C8
HEXIM1	3.81E-40	0.763196901	0.333	0.140	6.33E-36	CD8-C8
SNHG8	8.88E-40	0.401341416	0.546	0.554	1.48E-35	CD8-C8
POLR2A	1.46E-39	0.66431855	0.359	0.223	2.42E-35	CD8-C8
EIF5	2.07E-39	0.387699508	0.560	0.587	3.44E-35	CD8-C8
H3F3B	2.46E-39	0.419201617	0.981	0.990	4.09E-35	CD8-C8
MALAT1	4.65E-39	0.371272398	1.000	1.000	7.74E-35	CD8-C8
DNAJB1	4.76E-39	0.499583085	0.657	0.692	7.92E-35	CD8-C8
ARL4C	1.74E-38	0.362969669	0.603	0.663	2.89E-34	CD8-C8
CHD2	2.21E-38	0.383879378	0.390	0.360	3.68E-34	CD8-C8
H2AFX	3.23E-38	0.659470895	0.401	0.249	5.37E-34	CD8-C8
HIST2H2AA4	3.41E-38	0.875050275	0.434	0.224	5.67E-34	CD8-C8
DDX3X	1.26E-37	0.525284869	0.503	0.436	2.10E-33	CD8-C8
PPP1R10	4.28E-37	0.593415121	0.265	0.138	7.11E-33	CD8-C8
SOCS1	1.11E-36	0.619659012	0.368	0.228	1.85E-32	CD8-C8
FNBP1	2.13E-36	0.480368138	0.529	0.508	3.54E-32	CD8-C8
ZFP36L1	3.31E-36	0.621153496	0.710	0.643	5.51E-32	CD8-C8
IER5	3.93E-36	0.657352802	0.296	0.126	6.54E-32	CD8-C8
SAT1	1.11E-35	0.596124297	0.626	0.607	1.85E-31	CD8-C8
SCML4	4.42E-35	0.505630157	0.321	0.219	7.34E-31	CD8-C8
AC026979.2	3.23E-33	0.488250222	0.429	0.333	5.37E-29	CD8-C8
IFNG	8.27E-33	0.94500138	0.261	0.131	1.37E-28	CD8-C8
TAF1D	1.20E-32	0.373257064	0.407	0.388	1.99E-28	CD8-C8
SNHG12	2.32E-32	0.553345241	0.342	0.252	3.85E-28	CD8-C8
DNAAF2	3.32E-32	0.54918782	0.315	0.184	5.52E-28	CD8-C8
EIF4A3	3.75E-32	0.513189622	0.463	0.402	6.23E-28	CD8-C8
BTG1	3.96E-32	0.356139827	0.991	0.983	6.58E-28	CD8-C8
TRA2B	6.13E-32	0.366641601	0.599	0.615	1.02E-27	CD8-C8
CCL4L2	4.55E-31	2.134506171	0.258	0.146	7.56E-27	CD8-C8
ZFP36	1.38E-30	0.424288043	0.938	0.895	2.30E-26	CD8-C8
RGS1	4.16E-30	0.733648709	0.435	0.303	6.92E-26	CD8-C8
ANXA2R	1.17E-29	0.416899104	0.289	0.228	1.95E-25	CD8-C8
C12orf57	5.27E-29	0.459024134	0.741	0.841	8.77E-25	CD8-C8
MYLIP	1.77E-28	0.416045371	0.336	0.285	2.95E-24	CD8-C8
SERTAD1	3.25E-28	0.534781375	0.508	0.395	5.41E-24	CD8-C8
NEDD9	4.45E-28	0.42031148	0.334	0.248	7.40E-24	CD8-C8
ATF4	7.42E-28	0.351440923	0.333	0.301	1.23E-23	CD8-C8
BTG2	2.41E-27	0.454812633	0.529	0.482	4.01E-23	CD8-C8
HSPH1	3.46E-27	0.523928956	0.378	0.273	5.75E-23	CD8-C8
ZFAND5	1.05E-26	0.409175816	0.515	0.453	1.75E-22	CD8-C8
TOB1	1.32E-26	0.386487374	0.508	0.463	2.20E-22	CD8-C8
ILF3-DT	1.67E-26	0.480188862	0.285	0.180	2.78E-22	CD8-C8
GATA3	2.77E-26	0.451030255	0.498	0.481	4.60E-22	CD8-C8

JUNB	3.46E-26	0.484914273	0.915	0.879	5.76E-22	CD8-C8
WARS2	3.93E-26	0.488662813	0.194	0.079	6.54E-22	CD8-C8
MT-ATP8	3.43E-25	0.368661711	0.958	0.965	5.71E-21	CD8-C8
PPP1R15B	5.91E-25	0.387741671	0.252	0.166	9.82E-21	CD8-C8
IVNS1ABP	6.00E-25	0.436347826	0.376	0.310	9.98E-21	CD8-C8
CHMP1B	8.67E-25	0.483925283	0.294	0.190	1.44E-20	CD8-C8
RGS2	1.57E-24	0.418457778	0.312	0.236	2.61E-20	CD8-C8
CCL3	1.71E-24	0.986567263	0.175	0.078	2.84E-20	CD8-C8
INTS6	3.21E-24	0.381995381	0.180	0.094	5.33E-20	CD8-C8
KLF10	3.38E-24	0.378289255	0.352	0.311	5.62E-20	CD8-C8
CXCR4	3.59E-24	0.36380996	0.805	0.716	5.97E-20	CD8-C8
TLE4	5.04E-24	0.406785978	0.280	0.213	8.37E-20	CD8-C8
MT-ND1	9.89E-24	0.349442239	0.920	0.943	1.64E-19	CD8-C8
SNHG9	1.09E-23	0.346862624	0.346	0.333	1.81E-19	CD8-C8
XCL2	1.61E-23	0.898614795	0.199	0.151	2.68E-19	CD8-C8
ANKRD28	5.60E-23	0.465532734	0.213	0.118	9.31E-19	CD8-C8
XCL1	1.18E-22	1.048118375	0.229	0.126	1.96E-18	CD8-C8
ZBTB10	2.60E-22	0.411365688	0.157	0.052	4.33E-18	CD8-C8
TMEM107	3.51E-22	0.741528401	0.230	0.112	5.83E-18	CD8-C8
TSPYL2	3.70E-22	0.418989097	0.603	0.501	6.15E-18	CD8-C8
KDM2A	4.31E-22	0.364986572	0.353	0.326	7.17E-18	CD8-C8
AC087623.3	1.19E-21	0.430154357	0.133	0.037	1.98E-17	CD8-C8
SGK1	1.23E-21	0.534810276	0.209	0.100	2.05E-17	CD8-C8
NR4A1	2.09E-21	0.515472026	0.250	0.154	3.48E-17	CD8-C8
NUFIP2	3.43E-21	0.379936922	0.237	0.180	5.71E-17	CD8-C8
SMAD7	9.04E-21	0.37754769	0.237	0.183	1.50E-16	CD8-C8
AHR	1.22E-20	0.44148405	0.338	0.241	2.02E-16	CD8-C8
AC025164.1	1.34E-20	0.485112237	0.258	0.177	2.22E-16	CD8-C8
DYNLL1	1.64E-20	0.348668233	0.398	0.366	2.73E-16	CD8-C8
AC087239.1	1.80E-20	0.494100653	0.213	0.097	2.99E-16	CD8-C8
MID1IP1	9.09E-20	0.390551017	0.162	0.103	1.51E-15	CD8-C8
MYADM	1.56E-19	0.431997807	0.481	0.392	2.59E-15	CD8-C8
AC091271.1	1.64E-19	0.420144419	0.163	0.054	2.73E-15	CD8-C8
WDR74	1.78E-19	0.460515806	0.320	0.307	2.97E-15	CD8-C8
PMAIP1	3.79E-19	0.55750922	0.418	0.334	6.30E-15	CD8-C8
HSPA1B	7.47E-19	0.573128504	0.121	0.036	1.24E-14	CD8-C8
GPR183	8.81E-19	0.35259263	0.371	0.332	1.47E-14	CD8-C8
ARL4A	2.66E-18	0.369835651	0.373	0.300	4.43E-14	CD8-C8
AC020916.1	2.78E-18	0.357506589	0.119	0.031	4.63E-14	CD8-C8
DDIT3	3.66E-18	0.41183195	0.209	0.152	6.08E-14	CD8-C8
NFKBIZ	4.15E-18	0.419764496	0.355	0.299	6.91E-14	CD8-C8
CISH	5.73E-18	0.348261952	0.196	0.139	9.53E-14	CD8-C8
PTCH2	6.17E-18	0.495603039	0.129	0.045	1.03E-13	CD8-C8
PNP	1.00E-17	0.402192749	0.352	0.292	1.66E-13	CD8-C8
AC044849.1	1.54E-17	0.40525617	0.158	0.048	2.56E-13	CD8-C8
SOCS3	2.12E-17	0.375305923	0.225	0.165	3.53E-13	CD8-C8
AC245014.3	4.94E-16	0.52555898	0.136	0.094	8.21E-12	CD8-C8
AC103591.3	9.49E-16	0.648011314	0.210	0.172	1.58E-11	CD8-C8
AC007952.4	9.71E-16	0.554800964	0.160	0.073	1.61E-11	CD8-C8
TOB2	3.67E-15	0.363983146	0.195	0.119	6.11E-11	CD8-C8
CCL3L1	4.47E-15	0.781773515	0.162	0.065	7.43E-11	CD8-C8
UBE2S	5.47E-15	0.383601915	0.192	0.107	9.09E-11	CD8-C8

HIST2H2AA3	3.32E-14	0.42265668	0.174	0.079	5.52E-10	CD8-C8
THAP2	4.93E-14	0.351866872	0.231	0.147	8.21E-10	CD8-C8
CDKN1A	8.32E-14	0.468820523	0.312	0.256	1.38E-09	CD8-C8
TNFSF9	1.46E-13	0.408400615	0.140	0.068	2.43E-09	CD8-C8
GADD45G	1.01E-11	0.4336136	0.142	0.064	1.68E-07	CD8-C8
IER3	3.36E-11	0.413335309	0.119	0.047	5.59E-07	CD8-C8
MT-ND2	3.82E-100	0.71698629	0.881	0.914	6.36E-96	CD8-C9
RPS26	2.11E-98	0.560310326	0.842	0.973	3.50E-94	CD8-C9
CD2	1.40E-97	0.749856034	0.744	0.700	2.33E-93	CD8-C9
PAXX	2.69E-97	0.573705547	0.620	0.670	4.47E-93	CD8-C9
MT-CO1	1.11E-92	0.644688235	0.985	0.992	1.84E-88	CD8-C9
ZAP70	7.57E-92	0.369270797	0.494	0.640	1.26E-87	CD8-C9
MT-ND6	7.88E-90	0.384637591	0.391	0.513	1.31E-85	CD8-C9
ACAP1	1.98E-89	0.464630414	0.502	0.627	3.30E-85	CD8-C9
1-Sep	2.93E-89	0.564292976	0.528	0.587	4.88E-85	CD8-C9
RNF213	4.27E-88	0.516306441	0.427	0.546	7.10E-84	CD8-C9
KIAA1551	4.76E-88	0.721616253	0.460	0.420	7.91E-84	CD8-C9
MPHOSPH8	7.39E-87	0.569617727	0.376	0.396	1.23E-82	CD8-C9
TBC1D10C	1.13E-86	0.555090951	0.542	0.589	1.87E-82	CD8-C9
MT-ND5	3.93E-85	0.617798426	0.906	0.939	6.54E-81	CD8-C9
REX1BD	5.22E-85	0.427264682	0.410	0.517	8.69E-81	CD8-C9
N4BP2L2	2.91E-84	0.635107776	0.384	0.371	4.85E-80	CD8-C9
NEAT1	9.92E-84	0.829319884	0.501	0.477	1.65E-79	CD8-C9
NDUFA3	3.67E-82	0.388092113	0.372	0.494	6.10E-78	CD8-C9
SEM1	7.91E-82	0.394020213	0.315	0.382	1.32E-77	CD8-C9
STK4	5.51E-80	0.526878874	0.437	0.497	9.17E-76	CD8-C9
HLA-F	4.70E-79	0.463750921	0.515	0.618	7.82E-75	CD8-C9
MT-ND4	5.34E-77	0.546226541	0.783	0.831	8.87E-73	CD8-C9
TRABD	1.54E-76	0.40892966	0.381	0.478	2.56E-72	CD8-C9
MT-ATP8	1.55E-76	0.608836583	0.931	0.965	2.58E-72	CD8-C9
TC2N	2.39E-76	0.666291833	0.391	0.372	3.98E-72	CD8-C9
KTN1	2.40E-76	0.461127145	0.346	0.405	4.00E-72	CD8-C9
EVL	3.52E-76	0.535232657	0.687	0.723	5.86E-72	CD8-C9
DDX17	9.82E-76	0.699059634	0.376	0.345	1.63E-71	CD8-C9
TMC8	2.91E-75	0.46525357	0.369	0.423	4.84E-71	CD8-C9
WIPF1	5.82E-75	0.362336109	0.403	0.553	9.68E-71	CD8-C9
NKTR	1.34E-73	0.617222211	0.340	0.354	2.23E-69	CD8-C9
LCP2	4.87E-73	0.387012354	0.451	0.553	8.10E-69	CD8-C9
TAGAP	9.36E-73	0.74480513	0.431	0.467	1.56E-68	CD8-C9
ARHGAP15	1.31E-72	0.440416595	0.340	0.403	2.18E-68	CD8-C9
RASAL3	3.56E-72	0.431859077	0.384	0.463	5.92E-68	CD8-C9
AAK1	1.20E-71	0.539069284	0.312	0.345	2.00E-67	CD8-C9
GNAS	1.68E-71	0.382203068	0.338	0.460	2.79E-67	CD8-C9
ZRANB2	3.36E-71	0.389622716	0.226	0.319	5.59E-67	CD8-C9
NUCKS1	4.35E-70	0.372475867	0.296	0.389	7.23E-66	CD8-C9
CDK5RAP3	1.09E-69	0.429946038	0.235	0.273	1.81E-65	CD8-C9
LENG8	2.93E-69	0.409069429	0.272	0.349	4.86E-65	CD8-C9
MT-ND1	4.59E-69	0.48380673	0.884	0.944	7.64E-65	CD8-C9
ITGAL	5.65E-69	0.440085666	0.295	0.357	9.40E-65	CD8-C9
TCIRG1	6.08E-68	0.374479202	0.316	0.408	1.01E-63	CD8-C9
MT-ND3	1.31E-66	0.582567965	0.954	0.979	2.17E-62	CD8-C9
RHOT2	3.02E-65	0.371103841	0.273	0.346	5.02E-61	CD8-C9

TAF1D	4.68E-65	0.354141325	0.306	0.391	7.78E-61	CD8-C9
RCSD1	6.70E-65	0.384713733	0.292	0.372	1.11E-60	CD8-C9
APBB1IP	4.44E-64	0.409428106	0.332	0.393	7.38E-60	CD8-C9
SNRNP70	3.00E-63	0.35813931	0.236	0.335	4.99E-59	CD8-C9
OGT	7.84E-63	0.508198675	0.219	0.237	1.30E-58	CD8-C9
UTRN	9.51E-62	0.375157863	0.237	0.302	1.58E-57	CD8-C9
SYNRG	8.96E-61	0.50400949	0.250	0.233	1.49E-56	CD8-C9
MACF1	1.09E-60	0.506294021	0.281	0.319	1.82E-56	CD8-C9
SYNE1	5.77E-60	0.433273806	0.275	0.329	9.59E-56	CD8-C9
APOBEC3G	1.13E-59	0.427032788	0.401	0.438	1.88E-55	CD8-C9
GOLGA8B	2.70E-59	0.347630104	0.183	0.240	4.49E-55	CD8-C9
TRBC2	2.95E-59	0.51894531	0.519	0.592	4.90E-55	CD8-C9
LUC7L3	1.10E-58	0.361419583	0.212	0.259	1.84E-54	CD8-C9
TRAF3IP3	1.15E-58	0.400829426	0.394	0.459	1.91E-54	CD8-C9
CKLF	1.33E-58	0.545649769	0.374	0.354	2.21E-54	CD8-C9
PTPN7	2.56E-58	0.525498757	0.302	0.311	4.26E-54	CD8-C9
DOCK8	4.48E-58	0.515785438	0.273	0.281	7.46E-54	CD8-C9
PCM1	5.47E-58	0.446289992	0.247	0.291	9.10E-54	CD8-C9
CCDC107	5.97E-58	0.352950906	0.259	0.323	9.93E-54	CD8-C9
CD52	5.11E-57	0.375392117	0.845	0.937	8.51E-53	CD8-C9
OXNAD1	5.21E-57	0.584061461	0.304	0.262	8.66E-53	CD8-C9
NLRC5	1.21E-56	0.45719108	0.197	0.221	2.01E-52	CD8-C9
ARAP2	1.42E-56	0.417338742	0.255	0.291	2.36E-52	CD8-C9
PCSK7	1.76E-55	0.373206259	0.212	0.258	2.93E-51	CD8-C9
MT-ND4L	4.65E-55	0.483299727	0.965	0.983	7.73E-51	CD8-C9
CCL4	4.24E-52	1.453892122	0.513	0.383	7.05E-48	CD8-C9
IKZF3	4.25E-52	0.518934221	0.264	0.257	7.07E-48	CD8-C9
NT5C	6.72E-52	0.422928903	0.226	0.250	1.12E-47	CD8-C9
ANKRD44	9.86E-52	0.408408317	0.242	0.299	1.64E-47	CD8-C9
SAMD3	1.42E-51	0.605643673	0.311	0.279	2.37E-47	CD8-C9
GRK2	1.78E-51	0.534444426	0.198	0.182	2.96E-47	CD8-C9
IRF3	3.27E-51	0.390083422	0.168	0.190	5.45E-47	CD8-C9
ATM	4.48E-51	0.437702304	0.219	0.254	7.45E-47	CD8-C9
KMT2A	2.10E-50	0.363649977	0.167	0.224	3.50E-46	CD8-C9
AKAP9	3.51E-50	0.371768058	0.242	0.308	5.83E-46	CD8-C9
MALAT1	6.03E-50	0.618423785	0.997	1.000	1.00E-45	CD8-C9
AC004687.1	7.38E-50	0.60895865	0.254	0.236	1.23E-45	CD8-C9
TRAC	8.13E-50	0.679552051	0.328	0.268	1.35E-45	CD8-C9
ANXA2R	1.46E-49	0.545596568	0.256	0.230	2.43E-45	CD8-C9
ITGB2-AS1	1.76E-49	0.530958374	0.225	0.207	2.92E-45	CD8-C9
CTSC	2.33E-49	0.369128202	0.408	0.460	3.87E-45	CD8-C9
ERICH1	3.74E-49	0.448338135	0.212	0.223	6.22E-45	CD8-C9
NLRC3	1.22E-48	0.556572712	0.228	0.205	2.03E-44	CD8-C9
SCML4	2.27E-48	0.564771573	0.255	0.223	3.77E-44	CD8-C9
INPP4B	2.34E-48	0.61691431	0.252	0.204	3.90E-44	CD8-C9
RSBN1L	2.98E-48	0.369143887	0.182	0.203	4.96E-44	CD8-C9
ASH1L	4.05E-48	0.518720026	0.211	0.186	6.74E-44	CD8-C9
NCF1	1.80E-46	0.487301504	0.282	0.265	2.99E-42	CD8-C9
CD69	2.12E-46	0.410782602	0.620	0.743	3.53E-42	CD8-C9
SRSF1	9.35E-46	0.372502017	0.175	0.206	1.55E-41	CD8-C9
ATF7IP	4.35E-45	0.420343896	0.199	0.220	7.23E-41	CD8-C9
DPYD	6.14E-45	0.4013541	0.162	0.170	1.02E-40	CD8-C9

LINC00861	1.64E-44	0.40945319	0.246	0.292	2.73E-40	CD8-C9
GPR174	1.77E-44	0.529964462	0.195	0.168	2.94E-40	CD8-C9
SLFN5	4.93E-44	0.56937367	0.260	0.222	8.21E-40	CD8-C9
NSD1	1.24E-43	0.386822025	0.167	0.178	2.07E-39	CD8-C9
WNK1	5.53E-43	0.403820479	0.188	0.213	9.20E-39	CD8-C9
THEMIS	1.04E-42	0.476097042	0.249	0.235	1.73E-38	CD8-C9
CREBZF	8.24E-42	0.361285106	0.171	0.185	1.37E-37	CD8-C9
CD27	1.29E-41	0.418733677	0.273	0.293	2.14E-37	CD8-C9
WASHC1	3.02E-41	0.460191343	0.210	0.203	5.02E-37	CD8-C9
BCL2	3.66E-41	0.384002644	0.157	0.172	6.09E-37	CD8-C9
PLEC	5.25E-41	0.39681999	0.246	0.291	8.74E-37	CD8-C9
KMT2E-AS1	5.56E-41	0.448720759	0.197	0.211	9.25E-37	CD8-C9
UPF2	7.80E-41	0.383685056	0.146	0.159	1.30E-36	CD8-C9
DGKA	7.85E-41	0.373088345	0.180	0.208	1.31E-36	CD8-C9
ZNF276	1.69E-40	0.432084525	0.172	0.165	2.81E-36	CD8-C9
BTN3A2	2.06E-40	0.396817073	0.256	0.284	3.43E-36	CD8-C9
PARVG	2.21E-40	0.395230319	0.218	0.229	3.68E-36	CD8-C9
CISH	9.13E-40	0.73542118	0.247	0.139	1.52E-35	CD8-C9
ANKRD36	1.11E-39	0.409656679	0.132	0.124	1.85E-35	CD8-C9
VPS13C	3.78E-39	0.451072601	0.234	0.256	6.29E-35	CD8-C9
SIT1	7.51E-39	0.427283229	0.244	0.238	1.25E-34	CD8-C9
DENND2D	2.35E-38	0.413151548	0.262	0.271	3.90E-34	CD8-C9
ZBTB20	3.24E-38	0.377458607	0.171	0.184	5.38E-34	CD8-C9
ANKRD36C	3.46E-37	0.355880591	0.136	0.151	5.75E-33	CD8-C9
C6orf62	6.42E-37	0.37893034	0.166	0.166	1.07E-32	CD8-C9
TBCD	1.72E-36	0.379473385	0.174	0.177	2.86E-32	CD8-C9
SLF1	9.41E-36	0.469450225	0.163	0.126	1.56E-31	CD8-C9
STX16	1.65E-35	0.360101595	0.138	0.161	2.75E-31	CD8-C9
SLFN12L	1.91E-35	0.533380084	0.186	0.138	3.17E-31	CD8-C9
GIMAP4	2.82E-35	0.352845478	0.307	0.316	4.69E-31	CD8-C9
MT-CO2	4.83E-35	0.365841573	0.984	0.995	8.02E-31	CD8-C9
CCS	2.07E-32	0.356435398	0.156	0.163	3.44E-28	CD8-C9
ADCY7	3.67E-32	0.352780219	0.131	0.136	6.10E-28	CD8-C9
GIMAP7	5.44E-32	0.397939267	0.479	0.462	9.04E-28	CD8-C9
MT-CYB	4.75E-31	0.384739898	0.955	0.980	7.90E-27	CD8-C9
HIST2H2AA4	5.19E-31	0.395226317	0.202	0.234	8.62E-27	CD8-C9
UBXN11	9.54E-31	0.387750278	0.118	0.125	1.59E-26	CD8-C9
GZMA	1.94E-30	0.374720798	0.696	0.663	3.22E-26	CD8-C9
SLAMF1	1.19E-29	0.432185996	0.160	0.124	1.98E-25	CD8-C9
PTGDR	1.72E-29	0.418413848	0.180	0.154	2.85E-25	CD8-C9
PDE7A	2.55E-29	0.395692195	0.127	0.100	4.23E-25	CD8-C9
SENP7	9.96E-29	0.428191343	0.156	0.123	1.66E-24	CD8-C9
CHD9	1.15E-28	0.348084182	0.132	0.127	1.91E-24	CD8-C9
CD84	2.44E-28	0.361996308	0.124	0.112	4.06E-24	CD8-C9
LYST	3.03E-28	0.356301236	0.138	0.152	5.04E-24	CD8-C9
ANO9	3.24E-28	0.358511978	0.127	0.112	5.39E-24	CD8-C9
NBPF19	1.25E-26	0.358163435	0.128	0.116	2.07E-22	CD8-C9
CSKMT	1.54E-26	0.540714983	0.193	0.153	2.56E-22	CD8-C9
CCL4L2	2.33E-25	1.148634251	0.262	0.148	3.88E-21	CD8-C9
GZMK	1.66E-24	0.558585339	0.529	0.423	2.76E-20	CD8-C9
UBASH3A	7.77E-24	0.367692719	0.155	0.120	1.29E-19	CD8-C9
MIAT	1.03E-23	0.377231266	0.107	0.093	1.71E-19	CD8-C9

PILRB	9.69E-23	0.347804482	0.107	0.094	1.61E-18	CD8-C9
TRAF5	3.38E-21	0.363915888	0.142	0.122	5.61E-17	CD8-C9
CAPG	9.03E-21	0.361728502	0.200	0.180	1.50E-16	CD8-C9
TNF	9.79E-21	0.816204494	0.225	0.161	1.63E-16	CD8-C9
EPHA1	3.27E-18	0.357189584	0.116	0.072	5.45E-14	CD8-C9
FOS	9.16E-18	0.353298586	0.669	0.644	1.52E-13	CD8-C9
CXCR6	1.15E-17	0.409929627	0.120	0.072	1.92E-13	CD8-C9
IFNG	1.45E-15	0.43979441	0.149	0.137	2.41E-11	CD8-C9
XCL1	9.42E-11	0.357226558	0.163	0.130	1.57E-06	CD8-C9
EGR1	2.25E-09	0.455888549	0.103	0.074	3.74E-05	CD8-C9
