# Supplementary material for: Targeting pro-inflammatory T cells as a novel therapeutic approach to potentially resolve atherosclerosis in humans
Source: Cell Res. 2024 Mar 15;34(6):407–27. doi: 10.1038/s41422-024-00945-0 (PMC11143203; doi:10.1038/s41422-024-00945-0)
Supplement: Supplementary file 14 — Supplementary information, Table S2 [file 41422_2024_945_MOESM14_ESM.pdf]

Supplementary information, Table S2. Differentially expressed genes (DEGs) of identified T cell clusters in integrated T cell dataset.

Gene	*P* value	avg_logFC	pct.1	pct.2	adjusted *P* value	cluster
CCR7	2.052E-132	1.712349683	0.630	0.131	4.97E-128	Meta_CD4_C1
RPL32	1.30E-113	0.694201288	0.996	0.992	3.14E-109	Meta_CD4_C1
RPL11	9.19E-110	0.620197755	0.996	0.994	2.22E-105	Meta_CD4_C1
RPS8	6.70E-102	0.701601823	0.995	0.988	1.62E-97	Meta_CD4_C1
RPS13	2.36E-91	0.660038185	0.986	0.979	5.72E-87	Meta_CD4_C1
RPS3A	1.72E-85	0.715833646	0.987	0.977	4.17E-81	Meta_CD4_C1
RPS12	1.64E-81	0.637161201	0.995	0.994	3.98E-77	Meta_CD4_C1
EEF1B2	3.32E-78	0.687480168	0.936	0.896	8.04E-74	Meta_CD4_C1
RPL34	4.14E-78	0.613432267	0.995	0.993	1.00E-73	Meta_CD4_C1
RPL9	1.81E-76	0.661018359	0.984	0.972	4.37E-72	Meta_CD4_C1
RPL13	7.37E-76	0.591610443	0.997	0.997	1.78E-71	Meta_CD4_C1
RPL19	2.01E-75	0.538426273	0.995	0.994	4.88E-71	Meta_CD4_C1
PABPC1	8.41E-72	0.72948549	0.944	0.892	2.04E-67	Meta_CD4_C1
RPS5	9.97E-72	0.611995062	0.977	0.969	2.41E-67	Meta_CD4_C1
LEF1	1.54E-71	1.106953578	0.481	0.130	3.74E-67	Meta_CD4_C1
RPL22	5.07E-69	0.619978494	0.907	0.889	1.23E-64	Meta_CD4_C1
SELL	1.12E-68	1.408862786	0.502	0.163	2.71E-64	Meta_CD4_C1
RPS23	4.10E-67	0.53285466	0.986	0.978	9.93E-63	Meta_CD4_C1
RPL5	2.68E-66	0.595571673	0.978	0.960	6.50E-62	Meta_CD4_C1
RPL18	4.62E-65	0.471464336	0.990	0.985	1.12E-60	Meta_CD4_C1
RPS14	5.44E-64	0.528827152	0.993	0.995	1.32E-59	Meta_CD4_C1
RPS6	1.10E-63	0.717052503	0.995	0.992	2.67E-59	Meta_CD4_C1
RPS27A	1.54E-62	0.511205631	0.996	0.994	3.72E-58	Meta_CD4_C1
RPS18	1.10E-61	0.618624196	0.996	0.994	2.66E-57	Meta_CD4_C1
TCF7	5.88E-60	1.145740801	0.546	0.204	1.42E-55	Meta_CD4_C1
RPL3	6.01E-59	0.623642766	0.992	0.986	1.46E-54	Meta_CD4_C1
RPS4X	6.26E-59	0.494299623	0.995	0.992	1.52E-54	Meta_CD4_C1
RPL30	3.57E-58	0.531284925	0.992	0.987	8.65E-54	Meta_CD4_C1
RPL10	3.42E-57	0.448469327	0.998	0.998	8.28E-53	Meta_CD4_C1
RPL18A	2.68E-55	0.494905753	0.991	0.989	6.50E-51	Meta_CD4_C1
RPL36	1.78E-54	0.530029295	0.983	0.978	4.32E-50	Meta_CD4_C1
MAL	4.77E-53	0.910699295	0.417	0.111	1.15E-48	Meta_CD4_C1
RPL29	9.92E-53	0.466468066	0.981	0.978	2.40E-48	Meta_CD4_C1
RPL35A	1.98E-52	0.457753617	0.986	0.986	4.80E-48	Meta_CD4_C1
RPL12	1.83E-49	0.433126685	0.988	0.989	4.43E-45	Meta_CD4_C1
RPS3	8.16E-49	0.466933044	0.996	0.994	1.98E-44	Meta_CD4_C1
RPS25	3.87E-48	0.461899628	0.980	0.968	9.38E-44	Meta_CD4_C1

FXYD5	5.46E-47	0.588287955	0.920	0.850	1.32E-42	Meta_CD4_C1
SARAF	9.68E-46	0.553387622	0.962	0.924	2.34E-41	Meta_CD4_C1
RPLP2	1.24E-45	0.497924851	0.992	0.993	3.00E-41	Meta_CD4_C1
LDHB	2.98E-45	0.653847743	0.855	0.743	7.22E-41	Meta_CD4_C1
RPL8	1.41E-44	0.459535454	0.992	0.986	3.41E-40	Meta_CD4_C1
RPL4	1.47E-44	0.522122483	0.953	0.925	3.57E-40	Meta_CD4_C1
RPL21	4.22E-44	0.65395482	0.982	0.984	1.02E-39	Meta_CD4_C1
LTB	8.97E-41	0.476381548	0.848	0.599	2.17E-36	Meta_CD4_C1
RPS9	3.77E-39	0.458597194	0.986	0.979	9.12E-35	Meta_CD4_C1
ACTN1	1.07E-38	0.55811669	0.191	0.023	2.59E-34	Meta_CD4_C1
RPL10A	1.73E-38	0.51296652	0.980	0.975	4.19E-34	Meta_CD4_C1
SCML1	2.84E-38	0.509004713	0.169	0.018	6.89E-34	Meta_CD4_C1
RPL7	7.39E-38	0.664689966	0.958	0.935	1.79E-33	Meta_CD4_C1
EIF3E	8.16E-38	0.580510932	0.781	0.673	1.98E-33	Meta_CD4_C1
PASK	1.21E-37	0.825993003	0.328	0.121	2.92E-33	Meta_CD4_C1
RPL37	1.10E-36	0.446349672	0.975	0.971	2.66E-32	Meta_CD4_C1
RPS15A	1.64E-36	0.376330497	0.995	0.993	3.96E-32	Meta_CD4_C1
NACA	2.68E-36	0.37942901	0.964	0.955	6.49E-32	Meta_CD4_C1
IL7R	3.20E-35	0.547019573	0.829	0.557	7.76E-31	Meta_CD4_C1
RPL14	4.62E-35	0.399018399	0.985	0.979	1.12E-30	Meta_CD4_C1
RPS15	1.56E-34	0.324403401	0.993	0.993	3.77E-30	Meta_CD4_C1
TMEM123	9.86E-34	0.581973937	0.627	0.460	2.39E-29	Meta_CD4_C1
RPS16	2.01E-33	0.443618067	0.985	0.983	4.87E-29	Meta_CD4_C1
RPS28	4.04E-33	0.468710832	0.971	0.964	9.78E-29	Meta_CD4_C1
C1orf162	8.65E-32	0.717826978	0.266	0.064	2.10E-27	Meta_CD4_C1
TSHZ2	9.51E-32	0.601786078	0.196	0.047	2.30E-27	Meta_CD4_C1
RPL39	7.01E-31	0.491725621	0.892	0.894	1.70E-26	Meta_CD4_C1
RPSA	5.57E-30	0.387366222	0.971	0.961	1.35E-25	Meta_CD4_C1
RPL38	1.96E-29	0.467660703	0.936	0.931	4.76E-25	Meta_CD4_C1
S1PR1	1.05E-28	0.570997144	0.294	0.116	2.54E-24	Meta_CD4_C1
RPS27	4.28E-28	0.36642663	0.997	0.995	1.04E-23	Meta_CD4_C1
TPT1	4.94E-28	0.375273044	0.990	0.985	1.20E-23	Meta_CD4_C1
EEF2	6.47E-28	0.362975093	0.927	0.901	1.57E-23	Meta_CD4_C1
JUNB	7.29E-28	0.771642401	0.936	0.883	1.77E-23	Meta_CD4_C1
RPS21	1.19E-27	0.409744667	0.953	0.947	2.88E-23	Meta_CD4_C1
KLF2	3.26E-26	1.100828892	0.502	0.302	7.90E-22	Meta_CD4_C1
SOCS3	4.04E-26	0.771082569	0.496	0.303	9.79E-22	Meta_CD4_C1
SESN3	5.16E-26	0.621239497	0.234	0.096	1.25E-21	Meta_CD4_C1
NOSIP	1.05E-25	0.575719713	0.507	0.396	2.54E-21	Meta_CD4_C1
PLAC8	1.32E-25	0.580842399	0.300	0.132	3.20E-21	Meta_CD4_C1

NOP53	1.44E-25	0.40240028	0.705	0.697	3.49E-21	Meta_CD4_C1
CMTM8	9.96E-25	0.503721271	0.211	0.086	2.41E-20	Meta_CD4_C1
FAAH2	1.07E-24	0.490567359	0.179	0.049	2.60E-20	Meta_CD4_C1
RPL24	2.01E-24	0.343786626	0.971	0.968	4.88E-20	Meta_CD4_C1
TOMM7	1.80E-23	0.355640899	0.913	0.894	4.37E-19	Meta_CD4_C1
LDLRAP1	5.09E-23	0.503957609	0.301	0.145	1.23E-18	Meta_CD4_C1
ZFAS1	8.92E-23	0.386648015	0.741	0.667	2.16E-18	Meta_CD4_C1
SERINC5	1.13E-22	0.482970325	0.273	0.139	2.73E-18	Meta_CD4_C1
EEF1G	1.16E-22	0.525907658	0.679	0.647	2.82E-18	Meta_CD4_C1
SLC2A3	1.14E-21	0.676209507	0.717	0.641	2.76E-17	Meta_CD4_C1
TRABD2A	2.14E-21	0.418211917	0.171	0.047	5.18E-17	Meta_CD4_C1
RNASET2	5.00E-21	0.469526158	0.614	0.492	1.21E-16	Meta_CD4_C1
RPL6	1.63E-20	0.319254528	0.977	0.970	3.95E-16	Meta_CD4_C1
EIF4B	3.84E-20	0.389084719	0.684	0.604	9.29E-16	Meta_CD4_C1
EEF1A1	5.40E-20	0.443615859	0.921	0.911	1.31E-15	Meta_CD4_C1
SPINT2	1.25E-19	0.458279879	0.260	0.105	3.03E-15	Meta_CD4_C1
CXCR5	2.05E-19	0.332847374	0.107	0.029	4.97E-15	Meta_CD4_C1
RPS2	1.48E-18	0.469849602	0.991	0.983	3.59E-14	Meta_CD4_C1
MALAT1	2.20E-18	0.399341416	1.000	1.000	5.32E-14	Meta_CD4_C1
RPL13A	2.21E-18	0.634258111	0.987	0.988	5.34E-14	Meta_CD4_C1
IFNGR2	4.56E-18	0.332844829	0.137	0.033	1.10E-13	Meta_CD4_C1
GPR183	6.27E-18	0.702112529	0.574	0.398	1.52E-13	Meta_CD4_C1
LINC00402	9.71E-18	0.416719891	0.153	0.048	2.35E-13	Meta_CD4_C1
IL6R	1.52E-17	0.495562929	0.213	0.069	3.69E-13	Meta_CD4_C1
RPL23A	1.75E-17	0.329765213	0.986	0.988	4.23E-13	Meta_CD4_C1
RPS7	2.68E-17	0.25420085	0.986	0.984	6.48E-13	Meta_CD4_C1
FBLN7	4.10E-17	0.361280104	0.190	0.094	9.92E-13	Meta_CD4_C1
FCMR	5.91E-17	0.435854246	0.488	0.371	1.43E-12	Meta_CD4_C1
NDFIP1	2.11E-16	0.453772654	0.585	0.492	5.12E-12	Meta_CD4_C1
DGKA	4.88E-16	0.437479775	0.372	0.266	1.18E-11	Meta_CD4_C1
RPS20	6.60E-16	0.657276174	0.910	0.890	1.60E-11	Meta_CD4_C1
LYPD3	1.54E-15	0.308479169	0.115	0.024	3.72E-11	Meta_CD4_C1
RIC3	2.51E-15	0.378889465	0.178	0.072	6.07E-11	Meta_CD4_C1
RPL36A	6.92E-15	0.415651824	0.823	0.792	1.68E-10	Meta_CD4_C1
APP	8.66E-15	0.354560716	0.158	0.055	2.10E-10	Meta_CD4_C1
RPL35	1.32E-14	0.301332167	0.971	0.972	3.19E-10	Meta_CD4_C1
TIAM1	1.34E-14	0.296304493	0.137	0.048	3.25E-10	Meta_CD4_C1
ICAM2	2.37E-14	0.435487232	0.345	0.210	5.74E-10	Meta_CD4_C1
RPL26	2.99E-14	0.325704006	0.981	0.978	7.23E-10	Meta_CD4_C1
RIPOR2	3.68E-14	0.394088235	0.321	0.243	8.90E-10	Meta_CD4_C1

STAT3	3.79E-14	0.309469718	0.579	0.509	9.18E-10	Meta_CD4_C1
FOXP1	4.26E-14	0.477313498	0.516	0.363	1.03E-09	Meta_CD4_C1
PIK3IP1	4.68E-14	0.518180329	0.622	0.477	1.13E-09	Meta_CD4_C1
ADD3	5.16E-14	0.373482164	0.372	0.265	1.25E-09	Meta_CD4_C1
AP3M2	7.97E-14	0.424584313	0.240	0.131	1.93E-09	Meta_CD4_C1
IL6ST	8.32E-14	0.524822438	0.370	0.214	2.01E-09	Meta_CD4_C1
TXK	1.89E-13	0.382814311	0.203	0.100	4.57E-09	Meta_CD4_C1
RPL27	2.34E-13	0.375921151	0.954	0.954	5.66E-09	Meta_CD4_C1
AC119396.1	3.22E-13	0.351367793	0.150	0.049	7.81E-09	Meta_CD4_C1
CYSLTR1	3.47E-13	0.292792182	0.109	0.035	8.41E-09	Meta_CD4_C1
TMEM243	3.82E-13	0.324650562	0.420	0.333	9.25E-09	Meta_CD4_C1
FXYD7	4.76E-13	0.383749773	0.134	0.038	1.15E-08	Meta_CD4_C1
RPS29	9.93E-13	0.382674026	0.973	0.973	2.40E-08	Meta_CD4_C1
RPL31	1.27E-12	0.495903294	0.955	0.955	3.06E-08	Meta_CD4_C1
ARMH1	2.03E-12	0.405877508	0.180	0.077	4.92E-08	Meta_CD4_C1
SLC40A1	3.42E-12	0.295684056	0.112	0.030	8.28E-08	Meta_CD4_C1
FHIT	3.43E-12	0.297764812	0.105	0.020	8.31E-08	Meta_CD4_C1
RASGRP2	4.56E-12	0.396134952	0.303	0.190	1.10E-07	Meta_CD4_C1
LINC01550	5.02E-12	0.293816092	0.183	0.107	1.22E-07	Meta_CD4_C1
PFDN5	6.83E-12	0.284991553	0.949	0.937	1.65E-07	Meta_CD4_C1
EIF2S3	8.52E-12	0.313979578	0.474	0.394	2.06E-07	Meta_CD4_C1
AREG	1.02E-11	0.27178135	0.300	0.171	2.46E-07	Meta_CD4_C1
EEF1D	1.43E-11	0.259810854	0.967	0.962	3.46E-07	Meta_CD4_C1
UBA52	1.77E-11	0.263041915	0.964	0.966	4.28E-07	Meta_CD4_C1
CD55	2.28E-11	0.463031384	0.369	0.264	5.52E-07	Meta_CD4_C1
BEX2	3.06E-11	0.339891581	0.205	0.101	7.42E-07	Meta_CD4_C1
SF1	3.20E-11	0.342289827	0.710	0.667	7.76E-07	Meta_CD4_C1
RPLP0	8.98E-11	0.256233581	0.963	0.948	2.17E-06	Meta_CD4_C1
CTSL	1.00E-10	0.302261094	0.112	0.036	2.42E-06	Meta_CD4_C1
RPS11	1.70E-10	0.332901106	0.938	0.930	4.11E-06	Meta_CD4_C1
TESPA1	1.99E-10	0.305219	0.252	0.174	4.81E-06	Meta_CD4_C1
ITGA6	2.77E-10	0.252715481	0.124	0.055	6.71E-06	Meta_CD4_C1
MAML2	2.98E-10	0.264680771	0.185	0.112	7.21E-06	Meta_CD4_C1
RASA3	3.53E-10	0.341295486	0.294	0.189	8.55E-06	Meta_CD4_C1
KLF3	4.50E-10	0.366327016	0.275	0.167	1.09E-05	Meta_CD4_C1
BIRC3	8.50E-10	0.434197372	0.383	0.302	2.06E-05	Meta_CD4_C1
RPL37A	1.22E-09	0.299125965	0.962	0.964	2.95E-05	Meta_CD4_C1
CHMP7	1.79E-09	0.290056299	0.223	0.164	4.35E-05	Meta_CD4_C1
GLTSCR2	3.59E-09	0.676974902	0.170	0.106	8.69E-05	Meta_CD4_C1
RPL23	3.76E-09	0.340143727	0.830	0.824	9.10E-05	Meta_CD4_C1

C6orf48	4.31E-09	0.471003316	0.412	0.297	1.04E-04	Meta_CD4_C1
SNHG8	4.62E-09	0.369202816	0.635	0.540	1.12E-04	Meta_CD4_C1
ATM	5.12E-09	0.3936488	0.340	0.248	1.24E-04	Meta_CD4_C1
ABLIM1	9.80E-09	0.37896407	0.394	0.304	2.37E-04	Meta_CD4_C1
LEPROTL1	1.93E-08	0.263134926	0.760	0.725	4.67E-04	Meta_CD4_C1
BACH2	2.03E-08	0.275821159	0.172	0.084	4.91E-04	Meta_CD4_C1
SFXN1	2.18E-08	0.272136998	0.328	0.262	5.28E-04	Meta_CD4_C1
SH3YL1	2.18E-08	0.376410704	0.225	0.114	5.29E-04	Meta_CD4_C1
RPS4Y1	2.46E-08	0.291028839	0.546	0.538	5.96E-04	Meta_CD4_C1
CAMK4	4.26E-08	0.274413405	0.490	0.423	1.03E-03	Meta_CD4_C1
TXNIP	4.39E-08	0.369868431	0.817	0.749	1.06E-03	Meta_CD4_C1
ANP32B	4.48E-08	0.270583125	0.676	0.629	1.09E-03	Meta_CD4_C1
PHLDB3	5.35E-08	0.261479951	0.149	0.067	1.30E-03	Meta_CD4_C1
ANK3	6.08E-08	0.266920727	0.138	0.059	1.47E-03	Meta_CD4_C1
SGK1	7.73E-08	0.263167551	0.171	0.098	1.87E-03	Meta_CD4_C1
THEM4	8.66E-08	0.295214018	0.267	0.177	2.10E-03	Meta_CD4_C1
INPP4B	8.90E-08	0.359439917	0.337	0.248	2.15E-03	Meta_CD4_C1
RPL17	1.13E-07	0.295505509	0.873	0.859	2.75E-03	Meta_CD4_C1
CD28	1.45E-07	0.287639873	0.327	0.261	3.50E-03	Meta_CD4_C1
SORL1	1.48E-07	0.314872404	0.282	0.190	3.57E-03	Meta_CD4_C1
LINC00861	2.30E-07	0.332069438	0.288	0.208	5.56E-03	Meta_CD4_C1
SYPL1	3.35E-07	0.266040885	0.330	0.255	8.11E-03	Meta_CD4_C1
LY96	5.30E-07	0.303046473	0.123	0.056	1.28E-02	Meta_CD4_C1
RPS17	9.63E-07	0.65328451	0.767	0.737	2.33E-02	Meta_CD4_C1
CXCR4	1.08E-06	0.314227358	0.894	0.827	2.60E-02	Meta_CD4_C1
SATB1	1.15E-06	0.314359122	0.283	0.200	2.78E-02	Meta_CD4_C1
GNB2L1	1.43E-06	0.60543087	0.207	0.163	3.46E-02	Meta_CD4_C1
CD40LG	1.75E-06	0.340918821	0.329	0.206	4.25E-02	Meta_CD4_C1
SMDT1	1.97E-06	0.253795041	0.590	0.548	4.77E-02	Meta_CD4_C1
TNFAIP8	2.48E-06	0.266839021	0.484	0.428	6.00E-02	Meta_CD4_C1
RPS10	3.65E-06	0.500161802	0.770	0.736	8.85E-02	Meta_CD4_C1
RSL1D1	4.00E-06	0.257839855	0.525	0.470	9.69E-02	Meta_CD4_C1
BTG2	4.28E-06	0.37399842	0.678	0.609	1.04E-01	Meta_CD4_C1
FTH1	4.31E-06	0.287516483	0.979	0.982	1.04E-01	Meta_CD4_C1
MSL3	4.36E-06	0.274444387	0.217	0.151	1.06E-01	Meta_CD4_C1
CD4	4.47E-06	0.268789819	0.336	0.239	1.08E-01	Meta_CD4_C1
SC5D	6.16E-06	0.269121288	0.156	0.094	1.49E-01	Meta_CD4_C1
NELL2	6.80E-06	0.302527624	0.208	0.111	1.65E-01	Meta_CD4_C1
LPAR6	7.80E-06	0.373538375	0.158	0.074	1.89E-01	Meta_CD4_C1
EIF3L	1.01E-05	0.307742129	0.694	0.635	2.43E-01	Meta_CD4_C1

LSR	1.85E-05	0.252456588	0.168	0.097	4.49E-01	Meta_CD4_C1
RCAN3	2.35E-05	0.258916685	0.355	0.279	5.70E-01	Meta_CD4_C1
MYC	6.34E-05	0.331246045	0.260	0.174	1.00E+00	Meta_CD4_C1
NAP1L1	6.77E-05	0.345632966	0.622	0.573	1.00E+00	Meta_CD4_C1
TRAT1	9.04E-05	0.25404818	0.390	0.325	1.00E+00	Meta_CD4_C1
RPL27A	9.76E-05	0.467770935	0.940	0.938	1.00E+00	Meta_CD4_C1
ZNF331	1.29E-04	0.254583101	0.389	0.340	1.00E+00	Meta_CD4_C1
TNFSF8	1.20E-03	0.281742814	0.205	0.131	1.00E+00	Meta_CD4_C1
IGKC	4.02E-03	0.488417803	0.152	0.126	1.00E+00	Meta_CD4_C1
NBEAL1	9.14E-03	0.374362794	0.247	0.205	1.00E+00	Meta_CD4_C1
LTB	6.07E-69	0.956792398	0.878	0.600	1.47E-64	Meta_CD4_C2
RPL11	1.75E-52	0.31710617	0.999	0.994	4.23E-48	Meta_CD4_C2
KLF2	1.11E-51	1.034015941	0.587	0.299	2.68E-47	Meta_CD4_C2
IL7R	2.04E-50	0.658525938	0.844	0.559	4.93E-46	Meta_CD4_C2
RPL18A	4.73E-49	0.360000074	0.997	0.989	1.15E-44	Meta_CD4_C2
S100A4	6.33E-49	0.607831822	0.972	0.863	1.53E-44	Meta_CD4_C2
RPS8	3.94E-46	0.360308146	0.997	0.987	9.55E-42	Meta_CD4_C2
S1PR1	2.97E-45	0.567334578	0.343	0.115	7.19E-41	Meta_CD4_C2
TIMP1	3.48E-44	0.714046211	0.540	0.238	8.44E-40	Meta_CD4_C2
LDHB	4.29E-44	0.574895175	0.892	0.742	1.04E-39	Meta_CD4_C2
EMP3	1.67E-43	0.558009584	0.879	0.688	4.04E-39	Meta_CD4_C2
CD52	9.19E-43	0.430243685	0.987	0.930	2.23E-38	Meta_CD4_C2
CD40LG	8.79E-41	0.533824569	0.462	0.200	2.13E-36	Meta_CD4_C2
S100A11	9.22E-41	0.570896994	0.885	0.676	2.23E-36	Meta_CD4_C2
RPL19	3.68E-39	0.265342654	0.998	0.994	8.90E-35	Meta_CD4_C2
FXYD5	9.08E-39	0.533581195	0.945	0.850	2.20E-34	Meta_CD4_C2
RPL14	1.01E-38	0.273224696	0.992	0.979	2.45E-34	Meta_CD4_C2
RPL29	2.56E-38	0.31101236	0.989	0.977	6.20E-34	Meta_CD4_C2
AQP3	2.68E-38	0.691568235	0.608	0.319	6.48E-34	Meta_CD4_C2
RPL34	1.30E-36	0.298278295	0.997	0.993	3.14E-32	Meta_CD4_C2
RPL32	1.32E-36	0.30158939	0.998	0.992	3.19E-32	Meta_CD4_C2
RPL13	2.60E-36	0.286263976	1.000	0.997	6.29E-32	Meta_CD4_C2
RPL10	2.63E-36	0.281723222	1.000	0.998	6.38E-32	Meta_CD4_C2
RPLP0	5.42E-35	0.42076414	0.974	0.947	1.31E-30	Meta_CD4_C2
TRADD	1.29E-34	0.427332526	0.470	0.271	3.12E-30	Meta_CD4_C2
RPSA	1.72E-34	0.377865673	0.985	0.961	4.16E-30	Meta_CD4_C2
ANXA1	6.10E-34	0.411676764	0.851	0.657	1.48E-29	Meta_CD4_C2
RPS27A	1.64E-33	0.25619886	0.999	0.994	3.97E-29	Meta_CD4_C2
TPT1	4.46E-33	0.378460115	0.995	0.985	1.08E-28	Meta_CD4_C2
RPS12	5.07E-33	0.313064013	0.999	0.993	1.23E-28	Meta_CD4_C2

NOSIP	2.00E-32	0.74208339	0.624	0.390	4.84E-28	Meta_CD4_C2
RPS4X	7.34E-32	0.287750969	0.997	0.992	1.78E-27	Meta_CD4_C2
RPL18	1.33E-31	0.26113876	0.994	0.985	3.23E-27	Meta_CD4_C2
TMSB10	6.26E-31	0.384213049	0.999	0.992	1.52E-26	Meta_CD4_C2
CD4	6.60E-31	0.413551059	0.473	0.232	1.60E-26	Meta_CD4_C2
RPS13	2.76E-30	0.276883568	0.992	0.979	6.67E-26	Meta_CD4_C2
FLT3LG	2.95E-30	0.565570681	0.665	0.431	7.15E-26	Meta_CD4_C2
RPL6	5.10E-30	0.286627227	0.989	0.970	1.23E-25	Meta_CD4_C2
LEF1	8.59E-30	0.355328102	0.308	0.144	2.08E-25	Meta_CD4_C2
RPL5	7.79E-29	0.335621991	0.986	0.960	1.89E-24	Meta_CD4_C2
RPS3A	1.33E-28	0.316222539	0.994	0.977	3.21E-24	Meta_CD4_C2
RPS23	2.18E-28	0.275696317	0.992	0.978	5.28E-24	Meta_CD4_C2
RPL10A	5.31E-28	0.27908375	0.990	0.974	1.29E-23	Meta_CD4_C2
RASGRP2	6.83E-28	0.49151435	0.404	0.185	1.65E-23	Meta_CD4_C2
PLSCR3	1.37E-27	0.398986028	0.367	0.195	3.32E-23	Meta_CD4_C2
TMEM173	3.21E-27	0.43152103	0.520	0.300	7.78E-23	Meta_CD4_C2
S100A10	3.26E-27	0.449235508	0.930	0.798	7.91E-23	Meta_CD4_C2
ADD3	2.28E-26	0.347203176	0.440	0.262	5.53E-22	Meta_CD4_C2
EEF1B2	9.08E-25	0.338064251	0.941	0.896	2.20E-20	Meta_CD4_C2
TNFRSF25	2.33E-24	0.393924907	0.416	0.223	5.63E-20	Meta_CD4_C2
RPS18	2.41E-24	0.309536709	0.998	0.994	5.82E-20	Meta_CD4_C2
RPL22	5.87E-23	0.31330224	0.929	0.888	1.42E-18	Meta_CD4_C2
RPL8	6.22E-23	0.253604453	0.996	0.985	1.51E-18	Meta_CD4_C2
RPS6	7.39E-23	0.300051646	0.997	0.992	1.79E-18	Meta_CD4_C2
AES	3.56E-22	0.450106765	0.751	0.583	8.62E-18	Meta_CD4_C2
CCR7	3.72E-22	0.400237086	0.325	0.155	9.02E-18	Meta_CD4_C2
RPS5	6.17E-22	0.288384963	0.987	0.969	1.49E-17	Meta_CD4_C2
TCF7	8.98E-22	0.373537436	0.401	0.216	2.17E-17	Meta_CD4_C2
RPL4	2.12E-21	0.348222702	0.969	0.925	5.13E-17	Meta_CD4_C2
CORO1B	5.40E-21	0.38638144	0.587	0.396	1.31E-16	Meta_CD4_C2
EEF1A1	9.29E-21	0.394908411	0.936	0.910	2.25E-16	Meta_CD4_C2
EEF2	9.48E-21	0.357877541	0.950	0.900	2.29E-16	Meta_CD4_C2
FTH1	2.55E-20	0.289111559	0.992	0.981	6.16E-16	Meta_CD4_C2
RNASET2	7.11E-20	0.354930446	0.660	0.491	1.72E-15	Meta_CD4_C2
RACK1	7.87E-20	0.303364773	0.843	0.802	1.91E-15	Meta_CD4_C2
PPA1	8.87E-20	0.267311476	0.570	0.409	2.15E-15	Meta_CD4_C2
THEM4	1.72E-19	0.256451466	0.318	0.175	4.15E-15	Meta_CD4_C2
HINT1	1.70E-18	0.301282232	0.940	0.860	4.11E-14	Meta_CD4_C2
PABPC1	2.39E-18	0.313877966	0.946	0.893	5.80E-14	Meta_CD4_C2
RIPOR2	2.49E-18	0.489278194	0.422	0.238	6.03E-14	Meta_CD4_C2

RPS2	3.54E-18	0.326077246	0.996	0.983	8.57E-14	Meta_CD4_C2
NME2	6.04E-18	0.358231082	0.653	0.502	1.46E-13	Meta_CD4_C2
CDC25B	6.85E-18	0.437166243	0.356	0.186	1.66E-13	Meta_CD4_C2
RPL3	1.22E-17	0.284525718	0.995	0.986	2.96E-13	Meta_CD4_C2
ICAM2	2.56E-17	0.376664264	0.393	0.209	6.20E-13	Meta_CD4_C2
RPL39	2.66E-17	0.272687843	0.917	0.892	6.45E-13	Meta_CD4_C2
NSG1	6.86E-17	0.263680803	0.177	0.064	1.66E-12	Meta_CD4_C2
MAL	1.04E-16	0.343494522	0.286	0.122	2.53E-12	Meta_CD4_C2
SH3BP5	1.05E-16	0.363102277	0.290	0.130	2.55E-12	Meta_CD4_C2
LIME1	1.34E-16	0.26195082	0.525	0.364	3.24E-12	Meta_CD4_C2
S100A6	2.77E-16	0.307320161	0.958	0.884	6.70E-12	Meta_CD4_C2
KLF3	3.01E-16	0.265474367	0.297	0.167	7.29E-12	Meta_CD4_C2
CD28	4.79E-16	0.305219555	0.422	0.256	1.16E-11	Meta_CD4_C2
S1PR4	4.94E-16	0.320240939	0.417	0.272	1.20E-11	Meta_CD4_C2
TMEM123	5.57E-16	0.37692677	0.656	0.460	1.35E-11	Meta_CD4_C2
PXN	6.04E-16	0.277649694	0.266	0.131	1.46E-11	Meta_CD4_C2
EIF3E	6.98E-16	0.283197294	0.805	0.673	1.69E-11	Meta_CD4_C2
GPR183	7.20E-16	0.278606663	0.572	0.400	1.74E-11	Meta_CD4_C2
CTSB	8.48E-16	0.340178917	0.462	0.288	2.05E-11	Meta_CD4_C2
CRIP2	8.66E-16	0.301390626	0.129	0.045	2.10E-11	Meta_CD4_C2
IFITM2	1.44E-15	0.256499919	0.897	0.801	3.49E-11	Meta_CD4_C2
CD5	3.00E-15	0.288626612	0.572	0.419	7.25E-11	Meta_CD4_C2
APRT	3.64E-15	0.259799461	0.815	0.682	8.81E-11	Meta_CD4_C2
GSTK1	4.90E-15	0.270414363	0.850	0.745	1.19E-10	Meta_CD4_C2
CCR6	8.22E-13	0.30064541	0.279	0.144	1.99E-08	Meta_CD4_C2
GIMAP7	1.46E-12	0.284711243	0.765	0.618	3.53E-08	Meta_CD4_C2
IFITM1	1.50E-12	0.290301239	0.864	0.803	3.64E-08	Meta_CD4_C2
ANXA2	2.19E-12	0.261570601	0.620	0.484	5.29E-08	Meta_CD4_C2
CRIP1	1.18E-11	0.381249814	0.834	0.741	2.85E-07	Meta_CD4_C2
EEF1G	2.21E-11	0.430654682	0.722	0.645	5.35E-07	Meta_CD4_C2
KDSR	3.67E-11	0.280773361	0.315	0.177	8.89E-07	Meta_CD4_C2
GIMAP4	1.77E-10	0.293098858	0.670	0.534	4.28E-06	Meta_CD4_C2
TAGLN2	2.42E-10	0.274531976	0.873	0.764	5.86E-06	Meta_CD4_C2
ITGB1	5.65E-10	0.364141169	0.387	0.264	1.37E-05	Meta_CD4_C2
GPSM3	1.31E-09	0.258897527	0.821	0.696	3.18E-05	Meta_CD4_C2
SYTL1	1.56E-09	0.266761436	0.444	0.300	3.77E-05	Meta_CD4_C2
NPDC1	2.88E-09	0.294918842	0.190	0.090	6.97E-05	Meta_CD4_C2
ABRACL	5.85E-09	0.255032857	0.724	0.583	1.42E-04	Meta_CD4_C2
RPL17	2.61E-07	0.288411347	0.900	0.858	6.31E-03	Meta_CD4_C2
IL7R	2.08E-109	1.372247329	0.881	0.523	5.04E-105	Meta_CD4_C3

FOS	1.06E-90	1.362824723	0.928	0.735	2.57E-86	Meta_CD4_C3
ANXA1	1.39E-87	1.140019927	0.910	0.626	3.37E-83	Meta_CD4_C3
RPL32	1.74E-54	0.395787315	0.998	0.992	4.22E-50	Meta_CD4_C3
FOSB	2.10E-46	1.093180107	0.731	0.476	5.09E-42	Meta_CD4_C3
RPS3	6.93E-45	0.344203412	0.998	0.994	1.68E-40	Meta_CD4_C3
TPT1	5.88E-44	0.425765674	0.993	0.984	1.42E-39	Meta_CD4_C3
RPL10	1.34E-43	0.348165452	1.000	0.998	3.24E-39	Meta_CD4_C3
RPL34	8.41E-43	0.36976368	0.997	0.992	2.04E-38	Meta_CD4_C3
RPLP1	1.09E-42	0.415225544	0.999	0.998	2.63E-38	Meta_CD4_C3
EEF1B2	9.54E-41	0.471968609	0.940	0.891	2.31E-36	Meta_CD4_C3
RPL9	3.11E-39	0.344485648	0.986	0.971	7.54E-35	Meta_CD4_C3
RPS12	1.21E-38	0.35601649	0.998	0.993	2.93E-34	Meta_CD4_C3
RPS27A	1.86E-38	0.346490652	0.998	0.994	4.51E-34	Meta_CD4_C3
CD40LG	6.28E-38	0.959349345	0.463	0.172	1.52E-33	Meta_CD4_C3
DUSP1	8.97E-38	0.801655384	0.891	0.788	2.17E-33	Meta_CD4_C3
RPS14	4.16E-37	0.350376868	0.998	0.994	1.01E-32	Meta_CD4_C3
RPL30	1.05E-36	0.328873016	0.995	0.987	2.54E-32	Meta_CD4_C3
GPR183	4.37E-36	0.792051985	0.626	0.372	1.06E-31	Meta_CD4_C3
VIM	1.03E-35	0.71057263	0.953	0.888	2.50E-31	Meta_CD4_C3
RPL3	1.27E-34	0.346105039	0.994	0.985	3.08E-30	Meta_CD4_C3
RPLP0	1.50E-34	0.442700229	0.967	0.946	3.63E-30	Meta_CD4_C3
RPS3A	3.54E-33	0.369756748	0.989	0.976	8.57E-29	Meta_CD4_C3
KLRB1	1.09E-31	0.612237203	0.680	0.430	2.64E-27	Meta_CD4_C3
RPL11	2.69E-31	0.299073139	0.998	0.993	6.51E-27	Meta_CD4_C3
RPS13	4.97E-31	0.302857798	0.989	0.978	1.20E-26	Meta_CD4_C3
RPS25	5.35E-29	0.31002953	0.981	0.967	1.30E-24	Meta_CD4_C3
RPS23	6.94E-29	0.310770513	0.988	0.977	1.68E-24	Meta_CD4_C3
RPL13	5.80E-28	0.250974409	0.999	0.997	1.40E-23	Meta_CD4_C3
RPS18	1.26E-27	0.310602393	0.998	0.993	3.04E-23	Meta_CD4_C3
RPL39	1.41E-27	0.382794354	0.914	0.890	3.42E-23	Meta_CD4_C3
EEF1A1	1.66E-25	0.419108438	0.931	0.908	4.02E-21	Meta_CD4_C3
TNFAIP3	1.14E-24	0.71214119	0.853	0.760	2.75E-20	Meta_CD4_C3
S100A11	4.44E-24	0.473812437	0.814	0.665	1.07E-19	Meta_CD4_C3
PTGER4	1.25E-23	0.61412521	0.671	0.529	3.03E-19	Meta_CD4_C3
RPS6	3.32E-23	0.27023543	0.996	0.991	8.04E-19	Meta_CD4_C3
ZFP36L2	4.22E-23	0.48090628	0.929	0.861	1.02E-18	Meta_CD4_C3
RPL35A	6.24E-23	0.254059015	0.993	0.985	1.51E-18	Meta_CD4_C3
RPS5	7.05E-23	0.295264069	0.983	0.968	1.71E-18	Meta_CD4_C3
RPLP2	1.26E-22	0.268794645	0.997	0.992	3.05E-18	Meta_CD4_C3
S100A10	1.49E-22	0.524318396	0.896	0.790	3.61E-18	Meta_CD4_C3

AHNAK	1.03E-21	0.589291703	0.679	0.504	2.49E-17	Meta_CD4_C3
PTGER2	1.24E-21	0.498919128	0.372	0.220	3.01E-17	Meta_CD4_C3
KLF6	2.66E-21	0.669973955	0.925	0.850	6.44E-17	Meta_CD4_C3
RPL8	5.64E-20	0.262194875	0.992	0.985	1.37E-15	Meta_CD4_C3
RPS21	2.10E-19	0.353139162	0.962	0.945	5.09E-15	Meta_CD4_C3
FXYD5	3.11E-19	0.291088245	0.914	0.845	7.53E-15	Meta_CD4_C3
RPL36	6.64E-19	0.25558433	0.986	0.977	1.61E-14	Meta_CD4_C3
RPS8	7.46E-19	0.267052653	0.995	0.987	1.81E-14	Meta_CD4_C3
RGCC	8.06E-19	0.851142309	0.660	0.505	1.95E-14	Meta_CD4_C3
RPL5	9.92E-19	0.291917471	0.976	0.959	2.40E-14	Meta_CD4_C3
MYADM	1.34E-18	0.662547431	0.578	0.401	3.25E-14	Meta_CD4_C3
RPL35	1.94E-18	0.285536868	0.984	0.970	4.70E-14	Meta_CD4_C3
RPS28	3.59E-18	0.271703238	0.973	0.963	8.70E-14	Meta_CD4_C3
RPL38	4.01E-18	0.292753823	0.952	0.928	9.71E-14	Meta_CD4_C3
AC020571.1	1.22E-17	0.477607015	0.179	0.070	2.96E-13	Meta_CD4_C3
PLAC8	1.89E-17	0.385266502	0.257	0.123	4.57E-13	Meta_CD4_C3
CD4	4.07E-17	0.418384103	0.399	0.219	9.85E-13	Meta_CD4_C3
IL2	4.80E-17	0.693138141	0.173	0.047	1.16E-12	Meta_CD4_C3
PPP1R15A	7.19E-17	0.614719873	0.779	0.649	1.74E-12	Meta_CD4_C3
TSC22D3	1.09E-16	0.529351508	0.944	0.881	2.65E-12	Meta_CD4_C3
FTH1	3.22E-16	0.394592857	0.990	0.980	7.79E-12	Meta_CD4_C3
RPSA	9.21E-16	0.280136187	0.973	0.960	2.23E-11	Meta_CD4_C3
TOMM7	8.47E-15	0.275559687	0.920	0.891	2.05E-10	Meta_CD4_C3
DHRS3	1.57E-14	0.31444008	0.186	0.078	3.80E-10	Meta_CD4_C3
RPS16	1.77E-14	0.269420563	0.991	0.981	4.29E-10	Meta_CD4_C3
TIMP1	1.18E-13	0.392360502	0.381	0.233	2.86E-09	Meta_CD4_C3
CCR2	1.24E-13	0.2617212	0.134	0.053	3.00E-09	Meta_CD4_C3
TNFSF13B	2.34E-13	0.356143083	0.179	0.080	5.6702E-09	Meta_CD4_C3
CD69	2.59E-13	0.601211931	0.889	0.823	6.27E-09	Meta_CD4_C3
RPL36A	6.22E-13	0.366382493	0.853	0.784	1.51E-08	Meta_CD4_C3
RPL22	8.36E-13	0.278436669	0.914	0.886	2.02E-08	Meta_CD4_C3
LMNA	1.05E-12	0.97295561	0.386	0.257	2.55E-08	Meta_CD4_C3
SSBP2	1.60E-12	0.337188398	0.199	0.085	3.87E-08	Meta_CD4_C3
FLT3LG	1.90E-12	0.325282528	0.537	0.428	4.60E-08	Meta_CD4_C3
CDKN1A	3.11E-12	0.648598698	0.289	0.171	7.52E-08	Meta_CD4_C3
PNP	3.54E-12	0.413888098	0.390	0.284	8.57E-08	Meta_CD4_C3
JUND	5.96E-12	0.311488237	0.488	0.412	1.44E-07	Meta_CD4_C3
JUNB	9.15E-12	0.513227127	0.925	0.880	2.21E-07	Meta_CD4_C3
ZFP36	1.06E-11	0.569945972	0.890	0.812	2.58E-07	Meta_CD4_C3
BTG2	2.95E-11	0.489847373	0.673	0.603	7.14E-07	Meta_CD4_C3

CXCR4	7.14E-11	0.406993434	0.894	0.821	1.73E-06	Meta_CD4_C3
EEF1G	9.58E-11	0.418752688	0.730	0.636	2.32E-06	Meta_CD4_C3
JUN	9.88E-11	0.593283725	0.861	0.796	2.39E-06	Meta_CD4_C3
AC020916.1	1.57E-10	0.434656641	0.325	0.208	3.81E-06	Meta_CD4_C3
TAGLN2	1.82E-10	0.347230323	0.827	0.760	4.41E-06	Meta_CD4_C3
GADD45B	2.08E-10	0.33033599	0.558	0.506	5.04E-06	Meta_CD4_C3
CRIP1	2.42E-10	0.396603863	0.812	0.735	5.87E-06	Meta_CD4_C3
RPL17	2.52E-10	0.322744784	0.893	0.854	6.11E-06	Meta_CD4_C3
ARL4A	2.66E-10	0.428398214	0.358	0.227	6.44E-06	Meta_CD4_C3
IGFBP3	2.88E-10	0.279431903	0.124	0.042	6.98E-06	Meta_CD4_C3
GATA3	6.38E-10	0.39073974	0.482	0.398	1.54E-05	Meta_CD4_C3
ATP2B1	6.57E-10	0.30146405	0.285	0.227	1.59E-05	Meta_CD4_C3
DNAJB1	9.14E-10	0.398694072	0.802	0.755	2.21E-05	Meta_CD4_C3
NFKBIA	1.17E-09	0.560923425	0.805	0.696	2.82E-05	Meta_CD4_C3
SLC2A3	2.36E-09	0.356160151	0.712	0.635	5.71E-05	Meta_CD4_C3
EGR1	2.52E-09	0.914201035	0.254	0.156	6.11E-05	Meta_CD4_C3
IDS	2.99E-09	0.314623849	0.609	0.574	7.25E-05	Meta_CD4_C3
ERN1	4.21E-09	0.259838271	0.321	0.244	1.02E-04	Meta_CD4_C3
TUBA1A	5.68E-09	0.44404522	0.631	0.552	1.38E-04	Meta_CD4_C3
ITM2B	6.53E-09	0.296007015	0.922	0.898	1.58E-04	Meta_CD4_C3
ARHGAP15	7.92E-09	0.299070603	0.521	0.446	1.92E-04	Meta_CD4_C3
DYNLT3	1.05E-08	0.318684651	0.319	0.214	2.54E-04	Meta_CD4_C3
MYC	1.24E-08	0.446054618	0.251	0.167	3.00E-04	Meta_CD4_C3
THEM4	1.78E-08	0.283194763	0.258	0.170	4.30E-04	Meta_CD4_C3
MCL1	2.28E-08	0.322661223	0.762	0.720	5.52E-04	Meta_CD4_C3
TNF	5.23E-08	0.871311202	0.291	0.171	1.27E-03	Meta_CD4_C3
HIST1H4C	6.59E-08	0.256692951	0.630	0.558	1.60E-03	Meta_CD4_C3
LGALS3	6.88E-08	0.268761477	0.478	0.363	1.66E-03	Meta_CD4_C3
SORBS3	2.16E-07	0.271090782	0.194	0.101	5.22E-03	Meta_CD4_C3
CSRNP1	2.50E-07	0.447367407	0.609	0.488	6.06E-03	Meta_CD4_C3
MID1IP1	2.95E-07	0.258258805	0.177	0.112	7.15E-03	Meta_CD4_C3
RBMS1	5.25E-07	0.260985859	0.418	0.341	1.27E-02	Meta_CD4_C3
RORA	5.62E-07	0.285576971	0.533	0.440	1.36E-02	Meta_CD4_C3
TMEM123	1.58E-06	0.256252199	0.542	0.459	3.82E-02	Meta_CD4_C3
PERP	1.65E-06	0.354903989	0.311	0.198	4.00E-02	Meta_CD4_C3
PLK3	1.79E-06	0.252003292	0.304	0.247	4.33E-02	Meta_CD4_C3
SCML4	1.93E-06	0.315079573	0.439	0.350	4.67E-02	Meta_CD4_C3
NR4A1	1.96E-06	0.674356527	0.405	0.275	4.75E-02	Meta_CD4_C3
CITED2	2.27E-06	0.416750252	0.561	0.486	5.49E-02	Meta_CD4_C3
ICAM2	2.83E-06	0.252781272	0.298	0.205	6.86E-02	Meta_CD4_C3

TXNIP	2.97E-06	0.339753253	0.806	0.745	7.18E-02	Meta_CD4_C3
MAL	3.15E-06	0.278895179	0.218	0.117	7.62E-02	Meta_CD4_C3
SESN1	7.24E-06	0.311310481	0.243	0.146	1.75E-01	Meta_CD4_C3
DPP4	1.11E-05	0.27122599	0.190	0.103	2.68E-01	Meta_CD4_C3
IFNGR1	1.29E-05	0.321139293	0.333	0.237	3.11E-01	Meta_CD4_C3
TUBA1B	2.78E-05	0.282565727	0.666	0.579	6.73E-01	Meta_CD4_C3
SORL1	3.02E-05	0.271888485	0.278	0.182	7.32E-01	Meta_CD4_C3
TUBA1C	4.53E-05	0.259364149	0.328	0.262	1.00E+00	Meta_CD4_C3
PBX4	1.61E-04	0.26196369	0.302	0.237	1.00E+00	Meta_CD4_C3
CD44	2.91E-04	0.264623473	0.802	0.758	1.00E+00	Meta_CD4_C3
RBKS	3.35E-04	0.255050844	0.183	0.140	1.00E+00	Meta_CD4_C3
HSPA1B	1.16E-03	0.443397702	0.329	0.305	1.00E+00	Meta_CD4_C3
PER1	2.43E-03	0.346048965	0.265	0.217	1.00E+00	Meta_CD4_C3
TOB1	6.62E-03	0.270191572	0.275	0.263	1.00E+00	Meta_CD4_C3
KLRB1	7.10E-151	1.452615654	0.903	0.438	1.72E-146	Meta_CD4_C4
AQP3	3.77E-96	1.192423423	0.710	0.310	9.14E-92	Meta_CD4_C4
RPLP1	9.56E-88	0.544559346	1.000	0.998	2.31E-83	Meta_CD4_C4
SLC4A10	7.61E-77	0.818487788	0.272	0.007	1.84E-72	Meta_CD4_C4
IL7R	3.10E-76	1.208466287	0.882	0.555	7.50E-72	Meta_CD4_C4
CCR6	1.73E-70	1.039061398	0.497	0.128	4.19E-66	Meta_CD4_C4
IL4I1	1.76E-69	0.846189584	0.312	0.033	4.27E-65	Meta_CD4_C4
TRAV1-2	1.91E-69	1.187887823	0.279	0.031	4.61E-65	Meta_CD4_C4
IL23R	3.44E-69	0.775592023	0.280	0.022	8.32E-65	Meta_CD4_C4
RPLP0	5.70E-66	0.625133398	0.992	0.946	1.38E-61	Meta_CD4_C4
CA2	2.50E-58	0.585547551	0.180	0.006	6.05E-54	Meta_CD4_C4
RORC	4.41E-55	0.67290169	0.257	0.028	1.07E-50	Meta_CD4_C4
CEBPD	8.98E-54	1.012509741	0.406	0.130	2.17E-49	Meta_CD4_C4
NFKBIA	6.98E-47	1.133684576	0.904	0.699	1.69E-42	Meta_CD4_C4
TPT1	7.83E-46	0.428645069	0.998	0.984	1.90E-41	Meta_CD4_C4
LTB	8.59E-46	0.756304579	0.874	0.599	2.08E-41	Meta_CD4_C4
TNFAIP3	9.18E-46	0.893669799	0.925	0.763	2.22E-41	Meta_CD4_C4
DPP4	1.02E-45	0.693437291	0.359	0.100	2.47E-41	Meta_CD4_C4
S100A4	1.33E-43	0.516802739	0.984	0.861	3.21E-39	Meta_CD4_C4
TNFSF13B	7.38E-43	0.784759968	0.320	0.080	1.79E-38	Meta_CD4_C4
TLE1	8.76E-43	0.506180558	0.169	0.017	2.12E-38	Meta_CD4_C4
NCR3	2.59E-41	1.032256502	0.307	0.076	6.26E-37	Meta_CD4_C4
ERN1	2.65E-41	0.650872984	0.497	0.239	6.43E-37	Meta_CD4_C4
DUSP1	3.37E-39	0.982116142	0.926	0.795	8.16E-35	Meta_CD4_C4
EEF1A1	2.17E-37	0.481461684	0.976	0.907	5.26E-33	Meta_CD4_C4
RORA	3.76E-37	0.668443693	0.706	0.437	9.10E-33	Meta_CD4_C4

CTSH	9.71E-37	0.540592055	0.277	0.072	2.35E-32	Meta_CD4_C4
RPS12	1.27E-35	0.31140258	0.999	0.993	3.08E-31	Meta_CD4_C4
LST1	2.11E-33	0.704168356	0.233	0.051	5.11E-29	Meta_CD4_C4
SPOCK2	5.33E-33	0.600846066	0.874	0.674	1.29E-28	Meta_CD4_C4
TMIGD2	5.88E-33	0.534705272	0.338	0.139	1.42E-28	Meta_CD4_C4
ME1	7.44E-33	0.345929882	0.115	0.005	1.80E-28	Meta_CD4_C4
ZBTB16	1.85E-31	0.504767333	0.207	0.045	4.48E-27	Meta_CD4_C4
TNFRSF25	1.01E-30	0.613112036	0.454	0.219	2.43E-26	Meta_CD4_C4
RPL32	7.88E-29	0.280896702	0.999	0.992	1.91E-24	Meta_CD4_C4
PERP	2.15E-27	0.508982188	0.405	0.202	5.21E-23	Meta_CD4_C4
RPS13	3.78E-27	0.284966016	0.996	0.978	9.14E-23	Meta_CD4_C4
EEF1B2	3.20E-26	0.388981861	0.973	0.894	7.74E-22	Meta_CD4_C4
GYG1	3.42E-26	0.506774448	0.544	0.351	8.29E-22	Meta_CD4_C4
CCL20	6.94E-26	0.799718277	0.208	0.050	1.68E-21	Meta_CD4_C4
LTK	1.43E-25	0.333714578	0.117	0.008	3.45E-21	Meta_CD4_C4
IFNGR1	2.41E-25	0.595537436	0.468	0.237	5.85E-21	Meta_CD4_C4
CD40LG	2.49E-25	0.536625727	0.396	0.202	6.02E-21	Meta_CD4_C4
RUNX2	8.08E-25	0.394626626	0.265	0.113	1.96E-20	Meta_CD4_C4
KLRG1	3.03E-24	0.581503955	0.358	0.163	7.35E-20	Meta_CD4_C4
ANXA1	4.83E-24	0.403045609	0.860	0.655	1.17E-19	Meta_CD4_C4
RPL39	2.15E-23	0.263022285	0.968	0.889	5.21E-19	Meta_CD4_C4
MGAT4A	8.57E-23	0.482591475	0.578	0.374	2.07E-18	Meta_CD4_C4
FOS	1.33E-22	0.857960437	0.888	0.755	3.21E-18	Meta_CD4_C4
SESN1	1.34E-22	0.51558729	0.334	0.149	3.24E-18	Meta_CD4_C4
NR1D1	3.48E-22	0.40401857	0.168	0.042	8.43E-18	Meta_CD4_C4
FLT3LG	9.31E-22	0.499020597	0.651	0.430	2.26E-17	Meta_CD4_C4
ABCA1	1.02E-21	0.486153924	0.162	0.032	2.48E-17	Meta_CD4_C4
ZFP36L2	1.91E-21	0.400744248	0.952	0.866	4.64E-17	Meta_CD4_C4
RPS21	2.25E-21	0.254075034	0.987	0.945	5.45E-17	Meta_CD4_C4
IL17RE	2.66E-21	0.343769729	0.131	0.013	6.45E-17	Meta_CD4_C4
S100A6	4.26E-21	0.397312961	0.968	0.883	1.03E-16	Meta_CD4_C4
COLQ	6.13E-21	0.349842721	0.125	0.014	1.48E-16	Meta_CD4_C4
TTC39C	7.65E-21	0.591813152	0.618	0.408	1.85E-16	Meta_CD4_C4
MYBL1	1.40E-20	0.454056658	0.249	0.099	3.39E-16	Meta_CD4_C4
ARL4A	3.44E-20	0.435843475	0.403	0.236	8.32E-16	Meta_CD4_C4
PPP1R15A	5.34E-20	0.46007727	0.784	0.660	1.29E-15	Meta_CD4_C4
FOSB	6.91E-20	0.775373447	0.661	0.503	1.67E-15	Meta_CD4_C4
FKBP11	3.32E-19	0.443270007	0.583	0.370	8.03E-15	Meta_CD4_C4
VIM	4.59E-19	0.479410375	0.969	0.893	1.11E-14	Meta_CD4_C4
CERK	6.49E-19	0.406692143	0.333	0.178	1.57E-14	Meta_CD4_C4

PNP	8.88E-19	0.513822406	0.458	0.289	2.15E-14	Meta_CD4_C4
TSPAN15	1.29E-18	0.27029919	0.104	0.009	3.12E-14	Meta_CD4_C4
BHLHE40	1.40E-18	0.657363913	0.579	0.370	3.38E-14	Meta_CD4_C4
ZFP36L1	1.40E-18	0.4492063	0.818	0.670	3.40E-14	Meta_CD4_C4
RPSA	1.55E-18	0.250413625	0.989	0.960	3.75E-14	Meta_CD4_C4
IL2	4.90E-18	0.376924371	0.152	0.060	1.19E-13	Meta_CD4_C4
EEF1G	7.34E-18	0.523603472	0.793	0.640	1.78E-13	Meta_CD4_C4
YWHAQ	1.18E-17	0.394623303	0.726	0.557	2.86E-13	Meta_CD4_C4
TRBV6-1	1.80E-17	0.850089135	0.111	0.027	4.35E-13	Meta_CD4_C4
MT-ATP8	1.80E-17	0.255146154	0.913	0.785	4.36E-13	Meta_CD4_C4
TC2N	1.82E-17	0.345303773	0.565	0.398	4.41E-13	Meta_CD4_C4
CSRNP1	4.21E-17	0.46346038	0.673	0.495	1.02E-12	Meta_CD4_C4
RPL17	2.94E-16	0.352593579	0.945	0.854	7.13E-12	Meta_CD4_C4
RPL41	3.74E-16	0.264078531	0.976	0.910	9.06E-12	Meta_CD4_C4
PDE4D	5.01E-16	0.552783945	0.486	0.307	1.21E-11	Meta_CD4_C4
RBL2	9.59E-16	0.419001542	0.555	0.417	2.32E-11	Meta_CD4_C4
CDC42EP3	1.60E-15	0.397403182	0.478	0.309	3.89E-11	Meta_CD4_C4
ITM2B	1.61E-15	0.2719189	0.956	0.898	3.90E-11	Meta_CD4_C4
ATF7IP2	1.73E-15	0.332322624	0.259	0.144	4.18E-11	Meta_CD4_C4
RPL22	2.06E-15	0.25054678	0.967	0.885	5.00E-11	Meta_CD4_C4
PER1	5.64E-15	0.512329511	0.355	0.215	1.36E-10	Meta_CD4_C4
IL18RAP	2.01E-14	0.295071243	0.195	0.087	4.86E-10	Meta_CD4_C4
HSPA5	3.56E-14	0.397587994	0.766	0.638	8.61E-10	Meta_CD4_C4
LGALS3	5.31E-14	0.481550211	0.561	0.368	1.28E-09	Meta_CD4_C4
PDCD4	6.46E-14	0.396971637	0.728	0.574	1.56E-09	Meta_CD4_C4
TNF	6.53E-14	0.998606677	0.334	0.179	1.58E-09	Meta_CD4_C4
ELK3	9.77E-14	0.279586348	0.231	0.117	2.37E-09	Meta_CD4_C4
TTC39C-AS1	1.05E-13	0.261995747	0.117	0.035	2.53E-09	Meta_CD4_C4
JUN	1.31E-13	0.712518021	0.897	0.800	3.18E-09	Meta_CD4_C4
ABCB1	2.42E-13	0.34488168	0.240	0.106	5.87E-09	Meta_CD4_C4
MYADM	5.13E-13	0.531582072	0.558	0.418	1.24E-08	Meta_CD4_C4
AC245014.3	5.84E-13	0.368371407	0.133	0.041	1.41E-08	Meta_CD4_C4
RARA	1.12E-12	0.282804291	0.262	0.161	2.72E-08	Meta_CD4_C4
SATB1	5.77E-12	0.395309385	0.345	0.196	1.40E-07	Meta_CD4_C4
ADRB2	9.75E-12	0.341188509	0.183	0.071	2.36E-07	Meta_CD4_C4
DSE	1.33E-11	0.291565535	0.164	0.062	3.21E-07	Meta_CD4_C4
TPD52	1.39E-11	0.270906819	0.253	0.145	3.36E-07	Meta_CD4_C4
DYNLT3	3.12E-11	0.358636178	0.382	0.219	7.54E-07	Meta_CD4_C4
RBMS1	3.30E-11	0.314463299	0.482	0.344	7.99E-07	Meta_CD4_C4
CD69	5.13E-11	0.590864048	0.894	0.829	1.24E-06	Meta_CD4_C4

SYTL2	8.54E-11	0.263852825	0.288	0.181	2.07E-06	Meta_CD4_C4
GTF3C1	8.55E-11	0.293207247	0.181	0.105	2.07E-06	Meta_CD4_C4
PIM1	9.91E-11	0.387577444	0.565	0.435	2.40E-06	Meta_CD4_C4
JAML	1.65E-10	0.293408337	0.445	0.288	4.00E-06	Meta_CD4_C4
TNFAIP8	1.93E-10	0.25547035	0.540	0.425	4.66E-06	Meta_CD4_C4
ATF3	5.29E-10	0.505034633	0.235	0.139	1.28E-05	Meta_CD4_C4
YWHAH	6.96E-10	0.314286895	0.455	0.338	1.69E-05	Meta_CD4_C4
LINC01871	1.43E-09	0.263558987	0.457	0.303	3.46E-05	Meta_CD4_C4
CITED2	1.96E-09	0.541202698	0.612	0.489	4.74E-05	Meta_CD4_C4
ODF2L	2.10E-09	0.324069421	0.438	0.296	5.08E-05	Meta_CD4_C4
GDI1	3.84E-09	0.258611248	0.389	0.279	9.30E-05	Meta_CD4_C4
BZW1	6.27E-09	0.258236463	0.632	0.524	1.52E-04	Meta_CD4_C4
AC020916.1	7.05E-09	0.416930673	0.314	0.219	1.71E-04	Meta_CD4_C4
EGR1	1.02E-08	0.777391192	0.228	0.166	2.46E-04	Meta_CD4_C4
Z93241.1	1.28E-08	0.350348954	0.120	0.049	3.09E-04	Meta_CD4_C4
IVNS1ABP	1.39E-08	0.262472761	0.447	0.334	3.37E-04	Meta_CD4_C4
TRAF4	1.76E-08	0.338480922	0.172	0.109	4.25E-04	Meta_CD4_C4
PRNP	2.93E-08	0.339114178	0.457	0.297	7.09E-04	Meta_CD4_C4
NINJ1	5.07E-08	0.489386945	0.294	0.213	1.23E-03	Meta_CD4_C4
MYC	6.83E-08	0.313049215	0.273	0.173	1.65E-03	Meta_CD4_C4
FURIN	8.18E-08	0.296371395	0.181	0.097	1.98E-03	Meta_CD4_C4
ZFP36	8.65E-08	0.557454564	0.901	0.818	2.09E-03	Meta_CD4_C4
CCNH	1.08E-07	0.363553748	0.496	0.372	2.61E-03	Meta_CD4_C4
TSC22D3	1.42E-07	0.324774307	0.934	0.888	3.44E-03	Meta_CD4_C4
SLC7A5	1.80E-07	0.509618103	0.362	0.261	4.35E-03	Meta_CD4_C4
YIPF5	2.09E-07	0.291904949	0.362	0.265	5.06E-03	Meta_CD4_C4
TGFB1	2.53E-07	0.325645402	0.562	0.449	6.13E-03	Meta_CD4_C4
RGS2	2.67E-07	0.266426278	0.533	0.401	6.45E-03	Meta_CD4_C4
MBNL2	3.80E-07	0.253322669	0.223	0.139	9.20E-03	Meta_CD4_C4
IER2	4.20E-07	0.372972509	0.747	0.656	1.02E-02	Meta_CD4_C4
AHNAK	6.65E-07	0.290507665	0.663	0.521	1.61E-02	Meta_CD4_C4
HIST2H2AA4	9.01E-07	0.37904428	0.180	0.089	2.18E-02	Meta_CD4_C4
AL118516.1	9.14E-07	0.253843078	0.215	0.128	2.21E-02	Meta_CD4_C4
PBX4	1.30E-06	0.290444269	0.342	0.241	3.16E-02	Meta_CD4_C4
RPS10	1.41E-06	0.33450902	0.799	0.735	3.42E-02	Meta_CD4_C4
OSTF1	1.62E-06	0.296348	0.630	0.508	3.91E-02	Meta_CD4_C4
KLF6	2.88E-06	0.312087257	0.924	0.857	6.98E-02	Meta_CD4_C4
AC103591.3	4.18E-06	0.344862851	0.114	0.052	1.01E-01	Meta_CD4_C4
AC020571.1	4.99E-06	0.291619592	0.149	0.082	1.21E-01	Meta_CD4_C4
CCDC107	5.87E-06	0.256751243	0.500	0.378	1.42E-01	Meta_CD4_C4

ZC3H12A	1.18E-05	0.306610137	0.327	0.219	2.86E-01	Meta_CD4_C4
GCHFR	1.23E-05	0.250265409	0.327	0.246	2.99E-01	Meta_CD4_C4
TRBV20-1	1.27E-05	0.342512206	0.133	0.073	3.08E-01	Meta_CD4_C4
BTG2	1.71E-05	0.466458882	0.663	0.610	4.14E-01	Meta_CD4_C4
SPRY1	4.18E-05	0.259420662	0.218	0.137	1.00E+00	Meta_CD4_C4
CD83	5.22E-05	0.353851516	0.186	0.096	1.00E+00	Meta_CD4_C4
DDIT4	7.32E-05	0.332913271	0.660	0.584	1.00E+00	Meta_CD4_C4
NFKBIZ	1.15E-04	0.404649966	0.450	0.358	1.00E+00	Meta_CD4_C4
GPR65	5.80E-04	0.262802053	0.405	0.291	1.00E+00	Meta_CD4_C4
NR4A1	6.29E-04	0.501509944	0.365	0.290	1.00E+00	Meta_CD4_C4
KDM6B	2.41E-03	0.28280436	0.282	0.197	1.00E+00	Meta_CD4_C4
CDKN1A	5.63E-03	0.34520636	0.283	0.182	1.00E+00	Meta_CD4_C4
ACTB	1.64E-85	0.769820845	1.000	0.998	3.96E-81	Meta_CD4_C5
S100A4	6.93E-85	0.86360247	0.987	0.863	1.68E-80	Meta_CD4_C5
CTSH	3.62E-82	0.865047308	0.378	0.069	8.77E-78	Meta_CD4_C5
CD40LG	2.58E-72	1.007161706	0.579	0.195	6.26E-68	Meta_CD4_C5
PFN1	6.50E-71	0.74786098	0.996	0.956	1.57E-66	Meta_CD4_C5
LAPTM5	1.80E-67	0.62124475	0.978	0.887	4.37E-63	Meta_CD4_C5
SH3BGRL3	9.80E-64	0.548500786	0.998	0.958	2.37E-59	Meta_CD4_C5
SPOCK2	2.64E-60	0.657176234	0.907	0.674	6.39E-56	Meta_CD4_C5
SLAMF1	1.14E-58	0.716208057	0.595	0.251	2.76E-54	Meta_CD4_C5
CXCR6	1.68E-56	0.989741111	0.647	0.314	4.07E-52	Meta_CD4_C5
CD52	5.00E-56	0.618223915	0.988	0.930	1.21E-51	Meta_CD4_C5
MYL6	1.65E-54	0.55101248	0.996	0.944	4.00E-50	Meta_CD4_C5
GPR25	1.67E-54	0.821504078	0.257	0.042	4.04E-50	Meta_CD4_C5
CD4	1.40E-53	0.599550716	0.571	0.228	3.40E-49	Meta_CD4_C5
CORO1A	1.04E-52	0.669277971	0.973	0.870	2.51E-48	Meta_CD4_C5
PDCD1	1.77E-52	0.659850718	0.512	0.201	4.28E-48	Meta_CD4_C5
TNFRSF25	2.90E-52	0.734905085	0.575	0.216	7.01E-48	Meta_CD4_C5
ARPC1B	1.01E-51	0.710624551	0.924	0.719	2.45E-47	Meta_CD4_C5
CD82	7.50E-51	0.640084019	0.662	0.319	1.82E-46	Meta_CD4_C5
TMEM167A	1.95E-49	0.725793995	0.547	0.236	4.72E-45	Meta_CD4_C5
ARHGDIB	2.14E-49	0.486833207	0.989	0.942	5.17E-45	Meta_CD4_C5
ACTG1	2.96E-49	0.689702552	0.992	0.945	7.18E-45	Meta_CD4_C5
RORC	4.03E-49	0.434687704	0.217	0.033	9.76E-45	Meta_CD4_C5
COTL1	5.56E-49	0.640385875	0.936	0.744	1.35E-44	Meta_CD4_C5
CAPZB	8.90E-48	0.589854614	0.911	0.699	2.15E-43	Meta_CD4_C5
MIR4435-2HG	4.77E-47	0.625817514	0.602	0.273	1.15E-42	Meta_CD4_C5
ABRACL	4.12E-46	0.578457138	0.830	0.578	9.9799E-42	Meta_CD4_C5
CCR6	6.73E-46	0.734075923	0.390	0.138	1.6305E-41	Meta_CD4_C5

CD6	1.29542E-45	0.508388178	0.814	0.552	3.1369E-41	Meta_CD4_C5
TMSB10	9.58454E-45	0.609698761	0.999	0.992	2.3209E-40	Meta_CD4_C5
ADAM19	2.80E-44	0.593721543	0.396	0.112	6.79E-40	Meta_CD4_C5
TPM4	2.85E-44	0.6581599	0.701	0.386	6.89E-40	Meta_CD4_C5
ADA	3.29E-44	0.552683129	0.483	0.203	7.97E-40	Meta_CD4_C5
ARPC3	3.34E-44	0.482603153	0.957	0.819	8.09E-40	Meta_CD4_C5
PPP1CA	5.09E-44	0.501265108	0.871	0.634	1.23E-39	Meta_CD4_C5
TMEM173	6.48E-44	0.560991356	0.610	0.296	1.57E-39	Meta_CD4_C5
GAPDH	3.31E-43	0.464125961	0.998	0.974	8.01E-39	Meta_CD4_C5
CTSB	9.63E-42	0.57097237	0.611	0.281	2.33E-37	Meta_CD4_C5
KLRB1	1.08E-41	0.932268127	0.715	0.454	2.63E-37	Meta_CD4_C5
CCDC167	1.57E-41	0.421637577	0.487	0.228	3.81E-37	Meta_CD4_C5
CD2	2.22E-41	0.597732275	0.973	0.835	5.37E-37	Meta_CD4_C5
UCP2	4.00E-41	0.730055426	0.765	0.484	9.68E-37	Meta_CD4_C5
GBP5	1.12E-40	0.879563261	0.653	0.386	2.71E-36	Meta_CD4_C5
CFL1	2.60E-40	0.477489502	0.996	0.957	6.29E-36	Meta_CD4_C5
IL4I1	2.82E-40	0.478021929	0.217	0.041	6.84E-36	Meta_CD4_C5
C4orf48	1.40E-38	0.419715889	0.636	0.368	3.38E-34	Meta_CD4_C5
IL32	1.56E-38	0.464816622	0.985	0.936	3.79E-34	Meta_CD4_C5
CPNE7	6.81E-38	0.38285164	0.270	0.084	1.65E-33	Meta_CD4_C5
STAT1	9.03E-38	0.871787711	0.640	0.369	2.19E-33	Meta_CD4_C5
PSME2	5.20E-37	0.601153154	0.872	0.651	1.26E-32	Meta_CD4_C5
GALM	5.37E-37	0.456905429	0.510	0.250	1.30E-32	Meta_CD4_C5
PSME1	5.89E-37	0.396695129	0.960	0.844	1.43E-32	Meta_CD4_C5
GNA15	9.47E-37	0.455017959	0.263	0.069	2.29E-32	Meta_CD4_C5
OSTF1	2.76E-36	0.493748919	0.750	0.503	6.68E-32	Meta_CD4_C5
IL23R	4.29E-36	0.344423655	0.169	0.031	1.04E-31	Meta_CD4_C5
RORA	4.34E-36	0.513119777	0.719	0.439	1.05E-31	Meta_CD4_C5
CTLA4	4.75E-36	0.678412688	0.538	0.236	1.15E-31	Meta_CD4_C5
CYTOR	1.50E-35	0.53993982	0.677	0.415	3.64E-31	Meta_CD4_C5
ICOS	1.57E-35	0.558247069	0.649	0.364	3.80E-31	Meta_CD4_C5
MAF	2.20E-35	0.485376421	0.436	0.172	5.33E-31	Meta_CD4_C5
SH2D2A	4.18E-35	0.520202325	0.716	0.451	1.01E-30	Meta_CD4_C5
TYMP	4.47E-35	0.395635785	0.493	0.269	1.08E-30	Meta_CD4_C5
TNFRSF18	1.21E-34	0.557426924	0.437	0.195	2.92E-30	Meta_CD4_C5
RBPJ	4.20E-34	0.506806216	0.596	0.324	1.02E-29	Meta_CD4_C5
CD28	6.84E-34	0.466139188	0.521	0.251	1.66E-29	Meta_CD4_C5
SMCO4	7.76E-34	0.473926091	0.274	0.078	1.88E-29	Meta_CD4_C5
LIMS1	8.48E-34	0.644937367	0.622	0.318	2.05E-29	Meta_CD4_C5
PSMB8	8.75E-34	0.458153329	0.813	0.590	2.12E-29	Meta_CD4_C5

TNFRSF4	1.17E-33	0.657852603	0.422	0.170	2.83E-29	Meta_CD4_C5
GBP4	1.20E-33	0.51758696	0.455	0.213	2.91E-29	Meta_CD4_C5
RAC2	1.58E-33	0.444349564	0.949	0.824	3.82E-29	Meta_CD4_C5
LINC01871	3.91E-33	0.623701373	0.551	0.299	9.48E-29	Meta_CD4_C5
CCL20	1.84E-32	0.755944766	0.225	0.051	4.45E-28	Meta_CD4_C5
NPDC1	2.61E-32	0.424103309	0.290	0.086	6.32E-28	Meta_CD4_C5
CTSC	3.18E-32	0.35442455	0.785	0.546	7.71E-28	Meta_CD4_C5
TBXAS1	3.67E-32	0.369540933	0.198	0.037	8.88E-28	Meta_CD4_C5
TAP1	3.72E-32	0.554946072	0.734	0.484	9.00E-28	Meta_CD4_C5
AQP3	4.88E-32	0.511562281	0.570	0.322	1.18E-27	Meta_CD4_C5
UBE2L6	5.40E-32	0.488682115	0.714	0.462	1.31E-27	Meta_CD4_C5
LAT	9.45E-32	0.419300533	0.835	0.619	2.29E-27	Meta_CD4_C5
YWHAQ	9.59E-32	0.488949199	0.814	0.555	2.32E-27	Meta_CD4_C5
PKM	1.12E-31	0.473817939	0.860	0.646	2.72E-27	Meta_CD4_C5
JPT1	1.83E-31	0.444180676	0.620	0.361	4.42E-27	Meta_CD4_C5
CSF2	1.99E-31	1.360538559	0.157	0.021	4.81E-27	Meta_CD4_C5
GBP1	2.98E-31	0.603937933	0.500	0.253	7.21E-27	Meta_CD4_C5
S100A11	3.02E-31	0.513843541	0.884	0.677	7.31E-27	Meta_CD4_C5
LTB	3.36E-31	0.931372642	0.829	0.604	8.14E-27	Meta_CD4_C5
YWHAH	5.11E-31	0.50852467	0.593	0.332	1.24E-26	Meta_CD4_C5
ARPC2	5.63E-31	0.40947028	0.979	0.881	1.36E-26	Meta_CD4_C5
EPSTI1	6.72E-31	0.417163814	0.478	0.228	1.63E-26	Meta_CD4_C5
CAP1	7.56E-31	0.46782322	0.858	0.633	1.83E-26	Meta_CD4_C5
GMFG	1.79E-30	0.480977691	0.933	0.761	4.34E-26	Meta_CD4_C5
FURIN	2.33E-30	0.461793088	0.301	0.092	5.64E-26	Meta_CD4_C5
COL5A3	2.75E-30	0.296956029	0.129	0.010	6.65E-26	Meta_CD4_C5
ZNRF1	5.04E-30	0.359229528	0.256	0.075	1.22E-25	Meta_CD4_C5
HCLS1	7.97E-30	0.369511467	0.860	0.666	1.93E-25	Meta_CD4_C5
LCP1	1.20E-29	0.375224538	0.899	0.729	2.90E-25	Meta_CD4_C5
FKBP11	2.23E-29	0.370685276	0.621	0.371	5.40E-25	Meta_CD4_C5
DNPH1	3.01E-29	0.44657773	0.546	0.281	7.28E-25	Meta_CD4_C5
DBNL	4.61E-29	0.330162614	0.541	0.309	1.12E-24	Meta_CD4_C5
IDH2	5.20E-29	0.36987591	0.662	0.429	1.26E-24	Meta_CD4_C5
IL26	6.31E-29	0.276740053	0.109	0.008	1.53E-24	Meta_CD4_C5
DEF6	7.14E-29	0.314954208	0.666	0.436	1.73E-24	Meta_CD4_C5
MVP	8.62E-29	0.370071623	0.592	0.341	2.09E-24	Meta_CD4_C5
SERF2	9.89E-29	0.367636342	0.989	0.939	2.40E-24	Meta_CD4_C5
LCK	1.15E-28	0.390376738	0.904	0.740	2.80E-24	Meta_CD4_C5
SEPT1	1.40E-28	0.408169681	0.836	0.638	3.38E-24	Meta_CD4_C5
IL12RB1	1.58E-28	0.454626426	0.436	0.190	3.83E-24	Meta_CD4_C5

CLIC1	1.78E-28	0.423598129	0.957	0.818	4.32E-24	Meta_CD4_C5
PSMB2	1.80E-28	0.316183291	0.603	0.365	4.35E-24	Meta_CD4_C5
TALDO1	1.82E-28	0.367965449	0.632	0.379	4.40E-24	Meta_CD4_C5
PSMC5	2.03E-28	0.253156729	0.627	0.411	4.91E-24	Meta_CD4_C5
WDR1	2.20E-28	0.512109475	0.786	0.534	5.32E-24	Meta_CD4_C5
RASAL3	2.36E-28	0.374542997	0.673	0.436	5.71E-24	Meta_CD4_C5
PSMB3	2.40E-28	0.317133336	0.765	0.532	5.81E-24	Meta_CD4_C5
SIT1	3.42E-28	0.411521036	0.655	0.401	8.28E-24	Meta_CD4_C5
ITGB2	4.18E-28	0.385323968	0.819	0.601	1.01E-23	Meta_CD4_C5
ANXA5	5.10E-28	0.459652589	0.699	0.432	1.24E-23	Meta_CD4_C5
SASH3	7.15E-28	0.480043847	0.658	0.413	1.73E-23	Meta_CD4_C5
ATP5MC3	7.92E-28	0.446536387	0.775	0.553	1.92E-23	Meta_CD4_C5
MSN	1.33E-27	0.364496934	0.826	0.604	3.22E-23	Meta_CD4_C5
PPP4C	2.28E-27	0.316852816	0.681	0.443	5.52E-23	Meta_CD4_C5
IL21	2.29E-27	0.6328806	0.115	0.009	5.55E-23	Meta_CD4_C5
NDUFB2	2.86E-27	0.280278483	0.791	0.580	6.92E-23	Meta_CD4_C5
CD38	3.12E-27	0.59998524	0.369	0.159	7.54E-23	Meta_CD4_C5
SDF4	3.19E-27	0.277018723	0.610	0.391	7.72E-23	Meta_CD4_C5
VAMP5	3.24E-27	0.348587521	0.555	0.332	7.85E-23	Meta_CD4_C5
PSMA6	3.32E-27	0.329453155	0.640	0.414	8.03E-23	Meta_CD4_C5
COX5A	3.69E-27	0.266150524	0.776	0.553	8.93E-23	Meta_CD4_C5
ENO1	4.25E-27	0.296195505	0.874	0.693	1.03E-22	Meta_CD4_C5
PBXIP1	4.48E-27	0.379081287	0.733	0.485	1.08E-22	Meta_CD4_C5
GPSM3	7.00E-27	0.418742232	0.876	0.693	1.70E-22	Meta_CD4_C5
FERMT3	7.39E-27	0.321614884	0.625	0.385	1.79E-22	Meta_CD4_C5
TANK	1.07E-26	0.291472066	0.530	0.309	2.58E-22	Meta_CD4_C5
FKBP1A	1.18E-26	0.383386893	0.806	0.559	2.85E-22	Meta_CD4_C5
GTF3C6	1.28E-26	0.260945661	0.550	0.325	3.09E-22	Meta_CD4_C5
PSMB10	1.83E-26	0.546868519	0.743	0.498	4.44E-22	Meta_CD4_C5
BST2	1.88E-26	0.409218966	0.712	0.484	4.55E-22	Meta_CD4_C5
PDIA6	2.02E-26	0.335687062	0.672	0.440	4.89E-22	Meta_CD4_C5
TBCB	3.77E-26	0.286823377	0.722	0.497	9.13E-22	Meta_CD4_C5
TWF2	4.16E-26	0.314882362	0.595	0.373	1.01E-21	Meta_CD4_C5
ACAA2	5.48E-26	0.314502905	0.433	0.218	1.33E-21	Meta_CD4_C5
PHTF2	5.77E-26	0.342406386	0.415	0.201	1.40E-21	Meta_CD4_C5
PMVK	7.38E-26	0.328295251	0.405	0.188	1.79E-21	Meta_CD4_C5
PRDX3	9.83E-26	0.295590439	0.476	0.257	2.38E-21	Meta_CD4_C5
COX6C	9.97E-26	0.266924768	0.912	0.765	2.41E-21	Meta_CD4_C5
WAS	1.39E-25	0.324618701	0.701	0.475	3.37E-21	Meta_CD4_C5
PPP1R18	1.42E-25	0.393742965	0.753	0.519	3.43E-21	Meta_CD4_C5

RUNX2	1.98E-25	0.352903671	0.308	0.112	4.81E-21	Meta_CD4_C5
SYTL1	2.50E-25	0.339220744	0.525	0.296	6.06E-21	Meta_CD4_C5
ARL6IP5	2.57E-25	0.271834921	0.874	0.691	6.23E-21	Meta_CD4_C5
CHMP2A	2.84E-25	0.357116108	0.612	0.361	6.87E-21	Meta_CD4_C5
CEBPD	2.95E-25	0.675512594	0.306	0.139	7.14E-21	Meta_CD4_C5
SKA2	3.23E-25	0.261672282	0.339	0.157	7.82E-21	Meta_CD4_C5
CD3D	3.83E-25	0.327152527	0.978	0.886	9.27E-21	Meta_CD4_C5
IFITM2	3.85E-25	0.332114361	0.923	0.800	9.32E-21	Meta_CD4_C5
PSMB9	4.41E-25	0.402343598	0.858	0.677	1.07E-20	Meta_CD4_C5
CERK	4.62E-25	0.320563631	0.375	0.177	1.12E-20	Meta_CD4_C5
PSMA1	4.76E-25	0.270904304	0.645	0.424	1.15E-20	Meta_CD4_C5
NOP10	4.98E-25	0.283521227	0.710	0.483	1.21E-20	Meta_CD4_C5
COPE	5.07E-25	0.259349816	0.782	0.573	1.23E-20	Meta_CD4_C5
YWHAB	5.75E-25	0.341229522	0.935	0.805	1.39E-20	Meta_CD4_C5
CORO1B	6.12E-25	0.434547487	0.641	0.394	1.48E-20	Meta_CD4_C5
IL17A	6.71E-25	1.652039966	0.106	0.008	1.62E-20	Meta_CD4_C5
CD5	8.18E-25	0.381746464	0.650	0.415	1.98E-20	Meta_CD4_C5
ETV7	8.90E-25	0.307462454	0.189	0.058	2.15E-20	Meta_CD4_C5
TPI1	9.62E-25	0.272159945	0.884	0.708	2.33E-20	Meta_CD4_C5
VDAC1	1.02E-24	0.309609285	0.656	0.420	2.46E-20	Meta_CD4_C5
ACTR3	1.10E-24	0.444591754	0.823	0.611	2.67E-20	Meta_CD4_C5
TMSB4X	1.13E-24	0.293162591	1.000	0.999	2.74E-20	Meta_CD4_C5
UBE2N	1.36E-24	0.34108599	0.683	0.437	3.29E-20	Meta_CD4_C5
LSP1	1.69E-24	0.368333031	0.955	0.824	4.10E-20	Meta_CD4_C5
ATP5PB	2.47E-24	0.365829726	0.671	0.445	5.98E-20	Meta_CD4_C5
ATP2B4	2.98E-24	0.281147334	0.480	0.269	7.22E-20	Meta_CD4_C5
ATP5MF	3.32E-24	0.434490218	0.732	0.495	8.05E-20	Meta_CD4_C5
AP2S1	3.75E-24	0.285141238	0.584	0.342	9.09E-20	Meta_CD4_C5
CCDC107	3.95E-24	0.306451667	0.609	0.373	9.57E-20	Meta_CD4_C5
CKLF	4.45E-24	0.420893996	0.790	0.553	1.08E-19	Meta_CD4_C5
C9orf16	6.59E-24	0.344597764	0.820	0.609	1.60E-19	Meta_CD4_C5
GYG1	7.99E-24	0.286499233	0.567	0.352	1.93E-19	Meta_CD4_C5
RNF213	8.11E-24	0.383293981	0.788	0.574	1.96E-19	Meta_CD4_C5
PGAM1	8.32E-24	0.402345504	0.785	0.563	2.01E-19	Meta_CD4_C5
RHOA	1.13E-23	0.372426685	0.945	0.827	2.74E-19	Meta_CD4_C5
SEM1	1.31E-23	0.471967463	0.700	0.459	3.17E-19	Meta_CD4_C5
EIF3I	2.12E-23	0.255839243	0.647	0.430	5.13E-19	Meta_CD4_C5
CSK	2.25E-23	0.43106427	0.660	0.427	5.45E-19	Meta_CD4_C5
PRDX1	2.91E-23	0.348475843	0.757	0.527	7.05E-19	Meta_CD4_C5
CLTA	3.22E-23	0.263188014	0.600	0.383	7.79E-19	Meta_CD4_C5

EVL	3.44E-23	0.335242819	0.931	0.796	8.32E-19	Meta_CD4_C5
EMB	4.63E-23	0.273746511	0.682	0.466	1.12E-18	Meta_CD4_C5
SELENOT	4.93E-23	0.324145708	0.662	0.442	1.19E-18	Meta_CD4_C5
SQOR	5.10E-23	0.363734584	0.410	0.193	1.24E-18	Meta_CD4_C5
NDUFB3	6.06E-23	0.322356644	0.564	0.328	1.47E-18	Meta_CD4_C5
CMTM6	9.11E-23	0.250782403	0.576	0.359	2.21E-18	Meta_CD4_C5
TXN	9.57E-23	0.441638181	0.800	0.548	2.32E-18	Meta_CD4_C5
ARPC5	9.63E-23	0.365840898	0.759	0.529	2.33E-18	Meta_CD4_C5
RNF149	1.01E-22	0.38388149	0.667	0.443	2.43E-18	Meta_CD4_C5
NDUFV2	1.05E-22	0.420825312	0.724	0.493	2.54E-18	Meta_CD4_C5
FKBP8	1.08E-22	0.265990182	0.790	0.589	2.60E-18	Meta_CD4_C5
ANXA6	1.12E-22	0.367768635	0.751	0.521	2.70E-18	Meta_CD4_C5
TNFRSF14	1.20E-22	0.348245807	0.661	0.434	2.89E-18	Meta_CD4_C5
FBXW5	1.21E-22	0.251060103	0.502	0.294	2.93E-18	Meta_CD4_C5
IFI35	1.60E-22	0.256145042	0.455	0.245	3.87E-18	Meta_CD4_C5
COX8A	2.06E-22	0.312681137	0.874	0.689	4.98E-18	Meta_CD4_C5
CALM3	2.29E-22	0.334156864	0.773	0.561	5.54E-18	Meta_CD4_C5
RAP1B	3.35E-22	0.364761104	0.796	0.591	8.11E-18	Meta_CD4_C5
C12orf75	5.43E-22	0.297933604	0.512	0.315	1.32E-17	Meta_CD4_C5
PAK2	5.99E-22	0.287413047	0.655	0.448	1.45E-17	Meta_CD4_C5
M6PR	6.94E-22	0.341050437	0.585	0.372	1.68E-17	Meta_CD4_C5
ERN1	8.16E-22	0.284452523	0.443	0.245	1.98E-17	Meta_CD4_C5
EML4	8.34E-22	0.354153994	0.751	0.526	2.02E-17	Meta_CD4_C5
FKBP5	8.88E-22	0.284671474	0.477	0.283	2.15E-17	Meta_CD4_C5
CCR5	9.46E-22	0.266946485	0.281	0.118	2.29E-17	Meta_CD4_C5
GABARAP	1.22E-21	0.338892297	0.828	0.650	2.95E-17	Meta_CD4_C5
PPIB	1.61E-21	0.326019707	0.919	0.791	3.89E-17	Meta_CD4_C5
GNG5	1.84E-21	0.278948069	0.760	0.563	4.46E-17	Meta_CD4_C5
PPA1	1.88E-21	0.259680825	0.615	0.407	4.56E-17	Meta_CD4_C5
GBP2	2.13E-21	0.424590568	0.624	0.406	5.16E-17	Meta_CD4_C5
BRK1	2.91E-21	0.321689609	0.804	0.590	7.05E-17	Meta_CD4_C5
LDHB	2.93E-21	0.269703033	0.899	0.742	7.09E-17	Meta_CD4_C5
SUB1	3.06E-21	0.265305095	0.934	0.804	7.40E-17	Meta_CD4_C5
MYL12B	5.57E-21	0.316890546	0.964	0.852	1.35E-16	Meta_CD4_C5
ARPC4	6.82E-21	0.260695174	0.473	0.272	1.65E-16	Meta_CD4_C5
TNFSF10	8.08E-21	0.366931374	0.441	0.220	1.96E-16	Meta_CD4_C5
ATP5MC2	9.87E-21	0.321775091	0.864	0.739	2.39E-16	Meta_CD4_C5
PSMA5	1.03E-20	0.345820078	0.665	0.432	2.49E-16	Meta_CD4_C5
THEMIS	1.04E-20	0.294628855	0.522	0.318	2.51E-16	Meta_CD4_C5
FYB1	1.46E-20	0.508946708	0.806	0.625	3.54E-16	Meta_CD4_C5

GADD45G	1.81E-20	0.376662523	0.227	0.076	4.39E-16	Meta_CD4_C5
COPZ1	2.33E-20	0.275504266	0.529	0.309	5.65E-16	Meta_CD4_C5
HNRNPLL	2.39E-20	0.342398986	0.551	0.312	5.78E-16	Meta_CD4_C5
GIMAP4	2.75E-20	0.458008388	0.729	0.531	6.66E-16	Meta_CD4_C5
LMO4	3.05E-20	0.480414506	0.370	0.160	7.38E-16	Meta_CD4_C5
CD74	3.12E-20	0.322183346	0.959	0.870	7.56E-16	Meta_CD4_C5
SUSD3	3.94E-20	0.338371785	0.481	0.262	9.53E-16	Meta_CD4_C5
RNF167	5.60E-20	0.334325264	0.656	0.425	1.36E-15	Meta_CD4_C5
CASP4	7.38E-20	0.257690551	0.623	0.420	1.79E-15	Meta_CD4_C5
LY6E	7.61E-20	0.265824895	0.894	0.732	1.84E-15	Meta_CD4_C5
PSMC4	7.80E-20	0.291074474	0.510	0.296	1.89E-15	Meta_CD4_C5
DUSP5	7.92E-20	0.416891071	0.386	0.192	1.92E-15	Meta_CD4_C5
PNP	8.38E-20	0.266707999	0.495	0.289	2.03E-15	Meta_CD4_C5
S100A6	8.43E-20	0.360745939	0.968	0.884	2.04E-15	Meta_CD4_C5
PYCARD	9.11E-20	0.25899849	0.491	0.286	2.21E-15	Meta_CD4_C5
ADI1	1.20E-19	0.254942709	0.371	0.189	2.91E-15	Meta_CD4_C5
DYNLL1	1.30E-19	0.353699112	0.754	0.541	3.15E-15	Meta_CD4_C5
LGALS3BP	1.47E-19	0.288216166	0.251	0.103	3.56E-15	Meta_CD4_C5
ETHE1	1.55E-19	0.338974688	0.476	0.251	3.75E-15	Meta_CD4_C5
CNN2	2.02E-19	0.427234996	0.809	0.621	4.90E-15	Meta_CD4_C5
TMBIM6	2.20E-19	0.253258264	0.903	0.762	5.32E-15	Meta_CD4_C5
ITGA4	3.58E-19	0.476540104	0.655	0.420	8.66E-15	Meta_CD4_C5
MYL12A	4.48E-19	0.334262017	0.973	0.881	1.08E-14	Meta_CD4_C5
PRDX2	4.81E-19	0.288530967	0.686	0.459	1.16E-14	Meta_CD4_C5
PPM1M	5.04E-19	0.301810097	0.390	0.200	1.22E-14	Meta_CD4_C5
PSMD8	5.30E-19	0.279361378	0.723	0.498	1.28E-14	Meta_CD4_C5
GIMAP5	1.23E-18	0.316486789	0.399	0.212	2.97E-14	Meta_CD4_C5
ARF5	1.54E-18	0.304505579	0.752	0.540	3.72E-14	Meta_CD4_C5
GIMAP7	1.57E-18	0.381361828	0.799	0.617	3.79E-14	Meta_CD4_C5
ARF3	1.66E-18	0.260315632	0.393	0.208	4.02E-14	Meta_CD4_C5
RTRAF	2.15E-18	0.272651416	0.680	0.474	5.20E-14	Meta_CD4_C5
ATP5F1C	2.47E-18	0.386265064	0.679	0.443	5.98E-14	Meta_CD4_C5
ALOX5AP	2.59E-18	0.431916031	0.799	0.593	6.27E-14	Meta_CD4_C5
ATP5F1A	2.69E-18	0.278872434	0.654	0.457	6.51E-14	Meta_CD4_C5
PRELID1	2.94E-18	0.26725842	0.723	0.517	7.13E-14	Meta_CD4_C5
LPXN	3.12E-18	0.372361055	0.630	0.392	7.57E-14	Meta_CD4_C5
SLC9A3R1	3.16E-18	0.407265675	0.780	0.566	7.66E-14	Meta_CD4_C5
RHOG	3.30E-18	0.294481903	0.725	0.508	7.99E-14	Meta_CD4_C5
SELPLG	3.34E-18	0.324033887	0.662	0.451	8.09E-14	Meta_CD4_C5
IL2RG	3.90E-18	0.356062848	0.784	0.576	9.43E-14	Meta_CD4_C5

RGS19	3.97E-18	0.263965222	0.532	0.339	9.62E-14	Meta_CD4_C5
SEC61B	4.42E-18	0.264247112	0.828	0.644	1.07E-13	Meta_CD4_C5
ADSL	4.76E-18	0.255881498	0.438	0.241	1.15E-13	Meta_CD4_C5
MYH9	5.45E-18	0.268759832	0.821	0.645	1.32E-13	Meta_CD4_C5
FAM104A	6.34E-18	0.255066644	0.380	0.194	1.54E-13	Meta_CD4_C5
TADA3	6.67E-18	0.254714939	0.462	0.266	1.61E-13	Meta_CD4_C5
H2AFV	6.88E-18	0.276167021	0.728	0.526	1.67E-13	Meta_CD4_C5
FLT3LG	6.92E-18	0.343475634	0.657	0.432	1.68E-13	Meta_CD4_C5
TPM3	6.98E-18	0.295055838	0.912	0.766	1.69E-13	Meta_CD4_C5
LCP2	1.07E-17	0.365503823	0.791	0.586	2.60E-13	Meta_CD4_C5
HNRNPA3	1.08E-17	0.252489247	0.792	0.602	2.61E-13	Meta_CD4_C5
SLC25A5	1.23E-17	0.27554957	0.805	0.632	2.99E-13	Meta_CD4_C5
NOSIP	1.54E-17	0.312498584	0.595	0.393	3.74E-13	Meta_CD4_C5
ATP5F1E	2.04E-17	0.371962888	0.883	0.779	4.93E-13	Meta_CD4_C5
JAK3	3.23E-17	0.296606908	0.473	0.275	7.83E-13	Meta_CD4_C5
KDM5B	3.23E-17	0.26772319	0.307	0.146	7.83E-13	Meta_CD4_C5
GDE1	4.19E-17	0.267894842	0.311	0.149	1.01E-12	Meta_CD4_C5
TRAF3IP3	4.62E-17	0.316038389	0.727	0.515	1.12E-12	Meta_CD4_C5
ATP5PF	4.78E-17	0.339060523	0.687	0.467	1.16E-12	Meta_CD4_C5
ATP5MG	5.10E-17	0.369181013	0.870	0.745	1.24E-12	Meta_CD4_C5
ATP5IF1	5.81E-17	0.294037626	0.737	0.535	1.41E-12	Meta_CD4_C5
ATP5PD	7.04E-17	0.286819171	0.629	0.423	1.70E-12	Meta_CD4_C5
ITGB7	7.49E-17	0.250013022	0.557	0.370	1.81E-12	Meta_CD4_C5
PCBP1	8.34E-17	0.372085556	0.885	0.756	2.02E-12	Meta_CD4_C5
ELOB	1.41E-16	0.311544015	0.824	0.653	3.42E-12	Meta_CD4_C5
RAB1B	1.55E-16	0.256346485	0.595	0.389	3.77E-12	Meta_CD4_C5
FMNL1	1.89E-16	0.288886053	0.598	0.392	4.58E-12	Meta_CD4_C5
SEPT6	2.23E-16	0.255458697	0.769	0.570	5.39E-12	Meta_CD4_C5
BHLHE40	2.25E-16	0.318977014	0.583	0.372	5.46E-12	Meta_CD4_C5
NFKBIZ	2.42E-16	0.426809099	0.558	0.353	5.86E-12	Meta_CD4_C5
GSDMD	2.84E-16	0.274522198	0.512	0.301	6.88E-12	Meta_CD4_C5
TESC	3.19E-16	0.298719207	0.284	0.130	7.73E-12	Meta_CD4_C5
ZBTB7B	4.36E-16	0.260807801	0.214	0.072	1.06E-11	Meta_CD4_C5
SEPT9	4.50E-16	0.268780348	0.809	0.631	1.09E-11	Meta_CD4_C5
WIPF1	6.60E-16	0.255818183	0.789	0.602	1.60E-11	Meta_CD4_C5
GPRIN3	7.66E-16	0.322318058	0.544	0.337	1.85E-11	Meta_CD4_C5
TRIM69	1.09E-15	0.258233922	0.329	0.159	2.65E-11	Meta_CD4_C5
POLD4	1.10E-15	0.267707151	0.604	0.406	2.66E-11	Meta_CD4_C5
LAIR1	1.13E-15	0.323129456	0.363	0.182	2.75E-11	Meta_CD4_C5
TOX2	1.15E-15	0.257254154	0.198	0.071	2.79E-11	Meta_CD4_C5

SUMO2	1.42E-15	0.257004504	0.927	0.799	3.43E-11	Meta_CD4_C5
GNB2	2.09E-15	0.296951219	0.735	0.537	5.07E-11	Meta_CD4_C5
RPS6KA1	2.17E-15	0.252247883	0.371	0.194	5.25E-11	Meta_CD4_C5
ATP5F1B	2.43E-15	0.381224412	0.790	0.599	5.89E-11	Meta_CD4_C5
MTHFD2	2.85E-15	0.31464938	0.442	0.248	6.90E-11	Meta_CD4_C5
SAMHD1	3.22E-15	0.255185741	0.550	0.358	7.80E-11	Meta_CD4_C5
RGS10	3.38E-15	0.261404787	0.799	0.614	8.18E-11	Meta_CD4_C5
NCKAP1L	7.18E-15	0.269816741	0.456	0.261	1.74E-10	Meta_CD4_C5
MGAT4A	8.93E-15	0.296006341	0.582	0.376	2.16E-10	Meta_CD4_C5
CYBC1	1.14E-14	0.30900117	0.488	0.288	2.77E-10	Meta_CD4_C5
GPR34	1.25E-14	0.285224794	0.178	0.060	3.03E-10	Meta_CD4_C5
RTF2	1.30E-14	0.275248423	0.477	0.281	3.15E-10	Meta_CD4_C5
LIMD2	1.51E-14	0.397047346	0.851	0.709	3.67E-10	Meta_CD4_C5
ESYT1	1.93E-14	0.306245734	0.460	0.261	4.68E-10	Meta_CD4_C5
RHOF	4.85E-14	0.272682237	0.699	0.490	1.18E-09	Meta_CD4_C5
CAMK1	5.68E-14	0.298667963	0.156	0.038	1.38E-09	Meta_CD4_C5
RILPL2	7.25E-14	0.271723961	0.448	0.260	1.76E-09	Meta_CD4_C5
ITM2A	9.22E-14	0.311523598	0.789	0.601	2.23E-09	Meta_CD4_C5
ASB2	9.73E-14	0.430179817	0.342	0.163	2.36E-09	Meta_CD4_C5
RBL2	1.40E-13	0.345057818	0.621	0.416	3.40E-09	Meta_CD4_C5
BCL3	1.91E-13	0.253468825	0.330	0.186	4.63E-09	Meta_CD4_C5
CALCOCO2	2.20E-13	0.275943211	0.524	0.318	5.32E-09	Meta_CD4_C5
CRIP1	2.56E-13	0.290789683	0.870	0.740	6.20E-09	Meta_CD4_C5
RNASEK	5.46E-13	0.317044742	0.664	0.492	1.32E-08	Meta_CD4_C5
MAP1LC3A	8.96E-13	0.254910512	0.217	0.090	2.17E-08	Meta_CD4_C5
LBH	1.24E-12	0.276990271	0.667	0.478	2.99E-08	Meta_CD4_C5
S1PR4	1.34E-12	0.257512944	0.445	0.271	3.24E-08	Meta_CD4_C5
DUSP4	2.27E-12	0.264082098	0.545	0.366	5.48E-08	Meta_CD4_C5
RARRES3	3.13E-12	0.311759818	0.872	0.733	7.57E-08	Meta_CD4_C5
PPIA	4.97E-12	0.278951863	0.953	0.872	1.20E-07	Meta_CD4_C5
LIMA1	5.74E-12	0.261069969	0.237	0.095	1.39E-07	Meta_CD4_C5
NCDN	6.48E-11	0.271676414	0.184	0.061	1.57E-06	Meta_CD4_C5
PLSCR3	1.07E-10	0.272098116	0.362	0.196	2.60E-06	Meta_CD4_C5
ZFP36L1	1.72E-10	0.448255184	0.807	0.672	4.17E-06	Meta_CD4_C5
IRF1	6.09E-10	0.341480934	0.729	0.579	1.47E-05	Meta_CD4_C5
PHLDA1	8.64E-10	0.288400234	0.405	0.271	2.09E-05	Meta_CD4_C5
MT2A	5.37E-08	0.261372403	0.638	0.518	1.30E-03	Meta_CD4_C5
TIMP1	3.95E-06	0.276990926	0.379	0.248	9.58E-02	Meta_CD4_C5
LINC01943	5.68E-05	0.279375234	0.211	0.112	1.00E+00	Meta_CD4_C5
MIR155HG	1.41E-04	0.419076822	0.193	0.101	1.00E+00	Meta_CD4_C5

CXCL13	2.82E-03	1.137969106	0.110	0.066	1.00E+00	Meta_CD4_C5
ZFP36L2	6.58E-178	1.728887354	0.993	0.870	1.59E-173	Meta_CD4_C6
RPS10	8.86E-165	1.406085107	0.947	0.737	2.14E-160	Meta_CD4_C6
FTH1	3.95E-161	1.674038522	0.999	0.981	9.56E-157	Meta_CD4_C6
SLC7A5	3.77E-151	1.86256965	0.838	0.263	9.13E-147	Meta_CD4_C6
CDKN1A	1.53E-137	1.796893965	0.752	0.185	3.70E-133	Meta_CD4_C6
EIF1	1.78E-135	0.919529947	1.000	0.985	4.30E-131	Meta_CD4_C6
BTG1	9.56E-130	1.085320376	1.000	0.973	2.31E-125	Meta_CD4_C6
ELL2	8.03E-124	1.49306144	0.651	0.103	1.94E-119	Meta_CD4_C6
P2RY8	2.16E-121	1.474567478	0.741	0.225	5.24E-117	Meta_CD4_C6
CSRNP1	7.75E-118	1.407734266	0.925	0.503	1.88E-113	Meta_CD4_C6
DDIT4	3.15E-112	1.643078225	0.937	0.586	7.64E-108	Meta_CD4_C6
RPS16	7.69E-109	0.707763802	0.999	0.983	1.86E-104	Meta_CD4_C6
ZFP36	7.04E-107	1.412894156	0.980	0.823	1.71E-102	Meta_CD4_C6
NINJ1	3.67E-106	1.660098056	0.707	0.214	8.88E-102	Meta_CD4_C6
SNX9	6.72E-105	1.333861016	0.699	0.235	1.63E-100	Meta_CD4_C6
PTMA	3.26E-100	0.719542102	1.000	0.985	7.90E-96	Meta_CD4_C6
PBX4	1.94E-99	1.31304612	0.691	0.244	4.69E-95	Meta_CD4_C6
PER1	3.39E-98	1.366175638	0.703	0.220	8.20E-94	Meta_CD4_C6
UBC	6.62E-98	0.924668499	1.000	0.966	1.60E-93	Meta_CD4_C6
SPOCK2	3.72E-96	1.097857374	0.947	0.684	9.01E-92	Meta_CD4_C6
TRAF4	9.49E-96	1.389031677	0.563	0.110	2.30E-91	Meta_CD4_C6
TGFB1	2.22E-95	1.268898734	0.831	0.454	5.37E-91	Meta_CD4_C6
EPC1	1.18E-94	1.022555658	0.909	0.572	2.85E-90	Meta_CD4_C6
RPL30	2.38E-91	0.573073591	0.999	0.988	5.76E-87	Meta_CD4_C6
RNF125	1.91E-90	1.236747298	0.752	0.319	4.63E-86	Meta_CD4_C6
KDM6B	2.29E-90	1.336493442	0.659	0.199	5.55E-86	Meta_CD4_C6
HSPA5	2.85E-90	1.139066006	0.935	0.644	6.91E-86	Meta_CD4_C6
RPS4Y1	1.06E-88	0.731457223	0.935	0.536	2.56E-84	Meta_CD4_C6
GABARAPL1	1.05E-86	1.493042728	0.715	0.292	2.54E-82	Meta_CD4_C6
FAM177A1	1.01E-85	1.159366475	0.801	0.419	2.45E-81	Meta_CD4_C6
FXYD5	1.34E-85	0.818556083	0.982	0.854	3.24E-81	Meta_CD4_C6
BHLHE40	3.03E-84	1.327130071	0.790	0.380	7.33E-80	Meta_CD4_C6
RBM3	1.52E-76	0.861095339	0.926	0.697	3.67E-72	Meta_CD4_C6
H3F3B	1.81E-76	0.717865131	0.997	0.979	4.37E-72	Meta_CD4_C6
DNAJB6	2.94E-76	1.048837582	0.814	0.466	7.12E-72	Meta_CD4_C6
TNFAIP3	2.72E-75	0.859302125	0.984	0.772	6.58E-71	Meta_CD4_C6
ARID5A	3.89E-74	1.057400802	0.750	0.399	9.42E-70	Meta_CD4_C6
PABPC1	5.47E-74	0.909362232	0.995	0.895	1.33E-69	Meta_CD4_C6
DDX24	3.06E-73	0.940381811	0.902	0.648	7.41E-69	Meta_CD4_C6

RPS21	1.30E-72	0.561094493	1.000	0.947	3.15E-68	Meta_CD4_C6
RPS25	4.69E-72	0.498780285	0.999	0.969	1.13E-67	Meta_CD4_C6
ZNF331	7.44E-72	1.12414058	0.758	0.340	1.80E-67	Meta_CD4_C6
PTP4A1	5.34E-71	1.183679493	0.711	0.302	1.29E-66	Meta_CD4_C6
IRF2BP2	1.23E-70	1.08165422	0.670	0.268	2.99E-66	Meta_CD4_C6
HNRNPA0	1.58E-70	0.866264197	0.923	0.691	3.82E-66	Meta_CD4_C6
SRSF5	3.26E-70	0.7902844	0.967	0.759	7.88E-66	Meta_CD4_C6
LMNA	1.30E-69	0.761025605	0.715	0.273	3.16E-65	Meta_CD4_C6
SLC2A3	1.42E-69	1.059728033	0.914	0.644	3.45E-65	Meta_CD4_C6
RPL28	2.33E-68	0.455185481	1.000	0.995	5.64E-64	Meta_CD4_C6
RPS28	9.70E-68	0.511992031	0.999	0.964	2.35E-63	Meta_CD4_C6
PCBP2	5.37E-66	0.667843058	0.984	0.815	1.30E-61	Meta_CD4_C6
SLBP	7.02E-65	1.18961503	0.626	0.336	1.70E-60	Meta_CD4_C6
HNRNPH3	8.37E-65	0.924040644	0.770	0.440	2.03E-60	Meta_CD4_C6
LDHA	1.32E-64	0.851971625	0.962	0.788	3.20E-60	Meta_CD4_C6
ABCG1	1.79E-64	1.090445321	0.526	0.173	4.32E-60	Meta_CD4_C6
TIPARP	1.95E-64	1.094259083	0.627	0.243	4.73E-60	Meta_CD4_C6
UBE2D3	9.27E-64	0.725433889	0.935	0.769	2.24E-59	Meta_CD4_C6
TSC22D3	1.06E-62	0.795333	0.987	0.890	2.57E-58	Meta_CD4_C6
TUBA4A	1.48E-62	1.050435218	0.836	0.539	3.59E-58	Meta_CD4_C6
SRRM1	1.64E-62	0.885236372	0.867	0.584	3.96E-58	Meta_CD4_C6
PDE4B	3.14E-62	1.186381207	0.744	0.387	7.60E-58	Meta_CD4_C6
RPS27	3.75E-62	0.502240512	1.000	0.996	9.09E-58	Meta_CD4_C6
IDI1	1.22E-61	0.950658683	0.672	0.310	2.96E-57	Meta_CD4_C6
YIPF5	1.43E-61	1.081591219	0.614	0.268	3.45E-57	Meta_CD4_C6
PDE4D	1.48E-61	1.198207287	0.650	0.316	3.59E-57	Meta_CD4_C6
EML4	1.59E-60	0.915351414	0.835	0.536	3.86E-56	Meta_CD4_C6
RPL39	1.81E-60	0.514763735	0.991	0.893	4.37E-56	Meta_CD4_C6
YPEL5	1.94E-59	0.919223728	0.863	0.584	4.69E-55	Meta_CD4_C6
LAPTM5	1.49E-58	0.578505068	0.985	0.891	3.62E-54	Meta_CD4_C6
CEMIP2	3.91E-58	0.984801548	0.646	0.297	9.48E-54	Meta_CD4_C6
DDX3Y	5.57E-58	0.925273573	0.610	0.210	1.35E-53	Meta_CD4_C6
BACH2	1.06E-57	0.857377382	0.402	0.088	2.57E-53	Meta_CD4_C6
SBDS	6.95E-57	0.880838727	0.797	0.459	1.68E-52	Meta_CD4_C6
RPS27A	1.80E-56	0.402585574	0.999	0.994	4.35E-52	Meta_CD4_C6
RPL9	3.05E-56	0.440966541	0.999	0.973	7.39E-52	Meta_CD4_C6
FAU	1.22E-55	0.400766692	0.997	0.986	2.95E-51	Meta_CD4_C6
DAZAP2	1.23E-55	0.662850449	0.960	0.784	2.98E-51	Meta_CD4_C6
UPP1	2.11E-55	0.912594034	0.610	0.270	5.11E-51	Meta_CD4_C6
EEF1A1	2.87E-55	0.520900076	0.993	0.911	6.95E-51	Meta_CD4_C6

LITAF	3.51E-55	1.179434667	0.789	0.491	8.49E-51	Meta_CD4_C6
GNA15	1.40E-54	0.947147117	0.370	0.077	3.40E-50	Meta_CD4_C6
GRAMD1B	1.75E-54	0.825632338	0.374	0.098	4.25E-50	Meta_CD4_C6
MAP1LC3B	1.10E-53	0.754927957	0.823	0.565	2.66E-49	Meta_CD4_C6
RPS12	1.25E-53	0.430133164	1.000	0.994	3.02E-49	Meta_CD4_C6
RPS14	1.28E-53	0.375409886	0.999	0.995	3.11E-49	Meta_CD4_C6
RPS23	1.72E-53	0.407654821	0.997	0.979	4.18E-49	Meta_CD4_C6
ARL4C	2.23E-53	0.827096537	0.810	0.533	5.40E-49	Meta_CD4_C6
RPS7	2.38E-53	0.393661217	1.000	0.984	5.77E-49	Meta_CD4_C6
STK17B	2.00E-52	0.724133357	0.905	0.676	4.84E-48	Meta_CD4_C6
NFKB1	7.01E-52	0.883066521	0.576	0.257	1.70E-47	Meta_CD4_C6
TSPYL2	7.21E-52	0.829699338	0.705	0.326	1.75E-47	Meta_CD4_C6
RASA3	1.42E-51	0.925910402	0.536	0.194	3.43E-47	Meta_CD4_C6
FOXP1	1.93E-51	0.8372575	0.701	0.371	4.66E-47	Meta_CD4_C6
ZC3H12A	1.08E-50	0.881591961	0.542	0.223	2.62E-46	Meta_CD4_C6
YWHAZ	2.08E-50	0.622661803	0.982	0.830	5.0394E-46	Meta_CD4_C6
HAUS3	4.23E-50	0.796326299	0.499	0.177	1.0238E-45	Meta_CD4_C6
CD44	4.97E-50	0.625563704	0.943	0.763	1.2044E-45	Meta_CD4_C6
SMIM3	7.61E-50	0.895365733	0.375	0.094	1.84E-45	Meta_CD4_C6
RGS16	7.82E-50	1.054949217	0.373	0.071	1.89E-45	Meta_CD4_C6
SARAF	1.01E-49	0.643259474	0.996	0.926	2.44E-45	Meta_CD4_C6
MAFF	1.33E-49	1.018144923	0.411	0.104	3.21E-45	Meta_CD4_C6
RPL34	2.84E-49	0.371424864	0.999	0.993	6.88E-45	Meta_CD4_C6
RPL41	4.22E-49	0.571781608	0.993	0.913	1.02E-44	Meta_CD4_C6
CRYBG1	5.05E-49	0.920418929	0.522	0.190	1.22E-44	Meta_CD4_C6
RPL17	5.81E-49	0.52876655	0.984	0.859	1.41E-44	Meta_CD4_C6
S1PR1	1.11E-48	0.904316256	0.440	0.126	2.70E-44	Meta_CD4_C6
ETF1	2.41E-48	0.816203906	0.536	0.226	5.83E-44	Meta_CD4_C6
CCNH	1.84E-47	0.875258547	0.686	0.377	4.45E-43	Meta_CD4_C6
RPL37	7.25E-47	0.419591868	1.000	0.971	1.75E-42	Meta_CD4_C6
VIM	9.29E-47	0.619817893	0.992	0.897	2.25E-42	Meta_CD4_C6
PLK3	5.08E-46	0.837343302	0.584	0.253	1.23E-41	Meta_CD4_C6
JUN	5.66E-46	0.524942748	0.967	0.805	1.37E-41	Meta_CD4_C6
SRSF7	7.66E-46	0.612690887	0.955	0.804	1.85E-41	Meta_CD4_C6
CXCR3	1.12E-44	0.88930376	0.757	0.478	2.71E-40	Meta_CD4_C6
DUSP4	1.15E-44	0.901598882	0.686	0.373	2.77E-40	Meta_CD4_C6
JMJD1C	2.46E-44	0.872360713	0.653	0.354	5.97E-40	Meta_CD4_C6
NOP53	2.77E-44	0.658024937	0.917	0.696	6.70E-40	Meta_CD4_C6
KLF3	2.83E-44	0.828336011	0.465	0.172	6.86E-40	Meta_CD4_C6
WDR74	9.70E-44	0.855861286	0.513	0.198	2.35E-39	Meta_CD4_C6

IDS	9.79E-44	0.713189567	0.830	0.577	2.37E-39	Meta_CD4_C6
RBM39	2.34E-43	0.654501978	0.914	0.708	5.67E-39	Meta_CD4_C6
SRSF3	6.61E-43	0.656548771	0.873	0.615	1.60E-38	Meta_CD4_C6
TOB1	6.62E-43	1.013222206	0.573	0.263	1.60E-38	Meta_CD4_C6
CYTH1	7.82E-43	0.740149942	0.674	0.368	1.89E-38	Meta_CD4_C6
CEBPZ	1.71E-42	0.847931955	0.589	0.291	4.14E-38	Meta_CD4_C6
DUSP2	1.78E-42	0.614904444	0.911	0.635	4.31E-38	Meta_CD4_C6
RNMT	6.57E-42	0.718301564	0.572	0.268	1.59E-37	Meta_CD4_C6
PFKFB3	1.59E-41	0.786239834	0.457	0.183	3.84E-37	Meta_CD4_C6
MED10	1.68E-41	0.580793799	0.708	0.443	4.07E-37	Meta_CD4_C6
HMGB1	2.03E-41	0.580897368	0.983	0.872	4.93E-37	Meta_CD4_C6
PHF1	4.40E-41	0.707474835	0.663	0.343	1.07E-36	Meta_CD4_C6
RPS15A	4.53E-41	0.339532055	1.000	0.993	1.10E-36	Meta_CD4_C6
RELB	8.54E-41	0.800397788	0.618	0.316	2.07E-36	Meta_CD4_C6
SYNJ2	1.30E-40	0.708316909	0.346	0.095	3.15E-36	Meta_CD4_C6
NR1H2	1.78E-40	0.685698594	0.616	0.332	4.31E-36	Meta_CD4_C6
ARHGEF7	2.02E-40	0.945201457	0.499	0.186	4.89E-36	Meta_CD4_C6
RPL11	2.51E-40	0.321857204	1.000	0.994	6.07E-36	Meta_CD4_C6
TENT5C	3.51E-40	0.674765024	0.707	0.409	8.50E-36	Meta_CD4_C6
GADD45B	4.70E-40	0.673335416	0.799	0.512	1.14E-35	Meta_CD4_C6
MATR3.1	7.67E-40	0.721383747	0.713	0.412	1.86E-35	Meta_CD4_C6
RPL14	3.52E-39	0.366829444	0.999	0.979	8.53E-35	Meta_CD4_C6
RPL32	3.64E-39	0.340035314	1.000	0.993	8.80E-35	Meta_CD4_C6
RPLP0	6.59E-39	0.442555088	0.992	0.948	1.60E-34	Meta_CD4_C6
C9orf78	8.00E-39	0.674848331	0.774	0.518	1.94E-34	Meta_CD4_C6
ZFAND5	8.15E-39	0.6866237	0.657	0.371	1.97E-34	Meta_CD4_C6
PELI1	1.11E-38	0.8288111	0.487	0.198	2.69E-34	Meta_CD4_C6
RPS3	2.97E-38	0.310767169	0.999	0.994	7.19E-34	Meta_CD4_C6
AC016831.7	3.12E-38	0.665895874	0.708	0.442	7.56E-34	Meta_CD4_C6
SF1	4.30E-38	0.564700172	0.886	0.668	1.04E-33	Meta_CD4_C6
NFKBIA	6.88E-38	0.770440154	0.923	0.711	1.67E-33	Meta_CD4_C6
AL118516.1	7.06E-38	0.662839457	0.396	0.131	1.71E-33	Meta_CD4_C6
PDCL3	7.60E-38	0.808719654	0.563	0.298	1.84E-33	Meta_CD4_C6
IRS2	1.60E-37	0.641449167	0.303	0.065	3.88E-33	Meta_CD4_C6
SAP18	2.30E-37	0.539690056	0.897	0.698	5.58E-33	Meta_CD4_C6
PRNP	2.56E-37	0.705491659	0.597	0.305	6.20E-33	Meta_CD4_C6
ARF1	7.21E-37	0.606696885	0.905	0.730	1.75E-32	Meta_CD4_C6
RASSF5	1.03E-36	0.692660369	0.728	0.481	2.49E-32	Meta_CD4_C6
SUPT5H	1.53E-36	0.680700624	0.561	0.287	3.69E-32	Meta_CD4_C6
SAP30BP	1.62E-36	0.609700638	0.557	0.292	3.91E-32	Meta_CD4_C6

EEF1B2	3.20E-36	0.429148732	0.993	0.898	7.75E-32	Meta_CD4_C6
MANBA	4.78E-36	0.641835394	0.326	0.106	1.16E-31	Meta_CD4_C6
WBP11	5.59E-36	0.607742201	0.572	0.303	1.35E-31	Meta_CD4_C6
CD6	4.75E-35	0.707571617	0.814	0.564	1.15E-30	Meta_CD4_C6
SNHG15	4.87E-35	0.722089741	0.548	0.264	1.18E-30	Meta_CD4_C6
FAM102A	6.64E-35	0.721869032	0.604	0.335	1.61E-30	Meta_CD4_C6
CIRBP	1.81E-34	0.476362644	0.954	0.817	4.38E-30	Meta_CD4_C6
CKAP4	2.10E-34	0.565050583	0.231	0.031	5.08E-30	Meta_CD4_C6
LAPTM4A	2.25E-34	0.709318574	0.638	0.377	5.46E-30	Meta_CD4_C6
RPL35A	3.46E-34	0.308240703	0.997	0.986	8.39E-30	Meta_CD4_C6
EEF1G	5.27E-34	0.389614515	0.901	0.648	1.28E-29	Meta_CD4_C6
CDK17	9.49E-34	0.675632605	0.597	0.336	2.30E-29	Meta_CD4_C6
RPL7A	9.76E-34	0.330316501	0.997	0.974	2.36E-29	Meta_CD4_C6
SRSF10	1.21E-33	0.622597753	0.741	0.484	2.94E-29	Meta_CD4_C6
HSPA9	1.81E-33	0.658105835	0.664	0.417	4.39E-29	Meta_CD4_C6
PIK3R1	1.88E-33	0.680139115	0.674	0.423	4.54E-29	Meta_CD4_C6
RPL10	1.97E-33	0.295695227	1.000	0.998	4.76E-29	Meta_CD4_C6
SKIL	2.33E-33	0.729059629	0.480	0.216	5.64E-29	Meta_CD4_C6
ARRDC2	2.53E-33	0.592187541	0.350	0.143	6.12E-29	Meta_CD4_C6
RUNX3	2.73E-33	0.708774608	0.691	0.424	6.61E-29	Meta_CD4_C6
TNFRSF25	4.99E-33	0.638186902	0.481	0.232	1.21E-28	Meta_CD4_C6
CSNK1D	6.31E-33	0.785850581	0.581	0.300	1.53E-28	Meta_CD4_C6
IFITM1	1.17E-32	0.315330245	0.982	0.805	2.84E-28	Meta_CD4_C6
USP36	1.71E-32	0.634075394	0.456	0.212	4.14E-28	Meta_CD4_C6
ISCA1	2.61E-32	0.696184428	0.546	0.273	6.32E-28	Meta_CD4_C6
PCGF5	2.76E-32	0.644188215	0.547	0.260	6.67E-28	Meta_CD4_C6
RSRC2	2.94E-32	0.577230344	0.699	0.460	7.11E-28	Meta_CD4_C6
TPT1	3.46E-32	0.356028351	0.999	0.985	8.38E-28	Meta_CD4_C6
COG3	3.65E-32	0.610784742	0.412	0.162	8.84E-28	Meta_CD4_C6
DNTTIP2	9.98E-32	0.676687698	0.571	0.306	2.42E-27	Meta_CD4_C6
DBF4	1.04E-31	0.665777732	0.468	0.218	2.53E-27	Meta_CD4_C6
PIM3	1.81E-31	0.794235409	0.557	0.310	4.38E-27	Meta_CD4_C6
RHBDD2	3.17E-31	0.617039574	0.625	0.347	7.68E-27	Meta_CD4_C6
RPS8	3.52E-31	0.306464974	0.999	0.988	8.53E-27	Meta_CD4_C6
DDX21	3.65E-31	0.630014638	0.616	0.362	8.85E-27	Meta_CD4_C6
ARIH2	3.79E-31	0.620136733	0.596	0.327	9.17E-27	Meta_CD4_C6
RPL36A	4.00E-31	0.411250214	0.945	0.793	9.68E-27	Meta_CD4_C6
BCL9L	4.84E-31	0.676756551	0.358	0.117	1.17E-26	Meta_CD4_C6
UAP1	6.07E-31	0.623759348	0.347	0.112	1.47E-26	Meta_CD4_C6
NR4A2	6.42E-31	0.464513513	0.849	0.576	1.55E-26	Meta_CD4_C6

SELENOK	8.80E-31	0.642517416	0.761	0.521	2.13E-26	Meta_CD4_C6
HNRNPA1	1.38E-30	0.392194632	0.988	0.896	3.34E-26	Meta_CD4_C6
CALM1	1.39E-30	0.472944361	0.992	0.912	3.38E-26	Meta_CD4_C6
RPL24	1.40E-30	0.300745198	1.000	0.968	3.40E-26	Meta_CD4_C6
TOM1	1.72E-30	0.595297178	0.428	0.176	4.15E-26	Meta_CD4_C6
TOMM5	1.99E-30	0.602774032	0.629	0.434	4.83E-26	Meta_CD4_C6
RPS29	2.21E-30	0.317989471	0.995	0.973	5.34E-26	Meta_CD4_C6
TOMM20	2.34E-30	0.569131818	0.774	0.564	5.67E-26	Meta_CD4_C6
HIST2H2AA4	3.51E-30	0.575421163	0.283	0.093	8.50E-26	Meta_CD4_C6
RCC1	4.69E-30	0.542598645	0.412	0.186	1.14E-25	Meta_CD4_C6
SNRPA1	5.57E-30	0.582497401	0.569	0.312	1.35E-25	Meta_CD4_C6
EPB41L4A-AS1	9.65E-30	0.555233454	0.482	0.238	2.34E-25	Meta_CD4_C6
SLC39A8	1.20E-29	0.571292039	0.336	0.115	2.91E-25	Meta_CD4_C6
RPS19	1.25E-29	0.376453719	0.999	0.996	3.03E-25	Meta_CD4_C6
PDCD4	3.79E-29	0.620187601	0.797	0.582	9.18E-25	Meta_CD4_C6
PMEPA1	5.15E-29	0.58816875	0.259	0.049	1.25E-24	Meta_CD4_C6
SKI	6.99E-29	0.681173318	0.380	0.120	1.69E-24	Meta_CD4_C6
RPS13	7.00E-29	0.293270624	0.999	0.979	1.70E-24	Meta_CD4_C6
CCDC59	8.47E-29	0.522299836	0.587	0.350	2.05E-24	Meta_CD4_C6
ATP6V0C	9.07E-29	0.615253532	0.803	0.583	2.20E-24	Meta_CD4_C6
CD5	1.48E-28	0.696158167	0.682	0.425	3.60E-24	Meta_CD4_C6
PLP2	1.62E-28	0.484498958	0.808	0.560	3.93E-24	Meta_CD4_C6
EIF4G2	1.92E-28	0.57264519	0.838	0.660	4.64E-24	Meta_CD4_C6
MAPK1IP1L	2.01E-28	0.621011661	0.641	0.406	4.88E-24	Meta_CD4_C6
RSL24D1	2.21E-28	0.538517967	0.756	0.535	5.34E-24	Meta_CD4_C6
RPL19	2.31E-28	0.267249139	1.000	0.994	5.60E-24	Meta_CD4_C6
BCAS2	2.95E-28	0.504274594	0.609	0.382	7.14E-24	Meta_CD4_C6
RBM38	3.48E-28	0.682735245	0.483	0.239	8.43E-24	Meta_CD4_C6
TMEM123	3.73E-28	0.636157057	0.733	0.469	9.032E-24	Meta_CD4_C6
RPL26	5.54E-28	0.292378471	1.000	0.978	1.34E-23	Meta_CD4_C6
COPS2	5.81E-28	0.526486546	0.532	0.277	1.41E-23	Meta_CD4_C6
RPLP1	5.81E-28	0.324370741	1.000	0.998	1.41E-23	Meta_CD4_C6
STRAP	6.13E-28	0.595405854	0.664	0.428	1.48E-23	Meta_CD4_C6
MAP3K2	1.24E-27	0.501955311	0.388	0.161	3.00E-23	Meta_CD4_C6
RPL36	1.68E-27	0.296159249	0.996	0.978	4.06E-23	Meta_CD4_C6
RANGAP1	1.82E-27	0.544574297	0.320	0.115	4.41E-23	Meta_CD4_C6
RWDD1	2.52E-27	0.533753664	0.742	0.505	6.10E-23	Meta_CD4_C6
KLF10	3.77E-27	0.71431352	0.435	0.184	9.14E-23	Meta_CD4_C6
ATP1B1	6.07E-27	0.679805446	0.268	0.070	1.47E-22	Meta_CD4_C6
RPL18	6.97E-27	0.265690399	0.999	0.985	1.69E-22	Meta_CD4_C6

ITGAV	7.03E-27	0.570712475	0.239	0.060	1.70E-22	Meta_CD4_C6
CNOT1	9.99E-27	0.627675661	0.486	0.240	2.42E-22	Meta_CD4_C6
SLCO4A1	1.41E-26	0.436888315	0.181	0.036	3.40E-22	Meta_CD4_C6
PIK3IP1	1.93E-26	0.436117758	0.700	0.485	4.68E-22	Meta_CD4_C6
EIF4H	2.39E-26	0.563489737	0.696	0.456	5.79E-22	Meta_CD4_C6
SMAD7	2.98E-26	0.515865974	0.310	0.100	7.21E-22	Meta_CD4_C6
PLIN2	3.23E-26	0.623134357	0.457	0.247	7.82E-22	Meta_CD4_C6
NCBP2-AS2	4.15E-26	0.483216192	0.486	0.251	1.00E-21	Meta_CD4_C6
MACO1	4.57E-26	0.619926335	0.343	0.135	1.11E-21	Meta_CD4_C6
IFNGR1	5.97E-26	0.642021705	0.486	0.250	1.44E-21	Meta_CD4_C6
AC025164.1	1.83E-25	0.513561481	0.271	0.094	4.44E-21	Meta_CD4_C6
AQP3	2.94E-25	0.740263602	0.572	0.333	7.12E-21	Meta_CD4_C6
PRKRA	3.45E-25	0.527730476	0.371	0.166	8.36E-21	Meta_CD4_C6
RELA	7.24E-25	0.551257942	0.486	0.281	1.75E-20	Meta_CD4_C6
BZW1	7.82E-25	0.668264575	0.745	0.529	1.89E-20	Meta_CD4_C6
IL21R	2.61E-24	0.58403563	0.398	0.185	6.33E-20	Meta_CD4_C6
SRSF2	2.76E-24	0.551431362	0.812	0.634	6.68E-20	Meta_CD4_C6
RNF19A	5.16E-24	0.531975674	0.618	0.396	1.25E-19	Meta_CD4_C6
REL	7.68E-24	0.622036835	0.651	0.420	1.86E-19	Meta_CD4_C6
RANBP2	1.21E-23	0.598131606	0.518	0.317	2.92E-19	Meta_CD4_C6
RPL38	1.53E-23	0.279984886	0.985	0.931	3.71E-19	Meta_CD4_C6
TMEM173	3.26E-23	0.541173457	0.548	0.311	7.90E-19	Meta_CD4_C6
MYH9	4.68E-23	0.462498717	0.820	0.653	1.13E-18	Meta_CD4_C6
DHX9	7.50E-23	0.514095698	0.437	0.232	1.82E-18	Meta_CD4_C6
TRA2B	7.56E-23	0.491018201	0.692	0.465	1.83E-18	Meta_CD4_C6
SYAP1	8.03E-23	0.523952704	0.486	0.276	1.95E-18	Meta_CD4_C6
RPL5	8.94E-23	0.321039315	0.996	0.961	2.16E-18	Meta_CD4_C6
GGA2	9.62E-23	0.523002593	0.421	0.207	2.33E-18	Meta_CD4_C6
CLK1	1.12E-22	0.500443862	0.684	0.455	2.71E-18	Meta_CD4_C6
CTSL	1.32E-22	0.541799369	0.194	0.040	3.19E-18	Meta_CD4_C6
NR4A3	1.52E-22	0.505373429	0.402	0.184	3.69E-18	Meta_CD4_C6
CHRAC1	1.67E-22	0.490429886	0.408	0.214	4.05E-18	Meta_CD4_C6
GRASP	1.90E-22	0.302866583	0.118	0.013	4.60E-18	Meta_CD4_C6
DHRS3	1.92E-22	0.43032512	0.254	0.093	4.64E-18	Meta_CD4_C6
RARA	2.26E-22	0.583293721	0.379	0.166	5.48E-18	Meta_CD4_C6
DDX47	2.46E-22	0.419011909	0.248	0.075	5.96E-18	Meta_CD4_C6
SGK1	3.68E-22	0.421628013	0.279	0.102	8.91E-18	Meta_CD4_C6
DDX39A	4.97E-22	0.565836034	0.587	0.356	1.20E-17	Meta_CD4_C6
TPPP	5.15E-22	0.395281002	0.149	0.020	1.25E-17	Meta_CD4_C6
NUP58	5.20E-22	0.447711266	0.398	0.208	1.26E-17	Meta_CD4_C6

ABLIM1	5.47E-22	0.575708945	0.506	0.308	1.33E-17	Meta_CD4_C6
SMPD4	7.02E-22	0.37341459	0.275	0.117	1.70E-17	Meta_CD4_C6
PSMD7	8.16E-22	0.495016418	0.630	0.420	1.98E-17	Meta_CD4_C6
BPGM	8.90E-22	0.405842565	0.231	0.080	2.16E-17	Meta_CD4_C6
RNPS1	1.36E-21	0.450063828	0.712	0.511	3.30E-17	Meta_CD4_C6
TP53INP2	1.39E-21	0.435818587	0.199	0.048	3.36E-17	Meta_CD4_C6
KLF9	1.42E-21	0.469418513	0.321	0.135	3.44E-17	Meta_CD4_C6
KANSL2	1.75E-21	0.448618222	0.370	0.171	4.23E-17	Meta_CD4_C6
SNRK	1.92E-21	0.497983199	0.388	0.197	4.66E-17	Meta_CD4_C6
CCNL1	2.00E-21	0.497463844	0.749	0.559	4.85E-17	Meta_CD4_C6
CHD1	2.77E-21	0.519834808	0.487	0.293	6.70E-17	Meta_CD4_C6
RNF166	2.92E-21	0.499896657	0.560	0.349	7.08E-17	Meta_CD4_C6
JOSD1	3.64E-21	0.447226345	0.318	0.135	8.81E-17	Meta_CD4_C6
ARPC5L	3.71E-21	0.511049844	0.682	0.478	8.97E-17	Meta_CD4_C6
HIF1A	5.03E-21	0.453357549	0.502	0.340	1.22E-16	Meta_CD4_C6
MAPKAPK2	5.73E-21	0.44196623	0.289	0.123	1.39E-16	Meta_CD4_C6
CREM	6.93E-21	0.606121605	0.627	0.405	1.68E-16	Meta_CD4_C6
CIB1	7.04E-21	0.478407788	0.859	0.708	1.70E-16	Meta_CD4_C6
EPHA4	9.09E-21	0.379993894	0.201	0.055	2.20E-16	Meta_CD4_C6
STX11	1.21E-20	0.471311872	0.373	0.172	2.92E-16	Meta_CD4_C6
NR3C1	1.39E-20	0.514966247	0.684	0.501	3.38E-16	Meta_CD4_C6
GADD45A	1.60E-20	0.513707037	0.325	0.141	3.87E-16	Meta_CD4_C6
BAG3	2.65E-20	0.53220957	0.285	0.103	6.43E-16	Meta_CD4_C6
AGO2	2.82E-20	0.477941958	0.354	0.175	6.82E-16	Meta_CD4_C6
KRR1	3.12E-20	0.423630903	0.465	0.269	7.57E-16	Meta_CD4_C6
CD4	3.33E-20	0.312773346	0.420	0.244	8.07E-16	Meta_CD4_C6
TGFBR3	3.83E-20	0.461867108	0.264	0.100	9.28E-16	Meta_CD4_C6
LRRFIP1	4.62E-20	0.534516339	0.789	0.624	1.12E-15	Meta_CD4_C6
AKAP17A	5.71E-20	0.459291986	0.316	0.164	1.38E-15	Meta_CD4_C6
PRDM1	7.75E-20	0.406310844	0.560	0.339	1.88E-15	Meta_CD4_C6
LPAR2	8.13E-20	0.480794298	0.297	0.144	1.97E-15	Meta_CD4_C6
HNRNPUL1	9.28E-20	0.543977615	0.668	0.446	2.25E-15	Meta_CD4_C6
AC010422.3	9.39E-20	0.528692143	0.231	0.064	2.27E-15	Meta_CD4_C6
CCNK	2.50E-19	0.410378731	0.392	0.214	6.04E-15	Meta_CD4_C6
CDK5RAP1	2.81E-19	0.471079271	0.317	0.133	6.80E-15	Meta_CD4_C6
RCL1	2.87E-19	0.316669737	0.221	0.085	6.95E-15	Meta_CD4_C6
SIVA1	3.04E-19	0.442593823	0.634	0.423	7.37E-15	Meta_CD4_C6
LEPROTL1	3.11E-19	0.520042063	0.857	0.727	7.53E-15	Meta_CD4_C6
TP53BP2	3.82E-19	0.404044011	0.246	0.096	9.24E-15	Meta_CD4_C6
RBM8A	4.42E-19	0.464705679	0.804	0.630	1.07E-14	Meta_CD4_C6

ATP1B3	6.21E-19	0.603699367	0.436	0.244	1.50E-14	Meta_CD4_C6
IFRD1	6.37E-19	0.388485351	0.461	0.269	1.54E-14	Meta_CD4_C6
IGFBP4	6.38E-19	0.624907702	0.218	0.054	1.54E-14	Meta_CD4_C6
U2AF1	7.19E-19	0.453258947	0.308	0.124	1.74E-14	Meta_CD4_C6
MAGOH	7.35E-19	0.46112275	0.642	0.433	1.78E-14	Meta_CD4_C6
G3BP2	9.92E-19	0.501260908	0.692	0.477	2.40E-14	Meta_CD4_C6
VPS37B	1.11E-18	0.450761023	0.449	0.268	2.69E-14	Meta_CD4_C6
RNF138	1.52E-18	0.480046564	0.407	0.223	3.68E-14	Meta_CD4_C6
PMAIP1	1.54E-18	0.616513014	0.466	0.255	3.74E-14	Meta_CD4_C6
MORF4L1	1.67E-18	0.399611949	0.839	0.676	4.04E-14	Meta_CD4_C6
EEF1D	1.71E-18	0.259872063	0.995	0.962	4.14E-14	Meta_CD4_C6
CDC42	1.81E-18	0.338418309	0.815	0.663	4.38E-14	Meta_CD4_C6
RBM34	2.04E-18	0.331556243	0.218	0.084	4.94E-14	Meta_CD4_C6
DCP1A	2.14E-18	0.445373922	0.371	0.196	5.19E-14	Meta_CD4_C6
ETV3	2.57E-18	0.427681208	0.247	0.095	6.23E-14	Meta_CD4_C6
RBM17	3.69E-18	0.436771876	0.576	0.381	8.95E-14	Meta_CD4_C6
RORA	4.38E-18	0.455841532	0.658	0.452	1.06E-13	Meta_CD4_C6
RNF168	6.53E-18	0.472090715	0.345	0.156	1.58E-13	Meta_CD4_C6
YTHDF2	6.61E-18	0.430031028	0.511	0.342	1.60E-13	Meta_CD4_C6
YARS	6.66E-18	0.394540333	0.441	0.259	1.61E-13	Meta_CD4_C6
SAMSN1	7.39E-18	0.486884423	0.749	0.542	1.79E-13	Meta_CD4_C6
NOL7	8.32E-18	0.402306164	0.609	0.440	2.01E-13	Meta_CD4_C6
NUP98	9.63E-18	0.507625393	0.424	0.221	2.33E-13	Meta_CD4_C6
NFATC1	1.05E-17	0.4553823	0.306	0.133	2.55E-13	Meta_CD4_C6
HNRNPDL	1.21E-17	0.301147809	0.934	0.800	2.94E-13	Meta_CD4_C6
CACYBP	1.37E-17	0.440580481	0.667	0.503	3.31E-13	Meta_CD4_C6
SEC61G	1.50E-17	0.403391479	0.773	0.601	3.64E-13	Meta_CD4_C6
PDZD8	1.64E-17	0.467626603	0.276	0.120	3.97E-13	Meta_CD4_C6
VDAC2	1.73E-17	0.360460544	0.621	0.482	4.18E-13	Meta_CD4_C6
MFHAS1	2.50E-17	0.335103963	0.184	0.066	6.05E-13	Meta_CD4_C6
GPCPD1	3.52E-17	0.374978964	0.334	0.184	8.51E-13	Meta_CD4_C6
SLC5A6	3.66E-17	0.377621198	0.211	0.069	8.85E-13	Meta_CD4_C6
CBLL1	4.39E-17	0.346828278	0.283	0.145	1.06E-12	Meta_CD4_C6
USP3	4.51E-17	0.499517329	0.448	0.244	1.09E-12	Meta_CD4_C6
CTDP1	4.88E-17	0.413690913	0.255	0.104	1.18E-12	Meta_CD4_C6
SPTBN1	5.62E-17	0.456210149	0.292	0.146	1.36E-12	Meta_CD4_C6
AHNAK	5.81E-17	0.294020594	0.731	0.529	1.41E-12	Meta_CD4_C6
GPR183	7.14E-17	0.394753451	0.613	0.408	1.73E-12	Meta_CD4_C6
PELI2	7.57E-17	0.253292857	0.104	0.014	1.83E-12	Meta_CD4_C6
SLC25A36	8.99E-17	0.442362823	0.369	0.182	2.18E-12	Meta_CD4_C6

LTBP4	1.10E-16	0.461583145	0.303	0.135	2.67E-12	Meta_CD4_C6
UBE2B	1.69E-16	0.396777448	0.683	0.500	4.08E-12	Meta_CD4_C6
UBE2J1	2.63E-16	0.442664896	0.448	0.285	6.36E-12	Meta_CD4_C6
DSE	2.83E-16	0.377768906	0.192	0.068	6.85E-12	Meta_CD4_C6
GLUL	3.29E-16	0.487083647	0.312	0.142	7.98E-12	Meta_CD4_C6
SCAND1	3.33E-16	0.367737361	0.715	0.546	8.07E-12	Meta_CD4_C6
RAC1	3.48E-16	0.417593754	0.691	0.515	8.43E-12	Meta_CD4_C6
RPSA	4.42E-16	0.250299765	0.999	0.962	1.07E-11	Meta_CD4_C6
PERP	5.35E-16	0.436494322	0.386	0.213	1.29E-11	Meta_CD4_C6
MSL2	6.00E-16	0.406575857	0.285	0.138	1.45E-11	Meta_CD4_C6
GNAO1	6.04E-16	0.287130348	0.132	0.033	1.46E-11	Meta_CD4_C6
ANK3	6.20E-16	0.311127854	0.186	0.064	1.50E-11	Meta_CD4_C6
CXCL16	6.28E-16	0.295101155	0.131	0.025	1.52E-11	Meta_CD4_C6
MRPL9	6.46E-16	0.365443676	0.408	0.243	1.57E-11	Meta_CD4_C6
SELENOT	6.77E-16	0.447249239	0.625	0.452	1.64E-11	Meta_CD4_C6
SRGN	6.80E-16	0.434362063	0.983	0.956	1.65E-11	Meta_CD4_C6
IL4R	6.85E-16	0.387320425	0.299	0.156	1.66E-11	Meta_CD4_C6
RELL1	7.31E-16	0.411784811	0.264	0.105	1.77E-11	Meta_CD4_C6
SCML1	7.43E-16	0.297226257	0.122	0.028	1.80E-11	Meta_CD4_C6
HSP90AB1	9.88E-16	0.312152466	0.946	0.840	2.39E-11	Meta_CD4_C6
CCDC12	1.28E-15	0.361291773	0.572	0.409	3.11E-11	Meta_CD4_C6
CUL1	1.38E-15	0.404815305	0.343	0.184	3.35E-11	Meta_CD4_C6
OXCT1	1.66E-15	0.377377142	0.328	0.183	4.02E-11	Meta_CD4_C6
EHD4	1.91E-15	0.363900883	0.252	0.110	4.63E-11	Meta_CD4_C6
ZFP36L1	2.19E-15	0.32614264	0.840	0.678	5.31E-11	Meta_CD4_C6
TSN	2.26E-15	0.328030877	0.342	0.202	5.46E-11	Meta_CD4_C6
ITPKB	2.36E-15	0.4053509	0.243	0.107	5.71E-11	Meta_CD4_C6
CNOT6L	2.70E-15	0.362473015	0.433	0.282	6.53E-11	Meta_CD4_C6
JAZF1	2.71E-15	0.43005272	0.275	0.110	6.55E-11	Meta_CD4_C6
ICOS	3.47E-15	0.39263299	0.579	0.377	8.41E-11	Meta_CD4_C6
FOXO1	3.80E-15	0.346143378	0.291	0.152	9.21E-11	Meta_CD4_C6
DUSP10	3.88E-15	0.357297938	0.309	0.158	9.40E-11	Meta_CD4_C6
GLTP	4.71E-15	0.363595475	0.371	0.209	1.14E-10	Meta_CD4_C6
EIF5	4.87E-15	0.436331799	0.719	0.519	1.18E-10	Meta_CD4_C6
ZNF410	4.99E-15	0.299509624	0.244	0.131	1.21E-10	Meta_CD4_C6
JUNB	5.16E-15	0.305741466	0.970	0.886	1.25E-10	Meta_CD4_C6
TRMT10C	5.95E-15	0.422361193	0.350	0.183	1.44E-10	Meta_CD4_C6
LEMD3	6.05E-15	0.357918383	0.289	0.143	1.47E-10	Meta_CD4_C6
NXT1	6.13E-15	0.381511228	0.398	0.240	1.48E-10	Meta_CD4_C6
CAMK1D	6.30E-15	0.310041005	0.239	0.115	1.53E-10	Meta_CD4_C6

STAU1	6.35E-15	0.398411282	0.468	0.283	1.54E-10	Meta_CD4_C6
CMSS1	6.44E-15	0.365230982	0.215	0.076	1.56E-10	Meta_CD4_C6
ATP5MPL	6.53E-15	0.340047698	0.663	0.504	1.58E-10	Meta_CD4_C6
RAB5C	6.54E-15	0.422658682	0.622	0.426	1.58E-10	Meta_CD4_C6
HNRNPM	6.71E-15	0.321769179	0.650	0.499	1.62E-10	Meta_CD4_C6
RIOK3	6.86E-15	0.425625238	0.413	0.249	1.66E-10	Meta_CD4_C6
METAP2	7.77E-15	0.353176984	0.443	0.296	1.88E-10	Meta_CD4_C6
PIM1	9.51E-15	0.441443347	0.604	0.442	2.30E-10	Meta_CD4_C6
HIST1H1C	1.26E-14	0.482748516	0.303	0.154	3.04E-10	Meta_CD4_C6
DNAJC2	1.30E-14	0.416879922	0.406	0.222	3.15E-10	Meta_CD4_C6
FURIN	1.32E-14	0.389567795	0.213	0.102	3.19E-10	Meta_CD4_C6
PABPC4	1.39E-14	0.408590789	0.380	0.230	3.36E-10	Meta_CD4_C6
TSC22D2	1.40E-14	0.289599543	0.263	0.131	3.40E-10	Meta_CD4_C6
PPP1CB	1.66E-14	0.309666262	0.446	0.286	4.02E-10	Meta_CD4_C6
INSIG1	1.71E-14	0.410127452	0.343	0.207	4.14E-10	Meta_CD4_C6
BUD23	1.71E-14	0.379249682	0.400	0.255	4.15E-10	Meta_CD4_C6
SORBS3	2.06E-14	0.380387209	0.268	0.114	4.99E-10	Meta_CD4_C6
BRD1	2.32E-14	0.339299828	0.180	0.062	5.63E-10	Meta_CD4_C6
OSER1	2.50E-14	0.394615501	0.383	0.219	6.07E-10	Meta_CD4_C6
RAP2B	2.68E-14	0.429753545	0.281	0.137	6.49E-10	Meta_CD4_C6
TGIF1	3.00E-14	0.364949166	0.336	0.181	7.26E-10	Meta_CD4_C6
VDAC1	3.00E-14	0.403330593	0.594	0.431	7.27E-10	Meta_CD4_C6
CCNI	3.37E-14	0.31649561	0.956	0.841	8.16E-10	Meta_CD4_C6
CRY1	3.48E-14	0.400335328	0.336	0.165	8.43E-10	Meta_CD4_C6
SSH2	4.43E-14	0.376581706	0.456	0.284	1.07E-09	Meta_CD4_C6
ATG14	4.55E-14	0.355073798	0.221	0.090	1.10E-09	Meta_CD4_C6
GAB2	4.83E-14	0.289184347	0.124	0.024	1.17E-09	Meta_CD4_C6
ATP6V1G1	5.03E-14	0.301002523	0.845	0.721	1.22E-09	Meta_CD4_C6
MCUB	5.85E-14	0.395884785	0.605	0.418	1.42E-09	Meta_CD4_C6
USP12	8.16E-14	0.366036034	0.239	0.099	1.98E-09	Meta_CD4_C6
GRK5	1.10E-13	0.355778626	0.206	0.077	2.67E-09	Meta_CD4_C6
FUS	1.11E-13	0.289762406	0.849	0.700	2.70E-09	Meta_CD4_C6
CWC15	1.23E-13	0.305085327	0.486	0.305	2.98E-09	Meta_CD4_C6
TNFRSF10B	1.23E-13	0.278380258	0.161	0.062	2.98E-09	Meta_CD4_C6
BCCIP	1.27E-13	0.348605795	0.424	0.256	3.08E-09	Meta_CD4_C6
GSPT1	1.44E-13	0.445071248	0.445	0.294	3.50E-09	Meta_CD4_C6
AC103591.3	1.50E-13	0.252765675	0.160	0.055	3.64E-09	Meta_CD4_C6
ELOC	1.67E-13	0.338933362	0.543	0.385	4.03E-09	Meta_CD4_C6
WDR37	1.75E-13	0.334948987	0.214	0.098	4.24E-09	Meta_CD4_C6
OSBPL8	1.76E-13	0.462611357	0.542	0.348	4.27E-09	Meta_CD4_C6

IRF2BPL	1.97E-13	0.315685237	0.214	0.104	4.78E-09	Meta_CD4_C6
TWF1	2.31E-13	0.366789067	0.283	0.140	5.58E-09	Meta_CD4_C6
ILF2	2.33E-13	0.366370378	0.601	0.437	5.65E-09	Meta_CD4_C6
ANKHD1	2.73E-13	0.340392465	0.338	0.191	6.62E-09	Meta_CD4_C6
ARNTL	2.77E-13	0.34554814	0.309	0.178	6.70E-09	Meta_CD4_C6
PLEKHO1	2.96E-13	0.396362652	0.272	0.125	7.17E-09	Meta_CD4_C6
TBC1D15	3.12E-13	0.300537399	0.242	0.113	7.55E-09	Meta_CD4_C6
ZFAND2A	3.67E-13	0.314974356	0.334	0.179	8.89E-09	Meta_CD4_C6
PLEKHA2	3.79E-13	0.443794026	0.333	0.163	9.17E-09	Meta_CD4_C6
SRRT	3.90E-13	0.488009488	0.461	0.282	9.45E-09	Meta_CD4_C6
CENPC	4.38E-13	0.398200539	0.424	0.262	1.06E-08	Meta_CD4_C6
KDSR	4.93E-13	0.394992051	0.330	0.184	1.19E-08	Meta_CD4_C6
PTGER4	5.87E-13	0.294827489	0.720	0.548	1.42E-08	Meta_CD4_C6
BUD31	6.37E-13	0.327146183	0.565	0.385	1.54E-08	Meta_CD4_C6
TIMM23	7.96E-13	0.320625737	0.303	0.173	1.93E-08	Meta_CD4_C6
FXYD7	8.00E-13	0.320938055	0.153	0.043	1.94E-08	Meta_CD4_C6
SETD2	9.15E-13	0.458419933	0.408	0.216	2.22E-08	Meta_CD4_C6
USP38	9.33E-13	0.308445465	0.230	0.115	2.26E-08	Meta_CD4_C6
SF3B6	9.49E-13	0.28295628	0.663	0.493	2.30E-08	Meta_CD4_C6
PNO1	9.71E-13	0.348585593	0.250	0.110	2.35E-08	Meta_CD4_C6
CTR9	1.03E-12	0.404056953	0.350	0.217	2.50E-08	Meta_CD4_C6
NELL2	1.08E-12	0.309146301	0.254	0.117	2.61E-08	Meta_CD4_C6
AKIRIN1	1.15E-12	0.406190163	0.498	0.332	2.79E-08	Meta_CD4_C6
ANKRD12	1.22E-12	0.380400181	0.774	0.634	2.95E-08	Meta_CD4_C6
CDK11A	1.26E-12	0.325636043	0.321	0.180	3.04E-08	Meta_CD4_C6
UHRF1BP1L	1.30E-12	0.335202732	0.243	0.108	3.16E-08	Meta_CD4_C6
SLC38A1	1.38E-12	0.384174003	0.629	0.463	3.34E-08	Meta_CD4_C6
TOE1	1.45E-12	0.268164267	0.189	0.083	3.50E-08	Meta_CD4_C6
EMC7	1.65E-12	0.317695137	0.495	0.341	3.99E-08	Meta_CD4_C6
RNF114	1.67E-12	0.336178	0.491	0.326	4.04E-08	Meta_CD4_C6
CENPN	1.88E-12	0.330297114	0.176	0.052	4.55E-08	Meta_CD4_C6
PLXND1	1.93E-12	0.253371304	0.124	0.037	4.67E-08	Meta_CD4_C6
EZR	2.56E-12	0.404334909	0.768	0.640	6.20E-08	Meta_CD4_C6
MT2A	3.13E-12	0.703474241	0.617	0.523	7.58E-08	Meta_CD4_C6
HERPUD2	3.58E-12	0.378569558	0.510	0.365	8.66E-08	Meta_CD4_C6
CSDE1	3.83E-12	0.362083202	0.750	0.607	9.27E-08	Meta_CD4_C6
RALA	4.20E-12	0.391451119	0.410	0.254	1.02E-07	Meta_CD4_C6
RAB5A	4.26E-12	0.342539079	0.369	0.220	1.03E-07	Meta_CD4_C6
OASL	4.30E-12	0.557917333	0.359	0.214	1.04E-07	Meta_CD4_C6
YTHDC1	4.33E-12	0.312766364	0.456	0.312	1.05E-07	Meta_CD4_C6

AMD1	6.03E-12	0.343761876	0.524	0.361	1.46E-07	Meta_CD4_C6
BCLAF1	7.82E-12	0.443631323	0.625	0.453	1.89E-07	Meta_CD4_C6
MADD	8.18E-12	0.334324894	0.210	0.092	1.98E-07	Meta_CD4_C6
HERPUD1	8.62E-12	0.327542554	0.682	0.536	2.09E-07	Meta_CD4_C6
FAM133B	9.24E-12	0.363295297	0.417	0.265	2.24E-07	Meta_CD4_C6
AC026979.2	9.62E-12	0.329718946	0.301	0.172	2.33E-07	Meta_CD4_C6
ZNF326	9.80E-12	0.286439189	0.243	0.120	2.37E-07	Meta_CD4_C6
CNOT2	1.05E-11	0.3453094	0.469	0.315	2.55E-07	Meta_CD4_C6
CD55	1.22E-11	0.302369187	0.427	0.270	2.95E-07	Meta_CD4_C6
METRNL	1.33E-11	0.354237729	0.177	0.056	3.23E-07	Meta_CD4_C6
ID2	1.42E-11	0.284440724	0.752	0.595	3.44E-07	Meta_CD4_C6
RAMMET	1.52E-11	0.340316789	0.333	0.211	3.68E-07	Meta_CD4_C6
ZDHHC7	1.64E-11	0.347777195	0.271	0.139	3.96E-07	Meta_CD4_C6
ZBTB1	1.80E-11	0.437127296	0.410	0.242	4.37E-07	Meta_CD4_C6
BCL6	1.97E-11	0.258439277	0.133	0.039	4.76E-07	Meta_CD4_C6
FBXO34	2.05E-11	0.398381845	0.263	0.124	4.97E-07	Meta_CD4_C6
SKP1	2.06E-11	0.28081586	0.897	0.803	4.98E-07	Meta_CD4_C6
BNIP2	2.24E-11	0.356139507	0.464	0.309	5.44E-07	Meta_CD4_C6
SLC12A7	2.89E-11	0.291478798	0.140	0.039	6.99E-07	Meta_CD4_C6
TXNDC11	3.14E-11	0.320526365	0.313	0.170	7.60E-07	Meta_CD4_C6
SLC22A5	3.70E-11	0.293762765	0.168	0.052	8.97E-07	Meta_CD4_C6
SLC3A2	4.21E-11	0.391995379	0.680	0.507	1.02E-06	Meta_CD4_C6
RNF145	4.32E-11	0.321168117	0.394	0.266	1.05E-06	Meta_CD4_C6
ABCA1	4.37E-11	0.301188376	0.125	0.040	1.06E-06	Meta_CD4_C6
CAMLG	4.74E-11	0.314653139	0.494	0.330	1.15E-06	Meta_CD4_C6
OTULINL	5.50E-11	0.415098443	0.322	0.166	1.33E-06	Meta_CD4_C6
GNA13	6.01E-11	0.389224131	0.333	0.183	1.45E-06	Meta_CD4_C6
STARD7	6.51E-11	0.353007215	0.425	0.282	1.58E-06	Meta_CD4_C6
TOP1	6.81E-11	0.42825831	0.490	0.350	1.65E-06	Meta_CD4_C6
NOP58	7.08E-11	0.354076447	0.606	0.421	1.71E-06	Meta_CD4_C6
PTPMT1	7.43E-11	0.326324678	0.283	0.163	1.80E-06	Meta_CD4_C6
KRT10	7.72E-11	0.300516312	0.723	0.584	1.87E-06	Meta_CD4_C6
BRD9	8.33E-11	0.312599845	0.264	0.147	2.02E-06	Meta_CD4_C6
PTS	9.60E-11	0.313795708	0.268	0.127	2.33E-06	Meta_CD4_C6
UQCRFS1	1.01E-10	0.294064614	0.613	0.471	2.44E-06	Meta_CD4_C6
NLRP3	1.17E-10	0.258050697	0.128	0.030	2.84E-06	Meta_CD4_C6
PPP2CA	1.27E-10	0.336158033	0.556	0.386	3.06E-06	Meta_CD4_C6
NAMPT	1.29E-10	0.330690263	0.378	0.241	3.12E-06	Meta_CD4_C6
CDK16	1.31E-10	0.309271395	0.231	0.111	3.18E-06	Meta_CD4_C6
ZC3H15	1.40E-10	0.415430224	0.634	0.439	3.38E-06	Meta_CD4_C6

COPS8	1.44E-10	0.390631186	0.402	0.240	3.48E-06	Meta_CD4_C6
PRDX6	1.48E-10	0.317315578	0.621	0.487	3.57E-06	Meta_CD4_C6
BIRC2	1.64E-10	0.325911458	0.357	0.217	3.98E-06	Meta_CD4_C6
GTPBP4	1.74E-10	0.250359437	0.262	0.160	4.21E-06	Meta_CD4_C6
GET4	2.11E-10	0.345021445	0.308	0.178	5.11E-06	Meta_CD4_C6
PCIF1	2.27E-10	0.371694453	0.398	0.244	5.50E-06	Meta_CD4_C6
SLC6A6	2.47E-10	0.320456329	0.260	0.130	5.98E-06	Meta_CD4_C6
KMT2E	2.53E-10	0.304989122	0.749	0.611	6.13E-06	Meta_CD4_C6
NCF1	2.53E-10	0.301630174	0.449	0.335	6.13E-06	Meta_CD4_C6
NDRG1	2.74E-10	0.385228919	0.296	0.153	6.63E-06	Meta_CD4_C6
HIST1H1E	3.35E-10	0.449127304	0.273	0.171	8.12E-06	Meta_CD4_C6
EIF4A3	3.41E-10	0.364750081	0.499	0.331	8.26E-06	Meta_CD4_C6
ZEB1	3.54E-10	0.289708673	0.252	0.151	8.58E-06	Meta_CD4_C6
PTTG1IP	3.68E-10	0.353664262	0.295	0.159	8.90E-06	Meta_CD4_C6
DIABLO.1	3.77E-10	0.338368766	0.211	0.093	9.12E-06	Meta_CD4_C6
NFKBIZ	3.92E-10	0.34672189	0.518	0.362	9.49E-06	Meta_CD4_C6
NUP54	4.30E-10	0.300521777	0.262	0.149	1.04E-05	Meta_CD4_C6
POLB	4.91E-10	0.356753682	0.226	0.104	1.19E-05	Meta_CD4_C6
RB1CC1	5.57E-10	0.329736307	0.371	0.237	1.35E-05	Meta_CD4_C6
CIR1	5.75E-10	0.299520314	0.420	0.285	1.39E-05	Meta_CD4_C6
TCF25	7.65E-10	0.341857273	0.614	0.451	1.85E-05	Meta_CD4_C6
PPP3CC	7.68E-10	0.276855573	0.445	0.330	1.86E-05	Meta_CD4_C6
SLC25A3	7.95E-10	0.307300157	0.830	0.701	1.93E-05	Meta_CD4_C6
SYPL1	8.15E-10	0.358865273	0.404	0.259	1.97E-05	Meta_CD4_C6
BLOC1S2	9.03E-10	0.275365689	0.429	0.285	2.19E-05	Meta_CD4_C6
CGRRF1	9.62E-10	0.271825333	0.221	0.115	2.33E-05	Meta_CD4_C6
RNF11	9.69E-10	0.342317262	0.361	0.231	2.35E-05	Meta_CD4_C6
CCDC6	1.00E-09	0.286607296	0.231	0.112	2.43E-05	Meta_CD4_C6
RIC8A	1.10E-09	0.311273227	0.361	0.235	2.67E-05	Meta_CD4_C6
FBXO7	1.20E-09	0.295047586	0.490	0.358	2.90E-05	Meta_CD4_C6
MFSD14A	1.20E-09	0.315474709	0.354	0.225	2.91E-05	Meta_CD4_C6
UNC119	1.21E-09	0.275665958	0.284	0.174	2.93E-05	Meta_CD4_C6
EIF1AD	1.23E-09	0.268024802	0.185	0.080	2.99E-05	Meta_CD4_C6
PLEC	1.25E-09	0.357000056	0.361	0.205	3.04E-05	Meta_CD4_C6
EIF5A	1.27E-09	0.262761755	0.700	0.559	3.07E-05	Meta_CD4_C6
NGDN	1.42E-09	0.338362747	0.402	0.260	3.44E-05	Meta_CD4_C6
MAP2K3	1.52E-09	0.258276253	0.354	0.255	3.69E-05	Meta_CD4_C6
SLC1A5	1.61E-09	0.31685478	0.320	0.193	3.90E-05	Meta_CD4_C6
GPR132	1.62E-09	0.272365354	0.218	0.110	3.92E-05	Meta_CD4_C6
DENND3	1.73E-09	0.281111631	0.152	0.050	4.20E-05	Meta_CD4_C6

ARGLU1	2.03E-09	0.28803166	0.707	0.554	4.91E-05	Meta_CD4_C6
VAPA	2.21E-09	0.312296865	0.649	0.513	5.35E-05	Meta_CD4_C6
PLEKHM1	2.54E-09	0.284483418	0.218	0.112	6.15E-05	Meta_CD4_C6
HAUS2	2.60E-09	0.255540427	0.211	0.111	6.30E-05	Meta_CD4_C6
CNOT8	2.63E-09	0.297153207	0.353	0.241	6.38E-05	Meta_CD4_C6
PSMD6	2.73E-09	0.302781241	0.458	0.339	6.62E-05	Meta_CD4_C6
TUBB4B	2.74E-09	0.259377558	0.617	0.463	6.64E-05	Meta_CD4_C6
RBM48	2.89E-09	0.289437616	0.242	0.127	6.99E-05	Meta_CD4_C6
UBE2G1	3.52E-09	0.313173909	0.380	0.254	8.52E-05	Meta_CD4_C6
XBP1	3.61E-09	0.32700756	0.584	0.425	8.73E-05	Meta_CD4_C6
EHD1	3.80E-09	0.359042552	0.400	0.271	9.21E-05	Meta_CD4_C6
PSME4	4.55E-09	0.307488215	0.230	0.108	1.10E-04	Meta_CD4_C6
RAB2A	4.60E-09	0.302417441	0.576	0.405	1.12E-04	Meta_CD4_C6
SF3B1	4.75E-09	0.278400862	0.715	0.566	1.15E-04	Meta_CD4_C6
SF3B2	5.34E-09	0.258540306	0.713	0.572	1.29E-04	Meta_CD4_C6
RBM22	5.64E-09	0.327368288	0.400	0.258	1.37E-04	Meta_CD4_C6
DNAJC9	6.82E-09	0.308789628	0.332	0.192	1.65E-04	Meta_CD4_C6
ELL	6.99E-09	0.317300125	0.280	0.155	1.69E-04	Meta_CD4_C6
PRELID3B	7.59E-09	0.317459083	0.388	0.251	1.84E-04	Meta_CD4_C6
RFTN1	8.60E-09	0.264533494	0.306	0.208	2.08E-04	Meta_CD4_C6
BBC3	8.97E-09	0.263536505	0.230	0.129	2.17E-04	Meta_CD4_C6
AZIN1	1.04E-08	0.334946707	0.354	0.219	2.53E-04	Meta_CD4_C6
ITGB1	1.07E-08	0.274114604	0.402	0.270	2.58E-04	Meta_CD4_C6
DUSP12	1.11E-08	0.264080664	0.250	0.139	2.69E-04	Meta_CD4_C6
CMC2	1.13E-08	0.318328201	0.482	0.345	2.74E-04	Meta_CD4_C6
RABGGTB	1.14E-08	0.308017114	0.363	0.245	2.76E-04	Meta_CD4_C6
FAM32A	1.17E-08	0.272778309	0.400	0.292	2.84E-04	Meta_CD4_C6
ELMO1	1.30E-08	0.256052466	0.316	0.211	3.14E-04	Meta_CD4_C6
FOSL2	1.41E-08	0.351253571	0.325	0.179	3.42E-04	Meta_CD4_C6
HIST1H1D	1.51E-08	0.40419377	0.207	0.135	3.65E-04	Meta_CD4_C6
RSL1D1	1.63E-08	0.297158553	0.589	0.473	3.95E-04	Meta_CD4_C6
PSPC1	1.67E-08	0.262956273	0.295	0.178	4.04E-04	Meta_CD4_C6
C3orf58	1.70E-08	0.290639334	0.199	0.100	4.11E-04	Meta_CD4_C6
WDR43	1.71E-08	0.336719719	0.295	0.162	4.14E-04	Meta_CD4_C6
MTCH1	1.80E-08	0.256565513	0.458	0.342	4.36E-04	Meta_CD4_C6
RCE1	1.92E-08	0.270585204	0.267	0.152	4.65E-04	Meta_CD4_C6
TMEM87A	2.03E-08	0.299420719	0.395	0.247	4.93E-04	Meta_CD4_C6
SLC38A2	2.15E-08	0.335558853	0.458	0.315	5.20E-04	Meta_CD4_C6
AC245014.3	2.32E-08	0.316064885	0.129	0.046	5.61E-04	Meta_CD4_C6
WDR45B	2.48E-08	0.2911612	0.281	0.156	6.01E-04	Meta_CD4_C6

RFX2	3.02E-08	0.319968287	0.124	0.029	7.31E-04	Meta_CD4_C6
FNBP4	4.05E-08	0.257267497	0.474	0.340	9.81E-04	Meta_CD4_C6
MAPRE1	5.04E-08	0.250653998	0.441	0.332	1.22E-03	Meta_CD4_C6
SOD2	5.20E-08	0.368594792	0.396	0.247	1.26E-03	Meta_CD4_C6
PRRC2C	5.32E-08	0.282516782	0.760	0.629	1.29E-03	Meta_CD4_C6
MRM3	5.72E-08	0.256824783	0.186	0.093	1.38E-03	Meta_CD4_C6
RBM25	5.82E-08	0.269623048	0.625	0.493	1.41E-03	Meta_CD4_C6
TUT4	5.98E-08	0.326118683	0.338	0.226	1.45E-03	Meta_CD4_C6
JMJD6	6.73E-08	0.378028156	0.390	0.249	1.63E-03	Meta_CD4_C6
RIOK1	9.48E-08	0.295082913	0.264	0.150	2.30E-03	Meta_CD4_C6
STK17A	1.08E-07	0.277770312	0.828	0.727	2.62E-03	Meta_CD4_C6
OAT	1.09E-07	0.282417744	0.281	0.173	2.63E-03	Meta_CD4_C6
DCTN4	1.09E-07	0.275485095	0.272	0.168	2.63E-03	Meta_CD4_C6
TNFSF8	1.16E-07	0.297258979	0.247	0.135	2.80E-03	Meta_CD4_C6
WTAP	1.40E-07	0.251668233	0.526	0.409	3.40E-03	Meta_CD4_C6
PPP1R2	1.66E-07	0.268165759	0.734	0.591	4.01E-03	Meta_CD4_C6
SLC35E1	1.66E-07	0.253700906	0.165	0.071	4.02E-03	Meta_CD4_C6
CDC123	1.93E-07	0.267540752	0.410	0.285	4.66E-03	Meta_CD4_C6
AFF4	2.72E-07	0.283571242	0.367	0.248	6.58E-03	Meta_CD4_C6
YME1L1	2.72E-07	0.296466882	0.493	0.370	6.60E-03	Meta_CD4_C6
PPP1R16B	2.83E-07	0.291475673	0.312	0.215	6.84E-03	Meta_CD4_C6
U2AF2	3.03E-07	0.275573325	0.365	0.261	7.34E-03	Meta_CD4_C6
CTPS1	4.02E-07	0.308428816	0.188	0.077	9.74E-03	Meta_CD4_C6
CD83	4.33E-07	0.25271549	0.199	0.101	1.05E-02	Meta_CD4_C6
SUCO	4.74E-07	0.251959041	0.254	0.157	1.15E-02	Meta_CD4_C6
ASB6	5.32E-07	0.273035831	0.194	0.096	1.29E-02	Meta_CD4_C6
RNH1	6.48E-07	0.28129962	0.547	0.412	1.57E-02	Meta_CD4_C6
KCTD20	6.76E-07	0.274100677	0.243	0.139	1.64E-02	Meta_CD4_C6
DSTN	6.90356E-07	0.266027097	0.482	0.374	0.01671698	Meta_CD4_C6
SMNDC1	6.98732E-07	0.297990288	0.332	0.228	0.01691979	Meta_CD4_C6
PITHD1	8.52354E-07	0.261591726	0.421	0.293	0.02063976	Meta_CD4_C6
SAFB2	9.29701E-07	0.253219451	0.371	0.247	2.25E-02	Meta_CD4_C6
FXR1	1.02E-06	0.258077799	0.407	0.291	2.47E-02	Meta_CD4_C6
PAPOLA	1.09E-06	0.267483741	0.593	0.453	2.64E-02	Meta_CD4_C6
LDLRAD4	1.13E-06	0.28075643	0.375	0.248	2.75E-02	Meta_CD4_C6
DNAJC3	1.46E-06	0.312957254	0.386	0.250	3.53E-02	Meta_CD4_C6
SERINC1	1.47E-06	0.312111396	0.503	0.375	3.56E-02	Meta_CD4_C6
MGAT4A	1.76E-06	0.267436056	0.510	0.386	4.27E-02	Meta_CD4_C6
SPCS3	2.38E-06	0.310418066	0.498	0.369	5.75E-02	Meta_CD4_C6
EMB	2.39E-06	0.269738409	0.588	0.476	5.79E-02	Meta_CD4_C6

NFKB2	3.29E-06	0.260099277	0.301	0.210	7.97E-02	Meta_CD4_C6
SRRM2	3.66E-06	0.252983708	0.569	0.449	8.85E-02	Meta_CD4_C6
BCOR	3.66E-06	0.251420177	0.169	0.084	8.86E-02	Meta_CD4_C6
CDK11B	3.66E-06	0.266930594	0.281	0.173	8.87E-02	Meta_CD4_C6
BRIX1	3.84E-06	0.256455275	0.296	0.182	9.29E-02	Meta_CD4_C6
JAK1	3.90E-06	0.259538148	0.758	0.659	9.45E-02	Meta_CD4_C6
RCAN3	4.47E-06	0.255800409	0.392	0.284	1.08E-01	Meta_CD4_C6
LGALS8	5.37E-06	0.276508052	0.292	0.189	1.30E-01	Meta_CD4_C6
SELENOM	7.01E-06	0.295387337	0.346	0.212	1.70E-01	Meta_CD4_C6
PPP6C	9.86E-06	0.261488902	0.431	0.323	2.39E-01	Meta_CD4_C6
COX16	1.16E-05	0.261019042	0.410	0.294	2.81E-01	Meta_CD4_C6
DEGS1	1.36E-05	0.256181404	0.427	0.321	3.29E-01	Meta_CD4_C6
KAT6A	1.52E-05	0.290086491	0.293	0.179	3.69E-01	Meta_CD4_C6
TENT2	1.65E-05	0.261957025	0.255	0.159	4.00E-01	Meta_CD4_C6
BCL7B	1.69E-05	0.280435963	0.458	0.329	4.10E-01	Meta_CD4_C6
EIF1AX	1.82E-05	0.303285473	0.481	0.354	4.40E-01	Meta_CD4_C6
RALGAPA1	1.85E-05	0.262977173	0.363	0.259	4.47E-01	Meta_CD4_C6
PLEKHB2	1.86E-05	0.260197183	0.297	0.200	4.50E-01	Meta_CD4_C6
GATA3	2.73E-05	0.366190054	0.495	0.410	6.62E-01	Meta_CD4_C6
COPS3	4.04E-05	0.262215097	0.378	0.262	9.79E-01	Meta_CD4_C6
PARP8	5.07E-05	0.262617662	0.557	0.432	1.00E+00	Meta_CD4_C6
TMEM183A	9.15E-05	0.276044925	0.366	0.247	1.00E+00	Meta_CD4_C6
HIST1H4C	4.50E-04	0.250525249	0.668	0.568	1.00E+00	Meta_CD4_C6
ANKLE2	1.67E-03	0.277818448	0.291	0.184	1.00E+00	Meta_CD4_C6
TOX2	5.37E-76	1.281842746	0.410	0.066	1.30E-71	Meta_CD4_C7
PASK	2.53E-73	1.361652451	0.476	0.122	6.13E-69	Meta_CD4_C7
IL6ST	2.94E-65	1.159158735	0.564	0.212	7.12E-61	Meta_CD4_C7
TSHZ2	3.11E-58	0.995666768	0.322	0.047	7.53E-54	Meta_CD4_C7
IL6R	9.83E-57	0.660509344	0.308	0.070	2.38E-52	Meta_CD4_C7
FKBP5	1.07E-54	1.130249504	0.611	0.282	2.60E-50	Meta_CD4_C7
ICA1	5.16E-54	1.204972983	0.304	0.039	1.25E-49	Meta_CD4_C7
NR3C1	8.11E-45	0.966563396	0.736	0.494	1.96E-40	Meta_CD4_C7
TIAM1	8.26E-45	0.591000022	0.231	0.047	2.00E-40	Meta_CD4_C7
CXCR5	1.19E-43	0.80749082	0.235	0.026	2.88E-39	Meta_CD4_C7
TNFRSF4	8.09E-43	0.584455262	0.426	0.174	1.96E-38	Meta_CD4_C7
CD4	2.15E-42	0.731019988	0.521	0.236	5.20E-38	Meta_CD4_C7
CTLA4	6.48E-42	0.940740157	0.552	0.241	1.57E-37	Meta_CD4_C7
CORO1B	1.25E-39	0.762264709	0.623	0.398	3.03E-35	Meta_CD4_C7
SMCO4	9.99E-39	0.798505343	0.304	0.081	2.42E-34	Meta_CD4_C7
CXCL13	1.46E-38	2.741823141	0.288	0.060	3.53E-34	Meta_CD4_C7

TBC1D4	1.77E-38	0.763044614	0.337	0.094	4.29E-34	Meta_CD4_C7
PDCD1	8.01E-38	1.007248758	0.500	0.207	1.94E-33	Meta_CD4_C7
RNASET2	3.90E-37	0.665241679	0.692	0.493	9.45E-33	Meta_CD4_C7
ITM2A	8.30E-37	0.805838674	0.798	0.604	2.01E-32	Meta_CD4_C7
THADA	1.05E-36	0.797684102	0.261	0.078	2.53E-32	Meta_CD4_C7
LY96	1.49E-36	0.537908082	0.233	0.055	3.60E-32	Meta_CD4_C7
CD200	4.62E-36	0.760601259	0.182	0.013	1.12E-31	Meta_CD4_C7
TOX	6.13E-36	0.773312741	0.416	0.159	1.48E-31	Meta_CD4_C7
SRGN	1.00E-35	0.758784457	0.987	0.955	2.43E-31	Meta_CD4_C7
TIGIT	1.36E-34	0.865525581	0.582	0.323	3.30E-30	Meta_CD4_C7
ARID5B	3.92E-34	0.687625477	0.627	0.358	9.48E-30	Meta_CD4_C7
MAF	4.95E-34	0.736329426	0.430	0.177	1.20E-29	Meta_CD4_C7
SESN3	1.06E-33	0.777311327	0.321	0.097	2.56E-29	Meta_CD4_C7
SLC9A9	6.82E-33	0.568272606	0.272	0.084	1.65E-28	Meta_CD4_C7
CD40LG	2.18E-32	0.750323294	0.461	0.205	5.29E-28	Meta_CD4_C7
FBLN7	3.76E-32	0.67617736	0.307	0.093	9.11E-28	Meta_CD4_C7
BTLA	5.02E-31	0.697553381	0.260	0.059	1.21E-26	Meta_CD4_C7
AC004585.1	1.07E-29	0.460585969	0.171	0.030	2.58E-25	Meta_CD4_C7
LINC01281	2.85E-29	0.444852118	0.117	0.009	6.89E-25	Meta_CD4_C7
FXYD5	1.00E-28	0.440494983	0.921	0.852	2.43E-24	Meta_CD4_C7
TCF7	1.45E-28	0.855750135	0.447	0.218	3.51E-24	Meta_CD4_C7
DGKA	3.22E-28	0.562870999	0.463	0.266	7.79E-24	Meta_CD4_C7
ANKRD55	5.17E-28	0.425954467	0.124	0.011	1.25E-23	Meta_CD4_C7
CPM	5.97E-28	0.517877727	0.124	0.006	1.45E-23	Meta_CD4_C7
ICOS	8.96E-28	0.680654138	0.594	0.371	2.17E-23	Meta_CD4_C7
PPP1CC	2.72E-27	0.61966159	0.631	0.460	6.59E-23	Meta_CD4_C7
BATF	1.00E-26	0.571186817	0.555	0.336	2.43E-22	Meta_CD4_C7
LIMS1	1.81E-26	0.725073093	0.560	0.325	4.37E-22	Meta_CD4_C7
FAAH2	2.78E-26	0.519373949	0.214	0.051	6.72E-22	Meta_CD4_C7
ARMH1	4.18E-26	0.569577033	0.227	0.079	1.01E-21	Meta_CD4_C7
IGFBP4	2.99E-25	0.501473475	0.193	0.050	7.24E-21	Meta_CD4_C7
NMB	1.18E-24	1.017069436	0.185	0.035	2.85E-20	Meta_CD4_C7
MAGEH1	1.37E-24	0.696915442	0.274	0.093	3.31E-20	Meta_CD4_C7
LAT	2.81E-24	0.538559206	0.720	0.627	6.79E-20	Meta_CD4_C7
IL21	2.84E-24	0.486405188	0.108	0.011	6.89E-20	Meta_CD4_C7
CTSB	6.11E-24	0.564209927	0.512	0.290	1.48E-19	Meta_CD4_C7
CD84	9.00E-24	0.517887135	0.307	0.151	2.18E-19	Meta_CD4_C7
AC012645.3	1.56E-23	0.514411874	0.183	0.044	3.78E-19	Meta_CD4_C7
RNF19A	1.70E-23	0.655490338	0.606	0.390	4.11E-19	Meta_CD4_C7
GK	3.49E-23	0.588426794	0.238	0.070	8.46E-19	Meta_CD4_C7

CD28	3.08E-22	0.613897759	0.472	0.258	7.45E-18	Meta_CD4_C7
METTL8	4.00E-22	0.540485153	0.243	0.082	9.69E-18	Meta_CD4_C7
CCR7	4.47E-22	0.620390966	0.339	0.158	1.08E-17	Meta_CD4_C7
KLRB1	7.11E-22	0.549180999	0.663	0.460	1.72E-17	Meta_CD4_C7
CYSLTR1	1.29E-21	0.508637848	0.144	0.036	3.12E-17	Meta_CD4_C7
TMEM243	2.50E-21	0.471387258	0.503	0.333	6.06E-17	Meta_CD4_C7
TNFSF8	6.93E-21	0.579191121	0.314	0.129	1.68E-16	Meta_CD4_C7
LEF1	7.81E-21	0.517996688	0.339	0.147	1.89E-16	Meta_CD4_C7
ITGB2-AS1	1.10E-20	0.411025708	0.259	0.112	2.67E-16	Meta_CD4_C7
PTPN13	2.06E-20	0.42482138	0.112	0.018	4.98E-16	Meta_CD4_C7
TMEM123	3.34E-20	0.483445664	0.642	0.465	8.10E-16	Meta_CD4_C7
PTPRC	3.47E-20	0.408181727	0.932	0.893	8.40E-16	Meta_CD4_C7
SERINC5	3.74E-20	0.470017325	0.298	0.142	9.05E-16	Meta_CD4_C7
LIMS2	4.97E-20	0.27633527	0.102	0.016	1.20E-15	Meta_CD4_C7
RPL7	1.05E-19	0.412132682	0.968	0.936	2.54E-15	Meta_CD4_C7
TLK1	1.14E-19	0.442711446	0.435	0.261	2.76E-15	Meta_CD4_C7
NFATC1	1.15E-19	0.538189106	0.276	0.129	2.79E-15	Meta_CD4_C7
SH3TC1	8.12E-19	0.404437111	0.189	0.056	1.97E-14	Meta_CD4_C7
FYB1	1.29E-18	0.585479943	0.669	0.633	3.12E-14	Meta_CD4_C7
KIAA0319L	1.64E-18	0.410340496	0.217	0.104	3.98E-14	Meta_CD4_C7
ACTN1	2.10E-18	0.380669855	0.147	0.030	5.09E-14	Meta_CD4_C7
SFXN1	2.29E-18	0.483219195	0.434	0.261	5.55E-14	Meta_CD4_C7
TNFAIP8	2.71E-18	0.491953218	0.577	0.427	6.55E-14	Meta_CD4_C7
VOPP1	5.43E-18	0.325177499	0.399	0.278	1.32E-13	Meta_CD4_C7
POU2AF1	2.05E-17	0.330222286	0.102	0.010	4.96E-13	Meta_CD4_C7
RILPL2	2.11E-17	0.349195243	0.392	0.265	5.11E-13	Meta_CD4_C7
TBXAS1	2.31E-17	0.318990179	0.144	0.041	5.60E-13	Meta_CD4_C7
LIMA1	3.99E-17	0.34286163	0.221	0.098	9.66E-13	Meta_CD4_C7
NAP1L4	1.56E-16	0.615547326	0.590	0.414	3.77E-12	Meta_CD4_C7
AC090152.1	2.78E-16	0.310404548	0.196	0.085	6.72E-12	Meta_CD4_C7
CMTM8	4.72E-16	0.37648744	0.212	0.090	1.14E-11	Meta_CD4_C7
ST8SIA1	5.02E-16	0.366423762	0.156	0.037	1.22E-11	Meta_CD4_C7
NUDT16	8.72E-16	0.480686356	0.169	0.064	2.11E-11	Meta_CD4_C7
SPOCK2	9.95E-16	0.440142563	0.815	0.681	2.41E-11	Meta_CD4_C7
PHTF2	5.73E-15	0.383074157	0.359	0.207	1.39E-10	Meta_CD4_C7
EIF3E	1.02E-14	0.311040277	0.771	0.677	2.47E-10	Meta_CD4_C7
HINT1	1.16E-14	0.31773185	0.905	0.863	2.81E-10	Meta_CD4_C7
SOCS3	1.26E-14	0.44946247	0.454	0.311	3.06E-10	Meta_CD4_C7
CMTM7	1.59E-14	0.572333601	0.478	0.310	3.85E-10	Meta_CD4_C7
CNIH1	1.71E-14	0.461821987	0.467	0.308	4.14E-10	Meta_CD4_C7

C10orf54	2.04E-14	0.522415258	0.121	0.037	4.93E-10	Meta_CD4_C7
CD27	4.80E-14	0.308700612	0.648	0.483	1.16E-09	Meta_CD4_C7
ANP32B	7.39E-14	0.356392364	0.734	0.629	1.79E-09	Meta_CD4_C7
CHN1	7.70E-14	0.469253353	0.129	0.040	1.86E-09	Meta_CD4_C7
UCP2	1.24E-13	0.437287714	0.612	0.494	3.01E-09	Meta_CD4_C7
PIM2	1.25E-13	0.434836162	0.535	0.416	3.01E-09	Meta_CD4_C7
CCDC167	1.54E-13	0.291985119	0.356	0.238	3.73E-09	Meta_CD4_C7
PGM2L1	1.58E-13	0.527810314	0.194	0.084	3.82E-09	Meta_CD4_C7
PHACTR2	1.61E-13	0.368987638	0.238	0.107	3.90E-09	Meta_CD4_C7
SPINT2	2.03E-13	0.410539592	0.249	0.110	4.92E-09	Meta_CD4_C7
H2AFZ	2.27E-13	0.620318585	0.723	0.622	5.50E-09	Meta_CD4_C7
CCR4	2.94E-13	0.327507844	0.156	0.058	7.12E-09	Meta_CD4_C7
JUNB	3.00E-13	0.390432643	0.918	0.886	7.27E-09	Meta_CD4_C7
MYCBP2	3.67E-13	0.307340434	0.440	0.329	8.88E-09	Meta_CD4_C7
LRMP	4.00E-13	0.336772893	0.223	0.118	9.69E-09	Meta_CD4_C7
SLA	6.07E-13	0.565474459	0.544	0.376	1.47E-08	Meta_CD4_C7
ZNRF1	8.83E-13	0.310611296	0.182	0.081	2.14E-08	Meta_CD4_C7
PIK3IP1	2.17E-12	0.489640883	0.650	0.481	5.25E-08	Meta_CD4_C7
ETV6	2.29E-12	0.314114958	0.210	0.108	5.54E-08	Meta_CD4_C7
NDUFV2	2.48E-12	0.438051947	0.632	0.500	6.01E-08	Meta_CD4_C7
GMFG	2.94E-12	0.277111152	0.834	0.767	7.11E-08	Meta_CD4_C7
SEPT6	2.96E-12	0.441602549	0.696	0.576	7.16E-08	Meta_CD4_C7
C9orf16	3.80E-12	0.458907963	0.698	0.617	9.20E-08	Meta_CD4_C7
PIP4P2	5.54E-12	0.26653001	0.139	0.042	1.34E-07	Meta_CD4_C7
NPDC1	7.53E-12	0.276586107	0.186	0.093	1.82E-07	Meta_CD4_C7
TMEM173	8.83E-12	0.458821092	0.450	0.307	2.14E-07	Meta_CD4_C7
TESPA1	1.03E-11	0.25472736	0.281	0.176	2.49E-07	Meta_CD4_C7
SELL	1.18E-11	0.418077831	0.295	0.182	2.86E-07	Meta_CD4_C7
TNFRSF18	1.21E-11	0.320194333	0.328	0.203	2.94E-07	Meta_CD4_C7
MYL6B	1.29E-11	0.289051405	0.194	0.086	3.12E-07	Meta_CD4_C7
GPRIN3	1.65E-11	0.375538951	0.471	0.343	4.00E-07	Meta_CD4_C7
PBXIP1	2.88E-11	0.409728152	0.624	0.493	6.97E-07	Meta_CD4_C7
KIAA1324	3.05E-11	0.316259203	0.126	0.034	7.38E-07	Meta_CD4_C7
ITK	3.91E-11	0.37928518	0.478	0.358	9.47E-07	Meta_CD4_C7
LRRC8D	4.60E-11	0.338004915	0.193	0.082	1.11E-06	Meta_CD4_C7
TNIK	7.23E-11	0.354582414	0.350	0.222	1.75E-06	Meta_CD4_C7
CD5	7.49E-11	0.336640587	0.545	0.423	1.81E-06	Meta_CD4_C7
TP53INP1	1.05E-10	0.379955631	0.218	0.093	2.53E-06	Meta_CD4_C7
INPP4B	1.24E-10	0.384656875	0.389	0.249	3.00E-06	Meta_CD4_C7
AHI1	1.29E-10	0.327108761	0.167	0.074	3.11E-06	Meta_CD4_C7

ZNF281	1.40E-10	0.291848812	0.200	0.111	3.39E-06	Meta_CD4_C7
NINJ2	2.35E-10	0.292148561	0.153	0.077	5.70E-06	Meta_CD4_C7
RPL23	2.36E-10	0.298002167	0.875	0.823	5.71E-06	Meta_CD4_C7
APP	2.57E-10	0.251369174	0.144	0.059	6.22E-06	Meta_CD4_C7
SH2D1A	2.90E-10	0.492272167	0.472	0.321	7.02E-06	Meta_CD4_C7
SNX6	3.64E-10	0.317780499	0.498	0.398	8.81E-06	Meta_CD4_C7
HMGB2	3.75E-10	0.507131212	0.573	0.469	9.09E-06	Meta_CD4_C7
JAK3	7.38E-10	0.277428948	0.393	0.281	1.79E-05	Meta_CD4_C7
PPP1R2	1.04E-09	0.312157448	0.687	0.589	2.52E-05	Meta_CD4_C7
HMGN1	1.57E-09	0.285446596	0.787	0.701	3.80E-05	Meta_CD4_C7
MAL	1.88E-09	0.318145839	0.219	0.128	4.56E-05	Meta_CD4_C7
LDHB	1.94E-09	0.257306215	0.821	0.748	4.71E-05	Meta_CD4_C7
CLNS1A	2.13E-09	0.343257797	0.428	0.324	5.15E-05	Meta_CD4_C7
ELF1	2.75E-09	0.31281451	0.678	0.578	6.67E-05	Meta_CD4_C7
IFI16	2.85E-09	0.330712006	0.546	0.415	6.91E-05	Meta_CD4_C7
ADD3	3.77E-09	0.325601729	0.369	0.269	9.12E-05	Meta_CD4_C7
RAP1A	5.23E-09	0.310586656	0.605	0.499	1.27E-04	Meta_CD4_C7
LPAR6	5.45E-09	0.305417081	0.167	0.076	1.32E-04	Meta_CD4_C7
CCDC50	5.95E-09	0.361821019	0.172	0.068	1.44E-04	Meta_CD4_C7
IL16	6.49E-09	0.394798826	0.537	0.457	1.57E-04	Meta_CD4_C7
SARAF	7.25E-09	0.288410099	0.952	0.926	1.76E-04	Meta_CD4_C7
SNX9	7.76E-09	0.476774213	0.346	0.234	1.88E-04	Meta_CD4_C7
IPCEF1	8.43E-09	0.317837885	0.269	0.176	2.04E-04	Meta_CD4_C7
ADI1	8.45E-09	0.302611236	0.318	0.194	2.05E-04	Meta_CD4_C7
SMAP2	1.04E-08	0.546506999	0.463	0.309	2.51E-04	Meta_CD4_C7
SESN1	1.96E-08	0.573172409	0.261	0.157	4.74E-04	Meta_CD4_C7
PPP2R5C	2.03E-08	0.287593731	0.612	0.502	4.92E-04	Meta_CD4_C7
SOD1	2.04E-08	0.383468378	0.820	0.735	4.94E-04	Meta_CD4_C7
TRAT1	2.11E-08	0.288198873	0.427	0.326	5.12E-04	Meta_CD4_C7
RPL13A	3.04E-08	0.334636878	0.991	0.987	7.35E-04	Meta_CD4_C7
ELMO1	4.61E-08	0.390904607	0.342	0.207	1.12E-03	Meta_CD4_C7
N4BP2L2	5.34E-08	0.302143024	0.642	0.519	1.29E-03	Meta_CD4_C7
FYB	6.35E-08	0.751320923	0.182	0.085	1.54E-03	Meta_CD4_C7
EPSTI1	6.71E-08	0.297999902	0.348	0.237	1.63E-03	Meta_CD4_C7
STAT3	7.55E-08	0.356671854	0.625	0.509	1.83E-03	Meta_CD4_C7
FCMR	7.68E-08	0.51434025	0.497	0.375	1.86E-03	Meta_CD4_C7
ARID5A	8.79E-08	0.259112837	0.501	0.397	2.13E-03	Meta_CD4_C7
ISCU	1.01E-07	0.364245574	0.662	0.555	2.45E-03	Meta_CD4_C7
NAP1L1	1.44E-07	0.447824587	0.697	0.572	3.48E-03	Meta_CD4_C7
GNB2L1	1.55E-07	0.576851083	0.242	0.163	3.75E-03	Meta_CD4_C7

SOCS1	2.21E-07	0.307438006	0.472	0.377	5.35E-03	Meta_CD4_C7
YWHAB	2.39E-07	0.274159535	0.840	0.811	5.79E-03	Meta_CD4_C7
XIST	2.44E-07	0.259251826	0.183	0.110	5.90E-03	Meta_CD4_C7
GIMAP4	4.12E-07	0.26145599	0.619	0.539	9.97E-03	Meta_CD4_C7
IGKC	5.08E-07	0.859335324	0.200	0.125	1.23E-02	Meta_CD4_C7
GADD45G	6.94E-07	0.346166156	0.171	0.080	1.68E-02	Meta_CD4_C7
GLCCI1	7.41E-07	0.253446824	0.207	0.123	1.79E-02	Meta_CD4_C7
GYPC	8.16E-07	0.323998631	0.726	0.639	1.98E-02	Meta_CD4_C7
PEBP1	1.24E-06	0.309756851	0.677	0.589	3.01E-02	Meta_CD4_C7
GPX1	2.02E-06	0.385418285	0.116	0.046	4.90E-02	Meta_CD4_C7
SEC11A	2.09E-06	0.283098772	0.528	0.443	5.07E-02	Meta_CD4_C7
PRDX2	2.16E-06	0.253583666	0.569	0.467	5.24E-02	Meta_CD4_C7
RHOG	2.71E-06	0.264787378	0.611	0.516	6.57E-02	Meta_CD4_C7
FBXO32	3.19E-06	0.407132122	0.239	0.133	7.71E-02	Meta_CD4_C7
COA1	3.46E-06	0.294166438	0.287	0.187	8.38E-02	Meta_CD4_C7
RPS11	3.71E-06	0.259040499	0.946	0.930	8.99E-02	Meta_CD4_C7
GLTSCR2	3.76E-06	0.374442934	0.184	0.107	9.11E-02	Meta_CD4_C7
CYTH1	4.29E-06	0.285437211	0.477	0.366	1.04E-01	Meta_CD4_C7
SH3KBP1	4.69E-06	0.281467533	0.564	0.452	1.14E-01	Meta_CD4_C7
NABP1	4.74E-06	0.300904096	0.236	0.143	1.15E-01	Meta_CD4_C7
RPS17	4.93E-06	0.431825721	0.776	0.738	1.19E-01	Meta_CD4_C7
PNISR	5.26E-06	0.254597855	0.697	0.593	1.27E-01	Meta_CD4_C7
CHMP7	6.65E-06	0.292445739	0.260	0.164	1.61E-01	Meta_CD4_C7
ZFP36L1	8.03E-06	0.286387753	0.703	0.678	1.94E-01	Meta_CD4_C7
ST13	8.41E-06	0.272318255	0.603	0.508	2.04E-01	Meta_CD4_C7
NDUFB1	8.63E-06	0.278155356	0.490	0.388	2.09E-01	Meta_CD4_C7
SGK1	1.43E-05	0.25524188	0.169	0.100	0.34713373	Meta_CD4_C7
PPM1M	1.68E-05	0.26022691	0.289	0.207	4.08E-01	Meta_CD4_C7
FOXP1	1.80E-05	0.280788847	0.443	0.371	4.36E-01	Meta_CD4_C7
RBPJ	1.84E-05	0.406478927	0.437	0.335	4.45E-01	Meta_CD4_C7
ATP5L	1.96E-05	0.303621533	0.196	0.127	4.74E-01	Meta_CD4_C7
HIF1A	1.98E-05	0.264956289	0.422	0.338	4.80E-01	Meta_CD4_C7
DNPH1	2.17E-05	0.255601881	0.403	0.291	5.26E-01	Meta_CD4_C7
ATP5G2	2.45E-05	0.380076489	0.191	0.116	5.94E-01	Meta_CD4_C7
IGLC2	3.62E-05	0.655715897	0.102	0.055	8.77E-01	Meta_CD4_C7
PARP1	4.56E-05	0.250722434	0.441	0.343	1.00E+00	Meta_CD4_C7
MSI2	5.50E-05	0.276206582	0.218	0.133	1.00E+00	Meta_CD4_C7
ADSL	6.06E-05	0.306525133	0.347	0.248	1.00E+00	Meta_CD4_C7
DDX17	6.39E-05	0.309393972	0.559	0.462	1.00E+00	Meta_CD4_C7
CEMIP2	8.94E-05	0.307368601	0.401	0.296	1.00E+00	Meta_CD4_C7

ATP5E	9.37E-05	0.345112626	0.215	0.145	1.00E+00	Meta_CD4_C7
PLIN2	9.51E-05	0.293669811	0.337	0.246	1.00E+00	Meta_CD4_C7
ZNF331	1.60E-04	0.304749617	0.416	0.340	1.00E+00	Meta_CD4_C7
ETS1	1.99E-04	0.288967282	0.601	0.513	1.00E+00	Meta_CD4_C7
MTHFD2	2.04E-04	0.271849542	0.336	0.255	1.00E+00	Meta_CD4_C7
KIAA1551	2.15E-04	0.253292063	0.577	0.509	1.00E+00	Meta_CD4_C7
SIAH2	4.05E-04	0.274284298	0.279	0.204	1.00E+00	Meta_CD4_C7
P2RY10	4.31E-04	0.263998686	0.336	0.238	1.00E+00	Meta_CD4_C7
AKAP13	4.95E-04	0.269829147	0.657	0.547	1.00E+00	Meta_CD4_C7
VMP1	5.86E-04	0.285666062	0.419	0.315	1.00E+00	Meta_CD4_C7
NAB1	6.58E-04	0.265335453	0.168	0.074	1.00E+00	Meta_CD4_C7
BIRC3	7.28E-04	0.460709068	0.381	0.305	1.00E+00	Meta_CD4_C7
EVI2B	8.34E-04	0.265520389	0.511	0.413	1.00E+00	Meta_CD4_C7
TCEB2	9.14E-04	0.266156266	0.150	0.087	1.00E+00	Meta_CD4_C7
IKZF3	1.39E-03	0.252957697	0.413	0.353	1.00E+00	Meta_CD4_C7
CD59	1.50E-03	0.30289383	0.220	0.135	1.00E+00	Meta_CD4_C7
RABGAP1L	1.57E-03	0.253769381	0.434	0.345	1.00E+00	Meta_CD4_C7
RPS20	1.88E-03	0.28018466	0.901	0.891	1.00E+00	Meta_CD4_C7
IGHA1	2.05E-03	0.285249858	0.115	0.059	1.00E+00	Meta_CD4_C7
CYLD	2.54E-03	0.270742109	0.461	0.365	1.00E+00	Meta_CD4_C7
SYPL1	3.39E-03	0.307360894	0.373	0.256	1.00E+00	Meta_CD4_C7
NBEAL1	7.51E-03	0.254747186	0.266	0.206	1.00E+00	Meta_CD4_C7
GZMK	1.59E-248	2.039748399	0.938	0.368	3.85E-244	Meta_CD4_C8
GZMA	7.31E-120	0.879949623	0.948	0.577	1.77E-115	Meta_CD4_C8
CD27	9.70E-104	1.225385299	0.850	0.484	2.35E-99	Meta_CD4_C8
CST7	1.11E-89	0.978582068	0.931	0.694	2.68E-85	Meta_CD4_C8
HLA-A	5.55E-85	0.520109891	1.000	0.997	1.34E-80	Meta_CD4_C8
PFN1	1.80E-81	0.681882406	0.994	0.958	4.36E-77	Meta_CD4_C8
CNN2	4.50E-79	0.950543471	0.855	0.628	1.09E-74	Meta_CD4_C8
CD74	4.67E-79	0.826605656	0.972	0.873	1.13E-74	Meta_CD4_C8
PDCD1	6.77E-75	1.171769141	0.599	0.213	1.64E-70	Meta_CD4_C8
CORO1A	3.06E-71	0.698313419	0.969	0.875	7.41E-67	Meta_CD4_C8
CTLA4	7.43E-68	0.864190771	0.611	0.248	1.80E-63	Meta_CD4_C8
GIMAP7	8.28E-68	0.842614504	0.871	0.623	2.01E-63	Meta_CD4_C8
IL10	3.90E-63	1.307002934	0.263	0.020	9.45E-59	Meta_CD4_C8
GIMAP4	6.52E-63	0.926945111	0.808	0.539	1.58E-58	Meta_CD4_C8
MIR4435-2HG	2.52E-61	0.942597573	0.629	0.286	6.09E-57	Meta_CD4_C8
ITGB2	6.49E-61	0.740224042	0.862	0.609	1.57E-56	Meta_CD4_C8
CD38	4.84E-58	0.916843723	0.458	0.166	1.17E-53	Meta_CD4_C8
CYTOR	1.92E-56	0.939253056	0.730	0.425	4.65E-52	Meta_CD4_C8

RAC2	1.45E-53	0.622858192	0.940	0.829	3.51E-49	Meta_CD4_C8
PSMB9	1.63E-53	0.634231671	0.873	0.684	3.96E-49	Meta_CD4_C8
COTL1	4.61E-51	0.697967736	0.910	0.752	1.12E-46	Meta_CD4_C8
LAG3	2.41E-50	1.388319834	0.618	0.369	5.83E-46	Meta_CD4_C8
LIMD2	2.63E-50	0.701857555	0.872	0.715	6.36E-46	Meta_CD4_C8
CTSC	4.31E-49	0.698527284	0.810	0.556	1.04E-44	Meta_CD4_C8
DENND2D	5.35E-49	0.867593326	0.668	0.425	1.30E-44	Meta_CD4_C8
HAVCR2	3.62E-48	0.900723341	0.403	0.139	8.76E-44	Meta_CD4_C8
CXCR6	4.86E-48	0.942691758	0.623	0.328	1.18E-43	Meta_CD4_C8
F2R	1.16E-47	0.715828848	0.477	0.189	2.80E-43	Meta_CD4_C8
CAPZB	3.97E-46	0.603009379	0.876	0.708	9.61E-42	Meta_CD4_C8
ISG20	1.86E-45	0.703875364	0.810	0.628	4.51E-41	Meta_CD4_C8
ACTB	4.80E-45	0.495738217	1.000	0.998	1.16E-40	Meta_CD4_C8
ATP5F1E	2.24E-44	0.551383708	0.889	0.783	5.41E-40	Meta_CD4_C8
SIT1	7.89E-44	0.634595005	0.680	0.411	1.91E-39	Meta_CD4_C8
PSMB10	2.68E-43	0.670322713	0.721	0.508	6.50E-39	Meta_CD4_C8
ACTG1	1.32E-42	0.646515882	0.983	0.947	3.19E-38	Meta_CD4_C8
GMFG	3.23E-42	0.567955165	0.905	0.768	7.82E-38	Meta_CD4_C8
CCDC167	4.78E-42	0.689210153	0.496	0.239	1.16E-37	Meta_CD4_C8
ARHGDIB	5.32E-42	0.408870142	0.980	0.944	1.29E-37	Meta_CD4_C8
RARRES3	3.18E-40	0.633309187	0.895	0.738	7.71E-36	Meta_CD4_C8
CXCR3	4.02E-40	0.557029461	0.750	0.477	9.74E-36	Meta_CD4_C8
CYBA	3.57E-39	0.449173819	0.957	0.883	8.65E-35	Meta_CD4_C8
APOBEC3G	1.49E-38	0.606142562	0.721	0.443	3.60E-34	Meta_CD4_C8
TMSB4X	2.67E-38	0.306731696	1.000	0.999	6.46E-34	Meta_CD4_C8
HLA-C	5.41E-38	0.30510293	0.998	0.994	1.31E-33	Meta_CD4_C8
CLIC1	2.51E-37	0.49788316	0.936	0.824	6.07E-33	Meta_CD4_C8
PPM1G	3.86E-37	0.579390259	0.624	0.407	9.35E-33	Meta_CD4_C8
APOBEC3C	1.20E-36	0.609725547	0.527	0.281	2.90E-32	Meta_CD4_C8
LCK	6.19E-36	0.518342246	0.894	0.747	1.50E-31	Meta_CD4_C8
ARPC3	7.84E-36	0.460413543	0.932	0.825	1.90E-31	Meta_CD4_C8
C12orf75	3.36E-35	0.690441411	0.581	0.323	8.15E-31	Meta_CD4_C8
YWHAB	9.37E-35	0.463164079	0.915	0.811	2.27E-30	Meta_CD4_C8
TAP1	2.23E-34	0.582839682	0.698	0.495	5.39E-30	Meta_CD4_C8
CD28	2.26E-34	0.542681301	0.506	0.262	5.48E-30	Meta_CD4_C8
ATP5IF1	2.42E-34	0.587595694	0.730	0.543	5.85E-30	Meta_CD4_C8
CD3E	4.94E-34	0.352288247	0.985	0.923	1.20E-29	Meta_CD4_C8
PSMB8	5.37E-34	0.558766019	0.792	0.599	1.30E-29	Meta_CD4_C8
CHI3L2	1.98E-33	0.658831026	0.251	0.079	4.78E-29	Meta_CD4_C8
SUB1	8.49E-33	0.500786761	0.926	0.809	2.06E-28	Meta_CD4_C8

RNF213	1.27E-32	0.661142133	0.779	0.583	3.07E-28	Meta_CD4_C8
CD81	2.13E-32	0.811414861	0.348	0.131	5.16E-28	Meta_CD4_C8
PTPRCAP	2.29E-32	0.63050649	0.866	0.739	5.54E-28	Meta_CD4_C8
CFL1	2.37E-32	0.345972746	0.989	0.958	5.75E-28	Meta_CD4_C8
GBP5	2.50E-32	0.711065366	0.621	0.397	6.05E-28	Meta_CD4_C8
CARHSP1	2.64E-32	0.537294085	0.475	0.248	6.39E-28	Meta_CD4_C8
IKZF3	1.08E-31	0.5783295	0.584	0.353	2.62E-27	Meta_CD4_C8
CSTB	6.72E-31	0.484383661	0.778	0.613	1.63E-26	Meta_CD4_C8
FCMR	8.18E-31	0.723433757	0.607	0.376	1.98E-26	Meta_CD4_C8
CD3D	1.22E-30	0.384224055	0.977	0.890	2.96E-26	Meta_CD4_C8
SEM1	2.41E-30	0.597463439	0.690	0.469	5.85E-26	Meta_CD4_C8
EOMES	2.42E-30	0.558234932	0.302	0.098	5.85E-26	Meta_CD4_C8
TNFRSF1B	2.65E-30	0.55924202	0.630	0.397	6.41E-26	Meta_CD4_C8
IL32	4.47E-30	0.422870355	0.982	0.938	1.08E-25	Meta_CD4_C8
ARID5B	1.29E-29	0.532245504	0.610	0.365	3.14E-25	Meta_CD4_C8
HMOX1	1.40E-29	0.576656421	0.150	0.028	3.39E-25	Meta_CD4_C8
LYST	2.62E-29	0.637653295	0.456	0.219	6.35E-25	Meta_CD4_C8
TMSB10	4.89E-29	0.312811025	0.998	0.993	1.18E-24	Meta_CD4_C8
CD4	7.81E-29	0.601199955	0.471	0.243	1.89E-24	Meta_CD4_C8
HLA-E	1.20E-28	0.360376148	0.985	0.958	2.90E-24	Meta_CD4_C8
CSK	1.49E-28	0.57453013	0.661	0.437	3.60E-24	Meta_CD4_C8
RGS1	2.03E-28	0.425460585	0.799	0.618	4.92E-24	Meta_CD4_C8
GIMAP5	6.74E-28	0.559881369	0.422	0.220	1.63E-23	Meta_CD4_C8
CARD16	8.30E-28	0.353139017	0.599	0.396	2.01E-23	Meta_CD4_C8
WIPF1	1.42E-27	0.445948066	0.788	0.609	3.43E-23	Meta_CD4_C8
LIME1	2.21E-27	0.649424009	0.599	0.370	5.36E-23	Meta_CD4_C8
MYL6	2.95E-27	0.385216718	0.989	0.947	7.14E-23	Meta_CD4_C8
GBP4	3.10E-27	0.584303303	0.437	0.223	7.51E-23	Meta_CD4_C8
CORO1B	3.85E-27	0.611928068	0.603	0.404	9.33E-23	Meta_CD4_C8
UBE2L6	6.48E-27	0.527152232	0.699	0.472	1.57E-22	Meta_CD4_C8
CD2	7.89E-27	0.431609432	0.949	0.841	1.91E-22	Meta_CD4_C8
LINC00861	1.15E-26	0.571276033	0.430	0.211	2.79E-22	Meta_CD4_C8
GBP2	1.40E-26	0.433457685	0.612	0.415	3.40E-22	Meta_CD4_C8
LINC01943	1.86E-26	0.798035109	0.316	0.115	4.51E-22	Meta_CD4_C8
GBP1	3.35E-26	0.618123508	0.470	0.263	8.10E-22	Meta_CD4_C8
SUMO2	4.22E-26	0.421738558	0.905	0.805	1.02E-21	Meta_CD4_C8
JPT1	6.06E-26	0.57507536	0.603	0.372	1.47E-21	Meta_CD4_C8
TNFRSF14	6.55E-26	0.561326754	0.662	0.444	1.59E-21	Meta_CD4_C8
MAD1L1	7.97E-26	0.448038758	0.395	0.198	1.93E-21	Meta_CD4_C8
COX6B1	1.03E-25	0.36477708	0.872	0.755	2.49E-21	Meta_CD4_C8

PARK7	1.04E-25	0.357558419	0.790	0.629	2.53E-21	Meta_CD4_C8
TIMD4	1.20E-25	0.506059634	0.127	0.015	2.90E-21	Meta_CD4_C8
MYL6B	2.57E-25	0.384119076	0.250	0.088	6.21E-21	Meta_CD4_C8
CD79B	5.20E-25	0.373712156	0.272	0.104	1.26E-20	Meta_CD4_C8
DTHD1	5.47E-25	0.339027654	0.208	0.065	1.33E-20	Meta_CD4_C8
AC004585.1	6.44E-25	0.469323378	0.178	0.033	1.56E-20	Meta_CD4_C8
SELPLG	1.08E-24	0.507228105	0.629	0.460	2.62E-20	Meta_CD4_C8
RHOG	1.53E-24	0.446182335	0.687	0.518	3.70E-20	Meta_CD4_C8
SHISA5	1.57E-24	0.485356183	0.704	0.509	3.81E-20	Meta_CD4_C8
PPP1CA	6.68E-24	0.449134235	0.811	0.644	1.62E-19	Meta_CD4_C8
MAF	9.10E-24	0.488795049	0.375	0.184	2.20E-19	Meta_CD4_C8
DUSP4	9.36E-24	0.383700967	0.569	0.373	2.27E-19	Meta_CD4_C8
IFNG-AS1	3.56E-23	0.364723455	0.136	0.021	8.62E-19	Meta_CD4_C8
ATP5MC2	3.75E-23	0.479974147	0.861	0.745	9.07E-19	Meta_CD4_C8
PSME1	4.04E-23	0.346967362	0.931	0.849	9.77E-19	Meta_CD4_C8
LY6E	4.56E-23	0.464234168	0.862	0.739	1.11E-18	Meta_CD4_C8
EPSTI1	8.70E-23	0.420858238	0.427	0.238	2.11E-18	Meta_CD4_C8
PRF1	9.85E-23	0.571888713	0.614	0.415	2.38E-18	Meta_CD4_C8
OAZ1	1.11E-22	0.366145253	0.946	0.877	2.70E-18	Meta_CD4_C8
DYNLL1	1.28E-22	0.39399644	0.717	0.550	3.09E-18	Meta_CD4_C8
ARPC2	1.67E-22	0.350843759	0.946	0.885	4.04E-18	Meta_CD4_C8
IRF1	2.45E-22	0.578037451	0.739	0.585	5.94E-18	Meta_CD4_C8
ANXA5	2.66E-22	0.468761412	0.607	0.444	6.43E-18	Meta_CD4_C8
VAMP5	3.00E-22	0.409048139	0.524	0.341	7.26E-18	Meta_CD4_C8
PLEK	3.59E-22	0.486084288	0.280	0.103	8.69E-18	Meta_CD4_C8
S1PR4	4.07E-22	0.500175639	0.461	0.278	9.85E-18	Meta_CD4_C8
LAT	6.92E-22	0.436663696	0.796	0.628	1.68E-17	Meta_CD4_C8
SASH3	9.21E-22	0.510484679	0.611	0.423	2.23E-17	Meta_CD4_C8
ARPC1B	1.01E-21	0.417432635	0.870	0.728	2.44E-17	Meta_CD4_C8
RAB37	1.14E-21	0.433599638	0.309	0.142	2.75E-17	Meta_CD4_C8
CCR5	2.81E-21	0.450539093	0.296	0.124	6.81E-17	Meta_CD4_C8
STAT1	3.72E-21	0.408762568	0.548	0.381	9.00E-17	Meta_CD4_C8
SLC9A3R1	5.28E-21	0.468185621	0.750	0.575	1.28E-16	Meta_CD4_C8
CCR2	8.83E-21	0.481186629	0.206	0.063	2.14E-16	Meta_CD4_C8
PSMB3	1.10E-20	0.332256105	0.696	0.542	2.66E-16	Meta_CD4_C8
PYCARD	1.48E-20	0.388193547	0.483	0.294	3.57E-16	Meta_CD4_C8
LMNB1	1.85E-20	0.30223392	0.296	0.154	4.48E-16	Meta_CD4_C8
SCAMP2	2.34E-20	0.463891244	0.573	0.383	5.66E-16	Meta_CD4_C8
HCST	3.23E-20	0.287621925	0.935	0.842	7.83E-16	Meta_CD4_C8
PSME2	3.75E-20	0.416879918	0.815	0.660	9.07E-16	Meta_CD4_C8

CMTM7	3.89E-20	0.423725444	0.495	0.314	9.42E-16	Meta_CD4_C8
LIMS1	5.66E-20	0.396893666	0.532	0.331	1.37E-15	Meta_CD4_C8
GYPC	8.73E-20	0.476991922	0.796	0.640	2.11E-15	Meta_CD4_C8
ITM2A	1.03E-19	0.391475935	0.753	0.609	2.50E-15	Meta_CD4_C8
IQGAP2	1.36E-19	0.414179677	0.459	0.264	3.30E-15	Meta_CD4_C8
OPTN	1.51E-19	0.466507707	0.624	0.423	3.67E-15	Meta_CD4_C8
SERF2	4.71E-19	0.268723505	0.980	0.941	1.14E-14	Meta_CD4_C8
CASP4	6.17E-19	0.457475118	0.611	0.429	1.49E-14	Meta_CD4_C8
GALM	6.33E-19	0.478113773	0.443	0.261	1.53E-14	Meta_CD4_C8
WDR1	6.69E-19	0.442778724	0.728	0.545	1.62E-14	Meta_CD4_C8
LITAF	6.75E-19	0.398950425	0.674	0.491	1.63E-14	Meta_CD4_C8
AC017002.3	9.63E-19	0.459870454	0.171	0.046	2.33E-14	Meta_CD4_C8
SLFN5	1.49E-18	0.353474922	0.549	0.378	3.62E-14	Meta_CD4_C8
CTSB	1.75E-18	0.46955102	0.508	0.295	4.25E-14	Meta_CD4_C8
HSPB11	3.79E-18	0.396927905	0.463	0.272	9.18E-14	Meta_CD4_C8
AES	4.72E-18	0.450339826	0.740	0.591	1.14E-13	Meta_CD4_C8
PCED1B	5.51E-18	0.381433	0.407	0.234	1.34E-13	Meta_CD4_C8
CD70	6.45E-18	0.444416257	0.207	0.081	1.56E-13	Meta_CD4_C8
HNRNPLL	7.14E-18	0.436031727	0.498	0.322	1.73E-13	Meta_CD4_C8
IFITM1	8.92E-18	0.514652609	0.876	0.806	2.16E-13	Meta_CD4_C8
HLA-F	8.94E-18	0.367115208	0.753	0.605	2.17E-13	Meta_CD4_C8
PPP1R18	1.18E-17	0.437438884	0.701	0.529	2.85E-13	Meta_CD4_C8
FERMT3	1.53E-17	0.283597258	0.536	0.396	3.71E-13	Meta_CD4_C8
UBL5	1.62E-17	0.332250306	0.872	0.757	3.92E-13	Meta_CD4_C8
NINJ2	1.75E-17	0.390951149	0.221	0.078	4.24E-13	Meta_CD4_C8
ATP5MF	1.83E-17	0.468252087	0.700	0.505	4.44E-13	Meta_CD4_C8
FKBP1A	2.28E-17	0.43155342	0.737	0.570	5.52E-13	Meta_CD4_C8
HCLS1	2.49E-17	0.309914804	0.804	0.674	6.03E-13	Meta_CD4_C8
TMEM258	3.12E-17	0.404325118	0.742	0.614	7.55E-13	Meta_CD4_C8
SAMD3	3.30E-17	0.316278134	0.363	0.197	7.98E-13	Meta_CD4_C8
GABARAP	3.70E-17	0.417098698	0.796	0.658	8.97E-13	Meta_CD4_C8
AP2S1	4.10E-17	0.414085113	0.531	0.353	9.93E-13	Meta_CD4_C8
PRKCQ	4.75E-17	0.313241259	0.366	0.221	1.15E-12	Meta_CD4_C8
UQCR10	4.88E-17	0.350097684	0.771	0.638	1.18E-12	Meta_CD4_C8
IFI6	5.43E-17	0.457516878	0.475	0.313	1.31E-12	Meta_CD4_C8
CDC25B	7.12E-17	0.482434046	0.362	0.193	1.72E-12	Meta_CD4_C8
DECR1	7.23E-17	0.355260981	0.417	0.256	1.75E-12	Meta_CD4_C8
GPX7	8.21E-17	0.296160986	0.218	0.088	1.99E-12	Meta_CD4_C8
PPP4C	8.34E-17	0.319406166	0.608	0.453	2.02E-12	Meta_CD4_C8
AIP	8.62E-17	0.340609664	0.584	0.430	2.09E-12	Meta_CD4_C8

CLDND1	9.05E-17	0.550309755	0.574	0.461	2.19E-12	Meta_CD4_C8
AP3S1	1.93E-16	0.371786505	0.469	0.306	4.68E-12	Meta_CD4_C8
STX11	2.44E-16	0.373469864	0.341	0.171	5.92E-12	Meta_CD4_C8
TMBIM6	2.76E-16	0.375499188	0.875	0.768	6.69E-12	Meta_CD4_C8
SH2D1A	3.21E-16	0.372872398	0.500	0.325	7.77E-12	Meta_CD4_C8
RAB27A	4.37E-16	0.38431529	0.476	0.315	1.06E-11	Meta_CD4_C8
WNK1	4.84E-16	0.391866978	0.475	0.310	1.17E-11	Meta_CD4_C8
PRDX1	6.04E-16	0.314909309	0.693	0.537	1.46E-11	Meta_CD4_C8
RASAL3	6.13E-16	0.427365566	0.622	0.446	1.48E-11	Meta_CD4_C8
CAP1	6.18E-16	0.350250549	0.782	0.643	1.50E-11	Meta_CD4_C8
ATP5F1C	6.41E-16	0.399498446	0.618	0.453	1.55E-11	Meta_CD4_C8
PTMS	7.81E-16	0.554824359	0.384	0.227	1.89E-11	Meta_CD4_C8
GNB2	8.50E-16	0.406017926	0.714	0.545	2.06E-11	Meta_CD4_C8
DYNLRB1	9.31E-16	0.331578285	0.642	0.487	2.26E-11	Meta_CD4_C8
PSMA5	1.00E-15	0.373092328	0.607	0.443	2.42E-11	Meta_CD4_C8
MAT2B	1.04E-15	0.34511669	0.583	0.440	2.51E-11	Meta_CD4_C8
ITGB2-AS1	1.27E-15	0.341483045	0.249	0.116	3.08E-11	Meta_CD4_C8
PIK3R5	1.28E-15	0.272973485	0.291	0.167	3.11E-11	Meta_CD4_C8
AC243829.4	1.71E-15	0.27271699	0.125	0.041	4.14E-11	Meta_CD4_C8
PGAM1	1.92E-15	0.394603432	0.722	0.573	4.65E-11	Meta_CD4_C8
BATF	1.92E-15	0.2656074	0.523	0.342	4.66E-11	Meta_CD4_C8
BLVRA	2.27E-15	0.304867886	0.254	0.117	5.50E-11	Meta_CD4_C8
CALM3	2.31E-15	0.332565745	0.712	0.570	5.60E-11	Meta_CD4_C8
GIMAP1	2.58E-15	0.424346427	0.520	0.360	6.25E-11	Meta_CD4_C8
ITGAL	2.65E-15	0.398508728	0.498	0.333	6.41E-11	Meta_CD4_C8
CADM1	3.00E-15	0.365308675	0.101	0.022	7.27E-11	Meta_CD4_C8
IFI35	3.03E-15	0.281615392	0.408	0.254	7.34E-11	Meta_CD4_C8
HIGD2A	3.07E-15	0.413874977	0.812	0.671	7.44E-11	Meta_CD4_C8
PRDM1	3.54E-15	0.322080441	0.505	0.339	8.57E-11	Meta_CD4_C8
MVP	3.60E-15	0.303013194	0.504	0.352	8.72E-11	Meta_CD4_C8
SNX29	3.78E-15	0.285587479	0.194	0.076	9.15E-11	Meta_CD4_C8
JAK3	4.29E-15	0.332074098	0.442	0.283	1.04E-10	Meta_CD4_C8
GIMAP2	5.13E-15	0.36381671	0.439	0.274	1.24E-10	Meta_CD4_C8
TOX	5.23E-15	0.325401657	0.313	0.167	1.27E-10	Meta_CD4_C8
HDAC1	6.34E-15	0.301062776	0.459	0.309	1.53E-10	Meta_CD4_C8
TPM4	6.61E-15	0.438753983	0.528	0.400	1.60E-10	Meta_CD4_C8
CD5	6.62E-15	0.436799181	0.597	0.425	1.60E-10	Meta_CD4_C8
TIGIT	7.65E-15	0.288957109	0.490	0.331	1.85E-10	Meta_CD4_C8
CHST12	8.23E-15	0.319522792	0.520	0.336	1.99E-10	Meta_CD4_C8
TRIR	8.45E-15	0.402554397	0.791	0.647	2.05E-10	Meta_CD4_C8

GABARAPL2	1.15E-14	0.382507834	0.673	0.501	2.79E-10	Meta_CD4_C8
DOK2	1.22E-14	0.406548689	0.583	0.434	2.95E-10	Meta_CD4_C8
TWF2	1.38E-14	0.363774801	0.559	0.383	3.34E-10	Meta_CD4_C8
SQOR	1.39E-14	0.339404071	0.355	0.202	3.35E-10	Meta_CD4_C8
BIN1	1.40E-14	0.412289762	0.603	0.447	3.40E-10	Meta_CD4_C8
GPSM3	1.57E-14	0.363693129	0.838	0.701	3.80E-10	Meta_CD4_C8
BCL3	1.65E-14	0.374382779	0.325	0.192	4.00E-10	Meta_CD4_C8
TRAF3IP3	1.82E-14	0.354496567	0.683	0.524	4.40E-10	Meta_CD4_C8
SEPT9	1.87E-14	0.401410351	0.763	0.638	4.53E-10	Meta_CD4_C8
ATP5F1B	2.03E-14	0.324874711	0.729	0.607	4.92E-10	Meta_CD4_C8
LBH	2.33E-14	0.420064932	0.653	0.486	5.63E-10	Meta_CD4_C8
GK	2.36E-14	0.321293148	0.170	0.075	5.72E-10	Meta_CD4_C8
ACP5	2.39E-14	0.378926192	0.441	0.260	5.80E-10	Meta_CD4_C8
HNRNPF	2.57E-14	0.312472328	0.733	0.602	6.22E-10	Meta_CD4_C8
AC090152.1	3.62E-14	0.284577772	0.197	0.088	8.77E-10	Meta_CD4_C8
NDUFC1	4.65E-14	0.309686891	0.518	0.369	1.13E-09	Meta_CD4_C8
ELOVL5	4.72E-14	0.366994697	0.557	0.400	1.14E-09	Meta_CD4_C8
PYHIN1	5.23E-14	0.267985173	0.426	0.296	1.27E-09	Meta_CD4_C8
CD247	5.55E-14	0.318162537	0.784	0.648	1.34E-09	Meta_CD4_C8
DRAP1	6.01E-14	0.351384479	0.782	0.651	1.45E-09	Meta_CD4_C8
DNPH1	6.22E-14	0.358015677	0.451	0.293	1.51E-09	Meta_CD4_C8
ANXA11	6.76E-14	0.361362067	0.733	0.593	1.64E-09	Meta_CD4_C8
COX8A	8.23E-14	0.298030896	0.809	0.697	1.99E-09	Meta_CD4_C8
DBI	8.33E-14	0.291400454	0.721	0.575	2.02E-09	Meta_CD4_C8
ATP5MC3	1.01E-13	0.422089954	0.717	0.563	2.45E-09	Meta_CD4_C8
KIAA1551	1.02E-13	0.426307058	0.651	0.510	2.46E-09	Meta_CD4_C8
SEPT1	1.06E-13	0.351950419	0.779	0.647	2.56E-09	Meta_CD4_C8
ATP5MPL	1.52E-13	0.333411328	0.642	0.504	3.67E-09	Meta_CD4_C8
PSMD8	1.73E-13	0.285976749	0.658	0.508	4.18E-09	Meta_CD4_C8
TENT5C	1.75E-13	0.372674341	0.571	0.409	4.23E-09	Meta_CD4_C8
TRAFD1	1.88E-13	0.268666878	0.286	0.162	4.54E-09	Meta_CD4_C8
TRAPPC1	1.90E-13	0.294201325	0.720	0.570	4.61E-09	Meta_CD4_C8
PTTG1	2.09E-13	0.273740682	0.347	0.209	5.06E-09	Meta_CD4_C8
DUSP2	2.41E-13	0.390700468	0.747	0.636	5.83E-09	Meta_CD4_C8
CYBC1	2.54E-13	0.390546955	0.468	0.296	6.15E-09	Meta_CD4_C8
LAMTOR4	3.03E-13	0.307301622	0.670	0.536	7.33E-09	Meta_CD4_C8
MRPL10	4.12E-13	0.354058558	0.426	0.273	9.98E-09	Meta_CD4_C8
TSPO	4.54E-13	0.373501897	0.665	0.492	1.10E-08	Meta_CD4_C8
BICDL1	4.76E-13	0.251864145	0.180	0.076	1.15E-08	Meta_CD4_C8
EIF3G	4.94E-13	0.304892551	0.770	0.675	1.20E-08	Meta_CD4_C8

SLC1A4	5.24E-13	0.331794519	0.164	0.065	1.27E-08	Meta_CD4_C8
STK17A	5.86E-13	0.25281652	0.821	0.727	1.42E-08	Meta_CD4_C8
UCP2	6.03E-13	0.411757935	0.625	0.497	1.46E-08	Meta_CD4_C8
DGKZ	6.41E-13	0.30431826	0.349	0.219	1.55E-08	Meta_CD4_C8
PSMB2	7.44E-13	0.294555863	0.527	0.376	1.80E-08	Meta_CD4_C8
IRF4	9.12E-13	0.290283336	0.216	0.117	2.21E-08	Meta_CD4_C8
PIM2	9.68E-13	0.378030879	0.568	0.419	2.34E-08	Meta_CD4_C8
GPKOW	1.12E-12	0.287268724	0.205	0.088	2.70E-08	Meta_CD4_C8
CHCHD5	1.16E-12	0.289475059	0.374	0.224	2.80E-08	Meta_CD4_C8
NOP10	1.17E-12	0.30317881	0.638	0.493	2.84E-08	Meta_CD4_C8
LSP1	1.31E-12	0.306726612	0.886	0.830	3.17E-08	Meta_CD4_C8
BTN3A2	1.32E-12	0.324083521	0.513	0.354	3.19E-08	Meta_CD4_C8
PKM	1.34E-12	0.262879439	0.784	0.656	3.23E-08	Meta_CD4_C8
H3F3A	1.37E-12	0.257686925	0.898	0.823	3.32E-08	Meta_CD4_C8
RHOA	1.74E-12	0.269904597	0.903	0.832	4.20E-08	Meta_CD4_C8
ZNF706	2.28E-12	0.302050099	0.569	0.430	5.52E-08	Meta_CD4_C8
ELOB	2.30E-12	0.323479164	0.784	0.660	5.56E-08	Meta_CD4_C8
ATP5F1D	2.30E-12	0.357103552	0.721	0.585	5.57E-08	Meta_CD4_C8
RHOF	2.88E-12	0.314781179	0.632	0.499	6.98E-08	Meta_CD4_C8
NDUFA2	3.12E-12	0.261271302	0.552	0.406	7.55E-08	Meta_CD4_C8
TBCB	3.2447E-12	0.270982548	0.655	0.507	7.857E-08	Meta_CD4_C8
NUCB2	3.3443E-12	0.402149123	0.381	0.277	8.098E-08	Meta_CD4_C8
NCKAP1L	3.6534E-12	0.313254108	0.415	0.270	8.847E-08	Meta_CD4_C8
COX5A	3.8211E-12	0.318880301	0.692	0.563	9.253E-08	Meta_CD4_C8
PSMB6	4.2865E-12	0.255620041	0.624	0.500	1.038E-07	Meta_CD4_C8
CD6	4.3938E-12	0.347266462	0.713	0.564	1.064E-07	Meta_CD4_C8
FKBP8	4.4517E-12	0.346262323	0.725	0.598	1.078E-07	Meta_CD4_C8
MTRNR2L12	4.5877E-12	0.803365225	0.333	0.195	1.111E-07	Meta_CD4_C8
CLTA	4.8002E-12	0.333459919	0.552	0.392	1.162E-07	Meta_CD4_C8
CACYBP	4.9059E-12	0.311829241	0.631	0.503	1.188E-07	Meta_CD4_C8
TALDO1	5.5613E-12	0.294229676	0.548	0.390	1.347E-07	Meta_CD4_C8
LINC00426	5.8788E-12	0.347783833	0.234	0.101	1.424E-07	Meta_CD4_C8
ITGA4	7.1469E-12	0.377389881	0.566	0.430	1.731E-07	Meta_CD4_C8
NDUFA1	9.3077E-12	0.272690804	0.790	0.670	2.254E-07	Meta_CD4_C8
AC116366.3	9.4111E-12	0.340606322	0.376	0.241	2.279E-07	Meta_CD4_C8
PRR5L	9.9505E-12	0.273175516	0.143	0.044	2.41E-07	Meta_CD4_C8
ACTR3	1.0034E-11	0.253187785	0.737	0.620	2.43E-07	Meta_CD4_C8
ETS1	1.0591E-11	0.325706089	0.647	0.514	2.565E-07	Meta_CD4_C8
GTF3C6	1.1524E-11	0.287699741	0.473	0.335	2.791E-07	Meta_CD4_C8
C9orf16	1.1996E-11	0.276209254	0.744	0.618	2.905E-07	Meta_CD4_C8

SEC11A	1.2515E-11	0.316117436	0.591	0.445	3.031E-07	Meta_CD4_C8
NDUFB4	1.3008E-11	0.303123468	0.674	0.521	3.15E-07	Meta_CD4_C8
ARF5	1.3279E-11	0.285028881	0.670	0.550	3.215E-07	Meta_CD4_C8
SSR2	1.3646E-11	0.258415553	0.838	0.747	3.304E-07	Meta_CD4_C8
COX6C	1.4072E-11	0.250856141	0.872	0.772	3.407E-07	Meta_CD4_C8
HACD4	1.5834E-11	0.30677038	0.336	0.206	3.834E-07	Meta_CD4_C8
MDH2	1.7375E-11	0.320290746	0.648	0.509	4.207E-07	Meta_CD4_C8
SP110	1.7987E-11	0.25862939	0.448	0.312	4.356E-07	Meta_CD4_C8
PBXIP1	2.0529E-11	0.268740904	0.649	0.496	4.971E-07	Meta_CD4_C8
SH3BGRL	2.0742E-11	0.278110534	0.667	0.532	5.023E-07	Meta_CD4_C8
XAF1	2.2485E-11	0.257925587	0.333	0.217	5.445E-07	Meta_CD4_C8
TRPC4AP	2.5628E-11	0.299299058	0.324	0.195	6.206E-07	Meta_CD4_C8
RABGAP1L	3.0963E-11	0.353188525	0.491	0.347	7.498E-07	Meta_CD4_C8
IRF2	3.2359E-11	0.300668194	0.452	0.303	7.836E-07	Meta_CD4_C8
SKAP1	3.2974E-11	0.289886732	0.695	0.550	7.985E-07	Meta_CD4_C8
YARS	3.411E-11	0.323065348	0.405	0.258	8.26E-07	Meta_CD4_C8
APOL6	3.7578E-11	0.319611913	0.431	0.282	9.1E-07	Meta_CD4_C8
DEF6	4.3954E-11	0.321784075	0.599	0.446	1.064E-06	Meta_CD4_C8
CBLB	4.5227E-11	0.316673895	0.525	0.398	1.095E-06	Meta_CD4_C8
CUTA	5.0135E-11	0.312804068	0.721	0.590	1.214E-06	Meta_CD4_C8
JAKMIP1	5.1111E-11	0.304984967	0.247	0.116	1.238E-06	Meta_CD4_C8
CD47	5.1148E-11	0.272405132	0.567	0.429	1.239E-06	Meta_CD4_C8
RNF149	6.1583E-11	0.275436253	0.590	0.453	1.491E-06	Meta_CD4_C8
SLAMF1	6.6918E-11	0.362196739	0.423	0.267	1.62E-06	Meta_CD4_C8
ZYX	7.783E-11	0.36976488	0.687	0.548	1.885E-06	Meta_CD4_C8
DNM2	8.2073E-11	0.304315975	0.391	0.258	1.987E-06	Meta_CD4_C8
NELFCD	1.2023E-10	0.260838176	0.350	0.203	2.911E-06	Meta_CD4_C8
ARHGAP30	1.324E-10	0.257713254	0.527	0.383	3.206E-06	Meta_CD4_C8
NAP1L4	1.376E-10	0.30359428	0.573	0.419	3.332E-06	Meta_CD4_C8
ATP6V0E2	1.3809E-10	0.282053084	0.381	0.258	3.344E-06	Meta_CD4_C8
CDK2AP2	1.4564E-10	0.302423348	0.543	0.393	3.527E-06	Meta_CD4_C8
ATP5MG	1.6052E-10	0.302878134	0.868	0.750	3.887E-06	Meta_CD4_C8
MPG	1.6235E-10	0.2787185	0.435	0.293	3.931E-06	Meta_CD4_C8
MT1F	1.659E-10	0.272466036	0.279	0.170	4.017E-06	Meta_CD4_C8
PPP2R1A	1.7639E-10	0.253778578	0.546	0.428	4.271E-06	Meta_CD4_C8
ATP5PD	2.2178E-10	0.30731922	0.574	0.432	5.37E-06	Meta_CD4_C8
MT1X	2.3353E-10	0.301436791	0.428	0.296	5.655E-06	Meta_CD4_C8
SAMHD1	2.5922E-10	0.318189539	0.506	0.367	6.277E-06	Meta_CD4_C8
ANP32A	3.4455E-10	0.255200734	0.523	0.399	8.343E-06	Meta_CD4_C8
NDUFS2	3.7676E-10	0.257211095	0.394	0.271	9.123E-06	Meta_CD4_C8

TIMM17B	4.0356E-10	0.252013393	0.381	0.254	9.772E-06	Meta_CD4_C8
TKT	4.1419E-10	0.315010967	0.494	0.354	1.003E-05	Meta_CD4_C8
TRBC2	4.2776E-10	0.292601602	0.823	0.717	1.036E-05	Meta_CD4_C8
GIMAP6	5.1807E-10	0.253466402	0.382	0.262	1.255E-05	Meta_CD4_C8
ATP5MD	5.3429E-10	0.316356823	0.698	0.564	1.294E-05	Meta_CD4_C8
CD99	5.3523E-10	0.282771147	0.923	0.841	1.296E-05	Meta_CD4_C8
TBC1D10C	5.4784E-10	0.284207836	0.720	0.578	1.327E-05	Meta_CD4_C8
TC2N	5.7695E-10	0.330111981	0.560	0.406	1.397E-05	Meta_CD4_C8
PRKCB	5.7809E-10	0.255726752	0.291	0.165	1.4E-05	Meta_CD4_C8
HLA-DMA	5.8374E-10	0.257571886	0.326	0.207	1.414E-05	Meta_CD4_C8
YPEL3	6.0395E-10	0.284721392	0.435	0.292	1.462E-05	Meta_CD4_C8
RIPOR2	6.1021E-10	0.410928709	0.393	0.246	1.478E-05	Meta_CD4_C8
CCM2	7.8521E-10	0.302467874	0.389	0.256	1.901E-05	Meta_CD4_C8
SMCHD1	7.9838E-10	0.27083921	0.624	0.478	1.933E-05	Meta_CD4_C8
CD82	8.1669E-10	0.259599198	0.485	0.335	1.978E-05	Meta_CD4_C8
GRK6	8.5576E-10	0.365615149	0.452	0.305	2.072E-05	Meta_CD4_C8
CDC42SE1	8.758E-10	0.309930141	0.547	0.384	2.121E-05	Meta_CD4_C8
SLC25A5	9.221E-10	0.299573605	0.743	0.640	2.233E-05	Meta_CD4_C8
PAM	1.1386E-09	0.335820794	0.220	0.097	2.757E-05	Meta_CD4_C8
RASGRP1	1.341E-09	0.335261239	0.355	0.213	3.247E-05	Meta_CD4_C8
ATP5PF	1.381E-09	0.303458147	0.624	0.476	3.344E-05	Meta_CD4_C8
SYNE2	1.3912E-09	0.279533248	0.641	0.508	3.369E-05	Meta_CD4_C8
CD2BP2	1.421E-09	0.258151316	0.363	0.226	3.441E-05	Meta_CD4_C8
FYB1	1.5445E-09	0.32380248	0.774	0.633	3.74E-05	Meta_CD4_C8
RALY	1.6246E-09	0.266699146	0.661	0.533	3.934E-05	Meta_CD4_C8
NDUFB9	1.6335E-09	0.30380226	0.662	0.528	3.955E-05	Meta_CD4_C8
ARL4C	1.6821E-09	0.290534041	0.695	0.533	4.073E-05	Meta_CD4_C8
WAS	1.8418E-09	0.308801439	0.607	0.485	4.46E-05	Meta_CD4_C8
SYTL2	1.9039E-09	0.268972646	0.291	0.186	4.61E-05	Meta_CD4_C8
ZAP70	2.2004E-09	0.285473001	0.673	0.536	5.328E-05	Meta_CD4_C8
MTHFD2	2.2679E-09	0.287368252	0.393	0.257	5.492E-05	Meta_CD4_C8
ORAI1	2.9991E-09	0.262502091	0.380	0.256	7.262E-05	Meta_CD4_C8
ATP5F1A	3.005E-09	0.289420633	0.590	0.466	7.277E-05	Meta_CD4_C8
CASP1	3.2702E-09	0.313943833	0.441	0.303	7.919E-05	Meta_CD4_C8
POP4	4.0762E-09	0.297147828	0.279	0.179	9.87E-05	Meta_CD4_C8
PPIA	4.9676E-09	0.251737592	0.937	0.875	0.0001203	Meta_CD4_C8
BAX	5.1967E-09	0.338374044	0.618	0.492	0.0001258	Meta_CD4_C8
NFATC3	5.2333E-09	0.280222368	0.345	0.227	0.0001267	Meta_CD4_C8
ZBP1	5.6707E-09	0.2946645	0.298	0.176	0.0001373	Meta_CD4_C8
RCSD1	5.7521E-09	0.309256367	0.565	0.429	0.0001393	Meta_CD4_C8

SKA2	5.9347E-09	0.283961179	0.290	0.165	0.0001437	Meta_CD4_C8
ANXA6	6.0212E-09	0.265506521	0.683	0.531	0.0001458	Meta_CD4_C8
NDUFB11	6.4396E-09	0.261772025	0.748	0.639	0.0001559	Meta_CD4_C8
COX7A2	6.9546E-09	0.254403494	0.842	0.750	0.0001684	Meta_CD4_C8
GTPBP8	7.2415E-09	0.298703581	0.176	0.102	0.0001754	Meta_CD4_C8
CTSS	7.2978E-09	0.255810774	0.463	0.330	0.0001767	Meta_CD4_C8
UBALD2	7.9667E-09	0.305481648	0.306	0.188	0.0001929	Meta_CD4_C8
PSMA3	8.0178E-09	0.250969196	0.509	0.359	0.0001942	Meta_CD4_C8
PTPRJ	8.8456E-09	0.257417744	0.222	0.116	0.0002142	Meta_CD4_C8
ARPC5L	8.9624E-09	0.252099038	0.622	0.478	0.000217	Meta_CD4_C8
NAPA	9.3113E-09	0.265155529	0.541	0.403	0.0002255	Meta_CD4_C8
IDH2	1.1529E-08	0.313546203	0.592	0.439	0.0002792	Meta_CD4_C8
HINT1	1.1685E-08	0.272724051	0.926	0.864	0.000283	Meta_CD4_C8
RBX1	1.1789E-08	0.291015001	0.595	0.460	0.0002855	Meta_CD4_C8
AURKAIP1	1.2731E-08	0.255626197	0.557	0.430	0.0003083	Meta_CD4_C8
RNF214	1.2987E-08	0.276564503	0.254	0.136	0.0003145	Meta_CD4_C8
SYNRG	1.4264E-08	0.273636863	0.489	0.357	0.0003454	Meta_CD4_C8
RBCK1	1.4353E-08	0.293227245	0.527	0.375	0.0003476	Meta_CD4_C8
IFI27L2	1.5014E-08	0.282501583	0.500	0.365	0.0003636	Meta_CD4_C8
FMNL1	1.644E-08	0.309272202	0.537	0.401	0.0003981	Meta_CD4_C8
IRF7	1.6949E-08	0.270799795	0.401	0.267	0.0004104	Meta_CD4_C8
DENND1C	1.7319E-08	0.273174468	0.386	0.268	0.0004194	Meta_CD4_C8
MYO1F	1.7794E-08	0.278817861	0.456	0.323	0.0004309	Meta_CD4_C8
IL10RA	1.7924E-08	0.294988727	0.624	0.484	0.000434	Meta_CD4_C8
ARPC5	1.9041E-08	0.343998847	0.665	0.539	0.0004611	Meta_CD4_C8
GNG5	1.9738E-08	0.28702805	0.687	0.572	0.000478	Meta_CD4_C8
OGDH	2.0122E-08	0.266298592	0.275	0.168	0.0004873	Meta_CD4_C8
SLAMF6	2.172E-08	0.28908788	0.321	0.172	0.0005259	Meta_CD4_C8
IL16	2.4643E-08	0.265286326	0.561	0.458	0.0005967	Meta_CD4_C8
ARHGAP45	3.0679E-08	0.341595636	0.550	0.415	0.0007429	Meta_CD4_C8
PPP1CC	3.2345E-08	0.251657865	0.586	0.465	0.0007832	Meta_CD4_C8
SLC44A2	3.3064E-08	0.288194735	0.433	0.301	0.0008006	Meta_CD4_C8
C16orf87	3.6537E-08	0.275379865	0.267	0.154	0.0008847	Meta_CD4_C8
GSDMD	4.0316E-08	0.295090007	0.456	0.310	0.0009763	Meta_CD4_C8
TXNDC17	4.3316E-08	0.257296895	0.401	0.277	0.0010489	Meta_CD4_C8
IGBP1	5.4236E-08	0.268878661	0.463	0.342	0.0013133	Meta_CD4_C8
RAB29	5.9233E-08	0.271412963	0.311	0.192	0.0014343	Meta_CD4_C8
GPRIN3	6.6113E-08	0.309653118	0.480	0.346	0.0016009	Meta_CD4_C8
TECR	6.9798E-08	0.294925569	0.624	0.497	0.0016901	Meta_CD4_C8
MYH9	8.6927E-08	0.291293345	0.765	0.653	0.0021049	Meta_CD4_C8

RAC1	8.9165E-08	0.281052545	0.642	0.515	0.0021591	Meta_CD4_C8
NCOR1	8.957E-08	0.269557498	0.620	0.495	0.0021689	Meta_CD4_C8
RPS27L	9.8427E-08	0.258322158	0.649	0.546	0.0023834	Meta_CD4_C8
TMEM256	1.3192E-07	0.283690384	0.437	0.313	0.0031944	Meta_CD4_C8
EID1	2.2497E-07	0.262867814	0.713	0.607	0.0054475	Meta_CD4_C8
RBM38	2.3384E-07	0.262410466	0.355	0.239	0.0056624	Meta_CD4_C8
UBXN1	2.4333E-07	0.301337811	0.766	0.644	0.0058923	Meta_CD4_C8
FAM160B1	2.5309E-07	0.256989546	0.248	0.146	0.0061287	Meta_CD4_C8
RAB1B	2.9287E-07	0.272112681	0.516	0.399	0.007092	Meta_CD4_C8
R3HDM4	3.0902E-07	0.302304132	0.522	0.394	0.0074828	Meta_CD4_C8
CEBPD	3.2507E-07	0.304298205	0.224	0.146	0.0078716	Meta_CD4_C8
SEPT6	3.3954E-07	0.291691054	0.676	0.580	0.0082221	Meta_CD4_C8
PIK3CD	5.1944E-07	0.265862964	0.339	0.227	0.0125783	Meta_CD4_C8
RNASEH2B	5.6636E-07	0.315647636	0.389	0.261	0.0137145	Meta_CD4_C8
NFATC2	6.6791E-07	0.261529613	0.453	0.338	0.0161734	Meta_CD4_C8
C11orf98	6.785E-07	0.251952543	0.327	0.226	0.01643	Meta_CD4_C8
ATP2B4	7.88E-07	0.276800948	0.406	0.279	0.0190813	Meta_CD4_C8
RGS10	9.754E-07	0.253303487	0.719	0.622	0.0236194	Meta_CD4_C8
SUN2	9.8532E-07	0.279887419	0.654	0.524	0.0238595	Meta_CD4_C8
ETFB	1.38E-06	0.25406942	0.410	0.292	0.0334164	Meta_CD4_C8
FLNA	1.5785E-06	0.29877803	0.538	0.423	0.0382225	Meta_CD4_C8
LMAN2	2.07E-06	0.252787248	0.563	0.447	0.050126	Meta_CD4_C8
NRBP1	4.8367E-06	0.25781584	0.489	0.366	0.1171218	Meta_CD4_C8
APOBEC3H	4.8852E-06	0.265612465	0.220	0.128	0.1182958	Meta_CD4_C8
C8orf59	5.6842E-06	0.277122594	0.538	0.427	0.1376433	Meta_CD4_C8
RASGRP2	7.3876E-06	0.26851963	0.300	0.196	0.1788904	Meta_CD4_C8
RTRAF	7.9255E-06	0.265693778	0.601	0.483	0.1919151	Meta_CD4_C8
GLG1	1.6614E-05	0.250028453	0.416	0.307	0.4023135	Meta_CD4_C8
BTG3	2.2928E-05	0.259704514	0.401	0.285	0.5551998	Meta_CD4_C8
FABP5	0.00013412	0.373435885	0.293	0.199	1	Meta_CD4_C8
TNFSF9	0.00021269	0.324798124	0.206	0.136	1	Meta_CD4_C8
NEAT1	0.00155058	0.250995042	0.624	0.545	1	Meta_CD4_C8
PKM	1.665E-208	1.929468738	0.969	0.655	4.03E-204	Meta_CD4_C9
ENO1	1.18E-142	1.518081043	0.964	0.701	2.86E-138	Meta_CD4_C9
HSP90AB1	2.694E-132	1.324803043	0.989	0.840	6.52E-128	Meta_CD4_C9
TNFRSF18	2.35E-123	1.949986002	0.733	0.204	5.69E-119	Meta_CD4_C9
NME1	7.377E-118	1.390838212	0.617	0.101	1.79E-113	Meta_CD4_C9
RAN	2.426E-109	1.150088129	0.974	0.732	5.87E-105	Meta_CD4_C9
MIR155HG	1.841E-108	2.164548354	0.591	0.102	4.46E-104	Meta_CD4_C9
GAPDH	3.152E-108	1.141629383	1.000	0.975	7.63E-104	Meta_CD4_C9

TNFRSF4	2.56E-99	2.476052817	0.627	0.180	6.199E-95	Meta_CD4_C9
PRDX1	1.1845E-90	1.178597001	0.890	0.536	2.868E-86	Meta_CD4_C9
FABP5	2.6308E-90	1.995863363	0.643	0.197	6.37E-86	Meta_CD4_C9
EIF5A	3.0352E-90	1.07429508	0.893	0.558	7.35E-86	Meta_CD4_C9
RANBP1	1.1538E-86	1.076690461	0.809	0.355	2.794E-82	Meta_CD4_C9
YBX1	3.5456E-86	0.87379673	0.977	0.844	8.586E-82	Meta_CD4_C9
TPI1	6.5955E-86	1.197094787	0.934	0.716	1.597E-81	Meta_CD4_C9
PGAM1	7.8109E-84	1.302939554	0.840	0.573	1.891E-79	Meta_CD4_C9
NHP2	2.5775E-83	1.017323162	0.766	0.342	6.242E-79	Meta_CD4_C9
CTLA4	5.9297E-82	0.986942374	0.683	0.249	1.436E-77	Meta_CD4_C9
PRMT1	1.9932E-79	1.023500649	0.741	0.296	4.827E-75	Meta_CD4_C9
PARK7	4.9702E-78	1.038405745	0.926	0.629	1.204E-73	Meta_CD4_C9
PAICS	2.2891E-77	0.828029532	0.447	0.063	5.543E-73	Meta_CD4_C9
C1QBP	1.2624E-76	1.001411538	0.786	0.411	3.057E-72	Meta_CD4_C9
LDHA	1.6269E-73	0.973864726	0.964	0.788	3.94E-69	Meta_CD4_C9
BATF	5.9154E-73	1.101374967	0.756	0.341	1.432E-68	Meta_CD4_C9
DCTPP1	3.9849E-72	0.806184893	0.543	0.132	9.649E-68	Meta_CD4_C9
PPA1	1.2369E-69	1.013306887	0.793	0.416	2.995E-65	Meta_CD4_C9
DNPH1	3.4609E-69	0.920311024	0.711	0.292	8.381E-65	Meta_CD4_C9
MRTO4	3.6866E-68	0.68855372	0.464	0.091	8.927E-64	Meta_CD4_C9
EBNA1BP2	4.2584E-68	0.698116758	0.529	0.136	1.031E-63	Meta_CD4_C9
SNRPE	1.1915E-66	0.816209229	0.736	0.321	2.885E-62	Meta_CD4_C9
SERBP1	1.1728E-65	0.88778912	0.897	0.621	2.84E-61	Meta_CD4_C9
CCT5	7.2306E-65	0.826121716	0.713	0.314	1.751E-60	Meta_CD4_C9
TNFRSF9	5.9748E-64	1.542907706	0.369	0.055	1.447E-59	Meta_CD4_C9
SMS	9.926E-64	0.854874057	0.599	0.212	2.404E-59	Meta_CD4_C9
GTF3C6	1.1376E-63	0.625938598	0.730	0.334	2.755E-59	Meta_CD4_C9
BZW2	2.1893E-62	0.648581921	0.521	0.150	5.302E-58	Meta_CD4_C9
TXNDC17	5.9031E-62	0.725881072	0.684	0.276	1.429E-57	Meta_CD4_C9
PSMA7	9.4828E-62	0.74926127	0.947	0.739	2.296E-57	Meta_CD4_C9
NPM1	1.1867E-61	0.815446547	0.977	0.880	2.874E-57	Meta_CD4_C9
CALR	3.9845E-61	0.923878827	0.906	0.668	9.648E-57	Meta_CD4_C9
NOP16	7.3052E-61	0.795006313	0.457	0.105	1.769E-56	Meta_CD4_C9
DDX21	1.0917E-60	0.908095134	0.744	0.361	2.643E-56	Meta_CD4_C9
CHCHD2	2.5535E-60	0.610119138	0.964	0.838	6.183E-56	Meta_CD4_C9
VDR	5.416E-60	0.51218383	0.266	0.021	1.311E-55	Meta_CD4_C9
ATP1B3	1.3764E-59	0.722916483	0.631	0.243	3.333E-55	Meta_CD4_C9
NPM3	1.6752E-59	0.493931065	0.317	0.042	4.056E-55	Meta_CD4_C9
PDIA6	1.7829E-59	0.947445448	0.786	0.450	4.317E-55	Meta_CD4_C9
COX5A	2.7738E-59	0.717330722	0.883	0.562	6.717E-55	Meta_CD4_C9

TIMM13	4.0085E-59	0.867547592	0.604	0.205	9.706E-55	Meta_CD4_C9
SRM	4.2491E-59	1.161127686	0.576	0.217	1.029E-54	Meta_CD4_C9
VDAC1	6.0819E-59	0.813550873	0.813	0.430	1.473E-54	Meta_CD4_C9
HSPD1	3.9527E-58	1.047196112	0.786	0.440	9.571E-54	Meta_CD4_C9
PSME2	3.189E-57	0.841164822	0.917	0.660	7.722E-53	Meta_CD4_C9
SOD1	4.0419E-57	0.888221513	0.934	0.736	9.787E-53	Meta_CD4_C9
PFDN6	5.0076E-57	0.463313986	0.494	0.176	1.213E-52	Meta_CD4_C9
SNRPD1	5.608E-57	0.697350483	0.797	0.412	1.358E-52	Meta_CD4_C9
WARS	7.6214E-57	0.886228232	0.487	0.128	1.846E-52	Meta_CD4_C9
CCT3	7.7931E-57	0.866856585	0.771	0.379	1.887E-52	Meta_CD4_C9
DKC1	1.4003E-56	0.559538039	0.461	0.130	3.391E-52	Meta_CD4_C9
MIF	1.571E-56	0.855007226	0.921	0.799	3.804E-52	Meta_CD4_C9
NCL	5.0033E-56	0.907713137	0.930	0.717	1.212E-51	Meta_CD4_C9
SNRPG	1.5792E-55	0.735040312	0.856	0.536	3.824E-51	Meta_CD4_C9
FKBP1A	2.2823E-55	0.815425374	0.856	0.570	5.526E-51	Meta_CD4_C9
IMPDH2	2.9193E-55	0.662243038	0.564	0.208	7.069E-51	Meta_CD4_C9
MRPS12	3.1562E-55	0.576787169	0.579	0.221	7.643E-51	Meta_CD4_C9
AIMP2	4.7301E-55	0.48626701	0.374	0.080	1.145E-50	Meta_CD4_C9
NAMPT	7.8557E-55	0.717552146	0.611	0.239	1.902E-50	Meta_CD4_C9
ILF2	1.0424E-54	0.725604093	0.804	0.435	2.524E-50	Meta_CD4_C9
NDFIP2	1.1563E-54	0.674748184	0.491	0.140	2.8E-50	Meta_CD4_C9
POLD2	2.4727E-54	0.571040088	0.453	0.112	5.988E-50	Meta_CD4_C9
CCT2	3.7968E-54	0.777784984	0.733	0.349	9.194E-50	Meta_CD4_C9
PSMA3	7.1148E-54	0.619979421	0.727	0.359	1.723E-49	Meta_CD4_C9
MRPL4	1.095E-53	0.748648001	0.634	0.249	2.651E-49	Meta_CD4_C9
TOMM40	1.4924E-53	0.679097932	0.514	0.153	3.614E-49	Meta_CD4_C9
PHB	4.3656E-53	0.794445142	0.656	0.292	1.057E-48	Meta_CD4_C9
MTHFD2	5.351E-53	0.764967804	0.610	0.256	1.296E-48	Meta_CD4_C9
MRPL24	7.6583E-53	0.502023228	0.451	0.138	1.854E-48	Meta_CD4_C9
PRDX4	1.6158E-52	0.536710836	0.413	0.110	3.913E-48	Meta_CD4_C9
PSMB3	3.7863E-52	0.685173642	0.867	0.542	9.168E-48	Meta_CD4_C9
NDUFV2	4.1249E-52	0.795241381	0.817	0.503	9.989E-48	Meta_CD4_C9
GPATCH4	1.1982E-51	0.585558505	0.404	0.090	2.901E-47	Meta_CD4_C9
TIMM50	1.275E-51	0.439371329	0.459	0.137	3.087E-47	Meta_CD4_C9
CCT6A	2.6199E-51	0.765932169	0.716	0.353	6.344E-47	Meta_CD4_C9
PSMD8	3.5931E-51	0.726056075	0.846	0.508	8.701E-47	Meta_CD4_C9
MRPS7	3.9075E-51	0.512168469	0.640	0.280	9.462E-47	Meta_CD4_C9
SSSCA1	8.9665E-51	0.448919059	0.464	0.153	2.171E-46	Meta_CD4_C9
PSMB6	2.1832E-50	0.652122069	0.820	0.500	5.287E-46	Meta_CD4_C9
GADD45GIP1	3.4989E-50	0.549762107	0.741	0.383	8.473E-46	Meta_CD4_C9

ZNF593	3.7139E-50	0.5647009	0.431	0.115	8.993E-46	Meta_CD4_C9
GTPBP4	5.1689E-50	0.526012115	0.487	0.159	1.252E-45	Meta_CD4_C9
PSMA2	5.7876E-50	0.667709592	0.486	0.166	1.401E-45	Meta_CD4_C9
FKBP4	6.3901E-50	0.692228982	0.474	0.136	1.547E-45	Meta_CD4_C9
MRPL15	1.3175E-49	0.445970571	0.399	0.105	3.19E-45	Meta_CD4_C9
SEC61B	2.1366E-49	0.738744547	0.899	0.651	5.174E-45	Meta_CD4_C9
TXN	2.1791E-49	0.832862908	0.859	0.559	5.277E-45	Meta_CD4_C9
MRPL52	2.2901E-49	0.621671638	0.679	0.328	5.545E-45	Meta_CD4_C9
BANF1	2.6543E-49	0.642836055	0.713	0.356	6.427E-45	Meta_CD4_C9
NOLC1	3.9548E-49	0.693607978	0.494	0.166	9.577E-45	Meta_CD4_C9
CCDC86	4.7095E-49	0.449193845	0.310	0.047	1.14E-44	Meta_CD4_C9
TIMM23	4.7184E-49	0.427063012	0.496	0.172	1.143E-44	Meta_CD4_C9
SNRPB	9.7426E-49	0.64264224	0.894	0.603	2.359E-44	Meta_CD4_C9
TFRC	1.2539E-48	0.557277385	0.407	0.101	3.036E-44	Meta_CD4_C9
IFRD2	2.5882E-48	0.51251953	0.383	0.093	6.267E-44	Meta_CD4_C9
SNX9	3.044E-48	0.645037178	0.561	0.236	7.371E-44	Meta_CD4_C9
PES1	3.2561E-48	0.435607857	0.399	0.109	7.885E-44	Meta_CD4_C9
C19orf24	3.6722E-48	0.517613556	0.620	0.283	8.892E-44	Meta_CD4_C9
SDF2L1	7.4434E-48	0.798399923	0.656	0.299	1.802E-43	Meta_CD4_C9
NUTF2	1.0534E-47	0.580936883	0.654	0.305	2.551E-43	Meta_CD4_C9
ALDOA	1.309E-47	0.633873873	0.961	0.800	3.17E-43	Meta_CD4_C9
NDUFS6	1.4012E-47	0.675724684	0.737	0.386	3.393E-43	Meta_CD4_C9
POMP	1.7698E-47	0.771722595	0.869	0.550	4.285E-43	Meta_CD4_C9
RBPJ	1.9931E-47	0.589027889	0.676	0.336	4.826E-43	Meta_CD4_C9
RPIA	4.2592E-47	0.41990131	0.524	0.216	1.031E-42	Meta_CD4_C9
EIF6	4.3131E-47	0.611445974	0.683	0.307	1.044E-42	Meta_CD4_C9
MAP2K3	4.3582E-47	0.725796066	0.623	0.253	1.055E-42	Meta_CD4_C9
FARSA	6.4465E-47	0.471007241	0.491	0.158	1.561E-42	Meta_CD4_C9
MRPL3	9.9554E-47	0.616142791	0.569	0.214	2.411E-42	Meta_CD4_C9
COA6	1.0726E-46	0.383336756	0.439	0.168	2.597E-42	Meta_CD4_C9
TNFRSF25	1.2698E-46	0.588069545	0.553	0.232	3.075E-42	Meta_CD4_C9
PSMC2	1.9968E-46	0.316169754	0.551	0.253	4.835E-42	Meta_CD4_C9
GLRX3	2.004E-46	0.44495844	0.494	0.187	4.853E-42	Meta_CD4_C9
SEC11C	4.1219E-46	0.683253079	0.729	0.359	9.981E-42	Meta_CD4_C9
EIF3I	6.9E-46	0.674353049	0.793	0.439	1.671E-41	Meta_CD4_C9
RPA3	7.227E-46	0.507720744	0.586	0.248	1.75E-41	Meta_CD4_C9
SYNGR2	7.4843E-46	0.711978776	0.739	0.415	1.812E-41	Meta_CD4_C9
NME2	7.723E-46	0.993829816	0.724	0.509	1.87E-41	Meta_CD4_C9
DDX54	7.9499E-46	0.259725404	0.480	0.201	1.925E-41	Meta_CD4_C9
BOP1	9.281E-46	0.542734045	0.404	0.095	2.247E-41	Meta_CD4_C9

PSMD1	9.4787E-46	0.493534685	0.526	0.194	2.295E-41	Meta_CD4_C9
YIF1A	1.232E-45	0.463724451	0.527	0.198	2.983E-41	Meta_CD4_C9
PUM3	2.1622E-45	0.491199367	0.443	0.136	5.236E-41	Meta_CD4_C9
EXOSC5	3.0209E-45	0.446430649	0.369	0.096	7.315E-41	Meta_CD4_C9
TRAP1	3.8886E-45	0.539204454	0.414	0.109	9.416E-41	Meta_CD4_C9
LAMTOR5	4.4146E-45	0.439845071	0.757	0.403	1.069E-40	Meta_CD4_C9
PSMC4	5.5161E-45	0.545448768	0.667	0.305	1.336E-40	Meta_CD4_C9
TNIP2	7.1466E-45	0.560332672	0.553	0.228	1.731E-40	Meta_CD4_C9
SYNCRIP	7.4137E-45	0.607721889	0.597	0.257	1.795E-40	Meta_CD4_C9
EIF2S1	8.357E-45	0.498530333	0.527	0.198	2.024E-40	Meta_CD4_C9
SNRPF	9.1359E-45	0.598659607	0.790	0.437	2.212E-40	Meta_CD4_C9
GNL3	1.339E-44	0.542036801	0.466	0.147	3.242E-40	Meta_CD4_C9
TMEM147	1.5227E-44	0.4339661	0.591	0.272	3.687E-40	Meta_CD4_C9
RRP7A	1.5494E-44	0.486720693	0.551	0.226	3.752E-40	Meta_CD4_C9
GBP2	1.8563E-44	0.724832932	0.749	0.415	4.495E-40	Meta_CD4_C9
CDK4	2.2993E-44	0.536209834	0.477	0.144	5.568E-40	Meta_CD4_C9
COA4	4.7496E-44	0.409583847	0.481	0.181	1.15E-39	Meta_CD4_C9
PSME3	4.8707E-44	0.335965862	0.431	0.160	1.179E-39	Meta_CD4_C9
SNU13	7.3828E-44	0.554244271	0.877	0.595	1.788E-39	Meta_CD4_C9
SHMT2	7.5437E-44	0.577548477	0.517	0.184	1.827E-39	Meta_CD4_C9
NOP10	8.389E-44	0.5637157	0.816	0.493	2.031E-39	Meta_CD4_C9
PSMD14	9.4324E-44	0.455374679	0.561	0.242	2.284E-39	Meta_CD4_C9
RELB	9.569E-44	0.534571001	0.669	0.316	2.317E-39	Meta_CD4_C9
TRIB1	1.2166E-43	0.441904754	0.264	0.047	2.946E-39	Meta_CD4_C9
TIMM8B	1.8303E-43	0.552571842	0.560	0.225	4.432E-39	Meta_CD4_C9
MRPL11	1.8803E-43	0.339987965	0.534	0.227	4.553E-39	Meta_CD4_C9
MRPL14	2.2107E-43	0.509130174	0.540	0.204	5.353E-39	Meta_CD4_C9
TMEM208	2.8072E-43	0.42486531	0.516	0.191	6.798E-39	Meta_CD4_C9
RUVBL2	2.9743E-43	0.449840923	0.411	0.122	7.202E-39	Meta_CD4_C9
UQCRQ	5.1489E-43	0.562414694	0.803	0.517	1.247E-38	Meta_CD4_C9
RRP1	5.2897E-43	0.443413839	0.346	0.078	1.281E-38	Meta_CD4_C9
GABPB1	5.4112E-43	0.347144981	0.421	0.157	1.31E-38	Meta_CD4_C9
RRS1	6.6712E-43	0.404070444	0.333	0.080	1.615E-38	Meta_CD4_C9
NDUFAB1	9.8118E-43	0.642079439	0.747	0.405	2.376E-38	Meta_CD4_C9
PIM3	1.1067E-42	0.936122964	0.624	0.309	2.68E-38	Meta_CD4_C9
UQCC2	1.3799E-42	0.531315518	0.487	0.179	3.341E-38	Meta_CD4_C9
GGCT	1.6357E-42	0.347952338	0.419	0.130	3.961E-38	Meta_CD4_C9
HSPE1	1.6419E-42	0.736754638	0.853	0.578	3.976E-38	Meta_CD4_C9
RRP15	1.7936E-42	0.309091966	0.324	0.098	4.343E-38	Meta_CD4_C9
MRPS23	2.2604E-42	0.440069024	0.463	0.159	5.474E-38	Meta_CD4_C9

FTSJ1	2.3952E-42	0.286703327	0.419	0.156	5.8E-38	Meta_CD4_C9
FAM207A	2.4808E-42	0.442092621	0.411	0.127	6.007E-38	Meta_CD4_C9
DNAJB11	2.9064E-42	0.455420644	0.527	0.208	7.038E-38	Meta_CD4_C9
MRPL51	2.9775E-42	0.520913047	0.666	0.341	7.21E-38	Meta_CD4_C9
PSMB7	3.0809E-42	0.509311622	0.631	0.293	7.46E-38	Meta_CD4_C9
LYRM4	3.8628E-42	0.431240225	0.356	0.101	9.354E-38	Meta_CD4_C9
NAA20	4.2076E-42	0.376876188	0.537	0.221	1.019E-37	Meta_CD4_C9
HNRNPR	6.2476E-42	0.503324713	0.727	0.422	1.513E-37	Meta_CD4_C9
CD82	6.2734E-42	0.903361974	0.677	0.335	1.519E-37	Meta_CD4_C9
ZBTB32	6.3947E-42	0.635355599	0.239	0.027	1.548E-37	Meta_CD4_C9
YWHAE	7.126E-42	0.452673345	0.660	0.338	1.726E-37	Meta_CD4_C9
FARSB	8.9164E-42	0.415844512	0.357	0.091	2.159E-37	Meta_CD4_C9
AAMP	1.0523E-41	0.272993534	0.509	0.234	2.548E-37	Meta_CD4_C9
POLR2H	1.1196E-41	0.418348904	0.466	0.164	2.711E-37	Meta_CD4_C9
HSD17B10	1.2129E-41	0.414570717	0.537	0.236	2.937E-37	Meta_CD4_C9
STIP1	1.2631E-41	0.576458099	0.540	0.219	3.059E-37	Meta_CD4_C9
DCAF13	1.3688E-41	0.356755352	0.423	0.145	3.314E-37	Meta_CD4_C9
PSMD13	1.5923E-41	0.495030416	0.677	0.345	3.856E-37	Meta_CD4_C9
EIF5B	1.597E-41	0.642966526	0.671	0.303	3.867E-37	Meta_CD4_C9
PSMA4	1.669E-41	0.562557615	0.684	0.346	4.041E-37	Meta_CD4_C9
IARS	1.6994E-41	0.371192619	0.397	0.131	4.115E-37	Meta_CD4_C9
CYC1	2.159E-41	0.436410768	0.614	0.284	5.228E-37	Meta_CD4_C9
SLIRP	2.2103E-41	0.545362214	0.634	0.297	5.352E-37	Meta_CD4_C9
TIMM10	2.3894E-41	0.446514097	0.453	0.150	5.786E-37	Meta_CD4_C9
PA2G4	2.4375E-41	0.620969675	0.799	0.489	5.903E-37	Meta_CD4_C9
CTPS1	2.6061E-41	0.379459625	0.324	0.077	6.311E-37	Meta_CD4_C9
REXO2	2.6083E-41	0.390032774	0.551	0.241	6.316E-37	Meta_CD4_C9
ETF1	2.6131E-41	0.296346923	0.506	0.226	6.328E-37	Meta_CD4_C9
ISOC2	2.6298E-41	0.45561922	0.376	0.092	6.368E-37	Meta_CD4_C9
NOP14	3.1398E-41	0.437613717	0.394	0.116	7.603E-37	Meta_CD4_C9
ESF1	3.3007E-41	0.281283681	0.376	0.130	7.993E-37	Meta_CD4_C9
CAMK1	5.1032E-41	0.407625091	0.263	0.042	1.236E-36	Meta_CD4_C9
DESI1	5.6873E-41	0.401603279	0.419	0.143	1.377E-36	Meta_CD4_C9
RBX1	6.9433E-41	0.579794136	0.787	0.459	1.681E-36	Meta_CD4_C9
PSMC3	7.1931E-41	0.460673574	0.629	0.299	1.742E-36	Meta_CD4_C9
PRELID1	7.5535E-41	0.581505189	0.833	0.526	1.829E-36	Meta_CD4_C9
RANGAP1	7.7506E-41	0.284344252	0.354	0.115	1.877E-36	Meta_CD4_C9
MRPS26	8.1215E-41	0.35714418	0.467	0.190	1.967E-36	Meta_CD4_C9
ATIC	8.3386E-41	0.471041696	0.490	0.191	2.019E-36	Meta_CD4_C9
DDX56	8.5343E-41	0.264568108	0.383	0.139	2.067E-36	Meta_CD4_C9

NOP56	8.7363E-41	0.435969168	0.513	0.209	2.115E-36	Meta_CD4_C9
MANF	1.2662E-40	0.494863338	0.531	0.228	3.066E-36	Meta_CD4_C9
BTF3L4	1.5273E-40	0.305925582	0.531	0.245	3.698E-36	Meta_CD4_C9
TAF9	1.8527E-40	0.25037389	0.486	0.220	4.486E-36	Meta_CD4_C9
PPIA	1.9338E-40	0.656123479	0.944	0.876	4.683E-36	Meta_CD4_C9
NINJ1	1.9588E-40	0.641987994	0.533	0.216	4.743E-36	Meta_CD4_C9
EBI3	2.0157E-40	0.669775874	0.176	0.010	4.881E-36	Meta_CD4_C9
CD59	2.157E-40	0.600339799	0.424	0.136	5.223E-36	Meta_CD4_C9
MRPL47	2.4058E-40	0.416928234	0.576	0.254	5.826E-36	Meta_CD4_C9
DUSP4	3.6013E-40	0.670491195	0.704	0.373	8.721E-36	Meta_CD4_C9
PSMD11	4.3961E-40	0.442749377	0.579	0.266	1.065E-35	Meta_CD4_C9
MTFP1	5.2341E-40	0.254760178	0.410	0.171	1.267E-35	Meta_CD4_C9
GNG5	5.4558E-40	0.681613135	0.853	0.571	1.321E-35	Meta_CD4_C9
WDR77	5.583E-40	0.389803098	0.341	0.093	1.352E-35	Meta_CD4_C9
SLC3A2	7.9901E-40	0.620944965	0.799	0.507	1.935E-35	Meta_CD4_C9
SRPRB	9.5686E-40	0.430009917	0.427	0.148	2.317E-35	Meta_CD4_C9
GART	1.0076E-39	0.325632712	0.347	0.111	2.44E-35	Meta_CD4_C9
HPRT1	1.0247E-39	0.504825438	0.560	0.246	2.481E-35	Meta_CD4_C9
LAP3	1.0285E-39	0.324830956	0.521	0.236	2.491E-35	Meta_CD4_C9
PARVB	1.4123E-39	0.492895927	0.384	0.109	3.42E-35	Meta_CD4_C9
MRPL17	1.7977E-39	0.392698349	0.416	0.134	4.353E-35	Meta_CD4_C9
EIF4A1	1.8029E-39	0.506339686	0.693	0.399	4.366E-35	Meta_CD4_C9
MRPL13	1.8957E-39	0.3342362	0.450	0.182	4.591E-35	Meta_CD4_C9
TUFM	1.8997E-39	0.622816747	0.809	0.486	4.6E-35	Meta_CD4_C9
SNRPC	1.9964E-39	0.398952042	0.697	0.387	4.834E-35	Meta_CD4_C9
MYDGF	2.446E-39	0.588968017	0.706	0.369	5.923E-35	Meta_CD4_C9
PYCR1	2.4713E-39	0.279572856	0.154	0.004	5.984E-35	Meta_CD4_C9
PUF60	2.7686E-39	0.407878093	0.711	0.406	6.704E-35	Meta_CD4_C9
ZPR1	3.3274E-39	0.270682504	0.436	0.186	8.057E-35	Meta_CD4_C9
PMVK	3.4142E-39	0.433642953	0.484	0.197	8.268E-35	Meta_CD4_C9
EIF3J	3.5435E-39	0.444592254	0.526	0.214	8.581E-35	Meta_CD4_C9
PAK1IP1	4.2016E-39	0.404662128	0.316	0.078	1.017E-34	Meta_CD4_C9
MRPL36	4.2992E-39	0.418467022	0.469	0.181	1.041E-34	Meta_CD4_C9
CCDC47	4.9401E-39	0.354021339	0.444	0.173	1.196E-34	Meta_CD4_C9
SLC25A5	4.9728E-39	0.650177591	0.884	0.640	1.204E-34	Meta_CD4_C9
PSMB2	4.9843E-39	0.571248148	0.716	0.375	1.207E-34	Meta_CD4_C9
LINC01943	5.0137E-39	0.771883094	0.371	0.116	1.214E-34	Meta_CD4_C9
PDAP1	5.0328E-39	0.479039471	0.650	0.328	1.219E-34	Meta_CD4_C9
AK2	6.0834E-39	0.522130967	0.519	0.203	1.473E-34	Meta_CD4_C9
ATP5MC1	6.3153E-39	0.599148397	0.574	0.260	1.529E-34	Meta_CD4_C9

DPP3	6.3368E-39	0.333442969	0.354	0.107	1.534E-34	Meta_CD4_C9
TIMM17A	6.9117E-39	0.355788739	0.513	0.224	1.674E-34	Meta_CD4_C9
GNL2	8.11E-39	0.269487674	0.369	0.137	1.964E-34	Meta_CD4_C9
POLR2I	8.404E-39	0.305266618	0.564	0.287	2.035E-34	Meta_CD4_C9
HNRNPC	8.4471E-39	0.563115438	0.900	0.674	2.045E-34	Meta_CD4_C9
WDR18	9.4675E-39	0.325803645	0.399	0.127	2.293E-34	Meta_CD4_C9
AKR1A1	9.9197E-39	0.333807497	0.513	0.226	2.402E-34	Meta_CD4_C9
PDCD5	1.0082E-38	0.599559763	0.660	0.334	2.441E-34	Meta_CD4_C9
DCUN1D5	1.0584E-38	0.47837947	0.463	0.170	2.563E-34	Meta_CD4_C9
COPZ1	1.1513E-38	0.332262355	0.604	0.319	2.788E-34	Meta_CD4_C9
NFKB2	1.1913E-38	0.508477715	0.511	0.208	2.885E-34	Meta_CD4_C9
ITPA	1.4064E-38	0.407848492	0.481	0.200	3.406E-34	Meta_CD4_C9
MLEC	1.4478E-38	0.43868966	0.526	0.222	3.506E-34	Meta_CD4_C9
ALG3	1.5596E-38	0.37441764	0.410	0.134	3.777E-34	Meta_CD4_C9
MRPL20	1.8878E-38	0.5673381	0.690	0.367	4.571E-34	Meta_CD4_C9
KPNA2	2.0002E-38	0.457389812	0.473	0.171	4.843E-34	Meta_CD4_C9
PELI1	2.2443E-38	0.400154447	0.457	0.198	5.435E-34	Meta_CD4_C9
SDHB	2.4088E-38	0.343829483	0.563	0.271	5.833E-34	Meta_CD4_C9
PDCD1	2.5694E-38	0.458823903	0.497	0.215	6.222E-34	Meta_CD4_C9
ATAD3A	2.6106E-38	0.313958011	0.253	0.041	6.322E-34	Meta_CD4_C9
TCERG1	4.6374E-38	0.288029201	0.461	0.205	1.123E-33	Meta_CD4_C9
APEX1	5.0619E-38	0.50946853	0.613	0.295	1.226E-33	Meta_CD4_C9
AHSA1	5.6559E-38	0.490471095	0.564	0.245	1.37E-33	Meta_CD4_C9
PSMA6	6.5479E-38	0.641677997	0.706	0.424	1.586E-33	Meta_CD4_C9
PSMD3	6.8246E-38	0.397591998	0.526	0.222	1.653E-33	Meta_CD4_C9
TNFRSF1B	7.0756E-38	0.708844357	0.696	0.397	1.713E-33	Meta_CD4_C9
ADSL	7.4795E-38	0.494983657	0.570	0.249	1.811E-33	Meta_CD4_C9
SRP68	8.9332E-38	0.295691377	0.437	0.177	2.163E-33	Meta_CD4_C9
ANP32E	9.8607E-38	0.484603999	0.577	0.267	2.388E-33	Meta_CD4_C9
PSMD7	1.0175E-37	0.431635825	0.719	0.420	2.464E-33	Meta_CD4_C9
PRELID3B	1.0388E-37	0.308538997	0.536	0.250	2.515E-33	Meta_CD4_C9
BCCIP	1.0436E-37	0.444656448	0.574	0.255	2.527E-33	Meta_CD4_C9
ENY2	1.1554E-37	0.330179153	0.677	0.374	2.798E-33	Meta_CD4_C9
ARPP19	1.2375E-37	0.29889156	0.529	0.256	2.997E-33	Meta_CD4_C9
H2AFY	1.2601E-37	0.366615134	0.629	0.330	3.051E-33	Meta_CD4_C9
NOP2	1.2788E-37	0.250814119	0.334	0.113	3.097E-33	Meta_CD4_C9
CCT8	1.3708E-37	0.580606557	0.711	0.392	3.319E-33	Meta_CD4_C9
LRRC59	1.5601E-37	0.317225823	0.459	0.189	3.778E-33	Meta_CD4_C9
WDR43	1.7177E-37	0.354852577	0.414	0.161	4.16E-33	Meta_CD4_C9
CCT7	1.7266E-37	0.644125987	0.726	0.398	4.181E-33	Meta_CD4_C9

ERCC1	1.761E-37	0.393307176	0.480	0.188	4.264E-33	Meta_CD4_C9
DPM2	2.073E-37	0.283131046	0.384	0.139	5.02E-33	Meta_CD4_C9
SEH1L	2.2676E-37	0.295342062	0.271	0.060	5.491E-33	Meta_CD4_C9
DDX39A	2.3245E-37	0.470125999	0.677	0.356	5.629E-33	Meta_CD4_C9
GRWD1	2.4976E-37	0.330719925	0.349	0.106	6.048E-33	Meta_CD4_C9
EIF4G1	3.4846E-37	0.449949529	0.520	0.223	8.438E-33	Meta_CD4_C9
ATOX1	3.5224E-37	0.4244638	0.479	0.189	8.53E-33	Meta_CD4_C9
NDUFA2	4.0427E-37	0.270360787	0.661	0.406	9.789E-33	Meta_CD4_C9
WDR46	4.1299E-37	0.384700404	0.397	0.137	1E-32	Meta_CD4_C9
MRPL37	4.1871E-37	0.293312266	0.423	0.170	1.014E-32	Meta_CD4_C9
LSM6	4.4685E-37	0.330404284	0.589	0.305	1.082E-32	Meta_CD4_C9
PSMB1	4.6179E-37	0.590683064	0.871	0.609	1.118E-32	Meta_CD4_C9
TSTA3	5.5621E-37	0.307350106	0.487	0.215	1.347E-32	Meta_CD4_C9
STOML2	6.1427E-37	0.42657261	0.544	0.238	1.487E-32	Meta_CD4_C9
TMED9	7.4933E-37	0.434733389	0.723	0.405	1.814E-32	Meta_CD4_C9
COX20	8.255E-37	0.414854902	0.544	0.239	1.999E-32	Meta_CD4_C9
TMEM167A	8.8308E-37	0.507071101	0.551	0.250	2.138E-32	Meta_CD4_C9
COX6A1	9.6323E-37	0.483725974	0.934	0.770	2.332E-32	Meta_CD4_C9
ALG5	1.0631E-36	0.279638407	0.394	0.155	2.574E-32	Meta_CD4_C9
RPF2	1.2903E-36	0.337281962	0.303	0.078	3.124E-32	Meta_CD4_C9
BOLA3	1.3229E-36	0.356065117	0.491	0.208	3.204E-32	Meta_CD4_C9
MRPS34	1.3366E-36	0.48956772	0.664	0.359	3.237E-32	Meta_CD4_C9
CCT4	1.6153E-36	0.502691184	0.761	0.470	3.911E-32	Meta_CD4_C9
TYMP	1.6913E-36	0.704635446	0.594	0.278	4.096E-32	Meta_CD4_C9
HSPA4	1.7786E-36	0.41845042	0.494	0.211	4.307E-32	Meta_CD4_C9
HSPBP1	1.8277E-36	0.413543253	0.379	0.109	4.426E-32	Meta_CD4_C9
EEF1E1	2.231E-36	0.428395369	0.416	0.138	5.402E-32	Meta_CD4_C9
NDUFB7	2.5708E-36	0.402309636	0.713	0.416	6.225E-32	Meta_CD4_C9
RSL1D1	2.7086E-36	0.483916725	0.773	0.472	6.559E-32	Meta_CD4_C9
C9orf16	2.8838E-36	0.624414538	0.861	0.618	6.983E-32	Meta_CD4_C9
CCDC124	3.1261E-36	0.425973297	0.544	0.250	7.57E-32	Meta_CD4_C9
SNRPA	3.1553E-36	0.340543548	0.571	0.294	7.641E-32	Meta_CD4_C9
CDC123	3.1982E-36	0.373004003	0.591	0.284	7.745E-32	Meta_CD4_C9
RUVBL1	3.2075E-36	0.370877857	0.356	0.109	7.767E-32	Meta_CD4_C9
NUDT5	3.8435E-36	0.32864207	0.539	0.265	9.307E-32	Meta_CD4_C9
IMP4	3.904E-36	0.44439878	0.561	0.232	9.453E-32	Meta_CD4_C9
UBE2N	3.9515E-36	0.512592857	0.760	0.448	9.569E-32	Meta_CD4_C9
MRPL32	4.0392E-36	0.345742148	0.433	0.163	9.781E-32	Meta_CD4_C9
MOB1A	4.3697E-36	0.26294099	0.659	0.384	1.058E-31	Meta_CD4_C9
SEC61G	4.9499E-36	0.581291826	0.859	0.600	1.199E-31	Meta_CD4_C9

C20orf27	5.3075E-36	0.264708167	0.363	0.134	1.285E-31	Meta_CD4_C9
NOP58	5.5999E-36	0.448425067	0.721	0.421	1.356E-31	Meta_CD4_C9
ADRM1	5.7471E-36	0.507708013	0.717	0.393	1.392E-31	Meta_CD4_C9
SPG21	5.8906E-36	0.268149641	0.491	0.237	1.426E-31	Meta_CD4_C9
AHCY	6.6805E-36	0.389758878	0.346	0.095	1.618E-31	Meta_CD4_C9
CDV3	7.8674E-36	0.260588229	0.476	0.224	1.905E-31	Meta_CD4_C9
MRPS35	8.333E-36	0.298317001	0.496	0.215	2.018E-31	Meta_CD4_C9
TXN2	9.8599E-36	0.292856731	0.526	0.255	2.388E-31	Meta_CD4_C9
TXNL4A	9.9635E-36	0.348448861	0.534	0.247	2.413E-31	Meta_CD4_C9
TMEM70	1.0913E-35	0.309556681	0.429	0.157	2.643E-31	Meta_CD4_C9
MARS	1.4179E-35	0.256119167	0.387	0.155	3.433E-31	Meta_CD4_C9
ERH	1.453E-35	0.529038937	0.827	0.524	3.518E-31	Meta_CD4_C9
TSPAN17	1.4692E-35	0.437906092	0.356	0.116	3.558E-31	Meta_CD4_C9
SAR1B	1.5061E-35	0.264033417	0.447	0.196	3.647E-31	Meta_CD4_C9
ZC3H15	1.5384E-35	0.362271156	0.737	0.439	3.725E-31	Meta_CD4_C9
TMEM126A	1.8153E-35	0.300368195	0.419	0.167	4.396E-31	Meta_CD4_C9
HSBP1	1.8221E-35	0.376156385	0.511	0.225	4.412E-31	Meta_CD4_C9
ABCE1	1.9393E-35	0.349139189	0.427	0.169	4.696E-31	Meta_CD4_C9
ABCF1	2.2121E-35	0.36098535	0.501	0.221	5.357E-31	Meta_CD4_C9
RRP9	2.5748E-35	0.369689653	0.306	0.073	6.235E-31	Meta_CD4_C9
NDUFA11	2.6461E-35	0.270159637	0.590	0.331	6.408E-31	Meta_CD4_C9
EIF4E	2.8648E-35	0.303251255	0.547	0.261	6.937E-31	Meta_CD4_C9
DCPS	3.0354E-35	0.275308211	0.416	0.162	7.35E-31	Meta_CD4_C9
METAP2	3.1443E-35	0.343184019	0.581	0.295	7.614E-31	Meta_CD4_C9
PRDX3	3.3581E-35	0.52792272	0.564	0.266	8.132E-31	Meta_CD4_C9
PPIL1	3.5825E-35	0.322462605	0.269	0.059	8.675E-31	Meta_CD4_C9
XRCC5	3.721E-35	0.432902167	0.757	0.472	9.01E-31	Meta_CD4_C9
LYPLA1	3.7308E-35	0.340234762	0.511	0.244	9.034E-31	Meta_CD4_C9
SSBP1	3.8831E-35	0.541884994	0.760	0.462	9.403E-31	Meta_CD4_C9
SRSF9	4.1367E-35	0.404633255	0.667	0.362	1.002E-30	Meta_CD4_C9
SSRP1	4.6439E-35	0.381682663	0.497	0.216	1.125E-30	Meta_CD4_C9
MRPL57	4.7618E-35	0.350734145	0.683	0.391	1.153E-30	Meta_CD4_C9
MRPS16	4.907E-35	0.289378075	0.441	0.185	1.188E-30	Meta_CD4_C9
SDC4	5.2008E-35	0.43426294	0.210	0.027	1.259E-30	Meta_CD4_C9
GARS	5.6575E-35	0.355542159	0.476	0.204	1.37E-30	Meta_CD4_C9
TRAF1	6.024E-35	0.481265536	0.463	0.202	1.459E-30	Meta_CD4_C9
CD151	7.8616E-35	0.399846483	0.424	0.148	1.904E-30	Meta_CD4_C9
PHPT1	1.0105E-34	0.346954774	0.616	0.322	2.447E-30	Meta_CD4_C9
ZNF706	1.1206E-34	0.448210855	0.729	0.429	2.714E-30	Meta_CD4_C9
MRPL21	1.1353E-34	0.303515409	0.471	0.206	2.749E-30	Meta_CD4_C9

CD2BP2	1.1529E-34	0.291318788	0.494	0.226	2.792E-30	Meta_CD4_C9
MRPL27	1.3221E-34	0.313843514	0.423	0.168	3.201E-30	Meta_CD4_C9
MRPS15	1.3377E-34	0.323027613	0.551	0.267	3.239E-30	Meta_CD4_C9
SRA1	1.4224E-34	0.363015459	0.517	0.233	3.444E-30	Meta_CD4_C9
BRIX1	1.5513E-34	0.277675847	0.399	0.181	3.756E-30	Meta_CD4_C9
NFKB1	1.6759E-34	0.552683778	0.536	0.258	4.058E-30	Meta_CD4_C9
NSFL1C	1.7411E-34	0.263464137	0.461	0.220	4.216E-30	Meta_CD4_C9
NDUFB2	1.8703E-34	0.549134486	0.857	0.589	4.529E-30	Meta_CD4_C9
FBL	1.9847E-34	0.487207612	0.711	0.396	4.806E-30	Meta_CD4_C9
UTP4	2.1022E-34	0.327588827	0.304	0.091	5.09E-30	Meta_CD4_C9
EXOSC7	2.1415E-34	0.288543396	0.357	0.128	5.186E-30	Meta_CD4_C9
CYCS	2.3122E-34	0.556608921	0.809	0.516	5.599E-30	Meta_CD4_C9
PSMB5	2.3847E-34	0.406039764	0.440	0.164	5.775E-30	Meta_CD4_C9
HNRNPAB	2.641E-34	0.641337077	0.584	0.253	6.395E-30	Meta_CD4_C9
STX11	2.8256E-34	0.277060427	0.411	0.172	6.842E-30	Meta_CD4_C9
MTCH2	3.3963E-34	0.428929495	0.499	0.194	8.224E-30	Meta_CD4_C9
OSTC	3.7503E-34	0.444850102	0.689	0.391	9.081E-30	Meta_CD4_C9
CNIH1	3.8375E-34	0.394163035	0.601	0.312	9.293E-30	Meta_CD4_C9
GAR1	3.8791E-34	0.252051649	0.337	0.107	9.393E-30	Meta_CD4_C9
C19orf70	3.9236E-34	0.260307693	0.637	0.384	9.501E-30	Meta_CD4_C9
HSPA9	4.0844E-34	0.336933586	0.709	0.417	9.89E-30	Meta_CD4_C9
ANP32A	4.1663E-34	0.502112757	0.694	0.399	1.009E-29	Meta_CD4_C9
HNRNPD	4.2535E-34	0.263114685	0.544	0.273	1.03E-29	Meta_CD4_C9
UQCRFS1	4.2637E-34	0.324127266	0.744	0.470	1.032E-29	Meta_CD4_C9
NOC2L	4.9196E-34	0.399895488	0.451	0.169	1.191E-29	Meta_CD4_C9
SRP72	6.2425E-34	0.318429519	0.564	0.289	1.512E-29	Meta_CD4_C9
RPL22L1	6.3684E-34	0.679661427	0.793	0.511	1.542E-29	Meta_CD4_C9
DAZAP1	6.6019E-34	0.267474664	0.440	0.192	1.599E-29	Meta_CD4_C9
GSTO1	6.7172E-34	0.430162226	0.560	0.281	1.627E-29	Meta_CD4_C9
VCP	7.3715E-34	0.436445738	0.807	0.531	1.785E-29	Meta_CD4_C9
PDHA1	8.5283E-34	0.308032423	0.384	0.140	2.065E-29	Meta_CD4_C9
ORMDL2	8.8997E-34	0.25883349	0.490	0.221	2.155E-29	Meta_CD4_C9
NOB1	1.0227E-33	0.305660913	0.466	0.200	2.476E-29	Meta_CD4_C9
NCBP2	1.0272E-33	0.362587785	0.436	0.180	2.487E-29	Meta_CD4_C9
PSMD2	1.0777E-33	0.353507431	0.580	0.300	2.61E-29	Meta_CD4_C9
C19orf53	1.0831E-33	0.468483106	0.874	0.622	2.623E-29	Meta_CD4_C9
FUBP1	1.1377E-33	0.260873746	0.470	0.219	2.755E-29	Meta_CD4_C9
CFLAR	1.2247E-33	0.555729176	0.753	0.456	2.966E-29	Meta_CD4_C9
DNAJC2	1.2631E-33	0.278269181	0.489	0.222	3.058E-29	Meta_CD4_C9
MT2A	1.4568E-33	0.797882367	0.771	0.522	3.528E-29	Meta_CD4_C9

PFKP	1.5307E-33	0.445502747	0.507	0.215	3.707E-29	Meta_CD4_C9
CTSB	1.5611E-33	0.40120798	0.550	0.296	3.78E-29	Meta_CD4_C9
MTHFD1L	1.7443E-33	0.305668922	0.219	0.029	4.224E-29	Meta_CD4_C9
PSMG2	1.751E-33	0.305338635	0.656	0.368	4.24E-29	Meta_CD4_C9
COPS6	1.8161E-33	0.323074359	0.596	0.347	4.398E-29	Meta_CD4_C9
COX17	1.9499E-33	0.42777558	0.657	0.377	4.722E-29	Meta_CD4_C9
AURKAIP1	2.1291E-33	0.343572772	0.716	0.430	5.156E-29	Meta_CD4_C9
ZBED2	2.3328E-33	0.665404547	0.159	0.013	5.649E-29	Meta_CD4_C9
PSMC6	2.4011E-33	0.251653377	0.496	0.244	5.814E-29	Meta_CD4_C9
ARL5A	2.4604E-33	0.278562621	0.524	0.257	5.958E-29	Meta_CD4_C9
NDUFA13	3.0581E-33	0.399206879	0.741	0.476	7.405E-29	Meta_CD4_C9
DPH2	3.0641E-33	0.34976564	0.249	0.052	7.42E-29	Meta_CD4_C9
RNF181	3.0947E-33	0.354423357	0.659	0.356	7.494E-29	Meta_CD4_C9
SLC25A3	3.2693E-33	0.544572491	0.893	0.701	7.917E-29	Meta_CD4_C9
SF3A3	3.5275E-33	0.251409511	0.501	0.247	8.542E-29	Meta_CD4_C9
YARS	3.6424E-33	0.349220239	0.533	0.258	8.82E-29	Meta_CD4_C9
NASP	4.2716E-33	0.288319713	0.587	0.318	1.034E-28	Meta_CD4_C9
SLC35B1	4.6377E-33	0.285014415	0.354	0.129	1.123E-28	Meta_CD4_C9
IL2RA	4.7936E-33	0.893292831	0.303	0.080	1.161E-28	Meta_CD4_C9
PPM1G	5.3164E-33	0.404639042	0.690	0.407	1.287E-28	Meta_CD4_C9
TRUB2	5.4306E-33	0.265995327	0.289	0.082	1.315E-28	Meta_CD4_C9
GTF2A2	6.39E-33	0.269623157	0.647	0.378	1.547E-28	Meta_CD4_C9
VPS25	7.024E-33	0.296177045	0.403	0.171	1.701E-28	Meta_CD4_C9
CLNS1A	7.8487E-33	0.406409315	0.613	0.326	1.901E-28	Meta_CD4_C9
RTCB	8.2293E-33	0.265856292	0.433	0.192	1.993E-28	Meta_CD4_C9
QTRT1	8.3343E-33	0.359566984	0.400	0.139	2.018E-28	Meta_CD4_C9
PSMA5	8.5476E-33	0.575901532	0.740	0.442	2.07E-28	Meta_CD4_C9
ELP5	9.6444E-33	0.287378233	0.440	0.185	2.335E-28	Meta_CD4_C9
COPE	1.051E-32	0.31925264	0.837	0.582	2.545E-28	Meta_CD4_C9
SNRPA1	1.0518E-32	0.366102183	0.613	0.312	2.547E-28	Meta_CD4_C9
PWP1	1.2157E-32	0.281080048	0.476	0.220	2.944E-28	Meta_CD4_C9
POLR1C	1.3524E-32	0.347842555	0.349	0.110	3.275E-28	Meta_CD4_C9
HM13	1.3796E-32	0.431211445	0.657	0.376	3.341E-28	Meta_CD4_C9
CHCHD1	1.4311E-32	0.298385802	0.414	0.180	3.465E-28	Meta_CD4_C9
CHMP4B	1.4912E-32	0.404879558	0.477	0.209	3.611E-28	Meta_CD4_C9
KDELR2	1.508E-32	0.308640622	0.600	0.327	3.652E-28	Meta_CD4_C9
FDPS	1.5253E-32	0.335884722	0.523	0.261	3.693E-28	Meta_CD4_C9
PRPF40A	1.6371E-32	0.342407624	0.669	0.385	3.964E-28	Meta_CD4_C9
SNRNP70	1.6389E-32	0.34077284	0.639	0.345	3.969E-28	Meta_CD4_C9
COX7A2	1.8979E-32	0.388893375	0.937	0.750	4.596E-28	Meta_CD4_C9

NDUFAF8	2.1401E-32	0.340214607	0.501	0.239	5.182E-28	Meta_CD4_C9
SF3B4	2.2296E-32	0.286548001	0.601	0.335	5.399E-28	Meta_CD4_C9
LYPLA2	2.2457E-32	0.266787119	0.511	0.247	5.438E-28	Meta_CD4_C9
LAGE3	2.2882E-32	0.33975988	0.464	0.204	5.541E-28	Meta_CD4_C9
DIMT1	2.3604E-32	0.320948344	0.387	0.152	5.716E-28	Meta_CD4_C9
NDUFS3	2.3804E-32	0.285139725	0.544	0.276	5.764E-28	Meta_CD4_C9
PTMA	2.4435E-32	0.45586266	1.000	0.985	5.917E-28	Meta_CD4_C9
PFDN2	2.6511E-32	0.488499396	0.666	0.339	6.42E-28	Meta_CD4_C9
SFXN4	2.6614E-32	0.271979084	0.256	0.055	6.444E-28	Meta_CD4_C9
ELAVL1	2.8218E-32	0.318093521	0.537	0.258	6.833E-28	Meta_CD4_C9
SLC27A2	2.9461E-32	0.493359872	0.276	0.058	7.134E-28	Meta_CD4_C9
GK	3.5878E-32	0.440873117	0.291	0.075	8.688E-28	Meta_CD4_C9
CIAO1	3.6404E-32	0.256905936	0.454	0.208	8.815E-28	Meta_CD4_C9
TIGIT	3.6511E-32	0.590403297	0.621	0.331	8.841E-28	Meta_CD4_C9
TIAM1	3.6865E-32	0.301918701	0.217	0.053	8.927E-28	Meta_CD4_C9
MAGOH	4.0155E-32	0.370494107	0.729	0.433	9.724E-28	Meta_CD4_C9
TIMM9	5.1105E-32	0.270185545	0.373	0.148	1.237E-27	Meta_CD4_C9
PRPF19	5.2309E-32	0.382297398	0.439	0.171	1.267E-27	Meta_CD4_C9
TOMM22	5.6217E-32	0.547425208	0.701	0.400	1.361E-27	Meta_CD4_C9
C16orf87	5.682E-32	0.32240896	0.386	0.154	1.376E-27	Meta_CD4_C9
PSMD4	6.158E-32	0.396503093	0.634	0.339	1.491E-27	Meta_CD4_C9
PSMA1	6.2118E-32	0.425652249	0.704	0.434	1.504E-27	Meta_CD4_C9
FAM96B	6.2768E-32	0.498221407	0.770	0.478	1.52E-27	Meta_CD4_C9
CHMP2A	7.0913E-32	0.424114845	0.664	0.372	1.717E-27	Meta_CD4_C9
NIFK	7.3156E-32	0.363426828	0.489	0.216	1.771E-27	Meta_CD4_C9
MINOS1	9.7849E-32	0.271449913	0.707	0.445	2.369E-27	Meta_CD4_C9
CD320	1.0293E-31	0.271571063	0.419	0.183	2.493E-27	Meta_CD4_C9
MDH1	1.058E-31	0.393399956	0.683	0.397	2.562E-27	Meta_CD4_C9
PGD	1.073E-31	0.310494876	0.344	0.121	2.598E-27	Meta_CD4_C9
DNPEP	1.2146E-31	0.26639629	0.450	0.208	2.941E-27	Meta_CD4_C9
SUB1	1.3805E-31	0.464787326	0.947	0.810	3.343E-27	Meta_CD4_C9
HNRNPA3	1.4154E-31	0.480300115	0.844	0.611	3.427E-27	Meta_CD4_C9
PHLDA1	1.4362E-31	0.423853561	0.554	0.276	3.478E-27	Meta_CD4_C9
AGFG1	1.7525E-31	0.283935633	0.271	0.066	4.244E-27	Meta_CD4_C9
GNA15	2.0876E-31	0.327605735	0.266	0.078	5.055E-27	Meta_CD4_C9
MTHFD1	2.1317E-31	0.303233381	0.299	0.085	5.162E-27	Meta_CD4_C9
CHD4	2.1333E-31	0.323113217	0.440	0.193	5.166E-27	Meta_CD4_C9
NCLN	2.1972E-31	0.298746633	0.320	0.102	5.321E-27	Meta_CD4_C9
TCP1	2.329E-31	0.277980784	0.577	0.320	5.64E-27	Meta_CD4_C9
TRMT1	2.3622E-31	0.398993931	0.419	0.143	5.72E-27	Meta_CD4_C9

GDI2	2.3666E-31	0.339759514	0.719	0.448	5.731E-27	Meta_CD4_C9
SDF4	2.4743E-31	0.525678377	0.697	0.401	5.992E-27	Meta_CD4_C9
DNAJC15	2.5237E-31	0.332458188	0.587	0.320	6.111E-27	Meta_CD4_C9
RNASEH2C	2.9599E-31	0.29181697	0.499	0.238	7.167E-27	Meta_CD4_C9
PDIA4	3.0461E-31	0.38595321	0.491	0.238	7.376E-27	Meta_CD4_C9
USP14	3.2228E-31	0.268067385	0.447	0.199	7.804E-27	Meta_CD4_C9
NUDC	3.2506E-31	0.484800153	0.656	0.345	7.871E-27	Meta_CD4_C9
COX7B	3.7014E-31	0.459469641	0.851	0.582	8.963E-27	Meta_CD4_C9
HNRNPM	4.0086E-31	0.398600218	0.779	0.498	9.707E-27	Meta_CD4_C9
LTV1	5.3945E-31	0.315634905	0.337	0.119	1.306E-26	Meta_CD4_C9
LARP4	6.2276E-31	0.260020129	0.287	0.092	1.508E-26	Meta_CD4_C9
FUNDC2	6.266E-31	0.312997219	0.567	0.300	1.517E-26	Meta_CD4_C9
GSPT1	6.6521E-31	0.435570549	0.586	0.293	1.611E-26	Meta_CD4_C9
APRT	6.8261E-31	0.490048433	0.867	0.688	1.653E-26	Meta_CD4_C9
STRAP	7.9922E-31	0.292865684	0.689	0.428	1.935E-26	Meta_CD4_C9
MAP1LC3A	8.1083E-31	0.478074109	0.327	0.095	1.963E-26	Meta_CD4_C9
UBA2	9.0582E-31	0.356753705	0.579	0.303	2.193E-26	Meta_CD4_C9
PSMC1	9.3852E-31	0.29415225	0.544	0.284	2.273E-26	Meta_CD4_C9
PGK1	9.638E-31	0.529747521	0.927	0.755	2.334E-26	Meta_CD4_C9
ATP5MC3	1.0375E-30	0.685924197	0.727	0.563	2.512E-26	Meta_CD4_C9
G3BP1	1.1301E-30	0.508034183	0.609	0.315	2.736E-26	Meta_CD4_C9
UQCRC2	1.1318E-30	0.377677355	0.643	0.362	2.741E-26	Meta_CD4_C9
TUBB	1.1822E-30	0.793289642	0.781	0.548	2.863E-26	Meta_CD4_C9
NELFE	1.1967E-30	0.252439512	0.491	0.233	2.898E-26	Meta_CD4_C9
RPL26L1	1.3009E-30	0.307031512	0.360	0.128	3.15E-26	Meta_CD4_C9
METTL1	1.3325E-30	0.317209122	0.187	0.022	3.227E-26	Meta_CD4_C9
CCDC167	1.5513E-30	0.429705982	0.507	0.240	3.756E-26	Meta_CD4_C9
ARF6	1.5589E-30	0.295965479	0.759	0.527	3.775E-26	Meta_CD4_C9
NDUFB6	2.0475E-30	0.394218864	0.610	0.311	4.958E-26	Meta_CD4_C9
POLR2E	2.3871E-30	0.389224028	0.659	0.382	5.78E-26	Meta_CD4_C9
MRPS6	2.9125E-30	0.410930714	0.647	0.355	7.053E-26	Meta_CD4_C9
IL21R	2.9301E-30	0.414291575	0.426	0.185	7.095E-26	Meta_CD4_C9
GRPEL1	3.3096E-30	0.306433533	0.476	0.216	8.014E-26	Meta_CD4_C9
LSM7	3.5132E-30	0.465930778	0.809	0.526	8.507E-26	Meta_CD4_C9
RBFA	3.6918E-30	0.287382015	0.241	0.063	8.94E-26	Meta_CD4_C9
TOP1	3.8871E-30	0.416917261	0.647	0.349	9.413E-26	Meta_CD4_C9
WDR12	3.9453E-30	0.274792789	0.214	0.044	9.553E-26	Meta_CD4_C9
NDUFA6	3.9875E-30	0.39159449	0.731	0.461	9.656E-26	Meta_CD4_C9
SNRPB2	4.0427E-30	0.319667853	0.696	0.440	9.789E-26	Meta_CD4_C9
TUBA1C	4.1994E-30	0.402036528	0.536	0.270	1.017E-25	Meta_CD4_C9

BIRC3	4.6685E-30	0.484379033	0.546	0.306	1.13E-25	Meta_CD4_C9
RNH1	4.7425E-30	0.345786482	0.683	0.411	1.148E-25	Meta_CD4_C9
AC017002.3	5.1052E-30	0.433937251	0.214	0.046	1.236E-25	Meta_CD4_C9
SNRPD3	5.4455E-30	0.436827286	0.710	0.417	1.319E-25	Meta_CD4_C9
C4orf48	6.1924E-30	0.353136629	0.647	0.380	1.499E-25	Meta_CD4_C9
PRKAR1B	6.3857E-30	0.316282037	0.277	0.082	1.546E-25	Meta_CD4_C9
LSM4	6.7079E-30	0.252843365	0.553	0.297	1.624E-25	Meta_CD4_C9
SH2D2A	7.8668E-30	0.646093257	0.719	0.463	1.905E-25	Meta_CD4_C9
PNO1	8.3318E-30	0.402738205	0.347	0.109	2.018E-25	Meta_CD4_C9
MCTS1	8.4195E-30	0.313689084	0.437	0.192	2.039E-25	Meta_CD4_C9
NDUFAF2	8.6445E-30	0.252804361	0.317	0.105	2.093E-25	Meta_CD4_C9
POU2F2	8.8985E-30	0.410660773	0.336	0.113	2.155E-25	Meta_CD4_C9
LIMA1	9.3068E-30	0.252915933	0.289	0.101	2.254E-25	Meta_CD4_C9
DNAJC7	9.5214E-30	0.250326146	0.507	0.261	2.306E-25	Meta_CD4_C9
SNRNP40	1.0759E-29	0.259099868	0.494	0.244	2.605E-25	Meta_CD4_C9
MIR4435-2HG	1.1025E-29	0.613774587	0.581	0.288	2.67E-25	Meta_CD4_C9
ILF3	1.2008E-29	0.39486591	0.660	0.373	2.908E-25	Meta_CD4_C9
TBC1D4	1.2527E-29	0.462127621	0.304	0.102	3.033E-25	Meta_CD4_C9
SNHG15	1.3361E-29	0.439295758	0.530	0.265	3.235E-25	Meta_CD4_C9
IRF4	1.3521E-29	0.453591502	0.316	0.117	3.274E-25	Meta_CD4_C9
GRSF1	1.4906E-29	0.259788243	0.564	0.315	3.61E-25	Meta_CD4_C9
SF3B6	1.6061E-29	0.299134019	0.766	0.493	3.889E-25	Meta_CD4_C9
FKBP2	1.7486E-29	0.459511139	0.604	0.307	4.234E-25	Meta_CD4_C9
NDUFAF3	1.8362E-29	0.35036151	0.613	0.340	4.446E-25	Meta_CD4_C9
MRPS25	1.8597E-29	0.28974641	0.361	0.131	4.503E-25	Meta_CD4_C9
THOC7	2.114E-29	0.287281237	0.544	0.292	5.119E-25	Meta_CD4_C9
TRAF3	2.1657E-29	0.336097583	0.387	0.148	5.244E-25	Meta_CD4_C9
LSM5	2.3334E-29	0.343570587	0.589	0.308	5.65E-25	Meta_CD4_C9
CLPP	2.4343E-29	0.335006858	0.641	0.356	5.895E-25	Meta_CD4_C9
POLR3H	2.4825E-29	0.266303496	0.231	0.052	6.011E-25	Meta_CD4_C9
HBS1L	2.7314E-29	0.252411991	0.361	0.156	6.614E-25	Meta_CD4_C9
RNPS1	2.9627E-29	0.37478264	0.780	0.511	7.174E-25	Meta_CD4_C9
ARL3	3.1826E-29	0.305018325	0.304	0.106	7.707E-25	Meta_CD4_C9
RBM8A	3.9027E-29	0.381720046	0.863	0.630	9.45E-25	Meta_CD4_C9
TKT	3.9265E-29	0.492984724	0.653	0.354	9.508E-25	Meta_CD4_C9
CLTA	4.8338E-29	0.405422923	0.679	0.392	1.171E-24	Meta_CD4_C9
HSPA8	5.7819E-29	0.61834887	0.979	0.904	1.4E-24	Meta_CD4_C9
ARF4	5.8384E-29	0.28019436	0.604	0.349	1.414E-24	Meta_CD4_C9
PPP4C	6.3555E-29	0.287774604	0.719	0.453	1.539E-24	Meta_CD4_C9
MRPL28	6.9631E-29	0.33244017	0.479	0.217	1.686E-24	Meta_CD4_C9

ZMAT2	9.1733E-29	0.276622635	0.549	0.299	2.221E-24	Meta_CD4_C9
OLA1	9.5303E-29	0.316816038	0.539	0.276	2.308E-24	Meta_CD4_C9
NDUFB3	1.0594E-28	0.334413779	0.609	0.338	2.565E-24	Meta_CD4_C9
NDUFB4	1.1076E-28	0.271566793	0.753	0.521	2.682E-24	Meta_CD4_C9
ATXN10	1.1168E-28	0.282355544	0.517	0.274	2.704E-24	Meta_CD4_C9
PSMD6	1.1773E-28	0.25226446	0.607	0.338	2.851E-24	Meta_CD4_C9
UQCRC1	1.1785E-28	0.28266709	0.577	0.331	2.854E-24	Meta_CD4_C9
EIF2S2	1.272E-28	0.411653514	0.644	0.347	3.08E-24	Meta_CD4_C9
MRPL12	1.5526E-28	0.433502148	0.277	0.066	3.76E-24	Meta_CD4_C9
NOC4L	1.5786E-28	0.25525874	0.287	0.091	3.823E-24	Meta_CD4_C9
TMED3	1.6331E-28	0.260359466	0.403	0.181	3.955E-24	Meta_CD4_C9
TOMM5	1.6788E-28	0.499963059	0.720	0.434	4.065E-24	Meta_CD4_C9
MAF	1.7978E-28	0.318682532	0.423	0.184	4.353E-24	Meta_CD4_C9
FUOM	2.4345E-28	0.302354174	0.301	0.103	5.895E-24	Meta_CD4_C9
TSR1	2.7175E-28	0.278180298	0.277	0.081	6.58E-24	Meta_CD4_C9
TARS	2.7223E-28	0.322179677	0.367	0.142	6.592E-24	Meta_CD4_C9
AK6	2.8287E-28	0.342767607	0.343	0.117	6.85E-24	Meta_CD4_C9
RCC1	3.2292E-28	0.291677194	0.419	0.186	7.819E-24	Meta_CD4_C9
GTF3A	3.3283E-28	0.452683426	0.781	0.514	8.059E-24	Meta_CD4_C9
DARS	3.3879E-28	0.264345687	0.519	0.274	8.204E-24	Meta_CD4_C9
TM2D3	3.443E-28	0.336172348	0.466	0.228	8.337E-24	Meta_CD4_C9
RPN2	3.4579E-28	0.251095288	0.703	0.452	8.373E-24	Meta_CD4_C9
SF3B5	4.6743E-28	0.261511472	0.794	0.548	1.132E-23	Meta_CD4_C9
ARID5B	4.9356E-28	0.447162865	0.637	0.366	1.195E-23	Meta_CD4_C9
RAD23A	5.2517E-28	0.331176716	0.699	0.440	1.272E-23	Meta_CD4_C9
SRGN	5.4731E-28	0.476201048	0.989	0.956	1.325E-23	Meta_CD4_C9
NDUFC2	5.5528E-28	0.295820277	0.533	0.295	1.345E-23	Meta_CD4_C9
DDB1	5.6344E-28	0.263486853	0.454	0.219	1.364E-23	Meta_CD4_C9
ICOS	6.1724E-28	0.567030291	0.636	0.377	1.495E-23	Meta_CD4_C9
NAB2	6.9342E-28	0.328382386	0.219	0.035	1.679E-23	Meta_CD4_C9
HDAC2	7.4543E-28	0.340135926	0.460	0.203	1.805E-23	Meta_CD4_C9
SFPQ	7.6027E-28	0.507229644	0.784	0.525	1.841E-23	Meta_CD4_C9
DDX18	8.2483E-28	0.289267864	0.630	0.369	1.997E-23	Meta_CD4_C9
SLC1A5	8.4349E-28	0.274773813	0.433	0.192	2.043E-23	Meta_CD4_C9
UTP18	9.3132E-28	0.254003136	0.383	0.157	2.255E-23	Meta_CD4_C9
SLC25A39	1.2928E-27	0.301799371	0.520	0.281	3.13E-23	Meta_CD4_C9
RPN1	1.4558E-27	0.355761563	0.679	0.424	3.525E-23	Meta_CD4_C9
ENSA	1.5025E-27	0.30934814	0.741	0.482	3.638E-23	Meta_CD4_C9
CRELD2	1.7103E-27	0.321801342	0.449	0.207	4.142E-23	Meta_CD4_C9
RILPL2	1.7453E-27	0.505214622	0.531	0.268	4.226E-23	Meta_CD4_C9

LMAN1	1.7828E-27	0.377417831	0.540	0.273	4.317E-23	Meta_CD4_C9
YWHAQ	1.8178E-27	0.435979335	0.809	0.566	4.402E-23	Meta_CD4_C9
STAT5A	2.0469E-27	0.325409838	0.436	0.202	4.957E-23	Meta_CD4_C9
IGFLR1	2.0812E-27	0.302775607	0.404	0.189	5.04E-23	Meta_CD4_C9
IMMT	2.4181E-27	0.259887748	0.424	0.191	5.855E-23	Meta_CD4_C9
CMSS1	2.552E-27	0.3256276	0.264	0.076	6.18E-23	Meta_CD4_C9
SET	2.5655E-27	0.416358048	0.736	0.449	6.212E-23	Meta_CD4_C9
POLR2L	2.5786E-27	0.429275663	0.856	0.622	6.244E-23	Meta_CD4_C9
CANX	2.8552E-27	0.335615605	0.650	0.381	6.914E-23	Meta_CD4_C9
EMC4	3.0238E-27	0.261965972	0.627	0.370	7.322E-23	Meta_CD4_C9
COX6C	3.1747E-27	0.403594435	0.920	0.772	7.687E-23	Meta_CD4_C9
EWSR1	3.3448E-27	0.348355538	0.753	0.519	8.099E-23	Meta_CD4_C9
HSP90B1	3.3977E-27	0.641797149	0.823	0.582	8.228E-23	Meta_CD4_C9
XRCC6	3.6666E-27	0.437405071	0.826	0.584	8.879E-23	Meta_CD4_C9
NOL7	3.8844E-27	0.354746494	0.721	0.440	9.406E-23	Meta_CD4_C9
MRPL23	4.1121E-27	0.341070431	0.267	0.087	9.957E-23	Meta_CD4_C9
YDJC	4.1975E-27	0.269988223	0.393	0.175	1.016E-22	Meta_CD4_C9
ANXA7	4.2782E-27	0.284806413	0.641	0.381	1.036E-22	Meta_CD4_C9
NDUFS8	5.2796E-27	0.282394654	0.661	0.406	1.278E-22	Meta_CD4_C9
LMAN2	7.1062E-27	0.296089829	0.713	0.447	1.721E-22	Meta_CD4_C9
SURF4	7.1714E-27	0.351566386	0.706	0.444	1.737E-22	Meta_CD4_C9
SRI	7.6468E-27	0.348667957	0.733	0.454	1.852E-22	Meta_CD4_C9
NAP1L1	9.3128E-27	0.354605841	0.826	0.575	2.255E-22	Meta_CD4_C9
SRPK1	1.0029E-26	0.281463153	0.404	0.186	2.429E-22	Meta_CD4_C9
PHACTR2	1.0161E-26	0.287568941	0.307	0.110	2.46E-22	Meta_CD4_C9
XRN2	1.0556E-26	0.31560617	0.624	0.353	2.556E-22	Meta_CD4_C9
EMG1	1.1155E-26	0.274036778	0.297	0.112	2.701E-22	Meta_CD4_C9
KCNN4	1.2369E-26	0.444132422	0.277	0.069	2.995E-22	Meta_CD4_C9
HDDC2	1.3025E-26	0.268958767	0.413	0.192	3.154E-22	Meta_CD4_C9
IL27RA	1.3691E-26	0.251773893	0.540	0.307	3.315E-22	Meta_CD4_C9
AP2M1	1.6349E-26	0.267284955	0.697	0.449	3.959E-22	Meta_CD4_C9
SEC13	2.18E-26	0.353249882	0.560	0.290	5.279E-22	Meta_CD4_C9
GPR137B	2.6358E-26	0.276253797	0.359	0.152	6.382E-22	Meta_CD4_C9
CKS2	3.2285E-26	0.3460922	0.393	0.164	7.818E-22	Meta_CD4_C9
DYNLL1	3.52E-26	0.497512531	0.807	0.550	8.524E-22	Meta_CD4_C9
PIGT	3.782E-26	0.258652056	0.446	0.217	9.158E-22	Meta_CD4_C9
ROMO1	3.8175E-26	0.42930853	0.729	0.457	9.244E-22	Meta_CD4_C9
SNF8	4.1031E-26	0.284976138	0.447	0.215	9.936E-22	Meta_CD4_C9
BLVRA	4.5926E-26	0.251853106	0.313	0.117	1.112E-21	Meta_CD4_C9
PPIF	5.1961E-26	0.257523214	0.339	0.138	1.258E-21	Meta_CD4_C9

MRPS17	6.1003E-26	0.280464455	0.253	0.066	1.477E-21	Meta_CD4_C9
MAGOHB	6.6723E-26	0.257681572	0.291	0.089	1.616E-21	Meta_CD4_C9
DGUOK	7.134E-26	0.294528918	0.610	0.350	1.727E-21	Meta_CD4_C9
MYL6	8.343E-26	0.39977242	0.991	0.947	2.02E-21	Meta_CD4_C9
MRPL41	8.6593E-26	0.289369666	0.711	0.452	2.097E-21	Meta_CD4_C9
DNAJA2	8.7831E-26	0.324248396	0.593	0.346	2.127E-21	Meta_CD4_C9
MYL6B	9.1751E-26	0.272992066	0.277	0.088	2.222E-21	Meta_CD4_C9
GNG4	9.7165E-26	0.428762216	0.127	0.006	2.353E-21	Meta_CD4_C9
DDX10	1.0266E-25	0.253849515	0.250	0.073	2.486E-21	Meta_CD4_C9
SFXN1	1.0308E-25	0.260132769	0.491	0.265	2.496E-21	Meta_CD4_C9
MAT2A	1.1135E-25	0.647355982	0.487	0.231	2.696E-21	Meta_CD4_C9
RAB27A	1.1979E-25	0.380291501	0.560	0.315	2.901E-21	Meta_CD4_C9
PTBP1	1.2282E-25	0.282744211	0.607	0.357	2.974E-21	Meta_CD4_C9
SLC43A3	1.2622E-25	0.409376904	0.250	0.067	3.056E-21	Meta_CD4_C9
LAMTOR1	1.6722E-25	0.273482753	0.653	0.396	4.049E-21	Meta_CD4_C9
PDCD6	1.706E-25	0.405668698	0.670	0.385	4.131E-21	Meta_CD4_C9
TNIP3	1.7087E-25	0.461220036	0.283	0.094	4.138E-21	Meta_CD4_C9
JTB	1.853E-25	0.3530363	0.827	0.579	4.487E-21	Meta_CD4_C9
PTGES2	1.8562E-25	0.271042539	0.366	0.152	4.495E-21	Meta_CD4_C9
PTGES3	2.0719E-25	0.359596334	0.894	0.699	5.017E-21	Meta_CD4_C9
PDHB	2.296E-25	0.255695944	0.426	0.205	5.56E-21	Meta_CD4_C9
CDK6	2.3916E-25	0.323551715	0.393	0.182	5.791E-21	Meta_CD4_C9
PTPN7	2.4793E-25	0.432068192	0.696	0.425	6.004E-21	Meta_CD4_C9
RBM17	2.5387E-25	0.278761775	0.649	0.381	6.148E-21	Meta_CD4_C9
NDUFB8	3.1888E-25	0.409078312	0.391	0.184	7.722E-21	Meta_CD4_C9
SND1	3.7718E-25	0.255988094	0.457	0.231	9.133E-21	Meta_CD4_C9
KDM6B	3.859E-25	0.286733083	0.407	0.201	9.345E-21	Meta_CD4_C9
ANAPC11	3.9434E-25	0.264563086	0.677	0.445	9.549E-21	Meta_CD4_C9
CDK2AP2	4.1116E-25	0.546489465	0.659	0.393	9.956E-21	Meta_CD4_C9
ODC1	4.4167E-25	0.695020662	0.704	0.427	1.069E-20	Meta_CD4_C9
PFN1	4.7788E-25	0.47677992	0.993	0.958	1.157E-20	Meta_CD4_C9
MDH2	5.2492E-25	0.43290049	0.774	0.509	1.271E-20	Meta_CD4_C9
EIF3A	5.2587E-25	0.342528603	0.666	0.404	1.273E-20	Meta_CD4_C9
PPAN	6.093E-25	0.309755298	0.207	0.050	1.475E-20	Meta_CD4_C9
HNRNPU	7.057E-25	0.282082916	0.819	0.583	1.709E-20	Meta_CD4_C9
BUD23	8.9303E-25	0.288095142	0.501	0.254	2.162E-20	Meta_CD4_C9
NPDC1	9.1186E-25	0.269782762	0.289	0.095	2.208E-20	Meta_CD4_C9
NDUFA4	9.9195E-25	0.355983443	0.940	0.774	2.402E-20	Meta_CD4_C9
HYOU1	1.0088E-24	0.308092283	0.319	0.110	2.443E-20	Meta_CD4_C9
HNRNPK	1.0116E-24	0.404273285	0.931	0.768	2.45E-20	Meta_CD4_C9

SRSF3	1.1619E-24	0.400624453	0.854	0.616	2.814E-20	Meta_CD4_C9
SERF2	1.3387E-24	0.329528401	0.983	0.941	3.242E-20	Meta_CD4_C9
HSPA5	1.6725E-24	0.630688174	0.870	0.644	4.05E-20	Meta_CD4_C9
TXNL1	1.6936E-24	0.255991409	0.567	0.329	4.101E-20	Meta_CD4_C9
EIF1AX	1.7072E-24	0.325275298	0.616	0.353	4.134E-20	Meta_CD4_C9
CISD3	1.9529E-24	0.31407943	0.451	0.226	4.729E-20	Meta_CD4_C9
AP2S1	2.2957E-24	0.310048695	0.616	0.353	5.559E-20	Meta_CD4_C9
PARP1	2.4317E-24	0.258862056	0.569	0.345	5.888E-20	Meta_CD4_C9
RHOG	2.7912E-24	0.384819955	0.757	0.518	6.759E-20	Meta_CD4_C9
POLR2K	3.2069E-24	0.299303594	0.616	0.346	7.765E-20	Meta_CD4_C9
STAT3	3.6841E-24	0.284141892	0.737	0.512	8.921E-20	Meta_CD4_C9
P4HB	5.0277E-24	0.397913682	0.784	0.538	1.217E-19	Meta_CD4_C9
CALM2	5.56E-24	0.350255232	0.926	0.765	1.346E-19	Meta_CD4_C9
SERPINB9	5.965E-24	0.582182519	0.454	0.223	1.444E-19	Meta_CD4_C9
JARID2	7.0273E-24	0.27553816	0.297	0.113	1.702E-19	Meta_CD4_C9
SRSF2	8.3786E-24	0.37566734	0.876	0.634	2.029E-19	Meta_CD4_C9
LSM1	1.1951E-23	0.303232646	0.446	0.251	2.894E-19	Meta_CD4_C9
RBM3	1.9955E-23	0.435388	0.893	0.697	4.832E-19	Meta_CD4_C9
EIF3D	2.6341E-23	0.281379826	0.744	0.501	6.379E-19	Meta_CD4_C9
POLE4	2.7219E-23	0.29938283	0.444	0.244	6.591E-19	Meta_CD4_C9
PSMB8	2.9021E-23	0.371714761	0.814	0.600	7.027E-19	Meta_CD4_C9
HNRNPF	3.4015E-23	0.45767962	0.810	0.602	8.237E-19	Meta_CD4_C9
VASP	3.5061E-23	0.31967542	0.644	0.392	8.49E-19	Meta_CD4_C9
CACYBP	3.5342E-23	0.339654086	0.731	0.503	8.558E-19	Meta_CD4_C9
LSM3	3.7437E-23	0.263903583	0.567	0.317	9.065E-19	Meta_CD4_C9
NMB	4.1536E-23	0.567094448	0.159	0.040	1.006E-18	Meta_CD4_C9
NDUFC1	5.1675E-23	0.346074759	0.623	0.369	1.251E-18	Meta_CD4_C9
ATP6V0B	6.9124E-23	0.314858836	0.699	0.444	1.674E-18	Meta_CD4_C9
PAM	7.4431E-23	0.269354564	0.270	0.098	1.802E-18	Meta_CD4_C9
ECHS1	9.1421E-23	0.288929617	0.457	0.228	2.214E-18	Meta_CD4_C9
SUMO2	9.2548E-23	0.338070416	0.943	0.805	2.241E-18	Meta_CD4_C9
HNRNPDL	9.7149E-23	0.404674765	0.939	0.800	2.352E-18	Meta_CD4_C9
ZNF580	1.0014E-22	0.25960867	0.334	0.135	2.425E-18	Meta_CD4_C9
HIF1A	1.2113E-22	0.316426006	0.577	0.339	2.933E-18	Meta_CD4_C9
WDR83OS	1.2895E-22	0.277423314	0.819	0.573	3.123E-18	Meta_CD4_C9
SFT2D1	1.4548E-22	0.252084216	0.459	0.251	3.523E-18	Meta_CD4_C9
IL21	1.7176E-22	0.463420397	0.130	0.013	4.159E-18	Meta_CD4_C9
WDR36	1.7996E-22	0.274982743	0.286	0.098	4.358E-18	Meta_CD4_C9
NDUFS5	2.4813E-22	0.322837875	0.899	0.704	6.008E-18	Meta_CD4_C9
CD200	2.5761E-22	0.443738986	0.131	0.018	6.238E-18	Meta_CD4_C9

MAGEH1	2.7642E-22	0.266887988	0.270	0.098	6.693E-18	Meta_CD4_C9
SPCS2	3.0222E-22	0.313569075	0.753	0.498	7.318E-18	Meta_CD4_C9
GADD45G	4.9982E-22	0.31560679	0.251	0.083	1.21E-17	Meta_CD4_C9
ARPC2	5.0262E-22	0.383574011	0.969	0.885	1.217E-17	Meta_CD4_C9
FDFT1	5.382E-22	0.250080045	0.521	0.307	1.303E-17	Meta_CD4_C9
DUSP5	6.0075E-22	0.257173807	0.386	0.201	1.455E-17	Meta_CD4_C9
BZW1	6.6167E-22	0.381866378	0.776	0.529	1.602E-17	Meta_CD4_C9
F5	6.6968E-22	0.25948212	0.137	0.026	1.622E-17	Meta_CD4_C9
GALM	7.9882E-22	0.375059038	0.481	0.262	1.934E-17	Meta_CD4_C9
HNRNPA2B1	8.7247E-22	0.42839694	0.941	0.835	2.113E-17	Meta_CD4_C9
TNIP1	9.5125E-22	0.29603158	0.623	0.385	2.303E-17	Meta_CD4_C9
LSM2	1.2355E-21	0.322179655	0.607	0.356	2.992E-17	Meta_CD4_C9
NDFIP1	1.6829E-21	0.322138015	0.733	0.496	4.075E-17	Meta_CD4_C9
LTA	2.0255E-21	0.703244812	0.283	0.089	4.905E-17	Meta_CD4_C9
TPM4	2.0603E-21	0.339209547	0.610	0.400	4.989E-17	Meta_CD4_C9
TUBA1B	2.3938E-21	0.625983118	0.791	0.590	5.797E-17	Meta_CD4_C9
BCL2L1	2.4597E-21	0.418246804	0.373	0.164	5.956E-17	Meta_CD4_C9
CDC37	2.4831E-21	0.352656239	0.831	0.635	6.013E-17	Meta_CD4_C9
CALM3	2.7604E-21	0.312342556	0.793	0.570	6.684E-17	Meta_CD4_C9
NAA10	2.8447E-21	0.359837036	0.553	0.304	6.888E-17	Meta_CD4_C9
BST2	3.146E-21	0.26589213	0.703	0.494	7.618E-17	Meta_CD4_C9
SQLE	3.4938E-21	0.270430407	0.220	0.062	8.46E-17	Meta_CD4_C9
PPIB	4.6466E-21	0.463428715	0.919	0.797	1.125E-16	Meta_CD4_C9
LDHB	5.8174E-21	0.364177654	0.891	0.750	1.409E-16	Meta_CD4_C9
PRDX2	6.0268E-21	0.390249857	0.709	0.469	1.459E-16	Meta_CD4_C9
SUMO1	7.1936E-21	0.282485857	0.733	0.501	1.742E-16	Meta_CD4_C9
UBE2L3	7.3342E-21	0.316849342	0.700	0.452	1.776E-16	Meta_CD4_C9
PPP1CC	7.9844E-21	0.356945296	0.701	0.465	1.933E-16	Meta_CD4_C9
HNRNPA1	8.3545E-21	0.403548157	0.956	0.896	2.023E-16	Meta_CD4_C9
AGK	1.1031E-20	0.30037152	0.299	0.109	2.671E-16	Meta_CD4_C9
RNF19A	2.4645E-20	0.270983506	0.621	0.396	5.968E-16	Meta_CD4_C9
NDUFA12	2.4788E-20	0.30645582	0.706	0.453	6.003E-16	Meta_CD4_C9
CCND2	2.797E-20	0.361336316	0.521	0.318	6.773E-16	Meta_CD4_C9
GPX4	2.8938E-20	0.353241582	0.813	0.609	7.007E-16	Meta_CD4_C9
RPS17	2.9188E-20	0.539899982	0.883	0.738	7.068E-16	Meta_CD4_C9
SIAH2	2.9898E-20	0.438925015	0.427	0.205	7.24E-16	Meta_CD4_C9
WDR1	3.0733E-20	0.433902029	0.779	0.545	7.442E-16	Meta_CD4_C9
DBI	3.3891E-20	0.342491068	0.791	0.576	8.207E-16	Meta_CD4_C9
TOX	3.8413E-20	0.326906221	0.360	0.167	9.302E-16	Meta_CD4_C9
CCL20	4.3487E-20	1.707866145	0.191	0.059	1.053E-15	Meta_CD4_C9

DDOST	8.5437E-20	0.264123537	0.677	0.464	2.069E-15	Meta_CD4_C9
TAP1	9.6439E-20	0.278409499	0.700	0.496	2.335E-15	Meta_CD4_C9
ISCU	1.4607E-19	0.278148823	0.780	0.557	3.537E-15	Meta_CD4_C9
GHITM	1.6801E-19	0.284437752	0.713	0.482	4.068E-15	Meta_CD4_C9
CLEC2D	1.7331E-19	0.356130214	0.714	0.478	4.197E-15	Meta_CD4_C9
ANP32B	1.7727E-19	0.344987968	0.829	0.631	4.293E-15	Meta_CD4_C9
CD83	2.6942E-19	0.404504758	0.266	0.101	6.524E-15	Meta_CD4_C9
PTP4A3	3.4082E-19	0.425322352	0.189	0.060	8.253E-15	Meta_CD4_C9
PSMC5	3.4141E-19	0.449232019	0.696	0.421	8.267E-15	Meta_CD4_C9
REL	3.4641E-19	0.586882583	0.654	0.421	8.388E-15	Meta_CD4_C9
EDF1	4.5992E-19	0.372185735	0.914	0.765	1.114E-14	Meta_CD4_C9
SLC16A3	5.4626E-19	0.340713178	0.329	0.149	1.323E-14	Meta_CD4_C9
UQCRH	6.2762E-19	0.326315455	0.854	0.654	1.52E-14	Meta_CD4_C9
ATP5F1B	9.3502E-19	0.509397999	0.743	0.608	2.264E-14	Meta_CD4_C9
POU2AF1	1.0418E-18	0.255057755	0.103	0.013	2.523E-14	Meta_CD4_C9
UBE2D3	1.117E-18	0.272392187	0.911	0.769	2.705E-14	Meta_CD4_C9
GADD45A	1.3612E-18	0.471406755	0.329	0.141	3.296E-14	Meta_CD4_C9
IL1R2	1.8897E-18	0.40073662	0.103	0.018	4.576E-14	Meta_CD4_C9
TRMT112	2.1413E-18	0.352357085	0.860	0.650	5.185E-14	Meta_CD4_C9
MT1E	2.1811E-18	0.318024584	0.306	0.128	5.282E-14	Meta_CD4_C9
HSPB1	2.4381E-18	0.465525132	0.631	0.392	5.904E-14	Meta_CD4_C9
OAZ1	3.766E-18	0.337889819	0.973	0.877	9.119E-14	Meta_CD4_C9
NFKBID	4.2004E-18	0.547393545	0.259	0.107	1.017E-13	Meta_CD4_C9
ID3	4.601E-18	0.458922337	0.263	0.105	1.114E-13	Meta_CD4_C9
APOBEC3G	6.4717E-18	0.28107414	0.661	0.444	1.567E-13	Meta_CD4_C9
ARMH1	6.7595E-18	0.391619559	0.220	0.083	1.637E-13	Meta_CD4_C9
HMGN1	6.9511E-18	0.255644379	0.870	0.703	1.683E-13	Meta_CD4_C9
TMEM258	7.2473E-18	0.322694513	0.816	0.615	1.755E-13	Meta_CD4_C9
SEM1	8.3475E-18	0.489833613	0.651	0.470	2.021E-13	Meta_CD4_C9
GPI	8.7634E-18	0.349509858	0.680	0.459	2.122E-13	Meta_CD4_C9
TRAF4	1.011E-17	0.284549516	0.287	0.112	2.448E-13	Meta_CD4_C9
LAIR2	1.9113E-17	0.416622796	0.163	0.050	4.628E-13	Meta_CD4_C9
LIMS1	3.1049E-17	0.312490957	0.553	0.332	7.518E-13	Meta_CD4_C9
JPT1	3.4031E-17	0.297589863	0.567	0.373	8.241E-13	Meta_CD4_C9
TPM3	3.4832E-17	0.378874707	0.913	0.773	8.435E-13	Meta_CD4_C9
NFKBIA	3.485E-17	0.280179026	0.864	0.711	8.439E-13	Meta_CD4_C9
ATP5MF	4.8253E-17	0.401873348	0.667	0.506	1.168E-12	Meta_CD4_C9
PDIA3	4.8337E-17	0.461558615	0.847	0.663	1.17E-12	Meta_CD4_C9
COX6B1	4.9681E-17	0.324144424	0.910	0.755	1.203E-12	Meta_CD4_C9
ACTB	5.4501E-17	0.393959321	1.000	0.998	1.32E-12	Meta_CD4_C9

BTLA	6.1863E-17	0.259529127	0.204	0.065	1.498E-12	Meta_CD4_C9
ETV7	7.5071E-17	0.254861606	0.194	0.064	1.818E-12	Meta_CD4_C9
APOBEC3C	8.2508E-17	0.267232748	0.493	0.282	1.998E-12	Meta_CD4_C9
C17orf89	8.8658E-17	0.315938199	0.104	0.024	2.147E-12	Meta_CD4_C9
EIF3M	9.2215E-17	0.28459173	0.740	0.522	2.233E-12	Meta_CD4_C9
SH2D1A	1.2516E-16	0.320143198	0.520	0.325	3.031E-12	Meta_CD4_C9
PAK2	1.4709E-16	0.257469845	0.669	0.457	3.562E-12	Meta_CD4_C9
NDUFB9	1.7117E-16	0.294288152	0.750	0.528	4.145E-12	Meta_CD4_C9
IL2RG	2.2198E-16	0.26156833	0.783	0.585	5.375E-12	Meta_CD4_C9
PEBP1	2.2552E-16	0.311058736	0.784	0.591	5.461E-12	Meta_CD4_C9
TALDO1	2.3917E-16	0.270138583	0.600	0.390	5.791E-12	Meta_CD4_C9
COX8A	2.7185E-16	0.305319093	0.874	0.697	6.583E-12	Meta_CD4_C9
CHCHD10	2.7297E-16	0.309077952	0.604	0.392	6.61E-12	Meta_CD4_C9
ARPC1B	7.5373E-16	0.317539846	0.877	0.728	1.825E-11	Meta_CD4_C9
PHB2	8.6896E-16	0.318454966	0.710	0.483	2.104E-11	Meta_CD4_C9
RPL23	9.1482E-16	0.294386685	0.944	0.824	2.215E-11	Meta_CD4_C9
CTSC	1.2486E-15	0.281045012	0.759	0.557	3.023E-11	Meta_CD4_C9
NUDT1	1.3265E-15	0.26252677	0.313	0.172	3.212E-11	Meta_CD4_C9
ICA1	3.3745E-15	0.293186678	0.161	0.047	8.171E-11	Meta_CD4_C9
ACTR3	4.5824E-15	0.342798729	0.810	0.621	1.11E-10	Meta_CD4_C9
RTRAF	4.6524E-15	0.303430709	0.683	0.483	1.127E-10	Meta_CD4_C9
GEM	6.1071E-15	0.487579055	0.164	0.052	1.479E-10	Meta_CD4_C9
CYTOR	6.4351E-15	0.419915421	0.637	0.427	1.558E-10	Meta_CD4_C9
PPP1CA	9.2143E-15	0.262868424	0.834	0.645	2.231E-10	Meta_CD4_C9
ENTPD1	1.1434E-14	0.373152438	0.259	0.119	2.769E-10	Meta_CD4_C9
ATP5G1	1.1875E-14	0.417188721	0.117	0.036	2.876E-10	Meta_CD4_C9
COX5B	1.3391E-14	0.30504569	0.881	0.723	3.243E-10	Meta_CD4_C9
CSF2	2.6122E-14	1.035012727	0.103	0.027	6.325E-10	Meta_CD4_C9
IER3	3.482E-14	0.289920658	0.176	0.062	8.432E-10	Meta_CD4_C9
CRTAM	4.3014E-14	1.074391108	0.204	0.103	1.042E-09	Meta_CD4_C9
SKP1	4.9097E-14	0.258809725	0.936	0.803	1.189E-09	Meta_CD4_C9
IL4I1	5.0534E-14	0.431375286	0.173	0.049	1.224E-09	Meta_CD4_C9
LAG3	5.8037E-14	0.729838783	0.579	0.370	1.405E-09	Meta_CD4_C9
RHOA	2.2399E-13	0.28651042	0.934	0.832	5.424E-09	Meta_CD4_C9
AHI1	2.2734E-13	0.252829045	0.213	0.077	5.505E-09	Meta_CD4_C9
UCP2	5.0516E-13	0.402366644	0.674	0.497	1.223E-08	Meta_CD4_C9
RPLP0	5.3038E-13	0.316492616	0.984	0.949	1.284E-08	Meta_CD4_C9
ATP5F1A	6.7977E-13	0.363527655	0.649	0.466	1.646E-08	Meta_CD4_C9
ITM2A	7.3562E-13	0.387266441	0.779	0.609	1.781E-08	Meta_CD4_C9
NFATC1	8.3825E-13	0.332936934	0.276	0.133	2.03E-08	Meta_CD4_C9

CREM	1.3708E-12	0.304861592	0.591	0.405	3.319E-08	Meta_CD4_C9
ATP5F1C	1.8513E-12	0.315064151	0.636	0.454	4.483E-08	Meta_CD4_C9
ATP5PF	5.1663E-12	0.43784306	0.659	0.477	1.251E-07	Meta_CD4_C9
HMGA1	1.3992E-11	0.255834259	0.504	0.332	3.388E-07	Meta_CD4_C9
RGCC	2.9521E-11	0.338472789	0.699	0.527	7.148E-07	Meta_CD4_C9
BCL2A1	3.9946E-11	0.380455916	0.319	0.189	9.673E-07	Meta_CD4_C9
SELENOH	4.0763E-11	0.342091576	0.647	0.453	9.871E-07	Meta_CD4_C9
CD70	4.1545E-11	0.406094634	0.201	0.082	1.006E-06	Meta_CD4_C9
TCEB1	4.3697E-11	0.251710791	0.106	0.037	1.058E-06	Meta_CD4_C9
LINC01588	4.6036E-11	0.34745231	0.126	0.038	1.115E-06	Meta_CD4_C9
ABRACL	2.2415E-10	0.274317861	0.757	0.590	5.428E-06	Meta_CD4_C9
RPS2	4.1325E-10	0.259920857	0.994	0.984	1.001E-05	Meta_CD4_C9
MYC	4.1603E-10	0.361213737	0.329	0.178	1.007E-05	Meta_CD4_C9
ACTG1	6.8555E-10	0.300418647	0.977	0.947	1.66E-05	Meta_CD4_C9
USMG5	7.2429E-10	0.405700627	0.146	0.065	1.754E-05	Meta_CD4_C9
RGS16	8.885E-10	0.2905603	0.170	0.072	2.152E-05	Meta_CD4_C9
GZMB	1.1878E-09	1.681517293	0.319	0.299	2.876E-05	Meta_CD4_C9
CXCL13	1.2072E-09	1.87211295	0.183	0.068	2.923E-05	Meta_CD4_C9
ATP5G3	1.591E-09	0.530428953	0.173	0.072	3.853E-05	Meta_CD4_C9
C11orf31	1.5924E-09	0.256951157	0.116	0.047	3.856E-05	Meta_CD4_C9
ATP5MD	1.7911E-09	0.288736075	0.704	0.565	4.337E-05	Meta_CD4_C9
CTSH	2.6206E-09	0.34611488	0.187	0.084	6.346E-05	Meta_CD4_C9
ATP5J2	8.2463E-09	0.455884012	0.167	0.071	0.0001997	Meta_CD4_C9
AC133644.2	1.0255E-08	0.409017865	0.133	0.049	0.0002483	Meta_CD4_C9
TNFSF8	1.6415E-08	0.25855576	0.229	0.135	0.0003975	Meta_CD4_C9
ATP5D	6.1411E-08	0.32099001	0.157	0.074	0.0014871	Meta_CD4_C9
SHFM1	1.0962E-07	0.344639266	0.146	0.066	0.0026544	Meta_CD4_C9
ATP5B	4.4718E-07	0.481426925	0.159	0.062	0.0108285	Meta_CD4_C9
XCL2	4.5549E-07	1.442375313	0.177	0.145	0.0110297	Meta_CD4_C9
TNFSF14	5.032E-07	0.262791866	0.267	0.146	0.0121849	Meta_CD4_C9
COTL1	1.5567E-06	0.303736202	0.843	0.753	0.0376957	Meta_CD4_C9
LMNA	1.6735E-06	0.299257326	0.391	0.275	0.0405237	Meta_CD4_C9
CD7	2.7377E-06	0.294280558	0.849	0.762	0.0662923	Meta_CD4_C9
TCEB2	3.5425E-06	0.296988619	0.167	0.089	0.0857825	Meta_CD4_C9
EMP3	4.6239E-06	0.260606559	0.826	0.698	0.1119677	Meta_CD4_C9
XCL1	8.2548E-06	1.281494875	0.170	0.130	0.1998911	Meta_CD4_C9
ATP5G2	9.1912E-06	0.330082377	0.190	0.118	0.2225637	Meta_CD4_C9
ATP5J	1.0412E-05	0.356689771	0.137	0.061	0.2521284	Meta_CD4_C9
LINC00152	3.5858E-05	0.440023369	0.143	0.072	0.8682916	Meta_CD4_C9
C14orf2	0.00013579	0.313304677	0.147	0.079	1	Meta_CD4_C9

ATP5E	0.00063551	0.308793381	0.206	0.147	1	Meta_CD4_C9
GNB2L1	0.00835097	0.310379329	0.211	0.166	1	Meta_CD4_C9
FOXP3	1.643E-222	2.400403248	0.655	0.022	3.98E-218	Meta_CD4_C10
CTLA4	3.549E-132	1.732651514	0.710	0.210	8.59E-128	Meta_CD4_C10
BATF	3.264E-120	1.791145669	0.734	0.308	7.9E-116	Meta_CD4_C10
IL2RA	1.017E-117	2.034447947	0.474	0.045	2.46E-113	Meta_CD4_C10
TNFRSF4	2.294E-116	2.328158489	0.588	0.146	5.55E-112	Meta_CD4_C10
IL32	1.492E-113	1.191313113	0.990	0.934	3.61E-109	Meta_CD4_C10
TNFRSF18	8.264E-106	1.817611339	0.609	0.171	2E-101	Meta_CD4_C10
LTB	8.5025E-95	1.547986387	0.887	0.591	2.059E-90	Meta_CD4_C10
TBC1D4	4.0455E-93	1.368974317	0.475	0.069	9.796E-89	Meta_CD4_C10
TIGIT	2.4677E-88	1.323492384	0.696	0.299	5.976E-84	Meta_CD4_C10
RTKN2	3.2629E-87	1.404528844	0.352	0.023	7.901E-83	Meta_CD4_C10
MIR4435-2HG	4.7958E-78	1.346869503	0.627	0.259	1.161E-73	Meta_CD4_C10
SAT1	3.979E-72	1.337159557	0.836	0.625	9.635E-68	Meta_CD4_C10
PIM2	3.4533E-70	1.165835428	0.678	0.397	8.362E-66	Meta_CD4_C10
ARID5B	7.0885E-67	1.033619805	0.673	0.340	1.716E-62	Meta_CD4_C10
TNFRSF1B	7.2976E-66	1.314112815	0.661	0.375	1.767E-61	Meta_CD4_C10
LAIR2	2.952E-64	0.960642511	0.269	0.030	7.148E-60	Meta_CD4_C10
SELL	1.1961E-57	1.09673842	0.507	0.156	2.896E-53	Meta_CD4_C10
CARD16	1.9789E-57	1.100992879	0.646	0.376	4.792E-53	Meta_CD4_C10
LINC01943	9.0137E-57	1.212472188	0.356	0.095	2.183E-52	Meta_CD4_C10
UGP2	2.4137E-56	1.103062546	0.572	0.294	5.845E-52	Meta_CD4_C10
STAM	1.1593E-55	0.973562635	0.402	0.125	2.807E-51	Meta_CD4_C10
PBXIP1	6.1858E-53	0.954923118	0.727	0.476	1.498E-48	Meta_CD4_C10
GLRX	1.0187E-52	1.009069909	0.496	0.223	2.467E-48	Meta_CD4_C10
CORO1B	1.2277E-51	1.042573667	0.645	0.384	2.973E-47	Meta_CD4_C10
ICA1	7.7771E-51	0.84721988	0.232	0.031	1.883E-46	Meta_CD4_C10
HLA-A	7.8317E-49	0.50871145	0.998	0.996	1.896E-44	Meta_CD4_C10
B2M	2.3949E-48	0.382265637	1.000	0.999	5.799E-44	Meta_CD4_C10
MAST4	1.3568E-47	0.651070366	0.268	0.064	3.286E-43	Meta_CD4_C10
UCP2	4.8446E-47	0.870007816	0.675	0.482	1.173E-42	Meta_CD4_C10
IKZF2	2.5633E-46	0.807986585	0.305	0.058	6.207E-42	Meta_CD4_C10
AC017002.3	3.9212E-46	0.750937501	0.230	0.030	9.495E-42	Meta_CD4_C10
RGS1	1.0408E-45	0.82739465	0.823	0.602	2.52E-41	Meta_CD4_C10
CD27	1.5175E-45	1.024264387	0.770	0.463	3.675E-41	Meta_CD4_C10
CCR8	2.5918E-45	0.597552496	0.161	0.003	6.276E-41	Meta_CD4_C10
IL1R2	2.7365E-45	0.84964998	0.169	0.005	6.626E-41	Meta_CD4_C10
PELI1	3.4534E-45	0.99422966	0.446	0.177	8.362E-41	Meta_CD4_C10
CD4	7.1131E-45	0.742187627	0.496	0.223	1.722E-40	Meta_CD4_C10

DNPH1	7.739E-44	0.934794848	0.549	0.272	1.874E-39	Meta_CD4_C10
ZC2HC1A	1.3701E-42	0.630362937	0.187	0.021	3.318E-38	Meta_CD4_C10
CTSC	8.5834E-42	0.882415114	0.708	0.545	2.078E-37	Meta_CD4_C10
F5	1.7952E-41	0.627414891	0.180	0.013	4.347E-37	Meta_CD4_C10
FANK1	3.8837E-41	0.525468619	0.139	0.002	9.404E-37	Meta_CD4_C10
MAGEH1	7.7221E-41	1.18261182	0.316	0.080	1.87E-36	Meta_CD4_C10
ICOS	1.5758E-39	0.859590231	0.590	0.359	3.816E-35	Meta_CD4_C10
FAS	1.8147E-39	0.825188564	0.463	0.232	4.394E-35	Meta_CD4_C10
CD79B	3.139E-38	0.669351306	0.302	0.087	7.601E-34	Meta_CD4_C10
ADTRP	3.2161E-35	0.613766805	0.176	0.017	7.788E-31	Meta_CD4_C10
AC133644.2	7.1774E-35	1.107721803	0.201	0.036	1.738E-30	Meta_CD4_C10
S100A4	2.6589E-34	0.790634403	0.946	0.862	6.439E-30	Meta_CD4_C10
IL6R	3.7219E-34	0.579921076	0.241	0.063	9.013E-30	Meta_CD4_C10
RHOG	4.078E-34	0.589820079	0.664	0.506	9.875E-30	Meta_CD4_C10
ETV7	3.8672E-33	0.557443648	0.213	0.051	9.364E-29	Meta_CD4_C10
FCMR	8.8759E-33	0.872474895	0.567	0.362	2.149E-28	Meta_CD4_C10
SOD1	9.3086E-33	0.566359266	0.825	0.730	2.254E-28	Meta_CD4_C10
GK	3.435E-32	0.702271737	0.257	0.059	8.318E-28	Meta_CD4_C10
NAMPT	5.4475E-32	0.669073284	0.417	0.226	1.319E-27	Meta_CD4_C10
NCF4	5.6653E-32	0.656107218	0.360	0.146	1.372E-27	Meta_CD4_C10
SAMHD1	2.178E-31	0.658468608	0.524	0.354	5.274E-27	Meta_CD4_C10
LAYN	2.2643E-31	0.643421335	0.227	0.051	5.483E-27	Meta_CD4_C10
GBP2	6.3122E-31	0.856714339	0.641	0.397	1.528E-26	Meta_CD4_C10
IKZF4	1.7848E-30	0.474187743	0.148	0.017	4.322E-26	Meta_CD4_C10
PHTF2	3.7751E-30	0.669454313	0.400	0.195	9.141E-26	Meta_CD4_C10
CCR4	5.1325E-30	0.609744674	0.225	0.046	1.243E-25	Meta_CD4_C10
TMSB10	1.0044E-29	0.497681107	0.994	0.993	2.432E-25	Meta_CD4_C10
HTATIP2	1.6485E-29	0.523506042	0.333	0.178	3.992E-25	Meta_CD4_C10
SPOCK2	2.8091E-29	0.568297757	0.823	0.674	6.802E-25	Meta_CD4_C10
GADD45A	1.0622E-28	0.804780585	0.318	0.126	2.572E-24	Meta_CD4_C10
PMAIP1	1.0725E-28	0.902132435	0.456	0.238	2.597E-24	Meta_CD4_C10
RPS27L	1.3164E-28	0.697702593	0.695	0.533	3.188E-24	Meta_CD4_C10
GBP5	1.7548E-28	0.707373275	0.613	0.380	4.249E-24	Meta_CD4_C10
NDUFV2	4.6677E-28	0.563403379	0.642	0.492	1.13E-23	Meta_CD4_C10
CD28	7.8649E-28	0.603132265	0.435	0.250	1.904E-23	Meta_CD4_C10
SLAMF1	8.5062E-28	0.632830457	0.437	0.253	2.06E-23	Meta_CD4_C10
CMTM6	3.0075E-27	0.690977716	0.486	0.360	7.283E-23	Meta_CD4_C10
TOX2	3.1111E-27	0.409911421	0.207	0.066	7.533E-23	Meta_CD4_C10
HACD1	7.2062E-27	0.356086815	0.119	0.009	1.745E-22	Meta_CD4_C10
SUMO2	1.0532E-26	0.367219427	0.848	0.802	2.55E-22	Meta_CD4_C10

MAF	5.6694E-26	0.566318275	0.357	0.170	1.373E-21	Meta_CD4_C10
IL1R1	9.135E-26	0.389047681	0.110	0.006	2.212E-21	Meta_CD4_C10
ZC3H12D	2.2632E-25	0.53390069	0.245	0.087	5.48E-21	Meta_CD4_C10
TMEM173	6.4311E-24	0.604269392	0.509	0.294	1.557E-19	Meta_CD4_C10
PPM1G	1.302E-23	0.505857726	0.548	0.396	3.153E-19	Meta_CD4_C10
ISG20	1.3285E-23	0.610672544	0.762	0.618	3.217E-19	Meta_CD4_C10
TNFRSF9	6.7285E-23	0.629252356	0.191	0.045	1.629E-18	Meta_CD4_C10
IL10RA	8.4373E-23	0.653082293	0.631	0.472	2.043E-18	Meta_CD4_C10
USP15	1.17E-22	0.537705501	0.546	0.403	2.833E-18	Meta_CD4_C10
CALM3	1.6636E-22	0.512607091	0.689	0.561	4.029E-18	Meta_CD4_C10
YWHAB	1.7051E-22	0.46154066	0.859	0.808	4.129E-18	Meta_CD4_C10
RHBDD2	1.8505E-22	0.732229366	0.485	0.336	4.481E-18	Meta_CD4_C10
CD74	8.7738E-22	0.507757377	0.934	0.869	2.125E-17	Meta_CD4_C10
OAZ1	2.1833E-21	0.511002087	0.916	0.874	5.287E-17	Meta_CD4_C10
CACYBP	2.9731E-21	0.575784556	0.608	0.495	7.199E-17	Meta_CD4_C10
RAP1A	3.057E-21	0.476608353	0.627	0.492	7.402E-17	Meta_CD4_C10
BIRC3	3.6114E-21	0.781827905	0.497	0.290	8.745E-17	Meta_CD4_C10
PRDM1	6.4019E-21	0.610412838	0.514	0.325	1.55E-16	Meta_CD4_C10
CCNG2	6.6094E-21	0.473043801	0.192	0.054	1.6E-16	Meta_CD4_C10
ARPC1B	1.1849E-20	0.52681086	0.818	0.721	2.869E-16	Meta_CD4_C10
ENTPD1	1.3099E-20	0.47315695	0.256	0.107	3.172E-16	Meta_CD4_C10
SEPT6	1.4464E-20	0.359911907	0.649	0.574	3.503E-16	Meta_CD4_C10
TRIB1	2.3302E-20	0.410443473	0.164	0.038	5.643E-16	Meta_CD4_C10
DUSP4	2.5154E-20	0.552822319	0.547	0.359	6.091E-16	Meta_CD4_C10
BEX3	2.81E-20	0.368230359	0.119	0.028	6.804E-16	Meta_CD4_C10
EPSTI1	3.772E-20	0.525159823	0.395	0.226	9.134E-16	Meta_CD4_C10
DUSP16	4.6975E-20	0.514786965	0.268	0.105	1.137E-15	Meta_CD4_C10
SPATS2L	6.6801E-20	0.535667761	0.212	0.066	1.618E-15	Meta_CD4_C10
FXYD5	6.9827E-20	0.36378492	0.900	0.851	1.691E-15	Meta_CD4_C10
CCM2	9.2184E-20	0.501951055	0.396	0.245	2.232E-15	Meta_CD4_C10
CD247	1.2131E-19	0.519499449	0.738	0.641	2.938E-15	Meta_CD4_C10
HPGD	1.2643E-19	0.785641282	0.177	0.054	3.061E-15	Meta_CD4_C10
CMTM7	1.7927E-19	0.499335857	0.455	0.304	4.341E-15	Meta_CD4_C10
PRDX2	2.2008E-19	0.406783105	0.574	0.461	5.329E-15	Meta_CD4_C10
PRDX1	2.3969E-19	0.483583319	0.659	0.527	5.804E-15	Meta_CD4_C10
HS3ST3B1	3.3404E-19	0.490606434	0.213	0.069	8.089E-15	Meta_CD4_C10
AC017002.1	3.5815E-19	0.56576177	0.105	0.012	8.673E-15	Meta_CD4_C10
44623	5.085E-19	0.334148706	0.154	0.052	1.231E-14	Meta_CD4_C10
LY96	5.12E-19	0.418979022	0.167	0.051	1.24E-14	Meta_CD4_C10
TPP1	7.1675E-19	0.528387052	0.375	0.209	1.736E-14	Meta_CD4_C10

SOCS3	1.1831E-18	0.518245649	0.444	0.304	2.865E-14	Meta_CD4_C10
TLK1	2.0024E-18	0.499537067	0.409	0.254	4.849E-14	Meta_CD4_C10
FAM184A	2.6423E-18	0.28381555	0.104	0.017	6.398E-14	Meta_CD4_C10
SLC16A1	3.2032E-18	0.385127495	0.178	0.064	7.757E-14	Meta_CD4_C10
ACP5	3.6449E-18	0.561113253	0.422	0.247	8.826E-14	Meta_CD4_C10
ENO1	3.695E-18	0.553065783	0.774	0.696	8.947E-14	Meta_CD4_C10
FTL	3.9615E-18	0.394244338	0.975	0.967	9.593E-14	Meta_CD4_C10
PHACTR2	4.0109E-18	0.47805959	0.246	0.099	9.712E-14	Meta_CD4_C10
PSAP	7.38E-18	0.40197676	0.627	0.519	1.787E-13	Meta_CD4_C10
PTTG1	1.2163E-17	0.457058141	0.348	0.198	2.945E-13	Meta_CD4_C10
UBE2B	1.2597E-17	0.415712969	0.598	0.492	3.05E-13	Meta_CD4_C10
SH3BGRL	1.6928E-17	0.411927525	0.641	0.524	4.099E-13	Meta_CD4_C10
CUL9	1.6992E-17	0.420757223	0.139	0.031	4.115E-13	Meta_CD4_C10
POU2F2	1.7429E-17	0.44864964	0.237	0.104	4.22E-13	Meta_CD4_C10
MALAT1	1.9445E-17	0.596525377	0.999	1.000	4.709E-13	Meta_CD4_C10
SQSTM1	2.178E-17	0.401932916	0.647	0.563	5.274E-13	Meta_CD4_C10
CD59	3.1556E-17	0.541258414	0.283	0.125	7.641E-13	Meta_CD4_C10
SNX9	4.0112E-17	0.5432058	0.386	0.225	9.713E-13	Meta_CD4_C10
MYL6	6.3026E-17	0.351666441	0.959	0.946	1.526E-12	Meta_CD4_C10
TYMP	1.0688E-16	0.741680636	0.436	0.266	2.588E-12	Meta_CD4_C10
ARHGDIB	1.4998E-16	0.302957643	0.956	0.944	3.632E-12	Meta_CD4_C10
YPEL2	1.9686E-16	0.274100011	0.181	0.090	4.767E-12	Meta_CD4_C10
LIMA1	2.3563E-16	0.347656926	0.203	0.093	5.706E-12	Meta_CD4_C10
TRAC	2.413E-16	0.733377545	0.684	0.559	5.843E-12	Meta_CD4_C10
LTA	2.8694E-16	0.535952006	0.189	0.082	6.948E-12	Meta_CD4_C10
IL6ST	4.056E-16	0.411329474	0.349	0.212	9.822E-12	Meta_CD4_C10
BTG3	5.8058E-16	0.538409047	0.430	0.273	1.406E-11	Meta_CD4_C10
TTN	6.4297E-16	0.415922966	0.160	0.042	1.557E-11	Meta_CD4_C10
HOXB2	6.5656E-16	0.440750425	0.281	0.153	1.59E-11	Meta_CD4_C10
TNIK	6.7059E-16	0.462705263	0.360	0.214	1.624E-11	Meta_CD4_C10
HAPLN3	9.1598E-16	0.491480195	0.238	0.098	2.218E-11	Meta_CD4_C10
CTSB	1.2771E-15	0.369257684	0.415	0.287	3.092E-11	Meta_CD4_C10
C15orf53	1.2966E-15	0.311248199	0.129	0.034	3.14E-11	Meta_CD4_C10
PVT1	1.4052E-15	0.439037445	0.257	0.120	3.403E-11	Meta_CD4_C10
UQCRB	1.8632E-15	0.298998436	0.854	0.830	4.512E-11	Meta_CD4_C10
CREM	2.7432E-15	0.48507807	0.516	0.396	6.643E-11	Meta_CD4_C10
CNST	4.1969E-15	0.359241184	0.290	0.185	1.016E-10	Meta_CD4_C10
SRGN	4.5588E-15	0.328315207	0.958	0.956	1.104E-10	Meta_CD4_C10
SMAP2	5.3448E-15	0.43698171	0.437	0.303	1.294E-10	Meta_CD4_C10
INPP1	9.8047E-15	0.280567719	0.141	0.051	2.374E-10	Meta_CD4_C10

SETD7	1.1363E-14	0.32595117	0.129	0.042	2.752E-10	Meta_CD4_C10
PKM	1.155E-14	0.59758591	0.738	0.650	2.797E-10	Meta_CD4_C10
BACH1	1.3692E-14	0.437258352	0.215	0.094	3.315E-10	Meta_CD4_C10
NINJ2	1.418E-14	0.392541171	0.190	0.070	3.434E-10	Meta_CD4_C10
CUTA	1.5798E-14	0.404184195	0.680	0.584	3.825E-10	Meta_CD4_C10
RAB11FIP1	1.9833E-14	0.397293771	0.280	0.162	4.803E-10	Meta_CD4_C10
ACTA2	1.9933E-14	0.378640963	0.190	0.075	4.827E-10	Meta_CD4_C10
CASP1	2.7339E-14	0.434175846	0.419	0.294	6.62E-10	Meta_CD4_C10
CFLAR	4.0537E-14	0.398132794	0.558	0.449	9.816E-10	Meta_CD4_C10
TXN	4.1829E-14	0.487314822	0.627	0.555	1.013E-09	Meta_CD4_C10
ICAM3	5.7716E-14	0.358927598	0.715	0.630	1.398E-09	Meta_CD4_C10
LGALS3	7.1128E-14	0.562120067	0.454	0.373	1.722E-09	Meta_CD4_C10
REXO2	7.2754E-14	0.437159951	0.347	0.234	1.762E-09	Meta_CD4_C10
VMP1	1.3168E-13	0.44135494	0.428	0.308	3.189E-09	Meta_CD4_C10
SCAND1	1.4739E-13	0.370814062	0.625	0.540	3.569E-09	Meta_CD4_C10
CYTOR	1.7835E-13	0.543768763	0.533	0.419	4.319E-09	Meta_CD4_C10
FCRL3	2.3909E-13	0.305657033	0.118	0.028	5.79E-09	Meta_CD4_C10
THEM4	3.5368E-13	0.412035453	0.297	0.173	8.564E-09	Meta_CD4_C10
SGMS1	3.704E-13	0.425535426	0.255	0.121	8.969E-09	Meta_CD4_C10
CRADD	4.5911E-13	0.3764231	0.140	0.047	1.112E-08	Meta_CD4_C10
CTSA	4.8626E-13	0.477054568	0.448	0.309	1.177E-08	Meta_CD4_C10
TNIP1	5.8923E-13	0.33687338	0.471	0.379	1.427E-08	Meta_CD4_C10
FBLN7	6.1433E-13	0.381602857	0.217	0.089	1.488E-08	Meta_CD4_C10
KCNN4	6.1656E-13	0.352721059	0.167	0.062	1.493E-08	Meta_CD4_C10
TIAM1	6.5574E-13	0.293744821	0.143	0.045	1.588E-08	Meta_CD4_C10
ADI1	8.8756E-13	0.345169366	0.302	0.189	2.149E-08	Meta_CD4_C10
HNRNPLL	1.1184E-12	0.346600111	0.419	0.315	2.708E-08	Meta_CD4_C10
IL21R	1.4298E-12	0.356224719	0.275	0.178	3.462E-08	Meta_CD4_C10
DYNLRB1	1.4829E-12	0.355916334	0.573	0.481	3.591E-08	Meta_CD4_C10
CHST7	1.5839E-12	0.35256228	0.158	0.058	3.836E-08	Meta_CD4_C10
SKAP1	1.7074E-12	0.391377994	0.642	0.544	4.134E-08	Meta_CD4_C10
SDF4	2.1383E-12	0.398248472	0.500	0.394	5.178E-08	Meta_CD4_C10
MGST2	2.63E-12	0.294513673	0.111	0.023	6.369E-08	Meta_CD4_C10
IL2RB	2.7816E-12	0.454262328	0.563	0.439	6.736E-08	Meta_CD4_C10
OAS1	3.4379E-12	0.484457696	0.240	0.107	8.325E-08	Meta_CD4_C10
COX8A	3.6806E-12	0.262748438	0.747	0.694	8.912E-08	Meta_CD4_C10
SIRPG	4.0715E-12	0.456510797	0.476	0.345	9.859E-08	Meta_CD4_C10
IL12RB2	4.4387E-12	0.345783089	0.176	0.088	1.075E-07	Meta_CD4_C10
CD320	6.2627E-12	0.367763883	0.294	0.175	1.517E-07	Meta_CD4_C10
VAV3	6.5958E-12	0.293331628	0.129	0.048	1.597E-07	Meta_CD4_C10

SEC11C	7.867E-12	0.306904674	0.445	0.353	1.905E-07	Meta_CD4_C10
RNF213	8.9226E-12	0.477714011	0.644	0.580	2.161E-07	Meta_CD4_C10
CCDC167	9.632E-12	0.319989334	0.340	0.233	2.332E-07	Meta_CD4_C10
C4orf48	1.0485E-11	0.384326276	0.478	0.373	2.539E-07	Meta_CD4_C10
HPRT1	1.6002E-11	0.470560861	0.358	0.238	3.875E-07	Meta_CD4_C10
TMBIM6	1.8933E-11	0.265453859	0.803	0.766	4.585E-07	Meta_CD4_C10
FOXP1	2.8287E-11	0.419281948	0.433	0.368	6.85E-07	Meta_CD4_C10
SFT2D1	3.0806E-11	0.436242438	0.382	0.240	7.46E-07	Meta_CD4_C10
THADA	3.5252E-11	0.343196715	0.154	0.078	8.536E-07	Meta_CD4_C10
HERC5	4.7761E-11	0.330375363	0.168	0.102	1.157E-06	Meta_CD4_C10
APLP2	5.0397E-11	0.315178566	0.196	0.104	1.22E-06	Meta_CD4_C10
ARL6IP5	5.2248E-11	0.371229178	0.761	0.695	1.265E-06	Meta_CD4_C10
NEAT1	5.3563E-11	0.532688402	0.650	0.536	1.297E-06	Meta_CD4_C10
AKIP1	5.5818E-11	0.365769402	0.183	0.073	1.352E-06	Meta_CD4_C10
GTF3C6	5.8572E-11	0.378300747	0.442	0.327	1.418E-06	Meta_CD4_C10
NABP1	6.6127E-11	0.437193111	0.253	0.137	1.601E-06	Meta_CD4_C10
SMCHD1	1.2299E-10	0.352641773	0.533	0.475	2.978E-06	Meta_CD4_C10
PIK3IP1	1.3837E-10	0.333791557	0.592	0.477	3.351E-06	Meta_CD4_C10
IFI6	1.4885E-10	0.535738222	0.427	0.305	3.604E-06	Meta_CD4_C10
MAT2B	1.4987E-10	0.312268107	0.516	0.435	3.629E-06	Meta_CD4_C10
HLA-DRB1	1.6648E-10	0.460142627	0.559	0.425	4.031E-06	Meta_CD4_C10
EPHX2	1.6827E-10	0.301169485	0.132	0.049	4.075E-06	Meta_CD4_C10
DGKA	1.8209E-10	0.266797963	0.343	0.266	4.409E-06	Meta_CD4_C10
GMFG	1.8465E-10	0.311071921	0.820	0.765	4.471E-06	Meta_CD4_C10
SEC14L1	1.8507E-10	0.366197723	0.263	0.154	4.482E-06	Meta_CD4_C10
COX5A	2.5283E-10	0.288726285	0.613	0.560	6.122E-06	Meta_CD4_C10
CIRBP	2.957E-10	0.361706694	0.861	0.814	7.16E-06	Meta_CD4_C10
SMS	3.0099E-10	0.381820615	0.315	0.206	7.288E-06	Meta_CD4_C10
ABI1	3.0741E-10	0.353137582	0.420	0.312	7.444E-06	Meta_CD4_C10
ZNF101	4.0528E-10	0.321487497	0.214	0.122	9.814E-06	Meta_CD4_C10
INPP5F	4.1164E-10	0.29793203	0.135	0.046	9.968E-06	Meta_CD4_C10
UBC	4.2092E-10	0.351380501	0.968	0.966	1.019E-05	Meta_CD4_C10
NDUFC1	4.367E-10	0.344700688	0.475	0.361	1.057E-05	Meta_CD4_C10
SYNGR2	4.4296E-10	0.423830163	0.496	0.410	1.073E-05	Meta_CD4_C10
FAAH2	4.7046E-10	0.258321684	0.132	0.050	1.139E-05	Meta_CD4_C10
LINC00152	4.7472E-10	0.638922033	0.157	0.065	1.15E-05	Meta_CD4_C10
FRMD4B	4.9931E-10	0.375799178	0.228	0.117	1.209E-05	Meta_CD4_C10
IL2RG	5.4411E-10	0.442069096	0.656	0.580	1.318E-05	Meta_CD4_C10
TRAF3	5.7165E-10	0.335038107	0.242	0.141	1.384E-05	Meta_CD4_C10
GPX1	5.891E-10	0.584386267	0.123	0.041	1.427E-05	Meta_CD4_C10

COX17	6.4769E-10	0.348689891	0.464	0.371	1.568E-05	Meta_CD4_C10
ID3	6.7718E-10	0.566890139	0.197	0.097	1.64E-05	Meta_CD4_C10
METTL8	7.2305E-10	0.350566953	0.174	0.079	1.751E-05	Meta_CD4_C10
ICAM2	8.7475E-10	0.349119864	0.325	0.209	2.118E-05	Meta_CD4_C10
VAMP5	8.8687E-10	0.31453814	0.436	0.335	2.148E-05	Meta_CD4_C10
HLA-DMA	8.9987E-10	0.400943652	0.334	0.197	2.179E-05	Meta_CD4_C10
KDSR	9.5303E-10	0.269516557	0.272	0.177	2.308E-05	Meta_CD4_C10
HSDL2	1.1097E-09	0.250323197	0.141	0.069	2.687E-05	Meta_CD4_C10
SUSD3	1.1337E-09	0.323312956	0.365	0.265	2.745E-05	Meta_CD4_C10
FAM110A	1.1457E-09	0.374092708	0.240	0.121	2.774E-05	Meta_CD4_C10
LAPTM4A	1.1694E-09	0.413819028	0.462	0.372	2.832E-05	Meta_CD4_C10
C9orf16	1.4112E-09	0.349542262	0.679	0.614	3.417E-05	Meta_CD4_C10
ATP1B1	1.4879E-09	0.376575063	0.171	0.062	3.603E-05	Meta_CD4_C10
KAT2B	1.5452E-09	0.258123352	0.142	0.065	3.742E-05	Meta_CD4_C10
MX1	1.7006E-09	0.39154767	0.311	0.206	4.118E-05	Meta_CD4_C10
TXNDC11	1.8087E-09	0.250201036	0.247	0.165	4.38E-05	Meta_CD4_C10
MAD1L1	1.8769E-09	0.34422158	0.308	0.190	4.545E-05	Meta_CD4_C10
SESN3	2.0301E-09	0.266764326	0.185	0.098	4.916E-05	Meta_CD4_C10
ISCU	2.2197E-09	0.315462157	0.633	0.552	5.375E-05	Meta_CD4_C10
NDUFB1	2.2271E-09	0.338296073	0.480	0.383	5.393E-05	Meta_CD4_C10
LIMS1	2.7648E-09	0.305899941	0.417	0.326	6.695E-05	Meta_CD4_C10
UBE2D2	3.6191E-09	0.300127755	0.739	0.687	8.764E-05	Meta_CD4_C10
NDUFA4	3.9167E-09	0.276977097	0.809	0.771	9.484E-05	Meta_CD4_C10
SLC44A2	4.142E-09	0.279624776	0.376	0.295	0.0001003	Meta_CD4_C10
LRP10	4.4281E-09	0.300788798	0.466	0.382	0.0001072	Meta_CD4_C10
UXS1	4.5538E-09	0.35436861	0.272	0.150	0.0001103	Meta_CD4_C10
NDFIP1	5.4546E-09	0.338923879	0.590	0.489	0.0001321	Meta_CD4_C10
DOK2	6.0834E-09	0.343899179	0.517	0.428	0.0001473	Meta_CD4_C10
GLCCI1	6.2107E-09	0.308731343	0.210	0.119	0.0001504	Meta_CD4_C10
PMVK	7.208E-09	0.363150286	0.294	0.190	0.0001745	Meta_CD4_C10
NAB1	8.2166E-09	0.259460673	0.147	0.071	0.000199	Meta_CD4_C10
CPNE2	8.6769E-09	0.277321615	0.103	0.033	0.0002101	Meta_CD4_C10
ANTXR2	8.8794E-09	0.290077547	0.173	0.099	0.000215	Meta_CD4_C10
NCOA3	9.3826E-09	0.316665224	0.244	0.148	0.0002272	Meta_CD4_C10
P2RY10	9.4479E-09	0.368624898	0.339	0.233	0.0002288	Meta_CD4_C10
RHOH	9.8855E-09	0.384396615	0.640	0.545	0.0002394	Meta_CD4_C10
ZNF292	1.0032E-08	0.360416453	0.412	0.306	0.0002429	Meta_CD4_C10
ATP13A3	1.0319E-08	0.26644597	0.127	0.051	0.0002499	Meta_CD4_C10
CD70	1.1698E-08	0.498351134	0.162	0.076	0.0002833	Meta_CD4_C10
PIM3	1.3424E-08	0.467886673	0.411	0.302	0.0003251	Meta_CD4_C10

ZBTB38	1.3464E-08	0.434048319	0.410	0.279	0.000326	Meta_CD4_C10
BCL3	1.4218E-08	0.384890107	0.282	0.185	0.0003443	Meta_CD4_C10
CTSS	1.6641E-08	0.28206738	0.422	0.323	0.000403	Meta_CD4_C10
GOLGA8B	1.7798E-08	0.312218146	0.319	0.230	0.000431	Meta_CD4_C10
GALM	1.9156E-08	0.268243613	0.342	0.256	0.0004639	Meta_CD4_C10
PHLDA1	2.0147E-08	0.409453742	0.373	0.269	0.0004879	Meta_CD4_C10
VAMP8	2.3006E-08	0.338037657	0.647	0.566	0.0005571	Meta_CD4_C10
CSK	2.3358E-08	0.33357473	0.514	0.432	0.0005656	Meta_CD4_C10
COX5B	2.3577E-08	0.305896862	0.777	0.719	0.0005709	Meta_CD4_C10
NSD3	2.4859E-08	0.26253772	0.415	0.364	0.000602	Meta_CD4_C10
SGTB	2.7282E-08	0.275769401	0.187	0.099	0.0006606	Meta_CD4_C10
PAK2	2.923E-08	0.397734625	0.555	0.450	0.0007078	Meta_CD4_C10
ISG15	3.0405E-08	0.524307774	0.509	0.409	0.0007363	Meta_CD4_C10
MIIP	3.0487E-08	0.321302442	0.263	0.159	0.0007382	Meta_CD4_C10
LEF1	3.5525E-08	0.364207691	0.247	0.145	0.0008602	Meta_CD4_C10
SELPLG	4.0967E-08	0.394135429	0.539	0.455	0.000992	Meta_CD4_C10
TENT5C	4.1457E-08	0.286052555	0.462	0.406	0.0010039	Meta_CD4_C10
SYNE2	4.334E-08	0.408350457	0.578	0.503	0.0010495	Meta_CD4_C10
BAX	5.3014E-08	0.390787928	0.557	0.488	0.0012837	Meta_CD4_C10
UGCG	5.6862E-08	0.260383731	0.185	0.103	0.0013769	Meta_CD4_C10
FKBP1A	6.0084E-08	0.320912189	0.652	0.564	0.0014549	Meta_CD4_C10
ATP6V0E1	6.4368E-08	0.308193033	0.721	0.648	0.0015587	Meta_CD4_C10
SHMT2	6.6416E-08	0.33500908	0.277	0.178	0.0016083	Meta_CD4_C10
PCED1B	7.2209E-08	0.306988021	0.324	0.228	0.0017485	Meta_CD4_C10
OPTN	8.945E-08	0.263584056	0.491	0.419	0.002166	Meta_CD4_C10
LAX1	9.3723E-08	0.297947201	0.207	0.128	0.0022695	Meta_CD4_C10
BCL2	1.1344E-07	0.361638608	0.324	0.206	0.0027471	Meta_CD4_C10
TRBC1	1.3456E-07	0.565753083	0.519	0.455	0.0032584	Meta_CD4_C10
NOP10	1.3595E-07	0.369054217	0.588	0.486	0.0032921	Meta_CD4_C10
TNIP2	1.3623E-07	0.278456585	0.301	0.224	0.0032989	Meta_CD4_C10
IPCEF1	1.4368E-07	0.32210281	0.270	0.171	0.0034792	Meta_CD4_C10
TFRC	1.8432E-07	0.319424516	0.179	0.096	0.0044633	Meta_CD4_C10
CNIH1	1.868E-07	0.364860819	0.401	0.306	0.0045232	Meta_CD4_C10
HLA-DQB1	2.0462E-07	0.401133064	0.307	0.188	0.0049548	Meta_CD4_C10
NOP58	2.21E-07	0.373903157	0.486	0.417	0.0053515	Meta_CD4_C10
ZC3H7A	2.5091E-07	0.340037109	0.264	0.158	0.0060758	Meta_CD4_C10
ATOX1	2.5537E-07	0.335408501	0.279	0.183	0.0061837	Meta_CD4_C10
CD58	2.5911E-07	0.251769039	0.335	0.257	0.0062744	Meta_CD4_C10
OGDH	2.7644E-07	0.266416982	0.251	0.162	0.0066939	Meta_CD4_C10
ATPIF1	3.1248E-07	0.308540464	0.124	0.062	0.0075666	Meta_CD4_C10

STAT3	3.2625E-07	0.349568731	0.598	0.506	0.0079001	Meta_CD4_C10
HLA-DRB5	3.7312E-07	0.344243994	0.246	0.163	0.009035	Meta_CD4_C10
SLA	3.9232E-07	0.444571949	0.510	0.371	0.0095	Meta_CD4_C10
TMEM60	4.3514E-07	0.296037088	0.191	0.102	0.0105368	Meta_CD4_C10
C16orf87	5.5669E-07	0.281978483	0.241	0.147	0.0134802	Meta_CD4_C10
GRINA	5.6337E-07	0.251603507	0.274	0.198	0.0136419	Meta_CD4_C10
AKIRIN2	5.7004E-07	0.281510167	0.377	0.316	0.0138036	Meta_CD4_C10
STAT1	5.8884E-07	0.308774164	0.444	0.377	0.0142587	Meta_CD4_C10
TNFRSF25	6.2807E-07	0.253057048	0.312	0.227	0.0152086	Meta_CD4_C10
MAN1A2	6.5137E-07	0.268213316	0.253	0.176	0.0157728	Meta_CD4_C10
CMTM3	7.4912E-07	0.251136237	0.465	0.405	0.0181399	Meta_CD4_C10
ETS1	8.2344E-07	0.259839279	0.580	0.510	0.0199396	Meta_CD4_C10
FAM129A	8.3077E-07	0.323632407	0.350	0.250	0.0201172	Meta_CD4_C10
MYO5A	8.9656E-07	0.263133735	0.180	0.100	0.0217101	Meta_CD4_C10
RCAN3	1.0109E-06	0.27773337	0.362	0.277	0.0244793	Meta_CD4_C10
RASGRP1	1.0343E-06	0.296052797	0.294	0.208	0.0250458	Meta_CD4_C10
LYPLA1	1.2006E-06	0.260880845	0.319	0.239	0.0290727	Meta_CD4_C10
PTP4A3	1.2151E-06	0.317698123	0.119	0.055	0.029424	Meta_CD4_C10
PARK7	1.3214E-06	0.334685637	0.710	0.623	0.0319977	Meta_CD4_C10
TULP4	1.3221E-06	0.262355575	0.147	0.071	0.0320145	Meta_CD4_C10
R3HDM4	1.3222E-06	0.284050194	0.453	0.390	0.0320159	Meta_CD4_C10
NPC2	1.564E-06	0.342629578	0.360	0.252	0.0378734	Meta_CD4_C10
MRPS6	1.5757E-06	0.299274561	0.436	0.349	0.0381556	Meta_CD4_C10
ELOVL5	1.8372E-06	0.286534398	0.472	0.396	0.0444868	Meta_CD4_C10
CAST	1.907E-06	0.263095476	0.515	0.454	0.0461791	Meta_CD4_C10
CDKN1B	2.6461E-06	0.2576462	0.509	0.440	0.0640762	Meta_CD4_C10
UBE2N	2.898E-06	0.258691585	0.518	0.443	0.0701751	Meta_CD4_C10
NDUFA13	2.9958E-06	0.259601476	0.538	0.473	0.0725429	Meta_CD4_C10
TRIB2	3.5626E-06	0.337966479	0.266	0.167	0.0862693	Meta_CD4_C10
ATP5E	4.6354E-06	0.648102668	0.225	0.140	0.1122469	Meta_CD4_C10
S1PR4	5.5421E-06	0.311133586	0.348	0.274	0.1342008	Meta_CD4_C10
PSMB3	5.5516E-06	0.27615578	0.628	0.536	0.1344327	Meta_CD4_C10
IGFLR1	5.7841E-06	0.295247155	0.266	0.184	0.140062	Meta_CD4_C10
RAB10	8.0347E-06	0.258850333	0.348	0.265	0.1945608	Meta_CD4_C10
PRNP	8.802E-06	0.254286771	0.353	0.303	0.213141	Meta_CD4_C10
RNF145	9.2582E-06	0.259296119	0.334	0.261	0.2241873	Meta_CD4_C10
LINC00649	9.7501E-06	0.25096754	0.199	0.120	0.236098	Meta_CD4_C10
CHMP2A	1.0937E-05	0.252826694	0.446	0.367	0.2648327	Meta_CD4_C10
CISH	1.1023E-05	0.34343181	0.229	0.173	0.2669153	Meta_CD4_C10
TPR	1.1238E-05	0.360452967	0.496	0.427	0.2721332	Meta_CD4_C10

PAIP2	1.2435E-05	0.305134933	0.630	0.558	0.3011209	Meta_CD4_C10
TSPAN5	1.2744E-05	0.366243686	0.291	0.179	0.3085932	Meta_CD4_C10
MTHFD2	1.6674E-05	0.267881904	0.344	0.250	0.4037592	Meta_CD4_C10
AQP3	1.7201E-05	0.324797116	0.444	0.325	0.416527	Meta_CD4_C10
GADD45G	1.7513E-05	0.286863082	0.146	0.078	0.4240661	Meta_CD4_C10
STX11	2.1169E-05	0.284370526	0.266	0.165	0.5126095	Meta_CD4_C10
TACC3	2.1375E-05	0.302149281	0.277	0.174	0.5175933	Meta_CD4_C10
VOPP1	2.2635E-05	0.274931809	0.363	0.275	0.548116	Meta_CD4_C10
GCHFR	2.5338E-05	0.300885255	0.344	0.243	0.6135707	Meta_CD4_C10
HDAC7	2.6831E-05	0.27089387	0.265	0.183	0.6497155	Meta_CD4_C10
LAT	2.7893E-05	0.296395962	0.685	0.625	0.675419	Meta_CD4_C10
DDB2	3.1177E-05	0.258738507	0.229	0.145	0.7549615	Meta_CD4_C10
OTUD5	3.2995E-05	0.324072447	0.186	0.109	0.7989648	Meta_CD4_C10
TECR	4.049E-05	0.256648214	0.561	0.493	0.9804654	Meta_CD4_C10
EZR	4.4124E-05	0.276141199	0.697	0.635	1	Meta_CD4_C10
ITK	4.8627E-05	0.253673763	0.422	0.357	1	Meta_CD4_C10
ATP5I	5.0165E-05	0.266020955	0.112	0.057	1	Meta_CD4_C10
WSB1	5.6558E-05	0.353006211	0.484	0.401	1	Meta_CD4_C10
ZNRF1	5.8242E-05	0.255518136	0.157	0.078	1	Meta_CD4_C10
JAK1	6.3728E-05	0.28661521	0.713	0.655	1	Meta_CD4_C10
MAP1LC3A	6.5189E-05	0.278779556	0.160	0.090	1	Meta_CD4_C10
CLTA	6.9925E-05	0.254488799	0.473	0.387	1	Meta_CD4_C10
TNFRSF14	7.0318E-05	0.308236378	0.529	0.438	1	Meta_CD4_C10
TBCA	7.5393E-05	0.272252872	0.546	0.483	1	Meta_CD4_C10
CCR6	7.76E-05	0.308839523	0.229	0.144	1	Meta_CD4_C10
ASXL1	8.7337E-05	0.251069278	0.291	0.209	1	Meta_CD4_C10
GNB2L1	9.0927E-05	0.449031178	0.245	0.159	1	Meta_CD4_C10
YWHAH	9.0968E-05	0.275248004	0.406	0.339	1	Meta_CD4_C10
CASP8	9.7001E-05	0.273365768	0.381	0.301	1	Meta_CD4_C10
FOXN3	0.00010416	0.269763184	0.337	0.253	1	Meta_CD4_C10
ATP5O	0.00010477	0.280824725	0.123	0.063	1	Meta_CD4_C10
USP48	0.00010884	0.255355117	0.254	0.173	1	Meta_CD4_C10
PHPT1	0.00012582	0.31940802	0.393	0.318	1	Meta_CD4_C10
ANKRD12	0.00013041	0.293299116	0.680	0.630	1	Meta_CD4_C10
SNX5	0.00014856	0.275061984	0.322	0.250	1	Meta_CD4_C10
ANXA7	0.00018213	0.270356261	0.463	0.376	1	Meta_CD4_C10
GLTSCR2	0.00021395	0.305924299	0.176	0.104	1	Meta_CD4_C10
TIMP1	0.00021643	0.303844978	0.288	0.252	1	Meta_CD4_C10
C19orf43	0.00021972	0.254982294	0.148	0.083	1	Meta_CD4_C10
PGM2L1	0.00026657	0.257782629	0.140	0.084	1	Meta_CD4_C10

CSF1	0.00032162	0.335481641	0.183	0.090	1	Meta_CD4_C10
IFNAR2	0.00033446	0.301071494	0.161	0.080	1	Meta_CD4_C10
CRTAP	0.00034593	0.268990402	0.281	0.190	1	Meta_CD4_C10
ATP5G2	0.0003743	0.39459709	0.191	0.112	1	Meta_CD4_C10
NDUFB8	0.00038384	0.313265499	0.259	0.178	1	Meta_CD4_C10
FYB	0.00045808	0.292044075	0.145	0.083	1	Meta_CD4_C10
BCL2L1	0.00055945	0.257834162	0.218	0.161	1	Meta_CD4_C10
ITGB1	0.00056802	0.250754324	0.334	0.265	1	Meta_CD4_C10
TCEB2	0.00058904	0.393780496	0.159	0.083	1	Meta_CD4_C10
AP1S2	0.00117069	0.281013528	0.236	0.165	1	Meta_CD4_C10
C14orf2	0.00118836	0.266116636	0.137	0.074	1	Meta_CD4_C10
RAB33A	0.0012325	0.284146521	0.194	0.106	1	Meta_CD4_C10
SH3KBP1	0.00128327	0.288826337	0.534	0.449	1	Meta_CD4_C10
IGKC	0.00135351	0.304527366	0.187	0.122	1	Meta_CD4_C10
ZNF706	0.0020813	0.259011211	0.498	0.425	1	Meta_CD4_C10
ATP5L	0.00210013	0.388962974	0.198	0.123	1	Meta_CD4_C10
SNX20	0.00214814	0.270595714	0.247	0.166	1	Meta_CD4_C10
NECAP2	0.00265278	0.288393597	0.347	0.263	1	Meta_CD4_C10
TRAF1	0.00286489	0.352099847	0.285	0.196	1	Meta_CD4_C10
HLA-DQA1	0.00301456	0.313138084	0.211	0.144	1	Meta_CD4_C10
MIR155HG	0.00325334	0.319728911	0.141	0.102	1	Meta_CD4_C10
ARPP19	0.00386112	0.287451515	0.339	0.250	1	Meta_CD4_C10
NT5C	0.00431278	0.268164249	0.354	0.272	1	Meta_CD4_C10
TMEM154	0.00456487	0.304511748	0.182	0.098	1	Meta_CD4_C10
SIT1	0.0061519	0.287268602	0.494	0.407	1	Meta_CD4_C10
ATP5J2	0.00834683	0.280083995	0.126	0.066	1	Meta_CD4_C10
FOXP3	1.252E-119	1.647989731	0.837	0.074	3.03E-115	Meta_CD4_C11
TNFRSF18	9.073E-75	0.862293254	0.824	0.207	2.197E-70	Meta_CD4_C11
TBC1D4	4.7551E-73	0.88277411	0.692	0.102	1.151E-68	Meta_CD4_C11
CTLA4	1.1728E-71	1.291162564	0.891	0.250	2.84E-67	Meta_CD4_C11
IL2RA	9.7489E-63	0.801630323	0.570	0.080	2.361E-58	Meta_CD4_C11
KLRD1	4.9001E-62	0.333470506	0.796	0.252	1.187E-57	Meta_CD4_C11
TNFRSF4	5.7788E-61	0.943556235	0.738	0.182	1.399E-56	Meta_CD4_C11
TIGIT	2.9786E-60	0.862346566	0.928	0.331	7.213E-56	Meta_CD4_C11
BATF	8.3268E-60	0.985351897	0.928	0.343	2.016E-55	Meta_CD4_C11
IKZF2	4.4982E-58	0.677924165	0.552	0.078	1.089E-53	Meta_CD4_C11
SLAMF1	2.5152E-57	0.482288988	0.765	0.267	6.09E-53	Meta_CD4_C11
RHOC	1.3747E-56	0.471387278	0.801	0.280	3.329E-52	Meta_CD4_C11
CARD16	1.8575E-53	0.583033553	0.914	0.398	4.498E-49	Meta_CD4_C11
MIR4435-2HG	2.0158E-51	0.766918034	0.828	0.289	4.881E-47	Meta_CD4_C11

ENTPD1	5.0021E-51	0.604125384	0.584	0.119	1.211E-46	Meta_CD4_C11
DNPH1	2.345E-50	0.607711043	0.810	0.294	5.678E-46	Meta_CD4_C11
BCL2L1	5.2772E-50	0.419198072	0.624	0.165	1.278E-45	Meta_CD4_C11
PHTF2	8.7376E-49	0.420796794	0.674	0.211	2.116E-44	Meta_CD4_C11
CTSC	3.2383E-48	0.809300976	0.977	0.558	7.842E-44	Meta_CD4_C11
SIRPG	5.9398E-48	0.620776684	0.860	0.355	1.438E-43	Meta_CD4_C11
LAYN	3.1341E-47	0.448421671	0.443	0.065	7.589E-43	Meta_CD4_C11
CXCR6	5.6743E-46	0.540727473	0.828	0.330	1.374E-41	Meta_CD4_C11
PHLDA1	1.044E-45	0.866097807	0.783	0.277	2.528E-41	Meta_CD4_C11
PTTG1	1.0567E-45	0.407218476	0.638	0.210	2.559E-41	Meta_CD4_C11
STAM	1.1022E-45	0.397898136	0.552	0.147	2.669E-41	Meta_CD4_C11
NCF4	1.2579E-45	0.425446077	0.588	0.163	3.046E-41	Meta_CD4_C11
RTKN2	4.7276E-45	0.629919943	0.439	0.050	1.145E-40	Meta_CD4_C11
ABI3	5.2786E-45	0.534782075	0.828	0.342	1.278E-40	Meta_CD4_C11
AC243960.1	8.5579E-45	0.387105408	0.697	0.250	2.072E-40	Meta_CD4_C11
GBP5	2.2729E-44	0.586466083	0.896	0.399	5.504E-40	Meta_CD4_C11
ITGAE	4.2291E-44	0.364714163	0.787	0.330	1.024E-39	Meta_CD4_C11
ITGA1	5.4268E-44	0.365093132	0.647	0.219	1.314E-39	Meta_CD4_C11
LAIR2	6.0493E-44	0.522065372	0.394	0.050	1.465E-39	Meta_CD4_C11
CCDC69	6.9958E-44	0.320975306	0.738	0.295	1.694E-39	Meta_CD4_C11
ASB2	2.1708E-43	0.651190395	0.633	0.171	5.257E-39	Meta_CD4_C11
TOX	2.6452E-43	0.287089117	0.552	0.168	6.405E-39	Meta_CD4_C11
CSF1	2.781E-43	0.415394704	0.466	0.097	6.734E-39	Meta_CD4_C11
GLCCI1	3.0776E-43	0.407993206	0.507	0.125	7.452E-39	Meta_CD4_C11
GOLGA8B	3.4065E-43	0.285953083	0.643	0.236	8.249E-39	Meta_CD4_C11
FAM110A	4.0676E-43	0.380492552	0.534	0.130	9.85E-39	Meta_CD4_C11
UGP2	4.3619E-43	0.501154169	0.801	0.316	1.056E-38	Meta_CD4_C11
HOPX	4.5518E-43	0.376194678	0.796	0.346	1.102E-38	Meta_CD4_C11
CD27	1.566E-42	0.414384194	0.910	0.488	3.792E-38	Meta_CD4_C11
RNF187	2.5666E-42	0.325633797	0.729	0.303	6.215E-38	Meta_CD4_C11
FASLG	2.5691E-42	0.388861791	0.570	0.164	6.221E-38	Meta_CD4_C11
NDUFB5	3.4151E-42	0.252324751	0.701	0.286	8.27E-38	Meta_CD4_C11
CYBC1	4.8516E-42	0.298261749	0.706	0.297	1.175E-37	Meta_CD4_C11
CD160	5.2225E-42	0.572684078	0.484	0.089	1.265E-37	Meta_CD4_C11
CMTM3	7.5687E-42	0.324298106	0.855	0.409	1.833E-37	Meta_CD4_C11
ARID5B	2.1777E-41	0.285266371	0.805	0.367	5.273E-37	Meta_CD4_C11
TNFRSF1B	3.1014E-41	0.845830592	0.878	0.398	7.51E-37	Meta_CD4_C11
ACP5	5.8014E-41	0.511764909	0.715	0.261	1.405E-36	Meta_CD4_C11
IGFLR1	5.8975E-41	0.296044664	0.566	0.190	1.428E-36	Meta_CD4_C11
SSBP4	7.9348E-41	0.373382651	0.747	0.310	1.921E-36	Meta_CD4_C11

DENND1C	8.4683E-41	0.324745622	0.679	0.268	2.051E-36	Meta_CD4_C11
LAIR1	1.8813E-40	0.27603791	0.538	0.191	4.556E-36	Meta_CD4_C11
GPR174	2.9028E-40	0.377714048	0.661	0.250	7.029E-36	Meta_CD4_C11
CD28	8.6519E-40	0.356319888	0.661	0.264	2.095E-35	Meta_CD4_C11
NEDD9	9.2017E-40	0.337406004	0.638	0.238	2.228E-35	Meta_CD4_C11
PIM2	1.2865E-39	0.780222996	0.900	0.419	3.115E-35	Meta_CD4_C11
IL10RA	1.2962E-39	0.291607486	0.910	0.485	3.139E-35	Meta_CD4_C11
BCL2	1.5863E-39	0.32660651	0.624	0.215	3.841E-35	Meta_CD4_C11
SH3BP1	1.9199E-39	0.265323901	0.729	0.326	4.649E-35	Meta_CD4_C11
NAMPT	2.3178E-39	0.33793009	0.661	0.241	5.613E-35	Meta_CD4_C11
RIN3	5.9734E-39	0.258985567	0.471	0.131	1.446E-34	Meta_CD4_C11
SPRY1	6.0509E-39	0.485380264	0.529	0.141	1.465E-34	Meta_CD4_C11
ATP5MC1	1.0428E-38	0.270955104	0.633	0.261	2.525E-34	Meta_CD4_C11
PSMA5	1.2047E-38	0.280243954	0.855	0.444	2.917E-34	Meta_CD4_C11
GPR15	1.2053E-38	0.590714324	0.548	0.131	2.919E-34	Meta_CD4_C11
IL32	1.6383E-38	0.791793399	1.000	0.939	3.967E-34	Meta_CD4_C11
POU2F2	2.6493E-38	0.431574698	0.493	0.114	6.415E-34	Meta_CD4_C11
RBKS	3.1756E-38	0.51005667	0.552	0.146	7.69E-34	Meta_CD4_C11
RAB8B	3.3911E-38	0.255150588	0.652	0.272	8.211E-34	Meta_CD4_C11
STN1	3.6448E-38	0.438991232	0.638	0.212	8.826E-34	Meta_CD4_C11
PTPN22	3.8244E-38	0.312759409	0.801	0.377	9.261E-34	Meta_CD4_C11
RCSD1	5.0787E-38	0.278160012	0.828	0.430	1.23E-33	Meta_CD4_C11
CCND2	5.5304E-38	0.343712963	0.747	0.319	1.339E-33	Meta_CD4_C11
CDIP1	7.8735E-38	0.302633578	0.471	0.139	1.907E-33	Meta_CD4_C11
CSNK2B	1.1789E-37	0.300416934	0.855	0.433	2.855E-33	Meta_CD4_C11
GNAI2	1.3904E-37	0.313767084	0.742	0.332	3.367E-33	Meta_CD4_C11
HOXB2	1.5824E-37	0.276422524	0.520	0.163	3.832E-33	Meta_CD4_C11
CD8A	1.7857E-37	0.378526881	0.873	0.441	4.324E-33	Meta_CD4_C11
TLK1	1.9835E-37	0.284400606	0.674	0.266	4.803E-33	Meta_CD4_C11
CSK	2.175E-37	0.441881174	0.878	0.438	5.267E-33	Meta_CD4_C11
ZBTB38	2.2312E-37	0.339591632	0.710	0.289	5.403E-33	Meta_CD4_C11
AC017002.3	2.5942E-37	0.408771743	0.357	0.046	6.282E-33	Meta_CD4_C11
IL6R	2.6247E-37	0.29189636	0.385	0.078	6.356E-33	Meta_CD4_C11
TSPAN5	3.2276E-37	0.263556005	0.543	0.188	7.816E-33	Meta_CD4_C11
TRIB2	3.3522E-37	0.291240131	0.525	0.174	8.117E-33	Meta_CD4_C11
YPEL3	3.9848E-37	0.4478286	0.733	0.293	9.649E-33	Meta_CD4_C11
MAST4	9.6605E-37	0.287060545	0.416	0.081	2.339E-32	Meta_CD4_C11
FMNL1	1.022E-36	0.357653706	0.824	0.402	2.475E-32	Meta_CD4_C11
CORO1B	1.0774E-36	0.495663525	0.833	0.406	2.609E-32	Meta_CD4_C11
CASP1	1.0939E-36	0.349434608	0.733	0.304	2.649E-32	Meta_CD4_C11

TOX2	1.1831E-36	0.320529199	0.403	0.077	2.865E-32	Meta_CD4_C11
XCL1	1.2261E-36	0.84137868	0.516	0.130	2.969E-32	Meta_CD4_C11
LASP1	1.4853E-36	0.308652988	0.697	0.310	3.597E-32	Meta_CD4_C11
RAP1A	2.0333E-36	0.261416752	0.887	0.502	4.924E-32	Meta_CD4_C11
YWHAH	2.0682E-36	0.432050758	0.778	0.344	5.008E-32	Meta_CD4_C11
LIMS1	2.9178E-36	0.366456095	0.738	0.333	7.065E-32	Meta_CD4_C11
OLFM2	3.1054E-36	0.251980293	0.421	0.098	7.52E-32	Meta_CD4_C11
TRAF1	3.9511E-36	0.304185478	0.566	0.203	9.568E-32	Meta_CD4_C11
TMEM35B	4.4945E-36	0.280761489	0.620	0.253	1.088E-31	Meta_CD4_C11
MAPKAPK3	8.8448E-36	0.390122535	0.593	0.198	2.142E-31	Meta_CD4_C11
ATP5PB	9.3163E-36	0.251636248	0.837	0.456	2.256E-31	Meta_CD4_C11
F5	1.7506E-35	0.353546078	0.285	0.026	4.239E-31	Meta_CD4_C11
CCR6	2.2861E-35	0.316904159	0.493	0.151	5.536E-31	Meta_CD4_C11
GLRX	2.3314E-35	0.34031537	0.643	0.245	5.645E-31	Meta_CD4_C11
MAT2B	2.3492E-35	0.282480662	0.833	0.441	5.689E-31	Meta_CD4_C11
UBE2V1	2.5194E-35	0.257544666	0.724	0.353	6.101E-31	Meta_CD4_C11
CDK2AP2	2.5366E-35	0.285793931	0.805	0.394	6.142E-31	Meta_CD4_C11
SLA	2.6139E-35	0.293142846	0.792	0.381	6.329E-31	Meta_CD4_C11
ZC3H12D	2.7113E-35	0.337471534	0.416	0.099	6.566E-31	Meta_CD4_C11
BUB3	2.7121E-35	0.273593924	0.914	0.516	6.567E-31	Meta_CD4_C11
ICOS	3.399E-35	0.399699364	0.796	0.378	8.231E-31	Meta_CD4_C11
IKZF3	5.3527E-35	0.260380863	0.769	0.355	1.296E-30	Meta_CD4_C11
AC020916.1	5.4705E-35	0.424327516	0.620	0.224	1.325E-30	Meta_CD4_C11
SAMHD1	5.7428E-35	0.36672295	0.774	0.367	1.391E-30	Meta_CD4_C11
ASXL2	5.82E-35	0.288921651	0.484	0.151	1.409E-30	Meta_CD4_C11
SELPLG	6.6541E-35	0.253291162	0.846	0.461	1.611E-30	Meta_CD4_C11
IL2RB	8.5876E-35	0.531583704	0.882	0.448	2.079E-30	Meta_CD4_C11
PCED1B	8.7153E-35	0.335714221	0.629	0.235	2.11E-30	Meta_CD4_C11
RASGRP1	1.1012E-34	0.355345987	0.620	0.214	2.667E-30	Meta_CD4_C11
GBP2	1.1618E-34	0.491121398	0.860	0.416	2.813E-30	Meta_CD4_C11
NOP10	1.417E-34	0.261340662	0.887	0.494	3.431E-30	Meta_CD4_C11
GADD45A	2.3555E-34	0.304296132	0.489	0.142	5.704E-30	Meta_CD4_C11
BPTF	2.5305E-34	0.302676188	0.670	0.276	6.128E-30	Meta_CD4_C11
SET	2.5332E-34	0.303315615	0.842	0.450	6.134E-30	Meta_CD4_C11
LIMA1	2.5561E-34	0.304141262	0.421	0.102	6.19E-30	Meta_CD4_C11
LSM2	2.9472E-34	0.284350426	0.747	0.357	7.137E-30	Meta_CD4_C11
POLD4	3.8771E-34	0.318744725	0.810	0.416	9.388E-30	Meta_CD4_C11
SDHD	4.1368E-34	0.258762854	0.701	0.315	1.002E-29	Meta_CD4_C11
AD000671.2	5.9239E-34	0.371957609	0.443	0.104	1.434E-29	Meta_CD4_C11
PTPN6	6.1058E-34	0.417865734	0.801	0.397	1.479E-29	Meta_CD4_C11

PPP1R18	8.7208E-34	0.28751848	0.914	0.531	2.112E-29	Meta_CD4_C11
CTCF	9.0976E-34	0.289237679	0.507	0.163	2.203E-29	Meta_CD4_C11
CD7	9.804E-34	0.761127796	0.995	0.762	2.374E-29	Meta_CD4_C11
ZFP36L1	9.984E-34	0.818884467	0.959	0.678	2.418E-29	Meta_CD4_C11
CHURC1	1.1678E-33	0.344511159	0.842	0.438	2.828E-29	Meta_CD4_C11
OSTF1	1.2724E-33	0.327071787	0.905	0.515	3.081E-29	Meta_CD4_C11
PMVK	1.3484E-33	0.380985558	0.579	0.198	3.265E-29	Meta_CD4_C11
TPP1	2.3433E-33	0.259799927	0.575	0.222	5.674E-29	Meta_CD4_C11
MAP2K2	3.8477E-33	0.268064398	0.557	0.213	9.317E-29	Meta_CD4_C11
EPSTI1	5.1488E-33	0.2811713	0.602	0.240	1.247E-28	Meta_CD4_C11
TPM4	5.6993E-33	0.308343931	0.792	0.401	1.38E-28	Meta_CD4_C11
LPXN	5.8141E-33	0.423493458	0.796	0.403	1.408E-28	Meta_CD4_C11
TNFRSF14	6.2563E-33	0.268668908	0.824	0.445	1.515E-28	Meta_CD4_C11
SASH3	7.5975E-33	0.40005468	0.819	0.424	1.84E-28	Meta_CD4_C11
TSC22D4	1.1329E-32	0.268378566	0.805	0.411	2.743E-28	Meta_CD4_C11
ARPC1B	1.7546E-32	0.638098235	0.986	0.729	4.249E-28	Meta_CD4_C11
UCP2	2.0305E-32	0.614059226	0.905	0.497	4.917E-28	Meta_CD4_C11
TRAF3IP3	2.5253E-32	0.277739578	0.900	0.525	6.115E-28	Meta_CD4_C11
CAPG	2.5287E-32	0.276055049	0.643	0.281	6.123E-28	Meta_CD4_C11
RHOG	4.5103E-32	0.30093754	0.896	0.519	1.092E-27	Meta_CD4_C11
CALHM2	4.7546E-32	0.266199808	0.498	0.161	1.151E-27	Meta_CD4_C11
NSMCE3	4.9316E-32	0.268733372	0.656	0.278	1.194E-27	Meta_CD4_C11
TMIGD2	6.5167E-32	0.310529489	0.471	0.151	1.578E-27	Meta_CD4_C11
NABP1	6.8914E-32	0.255793791	0.475	0.146	1.669E-27	Meta_CD4_C11
GRK6	1.0361E-31	0.253043919	0.665	0.306	2.509E-27	Meta_CD4_C11
ADTRP	1.7141E-31	0.410477378	0.290	0.030	4.151E-27	Meta_CD4_C11
KLRC3	3.0994E-31	0.410775881	0.357	0.076	7.505E-27	Meta_CD4_C11
FKBP1A	3.2745E-31	0.390260187	0.946	0.571	7.929E-27	Meta_CD4_C11
JUND	4.5315E-31	0.266828943	0.824	0.423	1.097E-26	Meta_CD4_C11
ACTA2	4.9506E-31	0.271379928	0.362	0.084	1.199E-26	Meta_CD4_C11
NDFIP2	6.8186E-31	0.284503206	0.466	0.141	1.651E-26	Meta_CD4_C11
LINC01871	6.9997E-31	0.515619465	0.733	0.311	1.695E-26	Meta_CD4_C11
LTA	9.0513E-31	0.600792447	0.416	0.090	2.192E-26	Meta_CD4_C11
SELENOW	1.0705E-30	0.273028291	0.842	0.470	2.592E-26	Meta_CD4_C11
ECH1	1.4588E-30	0.282979791	0.819	0.438	3.532E-26	Meta_CD4_C11
PBXIP1	1.4591E-30	0.295655064	0.882	0.497	3.533E-26	Meta_CD4_C11
WNK1	1.5006E-30	0.295686237	0.683	0.311	3.634E-26	Meta_CD4_C11
DIAPH1	1.5375E-30	0.279891238	0.656	0.297	3.723E-26	Meta_CD4_C11
SOCS1	1.9771E-30	0.281702392	0.742	0.380	4.788E-26	Meta_CD4_C11
ROCK1	2.1837E-30	0.263181723	0.774	0.396	5.288E-26	Meta_CD4_C11

BIRC3	2.3999E-30	0.308683716	0.710	0.307	5.811E-26	Meta_CD4_C11
GLUD1	4.2015E-30	0.256903191	0.697	0.321	1.017E-25	Meta_CD4_C11
IL16	5.0515E-30	0.253206085	0.819	0.459	1.223E-25	Meta_CD4_C11
TNFRSF13B	7.5587E-30	0.344743221	0.208	0.008	1.83E-25	Meta_CD4_C11
RHOH	8.1882E-30	0.258599539	0.896	0.552	1.983E-25	Meta_CD4_C11
CLDND1	9.83E-30	0.496059724	0.873	0.462	2.38E-25	Meta_CD4_C11
RGS1	1.184E-29	0.525338688	0.950	0.620	2.867E-25	Meta_CD4_C11
MAF	1.2511E-29	0.323703175	0.548	0.185	3.03E-25	Meta_CD4_C11
H1FX	1.651E-29	0.261718475	0.629	0.280	3.998E-25	Meta_CD4_C11
SEM1	1.8209E-29	0.321719484	0.842	0.471	4.409E-25	Meta_CD4_C11
TNIK	1.8863E-29	0.251555945	0.570	0.225	4.568E-25	Meta_CD4_C11
LINC01943	2.0119E-29	0.532422381	0.462	0.116	4.872E-25	Meta_CD4_C11
LBH	2.1392E-29	0.397589372	0.864	0.487	5.18E-25	Meta_CD4_C11
SYNGR2	2.4166E-29	0.373187908	0.810	0.417	5.852E-25	Meta_CD4_C11
KIR2DL4	2.622E-29	0.418992582	0.330	0.065	6.349E-25	Meta_CD4_C11
CKLF	3.1169E-29	0.410393686	0.923	0.564	7.548E-25	Meta_CD4_C11
CCR8	4.0742E-29	0.275566675	0.213	0.016	9.866E-25	Meta_CD4_C11
MAP3K8	4.7097E-29	0.25670656	0.647	0.301	1.14E-24	Meta_CD4_C11
CREM	5.7954E-29	0.368876842	0.787	0.405	1.403E-24	Meta_CD4_C11
XCL2	7.3237E-29	0.687834121	0.493	0.145	1.773E-24	Meta_CD4_C11
CISH	7.5469E-29	0.394241825	0.534	0.177	1.827E-24	Meta_CD4_C11
PAK2	1.1193E-28	0.335509453	0.837	0.458	2.71E-24	Meta_CD4_C11
TNFSF9	1.2136E-28	0.448418904	0.489	0.136	2.939E-24	Meta_CD4_C11
FCMR	1.4204E-28	0.538483898	0.787	0.378	3.44E-24	Meta_CD4_C11
WASHC3	1.6183E-28	0.287509007	0.525	0.197	3.919E-24	Meta_CD4_C11
CMTM6	1.9373E-28	0.284080806	0.747	0.370	4.691E-24	Meta_CD4_C11
CYTOR	2.222E-28	0.496345368	0.819	0.428	5.38E-24	Meta_CD4_C11
PSMB10	2.3201E-28	0.322528298	0.873	0.509	5.618E-24	Meta_CD4_C11
LTB	3.08E-28	0.595217963	0.959	0.615	7.458E-24	Meta_CD4_C11
CAP1	3.1573E-28	0.347386326	0.968	0.644	7.646E-24	Meta_CD4_C11
ARHGAP9	3.5666E-28	0.339963833	0.878	0.509	8.637E-24	Meta_CD4_C11
RAB11FIP1	4.3249E-28	0.313418982	0.507	0.171	1.047E-23	Meta_CD4_C11
STAT1	7.3721E-28	0.309521859	0.733	0.382	1.785E-23	Meta_CD4_C11
PIM3	7.9721E-28	0.396373149	0.679	0.311	1.93E-23	Meta_CD4_C11
ZNF331	9.846E-28	0.534168846	0.738	0.342	2.384E-23	Meta_CD4_C11
IL1R2	1.2237E-27	0.277706857	0.222	0.018	2.963E-23	Meta_CD4_C11
MAGEH1	1.4435E-27	0.399927233	0.407	0.099	3.495E-23	Meta_CD4_C11
PTMS	1.5496E-27	0.357905163	0.579	0.228	3.752E-23	Meta_CD4_C11
ATP5MF	2.0547E-27	0.353482481	0.864	0.506	4.976E-23	Meta_CD4_C11
SH3BGRL	2.6625E-27	0.296785933	0.896	0.533	6.447E-23	Meta_CD4_C11

CDKN1B	3.3866E-27	0.26845409	0.814	0.445	8.201E-23	Meta_CD4_C11
WDR1	7.0466E-27	0.306771133	0.891	0.546	1.706E-22	Meta_CD4_C11
CALM3	7.6228E-27	0.401117409	0.919	0.571	1.846E-22	Meta_CD4_C11
NR4A2	8.239E-27	0.561417458	0.932	0.578	1.995E-22	Meta_CD4_C11
FYN	8.9002E-27	0.254331565	0.923	0.567	2.155E-22	Meta_CD4_C11
GK	1.0043E-26	0.298060327	0.348	0.075	2.432E-22	Meta_CD4_C11
PHACTR2	1.4948E-26	0.304303443	0.407	0.111	3.62E-22	Meta_CD4_C11
LCP2	1.8773E-26	0.572620846	0.928	0.596	4.546E-22	Meta_CD4_C11
RNASEK	2.274E-26	0.448926871	0.860	0.501	5.507E-22	Meta_CD4_C11
SAMSN1	2.3166E-26	0.410302951	0.900	0.543	5.61E-22	Meta_CD4_C11
CCR4	2.4935E-26	0.262031283	0.299	0.061	6.038E-22	Meta_CD4_C11
ANKRD44	3.8163E-26	0.2546187	0.715	0.363	9.241E-22	Meta_CD4_C11
TUBB	4.6295E-26	0.344981066	0.882	0.549	1.121E-21	Meta_CD4_C11
SLC9A3R1	4.7966E-26	0.276416083	0.910	0.577	1.161E-21	Meta_CD4_C11
ID3	5.6564E-26	0.340245114	0.371	0.105	1.37E-21	Meta_CD4_C11
APBB1IP	6.2965E-26	0.459155718	0.882	0.514	1.525E-21	Meta_CD4_C11
TNIP3	8.1958E-26	0.446752509	0.385	0.095	1.985E-21	Meta_CD4_C11
TRIP12	9.4411E-26	0.282883738	0.466	0.156	2.286E-21	Meta_CD4_C11
ZC2HC1A	1.184E-25	0.380820811	0.290	0.034	2.867E-21	Meta_CD4_C11
NFKBIZ	1.5496E-25	0.278552532	0.719	0.363	3.752E-21	Meta_CD4_C11
ZAP70	1.5739E-25	0.265955889	0.882	0.537	3.811E-21	Meta_CD4_C11
TRIB1	1.7543E-25	0.276565286	0.267	0.048	4.248E-21	Meta_CD4_C11
ACTR3	2.0559E-25	0.325688034	0.937	0.621	4.978E-21	Meta_CD4_C11
SH2D2A	2.4578E-25	0.462271529	0.846	0.464	5.952E-21	Meta_CD4_C11
KIAA1551	5.5419E-25	0.272009724	0.855	0.511	1.342E-20	Meta_CD4_C11
NR4A1	1.2449E-24	0.628276101	0.674	0.293	3.014E-20	Meta_CD4_C11
CD70	2.2552E-24	0.295919102	0.317	0.082	5.461E-20	Meta_CD4_C11
SEPT6	2.3048E-24	0.425843641	0.928	0.580	5.581E-20	Meta_CD4_C11
BTG2	4.2498E-24	0.28252027	0.932	0.613	1.029E-19	Meta_CD4_C11
SLC12A6	4.4635E-24	0.27132833	0.326	0.072	1.081E-19	Meta_CD4_C11
KLRC2	7.5047E-24	0.327275425	0.321	0.072	1.817E-19	Meta_CD4_C11
ARL6IP5	7.7483E-24	0.296179047	0.973	0.700	1.876E-19	Meta_CD4_C11
SAT1	9.3076E-24	0.473205647	0.955	0.642	2.254E-19	Meta_CD4_C11
SELENOH	1.431E-23	0.273689341	0.787	0.453	3.465E-19	Meta_CD4_C11
SERPINB9	1.4925E-23	0.275911951	0.529	0.224	3.614E-19	Meta_CD4_C11
ACTB	3.0353E-23	0.443370641	1.000	0.998	7.35E-19	Meta_CD4_C11
CCL4	4.8144E-23	0.709443784	0.792	0.431	1.166E-18	Meta_CD4_C11
GEM	8.7321E-23	0.417162023	0.271	0.052	2.114E-18	Meta_CD4_C11
ATP5MC3	1.1119E-22	0.273135369	0.882	0.564	2.693E-18	Meta_CD4_C11
C9orf16	1.574E-22	0.270783916	0.932	0.619	3.812E-18	Meta_CD4_C11

BCL3	2.3419E-22	0.512286973	0.552	0.193	5.671E-18	Meta_CD4_C11
EIF4G3	3.0616E-22	0.25946296	0.312	0.081	7.414E-18	Meta_CD4_C11
RAC2	3.9222E-22	0.493567249	0.986	0.830	9.498E-18	Meta_CD4_C11
LAG3	4.9269E-22	0.540700884	0.706	0.371	1.193E-17	Meta_CD4_C11
SKAP1	8.8828E-22	0.462245103	0.896	0.551	2.151E-17	Meta_CD4_C11
ATP5IF1	1.2141E-21	0.273901428	0.864	0.544	2.94E-17	Meta_CD4_C11
PRR13	3.3609E-21	0.269932219	0.955	0.661	8.139E-17	Meta_CD4_C11
SEPT1	4.9442E-21	0.36828438	0.941	0.648	1.197E-16	Meta_CD4_C11
ETV7	5.2431E-21	0.32094161	0.303	0.064	1.27E-16	Meta_CD4_C11
BRK1	1.1428E-20	0.253698606	0.905	0.600	2.767E-16	Meta_CD4_C11
PTPRCAP	1.6627E-20	0.568695008	0.919	0.740	4.026E-16	Meta_CD4_C11
RPS27L	2.257E-20	0.269251798	0.855	0.546	5.465E-16	Meta_CD4_C11
TTN	2.3402E-20	0.279653761	0.262	0.052	5.667E-16	Meta_CD4_C11
LAT	4.98E-20	0.25495164	0.923	0.629	1.206E-15	Meta_CD4_C11
LINC02446	6.7234E-20	0.446774368	0.353	0.109	1.628E-15	Meta_CD4_C11
IFNG	7.1949E-20	0.329424033	0.548	0.250	1.742E-15	Meta_CD4_C11
PGAM1	9.9601E-20	0.261760164	0.882	0.574	2.412E-15	Meta_CD4_C11
ACTG1	1.5745E-19	0.457262687	0.995	0.947	3.813E-15	Meta_CD4_C11
CCL4L2	3.0986E-19	0.910744917	0.493	0.176	7.503E-15	Meta_CD4_C11
JAK1	5.2036E-19	0.304541761	0.937	0.660	1.26E-14	Meta_CD4_C11
FYB1	6.1433E-19	0.615613441	0.887	0.634	1.488E-14	Meta_CD4_C11
CKS2	1.2969E-18	0.293208663	0.394	0.165	3.141E-14	Meta_CD4_C11
DUSP1	1.3511E-18	0.489942968	0.977	0.803	3.272E-14	Meta_CD4_C11
HCLS1	2.3251E-18	0.381332784	0.950	0.675	5.63E-14	Meta_CD4_C11
CD177	3.8177E-18	0.37913443	0.131	0.007	9.244E-14	Meta_CD4_C11
CD247	4.6483E-18	0.274269776	0.937	0.649	1.126E-13	Meta_CD4_C11
PKM	6.9168E-18	0.314815229	0.932	0.657	1.675E-13	Meta_CD4_C11
BEX3	1.8363E-17	0.283836292	0.213	0.035	4.447E-13	Meta_CD4_C11
DUSP2	2.1467E-17	0.253505191	0.905	0.637	5.198E-13	Meta_CD4_C11
LCK	2.1743E-17	0.311990273	0.968	0.748	5.265E-13	Meta_CD4_C11
TNFRSF9	3.4728E-17	0.459171761	0.276	0.057	8.409E-13	Meta_CD4_C11
CORO1A	4.7071E-17	0.3433834	0.995	0.875	1.14E-12	Meta_CD4_C11
GABARAP	6.0609E-17	0.376915514	0.923	0.659	1.468E-12	Meta_CD4_C11
TRGV3	6.2624E-17	0.319184093	0.317	0.096	1.516E-12	Meta_CD4_C11
PTPRC	2.0112E-16	0.369003271	1.000	0.894	4.87E-12	Meta_CD4_C11
PFN1	2.384E-16	0.350076216	1.000	0.958	5.773E-12	Meta_CD4_C11
TRGV4	2.4352E-16	0.508133948	0.276	0.057	5.897E-12	Meta_CD4_C11
EVL	3.5694E-16	0.312679106	0.982	0.802	8.643E-12	Meta_CD4_C11
OAZ1	6.2397E-16	0.261886827	0.995	0.878	1.511E-11	Meta_CD4_C11
ATP5F1D	9.6922E-16	0.317174198	0.873	0.586	2.347E-11	Meta_CD4_C11

INSIG1	3.7272E-15	0.255879126	0.452	0.207	9.025E-11	Meta_CD4_C11
HMGN2	5.6469E-15	0.260518653	0.928	0.688	1.367E-10	Meta_CD4_C11
LSP1	7.3402E-15	0.271167617	0.991	0.831	1.777E-10	Meta_CD4_C11
HSPA8	2.3841E-14	0.387339929	0.991	0.904	5.773E-10	Meta_CD4_C11
TRIR	3.2942E-14	0.296323766	0.905	0.648	7.977E-10	Meta_CD4_C11
PCBP1	3.4788E-14	0.401625912	0.946	0.762	8.424E-10	Meta_CD4_C11
CRIP1	1.4165E-13	0.355851038	0.946	0.746	3.43E-09	Meta_CD4_C11
HNRNPK	1.8339E-12	0.297650198	0.968	0.769	4.441E-08	Meta_CD4_C11
TRDC	2.0992E-12	0.404976206	0.208	0.056	5.083E-08	Meta_CD4_C11
IL10	3.1736E-12	0.62988201	0.145	0.022	7.685E-08	Meta_CD4_C11
TRAV29DV5	6.2704E-11	0.45501	0.199	0.055	1.518E-06	Meta_CD4_C11
CD3E	7.591E-11	0.288817936	0.995	0.924	1.838E-06	Meta_CD4_C11
CD69	9.0241E-11	0.277815233	0.973	0.833	2.185E-06	Meta_CD4_C11
TRBV28	2.8803E-10	0.453339904	0.208	0.061	6.975E-06	Meta_CD4_C11
TRGV5	4.2882E-10	0.47299456	0.181	0.056	1.038E-05	Meta_CD4_C11
ATP5MC2	4.737E-10	0.253187906	0.928	0.745	1.147E-05	Meta_CD4_C11
PNRC1	5.3272E-10	0.295001241	0.968	0.830	1.29E-05	Meta_CD4_C11
TRGV2	1.1067E-09	0.331989309	0.208	0.072	2.68E-05	Meta_CD4_C11
ATP5F1E	2.7683E-09	0.327297444	0.928	0.784	6.703E-05	Meta_CD4_C11
TRBV18	5.9817E-09	0.485852751	0.140	0.034	0.0001448	Meta_CD4_C11
CCL3L1	1.4562E-08	0.387474312	0.199	0.064	0.0003526	Meta_CD4_C11
H3F3A	9.4261E-08	0.291901858	0.950	0.823	0.0022825	Meta_CD4_C11
ATP5MG	4.2352E-07	0.253963849	0.919	0.751	0.0102556	Meta_CD4_C11
TRDV1	1.295E-06	0.34938309	0.122	0.034	0.0313596	Meta_CD4_C11
RAV38-2DV	1.4711E-06	0.635027004	0.109	0.027	0.035622	Meta_CD4_C11
TRAV17	1.0706E-05	0.3290589	0.136	0.044	0.2592453	Meta_CD4_C11
MT-ATP8	2.6015E-05	0.265390768	0.919	0.792	0.6299525	Meta_CD4_C11
TRBV19	3.0867E-05	0.455834177	0.140	0.048	0.74745	Meta_CD4_C11
TRBV5-1	3.6014E-05	0.74684886	0.118	0.047	0.8720773	Meta_CD4_C11
TRBV27	0.00023192	0.292630795	0.104	0.040	1	Meta_CD4_C11
TRBV20-1	0.00091653	0.752572056	0.158	0.077	1	Meta_CD4_C11
TRAV19	0.00125551	0.321283296	0.127	0.049	1	Meta_CD4_C11
TRAV8-4	0.00126649	0.34734179	0.104	0.037	1	Meta_CD4_C11
JUN	2.31E-149	2.201455709	0.938	0.803	5.59E-145	Meta_CD4_C12
FOSB	4.077E-118	1.936404351	0.778	0.508	9.87E-114	Meta_CD4_C12
IER2	1.965E-113	1.974311103	0.788	0.659	4.76E-109	Meta_CD4_C12
FOS	2.532E-104	1.877076701	0.903	0.761	6.13E-100	Meta_CD4_C12
SRSF7	2.259E-97	1.216619875	0.796	0.805	5.47E-93	Meta_CD4_C12
KLF6	8.1747E-97	1.471196548	0.872	0.861	1.979E-92	Meta_CD4_C12
PPP1R15A	2.7114E-95	1.394131951	0.722	0.667	6.566E-91	Meta_CD4_C12

BRD2	3.0578E-89	1.186781538	0.541	0.504	7.404E-85	Meta_CD4_C12
JUND	6.596E-87	1.300674735	0.502	0.422	1.597E-82	Meta_CD4_C12
GADD45B	8.7581E-83	2.049100066	0.693	0.510	2.121E-78	Meta_CD4_C12
RSRP1	2.6898E-75	0.838258672	0.579	0.588	6.513E-71	Meta_CD4_C12
FUS	5.8192E-75	0.711902222	0.643	0.702	1.409E-70	Meta_CD4_C12
DNAJA1	6.227E-75	0.961733539	0.604	0.623	1.508E-70	Meta_CD4_C12
SF1	1.92E-73	0.345998155	0.546	0.672	4.649E-69	Meta_CD4_C12
TUBB4B	2.4317E-73	1.610209931	0.561	0.462	5.888E-69	Meta_CD4_C12
SNHG8	1.1108E-71	0.599289731	0.457	0.548	2.69E-67	Meta_CD4_C12
VCP	2.3691E-71	0.288454142	0.388	0.535	5.737E-67	Meta_CD4_C12
CCNL1	2.2788E-70	0.800294619	0.552	0.561	5.518E-66	Meta_CD4_C12
CD44	2.5855E-68	0.595522067	0.685	0.766	6.261E-64	Meta_CD4_C12
MALAT1	5.8436E-68	0.814778659	0.999	1.000	1.415E-63	Meta_CD4_C12
RPL22L1	1.9712E-67	0.451195983	0.402	0.515	4.773E-63	Meta_CD4_C12
DUSP1	2.7153E-67	1.261039543	0.862	0.802	6.575E-63	Meta_CD4_C12
DDX3X	1.2965E-66	0.818992417	0.466	0.470	3.139E-62	Meta_CD4_C12
SQSTM1	2.8607E-66	0.517816744	0.417	0.573	6.927E-62	Meta_CD4_C12
SRSF2	2.4272E-65	0.63072128	0.585	0.637	5.877E-61	Meta_CD4_C12
SPSB3	7.1395E-65	0.38079263	0.331	0.421	1.729E-60	Meta_CD4_C12
SERTAD1	8.1994E-65	1.208269587	0.442	0.334	1.985E-60	Meta_CD4_C12
YME1L1	2.8704E-63	0.435527869	0.295	0.373	6.951E-59	Meta_CD4_C12
TUBA1A	6.2233E-63	1.130206169	0.567	0.564	1.507E-58	Meta_CD4_C12
RBM39	1.9275E-62	0.52315406	0.639	0.711	4.667E-58	Meta_CD4_C12
RBM8A	2.593E-62	0.30145213	0.490	0.634	6.279E-58	Meta_CD4_C12
RPP21	3.6458E-62	0.283008911	0.211	0.277	8.828E-58	Meta_CD4_C12
HNRNPA0	3.5444E-61	0.284538935	0.526	0.696	8.583E-57	Meta_CD4_C12
EGR1	3.9608E-61	2.242985928	0.409	0.166	9.591E-57	Meta_CD4_C12
FNBP1	5.0643E-61	0.344031277	0.400	0.538	1.226E-56	Meta_CD4_C12
EIF4A2	5.5354E-61	0.42542579	0.605	0.716	1.34E-56	Meta_CD4_C12
SRSF3	1.7433E-60	0.479595389	0.520	0.619	4.222E-56	Meta_CD4_C12
EIF5	4.3433E-60	0.517630259	0.443	0.522	1.052E-55	Meta_CD4_C12
TRA2B	4.8908E-60	0.548858714	0.418	0.467	1.184E-55	Meta_CD4_C12
NFKBIA	1.0014E-59	1.048944658	0.727	0.712	2.425E-55	Meta_CD4_C12
ARGLU1	1.1513E-59	0.396610333	0.470	0.557	2.788E-55	Meta_CD4_C12
ZFAS1	2.0057E-59	0.429624398	0.554	0.674	4.857E-55	Meta_CD4_C12
ATF4	2.645E-58	0.502648046	0.296	0.345	6.405E-54	Meta_CD4_C12
SRSF11	4.0436E-58	0.277204957	0.433	0.548	9.791E-54	Meta_CD4_C12
TAF1D	2.9971E-57	0.401943229	0.347	0.422	7.257E-53	Meta_CD4_C12
H3F3B	1.2089E-56	0.772705839	0.950	0.980	2.927E-52	Meta_CD4_C12
DNAJB1	1.8092E-56	1.055238104	0.776	0.761	4.381E-52	Meta_CD4_C12

CITED2	4.8182E-56	1.207789286	0.526	0.496	1.167E-51	Meta_CD4_C12
SLC38A2	1.2182E-55	0.930457119	0.379	0.315	2.95E-51	Meta_CD4_C12
TSC22D3	2.1964E-55	0.869842001	0.858	0.891	5.318E-51	Meta_CD4_C12
MT-ND2	2.4276E-55	0.868111546	0.936	0.946	5.879E-51	Meta_CD4_C12
AMD1	3.0153E-55	0.653657035	0.351	0.363	7.302E-51	Meta_CD4_C12
SNHG12	3.3212E-55	0.830566981	0.300	0.259	8.042E-51	Meta_CD4_C12
EIF4A1	4.3889E-55	0.362507295	0.313	0.402	1.063E-50	Meta_CD4_C12
TBCC	4.8023E-55	0.454092758	0.296	0.352	1.163E-50	Meta_CD4_C12
HNRNPDL	5.4856E-55	0.343744736	0.687	0.803	1.328E-50	Meta_CD4_C12
DDX17	6.0025E-55	0.263444427	0.331	0.468	1.453E-50	Meta_CD4_C12
PAXX	1.5974E-54	0.307042355	0.468	0.583	3.868E-50	Meta_CD4_C12
RGCC	1.6825E-54	1.141146627	0.520	0.528	4.074E-50	Meta_CD4_C12
CHD2	1.6985E-54	0.436830578	0.288	0.340	4.113E-50	Meta_CD4_C12
RBM25	2.3426E-54	0.363910816	0.394	0.496	5.673E-50	Meta_CD4_C12
NEU1	1.3106E-53	1.199756581	0.340	0.213	3.174E-49	Meta_CD4_C12
EIF4A3	1.5553E-53	0.775833993	0.323	0.332	3.766E-49	Meta_CD4_C12
UBB	1.734E-53	0.624363928	0.797	0.896	4.199E-49	Meta_CD4_C12
AKNA	3.9904E-53	0.340768493	0.317	0.411	9.663E-49	Meta_CD4_C12
IDS	8.058E-53	0.510410594	0.501	0.581	1.951E-48	Meta_CD4_C12
STK17B	9.7892E-53	0.357157388	0.564	0.679	2.37E-48	Meta_CD4_C12
ARHGAP4	1.086E-52	0.289371737	0.288	0.392	2.63E-48	Meta_CD4_C12
SNRNP70	2.5042E-52	0.283898409	0.250	0.349	6.064E-48	Meta_CD4_C12
RSRC2	4.7224E-52	0.350836258	0.374	0.464	1.144E-47	Meta_CD4_C12
TMC8	6.0048E-52	0.287238315	0.276	0.373	1.454E-47	Meta_CD4_C12
PARP8	7.3374E-52	0.309804065	0.350	0.434	1.777E-47	Meta_CD4_C12
MCL1	7.5891E-52	0.6061818	0.673	0.727	1.838E-47	Meta_CD4_C12
H2AFX	9.6226E-52	1.126256776	0.351	0.233	2.33E-47	Meta_CD4_C12
SLC2A3	1.5727E-51	0.691844843	0.564	0.648	3.808E-47	Meta_CD4_C12
SRSF10	1.7044E-51	0.390314143	0.400	0.487	4.127E-47	Meta_CD4_C12
TNF	2.0705E-51	2.251923917	0.395	0.184	5.014E-47	Meta_CD4_C12
BCLAF1	2.599E-51	0.295814914	0.341	0.456	6.294E-47	Meta_CD4_C12
ZC3HAV1	9.3893E-51	0.321132486	0.333	0.422	2.274E-46	Meta_CD4_C12
WSB1	2.3841E-50	0.609572906	0.378	0.409	5.773E-46	Meta_CD4_C12
ODC1	3.0424E-50	0.602198818	0.369	0.430	7.367E-46	Meta_CD4_C12
HSP90AB1	4.213E-50	0.524155842	0.743	0.843	1.02E-45	Meta_CD4_C12
ZFAND5	7.2099E-50	0.525014973	0.325	0.374	1.746E-45	Meta_CD4_C12
C12orf57	1.1566E-48	0.457204347	0.551	0.722	2.801E-44	Meta_CD4_C12
PPP2R5C	3.8801E-48	0.563672777	0.431	0.508	9.396E-44	Meta_CD4_C12
SRSF5	4.0979E-48	0.401748534	0.645	0.762	9.923E-44	Meta_CD4_C12
TUBA4A	4.4918E-48	0.702452928	0.479	0.542	1.088E-43	Meta_CD4_C12

ADGRE5	8.9459E-48	0.526288634	0.497	0.564	2.166E-43	Meta_CD4_C12
PNP	1.0018E-47	0.474901882	0.259	0.300	2.426E-43	Meta_CD4_C12
MT-ND1	1.207E-47	0.856956676	0.937	0.951	2.923E-43	Meta_CD4_C12
IFRD1	1.7031E-47	0.994634603	0.356	0.269	4.124E-43	Meta_CD4_C12
KMT2E-AS1	1.8615E-47	1.059484021	0.258	0.158	4.508E-43	Meta_CD4_C12
HNRNPH1	1.8728E-47	0.446340026	0.303	0.328	4.535E-43	Meta_CD4_C12
CLK1	1.9467E-47	0.510084238	0.406	0.457	4.714E-43	Meta_CD4_C12
MBNL1	8.7648E-47	0.315375367	0.575	0.705	2.122E-42	Meta_CD4_C12
TUBA1B	2.1785E-46	0.715873069	0.548	0.592	5.275E-42	Meta_CD4_C12
DDX5	2.7865E-46	0.359227248	0.846	0.926	6.748E-42	Meta_CD4_C12
HERPUD2	3.0255E-46	0.276268584	0.275	0.368	7.326E-42	Meta_CD4_C12
FNBP4	3.2601E-46	0.279160977	0.260	0.342	7.894E-42	Meta_CD4_C12
HIST1H4C	3.3324E-46	0.622106995	0.471	0.570	8.069E-42	Meta_CD4_C12
GLUD1	3.4883E-46	0.371776369	0.242	0.323	8.447E-42	Meta_CD4_C12
ARHGEF1	5.0861E-46	0.293619615	0.501	0.631	1.232E-41	Meta_CD4_C12
TC2N	8.3698E-46	0.392476601	0.347	0.409	2.027E-41	Meta_CD4_C12
PRPF38B	1.0944E-45	0.351348035	0.357	0.443	2.65E-41	Meta_CD4_C12
CDC42EP3	1.241E-45	0.401613764	0.283	0.320	3.005E-41	Meta_CD4_C12
CIRBP	2.6225E-45	0.322788708	0.712	0.820	6.35E-41	Meta_CD4_C12
EPC1	3.9531E-45	0.309516385	0.468	0.576	9.572E-41	Meta_CD4_C12
SBDS	4.3552E-45	0.289826355	0.359	0.464	1.055E-40	Meta_CD4_C12
GNG2	6.6141E-45	0.256418744	0.433	0.556	1.602E-40	Meta_CD4_C12
EHD1	1.1365E-44	0.347188043	0.214	0.273	2.752E-40	Meta_CD4_C12
TAGAP	1.964E-44	0.793649813	0.562	0.583	4.756E-40	Meta_CD4_C12
NFATC2	2.8605E-44	0.276545876	0.261	0.341	6.927E-40	Meta_CD4_C12
BTG2	4.5523E-44	0.91891191	0.581	0.614	1.102E-39	Meta_CD4_C12
ARL4C	5.9015E-44	0.363313873	0.429	0.537	1.429E-39	Meta_CD4_C12
TNFAIP3	6.4088E-44	0.795375504	0.723	0.774	1.552E-39	Meta_CD4_C12
HSPB1	9.1033E-44	0.278531623	0.263	0.396	2.204E-39	Meta_CD4_C12
SYNE2	1.4493E-43	0.321427856	0.408	0.511	3.51E-39	Meta_CD4_C12
PPP1R10	2.1765E-43	0.771478054	0.238	0.205	5.27E-39	Meta_CD4_C12
HSPA5	2.3275E-43	0.391702358	0.523	0.648	5.636E-39	Meta_CD4_C12
MAPRE2	3.0473E-43	0.55423754	0.301	0.339	7.379E-39	Meta_CD4_C12
AC016831.7	3.4596E-43	0.400872243	0.379	0.445	8.377E-39	Meta_CD4_C12
YPEL5	3.5994E-43	0.400420988	0.454	0.588	8.716E-39	Meta_CD4_C12
AAK1	5.043E-43	0.389087129	0.299	0.387	1.221E-38	Meta_CD4_C12
SAT1	6.596E-43	0.951563313	0.609	0.643	1.597E-38	Meta_CD4_C12
NASP	6.9709E-43	0.420562584	0.269	0.320	1.688E-38	Meta_CD4_C12
SMARCA5	7.1814E-43	0.287333324	0.241	0.310	1.739E-38	Meta_CD4_C12
PRPF4B	7.3154E-43	0.30040841	0.253	0.329	1.771E-38	Meta_CD4_C12

POLR2A	1.8068E-42	0.810708196	0.239	0.227	4.375E-38	Meta_CD4_C12
INPP4B	4.2586E-42	0.296721932	0.193	0.255	1.031E-37	Meta_CD4_C12
MT-ND5	4.6984E-42	0.846913867	0.871	0.887	1.138E-37	Meta_CD4_C12
UTRN	4.935E-42	0.352780928	0.226	0.276	1.195E-37	Meta_CD4_C12
CD69	6.4158E-42	0.965397337	0.804	0.834	1.554E-37	Meta_CD4_C12
PTGER4	7.8288E-42	0.282305626	0.422	0.552	1.896E-37	Meta_CD4_C12
GOLGA8B	1.3E-41	0.287190999	0.175	0.238	3.148E-37	Meta_CD4_C12
HSP90AA1	1.437E-41	0.858060929	0.843	0.903	3.48E-37	Meta_CD4_C12
JMJD1C	3.2563E-41	0.392808536	0.310	0.357	7.885E-37	Meta_CD4_C12
CSKMT	3.86E-41	1.192877703	0.225	0.115	9.347E-37	Meta_CD4_C12
RALGAPA1	5.0464E-41	0.397695364	0.226	0.260	1.222E-36	Meta_CD4_C12
NEAT1	7.0478E-41	0.708775676	0.575	0.545	1.707E-36	Meta_CD4_C12
PLK3	7.9079E-41	0.594874304	0.260	0.256	1.915E-36	Meta_CD4_C12
CSRNP1	1.071E-40	0.608047541	0.443	0.507	2.593E-36	Meta_CD4_C12
MYLIP	2.0118E-40	0.642456563	0.287	0.263	4.872E-36	Meta_CD4_C12
SCML4	2.0641E-40	0.386200589	0.281	0.365	4.998E-36	Meta_CD4_C12
CCNH	3.2605E-40	0.333183459	0.296	0.381	7.895E-36	Meta_CD4_C12
LENG8	4.3561E-40	0.398143971	0.198	0.218	1.055E-35	Meta_CD4_C12
NR4A1	6.1583E-40	1.264039359	0.380	0.293	1.491E-35	Meta_CD4_C12
MACF1	6.2198E-40	0.332325039	0.248	0.334	1.506E-35	Meta_CD4_C12
KLF2	8.706E-40	1.183800277	0.402	0.313	2.108E-35	Meta_CD4_C12
JUNB	1.0766E-39	0.720061314	0.887	0.887	2.607E-35	Meta_CD4_C12
HSPH1	1.9356E-39	0.903801952	0.326	0.285	4.687E-35	Meta_CD4_C12
TUBA1C	2.4166E-39	0.478251359	0.233	0.273	5.852E-35	Meta_CD4_C12
AKAP9	4.2617E-39	0.294641219	0.260	0.341	1.032E-34	Meta_CD4_C12
MT-ND3	4.4955E-39	0.882810214	0.940	0.950	1.089E-34	Meta_CD4_C12
TRA2A	5.6115E-39	0.459365076	0.269	0.300	1.359E-34	Meta_CD4_C12
MT-ND6	7.802E-39	0.641783347	0.512	0.605	1.889E-34	Meta_CD4_C12
SOCS1	8.1034E-39	0.497750831	0.317	0.382	1.962E-34	Meta_CD4_C12
ZFP36	1.1535E-38	0.971132298	0.819	0.824	2.793E-34	Meta_CD4_C12
HSPA1A	1.5145E-38	1.365942359	0.320	0.371	3.667E-34	Meta_CD4_C12
MT-ND4	4.2224E-38	0.683113476	0.886	0.928	1.022E-33	Meta_CD4_C12
MYADM	6.8424E-38	0.767390538	0.450	0.427	1.657E-33	Meta_CD4_C12
AC020916.1	9.046E-38	0.71315848	0.207	0.226	2.19E-33	Meta_CD4_C12
KDM2A	1.5321E-37	0.429350276	0.223	0.231	3.71E-33	Meta_CD4_C12
SNHG9	1.5327E-37	0.566902103	0.222	0.213	3.711E-33	Meta_CD4_C12
NFKBIZ	1.6489E-37	0.409867482	0.282	0.365	3.993E-33	Meta_CD4_C12
NKTR	2.4906E-37	0.423662762	0.293	0.328	6.031E-33	Meta_CD4_C12
RBM33	3.5335E-37	0.267638299	0.172	0.210	8.556E-33	Meta_CD4_C12
GLS	5.5847E-37	0.305373461	0.149	0.182	1.352E-32	Meta_CD4_C12

AHNAK	6.5347E-37	0.550395678	0.518	0.530	1.582E-32	Meta_CD4_C12
IVNS1ABP	7.8635E-37	0.351338038	0.268	0.343	1.904E-32	Meta_CD4_C12
ERN1	1.2151E-36	0.361270553	0.204	0.256	2.942E-32	Meta_CD4_C12
MT-CYB	1.3818E-36	0.875320198	0.968	0.977	3.346E-32	Meta_CD4_C12
RHOB	1.7372E-36	1.182236566	0.250	0.142	4.207E-32	Meta_CD4_C12
TP53I13	2.2387E-36	0.269863872	0.141	0.183	5.421E-32	Meta_CD4_C12
AHR	4.6425E-36	0.387198142	0.181	0.205	1.124E-31	Meta_CD4_C12
DNAAF2	6.8339E-36	0.486039973	0.199	0.194	1.655E-31	Meta_CD4_C12
AC004687.1	3.5979E-35	0.790231557	0.187	0.162	8.712E-31	Meta_CD4_C12
OGA	4.4733E-35	0.270365378	0.208	0.266	1.083E-30	Meta_CD4_C12
ARL4A	6.6296E-35	0.376118621	0.232	0.247	1.605E-30	Meta_CD4_C12
HSPA1B	1.4525E-34	1.42537945	0.413	0.306	3.517E-30	Meta_CD4_C12
CDK17	2.0304E-34	0.281435676	0.262	0.340	4.917E-30	Meta_CD4_C12
ZFP36L1	4.2978E-34	0.674906457	0.602	0.680	1.041E-29	Meta_CD4_C12
MARCKSL1	4.3531E-34	0.672472001	0.218	0.181	1.054E-29	Meta_CD4_C12
MT-CO3	4.9194E-34	0.786655305	0.983	0.988	1.191E-29	Meta_CD4_C12
CDKN2AIP	1.0134E-33	0.414951075	0.187	0.180	2.454E-29	Meta_CD4_C12
OTUD6B-AS1	1.155E-33	0.309255716	0.164	0.215	2.797E-29	Meta_CD4_C12
ATP2B1-AS1	1.2242E-33	1.093912711	0.204	0.114	2.964E-29	Meta_CD4_C12
TSPYL2	1.7968E-33	0.831161172	0.395	0.327	4.351E-29	Meta_CD4_C12
ZC3H12A	2.063E-33	0.611615571	0.240	0.225	4.996E-29	Meta_CD4_C12
PCF11	2.0969E-33	0.480081276	0.214	0.215	5.078E-29	Meta_CD4_C12
H1FX	2.9836E-33	0.29432681	0.189	0.282	7.225E-29	Meta_CD4_C12
TOB1	5.909E-33	0.662447971	0.302	0.264	1.431E-28	Meta_CD4_C12
MLLT3	5.9215E-33	0.25476394	0.139	0.184	1.434E-28	Meta_CD4_C12
NFKBIB	6.0598E-33	0.322664579	0.180	0.204	1.467E-28	Meta_CD4_C12
RCC1	1.3161E-32	0.299031323	0.159	0.188	3.187E-28	Meta_CD4_C12
MT-ND4L	1.6359E-32	0.917236807	0.838	0.839	3.961E-28	Meta_CD4_C12
OFD1	1.7546E-32	0.268998189	0.203	0.256	4.249E-28	Meta_CD4_C12
MKNK2	1.7589E-32	0.378476952	0.150	0.168	4.259E-28	Meta_CD4_C12
MIDN	3.3261E-32	0.462124095	0.191	0.179	8.054E-28	Meta_CD4_C12
IER5	6.35E-32	0.719352016	0.181	0.118	1.538E-27	Meta_CD4_C12
ID2	6.6883E-32	0.737377329	0.615	0.596	1.62E-27	Meta_CD4_C12
LINC00861	5.3976E-31	0.261127074	0.151	0.214	1.307E-26	Meta_CD4_C12
WDR74	7.8717E-31	0.728568892	0.196	0.200	1.906E-26	Meta_CD4_C12
DDIT3	1.3795E-30	0.511189805	0.179	0.172	3.34E-26	Meta_CD4_C12
ANXA2R	1.4646E-30	0.309341423	0.178	0.228	3.546E-26	Meta_CD4_C12
SLC20A1	2.6399E-30	0.434109335	0.183	0.180	6.392E-26	Meta_CD4_C12
ASXL1	6.2182E-30	0.256958309	0.159	0.217	1.506E-25	Meta_CD4_C12
SNHG15	7.0849E-30	0.424025412	0.250	0.267	1.716E-25	Meta_CD4_C12

ZFP36L2	9.5639E-30	0.547443983	0.831	0.872	2.316E-25	Meta_CD4_C12
RHOT2	1.7301E-29	0.258286256	0.172	0.216	4.189E-25	Meta_CD4_C12
NOP56	1.8516E-29	0.308674711	0.180	0.212	4.484E-25	Meta_CD4_C12
UBE2S	6.0538E-29	0.800807127	0.195	0.133	1.466E-24	Meta_CD4_C12
TLE4	9.0567E-29	0.58298891	0.217	0.200	2.193E-24	Meta_CD4_C12
PPP1R15B	1.2942E-28	0.361460119	0.139	0.130	3.134E-24	Meta_CD4_C12
C6orf62	1.349E-28	0.285021673	0.149	0.174	3.267E-24	Meta_CD4_C12
RBM6	2.8888E-28	0.34050955	0.163	0.189	6.995E-24	Meta_CD4_C12
SLC52A2	4.2449E-28	0.71133443	0.067	0.156	1.028E-23	Meta_CD4_C12
HEXIM1	8.1892E-28	0.929099812	0.244	0.135	1.983E-23	Meta_CD4_C12
PNRC1	8.2244E-28	0.261526123	0.739	0.832	1.992E-23	Meta_CD4_C12
AC026979.2	1.0489E-27	0.676710151	0.217	0.172	2.54E-23	Meta_CD4_C12
ABHD5	1.0918E-27	0.361812843	0.103	0.111	2.644E-23	Meta_CD4_C12
PKN2	1.5681E-27	0.310345318	0.138	0.146	3.797E-23	Meta_CD4_C12
TRIM28	1.7683E-27	0.333294877	0.137	0.146	4.282E-23	Meta_CD4_C12
SYNE1	3.585E-27	0.267944731	0.199	0.258	8.681E-23	Meta_CD4_C12
ATF3	5.7091E-27	1.072331612	0.227	0.144	1.382E-22	Meta_CD4_C12
CHMP1B	7.4443E-27	0.390392108	0.207	0.228	1.803E-22	Meta_CD4_C12
CCNL2	7.5886E-27	0.286496124	0.167	0.181	1.838E-22	Meta_CD4_C12
TOB2	9.814E-27	0.4130302	0.141	0.132	2.376E-22	Meta_CD4_C12
EIF1	1.0798E-26	0.620223847	0.965	0.986	2.615E-22	Meta_CD4_C12
NXF1	2.3771E-26	0.507160169	0.161	0.125	5.756E-22	Meta_CD4_C12
BORCS5	3.9353E-26	0.359424692	0.136	0.167	9.529E-22	Meta_CD4_C12
BTG1	4.5278E-26	0.433151043	0.938	0.974	1.096E-21	Meta_CD4_C12
Z93241.1	6.722E-26	1.264417501	0.158	0.051	1.628E-21	Meta_CD4_C12
ILF3-DT	6.724E-26	0.356689914	0.129	0.119	1.628E-21	Meta_CD4_C12
DDX3Y	7.203E-26	0.522998379	0.238	0.213	1.744E-21	Meta_CD4_C12
TSC22D2	1.0567E-25	0.444222726	0.150	0.132	2.559E-21	Meta_CD4_C12
ANKRD13D	1.4076E-25	0.264406577	0.110	0.134	3.408E-21	Meta_CD4_C12
MAPK8IP3	2.8484E-25	0.258045501	0.093	0.100	6.897E-21	Meta_CD4_C12
FAM53C	3.0929E-25	0.435011763	0.139	0.144	7.49E-21	Meta_CD4_C12
RGS2	4.2312E-25	0.768224003	0.383	0.410	1.025E-20	Meta_CD4_C12
KLF10	5.205E-25	0.585347386	0.219	0.185	1.26E-20	Meta_CD4_C12
MT-ATP6	5.2445E-25	0.797667286	0.949	0.968	1.27E-20	Meta_CD4_C12
ANKRD36	5.3419E-25	0.325451604	0.109	0.103	1.294E-20	Meta_CD4_C12
KDM6B	9.4964E-25	0.405952267	0.204	0.202	2.3E-20	Meta_CD4_C12
ZBTB20	1.2569E-24	0.329190824	0.169	0.187	3.044E-20	Meta_CD4_C12
PBX4	1.261E-24	0.293036385	0.203	0.248	3.053E-20	Meta_CD4_C12
SLC1A5	1.3649E-24	0.391828819	0.183	0.194	3.305E-20	Meta_CD4_C12
NR4A3	3.4665E-24	0.512292894	0.192	0.186	8.394E-20	Meta_CD4_C12

IRF2BP2	4.1413E-24	0.351332101	0.246	0.271	1.003E-19	Meta_CD4_C12
MT-ATP8	4.6905E-24	0.979949606	0.798	0.792	1.136E-19	Meta_CD4_C12
HIST2H2AA4	6.5641E-24	0.986827653	0.197	0.093	1.589E-19	Meta_CD4_C12
SKIL	6.71E-24	0.479109425	0.221	0.218	1.625E-19	Meta_CD4_C12
CXCR4	6.7516E-24	0.33019592	0.762	0.833	1.635E-19	Meta_CD4_C12
MT-CO2	9.6251E-24	0.705364398	0.985	0.992	2.331E-19	Meta_CD4_C12
TMEM107	1.7433E-23	0.742235408	0.143	0.114	4.221E-19	Meta_CD4_C12
NUFIP2	2.6334E-23	0.484950163	0.170	0.149	6.377E-19	Meta_CD4_C12
SFI1	3.2968E-23	0.270384587	0.126	0.144	7.983E-19	Meta_CD4_C12
AC007384.1	5.246E-23	0.483210902	0.120	0.119	1.27E-18	Meta_CD4_C12
TARSL2	6.1835E-23	0.317902467	0.161	0.180	1.497E-18	Meta_CD4_C12
ITGB2-AS1	1.2978E-22	0.316401784	0.113	0.118	3.143E-18	Meta_CD4_C12
ARIH1	2.4696E-22	0.303509872	0.135	0.145	5.98E-18	Meta_CD4_C12
NABP1	5.6192E-22	0.412833669	0.132	0.147	1.361E-17	Meta_CD4_C12
NR4A2	7.9609E-22	0.580069621	0.548	0.579	1.928E-17	Meta_CD4_C12
DUSP6	8.0667E-22	0.846489114	0.156	0.089	1.953E-17	Meta_CD4_C12
INTS6	1.3972E-21	0.569581805	0.157	0.147	3.383E-17	Meta_CD4_C12
TBX21	3.774E-21	0.261652977	0.158	0.187	9.139E-17	Meta_CD4_C12
TUBB2A	4.6857E-21	0.754884344	0.195	0.118	1.135E-16	Meta_CD4_C12
LUC7L	9.4614E-21	0.275237234	0.136	0.157	2.291E-16	Meta_CD4_C12
AC087239.1	1.2294E-20	0.591550191	0.111	0.056	2.977E-16	Meta_CD4_C12
PER1	1.4914E-20	0.360907854	0.201	0.224	3.611E-16	Meta_CD4_C12
AC245014.3	2.2203E-20	0.844665194	0.117	0.046	5.376E-16	Meta_CD4_C12
MT-CO1	2.2309E-20	0.746674523	0.990	0.995	5.402E-16	Meta_CD4_C12
NDRG1	2.554E-20	0.253793097	0.126	0.155	6.185E-16	Meta_CD4_C12
ANXA1	2.8655E-20	0.578975429	0.648	0.668	6.939E-16	Meta_CD4_C12
GABPB1-AS1	6.0742E-20	0.432069418	0.169	0.199	1.471E-15	Meta_CD4_C12
CDC14A	1.1419E-19	0.321617312	0.143	0.148	2.765E-15	Meta_CD4_C12
AL118516.1	1.9609E-19	0.554255044	0.172	0.133	4.748E-15	Meta_CD4_C12
MYBL1	3.5326E-19	0.290126839	0.098	0.108	8.554E-15	Meta_CD4_C12
ANKRD36B	5.5845E-19	0.339844287	0.117	0.102	1.352E-14	Meta_CD4_C12
NFKBID	7.1163E-19	0.292590698	0.095	0.108	1.723E-14	Meta_CD4_C12
HIST3H2A	1.3546E-18	0.252698754	0.102	0.132	3.28E-14	Meta_CD4_C12
DDIT4	2.8184E-18	0.352279152	0.541	0.590	6.825E-14	Meta_CD4_C12
ANKRD36C	3.8418E-18	0.337159924	0.114	0.104	9.303E-14	Meta_CD4_C12
MBNL2	9.6705E-18	0.293966728	0.128	0.144	2.342E-13	Meta_CD4_C12
AC025164.1	1.145E-17	0.479917066	0.117	0.095	2.772E-13	Meta_CD4_C12
C3orf58	1.6003E-17	0.300346279	0.100	0.101	3.875E-13	Meta_CD4_C12
HSPA8	2.1556E-17	0.395017771	0.846	0.906	5.22E-13	Meta_CD4_C12
DUSP2	3.1647E-17	0.347018987	0.583	0.638	7.663E-13	Meta_CD4_C12

RBKS	3.7056E-17	0.959915896	0.133	0.147	8.973E-13	Meta_CD4_C12
THAP2	4.3943E-17	0.316022692	0.109	0.085	1.064E-12	Meta_CD4_C12
ARRDC3	6.1451E-17	0.494643595	0.149	0.114	1.488E-12	Meta_CD4_C12
UBC	7.5947E-17	0.373035715	0.936	0.967	1.839E-12	Meta_CD4_C12
BCL9L	4.3719E-16	0.283377889	0.128	0.118	1.059E-11	Meta_CD4_C12
PMAIP1	4.4078E-16	0.510882949	0.247	0.257	1.067E-11	Meta_CD4_C12
EPM2AIP1	5.1215E-16	0.277957096	0.103	0.102	1.24E-11	Meta_CD4_C12
SMAD7	2.6646E-15	0.399954445	0.144	0.100	6.452E-11	Meta_CD4_C12
CDKN1A	2.8101E-15	0.391112292	0.174	0.189	6.805E-11	Meta_CD4_C12
LMNA	5.6227E-15	0.957337638	0.379	0.274	1.362E-10	Meta_CD4_C12
ADRB2	5.9258E-15	0.447843806	0.121	0.077	1.435E-10	Meta_CD4_C12
ANKRD37	6.2643E-15	0.282405312	0.094	0.136	1.517E-10	Meta_CD4_C12
FNIP1	4.7325E-14	0.282403811	0.108	0.117	1.146E-09	Meta_CD4_C12
MYC	4.9335E-14	0.287525224	0.144	0.180	1.195E-09	Meta_CD4_C12
ZNF276	7.4623E-14	0.295492929	0.120	0.123	1.807E-09	Meta_CD4_C12
CD83	1.7459E-13	0.300956613	0.110	0.102	4.228E-09	Meta_CD4_C12
IER3	7.1063E-13	0.756792345	0.115	0.062	1.721E-08	Meta_CD4_C12
FBXO32	1.9753E-12	0.278789404	0.133	0.137	4.783E-08	Meta_CD4_C12
KLRG1	2.6475E-12	0.277956561	0.170	0.176	6.411E-08	Meta_CD4_C12
ANKRD28	8.2373E-12	0.279655705	0.147	0.177	1.995E-07	Meta_CD4_C12
IL7R	1.015E-10	0.40496455	0.598	0.575	2.458E-06	Meta_CD4_C12
SGK1	1.288E-10	0.538533359	0.141	0.102	3.119E-06	Meta_CD4_C12
CNBD2	2.3587E-10	0.328923416	0.127	0.103	5.711E-06	Meta_CD4_C12
ID3	3.9451E-09	0.277107639	0.082	0.106	9.553E-05	Meta_CD4_C12
TNFSF9	5.1478E-07	0.354578926	0.139	0.137	0.0124654	Meta_CD4_C12
IFNG	6.4421E-06	0.468985387	0.234	0.251	0.1559953	Meta_CD4_C12
CCL4L2	1.5598E-05	0.802361248	0.177	0.176	0.3776956	Meta_CD4_C12
GZMK	0.00013986	0.307626619	0.454	0.372	1	Meta_CD4_C12
CCL4	0.00019391	0.737844651	0.430	0.432	1	Meta_CD4_C12
AREG	0.00320522	0.317819125	0.162	0.179	1	Meta_CD4_C12
CD8B	3.559E-119	1.012384158	0.859	0.369	8.62E-115	Meta_CD8_C1
KLF2	2.154E-117	1.701131922	0.782	0.308	5.22E-113	Meta_CD8_C1
CD8A	5.133E-113	0.490256047	0.885	0.435	1.24E-108	Meta_CD8_C1
NELL2	9.409E-103	1.124013225	0.554	0.111	2.278E-98	Meta_CD8_C1
CCR7	1.8433E-97	1.28956599	0.604	0.158	4.464E-93	Meta_CD8_C1
LEF1	1.575E-77	1.053870923	0.521	0.148	3.814E-73	Meta_CD8_C1
RPS12	1.0674E-74	0.543999867	1.000	0.994	2.585E-70	Meta_CD8_C1
TCF7	1.33E-61	0.974342433	0.583	0.221	3.221E-57	Meta_CD8_C1
LINC02446	2.8637E-60	0.893203491	0.409	0.105	6.934E-56	Meta_CD8_C1
SELL	5.6006E-60	1.280088989	0.552	0.180	1.356E-55	Meta_CD8_C1

RPL13	1.8269E-58	0.391241447	1.000	0.997	4.424E-54	Meta_CD8_C1
RPL32	1.3328E-57	0.436958082	0.999	0.993	3.227E-53	Meta_CD8_C1
RPS8	7.5867E-56	0.439894835	1.000	0.988	1.837E-51	Meta_CD8_C1
RPS23	6.6117E-55	0.461419442	0.996	0.979	1.601E-50	Meta_CD8_C1
RPS5	8.4334E-55	0.453371364	0.996	0.970	2.042E-50	Meta_CD8_C1
S1PR1	1.223E-53	0.785975382	0.435	0.123	2.961E-49	Meta_CD8_C1
RPL19	2.1193E-53	0.381427563	1.000	0.994	5.132E-49	Meta_CD8_C1
RPL5	1.5116E-52	0.45037408	0.989	0.961	3.66E-48	Meta_CD8_C1
RPL18A	1.2063E-51	0.394316146	0.997	0.989	2.921E-47	Meta_CD8_C1
RPS13	1.4606E-51	0.443155305	0.996	0.979	3.537E-47	Meta_CD8_C1
RPS3A	1.4888E-51	0.489028562	0.997	0.978	3.605E-47	Meta_CD8_C1
RPS14	4.929E-51	0.370463476	1.000	0.995	1.194E-46	Meta_CD8_C1
RPL34	3.9905E-49	0.359989988	1.000	0.993	9.663E-45	Meta_CD8_C1
RPL14	1.2217E-48	0.40680821	0.998	0.979	2.958E-44	Meta_CD8_C1
IL7R	5.3451E-48	0.407636789	0.876	0.571	1.294E-43	Meta_CD8_C1
RPS18	2.0192E-47	0.364447364	0.999	0.994	4.89E-43	Meta_CD8_C1
RPL30	2.4594E-47	0.3931561	0.998	0.988	5.955E-43	Meta_CD8_C1
RPL11	1.8134E-45	0.316952301	0.999	0.994	4.391E-41	Meta_CD8_C1
RPL10	8.9248E-45	0.291906302	1.000	0.998	2.161E-40	Meta_CD8_C1
CD55	1.9027E-44	0.784723602	0.565	0.267	4.607E-40	Meta_CD8_C1
RPL29	1.111E-43	0.351589057	0.998	0.978	2.69E-39	Meta_CD8_C1
RPS3	1.4996E-43	0.347559379	1.000	0.994	3.631E-39	Meta_CD8_C1
RPS4X	7.3092E-43	0.312961062	0.999	0.992	1.77E-38	Meta_CD8_C1
RPL18	1.7515E-42	0.34138159	0.999	0.985	4.241E-38	Meta_CD8_C1
RPS6	6.5278E-42	0.348743453	0.998	0.992	1.581E-37	Meta_CD8_C1
RPS27A	7.6859E-41	0.311959193	0.999	0.994	1.861E-36	Meta_CD8_C1
EEF1B2	1.1347E-35	0.471668263	0.966	0.897	2.748E-31	Meta_CD8_C1
PABPC1	1.2812E-35	0.508847658	0.965	0.895	3.103E-31	Meta_CD8_C1
YBX3	1.3751E-35	0.572001817	0.286	0.060	3.33E-31	Meta_CD8_C1
RPL10A	1.7747E-35	0.341720764	0.996	0.975	4.298E-31	Meta_CD8_C1
RPL3	2.1722E-35	0.328803084	0.998	0.986	5.26E-31	Meta_CD8_C1
RPL6	2.561E-35	0.324336954	0.993	0.970	6.201E-31	Meta_CD8_C1
RIPOR2	2.0298E-34	0.69195639	0.515	0.244	4.915E-30	Meta_CD8_C1
RPS28	1.5694E-33	0.397981951	0.994	0.964	3.8E-29	Meta_CD8_C1
RPL7A	3.1064E-33	0.319975834	0.995	0.974	7.522E-29	Meta_CD8_C1
ARMH1	1.8151E-32	0.578069595	0.276	0.081	4.395E-28	Meta_CD8_C1
RPL12	3.217E-32	0.294535391	0.996	0.989	7.79E-28	Meta_CD8_C1
RPS25	3.8057E-32	0.356477453	0.995	0.969	9.216E-28	Meta_CD8_C1
RPL35A	7.5191E-32	0.312699189	0.998	0.986	1.821E-27	Meta_CD8_C1
RPS15A	7.5743E-32	0.278640152	0.998	0.993	1.834E-27	Meta_CD8_C1

NACA	8.3282E-32	0.312721767	0.984	0.955	2.017E-27	Meta_CD8_C1
RPSA	3.1999E-31	0.329229349	0.994	0.962	7.749E-27	Meta_CD8_C1
ACTN1	4.4905E-31	0.495262063	0.185	0.032	1.087E-26	Meta_CD8_C1
RPS7	1.4601E-30	0.282660241	0.997	0.984	3.536E-26	Meta_CD8_C1
RPL36	2.9839E-30	0.312009815	0.999	0.978	7.226E-26	Meta_CD8_C1
RPS21	3.9824E-30	0.422917911	0.989	0.947	9.643E-26	Meta_CD8_C1
RPS24	5.2418E-28	0.268308984	0.997	0.981	1.269E-23	Meta_CD8_C1
RPL9	3.5452E-27	0.285907929	0.994	0.973	8.585E-23	Meta_CD8_C1
NOSIP	4.5097E-27	0.681252295	0.613	0.400	1.092E-22	Meta_CD8_C1
RASGRP2	1.3176E-26	0.525365983	0.406	0.194	3.191E-22	Meta_CD8_C1
RACK1	2.8988E-26	0.412812882	0.912	0.803	7.02E-22	Meta_CD8_C1
IFITM1	9.5237E-26	0.45489028	0.923	0.805	2.306E-21	Meta_CD8_C1
CDC25B	6.1273E-25	0.53399947	0.414	0.192	1.484E-20	Meta_CD8_C1
AIF1	1.0272E-24	0.442596452	0.218	0.055	2.487E-20	Meta_CD8_C1
C1orf162	5.5169E-24	0.444286683	0.239	0.075	1.336E-19	Meta_CD8_C1
RPL37	6.1037E-24	0.330715059	0.993	0.971	1.478E-19	Meta_CD8_C1
LDLRAP1	1.4872E-23	0.495940986	0.374	0.152	3.601E-19	Meta_CD8_C1
RPL22	1.506E-23	0.407634796	0.958	0.889	3.647E-19	Meta_CD8_C1
RPS9	7.4961E-23	0.266418083	0.992	0.980	1.815E-18	Meta_CD8_C1
EEF1A1	1.3703E-22	0.420718588	0.964	0.911	3.318E-18	Meta_CD8_C1
RPL4	3.4445E-22	0.312442775	0.976	0.926	8.341E-18	Meta_CD8_C1
KLF3	5.5081E-22	0.505411202	0.360	0.172	1.334E-17	Meta_CD8_C1
RPS27	6.5659E-22	0.29686879	1.000	0.995	1.59E-17	Meta_CD8_C1
RPL39	1.1852E-21	0.394613634	0.954	0.893	2.87E-17	Meta_CD8_C1
PASK	1.7742E-21	0.521610896	0.332	0.132	4.296E-17	Meta_CD8_C1
RPLP0	2.141E-21	0.297136175	0.989	0.948	5.185E-17	Meta_CD8_C1
NSG1	4.1546E-21	0.441841083	0.234	0.068	1.006E-16	Meta_CD8_C1
RPL23A	5.9E-21	0.252244562	0.998	0.987	1.429E-16	Meta_CD8_C1
CD248	1.484E-20	0.468304012	0.178	0.038	3.593E-16	Meta_CD8_C1
FLT3LG	1.4986E-20	0.40803767	0.640	0.441	3.629E-16	Meta_CD8_C1
LDHB	1.5766E-20	0.461869985	0.891	0.748	3.818E-16	Meta_CD8_C1
FXYD2	3.4932E-19	0.414641222	0.136	0.024	8.459E-15	Meta_CD8_C1
LSR	6.2802E-19	0.341272396	0.241	0.100	1.521E-14	Meta_CD8_C1
APBA2	3.1153E-18	0.301501916	0.198	0.070	7.544E-14	Meta_CD8_C1
PRMT2	3.548E-18	0.510741616	0.652	0.438	8.591E-14	Meta_CD8_C1
TRABD2A	4.9306E-18	0.291857883	0.160	0.053	1.194E-13	Meta_CD8_C1
BEX3	6.37E-18	0.250510269	0.139	0.034	1.542E-13	Meta_CD8_C1
RASA3	6.438E-18	0.420734817	0.384	0.193	1.559E-13	Meta_CD8_C1
BEX2	7.1441E-18	0.290252441	0.237	0.106	1.73E-13	Meta_CD8_C1
PCSK1N	9.2795E-18	0.405183818	0.130	0.022	2.247E-13	Meta_CD8_C1

PDE3B	1.7918E-17	0.389850702	0.416	0.238	4.339E-13	Meta_CD8_C1
BACH2	2.1654E-17	0.362966307	0.226	0.088	5.243E-13	Meta_CD8_C1
P2RY8	2.4225E-17	0.414077196	0.404	0.226	5.866E-13	Meta_CD8_C1
AES	3.2167E-17	0.460536409	0.768	0.590	7.789E-13	Meta_CD8_C1
FXYD7	7.2618E-17	0.347019584	0.158	0.042	1.758E-12	Meta_CD8_C1
NME2	1.5097E-16	0.409695595	0.680	0.508	3.656E-12	Meta_CD8_C1
NOP53	1.6517E-16	0.37676584	0.848	0.696	4E-12	Meta_CD8_C1
LYAR	3.3776E-16	0.483014755	0.485	0.316	8.179E-12	Meta_CD8_C1
AC119396.1	3.9197E-16	0.302417851	0.174	0.054	9.491E-12	Meta_CD8_C1
LIMD2	1.4571E-15	0.309179483	0.868	0.714	3.528E-11	Meta_CD8_C1
GYPC	1.8567E-15	0.299768665	0.790	0.639	4.496E-11	Meta_CD8_C1
ZNF683	5.2186E-15	0.37038024	0.216	0.096	1.264E-10	Meta_CD8_C1
PLAC8	1.1498E-14	0.405067669	0.318	0.140	2.784E-10	Meta_CD8_C1
EEF1G	1.4461E-14	0.421776915	0.723	0.648	3.502E-10	Meta_CD8_C1
MT-ND4L	2.0467E-14	0.330853205	0.929	0.838	4.956E-10	Meta_CD8_C1
RPS2	2.1028E-14	0.283146538	0.994	0.983	5.092E-10	Meta_CD8_C1
EEF2	2.2825E-14	0.260358602	0.963	0.902	5.527E-10	Meta_CD8_C1
STK38	4.2805E-14	0.321546603	0.322	0.183	1.037E-09	Meta_CD8_C1
SARAF	4.3444E-14	0.288137066	0.980	0.926	1.052E-09	Meta_CD8_C1
MYC	1.3385E-13	0.425242308	0.320	0.177	3.241E-09	Meta_CD8_C1
ZFP36L2	1.4288E-13	0.296867187	0.958	0.870	3.46E-09	Meta_CD8_C1
ABLIM1	1.6864E-13	0.417311677	0.483	0.307	4.084E-09	Meta_CD8_C1
TOB1	3.8858E-13	0.445864586	0.419	0.263	9.409E-09	Meta_CD8_C1
LITAF	1.0389E-12	0.414914449	0.694	0.490	2.516E-08	Meta_CD8_C1
TXK	1.2953E-12	0.283121386	0.231	0.105	3.137E-08	Meta_CD8_C1
EIF3E	2.5609E-12	0.254573469	0.801	0.678	6.201E-08	Meta_CD8_C1
TMEM123	3.4185E-12	0.3380084	0.646	0.469	8.278E-08	Meta_CD8_C1
SATB1	3.8504E-12	0.345695535	0.356	0.203	9.324E-08	Meta_CD8_C1
ATM	7.0715E-12	0.363940747	0.405	0.252	1.712E-07	Meta_CD8_C1
NUCB2	2.3606E-11	0.330653195	0.425	0.275	5.716E-07	Meta_CD8_C1
CARS	4.2E-11	0.282938151	0.270	0.159	1.017E-06	Meta_CD8_C1
NPM1	4.9232E-11	0.268209242	0.953	0.880	1.192E-06	Meta_CD8_C1
RPS4Y1	7.0214E-11	0.296518505	0.596	0.538	1.7E-06	Meta_CD8_C1
MCUB	7.3274E-11	0.354514761	0.595	0.417	1.774E-06	Meta_CD8_C1
LINC00861	9.9685E-11	0.321470947	0.362	0.211	2.414E-06	Meta_CD8_C1
LIME1	1.0057E-10	0.256673009	0.526	0.371	2.435E-06	Meta_CD8_C1
JUNB	7.1909E-10	0.263367329	0.948	0.886	1.741E-05	Meta_CD8_C1
EIF4B	1.3784E-09	0.259841956	0.722	0.608	3.338E-05	Meta_CD8_C1
LEPROTL1	3.5039E-09	0.281763391	0.842	0.726	8.485E-05	Meta_CD8_C1
SORL1	8.2018E-09	0.251533827	0.326	0.194	0.0001986	Meta_CD8_C1

MAL	1.0139E-08	0.304304079	0.260	0.130	0.0002455	Meta_CD8_C1
PXN	1.4755E-08	0.271144181	0.261	0.137	0.0003573	Meta_CD8_C1
IMPDH2	1.942E-08	0.291764215	0.342	0.208	0.0004703	Meta_CD8_C1
RPL17	4.1503E-08	0.285959997	0.920	0.859	0.001005	Meta_CD8_C1
TPST2	4.3889E-08	0.290716172	0.374	0.259	0.0010628	Meta_CD8_C1
PRKCB	5.4668E-07	0.288759655	0.267	0.165	0.0132377	Meta_CD8_C1
RPL41	5.0471E-06	0.410774167	0.962	0.913	0.1222161	Meta_CD8_C1
NDFIP1	7.0388E-06	0.30942254	0.631	0.496	0.1704436	Meta_CD8_C1
SLC2A3	1.0377E-05	0.280158637	0.753	0.644	0.2512764	Meta_CD8_C1
RPS26	0.00619683	0.277514179	0.951	0.918	1	Meta_CD8_C1
GZMK	1.874E-127	1.829534315	0.839	0.334	4.54E-123	Meta_CD8_C2
CCL5	6.4911E-85	0.624964426	0.988	0.732	1.572E-80	Meta_CD8_C2
NKG7	4.929E-77	0.888516084	0.912	0.545	1.194E-72	Meta_CD8_C2
CST7	2.1359E-70	1.070074469	0.856	0.683	5.172E-66	Meta_CD8_C2
CD8A	2.6252E-47	0.735009298	0.729	0.417	6.357E-43	Meta_CD8_C2
CMC1	2.7067E-46	1.218128202	0.394	0.217	6.554E-42	Meta_CD8_C2
DDX24	1.0434E-42	0.313040917	0.569	0.657	2.527E-38	Meta_CD8_C2
CCL4	1.6659E-40	1.169704485	0.739	0.405	4.034E-36	Meta_CD8_C2
TUBA4A	7.8307E-37	0.823555846	0.544	0.540	1.896E-32	Meta_CD8_C2
RBM39	1.5835E-36	0.359341087	0.648	0.715	3.835E-32	Meta_CD8_C2
GZMM	1.9519E-35	0.657506963	0.622	0.540	4.727E-31	Meta_CD8_C2
CD74	1.2714E-34	0.617871224	0.921	0.870	3.079E-30	Meta_CD8_C2
LITAF	9.5875E-33	0.587471292	0.538	0.490	2.322E-28	Meta_CD8_C2
SRSF7	9.8965E-33	0.473105805	0.760	0.809	2.396E-28	Meta_CD8_C2
DUSP2	1.1665E-32	0.790221025	0.697	0.632	2.825E-28	Meta_CD8_C2
SRSF5	2.3014E-32	0.327617558	0.678	0.767	5.573E-28	Meta_CD8_C2
SAP18	5.4666E-32	0.255920875	0.604	0.708	1.324E-27	Meta_CD8_C2
YPEL5	2.2817E-31	0.57447025	0.512	0.592	5.525E-27	Meta_CD8_C2
HLA-DPB1	8.8802E-31	0.850275757	0.646	0.429	2.15E-26	Meta_CD8_C2
GZMH	1.5705E-30	0.901168762	0.507	0.276	3.803E-26	Meta_CD8_C2
SRSF2	2.6356E-30	0.416460347	0.577	0.641	6.382E-26	Meta_CD8_C2
NFATC2	3.9982E-30	0.404688997	0.351	0.339	9.682E-26	Meta_CD8_C2
PIK3R1	7.5463E-30	0.356718016	0.393	0.427	1.827E-25	Meta_CD8_C2
ITM2C	8.725E-30	0.876604873	0.484	0.366	2.113E-25	Meta_CD8_C2
SRRT	1.8704E-29	0.814889081	0.319	0.280	4.529E-25	Meta_CD8_C2
CLDND1	3.0955E-29	0.27114279	0.378	0.470	7.496E-25	Meta_CD8_C2
TGFB1	6.7411E-29	0.412934405	0.404	0.461	1.632E-24	Meta_CD8_C2
TUBB4B	3.3496E-28	0.316968269	0.406	0.469	8.111E-24	Meta_CD8_C2
CSRNP1	3.2804E-27	0.262386197	0.416	0.514	7.943E-23	Meta_CD8_C2
SRRM1	3.2928E-27	0.29943823	0.521	0.592	7.973E-23	Meta_CD8_C2

ENC1	4.8996E-27	0.621859263	0.161	0.041	1.186E-22	Meta_CD8_C2
SRRM2	8.4847E-27	0.250136269	0.401	0.454	2.055E-22	Meta_CD8_C2
ADGRE5	1.4714E-26	0.280152184	0.509	0.568	3.563E-22	Meta_CD8_C2
PPP2R5C	2.8877E-26	0.389531333	0.478	0.509	6.992E-22	Meta_CD8_C2
HLA-F	8.6847E-26	0.256786431	0.531	0.613	2.103E-21	Meta_CD8_C2
FYN	1.3973E-25	0.329082391	0.533	0.571	3.383E-21	Meta_CD8_C2
HERPUD1	3.8611E-25	0.461019353	0.530	0.538	9.35E-21	Meta_CD8_C2
EZR	4.5903E-25	0.252704491	0.557	0.648	1.112E-20	Meta_CD8_C2
CMC2	6.7305E-25	0.312914424	0.308	0.349	1.63E-20	Meta_CD8_C2
SH2D1A	7.4703E-25	0.553694709	0.376	0.322	1.809E-20	Meta_CD8_C2
JMJD6	7.6983E-25	0.40774494	0.275	0.248	1.864E-20	Meta_CD8_C2
DKK3	1.1427E-24	0.59373506	0.115	0.020	2.767E-20	Meta_CD8_C2
RNF125	1.2708E-24	0.329002278	0.303	0.323	3.077E-20	Meta_CD8_C2
LDHA	2.9726E-24	0.370834439	0.692	0.797	7.198E-20	Meta_CD8_C2
DNAJB6	4.4208E-24	0.429751667	0.446	0.471	1.07E-19	Meta_CD8_C2
IDI1	1.0781E-23	0.280119194	0.285	0.315	2.611E-19	Meta_CD8_C2
CXCR4	1.3562E-23	0.533871413	0.854	0.830	3.284E-19	Meta_CD8_C2
APMAP	1.8497E-23	0.503198166	0.383	0.345	4.479E-19	Meta_CD8_C2
SBDS	2.8145E-23	0.258528778	0.416	0.466	6.815E-19	Meta_CD8_C2
CD8B	3.2609E-23	0.808718727	0.606	0.356	7.896E-19	Meta_CD8_C2
CD44	4.6102E-23	0.321129724	0.726	0.768	1.116E-18	Meta_CD8_C2
AKNA	5.7115E-23	0.306536401	0.382	0.412	1.383E-18	Meta_CD8_C2
PYHIN1	9.701E-23	0.356167766	0.273	0.300	2.349E-18	Meta_CD8_C2
SERTAD1	1.1714E-22	0.501609201	0.349	0.335	2.836E-18	Meta_CD8_C2
MALAT1	1.3986E-22	0.467407744	0.999	1.000	3.387E-18	Meta_CD8_C2
HERPUD2	1.687E-22	0.260912353	0.332	0.369	4.085E-18	Meta_CD8_C2
ARPC5L	3.3603E-22	0.279443748	0.437	0.483	8.137E-18	Meta_CD8_C2
HSPA5	3.4876E-22	0.434410641	0.579	0.651	8.445E-18	Meta_CD8_C2
BTN3A1	3.8259E-22	0.254397601	0.189	0.199	9.264E-18	Meta_CD8_C2
EOMES	4.6744E-22	0.780119875	0.248	0.087	1.132E-17	Meta_CD8_C2
RUNX3	5.1762E-22	0.380072764	0.422	0.426	1.253E-17	Meta_CD8_C2
TSPYL2	1.3014E-21	0.556451658	0.346	0.327	3.151E-17	Meta_CD8_C2
ZEB2	1.8216E-21	0.507640519	0.230	0.150	4.411E-17	Meta_CD8_C2
HLA-DPA1	2.5326E-21	0.690683901	0.624	0.463	6.133E-17	Meta_CD8_C2
ITGB2	2.969E-21	0.311328021	0.610	0.612	7.189E-17	Meta_CD8_C2
IDS	5.1519E-21	0.300970837	0.509	0.585	1.248E-16	Meta_CD8_C2
CEMIP2	5.5888E-21	0.407978019	0.306	0.299	1.353E-16	Meta_CD8_C2
IER2	6.1614E-21	0.413333535	0.626	0.665	1.492E-16	Meta_CD8_C2
AP3S1	6.5422E-21	0.289495968	0.289	0.310	1.584E-16	Meta_CD8_C2
LYAR	2.1185E-20	0.577539458	0.379	0.314	5.13E-16	Meta_CD8_C2

EIF1	6.9953E-20	0.430301966	0.969	0.987	1.694E-15	Meta_CD8_C2
GGA2	7.7148E-20	0.512033926	0.267	0.203	1.868E-15	Meta_CD8_C2
SAMD3	1.1686E-19	0.5503899	0.282	0.192	2.83E-15	Meta_CD8_C2
VAMP2	1.2434E-19	0.291123365	0.368	0.412	3.011E-15	Meta_CD8_C2
CXCR3	1.5722E-19	0.451903504	0.498	0.479	3.807E-15	Meta_CD8_C2
H3F3B	5.9824E-19	0.332640454	0.957	0.981	1.449E-14	Meta_CD8_C2
NCBP2-AS2	1.1277E-18	0.257904468	0.240	0.254	2.731E-14	Meta_CD8_C2
UBC	1.4697E-18	0.300838703	0.933	0.969	3.559E-14	Meta_CD8_C2
APOBEC3G	2.0869E-18	0.490731321	0.497	0.441	5.053E-14	Meta_CD8_C2
CCL4L2	2.1899E-18	1.34203995	0.332	0.163	5.303E-14	Meta_CD8_C2
MARCKSL1	2.8944E-18	0.412065511	0.215	0.179	7.009E-14	Meta_CD8_C2
PLEK	5.3976E-18	0.552245525	0.195	0.097	1.307E-13	Meta_CD8_C2
PLP2	6.2468E-18	0.31363746	0.531	0.564	1.513E-13	Meta_CD8_C2
B3GNT2	1.6467E-17	0.250801052	0.239	0.247	3.987E-13	Meta_CD8_C2
HLA-DRB1	1.7407E-17	0.629895889	0.622	0.420	4.215E-13	Meta_CD8_C2
ZDHHC7	1.8768E-17	0.280055543	0.159	0.139	4.545E-13	Meta_CD8_C2
DUSP4	2.147E-17	0.574340467	0.407	0.372	5.199E-13	Meta_CD8_C2
ARAP2	2.3306E-17	0.287301624	0.306	0.323	5.643E-13	Meta_CD8_C2
SYNE1	2.7919E-17	0.349234438	0.266	0.256	6.761E-13	Meta_CD8_C2
ARID5A	4.2286E-17	0.250156123	0.372	0.403	1.024E-12	Meta_CD8_C2
HLA-C	5.6618E-17	0.280950085	0.991	0.994	1.371E-12	Meta_CD8_C2
CD81	8.5518E-17	0.263427777	0.146	0.133	2.071E-12	Meta_CD8_C2
KLRG1	1.9334E-16	0.724655283	0.281	0.166	4.682E-12	Meta_CD8_C2
CRTAM	2.2195E-16	0.645377242	0.211	0.094	5.374E-12	Meta_CD8_C2
H2AFX	2.3163E-16	0.33817318	0.241	0.235	5.609E-12	Meta_CD8_C2
TBCD	3.2136E-16	0.307085653	0.198	0.185	7.782E-12	Meta_CD8_C2
HAUS3	1.2744E-15	0.267050684	0.190	0.178	3.086E-11	Meta_CD8_C2
IFRD1	1.9682E-15	0.408005736	0.278	0.270	4.766E-11	Meta_CD8_C2
SLC7A5	4.5584E-15	0.61648604	0.303	0.264	1.104E-10	Meta_CD8_C2
GABARAPL1	1.457E-14	0.341827711	0.300	0.294	3.528E-10	Meta_CD8_C2
RPL23A	1.5533E-14	0.251237047	0.974	0.988	3.761E-10	Meta_CD8_C2
PLA2G16	1.6273E-14	0.30727506	0.214	0.199	3.94E-10	Meta_CD8_C2
RPL27A	1.8697E-14	0.345316384	0.895	0.942	4.527E-10	Meta_CD8_C2
CLEC2B	2.2069E-14	0.26193326	0.578	0.634	5.344E-10	Meta_CD8_C2
LYST	3.372E-14	0.498533997	0.277	0.217	8.165E-10	Meta_CD8_C2
RPL41	3.7065E-14	0.304777303	0.871	0.917	8.975E-10	Meta_CD8_C2
BCL9L	9.6584E-14	0.2539306	0.144	0.116	2.339E-09	Meta_CD8_C2
PPP1R14B	1.9332E-13	0.464662365	0.153	0.100	4.681E-09	Meta_CD8_C2
HLA-B	2.7411E-13	0.275632938	0.995	0.997	6.638E-09	Meta_CD8_C2
MATK	2.8135E-13	0.281206484	0.294	0.276	6.813E-09	Meta_CD8_C2

SLAMF7	4.3592E-13	0.271159349	0.158	0.139	1.056E-08	Meta_CD8_C2
F2R	1.0852E-12	0.420038004	0.246	0.188	2.628E-08	Meta_CD8_C2
CTSW	1.4094E-12	0.450970751	0.545	0.427	3.413E-08	Meta_CD8_C2
PILRB	1.823E-12	0.294118709	0.114	0.092	4.414E-08	Meta_CD8_C2
DDX3Y	2.2866E-12	0.267394527	0.218	0.213	5.537E-08	Meta_CD8_C2
MT-ND2	3.5475E-12	0.261844348	0.908	0.949	8.59E-08	Meta_CD8_C2
HLA-DQB1	7.2821E-12	0.413887943	0.249	0.194	1.763E-07	Meta_CD8_C2
YBX3	9.8349E-12	0.32564833	0.108	0.060	2.382E-07	Meta_CD8_C2
MIDN	1.8458E-11	0.255792129	0.183	0.179	4.47E-07	Meta_CD8_C2
SLC1A5	3.3234E-11	0.303288032	0.201	0.193	8.048E-07	Meta_CD8_C2
AOAH	3.5339E-11	0.398797596	0.262	0.206	8.557E-07	Meta_CD8_C2
SERTAD3	3.7919E-11	0.277630779	0.123	0.094	9.182E-07	Meta_CD8_C2
TUBB2A	1.1404E-10	0.374596885	0.155	0.117	2.761E-06	Meta_CD8_C2
C1orf21	1.2671E-10	0.346182816	0.105	0.040	3.068E-06	Meta_CD8_C2
RGCC	1.6718E-10	0.282015331	0.457	0.534	4.048E-06	Meta_CD8_C2
P2RY8	6.2418E-10	0.332434224	0.252	0.227	1.511E-05	Meta_CD8_C2
DTHD1	6.7701E-10	0.479750309	0.138	0.060	1.639E-05	Meta_CD8_C2
HEXIM1	1.7677E-09	0.314487006	0.153	0.136	4.281E-05	Meta_CD8_C2
FABP5	3.023E-09	0.300226265	0.224	0.198	7.32E-05	Meta_CD8_C2
OASL	9.1967E-09	0.387840383	0.262	0.211	0.0002227	Meta_CD8_C2
PTGDR	1.5337E-08	0.259434804	0.106	0.079	0.0003714	Meta_CD8_C2
TNFSF9	2.3674E-08	0.579462508	0.194	0.132	0.0005733	Meta_CD8_C2
RPS27	3.0419E-08	0.25984305	0.990	0.996	0.0007366	Meta_CD8_C2
MIAT	7.2171E-08	0.292683743	0.141	0.135	0.0017476	Meta_CD8_C2
HLA-DMA	1.2137E-07	0.311486961	0.235	0.206	0.0029389	Meta_CD8_C2
PECAM1	1.3907E-07	0.260186637	0.132	0.101	0.0033676	Meta_CD8_C2
EIF4EBP1	2.3941E-07	0.269911689	0.159	0.141	0.0057972	Meta_CD8_C2
HLA-DQA1	4.7228E-07	0.408608687	0.222	0.144	0.0114362	Meta_CD8_C2
HLA-DRB5	1.7881E-06	0.40934546	0.223	0.166	0.0432998	Meta_CD8_C2
HIST2H2AA4	4.1142E-06	0.309045358	0.124	0.092	0.0996257	Meta_CD8_C2
RPL13A	5.9195E-06	0.278978906	0.976	0.988	0.1433414	Meta_CD8_C2
CCL3L1	5.3029E-05	0.470437874	0.107	0.060	1	Meta_CD8_C2
MTRNR2L12	0.00014977	0.388188748	0.215	0.195	1	Meta_CD8_C2
TRGC2	0.00026554	0.274500073	0.107	0.111	1	Meta_CD8_C2
HLA-DRA	0.0007543	0.310369897	0.369	0.302	1	Meta_CD8_C2
IGKC	0.00105499	0.652246387	0.150	0.125	1	Meta_CD8_C2
LMNA	0.00120916	0.46865793	0.301	0.273	1	Meta_CD8_C2
IFNG	0.00191433	0.403127119	0.281	0.248	1	Meta_CD8_C2
NKG7	5.263E-199	1.62168772	0.984	0.537	1.27E-194	Meta_CD8_C3
GZMK	6.929E-194	1.813974187	0.907	0.326	1.68E-189	Meta_CD8_C3

HLA-DRB1	3.786E-163	1.519442091	0.890	0.395	9.17E-159	Meta_CD8_C3
CCL5	2.744E-158	1.127348787	0.998	0.730	6.65E-154	Meta_CD8_C3
GZMH	2.722E-147	1.759455754	0.803	0.249	6.59E-143	Meta_CD8_C3
GZMA	7.407E-144	0.897676396	0.970	0.546	1.79E-139	Meta_CD8_C3
CD74	4.405E-140	1.307576457	0.979	0.865	1.07E-135	Meta_CD8_C3
CST7	1.866E-132	1.199492288	0.937	0.675	4.52E-128	Meta_CD8_C3
CCL4	1.766E-122	1.461441716	0.865	0.393	4.28E-118	Meta_CD8_C3
HLA-DPB1	2.887E-121	1.229438261	0.848	0.410	6.99E-117	Meta_CD8_C3
CD8A	3.192E-120	1.236177528	0.861	0.404	7.73E-116	Meta_CD8_C3
HLA-DPA1	7.596E-119	1.26413194	0.853	0.442	1.84E-114	Meta_CD8_C3
CD8B	6.3823E-96	1.135841431	0.772	0.340	1.545E-91	Meta_CD8_C3
HLA-DRA	9.403E-92	1.377369229	0.702	0.271	2.277E-87	Meta_CD8_C3
APOBEC3G	2.1176E-74	1.026487356	0.754	0.418	5.128E-70	Meta_CD8_C3
HLA-DQA1	1.5863E-60	1.161487152	0.455	0.122	3.841E-56	Meta_CD8_C3
HLA-DMA	1.7997E-53	0.798314133	0.462	0.186	4.358E-49	Meta_CD8_C3
CCL4L2	1.8646E-53	1.606258531	0.442	0.152	4.515E-49	Meta_CD8_C3
HLA-DQB1	3.2367E-53	0.97877781	0.490	0.172	7.838E-49	Meta_CD8_C3
CCL3	2.7372E-52	1.280937266	0.379	0.114	6.628E-48	Meta_CD8_C3
IFNG	2.4099E-50	1.055030454	0.529	0.226	5.836E-46	Meta_CD8_C3
HLA-DRB5	6.1734E-48	1.10945804	0.413	0.148	1.495E-43	Meta_CD8_C3
HLA-A	6.4312E-48	0.388889926	1.000	0.996	1.557E-43	Meta_CD8_C3
COTL1	7.9501E-48	0.758208279	0.864	0.743	1.925E-43	Meta_CD8_C3
GZMB	2.299E-46	1.046667658	0.578	0.274	5.567E-42	Meta_CD8_C3
EOMES	2.834E-46	0.797008954	0.339	0.078	6.863E-42	Meta_CD8_C3
PLEK	2.184E-45	0.766068217	0.337	0.084	5.289E-41	Meta_CD8_C3
ITGB2	8.2478E-43	0.627972088	0.791	0.596	1.997E-38	Meta_CD8_C3
HCST	3.5737E-41	0.529106345	0.933	0.835	8.654E-37	Meta_CD8_C3
CMC1	3.2968E-40	0.890516013	0.424	0.214	7.983E-36	Meta_CD8_C3
CTSW	2.1386E-39	0.698092794	0.687	0.414	5.179E-35	Meta_CD8_C3
SAMD3	5.295E-39	0.660490934	0.421	0.179	1.282E-34	Meta_CD8_C3
DUSP2	8.094E-39	0.893210912	0.789	0.624	1.96E-34	Meta_CD8_C3
HLA-C	1.3156E-37	0.365959753	0.999	0.993	3.186E-33	Meta_CD8_C3
GZMM	3.0704E-36	0.625477176	0.701	0.533	7.435E-32	Meta_CD8_C3
LAG3	7.1094E-36	0.779311647	0.604	0.351	1.722E-31	Meta_CD8_C3
FABP5	9.2328E-36	0.699113835	0.425	0.180	2.236E-31	Meta_CD8_C3
PRF1	1.7025E-35	0.867470391	0.625	0.399	4.123E-31	Meta_CD8_C3
OASL	9.0104E-35	0.583937915	0.416	0.197	2.182E-30	Meta_CD8_C3
HLA-B	4.5881E-33	0.358994403	0.999	0.997	1.111E-28	Meta_CD8_C3
LITAF	5.7308E-33	0.649835395	0.666	0.478	1.388E-28	Meta_CD8_C3
SH2D1A	8.9559E-32	0.716859593	0.547	0.307	2.169E-27	Meta_CD8_C3

FCRL6	3.8978E-31	0.426360648	0.189	0.060	9.438E-27	Meta_CD8_C3
RARRES3	2.6286E-30	0.630588367	0.837	0.731	6.365E-26	Meta_CD8_C3
ITM2C	3.0404E-30	0.849707103	0.545	0.360	7.362E-26	Meta_CD8_C3
CD27	1.7E-29	0.619951952	0.655	0.473	4.117E-25	Meta_CD8_C3
CCL3L1	6.6671E-27	0.698307052	0.194	0.052	1.614E-22	Meta_CD8_C3
CLEC2B	2.2111E-26	0.517109694	0.765	0.617	5.354E-22	Meta_CD8_C3
CRTAM	2.547E-26	0.676653766	0.277	0.088	6.168E-22	Meta_CD8_C3
SUB1	3.3771E-26	0.463207136	0.885	0.804	8.178E-22	Meta_CD8_C3
PSMB9	1.0064E-24	0.527823509	0.775	0.678	2.437E-20	Meta_CD8_C3
IDH2	4.7412E-24	0.540746913	0.576	0.428	1.148E-19	Meta_CD8_C3
CXCR3	8.5249E-24	0.504876286	0.640	0.466	2.064E-19	Meta_CD8_C3
AOAH	8.5737E-24	0.577864903	0.399	0.193	2.076E-19	Meta_CD8_C3
C12orf75	8.5805E-24	0.593137999	0.519	0.308	2.078E-19	Meta_CD8_C3
ENC1	1.5422E-22	0.564300401	0.180	0.039	3.734E-18	Meta_CD8_C3
F2R	1.608E-21	0.507698114	0.360	0.177	3.894E-17	Meta_CD8_C3
SLAMF7	6.7297E-21	0.477959237	0.289	0.128	1.63E-16	Meta_CD8_C3
LYST	1.7231E-19	0.659756388	0.425	0.203	4.172E-15	Meta_CD8_C3
KLRG1	9.1912E-19	0.620973373	0.328	0.162	2.226E-14	Meta_CD8_C3
CD99	1.5237E-18	0.301815459	0.893	0.837	3.69E-14	Meta_CD8_C3
AC243829.4	1.628E-18	0.353966479	0.139	0.033	3.942E-14	Meta_CD8_C3
PLA2G16	2.4951E-18	0.440345876	0.341	0.188	6.042E-14	Meta_CD8_C3
LIME1	2.5561E-18	0.433208047	0.501	0.362	6.19E-14	Meta_CD8_C3
TUBA4A	4.327E-18	0.690502138	0.627	0.533	1.048E-13	Meta_CD8_C3
APMAP	4.387E-18	0.465702394	0.483	0.336	1.062E-13	Meta_CD8_C3
DUSP4	1.1533E-17	0.682607361	0.548	0.359	2.793E-13	Meta_CD8_C3
HMGB1	1.6077E-17	0.284448426	0.897	0.870	3.893E-13	Meta_CD8_C3
PECAM1	2.5111E-17	0.506670033	0.256	0.089	6.081E-13	Meta_CD8_C3
DTHD1	4.5434E-17	0.428761159	0.177	0.056	1.1E-12	Meta_CD8_C3
PTMS	5.5903E-17	0.531842137	0.374	0.216	1.354E-12	Meta_CD8_C3
GIMAP4	9.8234E-17	0.420800101	0.631	0.534	2.379E-12	Meta_CD8_C3
CYTOR	1.2998E-16	0.492320115	0.576	0.415	3.147E-12	Meta_CD8_C3
PSME1	2.2063E-16	0.343604654	0.893	0.846	5.342E-12	Meta_CD8_C3
PFN1	9.9265E-16	0.298834888	0.977	0.957	2.404E-11	Meta_CD8_C3
SRRT	1.3799E-15	0.646657818	0.369	0.276	3.342E-11	Meta_CD8_C3
ARPC5L	1.6596E-15	0.420740323	0.605	0.468	4.019E-11	Meta_CD8_C3
STK17A	3.1423E-15	0.264553586	0.777	0.724	7.609E-11	Meta_CD8_C3
LYAR	3.5699E-15	0.346727004	0.431	0.309	8.644E-11	Meta_CD8_C3
CLIC1	7.5095E-15	0.366457011	0.888	0.819	1.818E-10	Meta_CD8_C3
CD38	7.5313E-15	0.496556664	0.282	0.159	1.824E-10	Meta_CD8_C3
CTSC	1.2016E-14	0.426966995	0.675	0.548	2.91E-10	Meta_CD8_C3

HLA-DQA2	1.7209E-14	0.420294169	0.102	0.023	4.167E-10	Meta_CD8_C3
PDLIM1	1.7221E-14	0.267323195	0.110	0.024	4.17E-10	Meta_CD8_C3
GGA2	2.1395E-14	0.450617384	0.320	0.198	5.181E-10	Meta_CD8_C3
PSME2	2.1599E-14	0.34599657	0.730	0.656	5.23E-10	Meta_CD8_C3
CHST12	3.4895E-14	0.456170997	0.464	0.326	8.45E-10	Meta_CD8_C3
PYCARD	4.3282E-14	0.291996426	0.370	0.290	1.048E-09	Meta_CD8_C3
VCAM1	5.9518E-14	0.438214905	0.111	0.020	1.441E-09	Meta_CD8_C3
PTPRCAP	6.9431E-14	0.481001357	0.758	0.739	1.681E-09	Meta_CD8_C3
CFL1	1.9125E-12	0.306505023	0.982	0.956	4.631E-08	Meta_CD8_C3
CNN2	1.9485E-12	0.446584999	0.712	0.623	4.718E-08	Meta_CD8_C3
ARPC2	2.3668E-12	0.262572063	0.927	0.882	5.731E-08	Meta_CD8_C3
PSMA7	3.1003E-12	0.341077561	0.809	0.735	7.507E-08	Meta_CD8_C3
RBCK1	3.4731E-12	0.288441832	0.459	0.369	8.41E-08	Meta_CD8_C3
GUK1	4.1718E-12	0.296237897	0.817	0.746	1.01E-07	Meta_CD8_C3
YARS	4.598E-12	0.274471411	0.342	0.253	1.113E-07	Meta_CD8_C3
RAB5IF	4.69E-12	0.2867681	0.458	0.393	1.136E-07	Meta_CD8_C3
MATK	6.187E-12	0.32781964	0.400	0.266	1.498E-07	Meta_CD8_C3
GLIPR2	6.3726E-12	0.300313543	0.392	0.308	1.543E-07	Meta_CD8_C3
APOBEC3C	8.4943E-12	0.555479388	0.429	0.271	2.057E-07	Meta_CD8_C3
CASP4	8.5086E-12	0.324426654	0.516	0.423	2.06E-07	Meta_CD8_C3
LY6E	9.2051E-12	0.362841441	0.812	0.734	2.229E-07	Meta_CD8_C3
MT2A	1.2205E-11	0.36794268	0.664	0.511	2.955E-07	Meta_CD8_C3
CYBA	1.2384E-11	0.288058372	0.909	0.881	2.999E-07	Meta_CD8_C3
JAKMIP1	1.2423E-11	0.370820018	0.232	0.107	3.008E-07	Meta_CD8_C3
ZEB2	1.5858E-11	0.416258117	0.284	0.145	3.84E-07	Meta_CD8_C3
UBC	1.5904E-11	0.323006877	0.980	0.965	3.851E-07	Meta_CD8_C3
EIF4EBP1	1.6648E-11	0.274706932	0.210	0.136	4.031E-07	Meta_CD8_C3
TNFSF9	1.8749E-11	0.671692255	0.254	0.127	4.54E-07	Meta_CD8_C3
PDCD1	2.2656E-11	0.402158895	0.329	0.207	5.486E-07	Meta_CD8_C3
HLA-DMB	2.4531E-11	0.346538099	0.155	0.056	5.94E-07	Meta_CD8_C3
CCR5	3.1863E-11	0.313950813	0.210	0.119	7.716E-07	Meta_CD8_C3
CAP1	3.8212E-11	0.303452811	0.711	0.639	9.253E-07	Meta_CD8_C3
GYG1	4.7372E-11	0.361271685	0.451	0.355	1.147E-06	Meta_CD8_C3
GBP5	6.4432E-11	0.383140395	0.487	0.392	1.56E-06	Meta_CD8_C3
LCP1	7.2243E-11	0.354299002	0.812	0.731	1.749E-06	Meta_CD8_C3
TPST2	1.2967E-10	0.258202499	0.332	0.255	3.14E-06	Meta_CD8_C3
AP3S1	1.3149E-10	0.323710271	0.388	0.301	3.184E-06	Meta_CD8_C3
ITM2A	1.4892E-10	0.418156673	0.697	0.603	3.606E-06	Meta_CD8_C3
AC116366.3	2.0129E-10	0.306610538	0.325	0.235	4.874E-06	Meta_CD8_C3
UBE2L6	2.6644E-10	0.340654336	0.555	0.467	6.452E-06	Meta_CD8_C3

SLC9A3R1	3.8861E-10	0.294895271	0.637	0.572	9.41E-06	Meta_CD8_C3
CHI3L2	4.3324E-10	0.351197514	0.166	0.073	1.049E-05	Meta_CD8_C3
NFATC2	4.8556E-10	0.365648247	0.441	0.331	1.176E-05	Meta_CD8_C3
HERPUD1	5.5534E-10	0.380841312	0.633	0.528	1.345E-05	Meta_CD8_C3
FYN	8.8982E-10	0.290789578	0.648	0.560	2.155E-05	Meta_CD8_C3
CORO1A	9.8091E-10	0.328261921	0.909	0.873	2.375E-05	Meta_CD8_C3
SYNE1	1.1735E-09	0.258563443	0.337	0.249	2.842E-05	Meta_CD8_C3
CLIC3	1.1753E-09	0.313541429	0.284	0.183	2.846E-05	Meta_CD8_C3
ARAP2	1.2836E-09	0.303989771	0.413	0.313	3.108E-05	Meta_CD8_C3
ANXA5	1.4218E-09	0.407570521	0.551	0.436	3.443E-05	Meta_CD8_C3
LIMD2	1.6656E-09	0.302156154	0.758	0.713	4.033E-05	Meta_CD8_C3
JMJD6	1.7965E-09	0.305598252	0.326	0.244	4.35E-05	Meta_CD8_C3
MSC	2.1184E-09	0.306902579	0.131	0.046	5.13E-05	Meta_CD8_C3
ZYX	2.2102E-09	0.294989767	0.606	0.544	5.352E-05	Meta_CD8_C3
FASLG	2.2155E-09	0.484959776	0.291	0.154	5.365E-05	Meta_CD8_C3
SLA2	2.2775E-09	0.405001942	0.399	0.276	5.515E-05	Meta_CD8_C3
PSMB8	2.3937E-09	0.323416183	0.675	0.594	5.796E-05	Meta_CD8_C3
WIPF1	3.1494E-09	0.274641012	0.676	0.605	7.626E-05	Meta_CD8_C3
PSMB10	4.9061E-09	0.356701405	0.572	0.505	0.0001188	Meta_CD8_C3
DRAP1	5.8335E-09	0.282603599	0.703	0.648	0.0001413	Meta_CD8_C3
RUNX3	7.3637E-09	0.335185304	0.528	0.416	0.0001783	Meta_CD8_C3
APOBEC3H	7.8095E-09	0.347405173	0.227	0.120	0.0001891	Meta_CD8_C3
LINC02446	9.098E-09	0.578737789	0.185	0.103	0.0002203	Meta_CD8_C3
TAP1	1.2141E-08	0.297255856	0.565	0.491	0.000294	Meta_CD8_C3
ABI3	1.2352E-08	0.372076939	0.453	0.333	0.0002991	Meta_CD8_C3
MT1F	1.4658E-08	0.36390353	0.268	0.163	0.0003549	Meta_CD8_C3
ADGRE5	2.462E-08	0.34452764	0.651	0.555	0.0005962	Meta_CD8_C3
SRSF2	2.7824E-08	0.276668759	0.668	0.633	0.0006738	Meta_CD8_C3
HERPUD2	3.2846E-08	0.284318517	0.419	0.362	0.0007954	Meta_CD8_C3
CMC2	3.8422E-08	0.298534383	0.409	0.340	0.0009304	Meta_CD8_C3
TERF2IP	3.8567E-08	0.270449767	0.630	0.566	0.0009339	Meta_CD8_C3
XCL2	4.2528E-08	0.424671834	0.247	0.136	0.0010298	Meta_CD8_C3
DBI	4.4866E-08	0.258909228	0.645	0.571	0.0010864	Meta_CD8_C3
ISG20	4.9971E-08	0.299122753	0.683	0.625	0.00121	Meta_CD8_C3
RAB27A	8.6853E-08	0.326218768	0.408	0.308	0.0021031	Meta_CD8_C3
CALR	1.0733E-07	0.295023743	0.718	0.665	0.002599	Meta_CD8_C3
MT1E	1.1565E-07	0.318626273	0.211	0.122	0.0028005	Meta_CD8_C3
PPP1CA	1.1693E-07	0.267291375	0.709	0.641	0.0028314	Meta_CD8_C3
B3GNT2	1.3332E-07	0.327007608	0.337	0.238	0.0032282	Meta_CD8_C3
POMP	1.4911E-07	0.288229705	0.627	0.545	0.0036107	Meta_CD8_C3

GSTP1	1.5364E-07	0.276955067	0.622	0.523	0.0037205	Meta_CD8_C3
DNAJB6	1.905E-07	0.353102207	0.530	0.463	0.0046129	Meta_CD8_C3
SEPT7	2.2331E-07	0.267000441	0.758	0.699	0.0054074	Meta_CD8_C3
UBB	2.5394E-07	0.259361436	0.934	0.891	0.0061493	Meta_CD8_C3
GNG2	2.8248E-07	0.276307837	0.616	0.548	0.0068403	Meta_CD8_C3
PPP1R18	7.184E-07	0.286390511	0.588	0.526	0.0173959	Meta_CD8_C3
MCOLN2	8.9201E-07	0.255224655	0.175	0.092	0.0216	Meta_CD8_C3
HAVCR2	1.0663E-06	0.464237565	0.218	0.135	0.0258203	Meta_CD8_C3
ANXA6	1.2153E-06	0.253853523	0.589	0.528	0.0294293	Meta_CD8_C3
THEMIS	1.2547E-06	0.27201559	0.397	0.323	0.0303823	Meta_CD8_C3
SIRT2	1.9012E-06	0.272350405	0.282	0.200	0.046038	Meta_CD8_C3
ALOX5AP	2.2149E-06	0.266419994	0.627	0.602	0.0536346	Meta_CD8_C3
ZBP1	3.518E-06	0.272005997	0.262	0.170	0.0851874	Meta_CD8_C3
LSP1	4.0665E-06	0.263178577	0.872	0.827	0.0984698	Meta_CD8_C3
LDHA	5.4208E-06	0.342084397	0.810	0.787	0.1312641	Meta_CD8_C3
KPNA2	5.4293E-06	0.263882916	0.227	0.168	0.131471	Meta_CD8_C3
PYHIN1	6.0947E-06	0.345433713	0.394	0.289	0.1475825	Meta_CD8_C3
GBP1	1.0204E-05	0.251445609	0.323	0.260	0.2470973	Meta_CD8_C3
CEMIP2	1.049E-05	0.394701241	0.368	0.293	0.2540037	Meta_CD8_C3
TNIP3	1.345E-05	0.297001515	0.166	0.089	0.3256877	Meta_CD8_C3
PRKCH	1.3676E-05	0.259105464	0.560	0.491	0.3311604	Meta_CD8_C3
SLC7A5	1.5458E-05	0.330720281	0.325	0.262	0.3743227	Meta_CD8_C3
RNF213	1.5513E-05	0.261739973	0.651	0.579	0.3756443	Meta_CD8_C3
STX11	2.0385E-05	0.25404421	0.248	0.167	0.493631	Meta_CD8_C3
TBCD	2.1282E-05	0.295236555	0.266	0.179	0.5153472	Meta_CD8_C3
SERTAD1	2.3232E-05	0.254453973	0.371	0.333	0.562572	Meta_CD8_C3
PSTPIP1	2.543E-05	0.278440183	0.420	0.339	0.6157758	Meta_CD8_C3
MRPL10	2.6428E-05	0.260421438	0.354	0.268	0.6399529	Meta_CD8_C3
KLRD1	2.8314E-05	0.283072292	0.347	0.244	0.6856328	Meta_CD8_C3
NELFCD	3.8772E-05	0.271522922	0.272	0.198	0.9388542	Meta_CD8_C3
TOX	0.00012629	0.316815491	0.253	0.161	1	Meta_CD8_C3
LGALS1	0.00016243	0.253276752	0.488	0.434	1	Meta_CD8_C3
IFI27L2	0.0001712	0.306563847	0.450	0.359	1	Meta_CD8_C3
IKZF3	0.00027274	0.299317594	0.447	0.347	1	Meta_CD8_C3
HSPA5	0.00029639	0.30136513	0.659	0.644	1	Meta_CD8_C3
BTN3A2	0.00044142	0.265547304	0.429	0.349	1	Meta_CD8_C3
LMNB1	0.00055575	0.250796194	0.217	0.150	1	Meta_CD8_C3
PPP2R2B	0.00157048	0.296185246	0.209	0.115	1	Meta_CD8_C3
CXCR6	0.00184485	0.388411006	0.410	0.324	1	Meta_CD8_C3
PIK3R1	0.00501763	0.274031532	0.480	0.419	1	Meta_CD8_C3

SLF1	0.00504685	0.262233778	0.255	0.179	1	Meta_CD8_C3
CD8A	2.371E-158	1.422338664	0.890	0.375	5.74E-154	Meta_CD8_C4
CCL5	3.811E-149	1.216068397	0.997	0.716	9.23E-145	Meta_CD8_C4
NKG7	2.646E-133	1.060497637	0.957	0.516	6.41E-129	Meta_CD8_C4
CD8B	1.723E-114	1.189414106	0.817	0.310	4.17E-110	Meta_CD8_C4
GZMA	1.2399E-95	1.118011653	0.900	0.534	3.002E-91	Meta_CD8_C4
GZMB	6.492E-86	1.974418847	0.714	0.237	1.572E-81	Meta_CD8_C4
SH3BGRL3	4.8897E-81	0.674928548	0.994	0.955	1.184E-76	Meta_CD8_C4
KLRD1	1.7487E-78	1.112020735	0.626	0.197	4.235E-74	Meta_CD8_C4
LAG3	2.4074E-67	0.984818845	0.700	0.322	5.829E-63	Meta_CD8_C4
CCL4	8.3742E-67	1.127484865	0.777	0.380	2.028E-62	Meta_CD8_C4
GAPDH	8.6907E-65	0.682603771	0.997	0.972	2.104E-60	Meta_CD8_C4
ZNF683	2.0784E-56	1.252377944	0.355	0.059	5.033E-52	Meta_CD8_C4
CD7	8.0133E-56	0.813501186	0.920	0.739	1.94E-51	Meta_CD8_C4
PRF1	8.3877E-56	1.064583665	0.725	0.372	2.031E-51	Meta_CD8_C4
GZMH	2.9984E-52	0.968749102	0.605	0.248	7.261E-48	Meta_CD8_C4
CD52	3.9613E-51	0.628028105	0.984	0.926	9.592E-47	Meta_CD8_C4
IFNG	9.5935E-51	1.082446332	0.542	0.207	2.323E-46	Meta_CD8_C4
CTSW	1.2986E-47	0.870580773	0.690	0.398	3.145E-43	Meta_CD8_C4
LINC02446	2.2091E-46	1.631943408	0.344	0.074	5.349E-42	Meta_CD8_C4
LSP1	1.6381E-45	0.563864728	0.930	0.816	3.967E-41	Meta_CD8_C4
COTL1	4.4319E-45	0.805220893	0.872	0.736	1.073E-40	Meta_CD8_C4
FKBP1A	9.0501E-45	0.624483846	0.774	0.541	2.191E-40	Meta_CD8_C4
ACTB	2.3855E-44	0.510581237	1.000	0.997	5.776E-40	Meta_CD8_C4
ITGAE	2.6698E-44	0.8675395	0.614	0.288	6.465E-40	Meta_CD8_C4
ITGA1	3.3906E-44	0.625767886	0.460	0.183	8.21E-40	Meta_CD8_C4
CFL1	7.0878E-41	0.433028714	0.988	0.954	1.716E-36	Meta_CD8_C4
HLA-DRB1	1.1376E-40	0.743603806	0.704	0.396	2.755E-36	Meta_CD8_C4
CLIC1	7.74E-40	0.522862309	0.930	0.809	1.874E-35	Meta_CD8_C4
ARPC2	2.4547E-38	0.478040471	0.954	0.875	5.944E-34	Meta_CD8_C4
ALOX5AP	2.8385E-37	0.861893682	0.808	0.573	6.873E-33	Meta_CD8_C4
LINC01871	8.9315E-37	0.869598747	0.559	0.275	2.163E-32	Meta_CD8_C4
CXCR6	1.0485E-36	0.672450302	0.553	0.298	2.539E-32	Meta_CD8_C4
CD63	1.7598E-36	0.733307109	0.707	0.436	4.261E-32	Meta_CD8_C4
HAVCR2	8.8544E-35	0.891584775	0.347	0.112	2.144E-30	Meta_CD8_C4
TMSB4X	1.0096E-34	0.371171	1.000	0.999	2.445E-30	Meta_CD8_C4
CLIC3	1.1347E-34	0.705449888	0.426	0.157	2.748E-30	Meta_CD8_C4
CD3D	4.6074E-34	0.609892208	0.967	0.880	1.116E-29	Meta_CD8_C4
HCST	3.4591E-33	0.50300486	0.950	0.827	8.376E-29	Meta_CD8_C4
HOPX	4.0378E-33	0.818024438	0.604	0.309	9.777E-29	Meta_CD8_C4

FASLG	5.3609E-33	0.698476538	0.379	0.133	1.298E-28	Meta_CD8_C4
CCL3	1.4509E-32	1.291692	0.322	0.108	3.513E-28	Meta_CD8_C4
MYL6	1.6489E-32	0.363008028	0.985	0.941	3.993E-28	Meta_CD8_C4
PTMS	2.6303E-32	0.64557924	0.452	0.196	6.369E-28	Meta_CD8_C4
ITM2C	4.3718E-32	0.591277818	0.607	0.340	1.059E-27	Meta_CD8_C4
CD248	4.7799E-31	0.441647865	0.169	0.020	1.157E-26	Meta_CD8_C4
ACTG1	3.544E-30	0.524997867	0.981	0.942	8.582E-26	Meta_CD8_C4
ZYX	2.0263E-29	0.513363562	0.704	0.526	4.907E-25	Meta_CD8_C4
GPR34	5.2992E-29	0.464714845	0.208	0.045	1.283E-24	Meta_CD8_C4
ID2	8.4055E-29	0.655706709	0.824	0.562	2.035E-24	Meta_CD8_C4
XCL2	8.7127E-29	0.980748623	0.313	0.121	2.11E-24	Meta_CD8_C4
PFN1	3.1938E-28	0.437341774	0.982	0.955	7.734E-24	Meta_CD8_C4
SIT1	5.1941E-28	0.412393215	0.589	0.388	1.258E-23	Meta_CD8_C4
ABI3	9.6929E-28	0.673868566	0.591	0.306	2.347E-23	Meta_CD8_C4
PTPN22	1.5077E-27	0.53552912	0.586	0.347	3.651E-23	Meta_CD8_C4
APOBEC3G	1.1251E-26	0.612278589	0.669	0.412	2.724E-22	Meta_CD8_C4
JAML	3.5473E-26	0.569735308	0.490	0.269	8.59E-22	Meta_CD8_C4
GNLY	1.0222E-25	1.504641947	0.406	0.180	2.475E-21	Meta_CD8_C4
HLA-DPA1	1.0324E-25	0.522724142	0.698	0.442	2.5E-21	Meta_CD8_C4
IDH2	1.1004E-25	0.532265328	0.642	0.410	2.665E-21	Meta_CD8_C4
CORO1A	1.3153E-25	0.465684838	0.937	0.866	3.185E-21	Meta_CD8_C4
CHST12	2.7349E-25	0.424365457	0.530	0.309	6.622E-21	Meta_CD8_C4
PECAM1	2.9065E-25	0.427649469	0.260	0.080	7.038E-21	Meta_CD8_C4
CD160	3.7659E-25	0.447776331	0.217	0.071	9.119E-21	Meta_CD8_C4
RARRES3	5.0182E-25	0.543731271	0.844	0.725	1.215E-20	Meta_CD8_C4
CKLF	5.5662E-25	0.504128717	0.727	0.541	1.348E-20	Meta_CD8_C4
CD2	1.1885E-24	0.438674546	0.937	0.828	2.878E-20	Meta_CD8_C4
TPI1	1.5032E-24	0.54373374	0.853	0.697	3.64E-20	Meta_CD8_C4
PSMB9	3.7288E-24	0.445309749	0.826	0.665	9.029E-20	Meta_CD8_C4
C12orf75	3.9777E-24	0.503491834	0.534	0.294	9.632E-20	Meta_CD8_C4
HLA-DRA	4.5549E-24	0.750779253	0.514	0.276	1.103E-19	Meta_CD8_C4
LGALS1	6.9697E-24	0.702511521	0.630	0.409	1.688E-19	Meta_CD8_C4
SPRY1	7.4184E-24	0.449952049	0.286	0.120	1.796E-19	Meta_CD8_C4
RAC2	1.252E-23	0.41866378	0.906	0.819	3.032E-19	Meta_CD8_C4
PGK1	1.2692E-23	0.390880021	0.864	0.740	3.073E-19	Meta_CD8_C4
HLA-DPB1	3.6E-23	0.462042508	0.655	0.415	8.717E-19	Meta_CD8_C4
RHOA	1.061E-22	0.300045494	0.902	0.822	2.569E-18	Meta_CD8_C4
GABARAPL1	1.1102E-22	0.257959215	0.467	0.269	2.688E-18	Meta_CD8_C4
THEMIS	1.4018E-22	0.45279847	0.516	0.301	3.394E-18	Meta_CD8_C4
XCL1	1.5145E-22	1.043937722	0.289	0.107	3.667E-18	Meta_CD8_C4

CD244	2.791E-22	0.381428881	0.252	0.077	6.758E-18	Meta_CD8_C4
BCAS4	2.9476E-22	0.37199348	0.294	0.124	7.138E-18	Meta_CD8_C4
ENTPD1	4.4669E-22	0.610167116	0.265	0.098	1.082E-17	Meta_CD8_C4
SLA2	5.6373E-22	0.430978897	0.482	0.256	1.365E-17	Meta_CD8_C4
IFI27L2	5.8784E-22	0.426199606	0.554	0.338	1.423E-17	Meta_CD8_C4
DRAP1	7.0123E-22	0.378781252	0.799	0.630	1.698E-17	Meta_CD8_C4
WDR1	7.5967E-22	0.343183648	0.687	0.526	1.84E-17	Meta_CD8_C4
ZEB2	7.7328E-22	0.355148854	0.306	0.134	1.872E-17	Meta_CD8_C4
GZMM	7.9142E-22	0.338950429	0.711	0.522	1.916E-17	Meta_CD8_C4
BIN1	9.5774E-22	0.26631204	0.587	0.428	2.319E-17	Meta_CD8_C4
CST7	1.2611E-21	0.304751706	0.860	0.673	3.054E-17	Meta_CD8_C4
CD38	1.6792E-21	0.503499017	0.334	0.145	4.066E-17	Meta_CD8_C4
CD74	5.8355E-21	0.427977201	0.938	0.865	1.413E-16	Meta_CD8_C4
TRG-AS1	7.4318E-21	0.44708687	0.330	0.147	1.8E-16	Meta_CD8_C4
MSN	9.2509E-21	0.355756531	0.752	0.595	2.24E-16	Meta_CD8_C4
CAP1	1.9002E-20	0.403373807	0.784	0.624	4.601E-16	Meta_CD8_C4
PPP1CA	2.0473E-20	0.398573986	0.791	0.625	4.958E-16	Meta_CD8_C4
CAPZB	2.9406E-20	0.366763641	0.834	0.691	7.121E-16	Meta_CD8_C4
CD3G	3.5149E-20	0.424750501	0.839	0.693	8.511E-16	Meta_CD8_C4
GSTP1	4.9692E-20	0.359151414	0.703	0.506	1.203E-15	Meta_CD8_C4
CLEC2B	8.9951E-20	0.488653993	0.807	0.603	2.178E-15	Meta_CD8_C4
TWF2	9.7971E-20	0.299659796	0.530	0.363	2.372E-15	Meta_CD8_C4
CTSD	1.022E-19	0.597033508	0.435	0.248	2.475E-15	Meta_CD8_C4
RABAC1	1.0367E-19	0.327552234	0.755	0.587	2.51E-15	Meta_CD8_C4
BST2	1.3474E-19	0.313566487	0.640	0.474	3.263E-15	Meta_CD8_C4
TESC	1.3791E-19	0.434669688	0.304	0.113	3.339E-15	Meta_CD8_C4
EVL	1.571E-19	0.417347513	0.896	0.789	3.804E-15	Meta_CD8_C4
APOBEC3C	1.7295E-19	0.467370623	0.460	0.257	4.188E-15	Meta_CD8_C4
AOAH	2.1492E-19	0.372553539	0.392	0.183	5.204E-15	Meta_CD8_C4
ARPC5	3.1498E-19	0.343540253	0.689	0.518	7.627E-15	Meta_CD8_C4
CD96	6.1994E-19	0.388846709	0.761	0.576	1.501E-14	Meta_CD8_C4
TAP1	6.5603E-19	0.293359842	0.630	0.477	1.589E-14	Meta_CD8_C4
GALNT2	6.9237E-19	0.457344874	0.263	0.111	1.677E-14	Meta_CD8_C4
DAPK2	2.0866E-18	0.39429214	0.234	0.067	5.053E-14	Meta_CD8_C4
CXCL13	2.3577E-18	1.105382084	0.162	0.054	5.709E-14	Meta_CD8_C4
LY6E	2.7475E-18	0.389944678	0.856	0.723	6.653E-14	Meta_CD8_C4
CARS	2.8727E-18	0.395779785	0.294	0.141	6.956E-14	Meta_CD8_C4
PARK7	5.7406E-18	0.366107092	0.761	0.611	1.39E-13	Meta_CD8_C4
GRAP2	6.7378E-18	0.411804842	0.442	0.244	1.632E-13	Meta_CD8_C4
CD9	7.0978E-18	0.432926094	0.191	0.064	1.719E-13	Meta_CD8_C4

RAB27A	1.6001E-17	0.537658515	0.522	0.286	3.875E-13	Meta_CD8_C4
ASB2	1.7268E-17	0.36431283	0.310	0.151	4.181E-13	Meta_CD8_C4
LCP1	1.7392E-17	0.348421137	0.838	0.723	4.211E-13	Meta_CD8_C4
PSME2	1.7589E-17	0.428249504	0.797	0.642	4.259E-13	Meta_CD8_C4
PLA2G16	2.0755E-17	0.267275915	0.340	0.179	5.026E-13	Meta_CD8_C4
GYG1	2.1155E-17	0.316800154	0.527	0.338	5.123E-13	Meta_CD8_C4
FKBP11	2.606E-17	0.325079286	0.542	0.360	6.31E-13	Meta_CD8_C4
TIGIT	2.7228E-17	0.422242558	0.510	0.306	6.593E-13	Meta_CD8_C4
LSM2	3.0663E-17	0.310093951	0.508	0.335	7.425E-13	Meta_CD8_C4
PYCARD	4.2179E-17	0.318456635	0.446	0.274	1.021E-12	Meta_CD8_C4
RUNX3	6.0624E-17	0.357059634	0.600	0.400	1.468E-12	Meta_CD8_C4
CCL4L2	6.7717E-17	0.972738945	0.332	0.153	1.64E-12	Meta_CD8_C4
PGAM1	7.5189E-17	0.352254808	0.693	0.557	1.821E-12	Meta_CD8_C4
ANXA5	9.248E-17	0.431247018	0.621	0.419	2.239E-12	Meta_CD8_C4
TSEN54	9.6631E-17	0.265263034	0.455	0.295	2.34E-12	Meta_CD8_C4
RNF167	1.0039E-16	0.332264392	0.584	0.415	2.431E-12	Meta_CD8_C4
CDK2AP2	1.0774E-16	0.445466125	0.555	0.371	2.609E-12	Meta_CD8_C4
MIR4435-2H[illegible]	1.1564E-16	0.251171055	0.445	0.266	2.8E-12	Meta_CD8_C4
DUSP4	1.4219E-16	0.425783878	0.558	0.348	3.443E-12	Meta_CD8_C4
CBLB	1.5587E-16	0.438022293	0.566	0.375	3.774E-12	Meta_CD8_C4
SLC27A2	1.6095E-16	0.267038687	0.144	0.047	3.897E-12	Meta_CD8_C4
ARPC3	2.368E-16	0.353521087	0.902	0.815	5.734E-12	Meta_CD8_C4
FABP5	3.2732E-16	0.362830469	0.343	0.178	7.926E-12	Meta_CD8_C4
ENO1	4.0769E-16	0.338688015	0.810	0.687	9.872E-12	Meta_CD8_C4
KLRC1	4.2632E-16	0.468850636	0.164	0.055	1.032E-11	Meta_CD8_C4
COX5A	5.6315E-16	0.280864148	0.698	0.544	1.364E-11	Meta_CD8_C4
MATK	6.6195E-16	0.288188018	0.434	0.254	1.603E-11	Meta_CD8_C4
PSMB8	8.1292E-16	0.378454156	0.740	0.580	1.968E-11	Meta_CD8_C4
CLECL1	8.6944E-16	0.369721895	0.174	0.049	2.105E-11	Meta_CD8_C4
ACP5	9.2329E-16	0.534814618	0.446	0.234	2.236E-11	Meta_CD8_C4
PTTG1	1.1697E-15	0.310241755	0.346	0.190	2.832E-11	Meta_CD8_C4
LCK	1.3029E-15	0.320775016	0.850	0.734	3.155E-11	Meta_CD8_C4
RGS1	1.3354E-15	0.417962976	0.766	0.598	3.234E-11	Meta_CD8_C4
ITGB7	2.0007E-15	0.361911893	0.541	0.356	4.845E-11	Meta_CD8_C4
TBC1D10C	2.5093E-15	0.275274083	0.712	0.560	6.076E-11	Meta_CD8_C4
PAG1	2.6081E-15	0.259443319	0.427	0.277	6.316E-11	Meta_CD8_C4
CTSC	2.6284E-15	0.310098795	0.723	0.534	6.365E-11	Meta_CD8_C4
HLA-DRB5	2.9131E-15	0.357450488	0.286	0.153	7.054E-11	Meta_CD8_C4
HLA-DMA	3.462E-15	0.322689748	0.353	0.187	8.383E-11	Meta_CD8_C4
ARRB2	3.5475E-15	0.251384346	0.562	0.403	8.59E-11	Meta_CD8_C4

ISG15	4.3748E-15	0.314286504	0.569	0.394	1.059E-10	Meta_CD8_C4
PKM	1.4107E-14	0.387366092	0.771	0.640	3.416E-10	Meta_CD8_C4
ACTR3	1.7903E-14	0.312890997	0.746	0.603	4.335E-10	Meta_CD8_C4
POMP	1.8983E-14	0.313162288	0.685	0.532	4.597E-10	Meta_CD8_C4
CALCOCO2	2.0055E-14	0.279813714	0.474	0.307	4.856E-10	Meta_CD8_C4
PLEKHF1	2.0077E-14	0.334219863	0.388	0.233	4.862E-10	Meta_CD8_C4
RGL4	2.5127E-14	0.28711137	0.420	0.262	6.085E-10	Meta_CD8_C4
PSMB3	2.7806E-14	0.266320356	0.674	0.525	6.733E-10	Meta_CD8_C4
OSTF1	2.7807E-14	0.316345389	0.648	0.496	6.734E-10	Meta_CD8_C4
ATP5MC3	2.9972E-14	0.2863205	0.666	0.549	7.258E-10	Meta_CD8_C4
TRGC2	5.2573E-14	0.443382344	0.215	0.095	1.273E-09	Meta_CD8_C4
DBI	5.5406E-14	0.341921182	0.727	0.555	1.342E-09	Meta_CD8_C4
RBX1	5.8365E-14	0.277243915	0.595	0.441	1.413E-09	Meta_CD8_C4
NDUFB3	1.2723E-13	0.253487837	0.468	0.321	3.081E-09	Meta_CD8_C4
PRELID1	1.4838E-13	0.292518553	0.659	0.508	3.593E-09	Meta_CD8_C4
ENSA	2.0524E-13	0.267116699	0.618	0.463	4.97E-09	Meta_CD8_C4
DYNLL1	2.6088E-13	0.341123976	0.693	0.530	6.317E-09	Meta_CD8_C4
NDFIP2	2.6127E-13	0.396346175	0.258	0.124	6.327E-09	Meta_CD8_C4
PRDX5	3.3302E-13	0.414742557	0.697	0.508	8.064E-09	Meta_CD8_C4
TNIP3	4.0336E-13	0.377197622	0.210	0.078	9.767E-09	Meta_CD8_C4
BUB3	4.4527E-13	0.266134524	0.660	0.495	1.078E-08	Meta_CD8_C4
FIBP	4.6059E-13	0.251891142	0.401	0.252	1.115E-08	Meta_CD8_C4
FAM3C	7.7354E-13	0.292557852	0.217	0.105	1.873E-08	Meta_CD8_C4
CAPG	8.1601E-13	0.453111896	0.408	0.263	1.976E-08	Meta_CD8_C4
HLA-DQA1	8.7835E-13	0.352003427	0.255	0.134	2.127E-08	Meta_CD8_C4
TRAC	9.2186E-13	0.549840303	0.704	0.549	2.232E-08	Meta_CD8_C4
CYTOR	9.2452E-13	0.341450809	0.588	0.404	2.239E-08	Meta_CD8_C4
CRTAM	1.124E-12	0.310175272	0.204	0.088	2.722E-08	Meta_CD8_C4
SIRPG	1.5984E-12	0.383394711	0.487	0.336	3.87E-08	Meta_CD8_C4
RGS10	2.0889E-12	0.313245012	0.736	0.607	5.058E-08	Meta_CD8_C4
MYL12A	2.9683E-12	0.268884965	0.940	0.877	7.188E-08	Meta_CD8_C4
SLC9A3R1	3.3447E-12	0.324831945	0.701	0.559	8.099E-08	Meta_CD8_C4
HMGA1	3.4465E-12	0.284202325	0.456	0.315	8.346E-08	Meta_CD8_C4
SASH3	4.0935E-12	0.286976658	0.562	0.405	9.912E-08	Meta_CD8_C4
ACTN4	4.4018E-12	0.270079791	0.536	0.371	1.066E-07	Meta_CD8_C4
BLOC1S1	5.6724E-12	0.371032508	0.405	0.255	1.374E-07	Meta_CD8_C4
ARPC1B	5.8865E-12	0.302759968	0.831	0.714	1.425E-07	Meta_CD8_C4
LASP1	7.4842E-12	0.270484716	0.449	0.290	1.812E-07	Meta_CD8_C4
SYTL3	7.6925E-12	0.255223374	0.558	0.395	1.863E-07	Meta_CD8_C4
OASL	8.2083E-12	0.250622346	0.339	0.197	1.988E-07	Meta_CD8_C4

MT1F	9.9201E-12	0.263863983	0.288	0.154	2.402E-07	Meta_CD8_C4
APOBEC3H	1.0026E-11	0.279676991	0.233	0.114	2.428E-07	Meta_CD8_C4
PTPN6	1.3394E-11	0.299190309	0.526	0.378	3.243E-07	Meta_CD8_C4
TMIGD2	1.3455E-11	0.338519014	0.256	0.136	3.258E-07	Meta_CD8_C4
PRDX6	1.6736E-11	0.307928931	0.635	0.466	4.053E-07	Meta_CD8_C4
PSMA4	2.0166E-11	0.284305688	0.481	0.328	4.883E-07	Meta_CD8_C4
TPM4	2.4816E-11	0.365290275	0.537	0.382	6.009E-07	Meta_CD8_C4
RBPJ	3.145E-11	0.587981988	0.489	0.316	7.616E-07	Meta_CD8_C4
TRAPPC1	3.3006E-11	0.256391713	0.698	0.552	7.992E-07	Meta_CD8_C4
AKAP5	4.4867E-11	0.269041004	0.126	0.037	1.086E-06	Meta_CD8_C4
RGS2	4.8073E-11	0.374733198	0.554	0.387	1.164E-06	Meta_CD8_C4
CLEC2D	4.8574E-11	0.371610127	0.620	0.459	1.176E-06	Meta_CD8_C4
ANKRD28	7.1682E-11	0.363235931	0.275	0.162	1.736E-06	Meta_CD8_C4
UBE2L6	8.9036E-11	0.315188271	0.625	0.452	2.156E-06	Meta_CD8_C4
SLC25A5	9.6617E-11	0.270369926	0.740	0.626	2.34E-06	Meta_CD8_C4
NOP10	9.9888E-11	0.256315053	0.625	0.476	2.419E-06	Meta_CD8_C4
SEPT1	1.0036E-10	0.253507524	0.754	0.633	2.43E-06	Meta_CD8_C4
KLRC2	1.0519E-10	0.290506498	0.143	0.062	2.547E-06	Meta_CD8_C4
PDIA6	1.1361E-10	0.279972011	0.581	0.432	2.751E-06	Meta_CD8_C4
PPP1R18	1.4313E-10	0.367465011	0.671	0.510	3.466E-06	Meta_CD8_C4
CD2BP2	1.4642E-10	0.271567879	0.343	0.211	3.545E-06	Meta_CD8_C4
PTPN7	1.9903E-10	0.309617156	0.562	0.407	4.819E-06	Meta_CD8_C4
TLN1	2.4964E-10	0.259452809	0.478	0.326	6.045E-06	Meta_CD8_C4
PSMB10	2.6733E-10	0.36006865	0.646	0.490	6.473E-06	Meta_CD8_C4
ADA	2.8776E-10	0.259975656	0.350	0.198	6.968E-06	Meta_CD8_C4
CYBA	2.8835E-10	0.271282111	0.939	0.875	6.982E-06	Meta_CD8_C4
VASP	3.49E-10	0.285868818	0.537	0.372	8.451E-06	Meta_CD8_C4
SH2D2A	3.4934E-10	0.264617546	0.584	0.447	8.459E-06	Meta_CD8_C4
POLR2G	4.9363E-10	0.257424008	0.496	0.348	1.195E-05	Meta_CD8_C4
ARHGAP9	4.9743E-10	0.27459239	0.654	0.489	1.205E-05	Meta_CD8_C4
SRGAP3	5.3684E-10	0.272233154	0.101	0.036	1.3E-05	Meta_CD8_C4
MT1E	1.673E-09	0.251483246	0.215	0.116	4.051E-05	Meta_CD8_C4
GPI	1.8552E-09	0.287135035	0.582	0.442	4.492E-05	Meta_CD8_C4
SH3BP1	1.9222E-09	0.319501624	0.475	0.304	4.655E-05	Meta_CD8_C4
RAB1B	1.9649E-09	0.275645205	0.537	0.379	4.758E-05	Meta_CD8_C4
BRK1	1.9956E-09	0.264737644	0.726	0.582	4.832E-05	Meta_CD8_C4
AC092580.4	2.2769E-09	0.796097371	0.109	0.036	5.513E-05	Meta_CD8_C4
SRI	4.4374E-09	0.263884929	0.585	0.437	0.0001075	Meta_CD8_C4
TYMP	4.5008E-09	0.290471634	0.394	0.263	0.000109	Meta_CD8_C4
SEPT7	5.2205E-09	0.297821537	0.823	0.686	0.0001264	Meta_CD8_C4

PSME1	6.4481E-09	0.279382603	0.913	0.841	0.0001561	Meta_CD8_C4
CALR	6.477E-09	0.380555096	0.777	0.653	0.0001568	Meta_CD8_C4
KLRC4	9.3403E-09	0.253747479	0.119	0.035	0.0002262	Meta_CD8_C4
PTPRCAP	1.0098E-08	0.403615415	0.771	0.736	0.0002445	Meta_CD8_C4
CNN2	1.1403E-08	0.293228587	0.725	0.617	0.0002761	Meta_CD8_C4
PHLDA1	1.5627E-08	0.269759478	0.389	0.261	0.0003784	Meta_CD8_C4
HMGB1	1.9449E-08	0.274770277	0.921	0.865	0.000471	Meta_CD8_C4
PLSCR1	2.2667E-08	0.254495779	0.275	0.158	0.0005489	Meta_CD8_C4
LPXN	2.744E-08	0.259863416	0.538	0.384	0.0006645	Meta_CD8_C4
LCP2	2.8368E-08	0.267386884	0.698	0.581	0.0006869	Meta_CD8_C4
PRKAR1B	6.8723E-08	0.262833744	0.177	0.070	0.0016641	Meta_CD8_C4
PDCD1	1.2244E-07	0.310259056	0.300	0.205	0.0029648	Meta_CD8_C4
LAT	1.233E-07	0.270270302	0.729	0.615	0.0029856	Meta_CD8_C4
GNG5	2.605E-07	0.252854823	0.695	0.555	0.0063081	Meta_CD8_C4
FYB1	6.2908E-07	0.312581947	0.708	0.623	0.0152332	Meta_CD8_C4
COX8A	1.2349E-06	0.257617099	0.804	0.682	0.0299032	Meta_CD8_C4
LAYN	1.2973E-06	0.2857223	0.130	0.056	0.0314152	Meta_CD8_C4
TRGV10	1.2989E-06	0.392622051	0.172	0.094	0.031454	Meta_CD8_C4
MT2A	1.3323E-06	0.363799199	0.636	0.507	0.0322606	Meta_CD8_C4
TRGV9	1.5599E-06	0.290611735	0.109	0.045	0.0377739	Meta_CD8_C4
TRBC2	2.4846E-06	0.370215074	0.822	0.703	0.0601652	Meta_CD8_C4
BCL2L11	2.9731E-06	0.259365197	0.224	0.117	0.0719929	Meta_CD8_C4
IFI6	2.99E-06	0.26215967	0.426	0.298	0.0724035	Meta_CD8_C4
SP140	3.0202E-06	0.259924137	0.432	0.290	0.0731352	Meta_CD8_C4
LINC00152	1.4589E-05	0.351134224	0.114	0.067	0.3532628	Meta_CD8_C4
LYST	1.6688E-05	0.267497843	0.325	0.206	0.4041068	Meta_CD8_C4
TNFRSF9	0.00024271	0.329947736	0.105	0.050	1	Meta_CD8_C4
TNFSF9	0.00029707	0.342351615	0.220	0.125	1	Meta_CD8_C4
NKG7	3.434E-201	1.602553849	0.990	0.567	8.32E-197	Meta_CD8_C5
GZMH	6.978E-125	1.601066251	0.774	0.287	1.69E-120	Meta_CD8_C5
FGFBP2	2.574E-105	2.282150939	0.394	0.032	6.23E-101	Meta_CD8_C5
CCL5	8.1342E-91	0.720597371	0.989	0.748	1.97E-86	Meta_CD8_C5
GZMB	2.2408E-84	0.982649898	0.701	0.292	5.426E-80	Meta_CD8_C5
GNLY	1.2913E-73	1.579700584	0.565	0.203	3.127E-69	Meta_CD8_C5
CTSW	1.463E-62	0.88588719	0.756	0.431	3.543E-58	Meta_CD8_C5
KLRD1	8.5603E-60	1.230122752	0.589	0.247	2.073E-55	Meta_CD8_C5
CST7	1.5296E-58	0.707712552	0.881	0.694	3.704E-54	Meta_CD8_C5
CD8A	9.6315E-57	0.721756048	0.780	0.436	2.332E-52	Meta_CD8_C5
CD8B	2.7506E-56	0.95467729	0.719	0.370	6.661E-52	Meta_CD8_C5
LITAF	4.2893E-49	0.777624327	0.742	0.489	1.039E-44	Meta_CD8_C5

CCL4	4.7475E-49	0.374039109	0.742	0.426	1.15E-44	Meta_CD8_C5
CD52	5.6982E-48	0.64773942	0.971	0.933	1.38E-43	Meta_CD8_C5
C12orf75	1.2927E-45	0.862110523	0.617	0.321	3.13E-41	Meta_CD8_C5
PRF1	4.7534E-45	1.065710146	0.680	0.413	1.151E-40	Meta_CD8_C5
TMSB10	3.1862E-43	0.405990436	0.997	0.993	7.715E-39	Meta_CD8_C5
LYAR	1.8767E-41	0.862680884	0.578	0.315	4.544E-37	Meta_CD8_C5
CLIC3	9.0744E-41	1.000372638	0.421	0.188	2.197E-36	Meta_CD8_C5
FLNA	3.5773E-37	0.803500055	0.652	0.420	8.662E-33	Meta_CD8_C5
SH3BGRL3	8.521E-37	0.372806759	0.968	0.960	2.063E-32	Meta_CD8_C5
PXN	2.9962E-36	0.531800567	0.323	0.135	7.255E-32	Meta_CD8_C5
FCGR3A	4.2469E-36	0.967427613	0.173	0.032	1.028E-31	Meta_CD8_C5
KLF2	5.666E-36	0.736432604	0.586	0.310	1.372E-31	Meta_CD8_C5
HLA-DPB1	9.3508E-35	0.598635056	0.684	0.443	2.264E-30	Meta_CD8_C5
ZNF683	1.7211E-33	1.042719066	0.279	0.094	4.168E-29	Meta_CD8_C5
SAMD3	1.2473E-31	0.666720234	0.437	0.195	3.02E-27	Meta_CD8_C5
ITGB2	8.9101E-31	0.62709027	0.774	0.609	2.158E-26	Meta_CD8_C5
S100A6	3.0858E-30	0.472383412	0.941	0.887	7.472E-26	Meta_CD8_C5
CDC25B	9.1887E-30	0.6817468	0.409	0.191	2.225E-25	Meta_CD8_C5
EMP3	1.0447E-27	0.535136064	0.811	0.697	2.53E-23	Meta_CD8_C5
C1orf21	2.5147E-26	0.56335484	0.187	0.043	6.089E-22	Meta_CD8_C5
HLA-DRB1	3.0423E-26	0.442704335	0.651	0.433	7.367E-22	Meta_CD8_C5
BIN2	3.9311E-25	0.671342899	0.612	0.441	9.519E-21	Meta_CD8_C5
CD74	3.3929E-24	0.424053025	0.879	0.874	8.216E-20	Meta_CD8_C5
S1PR5	3.6176E-24	0.670756267	0.137	0.019	8.76E-20	Meta_CD8_C5
KLF3	3.2957E-23	0.567887894	0.357	0.171	7.98E-19	Meta_CD8_C5
GZMK	3.7817E-23	0.392197751	0.590	0.370	9.157E-19	Meta_CD8_C5
PLEK	4.3439E-23	0.618716318	0.258	0.103	1.052E-18	Meta_CD8_C5
MATK	1.0324E-22	0.503800063	0.440	0.275	2.5E-18	Meta_CD8_C5
HLA-C	1.4299E-22	0.303544006	0.998	0.994	3.463E-18	Meta_CD8_C5
CNN2	3.0765E-22	0.446468479	0.671	0.630	7.45E-18	Meta_CD8_C5
MYO1F	1.2701E-21	0.619397052	0.488	0.321	3.076E-17	Meta_CD8_C5
AES	1.4754E-21	0.484195645	0.678	0.591	3.573E-17	Meta_CD8_C5
SH3BP5	1.6637E-21	0.57013328	0.306	0.136	4.029E-17	Meta_CD8_C5
FCRL6	4.154E-21	0.542704412	0.198	0.069	1.006E-16	Meta_CD8_C5
LGALS1	4.8486E-21	0.704540938	0.632	0.435	1.174E-16	Meta_CD8_C5
HLA-DRA	1.6895E-20	0.532698142	0.498	0.304	4.091E-16	Meta_CD8_C5
HLA-DPA1	2.3186E-20	0.42305136	0.635	0.473	5.614E-16	Meta_CD8_C5
GZMM	2.5095E-20	0.503448801	0.696	0.544	6.077E-16	Meta_CD8_C5
LINC02446	3.3888E-20	0.777786147	0.262	0.107	8.206E-16	Meta_CD8_C5
SPON2	1.1342E-19	0.970468197	0.245	0.115	2.747E-15	Meta_CD8_C5

MYO1G	1.8887E-19	0.443286508	0.453	0.327	4.573E-15	Meta_CD8_C5
CD99	2.7042E-19	0.298612879	0.882	0.841	6.548E-15	Meta_CD8_C5
S100A4	4.1496E-19	0.291253009	0.946	0.868	1.005E-14	Meta_CD8_C5
TPST2	4.3522E-19	0.563865393	0.404	0.259	1.054E-14	Meta_CD8_C5
APOBEC3G	1.0064E-18	0.503678809	0.634	0.443	2.437E-14	Meta_CD8_C5
S1PR4	2.0265E-18	0.631102035	0.435	0.277	4.907E-14	Meta_CD8_C5
FGR	5.5998E-18	0.405562327	0.102	0.021	1.356E-13	Meta_CD8_C5
S1PR1	2.8418E-17	0.403560997	0.271	0.125	6.882E-13	Meta_CD8_C5
PRKCB	8.3882E-17	0.466584827	0.318	0.163	2.031E-12	Meta_CD8_C5
YBX3	2.0826E-16	0.322934193	0.169	0.062	5.043E-12	Meta_CD8_C5
ZEB2	4.6095E-16	0.661020927	0.309	0.153	1.116E-11	Meta_CD8_C5
LIME1	6.3189E-16	0.392913524	0.487	0.371	1.53E-11	Meta_CD8_C5
HLA-DQA1	1.5492E-15	0.31374573	0.263	0.148	3.751E-11	Meta_CD8_C5
KLRG1	1.7398E-15	0.594352175	0.318	0.173	4.213E-11	Meta_CD8_C5
NEAT1	4.3255E-15	0.510702858	0.658	0.544	1.047E-10	Meta_CD8_C5
P2RY8	3.6237E-14	0.50351244	0.383	0.226	8.775E-10	Meta_CD8_C5
RASA3	6.6893E-14	0.377696273	0.340	0.194	1.62E-09	Meta_CD8_C5
S100A10	7.6076E-14	0.38088123	0.864	0.804	1.842E-09	Meta_CD8_C5
YWHAQ	4.7507E-13	0.315679017	0.637	0.567	1.15E-08	Meta_CD8_C5
RAB29	5.5902E-13	0.383128263	0.310	0.192	1.354E-08	Meta_CD8_C5
CMC1	5.6537E-13	0.691405347	0.311	0.230	1.369E-08	Meta_CD8_C5
ADGRG1	7.298E-13	0.457693796	0.135	0.059	1.767E-08	Meta_CD8_C5
ITGB1	9.3864E-13	0.45163432	0.372	0.269	2.273E-08	Meta_CD8_C5
RIPOR2	9.8197E-13	0.338853585	0.361	0.246	2.378E-08	Meta_CD8_C5
CCND3	1.6288E-12	0.422658318	0.639	0.544	3.944E-08	Meta_CD8_C5
CYBA	9.612E-12	0.329228434	0.911	0.883	2.328E-07	Meta_CD8_C5
LIMD2	2.1949E-11	0.304843298	0.727	0.717	5.315E-07	Meta_CD8_C5
RPL13A	3.6323E-11	0.332162027	0.998	0.987	8.796E-07	Meta_CD8_C5
PPP2R5C	4.5963E-11	0.436375843	0.614	0.504	1.113E-06	Meta_CD8_C5
HOPX	5.1011E-11	0.412183241	0.494	0.345	1.235E-06	Meta_CD8_C5
BIN1	5.7777E-11	0.340969019	0.528	0.447	1.399E-06	Meta_CD8_C5
HLA-DQB1	9.7202E-11	0.279921016	0.312	0.196	2.354E-06	Meta_CD8_C5
MT1E	1.1234E-10	0.437897066	0.221	0.128	2.72E-06	Meta_CD8_C5
TGFBR3	1.168E-10	0.322779943	0.202	0.100	2.828E-06	Meta_CD8_C5
EFHD2	1.2417E-10	0.453124087	0.355	0.255	3.007E-06	Meta_CD8_C5
HLA-DRB5	1.7805E-10	0.353390793	0.294	0.168	4.312E-06	Meta_CD8_C5
EOMES	3.0199E-10	0.255385069	0.188	0.098	7.313E-06	Meta_CD8_C5
RASGRP2	3.3008E-10	0.389217057	0.313	0.195	7.993E-06	Meta_CD8_C5
HCST	8.0601E-10	0.281784516	0.889	0.842	1.952E-05	Meta_CD8_C5
GLUL	1.6881E-09	0.385852339	0.252	0.142	4.088E-05	Meta_CD8_C5

DSTN	2.3827E-09	0.446950208	0.480	0.373	5.77E-05	Meta_CD8_C5
CDKN2D	3.2951E-09	0.339499264	0.261	0.163	7.979E-05	Meta_CD8_C5
LINC00861	4.1523E-09	0.39623064	0.339	0.211	0.0001005	Meta_CD8_C5
ANXA5	5.7244E-09	0.276832445	0.513	0.445	0.0001386	Meta_CD8_C5
ISG20	7.5063E-09	0.273326486	0.672	0.629	0.0001818	Meta_CD8_C5
ARRB2	1.1559E-08	0.2691683	0.483	0.423	0.0002799	Meta_CD8_C5
CAPN2	1.5824E-08	0.332963884	0.493	0.389	0.0003832	Meta_CD8_C5
DOK2	2.7129E-08	0.320799082	0.502	0.435	0.0006569	Meta_CD8_C5
TSPAN2	2.949E-08	0.261943436	0.106	0.038	0.0007141	Meta_CD8_C5
NOSIP	5.0379E-08	0.26617326	0.433	0.403	0.0012199	Meta_CD8_C5
TRGC2	5.9316E-08	0.553732137	0.187	0.109	0.0014363	Meta_CD8_C5
TSPAN32	8.0224E-08	0.304758548	0.138	0.058	0.0019426	Meta_CD8_C5
GNB2L1	1.3081E-07	0.562287863	0.252	0.165	0.0031676	Meta_CD8_C5
MED15	1.9655E-07	0.314713387	0.336	0.242	0.0047594	Meta_CD8_C5
GSTP1	2.0585E-07	0.330458188	0.633	0.530	0.0049846	Meta_CD8_C5
BHLHE40	3.2754E-07	0.307862788	0.463	0.382	0.0079314	Meta_CD8_C5
CEP78	3.9128E-07	0.256763195	0.116	0.063	0.0094749	Meta_CD8_C5
PATL2	5.8601E-07	0.381586536	0.162	0.082	0.0141903	Meta_CD8_C5
SYNE1	6.5427E-07	0.28554866	0.344	0.255	0.0158432	Meta_CD8_C5
STK38	7.7551E-07	0.273569553	0.276	0.184	0.0187791	Meta_CD8_C5
SELPLG	8.6321E-07	0.288564589	0.502	0.461	0.0209026	Meta_CD8_C5
CHST12	8.9514E-07	0.323861289	0.429	0.336	0.0216758	Meta_CD8_C5
MT-ND4	1.1416E-06	0.336481418	0.960	0.926	0.0276436	Meta_CD8_C5
CD47	1.219E-06	0.321399952	0.486	0.429	0.0295171	Meta_CD8_C5
APMAP	1.3614E-06	0.339117651	0.424	0.347	0.0329675	Meta_CD8_C5
RNF166	3.3663E-06	0.340702363	0.445	0.349	0.0815147	Meta_CD8_C5
PLAC8	4.0844E-06	0.42979105	0.188	0.142	0.0989037	Meta_CD8_C5
RPL27A	4.5725E-06	0.324798418	0.959	0.938	0.110723	Meta_CD8_C5
PYHIN1	5.1653E-06	0.344932568	0.366	0.297	0.1250779	Meta_CD8_C5
KLF13	6.807E-06	0.304020422	0.293	0.200	0.1648305	Meta_CD8_C5
UCP2	8.3732E-06	0.286044871	0.535	0.498	0.2027567	Meta_CD8_C5
CEBPD	8.956E-06	0.420447918	0.210	0.146	0.2168695	Meta_CD8_C5
CD300A	9.6964E-06	0.339734128	0.179	0.105	0.2347976	Meta_CD8_C5
TTC38	1.6345E-05	0.321294301	0.106	0.066	0.3957874	Meta_CD8_C5
PRMT2	2.3513E-05	0.310560357	0.524	0.440	0.5693761	Meta_CD8_C5
TBX21	3.7228E-05	0.336433149	0.279	0.185	0.9014855	Meta_CD8_C5
ATP5G2	3.9433E-05	0.310253484	0.160	0.118	0.9548814	Meta_CD8_C5
CD55	9.1643E-05	0.276009058	0.352	0.270	1	Meta_CD8_C5
CDC42SE1	0.00010016	0.292127762	0.426	0.385	1	Meta_CD8_C5
ADRB2	0.00013265	0.349391976	0.147	0.077	1	Meta_CD8_C5

GLIPR2	0.0002044	0.258382075	0.384	0.314	1	Meta_CD8_C5
RPS17	0.00020666	0.326014885	0.759	0.739	1	Meta_CD8_C5
AHNAK	0.00021926	0.275426837	0.603	0.529	1	Meta_CD8_C5
GABARAPL1	0.00073595	0.271255561	0.389	0.293	1	Meta_CD8_C5
VAMP2	0.00081144	0.350298219	0.469	0.408	1	Meta_CD8_C5
TXNIP	0.00098123	0.337446952	0.771	0.754	1	Meta_CD8_C5
GLTSCR2	0.00154573	0.311792537	0.158	0.109	1	Meta_CD8_C5
TGFB1	0.00162682	0.337484433	0.552	0.455	1	Meta_CD8_C5
C19orf43	0.00179861	0.383823553	0.130	0.088	1	Meta_CD8_C5
SUPT4H1	0.00180815	0.250627464	0.354	0.331	1	Meta_CD8_C5
PIP4K2A	0.00448447	0.253616487	0.216	0.174	1	Meta_CD8_C5
CD8A	7.717E-112	1.003597579	0.896	0.424	1.87E-107	Meta_CD8_C6
CD8B	2.382E-108	1.075118379	0.824	0.359	5.77E-104	Meta_CD8_C6
CCL5	4.359E-102	0.634952487	0.998	0.743	1.056E-97	Meta_CD8_C6
XCL1	4.428E-48	1.463256105	0.398	0.120	1.072E-43	Meta_CD8_C6
XCL2	1.3102E-37	1.166489272	0.390	0.136	3.173E-33	Meta_CD8_C6
KLRD1	3.0393E-35	0.725578987	0.536	0.241	7.36E-31	Meta_CD8_C6
ANXA1	6.1376E-34	0.637879189	0.873	0.659	1.486E-29	Meta_CD8_C6
HOPX	1.4855E-33	0.583358903	0.602	0.337	3.597E-29	Meta_CD8_C6
ZNF683	5.0376E-32	0.798981661	0.293	0.090	1.22E-27	Meta_CD8_C6
FOS	1.3881E-31	0.706759012	0.887	0.759	3.361E-27	Meta_CD8_C6
FOSB	1.4357E-30	0.770951237	0.715	0.505	3.477E-26	Meta_CD8_C6
CTSW	1.7659E-28	0.47790338	0.671	0.427	4.276E-24	Meta_CD8_C6
FXYD2	2.0181E-27	0.479533294	0.131	0.022	4.887E-23	Meta_CD8_C6
LINC02446	1.8626E-25	0.606824806	0.288	0.102	4.51E-21	Meta_CD8_C6
ZFP36L2	1.1554E-24	0.520649834	0.919	0.869	2.798E-20	Meta_CD8_C6
CD55	2.191E-23	0.525267607	0.436	0.265	5.305E-19	Meta_CD8_C6
NT5E	2.9157E-23	0.298859187	0.118	0.025	7.06E-19	Meta_CD8_C6
IL7R	5.3304E-23	0.467748306	0.749	0.569	1.291E-18	Meta_CD8_C6
ITM2C	2.0679E-22	0.45845345	0.601	0.366	5.007E-18	Meta_CD8_C6
SPRY1	3.6651E-22	0.676171805	0.293	0.136	8.875E-18	Meta_CD8_C6
CCL4	5.3463E-20	0.868997478	0.638	0.424	1.295E-15	Meta_CD8_C6
ZFP36	7.494E-20	0.513339525	0.885	0.821	1.815E-15	Meta_CD8_C6
SCML4	8.0213E-20	0.501281693	0.504	0.358	1.942E-15	Meta_CD8_C6
PTGER2	5.8461E-19	0.515222619	0.410	0.236	1.416E-14	Meta_CD8_C6
VIM	1.292E-18	0.548326016	0.948	0.896	3.129E-14	Meta_CD8_C6
NR4A2	1.5518E-15	0.585560906	0.691	0.574	3.758E-11	Meta_CD8_C6
PARP8	2.6276E-15	0.339415462	0.539	0.428	6.363E-11	Meta_CD8_C6
NR4A1	6.2686E-15	0.636168384	0.429	0.289	1.518E-10	Meta_CD8_C6
CD69	8.0639E-15	0.391517639	0.880	0.831	1.953E-10	Meta_CD8_C6

KLF6	1.3036E-14	0.414115109	0.905	0.860	3.157E-10	Meta_CD8_C6
LYAR	5.0876E-14	0.385311427	0.439	0.314	1.232E-09	Meta_CD8_C6
PTGER4	8.822E-14	0.461500612	0.655	0.545	2.136E-09	Meta_CD8_C6
SPINK2	2.4789E-13	0.293120868	0.135	0.045	6.003E-09	Meta_CD8_C6
GABARAPL1	3.9164E-13	0.42526494	0.442	0.289	9.483E-09	Meta_CD8_C6
AC020916.1	6.4684E-13	0.415548065	0.330	0.221	1.566E-08	Meta_CD8_C6
KLRC1	9.9962E-13	0.561849231	0.157	0.066	2.421E-08	Meta_CD8_C6
GPR15	1.9733E-12	0.30764789	0.225	0.128	4.778E-08	Meta_CD8_C6
ANKRD28	2.5778E-12	0.510005254	0.286	0.172	6.242E-08	Meta_CD8_C6
MYADM	3.715E-12	0.539546613	0.567	0.422	8.996E-08	Meta_CD8_C6
ITGA1	5.0154E-12	0.425553121	0.358	0.214	1.214E-07	Meta_CD8_C6
CAPG	6.9171E-12	0.386287992	0.413	0.276	1.675E-07	Meta_CD8_C6
CD9	2.0845E-11	0.324898068	0.178	0.076	5.048E-07	Meta_CD8_C6
S100A10	1.6716E-10	0.365116284	0.886	0.802	4.048E-06	Meta_CD8_C6
JUN	2.8368E-10	0.417138293	0.877	0.803	6.869E-06	Meta_CD8_C6
SMIM3	2.9735E-10	0.297004004	0.180	0.093	7.2E-06	Meta_CD8_C6
S100A6	3.829E-10	0.265928628	0.948	0.886	9.272E-06	Meta_CD8_C6
CD248	1.8335E-09	0.279311704	0.120	0.037	4.44E-05	Meta_CD8_C6
YBX3	2.709E-09	0.364227439	0.165	0.060	6.56E-05	Meta_CD8_C6
RASGEF1B	3.2369E-09	0.331775604	0.218	0.128	7.838E-05	Meta_CD8_C6
GADD45B	4.798E-09	0.389362452	0.601	0.510	0.0001162	Meta_CD8_C6
TUBA1A	5.0855E-09	0.378075517	0.654	0.560	0.0001231	Meta_CD8_C6
CD63	5.6214E-09	0.329563101	0.590	0.467	0.0001361	Meta_CD8_C6
TOB1	6.8223E-09	0.562476888	0.332	0.262	0.0001652	Meta_CD8_C6
TSC22D3	7.7036E-09	0.412088154	0.937	0.889	0.0001865	Meta_CD8_C6
DUSP1	3.073E-08	0.321681606	0.862	0.801	0.0007441	Meta_CD8_C6
MT-ND4L	3.9665E-08	0.2504979	0.847	0.839	0.0009605	Meta_CD8_C6
KLRK1	5.9597E-08	0.2898443	0.177	0.075	0.0014431	Meta_CD8_C6
PERP	6.7888E-08	0.298809667	0.302	0.211	0.0016439	Meta_CD8_C6
EGR1	9.0726E-08	0.436103797	0.271	0.166	0.0021969	Meta_CD8_C6
PPP1R15A	1.5297E-07	0.330129261	0.742	0.665	0.0037043	Meta_CD8_C6
PTPN22	2.0953E-07	0.279046759	0.461	0.375	0.0050738	Meta_CD8_C6
NR4A3	3.0724E-07	0.373785317	0.284	0.182	0.0074399	Meta_CD8_C6
RGCC	4.4488E-07	0.373960172	0.579	0.526	0.0107727	Meta_CD8_C6
TNFAIP3	5.1381E-07	0.312179784	0.825	0.771	0.012442	Meta_CD8_C6
MT-ND4	5.9588E-07	0.26179928	0.946	0.926	0.0144292	Meta_CD8_C6
MYBL1	6.7398E-07	0.311184092	0.196	0.105	0.0163204	Meta_CD8_C6
GLUL	7.2839E-07	0.49682739	0.235	0.140	0.0176379	Meta_CD8_C6
JAML	1.1128E-06	0.355173136	0.402	0.293	0.0269458	Meta_CD8_C6
BTG2	2.6722E-06	0.291229767	0.681	0.611	0.0647082	Meta_CD8_C6

CXCR4	3.2572E-06	0.276059275	0.896	0.829	0.078874	Meta_CD8_C6
ID2	5.9081E-06	0.28048541	0.694	0.592	0.143065	Meta_CD8_C6
CITED2	7.5611E-06	0.423024404	0.573	0.494	0.183091	Meta_CD8_C6
RGS2	8.7037E-06	0.257978369	0.467	0.407	0.2107602	Meta_CD8_C6
TNFSF9	8.7926E-06	0.318614078	0.208	0.134	0.2129132	Meta_CD8_C6
JUND	2.3113E-05	0.251460922	0.492	0.421	0.5596693	Meta_CD8_C6
STOM	2.4355E-05	0.262307698	0.496	0.404	0.5897506	Meta_CD8_C6
PLP2	2.5805E-05	0.268356896	0.654	0.558	0.6248779	Meta_CD8_C6
CSRNP1	2.8213E-05	0.325348626	0.593	0.502	0.6831667	Meta_CD8_C6
RUNX3	2.9041E-05	0.280386435	0.523	0.422	0.703237	Meta_CD8_C6
TRGV10	4.4318E-05	0.263984051	0.157	0.102	1	Meta_CD8_C6
ATF3	5.0046E-05	0.253536278	0.183	0.144	1	Meta_CD8_C6
LMNA	0.00010578	0.502932743	0.349	0.273	1	Meta_CD8_C6
AREG	0.00017862	0.370709323	0.248	0.176	1	Meta_CD8_C6
MYC	0.00021185	0.304321809	0.235	0.177	1	Meta_CD8_C6
ITGAE	0.00119161	0.259814952	0.419	0.327	1	Meta_CD8_C6
HIST1H4C	0.00126681	0.25082148	0.625	0.566	1	Meta_CD8_C6
CCL4L2	0.00597821	0.765390904	0.244	0.174	1	Meta_CD8_C6
ISG15	1.242E-253	3.089018106	0.926	0.409	3.01E-249	Meta_CD8_C7
LY6E	2.376E-243	1.806128686	0.957	0.737	5.75E-239	Meta_CD8_C7
IFI6	9.603E-227	2.595361012	0.847	0.307	2.33E-222	Meta_CD8_C7
MX1	7.213E-225	2.628667224	0.814	0.206	1.75E-220	Meta_CD8_C7
ISG20	1.348E-195	1.918390767	0.920	0.626	3.26E-191	Meta_CD8_C7
IFIT3	3.587E-149	2.289911179	0.557	0.051	8.69E-145	Meta_CD8_C7
OAS1	1.141E-147	1.986270534	0.607	0.111	2.76E-143	Meta_CD8_C7
IFI44L	1.967E-144	2.21899864	0.576	0.086	4.76E-140	Meta_CD8_C7
IFIT1	9.544E-141	2.083622328	0.477	0.024	2.31E-136	Meta_CD8_C7
PSMB9	1.173E-139	1.247169829	0.884	0.683	2.84E-135	Meta_CD8_C7
RSAD2	6.656E-135	1.962425608	0.502	0.042	1.61E-130	Meta_CD8_C7
UBE2L6	1.97E-123	1.403290622	0.758	0.470	4.77E-119	Meta_CD8_C7
GZMB	1.666E-121	2.068783888	0.723	0.293	4.03E-117	Meta_CD8_C7
MT2A	1.757E-114	1.670898664	0.839	0.519	4.26E-110	Meta_CD8_C7
EPSTI1	5.288E-110	1.621698122	0.641	0.234	1.28E-105	Meta_CD8_C7
HERC5	1.214E-106	1.618955611	0.502	0.102	2.94E-102	Meta_CD8_C7
LAG3	9.33E-106	1.576575285	0.742	0.366	2.26E-101	Meta_CD8_C7
BST2	2.37E-104	1.272057854	0.747	0.492	5.74E-100	Meta_CD8_C7
IFITM1	1.354E-99	0.867729296	0.618	0.809	3.279E-95	Meta_CD8_C7
IFI16	9.04E-99	1.596497606	0.770	0.414	2.189E-94	Meta_CD8_C7
OASL	6.7065E-97	1.623394034	0.606	0.209	1.624E-92	Meta_CD8_C7
PLSCR1	2.5211E-95	1.649702216	0.551	0.168	6.105E-91	Meta_CD8_C7

HLA-A	8.6863E-93	0.619689225	0.998	0.997	2.103E-88	Meta_CD8_C7
XAF1	9.6177E-93	1.476435885	0.606	0.212	2.329E-88	Meta_CD8_C7
PRF1	8.3445E-91	1.45946757	0.694	0.413	2.021E-86	Meta_CD8_C7
IFI35	6.6455E-90	1.549941575	0.612	0.250	1.609E-85	Meta_CD8_C7
STAT1	9.5638E-89	1.301132665	0.693	0.378	2.316E-84	Meta_CD8_C7
GZMA	1.1638E-87	1.015054614	0.880	0.577	2.818E-83	Meta_CD8_C7
DRAP1	1.1768E-85	1.043858716	0.797	0.650	2.85E-81	Meta_CD8_C7
B2M	3.6223E-85	0.502051093	1.000	1.000	8.771E-81	Meta_CD8_C7
NT5C3A	6.8453E-81	1.545393563	0.541	0.245	1.658E-76	Meta_CD8_C7
NKG7	6.288E-80	1.377372242	0.846	0.570	1.523E-75	Meta_CD8_C7
TYMP	1.299E-76	1.420187946	0.619	0.275	3.146E-72	Meta_CD8_C7
SP100	4.5154E-76	1.087616279	0.748	0.548	1.093E-71	Meta_CD8_C7
IRF7	6.0809E-76	1.396373443	0.594	0.263	1.472E-71	Meta_CD8_C7
CXCL13	6.6736E-76	2.623110736	0.351	0.064	1.616E-71	Meta_CD8_C7
SAMD9L	7.51E-76	1.543496924	0.509	0.175	1.819E-71	Meta_CD8_C7
RARRES3	4.7695E-74	0.860277417	0.843	0.739	1.155E-69	Meta_CD8_C7
OAS3	1.1369E-73	1.200301	0.363	0.065	2.753E-69	Meta_CD8_C7
IFI44	1.8154E-72	1.440248121	0.400	0.096	4.396E-68	Meta_CD8_C7
GBP1	4.9214E-72	1.342069579	0.585	0.261	1.192E-67	Meta_CD8_C7
GZMH	8.8527E-72	1.261285705	0.658	0.289	2.144E-67	Meta_CD8_C7
CD38	5.1802E-70	1.422678216	0.490	0.165	1.254E-65	Meta_CD8_C7
EIF2AK2	1.2093E-68	1.360583139	0.545	0.212	2.928E-64	Meta_CD8_C7
GNLY	3.9688E-67	2.095990138	0.518	0.205	9.611E-63	Meta_CD8_C7
TAP1	2.0977E-66	1.009283828	0.692	0.494	5.08E-62	Meta_CD8_C7
CCL5	2.569E-66	0.869270415	0.943	0.750	6.221E-62	Meta_CD8_C7
RNF213	4.1622E-66	0.966701971	0.739	0.583	1.008E-61	Meta_CD8_C7
TNFSF10	1.3124E-64	1.517440121	0.529	0.227	3.178E-60	Meta_CD8_C7
CMPK2	4.0665E-63	1.197071144	0.345	0.052	9.847E-59	Meta_CD8_C7
SAMD9	1.3519E-62	1.385451424	0.498	0.224	3.274E-58	Meta_CD8_C7
PSME2	1.7673E-62	0.804584638	0.780	0.660	4.28E-58	Meta_CD8_C7
HAVCR2	5.1292E-62	1.538366184	0.440	0.138	1.242E-57	Meta_CD8_C7
IFIT2	8.0719E-60	1.506066644	0.299	0.041	1.955E-55	Meta_CD8_C7
IFITM2	4.4936E-59	0.817093413	0.897	0.805	1.088E-54	Meta_CD8_C7
HLA-C	1.3767E-58	0.483216305	0.998	0.994	3.334E-54	Meta_CD8_C7
TMSB10	1.5689E-58	0.529744753	0.997	0.993	3.799E-54	Meta_CD8_C7
CD74	2.8905E-58	0.666406446	0.929	0.873	6.999E-54	Meta_CD8_C7
PSMB8	1.3694E-56	0.877784667	0.739	0.599	3.316E-52	Meta_CD8_C7
PSME1	1.5205E-56	0.505281172	0.848	0.850	3.682E-52	Meta_CD8_C7
MX2	8.5826E-54	1.225379828	0.406	0.149	2.078E-49	Meta_CD8_C7
LGALS9	3.2214E-53	1.090429203	0.377	0.126	7.801E-49	Meta_CD8_C7

USP18	1.2845E-51	0.706642348	0.257	0.036	3.11E-47	Meta_CD8_C7
ADAR	5.3706E-51	0.935257947	0.556	0.376	1.3E-46	Meta_CD8_C7
CHST12	1.3403E-50	1.111082718	0.577	0.334	3.245E-46	Meta_CD8_C7
PARP14	1.8596E-50	1.113300046	0.431	0.180	4.503E-46	Meta_CD8_C7
IFI27	1.8921E-49	2.584927049	0.250	0.041	4.582E-45	Meta_CD8_C7
NMI	9.5126E-49	0.980474585	0.423	0.202	2.303E-44	Meta_CD8_C7
TMSB4X	7.0635E-48	0.420030834	1.000	0.999	1.71E-43	Meta_CD8_C7
LAP3	1.2711E-47	0.986145334	0.462	0.234	3.078E-43	Meta_CD8_C7
ALOX5AP	4.9896E-45	1.009278561	0.668	0.603	1.208E-40	Meta_CD8_C7
TRIM22	5.8732E-45	0.955815865	0.616	0.426	1.422E-40	Meta_CD8_C7
HLA-E	6.0351E-45	0.496794731	0.958	0.958	1.461E-40	Meta_CD8_C7
DDX60	3.6321E-44	0.904137203	0.307	0.092	8.795E-40	Meta_CD8_C7
CCL4	1.5105E-43	0.614924112	0.731	0.427	3.658E-39	Meta_CD8_C7
GBP2	1.5368E-43	0.848325827	0.601	0.414	3.721E-39	Meta_CD8_C7
CLEC2B	9.7096E-43	0.808475313	0.747	0.628	2.351E-38	Meta_CD8_C7
LSP1	1.6767E-41	0.457277997	0.824	0.831	4.06E-37	Meta_CD8_C7
APOBEC3G	8.8933E-40	0.979569632	0.651	0.443	2.154E-35	Meta_CD8_C7
GAPDH	1.7183E-39	0.624523285	0.981	0.975	4.161E-35	Meta_CD8_C7
ZBP1	2.639E-39	0.907008018	0.410	0.174	6.39E-35	Meta_CD8_C7
HLA-DRA	1.1634E-37	1.13458655	0.542	0.303	2.817E-33	Meta_CD8_C7
HELZ2	1.6756E-37	0.936998212	0.291	0.083	4.058E-33	Meta_CD8_C7
XIST	4.5155E-37	1.069445181	0.284	0.110	1.093E-32	Meta_CD8_C7
HLA-DRB1	1.519E-36	0.967782768	0.674	0.433	3.678E-32	Meta_CD8_C7
TRAC	2.6115E-36	1.144863396	0.730	0.567	6.324E-32	Meta_CD8_C7
LIMD2	6.135E-36	0.360901667	0.692	0.717	1.486E-31	Meta_CD8_C7
PARP9	7.8912E-36	0.936412845	0.403	0.176	1.911E-31	Meta_CD8_C7
PHF11	8.0317E-36	0.792348578	0.462	0.305	1.945E-31	Meta_CD8_C7
CD2	3.6993E-35	0.644302476	0.907	0.841	8.958E-31	Meta_CD8_C7
MYL12A	4.2865E-35	0.590882449	0.938	0.885	1.038E-30	Meta_CD8_C7
OAS2	1.107E-34	0.867916468	0.370	0.156	2.681E-30	Meta_CD8_C7
GBP5	1.2214E-34	0.949615236	0.568	0.397	2.958E-30	Meta_CD8_C7
CD3D	1.8636E-34	0.589524714	0.940	0.890	4.513E-30	Meta_CD8_C7
CCL3	4.2383E-34	1.458281557	0.347	0.133	1.026E-29	Meta_CD8_C7
GZMK	1.1202E-33	0.822594304	0.636	0.370	2.713E-29	Meta_CD8_C7
HLA-B	2.0863E-33	0.354351894	0.999	0.997	5.052E-29	Meta_CD8_C7
IFITM3	1.23E-32	0.950326587	0.338	0.158	2.979E-28	Meta_CD8_C7
GBP4	1.2427E-32	0.98168341	0.446	0.222	3.009E-28	Meta_CD8_C7
SP110	1.7021E-32	0.733406261	0.480	0.312	4.122E-28	Meta_CD8_C7
COX5A	2.9823E-32	0.782554422	0.644	0.563	7.222E-28	Meta_CD8_C7
PPM1K	8.166E-32	0.878083492	0.419	0.250	1.977E-27	Meta_CD8_C7

IDH2	1.4611E-31	0.727301214	0.587	0.438	3.538E-27	Meta_CD8_C7
CHMP5	1.7122E-31	0.771774823	0.403	0.268	4.146E-27	Meta_CD8_C7
IRF1	1.735E-31	0.726175495	0.625	0.586	4.201E-27	Meta_CD8_C7
GIMAP7	2.6642E-31	0.596577687	0.707	0.625	6.451E-27	Meta_CD8_C7
CLEC2D	3.1835E-31	0.878042033	0.595	0.478	7.709E-27	Meta_CD8_C7
TPI1	3.2785E-31	0.660849318	0.753	0.716	7.939E-27	Meta_CD8_C7
RBPJ	6.7503E-31	1.093712273	0.525	0.336	1.635E-26	Meta_CD8_C7
LYST	1.5695E-30	0.833434397	0.415	0.219	3.8E-26	Meta_CD8_C7
MYL12B	3.9501E-30	0.353263841	0.837	0.858	9.565E-26	Meta_CD8_C7
RBCK1	6.6945E-30	0.715181407	0.515	0.375	1.621E-25	Meta_CD8_C7
STAT2	9.4295E-30	0.748729344	0.280	0.103	2.283E-25	Meta_CD8_C7
C19orf66	1.0577E-29	0.799248696	0.540	0.393	2.561E-25	Meta_CD8_C7
MYL6	6.7226E-29	0.333620903	0.944	0.947	1.628E-24	Meta_CD8_C7
HLA-DPA1	1.9112E-28	0.831734113	0.625	0.473	4.628E-24	Meta_CD8_C7
HLA-DPB1	2.8135E-28	0.781233219	0.626	0.444	6.813E-24	Meta_CD8_C7
SUB1	3.5321E-28	0.520647287	0.837	0.810	8.553E-24	Meta_CD8_C7
IFNG	3.8726E-28	0.869543918	0.466	0.248	9.377E-24	Meta_CD8_C7
GNB2L1	5.2973E-28	0.58321985	0.347	0.163	1.283E-23	Meta_CD8_C7
SAT1	3.1966E-27	0.803248906	0.740	0.641	7.741E-23	Meta_CD8_C7
HSPB1	3.78E-27	0.842733675	0.383	0.394	9.153E-23	Meta_CD8_C7
DDX60L	4.1296E-27	0.680410592	0.250	0.102	1E-22	Meta_CD8_C7
POMP	4.2062E-27	0.533143346	0.626	0.550	1.019E-22	Meta_CD8_C7
C4orf3	5.6679E-27	0.51331077	0.630	0.596	1.372E-22	Meta_CD8_C7
GNG5	5.8146E-27	0.54198507	0.617	0.572	1.408E-22	Meta_CD8_C7
CYBA	8.8729E-27	0.3640482	0.880	0.884	2.149E-22	Meta_CD8_C7
PSMA4	2.2477E-26	0.693893196	0.485	0.346	5.443E-22	Meta_CD8_C7
ARPC2	3.3605E-26	0.343053908	0.878	0.886	8.138E-22	Meta_CD8_C7
HSP90AA1	4.0626E-26	0.764204835	0.928	0.902	9.838E-22	Meta_CD8_C7
SPATS2L	6.5505E-26	0.65968734	0.218	0.077	1.586E-21	Meta_CD8_C7
CFL1	6.9526E-26	0.321190446	0.952	0.959	1.684E-21	Meta_CD8_C7
CCR1	1.0743E-25	0.655017035	0.170	0.037	2.601E-21	Meta_CD8_C7
CLIC1	2.4626E-25	0.433612893	0.837	0.825	5.963E-21	Meta_CD8_C7
APOL6	2.5631E-25	0.691773622	0.441	0.281	6.206E-21	Meta_CD8_C7
IFIH1	2.9867E-25	0.704603529	0.231	0.076	7.232E-21	Meta_CD8_C7
FYB	6.2936E-25	0.936722123	0.222	0.086	1.524E-20	Meta_CD8_C7
NEAT1	1.7421E-24	0.775409457	0.684	0.544	4.218E-20	Meta_CD8_C7
HSH2D	2.7969E-24	0.798689876	0.325	0.140	6.773E-20	Meta_CD8_C7
CD164	4.0791E-24	0.689473249	0.584	0.485	9.878E-20	Meta_CD8_C7
ATP5E	4.244E-24	0.843339006	0.337	0.144	1.028E-19	Meta_CD8_C7
CD27	2.6639E-23	0.587856293	0.595	0.487	6.451E-19	Meta_CD8_C7

DDX58	3.2439E-23	0.726998823	0.249	0.107	7.855E-19	Meta_CD8_C7
APOBEC3C	9.8175E-23	0.725297712	0.418	0.282	2.377E-18	Meta_CD8_C7
TAPBP	1.1577E-22	0.340859397	0.604	0.617	2.803E-18	Meta_CD8_C7
CARD16	2.0242E-22	0.757144919	0.520	0.397	4.902E-18	Meta_CD8_C7
HM13	3.2595E-22	0.528938131	0.434	0.377	7.893E-18	Meta_CD8_C7
PSMA5	6.351E-22	0.474973323	0.513	0.443	1.538E-17	Meta_CD8_C7
RTP4	1.3991E-21	0.595493703	0.219	0.077	3.388E-17	Meta_CD8_C7
CST7	1.5485E-21	0.454585303	0.768	0.696	3.75E-17	Meta_CD8_C7
XRN1	1.9943E-21	0.691636293	0.355	0.236	4.829E-17	Meta_CD8_C7
PNPT1	3.0265E-21	0.562025139	0.187	0.089	7.329E-17	Meta_CD8_C7
VAMP5	3.1653E-21	0.632816561	0.451	0.342	7.665E-17	Meta_CD8_C7
SHISA5	3.3061E-21	0.381458275	0.532	0.511	8.006E-17	Meta_CD8_C7
GIMAP4	3.8646E-21	0.570553177	0.642	0.540	9.358E-17	Meta_CD8_C7
ATP5L	8.0326E-21	0.841185081	0.288	0.127	1.945E-16	Meta_CD8_C7
SHFM1	8.4448E-21	0.779315281	0.169	0.065	2.045E-16	Meta_CD8_C7
COX7A2	9.1832E-21	0.386052731	0.742	0.751	2.224E-16	Meta_CD8_C7
TRBC1	1.3078E-20	0.831013873	0.540	0.459	3.167E-16	Meta_CD8_C7
SLFN5	1.3149E-20	0.609913628	0.480	0.378	3.184E-16	Meta_CD8_C7
SEPT7	1.9979E-20	0.489470944	0.714	0.704	4.838E-16	Meta_CD8_C7
SP140	2.9009E-20	0.676399624	0.447	0.307	7.025E-16	Meta_CD8_C7
ARPC3	5.1742E-20	0.26033468	0.788	0.826	1.253E-15	Meta_CD8_C7
RBX1	5.5277E-20	0.506770444	0.519	0.461	1.339E-15	Meta_CD8_C7
KLRD1	6.8721E-20	0.847428797	0.410	0.250	1.664E-15	Meta_CD8_C7
EVL	9.3077E-20	0.454500741	0.848	0.802	2.254E-15	Meta_CD8_C7
MT1E	1.5168E-19	0.62685705	0.246	0.127	3.673E-15	Meta_CD8_C7
TRIM25	2.0549E-19	0.47239424	0.186	0.063	4.976E-15	Meta_CD8_C7
ZNFX1	2.581E-19	0.527372189	0.322	0.202	6.25E-15	Meta_CD8_C7
HSPA1A	2.9629E-19	0.942263824	0.505	0.368	7.175E-15	Meta_CD8_C7
C5orf56	3.2087E-19	0.808861428	0.115	0.023	7.77E-15	Meta_CD8_C7
ATP5G3	3.2805E-19	0.783131487	0.180	0.071	7.944E-15	Meta_CD8_C7
SMCHD1	3.4575E-19	0.542369046	0.553	0.478	8.372E-15	Meta_CD8_C7
ATP5J2	3.7442E-19	0.7579926	0.173	0.070	9.067E-15	Meta_CD8_C7
ELF1	4.1949E-19	0.651896523	0.659	0.580	1.016E-14	Meta_CD8_C7
LPIN2	8.127E-19	0.719314958	0.395	0.262	1.968E-14	Meta_CD8_C7
DYNLT1	8.443E-19	0.58580798	0.404	0.340	2.044E-14	Meta_CD8_C7
PARP10	1.0704E-18	0.589763121	0.348	0.213	2.592E-14	Meta_CD8_C7
S100A11	1.0718E-18	0.480539716	0.740	0.686	2.595E-14	Meta_CD8_C7
CD8A	1.1786E-18	0.548028317	0.629	0.439	2.854E-14	Meta_CD8_C7
CD2BP2	1.2071E-18	0.512162	0.291	0.227	2.923E-14	Meta_CD8_C7
DTX3L	1.4376E-18	0.50248024	0.252	0.128	3.481E-14	Meta_CD8_C7

TRAFD1	1.5666E-18	0.570412092	0.303	0.161	3.793E-14	Meta_CD8_C7
CTSW	1.5719E-18	0.74318464	0.600	0.434	3.806E-14	Meta_CD8_C7
PSMA7	1.7292E-18	0.322737358	0.720	0.741	4.187E-14	Meta_CD8_C7
ETV7	3.083E-18	0.590649405	0.187	0.063	7.466E-14	Meta_CD8_C7
ENO1	4.0125E-18	0.541246928	0.703	0.703	9.716E-14	Meta_CD8_C7
IGKC	5.6667E-18	0.399903045	0.272	0.125	1.372E-13	Meta_CD8_C7
PKM	5.7049E-18	0.404315623	0.665	0.657	1.381E-13	Meta_CD8_C7
NUB1	6.5082E-18	0.611400189	0.364	0.256	1.576E-13	Meta_CD8_C7
WIPF1	9.0735E-18	0.388766226	0.631	0.611	2.197E-13	Meta_CD8_C7
GPBP1	1.0323E-17	0.709089952	0.490	0.424	2.5E-13	Meta_CD8_C7
CAPZB	1.0426E-17	0.316311313	0.692	0.710	2.525E-13	Meta_CD8_C7
ATP5G2	1.3328E-17	0.746219051	0.244	0.116	3.227E-13	Meta_CD8_C7
IRF9	1.7097E-17	0.536320096	0.202	0.105	4.14E-13	Meta_CD8_C7
CASP4	2.557E-17	0.429946442	0.492	0.430	6.192E-13	Meta_CD8_C7
LINC00152	5.3571E-17	0.785888879	0.182	0.071	1.297E-12	Meta_CD8_C7
LDHA	1.086E-16	0.318755745	0.783	0.789	2.63E-12	Meta_CD8_C7
PGK1	1.4063E-16	0.254796058	0.705	0.757	3.405E-12	Meta_CD8_C7
MALAT1	2.082E-16	0.457613487	0.999	1.000	5.042E-12	Meta_CD8_C7
IFIT5	2.2789E-16	0.536930166	0.206	0.078	5.518E-12	Meta_CD8_C7
ANXA5	3.7672E-16	0.397769632	0.484	0.445	9.122E-12	Meta_CD8_C7
HAPLN3	3.8703E-16	0.512214038	0.235	0.108	9.372E-12	Meta_CD8_C7
ATP5J	4.7102E-16	0.580211983	0.131	0.060	1.141E-11	Meta_CD8_C7
CNP	8.26E-16	0.621979601	0.231	0.092	2E-11	Meta_CD8_C7
PSMB10	9.1578E-16	0.344538577	0.510	0.510	2.218E-11	Meta_CD8_C7
RGS1	1.2397E-15	0.799475171	0.732	0.619	3.002E-11	Meta_CD8_C7
EOMES	1.7938E-15	0.414936097	0.196	0.099	4.344E-11	Meta_CD8_C7
CD8B	2.0942E-15	0.648383162	0.545	0.374	5.071E-11	Meta_CD8_C7
UBB	2.2223E-15	0.436368913	0.907	0.894	5.381E-11	Meta_CD8_C7
RAB27A	2.7788E-15	0.575235471	0.419	0.315	6.729E-11	Meta_CD8_C7
SOX4	3.422E-15	0.523375099	0.106	0.030	8.286E-11	Meta_CD8_C7
REEP5	3.9428E-15	0.336192046	0.543	0.517	9.548E-11	Meta_CD8_C7
PSMB3	4.3817E-15	0.345621332	0.567	0.544	1.061E-10	Meta_CD8_C7
HCST	5.2269E-15	0.41253897	0.859	0.843	1.266E-10	Meta_CD8_C7
GALM	5.3363E-15	0.646369589	0.395	0.261	1.292E-10	Meta_CD8_C7
ACP5	7.1127E-15	0.603306511	0.373	0.260	1.722E-10	Meta_CD8_C7
ARPC5	7.4476E-15	0.390715216	0.558	0.540	1.803E-10	Meta_CD8_C7
PML	1.0264E-14	0.471380863	0.259	0.167	2.486E-10	Meta_CD8_C7
TIGIT	1.1309E-14	0.672170466	0.439	0.331	2.738E-10	Meta_CD8_C7
LGALS1	2.2772E-14	0.613699955	0.538	0.437	5.514E-10	Meta_CD8_C7
C9orf142	3.5575E-14	0.53418704	0.115	0.054	8.614E-10	Meta_CD8_C7

LRBA	4.0628E-14	0.459428994	0.262	0.212	9.838E-10	Meta_CD8_C7
CHMP4A	4.2542E-14	0.386943175	0.264	0.264	1.03E-09	Meta_CD8_C7
APOBEC3H	4.6106E-14	0.483255775	0.242	0.128	1.116E-09	Meta_CD8_C7
RNF181	5.3983E-14	0.402330906	0.410	0.357	1.307E-09	Meta_CD8_C7
TAP2	6.5463E-14	0.544204408	0.140	0.065	1.585E-09	Meta_CD8_C7
TANK	6.7051E-14	0.499130673	0.383	0.320	1.624E-09	Meta_CD8_C7
CTSS	7.0127E-14	0.475453151	0.402	0.330	1.698E-09	Meta_CD8_C7
TIMD4	7.3999E-14	0.410434873	0.100	0.015	1.792E-09	Meta_CD8_C7
CCDC85B	8.8243E-14	0.357608032	0.543	0.541	2.137E-09	Meta_CD8_C7
CALCOCO2	9.7845E-14	0.421057342	0.396	0.328	2.369E-09	Meta_CD8_C7
PDCD1	1.0385E-13	0.674420581	0.314	0.216	2.515E-09	Meta_CD8_C7
CXCR6	1.0744E-13	0.559834954	0.454	0.330	2.602E-09	Meta_CD8_C7
COPE	1.2715E-13	0.32638102	0.578	0.584	3.079E-09	Meta_CD8_C7
RGS2	1.272E-13	0.677630875	0.483	0.408	3.08E-09	Meta_CD8_C7
GALNT2	1.5266E-13	0.534764614	0.204	0.130	3.697E-09	Meta_CD8_C7
N4BP2L2	1.8169E-13	0.432916351	0.538	0.523	4.4E-09	Meta_CD8_C7
AC092580.4	2.7469E-13	0.589114742	0.126	0.044	6.652E-09	Meta_CD8_C7
TRIM38	2.9131E-13	0.522067492	0.251	0.171	7.054E-09	Meta_CD8_C7
TMX1	3.4467E-13	0.41188714	0.315	0.269	8.346E-09	Meta_CD8_C7
BTN3A2	3.7896E-13	0.445752021	0.442	0.354	9.176E-09	Meta_CD8_C7
VAMP8	5.8668E-13	0.363181292	0.571	0.573	1.421E-08	Meta_CD8_C7
POLR2K	5.8819E-13	0.271786227	0.327	0.348	1.424E-08	Meta_CD8_C7
RABAC1	7.451E-13	0.360194118	0.604	0.609	1.804E-08	Meta_CD8_C7
PMF1	7.7675E-13	0.553456228	0.138	0.066	1.881E-08	Meta_CD8_C7
HSPE1	9.0222E-13	0.361333482	0.543	0.580	2.185E-08	Meta_CD8_C7
ARF6	9.7029E-13	0.308815901	0.535	0.528	2.35E-08	Meta_CD8_C7
WARS	1.0384E-12	0.460747783	0.219	0.129	2.515E-08	Meta_CD8_C7
PARP12	1.0391E-12	0.368125169	0.231	0.128	2.516E-08	Meta_CD8_C7
CASP1	1.1056E-12	0.534317321	0.365	0.304	2.677E-08	Meta_CD8_C7
C14orf2	1.2337E-12	0.68156458	0.164	0.078	2.987E-08	Meta_CD8_C7
ATPIF1	1.3349E-12	0.653237537	0.150	0.066	3.232E-08	Meta_CD8_C7
HMGB2	1.6671E-12	0.44130582	0.498	0.472	4.037E-08	Meta_CD8_C7
ID2	1.6816E-12	0.536664823	0.670	0.595	4.072E-08	Meta_CD8_C7
CTSD	1.7335E-12	0.577922826	0.342	0.272	4.198E-08	Meta_CD8_C7
IRF2	2.2327E-12	0.451077843	0.353	0.304	5.406E-08	Meta_CD8_C7
CD53	2.3694E-12	0.422842423	0.690	0.666	5.737E-08	Meta_CD8_C7
CFLAR	2.4289E-12	0.46712178	0.527	0.457	5.882E-08	Meta_CD8_C7
ATP5C1	2.5151E-12	0.529784556	0.109	0.047	6.09E-08	Meta_CD8_C7
MT1F	2.679E-12	0.489617832	0.242	0.170	6.487E-08	Meta_CD8_C7
PSMB1	3.4947E-12	0.272470126	0.603	0.611	8.462E-08	Meta_CD8_C7

PNISR	4.1843E-12	0.320594193	0.590	0.597	1.013E-07	Meta_CD8_C7
TRIM56	4.7333E-12	0.457424523	0.206	0.132	1.146E-07	Meta_CD8_C7
FBXO6	5.1215E-12	0.479707925	0.225	0.110	1.24E-07	Meta_CD8_C7
NDUFB8	5.3912E-12	0.493735758	0.216	0.185	1.305E-07	Meta_CD8_C7
DBI	6.8756E-12	0.268024884	0.567	0.577	1.665E-07	Meta_CD8_C7
KPNB1	9.5778E-12	0.382866503	0.452	0.405	2.319E-07	Meta_CD8_C7
NEDD8	9.6398E-12	0.323504491	0.546	0.554	2.334E-07	Meta_CD8_C7
VCAM1	9.8765E-12	0.734459712	0.103	0.027	2.392E-07	Meta_CD8_C7
PPP1CC	1.1027E-11	0.322439943	0.470	0.466	2.67E-07	Meta_CD8_C7
TCEB2	1.1075E-11	0.573346702	0.173	0.088	2.682E-07	Meta_CD8_C7
SEPW1	1.1446E-11	0.62056315	0.124	0.052	2.772E-07	Meta_CD8_C7
NOP10	1.4596E-11	0.323996035	0.512	0.495	3.534E-07	Meta_CD8_C7
MTRNR2L12	1.5645E-11	1.019390421	0.350	0.194	3.788E-07	Meta_CD8_C7
ATP5B	1.6257E-11	0.575448682	0.136	0.062	3.937E-07	Meta_CD8_C7
SCAMP2	2.1547E-11	0.272592982	0.396	0.385	5.218E-07	Meta_CD8_C7
HELB	2.3088E-11	0.439450371	0.256	0.164	5.591E-07	Meta_CD8_C7
FKBP5	2.3719E-11	0.562579615	0.375	0.292	5.744E-07	Meta_CD8_C7
ACAP1	3.1945E-11	0.348722623	0.681	0.678	7.735E-07	Meta_CD8_C7
CTSC	3.8664E-11	0.428239143	0.617	0.558	9.362E-07	Meta_CD8_C7
TSPO	4.4293E-11	0.296550159	0.515	0.493	1.073E-06	Meta_CD8_C7
PRDX5	4.6159E-11	0.381019803	0.556	0.532	1.118E-06	Meta_CD8_C7
WHSC1L1	5.0516E-11	0.631949517	0.107	0.038	1.223E-06	Meta_CD8_C7
HLA-F	5.4874E-11	0.277669492	0.599	0.606	1.329E-06	Meta_CD8_C7
SLA	6.4218E-11	0.466580059	0.431	0.382	1.555E-06	Meta_CD8_C7
HN1	6.496E-11	0.54600812	0.112	0.046	1.573E-06	Meta_CD8_C7
HERC6	7.0415E-11	0.482516439	0.137	0.050	1.705E-06	Meta_CD8_C7
AC133644.2	7.1447E-11	0.522411562	0.102	0.049	1.73E-06	Meta_CD8_C7
CD48	8.1927E-11	0.323400971	0.701	0.717	1.984E-06	Meta_CD8_C7
ARPC4	9.7278E-11	0.374758491	0.302	0.282	2.356E-06	Meta_CD8_C7
DHX58	9.829E-11	0.380442804	0.119	0.043	2.38E-06	Meta_CD8_C7
OAZ1	1.1071E-10	0.252070467	0.866	0.878	2.681E-06	Meta_CD8_C7
PYHIN1	1.3695E-10	0.597183314	0.396	0.296	3.316E-06	Meta_CD8_C7
TMEM140	1.4065E-10	0.439510087	0.209	0.115	3.406E-06	Meta_CD8_C7
USMG5	1.7156E-10	0.44863001	0.119	0.065	4.154E-06	Meta_CD8_C7
BLOC1S1	1.789E-10	0.54259183	0.342	0.274	4.332E-06	Meta_CD8_C7
MIAT	1.8968E-10	0.539170545	0.211	0.134	4.593E-06	Meta_CD8_C7
GSDMD	2.2173E-10	0.481412763	0.399	0.310	5.369E-06	Meta_CD8_C7
CLIC3	2.2678E-10	0.529488535	0.308	0.190	5.491E-06	Meta_CD8_C7
CPNE7	2.295E-10	0.492885535	0.195	0.092	5.557E-06	Meta_CD8_C7
PTMS	2.4184E-10	0.464548434	0.342	0.227	5.856E-06	Meta_CD8_C7

NAPA	2.6149E-10	0.366836817	0.456	0.404	6.332E-06	Meta_CD8_C7
AZI2	2.6388E-10	0.413092968	0.221	0.138	6.39E-06	Meta_CD8_C7
ATP5A1	2.721E-10	0.508036517	0.113	0.057	6.589E-06	Meta_CD8_C7
MSN	3.0831E-10	0.29837678	0.620	0.616	7.466E-06	Meta_CD8_C7
SRI	3.0836E-10	0.313243647	0.451	0.456	7.467E-06	Meta_CD8_C7
GSTO1	3.2697E-10	0.473923597	0.335	0.282	7.918E-06	Meta_CD8_C7
LUC7L3	4.0903E-10	0.356965261	0.333	0.319	9.905E-06	Meta_CD8_C7
C19orf43	4.6399E-10	0.645603836	0.179	0.087	1.124E-05	Meta_CD8_C7
PSMA3	4.7704E-10	0.353776468	0.387	0.361	1.155E-05	Meta_CD8_C7
RTF1	6.1214E-10	0.353670509	0.382	0.335	1.482E-05	Meta_CD8_C7
ODF2L	7.2141E-10	0.471411958	0.353	0.305	1.747E-05	Meta_CD8_C7
MVP	7.3218E-10	0.328587425	0.402	0.353	1.773E-05	Meta_CD8_C7
STK4	7.3906E-10	0.28246126	0.603	0.614	1.79E-05	Meta_CD8_C7
SNAP47	9.3137E-10	0.37478928	0.163	0.137	2.255E-05	Meta_CD8_C7
THRAP3	1.5677E-09	0.284096949	0.457	0.451	3.796E-05	Meta_CD8_C7
C14orf166	1.9209E-09	0.521978413	0.115	0.056	4.651E-05	Meta_CD8_C7
ATP6V1E1	1.9549E-09	0.331200176	0.219	0.212	4.734E-05	Meta_CD8_C7
SERPINB1	2.7987E-09	0.394530814	0.441	0.404	6.777E-05	Meta_CD8_C7
CCR5	2.9189E-09	0.41999337	0.201	0.125	7.068E-05	Meta_CD8_C7
GBP3	3.0431E-09	0.48750554	0.198	0.103	7.369E-05	Meta_CD8_C7
MYD88	3.124E-09	0.329034887	0.263	0.197	7.565E-05	Meta_CD8_C7
ATP5O	3.2651E-09	0.544221807	0.140	0.067	7.906E-05	Meta_CD8_C7
HSPB11	3.5169E-09	0.318349651	0.313	0.274	8.516E-05	Meta_CD8_C7
SELK	4.0728E-09	0.394241725	0.101	0.051	9.862E-05	Meta_CD8_C7
TRANK1	5.5774E-09	0.410689249	0.237	0.139	0.0001351	Meta_CD8_C7
IL2RG	6.0971E-09	0.381829647	0.563	0.587	0.0001476	Meta_CD8_C7
ARAP2	6.907E-09	0.315728954	0.369	0.321	0.0001673	Meta_CD8_C7
GPR171	7.9912E-09	0.483290972	0.341	0.253	0.0001935	Meta_CD8_C7
DNAJA1	1.0322E-08	0.33482225	0.631	0.622	0.0002499	Meta_CD8_C7
CNDP2	1.0384E-08	0.322527492	0.232	0.186	0.0002515	Meta_CD8_C7
TRGC2	1.1441E-08	0.54557408	0.133	0.110	0.000277	Meta_CD8_C7
USP15	1.1758E-08	0.345151948	0.460	0.414	0.0002847	Meta_CD8_C7
ENTPD1	1.3378E-08	0.36674013	0.138	0.119	0.0003239	Meta_CD8_C7
MKRN1	1.4343E-08	0.305265901	0.234	0.231	0.0003473	Meta_CD8_C7
RPL27A	1.6125E-08	0.301890153	0.944	0.938	0.0003905	Meta_CD8_C7
OGFR	1.7246E-08	0.347437035	0.341	0.272	0.0004176	Meta_CD8_C7
OPTN	1.7718E-08	0.32602799	0.469	0.425	0.000429	Meta_CD8_C7
HERPUD1	1.8209E-08	0.384990237	0.568	0.537	0.0004409	Meta_CD8_C7
CD63	1.9066E-08	0.436585512	0.507	0.471	0.0004617	Meta_CD8_C7
TRIM21	1.9478E-08	0.322493315	0.210	0.133	0.0004716	Meta_CD8_C7

TBC1D1	2.2996E-08	0.296188746	0.244	0.179	0.0005568	Meta_CD8_C7
SLA2	2.4077E-08	0.419063274	0.354	0.285	0.000583	Meta_CD8_C7
ATP6V0E1	2.4292E-08	0.279743111	0.625	0.655	0.0005882	Meta_CD8_C7
CEBPD	2.5137E-08	0.370008323	0.255	0.145	0.0006087	Meta_CD8_C7
BISPR	2.5297E-08	0.283906046	0.151	0.082	0.0006126	Meta_CD8_C7
GTF2H5	2.8214E-08	0.359887546	0.212	0.183	0.0006832	Meta_CD8_C7
FIBP	3.0477E-08	0.302511726	0.300	0.271	0.000738	Meta_CD8_C7
PTTG1	3.3259E-08	0.288411335	0.217	0.210	0.0008054	Meta_CD8_C7
CCND2	3.3956E-08	0.25848782	0.292	0.320	0.0008222	Meta_CD8_C7
GIMAP2	3.6243E-08	0.382816812	0.328	0.275	0.0008776	Meta_CD8_C7
TRAT1	3.803E-08	0.250228005	0.291	0.330	0.0009209	Meta_CD8_C7
NDUFS6	3.8471E-08	0.358332163	0.425	0.388	0.0009316	Meta_CD8_C7
PLA2G16	3.9306E-08	0.342483841	0.245	0.200	0.0009518	Meta_CD8_C7
ATP5D	4.011E-08	0.554569486	0.145	0.074	0.0009713	Meta_CD8_C7
44819	4.3563E-08	0.437099109	0.110	0.053	0.0010549	Meta_CD8_C7
N4BP1	4.7088E-08	0.428698926	0.245	0.142	0.0011402	Meta_CD8_C7
PATL2	4.7562E-08	0.408513156	0.123	0.083	0.0011517	Meta_CD8_C7
HSPH1	5.3424E-08	0.380229505	0.274	0.286	0.0012937	Meta_CD8_C7
ARHGEF3	5.3809E-08	0.302116418	0.327	0.287	0.001303	Meta_CD8_C7
PI4K2B	5.7819E-08	0.276328362	0.109	0.056	0.0014001	Meta_CD8_C7
SH2D1A	5.9832E-08	0.334195469	0.388	0.326	0.0014488	Meta_CD8_C7
CCL4L2	6.3373E-08	0.381691065	0.259	0.175	0.0015346	Meta_CD8_C7
PTPN7	7.6521E-08	0.32274273	0.437	0.427	0.0018529	Meta_CD8_C7
CAPN2	8.1342E-08	0.396889327	0.443	0.390	0.0019697	Meta_CD8_C7
TNIP3	9.0016E-08	0.349291524	0.121	0.095	0.0021797	Meta_CD8_C7
HNRNPD	9.3931E-08	0.261086552	0.280	0.275	0.0022745	Meta_CD8_C7
STOM	9.9392E-08	0.29359578	0.430	0.407	0.0024068	Meta_CD8_C7
ITM2C	1.0605E-07	0.256536903	0.392	0.375	0.002568	Meta_CD8_C7
GLTSCR2	1.0624E-07	0.328994003	0.182	0.109	0.0025727	Meta_CD8_C7
SCAF11	1.0791E-07	0.25560917	0.417	0.428	0.0026131	Meta_CD8_C7
SELT	1.0997E-07	0.439214357	0.107	0.051	0.002663	Meta_CD8_C7
SETX	1.1541E-07	0.295555907	0.242	0.212	0.0027946	Meta_CD8_C7
CAPZA1	1.29E-07	0.273126741	0.441	0.455	0.0031238	Meta_CD8_C7
CD47	1.3881E-07	0.295646922	0.447	0.430	0.0033612	Meta_CD8_C7
PSMA2	1.9962E-07	0.289805965	0.159	0.168	0.0048337	Meta_CD8_C7
ANKRD28	2.3906E-07	0.457659163	0.195	0.176	0.0057889	Meta_CD8_C7
DYNLL1	2.5039E-07	0.365195411	0.565	0.551	0.0060632	Meta_CD8_C7
SLFN12L	2.5083E-07	0.369756273	0.271	0.185	0.0060738	Meta_CD8_C7
ARRDC3	2.5583E-07	0.28982529	0.131	0.115	0.0061949	Meta_CD8_C7
ODF3B	2.7327E-07	0.278490705	0.176	0.102	0.0066172	Meta_CD8_C7

LGALS3BP	2.8473E-07	0.304418239	0.162	0.110	0.0068948	Meta_CD8_C7
IFI27L2	3.074E-07	0.374771488	0.432	0.365	0.0074436	Meta_CD8_C7
RSRC2	3.0809E-07	0.260567056	0.449	0.462	0.0074604	Meta_CD8_C7
TMEM165	3.2619E-07	0.278559807	0.251	0.243	0.0078987	Meta_CD8_C7
COMMD3	3.4327E-07	0.318544246	0.116	0.097	0.0083123	Meta_CD8_C7
SIT1	3.5113E-07	0.345772065	0.460	0.413	0.0085026	Meta_CD8_C7
ENY2	4E-07	0.272623783	0.368	0.376	0.0096861	Meta_CD8_C7
PYCARD	4.0012E-07	0.347918691	0.362	0.296	0.0096888	Meta_CD8_C7
LAMTOR5	4.7205E-07	0.338356143	0.424	0.405	0.0114306	Meta_CD8_C7
FUT8	5.2723E-07	0.326727903	0.170	0.111	0.0127669	Meta_CD8_C7
CD84	5.2781E-07	0.31570182	0.176	0.156	0.0127809	Meta_CD8_C7
ATP5H	5.5577E-07	0.46413576	0.100	0.050	0.0134579	Meta_CD8_C7
SUPT3H	5.7423E-07	0.284183481	0.212	0.158	0.0139049	Meta_CD8_C7
PAG1	5.7718E-07	0.369727279	0.351	0.296	0.0139763	Meta_CD8_C7
PPP2R5C	7.1195E-07	0.380074295	0.529	0.506	0.0172398	Meta_CD8_C7
PPP2R2A	7.9709E-07	0.338947992	0.246	0.207	0.0193015	Meta_CD8_C7
SAMD3	8.9517E-07	0.275881334	0.277	0.198	0.0216766	Meta_CD8_C7
MTHFD2	9.2913E-07	0.428727676	0.342	0.257	0.0224989	Meta_CD8_C7
PSMB2	1.0811E-06	0.308059878	0.398	0.377	0.0261782	Meta_CD8_C7
SAMSN1	1.0912E-06	0.314200384	0.549	0.544	0.0264246	Meta_CD8_C7
DHX36	1.1229E-06	0.272696505	0.339	0.321	0.0271902	Meta_CD8_C7
HIF1A	1.1372E-06	0.282510667	0.371	0.340	0.0275377	Meta_CD8_C7
SYTL3	1.1856E-06	0.312046408	0.436	0.416	0.0287087	Meta_CD8_C7
APOL3	1.1872E-06	0.330776957	0.219	0.158	0.0287491	Meta_CD8_C7
SMC4	1.2518E-06	0.389771179	0.217	0.167	0.0303132	Meta_CD8_C7
RICTOR	1.2641E-06	0.292737931	0.215	0.190	0.0306107	Meta_CD8_C7
SLC38A5	1.279E-06	0.314333749	0.129	0.075	0.0309718	Meta_CD8_C7
BAG1	1.3243E-06	0.32721591	0.301	0.249	0.0320686	Meta_CD8_C7
CASP3	1.3649E-06	0.252374301	0.175	0.135	0.0330505	Meta_CD8_C7
CKLF	1.5368E-06	0.257925972	0.531	0.565	0.0372137	Meta_CD8_C7
CMTR1	1.7464E-06	0.25867693	0.147	0.078	0.0422891	Meta_CD8_C7
LAMTOR2	1.7799E-06	0.285342644	0.305	0.263	0.0430997	Meta_CD8_C7
NAP1L4	1.8413E-06	0.255499257	0.430	0.420	0.0445877	Meta_CD8_C7
TBX21	2.1861E-06	0.321009514	0.275	0.185	0.0529367	Meta_CD8_C7
RSBN1L	2.4382E-06	0.362955711	0.310	0.291	0.0590404	Meta_CD8_C7
ARGLU1	2.5347E-06	0.254349802	0.537	0.555	0.0613779	Meta_CD8_C7
NDUFS4	2.751E-06	0.318459033	0.205	0.170	0.0666163	Meta_CD8_C7
VAMP2	2.8012E-06	0.273060047	0.404	0.409	0.06783	Meta_CD8_C7
EED	2.9541E-06	0.251837065	0.161	0.142	0.0715332	Meta_CD8_C7
UBE2F	2.9564E-06	0.297649515	0.117	0.094	0.0715896	Meta_CD8_C7

PRKD2	2.9812E-06	0.279641394	0.233	0.199	0.0721902	Meta_CD8_C7
PSMD4	3.1476E-06	0.252539724	0.353	0.341	0.0762202	Meta_CD8_C7
PSMB7	3.3583E-06	0.336238221	0.327	0.295	0.0813206	Meta_CD8_C7
APOL2	3.7631E-06	0.305078361	0.154	0.092	0.0911238	Meta_CD8_C7
CD82	4.5724E-06	0.354783673	0.389	0.336	0.1107201	Meta_CD8_C7
PSMA1	4.7237E-06	0.265793895	0.425	0.435	0.1143839	Meta_CD8_C7
TRIM69	5.2743E-06	0.302518731	0.194	0.168	0.1277172	Meta_CD8_C7
TMEM109	5.825E-06	0.328575173	0.335	0.306	0.141053	Meta_CD8_C7
COPZ1	7.4293E-06	0.254392332	0.332	0.320	0.1798999	Meta_CD8_C7
USP30-AS1	7.7949E-06	0.340296321	0.113	0.073	0.1887543	Meta_CD8_C7
YARS	8.2772E-06	0.323682274	0.325	0.259	0.2004334	Meta_CD8_C7
IKZF3	9.2279E-06	0.301533461	0.395	0.355	0.2234533	Meta_CD8_C7
JOSD2	9.4026E-06	0.286111297	0.210	0.188	0.227684	Meta_CD8_C7
BIN1	1.1689E-05	0.254510631	0.448	0.448	0.2830584	Meta_CD8_C7
PTPN22	1.5323E-05	0.254903815	0.394	0.378	0.3710554	Meta_CD8_C7
C1GALT1	1.5985E-05	0.303373427	0.248	0.192	0.3870781	Meta_CD8_C7
FNIP1	1.7077E-05	0.278789521	0.136	0.116	0.4135299	Meta_CD8_C7
KIF20B	1.7287E-05	0.250195477	0.149	0.130	0.4186115	Meta_CD8_C7
CBLB	1.8456E-05	0.266809772	0.426	0.399	0.4469192	Meta_CD8_C7
STMN1	2.0343E-05	0.339197901	0.154	0.140	0.4926018	Meta_CD8_C7
TTC14	2.1159E-05	0.331627744	0.244	0.216	0.5123703	Meta_CD8_C7
FASLG	2.1318E-05	0.347677194	0.221	0.164	0.5162168	Meta_CD8_C7
FKBP1A	2.1459E-05	0.274054156	0.573	0.572	0.5196233	Meta_CD8_C7
CASP7	2.2114E-05	0.276926008	0.181	0.116	0.5354937	Meta_CD8_C7
PCGF5	2.3116E-05	0.25923264	0.288	0.262	0.5597581	Meta_CD8_C7
ZC3HAV1	2.7691E-05	0.271906669	0.439	0.420	0.6705297	Meta_CD8_C7
PHPT1	2.9592E-05	0.307362298	0.349	0.324	0.7165788	Meta_CD8_C7
TOX	3.1463E-05	0.254229368	0.182	0.168	0.7618705	Meta_CD8_C7
MIS18BP1	3.6854E-05	0.304691374	0.226	0.196	0.8924095	Meta_CD8_C7
KRAS	3.7293E-05	0.251773084	0.366	0.335	0.9030467	Meta_CD8_C7
SUSD3	4.6983E-05	0.308083806	0.301	0.273	1	Meta_CD8_C7
SH3GLB1	6.3738E-05	0.318919354	0.361	0.334	1	Meta_CD8_C7
ORMDL3	6.5263E-05	0.366121934	0.275	0.222	1	Meta_CD8_C7
TRAF5	6.5747E-05	0.259732658	0.193	0.170	1	Meta_CD8_C7
C12orf75	8.1775E-05	0.277450946	0.378	0.325	1	Meta_CD8_C7
DNAJC7	9.8556E-05	0.265275985	0.280	0.262	1	Meta_CD8_C7
PRDM1	0.00010991	0.279302572	0.378	0.340	1	Meta_CD8_C7
MYO1F	0.00013094	0.336447162	0.377	0.323	1	Meta_CD8_C7
PMAIP1	0.00013905	0.455364864	0.333	0.256	1	Meta_CD8_C7
HSPA1B	0.00017997	0.282901333	0.349	0.308	1	Meta_CD8_C7

TRBC2	0.00018116	0.344343626	0.723	0.718	1	Meta_CD8_C7
PPP1R12A	0.00018159	0.269223721	0.430	0.403	1	Meta_CD8_C7
ID3	0.0002007	0.266538973	0.097	0.106	1	Meta_CD8_C7
GPR155	0.00032525	0.304853039	0.136	0.108	1	Meta_CD8_C7
GCH1	0.00034528	0.275069057	0.217	0.147	1	Meta_CD8_C7
VMP1	0.00047807	0.331668347	0.331	0.318	1	Meta_CD8_C7
DUSP4	0.00078582	0.348525416	0.449	0.374	1	Meta_CD8_C7
BCL2L11	0.00109486	0.326657018	0.211	0.130	1	Meta_CD8_C7
HSD17B10	0.0011534	0.254113929	0.266	0.238	1	Meta_CD8_C7
TRIM14	0.00127013	0.283006405	0.186	0.134	1	Meta_CD8_C7
EIF4EBP1	0.00127957	0.281309836	0.209	0.142	1	Meta_CD8_C7
SLF1	0.00170142	0.290646641	0.228	0.184	1	Meta_CD8_C7
CARS	0.00216087	0.266980605	0.207	0.160	1	Meta_CD8_C7
BTG3	0.00308373	0.258398175	0.304	0.286	1	Meta_CD8_C7
MCOLN2	0.00372368	0.252528636	0.148	0.099	1	Meta_CD8_C7
ARPC5L	0.00373416	0.255031228	0.506	0.479	1	Meta_CD8_C7
TBCD	0.00419482	0.253873199	0.214	0.186	1	Meta_CD8_C7
CD70	0.00551216	0.265396119	0.125	0.082	1	Meta_CD8_C7
LMO4	0.00756524	0.251204651	0.223	0.170	1	Meta_CD8_C7
EHD4	0.00981492	0.252773907	0.147	0.111	1	Meta_CD8_C7
KIR2DL4	2.594E-219	2.192316801	0.637	0.019	6.28E-215	Meta_γδT
KLRC3	1.738E-169	1.796451599	0.589	0.035	4.21E-165	Meta_γδT
KLRD1	1.977E-166	1.688189577	0.842	0.205	4.79E-162	Meta_γδT
CD7	5.639E-164	1.533940487	0.963	0.746	1.37E-159	Meta_γδT
KLRC2	9.214E-154	1.759149336	0.568	0.032	2.23E-149	Meta_γδT
HOPX	1.652E-142	1.683379395	0.829	0.308	4E-138	Meta_γδT
LAT2	1.7E-125	1.265128546	0.466	0.023	4.12E-121	Meta_γδT
TRDC	2.186E-119	1.843406668	0.446	0.024	5.29E-115	Meta_γδT
TYROBP	5.158E-118	2.264079832	0.474	0.039	1.25E-113	Meta_γδT
FCER1G	1.174E-113	2.023467688	0.443	0.024	2.84E-109	Meta_γδT
NKG7	8.506E-111	0.837264281	0.955	0.543	2.06E-106	Meta_γδT
CD160	2.044E-109	1.418787255	0.489	0.057	4.95E-105	Meta_γδT
CCL5	2.132E-108	1.019440095	0.968	0.735	5.16E-104	Meta_γδT
TMIGD2	3.965E-103	1.390937038	0.557	0.118	9.6E-99	Meta_γδT
ATP8B4	1.937E-101	0.922553065	0.363	0.023	4.691E-97	Meta_γδT
AC068775.1	6.2345E-98	1.06078534	0.388	0.014	1.51E-93	Meta_γδT
CD63	7.2417E-96	1.167061331	0.841	0.442	1.754E-91	Meta_γδT
GNLY	3.0887E-89	2.137352536	0.606	0.177	7.479E-85	Meta_γδT
AOAH	3.0483E-81	0.935162023	0.564	0.181	7.382E-77	Meta_γδT
IL2RB	3.2046E-76	1.032875533	0.741	0.426	7.76E-72	Meta_γδT

ITGA1	3.887E-74	1.185294703	0.574	0.191	9.412E-70	Meta_γδT
GZMA	2.2201E-73	1.974213371	0.836	0.561	5.376E-69	Meta_γδT
NCR1	2.2903E-73	0.639038038	0.247	0.007	5.546E-69	Meta_γδT
XCL2	6.6762E-73	1.531136559	0.484	0.118	1.617E-68	Meta_γδT
GZMB	8.2264E-71	0.735620943	0.654	0.271	1.992E-66	Meta_γδT
CXXC5	4.5731E-70	0.735516803	0.280	0.016	1.107E-65	Meta_γδT
SPRY1	6.2617E-70	1.046703059	0.469	0.115	1.516E-65	Meta_γδT
LYN	1.5082E-68	0.718410477	0.296	0.017	3.652E-64	Meta_γδT
TRGV4	7.5593E-68	1.47277184	0.314	0.036	1.83E-63	Meta_γδT
SH2D1B	4.399E-66	0.847660541	0.225	0.002	1.065E-61	Meta_γδT
CD96	1.506E-65	0.825137571	0.783	0.585	3.647E-61	Meta_γδT
MATK	9.1377E-64	0.897681895	0.604	0.251	2.213E-59	Meta_γδT
KLRC1	3.0253E-63	1.732554749	0.325	0.049	7.326E-59	Meta_γδT
GSTP1	8.285E-63	0.866412158	0.781	0.511	2.006E-58	Meta_γδT
RHOC	4.6434E-61	0.932817106	0.600	0.255	1.124E-56	Meta_γδT
TRGC2	4.2615E-59	0.685862818	0.348	0.091	1.032E-54	Meta_γδT
ITGAE	2.2707E-57	0.957837448	0.632	0.306	5.499E-53	Meta_γδT
CD244	3.5234E-55	0.703318576	0.365	0.079	8.532E-51	Meta_γδT
XCL1	1.656E-54	1.509031207	0.396	0.109	4.01E-50	Meta_γδT
RIN3	2.4288E-54	0.771166177	0.417	0.108	5.881E-50	Meta_γδT
PRF1	1.2546E-53	0.813359633	0.732	0.392	3.038E-49	Meta_γδT
FASLG	2.0942E-52	0.997412932	0.464	0.141	5.071E-48	Meta_γδT
CTSW	4.3689E-52	1.03667489	0.746	0.411	1.058E-47	Meta_γδT
MAP3K8	6.2546E-51	0.987293091	0.590	0.278	1.515E-46	Meta_γδT
ADGRG1	1.9347E-49	0.665258665	0.282	0.043	4.685E-45	Meta_γδT
ADAM28	7.2348E-49	0.516506894	0.184	0.006	1.752E-44	Meta_γδT
FES	1.6614E-48	0.486268725	0.186	0.011	4.023E-44	Meta_γδT
TXK	3.9814E-48	0.816011991	0.363	0.086	9.641E-44	Meta_γδT
TRDV1	8.9343E-48	2.07672419	0.224	0.019	2.163E-43	Meta_γδT
IKZF2	7.1925E-47	0.684681372	0.332	0.058	1.742E-42	Meta_γδT
NMUR1	6.02E-44	0.476557332	0.206	0.027	1.458E-39	Meta_γδT
GNPTAB	1.6345E-43	0.697263485	0.423	0.154	3.958E-39	Meta_γδT
MT-CO2	4.0182E-43	0.574836755	0.993	0.992	9.73E-39	Meta_γδT
MT-CO1	4.3583E-43	0.625811358	0.996	0.995	1.055E-38	Meta_γδT
DBN1	6.4115E-42	0.589010697	0.251	0.041	1.553E-37	Meta_γδT
BCAS4	2.2238E-41	0.528790012	0.353	0.130	5.385E-37	Meta_γδT
MT-ND4	7.9442E-41	0.659253091	0.974	0.923	1.924E-36	Meta_γδT
PTPN22	1.7078E-40	0.770291418	0.626	0.358	4.135E-36	Meta_γδT
ABI3	2.1075E-40	0.768022917	0.589	0.323	5.103E-36	Meta_γδT
MT-ND5	3.0992E-40	0.613200187	0.917	0.884	7.505E-36	Meta_γδT

MYO1E	3.2196E-40	0.503633844	0.200	0.018	7.796E-36	Meta_γδT
KIR3DL2	8.2004E-40	0.722494642	0.171	0.011	1.986E-35	Meta_γδT
SLAMF7	9.2084E-40	0.512012943	0.344	0.124	2.23E-35	Meta_γδT
FAM3C	9.9851E-40	0.799681407	0.387	0.098	2.418E-35	Meta_γδT
LDLRAD4	2.0312E-39	0.93640033	0.542	0.225	4.919E-35	Meta_γδT
PLPP1	3.4629E-39	0.789362329	0.310	0.079	8.386E-35	Meta_γδT
GPR65	5.9056E-38	0.766830408	0.545	0.278	1.43E-33	Meta_γδT
AREG	2.9421E-37	1.683982456	0.394	0.162	7.124E-33	Meta_γδT
HCST	4.7852E-37	0.434810979	0.939	0.835	1.159E-32	Meta_γδT
NR4A2	1.2173E-36	0.958362869	0.746	0.565	2.948E-32	Meta_γδT
KRT86	5.6996E-36	0.715302614	0.181	0.012	1.38E-31	Meta_γδT
SLC16A3	6.8639E-36	0.700584289	0.377	0.131	1.662E-31	Meta_γδT
PLCG2	7.981E-36	0.446691516	0.214	0.039	1.933E-31	Meta_γδT
LINC01871	1.0341E-35	0.906021447	0.558	0.292	2.504E-31	Meta_γδT
GPR15	2.468E-35	0.649682721	0.359	0.114	5.976E-31	Meta_γδT
PTPN6	5.1913E-35	0.715288375	0.617	0.380	1.257E-30	Meta_γδT
B3GNT7	9.2886E-35	0.352590744	0.119	0.002	2.249E-30	Meta_γδT
MT-CYB	1.4781E-34	0.5612338	0.979	0.977	3.579E-30	Meta_γδT
SPRY2	1.6051E-34	0.418548229	0.141	0.005	3.887E-30	Meta_γδT
CLIC3	1.8581E-34	0.834071723	0.414	0.174	4.499E-30	Meta_γδT
UBASH3B	3.1003E-34	0.592082996	0.295	0.078	7.507E-30	Meta_γδT
CAPN12	6.2318E-34	0.526774964	0.259	0.059	1.509E-29	Meta_γδT
MT-ATP6	6.4349E-34	0.527211833	0.977	0.967	1.558E-29	Meta_γδT
MT-ND4L	7.9112E-34	0.650722701	0.863	0.837	1.916E-29	Meta_γδT
ID3	8.2006E-34	0.847907916	0.330	0.087	1.986E-29	Meta_γδT
SLA2	1.2748E-33	0.632032398	0.524	0.266	3.087E-29	Meta_γδT
MT-ND3	2.0727E-33	0.590654438	0.960	0.949	5.019E-29	Meta_γδT
GEM	3.7689E-33	0.923087727	0.235	0.038	9.126E-29	Meta_γδT
CAPG	1.0797E-32	0.724649082	0.527	0.262	2.615E-28	Meta_γδT
TNFRSF18	1.6062E-31	0.648638008	0.435	0.189	3.889E-27	Meta_γδT
ACP5	1.7191E-31	0.609191708	0.472	0.245	4.163E-27	Meta_γδT
CKLF	2.2599E-31	0.60324181	0.735	0.551	5.472E-27	Meta_γδT
TIGIT	3.4912E-31	0.548227162	0.582	0.312	8.454E-27	Meta_γδT
RBKS	3.9683E-31	0.788236857	0.333	0.131	9.609E-27	Meta_γδT
CCRL2	5.094E-31	0.43980067	0.199	0.034	1.234E-26	Meta_γδT
ABCB1	6.7418E-31	0.526171486	0.315	0.098	1.633E-26	Meta_γδT
TRG-AS1	1.3452E-30	0.668384599	0.382	0.154	3.257E-26	Meta_γδT
KIFC3	3.0241E-30	0.345417569	0.148	0.013	7.323E-26	Meta_γδT
SSBP4	5.426E-30	0.477663846	0.487	0.297	1.314E-25	Meta_γδT
ENTPD1	7.368E-30	0.594126167	0.335	0.102	1.784E-25	Meta_γδT

LAYN	2.1772E-29	0.522715913	0.242	0.051	5.272E-25	Meta_γδT
CD9	2.2165E-29	0.407153286	0.222	0.069	5.367E-25	Meta_γδT
CRACR2B	2.6278E-29	0.34029267	0.147	0.014	6.363E-25	Meta_γδT
HES4	1.2109E-28	0.542925986	0.153	0.018	2.932E-24	Meta_γδT
TRGV3	2.8282E-28	0.682479303	0.239	0.085	6.849E-24	Meta_γδT
ARHGAP9	2.9304E-28	0.598028275	0.710	0.494	7.096E-24	Meta_γδT
PLA2G16	3.0904E-28	0.44899425	0.401	0.184	7.483E-24	Meta_γδT
SERPINB6	3.2155E-28	0.379217142	0.234	0.077	7.786E-24	Meta_γδT
MT-ATP8	5.9101E-28	0.672053774	0.821	0.790	1.431E-23	Meta_γδT
CST7	7.0684E-28	0.323465053	0.888	0.682	1.712E-23	Meta_γδT
CDIP1	4.6035E-27	0.529820739	0.352	0.123	1.115E-22	Meta_γδT
ISYNA1	6.2239E-27	0.463597359	0.242	0.060	1.507E-22	Meta_γδT
CCDC69	8.4375E-27	0.630662515	0.529	0.277	2.043E-22	Meta_γδT
PTGER2	1.1314E-26	0.447268259	0.416	0.228	2.74E-22	Meta_γδT
FCRL6	2.4112E-26	0.383280415	0.208	0.060	5.839E-22	Meta_γδT
PRDX5	3.6864E-26	0.451293791	0.698	0.519	8.927E-22	Meta_γδT
HIC1	9.5312E-26	0.410205703	0.207	0.057	2.308E-21	Meta_γδT
FTL	1.8586E-25	0.358607546	0.986	0.967	4.501E-21	Meta_γδT
KIR2DL3	1.9279E-25	0.543052503	0.101	0.004	4.668E-21	Meta_γδT
MT-CO3	3.4537E-25	0.504998094	0.988	0.987	8.363E-21	Meta_γδT
GFOD1	3.8949E-25	0.518644199	0.233	0.055	9.431E-21	Meta_γδT
KLRK1	4.1839E-25	0.380431277	0.220	0.067	1.013E-20	Meta_γδT
TRGV2	4.797E-25	1.217474974	0.176	0.064	1.162E-20	Meta_γδT
NR4A1	5.2322E-25	0.759775115	0.502	0.277	1.267E-20	Meta_γδT
ITGB7	5.7846E-25	0.394488662	0.536	0.367	1.401E-20	Meta_γδT
MT-ND2	9.4475E-25	0.450277516	0.971	0.943	2.288E-20	Meta_γδT
ASB2	1.459E-24	0.499329752	0.366	0.156	3.533E-20	Meta_γδT
MPST	3.2446E-24	0.406523473	0.283	0.121	7.857E-20	Meta_γδT
FAM173A	1.7905E-23	0.40111867	0.350	0.165	4.336E-19	Meta_γδT
LSP1	7.9224E-23	0.398983804	0.910	0.825	1.918E-18	Meta_γδT
CCL4	1.0728E-22	0.291926174	0.652	0.414	2.598E-18	Meta_γδT
PITPNC1	1.3592E-22	0.413070259	0.470	0.286	3.291E-18	Meta_γδT
CHN2	1.5928E-22	0.321728773	0.175	0.041	3.857E-18	Meta_γδT
TRGC1	3.5489E-22	0.419462124	0.152	0.029	8.594E-18	Meta_γδT
TMSB4X	4.852E-22	0.28067109	1.000	0.999	1.175E-17	Meta_γδT
CD8A	7.5186E-22	0.277159278	0.624	0.427	1.821E-17	Meta_γδT
APOBR	7.8117E-22	0.348337149	0.187	0.055	1.892E-17	Meta_γδT
LINC00996	1.0442E-21	0.402203149	0.103	0.008	2.528E-17	Meta_γδT
P2RY11	1.1267E-21	0.390952256	0.197	0.050	2.728E-17	Meta_γδT
ADRB1	1.5615E-21	0.264459228	0.107	0.004	3.781E-17	Meta_γδT

RASSF1	1.6606E-21	0.530722586	0.469	0.244	4.021E-17	Meta_γδT
MT-ND1	1.8155E-21	0.370758181	0.958	0.950	4.396E-17	Meta_γδT
DENND1B	1.8398E-21	0.313048393	0.316	0.172	4.455E-17	Meta_γδT
EVL	1.9855E-21	0.406616284	0.883	0.796	4.808E-17	Meta_γδT
CHCHD10	3.1017E-21	0.420440038	0.538	0.381	7.511E-17	Meta_γδT
CD247	3.4039E-21	0.560489756	0.786	0.638	8.243E-17	Meta_γδT
AKAP5	3.9139E-21	0.388641596	0.181	0.038	9.478E-17	Meta_γδT
ID2	3.9309E-21	0.510107152	0.802	0.580	9.519E-17	Meta_γδT
TPI1	7.9225E-21	0.490925763	0.856	0.706	1.918E-16	Meta_γδT
NEDD9	1.1595E-20	0.436454599	0.416	0.224	2.808E-16	Meta_γδT
SEPT7	1.9801E-20	0.353940048	0.827	0.694	4.795E-16	Meta_γδT
FOSL2	2.1327E-20	0.487017343	0.364	0.165	5.164E-16	Meta_γδT
TRGV8	2.3255E-20	0.705522526	0.151	0.028	5.631E-16	Meta_γδT
CD151	2.414E-20	0.406748492	0.311	0.137	5.846E-16	Meta_γδT
CA10	2.6208E-20	0.282495303	0.123	0.014	6.346E-16	Meta_γδT
CLDND1	5.1047E-20	0.461544448	0.647	0.448	1.236E-15	Meta_γδT
TRGV5	5.1762E-20	1.144278693	0.144	0.049	1.253E-15	Meta_γδT
PABPC1	6.1085E-20	0.381750509	0.908	0.895	1.479E-15	Meta_γδT
SRGAP3	6.4958E-20	0.36387023	0.140	0.036	1.573E-15	Meta_γδT
RAB3GAP1	2.0364E-19	0.475237023	0.316	0.141	4.931E-15	Meta_γδT
HAGHL	2.1806E-19	0.35459193	0.197	0.059	5.28E-15	Meta_γδT
SIRPG	3.4695E-19	0.520953522	0.532	0.342	8.401E-15	Meta_γδT
CCND2	3.7437E-19	0.483196768	0.480	0.306	9.065E-15	Meta_γδT
PIK3AP1	4.4062E-19	0.357226383	0.178	0.042	1.067E-14	Meta_γδT
HMGN3	5.3602E-19	0.344134161	0.467	0.310	1.298E-14	Meta_γδT
ABHD15	6.1294E-19	0.281366891	0.155	0.042	1.484E-14	Meta_γδT
FYN	6.2912E-19	0.442242258	0.730	0.554	1.523E-14	Meta_γδT
SYTL3	7.1679E-19	0.537135201	0.614	0.400	1.736E-14	Meta_γδT
TOX	1.1882E-18	0.297358826	0.316	0.156	2.877E-14	Meta_γδT
CCDC50	1.2579E-18	0.308678224	0.195	0.062	3.046E-14	Meta_γδT
CCL4L2	1.3127E-18	0.46991723	0.352	0.162	3.179E-14	Meta_γδT
PDE4A	1.3601E-18	0.312881963	0.190	0.063	3.294E-14	Meta_γδT
TTC38	1.4456E-18	0.354586159	0.193	0.057	3.5E-14	Meta_γδT
PRKACB	2.2504E-18	0.391421319	0.454	0.281	5.449E-14	Meta_γδT
PTGDR	2.8141E-18	0.25020436	0.168	0.074	6.814E-14	Meta_γδT
DHRS7	9.6031E-18	0.340985868	0.624	0.463	2.325E-13	Meta_γδT
IRF8	1.0191E-17	0.306628459	0.133	0.027	2.468E-13	Meta_γδT
CBLB	1.3355E-17	0.396132996	0.565	0.386	3.234E-13	Meta_γδT
SCML4	2.2611E-17	0.471626749	0.544	0.348	5.475E-13	Meta_γδT
OSTF1	2.2898E-17	0.409068673	0.671	0.503	5.545E-13	Meta_γδT

ADD1	2.3726E-17	0.316832118	0.329	0.184	5.745E-13	Meta_γδT
LSM2	3.6489E-17	0.315504836	0.502	0.346	8.836E-13	Meta_γδT
NBL1	4.6022E-17	0.363567157	0.243	0.104	1.114E-12	Meta_γδT
SKAP2	4.8961E-17	0.371860651	0.234	0.076	1.186E-12	Meta_γδT
MAPK1	5.5965E-17	0.461825351	0.383	0.206	1.355E-12	Meta_γδT
ZFP36	7.8663E-17	0.40478662	0.881	0.819	1.905E-12	Meta_γδT
RUNX3	1.2293E-16	0.415473115	0.602	0.411	2.977E-12	Meta_γδT
NR4A3	1.3266E-16	0.472159214	0.343	0.173	3.212E-12	Meta_γδT
CDK2AP2	2.0804E-16	0.433231856	0.559	0.381	5.038E-12	Meta_γδT
ADAM8	2.8587E-16	0.339379312	0.405	0.247	6.922E-12	Meta_γδT
STIP1	2.9927E-16	0.253573232	0.332	0.212	7.247E-12	Meta_γδT
PAXX	3.3535E-16	0.417101497	0.705	0.571	8.12E-12	Meta_γδT
DAPK2	4.3631E-16	0.340841348	0.211	0.079	1.057E-11	Meta_γδT
YES1	4.3855E-16	0.34238777	0.208	0.076	1.062E-11	Meta_γδT
GPR68	5.0047E-16	0.399546687	0.253	0.091	1.212E-11	Meta_γδT
MCTP2	5.1465E-16	0.375329999	0.191	0.064	1.246E-11	Meta_γδT
GPR18	6.431E-16	0.326772147	0.220	0.094	1.557E-11	Meta_γδT
TNFSF14	6.6045E-16	0.315134296	0.272	0.136	1.599E-11	Meta_γδT
SYTL2	1.4758E-15	0.40056615	0.335	0.175	3.574E-11	Meta_γδT
ALOX5AP	1.6992E-15	0.299528121	0.762	0.591	4.115E-11	Meta_γδT
SYNGR1	1.8354E-15	0.26571292	0.107	0.023	4.444E-11	Meta_γδT
FUCA1	2.0212E-15	0.270997999	0.161	0.052	4.894E-11	Meta_γδT
DZIP3	2.6756E-15	0.334079798	0.237	0.095	6.479E-11	Meta_γδT
AUTS2	3.3497E-15	0.385232829	0.268	0.106	8.111E-11	Meta_γδT
RNF130	3.4657E-15	0.310186274	0.176	0.062	8.392E-11	Meta_γδT
USP11	3.5454E-15	0.256291058	0.311	0.179	8.585E-11	Meta_γδT
EPS8L2	3.8518E-15	0.308478312	0.232	0.104	9.327E-11	Meta_γδT
DCXR	4.5048E-15	0.38009931	0.500	0.337	1.091E-10	Meta_γδT
GABARAPL1	8.3506E-15	0.320424879	0.477	0.280	2.022E-10	Meta_γδT
MMP25-AS1	8.4751E-15	0.255964377	0.204	0.097	2.052E-10	Meta_γδT
PTPN4	1.0797E-14	0.386311605	0.424	0.246	2.614E-10	Meta_γδT
HAVCR2	1.0944E-14	0.397231252	0.255	0.133	2.65E-10	Meta_γδT
SLC12A6	1.1957E-14	0.275210732	0.181	0.064	2.895E-10	Meta_γδT
PRMT9	1.2946E-14	0.359214519	0.336	0.184	3.135E-10	Meta_γδT
MAFF	1.7123E-14	0.361842898	0.233	0.096	4.146E-10	Meta_γδT
ECH1	2.0124E-14	0.280630851	0.572	0.428	4.873E-10	Meta_γδT
ITPK1	2.1934E-14	0.262940571	0.190	0.078	5.311E-10	Meta_γδT
BUB3	2.7999E-14	0.28930484	0.650	0.506	6.78E-10	Meta_γδT
CD101	3.0408E-14	0.326121009	0.200	0.062	7.363E-10	Meta_γδT
UBAC2	3.1767E-14	0.27231737	0.453	0.316	7.692E-10	Meta_γδT

LIMK1	3.5618E-14	0.254568251	0.143	0.043	8.625E-10	Meta_γδT
RGS1	3.7344E-14	0.423007774	0.753	0.609	9.043E-10	Meta_γδT
PIK3R1	6.6811E-14	0.480802057	0.602	0.410	1.618E-09	Meta_γδT
NCALD	1.0943E-13	0.255845521	0.173	0.065	2.65E-09	Meta_γδT
AP2A1	1.1597E-13	0.302035387	0.287	0.149	2.808E-09	Meta_γδT
STOM	1.1896E-13	0.374096011	0.581	0.394	2.881E-09	Meta_γδT
DGKD	1.3144E-13	0.361469354	0.231	0.082	3.183E-09	Meta_γδT
RASGEF1B	1.5831E-13	0.365952938	0.259	0.121	3.833E-09	Meta_γδT
IL12RB2	1.8098E-13	0.33601785	0.216	0.085	4.382E-09	Meta_γδT
PTK2B	2.6685E-13	0.262454787	0.400	0.269	6.462E-09	Meta_γδT
STARD3NL	3.3485E-13	0.406119359	0.307	0.163	8.109E-09	Meta_γδT
TRGV9	3.9082E-13	0.50442378	0.137	0.046	9.464E-09	Meta_γδT
TCIRG1	3.9824E-13	0.271052965	0.439	0.302	9.643E-09	Meta_γδT
TLN1	8.95E-13	0.353100344	0.493	0.334	2.167E-08	Meta_γδT
GLIPR1	1.2533E-12	0.299537777	0.575	0.434	3.035E-08	Meta_γδT
PDLIM7	1.295E-12	0.303379101	0.187	0.061	3.136E-08	Meta_γδT
PHYKPL	1.5617E-12	0.491543817	0.449	0.280	3.782E-08	Meta_γδT
CLEC2B	1.6673E-12	0.285063666	0.771	0.618	4.037E-08	Meta_γδT
ACTR3	1.769E-12	0.282513354	0.723	0.614	4.284E-08	Meta_γδT
SLC9A3R1	4.5548E-12	0.286790899	0.694	0.568	1.103E-07	Meta_γδT
AC022706.1	7.0038E-12	0.278274063	0.194	0.076	1.696E-07	Meta_γδT
NSMCE1	8.0349E-12	0.372533847	0.371	0.224	1.946E-07	Meta_γδT
ABHD17A	8.2966E-12	0.343265599	0.349	0.195	2.009E-07	Meta_γδT
TRBV28	8.6396E-12	1.466657438	0.169	0.053	2.092E-07	Meta_γδT
ZNF331	1.0115E-11	0.656639321	0.491	0.331	2.449E-07	Meta_γδT
NCR3	1.4799E-11	0.32631409	0.198	0.082	3.584E-07	Meta_γδT
RHOB	1.5106E-11	0.439454626	0.284	0.133	3.658E-07	Meta_γδT
MBP	1.8577E-11	0.350025389	0.602	0.444	4.498E-07	Meta_γδT
CCL3	1.8744E-11	0.822206913	0.237	0.128	4.539E-07	Meta_γδT
PSD4	2.0672E-11	0.273662683	0.302	0.172	5.006E-07	Meta_γδT
MT-ND6	2.1294E-11	0.465956566	0.701	0.595	5.156E-07	Meta_γδT
SSH1	3.2469E-11	0.274004283	0.194	0.073	7.862E-07	Meta_γδT
CEBPD	5.0124E-11	0.318685787	0.261	0.138	1.214E-06	Meta_γδT
NUDT14	5.8546E-11	0.265577721	0.229	0.110	1.418E-06	Meta_γδT
ZFP36L1	7.5654E-11	0.490382295	0.780	0.671	1.832E-06	Meta_γδT
MSH3	8.224E-11	0.282860134	0.218	0.098	1.991E-06	Meta_γδT
ECI2	8.3569E-11	0.361015086	0.296	0.143	2.024E-06	Meta_γδT
VASP	8.4735E-11	0.341108572	0.530	0.382	2.052E-06	Meta_γδT
TSEN54	8.8345E-11	0.271991006	0.450	0.305	2.139E-06	Meta_γδT
CHPT1	8.8424E-11	0.299689499	0.178	0.060	2.141E-06	Meta_γδT

SLC25A39	9.1746E-11	0.347840205	0.424	0.271	2.222E-06	Meta_γδT
HENMT1	9.2409E-11	0.258042015	0.213	0.105	2.238E-06	Meta_γδT
MAD2L2	9.3336E-11	0.33024479	0.355	0.210	2.26E-06	Meta_γδT
PDCD4	1.2913E-10	0.344615407	0.710	0.573	3.127E-06	Meta_γδT
ATP8A1	1.7824E-10	0.261435031	0.286	0.160	4.316E-06	Meta_γδT
RAB24	1.8368E-10	0.303119401	0.310	0.179	4.448E-06	Meta_γδT
SH3BP1	1.8866E-10	0.305678379	0.455	0.316	4.569E-06	Meta_γδT
ST3GAL1	2.0138E-10	0.291467454	0.429	0.295	4.876E-06	Meta_γδT
RARRES3	3.1702E-10	0.261804949	0.826	0.733	7.677E-06	Meta_γδT
TNIP3	3.1889E-10	0.268091109	0.205	0.086	7.722E-06	Meta_γδT
PRKAR1A	4.8113E-10	0.360491125	0.525	0.370	1.165E-05	Meta_γδT
ARF6	5.3906E-10	0.259509297	0.648	0.519	1.305E-05	Meta_γδT
TMEM14C	5.6537E-10	0.268526889	0.412	0.281	1.369E-05	Meta_γδT
IL18RAP	6.0212E-10	0.308583874	0.204	0.085	1.458E-05	Meta_γδT
GFI1	6.3244E-10	0.266559921	0.246	0.129	1.531E-05	Meta_γδT
TTC1	6.6453E-10	0.257017376	0.342	0.216	1.609E-05	Meta_γδT
SURF4	7.4969E-10	0.289190443	0.571	0.435	1.815E-05	Meta_γδT
HSH2D	8.1762E-10	0.267454604	0.239	0.135	1.98E-05	Meta_γδT
CHST12	9.8458E-10	0.336220537	0.485	0.326	2.384E-05	Meta_γδT
HSPA1A	1.1307E-09	0.283999496	0.513	0.358	2.738E-05	Meta_γδT
PRKCH	1.166E-09	0.37039704	0.636	0.485	2.823E-05	Meta_γδT
STK17A	1.4268E-09	0.279696179	0.825	0.720	3.455E-05	Meta_γδT
ZBTB7A	2.0154E-09	0.277873118	0.279	0.150	4.88E-05	Meta_γδT
SOCS1	2.1258E-09	0.436530153	0.525	0.369	5.148E-05	Meta_γδT
LITAF	2.9366E-09	0.279422164	0.614	0.484	7.111E-05	Meta_γδT
HNRNPAB	5.0848E-09	0.352039813	0.400	0.244	0.0001231	Meta_γδT
P2RX4	8.425E-09	0.259277408	0.210	0.096	0.000204	Meta_γδT
ILK	1.9204E-08	0.251281295	0.388	0.263	0.000465	Meta_γδT
JAK1	2.5639E-08	0.321649994	0.765	0.652	0.0006209	Meta_γδT
SELENOH	3.8419E-08	0.325284081	0.576	0.444	0.0009303	Meta_γδT
HIST1H4C	6.0513E-08	0.32170039	0.665	0.561	0.0014653	Meta_γδT
PPDPF	8.7013E-08	0.254994254	0.903	0.852	0.002107	Meta_γδT
SAMSN1	1.2597E-07	0.408839473	0.671	0.534	0.0030503	Meta_γδT
GPR174	1.2908E-07	0.251129289	0.363	0.242	0.0031256	Meta_γδT
LNPEP	4.0376E-07	0.289768213	0.426	0.288	0.0097771	Meta_γδT
SET	4.2141E-07	0.253467388	0.565	0.441	0.0102044	Meta_γδT
REL	5.3541E-07	0.290434326	0.556	0.411	0.0129648	Meta_γδT
ZEB2	8.5204E-07	0.27584255	0.272	0.147	0.0206323	Meta_γδT
NDFIP2	1.0225E-06	0.364383268	0.253	0.133	0.024759	Meta_γδT
RNF187	1.5362E-06	0.271027572	0.420	0.294	0.0371998	Meta_γδT

FLOT1	1.5485E-06	0.284463509	0.373	0.245	0.0374959	Meta_γδT
IVNS1ABP	2.0148E-06	0.277487707	0.458	0.332	0.0487886	Meta_γδT
LPXN	4.4111E-06	0.294217116	0.521	0.394	0.1068147	Meta_γδT
IFITM3	4.9885E-06	0.382937733	0.234	0.155	0.1207969	Meta_γδT
CTSD	6.4014E-06	0.394195201	0.377	0.264	0.1550105	Meta_γδT
PTP4A1	1.1293E-05	0.25560903	0.421	0.295	0.2734678	Meta_γδT
NARF	1.2501E-05	0.258377412	0.407	0.279	0.3027159	Meta_γδT
NAP1L4	2.6371E-05	0.265550111	0.539	0.411	0.6385622	Meta_γδT
CSF1	2.9863E-05	0.274368221	0.156	0.093	0.7231319	Meta_γδT
RANBP2	3.0492E-05	0.281636178	0.417	0.310	0.7383569	Meta_γδT
TRGV10	5.2269E-05	0.4996644	0.201	0.097	1	Meta_γδT
POLR2L	7.4517E-05	0.264622861	0.717	0.616	1	Meta_γδT
CYTIP	0.00012844	0.257759643	0.795	0.710	1	Meta_γδT
ATP6V0A2	0.00014977	0.25487286	0.209	0.107	1	Meta_γδT
HSPE1	0.00025244	0.278627306	0.676	0.572	1	Meta_γδT
ATF3	0.00070957	0.281628739	0.213	0.140	1	Meta_γδT
CREM	0.0008037	0.257968355	0.502	0.398	1	Meta_γδT
CMC1	0.00144698	0.480016009	0.275	0.227	1	Meta_γδT
