# Supplementary material for: Targeting pro-inflammatory T cells as a novel therapeutic approach to potentially resolve atherosclerosis in humans
Source: Cell Res. 2024 Mar 15;34(6):407–27. doi: 10.1038/s41422-024-00945-0 (PMC11143203; doi:10.1038/s41422-024-00945-0)
Supplement: Supplementary file 15 — Supplementary information, Table S3 [file 41422_2024_945_MOESM15_ESM.pdf]

Supplementary information, Table S3. Antibody staining panels for CyTOF analyses.

T cell-specific antibody panel

Elemental Isotope	Antigen Target	Clone Number	Supplier	Staining Step
89Y	CD45	HI30	BioLegend	Surface
115In	CD3	UCHT1	BioXcell	Surface
139La	CD47	CC2C6	BioLegend	Surface
141Pr	CD56	NCAM16.2	BD Biosciences	Surface
142Nd	TCRγδ	5A6.E9	In-house	Surface
142Nd	CD19	HIB19	BioLegend	Surface
143Nd	CD196 (CCR6)	G034E3	BioLegend	Surface
145Nd	CD95 (Fas)	DX2	BioLegend	Surface
146Nd	CD123 (IL-3Rα)	6H6	BioLegend	Surface
147Sm	CD66b	G10F5	BioLegend	Surface
148Nd	CD33	WM53	BioLegend	Surface
149Sm	CD25	24212	R&D Systems	Surface
150Nd	CD14	M5E2	BioLegend	Surface
151Eu	CD38	HIT2	BioLegend	Surface
152Sm	CD39	A1	BioLegend	Surface
153Eu	CD274 (PD-L1)	29E.2A3	BioLegend	Surface
154Sm	Ki67	SolA15	eBioscience	Intracellular
155Gd	CD45RA	HI100	BioLegend	Surface
156Gd	CD11c	BU15	BioLegend	Surface
157Gd	CD68	Y1/82A	BioLegend	Intracellular
158Gd	CD197 (CCR7)	G043H7	BioLegend	Surface
159Tb	CD357 (GITR)	621	BioLegend	Surface
160Gd	CD28	CD28.2	BioLegend	Surface
161Dy	CD152 (CTLA-4)	14D3	eBioscience	Intracellular
162Dy	FoxP3	PCH101	eBioscience	Intracellular
163Dy	CD183 (CXCR3)	G025H7	BioLegend	Surface
164Dy	RORγ	600214	R&D Systems	Intracellular
165Ho	CD161	HP-3G10	BioLegend	Surface
166Er	CD27	O323	BioLegend	Surface
167Er	CD278 (ICOS)	C398.4A	BioLegend	Surface
168Er	T-bet	4B10	BioLegend	Intracellular
169Tm	CD15 (SSEA-1)	W6D3	BioLegend	Surface
170Er	CD127 (IL-7Rα)	A019D5	BioLegend	Surface
171Yb	GATA3	TWAJ	eBioscience	Intracellular
172Yb	CD272 (BTLA)	MIH26	BioLegend	Surface

173Yb	Granzyme B	GB11	Fluidigm	Intracellular
174Yb	CD279 (PD-1)	EH12.2H7	BioLegend	Surface
175Lu	CD16	3G8	BioLegend	Surface
176Yb	HLA-DR	L243	BioLegend	Surface
197Au	CD4	RPA-T4	BioLegend	Surface
198Pt	CD8a	RPA-T8	BioLegend	Surface
209Bi	CD11b	M1/70	BioLegend	Surface

**T-cell activation and exhaustion panel**				
**Elemental Isotope**	**Antigen Target**	**Clone Number**	**Supplier**	**Staining Step**
89Y	CD45	HI30	BioLegend	Surface
115In	CD3	UCHT1	BioXcell	Surface
142Nd	TCRγδ	5A6.E9	In-house	Surface
143Nd	CD196 (CCR6)	G034E3	BioLegend	Surface
145Nd	CD69	FN50	BioLegend	Intracellular
146Nd	CD7	CD7-6B7	BioLegend	Surface
147Sm	CD197 (CCR7)	G043H7	BioLegend	Surface
148Nd	TIGIT	A15153G	BioLegend	Surface
149Sm	CD25	24212	R&D Systems	Surface
151Eu	CD38	HIT2	BioLegend	Surface
152Sm	CD103	B-Ly7	eBioscience	Surface
153Eu	CD161	HP-3G10	BioLegend	Surface
156Gd	CD194(CCR4)	L291H4	BioLegend	Surface
157Gd	CD28	CD28.2	BioLegend	Surface
158Gd	CD127(IL-7Ra)	A019D5	BioLegend	Surface
161Dy	CD152(CTLA-4)	14D3	eBioscience	Intracellular
162Dy	Foxp3	PCH101	eBioscience	Intracellular
163Dy	CD137(4-1BB)	4B4-1	BioLegend	Surface
164Dy	CD45RA	HI100	BioLegend	Surface
166Er	Perforin	B-D48	Abcam	Intracellular
167Er	CD27	O323	BioLegend	Surface
170Er	CD39	A1	BioLegend	Surface
171Yb	CD279(PD-1)	EH12.2H7	BioLegend	Surface
172Yb	CD366(Tim-3)	F38-2E2	BioLegend	Surface
173Yb	Granzyme B	GB11	Fluidigm	Intracellular
174Yb	CD223(LAG-3)	874501	R&D Systems	Surface
176Yb	HLA-DR	L243	BioLegend	Surface
197Au	CD4	RPA-T4	BioLegend	Surface
198Pt	CD8a	RPA-T8	BioLegend	Surface

**Myeloid cell-specific antibody panel**				
**Elemental Isotope**	**Antigen Target**	**Clone Number**	**Supplier**	**Staining Step**
89Y	CD45	HI30	BioLegend	Surface
115In	CD3	UCHT1	BioXcell	Surface

139La	CD47	CC2C6	BioLegend	Surface
141Pr	CD56	NCAM16.2	BD Biosciences	Surface
142Nd	CD19	HIB19	BioLegend	Surface
143Nd	CD184 (CXCR4)	12G5	BioLegend	Surface
144Nd	CD38	HIT2	BioLegend	Surface
145Nd	CD115 (CSF-1R)	9-4D2-1E4	BioLegend	Surface
146Nd	CD54 (ICAM-1)	HA58	BioLegend	Surface
147Sm	CD15 (SSEA-1)	W6D3	BioLegend	Surface
148Nd	CD33	WM53	BioLegend	Surface
149Sm	CD169 (Siglec-1)	7-239	BioLegend	Surface
150Nd	CD14	M5E2	BD Biosciences	Surface
151Eu	CD36L1 (SR-BI)	m1B9	BioLegend	Surface
152Sm	FcεRIα	AER-37[CRA-1]	BioLegend	Surface
153Eu	CD274 (PD-L1)	29E.2A3	BioLegend	Surface
154Sm	CD163	GHI/61	BioLegend	Surface
155Gd	CD206 (MMR)	15-2	BioLegend	Surface
156Gd	CD24	ML5	BioLegend	Surface
157Gd	CD172a (SIRPα)	SE5A5	BioLegend	Surface
158Gd	CD204 (SR-AI)	351615	R&D Systems	Surface
159Tb	CD11c	BU15	BioLegend	Surface
160Gd	CD319 (Slamf7)	162.1	BioLegend	Surface
161Dy	CD66b	G10F5	BioLegend	Surface
162Dy	CD32 (FcγRII)	Fun-2	BioLegend	Surface
163Dy	CD68	Y1/82A	BioLegend	Intracellular
164Dy	CD192 (CCR2)	K036C2	BioLegend	Surface
165Ho	ProMBP-1	J175-7D4	BioLegend	Intracellular
166Er	CX3CR1	K0124E1	BioLegend	Surface
167Er	CD36	5-271	BioLegend	Surface
168Er	CD95 (Fas)	DX2	BioLegend	Surface
169Tm	CD40	82111	R&D Systems	Surface
170Er	CD86	Fun-1	BD Biosciences	Surface
171Yb	CD64 (FcγRI)	10.1	BioLegend	Surface
172Yb	CD117 (C-kit)	104D2	BioLegend	Surface
173Yb	Siglec-8	7C9	BioLegend	Surface
174Yb	CD279 (PD-1)	EH12.2H7	BioLegend	Surface
175Lu	CD16 (FcγRIII)	3G8	BioLegend	Surface
176Yb	HLA-DR	L243	BioLegend	Surface
198Pt	Ki67	SolA15	eBioscience	Intracellular
209Bi	CD11b	M1/70	BioLegend	Surface
