# Supplementary material for: Targeting pro-inflammatory T cells as a novel therapeutic approach to potentially resolve atherosclerosis in humans
Source: Cell Res. 2024 Mar 15;34(6):407–27. doi: 10.1038/s41422-024-00945-0 (PMC11143203; doi:10.1038/s41422-024-00945-0)
Supplement: Supplementary file 16 — Supplementary information, Table S4 [file 41422_2024_945_MOESM16_ESM.pdf]

Supplementary information, Table S4. Differentially expressed genes (DEGs) of identified T cell clusters in Fernandez et al.

Gene	*P* value	avg_logFC	pct.1	pct.2	adjusted *P* value	cluster
IL7R	1.80021E-98	0.00102733	0.849	0.551	1.80021E-94	F_C0
LTB	6.10481E-84	0.000861184	0.706	0.405	6.10481E-80	F_C0
S100A4	1.509E-70	0.001442574	0.960	0.854	1.509E-66	F_C0
NFKBIA	1.03937E-68	0.001126318	0.810	0.607	1.03937E-64	F_C0
FOS	8.44966E-63	0.001147803	0.732	0.505	8.44966E-59	F_C0
TPT1	5.822E-59	0.001398667	0.992	0.964	5.822E-55	F_C0
KLRB1	8.20387E-51	0.000924689	0.481	0.277	8.20387E-47	F_C0
CD40LG	7.2671E-50	0.000183264	0.273	0.090	7.2671E-46	F_C0
JUNB	5.01475E-41	0.00121244	0.877	0.752	5.01475E-37	F_C0
S100A11	1.24882E-34	0.000439689	0.718	0.535	1.24882E-30	F_C0
BIRC3	3.77943E-34	0.000426278	0.480	0.292	3.77943E-30	F_C0
VIM	1.27067E-32	0.000633331	0.832	0.724	1.27067E-28	F_C0
TIMP1	2.91441E-31	0.000184547	0.370	0.196	2.91441E-27	F_C0
KLF6	5.6287E-30	0.000538254	0.745	0.592	5.6287E-26	F_C0
TSC22D3	2.32961E-29	0.00075853	0.897	0.820	2.32961E-25	F_C0
ANXA1	4.98843E-29	0.000573352	0.730	0.581	4.98843E-25	F_C0
CD69	3.19172E-28	0.000711634	0.745	0.615	3.19172E-24	F_C0
ZFP36L2	4.65759E-28	0.000752609	0.924	0.893	4.65759E-24	F_C0
TNFAIP3	5.74778E-28	0.000418314	0.492	0.333	5.74778E-24	F_C0
PABPC1	7.8563E-28	0.000460247	0.783	0.638	7.8563E-24	F_C0
SOCS3	8.87533E-28	0.000137197	0.258	0.118	8.87533E-24	F_C0
FXYD5	6.53047E-27	0.000418495	0.788	0.651	6.53047E-23	F_C0
DUSP1	4.18632E-26	0.000644649	0.722	0.581	4.18632E-22	F_C0
PPP1R15A	5.74069E-26	0.000290153	0.500	0.330	5.74069E-22	F_C0
LDHB	4.80058E-24	0.000376538	0.702	0.558	4.80058E-20	F_C0
ZFP36	1.91101E-23	0.000599225	0.763	0.649	1.91101E-19	F_C0
TNFRSF25	4.40669E-23	0.000104867	0.198	0.085	4.40669E-19	F_C0
JUN	4.53283E-22	0.000687271	0.780	0.657	4.53283E-18	F_C0
TMEM123	5.66962E-22	0.000147303	0.392	0.241	5.66962E-18	F_C0
EEF1A1	1.42709E-21	0.000963072	0.997	0.995	1.42709E-17	F_C0
EEF1B2	3.9123E-21	0.000509834	0.859	0.767	3.9123E-17	F_C0
AQP3	1.34448E-20	0.000100704	0.215	0.103	1.34448E-16	F_C0
TRADD	1.49563E-20	0.000128288	0.336	0.198	1.49563E-16	F_C0
CD52	5.13853E-20	0.000673433	0.959	0.917	5.13853E-16	F_C0
ANKRD28	6.77744E-20	0.000160938	0.226	0.113	6.77744E-16	F_C0
FTH1	1.14571E-19	0.000613545	0.974	0.945	1.14571E-15	F_C0
TMEM173	1.7622E-19	0.000163108	0.385	0.245	1.7622E-15	F_C0

RP11-138A9.2	4.63174E-18	0.00018667	0.322	0.196	4.63174E-14	F_C0
GPR183	5.4014E-18	0.000186223	0.417	0.279	5.4014E-14	F_C0
DDIT4	6.30857E-17	0.000391247	0.730	0.617	6.30857E-13	F_C0
TOB1	6.72633E-17	0.00014976	0.353	0.226	6.72633E-13	F_C0
NBEAL1	2.36372E-16	0.000361722	0.769	0.662	2.36372E-12	F_C0
LMNA	4.432E-16	0.000181662	0.252	0.145	4.432E-12	F_C0
CORO1B	5.20526E-16	0.000129066	0.337	0.214	5.20526E-12	F_C0
RP11-138A9.1	1.88035E-15	0.000152923	0.342	0.222	1.88035E-11	F_C0
PIM1	4.93823E-15	0.000107847	0.314	0.198	4.93823E-11	F_C0
S100A10	6.06257E-15	0.00034351	0.785	0.690	6.06257E-11	F_C0
RORA	1.15728E-14	0.000137098	0.362	0.242	1.15728E-10	F_C0
S100A6	2.07402E-14	0.000423378	0.907	0.841	2.07402E-10	F_C0
UBA52	2.60969E-14	0.000479441	0.973	0.942	2.60969E-10	F_C0
C10orf54	4.02678E-14	0.000116877	0.350	0.234	4.02678E-10	F_C0
PTGER4	5.49801E-14	0.000117741	0.335	0.221	5.49801E-10	F_C0
EIF3E	8.79825E-14	0.000244511	0.674	0.557	8.79825E-10	F_C0
LGALS3	1.36611E-13	0.00010736	0.231	0.135	1.36611E-09	F_C0
PTMA	1.37049E-13	0.000531157	0.990	0.976	1.37049E-09	F_C0
RBPJ	1.45443E-13	0.000110274	0.256	0.156	1.45443E-09	F_C0
RNASET2	2.79518E-13	0.000135268	0.400	0.283	2.79518E-09	F_C0
FKBP11	1.12101E-12	0.000119813	0.384	0.270	1.12101E-08	F_C0
GPR171	1.52877E-12	0.000157496	0.375	0.264	1.52877E-08	F_C0
BTG2	3.05393E-12	0.000146722	0.349	0.242	3.05393E-08	F_C0
EEF2	1.2772E-11	0.000312918	0.771	0.702	1.2772E-07	F_C0
NACA	2.28696E-11	0.000330353	0.919	0.857	2.28696E-07	F_C0
FOSB	4.33257E-11	0.000172467	0.390	0.285	4.33257E-07	F_C0
ODF2L	8.18781E-11	0.000108155	0.291	0.196	8.18781E-07	F_C0
AC090498.1	8.32829E-11	0.000153125	0.445	0.337	8.32829E-07	F_C0
GNB2L1	1.55056E-10	0.00038828	0.953	0.924	1.55056E-06	F_C0
NOSIP	1.64865E-10	0.000105768	0.320	0.223	1.64865E-06	F_C0
NHSL2	2.20824E-10	0.000115978	0.340	0.242	2.20824E-06	F_C0
SPOCK2	8.82085E-10	0.000103524	0.436	0.332	8.82085E-06	F_C0
CREM	9.59468E-10	0.000104763	0.276	0.187	9.59468E-06	F_C0
GTF3A	1.07076E-09	0.00013178	0.546	0.441	1.07076E-05	F_C0
PPP1CB	1.14719E-09	0.000104547	0.325	0.231	1.14719E-05	F_C0
CITED2	1.2168E-09	0.00015839	0.391	0.292	1.2168E-05	F_C0
MCL1	1.62211E-09	0.000136847	0.428	0.328	1.62211E-05	F_C0
RHOH	2.71155E-09	0.000113574	0.418	0.319	2.71155E-05	F_C0
NPM1	2.85761E-09	0.000259559	0.820	0.750	2.85761E-05	F_C0
NAP1L1	3.51333E-09	0.000151692	0.634	0.533	3.51333E-05	F_C0

CRIP1	7.89873E-09	0.000180429	0.460	0.368	7.89873E-05	F_C0
CKLF	1.05693E-08	0.000161679	0.503	0.409	0.000105693	F_C0
COMMD6	1.26228E-08	0.000215357	0.807	0.730	0.000126228	F_C0
TOMM7	2.66778E-08	0.000268807	0.909	0.857	0.000266778	F_C0
HSP90AB1	3.20182E-08	0.000160593	0.664	0.571	0.000320182	F_C0
HNRNPA1	3.39317E-08	0.00026461	0.855	0.803	0.000339317	F_C0
ZFAS1	3.66219E-08	0.000139423	0.596	0.500	0.000366219	F_C0
YWHAQ	4.58599E-08	0.000108989	0.406	0.315	0.000458599	F_C0
GSTK1	4.76419E-08	0.000141297	0.578	0.485	0.000476419	F_C0
STK17B	5.1476E-08	0.000104372	0.492	0.397	0.00051476	F_C0
OCIAD2	5.91295E-08	0.000111325	0.489	0.396	0.000591295	F_C0
HINT1	6.2874E-08	0.000189834	0.738	0.656	0.00062874	F_C0
MIF	6.66834E-08	0.000141903	0.511	0.419	0.000666834	F_C0
IFITM2	1.04925E-07	0.000170801	0.617	0.528	0.001049251	F_C0
LDHA	1.06943E-07	0.000142539	0.599	0.507	0.001069434	F_C0
ABRACL	2.00952E-07	0.000101465	0.456	0.367	0.002009519	F_C0
APRT	2.29175E-07	0.000123871	0.591	0.501	0.002291755	F_C0
PLP2	2.4868E-07	0.00012412	0.545	0.455	0.002486804	F_C0
NDUFS5	2.88493E-07	0.000128336	0.570	0.481	0.002884927	F_C0
ZFP36L1	3.1985E-07	0.000107263	0.371	0.287	0.0031985	F_C0
SARAF	3.20593E-07	0.000221345	0.794	0.726	0.003205934	F_C0
EIF1	3.48925E-07	0.000316232	0.979	0.959	0.003489247	F_C0
TAGLN2	4.79038E-07	0.000133942	0.414	0.333	0.004790385	F_C0
DNAJA1	6.25872E-07	0.000119365	0.428	0.342	0.006258715	F_C0
TMSB10	7.8816E-07	0.000443913	0.995	0.994	0.007881603	F_C0
HSPA8	8.4633E-07	0.000174648	0.732	0.654	0.008463304	F_C0
FAU	1.08967E-06	0.000338897	0.988	0.980	0.010896732	F_C0
DNAJB1	1.09504E-06	0.00021169	0.559	0.477	0.010950385	F_C0
PFDN5	1.21346E-06	0.000262852	0.927	0.896	0.012134553	F_C0
IER2	1.25286E-06	0.000173073	0.543	0.462	0.012528601	F_C0
EEF1D	1.31328E-06	0.00027061	0.922	0.903	0.01313276	F_C0
GLTSCR2	2.25409E-06	0.000225628	0.744	0.685	0.022540933	F_C0
ATP5L	2.27794E-06	0.000121866	0.840	0.773	0.022779377	F_C0
RGCC	2.49862E-06	0.000116843	0.353	0.276	0.02498616	F_C0
CD44	5.22164E-06	0.000149185	0.705	0.630	0.052216409	F_C0
SLC25A6	7.73447E-06	0.000179106	0.791	0.728	0.077344678	F_C0
YBX1	1.27525E-05	0.000141841	0.737	0.667	0.127524966	F_C0
CCNI	1.44851E-05	0.000118019	0.602	0.526	0.144850744	F_C0
SERP1	2.12036E-05	0.000109994	0.514	0.439	0.212036172	F_C0
GAPDH	4.24767E-05	0.000113622	0.896	0.846	0.424767406	F_C0

HSPA1B	6.88239E-05	0.000119864	0.323	0.257	0.688238618	F_C0
COX6C	8.19253E-05	0.000126584	0.695	0.630	0.819252547	F_C0
COX7C	0.000108322	0.000183467	0.869	0.827	1	F_C0
LGALS1	0.000191964	0.000175305	0.497	0.436	1	F_C0
OST4	0.000192378	0.000133912	0.807	0.753	1	F_C0
BTF3	0.000224761	0.000156702	0.874	0.830	1	F_C0
LAPTM5	0.000249574	0.000130453	0.705	0.646	1	F_C0
ENO1	0.000284637	0.000116717	0.586	0.523	1	F_C0
TXNIP	0.000452557	0.000239335	0.885	0.848	1	F_C0
PNRC1	0.000654536	0.000119665	0.592	0.534	1	F_C0
ITM2B	0.001434184	0.000134044	0.826	0.784	1	F_C0
FTL	0.00226883	0.000202418	0.914	0.887	1	F_C0
MYL6	0.007479977	0.000103233	0.871	0.835	1	F_C0
BTG1	0.009857705	0.000209388	0.959	0.956	1	F_C0
GZMK	4.6929E-124	0.001783617	0.781	0.464	4.6929E-120	F_C1
CCL5	1.9676E-102	0.001975044	0.937	0.719	1.96764E-98	F_C1
NKG7	1.2524E-61	0.00065342	0.771	0.498	1.2524E-57	F_C1
HLA-A	1.75565E-58	0.001142056	0.981	0.990	1.75565E-54	F_C1
GZMA	9.7204E-57	0.001000806	0.848	0.648	9.7204E-53	F_C1
CD8A	2.99343E-54	0.00048782	0.465	0.225	2.99343E-50	F_C1
CD74	4.01055E-45	0.000772378	0.705	0.590	4.01055E-41	F_C1
CD8B	6.14129E-44	0.000356242	0.392	0.183	6.14129E-40	F_C1
MALAT1	9.4974E-38	0.004140176	0.999	1.000	9.4974E-34	F_C1
HLA-C	1.00039E-37	0.000820911	0.979	0.992	1.00039E-33	F_C1
CCL4	2.97908E-37	0.000411928	0.771	0.572	2.97908E-33	F_C1
CST7	2.27778E-31	0.000515762	0.666	0.493	2.27778E-27	F_C1
B2M	1.24839E-29	0.00167655	0.999	1.000	1.24839E-25	F_C1
HLA-B	5.29055E-29	0.00089635	0.990	0.995	5.29055E-25	F_C1
CMC1	4.20335E-27	0.00044605	0.398	0.250	4.20335E-23	F_C1
HLA-DPB1	5.39727E-26	0.000372565	0.573	0.414	5.39727E-22	F_C1
LYAR	6.6418E-17	0.000252713	0.474	0.341	6.6418E-13	F_C1
TMSB4X	8.42698E-17	0.001172471	0.999	1.000	8.42698E-13	F_C1
ITM2C	1.74915E-16	0.000218951	0.320	0.202	1.74915E-12	F_C1
CXCR6	4.59554E-15	0.000142559	0.229	0.127	4.59554E-11	F_C1
HLA-DRB1	1.4805E-14	0.000275203	0.460	0.347	1.4805E-10	F_C1
HLA-E	1.29915E-13	0.000333324	0.893	0.898	1.29915E-09	F_C1
GZMH	2.13566E-13	0.000205506	0.462	0.338	2.13566E-09	F_C1
APOBEC3G	8.28641E-13	0.000252223	0.471	0.370	8.28641E-09	F_C1
HLA-DPA1	1.95093E-12	0.00023268	0.430	0.327	1.95093E-08	F_C1
HCST	9.84389E-12	0.000357991	0.830	0.788	9.84389E-08	F_C1

MYO1F	5.7237E-11	0.000194427	0.383	0.287	5.7237E-07	F_C1
OAZ1	8.16346E-11	0.000108465	0.779	0.831	8.16346E-07	F_C1
CD27	2.17409E-10	0.000207979	0.388	0.299	2.17409E-06	F_C1
CORO1A	1.20582E-09	0.000222679	0.798	0.814	1.20582E-05	F_C1
TRAC	1.86331E-09	0.000352454	0.815	0.802	1.86331E-05	F_C1
CYBA	2.30461E-09	0.000300594	0.854	0.844	2.30461E-05	F_C1
PTPRC	2.89554E-09	0.000214831	0.757	0.772	2.89554E-05	F_C1
HLA-DRB5	2.98713E-09	0.000141895	0.245	0.166	2.98713E-05	F_C1
ATP5E	3.04235E-09	0.0001023	0.862	0.906	3.04235E-05	F_C1
ITM2A	3.17323E-09	0.000237626	0.488	0.440	3.17323E-05	F_C1
CD3D	6.99221E-09	0.000292582	0.856	0.842	6.99221E-05	F_C1
KLRG1	1.04307E-07	0.000152531	0.338	0.256	0.001043065	F_C1
CD2	1.5875E-07	0.000191749	0.775	0.792	0.001587505	F_C1
ARHGDIB	2.00416E-07	0.000102945	0.834	0.872	0.002004157	F_C1
EVL	2.53733E-07	0.000164404	0.614	0.619	0.002537327	F_C1
TRBC1	2.68185E-07	0.000189782	0.443	0.454	0.002681848	F_C1
GIMAP7	3.07665E-07	0.000212468	0.561	0.494	0.003076648	F_C1
HLA-F	1.04418E-06	0.000181469	0.510	0.457	0.010441829	F_C1
ACAP1	1.44769E-06	0.000138028	0.550	0.566	0.014476896	F_C1
STK17A	1.50912E-06	0.000161538	0.520	0.511	0.015091178	F_C1
HLA-DRA	2.15384E-06	0.000173453	0.300	0.249	0.021538416	F_C1
CD99	4.49576E-06	0.000168565	0.656	0.648	0.044957558	F_C1
CXCR3	5.32176E-06	0.000102451	0.293	0.223	0.053217637	F_C1
N4BP2L2	9.66698E-06	0.000164562	0.512	0.498	0.096669766	F_C1
GIMAP4	1.35966E-05	0.000164502	0.481	0.434	0.135965638	F_C1
IGKC	2.01237E-05	0.000161173	0.506	0.505	0.201237437	F_C1
IL32	2.48178E-05	0.000226265	0.905	0.916	0.248178402	F_C1
RARRES3	2.79529E-05	0.000147623	0.573	0.566	0.279529128	F_C1
FYN	3.26423E-05	0.000150363	0.429	0.384	0.326422849	F_C1
PNISR	3.45761E-05	0.000165816	0.507	0.467	0.345761262	F_C1
PSMB9	5.82044E-05	0.000154924	0.563	0.533	0.582044494	F_C1
PYHIN1	7.74608E-05	0.00011756	0.316	0.257	0.774607896	F_C1
CD3E	0.000171196	0.000154407	0.684	0.668	1	F_C1
CHST12	0.000193499	0.000114697	0.283	0.235	1	F_C1
HMGB1	0.000202966	0.000129537	0.810	0.826	1	F_C1
GIMAP1	0.000247718	0.00010194	0.309	0.255	1	F_C1
CLEC2B	0.000292408	0.000120622	0.372	0.319	1	F_C1
TRBC2	0.000363165	0.000146709	0.656	0.663	1	F_C1
TXNIP	0.000450819	0.000282987	0.876	0.853	1	F_C1
GZMM	0.001566807	0.000108942	0.395	0.348	1	F_C1

NKTR	0.001957062	0.000104323	0.368	0.321	1	F_C1
DDX17	0.002877617	0.000100114	0.325	0.316	1	F_C1
LIMD2	0.003143709	0.000107997	0.477	0.466	1	F_C1
LINC00152	0.003593881	0.000105151	0.361	0.318	1	F_C1
TRAF3IP3	0.004527274	0.000103674	0.385	0.360	1	F_C1
KIAA1551	0.004799223	0.000105926	0.386	0.354	1	F_C1
CCL4	1.9613E-159	0.004776235	0.925	0.585	1.9613E-155	F_C2
CXCR4	4.0107E-106	0.002484039	0.978	0.903	4.0107E-102	F_C2
DUSP2	3.02032E-81	0.001358242	0.772	0.447	3.02032E-77	F_C2
CCL4L2	1.09201E-75	0.001647092	0.606	0.268	1.09201E-71	F_C2
DUSP1	9.92318E-51	0.001297876	0.846	0.593	9.92318E-47	F_C2
JUNB	1.40821E-50	0.001920835	0.882	0.779	1.40821E-46	F_C2
CCL5	6.0951E-50	0.001708836	0.966	0.755	6.0951E-46	F_C2
NR4A2	1.07813E-48	0.000595112	0.459	0.178	1.07813E-44	F_C2
ZFP36	2.85184E-39	0.001125479	0.826	0.665	2.85184E-35	F_C2
RGS1	9.52688E-39	0.001291758	0.695	0.505	9.52688E-35	F_C2
GZMK	1.12157E-37	0.001175618	0.790	0.522	1.12157E-33	F_C2
DNAJB1	2.13954E-35	0.000860511	0.708	0.472	2.13954E-31	F_C2
HSP90AA1	3.41213E-34	0.001009338	0.851	0.766	3.41213E-30	F_C2
CD69	1.584E-33	0.001115105	0.810	0.634	1.584E-29	F_C2
DNAJA1	6.24293E-33	0.000565006	0.577	0.338	6.24293E-29	F_C2
GZMA	1.04389E-31	0.000837572	0.896	0.678	1.04389E-27	F_C2
IFNG	8.32927E-31	0.000527587	0.269	0.089	8.32927E-27	F_C2
GZMH	3.84308E-28	0.000459254	0.591	0.341	3.84308E-24	F_C2
TSC22D3	3.94283E-25	0.00097081	0.898	0.837	3.94283E-21	F_C2
NKG7	1.08472E-24	0.000584902	0.776	0.548	1.08472E-20	F_C2
H3F3B	1.14522E-23	0.000824363	0.930	0.893	1.14522E-19	F_C2
RGCC	8.63372E-23	0.000488703	0.462	0.277	8.63372E-19	F_C2
SRGN	5.85056E-22	0.000754507	0.905	0.854	5.85056E-18	F_C2
UBB	1.99003E-21	0.000635367	0.873	0.818	1.99003E-17	F_C2
EIF1	3.73057E-21	0.000777054	0.970	0.965	3.73057E-17	F_C2
JUN	3.47089E-20	0.001001835	0.792	0.682	3.47089E-16	F_C2
CST7	4.24393E-18	0.000397164	0.715	0.518	4.24393E-14	F_C2
CRTAM	4.28773E-18	0.000198152	0.190	0.066	4.28773E-14	F_C2
DUSP4	8.4839E-18	0.000260799	0.254	0.112	8.4839E-14	F_C2
HERPUD1	3.42491E-17	0.000401755	0.570	0.403	3.42491E-13	F_C2
MYADM	9.47335E-17	0.000201311	0.276	0.129	9.47335E-13	F_C2
FOS	1.06505E-16	0.000858234	0.703	0.560	1.06505E-12	F_C2
UBC	1.26705E-16	0.000608945	0.901	0.845	1.26705E-12	F_C2
CCL3L3	2.51469E-15	0.000302996	0.226	0.106	2.51469E-11	F_C2

SRSF7	1.25466E-14	0.000452663	0.701	0.569	1.25466E-10	F_C2
SERTAD1	1.4439E-14	0.000171505	0.228	0.102	1.4439E-10	F_C2
NFKBIA	1.60021E-14	0.000739158	0.747	0.662	1.60021E-10	F_C2
BRD2	2.03055E-14	0.000255045	0.407	0.248	2.03055E-10	F_C2
ID2	4.17361E-14	0.000627132	0.735	0.648	4.17361E-10	F_C2
ZNF331	4.4507E-13	0.000254729	0.267	0.144	4.4507E-09	F_C2
CD8A	1.78678E-12	0.000240746	0.432	0.275	1.78678E-08	F_C2
PNRC1	1.80607E-12	0.000349862	0.677	0.534	1.80607E-08	F_C2
CTD-3252C9.4	2.65879E-12	0.000127004	0.167	0.067	2.65879E-08	F_C2
REL	3.20779E-12	0.000230419	0.312	0.182	3.20779E-08	F_C2
FOSB	4.60728E-12	0.000291909	0.448	0.299	4.60728E-08	F_C2
NFKBID	9.00332E-12	0.000138769	0.183	0.080	9.00332E-08	F_C2
CITED2	1.14007E-11	0.000308795	0.448	0.305	1.14007E-07	F_C2
YPEL5	1.1548E-11	0.000257534	0.382	0.250	1.1548E-07	F_C2
TMEM2	2.32636E-11	0.000165155	0.267	0.145	2.32636E-07	F_C2
GADD45B	3.00388E-11	0.000296433	0.394	0.260	3.00388E-07	F_C2
INTS6	5.67684E-11	0.000243166	0.353	0.223	5.67684E-07	F_C2
HLA-B	4.0185E-10	0.00071597	1.000	0.993	4.0185E-06	F_C2
ZFP36L2	5.07499E-10	0.000584864	0.914	0.901	5.07499E-06	F_C2
IER2	1.30004E-09	0.000362032	0.582	0.474	1.30004E-05	F_C2
ZEB2	1.52673E-09	0.000117461	0.213	0.111	1.52673E-05	F_C2
PPP1R15A	3.27999E-09	0.000293795	0.491	0.369	3.27999E-05	F_C2
CLK1	5.6262E-09	0.000189206	0.389	0.264	5.6262E-05	F_C2
FABP5	5.75002E-09	0.000128853	0.201	0.105	5.75002E-05	F_C2
HLA-DPB1	1.25265E-08	0.000240704	0.575	0.443	0.000125265	F_C2
HSPA1B	1.7135E-08	0.000338837	0.371	0.264	0.00017135	F_C2
TNFAIP3	2.01483E-08	0.000289472	0.493	0.368	0.000201483	F_C2
CCL3	2.20449E-08	0.000352892	0.213	0.138	0.000220449	F_C2
METTL15	3.84907E-08	0.000118656	0.167	0.084	0.000384907	F_C2
BTG1	4.97818E-08	0.000564137	0.968	0.956	0.000497818	F_C2
CREM	5.44232E-08	0.000214245	0.305	0.202	0.000544232	F_C2
DYNLL1	6.20275E-08	0.000256806	0.461	0.367	0.000620275	F_C2
SLC38A2	7.76348E-08	0.00015916	0.303	0.196	0.000776348	F_C2
XCL2	1.08046E-07	0.00017106	0.201	0.115	0.001080461	F_C2
DDX5	1.39966E-07	0.00031198	0.855	0.862	0.001399665	F_C2
B2M	1.45563E-07	0.001110342	1.000	1.000	0.001455629	F_C2
TMSB4X	1.50476E-07	0.000988282	0.998	1.000	0.00150476	F_C2
TUBA1A	2.44717E-07	0.000182899	0.353	0.248	0.002447172	F_C2
CALM2	5.99481E-07	0.000283176	0.711	0.656	0.005994811	F_C2
ITM2C	6.1214E-07	0.000173405	0.326	0.223	0.006121405	F_C2

MT1E	6.16808E-07	0.000111056	0.133	0.065	0.006168082	F_C2
TSPYL2	7.44329E-07	0.000167919	0.299	0.201	0.007443293	F_C2
IFRD1	7.75837E-07	0.000121811	0.220	0.133	0.007758372	F_C2
SRSF3	9.03247E-07	0.000269094	0.582	0.517	0.009032466	F_C2
FAM46C	9.05024E-07	0.000103959	0.170	0.094	0.009050244	F_C2
SAMSN1	1.15452E-06	0.000194382	0.419	0.316	0.011545249	F_C2
TNF	1.25777E-06	0.00022821	0.220	0.141	0.012577738	F_C2
KLF6	1.61152E-06	0.000352286	0.679	0.637	0.016115237	F_C2
MCL1	2.63499E-06	0.00021987	0.434	0.349	0.026349927	F_C2
HLA-DQB1	3.18805E-06	0.000121147	0.297	0.201	0.031880535	F_C2
RGS2	3.76296E-06	0.000171833	0.258	0.174	0.037629618	F_C2
HLA-DRB1	6.3178E-06	0.000191884	0.471	0.366	0.063178001	F_C2
SRSF2	8.51605E-06	0.000230989	0.509	0.439	0.085160517	F_C2
HSPA1A	8.6037E-06	0.000206634	0.283	0.196	0.086037041	F_C2
RBM39	1.04297E-05	0.000241583	0.663	0.594	0.104297283	F_C2
CD8B	1.08245E-05	0.000144874	0.328	0.232	0.108244732	F_C2
ELF1	1.24062E-05	0.000195122	0.452	0.365	0.124062104	F_C2
AKIRIN2	1.38873E-05	0.000102095	0.246	0.162	0.138873349	F_C2
LMNA	1.56214E-05	0.000144141	0.253	0.169	0.15621396	F_C2
HLA-DPA1	1.76798E-05	0.000187565	0.439	0.345	0.176798366	F_C2
JUND	1.88609E-05	0.000150054	0.294	0.210	0.188609017	F_C2
HSP90AB1	2.16419E-05	0.000255554	0.649	0.594	0.216418979	F_C2
MT2A	3.44984E-05	0.000284873	0.459	0.425	0.344983708	F_C2
ITM2A	4.70172E-05	0.000218734	0.527	0.443	0.47017248	F_C2
ACTB	5.76476E-05	0.000565505	0.993	0.984	0.57647616	F_C2
TAGAP	0.000115767	0.000138689	0.267	0.196	1	F_C2
SH2D1A	0.00016747	0.000117323	0.310	0.230	1	F_C2
TUBB4B	0.000186213	0.000114587	0.229	0.161	1	F_C2
FTH1	0.000239319	0.000344154	0.953	0.954	1	F_C2
APOBEC3G	0.000242517	0.00016624	0.473	0.389	1	F_C2
HSPA8	0.000321778	0.000230628	0.724	0.673	1	F_C2
CD74	0.000322066	0.000278851	0.685	0.615	1	F_C2
EIF4A3	0.000345577	0.000102383	0.204	0.141	1	F_C2
MT1X	0.000360391	0.000175842	0.335	0.276	1	F_C2
BTG2	0.000405061	0.000145019	0.341	0.267	1	F_C2
GPR183	0.000413755	0.000184478	0.369	0.317	1	F_C2
DDIT4	0.000451554	0.000264697	0.686	0.649	1	F_C2
RUNX3	0.000532258	0.000103424	0.355	0.275	1	F_C2
HLA-A	0.000543093	0.000388008	0.987	0.988	1	F_C2
ANXA1	0.000662378	0.00028696	0.674	0.623	1	F_C2

CLEC2B	0.000695703	0.000153876	0.394	0.325	1	F_C2
TGIF1	0.001067061	0.000102709	0.235	0.172	1	F_C2
HNRNPDL	0.001088969	0.000184619	0.658	0.601	1	F_C2
LINC00152	0.001116956	0.000133446	0.396	0.320	1	F_C2
RSRP1	0.001530555	0.000149056	0.439	0.369	1	F_C2
CMC1	0.00172053	0.000141248	0.358	0.284	1	F_C2
CD44	0.001760307	0.000203887	0.677	0.651	1	F_C2
HCST	0.002570693	0.000233289	0.833	0.795	1	F_C2
RNF19A	0.003409166	0.000110179	0.262	0.204	1	F_C2
CYTIP	0.00465923	0.000161206	0.586	0.547	1	F_C2
CCNH	0.006390036	0.000151938	0.416	0.377	1	F_C2
PIP4K2A	0.00754902	0.000110862	0.400	0.337	1	F_C2
EIF5	0.008824154	0.000126308	0.344	0.309	1	F_C2
TERF2IP	0.008924787	0.000123325	0.418	0.366	1	F_C2
NKG7	1.359E-200	0.003174511	0.973	0.523	1.359E-196	F_C3
GNLY	3.9221E-165	0.003372152	0.749	0.208	3.9221E-161	F_C3
FGFBP2	4.7907E-143	0.00097368	0.457	0.036	4.7907E-139	F_C3
KLRD1	1.5915E-131	0.001066289	0.640	0.131	1.5915E-127	F_C3
GZMB	5.8234E-124	0.001309552	0.654	0.162	5.8234E-120	F_C3
GZMH	9.75411E-89	0.001540112	0.732	0.325	9.75411E-85	F_C3
PRF1	3.19539E-77	0.00075732	0.617	0.218	3.19539E-73	F_C3
KLRF1	1.3834E-76	0.000273119	0.276	0.023	1.3834E-72	F_C3
FCGR3A	1.55426E-68	0.000236874	0.239	0.018	1.55426E-64	F_C3
TYROBP	2.26357E-65	0.000515658	0.346	0.064	2.26357E-61	F_C3
TRDC	9.20675E-60	0.000267766	0.241	0.025	9.20675E-56	F_C3
ADGRG1	3.12569E-53	0.000158913	0.198	0.017	3.12569E-49	F_C3
CCL4	2.38298E-47	0.000973409	0.897	0.593	2.38298E-43	F_C3
FGR	3.35963E-47	0.000138362	0.187	0.018	3.35963E-43	F_C3
CST7	5.56326E-47	0.000799171	0.817	0.506	5.56326E-43	F_C3
TRGC2	1.28164E-46	0.000427814	0.447	0.154	1.28164E-42	F_C3
CTSW	6.35846E-45	0.000644145	0.605	0.296	6.35846E-41	F_C3
CCL5	3.16331E-43	0.001578603	0.967	0.758	3.16331E-39	F_C3
S1PR5	3.06307E-39	0.000141949	0.173	0.021	3.06307E-35	F_C3
C1orf21	7.02074E-38	0.000147872	0.237	0.049	7.02074E-34	F_C3
HOPX	7.69464E-38	0.000381166	0.533	0.243	7.69464E-34	F_C3
PRSS23	4.25397E-35	0.000117404	0.138	0.013	4.25397E-31	F_C3
ZEB2	4.51169E-34	0.000238131	0.315	0.098	4.51169E-30	F_C3
FCER1G	1.54985E-33	0.000207164	0.210	0.043	1.54985E-29	F_C3
SPON2	5.50063E-33	0.000290743	0.319	0.105	5.50063E-29	F_C3
EFHD2	1.19984E-32	0.000208606	0.331	0.111	1.19984E-28	F_C3

MATK	4.65302E-31	0.000203207	0.385	0.152	4.65302E-27	F_C3
PLAC8	6.30286E-30	0.000304232	0.453	0.207	6.30286E-26	F_C3
FCRL6	1.7279E-29	0.000106803	0.179	0.034	1.7279E-25	F_C3
CLIC3	4.38384E-27	0.000193141	0.268	0.086	4.38384E-23	F_C3
CCL3	7.50796E-26	0.000456705	0.319	0.124	7.50796E-22	F_C3
KLRC1	4.71837E-24	0.000165729	0.171	0.039	4.71837E-20	F_C3
MAPK1	8.1488E-24	0.000144001	0.290	0.109	8.1488E-20	F_C3
GZMA	1.81915E-23	0.000508677	0.885	0.682	1.81915E-19	F_C3
GZMM	1.84052E-23	0.000291452	0.568	0.333	1.84052E-19	F_C3
C12orf75	2.08158E-22	0.000229916	0.502	0.277	2.08158E-18	F_C3
ID2	2.88996E-20	0.000328048	0.835	0.635	2.88996E-16	F_C3
GSTP1	1.12227E-19	0.00027447	0.529	0.318	1.12227E-15	F_C3
TRGC1	7.59333E-19	0.000100229	0.156	0.041	7.59333E-15	F_C3
PLEK	8.10179E-19	0.000110543	0.224	0.081	8.10179E-15	F_C3
KLRG1	1.22903E-18	0.000256245	0.455	0.256	1.22903E-14	F_C3
CMC1	8.91296E-18	0.000430309	0.461	0.270	8.91296E-14	F_C3
EOMES	2.47366E-17	0.000104397	0.237	0.094	2.47366E-13	F_C3
AOAH	5.67847E-17	0.000105539	0.208	0.076	5.67847E-13	F_C3
ITGB2	1.34938E-16	0.000303459	0.660	0.464	1.34938E-12	F_C3
PYHIN1	1.43254E-16	0.000183838	0.438	0.251	1.43254E-12	F_C3
CCL4L2	2.28332E-16	0.000424389	0.481	0.289	2.28332E-12	F_C3
PLEKHF1	2.55127E-16	0.000132535	0.286	0.132	2.55127E-12	F_C3
CYBA	2.14333E-15	0.000591452	0.911	0.838	2.14333E-11	F_C3
CD63	2.82047E-15	0.000228464	0.508	0.322	2.82047E-11	F_C3
CD7	3.35384E-15	0.000346357	0.568	0.385	3.35384E-11	F_C3
XCL1	7.6892E-15	0.000147027	0.232	0.099	7.6892E-11	F_C3
TPST2	1.3952E-13	0.000104354	0.261	0.124	1.3952E-09	F_C3
FLNA	3.00642E-13	0.000157803	0.352	0.198	3.00642E-09	F_C3
TBX21	7.10962E-13	0.000100625	0.191	0.078	7.10962E-09	F_C3
BIN2	3.22434E-12	0.000170306	0.543	0.373	3.22434E-08	F_C3
LYAR	4.65702E-12	0.000201714	0.527	0.360	4.65702E-08	F_C3
HCST	7.57188E-12	0.000467737	0.889	0.788	7.57188E-08	F_C3
CHST12	1.53671E-11	0.0001027	0.379	0.231	1.53671E-07	F_C3
APOBEC3G	8.89981E-11	0.000171588	0.539	0.380	8.89981E-07	F_C3
IFITM2	9.57923E-11	0.000307658	0.687	0.539	9.57923E-07	F_C3
AKNA	1.28796E-10	0.000151198	0.426	0.277	1.28796E-06	F_C3
HLA-C	4.02606E-10	0.000665663	0.998	0.987	4.02606E-06	F_C3
CD247	9.02847E-10	0.000177281	0.463	0.318	9.02847E-06	F_C3
CLIC1	1.08234E-09	0.00025342	0.802	0.667	1.08234E-05	F_C3
SUN2	1.94209E-08	0.000121414	0.366	0.239	0.000194209	F_C3

XCL2	2.52161E-08	0.000144913	0.212	0.114	0.000252161	F_C3
ARPC2	3.72595E-08	0.000319208	0.825	0.725	0.000372595	F_C3
SYTL3	4.26036E-08	0.000113497	0.352	0.230	0.000426036	F_C3
MYO1F	4.35514E-08	0.000139954	0.430	0.300	0.000435514	F_C3
ITGB1	8.15068E-08	0.000166473	0.469	0.339	0.000815068	F_C3
C9orf142	8.93683E-08	0.000133423	0.492	0.360	0.000893683	F_C3
CTSC	1.60329E-07	0.000143249	0.428	0.303	0.001603294	F_C3
LITAF	1.67746E-07	0.000142387	0.473	0.345	0.001677457	F_C3
HLA-DPB1	1.83346E-07	0.000167974	0.576	0.445	0.001833462	F_C3
LGALS1	6.29939E-07	0.000227689	0.566	0.441	0.006299391	F_C3
UCP2	1.61342E-06	0.000125607	0.434	0.317	0.016134233	F_C3
TBC1D10C	1.73729E-06	0.000107723	0.558	0.438	0.017372917	F_C3
VAMP8	3.44406E-06	0.00012468	0.537	0.419	0.034440575	F_C3
HLA-F	3.76515E-06	0.000137372	0.576	0.458	0.037651472	F_C3
SAMD3	6.53383E-06	0.000107668	0.298	0.200	0.065338321	F_C3
LSP1	9.11196E-06	0.000121936	0.644	0.532	0.091119642	F_C3
CCND3	1.36299E-05	0.000106914	0.518	0.407	0.136299464	F_C3
MSN	1.9401E-05	0.000104246	0.407	0.303	0.194010458	F_C3
RARRES3	3.64644E-05	0.000128289	0.660	0.555	0.364643793	F_C3
LCP1	6.45896E-05	0.000100709	0.516	0.413	0.645896172	F_C3
BTN3A2	6.77589E-05	0.000102126	0.372	0.276	0.677588601	F_C3
LY6E	7.46978E-05	0.000129596	0.584	0.481	0.746977618	F_C3
SEPT7	0.000115285	0.000139282	0.660	0.562	1	F_C3
PSMB9	0.000184952	0.000105086	0.626	0.530	1	F_C3
CD99	0.00022711	0.00013303	0.730	0.639	1	F_C3
S100A4	0.000261563	0.000264161	0.938	0.882	1	F_C3
ARPC3	0.000447436	0.000158621	0.741	0.659	1	F_C3
C19orf43	0.00080484	0.000118211	0.621	0.533	1	F_C3
HLA-E	0.001230449	0.000188075	0.938	0.891	1	F_C3
EMP3	0.001321687	0.000134577	0.702	0.621	1	F_C3
CALM1	0.001656537	0.000156577	0.829	0.761	1	F_C3
MYL6	0.002124185	0.000139795	0.897	0.840	1	F_C3
CFL1	0.002706292	0.000151672	0.920	0.870	1	F_C3
SERF2	0.003927841	0.000206913	0.922	0.887	1	F_C3
PFN1	0.004331223	0.000274438	0.938	0.912	1	F_C3
SELL	3.7383E-110	0.001149892	0.764	0.142	3.7383E-106	F_C4
CCR7	5.93054E-90	0.000907038	0.698	0.140	5.93054E-86	F_C4
TSHZ2	1.01425E-78	0.0005263	0.436	0.037	1.01425E-74	F_C4
EEF1B2	2.29044E-68	0.001820267	0.953	0.786	2.29044E-64	F_C4
NACA	2.66412E-54	0.001573064	0.982	0.870	2.66412E-50	F_C4

LDHB	5.7034E-49	0.001163086	0.887	0.585	5.7034E-45	F_C4
TPT1	7.86486E-43	0.002248986	0.996	0.971	7.86486E-39	F_C4
LEF1	2.03083E-42	0.000362716	0.444	0.102	2.03083E-38	F_C4
PABPC1	5.01812E-41	0.001207302	0.891	0.670	5.01812E-37	F_C4
EEF1A1	6.98109E-41	0.002507584	1.000	0.995	6.98109E-37	F_C4
SOCS3	6.17598E-35	0.000413096	0.473	0.142	6.17598E-31	F_C4
MAL	8.51864E-35	0.000264483	0.338	0.067	8.51864E-31	F_C4
UBA52	6.98869E-34	0.00142304	0.996	0.949	6.98869E-30	F_C4
EIF3E	7.05141E-33	0.000908434	0.836	0.578	7.05141E-29	F_C4
NPM1	8.09678E-33	0.001027786	0.895	0.764	8.09678E-29	F_C4
GNB2L1	8.68387E-33	0.001313073	0.993	0.929	8.68387E-29	F_C4
NBEAL1	1.15795E-31	0.00105662	0.905	0.683	1.15795E-27	F_C4
BTF3	3.20759E-31	0.001047603	0.960	0.836	3.20759E-27	F_C4
GLTSCR2	3.81719E-31	0.001045482	0.895	0.691	3.81719E-27	F_C4
IL6ST	2.17811E-30	0.000224733	0.356	0.087	2.17811E-26	F_C4
TXK	2.59901E-30	0.000290301	0.356	0.088	2.59901E-26	F_C4
LTB	9.50467E-29	0.000925147	0.811	0.482	9.50467E-25	F_C4
EEF2	6.51821E-28	0.000963909	0.884	0.713	6.51821E-24	F_C4
FAU	2.68616E-26	0.001313138	0.996	0.981	2.68616E-22	F_C4
TMEM123	7.33829E-26	0.000439586	0.585	0.270	7.33829E-22	F_C4
PFDN5	7.46585E-26	0.001019527	0.989	0.900	7.46585E-22	F_C4
MYC	8.56611E-25	0.000276248	0.356	0.105	8.56611E-21	F_C4
TCF7	3.9032E-23	0.00030169	0.480	0.196	3.9032E-19	F_C4
SATB1	7.05124E-20	0.000193967	0.269	0.073	7.05124E-16	F_C4
PRKCA	4.11239E-19	0.000126597	0.222	0.051	4.11239E-15	F_C4
EEF1D	6.88415E-19	0.000892511	0.975	0.904	6.88415E-15	F_C4
PIK3IP1	2.22641E-18	0.000503061	0.735	0.473	2.22641E-14	F_C4
RIC3	4.876E-18	0.000170715	0.255	0.071	4.876E-14	F_C4
CMTM8	8.09796E-18	0.000101036	0.160	0.026	8.09796E-14	F_C4
C6orf48	1.26468E-17	0.00044317	0.651	0.389	1.26468E-13	F_C4
NOSIP	1.68694E-17	0.000384788	0.484	0.238	1.68694E-13	F_C4
COX4I1	1.88635E-17	0.000723254	0.905	0.794	1.88635E-13	F_C4
PLAC8	3.70834E-17	0.000262573	0.469	0.221	3.70834E-13	F_C4
SNHG8	3.9957E-17	0.000375736	0.636	0.372	3.9957E-13	F_C4
ZFAS1	4.71916E-17	0.000568113	0.745	0.516	4.71916E-13	F_C4
SARAF	6.51327E-17	0.000710954	0.902	0.737	6.51327E-13	F_C4
PASK	1.36397E-16	0.000162172	0.211	0.053	1.36397E-12	F_C4
C1orf162	1.9689E-16	0.000160841	0.291	0.099	1.9689E-12	F_C4
LPAR6	2.1279E-16	0.000191606	0.291	0.099	2.1279E-12	F_C4
SERINC5	1.02515E-15	0.000214623	0.291	0.102	1.02515E-11	F_C4

RSL1D1	1.32945E-15	0.000303251	0.538	0.293	1.32945E-11	F_C4
NACA2	7.03882E-15	0.000298562	0.520	0.282	7.03882E-11	F_C4
NAP1L1	8.72386E-15	0.00055088	0.749	0.553	8.72386E-11	F_C4
ZNF90	1.02472E-14	0.000390965	0.684	0.442	1.02472E-10	F_C4
TOMM7	1.06659E-14	0.000766001	0.938	0.870	1.06659E-10	F_C4
FBXO32	1.76068E-14	0.0001492	0.258	0.087	1.76068E-10	F_C4
EIF3L	2.81243E-14	0.000404937	0.647	0.417	2.81243E-10	F_C4
IL7R	6.88739E-14	0.000704792	0.840	0.634	6.88739E-10	F_C4
UQCRB	1.58758E-13	0.000642095	0.916	0.792	1.58758E-09	F_C4
ITK	3.3613E-13	0.000211602	0.404	0.196	3.3613E-09	F_C4
EIF2S3	5.21547E-13	0.000202211	0.389	0.187	5.21547E-09	F_C4
BIRC3	6.15623E-13	0.000300414	0.567	0.339	6.15623E-09	F_C4
RNASET2	6.93894E-13	0.000266724	0.531	0.306	6.93894E-09	F_C4
EIF3H	7.87437E-13	0.000467496	0.705	0.517	7.87437E-09	F_C4
HINT1	8.08732E-12	0.000511085	0.836	0.672	8.08732E-08	F_C4
NDFIP1	8.56618E-12	0.000275638	0.513	0.302	8.56618E-08	F_C4
SRSF5	1.02604E-11	0.000498221	0.844	0.681	1.02604E-07	F_C4
AIF1	1.37569E-11	0.000112925	0.156	0.041	1.37569E-07	F_C4
FYB	1.99172E-11	0.00042497	0.633	0.440	1.99172E-07	F_C4
COMMD6	4.27454E-11	0.00053261	0.880	0.747	4.27454E-07	F_C4
COX7C	4.73442E-11	0.000617495	0.916	0.836	4.73442E-07	F_C4
SH3YL1	1.22601E-10	0.000108919	0.215	0.078	1.22601E-06	F_C4
TIPIN	1.91575E-10	0.000250757	0.458	0.265	1.91575E-06	F_C4
LDLRAP1	3.62328E-10	0.000140991	0.280	0.125	3.62328E-06	F_C4
FTL	4.39378E-10	0.000718274	0.971	0.890	4.39378E-06	F_C4
ATXN8OS	4.66479E-10	0.000223665	0.433	0.245	4.66479E-06	F_C4
SMDT1	5.19539E-10	0.000281581	0.593	0.394	5.19539E-06	F_C4
DGKA	6.71573E-10	0.000100455	0.222	0.086	6.71573E-06	F_C4
FOXP1	1.10083E-09	0.000344441	0.575	0.393	1.10083E-05	F_C4
AP3M2	1.21524E-09	0.000113013	0.207	0.078	1.21524E-05	F_C4
KLF2	1.33715E-09	0.000235405	0.469	0.281	1.33715E-05	F_C4
FAM65B	1.57951E-09	0.000195797	0.447	0.262	1.57951E-05	F_C4
C10orf54	1.96011E-09	0.000146792	0.444	0.260	1.96011E-05	F_C4
PPA1	3.07782E-09	0.000192291	0.451	0.269	3.07782E-05	F_C4
RP11-255M2.3	4.45035E-09	0.000123903	0.273	0.127	4.45035E-05	F_C4
EIF4B	5.76317E-09	0.000253547	0.524	0.340	5.76317E-05	F_C4
FBL	6.07646E-09	0.000167195	0.407	0.234	6.07646E-05	F_C4
CD55	7.81405E-09	0.000176403	0.284	0.137	7.81405E-05	F_C4
SLC25A6	8.8062E-09	0.000470696	0.865	0.740	8.8062E-05	F_C4
AC090498.1	1.12327E-08	0.000315102	0.538	0.361	0.000112327	F_C4

HNRNPA1	1.21306E-08	0.000527681	0.895	0.814	0.000121306	F_C4
UXT	1.28815E-08	0.000289891	0.607	0.428	0.000128815	F_C4
CCNI	1.33344E-08	0.00032442	0.713	0.539	0.000133344	F_C4
SERP1	1.3419E-08	0.000249759	0.636	0.452	0.00013419	F_C4
TMEM243	1.45255E-08	0.000152393	0.364	0.201	0.000145255	F_C4
CAMK4	1.47008E-08	0.000132287	0.284	0.138	0.000147008	F_C4
ADD3	1.79647E-08	0.000148515	0.327	0.173	0.000179647	F_C4
ARID5B	2.16924E-08	0.00023891	0.462	0.288	0.000216924	F_C4
TXNIP	3.08411E-08	0.000633813	0.953	0.853	0.000308411	F_C4
EIF2A	3.49497E-08	0.000132901	0.320	0.169	0.000349497	F_C4
COX6C	8.33048E-08	0.00028733	0.800	0.641	0.000833048	F_C4
CD7	1.12589E-07	0.000223138	0.571	0.396	0.001125888	F_C4
UGP2	1.17531E-07	0.000173147	0.447	0.281	0.001175307	F_C4
C1QBP	1.18209E-07	0.00014966	0.411	0.249	0.001182088	F_C4
AES	1.50033E-07	0.000232963	0.578	0.407	0.001500332	F_C4
PNISR	1.70511E-07	0.00023707	0.640	0.467	0.001705105	F_C4
FXYD5	1.86896E-07	0.000372391	0.825	0.687	0.001868956	F_C4
CLEC2D	1.98084E-07	0.000266837	0.651	0.482	0.001980839	F_C4
BCL11B	2.11511E-07	0.000152942	0.349	0.200	0.002115114	F_C4
LINC00861	2.19658E-07	0.000154467	0.276	0.143	0.002196575	F_C4
NAP1L4	2.30948E-07	0.000134444	0.385	0.231	0.002309481	F_C4
LSM5	2.5268E-07	0.000158022	0.389	0.234	0.002526799	F_C4
GIMAP7	3.39973E-07	0.000371759	0.651	0.504	0.003399731	F_C4
APEX1	4.32825E-07	0.000104562	0.305	0.167	0.004328251	F_C4
DDT	5.72889E-07	0.000141231	0.393	0.241	0.005728887	F_C4
SOD1	6.86819E-07	0.000293415	0.662	0.512	0.006868195	F_C4
CD48	7.33685E-07	0.000320719	0.691	0.543	0.007336855	F_C4
PRMT2	8.09277E-07	0.000150372	0.404	0.252	0.008092767	F_C4
GIMAP4	9.94503E-07	0.000214334	0.600	0.437	0.009945035	F_C4
ZCCHC11	1.01764E-06	0.000106133	0.295	0.162	0.010176419	F_C4
CCDC109B	1.02185E-06	0.000180411	0.422	0.271	0.010218465	F_C4
ANP32B	1.02286E-06	0.000216712	0.513	0.356	0.010228614	F_C4
NHSL2	1.07688E-06	0.000178338	0.415	0.264	0.010768768	F_C4
EIF3K	1.18275E-06	0.000259751	0.764	0.614	0.011827458	F_C4
TOMM20	1.62623E-06	0.000153641	0.444	0.291	0.016262286	F_C4
SNHG7	2.17376E-06	0.000125828	0.309	0.177	0.021737573	F_C4
SLC25A45	2.2882E-06	0.000108156	0.182	0.082	0.022882019	F_C4
PTMA	2.34789E-06	0.000649537	0.996	0.979	0.023478921	F_C4
ST13	2.42168E-06	0.000171645	0.455	0.304	0.024216813	F_C4
SFXN1	2.45373E-06	0.000112931	0.269	0.146	0.024537297	F_C4

EIF3F	2.8559E-06	0.000311759	0.705	0.573	0.028559016	F_C4
APRT	3.15544E-06	0.000251536	0.669	0.520	0.03155445	F_C4
STAT3	4.60748E-06	0.000157554	0.415	0.271	0.046074783	F_C4
NSA2	5.05242E-06	0.000140626	0.360	0.224	0.05052424	F_C4
EIF3D	5.50358E-06	0.000127682	0.513	0.362	0.05503584	F_C4
FCMR	6.06003E-06	0.000113302	0.287	0.164	0.060600251	F_C4
HSPD1	6.30161E-06	0.000122022	0.327	0.197	0.063016054	F_C4
EIF4A2	7.61538E-06	0.00022212	0.713	0.568	0.076153824	F_C4
ERP29	9.61256E-06	0.000177513	0.615	0.465	0.096125605	F_C4
PEBP1	1.00379E-05	0.000160629	0.498	0.352	0.100378708	F_C4
PIM1	1.02026E-05	0.000128495	0.360	0.227	0.102026035	F_C4
GABPB1-AS1	1.02384E-05	0.000138469	0.455	0.312	0.102383763	F_C4
SNRPD2	1.33943E-05	0.000266444	0.815	0.691	0.133943102	F_C4
TRAF3IP3	1.42171E-05	0.000119709	0.502	0.358	0.142171437	F_C4
DDX21	1.62572E-05	0.000109688	0.324	0.199	0.162572125	F_C4
TMEM256	1.79544E-05	0.00010044	0.345	0.218	0.17954433	F_C4
HIST1H4C	1.81196E-05	0.000103196	0.353	0.224	0.181195841	F_C4
CNBP	2.31119E-05	0.000185571	0.665	0.524	0.231119319	F_C4
ALKBH7	2.33401E-05	0.000115478	0.287	0.171	0.233401052	F_C4
NDUFB9	2.5252E-05	0.000134234	0.531	0.389	0.252520033	F_C4
HSPE1	2.71801E-05	0.00018939	0.578	0.436	0.271801252	F_C4
RASGRP2	3.40027E-05	0.000101103	0.265	0.154	0.340027283	F_C4
NDUFB1	3.92983E-05	0.000147579	0.465	0.330	0.392983221	F_C4
SNRPF	4.25614E-05	0.000113024	0.476	0.340	0.425613598	F_C4
NDUFS5	4.32426E-05	0.000158096	0.640	0.501	0.432425924	F_C4
YBX1	4.35708E-05	0.000328002	0.778	0.684	0.435708102	F_C4
COX7A2L	4.50465E-05	0.000116989	0.476	0.341	0.450465336	F_C4
CD27	6.17887E-05	0.000111194	0.447	0.317	0.617887137	F_C4
C19orf53	7.29014E-05	0.000173197	0.556	0.422	0.729014164	F_C4
SF1	7.43666E-05	0.000165147	0.527	0.393	0.743665523	F_C4
MPHOSPH8	8.26271E-05	0.000127651	0.451	0.321	0.826271147	F_C4
TPR	8.44825E-05	0.00011376	0.396	0.271	0.844825327	F_C4
WASF2	0.000120646	0.000107628	0.327	0.213	1	F_C4
PRDX2	0.000150675	0.000151241	0.465	0.340	1	F_C4
PPP1R2	0.0001761	0.000114302	0.495	0.367	1	F_C4
HSP90AB1	0.000194393	0.000200032	0.716	0.593	1	F_C4
EIF3M	0.000266933	0.000138633	0.429	0.310	1	F_C4
C12orf57	0.000267755	0.000170036	0.520	0.396	1	F_C4
SERPINB1	0.000284128	0.000104208	0.324	0.215	1	F_C4
EDF1	0.000286889	0.000135446	0.720	0.600	1	F_C4

TBCA	0.000304509	0.000110543	0.465	0.343	1	F_C4
CD37	0.000319031	0.000211562	0.724	0.609	1	F_C4
SKP1	0.000356244	0.000142857	0.724	0.605	1	F_C4
UQCRH	0.000368372	0.000170062	0.593	0.471	1	F_C4
C4orf3	0.000386726	0.000114679	0.531	0.408	1	F_C4
USP15	0.000392334	0.000102846	0.349	0.239	1	F_C4
RAN	0.000396184	0.000183931	0.604	0.485	1	F_C4
STK4	0.000408633	0.000110293	0.629	0.508	1	F_C4
ATP5L	0.000411715	0.000273806	0.873	0.789	1	F_C4
CUTA	0.00041281	0.000171474	0.589	0.468	1	F_C4
HMGN1	0.000518565	0.000116544	0.571	0.450	1	F_C4
TOB1	0.000521401	0.000125787	0.371	0.260	1	F_C4
ATP5G2	0.00057884	0.000257024	0.771	0.676	1	F_C4
SRRM1	0.000605182	0.000159978	0.516	0.400	1	F_C4
MIF	0.000646164	0.000118331	0.560	0.441	1	F_C4
RSL24D1	0.000659476	0.00016248	0.487	0.374	1	F_C4
LIMD2	0.000673278	0.000176537	0.578	0.461	1	F_C4
TRADD	0.000692181	0.000106352	0.342	0.236	1	F_C4
IFITM1	0.001049653	0.000115688	0.265	0.173	1	F_C4
BTG1	0.001150431	0.000206575	0.993	0.955	1	F_C4
C14orf166	0.00142599	0.000109858	0.418	0.311	1	F_C4
NDUFB11	0.001448802	0.000117871	0.524	0.412	1	F_C4
GYPC	0.001518811	0.000142615	0.527	0.417	1	F_C4
GSTK1	0.001721688	0.000144953	0.618	0.508	1	F_C4
GTF3A	0.001744139	0.000167959	0.575	0.468	1	F_C4
ATP6V1G1	0.001832362	0.000155176	0.611	0.503	1	F_C4
ARL6IP4	0.002042097	0.000102511	0.545	0.436	1	F_C4
C11orf31	0.003224966	0.000101254	0.396	0.297	1	F_C4
LEPROTL1	0.00389059	0.00012394	0.571	0.467	1	F_C4
ITM2B	0.004866944	0.000174758	0.869	0.792	1	F_C4
EIF3G	0.005094985	0.000130572	0.560	0.460	1	F_C4
SLC25A3	0.00632778	0.000106417	0.509	0.411	1	F_C4
ATP5O	0.0071248	0.000134653	0.505	0.411	1	F_C4
IL2RA	1.33541E-48	0.000601634	0.464	0.029	1.33541E-44	F_C5
ICA1	9.26256E-47	0.000316601	0.360	0.011	9.26256E-43	F_C5
TNFRSF18	1.99966E-45	0.000776747	0.536	0.058	1.99966E-41	F_C5
TNFRSF4	5.01489E-39	0.000852499	0.504	0.064	5.01489E-35	F_C5
RTKN2	7.27135E-35	0.00034944	0.336	0.020	7.27135E-31	F_C5
TIGIT	1.74083E-34	0.000644161	0.584	0.113	1.74083E-30	F_C5
CTLA4	3.96186E-33	0.0003229	0.352	0.026	3.96186E-29	F_C5

ZC2HC1A	8.9888E-31	0.000199141	0.248	0.008	8.9888E-27	F_C5
PMAIP1	1.54779E-29	0.000576213	0.520	0.100	1.54779E-25	F_C5
BATF	1.67099E-27	0.000577767	0.616	0.167	1.67099E-23	F_C5
AC002331.1	5.85671E-27	0.000245522	0.264	0.015	5.85671E-23	F_C5
ENTPD1	9.14143E-24	0.000185653	0.272	0.023	9.14143E-20	F_C5
GADD45A	2.0796E-22	0.000306309	0.392	0.069	2.0796E-18	F_C5
IL32	1.73176E-21	0.001715764	0.968	0.911	1.73176E-17	F_C5
TBC1D4	1.08314E-18	0.000186145	0.312	0.050	1.08314E-14	F_C5
ARID5B	1.26953E-18	0.000603488	0.680	0.287	1.26953E-14	F_C5
P11-1399P15	1.08909E-17	0.000273675	0.272	0.038	1.08909E-13	F_C5
SLAMF1	1.56338E-15	0.000208253	0.344	0.078	1.56338E-11	F_C5
UGP2	1.17911E-14	0.000502087	0.624	0.282	1.17911E-10	F_C5
RGS1	2.40881E-14	0.001457202	0.816	0.521	2.40881E-10	F_C5
CREM	3.01782E-14	0.00051708	0.528	0.206	3.01782E-10	F_C5
GPX1	3.48351E-14	0.000336861	0.544	0.216	3.48351E-10	F_C5
PIM2	3.72264E-14	0.000340746	0.432	0.138	3.72264E-10	F_C5
CARD16	2.07429E-13	0.00044195	0.568	0.247	2.07429E-09	F_C5
LTB	2.46121E-13	0.001061139	0.792	0.494	2.46121E-09	F_C5
CORO1B	2.52239E-13	0.00040785	0.568	0.244	2.52239E-09	F_C5
RP11-138A9.1	3.8671E-13	0.000349999	0.576	0.251	3.8671E-09	F_C5
LAIR2	5.80527E-13	0.00016695	0.216	0.033	5.80527E-09	F_C5
HPGD	7.47352E-13	0.00027185	0.232	0.040	7.47352E-09	F_C5
DUSP4	8.52188E-13	0.000331092	0.392	0.123	8.52188E-09	F_C5
BIRC3	9.54371E-13	0.000650929	0.672	0.344	9.54371E-09	F_C5
MIR4435-2HG	1.07311E-12	0.000174673	0.384	0.119	1.07311E-08	F_C5
TYMP	2.16776E-12	0.000195084	0.392	0.126	2.16776E-08	F_C5
SIRPG	4.96938E-12	0.000144517	0.304	0.078	4.96938E-08	F_C5
NAMPT	5.55811E-12	0.000142059	0.264	0.058	5.55811E-08	F_C5
GATA3	5.8712E-12	0.000355016	0.424	0.153	5.8712E-08	F_C5
AC133644.2	6.16268E-12	0.00019948	0.264	0.058	6.16268E-08	F_C5
TNFRSF1B	2.55249E-11	0.000239779	0.440	0.167	2.55249E-07	F_C5
PHLDA2	2.86619E-11	0.000118892	0.168	0.022	2.86619E-07	F_C5
DNPH1	3.66939E-11	0.000197178	0.352	0.111	3.66939E-07	F_C5
ICOS	5.12891E-11	0.000173155	0.296	0.080	5.12891E-07	F_C5
GBP2	5.63375E-11	0.000269383	0.480	0.200	5.63375E-07	F_C5
CCNG2	7.29086E-11	0.000100326	0.152	0.018	7.29086E-07	F_C5
PHLDA1	1.12337E-10	0.000208356	0.288	0.078	1.12337E-06	F_C5
USP15	1.98089E-10	0.000240405	0.520	0.238	1.98089E-06	F_C5
RHOH	2.2437E-10	0.000312705	0.640	0.342	2.2437E-06	F_C5
GLRX	2.75211E-10	0.000414801	0.528	0.255	2.75211E-06	F_C5

UCP2	5.66831E-10	0.000386934	0.608	0.323	5.66831E-06	F_C5
CD58	8.80175E-10	0.000137603	0.376	0.139	8.80175E-06	F_C5
FAS	1.69053E-09	0.00012405	0.264	0.073	1.69053E-05	F_C5
BTG3	1.8088E-09	0.000180858	0.312	0.101	1.8088E-05	F_C5
PELI1	3.46914E-09	0.000127979	0.176	0.032	3.46914E-05	F_C5
PKM	4.61765E-09	0.000483545	0.592	0.341	4.61765E-05	F_C5
HLA-DQB1	6.42125E-09	0.000229266	0.456	0.206	6.42125E-05	F_C5
SAT1	6.84071E-09	0.000654659	0.664	0.431	6.84071E-05	F_C5
MAGEH1	7.02624E-09	0.000127962	0.208	0.048	7.02624E-05	F_C5
PTP4A3	1.35076E-08	0.000103815	0.160	0.028	0.000135076	F_C5
CD4	4.05728E-08	0.000100211	0.288	0.098	0.000405728	F_C5
RP11-138A9.2	4.21645E-08	0.000264824	0.472	0.230	0.000421645	F_C5
TRAC	5.85864E-08	0.000906717	0.928	0.802	0.000585864	F_C5
PIM3	6.61625E-08	0.000138542	0.304	0.110	0.000661625	F_C5
SMS	8.53708E-08	0.000170264	0.320	0.122	0.000853708	F_C5
CD79B	8.61658E-08	0.000108432	0.272	0.091	0.000861658	F_C5
CD27	9.26291E-08	0.000348494	0.568	0.318	0.000926291	F_C5
RHOG	9.6904E-08	0.000288666	0.512	0.271	0.00096904	F_C5
SQSTM1	1.4397E-07	0.000136882	0.504	0.266	0.001439696	F_C5
DYNLL1	1.44085E-07	0.00027192	0.624	0.372	0.001440846	F_C5
HLA-DQA1	2.02704E-07	0.000146101	0.280	0.100	0.002027038	F_C5
STAT3	2.04315E-07	0.000199197	0.512	0.273	0.002043147	F_C5
PFKL	2.13335E-07	0.000112185	0.256	0.085	0.002133345	F_C5
ATP5J2	2.74981E-07	0.000168477	0.632	0.387	0.00274981	F_C5
C4orf48	2.78313E-07	0.000138353	0.392	0.179	0.002783132	F_C5
TPI1	2.9989E-07	0.000286663	0.680	0.433	0.002998899	F_C5
MTHFD2	3.01536E-07	0.000107086	0.224	0.068	0.003015356	F_C5
ANXA7	4.37758E-07	0.000170491	0.448	0.225	0.004377579	F_C5
RHBDD2	4.65813E-07	0.000143736	0.296	0.113	0.00465813	F_C5
LIMS1	4.68988E-07	0.000127912	0.320	0.130	0.004689878	F_C5
LAPTM4A	5.06466E-07	0.000137391	0.400	0.189	0.005064658	F_C5
FKBP1A	5.25623E-07	0.000203297	0.528	0.294	0.005256234	F_C5
CTSC	5.66211E-07	0.000320441	0.544	0.311	0.005662114	F_C5
DUSP10	5.8291E-07	0.000144662	0.232	0.075	0.005829105	F_C5
NR3C1	6.94407E-07	0.000180446	0.360	0.160	0.006944065	F_C5
HLA-DRB1	8.05105E-07	0.000388178	0.608	0.373	0.008051051	F_C5
SOCS3	1.14321E-06	0.000141884	0.352	0.158	0.011432059	F_C5
SRGN	1.21256E-06	0.000788677	0.952	0.858	0.01212556	F_C5
HLA-DRB5	1.6465E-06	0.00017617	0.384	0.183	0.016465013	F_C5
SNU13	1.68693E-06	0.000147527	0.592	0.363	0.016869284	F_C5

COX5A	1.72791E-06	0.000132075	0.504	0.283	0.017279102	F_C5
EIF3D	1.87642E-06	0.000136267	0.592	0.365	0.018764181	F_C5
HLA-DMA	1.97267E-06	0.000126867	0.288	0.115	0.019726695	F_C5
COX17	2.69708E-06	0.000189112	0.432	0.224	0.026970774	F_C5
GAPDH	3.6246E-06	0.000682995	0.960	0.860	0.036245954	F_C5
PPM1K	4.18934E-06	0.000102561	0.336	0.154	0.04189342	F_C5
PBXIP1	4.3215E-06	0.000236412	0.488	0.275	0.043214996	F_C5
IL10RA	4.63136E-06	0.000152751	0.480	0.269	0.04631356	F_C5
CNIH1	4.71769E-06	0.000128825	0.320	0.142	0.047176894	F_C5
CMTM3	4.95908E-06	0.000115334	0.376	0.185	0.049590836	F_C5
SH3KBP1	5.14853E-06	0.000163547	0.488	0.276	0.051485297	F_C5
HSPB1	5.16364E-06	0.000132781	0.384	0.191	0.051636431	F_C5
ENO1	5.30782E-06	0.000424873	0.736	0.538	0.053078215	F_C5
ARPC1B	6.47396E-06	0.000357163	0.776	0.570	0.0647396	F_C5
HLA-DPA1	6.56062E-06	0.000203503	0.568	0.351	0.065606159	F_C5
HN1	6.86786E-06	0.0001194	0.416	0.219	0.068678599	F_C5
YWHAB	7.58732E-06	0.000331247	0.736	0.526	0.075873194	F_C5
SLA	9.05366E-06	0.000174709	0.560	0.346	0.09053659	F_C5
LINC00152	9.52981E-06	0.000277126	0.536	0.324	0.095298089	F_C5
CSRNP1	1.36361E-05	0.000105556	0.296	0.131	0.136360662	F_C5
TSPO	1.42337E-05	0.00012744	0.632	0.423	0.142337372	F_C5
RAP1A	1.47308E-05	0.000205503	0.528	0.320	0.147308448	F_C5
ATP6V1F	1.61794E-05	0.000139801	0.496	0.293	0.1617943	F_C5
COX8A	1.65895E-05	0.000207207	0.680	0.469	0.16589476	F_C5
UQCRH	1.73045E-05	0.000151245	0.680	0.473	0.173045282	F_C5
VMP1	2.09391E-05	0.000113222	0.376	0.195	0.209390952	F_C5
FNBP1	2.39998E-05	0.00012546	0.544	0.342	0.239998068	F_C5
JUNB	2.43387E-05	0.00073305	0.936	0.789	0.243387332	F_C5
TNFAIP3	2.54213E-05	0.000240487	0.584	0.379	0.254212618	F_C5
SLC25A3	2.99937E-05	0.000246323	0.616	0.411	0.299936937	F_C5
ITM2A	3.41915E-05	0.000357353	0.648	0.448	0.34191482	F_C5
EEF2	3.52356E-05	0.000294954	0.888	0.719	0.352355891	F_C5
COX5B	3.5639E-05	0.00018977	0.720	0.519	0.35638965	F_C5
FCMR	3.70998E-05	0.000105046	0.336	0.167	0.370998359	F_C5
PRDX1	3.71236E-05	0.000154887	0.504	0.307	0.371236054	F_C5
RSL1D1	4.21931E-05	0.000115156	0.496	0.303	0.421930518	F_C5
NEAT1	4.966E-05	0.000266643	0.824	0.642	0.496600353	F_C5
FAM46C	5.02712E-05	0.000100064	0.240	0.100	0.502712282	F_C5
PSMB3	5.4663E-05	0.00016172	0.568	0.370	0.546630149	F_C5
OAZ1	5.54479E-05	0.000365584	0.944	0.811	0.554479221	F_C5

SRSF9	5.56952E-05	0.000103056	0.416	0.235	0.556952473	F_C5
STK17B	8.26817E-05	0.000161386	0.616	0.422	0.826816967	F_C5
SOD1	8.48884E-05	0.000339676	0.696	0.517	0.848883966	F_C5
ATP5G2	8.68562E-05	0.000287089	0.848	0.677	0.868562071	F_C5
MRFAP1	8.70525E-05	0.000133587	0.352	0.185	0.870525053	F_C5
CALM3	9.86942E-05	0.000203351	0.456	0.274	0.986941825	F_C5
RAN	9.90931E-05	0.000210555	0.680	0.487	0.990931202	F_C5
PPA1	0.000117362	0.000122672	0.456	0.275	1	F_C5
FAM129A	0.000138456	0.000101486	0.368	0.203	1	F_C5
PSMB8	0.000139235	0.000108371	0.552	0.367	1	F_C5
UBC	0.000141835	0.000510683	0.952	0.850	1	F_C5
GNG5	0.000156552	0.000121718	0.456	0.278	1	F_C5
HSPA8	0.000173896	0.000303432	0.840	0.675	1	F_C5
MTDH	0.000178345	0.000131961	0.544	0.360	1	F_C5
SUMO2	0.000188404	0.000300951	0.776	0.605	1	F_C5
CD74	0.000188641	0.00039849	0.792	0.619	1	F_C5
SPOCK2	0.000193848	0.000232075	0.544	0.361	1	F_C5
PFDN2	0.000195144	0.000109888	0.352	0.192	1	F_C5
ARL6IP4	0.000200824	0.000169849	0.624	0.438	1	F_C5
HLA-DRA	0.000207415	0.000188117	0.432	0.259	1	F_C5
ARPC2	0.000215229	0.00014483	0.880	0.732	1	F_C5
ZNF331	0.000229903	0.000111127	0.304	0.156	1	F_C5
TAX1BP1	0.000230966	0.000146781	0.456	0.281	1	F_C5
ELF1	0.000252708	0.000141396	0.552	0.371	1	F_C5
GPR183	0.00028506	0.000218033	0.496	0.319	1	F_C5
DUSP1	0.000285979	0.000384088	0.792	0.622	1	F_C5
ATP5O	0.000295477	0.000127451	0.592	0.412	1	F_C5
CACYBP	0.000299226	0.000106001	0.384	0.222	1	F_C5
WHSC1L1	0.000307261	0.000124121	0.432	0.263	1	F_C5
ANAPC16	0.00031075	0.000102886	0.760	0.593	1	F_C5
PRDX5	0.000321638	0.00012271	0.440	0.270	1	F_C5
PGAM1	0.000354024	0.000170054	0.456	0.285	1	F_C5
SELK	0.00035986	0.000102828	0.472	0.301	1	F_C5
RHOC	0.00040505	0.000107192	0.368	0.211	1	F_C5
CCNI	0.000409212	0.000203318	0.720	0.545	1	F_C5
PPP1R15A	0.000475481	0.000141188	0.552	0.380	1	F_C5
NPM1	0.000508853	0.000269829	0.904	0.769	1	F_C5
PGM2L1	0.000509502	0.0001076	0.168	0.065	1	F_C5
H2AFY	0.000518961	0.000121155	0.360	0.206	1	F_C5
DDX5	0.000564303	0.000107536	0.960	0.858	1	F_C5

SOCS1	0.000591687	0.000137639	0.424	0.262	1	F_C5
LINC00936	0.000600131	0.000107315	0.264	0.132	1	F_C5
REL	0.000631522	0.000141169	0.344	0.195	1	F_C5
CYCS	0.000632432	0.000106423	0.496	0.329	1	F_C5
CCND2	0.000648437	0.000105309	0.256	0.127	1	F_C5
SIT1	0.00072482	0.000112304	0.424	0.265	1	F_C5
NAP1L1	0.000728313	0.000205231	0.728	0.561	1	F_C5
BAX	0.000739305	0.000109427	0.456	0.294	1	F_C5
PGK1	0.000775441	0.000135604	0.592	0.422	1	F_C5
UQCR10	0.000826214	0.000141931	0.576	0.406	1	F_C5
EIF3E	0.000880299	0.000201444	0.752	0.590	1	F_C5
UBXN1	0.001024119	0.000105034	0.592	0.428	1	F_C5
CD247	0.001033452	0.0002366	0.488	0.331	1	F_C5
LMNA	0.001093412	0.000239791	0.312	0.176	1	F_C5
S100A4	0.001100292	0.000657019	0.968	0.886	1	F_C5
HNRNPA2B1	0.001126299	0.000179224	0.816	0.667	1	F_C5
HERPUD1	0.001127114	0.000136593	0.584	0.420	1	F_C5
ARPC4	0.001170649	0.000106366	0.496	0.335	1	F_C5
COX7C	0.001176256	0.000131397	0.944	0.838	1	F_C5
NDUFB5	0.001222434	0.00010166	0.336	0.194	1	F_C5
EIF3H	0.001236737	0.000198174	0.688	0.525	1	F_C5
ARPC3	0.001479742	0.000122259	0.808	0.665	1	F_C5
ANXA2	0.001786678	0.000126981	0.576	0.417	1	F_C5
YBX1	0.001847711	0.000280511	0.824	0.686	1	F_C5
HNRNPDL	0.001941678	0.000117798	0.752	0.604	1	F_C5
UBE2B	0.001964128	0.00010516	0.368	0.226	1	F_C5
TRBC2	0.002029075	0.000336624	0.800	0.657	1	F_C5
EIF4G2	0.002124153	0.000138093	0.496	0.341	1	F_C5
NDUFB1	0.002166223	0.000122429	0.488	0.334	1	F_C5
PRDM1	0.002269568	0.000179166	0.488	0.334	1	F_C5
ALDOA	0.002338128	0.000151406	0.736	0.586	1	F_C5
HNRNPM	0.002364249	0.000106713	0.472	0.321	1	F_C5
TRAPPC1	0.002693882	0.000105956	0.528	0.375	1	F_C5
C9orf16	0.002851042	0.000191579	0.568	0.414	1	F_C5
DOK2	0.003317703	0.000135044	0.368	0.231	1	F_C5
STK4	0.004991954	0.000237543	0.656	0.512	1	F_C5
LDHB	0.005457841	0.000118212	0.736	0.601	1	F_C5
IL2RG	0.005998371	0.000125248	0.640	0.497	1	F_C5
CLEC2D	0.006159834	0.00020148	0.632	0.489	1	F_C5
UBE2D2	0.006316414	0.000127363	0.552	0.408	1	F_C5

CKLF	0.006963493	0.000104532	0.576	0.436	1	F_C5
MYL6	0.006989117	0.000260819	0.936	0.844	1	F_C5
EIF3L	0.007126996	0.00010875	0.568	0.428	1	F_C5
KLF6	0.008434378	0.000200222	0.768	0.638	1	F_C5
SARAF	0.008454475	0.000112829	0.856	0.745	1	F_C5
H3F3A	0.008640875	0.000203178	0.872	0.761	1	F_C5
FTH1	0.0093184	0.000447803	0.992	0.953	1	F_C5
MKI67	1.73995E-69	0.000569815	0.614	0.004	1.73995E-65	F_C6
BIRC5	6.43555E-69	0.00048485	0.614	0.004	6.43555E-65	F_C6
UBE2C	5.54316E-66	0.0005295	0.586	0.004	5.54316E-62	F_C6
STMN1	4.40968E-63	0.00199639	0.929	0.093	4.40968E-59	F_C6
KIAA0101	1.02444E-57	0.000686574	0.714	0.026	1.02444E-53	F_C6
TYMS	1.04207E-53	0.000432143	0.514	0.005	1.04207E-49	F_C6
NUSAP1	2.88876E-50	0.000434958	0.571	0.013	2.88876E-46	F_C6
CKS1B	1.31734E-47	0.000548852	0.671	0.035	1.31734E-43	F_C6
CENPF	3.84308E-43	0.000427588	0.543	0.018	3.84308E-39	F_C6
TUBA1B	2.7892E-41	0.002080397	0.943	0.405	2.7892E-37	F_C6
TROAP	5.46406E-39	0.000234934	0.429	0.008	5.46406E-35	F_C6
CDKN3	3.68832E-38	0.000264548	0.414	0.007	3.68832E-34	F_C6
CENPU	2.32364E-37	0.000171101	0.429	0.009	2.32364E-33	F_C6
SMC4	1.30568E-34	0.000620774	0.757	0.109	1.30568E-30	F_C6
SMC2	4.99294E-34	0.000260463	0.543	0.034	4.99294E-30	F_C6
HMGB2	4.82959E-33	0.001765964	0.943	0.425	4.82959E-29	F_C6
CLSPN	1.69699E-32	0.000189041	0.443	0.017	1.69699E-28	F_C6
UBE2T	1.92391E-31	0.000188582	0.429	0.016	1.92391E-27	F_C6
PCNA	7.09348E-31	0.000557796	0.657	0.083	7.09348E-27	F_C6
PHF19	7.39389E-31	0.000199802	0.486	0.029	7.39389E-27	F_C6
DTYMK	2.47945E-30	0.000209828	0.543	0.045	2.47945E-26	F_C6
HMGB3	4.0448E-30	0.000174257	0.343	0.007	4.0448E-26	F_C6
TUBB	1.50404E-29	0.001438282	0.829	0.254	1.50404E-25	F_C6
LMNB1	4.56559E-27	0.000207457	0.486	0.039	4.56559E-23	F_C6
CENPM	2.02067E-26	0.00020819	0.471	0.037	2.02067E-22	F_C6
PTTG1	2.46164E-26	0.000599603	0.629	0.093	2.46164E-22	F_C6
CKS2	5.58379E-26	0.000421279	0.614	0.088	5.58379E-22	F_C6
RACGAP1	6.3931E-25	0.000102971	0.271	0.004	6.3931E-21	F_C6
MAD2L1	8.68562E-25	0.000214207	0.486	0.046	8.68562E-21	F_C6
TUBB4B	2.36559E-24	0.000557155	0.729	0.160	2.36559E-20	F_C6
RANBP1	1.34896E-23	0.000532273	0.857	0.267	1.34896E-19	F_C6
CENPK	2.14569E-23	0.000168016	0.471	0.047	2.14569E-19	F_C6
H2AFZ	2.3199E-23	0.00126698	0.914	0.410	2.3199E-19	F_C6

DEK	7.65504E-23	0.000611151	0.886	0.304	7.65504E-19	F_C6
UBE2S	1.15973E-22	0.000270072	0.557	0.082	1.15973E-18	F_C6
ACOT7	1.22957E-22	0.000126395	0.357	0.020	1.22957E-18	F_C6
ATAD2	3.79259E-22	0.000211806	0.429	0.038	3.79259E-18	F_C6
KPNA2	9.41219E-22	0.000335149	0.500	0.064	9.41219E-18	F_C6
SKA2	1.0572E-21	0.000219942	0.543	0.081	1.0572E-17	F_C6
DHFR	1.19077E-21	0.000133303	0.343	0.019	1.19077E-17	F_C6
CARHSP1	5.03766E-21	0.000322134	0.657	0.144	5.03766E-17	F_C6
H2AFV	1.07777E-20	0.000646966	0.829	0.277	1.07777E-16	F_C6
NDC80	1.08063E-20	0.000128512	0.343	0.021	1.08063E-16	F_C6
EZH2	4.13677E-20	0.000142973	0.386	0.033	4.13677E-16	F_C6
TFDP1	5.62251E-20	0.00016876	0.471	0.062	5.62251E-16	F_C6
COX8A	7.13384E-20	0.00069665	0.971	0.467	7.13384E-16	F_C6
H2AFX	7.1575E-20	0.00018988	0.486	0.068	7.1575E-16	F_C6
NUCB2	7.69839E-20	0.000286515	0.686	0.174	7.69839E-16	F_C6
KIF22	1.3852E-19	0.000177688	0.457	0.058	1.3852E-15	F_C6
TUBA1C	1.62295E-19	0.000313064	0.500	0.075	1.62295E-15	F_C6
DDX39A	1.75714E-19	0.000237406	0.614	0.133	1.75714E-15	F_C6
HMGN2	2.52236E-19	0.001236963	0.886	0.467	2.52236E-15	F_C6
AP2S1	2.79946E-19	0.000268544	0.714	0.199	2.79946E-15	F_C6
NUDT1	3.57662E-19	0.000173624	0.557	0.105	3.57662E-15	F_C6
MZT1	5.42533E-19	0.000148962	0.486	0.073	5.42533E-15	F_C6
PSMB2	7.01758E-19	0.000269969	0.700	0.193	7.01758E-15	F_C6
SGOL2	9.57238E-19	0.000155149	0.314	0.020	9.57238E-15	F_C6
DLEU2	1.87114E-18	0.000134491	0.314	0.020	1.87114E-14	F_C6
HELLS	3.53827E-18	0.000167064	0.357	0.032	3.53827E-14	F_C6
EBP	3.98439E-18	0.000195129	0.600	0.136	3.98439E-14	F_C6
SNRNP25	4.08256E-18	0.000118293	0.371	0.036	4.08256E-14	F_C6
LSM4	4.9869E-18	0.000201017	0.686	0.193	4.9869E-14	F_C6
H2AFY	7.38423E-18	0.000334548	0.700	0.203	7.38423E-14	F_C6
TUBA1A	7.41434E-18	0.000248833	0.757	0.253	7.41434E-14	F_C6
SAE1	1.10566E-17	0.0001573	0.514	0.094	1.10566E-13	F_C6
TMEM106C	1.3227E-17	0.000154883	0.429	0.058	1.3227E-13	F_C6
USP1	1.55472E-17	0.000174705	0.543	0.109	1.55472E-13	F_C6
RALY	2.25662E-17	0.000334764	0.714	0.219	2.25662E-13	F_C6
ATP5G3	2.69832E-17	0.000414597	0.886	0.381	2.69832E-13	F_C6
GMNN	4.08836E-17	0.000127577	0.314	0.024	4.08836E-13	F_C6
MCM3	4.81299E-17	0.000224894	0.443	0.066	4.81299E-13	F_C6
YWHAH	6.22954E-17	0.000249755	0.543	0.114	6.22954E-13	F_C6
MCM7	7.18058E-17	0.000196277	0.386	0.046	7.18058E-13	F_C6

HPRT1	8.88072E-17	0.000194556	0.529	0.108	8.88072E-13	F_C6
SLBP	9.39173E-17	0.00028943	0.571	0.130	9.39173E-13	F_C6
CBX5	9.73545E-17	0.000135333	0.486	0.087	9.73545E-13	F_C6
DUT	1.02771E-16	0.00073588	0.757	0.272	1.02771E-12	F_C6
EMC9	1.33603E-16	0.000109715	0.329	0.029	1.33603E-12	F_C6
DCTN3	1.43111E-16	0.00033098	0.686	0.207	1.43111E-12	F_C6
DNAJC9	1.81382E-16	0.000189243	0.486	0.089	1.81382E-12	F_C6
CDK2AP2	1.87265E-16	0.000243942	0.586	0.142	1.87265E-12	F_C6
HIST1H4C	2.0306E-16	0.000899201	0.671	0.225	2.0306E-12	F_C6
IDH2	2.05367E-16	0.000386393	0.729	0.241	2.05367E-12	F_C6
ARPC5	4.04857E-16	0.000515207	0.914	0.437	4.04857E-12	F_C6
ANXA5	4.1237E-16	0.000356927	0.729	0.245	4.1237E-12	F_C6
C12orf75	4.73039E-16	0.000373325	0.786	0.297	4.73039E-12	F_C6
ANP32E	4.9553E-16	0.000357648	0.629	0.173	4.9553E-12	F_C6
RP11-620J15.[illegible]	6.97981E-16	0.000114522	0.314	0.028	6.97981E-12	F_C6
MCM5	8.16903E-16	0.00018465	0.371	0.046	8.16903E-12	F_C6
NONO	8.73025E-16	0.000172878	0.614	0.168	8.73025E-12	F_C6
ANP32B	1.02558E-15	0.000433745	0.843	0.358	1.02558E-11	F_C6
SNRPG	1.14561E-15	0.000407172	0.857	0.375	1.14561E-11	F_C6
RPA3	1.31652E-15	0.000230523	0.586	0.151	1.31652E-11	F_C6
ITGB3BP	1.55475E-15	0.000101772	0.357	0.043	1.55475E-11	F_C6
TPM4	2.58057E-15	0.000338438	0.629	0.181	2.58057E-11	F_C6
ILF2	2.9728E-15	0.000223088	0.643	0.193	2.9728E-11	F_C6
ITGB1BP1	4.16931E-15	0.000156298	0.600	0.167	4.16931E-11	F_C6
PMVK	4.98689E-15	0.000118867	0.500	0.108	4.98689E-11	F_C6
GLRX5	6.45022E-15	0.000105317	0.429	0.074	6.45022E-11	F_C6
SLC25A5	7.65447E-15	0.000545478	0.843	0.373	7.65447E-11	F_C6
MAPRE1	8.25261E-15	0.000136554	0.557	0.142	8.25261E-11	F_C6
EZR	9.01331E-15	0.000215426	0.757	0.296	9.01331E-11	F_C6
ARPP19	1.32505E-14	0.000146564	0.514	0.120	1.32505E-10	F_C6
NAA38	1.6836E-14	0.000221278	0.643	0.202	1.6836E-10	F_C6
FABP5	1.72772E-14	0.000231385	0.500	0.112	1.72772E-10	F_C6
HIST1H1C	2.06022E-14	0.000150563	0.357	0.048	2.06022E-10	F_C6
TACC3	2.44985E-14	0.000141129	0.443	0.084	2.44985E-10	F_C6
RBBP7	2.46671E-14	0.000192561	0.543	0.137	2.46671E-10	F_C6
IER3IP1	4.17594E-14	0.000109619	0.543	0.142	4.17594E-10	F_C6
HNRNPR	5.73139E-14	0.000167553	0.686	0.245	5.73139E-10	F_C6
SNRPA	6.26336E-14	0.000119551	0.457	0.095	6.26336E-10	F_C6
PSMD13	7.98792E-14	0.00016217	0.600	0.181	7.98792E-10	F_C6
PGAM1	1.26842E-13	0.000239352	0.729	0.283	1.26842E-09	F_C6

STRA13	1.30173E-13	0.000184822	0.486	0.112	1.30173E-09	F_C6
ATP5C1	1.34669E-13	0.000245428	0.729	0.283	1.34669E-09	F_C6
PSMB6	1.51299E-13	0.00019011	0.671	0.238	1.51299E-09	F_C6
RAD21	1.8226E-13	0.000172693	0.586	0.176	1.8226E-09	F_C6
ACAA2	1.85952E-13	0.000154945	0.443	0.091	1.85952E-09	F_C6
CALM3	1.8596E-13	0.000510894	0.714	0.272	1.8596E-09	F_C6
WDR76	2.28256E-13	0.000149608	0.271	0.025	2.28256E-09	F_C6
TXN	3.14602E-13	0.000343009	0.843	0.402	3.14602E-09	F_C6
UQCRC1	4.01976E-13	0.00011418	0.486	0.117	4.01976E-09	F_C6
KIF20B	4.04719E-13	0.00016905	0.386	0.067	4.04719E-09	F_C6
PDCD10	4.27716E-13	0.000106038	0.557	0.163	4.27716E-09	F_C6
ANAPC11	4.38185E-13	0.000271799	0.657	0.230	4.38185E-09	F_C6
COX20	5.01884E-13	0.000128433	0.486	0.118	5.01884E-09	F_C6
PSMB8	7.2759E-13	0.00025776	0.800	0.365	7.2759E-09	F_C6
UBALD2	9.12354E-13	0.000237485	0.571	0.172	9.12354E-09	F_C6
NDUFA2	1.1941E-12	0.000246322	0.700	0.272	1.1941E-08	F_C6
SNRPD1	1.22523E-12	0.000260008	0.657	0.237	1.22523E-08	F_C6
PRDX3	1.24652E-12	0.000130013	0.429	0.091	1.24652E-08	F_C6
NDUFA1	1.30423E-12	0.000189502	0.914	0.517	1.30423E-08	F_C6
SMS	1.35275E-12	0.000170305	0.486	0.121	1.35275E-08	F_C6
COPS3	1.72879E-12	0.000122222	0.443	0.099	1.72879E-08	F_C6
COX17	2.02595E-12	0.000138994	0.629	0.224	2.02595E-08	F_C6
ENSA	2.27429E-12	0.000127373	0.714	0.298	2.27429E-08	F_C6
HMGN1	2.30596E-12	0.000391398	0.871	0.451	2.30596E-08	F_C6
HN1	2.43761E-12	0.000384343	0.629	0.218	2.43761E-08	F_C6
EIF4A3	2.68285E-12	0.000142464	0.514	0.143	2.68285E-08	F_C6
VRK1	2.71286E-12	0.000100558	0.371	0.067	2.71286E-08	F_C6
TSTD1	2.85238E-12	0.000116826	0.757	0.344	2.85238E-08	F_C6
LSM3	3.00656E-12	0.000216021	0.643	0.233	3.00656E-08	F_C6
PSMA2	3.54678E-12	0.000153471	0.657	0.248	3.54678E-08	F_C6
CKAP2	3.65668E-12	0.000108773	0.329	0.050	3.65668E-08	F_C6
SQRDL	4.16792E-12	0.000104768	0.471	0.119	4.16792E-08	F_C6
FAM111A	4.2117E-12	0.00010916	0.386	0.075	4.2117E-08	F_C6
COX7B	4.44249E-12	0.000297614	0.843	0.424	4.44249E-08	F_C6
HSPB11	5.53202E-12	0.000177356	0.600	0.205	5.53202E-08	F_C6
HNRNPA3	5.56426E-12	0.000339426	0.843	0.425	5.56426E-08	F_C6
CYCS	5.66473E-12	0.000206274	0.743	0.327	5.66473E-08	F_C6
HLA-DRB1	5.91255E-12	0.00028008	0.786	0.373	5.91255E-08	F_C6
TPRKB	7.57325E-12	0.000119267	0.486	0.130	7.57325E-08	F_C6
MZT2B	7.57378E-12	0.000345548	0.786	0.365	7.57378E-08	F_C6

TMEM160	7.73248E-12	0.000106553	0.600	0.210	7.73248E-08	F_C6
NDUFB6	7.79081E-12	0.000132714	0.529	0.157	7.79081E-08	F_C6
PARP1	8.30034E-12	0.000106493	0.571	0.188	8.30034E-08	F_C6
RNASEH2C	8.85411E-12	0.000144907	0.471	0.122	8.85411E-08	F_C6
LIG1	9.4002E-12	0.000100115	0.271	0.032	9.4002E-08	F_C6
NUCKS1	9.58601E-12	0.000413373	0.743	0.323	9.58601E-08	F_C6
CCT5	1.03187E-11	0.000132988	0.514	0.149	1.03187E-07	F_C6
ERH	1.05828E-11	0.000313611	0.814	0.398	1.05828E-07	F_C6
BLOC1S1	1.14336E-11	0.000256393	0.700	0.288	1.14336E-07	F_C6
ATP5F1	1.18873E-11	0.000126187	0.700	0.296	1.18873E-07	F_C6
UBE2A	1.34969E-11	0.000135298	0.457	0.116	1.34969E-07	F_C6
RBX1	1.39424E-11	0.000283254	0.757	0.342	1.39424E-07	F_C6
CD59	1.45508E-11	0.000159938	0.414	0.092	1.45508E-07	F_C6
MAGOH	1.50956E-11	0.000112128	0.671	0.273	1.50956E-07	F_C6
SHFM1	1.58261E-11	0.000228125	0.771	0.361	1.58261E-07	F_C6
TMPO	1.66966E-11	0.000125249	0.429	0.101	1.66966E-07	F_C6
MPC2	1.89788E-11	0.000142864	0.571	0.191	1.89788E-07	F_C6
CHMP2A	1.90038E-11	0.000110703	0.614	0.226	1.90038E-07	F_C6
SRSF2	1.96101E-11	0.00027959	0.843	0.441	1.96101E-07	F_C6
SET	2.28843E-11	0.000167586	0.757	0.358	2.28843E-07	F_C6
RTFDC1	2.73422E-11	0.000127277	0.514	0.154	2.73422E-07	F_C6
PSMC3	2.77656E-11	0.00013332	0.500	0.145	2.77656E-07	F_C6
TXNDC17	2.86381E-11	0.000124221	0.443	0.111	2.86381E-07	F_C6
DYNLL1	2.96476E-11	0.000187395	0.771	0.373	2.96476E-07	F_C6
PDIA6	3.2981E-11	0.000148933	0.643	0.251	3.2981E-07	F_C6
FAM110A	3.30864E-11	0.000100953	0.300	0.044	3.30864E-07	F_C6
LSM5	3.30902E-11	0.000187198	0.629	0.238	3.30902E-07	F_C6
COX5A	3.37776E-11	0.00025261	0.686	0.283	3.37776E-07	F_C6
CBX3	3.39021E-11	0.000209615	0.643	0.249	3.39021E-07	F_C6
TMEM50A	3.80813E-11	0.000114175	0.714	0.320	3.80813E-07	F_C6
ATP5J	4.07505E-11	0.000269223	0.800	0.396	4.07505E-07	F_C6
SMC3	4.19377E-11	0.000159758	0.529	0.166	4.19377E-07	F_C6
FAM96B	4.42296E-11	0.000119114	0.643	0.256	4.42296E-07	F_C6
CKLF	4.49951E-11	0.000110746	0.814	0.434	4.49951E-07	F_C6
NDUFB1	4.57436E-11	0.000167513	0.729	0.332	4.57436E-07	F_C6
HSPA5	4.57758E-11	0.000157617	0.714	0.321	4.57758E-07	F_C6
SRP9	4.94061E-11	0.000216102	0.729	0.327	4.94061E-07	F_C6
COMMD4	5.02416E-11	0.00011853	0.386	0.083	5.02416E-07	F_C6
DECR1	5.30791E-11	0.000131405	0.529	0.168	5.30791E-07	F_C6
CACYBP	5.50836E-11	0.000150131	0.600	0.220	5.50836E-07	F_C6

EIF5A	7.36694E-11	0.000224419	0.600	0.219	7.36694E-07	F_C6
KPNB1	7.55017E-11	0.000140784	0.571	0.201	7.55017E-07	F_C6
IFI27L2	7.67463E-11	0.000189355	0.586	0.209	7.67463E-07	F_C6
KHDRBS1	7.96722E-11	0.000126367	0.614	0.237	7.96722E-07	F_C6
LRRFIP1	8.7816E-11	0.000196865	0.771	0.380	8.7816E-07	F_C6
ARL6IP1	9.13014E-11	0.000387068	0.629	0.241	9.13014E-07	F_C6
PKM	9.32206E-11	0.000358082	0.743	0.342	9.32206E-07	F_C6
HMGA1	9.33508E-11	0.000139551	0.471	0.133	9.33508E-07	F_C6
CAPZA1	9.53956E-11	0.000147971	0.729	0.340	9.53956E-07	F_C6
PSMA4	1.07816E-10	0.000188054	0.671	0.282	1.07816E-06	F_C6
NDUFS6	1.1195E-10	0.000242679	0.643	0.255	1.1195E-06	F_C6
BANF1	1.23647E-10	0.000183282	0.571	0.202	1.23647E-06	F_C6
SNRPF	1.26192E-10	0.000124711	0.729	0.343	1.26192E-06	F_C6
SEC61G	1.27713E-10	0.000176793	0.843	0.464	1.27713E-06	F_C6
RHEB	1.40117E-10	0.000165028	0.500	0.153	1.40117E-06	F_C6
WDR1	1.44264E-10	0.000201721	0.643	0.259	1.44264E-06	F_C6
HSP90B1	1.57004E-10	0.000190089	0.700	0.313	1.57004E-06	F_C6
DCXR	1.57139E-10	0.000100292	0.457	0.128	1.57139E-06	F_C6
NASP	1.70728E-10	0.000206556	0.500	0.154	1.70728E-06	F_C6
LINC00152	1.90424E-10	0.000141559	0.700	0.324	1.90424E-06	F_C6
CRIP1	2.00501E-10	0.000415778	0.786	0.391	2.00501E-06	F_C6
ATP5B	2.0273E-10	0.000196277	0.743	0.353	2.0273E-06	F_C6
ITGAE	2.08123E-10	0.00013706	0.471	0.138	2.08123E-06	F_C6
YWHAQ	2.41981E-10	0.000269014	0.729	0.338	2.41981E-06	F_C6
ARPC4	2.72871E-10	0.000137511	0.714	0.334	2.72871E-06	F_C6
ANAPC15	2.87201E-10	0.000105463	0.414	0.106	2.87201E-06	F_C6
PSMA1	2.87961E-10	0.000141488	0.671	0.293	2.87961E-06	F_C6
TRAPPC1	2.87987E-10	0.00018058	0.757	0.373	2.87987E-06	F_C6
PCNP	3.10454E-10	0.000170888	0.629	0.254	3.10454E-06	F_C6
IFI16	3.15931E-10	0.000195341	0.800	0.422	3.15931E-06	F_C6
SDF2L1	3.2944E-10	0.000137713	0.514	0.168	3.2944E-06	F_C6
PNRC2	3.37671E-10	0.000122332	0.486	0.150	3.37671E-06	F_C6
H1FX	3.41624E-10	0.000150149	0.486	0.150	3.41624E-06	F_C6
ACTR3	3.54629E-10	0.000268303	0.786	0.400	3.54629E-06	F_C6
HNRNPC	3.71467E-10	0.000199963	0.829	0.455	3.71467E-06	F_C6
YBX1	3.83509E-10	0.000456348	0.986	0.685	3.83509E-06	F_C6
TPM3	3.84988E-10	0.000287928	0.857	0.483	3.84988E-06	F_C6
PSME2	4.06677E-10	0.000350271	0.800	0.413	4.06677E-06	F_C6
VDAC3	4.17977E-10	0.000128375	0.529	0.180	4.17977E-06	F_C6
BSG	4.24685E-10	0.00010915	0.657	0.286	4.24685E-06	F_C6

MAD2L2	4.6736E-10	0.000102247	0.386	0.092	4.6736E-06	F_C6
TAGLN2	5.07298E-10	0.000168089	0.729	0.353	5.07298E-06	F_C6
TALDO1	5.16996E-10	0.000148458	0.557	0.201	5.16996E-06	F_C6
OSTC	5.35084E-10	0.000102701	0.600	0.239	5.35084E-06	F_C6
NUDT21	5.42864E-10	0.000103763	0.414	0.109	5.42864E-06	F_C6
EEF1E1	5.49705E-10	0.000104415	0.400	0.101	5.49705E-06	F_C6
PCMT1	5.63046E-10	0.000120835	0.543	0.193	5.63046E-06	F_C6
CRELD2	6.50141E-10	0.000107563	0.429	0.118	6.50141E-06	F_C6
SFPQ	6.59249E-10	0.000174273	0.657	0.286	6.59249E-06	F_C6
HLA-DRA	7.05288E-10	0.000336979	0.629	0.258	7.05288E-06	F_C6
MDH1	7.76666E-10	0.000111067	0.557	0.206	7.76666E-06	F_C6
TMBIM6	8.28777E-10	0.000181355	0.857	0.500	8.28777E-06	F_C6
PSMC4	8.86887E-10	0.000106956	0.471	0.146	8.86887E-06	F_C6
SNRPB	9.52494E-10	0.000275101	0.771	0.392	9.52494E-06	F_C6
SSNA1	9.60284E-10	0.000109496	0.543	0.197	9.60284E-06	F_C6
PRDX1	9.74476E-10	0.000106933	0.671	0.307	9.74476E-06	F_C6
ARL6IP6	1.16697E-09	0.000114145	0.400	0.104	1.16697E-05	F_C6
PSMB3	1.28078E-09	0.000199465	0.743	0.369	1.28078E-05	F_C6
GSTO1	1.48129E-09	0.000103471	0.543	0.200	1.48129E-05	F_C6
OSTF1	1.50329E-09	0.000120364	0.629	0.269	1.50329E-05	F_C6
SIVA1	1.56738E-09	0.00026946	0.586	0.228	1.56738E-05	F_C6
PCBP1	1.64929E-09	0.00012251	0.729	0.365	1.64929E-05	F_C6
LDHA	1.79534E-09	0.000395444	0.886	0.531	1.79534E-05	F_C6
GNAS	1.86756E-09	0.000103869	0.700	0.341	1.86756E-05	F_C6
RNPS1	1.95888E-09	0.00013032	0.629	0.271	1.95888E-05	F_C6
PSMD8	1.9653E-09	0.000161696	0.643	0.281	1.9653E-05	F_C6
LSM2	1.98595E-09	0.000141311	0.471	0.150	1.98595E-05	F_C6
CSNK2B	2.10328E-09	0.00018113	0.586	0.232	2.10328E-05	F_C6
SNRPE	2.16873E-09	0.00017578	0.643	0.281	2.16873E-05	F_C6
IFNG	2.17922E-09	0.000343662	0.400	0.108	2.17922E-05	F_C6
COTL1	2.32203E-09	0.000525016	0.857	0.497	2.32203E-05	F_C6
HP1BP3	2.53814E-09	0.000121375	0.586	0.238	2.53814E-05	F_C6
HLA-DMA	2.53831E-09	0.00017966	0.414	0.115	2.53831E-05	F_C6
IFI6	2.72501E-09	0.000117874	0.500	0.174	2.72501E-05	F_C6
SPCS2	2.77761E-09	0.000144374	0.771	0.412	2.77761E-05	F_C6
ANXA2	2.8061E-09	0.000401592	0.786	0.415	2.8061E-05	F_C6
SUMO3	2.8972E-09	0.000143666	0.500	0.172	2.8972E-05	F_C6
PIN1	2.98121E-09	0.000128278	0.557	0.215	2.98121E-05	F_C6
COX6A1	3.07005E-09	0.000212169	0.857	0.509	3.07005E-05	F_C6
RAN	3.60374E-09	0.000487048	0.843	0.487	3.60374E-05	F_C6

LCP1	3.7129E-09	0.000318778	0.786	0.419	3.7129E-05	F_C6
NDUFA6	4.17889E-09	0.000109929	0.586	0.241	4.17889E-05	F_C6
MZT2A	4.5995E-09	0.000167542	0.700	0.341	4.5995E-05	F_C6
DBI	4.98754E-09	0.000243882	0.729	0.365	4.98754E-05	F_C6
GNG5	5.00082E-09	0.000145499	0.629	0.277	5.00082E-05	F_C6
CAP1	5.23994E-09	0.000287557	0.814	0.456	5.23994E-05	F_C6
NDUFAB1	5.47391E-09	0.00011628	0.543	0.208	5.47391E-05	F_C6
GNAI2	5.60189E-09	0.00013899	0.557	0.218	5.60189E-05	F_C6
RBM3	5.75123E-09	0.000281464	0.900	0.567	5.75123E-05	F_C6
MT2A	6.17466E-09	0.000478467	0.786	0.424	6.17466E-05	F_C6
ATP5J2	6.34783E-09	0.000155263	0.743	0.388	6.34783E-05	F_C6
NDUFB3	6.56805E-09	0.000131187	0.571	0.232	6.56805E-05	F_C6
MIR4435-2HG	7.80848E-09	0.00013957	0.414	0.122	7.80848E-05	F_C6
SUB1	7.95551E-09	0.000445627	0.929	0.609	7.95551E-05	F_C6
CAPN2	8.03986E-09	0.000127525	0.571	0.233	8.03986E-05	F_C6
BUB3	8.51221E-09	0.000204424	0.643	0.291	8.51221E-05	F_C6
PSMD11	8.71003E-09	0.000100306	0.386	0.106	8.71003E-05	F_C6
PLP2	8.82256E-09	0.000296951	0.829	0.478	8.82256E-05	F_C6
CCDC167	1.06458E-08	0.000128915	0.529	0.202	0.000106458	F_C6
NDUFB7	1.1479E-08	0.000169641	0.657	0.308	0.00011479	F_C6
VPS29	1.15665E-08	0.00013631	0.600	0.260	0.000115665	F_C6
HLA-DQB1	1.28778E-08	0.000118211	0.529	0.208	0.000128778	F_C6
SRRM1	1.29916E-08	0.000114258	0.743	0.402	0.000129916	F_C6
PSMB9	1.51895E-08	0.000316095	0.871	0.536	0.000151895	F_C6
THOC7	1.55594E-08	0.000129916	0.500	0.183	0.000155594	F_C6
PPP1CA	1.59808E-08	0.000274122	0.786	0.435	0.000159808	F_C6
LBR	1.60355E-08	0.000142302	0.443	0.144	0.000160355	F_C6
POMP	1.63007E-08	0.000249783	0.729	0.375	0.000163007	F_C6
LGALS1	1.67137E-08	0.000721062	0.800	0.450	0.000167137	F_C6
UQCRQ	1.94516E-08	0.000228862	0.743	0.393	0.000194516	F_C6
ATP1B3	2.02182E-08	0.000157493	0.486	0.174	0.000202182	F_C6
PYCARD	2.1635E-08	0.000106813	0.486	0.176	0.00021635	F_C6
TCEB2	2.49481E-08	0.000240039	0.900	0.585	0.000249481	F_C6
HNRNPF	2.7959E-08	0.000260297	0.714	0.365	0.00027959	F_C6
GSTP1	2.94162E-08	0.000267415	0.686	0.338	0.000294162	F_C6
ARPC5L	3.14571E-08	0.000143576	0.629	0.293	0.000314571	F_C6
RAC1	3.38921E-08	0.000142555	0.657	0.320	0.000338921	F_C6
CAPZB	3.39296E-08	0.00015491	0.829	0.504	0.000339296	F_C6
SCP2	3.43299E-08	0.000121218	0.614	0.282	0.000343299	F_C6
ARL6IP4	3.49451E-08	0.000147016	0.771	0.438	0.000349451	F_C6

RHOA	3.76325E-08	0.000288727	0.786	0.442	0.000376325	F_C6
TPI1	4.01882E-08	0.000524263	0.771	0.435	0.000401882	F_C6
ARHGDIA	4.18739E-08	0.000189956	0.671	0.331	0.000418739	F_C6
HLA-DPA1	4.61847E-08	0.000183521	0.686	0.352	0.000461847	F_C6
CCT6A	5.00575E-08	0.000126504	0.486	0.181	0.000500575	F_C6
SEPT7	5.31784E-08	0.000118815	0.871	0.569	0.000531784	F_C6
S100A10	6.07539E-08	0.000237676	0.971	0.717	0.000607539	F_C6
CNN2	6.23868E-08	0.000103732	0.514	0.206	0.000623868	F_C6
PSMB7	6.54223E-08	0.000101779	0.529	0.217	0.000654223	F_C6
NAP1L1	7.02953E-08	0.000223612	0.871	0.561	0.000702953	F_C6
APOBEC3G	7.17823E-08	0.000105193	0.714	0.395	0.000717823	F_C6
DNMT1	7.32907E-08	0.000136381	0.429	0.144	0.000732907	F_C6
NDUFC2	8.92415E-08	0.00011898	0.600	0.278	0.000892415	F_C6
PARK7	1.01746E-07	0.000155066	0.771	0.447	0.001017456	F_C6
EIF3I	1.07932E-07	0.000111489	0.571	0.255	0.001079315	F_C6
MIF	1.10029E-07	0.000224204	0.771	0.443	0.00110029	F_C6
ANXA6	1.12271E-07	0.00011097	0.629	0.307	0.001122708	F_C6
SERBP1	1.14086E-07	0.000137711	0.686	0.359	0.001140861	F_C6
PGK1	1.16549E-07	0.000119117	0.743	0.422	0.001165488	F_C6
GNG2	1.23561E-07	0.000107789	0.471	0.178	0.001235608	F_C6
PHPT1	1.33032E-07	0.000153209	0.500	0.198	0.001330319	F_C6
VDAC1	1.37048E-07	0.000110252	0.486	0.189	0.001370479	F_C6
PTGES3	1.39576E-07	0.00014396	0.729	0.404	0.001395763	F_C6
VIM	1.40943E-07	0.000905017	0.971	0.756	0.001409428	F_C6
NDUFB2	1.4348E-07	0.000208489	0.700	0.370	0.001434797	F_C6
ITM2C	1.46142E-07	0.000251473	0.543	0.231	0.001461419	F_C6
FKBP1A	1.78014E-07	0.00013794	0.614	0.295	0.001780144	F_C6
XRCC5	1.83685E-07	0.000158991	0.600	0.281	0.001836849	F_C6
C11orf31	2.07159E-07	0.000129492	0.614	0.298	0.002071586	F_C6
CAPG	2.07314E-07	0.000153161	0.414	0.141	0.002073141	F_C6
C14orf2	2.13773E-07	0.00015544	0.800	0.488	0.002137734	F_C6
EIF4G2	2.15448E-07	0.00011804	0.657	0.340	0.002154484	F_C6
CD99	2.59365E-07	0.000129702	0.914	0.646	0.002593652	F_C6
GAPDH	2.68372E-07	0.001050621	0.971	0.861	0.002683718	F_C6
SEC61B	2.79438E-07	0.000123225	0.757	0.446	0.002794384	F_C6
TMEM59	3.13396E-07	0.000102154	0.757	0.449	0.003133958	F_C6
SRSF3	3.23782E-07	0.000233301	0.829	0.521	0.003237821	F_C6
RBM8A	3.78929E-07	0.000124959	0.686	0.373	0.003789294	F_C6
ZNF706	3.85423E-07	0.000105556	0.529	0.231	0.003854228	F_C6
DNAJA1	4.88202E-07	0.000150064	0.671	0.365	0.004882022	F_C6

POLR2L	5.38921E-07	0.000163094	0.757	0.448	0.005389214	F_C6
PCBP2	5.61056E-07	0.000124438	0.757	0.453	0.005610557	F_C6
UQCRH	5.67972E-07	0.00010077	0.771	0.474	0.00567972	F_C6
PA2G4	5.70642E-07	0.000103735	0.643	0.339	0.005706425	F_C6
HNRNPA2B1	6.21753E-07	0.000522844	0.929	0.667	0.006217529	F_C6
EMP3	6.62987E-07	0.000212626	0.900	0.627	0.006629871	F_C6
CCT4	6.707E-07	0.00010707	0.514	0.224	0.006706999	F_C6
CD74	7.11561E-07	0.000415179	0.900	0.620	0.00711561	F_C6
COX7A2	8.44379E-07	0.000179207	0.886	0.609	0.008443792	F_C6
CCL3	1.14213E-06	0.000313923	0.400	0.143	0.011421317	F_C6
PPP4C	1.4269E-06	0.000112639	0.543	0.254	0.014269042	F_C6
ENO1	1.65191E-06	0.000472564	0.829	0.539	0.016519126	F_C6
HMGB1	1.8783E-06	0.000794946	0.971	0.819	0.018783021	F_C6
CALM2	2.26763E-06	0.000483621	0.914	0.659	0.022676271	F_C6
ATP5O	3.01048E-06	0.000105443	0.700	0.413	0.030104823	F_C6
TUBA4A	3.05402E-06	0.000173727	0.757	0.468	0.030540218	F_C6
NDUFA4	3.08067E-06	0.000204112	0.886	0.621	0.030806707	F_C6
BUD31	3.14043E-06	0.000103299	0.486	0.215	0.031404345	F_C6
PET100	3.47429E-06	0.00011039	0.557	0.276	0.034742879	F_C6
HSPA8	4.76207E-06	0.000177644	0.914	0.676	0.047620695	F_C6
ATPIF1	5.04644E-06	0.000106048	0.729	0.448	0.050464355	F_C6
PPIA	1.09709E-05	0.000401397	0.929	0.696	0.109708804	F_C6
COX5B	1.11078E-05	0.000117024	0.786	0.521	0.111078005	F_C6
SLC25A3	1.18116E-05	0.000114033	0.686	0.413	0.118115771	F_C6
COPE	1.32975E-05	0.000157303	0.700	0.423	0.132974865	F_C6
PSIP1	1.40912E-05	0.000122696	0.500	0.240	0.140912033	F_C6
RBMX	1.64249E-05	0.000114937	0.543	0.279	0.164248664	F_C6
COX6C	3.06951E-05	0.00025066	0.886	0.648	0.306950949	F_C6
HEXIM1	3.22207E-05	0.000103497	0.329	0.120	0.322206799	F_C6
H3F3A	3.5322E-05	0.000415299	0.957	0.761	0.353219835	F_C6
NDUFB4	3.6078E-05	0.000137297	0.629	0.365	0.360779679	F_C6
CHCHD2	3.6294E-05	0.00028271	0.900	0.668	0.362940209	F_C6
UBL5	5.5738E-05	0.000120938	0.829	0.593	0.557379581	F_C6
CALM1	5.72033E-05	0.000272163	0.957	0.766	0.572032833	F_C6
PSMA7	5.82322E-05	0.000104588	0.743	0.496	0.582322047	F_C6
PRDX2	6.32287E-05	0.000137353	0.600	0.344	0.632286971	F_C6
HINT1	6.3305E-05	0.000248254	0.900	0.679	0.633050303	F_C6
CCL4L2	7.24907E-05	0.000115905	0.543	0.309	0.724907439	F_C6
NDUFA13	7.60016E-05	0.00010053	0.571	0.323	0.760015695	F_C6
CLIC1	8.63364E-05	0.000335785	0.900	0.680	0.863364069	F_C6

CFL1	8.90426E-05	0.000697758	0.986	0.874	0.890425929	F_C6
ARPC2	0.000161009	0.000392224	0.929	0.734	1	F_C6
SUMO2	0.000262402	0.000185104	0.829	0.607	1	F_C6
ARPC3	0.000270234	0.000160444	0.871	0.665	1	F_C6
MYL6	0.000294537	0.000332632	0.986	0.845	1	F_C6
HSP90AA1	0.000510162	0.000239725	0.943	0.774	1	F_C6
ARPC1B	0.000620518	0.000157982	0.786	0.572	1	F_C6
PPDPF	0.000949158	0.000261653	0.914	0.737	1	F_C6
OAZ1	0.001089403	0.000136123	0.957	0.813	1	F_C6
GZMA	0.001615044	0.000153901	0.871	0.705	1	F_C6
ANKRD28	0.001860419	0.000110574	0.314	0.147	1	F_C6
RAC2	0.002286914	0.000338508	0.771	0.575	1	F_C6
UBB	0.002905962	0.000214186	0.957	0.823	1	F_C6
CORO1A	0.003948713	0.000187322	0.943	0.807	1	F_C6
CD52	2.4283E-09	0.002351976	0.933	0.931	2.4283E-05	F_C7
PREX1	5.36372E-05	0.000672903	0.400	0.111	0.536372193	F_C7
PKN2	0.000113572	0.00027317	0.333	0.068	1	F_C7
GZMB	0.000217468	0.000719052	0.567	0.220	1	F_C7
MALAT1	0.000364507	0.007036836	1.000	1.000	1	F_C7
TTLL7	0.000459454	0.00027326	0.300	0.064	1	F_C7
SPPL2B	0.002478656	0.000105466	0.167	0.022	1	F_C7
SRSF11	0.003946993	0.00059385	0.433	0.357	1	F_C7
EXTL3	0.005081078	0.000250704	0.133	0.015	1	F_C7
HLA-B	0.005263854	0.000539734	0.933	0.994	1	F_C7
RFX5	0.005299431	0.000129379	0.167	0.026	1	F_C7
ATXN3	0.006003396	0.000424318	0.267	0.083	1	F_C7
PTPN4	0.00638759	0.000374321	0.433	0.186	1	F_C7
ZNF683	0.007648656	0.000208019	0.200	0.043	1	F_C7
WIPF1	0.00778326	0.000384307	0.300	0.334	1	F_C7
ZNF432	0.008833154	0.000107551	0.133	0.018	1	F_C7
KLRD1	0.009522949	0.000529627	0.433	0.192	1	F_C7
