# Supplementary material for: Targeting pro-inflammatory T cells as a novel therapeutic approach to potentially resolve atherosclerosis in humans
Source: Cell Res. 2024 Mar 15;34(6):407–27. doi: 10.1038/s41422-024-00945-0 (PMC11143203; doi:10.1038/s41422-024-00945-0)
Supplement: Supplementary file 17 — Supplementary information, Table S5 [file 41422_2024_945_MOESM17_ESM.pdf]

Supplementary information, Table S5. Differentially accessible peaks (DAPs) of identified T cell clusters in snATAC-seq dataset.

Differentially accessible peaks (DAPs) of T cell clusters in AS plaque.

Peak	*P* value	avg_logFC	pct.1	pct.2	adjusted *P* value	cluster
chr21-31354162-31355261	9.53E-46	0.721616807	0.383	0.084	6.31E-41	C1_CD8_Tres
chr5-82356754-82357721	2.17E-35	0.624343312	0.365	0.102	1.43E-30	C1_CD8_Tres
chr8-80864501-80865161	8.40E-32	0.620182413	0.293	0.07	5.56E-27	C1_CD8_Tres
chr21-36288508-36289609	1.04E-30	0.588002231	0.284	0.079	6.87E-26	C1_CD8_Tres
chr10-8898769-8899165	4.63E-29	0.557626586	0.099	0.004	3.06E-24	C1_CD8_Tres
chr11-13868785-13869542	9.99E-28	0.56756676	0.263	0.063	6.61E-23	C1_CD8_Tres
chr2-160417640-160418130	4.30E-27	0.571275875	0.168	0.025	2.85E-22	C1_CD8_Tres
chr11-46331145-46331748	3.82E-26	0.554822845	0.153	0.021	2.53E-21	C1_CD8_Tres
chr11-129846333-129846856	8.98E-26	0.536234896	0.129	0.013	5.95E-21	C1_CD8_Tres
chr8-19496478-19497608	1.07E-25	0.51054402	0.404	0.153	7.08E-21	C1_CD8_Tres
chr20-5736988-5737733	4.10E-25	0.547008611	0.219	0.046	2.71E-20	C1_CD8_Tres
chr21-31558627-31559975	1.24E-24	0.507416273	0.323	0.111	8.24E-20	C1_CD8_Tres
chr17-82126395-82127458	2.46E-24	0.455969183	0.449	0.2	1.63E-19	C1_CD8_Tres
chr4-52588534-52589208	5.04E-24	0.555030723	0.141	0.017	3.33E-19	C1_CD8_Tres
chr2-149069078-149069733	1.03E-23	0.503801631	0.108	0.008	6.81E-19	C1_CD8_Tres
chr3-112074691-112075380	2.07E-23	0.523557428	0.132	0.017	1.37E-18	C1_CD8_Tres
chr11-128840076-128840452	1.06E-22	0.477031899	0.114	0.012	6.99E-18	C1_CD8_Tres
chr11-129853443-129854439	2.45E-22	0.526511377	0.18	0.036	1.62E-17	C1_CD8_Tres
chr7-38433669-38434193	2.72E-22	0.487926838	0.102	0.008	1.80E-17	C1_CD8_Tres
chr17-2814110-2815366	7.92E-22	0.410789209	0.497	0.259	5.24E-17	C1_CD8_Tres
chr2-206530475-206531411	8.83E-22	0.492033839	0.129	0.016	5.85E-17	C1_CD8_Tres
chr4-108107558-108108443	9.43E-22	0.497900958	0.246	0.075	6.25E-17	C1_CD8_Tres

chr2-147449266-147450025	2.15E-21	0.473499641	0.129	0.019	1.42E-16	C1_CD8_Tres
chr2-201448194-201448990	3.64E-21	0.487444486	0.195	0.045	2.41E-16	C1_CD8_Tres
chr5-56058012-56059238	4.53E-21	0.484032327	0.251	0.076	3.00E-16	C1_CD8_Tres
chr22-24305022-24305671	5.01E-21	0.472396929	0.141	0.024	3.32E-16	C1_CD8_Tres
chr15-88904290-88905053	1.00E-20	0.449853959	0.353	0.146	6.63E-16	C1_CD8_Tres
chr3-7876673-7877294	1.19E-20	0.478080221	0.102	0.012	7.88E-16	C1_CD8_Tres
chr1-83508052-83508796	1.65E-20	0.485690125	0.129	0.018	1.09E-15	C1_CD8_Tres
chr12-109981321-109981669	2.24E-20	0.478577343	0.114	0.013	1.48E-15	C1_CD8_Tres
chr21-31181419-31182555	6.30E-20	0.476431441	0.213	0.059	4.17E-15	C1_CD8_Tres
chr10-43331916-43332565	1.00E-19	0.470653416	0.141	0.027	6.62E-15	C1_CD8_Tres
chr10-8897187-8897555	1.12E-19	0.382233317	0.06	0.002	7.43E-15	C1_CD8_Tres
chr3-15685900-15686174	1.25E-19	0.413295739	0.057	0.001	8.27E-15	C1_CD8_Tres
chr11-95688151-95689305	1.73E-19	0.486716829	0.159	0.033	1.14E-14	C1_CD8_Tres
chr17-76772497-76773639	2.02E-19	0.430145549	0.332	0.143	1.34E-14	C1_CD8_Tres
chr4-122156762-122157172	2.15E-19	0.466060744	0.141	0.026	1.43E-14	C1_CD8_Tres
chr11-68962496-68963150	2.45E-19	0.442099885	0.081	0.006	1.62E-14	C1_CD8_Tres
chr2-113876400-113877141	2.88E-19	0.476183238	0.204	0.055	1.91E-14	C1_CD8_Tres
chr17-75036583-75037040	5.01E-19	0.440601955	0.308	0.129	3.32E-14	C1_CD8_Tres
chr15-63446190-63446645	5.74E-19	0.430549752	0.087	0.008	3.80E-14	C1_CD8_Tres
chr19-49567775-49568531	6.38E-19	0.442959757	0.24	0.085	4.23E-14	C1_CD8_Tres
chr12-3962350-3963101	7.49E-19	0.468873415	0.147	0.03	4.96E-14	C1_CD8_Tres
chr3-112128704-112129279	7.71E-19	0.467849801	0.168	0.039	5.11E-14	C1_CD8_Tres
chr12-104498240-104498577	9.05E-19	0.434665965	0.114	0.018	5.99E-14	C1_CD8_Tres
chr18-58605329-58605745	1.19E-18	0.450776737	0.105	0.013	7.87E-14	C1_CD8_Tres
chr4-87103173-87104125	1.40E-18	0.452624512	0.159	0.042	9.26E-14	C1_CD8_Tres
chr14-31937565-31938397	1.96E-18	0.476841385	0.201	0.059	1.30E-13	C1_CD8_Tres
chr1-35505676-35506798	2.15E-18	0.430393566	0.335	0.138	1.42E-13	C1_CD8_Tres

chr16-27195818-27196446	3.31E-18	0.450607683	0.192	0.061	2.19E-13	C1_CD8_Tres
chr2-96962262-96963250	4.59E-18	0.41571145	0.302	0.124	3.04E-13	C1_CD8_Tres
chr2-230254210-230254912	4.85E-18	0.436258124	0.111	0.016	3.21E-13	C1_CD8_Tres
chr11-133927455-133928846	4.86E-18	0.451080535	0.183	0.054	3.22E-13	C1_CD8_Tres
chr2-73978805-73979404	6.30E-18	0.447477803	0.195	0.06	4.17E-13	C1_CD8_Tres
chr7-74451939-74452898	9.02E-18	0.403017188	0.365	0.167	5.98E-13	C1_CD8_Tres
chr3-33023056-33023996	1.51E-17	0.440630499	0.153	0.034	1.00E-12	C1_CD8_Tres
chr19-51354750-51355094	1.82E-17	0.388419956	0.063	0.003	1.21E-12	C1_CD8_Tres
chrX-46320257-46320809	1.89E-17	0.412659601	0.093	0.01	1.25E-12	C1_CD8_Tres
chr16-15509172-15509969	2.41E-17	0.434741179	0.108	0.017	1.60E-12	C1_CD8_Tres
chr9-33415251-33415946	2.65E-17	0.429675115	0.231	0.08	1.75E-12	C1_CD8_Tres
chr5-98773002-98774550	7.82E-17	0.400003602	0.296	0.133	5.18E-12	C1_CD8_Tres
chrX-123803310-123803865	9.04E-17	0.415763876	0.099	0.013	5.99E-12	C1_CD8_Tres
chr1-33128184-33128619	1.00E-16	0.423415633	0.159	0.041	6.62E-12	C1_CD8_Tres
chr15-59803380-59804029	1.41E-16	0.430675696	0.123	0.026	9.31E-12	C1_CD8_Tres
chr9-79581567-79582252	1.50E-16	0.416413781	0.105	0.018	9.90E-12	C1_CD8_Tres
chr22-40711144-40711920	1.73E-16	0.420539447	0.204	0.07	1.14E-11	C1_CD8_Tres
chr5-56479752-56481429	1.75E-16	0.423157766	0.117	0.022	1.16E-11	C1_CD8_Tres
chr4-108071859-108072789	1.77E-16	0.439694115	0.165	0.043	1.17E-11	C1_CD8_Tres
chr2-147288076-147288514	2.44E-16	0.434798098	0.108	0.017	1.62E-11	C1_CD8_Tres
chr21-45642617-45643885	2.59E-16	0.368796626	0.392	0.201	1.72E-11	C1_CD8_Tres
chr3-31213468-31214195	3.04E-16	0.40289421	0.135	0.03	2.01E-11	C1_CD8_Tres
chr9-89456643-89457725	3.60E-16	0.415534351	0.18	0.058	2.38E-11	C1_CD8_Tres
chr14-98983929-98985162	3.88E-16	0.401514325	0.243	0.086	2.57E-11	C1_CD8_Tres
chr22-31278702-31279429	3.98E-16	0.421034576	0.165	0.046	2.63E-11	C1_CD8_Tres
chr12-56291386-56292107	6.16E-16	0.421580259	0.135	0.03	4.08E-11	C1_CD8_Tres
chr11-133944712-133945688	6.75E-16	0.357961486	0.437	0.253	4.47E-11	C1_CD8_Tres

chr19-50328720-50329660	6.98E-16	0.363165942	0.395	0.209	4.62E-11	C1_CD8_Tres
chr12-31736324-31736891	9.14E-16	0.41087356	0.251	0.094	6.06E-11	C1_CD8_Tres
chr2-147297569-147298175	1.06E-15	0.397714694	0.102	0.018	7.01E-11	C1_CD8_Tres
chrX-8987543-8988639	1.47E-15	0.412733453	0.114	0.021	9.70E-11	C1_CD8_Tres
chr1-235206595-235207509	1.48E-15	0.400374015	0.237	0.098	9.82E-11	C1_CD8_Tres
chr4-38160544-38161718	1.65E-15	0.410656685	0.219	0.08	1.10E-10	C1_CD8_Tres
chr17-80791257-80792212	1.98E-15	0.398169922	0.12	0.025	1.31E-10	C1_CD8_Tres
chr5-137737372-137738112	3.42E-15	0.40983031	0.129	0.029	2.26E-10	C1_CD8_Tres
chr5-174454197-174454784	3.79E-15	0.366321889	0.069	0.006	2.51E-10	C1_CD8_Tres
chr14-54773156-54774273	3.85E-15	0.397139848	0.269	0.108	2.55E-10	C1_CD8_Tres
chr3-33022269-33022699	4.19E-15	0.362932554	0.063	0.005	2.77E-10	C1_CD8_Tres
chr21-31326510-31327226	4.79E-15	0.385287467	0.081	0.01	3.17E-10	C1_CD8_Tres
chr15-82286287-82287632	5.10E-15	0.379867854	0.099	0.015	3.38E-10	C1_CD8_Tres
chr3-31377041-31377469	6.87E-15	0.390106549	0.084	0.012	4.55E-10	C1_CD8_Tres
chr8-27897108-27897853	1.43E-14	0.376987469	0.096	0.015	9.48E-10	C1_CD8_Tres
chr4-108166096-108167509	1.83E-14	0.383134564	0.228	0.083	1.21E-09	C1_CD8_Tres
chr6-73391601-73391958	1.90E-14	0.363326488	0.081	0.009	1.26E-09	C1_CD8_Tres
chr10-67612614-67613590	1.97E-14	0.402960871	0.234	0.092	1.30E-09	C1_CD8_Tres
chr10-11684325-11685479	2.27E-14	0.391993875	0.171	0.055	1.50E-09	C1_CD8_Tres
chr9-120846384-120846831	2.71E-14	0.395531059	0.171	0.053	1.80E-09	C1_CD8_Tres
chr2-206442859-206444018	2.73E-14	0.374960386	0.093	0.013	1.80E-09	C1_CD8_Tres
chr17-72592318-72592952	2.82E-14	0.342012082	0.06	0.006	1.87E-09	C1_CD8_Tres
chr2-95022886-95023505	2.92E-14	0.386311242	0.12	0.027	1.93E-09	C1_CD8_Tres
chr6-134529945-134530292	3.36E-14	0.373108682	0.075	0.007	2.23E-09	C1_CD8_Tres
chr3-39361287-39361885	3.45E-14	0.356096416	0.066	0.006	2.28E-09	C1_CD8_Tres
chr22-19022702-19023241	4.89E-14	0.396685099	0.102	0.018	3.24E-09	C1_CD8_Tres
chr4-102630512-102631095	4.94E-14	0.389487407	0.132	0.034	3.27E-09	C1_CD8_Tres

chr1-37673680-37674333	6.29E-14	0.384297385	0.096	0.019	4.17E-09	C1_CD8_Tres
chr10-23340977-23341304	6.58E-14	0.359563161	0.075	0.01	4.36E-09	C1_CD8_Tres
chr3-187028971-187029250	8.18E-14	0.375007266	0.105	0.024	5.42E-09	C1_CD8_Tres
chr16-15672337-15673212	8.29E-14	0.392334543	0.165	0.049	5.49E-09	C1_CD8_Tres
chr17-72617949-72618607	8.57E-14	0.326229634	0.054	0.004	5.67E-09	C1_CD8_Tres
chr6-3370286-3371101	8.71E-14	0.351451602	0.075	0.009	5.77E-09	C1_CD8_Tres
chr15-97959959-97961285	8.85E-14	0.364677878	0.326	0.155	5.86E-09	C1_CD8_Tres
chr10-9097450-9097902	9.30E-14	0.364158758	0.081	0.013	6.16E-09	C1_CD8_Tres
chr5-134103456-134103722	1.20E-13	0.356222161	0.084	0.013	7.97E-09	C1_CD8_Tres
chr2-207165058-207167063	1.47E-13	0.347194607	0.35	0.169	9.74E-09	C1_CD8_Tres
chr15-63481072-63481443	1.51E-13	0.368990695	0.093	0.017	1.00E-08	C1_CD8_Tres
chr3-187006868-187007491	1.58E-13	0.374722692	0.084	0.012	1.05E-08	C1_CD8_Tres
chr6-45585934-45587171	1.77E-13	0.384003834	0.204	0.08	1.17E-08	C1_CD8_Tres
chr17-48194005-48194522	2.07E-13	0.334948176	0.054	0.004	1.37E-08	C1_CD8_Tres
chr11-128379351-128380164	2.21E-13	0.377012041	0.171	0.056	1.46E-08	C1_CD8_Tres
chr5-154829296-154830233	2.23E-13	0.387096294	0.15	0.038	1.48E-08	C1_CD8_Tres
chr10-470188-471014	2.26E-13	0.389134944	0.138	0.037	1.49E-08	C1_CD8_Tres
chr5-132055757-132056644	2.31E-13	0.381665913	0.207	0.079	1.53E-08	C1_CD8_Tres
chr11-129836518-129836864	2.43E-13	0.342723166	0.06	0.005	1.61E-08	C1_CD8_Tres
chr3-151282893-151283705	2.81E-13	0.360990483	0.114	0.029	1.86E-08	C1_CD8_Tres
chr17-51820146-51820617	2.92E-13	0.363620619	0.075	0.009	1.93E-08	C1_CD8_Tres
chr5-175517488-175518193	3.86E-13	0.364669497	0.171	0.06	2.56E-08	C1_CD8_Tres
chr4-98688335-98689318	4.05E-13	0.373286124	0.105	0.021	2.68E-08	C1_CD8_Tres
chr7-23020655-23021404	4.64E-13	0.38616508	0.171	0.059	3.07E-08	C1_CD8_Tres
chr1-101295211-101295547	4.77E-13	0.342560281	0.075	0.009	3.16E-08	C1_CD8_Tres
chr7-77393208-77393730	5.31E-13	0.390385199	0.138	0.038	3.52E-08	C1_CD8_Tres
chr2-106057215-106057529	5.42E-13	0.373400422	0.108	0.024	3.59E-08	C1_CD8_Tres

chr6-90456933-90457172	6.04E-13	0.307983562	0.054	0.004	4.00E-08	C1_CD8_Tres
chr21-31132453-31133490	7.33E-13	0.353937582	0.129	0.036	4.85E-08	C1_CD8_Tres
chr20-3122892-3123357	7.59E-13	0.36706854	0.099	0.02	5.02E-08	C1_CD8_Tres
chr14-75565888-75566746	7.69E-13	0.347784569	0.308	0.162	5.09E-08	C1_CD8_Tres
chr2-197052280-197052589	9.40E-13	0.308346986	0.054	0.004	6.23E-08	C1_CD8_Tres
chr7-157502480-157503684	1.00E-12	0.351200523	0.26	0.125	6.64E-08	C1_CD8_Tres
chr17-41403563-41404364	1.03E-12	0.363310149	0.165	0.056	6.84E-08	C1_CD8_Tres
chr18-58321858-58322325	1.03E-12	0.368025109	0.075	0.01	6.85E-08	C1_CD8_Tres
chr7-75920383-75920747	1.12E-12	0.364903803	0.081	0.014	7.44E-08	C1_CD8_Tres
chr3-48268119-48269543	1.23E-12	0.346476432	0.296	0.15	8.13E-08	C1_CD8_Tres
chr11-129813909-129814861	1.24E-12	0.373126234	0.12	0.03	8.18E-08	C1_CD8_Tres
chr5-35129908-35130508	1.40E-12	0.333324079	0.069	0.009	9.27E-08	C1_CD8_Tres
chr5-77155202-77155772	1.60E-12	0.344625279	0.081	0.012	1.06E-07	C1_CD8_Tres
chr13-73058336-73059823	1.91E-12	0.325817462	0.344	0.178	1.27E-07	C1_CD8_Tres
chr8-121724353-121724881	2.15E-12	0.327400529	0.063	0.006	1.42E-07	C1_CD8_Tres
chr11-64899995-64900620	2.33E-12	0.353868624	0.21	0.087	1.54E-07	C1_CD8_Tres
chr10-3668289-3669209	2.48E-12	0.348774367	0.195	0.075	1.64E-07	C1_CD8_Tres
chr1-100423433-100424332	2.76E-12	0.357690595	0.153	0.05	1.83E-07	C1_CD8_Tres
chr19-35346361-35347695	2.79E-12	0.353303832	0.144	0.046	1.85E-07	C1_CD8_Tres
chr7-5688922-5689525	2.81E-12	0.357742457	0.12	0.031	1.86E-07	C1_CD8_Tres
chr12-102059274-102059663	2.92E-12	0.364727872	0.15	0.047	1.94E-07	C1_CD8_Tres
chr10-6083475-6084035	2.99E-12	0.353592233	0.105	0.024	1.98E-07	C1_CD8_Tres
chr5-81006557-81008275	3.91E-12	0.349286887	0.189	0.067	2.59E-07	C1_CD8_Tres
chr12-89244467-89245416	4.39E-12	0.366094209	0.189	0.065	2.91E-07	C1_CD8_Tres
chr18-48455837-48456529	4.67E-12	0.332547606	0.063	0.007	3.09E-07	C1_CD8_Tres
chr3-188322747-188323755	4.86E-12	0.354539109	0.192	0.071	3.22E-07	C1_CD8_Tres
chr17-76198813-76199833	4.99E-12	0.345537729	0.177	0.066	3.30E-07	C1_CD8_Tres

chr8-11820144-11820848	5.04E-12	0.366041421	0.108	0.026	3.34E-07	C1_CD8_Tres
chr14-61569748-61570185	5.66E-12	0.35604854	0.111	0.027	3.75E-07	C1_CD8_Tres
chr22-19891000-19892614	7.40E-12	0.302472141	0.38	0.222	4.90E-07	C1_CD8_Tres
chr12-3896836-3897918	8.24E-12	0.345467508	0.108	0.028	5.45E-07	C1_CD8_Tres
chr12-15546160-15546740	9.02E-12	0.341227673	0.075	0.013	5.97E-07	C1_CD8_Tres
chr3-13879587-13880852	9.32E-12	0.350717979	0.168	0.056	6.17E-07	C1_CD8_Tres
chr1-223229945-223230727	9.78E-12	0.306049564	0.051	0.005	6.48E-07	C1_CD8_Tres
chr1-63651066-63651653	1.02E-11	0.316595441	0.066	0.007	6.73E-07	C1_CD8_Tres
chr18-62339492-62339920	1.04E-11	0.31639089	0.057	0.007	6.87E-07	C1_CD8_Tres
chr1-45246430-45247084	1.07E-11	0.321387572	0.069	0.01	7.09E-07	C1_CD8_Tres
chr17-57879920-57880394	1.12E-11	0.335429437	0.072	0.011	7.43E-07	C1_CD8_Tres
chr15-33125871-33127048	1.28E-11	0.336070956	0.102	0.023	8.49E-07	C1_CD8_Tres
chr17-77666443-77667741	1.30E-11	0.312158817	0.266	0.138	8.63E-07	C1_CD8_Tres
chr12-120248506-120248818	1.37E-11	0.337526834	0.087	0.02	9.05E-07	C1_CD8_Tres
chr4-142845779-142847546	1.60E-11	0.338388106	0.168	0.064	1.06E-06	C1_CD8_Tres
chr1-111354586-111354949	1.61E-11	0.299875836	0.051	0.004	1.07E-06	C1_CD8_Tres
chr15-63469133-63469588	1.75E-11	0.335757927	0.081	0.015	1.16E-06	C1_CD8_Tres
chr6-139160917-139162291	1.77E-11	0.307963704	0.377	0.217	1.17E-06	C1_CD8_Tres
chr16-28990309-28990992	1.80E-11	0.337291688	0.183	0.075	1.19E-06	C1_CD8_Tres
chr11-133946903-133948357	1.86E-11	0.340953744	0.186	0.071	1.23E-06	C1_CD8_Tres
chr2-189580239-189581447	1.87E-11	0.357060352	0.132	0.039	1.24E-06	C1_CD8_Tres
chr19-35207312-35207769	1.91E-11	0.33477183	0.174	0.067	1.27E-06	C1_CD8_Tres
chr20-13220538-13222122	2.00E-11	0.345490167	0.144	0.047	1.33E-06	C1_CD8_Tres
chr8-38368188-38368884	2.09E-11	0.342824201	0.129	0.036	1.39E-06	C1_CD8_Tres
chr10-6593172-6593523	2.19E-11	0.307385725	0.057	0.007	1.45E-06	C1_CD8_Tres
chr8-17026652-17027754	2.30E-11	0.337321211	0.126	0.035	1.52E-06	C1_CD8_Tres
chr20-44552832-44553759	2.36E-11	0.349476248	0.102	0.023	1.56E-06	C1_CD8_Tres

chr15-60580112-60580970	2.37E-11	0.319570729	0.249	0.126	1.57E-06	C1_CD8_Tres
chr4-185254344-185254839	2.55E-11	0.3300167	0.135	0.044	1.69E-06	C1_CD8_Tres
chr21-26169083-26171127	2.58E-11	0.333414638	0.222	0.098	1.71E-06	C1_CD8_Tres
chr7-54995679-54996310	2.62E-11	0.299560171	0.054	0.006	1.73E-06	C1_CD8_Tres
chr21-31184513-31184764	2.63E-11	0.339183338	0.087	0.017	1.74E-06	C1_CD8_Tres
chr3-115791932-115792900	2.65E-11	0.337929325	0.225	0.104	1.75E-06	C1_CD8_Tres
chr5-132687864-132689114	2.74E-11	0.338985741	0.147	0.045	1.81E-06	C1_CD8_Tres
chr5-157544451-157545007	2.80E-11	0.332658758	0.126	0.038	1.86E-06	C1_CD8_Tres
chr5-133170668-133171170	3.15E-11	0.328428158	0.069	0.011	2.09E-06	C1_CD8_Tres
chr7-50206183-50206951	3.64E-11	0.329591005	0.237	0.116	2.41E-06	C1_CD8_Tres
chr7-150478861-150479476	4.23E-11	0.337352518	0.159	0.063	2.80E-06	C1_CD8_Tres
chr6-37289803-37290395	4.49E-11	0.317314357	0.081	0.016	2.97E-06	C1_CD8_Tres
chr22-40224483-40225060	4.53E-11	0.339344054	0.087	0.019	3.00E-06	C1_CD8_Tres
chr2-174778243-174779056	4.97E-11	0.328175777	0.195	0.084	3.29E-06	C1_CD8_Tres
chr10-17425918-17426234	5.00E-11	0.326839081	0.075	0.013	3.31E-06	C1_CD8_Tres
chr7-134710579-134711526	5.05E-11	0.341455232	0.201	0.084	3.34E-06	C1_CD8_Tres
chr6-35718831-35719415	5.07E-11	0.310592063	0.069	0.012	3.36E-06	C1_CD8_Tres
chr5-56736925-56737623	5.17E-11	0.296286331	0.066	0.01	3.42E-06	C1_CD8_Tres
chr5-142088214-142089105	5.17E-11	0.326543241	0.105	0.028	3.42E-06	C1_CD8_Tres
chr5-134964405-134964998	5.33E-11	0.298762779	0.054	0.006	3.53E-06	C1_CD8_Tres
chr6-166942461-166943155	6.29E-11	0.330140965	0.078	0.015	4.16E-06	C1_CD8_Tres
chr2-135960140-135961219	6.79E-11	0.315096315	0.09	0.021	4.50E-06	C1_CD8_Tres
chr3-52982868-52983957	6.94E-11	0.321001344	0.141	0.049	4.59E-06	C1_CD8_Tres
chr19-12289014-12289628	7.17E-11	0.33373532	0.174	0.073	4.75E-06	C1_CD8_Tres
chr3-112071979-112072769	7.39E-11	0.324506657	0.096	0.024	4.89E-06	C1_CD8_Tres
chrX-48714555-48715412	7.63E-11	0.327556022	0.18	0.073	5.05E-06	C1_CD8_Tres
chr9-107243581-107244128	7.79E-11	0.324829228	0.102	0.029	5.16E-06	C1_CD8_Tres

chr13-73984811-73986085	8.07E-11	0.336055945	0.126	0.036	5.34E-06	C1_CD8_Tres
chr2-43508174-43508655	9.67E-11	0.330857851	0.099	0.025	6.40E-06	C1_CD8_Tres
chr17-66330821-66331717	9.92E-11	0.326107429	0.084	0.017	6.57E-06	C1_CD8_Tres
chr13-74069847-74070979	1.03E-10	0.336530462	0.171	0.06	6.80E-06	C1_CD8_Tres
chr17-65522837-65523959	1.05E-10	0.321842627	0.111	0.03	6.95E-06	C1_CD8_Tres
chr1-116933262-116934132	1.17E-10	0.329585514	0.138	0.046	7.72E-06	C1_CD8_Tres
chr5-65796812-65797221	1.17E-10	0.296017257	0.057	0.006	7.74E-06	C1_CD8_Tres
chr2-112187547-112188686	1.17E-10	0.282433138	0.389	0.24	7.78E-06	C1_CD8_Tres
chr1-181395947-181396693	1.19E-10	0.3248826	0.108	0.028	7.89E-06	C1_CD8_Tres
chr9-35844908-35845230	1.22E-10	0.301298878	0.075	0.013	8.11E-06	C1_CD8_Tres
chr11-128479179-128479398	1.23E-10	0.322620217	0.105	0.03	8.17E-06	C1_CD8_Tres
chr2-147843718-147845658	1.36E-10	0.297675881	0.275	0.142	9.04E-06	C1_CD8_Tres
chr11-122697418-122698052	1.41E-10	0.334346469	0.132	0.049	9.32E-06	C1_CD8_Tres
chr1-87449314-87449987	1.45E-10	0.314069089	0.06	0.008	9.57E-06	C1_CD8_Tres
chr10-8136831-8138196	1.57E-10	0.32038441	0.117	0.035	1.04E-05	C1_CD8_Tres
chr15-92813428-92813750	1.58E-10	0.328248375	0.141	0.049	1.05E-05	C1_CD8_Tres
chr6-3869841-3870317	1.61E-10	0.321365648	0.075	0.014	1.07E-05	C1_CD8_Tres
chr15-90061916-90062642	1.67E-10	0.313522371	0.075	0.015	1.11E-05	C1_CD8_Tres
chr12-51024366-51025339	1.79E-10	0.309779253	0.228	0.105	1.19E-05	C1_CD8_Tres
chr10-33008542-33009501	1.85E-10	0.327204429	0.186	0.077	1.22E-05	C1_CD8_Tres
chr12-124636086-124636573	2.00E-10	0.328773581	0.093	0.023	1.33E-05	C1_CD8_Tres
chr10-9010061-9010417	2.01E-10	0.287472461	0.057	0.007	1.33E-05	C1_CD8_Tres
chr17-1871661-1872163	2.02E-10	0.311370015	0.087	0.02	1.34E-05	C1_CD8_Tres
chr15-66911780-66912419	2.08E-10	0.307312367	0.075	0.013	1.38E-05	C1_CD8_Tres
chr4-8228336-8229641	2.42E-10	0.327054814	0.192	0.081	1.60E-05	C1_CD8_Tres
chr17-74012406-74012712	2.52E-10	0.283973742	0.054	0.006	1.67E-05	C1_CD8_Tres
chr12-124469301-124469836	2.61E-10	0.323023036	0.132	0.043	1.73E-05	C1_CD8_Tres

chr11-121459423-121459742	2.80E-10	0.289465286	0.057	0.008	1.86E-05	C1_CD8_Tres
chr10-74194549-74194880	3.01E-10	0.322265208	0.084	0.02	1.99E-05	C1_CD8_Tres
chr11-102360573-102362042	3.47E-10	0.316835862	0.135	0.046	2.29E-05	C1_CD8_Tres
chr2-147182124-147182640	3.89E-10	0.327605625	0.096	0.024	2.58E-05	C1_CD8_Tres
chr3-5019459-5019917	4.05E-10	0.31289961	0.156	0.061	2.68E-05	C1_CD8_Tres
chr1-92951077-92951575	4.28E-10	0.318260359	0.081	0.018	2.83E-05	C1_CD8_Tres
chr2-12492984-12493465	4.39E-10	0.291127878	0.057	0.008	2.91E-05	C1_CD8_Tres
chr9-75889731-75891611	4.40E-10	0.325201631	0.153	0.058	2.91E-05	C1_CD8_Tres
chr12-124599025-124599586	4.44E-10	0.295142044	0.06	0.009	2.94E-05	C1_CD8_Tres
chr5-56328552-56329400	4.53E-10	0.310198928	0.096	0.024	3.00E-05	C1_CD8_Tres
chr7-63925380-63926794	4.58E-10	0.296874119	0.314	0.177	3.03E-05	C1_CD8_Tres
chr14-64698882-64699230	4.77E-10	0.2948807	0.066	0.011	3.16E-05	C1_CD8_Tres
chr2-37243101-37244262	6.13E-10	0.295064103	0.066	0.011	4.06E-05	C1_CD8_Tres
chr4-184792913-184793389	6.37E-10	0.322083812	0.099	0.028	4.22E-05	C1_CD8_Tres
chrX-150574856-150575791	6.37E-10	0.313334849	0.129	0.051	4.22E-05	C1_CD8_Tres
chr10-97851783-97851989	6.44E-10	0.285492677	0.069	0.013	4.26E-05	C1_CD8_Tres
chr2-207143134-207144070	6.48E-10	0.304888192	0.093	0.023	4.29E-05	C1_CD8_Tres
chr2-109613514-109614698	6.68E-10	0.318794198	0.153	0.051	4.43E-05	C1_CD8_Tres
chr2-38387732-38388827	7.13E-10	0.303652372	0.093	0.025	4.72E-05	C1_CD8_Tres
chr9-38620001-38622123	7.34E-10	0.307067721	0.162	0.067	4.86E-05	C1_CD8_Tres
chr3-112134742-112135071	7.52E-10	0.310130978	0.078	0.016	4.98E-05	C1_CD8_Tres
chr14-32102108-32102841	7.56E-10	0.309491189	0.084	0.021	5.01E-05	C1_CD8_Tres
chr17-64929501-64930067	7.64E-10	0.327955845	0.129	0.042	5.06E-05	C1_CD8_Tres
chr16-29827065-29827442	8.78E-10	0.291398318	0.072	0.014	5.82E-05	C1_CD8_Tres
chr16-50708575-50709338	9.63E-10	0.297840365	0.069	0.014	6.38E-05	C1_CD8_Tres
chr3-187027208-187027918	9.66E-10	0.308263056	0.186	0.084	6.39E-05	C1_CD8_Tres
chr1-168419981-168420510	9.85E-10	0.305297469	0.099	0.029	6.52E-05	C1_CD8_Tres

chr13-98998744-98999348	1.03E-09	0.303987757	0.111	0.031	6.85E-05	C1_CD8_Tres
chr8-10332567-10335076	1.07E-09	0.308604212	0.186	0.077	7.08E-05	C1_CD8_Tres
chr13-45395720-45396421	1.10E-09	0.312303075	0.105	0.032	7.30E-05	C1_CD8_Tres
chr17-51427266-51428214	1.16E-09	0.305951712	0.099	0.029	7.68E-05	C1_CD8_Tres
chr5-14413293-14413754	1.16E-09	0.30263513	0.099	0.025	7.69E-05	C1_CD8_Tres
chr1-40014173-40014678	1.20E-09	0.301555372	0.096	0.025	7.95E-05	C1_CD8_Tres
chr13-109779026-109779436	1.20E-09	0.290269262	0.084	0.019	7.97E-05	C1_CD8_Tres
chr4-84582300-84583857	1.26E-09	0.304464173	0.228	0.104	8.33E-05	C1_CD8_Tres
chr1-61736160-61736819	1.26E-09	0.288656773	0.072	0.013	8.34E-05	C1_CD8_Tres
chr4-8221373-8221947	1.29E-09	0.303165094	0.123	0.041	8.54E-05	C1_CD8_Tres
chr17-14200930-14201936	1.31E-09	0.304662691	0.219	0.108	8.70E-05	C1_CD8_Tres
chr14-104389873-104390222	1.32E-09	0.275780734	0.06	0.009	8.74E-05	C1_CD8_Tres
chr2-95022314-95022602	1.41E-09	0.315239141	0.099	0.026	9.32E-05	C1_CD8_Tres
chr6-143549556-143549930	1.60E-09	0.306345696	0.096	0.026	0.000105877	C1_CD8_Tres
chr12-104485330-104486113	1.61E-09	0.309805335	0.159	0.068	0.000106526	C1_CD8_Tres
chr4-108433425-108434172	1.61E-09	0.283520677	0.069	0.014	0.000106794	C1_CD8_Tres
chr1-67674051-67674973	1.63E-09	0.301466661	0.102	0.027	0.000108036	C1_CD8_Tres
chr12-123447987-123448777	1.64E-09	0.295150673	0.231	0.115	0.000108616	C1_CD8_Tres
chr9-99112799-99113629	1.74E-09	0.30959464	0.123	0.045	0.000115521	C1_CD8_Tres
chr9-35910837-35911183	1.79E-09	0.282629206	0.063	0.012	0.000118285	C1_CD8_Tres
chr12-21656738-21658116	1.86E-09	0.25213382	0.437	0.294	0.00012336	C1_CD8_Tres
chr4-108165590-108165834	1.88E-09	0.288108791	0.081	0.02	0.000124422	C1_CD8_Tres
chr2-147293737-147294390	1.92E-09	0.265456648	0.06	0.011	0.000127298	C1_CD8_Tres
chr10-118698094-118698841	1.93E-09	0.299718388	0.228	0.113	0.00012791	C1_CD8_Tres
chr22-35551999-35552992	1.98E-09	0.29593426	0.111	0.035	0.000131332	C1_CD8_Tres
chr1-169692366-169693382	2.58E-09	0.298169009	0.207	0.098	0.000170548	C1_CD8_Tres
chr6-149488799-149489139	2.90E-09	0.304119793	0.111	0.035	0.00019213	C1_CD8_Tres

chr11-13940393-13940985	2.94E-09	0.285674851	0.06	0.012	0.000194953	C1_CD8_Tres
chr18-62329615-62329886	3.02E-09	0.279778183	0.054	0.008	0.00020011	C1_CD8_Tres
chr15-60720228-60721993	3.19E-09	0.284195753	0.266	0.139	0.000211463	C1_CD8_Tres
chr3-172530163-172530949	3.20E-09	0.303113109	0.096	0.024	0.000211591	C1_CD8_Tres
chr3-32313450-32314223	3.47E-09	0.289083179	0.09	0.025	0.00023011	C1_CD8_Tres
chr6-16723769-16724222	3.70E-09	0.288869706	0.093	0.027	0.000244841	C1_CD8_Tres
chr22-40728755-40729037	3.86E-09	0.269159411	0.06	0.012	0.000255435	C1_CD8_Tres
chr13-40272434-40273062	4.00E-09	0.278075876	0.069	0.014	0.000264743	C1_CD8_Tres
chr13-30654090-30654855	4.12E-09	0.300493846	0.111	0.037	0.000273084	C1_CD8_Tres
chr4-153205070-153206058	4.13E-09	0.291165444	0.078	0.016	0.000273526	C1_CD8_Tres
chr22-30104471-30104817	4.18E-09	0.258937807	0.054	0.008	0.00027706	C1_CD8_Tres
chr17-82200599-82201168	4.23E-09	0.292912884	0.189	0.089	0.000280008	C1_CD8_Tres
chr1-240666520-240666960	4.27E-09	0.265565591	0.051	0.008	0.000282517	C1_CD8_Tres
chr12-21615425-21615792	4.58E-09	0.283688877	0.069	0.014	0.000303391	C1_CD8_Tres
chr11-4090275-4090505	4.70E-09	0.293643019	0.081	0.018	0.000311097	C1_CD8_Tres
chr19-41774440-41775003	4.83E-09	0.281699782	0.087	0.023	0.000319701	C1_CD8_Tres
chr2-157897177-157899524	4.94E-09	0.277130766	0.222	0.113	0.000327128	C1_CD8_Tres
chr1-235734155-235734667	4.98E-09	0.285407344	0.072	0.018	0.00032977	C1_CD8_Tres
chr2-39216328-39217463	5.32E-09	0.275806726	0.293	0.163	0.000352246	C1_CD8_Tres
chr2-239326744-239327330	5.35E-09	0.299254087	0.153	0.062	0.000353999	C1_CD8_Tres
chr2-230905310-230905853	5.49E-09	0.300424154	0.123	0.044	0.000363444	C1_CD8_Tres
chr11-44596293-44597264	5.53E-09	0.293558206	0.147	0.055	0.00036631	C1_CD8_Tres
chr2-191739699-191740487	5.76E-09	0.303116944	0.165	0.07	0.000381309	C1_CD8_Tres
chr12-3947462-3948089	5.78E-09	0.267227119	0.066	0.014	0.000383087	C1_CD8_Tres
chr3-25911126-25911920	5.81E-09	0.280758923	0.057	0.009	0.000384697	C1_CD8_Tres
chr6-90239368-90239862	6.10E-09	0.278104609	0.072	0.016	0.000404084	C1_CD8_Tres
chr10-5548637-5549246	6.22E-09	0.303246675	0.117	0.04	0.000411919	C1_CD8_Tres

chr17-35238762-35239105	6.51E-09	0.28742887	0.108	0.038	0.000431106	C1_CD8_Tres
chr2-63855841-63856696	6.71E-09	0.282101884	0.213	0.108	0.000444626	C1_CD8_Tres
chr3-115790540-115791139	6.88E-09	0.290946397	0.198	0.092	0.000455391	C1_CD8_Tres
chr13-48653150-48654064	7.15E-09	0.290478829	0.171	0.077	0.000473287	C1_CD8_Tres
chr8-127196582-127197223	7.23E-09	0.293129533	0.09	0.024	0.000478466	C1_CD8_Tres
chr15-43151602-43152007	7.37E-09	0.283016136	0.075	0.016	0.000487778	C1_CD8_Tres
chr5-111530075-111530703	7.65E-09	0.284952397	0.201	0.1	0.000506367	C1_CD8_Tres
chr3-16468233-16468682	7.72E-09	0.253350377	0.051	0.006	0.000511475	C1_CD8_Tres
chr3-189355348-189356273	8.28E-09	0.286715932	0.225	0.111	0.000548486	C1_CD8_Tres
chr2-143123982-143124199	8.30E-09	0.261168555	0.054	0.008	0.000549806	C1_CD8_Tres
chr15-65460892-65461294	8.62E-09	0.26608737	0.051	0.009	0.000570951	C1_CD8_Tres
chr6-37209460-37210717	9.10E-09	0.275775273	0.159	0.079	0.000602924	C1_CD8_Tres
chr15-85316161-85316734	9.32E-09	0.283096986	0.087	0.023	0.00061693	C1_CD8_Tres
chr3-31212741-31213107	9.67E-09	0.29091597	0.075	0.017	0.000640346	C1_CD8_Tres
chr2-12554705-12555502	9.79E-09	0.291458061	0.105	0.034	0.000648554	C1_CD8_Tres
chr7-5833513-5834769	1.04E-08	0.285706933	0.072	0.016	0.000687564	C1_CD8_Tres
chr5-35830501-35830963	1.06E-08	0.268312441	0.072	0.016	0.000704708	C1_CD8_Tres
chr1-210446098-210447006	1.11E-08	0.290733497	0.192	0.092	0.000734878	C1_CD8_Tres
chr5-80140182-80140705	1.16E-08	0.2588144	0.057	0.01	0.000765231	C1_CD8_Tres
chr11-35216770-35217513	1.21E-08	0.273891241	0.21	0.105	0.000801823	C1_CD8_Tres
chr3-150722776-150723110	1.22E-08	0.275940325	0.075	0.02	0.000806197	C1_CD8_Tres
chr3-112132950-112133478	1.25E-08	0.28500721	0.108	0.033	0.000828771	C1_CD8_Tres
chr6-45488766-45489199	1.28E-08	0.272298836	0.066	0.014	0.000849328	C1_CD8_Tres
chr2-226423204-226423867	1.36E-08	0.261546101	0.054	0.01	0.000898587	C1_CD8_Tres
chr1-61782618-61783124	1.38E-08	0.283453854	0.108	0.04	0.000912584	C1_CD8_Tres
chr6-35331999-35332596	1.55E-08	0.295440152	0.165	0.071	0.001028539	C1_CD8_Tres
chr11-47234057-47235190	1.56E-08	0.287879886	0.138	0.058	0.001032875	C1_CD8_Tres

chr1-23148051-23148443	1.64E-08	0.263298395	0.063	0.013	0.00108305	C1_CD8_Tres
chr7-1046744-1046983	1.65E-08	0.267365597	0.066	0.014	0.001095616	C1_CD8_Tres
chr1-8700968-8701774	1.72E-08	0.270975774	0.21	0.11	0.001136177	C1_CD8_Tres
chr3-195524112-195524916	1.72E-08	0.274843518	0.093	0.028	0.001141437	C1_CD8_Tres
chr1-223363515-223364829	1.73E-08	0.286412271	0.105	0.031	0.001148018	C1_CD8_Tres
chr5-77379400-77380032	1.76E-08	0.277856699	0.075	0.016	0.001165416	C1_CD8_Tres
chr5-80487743-80488360	1.95E-08	0.26066504	0.075	0.018	0.001289665	C1_CD8_Tres
chr14-31947518-31948078	2.01E-08	0.262159104	0.054	0.01	0.001330115	C1_CD8_Tres
chr21-36145297-36145547	2.03E-08	0.261912031	0.06	0.012	0.001342909	C1_CD8_Tres
chr22-24284770-24285679	2.04E-08	0.272655428	0.099	0.035	0.001353975	C1_CD8_Tres
chr14-76977783-76978337	2.23E-08	0.288229888	0.111	0.044	0.001474127	C1_CD8_Tres
chr12-12006921-12007387	2.44E-08	0.279692203	0.246	0.133	0.001615242	C1_CD8_Tres
chr12-122646630-122647161	2.48E-08	0.278829135	0.102	0.04	0.001640041	C1_CD8_Tres
chr11-128346831-128347091	2.53E-08	0.269952829	0.078	0.022	0.001677886	C1_CD8_Tres
chr11-2076881-2077764	2.65E-08	0.275863941	0.102	0.032	0.00175244	C1_CD8_Tres
chr15-33091957-33092571	2.66E-08	0.263269631	0.057	0.011	0.0017642	C1_CD8_Tres
chr11-6693875-6694286	2.76E-08	0.278860698	0.096	0.033	0.00182854	C1_CD8_Tres
chr10-33248292-33248999	2.90E-08	0.275434455	0.183	0.085	0.001921508	C1_CD8_Tres
chr20-47727109-47727940	3.00E-08	0.293693857	0.153	0.066	0.001987754	C1_CD8_Tres
chr12-91978021-91978778	3.10E-08	0.279799434	0.099	0.032	0.002055154	C1_CD8_Tres
chr2-239164246-239164821	3.21E-08	0.26309386	0.06	0.013	0.002129014	C1_CD8_Tres
chr17-41338688-41339538	3.22E-08	0.264565752	0.249	0.143	0.002130776	C1_CD8_Tres
chr10-33114380-33114979	3.22E-08	0.278925197	0.168	0.079	0.002135646	C1_CD8_Tres
chr20-13230872-13231669	3.27E-08	0.276369869	0.072	0.016	0.002166286	C1_CD8_Tres
chr10-88193938-88194699	3.32E-08	0.283036274	0.114	0.041	0.002198178	C1_CD8_Tres
chr18-70781711-70782229	3.37E-08	0.271151769	0.066	0.015	0.002232813	C1_CD8_Tres
chr1-12510196-12511175	3.65E-08	0.272750393	0.219	0.121	0.002419754	C1_CD8_Tres

chr1-154418172-154418597	3.93E-08	0.250375566	0.06	0.012	0.002603802	C1_CD8_Tres
chr22-20557024-20558072	3.95E-08	0.271330955	0.183	0.094	0.002617037	C1_CD8_Tres
chr3-31076939-31077427	3.97E-08	0.260479874	0.06	0.011	0.002626048	C1_CD8_Tres
chr2-170651625-170652027	3.99E-08	0.275405752	0.063	0.014	0.00264291	C1_CD8_Tres
chr2-69600468-69601507	4.03E-08	0.282976123	0.117	0.044	0.002667082	C1_CD8_Tres
chr2-171318734-171319646	4.32E-08	0.281347584	0.144	0.054	0.002860362	C1_CD8_Tres
chr12-104497402-104497980	4.44E-08	0.259990779	0.078	0.021	0.002939951	C1_CD8_Tres
chr4-102627761-102628377	4.57E-08	0.28030683	0.117	0.044	0.00302498	C1_CD8_Tres
chr7-45013349-45013764	4.70E-08	0.252242538	0.051	0.009	0.003112814	C1_CD8_Tres
chr12-89663546-89664413	4.84E-08	0.277503569	0.126	0.049	0.003204066	C1_CD8_Tres
chr1-28971976-28972791	4.85E-08	0.277102678	0.111	0.041	0.003210163	C1_CD8_Tres
chrX-17736504-17738390	5.11E-08	0.270721002	0.171	0.08	0.003385311	C1_CD8_Tres
chr16-75380194-75380976	5.18E-08	0.266336583	0.072	0.016	0.003428378	C1_CD8_Tres
chr10-103803952-103804323	5.32E-08	0.266047973	0.084	0.024	0.003524461	C1_CD8_Tres
chr20-36988410-36988905	5.48E-08	0.258157777	0.057	0.014	0.003626462	C1_CD8_Tres
chr15-65828358-65829440	5.60E-08	0.258305776	0.186	0.091	0.003706188	C1_CD8_Tres
chr1-169708021-169708604	5.73E-08	0.253020397	0.054	0.011	0.003794155	C1_CD8_Tres
chr5-67161007-67161300	6.07E-08	0.27154418	0.093	0.029	0.004016837	C1_CD8_Tres
chr1-1371151-1372325	6.23E-08	0.254811679	0.228	0.134	0.004128522	C1_CD8_Tres
chr18-24075543-24076605	6.24E-08	0.261012374	0.272	0.176	0.004134984	C1_CD8_Tres
chr4-122577849-122579037	6.27E-08	0.267096403	0.24	0.13	0.004155462	C1_CD8_Tres
chr13-76884967-76886921	6.57E-08	0.264264274	0.201	0.097	0.004350372	C1_CD8_Tres
chr7-151381343-151382371	6.65E-08	0.270578783	0.144	0.058	0.004400771	C1_CD8_Tres
chr8-122798766-122799403	6.77E-08	0.27875227	0.162	0.074	0.004481722	C1_CD8_Tres
chr2-207123347-207123803	6.85E-08	0.257786362	0.066	0.015	0.004536478	C1_CD8_Tres
chr6-134287862-134289224	6.98E-08	0.273170533	0.129	0.049	0.004623319	C1_CD8_Tres
chr22-29994026-29994715	6.99E-08	0.274298134	0.087	0.026	0.00463213	C1_CD8_Tres

chr14-64673394-64674157	7.16E-08	0.275037811	0.108	0.036	0.004743883	C1_CD8_Tres
chr1-181100535-181100791	7.32E-08	0.253238051	0.072	0.018	0.004846425	C1_CD8_Tres
chr4-122221248-122221713	7.48E-08	0.262814687	0.069	0.016	0.004956676	C1_CD8_Tres
chr14-95462999-95463674	7.55E-08	0.2688587	0.096	0.029	0.005002765	C1_CD8_Tres
chr7-129906470-129907126	7.76E-08	0.251809351	0.063	0.014	0.005137777	C1_CD8_Tres
chr14-31944184-31945344	7.76E-08	0.268999492	0.072	0.017	0.005139351	C1_CD8_Tres
chr2-98807095-98807581	8.06E-08	0.273604642	0.099	0.034	0.005338215	C1_CD8_Tres
chr6-157860198-157860792	8.43E-08	0.26627343	0.105	0.037	0.005582767	C1_CD8_Tres
chr14-76844045-76844599	8.66E-08	0.266945609	0.078	0.022	0.00573504	C1_CD8_Tres
chr20-62501785-62502282	8.69E-08	0.279993163	0.153	0.063	0.005754404	C1_CD8_Tres
chr4-108118459-108118922	8.73E-08	0.272721145	0.138	0.057	0.00578118	C1_CD8_Tres
chr16-29007977-29008363	8.75E-08	0.266902157	0.123	0.05	0.005791837	C1_CD8_Tres
chr22-36887085-36887532	8.88E-08	0.251259594	0.057	0.011	0.005879959	C1_CD8_Tres
chr1-89607200-89607466	9.03E-08	0.267721037	0.153	0.065	0.005978163	C1_CD8_Tres
chr14-91980024-91980540	9.03E-08	0.275302536	0.087	0.026	0.005979801	C1_CD8_Tres
chr1-43453152-43454832	9.36E-08	0.252399399	0.189	0.1	0.006197362	C1_CD8_Tres
chr4-122507988-122508404	9.37E-08	0.254287322	0.057	0.011	0.006204474	C1_CD8_Tres
chr1-89870891-89871236	9.48E-08	0.265398117	0.063	0.013	0.00627596	C1_CD8_Tres
chr9-120890146-120890767	9.80E-08	0.272933319	0.15	0.068	0.006488035	C1_CD8_Tres
chr11-122580325-122581444	1.00E-07	0.25744521	0.171	0.087	0.006644484	C1_CD8_Tres
chr1-223248405-223249194	1.03E-07	0.275454798	0.066	0.017	0.006829597	C1_CD8_Tres
chrX-39309110-39310066	1.03E-07	0.261051316	0.084	0.027	0.006840988	C1_CD8_Tres
chr18-77140699-77141089	1.05E-07	0.252782841	0.063	0.013	0.006924654	C1_CD8_Tres
chr17-66667209-66667775	1.08E-07	0.256963757	0.084	0.026	0.007120655	C1_CD8_Tres
chr17-36673842-36674562	1.08E-07	0.250736885	0.072	0.02	0.007181606	C1_CD8_Tres
chr4-99507514-99508118	1.09E-07	0.259515522	0.06	0.013	0.007221213	C1_CD8_Tres
chr10-14506919-14507504	1.11E-07	0.261761318	0.078	0.025	0.007329087	C1_CD8_Tres

chr11-62853215-62853866	1.13E-07	0.257469008	0.144	0.073	0.007494667	C1_CD8_Tres
chr2-157463896-157464634	1.18E-07	0.263826312	0.153	0.068	0.007804596	C1_CD8_Tres
chr3-113005299-113006026	1.19E-07	0.262257757	0.099	0.035	0.007857703	C1_CD8_Tres
chr11-6733322-6733997	1.22E-07	0.267524094	0.144	0.066	0.008084592	C1_CD8_Tres
chr19-12635499-12635851	1.22E-07	0.260953706	0.081	0.025	0.008088176	C1_CD8_Tres
chr1-24506618-24507685	1.29E-07	0.258733961	0.174	0.086	0.008510238	C1_CD8_Tres
chr2-197869407-197870296	1.29E-07	0.272590707	0.108	0.037	0.008528245	C1_CD8_Tres
chr11-118877972-118878403	1.34E-07	0.255986182	0.081	0.024	0.008850638	C1_CD8_Tres
chr8-19438281-19438790	1.38E-07	0.253714175	0.066	0.017	0.009113578	C1_CD8_Tres
chrX-21373624-21375029	1.41E-07	0.258484059	0.099	0.035	0.009308686	C1_CD8_Tres
chr16-11263657-11264190	1.44E-07	0.256933321	0.084	0.026	0.009530354	C1_CD8_Tres
chr1-89855439-89856103	1.45E-07	0.26351894	0.138	0.059	0.009596152	C1_CD8_Tres
chr11-32420625-32421412	1.46E-07	0.254909768	0.063	0.014	0.009650821	C1_CD8_Tres
chr21-42002368-42003339	1.50E-07	0.269450433	0.15	0.065	0.009963822	C1_CD8_Tres
chr3-161349586-161350276	1.57E-07	0.266283791	0.132	0.058	0.010424725	C1_CD8_Tres
chr1-232923575-232924398	1.59E-07	0.268834092	0.12	0.052	0.010557335	C1_CD8_Tres
chr11-11149350-11150552	1.60E-07	0.266368098	0.231	0.118	0.010599823	C1_CD8_Tres
chr16-996722-998093	1.62E-07	0.261029024	0.171	0.084	0.010709837	C1_CD8_Tres
chr8-125272893-125274165	1.63E-07	0.265436511	0.183	0.086	0.010761756	C1_CD8_Tres
chr7-76153463-76153892	1.68E-07	0.256920152	0.063	0.015	0.011097083	C1_CD8_Tres
chr15-41970026-41970356	1.72E-07	0.251570711	0.078	0.024	0.011412579	C1_CD8_Tres
chr14-91310500-91311575	1.78E-07	0.264548287	0.177	0.087	0.011806407	C1_CD8_Tres
chr10-5563994-5565107	1.79E-07	0.264540553	0.108	0.04	0.011879015	C1_CD8_Tres
chr5-14415267-14416028	1.81E-07	0.262584692	0.093	0.032	0.012008932	C1_CD8_Tres
chr8-60792613-60793213	1.85E-07	0.254922103	0.171	0.082	0.012282443	C1_CD8_Tres
chr1-31771868-31772831	1.89E-07	0.260978842	0.189	0.09	0.012496424	C1_CD8_Tres
chr10-110750898-110751288	1.89E-07	0.250483374	0.06	0.013	0.012540434	C1_CD8_Tres

chr13-102779324-102780127	1.96E-07	0.258400778	0.093	0.031	0.012955491	C1_CD8_Tres
chr13-40186805-40188189	1.96E-07	0.251008466	0.231	0.128	0.01300417	C1_CD8_Tres
chr5-173781685-173782434	2.10E-07	0.267049188	0.135	0.055	0.013903914	C1_CD8_Tres
chr6-135013920-135014846	2.10E-07	0.263485249	0.165	0.07	0.013915991	C1_CD8_Tres
chr14-102527467-102527755	2.11E-07	0.253110495	0.063	0.015	0.014002318	C1_CD8_Tres
chr4-23333542-23334547	2.27E-07	0.261781779	0.195	0.105	0.015022898	C1_CD8_Tres
chr9-93091520-93091803	2.27E-07	0.268790117	0.09	0.031	0.015046768	C1_CD8_Tres
chr2-101311084-101311634	2.28E-07	0.25761217	0.099	0.039	0.015072952	C1_CD8_Tres
chr15-85357893-85358269	2.32E-07	0.257598342	0.072	0.02	0.015336138	C1_CD8_Tres
chr8-119638361-119639273	2.39E-07	0.260020101	0.21	0.109	0.015830528	C1_CD8_Tres
chr4-38949216-38950048	2.43E-07	0.263433852	0.147	0.071	0.016113645	C1_CD8_Tres
chr4-109648192-109649022	2.46E-07	0.250882921	0.183	0.097	0.016316312	C1_CD8_Tres
chr12-50597329-50597819	2.47E-07	0.266555788	0.102	0.04	0.0163324	C1_CD8_Tres
chr5-143434366-143435536	2.50E-07	0.259005645	0.156	0.068	0.016579892	C1_CD8_Tres
chr21-33268575-33268995	2.56E-07	0.25577453	0.072	0.02	0.016939594	C1_CD8_Tres
chr5-1105490-1106169	2.58E-07	0.26031055	0.162	0.079	0.017063126	C1_CD8_Tres
chr12-4506471-4507325	2.74E-07	0.263955557	0.195	0.093	0.018144527	C1_CD8_Tres
chr5-175895298-175896056	2.80E-07	0.2566795	0.096	0.033	0.018560424	C1_CD8_Tres
chr7-138903069-138903853	2.94E-07	0.262852736	0.123	0.046	0.019443066	C1_CD8_Tres
chr17-78772479-78773053	3.00E-07	0.259039154	0.12	0.053	0.019876009	C1_CD8_Tres
chr15-85318007-85318839	3.03E-07	0.266782751	0.135	0.056	0.020042431	C1_CD8_Tres
chr9-109110153-109111048	3.08E-07	0.258952	0.15	0.071	0.020391366	C1_CD8_Tres
chr2-109143616-109144570	3.18E-07	0.259152561	0.102	0.035	0.021073107	C1_CD8_Tres
chr1-169604630-169605193	3.32E-07	0.254795176	0.072	0.02	0.021973737	C1_CD8_Tres
chr20-56411601-56412587	3.38E-07	0.257594727	0.162	0.07	0.022396604	C1_CD8_Tres
chr19-16368879-16369329	3.62E-07	0.255062026	0.165	0.081	0.023985509	C1_CD8_Tres
chr10-60727760-60728404	3.62E-07	0.258357447	0.147	0.065	0.023988455	C1_CD8_Tres

chrX-16889110-16889721	3.92E-07	0.253238421	0.114	0.046	0.025971532	C1_CD8_Tres
chr3-32237980-32239111	3.95E-07	0.254435482	0.171	0.086	0.02614165	C1_CD8_Tres
chrX-1205702-1206264	4.18E-07	0.250472196	0.084	0.027	0.027691428	C1_CD8_Tres
chr11-121467993-121468350	4.19E-07	0.259112162	0.126	0.053	0.027767703	C1_CD8_Tres
chr8-78515522-78516693	4.24E-07	0.251158184	0.189	0.093	0.028058042	C1_CD8_Tres
chr12-68932468-68933453	4.38E-07	0.255250712	0.126	0.055	0.029031184	C1_CD8_Tres
chr3-120344171-120344675	4.40E-07	0.258194867	0.174	0.09	0.029130576	C1_CD8_Tres
chr1-1098814-1099840	4.57E-07	0.251000807	0.093	0.031	0.030282563	C1_CD8_Tres
chr5-132059009-132059400	4.82E-07	0.263369871	0.108	0.037	0.031907286	C1_CD8_Tres
chr4-110164878-110165829	5.04E-07	0.254626207	0.15	0.074	0.033396163	C1_CD8_Tres
chr14-49911425-49911963	5.08E-07	0.250855722	0.132	0.062	0.033668399	C1_CD8_Tres
chr15-92849056-92849741	5.69E-07	0.250006887	0.138	0.066	0.037681687	C1_CD8_Tres
chr13-74230618-74231534	5.76E-07	0.251507596	0.132	0.061	0.038146555	C1_CD8_Tres
chr2-239369569-239370122	5.94E-07	0.25364041	0.156	0.07	0.039362878	C1_CD8_Tres
chr8-78525040-78526111	6.10E-07	0.250938859	0.105	0.04	0.040408965	C1_CD8_Tres
chr10-23362927-23363862	6.61E-07	0.255208859	0.123	0.049	0.0438056	C1_CD8_Tres
chr7-36285846-36286567	7.65E-07	0.250789135	0.114	0.047	0.050676483	C1_CD8_Tres
chr8-143966180-143966904	9.57E-07	0.250077276	0.093	0.032	0.063364859	C1_CD8_Tres
chr12-92055807-92056207	1.03E-06	0.250166352	0.153	0.076	0.067977008	C1_CD8_Tres
chr16-29679437-29680044	1.07E-06	0.252222763	0.135	0.059	0.070552253	C1_CD8_Tres
chr2-54903797-54904466	1.07E-06	0.252423097	0.123	0.054	0.071116871	C1_CD8_Tres
chr16-29012895-29013446	1.27E-06	0.251088665	0.105	0.044	0.084176144	C1_CD8_Tres
chr21-45642617-45643885	2.59E-16	0.368796626	0.392	0.201	1.72E-11	C1_CD8_Tres
chr19-50328720-50329660	6.98E-16	0.363165942	0.395	0.209	4.62E-11	C1_CD8_Tres
chr5-173781685-173782434	2.10E-07	0.267049188	0.135	0.055	0.013903914	C1_CD8_Tres
chr14-75565888-75566746	7.69E-13	0.347784569	0.308	0.162	5.09E-08	C1_CD8_Tres
chr14-31937565-31938397	1.96E-18	0.476841385	0.201	0.059	1.30E-13	C1_CD8_Tres

chr2-43508174-43508655	9.67E-11	0.330857851	0.099	0.025	6.40E-06	C1_CD8_Tres
chr21-33268575-33268995	2.56E-07	0.25577453	0.072	0.02	0.016939594	C1_CD8_Tres
chr12-102059274-102059663	2.92E-12	0.364727872	0.15	0.047	1.94E-07	C1_CD8_Tres
chr20-62501785-62502282	8.69E-08	0.279993163	0.153	0.063	0.005754404	C1_CD8_Tres
chr4-108118459-108118922	8.73E-08	0.272721145	0.138	0.057	0.00578118	C1_CD8_Tres
chr2-12554705-12555502	9.79E-09	0.291458061	0.105	0.034	0.000648554	C1_CD8_Tres
chr2-197869407-197870296	1.29E-07	0.272590707	0.108	0.037	0.008528245	C1_CD8_Tres
chr17-51820146-51820617	2.92E-13	0.363620619	0.075	0.009	1.93E-08	C1_CD8_Tres
chr2-201448194-201448990	3.64E-21	0.487444486	0.195	0.045	2.41E-16	C1_CD8_Tres
chr22-36887085-36887532	8.88E-08	0.251259594	0.057	0.011	0.005879959	C1_CD8_Tres
chr15-85318007-85318839	3.03E-07	0.266782751	0.135	0.056	0.020042431	C1_CD8_Tres
chrX-21373624-21375029	1.41E-07	0.258484059	0.099	0.035	0.009308686	C1_CD8_Tres
chr4-23333542-23334547	2.27E-07	0.261781779	0.195	0.105	0.015022898	C1_CD8_Tres
chr15-88904290-88905053	1.00E-20	0.449853959	0.353	0.146	6.63E-16	C1_CD8_Tres
chr22-40711144-40711920	1.73E-16	0.420539447	0.204	0.07	1.14E-11	C1_CD8_Tres
chr12-92055807-92056207	1.03E-06	0.250166352	0.153	0.076	0.067977008	C1_CD8_Tres
chr12-4506471-4507325	2.74E-07	0.263955557	0.195	0.093	0.018144527	C1_CD8_Tres
chr11-64899995-64900620	2.33E-12	0.353868624	0.21	0.087	1.54E-07	C1_CD8_Tres
chr5-142088214-142089105	5.17E-11	0.326543241	0.105	0.028	3.42E-06	C1_CD8_Tres
chr1-154418172-154418597	3.93E-08	0.250375566	0.06	0.012	0.002603802	C1_CD8_Tres
chr2-239369569-239370122	5.94E-07	0.25364041	0.156	0.07	0.039362878	C1_CD8_Tres
chr1-40014173-40014678	1.20E-09	0.301555372	0.096	0.025	7.95E-05	C1_CD8_Tres
chr22-19022702-19023241	4.89E-14	0.396685099	0.102	0.018	3.24E-09	C1_CD8_Tres
chr1-100423433-100424332	2.76E-12	0.357690595	0.153	0.05	1.83E-07	C1_CD8_Tres
chr3-187028971-187029250	8.18E-14	0.375007266	0.105	0.024	5.42E-09	C1_CD8_Tres
chr17-74012406-74012712	2.52E-10	0.283973742	0.054	0.006	1.67E-05	C1_CD8_Tres
chr15-97959959-97961285	8.85E-14	0.364677878	0.326	0.155	5.86E-09	C1_CD8_Tres

chr17-2814110-2815366	7.92E-22	0.410789209	0.497	0.259	5.24E-17	C1_CD8_Tres
chr12-51024366-51025339	1.79E-10	0.309779253	0.228	0.105	1.19E-05	C1_CD8_Tres
chr8-125272893-125274165	1.63E-07	0.265436511	0.183	0.086	0.010761756	C1_CD8_Tres
chr8-78515522-78516693	4.24E-07	0.251158184	0.189	0.093	0.028058042	C1_CD8_Tres
chr12-124469301-124469836	2.61E-10	0.323023036	0.132	0.043	1.73E-05	C1_CD8_Tres
chr21-31184513-31184764	2.63E-11	0.339183338	0.087	0.017	1.74E-06	C1_CD8_Tres
chr9-109110153-109111048	3.08E-07	0.258952	0.15	0.071	0.020391366	C1_CD8_Tres
chr5-56058012-56059238	4.53E-21	0.484032327	0.251	0.076	3.00E-16	C1_CD8_Tres
chr12-123447987-123448777	1.64E-09	0.295150673	0.231	0.115	0.000108616	C1_CD8_Tres
chr12-31736324-31736891	9.14E-16	0.41087356	0.251	0.094	6.06E-11	C1_CD8_Tres
chr5-14413293-14413754	1.16E-09	0.30263513	0.099	0.025	7.69E-05	C1_CD8_Tres
chr11-95688151-95689305	1.73E-19	0.486716829	0.159	0.033	1.14E-14	C1_CD8_Tres
chr9-120846384-120846831	2.71E-14	0.395531059	0.171	0.053	1.80E-09	C1_CD8_Tres
chr16-29012895-29013446	1.27E-06	0.251088665	0.105	0.044	0.084176144	C1_CD8_Tres
chr13-73984811-73986085	8.07E-11	0.336055945	0.126	0.036	5.34E-06	C1_CD8_Tres
chr15-97959959-97961285	8.85E-14	0.364677878	0.326	0.155	5.86E-09	C1_CD8_Tres
chr21-45642617-45643885	2.59E-16	0.368796626	0.392	0.201	1.72E-11	C1_CD8_Tres
chr2-113876400-113877141	2.88E-19	0.476183238	0.204	0.055	1.91E-14	C1_CD8_Tres
chr5-77379400-77380032	1.76E-08	0.277856699	0.075	0.016	0.001165416	C1_CD8_Tres
chr9-75889731-75891611	4.40E-10	0.325201631	0.153	0.058	2.91E-05	C1_CD8_Tres
chr17-82126395-82127458	2.46E-24	0.455969183	0.449	0.2	1.63E-19	C1_CD8_Tres
chr11-13868785-13869542	9.99E-28	0.56756676	0.263	0.063	6.61E-23	C1_CD8_Tres
chr11-102360573-102362042	3.47E-10	0.316835862	0.135	0.046	2.29E-05	C1_CD8_Tres
chr4-108071859-108072789	1.77E-16	0.439694115	0.165	0.043	1.17E-11	C1_CD8_Tres
chr1-83508052-83508796	1.65E-20	0.485690125	0.129	0.018	1.09E-15	C1_CD8_Tres
chr11-11149350-11150552	1.60E-07	0.266368098	0.231	0.118	0.010599823	C1_CD8_Tres
chr21-31354162-31355261	9.53E-46	0.721616807	0.383	0.084	6.31E-41	C1_CD8_Tres

chr8-122798766-122799403	6.77E-08	0.27875227	0.162	0.074	0.004481722	C1_CD8_Tres
chr1-1098814-1099840	4.57E-07	0.251000807	0.093	0.031	0.030282563	C1_CD8_Tres
chr12-15546160-15546740	9.02E-12	0.341227673	0.075	0.013	5.97E-07	C1_CD8_Tres
chr13-40186805-40188189	1.96E-07	0.251008466	0.231	0.128	0.01300417	C1_CD8_Tres
chr5-77155202-77155772	1.60E-12	0.344625279	0.081	0.012	1.06E-07	C1_CD8_Tres
chr15-88904290-88905053	1.00E-20	0.449853959	0.353	0.146	6.63E-16	C1_CD8_Tres
chr16-29827065-29827442	8.78E-10	0.291398318	0.072	0.014	5.82E-05	C1_CD8_Tres
chr9-120846384-120846831	2.71E-14	0.395531059	0.171	0.053	1.80E-09	C1_CD8_Tres
chr21-36288508-36289609	1.04E-30	0.588002231	0.284	0.079	6.87E-26	C1_CD8_Tres
chr2-207143134-207144070	6.48E-10	0.304888192	0.093	0.023	4.29E-05	C1_CD8_Tres
chr8-19496478-19497608	1.07E-25	0.51054402	0.404	0.153	7.08E-21	C1_CD8_Tres
chr2-239369569-239370122	5.94E-07	0.25364041	0.156	0.07	0.039362878	C1_CD8_Tres
chr12-3962350-3963101	7.49E-19	0.468873415	0.147	0.03	4.96E-14	C1_CD8_Tres
chr2-207165058-207167063	1.47E-13	0.347194607	0.35	0.169	9.74E-09	C1_CD8_Tres
chr21-31558627-31559975	1.24E-24	0.507416273	0.323	0.111	8.24E-20	C1_CD8_Tres
chr8-119638361-119639273	2.39E-07	0.260020101	0.21	0.109	0.015830528	C1_CD8_Tres
chr2-96962262-96963250	4.59E-18	0.41571145	0.302	0.124	3.04E-13	C1_CD8_Tres
chr17-75036583-75037040	5.01E-19	0.440601955	0.308	0.129	3.32E-14	C1_CD8_Tres
chr11-129853443-129854439	2.45E-22	0.526511377	0.18	0.036	1.62E-17	C1_CD8_Tres
chr3-112071979-112072769	7.39E-11	0.324506657	0.096	0.024	4.89E-06	C1_CD8_Tres
chr10-9097450-9097902	9.30E-14	0.364158758	0.081	0.013	6.16E-09	C1_CD8_Tres
chr8-60792613-60793213	1.85E-07	0.254922103	0.171	0.082	0.012282443	C1_CD8_Tres
chr17-76198813-76199833	4.99E-12	0.345537729	0.177	0.066	3.30E-07	C1_CD8_Tres
chr17-66330821-66331717	9.92E-11	0.326107429	0.084	0.017	6.57E-06	C1_CD8_Tres
chr15-92813428-92813750	1.58E-10	0.328248375	0.141	0.049	1.05E-05	C1_CD8_Tres
chr21-26169083-26171127	2.58E-11	0.333414638	0.222	0.098	1.71E-06	C1_CD8_Tres
chr11-46331145-46331748	3.82E-26	0.554822845	0.153	0.021	2.53E-21	C1_CD8_Tres

chr21-31132453-31133490	7.33E-13	0.353937582	0.129	0.036	4.85E-08	C1_CD8_Tres
chr11-95688151-95689305	1.73E-19	0.486716829	0.159	0.033	1.14E-14	C1_CD8_Tres
chr5-98773002-98774550	7.82E-17	0.400003602	0.296	0.133	5.18E-12	C1_CD8_Tres
chr4-23333542-23334547	2.27E-07	0.261781779	0.195	0.105	0.015022898	C1_CD8_Tres
chr16-27195818-27196446	3.31E-18	0.450607683	0.192	0.061	2.19E-13	C1_CD8_Tres
chr16-29012895-29013446	1.27E-06	0.251088665	0.105	0.044	0.084176144	C1_CD8_Tres
chr21-31181419-31182555	6.30E-20	0.476431441	0.213	0.059	4.17E-15	C1_CD8_Tres
chr10-11684325-11685479	2.27E-14	0.391993875	0.171	0.055	1.50E-09	C1_CD8_Tres
chr19-16368879-16369329	3.62E-07	0.255062026	0.165	0.081	0.023985509	C1_CD8_Tres
chr1-67674051-67674973	1.63E-09	0.301466661	0.102	0.027	0.000108036	C1_CD8_Tres
chr1-181395947-181396693	1.19E-10	0.3248826	0.108	0.028	7.89E-06	C1_CD8_Tres
chr2-95022314-95022602	1.41E-09	0.315239141	0.099	0.026	9.32E-05	C1_CD8_Tres
chr2-197869407-197870296	1.29E-07	0.272590707	0.108	0.037	0.008528245	C1_CD8_Tres
chr17-1871661-1872163	2.02E-10	0.311370015	0.087	0.02	1.34E-05	C1_CD8_Tres
chr2-170651625-170652027	3.99E-08	0.275405752	0.063	0.014	0.00264291	C1_CD8_Tres
chr13-109779026-109779436	1.20E-09	0.290269262	0.084	0.019	7.97E-05	C1_CD8_Tres
chr5-82356754-82357721	2.17E-35	0.624343312	0.365	0.102	1.43E-30	C1_CD8_Tres
chr3-187027208-187027918	9.66E-10	0.308263056	0.186	0.084	6.39E-05	C1_CD8_Tres
chr4-38949216-38950048	2.43E-07	0.263433852	0.147	0.071	0.016113645	C1_CD8_Tres
chr12-3947462-3948089	5.78E-09	0.267227119	0.066	0.014	0.000383087	C1_CD8_Tres
chr3-187028971-187029250	8.18E-14	0.375007266	0.105	0.024	5.42E-09	C1_CD8_Tres
chr13-74230618-74231534	5.76E-07	0.251507596	0.132	0.061	0.038146555	C1_CD8_Tres
chr11-133946903-133948357	1.86E-11	0.340953744	0.186	0.071	1.23E-06	C1_CD8_Tres
chr4-108107558-108108443	9.43E-22	0.497900958	0.246	0.075	6.25E-17	C1_CD8_Tres
chr11-129813909-129814861	1.24E-12	0.373126234	0.12	0.03	8.18E-08	C1_CD8_Tres
chr4-108166096-108167509	1.83E-14	0.383134564	0.228	0.083	1.21E-09	C1_CD8_Tres
chr5-134103456-134103722	1.20E-13	0.356222161	0.084	0.013	7.97E-09	C1_CD8_Tres

chr6-35718831-35719415	5.07E-11	0.310592063	0.069	0.012	3.36E-06	C1_CD8_Tres
chr4-142845779-142847546	1.60E-11	0.338388106	0.168	0.064	1.06E-06	C1_CD8_Tres
chr3-48268119-48269543	1.23E-12	0.346476432	0.296	0.15	8.13E-08	C1_CD8_Tres
chr3-113005299-113006026	1.19E-07	0.262257757	0.099	0.035	0.007857703	C1_CD8_Tres
chr17-2814110-2815366	7.92E-22	0.410789209	0.497	0.259	5.24E-17	C1_CD8_Tres
chr8-17026652-17027754	2.30E-11	0.337321211	0.126	0.035	1.52E-06	C1_CD8_Tres
chr14-104389873-104390222	1.32E-09	0.275780734	0.06	0.009	8.74E-05	C1_CD8_Tres
chr20-3122892-3123357	7.59E-13	0.36706854	0.099	0.02	5.02E-08	C1_CD8_Tres
chr15-92849056-92849741	5.69E-07	0.250006887	0.138	0.066	0.037681687	C1_CD8_Tres
chr13-76884967-76886921	6.57E-08	0.264264274	0.201	0.097	0.004350372	C1_CD8_Tres
chr22-31278702-31279429	3.98E-16	0.421034576	0.165	0.046	2.63E-11	C1_CD8_Tres
chrX-8987543-8988639	1.47E-15	0.412733453	0.114	0.021	9.70E-11	C1_CD8_Tres
chr4-122577849-122579037	6.27E-08	0.267096403	0.24	0.13	0.004155462	C1_CD8_Tres
chr19-12289014-12289628	7.17E-11	0.33373532	0.174	0.073	4.75E-06	C1_CD8_Tres
chr1-223363515-223364829	1.73E-08	0.286412271	0.105	0.031	0.001148018	C1_CD8_Tres
chrX-17736504-17738390	5.11E-08	0.270721002	0.171	0.08	0.003385311	C1_CD8_Tres
chr15-59803380-59804029	1.41E-16	0.430675696	0.123	0.026	9.31E-12	C1_CD8_Tres
chr14-76844045-76844599	8.66E-08	0.266945609	0.078	0.022	0.00573504	C1_CD8_Tres
chr8-127196582-127197223	7.23E-09	0.293129533	0.09	0.024	0.000478466	C1_CD8_Tres
chr5-14413293-14413754	1.16E-09	0.30263513	0.099	0.025	7.69E-05	C1_CD8_Tres
chr5-134964405-134964998	5.33E-11	0.298762779	0.054	0.006	3.53E-06	C1_CD8_Tres
chr19-12635499-12635851	1.22E-07	0.260953706	0.081	0.025	0.008088176	C1_CD8_Tres
chr4-108433425-108434172	1.61E-09	0.283520677	0.069	0.014	0.000106794	C1_CD8_Tres
chr17-66667209-66667775	1.08E-07	0.256963757	0.084	0.026	0.007120655	C1_CD8_Tres
chr5-56058012-56059238	4.53E-21	0.484032327	0.251	0.076	3.00E-16	C1_CD8_Tres
chr1-169604630-169605193	3.32E-07	0.254795176	0.072	0.02	0.021973737	C1_CD8_Tres
chr2-95022886-95023505	2.92E-14	0.386311242	0.12	0.027	1.93E-09	C1_CD8_Tres

chr3-13879587-13880852	9.32E-12	0.350717979	0.168	0.056	6.17E-07	C1_CD8_Tres
chr3-112132950-112133478	1.25E-08	0.28500721	0.108	0.033	0.000828771	C1_CD8_Tres
chr8-80864501-80865161	8.40E-32	0.620182413	0.293	0.07	5.56E-27	C1_CD8_Tres
chr3-112128704-112129279	7.71E-19	0.467849801	0.168	0.039	5.11E-14	C1_CD8_Tres
chr7-138903069-138903853	2.94E-07	0.262852736	0.123	0.046	0.019443066	C1_CD8_Tres
chr7-134710579-134711526	5.05E-11	0.341455232	0.201	0.084	3.34E-06	C1_CD8_Tres
chr1-61782618-61783124	1.38E-08	0.283453854	0.108	0.04	0.000912584	C1_CD8_Tres
chr5-137737372-137738112	3.42E-15	0.40983031	0.129	0.029	2.26E-10	C1_CD8_Tres
chr22-40224483-40225060	4.53E-11	0.339344054	0.087	0.019	3.00E-06	C1_CD8_Tres
chr9-33415251-33415946	2.65E-17	0.429675115	0.231	0.08	1.75E-12	C1_CD8_Tres
chr21-31326510-31327226	4.79E-15	0.385287467	0.081	0.01	3.17E-10	C1_CD8_Tres
chr7-5833513-5834769	1.04E-08	0.285706933	0.072	0.016	0.000687564	C1_CD8_Tres
chr1-169692366-169693382	2.58E-09	0.298169009	0.207	0.098	0.000170548	C1_CD8_Tres
chr11-122697418-122698052	1.41E-10	0.334346469	0.132	0.049	9.32E-06	C1_CD8_Tres
chr2-109143616-109144570	3.18E-07	0.259152561	0.102	0.035	0.021073107	C1_CD8_Tres
chr4-109648192-109649022	2.46E-07	0.250882921	0.183	0.097	0.016316312	C1_CD8_Tres
chr20-13230872-13231669	3.27E-08	0.276369869	0.072	0.016	0.002166286	C1_CD8_Tres
chr5-133170668-133171170	3.15E-11	0.328428158	0.069	0.011	2.09E-06	C1_CD8_Tres
chr3-7876673-7877294	1.19E-20	0.478080221	0.102	0.012	7.88E-16	C1_CD8_Tres
chrX-1205702-1206264	4.18E-07	0.250472196	0.084	0.027	0.027691428	C1_CD8_Tres
chr16-15672337-15673212	8.29E-14	0.392334543	0.165	0.049	5.49E-09	C1_CD8_Tres
chr1-12510196-12511175	3.65E-08	0.272750393	0.219	0.121	0.002419754	C1_CD8_Tres
chr5-81006557-81008275	3.91E-12	0.349286887	0.189	0.067	2.59E-07	C1_CD8_Tres
chr7-74451939-74452898	9.02E-18	0.403017188	0.365	0.167	5.98E-13	C1_CD8_Tres
chr4-84582300-84583857	1.26E-09	0.304464173	0.228	0.104	8.33E-05	C1_CD8_Tres
chr15-60580112-60580970	2.37E-11	0.319570729	0.249	0.126	1.57E-06	C1_CD8_Tres
chrX-39309110-39310066	1.03E-07	0.261051316	0.084	0.027	0.006840988	C1_CD8_Tres

chr2-230905310-230905853	5.49E-09	0.300424154	0.123	0.044	0.000363444	C1_CD8_Tres
chrX-16889110-16889721	3.92E-07	0.253238421	0.114	0.046	0.025971532	C1_CD8_Tres
chr11-118877972-118878403	1.34E-07	0.255986182	0.081	0.024	0.008850638	C1_CD8_Tres
chr7-5688922-5689525	2.81E-12	0.357742457	0.12	0.031	1.86E-07	C1_CD8_Tres
chr2-206530475-206531411	8.83E-22	0.492033839	0.129	0.016	5.85E-17	C1_CD8_Tres
chr18-70781711-70782229	3.37E-08	0.271151769	0.066	0.015	0.002232813	C1_CD8_Tres
chr5-111530075-111530703	7.65E-09	0.284952397	0.201	0.1	0.000506367	C1_CD8_Tres
chr5-80487743-80488360	1.95E-08	0.26066504	0.075	0.018	0.001289665	C1_CD8_Tres
chr20-36988410-36988905	5.48E-08	0.258157777	0.057	0.014	0.003626462	C1_CD8_Tres
chr6-166942461-166943155	6.29E-11	0.330140965	0.078	0.015	4.16E-06	C1_CD8_Tres
chr5-1105490-1106169	2.58E-07	0.26031055	0.162	0.079	0.017063126	C1_CD8_Tres
chr10-3668289-3669209	2.48E-12	0.348774367	0.195	0.075	1.64E-07	C1_CD8_Tres
chr22-19891000-19892614	7.40E-12	0.302472141	0.38	0.222	4.90E-07	C1_CD8_Tres
chr10-23362927-23363862	6.61E-07	0.255208859	0.123	0.049	0.0438056	C1_CD8_Tres
chr7-150478861-150479476	4.23E-11	0.337352518	0.159	0.063	2.80E-06	C1_CD8_Tres
chr9-99112799-99113629	1.74E-09	0.30959464	0.123	0.045	0.000115521	C1_CD8_Tres
chr16-29007977-29008363	8.75E-08	0.266902157	0.123	0.05	0.005791837	C1_CD8_Tres
chr9-109110153-109111048	3.08E-07	0.258952	0.15	0.071	0.020391366	C1_CD8_Tres
chr1-235206595-235207509	1.48E-15	0.400374015	0.237	0.098	9.82E-11	C1_CD8_Tres
chr10-470188-471014	2.26E-13	0.389134944	0.138	0.037	1.49E-08	C1_CD8_Tres
chr10-118698094-118698841	1.93E-09	0.299718388	0.228	0.113	0.00012791	C1_CD8_Tres
chr2-37243101-37244262	6.13E-10	0.295064103	0.066	0.011	4.06E-05	C1_CD8_Tres
chr1-232923575-232924398	1.59E-07	0.268834092	0.12	0.052	0.010557335	C1_CD8_Tres
chr20-56411601-56412587	3.38E-07	0.257594727	0.162	0.07	0.022396604	C1_CD8_Tres
chr11-4090275-4090505	4.70E-09	0.293643019	0.081	0.018	0.000311097	C1_CD8_Tres
chr1-8700968-8701774	1.72E-08	0.270975774	0.21	0.11	0.001136177	C1_CD8_Tres
chr22-40728755-40729037	3.86E-09	0.269159411	0.06	0.012	0.000255435	C1_CD8_Tres

chr10-6083475-6084035	2.99E-12	0.353592233	0.105	0.024	1.98E-07	C1_CD8_Tres
chr20-5736988-5737733	4.10E-25	0.547008611	0.219	0.046	2.71E-20	C1_CD8_Tres
chr17-51427266-51428214	1.16E-09	0.305951712	0.099	0.029	7.68E-05	C1_CD8_Tres
chr2-239326744-239327330	5.35E-09	0.299254087	0.153	0.062	0.000353999	C1_CD8_Tres
chr7-76153463-76153892	1.68E-07	0.256920152	0.063	0.015	0.011097083	C1_CD8_Tres
chr12-124636086-124636573	2.00E-10	0.328773581	0.093	0.023	1.33E-05	C1_CD8_Tres
chr15-63446190-63446645	5.74E-19	0.430549752	0.087	0.008	3.80E-14	C1_CD8_Tres
chr2-109613514-109614698	6.68E-10	0.318794198	0.153	0.051	4.43E-05	C1_CD8_Tres
chr10-9010061-9010417	2.01E-10	0.287472461	0.057	0.007	1.33E-05	C1_CD8_Tres
chr22-35551999-35552992	1.98E-09	0.29593426	0.111	0.035	0.000131332	C1_CD8_Tres
chr13-73058336-73059823	1.91E-12	0.325817462	0.344	0.178	1.27E-07	C1_CD8_Tres
chr8-27897108-27897853	1.43E-14	0.376987469	0.096	0.015	9.48E-10	C1_CD8_Tres
chr13-98998744-98999348	1.03E-09	0.303987757	0.111	0.031	6.85E-05	C1_CD8_Tres
chr7-75920383-75920747	1.12E-12	0.364903803	0.081	0.014	7.44E-08	C1_CD8_Tres
chr2-191739699-191740487	5.76E-09	0.303116944	0.165	0.07	0.000381309	C1_CD8_Tres
chr8-143966180-143966904	9.57E-07	0.250077276	0.093	0.032	0.063364859	C1_CD8_Tres
chr4-8228336-8229641	2.42E-10	0.327054814	0.192	0.081	1.60E-05	C1_CD8_Tres
chr10-14506919-14507504	1.11E-07	0.261761318	0.078	0.025	0.007329087	C1_CD8_Tres
chr9-89456643-89457725	3.60E-16	0.415534351	0.18	0.058	2.38E-11	C1_CD8_Tres
chr6-149488799-149489139	2.90E-09	0.304119793	0.111	0.035	0.00019213	C1_CD8_Tres
chr17-41338688-41339538	3.22E-08	0.264565752	0.249	0.143	0.002130776	C1_CD8_Tres
chr18-77140699-77141089	1.05E-07	0.252782841	0.063	0.013	0.006924654	C1_CD8_Tres
chr8-11820144-11820848	5.04E-12	0.366041421	0.108	0.026	3.34E-07	C1_CD8_Tres
chr4-102630512-102631095	4.94E-14	0.389487407	0.132	0.034	3.27E-09	C1_CD8_Tres
chr16-29679437-29680044	1.07E-06	0.252222763	0.135	0.059	0.070552253	C1_CD8_Tres
chr4-99507514-99508118	1.09E-07	0.259515522	0.06	0.013	0.007221213	C1_CD8_Tres
chr12-91978021-91978778	3.10E-08	0.279799434	0.099	0.032	0.002055154	C1_CD8_Tres

chr3-32237980-32239111	3.95E-07	0.254435482	0.171	0.086	0.02614165	C1_CD8_Tres
chr3-52982868-52983957	6.94E-11	0.321001344	0.141	0.049	4.59E-06	C1_CD8_Tres
chr6-37209460-37210717	9.10E-09	0.275775273	0.159	0.079	0.000602924	C1_CD8_Tres
chr9-79581567-79582252	1.50E-16	0.416413781	0.105	0.018	9.90E-12	C1_CD8_Tres
chr2-112187547-112188686	1.17E-10	0.282433138	0.389	0.24	7.78E-06	C1_CD8_Tres
chr7-50206183-50206951	3.64E-11	0.329591005	0.237	0.116	2.41E-06	C1_CD8_Tres
chr3-188322747-188323755	4.86E-12	0.354539109	0.192	0.071	3.22E-07	C1_CD8_Tres
chr6-35331999-35332596	1.55E-08	0.295440152	0.165	0.071	0.001028539	C1_CD8_Tres
chr4-87103173-87104125	1.40E-18	0.452624512	0.159	0.042	9.26E-14	C1_CD8_Tres
chr1-169708021-169708604	5.73E-08	0.253020397	0.054	0.011	0.003794155	C1_CD8_Tres
chr12-31736324-31736891	9.14E-16	0.41087356	0.251	0.094	6.06E-11	C1_CD8_Tres
chr17-65522837-65523959	1.05E-10	0.321842627	0.111	0.03	6.95E-06	C1_CD8_Tres
chrX-21373624-21375029	1.41E-07	0.258484059	0.099	0.035	0.009308686	C1_CD8_Tres
chr10-5563994-5565107	1.79E-07	0.264540553	0.108	0.04	0.011879015	C1_CD8_Tres
chr18-58321858-58322325	1.03E-12	0.368025109	0.075	0.01	6.85E-08	C1_CD8_Tres
chr11-13940393-13940985	2.94E-09	0.285674851	0.06	0.012	0.000194953	C1_CD8_Tres
chr4-185254344-185254839	2.55E-11	0.3300167	0.135	0.044	1.69E-06	C1_CD8_Tres
chr2-39216328-39217463	5.32E-09	0.275806726	0.293	0.163	0.000352246	C1_CD8_Tres
chr7-63925380-63926794	4.58E-10	0.296874119	0.314	0.177	3.03E-05	C1_CD8_Tres
chr22-20557024-20558072	3.95E-08	0.271330955	0.183	0.094	0.002617037	C1_CD8_Tres
chr5-35129908-35130508	1.40E-12	0.333324079	0.069	0.009	9.27E-08	C1_CD8_Tres
chr6-157860198-157860792	8.43E-08	0.26627343	0.105	0.037	0.005582767	C1_CD8_Tres
chr12-109981321-109981669	2.24E-20	0.478577343	0.114	0.013	1.48E-15	C1_CD8_Tres
chr21-31184513-31184764	2.63E-11	0.339183338	0.087	0.017	1.74E-06	C1_CD8_Tres
chr8-78515522-78516693	4.24E-07	0.251158184	0.189	0.093	0.028058042	C1_CD8_Tres
chr15-65828358-65829440	5.60E-08	0.258305776	0.186	0.091	0.003706188	C1_CD8_Tres
chr17-82200599-82201168	4.23E-09	0.292912884	0.189	0.089	0.000280008	C1_CD8_Tres

chr1-223248405-223249194	1.03E-07	0.275454798	0.066	0.017	0.006829597	C1_CD8_Tres
chr1-63651066-63651653	1.02E-11	0.316595441	0.066	0.007	6.73E-07	C1_CD8_Tres
chr18-48455837-48456529	4.67E-12	0.332547606	0.063	0.007	3.09E-07	C1_CD8_Tres
chr12-4506471-4507325	2.74E-07	0.263955557	0.195	0.093	0.018144527	C1_CD8_Tres
chr6-134529945-134530292	3.36E-14	0.373108682	0.075	0.007	2.23E-09	C1_CD8_Tres
chr1-33128184-33128619	1.00E-16	0.423415633	0.159	0.041	6.62E-12	C1_CD8_Tres
chr3-161349586-161350276	1.57E-07	0.266283791	0.132	0.058	0.010424725	C1_CD8_Tres
chr9-38620001-38622123	7.34E-10	0.307067721	0.162	0.067	4.86E-05	C1_CD8_Tres
chrX-46320257-46320809	1.89E-17	0.412659601	0.093	0.01	1.25E-12	C1_CD8_Tres
chr14-32102108-32102841	7.56E-10	0.309491189	0.084	0.021	5.01E-05	C1_CD8_Tres
chr12-102059274-102059663	2.92E-12	0.364727872	0.15	0.047	1.94E-07	C1_CD8_Tres
chr14-31944184-31945344	7.76E-08	0.268999492	0.072	0.017	0.005139351	C1_CD8_Tres
chr1-89607200-89607466	9.03E-08	0.267721037	0.153	0.065	0.005978163	C1_CD8_Tres
chr5-35830501-35830963	1.06E-08	0.268312441	0.072	0.016	0.000704708	C1_CD8_Tres
chrX-123803310-123803865	9.04E-17	0.415763876	0.099	0.013	5.99E-12	C1_CD8_Tres
chr14-61569748-61570185	5.66E-12	0.35604854	0.111	0.027	3.75E-07	C1_CD8_Tres
chr14-95462999-95463674	7.55E-08	0.2688587	0.096	0.029	0.005002765	C1_CD8_Tres
chr11-121467993-121468350	4.19E-07	0.259112162	0.126	0.053	0.027767703	C1_CD8_Tres
chr16-28990309-28990992	1.80E-11	0.337291688	0.183	0.075	1.19E-06	C1_CD8_Tres
chr2-174778243-174779056	4.97E-11	0.328175777	0.195	0.084	3.29E-06	C1_CD8_Tres
chr20-13220538-13222122	2.00E-11	0.345490167	0.144	0.047	1.33E-06	C1_CD8_Tres
chr7-38433669-38434193	2.72E-22	0.487926838	0.102	0.008	1.80E-17	C1_CD8_Tres
chr19-49567775-49568531	6.38E-19	0.442959757	0.24	0.085	4.23E-14	C1_CD8_Tres
chr13-48653150-48654064	7.15E-09	0.290478829	0.171	0.077	0.000473287	C1_CD8_Tres
chr2-63855841-63856696	6.71E-09	0.282101884	0.213	0.108	0.000444626	C1_CD8_Tres
chr4-110164878-110165829	5.04E-07	0.254626207	0.15	0.074	0.033396163	C1_CD8_Tres
chr11-35216770-35217513	1.21E-08	0.273891241	0.21	0.105	0.000801823	C1_CD8_Tres

chr12-92055807-92056207	1.03E-06	0.250166352	0.153	0.076	0.067977008	C1_CD8_Tres
chr5-67161007-67161300	6.07E-08	0.27154418	0.093	0.029	0.004016837	C1_CD8_Tres
chr3-5019459-5019917	4.05E-10	0.31289961	0.156	0.061	2.68E-05	C1_CD8_Tres
chr8-38368188-38368884	2.09E-11	0.342824201	0.129	0.036	1.39E-06	C1_CD8_Tres
chr11-47234057-47235190	1.56E-08	0.287879886	0.138	0.058	0.001032875	C1_CD8_Tres
chr5-56479752-56481429	1.75E-16	0.423157766	0.117	0.022	1.16E-11	C1_CD8_Tres
chr10-74194549-74194880	3.01E-10	0.322265208	0.084	0.02	1.99E-05	C1_CD8_Tres
chr1-43453152-43454832	9.36E-08	0.252399399	0.189	0.1	0.006197362	C1_CD8_Tres
chr11-64899995-64900620	2.33E-12	0.353868624	0.21	0.087	1.54E-07	C1_CD8_Tres
chr2-160417640-160418130	4.30E-27	0.571275875	0.168	0.025	2.85E-22	C1_CD8_Tres
chr1-24506618-24507685	1.29E-07	0.258733961	0.174	0.086	0.008510238	C1_CD8_Tres
chr10-88193938-88194699	3.32E-08	0.283036274	0.114	0.041	0.002198178	C1_CD8_Tres
chr3-115791932-115792900	2.65E-11	0.337929325	0.225	0.104	1.75E-06	C1_CD8_Tres
chr12-12006921-12007387	2.44E-08	0.279692203	0.246	0.133	0.001615242	C1_CD8_Tres
chr5-132055757-132056644	2.31E-13	0.381665913	0.207	0.079	1.53E-08	C1_CD8_Tres
chr3-115790540-115791139	6.88E-09	0.290946397	0.198	0.092	0.000455391	C1_CD8_Tres
chr2-157463896-157464634	1.18E-07	0.263826312	0.153	0.068	0.007804596	C1_CD8_Tres
chr13-45395720-45396421	1.10E-09	0.312303075	0.105	0.032	7.30E-05	C1_CD8_Tres
chr2-239326744-239327330	5.35E-09	0.299254087	0.153	0.062	0.000353999	C1_CD8_Tres
chr12-124469301-124469836	2.61E-10	0.323023036	0.132	0.043	1.73E-05	C1_CD8_Tres
chr11-121467993-121468350	4.19E-07	0.259112162	0.126	0.053	0.027767703	C1_CD8_Tres
chr16-15509172-15509969	2.41E-17	0.434741179	0.108	0.017	1.60E-12	C1_CD8_Tres
chr1-168419981-168420510	9.85E-10	0.305297469	0.099	0.029	6.52E-05	C1_CD8_Tres
chr7-77393208-77393730	5.31E-13	0.390385199	0.138	0.038	3.52E-08	C1_CD8_Tres
chr16-996722-998093	1.62E-07	0.261029024	0.171	0.084	0.010709837	C1_CD8_Tres
chr17-76772497-76773639	2.02E-19	0.430145549	0.332	0.143	1.34E-14	C1_CD8_Tres
chr1-1371151-1372325	6.23E-08	0.254811679	0.228	0.134	0.004128522	C1_CD8_Tres

chr8-143966180-143966904	9.57E-07	0.250077276	0.093	0.032	0.063364859	C1_CD8_Tres
chr2-157897177-157899524	4.94E-09	0.277130766	0.222	0.113	0.000327128	C1_CD8_Tres
chr1-12510196-12511175	3.65E-08	0.272750393	0.219	0.121	0.002419754	C1_CD8_Tres
chr18-24075543-24076605	6.24E-08	0.261012374	0.272	0.176	0.004134984	C1_CD8_Tres
chr7-23020655-23021404	4.64E-13	0.38616508	0.171	0.059	3.07E-08	C1_CD8_Tres
chr2-174778243-174779056	4.97E-11	0.328175777	0.195	0.084	3.29E-06	C1_CD8_Tres
chr6-139160917-139162291	1.77E-11	0.307963704	0.377	0.217	1.17E-06	C1_CD8_Tres
chr6-134287862-134289224	6.98E-08	0.273170533	0.129	0.049	0.004623319	C1_CD8_Tres
chr7-50206183-50206951	3.64E-11	0.329591005	0.237	0.116	2.41E-06	C1_CD8_Tres
chr3-52982868-52983957	6.94E-11	0.321001344	0.141	0.049	4.59E-06	C1_CD8_Tres
chr15-60720228-60721993	3.19E-09	0.284195753	0.266	0.139	0.000211463	C1_CD8_Tres
chr4-184792913-184793389	6.37E-10	0.322083812	0.099	0.028	4.22E-05	C1_CD8_Tres
chr6-157860198-157860792	8.43E-08	0.26627343	0.105	0.037	0.005582767	C1_CD8_Tres
chr16-28990309-28990992	1.80E-11	0.337291688	0.183	0.075	1.19E-06	C1_CD8_Tres
chr20-62501785-62502282	8.69E-08	0.279993163	0.153	0.063	0.005754404	C1_CD8_Tres
chr12-122646630-122647161	2.48E-08	0.278829135	0.102	0.04	0.001640041	C1_CD8_Tres
chr10-33248292-33248999	2.90E-08	0.275434455	0.183	0.085	0.001921508	C1_CD8_Tres
chr3-188322747-188323755	4.86E-12	0.354539109	0.192	0.071	3.22E-07	C1_CD8_Tres
chr1-232923575-232924398	1.59E-07	0.268834092	0.12	0.052	0.010557335	C1_CD8_Tres
chr9-89456643-89457725	3.60E-16	0.415534351	0.18	0.058	2.38E-11	C1_CD8_Tres
chr1-235206595-235207509	1.48E-15	0.400374015	0.237	0.098	9.82E-11	C1_CD8_Tres
chr6-37209460-37210717	9.10E-09	0.275775273	0.159	0.079	0.000602924	C1_CD8_Tres
chrX-48714555-48715412	7.63E-11	0.327556022	0.18	0.073	5.05E-06	C1_CD8_Tres
chr15-60580112-60580970	2.37E-11	0.319570729	0.249	0.126	1.57E-06	C1_CD8_Tres
chr3-188322747-188323755	4.86E-12	0.354539109	0.192	0.071	3.22E-07	C1_CD8_Tres
chr5-132687864-132689114	2.74E-11	0.338985741	0.147	0.045	1.81E-06	C1_CD8_Tres
chr5-132055757-132056644	2.31E-13	0.381665913	0.207	0.079	1.53E-08	C1_CD8_Tres

chr8-100415528-100417352	4.77E-18	0.014690835	0.197	0.316	3.16E-13	C2_CD8_Tem
chr19-41935809-41936940	2.82E-17	0.031039493	0.189	0.294	1.87E-12	C2_CD8_Tem
chr9-89501607-89502643	1.36E-15	0.051175517	0.246	0.367	9.01E-11	C2_CD8_Tem
chr1-201170280-201171791	8.99E-15	0.029395885	0.193	0.293	5.95E-10	C2_CD8_Tem
chr12-22409066-22410762	1.14E-14	0.032672379	0.248	0.394	7.56E-10	C2_CD8_Tem
chr3-46359594-46360492	1.41E-13	0.008223882	0.136	0.222	9.33E-09	C2_CD8_Tem
chr8-29990174-29991436	4.35E-13	0.023742296	0.153	0.232	2.88E-08	C2_CD8_Tem
chr16-68752400-68753017	1.76E-12	0.041757143	0.101	0.139	1.17E-07	C2_CD8_Tem
chr1-91555389-91556947	3.71E-12	0.022463679	0.278	0.448	2.46E-07	C2_CD8_Tem
chr1-91124279-91125427	1.37E-11	0.001013806	0.114	0.179	9.09E-07	C2_CD8_Tem
chr2-223759699-223762024	6.27E-11	0.072167982	0.175	0.245	4.15E-06	C2_CD8_Tem
chr8-100409389-100410844	1.67E-10	0.04555802	0.24	0.361	1.11E-05	C2_CD8_Tem
chr16-80569205-80570422	2.04E-10	0.012874838	0.125	0.196	1.35E-05	C2_CD8_Tem
chr6-215328-216209	2.07E-10	0.039467147	0.164	0.256	1.37E-05	C2_CD8_Tem
chr5-107485956-107486918	2.36E-10	0.021465371	0.079	0.117	1.56E-05	C2_CD8_Tem
chr19-13043761-13044879	3.30E-10	0.014091004	0.134	0.202	2.19E-05	C2_CD8_Tem
chr4-77586502-77587521	4.55E-10	0.058090821	0.139	0.198	3.02E-05	C2_CD8_Tem
chr2-9655145-9656958	1.15E-09	0.002587755	0.162	0.264	7.61E-05	C2_CD8_Tem
chr11-45092887-45093822	1.30E-09	0.03453028	0.105	0.148	8.58E-05	C2_CD8_Tem
chr7-36686279-36686888	2.04E-09	0.010648005	0.072	0.111	0.000135241	C2_CD8_Tem
chr3-20054241-20055644	3.07E-09	0.008750703	0.218	0.358	0.000203353	C2_CD8_Tem
chr1-226662281-226663643	7.36E-09	0.027269806	0.383	0.615	0.000487712	C2_CD8_Tem
chr14-80929988-80931686	9.65E-09	0.00999377	0.198	0.334	0.000638725	C2_CD8_Tem
chr8-27361638-27362548	4.12E-08	0.010017577	0.224	0.376	0.002725097	C2_CD8_Tem
chr14-74298870-74299288	5.47E-08	0.025615807	0.044	0.055	0.003624654	C2_CD8_Tem
chr6-135537548-135538735	6.36E-08	0.053988222	0.094	0.123	0.00421087	C2_CD8_Tem
chr2-96556544-96557329	1.89E-07	0.004931515	0.108	0.169	0.012524419	C2_CD8_Tem

chr10-104328762-104329932	3.33E-07	0.016242719	0.263	0.441	0.022028884	C2_CD8_Tem
chr1-93817483-93818780	3.52E-07	0.032361032	0.098	0.143	0.023319672	C2_CD8_Tem
chr19-41327145-41328838	5.14E-07	0.031267031	0.493	0.721	0.034066008	C2_CD8_Tem
chr6-155223690-155224508	6.13E-07	0.010925521	0.121	0.187	0.040623383	C2_CD8_Tem
chr15-51727470-51727966	6.86E-07	0.012512345	0.056	0.081	0.045418373	C2_CD8_Tem
chr11-9759348-9760157	1.10E-06	0.001277076	0.188	0.309	0.072884436	C2_CD8_Tem
chr1-40628211-40628877	1.26E-06	0.044814522	0.089	0.115	0.083388884	C2_CD8_Tem
chr7-126700963-126701509	2.15E-06	0.026564471	0.056	0.076	0.142105723	C2_CD8_Tem
chr6-159929539-159930247	2.34E-06	0.006509378	0.044	0.062	0.154917581	C2_CD8_Tem
chr9-91798086-91799208	2.82E-06	0.020315779	0.107	0.163	0.186441518	C2_CD8_Tem
chr10-132427249-132428487	3.67E-06	0.015257823	0.063	0.086	0.242744783	C2_CD8_Tem
chr2-113240392-113241310	1.29E-05	0.003834604	0.048	0.069	0.853146387	C2_CD8_Tem
chr4-122635027-122635580	7.63E-05	0.001057459	0.034	0.05	1	C2_CD8_Tem
chr19-40465090-40467143	8.66E-05	0.02739234	0.507	0.765	1	C2_CD8_Tem
chr5-911844-913044	0.000114058	0.022933684	0.2	0.29	1	C2_CD8_Tem
chr12-24838485-24839770	0.000147539	0.012373954	0.069	0.095	1	C2_CD8_Tem
chr1-200880056-200880647	0.000212297	0.003833492	0.051	0.076	1	C2_CD8_Tem
chr10-114495192-114495890	0.000240264	0.002374397	0.048	0.067	1	C2_CD8_Tem
chr7-94656122-94658687	0.002116167	0.03211501	0.356	0.581	1	C2_CD8_Tem
chr1-227934560-227935455	0.049288277	0.00914237	0.1	0.144	1	C2_CD8_Tem
chr2-64749972-64752034	0.05017438	0.010838095	0.511	0.798	1	C2_CD8_Tem
chr3-130110724-130112645	0.058510345	0.061327667	0.513	0.773	1	C2_CD8_Tem
chr4-3954555-3956401	0.067824538	0.006754577	0.465	0.75	1	C2_CD8_Tem
chr4-8408869-8410058	0.157297471	0.006052909	0.205	0.324	1	C2_CD8_Tem
chr3-125915247-125917232	0.368110702	0.037848913	0.535	0.788	1	C2_CD8_Tem
chr2-86785384-86786603	9.54E-94	0.512922872	0.73	0.266	6.32E-89	C3_CD8_Tem
chr20-51553758-51554917	6.14E-72	0.616741585	0.356	0.066	4.07E-67	C3_CD8_Tem

chr16-23482470-23483873	1.81E-71	0.514220615	0.568	0.183	1.20E-66	C3_CD8_Tem
chr2-8551423-8552687	8.05E-70	0.612928779	0.311	0.052	5.33E-65	C3_CD8_Tem
chr19-13043761-13044879	1.20E-65	0.532305291	0.473	0.134	7.98E-61	C3_CD8_Tem
chr10-124650100-124651550	1.83E-65	0.488205685	0.616	0.224	1.21E-60	C3_CD8_Tem
chr2-86825469-86826726	2.90E-63	0.524982874	0.51	0.15	1.92E-58	C3_CD8_Tem
chr11-122838103-122838816	6.86E-63	0.530195152	0.483	0.138	4.54E-58	C3_CD8_Tem
chr11-36420374-36421610	2.33E-62	0.532998647	0.491	0.147	1.55E-57	C3_CD8_Tem
chr2-230877055-230878272	4.30E-62	0.46934429	0.609	0.217	2.85E-57	C3_CD8_Tem
chr2-238290100-238290499	1.85E-59	0.568473957	0.332	0.068	1.23E-54	C3_CD8_Tem
chr1-3786925-3788055	6.37E-57	0.494023052	0.513	0.167	4.22E-52	C3_CD8_Tem
chr17-76714931-76716698	1.64E-56	0.4002413	0.733	0.326	1.08E-51	C3_CD8_Tem
chr20-5038646-5039755	4.70E-55	0.537415596	0.356	0.086	3.11E-50	C3_CD8_Tem
chr1-91124279-91125427	4.32E-54	0.502427996	0.41	0.119	2.86E-49	C3_CD8_Tem
chr20-57604829-57606180	1.60E-53	0.511767611	0.421	0.116	1.06E-48	C3_CD8_Tem
chr17-10114141-10115557	2.18E-53	0.420008431	0.622	0.245	1.44E-48	C3_CD8_Tem
chr19-41935809-41936940	2.94E-53	0.451628491	0.572	0.215	1.95E-48	C3_CD8_Tem
chr1-107936381-107937456	2.20E-52	0.485755912	0.48	0.157	1.45E-47	C3_CD8_Tem
chr17-3794854-3797125	3.96E-50	0.31978906	0.826	0.443	2.62E-45	C3_CD8_Tem
chr17-36188180-36189273	4.67E-50	0.416924784	0.624	0.245	3.09E-45	C3_CD8_Tem
chr22-43376194-43378148	5.44E-50	0.45579932	0.53	0.192	3.60E-45	C3_CD8_Tem
chr8-29990174-29991436	1.94E-48	0.455861169	0.474	0.167	1.28E-43	C3_CD8_Tem
chr6-13453743-13455250	6.90E-47	0.401473649	0.635	0.271	4.57E-42	C3_CD8_Tem
chr6-152565755-152566727	7.49E-47	0.491321908	0.36	0.096	4.96E-42	C3_CD8_Tem
chr1-8495849-8497059	9.28E-47	0.455915099	0.399	0.132	6.15E-42	C3_CD8_Tem
chr8-100409389-100410844	1.12E-46	0.38824773	0.649	0.276	7.41E-42	C3_CD8_Tem
chr12-121557117-121558231	3.45E-46	0.435986111	0.486	0.173	2.28E-41	C3_CD8_Tem
chr8-100415528-100417352	9.27E-46	0.421102122	0.594	0.234	6.14E-41	C3_CD8_Tem

chr4-6180491-6181411	1.00E-45	0.429582859	0.536	0.205	6.64E-41	C3_CD8_Tem
chr16-75620163-75620836	1.19E-45	0.463922187	0.457	0.147	7.89E-41	C3_CD8_Tem
chr6-32945173-32945747	2.07E-45	0.511800555	0.262	0.052	1.37E-40	C3_CD8_Tem
chr16-57028997-57030129	2.21E-45	0.513091341	0.248	0.049	1.46E-40	C3_CD8_Tem
chr11-122842844-122844041	1.12E-44	0.440595383	0.474	0.168	7.45E-40	C3_CD8_Tem
chr18-48868097-48869427	1.20E-44	0.453947307	0.431	0.147	7.92E-40	C3_CD8_Tem
chr6-2861395-2861985	2.79E-44	0.481999437	0.373	0.104	1.85E-39	C3_CD8_Tem
chr7-105675523-105676465	3.97E-44	0.457700644	0.44	0.149	2.63E-39	C3_CD8_Tem
chr1-39525182-39526389	1.00E-43	0.45341987	0.448	0.144	6.65E-39	C3_CD8_Tem
chr16-68752400-68753017	1.48E-43	0.47304238	0.333	0.092	9.83E-39	C3_CD8_Tem
chr6-152635971-152637143	1.77E-43	0.453681288	0.416	0.133	1.17E-38	C3_CD8_Tem
chr6-215328-216209	7.60E-43	0.423977477	0.509	0.185	5.03E-38	C3_CD8_Tem
chr12-10394483-10395812	9.93E-43	0.444058167	0.433	0.146	6.58E-38	C3_CD8_Tem
chr6-105458118-105459658	1.09E-42	0.416165735	0.55	0.217	7.19E-38	C3_CD8_Tem
chr20-24959621-24961536	1.18E-42	0.418762161	0.54	0.216	7.78E-38	C3_CD8_Tem
chr2-241867876-241869439	1.59E-42	0.454576397	0.458	0.16	1.05E-37	C3_CD8_Tem
chr1-201170280-201171791	1.92E-42	0.401253707	0.525	0.225	1.27E-37	C3_CD8_Tem
chr1-202197442-202198255	3.72E-42	0.454534451	0.35	0.106	2.47E-37	C3_CD8_Tem
chr1-225460124-225461145	6.02E-42	0.446481525	0.431	0.143	3.99E-37	C3_CD8_Tem
chr9-89501607-89502643	6.78E-42	0.369161445	0.644	0.285	4.49E-37	C3_CD8_Tem
chr14-92530938-92532058	6.87E-42	0.414754601	0.521	0.199	4.55E-37	C3_CD8_Tem
chr5-76732397-76733449	2.17E-41	0.391823243	0.611	0.266	1.44E-36	C3_CD8_Tem
chr22-39115709-39118155	2.34E-41	0.333202255	0.77	0.403	1.55E-36	C3_CD8_Tem
chr1-172818339-172819563	1.16E-39	0.458916816	0.363	0.11	7.71E-35	C3_CD8_Tem
chr15-38576418-38577719	7.10E-39	0.453564338	0.272	0.072	4.70E-34	C3_CD8_Tem
chr4-970184-971071	7.12E-39	0.466223479	0.309	0.078	4.71E-34	C3_CD8_Tem
chr15-65512623-65513362	1.29E-38	0.444721203	0.349	0.108	8.55E-34	C3_CD8_Tem

chr9-123304331-123305414	1.31E-38	0.427704352	0.435	0.155	8.66E-34	C3_CD8_Tem
chr5-142924201-142924891	1.95E-38	0.471266569	0.275	0.067	1.29E-33	C3_CD8_Tem
chr6-34484371-34485456	2.22E-38	0.436489678	0.403	0.14	1.47E-33	C3_CD8_Tem
chr17-38900699-38901837	2.51E-38	0.441933498	0.439	0.149	1.66E-33	C3_CD8_Tem
chr9-123321919-123323358	2.94E-38	0.385854652	0.562	0.241	1.94E-33	C3_CD8_Tem
chr17-83083303-83084182	4.80E-38	0.35499318	0.626	0.292	3.18E-33	C3_CD8_Tem
chr1-25064358-25066301	5.55E-38	0.433033475	0.41	0.137	3.67E-33	C3_CD8_Tem
chr17-5220631-5221894	4.33E-37	0.468276948	0.266	0.069	2.87E-32	C3_CD8_Tem
chr9-20403156-20403890	1.25E-36	0.44824434	0.219	0.048	8.29E-32	C3_CD8_Tem
chr1-91116594-91117319	2.42E-36	0.461154596	0.226	0.05	1.60E-31	C3_CD8_Tem
chr8-29472111-29472994	2.70E-36	0.458383149	0.21	0.045	1.79E-31	C3_CD8_Tem
chr20-51499996-51501369	6.06E-36	0.343950349	0.656	0.318	4.02E-31	C3_CD8_Tem
chr9-131627343-131628902	6.83E-36	0.364028761	0.591	0.258	4.52E-31	C3_CD8_Tem
chr1-221839379-221840266	7.56E-36	0.367484178	0.549	0.237	5.00E-31	C3_CD8_Tem
chr11-35334234-35335006	8.70E-36	0.440834746	0.297	0.082	5.76E-31	C3_CD8_Tem
chr9-114369205-114370071	1.39E-35	0.37048113	0.545	0.236	9.19E-31	C3_CD8_Tem
chr2-221517255-221519122	1.47E-35	0.315146339	0.675	0.334	9.75E-31	C3_CD8_Tem
chr19-44760126-44761340	1.86E-35	0.431430522	0.374	0.127	1.23E-30	C3_CD8_Tem
chr16-23504007-23505334	2.80E-35	0.367561795	0.587	0.258	1.85E-30	C3_CD8_Tem
chr1-38052567-38053460	5.05E-35	0.449905845	0.237	0.057	3.34E-30	C3_CD8_Tem
chr2-144323881-144325643	8.79E-35	0.36153022	0.601	0.274	5.82E-30	C3_CD8_Tem
chr2-135244873-135245614	1.00E-34	0.436410585	0.176	0.034	6.64E-30	C3_CD8_Tem
chr10-22634856-22635355	1.39E-34	0.441095361	0.341	0.102	9.18E-30	C3_CD8_Tem
chr14-103908297-103909068	1.56E-34	0.453817813	0.224	0.05	1.03E-29	C3_CD8_Tem
chr15-70299789-70301094	2.90E-34	0.345006839	0.607	0.286	1.92E-29	C3_CD8_Tem
chr2-144459392-144460394	4.85E-34	0.383977126	0.48	0.192	3.21E-29	C3_CD8_Tem
chr3-46958859-46959827	5.35E-34	0.403673861	0.406	0.154	3.54E-29	C3_CD8_Tem

chr1-168509786-168510939	6.11E-34	0.422557305	0.362	0.118	4.05E-29	C3_CD8_Tem
chr22-43085813-43086551	1.25E-33	0.447764462	0.255	0.062	8.26E-29	C3_CD8_Tem
chr12-681735-682825	1.51E-33	0.450206663	0.229	0.052	9.97E-29	C3_CD8_Tem
chr7-35722248-35723372	1.77E-33	0.386760073	0.51	0.212	1.17E-28	C3_CD8_Tem
chr16-87979501-87980612	1.85E-33	0.430727123	0.288	0.085	1.22E-28	C3_CD8_Tem
chr6-150310284-150311807	2.53E-33	0.401673393	0.395	0.144	1.68E-28	C3_CD8_Tem
chr13-48504671-48506020	4.02E-33	0.401950564	0.403	0.148	2.66E-28	C3_CD8_Tem
chr3-183349015-183350695	5.19E-33	0.390051118	0.444	0.172	3.44E-28	C3_CD8_Tem
chr16-84593536-84595332	6.14E-33	0.354450413	0.561	0.26	4.06E-28	C3_CD8_Tem
chr10-110764818-110765711	6.79E-33	0.409551552	0.108	0.01	4.50E-28	C3_CD8_Tem
chr9-134301405-134302179	7.18E-33	0.432466989	0.352	0.115	4.75E-28	C3_CD8_Tem
chr8-133138417-133139840	1.08E-32	0.390996469	0.492	0.198	7.15E-28	C3_CD8_Tem
chr15-74399958-74401241	1.10E-32	0.443191314	0.322	0.098	7.32E-28	C3_CD8_Tem
chr9-133369546-133371849	1.79E-32	0.33146063	0.668	0.334	1.18E-27	C3_CD8_Tem
chr2-144505188-144505875	2.26E-32	0.434748079	0.284	0.079	1.50E-27	C3_CD8_Tem
chr3-115164316-115166011	3.10E-32	0.391551597	0.439	0.174	2.05E-27	C3_CD8_Tem
chr1-160751298-160751835	3.20E-32	0.442304247	0.305	0.085	2.12E-27	C3_CD8_Tem
chr2-161233994-161234768	4.31E-32	0.429300868	0.257	0.072	2.85E-27	C3_CD8_Tem
chr5-142799721-142801418	4.67E-32	0.395840386	0.449	0.176	3.09E-27	C3_CD8_Tem
chr19-38864390-38866046	5.76E-32	0.332257728	0.636	0.311	3.81E-27	C3_CD8_Tem
chr16-84603038-84603523	7.13E-32	0.441817944	0.235	0.058	4.72E-27	C3_CD8_Tem
chr11-57546909-57548228	1.02E-31	0.428892043	0.169	0.03	6.74E-27	C3_CD8_Tem
chr11-11987445-11988281	1.18E-31	0.425519203	0.2	0.046	7.84E-27	C3_CD8_Tem
chr7-100182171-100183142	2.65E-31	0.42460428	0.213	0.053	1.75E-26	C3_CD8_Tem
chr19-2223703-2224311	3.29E-31	0.407532433	0.143	0.027	2.18E-26	C3_CD8_Tem
chr2-85862138-85862826	3.41E-31	0.422210421	0.311	0.1	2.26E-26	C3_CD8_Tem
chr6-105452630-105453843	3.64E-31	0.404992631	0.388	0.139	2.41E-26	C3_CD8_Tem

chr5-154384422-154385470	4.18E-31	0.425890166	0.23	0.057	2.77E-26	C3_CD8_Tem
chr2-232309525-232310661	8.27E-31	0.424750293	0.243	0.069	5.48E-26	C3_CD8_Tem
chr1-32798709-32799795	1.28E-30	0.369541777	0.537	0.227	8.47E-26	C3_CD8_Tem
chr17-3764719-3765887	1.45E-30	0.34554844	0.569	0.264	9.62E-26	C3_CD8_Tem
chr1-66246411-66248118	1.52E-30	0.398339401	0.385	0.152	1.01E-25	C3_CD8_Tem
chr5-1488691-1489680	2.10E-30	0.427019126	0.287	0.084	1.39E-25	C3_CD8_Tem
chr8-27394817-27395510	2.78E-30	0.412760096	0.387	0.135	1.84E-25	C3_CD8_Tem
chr21-45227809-45228640	2.84E-30	0.349452367	0.539	0.238	1.88E-25	C3_CD8_Tem
chr13-30147654-30148998	2.90E-30	0.363626774	0.45	0.19	1.92E-25	C3_CD8_Tem
chr18-49958736-49959702	3.82E-30	0.408256734	0.221	0.057	2.53E-25	C3_CD8_Tem
chr16-30767493-30768294	4.33E-30	0.415809236	0.265	0.074	2.87E-25	C3_CD8_Tem
chr5-74631355-74632512	5.38E-30	0.399668189	0.281	0.083	3.56E-25	C3_CD8_Tem
chr17-47220233-47221044	6.73E-30	0.418259689	0.267	0.077	4.46E-25	C3_CD8_Tem
chr12-10369630-10370340	7.51E-30	0.421433434	0.234	0.061	4.98E-25	C3_CD8_Tem
chr1-167373983-167374849	9.06E-30	0.423695745	0.194	0.043	6.00E-25	C3_CD8_Tem
chr11-115217768-115218322	9.08E-30	0.423876878	0.167	0.03	6.01E-25	C3_CD8_Tem
chr10-73758806-73760041	1.04E-29	0.382899159	0.419	0.158	6.86E-25	C3_CD8_Tem
chr1-160744309-160744754	1.13E-29	0.425769803	0.216	0.056	7.46E-25	C3_CD8_Tem
chr2-241762258-241764409	1.19E-29	0.284367306	0.758	0.398	7.91E-25	C3_CD8_Tem
chr2-181145933-181146699	1.28E-29	0.401658	0.337	0.116	8.50E-25	C3_CD8_Tem
chr4-121776309-121777398	1.43E-29	0.405839111	0.15	0.028	9.48E-25	C3_CD8_Tem
chr8-141156903-141157559	1.59E-29	0.411291695	0.25	0.07	1.05E-24	C3_CD8_Tem
chr19-40756246-40757119	1.71E-29	0.404217512	0.306	0.103	1.13E-24	C3_CD8_Tem
chr9-4653657-4654532	2.07E-29	0.410156063	0.176	0.038	1.37E-24	C3_CD8_Tem
chr2-230781194-230782105	2.15E-29	0.373933908	0.455	0.18	1.43E-24	C3_CD8_Tem
chr20-38839996-38841506	2.31E-29	0.360709465	0.516	0.221	1.53E-24	C3_CD8_Tem
chr7-105848750-105849993	2.74E-29	0.374590124	0.447	0.18	1.81E-24	C3_CD8_Tem

chr16-81442580-81443286	3.16E-29	0.411598157	0.312	0.1	2.09E-24	C3_CD8_Tem
chr21-46423897-46425970	5.17E-29	0.393416485	0.413	0.153	3.42E-24	C3_CD8_Tem
chr12-25333537-25334249	5.60E-29	0.414691217	0.316	0.1	3.71E-24	C3_CD8_Tem
chr17-47829370-47831773	6.67E-29	0.274226406	0.76	0.436	4.42E-24	C3_CD8_Tem
chr19-45087196-45087984	7.64E-29	0.359524422	0.496	0.213	5.06E-24	C3_CD8_Tem
chr2-86807185-86808995	8.80E-29	0.357884894	0.509	0.217	5.83E-24	C3_CD8_Tem
chr5-172132817-172133989	1.02E-28	0.407532469	0.371	0.126	6.79E-24	C3_CD8_Tem
chr10-70578066-70578810	1.13E-28	0.377173546	0.465	0.188	7.45E-24	C3_CD8_Tem
chr5-74630330-74631054	1.17E-28	0.403077818	0.234	0.062	7.73E-24	C3_CD8_Tem
chr4-6198149-6201381	1.37E-28	0.310498365	0.67	0.343	9.10E-24	C3_CD8_Tem
chr22-44454921-44456171	1.38E-28	0.34501323	0.556	0.259	9.14E-24	C3_CD8_Tem
chr19-19419983-19421059	1.41E-28	0.390920055	0.362	0.134	9.32E-24	C3_CD8_Tem
chr1-25795882-25797196	1.50E-28	0.390962974	0.375	0.144	9.95E-24	C3_CD8_Tem
chrX-155997179-155997901	1.64E-28	0.392014888	0.38	0.143	1.09E-23	C3_CD8_Tem
chr9-130106807-130108214	1.79E-28	0.373937373	0.454	0.187	1.18E-23	C3_CD8_Tem
chr7-152027350-152028282	2.04E-28	0.403709223	0.296	0.094	1.35E-23	C3_CD8_Tem
chr3-31502345-31503474	2.23E-28	0.389215892	0.249	0.074	1.48E-23	C3_CD8_Tem
chr5-148844559-148845169	3.01E-28	0.386396086	0.37	0.136	1.99E-23	C3_CD8_Tem
chr2-86787734-86787992	3.20E-28	0.413166068	0.176	0.036	2.12E-23	C3_CD8_Tem
chr3-27900129-27900802	4.50E-28	0.394761064	0.285	0.088	2.98E-23	C3_CD8_Tem
chr8-58973209-58974410	6.29E-28	0.406309828	0.184	0.042	4.17E-23	C3_CD8_Tem
chr11-122849806-122850635	6.41E-28	0.394173484	0.288	0.095	4.24E-23	C3_CD8_Tem
chr16-9081917-9082880	7.10E-28	0.402989346	0.188	0.044	4.70E-23	C3_CD8_Tem
chr10-6491805-6492593	7.94E-28	0.338908704	0.571	0.255	5.26E-23	C3_CD8_Tem
chr2-136305136-136305972	7.94E-28	0.386165787	0.339	0.115	5.26E-23	C3_CD8_Tem
chr12-68118976-68120277	7.97E-28	0.374996837	0.413	0.161	5.28E-23	C3_CD8_Tem
chr9-63816618-63818431	8.63E-28	0.338257044	0.549	0.256	5.71E-23	C3_CD8_Tem

chr13-113930452-113931666	1.01E-27	0.399042401	0.322	0.109	6.70E-23	C3_CD8_Tem
chr1-173409657-173411119	1.30E-27	0.367791949	0.403	0.157	8.61E-23	C3_CD8_Tem
chr12-10384862-10385642	1.87E-27	0.412465103	0.23	0.064	1.24E-22	C3_CD8_Tem
chr8-29060438-29061827	2.25E-27	0.370211966	0.431	0.173	1.49E-22	C3_CD8_Tem
chr21-44064889-44066026	2.28E-27	0.323997428	0.578	0.276	1.51E-22	C3_CD8_Tem
chr19-54209936-54211289	2.49E-27	0.288755243	0.659	0.341	1.65E-22	C3_CD8_Tem
chr1-160755624-160757066	2.73E-27	0.399878784	0.317	0.111	1.81E-22	C3_CD8_Tem
chr16-89784179-89785008	3.22E-27	0.392923513	0.24	0.074	2.14E-22	C3_CD8_Tem
chr2-86790522-86791567	3.24E-27	0.391150585	0.325	0.11	2.14E-22	C3_CD8_Tem
chr2-73830489-73831535	4.71E-27	0.349584131	0.499	0.221	3.12E-22	C3_CD8_Tem
chr1-225454008-225455207	5.00E-27	0.382269244	0.342	0.125	3.31E-22	C3_CD8_Tem
chr3-45957881-45958880	5.16E-27	0.410714769	0.219	0.057	3.42E-22	C3_CD8_Tem
chr2-86815456-86815995	8.45E-27	0.400444385	0.149	0.027	5.60E-22	C3_CD8_Tem
chr18-3511535-3512424	9.03E-27	0.391494761	0.323	0.113	5.98E-22	C3_CD8_Tem
chr14-106506652-106507782	1.00E-26	0.308199789	0.633	0.31	6.64E-22	C3_CD8_Tem
chr3-122974779-122976329	1.02E-26	0.380523885	0.287	0.096	6.75E-22	C3_CD8_Tem
chr3-123491916-123492608	1.36E-26	0.394801697	0.173	0.039	8.98E-22	C3_CD8_Tem
chr1-168519863-168520828	1.56E-26	0.400082623	0.297	0.096	1.03E-21	C3_CD8_Tem
chr2-144508210-144508577	1.83E-26	0.390843979	0.222	0.062	1.21E-21	C3_CD8_Tem
chr4-36281030-36281699	1.97E-26	0.399794995	0.311	0.099	1.30E-21	C3_CD8_Tem
chr10-30746968-30747990	2.48E-26	0.399456158	0.216	0.061	1.64E-21	C3_CD8_Tem
chr4-2936723-2937154	2.51E-26	0.388402528	0.345	0.125	1.66E-21	C3_CD8_Tem
chr14-105993495-105994314	3.29E-26	0.389966613	0.177	0.043	2.18E-21	C3_CD8_Tem
chr15-69679407-69680170	4.20E-26	0.404644294	0.209	0.055	2.78E-21	C3_CD8_Tem
chr6-224832-225792	4.24E-26	0.351403389	0.437	0.183	2.81E-21	C3_CD8_Tem
chr6-135537548-135538735	4.63E-26	0.366481101	0.265	0.089	3.07E-21	C3_CD8_Tem
chr12-6426603-6427592	4.64E-26	0.370354492	0.417	0.165	3.08E-21	C3_CD8_Tem

chr2-96167209-96167689	4.82E-26	0.39548617	0.149	0.03	3.19E-21	C3_CD8_Tem
chr2-86037206-86039136	4.83E-26	0.272752524	0.716	0.399	3.20E-21	C3_CD8_Tem
chr17-3737410-3739041	4.85E-26	0.369655757	0.393	0.155	3.21E-21	C3_CD8_Tem
chr8-58790453-58791432	5.14E-26	0.387735398	0.25	0.075	3.40E-21	C3_CD8_Tem
chr12-52361402-52361943	6.45E-26	0.389509429	0.146	0.028	4.27E-21	C3_CD8_Tem
chr3-28049995-28051257	6.50E-26	0.399861476	0.252	0.072	4.30E-21	C3_CD8_Tem
chr2-68378735-68379810	6.82E-26	0.399492165	0.254	0.076	4.51E-21	C3_CD8_Tem
chr7-106021478-106022922	7.03E-26	0.314524733	0.56	0.272	4.66E-21	C3_CD8_Tem
chr3-111608073-111608709	8.26E-26	0.378462145	0.333	0.119	5.47E-21	C3_CD8_Tem
chr1-100712436-100713975	1.05E-25	0.379773806	0.275	0.084	6.97E-21	C3_CD8_Tem
chr15-74383546-74385446	1.07E-25	0.37353857	0.381	0.142	7.05E-21	C3_CD8_Tem
chr2-29010912-29012188	1.22E-25	0.303148088	0.611	0.311	8.06E-21	C3_CD8_Tem
chr11-122856416-122857078	1.23E-25	0.382771063	0.253	0.079	8.13E-21	C3_CD8_Tem
chr1-51520040-51521153	1.40E-25	0.355988108	0.428	0.178	9.29E-21	C3_CD8_Tem
chr21-34086507-34087276	1.41E-25	0.378901592	0.207	0.057	9.35E-21	C3_CD8_Tem
chr10-50527062-50527918	1.81E-25	0.39179457	0.209	0.058	1.20E-20	C3_CD8_Tem
chr6-30568132-30568616	1.81E-25	0.403488562	0.26	0.077	1.20E-20	C3_CD8_Tem
chr8-29407902-29408865	2.14E-25	0.314735912	0.586	0.28	1.42E-20	C3_CD8_Tem
chr14-106004855-106006329	2.16E-25	0.338420052	0.512	0.23	1.43E-20	C3_CD8_Tem
chr19-6521764-6522766	2.22E-25	0.362325047	0.391	0.155	1.47E-20	C3_CD8_Tem
chr3-114323531-114324200	2.26E-25	0.395711916	0.178	0.042	1.50E-20	C3_CD8_Tem
chr1-53561863-53562889	2.51E-25	0.384543369	0.152	0.03	1.66E-20	C3_CD8_Tem
chr17-36198897-36199460	2.57E-25	0.373812919	0.355	0.125	1.70E-20	C3_CD8_Tem
chr12-52558738-52559814	2.58E-25	0.379609732	0.224	0.065	1.71E-20	C3_CD8_Tem
chr2-98835548-98836629	2.73E-25	0.351327571	0.436	0.185	1.81E-20	C3_CD8_Tem
chr20-37189590-37190679	3.06E-25	0.384343207	0.149	0.029	2.02E-20	C3_CD8_Tem
chr16-57040964-57042553	3.07E-25	0.292502224	0.683	0.361	2.03E-20	C3_CD8_Tem

chr17-36081820-36083037	3.27E-25	0.340027206	0.447	0.192	2.17E-20	C3_CD8_Tem
chr12-22409066-22410762	3.62E-25	0.287264739	0.613	0.317	2.40E-20	C3_CD8_Tem
chr13-29934808-29936565	3.93E-25	0.317144328	0.526	0.247	2.60E-20	C3_CD8_Tem
chr14-101712387-101713961	4.40E-25	0.331055306	0.465	0.196	2.91E-20	C3_CD8_Tem
chr19-51372441-51373731	4.52E-25	0.330227057	0.52	0.232	2.99E-20	C3_CD8_Tem
chr11-35247140-35248293	4.62E-25	0.312274251	0.519	0.246	3.06E-20	C3_CD8_Tem
chr9-97910196-97910809	7.11E-25	0.392881277	0.217	0.06	4.71E-20	C3_CD8_Tem
chr2-96157488-96158263	1.01E-24	0.363602957	0.4	0.162	6.67E-20	C3_CD8_Tem
chr6-16487460-16488540	1.03E-24	0.372734058	0.348	0.133	6.83E-20	C3_CD8_Tem
chr1-7930547-7931699	1.05E-24	0.373416631	0.363	0.138	6.93E-20	C3_CD8_Tem
chr16-71855751-71856725	1.17E-24	0.38452312	0.197	0.054	7.72E-20	C3_CD8_Tem
chr4-6855496-6856344	1.22E-24	0.332225289	0.484	0.221	8.11E-20	C3_CD8_Tem
chr9-20041689-20043404	1.25E-24	0.386952193	0.202	0.053	8.26E-20	C3_CD8_Tem
chr2-121745929-121746674	2.02E-24	0.358843216	0.138	0.03	1.34E-19	C3_CD8_Tem
chr16-72013109-72013601	2.02E-24	0.372246342	0.13	0.025	1.34E-19	C3_CD8_Tem
chr15-77021514-77022455	2.03E-24	0.376268548	0.378	0.148	1.34E-19	C3_CD8_Tem
chr1-160738839-160739406	2.56E-24	0.388545822	0.311	0.103	1.69E-19	C3_CD8_Tem
chr12-49043678-49044518	2.74E-24	0.383391265	0.212	0.062	1.81E-19	C3_CD8_Tem
chr19-17124948-17126597	2.87E-24	0.271071233	0.658	0.363	1.90E-19	C3_CD8_Tem
chr11-128550976-128552304	2.88E-24	0.357412368	0.402	0.17	1.90E-19	C3_CD8_Tem
chr4-36278538-36279126	2.92E-24	0.390739069	0.18	0.044	1.93E-19	C3_CD8_Tem
chr2-112920708-112921333	3.26E-24	0.367370142	0.153	0.039	2.16E-19	C3_CD8_Tem
chr19-38559109-38560093	3.31E-24	0.371524061	0.392	0.156	2.19E-19	C3_CD8_Tem
chr1-42936414-42937495	3.79E-24	0.358301761	0.415	0.165	2.51E-19	C3_CD8_Tem
chr7-6240465-6242175	4.53E-24	0.379682979	0.213	0.06	3.00E-19	C3_CD8_Tem
chr12-9906361-9907281	4.72E-24	0.375287804	0.328	0.117	3.12E-19	C3_CD8_Tem
chr11-64331142-64332138	5.31E-24	0.369313219	0.355	0.132	3.51E-19	C3_CD8_Tem

chr14-93065704-93066578	5.93E-24	0.369155744	0.321	0.115	3.93E-19	C3_CD8_Tem
chr17-64392409-64393697	6.20E-24	0.379405374	0.177	0.045	4.11E-19	C3_CD8_Tem
chr1-235862718-235863357	6.64E-24	0.390890633	0.253	0.078	4.40E-19	C3_CD8_Tem
chr14-74617453-74618817	6.86E-24	0.301039696	0.604	0.311	4.54E-19	C3_CD8_Tem
chr19-58326425-58328187	7.59E-24	0.303097232	0.582	0.304	5.02E-19	C3_CD8_Tem
chr20-38872677-38873721	7.69E-24	0.287861607	0.618	0.321	5.09E-19	C3_CD8_Tem
chr17-10149804-10150604	9.30E-24	0.380023993	0.135	0.025	6.16E-19	C3_CD8_Tem
chr17-35217998-35218860	9.59E-24	0.357411739	0.407	0.167	6.35E-19	C3_CD8_Tem
chr3-45980223-45980831	1.12E-23	0.381794647	0.303	0.109	7.39E-19	C3_CD8_Tem
chr1-38027955-38028570	1.15E-23	0.393330278	0.198	0.052	7.62E-19	C3_CD8_Tem
chr12-52566301-52567390	1.27E-23	0.366220251	0.231	0.074	8.40E-19	C3_CD8_Tem
chrX-107449624-107451395	1.32E-23	0.3690876	0.26	0.083	8.74E-19	C3_CD8_Tem
chr7-100884591-100885134	1.65E-23	0.376571792	0.272	0.093	1.09E-18	C3_CD8_Tem
chr1-154790841-154792148	1.66E-23	0.353665017	0.438	0.184	1.10E-18	C3_CD8_Tem
chr13-41492087-41493279	1.73E-23	0.368393389	0.354	0.137	1.14E-18	C3_CD8_Tem
chr7-925609-926009	1.78E-23	0.379452453	0.289	0.1	1.18E-18	C3_CD8_Tem
chr17-35519463-35520065	2.27E-23	0.395364217	0.186	0.046	1.50E-18	C3_CD8_Tem
chrX-107479597-107480657	2.54E-23	0.373371477	0.15	0.033	1.68E-18	C3_CD8_Tem
chr10-7483654-7485358	2.73E-23	0.357225138	0.292	0.108	1.81E-18	C3_CD8_Tem
chr3-188553262-188554692	2.75E-23	0.368303367	0.315	0.114	1.82E-18	C3_CD8_Tem
chr18-13453621-13454061	3.40E-23	0.325907379	0.084	0.01	2.25E-18	C3_CD8_Tem
chrX-107451771-107452071	3.85E-23	0.342319801	0.105	0.016	2.55E-18	C3_CD8_Tem
chr12-110577395-110579067	3.95E-23	0.30213134	0.581	0.293	2.62E-18	C3_CD8_Tem
chr22-22787701-22788489	4.08E-23	0.385223346	0.237	0.072	2.70E-18	C3_CD8_Tem
chr3-47037543-47038612	4.11E-23	0.267813243	0.644	0.342	2.72E-18	C3_CD8_Tem
chr18-12920744-12921812	4.19E-23	0.349518852	0.278	0.101	2.77E-18	C3_CD8_Tem
chr16-87853497-87854843	4.52E-23	0.279800603	0.638	0.347	2.99E-18	C3_CD8_Tem

chr17-82775027-82775870	5.76E-23	0.376472486	0.173	0.043	3.81E-18	C3_CD8_Tem
chr5-1518362-1519073	5.77E-23	0.369287916	0.273	0.087	3.82E-18	C3_CD8_Tem
chr19-5077800-5078647	7.03E-23	0.356257812	0.144	0.033	4.66E-18	C3_CD8_Tem
chr6-193662-194430	8.75E-23	0.365903248	0.352	0.13	5.79E-18	C3_CD8_Tem
chr5-171957368-171958385	8.75E-23	0.360426516	0.233	0.08	5.79E-18	C3_CD8_Tem
chr2-105863707-105864687	8.80E-23	0.374377827	0.195	0.052	5.83E-18	C3_CD8_Tem
chr2-136310865-136311505	9.70E-23	0.384268882	0.195	0.05	6.42E-18	C3_CD8_Tem
chr2-24739351-24739990	9.93E-23	0.35315211	0.131	0.03	6.58E-18	C3_CD8_Tem
chr5-107476616-107477202	1.11E-22	0.358656448	0.285	0.097	7.33E-18	C3_CD8_Tem
chr19-45667368-45669445	1.14E-22	0.305924631	0.53	0.265	7.55E-18	C3_CD8_Tem
chr3-27905361-27906046	1.17E-22	0.382435358	0.24	0.067	7.72E-18	C3_CD8_Tem
chr6-130033947-130034987	1.33E-22	0.375607786	0.285	0.099	8.81E-18	C3_CD8_Tem
chr16-80569205-80570422	1.36E-22	0.342394518	0.366	0.146	8.99E-18	C3_CD8_Tem
chr1-91947948-91949312	1.37E-22	0.335226166	0.443	0.186	9.10E-18	C3_CD8_Tem
chr8-29049783-29050546	1.54E-22	0.368428451	0.222	0.065	1.02E-17	C3_CD8_Tem
chr3-47056584-47057721	1.64E-22	0.329772056	0.471	0.215	1.09E-17	C3_CD8_Tem
chr6-127902865-127903304	1.86E-22	0.377508782	0.194	0.049	1.23E-17	C3_CD8_Tem
chr11-118051199-118052134	2.02E-22	0.309313193	0.468	0.225	1.34E-17	C3_CD8_Tem
chr7-35729052-35730816	2.32E-22	0.333740299	0.439	0.185	1.54E-17	C3_CD8_Tem
chr2-64880665-64881690	2.62E-22	0.352434832	0.154	0.038	1.74E-17	C3_CD8_Tem
chr19-10523026-10523671	3.62E-22	0.362601431	0.249	0.081	2.40E-17	C3_CD8_Tem
chr11-45092887-45093822	3.78E-22	0.359180364	0.3	0.108	2.50E-17	C3_CD8_Tem
chr2-10497665-10498228	3.81E-22	0.373986992	0.219	0.068	2.52E-17	C3_CD8_Tem
chr1-228136881-228137236	4.03E-22	0.365633787	0.132	0.025	2.67E-17	C3_CD8_Tem
chr8-133068040-133069388	4.50E-22	0.285323379	0.614	0.326	2.98E-17	C3_CD8_Tem
chr7-150665882-150666234	5.21E-22	0.367167393	0.23	0.069	3.45E-17	C3_CD8_Tem
chr5-61327800-61328385	5.42E-22	0.381458844	0.208	0.058	3.59E-17	C3_CD8_Tem

chr2-15178578-15179277	5.50E-22	0.359982491	0.152	0.036	3.64E-17	C3_CD8_Tem
chr11-118692156-118692907	5.77E-22	0.353788218	0.338	0.134	3.82E-17	C3_CD8_Tem
chr22-50018577-50019498	6.14E-22	0.387622791	0.213	0.06	4.07E-17	C3_CD8_Tem
chr5-32537250-32538363	6.41E-22	0.360493124	0.292	0.107	4.25E-17	C3_CD8_Tem
chr7-100353226-100354065	6.42E-22	0.376434593	0.179	0.044	4.25E-17	C3_CD8_Tem
chr16-57601346-57602512	7.32E-22	0.329234155	0.449	0.203	4.85E-17	C3_CD8_Tem
chr2-68771057-68771646	8.83E-22	0.368900662	0.276	0.1	5.85E-17	C3_CD8_Tem
chr22-27875514-27876372	9.03E-22	0.35920888	0.171	0.045	5.98E-17	C3_CD8_Tem
chr6-105645959-105646893	9.41E-22	0.320038296	0.459	0.203	6.23E-17	C3_CD8_Tem
chr9-20358104-20358641	1.03E-21	0.354482594	0.134	0.029	6.83E-17	C3_CD8_Tem
chr20-51530978-51531758	1.05E-21	0.272450294	0.62	0.319	6.94E-17	C3_CD8_Tem
chr1-156217552-156218363	1.13E-21	0.362320141	0.382	0.151	7.45E-17	C3_CD8_Tem
chr5-176653148-176653757	1.13E-21	0.364241561	0.253	0.083	7.46E-17	C3_CD8_Tem
chr16-88619267-88620766	1.15E-21	0.275143889	0.638	0.336	7.60E-17	C3_CD8_Tem
chr20-32284843-32286307	1.31E-21	0.333058728	0.472	0.221	8.69E-17	C3_CD8_Tem
chr1-236156236-236157125	1.44E-21	0.341686796	0.361	0.141	9.51E-17	C3_CD8_Tem
chr7-36787715-36788380	1.47E-21	0.360292294	0.304	0.112	9.73E-17	C3_CD8_Tem
chr11-36384958-36385584	1.48E-21	0.370075875	0.253	0.08	9.82E-17	C3_CD8_Tem
chr2-219247439-219248340	1.49E-21	0.304657725	0.495	0.238	9.89E-17	C3_CD8_Tem
chr1-26275597-26276590	1.68E-21	0.292507929	0.586	0.305	1.11E-16	C3_CD8_Tem
chr13-99403154-99403966	1.85E-21	0.355522259	0.209	0.065	1.23E-16	C3_CD8_Tem
chr22-22737509-22738033	1.90E-21	0.364490198	0.147	0.032	1.26E-16	C3_CD8_Tem
chr1-192550681-192551572	2.18E-21	0.348306493	0.286	0.108	1.44E-16	C3_CD8_Tem
chr6-10732633-10733726	2.19E-21	0.357141717	0.154	0.038	1.45E-16	C3_CD8_Tem
chr2-169682683-169683766	2.52E-21	0.350050641	0.315	0.12	1.67E-16	C3_CD8_Tem
chr5-143001776-143003351	2.61E-21	0.272678784	0.62	0.328	1.73E-16	C3_CD8_Tem
chr11-12215903-12216717	2.74E-21	0.339893082	0.142	0.035	1.82E-16	C3_CD8_Tem

chr3-98563422-98564975	2.87E-21	0.307567671	0.471	0.229	1.90E-16	C3_CD8_Tem
chr8-90466949-90468031	3.14E-21	0.359600618	0.163	0.043	2.08E-16	C3_CD8_Tem
chr16-31421353-31422176	3.53E-21	0.370668518	0.252	0.081	2.34E-16	C3_CD8_Tem
chr17-12981952-12982730	3.67E-21	0.361095882	0.195	0.058	2.43E-16	C3_CD8_Tem
chr16-89096594-89097599	3.70E-21	0.320491422	0.413	0.185	2.45E-16	C3_CD8_Tem
chr1-110879494-110881374	3.87E-21	0.258719737	0.696	0.397	2.57E-16	C3_CD8_Tem
chr3-46359594-46360492	3.94E-21	0.331783905	0.395	0.168	2.61E-16	C3_CD8_Tem
chr17-80265206-80265894	4.33E-21	0.342053573	0.403	0.172	2.86E-16	C3_CD8_Tem
chr20-49805033-49805682	4.42E-21	0.348738596	0.288	0.106	2.92E-16	C3_CD8_Tem
chr1-25099271-25100697	4.51E-21	0.367066614	0.286	0.101	2.99E-16	C3_CD8_Tem
chr14-103909899-103910613	4.60E-21	0.373240631	0.237	0.075	3.04E-16	C3_CD8_Tem
chr17-74470999-74471668	5.10E-21	0.369196458	0.191	0.055	3.38E-16	C3_CD8_Tem
chr1-234699694-234700645	5.45E-21	0.343335605	0.254	0.088	3.61E-16	C3_CD8_Tem
chr2-231425680-231426544	5.68E-21	0.344401339	0.197	0.064	3.76E-16	C3_CD8_Tem
chr22-20550440-20552163	6.31E-21	0.345961405	0.374	0.157	4.18E-16	C3_CD8_Tem
chr9-71782690-71783986	6.33E-21	0.251068861	0.669	0.368	4.19E-16	C3_CD8_Tem
chr1-26377507-26378830	6.34E-21	0.258385109	0.638	0.347	4.20E-16	C3_CD8_Tem
chr14-106184342-106186119	6.45E-21	0.321910755	0.48	0.221	4.27E-16	C3_CD8_Tem
chr20-49668267-49670238	6.72E-21	0.313819911	0.501	0.239	4.45E-16	C3_CD8_Tem
chr2-203763279-203764214	1.00E-20	0.354424902	0.18	0.05	6.65E-16	C3_CD8_Tem
chr3-39267373-39268496	1.01E-20	0.325331127	0.404	0.17	6.68E-16	C3_CD8_Tem
chr9-89448381-89448821	1.04E-20	0.370564281	0.19	0.053	6.89E-16	C3_CD8_Tem
chr20-62977422-62978333	1.09E-20	0.34138829	0.354	0.143	7.20E-16	C3_CD8_Tem
chr1-53412950-53413955	1.09E-20	0.317416739	0.463	0.219	7.25E-16	C3_CD8_Tem
chr16-10876338-10877689	1.18E-20	0.332769759	0.418	0.184	7.83E-16	C3_CD8_Tem
chr7-6220925-6222411	1.28E-20	0.316811857	0.377	0.167	8.49E-16	C3_CD8_Tem
chr5-68344268-68345273	1.32E-20	0.351168387	0.218	0.072	8.73E-16	C3_CD8_Tem

chr15-38578506-38578944	1.47E-20	0.348149122	0.128	0.028	9.75E-16	C3_CD8_Tem
chr14-97380434-97381041	1.64E-20	0.351659727	0.22	0.071	1.08E-15	C3_CD8_Tem
chr13-99289490-99290186	1.64E-20	0.342869047	0.296	0.11	1.09E-15	C3_CD8_Tem
chr12-6069234-6069891	1.99E-20	0.357593272	0.205	0.062	1.32E-15	C3_CD8_Tem
chr11-1568276-1568696	2.24E-20	0.357545817	0.206	0.058	1.48E-15	C3_CD8_Tem
chr1-25642076-25643133	2.25E-20	0.334055453	0.409	0.177	1.49E-15	C3_CD8_Tem
chr10-50508093-50508803	2.26E-20	0.333574884	0.354	0.148	1.49E-15	C3_CD8_Tem
chr19-51393858-51395772	2.29E-20	0.275094357	0.653	0.357	1.51E-15	C3_CD8_Tem
chr3-149009270-149010325	2.34E-20	0.317586123	0.408	0.175	1.55E-15	C3_CD8_Tem
chr3-141243005-141243725	2.80E-20	0.34615908	0.158	0.042	1.86E-15	C3_CD8_Tem
chr6-16699041-16699777	3.12E-20	0.332245122	0.44	0.191	2.07E-15	C3_CD8_Tem
chr2-37623512-37624861	3.32E-20	0.338186174	0.384	0.163	2.20E-15	C3_CD8_Tem
chr8-664759-665967	3.33E-20	0.343537853	0.155	0.042	2.21E-15	C3_CD8_Tem
chr5-134111599-134112485	3.40E-20	0.339824601	0.342	0.142	2.25E-15	C3_CD8_Tem
chr17-40479856-40481224	3.64E-20	0.256139059	0.633	0.343	2.41E-15	C3_CD8_Tem
chr6-188656-189699	4.05E-20	0.35811146	0.273	0.098	2.68E-15	C3_CD8_Tem
chr20-38874619-38875891	4.14E-20	0.315410152	0.468	0.217	2.74E-15	C3_CD8_Tem
chr17-27471322-27472502	4.31E-20	0.301049507	0.49	0.241	2.86E-15	C3_CD8_Tem
chr14-50789772-50790133	4.33E-20	0.341753264	0.103	0.017	2.87E-15	C3_CD8_Tem
chr16-391649-392432	4.36E-20	0.318593738	0.393	0.171	2.89E-15	C3_CD8_Tem
chr5-55036843-55037992	5.06E-20	0.364184658	0.274	0.095	3.35E-15	C3_CD8_Tem
chr3-27720978-27722529	5.06E-20	0.328955346	0.436	0.191	3.35E-15	C3_CD8_Tem
chr21-33937726-33938566	5.08E-20	0.347488819	0.311	0.118	3.37E-15	C3_CD8_Tem
chr12-10595665-10596319	5.08E-20	0.322726963	0.092	0.015	3.37E-15	C3_CD8_Tem
chr15-67106490-67107471	5.39E-20	0.358704513	0.266	0.093	3.57E-15	C3_CD8_Tem
chr9-131310396-131311231	5.40E-20	0.353613802	0.243	0.084	3.58E-15	C3_CD8_Tem
chr5-143218472-143219003	5.69E-20	0.333074104	0.129	0.03	3.77E-15	C3_CD8_Tem

chr6-143544221-143544640	6.09E-20	0.341272425	0.128	0.03	4.03E-15	C3_CD8_Tem
chr12-10721588-10723505	6.21E-20	0.337191809	0.367	0.141	4.12E-15	C3_CD8_Tem
chr2-203992358-203993938	6.28E-20	0.337453133	0.298	0.121	4.16E-15	C3_CD8_Tem
chr19-35712568-35713417	6.46E-20	0.325308085	0.414	0.186	4.28E-15	C3_CD8_Tem
chr16-85935569-85937124	6.55E-20	0.329051168	0.432	0.194	4.34E-15	C3_CD8_Tem
chr8-133498177-133500468	6.60E-20	0.261722262	0.657	0.372	4.37E-15	C3_CD8_Tem
chr1-32801060-32801651	6.77E-20	0.330863487	0.384	0.167	4.48E-15	C3_CD8_Tem
chr3-66298568-66298893	6.93E-20	0.348984035	0.139	0.034	4.59E-15	C3_CD8_Tem
chr18-46536388-46536937	7.36E-20	0.333134482	0.113	0.023	4.87E-15	C3_CD8_Tem
chr22-21766646-21767539	7.62E-20	0.31765027	0.439	0.193	5.05E-15	C3_CD8_Tem
chr1-8873543-8873917	7.64E-20	0.364375056	0.321	0.121	5.06E-15	C3_CD8_Tem
chr12-57222735-57223690	7.67E-20	0.333545829	0.322	0.125	5.08E-15	C3_CD8_Tem
chr3-183265877-183266583	8.00E-20	0.347794591	0.135	0.03	5.30E-15	C3_CD8_Tem
chr5-155766982-155767882	8.14E-20	0.337324818	0.177	0.052	5.39E-15	C3_CD8_Tem
chr20-51529421-51530098	8.29E-20	0.322766396	0.413	0.183	5.49E-15	C3_CD8_Tem
chr12-52327151-52328001	8.58E-20	0.35141788	0.277	0.1	5.68E-15	C3_CD8_Tem
chr22-20519718-20520552	8.80E-20	0.363361485	0.216	0.064	5.83E-15	C3_CD8_Tem
chr5-107485956-107486918	9.14E-20	0.342203725	0.242	0.084	6.05E-15	C3_CD8_Tem
chr3-45975843-45977120	9.17E-20	0.324419757	0.4	0.174	6.08E-15	C3_CD8_Tem
chr10-14007906-14010051	9.34E-20	0.310995816	0.44	0.208	6.19E-15	C3_CD8_Tem
chr1-24910625-24911762	1.07E-19	0.250888857	0.678	0.369	7.08E-15	C3_CD8_Tem
chr16-87895386-87896667	1.13E-19	0.299908999	0.483	0.233	7.50E-15	C3_CD8_Tem
chr1-26799587-26800842	1.14E-19	0.369433958	0.273	0.094	7.58E-15	C3_CD8_Tem
chr7-138875436-138876315	1.20E-19	0.290661369	0.47	0.229	7.95E-15	C3_CD8_Tem
chr10-100370276-100371692	1.21E-19	0.29142132	0.549	0.278	8.03E-15	C3_CD8_Tem
chr5-107474537-107475439	1.28E-19	0.345533659	0.274	0.097	8.46E-15	C3_CD8_Tem
chr21-34800971-34801962	1.30E-19	0.342458911	0.184	0.054	8.61E-15	C3_CD8_Tem

chr17-3763177-3763727	1.41E-19	0.344452314	0.374	0.148	9.35E-15	C3_CD8_Tem
chr9-114366219-114366991	1.41E-19	0.325904713	0.438	0.196	9.36E-15	C3_CD8_Tem
chr1-107785657-107786884	1.46E-19	0.328962083	0.33	0.129	9.64E-15	C3_CD8_Tem
chr16-27449378-27451209	1.46E-19	0.311707592	0.462	0.213	9.65E-15	C3_CD8_Tem
chr15-38251201-38253230	1.51E-19	0.337768129	0.295	0.112	1.00E-14	C3_CD8_Tem
chr1-100714573-100714958	1.52E-19	0.328828738	0.131	0.031	1.00E-14	C3_CD8_Tem
chr7-26166549-26168204	1.73E-19	0.354570297	0.316	0.116	1.15E-14	C3_CD8_Tem
chr8-22443319-22444348	1.76E-19	0.292610992	0.554	0.28	1.17E-14	C3_CD8_Tem
chr18-80171880-80172411	1.80E-19	0.348174815	0.184	0.054	1.19E-14	C3_CD8_Tem
chr2-86820019-86820947	1.82E-19	0.351125942	0.149	0.035	1.21E-14	C3_CD8_Tem
chr12-121033338-121034295	1.99E-19	0.299293241	0.543	0.274	1.32E-14	C3_CD8_Tem
chr2-230793603-230794832	2.17E-19	0.303183108	0.497	0.251	1.43E-14	C3_CD8_Tem
chr5-163461335-163462593	2.19E-19	0.277097752	0.565	0.293	1.45E-14	C3_CD8_Tem
chr10-14118784-14119354	2.27E-19	0.321785827	0.105	0.019	1.50E-14	C3_CD8_Tem
chr3-71421672-71422664	2.33E-19	0.356310533	0.285	0.105	1.54E-14	C3_CD8_Tem
chr16-56261625-56262745	2.43E-19	0.311111774	0.437	0.199	1.61E-14	C3_CD8_Tem
chr5-170269473-170269949	2.58E-19	0.309942183	0.454	0.202	1.71E-14	C3_CD8_Tem
chr1-221829155-221830135	2.61E-19	0.308853757	0.443	0.199	1.73E-14	C3_CD8_Tem
chr11-63553371-63554243	2.64E-19	0.349893849	0.283	0.106	1.75E-14	C3_CD8_Tem
chr5-150376673-150377594	3.08E-19	0.349414139	0.147	0.037	2.04E-14	C3_CD8_Tem
chr2-62208366-62209144	3.19E-19	0.349666192	0.212	0.065	2.11E-14	C3_CD8_Tem
chr4-77586502-77587521	3.53E-19	0.308595698	0.361	0.152	2.33E-14	C3_CD8_Tem
chr1-156214299-156214702	3.72E-19	0.371661004	0.224	0.07	2.47E-14	C3_CD8_Tem
chr13-33015915-33017891	3.91E-19	0.331826732	0.127	0.031	2.59E-14	C3_CD8_Tem
chr20-62141216-62142022	4.10E-19	0.355751585	0.218	0.068	2.72E-14	C3_CD8_Tem
chr12-10672135-10673007	4.15E-19	0.33932206	0.252	0.089	2.75E-14	C3_CD8_Tem
chr2-168194674-168195463	4.51E-19	0.32731491	0.286	0.112	2.98E-14	C3_CD8_Tem

chr1-172646674-172647603	4.98E-19	0.350701489	0.212	0.064	3.30E-14	C3_CD8_Tem
chr5-102853536-102854097	5.45E-19	0.349816846	0.15	0.039	3.61E-14	C3_CD8_Tem
chr12-94243950-94245220	5.48E-19	0.34107432	0.201	0.065	3.63E-14	C3_CD8_Tem
chr10-7028076-7028824	5.55E-19	0.341592001	0.158	0.043	3.68E-14	C3_CD8_Tem
chr22-43262997-43264495	5.78E-19	0.305171869	0.521	0.255	3.83E-14	C3_CD8_Tem
chr17-80891264-80893233	5.86E-19	0.261647599	0.658	0.374	3.88E-14	C3_CD8_Tem
chr9-89426299-89428064	6.85E-19	0.293416361	0.509	0.249	4.53E-14	C3_CD8_Tem
chr22-22721908-22722289	7.42E-19	0.338468428	0.121	0.025	4.92E-14	C3_CD8_Tem
chr5-143228855-143230545	7.47E-19	0.307694205	0.45	0.208	4.94E-14	C3_CD8_Tem
chr15-78046823-78047423	7.66E-19	0.318131294	0.386	0.168	5.07E-14	C3_CD8_Tem
chr5-139925797-139926640	7.98E-19	0.327748489	0.138	0.034	5.28E-14	C3_CD8_Tem
chr4-10095455-10097209	8.34E-19	0.27499645	0.568	0.301	5.52E-14	C3_CD8_Tem
chr8-27386227-27386923	8.58E-19	0.338650194	0.139	0.034	5.68E-14	C3_CD8_Tem
chr19-4104223-4105353	8.93E-19	0.281197098	0.55	0.279	5.92E-14	C3_CD8_Tem
chr2-62304674-62306185	9.42E-19	0.346547714	0.261	0.091	6.24E-14	C3_CD8_Tem
chr5-87120180-87121626	9.44E-19	0.306879157	0.444	0.205	6.25E-14	C3_CD8_Tem
chr6-10726322-10726976	9.46E-19	0.317089163	0.111	0.026	6.26E-14	C3_CD8_Tem
chr18-13611875-13612700	9.73E-19	0.260583255	0.562	0.296	6.44E-14	C3_CD8_Tem
chr16-70737036-70738164	1.00E-18	0.338683295	0.227	0.078	6.64E-14	C3_CD8_Tem
chr18-80174997-80175974	1.09E-18	0.279960023	0.459	0.217	7.23E-14	C3_CD8_Tem
chr8-29489883-29490946	1.12E-18	0.33098713	0.328	0.132	7.42E-14	C3_CD8_Tem
chr14-24206945-24207575	1.14E-18	0.317190718	0.103	0.02	7.58E-14	C3_CD8_Tem
chr19-4108531-4109469	1.16E-18	0.314097828	0.354	0.151	7.65E-14	C3_CD8_Tem
chr16-80580013-80580858	1.16E-18	0.331357297	0.315	0.124	7.68E-14	C3_CD8_Tem
chr17-75241875-75243049	1.18E-18	0.344326374	0.303	0.12	7.84E-14	C3_CD8_Tem
chr16-53537470-53538074	1.20E-18	0.338230582	0.155	0.042	7.95E-14	C3_CD8_Tem
chr8-58850557-58851235	1.21E-18	0.322738408	0.108	0.022	8.02E-14	C3_CD8_Tem

chr5-76542471-76543686	1.33E-18	0.254783694	0.582	0.322	8.84E-14	C3_CD8_Tem
chr6-42417154-42418179	1.39E-18	0.357594711	0.237	0.077	9.18E-14	C3_CD8_Tem
chr1-93817483-93818780	1.57E-18	0.321026242	0.272	0.107	1.04E-13	C3_CD8_Tem
chr9-114348407-114349465	1.59E-18	0.330673679	0.381	0.158	1.05E-13	C3_CD8_Tem
chr20-48757937-48758583	1.64E-18	0.347472543	0.249	0.085	1.09E-13	C3_CD8_Tem
chr1-221793265-221794674	1.70E-18	0.316785209	0.286	0.109	1.13E-13	C3_CD8_Tem
chr15-38308208-38309253	1.71E-18	0.341946996	0.129	0.03	1.13E-13	C3_CD8_Tem
chr3-188974768-188975646	1.74E-18	0.342759307	0.184	0.055	1.15E-13	C3_CD8_Tem
chr4-7831165-7831725	1.86E-18	0.355874708	0.233	0.077	1.23E-13	C3_CD8_Tem
chr16-21540504-21541478	1.89E-18	0.299753338	0.361	0.158	1.25E-13	C3_CD8_Tem
chr22-44180249-44182890	1.91E-18	0.294148173	0.527	0.277	1.26E-13	C3_CD8_Tem
chr20-36627671-36628516	2.08E-18	0.336206318	0.288	0.104	1.38E-13	C3_CD8_Tem
chr13-112768160-112769367	2.17E-18	0.339321401	0.164	0.046	1.44E-13	C3_CD8_Tem
chr13-30371414-30372280	2.22E-18	0.303202417	0.487	0.235	1.47E-13	C3_CD8_Tem
chr6-111803953-111806086	2.27E-18	0.282512781	0.585	0.303	1.50E-13	C3_CD8_Tem
chr7-105806371-105806968	2.36E-18	0.346478639	0.204	0.065	1.57E-13	C3_CD8_Tem
chr7-2037211-2038557	2.38E-18	0.345271934	0.195	0.06	1.57E-13	C3_CD8_Tem
chr17-1823106-1823993	2.41E-18	0.312986376	0.358	0.156	1.60E-13	C3_CD8_Tem
chr2-134669904-134670799	2.70E-18	0.338860332	0.297	0.109	1.79E-13	C3_CD8_Tem
chr17-5241943-5243263	2.76E-18	0.339419567	0.166	0.049	1.83E-13	C3_CD8_Tem
chr18-51191044-51191652	2.93E-18	0.339014905	0.271	0.1	1.94E-13	C3_CD8_Tem
chr17-10198118-10199316	2.93E-18	0.330711004	0.229	0.081	1.94E-13	C3_CD8_Tem
chr7-36686279-36686888	2.95E-18	0.344426857	0.233	0.078	1.96E-13	C3_CD8_Tem
chr14-99248859-99249767	2.98E-18	0.354460459	0.281	0.099	1.97E-13	C3_CD8_Tem
chr14-68733905-68734895	3.04E-18	0.315146357	0.369	0.157	2.02E-13	C3_CD8_Tem
chr1-92537995-92539149	3.23E-18	0.314064358	0.432	0.19	2.14E-13	C3_CD8_Tem
chr3-111695343-111696101	3.27E-18	0.345756959	0.199	0.062	2.17E-13	C3_CD8_Tem

chr20-5056444-5057393	3.39E-18	0.308475238	0.375	0.165	2.24E-13	C3_CD8_Tem
chr1-40058897-40060781	3.60E-18	0.346913948	0.344	0.136	2.39E-13	C3_CD8_Tem
chr7-36784623-36785346	4.51E-18	0.298230775	0.413	0.188	2.98E-13	C3_CD8_Tem
chr17-42266518-42267454	4.56E-18	0.264771569	0.579	0.316	3.02E-13	C3_CD8_Tem
chr17-64384876-64385574	4.63E-18	0.325991638	0.145	0.038	3.07E-13	C3_CD8_Tem
chr3-4587762-4588686	4.64E-18	0.311740574	0.098	0.018	3.07E-13	C3_CD8_Tem
chr20-36644017-36645047	4.64E-18	0.333276443	0.38	0.164	3.08E-13	C3_CD8_Tem
chr6-127971429-127973112	5.69E-18	0.266397461	0.556	0.29	3.77E-13	C3_CD8_Tem
chr16-80725655-80726057	5.83E-18	0.269138376	0.055	0.006	3.86E-13	C3_CD8_Tem
chr17-36066120-36067103	5.96E-18	0.318237369	0.273	0.104	3.95E-13	C3_CD8_Tem
chr1-53600208-53600946	6.00E-18	0.328170646	0.168	0.052	3.98E-13	C3_CD8_Tem
chr5-74974290-74975239	6.17E-18	0.334400863	0.212	0.074	4.08E-13	C3_CD8_Tem
chr13-46295121-46296637	6.25E-18	0.251466526	0.583	0.317	4.14E-13	C3_CD8_Tem
chr1-117663947-117664983	6.52E-18	0.274260578	0.593	0.312	4.32E-13	C3_CD8_Tem
chr14-97393042-97394054	6.55E-18	0.334448808	0.152	0.041	4.34E-13	C3_CD8_Tem
chr6-41531602-41532559	6.72E-18	0.323177271	0.314	0.124	4.45E-13	C3_CD8_Tem
chr17-36089888-36090742	7.29E-18	0.335586743	0.237	0.083	4.83E-13	C3_CD8_Tem
chr3-183328180-183329087	7.39E-18	0.30323466	0.079	0.011	4.89E-13	C3_CD8_Tem
chr20-51480265-51481961	7.55E-18	0.333289629	0.331	0.138	5.00E-13	C3_CD8_Tem
chrX-39859781-39860844	7.69E-18	0.291851179	0.462	0.216	5.09E-13	C3_CD8_Tem
chr1-248853194-248854037	7.94E-18	0.346411768	0.176	0.05	5.26E-13	C3_CD8_Tem
chr1-174153151-174153920	8.82E-18	0.346776212	0.224	0.078	5.84E-13	C3_CD8_Tem
chrX-107454096-107454662	9.09E-18	0.347997413	0.179	0.051	6.02E-13	C3_CD8_Tem
chr1-7948570-7949548	9.16E-18	0.321958697	0.356	0.154	6.07E-13	C3_CD8_Tem
chr16-67555402-67556295	9.26E-18	0.346986347	0.294	0.112	6.13E-13	C3_CD8_Tem
chr7-2698362-2699465	9.39E-18	0.332212239	0.304	0.12	6.22E-13	C3_CD8_Tem
chr20-57716215-57716565	9.63E-18	0.303121801	0.083	0.013	6.38E-13	C3_CD8_Tem

chr2-128381991-128382821	9.69E-18	0.321474849	0.122	0.028	6.42E-13	C3_CD8_Tem
chr1-172670231-172671127	9.72E-18	0.344416545	0.311	0.118	6.44E-13	C3_CD8_Tem
chr9-131732826-131734540	9.84E-18	0.279713608	0.552	0.281	6.51E-13	C3_CD8_Tem
chr3-28326120-28326988	9.91E-18	0.301019936	0.437	0.202	6.56E-13	C3_CD8_Tem
chr12-4806518-4808422	1.01E-17	0.339761483	0.251	0.093	6.69E-13	C3_CD8_Tem
chr7-143375135-143375888	1.07E-17	0.318312327	0.399	0.173	7.09E-13	C3_CD8_Tem
chr12-121036272-121037192	1.08E-17	0.328128376	0.399	0.176	7.13E-13	C3_CD8_Tem
chr16-68756090-68757132	1.10E-17	0.317731536	0.27	0.107	7.26E-13	C3_CD8_Tem
chr14-80929988-80931686	1.13E-17	0.260198145	0.509	0.268	7.46E-13	C3_CD8_Tem
chr1-160812949-160813628	1.13E-17	0.337257218	0.243	0.088	7.46E-13	C3_CD8_Tem
chr8-115218449-115219325	1.13E-17	0.258739045	0.487	0.248	7.48E-13	C3_CD8_Tem
chr6-34181890-34183339	1.15E-17	0.295552568	0.398	0.184	7.62E-13	C3_CD8_Tem
chr4-98446314-98447895	1.15E-17	0.316385787	0.393	0.172	7.64E-13	C3_CD8_Tem
chr2-239288791-239289933	1.18E-17	0.338085294	0.254	0.094	7.80E-13	C3_CD8_Tem
chr19-10963238-10963842	1.36E-17	0.338987601	0.248	0.09	9.03E-13	C3_CD8_Tem
chr2-234422145-234423481	1.39E-17	0.301056353	0.458	0.211	9.22E-13	C3_CD8_Tem
chr3-177198442-177199536	1.46E-17	0.327390583	0.311	0.125	9.64E-13	C3_CD8_Tem
chr1-53409902-53410451	1.47E-17	0.336236987	0.172	0.049	9.73E-13	C3_CD8_Tem
chr19-543224-544491	1.48E-17	0.310445792	0.424	0.189	9.82E-13	C3_CD8_Tem
chr3-28053526-28054012	1.53E-17	0.302990213	0.087	0.015	1.01E-12	C3_CD8_Tem
chr4-40305228-40305662	1.58E-17	0.348958709	0.19	0.058	1.05E-12	C3_CD8_Tem
chr20-5719626-5720622	1.59E-17	0.344589935	0.239	0.081	1.05E-12	C3_CD8_Tem
chr15-78049659-78051221	1.65E-17	0.257318704	0.613	0.318	1.09E-12	C3_CD8_Tem
chr1-38029862-38030477	1.68E-17	0.336291873	0.184	0.058	1.11E-12	C3_CD8_Tem
chr1-2305042-2305594	1.76E-17	0.315693395	0.124	0.033	1.17E-12	C3_CD8_Tem
chr12-52351332-52352107	1.84E-17	0.330891331	0.224	0.076	1.22E-12	C3_CD8_Tem
chr15-69718935-69719905	1.92E-17	0.310042022	0.43	0.197	1.27E-12	C3_CD8_Tem

chr19-35462181-35463328	2.02E-17	0.309744651	0.419	0.193	1.34E-12	C3_CD8_Tem
chr15-38670069-38671174	2.15E-17	0.287066192	0.498	0.249	1.42E-12	C3_CD8_Tem
chr22-46370317-46371312	2.19E-17	0.336796695	0.19	0.058	1.45E-12	C3_CD8_Tem
chr2-136052421-136053254	2.25E-17	0.345535401	0.221	0.07	1.49E-12	C3_CD8_Tem
chr4-36189361-36190203	2.44E-17	0.339511152	0.226	0.077	1.62E-12	C3_CD8_Tem
chr11-61110341-61111058	2.48E-17	0.337024114	0.218	0.078	1.64E-12	C3_CD8_Tem
chr16-29626615-29627455	2.66E-17	0.281776193	0.411	0.195	1.76E-12	C3_CD8_Tem
chr6-142842442-142843309	2.84E-17	0.284188891	0.527	0.263	1.88E-12	C3_CD8_Tem
chr2-181393318-181395118	3.05E-17	0.319350652	0.312	0.125	2.02E-12	C3_CD8_Tem
chr19-2607565-2608631	3.05E-17	0.31179903	0.468	0.221	2.02E-12	C3_CD8_Tem
chr10-104333173-104333562	3.11E-17	0.329641239	0.213	0.073	2.06E-12	C3_CD8_Tem
chr10-84330139-84330644	3.31E-17	0.274338983	0.455	0.226	2.19E-12	C3_CD8_Tem
chr16-56938222-56939605	3.61E-17	0.326778475	0.336	0.139	2.39E-12	C3_CD8_Tem
chr7-2417689-2418570	3.85E-17	0.32414492	0.303	0.122	2.55E-12	C3_CD8_Tem
chr9-134324251-134324675	3.97E-17	0.344310787	0.217	0.069	2.63E-12	C3_CD8_Tem
chr6-4139347-4139677	4.21E-17	0.326667298	0.114	0.023	2.79E-12	C3_CD8_Tem
chr6-41533293-41533722	4.28E-17	0.325513608	0.154	0.043	2.83E-12	C3_CD8_Tem
chr11-72736269-72737114	4.66E-17	0.309129555	0.38	0.169	3.08E-12	C3_CD8_Tem
chr8-96231211-96231855	4.82E-17	0.332246233	0.212	0.07	3.19E-12	C3_CD8_Tem
chr22-44213017-44214167	4.82E-17	0.32279316	0.123	0.029	3.19E-12	C3_CD8_Tem
chr18-13455552-13456423	4.89E-17	0.32768521	0.141	0.038	3.24E-12	C3_CD8_Tem
chr20-62739367-62741172	5.00E-17	0.250297882	0.63	0.353	3.31E-12	C3_CD8_Tem
chr20-48759795-48761687	5.01E-17	0.251909349	0.629	0.355	3.32E-12	C3_CD8_Tem
chr10-50539964-50540810	5.05E-17	0.32102582	0.163	0.048	3.35E-12	C3_CD8_Tem
chr5-155768313-155768661	5.18E-17	0.314730816	0.113	0.026	3.43E-12	C3_CD8_Tem
chr21-25396071-25396681	5.21E-17	0.317127022	0.128	0.032	3.45E-12	C3_CD8_Tem
chr1-172638263-172639731	5.45E-17	0.262989259	0.558	0.297	3.61E-12	C3_CD8_Tem

chr20-43983420-43984372	5.92E-17	0.335650075	0.139	0.033	3.92E-12	C3_CD8_Tem
chr17-36212926-36213380	6.13E-17	0.331956027	0.157	0.042	4.06E-12	C3_CD8_Tem
chr5-138979252-138980220	6.19E-17	0.31108587	0.124	0.032	4.10E-12	C3_CD8_Tem
chr16-57128610-57129150	6.37E-17	0.3174271	0.172	0.057	4.22E-12	C3_CD8_Tem
chr20-53750872-53751930	6.48E-17	0.329026484	0.221	0.077	4.29E-12	C3_CD8_Tem
chr12-52349177-52349581	6.57E-17	0.328684696	0.111	0.022	4.35E-12	C3_CD8_Tem
chr13-114142248-114143533	6.58E-17	0.274908541	0.485	0.251	4.36E-12	C3_CD8_Tem
chr11-19280863-19281397	6.67E-17	0.315152732	0.129	0.034	4.42E-12	C3_CD8_Tem
chr4-121777655-121778050	6.94E-17	0.280092037	0.072	0.01	4.60E-12	C3_CD8_Tem
chr1-150615613-150616066	7.07E-17	0.331115787	0.194	0.062	4.68E-12	C3_CD8_Tem
chr10-124688200-124689359	7.20E-17	0.296978217	0.443	0.213	4.77E-12	C3_CD8_Tem
chr17-82583520-82584286	7.96E-17	0.317163981	0.187	0.063	5.27E-12	C3_CD8_Tem
chr2-161237113-161238201	8.14E-17	0.323899496	0.233	0.086	5.39E-12	C3_CD8_Tem
chr14-61384870-61385754	8.25E-17	0.317440482	0.345	0.153	5.46E-12	C3_CD8_Tem
chr1-12201232-12202096	8.35E-17	0.327482148	0.206	0.066	5.53E-12	C3_CD8_Tem
chr1-24984496-24985576	9.05E-17	0.342182996	0.237	0.083	5.99E-12	C3_CD8_Tem
chr15-20353048-20354546	9.15E-17	0.321601588	0.314	0.126	6.06E-12	C3_CD8_Tem
chr5-143221788-143223304	9.39E-17	0.252987727	0.584	0.316	6.22E-12	C3_CD8_Tem
chr5-54609889-54611092	9.56E-17	0.316984429	0.263	0.099	6.33E-12	C3_CD8_Tem
chr1-192520140-192520418	9.87E-17	0.302671643	0.089	0.016	6.53E-12	C3_CD8_Tem
chr1-112344441-112345311	1.00E-16	0.288611925	0.08	0.015	6.65E-12	C3_CD8_Tem
chr14-106163482-106164277	1.02E-16	0.303348394	0.099	0.022	6.79E-12	C3_CD8_Tem
chr7-55240414-55240993	1.07E-16	0.269530595	0.064	0.01	7.07E-12	C3_CD8_Tem
chr19-6663130-6664168	1.15E-16	0.30826542	0.356	0.154	7.61E-12	C3_CD8_Tem
chr1-172697419-172699598	1.16E-16	0.283008087	0.542	0.279	7.70E-12	C3_CD8_Tem
chr9-96856664-96857311	1.21E-16	0.328041359	0.267	0.104	8.04E-12	C3_CD8_Tem
chr8-30137089-30137602	1.22E-16	0.30811051	0.317	0.134	8.07E-12	C3_CD8_Tem

chr12-104426834-104427931	1.33E-16	0.316409715	0.16	0.05	8.79E-12	C3_CD8_Tem
chr22-38873763-38874357	1.39E-16	0.296612656	0.439	0.212	9.21E-12	C3_CD8_Tem
chr6-143396717-143398041	1.44E-16	0.299756567	0.271	0.109	9.52E-12	C3_CD8_Tem
chr2-61662962-61663425	1.62E-16	0.31924819	0.117	0.027	1.08E-11	C3_CD8_Tem
chr17-77440699-77442224	1.69E-16	0.276689825	0.55	0.291	1.12E-11	C3_CD8_Tem
chr3-126607119-126607778	1.73E-16	0.31078065	0.164	0.05	1.15E-11	C3_CD8_Tem
chr1-235104198-235105982	1.74E-16	0.305160727	0.395	0.183	1.15E-11	C3_CD8_Tem
chr21-33975074-33977016	1.92E-16	0.28637367	0.487	0.237	1.27E-11	C3_CD8_Tem
chr3-46953178-46953995	2.19E-16	0.333667987	0.195	0.062	1.45E-11	C3_CD8_Tem
chr10-124640818-124641704	2.30E-16	0.327858527	0.252	0.092	1.53E-11	C3_CD8_Tem
chr13-113875597-113877300	2.41E-16	0.310796629	0.306	0.129	1.60E-11	C3_CD8_Tem
chr8-58841995-58842404	2.42E-16	0.315485393	0.131	0.035	1.60E-11	C3_CD8_Tem
chr1-221800726-221801674	2.53E-16	0.304737847	0.226	0.086	1.68E-11	C3_CD8_Tem
chr14-106510787-106511672	2.56E-16	0.319474442	0.332	0.147	1.70E-11	C3_CD8_Tem
chr2-223759699-223762024	2.68E-16	0.27363561	0.406	0.197	1.77E-11	C3_CD8_Tem
chr2-144659399-144660199	2.74E-16	0.293491407	0.319	0.136	1.81E-11	C3_CD8_Tem
chr16-57577489-57578337	3.22E-16	0.330973926	0.162	0.045	2.13E-11	C3_CD8_Tem
chr2-239297658-239298926	3.26E-16	0.268576673	0.536	0.277	2.16E-11	C3_CD8_Tem
chr2-105855655-105857360	3.31E-16	0.305791529	0.413	0.194	2.19E-11	C3_CD8_Tem
chr22-24409682-24410607	3.41E-16	0.329648243	0.186	0.058	2.26E-11	C3_CD8_Tem
chr1-53581990-53582744	3.51E-16	0.318184182	0.165	0.049	2.33E-11	C3_CD8_Tem
chr10-70610194-70611322	3.78E-16	0.313360208	0.228	0.082	2.50E-11	C3_CD8_Tem
chr3-141403417-141403956	3.94E-16	0.33402031	0.172	0.052	2.61E-11	C3_CD8_Tem
chr11-36352501-36353303	3.95E-16	0.309569792	0.307	0.126	2.62E-11	C3_CD8_Tem
chr2-105859090-105859483	3.99E-16	0.332532179	0.194	0.064	2.64E-11	C3_CD8_Tem
chr6-13375080-13375836	4.16E-16	0.314509152	0.15	0.042	2.75E-11	C3_CD8_Tem
chr22-39080163-39080852	4.21E-16	0.327873614	0.231	0.081	2.79E-11	C3_CD8_Tem

chr4-2290508-2291625	4.65E-16	0.28163291	0.464	0.227	3.08E-11	C3_CD8_Tem
chr18-78642705-78643359	4.70E-16	0.296073551	0.091	0.018	3.11E-11	C3_CD8_Tem
chr20-59159150-59160047	5.01E-16	0.27041589	0.487	0.247	3.32E-11	C3_CD8_Tem
chr6-141326181-141326964	5.28E-16	0.288359202	0.337	0.15	3.49E-11	C3_CD8_Tem
chr6-46325308-46326080	5.31E-16	0.313422112	0.156	0.047	3.52E-11	C3_CD8_Tem
chr12-51970877-51973208	5.31E-16	0.287383609	0.494	0.247	3.52E-11	C3_CD8_Tem
chr15-38538133-38539290	6.24E-16	0.330597575	0.193	0.063	4.13E-11	C3_CD8_Tem
chr2-8514801-8516488	6.30E-16	0.298082711	0.413	0.189	4.17E-11	C3_CD8_Tem
chr15-85618627-85619422	6.56E-16	0.323251348	0.332	0.135	4.34E-11	C3_CD8_Tem
chr5-142821723-142822399	6.81E-16	0.334936985	0.232	0.079	4.51E-11	C3_CD8_Tem
chr14-99240565-99241669	7.56E-16	0.325281419	0.345	0.154	5.01E-11	C3_CD8_Tem
chr8-71843261-71844636	7.59E-16	0.288472447	0.315	0.137	5.03E-11	C3_CD8_Tem
chr14-24051638-24053567	8.14E-16	0.297056489	0.392	0.184	5.39E-11	C3_CD8_Tem
chr16-57070100-57072258	8.33E-16	0.313104055	0.31	0.128	5.52E-11	C3_CD8_Tem
chr3-46440680-46441475	8.65E-16	0.315005105	0.253	0.099	5.73E-11	C3_CD8_Tem
chr20-53842071-53843061	9.92E-16	0.3149504	0.304	0.127	6.57E-11	C3_CD8_Tem
chr19-16449052-16450086	1.01E-15	0.30419079	0.31	0.138	6.67E-11	C3_CD8_Tem
chr21-45266014-45267006	1.03E-15	0.319305262	0.215	0.073	6.85E-11	C3_CD8_Tem
chr11-128058615-128059286	1.07E-15	0.302233115	0.114	0.027	7.07E-11	C3_CD8_Tem
chr7-149620902-149622091	1.09E-15	0.297993996	0.369	0.166	7.22E-11	C3_CD8_Tem
chr5-150045602-150046413	1.10E-15	0.317815643	0.175	0.058	7.30E-11	C3_CD8_Tem
chr6-16322506-16323867	1.11E-15	0.265546462	0.542	0.285	7.35E-11	C3_CD8_Tem
chr12-10554098-10555445	1.12E-15	0.304838191	0.197	0.071	7.42E-11	C3_CD8_Tem
chr11-118240692-118241853	1.15E-15	0.278067416	0.481	0.253	7.62E-11	C3_CD8_Tem
chr1-3889848-3891382	1.17E-15	0.328674113	0.221	0.076	7.73E-11	C3_CD8_Tem
chr11-85755749-85756689	1.17E-15	0.333137581	0.249	0.093	7.74E-11	C3_CD8_Tem
chr12-68189707-68190475	1.19E-15	0.324503566	0.249	0.093	7.91E-11	C3_CD8_Tem

chr3-52252608-52254259	1.30E-15	0.260371116	0.52	0.282	8.60E-11	C3_CD8_Tem
chr11-96204605-96205684	1.30E-15	0.314860697	0.282	0.115	8.62E-11	C3_CD8_Tem
chr19-57192947-57193349	1.34E-15	0.311115386	0.128	0.035	8.86E-11	C3_CD8_Tem
chr1-52636599-52637735	1.35E-15	0.274191386	0.506	0.264	8.91E-11	C3_CD8_Tem
chr1-200154033-200154586	1.36E-15	0.291405767	0.288	0.115	9.01E-11	C3_CD8_Tem
chr15-28984650-28985528	1.42E-15	0.315713791	0.186	0.064	9.42E-11	C3_CD8_Tem
chr17-75864256-75865335	1.56E-15	0.310979547	0.325	0.136	1.03E-10	C3_CD8_Tem
chr1-202201227-202201590	1.57E-15	0.308227084	0.123	0.033	1.04E-10	C3_CD8_Tem
chr19-8573198-8573976	1.58E-15	0.30605099	0.348	0.146	1.05E-10	C3_CD8_Tem
chr15-31341177-31342073	1.67E-15	0.311379023	0.271	0.108	1.11E-10	C3_CD8_Tem
chr10-96670477-96671823	1.71E-15	0.30565686	0.204	0.075	1.13E-10	C3_CD8_Tem
chr20-59151787-59152239	1.83E-15	0.322988451	0.228	0.08	1.21E-10	C3_CD8_Tem
chr2-219249317-219249833	1.87E-15	0.334396935	0.228	0.081	1.24E-10	C3_CD8_Tem
chr17-49714993-49716355	1.92E-15	0.255654534	0.567	0.308	1.27E-10	C3_CD8_Tem
chr8-29035430-29036887	1.99E-15	0.326588547	0.185	0.061	1.32E-10	C3_CD8_Tem
chr16-87761941-87762604	2.04E-15	0.291300809	0.256	0.106	1.35E-10	C3_CD8_Tem
chrX-79169280-79170245	2.05E-15	0.336028225	0.174	0.052	1.36E-10	C3_CD8_Tem
chr16-57592479-57593323	2.06E-15	0.309601995	0.34	0.144	1.37E-10	C3_CD8_Tem
chr17-36192338-36192639	2.16E-15	0.326772772	0.205	0.068	1.43E-10	C3_CD8_Tem
chr3-114315466-114316209	2.20E-15	0.317796576	0.15	0.044	1.46E-10	C3_CD8_Tem
chr8-140571963-140572770	2.25E-15	0.305007405	0.403	0.185	1.49E-10	C3_CD8_Tem
chr2-68769927-68770619	2.28E-15	0.323544093	0.312	0.135	1.51E-10	C3_CD8_Tem
chr18-59902107-59902595	2.34E-15	0.309928059	0.298	0.124	1.55E-10	C3_CD8_Tem
chr13-111182833-111183595	2.35E-15	0.317005734	0.155	0.048	1.56E-10	C3_CD8_Tem
chr7-29194356-29195707	2.35E-15	0.325928457	0.209	0.071	1.56E-10	C3_CD8_Tem
chr16-81872605-81874152	2.61E-15	0.324883134	0.147	0.038	1.73E-10	C3_CD8_Tem
chr8-100442607-100443600	2.73E-15	0.304007008	0.371	0.168	1.81E-10	C3_CD8_Tem

chr11-118045208-118047222	2.76E-15	0.300904472	0.366	0.163	1.83E-10	C3_CD8_Tem
chr5-74639813-74641625	2.89E-15	0.288940145	0.347	0.155	1.91E-10	C3_CD8_Tem
chr10-79025064-79025989	2.92E-15	0.297463897	0.23	0.085	1.93E-10	C3_CD8_Tem
chr15-93164162-93164791	2.94E-15	0.277318284	0.079	0.016	1.95E-10	C3_CD8_Tem
chr7-2720060-2720398	3.02E-15	0.312768796	0.11	0.025	2.00E-10	C3_CD8_Tem
chr16-70403681-70404804	3.02E-15	0.327983902	0.26	0.098	2.00E-10	C3_CD8_Tem
chr3-98557230-98557783	3.09E-15	0.316026627	0.24	0.094	2.05E-10	C3_CD8_Tem
chr1-3809711-3810580	3.10E-15	0.291488146	0.114	0.029	2.05E-10	C3_CD8_Tem
chr14-106176911-106177756	3.15E-15	0.29969122	0.361	0.158	2.09E-10	C3_CD8_Tem
chrX-1507227-1508353	3.15E-15	0.305828242	0.22	0.083	2.09E-10	C3_CD8_Tem
chr5-172147762-172148141	3.18E-15	0.297380198	0.105	0.023	2.10E-10	C3_CD8_Tem
chr1-16122578-16124085	3.20E-15	0.308181314	0.167	0.055	2.12E-10	C3_CD8_Tem
chr5-76738479-76739611	3.21E-15	0.250975125	0.541	0.291	2.12E-10	C3_CD8_Tem
chr2-230575316-230576082	3.41E-15	0.314804303	0.2	0.071	2.26E-10	C3_CD8_Tem
chr16-81615639-81617046	3.59E-15	0.263258361	0.509	0.262	2.38E-10	C3_CD8_Tem
chr8-29469060-29469536	3.66E-15	0.30559718	0.112	0.028	2.43E-10	C3_CD8_Tem
chr6-42356959-42358089	3.80E-15	0.320361236	0.221	0.08	2.52E-10	C3_CD8_Tem
chr12-122227099-122227757	3.98E-15	0.317722025	0.217	0.078	2.63E-10	C3_CD8_Tem
chr20-62117493-62118052	4.28E-15	0.31369938	0.304	0.127	2.84E-10	C3_CD8_Tem
chr6-105462528-105462777	4.38E-15	0.279910361	0.081	0.015	2.90E-10	C3_CD8_Tem
chr15-74869524-74870313	4.41E-15	0.313359847	0.196	0.067	2.92E-10	C3_CD8_Tem
chr17-74473957-74474793	4.48E-15	0.31602336	0.165	0.049	2.97E-10	C3_CD8_Tem
chr6-149053939-149054961	4.49E-15	0.294030277	0.108	0.027	2.97E-10	C3_CD8_Tem
chr19-10111953-10113270	4.56E-15	0.277720507	0.479	0.232	3.02E-10	C3_CD8_Tem
chr3-183377312-183378294	4.72E-15	0.297451915	0.119	0.029	3.12E-10	C3_CD8_Tem
chr4-40254631-40255993	4.77E-15	0.334180178	0.231	0.081	3.16E-10	C3_CD8_Tem
chr1-113783097-113784687	4.83E-15	0.271414135	0.44	0.232	3.20E-10	C3_CD8_Tem

chr9-96874301-96876019	4.88E-15	0.299203215	0.332	0.148	3.23E-10	C3_CD8_Tem
chr13-30373890-30374880	5.11E-15	0.280689732	0.427	0.211	3.38E-10	C3_CD8_Tem
chr8-58800776-58801834	5.23E-15	0.280678992	0.44	0.211	3.47E-10	C3_CD8_Tem
chr3-58470866-58471787	5.76E-15	0.314580907	0.188	0.064	3.81E-10	C3_CD8_Tem
chr17-8958263-8958907	5.78E-15	0.332340533	0.27	0.101	3.83E-10	C3_CD8_Tem
chr1-24922243-24922632	5.86E-15	0.310033276	0.353	0.156	3.88E-10	C3_CD8_Tem
chr1-198167012-198168407	6.01E-15	0.297665392	0.296	0.128	3.98E-10	C3_CD8_Tem
chr11-36375978-36377906	6.06E-15	0.304990661	0.386	0.167	4.01E-10	C3_CD8_Tem
chr17-77783640-77784842	6.29E-15	0.281308734	0.385	0.182	4.16E-10	C3_CD8_Tem
chr2-136225503-136226572	6.41E-15	0.308262946	0.154	0.05	4.24E-10	C3_CD8_Tem
chr17-2192183-2193154	6.50E-15	0.323821716	0.271	0.104	4.31E-10	C3_CD8_Tem
chr3-114328320-114328705	6.63E-15	0.305126982	0.144	0.044	4.39E-10	C3_CD8_Tem
chr1-25549312-25550465	6.65E-15	0.30451488	0.358	0.16	4.40E-10	C3_CD8_Tem
chr22-41858957-41859826	6.72E-15	0.310547899	0.34	0.147	4.45E-10	C3_CD8_Tem
chr4-1176639-1177899	7.19E-15	0.314598375	0.219	0.077	4.76E-10	C3_CD8_Tem
chr19-55420941-55421695	7.30E-15	0.326015532	0.182	0.059	4.83E-10	C3_CD8_Tem
chr9-19999130-19999982	7.37E-15	0.30040724	0.154	0.047	4.88E-10	C3_CD8_Tem
chr19-38683391-38684667	7.49E-15	0.29083139	0.44	0.218	4.96E-10	C3_CD8_Tem
chr22-37185725-37186995	7.54E-15	0.298658157	0.305	0.134	4.99E-10	C3_CD8_Tem
chr19-14475801-14477045	7.83E-15	0.28550312	0.297	0.125	5.19E-10	C3_CD8_Tem
chr8-29445670-29446425	8.16E-15	0.285117035	0.091	0.021	5.40E-10	C3_CD8_Tem
chr18-22281989-22283234	8.25E-15	0.296227148	0.249	0.1	5.46E-10	C3_CD8_Tem
chr10-84177301-84178035	8.58E-15	0.28732399	0.136	0.042	5.68E-10	C3_CD8_Tem
chr12-44847506-44848934	8.64E-15	0.297488565	0.352	0.156	5.72E-10	C3_CD8_Tem
chr2-144510298-144511025	8.75E-15	0.291066952	0.327	0.142	5.79E-10	C3_CD8_Tem
chr9-126329407-126330214	9.88E-15	0.310227972	0.146	0.045	6.54E-10	C3_CD8_Tem
chrX-20414059-20415096	9.99E-15	0.296301204	0.101	0.024	6.62E-10	C3_CD8_Tem

chr12-10602523-10603301	1.00E-14	0.305020708	0.13	0.036	6.64E-10	C3_CD8_Tem
chr1-174157160-174157487	1.08E-14	0.312067576	0.151	0.046	7.17E-10	C3_CD8_Tem
chr12-31722842-31723776	1.08E-14	0.28684634	0.199	0.076	7.17E-10	C3_CD8_Tem
chr1-198120924-198121694	1.08E-14	0.281068119	0.095	0.02	7.18E-10	C3_CD8_Tem
chr9-126396589-126397526	1.09E-14	0.292301437	0.343	0.155	7.25E-10	C3_CD8_Tem
chr6-26309496-26310055	1.13E-14	0.321118909	0.215	0.077	7.48E-10	C3_CD8_Tem
chr22-22720135-22721253	1.14E-14	0.320153624	0.193	0.063	7.52E-10	C3_CD8_Tem
chr10-32981817-32982654	1.14E-14	0.314816067	0.157	0.047	7.54E-10	C3_CD8_Tem
chr1-161545114-161545652	1.16E-14	0.299469675	0.12	0.033	7.71E-10	C3_CD8_Tem
chr16-56278591-56279579	1.18E-14	0.311332413	0.261	0.101	7.82E-10	C3_CD8_Tem
chr2-15141844-15142760	1.22E-14	0.290650542	0.2	0.078	8.05E-10	C3_CD8_Tem
chr2-62210745-62211017	1.22E-14	0.252124732	0.057	0.008	8.05E-10	C3_CD8_Tem
chr10-131896262-131896784	1.22E-14	0.309666454	0.244	0.097	8.07E-10	C3_CD8_Tem
chr3-27451226-27452196	1.22E-14	0.302495364	0.121	0.03	8.08E-10	C3_CD8_Tem
chr12-70049371-70050399	1.24E-14	0.280967823	0.208	0.08	8.18E-10	C3_CD8_Tem
chr11-88353301-88354463	1.28E-14	0.315724785	0.161	0.052	8.47E-10	C3_CD8_Tem
chr6-16654399-16655089	1.32E-14	0.317180844	0.22	0.081	8.72E-10	C3_CD8_Tem
chr10-119119561-119120834	1.38E-14	0.294371769	0.238	0.095	9.14E-10	C3_CD8_Tem
chr10-133388958-133390448	1.42E-14	0.285890164	0.481	0.238	9.38E-10	C3_CD8_Tem
chr10-124719017-124719726	1.43E-14	0.292581613	0.453	0.219	9.45E-10	C3_CD8_Tem
chr1-206944539-206945182	1.46E-14	0.281059945	0.102	0.025	9.65E-10	C3_CD8_Tem
chr8-123727616-123728227	1.47E-14	0.28656786	0.106	0.025	9.73E-10	C3_CD8_Tem
chr19-7772281-7773074	1.47E-14	0.299410846	0.135	0.041	9.73E-10	C3_CD8_Tem
chr1-27521329-27523346	1.49E-14	0.280469765	0.406	0.202	9.84E-10	C3_CD8_Tem
chr11-11995396-11996007	1.49E-14	0.289835809	0.109	0.027	9.84E-10	C3_CD8_Tem
chr14-106275951-106277107	1.61E-14	0.307011599	0.162	0.052	1.07E-09	C3_CD8_Tem
chr16-30873288-30873655	1.64E-14	0.317514599	0.157	0.047	1.08E-09	C3_CD8_Tem

chr10-6493437-6493865	1.66E-14	0.315042055	0.282	0.117	1.10E-09	C3_CD8_Tem
chr9-92664259-92665110	1.67E-14	0.302966927	0.168	0.056	1.11E-09	C3_CD8_Tem
chr20-49794549-49795270	1.69E-14	0.305170652	0.155	0.049	1.12E-09	C3_CD8_Tem
chr6-105483343-105483922	1.69E-14	0.306151625	0.131	0.035	1.12E-09	C3_CD8_Tem
chr21-35138340-35139519	1.84E-14	0.297472014	0.133	0.04	1.22E-09	C3_CD8_Tem
chr1-184892030-184892864	1.84E-14	0.306454695	0.323	0.137	1.22E-09	C3_CD8_Tem
chr10-119332916-119333577	1.88E-14	0.307173354	0.231	0.087	1.24E-09	C3_CD8_Tem
chr12-1993185-1994540	1.92E-14	0.304318852	0.305	0.129	1.27E-09	C3_CD8_Tem
chr13-33743565-33744407	1.96E-14	0.29164411	0.141	0.043	1.30E-09	C3_CD8_Tem
chr11-57649096-57650233	1.98E-14	0.274568944	0.425	0.213	1.31E-09	C3_CD8_Tem
chr20-31602810-31603204	1.99E-14	0.313570008	0.24	0.088	1.32E-09	C3_CD8_Tem
chr5-132456192-132458945	2.00E-14	0.277484094	0.451	0.225	1.33E-09	C3_CD8_Tem
chr9-281823-282760	2.16E-14	0.310039441	0.248	0.102	1.43E-09	C3_CD8_Tem
chr11-118911875-118912806	2.16E-14	0.3033642	0.353	0.161	1.43E-09	C3_CD8_Tem
chr12-108635167-108635848	2.18E-14	0.305821572	0.288	0.118	1.44E-09	C3_CD8_Tem
chr7-150665070-150665525	2.19E-14	0.318195075	0.16	0.046	1.45E-09	C3_CD8_Tem
chr2-144516796-144517798	2.24E-14	0.298698154	0.353	0.154	1.48E-09	C3_CD8_Tem
chr19-55276055-55276993	2.26E-14	0.31408505	0.198	0.07	1.49E-09	C3_CD8_Tem
chr10-14587622-14588809	2.37E-14	0.279466983	0.464	0.228	1.57E-09	C3_CD8_Tem
chr6-2853632-2854305	2.37E-14	0.305962656	0.361	0.163	1.57E-09	C3_CD8_Tem
chr2-30267026-30267904	2.50E-14	0.31359595	0.208	0.075	1.66E-09	C3_CD8_Tem
chrX-53085540-53086085	2.52E-14	0.312507469	0.158	0.052	1.67E-09	C3_CD8_Tem
chr7-139084116-139085810	2.74E-14	0.313342228	0.287	0.113	1.81E-09	C3_CD8_Tem
chr12-47211956-47212706	2.82E-14	0.314199313	0.333	0.145	1.87E-09	C3_CD8_Tem
chr3-66303282-66304068	2.82E-14	0.276626694	0.427	0.209	1.87E-09	C3_CD8_Tem
chr6-20836832-20837591	2.83E-14	0.288976276	0.11	0.028	1.88E-09	C3_CD8_Tem
chr11-61164059-61164543	2.93E-14	0.285395815	0.111	0.03	1.94E-09	C3_CD8_Tem

chr12-123916635-123917094	3.01E-14	0.303044972	0.151	0.046	1.99E-09	C3_CD8_Tem
chr10-109912877-109913260	3.06E-14	0.291183463	0.089	0.018	2.03E-09	C3_CD8_Tem
chr10-114808541-114809335	3.07E-14	0.294663542	0.136	0.04	2.03E-09	C3_CD8_Tem
chr17-1200824-1201683	3.09E-14	0.302154078	0.204	0.077	2.04E-09	C3_CD8_Tem
chr18-45686597-45689178	3.56E-14	0.265451991	0.46	0.238	2.36E-09	C3_CD8_Tem
chr17-80876809-80877767	3.67E-14	0.321260958	0.231	0.086	2.43E-09	C3_CD8_Tem
chr2-190695531-190696245	3.71E-14	0.277189608	0.131	0.041	2.45E-09	C3_CD8_Tem
chr8-130286360-130287025	3.75E-14	0.277335769	0.083	0.016	2.48E-09	C3_CD8_Tem
chr13-114339713-114340650	3.75E-14	0.309659574	0.332	0.141	2.48E-09	C3_CD8_Tem
chr1-202203369-202204084	3.81E-14	0.28657586	0.113	0.03	2.52E-09	C3_CD8_Tem
chr5-142786694-142787240	3.84E-14	0.295767724	0.261	0.106	2.54E-09	C3_CD8_Tem
chr11-116953413-116954230	3.92E-14	0.312114543	0.163	0.049	2.59E-09	C3_CD8_Tem
chr17-36196490-36197498	3.94E-14	0.287380923	0.296	0.127	2.61E-09	C3_CD8_Tem
chr6-16419990-16421696	3.99E-14	0.271800656	0.505	0.252	2.64E-09	C3_CD8_Tem
chr1-51501021-51501671	4.06E-14	0.312923862	0.187	0.067	2.69E-09	C3_CD8_Tem
chr14-106174117-106174932	4.08E-14	0.273911456	0.403	0.192	2.70E-09	C3_CD8_Tem
chr3-46371636-46372715	4.20E-14	0.317373561	0.227	0.083	2.78E-09	C3_CD8_Tem
chr2-181162826-181163908	4.23E-14	0.300321358	0.304	0.131	2.80E-09	C3_CD8_Tem
chr19-10516551-10517154	4.41E-14	0.296845319	0.118	0.035	2.92E-09	C3_CD8_Tem
chr11-117827963-117829091	4.52E-14	0.291620738	0.365	0.168	2.99E-09	C3_CD8_Tem
chr7-8125259-8126551	4.85E-14	0.252609372	0.501	0.258	3.21E-09	C3_CD8_Tem
chr6-157143009-157143795	5.26E-14	0.305627524	0.212	0.08	3.48E-09	C3_CD8_Tem
chr8-125125285-125126199	5.28E-14	0.301909528	0.191	0.066	3.50E-09	C3_CD8_Tem
chr1-161626522-161627073	5.34E-14	0.294309079	0.116	0.029	3.53E-09	C3_CD8_Tem
chr16-4465340-4466660	5.66E-14	0.295642982	0.312	0.133	3.75E-09	C3_CD8_Tem
chr5-142783441-142784189	5.86E-14	0.317290009	0.257	0.096	3.88E-09	C3_CD8_Tem
chr11-1771996-1772953	5.90E-14	0.295385461	0.307	0.136	3.91E-09	C3_CD8_Tem

chr18-75070379-75071623	6.10E-14	0.264586348	0.427	0.213	4.04E-09	C3_CD8_Tem
chr2-7725167-7726015	6.29E-14	0.29396359	0.353	0.16	4.17E-09	C3_CD8_Tem
chr17-40283257-40284077	6.61E-14	0.296111143	0.356	0.162	4.38E-09	C3_CD8_Tem
chr1-44801637-44802432	7.13E-14	0.304923606	0.271	0.119	4.72E-09	C3_CD8_Tem
chr16-75617604-75617832	7.47E-14	0.269668046	0.08	0.017	4.95E-09	C3_CD8_Tem
chr13-99314427-99315476	7.67E-14	0.285206845	0.382	0.18	5.08E-09	C3_CD8_Tem
chr13-29901673-29902832	7.69E-14	0.293280014	0.262	0.106	5.09E-09	C3_CD8_Tem
chr1-43581747-43582584	7.70E-14	0.270058841	0.109	0.03	5.10E-09	C3_CD8_Tem
chr12-10497396-10498070	8.06E-14	0.292194153	0.142	0.046	5.34E-09	C3_CD8_Tem
chr2-46239011-46240224	8.07E-14	0.292174301	0.32	0.136	5.34E-09	C3_CD8_Tem
chr16-50160451-50161037	8.26E-14	0.30724444	0.177	0.058	5.47E-09	C3_CD8_Tem
chrX-11792469-11793440	8.48E-14	0.29034777	0.178	0.064	5.61E-09	C3_CD8_Tem
chr3-187993777-187994654	8.50E-14	0.296192127	0.273	0.114	5.63E-09	C3_CD8_Tem
chr11-67292914-67293862	8.64E-14	0.306103556	0.348	0.157	5.72E-09	C3_CD8_Tem
chr9-62800737-62802770	8.71E-14	0.285712847	0.41	0.197	5.77E-09	C3_CD8_Tem
chr17-68237275-68238243	8.93E-14	0.267337388	0.41	0.207	5.92E-09	C3_CD8_Tem
chr17-19502291-19502855	9.14E-14	0.303180694	0.155	0.049	6.06E-09	C3_CD8_Tem
chr9-114363274-114363843	9.18E-14	0.309387839	0.195	0.068	6.08E-09	C3_CD8_Tem
chr3-105746112-105747336	9.43E-14	0.287536209	0.361	0.165	6.24E-09	C3_CD8_Tem
chr16-57539002-57539356	9.55E-14	0.325262375	0.224	0.081	6.32E-09	C3_CD8_Tem
chr3-4350816-4351893	9.55E-14	0.298023894	0.262	0.109	6.33E-09	C3_CD8_Tem
chr5-151138721-151139887	9.63E-14	0.266781573	0.319	0.145	6.38E-09	C3_CD8_Tem
chr18-44729308-44730171	1.02E-13	0.28652906	0.212	0.08	6.73E-09	C3_CD8_Tem
chr1-160861979-160863054	1.03E-13	0.304605046	0.177	0.06	6.85E-09	C3_CD8_Tem
chr3-47016683-47017556	1.04E-13	0.290869132	0.353	0.16	6.91E-09	C3_CD8_Tem
chr16-57815530-57816711	1.05E-13	0.303800074	0.243	0.096	6.94E-09	C3_CD8_Tem
chr6-20708291-20708750	1.05E-13	0.291371228	0.123	0.034	6.95E-09	C3_CD8_Tem

chr2-68385217-68385954	1.08E-13	0.298419457	0.187	0.067	7.13E-09	C3_CD8_Tem
chr10-22246134-22247397	1.08E-13	0.271093922	0.443	0.216	7.13E-09	C3_CD8_Tem
chr7-143385779-143386618	1.09E-13	0.285072141	0.322	0.145	7.24E-09	C3_CD8_Tem
chr16-87963035-87963862	1.11E-13	0.297207553	0.312	0.133	7.34E-09	C3_CD8_Tem
chr14-61388042-61389518	1.15E-13	0.270443459	0.453	0.232	7.62E-09	C3_CD8_Tem
chr15-94306679-94307364	1.16E-13	0.308109895	0.18	0.057	7.71E-09	C3_CD8_Tem
chr17-2353063-2354351	1.17E-13	0.309990323	0.312	0.136	7.76E-09	C3_CD8_Tem
chr5-139389577-139390346	1.20E-13	0.269309629	0.428	0.216	7.94E-09	C3_CD8_Tem
chr3-189071290-189071816	1.21E-13	0.27767321	0.099	0.025	8.00E-09	C3_CD8_Tem
chr4-121706102-121707612	1.22E-13	0.288363081	0.242	0.094	8.05E-09	C3_CD8_Tem
chr19-14521961-14522806	1.22E-13	0.267773342	0.472	0.242	8.09E-09	C3_CD8_Tem
chr1-200862003-200863385	1.23E-13	0.273232642	0.367	0.173	8.12E-09	C3_CD8_Tem
chr17-40607524-40608588	1.23E-13	0.270520911	0.455	0.232	8.17E-09	C3_CD8_Tem
chr2-191206130-191207147	1.26E-13	0.283445411	0.339	0.149	8.32E-09	C3_CD8_Tem
chr2-105749545-105750333	1.29E-13	0.295175151	0.277	0.119	8.55E-09	C3_CD8_Tem
chr6-155223690-155224508	1.32E-13	0.271009521	0.32	0.145	8.74E-09	C3_CD8_Tem
chr3-46279866-46280683	1.33E-13	0.316464106	0.265	0.105	8.83E-09	C3_CD8_Tem
chr10-97414740-97415235	1.34E-13	0.311681131	0.213	0.08	8.88E-09	C3_CD8_Tem
chr7-35727649-35727934	1.35E-13	0.304344328	0.179	0.06	8.91E-09	C3_CD8_Tem
chr8-133152638-133153228	1.36E-13	0.281802867	0.103	0.027	9.01E-09	C3_CD8_Tem
chr1-53585160-53585509	1.38E-13	0.290185458	0.109	0.03	9.12E-09	C3_CD8_Tem
chr10-3739840-3740818	1.39E-13	0.310082396	0.255	0.099	9.22E-09	C3_CD8_Tem
chr16-81841425-81842485	1.48E-13	0.291568331	0.144	0.045	9.80E-09	C3_CD8_Tem
chr5-1447801-1449146	1.49E-13	0.300032555	0.131	0.036	9.84E-09	C3_CD8_Tem
chr9-114690589-114692004	1.64E-13	0.277864317	0.432	0.216	1.09E-08	C3_CD8_Tem
chr20-58980863-58983206	1.68E-13	0.263414435	0.536	0.288	1.11E-08	C3_CD8_Tem
chrX-156000197-156000775	1.71E-13	0.294136942	0.157	0.052	1.13E-08	C3_CD8_Tem

chr21-34935382-34936555	1.75E-13	0.3043521	0.25	0.102	1.16E-08	C3_CD8_Tem
chr17-59828800-59829700	1.82E-13	0.273460064	0.432	0.216	1.20E-08	C3_CD8_Tem
chr10-128063775-128065199	1.83E-13	0.274088124	0.417	0.202	1.21E-08	C3_CD8_Tem
chr10-69054683-69055615	1.84E-13	0.312111106	0.189	0.065	1.22E-08	C3_CD8_Tem
chr8-58991013-58992231	2.00E-13	0.290292571	0.171	0.06	1.33E-08	C3_CD8_Tem
chr11-115222115-115223260	2.05E-13	0.291488082	0.202	0.076	1.36E-08	C3_CD8_Tem
chr16-11562843-11563505	2.05E-13	0.296395636	0.143	0.043	1.36E-08	C3_CD8_Tem
chr19-37209830-37210881	2.08E-13	0.276512906	0.466	0.243	1.38E-08	C3_CD8_Tem
chrX-13691803-13692928	2.08E-13	0.305616825	0.188	0.065	1.38E-08	C3_CD8_Tem
chr14-91354247-91354655	2.14E-13	0.28132722	0.123	0.037	1.42E-08	C3_CD8_Tem
chr1-235998615-235999965	2.23E-13	0.250960428	0.523	0.279	1.47E-08	C3_CD8_Tem
chr2-144382526-144383320	2.27E-13	0.290292596	0.22	0.085	1.50E-08	C3_CD8_Tem
chr6-15022680-15023793	2.43E-13	0.281589654	0.198	0.076	1.61E-08	C3_CD8_Tem
chr1-20931867-20933023	2.52E-13	0.297103027	0.147	0.046	1.67E-08	C3_CD8_Tem
chr8-124649586-124650602	2.61E-13	0.302950958	0.185	0.063	1.73E-08	C3_CD8_Tem
chr11-86047054-86047932	2.64E-13	0.317001779	0.213	0.075	1.75E-08	C3_CD8_Tem
chr9-129236636-129237468	2.68E-13	0.316059313	0.175	0.056	1.77E-08	C3_CD8_Tem
chr4-121216247-121216803	2.69E-13	0.265030625	0.083	0.019	1.78E-08	C3_CD8_Tem
chr10-27224711-27226309	2.73E-13	0.296578576	0.183	0.067	1.81E-08	C3_CD8_Tem
chr12-52358505-52359097	2.74E-13	0.292950925	0.135	0.04	1.81E-08	C3_CD8_Tem
chr3-73028827-73029647	2.80E-13	0.284680323	0.212	0.084	1.85E-08	C3_CD8_Tem
chr2-181470737-181472187	2.86E-13	0.299106647	0.342	0.153	1.89E-08	C3_CD8_Tem
chr13-50365336-50366325	2.93E-13	0.307889033	0.259	0.102	1.94E-08	C3_CD8_Tem
chr12-757930-758385	3.02E-13	0.294514916	0.127	0.035	2.00E-08	C3_CD8_Tem
chr10-32982979-32983531	3.07E-13	0.297035316	0.157	0.051	2.03E-08	C3_CD8_Tem
chr14-75229715-75230721	3.09E-13	0.307742121	0.338	0.145	2.04E-08	C3_CD8_Tem
chr2-173145274-173145781	3.24E-13	0.27720815	0.102	0.027	2.14E-08	C3_CD8_Tem

chr2-68423586-68424502	3.34E-13	0.305569841	0.187	0.065	2.21E-08	C3_CD8_Tem
chr2-127080441-127082110	3.41E-13	0.26395076	0.469	0.244	2.26E-08	C3_CD8_Tem
chr13-30396394-30396829	3.49E-13	0.297364532	0.135	0.04	2.31E-08	C3_CD8_Tem
chr3-27960127-27960527	3.49E-13	0.260195265	0.067	0.012	2.31E-08	C3_CD8_Tem
chr14-24673052-24674691	3.77E-13	0.252283253	0.491	0.26	2.50E-08	C3_CD8_Tem
chr8-101155516-101156372	3.78E-13	0.288289189	0.157	0.055	2.50E-08	C3_CD8_Tem
chr17-67433918-67435509	3.87E-13	0.258727361	0.517	0.268	2.56E-08	C3_CD8_Tem
chr3-31461768-31463020	3.91E-13	0.273077137	0.286	0.129	2.59E-08	C3_CD8_Tem
chr15-77012654-77014551	3.95E-13	0.25118743	0.518	0.277	2.61E-08	C3_CD8_Tem
chr3-197793259-197793604	4.12E-13	0.31092266	0.226	0.084	2.73E-08	C3_CD8_Tem
chr17-1890533-1891414	4.13E-13	0.295439552	0.263	0.106	2.73E-08	C3_CD8_Tem
chr11-35337134-35337825	4.25E-13	0.290702903	0.132	0.039	2.82E-08	C3_CD8_Tem
chr9-127426583-127427109	4.36E-13	0.311107887	0.201	0.072	2.89E-08	C3_CD8_Tem
chr1-12438275-12438835	4.54E-13	0.313902102	0.208	0.076	3.01E-08	C3_CD8_Tem
chr18-22260170-22260899	4.68E-13	0.263661221	0.08	0.018	3.10E-08	C3_CD8_Tem
chr11-14577367-14579078	4.69E-13	0.254657626	0.494	0.259	3.11E-08	C3_CD8_Tem
chr11-118699279-118700427	4.70E-13	0.285269613	0.259	0.109	3.11E-08	C3_CD8_Tem
chr7-2720739-2721214	4.76E-13	0.319808905	0.19	0.062	3.15E-08	C3_CD8_Tem
chr16-75616348-75616998	4.78E-13	0.289692026	0.149	0.048	3.16E-08	C3_CD8_Tem
chr5-94793019-94794231	4.95E-13	0.272164588	0.34	0.157	3.28E-08	C3_CD8_Tem
chr7-24907220-24907930	4.96E-13	0.282268581	0.359	0.164	3.29E-08	C3_CD8_Tem
chr6-13431895-13432172	5.03E-13	0.296373667	0.155	0.05	3.33E-08	C3_CD8_Tem
chr17-35900277-35901096	5.17E-13	0.300358006	0.224	0.087	3.42E-08	C3_CD8_Tem
chr17-42252408-42253147	5.17E-13	0.303884409	0.156	0.049	3.42E-08	C3_CD8_Tem
chr16-27446485-27447081	5.31E-13	0.274430222	0.345	0.164	3.51E-08	C3_CD8_Tem
chr10-114495192-114495890	5.45E-13	0.275210988	0.144	0.047	3.61E-08	C3_CD8_Tem
chr4-6182446-6182666	5.47E-13	0.274628272	0.087	0.019	3.62E-08	C3_CD8_Tem

chr5-132662966-132664463	5.50E-13	0.301207492	0.219	0.085	3.64E-08	C3_CD8_Tem
chr5-142977014-142978026	5.85E-13	0.289093989	0.365	0.169	3.87E-08	C3_CD8_Tem
chr12-10540546-10541263	5.87E-13	0.276215421	0.099	0.025	3.89E-08	C3_CD8_Tem
chr16-89602051-89603061	5.93E-13	0.315424402	0.222	0.082	3.93E-08	C3_CD8_Tem
chr19-41947056-41947370	6.03E-13	0.29958636	0.14	0.039	3.99E-08	C3_CD8_Tem
chr3-189131723-189132776	6.03E-13	0.259167957	0.476	0.24	4.00E-08	C3_CD8_Tem
chr2-169107254-169108739	6.23E-13	0.268325621	0.311	0.144	4.12E-08	C3_CD8_Tem
chr22-26641977-26643804	6.25E-13	0.286544255	0.325	0.148	4.14E-08	C3_CD8_Tem
chr5-109852127-109852711	6.33E-13	0.280823356	0.123	0.036	4.19E-08	C3_CD8_Tem
chr2-37201234-37202142	6.36E-13	0.302458128	0.165	0.053	4.21E-08	C3_CD8_Tem
chr3-141489216-141489536	6.50E-13	0.29851219	0.13	0.039	4.30E-08	C3_CD8_Tem
chr13-44258746-44259999	6.61E-13	0.273024903	0.325	0.149	4.38E-08	C3_CD8_Tem
chr2-101328201-101329516	6.79E-13	0.287071945	0.285	0.121	4.49E-08	C3_CD8_Tem
chr21-34192430-34193200	7.28E-13	0.262913314	0.094	0.025	4.82E-08	C3_CD8_Tem
chr2-37851387-37852354	7.32E-13	0.286828729	0.152	0.053	4.85E-08	C3_CD8_Tem
chr7-36776701-36777574	7.39E-13	0.274899733	0.301	0.136	4.89E-08	C3_CD8_Tem
chr2-38696807-38697971	7.45E-13	0.279707141	0.371	0.174	4.93E-08	C3_CD8_Tem
chr2-135953947-135954581	7.55E-13	0.284966736	0.143	0.046	5.00E-08	C3_CD8_Tem
chr18-13608087-13608517	7.68E-13	0.29732638	0.2	0.075	5.09E-08	C3_CD8_Tem
chr10-62732636-62734131	7.70E-13	0.26115134	0.435	0.22	5.10E-08	C3_CD8_Tem
chr1-206770437-206770829	7.74E-13	0.27908432	0.106	0.029	5.12E-08	C3_CD8_Tem
chr20-38870531-38871035	7.82E-13	0.314078644	0.206	0.073	5.18E-08	C3_CD8_Tem
chr12-9868825-9870030	8.01E-13	0.273991904	0.406	0.203	5.30E-08	C3_CD8_Tem
chr20-10350624-10351911	8.08E-13	0.273040706	0.261	0.117	5.35E-08	C3_CD8_Tem
chr12-68159276-68160057	8.08E-13	0.281675404	0.34	0.16	5.35E-08	C3_CD8_Tem
chr10-50552664-50553397	8.30E-13	0.293595865	0.157	0.051	5.50E-08	C3_CD8_Tem
chr2-37599387-37600389	8.38E-13	0.26830567	0.378	0.183	5.55E-08	C3_CD8_Tem

chr17-31493821-31494998	8.45E-13	0.284530025	0.377	0.183	5.60E-08	C3_CD8_Tem
chr12-47390904-47392148	8.59E-13	0.287977682	0.259	0.108	5.69E-08	C3_CD8_Tem
chr13-40221169-40222360	8.89E-13	0.257645093	0.414	0.208	5.88E-08	C3_CD8_Tem
chr18-58541077-58542576	9.12E-13	0.293897681	0.323	0.144	6.04E-08	C3_CD8_Tem
chr10-69179181-69181157	9.13E-13	0.254362035	0.531	0.294	6.04E-08	C3_CD8_Tem
chr6-127933636-127934859	9.36E-13	0.295547857	0.259	0.109	6.20E-08	C3_CD8_Tem
chr13-46751365-46752411	9.37E-13	0.273274328	0.29	0.128	6.21E-08	C3_CD8_Tem
chr15-69755310-69755977	9.96E-13	0.28816233	0.361	0.169	6.60E-08	C3_CD8_Tem
chr9-41234540-41235024	1.03E-12	0.297470825	0.199	0.076	6.80E-08	C3_CD8_Tem
chr17-3788051-3788536	1.04E-12	0.296400083	0.176	0.06	6.91E-08	C3_CD8_Tem
chr17-68182449-68183538	1.08E-12	0.266863302	0.468	0.239	7.15E-08	C3_CD8_Tem
chr1-28199130-28200262	1.09E-12	0.28212894	0.34	0.162	7.20E-08	C3_CD8_Tem
chr3-15309624-15311172	1.12E-12	0.270348274	0.363	0.173	7.39E-08	C3_CD8_Tem
chr20-50109921-50110653	1.17E-12	0.286381704	0.314	0.136	7.76E-08	C3_CD8_Tem
chr1-203320345-203321303	1.18E-12	0.283707141	0.337	0.155	7.79E-08	C3_CD8_Tem
chr3-52310774-52311322	1.19E-12	0.271145319	0.113	0.035	7.86E-08	C3_CD8_Tem
chr3-126613373-126614080	1.20E-12	0.264917684	0.096	0.024	7.96E-08	C3_CD8_Tem
chr7-55569466-55570515	1.22E-12	0.293827793	0.286	0.123	8.07E-08	C3_CD8_Tem
chr20-23366409-23367211	1.28E-12	0.301771771	0.224	0.088	8.49E-08	C3_CD8_Tem
chr19-16080077-16080425	1.29E-12	0.291373317	0.253	0.109	8.57E-08	C3_CD8_Tem
chr18-24018330-24018559	1.30E-12	0.285418282	0.112	0.031	8.64E-08	C3_CD8_Tem
chr15-69713592-69714665	1.31E-12	0.262223333	0.491	0.25	8.65E-08	C3_CD8_Tem
chr18-22342578-22343028	1.37E-12	0.288494982	0.151	0.05	9.10E-08	C3_CD8_Tem
chr3-27880867-27881933	1.43E-12	0.285569044	0.227	0.093	9.49E-08	C3_CD8_Tem
chr2-85852464-85854151	1.44E-12	0.271628156	0.446	0.227	9.56E-08	C3_CD8_Tem
chr14-50521142-50522337	1.51E-12	0.299403221	0.301	0.129	1.00E-07	C3_CD8_Tem
chr17-8915529-8916423	1.51E-12	0.290759784	0.145	0.046	1.00E-07	C3_CD8_Tem

chr21-46368226-46369182	1.53E-12	0.297719042	0.204	0.076	1.01E-07	C3_CD8_Tem
chr12-22130321-22131038	1.54E-12	0.255119662	0.074	0.015	1.02E-07	C3_CD8_Tem
chr12-10558323-10558663	1.54E-12	0.27558603	0.084	0.018	1.02E-07	C3_CD8_Tem
chr1-25035751-25036614	1.56E-12	0.269513856	0.259	0.113	1.03E-07	C3_CD8_Tem
chr4-152099735-152101089	1.57E-12	0.25392276	0.396	0.202	1.04E-07	C3_CD8_Tem
chr3-177337230-177338254	1.58E-12	0.284640075	0.253	0.103	1.05E-07	C3_CD8_Tem
chr6-152634627-152635124	1.58E-12	0.285626062	0.196	0.075	1.05E-07	C3_CD8_Tem
chrX-124342178-124343394	1.59E-12	0.291591068	0.279	0.12	1.05E-07	C3_CD8_Tem
chr17-35588392-35588820	1.63E-12	0.298318583	0.14	0.043	1.08E-07	C3_CD8_Tem
chr12-124919347-124920368	1.66E-12	0.304162851	0.322	0.145	1.10E-07	C3_CD8_Tem
chr6-107715618-107717134	1.67E-12	0.264626441	0.406	0.202	1.11E-07	C3_CD8_Tem
chr5-150398547-150399063	1.73E-12	0.279039579	0.096	0.022	1.15E-07	C3_CD8_Tem
chr6-17632973-17634086	1.73E-12	0.301077952	0.184	0.066	1.15E-07	C3_CD8_Tem
chr9-20319465-20320310	1.77E-12	0.290502189	0.165	0.058	1.17E-07	C3_CD8_Tem
chr6-13427444-13428661	1.81E-12	0.27470993	0.286	0.12	1.20E-07	C3_CD8_Tem
chr2-176931691-176932544	1.83E-12	0.279315375	0.231	0.097	1.21E-07	C3_CD8_Tem
chr3-46406499-46407661	1.86E-12	0.289606254	0.22	0.088	1.23E-07	C3_CD8_Tem
chrX-119647525-119649045	1.90E-12	0.258243694	0.396	0.192	1.26E-07	C3_CD8_Tem
chr12-10378752-10379115	1.92E-12	0.278963727	0.083	0.017	1.27E-07	C3_CD8_Tem
chr22-22804819-22805444	1.93E-12	0.265139585	0.083	0.018	1.28E-07	C3_CD8_Tem
chr14-89773254-89774387	2.02E-12	0.274944127	0.233	0.101	1.34E-07	C3_CD8_Tem
chr11-48011439-48012430	2.07E-12	0.250328018	0.449	0.231	1.37E-07	C3_CD8_Tem
chr10-22626753-22627429	2.08E-12	0.311914547	0.188	0.069	1.38E-07	C3_CD8_Tem
chr2-27391044-27392222	2.13E-12	0.294279471	0.221	0.089	1.41E-07	C3_CD8_Tem
chr17-68234882-68235293	2.17E-12	0.299404447	0.281	0.119	1.43E-07	C3_CD8_Tem
chr14-105986190-105987143	2.18E-12	0.291154741	0.267	0.116	1.44E-07	C3_CD8_Tem
chrX-124225919-124226446	2.36E-12	0.253877327	0.075	0.017	1.56E-07	C3_CD8_Tem

chr1-235855783-235856376	2.37E-12	0.274675668	0.094	0.023	1.57E-07	C3_CD8_Tem
chr10-72369720-72370856	2.40E-12	0.250812974	0.391	0.201	1.59E-07	C3_CD8_Tem
chr16-29028776-29029693	2.40E-12	0.292200895	0.212	0.083	1.59E-07	C3_CD8_Tem
chr13-50427239-50427876	2.41E-12	0.273011466	0.129	0.041	1.59E-07	C3_CD8_Tem
chr1-24915001-24915963	2.47E-12	0.287700171	0.244	0.097	1.64E-07	C3_CD8_Tem
chr4-3201683-3203141	2.47E-12	0.274911009	0.312	0.147	1.64E-07	C3_CD8_Tem
chr8-130235971-130236839	2.53E-12	0.294994799	0.163	0.055	1.67E-07	C3_CD8_Tem
chr14-73503631-73504001	2.53E-12	0.251859066	0.075	0.017	1.68E-07	C3_CD8_Tem
chr12-53102878-53103402	2.57E-12	0.293806941	0.199	0.077	1.70E-07	C3_CD8_Tem
chr8-130041775-130042771	2.76E-12	0.300064815	0.259	0.104	1.83E-07	C3_CD8_Tem
chr12-674054-674862	2.76E-12	0.255100762	0.441	0.224	1.83E-07	C3_CD8_Tem
chr3-111571316-111572399	2.94E-12	0.283566566	0.172	0.065	1.95E-07	C3_CD8_Tem
chr5-102865364-102866182	2.94E-12	0.289551474	0.166	0.058	1.95E-07	C3_CD8_Tem
chr7-38218456-38218770	3.03E-12	0.29008122	0.108	0.027	2.01E-07	C3_CD8_Tem
chr22-23137252-23138634	3.05E-12	0.291712609	0.3	0.129	2.02E-07	C3_CD8_Tem
chr16-81778764-81779995	3.17E-12	0.301535562	0.259	0.104	2.10E-07	C3_CD8_Tem
chr5-65021146-65022063	3.20E-12	0.284093009	0.163	0.056	2.12E-07	C3_CD8_Tem
chr1-52139610-52140503	3.24E-12	0.29161342	0.304	0.132	2.15E-07	C3_CD8_Tem
chr14-91377071-91377696	3.33E-12	0.275301417	0.152	0.054	2.21E-07	C3_CD8_Tem
chr8-133054632-133055107	3.42E-12	0.304147575	0.286	0.119	2.27E-07	C3_CD8_Tem
chr21-42198955-42200215	3.44E-12	0.292277705	0.245	0.101	2.28E-07	C3_CD8_Tem
chr3-114299448-114300373	3.47E-12	0.278493662	0.144	0.048	2.29E-07	C3_CD8_Tem
chr17-27568723-27569557	3.50E-12	0.276850581	0.138	0.045	2.32E-07	C3_CD8_Tem
chrX-1471671-1472239	3.62E-12	0.269386283	0.242	0.104	2.40E-07	C3_CD8_Tem
chr3-119437547-119438330	3.63E-12	0.257432852	0.086	0.022	2.40E-07	C3_CD8_Tem
chr3-129576399-129577446	3.64E-12	0.263151494	0.311	0.146	2.41E-07	C3_CD8_Tem
chr2-230893993-230894713	3.66E-12	0.260430917	0.08	0.018	2.42E-07	C3_CD8_Tem

chrX-9351156-9352959	3.77E-12	0.276420053	0.312	0.144	2.50E-07	C3_CD8_Tem
chr4-77594956-77595777	3.79E-12	0.275933484	0.109	0.03	2.51E-07	C3_CD8_Tem
chr3-71492582-71493788	3.82E-12	0.276292988	0.378	0.179	2.53E-07	C3_CD8_Tem
chr19-19451245-19452586	3.83E-12	0.287746658	0.195	0.071	2.53E-07	C3_CD8_Tem
chr5-170253640-170254783	3.94E-12	0.251598084	0.463	0.246	2.61E-07	C3_CD8_Tem
chr10-50503586-50504280	3.95E-12	0.258974267	0.101	0.028	2.62E-07	C3_CD8_Tem
chr11-118919418-118920066	3.97E-12	0.271616074	0.364	0.174	2.63E-07	C3_CD8_Tem
chr2-230973545-230974871	3.99E-12	0.25436873	0.414	0.217	2.64E-07	C3_CD8_Tem
chr1-192559117-192559888	4.00E-12	0.290613853	0.176	0.061	2.65E-07	C3_CD8_Tem
chr3-14142106-14143151	4.15E-12	0.269670462	0.392	0.196	2.75E-07	C3_CD8_Tem
chr15-28937881-28938648	4.20E-12	0.288446037	0.14	0.045	2.78E-07	C3_CD8_Tem
chr4-4268731-4270272	4.22E-12	0.279242054	0.351	0.161	2.80E-07	C3_CD8_Tem
chr12-42094064-42094682	4.24E-12	0.288016446	0.19	0.071	2.81E-07	C3_CD8_Tem
chr2-203781820-203782629	4.27E-12	0.295677554	0.205	0.078	2.83E-07	C3_CD8_Tem
chr14-51869345-51869756	4.43E-12	0.287652972	0.26	0.109	2.93E-07	C3_CD8_Tem
chr11-117984168-117984752	4.50E-12	0.294631314	0.161	0.053	2.98E-07	C3_CD8_Tem
chr2-61670277-61671446	4.50E-12	0.273861378	0.097	0.023	2.98E-07	C3_CD8_Tem
chr15-62856936-62857867	4.51E-12	0.251915682	0.084	0.02	2.99E-07	C3_CD8_Tem
chr4-38131084-38132672	4.59E-12	0.251464521	0.493	0.258	3.04E-07	C3_CD8_Tem
chr16-81717582-81718106	5.04E-12	0.286297629	0.139	0.047	3.34E-07	C3_CD8_Tem
chr19-6535918-6536472	5.31E-12	0.281400658	0.224	0.091	3.52E-07	C3_CD8_Tem
chr6-111812459-111813455	5.56E-12	0.304171925	0.151	0.048	3.68E-07	C3_CD8_Tem
chr6-127879604-127880774	5.91E-12	0.305173282	0.179	0.059	3.92E-07	C3_CD8_Tem
chr2-202277816-202278699	6.03E-12	0.288528182	0.191	0.072	4.00E-07	C3_CD8_Tem
chr22-30434891-30436225	6.26E-12	0.271646735	0.363	0.17	4.15E-07	C3_CD8_Tem
chr2-159225953-159226543	6.26E-12	0.277930204	0.19	0.071	4.15E-07	C3_CD8_Tem
chr1-161182037-161182623	6.37E-12	0.283363589	0.297	0.136	4.22E-07	C3_CD8_Tem

chr20-62087952-62088847	6.59E-12	0.28478533	0.172	0.063	4.36E-07	C3_CD8_Tem
chr1-114340814-114342699	6.78E-12	0.252558639	0.425	0.214	4.49E-07	C3_CD8_Tem
chr5-52743033-52744272	6.83E-12	0.278685059	0.3	0.135	4.52E-07	C3_CD8_Tem
chr16-89785283-89785996	6.84E-12	0.292537115	0.152	0.051	4.53E-07	C3_CD8_Tem
chr7-99631245-99631953	7.05E-12	0.258849635	0.091	0.026	4.67E-07	C3_CD8_Tem
chr1-160567398-160568742	7.08E-12	0.257843964	0.424	0.215	4.69E-07	C3_CD8_Tem
chr9-124873648-124874001	7.08E-12	0.279269045	0.157	0.054	4.69E-07	C3_CD8_Tem
chr19-1030247-1031629	7.09E-12	0.251777744	0.508	0.274	4.69E-07	C3_CD8_Tem
chr17-78133905-78134571	7.26E-12	0.272193104	0.332	0.157	4.81E-07	C3_CD8_Tem
chr12-12430729-12431533	7.29E-12	0.285150942	0.139	0.045	4.83E-07	C3_CD8_Tem
chr17-4902860-4903824	7.44E-12	0.294339721	0.287	0.122	4.92E-07	C3_CD8_Tem
chr1-116537182-116537858	7.54E-12	0.287867546	0.176	0.064	4.99E-07	C3_CD8_Tem
chr2-208100304-208101691	7.81E-12	0.278487298	0.133	0.041	5.17E-07	C3_CD8_Tem
chr5-126747807-126748494	8.10E-12	0.285404595	0.276	0.119	5.37E-07	C3_CD8_Tem
chr3-136142213-136142946	8.56E-12	0.270288294	0.136	0.046	5.67E-07	C3_CD8_Tem
chr7-99623245-99624899	8.58E-12	0.278563541	0.249	0.103	5.68E-07	C3_CD8_Tem
chr9-126982085-126982722	9.00E-12	0.278124711	0.213	0.086	5.96E-07	C3_CD8_Tem
chr20-433170-434015	9.01E-12	0.274201556	0.296	0.133	5.97E-07	C3_CD8_Tem
chr12-666650-667978	9.07E-12	0.297457545	0.173	0.06	6.01E-07	C3_CD8_Tem
chr14-61502498-61503850	9.10E-12	0.291953106	0.323	0.144	6.03E-07	C3_CD8_Tem
chr14-61328202-61329221	9.39E-12	0.287150006	0.328	0.152	6.22E-07	C3_CD8_Tem
chr16-88636781-88637627	9.54E-12	0.277595035	0.327	0.145	6.32E-07	C3_CD8_Tem
chr6-118725705-118726394	1.01E-11	0.277340684	0.274	0.123	6.69E-07	C3_CD8_Tem
chr20-51413798-51414278	1.01E-11	0.284685391	0.212	0.084	6.71E-07	C3_CD8_Tem
chr8-21938183-21938667	1.02E-11	0.268680065	0.095	0.023	6.74E-07	C3_CD8_Tem
chr9-131406707-131408148	1.02E-11	0.296792954	0.286	0.122	6.77E-07	C3_CD8_Tem
chr3-46108784-46109849	1.05E-11	0.279836066	0.298	0.136	6.93E-07	C3_CD8_Tem

chr2-97720946-97721561	1.07E-11	0.287447889	0.202	0.077	7.10E-07	C3_CD8_Tem
chr1-9626753-9627649	1.17E-11	0.259863935	0.501	0.268	7.74E-07	C3_CD8_Tem
chr13-42368871-42369737	1.22E-11	0.289037752	0.15	0.047	8.05E-07	C3_CD8_Tem
chr16-87865820-87867009	1.24E-11	0.254895679	0.411	0.217	8.20E-07	C3_CD8_Tem
chr22-20549056-20549841	1.27E-11	0.291152354	0.189	0.069	8.44E-07	C3_CD8_Tem
chr10-112389090-112389930	1.30E-11	0.278051576	0.234	0.097	8.61E-07	C3_CD8_Tem
chr3-27719890-27720151	1.31E-11	0.270644699	0.113	0.032	8.70E-07	C3_CD8_Tem
chr16-3170887-3172150	1.36E-11	0.293476526	0.244	0.097	9.00E-07	C3_CD8_Tem
chrX-47634583-47635540	1.37E-11	0.271971	0.141	0.047	9.05E-07	C3_CD8_Tem
chr6-89952038-89953204	1.37E-11	0.26188083	0.34	0.161	9.10E-07	C3_CD8_Tem
chr17-49217767-49218568	1.39E-11	0.273113856	0.312	0.145	9.20E-07	C3_CD8_Tem
chr12-128795987-128796930	1.43E-11	0.278969027	0.207	0.079	9.45E-07	C3_CD8_Tem
chr14-63524317-63525177	1.44E-11	0.267918826	0.117	0.036	9.55E-07	C3_CD8_Tem
chr16-84514398-84515334	1.47E-11	0.257729612	0.377	0.188	9.73E-07	C3_CD8_Tem
chr19-43764828-43766041	1.50E-11	0.273349812	0.396	0.196	9.94E-07	C3_CD8_Tem
chr20-53835364-53835846	1.53E-11	0.288302751	0.233	0.096	1.02E-06	C3_CD8_Tem
chr6-159061930-159063226	1.57E-11	0.257224883	0.422	0.21	1.04E-06	C3_CD8_Tem
chr11-36393948-36394445	1.60E-11	0.287170818	0.129	0.038	1.06E-06	C3_CD8_Tem
chr20-53892841-53893753	1.62E-11	0.284801531	0.216	0.086	1.07E-06	C3_CD8_Tem
chr11-69296852-69298704	1.64E-11	0.251227552	0.499	0.268	1.09E-06	C3_CD8_Tem
chr15-59482258-59483059	1.70E-11	0.268851938	0.123	0.038	1.12E-06	C3_CD8_Tem
chr3-108805888-108806824	1.71E-11	0.271094214	0.27	0.12	1.14E-06	C3_CD8_Tem
chrX-71619070-71619528	1.75E-11	0.284746003	0.284	0.125	1.16E-06	C3_CD8_Tem
chr15-38555833-38556362	1.78E-11	0.282159578	0.271	0.113	1.18E-06	C3_CD8_Tem
chr3-149003738-149004386	1.80E-11	0.278210872	0.206	0.078	1.19E-06	C3_CD8_Tem
chr17-58352950-58353766	1.87E-11	0.292057856	0.194	0.073	1.24E-06	C3_CD8_Tem
chrX-3707649-3708106	1.89E-11	0.267977302	0.136	0.046	1.25E-06	C3_CD8_Tem

chr21-44343509-44343886	1.89E-11	0.28097438	0.118	0.033	1.25E-06	C3_CD8_Tem
chr14-99234956-99235814	1.98E-11	0.294038553	0.35	0.164	1.31E-06	C3_CD8_Tem
chr3-28243428-28244017	2.04E-11	0.285543809	0.182	0.068	1.35E-06	C3_CD8_Tem
chr2-106094047-106094928	2.06E-11	0.257310826	0.336	0.167	1.36E-06	C3_CD8_Tem
chr10-24887229-24888162	2.12E-11	0.265232276	0.26	0.115	1.41E-06	C3_CD8_Tem
chr3-46441790-46442333	2.15E-11	0.261360091	0.114	0.035	1.42E-06	C3_CD8_Tem
chr20-44019430-44020393	2.17E-11	0.288199272	0.188	0.066	1.44E-06	C3_CD8_Tem
chr1-202295192-202296111	2.21E-11	0.278057545	0.267	0.12	1.46E-06	C3_CD8_Tem
chr10-69203724-69204463	2.28E-11	0.280983837	0.245	0.105	1.51E-06	C3_CD8_Tem
chr10-50511142-50512258	2.29E-11	0.285579656	0.233	0.095	1.52E-06	C3_CD8_Tem
chr3-66297408-66298303	2.35E-11	0.282030083	0.186	0.069	1.56E-06	C3_CD8_Tem
chr1-235872805-235873637	2.37E-11	0.271462644	0.186	0.07	1.57E-06	C3_CD8_Tem
chr6-144353544-144354425	2.39E-11	0.275308526	0.27	0.119	1.58E-06	C3_CD8_Tem
chr1-192580320-192581740	2.43E-11	0.291126815	0.216	0.081	1.61E-06	C3_CD8_Tem
chr3-46557742-46558819	2.44E-11	0.290695184	0.273	0.115	1.62E-06	C3_CD8_Tem
chr6-31354130-31354531	2.46E-11	0.299362737	0.197	0.073	1.63E-06	C3_CD8_Tem
chr14-61351428-61351787	2.47E-11	0.266062127	0.111	0.031	1.64E-06	C3_CD8_Tem
chr13-99366761-99367637	2.48E-11	0.281752899	0.146	0.048	1.64E-06	C3_CD8_Tem
chr3-14175481-14175997	2.55E-11	0.290612226	0.226	0.091	1.69E-06	C3_CD8_Tem
chr8-41507466-41508702	2.60E-11	0.278403701	0.135	0.042	1.72E-06	C3_CD8_Tem
chr17-1119934-1121063	2.63E-11	0.251058011	0.101	0.032	1.74E-06	C3_CD8_Tem
chr5-112923297-112923942	2.65E-11	0.273216725	0.213	0.089	1.75E-06	C3_CD8_Tem
chr10-132427249-132428487	2.65E-11	0.257729908	0.168	0.065	1.75E-06	C3_CD8_Tem
chr1-156524169-156524901	2.68E-11	0.273568431	0.135	0.045	1.78E-06	C3_CD8_Tem
chr18-74995694-74996447	2.78E-11	0.283727722	0.143	0.045	1.84E-06	C3_CD8_Tem
chr15-93141127-93141850	2.82E-11	0.268619428	0.14	0.047	1.87E-06	C3_CD8_Tem
chr5-1535643-1537104	2.90E-11	0.284069418	0.329	0.15	1.92E-06	C3_CD8_Tem

chr2-38587698-38588105	2.97E-11	0.250715355	0.11	0.036	1.97E-06	C3_CD8_Tem
chr5-107387910-107389582	2.98E-11	0.260611066	0.136	0.046	1.98E-06	C3_CD8_Tem
chr10-124694546-124695322	3.01E-11	0.282549007	0.174	0.067	2.00E-06	C3_CD8_Tem
chr14-99055289-99056326	3.09E-11	0.27022848	0.215	0.087	2.05E-06	C3_CD8_Tem
chrX-153966946-153967202	3.11E-11	0.268188107	0.1	0.028	2.06E-06	C3_CD8_Tem
chr19-29610167-29611018	3.13E-11	0.288641039	0.256	0.109	2.07E-06	C3_CD8_Tem
chr8-29405399-29405923	3.18E-11	0.274473022	0.174	0.068	2.11E-06	C3_CD8_Tem
chr6-125299184-125299677	3.22E-11	0.269861551	0.191	0.077	2.14E-06	C3_CD8_Tem
chr3-113741828-113742303	3.25E-11	0.296861365	0.168	0.058	2.15E-06	C3_CD8_Tem
chr17-77836360-77837135	3.36E-11	0.275790199	0.227	0.097	2.22E-06	C3_CD8_Tem
chr22-44671794-44672138	3.42E-11	0.26686368	0.193	0.077	2.26E-06	C3_CD8_Tem
chr2-62287706-62288301	3.47E-11	0.251038709	0.081	0.02	2.30E-06	C3_CD8_Tem
chr2-233099908-233101037	3.52E-11	0.286802165	0.217	0.087	2.33E-06	C3_CD8_Tem
chr6-159978466-159979502	3.56E-11	0.268830132	0.328	0.158	2.36E-06	C3_CD8_Tem
chr12-52645010-52646024	3.58E-11	0.262280533	0.186	0.076	2.37E-06	C3_CD8_Tem
chr17-49208732-49209807	3.59E-11	0.285231231	0.206	0.081	2.38E-06	C3_CD8_Tem
chr17-39757813-39758477	3.59E-11	0.278447733	0.365	0.173	2.38E-06	C3_CD8_Tem
chr14-92585589-92587275	3.71E-11	0.279512176	0.347	0.16	2.46E-06	C3_CD8_Tem
chr11-117982764-117983041	3.92E-11	0.259227258	0.092	0.025	2.60E-06	C3_CD8_Tem
chr19-35445789-35446564	3.95E-11	0.270214133	0.131	0.043	2.62E-06	C3_CD8_Tem
chr3-112642929-112644237	4.14E-11	0.284103076	0.211	0.088	2.74E-06	C3_CD8_Tem
chr14-50085654-50086264	4.24E-11	0.295342896	0.202	0.077	2.81E-06	C3_CD8_Tem
chr13-40612805-40613852	4.34E-11	0.252685658	0.327	0.163	2.87E-06	C3_CD8_Tem
chr7-50367163-50367968	4.47E-11	0.275621261	0.248	0.11	2.96E-06	C3_CD8_Tem
chr17-67431290-67431893	4.56E-11	0.294672437	0.251	0.103	3.02E-06	C3_CD8_Tem
chr4-1203540-1204028	4.60E-11	0.284241496	0.196	0.075	3.05E-06	C3_CD8_Tem
chr7-36723790-36724702	4.64E-11	0.254980846	0.295	0.137	3.08E-06	C3_CD8_Tem

chr6-158995221-158995663	4.72E-11	0.277596982	0.149	0.051	3.12E-06	C3_CD8_Tem
chr14-24633431-24634610	4.78E-11	0.265266542	0.267	0.119	3.17E-06	C3_CD8_Tem
chr18-13614770-13615865	4.83E-11	0.273976088	0.307	0.14	3.20E-06	C3_CD8_Tem
chr9-129956419-129957185	5.09E-11	0.282144082	0.176	0.065	3.37E-06	C3_CD8_Tem
chr5-178113100-178115384	5.10E-11	0.25059096	0.413	0.216	3.38E-06	C3_CD8_Tem
chr6-26240015-26241308	5.13E-11	0.270453305	0.363	0.178	3.40E-06	C3_CD8_Tem
chr16-87874680-87875518	5.21E-11	0.283991996	0.205	0.081	3.45E-06	C3_CD8_Tem
chr12-10673283-10674412	5.21E-11	0.282136964	0.266	0.114	3.45E-06	C3_CD8_Tem
chr1-168512608-168513639	5.26E-11	0.274372922	0.175	0.063	3.48E-06	C3_CD8_Tem
chr19-4914755-4915456	5.29E-11	0.266757231	0.35	0.171	3.50E-06	C3_CD8_Tem
chr10-79322827-79323611	5.29E-11	0.2815337	0.322	0.149	3.50E-06	C3_CD8_Tem
chr2-113240392-113241310	5.34E-11	0.262898593	0.142	0.05	3.54E-06	C3_CD8_Tem
chr1-8520336-8520883	5.35E-11	0.252421425	0.102	0.034	3.54E-06	C3_CD8_Tem
chr5-143120638-143121433	5.41E-11	0.263804326	0.123	0.039	3.58E-06	C3_CD8_Tem
chr14-54435240-54436060	5.42E-11	0.272121646	0.264	0.119	3.59E-06	C3_CD8_Tem
chr15-55267043-55268079	5.46E-11	0.251299569	0.433	0.223	3.61E-06	C3_CD8_Tem
chr13-30157033-30157717	5.77E-11	0.261222373	0.105	0.028	3.82E-06	C3_CD8_Tem
chr3-48989165-48991309	6.11E-11	0.265904461	0.377	0.194	4.05E-06	C3_CD8_Tem
chr12-12384393-12385643	6.24E-11	0.271816155	0.249	0.109	4.13E-06	C3_CD8_Tem
chr3-43477309-43478624	6.28E-11	0.254820529	0.311	0.149	4.16E-06	C3_CD8_Tem
chr3-112667615-112667952	6.74E-11	0.253526585	0.076	0.017	4.47E-06	C3_CD8_Tem
chr2-110674997-110676078	6.93E-11	0.263584345	0.298	0.135	4.59E-06	C3_CD8_Tem
chr22-42316743-42317483	7.01E-11	0.277109573	0.204	0.082	4.64E-06	C3_CD8_Tem
chr18-23872449-23873091	7.25E-11	0.269622205	0.174	0.064	4.80E-06	C3_CD8_Tem
chr17-35081980-35082629	7.34E-11	0.29601339	0.205	0.08	4.86E-06	C3_CD8_Tem
chr17-3770352-3771391	7.47E-11	0.260779603	0.144	0.05	4.95E-06	C3_CD8_Tem
chr17-14359049-14359741	7.50E-11	0.255919839	0.14	0.053	4.97E-06	C3_CD8_Tem

chr22-22753064-22753864	7.57E-11	0.276354186	0.164	0.06	5.01E-06	C3_CD8_Tem
chr19-996309-996912	8.02E-11	0.265239841	0.345	0.165	5.31E-06	C3_CD8_Tem
chr8-133614979-133615492	8.15E-11	0.272625209	0.112	0.032	5.39E-06	C3_CD8_Tem
chr22-36356813-36358113	8.15E-11	0.266583679	0.334	0.16	5.40E-06	C3_CD8_Tem
chr17-17391466-17392458	8.15E-11	0.269767602	0.256	0.114	5.40E-06	C3_CD8_Tem
chr19-3357402-3358618	8.34E-11	0.277008275	0.219	0.089	5.52E-06	C3_CD8_Tem
chr13-50421534-50422621	8.34E-11	0.266560904	0.125	0.04	5.52E-06	C3_CD8_Tem
chr5-132099000-132099509	8.47E-11	0.266943378	0.337	0.156	5.61E-06	C3_CD8_Tem
chr16-56999335-57000938	8.48E-11	0.278642489	0.319	0.147	5.61E-06	C3_CD8_Tem
chr5-96793985-96795199	8.62E-11	0.281499038	0.238	0.099	5.71E-06	C3_CD8_Tem
chr11-118227514-118228683	8.63E-11	0.282074668	0.331	0.155	5.71E-06	C3_CD8_Tem
chr3-188339243-188340186	8.63E-11	0.271056748	0.216	0.092	5.71E-06	C3_CD8_Tem
chr12-47229198-47229917	8.77E-11	0.289679368	0.228	0.091	5.81E-06	C3_CD8_Tem
chr1-107820641-107821706	8.82E-11	0.268692373	0.13	0.042	5.84E-06	C3_CD8_Tem
chr12-124921145-124921808	8.92E-11	0.261103212	0.371	0.182	5.90E-06	C3_CD8_Tem
chr17-8388212-8388549	8.99E-11	0.256885174	0.091	0.025	5.96E-06	C3_CD8_Tem
chr2-239630043-239630854	9.03E-11	0.263425074	0.107	0.032	5.98E-06	C3_CD8_Tem
chr7-92809356-92810607	9.10E-11	0.274629497	0.359	0.169	6.03E-06	C3_CD8_Tem
chr17-15958802-15959638	9.17E-11	0.26546612	0.332	0.155	6.07E-06	C3_CD8_Tem
chr22-20566372-20567525	9.30E-11	0.268381603	0.318	0.148	6.16E-06	C3_CD8_Tem
chr9-128032997-128033511	9.46E-11	0.255434128	0.407	0.205	6.26E-06	C3_CD8_Tem
chr17-35889125-35891509	9.75E-11	0.264836981	0.372	0.178	6.46E-06	C3_CD8_Tem
chr16-79380583-79382128	9.78E-11	0.26807118	0.188	0.074	6.48E-06	C3_CD8_Tem
chr6-16676897-16677812	9.89E-11	0.259976398	0.095	0.025	6.55E-06	C3_CD8_Tem
chr16-57609271-57610284	9.92E-11	0.268301747	0.264	0.115	6.57E-06	C3_CD8_Tem
chr20-5077780-5078868	1.00E-10	0.255058229	0.354	0.178	6.65E-06	C3_CD8_Tem
chr22-39919971-39921390	1.01E-10	0.255716203	0.438	0.23	6.70E-06	C3_CD8_Tem

chr11-76494838-76495403	1.03E-10	0.261163985	0.086	0.021	6.83E-06	C3_CD8_Tem
chr6-31351263-31351901	1.04E-10	0.278371798	0.178	0.068	6.88E-06	C3_CD8_Tem
chr15-40098171-40099143	1.05E-10	0.256674674	0.321	0.153	6.96E-06	C3_CD8_Tem
chr19-19039612-19040323	1.05E-10	0.272221984	0.13	0.039	6.97E-06	C3_CD8_Tem
chr2-112624045-112624907	1.06E-10	0.267029546	0.256	0.113	7.03E-06	C3_CD8_Tem
chr1-226682729-226683203	1.07E-10	0.272306015	0.193	0.078	7.06E-06	C3_CD8_Tem
chr12-64450790-64451046	1.08E-10	0.250044258	0.077	0.019	7.13E-06	C3_CD8_Tem
chr17-57982290-57982826	1.09E-10	0.267975456	0.182	0.071	7.24E-06	C3_CD8_Tem
chr6-33009042-33009791	1.10E-10	0.268499215	0.143	0.048	7.26E-06	C3_CD8_Tem
chr4-26308466-26308909	1.12E-10	0.266108566	0.122	0.037	7.42E-06	C3_CD8_Tem
chr1-192546054-192547091	1.15E-10	0.283706128	0.223	0.091	7.59E-06	C3_CD8_Tem
chr3-56789605-56790716	1.16E-10	0.275490456	0.307	0.141	7.65E-06	C3_CD8_Tem
chr17-42483623-42484911	1.16E-10	0.271394003	0.323	0.153	7.67E-06	C3_CD8_Tem
chr5-55023801-55024381	1.17E-10	0.271111923	0.16	0.056	7.76E-06	C3_CD8_Tem
chr19-13790098-13790664	1.21E-10	0.265079942	0.182	0.071	8.00E-06	C3_CD8_Tem
chr12-32134163-32134935	1.24E-10	0.268898261	0.186	0.073	8.19E-06	C3_CD8_Tem
chr21-43733027-43734456	1.24E-10	0.27907341	0.226	0.096	8.23E-06	C3_CD8_Tem
chr12-48824087-48824575	1.28E-10	0.255874886	0.11	0.032	8.47E-06	C3_CD8_Tem
chr1-107963271-107965797	1.28E-10	0.282611333	0.281	0.128	8.47E-06	C3_CD8_Tem
chr3-69281817-69282554	1.28E-10	0.252809474	0.207	0.086	8.50E-06	C3_CD8_Tem
chr10-30436827-30437501	1.28E-10	0.275411409	0.285	0.132	8.50E-06	C3_CD8_Tem
chr19-16586712-16587917	1.29E-10	0.257293176	0.43	0.224	8.56E-06	C3_CD8_Tem
chr18-45669987-45670872	1.31E-10	0.267652496	0.254	0.115	8.66E-06	C3_CD8_Tem
chr10-14564173-14564801	1.33E-10	0.265330661	0.363	0.182	8.79E-06	C3_CD8_Tem
chr1-172674901-172675949	1.33E-10	0.278776672	0.206	0.081	8.80E-06	C3_CD8_Tem
chr1-75784374-75785133	1.33E-10	0.257262753	0.15	0.054	8.81E-06	C3_CD8_Tem
chr7-50350119-50350963	1.33E-10	0.254186647	0.375	0.197	8.84E-06	C3_CD8_Tem

chr14-68719307-68720269	1.34E-10	0.279276537	0.273	0.118	8.90E-06	C3_CD8_Tem
chr21-44083156-44084366	1.35E-10	0.274456016	0.378	0.184	8.97E-06	C3_CD8_Tem
chr8-43141786-43142639	1.36E-10	0.292561884	0.193	0.073	9.02E-06	C3_CD8_Tem
chr10-3892115-3893070	1.38E-10	0.267718762	0.339	0.161	9.12E-06	C3_CD8_Tem
chr2-96343712-96344471	1.38E-10	0.260298961	0.318	0.158	9.12E-06	C3_CD8_Tem
chr14-92529831-92530135	1.38E-10	0.260438973	0.092	0.024	9.13E-06	C3_CD8_Tem
chr3-46102483-46103018	1.39E-10	0.257701327	0.096	0.027	9.23E-06	C3_CD8_Tem
chr15-69721380-69722147	1.40E-10	0.280369822	0.207	0.084	9.30E-06	C3_CD8_Tem
chr15-50259969-50260854	1.44E-10	0.279423774	0.19	0.072	9.52E-06	C3_CD8_Tem
chr4-40264977-40266011	1.48E-10	0.278518835	0.164	0.058	9.82E-06	C3_CD8_Tem
chr1-100396516-100398052	1.50E-10	0.269443473	0.27	0.117	9.93E-06	C3_CD8_Tem
chr4-6183702-6184044	1.52E-10	0.261283115	0.1	0.027	1.00E-05	C3_CD8_Tem
chr20-49775927-49776688	1.54E-10	0.281296631	0.171	0.061	1.02E-05	C3_CD8_Tem
chr1-40241380-40242184	1.54E-10	0.281582294	0.262	0.116	1.02E-05	C3_CD8_Tem
chr6-30227452-30228309	1.58E-10	0.256815081	0.111	0.035	1.05E-05	C3_CD8_Tem
chr6-26360593-26361302	1.59E-10	0.261048035	0.176	0.069	1.05E-05	C3_CD8_Tem
chr3-111711950-111712785	1.61E-10	0.251737323	0.142	0.053	1.07E-05	C3_CD8_Tem
chr19-44164407-44165493	1.67E-10	0.260870992	0.322	0.154	1.10E-05	C3_CD8_Tem
chr7-157306434-157306782	1.70E-10	0.26364408	0.112	0.032	1.13E-05	C3_CD8_Tem
chr11-64864425-64866374	1.76E-10	0.277409589	0.411	0.208	1.17E-05	C3_CD8_Tem
chr12-130796604-130797605	1.77E-10	0.270376076	0.215	0.087	1.17E-05	C3_CD8_Tem
chr14-50821248-50823376	1.82E-10	0.276764094	0.223	0.093	1.20E-05	C3_CD8_Tem
chr17-3791506-3791870	1.85E-10	0.295127769	0.197	0.074	1.23E-05	C3_CD8_Tem
chr3-177201295-177202118	1.87E-10	0.252332015	0.348	0.18	1.24E-05	C3_CD8_Tem
chr1-31826680-31827733	1.88E-10	0.269711312	0.257	0.115	1.25E-05	C3_CD8_Tem
chr10-71698226-71699001	1.98E-10	0.255477324	0.123	0.044	1.31E-05	C3_CD8_Tem
chr11-359233-359635	2.07E-10	0.275808723	0.163	0.057	1.37E-05	C3_CD8_Tem

chr4-10108841-10109483	2.11E-10	0.260218355	0.306	0.145	1.40E-05	C3_CD8_Tem
chr11-62557935-62558490	2.15E-10	0.267247929	0.283	0.13	1.42E-05	C3_CD8_Tem
chrX-71992055-71993150	2.17E-10	0.251354104	0.218	0.093	1.44E-05	C3_CD8_Tem
chr11-35214696-35215174	2.17E-10	0.277931385	0.237	0.1	1.44E-05	C3_CD8_Tem
chr2-200466377-200467579	2.18E-10	0.252720402	0.243	0.112	1.44E-05	C3_CD8_Tem
chr7-17260841-17261797	2.28E-10	0.251024811	0.289	0.132	1.51E-05	C3_CD8_Tem
chr11-3784763-3785789	2.33E-10	0.262116505	0.348	0.169	1.54E-05	C3_CD8_Tem
chr12-25054728-25055038	2.35E-10	0.278366935	0.183	0.068	1.56E-05	C3_CD8_Tem
chr17-35536587-35537227	2.36E-10	0.273790538	0.238	0.103	1.57E-05	C3_CD8_Tem
chr6-16711472-16713474	2.39E-10	0.254519247	0.419	0.215	1.59E-05	C3_CD8_Tem
chr5-160486133-160486893	2.44E-10	0.255087185	0.132	0.046	1.61E-05	C3_CD8_Tem
chr2-174752335-174753609	2.45E-10	0.254405464	0.294	0.142	1.62E-05	C3_CD8_Tem
chr17-35518415-35518747	2.48E-10	0.269100152	0.098	0.025	1.64E-05	C3_CD8_Tem
chr8-29664761-29665744	2.54E-10	0.273517859	0.198	0.08	1.68E-05	C3_CD8_Tem
chr2-218230831-218231380	2.71E-10	0.275881482	0.229	0.097	1.80E-05	C3_CD8_Tem
chr21-42417508-42417844	2.72E-10	0.268489791	0.124	0.041	1.80E-05	C3_CD8_Tem
chr3-111717329-111717925	2.72E-10	0.267117778	0.139	0.05	1.80E-05	C3_CD8_Tem
chr20-51410666-51411119	2.79E-10	0.267907735	0.139	0.047	1.85E-05	C3_CD8_Tem
chr1-66274420-66275430	2.83E-10	0.263927943	0.272	0.126	1.87E-05	C3_CD8_Tem
chr1-221801931-221802412	2.96E-10	0.265768323	0.116	0.032	1.96E-05	C3_CD8_Tem
chrX-322612-323594	2.99E-10	0.250045936	0.351	0.177	1.98E-05	C3_CD8_Tem
chr7-105690868-105692323	3.11E-10	0.264435324	0.362	0.175	2.06E-05	C3_CD8_Tem
chr12-10293146-10293990	3.24E-10	0.289748218	0.167	0.057	2.15E-05	C3_CD8_Tem
chr14-68663077-68664455	3.32E-10	0.273829959	0.178	0.066	2.20E-05	C3_CD8_Tem
chr9-99052671-99053378	3.37E-10	0.256863971	0.101	0.028	2.23E-05	C3_CD8_Tem
chr17-47809886-47810847	3.37E-10	0.269346378	0.268	0.12	2.23E-05	C3_CD8_Tem
chr3-56732303-56733288	3.42E-10	0.2742741	0.209	0.084	2.26E-05	C3_CD8_Tem

chr15-78044354-78044820	3.47E-10	0.263806929	0.224	0.096	2.30E-05	C3_CD8_Tem
chr17-38727667-38728769	3.49E-10	0.257254455	0.312	0.15	2.31E-05	C3_CD8_Tem
chr5-39271889-39272647	3.54E-10	0.261302039	0.112	0.034	2.34E-05	C3_CD8_Tem
chr14-61360723-61361411	3.69E-10	0.265950881	0.111	0.032	2.44E-05	C3_CD8_Tem
chr3-47019209-47019536	3.69E-10	0.27910412	0.161	0.057	2.45E-05	C3_CD8_Tem
chrX-154733786-154734747	3.83E-10	0.272573082	0.194	0.077	2.54E-05	C3_CD8_Tem
chr19-6674620-6675357	3.93E-10	0.26600727	0.369	0.183	2.60E-05	C3_CD8_Tem
chr14-99259303-99259653	3.94E-10	0.260034514	0.293	0.136	2.61E-05	C3_CD8_Tem
chr10-3960681-3961472	3.97E-10	0.259562368	0.157	0.06	2.63E-05	C3_CD8_Tem
chr16-525341-525980	4.14E-10	0.280586733	0.292	0.125	2.74E-05	C3_CD8_Tem
chr17-16418759-16419440	4.16E-10	0.274091086	0.244	0.103	2.75E-05	C3_CD8_Tem
chr8-81106233-81107262	4.21E-10	0.255203243	0.337	0.17	2.79E-05	C3_CD8_Tem
chr6-16435696-16436919	4.25E-10	0.267767003	0.207	0.087	2.82E-05	C3_CD8_Tem
chrX-154541768-154543075	4.31E-10	0.273670653	0.151	0.051	2.85E-05	C3_CD8_Tem
chr11-44611238-44612315	4.33E-10	0.265101029	0.303	0.137	2.87E-05	C3_CD8_Tem
chr11-128628084-128629047	4.68E-10	0.263230843	0.249	0.108	3.10E-05	C3_CD8_Tem
chr19-38556839-38557697	4.74E-10	0.275790552	0.178	0.07	3.14E-05	C3_CD8_Tem
chr3-46284941-46285553	4.82E-10	0.279767914	0.266	0.116	3.19E-05	C3_CD8_Tem
chr3-129282640-129283094	4.96E-10	0.291935741	0.245	0.103	3.28E-05	C3_CD8_Tem
chr2-136212448-136213283	5.02E-10	0.273666771	0.206	0.083	3.32E-05	C3_CD8_Tem
chrX-42948523-42949293	5.12E-10	0.26575861	0.161	0.061	3.39E-05	C3_CD8_Tem
chr3-177679427-177680700	5.22E-10	0.263836313	0.164	0.059	3.46E-05	C3_CD8_Tem
chr9-5841326-5842455	5.49E-10	0.260768695	0.235	0.104	3.63E-05	C3_CD8_Tem
chrY-13474928-13475281	5.49E-10	0.254327214	0.103	0.032	3.64E-05	C3_CD8_Tem
chr13-24617927-24619031	5.95E-10	0.252205019	0.294	0.135	3.94E-05	C3_CD8_Tem
chr8-100501616-100503037	5.97E-10	0.26359668	0.283	0.126	3.95E-05	C3_CD8_Tem
chr5-52775599-52776556	6.11E-10	0.259784933	0.217	0.087	4.05E-05	C3_CD8_Tem

chr14-51877793-51878162	6.16E-10	0.255108252	0.1	0.03	4.08E-05	C3_CD8_Tem
chr11-35272216-35273053	6.17E-10	0.27280535	0.245	0.103	4.08E-05	C3_CD8_Tem
chr1-206924605-206925631	6.27E-10	0.27264395	0.221	0.094	4.15E-05	C3_CD8_Tem
chr10-7029813-7030789	6.38E-10	0.251538949	0.156	0.06	4.22E-05	C3_CD8_Tem
chrX-72272557-72273155	6.38E-10	0.258611626	0.099	0.028	4.23E-05	C3_CD8_Tem
chr15-92907405-92907607	6.41E-10	0.277316135	0.183	0.067	4.25E-05	C3_CD8_Tem
chr2-203740503-203741793	6.43E-10	0.267348928	0.27	0.123	4.26E-05	C3_CD8_Tem
chr10-33136326-33137128	6.45E-10	0.259368005	0.253	0.116	4.27E-05	C3_CD8_Tem
chr11-44983333-44984077	6.81E-10	0.25060401	0.09	0.024	4.51E-05	C3_CD8_Tem
chr5-159341175-159342321	7.02E-10	0.260838981	0.212	0.09	4.65E-05	C3_CD8_Tem
chr2-230864386-230865285	7.21E-10	0.259381327	0.363	0.179	4.77E-05	C3_CD8_Tem
chr21-44927273-44927898	7.90E-10	0.258075788	0.362	0.18	5.23E-05	C3_CD8_Tem
chr16-78794405-78795284	7.91E-10	0.253085057	0.15	0.058	5.24E-05	C3_CD8_Tem
chr2-144194300-144194976	7.91E-10	0.253289028	0.102	0.03	5.24E-05	C3_CD8_Tem
chrY-7279691-7280684	8.01E-10	0.259220246	0.276	0.127	5.30E-05	C3_CD8_Tem
chr17-5237381-5238054	8.17E-10	0.255523234	0.107	0.033	5.41E-05	C3_CD8_Tem
chr2-43139720-43140267	8.40E-10	0.269778538	0.186	0.075	5.56E-05	C3_CD8_Tem
chr11-118216313-118217830	8.64E-10	0.267750793	0.257	0.12	5.72E-05	C3_CD8_Tem
chr22-19419104-19420340	8.89E-10	0.254519912	0.237	0.106	5.89E-05	C3_CD8_Tem
chr2-203733674-203733997	8.95E-10	0.252772416	0.092	0.026	5.93E-05	C3_CD8_Tem
chr7-36677716-36678821	9.15E-10	0.265448024	0.142	0.047	6.06E-05	C3_CD8_Tem
chr8-115429082-115429651	9.42E-10	0.254173833	0.11	0.033	6.24E-05	C3_CD8_Tem
chr6-37514251-37514771	9.47E-10	0.259776712	0.278	0.129	6.27E-05	C3_CD8_Tem
chr22-50027654-50028185	9.56E-10	0.263451533	0.163	0.062	6.33E-05	C3_CD8_Tem
chr6-129671652-129673596	9.81E-10	0.255569803	0.384	0.19	6.49E-05	C3_CD8_Tem
chr17-35160382-35161809	9.81E-10	0.28320615	0.285	0.129	6.50E-05	C3_CD8_Tem
chr3-46536702-46537036	1.01E-09	0.267233242	0.107	0.03	6.71E-05	C3_CD8_Tem

chr15-78054999-78055307	1.02E-09	0.26146476	0.158	0.062	6.73E-05	C3_CD8_Tem
chr1-223831622-223832839	1.03E-09	0.263099587	0.341	0.164	6.80E-05	C3_CD8_Tem
chr9-123338822-123340686	1.03E-09	0.252663628	0.364	0.175	6.84E-05	C3_CD8_Tem
chr15-63203399-63204113	1.06E-09	0.268105399	0.217	0.089	7.05E-05	C3_CD8_Tem
chr19-52715420-52716518	1.07E-09	0.271291106	0.158	0.055	7.10E-05	C3_CD8_Tem
chr11-134228632-134229052	1.07E-09	0.250072987	0.13	0.046	7.11E-05	C3_CD8_Tem
chr7-131238148-131238849	1.08E-09	0.262341393	0.176	0.07	7.17E-05	C3_CD8_Tem
chr20-53884739-53885569	1.10E-09	0.258336438	0.133	0.045	7.27E-05	C3_CD8_Tem
chr22-46603904-46604701	1.12E-09	0.253272288	0.121	0.042	7.40E-05	C3_CD8_Tem
chr12-91851937-91852462	1.13E-09	0.260735262	0.098	0.029	7.52E-05	C3_CD8_Tem
chr19-1939203-1939466	1.14E-09	0.264922066	0.138	0.049	7.52E-05	C3_CD8_Tem
chr10-124648564-124649005	1.15E-09	0.269627161	0.153	0.054	7.63E-05	C3_CD8_Tem
chr10-30783278-30785406	1.19E-09	0.269733764	0.245	0.109	7.88E-05	C3_CD8_Tem
chr3-66306992-66307313	1.20E-09	0.270014108	0.151	0.053	7.94E-05	C3_CD8_Tem
chr1-158978573-158979257	1.21E-09	0.252782967	0.128	0.043	8.01E-05	C3_CD8_Tem
chr3-105805902-105806733	1.21E-09	0.268234444	0.303	0.142	8.04E-05	C3_CD8_Tem
chr19-38538269-38539464	1.23E-09	0.267409094	0.314	0.148	8.12E-05	C3_CD8_Tem
chr6-42367126-42368498	1.27E-09	0.282587569	0.216	0.087	8.43E-05	C3_CD8_Tem
chr8-23230029-23231334	1.28E-09	0.253378599	0.391	0.201	8.51E-05	C3_CD8_Tem
chr16-56202633-56203391	1.38E-09	0.274359896	0.171	0.067	9.14E-05	C3_CD8_Tem
chr22-44190923-44191455	1.38E-09	0.2517491	0.105	0.03	9.17E-05	C3_CD8_Tem
chr11-96317764-96318513	1.41E-09	0.252540781	0.114	0.038	9.33E-05	C3_CD8_Tem
chr14-106013425-106014366	1.42E-09	0.258114698	0.254	0.117	9.37E-05	C3_CD8_Tem
chr10-62689722-62691033	1.42E-09	0.255157396	0.16	0.059	9.41E-05	C3_CD8_Tem
chr1-24966076-24967007	1.44E-09	0.2548147	0.31	0.151	9.53E-05	C3_CD8_Tem
chr5-39174853-39175558	1.49E-09	0.271983776	0.217	0.087	9.84E-05	C3_CD8_Tem
chr3-33047436-33048236	1.60E-09	0.265291741	0.304	0.139	0.000105851	C3_CD8_Tem

chrX-107711845-107713138	1.61E-09	0.266085701	0.309	0.146	0.000106883	C3_CD8_Tem
chr2-238287604-238287946	1.63E-09	0.266266449	0.154	0.056	0.000107848	C3_CD8_Tem
chr6-42389768-42390868	1.70E-09	0.255593085	0.301	0.144	0.000112284	C3_CD8_Tem
chr9-126523558-126524156	1.79E-09	0.274821449	0.201	0.079	0.000118495	C3_CD8_Tem
chr19-34225149-34226425	1.84E-09	0.260942107	0.131	0.044	0.000121931	C3_CD8_Tem
chrX-1480086-1480445	1.89E-09	0.272976684	0.135	0.044	0.000125397	C3_CD8_Tem
chr13-99273061-99273690	1.92E-09	0.269450005	0.182	0.071	0.000126962	C3_CD8_Tem
chr6-105644007-105644691	1.96E-09	0.254583716	0.12	0.041	0.000129773	C3_CD8_Tem
chr22-21762781-21763602	2.06E-09	0.25680902	0.2	0.083	0.000136641	C3_CD8_Tem
chr20-31738754-31740685	2.12E-09	0.255802851	0.345	0.173	0.000140274	C3_CD8_Tem
chr1-230208534-230209331	2.16E-09	0.253736715	0.13	0.047	0.00014306	C3_CD8_Tem
chr20-1660064-1660606	2.20E-09	0.251087358	0.252	0.115	0.000145368	C3_CD8_Tem
chr2-241965768-241966490	2.24E-09	0.251570415	0.139	0.052	0.000148606	C3_CD8_Tem
chr4-121256077-121257171	2.44E-09	0.252348774	0.3	0.145	0.000161769	C3_CD8_Tem
chr1-24191815-24192234	2.58E-09	0.25722293	0.14	0.051	0.000170936	C3_CD8_Tem
chr2-27637663-27639040	2.60E-09	0.272099706	0.235	0.096	0.000172266	C3_CD8_Tem
chr6-42386982-42387699	2.62E-09	0.273884917	0.171	0.065	0.000173759	C3_CD8_Tem
chr2-68726571-68727569	2.86E-09	0.258092428	0.33	0.159	0.000189662	C3_CD8_Tem
chr7-139043878-139044824	2.89E-09	0.252149696	0.33	0.158	0.000191572	C3_CD8_Tem
chr1-223716319-223717014	2.92E-09	0.254567604	0.265	0.12	0.000193346	C3_CD8_Tem
chr17-14205803-14206381	3.01E-09	0.265463113	0.264	0.122	0.00019904	C3_CD8_Tem
chr7-36780816-36781372	3.17E-09	0.256858594	0.177	0.073	0.00020962	C3_CD8_Tem
chr7-43625003-43626369	3.22E-09	0.265581113	0.235	0.108	0.00021309	C3_CD8_Tem
chr1-150613223-150614328	3.54E-09	0.266633364	0.376	0.191	0.000234114	C3_CD8_Tem
chr11-35160155-35160602	3.70E-09	0.254702248	0.14	0.052	0.000245099	C3_CD8_Tem
chr9-132451050-132451675	3.80E-09	0.263277118	0.16	0.058	0.000251641	C3_CD8_Tem
chr17-35898230-35898645	3.85E-09	0.259657131	0.172	0.067	0.00025478	C3_CD8_Tem

chr17-35530585-35531456	3.90E-09	0.263696191	0.158	0.06	0.00025831	C3_CD8_Tem
chr5-68343379-68343949	4.00E-09	0.25722865	0.15	0.055	0.000265038	C3_CD8_Tem
chr5-107570845-107572433	4.02E-09	0.257090768	0.183	0.073	0.000266173	C3_CD8_Tem
chr17-8939402-8939794	4.17E-09	0.269466267	0.194	0.08	0.00027619	C3_CD8_Tem
chr19-19410709-19411230	4.18E-09	0.255535372	0.144	0.054	0.000276662	C3_CD8_Tem
chr10-71742093-71742966	4.25E-09	0.255388676	0.178	0.076	0.000281258	C3_CD8_Tem
chr1-203606826-203607690	4.25E-09	0.257801571	0.366	0.181	0.000281595	C3_CD8_Tem
chr5-176660444-176661145	4.31E-09	0.272615306	0.175	0.069	0.000285661	C3_CD8_Tem
chr12-54933126-54933791	4.32E-09	0.262016134	0.183	0.071	0.000285897	C3_CD8_Tem
chr2-112749484-112750559	4.50E-09	0.260918349	0.163	0.066	0.000297904	C3_CD8_Tem
chr5-170266528-170268125	4.64E-09	0.250690705	0.292	0.15	0.000307543	C3_CD8_Tem
chr8-66615249-66615541	4.71E-09	0.259076956	0.136	0.049	0.000311844	C3_CD8_Tem
chr10-6507024-6507707	4.85E-09	0.266549808	0.146	0.053	0.000321342	C3_CD8_Tem
chr22-39077912-39078712	4.86E-09	0.255220116	0.152	0.058	0.000321809	C3_CD8_Tem
chr10-3197279-3198114	4.91E-09	0.266187455	0.274	0.126	0.000325388	C3_CD8_Tem
chr2-144519981-144520278	5.11E-09	0.253106812	0.135	0.046	0.000338417	C3_CD8_Tem
chr8-29525976-29526636	5.13E-09	0.263580879	0.121	0.037	0.000339891	C3_CD8_Tem
chr8-142844399-142844984	5.15E-09	0.266239737	0.188	0.079	0.000340777	C3_CD8_Tem
chr22-26658138-26659244	5.16E-09	0.252580596	0.385	0.194	0.000341382	C3_CD8_Tem
chr2-96131228-96131850	5.20E-09	0.259522982	0.127	0.042	0.000344419	C3_CD8_Tem
chr12-68163405-68164141	5.29E-09	0.255187317	0.186	0.075	0.000350487	C3_CD8_Tem
chr1-158969126-158970202	5.44E-09	0.264654079	0.172	0.065	0.000360056	C3_CD8_Tem
chr10-50417507-50418611	5.45E-09	0.259535141	0.244	0.109	0.000360688	C3_CD8_Tem
chr16-57628103-57628945	5.45E-09	0.253929378	0.15	0.054	0.000360772	C3_CD8_Tem
chr14-89507455-89508275	5.58E-09	0.258996837	0.189	0.077	0.000369667	C3_CD8_Tem
chrX-1480711-1481297	5.63E-09	0.258709738	0.122	0.041	0.000372581	C3_CD8_Tem
chr3-45880671-45881550	5.96E-09	0.254568447	0.242	0.111	0.000394838	C3_CD8_Tem

chr1-160705280-160706149	6.05E-09	0.260389188	0.173	0.07	0.000400963	C3_CD8_Tem
chr12-116559070-116560159	6.37E-09	0.251396411	0.265	0.126	0.000421749	C3_CD8_Tem
chr9-122991242-122991831	6.46E-09	0.262370283	0.199	0.084	0.000427877	C3_CD8_Tem
chr10-109914741-109915379	6.60E-09	0.26893274	0.271	0.123	0.00043676	C3_CD8_Tem
chr8-133500723-133500961	6.92E-09	0.25714095	0.153	0.058	0.000458387	C3_CD8_Tem
chr20-53642262-53643481	7.01E-09	0.2536744	0.321	0.154	0.00046444	C3_CD8_Tem
chr3-197393766-197395033	7.52E-09	0.256312722	0.198	0.083	0.000497894	C3_CD8_Tem
chr1-10524905-10525628	7.60E-09	0.251598953	0.163	0.063	0.000503322	C3_CD8_Tem
chr6-41705906-41707149	7.64E-09	0.25094555	0.19	0.079	0.000505673	C3_CD8_Tem
chr2-105738026-105739249	7.67E-09	0.259881098	0.319	0.161	0.000507861	C3_CD8_Tem
chr22-49825741-49826340	7.72E-09	0.268087421	0.22	0.093	0.00051142	C3_CD8_Tem
chr9-99811500-99812222	7.73E-09	0.254941097	0.11	0.034	0.000511788	C3_CD8_Tem
chr15-28985929-28986480	7.94E-09	0.254509182	0.144	0.053	0.000525704	C3_CD8_Tem
chr3-105823079-105823914	8.02E-09	0.260197415	0.233	0.1	0.000531301	C3_CD8_Tem
chr6-26568122-26569395	8.11E-09	0.266703451	0.343	0.169	0.000537272	C3_CD8_Tem
chr10-62855732-62856589	8.33E-09	0.255602871	0.157	0.063	0.000551586	C3_CD8_Tem
chr3-32432446-32433567	8.49E-09	0.254985748	0.256	0.118	0.000561924	C3_CD8_Tem
chr1-117001254-117001952	8.96E-09	0.260886633	0.136	0.048	0.00059356	C3_CD8_Tem
chr13-99274870-99275424	9.02E-09	0.261014881	0.183	0.07	0.000597543	C3_CD8_Tem
chr12-12736303-12737402	9.20E-09	0.266574449	0.292	0.138	0.000608986	C3_CD8_Tem
chr14-97456834-97457349	9.30E-09	0.259795283	0.209	0.087	0.000616202	C3_CD8_Tem
chr14-91377987-91378617	9.42E-09	0.267139594	0.24	0.102	0.000623985	C3_CD8_Tem
chr5-109857035-109858029	9.90E-09	0.258581923	0.172	0.068	0.000655651	C3_CD8_Tem
chr22-39094669-39094965	9.93E-09	0.257559664	0.217	0.092	0.000657603	C3_CD8_Tem
chr7-50363705-50364024	1.04E-08	0.271406751	0.19	0.076	0.000687668	C3_CD8_Tem
chr14-74620541-74620955	1.04E-08	0.266130363	0.2	0.081	0.000687956	C3_CD8_Tem
chr17-58331169-58331614	1.06E-08	0.253388762	0.309	0.145	0.000700091	C3_CD8_Tem

chr18-50356204-50357547	1.15E-08	0.265514992	0.146	0.055	0.000761366	C3_CD8_Tem
chr6-129686918-129687735	1.15E-08	0.271071276	0.263	0.115	0.000763045	C3_CD8_Tem
chr10-89337454-89337853	1.16E-08	0.256827699	0.131	0.046	0.00076795	C3_CD8_Tem
chr16-2663483-2664349	1.25E-08	0.258786661	0.227	0.098	0.000825144	C3_CD8_Tem
chr6-158645662-158645997	1.25E-08	0.258252771	0.182	0.073	0.000825265	C3_CD8_Tem
chr19-40143503-40144657	1.26E-08	0.256611192	0.182	0.074	0.000836321	C3_CD8_Tem
chr7-5629850-5631088	1.30E-08	0.250368742	0.252	0.119	0.000862402	C3_CD8_Tem
chr8-70217016-70218279	1.39E-08	0.253545612	0.23	0.107	0.000919732	C3_CD8_Tem
chr2-28423746-28425144	1.40E-08	0.261947712	0.179	0.072	0.000924647	C3_CD8_Tem
chr11-63866063-63866613	1.40E-08	0.265684062	0.177	0.072	0.000926612	C3_CD8_Tem
chr1-89721182-89721691	1.47E-08	0.260566621	0.202	0.085	0.000974984	C3_CD8_Tem
chr13-46202059-46202834	1.50E-08	0.253935372	0.133	0.048	0.000992788	C3_CD8_Tem
chr9-114364129-114364520	1.63E-08	0.258790124	0.155	0.059	0.001078573	C3_CD8_Tem
chr5-111230124-111230769	1.71E-08	0.250874142	0.191	0.08	0.001131493	C3_CD8_Tem
chr11-118229942-118230412	1.78E-08	0.254099765	0.165	0.067	0.001181211	C3_CD8_Tem
chr3-33061515-33062128	1.85E-08	0.25273953	0.219	0.099	0.001223258	C3_CD8_Tem
chr15-55253521-55253894	1.88E-08	0.26528584	0.124	0.04	0.001244601	C3_CD8_Tem
chr2-38611028-38611740	1.92E-08	0.25090098	0.12	0.041	0.00127103	C3_CD8_Tem
chr1-84156143-84156635	1.95E-08	0.256201845	0.139	0.051	0.00129395	C3_CD8_Tem
chr17-67359862-67360587	2.06E-08	0.26198757	0.27	0.127	0.001363859	C3_CD8_Tem
chr5-142866687-142867319	2.08E-08	0.253613565	0.141	0.051	0.001376956	C3_CD8_Tem
chr13-46320538-46321976	2.10E-08	0.264000479	0.185	0.073	0.001390168	C3_CD8_Tem
chr7-29196076-29197235	2.15E-08	0.254171205	0.172	0.066	0.001420842	C3_CD8_Tem
chr1-167445973-167446410	2.20E-08	0.252095342	0.14	0.051	0.00145701	C3_CD8_Tem
chrX-71542903-71543722	2.35E-08	0.264890811	0.239	0.101	0.001559147	C3_CD8_Tem
chr17-36107297-36107972	2.45E-08	0.254517085	0.166	0.063	0.001623801	C3_CD8_Tem
chr2-201127374-201127900	2.46E-08	0.255815312	0.283	0.132	0.001626529	C3_CD8_Tem

chr11-117072477-117073131	2.61E-08	0.264084653	0.222	0.096	0.001730418	C3_CD8_Tem
chr4-82905147-82905608	2.67E-08	0.251523605	0.18	0.074	0.001771206	C3_CD8_Tem
chr9-137445051-137445626	2.68E-08	0.266530529	0.189	0.081	0.001772984	C3_CD8_Tem
chr22-35371393-35372311	2.71E-08	0.257678841	0.222	0.099	0.001793216	C3_CD8_Tem
chr10-6498392-6499278	2.76E-08	0.251991746	0.321	0.158	0.001826027	C3_CD8_Tem
chr20-53839263-53839854	2.80E-08	0.257141146	0.195	0.082	0.001851843	C3_CD8_Tem
chr6-127907950-127908588	2.85E-08	0.260555215	0.117	0.038	0.001885996	C3_CD8_Tem
chr3-5016068-5016933	2.94E-08	0.254506609	0.26	0.118	0.001947632	C3_CD8_Tem
chr20-32386956-32387712	3.04E-08	0.255459415	0.205	0.088	0.002011527	C3_CD8_Tem
chr3-71393008-71393466	3.06E-08	0.252168994	0.235	0.107	0.002026042	C3_CD8_Tem
chr6-2899573-2899964	3.09E-08	0.261370782	0.145	0.051	0.002048639	C3_CD8_Tem
chr9-114382742-114383228	3.20E-08	0.255858852	0.223	0.1	0.002120821	C3_CD8_Tem
chr20-51424047-51425672	3.21E-08	0.266286602	0.259	0.116	0.002122607	C3_CD8_Tem
chr1-161621077-161622355	3.29E-08	0.257390002	0.245	0.113	0.002181213	C3_CD8_Tem
chr2-234278419-234279269	3.42E-08	0.25660617	0.186	0.076	0.002267966	C3_CD8_Tem
chr1-149843082-149844483	3.86E-08	0.263390925	0.199	0.087	0.002556895	C3_CD8_Tem
chr3-69085965-69086482	3.87E-08	0.259988592	0.169	0.068	0.002563228	C3_CD8_Tem
chr15-40046796-40048177	4.43E-08	0.254420955	0.205	0.086	0.002934162	C3_CD8_Tem
chr2-96156173-96156850	4.47E-08	0.258668853	0.207	0.086	0.002957953	C3_CD8_Tem
chr2-43425849-43426855	4.94E-08	0.250660791	0.199	0.091	0.003272973	C3_CD8_Tem
chr7-102431570-102432147	5.01E-08	0.262902884	0.216	0.094	0.003316955	C3_CD8_Tem
chr15-90418964-90420167	5.09E-08	0.252596087	0.2	0.09	0.003369656	C3_CD8_Tem
chr14-89429419-89429943	5.34E-08	0.256499682	0.11	0.033	0.003533226	C3_CD8_Tem
chr1-42938289-42938669	5.71E-08	0.258613485	0.156	0.058	0.003780385	C3_CD8_Tem
chr18-3602484-3603755	6.07E-08	0.269963541	0.275	0.132	0.004016453	C3_CD8_Tem
chr22-39316316-39316694	6.73E-08	0.268277111	0.262	0.118	0.00445754	C3_CD8_Tem
chr19-8575400-8576840	6.78E-08	0.26514789	0.301	0.142	0.004487289	C3_CD8_Tem

chr1-160519108-160520387	6.88E-08	0.257570553	0.222	0.102	0.004556052	C3_CD8_Tem
chr2-317260-318382	7.12E-08	0.252927835	0.204	0.086	0.004716248	C3_CD8_Tem
chr16-57083692-57084747	7.66E-08	0.256138359	0.178	0.075	0.005069534	C3_CD8_Tem
chr17-39164591-39165320	7.68E-08	0.26597874	0.197	0.083	0.005087056	C3_CD8_Tem
chr16-84553413-84554797	7.77E-08	0.250912536	0.165	0.065	0.005148529	C3_CD8_Tem
chr3-32428175-32428991	9.03E-08	0.253644269	0.144	0.055	0.005976629	C3_CD8_Tem
chr2-68716561-68717629	1.16E-07	0.250977731	0.167	0.071	0.007657405	C3_CD8_Tem
chr14-92551174-92551791	1.23E-07	0.25322306	0.14	0.05	0.00815519	C3_CD8_Tem
chr2-25794547-25795236	1.49E-07	0.259560863	0.171	0.067	0.009880971	C3_CD8_Tem
chr6-137233676-137234822	1.52E-07	0.250992615	0.156	0.062	0.010090561	C3_CD8_Tem
chr3-46370779-46371182	1.60E-07	0.252560463	0.209	0.091	0.010625523	C3_CD8_Tem
chr19-58580899-58581493	1.82E-07	0.250276693	0.197	0.084	0.012083986	C3_CD8_Tem
chr1-172894404-172895480	1.95E-07	0.251254318	0.161	0.063	0.012893667	C3_CD8_Tem
chr19-3176839-3177481	2.08E-07	0.25555478	0.268	0.124	0.013769362	C3_CD8_Tem
chr20-51386002-51386927	2.29E-07	0.251100735	0.183	0.076	0.01513847	C3_CD8_Tem
chr6-30527513-30528524	2.90E-07	0.257955288	0.234	0.108	0.019221491	C3_CD8_Tem
chr20-51416552-51418147	3.32E-07	0.250241716	0.268	0.126	0.022003254	C3_CD8_Tem
chr6-16668952-16669732	5.66E-07	0.252505415	0.219	0.097	0.03750461	C3_CD8_Tem
chr17-77341950-77342944	6.24E-07	0.25305944	0.183	0.077	0.041349017	C3_CD8_Tem
chr2-241803139-241805153	7.46E-07	0.254448746	0.205	0.088	0.049383254	C3_CD8_Tem
chr16-85610108-85610823	1.92E-06	0.255555068	0.268	0.126	0.127266244	C3_CD8_Tem
chr17-47719285-47720048	7.76E-06	0.251619956	0.229	0.1	0.514111321	C3_CD8_Tem
chr12-10673283-10674412	5.21E-11	0.282136964	0.266	0.114	3.45E-06	C3_CD8_Tem
chr17-36198897-36199460	2.57E-25	0.373812919	0.355	0.125	1.70E-20	C3_CD8_Tem
chr17-36196490-36197498	3.94E-14	0.287380923	0.296	0.127	2.61E-09	C3_CD8_Tem
chr3-46359594-46360492	3.94E-21	0.331783905	0.395	0.168	2.61E-16	C3_CD8_Tem
chr6-155223690-155224508	1.32E-13	0.271009521	0.32	0.145	8.74E-09	C3_CD8_Tem

chr2-203992358-203993938	6.28E-20	0.337453133	0.298	0.121	4.16E-15	C3_CD8_Tem
chr2-181470737-181472187	2.86E-13	0.299106647	0.342	0.153	1.89E-08	C3_CD8_Tem
chr20-51413798-51414278	1.01E-11	0.284685391	0.212	0.084	6.71E-07	C3_CD8_Tem
chr11-3784763-3785789	2.33E-10	0.262116505	0.348	0.169	1.54E-05	C3_CD8_Tem
chr17-14205803-14206381	3.01E-09	0.265463113	0.264	0.122	0.00019904	C3_CD8_Tem
chr22-44454921-44456171	1.38E-28	0.34501323	0.556	0.259	9.14E-24	C3_CD8_Tem
chr8-133054632-133055107	3.42E-12	0.304147575	0.286	0.119	2.27E-07	C3_CD8_Tem
chr16-56261625-56262745	2.43E-19	0.311111774	0.437	0.199	1.61E-14	C3_CD8_Tem
chr19-16449052-16450086	1.01E-15	0.30419079	0.31	0.138	6.67E-11	C3_CD8_Tem
chr5-87120180-87121626	9.44E-19	0.306879157	0.444	0.205	6.25E-14	C3_CD8_Tem
chr12-1993185-1994540	1.92E-14	0.304318852	0.305	0.129	1.27E-09	C3_CD8_Tem
chr14-106506652-106507782	1.00E-26	0.308199789	0.633	0.31	6.64E-22	C3_CD8_Tem
chr10-124688200-124689359	7.20E-17	0.296978217	0.443	0.213	4.77E-12	C3_CD8_Tem
chr1-66246411-66248118	1.52E-30	0.398339401	0.385	0.152	1.01E-25	C3_CD8_Tem
chr6-224832-225792	4.24E-26	0.351403389	0.437	0.183	2.81E-21	C3_CD8_Tem
chr13-40221169-40222360	8.89E-13	0.257645093	0.414	0.208	5.88E-08	C3_CD8_Tem
chr3-115164316-115166011	3.10E-32	0.391551597	0.439	0.174	2.05E-27	C3_CD8_Tem
chr16-78794405-78795284	7.91E-10	0.253085057	0.15	0.058	5.24E-05	C3_CD8_Tem
chr3-46284941-46285553	4.82E-10	0.279767914	0.266	0.116	3.19E-05	C3_CD8_Tem
chr7-17260841-17261797	2.28E-10	0.251024811	0.289	0.132	1.51E-05	C3_CD8_Tem
chr8-100415528-100417352	9.27E-46	0.421102122	0.594	0.234	6.14E-41	C3_CD8_Tem
chr5-143228855-143230545	7.47E-19	0.307694205	0.45	0.208	4.94E-14	C3_CD8_Tem
chr17-67359862-67360587	2.06E-08	0.26198757	0.27	0.127	0.001363859	C3_CD8_Tem
chr1-51520040-51521153	1.40E-25	0.355988108	0.428	0.178	9.29E-21	C3_CD8_Tem
chr20-23366409-23367211	1.28E-12	0.301771771	0.224	0.088	8.49E-08	C3_CD8_Tem
chr4-6855496-6856344	1.22E-24	0.332225289	0.484	0.221	8.11E-20	C3_CD8_Tem
chr5-170269473-170269949	2.58E-19	0.309942183	0.454	0.202	1.71E-14	C3_CD8_Tem

chr10-6498392-6499278	2.76E-08	0.251991746	0.321	0.158	0.001826027	C3_CD8_Tem
chr12-68118976-68120277	7.97E-28	0.374996837	0.413	0.161	5.28E-23	C3_CD8_Tem
chr7-92809356-92810607	9.10E-11	0.274629497	0.359	0.169	6.03E-06	C3_CD8_Tem
chr3-27451226-27452196	1.22E-14	0.302495364	0.121	0.03	8.08E-10	C3_CD8_Tem
chr1-32801060-32801651	6.77E-20	0.330863487	0.384	0.167	4.48E-15	C3_CD8_Tem
chr7-24907220-24907930	4.96E-13	0.282268581	0.359	0.164	3.29E-08	C3_CD8_Tem
chr18-58541077-58542576	9.12E-13	0.293897681	0.323	0.144	6.04E-08	C3_CD8_Tem
chr19-38538269-38539464	1.23E-09	0.267409094	0.314	0.148	8.12E-05	C3_CD8_Tem
chr8-29990174-29991436	1.94E-48	0.455861169	0.474	0.167	1.28E-43	C3_CD8_Tem
chr13-99314427-99315476	7.67E-14	0.285206845	0.382	0.18	5.08E-09	C3_CD8_Tem
chr1-235104198-235105982	1.74E-16	0.305160727	0.395	0.183	1.15E-11	C3_CD8_Tem
chr15-40098171-40099143	1.05E-10	0.256674674	0.321	0.153	6.96E-06	C3_CD8_Tem
chr2-38611028-38611740	1.92E-08	0.25090098	0.12	0.041	0.00127103	C3_CD8_Tem
chrX-71542903-71543722	2.35E-08	0.264890811	0.239	0.101	0.001559147	C3_CD8_Tem
chr18-45669987-45670872	1.31E-10	0.267652496	0.254	0.115	8.66E-06	C3_CD8_Tem
chr19-16586712-16587917	1.29E-10	0.257293176	0.43	0.224	8.56E-06	C3_CD8_Tem
chr14-68719307-68720269	1.34E-10	0.279276537	0.273	0.118	8.90E-06	C3_CD8_Tem
chr17-31493821-31494998	8.45E-13	0.284530025	0.377	0.183	5.60E-08	C3_CD8_Tem
chr13-44258746-44259999	6.61E-13	0.273024903	0.325	0.149	4.38E-08	C3_CD8_Tem
chr9-114364129-114364520	1.63E-08	0.258790124	0.155	0.059	0.001078573	C3_CD8_Tem
chr13-41492087-41493279	1.73E-23	0.368393389	0.354	0.137	1.14E-18	C3_CD8_Tem
chr10-33136326-33137128	6.45E-10	0.259368005	0.253	0.116	4.27E-05	C3_CD8_Tem
chr3-73028827-73029647	2.80E-13	0.284680323	0.212	0.084	1.85E-08	C3_CD8_Tem
chr19-35712568-35713417	6.46E-20	0.325308085	0.414	0.186	4.28E-15	C3_CD8_Tem
chr22-41858957-41859826	6.72E-15	0.310547899	0.34	0.147	4.45E-10	C3_CD8_Tem
chr2-144194300-144194976	7.91E-10	0.253289028	0.102	0.03	5.24E-05	C3_CD8_Tem
chr3-66303282-66304068	2.82E-14	0.276626694	0.427	0.209	1.87E-09	C3_CD8_Tem

chr1-234699694-234700645	5.45E-21	0.343335605	0.254	0.088	3.61E-16	C3_CD8_Tem
chr3-46371636-46372715	4.20E-14	0.317373561	0.227	0.083	2.78E-09	C3_CD8_Tem
chr8-30137089-30137602	1.22E-16	0.30811051	0.317	0.134	8.07E-12	C3_CD8_Tem
chr15-85618627-85619422	6.56E-16	0.323251348	0.332	0.135	4.34E-11	C3_CD8_Tem
chr12-68159276-68160057	8.08E-13	0.281675404	0.34	0.16	5.35E-08	C3_CD8_Tem
chr1-116537182-116537858	7.54E-12	0.287867546	0.176	0.064	4.99E-07	C3_CD8_Tem
chr10-119332916-119333577	1.88E-14	0.307173354	0.231	0.087	1.24E-09	C3_CD8_Tem
chr15-50259969-50260854	1.44E-10	0.279423774	0.19	0.072	9.52E-06	C3_CD8_Tem
chr12-68163405-68164141	5.29E-09	0.255187317	0.186	0.075	0.000350487	C3_CD8_Tem
chr20-32386956-32387712	3.04E-08	0.255459415	0.205	0.088	0.002011527	C3_CD8_Tem
chr12-68189707-68190475	1.19E-15	0.324503566	0.249	0.093	7.91E-11	C3_CD8_Tem
chr13-46202059-46202834	1.50E-08	0.253935372	0.133	0.048	0.000992788	C3_CD8_Tem
chr8-66615249-66615541	4.71E-09	0.259076956	0.136	0.049	0.000311844	C3_CD8_Tem
chr2-317260-318382	7.12E-08	0.252927835	0.204	0.086	0.004716248	C3_CD8_Tem
chr9-114366219-114366991	1.41E-19	0.325904713	0.438	0.196	9.36E-15	C3_CD8_Tem
chr7-139043878-139044824	2.89E-09	0.252149696	0.33	0.158	0.000191572	C3_CD8_Tem
chr1-66274420-66275430	2.83E-10	0.263927943	0.272	0.126	1.87E-05	C3_CD8_Tem
chr1-156214299-156214702	3.72E-19	0.371661004	0.224	0.07	2.47E-14	C3_CD8_Tem
chr5-32537250-32538363	6.41E-22	0.360493124	0.292	0.107	4.25E-17	C3_CD8_Tem
chr14-54435240-54436060	5.42E-11	0.272121646	0.264	0.119	3.59E-06	C3_CD8_Tem
chr8-96231211-96231855	4.82E-17	0.332246233	0.212	0.07	3.19E-12	C3_CD8_Tem
chr11-67292914-67293862	8.64E-14	0.306103556	0.348	0.157	5.72E-09	C3_CD8_Tem
chr1-160812949-160813628	1.13E-17	0.337257218	0.243	0.088	7.46E-13	C3_CD8_Tem
chr3-71393008-71393466	3.06E-08	0.252168994	0.235	0.107	0.002026042	C3_CD8_Tem
chr17-67431290-67431893	4.56E-11	0.294672437	0.251	0.103	3.02E-06	C3_CD8_Tem
chr1-52139610-52140503	3.24E-12	0.29161342	0.304	0.132	2.15E-07	C3_CD8_Tem
chr10-6507024-6507707	4.85E-09	0.266549808	0.146	0.053	0.000321342	C3_CD8_Tem

chr17-3791506-3791870	1.85E-10	0.295127769	0.197	0.074	1.23E-05	C3_CD8_Tem
chr14-106013425-106014366	1.42E-09	0.258114698	0.254	0.117	9.37E-05	C3_CD8_Tem
chr16-56278591-56279579	1.18E-14	0.311332413	0.261	0.101	7.82E-10	C3_CD8_Tem
chr1-40058897-40060781	3.60E-18	0.346913948	0.344	0.136	2.39E-13	C3_CD8_Tem
chr11-64864425-64866374	1.76E-10	0.277409589	0.411	0.208	1.17E-05	C3_CD8_Tem
chr2-30267026-30267904	2.50E-14	0.31359595	0.208	0.075	1.66E-09	C3_CD8_Tem
chr21-42198955-42200215	3.44E-12	0.292277705	0.245	0.101	2.28E-07	C3_CD8_Tem
chr2-201127374-201127900	2.46E-08	0.255815312	0.283	0.132	0.001626529	C3_CD8_Tem
chr11-36384958-36385584	1.48E-21	0.370075875	0.253	0.08	9.82E-17	C3_CD8_Tem
chr10-22626753-22627429	2.08E-12	0.311914547	0.188	0.069	1.38E-07	C3_CD8_Tem
chr1-192546054-192547091	1.15E-10	0.283706128	0.223	0.091	7.59E-06	C3_CD8_Tem
chr22-50027654-50028185	9.56E-10	0.263451533	0.163	0.062	6.33E-05	C3_CD8_Tem
chr3-33061515-33062128	1.85E-08	0.25273953	0.219	0.099	0.001223258	C3_CD8_Tem
chr22-35371393-35372311	2.71E-08	0.257678841	0.222	0.099	0.001793216	C3_CD8_Tem
chr2-43425849-43426855	4.94E-08	0.250660791	0.199	0.091	0.003272973	C3_CD8_Tem
chr3-197793259-197793604	4.12E-13	0.31092266	0.226	0.084	2.73E-08	C3_CD8_Tem
chr17-35081980-35082629	7.34E-11	0.29601339	0.205	0.08	4.86E-06	C3_CD8_Tem
chr7-6220925-6222411	1.28E-20	0.316811857	0.377	0.167	8.49E-16	C3_CD8_Tem
chr3-149009270-149010325	2.34E-20	0.317586123	0.408	0.175	1.55E-15	C3_CD8_Tem
chr3-149003738-149004386	1.80E-11	0.278210872	0.206	0.078	1.19E-06	C3_CD8_Tem
chr3-129576399-129577446	3.64E-12	0.263151494	0.311	0.146	2.41E-07	C3_CD8_Tem
chr18-23872449-23873091	7.25E-11	0.269622205	0.174	0.064	4.80E-06	C3_CD8_Tem
chr12-52566301-52567390	1.27E-23	0.366220251	0.231	0.074	8.40E-19	C3_CD8_Tem
chr19-14475801-14477045	7.83E-15	0.28550312	0.297	0.125	5.19E-10	C3_CD8_Tem
chr13-24617927-24619031	5.95E-10	0.252205019	0.294	0.135	3.94E-05	C3_CD8_Tem
chr1-10524905-10525628	7.60E-09	0.251598953	0.163	0.063	0.000503322	C3_CD8_Tem
chr18-44729308-44730171	1.02E-13	0.28652906	0.212	0.08	6.73E-09	C3_CD8_Tem

chr10-96670477-96671823	1.71E-15	0.30565686	0.204	0.075	1.13E-10	C3_CD8_Tem
chr5-107474537-107475439	1.28E-19	0.345533659	0.274	0.097	8.46E-15	C3_CD8_Tem
chr4-970184-971071	7.12E-39	0.466223479	0.309	0.078	4.71E-34	C3_CD8_Tem
chr12-121557117-121558231	3.45E-46	0.435986111	0.486	0.173	2.28E-41	C3_CD8_Tem
chr17-1119934-1121063	2.63E-11	0.251058011	0.101	0.032	1.74E-06	C3_CD8_Tem
chr1-202197442-202198255	3.72E-42	0.454534451	0.35	0.106	2.47E-37	C3_CD8_Tem
chr3-126607119-126607778	1.73E-16	0.31078065	0.164	0.05	1.15E-11	C3_CD8_Tem
chr14-91354247-91354655	2.14E-13	0.28132722	0.123	0.037	1.42E-08	C3_CD8_Tem
chr6-157143009-157143795	5.26E-14	0.305627524	0.212	0.08	3.48E-09	C3_CD8_Tem
chr16-87761941-87762604	2.04E-15	0.291300809	0.256	0.106	1.35E-10	C3_CD8_Tem
chrX-1507227-1508353	3.15E-15	0.305828242	0.22	0.083	2.09E-10	C3_CD8_Tem
chr12-57222735-57223690	7.67E-20	0.333545829	0.322	0.125	5.08E-15	C3_CD8_Tem
chr5-139925797-139926640	7.98E-19	0.327748489	0.138	0.034	5.28E-14	C3_CD8_Tem
chr12-52558738-52559814	2.58E-25	0.379609732	0.224	0.065	1.71E-20	C3_CD8_Tem
chr14-91377071-91377696	3.33E-12	0.275301417	0.152	0.054	2.21E-07	C3_CD8_Tem
chr8-141156903-141157559	1.59E-29	0.411291695	0.25	0.07	1.05E-24	C3_CD8_Tem
chr14-24633431-24634610	4.78E-11	0.265266542	0.267	0.119	3.17E-06	C3_CD8_Tem
chr21-46423897-46425970	5.17E-29	0.393416485	0.413	0.153	3.42E-24	C3_CD8_Tem
chr12-70049371-70050399	1.24E-14	0.280967823	0.208	0.08	8.18E-10	C3_CD8_Tem
chr19-44760126-44761340	1.86E-35	0.431430522	0.374	0.127	1.23E-30	C3_CD8_Tem
chr10-70610194-70611322	3.78E-16	0.313360208	0.228	0.082	2.50E-11	C3_CD8_Tem
chr20-36627671-36628516	2.08E-18	0.336206318	0.288	0.104	1.38E-13	C3_CD8_Tem
chr17-10114141-10115557	2.18E-53	0.420008431	0.622	0.245	1.44E-48	C3_CD8_Tem
chr4-1176639-1177899	7.19E-15	0.314598375	0.219	0.077	4.76E-10	C3_CD8_Tem
chr12-128795987-128796930	1.43E-11	0.278969027	0.207	0.079	9.45E-07	C3_CD8_Tem
chr8-124649586-124650602	2.61E-13	0.302950958	0.185	0.063	1.73E-08	C3_CD8_Tem
chr1-25064358-25066301	5.55E-38	0.433033475	0.41	0.137	3.67E-33	C3_CD8_Tem

chr2-144659399-144660199	2.74E-16	0.293491407	0.319	0.136	1.81E-11	C3_CD8_Tem
chr14-101712387-101713961	4.40E-25	0.331055306	0.465	0.196	2.91E-20	C3_CD8_Tem
chr19-10523026-10523671	3.62E-22	0.362601431	0.249	0.081	2.40E-17	C3_CD8_Tem
chr17-36066120-36067103	5.96E-18	0.318237369	0.273	0.104	3.95E-13	C3_CD8_Tem
chr10-79025064-79025989	2.92E-15	0.297463897	0.23	0.085	1.93E-10	C3_CD8_Tem
chr1-225460124-225461145	6.02E-42	0.446481525	0.431	0.143	3.99E-37	C3_CD8_Tem
chr2-64880665-64881690	2.62E-22	0.352434832	0.154	0.038	1.74E-17	C3_CD8_Tem
chr1-100712436-100713975	1.05E-25	0.379773806	0.275	0.084	6.97E-21	C3_CD8_Tem
chr10-124650100-124651550	1.83E-65	0.488205685	0.616	0.224	1.21E-60	C3_CD8_Tem
chr22-21762781-21763602	2.06E-09	0.25680902	0.2	0.083	0.000136641	C3_CD8_Tem
chr1-75784374-75785133	1.33E-10	0.257262753	0.15	0.054	8.81E-06	C3_CD8_Tem
chr19-4108531-4109469	1.16E-18	0.314097828	0.354	0.151	7.65E-14	C3_CD8_Tem
chr13-30147654-30148998	2.90E-30	0.363626774	0.45	0.19	1.92E-25	C3_CD8_Tem
chr11-115222115-115223260	2.05E-13	0.291488082	0.202	0.076	1.36E-08	C3_CD8_Tem
chr6-42356959-42358089	3.80E-15	0.320361236	0.221	0.08	2.52E-10	C3_CD8_Tem
chr7-35729052-35730816	2.32E-22	0.333740299	0.439	0.185	1.54E-17	C3_CD8_Tem
chr5-107476616-107477202	1.11E-22	0.358656448	0.285	0.097	7.33E-18	C3_CD8_Tem
chr11-134228632-134229052	1.07E-09	0.250072987	0.13	0.046	7.11E-05	C3_CD8_Tem
chr7-29196076-29197235	2.15E-08	0.254171205	0.172	0.066	0.001420842	C3_CD8_Tem
chr4-3201683-3203141	2.47E-12	0.274911009	0.312	0.147	1.64E-07	C3_CD8_Tem
chr1-39525182-39526389	1.00E-43	0.45341987	0.448	0.144	6.65E-39	C3_CD8_Tem
chr7-2417689-2418570	3.85E-17	0.32414492	0.303	0.122	2.55E-12	C3_CD8_Tem
chr4-4268731-4270272	4.22E-12	0.279242054	0.351	0.161	2.80E-07	C3_CD8_Tem
chr16-4465340-4466660	5.66E-14	0.295642982	0.312	0.133	3.75E-09	C3_CD8_Tem
chr14-92530938-92532058	6.87E-42	0.414754601	0.521	0.199	4.55E-37	C3_CD8_Tem
chr17-1890533-1891414	4.13E-13	0.295439552	0.263	0.106	2.73E-08	C3_CD8_Tem
chr2-223759699-223762024	2.68E-16	0.27363561	0.406	0.197	1.77E-11	C3_CD8_Tem

chr6-13427444-13428661	1.81E-12	0.27470993	0.286	0.12	1.20E-07	C3_CD8_Tem
chr18-12920744-12921812	4.19E-23	0.349518852	0.278	0.101	2.77E-18	C3_CD8_Tem
chr2-68378735-68379810	6.82E-26	0.399492165	0.254	0.076	4.51E-21	C3_CD8_Tem
chr12-52645010-52646024	3.58E-11	0.262280533	0.186	0.076	2.37E-06	C3_CD8_Tem
chr16-10876338-10877689	1.18E-20	0.332769759	0.418	0.184	7.83E-16	C3_CD8_Tem
chr4-121706102-121707612	1.22E-13	0.288363081	0.242	0.094	8.05E-09	C3_CD8_Tem
chr13-30157033-30157717	5.77E-11	0.261222373	0.105	0.028	3.82E-06	C3_CD8_Tem
chr3-69281817-69282554	1.28E-10	0.252809474	0.207	0.086	8.50E-06	C3_CD8_Tem
chr15-65512623-65513362	1.29E-38	0.444721203	0.349	0.108	8.55E-34	C3_CD8_Tem
chr5-155766982-155767882	8.14E-20	0.337324818	0.177	0.052	5.39E-15	C3_CD8_Tem
chr2-144505188-144505875	2.26E-32	0.434748079	0.284	0.079	1.50E-27	C3_CD8_Tem
chr9-126982085-126982722	9.00E-12	0.278124711	0.213	0.086	5.96E-07	C3_CD8_Tem
chr5-1518362-1519073	5.77E-23	0.369287916	0.273	0.087	3.82E-18	C3_CD8_Tem
chr1-3809711-3810580	3.10E-15	0.291488146	0.114	0.029	2.05E-10	C3_CD8_Tem
chr20-57604829-57606180	1.60E-53	0.511767611	0.421	0.116	1.06E-48	C3_CD8_Tem
chr2-85852464-85854151	1.44E-12	0.271628156	0.446	0.227	9.56E-08	C3_CD8_Tem
chrX-47634583-47635540	1.37E-11	0.271971	0.141	0.047	9.05E-07	C3_CD8_Tem
chr1-16122578-16124085	3.20E-15	0.308181314	0.167	0.055	2.12E-10	C3_CD8_Tem
chr3-39267373-39268496	1.01E-20	0.325331127	0.404	0.17	6.68E-16	C3_CD8_Tem
chr17-3794854-3797125	3.96E-50	0.31978906	0.826	0.443	2.62E-45	C3_CD8_Tem
chr2-24739351-24739990	9.93E-23	0.35315211	0.131	0.03	6.58E-18	C3_CD8_Tem
chr1-53581990-53582744	3.51E-16	0.318184182	0.165	0.049	2.33E-11	C3_CD8_Tem
chr16-21540504-21541478	1.89E-18	0.299753338	0.361	0.158	1.25E-13	C3_CD8_Tem
chr8-101155516-101156372	3.78E-13	0.288289189	0.157	0.055	2.50E-08	C3_CD8_Tem
chr5-54609889-54611092	9.56E-17	0.316984429	0.263	0.099	6.33E-12	C3_CD8_Tem
chr10-124640818-124641704	2.30E-16	0.327858527	0.252	0.092	1.53E-11	C3_CD8_Tem
chr19-4104223-4105353	8.93E-19	0.281197098	0.55	0.279	5.92E-14	C3_CD8_Tem

chr21-34800971-34801962	1.30E-19	0.342458911	0.184	0.054	8.61E-15	C3_CD8_Tem
chr6-10726322-10726976	9.46E-19	0.317089163	0.111	0.026	6.26E-14	C3_CD8_Tem
chr18-80174997-80175974	1.09E-18	0.279960023	0.459	0.217	7.23E-14	C3_CD8_Tem
chr6-152634627-152635124	1.58E-12	0.285626062	0.196	0.075	1.05E-07	C3_CD8_Tem
chr1-225454008-225455207	5.00E-27	0.382269244	0.342	0.125	3.31E-22	C3_CD8_Tem
chr12-681735-682825	1.51E-33	0.450206663	0.229	0.052	9.97E-29	C3_CD8_Tem
chr17-1823106-1823993	2.41E-18	0.312986376	0.358	0.156	1.60E-13	C3_CD8_Tem
chr17-38900699-38901837	2.51E-38	0.441933498	0.439	0.149	1.66E-33	C3_CD8_Tem
chr10-71698226-71699001	1.98E-10	0.255477324	0.123	0.044	1.31E-05	C3_CD8_Tem
chr12-31722842-31723776	1.08E-14	0.28684634	0.199	0.076	7.17E-10	C3_CD8_Tem
chr17-5237381-5238054	8.17E-10	0.255523234	0.107	0.033	5.41E-05	C3_CD8_Tem
chr9-131627343-131628902	6.83E-36	0.364028761	0.591	0.258	4.52E-31	C3_CD8_Tem
chr17-36081820-36083037	3.27E-25	0.340027206	0.447	0.192	2.17E-20	C3_CD8_Tem
chr17-1200824-1201683	3.09E-14	0.302154078	0.204	0.077	2.04E-09	C3_CD8_Tem
chr8-29489883-29490946	1.12E-18	0.33098713	0.328	0.132	7.42E-14	C3_CD8_Tem
chr19-51372441-51373731	4.52E-25	0.330227057	0.52	0.232	2.99E-20	C3_CD8_Tem
chr3-56732303-56733288	3.42E-10	0.2742741	0.209	0.084	2.26E-05	C3_CD8_Tem
chr5-132662966-132664463	5.50E-13	0.301207492	0.219	0.085	3.64E-08	C3_CD8_Tem
chr4-40264977-40266011	1.48E-10	0.278518835	0.164	0.058	9.82E-06	C3_CD8_Tem
chr11-118699279-118700427	4.70E-13	0.285269613	0.259	0.109	3.11E-08	C3_CD8_Tem
chr2-68385217-68385954	1.08E-13	0.298419457	0.187	0.067	7.13E-09	C3_CD8_Tem
chr7-150665882-150666234	5.21E-22	0.367167393	0.23	0.069	3.45E-17	C3_CD8_Tem
chr16-57070100-57072258	8.33E-16	0.313104055	0.31	0.128	5.52E-11	C3_CD8_Tem
chr3-52310774-52311322	1.19E-12	0.271145319	0.113	0.035	7.86E-08	C3_CD8_Tem
chr5-74631355-74632512	5.38E-30	0.399668189	0.281	0.083	3.56E-25	C3_CD8_Tem
chr2-144510298-144511025	8.75E-15	0.291066952	0.327	0.142	5.79E-10	C3_CD8_Tem
chr20-51553758-51554917	6.14E-72	0.616741585	0.356	0.066	4.07E-67	C3_CD8_Tem

chr9-114369205-114370071	1.39E-35	0.37048113	0.545	0.236	9.19E-31	C3_CD8_Tem
chr1-25099271-25100697	4.51E-21	0.367066614	0.286	0.101	2.99E-16	C3_CD8_Tem
chr1-107936381-107937456	2.20E-52	0.485755912	0.48	0.157	1.45E-47	C3_CD8_Tem
chr16-391649-392432	4.36E-20	0.318593738	0.393	0.171	2.89E-15	C3_CD8_Tem
chr16-71855751-71856725	1.17E-24	0.38452312	0.197	0.054	7.72E-20	C3_CD8_Tem
chr22-19419104-19420340	8.89E-10	0.254519912	0.237	0.106	5.89E-05	C3_CD8_Tem
chr10-7029813-7030789	6.38E-10	0.251538949	0.156	0.06	4.22E-05	C3_CD8_Tem
chr2-191206130-191207147	1.26E-13	0.283445411	0.339	0.149	8.32E-09	C3_CD8_Tem
chr7-43625003-43626369	3.22E-09	0.265581113	0.235	0.108	0.00021309	C3_CD8_Tem
chr5-150045602-150046413	1.10E-15	0.317815643	0.175	0.058	7.30E-11	C3_CD8_Tem
chr19-13043761-13044879	1.20E-65	0.532305291	0.473	0.134	7.98E-61	C3_CD8_Tem
chr2-110674997-110676078	6.93E-11	0.263584345	0.298	0.135	4.59E-06	C3_CD8_Tem
chr2-233099908-233101037	3.52E-11	0.286802165	0.217	0.087	2.33E-06	C3_CD8_Tem
chr9-131406707-131408148	1.02E-11	0.296792954	0.286	0.122	6.77E-07	C3_CD8_Tem
chr17-5220631-5221894	4.33E-37	0.468276948	0.266	0.069	2.87E-32	C3_CD8_Tem
chr19-6521764-6522766	2.22E-25	0.362325047	0.391	0.155	1.47E-20	C3_CD8_Tem
chr5-1488691-1489680	2.10E-30	0.427019126	0.287	0.084	1.39E-25	C3_CD8_Tem
chr16-81442580-81443286	3.16E-29	0.411598157	0.312	0.1	2.09E-24	C3_CD8_Tem
chr14-99055289-99056326	3.09E-11	0.27022848	0.215	0.087	2.05E-06	C3_CD8_Tem
chr6-15022680-15023793	2.43E-13	0.281589654	0.198	0.076	1.61E-08	C3_CD8_Tem
chr7-105848750-105849993	2.74E-29	0.374590124	0.447	0.18	1.81E-24	C3_CD8_Tem
chr10-27224711-27226309	2.73E-13	0.296578576	0.183	0.067	1.81E-08	C3_CD8_Tem
chr16-11562843-11563505	2.05E-13	0.296395636	0.143	0.043	1.36E-08	C3_CD8_Tem
chr20-24959621-24961536	1.18E-42	0.418762161	0.54	0.216	7.78E-38	C3_CD8_Tem
chrX-71992055-71993150	2.17E-10	0.251354104	0.218	0.093	1.44E-05	C3_CD8_Tem
chr6-159978466-159979502	3.56E-11	0.268830132	0.328	0.158	2.36E-06	C3_CD8_Tem
chr11-1568276-1568696	2.24E-20	0.357545817	0.206	0.058	1.48E-15	C3_CD8_Tem

chr1-24915001-24915963	2.47E-12	0.287700171	0.244	0.097	1.64E-07	C3_CD8_Tem
chr17-36188180-36189273	4.67E-50	0.416924784	0.624	0.245	3.09E-45	C3_CD8_Tem
chr7-131238148-131238849	1.08E-09	0.262341393	0.176	0.07	7.17E-05	C3_CD8_Tem
chr16-88636781-88637627	9.54E-12	0.277595035	0.327	0.145	6.32E-07	C3_CD8_Tem
chr2-144508210-144508577	1.83E-26	0.390843979	0.222	0.062	1.21E-21	C3_CD8_Tem
chr19-58326425-58328187	7.59E-24	0.303097232	0.582	0.304	5.02E-19	C3_CD8_Tem
chr9-89501607-89502643	6.78E-42	0.369161445	0.644	0.285	4.49E-37	C3_CD8_Tem
chr1-160755624-160757066	2.73E-27	0.399878784	0.317	0.111	1.81E-22	C3_CD8_Tem
chr16-29626615-29627455	2.66E-17	0.281776193	0.411	0.195	1.76E-12	C3_CD8_Tem
chr6-46325308-46326080	5.31E-16	0.313422112	0.156	0.047	3.52E-11	C3_CD8_Tem
chr1-12201232-12202096	8.35E-17	0.327482148	0.206	0.066	5.53E-12	C3_CD8_Tem
chr12-52361402-52361943	6.45E-26	0.389509429	0.146	0.028	4.27E-21	C3_CD8_Tem
chr1-202203369-202204084	3.81E-14	0.28657586	0.113	0.03	2.52E-09	C3_CD8_Tem
chr1-107785657-107786884	1.46E-19	0.328962083	0.33	0.129	9.64E-15	C3_CD8_Tem
chr19-10516551-10517154	4.41E-14	0.296845319	0.118	0.035	2.92E-09	C3_CD8_Tem
chr7-925609-926009	1.78E-23	0.379452453	0.289	0.1	1.18E-18	C3_CD8_Tem
chr12-68118976-68120277	7.97E-28	0.374996837	0.413	0.161	5.28E-23	C3_CD8_Tem
chr1-25035751-25036614	1.56E-12	0.269513856	0.259	0.113	1.03E-07	C3_CD8_Tem
chr5-107485956-107486918	9.14E-20	0.342203725	0.242	0.084	6.05E-15	C3_CD8_Tem
chr2-96131228-96131850	5.20E-09	0.259522982	0.127	0.042	0.000344419	C3_CD8_Tem
chr20-10350624-10351911	8.08E-13	0.273040706	0.261	0.117	5.35E-08	C3_CD8_Tem
chr5-1447801-1449146	1.49E-13	0.300032555	0.131	0.036	9.84E-09	C3_CD8_Tem
chr1-158978573-158979257	1.21E-09	0.252782967	0.128	0.043	8.01E-05	C3_CD8_Tem
chr7-2698362-2699465	9.39E-18	0.332212239	0.304	0.12	6.22E-13	C3_CD8_Tem
chr17-10198118-10199316	2.93E-18	0.330711004	0.229	0.081	1.94E-13	C3_CD8_Tem
chr2-144459392-144460394	4.85E-34	0.383977126	0.48	0.192	3.21E-29	C3_CD8_Tem
chr13-48504671-48506020	4.02E-33	0.401950564	0.403	0.148	2.66E-28	C3_CD8_Tem

chr19-8573198-8573976	1.58E-15	0.30605099	0.348	0.146	1.05E-10	C3_CD8_Tem
chr5-74630330-74631054	1.17E-28	0.403077818	0.234	0.062	7.73E-24	C3_CD8_Tem
chr3-43477309-43478624	6.28E-11	0.254820529	0.311	0.149	4.16E-06	C3_CD8_Tem
chr6-42417154-42418179	1.39E-18	0.357594711	0.237	0.077	9.18E-14	C3_CD8_Tem
chr2-241867876-241869439	1.59E-42	0.454576397	0.458	0.16	1.05E-37	C3_CD8_Tem
chr12-10497396-10498070	8.06E-14	0.292194153	0.142	0.046	5.34E-09	C3_CD8_Tem
chr9-89448381-89448821	1.04E-20	0.370564281	0.19	0.053	6.89E-16	C3_CD8_Tem
chr3-126613373-126614080	1.20E-12	0.264917684	0.096	0.024	7.96E-08	C3_CD8_Tem
chr1-20931867-20933023	2.52E-13	0.297103027	0.147	0.046	1.67E-08	C3_CD8_Tem
chr17-12981952-12982730	3.67E-21	0.361095882	0.195	0.058	2.43E-16	C3_CD8_Tem
chr9-132451050-132451675	3.80E-09	0.263277118	0.16	0.058	0.000251641	C3_CD8_Tem
chr11-118692156-118692907	5.77E-22	0.353788218	0.338	0.134	3.82E-17	C3_CD8_Tem
chr20-62117493-62118052	4.28E-15	0.31369938	0.304	0.127	2.84E-10	C3_CD8_Tem
chr12-122227099-122227757	3.98E-15	0.317722025	0.217	0.078	2.63E-10	C3_CD8_Tem
chr20-50109921-50110653	1.17E-12	0.286381704	0.314	0.136	7.76E-08	C3_CD8_Tem
chr15-59482258-59483059	1.70E-11	0.268851938	0.123	0.038	1.12E-06	C3_CD8_Tem
chr5-112923297-112923942	2.65E-11	0.273216725	0.213	0.089	1.75E-06	C3_CD8_Tem
chr2-127080441-127082110	3.41E-13	0.26395076	0.469	0.244	2.26E-08	C3_CD8_Tem
chr19-55276055-55276993	2.26E-14	0.31408505	0.198	0.07	1.49E-09	C3_CD8_Tem
chr1-28199130-28200262	1.09E-12	0.28212894	0.34	0.162	7.20E-08	C3_CD8_Tem
chr8-41507466-41508702	2.60E-11	0.278403701	0.135	0.042	1.72E-06	C3_CD8_Tem
chr22-26641977-26643804	6.25E-13	0.286544255	0.325	0.148	4.14E-08	C3_CD8_Tem
chr18-22281989-22283234	8.25E-15	0.296227148	0.249	0.1	5.46E-10	C3_CD8_Tem
chr1-100396516-100398052	1.50E-10	0.269443473	0.27	0.117	9.93E-06	C3_CD8_Tem
chr12-49043678-49044518	2.74E-24	0.383391265	0.212	0.062	1.81E-19	C3_CD8_Tem
chr14-105993495-105994314	3.29E-26	0.389966613	0.177	0.043	2.18E-21	C3_CD8_Tem
chr9-123304331-123305414	1.31E-38	0.427704352	0.435	0.155	8.66E-34	C3_CD8_Tem

chr19-10111953-10113270	4.56E-15	0.277720507	0.479	0.232	3.02E-10	C3_CD8_Tem
chr1-43581747-43582584	7.70E-14	0.270058841	0.109	0.03	5.10E-09	C3_CD8_Tem
chr22-21766646-21767539	7.62E-20	0.31765027	0.439	0.193	5.05E-15	C3_CD8_Tem
chr16-23482470-23483873	1.81E-71	0.514220615	0.568	0.183	1.20E-66	C3_CD8_Tem
chr6-105452630-105453843	3.64E-31	0.404992631	0.388	0.139	2.41E-26	C3_CD8_Tem
chr1-160861979-160863054	1.03E-13	0.304605046	0.177	0.06	6.85E-09	C3_CD8_Tem
chr2-97720946-97721561	1.07E-11	0.287447889	0.202	0.077	7.10E-07	C3_CD8_Tem
chr17-80876809-80877767	3.67E-14	0.321260958	0.231	0.086	2.43E-09	C3_CD8_Tem
chr5-107570845-107572433	4.02E-09	0.257090768	0.183	0.073	0.000266173	C3_CD8_Tem
chr10-24887229-24888162	2.12E-11	0.265232276	0.26	0.115	1.41E-06	C3_CD8_Tem
chr5-102853536-102854097	5.45E-19	0.349816846	0.15	0.039	3.61E-14	C3_CD8_Tem
chr16-57577489-57578337	3.22E-16	0.330973926	0.162	0.045	2.13E-11	C3_CD8_Tem
chr14-92585589-92587275	3.71E-11	0.279512176	0.347	0.16	2.46E-06	C3_CD8_Tem
chr15-78044354-78044820	3.47E-10	0.263806929	0.224	0.096	2.30E-05	C3_CD8_Tem
chr6-42389768-42390868	1.70E-09	0.255593085	0.301	0.144	0.000112284	C3_CD8_Tem
chr14-91377987-91378617	9.42E-09	0.267139594	0.24	0.102	0.000623985	C3_CD8_Tem
chr15-69718935-69719905	1.92E-17	0.310042022	0.43	0.197	1.27E-12	C3_CD8_Tem
chr11-122856416-122857078	1.23E-25	0.382771063	0.253	0.079	8.13E-21	C3_CD8_Tem
chr1-200154033-200154586	1.36E-15	0.291405767	0.288	0.115	9.01E-11	C3_CD8_Tem
chr1-161626522-161627073	5.34E-14	0.294309079	0.116	0.029	3.53E-09	C3_CD8_Tem
chr2-181393318-181395118	3.05E-17	0.319350652	0.312	0.125	2.02E-12	C3_CD8_Tem
chr12-10602523-10603301	1.00E-14	0.305020708	0.13	0.036	6.64E-10	C3_CD8_Tem
chr17-36089888-36090742	7.29E-18	0.335586743	0.237	0.083	4.83E-13	C3_CD8_Tem
chr18-51191044-51191652	2.93E-18	0.339014905	0.271	0.1	1.94E-13	C3_CD8_Tem
chr16-87979501-87980612	1.85E-33	0.430727123	0.288	0.085	1.22E-28	C3_CD8_Tem
chr19-3357402-3358618	8.34E-11	0.277008275	0.219	0.089	5.52E-06	C3_CD8_Tem
chr12-10293146-10293990	3.24E-10	0.289748218	0.167	0.057	2.15E-05	C3_CD8_Tem

chrX-1480086-1480445	1.89E-09	0.272976684	0.135	0.044	0.000125397	C3_CD8_Tem
chr5-142786694-142787240	3.84E-14	0.295767724	0.261	0.106	2.54E-09	C3_CD8_Tem
chr10-32981817-32982654	1.14E-14	0.314816067	0.157	0.047	7.54E-10	C3_CD8_Tem
chr19-29610167-29611018	3.13E-11	0.288641039	0.256	0.109	2.07E-06	C3_CD8_Tem
chrX-107449624-107451395	1.32E-23	0.3690876	0.26	0.083	8.74E-19	C3_CD8_Tem
chr10-32982979-32983531	3.07E-13	0.297035316	0.157	0.051	2.03E-08	C3_CD8_Tem
chr3-188553262-188554692	2.75E-23	0.368303367	0.315	0.114	1.82E-18	C3_CD8_Tem
chr14-89507455-89508275	5.58E-09	0.258996837	0.189	0.077	0.000369667	C3_CD8_Tem
chr17-36107297-36107972	2.45E-08	0.254517085	0.166	0.063	0.001623801	C3_CD8_Tem
chr3-27900129-27900802	4.50E-28	0.394761064	0.285	0.088	2.98E-23	C3_CD8_Tem
chr19-13790098-13790664	1.21E-10	0.265079942	0.182	0.071	8.00E-06	C3_CD8_Tem
chr7-36723790-36724702	4.64E-11	0.254980846	0.295	0.137	3.08E-06	C3_CD8_Tem
chr19-19419983-19421059	1.41E-28	0.390920055	0.362	0.134	9.32E-24	C3_CD8_Tem
chr22-23137252-23138634	3.05E-12	0.291712609	0.3	0.129	2.02E-07	C3_CD8_Tem
chr16-57609271-57610284	9.92E-11	0.268301747	0.264	0.115	6.57E-06	C3_CD8_Tem
chr1-168519863-168520828	1.56E-26	0.400082623	0.297	0.096	1.03E-21	C3_CD8_Tem
chr5-155768313-155768661	5.18E-17	0.314730816	0.113	0.026	3.43E-12	C3_CD8_Tem
chr1-53600208-53600946	6.00E-18	0.328170646	0.168	0.052	3.98E-13	C3_CD8_Tem
chr11-11987445-11988281	1.18E-31	0.425519203	0.2	0.046	7.84E-27	C3_CD8_Tem
chr13-113875597-113877300	2.41E-16	0.310796629	0.306	0.129	1.60E-11	C3_CD8_Tem
chr22-46370317-46371312	2.19E-17	0.336796695	0.19	0.058	1.45E-12	C3_CD8_Tem
chr2-68771057-68771646	8.83E-22	0.368900662	0.276	0.1	5.85E-17	C3_CD8_Tem
chr7-105806371-105806968	2.36E-18	0.346478639	0.204	0.065	1.57E-13	C3_CD8_Tem
chr2-176931691-176932544	1.83E-12	0.279315375	0.231	0.097	1.21E-07	C3_CD8_Tem
chr1-206924605-206925631	6.27E-10	0.27264395	0.221	0.094	4.15E-05	C3_CD8_Tem
chr19-19410709-19411230	4.18E-09	0.255535372	0.144	0.054	0.000276662	C3_CD8_Tem
chr5-142783441-142784189	5.86E-14	0.317290009	0.257	0.096	3.88E-09	C3_CD8_Tem

chr11-45092887-45093822	3.78E-22	0.359180364	0.3	0.108	2.50E-17	C3_CD8_Tem
chr18-80171880-80172411	1.80E-19	0.348174815	0.184	0.054	1.19E-14	C3_CD8_Tem
chr18-74995694-74996447	2.78E-11	0.283727722	0.143	0.045	1.84E-06	C3_CD8_Tem
chr6-41705906-41707149	7.64E-09	0.25094555	0.19	0.079	0.000505673	C3_CD8_Tem
chr8-58790453-58791432	5.14E-26	0.387735398	0.25	0.075	3.40E-21	C3_CD8_Tem
chr5-107387910-107389582	2.98E-11	0.260611066	0.136	0.046	1.98E-06	C3_CD8_Tem
chr16-81841425-81842485	1.48E-13	0.291568331	0.144	0.045	9.80E-09	C3_CD8_Tem
chr15-94306679-94307364	1.16E-13	0.308109895	0.18	0.057	7.71E-09	C3_CD8_Tem
chr2-231425680-231426544	5.68E-21	0.344401339	0.197	0.064	3.76E-16	C3_CD8_Tem
chr3-188974768-188975646	1.74E-18	0.342759307	0.184	0.055	1.15E-13	C3_CD8_Tem
chr2-190695531-190696245	3.71E-14	0.277189608	0.131	0.041	2.45E-09	C3_CD8_Tem
chr3-114328320-114328705	6.63E-15	0.305126982	0.144	0.044	4.39E-10	C3_CD8_Tem
chr16-68756090-68757132	1.10E-17	0.317731536	0.27	0.107	7.26E-13	C3_CD8_Tem
chr2-27391044-27392222	2.13E-12	0.294279471	0.221	0.089	1.41E-07	C3_CD8_Tem
chr3-114315466-114316209	2.20E-15	0.317796576	0.15	0.044	1.46E-10	C3_CD8_Tem
chr8-29405399-29405923	3.18E-11	0.274473022	0.174	0.068	2.11E-06	C3_CD8_Tem
chr16-57128610-57129150	6.37E-17	0.3174271	0.172	0.057	4.22E-12	C3_CD8_Tem
chr1-107963271-107965797	1.28E-10	0.282611333	0.281	0.128	8.47E-06	C3_CD8_Tem
chr21-34192430-34193200	7.28E-13	0.262913314	0.094	0.025	4.82E-08	C3_CD8_Tem
chr8-133152638-133153228	1.36E-13	0.281802867	0.103	0.027	9.01E-09	C3_CD8_Tem
chr1-107820641-107821706	8.82E-11	0.268692373	0.13	0.042	5.84E-06	C3_CD8_Tem
chr3-45980223-45980831	1.12E-23	0.381794647	0.303	0.109	7.39E-19	C3_CD8_Tem
chr5-160486133-160486893	2.44E-10	0.255087185	0.132	0.046	1.61E-05	C3_CD8_Tem
chr11-115222115-115223260	2.05E-13	0.291488082	0.202	0.076	1.36E-08	C3_CD8_Tem
chr4-152099735-152101089	1.57E-12	0.25392276	0.396	0.202	1.04E-07	C3_CD8_Tem
chr12-52645010-52646024	3.58E-11	0.262280533	0.186	0.076	2.37E-06	C3_CD8_Tem
chr8-133498177-133500468	6.60E-20	0.261722262	0.657	0.372	4.37E-15	C3_CD8_Tem

chr1-27521329-27523346	1.49E-14	0.280469765	0.406	0.202	9.84E-10	C3_CD8_Tem
chr2-203740503-203741793	6.43E-10	0.267348928	0.27	0.123	4.26E-05	C3_CD8_Tem
chrX-1471671-1472239	3.62E-12	0.269386283	0.242	0.104	2.40E-07	C3_CD8_Tem
chr21-34935382-34936555	1.75E-13	0.3043521	0.25	0.102	1.16E-08	C3_CD8_Tem
chr20-53884739-53885569	1.10E-09	0.258336438	0.133	0.045	7.27E-05	C3_CD8_Tem
chr3-197393766-197395033	7.52E-09	0.256312722	0.198	0.083	0.000497894	C3_CD8_Tem
chr1-230208534-230209331	2.16E-09	0.253736715	0.13	0.047	0.00014306	C3_CD8_Tem
chr6-224832-225792	4.24E-26	0.351403389	0.437	0.183	2.81E-21	C3_CD8_Tem
chr2-27637663-27639040	2.60E-09	0.272099706	0.235	0.096	0.000172266	C3_CD8_Tem
chr17-2192183-2193154	6.50E-15	0.323821716	0.271	0.104	4.31E-10	C3_CD8_Tem
chr20-36627671-36628516	2.08E-18	0.336206318	0.288	0.104	1.38E-13	C3_CD8_Tem
chr15-74383546-74385446	1.07E-25	0.37353857	0.381	0.142	7.05E-21	C3_CD8_Tem
chr12-10554098-10555445	1.12E-15	0.304838191	0.197	0.071	7.42E-11	C3_CD8_Tem
chr12-10384862-10385642	1.87E-27	0.412465103	0.23	0.064	1.24E-22	C3_CD8_Tem
chr2-86825469-86826726	2.90E-63	0.524982874	0.51	0.15	1.92E-58	C3_CD8_Tem
chr2-86785384-86786603	9.54E-94	0.512922872	0.73	0.266	6.32E-89	C3_CD8_Tem
chr14-24633431-24634610	4.78E-11	0.265266542	0.267	0.119	3.17E-06	C3_CD8_Tem
chr14-92530938-92532058	6.87E-42	0.414754601	0.521	0.199	4.55E-37	C3_CD8_Tem
chr2-62304674-62306185	9.42E-19	0.346547714	0.261	0.091	6.24E-14	C3_CD8_Tem
chr3-5016068-5016933	2.94E-08	0.254506609	0.26	0.118	0.001947632	C3_CD8_Tem
chr22-20550440-20552163	6.31E-21	0.345961405	0.374	0.157	4.18E-16	C3_CD8_Tem
chr7-152027350-152028282	2.04E-28	0.403709223	0.296	0.094	1.35E-23	C3_CD8_Tem
chr1-221800726-221801674	2.53E-16	0.304737847	0.226	0.086	1.68E-11	C3_CD8_Tem
chr1-117001254-117001952	8.96E-09	0.260886633	0.136	0.048	0.00059356	C3_CD8_Tem
chr12-10540546-10541263	5.87E-13	0.276215421	0.099	0.025	3.89E-08	C3_CD8_Tem
chr17-3794854-3797125	3.96E-50	0.31978906	0.826	0.443	2.62E-45	C3_CD8_Tem
chr9-89426299-89428064	6.85E-19	0.293416361	0.509	0.249	4.53E-14	C3_CD8_Tem

chr16-31421353-31422176	3.53E-21	0.370668518	0.252	0.081	2.34E-16	C3_CD8_Tem
chr12-44847506-44848934	8.64E-15	0.297488565	0.352	0.156	5.72E-10	C3_CD8_Tem
chr15-74399958-74401241	1.10E-32	0.443191314	0.322	0.098	7.32E-28	C3_CD8_Tem
chr17-27471322-27472502	4.31E-20	0.301049507	0.49	0.241	2.86E-15	C3_CD8_Tem
chr2-98835548-98836629	2.73E-25	0.351327571	0.436	0.185	1.81E-20	C3_CD8_Tem
chr2-230973545-230974871	3.99E-12	0.25436873	0.414	0.217	2.64E-07	C3_CD8_Tem
chr2-174752335-174753609	2.45E-10	0.254405464	0.294	0.142	1.62E-05	C3_CD8_Tem
chr3-47016683-47017556	1.04E-13	0.290869132	0.353	0.16	6.91E-09	C3_CD8_Tem
chr20-50109921-50110653	1.17E-12	0.286381704	0.314	0.136	7.76E-08	C3_CD8_Tem
chr17-3764719-3765887	1.45E-30	0.34554844	0.569	0.264	9.62E-26	C3_CD8_Tem
chr14-99055289-99056326	3.09E-11	0.27022848	0.215	0.087	2.05E-06	C3_CD8_Tem
chr14-92529831-92530135	1.38E-10	0.260438973	0.092	0.024	9.13E-06	C3_CD8_Tem
chr20-5719626-5720622	1.59E-17	0.344589935	0.239	0.081	1.05E-12	C3_CD8_Tem
chr9-63816618-63818431	8.63E-28	0.338257044	0.549	0.256	5.71E-23	C3_CD8_Tem
chr17-83083303-83084182	4.80E-38	0.35499318	0.626	0.292	3.18E-33	C3_CD8_Tem
chr15-55253521-55253894	1.88E-08	0.26528584	0.124	0.04	0.001244601	C3_CD8_Tem
chr2-144659399-144660199	2.74E-16	0.293491407	0.319	0.136	1.81E-11	C3_CD8_Tem
chr6-13453743-13455250	6.90E-47	0.401473649	0.635	0.271	4.57E-42	C3_CD8_Tem
chr3-46108784-46109849	1.05E-11	0.279836066	0.298	0.136	6.93E-07	C3_CD8_Tem
chr1-52636599-52637735	1.35E-15	0.274191386	0.506	0.264	8.91E-11	C3_CD8_Tem
chr3-105823079-105823914	8.02E-09	0.260197415	0.233	0.1	0.000531301	C3_CD8_Tem
chr16-391649-392432	4.36E-20	0.318593738	0.393	0.171	2.89E-15	C3_CD8_Tem
chr12-10497396-10498070	8.06E-14	0.292194153	0.142	0.046	5.34E-09	C3_CD8_Tem
chr12-53102878-53103402	2.57E-12	0.293806941	0.199	0.077	1.70E-07	C3_CD8_Tem
chr12-10394483-10395812	9.93E-43	0.444058167	0.433	0.146	6.58E-38	C3_CD8_Tem
chr10-3197279-3198114	4.91E-09	0.266187455	0.274	0.126	0.000325388	C3_CD8_Tem
chr20-53835364-53835846	1.53E-11	0.288302751	0.233	0.096	1.02E-06	C3_CD8_Tem

chr2-8551423-8552687	8.05E-70	0.612928779	0.311	0.052	5.33E-65	C3_CD8_Tem
chr19-52715420-52716518	1.07E-09	0.271291106	0.158	0.055	7.10E-05	C3_CD8_Tem
chr7-5629850-5631088	1.30E-08	0.250368742	0.252	0.119	0.000862402	C3_CD8_Tem
chr10-97414740-97415235	1.34E-13	0.311681131	0.213	0.08	8.88E-09	C3_CD8_Tem
chr11-64331142-64332138	5.31E-24	0.369313219	0.355	0.132	3.51E-19	C3_CD8_Tem
chr22-20519718-20520552	8.80E-20	0.363361485	0.216	0.064	5.83E-15	C3_CD8_Tem
chr16-87853497-87854843	4.52E-23	0.279800603	0.638	0.347	2.99E-18	C3_CD8_Tem
chr16-56938222-56939605	3.61E-17	0.326778475	0.336	0.139	2.39E-12	C3_CD8_Tem
chr19-41935809-41936940	2.94E-53	0.451628491	0.572	0.215	1.95E-48	C3_CD8_Tem
chr10-70578066-70578810	1.13E-28	0.377173546	0.465	0.188	7.45E-24	C3_CD8_Tem
chr1-39525182-39526389	1.00E-43	0.45341987	0.448	0.144	6.65E-39	C3_CD8_Tem
chr6-127933636-127934859	9.36E-13	0.295547857	0.259	0.109	6.20E-08	C3_CD8_Tem
chr8-130041775-130042771	2.76E-12	0.300064815	0.259	0.104	1.83E-07	C3_CD8_Tem
chr11-117827963-117829091	4.52E-14	0.291620738	0.365	0.168	2.99E-09	C3_CD8_Tem
chr12-47211956-47212706	2.82E-14	0.314199313	0.333	0.145	1.87E-09	C3_CD8_Tem
chr18-23872449-23873091	7.25E-11	0.269622205	0.174	0.064	4.80E-06	C3_CD8_Tem
chr19-51372441-51373731	4.52E-25	0.330227057	0.52	0.232	2.99E-20	C3_CD8_Tem
chr12-57222735-57223690	7.67E-20	0.333545829	0.322	0.125	5.08E-15	C3_CD8_Tem
chr16-89096594-89097599	3.70E-21	0.320491422	0.413	0.185	2.45E-16	C3_CD8_Tem
chr16-29028776-29029693	2.40E-12	0.292200895	0.212	0.083	1.59E-07	C3_CD8_Tem
chr2-230781194-230782105	2.15E-29	0.373933908	0.455	0.18	1.43E-24	C3_CD8_Tem
chr3-149009270-149010325	2.34E-20	0.317586123	0.408	0.175	1.55E-15	C3_CD8_Tem
chr3-28326120-28326988	9.91E-18	0.301019936	0.437	0.202	6.56E-13	C3_CD8_Tem
chr9-62800737-62802770	8.71E-14	0.285712847	0.41	0.197	5.77E-09	C3_CD8_Tem
chr5-143221788-143223304	9.39E-17	0.252987727	0.584	0.316	6.22E-12	C3_CD8_Tem
chr12-10558323-10558663	1.54E-12	0.27558603	0.084	0.018	1.02E-07	C3_CD8_Tem
chr14-74620541-74620955	1.04E-08	0.266130363	0.2	0.081	0.000687956	C3_CD8_Tem

chr6-2861395-2861985	2.79E-44	0.481999437	0.373	0.104	1.85E-39	C3_CD8_Tem
chr11-44611238-44612315	4.33E-10	0.265101029	0.303	0.137	2.87E-05	C3_CD8_Tem
chr2-86807185-86808995	8.80E-29	0.357884894	0.509	0.217	5.83E-24	C3_CD8_Tem
chr3-46359594-46360492	3.94E-21	0.331783905	0.395	0.168	2.61E-16	C3_CD8_Tem
chr7-139084116-139085810	2.74E-14	0.313342228	0.287	0.113	1.81E-09	C3_CD8_Tem
chr17-35898230-35898645	3.85E-09	0.259657131	0.172	0.067	0.00025478	C3_CD8_Tem
chr7-925609-926009	1.78E-23	0.379452453	0.289	0.1	1.18E-18	C3_CD8_Tem
chr19-8573198-8573976	1.58E-15	0.30605099	0.348	0.146	1.05E-10	C3_CD8_Tem
chr9-130106807-130108214	1.79E-28	0.373937373	0.454	0.187	1.18E-23	C3_CD8_Tem
chr21-42198955-42200215	3.44E-12	0.292277705	0.245	0.101	2.28E-07	C3_CD8_Tem
chr4-10108841-10109483	2.11E-10	0.260218355	0.306	0.145	1.40E-05	C3_CD8_Tem
chr1-92537995-92539149	3.23E-18	0.314064358	0.432	0.19	2.14E-13	C3_CD8_Tem
chr5-142786694-142787240	3.84E-14	0.295767724	0.261	0.106	2.54E-09	C3_CD8_Tem
chr1-160738839-160739406	2.56E-24	0.388545822	0.311	0.103	1.69E-19	C3_CD8_Tem
chr2-37201234-37202142	6.36E-13	0.302458128	0.165	0.053	4.21E-08	C3_CD8_Tem
chr1-24966076-24967007	1.44E-09	0.2548147	0.31	0.151	9.53E-05	C3_CD8_Tem
chr17-3763177-3763727	1.41E-19	0.344452314	0.374	0.148	9.35E-15	C3_CD8_Tem
chrX-72272557-72273155	6.38E-10	0.258611626	0.099	0.028	4.23E-05	C3_CD8_Tem
chr2-127080441-127082110	3.41E-13	0.26395076	0.469	0.244	2.26E-08	C3_CD8_Tem
chr1-173409657-173411119	1.30E-27	0.367791949	0.403	0.157	8.61E-23	C3_CD8_Tem
chr6-129686918-129687735	1.15E-08	0.271071276	0.263	0.115	0.000763045	C3_CD8_Tem
chr9-114369205-114370071	1.39E-35	0.37048113	0.545	0.236	9.19E-31	C3_CD8_Tem
chr16-57539002-57539356	9.55E-14	0.325262375	0.224	0.081	6.32E-09	C3_CD8_Tem
chr11-118045208-118047222	2.76E-15	0.300904472	0.366	0.163	1.83E-10	C3_CD8_Tem
chr12-10378752-10379115	1.92E-12	0.278963727	0.083	0.017	1.27E-07	C3_CD8_Tem
chr2-10497665-10498228	3.81E-22	0.373986992	0.219	0.068	2.52E-17	C3_CD8_Tem
chr14-92585589-92587275	3.71E-11	0.279512176	0.347	0.16	2.46E-06	C3_CD8_Tem

chr10-22246134-22247397	1.08E-13	0.271093922	0.443	0.216	7.13E-09	C3_CD8_Tem
chr1-200862003-200863385	1.23E-13	0.273232642	0.367	0.173	8.12E-09	C3_CD8_Tem
chr16-525341-525980	4.14E-10	0.280586733	0.292	0.125	2.74E-05	C3_CD8_Tem
chr2-230793603-230794832	2.17E-19	0.303183108	0.497	0.251	1.43E-14	C3_CD8_Tem
chr3-115164316-115166011	3.10E-32	0.391551597	0.439	0.174	2.05E-27	C3_CD8_Tem
chr11-35272216-35273053	6.17E-10	0.27280535	0.245	0.103	4.08E-05	C3_CD8_Tem
chr3-15309624-15311172	1.12E-12	0.270348274	0.363	0.173	7.39E-08	C3_CD8_Tem
chr13-99273061-99273690	1.92E-09	0.269450005	0.182	0.071	0.000126962	C3_CD8_Tem
chr10-6493437-6493865	1.66E-14	0.315042055	0.282	0.117	1.10E-09	C3_CD8_Tem
chr7-143385779-143386618	1.09E-13	0.285072141	0.322	0.145	7.24E-09	C3_CD8_Tem
chr12-10369630-10370340	7.51E-30	0.421433434	0.234	0.061	4.98E-25	C3_CD8_Tem
chr1-201170280-201171791	1.92E-42	0.401253707	0.525	0.225	1.27E-37	C3_CD8_Tem
chr7-143375135-143375888	1.07E-17	0.318312327	0.399	0.173	7.09E-13	C3_CD8_Tem
chr2-223759699-223762024	2.68E-16	0.27363561	0.406	0.197	1.77E-11	C3_CD8_Tem
chr7-2417689-2418570	3.85E-17	0.32414492	0.303	0.122	2.55E-12	C3_CD8_Tem
chr17-47719285-47720048	7.76E-06	0.251619956	0.229	0.1	0.514111321	C3_CD8_Tem
chr10-72369720-72370856	2.40E-12	0.250812974	0.391	0.201	1.59E-07	C3_CD8_Tem
chr13-46751365-46752411	9.37E-13	0.273274328	0.29	0.128	6.21E-08	C3_CD8_Tem
chr5-87120180-87121626	9.44E-19	0.306879157	0.444	0.205	6.25E-14	C3_CD8_Tem
chr9-126523558-126524156	1.79E-09	0.274821449	0.201	0.079	0.000118495	C3_CD8_Tem
chr6-130033947-130034987	1.33E-22	0.375607786	0.285	0.099	8.81E-18	C3_CD8_Tem
chr2-113240392-113241310	5.34E-11	0.262898593	0.142	0.05	3.54E-06	C3_CD8_Tem
chr6-193662-194430	8.75E-23	0.365903248	0.352	0.13	5.79E-18	C3_CD8_Tem
chr5-142799721-142801418	4.67E-32	0.395840386	0.449	0.176	3.09E-27	C3_CD8_Tem
chr12-9906361-9907281	4.72E-24	0.375287804	0.328	0.117	3.12E-19	C3_CD8_Tem
chr20-5077780-5078868	1.00E-10	0.255058229	0.354	0.178	6.65E-06	C3_CD8_Tem
chr5-139389577-139390346	1.20E-13	0.269309629	0.428	0.216	7.94E-09	C3_CD8_Tem

chr5-52743033-52744272	6.83E-12	0.278685059	0.3	0.135	4.52E-07	C3_CD8_Tem
chr11-45092887-45093822	3.78E-22	0.359180364	0.3	0.108	2.50E-17	C3_CD8_Tem
chr9-114690589-114692004	1.64E-13	0.277864317	0.432	0.216	1.09E-08	C3_CD8_Tem
chr1-25549312-25550465	6.65E-15	0.30451488	0.358	0.16	4.40E-10	C3_CD8_Tem
chr16-27449378-27451209	1.46E-19	0.311707592	0.462	0.213	9.65E-15	C3_CD8_Tem
chr9-126396589-126397526	1.09E-14	0.292301437	0.343	0.155	7.25E-10	C3_CD8_Tem
chr12-10721588-10723505	6.21E-20	0.337191809	0.367	0.141	4.12E-15	C3_CD8_Tem
chr14-92551174-92551791	1.23E-07	0.25322306	0.14	0.05	0.00815519	C3_CD8_Tem
chr17-39757813-39758477	3.59E-11	0.278447733	0.365	0.173	2.38E-06	C3_CD8_Tem
chr14-89773254-89774387	2.02E-12	0.274944127	0.233	0.101	1.34E-07	C3_CD8_Tem
chr2-159225953-159226543	6.26E-12	0.277930204	0.19	0.071	4.15E-07	C3_CD8_Tem
chr6-42367126-42368498	1.27E-09	0.282587569	0.216	0.087	8.43E-05	C3_CD8_Tem
chr14-106174117-106174932	4.08E-14	0.273911456	0.403	0.192	2.70E-09	C3_CD8_Tem
chr16-57815530-57816711	1.05E-13	0.303800074	0.243	0.096	6.94E-09	C3_CD8_Tem
chr22-43376194-43378148	5.44E-50	0.45579932	0.53	0.192	3.60E-45	C3_CD8_Tem
chr1-172670231-172671127	9.72E-18	0.344416545	0.311	0.118	6.44E-13	C3_CD8_Tem
chr1-26799587-26800842	1.14E-19	0.369433958	0.273	0.094	7.58E-15	C3_CD8_Tem
chr6-159978466-159979502	3.56E-11	0.268830132	0.328	0.158	2.36E-06	C3_CD8_Tem
chr17-47809886-47810847	3.37E-10	0.269346378	0.268	0.12	2.23E-05	C3_CD8_Tem
chr17-3791506-3791870	1.85E-10	0.295127769	0.197	0.074	1.23E-05	C3_CD8_Tem
chr5-96793985-96795199	8.62E-11	0.281499038	0.238	0.099	5.71E-06	C3_CD8_Tem
chr11-57649096-57650233	1.98E-14	0.274568944	0.425	0.213	1.31E-09	C3_CD8_Tem
chr11-35214696-35215174	2.17E-10	0.277931385	0.237	0.1	1.44E-05	C3_CD8_Tem
chr12-124921145-124921808	8.92E-11	0.261103212	0.371	0.182	5.90E-06	C3_CD8_Tem
chr10-50511142-50512258	2.29E-11	0.285579656	0.233	0.095	1.52E-06	C3_CD8_Tem
chr1-221801931-221802412	2.96E-10	0.265768323	0.116	0.032	1.96E-05	C3_CD8_Tem
chr11-118216313-118217830	8.64E-10	0.267750793	0.257	0.12	5.72E-05	C3_CD8_Tem

chr3-98557230-98557783	3.09E-15	0.316026627	0.24	0.094	2.05E-10	C3_CD8_Tem
chr2-97720946-97721561	1.07E-11	0.287447889	0.202	0.077	7.10E-07	C3_CD8_Tem
chr11-117984168-117984752	4.50E-12	0.294631314	0.161	0.053	2.98E-07	C3_CD8_Tem
chr22-44671794-44672138	3.42E-11	0.26686368	0.193	0.077	2.26E-06	C3_CD8_Tem
chr15-90418964-90420167	5.09E-08	0.252596087	0.2	0.09	0.003369656	C3_CD8_Tem
chr4-1176639-1177899	7.19E-15	0.314598375	0.219	0.077	4.76E-10	C3_CD8_Tem
chr9-4653657-4654532	2.07E-29	0.410156063	0.176	0.038	1.37E-24	C3_CD8_Tem
chr7-2698362-2699465	9.39E-18	0.332212239	0.304	0.12	6.22E-13	C3_CD8_Tem
chr8-140571963-140572770	2.25E-15	0.305007405	0.403	0.185	1.49E-10	C3_CD8_Tem
chr1-52139610-52140503	3.24E-12	0.29161342	0.304	0.132	2.15E-07	C3_CD8_Tem
chr1-116537182-116537858	7.54E-12	0.287867546	0.176	0.064	4.99E-07	C3_CD8_Tem
chr16-27446485-27447081	5.31E-13	0.274430222	0.345	0.164	3.51E-08	C3_CD8_Tem
chr1-32801060-32801651	6.77E-20	0.330863487	0.384	0.167	4.48E-15	C3_CD8_Tem
chr6-150310284-150311807	2.53E-33	0.401673393	0.395	0.144	1.68E-28	C3_CD8_Tem
chr2-317260-318382	7.12E-08	0.252927835	0.204	0.086	0.004716248	C3_CD8_Tem
chr7-2037211-2038557	2.38E-18	0.345271934	0.195	0.06	1.57E-13	C3_CD8_Tem
chr1-154790841-154792148	1.66E-23	0.353665017	0.438	0.184	1.10E-18	C3_CD8_Tem
chr14-61328202-61329221	9.39E-12	0.287150006	0.328	0.152	6.22E-07	C3_CD8_Tem
chr2-241965768-241966490	2.24E-09	0.251570415	0.139	0.052	0.000148606	C3_CD8_Tem
chr20-48757937-48758583	1.64E-18	0.347472543	0.249	0.085	1.09E-13	C3_CD8_Tem
chr7-149620902-149622091	1.09E-15	0.297993996	0.369	0.166	7.22E-11	C3_CD8_Tem
chr2-86790522-86791567	3.24E-27	0.391150585	0.325	0.11	2.14E-22	C3_CD8_Tem
chr17-14359049-14359741	7.50E-11	0.255919839	0.14	0.053	4.97E-06	C3_CD8_Tem
chr1-25035751-25036614	1.56E-12	0.269513856	0.259	0.113	1.03E-07	C3_CD8_Tem
chr2-7725167-7726015	6.29E-14	0.29396359	0.353	0.16	4.17E-09	C3_CD8_Tem
chr8-43141786-43142639	1.36E-10	0.292561884	0.193	0.073	9.02E-06	C3_CD8_Tem
chr1-172818339-172819563	1.16E-39	0.458916816	0.363	0.11	7.71E-35	C3_CD8_Tem

chr2-46239011-46240224	8.07E-14	0.292174301	0.32	0.136	5.34E-09	C3_CD8_Tem
chr9-134301405-134302179	7.18E-33	0.432466989	0.352	0.115	4.75E-28	C3_CD8_Tem
chr7-50367163-50367968	4.47E-11	0.275621261	0.248	0.11	2.96E-06	C3_CD8_Tem
chr2-233099908-233101037	3.52E-11	0.286802165	0.217	0.087	2.33E-06	C3_CD8_Tem
chr1-168509786-168510939	6.11E-34	0.422557305	0.362	0.118	4.05E-29	C3_CD8_Tem
chr1-42938289-42938669	5.71E-08	0.258613485	0.156	0.058	0.003780385	C3_CD8_Tem
chr12-48824087-48824575	1.28E-10	0.255874886	0.11	0.032	8.47E-06	C3_CD8_Tem
chrX-156000197-156000775	1.71E-13	0.294136942	0.157	0.052	1.13E-08	C3_CD8_Tem
chr21-35138340-35139519	1.84E-14	0.297472014	0.133	0.04	1.22E-09	C3_CD8_Tem
chr22-24409682-24410607	3.41E-16	0.329648243	0.186	0.058	2.26E-11	C3_CD8_Tem
chr20-53892841-53893753	1.62E-11	0.284801531	0.216	0.086	1.07E-06	C3_CD8_Tem
chr13-30396394-30396829	3.49E-13	0.297364532	0.135	0.04	2.31E-08	C3_CD8_Tem
chr9-114364129-114364520	1.63E-08	0.258790124	0.155	0.059	0.001078573	C3_CD8_Tem
chr17-67431290-67431893	4.56E-11	0.294672437	0.251	0.103	3.02E-06	C3_CD8_Tem
chr20-49775927-49776688	1.54E-10	0.281296631	0.171	0.061	1.02E-05	C3_CD8_Tem
chr19-57192947-57193349	1.34E-15	0.311115386	0.128	0.035	8.86E-11	C3_CD8_Tem
chr1-160751298-160751835	3.20E-32	0.442304247	0.305	0.085	2.12E-27	C3_CD8_Tem
chr6-26309496-26310055	1.13E-14	0.321118909	0.215	0.077	7.48E-10	C3_CD8_Tem
chr1-160861979-160863054	1.03E-13	0.304605046	0.177	0.06	6.85E-09	C3_CD8_Tem
chr21-46368226-46369182	1.53E-12	0.297719042	0.204	0.076	1.01E-07	C3_CD8_Tem
chr1-149843082-149844483	3.86E-08	0.263390925	0.199	0.087	0.002556895	C3_CD8_Tem
chr17-80876809-80877767	3.67E-14	0.321260958	0.231	0.086	2.43E-09	C3_CD8_Tem
chr17-77836360-77837135	3.36E-11	0.275790199	0.227	0.097	2.22E-06	C3_CD8_Tem
chr17-36192338-36192639	2.16E-15	0.326772772	0.205	0.068	1.43E-10	C3_CD8_Tem
chr3-188339243-188340186	8.63E-11	0.271056748	0.216	0.092	5.71E-06	C3_CD8_Tem
chr16-89785283-89785996	6.84E-12	0.292537115	0.152	0.051	4.53E-07	C3_CD8_Tem
chr2-203781820-203782629	4.27E-12	0.295677554	0.205	0.078	2.83E-07	C3_CD8_Tem

chr15-63203399-63204113	1.06E-09	0.268105399	0.217	0.089	7.05E-05	C3_CD8_Tem
chr2-105859090-105859483	3.99E-16	0.332532179	0.194	0.064	2.64E-11	C3_CD8_Tem
chr17-8958263-8958907	5.78E-15	0.332340533	0.27	0.101	3.83E-10	C3_CD8_Tem
chr7-152027350-152028282	2.04E-28	0.403709223	0.296	0.094	1.35E-23	C3_CD8_Tem
chr10-96670477-96671823	1.71E-15	0.30565686	0.204	0.075	1.13E-10	C3_CD8_Tem
chr2-62208366-62209144	3.19E-19	0.349666192	0.212	0.065	2.11E-14	C3_CD8_Tem
chr15-74399958-74401241	1.10E-32	0.443191314	0.322	0.098	7.32E-28	C3_CD8_Tem
chr17-74473957-74474793	4.48E-15	0.31602336	0.165	0.049	2.97E-10	C3_CD8_Tem
chr16-9081917-9082880	7.10E-28	0.402989346	0.188	0.044	4.70E-23	C3_CD8_Tem
chr9-97910196-97910809	7.11E-25	0.392881277	0.217	0.06	4.71E-20	C3_CD8_Tem
chr19-52715420-52716518	1.07E-09	0.271291106	0.158	0.055	7.10E-05	C3_CD8_Tem
chr6-34484371-34485456	2.22E-38	0.436489678	0.403	0.14	1.47E-33	C3_CD8_Tem
chr17-77836360-77837135	3.36E-11	0.275790199	0.227	0.097	2.22E-06	C3_CD8_Tem
chr1-225454008-225455207	5.00E-27	0.382269244	0.342	0.125	3.31E-22	C3_CD8_Tem
chr22-22720135-22721253	1.14E-14	0.320153624	0.193	0.063	7.52E-10	C3_CD8_Tem
chr1-167445973-167446410	2.20E-08	0.252095342	0.14	0.051	0.00145701	C3_CD8_Tem
chr12-57222735-57223690	7.67E-20	0.333545829	0.322	0.125	5.08E-15	C3_CD8_Tem
chr17-1890533-1891414	4.13E-13	0.295439552	0.263	0.106	2.73E-08	C3_CD8_Tem
chr18-22281989-22283234	8.25E-15	0.296227148	0.249	0.1	5.46E-10	C3_CD8_Tem
chr2-37201234-37202142	6.36E-13	0.302458128	0.165	0.053	4.21E-08	C3_CD8_Tem
chr16-89784179-89785008	3.22E-27	0.392923513	0.24	0.074	2.14E-22	C3_CD8_Tem
chr3-188553262-188554692	2.75E-23	0.368303367	0.315	0.114	1.82E-18	C3_CD8_Tem
chr12-121557117-121558231	3.45E-46	0.435986111	0.486	0.173	2.28E-41	C3_CD8_Tem
chr2-169682683-169683766	2.52E-21	0.350050641	0.315	0.12	1.67E-16	C3_CD8_Tem
chr17-36196490-36197498	3.94E-14	0.287380923	0.296	0.127	2.61E-09	C3_CD8_Tem
chr16-81841425-81842485	1.48E-13	0.291568331	0.144	0.045	9.80E-09	C3_CD8_Tem
chr10-73758806-73760041	1.04E-29	0.382899159	0.419	0.158	6.86E-25	C3_CD8_Tem

chr14-92530938-92532058	6.87E-42	0.414754601	0.521	0.199	4.55E-37	C3_CD8_Tem
chrX-124225919-124226446	2.36E-12	0.253877327	0.075	0.017	1.56E-07	C3_CD8_Tem
chr5-1518362-1519073	5.77E-23	0.369287916	0.273	0.087	3.82E-18	C3_CD8_Tem
chr11-35272216-35273053	6.17E-10	0.27280535	0.245	0.103	4.08E-05	C3_CD8_Tem
chr16-89096594-89097599	3.70E-21	0.320491422	0.413	0.185	2.45E-16	C3_CD8_Tem
chr1-52636599-52637735	1.35E-15	0.274191386	0.506	0.264	8.91E-11	C3_CD8_Tem
chr17-38900699-38901837	2.51E-38	0.441933498	0.439	0.149	1.66E-33	C3_CD8_Tem
chr5-132099000-132099509	8.47E-11	0.266943378	0.337	0.156	5.61E-06	C3_CD8_Tem
chr17-35900277-35901096	5.17E-13	0.300358006	0.224	0.087	3.42E-08	C3_CD8_Tem
chr17-14359049-14359741	7.50E-11	0.255919839	0.14	0.053	4.97E-06	C3_CD8_Tem
chr12-10554098-10555445	1.12E-15	0.304838191	0.197	0.071	7.42E-11	C3_CD8_Tem
chr17-36192338-36192639	2.16E-15	0.326772772	0.205	0.068	1.43E-10	C3_CD8_Tem
chr22-43376194-43378148	5.44E-50	0.45579932	0.53	0.192	3.60E-45	C3_CD8_Tem
chr13-99403154-99403966	1.85E-21	0.355522259	0.209	0.065	1.23E-16	C3_CD8_Tem
chr9-62800737-62802770	8.71E-14	0.285712847	0.41	0.197	5.77E-09	C3_CD8_Tem
chr15-94306679-94307364	1.16E-13	0.308109895	0.18	0.057	7.71E-09	C3_CD8_Tem
chr2-28423746-28425144	1.40E-08	0.261947712	0.179	0.072	0.000924647	C3_CD8_Tem
chr5-1488691-1489680	2.10E-30	0.427019126	0.287	0.084	1.39E-25	C3_CD8_Tem
chr16-57028997-57030129	2.21E-45	0.513091341	0.248	0.049	1.46E-40	C3_CD8_Tem
chr20-36627671-36628516	2.08E-18	0.336206318	0.288	0.104	1.38E-13	C3_CD8_Tem
chr17-10198118-10199316	2.93E-18	0.330711004	0.229	0.081	1.94E-13	C3_CD8_Tem
chr17-10114141-10115557	2.18E-53	0.420008431	0.622	0.245	1.44E-48	C3_CD8_Tem
chrX-156000197-156000775	1.71E-13	0.294136942	0.157	0.052	1.13E-08	C3_CD8_Tem
chr19-51372441-51373731	4.52E-25	0.330227057	0.52	0.232	2.99E-20	C3_CD8_Tem
chr6-31354130-31354531	2.46E-11	0.299362737	0.197	0.073	1.63E-06	C3_CD8_Tem
chr14-74620541-74620955	1.04E-08	0.266130363	0.2	0.081	0.000687956	C3_CD8_Tem
chr16-75617604-75617832	7.47E-14	0.269668046	0.08	0.017	4.95E-09	C3_CD8_Tem

chr19-6674620-6675357	3.93E-10	0.26600727	0.369	0.183	2.60E-05	C3_CD8_Tem
chr18-22342578-22343028	1.37E-12	0.288494982	0.151	0.05	9.10E-08	C3_CD8_Tem
chr2-136212448-136213283	5.02E-10	0.273666771	0.206	0.083	3.32E-05	C3_CD8_Tem
chr16-75616348-75616998	4.78E-13	0.289692026	0.149	0.048	3.16E-08	C3_CD8_Tem
chr5-142786694-142787240	3.84E-14	0.295767724	0.261	0.106	2.54E-09	C3_CD8_Tem
chr10-124650100-124651550	1.83E-65	0.488205685	0.616	0.224	1.21E-60	C3_CD8_Tem
chr17-5241943-5243263	2.76E-18	0.339419567	0.166	0.049	1.83E-13	C3_CD8_Tem
chr14-93065704-93066578	5.93E-24	0.369155744	0.321	0.115	3.93E-19	C3_CD8_Tem
chr20-49805033-49805682	4.42E-21	0.348738596	0.288	0.106	2.92E-16	C3_CD8_Tem
chr4-970184-971071	7.12E-39	0.466223479	0.309	0.078	4.71E-34	C3_CD8_Tem
chr20-24959621-24961536	1.18E-42	0.418762161	0.54	0.216	7.78E-38	C3_CD8_Tem
chr2-233099908-233101037	3.52E-11	0.286802165	0.217	0.087	2.33E-06	C3_CD8_Tem
chr6-150310284-150311807	2.53E-33	0.401673393	0.395	0.144	1.68E-28	C3_CD8_Tem
chr13-29934808-29936565	3.93E-25	0.317144328	0.526	0.247	2.60E-20	C3_CD8_Tem
chr10-50508093-50508803	2.26E-20	0.333574884	0.354	0.148	1.49E-15	C3_CD8_Tem
chr2-230877055-230878272	4.30E-62	0.46934429	0.609	0.217	2.85E-57	C3_CD8_Tem
chr5-132456192-132458945	2.00E-14	0.277484094	0.451	0.225	1.33E-09	C3_CD8_Tem
chr17-47809886-47810847	3.37E-10	0.269346378	0.268	0.12	2.23E-05	C3_CD8_Tem
chr20-53835364-53835846	1.53E-11	0.288302751	0.233	0.096	1.02E-06	C3_CD8_Tem
chr14-89773254-89774387	2.02E-12	0.274944127	0.233	0.101	1.34E-07	C3_CD8_Tem
chr15-74383546-74385446	1.07E-25	0.37353857	0.381	0.142	7.05E-21	C3_CD8_Tem
chr2-223759699-223762024	2.68E-16	0.27363561	0.406	0.197	1.77E-11	C3_CD8_Tem
chr13-99289490-99290186	1.64E-20	0.342869047	0.296	0.11	1.09E-15	C3_CD8_Tem
chr3-149003738-149004386	1.80E-11	0.278210872	0.206	0.078	1.19E-06	C3_CD8_Tem
chr1-221801931-221802412	2.96E-10	0.265768323	0.116	0.032	1.96E-05	C3_CD8_Tem
chr19-10523026-10523671	3.62E-22	0.362601431	0.249	0.081	2.40E-17	C3_CD8_Tem
chr12-10293146-10293990	3.24E-10	0.289748218	0.167	0.057	2.15E-05	C3_CD8_Tem

chr1-42936414-42937495	3.79E-24	0.358301761	0.415	0.165	2.51E-19	C3_CD8_Tem
chr16-27449378-27451209	1.46E-19	0.311707592	0.462	0.213	9.65E-15	C3_CD8_Tem
chr10-100370276-100371692	1.21E-19	0.29142132	0.549	0.278	8.03E-15	C3_CD8_Tem
chr6-42367126-42368498	1.27E-09	0.282587569	0.216	0.087	8.43E-05	C3_CD8_Tem
chr14-106506652-106507782	1.00E-26	0.308199789	0.633	0.31	6.64E-22	C3_CD8_Tem
chr18-58541077-58542576	9.12E-13	0.293897681	0.323	0.144	6.04E-08	C3_CD8_Tem
chr13-46320538-46321976	2.10E-08	0.264000479	0.185	0.073	0.001390168	C3_CD8_Tem
chr5-132662966-132664463	5.50E-13	0.301207492	0.219	0.085	3.64E-08	C3_CD8_Tem
chr1-221793265-221794674	1.70E-18	0.316785209	0.286	0.109	1.13E-13	C3_CD8_Tem
chr11-64331142-64332138	5.31E-24	0.369313219	0.355	0.132	3.51E-19	C3_CD8_Tem
chr14-50789772-50790133	4.33E-20	0.341753264	0.103	0.017	2.87E-15	C3_CD8_Tem
chr3-149009270-149010325	2.34E-20	0.317586123	0.408	0.175	1.55E-15	C3_CD8_Tem
chr3-56732303-56733288	3.42E-10	0.2742741	0.209	0.084	2.26E-05	C3_CD8_Tem
chr11-72736269-72737114	4.66E-17	0.309129555	0.38	0.169	3.08E-12	C3_CD8_Tem
chr12-128795987-128796930	1.43E-11	0.278969027	0.207	0.079	9.45E-07	C3_CD8_Tem
chr16-56278591-56279579	1.18E-14	0.311332413	0.261	0.101	7.82E-10	C3_CD8_Tem
chr1-25064358-25066301	5.55E-38	0.433033475	0.41	0.137	3.67E-33	C3_CD8_Tem
chr10-104333173-104333562	3.11E-17	0.329641239	0.213	0.073	2.06E-12	C3_CD8_Tem
chr19-40143503-40144657	1.26E-08	0.256611192	0.182	0.074	0.000836321	C3_CD8_Tem
chr3-183349015-183350695	5.19E-33	0.390051118	0.444	0.172	3.44E-28	C3_CD8_Tem
chr9-114382742-114383228	3.20E-08	0.255858852	0.223	0.1	0.002120821	C3_CD8_Tem
chr20-49775927-49776688	1.54E-10	0.281296631	0.171	0.061	1.02E-05	C3_CD8_Tem
chr20-38872677-38873721	7.69E-24	0.287861607	0.618	0.321	5.09E-19	C3_CD8_Tem
chr16-87853497-87854843	4.52E-23	0.279800603	0.638	0.347	2.99E-18	C3_CD8_Tem
chr14-61351428-61351787	2.47E-11	0.266062127	0.111	0.031	1.64E-06	C3_CD8_Tem
chr14-63524317-63525177	1.44E-11	0.267918826	0.117	0.036	9.55E-07	C3_CD8_Tem
chr6-129671652-129673596	9.81E-10	0.255569803	0.384	0.19	6.49E-05	C3_CD8_Tem

chr5-139389577-139390346	1.20E-13	0.269309629	0.428	0.216	7.94E-09	C3_CD8_Tem
chr7-36787715-36788380	1.47E-21	0.360292294	0.304	0.112	9.73E-17	C3_CD8_Tem
chr14-24633431-24634610	4.78E-11	0.265266542	0.267	0.119	3.17E-06	C3_CD8_Tem
chr11-118227514-118228683	8.63E-11	0.282074668	0.331	0.155	5.71E-06	C3_CD8_Tem
chr20-51499996-51501369	6.06E-36	0.343950349	0.656	0.318	4.02E-31	C3_CD8_Tem
chr6-2861395-2861985	2.79E-44	0.481999437	0.373	0.104	1.85E-39	C3_CD8_Tem
chr3-183377312-183378294	4.72E-15	0.297451915	0.119	0.029	3.12E-10	C3_CD8_Tem
chr18-3511535-3512424	9.03E-27	0.391494761	0.323	0.113	5.98E-22	C3_CD8_Tem
chr7-35722248-35723372	1.77E-33	0.386760073	0.51	0.212	1.17E-28	C3_CD8_Tem
chr19-4108531-4109469	1.16E-18	0.314097828	0.354	0.151	7.65E-14	C3_CD8_Tem
chr1-160751298-160751835	3.20E-32	0.442304247	0.305	0.085	2.12E-27	C3_CD8_Tem
chr1-16122578-16124085	3.20E-15	0.308181314	0.167	0.055	2.12E-10	C3_CD8_Tem
chrX-13691803-13692928	2.08E-13	0.305616825	0.188	0.065	1.38E-08	C3_CD8_Tem
chr17-74470999-74471668	5.10E-21	0.369196458	0.191	0.055	3.38E-16	C3_CD8_Tem
chr6-41531602-41532559	6.72E-18	0.323177271	0.314	0.124	4.45E-13	C3_CD8_Tem
chr18-44729308-44730171	1.02E-13	0.28652906	0.212	0.08	6.73E-09	C3_CD8_Tem
chr22-27875514-27876372	9.03E-22	0.35920888	0.171	0.045	5.98E-17	C3_CD8_Tem
chr22-39080163-39080852	4.21E-16	0.327873614	0.231	0.081	2.79E-11	C3_CD8_Tem
chr1-248853194-248854037	7.94E-18	0.346411768	0.176	0.05	5.26E-13	C3_CD8_Tem
chr2-97720946-97721561	1.07E-11	0.287447889	0.202	0.077	7.10E-07	C3_CD8_Tem
chr20-32284843-32286307	1.31E-21	0.333058728	0.472	0.221	8.69E-17	C3_CD8_Tem
chr11-35334234-35335006	8.70E-36	0.440834746	0.297	0.082	5.76E-31	C3_CD8_Tem
chr20-38874619-38875891	4.14E-20	0.315410152	0.468	0.217	2.74E-15	C3_CD8_Tem
chr17-3794854-3797125	3.96E-50	0.31978906	0.826	0.443	2.62E-45	C3_CD8_Tem
chr1-206944539-206945182	1.46E-14	0.281059945	0.102	0.025	9.65E-10	C3_CD8_Tem
chr1-3809711-3810580	3.10E-15	0.291488146	0.114	0.029	2.05E-10	C3_CD8_Tem
chr22-22737509-22738033	1.90E-21	0.364490198	0.147	0.032	1.26E-16	C3_CD8_Tem

chr19-4104223-4105353	8.93E-19	0.281197098	0.55	0.279	5.92E-14	C3_CD8_Tem
chr19-44760126-44761340	1.86E-35	0.431430522	0.374	0.127	1.23E-30	C3_CD8_Tem
chr9-99052671-99053378	3.37E-10	0.256863971	0.101	0.028	2.23E-05	C3_CD8_Tem
chr1-84156143-84156635	1.95E-08	0.256201845	0.139	0.051	0.00129395	C3_CD8_Tem
chr20-51530978-51531758	1.05E-21	0.272450294	0.62	0.319	6.94E-17	C3_CD8_Tem
chr17-36089888-36090742	7.29E-18	0.335586743	0.237	0.083	4.83E-13	C3_CD8_Tem
chr6-143396717-143398041	1.44E-16	0.299756567	0.271	0.109	9.52E-12	C3_CD8_Tem
chr2-127080441-127082110	3.41E-13	0.26395076	0.469	0.244	2.26E-08	C3_CD8_Tem
chr17-36188180-36189273	4.67E-50	0.416924784	0.624	0.245	3.09E-45	C3_CD8_Tem
chr3-73028827-73029647	2.80E-13	0.284680323	0.212	0.084	1.85E-08	C3_CD8_Tem
chr2-230893993-230894713	3.66E-12	0.260430917	0.08	0.018	2.42E-07	C3_CD8_Tem
chr5-148844559-148845169	3.01E-28	0.386396086	0.37	0.136	1.99E-23	C3_CD8_Tem
chr18-24018330-24018559	1.30E-12	0.285418282	0.112	0.031	8.64E-08	C3_CD8_Tem
chr21-44064889-44066026	2.28E-27	0.323997428	0.578	0.276	1.51E-22	C3_CD8_Tem
chr19-45667368-45669445	1.14E-22	0.305924631	0.53	0.265	7.55E-18	C3_CD8_Tem
chr18-22260170-22260899	4.68E-13	0.263661221	0.08	0.018	3.10E-08	C3_CD8_Tem
chr6-157143009-157143795	5.26E-14	0.305627524	0.212	0.08	3.48E-09	C3_CD8_Tem
chr15-77012654-77014551	3.95E-13	0.25118743	0.518	0.277	2.61E-08	C3_CD8_Tem
chr1-91124279-91125427	4.32E-54	0.502427996	0.41	0.119	2.86E-49	C3_CD8_Tem
chr19-43764828-43766041	1.50E-11	0.273349812	0.396	0.196	9.94E-07	C3_CD8_Tem
chr9-114363274-114363843	9.18E-14	0.309387839	0.195	0.068	6.08E-09	C3_CD8_Tem
chr22-38873763-38874357	1.39E-16	0.296612656	0.439	0.212	9.21E-12	C3_CD8_Tem
chr12-70049371-70050399	1.24E-14	0.280967823	0.208	0.08	8.18E-10	C3_CD8_Tem
chr2-203763279-203764214	1.00E-20	0.354424902	0.18	0.05	6.65E-16	C3_CD8_Tem
chr7-925609-926009	1.78E-23	0.379452453	0.289	0.1	1.18E-18	C3_CD8_Tem
chr2-46239011-46240224	8.07E-14	0.292174301	0.32	0.136	5.34E-09	C3_CD8_Tem
chr16-87874680-87875518	5.21E-11	0.283991996	0.205	0.081	3.45E-06	C3_CD8_Tem

chr19-8573198-8573976	1.58E-15	0.30605099	0.348	0.146	1.05E-10	C3_CD8_Tem
chr19-8575400-8576840	6.78E-08	0.26514789	0.301	0.142	0.004487289	C3_CD8_Tem
chr11-134228632-134229052	1.07E-09	0.250072987	0.13	0.046	7.11E-05	C3_CD8_Tem
chr3-46441790-46442333	2.15E-11	0.261360091	0.114	0.035	1.42E-06	C3_CD8_Tem
chr1-200154033-200154586	1.36E-15	0.291405767	0.288	0.115	9.01E-11	C3_CD8_Tem
chr3-58470866-58471787	5.76E-15	0.314580907	0.188	0.064	3.81E-10	C3_CD8_Tem
chr1-3786925-3788055	6.37E-57	0.494023052	0.513	0.167	4.22E-52	C3_CD8_Tem
chr12-68118976-68120277	7.97E-28	0.374996837	0.413	0.161	5.28E-23	C3_CD8_Tem
chr19-13043761-13044879	1.20E-65	0.532305291	0.473	0.134	7.98E-61	C3_CD8_Tem
chr10-50511142-50512258	2.29E-11	0.285579656	0.233	0.095	1.52E-06	C3_CD8_Tem
chr10-22634856-22635355	1.39E-34	0.441095361	0.341	0.102	9.18E-30	C3_CD8_Tem
chr18-51191044-51191652	2.93E-18	0.339014905	0.271	0.1	1.94E-13	C3_CD8_Tem
chrX-154541768-154543075	4.31E-10	0.273670653	0.151	0.051	2.85E-05	C3_CD8_Tem
chr7-36723790-36724702	4.64E-11	0.254980846	0.295	0.137	3.08E-06	C3_CD8_Tem
chr3-188339243-188340186	8.63E-11	0.271056748	0.216	0.092	5.71E-06	C3_CD8_Tem
chr6-20708291-20708750	1.05E-13	0.291371228	0.123	0.034	6.95E-09	C3_CD8_Tem
chr14-101712387-101713961	4.40E-25	0.331055306	0.465	0.196	2.91E-20	C3_CD8_Tem
chr12-10602523-10603301	1.00E-14	0.305020708	0.13	0.036	6.64E-10	C3_CD8_Tem
chr9-89501607-89502643	6.78E-42	0.369161445	0.644	0.285	4.49E-37	C3_CD8_Tem
chr2-203781820-203782629	4.27E-12	0.295677554	0.205	0.078	2.83E-07	C3_CD8_Tem
chr5-52775599-52776556	6.11E-10	0.259784933	0.217	0.087	4.05E-05	C3_CD8_Tem
chr17-83083303-83084182	4.80E-38	0.35499318	0.626	0.292	3.18E-33	C3_CD8_Tem
chr7-6220925-6222411	1.28E-20	0.316811857	0.377	0.167	8.49E-16	C3_CD8_Tem
chr8-29060438-29061827	2.25E-27	0.370211966	0.431	0.173	1.49E-22	C3_CD8_Tem
chr9-114369205-114370071	1.39E-35	0.37048113	0.545	0.236	9.19E-31	C3_CD8_Tem
chr3-126607119-126607778	1.73E-16	0.31078065	0.164	0.05	1.15E-11	C3_CD8_Tem
chr20-5719626-5720622	1.59E-17	0.344589935	0.239	0.081	1.05E-12	C3_CD8_Tem

chrX-39859781-39860844	7.69E-18	0.291851179	0.462	0.216	5.09E-13	C3_CD8_Tem
chr9-114364129-114364520	1.63E-08	0.258790124	0.155	0.059	0.001078573	C3_CD8_Tem
chr2-98835548-98836629	2.73E-25	0.351327571	0.436	0.185	1.81E-20	C3_CD8_Tem
chr7-105848750-105849993	2.74E-29	0.374590124	0.447	0.18	1.81E-24	C3_CD8_Tem
chr14-24206945-24207575	1.14E-18	0.317190718	0.103	0.02	7.58E-14	C3_CD8_Tem
chr8-100415528-100417352	9.27E-46	0.421102122	0.594	0.234	6.14E-41	C3_CD8_Tem
chr17-2192183-2193154	6.50E-15	0.323821716	0.271	0.104	4.31E-10	C3_CD8_Tem
chr11-1771996-1772953	5.90E-14	0.295385461	0.307	0.136	3.91E-09	C3_CD8_Tem
chr15-31341177-31342073	1.67E-15	0.311379023	0.271	0.108	1.11E-10	C3_CD8_Tem
chr20-62087952-62088847	6.59E-12	0.28478533	0.172	0.063	4.36E-07	C3_CD8_Tem
chr3-27905361-27906046	1.17E-22	0.382435358	0.24	0.067	7.72E-18	C3_CD8_Tem
chr8-29407902-29408865	2.14E-25	0.314735912	0.586	0.28	1.42E-20	C3_CD8_Tem
chr22-22721908-22722289	7.42E-19	0.338468428	0.121	0.025	4.92E-14	C3_CD8_Tem
chr2-86037206-86039136	4.83E-26	0.272752524	0.716	0.399	3.20E-21	C3_CD8_Tem
chr3-46557742-46558819	2.44E-11	0.290695184	0.273	0.115	1.62E-06	C3_CD8_Tem
chr16-81615639-81617046	3.59E-15	0.263258361	0.509	0.262	2.38E-10	C3_CD8_Tem
chr5-155768313-155768661	5.18E-17	0.314730816	0.113	0.026	3.43E-12	C3_CD8_Tem
chr17-80265206-80265894	4.33E-21	0.342053573	0.403	0.172	2.86E-16	C3_CD8_Tem
chr2-37599387-37600389	8.38E-13	0.26830567	0.378	0.183	5.55E-08	C3_CD8_Tem
chr12-94243950-94245220	5.48E-19	0.34107432	0.201	0.065	3.63E-14	C3_CD8_Tem
chrX-9351156-9352959	3.77E-12	0.276420053	0.312	0.144	2.50E-07	C3_CD8_Tem
chr2-144382526-144383320	2.27E-13	0.290292596	0.22	0.085	1.50E-08	C3_CD8_Tem
chr7-100353226-100354065	6.42E-22	0.376434593	0.179	0.044	4.25E-17	C3_CD8_Tem
chr17-8958263-8958907	5.78E-15	0.332340533	0.27	0.101	3.83E-10	C3_CD8_Tem
chr1-221829155-221830135	2.61E-19	0.308853757	0.443	0.199	1.73E-14	C3_CD8_Tem
chr9-123304331-123305414	1.31E-38	0.427704352	0.435	0.155	8.66E-34	C3_CD8_Tem
chr11-11995396-11996007	1.49E-14	0.289835809	0.109	0.027	9.84E-10	C3_CD8_Tem

chr17-49217767-49218568	1.39E-11	0.273113856	0.312	0.145	9.20E-07	C3_CD8_Tem
chr16-87865820-87867009	1.24E-11	0.254895679	0.411	0.217	8.20E-07	C3_CD8_Tem
chr19-16586712-16587917	1.29E-10	0.257293176	0.43	0.224	8.56E-06	C3_CD8_Tem
chr3-56789605-56790716	1.16E-10	0.275490456	0.307	0.141	7.65E-06	C3_CD8_Tem
chr1-154790841-154792148	1.66E-23	0.353665017	0.438	0.184	1.10E-18	C3_CD8_Tem
chr1-100712436-100713975	1.05E-25	0.379773806	0.275	0.084	6.97E-21	C3_CD8_Tem
chr17-27471322-27472502	4.31E-20	0.301049507	0.49	0.241	2.86E-15	C3_CD8_Tem
chr11-118911875-118912806	2.16E-14	0.3033642	0.353	0.161	1.43E-09	C3_CD8_Tem
chr10-79025064-79025989	2.92E-15	0.297463897	0.23	0.085	1.93E-10	C3_CD8_Tem
chr19-35445789-35446564	3.95E-11	0.270214133	0.131	0.043	2.62E-06	C3_CD8_Tem
chr19-10516551-10517154	4.41E-14	0.296845319	0.118	0.035	2.92E-09	C3_CD8_Tem
chr2-144659399-144660199	2.74E-16	0.293491407	0.319	0.136	1.81E-11	C3_CD8_Tem
chr14-24673052-24674691	3.77E-13	0.252283253	0.491	0.26	2.50E-08	C3_CD8_Tem
chr5-68343379-68343949	4.00E-09	0.25722865	0.15	0.055	0.000265038	C3_CD8_Tem
chr14-61502498-61503850	9.10E-12	0.291953106	0.323	0.144	6.03E-07	C3_CD8_Tem
chr17-49714993-49716355	1.92E-15	0.255654534	0.567	0.308	1.27E-10	C3_CD8_Tem
chr9-133369546-133371849	1.79E-32	0.33146063	0.668	0.334	1.18E-27	C3_CD8_Tem
chr3-47016683-47017556	1.04E-13	0.290869132	0.353	0.16	6.91E-09	C3_CD8_Tem
chr6-152634627-152635124	1.58E-12	0.285626062	0.196	0.075	1.05E-07	C3_CD8_Tem
chr16-56938222-56939605	3.61E-17	0.326778475	0.336	0.139	2.39E-12	C3_CD8_Tem
chr11-359233-359635	2.07E-10	0.275808723	0.163	0.057	1.37E-05	C3_CD8_Tem
chr18-50356204-50357547	1.15E-08	0.265514992	0.146	0.055	0.000761366	C3_CD8_Tem
chr7-2698362-2699465	9.39E-18	0.332212239	0.304	0.12	6.22E-13	C3_CD8_Tem
chr1-53600208-53600946	6.00E-18	0.328170646	0.168	0.052	3.98E-13	C3_CD8_Tem
chr6-32945173-32945747	2.07E-45	0.511800555	0.262	0.052	1.37E-40	C3_CD8_Tem
chr22-44180249-44182890	1.91E-18	0.294148173	0.527	0.277	1.26E-13	C3_CD8_Tem
chr17-75864256-75865335	1.56E-15	0.310979547	0.325	0.136	1.03E-10	C3_CD8_Tem

chr1-221800726-221801674	2.53E-16	0.304737847	0.226	0.086	1.68E-11	C3_CD8_Tem
chr2-241762258-241764409	1.19E-29	0.284367306	0.758	0.398	7.91E-25	C3_CD8_Tem
chr17-35519463-35520065	2.27E-23	0.395364217	0.186	0.046	1.50E-18	C3_CD8_Tem
chrX-155997179-155997901	1.64E-28	0.392014888	0.38	0.143	1.09E-23	C3_CD8_Tem
chr20-51410666-51411119	2.79E-10	0.267907735	0.139	0.047	1.85E-05	C3_CD8_Tem
chr8-29035430-29036887	1.99E-15	0.326588547	0.185	0.061	1.32E-10	C3_CD8_Tem
chr7-29196076-29197235	2.15E-08	0.254171205	0.172	0.066	0.001420842	C3_CD8_Tem
chr18-12920744-12921812	4.19E-23	0.349518852	0.278	0.101	2.77E-18	C3_CD8_Tem
chr12-6069234-6069891	1.99E-20	0.357593272	0.205	0.062	1.32E-15	C3_CD8_Tem
chr10-70610194-70611322	3.78E-16	0.313360208	0.228	0.082	2.50E-11	C3_CD8_Tem
chr12-10497396-10498070	8.06E-14	0.292194153	0.142	0.046	5.34E-09	C3_CD8_Tem
chr1-172697419-172699598	1.16E-16	0.283008087	0.542	0.279	7.70E-12	C3_CD8_Tem
chr19-38683391-38684667	7.49E-15	0.29083139	0.44	0.218	4.96E-10	C3_CD8_Tem
chr17-35160382-35161809	9.81E-10	0.28320615	0.285	0.129	6.50E-05	C3_CD8_Tem
chr13-30157033-30157717	5.77E-11	0.261222373	0.105	0.028	3.82E-06	C3_CD8_Tem
chr15-50259969-50260854	1.44E-10	0.279423774	0.19	0.072	9.52E-06	C3_CD8_Tem
chr5-142977014-142978026	5.85E-13	0.289093989	0.365	0.169	3.87E-08	C3_CD8_Tem
chr22-23137252-23138634	3.05E-12	0.291712609	0.3	0.129	2.02E-07	C3_CD8_Tem
chr9-123338822-123340686	1.03E-09	0.252663628	0.364	0.175	6.84E-05	C3_CD8_Tem
chr20-51480265-51481961	7.55E-18	0.333289629	0.331	0.138	5.00E-13	C3_CD8_Tem
chr15-78044354-78044820	3.47E-10	0.263806929	0.224	0.096	2.30E-05	C3_CD8_Tem
chr19-1939203-1939466	1.14E-09	0.264922066	0.138	0.049	7.52E-05	C3_CD8_Tem
chr1-206924605-206925631	6.27E-10	0.27264395	0.221	0.094	4.15E-05	C3_CD8_Tem
chr6-17632973-17634086	1.73E-12	0.301077952	0.184	0.066	1.15E-07	C3_CD8_Tem
chr15-69713592-69714665	1.31E-12	0.262223333	0.491	0.25	8.65E-08	C3_CD8_Tem
chr19-10963238-10963842	1.36E-17	0.338987601	0.248	0.09	9.03E-13	C3_CD8_Tem
chr7-149620902-149622091	1.09E-15	0.297993996	0.369	0.166	7.22E-11	C3_CD8_Tem

chr5-142866687-142867319	2.08E-08	0.253613565	0.141	0.051	0.001376956	C3_CD8_Tem
chr11-122842844-122844041	1.12E-44	0.440595383	0.474	0.168	7.45E-40	C3_CD8_Tem
chr17-27568723-27569557	3.50E-12	0.276850581	0.138	0.045	2.32E-07	C3_CD8_Tem
chr6-193662-194430	8.75E-23	0.365903248	0.352	0.13	5.79E-18	C3_CD8_Tem
chr11-35247140-35248293	4.62E-25	0.312274251	0.519	0.246	3.06E-20	C3_CD8_Tem
chr4-10108841-10109483	2.11E-10	0.260218355	0.306	0.145	1.40E-05	C3_CD8_Tem
chr9-131627343-131628902	6.83E-36	0.364028761	0.591	0.258	4.52E-31	C3_CD8_Tem
chr1-221839379-221840266	7.56E-36	0.367484178	0.549	0.237	5.00E-31	C3_CD8_Tem
chr20-48757937-48758583	1.64E-18	0.347472543	0.249	0.085	1.09E-13	C3_CD8_Tem
chr15-20353048-20354546	9.15E-17	0.321601588	0.314	0.126	6.06E-12	C3_CD8_Tem
chr6-13453743-13455250	6.90E-47	0.401473649	0.635	0.271	4.57E-42	C3_CD8_Tem
chr19-51393858-51395772	2.29E-20	0.275094357	0.653	0.357	1.51E-15	C3_CD8_Tem
chr3-15309624-15311172	1.12E-12	0.270348274	0.363	0.173	7.39E-08	C3_CD8_Tem
chr1-26377507-26378830	6.34E-21	0.258385109	0.638	0.347	4.20E-16	C3_CD8_Tem
chr7-36686279-36686888	2.95E-18	0.344426857	0.233	0.078	1.96E-13	C3_CD8_Tem
chr2-68378735-68379810	6.82E-26	0.399492165	0.254	0.076	4.51E-21	C3_CD8_Tem
chr20-5056444-5057393	3.39E-18	0.308475238	0.375	0.165	2.24E-13	C3_CD8_Tem
chr2-144519981-144520278	5.11E-09	0.253106812	0.135	0.046	0.000338417	C3_CD8_Tem
chr12-10378752-10379115	1.92E-12	0.278963727	0.083	0.017	1.27E-07	C3_CD8_Tem
chr20-50109921-50110653	1.17E-12	0.286381704	0.314	0.136	7.76E-08	C3_CD8_Tem
chr11-61164059-61164543	2.93E-14	0.285395815	0.111	0.03	1.94E-09	C3_CD8_Tem
chr1-235998615-235999965	2.23E-13	0.250960428	0.523	0.279	1.47E-08	C3_CD8_Tem
chr20-36644017-36645047	4.64E-18	0.333276443	0.38	0.164	3.08E-13	C3_CD8_Tem
chr17-12981952-12982730	3.67E-21	0.361095882	0.195	0.058	2.43E-16	C3_CD8_Tem
chr14-54435240-54436060	5.42E-11	0.272121646	0.264	0.119	3.59E-06	C3_CD8_Tem
chr19-6521764-6522766	2.22E-25	0.362325047	0.391	0.155	1.47E-20	C3_CD8_Tem
chr19-41935809-41936940	2.94E-53	0.451628491	0.572	0.215	1.95E-48	C3_CD8_Tem

chr1-174153151-174153920	8.82E-18	0.346776212	0.224	0.078	5.84E-13	C3_CD8_Tem
chr7-55569466-55570515	1.22E-12	0.293827793	0.286	0.123	8.07E-08	C3_CD8_Tem
chr9-126329407-126330214	9.88E-15	0.310227972	0.146	0.045	6.54E-10	C3_CD8_Tem
chr16-75620163-75620836	1.19E-45	0.463922187	0.457	0.147	7.89E-41	C3_CD8_Tem
chr2-61662962-61663425	1.62E-16	0.31924819	0.117	0.027	1.08E-11	C3_CD8_Tem
chr1-114340814-114342699	6.78E-12	0.252558639	0.425	0.214	4.49E-07	C3_CD8_Tem
chr6-42417154-42418179	1.39E-18	0.357594711	0.237	0.077	9.18E-14	C3_CD8_Tem
chr11-64864425-64866374	1.76E-10	0.277409589	0.411	0.208	1.17E-05	C3_CD8_Tem
chr19-10111953-10113270	4.56E-15	0.277720507	0.479	0.232	3.02E-10	C3_CD8_Tem
chr3-47019209-47019536	3.69E-10	0.27910412	0.161	0.057	2.45E-05	C3_CD8_Tem
chr3-39267373-39268496	1.01E-20	0.325331127	0.404	0.17	6.68E-16	C3_CD8_Tem
chr6-159061930-159063226	1.57E-11	0.257224883	0.422	0.21	1.04E-06	C3_CD8_Tem
chr21-34800971-34801962	1.30E-19	0.342458911	0.184	0.054	8.61E-15	C3_CD8_Tem
chr20-49794549-49795270	1.69E-14	0.305170652	0.155	0.049	1.12E-09	C3_CD8_Tem
chr4-1176639-1177899	7.19E-15	0.314598375	0.219	0.077	4.76E-10	C3_CD8_Tem
chr2-159225953-159226543	6.26E-12	0.277930204	0.19	0.071	4.15E-07	C3_CD8_Tem
chr19-1030247-1031629	7.09E-12	0.251777744	0.508	0.274	4.69E-07	C3_CD8_Tem
chr3-46359594-46360492	3.94E-21	0.331783905	0.395	0.168	2.61E-16	C3_CD8_Tem
chr17-3770352-3771391	7.47E-11	0.260779603	0.144	0.05	4.95E-06	C3_CD8_Tem
chr2-43139720-43140267	8.40E-10	0.269778538	0.186	0.075	5.56E-05	C3_CD8_Tem
chr16-30873288-30873655	1.64E-14	0.317514599	0.157	0.047	1.08E-09	C3_CD8_Tem
chr17-3763177-3763727	1.41E-19	0.344452314	0.374	0.148	9.35E-15	C3_CD8_Tem
chr7-50367163-50367968	4.47E-11	0.275621261	0.248	0.11	2.96E-06	C3_CD8_Tem
chr19-45087196-45087984	7.64E-29	0.359524422	0.496	0.213	5.06E-24	C3_CD8_Tem
chr7-2037211-2038557	2.38E-18	0.345271934	0.195	0.06	1.57E-13	C3_CD8_Tem
chr17-2353063-2354351	1.17E-13	0.309990323	0.312	0.136	7.76E-09	C3_CD8_Tem
chr16-57539002-57539356	9.55E-14	0.325262375	0.224	0.081	6.32E-09	C3_CD8_Tem

chr7-35729052-35730816	2.32E-22	0.333740299	0.439	0.185	1.54E-17	C3_CD8_Tem
chr14-106184342-106186119	6.45E-21	0.321910755	0.48	0.221	4.27E-16	C3_CD8_Tem
chr1-25035751-25036614	1.56E-12	0.269513856	0.259	0.113	1.03E-07	C3_CD8_Tem
chr4-36281030-36281699	1.97E-26	0.399794995	0.311	0.099	1.30E-21	C3_CD8_Tem
chr21-34086507-34087276	1.41E-25	0.378901592	0.207	0.057	9.35E-21	C3_CD8_Tem
chr17-77783640-77784842	6.29E-15	0.281308734	0.385	0.182	4.16E-10	C3_CD8_Tem
chr9-63816618-63818431	8.63E-28	0.338257044	0.549	0.256	5.71E-23	C3_CD8_Tem
chr10-84330139-84330644	3.31E-17	0.274338983	0.455	0.226	2.19E-12	C3_CD8_Tem
chr3-98563422-98564975	2.87E-21	0.307567671	0.471	0.229	1.90E-16	C3_CD8_Tem
chr1-160755624-160757066	2.73E-27	0.399878784	0.317	0.111	1.81E-22	C3_CD8_Tem
chr17-35889125-35891509	9.75E-11	0.264836981	0.372	0.178	6.46E-06	C3_CD8_Tem
chr2-29010912-29012188	1.22E-25	0.303148088	0.611	0.311	8.06E-21	C3_CD8_Tem
chr3-4350816-4351893	9.55E-14	0.298023894	0.262	0.109	6.33E-09	C3_CD8_Tem
chr8-100501616-100503037	5.97E-10	0.26359668	0.283	0.126	3.95E-05	C3_CD8_Tem
chr21-46368226-46369182	1.53E-12	0.297719042	0.204	0.076	1.01E-07	C3_CD8_Tem
chr8-29664761-29665744	2.54E-10	0.273517859	0.198	0.08	1.68E-05	C3_CD8_Tem
chr7-24907220-24907930	4.96E-13	0.282268581	0.359	0.164	3.29E-08	C3_CD8_Tem
chr1-53581990-53582744	3.51E-16	0.318184182	0.165	0.049	2.33E-11	C3_CD8_Tem
chr1-53409902-53410451	1.47E-17	0.336236987	0.172	0.049	9.73E-13	C3_CD8_Tem
chr8-125125285-125126199	5.28E-14	0.301909528	0.191	0.066	3.50E-09	C3_CD8_Tem
chr5-107476616-107477202	1.11E-22	0.358656448	0.285	0.097	7.33E-18	C3_CD8_Tem
chr18-48868097-48869427	1.20E-44	0.453947307	0.431	0.147	7.92E-40	C3_CD8_Tem
chr6-161241983-161242489	6.20E-77	0.898000685	0.219	0.005	4.11E-72	C4_CD8_Teff
chr4-15961711-15962460	1.13E-52	0.754217531	0.219	0.018	7.48E-48	C4_CD8_Teff
chr1-6294018-6295206	2.67E-51	0.686733797	0.138	0.003	1.77E-46	C4_CD8_Teff
chr7-33707213-33707881	3.17E-50	0.711431595	0.18	0.009	2.10E-45	C4_CD8_Teff
chr19-10515556-10516304	6.52E-50	0.736091231	0.32	0.055	4.32E-45	C4_CD8_Teff

chr22-46289074-46289906	2.36E-49	0.733182116	0.195	0.011	1.56E-44	C4_CD8_Teff
chr4-15962971-15963912	1.03E-48	0.726301049	0.276	0.038	6.81E-44	C4_CD8_Teff
chr15-58614423-58615291	1.37E-47	0.704313767	0.161	0.007	9.10E-43	C4_CD8_Teff
chr6-161242937-161243613	1.62E-47	0.708015296	0.221	0.02	1.07E-42	C4_CD8_Teff
chr1-184386329-184388089	2.62E-47	0.634592725	0.518	0.158	1.74E-42	C4_CD8_Teff
chr2-36800224-36800983	1.46E-45	0.714318495	0.258	0.033	9.65E-41	C4_CD8_Teff
chr2-110846345-110847414	2.69E-45	0.697561137	0.315	0.065	1.78E-40	C4_CD8_Teff
chr11-134582476-134582776	3.74E-45	0.656400009	0.133	0.003	2.48E-40	C4_CD8_Teff
chr16-57646598-57647057	3.97E-45	0.677144685	0.177	0.012	2.63E-40	C4_CD8_Teff
chr4-1087278-1087798	4.46E-45	0.68478194	0.151	0.007	2.95E-40	C4_CD8_Teff
chr20-20215934-20216617	6.64E-45	0.696152012	0.188	0.014	4.40E-40	C4_CD8_Teff
chr9-131621481-131622102	8.24E-45	0.680894066	0.146	0.006	5.46E-40	C4_CD8_Teff
chr1-54807085-54807644	1.08E-44	0.685116423	0.167	0.01	7.13E-40	C4_CD8_Teff
chr8-25198436-25199354	1.37E-44	0.68473946	0.286	0.046	9.07E-40	C4_CD8_Teff
chr3-142269739-142270886	4.32E-44	0.68907647	0.297	0.052	2.86E-39	C4_CD8_Teff
chr10-77877596-77878312	1.09E-43	0.690848049	0.286	0.051	7.21E-39	C4_CD8_Teff
chr10-124641962-124642445	1.06E-42	0.681073516	0.167	0.011	7.00E-38	C4_CD8_Teff
chr9-136315890-136316920	1.76E-42	0.663730079	0.31	0.063	1.17E-37	C4_CD8_Teff
chr4-8408869-8410058	9.62E-41	0.531382313	0.612	0.278	6.37E-36	C4_CD8_Teff
chr4-7649400-7650381	3.92E-38	0.571318053	0.482	0.169	2.60E-33	C4_CD8_Teff
chr12-108332684-108333036	8.37E-38	0.586051625	0.112	0.003	5.54E-33	C4_CD8_Teff
chr12-124423792-124424175	1.09E-37	0.556436344	0.099	0.001	7.21E-33	C4_CD8_Teff
chr18-44700560-44700996	1.45E-37	0.638251565	0.221	0.032	9.63E-33	C4_CD8_Teff
chr22-46280826-46281500	2.67E-37	0.637610293	0.237	0.04	1.77E-32	C4_CD8_Teff
chr8-55843559-55845375	1.26E-36	0.622259768	0.289	0.062	8.33E-32	C4_CD8_Teff
chr3-196338092-196338771	5.09E-36	0.596888821	0.133	0.007	3.37E-31	C4_CD8_Teff
chr16-29111778-29112539	9.80E-36	0.631460868	0.216	0.031	6.49E-31	C4_CD8_Teff

chr2-27572153-27573519	1.06E-35	0.613364547	0.208	0.029	7.04E-31	C4_CD8_Teff
chr14-23663092-23663505	1.21E-35	0.550846434	0.094	0.001	7.99E-31	C4_CD8_Teff
chr16-30350358-30350838	1.02E-34	0.618554367	0.237	0.042	6.73E-30	C4_CD8_Teff
chr1-11335432-11335881	2.32E-34	0.53142997	0.089	0.001	1.54E-29	C4_CD8_Teff
chr16-82936709-82937497	2.89E-34	0.597676141	0.146	0.011	1.91E-29	C4_CD8_Teff
chr12-52871343-52871596	6.98E-34	0.565333757	0.117	0.006	4.62E-29	C4_CD8_Teff
chr20-18738034-18738493	2.11E-33	0.587790064	0.138	0.009	1.40E-28	C4_CD8_Teff
chr20-24156928-24157560	2.44E-33	0.600839954	0.185	0.024	1.62E-28	C4_CD8_Teff
chr11-86486452-86487087	3.23E-33	0.594464189	0.169	0.018	2.14E-28	C4_CD8_Teff
chr19-40687552-40687751	3.78E-33	0.553690422	0.109	0.004	2.50E-28	C4_CD8_Teff
chr10-128042170-128042547	8.88E-33	0.577506963	0.128	0.009	5.88E-28	C4_CD8_Teff
chr11-104163407-104164411	9.16E-33	0.595581315	0.201	0.028	6.07E-28	C4_CD8_Teff
chr20-24148061-24148691	9.96E-33	0.533679322	0.102	0.004	6.60E-28	C4_CD8_Teff
chr8-141152316-141153097	3.02E-32	0.586180345	0.193	0.029	2.00E-27	C4_CD8_Teff
chr7-121530116-121531141	4.44E-32	0.536595767	0.417	0.149	2.94E-27	C4_CD8_Teff
chr7-36863792-36865390	1.05E-31	0.568951444	0.268	0.065	6.96E-27	C4_CD8_Teff
chr1-36400534-36401245	2.17E-31	0.581243499	0.156	0.017	1.44E-26	C4_CD8_Teff
chr8-73168114-73168779	2.41E-31	0.56732201	0.151	0.015	1.59E-26	C4_CD8_Teff
chr11-2270658-2271271	2.51E-31	0.557167435	0.13	0.012	1.66E-26	C4_CD8_Teff
chr19-54905787-54906383	2.65E-31	0.546045501	0.109	0.006	1.76E-26	C4_CD8_Teff
chr22-20556585-20556784	3.65E-31	0.552309394	0.12	0.009	2.42E-26	C4_CD8_Teff
chr3-50465936-50466510	4.21E-31	0.55735968	0.112	0.006	2.79E-26	C4_CD8_Teff
chr9-126636966-126637873	8.06E-31	0.569231126	0.331	0.086	5.34E-26	C4_CD8_Teff
chr16-81816756-81818369	1.13E-30	0.571022834	0.167	0.021	7.47E-26	C4_CD8_Teff
chr4-158895825-158897132	1.32E-30	0.562912411	0.148	0.015	8.75E-26	C4_CD8_Teff
chr6-163750735-163751431	2.50E-30	0.525150126	0.102	0.004	1.66E-25	C4_CD8_Teff
chr18-45106137-45106769	3.42E-30	0.551608827	0.141	0.013	2.26E-25	C4_CD8_Teff

chr3-39234573-39235058	3.68E-30	0.572095806	0.177	0.025	2.44E-25	C4_CD8_Teff
chr19-3788955-3789725	3.96E-30	0.560621927	0.206	0.036	2.62E-25	C4_CD8_Teff
chr2-195649750-195650636	5.21E-30	0.55519389	0.188	0.031	3.45E-25	C4_CD8_Teff
chr1-53084389-53084876	5.31E-30	0.504061894	0.083	0.002	3.51E-25	C4_CD8_Teff
chr8-60409105-60409515	8.84E-30	0.50754606	0.089	0.003	5.86E-25	C4_CD8_Teff
chr16-17342801-17344141	1.63E-29	0.538428473	0.331	0.1	1.08E-24	C4_CD8_Teff
chr18-23422625-23423422	2.94E-29	0.550408795	0.12	0.01	1.95E-24	C4_CD8_Teff
chr1-53135352-53136106	4.30E-29	0.546020854	0.135	0.014	2.85E-24	C4_CD8_Teff
chr2-176491158-176491662	7.97E-29	0.551237888	0.125	0.012	5.28E-24	C4_CD8_Teff
chr15-94272057-94272936	9.80E-29	0.552895456	0.214	0.044	6.49E-24	C4_CD8_Teff
chr7-3107460-3107702	1.04E-28	0.505064919	0.094	0.004	6.90E-24	C4_CD8_Teff
chr12-128782326-128783599	2.58E-28	0.528596469	0.307	0.09	1.71E-23	C4_CD8_Teff
chr19-54550831-54551646	2.62E-28	0.548811586	0.151	0.02	1.74E-23	C4_CD8_Teff
chr2-85693753-85694481	3.00E-28	0.534074177	0.151	0.018	1.98E-23	C4_CD8_Teff
chr3-196357886-196358280	3.48E-28	0.524695745	0.096	0.005	2.31E-23	C4_CD8_Teff
chr5-155834127-155834990	3.85E-28	0.5236384	0.112	0.008	2.55E-23	C4_CD8_Teff
chr16-57889406-57890119	4.24E-28	0.477518393	0.086	0.003	2.81E-23	C4_CD8_Teff
chr19-12983457-12985835	6.94E-28	0.48812849	0.372	0.151	4.60E-23	C4_CD8_Teff
chr19-29263284-29263735	7.87E-28	0.513439033	0.112	0.008	5.21E-23	C4_CD8_Teff
chr7-134640813-134641492	1.27E-27	0.549362244	0.19	0.032	8.40E-23	C4_CD8_Teff
chr18-77073540-77074063	1.82E-27	0.537439645	0.198	0.034	1.21E-22	C4_CD8_Teff
chr20-17612090-17612811	2.91E-27	0.540502713	0.172	0.029	1.93E-22	C4_CD8_Teff
chr10-70601791-70603858	3.96E-27	0.46654361	0.422	0.182	2.63E-22	C4_CD8_Teff
chr7-28203798-28204143	4.65E-27	0.500447975	0.099	0.006	3.08E-22	C4_CD8_Teff
chr1-53090595-53091098	8.10E-27	0.446827011	0.073	0.001	5.36E-22	C4_CD8_Teff
chr5-157030656-157031080	9.49E-27	0.524485952	0.125	0.012	6.28E-22	C4_CD8_Teff
chr11-134348881-134349933	1.48E-26	0.513325677	0.146	0.019	9.79E-22	C4_CD8_Teff

chr10-79027120-79028053	1.64E-26	0.524587261	0.273	0.074	1.09E-21	C4_CD8_Teff
chr6-168905458-168906167	1.71E-26	0.524690589	0.141	0.018	1.13E-21	C4_CD8_Teff
chr16-87724547-87725360	1.78E-26	0.527615171	0.148	0.02	1.18E-21	C4_CD8_Teff
chr20-2865582-2866467	1.97E-26	0.504843618	0.104	0.007	1.31E-21	C4_CD8_Teff
chr17-47634861-47635166	2.00E-26	0.426454627	0.065	0.001	1.33E-21	C4_CD8_Teff
chr2-159144828-159145758	2.17E-26	0.522548627	0.151	0.02	1.44E-21	C4_CD8_Teff
chr22-20122665-20123654	2.30E-26	0.528011742	0.156	0.023	1.52E-21	C4_CD8_Teff
chr2-60954055-60954421	2.58E-26	0.516097418	0.128	0.015	1.71E-21	C4_CD8_Teff
chr22-23263385-23264547	5.32E-26	0.483286159	0.362	0.147	3.52E-21	C4_CD8_Teff
chr19-14459391-14460097	5.72E-26	0.524454138	0.195	0.04	3.79E-21	C4_CD8_Teff
chr9-86106623-86107344	6.02E-26	0.530144407	0.271	0.074	3.99E-21	C4_CD8_Teff
chr8-73167236-73167579	6.18E-26	0.504721678	0.117	0.01	4.09E-21	C4_CD8_Teff
chr20-61989751-61990109	6.31E-26	0.451604111	0.076	0.002	4.18E-21	C4_CD8_Teff
chr19-54532364-54532870	8.13E-26	0.494567229	0.102	0.007	5.38E-21	C4_CD8_Teff
chr11-120908141-120908977	1.09E-25	0.479790318	0.091	0.005	7.21E-21	C4_CD8_Teff
chr7-28200374-28200895	1.23E-25	0.467658511	0.083	0.004	8.12E-21	C4_CD8_Teff
chr8-125729895-125730351	1.33E-25	0.469756985	0.091	0.005	8.82E-21	C4_CD8_Teff
chr6-46174902-46175839	1.90E-25	0.514000017	0.253	0.064	1.26E-20	C4_CD8_Teff
chr19-49613385-49614834	1.98E-25	0.514263328	0.203	0.046	1.31E-20	C4_CD8_Teff
chr17-46019786-46020825	2.22E-25	0.494004193	0.299	0.102	1.47E-20	C4_CD8_Teff
chr12-10307683-10307956	2.39E-25	0.503233313	0.122	0.012	1.58E-20	C4_CD8_Teff
chr12-10305448-10306148	3.21E-25	0.504139537	0.138	0.018	2.13E-20	C4_CD8_Teff
chr21-33227850-33228121	3.39E-25	0.501437861	0.112	0.011	2.25E-20	C4_CD8_Teff
chr15-58517225-58518085	3.45E-25	0.493224944	0.112	0.009	2.29E-20	C4_CD8_Teff
chr4-38677485-38677764	4.69E-25	0.454487469	0.089	0.006	3.11E-20	C4_CD8_Teff
chr9-78238064-78238326	5.12E-25	0.500671737	0.125	0.013	3.39E-20	C4_CD8_Teff
chr1-27624800-27625617	5.38E-25	0.530011685	0.172	0.03	3.56E-20	C4_CD8_Teff

chr16-31364431-31365532	5.66E-25	0.524868809	0.203	0.04	3.75E-20	C4_CD8_Teff
chr2-233385348-233386049	5.91E-25	0.511132321	0.159	0.029	3.91E-20	C4_CD8_Teff
chr11-115267533-115268247	6.04E-25	0.480100275	0.107	0.008	4.00E-20	C4_CD8_Teff
chr5-54646159-54647075	6.69E-25	0.50540037	0.234	0.065	4.43E-20	C4_CD8_Teff
chr7-37657024-37657499	7.38E-25	0.431213552	0.07	0.001	4.89E-20	C4_CD8_Teff
chr12-108334194-108334419	8.42E-25	0.421037457	0.065	0.001	5.57E-20	C4_CD8_Teff
chr11-86800116-86800797	1.03E-24	0.500235757	0.146	0.02	6.80E-20	C4_CD8_Teff
chr15-58518658-58518888	1.16E-24	0.445410935	0.07	0.002	7.67E-20	C4_CD8_Teff
chr15-58331692-58332623	1.25E-24	0.488598378	0.318	0.111	8.28E-20	C4_CD8_Teff
chr5-73693614-73694468	1.60E-24	0.506765365	0.143	0.021	1.06E-19	C4_CD8_Teff
chr19-54803320-54803853	1.97E-24	0.449682476	0.083	0.004	1.30E-19	C4_CD8_Teff
chr2-144474986-144475414	2.42E-24	0.497763763	0.13	0.016	1.60E-19	C4_CD8_Teff
chr2-36798760-36799012	2.60E-24	0.437510145	0.078	0.003	1.72E-19	C4_CD8_Teff
chr12-10249665-10250337	4.43E-24	0.479243699	0.122	0.014	2.93E-19	C4_CD8_Teff
chr8-129993546-129995502	4.49E-24	0.482067471	0.294	0.103	2.97E-19	C4_CD8_Teff
chr2-144598045-144598443	4.61E-24	0.452546738	0.089	0.005	3.05E-19	C4_CD8_Teff
chr1-159825859-159827880	5.32E-24	0.486689686	0.245	0.065	3.53E-19	C4_CD8_Teff
chr17-8925655-8926140	6.22E-24	0.463517841	0.089	0.006	4.12E-19	C4_CD8_Teff
chr6-40436496-40437057	7.81E-24	0.506265669	0.143	0.02	5.17E-19	C4_CD8_Teff
chr7-74093222-74095067	1.17E-23	0.42068909	0.432	0.213	7.77E-19	C4_CD8_Teff
chr1-53127345-53127787	1.66E-23	0.432331084	0.076	0.003	1.10E-18	C4_CD8_Teff
chr12-94941416-94941993	2.18E-23	0.482769398	0.318	0.106	1.45E-18	C4_CD8_Teff
chr10-22505731-22506455	2.81E-23	0.503567229	0.18	0.034	1.86E-18	C4_CD8_Teff
chr8-25345238-25345791	2.97E-23	0.476357696	0.135	0.019	1.97E-18	C4_CD8_Teff
chr14-23660023-23660690	3.09E-23	0.469857941	0.096	0.008	2.05E-18	C4_CD8_Teff
chr5-177327484-177328163	3.61E-23	0.483007599	0.138	0.02	2.39E-18	C4_CD8_Teff
chr4-121253639-121254863	3.71E-23	0.450784769	0.375	0.146	2.46E-18	C4_CD8_Teff

chr11-115502847-115505075	4.02E-23	0.452073285	0.37	0.142	2.66E-18	C4_CD8_Teff
chr16-89821523-89821914	4.68E-23	0.488855321	0.18	0.038	3.10E-18	C4_CD8_Teff
chr6-158717301-158718256	8.65E-23	0.478113141	0.24	0.073	5.73E-18	C4_CD8_Teff
chr7-38242795-38243176	9.82E-23	0.501211033	0.182	0.04	6.50E-18	C4_CD8_Teff
chr8-124757086-124757833	1.54E-22	0.476359639	0.135	0.018	1.02E-17	C4_CD8_Teff
chr1-243507985-243508809	1.55E-22	0.479930479	0.143	0.024	1.03E-17	C4_CD8_Teff
chr11-115214665-115215206	2.14E-22	0.471149467	0.117	0.014	1.42E-17	C4_CD8_Teff
chr3-185582820-185583272	2.21E-22	0.488166207	0.177	0.036	1.46E-17	C4_CD8_Teff
chr19-10517383-10518508	2.77E-22	0.460594631	0.273	0.091	1.84E-17	C4_CD8_Teff
chr18-22465727-22466370	3.18E-22	0.48647735	0.148	0.027	2.11E-17	C4_CD8_Teff
chr19-10797000-10798246	3.51E-22	0.418848858	0.417	0.19	2.33E-17	C4_CD8_Teff
chr2-231529810-231530789	4.41E-22	0.463932109	0.24	0.084	2.92E-17	C4_CD8_Teff
chr8-25171308-25171704	4.76E-22	0.412592444	0.07	0.003	3.15E-17	C4_CD8_Teff
chr3-3109424-3110729	5.37E-22	0.484234662	0.224	0.059	3.56E-17	C4_CD8_Teff
chr20-45945960-45946761	5.86E-22	0.485848065	0.203	0.05	3.88E-17	C4_CD8_Teff
chr10-133278484-133279189	6.26E-22	0.467962852	0.245	0.079	4.14E-17	C4_CD8_Teff
chr2-85704799-85705330	7.04E-22	0.47684009	0.141	0.022	4.66E-17	C4_CD8_Teff
chr6-10751519-10752026	9.75E-22	0.485051601	0.143	0.025	6.46E-17	C4_CD8_Teff
chr6-40477130-40478225	1.00E-21	0.460187099	0.292	0.099	6.62E-17	C4_CD8_Teff
chr16-326942-327829	1.98E-21	0.413357986	0.406	0.191	1.31E-16	C4_CD8_Teff
chr12-128763410-128763767	2.31E-21	0.466823538	0.112	0.013	1.53E-16	C4_CD8_Teff
chr6-5506844-5507645	2.66E-21	0.421338494	0.078	0.005	1.76E-16	C4_CD8_Teff
chr16-57648569-57649327	3.13E-21	0.451053614	0.203	0.058	2.07E-16	C4_CD8_Teff
chr16-57667384-57668198	3.17E-21	0.399179234	0.073	0.004	2.10E-16	C4_CD8_Teff
chr2-237898491-237899081	4.35E-21	0.462151552	0.107	0.013	2.88E-16	C4_CD8_Teff
chr6-40409221-40409430	4.39E-21	0.381384091	0.057	0.001	2.90E-16	C4_CD8_Teff
chr3-13040011-13040691	4.47E-21	0.472458203	0.164	0.034	2.96E-16	C4_CD8_Teff

chr14-24678582-24680022	5.29E-21	0.442340007	0.266	0.096	3.50E-16	C4_CD8_Teff
chr1-167430678-167431750	5.33E-21	0.466086193	0.135	0.023	3.53E-16	C4_CD8_Teff
chr4-168816422-168817018	6.47E-21	0.41152624	0.073	0.004	4.28E-16	C4_CD8_Teff
chr8-141172247-141172549	8.65E-21	0.417931011	0.073	0.004	5.73E-16	C4_CD8_Teff
chr3-50428075-50428409	1.01E-20	0.428983991	0.094	0.01	6.68E-16	C4_CD8_Teff
chr18-23306676-23307338	1.11E-20	0.471603112	0.159	0.032	7.38E-16	C4_CD8_Teff
chr3-48931087-48931615	1.57E-20	0.442566904	0.112	0.015	1.04E-15	C4_CD8_Teff
chr13-113176236-113176601	1.84E-20	0.454758786	0.125	0.02	1.22E-15	C4_CD8_Teff
chr1-24856950-24857412	1.85E-20	0.448361121	0.099	0.011	1.22E-15	C4_CD8_Teff
chr3-12915858-12916481	2.22E-20	0.436649237	0.096	0.01	1.47E-15	C4_CD8_Teff
chr8-59655615-59655962	2.37E-20	0.35912991	0.057	0.001	1.57E-15	C4_CD8_Teff
chr15-89936034-89936904	2.62E-20	0.436185014	0.099	0.011	1.74E-15	C4_CD8_Teff
chr7-37657812-37658119	2.65E-20	0.380341204	0.06	0.002	1.76E-15	C4_CD8_Teff
chr12-128810142-128810932	3.67E-20	0.462867865	0.146	0.029	2.43E-15	C4_CD8_Teff
chr4-158873881-158874451	3.77E-20	0.444672677	0.102	0.011	2.50E-15	C4_CD8_Teff
chr22-23185555-23186129	3.79E-20	0.458595876	0.13	0.023	2.51E-15	C4_CD8_Teff
chr6-132612264-132612817	4.44E-20	0.402394973	0.073	0.005	2.94E-15	C4_CD8_Teff
chr1-15411633-15412455	5.08E-20	0.399354954	0.414	0.195	3.37E-15	C4_CD8_Teff
chr20-2851769-2852368	5.12E-20	0.460093528	0.148	0.029	3.39E-15	C4_CD8_Teff
chr15-94304939-94305540	5.33E-20	0.454993984	0.185	0.045	3.53E-15	C4_CD8_Teff
chr18-44678863-44681010	5.88E-20	0.41928062	0.326	0.132	3.90E-15	C4_CD8_Teff
chr16-17224454-17225255	6.57E-20	0.432211641	0.104	0.013	4.35E-15	C4_CD8_Teff
chr17-1286281-1287032	7.34E-20	0.37943851	0.065	0.003	4.86E-15	C4_CD8_Teff
chr7-98514235-98515138	7.92E-20	0.45054618	0.141	0.024	5.25E-15	C4_CD8_Teff
chr10-17853977-17854653	9.25E-20	0.445227849	0.104	0.013	6.13E-15	C4_CD8_Teff
chr17-48472024-48472803	1.03E-19	0.441184878	0.117	0.017	6.80E-15	C4_CD8_Teff
chr14-99286735-99287781	1.22E-19	0.443574107	0.174	0.041	8.05E-15	C4_CD8_Teff

chr18-79156679-79157477	1.37E-19	0.453514668	0.156	0.033	9.05E-15	C4_CD8_Teff
chr5-14108596-14110068	1.39E-19	0.442035414	0.117	0.016	9.21E-15	C4_CD8_Teff
chr20-24953770-24954170	1.69E-19	0.453435415	0.164	0.037	1.12E-14	C4_CD8_Teff
chr9-136572519-136572836	1.70E-19	0.360984765	0.055	0.001	1.12E-14	C4_CD8_Teff
chr3-16789235-16789741	1.74E-19	0.41474954	0.094	0.009	1.15E-14	C4_CD8_Teff
chr10-72941410-72942103	1.86E-19	0.429729577	0.099	0.011	1.23E-14	C4_CD8_Teff
chr1-19045071-19045791	1.93E-19	0.444495279	0.122	0.02	1.28E-14	C4_CD8_Teff
chr1-41728547-41729134	2.79E-19	0.44392428	0.125	0.02	1.84E-14	C4_CD8_Teff
chr11-86503853-86504466	2.80E-19	0.436512142	0.112	0.016	1.86E-14	C4_CD8_Teff
chr13-114108073-114108397	2.81E-19	0.447840666	0.112	0.016	1.86E-14	C4_CD8_Teff
chr9-96435774-96436570	2.96E-19	0.450393724	0.201	0.054	1.96E-14	C4_CD8_Teff
chr4-158870854-158871126	3.34E-19	0.385733134	0.062	0.003	2.21E-14	C4_CD8_Teff
chr1-55138744-55139160	3.41E-19	0.419592447	0.091	0.01	2.26E-14	C4_CD8_Teff
chr3-50429657-50430497	3.77E-19	0.401334625	0.081	0.007	2.49E-14	C4_CD8_Teff
chr2-85701487-85701818	6.34E-19	0.425207389	0.117	0.017	4.20E-14	C4_CD8_Teff
chr16-85569565-85571266	6.59E-19	0.419504794	0.255	0.092	4.37E-14	C4_CD8_Teff
chr10-110441270-110442167	6.89E-19	0.448410814	0.18	0.047	4.56E-14	C4_CD8_Teff
chr12-10303912-10304558	6.94E-19	0.44101507	0.177	0.044	4.59E-14	C4_CD8_Teff
chr15-94263108-94264045	7.80E-19	0.406690728	0.081	0.007	5.16E-14	C4_CD8_Teff
chr17-1607193-1607853	8.15E-19	0.429701142	0.117	0.019	5.40E-14	C4_CD8_Teff
chr2-239137391-239138194	8.30E-19	0.423001158	0.094	0.009	5.50E-14	C4_CD8_Teff
chr8-38768272-38770666	9.09E-19	0.337550876	0.555	0.337	6.02E-14	C4_CD8_Teff
chr6-161226089-161227237	9.22E-19	0.421994793	0.117	0.019	6.11E-14	C4_CD8_Teff
chr9-37512116-37512807	9.47E-19	0.448152946	0.167	0.036	6.27E-14	C4_CD8_Teff
chr1-244325052-244325789	9.61E-19	0.427302496	0.273	0.097	6.36E-14	C4_CD8_Teff
chr19-1896931-1898310	1.05E-18	0.39503309	0.354	0.162	6.94E-14	C4_CD8_Teff
chr1-53106453-53106790	1.19E-18	0.386015942	0.068	0.003	7.87E-14	C4_CD8_Teff

chr6-168877798-168878184	1.42E-18	0.35232479	0.055	0.002	9.39E-14	C4_CD8_Teff
chr2-119338871-119339599	1.43E-18	0.441883742	0.13	0.024	9.47E-14	C4_CD8_Teff
chr17-83080706-83081898	1.62E-18	0.395063411	0.328	0.141	1.07E-13	C4_CD8_Teff
chr2-144456679-144456927	1.74E-18	0.442832597	0.154	0.032	1.15E-13	C4_CD8_Teff
chr17-2792476-2793160	2.21E-18	0.437895688	0.214	0.064	1.46E-13	C4_CD8_Teff
chr2-191049861-191050167	3.51E-18	0.408593397	0.094	0.012	2.32E-13	C4_CD8_Teff
chr19-16256510-16257138	3.97E-18	0.427155406	0.112	0.019	2.63E-13	C4_CD8_Teff
chr14-101726547-101726808	4.93E-18	0.436011711	0.138	0.032	3.27E-13	C4_CD8_Teff
chr14-93036187-93036733	5.20E-18	0.426338578	0.112	0.017	3.44E-13	C4_CD8_Teff
chr10-22596797-22597505	5.54E-18	0.432369205	0.18	0.05	3.67E-13	C4_CD8_Teff
chr17-82257065-82257733	5.73E-18	0.434386741	0.172	0.048	3.80E-13	C4_CD8_Teff
chr22-50780259-50780463	6.13E-18	0.354460197	0.055	0.002	4.06E-13	C4_CD8_Teff
chr14-94945532-94946593	7.07E-18	0.440400511	0.164	0.04	4.68E-13	C4_CD8_Teff
chr11-130388522-130389781	7.21E-18	0.428316394	0.12	0.022	4.77E-13	C4_CD8_Teff
chr1-184491300-184492350	7.54E-18	0.427038624	0.214	0.067	4.99E-13	C4_CD8_Teff
chr19-40826622-40827364	7.69E-18	0.424752174	0.214	0.072	5.10E-13	C4_CD8_Teff
chr1-158924181-158925048	9.65E-18	0.428307417	0.154	0.036	6.39E-13	C4_CD8_Teff
chr5-157024936-157025759	9.65E-18	0.430179214	0.198	0.06	6.39E-13	C4_CD8_Teff
chr16-3818357-3819464	1.04E-17	0.407295306	0.104	0.015	6.89E-13	C4_CD8_Teff
chr17-5184249-5184741	1.13E-17	0.416254893	0.117	0.021	7.48E-13	C4_CD8_Teff
chr5-180367049-180367740	1.27E-17	0.419056775	0.138	0.028	8.42E-13	C4_CD8_Teff
chr16-85099230-85099696	1.34E-17	0.421305806	0.128	0.026	8.87E-13	C4_CD8_Teff
chr8-19461656-19462271	1.35E-17	0.429374385	0.122	0.023	8.91E-13	C4_CD8_Teff
chr1-94017157-94017843	1.38E-17	0.41839084	0.112	0.019	9.15E-13	C4_CD8_Teff
chr19-54904312-54904529	1.39E-17	0.329855947	0.052	0.002	9.18E-13	C4_CD8_Teff
chr19-51131882-51132291	1.56E-17	0.401860168	0.086	0.011	1.04E-12	C4_CD8_Teff
chr2-46369738-46370174	1.76E-17	0.37023054	0.065	0.005	1.17E-12	C4_CD8_Teff

chr8-124637275-124637986	2.60E-17	0.374109245	0.393	0.186	1.72E-12	C4_CD8_Teff
chr16-29640627-29641310	2.64E-17	0.420623278	0.128	0.025	1.75E-12	C4_CD8_Teff
chr1-175514328-175515104	2.72E-17	0.387670628	0.083	0.01	1.80E-12	C4_CD8_Teff
chr7-2902489-2903871	3.13E-17	0.420395734	0.146	0.033	2.07E-12	C4_CD8_Teff
chr2-12064177-12064536	3.56E-17	0.387147595	0.076	0.007	2.36E-12	C4_CD8_Teff
chr1-108653295-108653805	3.56E-17	0.393922002	0.099	0.015	2.36E-12	C4_CD8_Teff
chr2-207423798-207424142	3.64E-17	0.348935602	0.055	0.002	2.41E-12	C4_CD8_Teff
chr12-96473063-96473740	3.69E-17	0.421197102	0.115	0.02	2.44E-12	C4_CD8_Teff
chr12-101743658-101744460	3.69E-17	0.420742706	0.13	0.029	2.45E-12	C4_CD8_Teff
chr6-75781491-75782175	3.76E-17	0.41158291	0.112	0.018	2.49E-12	C4_CD8_Teff
chr19-52833277-52834042	3.78E-17	0.42994913	0.13	0.026	2.50E-12	C4_CD8_Teff
chr3-57671133-57671708	3.90E-17	0.41758737	0.198	0.062	2.58E-12	C4_CD8_Teff
chr4-6825019-6825950	4.08E-17	0.406901431	0.115	0.022	2.70E-12	C4_CD8_Teff
chr1-27990940-27991227	4.29E-17	0.36123991	0.06	0.003	2.84E-12	C4_CD8_Teff
chr22-46288226-46288840	4.67E-17	0.397447716	0.102	0.016	3.09E-12	C4_CD8_Teff
chr12-10285702-10286335	4.74E-17	0.411939833	0.115	0.02	3.14E-12	C4_CD8_Teff
chr5-155773428-155774034	4.99E-17	0.403711804	0.109	0.017	3.30E-12	C4_CD8_Teff
chr11-110524194-110524644	5.40E-17	0.410449302	0.117	0.022	3.58E-12	C4_CD8_Teff
chr2-144430473-144431724	5.65E-17	0.415980488	0.154	0.037	3.74E-12	C4_CD8_Teff
chr22-39960633-39961820	6.31E-17	0.375792401	0.352	0.161	4.18E-12	C4_CD8_Teff
chr3-50472751-50473378	6.42E-17	0.405279039	0.107	0.019	4.25E-12	C4_CD8_Teff
chr21-44626134-44626560	6.60E-17	0.37634779	0.078	0.008	4.37E-12	C4_CD8_Teff
chr2-98874931-98875765	6.95E-17	0.419235291	0.135	0.027	4.60E-12	C4_CD8_Teff
chr1-91713031-91713897	7.15E-17	0.417181217	0.18	0.052	4.74E-12	C4_CD8_Teff
chr14-102500096-102500678	7.36E-17	0.3960163	0.086	0.011	4.87E-12	C4_CD8_Teff
chr13-25737866-25738508	7.63E-17	0.360079532	0.062	0.004	5.05E-12	C4_CD8_Teff
chr1-182259000-182259827	7.87E-17	0.400624157	0.245	0.089	5.21E-12	C4_CD8_Teff

chr10-44310373-44311620	8.32E-17	0.408287335	0.164	0.044	5.51E-12	C4_CD8_Teff
chr16-123924-124278	8.96E-17	0.371014407	0.06	0.004	5.93E-12	C4_CD8_Teff
chr2-12948190-12949287	9.17E-17	0.414615233	0.141	0.03	6.07E-12	C4_CD8_Teff
chr16-58264802-58265790	9.96E-17	0.395433316	0.102	0.015	6.60E-12	C4_CD8_Teff
chr1-100028356-100029206	1.25E-16	0.418953192	0.174	0.047	8.26E-12	C4_CD8_Teff
chr12-55667101-55667497	1.28E-16	0.361033971	0.065	0.004	8.48E-12	C4_CD8_Teff
chr15-58313319-58313881	1.39E-16	0.394891788	0.102	0.015	9.18E-12	C4_CD8_Teff
chr15-72805422-72805797	1.41E-16	0.384148999	0.081	0.009	9.36E-12	C4_CD8_Teff
chr12-124452954-124453904	1.56E-16	0.289566423	0.646	0.415	1.03E-11	C4_CD8_Teff
chr6-40587005-40588094	1.62E-16	0.410705934	0.151	0.036	1.07E-11	C4_CD8_Teff
chr4-168877670-168878512	1.71E-16	0.368206048	0.07	0.007	1.13E-11	C4_CD8_Teff
chr19-42352176-42352961	1.88E-16	0.381177912	0.078	0.01	1.25E-11	C4_CD8_Teff
chr14-24631806-24632062	1.97E-16	0.376214848	0.081	0.008	1.30E-11	C4_CD8_Teff
chr20-5996653-5997169	2.16E-16	0.410609307	0.135	0.032	1.43E-11	C4_CD8_Teff
chr13-113185991-113186856	2.24E-16	0.397935007	0.117	0.024	1.48E-11	C4_CD8_Teff
chr2-88239366-88240177	2.31E-16	0.397020706	0.117	0.022	1.53E-11	C4_CD8_Teff
chr11-3099202-3099527	2.33E-16	0.365952022	0.068	0.005	1.54E-11	C4_CD8_Teff
chr17-17662891-17664024	2.40E-16	0.34581472	0.451	0.243	1.59E-11	C4_CD8_Teff
chr5-112417854-112418360	2.44E-16	0.417210322	0.128	0.025	1.62E-11	C4_CD8_Teff
chr18-77080211-77081050	2.62E-16	0.406987879	0.146	0.035	1.73E-11	C4_CD8_Teff
chr2-74031888-74032866	2.78E-16	0.414005478	0.164	0.05	1.84E-11	C4_CD8_Teff
chr11-13550683-13551063	2.94E-16	0.344167617	0.057	0.003	1.94E-11	C4_CD8_Teff
chr7-70660047-70660932	3.09E-16	0.40143846	0.216	0.072	2.04E-11	C4_CD8_Teff
chr12-54017291-54018413	3.24E-16	0.393901885	0.247	0.095	2.15E-11	C4_CD8_Teff
chr19-32498449-32499151	3.68E-16	0.383261049	0.107	0.016	2.44E-11	C4_CD8_Teff
chr3-129633797-129634347	3.72E-16	0.329824255	0.052	0.003	2.46E-11	C4_CD8_Teff
chr20-43781031-43781614	3.89E-16	0.367556514	0.086	0.01	2.57E-11	C4_CD8_Teff

chr20-38776288-38776623	4.01E-16	0.370321601	0.078	0.008	2.66E-11	C4_CD8_Teff
chr9-131405174-131405600	4.39E-16	0.404625956	0.115	0.023	2.91E-11	C4_CD8_Teff
chr1-55127628-55128847	4.94E-16	0.399740499	0.234	0.08	3.27E-11	C4_CD8_Teff
chr1-91731336-91732897	5.15E-16	0.401238478	0.211	0.07	3.41E-11	C4_CD8_Teff
chr19-29302526-29302855	5.31E-16	0.333211177	0.055	0.003	3.52E-11	C4_CD8_Teff
chr1-198556590-198557308	5.77E-16	0.396365283	0.185	0.056	3.82E-11	C4_CD8_Teff
chr2-8315816-8316099	6.99E-16	0.365092258	0.076	0.007	4.63E-11	C4_CD8_Teff
chr6-135624071-135624885	7.98E-16	0.408852544	0.167	0.046	5.28E-11	C4_CD8_Teff
chr15-40776599-40777186	8.73E-16	0.396897541	0.159	0.044	5.78E-11	C4_CD8_Teff
chr11-61834389-61835002	1.00E-15	0.363570566	0.086	0.011	6.64E-11	C4_CD8_Teff
chrX-124641758-124642302	1.00E-15	0.342926994	0.057	0.004	6.65E-11	C4_CD8_Teff
chr5-1503317-1503721	1.23E-15	0.384547881	0.104	0.018	8.12E-11	C4_CD8_Teff
chr11-2217156-2217452	1.43E-15	0.338076206	0.057	0.003	9.47E-11	C4_CD8_Teff
chr12-94675699-94676685	1.44E-15	0.397833776	0.195	0.06	9.56E-11	C4_CD8_Teff
chr19-17679895-17680547	1.45E-15	0.392432399	0.125	0.025	9.57E-11	C4_CD8_Teff
chr2-100278936-100279351	1.46E-15	0.365576167	0.078	0.009	9.68E-11	C4_CD8_Teff
chr8-28781891-28782316	1.52E-15	0.328483	0.055	0.003	1.01E-10	C4_CD8_Teff
chr17-83199175-83200859	1.65E-15	0.338068846	0.396	0.213	1.09E-10	C4_CD8_Teff
chr16-11613566-11614218	1.71E-15	0.381115909	0.096	0.014	1.13E-10	C4_CD8_Teff
chr7-16346063-16346786	1.77E-15	0.363626366	0.083	0.012	1.17E-10	C4_CD8_Teff
chrX-47361618-47362377	1.93E-15	0.397843238	0.195	0.063	1.28E-10	C4_CD8_Teff
chr8-124604547-124605442	2.25E-15	0.389357806	0.138	0.037	1.49E-10	C4_CD8_Teff
chr5-32241013-32241763	2.36E-15	0.38925587	0.115	0.022	1.56E-10	C4_CD8_Teff
chr2-69770122-69770552	2.51E-15	0.388414102	0.12	0.024	1.66E-10	C4_CD8_Teff
chr8-55864340-55864719	2.52E-15	0.337420427	0.057	0.003	1.67E-10	C4_CD8_Teff
chr2-28547802-28548208	2.69E-15	0.386361126	0.112	0.023	1.78E-10	C4_CD8_Teff
chr7-133483833-133484470	2.80E-15	0.406150109	0.143	0.039	1.86E-10	C4_CD8_Teff

chr6-157146935-157147209	2.88E-15	0.360316981	0.081	0.01	1.91E-10	C4_CD8_Teff
chr14-92615207-92615959	2.97E-15	0.395164613	0.208	0.073	1.97E-10	C4_CD8_Teff
chr1-41916078-41916434	3.08E-15	0.390994433	0.128	0.028	2.04E-10	C4_CD8_Teff
chr5-126816486-126816991	3.17E-15	0.368738465	0.081	0.011	2.10E-10	C4_CD8_Teff
chr10-124341685-124342217	3.28E-15	0.403882497	0.109	0.021	2.17E-10	C4_CD8_Teff
chr17-10077500-10078331	3.41E-15	0.389661785	0.125	0.025	2.26E-10	C4_CD8_Teff
chr16-81438489-81438728	4.46E-15	0.37031784	0.081	0.011	2.95E-10	C4_CD8_Teff
chr19-50003761-50004288	4.52E-15	0.357493348	0.094	0.015	3.00E-10	C4_CD8_Teff
chr10-22912663-22913849	4.85E-15	0.388417661	0.125	0.026	3.22E-10	C4_CD8_Teff
chr5-112418813-112420570	5.25E-15	0.383801741	0.13	0.03	3.48E-10	C4_CD8_Teff
chr7-159198281-159198619	5.32E-15	0.3851807	0.109	0.02	3.53E-10	C4_CD8_Teff
chr8-38936586-38938972	5.52E-15	0.284653217	0.576	0.374	3.66E-10	C4_CD8_Teff
chr2-9680883-9681755	5.65E-15	0.385680126	0.133	0.033	3.74E-10	C4_CD8_Teff
chr10-103053813-103054760	5.82E-15	0.386920903	0.208	0.076	3.86E-10	C4_CD8_Teff
chr20-23456455-23457109	5.99E-15	0.385339687	0.099	0.016	3.97E-10	C4_CD8_Teff
chr7-2049612-2050595	6.35E-15	0.350113566	0.068	0.007	4.21E-10	C4_CD8_Teff
chr2-216349913-216350784	6.43E-15	0.379151701	0.263	0.114	4.26E-10	C4_CD8_Teff
chr9-91156617-91157212	6.66E-15	0.36633309	0.081	0.012	4.41E-10	C4_CD8_Teff
chr4-169015460-169016498	7.39E-15	0.379500769	0.146	0.04	4.89E-10	C4_CD8_Teff
chr19-29293972-29294697	7.99E-15	0.334996103	0.06	0.005	5.29E-10	C4_CD8_Teff
chr22-25054537-25055508	8.10E-15	0.386689932	0.156	0.044	5.36E-10	C4_CD8_Teff
chr1-25989958-25991332	8.30E-15	0.381921793	0.154	0.046	5.50E-10	C4_CD8_Teff
chr21-33078857-33079331	8.49E-15	0.385810239	0.112	0.021	5.62E-10	C4_CD8_Teff
chr1-171805282-171806053	8.65E-15	0.359265717	0.086	0.013	5.73E-10	C4_CD8_Teff
chr3-41419153-41419716	8.67E-15	0.368059377	0.081	0.01	5.74E-10	C4_CD8_Teff
chr1-159799549-159800580	8.70E-15	0.375990328	0.12	0.026	5.76E-10	C4_CD8_Teff
chr1-101338011-101339141	9.11E-15	0.388000026	0.172	0.05	6.03E-10	C4_CD8_Teff

chr17-82548700-82548930	9.67E-15	0.328175634	0.052	0.003	6.41E-10	C4_CD8_Teff
chr10-30502118-30502877	9.77E-15	0.380404304	0.117	0.025	6.47E-10	C4_CD8_Teff
chr2-231554181-231554979	9.83E-15	0.34420496	0.07	0.007	6.51E-10	C4_CD8_Teff
chr20-24032404-24033610	1.02E-14	0.373885162	0.112	0.022	6.75E-10	C4_CD8_Teff
chr22-21689281-21690526	1.12E-14	0.382001514	0.122	0.029	7.44E-10	C4_CD8_Teff
chr14-100408099-100408812	1.14E-14	0.385140079	0.146	0.037	7.57E-10	C4_CD8_Teff
chr12-132818116-132818950	1.24E-14	0.384519061	0.125	0.031	8.18E-10	C4_CD8_Teff
chr18-44723355-44723585	1.24E-14	0.343095827	0.065	0.006	8.22E-10	C4_CD8_Teff
chr17-10081483-10082006	1.28E-14	0.367552043	0.107	0.021	8.47E-10	C4_CD8_Teff
chr17-75534867-75535588	1.42E-14	0.380019217	0.138	0.04	9.43E-10	C4_CD8_Teff
chr11-110565415-110566163	1.46E-14	0.349779212	0.073	0.008	9.67E-10	C4_CD8_Teff
chr16-81522064-81522744	1.58E-14	0.353474609	0.305	0.14	1.04E-09	C4_CD8_Teff
chr9-85743374-85743622	1.60E-14	0.377952008	0.094	0.016	1.06E-09	C4_CD8_Teff
chr9-99041176-99041801	1.70E-14	0.344728283	0.073	0.007	1.13E-09	C4_CD8_Teff
chr3-56753188-56753488	1.78E-14	0.366403551	0.094	0.015	1.18E-09	C4_CD8_Teff
chr2-8345685-8347031	1.80E-14	0.371235978	0.102	0.021	1.19E-09	C4_CD8_Teff
chr14-92568344-92569790	1.82E-14	0.377132551	0.169	0.052	1.20E-09	C4_CD8_Teff
chr19-13886722-13887896	2.02E-14	0.35464659	0.323	0.14	1.34E-09	C4_CD8_Teff
chr20-45944148-45944437	2.21E-14	0.384417277	0.13	0.031	1.46E-09	C4_CD8_Teff
chr6-43813207-43813511	2.27E-14	0.318108114	0.057	0.004	1.50E-09	C4_CD8_Teff
chr16-28309407-28310371	2.29E-14	0.354355906	0.32	0.152	1.51E-09	C4_CD8_Teff
chr6-168968129-168968581	2.37E-14	0.360691576	0.091	0.015	1.57E-09	C4_CD8_Teff
chr10-109933640-109934562	2.53E-14	0.381259363	0.167	0.05	1.68E-09	C4_CD8_Teff
chr19-38928395-38928678	2.55E-14	0.374144887	0.104	0.02	1.69E-09	C4_CD8_Teff
chr5-148887182-148887836	3.44E-14	0.333676338	0.068	0.007	2.28E-09	C4_CD8_Teff
chr9-38226982-38227814	3.46E-14	0.322261504	0.062	0.006	2.29E-09	C4_CD8_Teff
chr17-18025565-18026714	3.61E-14	0.375232458	0.164	0.05	2.39E-09	C4_CD8_Teff

chr8-56999227-56999847	3.61E-14	0.35675453	0.099	0.018	2.39E-09	C4_CD8_Teff
chr22-23278801-23279304	3.88E-14	0.374832954	0.146	0.039	2.57E-09	C4_CD8_Teff
chr14-91390079-91390524	3.91E-14	0.358638397	0.102	0.021	2.59E-09	C4_CD8_Teff
chr18-23570812-23571367	3.99E-14	0.36193858	0.104	0.022	2.64E-09	C4_CD8_Teff
chr7-132485235-132486067	4.01E-14	0.378503506	0.185	0.06	2.65E-09	C4_CD8_Teff
chr10-124670990-124672127	4.05E-14	0.370980851	0.128	0.03	2.68E-09	C4_CD8_Teff
chr9-76576235-76576755	4.09E-14	0.373488058	0.219	0.087	2.71E-09	C4_CD8_Teff
chr7-2413390-2414104	4.82E-14	0.367162552	0.245	0.09	3.19E-09	C4_CD8_Teff
chr20-24108764-24109803	4.95E-14	0.368902891	0.133	0.03	3.28E-09	C4_CD8_Teff
chr5-142929335-142929995	5.07E-14	0.369951225	0.138	0.037	3.36E-09	C4_CD8_Teff
chr17-74730383-74731073	5.31E-14	0.363378644	0.227	0.092	3.52E-09	C4_CD8_Teff
chr4-145623780-145624155	5.41E-14	0.372231463	0.122	0.029	3.58E-09	C4_CD8_Teff
chr16-85104759-85105382	5.79E-14	0.365953037	0.107	0.023	3.83E-09	C4_CD8_Teff
chr12-10311003-10311510	6.40E-14	0.325984291	0.065	0.007	4.24E-09	C4_CD8_Teff
chr12-128569992-128570692	6.45E-14	0.374176407	0.13	0.033	4.27E-09	C4_CD8_Teff
chr1-160480584-160481100	6.50E-14	0.370318341	0.091	0.016	4.30E-09	C4_CD8_Teff
chr11-6228448-6228797	6.59E-14	0.30868889	0.06	0.005	4.37E-09	C4_CD8_Teff
chr10-110156454-110157399	6.80E-14	0.372810888	0.13	0.037	4.50E-09	C4_CD8_Teff
chr6-30963694-30964716	6.88E-14	0.375843438	0.128	0.029	4.56E-09	C4_CD8_Teff
chr7-77428850-77429150	6.95E-14	0.326831484	0.06	0.005	4.60E-09	C4_CD8_Teff
chr22-37993882-37994570	7.13E-14	0.348281712	0.089	0.015	4.72E-09	C4_CD8_Teff
chr14-92726062-92726627	7.32E-14	0.3715253	0.125	0.03	4.85E-09	C4_CD8_Teff
chr17-80471993-80472202	7.34E-14	0.321264751	0.055	0.004	4.86E-09	C4_CD8_Teff
chr11-134402464-134402811	7.64E-14	0.342143988	0.068	0.006	5.06E-09	C4_CD8_Teff
chr14-54669599-54670304	7.93E-14	0.366951636	0.099	0.02	5.25E-09	C4_CD8_Teff
chr7-2102032-2102670	8.12E-14	0.344239124	0.078	0.011	5.38E-09	C4_CD8_Teff
chr18-45937914-45938992	8.87E-14	0.358100784	0.24	0.094	5.88E-09	C4_CD8_Teff

chr22-45254512-45255367	8.91E-14	0.370862491	0.156	0.049	5.90E-09	C4_CD8_Teff
chr19-38924134-38924793	9.02E-14	0.373219474	0.138	0.04	5.97E-09	C4_CD8_Teff
chr3-14377448-14378683	9.51E-14	0.366068557	0.193	0.066	6.30E-09	C4_CD8_Teff
chr7-133480899-133481198	9.59E-14	0.332013344	0.057	0.005	6.35E-09	C4_CD8_Teff
chr2-70851627-70852078	9.96E-14	0.341531	0.086	0.014	6.60E-09	C4_CD8_Teff
chr14-24329575-24330083	1.01E-13	0.359986369	0.229	0.096	6.66E-09	C4_CD8_Teff
chr7-128422793-128423306	1.02E-13	0.363922896	0.112	0.023	6.75E-09	C4_CD8_Teff
chr8-124673311-124673644	1.03E-13	0.322623107	0.057	0.005	6.83E-09	C4_CD8_Teff
chr2-233386461-233386779	1.11E-13	0.333220687	0.062	0.006	7.37E-09	C4_CD8_Teff
chr14-89466207-89467147	1.12E-13	0.369872605	0.174	0.055	7.41E-09	C4_CD8_Teff
chr7-132450906-132451511	1.15E-13	0.362332277	0.117	0.028	7.61E-09	C4_CD8_Teff
chr5-115209163-115209734	1.18E-13	0.374639354	0.151	0.043	7.79E-09	C4_CD8_Teff
chr15-76336395-76336825	1.27E-13	0.358297873	0.104	0.02	8.41E-09	C4_CD8_Teff
chr16-54928338-54929868	1.33E-13	0.334039484	0.341	0.174	8.81E-09	C4_CD8_Teff
chr10-120683857-120684576	1.33E-13	0.324820439	0.06	0.006	8.82E-09	C4_CD8_Teff
chr11-126329264-126329992	1.37E-13	0.374691547	0.151	0.038	9.08E-09	C4_CD8_Teff
chr17-43701215-43701675	1.43E-13	0.349860234	0.094	0.02	9.49E-09	C4_CD8_Teff
chr7-159195482-159196150	1.48E-13	0.368003685	0.148	0.044	9.83E-09	C4_CD8_Teff
chr17-3870861-3871325	1.53E-13	0.298644749	0.052	0.004	1.02E-08	C4_CD8_Teff
chr1-32627513-32628086	1.55E-13	0.362089899	0.13	0.035	1.02E-08	C4_CD8_Teff
chr2-144489998-144490418	1.58E-13	0.372816348	0.141	0.038	1.05E-08	C4_CD8_Teff
chr9-113516940-113517550	1.72E-13	0.361273321	0.201	0.074	1.14E-08	C4_CD8_Teff
chr8-141089450-141090133	1.84E-13	0.332154724	0.07	0.009	1.22E-08	C4_CD8_Teff
chr2-201656079-201656495	1.90E-13	0.35658176	0.096	0.018	1.26E-08	C4_CD8_Teff
chr17-10046365-10047468	1.95E-13	0.34024245	0.086	0.015	1.29E-08	C4_CD8_Teff
chr4-3879513-3880885	1.97E-13	0.357192237	0.115	0.029	1.31E-08	C4_CD8_Teff
chr2-135112138-135112811	2.08E-13	0.343115508	0.081	0.013	1.38E-08	C4_CD8_Teff

chr1-184388426-184388636	2.12E-13	0.35437909	0.089	0.016	1.40E-08	C4_CD8_Teff
chr18-11818493-11818843	2.30E-13	0.305044076	0.052	0.003	1.53E-08	C4_CD8_Teff
chr22-39351636-39352097	2.36E-13	0.335520136	0.07	0.009	1.56E-08	C4_CD8_Teff
chr19-34184824-34185469	2.37E-13	0.365948116	0.122	0.031	1.57E-08	C4_CD8_Teff
chr17-31654867-31655258	2.58E-13	0.33706777	0.065	0.007	1.71E-08	C4_CD8_Teff
chr15-50003277-50004048	2.67E-13	0.35585847	0.091	0.017	1.77E-08	C4_CD8_Teff
chr11-115256133-115257162	2.84E-13	0.360077085	0.177	0.056	1.88E-08	C4_CD8_Teff
chr17-67407593-67408095	3.12E-13	0.334508753	0.086	0.014	2.06E-08	C4_CD8_Teff
chr11-70424100-70424502	3.25E-13	0.327934522	0.068	0.007	2.15E-08	C4_CD8_Teff
chr8-20251411-20252448	3.35E-13	0.328723658	0.344	0.183	2.22E-08	C4_CD8_Teff
chr14-91385208-91385523	3.52E-13	0.362533348	0.109	0.026	2.33E-08	C4_CD8_Teff
chr7-1011025-1011836	3.71E-13	0.353145693	0.099	0.02	2.46E-08	C4_CD8_Teff
chr14-60753105-60754193	3.72E-13	0.334168594	0.326	0.155	2.47E-08	C4_CD8_Teff
chr13-80399322-80400669	3.74E-13	0.355803927	0.133	0.035	2.48E-08	C4_CD8_Teff
chr12-122161588-122162025	4.03E-13	0.348718588	0.094	0.018	2.67E-08	C4_CD8_Teff
chr14-67875192-67875771	4.56E-13	0.331381293	0.076	0.011	3.02E-08	C4_CD8_Teff
chr2-111619014-111619601	4.60E-13	0.303109766	0.055	0.005	3.05E-08	C4_CD8_Teff
chr8-25319299-25320014	5.47E-13	0.354336216	0.104	0.022	3.62E-08	C4_CD8_Teff
chr10-70575859-70576317	5.52E-13	0.358194094	0.128	0.037	3.66E-08	C4_CD8_Teff
chr10-33117430-33118163	5.63E-13	0.34943653	0.232	0.097	3.73E-08	C4_CD8_Teff
chr15-58369239-58369703	5.65E-13	0.321434259	0.057	0.006	3.74E-08	C4_CD8_Teff
chr14-55337435-55338199	6.73E-13	0.293490566	0.453	0.267	4.45E-08	C4_CD8_Teff
chr14-24609461-24610202	6.99E-13	0.355278619	0.188	0.063	4.63E-08	C4_CD8_Teff
chr11-60978562-60979361	7.55E-13	0.356042172	0.193	0.073	5.00E-08	C4_CD8_Teff
chr7-7596178-7596484	7.60E-13	0.319660407	0.06	0.007	5.03E-08	C4_CD8_Teff
chr5-1501091-1502379	7.71E-13	0.35901516	0.169	0.054	5.11E-08	C4_CD8_Teff
chr8-101967543-101968103	7.72E-13	0.334918653	0.078	0.011	5.11E-08	C4_CD8_Teff

chr12-64657630-64658285	7.97E-13	0.362042079	0.138	0.038	5.28E-08	C4_CD8_Teff
chr5-59123589-59123997	8.05E-13	0.355089663	0.138	0.039	5.33E-08	C4_CD8_Teff
chr18-69895209-69896744	8.36E-13	0.346461737	0.26	0.112	5.54E-08	C4_CD8_Teff
chr12-6613453-6614299	8.41E-13	0.350055524	0.19	0.071	5.57E-08	C4_CD8_Teff
chr5-139746103-139748157	8.70E-13	0.321617812	0.312	0.159	5.76E-08	C4_CD8_Teff
chr12-52835962-52836393	9.38E-13	0.339019658	0.081	0.013	6.21E-08	C4_CD8_Teff
chr15-39426779-39427607	9.59E-13	0.361629048	0.208	0.075	6.35E-08	C4_CD8_Teff
chr2-10029018-10029625	9.78E-13	0.334032325	0.07	0.012	6.48E-08	C4_CD8_Teff
chr18-48813554-48815949	1.01E-12	0.304846957	0.411	0.236	6.68E-08	C4_CD8_Teff
chr14-24627126-24627626	1.03E-12	0.311620791	0.065	0.007	6.82E-08	C4_CD8_Teff
chr5-152113146-152113770	1.05E-12	0.3382868	0.083	0.013	6.94E-08	C4_CD8_Teff
chr17-76681255-76681992	1.08E-12	0.345890327	0.102	0.023	7.18E-08	C4_CD8_Teff
chr15-75042673-75043371	1.09E-12	0.352638404	0.13	0.035	7.20E-08	C4_CD8_Teff
chr10-17509096-17509486	1.10E-12	0.354855586	0.117	0.03	7.27E-08	C4_CD8_Teff
chr22-46373246-46373567	1.14E-12	0.328257898	0.073	0.012	7.55E-08	C4_CD8_Teff
chr16-57590753-57591676	1.16E-12	0.34119272	0.255	0.122	7.68E-08	C4_CD8_Teff
chr7-132430566-132431673	1.20E-12	0.355037889	0.224	0.087	7.92E-08	C4_CD8_Teff
chr20-63567117-63568346	1.21E-12	0.345842288	0.135	0.043	8.00E-08	C4_CD8_Teff
chr5-73692088-73692395	1.23E-12	0.339350995	0.076	0.012	8.13E-08	C4_CD8_Teff
chr3-52459189-52459423	1.27E-12	0.334728598	0.081	0.013	8.43E-08	C4_CD8_Teff
chr20-35069488-35070404	1.29E-12	0.350077802	0.094	0.021	8.53E-08	C4_CD8_Teff
chr5-143205478-143206795	1.29E-12	0.352620195	0.161	0.051	8.54E-08	C4_CD8_Teff
chr9-85715559-85716785	1.35E-12	0.346608262	0.128	0.036	8.91E-08	C4_CD8_Teff
chr19-16136628-16136952	1.48E-12	0.292620527	0.052	0.004	9.83E-08	C4_CD8_Teff
chr14-24680549-24681263	1.75E-12	0.328433633	0.081	0.013	1.16E-07	C4_CD8_Teff
chr19-39828147-39828699	1.81E-12	0.347149104	0.135	0.037	1.20E-07	C4_CD8_Teff
chr15-95864358-95864855	1.91E-12	0.328726925	0.07	0.01	1.27E-07	C4_CD8_Teff

chr1-91548286-91549627	2.15E-12	0.348315004	0.167	0.058	1.42E-07	C4_CD8_Teff
chr7-21383070-21384220	2.16E-12	0.339294866	0.104	0.021	1.43E-07	C4_CD8_Teff
chr7-3106217-3106891	2.24E-12	0.353873905	0.135	0.041	1.48E-07	C4_CD8_Teff
chr18-22237893-22238540	2.27E-12	0.32265492	0.068	0.011	1.50E-07	C4_CD8_Teff
chr16-27491703-27492455	2.28E-12	0.316643463	0.065	0.008	1.51E-07	C4_CD8_Teff
chr7-3103636-3103889	2.34E-12	0.320747824	0.068	0.009	1.55E-07	C4_CD8_Teff
chr6-75495812-75496742	2.36E-12	0.347435398	0.198	0.077	1.56E-07	C4_CD8_Teff
chr7-1042157-1042471	2.41E-12	0.332878514	0.078	0.012	1.60E-07	C4_CD8_Teff
chr15-28974053-28974483	2.46E-12	0.337335099	0.086	0.017	1.63E-07	C4_CD8_Teff
chr2-8281696-8283299	2.57E-12	0.34382355	0.154	0.045	1.70E-07	C4_CD8_Teff
chrX-1657222-1658293	2.60E-12	0.337045946	0.229	0.104	1.72E-07	C4_CD8_Teff
chr8-55879186-55880429	2.61E-12	0.325690876	0.081	0.015	1.73E-07	C4_CD8_Teff
chr5-146751512-146752354	2.62E-12	0.347678709	0.208	0.082	1.74E-07	C4_CD8_Teff
chr19-51128884-51129150	2.96E-12	0.3132597	0.062	0.008	1.96E-07	C4_CD8_Teff
chr4-15970971-15971455	2.97E-12	0.328253482	0.07	0.011	1.96E-07	C4_CD8_Teff
chr4-25687707-25688167	3.07E-12	0.310389439	0.062	0.007	2.03E-07	C4_CD8_Teff
chr19-29587087-29588120	3.10E-12	0.315408962	0.385	0.189	2.06E-07	C4_CD8_Teff
chr17-49403620-49404641	3.23E-12	0.332792462	0.091	0.02	2.14E-07	C4_CD8_Teff
chr1-221521581-221522129	3.27E-12	0.304854651	0.057	0.007	2.17E-07	C4_CD8_Teff
chr2-16663941-16664571	3.59E-12	0.325029087	0.086	0.017	2.38E-07	C4_CD8_Teff
chr17-31051892-31052728	3.62E-12	0.329078206	0.081	0.015	2.40E-07	C4_CD8_Teff
chr2-110852222-110853029	3.92E-12	0.344986777	0.151	0.052	2.60E-07	C4_CD8_Teff
chr7-130145661-130145954	3.93E-12	0.303354784	0.055	0.006	2.60E-07	C4_CD8_Teff
chr16-89020070-89020713	4.03E-12	0.342997218	0.117	0.03	2.67E-07	C4_CD8_Teff
chr6-170147237-170148491	4.08E-12	0.343822966	0.182	0.07	2.70E-07	C4_CD8_Teff
chr1-45393590-45394814	4.09E-12	0.346337803	0.138	0.044	2.71E-07	C4_CD8_Teff
chr3-66311880-66312444	4.21E-12	0.337932016	0.099	0.022	2.79E-07	C4_CD8_Teff

chr5-147804838-147805626	4.89E-12	0.340359397	0.146	0.044	3.24E-07	C4_CD8_Teff
chr19-33301992-33303242	5.03E-12	0.327413314	0.219	0.094	3.33E-07	C4_CD8_Teff
chr11-86805817-86806351	5.17E-12	0.337095788	0.094	0.019	3.42E-07	C4_CD8_Teff
chr5-76763113-76763461	5.21E-12	0.310857046	0.065	0.008	3.45E-07	C4_CD8_Teff
chr11-3216973-3217537	5.31E-12	0.334220798	0.104	0.025	3.52E-07	C4_CD8_Teff
chr18-23546087-23546614	5.40E-12	0.326070306	0.073	0.013	3.58E-07	C4_CD8_Teff
chr8-72000196-72000797	5.67E-12	0.345240575	0.169	0.059	3.75E-07	C4_CD8_Teff
chr16-27463504-27464259	5.67E-12	0.337978824	0.195	0.078	3.75E-07	C4_CD8_Teff
chr1-212131880-212132366	5.76E-12	0.302830108	0.055	0.006	3.81E-07	C4_CD8_Teff
chr19-5086165-5086940	6.16E-12	0.332648088	0.094	0.022	4.08E-07	C4_CD8_Teff
chr1-200129096-200129980	6.28E-12	0.340979654	0.167	0.059	4.16E-07	C4_CD8_Teff
chr3-153242772-153243234	6.34E-12	0.335981748	0.104	0.024	4.20E-07	C4_CD8_Teff
chr19-41120609-41121507	6.35E-12	0.296850267	0.391	0.225	4.21E-07	C4_CD8_Teff
chr7-105346261-105347149	6.52E-12	0.344168448	0.133	0.04	4.32E-07	C4_CD8_Teff
chr6-36118475-36118945	6.66E-12	0.33605457	0.25	0.11	4.41E-07	C4_CD8_Teff
chr6-37051112-37051757	6.72E-12	0.319978767	0.263	0.131	4.45E-07	C4_CD8_Teff
chr13-75489460-75490288	6.79E-12	0.324575625	0.086	0.016	4.50E-07	C4_CD8_Teff
chr17-67486557-67487413	6.85E-12	0.342761132	0.161	0.054	4.54E-07	C4_CD8_Teff
chr12-94649474-94650756	7.01E-12	0.339190648	0.141	0.045	4.64E-07	C4_CD8_Teff
chr5-107670912-107671673	7.06E-12	0.320653045	0.078	0.015	4.67E-07	C4_CD8_Teff
chr16-85553330-85554440	7.18E-12	0.313165305	0.299	0.146	4.75E-07	C4_CD8_Teff
chr13-80477733-80478369	7.31E-12	0.31694785	0.081	0.013	4.84E-07	C4_CD8_Teff
chr22-39353348-39353661	7.63E-12	0.302715989	0.055	0.006	5.05E-07	C4_CD8_Teff
chr7-75727549-75728624	7.98E-12	0.315523227	0.286	0.145	5.28E-07	C4_CD8_Teff
chr4-145626300-145626817	8.20E-12	0.306438231	0.073	0.012	5.43E-07	C4_CD8_Teff
chr6-42292026-42292365	8.68E-12	0.317748333	0.073	0.012	5.75E-07	C4_CD8_Teff
chr4-2922640-2923007	8.78E-12	0.301978388	0.06	0.007	5.81E-07	C4_CD8_Teff

chr14-92460887-92461592	9.17E-12	0.331784676	0.102	0.024	6.07E-07	C4_CD8_Teff
chr10-129517368-129518421	9.23E-12	0.304242356	0.076	0.012	6.11E-07	C4_CD8_Teff
chr6-116136323-116137365	9.55E-12	0.338612901	0.112	0.03	6.32E-07	C4_CD8_Teff
chr11-72827802-72828019	9.83E-12	0.305669351	0.062	0.009	6.51E-07	C4_CD8_Teff
chr10-112369032-112369824	1.02E-11	0.329602282	0.224	0.098	6.73E-07	C4_CD8_Teff
chr17-82350306-82350899	1.02E-11	0.323143356	0.081	0.016	6.76E-07	C4_CD8_Teff
chr5-14208019-14208476	1.02E-11	0.291209571	0.055	0.006	6.79E-07	C4_CD8_Teff
chr1-41888593-41888878	1.05E-11	0.312794605	0.062	0.007	6.94E-07	C4_CD8_Teff
chr12-1740319-1740894	1.05E-11	0.291434527	0.055	0.006	6.95E-07	C4_CD8_Teff
chr16-57588877-57589235	1.06E-11	0.32645014	0.102	0.021	7.02E-07	C4_CD8_Teff
chr17-49387502-49388620	1.08E-11	0.328797974	0.234	0.105	7.17E-07	C4_CD8_Teff
chr1-11692749-11693567	1.15E-11	0.336514749	0.198	0.078	7.59E-07	C4_CD8_Teff
chrX-149829497-149830713	1.17E-11	0.338487023	0.151	0.05	7.77E-07	C4_CD8_Teff
chr3-46955717-46956196	1.20E-11	0.337656124	0.138	0.044	7.94E-07	C4_CD8_Teff
chr19-38675547-38675922	1.22E-11	0.319755338	0.086	0.018	8.08E-07	C4_CD8_Teff
chr17-10040416-10040857	1.30E-11	0.288191705	0.055	0.006	8.59E-07	C4_CD8_Teff
chr5-139635806-139637900	1.35E-11	0.300805279	0.349	0.192	8.96E-07	C4_CD8_Teff
chr3-39249348-39249908	1.43E-11	0.316191727	0.081	0.014	9.45E-07	C4_CD8_Teff
chr18-8601407-8602044	1.61E-11	0.309768791	0.068	0.01	1.07E-06	C4_CD8_Teff
chr5-32283680-32284051	1.65E-11	0.337915994	0.096	0.023	1.09E-06	C4_CD8_Teff
chr12-54052841-54054342	1.76E-11	0.328671052	0.172	0.066	1.17E-06	C4_CD8_Teff
chr16-87764489-87766170	1.76E-11	0.250937903	0.521	0.351	1.17E-06	C4_CD8_Teff
chr7-150556371-150557079	1.78E-11	0.330210265	0.117	0.035	1.18E-06	C4_CD8_Teff
chr5-138565000-138565905	1.89E-11	0.299629225	0.32	0.176	1.25E-06	C4_CD8_Teff
chr5-150155451-150156051	1.93E-11	0.318451775	0.094	0.021	1.28E-06	C4_CD8_Teff
chr18-77114463-77115020	2.02E-11	0.293448378	0.062	0.008	1.34E-06	C4_CD8_Teff
chr8-123534342-123534906	2.04E-11	0.328743154	0.185	0.071	1.35E-06	C4_CD8_Teff

chr16-31354297-31355200	2.06E-11	0.319330413	0.094	0.02	1.36E-06	C4_CD8_Teff
chr19-41993402-41994734	2.12E-11	0.310677117	0.286	0.145	1.41E-06	C4_CD8_Teff
chr16-67542375-67542823	2.17E-11	0.330929369	0.18	0.075	1.44E-06	C4_CD8_Teff
chr2-97728068-97728649	2.23E-11	0.33208417	0.112	0.028	1.48E-06	C4_CD8_Teff
chr13-84126553-84127232	2.29E-11	0.320193035	0.083	0.018	1.52E-06	C4_CD8_Teff
chr7-55308550-55310016	2.44E-11	0.315552856	0.258	0.131	1.62E-06	C4_CD8_Teff
chr17-44556201-44558143	2.80E-11	0.326313003	0.154	0.054	1.86E-06	C4_CD8_Teff
chr6-37868224-37868806	2.84E-11	0.312270068	0.078	0.015	1.88E-06	C4_CD8_Teff
chr11-3164456-3165499	2.93E-11	0.320438982	0.099	0.024	1.94E-06	C4_CD8_Teff
chr2-97745413-97745809	2.94E-11	0.327723243	0.18	0.074	1.95E-06	C4_CD8_Teff
chr9-93624047-93625281	3.21E-11	0.323651986	0.104	0.025	2.12E-06	C4_CD8_Teff
chr5-74326657-74327326	3.26E-11	0.318404568	0.099	0.023	2.16E-06	C4_CD8_Teff
chr5-59098804-59099932	3.27E-11	0.298918898	0.346	0.18	2.17E-06	C4_CD8_Teff
chr6-36123502-36124155	3.28E-11	0.29747466	0.318	0.178	2.17E-06	C4_CD8_Teff
chr3-43036774-43037170	3.29E-11	0.295151768	0.055	0.007	2.18E-06	C4_CD8_Teff
chr17-2795538-2796544	3.38E-11	0.325657068	0.208	0.085	2.24E-06	C4_CD8_Teff
chr11-364121-364750	3.43E-11	0.332718255	0.151	0.055	2.27E-06	C4_CD8_Teff
chr15-93047493-93048020	3.45E-11	0.3085056	0.081	0.016	2.28E-06	C4_CD8_Teff
chr12-31931912-31932717	3.50E-11	0.328762132	0.133	0.043	2.32E-06	C4_CD8_Teff
chr3-170276876-170278095	3.58E-11	0.320321844	0.224	0.102	2.37E-06	C4_CD8_Teff
chr8-124629483-124630102	3.69E-11	0.329403791	0.112	0.031	2.44E-06	C4_CD8_Teff
chr10-122421894-122422637	3.70E-11	0.325618185	0.115	0.031	2.45E-06	C4_CD8_Teff
chr11-128189888-128190647	3.81E-11	0.288834704	0.06	0.008	2.53E-06	C4_CD8_Teff
chr10-96651690-96652627	3.89E-11	0.331069333	0.143	0.045	2.58E-06	C4_CD8_Teff
chr3-15303830-15304820	4.23E-11	0.319676694	0.112	0.031	2.80E-06	C4_CD8_Teff
chrY-7040598-7041260	4.43E-11	0.322876517	0.096	0.025	2.93E-06	C4_CD8_Teff
chr1-184110280-184111219	4.47E-11	0.286214303	0.052	0.006	2.96E-06	C4_CD8_Teff

chr7-3119492-3119792	4.67E-11	0.302488937	0.07	0.012	3.09E-06	C4_CD8_Teff
chr3-125119401-125120169	4.84E-11	0.31104383	0.089	0.02	3.21E-06	C4_CD8_Teff
chr6-135567967-135568722	4.92E-11	0.32960364	0.122	0.037	3.26E-06	C4_CD8_Teff
chr4-71107300-71108096	5.09E-11	0.320364036	0.117	0.031	3.37E-06	C4_CD8_Teff
chr2-86079669-86080996	5.15E-11	0.29017996	0.352	0.194	3.41E-06	C4_CD8_Teff
chr6-114989354-114989979	5.18E-11	0.295567768	0.068	0.009	3.43E-06	C4_CD8_Teff
chr2-151669574-151670532	5.18E-11	0.320355783	0.086	0.018	3.43E-06	C4_CD8_Teff
chr20-32569246-32569647	5.40E-11	0.279190702	0.057	0.007	3.58E-06	C4_CD8_Teff
chr16-4064883-4065411	5.49E-11	0.299819303	0.081	0.016	3.64E-06	C4_CD8_Teff
chr3-172106905-172108757	5.60E-11	0.286228063	0.375	0.218	3.71E-06	C4_CD8_Teff
chr10-4234389-4235123	5.92E-11	0.315637482	0.201	0.086	3.92E-06	C4_CD8_Teff
chr2-12057192-12057864	6.20E-11	0.298805025	0.065	0.01	4.10E-06	C4_CD8_Teff
chr1-91730812-91731065	6.63E-11	0.305823958	0.073	0.013	4.39E-06	C4_CD8_Teff
chr2-9702670-9703998	6.69E-11	0.278014566	0.419	0.251	4.43E-06	C4_CD8_Teff
chr4-2689725-2690225	6.81E-11	0.293875718	0.065	0.011	4.51E-06	C4_CD8_Teff
chr3-135965264-135966458	6.84E-11	0.322200549	0.135	0.047	4.53E-06	C4_CD8_Teff
chr12-120885638-120886642	6.95E-11	0.312541319	0.081	0.016	4.60E-06	C4_CD8_Teff
chr2-8303091-8303846	6.99E-11	0.321881348	0.19	0.074	4.63E-06	C4_CD8_Teff
chr17-80708430-80709030	7.23E-11	0.323508987	0.096	0.022	4.79E-06	C4_CD8_Teff
chr12-52555640-52555864	7.44E-11	0.285953532	0.06	0.009	4.93E-06	C4_CD8_Teff
chr10-98408778-98409438	7.50E-11	0.305394574	0.076	0.013	4.97E-06	C4_CD8_Teff
chr7-2953975-2955117	7.53E-11	0.319284336	0.112	0.032	4.98E-06	C4_CD8_Teff
chr19-52745839-52746310	7.89E-11	0.318897665	0.099	0.024	5.23E-06	C4_CD8_Teff
chr12-128794493-128795204	8.00E-11	0.318348042	0.156	0.058	5.30E-06	C4_CD8_Teff
chr15-72809259-72809634	8.02E-11	0.286190762	0.057	0.007	5.31E-06	C4_CD8_Teff
chr9-86200569-86201022	8.08E-11	0.285718898	0.057	0.007	5.35E-06	C4_CD8_Teff
chr1-26384099-26384411	8.10E-11	0.300470019	0.076	0.015	5.36E-06	C4_CD8_Teff

chr20-50048433-50048760	8.23E-11	0.278330922	0.052	0.006	5.45E-06	C4_CD8_Teff
chr5-146764759-146765838	9.07E-11	0.315252907	0.112	0.031	6.01E-06	C4_CD8_Teff
chr11-59728697-59729209	9.13E-11	0.301875143	0.073	0.012	6.05E-06	C4_CD8_Teff
chr7-2849879-2850916	9.34E-11	0.318358547	0.117	0.034	6.18E-06	C4_CD8_Teff
chr16-48457954-48458704	9.54E-11	0.318068984	0.193	0.076	6.32E-06	C4_CD8_Teff
chr20-57433604-57434218	1.00E-10	0.310225874	0.094	0.023	6.65E-06	C4_CD8_Teff
chr6-36099343-36100456	1.04E-10	0.314191631	0.208	0.088	6.92E-06	C4_CD8_Teff
chr18-22385697-22386058	1.08E-10	0.302475218	0.07	0.011	7.16E-06	C4_CD8_Teff
chr16-29118468-29118906	1.10E-10	0.296325261	0.068	0.012	7.29E-06	C4_CD8_Teff
chr11-128038873-128040012	1.13E-10	0.283829544	0.359	0.213	7.51E-06	C4_CD8_Teff
chr12-64714100-64714890	1.14E-10	0.318450536	0.112	0.031	7.57E-06	C4_CD8_Teff
chr6-57137355-57137787	1.14E-10	0.290182125	0.057	0.007	7.58E-06	C4_CD8_Teff
chr8-60925648-60926053	1.16E-10	0.320877195	0.104	0.027	7.69E-06	C4_CD8_Teff
chr13-31928089-31928362	1.22E-10	0.282194762	0.052	0.006	8.09E-06	C4_CD8_Teff
chr19-16446958-16447229	1.23E-10	0.315628187	0.102	0.025	8.13E-06	C4_CD8_Teff
chr2-96129524-96129845	1.26E-10	0.313873956	0.102	0.027	8.38E-06	C4_CD8_Teff
chr3-50588289-50588897	1.27E-10	0.312361434	0.237	0.108	8.38E-06	C4_CD8_Teff
chr9-131644930-131645570	1.27E-10	0.311190389	0.086	0.02	8.38E-06	C4_CD8_Teff
chr22-40128929-40129311	1.33E-10	0.281484612	0.057	0.008	8.82E-06	C4_CD8_Teff
chr13-46679299-46680143	1.36E-10	0.322419713	0.138	0.046	8.99E-06	C4_CD8_Teff
chr1-111215714-111216491	1.39E-10	0.265068098	0.438	0.265	9.19E-06	C4_CD8_Teff
chr2-237998316-237998921	1.44E-10	0.318821224	0.138	0.046	9.51E-06	C4_CD8_Teff
chr9-71774703-71775317	1.58E-10	0.31745013	0.109	0.031	1.05E-05	C4_CD8_Teff
chr2-208358913-208359863	1.62E-10	0.295735178	0.076	0.014	1.07E-05	C4_CD8_Teff
chr19-16343147-16343471	1.68E-10	0.296308404	0.083	0.016	1.11E-05	C4_CD8_Teff
chr19-51386736-51387180	1.80E-10	0.286705156	0.065	0.012	1.19E-05	C4_CD8_Teff
chr3-50111648-50112098	1.89E-10	0.281116454	0.055	0.008	1.25E-05	C4_CD8_Teff

chr20-50385704-50386777	1.94E-10	0.30008531	0.273	0.142	1.28E-05	C4_CD8_Teff
chr7-24441740-24442645	1.96E-10	0.27932739	0.055	0.007	1.30E-05	C4_CD8_Teff
chr2-121309656-121310145	1.99E-10	0.28967429	0.065	0.011	1.32E-05	C4_CD8_Teff
chr17-78474402-78475194	2.00E-10	0.295773366	0.078	0.017	1.32E-05	C4_CD8_Teff
chr7-123770266-123770758	2.04E-10	0.291706885	0.065	0.011	1.35E-05	C4_CD8_Teff
chr8-73867513-73868827	2.19E-10	0.313311351	0.112	0.031	1.45E-05	C4_CD8_Teff
chr1-11062947-11063255	2.29E-10	0.316231952	0.133	0.046	1.51E-05	C4_CD8_Teff
chr3-12870921-12871267	2.37E-10	0.305303071	0.086	0.022	1.57E-05	C4_CD8_Teff
chr19-6668578-6670466	2.42E-10	0.255402275	0.435	0.287	1.60E-05	C4_CD8_Teff
chr1-64882580-64883097	2.46E-10	0.28545392	0.055	0.007	1.63E-05	C4_CD8_Teff
chr18-79326345-79328199	2.48E-10	0.31306906	0.117	0.031	1.64E-05	C4_CD8_Teff
chr19-2081763-2082541	2.68E-10	0.28751316	0.307	0.161	1.78E-05	C4_CD8_Teff
chr6-11203000-11203242	2.73E-10	0.285017397	0.065	0.011	1.81E-05	C4_CD8_Teff
chr1-15341895-15342321	2.73E-10	0.310175352	0.12	0.04	1.81E-05	C4_CD8_Teff
chr7-2862813-2864025	2.76E-10	0.304386676	0.208	0.097	1.83E-05	C4_CD8_Teff
chr14-70926337-70926785	2.85E-10	0.276544312	0.062	0.011	1.89E-05	C4_CD8_Teff
chr11-66272627-66272942	2.93E-10	0.303163953	0.109	0.032	1.94E-05	C4_CD8_Teff
chr4-8046797-8047337	2.94E-10	0.304741008	0.089	0.022	1.95E-05	C4_CD8_Teff
chr8-47597847-47598471	2.98E-10	0.312838276	0.125	0.04	1.97E-05	C4_CD8_Teff
chr9-137000190-137000739	3.05E-10	0.307471797	0.151	0.059	2.02E-05	C4_CD8_Teff
chr12-6337143-6337559	3.18E-10	0.296478017	0.232	0.117	2.10E-05	C4_CD8_Teff
chr3-20080956-20081545	3.18E-10	0.311975524	0.112	0.033	2.10E-05	C4_CD8_Teff
chr2-144452098-144452708	3.24E-10	0.306103388	0.112	0.031	2.14E-05	C4_CD8_Teff
chr3-13233775-13234999	3.34E-10	0.301210335	0.083	0.018	2.21E-05	C4_CD8_Teff
chr14-35332999-35334752	3.48E-10	0.311409381	0.159	0.057	2.30E-05	C4_CD8_Teff
chr17-78789249-78789852	3.63E-10	0.2976909	0.078	0.018	2.40E-05	C4_CD8_Teff
chr5-1374037-1374398	3.82E-10	0.281250453	0.055	0.009	2.53E-05	C4_CD8_Teff

chr3-39222454-39222817	3.88E-10	0.28706904	0.073	0.015	2.57E-05	C4_CD8_Teff
chr5-155728080-155729632	3.89E-10	0.287085394	0.07	0.013	2.58E-05	C4_CD8_Teff
chr16-57529680-57530548	3.98E-10	0.298011886	0.25	0.121	2.63E-05	C4_CD8_Teff
chr9-99076203-99077051	3.99E-10	0.307430929	0.141	0.048	2.64E-05	C4_CD8_Teff
chr13-114104456-114104877	4.04E-10	0.283831479	0.062	0.011	2.68E-05	C4_CD8_Teff
chr1-198788484-198788980	4.06E-10	0.298750375	0.083	0.018	2.69E-05	C4_CD8_Teff
chr10-124616109-124616310	4.14E-10	0.294755113	0.068	0.013	2.74E-05	C4_CD8_Teff
chr5-73625647-73626314	4.17E-10	0.307215686	0.107	0.031	2.76E-05	C4_CD8_Teff
chr1-8112997-8113548	4.17E-10	0.275214161	0.055	0.008	2.76E-05	C4_CD8_Teff
chr7-128459021-128459612	4.23E-10	0.303840303	0.198	0.09	2.80E-05	C4_CD8_Teff
chr6-4607340-4608186	4.35E-10	0.269536367	0.055	0.008	2.88E-05	C4_CD8_Teff
chr16-57578842-57579319	4.44E-10	0.28948025	0.07	0.014	2.94E-05	C4_CD8_Teff
chr22-39173749-39174989	4.45E-10	0.279580975	0.326	0.176	2.95E-05	C4_CD8_Teff
chr1-33183071-33183306	4.56E-10	0.289308969	0.065	0.012	3.02E-05	C4_CD8_Teff
chr11-72743763-72744303	4.56E-10	0.306943876	0.102	0.03	3.02E-05	C4_CD8_Teff
chr11-65902073-65902532	4.73E-10	0.303734046	0.128	0.041	3.14E-05	C4_CD8_Teff
chr16-88264744-88265942	4.82E-10	0.310730435	0.188	0.076	3.19E-05	C4_CD8_Teff
chr12-126771657-126772858	4.83E-10	0.279387844	0.336	0.181	3.20E-05	C4_CD8_Teff
chr10-124611652-124613168	4.83E-10	0.300701015	0.219	0.098	3.20E-05	C4_CD8_Teff
chr19-41302554-41303090	4.98E-10	0.298798913	0.203	0.093	3.30E-05	C4_CD8_Teff
chr19-52778593-52779552	5.05E-10	0.300437777	0.102	0.03	3.34E-05	C4_CD8_Teff
chr19-43738375-43739147	5.23E-10	0.280018851	0.065	0.012	3.46E-05	C4_CD8_Teff
chr17-68356169-68356937	5.41E-10	0.290631826	0.289	0.146	3.58E-05	C4_CD8_Teff
chr7-128320093-128321361	5.47E-10	0.307009711	0.125	0.04	3.62E-05	C4_CD8_Teff
chr1-158930166-158931646	5.49E-10	0.294552251	0.237	0.116	3.63E-05	C4_CD8_Teff
chr6-145844988-145845928	5.53E-10	0.300085218	0.096	0.026	3.66E-05	C4_CD8_Teff
chr12-31721754-31722499	5.72E-10	0.31217943	0.154	0.058	3.79E-05	C4_CD8_Teff

chr11-67267554-67268608	5.93E-10	0.260170654	0.391	0.243	3.93E-05	C4_CD8_Teff
chr15-60541831-60542313	6.00E-10	0.299678295	0.086	0.021	3.97E-05	C4_CD8_Teff
chr3-196144186-196145064	6.24E-10	0.301216237	0.107	0.032	4.13E-05	C4_CD8_Teff
chr3-118851499-118852175	6.25E-10	0.305505155	0.107	0.03	4.14E-05	C4_CD8_Teff
chr16-4059692-4060452	6.26E-10	0.266935494	0.055	0.008	4.14E-05	C4_CD8_Teff
chr15-89136591-89137119	6.89E-10	0.27149409	0.06	0.009	4.56E-05	C4_CD8_Teff
chr12-94943633-94944066	6.92E-10	0.296713404	0.086	0.021	4.58E-05	C4_CD8_Teff
chr20-23919665-23920428	7.05E-10	0.294965267	0.076	0.017	4.67E-05	C4_CD8_Teff
chr15-58607966-58608612	7.10E-10	0.289335461	0.078	0.017	4.70E-05	C4_CD8_Teff
chr10-96719602-96721303	7.16E-10	0.285491404	0.284	0.151	4.74E-05	C4_CD8_Teff
chr12-9009852-9010522	7.20E-10	0.306733907	0.174	0.067	4.77E-05	C4_CD8_Teff
chr1-160855164-160856067	7.27E-10	0.297457839	0.091	0.024	4.81E-05	C4_CD8_Teff
chr7-929947-930407	7.56E-10	0.306876219	0.125	0.041	5.01E-05	C4_CD8_Teff
chr14-67668585-67668883	7.61E-10	0.280298693	0.065	0.011	5.04E-05	C4_CD8_Teff
chr2-28406519-28407079	7.76E-10	0.291571958	0.247	0.121	5.14E-05	C4_CD8_Teff
chrX-2897772-2898328	7.89E-10	0.260654984	0.052	0.007	5.23E-05	C4_CD8_Teff
chr1-27618132-27618944	7.96E-10	0.307354928	0.159	0.066	5.27E-05	C4_CD8_Teff
chr8-124727130-124728792	8.17E-10	0.295168632	0.12	0.038	5.41E-05	C4_CD8_Teff
chr17-67449711-67450561	8.44E-10	0.300643986	0.12	0.042	5.59E-05	C4_CD8_Teff
chr11-134406573-134406855	8.51E-10	0.284794116	0.07	0.015	5.63E-05	C4_CD8_Teff
chr5-157292655-157292949	8.72E-10	0.288255398	0.076	0.016	5.78E-05	C4_CD8_Teff
chr2-28508089-28508845	8.77E-10	0.308907123	0.167	0.066	5.80E-05	C4_CD8_Teff
chr11-9510547-9511363	8.89E-10	0.288593774	0.073	0.016	5.89E-05	C4_CD8_Teff
chr1-157697041-157697468	9.17E-10	0.29006909	0.068	0.013	6.07E-05	C4_CD8_Teff
chr2-136281055-136281929	9.23E-10	0.300818019	0.169	0.072	6.11E-05	C4_CD8_Teff
chr22-21772845-21773238	9.62E-10	0.287844613	0.068	0.013	6.37E-05	C4_CD8_Teff
chr8-2133830-2134213	9.79E-10	0.303184265	0.104	0.03	6.48E-05	C4_CD8_Teff

chr18-77107901-77108702	9.97E-10	0.303267992	0.128	0.042	6.60E-05	C4_CD8_Teff
chr1-54798844-54799644	1.02E-09	0.299422892	0.117	0.038	6.74E-05	C4_CD8_Teff
chr1-84365612-84366007	1.04E-09	0.271559282	0.057	0.009	6.87E-05	C4_CD8_Teff
chr7-8152189-8153125	1.07E-09	0.295284347	0.086	0.02	7.05E-05	C4_CD8_Teff
chr6-17014498-17016282	1.09E-09	0.291828951	0.245	0.114	7.22E-05	C4_CD8_Teff
chr2-144596270-144596856	1.12E-09	0.271318874	0.062	0.01	7.40E-05	C4_CD8_Teff
chr5-42961865-42962585	1.13E-09	0.29123071	0.083	0.02	7.47E-05	C4_CD8_Teff
chr4-3192121-3192568	1.14E-09	0.263073084	0.055	0.009	7.55E-05	C4_CD8_Teff
chr12-128797250-128797743	1.15E-09	0.301722417	0.109	0.032	7.59E-05	C4_CD8_Teff
chr18-44507552-44508282	1.15E-09	0.279661986	0.065	0.012	7.60E-05	C4_CD8_Teff
chr1-192604954-192605306	1.16E-09	0.292695965	0.083	0.022	7.65E-05	C4_CD8_Teff
chr17-39094185-39094947	1.19E-09	0.298039076	0.13	0.047	7.86E-05	C4_CD8_Teff
chr6-41732483-41733264	1.20E-09	0.28498603	0.281	0.149	7.96E-05	C4_CD8_Teff
chr5-147781983-147783016	1.23E-09	0.30158589	0.206	0.089	8.15E-05	C4_CD8_Teff
chr13-29529696-29530077	1.27E-09	0.282001051	0.065	0.012	8.40E-05	C4_CD8_Teff
chr2-235669088-235669666	1.29E-09	0.278084203	0.07	0.014	8.51E-05	C4_CD8_Teff
chr10-110085686-110086736	1.35E-09	0.297370282	0.156	0.063	8.92E-05	C4_CD8_Teff
chr15-58544853-58545667	1.36E-09	0.280244727	0.073	0.016	8.98E-05	C4_CD8_Teff
chr15-80159704-80160975	1.36E-09	0.300176247	0.125	0.043	9.01E-05	C4_CD8_Teff
chr12-68364740-68365760	1.43E-09	0.259667124	0.383	0.236	9.48E-05	C4_CD8_Teff
chr3-129567238-129568223	1.44E-09	0.258108286	0.055	0.008	9.52E-05	C4_CD8_Teff
chr1-92840417-92840927	1.47E-09	0.280562224	0.07	0.013	9.76E-05	C4_CD8_Teff
chr20-57627621-57628703	1.47E-09	0.289040263	0.221	0.113	9.76E-05	C4_CD8_Teff
chr16-2841950-2842989	1.49E-09	0.293443218	0.141	0.054	9.86E-05	C4_CD8_Teff
chr20-2729134-2729727	1.55E-09	0.294926643	0.086	0.022	0.00010235	C4_CD8_Teff
chr19-11196914-11197992	1.59E-09	0.288643125	0.198	0.091	0.000105052	C4_CD8_Teff
chr9-91163890-91165114	1.59E-09	0.292645901	0.234	0.11	0.000105421	C4_CD8_Teff

chr4-7539428-7540144	1.64E-09	0.298550468	0.104	0.029	0.000108284	C4_CD8_Teff
chr22-42828365-42828750	1.64E-09	0.278640715	0.07	0.014	0.000108321	C4_CD8_Teff
chr12-128817445-128817965	1.66E-09	0.26567859	0.057	0.01	0.000110055	C4_CD8_Teff
chr6-2939870-2940839	1.71E-09	0.27338394	0.307	0.175	0.000113252	C4_CD8_Teff
chr11-6196303-6196651	1.72E-09	0.27443443	0.06	0.01	0.000113803	C4_CD8_Teff
chr3-4748585-4749193	1.74E-09	0.277771069	0.065	0.012	0.000115064	C4_CD8_Teff
chr18-22344517-22345574	1.75E-09	0.297595204	0.172	0.068	0.000116115	C4_CD8_Teff
chr5-95973693-95974337	1.82E-09	0.286193559	0.083	0.02	0.000120592	C4_CD8_Teff
chr10-17511122-17511899	1.93E-09	0.295494528	0.12	0.038	0.000128139	C4_CD8_Teff
chr12-122166437-122167574	1.99E-09	0.292558196	0.091	0.026	0.00013183	C4_CD8_Teff
chr12-128388516-128388978	2.02E-09	0.257651367	0.052	0.007	0.000133912	C4_CD8_Teff
chr5-91389941-91390685	2.14E-09	0.289262654	0.096	0.026	0.000141892	C4_CD8_Teff
chr7-70669706-70670633	2.18E-09	0.300965089	0.133	0.044	0.00014469	C4_CD8_Teff
chr1-41891254-41892549	2.20E-09	0.285000471	0.229	0.118	0.000145759	C4_CD8_Teff
chr16-29115331-29116025	2.20E-09	0.276827575	0.073	0.017	0.000145983	C4_CD8_Teff
chr15-63905584-63906342	2.22E-09	0.284104741	0.086	0.021	0.000146851	C4_CD8_Teff
chr7-2119780-2120651	2.22E-09	0.295393138	0.128	0.044	0.0001471	C4_CD8_Teff
chr3-36876054-36876980	2.25E-09	0.282430485	0.268	0.139	0.000148844	C4_CD8_Teff
chr14-105290544-105290893	2.27E-09	0.2683506	0.055	0.01	0.000150629	C4_CD8_Teff
chr18-3525710-3527203	2.31E-09	0.298544381	0.156	0.064	0.000153089	C4_CD8_Teff
chr1-157702323-157702695	2.33E-09	0.284905574	0.076	0.018	0.000153998	C4_CD8_Teff
chr10-62631851-62632804	2.34E-09	0.28085215	0.271	0.145	0.00015496	C4_CD8_Teff
chr1-12132673-12133385	2.37E-09	0.293185113	0.109	0.035	0.000156832	C4_CD8_Teff
chr11-44870262-44870984	2.39E-09	0.280176872	0.073	0.015	0.000158588	C4_CD8_Teff
chr2-191127857-191128869	2.52E-09	0.289907708	0.188	0.086	0.000167152	C4_CD8_Teff
chr21-46052450-46053732	2.55E-09	0.295996052	0.117	0.039	0.000169184	C4_CD8_Teff
chr2-144636916-144637241	2.62E-09	0.254772613	0.052	0.007	0.000173397	C4_CD8_Teff

chr17-47740627-47742073	2.63E-09	0.285515169	0.203	0.093	0.000173942	C4_CD8_Teff
chr6-112285701-112286474	2.65E-09	0.296855888	0.122	0.042	0.000175374	C4_CD8_Teff
chr7-141053492-141054063	2.79E-09	0.287150421	0.083	0.022	0.000184996	C4_CD8_Teff
chr3-129017013-129017459	2.86E-09	0.275149761	0.07	0.016	0.00018908	C4_CD8_Teff
chr6-111753571-111753883	2.89E-09	0.278652213	0.086	0.022	0.000191222	C4_CD8_Teff
chr17-32523919-32524387	2.96E-09	0.290985514	0.112	0.032	0.000196309	C4_CD8_Teff
chr7-77351150-77352148	2.97E-09	0.284431731	0.234	0.118	0.000196902	C4_CD8_Teff
chr19-38726721-38727016	2.98E-09	0.268727145	0.06	0.012	0.000197535	C4_CD8_Teff
chr12-8987598-8988061	3.03E-09	0.291689917	0.115	0.035	0.000200675	C4_CD8_Teff
chr19-7185705-7186758	3.05E-09	0.289506866	0.122	0.039	0.000202017	C4_CD8_Teff
chr8-124751393-124752265	3.05E-09	0.290803826	0.115	0.038	0.000202034	C4_CD8_Teff
chr2-30421510-30422839	3.05E-09	0.286034736	0.266	0.136	0.000202184	C4_CD8_Teff
chr2-98438618-98439131	3.06E-09	0.295414872	0.096	0.029	0.000202338	C4_CD8_Teff
chrX-1481614-1482542	3.06E-09	0.263142075	0.32	0.193	0.00020278	C4_CD8_Teff
chr19-48492745-48493941	3.10E-09	0.250116079	0.37	0.249	0.000205107	C4_CD8_Teff
chr19-51122443-51122842	3.14E-09	0.25471866	0.052	0.007	0.000207637	C4_CD8_Teff
chr19-18374273-18374694	3.21E-09	0.26763529	0.062	0.013	0.000212571	C4_CD8_Teff
chr19-23378845-23379406	3.26E-09	0.271826521	0.06	0.011	0.000215689	C4_CD8_Teff
chr16-17448620-17449668	3.39E-09	0.291741331	0.172	0.077	0.000224619	C4_CD8_Teff
chr21-36297515-36298548	3.47E-09	0.282286915	0.232	0.117	0.000229636	C4_CD8_Teff
chr20-17504601-17506502	3.47E-09	0.273067342	0.268	0.143	0.000229882	C4_CD8_Teff
chr7-70693239-70694833	3.55E-09	0.274811268	0.286	0.156	0.000234886	C4_CD8_Teff
chr3-41117035-41117826	3.58E-09	0.289425412	0.156	0.064	0.000237158	C4_CD8_Teff
chr22-50444816-50445517	3.64E-09	0.280406938	0.078	0.019	0.000241252	C4_CD8_Teff
chr14-24665356-24666033	3.70E-09	0.295853106	0.13	0.046	0.000244766	C4_CD8_Teff
chr12-128811579-128812024	3.81E-09	0.270735974	0.062	0.013	0.000252269	C4_CD8_Teff
chr7-66033509-66034026	3.96E-09	0.27246784	0.06	0.012	0.000262309	C4_CD8_Teff

chr21-37225366-37225847	3.98E-09	0.29127122	0.122	0.046	0.000263695	C4_CD8_Teff
chr5-102295715-102297223	4.16E-09	0.289448642	0.195	0.089	0.000275681	C4_CD8_Teff
chr5-142996190-142996868	4.18E-09	0.290139366	0.096	0.03	0.000277027	C4_CD8_Teff
chr12-89762527-89762793	4.22E-09	0.26706315	0.062	0.011	0.000279487	C4_CD8_Teff
chr2-192672-193512	4.27E-09	0.279888878	0.104	0.032	0.000282742	C4_CD8_Teff
chr19-10101748-10102490	4.35E-09	0.282879879	0.216	0.107	0.000287931	C4_CD8_Teff
chr1-203655668-203656081	4.43E-09	0.285699215	0.096	0.029	0.000293205	C4_CD8_Teff
chr7-29255737-29256426	4.44E-09	0.296134692	0.102	0.03	0.000294308	C4_CD8_Teff
chr6-11260229-11260909	4.55E-09	0.281175162	0.083	0.024	0.00030104	C4_CD8_Teff
chr9-128550269-128550488	4.60E-09	0.270736983	0.065	0.012	0.000304371	C4_CD8_Teff
chr1-24847748-24848975	4.64E-09	0.25748742	0.32	0.19	0.000307552	C4_CD8_Teff
chr12-116916295-116917114	4.78E-09	0.284373561	0.232	0.106	0.000316602	C4_CD8_Teff
chr6-40788609-40789502	4.81E-09	0.273332568	0.065	0.013	0.000318562	C4_CD8_Teff
chr4-7324150-7325121	5.09E-09	0.277986643	0.094	0.027	0.000337337	C4_CD8_Teff
chr19-14042576-14043274	5.20E-09	0.277223261	0.099	0.029	0.000344334	C4_CD8_Teff
chr21-33036330-33036836	5.28E-09	0.270228983	0.068	0.015	0.000349901	C4_CD8_Teff
chr17-68310074-68310473	5.30E-09	0.272243721	0.068	0.016	0.000350889	C4_CD8_Teff
chr19-10812876-10813525	5.38E-09	0.274110924	0.076	0.02	0.000356507	C4_CD8_Teff
chr1-157723000-157723500	5.56E-09	0.26927497	0.062	0.012	0.000368337	C4_CD8_Teff
chr14-64447910-64448586	5.76E-09	0.281274594	0.081	0.02	0.000381228	C4_CD8_Teff
chr2-16665304-16666092	5.94E-09	0.288075669	0.12	0.04	0.000393555	C4_CD8_Teff
chr14-97705920-97706320	6.05E-09	0.290928252	0.133	0.049	0.000400872	C4_CD8_Teff
chr1-39327936-39328534	6.13E-09	0.278506959	0.083	0.023	0.000405646	C4_CD8_Teff
chr4-16000779-16001705	6.16E-09	0.275858983	0.073	0.018	0.000407708	C4_CD8_Teff
chr9-74496529-74497941	6.38E-09	0.27060913	0.086	0.022	0.000422771	C4_CD8_Teff
chr12-94704331-94704803	6.54E-09	0.266902457	0.065	0.012	0.000432865	C4_CD8_Teff
chr11-68412879-68413302	6.71E-09	0.252561586	0.055	0.009	0.000444565	C4_CD8_Teff

chr7-1289898-1290656	6.95E-09	0.264377679	0.06	0.013	0.00046012	C4_CD8_Teff
chr16-53453967-53454220	7.24E-09	0.288534663	0.115	0.039	0.000479572	C4_CD8_Teff
chr1-101136673-101137574	7.27E-09	0.287423731	0.117	0.043	0.000481389	C4_CD8_Teff
chr8-133575431-133575978	7.28E-09	0.284155508	0.221	0.1	0.000482357	C4_CD8_Teff
chr7-38240794-38241149	7.35E-09	0.278675506	0.078	0.02	0.000486426	C4_CD8_Teff
chr19-29276950-29277477	7.50E-09	0.285422775	0.102	0.03	0.000496547	C4_CD8_Teff
chr2-47888511-47889153	7.61E-09	0.285330148	0.115	0.039	0.000503691	C4_CD8_Teff
chr19-18044590-18045578	7.64E-09	0.281900874	0.172	0.078	0.000505969	C4_CD8_Teff
chr9-33202181-33202843	7.73E-09	0.260713397	0.06	0.013	0.000511975	C4_CD8_Teff
chr17-42015625-42015831	7.84E-09	0.27335531	0.081	0.021	0.00051913	C4_CD8_Teff
chr16-82945391-82946046	7.89E-09	0.262772116	0.073	0.016	0.000522698	C4_CD8_Teff
chr15-94324887-94325647	7.90E-09	0.288710306	0.148	0.051	0.000523282	C4_CD8_Teff
chr17-76700368-76700924	8.09E-09	0.271578536	0.07	0.017	0.000535936	C4_CD8_Teff
chr3-125110445-125111081	8.16E-09	0.264350251	0.06	0.012	0.000540629	C4_CD8_Teff
chr11-36426147-36426816	8.17E-09	0.284691573	0.091	0.026	0.000541277	C4_CD8_Teff
chr1-54886509-54887574	8.30E-09	0.288171781	0.161	0.066	0.000549758	C4_CD8_Teff
chr12-48409863-48410272	8.44E-09	0.26998115	0.065	0.013	0.000559101	C4_CD8_Teff
chr5-32280624-32281055	8.86E-09	0.280559342	0.089	0.022	0.00058705	C4_CD8_Teff
chr16-81831426-81832215	8.93E-09	0.285518044	0.099	0.031	0.000591439	C4_CD8_Teff
chr2-101223643-101224315	8.93E-09	0.284001058	0.107	0.035	0.000591553	C4_CD8_Teff
chr7-129535854-129536653	9.07E-09	0.274997086	0.091	0.026	0.00060059	C4_CD8_Teff
chr2-233409095-233409966	9.24E-09	0.283510274	0.117	0.043	0.00061202	C4_CD8_Teff
chr1-27875221-27875516	9.38E-09	0.278007157	0.083	0.022	0.000620993	C4_CD8_Teff
chr5-54620202-54620773	9.51E-09	0.254550074	0.055	0.01	0.000630079	C4_CD8_Teff
chr17-68346266-68346887	9.54E-09	0.284105664	0.148	0.062	0.000631472	C4_CD8_Teff
chr5-148848399-148848913	9.59E-09	0.283099357	0.138	0.056	0.000635051	C4_CD8_Teff
chr7-36620262-36620682	9.77E-09	0.260622838	0.057	0.01	0.000647176	C4_CD8_Teff

chr15-39623106-39624019	9.80E-09	0.258894352	0.328	0.189	0.000649295	C4_CD8_Teff
chr18-44725329-44726023	9.86E-09	0.279645142	0.219	0.106	0.000653066	C4_CD8_Teff
chr17-78630966-78632304	9.92E-09	0.278835235	0.219	0.11	0.000656801	C4_CD8_Teff
chr2-98637417-98638125	9.96E-09	0.26658667	0.276	0.152	0.000659707	C4_CD8_Teff
chr4-38666967-38667483	9.99E-09	0.272541369	0.271	0.136	0.00066172	C4_CD8_Teff
chr14-50559775-50561244	1.03E-08	0.283327901	0.13	0.052	0.000681936	C4_CD8_Teff
chr13-20890966-20892181	1.05E-08	0.281346546	0.107	0.036	0.000692826	C4_CD8_Teff
chr14-97697579-97698091	1.06E-08	0.26507081	0.068	0.014	0.000698931	C4_CD8_Teff
chr2-241871170-241871542	1.06E-08	0.283355969	0.125	0.044	0.000703341	C4_CD8_Teff
chr16-11556927-11557611	1.06E-08	0.282940533	0.12	0.043	0.000704423	C4_CD8_Teff
chr8-56006294-56006842	1.08E-08	0.259628406	0.06	0.012	0.000717116	C4_CD8_Teff
chr19-12784493-12785357	1.12E-08	0.263821507	0.263	0.149	0.000744152	C4_CD8_Teff
chr17-35879423-35880716	1.12E-08	0.253495211	0.336	0.198	0.000744888	C4_CD8_Teff
chr2-170177616-170178293	1.15E-08	0.251590082	0.055	0.009	0.000760868	C4_CD8_Teff
chr1-33045281-33046369	1.17E-08	0.278233456	0.193	0.09	0.00077781	C4_CD8_Teff
chr11-118704694-118705470	1.18E-08	0.279343312	0.089	0.024	0.000780479	C4_CD8_Teff
chr16-57801911-57803045	1.20E-08	0.271136859	0.195	0.096	0.000791746	C4_CD8_Teff
chr11-2268923-2269555	1.22E-08	0.281394751	0.117	0.038	0.000809772	C4_CD8_Teff
chr2-85702201-85702450	1.24E-08	0.262905879	0.073	0.017	0.000818712	C4_CD8_Teff
chr15-90889392-90889924	1.26E-08	0.274965138	0.081	0.019	0.000834124	C4_CD8_Teff
chr12-123994903-123995445	1.31E-08	0.272172783	0.07	0.016	0.000865518	C4_CD8_Teff
chr3-168094718-168096447	1.31E-08	0.284178147	0.125	0.04	0.000867964	C4_CD8_Teff
chr6-101845117-101846042	1.34E-08	0.274629321	0.086	0.024	0.000887833	C4_CD8_Teff
chr8-124660198-124660781	1.40E-08	0.272011443	0.078	0.021	0.000927546	C4_CD8_Teff
chr9-88178949-88179465	1.41E-08	0.250124271	0.052	0.009	0.000931064	C4_CD8_Teff
chr14-50736821-50737348	1.41E-08	0.282254777	0.091	0.026	0.000936795	C4_CD8_Teff
chr6-90424262-90425736	1.42E-08	0.277958584	0.164	0.071	0.000943673	C4_CD8_Teff

chr5-107501861-107502630	1.43E-08	0.28138723	0.109	0.036	0.000946611	C4_CD8_Teff
chr19-14274356-14275171	1.44E-08	0.275844461	0.169	0.075	0.00095603	C4_CD8_Teff
chr5-148849334-148849802	1.46E-08	0.272797442	0.221	0.107	0.000963765	C4_CD8_Teff
chr17-78242332-78242988	1.46E-08	0.257976946	0.057	0.012	0.000964261	C4_CD8_Teff
chr4-6169995-6171428	1.46E-08	0.260219089	0.07	0.015	0.00096942	C4_CD8_Teff
chr15-93050084-93050524	1.47E-08	0.272428976	0.076	0.019	0.000976766	C4_CD8_Teff
chr22-25076110-25076689	1.54E-08	0.278887893	0.107	0.034	0.001019011	C4_CD8_Teff
chr12-121614850-121615377	1.55E-08	0.261188547	0.065	0.015	0.00102562	C4_CD8_Teff
chr4-82797899-82799412	1.58E-08	0.27700016	0.203	0.091	0.001045634	C4_CD8_Teff
chr19-13181768-13182417	1.58E-08	0.259868472	0.073	0.017	0.001047649	C4_CD8_Teff
chr17-68349235-68349445	1.62E-08	0.253484896	0.057	0.012	0.001073789	C4_CD8_Teff
chr17-45282907-45283414	1.62E-08	0.25007515	0.052	0.009	0.001075588	C4_CD8_Teff
chr9-130834161-130835912	1.69E-08	0.250987941	0.32	0.188	0.001116228	C4_CD8_Teff
chr20-50450224-50451398	1.69E-08	0.271301416	0.081	0.025	0.001118136	C4_CD8_Teff
chr3-27860990-27861905	1.69E-08	0.273178907	0.099	0.032	0.001122086	C4_CD8_Teff
chr16-57764361-57765161	1.71E-08	0.268162614	0.086	0.023	0.001130352	C4_CD8_Teff
chr3-112672212-112673385	1.71E-08	0.270018332	0.221	0.114	0.001135634	C4_CD8_Teff
chr6-168884159-168884959	1.78E-08	0.270162164	0.094	0.03	0.001177041	C4_CD8_Teff
chr5-40377872-40379015	1.84E-08	0.273207207	0.206	0.103	0.001221336	C4_CD8_Teff
chr2-88269746-88270191	1.86E-08	0.250468732	0.055	0.01	0.001233183	C4_CD8_Teff
chr14-91402433-91402691	1.92E-08	0.269751182	0.086	0.027	0.001271965	C4_CD8_Teff
chrX-154347024-154347937	1.93E-08	0.279277487	0.13	0.051	0.001275985	C4_CD8_Teff
chr5-138458834-138459668	1.95E-08	0.263683215	0.263	0.141	0.001293276	C4_CD8_Teff
chr13-80338896-80339381	1.95E-08	0.250005672	0.06	0.012	0.001293984	C4_CD8_Teff
chr18-79138867-79139524	1.96E-08	0.271490255	0.094	0.03	0.00129946	C4_CD8_Teff
chr14-74573789-74574567	1.99E-08	0.276094086	0.193	0.089	0.001317212	C4_CD8_Teff
chr16-8663235-8664048	2.04E-08	0.260424888	0.06	0.013	0.001352956	C4_CD8_Teff

chr19-2621692-2622070	2.06E-08	0.27558436	0.143	0.057	0.001366655	C4_CD8_Teff
chr17-35895690-35896610	2.08E-08	0.267896586	0.214	0.11	0.001378168	C4_CD8_Teff
chr14-51930014-51930928	2.09E-08	0.261939123	0.068	0.016	0.001381923	C4_CD8_Teff
chr4-101334861-101335409	2.13E-08	0.25629421	0.06	0.014	0.001410378	C4_CD8_Teff
chr11-112961079-112962491	2.17E-08	0.251821697	0.06	0.013	0.001434977	C4_CD8_Teff
chr20-60058205-60058753	2.21E-08	0.265308716	0.073	0.017	0.001464123	C4_CD8_Teff
chr2-239609499-239610074	2.23E-08	0.260396168	0.065	0.016	0.00147771	C4_CD8_Teff
chr8-129885454-129886395	2.30E-08	0.275758286	0.128	0.048	0.001525039	C4_CD8_Teff
chr20-20602740-20603855	2.31E-08	0.281732015	0.164	0.072	0.001528965	C4_CD8_Teff
chr11-113867604-113868489	2.32E-08	0.251518308	0.06	0.012	0.00153502	C4_CD8_Teff
chr4-71186263-71188267	2.52E-08	0.267204839	0.224	0.11	0.001669694	C4_CD8_Teff
chr3-5037775-5038498	2.54E-08	0.267463919	0.086	0.025	0.001681372	C4_CD8_Teff
chr9-95462185-95463306	2.56E-08	0.276031858	0.193	0.092	0.001692333	C4_CD8_Teff
chr1-2234180-2234998	2.58E-08	0.254848214	0.286	0.168	0.001709792	C4_CD8_Teff
chr1-180189943-180190446	2.60E-08	0.253336944	0.057	0.013	0.00172317	C4_CD8_Teff
chr10-7045828-7046931	2.67E-08	0.27626582	0.151	0.059	0.0017653	C4_CD8_Teff
chr10-109945534-109946158	2.74E-08	0.26641886	0.078	0.021	0.001815257	C4_CD8_Teff
chr11-72519311-72520475	2.77E-08	0.279720967	0.141	0.055	0.001832673	C4_CD8_Teff
chr9-136960244-136960558	2.77E-08	0.250797191	0.062	0.014	0.001835926	C4_CD8_Teff
chr12-6949307-6949550	2.86E-08	0.275391134	0.154	0.071	0.001894692	C4_CD8_Teff
chrX-153906115-153906714	2.88E-08	0.264599414	0.091	0.026	0.001909198	C4_CD8_Teff
chr17-17792269-17793223	2.89E-08	0.272283914	0.18	0.085	0.001914934	C4_CD8_Teff
chr14-64445154-64445830	2.90E-08	0.273702354	0.102	0.036	0.001920437	C4_CD8_Teff
chr4-36192892-36193228	2.95E-08	0.265071868	0.083	0.023	0.001956551	C4_CD8_Teff
chr7-102415181-102416114	3.04E-08	0.277574486	0.164	0.077	0.002013864	C4_CD8_Teff
chr21-37257261-37258716	3.08E-08	0.26490515	0.214	0.113	0.002039929	C4_CD8_Teff
chr1-27891480-27891826	3.09E-08	0.268434483	0.091	0.027	0.002047068	C4_CD8_Teff

chr2-144515555-144516329	3.13E-08	0.267057291	0.208	0.103	0.002069838	C4_CD8_Teff
chr13-99230475-99231286	3.14E-08	0.267236838	0.081	0.022	0.00208223	C4_CD8_Teff
chr16-82431989-82432632	3.35E-08	0.27217949	0.096	0.027	0.002220906	C4_CD8_Teff
chr12-110661278-110661951	3.39E-08	0.270280935	0.104	0.033	0.00224221	C4_CD8_Teff
chr2-7748717-7749360	3.43E-08	0.269463988	0.135	0.055	0.002273427	C4_CD8_Teff
chr1-185420445-185421422	3.47E-08	0.262307763	0.089	0.026	0.00229835	C4_CD8_Teff
chr8-59141660-59142418	3.47E-08	0.253347438	0.062	0.013	0.002299703	C4_CD8_Teff
chr8-133898919-133899663	3.55E-08	0.264085607	0.076	0.02	0.002354079	C4_CD8_Teff
chr4-42647000-42647252	3.59E-08	0.251753054	0.068	0.015	0.002377997	C4_CD8_Teff
chr15-28963330-28964020	3.60E-08	0.265570816	0.083	0.023	0.002387058	C4_CD8_Teff
chr11-6200930-6201816	3.66E-08	0.270734071	0.096	0.031	0.002421385	C4_CD8_Teff
chr10-124695883-124696949	3.72E-08	0.271171546	0.161	0.075	0.002465941	C4_CD8_Teff
chr7-30162240-30163768	3.73E-08	0.275260504	0.154	0.065	0.002470139	C4_CD8_Teff
chr11-66327742-66328683	3.74E-08	0.266151959	0.172	0.082	0.002474334	C4_CD8_Teff
chr18-51211099-51212009	3.74E-08	0.273420146	0.086	0.028	0.002475439	C4_CD8_Teff
chr22-26596770-26597398	3.77E-08	0.258660923	0.081	0.02	0.00249661	C4_CD8_Teff
chr15-89129195-89130578	3.79E-08	0.273960992	0.128	0.046	0.002507281	C4_CD8_Teff
chr8-21857822-21858865	3.79E-08	0.277728771	0.146	0.059	0.002511796	C4_CD8_Teff
chr5-178116284-178116862	3.89E-08	0.270524287	0.188	0.089	0.002577063	C4_CD8_Teff
chr15-63892374-63893307	4.05E-08	0.265767557	0.232	0.115	0.002683231	C4_CD8_Teff
chr11-87173212-87174046	4.07E-08	0.269522744	0.094	0.03	0.002695745	C4_CD8_Teff
chr10-69027233-69027964	4.07E-08	0.27213027	0.104	0.033	0.002696206	C4_CD8_Teff
chr13-48749044-48750162	4.11E-08	0.266262772	0.247	0.13	0.00272098	C4_CD8_Teff
chr15-90407769-90408558	4.25E-08	0.263183735	0.219	0.111	0.002815279	C4_CD8_Teff
chr8-142838261-142838584	4.25E-08	0.272956888	0.12	0.044	0.002817093	C4_CD8_Teff
chr8-134277771-134278663	4.28E-08	0.274914969	0.143	0.052	0.002835131	C4_CD8_Teff
chr12-109944485-109945124	4.58E-08	0.254008746	0.057	0.011	0.00303094	C4_CD8_Teff

chr22-17842110-17842749	4.65E-08	0.267184652	0.089	0.027	0.00307935	C4_CD8_Teff
chr9-76577241-76578191	4.65E-08	0.2698139	0.172	0.078	0.003082365	C4_CD8_Teff
chr12-10397299-10397643	4.74E-08	0.269535217	0.102	0.035	0.003137379	C4_CD8_Teff
chr10-74078013-74078502	4.75E-08	0.272014313	0.169	0.082	0.003147397	C4_CD8_Teff
chr5-14144550-14145397	4.85E-08	0.266593598	0.096	0.032	0.003212266	C4_CD8_Teff
chr3-187828673-187829471	4.90E-08	0.252152015	0.062	0.013	0.003241916	C4_CD8_Teff
chr15-40313542-40313961	4.90E-08	0.267238114	0.115	0.039	0.003243148	C4_CD8_Teff
chr4-152485570-152486277	5.09E-08	0.264624198	0.24	0.126	0.00337348	C4_CD8_Teff
chr16-17026504-17027203	5.10E-08	0.264972859	0.115	0.046	0.003378872	C4_CD8_Teff
chr14-53148606-53148980	5.26E-08	0.274590231	0.115	0.044	0.003482169	C4_CD8_Teff
chr4-38128861-38129538	5.27E-08	0.264686118	0.234	0.123	0.00349281	C4_CD8_Teff
chr15-67056025-67056759	5.33E-08	0.251391846	0.07	0.017	0.003527345	C4_CD8_Teff
chr16-3962590-3963461	5.41E-08	0.26360043	0.203	0.102	0.003585707	C4_CD8_Teff
chr19-16341979-16342646	5.54E-08	0.26557334	0.112	0.037	0.003666408	C4_CD8_Teff
chr14-89385981-89386791	5.69E-08	0.258833304	0.065	0.016	0.003765575	C4_CD8_Teff
chr15-29031439-29032244	5.72E-08	0.266756629	0.102	0.033	0.003790971	C4_CD8_Teff
chr1-64863926-64864532	5.74E-08	0.266981143	0.169	0.077	0.003800966	C4_CD8_Teff
chr19-13121941-13122660	5.80E-08	0.267073071	0.096	0.028	0.003840167	C4_CD8_Teff
chr2-97742298-97742710	5.81E-08	0.256924577	0.086	0.025	0.003844605	C4_CD8_Teff
chr8-102106398-102107034	6.05E-08	0.265577555	0.122	0.048	0.004003444	C4_CD8_Teff
chr3-50502718-50503940	6.05E-08	0.260123333	0.214	0.108	0.004009616	C4_CD8_Teff
chr17-82847888-82848563	6.07E-08	0.26915363	0.096	0.035	0.004020496	C4_CD8_Teff
chr7-29337189-29338067	6.19E-08	0.268082212	0.115	0.042	0.004102445	C4_CD8_Teff
chr2-176852334-176853132	6.47E-08	0.264777182	0.117	0.043	0.004286879	C4_CD8_Teff
chr10-110460102-110461221	6.50E-08	0.261684529	0.086	0.027	0.004305286	C4_CD8_Teff
chr11-82955716-82956337	6.81E-08	0.269976945	0.122	0.048	0.004510848	C4_CD8_Teff
chr1-172658784-172659555	6.84E-08	0.2571053	0.227	0.118	0.00452966	C4_CD8_Teff

chr10-123771922-123773586	6.89E-08	0.261772025	0.089	0.024	0.004563259	C4_CD8_Teff
chr13-48402346-48403628	6.92E-08	0.27089326	0.151	0.064	0.004581112	C4_CD8_Teff
chr1-167450803-167451067	7.10E-08	0.26165787	0.091	0.031	0.004703572	C4_CD8_Teff
chr1-27595107-27595923	7.18E-08	0.266290039	0.109	0.04	0.004751516	C4_CD8_Teff
chr8-134619814-134620447	7.18E-08	0.250407028	0.06	0.014	0.004752675	C4_CD8_Teff
chr15-40256386-40256899	7.19E-08	0.260508314	0.086	0.027	0.00476201	C4_CD8_Teff
chr4-2811710-2814312	7.23E-08	0.252633392	0.211	0.115	0.004789141	C4_CD8_Teff
chr4-38654125-38654687	7.34E-08	0.270776715	0.122	0.046	0.004858725	C4_CD8_Teff
chr7-70679344-70680043	7.38E-08	0.264593255	0.102	0.037	0.004886202	C4_CD8_Teff
chr12-120480248-120481561	7.42E-08	0.262220059	0.167	0.079	0.004912293	C4_CD8_Teff
chr6-151374675-151375151	7.84E-08	0.267989402	0.135	0.055	0.00518953	C4_CD8_Teff
chr14-72594028-72594869	7.89E-08	0.266352291	0.117	0.044	0.005221688	C4_CD8_Teff
chr14-97924862-97925737	7.92E-08	0.262467412	0.096	0.034	0.005247678	C4_CD8_Teff
chr2-195535842-195536527	8.03E-08	0.261960276	0.193	0.097	0.0053161	C4_CD8_Teff
chr4-38654965-38655565	8.15E-08	0.265527714	0.154	0.069	0.005399832	C4_CD8_Teff
chr17-74738099-74738599	8.63E-08	0.261701575	0.188	0.095	0.005712623	C4_CD8_Teff
chr12-104569012-104570380	8.81E-08	0.253891387	0.25	0.139	0.005837441	C4_CD8_Teff
chr2-173358704-173359585	8.88E-08	0.258971878	0.089	0.03	0.005880236	C4_CD8_Teff
chr1-92029675-92030632	9.30E-08	0.255345369	0.081	0.026	0.006158155	C4_CD8_Teff
chr9-96425291-96426243	9.33E-08	0.259021279	0.115	0.043	0.006177122	C4_CD8_Teff
chr22-48097850-48099083	9.33E-08	0.26103386	0.135	0.053	0.006177676	C4_CD8_Teff
chr17-8195873-8196405	1.01E-07	0.267403203	0.12	0.046	0.00666313	C4_CD8_Teff
chr20-24027145-24027791	1.01E-07	0.264761908	0.151	0.066	0.006665638	C4_CD8_Teff
chr17-49968095-49969213	1.05E-07	0.259266929	0.146	0.068	0.006925924	C4_CD8_Teff
chr18-63788477-63789018	1.05E-07	0.262642006	0.083	0.025	0.006964057	C4_CD8_Teff
chr19-7690692-7691941	1.05E-07	0.259436847	0.195	0.093	0.006971533	C4_CD8_Teff
chr2-136015518-136016116	1.06E-07	0.264062033	0.102	0.036	0.007013883	C4_CD8_Teff

chr20-23415656-23416294	1.06E-07	0.260022493	0.12	0.045	0.007044353	C4_CD8_Teff
chr18-22391357-22392033	1.07E-07	0.264695872	0.112	0.041	0.007095703	C4_CD8_Teff
chr13-114226172-114227229	1.10E-07	0.259612267	0.117	0.048	0.007293576	C4_CD8_Teff
chr19-6551597-6552336	1.12E-07	0.263561528	0.141	0.058	0.007438855	C4_CD8_Teff
chr10-62852926-62854146	1.20E-07	0.266695401	0.146	0.058	0.007929313	C4_CD8_Teff
chr12-128829708-128830511	1.20E-07	0.251133157	0.068	0.018	0.007946053	C4_CD8_Teff
chr9-131233206-131234405	1.23E-07	0.259964616	0.156	0.069	0.008172868	C4_CD8_Teff
chr3-50450069-50451178	1.28E-07	0.263280206	0.161	0.076	0.00844979	C4_CD8_Teff
chr19-29622058-29622716	1.31E-07	0.262538544	0.161	0.073	0.008690611	C4_CD8_Teff
chr15-67085167-67086102	1.33E-07	0.264106569	0.133	0.057	0.008786332	C4_CD8_Teff
chr1-157700551-157701070	1.35E-07	0.255700167	0.07	0.018	0.008936607	C4_CD8_Teff
chr5-111757075-111758261	1.37E-07	0.259899369	0.122	0.049	0.009087151	C4_CD8_Teff
chr10-33109506-33110292	1.38E-07	0.255541143	0.109	0.039	0.009109265	C4_CD8_Teff
chr2-8519077-8519504	1.38E-07	0.259137564	0.076	0.022	0.009140272	C4_CD8_Teff
chr7-8197959-8198778	1.39E-07	0.258879701	0.104	0.037	0.009208695	C4_CD8_Teff
chr17-76492438-76494209	1.40E-07	0.255973675	0.201	0.104	0.009245495	C4_CD8_Teff
chr20-49661394-49662084	1.44E-07	0.253226419	0.078	0.023	0.009509812	C4_CD8_Teff
chr16-89025805-89026546	1.45E-07	0.25589577	0.198	0.098	0.009596109	C4_CD8_Teff
chr10-72326543-72327272	1.45E-07	0.261071355	0.146	0.062	0.00963063	C4_CD8_Teff
chr13-48214433-48215171	1.54E-07	0.264684853	0.138	0.058	0.010200074	C4_CD8_Teff
chr2-118185496-118186673	1.62E-07	0.252485763	0.203	0.106	0.0107122	C4_CD8_Teff
chr11-125495128-125496476	1.62E-07	0.257306854	0.094	0.03	0.010738401	C4_CD8_Teff
chr10-89367247-89367952	1.64E-07	0.262429855	0.141	0.056	0.010831786	C4_CD8_Teff
chr14-99319986-99321155	1.65E-07	0.256105522	0.107	0.036	0.010917755	C4_CD8_Teff
chr8-66445743-66446442	1.68E-07	0.253137056	0.094	0.028	0.011105163	C4_CD8_Teff
chr22-50561408-50562188	1.73E-07	0.253385567	0.109	0.037	0.01142831	C4_CD8_Teff
chr8-65954414-65954759	1.75E-07	0.257659955	0.109	0.04	0.011595538	C4_CD8_Teff

chrX-149513423-149514464	1.77E-07	0.260427863	0.141	0.058	0.01173014	C4_CD8_Teff
chr16-11621593-11622551	1.77E-07	0.257930401	0.104	0.038	0.011730382	C4_CD8_Teff
chr17-30975014-30975406	1.78E-07	0.251462993	0.081	0.022	0.011758219	C4_CD8_Teff
chr7-150999190-151000200	1.82E-07	0.252757342	0.081	0.024	0.012077902	C4_CD8_Teff
chr21-32538212-32538825	1.83E-07	0.251920176	0.076	0.023	0.012115724	C4_CD8_Teff
chr9-94860534-94861729	1.84E-07	0.255445251	0.169	0.082	0.012200519	C4_CD8_Teff
chr7-99997237-99997915	1.85E-07	0.257270353	0.151	0.063	0.012267275	C4_CD8_Teff
chr20-17530505-17531516	1.94E-07	0.2536315	0.117	0.045	0.012833531	C4_CD8_Teff
chr1-206768971-206769676	1.94E-07	0.256906409	0.109	0.044	0.012868936	C4_CD8_Teff
chr14-24694575-24695679	1.95E-07	0.257257048	0.159	0.071	0.012925139	C4_CD8_Teff
chr5-1494704-1495740	2.07E-07	0.257653715	0.104	0.04	0.013701921	C4_CD8_Teff
chr19-4057661-4058571	2.08E-07	0.255215758	0.161	0.072	0.013759399	C4_CD8_Teff
chr5-67514805-67516138	2.09E-07	0.256829441	0.148	0.066	0.013836363	C4_CD8_Teff
chr7-129553822-129554510	2.18E-07	0.259621486	0.12	0.044	0.014411915	C4_CD8_Teff
chr10-70579662-70580033	2.20E-07	0.252741036	0.091	0.031	0.014545019	C4_CD8_Teff
chr9-96149625-96150847	2.24E-07	0.2565684	0.182	0.089	0.01485138	C4_CD8_Teff
chr1-198101728-198102973	2.25E-07	0.258302161	0.141	0.06	0.014878954	C4_CD8_Teff
chr1-15252568-15253223	2.25E-07	0.253670664	0.089	0.028	0.014897645	C4_CD8_Teff
chr1-224205410-224205763	2.31E-07	0.264205859	0.117	0.05	0.015287032	C4_CD8_Teff
chr1-207882657-207883680	2.31E-07	0.257800895	0.143	0.065	0.015289425	C4_CD8_Teff
chr10-95290491-95291334	2.34E-07	0.252581685	0.089	0.029	0.015494668	C4_CD8_Teff
chr3-106068461-106069158	2.38E-07	0.254447076	0.122	0.048	0.01577604	C4_CD8_Teff
chr8-130006613-130007340	2.54E-07	0.256438631	0.115	0.047	0.016843091	C4_CD8_Teff
chr12-123937569-123938541	2.58E-07	0.255155393	0.122	0.049	0.017112526	C4_CD8_Teff
chr8-124687474-124688295	2.59E-07	0.255174198	0.12	0.048	0.017160215	C4_CD8_Teff
chr12-121649323-121650217	2.64E-07	0.25653654	0.107	0.043	0.017467222	C4_CD8_Teff
chr14-61444115-61444716	3.00E-07	0.254181947	0.148	0.073	0.019886858	C4_CD8_Teff

chr10-128102928-128103793	3.05E-07	0.250262548	0.099	0.037	0.020228058	C4_CD8_Teff
chr18-80246442-80247812	3.11E-07	0.250770879	0.185	0.094	0.020599987	C4_CD8_Teff
chr5-40479909-40480810	3.51E-07	0.25319956	0.133	0.055	0.023262768	C4_CD8_Teff
chr11-46127081-46127547	3.54E-07	0.257210006	0.133	0.055	0.023449937	C4_CD8_Teff
chr5-115068359-115069186	4.44E-07	0.250840887	0.099	0.036	0.029409302	C4_CD8_Teff
chr9-114696737-114697683	4.47E-07	0.25324716	0.156	0.081	0.029603459	C4_CD8_Teff
chr3-15635835-15636753	5.88E-07	0.250494742	0.107	0.042	0.038943253	C4_CD8_Teff
chr18-79156679-79157477	1.37E-19	0.453514668	0.156	0.033	9.05E-15	C4_CD8_Teff
chr6-36118475-36118945	6.66E-12	0.33605457	0.25	0.11	4.41E-07	C4_CD8_Teff
chr15-75042673-75043371	1.09E-12	0.352638404	0.13	0.035	7.20E-08	C4_CD8_Teff
chr17-78789249-78789852	3.63E-10	0.2976909	0.078	0.018	2.40E-05	C4_CD8_Teff
chr19-16341979-16342646	5.54E-08	0.26557334	0.112	0.037	0.003666408	C4_CD8_Teff
chr4-38654965-38655565	8.15E-08	0.265527714	0.154	0.069	0.005399832	C4_CD8_Teff
chr16-3962590-3963461	5.41E-08	0.26360043	0.203	0.102	0.003585707	C4_CD8_Teff
chr5-111757075-111758261	1.37E-07	0.259899369	0.122	0.049	0.009087151	C4_CD8_Teff
chr20-24027145-24027791	1.01E-07	0.264761908	0.151	0.066	0.006665638	C4_CD8_Teff
chr20-2729134-2729727	1.55E-09	0.294926643	0.086	0.022	0.00010235	C4_CD8_Teff
chr21-36297515-36298548	3.47E-09	0.282286915	0.232	0.117	0.000229636	C4_CD8_Teff
chr8-124687474-124688295	2.59E-07	0.255174198	0.12	0.048	0.017160215	C4_CD8_Teff
chr9-95462185-95463306	2.56E-08	0.276031858	0.193	0.092	0.001692333	C4_CD8_Teff
chr18-69895209-69896744	8.36E-13	0.346461737	0.26	0.112	5.54E-08	C4_CD8_Teff
chr19-13886722-13887896	2.02E-14	0.35464659	0.323	0.14	1.34E-09	C4_CD8_Teff
chr1-11692749-11693567	1.15E-11	0.336514749	0.198	0.078	7.59E-07	C4_CD8_Teff
chr9-96149625-96150847	2.24E-07	0.2565684	0.182	0.089	0.01485138	C4_CD8_Teff
chr8-20251411-20252448	3.35E-13	0.328723658	0.344	0.183	2.22E-08	C4_CD8_Teff
chr2-69770122-69770552	2.51E-15	0.388414102	0.12	0.024	1.66E-10	C4_CD8_Teff
chr19-13121941-13122660	5.80E-08	0.267073071	0.096	0.028	0.003840167	C4_CD8_Teff

chr20-50450224-50451398	1.69E-08	0.271301416	0.081	0.025	0.001118136	C4_CD8_Teff
chr8-25198436-25199354	1.37E-44	0.68473946	0.286	0.046	9.07E-40	C4_CD8_Teff
chr3-168094718-168096447	1.31E-08	0.284178147	0.125	0.04	0.000867964	C4_CD8_Teff
chr19-49613385-49614834	1.98E-25	0.514263328	0.203	0.046	1.31E-20	C4_CD8_Teff
chr16-17224454-17225255	6.57E-20	0.432211641	0.104	0.013	4.35E-15	C4_CD8_Teff
chr14-24631806-24632062	1.97E-16	0.376214848	0.081	0.008	1.30E-11	C4_CD8_Teff
chr8-38936586-38938972	5.52E-15	0.284653217	0.576	0.374	3.66E-10	C4_CD8_Teff
chr9-130834161-130835912	1.69E-08	0.250987941	0.32	0.188	0.001116228	C4_CD8_Teff
chr4-7649400-7650381	3.92E-38	0.571318053	0.482	0.169	2.60E-33	C4_CD8_Teff
chr12-10303912-10304558	6.94E-19	0.44101507	0.177	0.044	4.59E-14	C4_CD8_Teff
chr7-132430566-132431673	1.20E-12	0.355037889	0.224	0.087	7.92E-08	C4_CD8_Teff
chr8-142838261-142838584	4.25E-08	0.272956888	0.12	0.044	0.002817093	C4_CD8_Teff
chr17-35895690-35896610	2.08E-08	0.267896586	0.214	0.11	0.001378168	C4_CD8_Teff
chr9-76576235-76576755	4.09E-14	0.373488058	0.219	0.087	2.71E-09	C4_CD8_Teff
chr9-114696737-114697683	4.47E-07	0.25324716	0.156	0.081	0.029603459	C4_CD8_Teff
chr9-91163890-91165114	1.59E-09	0.292645901	0.234	0.11	0.000105421	C4_CD8_Teff
chr16-17342801-17344141	1.63E-29	0.538428473	0.331	0.1	1.08E-24	C4_CD8_Teff
chr7-2413390-2414104	4.82E-14	0.367162552	0.245	0.09	3.19E-09	C4_CD8_Teff
chr14-24329575-24330083	1.01E-13	0.359986369	0.229	0.096	6.66E-09	C4_CD8_Teff
chr21-37225366-37225847	3.98E-09	0.29127122	0.122	0.046	0.000263695	C4_CD8_Teff
chr8-25345238-25345791	2.97E-23	0.476357696	0.135	0.019	1.97E-18	C4_CD8_Teff
chr3-172106905-172108757	5.60E-11	0.286228063	0.375	0.218	3.71E-06	C4_CD8_Teff
chr10-70601791-70603858	3.96E-27	0.46654361	0.422	0.182	2.63E-22	C4_CD8_Teff
chr7-3106217-3106891	2.24E-12	0.353873905	0.135	0.041	1.48E-07	C4_CD8_Teff
chr15-80159704-80160975	1.36E-09	0.300176247	0.125	0.043	9.01E-05	C4_CD8_Teff
chr3-56753188-56753488	1.78E-14	0.366403551	0.094	0.015	1.18E-09	C4_CD8_Teff
chr22-39960633-39961820	6.31E-17	0.375792401	0.352	0.161	4.18E-12	C4_CD8_Teff

chr14-97924862-97925737	7.92E-08	0.262467412	0.096	0.034	0.005247678	C4_CD8_Teff
chr9-76577241-76578191	4.65E-08	0.2698139	0.172	0.078	0.003082365	C4_CD8_Teff
chr14-60753105-60754193	3.72E-13	0.334168594	0.326	0.155	2.47E-08	C4_CD8_Teff
chr21-32538212-32538825	1.83E-07	0.251920176	0.076	0.023	0.012115724	C4_CD8_Teff
chr11-126329264-126329992	1.37E-13	0.374691547	0.151	0.038	9.08E-09	C4_CD8_Teff
chr2-28406519-28407079	7.76E-10	0.291571958	0.247	0.121	5.14E-05	C4_CD8_Teff
chr8-25319299-25320014	5.47E-13	0.354336216	0.104	0.022	3.62E-08	C4_CD8_Teff
chr3-15635835-15636753	5.88E-07	0.250494742	0.107	0.042	0.038943253	C4_CD8_Teff
chr12-10397299-10397643	4.74E-08	0.269535217	0.102	0.035	0.003137379	C4_CD8_Teff
chr19-29587087-29588120	3.10E-12	0.315408962	0.385	0.189	2.06E-07	C4_CD8_Teff
chr9-96435774-96436570	2.96E-19	0.450393724	0.201	0.054	1.96E-14	C4_CD8_Teff
chr5-59098804-59099932	3.27E-11	0.298918898	0.346	0.18	2.17E-06	C4_CD8_Teff
chr21-46052450-46053732	2.55E-09	0.295996052	0.117	0.039	0.000169184	C4_CD8_Teff
chr15-28974053-28974483	2.46E-12	0.337335099	0.086	0.017	1.63E-07	C4_CD8_Teff
chr14-24694575-24695679	1.95E-07	0.257257048	0.159	0.071	0.012925139	C4_CD8_Teff
chr17-17662891-17664024	2.40E-16	0.34581472	0.451	0.243	1.59E-11	C4_CD8_Teff
chr7-1042157-1042471	2.41E-12	0.332878514	0.078	0.012	1.60E-07	C4_CD8_Teff
chr2-7748717-7749360	3.43E-08	0.269463988	0.135	0.055	0.002273427	C4_CD8_Teff
chr14-94945532-94946593	7.07E-18	0.440400511	0.164	0.04	4.68E-13	C4_CD8_Teff
chr2-28508089-28508845	8.77E-10	0.308907123	0.167	0.066	5.80E-05	C4_CD8_Teff
chr3-5037775-5038498	2.54E-08	0.267463919	0.086	0.025	0.001681372	C4_CD8_Teff
chr18-51211099-51212009	3.74E-08	0.273420146	0.086	0.028	0.002475439	C4_CD8_Teff
chr20-45945960-45946761	5.86E-22	0.485848065	0.203	0.05	3.88E-17	C4_CD8_Teff
chr17-74730383-74731073	5.31E-14	0.363378644	0.227	0.092	3.52E-09	C4_CD8_Teff
chr8-133575431-133575978	7.28E-09	0.284155508	0.221	0.1	0.000482357	C4_CD8_Teff
chr6-30963694-30964716	6.88E-14	0.375843438	0.128	0.029	4.56E-09	C4_CD8_Teff
chr7-8197959-8198778	1.39E-07	0.258879701	0.104	0.037	0.009208695	C4_CD8_Teff

chr16-326942-327829	1.98E-21	0.413357986	0.406	0.191	1.31E-16	C4_CD8_Teff
chr13-84126553-84127232	2.29E-11	0.320193035	0.083	0.018	1.52E-06	C4_CD8_Teff
chr2-85701487-85701818	6.34E-19	0.425207389	0.117	0.017	4.20E-14	C4_CD8_Teff
chr15-75042673-75043371	1.09E-12	0.352638404	0.13	0.035	7.20E-08	C4_CD8_Teff
chr15-94304939-94305540	5.33E-20	0.454993984	0.185	0.045	3.53E-15	C4_CD8_Teff
chr2-85702201-85702450	1.24E-08	0.262905879	0.073	0.017	0.000818712	C4_CD8_Teff
chr4-82797899-82799412	1.58E-08	0.27700016	0.203	0.091	0.001045634	C4_CD8_Teff
chr7-3103636-3103889	2.34E-12	0.320747824	0.068	0.009	1.55E-07	C4_CD8_Teff
chr8-124751393-124752265	3.05E-09	0.290803826	0.115	0.038	0.000202034	C4_CD8_Teff
chr15-28963330-28964020	3.60E-08	0.265570816	0.083	0.023	0.002387058	C4_CD8_Teff
chr1-55127628-55128847	4.94E-16	0.399740499	0.234	0.08	3.27E-11	C4_CD8_Teff
chr1-172658784-172659555	6.84E-08	0.2571053	0.227	0.118	0.00452966	C4_CD8_Teff
chr2-85693753-85694481	3.00E-28	0.534074177	0.151	0.018	1.98E-23	C4_CD8_Teff
chr7-132450906-132451511	1.15E-13	0.362332277	0.117	0.028	7.61E-09	C4_CD8_Teff
chr1-27875221-27875516	9.38E-09	0.278007157	0.083	0.022	0.000620993	C4_CD8_Teff
chr12-121649323-121650217	2.64E-07	0.25653654	0.107	0.043	0.017467222	C4_CD8_Teff
chr2-216349913-216350784	6.43E-15	0.379151701	0.263	0.114	4.26E-10	C4_CD8_Teff
chr11-364121-364750	3.43E-11	0.332718255	0.151	0.055	2.27E-06	C4_CD8_Teff
chr14-74573789-74574567	1.99E-08	0.276094086	0.193	0.089	0.001317212	C4_CD8_Teff
chr14-92615207-92615959	2.97E-15	0.395164613	0.208	0.073	1.97E-10	C4_CD8_Teff
chr19-41302554-41303090	4.98E-10	0.298798913	0.203	0.093	3.30E-05	C4_CD8_Teff
chr14-35332999-35334752	3.48E-10	0.311409381	0.159	0.057	2.30E-05	C4_CD8_Teff
chr6-112285701-112286474	2.65E-09	0.296855888	0.122	0.042	0.000175374	C4_CD8_Teff
chr20-17530505-17531516	1.94E-07	0.2536315	0.117	0.045	0.012833531	C4_CD8_Teff
chr11-6200930-6201816	3.66E-08	0.270734071	0.096	0.031	0.002421385	C4_CD8_Teff
chr12-128782326-128783599	2.58E-28	0.528596469	0.307	0.09	1.71E-23	C4_CD8_Teff
chr1-167430678-167431750	5.33E-21	0.466086193	0.135	0.023	3.53E-16	C4_CD8_Teff

chr19-13886722-13887896	2.02E-14	0.35464659	0.323	0.14	1.34E-09	C4_CD8_Teff
chr10-133278484-133279189	6.26E-22	0.467962852	0.245	0.079	4.14E-17	C4_CD8_Teff
chr12-10303912-10304558	6.94E-19	0.44101507	0.177	0.044	4.59E-14	C4_CD8_Teff
chr8-133575431-133575978	7.28E-09	0.284155508	0.221	0.1	0.000482357	C4_CD8_Teff
chr22-21772845-21773238	9.62E-10	0.287844613	0.068	0.013	6.37E-05	C4_CD8_Teff
chr10-96651690-96652627	3.89E-11	0.331069333	0.143	0.045	2.58E-06	C4_CD8_Teff
chr8-129993546-129995502	4.49E-24	0.482067471	0.294	0.103	2.97E-19	C4_CD8_Teff
chr9-37512116-37512807	9.47E-19	0.448152946	0.167	0.036	6.27E-14	C4_CD8_Teff
chr18-69895209-69896744	8.36E-13	0.346461737	0.26	0.112	5.54E-08	C4_CD8_Teff
chr11-126329264-126329992	1.37E-13	0.374691547	0.151	0.038	9.08E-09	C4_CD8_Teff
chr16-31364431-31365532	5.66E-25	0.524868809	0.203	0.04	3.75E-20	C4_CD8_Teff
chr1-33045281-33046369	1.17E-08	0.278233456	0.193	0.09	0.00077781	C4_CD8_Teff
chr20-50450224-50451398	1.69E-08	0.271301416	0.081	0.025	0.001118136	C4_CD8_Teff
chr7-121530116-121531141	4.44E-32	0.536595767	0.417	0.149	2.94E-27	C4_CD8_Teff
chr16-89020070-89020713	4.03E-12	0.342997218	0.117	0.03	2.67E-07	C4_CD8_Teff
chr7-2862813-2864025	2.76E-10	0.304386676	0.208	0.097	1.83E-05	C4_CD8_Teff
chr10-70601791-70603858	3.96E-27	0.46654361	0.422	0.182	2.63E-22	C4_CD8_Teff
chr18-79326345-79328199	2.48E-10	0.31306906	0.117	0.031	1.64E-05	C4_CD8_Teff
chr7-132430566-132431673	1.20E-12	0.355037889	0.224	0.087	7.92E-08	C4_CD8_Teff
chr6-170147237-170148491	4.08E-12	0.343822966	0.182	0.07	2.70E-07	C4_CD8_Teff
chr18-22237893-22238540	2.27E-12	0.32265492	0.068	0.011	1.50E-07	C4_CD8_Teff
chr2-195649750-195650636	5.21E-30	0.55519389	0.188	0.031	3.45E-25	C4_CD8_Teff
chr7-128422793-128423306	1.02E-13	0.363922896	0.112	0.023	6.75E-09	C4_CD8_Teff
chr7-38240794-38241149	7.35E-09	0.278675506	0.078	0.02	0.000486426	C4_CD8_Teff
chr10-44310373-44311620	8.32E-17	0.408287335	0.164	0.044	5.51E-12	C4_CD8_Teff
chr10-62852926-62854146	1.20E-07	0.266695401	0.146	0.058	0.007929313	C4_CD8_Teff
chr18-22344517-22345574	1.75E-09	0.297595204	0.172	0.068	0.000116115	C4_CD8_Teff

chr1-184386329-184388089	2.62E-47	0.634592725	0.518	0.158	1.74E-42	C4_CD8_Teff
chr5-42961865-42962585	1.13E-09	0.29123071	0.083	0.02	7.47E-05	C4_CD8_Teff
chr12-104569012-104570380	8.81E-08	0.253891387	0.25	0.139	0.005837441	C4_CD8_Teff
chr12-121649323-121650217	2.64E-07	0.25653654	0.107	0.043	0.017467222	C4_CD8_Teff
chr11-6196303-6196651	1.72E-09	0.27443443	0.06	0.01	0.000113803	C4_CD8_Teff
chr10-124611652-124613168	4.83E-10	0.300701015	0.219	0.098	3.20E-05	C4_CD8_Teff
chr12-128794493-128795204	8.00E-11	0.318348042	0.156	0.058	5.30E-06	C4_CD8_Teff
chr19-34184824-34185469	2.37E-13	0.365948116	0.122	0.031	1.57E-08	C4_CD8_Teff
chr12-128569992-128570692	6.45E-14	0.374176407	0.13	0.033	4.27E-09	C4_CD8_Teff
chrX-47361618-47362377	1.93E-15	0.397843238	0.195	0.063	1.28E-10	C4_CD8_Teff
chr1-45393590-45394814	4.09E-12	0.346337803	0.138	0.044	2.71E-07	C4_CD8_Teff
chr15-94304939-94305540	5.33E-20	0.454993984	0.185	0.045	3.53E-15	C4_CD8_Teff
chr17-1607193-1607853	8.15E-19	0.429701142	0.117	0.019	5.40E-14	C4_CD8_Teff
chr14-35332999-35334752	3.48E-10	0.311409381	0.159	0.057	2.30E-05	C4_CD8_Teff
chr11-66272627-66272942	2.93E-10	0.303163953	0.109	0.032	1.94E-05	C4_CD8_Teff
chr8-21857822-21858865	3.79E-08	0.277728771	0.146	0.059	0.002511796	C4_CD8_Teff
chr19-14459391-14460097	5.72E-26	0.524454138	0.195	0.04	3.79E-21	C4_CD8_Teff
chr9-130834161-130835912	1.69E-08	0.250987941	0.32	0.188	0.001116228	C4_CD8_Teff
chr12-8987598-8988061	3.03E-09	0.291689917	0.115	0.035	0.000200675	C4_CD8_Teff
chr15-95864358-95864855	1.91E-12	0.328726925	0.07	0.01	1.27E-07	C4_CD8_Teff
chr5-139635806-139637900	1.35E-11	0.300805279	0.349	0.192	8.96E-07	C4_CD8_Teff
chr22-25054537-25055508	8.10E-15	0.386689932	0.156	0.044	5.36E-10	C4_CD8_Teff
chr3-50588289-50588897	1.27E-10	0.312361434	0.237	0.108	8.38E-06	C4_CD8_Teff
chr1-167450803-167451067	7.10E-08	0.26165787	0.091	0.031	0.004703572	C4_CD8_Teff
chr10-79027120-79028053	1.64E-26	0.524587261	0.273	0.074	1.09E-21	C4_CD8_Teff
chr16-81831426-81832215	8.93E-09	0.285518044	0.099	0.031	0.000591439	C4_CD8_Teff
chr22-46280826-46281500	2.67E-37	0.637610293	0.237	0.04	1.77E-32	C4_CD8_Teff

chr5-1494704-1495740	2.07E-07	0.257653715	0.104	0.04	0.013701921	C4_CD8_Teff
chr5-95973693-95974337	1.82E-09	0.286193559	0.083	0.02	0.000120592	C4_CD8_Teff
chr9-91163890-91165114	1.59E-09	0.292645901	0.234	0.11	0.000105421	C4_CD8_Teff
chr7-36863792-36865390	1.05E-31	0.568951444	0.268	0.065	6.96E-27	C4_CD8_Teff
chr16-82945391-82946046	7.89E-09	0.262772116	0.073	0.016	0.000522698	C4_CD8_Teff
chr16-31354297-31355200	2.06E-11	0.319330413	0.094	0.02	1.36E-06	C4_CD8_Teff
chr1-91731336-91732897	5.15E-16	0.401238478	0.211	0.07	3.41E-11	C4_CD8_Teff
chr1-41728547-41729134	2.79E-19	0.44392428	0.125	0.02	1.84E-14	C4_CD8_Teff
chr19-52778593-52779552	5.05E-10	0.300437777	0.102	0.03	3.34E-05	C4_CD8_Teff
chr7-29337189-29338067	6.19E-08	0.268082212	0.115	0.042	0.004102445	C4_CD8_Teff
chr18-22391357-22392033	1.07E-07	0.264695872	0.112	0.041	0.007095703	C4_CD8_Teff
chr9-136572519-136572836	1.70E-19	0.360984765	0.055	0.001	1.12E-14	C4_CD8_Teff
chr19-13181768-13182417	1.58E-08	0.259868472	0.073	0.017	0.001047649	C4_CD8_Teff
chr7-70679344-70680043	7.38E-08	0.264593255	0.102	0.037	0.004886202	C4_CD8_Teff
chr3-50450069-50451178	1.28E-07	0.263280206	0.161	0.076	0.00844979	C4_CD8_Teff
chr7-132485235-132486067	4.01E-14	0.378503506	0.185	0.06	2.65E-09	C4_CD8_Teff
chrX-1481614-1482542	3.06E-09	0.263142075	0.32	0.193	0.00020278	C4_CD8_Teff
chr1-184491300-184492350	7.54E-18	0.427038624	0.214	0.067	4.99E-13	C4_CD8_Teff
chr2-70851627-70852078	9.96E-14	0.341531	0.086	0.014	6.60E-09	C4_CD8_Teff
chr8-60925648-60926053	1.16E-10	0.320877195	0.104	0.027	7.69E-06	C4_CD8_Teff
chr17-83199175-83200859	1.65E-15	0.338068846	0.396	0.213	1.09E-10	C4_CD8_Teff
chr12-10249665-10250337	4.43E-24	0.479243699	0.122	0.014	2.93E-19	C4_CD8_Teff
chr19-52833277-52834042	3.78E-17	0.42994913	0.13	0.026	2.50E-12	C4_CD8_Teff
chr22-50561408-50562188	1.73E-07	0.253385567	0.109	0.037	0.01142831	C4_CD8_Teff
chr15-90407769-90408558	4.25E-08	0.263183735	0.219	0.111	0.002815279	C4_CD8_Teff
chr22-23263385-23264547	5.32E-26	0.483286159	0.362	0.147	3.52E-21	C4_CD8_Teff
chr7-70660047-70660932	3.09E-16	0.40143846	0.216	0.072	2.04E-11	C4_CD8_Teff

chr15-63892374-63893307	4.05E-08	0.265767557	0.232	0.115	0.002683231	C4_CD8_Teff
chr6-158717301-158718256	8.65E-23	0.478113141	0.24	0.073	5.73E-18	C4_CD8_Teff
chr13-80399322-80400669	3.74E-13	0.355803927	0.133	0.035	2.48E-08	C4_CD8_Teff
chr15-39623106-39624019	9.80E-09	0.258894352	0.328	0.189	0.000649295	C4_CD8_Teff
chr17-82257065-82257733	5.73E-18	0.434386741	0.172	0.048	3.80E-13	C4_CD8_Teff
chr9-96435774-96436570	2.96E-19	0.450393724	0.201	0.054	1.96E-14	C4_CD8_Teff
chr11-104163407-104164411	9.16E-33	0.595581315	0.201	0.028	6.07E-28	C4_CD8_Teff
chr2-110846345-110847414	2.69E-45	0.697561137	0.315	0.065	1.78E-40	C4_CD8_Teff
chr3-57671133-57671708	3.90E-17	0.41758737	0.198	0.062	2.58E-12	C4_CD8_Teff
chr7-55308550-55310016	2.44E-11	0.315552856	0.258	0.131	1.62E-06	C4_CD8_Teff
chr2-233385348-233386049	5.91E-25	0.511132321	0.159	0.029	3.91E-20	C4_CD8_Teff
chr13-99230475-99231286	3.14E-08	0.267236838	0.081	0.022	0.00208223	C4_CD8_Teff
chr8-56999227-56999847	3.61E-14	0.35675453	0.099	0.018	2.39E-09	C4_CD8_Teff
chr2-28406519-28407079	7.76E-10	0.291571958	0.247	0.121	5.14E-05	C4_CD8_Teff
chr8-38936586-38938972	5.52E-15	0.284653217	0.576	0.374	3.66E-10	C4_CD8_Teff
chr12-128829708-128830511	1.20E-07	0.251133157	0.068	0.018	0.007946053	C4_CD8_Teff
chr3-125110445-125111081	8.16E-09	0.264350251	0.06	0.012	0.000540629	C4_CD8_Teff
chr9-137000190-137000739	3.05E-10	0.307471797	0.151	0.059	2.02E-05	C4_CD8_Teff
chr19-4057661-4058571	2.08E-07	0.255215758	0.161	0.072	0.013759399	C4_CD8_Teff
chr12-101743658-101744460	3.69E-17	0.420742706	0.13	0.029	2.45E-12	C4_CD8_Teff
chr6-161242937-161243613	1.62E-47	0.708015296	0.221	0.02	1.07E-42	C4_CD8_Teff
chr19-10101748-10102490	4.35E-09	0.282879879	0.216	0.107	0.000287931	C4_CD8_Teff
chr19-10515556-10516304	6.52E-50	0.736091231	0.32	0.055	4.32E-45	C4_CD8_Teff
chr2-7748717-7749360	3.43E-08	0.269463988	0.135	0.055	0.002273427	C4_CD8_Teff
chr7-2902489-2903871	3.13E-17	0.420395734	0.146	0.033	2.07E-12	C4_CD8_Teff
chr2-121309656-121310145	1.99E-10	0.28967429	0.065	0.011	1.32E-05	C4_CD8_Teff
chr14-99286735-99287781	1.22E-19	0.443574107	0.174	0.041	8.05E-15	C4_CD8_Teff

chr16-17342801-17344141	1.63E-29	0.538428473	0.331	0.1	1.08E-24	C4_CD8_Teff
chr5-59098804-59099932	3.27E-11	0.298918898	0.346	0.18	2.17E-06	C4_CD8_Teff
chr19-16446958-16447229	1.23E-10	0.315628187	0.102	0.025	8.13E-06	C4_CD8_Teff
chr15-58331692-58332623	1.25E-24	0.488598378	0.318	0.111	8.28E-20	C4_CD8_Teff
chr18-22465727-22466370	3.18E-22	0.48647735	0.148	0.027	2.11E-17	C4_CD8_Teff
chr10-62631851-62632804	2.34E-09	0.28085215	0.271	0.145	0.00015496	C4_CD8_Teff
chr8-134277771-134278663	4.28E-08	0.274914969	0.143	0.052	0.002835131	C4_CD8_Teff
chr3-106068461-106069158	2.38E-07	0.254447076	0.122	0.048	0.01577604	C4_CD8_Teff
chr7-38242795-38243176	9.82E-23	0.501211033	0.182	0.04	6.50E-18	C4_CD8_Teff
chr7-74093222-74095067	1.17E-23	0.42068909	0.432	0.213	7.77E-19	C4_CD8_Teff
chr5-112417854-112418360	2.44E-16	0.417210322	0.128	0.025	1.62E-11	C4_CD8_Teff
chr11-3216973-3217537	5.31E-12	0.334220798	0.104	0.025	3.52E-07	C4_CD8_Teff
chr13-113176236-113176601	1.84E-20	0.454758786	0.125	0.02	1.22E-15	C4_CD8_Teff
chr2-110852222-110853029	3.92E-12	0.344986777	0.151	0.052	2.60E-07	C4_CD8_Teff
chr18-44678863-44681010	5.88E-20	0.41928062	0.326	0.132	3.90E-15	C4_CD8_Teff
chr12-128763410-128763767	2.31E-21	0.466823538	0.112	0.013	1.53E-16	C4_CD8_Teff
chr17-78474402-78475194	2.00E-10	0.295773366	0.078	0.017	1.32E-05	C4_CD8_Teff
chr18-48813554-48815949	1.01E-12	0.304846957	0.411	0.236	6.68E-08	C4_CD8_Teff
chr10-103053813-103054760	5.82E-15	0.386920903	0.208	0.076	3.86E-10	C4_CD8_Teff
chr14-60753105-60754193	3.72E-13	0.334168594	0.326	0.155	2.47E-08	C4_CD8_Teff
chr6-161226089-161227237	9.22E-19	0.421994793	0.117	0.019	6.11E-14	C4_CD8_Teff
chr12-10285702-10286335	4.74E-17	0.411939833	0.115	0.02	3.14E-12	C4_CD8_Teff
chr16-89025805-89026546	1.45E-07	0.25589577	0.198	0.098	0.009596109	C4_CD8_Teff
chr20-2851769-2852368	5.12E-20	0.460093528	0.148	0.029	3.39E-15	C4_CD8_Teff
chr2-30421510-30422839	3.05E-09	0.286034736	0.266	0.136	0.000202184	C4_CD8_Teff
chr2-98637417-98638125	9.96E-09	0.26658667	0.276	0.152	0.000659707	C4_CD8_Teff
chr19-12983457-12985835	6.94E-28	0.48812849	0.372	0.151	4.60E-23	C4_CD8_Teff

chr2-86079669-86080996	5.15E-11	0.29017996	0.352	0.194	3.41E-06	C4_CD8_Teff
chr7-129535854-129536653	9.07E-09	0.274997086	0.091	0.026	0.00060059	C4_CD8_Teff
chr10-30502118-30502877	9.77E-15	0.380404304	0.117	0.025	6.47E-10	C4_CD8_Teff
chr15-60541831-60542313	6.00E-10	0.299678295	0.086	0.021	3.97E-05	C4_CD8_Teff
chr17-17792269-17793223	2.89E-08	0.272283914	0.18	0.085	0.001914934	C4_CD8_Teff
chr22-25076110-25076689	1.54E-08	0.278887893	0.107	0.034	0.001019011	C4_CD8_Teff
chr11-125495128-125496476	1.62E-07	0.257306854	0.094	0.03	0.010738401	C4_CD8_Teff
chrY-7040598-7041260	4.43E-11	0.322876517	0.096	0.025	2.93E-06	C4_CD8_Teff
chr10-74078013-74078502	4.75E-08	0.272014313	0.169	0.082	0.003147397	C4_CD8_Teff
chr15-94272057-94272936	9.80E-29	0.552895456	0.214	0.044	6.49E-24	C4_CD8_Teff
chr19-2621692-2622070	2.06E-08	0.27558436	0.143	0.057	0.001366655	C4_CD8_Teff
chr4-7324150-7325121	5.09E-09	0.277986643	0.094	0.027	0.000337337	C4_CD8_Teff
chr16-89821523-89821914	4.68E-23	0.488855321	0.18	0.038	3.10E-18	C4_CD8_Teff
chr21-37225366-37225847	3.98E-09	0.29127122	0.122	0.046	0.000263695	C4_CD8_Teff
chr22-39960633-39961820	6.31E-17	0.375792401	0.352	0.161	4.18E-12	C4_CD8_Teff
chr16-326942-327829	1.98E-21	0.413357986	0.406	0.191	1.31E-16	C4_CD8_Teff
chr15-90889392-90889924	1.26E-08	0.274965138	0.081	0.019	0.000834124	C4_CD8_Teff
chr6-40477130-40478225	1.00E-21	0.460187099	0.292	0.099	6.62E-17	C4_CD8_Teff
chr1-160855164-160856067	7.27E-10	0.297457839	0.091	0.024	4.81E-05	C4_CD8_Teff
chr2-216349913-216350784	6.43E-15	0.379151701	0.263	0.114	4.26E-10	C4_CD8_Teff
chr2-27572153-27573519	1.06E-35	0.613364547	0.208	0.029	7.04E-31	C4_CD8_Teff
chr19-10517383-10518508	2.77E-22	0.460594631	0.273	0.091	1.84E-17	C4_CD8_Teff
chr18-51211099-51212009	3.74E-08	0.273420146	0.086	0.028	0.002475439	C4_CD8_Teff
chr22-39353348-39353661	7.63E-12	0.302715989	0.055	0.006	5.05E-07	C4_CD8_Teff
chr19-17679895-17680547	1.45E-15	0.392432399	0.125	0.025	9.57E-11	C4_CD8_Teff
chr6-116136323-116137365	9.55E-12	0.338612901	0.112	0.03	6.32E-07	C4_CD8_Teff
chr20-24953770-24954170	1.69E-19	0.453435415	0.164	0.037	1.12E-14	C4_CD8_Teff

chr5-32283680-32284051	1.65E-11	0.337915994	0.096	0.023	1.09E-06	C4_CD8_Teff
chr6-46174902-46175839	1.90E-25	0.514000017	0.253	0.064	1.26E-20	C4_CD8_Teff
chr14-24665356-24666033	3.70E-09	0.295853106	0.13	0.046	0.000244766	C4_CD8_Teff
chr7-123770266-123770758	2.04E-10	0.291706885	0.065	0.011	1.35E-05	C4_CD8_Teff
chr5-126816486-126816991	3.17E-15	0.368738465	0.081	0.011	2.10E-10	C4_CD8_Teff
chr17-76492438-76494209	1.40E-07	0.255973675	0.201	0.104	0.009245495	C4_CD8_Teff
chr13-113185991-113186856	2.24E-16	0.397935007	0.117	0.024	1.48E-11	C4_CD8_Teff
chr5-91389941-91390685	2.14E-09	0.289262654	0.096	0.026	0.000141892	C4_CD8_Teff
chr2-159144828-159145758	2.17E-26	0.522548627	0.151	0.02	1.44E-21	C4_CD8_Teff
chr4-7649400-7650381	3.92E-38	0.571318053	0.482	0.169	2.60E-33	C4_CD8_Teff
chr19-10812876-10813525	5.38E-09	0.274110924	0.076	0.02	0.000356507	C4_CD8_Teff
chr2-231554181-231554979	9.83E-15	0.34420496	0.07	0.007	6.51E-10	C4_CD8_Teff
chr16-85569565-85571266	6.59E-19	0.419504794	0.255	0.092	4.37E-14	C4_CD8_Teff
chr14-89466207-89467147	1.12E-13	0.369872605	0.174	0.055	7.41E-09	C4_CD8_Teff
chr11-66327742-66328683	3.74E-08	0.266151959	0.172	0.082	0.002474334	C4_CD8_Teff
chr17-74730383-74731073	5.31E-14	0.363378644	0.227	0.092	3.52E-09	C4_CD8_Teff
chr14-97705920-97706320	6.05E-09	0.290928252	0.133	0.049	0.000400872	C4_CD8_Teff
chr18-77073540-77074063	1.82E-27	0.537439645	0.198	0.034	1.21E-22	C4_CD8_Teff
chr12-128388516-128388978	2.02E-09	0.257651367	0.052	0.007	0.000133912	C4_CD8_Teff
chr17-47740627-47742073	2.63E-09	0.285515169	0.203	0.093	0.000173942	C4_CD8_Teff
chr5-142996190-142996868	4.18E-09	0.290139366	0.096	0.03	0.000277027	C4_CD8_Teff
chr22-23185555-23186129	3.79E-20	0.458595876	0.13	0.023	2.51E-15	C4_CD8_Teff
chr16-82431989-82432632	3.35E-08	0.27217949	0.096	0.027	0.002220906	C4_CD8_Teff
chr3-142269739-142270886	4.32E-44	0.68907647	0.297	0.052	2.86E-39	C4_CD8_Teff
chr6-36118475-36118945	6.66E-12	0.33605457	0.25	0.11	4.41E-07	C4_CD8_Teff
chr3-153242772-153243234	6.34E-12	0.335981748	0.104	0.024	4.20E-07	C4_CD8_Teff
chr8-25198436-25199354	1.37E-44	0.68473946	0.286	0.046	9.07E-40	C4_CD8_Teff

chr13-29885997-29887364	9.12E-49	1.020629839	0.448	0.052	6.04E-44	C5_CD8_Tem
chr10-71730365-71730636	4.26E-27	0.795457923	0.23	0.018	2.82E-22	C5_CD8_Tem
chr20-25223184-25223558	1.12E-23	0.726306945	0.164	0.008	7.43E-19	C5_CD8_Tem
chr4-1928299-1928994	1.99E-23	0.741149005	0.202	0.014	1.32E-18	C5_CD8_Tem
chr3-151135437-151136317	5.45E-21	0.691689656	0.279	0.044	3.61E-16	C5_CD8_Tem
chr11-1728861-1729725	3.60E-20	0.668750559	0.169	0.011	2.38E-15	C5_CD8_Tem
chr9-133406665-133407377	5.62E-19	0.596844108	0.459	0.141	3.72E-14	C5_CD8_Tem
chr3-106215035-106215385	6.39E-19	0.63832839	0.142	0.009	4.23E-14	C5_CD8_Tem
chr13-29961481-29962153	2.06E-18	0.659900329	0.219	0.029	1.36E-13	C5_CD8_Tem
chr12-56581086-56582251	2.39E-18	0.623709026	0.388	0.093	1.58E-13	C5_CD8_Tem
chr3-105792056-105792856	3.57E-18	0.652598128	0.262	0.042	2.36E-13	C5_CD8_Tem
chr12-9362885-9363449	5.30E-18	0.653005124	0.208	0.026	3.51E-13	C5_CD8_Tem
chr2-85691782-85692343	3.31E-17	0.612285821	0.153	0.013	2.19E-12	C5_CD8_Tem
chr8-142841650-142842354	3.47E-17	0.616484141	0.317	0.073	2.30E-12	C5_CD8_Tem
chr14-105069895-105071142	6.37E-17	0.590930161	0.383	0.106	4.22E-12	C5_CD8_Tem
chr12-75635603-75636274	1.85E-16	0.616933413	0.18	0.02	1.23E-11	C5_CD8_Tem
chr16-87457196-87458130	2.08E-16	0.620724441	0.235	0.039	1.38E-11	C5_CD8_Tem
chr20-49595624-49596522	2.30E-16	0.607866279	0.268	0.059	1.52E-11	C5_CD8_Tem
chr1-234866088-234866705	2.71E-16	0.60790998	0.164	0.019	1.80E-11	C5_CD8_Tem
chr8-73356217-73356774	5.42E-16	0.611694762	0.213	0.034	3.59E-11	C5_CD8_Tem
chr4-123420121-123420439	8.02E-16	0.581659337	0.137	0.01	5.31E-11	C5_CD8_Tem
chr3-17774268-17775421	8.12E-16	0.596755375	0.262	0.06	5.37E-11	C5_CD8_Tem
chr17-81094547-81095689	8.70E-16	0.476690542	0.585	0.252	5.76E-11	C5_CD8_Tem
chr6-158031349-158031795	9.64E-16	0.604364062	0.202	0.028	6.39E-11	C5_CD8_Tem
chr4-123488920-123489348	1.56E-15	0.56543896	0.12	0.008	1.03E-10	C5_CD8_Tem
chr2-54040733-54041636	1.95E-15	0.580655485	0.344	0.088	1.29E-10	C5_CD8_Tem
chr1-207335669-207337433	2.09E-15	0.535382018	0.421	0.136	1.38E-10	C5_CD8_Tem

chr5-80759910-80760514	3.55E-15	0.588362845	0.169	0.019	2.35E-10	C5_CD8_Tem
chr5-157389806-157391016	6.55E-15	0.498331459	0.508	0.184	4.34E-10	C5_CD8_Tem
chr12-101781478-101782561	7.99E-15	0.584674314	0.175	0.022	5.29E-10	C5_CD8_Tem
chr12-31732958-31733460	1.07E-14	0.589515499	0.24	0.044	7.11E-10	C5_CD8_Tem
chr12-6167276-6168190	1.09E-14	0.579163465	0.24	0.05	7.23E-10	C5_CD8_Tem
chr11-587475-588457	1.56E-14	0.445690635	0.612	0.269	1.03E-09	C5_CD8_Tem
chr9-248582-249420	1.86E-14	0.574511792	0.164	0.021	1.23E-09	C5_CD8_Tem
chr9-30075103-30075874	2.47E-14	0.573626535	0.18	0.025	1.64E-09	C5_CD8_Tem
chr20-58312816-58313696	3.17E-14	0.572662499	0.268	0.058	2.10E-09	C5_CD8_Tem
chr1-200877869-200878550	5.79E-14	0.540430727	0.35	0.101	3.83E-09	C5_CD8_Tem
chr16-89319915-89320538	6.55E-14	0.541819959	0.366	0.108	4.34E-09	C5_CD8_Tem
chr17-80694963-80695546	6.58E-14	0.522794167	0.104	0.006	4.36E-09	C5_CD8_Tem
chr15-50137275-50137951	7.83E-14	0.568033625	0.208	0.036	5.18E-09	C5_CD8_Tem
chr10-16948102-16948793	8.51E-14	0.549014945	0.295	0.073	5.64E-09	C5_CD8_Tem
chr1-244341376-244342138	1.01E-13	0.564810849	0.175	0.024	6.71E-09	C5_CD8_Tem
chr11-34161491-34162212	1.69E-13	0.53524831	0.284	0.078	1.12E-08	C5_CD8_Tem
chr4-123397421-123399528	2.00E-13	0.514992813	0.393	0.111	1.32E-08	C5_CD8_Tem
chr9-127578626-127579792	2.92E-13	0.520638107	0.366	0.117	1.93E-08	C5_CD8_Tem
chr15-29050728-29051366	4.37E-13	0.545971894	0.23	0.045	2.89E-08	C5_CD8_Tem
chr2-7744369-7745278	8.53E-13	0.484696547	0.454	0.156	5.65E-08	C5_CD8_Tem
chr1-16727130-16727940	8.93E-13	0.534829372	0.301	0.077	5.92E-08	C5_CD8_Tem
chr17-2239905-2240417	9.87E-13	0.532632686	0.148	0.017	6.54E-08	C5_CD8_Tem
chr11-66336026-66337171	1.00E-12	0.509736545	0.377	0.11	6.63E-08	C5_CD8_Tem
chr13-30576523-30577824	1.05E-12	0.501376381	0.41	0.133	6.94E-08	C5_CD8_Tem
chr21-14569769-14570196	1.75E-12	0.496023261	0.109	0.009	1.16E-07	C5_CD8_Tem
chr6-11277458-11278552	2.05E-12	0.513053799	0.35	0.104	1.36E-07	C5_CD8_Tem
chr1-230084247-230084928	2.52E-12	0.53059237	0.202	0.039	1.67E-07	C5_CD8_Tem

chr16-81493560-81493983	3.38E-12	0.522360755	0.142	0.017	2.24E-07	C5_CD8_Tem
chr1-156126095-156127656	6.21E-12	0.350101716	0.727	0.404	4.11E-07	C5_CD8_Tem
chr1-173195242-173196174	6.51E-12	0.517087067	0.186	0.035	4.31E-07	C5_CD8_Tem
chr20-62715744-62716699	6.64E-12	0.498740177	0.317	0.099	4.40E-07	C5_CD8_Tem
chr3-196246689-196247325	8.71E-12	0.492255953	0.12	0.012	5.77E-07	C5_CD8_Tem
chr6-130368653-130368926	9.00E-12	0.511004515	0.131	0.017	5.96E-07	C5_CD8_Tem
chr2-127401273-127402245	1.06E-11	0.515460243	0.18	0.032	7.01E-07	C5_CD8_Tem
chr7-157411368-157412466	1.26E-11	0.432625224	0.481	0.203	8.37E-07	C5_CD8_Tem
chr22-37082324-37082607	1.58E-11	0.481015978	0.109	0.01	1.05E-06	C5_CD8_Tem
chr19-2089816-2090730	1.87E-11	0.472506418	0.388	0.136	1.24E-06	C5_CD8_Tem
chr19-13850407-13851612	1.89E-11	0.426228849	0.503	0.22	1.25E-06	C5_CD8_Tem
chr15-29012767-29013642	2.08E-11	0.511752104	0.164	0.026	1.38E-06	C5_CD8_Tem
chr12-10453107-10453564	2.10E-11	0.466051169	0.093	0.007	1.39E-06	C5_CD8_Tem
chr15-75107938-75108888	2.69E-11	0.502494034	0.164	0.024	1.78E-06	C5_CD8_Tem
chr8-22327004-22327768	3.18E-11	0.468141705	0.104	0.01	2.11E-06	C5_CD8_Tem
chr12-10405331-10405627	3.77E-11	0.490771584	0.137	0.019	2.50E-06	C5_CD8_Tem
chr6-159984234-159984684	4.74E-11	0.504189134	0.164	0.025	3.14E-06	C5_CD8_Tem
chr22-18151993-18152258	5.28E-11	0.46607755	0.104	0.01	3.49E-06	C5_CD8_Tem
chr10-45420434-45420818	5.38E-11	0.493009133	0.131	0.017	3.57E-06	C5_CD8_Tem
chr2-231397112-231397311	5.41E-11	0.433347548	0.077	0.004	3.58E-06	C5_CD8_Tem
chr21-42683916-42684968	5.89E-11	0.496066593	0.197	0.042	3.90E-06	C5_CD8_Tem
chr3-156535163-156535577	7.29E-11	0.433132588	0.077	0.005	4.83E-06	C5_CD8_Tem
chr10-97413677-97414299	1.00E-10	0.49118514	0.202	0.042	6.65E-06	C5_CD8_Tem
chr4-76200951-76201207	1.04E-10	0.410894046	0.066	0.003	6.86E-06	C5_CD8_Tem
chr3-48564015-48564331	1.09E-10	0.469164199	0.115	0.014	7.22E-06	C5_CD8_Tem
chr11-1727091-1727419	1.12E-10	0.455030121	0.093	0.008	7.45E-06	C5_CD8_Tem
chr11-118232602-118233067	1.26E-10	0.469085995	0.317	0.104	8.36E-06	C5_CD8_Tem

chr17-2069997-2070910	1.87E-10	0.475848263	0.251	0.075	1.24E-05	C5_CD8_Tem
chr6-31792847-31793216	2.13E-10	0.473784073	0.137	0.023	1.41E-05	C5_CD8_Tem
chr22-44309084-44309491	2.25E-10	0.459488881	0.109	0.011	1.49E-05	C5_CD8_Tem
chr20-47543122-47543458	2.31E-10	0.444448568	0.098	0.009	1.53E-05	C5_CD8_Tem
chr16-17602709-17602973	2.32E-10	0.424380269	0.077	0.005	1.53E-05	C5_CD8_Tem
chr4-123479914-123480190	2.45E-10	0.429514288	0.082	0.006	1.62E-05	C5_CD8_Tem
chr5-80777440-80778136	2.46E-10	0.467951533	0.148	0.026	1.63E-05	C5_CD8_Tem
chr12-101803950-101804293	2.51E-10	0.433111991	0.093	0.007	1.66E-05	C5_CD8_Tem
chr2-97747173-97747655	2.73E-10	0.4792975	0.148	0.028	1.81E-05	C5_CD8_Tem
chr13-110677706-110677951	2.93E-10	0.460492586	0.104	0.012	1.94E-05	C5_CD8_Tem
chr10-43781497-43782356	3.06E-10	0.48374374	0.219	0.047	2.03E-05	C5_CD8_Tem
chr2-43130912-43131861	3.12E-10	0.294573448	0.76	0.461	2.07E-05	C5_CD8_Tem
chr3-45950340-45951392	3.16E-10	0.44476118	0.35	0.131	2.10E-05	C5_CD8_Tem
chr2-191114311-191116116	3.25E-10	0.330064995	0.71	0.386	2.15E-05	C5_CD8_Tem
chr22-44709731-44711179	3.32E-10	0.470775883	0.219	0.049	2.20E-05	C5_CD8_Tem
chr7-70634427-70635183	3.51E-10	0.479374866	0.175	0.035	2.32E-05	C5_CD8_Tem
chr10-22681345-22683701	3.67E-10	0.301353528	0.76	0.463	2.43E-05	C5_CD8_Tem
chr2-241739674-241741386	3.86E-10	0.456322402	0.339	0.111	2.55E-05	C5_CD8_Tem
chr7-99273963-99274662	4.05E-10	0.454678906	0.104	0.012	2.68E-05	C5_CD8_Tem
chr14-73567787-73568552	4.39E-10	0.482071115	0.191	0.041	2.91E-05	C5_CD8_Tem
chr11-72140775-72141464	4.49E-10	0.470519349	0.148	0.026	2.98E-05	C5_CD8_Tem
chr9-4725663-4726129	4.58E-10	0.463638914	0.126	0.016	3.03E-05	C5_CD8_Tem
chr19-1363406-1364606	4.75E-10	0.45091176	0.12	0.016	3.15E-05	C5_CD8_Tem
chr4-47531211-47532101	4.96E-10	0.458258985	0.257	0.08	3.28E-05	C5_CD8_Tem
chrX-78073005-78073926	5.18E-10	0.468919713	0.18	0.04	3.43E-05	C5_CD8_Tem
chr8-38404870-38405525	5.82E-10	0.482802333	0.219	0.052	3.86E-05	C5_CD8_Tem
chr11-121514259-121514960	6.19E-10	0.437789716	0.104	0.01	4.10E-05	C5_CD8_Tem

chr5-415603-416613	6.20E-10	0.464263975	0.153	0.026	4.11E-05	C5_CD8_Tem
chr17-82985881-82986429	6.62E-10	0.470239534	0.197	0.052	4.39E-05	C5_CD8_Tem
chrX-41695340-41695863	6.96E-10	0.469420123	0.169	0.031	4.61E-05	C5_CD8_Tem
chr11-118200748-118201309	7.71E-10	0.468449085	0.262	0.076	5.10E-05	C5_CD8_Tem
chr12-6332545-6332809	9.52E-10	0.469801522	0.169	0.034	6.31E-05	C5_CD8_Tem
chr2-127084518-127085050	1.00E-09	0.463350822	0.23	0.063	6.62E-05	C5_CD8_Tem
chr8-30655795-30656170	1.01E-09	0.47156619	0.202	0.046	6.67E-05	C5_CD8_Tem
chr4-101015825-101016276	1.06E-09	0.466362544	0.23	0.061	7.02E-05	C5_CD8_Tem
chr2-224909784-224910475	1.22E-09	0.463935941	0.186	0.039	8.05E-05	C5_CD8_Tem
chr19-38841624-38842824	1.24E-09	0.422014285	0.404	0.163	8.21E-05	C5_CD8_Tem
chr4-123477612-123478227	1.30E-09	0.457241408	0.148	0.027	8.63E-05	C5_CD8_Tem
chr19-16160548-16161222	1.32E-09	0.441490113	0.109	0.013	8.73E-05	C5_CD8_Tem
chr16-81520797-81521704	1.57E-09	0.402575521	0.426	0.193	0.000103709	C5_CD8_Tem
chr12-10503277-10503827	1.94E-09	0.452631317	0.142	0.023	0.000128403	C5_CD8_Tem
chr14-49902642-49902931	2.21E-09	0.424372491	0.093	0.011	0.000146491	C5_CD8_Tem
chr22-36328686-36329793	2.31E-09	0.394628962	0.47	0.206	0.000153108	C5_CD8_Tem
chr11-121472323-121472641	2.40E-09	0.427921635	0.109	0.014	0.000158826	C5_CD8_Tem
chr2-85680967-85681188	2.45E-09	0.402352086	0.071	0.006	0.000162224	C5_CD8_Tem
chr9-224587-225420	2.47E-09	0.460738864	0.158	0.029	0.00016361	C5_CD8_Tem
chr17-81097350-81097584	2.55E-09	0.462863188	0.164	0.033	0.000168766	C5_CD8_Tem
chr12-123043299-123043974	2.83E-09	0.459031316	0.191	0.047	0.000187114	C5_CD8_Tem
chr13-111166524-111167378	2.86E-09	0.453246478	0.169	0.036	0.000189262	C5_CD8_Tem
chr8-143950810-143951158	2.86E-09	0.453615631	0.235	0.065	0.000189387	C5_CD8_Tem
chr14-52324842-52325579	3.86E-09	0.444298808	0.262	0.081	0.000255365	C5_CD8_Tem
chr2-85405309-85406046	3.95E-09	0.446540209	0.257	0.081	0.000261418	C5_CD8_Tem
chr15-64697750-64698345	3.95E-09	0.446956561	0.131	0.021	0.000261753	C5_CD8_Tem
chr5-177471356-177472019	4.48E-09	0.444223438	0.142	0.026	0.000296516	C5_CD8_Tem

chr6-106107268-106107521	4.69E-09	0.447107803	0.137	0.023	0.000310368	C5_CD8_Tem
chr11-35289042-35289436	4.70E-09	0.396462573	0.077	0.006	0.000311231	C5_CD8_Tem
chr7-22179415-22179871	5.12E-09	0.395596868	0.077	0.006	0.00033933	C5_CD8_Tem
chr4-123421504-123422425	5.60E-09	0.446972825	0.158	0.033	0.000370841	C5_CD8_Tem
chr11-47797435-47798033	5.68E-09	0.445493892	0.142	0.03	0.000376024	C5_CD8_Tem
chr11-1850299-1851392	5.85E-09	0.345244545	0.525	0.281	0.0003876	C5_CD8_Tem
chr1-152047400-152048717	5.90E-09	0.311212174	0.65	0.383	0.000390944	C5_CD8_Tem
chr1-23110298-23111268	6.31E-09	0.380258045	0.475	0.213	0.000418004	C5_CD8_Tem
chr22-37275602-37275923	6.62E-09	0.402496976	0.082	0.007	0.000438478	C5_CD8_Tem
chr15-74396914-74397281	6.65E-09	0.453678529	0.169	0.035	0.000440399	C5_CD8_Tem
chr3-106185921-106186192	6.76E-09	0.378395604	0.071	0.004	0.000447758	C5_CD8_Tem
chr1-1344800-1345081	6.90E-09	0.43905832	0.131	0.024	0.00045725	C5_CD8_Tem
chr1-150569880-150570355	7.58E-09	0.444075095	0.224	0.068	0.000502066	C5_CD8_Tem
chr2-127064455-127065286	7.70E-09	0.433644106	0.257	0.092	0.00050993	C5_CD8_Tem
chr1-109793573-109794180	7.80E-09	0.432659818	0.235	0.071	0.000516424	C5_CD8_Tem
chr18-13578481-13579195	8.00E-09	0.429104543	0.126	0.022	0.000529853	C5_CD8_Tem
chr11-77141062-77141430	8.38E-09	0.369256135	0.06	0.004	0.000555247	C5_CD8_Tem
chr11-34250945-34251468	8.72E-09	0.422854605	0.284	0.1	0.000577243	C5_CD8_Tem
chr9-114516844-114517273	9.11E-09	0.401319923	0.093	0.01	0.000603468	C5_CD8_Tem
chr2-98736254-98736951	9.50E-09	0.439955779	0.246	0.076	0.000628843	C5_CD8_Tem
chr2-234286306-234286606	1.01E-08	0.445281751	0.169	0.032	0.000666439	C5_CD8_Tem
chr3-106333820-106334528	1.02E-08	0.442526231	0.202	0.052	0.00067734	C5_CD8_Tem
chr5-71600876-71602029	1.09E-08	0.323239388	0.601	0.319	0.000721929	C5_CD8_Tem
chr5-80745473-80745863	1.18E-08	0.410970499	0.093	0.011	0.000780346	C5_CD8_Tem
chr15-33201563-33202199	1.22E-08	0.43614268	0.175	0.041	0.000807307	C5_CD8_Tem
chr2-8543120-8545542	1.26E-08	0.338380441	0.579	0.303	0.000834153	C5_CD8_Tem
chr6-137717199-137718049	1.31E-08	0.369661074	0.459	0.218	0.000866031	C5_CD8_Tem

chr20-48738763-48739779	1.31E-08	0.385396625	0.426	0.189	0.00086658	C5_CD8_Tem
chr4-75555423-75556172	1.39E-08	0.429441163	0.131	0.027	0.000917891	C5_CD8_Tem
chr16-90057592-90058118	1.42E-08	0.422122356	0.126	0.021	0.000943134	C5_CD8_Tem
chr8-133302840-133303865	1.43E-08	0.37984799	0.448	0.196	0.000945438	C5_CD8_Tem
chr11-225787-226040	1.49E-08	0.353317166	0.055	0.003	0.000985996	C5_CD8_Tem
chr9-114503729-114505786	1.50E-08	0.358883182	0.508	0.248	0.000996371	C5_CD8_Tem
chr2-196234489-196235162	1.69E-08	0.425791362	0.29	0.098	0.001120897	C5_CD8_Tem
chr21-31656217-31656445	1.71E-08	0.427539471	0.109	0.017	0.001130065	C5_CD8_Tem
chr10-73857235-73858101	1.78E-08	0.438575794	0.202	0.055	0.001181879	C5_CD8_Tem
chr19-38838538-38838914	1.87E-08	0.435742036	0.191	0.046	0.001235472	C5_CD8_Tem
chr1-9719099-9719404	2.02E-08	0.427190495	0.262	0.084	0.001336666	C5_CD8_Tem
chr5-52724672-52725892	2.02E-08	0.362542939	0.454	0.217	0.001340369	C5_CD8_Tem
chr16-1002720-1003121	2.04E-08	0.441022049	0.175	0.039	0.001349116	C5_CD8_Tem
chr15-101124498-101125045	2.07E-08	0.42506484	0.131	0.022	0.001374069	C5_CD8_Tem
chr4-123415989-123416490	2.13E-08	0.408464406	0.109	0.017	0.001413696	C5_CD8_Tem
chr18-69897668-69898750	2.18E-08	0.431147593	0.224	0.066	0.001446247	C5_CD8_Tem
chrX-101429872-101430825	2.27E-08	0.421915682	0.131	0.026	0.001501892	C5_CD8_Tem
chr17-82316814-82317752	2.40E-08	0.421507037	0.268	0.086	0.001590197	C5_CD8_Tem
chr2-196278382-196278703	2.49E-08	0.384576506	0.082	0.008	0.001649156	C5_CD8_Tem
chr12-101793377-101793716	2.65E-08	0.431987522	0.148	0.028	0.001754821	C5_CD8_Tem
chr10-110425756-110426445	2.78E-08	0.425892569	0.18	0.045	0.001844235	C5_CD8_Tem
chr3-105700499-105700953	2.92E-08	0.4270266	0.131	0.023	0.001931727	C5_CD8_Tem
chr7-3067216-3067986	2.99E-08	0.427926998	0.273	0.092	0.001983213	C5_CD8_Tem
chr4-123616807-123617351	3.03E-08	0.411831605	0.158	0.04	0.002003803	C5_CD8_Tem
chr19-35247585-35249208	3.05E-08	0.39341789	0.366	0.149	0.002017483	C5_CD8_Tem
chr1-235910363-235910983	3.06E-08	0.427873049	0.148	0.03	0.002028624	C5_CD8_Tem
chr2-234313625-234313942	3.13E-08	0.384580024	0.082	0.009	0.002070952	C5_CD8_Tem

chr1-52658711-52659613	3.26E-08	0.430163013	0.164	0.036	0.00215802	C5_CD8_Tem
chr1-117006886-117007868	3.27E-08	0.427521252	0.224	0.061	0.002168246	C5_CD8_Tem
chr1-58922254-58922886	3.29E-08	0.416170278	0.153	0.035	0.002177092	C5_CD8_Tem
chr1-61452542-61453693	3.38E-08	0.396468199	0.306	0.12	0.002239775	C5_CD8_Tem
chr18-79681487-79682034	3.48E-08	0.423065935	0.219	0.07	0.002304425	C5_CD8_Tem
chr1-84998254-84999298	3.50E-08	0.405427955	0.311	0.114	0.002318454	C5_CD8_Tem
chr7-92641656-92642569	3.63E-08	0.409512293	0.295	0.106	0.002403436	C5_CD8_Tem
chr10-17238002-17239250	3.80E-08	0.437618316	0.202	0.049	0.002516839	C5_CD8_Tem
chr1-230109865-230110443	3.89E-08	0.409719705	0.115	0.021	0.002577657	C5_CD8_Tem
chr15-34331685-34332149	4.08E-08	0.427442587	0.186	0.05	0.002703368	C5_CD8_Tem
chr9-33748543-33749512	4.28E-08	0.420199329	0.235	0.07	0.002833743	C5_CD8_Tem
chr17-68381070-68381460	4.67E-08	0.379275649	0.082	0.008	0.003094365	C5_CD8_Tem
chr14-55097805-55098613	4.88E-08	0.416668236	0.235	0.076	0.003233226	C5_CD8_Tem
chr15-85645630-85647197	4.94E-08	0.301938499	0.601	0.351	0.003268565	C5_CD8_Tem
chr2-20595436-20596528	5.05E-08	0.367858843	0.077	0.008	0.003343209	C5_CD8_Tem
chr15-84604721-84605484	5.05E-08	0.42044273	0.191	0.054	0.003343824	C5_CD8_Tem
chr11-119217855-119218824	5.11E-08	0.41380327	0.131	0.028	0.00338133	C5_CD8_Tem
chr17-2218522-2218736	5.37E-08	0.391141489	0.087	0.012	0.003556758	C5_CD8_Tem
chr19-18477201-18477605	5.39E-08	0.407294645	0.109	0.018	0.003568513	C5_CD8_Tem
chr9-127555909-127556449	5.72E-08	0.4192915	0.158	0.041	0.003785493	C5_CD8_Tem
chr9-91795771-91796794	5.90E-08	0.407270641	0.257	0.092	0.003909314	C5_CD8_Tem
chr2-11753459-11754635	6.48E-08	0.305458697	0.612	0.356	0.004292087	C5_CD8_Tem
chr14-71697800-71698396	6.53E-08	0.387833188	0.093	0.011	0.004325012	C5_CD8_Tem
chr18-49690202-49690676	6.57E-08	0.402734148	0.12	0.02	0.004350188	C5_CD8_Tem
chr5-177494324-177494861	6.77E-08	0.410878857	0.142	0.028	0.00448579	C5_CD8_Tem
chr5-378613-378982	6.97E-08	0.392918515	0.098	0.014	0.004616472	C5_CD8_Tem
chr6-90368845-90369489	7.64E-08	0.413232109	0.18	0.051	0.005061493	C5_CD8_Tem

chr15-48928827-48929149	7.70E-08	0.347285261	0.06	0.004	0.005097516	C5_CD8_Tem
chr9-75151671-75152898	8.60E-08	0.38455988	0.35	0.143	0.005692927	C5_CD8_Tem
chr13-40189468-40189790	8.68E-08	0.407213742	0.142	0.029	0.005745752	C5_CD8_Tem
chr6-157763876-157764988	8.72E-08	0.314328815	0.563	0.307	0.005773357	C5_CD8_Tem
chr2-96556544-96557329	8.83E-08	0.377423986	0.366	0.15	0.005849136	C5_CD8_Tem
chr11-121465916-121466273	9.62E-08	0.406990555	0.158	0.037	0.006372138	C5_CD8_Tem
chr15-70141625-70142758	9.95E-08	0.413103176	0.148	0.034	0.006586788	C5_CD8_Tem
chr21-34807265-34808813	1.03E-07	0.332561036	0.514	0.258	0.006853628	C5_CD8_Tem
chr3-183513151-183513641	1.05E-07	0.415976015	0.169	0.041	0.006984849	C5_CD8_Tem
chr1-156113857-156115386	1.06E-07	0.273047314	0.732	0.426	0.007026514	C5_CD8_Tem
chr1-24954700-24955506	1.10E-07	0.357235161	0.459	0.208	0.0073108	C5_CD8_Tem
chr1-19643673-19645030	1.11E-07	0.396079999	0.317	0.126	0.007337307	C5_CD8_Tem
chr16-89422342-89423633	1.11E-07	0.388111571	0.355	0.144	0.007381028	C5_CD8_Tem
chr2-42353580-42354147	1.12E-07	0.39341703	0.104	0.016	0.007441237	C5_CD8_Tem
chr22-23242630-23243581	1.13E-07	0.39855326	0.284	0.102	0.007457339	C5_CD8_Tem
chr2-234273944-234274588	1.19E-07	0.407468143	0.23	0.073	0.007908899	C5_CD8_Tem
chr2-54421817-54422540	1.30E-07	0.379955999	0.098	0.015	0.008594508	C5_CD8_Tem
chr20-3994987-3995699	1.45E-07	0.403339476	0.131	0.028	0.009632179	C5_CD8_Tem
chr3-151209427-151210045	1.58E-07	0.399601376	0.175	0.047	0.010494996	C5_CD8_Tem
chr6-106106396-106106752	1.75E-07	0.384549471	0.104	0.017	0.011562962	C5_CD8_Tem
chr10-71822086-71822766	1.86E-07	0.395666738	0.12	0.027	0.012343891	C5_CD8_Tem
chr10-73859195-73860037	1.95E-07	0.41007272	0.169	0.041	0.012915605	C5_CD8_Tem
chr3-4983523-4984406	2.00E-07	0.324622148	0.525	0.27	0.013277517	C5_CD8_Tem
chr11-128422224-128423073	2.10E-07	0.378010947	0.322	0.128	0.013882384	C5_CD8_Tem
chr8-143939083-143940463	2.12E-07	0.312226935	0.552	0.31	0.014011538	C5_CD8_Tem
chr8-30059433-30060536	2.19E-07	0.387502906	0.295	0.111	0.014524072	C5_CD8_Tem
chr10-12405009-12405527	2.19E-07	0.340843624	0.066	0.007	0.014528951	C5_CD8_Tem

chr18-69916359-69916954	2.21E-07	0.386585149	0.109	0.021	0.01461129	C5_CD8_Tem
chr16-84518295-84519273	2.27E-07	0.370939263	0.361	0.151	0.015030938	C5_CD8_Tem
chr9-127519356-127519689	2.40E-07	0.355472272	0.071	0.007	0.015882718	C5_CD8_Tem
chr7-50197180-50197973	2.41E-07	0.403212423	0.142	0.033	0.015943051	C5_CD8_Tem
chr19-19403375-19403706	2.63E-07	0.402977392	0.213	0.061	0.01739043	C5_CD8_Tem
chr3-189334199-189334786	2.83E-07	0.386853998	0.109	0.02	0.018761659	C5_CD8_Tem
chr10-11157328-11157601	2.89E-07	0.360498174	0.077	0.008	0.019142046	C5_CD8_Tem
chr21-45224281-45224986	2.99E-07	0.324141334	0.497	0.246	0.019814299	C5_CD8_Tem
chr13-113187231-113187637	3.30E-07	0.401862379	0.142	0.03	0.021872719	C5_CD8_Tem
chr6-46010877-46011813	3.35E-07	0.334811364	0.421	0.218	0.022198246	C5_CD8_Tem
chr4-123443431-123444508	3.35E-07	0.371732425	0.104	0.017	0.022211898	C5_CD8_Tem
chr13-99321864-99323139	3.47E-07	0.355443337	0.421	0.193	0.02297133	C5_CD8_Tem
chr4-83535346-83536610	3.82E-07	0.36614479	0.333	0.138	0.025279151	C5_CD8_Tem
chr18-24054062-24054904	4.03E-07	0.401039102	0.186	0.05	0.026708978	C5_CD8_Tem
chr13-22114262-22115202	4.17E-07	0.37677493	0.262	0.093	0.027591864	C5_CD8_Tem
chr20-35305679-35306054	4.27E-07	0.39275319	0.137	0.03	0.028269232	C5_CD8_Tem
chr10-132904293-132905104	4.37E-07	0.317175878	0.055	0.004	0.028945263	C5_CD8_Tem
chr6-42278040-42278490	4.41E-07	0.391948329	0.153	0.036	0.029179671	C5_CD8_Tem
chr3-106184034-106185385	4.41E-07	0.393288021	0.142	0.034	0.029199474	C5_CD8_Tem
chr1-91941494-91942178	4.43E-07	0.379814547	0.126	0.03	0.029308014	C5_CD8_Tem
chr2-234071681-234072045	4.48E-07	0.369641553	0.093	0.014	0.0296668	C5_CD8_Tem
chr18-9016724-9017791	4.62E-07	0.387138484	0.219	0.07	0.030624594	C5_CD8_Tem
chr21-36429704-36430483	4.88E-07	0.38413979	0.246	0.09	0.032288198	C5_CD8_Tem
chr2-195533802-195534618	4.91E-07	0.37157986	0.339	0.137	0.032515979	C5_CD8_Tem
chr13-99433974-99434864	5.04E-07	0.259458232	0.678	0.408	0.033404849	C5_CD8_Tem
chr12-89039825-89040764	5.07E-07	0.377535244	0.235	0.083	0.033568692	C5_CD8_Tem
chr2-127397933-127398589	5.11E-07	0.393890667	0.175	0.053	0.033862743	C5_CD8_Tem

chr13-114160955-114161300	5.41E-07	0.353459745	0.082	0.011	0.035801703	C5_CD8_Tem
chr22-26493542-26493983	5.49E-07	0.378126119	0.126	0.029	0.036338768	C5_CD8_Tem
chr1-116514824-116516096	5.75E-07	0.333566817	0.454	0.227	0.038049462	C5_CD8_Tem
chr10-99993940-99994675	5.86E-07	0.388191163	0.131	0.032	0.038808894	C5_CD8_Tem
chr2-239293943-239294333	5.95E-07	0.387414245	0.246	0.092	0.039425753	C5_CD8_Tem
chr2-43129845-43130196	6.11E-07	0.384923551	0.175	0.056	0.040480278	C5_CD8_Tem
chr6-16766246-16766452	6.28E-07	0.391881844	0.153	0.037	0.041573071	C5_CD8_Tem
chr15-69757166-69757507	6.56E-07	0.39261766	0.202	0.058	0.043419203	C5_CD8_Tem
chr10-30701202-30702109	6.67E-07	0.325414054	0.443	0.231	0.044155557	C5_CD8_Tem
chr22-40401142-40402360	6.77E-07	0.388733791	0.235	0.078	0.044830905	C5_CD8_Tem
chr1-9686695-9687821	6.99E-07	0.353991335	0.366	0.168	0.046295089	C5_CD8_Tem
chr12-111438406-111438781	7.16E-07	0.388137802	0.131	0.03	0.047433954	C5_CD8_Tem
chr18-23870990-23871741	7.23E-07	0.366265907	0.306	0.134	0.047900475	C5_CD8_Tem
chr17-39929217-39929496	7.36E-07	0.380998989	0.137	0.031	0.048717744	C5_CD8_Tem
chr14-93994905-93995321	7.37E-07	0.387657085	0.131	0.029	0.048829956	C5_CD8_Tem
chr1-13809359-13809883	7.87E-07	0.372798557	0.115	0.022	0.052124011	C5_CD8_Tem
chr6-154296-154947	7.89E-07	0.378879071	0.131	0.028	0.052257714	C5_CD8_Tem
chr16-67326404-67327082	8.23E-07	0.37582377	0.12	0.026	0.054498888	C5_CD8_Tem
chr8-143948399-143948748	8.28E-07	0.358514688	0.366	0.171	0.05481787	C5_CD8_Tem
chr15-64633938-64635273	8.66E-07	0.3852097	0.224	0.078	0.057376427	C5_CD8_Tem
chr8-29249642-29250330	8.86E-07	0.369784747	0.29	0.115	0.058702474	C5_CD8_Tem
chr2-102434523-102434888	8.89E-07	0.345966005	0.077	0.009	0.058890667	C5_CD8_Tem
chr12-132812423-132812964	9.00E-07	0.36715022	0.109	0.022	0.059614393	C5_CD8_Tem
chr1-203649404-203650627	9.83E-07	0.311246527	0.541	0.276	0.065105438	C5_CD8_Tem
chr16-3020156-3022540	9.94E-07	0.257642146	0.689	0.446	0.065853943	C5_CD8_Tem
chr8-93935396-93936515	1.02E-06	0.38745713	0.186	0.053	0.067345194	C5_CD8_Tem
chr15-64246661-64247093	1.02E-06	0.383315571	0.164	0.043	0.067467878	C5_CD8_Tem

chr1-185044541-185046123	1.02E-06	0.316642128	0.464	0.25	0.067584869	C5_CD8_Tem
chr21-45152222-45152641	1.05E-06	0.37608564	0.131	0.03	0.069260331	C5_CD8_Tem
chr1-156926098-156926567	1.10E-06	0.362504925	0.093	0.016	0.072989419	C5_CD8_Tem
chr15-50134784-50135559	1.11E-06	0.369447563	0.137	0.034	0.07340212	C5_CD8_Tem
chr1-85297648-85298665	1.12E-06	0.30627986	0.492	0.251	0.073999202	C5_CD8_Tem
chr20-46552593-46553362	1.14E-06	0.358345324	0.098	0.019	0.075625039	C5_CD8_Tem
chr2-230924249-230925362	1.16E-06	0.379876032	0.208	0.073	0.076772924	C5_CD8_Tem
chr5-80761968-80762198	1.17E-06	0.309116772	0.055	0.005	0.077397678	C5_CD8_Tem
chr1-200925693-200926392	1.19E-06	0.375078979	0.126	0.028	0.079105513	C5_CD8_Tem
chr6-454787-455350	1.28E-06	0.358428474	0.093	0.014	0.084803258	C5_CD8_Tem
chr18-35616312-35617324	1.35E-06	0.346538749	0.328	0.143	0.089631472	C5_CD8_Tem
chr1-9705316-9706004	1.38E-06	0.367618972	0.098	0.022	0.091311696	C5_CD8_Tem
chr9-91162159-91163600	1.38E-06	0.381062775	0.235	0.077	0.091510415	C5_CD8_Tem
chr6-158720110-158721260	1.41E-06	0.334744859	0.432	0.202	0.093690347	C5_CD8_Tem
chr8-38404129-38404397	1.43E-06	0.309479384	0.055	0.004	0.094932962	C5_CD8_Tem
chr22-50361213-50362026	1.45E-06	0.368317376	0.126	0.03	0.095859373	C5_CD8_Tem
chr15-60507462-60508748	1.47E-06	0.368726515	0.268	0.112	0.097130063	C5_CD8_Tem
chr20-1357785-1358576	1.47E-06	0.364681862	0.12	0.027	0.097465958	C5_CD8_Tem
chr17-77834338-77834812	1.48E-06	0.380501304	0.202	0.059	0.097858959	C5_CD8_Tem
chr17-46108887-46109131	1.48E-06	0.3133107	0.055	0.007	0.098054766	C5_CD8_Tem
chr2-106159784-106161249	1.49E-06	0.346031098	0.361	0.165	0.098750444	C5_CD8_Tem
chr17-49337948-49338151	1.55E-06	0.350790624	0.082	0.012	0.102905228	C5_CD8_Tem
chr13-29923888-29924434	1.62E-06	0.376268414	0.24	0.077	0.107064097	C5_CD8_Tem
chr10-6274704-6275756	1.62E-06	0.275474914	0.596	0.338	0.107557396	C5_CD8_Tem
chr1-168360097-168360457	1.69E-06	0.377822196	0.169	0.05	0.111718441	C5_CD8_Tem
chr6-90458163-90458907	1.76E-06	0.367593935	0.191	0.054	0.11622424	C5_CD8_Tem
chr14-71118875-71119413	1.77E-06	0.36494219	0.12	0.03	0.116902916	C5_CD8_Tem

chr10-33252703-33253050	1.81E-06	0.351171685	0.087	0.014	0.119661619	C5_CD8_Tem
chr3-11251301-11251791	1.84E-06	0.326875007	0.066	0.009	0.121604691	C5_CD8_Tem
chr6-155170763-155171927	1.93E-06	0.268525556	0.607	0.346	0.128059233	C5_CD8_Tem
chr4-147942690-147943553	1.95E-06	0.364625799	0.142	0.04	0.129229133	C5_CD8_Tem
chr2-98757844-98758431	1.96E-06	0.372317132	0.153	0.046	0.129527163	C5_CD8_Tem
chr10-97422855-97423133	2.02E-06	0.368430435	0.131	0.029	0.134093528	C5_CD8_Tem
chr21-38804430-38806699	2.04E-06	0.324281899	0.443	0.213	0.134862339	C5_CD8_Tem
chr15-31443576-31444114	2.08E-06	0.361245036	0.126	0.027	0.137996145	C5_CD8_Tem
chr11-96357836-96358113	2.10E-06	0.304588598	0.055	0.005	0.138737545	C5_CD8_Tem
chr9-129862954-129863468	2.17E-06	0.36246172	0.273	0.111	0.14378681	C5_CD8_Tem
chr3-141017374-141018166	2.21E-06	0.36087961	0.12	0.029	0.146483672	C5_CD8_Tem
chr1-206503839-206504385	2.23E-06	0.360550169	0.115	0.028	0.147806822	C5_CD8_Tem
chr16-30715390-30716124	2.27E-06	0.347905705	0.35	0.147	0.150640821	C5_CD8_Tem
chr12-9364265-9365196	2.28E-06	0.36945811	0.186	0.056	0.15113888	C5_CD8_Tem
chr21-43706359-43707102	2.29E-06	0.363184105	0.18	0.058	0.151378868	C5_CD8_Tem
chr5-67003640-67004819	2.34E-06	0.279212185	0.568	0.33	0.154981986	C5_CD8_Tem
chr16-57614601-57614894	2.34E-06	0.327459501	0.077	0.009	0.155133796	C5_CD8_Tem
chr9-3528393-3528865	2.37E-06	0.302077754	0.492	0.257	0.156636189	C5_CD8_Tem
chr3-151086027-151086998	2.37E-06	0.351669865	0.104	0.023	0.156731011	C5_CD8_Tem
chr12-6635240-6636420	2.45E-06	0.361361106	0.202	0.072	0.16257032	C5_CD8_Tem
chr1-27632239-27633225	2.46E-06	0.368311614	0.197	0.066	0.163162539	C5_CD8_Tem
chr2-239548428-239548938	2.47E-06	0.367311236	0.175	0.054	0.163476453	C5_CD8_Tem
chr16-46961345-46962085	2.48E-06	0.370350973	0.219	0.074	0.163997749	C5_CD8_Tem
chr19-610106-611951	2.48E-06	0.310901832	0.481	0.238	0.164435468	C5_CD8_Tem
chr3-106457265-106457591	2.49E-06	0.301119109	0.055	0.005	0.164894638	C5_CD8_Tem
chr1-184930291-184931068	2.53E-06	0.363123343	0.169	0.045	0.167403692	C5_CD8_Tem
chr10-4651547-4652513	2.58E-06	0.352777043	0.115	0.026	0.170563489	C5_CD8_Tem

chr6-36514876-36515410	2.63E-06	0.301135584	0.055	0.005	0.174034985	C5_CD8_Tem
chr10-6513052-6513898	2.63E-06	0.376239542	0.202	0.061	0.174295167	C5_CD8_Tem
chr3-16294900-16295224	2.69E-06	0.34781306	0.093	0.02	0.177964872	C5_CD8_Tem
chr16-85615088-85615287	2.69E-06	0.369822346	0.142	0.033	0.178182846	C5_CD8_Tem
chr8-133526111-133527173	2.76E-06	0.343111342	0.339	0.149	0.182610926	C5_CD8_Tem
chr10-3720283-3720811	2.77E-06	0.324760208	0.071	0.01	0.183577336	C5_CD8_Tem
chr10-35149047-35149968	2.82E-06	0.35966437	0.246	0.092	0.186779823	C5_CD8_Tem
chr20-59253037-59254373	2.82E-06	0.344796761	0.311	0.144	0.186779939	C5_CD8_Tem
chr1-100414408-100415159	2.86E-06	0.363843383	0.186	0.064	0.18938523	C5_CD8_Tem
chr5-88250561-88251483	2.88E-06	0.360708363	0.202	0.067	0.190932194	C5_CD8_Tem
chr11-44861726-44862897	2.97E-06	0.333480141	0.344	0.16	0.196761051	C5_CD8_Tem
chr13-30429999-30430725	2.97E-06	0.36727618	0.257	0.09	0.196856575	C5_CD8_Tem
chr1-26358461-26359382	3.02E-06	0.34077937	0.372	0.164	0.1999512	C5_CD8_Tem
chr1-243842804-243843450	3.07E-06	0.357528148	0.109	0.026	0.203440789	C5_CD8_Tem
chr6-106163048-106164775	3.09E-06	0.323717447	0.404	0.185	0.204454799	C5_CD8_Tem
chr20-50391868-50392497	3.18E-06	0.354843418	0.12	0.033	0.210716685	C5_CD8_Tem
chrX-72107315-72107683	3.20E-06	0.36152142	0.131	0.032	0.21171187	C5_CD8_Tem
chr1-230123718-230124051	3.27E-06	0.313717367	0.06	0.007	0.216440689	C5_CD8_Tem
chr4-77031149-77031929	3.34E-06	0.360931732	0.126	0.032	0.221093372	C5_CD8_Tem
chr17-50909211-50909589	3.37E-06	0.366933785	0.142	0.034	0.222950524	C5_CD8_Tem
chr7-17138775-17139518	3.39E-06	0.366092946	0.24	0.084	0.224621519	C5_CD8_Tem
chr11-123456276-123457499	3.44E-06	0.361420411	0.169	0.051	0.227639402	C5_CD8_Tem
chr11-9759348-9760157	3.45E-06	0.286483011	0.514	0.277	0.228169746	C5_CD8_Tem
chr15-70526465-70526854	3.58E-06	0.341725919	0.093	0.018	0.237046743	C5_CD8_Tem
chr15-94230822-94231909	3.62E-06	0.3074615	0.454	0.236	0.239836238	C5_CD8_Tem
chr15-48796666-48797346	3.71E-06	0.353340981	0.109	0.025	0.245505325	C5_CD8_Tem
chr19-48418715-48419552	3.72E-06	0.365561216	0.202	0.074	0.246678386	C5_CD8_Tem

chr6-144197790-144198476	3.73E-06	0.354336932	0.131	0.031	0.24687748	C5_CD8_Tem
chr19-2503181-2504126	3.77E-06	0.32399051	0.393	0.186	0.249428765	C5_CD8_Tem
chr9-127437097-127438230	3.89E-06	0.361385872	0.175	0.06	0.257405392	C5_CD8_Tem
chr1-153490519-153491082	4.03E-06	0.343128054	0.093	0.022	0.266866812	C5_CD8_Tem
chr1-230104630-230105094	4.21E-06	0.360192041	0.12	0.031	0.279035847	C5_CD8_Tem
chr2-240655989-240656548	4.60E-06	0.34757915	0.098	0.021	0.304860435	C5_CD8_Tem
chr16-4675942-4677022	4.66E-06	0.352613804	0.23	0.091	0.308363271	C5_CD8_Tem
chr6-3022986-3024354	4.80E-06	0.306548852	0.421	0.234	0.317909886	C5_CD8_Tem
chr6-44642405-44643221	4.87E-06	0.336167763	0.098	0.02	0.322571794	C5_CD8_Tem
chr17-50909873-50910921	4.91E-06	0.335369773	0.311	0.147	0.325388491	C5_CD8_Tem
chr3-106533813-106534695	4.92E-06	0.352574232	0.131	0.035	0.325728147	C5_CD8_Tem
chr6-109461822-109462412	4.98E-06	0.359837234	0.142	0.04	0.32951742	C5_CD8_Tem
chr20-35506879-35507165	5.09E-06	0.300004839	0.055	0.005	0.337101621	C5_CD8_Tem
chr1-42940851-42941276	5.11E-06	0.358632218	0.137	0.037	0.338102653	C5_CD8_Tem
chr6-30768952-30769914	5.18E-06	0.361309787	0.202	0.069	0.343133984	C5_CD8_Tem
chr1-40413722-40414567	5.36E-06	0.324630735	0.082	0.015	0.355166677	C5_CD8_Tem
chr1-221689072-221689471	5.42E-06	0.339954995	0.093	0.016	0.358791631	C5_CD8_Tem
chr19-39297678-39299817	5.43E-06	0.327253607	0.344	0.162	0.35965654	C5_CD8_Tem
chr7-151038980-151039621	5.59E-06	0.353165634	0.126	0.028	0.370474299	C5_CD8_Tem
chr3-56980681-56981362	5.61E-06	0.347320271	0.109	0.025	0.37182079	C5_CD8_Tem
chr2-7516543-7517446	5.63E-06	0.358802165	0.142	0.035	0.373075309	C5_CD8_Tem
chr3-18556003-18556909	5.78E-06	0.350007817	0.202	0.068	0.38254343	C5_CD8_Tem
chr10-103907912-103908644	5.99E-06	0.3400861	0.322	0.151	0.396593279	C5_CD8_Tem
chr12-12441537-12442794	5.99E-06	0.283942592	0.508	0.285	0.396833777	C5_CD8_Tem
chr1-31957653-31958126	6.08E-06	0.336131957	0.087	0.016	0.402664085	C5_CD8_Tem
chr20-48722814-48723050	6.09E-06	0.340649577	0.087	0.015	0.40313973	C5_CD8_Tem
chr10-60237982-60238713	6.15E-06	0.345401657	0.115	0.03	0.40742295	C5_CD8_Tem

chr5-80757363-80757600	6.22E-06	0.327106269	0.077	0.011	0.411692241	C5_CD8_Tem
chr10-29658562-29659853	6.27E-06	0.283377933	0.508	0.282	0.415382115	C5_CD8_Tem
chr16-87897428-87897985	6.29E-06	0.355450912	0.213	0.082	0.416250807	C5_CD8_Tem
chr15-25849848-25851079	6.39E-06	0.337376175	0.306	0.133	0.422930543	C5_CD8_Tem
chr10-8365800-8366409	6.42E-06	0.350764439	0.131	0.035	0.424889243	C5_CD8_Tem
chr3-111594253-111594507	6.42E-06	0.314579995	0.071	0.01	0.424895416	C5_CD8_Tem
chr8-20302981-20304211	6.52E-06	0.349170981	0.268	0.11	0.431552945	C5_CD8_Tem
chr5-96701055-96701439	6.71E-06	0.351656805	0.153	0.044	0.444046995	C5_CD8_Tem
chr2-237687387-237688046	6.71E-06	0.261812347	0.563	0.337	0.444548955	C5_CD8_Tem
chr12-6335421-6336883	6.77E-06	0.275757424	0.53	0.31	0.448368599	C5_CD8_Tem
chr15-70236268-70237040	6.80E-06	0.322475602	0.082	0.014	0.450325399	C5_CD8_Tem
chr3-71226241-71227700	6.80E-06	0.264978092	0.53	0.317	0.450569347	C5_CD8_Tem
chr15-40763276-40765752	6.81E-06	0.273220494	0.557	0.335	0.45108907	C5_CD8_Tem
chr2-134217364-134218430	6.84E-06	0.323764197	0.344	0.164	0.453262905	C5_CD8_Tem
chr2-190759682-190760249	6.85E-06	0.31264102	0.071	0.01	0.453642485	C5_CD8_Tem
chr11-121481647-121482948	6.91E-06	0.282592405	0.47	0.257	0.457431164	C5_CD8_Tem
chr3-4820695-4822472	6.91E-06	0.295646615	0.481	0.252	0.457917628	C5_CD8_Tem
chr10-3945681-3946260	7.01E-06	0.343917558	0.306	0.131	0.464195069	C5_CD8_Tem
chr1-248926475-248927068	7.30E-06	0.329302459	0.082	0.014	0.483526479	C5_CD8_Tem
chr3-157081113-157082069	7.31E-06	0.31475556	0.415	0.201	0.483801838	C5_CD8_Tem
chr20-49594045-49594705	7.36E-06	0.356405413	0.186	0.053	0.487383984	C5_CD8_Tem
chr1-244319341-244320052	7.40E-06	0.326024408	0.339	0.156	0.489935735	C5_CD8_Tem
chr20-63635056-63636218	7.43E-06	0.276251609	0.546	0.309	0.491850786	C5_CD8_Tem
chr6-139187398-139187789	7.52E-06	0.349603016	0.104	0.02	0.498043771	C5_CD8_Tem
chr15-74545909-74546665	7.67E-06	0.332392535	0.366	0.158	0.50804836	C5_CD8_Tem
chr3-157127893-157129028	7.77E-06	0.310853405	0.366	0.189	0.514500944	C5_CD8_Tem
chr10-80195757-80196922	7.95E-06	0.328302059	0.311	0.132	0.526483283	C5_CD8_Tem

chr6-156588-157425	7.99E-06	0.343791755	0.109	0.023	0.529221767	C5_CD8_Tem
chr11-62542803-62543577	8.03E-06	0.295675963	0.486	0.266	0.531454205	C5_CD8_Tem
chr15-50066174-50066563	8.04E-06	0.334377724	0.093	0.018	0.532450756	C5_CD8_Tem
chr19-2598750-2599757	8.13E-06	0.277490582	0.536	0.304	0.538086024	C5_CD8_Tem
chr6-431531-432455	8.19E-06	0.356237294	0.169	0.059	0.542550349	C5_CD8_Tem
chr4-87058003-87058619	8.31E-06	0.334835148	0.109	0.026	0.550168353	C5_CD8_Tem
chr15-55282008-55282801	8.48E-06	0.336590799	0.284	0.122	0.561719618	C5_CD8_Tem
chr1-58754179-58755866	8.60E-06	0.326228718	0.377	0.162	0.569550706	C5_CD8_Tem
chr2-69675442-69675785	8.65E-06	0.32360219	0.087	0.015	0.572976388	C5_CD8_Tem
chr1-15919300-15919680	8.69E-06	0.350575945	0.24	0.094	0.575380216	C5_CD8_Tem
chr12-120959565-120960303	9.22E-06	0.346250759	0.175	0.063	0.610377424	C5_CD8_Tem
chr2-162058983-162059673	9.24E-06	0.357708126	0.186	0.064	0.611978773	C5_CD8_Tem
chr15-65217468-65217920	9.40E-06	0.299082793	0.06	0.007	0.622611371	C5_CD8_Tem
chr1-248924638-248925555	9.57E-06	0.337680599	0.273	0.115	0.633796462	C5_CD8_Tem
chr11-316938-317527	9.90E-06	0.353784071	0.24	0.094	0.65529103	C5_CD8_Tem
chr10-122515512-122516257	9.92E-06	0.346540563	0.158	0.043	0.656941662	C5_CD8_Tem
chr19-13167290-13168030	1.00E-05	0.267349435	0.563	0.315	0.663997189	C5_CD8_Tem
chr5-177478921-177479495	1.01E-05	0.33222725	0.093	0.017	0.667461321	C5_CD8_Tem
chr3-98508509-98508910	1.01E-05	0.29938624	0.06	0.01	0.671028513	C5_CD8_Tem
chr14-60721309-60722140	1.04E-05	0.339106149	0.109	0.03	0.68652892	C5_CD8_Tem
chr18-69934888-69936458	1.05E-05	0.296555018	0.443	0.23	0.695222393	C5_CD8_Tem
chr8-66534684-66536138	1.05E-05	0.326358186	0.355	0.163	0.696780448	C5_CD8_Tem
chr6-90110865-90111691	1.05E-05	0.346446604	0.142	0.039	0.696873053	C5_CD8_Tem
chr16-89324221-89324605	1.07E-05	0.347001536	0.126	0.028	0.709440577	C5_CD8_Tem
chr6-2110667-2111576	1.07E-05	0.34627269	0.115	0.031	0.711488341	C5_CD8_Tem
chr10-62636875-62638307	1.08E-05	0.287791685	0.443	0.249	0.714144063	C5_CD8_Tem
chr9-96008395-96010381	1.09E-05	0.329328072	0.311	0.134	0.720627143	C5_CD8_Tem

chr13-114065002-114066732	1.09E-05	0.292347412	0.481	0.258	0.724749989	C5_CD8_Tem
chr3-16299886-16301388	1.10E-05	0.307232205	0.404	0.197	0.730130881	C5_CD8_Tem
chr20-45419526-45419933	1.11E-05	0.322378722	0.098	0.02	0.731906628	C5_CD8_Tem
chr12-105357087-105357992	1.12E-05	0.335920799	0.12	0.032	0.739266211	C5_CD8_Tem
chr8-29251858-29252392	1.12E-05	0.349590166	0.142	0.037	0.742679541	C5_CD8_Tem
chr5-143601982-143602406	1.12E-05	0.28505232	0.055	0.007	0.744023921	C5_CD8_Tem
chr3-46516609-46517697	1.13E-05	0.344646713	0.131	0.033	0.747026855	C5_CD8_Tem
chr3-59783442-59784017	1.15E-05	0.297955336	0.06	0.008	0.763832317	C5_CD8_Tem
chr13-99355947-99357621	1.16E-05	0.2629937	0.546	0.326	0.767649979	C5_CD8_Tem
chr2-65053293-65053498	1.18E-05	0.333272684	0.115	0.03	0.780253376	C5_CD8_Tem
chr2-102233971-102234562	1.19E-05	0.349283802	0.126	0.029	0.786521362	C5_CD8_Tem
chr9-21559043-21560315	1.22E-05	0.341793774	0.148	0.045	0.805968112	C5_CD8_Tem
chr14-65637567-65638190	1.22E-05	0.343586558	0.169	0.054	0.80615858	C5_CD8_Tem
chr10-4243638-4243991	1.22E-05	0.314386724	0.071	0.011	0.809536646	C5_CD8_Tem
chr16-30491852-30492398	1.23E-05	0.343969624	0.126	0.034	0.812985588	C5_CD8_Tem
chr17-41678495-41679547	1.30E-05	0.32840715	0.333	0.146	0.860090687	C5_CD8_Tem
chr1-151989519-151989782	1.31E-05	0.35355887	0.137	0.035	0.869188549	C5_CD8_Tem
chr10-14659217-14660171	1.34E-05	0.33832621	0.23	0.094	0.888106673	C5_CD8_Tem
chr5-40391649-40391964	1.34E-05	0.320112251	0.087	0.018	0.889795451	C5_CD8_Tem
chr20-5947322-5947582	1.35E-05	0.313381867	0.077	0.013	0.893390164	C5_CD8_Tem
chr17-4721449-4722079	1.35E-05	0.332750129	0.098	0.023	0.896747702	C5_CD8_Tem
chr10-132446791-132448041	1.36E-05	0.277689096	0.503	0.281	0.89872048	C5_CD8_Tem
chr13-99283464-99283788	1.36E-05	0.337393102	0.148	0.042	0.903130603	C5_CD8_Tem
chr17-50918950-50920059	1.37E-05	0.33298231	0.24	0.102	0.905822165	C5_CD8_Tem
chr1-24952316-24952863	1.40E-05	0.345175766	0.186	0.064	0.926491748	C5_CD8_Tem
chr15-64009239-64011234	1.43E-05	0.254786171	0.563	0.338	0.949958776	C5_CD8_Tem
chr7-73717579-73719884	1.44E-05	0.285189446	0.492	0.273	0.954860837	C5_CD8_Tem

chr20-48736257-48736514	1.45E-05	0.310596833	0.077	0.013	0.958099758	C5_CD8_Tem
chr4-47482150-47482684	1.46E-05	0.340827401	0.098	0.02	0.965528268	C5_CD8_Tem
chr2-143086990-143087281	1.46E-05	0.282122262	0.055	0.006	0.969439768	C5_CD8_Tem
chr19-39893399-39894033	1.48E-05	0.317560424	0.082	0.016	0.981958482	C5_CD8_Tem
chr3-11238758-11239264	1.49E-05	0.325512808	0.087	0.017	0.988039341	C5_CD8_Tem
chr3-196181445-196182175	1.49E-05	0.347170909	0.197	0.069	0.988448883	C5_CD8_Tem
chr14-105989699-105990239	1.51E-05	0.345812605	0.208	0.072	0.998726327	C5_CD8_Tem
chr14-24670539-24670969	1.52E-05	0.342183402	0.197	0.07	1	C5_CD8_Tem
chr19-13172174-13172899	1.54E-05	0.345332316	0.169	0.059	1	C5_CD8_Tem
chr12-9340452-9341540	1.55E-05	0.317408845	0.093	0.024	1	C5_CD8_Tem
chr8-29230626-29231599	1.55E-05	0.343067286	0.175	0.052	1	C5_CD8_Tem
chr2-25301275-25302024	1.57E-05	0.325241929	0.306	0.141	1	C5_CD8_Tem
chr12-92405803-92406159	1.58E-05	0.298483689	0.066	0.01	1	C5_CD8_Tem
chr14-89586293-89586620	1.60E-05	0.291442985	0.06	0.008	1	C5_CD8_Tem
chr6-159988907-159989533	1.62E-05	0.328073359	0.115	0.028	1	C5_CD8_Tem
chr3-27669455-27669837	1.66E-05	0.300003261	0.06	0.011	1	C5_CD8_Tem
chr6-108904717-108905087	1.67E-05	0.291563198	0.06	0.008	1	C5_CD8_Tem
chr13-45398836-45399263	1.67E-05	0.32739299	0.104	0.025	1	C5_CD8_Tem
chr9-88303778-88304430	1.67E-05	0.304040488	0.071	0.014	1	C5_CD8_Tem
chr14-61537146-61537848	1.70E-05	0.344951886	0.169	0.052	1	C5_CD8_Tem
chr1-115073318-115073833	1.71E-05	0.288152305	0.06	0.007	1	C5_CD8_Tem
chr10-12412623-12413670	1.72E-05	0.318397524	0.279	0.115	1	C5_CD8_Tem
chr6-130602179-130602871	1.74E-05	0.326804458	0.126	0.033	1	C5_CD8_Tem
chr16-81626171-81627260	1.74E-05	0.330259401	0.115	0.032	1	C5_CD8_Tem
chrX-10003255-10004199	1.77E-05	0.338251946	0.126	0.038	1	C5_CD8_Tem
chr18-62585565-62586431	1.83E-05	0.338124523	0.142	0.041	1	C5_CD8_Tem
chr6-109458613-109459110	1.88E-05	0.32745394	0.29	0.134	1	C5_CD8_Tem

chr12-94621298-94622082	1.91E-05	0.342853938	0.202	0.067	1	C5_CD8_Tem
chr5-379399-379715	1.98E-05	0.309565878	0.071	0.011	1	C5_CD8_Tem
chr17-57612451-57613050	2.00E-05	0.336039787	0.219	0.079	1	C5_CD8_Tem
chr3-48478213-48479727	2.00E-05	0.286499463	0.437	0.238	1	C5_CD8_Tem
chr2-135819320-135820567	2.02E-05	0.331650863	0.164	0.062	1	C5_CD8_Tem
chr1-184889903-184891184	2.02E-05	0.258754132	0.53	0.314	1	C5_CD8_Tem
chr17-49695220-49696615	2.05E-05	0.334906389	0.23	0.086	1	C5_CD8_Tem
chr14-105024777-105025155	2.06E-05	0.310877496	0.383	0.186	1	C5_CD8_Tem
chr14-71004347-71005898	2.12E-05	0.340200628	0.131	0.035	1	C5_CD8_Tem
chr22-24433931-24435044	2.16E-05	0.267311691	0.486	0.295	1	C5_CD8_Tem
chr10-119642274-119643012	2.21E-05	0.328180725	0.202	0.076	1	C5_CD8_Tem
chr2-203492419-203493372	2.24E-05	0.333408547	0.158	0.057	1	C5_CD8_Tem
chr2-38394798-38395160	2.27E-05	0.280080956	0.055	0.008	1	C5_CD8_Tem
chr3-101950427-101950936	2.30E-05	0.280632226	0.055	0.008	1	C5_CD8_Tem
chr19-6667304-6667600	2.31E-05	0.340966826	0.153	0.047	1	C5_CD8_Tem
chr5-157582713-157583330	2.31E-05	0.284278796	0.06	0.009	1	C5_CD8_Tem
chr2-148815134-148815344	2.34E-05	0.277611408	0.055	0.007	1	C5_CD8_Tem
chr1-206589094-206590676	2.38E-05	0.275900712	0.481	0.257	1	C5_CD8_Tem
chr11-118295276-118296138	2.45E-05	0.343809827	0.175	0.057	1	C5_CD8_Tem
chr11-57311019-57311709	2.48E-05	0.331248581	0.115	0.028	1	C5_CD8_Tem
chr3-98555296-98556764	2.49E-05	0.26751112	0.475	0.265	1	C5_CD8_Tem
chrX-71623735-71624082	2.50E-05	0.325791377	0.126	0.035	1	C5_CD8_Tem
chr6-90222669-90222949	2.51E-05	0.321216115	0.098	0.024	1	C5_CD8_Tem
chr7-131013200-131013528	2.55E-05	0.326668054	0.126	0.033	1	C5_CD8_Tem
chr7-121442756-121443205	2.55E-05	0.321425048	0.131	0.042	1	C5_CD8_Tem
chr10-132684279-132684760	2.56E-05	0.314100469	0.093	0.018	1	C5_CD8_Tem
chr1-11718904-11720037	2.60E-05	0.291828116	0.426	0.232	1	C5_CD8_Tem

chr10-12418885-12419856	2.60E-05	0.335285591	0.213	0.078	1	C5_CD8_Tem
chr22-46013044-46014383	2.60E-05	0.289975882	0.404	0.219	1	C5_CD8_Tem
chr19-13872618-13873201	2.62E-05	0.322230511	0.098	0.021	1	C5_CD8_Tem
chr3-98538418-98538933	2.68E-05	0.331127003	0.164	0.058	1	C5_CD8_Tem
chr12-10301111-10301472	2.70E-05	0.297945508	0.071	0.012	1	C5_CD8_Tem
chr10-84156586-84157173	2.72E-05	0.326230793	0.142	0.045	1	C5_CD8_Tem
chr2-74919792-74920335	2.73E-05	0.324558439	0.115	0.034	1	C5_CD8_Tem
chr2-196210936-196211868	2.77E-05	0.272744049	0.47	0.271	1	C5_CD8_Tem
chr1-207825446-207826308	2.78E-05	0.260768006	0.514	0.294	1	C5_CD8_Tem
chr15-28950071-28950406	2.85E-05	0.32334058	0.087	0.019	1	C5_CD8_Tem
chr22-38984750-38985599	2.88E-05	0.302999462	0.383	0.184	1	C5_CD8_Tem
chr9-136384932-136385369	2.91E-05	0.32743293	0.131	0.043	1	C5_CD8_Tem
chr6-33204302-33205948	2.93E-05	0.283083863	0.443	0.245	1	C5_CD8_Tem
chr7-29207195-29207901	2.94E-05	0.304886382	0.087	0.017	1	C5_CD8_Tem
chr5-142826599-142827411	2.95E-05	0.334443144	0.208	0.071	1	C5_CD8_Tem
chr15-42607854-42608451	2.98E-05	0.319682902	0.098	0.022	1	C5_CD8_Tem
chr17-45445851-45446301	3.02E-05	0.327911806	0.126	0.035	1	C5_CD8_Tem
chrX-119955576-119956742	3.02E-05	0.326311796	0.219	0.075	1	C5_CD8_Tem
chr16-72172180-72172725	3.04E-05	0.292780364	0.066	0.011	1	C5_CD8_Tem
chr8-125513004-125513480	3.05E-05	0.331367959	0.148	0.043	1	C5_CD8_Tem
chr17-55413904-55414432	3.06E-05	0.286059022	0.06	0.008	1	C5_CD8_Tem
chr17-1258454-1260011	3.07E-05	0.331948833	0.219	0.084	1	C5_CD8_Tem
chr12-108616875-108618370	3.08E-05	0.274822494	0.426	0.262	1	C5_CD8_Tem
chr10-63236126-63236729	3.09E-05	0.341688134	0.202	0.072	1	C5_CD8_Tem
chr3-194715029-194715816	3.10E-05	0.326390251	0.126	0.035	1	C5_CD8_Tem
chr9-134381409-134382506	3.12E-05	0.32459263	0.208	0.082	1	C5_CD8_Tem
chr1-224387248-224387840	3.19E-05	0.310109684	0.087	0.022	1	C5_CD8_Tem

chr3-45943007-45943699	3.19E-05	0.319842838	0.295	0.137	1	C5_CD8_Tem
chr6-166258549-166259778	3.24E-05	0.321372442	0.257	0.112	1	C5_CD8_Tem
chr15-85413223-85413841	3.26E-05	0.327902994	0.235	0.104	1	C5_CD8_Tem
chr6-42031089-42031503	3.29E-05	0.318428495	0.12	0.031	1	C5_CD8_Tem
chr22-43343206-43343617	3.30E-05	0.32502519	0.104	0.025	1	C5_CD8_Tem
chr12-31733746-31733978	3.39E-05	0.322824939	0.115	0.027	1	C5_CD8_Tem
chr8-73346121-73347130	3.40E-05	0.314543737	0.246	0.102	1	C5_CD8_Tem
chr13-30766918-30767586	3.41E-05	0.318204125	0.109	0.03	1	C5_CD8_Tem
chr3-71415466-71417239	3.42E-05	0.337898358	0.202	0.07	1	C5_CD8_Tem
chr6-131524298-131525162	3.44E-05	0.303039839	0.12	0.034	1	C5_CD8_Tem
chr10-6269791-6270840	3.45E-05	0.287195615	0.404	0.212	1	C5_CD8_Tem
chr3-32113428-32115253	3.46E-05	0.258944996	0.519	0.296	1	C5_CD8_Tem
chr11-48006136-48006998	3.47E-05	0.313241788	0.333	0.157	1	C5_CD8_Tem
chr1-36109067-36109352	3.48E-05	0.284513464	0.06	0.009	1	C5_CD8_Tem
chr1-224377207-224378012	3.51E-05	0.331834463	0.142	0.043	1	C5_CD8_Tem
chr2-65366422-65366934	3.51E-05	0.324160464	0.126	0.035	1	C5_CD8_Tem
chr11-128318745-128319321	3.52E-05	0.312356669	0.279	0.125	1	C5_CD8_Tem
chr22-43381675-43382132	3.57E-05	0.327977375	0.131	0.04	1	C5_CD8_Tem
chr6-166418699-166419622	3.58E-05	0.323428067	0.251	0.11	1	C5_CD8_Tem
chr19-55372632-55373022	3.59E-05	0.2974196	0.077	0.015	1	C5_CD8_Tem
chr11-57457205-57459446	3.63E-05	0.323913614	0.268	0.115	1	C5_CD8_Tem
chr2-10538359-10538847	3.64E-05	0.291215018	0.066	0.011	1	C5_CD8_Tem
chr15-59298225-59298611	3.65E-05	0.304274843	0.082	0.017	1	C5_CD8_Tem
chr14-65208086-65208324	3.65E-05	0.289846445	0.066	0.01	1	C5_CD8_Tem
chr1-247415390-247416311	3.67E-05	0.313898809	0.137	0.042	1	C5_CD8_Tem
chr17-67390201-67391379	3.71E-05	0.309200467	0.311	0.146	1	C5_CD8_Tem
chr5-91332076-91332926	3.81E-05	0.322176163	0.148	0.051	1	C5_CD8_Tem

chr18-79245127-79246012	3.81E-05	0.284931604	0.295	0.153	1	C5_CD8_Tem
chr10-62727924-62728831	3.84E-05	0.326632151	0.164	0.055	1	C5_CD8_Tem
chr1-160641440-160641922	3.87E-05	0.33317067	0.186	0.072	1	C5_CD8_Tem
chr1-247406912-247407731	3.90E-05	0.314244258	0.284	0.119	1	C5_CD8_Tem
chr18-13586088-13586608	3.92E-05	0.286934826	0.066	0.012	1	C5_CD8_Tem
chr1-223702996-223703441	3.94E-05	0.287345788	0.066	0.01	1	C5_CD8_Tem
chr16-2163039-2163533	3.99E-05	0.276627637	0.06	0.008	1	C5_CD8_Tem
chr11-96199138-96199970	4.00E-05	0.322598746	0.24	0.097	1	C5_CD8_Tem
chr1-24964452-24965515	4.04E-05	0.250240735	0.546	0.322	1	C5_CD8_Tem
chr5-35672850-35673342	4.04E-05	0.326075344	0.109	0.027	1	C5_CD8_Tem
chr6-85464185-85464494	4.15E-05	0.282045623	0.06	0.01	1	C5_CD8_Tem
chr11-77141936-77142504	4.16E-05	0.306415792	0.098	0.023	1	C5_CD8_Tem
chr6-37164365-37164849	4.18E-05	0.311553591	0.098	0.023	1	C5_CD8_Tem
chr16-21646703-21647667	4.21E-05	0.301496166	0.35	0.163	1	C5_CD8_Tem
chr1-31939487-31940290	4.21E-05	0.272402349	0.443	0.259	1	C5_CD8_Tem
chr6-37535730-37536631	4.22E-05	0.294298271	0.071	0.012	1	C5_CD8_Tem
chr15-90648073-90648855	4.23E-05	0.326524862	0.235	0.092	1	C5_CD8_Tem
chr1-150922478-150922884	4.24E-05	0.331140614	0.197	0.074	1	C5_CD8_Tem
chr13-44088603-44089750	4.24E-05	0.280765625	0.071	0.013	1	C5_CD8_Tem
chr17-8934619-8935587	4.25E-05	0.312825377	0.279	0.125	1	C5_CD8_Tem
chr7-151727523-151728027	4.35E-05	0.28662915	0.066	0.012	1	C5_CD8_Tem
chr14-101817409-101818097	4.38E-05	0.300344267	0.333	0.164	1	C5_CD8_Tem
chr21-14686474-14687543	4.40E-05	0.29379749	0.082	0.016	1	C5_CD8_Tem
chr6-130467135-130467798	4.43E-05	0.301998605	0.077	0.015	1	C5_CD8_Tem
chr2-46985041-46985813	4.51E-05	0.322738883	0.126	0.034	1	C5_CD8_Tem
chr6-150072213-150072509	4.51E-05	0.326557179	0.12	0.034	1	C5_CD8_Tem
chr6-159989789-159990428	4.52E-05	0.326040436	0.109	0.025	1	C5_CD8_Tem

chr18-13566916-13568052	4.55E-05	0.327133335	0.169	0.053	1	C5_CD8_Tem
chr22-43345695-43346691	4.62E-05	0.327277724	0.169	0.059	1	C5_CD8_Tem
chr1-42943071-42943442	4.63E-05	0.324451198	0.158	0.054	1	C5_CD8_Tem
chr1-15352418-15352854	4.67E-05	0.325201115	0.153	0.057	1	C5_CD8_Tem
chr1-111515645-111516492	4.68E-05	0.313357685	0.087	0.018	1	C5_CD8_Tem
chr2-71082487-71083221	4.75E-05	0.296389529	0.087	0.02	1	C5_CD8_Tem
chr2-37585777-37586597	4.77E-05	0.282274089	0.443	0.239	1	C5_CD8_Tem
chr15-44394628-44395398	4.79E-05	0.288759897	0.071	0.013	1	C5_CD8_Tem
chr11-66109149-66109597	4.80E-05	0.286146764	0.066	0.01	1	C5_CD8_Tem
chr1-12436964-12437256	4.90E-05	0.314592605	0.104	0.028	1	C5_CD8_Tem
chr21-45255154-45256068	4.94E-05	0.31368997	0.109	0.031	1	C5_CD8_Tem
chr19-47539830-47540182	4.94E-05	0.276549671	0.066	0.011	1	C5_CD8_Tem
chr1-221855985-221856710	5.04E-05	0.315095635	0.126	0.037	1	C5_CD8_Tem
chr2-62332611-62333314	5.05E-05	0.314106972	0.126	0.037	1	C5_CD8_Tem
chr21-38768417-38769000	5.07E-05	0.320812567	0.164	0.056	1	C5_CD8_Tem
chr5-178238844-178239484	5.16E-05	0.319665991	0.169	0.059	1	C5_CD8_Tem
chr7-92664250-92665026	5.26E-05	0.289521549	0.082	0.017	1	C5_CD8_Tem
chr15-59419922-59420462	5.27E-05	0.272262954	0.055	0.008	1	C5_CD8_Tem
chr1-211334681-211336094	5.36E-05	0.321351364	0.24	0.094	1	C5_CD8_Tem
chr22-37273467-37273731	5.39E-05	0.264116136	0.055	0.006	1	C5_CD8_Tem
chr1-153805020-153806041	5.40E-05	0.275846051	0.437	0.235	1	C5_CD8_Tem
chr10-46022955-46023765	5.44E-05	0.30781063	0.328	0.153	1	C5_CD8_Tem
chr4-56757249-56758410	5.53E-05	0.306784659	0.262	0.11	1	C5_CD8_Tem
chr11-13216334-13216796	5.54E-05	0.271088077	0.06	0.011	1	C5_CD8_Tem
chr11-117875104-117876435	5.58E-05	0.295636292	0.082	0.018	1	C5_CD8_Tem
chr11-118245898-118246180	5.60E-05	0.313890731	0.104	0.028	1	C5_CD8_Tem
chr9-114718278-114719263	5.63E-05	0.308267655	0.262	0.117	1	C5_CD8_Tem

chr10-129036423-129037248	5.65E-05	0.293772378	0.087	0.02	1	C5_CD8_Tem
chr16-68270063-68270730	5.68E-05	0.324411718	0.169	0.061	1	C5_CD8_Tem
chr17-48456624-48457598	5.70E-05	0.322325071	0.235	0.095	1	C5_CD8_Tem
chr22-36161765-36162643	5.71E-05	0.298200085	0.333	0.158	1	C5_CD8_Tem
chr3-71496056-71496530	5.77E-05	0.325300432	0.158	0.052	1	C5_CD8_Tem
chr8-52764202-52764760	5.78E-05	0.315801432	0.164	0.06	1	C5_CD8_Tem
chr22-45608691-45609186	5.80E-05	0.275691798	0.06	0.01	1	C5_CD8_Tem
chr19-38657711-38658115	5.81E-05	0.32415699	0.208	0.076	1	C5_CD8_Tem
chr15-45521754-45523159	5.83E-05	0.306570642	0.273	0.127	1	C5_CD8_Tem
chr12-94644054-94644939	5.86E-05	0.316904165	0.115	0.032	1	C5_CD8_Tem
chr17-67650490-67651169	5.88E-05	0.318755641	0.137	0.043	1	C5_CD8_Tem
chr9-6566384-6567492	5.97E-05	0.297693985	0.093	0.02	1	C5_CD8_Tem
chr6-341888-342273	5.99E-05	0.285789236	0.066	0.011	1	C5_CD8_Tem
chr17-8963632-8963924	6.10E-05	0.313563199	0.12	0.037	1	C5_CD8_Tem
chr11-96020238-96020646	6.15E-05	0.284054542	0.071	0.013	1	C5_CD8_Tem
chr10-12180921-12181821	6.24E-05	0.271309127	0.399	0.218	1	C5_CD8_Tem
chr2-134683415-134683947	6.39E-05	0.299453406	0.087	0.02	1	C5_CD8_Tem
chr17-59762152-59762770	6.40E-05	0.300909118	0.087	0.021	1	C5_CD8_Tem
chr22-40534893-40535324	6.47E-05	0.292364177	0.077	0.014	1	C5_CD8_Tem
chr2-216348988-216349622	6.58E-05	0.319322671	0.18	0.066	1	C5_CD8_Tem
chr22-40239052-40239327	6.66E-05	0.3004926	0.082	0.019	1	C5_CD8_Tem
chr6-108874179-108875227	6.66E-05	0.308228022	0.251	0.111	1	C5_CD8_Tem
chr19-41297646-41298585	6.67E-05	0.32068963	0.191	0.08	1	C5_CD8_Tem
chr1-222653429-222653799	6.67E-05	0.274122854	0.06	0.009	1	C5_CD8_Tem
chr7-24898455-24899158	6.77E-05	0.317508283	0.213	0.084	1	C5_CD8_Tem
chr8-20281983-20282492	6.79E-05	0.299615755	0.087	0.021	1	C5_CD8_Tem
chr6-44237157-44238419	6.90E-05	0.28271498	0.432	0.226	1	C5_CD8_Tem

chr17-4714902-4715337	6.92E-05	0.322922359	0.158	0.053	1	C5_CD8_Tem
chr18-67301515-67302327	6.95E-05	0.266604408	0.06	0.01	1	C5_CD8_Tem
chr3-11288675-11289452	6.96E-05	0.31979369	0.158	0.055	1	C5_CD8_Tem
chr12-75978694-75979577	6.96E-05	0.294892487	0.333	0.155	1	C5_CD8_Tem
chr22-36374698-36374934	7.01E-05	0.323991276	0.164	0.055	1	C5_CD8_Tem
chr15-48940397-48940885	7.08E-05	0.280190718	0.071	0.014	1	C5_CD8_Tem
chr18-69885499-69886408	7.20E-05	0.311943321	0.12	0.035	1	C5_CD8_Tem
chr5-157252467-157253643	7.25E-05	0.252858721	0.475	0.292	1	C5_CD8_Tem
chr13-30049589-30050211	7.46E-05	0.268837139	0.06	0.01	1	C5_CD8_Tem
chr4-87390617-87391620	7.51E-05	0.287214087	0.328	0.171	1	C5_CD8_Tem
chr18-13577194-13577707	7.62E-05	0.301407753	0.098	0.026	1	C5_CD8_Tem
chr22-30648942-30649975	7.64E-05	0.29892452	0.311	0.147	1	C5_CD8_Tem
chr1-210394231-210394749	7.71E-05	0.295777431	0.087	0.021	1	C5_CD8_Tem
chr12-94618812-94619272	7.74E-05	0.304453697	0.115	0.029	1	C5_CD8_Tem
chr3-12948893-12949899	7.74E-05	0.30828807	0.126	0.036	1	C5_CD8_Tem
chr17-67257330-67259176	7.74E-05	0.321283082	0.202	0.076	1	C5_CD8_Tem
chr5-40436392-40437905	7.88E-05	0.268332228	0.443	0.237	1	C5_CD8_Tem
chr3-111609809-111611067	7.94E-05	0.265952302	0.426	0.247	1	C5_CD8_Tem
chr6-34207591-34208351	7.96E-05	0.286375411	0.071	0.013	1	C5_CD8_Tem
chr11-14336812-14337472	8.02E-05	0.317487598	0.153	0.052	1	C5_CD8_Tem
chr2-134216488-134216895	8.06E-05	0.307577186	0.104	0.026	1	C5_CD8_Tem
chr5-98880087-98880527	8.10E-05	0.288385203	0.077	0.015	1	C5_CD8_Tem
chr1-148371452-148373872	8.10E-05	0.293369422	0.361	0.175	1	C5_CD8_Tem
chr21-43370266-43370647	8.14E-05	0.268389352	0.055	0.009	1	C5_CD8_Tem
chr6-4941776-4942921	8.21E-05	0.300815523	0.268	0.122	1	C5_CD8_Tem
chr16-23874451-23874884	8.32E-05	0.276124348	0.06	0.011	1	C5_CD8_Tem
chr6-34242824-34243525	8.36E-05	0.321089905	0.142	0.043	1	C5_CD8_Tem

chr1-168557579-168558101	8.69E-05	0.30640131	0.12	0.033	1	C5_CD8_Tem
chr7-36683919-36684260	8.79E-05	0.299982053	0.093	0.02	1	C5_CD8_Tem
chr18-79962870-79963594	8.80E-05	0.312281486	0.219	0.092	1	C5_CD8_Tem
chr20-47544264-47544561	8.82E-05	0.290638915	0.093	0.02	1	C5_CD8_Tem
chr6-107657494-107658235	9.12E-05	0.302787736	0.104	0.029	1	C5_CD8_Tem
chr14-22524050-22525004	9.34E-05	0.315892305	0.197	0.076	1	C5_CD8_Tem
chr21-14550247-14550530	9.41E-05	0.292776488	0.077	0.018	1	C5_CD8_Tem
chr20-53685860-53686390	9.43E-05	0.296572163	0.104	0.028	1	C5_CD8_Tem
chr5-150783282-150783993	9.57E-05	0.302658237	0.311	0.143	1	C5_CD8_Tem
chr19-4088649-4090008	9.97E-05	0.29679125	0.306	0.146	1	C5_CD8_Tem
chr17-76565170-76565564	0.000100491	0.310094998	0.148	0.05	1	C5_CD8_Tem
chr4-169604290-169604869	0.000100696	0.308546756	0.126	0.032	1	C5_CD8_Tem
chr22-20523486-20524493	0.000104991	0.319734207	0.131	0.038	1	C5_CD8_Tem
chr14-52328277-52328528	0.000105744	0.284858187	0.071	0.013	1	C5_CD8_Tem
chr2-30366296-30366719	0.000107291	0.267887624	0.055	0.008	1	C5_CD8_Tem
chr9-33452827-33453458	0.000107969	0.301374622	0.098	0.026	1	C5_CD8_Tem
chr5-134138317-134138717	0.000108428	0.308322578	0.126	0.043	1	C5_CD8_Tem
chr12-47766791-47767432	0.000109891	0.306406027	0.098	0.026	1	C5_CD8_Tem
chr1-40662835-40663258	0.000110586	0.297251739	0.098	0.026	1	C5_CD8_Tem
chr14-74875859-74876336	0.000111118	0.263301163	0.055	0.008	1	C5_CD8_Tem
chr16-48556275-48557251	0.000111413	0.312976456	0.197	0.077	1	C5_CD8_Tem
chr1-89671786-89672473	0.000112325	0.311308061	0.12	0.037	1	C5_CD8_Tem
chr1-93878188-93879401	0.000112443	0.266257758	0.443	0.248	1	C5_CD8_Tem
chr6-117677515-117677862	0.0001128	0.307881753	0.12	0.039	1	C5_CD8_Tem
chr9-86340353-86340874	0.0001129	0.304410995	0.109	0.026	1	C5_CD8_Tem
chr5-154202669-154203262	0.000113556	0.314393217	0.131	0.044	1	C5_CD8_Tem
chr12-76158378-76159169	0.000115548	0.309110109	0.142	0.047	1	C5_CD8_Tem

chr11-120431677-120432428	0.000117215	0.275334671	0.071	0.016	1	C5_CD8_Tem
chr4-80127300-80127706	0.000118428	0.315326979	0.197	0.079	1	C5_CD8_Tem
chr10-50325355-50325742	0.00012047	0.265809529	0.06	0.011	1	C5_CD8_Tem
chr6-130364425-130366530	0.000120515	0.30563546	0.257	0.116	1	C5_CD8_Tem
chr5-67165478-67166941	0.000124638	0.257675517	0.426	0.244	1	C5_CD8_Tem
chr15-63494425-63494723	0.000124904	0.311530569	0.12	0.034	1	C5_CD8_Tem
chr6-223861-224130	0.00012491	0.28741117	0.093	0.02	1	C5_CD8_Tem
chr6-85464746-85465136	0.000125611	0.284301202	0.093	0.023	1	C5_CD8_Tem
chr20-62602400-62604005	0.000125672	0.308401028	0.213	0.088	1	C5_CD8_Tem
chr15-45454650-45455247	0.000126059	0.306941463	0.251	0.11	1	C5_CD8_Tem
chr9-136700803-136701844	0.000126643	0.297794039	0.257	0.115	1	C5_CD8_Tem
chr9-133127090-133127894	0.000126889	0.295384807	0.235	0.108	1	C5_CD8_Tem
chr12-123075421-123076983	0.00012723	0.268892386	0.383	0.214	1	C5_CD8_Tem
chr12-52066419-52066849	0.000127252	0.298144952	0.148	0.046	1	C5_CD8_Tem
chr9-126395543-126395742	0.000129247	0.274028484	0.066	0.013	1	C5_CD8_Tem
chr16-89408918-89409734	0.000131267	0.253515103	0.464	0.26	1	C5_CD8_Tem
chr12-75984601-75985454	0.000131272	0.285910671	0.071	0.014	1	C5_CD8_Tem
chr19-1746878-1747789	0.000133771	0.310390726	0.131	0.046	1	C5_CD8_Tem
chr7-90178650-90179227	0.000140747	0.288130724	0.087	0.019	1	C5_CD8_Tem
chr6-118724237-118724481	0.000141217	0.290467487	0.087	0.02	1	C5_CD8_Tem
chr3-56758466-56758949	0.000141945	0.305075611	0.158	0.052	1	C5_CD8_Tem
chr12-89468651-89469769	0.000142058	0.295854574	0.24	0.103	1	C5_CD8_Tem
chr17-80780406-80781198	0.000144106	0.302845599	0.115	0.036	1	C5_CD8_Tem
chr5-35835883-35836351	0.000144486	0.28430276	0.077	0.015	1	C5_CD8_Tem
chr4-123396553-123396886	0.000146841	0.302112173	0.153	0.051	1	C5_CD8_Tem
chr19-44689117-44689471	0.000147894	0.291638597	0.077	0.016	1	C5_CD8_Tem
chr4-81724054-81725386	0.000148354	0.305083003	0.164	0.06	1	C5_CD8_Tem

chr17-68085683-68087588	0.00015005	0.302740023	0.131	0.043	1	C5_CD8_Tem
chr21-42267024-42268470	0.000151851	0.299075255	0.148	0.052	1	C5_CD8_Tem
chrX-78109464-78109773	0.000153334	0.291750775	0.098	0.023	1	C5_CD8_Tem
chr15-39774449-39775028	0.000154362	0.286058537	0.082	0.023	1	C5_CD8_Tem
chr17-76968812-76969670	0.000156558	0.296678639	0.093	0.024	1	C5_CD8_Tem
chr4-4334938-4335552	0.000156941	0.303441147	0.137	0.044	1	C5_CD8_Tem
chr1-227934560-227935455	0.000157335	0.272544732	0.29	0.13	1	C5_CD8_Tem
chr1-193070912-193071299	0.000157944	0.269019091	0.066	0.014	1	C5_CD8_Tem
chr11-85673210-85673769	0.000158514	0.265633822	0.06	0.011	1	C5_CD8_Tem
chr5-102778386-102778928	0.000160041	0.295015592	0.12	0.035	1	C5_CD8_Tem
chr19-14431799-14432186	0.00016169	0.30736425	0.142	0.048	1	C5_CD8_Tem
chr12-47085201-47086075	0.000163336	0.30377749	0.202	0.084	1	C5_CD8_Tem
chr2-113167035-113167587	0.000163591	0.283459484	0.087	0.021	1	C5_CD8_Tem
chr9-131634958-131635846	0.000165115	0.297684174	0.251	0.119	1	C5_CD8_Tem
chr9-95981554-95982046	0.000165962	0.276675032	0.087	0.02	1	C5_CD8_Tem
chr6-160015668-160016753	0.000166277	0.301626064	0.126	0.045	1	C5_CD8_Tem
chr7-142383916-142384929	0.000166517	0.278638145	0.087	0.024	1	C5_CD8_Tem
chr16-56679874-56680154	0.000166702	0.287694009	0.093	0.022	1	C5_CD8_Tem
chr1-231376704-231377518	0.000166797	0.272158473	0.071	0.014	1	C5_CD8_Tem
chr13-79392649-79394106	0.000166903	0.297289291	0.235	0.102	1	C5_CD8_Tem
chr11-9361525-9361930	0.000167762	0.309996925	0.153	0.05	1	C5_CD8_Tem
chr1-148363409-148363721	0.000169156	0.286116408	0.082	0.018	1	C5_CD8_Tem
chr6-16741003-16741696	0.000170753	0.299795405	0.148	0.051	1	C5_CD8_Tem
chr6-170087914-170088908	0.000173117	0.301708572	0.148	0.056	1	C5_CD8_Tem
chr15-74402410-74403487	0.00017454	0.306083641	0.18	0.065	1	C5_CD8_Tem
chr8-144402609-144402951	0.000174626	0.275179177	0.071	0.015	1	C5_CD8_Tem
chr1-1063692-1064603	0.000174671	0.25142375	0.464	0.282	1	C5_CD8_Tem

chr17-47714317-47714552	0.00017531	0.268083513	0.066	0.011	1	C5_CD8_Tem
chr1-200905330-200906209	0.00017591	0.266206912	0.377	0.206	1	C5_CD8_Tem
chr12-108611892-108612466	0.000180271	0.305151208	0.131	0.045	1	C5_CD8_Tem
chr3-198071596-198072277	0.000180671	0.29226405	0.191	0.081	1	C5_CD8_Tem
chr10-45418849-45419677	0.000182461	0.292746075	0.098	0.026	1	C5_CD8_Tem
chr12-51389393-51389860	0.000183174	0.297772579	0.126	0.045	1	C5_CD8_Tem
chr16-81632355-81633406	0.000187119	0.278427197	0.355	0.186	1	C5_CD8_Tem
chr3-182795144-182795765	0.000187788	0.29063156	0.23	0.109	1	C5_CD8_Tem
chr15-63763286-63763812	0.000190979	0.273072881	0.082	0.02	1	C5_CD8_Tem
chr5-55997774-55998031	0.000192021	0.302202344	0.12	0.038	1	C5_CD8_Tem
chr14-55330264-55330678	0.000197667	0.310175893	0.175	0.059	1	C5_CD8_Tem
chr5-783299-785042	0.000200407	0.275938462	0.361	0.193	1	C5_CD8_Tem
chr5-95823670-95824747	0.000201059	0.285772652	0.306	0.154	1	C5_CD8_Tem
chr15-25860819-25861066	0.00020247	0.283263451	0.082	0.02	1	C5_CD8_Tem
chr14-74860530-74861233	0.000203473	0.285077357	0.093	0.025	1	C5_CD8_Tem
chr2-232315107-232316163	0.000204059	0.270725343	0.35	0.185	1	C5_CD8_Tem
chr12-108837809-108838991	0.000204143	0.29043807	0.262	0.125	1	C5_CD8_Tem
chr21-45208406-45208978	0.000204221	0.284316656	0.093	0.023	1	C5_CD8_Tem
chr5-141681599-141682444	0.000204397	0.30204677	0.137	0.045	1	C5_CD8_Tem
chr1-168439207-168440119	0.000205516	0.299797112	0.219	0.09	1	C5_CD8_Tem
chr22-46592129-46592873	0.000206231	0.300867332	0.137	0.045	1	C5_CD8_Tem
chr15-70475008-70475427	0.000209716	0.298080476	0.12	0.037	1	C5_CD8_Tem
chr1-116595585-116596581	0.000211492	0.290629961	0.098	0.027	1	C5_CD8_Tem
chr16-66474556-66475090	0.000212251	0.293197546	0.158	0.061	1	C5_CD8_Tem
chr19-39073226-39074029	0.000214218	0.26024531	0.377	0.216	1	C5_CD8_Tem
chr12-111443552-111443831	0.000217054	0.287028792	0.093	0.024	1	C5_CD8_Tem
chr9-21334538-21335635	0.000217902	0.270547967	0.35	0.193	1	C5_CD8_Tem

chrX-38811629-38812393	0.000218222	0.294562402	0.12	0.038	1	C5_CD8_Tem
chr1-148426851-148427257	0.000219014	0.280379302	0.082	0.018	1	C5_CD8_Tem
chr9-137611644-137612094	0.000223397	0.291078672	0.098	0.027	1	C5_CD8_Tem
chrX-79181944-79182308	0.000225392	0.286208938	0.087	0.019	1	C5_CD8_Tem
chr5-131064641-131065510	0.000226782	0.278118428	0.104	0.031	1	C5_CD8_Tem
chr16-17272956-17273413	0.000227043	0.277204586	0.082	0.017	1	C5_CD8_Tem
chr3-197428016-197428695	0.000229437	0.26116942	0.066	0.012	1	C5_CD8_Tem
chr15-55279537-55280295	0.000230933	0.271162506	0.355	0.194	1	C5_CD8_Tem
chr9-37408810-37409703	0.000231938	0.27727498	0.339	0.171	1	C5_CD8_Tem
chr13-51793403-51793840	0.000232103	0.277948973	0.082	0.022	1	C5_CD8_Tem
chr6-130183456-130184310	0.000233943	0.289937879	0.23	0.101	1	C5_CD8_Tem
chr12-48977229-48978329	0.000234873	0.2608726	0.41	0.229	1	C5_CD8_Tem
chr1-211514906-211515844	0.000235089	0.297430224	0.213	0.093	1	C5_CD8_Tem
chr6-36691075-36691365	0.000236109	0.264072082	0.066	0.013	1	C5_CD8_Tem
chr20-36642111-36642442	0.000236356	0.290946423	0.104	0.027	1	C5_CD8_Tem
chr15-79872390-79873039	0.000237203	0.287512668	0.104	0.031	1	C5_CD8_Tem
chr15-29045608-29046026	0.000239479	0.289437489	0.093	0.021	1	C5_CD8_Tem
chr10-14598998-14599469	0.0002398	0.276696178	0.098	0.028	1	C5_CD8_Tem
chr12-75977484-75978355	0.000241684	0.251955469	0.448	0.245	1	C5_CD8_Tem
chr19-57283248-57283503	0.000242049	0.284764049	0.104	0.028	1	C5_CD8_Tem
chr3-46112278-46112563	0.000247666	0.287005473	0.109	0.028	1	C5_CD8_Tem
chr17-57459149-57459775	0.000248182	0.285998965	0.104	0.032	1	C5_CD8_Tem
chr8-101198477-101198836	0.000249504	0.263276749	0.066	0.012	1	C5_CD8_Tem
chr8-66589185-66590031	0.000250133	0.28470095	0.115	0.035	1	C5_CD8_Tem
chr3-98545962-98546296	0.000251511	0.27425759	0.082	0.024	1	C5_CD8_Tem
chr12-6199948-6200774	0.000252319	0.279845332	0.082	0.018	1	C5_CD8_Tem
chr20-32509296-32510058	0.000252423	0.290098688	0.131	0.049	1	C5_CD8_Tem

chr6-144054635-144055814	0.000252871	0.287436302	0.251	0.121	1	C5_CD8_Tem
chr20-46544742-46545579	0.000258337	0.275671412	0.098	0.024	1	C5_CD8_Tem
chr4-4387607-4387952	0.00026455	0.266291925	0.066	0.014	1	C5_CD8_Tem
chr5-178249065-178249945	0.000265349	0.28489679	0.191	0.075	1	C5_CD8_Tem
chr5-150846177-150847377	0.000265779	0.284828449	0.284	0.133	1	C5_CD8_Tem
chr7-156908716-156909284	0.000274178	0.288123404	0.137	0.046	1	C5_CD8_Tem
chrX-78075914-78076317	0.000277393	0.264474824	0.071	0.014	1	C5_CD8_Tem
chr14-55102080-55103535	0.000278555	0.257082382	0.399	0.223	1	C5_CD8_Tem
chr6-422583-423140	0.000278832	0.295451406	0.137	0.045	1	C5_CD8_Tem
chr14-103763640-103764089	0.000283543	0.253328107	0.06	0.013	1	C5_CD8_Tem
chr12-9644973-9645243	0.000283891	0.278621137	0.082	0.022	1	C5_CD8_Tem
chr15-85443156-85444077	0.000284923	0.281431734	0.098	0.033	1	C5_CD8_Tem
chr18-23828321-23828821	0.000287582	0.282057574	0.279	0.131	1	C5_CD8_Tem
chr9-33818659-33819261	0.000289411	0.299155629	0.213	0.087	1	C5_CD8_Tem
chr6-42281468-42281962	0.000289425	0.296190643	0.131	0.044	1	C5_CD8_Tem
chr4-121690622-121691117	0.00029161	0.284228899	0.093	0.025	1	C5_CD8_Tem
chr6-42294683-42295084	0.000293301	0.281096781	0.093	0.023	1	C5_CD8_Tem
chr10-69222060-69223068	0.000293944	0.272623894	0.333	0.178	1	C5_CD8_Tem
chr1-229692276-229692596	0.000294515	0.272749291	0.082	0.021	1	C5_CD8_Tem
chr2-201150932-201151979	0.000300102	0.293165409	0.126	0.038	1	C5_CD8_Tem
chr15-48966117-48966974	0.000304928	0.290829403	0.164	0.062	1	C5_CD8_Tem
chr5-163434342-163434752	0.000307112	0.288541008	0.12	0.036	1	C5_CD8_Tem
chr10-80471535-80472626	0.00030857	0.275754285	0.322	0.164	1	C5_CD8_Tem
chr3-57229057-57229592	0.000309825	0.295998166	0.169	0.063	1	C5_CD8_Tem
chr14-50343790-50344643	0.000316387	0.284346842	0.18	0.07	1	C5_CD8_Tem
chr9-129490142-129490580	0.000317473	0.291071796	0.175	0.073	1	C5_CD8_Tem
chr4-80125134-80126169	0.000319593	0.280979987	0.284	0.145	1	C5_CD8_Tem

chr12-120231020-120231923	0.00032093	0.276693344	0.35	0.179	1	C5_CD8_Tem
chr8-140575437-140575725	0.000321408	0.287697903	0.12	0.04	1	C5_CD8_Tem
chr6-106528021-106528285	0.000322463	0.277367456	0.082	0.018	1	C5_CD8_Tem
chr8-144766922-144767798	0.000322562	0.280127374	0.098	0.028	1	C5_CD8_Tem
chr11-2933126-2933921	0.000324682	0.28883785	0.126	0.042	1	C5_CD8_Tem
chr20-62703388-62703805	0.00032778	0.279792946	0.093	0.028	1	C5_CD8_Tem
chr15-39624654-39625209	0.000328088	0.262794654	0.328	0.182	1	C5_CD8_Tem
chr7-38217383-38218034	0.00032878	0.291524851	0.175	0.065	1	C5_CD8_Tem
chr1-113975402-113975903	0.000334571	0.266225512	0.082	0.022	1	C5_CD8_Tem
chr5-172806889-172807685	0.00033752	0.279050328	0.268	0.127	1	C5_CD8_Tem
chr16-9072726-9073306	0.000338525	0.269525788	0.082	0.019	1	C5_CD8_Tem
chr7-100545443-100547197	0.00034149	0.269383319	0.404	0.204	1	C5_CD8_Tem
chr2-241035419-241037030	0.000343013	0.27036012	0.295	0.137	1	C5_CD8_Tem
chr16-27245607-27246210	0.000344989	0.28765937	0.093	0.021	1	C5_CD8_Tem
chr22-37059903-37060369	0.000353863	0.253753351	0.066	0.016	1	C5_CD8_Tem
chr3-156160373-156161595	0.000355233	0.278782143	0.148	0.058	1	C5_CD8_Tem
chr16-4660196-4660940	0.000355862	0.29120354	0.142	0.046	1	C5_CD8_Tem
chr2-157403890-157404794	0.000362399	0.269378726	0.339	0.181	1	C5_CD8_Tem
chr1-172663613-172664007	0.00036591	0.283528005	0.126	0.043	1	C5_CD8_Tem
chr1-173205171-173206088	0.000367956	0.288479293	0.142	0.055	1	C5_CD8_Tem
chr6-108068389-108069053	0.000368557	0.282467457	0.109	0.038	1	C5_CD8_Tem
chr17-3804270-3804876	0.000368675	0.274822331	0.082	0.019	1	C5_CD8_Tem
chr1-93041963-93043006	0.000369761	0.269524173	0.301	0.162	1	C5_CD8_Tem
chr15-86624254-86625061	0.000379989	0.27614971	0.197	0.085	1	C5_CD8_Tem
chr14-49999129-49999675	0.000382462	0.258399767	0.393	0.214	1	C5_CD8_Tem
chr2-85413710-85414137	0.000383499	0.262538146	0.071	0.016	1	C5_CD8_Tem
chr9-113578585-113579843	0.000383809	0.26315481	0.361	0.192	1	C5_CD8_Tem

chr11-628190-628929	0.00038691	0.285444285	0.158	0.059	1	C5_CD8_Tem
chr17-66548009-66548535	0.000388483	0.271101318	0.098	0.031	1	C5_CD8_Tem
chr9-120909664-120910272	0.000390095	0.285696629	0.186	0.076	1	C5_CD8_Tem
chr5-39231574-39232462	0.000391071	0.291026391	0.153	0.051	1	C5_CD8_Tem
chr16-69166142-69166849	0.000394368	0.289733038	0.104	0.03	1	C5_CD8_Tem
chr9-110015739-110016098	0.000395296	0.251099326	0.055	0.011	1	C5_CD8_Tem
chr5-72138841-72139361	0.000396411	0.274448876	0.104	0.03	1	C5_CD8_Tem
chr17-48816624-48817404	0.000396729	0.281110134	0.268	0.129	1	C5_CD8_Tem
chr1-12603940-12604720	0.000397824	0.284731106	0.29	0.138	1	C5_CD8_Tem
chr10-3900361-3900730	0.000400266	0.25383095	0.06	0.012	1	C5_CD8_Tem
chr6-106339167-106339683	0.000406608	0.28345383	0.12	0.045	1	C5_CD8_Tem
chr2-85840011-85840493	0.000409332	0.260362433	0.077	0.017	1	C5_CD8_Tem
chr6-29015754-29017369	0.000416736	0.28256938	0.208	0.099	1	C5_CD8_Tem
chr11-45180179-45181153	0.000419854	0.279731349	0.104	0.033	1	C5_CD8_Tem
chr10-119494298-119494864	0.000420771	0.268816288	0.077	0.019	1	C5_CD8_Tem
chr6-31734614-31735044	0.00042206	0.283895798	0.257	0.13	1	C5_CD8_Tem
chr2-224973676-224974027	0.000422321	0.258729265	0.071	0.016	1	C5_CD8_Tem
chr14-101878156-101879203	0.00043011	0.288813072	0.191	0.086	1	C5_CD8_Tem
chr21-34986540-34987610	0.00043117	0.274778071	0.317	0.158	1	C5_CD8_Tem
chr2-162073203-162074424	0.000431828	0.29057038	0.164	0.061	1	C5_CD8_Tem
chr6-37202080-37203132	0.000434826	0.296037827	0.142	0.048	1	C5_CD8_Tem
chr17-27473422-27474209	0.000435066	0.267890863	0.366	0.187	1	C5_CD8_Tem
chr8-100218387-100219229	0.000436111	0.284864901	0.164	0.062	1	C5_CD8_Tem
chr6-170089208-170089900	0.000436305	0.267017988	0.087	0.025	1	C5_CD8_Tem
chr20-40961405-40962056	0.00043694	0.266188113	0.077	0.019	1	C5_CD8_Tem
chr14-92516932-92517295	0.000447801	0.26236124	0.087	0.023	1	C5_CD8_Tem
chr2-215982369-215982872	0.000447824	0.267926545	0.093	0.026	1	C5_CD8_Tem

chr5-35877615-35878003	0.000461066	0.264382877	0.077	0.017	1	C5_CD8_Tem
chr3-47030807-47031447	0.000462866	0.281705613	0.12	0.034	1	C5_CD8_Tem
chr7-23272869-23273592	0.000463004	0.288156233	0.208	0.084	1	C5_CD8_Tem
chr22-31692050-31692753	0.000463147	0.275516585	0.093	0.028	1	C5_CD8_Tem
chr1-35078658-35079882	0.000463604	0.275879013	0.311	0.159	1	C5_CD8_Tem
chr12-122108513-122109047	0.000463785	0.26855716	0.082	0.02	1	C5_CD8_Tem
chr8-128240080-128240774	0.000464447	0.285247703	0.169	0.064	1	C5_CD8_Tem
chr2-70084915-70085393	0.000466917	0.281915266	0.224	0.106	1	C5_CD8_Tem
chr6-89609618-89610380	0.000469933	0.259136005	0.071	0.018	1	C5_CD8_Tem
chr20-47316682-47317752	0.000472027	0.276672758	0.104	0.031	1	C5_CD8_Tem
chr8-142614135-142615009	0.000475925	0.284787336	0.23	0.104	1	C5_CD8_Tem
chr13-79385738-79386850	0.00047706	0.285407983	0.273	0.12	1	C5_CD8_Tem
chr1-38977213-38978268	0.000479777	0.282872304	0.175	0.074	1	C5_CD8_Tem
chrX-45710000-45710804	0.00048473	0.266781568	0.087	0.024	1	C5_CD8_Tem
chr18-77065187-77065764	0.000491059	0.280888317	0.251	0.11	1	C5_CD8_Tem
chr1-182629294-182630171	0.000493909	0.275058026	0.219	0.098	1	C5_CD8_Tem
chr12-10559168-10559496	0.000498775	0.265979535	0.093	0.029	1	C5_CD8_Tem
chr11-62553211-62553659	0.000501602	0.283474752	0.219	0.092	1	C5_CD8_Tem
chr17-1707041-1707494	0.000501834	0.267504171	0.077	0.021	1	C5_CD8_Tem
chr3-183551984-183552247	0.0005043	0.264052813	0.082	0.024	1	C5_CD8_Tem
chr17-57454840-57455533	0.000505225	0.262354081	0.087	0.026	1	C5_CD8_Tem
chr11-6650892-6651177	0.0005071	0.259614703	0.071	0.016	1	C5_CD8_Tem
chr2-130370517-130370961	0.000512402	0.273028308	0.115	0.039	1	C5_CD8_Tem
chr16-89840899-89841685	0.000518409	0.272772559	0.23	0.106	1	C5_CD8_Tem
chr10-97406979-97407765	0.000519071	0.285192264	0.273	0.124	1	C5_CD8_Tem
chr11-77151854-77152477	0.000519902	0.268858609	0.109	0.035	1	C5_CD8_Tem
chr2-70083311-70083646	0.000522177	0.290579947	0.169	0.064	1	C5_CD8_Tem

chr1-23100057-23100663	0.000524433	0.289348984	0.148	0.052	1	C5_CD8_Tem
chr2-174023893-174024536	0.000526893	0.262800594	0.082	0.019	1	C5_CD8_Tem
chr6-31776592-31777577	0.000527274	0.283315859	0.224	0.098	1	C5_CD8_Tem
chr2-233413596-233413842	0.000533419	0.255601096	0.082	0.024	1	C5_CD8_Tem
chr9-123224905-123225788	0.000534319	0.279096783	0.191	0.078	1	C5_CD8_Tem
chr3-15794256-15794531	0.000535174	0.274184878	0.126	0.042	1	C5_CD8_Tem
chr6-42022201-42023198	0.000540551	0.279994345	0.224	0.107	1	C5_CD8_Tem
chr1-206489950-206490412	0.000542862	0.265709736	0.082	0.023	1	C5_CD8_Tem
chr22-18958711-18959168	0.000546791	0.253195977	0.071	0.018	1	C5_CD8_Tem
chr22-43003950-43004637	0.000556821	0.252614145	0.071	0.019	1	C5_CD8_Tem
chr3-195608932-195610025	0.000559675	0.278666239	0.115	0.036	1	C5_CD8_Tem
chr9-126519472-126519967	0.000560361	0.282588003	0.137	0.053	1	C5_CD8_Tem
chr10-29657489-29658251	0.000560874	0.282898484	0.208	0.084	1	C5_CD8_Tem
chr12-47394130-47394870	0.000571183	0.27395102	0.18	0.077	1	C5_CD8_Tem
chr20-25237430-25238190	0.000573418	0.283455256	0.158	0.06	1	C5_CD8_Tem
chrX-20454054-20454453	0.000575892	0.265481165	0.082	0.019	1	C5_CD8_Tem
chr10-7354335-7354992	0.000580922	0.266341139	0.077	0.02	1	C5_CD8_Tem
chr15-34018003-34018891	0.000581155	0.268049481	0.093	0.028	1	C5_CD8_Tem
chr2-127415685-127416883	0.000581161	0.257208546	0.404	0.216	1	C5_CD8_Tem
chr1-212260783-212261338	0.000585032	0.270379761	0.115	0.034	1	C5_CD8_Tem
chr11-93737922-93738486	0.000590742	0.285310923	0.219	0.094	1	C5_CD8_Tem
chr10-50260656-50261222	0.000592682	0.260694518	0.098	0.027	1	C5_CD8_Tem
chr1-210373706-210374813	0.000600775	0.279670312	0.175	0.074	1	C5_CD8_Tem
chrX-154712569-154713347	0.000605664	0.268283311	0.268	0.129	1	C5_CD8_Tem
chr12-47807904-47808773	0.000610464	0.274189566	0.208	0.098	1	C5_CD8_Tem
chr3-106338268-106339082	0.00061158	0.267082495	0.12	0.039	1	C5_CD8_Tem
chr6-142850610-142850991	0.000615171	0.282374164	0.169	0.07	1	C5_CD8_Tem

chr9-127915784-127916078	0.000619679	0.261820249	0.077	0.017	1	C5_CD8_Tem
chr4-139266323-139267249	0.000621585	0.270906185	0.251	0.126	1	C5_CD8_Tem
chr22-37432266-37432691	0.00063168	0.262602744	0.087	0.025	1	C5_CD8_Tem
chr14-52318842-52319075	0.000635119	0.258208455	0.082	0.024	1	C5_CD8_Tem
chr22-40054988-40055943	0.000635856	0.257626371	0.355	0.19	1	C5_CD8_Tem
chr2-8608295-8608995	0.00064969	0.265637327	0.104	0.029	1	C5_CD8_Tem
chr1-160689426-160689875	0.000653332	0.257867396	0.077	0.02	1	C5_CD8_Tem
chr13-51821533-51822768	0.000658653	0.283332881	0.158	0.065	1	C5_CD8_Tem
chr2-84897727-84898761	0.000659615	0.282727244	0.224	0.097	1	C5_CD8_Tem
chr3-172132005-172132555	0.000660829	0.272178411	0.12	0.039	1	C5_CD8_Tem
chr1-168082136-168082563	0.000663887	0.254681502	0.071	0.018	1	C5_CD8_Tem
chr5-132100768-132101111	0.000667079	0.28329446	0.131	0.043	1	C5_CD8_Tem
chr10-8402440-8403212	0.000667245	0.274725806	0.175	0.074	1	C5_CD8_Tem
chr1-24963544-24963800	0.000668137	0.280006756	0.153	0.052	1	C5_CD8_Tem
chr7-55570903-55571165	0.000669255	0.286813912	0.142	0.049	1	C5_CD8_Tem
chr10-63554088-63555044	0.000676191	0.257684707	0.082	0.021	1	C5_CD8_Tem
chr19-32690085-32690457	0.00068142	0.277083532	0.109	0.033	1	C5_CD8_Tem
chr9-136532904-136534153	0.000682224	0.270931782	0.284	0.145	1	C5_CD8_Tem
chr6-90103000-90103736	0.000684831	0.286312524	0.175	0.069	1	C5_CD8_Tem
chr15-90665335-90665984	0.000689111	0.267058864	0.087	0.026	1	C5_CD8_Tem
chr11-64787448-64787892	0.000696706	0.251487803	0.066	0.014	1	C5_CD8_Tem
chr10-72292208-72292480	0.000700018	0.261889632	0.082	0.022	1	C5_CD8_Tem
chr2-237427863-237428595	0.000702656	0.27703493	0.158	0.06	1	C5_CD8_Tem
chr8-120731234-120733420	0.000711479	0.273469126	0.257	0.121	1	C5_CD8_Tem
chr20-51470590-51471370	0.000711573	0.260873023	0.087	0.021	1	C5_CD8_Tem
chr20-58906829-58907167	0.00071294	0.252235731	0.066	0.014	1	C5_CD8_Tem
chr3-69093885-69094950	0.000714428	0.284107948	0.164	0.063	1	C5_CD8_Tem

chr6-25026839-25027916	0.000718219	0.256980448	0.301	0.155	1	C5_CD8_Tem
chr17-50916035-50916411	0.000720809	0.273603039	0.12	0.042	1	C5_CD8_Tem
chr9-296832-297928	0.00072294	0.271305209	0.109	0.032	1	C5_CD8_Tem
chr3-52770412-52771385	0.000725021	0.280027013	0.197	0.09	1	C5_CD8_Tem
chr18-34978305-34979493	0.00072688	0.265330525	0.301	0.153	1	C5_CD8_Tem
chr2-231611737-231612866	0.000726887	0.270320094	0.284	0.142	1	C5_CD8_Tem
chr2-196268028-196269905	0.000727672	0.280730432	0.191	0.078	1	C5_CD8_Tem
chr14-97712947-97713426	0.000728318	0.260835774	0.082	0.025	1	C5_CD8_Tem
chr1-156119599-156119848	0.000730101	0.283012846	0.142	0.055	1	C5_CD8_Tem
chr1-84782177-84782778	0.000738035	0.260989943	0.093	0.028	1	C5_CD8_Tem
chr19-4051733-4052109	0.000741766	0.250216634	0.071	0.015	1	C5_CD8_Tem
chr6-166257734-166258201	0.000746623	0.265028201	0.219	0.1	1	C5_CD8_Tem
chr2-222707461-222708488	0.000753115	0.26230962	0.098	0.034	1	C5_CD8_Tem
chr10-5683052-5683534	0.000756437	0.274231251	0.142	0.054	1	C5_CD8_Tem
chr1-24149753-24150805	0.000757879	0.27812182	0.191	0.082	1	C5_CD8_Tem
chr5-40831877-40832183	0.00075874	0.266986754	0.109	0.033	1	C5_CD8_Tem
chr2-172084668-172085596	0.000760839	0.280904061	0.148	0.057	1	C5_CD8_Tem
chr4-1186917-1187545	0.000761733	0.274557791	0.224	0.102	1	C5_CD8_Tem
chr2-113174618-113175554	0.000781906	0.262774751	0.279	0.147	1	C5_CD8_Tem
chr17-67503146-67503529	0.000782347	0.255484314	0.077	0.019	1	C5_CD8_Tem
chr10-24920921-24921693	0.000784172	0.273726862	0.131	0.049	1	C5_CD8_Tem
chr1-64876955-64877577	0.000785996	0.276774343	0.208	0.095	1	C5_CD8_Tem
chrX-129786960-129787957	0.000786211	0.279095751	0.148	0.052	1	C5_CD8_Tem
chr19-51375468-51375720	0.000787289	0.253441479	0.077	0.021	1	C5_CD8_Tem
chr13-42377337-42378132	0.000788301	0.271997235	0.202	0.095	1	C5_CD8_Tem
chr1-200866527-200866888	0.000792271	0.265025671	0.098	0.029	1	C5_CD8_Tem
chr15-41831350-41831567	0.000793934	0.262331645	0.082	0.022	1	C5_CD8_Tem

chr2-224943418-224943979	0.000799253	0.280252336	0.169	0.063	1	C5_CD8_Tem
chr10-102452786-102453221	0.000803395	0.272643403	0.131	0.048	1	C5_CD8_Tem
chr12-6345309-6346049	0.000811877	0.288505833	0.158	0.061	1	C5_CD8_Tem
chr15-55237336-55238121	0.000813739	0.264897371	0.087	0.022	1	C5_CD8_Tem
chr2-203802348-203803146	0.000817777	0.27360505	0.131	0.045	1	C5_CD8_Tem
chr4-169621656-169622489	0.000818383	0.267388296	0.284	0.145	1	C5_CD8_Tem
chr12-91687106-91687705	0.000820014	0.258789691	0.093	0.03	1	C5_CD8_Tem
chrX-136750323-136750722	0.000821323	0.265076158	0.087	0.027	1	C5_CD8_Tem
chr2-160665844-160666780	0.000826439	0.264106836	0.109	0.04	1	C5_CD8_Tem
chrX-129677626-129678878	0.000826727	0.274429243	0.175	0.069	1	C5_CD8_Tem
chr2-143263101-143263523	0.000827424	0.265852853	0.109	0.032	1	C5_CD8_Tem
chr5-50699466-50700075	0.000828283	0.272485047	0.148	0.055	1	C5_CD8_Tem
chr22-38696516-38696880	0.000830912	0.283607301	0.191	0.077	1	C5_CD8_Tem
chr17-17819712-17819913	0.000843937	0.252889532	0.071	0.017	1	C5_CD8_Tem
chr1-9413008-9413735	0.000845372	0.271106162	0.109	0.038	1	C5_CD8_Tem
chr5-136019960-136020824	0.000853304	0.253767897	0.093	0.029	1	C5_CD8_Tem
chr22-37238031-37238921	0.00085385	0.257322967	0.322	0.173	1	C5_CD8_Tem
chr15-52013048-52013672	0.000859909	0.252426367	0.077	0.017	1	C5_CD8_Tem
chr3-43345675-43346295	0.000860021	0.26002372	0.071	0.019	1	C5_CD8_Tem
chr5-170338258-170339186	0.000873004	0.274305539	0.164	0.066	1	C5_CD8_Tem
chr6-142832768-142833393	0.000876629	0.27632428	0.169	0.069	1	C5_CD8_Tem
chr5-111232438-111233250	0.000888767	0.269926702	0.202	0.085	1	C5_CD8_Tem
chr2-112691995-112692852	0.000895132	0.269118665	0.197	0.085	1	C5_CD8_Tem
chr3-45105377-45106150	0.000896194	0.257093312	0.098	0.033	1	C5_CD8_Tem
chr3-18757597-18758425	0.000898191	0.271333111	0.18	0.084	1	C5_CD8_Tem
chr5-16467104-16467660	0.000917835	0.257677197	0.093	0.028	1	C5_CD8_Tem
chr2-181465701-181466268	0.000929891	0.281489331	0.175	0.071	1	C5_CD8_Tem

chr5-52786851-52788508	0.000942952	0.269227441	0.29	0.136	1	C5_CD8_Tem
chr16-3445093-3445351	0.000943676	0.258744167	0.077	0.018	1	C5_CD8_Tem
chr14-89959911-89960313	0.000950114	0.261337303	0.093	0.03	1	C5_CD8_Tem
chr18-3380313-3380993	0.000951172	0.272260421	0.158	0.068	1	C5_CD8_Tem
chr7-1417277-1417829	0.00095132	0.270025437	0.246	0.115	1	C5_CD8_Tem
chr10-72295981-72296382	0.000952915	0.272324322	0.23	0.109	1	C5_CD8_Tem
chr5-142432040-142433340	0.000955319	0.252337836	0.29	0.156	1	C5_CD8_Tem
chr19-16118440-16120399	0.000963177	0.269593616	0.251	0.106	1	C5_CD8_Tem
chr5-91281465-91281787	0.000966091	0.253871908	0.082	0.024	1	C5_CD8_Tem
chr12-9673280-9674565	0.000974736	0.257164214	0.301	0.163	1	C5_CD8_Tem
chr3-52934315-52934990	0.001009758	0.258852663	0.087	0.026	1	C5_CD8_Tem
chr9-127608067-127608694	0.001012408	0.274160464	0.153	0.062	1	C5_CD8_Tem
chr2-105798323-105798964	0.001013842	0.251805157	0.322	0.168	1	C5_CD8_Tem
chr17-1114718-1115613	0.001016044	0.274858689	0.18	0.075	1	C5_CD8_Tem
chr6-108781749-108782677	0.001023662	0.27354954	0.186	0.084	1	C5_CD8_Tem
chr9-113511729-113512750	0.001033787	0.262863077	0.262	0.134	1	C5_CD8_Tem
chr1-52919278-52919777	0.001048386	0.252340908	0.098	0.028	1	C5_CD8_Tem
chr13-45511411-45512415	0.00105468	0.274529507	0.12	0.04	1	C5_CD8_Tem
chr15-42056918-42057805	0.001057658	0.264084378	0.153	0.059	1	C5_CD8_Tem
chr8-46917486-46918178	0.001073704	0.250267276	0.126	0.044	1	C5_CD8_Tem
chr1-206736958-206737941	0.001077321	0.257268471	0.279	0.135	1	C5_CD8_Tem
chr12-47809002-47809208	0.0010808	0.267767422	0.12	0.041	1	C5_CD8_Tem
chr7-105267968-105269375	0.001088728	0.267702298	0.23	0.11	1	C5_CD8_Tem
chr17-77645029-77646016	0.001097721	0.275043655	0.219	0.099	1	C5_CD8_Tem
chr16-89357028-89357309	0.001106299	0.250506642	0.082	0.023	1	C5_CD8_Tem
chr12-75640883-75641454	0.001106491	0.262861409	0.131	0.05	1	C5_CD8_Tem
chr2-241788188-241788954	0.001113615	0.264009838	0.109	0.038	1	C5_CD8_Tem

chrX-72273746-72274052	0.001122665	0.26292979	0.104	0.036	1	C5_CD8_Tem
chr5-79990129-79991430	0.001126009	0.251178991	0.344	0.175	1	C5_CD8_Tem
chr6-130517322-130517835	0.001138626	0.267460216	0.175	0.073	1	C5_CD8_Tem
chr3-4980287-4980515	0.001138976	0.266823077	0.279	0.132	1	C5_CD8_Tem
chr17-7579033-7579748	0.001146071	0.272100753	0.202	0.094	1	C5_CD8_Tem
chr16-38646-39563	0.001151432	0.263828787	0.137	0.051	1	C5_CD8_Tem
chr3-151268369-151269265	0.001152803	0.251622397	0.087	0.027	1	C5_CD8_Tem
chrX-120457647-120458143	0.001160699	0.253843074	0.104	0.034	1	C5_CD8_Tem
chr5-170273865-170274623	0.001171582	0.261816579	0.268	0.131	1	C5_CD8_Tem
chr2-237731105-237731958	0.001175449	0.272298375	0.169	0.071	1	C5_CD8_Tem
chr6-106125759-106126810	0.001177516	0.272513647	0.131	0.053	1	C5_CD8_Tem
chr14-105537601-105538656	0.001177866	0.275193282	0.208	0.09	1	C5_CD8_Tem
chr11-63871881-63872355	0.001183528	0.266689085	0.115	0.037	1	C5_CD8_Tem
chr16-1645950-1646578	0.001185414	0.258494385	0.098	0.032	1	C5_CD8_Tem
chr14-61419862-61420659	0.001187608	0.271835701	0.158	0.064	1	C5_CD8_Tem
chrX-79182553-79183018	0.001202957	0.264310996	0.12	0.041	1	C5_CD8_Tem
chr2-39128011-39128379	0.001205296	0.258690376	0.175	0.076	1	C5_CD8_Tem
chr11-3954473-3954810	0.001211271	0.254902373	0.077	0.02	1	C5_CD8_Tem
chr1-206568182-206569104	0.001229054	0.258998487	0.109	0.036	1	C5_CD8_Tem
chr7-139133244-139134026	0.001229623	0.268070595	0.23	0.115	1	C5_CD8_Tem
chr8-81061025-81061633	0.001239405	0.253994275	0.098	0.038	1	C5_CD8_Tem
chr2-7743584-7743968	0.001261533	0.255403578	0.087	0.026	1	C5_CD8_Tem
chr1-221707989-221708533	0.001273118	0.264574693	0.23	0.103	1	C5_CD8_Tem
chr17-77420572-77421454	0.001274146	0.250631407	0.093	0.029	1	C5_CD8_Tem
chr2-143175338-143175833	0.001277452	0.258681521	0.104	0.033	1	C5_CD8_Tem
chr8-30141130-30142087	0.001278266	0.251714905	0.311	0.165	1	C5_CD8_Tem
chr16-5092123-5092554	0.001278468	0.25888166	0.104	0.034	1	C5_CD8_Tem

chr15-55221062-55222117	0.001283074	0.260538065	0.175	0.084	1	C5_CD8_Tem
chr19-47243916-47244544	0.001286673	0.250088152	0.339	0.183	1	C5_CD8_Tem
chr10-91944533-91945369	0.001290191	0.257968233	0.126	0.05	1	C5_CD8_Tem
chr19-55255685-55256276	0.001297201	0.273220627	0.24	0.117	1	C5_CD8_Tem
chr21-34983085-34984085	0.001299585	0.25314005	0.317	0.166	1	C5_CD8_Tem
chr15-31387099-31387938	0.001301887	0.260830173	0.246	0.119	1	C5_CD8_Tem
chr10-133159871-133160517	0.001303209	0.259237794	0.109	0.038	1	C5_CD8_Tem
chr8-128554733-128555434	0.001310939	0.268726007	0.131	0.05	1	C5_CD8_Tem
chr15-38695343-38696754	0.001317139	0.253715191	0.295	0.16	1	C5_CD8_Tem
chr1-184035858-184037957	0.001319662	0.25963148	0.197	0.091	1	C5_CD8_Tem
chr21-42614338-42615516	0.001325577	0.260302817	0.137	0.055	1	C5_CD8_Tem
chr15-77005256-77005671	0.001326614	0.265131772	0.104	0.033	1	C5_CD8_Tem
chr2-136242523-136243061	0.001327924	0.265788225	0.164	0.07	1	C5_CD8_Tem
chr3-37212890-37213342	0.001346918	0.264809059	0.12	0.041	1	C5_CD8_Tem
chr2-191148445-191149213	0.001347395	0.268046807	0.219	0.103	1	C5_CD8_Tem
chr12-51820315-51821948	0.001352973	0.252969537	0.306	0.166	1	C5_CD8_Tem
chr11-83044715-83045373	0.001357055	0.263222915	0.175	0.073	1	C5_CD8_Tem
chr17-35460247-35460925	0.001367662	0.259573206	0.142	0.06	1	C5_CD8_Tem
chr2-112706040-112706537	0.001371519	0.255790919	0.098	0.034	1	C5_CD8_Tem
chr9-126398515-126398758	0.001378577	0.251960968	0.087	0.025	1	C5_CD8_Tem
chr5-143386724-143387037	0.001380928	0.250708613	0.087	0.024	1	C5_CD8_Tem
chr14-90969687-90970462	0.001381254	0.259054212	0.12	0.041	1	C5_CD8_Tem
chr12-6764100-6764667	0.001403414	0.257463712	0.208	0.101	1	C5_CD8_Tem
chr5-180352482-180353953	0.00140451	0.257195263	0.29	0.146	1	C5_CD8_Tem
chr17-29219187-29219776	0.001405037	0.259218506	0.082	0.025	1	C5_CD8_Tem
chr6-30813935-30814547	0.001413553	0.253982611	0.087	0.027	1	C5_CD8_Tem
chr3-56724793-56725668	0.001426144	0.261488143	0.153	0.067	1	C5_CD8_Tem

chr7-513475-514044	0.001426614	0.254217799	0.077	0.02	1	C5_CD8_Tem
chr15-65612487-65612843	0.001427522	0.261028207	0.098	0.032	1	C5_CD8_Tem
chr19-16594044-16595422	0.00143132	0.263638338	0.23	0.111	1	C5_CD8_Tem
chr6-137827871-137828576	0.001452128	0.261808124	0.12	0.05	1	C5_CD8_Tem
chr2-172437422-172438372	0.00145683	0.259426024	0.213	0.109	1	C5_CD8_Tem
chr6-42774374-42775459	0.001475398	0.261735629	0.257	0.131	1	C5_CD8_Tem
chr10-58266284-58266702	0.001485967	0.258446542	0.268	0.132	1	C5_CD8_Tem
chr9-91253399-91253821	0.001488325	0.260566448	0.104	0.034	1	C5_CD8_Tem
chr8-29289137-29289880	0.001492588	0.265814655	0.12	0.041	1	C5_CD8_Tem
chr20-32406955-32407744	0.001494436	0.260897153	0.142	0.057	1	C5_CD8_Tem
chr11-122852520-122852784	0.001498914	0.250503087	0.093	0.032	1	C5_CD8_Tem
chr1-16728179-16728666	0.00151156	0.257372944	0.093	0.027	1	C5_CD8_Tem
chr15-92907860-92908224	0.001513872	0.261661257	0.262	0.119	1	C5_CD8_Tem
chr1-86714200-86714969	0.001517642	0.26468049	0.164	0.062	1	C5_CD8_Tem
chr9-96217808-96218524	0.001536335	0.255106046	0.098	0.036	1	C5_CD8_Tem
chr5-1593845-1594800	0.001539161	0.268591167	0.18	0.075	1	C5_CD8_Tem
chr9-109117826-109118371	0.001541252	0.270459686	0.175	0.072	1	C5_CD8_Tem
chr11-286876-287442	0.001548745	0.264112078	0.158	0.065	1	C5_CD8_Tem
chr14-99252967-99253796	0.001563319	0.253142616	0.268	0.133	1	C5_CD8_Tem
chr19-1089915-1090278	0.001568929	0.255097887	0.115	0.042	1	C5_CD8_Tem
chr2-28366289-28367182	0.001577979	0.258148508	0.158	0.062	1	C5_CD8_Tem
chr10-133258920-133259596	0.001591152	0.252105583	0.339	0.179	1	C5_CD8_Tem
chr5-157216753-157217563	0.001601714	0.270622133	0.164	0.069	1	C5_CD8_Tem
chr2-54570975-54571340	0.001631318	0.258800593	0.164	0.072	1	C5_CD8_Tem
chr12-28221867-28222442	0.001635574	0.253062714	0.098	0.032	1	C5_CD8_Tem
chr11-18763777-18764591	0.001645103	0.259494593	0.148	0.058	1	C5_CD8_Tem
chr1-64897939-64898683	0.001679351	0.264562679	0.164	0.063	1	C5_CD8_Tem

chr14-35390796-35391419	0.0017164	0.254074809	0.126	0.046	1	C5_CD8_Tem
chr12-124774146-124775146	0.001717496	0.255899418	0.197	0.09	1	C5_CD8_Tem
chr9-36148406-36148940	0.001730586	0.259059398	0.12	0.051	1	C5_CD8_Tem
chr1-162822416-162823809	0.001736664	0.253060262	0.126	0.052	1	C5_CD8_Tem
chr16-9127432-9128196	0.001741633	0.256046906	0.142	0.058	1	C5_CD8_Tem
chr17-50482514-50482793	0.001760516	0.258750926	0.098	0.03	1	C5_CD8_Tem
chr3-15275103-15275652	0.001767356	0.253528892	0.131	0.05	1	C5_CD8_Tem
chr6-30736618-30737898	0.001772195	0.258444153	0.197	0.085	1	C5_CD8_Tem
chr5-50634520-50635429	0.001817242	0.255772052	0.137	0.052	1	C5_CD8_Tem
chr6-106141232-106142302	0.001818818	0.257742901	0.191	0.082	1	C5_CD8_Tem
chr12-117133690-117134542	0.001853049	0.252798436	0.126	0.048	1	C5_CD8_Tem
chr15-64596743-64597607	0.001853079	0.258420813	0.098	0.033	1	C5_CD8_Tem
chr7-127856358-127857218	0.001856471	0.259745081	0.153	0.059	1	C5_CD8_Tem
chr13-49499965-49500458	0.001874728	0.257773645	0.115	0.038	1	C5_CD8_Tem
chr11-1757573-1758639	0.00188877	0.259567035	0.169	0.073	1	C5_CD8_Tem
chr4-150885065-150885846	0.001889504	0.257239184	0.153	0.061	1	C5_CD8_Tem
chr12-49342301-49343661	0.001899647	0.250315211	0.246	0.135	1	C5_CD8_Tem
chr3-183447143-183448018	0.001922861	0.259632532	0.153	0.059	1	C5_CD8_Tem
chr14-70942424-70943030	0.001926468	0.261748814	0.131	0.048	1	C5_CD8_Tem
chr18-63199360-63200321	0.001943622	0.250569591	0.224	0.115	1	C5_CD8_Tem
chr14-51867363-51868142	0.001947454	0.251726302	0.251	0.126	1	C5_CD8_Tem
chrX-150478594-150479457	0.001983157	0.253172425	0.104	0.038	1	C5_CD8_Tem
chr1-92365339-92366361	0.001999743	0.254165182	0.169	0.08	1	C5_CD8_Tem
chr6-31680392-31681663	0.002009143	0.262026688	0.153	0.062	1	C5_CD8_Tem
chr1-203325069-203325350	0.002070915	0.263599862	0.164	0.064	1	C5_CD8_Tem
chr21-36141149-36141916	0.00212028	0.253124895	0.213	0.099	1	C5_CD8_Tem
chr11-64186698-64187370	0.002134604	0.265168985	0.224	0.109	1	C5_CD8_Tem

chr3-50610261-50610470	0.002142154	0.250422415	0.104	0.037	1	C5_CD8_Tem
chr2-241842369-241843301	0.0021763	0.256614791	0.202	0.095	1	C5_CD8_Tem
chr16-27407846-27408326	0.002182232	0.25976826	0.164	0.066	1	C5_CD8_Tem
chr5-177472493-177473807	0.002204914	0.263685349	0.164	0.067	1	C5_CD8_Tem
chr9-121156145-121156774	0.002210585	0.258610572	0.197	0.089	1	C5_CD8_Tem
chr17-39209340-39210210	0.002240744	0.255149168	0.219	0.1	1	C5_CD8_Tem
chr1-161730056-161730515	0.002277016	0.261294963	0.153	0.058	1	C5_CD8_Tem
chr3-9808241-9810409	0.002284926	0.252586607	0.268	0.138	1	C5_CD8_Tem
chr7-101126248-101127193	0.002293821	0.250425633	0.115	0.04	1	C5_CD8_Tem
chr5-140533539-140534392	0.00231545	0.257916503	0.12	0.041	1	C5_CD8_Tem
chr5-109411652-109411932	0.002358256	0.25651736	0.158	0.065	1	C5_CD8_Tem
chr5-14675439-14676666	0.002421364	0.258413444	0.142	0.053	1	C5_CD8_Tem
chr14-91317363-91318247	0.002463203	0.25367329	0.235	0.118	1	C5_CD8_Tem
chr19-48836776-48837444	0.002476318	0.259554438	0.153	0.059	1	C5_CD8_Tem
chr1-211416240-211417412	0.002488284	0.253671954	0.197	0.089	1	C5_CD8_Tem
chr12-122081332-122081980	0.002580858	0.253481664	0.131	0.053	1	C5_CD8_Tem
chr2-43293521-43294473	0.002583432	0.254981527	0.153	0.069	1	C5_CD8_Tem
chr6-34244522-34244801	0.002629292	0.258589688	0.137	0.048	1	C5_CD8_Tem
chr4-101038995-101039746	0.002660134	0.250500586	0.257	0.119	1	C5_CD8_Tem
chr10-3227519-3228422	0.002681986	0.250999871	0.197	0.088	1	C5_CD8_Tem
chr2-68831823-68832854	0.002694829	0.261202958	0.153	0.058	1	C5_CD8_Tem
chr11-1870346-1871400	0.002696203	0.251788407	0.131	0.053	1	C5_CD8_Tem
chr9-135939051-135939861	0.002698283	0.252333247	0.137	0.046	1	C5_CD8_Tem
chr6-35606079-35606487	0.002698329	0.256894867	0.18	0.086	1	C5_CD8_Tem
chr17-75749706-75750232	0.002709832	0.259408043	0.142	0.054	1	C5_CD8_Tem
chr3-196183286-196183793	0.002771654	0.260487321	0.137	0.054	1	C5_CD8_Tem
chr1-9715484-9715789	0.002920161	0.253830844	0.148	0.061	1	C5_CD8_Tem

chr4-159175251-159175845	0.003062006	0.251710048	0.126	0.049	1	C5_CD8_Tem
chr4-80173030-80173733	0.003112711	0.251817687	0.191	0.09	1	C5_CD8_Tem
chr8-143955319-143955620	0.003165318	0.259757956	0.142	0.052	1	C5_CD8_Tem
chr7-150738216-150738966	0.003349292	0.257301783	0.202	0.09	1	C5_CD8_Tem
chr7-150753922-150754626	0.00339914	0.251477975	0.153	0.064	1	C5_CD8_Tem
chr2-197299946-197300457	0.003413181	0.250360326	0.142	0.058	1	C5_CD8_Tem
chr10-22652872-22653409	0.003496126	0.256029192	0.153	0.067	1	C5_CD8_Tem
chr19-29623462-29624943	0.003750178	0.25299458	0.169	0.075	1	C5_CD8_Tem
chr16-67052469-67054173	0.003813765	0.254714697	0.219	0.104	1	C5_CD8_Tem
chr19-4379281-4380728	0.003843614	0.254221997	0.224	0.107	1	C5_CD8_Tem
chr11-114166610-114167398	0.003846281	0.250180125	0.18	0.084	1	C5_CD8_Tem
chr19-41521323-41522354	0.005003122	0.253124217	0.126	0.05	1	C5_CD8_Tem
chr20-58312816-58313696	3.17E-14	0.572662499	0.268	0.058	2.10E-09	C5_CD8_Tem
chr12-10453107-10453564	2.10E-11	0.466051169	0.093	0.007	1.39E-06	C5_CD8_Tem
chr1-207335669-207337433	2.09E-15	0.535382018	0.421	0.136	1.38E-10	C5_CD8_Tem
chr10-50260656-50261222	0.000592682	0.260694518	0.098	0.027	1	C5_CD8_Tem
chr2-43293521-43294473	0.002583432	0.254981527	0.153	0.069	1	C5_CD8_Tem
chr1-23100057-23100663	0.000524433	0.289348984	0.148	0.052	1	C5_CD8_Tem
chr15-74402410-74403487	0.00017454	0.306083641	0.18	0.065	1	C5_CD8_Tem
chr14-52324842-52325579	3.86E-09	0.444298808	0.262	0.081	0.000255365	C5_CD8_Tem
chr21-34807265-34808813	1.03E-07	0.332561036	0.514	0.258	0.006853628	C5_CD8_Tem
chr10-14598998-14599469	0.0002398	0.276696178	0.098	0.028	1	C5_CD8_Tem
chr21-34986540-34987610	0.00043117	0.274778071	0.317	0.158	1	C5_CD8_Tem
chr8-142841650-142842354	3.47E-17	0.616484141	0.317	0.073	2.30E-12	C5_CD8_Tem
chr7-70634427-70635183	3.51E-10	0.479374866	0.175	0.035	2.32E-05	C5_CD8_Tem
chr11-114166610-114167398	0.003846281	0.250180125	0.18	0.084	1	C5_CD8_Tem
chr14-65637567-65638190	1.22E-05	0.343586558	0.169	0.054	0.80615858	C5_CD8_Tem

chr11-44861726-44862897	2.97E-06	0.333480141	0.344	0.16	0.196761051	C5_CD8_Tem
chr17-67257330-67259176	7.74E-05	0.321283082	0.202	0.076	1	C5_CD8_Tem
chr12-56581086-56582251	2.39E-18	0.623709026	0.388	0.093	1.58E-13	C5_CD8_Tem
chr12-10301111-10301472	2.70E-05	0.297945508	0.071	0.012	1	C5_CD8_Tem
chr10-6269791-6270840	3.45E-05	0.287195615	0.404	0.212	1	C5_CD8_Tem
chr13-111166524-111167378	2.86E-09	0.453246478	0.169	0.036	0.000189262	C5_CD8_Tem
chr6-33204302-33205948	2.93E-05	0.283083863	0.443	0.245	1	C5_CD8_Tem
chr16-87897428-87897985	6.29E-06	0.355450912	0.213	0.082	0.416250807	C5_CD8_Tem
chr16-9072726-9073306	0.000338525	0.269525788	0.082	0.019	1	C5_CD8_Tem
chr16-81520797-81521704	1.57E-09	0.402575521	0.426	0.193	0.000103709	C5_CD8_Tem
chr22-36328686-36329793	2.31E-09	0.394628962	0.47	0.206	0.000153108	C5_CD8_Tem
chr11-72140775-72141464	4.49E-10	0.470519349	0.148	0.026	2.98E-05	C5_CD8_Tem
chr17-39209340-39210210	0.002240744	0.255149168	0.219	0.1	1	C5_CD8_Tem
chr16-3020156-3022540	9.94E-07	0.257642146	0.689	0.446	0.065853943	C5_CD8_Tem
chr1-168557579-168558101	8.69E-05	0.30640131	0.12	0.033	1	C5_CD8_Tem
chr16-67052469-67054173	0.003813765	0.254714697	0.219	0.104	1	C5_CD8_Tem
chr2-25301275-25302024	1.57E-05	0.325241929	0.306	0.141	1	C5_CD8_Tem
chr20-46544742-46545579	0.000258337	0.275671412	0.098	0.024	1	C5_CD8_Tem
chr13-30766918-30767586	3.41E-05	0.318204125	0.109	0.03	1	C5_CD8_Tem
chr3-98538418-98538933	2.68E-05	0.331127003	0.164	0.058	1	C5_CD8_Tem
chr8-22327004-22327768	3.18E-11	0.468141705	0.104	0.01	2.11E-06	C5_CD8_Tem
chr5-142432040-142433340	0.000955319	0.252337836	0.29	0.156	1	C5_CD8_Tem
chr11-121481647-121482948	6.91E-06	0.282592405	0.47	0.257	0.457431164	C5_CD8_Tem
chr1-150569880-150570355	7.58E-09	0.444075095	0.224	0.068	0.000502066	C5_CD8_Tem
chr3-183513151-183513641	1.05E-07	0.415976015	0.169	0.041	0.006984849	C5_CD8_Tem
chr19-38838538-38838914	1.87E-08	0.435742036	0.191	0.046	0.001235472	C5_CD8_Tem
chr2-7744369-7745278	8.53E-13	0.484696547	0.454	0.156	5.65E-08	C5_CD8_Tem

chr9-96008395-96010381	1.09E-05	0.329328072	0.311	0.134	0.720627143	C5_CD8_Tem
chr16-89408918-89409734	0.000131267	0.253515103	0.464	0.26	1	C5_CD8_Tem
chr6-166258549-166259778	3.24E-05	0.321372442	0.257	0.112	1	C5_CD8_Tem
chr19-39073226-39074029	0.000214218	0.26024531	0.377	0.216	1	C5_CD8_Tem
chr3-48478213-48479727	2.00E-05	0.286499463	0.437	0.238	1	C5_CD8_Tem
chr19-2503181-2504126	3.77E-06	0.32399051	0.393	0.186	0.249428765	C5_CD8_Tem
chr9-113511729-113512750	0.001033787	0.262863077	0.262	0.134	1	C5_CD8_Tem
chrX-41695340-41695863	6.96E-10	0.469420123	0.169	0.031	4.61E-05	C5_CD8_Tem
chr6-42278040-42278490	4.41E-07	0.391948329	0.153	0.036	0.029179671	C5_CD8_Tem
chr1-1063692-1064603	0.000174671	0.25142375	0.464	0.282	1	C5_CD8_Tem
chr14-92516932-92517295	0.000447801	0.26236124	0.087	0.023	1	C5_CD8_Tem
chr2-136242523-136243061	0.001327924	0.265788225	0.164	0.07	1	C5_CD8_Tem
chr11-118295276-118296138	2.45E-05	0.343809827	0.175	0.057	1	C5_CD8_Tem
chr1-168360097-168360457	1.69E-06	0.377822196	0.169	0.05	0.111718441	C5_CD8_Tem
chr14-101878156-101879203	0.00043011	0.288813072	0.191	0.086	1	C5_CD8_Tem
chr2-195533802-195534618	4.91E-07	0.37157986	0.339	0.137	0.032515979	C5_CD8_Tem
chr22-43381675-43382132	3.57E-05	0.327977375	0.131	0.04	1	C5_CD8_Tem
chr12-6332545-6332809	9.52E-10	0.469801522	0.169	0.034	6.31E-05	C5_CD8_Tem
chr6-154296-154947	7.89E-07	0.378879071	0.131	0.028	0.052257714	C5_CD8_Tem
chr2-85840011-85840493	0.000409332	0.260362433	0.077	0.017	1	C5_CD8_Tem
chr12-111438406-111438781	7.16E-07	0.388137802	0.131	0.03	0.047433954	C5_CD8_Tem
chr11-587475-588457	1.56E-14	0.445690635	0.612	0.269	1.03E-09	C5_CD8_Tem
chr3-17774268-17775421	8.12E-16	0.596755375	0.262	0.06	5.37E-11	C5_CD8_Tem
chr3-151268369-151269265	0.001152803	0.251622397	0.087	0.027	1	C5_CD8_Tem
chr1-148426851-148427257	0.000219014	0.280379302	0.082	0.018	1	C5_CD8_Tem
chr5-177471356-177472019	4.48E-09	0.444223438	0.142	0.026	0.000296516	C5_CD8_Tem
chr9-123224905-123225788	0.000534319	0.279096783	0.191	0.078	1	C5_CD8_Tem

chr12-120231020-120231923	0.00032093	0.276693344	0.35	0.179	1	C5_CD8_Tem
chr1-92365339-92366361	0.001999743	0.254165182	0.169	0.08	1	C5_CD8_Tem
chr11-123456276-123457499	3.44E-06	0.361420411	0.169	0.051	0.227639402	C5_CD8_Tem
chr9-37408810-37409703	0.000231938	0.27727498	0.339	0.171	1	C5_CD8_Tem
chr1-206736958-206737941	0.001077321	0.257268471	0.279	0.135	1	C5_CD8_Tem
chr12-10503277-10503827	1.94E-09	0.452631317	0.142	0.023	0.000128403	C5_CD8_Tem
chr1-58754179-58755866	8.60E-06	0.326228718	0.377	0.162	0.569550706	C5_CD8_Tem
chr6-106163048-106164775	3.09E-06	0.323717447	0.404	0.185	0.204454799	C5_CD8_Tem
chr16-89422342-89423633	1.11E-07	0.388111571	0.355	0.144	0.007381028	C5_CD8_Tem
chr19-18477201-18477605	5.39E-08	0.407294645	0.109	0.018	0.003568513	C5_CD8_Tem
chr16-4660196-4660940	0.000355862	0.29120354	0.142	0.046	1	C5_CD8_Tem
chr15-55221062-55222117	0.001283074	0.260538065	0.175	0.084	1	C5_CD8_Tem
chr17-8963632-8963924	6.10E-05	0.313563199	0.12	0.037	1	C5_CD8_Tem
chr9-121156145-121156774	0.002210585	0.258610572	0.197	0.089	1	C5_CD8_Tem
chr11-1850299-1851392	5.85E-09	0.345244545	0.525	0.281	0.0003876	C5_CD8_Tem
chr6-4941776-4942921	8.21E-05	0.300815523	0.268	0.122	1	C5_CD8_Tem
chr2-102233971-102234562	1.19E-05	0.349283802	0.126	0.029	0.786521362	C5_CD8_Tem
chr2-70083311-70083646	0.000522177	0.290579947	0.169	0.064	1	C5_CD8_Tem
chr1-116514824-116516096	5.75E-07	0.333566817	0.454	0.227	0.038049462	C5_CD8_Tem
chr20-32509296-32510058	0.000252423	0.290098688	0.131	0.049	1	C5_CD8_Tem
chr19-41297646-41298585	6.67E-05	0.32068963	0.191	0.08	1	C5_CD8_Tem
chr1-100414408-100415159	2.86E-06	0.363843383	0.186	0.064	0.18938523	C5_CD8_Tem
chr3-45943007-45943699	3.19E-05	0.319842838	0.295	0.137	1	C5_CD8_Tem
chr3-11288675-11289452	6.96E-05	0.31979369	0.158	0.055	1	C5_CD8_Tem
chr13-29923888-29924434	1.62E-06	0.376268414	0.24	0.077	0.107064097	C5_CD8_Tem
chr2-216348988-216349622	6.58E-05	0.319322671	0.18	0.066	1	C5_CD8_Tem
chr6-117677515-117677862	0.0001128	0.307881753	0.12	0.039	1	C5_CD8_Tem

chr16-66474556-66475090	0.000212251	0.293197546	0.158	0.061	1	C5_CD8_Tem
chr6-36514876-36515410	2.63E-06	0.301135584	0.055	0.005	0.174034985	C5_CD8_Tem
chr19-610106-611951	2.48E-06	0.310901832	0.481	0.238	0.164435468	C5_CD8_Tem
chr12-6335421-6336883	6.77E-06	0.275757424	0.53	0.31	0.448368599	C5_CD8_Tem
chr20-53685860-53686390	9.43E-05	0.296572163	0.104	0.028	1	C5_CD8_Tem
chr11-57457205-57459446	3.63E-05	0.323913614	0.268	0.115	1	C5_CD8_Tem
chr4-47531211-47532101	4.96E-10	0.458258985	0.257	0.08	3.28E-05	C5_CD8_Tem
chr12-9340452-9341540	1.55E-05	0.317408845	0.093	0.024	1	C5_CD8_Tem
chr19-4379281-4380728	0.003843614	0.254221997	0.224	0.107	1	C5_CD8_Tem
chr9-133406665-133407377	5.62E-19	0.596844108	0.459	0.141	3.72E-14	C5_CD8_Tem
chr2-62332611-62333314	5.05E-05	0.314106972	0.126	0.037	1	C5_CD8_Tem
chr8-133526111-133527173	2.76E-06	0.343111342	0.339	0.149	0.182610926	C5_CD8_Tem
chr20-50391868-50392497	3.18E-06	0.354843418	0.12	0.033	0.210716685	C5_CD8_Tem
chr12-6635240-6636420	2.45E-06	0.361361106	0.202	0.072	0.16257032	C5_CD8_Tem
chr1-162822416-162823809	0.001736664	0.253060262	0.126	0.052	1	C5_CD8_Tem
chr11-34161491-34162212	1.69E-13	0.53524831	0.284	0.078	1.12E-08	C5_CD8_Tem
chr22-30648942-30649975	7.64E-05	0.29892452	0.311	0.147	1	C5_CD8_Tem
chr7-36683919-36684260	8.79E-05	0.299982053	0.093	0.02	1	C5_CD8_Tem
chr2-54040733-54041636	1.95E-15	0.580655485	0.344	0.088	1.29E-10	C5_CD8_Tem
chr1-23110298-23111268	6.31E-09	0.380258045	0.475	0.213	0.000418004	C5_CD8_Tem
chr19-32690085-32690457	0.00068142	0.277083532	0.109	0.033	1	C5_CD8_Tem
chr19-38841624-38842824	1.24E-09	0.422014285	0.404	0.163	8.21E-05	C5_CD8_Tem
chr20-63635056-63636218	7.43E-06	0.276251609	0.546	0.309	0.491850786	C5_CD8_Tem
chr12-75978694-75979577	6.96E-05	0.294892487	0.333	0.155	1	C5_CD8_Tem
chr20-49595624-49596522	2.30E-16	0.607866279	0.268	0.059	1.52E-11	C5_CD8_Tem
chr9-33748543-33749512	4.28E-08	0.420199329	0.235	0.07	0.002833743	C5_CD8_Tem
chr13-79392649-79394106	0.000166903	0.297289291	0.235	0.102	1	C5_CD8_Tem

chr9-136384932-136385369	2.91E-05	0.32743293	0.131	0.043	1	C5_CD8_Tem
chr3-141017374-141018166	2.21E-06	0.36087961	0.12	0.029	0.146483672	C5_CD8_Tem
chr1-12603940-12604720	0.000397824	0.284731106	0.29	0.138	1	C5_CD8_Tem
chr17-67390201-67391379	3.71E-05	0.309200467	0.311	0.146	1	C5_CD8_Tem
chr16-21646703-21647667	4.21E-05	0.301496166	0.35	0.163	1	C5_CD8_Tem
chr1-42943071-42943442	4.63E-05	0.324451198	0.158	0.054	1	C5_CD8_Tem
chr8-133302840-133303865	1.43E-08	0.37984799	0.448	0.196	0.000945438	C5_CD8_Tem
chr8-66589185-66590031	0.000250133	0.28470095	0.115	0.035	1	C5_CD8_Tem
chr15-39624654-39625209	0.000328088	0.262794654	0.328	0.182	1	C5_CD8_Tem
chr1-113975402-113975903	0.000334571	0.266225512	0.082	0.022	1	C5_CD8_Tem
chr16-9127432-9128196	0.001741633	0.256046906	0.142	0.058	1	C5_CD8_Tem
chr3-15275103-15275652	0.001767356	0.253528892	0.131	0.05	1	C5_CD8_Tem
chr2-239548428-239548938	2.47E-06	0.367311236	0.175	0.054	0.163476453	C5_CD8_Tem
chr12-124774146-124775146	0.001717496	0.255899418	0.197	0.09	1	C5_CD8_Tem
chr16-30715390-30716124	2.27E-06	0.347905705	0.35	0.147	0.150640821	C5_CD8_Tem
chr10-80195757-80196922	7.95E-06	0.328302059	0.311	0.132	0.526483283	C5_CD8_Tem
chr16-85615088-85615287	2.69E-06	0.369822346	0.142	0.033	0.178182846	C5_CD8_Tem
chr2-97747173-97747655	2.73E-10	0.4792975	0.148	0.028	1.81E-05	C5_CD8_Tem
chr1-38977213-38978268	0.000479777	0.282872304	0.175	0.074	1	C5_CD8_Tem
chr18-49690202-49690676	6.57E-08	0.402734148	0.12	0.02	0.004350188	C5_CD8_Tem
chr7-121442756-121443205	2.55E-05	0.321425048	0.131	0.042	1	C5_CD8_Tem
chr12-123075421-123076983	0.00012723	0.268892386	0.383	0.214	1	C5_CD8_Tem
chr2-134217364-134218430	6.84E-06	0.323764197	0.344	0.164	0.453262905	C5_CD8_Tem
chr9-134381409-134382506	3.12E-05	0.32459263	0.208	0.082	1	C5_CD8_Tem
chr3-4983523-4984406	2.00E-07	0.324622148	0.525	0.27	0.013277517	C5_CD8_Tem
chr12-48977229-48978329	0.000234873	0.2608726	0.41	0.229	1	C5_CD8_Tem
chr9-3528393-3528865	2.37E-06	0.302077754	0.492	0.257	0.156636189	C5_CD8_Tem

chr1-15352418-15352854	4.67E-05	0.325201115	0.153	0.057	1	C5_CD8_Tem
chr3-56724793-56725668	0.001426144	0.261488143	0.153	0.067	1	C5_CD8_Tem
chr8-100218387-100219229	0.000436111	0.284864901	0.164	0.062	1	C5_CD8_Tem
chr16-89840899-89841685	0.000518409	0.272772559	0.23	0.106	1	C5_CD8_Tem
chr21-31656217-31656445	1.71E-08	0.427539471	0.109	0.017	0.001130065	C5_CD8_Tem
chr1-52658711-52659613	3.26E-08	0.430163013	0.164	0.036	0.00215802	C5_CD8_Tem
chr15-77005256-77005671	0.001326614	0.265131772	0.104	0.033	1	C5_CD8_Tem
chr8-30059433-30060536	2.19E-07	0.387502906	0.295	0.111	0.014524072	C5_CD8_Tem
chr6-166418699-166419622	3.58E-05	0.323428067	0.251	0.11	1	C5_CD8_Tem
chr3-157127893-157129028	7.77E-06	0.310853405	0.366	0.189	0.514500944	C5_CD8_Tem
chr5-136019960-136020824	0.000853304	0.253767897	0.093	0.029	1	C5_CD8_Tem
chr2-70084915-70085393	0.000466917	0.281915266	0.224	0.106	1	C5_CD8_Tem
chr2-191114311-191116116	3.25E-10	0.330064995	0.71	0.386	2.15E-05	C5_CD8_Tem
chr20-35305679-35306054	4.27E-07	0.39275319	0.137	0.03	0.028269232	C5_CD8_Tem
chr2-85405309-85406046	3.95E-09	0.446540209	0.257	0.081	0.000261418	C5_CD8_Tem
chr14-50343790-50344643	0.000316387	0.284346842	0.18	0.07	1	C5_CD8_Tem
chr6-130183456-130184310	0.000233943	0.289937879	0.23	0.101	1	C5_CD8_Tem
chr11-62542803-62543577	8.03E-06	0.295675963	0.486	0.266	0.531454205	C5_CD8_Tem
chr3-4980287-4980515	0.001138976	0.266823077	0.279	0.132	1	C5_CD8_Tem
chr6-46010877-46011813	3.35E-07	0.334811364	0.421	0.218	0.022198246	C5_CD8_Tem
chr17-82985881-82986429	6.62E-10	0.470239534	0.197	0.052	4.39E-05	C5_CD8_Tem
chr1-248926475-248927068	7.30E-06	0.329302459	0.082	0.014	0.483526479	C5_CD8_Tem
chr3-194715029-194715816	3.10E-05	0.326390251	0.126	0.035	1	C5_CD8_Tem
chr1-207825446-207826308	2.78E-05	0.260768006	0.514	0.294	1	C5_CD8_Tem
chr1-172663613-172664007	0.00036591	0.283528005	0.126	0.043	1	C5_CD8_Tem
chr6-90110865-90111691	1.05E-05	0.346446604	0.142	0.039	0.696873053	C5_CD8_Tem
chr5-111232438-111233250	0.000888767	0.269926702	0.202	0.085	1	C5_CD8_Tem

chr6-42281468-42281962	0.000289425	0.296190643	0.131	0.044	1	C5_CD8_Tem
chr1-93878188-93879401	0.000112443	0.266257758	0.443	0.248	1	C5_CD8_Tem
chr2-207768376-207770365	1.74E-42	0.447742105	0.374	0.143	1.16E-37	C6_CD8_Tact
chr19-46713727-46714699	5.84E-40	0.403603636	0.48	0.223	3.87E-35	C6_CD8_Tact
chr5-132495465-132497241	8.28E-40	0.363942995	0.575	0.305	5.48E-35	C6_CD8_Tact
chr6-33276673-33278192	3.63E-39	0.419808304	0.421	0.173	2.40E-34	C6_CD8_Tact
chr16-30893383-30894900	1.55E-38	0.441661278	0.344	0.123	1.03E-33	C6_CD8_Tact
chr8-102238244-102239905	6.52E-38	0.319749204	0.666	0.402	4.31E-33	C6_CD8_Tact
chr6-31826481-31827934	1.32E-37	0.37228117	0.518	0.272	8.75E-33	C6_CD8_Tact
chr20-63651626-63653928	1.57E-37	0.417369081	0.391	0.167	1.04E-32	C6_CD8_Tact
chr10-30432933-30434884	4.99E-37	0.371286087	0.52	0.262	3.31E-32	C6_CD8_Tact
chr3-184361417-184363699	7.43E-37	0.360917747	0.535	0.289	4.92E-32	C6_CD8_Tact
chr3-51982450-51983827	9.56E-37	0.423777191	0.365	0.146	6.33E-32	C6_CD8_Tact
chr6-30742291-30744729	2.62E-36	0.400522838	0.431	0.188	1.74E-31	C6_CD8_Tact
chr6-142944225-142947754	1.08E-35	0.26821213	0.772	0.528	7.16E-31	C6_CD8_Tact
chr6-31814371-31815752	3.85E-35	0.421303093	0.331	0.127	2.55E-30	C6_CD8_Tact
chr19-12789770-12790418	5.67E-35	0.361911025	0.508	0.263	3.76E-30	C6_CD8_Tact
chr11-8688214-8689343	8.29E-35	0.422910751	0.328	0.119	5.49E-30	C6_CD8_Tact
chr11-65859060-65860834	1.97E-34	0.296377682	0.69	0.435	1.30E-29	C6_CD8_Tact
chr11-62611783-62613435	4.05E-34	0.348272417	0.55	0.291	2.68E-29	C6_CD8_Tact
chr9-15306110-15307657	2.64E-33	0.395704707	0.382	0.161	1.75E-28	C6_CD8_Tact
chr19-18436896-18438302	2.93E-33	0.398430014	0.372	0.156	1.94E-28	C6_CD8_Tact
chr16-67536621-67538493	3.37E-33	0.403180488	0.353	0.144	2.23E-28	C6_CD8_Tact
chr3-38029485-38029944	4.51E-33	0.432207941	0.171	0.036	2.98E-28	C6_CD8_Tact
chr22-46149077-46151438	5.38E-33	0.347310005	0.535	0.285	3.56E-28	C6_CD8_Tact
chr12-6946091-6946805	5.98E-33	0.421048166	0.274	0.097	3.96E-28	C6_CD8_Tact
chr1-243848701-243851280	1.38E-32	0.318795788	0.581	0.349	9.13E-28	C6_CD8_Tact

chr12-55727869-55730439	1.43E-32	0.383445245	0.418	0.187	9.49E-28	C6_CD8_Tact
chr5-140690631-140691801	1.94E-32	0.394157018	0.365	0.158	1.28E-27	C6_CD8_Tact
chr17-1484674-1486134	6.16E-32	0.389856441	0.374	0.163	4.08E-27	C6_CD8_Tact
chr6-30686340-30687622	9.28E-32	0.365814521	0.446	0.222	6.15E-27	C6_CD8_Tact
chr6-142819455-142820378	3.57E-31	0.415182361	0.226	0.067	2.37E-26	C6_CD8_Tact
chr3-50340534-50341345	3.89E-31	0.403216823	0.295	0.108	2.58E-26	C6_CD8_Tact
chr1-111203560-111204718	5.49E-31	0.375555952	0.389	0.174	3.64E-26	C6_CD8_Tact
chr2-84969532-84972224	9.17E-31	0.338111	0.523	0.285	6.07E-26	C6_CD8_Tact
chr2-10302069-10304210	1.27E-30	0.338483658	0.507	0.276	8.42E-26	C6_CD8_Tact
chr10-43395787-43397522	2.24E-30	0.372230514	0.405	0.185	1.48E-25	C6_CD8_Tact
chr1-155322816-155324703	3.49E-30	0.2848083	0.667	0.421	2.31E-25	C6_CD8_Tact
chr9-133478560-133479671	4.30E-30	0.378251146	0.362	0.164	2.84E-25	C6_CD8_Tact
chr22-19177950-19179646	6.58E-30	0.364915317	0.418	0.198	4.36E-25	C6_CD8_Tact
chr2-178480079-178481082	7.43E-30	0.385969178	0.334	0.137	4.92E-25	C6_CD8_Tact
chr8-144077810-144079173	9.74E-30	0.393943571	0.31	0.116	6.45E-25	C6_CD8_Tact
chr16-69329628-69331039	1.51E-29	0.341503847	0.499	0.255	1.00E-24	C6_CD8_Tact
chr1-1778025-1779266	1.59E-29	0.359239162	0.437	0.211	1.05E-24	C6_CD8_Tact
chr16-726772-728613	3.16E-29	0.369537547	0.383	0.17	2.09E-24	C6_CD8_Tact
chr1-16498659-16499805	9.18E-29	0.383496776	0.317	0.132	6.08E-24	C6_CD8_Tact
chr3-10010525-10011530	1.23E-28	0.400106017	0.245	0.08	8.11E-24	C6_CD8_Tact
chr1-212698911-212700864	1.57E-28	0.380881184	0.326	0.138	1.04E-23	C6_CD8_Tact
chr11-1573405-1574901	2.23E-28	0.370534905	0.359	0.164	1.47E-23	C6_CD8_Tact
chr1-226121232-226123054	2.51E-28	0.336710684	0.473	0.247	1.66E-23	C6_CD8_Tact
chr3-13479330-13480843	4.91E-28	0.290522711	0.613	0.375	3.25E-23	C6_CD8_Tact
chr11-61890599-61892308	5.66E-28	0.361075874	0.395	0.179	3.75E-23	C6_CD8_Tact
chr4-148444349-148445940	6.31E-28	0.340559503	0.446	0.236	4.18E-23	C6_CD8_Tact
chr17-81514611-81515087	6.82E-28	0.375794021	0.341	0.138	4.52E-23	C6_CD8_Tact

chr5-56909065-56910476	7.71E-28	0.31495438	0.526	0.295	5.10E-23	C6_CD8_Tact
chr2-238425962-238427664	7.72E-28	0.337729214	0.457	0.235	5.12E-23	C6_CD8_Tact
chr18-12657090-12658699	8.24E-28	0.396562401	0.223	0.072	5.46E-23	C6_CD8_Tact
chr22-37638364-37639792	8.50E-28	0.380585651	0.309	0.126	5.63E-23	C6_CD8_Tact
chr11-66545578-66546779	8.87E-28	0.346310627	0.438	0.216	5.88E-23	C6_CD8_Tact
chr8-93916110-93917978	2.31E-27	0.264700782	0.671	0.434	1.53E-22	C6_CD8_Tact
chr22-30289044-30290219	3.71E-27	0.339851276	0.446	0.232	2.46E-22	C6_CD8_Tact
chr19-51366967-51369046	4.20E-27	0.287166956	0.612	0.366	2.78E-22	C6_CD8_Tact
chr19-42198721-42200220	5.11E-27	0.352045655	0.384	0.175	3.39E-22	C6_CD8_Tact
chr16-720276-722254	5.17E-27	0.370561044	0.322	0.134	3.42E-22	C6_CD8_Tact
chr1-169105570-169106823	8.32E-27	0.328381243	0.45	0.243	5.51E-22	C6_CD8_Tact
chr6-125789681-125791871	9.70E-27	0.312076515	0.508	0.292	6.42E-22	C6_CD8_Tact
chr14-24093681-24095696	1.02E-26	0.354773038	0.389	0.177	6.77E-22	C6_CD8_Tact
chr8-98825111-98826274	1.08E-26	0.364767972	0.34	0.145	7.13E-22	C6_CD8_Tact
chr4-99946041-99947193	1.15E-26	0.337711816	0.419	0.213	7.60E-22	C6_CD8_Tact
chr18-74495552-74496800	1.41E-26	0.298381875	0.557	0.324	9.36E-22	C6_CD8_Tact
chr12-50084515-50085848	1.70E-26	0.335628774	0.444	0.224	1.13E-21	C6_CD8_Tact
chr5-140556996-140557788	2.70E-26	0.383887704	0.255	0.092	1.79E-21	C6_CD8_Tact
chr5-138032099-138033515	2.94E-26	0.324802328	0.487	0.258	1.95E-21	C6_CD8_Tact
chr4-128060596-128062470	3.37E-26	0.304146829	0.524	0.305	2.23E-21	C6_CD8_Tact
chr16-29811239-29811591	3.49E-26	0.379535707	0.253	0.094	2.31E-21	C6_CD8_Tact
chr6-158999101-159000396	5.86E-26	0.342028047	0.394	0.195	3.88E-21	C6_CD8_Tact
chr9-32525069-32526759	5.92E-26	0.352407259	0.366	0.169	3.92E-21	C6_CD8_Tact
chr10-13299464-13300285	1.04E-25	0.364040953	0.317	0.134	6.91E-21	C6_CD8_Tact
chr16-641538-642642	1.05E-25	0.386555281	0.197	0.058	6.96E-21	C6_CD8_Tact
chr17-40323676-40324211	1.09E-25	0.363156108	0.313	0.129	7.19E-21	C6_CD8_Tact
chr17-36182330-36184239	1.36E-25	0.369553179	0.246	0.097	8.98E-21	C6_CD8_Tact

chr10-100996456-100999883	1.38E-25	0.314542206	0.49	0.278	9.15E-21	C6_CD8_Tact
chr13-91346864-91349276	2.43E-25	0.270821372	0.619	0.385	1.61E-20	C6_CD8_Tact
chr12-48817949-48819074	2.73E-25	0.359902962	0.311	0.137	1.81E-20	C6_CD8_Tact
chr3-113947264-113948763	2.76E-25	0.36029122	0.313	0.132	1.83E-20	C6_CD8_Tact
chr17-50148438-50149449	4.68E-25	0.36684227	0.292	0.121	3.10E-20	C6_CD8_Tact
chr3-184259848-184262199	5.81E-25	0.352773455	0.335	0.156	3.85E-20	C6_CD8_Tact
chr20-64079194-64080474	6.16E-25	0.349710396	0.35	0.155	4.08E-20	C6_CD8_Tact
chr7-39622969-39624261	6.45E-25	0.328121745	0.419	0.215	4.27E-20	C6_CD8_Tact
chr9-126914197-126915693	7.34E-25	0.323080353	0.442	0.234	4.86E-20	C6_CD8_Tact
chr1-43990990-43992232	7.38E-25	0.361266506	0.289	0.125	4.89E-20	C6_CD8_Tact
chr3-50573508-50574737	8.88E-25	0.363745843	0.259	0.103	5.88E-20	C6_CD8_Tact
chr1-43366852-43368554	9.34E-25	0.327147156	0.431	0.227	6.19E-20	C6_CD8_Tact
chr1-25615626-25617392	1.22E-24	0.315768474	0.468	0.254	8.10E-20	C6_CD8_Tact
chr9-35748323-35750075	1.24E-24	0.344001528	0.375	0.175	8.23E-20	C6_CD8_Tact
chr6-42048183-42049093	1.32E-24	0.331602526	0.416	0.205	8.74E-20	C6_CD8_Tact
chr1-43389269-43390246	1.63E-24	0.342372299	0.379	0.182	1.08E-19	C6_CD8_Tact
chr1-233326728-233328663	1.68E-24	0.309339484	0.479	0.256	1.12E-19	C6_CD8_Tact
chr11-615145-616232	1.73E-24	0.32727759	0.428	0.215	1.14E-19	C6_CD8_Tact
chr9-120928393-120929264	1.81E-24	0.36221631	0.282	0.112	1.20E-19	C6_CD8_Tact
chr1-246565580-246567120	1.87E-24	0.293545705	0.549	0.315	1.24E-19	C6_CD8_Tact
chr4-51842275-51844929	2.08E-24	0.260518689	0.628	0.412	1.38E-19	C6_CD8_Tact
chr17-28370788-28372546	2.62E-24	0.328187255	0.408	0.211	1.73E-19	C6_CD8_Tact
chr10-133378126-133379742	2.69E-24	0.350872064	0.325	0.147	1.78E-19	C6_CD8_Tact
chr4-3292015-3293929	3.60E-24	0.30514011	0.495	0.279	2.38E-19	C6_CD8_Tact
chr15-84715385-84716924	3.64E-24	0.292350143	0.542	0.313	2.41E-19	C6_CD8_Tact
chr2-9554847-9556403	4.22E-24	0.327889155	0.418	0.21	2.80E-19	C6_CD8_Tact
chr12-6866923-6868214	5.52E-24	0.339034253	0.36	0.173	3.66E-19	C6_CD8_Tact

chr16-4314920-4316479	6.37E-24	0.375306849	0.187	0.058	4.22E-19	C6_CD8_Tact
chr16-88633480-88635103	8.09E-24	0.344333029	0.317	0.143	5.36E-19	C6_CD8_Tact
chr6-13485898-13488475	9.51E-24	0.318157288	0.43	0.223	6.30E-19	C6_CD8_Tact
chr7-56050581-56052237	1.00E-23	0.293965338	0.521	0.303	6.65E-19	C6_CD8_Tact
chr11-116787418-116788407	1.40E-23	0.349458686	0.322	0.145	9.26E-19	C6_CD8_Tact
chr20-32535941-32537492	1.48E-23	0.294281898	0.524	0.296	9.81E-19	C6_CD8_Tact
chr15-80999671-81002107	1.76E-23	0.300789758	0.501	0.277	1.16E-18	C6_CD8_Tact
chr12-52022835-52024185	1.79E-23	0.303346037	0.494	0.27	1.18E-18	C6_CD8_Tact
chr7-100603693-100605892	2.08E-23	0.303445292	0.479	0.267	1.38E-18	C6_CD8_Tact
chr22-37674895-37675690	2.39E-23	0.366497751	0.186	0.058	1.58E-18	C6_CD8_Tact
chr14-21097719-21098793	2.60E-23	0.368158431	0.174	0.051	1.72E-18	C6_CD8_Tact
chr17-80260807-80261703	3.74E-23	0.341153729	0.333	0.149	2.48E-18	C6_CD8_Tact
chr7-2518888-2520006	4.03E-23	0.320531804	0.416	0.21	2.67E-18	C6_CD8_Tact
chr8-143290609-143291678	4.06E-23	0.360307207	0.233	0.087	2.69E-18	C6_CD8_Tact
chr12-6953497-6954217	4.38E-23	0.344856914	0.297	0.131	2.90E-18	C6_CD8_Tact
chr6-29722858-29724377	4.64E-23	0.299887568	0.488	0.271	3.07E-18	C6_CD8_Tact
chr6-137873204-137873589	4.81E-23	0.360443084	0.169	0.048	3.18E-18	C6_CD8_Tact
chr8-12753796-12756152	4.94E-23	0.262472006	0.617	0.388	3.27E-18	C6_CD8_Tact
chr11-66855785-66857487	5.05E-23	0.30361497	0.474	0.252	3.35E-18	C6_CD8_Tact
chr8-27310187-27311769	5.42E-23	0.300572374	0.489	0.273	3.59E-18	C6_CD8_Tact
chr7-6636673-6638112	5.99E-23	0.358252843	0.247	0.097	3.97E-18	C6_CD8_Tact
chr15-81323579-81324744	6.15E-23	0.286750941	0.519	0.297	4.07E-18	C6_CD8_Tact
chr5-149344620-149346099	6.17E-23	0.349248166	0.288	0.124	4.09E-18	C6_CD8_Tact
chr17-38798747-38800330	6.86E-23	0.272305401	0.572	0.352	4.54E-18	C6_CD8_Tact
chr17-7884807-7888472	8.19E-23	0.272954487	0.566	0.357	5.42E-18	C6_CD8_Tact
chr10-32957372-32958966	8.64E-23	0.309170011	0.434	0.233	5.72E-18	C6_CD8_Tact
chr6-153002009-153003499	9.46E-23	0.317382378	0.401	0.209	6.27E-18	C6_CD8_Tact

chr12-459369-460775	1.08E-22	0.356202689	0.254	0.104	7.18E-18	C6_CD8_Tact
chr1-11803078-11803800	1.20E-22	0.355764015	0.23	0.083	7.92E-18	C6_CD8_Tact
chr21-44299162-44300787	1.37E-22	0.349104502	0.285	0.122	9.07E-18	C6_CD8_Tact
chr2-39119851-39121403	1.51E-22	0.315285073	0.411	0.212	9.99E-18	C6_CD8_Tact
chr2-219497703-219499037	1.66E-22	0.306296094	0.439	0.24	1.10E-17	C6_CD8_Tact
chr16-30406900-30407856	1.77E-22	0.32535043	0.36	0.181	1.17E-17	C6_CD8_Tact
chr1-61741772-61743003	1.79E-22	0.343444163	0.284	0.126	1.19E-17	C6_CD8_Tact
chr5-181246129-181247128	1.84E-22	0.355244143	0.24	0.094	1.22E-17	C6_CD8_Tact
chr19-13794570-13795881	2.46E-22	0.32672179	0.366	0.177	1.63E-17	C6_CD8_Tact
chr20-34708611-34710208	2.60E-22	0.283416647	0.535	0.316	1.72E-17	C6_CD8_Tact
chr9-112750023-112751784	2.61E-22	0.32024931	0.383	0.194	1.73E-17	C6_CD8_Tact
chr2-74464582-74466049	2.99E-22	0.332497505	0.352	0.168	1.98E-17	C6_CD8_Tact
chr12-8948871-8950434	3.02E-22	0.271405764	0.557	0.335	2.00E-17	C6_CD8_Tact
chr3-47802284-47803647	3.34E-22	0.309363715	0.439	0.236	2.21E-17	C6_CD8_Tact
chr8-143024017-143024693	3.41E-22	0.354823494	0.172	0.054	2.26E-17	C6_CD8_Tact
chr15-55288935-55290754	3.71E-22	0.28394795	0.519	0.309	2.45E-17	C6_CD8_Tact
chr6-33313370-33314762	4.02E-22	0.300787512	0.456	0.249	2.67E-17	C6_CD8_Tact
chr1-37477215-37477583	4.06E-22	0.338294589	0.327	0.137	2.69E-17	C6_CD8_Tact
chr15-50764438-50765979	4.33E-22	0.315098586	0.4	0.203	2.87E-17	C6_CD8_Tact
chr1-154324437-154326474	4.79E-22	0.316773107	0.411	0.209	3.17E-17	C6_CD8_Tact
chr11-804748-805544	5.12E-22	0.341379351	0.284	0.12	3.39E-17	C6_CD8_Tact
chr14-39102391-39103859	5.36E-22	0.319683112	0.383	0.192	3.55E-17	C6_CD8_Tact
chr6-20402992-20404791	5.46E-22	0.284373877	0.507	0.295	3.61E-17	C6_CD8_Tact
chr4-173168002-173170237	5.57E-22	0.323714391	0.35	0.17	3.69E-17	C6_CD8_Tact
chr3-15858828-15860492	5.87E-22	0.310565412	0.415	0.222	3.89E-17	C6_CD8_Tact
chr6-32189849-32190531	6.50E-22	0.347674296	0.241	0.095	4.30E-17	C6_CD8_Tact
chr19-35898736-35900714	6.71E-22	0.28087377	0.529	0.318	4.44E-17	C6_CD8_Tact

chr14-105473621-105474897	6.74E-22	0.3294385	0.337	0.155	4.47E-17	C6_CD8_Tact
chr17-77355940-77357165	6.90E-22	0.345766565	0.109	0.023	4.57E-17	C6_CD8_Tact
chr7-128409185-128410501	6.92E-22	0.306708564	0.432	0.234	4.58E-17	C6_CD8_Tact
chr15-80151933-80153570	7.44E-22	0.295371483	0.464	0.262	4.93E-17	C6_CD8_Tact
chr6-44263227-44263662	7.74E-22	0.346894508	0.122	0.028	5.12E-17	C6_CD8_Tact
chr16-68236897-68238112	8.25E-22	0.304961369	0.443	0.236	5.46E-17	C6_CD8_Tact
chr6-133951708-133953653	8.49E-22	0.286712387	0.479	0.281	5.62E-17	C6_CD8_Tact
chr21-44011696-44014001	8.77E-22	0.292379337	0.489	0.278	5.81E-17	C6_CD8_Tact
chr16-30526138-30527797	1.01E-21	0.298650561	0.464	0.257	6.70E-17	C6_CD8_Tact
chr12-93569400-93572169	1.07E-21	0.27110391	0.548	0.334	7.12E-17	C6_CD8_Tact
chr3-69012889-69014099	1.16E-21	0.328519029	0.326	0.155	7.69E-17	C6_CD8_Tact
chr7-601351-602853	1.24E-21	0.33015737	0.335	0.159	8.22E-17	C6_CD8_Tact
chr10-112949661-112950427	1.62E-21	0.347219635	0.214	0.08	1.08E-16	C6_CD8_Tact
chr6-158644054-158645149	1.70E-21	0.305457851	0.409	0.219	1.12E-16	C6_CD8_Tact
chr2-238239123-238240984	1.72E-21	0.331878923	0.333	0.156	1.14E-16	C6_CD8_Tact
chr3-50611260-50612373	1.81E-21	0.262715197	0.57	0.354	1.20E-16	C6_CD8_Tact
chr17-63699169-63701070	1.94E-21	0.27462603	0.539	0.322	1.28E-16	C6_CD8_Tact
chr5-163436787-163438170	1.94E-21	0.299718231	0.437	0.235	1.28E-16	C6_CD8_Tact
chr12-6927299-6928332	2.04E-21	0.351410076	0.177	0.059	1.35E-16	C6_CD8_Tact
chr12-123435626-123437036	2.26E-21	0.300608239	0.445	0.237	1.50E-16	C6_CD8_Tact
chr19-55640682-55641961	2.27E-21	0.351712821	0.221	0.082	1.50E-16	C6_CD8_Tact
chr7-99374040-99375647	2.39E-21	0.300059706	0.443	0.244	1.58E-16	C6_CD8_Tact
chr10-80453341-80455125	2.45E-21	0.285521623	0.488	0.275	1.62E-16	C6_CD8_Tact
chr2-62195386-62197568	2.84E-21	0.272223541	0.53	0.323	1.88E-16	C6_CD8_Tact
chr8-133571611-133572454	2.99E-21	0.300569986	0.43	0.233	1.98E-16	C6_CD8_Tact
chr4-18020299-18022490	3.26E-21	0.253059722	0.581	0.375	2.16E-16	C6_CD8_Tact
chr1-7953678-7954908	3.38E-21	0.341303198	0.26	0.104	2.24E-16	C6_CD8_Tact

chr4-70704074-70705948	4.19E-21	0.289648477	0.469	0.267	2.77E-16	C6_CD8_Tact
chr14-105092634-105094107	4.21E-21	0.338279209	0.272	0.115	2.79E-16	C6_CD8_Tact
chr12-64608848-64611307	4.77E-21	0.272645638	0.524	0.324	3.16E-16	C6_CD8_Tact
chr11-62621418-62622563	5.86E-21	0.26635968	0.557	0.339	3.88E-16	C6_CD8_Tact
chr14-70808124-70810053	6.32E-21	0.258379983	0.567	0.361	4.18E-16	C6_CD8_Tact
chr7-26151797-26153170	6.36E-21	0.324301077	0.332	0.164	4.21E-16	C6_CD8_Tact
chr4-25861636-25863362	1.07E-20	0.334860573	0.282	0.121	7.07E-16	C6_CD8_Tact
chr12-66301862-66302894	1.09E-20	0.291783202	0.445	0.244	7.21E-16	C6_CD8_Tact
chr11-6473289-6474705	1.16E-20	0.332706627	0.288	0.123	7.71E-16	C6_CD8_Tact
chr10-27240145-27241292	1.24E-20	0.327943546	0.313	0.145	8.23E-16	C6_CD8_Tact
chr11-64234075-64235184	1.35E-20	0.314244692	0.37	0.184	8.94E-16	C6_CD8_Tact
chr6-129709507-129710474	1.46E-20	0.341872416	0.205	0.076	9.64E-16	C6_CD8_Tact
chr15-75647022-75648101	1.75E-20	0.34639589	0.198	0.07	1.16E-15	C6_CD8_Tact
chr20-58890285-58892757	1.82E-20	0.275441939	0.527	0.312	1.21E-15	C6_CD8_Tact
chr2-180979693-180981439	1.92E-20	0.29907171	0.39	0.216	1.27E-15	C6_CD8_Tact
chr10-31927349-31930623	2.05E-20	0.279811167	0.488	0.287	1.36E-15	C6_CD8_Tact
chr12-57754156-57755125	2.19E-20	0.310305063	0.381	0.188	1.45E-15	C6_CD8_Tact
chr2-208253862-208255652	2.28E-20	0.283529991	0.469	0.282	1.51E-15	C6_CD8_Tact
chr1-12166676-12167396	2.59E-20	0.339469654	0.221	0.087	1.71E-15	C6_CD8_Tact
chr2-159903401-159905220	2.60E-20	0.273451258	0.483	0.289	1.72E-15	C6_CD8_Tact
chr1-112706441-112707537	3.09E-20	0.338852743	0.226	0.085	2.04E-15	C6_CD8_Tact
chr1-145960462-145960684	3.39E-20	0.335353186	0.124	0.031	2.24E-15	C6_CD8_Tact
chr3-126083456-126084500	3.47E-20	0.305524249	0.363	0.186	2.30E-15	C6_CD8_Tact
chr12-6451125-6453127	3.53E-20	0.313718012	0.352	0.178	2.34E-15	C6_CD8_Tact
chr2-70553163-70554435	3.56E-20	0.328950327	0.279	0.128	2.36E-15	C6_CD8_Tact
chr3-50568732-50569742	3.77E-20	0.301587967	0.408	0.219	2.49E-15	C6_CD8_Tact
chr3-50226093-50228150	4.23E-20	0.262703246	0.547	0.338	2.80E-15	C6_CD8_Tact

chr3-32391257-32392948	4.33E-20	0.317921485	0.325	0.154	2.87E-15	C6_CD8_Tact
chr17-39196884-39197942	4.84E-20	0.337696268	0.245	0.103	3.21E-15	C6_CD8_Tact
chr2-46296539-46298086	5.15E-20	0.306604543	0.388	0.193	3.41E-15	C6_CD8_Tact
chr19-45423318-45424645	5.23E-20	0.321326162	0.314	0.15	3.46E-15	C6_CD8_Tact
chr20-62804012-62805585	5.45E-20	0.319845927	0.341	0.163	3.61E-15	C6_CD8_Tact
chr15-67065019-67066835	5.75E-20	0.315921928	0.351	0.174	3.81E-15	C6_CD8_Tact
chr17-1490964-1491865	7.26E-20	0.332453045	0.248	0.099	4.81E-15	C6_CD8_Tact
chr19-58575139-58576053	7.57E-20	0.314288535	0.345	0.17	5.01E-15	C6_CD8_Tact
chr6-85592821-85594764	7.88E-20	0.279693805	0.468	0.278	5.22E-15	C6_CD8_Tact
chr14-54509250-54510447	8.86E-20	0.275460247	0.482	0.276	5.86E-15	C6_CD8_Tact
chr15-101250954-101252569	9.31E-20	0.299203807	0.395	0.217	6.17E-15	C6_CD8_Tact
chr1-89820487-89821992	9.97E-20	0.310657469	0.35	0.173	6.60E-15	C6_CD8_Tact
chr1-21289749-21290485	1.03E-19	0.338900091	0.171	0.057	6.82E-15	C6_CD8_Tact
chr3-51973518-51975688	1.03E-19	0.293845115	0.432	0.235	6.85E-15	C6_CD8_Tact
chr7-140923628-140925317	1.05E-19	0.256074551	0.548	0.34	6.97E-15	C6_CD8_Tact
chr2-65431963-65433020	1.08E-19	0.316436165	0.315	0.145	7.17E-15	C6_CD8_Tact
chr17-36534098-36535825	1.12E-19	0.286836783	0.458	0.259	7.39E-15	C6_CD8_Tact
chr1-116372634-116374312	1.17E-19	0.258849642	0.527	0.338	7.77E-15	C6_CD8_Tact
chr1-154960825-154962501	1.27E-19	0.292503389	0.422	0.23	8.38E-15	C6_CD8_Tact
chr14-23551027-23551925	1.33E-19	0.331715109	0.242	0.099	8.78E-15	C6_CD8_Tact
chr17-21125883-21127576	1.35E-19	0.298897576	0.408	0.223	8.96E-15	C6_CD8_Tact
chr12-106773469-106775357	1.45E-19	0.316054301	0.295	0.138	9.63E-15	C6_CD8_Tact
chr2-120012502-120013559	1.65E-19	0.331308862	0.233	0.092	1.09E-14	C6_CD8_Tact
chr17-7393577-7395098	1.70E-19	0.2940794	0.42	0.226	1.12E-14	C6_CD8_Tact
chr1-64744269-64746053	1.72E-19	0.331286528	0.212	0.088	1.14E-14	C6_CD8_Tact
chr13-113489995-113491280	1.82E-19	0.325007698	0.283	0.127	1.20E-14	C6_CD8_Tact
chr17-76271507-76272748	1.85E-19	0.330029509	0.248	0.104	1.22E-14	C6_CD8_Tact

chr16-526778-528971	1.87E-19	0.258661924	0.554	0.346	1.24E-14	C6_CD8_Tact
chr1-231039197-231041023	1.90E-19	0.320524175	0.285	0.135	1.26E-14	C6_CD8_Tact
chr12-53219311-53221893	1.90E-19	0.259842009	0.532	0.33	1.26E-14	C6_CD8_Tact
chrX-110001824-110003290	2.10E-19	0.294908296	0.369	0.196	1.39E-14	C6_CD8_Tact
chr2-32356124-32358159	2.14E-19	0.282719347	0.453	0.251	1.42E-14	C6_CD8_Tact
chr8-66428239-66430292	2.38E-19	0.287236609	0.436	0.248	1.58E-14	C6_CD8_Tact
chr18-62714675-62716380	2.60E-19	0.255246737	0.557	0.34	1.72E-14	C6_CD8_Tact
chr2-174485884-174487602	2.71E-19	0.258427278	0.537	0.326	1.79E-14	C6_CD8_Tact
chr2-73828347-73829488	2.78E-19	0.307234053	0.366	0.184	1.84E-14	C6_CD8_Tact
chr1-32200179-32201748	2.78E-19	0.298255037	0.382	0.206	1.84E-14	C6_CD8_Tact
chr1-155977133-155978922	2.88E-19	0.31199134	0.333	0.164	1.91E-14	C6_CD8_Tact
chrX-154485992-154487109	2.92E-19	0.331908296	0.22	0.083	1.93E-14	C6_CD8_Tact
chr6-109440155-109441684	3.01E-19	0.291071448	0.408	0.227	1.99E-14	C6_CD8_Tact
chr2-36355308-36356642	3.06E-19	0.322633272	0.224	0.091	2.03E-14	C6_CD8_Tact
chr1-175191587-175193257	3.14E-19	0.311829875	0.327	0.159	2.08E-14	C6_CD8_Tact
chr10-3874847-3875904	3.27E-19	0.301441577	0.359	0.184	2.17E-14	C6_CD8_Tact
chr19-55353370-55354516	3.32E-19	0.320218766	0.289	0.131	2.20E-14	C6_CD8_Tact
chrX-10014427-10016041	3.38E-19	0.309166355	0.327	0.163	2.24E-14	C6_CD8_Tact
chr3-52245211-52246586	3.76E-19	0.315734857	0.308	0.151	2.49E-14	C6_CD8_Tact
chr12-7129523-7131831	3.87E-19	0.274751756	0.465	0.279	2.56E-14	C6_CD8_Tact
chr12-52032483-52033368	3.99E-19	0.3010441	0.374	0.187	2.64E-14	C6_CD8_Tact
chr1-41240891-41242418	4.27E-19	0.329216193	0.235	0.099	2.83E-14	C6_CD8_Tact
chr7-76047339-76048773	4.47E-19	0.305901618	0.354	0.177	2.96E-14	C6_CD8_Tact
chr11-64230092-64230815	4.91E-19	0.300372181	0.374	0.192	3.25E-14	C6_CD8_Tact
chr11-64225534-64227022	5.37E-19	0.297321673	0.399	0.209	3.56E-14	C6_CD8_Tact
chr20-62928735-62929488	5.51E-19	0.334311266	0.168	0.054	3.65E-14	C6_CD8_Tact
chr11-66371010-66372600	5.96E-19	0.303949976	0.334	0.17	3.95E-14	C6_CD8_Tact

chr2-73984462-73986645	6.10E-19	0.302826563	0.358	0.186	4.04E-14	C6_CD8_Tact
chr16-2473561-2475814	6.17E-19	0.31677442	0.301	0.143	4.09E-14	C6_CD8_Tact
chr2-157875354-157877189	6.55E-19	0.265629526	0.501	0.303	4.34E-14	C6_CD8_Tact
chr20-2692255-2693746	6.93E-19	0.332298422	0.189	0.069	4.59E-14	C6_CD8_Tact
chr1-51517959-51519581	7.13E-19	0.273011885	0.47	0.275	4.72E-14	C6_CD8_Tact
chr11-62770692-62771883	7.50E-19	0.280900519	0.443	0.257	4.97E-14	C6_CD8_Tact
chr2-158456001-158458296	8.84E-19	0.250464906	0.523	0.335	5.85E-14	C6_CD8_Tact
chr8-144852209-144853570	9.13E-19	0.320042464	0.274	0.121	6.05E-14	C6_CD8_Tact
chr11-64809760-64811178	9.64E-19	0.304070456	0.348	0.174	6.38E-14	C6_CD8_Tact
chr17-10696715-10698313	1.02E-18	0.300316113	0.362	0.183	6.73E-14	C6_CD8_Tact
chr1-147171641-147173268	1.03E-18	0.266568325	0.511	0.3	6.79E-14	C6_CD8_Tact
chr2-74528976-74531152	1.11E-18	0.260722925	0.541	0.318	7.38E-14	C6_CD8_Tact
chr19-38361456-38362765	1.17E-18	0.320564509	0.278	0.122	7.75E-14	C6_CD8_Tact
chr19-44112769-44113859	1.20E-18	0.323181102	0.236	0.1	7.92E-14	C6_CD8_Tact
chr2-118087890-118089532	1.24E-18	0.307243885	0.313	0.151	8.22E-14	C6_CD8_Tact
chr11-71447770-71448730	1.25E-18	0.289906947	0.403	0.22	8.31E-14	C6_CD8_Tact
chr1-31937300-31938784	1.33E-18	0.257516211	0.508	0.321	8.79E-14	C6_CD8_Tact
chr8-119855224-119856388	1.40E-18	0.327169854	0.226	0.092	9.30E-14	C6_CD8_Tact
chr12-53498927-53499918	1.41E-18	0.32042094	0.266	0.113	9.36E-14	C6_CD8_Tact
chr3-11718677-11720827	1.54E-18	0.286091263	0.402	0.225	1.02E-13	C6_CD8_Tact
chr8-123072126-123073518	1.56E-18	0.33014651	0.186	0.068	1.03E-13	C6_CD8_Tact
chr10-14952933-14954540	1.57E-18	0.275653056	0.438	0.252	1.04E-13	C6_CD8_Tact
chr10-31603153-31603860	1.68E-18	0.324077675	0.154	0.048	1.11E-13	C6_CD8_Tact
chr3-47577817-47579311	1.69E-18	0.32772831	0.169	0.059	1.12E-13	C6_CD8_Tact
chr12-106954861-106956940	1.75E-18	0.26276712	0.494	0.301	1.16E-13	C6_CD8_Tact
chr2-169732916-169734488	1.85E-18	0.321687667	0.149	0.049	1.22E-13	C6_CD8_Tact
chr2-61693804-61695177	1.96E-18	0.310041526	0.277	0.133	1.30E-13	C6_CD8_Tact

chr19-1941778-1942968	1.98E-18	0.300772442	0.334	0.165	1.31E-13	C6_CD8_Tact
chr1-26335932-26336975	2.01E-18	0.301741291	0.337	0.169	1.33E-13	C6_CD8_Tact
chr11-45804023-45805564	2.02E-18	0.259776621	0.519	0.309	1.34E-13	C6_CD8_Tact
chr8-143557949-143558849	2.12E-18	0.32840046	0.208	0.078	1.41E-13	C6_CD8_Tact
chr10-102119409-102120919	2.18E-18	0.270890845	0.458	0.275	1.44E-13	C6_CD8_Tact
chr6-3162354-3163391	2.22E-18	0.311189563	0.29	0.136	1.47E-13	C6_CD8_Tact
chr14-50829653-50831731	2.37E-18	0.271251278	0.451	0.267	1.57E-13	C6_CD8_Tact
chr1-9128564-9129983	2.38E-18	0.272166817	0.464	0.27	1.57E-13	C6_CD8_Tact
chr14-73886115-73887435	2.41E-18	0.287504745	0.401	0.224	1.60E-13	C6_CD8_Tact
chr1-27360071-27360710	2.41E-18	0.32031082	0.124	0.034	1.60E-13	C6_CD8_Tact
chr6-33427664-33428771	2.51E-18	0.310497547	0.304	0.141	1.67E-13	C6_CD8_Tact
chr19-13162289-13163546	2.59E-18	0.2929513	0.366	0.194	1.72E-13	C6_CD8_Tact
chr11-124799075-124801168	2.68E-18	0.298502772	0.36	0.187	1.78E-13	C6_CD8_Tact
chr11-6603063-6604607	2.73E-18	0.288273082	0.389	0.212	1.81E-13	C6_CD8_Tact
chr12-56221270-56222447	2.87E-18	0.285589715	0.411	0.228	1.90E-13	C6_CD8_Tact
chr5-132410332-132411945	3.12E-18	0.279791919	0.446	0.24	2.07E-13	C6_CD8_Tact
chr6-117481855-117483892	3.89E-18	0.312311275	0.261	0.122	2.58E-13	C6_CD8_Tact
chr3-4867674-4869343	3.94E-18	0.291299222	0.379	0.196	2.61E-13	C6_CD8_Tact
chr19-18291492-18291889	3.95E-18	0.320982833	0.192	0.071	2.62E-13	C6_CD8_Tact
chr1-201480185-201481020	4.23E-18	0.311232684	0.274	0.126	2.80E-13	C6_CD8_Tact
chr19-2479234-2479433	4.44E-18	0.3171885	0.104	0.025	2.94E-13	C6_CD8_Tact
chr15-72230295-72232410	4.66E-18	0.262883619	0.502	0.3	3.09E-13	C6_CD8_Tact
chr12-57845042-57847279	4.79E-18	0.28158063	0.412	0.233	3.17E-13	C6_CD8_Tact
chr2-86622588-86624095	5.36E-18	0.28618701	0.394	0.214	3.55E-13	C6_CD8_Tact
chr6-29627610-29628904	5.53E-18	0.324924743	0.198	0.076	3.66E-13	C6_CD8_Tact
chr14-100305217-100307008	5.87E-18	0.309135079	0.291	0.143	3.89E-13	C6_CD8_Tact
chr10-88879633-88881119	7.35E-18	0.285948652	0.365	0.197	4.87E-13	C6_CD8_Tact

chr1-36322814-36323926	7.55E-18	0.325681441	0.168	0.057	5.00E-13	C6_CD8_Tact
chr17-45131976-45133435	7.73E-18	0.296608157	0.365	0.188	5.12E-13	C6_CD8_Tact
chr17-51165746-51167332	7.98E-18	0.306387449	0.325	0.165	5.28E-13	C6_CD8_Tact
chr17-80260026-80260513	8.39E-18	0.304622102	0.308	0.147	5.56E-13	C6_CD8_Tact
chr12-105329965-105332030	8.63E-18	0.259084635	0.482	0.292	5.72E-13	C6_CD8_Tact
chr2-105744403-105745783	9.05E-18	0.301664109	0.321	0.161	5.99E-13	C6_CD8_Tact
chr19-13839026-13840684	9.20E-18	0.252494038	0.521	0.315	6.09E-13	C6_CD8_Tact
chr12-2690663-2692455	9.62E-18	0.305978822	0.28	0.134	6.37E-13	C6_CD8_Tact
chr6-150068143-150069469	9.82E-18	0.3151853	0.246	0.106	6.50E-13	C6_CD8_Tact
chr12-89353306-89354457	9.93E-18	0.306394022	0.252	0.113	6.57E-13	C6_CD8_Tact
chr2-159285926-159287285	1.04E-17	0.250055652	0.508	0.314	6.87E-13	C6_CD8_Tact
chr15-40470409-40472048	1.18E-17	0.316251775	0.242	0.107	7.83E-13	C6_CD8_Tact
chr19-14384139-14384560	1.28E-17	0.316378674	0.233	0.102	8.47E-13	C6_CD8_Tact
chr19-4059467-4060321	1.37E-17	0.307365976	0.235	0.102	9.06E-13	C6_CD8_Tact
chr6-142926274-142927301	1.51E-17	0.315318595	0.242	0.104	1.00E-12	C6_CD8_Tact
chr8-90644724-90646533	1.60E-17	0.265057683	0.443	0.265	1.06E-12	C6_CD8_Tact
chr2-95159003-95160237	1.78E-17	0.30316114	0.321	0.155	1.18E-12	C6_CD8_Tact
chr13-42047524-42049607	1.82E-17	0.302822384	0.292	0.138	1.21E-12	C6_CD8_Tact
chr1-228405965-228407359	1.88E-17	0.274286818	0.425	0.241	1.25E-12	C6_CD8_Tact
chr19-45509291-45509545	2.00E-17	0.295360658	0.082	0.016	1.33E-12	C6_CD8_Tact
chr10-21525410-21527451	2.03E-17	0.295111862	0.327	0.171	1.35E-12	C6_CD8_Tact
chr6-107114087-107116131	2.08E-17	0.305840817	0.251	0.115	1.38E-12	C6_CD8_Tact
chr3-52052835-52053043	2.12E-17	0.301111059	0.087	0.018	1.41E-12	C6_CD8_Tact
chr9-120875425-120877766	2.18E-17	0.275480312	0.424	0.239	1.44E-12	C6_CD8_Tact
chr17-82230660-82231481	2.21E-17	0.314603062	0.229	0.096	1.46E-12	C6_CD8_Tact
chr20-25623074-25624542	2.41E-17	0.300592179	0.31	0.152	1.60E-12	C6_CD8_Tact
chr10-104353154-104353903	2.53E-17	0.285872815	0.369	0.195	1.68E-12	C6_CD8_Tact

chr14-91253155-91253859	2.60E-17	0.315296057	0.177	0.066	1.72E-12	C6_CD8_Tact
chr9-126721685-126723770	2.67E-17	0.272714119	0.427	0.238	1.77E-12	C6_CD8_Tact
chrX-153934364-153935742	2.68E-17	0.318467836	0.18	0.064	1.78E-12	C6_CD8_Tact
chr12-47079379-47080984	2.83E-17	0.307340462	0.257	0.125	1.87E-12	C6_CD8_Tact
chr7-121395411-121396657	2.84E-17	0.292997566	0.328	0.166	1.88E-12	C6_CD8_Tact
chr20-18587227-18588594	2.89E-17	0.297984821	0.316	0.153	1.92E-12	C6_CD8_Tact
chr6-31399468-31400835	2.92E-17	0.297675316	0.333	0.171	1.93E-12	C6_CD8_Tact
chr1-37807351-37808737	2.95E-17	0.280700629	0.394	0.22	1.95E-12	C6_CD8_Tact
chr3-32106020-32107184	3.27E-17	0.27454116	0.397	0.215	2.17E-12	C6_CD8_Tact
chr5-16464937-16466305	3.38E-17	0.292994903	0.337	0.172	2.24E-12	C6_CD8_Tact
chr10-46284125-46285130	3.39E-17	0.303296035	0.251	0.111	2.24E-12	C6_CD8_Tact
chr16-1983418-1984537	3.65E-17	0.312687255	0.22	0.093	2.42E-12	C6_CD8_Tact
chr7-43834364-43835191	3.94E-17	0.309353509	0.252	0.114	2.61E-12	C6_CD8_Tact
chr8-100385956-100386660	4.06E-17	0.308474293	0.124	0.036	2.69E-12	C6_CD8_Tact
chr19-10085449-10087246	4.07E-17	0.286715046	0.381	0.201	2.69E-12	C6_CD8_Tact
chr19-46716784-46718666	4.12E-17	0.283567264	0.393	0.214	2.73E-12	C6_CD8_Tact
chr19-42279876-42281063	4.33E-17	0.285188855	0.374	0.203	2.86E-12	C6_CD8_Tact
chr1-3771946-3773107	4.37E-17	0.290165763	0.335	0.174	2.90E-12	C6_CD8_Tact
chr11-8963481-8965209	4.46E-17	0.287553762	0.348	0.184	2.95E-12	C6_CD8_Tact
chr12-14366953-14367593	4.84E-17	0.306867082	0.22	0.095	3.20E-12	C6_CD8_Tact
chr8-143552974-143554060	5.91E-17	0.315953154	0.192	0.075	3.91E-12	C6_CD8_Tact
chr5-157265425-157266879	6.27E-17	0.258415537	0.467	0.284	4.15E-12	C6_CD8_Tact
chr8-22694156-22695920	7.11E-17	0.272102579	0.437	0.247	4.71E-12	C6_CD8_Tact
chr3-39177697-39178190	7.28E-17	0.304774226	0.121	0.034	4.82E-12	C6_CD8_Tact
chr16-67173832-67174607	7.61E-17	0.312765979	0.205	0.08	5.04E-12	C6_CD8_Tact
chr19-17950357-17950871	7.87E-17	0.30861794	0.135	0.042	5.21E-12	C6_CD8_Tact
chr15-64841047-64842114	7.94E-17	0.255077452	0.479	0.279	5.26E-12	C6_CD8_Tact

chr14-102508611-102510518	8.15E-17	0.274225993	0.412	0.231	5.40E-12	C6_CD8_Tact
chr4-145732749-145733600	8.56E-17	0.293159313	0.315	0.158	5.67E-12	C6_CD8_Tact
chr7-92244937-92246884	8.82E-17	0.264025614	0.433	0.26	5.84E-12	C6_CD8_Tact
chr12-120902989-120904724	8.98E-17	0.258715838	0.486	0.29	5.95E-12	C6_CD8_Tact
chr7-100171115-100172114	9.28E-17	0.308131232	0.136	0.043	6.14E-12	C6_CD8_Tact
chr5-178231750-178233036	9.34E-17	0.294467159	0.322	0.159	6.19E-12	C6_CD8_Tact
chr12-11648747-11650389	9.51E-17	0.284752441	0.368	0.19	6.30E-12	C6_CD8_Tact
chr4-109702266-109703744	1.01E-16	0.285678438	0.346	0.186	6.66E-12	C6_CD8_Tact
chr5-94617879-94619364	1.04E-16	0.272455188	0.402	0.234	6.92E-12	C6_CD8_Tact
chr19-40420520-40421425	1.08E-16	0.302175754	0.249	0.115	7.13E-12	C6_CD8_Tact
chr2-24049157-24050133	1.13E-16	0.313079779	0.174	0.064	7.48E-12	C6_CD8_Tact
chr7-142853731-142855576	1.14E-16	0.286520562	0.337	0.179	7.52E-12	C6_CD8_Tact
chr10-133372594-133373761	1.18E-16	0.283895845	0.366	0.195	7.84E-12	C6_CD8_Tact
chr1-151057099-151058135	1.23E-16	0.294818421	0.308	0.154	8.15E-12	C6_CD8_Tact
chr16-29662574-29665516	1.23E-16	0.284168143	0.34	0.185	8.15E-12	C6_CD8_Tact
chr10-119165045-119166114	1.27E-16	0.277826748	0.387	0.207	8.39E-12	C6_CD8_Tact
chr1-40665088-40666026	1.28E-16	0.289041842	0.353	0.179	8.48E-12	C6_CD8_Tact
chr9-133141698-133142646	1.42E-16	0.289711188	0.329	0.165	9.42E-12	C6_CD8_Tact
chr2-156435425-156437010	1.43E-16	0.304071235	0.232	0.1	9.50E-12	C6_CD8_Tact
chr10-119102962-119104790	1.55E-16	0.283869071	0.357	0.192	1.02E-11	C6_CD8_Tact
chr19-1258880-1259618	1.58E-16	0.303159905	0.252	0.114	1.05E-11	C6_CD8_Tact
chr13-40788890-40789930	1.70E-16	0.307326797	0.204	0.083	1.12E-11	C6_CD8_Tact
chr11-65516180-65517143	1.72E-16	0.284504221	0.082	0.017	1.14E-11	C6_CD8_Tact
chr16-3058195-3059724	1.91E-16	0.278532838	0.381	0.207	1.26E-11	C6_CD8_Tact
chr10-35335673-35337469	1.94E-16	0.285451674	0.341	0.176	1.29E-11	C6_CD8_Tact
chr9-35664495-35665480	2.02E-16	0.30757277	0.178	0.069	1.34E-11	C6_CD8_Tact
chr1-46615763-46617076	2.24E-16	0.300380714	0.257	0.12	1.48E-11	C6_CD8_Tact

chr5-170302476-170303853	2.25E-16	0.300044293	0.263	0.119	1.49E-11	C6_CD8_Tact
chr5-172283006-172284568	2.41E-16	0.2802342	0.359	0.196	1.59E-11	C6_CD8_Tact
chr9-37784299-37785767	2.47E-16	0.290195982	0.321	0.165	1.63E-11	C6_CD8_Tact
chr2-101696869-101698392	2.56E-16	0.280937293	0.357	0.19	1.69E-11	C6_CD8_Tact
chr12-51269336-51271151	2.68E-16	0.305291249	0.237	0.102	1.78E-11	C6_CD8_Tact
chr12-123388261-123389996	2.69E-16	0.272229771	0.42	0.235	1.78E-11	C6_CD8_Tact
chr3-49357145-49359106	2.79E-16	0.295665371	0.295	0.148	1.85E-11	C6_CD8_Tact
chr16-30394871-30395903	2.88E-16	0.311998199	0.193	0.075	1.91E-11	C6_CD8_Tact
chr1-154999143-154999912	2.95E-16	0.306143634	0.184	0.072	1.95E-11	C6_CD8_Tact
chr19-40609530-40610705	3.00E-16	0.273465212	0.377	0.209	1.99E-11	C6_CD8_Tact
chr22-41412717-41414533	3.04E-16	0.250944033	0.487	0.304	2.01E-11	C6_CD8_Tact
chr1-244460889-244461855	3.08E-16	0.301568275	0.21	0.091	2.04E-11	C6_CD8_Tact
chr17-28710731-28712526	3.08E-16	0.267008434	0.421	0.248	2.04E-11	C6_CD8_Tact
chr15-78130668-78131836	3.36E-16	0.302367813	0.247	0.112	2.23E-11	C6_CD8_Tact
chr1-226870121-226871098	3.36E-16	0.30488436	0.153	0.056	2.23E-11	C6_CD8_Tact
chr1-110673499-110675111	3.67E-16	0.286897785	0.313	0.155	2.43E-11	C6_CD8_Tact
chr11-65418746-65419322	3.91E-16	0.291829902	0.296	0.147	2.59E-11	C6_CD8_Tact
chr2-200509120-200510412	4.31E-16	0.276470855	0.37	0.203	2.86E-11	C6_CD8_Tact
chr17-7351097-7352243	4.53E-16	0.287546419	0.323	0.164	3.00E-11	C6_CD8_Tact
chr12-51391065-51391943	4.89E-16	0.303148902	0.226	0.099	3.24E-11	C6_CD8_Tact
chr1-145978888-145980525	4.96E-16	0.277839618	0.351	0.192	3.28E-11	C6_CD8_Tact
chr13-44141327-44142627	4.96E-16	0.267577104	0.395	0.221	3.29E-11	C6_CD8_Tact
chr16-67968030-67969395	5.17E-16	0.271887782	0.394	0.211	3.42E-11	C6_CD8_Tact
chr19-47239311-47240012	5.25E-16	0.304666021	0.184	0.069	3.48E-11	C6_CD8_Tact
chr9-93575708-93577359	5.30E-16	0.301180037	0.246	0.117	3.51E-11	C6_CD8_Tact
chr9-137027768-137029607	5.32E-16	0.269765054	0.4	0.231	3.53E-11	C6_CD8_Tact
chr7-87151791-87153598	5.39E-16	0.251077914	0.468	0.284	3.57E-11	C6_CD8_Tact

chr22-31943733-31945717	5.80E-16	0.272648847	0.401	0.229	3.84E-11	C6_CD8_Tact
chr6-148746057-148748592	5.80E-16	0.28305895	0.308	0.162	3.84E-11	C6_CD8_Tact
chr11-33038838-33040723	5.90E-16	0.256540703	0.44	0.264	3.91E-11	C6_CD8_Tact
chr2-9842844-9844222	5.96E-16	0.275687285	0.379	0.204	3.94E-11	C6_CD8_Tact
chr22-31080671-31081454	6.49E-16	0.283731223	0.334	0.168	4.30E-11	C6_CD8_Tact
chr4-110195939-110198156	6.65E-16	0.287828008	0.264	0.13	4.40E-11	C6_CD8_Tact
chr7-100583287-100584031	6.71E-16	0.303621166	0.2	0.08	4.44E-11	C6_CD8_Tact
chr2-174395213-174396277	6.84E-16	0.29495066	0.264	0.121	4.53E-11	C6_CD8_Tact
chr4-141635820-141637210	6.93E-16	0.297206575	0.193	0.082	4.59E-11	C6_CD8_Tact
chr8-22088651-22090238	7.50E-16	0.293892308	0.28	0.132	4.97E-11	C6_CD8_Tact
chr1-113389521-113391431	7.92E-16	0.272623885	0.363	0.199	5.25E-11	C6_CD8_Tact
chr20-48920890-48922409	8.01E-16	0.272004549	0.387	0.213	5.30E-11	C6_CD8_Tact
chr17-62477720-62479471	8.09E-16	0.271208497	0.387	0.212	5.36E-11	C6_CD8_Tact
chr10-6144253-6145273	8.44E-16	0.267910793	0.4	0.231	5.59E-11	C6_CD8_Tact
chr3-57227173-57228725	8.74E-16	0.284590067	0.309	0.158	5.79E-11	C6_CD8_Tact
chr3-49017734-49018710	8.82E-16	0.297402359	0.247	0.116	5.84E-11	C6_CD8_Tact
chr16-84647951-84649451	8.82E-16	0.284271859	0.314	0.163	5.84E-11	C6_CD8_Tact
chr1-166838970-166840309	9.67E-16	0.266453489	0.399	0.232	6.41E-11	C6_CD8_Tact
chr1-228212713-228214191	1.08E-15	0.268274875	0.374	0.212	7.14E-11	C6_CD8_Tact
chr22-21657108-21658351	1.08E-15	0.285828824	0.294	0.157	7.17E-11	C6_CD8_Tact
chr9-21993782-21995959	1.29E-15	0.252965892	0.446	0.267	8.51E-11	C6_CD8_Tact
chr15-90200751-90201846	1.36E-15	0.298151632	0.223	0.096	9.02E-11	C6_CD8_Tact
chr14-75247053-75247940	1.39E-15	0.285072625	0.304	0.146	9.24E-11	C6_CD8_Tact
chr3-196286779-196288272	1.51E-15	0.289553126	0.282	0.147	9.98E-11	C6_CD8_Tact
chr5-176542931-176544183	1.68E-15	0.274349659	0.354	0.192	1.11E-10	C6_CD8_Tact
chr2-231613398-231614526	1.72E-15	0.30343044	0.191	0.076	1.14E-10	C6_CD8_Tact
chr20-63978369-63979000	1.84E-15	0.30327291	0.172	0.062	1.22E-10	C6_CD8_Tact

chr3-33096044-33097562	1.86E-15	0.287049396	0.274	0.139	1.23E-10	C6_CD8_Tact
chr2-53969976-53971409	2.02E-15	0.267300899	0.379	0.22	1.34E-10	C6_CD8_Tact
chr1-65066469-65068293	2.04E-15	0.268669365	0.366	0.206	1.35E-10	C6_CD8_Tact
chr3-52533164-52534067	2.05E-15	0.30060874	0.214	0.089	1.36E-10	C6_CD8_Tact
chr18-75207987-75209791	2.07E-15	0.264366552	0.385	0.225	1.37E-10	C6_CD8_Tact
chr22-45240129-45241609	2.10E-15	0.259782777	0.432	0.242	1.39E-10	C6_CD8_Tact
chr5-55172823-55173711	2.11E-15	0.296880179	0.198	0.078	1.40E-10	C6_CD8_Tact
chr12-111429968-111431098	2.14E-15	0.296411457	0.218	0.093	1.42E-10	C6_CD8_Tact
chr17-82228186-82229194	2.35E-15	0.289994048	0.284	0.14	1.56E-10	C6_CD8_Tact
chr7-140672263-140673783	2.44E-15	0.281013419	0.316	0.162	1.62E-10	C6_CD8_Tact
chr10-132396120-132398011	2.47E-15	0.274998004	0.37	0.198	1.63E-10	C6_CD8_Tact
chr3-113018559-113019947	2.52E-15	0.254847733	0.408	0.243	1.67E-10	C6_CD8_Tact
chr12-112937834-112938693	2.58E-15	0.297437603	0.183	0.072	1.71E-10	C6_CD8_Tact
chr14-91397046-91397830	2.72E-15	0.297952578	0.169	0.066	1.80E-10	C6_CD8_Tact
chr11-2444121-2445633	2.82E-15	0.298296871	0.212	0.093	1.87E-10	C6_CD8_Tact
chr15-52567968-52569459	2.82E-15	0.273672261	0.333	0.182	1.87E-10	C6_CD8_Tact
chr11-111765381-111766834	2.98E-15	0.272731541	0.368	0.198	1.97E-10	C6_CD8_Tact
chr11-65574064-65575126	3.07E-15	0.252054022	0.447	0.27	2.03E-10	C6_CD8_Tact
chr17-50055067-50056740	3.18E-15	0.291721234	0.265	0.129	2.11E-10	C6_CD8_Tact
chr7-66114059-66115608	3.32E-15	0.259138887	0.408	0.238	2.20E-10	C6_CD8_Tact
chr7-129824883-129826114	3.43E-15	0.276969278	0.322	0.171	2.27E-10	C6_CD8_Tact
chr16-15948749-15950636	3.60E-15	0.272493578	0.359	0.202	2.38E-10	C6_CD8_Tact
chr12-104456423-104458157	3.60E-15	0.266210027	0.379	0.215	2.39E-10	C6_CD8_Tact
chr11-288561-289553	3.64E-15	0.296305595	0.222	0.096	2.41E-10	C6_CD8_Tact
chr1-84995970-84997842	3.64E-15	0.270724405	0.322	0.173	2.41E-10	C6_CD8_Tact
chr8-89757000-89758344	3.65E-15	0.292210212	0.197	0.086	2.42E-10	C6_CD8_Tact
chr3-51966947-51968297	3.71E-15	0.298481856	0.203	0.081	2.45E-10	C6_CD8_Tact

chr9-127568320-127570279	3.87E-15	0.257170378	0.433	0.248	2.56E-10	C6_CD8_Tact
chr6-157980900-157982444	3.96E-15	0.255929074	0.406	0.239	2.62E-10	C6_CD8_Tact
chr19-29664885-29666172	4.04E-15	0.276171604	0.351	0.186	2.67E-10	C6_CD8_Tact
chr2-85611350-85612277	4.13E-15	0.29588685	0.26	0.118	2.74E-10	C6_CD8_Tact
chr16-30185375-30186290	4.26E-15	0.262149937	0.379	0.217	2.82E-10	C6_CD8_Tact
chr12-53046310-53047921	4.42E-15	0.25264244	0.438	0.266	2.93E-10	C6_CD8_Tact
chr8-37898628-37899855	4.44E-15	0.252972233	0.44	0.257	2.94E-10	C6_CD8_Tact
chr18-54268462-54270540	4.46E-15	0.251632903	0.43	0.255	2.95E-10	C6_CD8_Tact
chr11-90222051-90223528	4.77E-15	0.262216842	0.357	0.193	3.16E-10	C6_CD8_Tact
chr2-203933790-203934551	5.31E-15	0.288695289	0.115	0.034	3.52E-10	C6_CD8_Tact
chr11-62545204-62546912	5.38E-15	0.274103995	0.339	0.188	3.56E-10	C6_CD8_Tact
chr19-39030980-39032179	5.39E-15	0.291297542	0.228	0.103	3.57E-10	C6_CD8_Tact
chr17-47703115-47703807	5.45E-15	0.295218376	0.141	0.049	3.61E-10	C6_CD8_Tact
chrX-48890686-48892004	5.79E-15	0.285648038	0.279	0.14	3.83E-10	C6_CD8_Tact
chr12-111597488-111600018	6.04E-15	0.257421651	0.411	0.241	4.00E-10	C6_CD8_Tact
chr9-131276411-131278990	6.45E-15	0.268004734	0.377	0.212	4.27E-10	C6_CD8_Tact
chr6-35451745-35452796	6.52E-15	0.275206949	0.31	0.163	4.32E-10	C6_CD8_Tact
chr2-24919063-24920829	6.91E-15	0.266847071	0.368	0.207	4.58E-10	C6_CD8_Tact
chr19-1265826-1267154	7.09E-15	0.252028298	0.449	0.261	4.69E-10	C6_CD8_Tact
chr6-145734604-145736153	7.18E-15	0.284586053	0.235	0.109	4.75E-10	C6_CD8_Tact
chr6-108560422-108561005	7.46E-15	0.288874168	0.222	0.099	4.94E-10	C6_CD8_Tact
chr7-13989740-13991801	8.49E-15	0.285248521	0.195	0.081	5.62E-10	C6_CD8_Tact
chr12-53324144-53325219	1.01E-14	0.261780399	0.378	0.216	6.71E-10	C6_CD8_Tact
chr11-10750642-10752061	1.05E-14	0.26626053	0.354	0.192	6.95E-10	C6_CD8_Tact
chr9-136799425-136800618	1.07E-14	0.275619097	0.304	0.157	7.06E-10	C6_CD8_Tact
chr3-122793315-122795433	1.07E-14	0.250612075	0.43	0.257	7.08E-10	C6_CD8_Tact
chr1-161038262-161039427	1.09E-14	0.263937754	0.363	0.206	7.25E-10	C6_CD8_Tact

chr22-42269346-42270914	1.10E-14	0.260795161	0.384	0.224	7.27E-10	C6_CD8_Tact
chr14-77032747-77034247	1.11E-14	0.264368701	0.362	0.204	7.34E-10	C6_CD8_Tact
chr11-76860250-76861423	1.13E-14	0.283590986	0.237	0.114	7.48E-10	C6_CD8_Tact
chr8-22565061-22566440	1.26E-14	0.29289594	0.166	0.063	8.37E-10	C6_CD8_Tact
chr7-76379505-76380420	1.39E-14	0.273612322	0.314	0.16	9.22E-10	C6_CD8_Tact
chr1-156746533-156747458	1.49E-14	0.291609698	0.184	0.075	9.86E-10	C6_CD8_Tact
chr15-63276829-63277958	2.05E-14	0.270047275	0.334	0.176	1.36E-09	C6_CD8_Tact
chr16-4768185-4769122	2.10E-14	0.289546396	0.193	0.083	1.39E-09	C6_CD8_Tact
chr12-96906335-96908455	2.15E-14	0.266059205	0.326	0.18	1.43E-09	C6_CD8_Tact
chr1-16612595-16613967	2.20E-14	0.280079406	0.309	0.155	1.46E-09	C6_CD8_Tact
chr1-167454829-167456313	2.22E-14	0.270047846	0.311	0.165	1.47E-09	C6_CD8_Tact
chr6-43687136-43688107	2.28E-14	0.292973221	0.196	0.08	1.51E-09	C6_CD8_Tact
chr22-50529937-50530420	2.30E-14	0.291985631	0.196	0.081	1.52E-09	C6_CD8_Tact
chr9-93057735-93059337	2.36E-14	0.276475172	0.267	0.134	1.56E-09	C6_CD8_Tact
chr16-85556610-85557340	2.36E-14	0.290268177	0.215	0.092	1.56E-09	C6_CD8_Tact
chr22-39501456-39502723	2.45E-14	0.274350924	0.296	0.156	1.62E-09	C6_CD8_Tact
chr6-33577967-33578991	2.45E-14	0.290895016	0.156	0.059	1.62E-09	C6_CD8_Tact
chr1-227728015-227728992	2.48E-14	0.288864749	0.186	0.076	1.64E-09	C6_CD8_Tact
chr4-121221404-121222078	2.53E-14	0.26355468	0.074	0.018	1.68E-09	C6_CD8_Tact
chr7-151086035-151087614	2.89E-14	0.288030869	0.241	0.112	1.92E-09	C6_CD8_Tact
chr19-3358962-3360781	2.91E-14	0.269371282	0.332	0.178	1.93E-09	C6_CD8_Tact
chr11-67443403-67444382	2.95E-14	0.27980404	0.273	0.137	1.95E-09	C6_CD8_Tact
chr6-16700313-16701163	2.96E-14	0.285136233	0.199	0.086	1.96E-09	C6_CD8_Tact
chrX-129930965-129932751	3.08E-14	0.25780158	0.379	0.209	2.04E-09	C6_CD8_Tact
chr3-43015524-43016642	3.16E-14	0.278901429	0.154	0.061	2.10E-09	C6_CD8_Tact
chr6-25278635-25279938	3.18E-14	0.282935406	0.224	0.103	2.10E-09	C6_CD8_Tact
chr16-2964828-2965262	3.21E-14	0.29081956	0.147	0.055	2.12E-09	C6_CD8_Tact

chr16-11586065-11587498	3.37E-14	0.282726302	0.214	0.1	2.23E-09	C6_CD8_Tact
chr17-63445474-63447526	3.42E-14	0.276028472	0.289	0.148	2.26E-09	C6_CD8_Tact
chr4-1683345-1684753	3.45E-14	0.258871171	0.375	0.213	2.28E-09	C6_CD8_Tact
chr15-74432984-74434201	3.62E-14	0.2704327	0.309	0.159	2.40E-09	C6_CD8_Tact
chr16-19717536-19718622	3.80E-14	0.273252707	0.3	0.155	2.52E-09	C6_CD8_Tact
chr6-33425330-33426181	3.94E-14	0.263725074	0.345	0.188	2.61E-09	C6_CD8_Tact
chr14-34461547-34462948	3.96E-14	0.250511558	0.405	0.241	2.62E-09	C6_CD8_Tact
chr9-136107033-136107665	4.09E-14	0.284863162	0.126	0.043	2.71E-09	C6_CD8_Tact
chr20-57389260-57390238	4.09E-14	0.288695252	0.195	0.084	2.71E-09	C6_CD8_Tact
chr6-158813911-158814247	4.12E-14	0.285217136	0.119	0.038	2.73E-09	C6_CD8_Tact
chr12-118060245-118062137	4.13E-14	0.284700109	0.234	0.107	2.74E-09	C6_CD8_Tact
chr20-33673705-33674716	4.32E-14	0.287330873	0.142	0.051	2.86E-09	C6_CD8_Tact
chr19-14432985-14434368	4.46E-14	0.28499807	0.232	0.107	2.96E-09	C6_CD8_Tact
chr17-1487928-1488491	4.49E-14	0.281297384	0.245	0.119	2.97E-09	C6_CD8_Tact
chr17-8162672-8164627	4.70E-14	0.250605924	0.419	0.244	3.11E-09	C6_CD8_Tact
chr2-202375051-202376933	4.83E-14	0.25344913	0.397	0.228	3.20E-09	C6_CD8_Tact
chr17-4986322-4988178	5.04E-14	0.2588453	0.379	0.217	3.34E-09	C6_CD8_Tact
chr1-2554931-2557236	5.11E-14	0.256920199	0.374	0.22	3.38E-09	C6_CD8_Tact
chr6-149647789-149648971	5.24E-14	0.265734046	0.323	0.179	3.47E-09	C6_CD8_Tact
chr11-67403634-67404246	5.32E-14	0.26777562	0.308	0.163	3.52E-09	C6_CD8_Tact
chr5-172114826-172115850	5.53E-14	0.280355777	0.212	0.097	3.66E-09	C6_CD8_Tact
chr10-43228575-43229999	5.56E-14	0.275944092	0.254	0.134	3.68E-09	C6_CD8_Tact
chr19-8208569-8210120	5.65E-14	0.251951008	0.385	0.23	3.74E-09	C6_CD8_Tact
chr20-49712723-49714238	5.66E-14	0.279331728	0.285	0.143	3.75E-09	C6_CD8_Tact
chr11-67485934-67486279	6.02E-14	0.277719386	0.112	0.034	3.99E-09	C6_CD8_Tact
chr2-112541462-112543312	6.07E-14	0.260646778	0.364	0.206	4.02E-09	C6_CD8_Tact
chr1-92298266-92299365	6.16E-14	0.262992338	0.32	0.175	4.08E-09	C6_CD8_Tact

chr9-35161379-35162767	6.17E-14	0.284718789	0.174	0.073	4.09E-09	C6_CD8_Tact
chr16-30354557-30355953	6.29E-14	0.262355409	0.358	0.193	4.16E-09	C6_CD8_Tact
chr1-41661329-41663076	6.30E-14	0.256503266	0.372	0.215	4.18E-09	C6_CD8_Tact
chr1-31154538-31155694	6.38E-14	0.270806687	0.294	0.152	4.23E-09	C6_CD8_Tact
chr12-55742617-55744007	6.56E-14	0.270008498	0.303	0.161	4.35E-09	C6_CD8_Tact
chr1-211326296-211327762	6.63E-14	0.284345974	0.208	0.096	4.39E-09	C6_CD8_Tact
chr12-6773803-6775186	6.81E-14	0.277450264	0.228	0.108	4.51E-09	C6_CD8_Tact
chr1-145962459-145962819	6.87E-14	0.286261201	0.147	0.052	4.55E-09	C6_CD8_Tact
chr9-133991665-133993655	7.09E-14	0.273745068	0.285	0.147	4.70E-09	C6_CD8_Tact
chr2-241636283-241638290	7.10E-14	0.259459353	0.381	0.22	4.70E-09	C6_CD8_Tact
chr6-44255883-44257787	7.56E-14	0.270409984	0.307	0.162	5.01E-09	C6_CD8_Tact
chr22-31629550-31631021	7.61E-14	0.251939793	0.395	0.231	5.04E-09	C6_CD8_Tact
chr13-30421082-30422809	7.64E-14	0.264997746	0.311	0.17	5.06E-09	C6_CD8_Tact
chr3-129605269-129607205	7.70E-14	0.266840535	0.317	0.169	5.10E-09	C6_CD8_Tact
chr3-197002403-197003709	8.00E-14	0.266110506	0.331	0.181	5.30E-09	C6_CD8_Tact
chr3-119240073-119241103	8.40E-14	0.282190398	0.203	0.094	5.56E-09	C6_CD8_Tact
chr17-47693383-47695351	8.42E-14	0.27123387	0.314	0.161	5.58E-09	C6_CD8_Tact
chr5-132464242-132464683	8.88E-14	0.263484861	0.082	0.022	5.88E-09	C6_CD8_Tact
chr15-78564901-78566307	9.31E-14	0.283965268	0.184	0.075	6.16E-09	C6_CD8_Tact
chr16-2603108-2603937	9.32E-14	0.28834564	0.143	0.05	6.17E-09	C6_CD8_Tact
chr15-40941105-40942544	9.81E-14	0.281970291	0.234	0.108	6.50E-09	C6_CD8_Tact
chr3-32501820-32503160	1.03E-13	0.260973762	0.338	0.19	6.83E-09	C6_CD8_Tact
chr22-38176245-38176711	1.07E-13	0.279988836	0.123	0.039	7.11E-09	C6_CD8_Tact
chr11-554685-555660	1.12E-13	0.275171032	0.266	0.133	7.43E-09	C6_CD8_Tact
chr17-75393404-75394247	1.17E-13	0.274646034	0.267	0.134	7.74E-09	C6_CD8_Tact
chr9-137076889-137078210	1.18E-13	0.283243885	0.193	0.077	7.84E-09	C6_CD8_Tact
chr16-66879902-66881127	1.23E-13	0.264435504	0.315	0.168	8.16E-09	C6_CD8_Tact

chr14-105180499-105181804	1.31E-13	0.266943568	0.314	0.162	8.67E-09	C6_CD8_Tact
chr2-11343508-11345322	1.35E-13	0.27149664	0.266	0.138	8.96E-09	C6_CD8_Tact
chr6-31729068-31730432	1.44E-13	0.261596488	0.356	0.195	9.56E-09	C6_CD8_Tact
chr10-3066673-3069173	1.44E-13	0.258815114	0.325	0.186	9.56E-09	C6_CD8_Tact
chr12-122078079-122079180	1.48E-13	0.259371293	0.353	0.195	9.80E-09	C6_CD8_Tact
chr2-219597271-219598339	1.54E-13	0.260445566	0.334	0.185	1.02E-08	C6_CD8_Tact
chr12-48350161-48351374	1.55E-13	0.250424617	0.374	0.209	1.03E-08	C6_CD8_Tact
chr7-87933506-87934898	1.60E-13	0.264859886	0.267	0.134	1.06E-08	C6_CD8_Tact
chr9-35079115-35080248	1.66E-13	0.268185038	0.308	0.159	1.10E-08	C6_CD8_Tact
chr13-99075315-99076269	1.68E-13	0.278938397	0.15	0.059	1.11E-08	C6_CD8_Tact
chr12-52036453-52037479	1.73E-13	0.253904476	0.363	0.202	1.15E-08	C6_CD8_Tact
chr15-90001169-90002683	1.75E-13	0.271607324	0.279	0.145	1.16E-08	C6_CD8_Tact
chr8-125090929-125092530	1.77E-13	0.250641635	0.375	0.224	1.17E-08	C6_CD8_Tact
chr7-105387739-105389417	1.85E-13	0.277486896	0.237	0.115	1.23E-08	C6_CD8_Tact
chr11-10302558-10303091	1.85E-13	0.279515072	0.144	0.056	1.23E-08	C6_CD8_Tact
chr6-33633218-33634105	1.92E-13	0.281115409	0.218	0.097	1.27E-08	C6_CD8_Tact
chr19-49113709-49114723	2.03E-13	0.251681753	0.391	0.221	1.34E-08	C6_CD8_Tact
chr5-138752457-138754386	2.03E-13	0.276202616	0.229	0.11	1.34E-08	C6_CD8_Tact
chr16-31487250-31488471	2.06E-13	0.257355788	0.329	0.184	1.36E-08	C6_CD8_Tact
chr19-39409471-39410304	2.16E-13	0.254874263	0.363	0.196	1.43E-08	C6_CD8_Tact
chr18-8704402-8705696	2.21E-13	0.259704973	0.325	0.178	1.47E-08	C6_CD8_Tact
chr1-91884467-91886454	2.32E-13	0.259341693	0.321	0.175	1.53E-08	C6_CD8_Tact
chr17-36210420-36211235	2.32E-13	0.275220923	0.171	0.074	1.54E-08	C6_CD8_Tact
chr1-2198970-2199576	2.38E-13	0.273833612	0.215	0.095	1.58E-08	C6_CD8_Tact
chr2-96163559-96165058	2.43E-13	0.257975742	0.29	0.158	1.61E-08	C6_CD8_Tact
chr9-89497883-89498566	2.61E-13	0.280224962	0.196	0.083	1.73E-08	C6_CD8_Tact
chr4-165206714-165208350	2.64E-13	0.268874386	0.252	0.13	1.75E-08	C6_CD8_Tact

chr14-67673810-67675171	2.74E-13	0.259833132	0.338	0.187	1.81E-08	C6_CD8_Tact
chr8-143334277-143335241	2.88E-13	0.277683641	0.234	0.113	1.91E-08	C6_CD8_Tact
chr12-642512-644005	2.91E-13	0.262172713	0.314	0.172	1.93E-08	C6_CD8_Tact
chr1-226109605-226110914	2.99E-13	0.270918111	0.237	0.117	1.98E-08	C6_CD8_Tact
chr4-81490679-81491064	3.00E-13	0.261367779	0.086	0.023	1.99E-08	C6_CD8_Tact
chr11-65719854-65720940	3.17E-13	0.270035092	0.277	0.14	2.10E-08	C6_CD8_Tact
chr6-30883840-30885037	3.44E-13	0.280283293	0.161	0.062	2.28E-08	C6_CD8_Tact
chr1-155261430-155262637	3.73E-13	0.252947468	0.353	0.203	2.47E-08	C6_CD8_Tact
chr12-94147811-94149784	3.94E-13	0.262591977	0.278	0.151	2.61E-08	C6_CD8_Tact
chr10-109922654-109923757	4.00E-13	0.256163421	0.327	0.179	2.65E-08	C6_CD8_Tact
chr22-26511654-26512784	4.18E-13	0.27881037	0.19	0.08	2.77E-08	C6_CD8_Tact
chr17-38703898-38704149	4.23E-13	0.281153125	0.149	0.057	2.80E-08	C6_CD8_Tact
chr7-149873193-149874354	4.57E-13	0.259488862	0.321	0.175	3.02E-08	C6_CD8_Tact
chr6-33426602-33427294	4.84E-13	0.272183097	0.229	0.106	3.21E-08	C6_CD8_Tact
chr1-11654182-11655112	4.86E-13	0.272382848	0.237	0.115	3.22E-08	C6_CD8_Tact
chr2-218398019-218398726	4.93E-13	0.269476608	0.221	0.098	3.26E-08	C6_CD8_Tact
chr12-4273558-4273828	4.98E-13	0.274214711	0.186	0.082	3.30E-08	C6_CD8_Tact
chr1-41366003-41366738	5.17E-13	0.271964363	0.121	0.041	3.42E-08	C6_CD8_Tact
chr3-38137886-38139147	5.20E-13	0.256769319	0.326	0.178	3.44E-08	C6_CD8_Tact
chr1-153963160-153963849	5.21E-13	0.260851907	0.327	0.174	3.45E-08	C6_CD8_Tact
chr2-223836861-223838108	5.43E-13	0.265178633	0.261	0.134	3.60E-08	C6_CD8_Tact
chr17-81345329-81346548	5.66E-13	0.266460751	0.292	0.15	3.75E-08	C6_CD8_Tact
chr1-2314402-2315380	5.67E-13	0.267643184	0.24	0.121	3.76E-08	C6_CD8_Tact
chr15-43133045-43134464	5.68E-13	0.272593506	0.255	0.125	3.76E-08	C6_CD8_Tact
chr5-177300210-177300541	5.87E-13	0.272114908	0.112	0.037	3.89E-08	C6_CD8_Tact
chr16-25106519-25107336	6.25E-13	0.276537531	0.168	0.072	4.14E-08	C6_CD8_Tact
chr11-44949131-44951335	6.28E-13	0.26646498	0.268	0.14	4.16E-08	C6_CD8_Tact

chr17-18257984-18258852	6.49E-13	0.26942037	0.255	0.13	4.29E-08	C6_CD8_Tact
chr14-104795045-104795909	6.51E-13	0.277976184	0.221	0.102	4.31E-08	C6_CD8_Tact
chr1-154328273-154328906	6.66E-13	0.260928579	0.303	0.167	4.41E-08	C6_CD8_Tact
chr7-26156316-26156663	6.74E-13	0.272267034	0.131	0.046	4.46E-08	C6_CD8_Tact
chr2-230865592-230867477	6.96E-13	0.268821639	0.233	0.118	4.61E-08	C6_CD8_Tact
chr15-40906210-40907676	7.07E-13	0.26797286	0.228	0.109	4.68E-08	C6_CD8_Tact
chr4-6574480-6575631	7.27E-13	0.263360194	0.272	0.141	4.82E-08	C6_CD8_Tact
chr9-129641182-129642574	7.36E-13	0.255193106	0.346	0.195	4.87E-08	C6_CD8_Tact
chrX-129980041-129980365	7.42E-13	0.255164236	0.08	0.02	4.92E-08	C6_CD8_Tact
chrX-21940251-21941596	7.58E-13	0.270935279	0.177	0.075	5.02E-08	C6_CD8_Tact
chr12-53231784-53233177	7.79E-13	0.2631232	0.286	0.153	5.16E-08	C6_CD8_Tact
chr19-56566878-56568150	8.19E-13	0.271047805	0.228	0.111	5.43E-08	C6_CD8_Tact
chr17-76382870-76385331	8.22E-13	0.250895426	0.334	0.195	5.44E-08	C6_CD8_Tact
chr2-190319033-190320159	8.33E-13	0.263175313	0.277	0.145	5.52E-08	C6_CD8_Tact
chr5-172833503-172834641	8.45E-13	0.273623167	0.209	0.091	5.60E-08	C6_CD8_Tact
chr19-1237771-1238253	8.59E-13	0.269359038	0.112	0.036	5.69E-08	C6_CD8_Tact
chr17-76239554-76241153	8.78E-13	0.26138095	0.292	0.153	5.81E-08	C6_CD8_Tact
chr21-42513167-42514643	9.29E-13	0.277829929	0.184	0.08	6.15E-08	C6_CD8_Tact
chrX-135421334-135422448	9.52E-13	0.256362109	0.274	0.141	6.31E-08	C6_CD8_Tact
chr11-2384117-2384734	9.67E-13	0.270979223	0.153	0.062	6.40E-08	C6_CD8_Tact
chrX-38803720-38804733	9.77E-13	0.268038018	0.125	0.046	6.47E-08	C6_CD8_Tact
chr2-230411165-230412154	9.78E-13	0.270793934	0.246	0.118	6.48E-08	C6_CD8_Tact
chr21-34072783-34074254	1.00E-12	0.250014751	0.366	0.209	6.65E-08	C6_CD8_Tact
chr22-37632797-37633982	1.03E-12	0.268856981	0.204	0.094	6.82E-08	C6_CD8_Tact
chr9-127903541-127903792	1.04E-12	0.250179272	0.074	0.018	6.86E-08	C6_CD8_Tact
chr8-60678134-60680589	1.07E-12	0.250893967	0.353	0.197	7.10E-08	C6_CD8_Tact
chr22-39097035-39097399	1.08E-12	0.273865879	0.137	0.051	7.17E-08	C6_CD8_Tact

chr20-36950173-36951991	1.09E-12	0.257770165	0.31	0.172	7.24E-08	C6_CD8_Tact
chr2-27123183-27124022	1.12E-12	0.274605202	0.165	0.063	7.44E-08	C6_CD8_Tact
chr11-72781306-72782293	1.14E-12	0.273674402	0.187	0.08	7.53E-08	C6_CD8_Tact
chr1-235366909-235367858	1.22E-12	0.266941833	0.243	0.116	8.11E-08	C6_CD8_Tact
chr6-2841129-2842180	1.23E-12	0.264437079	0.257	0.131	8.16E-08	C6_CD8_Tact
chr2-9003021-9004414	1.30E-12	0.266565834	0.232	0.111	8.63E-08	C6_CD8_Tact
chr2-55419188-55420475	1.33E-12	0.250505569	0.329	0.183	8.84E-08	C6_CD8_Tact
chr7-143361703-143363014	1.39E-12	0.273849236	0.184	0.079	9.24E-08	C6_CD8_Tact
chr12-101876691-101878254	1.54E-12	0.258914252	0.279	0.149	1.02E-07	C6_CD8_Tact
chr6-35496459-35498137	1.55E-12	0.273742199	0.212	0.102	1.02E-07	C6_CD8_Tact
chr10-26696835-26698080	1.60E-12	0.269351044	0.218	0.106	1.06E-07	C6_CD8_Tact
chr6-30690622-30691167	1.73E-12	0.272276667	0.189	0.083	1.14E-07	C6_CD8_Tact
chr12-3076664-3077995	1.78E-12	0.267309617	0.232	0.116	1.18E-07	C6_CD8_Tact
chr8-22164346-22165486	1.86E-12	0.260019271	0.288	0.149	1.23E-07	C6_CD8_Tact
chr11-64851077-64851535	1.86E-12	0.267621499	0.116	0.041	1.23E-07	C6_CD8_Tact
chr8-96644633-96645901	1.89E-12	0.260261038	0.223	0.106	1.25E-07	C6_CD8_Tact
chr14-105168380-105169341	1.93E-12	0.266575246	0.254	0.127	1.28E-07	C6_CD8_Tact
chr5-141849044-141850867	2.03E-12	0.267602776	0.217	0.11	1.35E-07	C6_CD8_Tact
chr19-14368052-14368460	2.12E-12	0.27015102	0.179	0.084	1.40E-07	C6_CD8_Tact
chr1-210328520-210329457	2.13E-12	0.265891666	0.15	0.061	1.41E-07	C6_CD8_Tact
chr19-41882578-41883253	2.14E-12	0.26853157	0.147	0.063	1.42E-07	C6_CD8_Tact
chr17-40062446-40063731	2.19E-12	0.260710489	0.279	0.15	1.45E-07	C6_CD8_Tact
chr10-114303987-114305345	2.19E-12	0.264916052	0.167	0.075	1.45E-07	C6_CD8_Tact
chr11-8910400-8911820	2.22E-12	0.262270558	0.221	0.111	1.47E-07	C6_CD8_Tact
chr22-41444135-41444739	2.28E-12	0.266241357	0.228	0.112	1.51E-07	C6_CD8_Tact
chr22-37848804-37849797	2.33E-12	0.257650746	0.292	0.153	1.54E-07	C6_CD8_Tact
chr4-88522854-88524048	2.34E-12	0.268284495	0.183	0.08	1.55E-07	C6_CD8_Tact

chr12-57590494-57591775	2.37E-12	0.26474967	0.251	0.124	1.57E-07	C6_CD8_Tact
chr1-23558921-23559757	2.39E-12	0.257872855	0.295	0.156	1.58E-07	C6_CD8_Tact
chr7-5531949-5532148	2.42E-12	0.26518235	0.122	0.044	1.60E-07	C6_CD8_Tact
chr3-187739332-187740236	2.47E-12	0.25794568	0.271	0.146	1.64E-07	C6_CD8_Tact
chr10-133393879-133394520	2.51E-12	0.250575369	0.326	0.186	1.66E-07	C6_CD8_Tact
chr11-10293478-10294405	2.52E-12	0.27102199	0.147	0.062	1.67E-07	C6_CD8_Tact
chr3-49929186-49930079	2.61E-12	0.265045934	0.249	0.124	1.73E-07	C6_CD8_Tact
chr1-232629425-232630954	2.62E-12	0.264135596	0.242	0.122	1.73E-07	C6_CD8_Tact
chr17-7293886-7295166	2.64E-12	0.269357625	0.193	0.089	1.75E-07	C6_CD8_Tact
chr5-131278120-131278571	2.65E-12	0.25847005	0.095	0.029	1.75E-07	C6_CD8_Tact
chr7-128405574-128406293	2.71E-12	0.268961252	0.211	0.099	1.80E-07	C6_CD8_Tact
chr1-2577382-2578687	2.82E-12	0.254136376	0.301	0.159	1.87E-07	C6_CD8_Tact
chr10-73865066-73866302	3.10E-12	0.261906277	0.119	0.041	2.05E-07	C6_CD8_Tact
chr9-35616702-35617418	3.16E-12	0.268885201	0.184	0.078	2.09E-07	C6_CD8_Tact
chr9-5449661-5450726	3.38E-12	0.264832007	0.229	0.112	2.24E-07	C6_CD8_Tact
chr17-49494834-49495506	3.40E-12	0.271009011	0.16	0.063	2.25E-07	C6_CD8_Tact
chr19-4831214-4831969	3.40E-12	0.267337683	0.211	0.095	2.25E-07	C6_CD8_Tact
chr6-131807380-131808450	3.54E-12	0.257341778	0.135	0.053	2.34E-07	C6_CD8_Tact
chr9-124852755-124853916	4.03E-12	0.254126683	0.292	0.158	2.67E-07	C6_CD8_Tact
chr11-65575480-65576126	4.12E-12	0.256530702	0.298	0.153	2.73E-07	C6_CD8_Tact
chr12-52005844-52007931	4.63E-12	0.25243865	0.303	0.167	3.07E-07	C6_CD8_Tact
chr3-100334060-100335293	4.66E-12	0.253740532	0.288	0.157	3.08E-07	C6_CD8_Tact
chr12-112130358-112130926	4.98E-12	0.266016019	0.153	0.059	3.30E-07	C6_CD8_Tact
chr16-569378-570593	5.09E-12	0.260224035	0.247	0.13	3.37E-07	C6_CD8_Tact
chr9-127783043-127783460	5.12E-12	0.258394308	0.216	0.109	3.39E-07	C6_CD8_Tact
chr8-144786371-144787514	5.29E-12	0.251638251	0.308	0.175	3.50E-07	C6_CD8_Tact
chr1-62687308-62688696	5.31E-12	0.264912366	0.141	0.059	3.52E-07	C6_CD8_Tact

chr5-141650564-141651733	5.55E-12	0.253968803	0.279	0.15	3.67E-07	C6_CD8_Tact
chr8-144443765-144445052	5.76E-12	0.25833814	0.305	0.163	3.81E-07	C6_CD8_Tact
chr11-63812892-63814226	5.77E-12	0.263853335	0.252	0.121	3.82E-07	C6_CD8_Tact
chr11-18698079-18699442	5.85E-12	0.254208449	0.29	0.155	3.88E-07	C6_CD8_Tact
chr2-65435734-65436719	5.90E-12	0.267172868	0.132	0.049	3.90E-07	C6_CD8_Tact
chr7-150449686-150451737	5.96E-12	0.255677355	0.278	0.154	3.95E-07	C6_CD8_Tact
chr11-355081-356446	6.27E-12	0.26115405	0.233	0.115	4.15E-07	C6_CD8_Tact
chr13-95300727-95301981	6.31E-12	0.260451015	0.185	0.085	4.18E-07	C6_CD8_Tact
chr1-230867790-230869013	6.44E-12	0.259380381	0.253	0.128	4.27E-07	C6_CD8_Tact
chr4-37890442-37891920	6.66E-12	0.256547999	0.247	0.132	4.41E-07	C6_CD8_Tact
chr19-38263762-38264979	7.04E-12	0.262236348	0.206	0.094	4.66E-07	C6_CD8_Tact
chr8-11867196-11868456	7.18E-12	0.265485461	0.185	0.085	4.75E-07	C6_CD8_Tact
chr15-41493081-41494470	7.31E-12	0.257619383	0.268	0.139	4.84E-07	C6_CD8_Tact
chr10-101042386-101043272	7.36E-12	0.265844908	0.179	0.083	4.88E-07	C6_CD8_Tact
chr14-103520605-103522114	7.47E-12	0.259904826	0.259	0.137	4.95E-07	C6_CD8_Tact
chr16-56657125-56658052	7.58E-12	0.261874068	0.15	0.061	5.02E-07	C6_CD8_Tact
chr17-1724276-1725251	8.35E-12	0.255100428	0.294	0.158	5.53E-07	C6_CD8_Tact
chr17-43021162-43022517	8.79E-12	0.265721039	0.203	0.093	5.82E-07	C6_CD8_Tact
chr22-36028178-36029353	9.10E-12	0.26668284	0.163	0.067	6.02E-07	C6_CD8_Tact
chr6-73523299-73523931	9.57E-12	0.2554903	0.253	0.128	6.34E-07	C6_CD8_Tact
chr22-37905146-37907163	1.13E-11	0.259298606	0.248	0.126	7.46E-07	C6_CD8_Tact
chr2-96116234-96117691	1.19E-11	0.250768672	0.301	0.161	7.87E-07	C6_CD8_Tact
chr11-2301121-2302686	1.21E-11	0.251639931	0.272	0.147	8.04E-07	C6_CD8_Tact
chr14-23057190-23058337	1.22E-11	0.256851397	0.268	0.142	8.07E-07	C6_CD8_Tact
chr19-1027164-1028507	1.29E-11	0.256774575	0.254	0.131	8.57E-07	C6_CD8_Tact
chr11-65422098-65422470	1.42E-11	0.252254447	0.249	0.124	9.41E-07	C6_CD8_Tact
chr2-11129451-11130224	1.47E-11	0.2519718	0.241	0.125	9.76E-07	C6_CD8_Tact

chr10-101839699-101840979	1.49E-11	0.262803922	0.233	0.112	9.87E-07	C6_CD8_Tact
chr10-13115822-13116796	1.53E-11	0.260957651	0.149	0.062	1.01E-06	C6_CD8_Tact
chr2-68774382-68774988	1.68E-11	0.257946705	0.156	0.064	1.12E-06	C6_CD8_Tact
chr9-128072120-128072779	1.73E-11	0.259287104	0.121	0.042	1.15E-06	C6_CD8_Tact
chr3-114294917-114295242	1.74E-11	0.254257799	0.125	0.05	1.15E-06	C6_CD8_Tact
chr5-170275326-170275657	1.79E-11	0.259260908	0.154	0.062	1.19E-06	C6_CD8_Tact
chr2-39436653-39438222	1.81E-11	0.257777859	0.205	0.094	1.20E-06	C6_CD8_Tact
chr17-38706879-38707160	1.90E-11	0.252556242	0.093	0.029	1.26E-06	C6_CD8_Tact
chr18-31684350-31686064	2.05E-11	0.254427341	0.158	0.068	1.36E-06	C6_CD8_Tact
chr17-14068753-14070018	2.07E-11	0.2578501	0.242	0.122	1.37E-06	C6_CD8_Tact
chr17-15562979-15563886	2.09E-11	0.255453022	0.247	0.125	1.38E-06	C6_CD8_Tact
chr2-190343131-190344552	2.18E-11	0.255787642	0.163	0.071	1.45E-06	C6_CD8_Tact
chr17-7219666-7221173	2.42E-11	0.251399177	0.276	0.148	1.60E-06	C6_CD8_Tact
chr10-46282444-46283229	2.52E-11	0.254603106	0.143	0.059	1.67E-06	C6_CD8_Tact
chr22-17773640-17774627	2.55E-11	0.257517974	0.218	0.105	1.69E-06	C6_CD8_Tact
chr4-184534466-184534899	2.58E-11	0.252445725	0.099	0.035	1.71E-06	C6_CD8_Tact
chr9-38392247-38393083	2.61E-11	0.255035347	0.15	0.061	1.73E-06	C6_CD8_Tact
chr22-50269932-50271021	2.80E-11	0.251804105	0.284	0.146	1.85E-06	C6_CD8_Tact
chr11-67408492-67409504	2.84E-11	0.253304317	0.177	0.085	1.88E-06	C6_CD8_Tact
chr16-68079490-68080256	2.86E-11	0.254311968	0.146	0.062	1.89E-06	C6_CD8_Tact
chr17-45430874-45432158	2.98E-11	0.261640032	0.195	0.089	1.98E-06	C6_CD8_Tact
chr19-1068693-1069545	3.15E-11	0.253669955	0.223	0.113	2.09E-06	C6_CD8_Tact
chr19-45498125-45499625	3.18E-11	0.250815529	0.226	0.117	2.11E-06	C6_CD8_Tact
chr7-23596968-23598069	3.52E-11	0.252620431	0.224	0.111	2.33E-06	C6_CD8_Tact
chr2-174750764-174751377	3.55E-11	0.252606699	0.112	0.04	2.35E-06	C6_CD8_Tact
chr7-149771328-149772417	3.74E-11	0.257800501	0.226	0.107	2.48E-06	C6_CD8_Tact
chr11-19240373-19241781	3.96E-11	0.253033207	0.217	0.112	2.62E-06	C6_CD8_Tact

chr16-67013425-67014421	4.17E-11	0.256984363	0.212	0.097	2.76E-06	C6_CD8_Tact
chr2-55268514-55269492	4.24E-11	0.254186668	0.204	0.097	2.81E-06	C6_CD8_Tact
chr16-31476114-31477695	4.33E-11	0.250493432	0.226	0.113	2.87E-06	C6_CD8_Tact
chr14-104864380-104865968	4.84E-11	0.256660758	0.171	0.077	3.21E-06	C6_CD8_Tact
chr19-19661392-19662269	4.96E-11	0.250304465	0.205	0.098	3.28E-06	C6_CD8_Tact
chr17-1267468-1268654	5.61E-11	0.261164661	0.167	0.073	3.72E-06	C6_CD8_Tact
chr19-35901898-35902570	5.77E-11	0.253712623	0.171	0.075	3.82E-06	C6_CD8_Tact
chr19-44093851-44095497	5.80E-11	0.25018285	0.252	0.135	3.84E-06	C6_CD8_Tact
chr12-122980038-122981450	6.14E-11	0.256556129	0.209	0.102	4.07E-06	C6_CD8_Tact
chr7-55964014-55965216	6.73E-11	0.250770799	0.252	0.132	4.46E-06	C6_CD8_Tact
chr2-241217364-241218918	7.07E-11	0.254561958	0.137	0.055	4.68E-06	C6_CD8_Tact
chr1-25874816-25876153	7.10E-11	0.255993793	0.174	0.082	4.70E-06	C6_CD8_Tact
chr9-503890-505011	7.39E-11	0.251742935	0.227	0.11	4.89E-06	C6_CD8_Tact
chr20-3777452-3778316	7.70E-11	0.25225375	0.111	0.04	5.10E-06	C6_CD8_Tact
chr6-34237985-34238309	8.26E-11	0.254145843	0.153	0.065	5.47E-06	C6_CD8_Tact
chr16-85612750-85613372	9.58E-11	0.254863889	0.191	0.087	6.34E-06	C6_CD8_Tact
chr11-67302905-67304269	1.33E-10	0.254177404	0.132	0.054	8.83E-06	C6_CD8_Tact
chr11-44095264-44096543	1.68E-10	0.25180173	0.185	0.091	1.11E-05	C6_CD8_Tact
chr12-53097620-53098859	1.79E-10	0.250510871	0.166	0.074	1.19E-05	C6_CD8_Tact
chr11-75561600-75562901	1.84E-10	0.250577178	0.206	0.101	1.22E-05	C6_CD8_Tact
chr1-248862745-248863531	2.07E-10	0.251533456	0.193	0.095	1.37E-05	C6_CD8_Tact
chr19-19661392-19662269	4.96E-11	0.250304465	0.205	0.098	3.28E-06	C6_CD8_Tact
chr19-4059467-4060321	1.37E-17	0.307365976	0.235	0.102	9.06E-13	C6_CD8_Tact
chr7-87933506-87934898	1.60E-13	0.264859886	0.267	0.134	1.06E-08	C6_CD8_Tact
chr17-81514611-81515087	6.82E-28	0.375794021	0.341	0.138	4.52E-23	C6_CD8_Tact
chr1-31937300-31938784	1.33E-18	0.257516211	0.508	0.321	8.79E-14	C6_CD8_Tact
chr20-58890285-58892757	1.82E-20	0.275441939	0.527	0.312	1.21E-15	C6_CD8_Tact

chr8-102238244-102239905	6.52E-38	0.319749204	0.666	0.402	4.31E-33	C6_CD8_Tact
chr2-238425962-238427664	7.72E-28	0.337729214	0.457	0.235	5.12E-23	C6_CD8_Tact
chr17-63699169-63701070	1.94E-21	0.27462603	0.539	0.322	1.28E-16	C6_CD8_Tact
chr6-142944225-142947754	1.08E-35	0.26821213	0.772	0.528	7.16E-31	C6_CD8_Tact
chr21-34072783-34074254	1.00E-12	0.250014751	0.366	0.209	6.65E-08	C6_CD8_Tact
chr19-12789770-12790418	5.67E-35	0.361911025	0.508	0.263	3.76E-30	C6_CD8_Tact
chr9-126914197-126915693	7.34E-25	0.323080353	0.442	0.234	4.86E-20	C6_CD8_Tact
chr12-53046310-53047921	4.42E-15	0.25264244	0.438	0.266	2.93E-10	C6_CD8_Tact
chr22-19177950-19179646	6.58E-30	0.364915317	0.418	0.198	4.36E-25	C6_CD8_Tact
chr17-1484674-1486134	6.16E-32	0.389856441	0.374	0.163	4.08E-27	C6_CD8_Tact
chr11-65574064-65575126	3.07E-15	0.252054022	0.447	0.27	2.03E-10	C6_CD8_Tact
chr1-25615626-25617392	1.22E-24	0.315768474	0.468	0.254	8.10E-20	C6_CD8_Tact
chr17-80260026-80260513	8.39E-18	0.304622102	0.308	0.147	5.56E-13	C6_CD8_Tact
chr13-113489995-113491280	1.82E-19	0.325007698	0.283	0.127	1.20E-14	C6_CD8_Tact
chr19-47239311-47240012	5.25E-16	0.304666021	0.184	0.069	3.48E-11	C6_CD8_Tact
chr6-33427664-33428771	2.51E-18	0.310497547	0.304	0.141	1.67E-13	C6_CD8_Tact
chr8-144077810-144079173	9.74E-30	0.393943571	0.31	0.116	6.45E-25	C6_CD8_Tact
chr2-207768376-207770365	1.74E-42	0.447742105	0.374	0.143	1.16E-37	C6_CD8_Tact
chr1-16498659-16499805	9.18E-29	0.383496776	0.317	0.132	6.08E-24	C6_CD8_Tact
chr12-52036453-52037479	1.73E-13	0.253904476	0.363	0.202	1.15E-08	C6_CD8_Tact
chr8-98825111-98826274	1.08E-26	0.364767972	0.34	0.145	7.13E-22	C6_CD8_Tact
chr3-113947264-113948763	2.76E-25	0.36029122	0.313	0.132	1.83E-20	C6_CD8_Tact
chr1-111203560-111204718	5.49E-31	0.375555952	0.389	0.174	3.64E-26	C6_CD8_Tact
chr1-23558921-23559757	2.39E-12	0.257872855	0.295	0.156	1.58E-07	C6_CD8_Tact
chr12-52005844-52007931	4.63E-12	0.25243865	0.303	0.167	3.07E-07	C6_CD8_Tact
chr11-355081-356446	6.27E-12	0.26115405	0.233	0.115	4.15E-07	C6_CD8_Tact
chr11-2444121-2445633	2.82E-15	0.298296871	0.212	0.093	1.87E-10	C6_CD8_Tact

chr2-174395213-174396277	6.84E-16	0.29495066	0.264	0.121	4.53E-11	C6_CD8_Tact
chr6-148746057-148748592	5.80E-16	0.28305895	0.308	0.162	3.84E-11	C6_CD8_Tact
chr17-7219666-7221173	2.42E-11	0.251399177	0.276	0.148	1.60E-06	C6_CD8_Tact
chr17-36210420-36211235	2.32E-13	0.275220923	0.171	0.074	1.54E-08	C6_CD8_Tact
chr17-63445474-63447526	3.42E-14	0.276028472	0.289	0.148	2.26E-09	C6_CD8_Tact
chr15-74432984-74434201	3.62E-14	0.2704327	0.309	0.159	2.40E-09	C6_CD8_Tact
chr17-47693383-47695351	8.42E-14	0.27123387	0.314	0.161	5.58E-09	C6_CD8_Tact
chr19-18291492-18291889	3.95E-18	0.320982833	0.192	0.071	2.62E-13	C6_CD8_Tact
chr9-35616702-35617418	3.16E-12	0.268885201	0.184	0.078	2.09E-07	C6_CD8_Tact
chr12-55727869-55730439	1.43E-32	0.383445245	0.418	0.187	9.49E-28	C6_CD8_Tact
chr11-44949131-44951335	6.28E-13	0.26646498	0.268	0.14	4.16E-08	C6_CD8_Tact
chr14-100237943-100240736	3.43E-47	0.376923149	0.731	0.61	2.27E-42	C7_CD8_Tact
chr19-17947097-17948730	1.00E-44	0.462303618	0.487	0.324	6.63E-40	C7_CD8_Tact
chr8-102653613-102656504	3.55E-39	0.347259626	0.751	0.648	2.35E-34	C7_CD8_Tact
chr22-49852895-49854776	4.72E-37	0.410159312	0.55	0.405	3.12E-32	C7_CD8_Tact
chr9-135906639-135908149	3.63E-32	0.362682097	0.499	0.382	2.41E-27	C7_CD8_Tact
chr4-1247385-1249993	3.67E-32	0.348137449	0.544	0.429	2.43E-27	C7_CD8_Tact
chr4-7042412-7044172	1.60E-31	0.379170211	0.467	0.33	1.06E-26	C7_CD8_Tact
chr17-7239248-7239916	8.32E-31	0.391133851	0.47	0.337	5.51E-26	C7_CD8_Tact
chr14-102776673-102778679	5.41E-30	0.37816122	0.453	0.34	3.59E-25	C7_CD8_Tact
chr9-136943736-136944988	7.73E-30	0.374091954	0.351	0.237	5.12E-25	C7_CD8_Tact
chr12-57521945-57523335	1.23E-29	0.369898649	0.493	0.356	8.14E-25	C7_CD8_Tact
chr11-2399673-2400907	1.14E-27	0.313893437	0.603	0.502	7.52E-23	C7_CD8_Tact
chr19-4064667-4066448	1.17E-27	0.315521369	0.601	0.509	7.76E-23	C7_CD8_Tact
chr19-18279436-18282213	2.95E-27	0.251146312	0.779	0.713	1.95E-22	C7_CD8_Tact
chr19-1248095-1249938	3.32E-26	0.266153496	0.635	0.578	2.20E-21	C7_CD8_Tact
chr12-123971674-123973861	5.32E-26	0.315252363	0.538	0.446	3.52E-21	C7_CD8_Tact

chr5-6712113-6714770	1.66E-25	0.316977959	0.527	0.436	1.10E-20	C7_CD8_Tact
chr4-10115429-10117494	3.52E-25	0.332090798	0.496	0.394	2.33E-20	C7_CD8_Tact
chr14-35403164-35405103	5.85E-25	0.296925092	0.609	0.52	3.87E-20	C7_CD8_Tact
chr19-10652745-10654934	6.86E-25	0.272417928	0.697	0.615	4.54E-20	C7_CD8_Tact
chr20-23361384-23362854	9.88E-25	0.308170042	0.564	0.473	6.55E-20	C7_CD8_Tact
chr8-1762756-1764585	1.25E-24	0.335788463	0.363	0.256	8.27E-20	C7_CD8_Tact
chr6-33409564-33411481	2.94E-24	0.269471245	0.646	0.589	1.94E-19	C7_CD8_Tact
chr7-74656546-74658770	8.67E-24	0.288694859	0.459	0.389	5.74E-19	C7_CD8_Tact
chr2-60880597-60882541	1.66E-23	0.314405645	0.496	0.398	1.10E-18	C7_CD8_Tact
chr8-143951762-143953420	2.24E-23	0.314605513	0.416	0.328	1.48E-18	C7_CD8_Tact
chr18-23502707-23504374	2.60E-22	0.333122733	0.448	0.35	1.72E-17	C7_CD8_Tact
chr18-79068262-79069771	7.80E-22	0.270640629	0.62	0.535	5.16E-17	C7_CD8_Tact
chr11-567574-569100	2.12E-21	0.294741236	0.456	0.377	1.40E-16	C7_CD8_Tact
chr19-35267316-35269830	2.67E-21	0.275645779	0.391	0.318	1.77E-16	C7_CD8_Tact
chr16-2267434-2269155	4.58E-21	0.265049306	0.442	0.362	3.04E-16	C7_CD8_Tact
chr1-633460-634698	5.50E-21	0.295368339	0.788	0.687	3.65E-16	C7_CD8_Tact
chr19-47256074-47257515	6.47E-21	0.262620862	0.567	0.526	4.28E-16	C7_CD8_Tact
chr20-62121258-62123251	6.91E-21	0.267272979	0.552	0.505	4.58E-16	C7_CD8_Tact
chr11-64269141-64270828	9.10E-21	0.260685722	0.518	0.45	6.03E-16	C7_CD8_Tact
chr22-49961357-49962799	1.26E-20	0.312345395	0.436	0.336	8.35E-16	C7_CD8_Tact
chr16-29973029-29974966	1.44E-20	0.280598326	0.363	0.286	9.55E-16	C7_CD8_Tact
chr8-42895767-42897713	2.46E-20	0.275880196	0.476	0.414	1.63E-15	C7_CD8_Tact
chr6-4774879-4777420	3.79E-20	0.294712506	0.479	0.395	2.51E-15	C7_CD8_Tact
chr17-81890937-81892221	8.36E-20	0.277798263	0.425	0.344	5.53E-15	C7_CD8_Tact
chr1-6612674-6615163	9.50E-20	0.252030791	0.55	0.49	6.29E-15	C7_CD8_Tact
chr19-39403386-39404168	1.10E-19	0.284528253	0.453	0.373	7.28E-15	C7_CD8_Tact
chr16-81444229-81445724	1.18E-19	0.309859859	0.357	0.273	7.80E-15	C7_CD8_Tact

chr2-10042529-10044676	1.85E-19	0.275209765	0.533	0.47	1.22E-14	C7_CD8_Tact
chr7-100675109-100676338	3.88E-19	0.281392074	0.482	0.405	2.57E-14	C7_CD8_Tact
chr9-129412927-129414624	4.04E-19	0.259855181	0.476	0.406	2.68E-14	C7_CD8_Tact
chr22-49959836-49960892	4.11E-19	0.27268384	0.414	0.348	2.72E-14	C7_CD8_Tact
chr2-43225862-43227899	4.14E-19	0.270758596	0.541	0.458	2.74E-14	C7_CD8_Tact
chr22-43186232-43187921	4.34E-19	0.273267226	0.411	0.343	2.87E-14	C7_CD8_Tact
chr19-14089445-14091769	5.00E-19	0.265804794	0.382	0.338	3.31E-14	C7_CD8_Tact
chr14-102086238-102088760	5.10E-19	0.252369966	0.507	0.435	3.38E-14	C7_CD8_Tact
chr7-152435292-152437030	5.57E-19	0.267253227	0.425	0.338	3.69E-14	C7_CD8_Tact
chr7-73005202-73006292	9.84E-19	0.316697144	0.331	0.243	6.52E-14	C7_CD8_Tact
chr20-63738939-63740757	1.70E-18	0.288838632	0.312	0.236	1.13E-13	C7_CD8_Tact
chr3-23944778-23946586	4.03E-18	0.252477601	0.572	0.54	2.67E-13	C7_CD8_Tact
chr8-140457097-140458476	7.08E-18	0.262169799	0.388	0.326	4.69E-13	C7_CD8_Tact
chr12-132488622-132490598	7.58E-18	0.259905981	0.518	0.462	5.02E-13	C7_CD8_Tact
chr2-237691410-237692495	8.71E-18	0.262927656	0.51	0.456	5.77E-13	C7_CD8_Tact
chr11-67288028-67289598	9.19E-18	0.253754242	0.456	0.401	6.09E-13	C7_CD8_Tact
chr6-17705567-17707152	9.44E-18	0.254702555	0.555	0.48	6.25E-13	C7_CD8_Tact
chr1-2227235-2228174	1.46E-17	0.25608571	0.518	0.461	9.68E-13	C7_CD8_Tact
chr22-37745309-37746600	9.77E-17	0.259287604	0.312	0.253	6.47E-12	C7_CD8_Tact
chr7-28179206-28181164	1.27E-16	0.297142464	0.317	0.239	8.39E-12	C7_CD8_Tact
chr11-61160516-61161935	1.28E-16	0.262787701	0.416	0.358	8.46E-12	C7_CD8_Tact
chr9-88387095-88389308	8.10E-16	0.250084803	0.348	0.297	5.36E-11	C7_CD8_Tact
chr2-234496134-234497622	1.22E-15	0.258410898	0.354	0.278	8.09E-11	C7_CD8_Tact
chr16-20899501-20901395	2.56E-15	0.258390417	0.382	0.315	1.69E-10	C7_CD8_Tact
chr5-40679057-40680115	1.18E-14	0.260721267	0.433	0.363	7.82E-10	C7_CD8_Tact
chr2-32009184-32011503	2.47E-14	0.257349526	0.351	0.29	1.64E-09	C7_CD8_Tact
chr7-158595018-158595361	1.41E-11	0.283635573	0.139	0.081	9.34E-07	C7_CD8_Tact

chr9-137449890-137450919	3.34E-11	0.261387683	0.102	0.051	2.21E-06	C7_CD8_Tact
chr17-36103111-36103959	1.19E-10	0.269505093	0.164	0.095	7.86E-06	C7_CD8_Tact
chr17-36103111-36103959	1.19E-10	0.269505093	0.164	0.095	7.86E-06	C7_CD8_Tact
chr9-129412927-129414624	4.04E-19	0.259855181	0.476	0.406	2.68E-14	C7_CD8_Tact
chr16-29973029-29974966	1.44E-20	0.280598326	0.363	0.286	9.55E-16	C7_CD8_Tact
chr4-108355270-108355972	8.22E-66	0.998287542	0.25	0.007	5.44E-61	C8_CD4_Tres
chr22-19596982-19597769	6.49E-63	0.960981725	0.257	0.009	4.30E-58	C8_CD4_Tres
chr19-43470213-43470531	9.84E-58	0.935082528	0.243	0.008	6.51E-53	C8_CD4_Tres
chr5-134086388-134087356	1.57E-57	0.937632112	0.336	0.029	1.04E-52	C8_CD4_Tres
chr8-143515975-143516723	1.80E-57	0.934654066	0.231	0.008	1.19E-52	C8_CD4_Tres
chr5-75059857-75060218	1.98E-50	0.897374077	0.224	0.008	1.31E-45	C8_CD4_Tres
chr5-109921458-109922889	4.43E-50	0.880773166	0.291	0.026	2.94E-45	C8_CD4_Tres
chr17-21419160-21420017	1.01E-49	0.831617696	0.179	0.004	6.71E-45	C8_CD4_Tres
chr4-108036259-108036605	1.57E-48	0.852951497	0.198	0.006	1.04E-43	C8_CD4_Tres
chr11-6413706-6414075	1.89E-47	0.866486522	0.239	0.015	1.25E-42	C8_CD4_Tres
chr20-53001032-53002102	9.64E-47	0.82638437	0.201	0.008	6.38E-42	C8_CD4_Tres
chr16-1855160-1855740	1.33E-46	0.843185491	0.209	0.009	8.80E-42	C8_CD4_Tres
chr11-61120300-61121079	4.72E-46	0.858126188	0.261	0.021	3.12E-41	C8_CD4_Tres
chr17-76752882-76753875	2.12E-45	0.808996591	0.347	0.059	1.40E-40	C8_CD4_Tres
chr9-101569910-101570464	4.54E-45	0.786883932	0.175	0.004	3.01E-40	C8_CD4_Tres
chr3-71372862-71373398	8.13E-45	0.834788215	0.25	0.018	5.39E-40	C8_CD4_Tres
chr21-38306091-38306703	8.86E-45	0.804186281	0.183	0.005	5.87E-40	C8_CD4_Tres
chr11-123680958-123681452	1.44E-44	0.816904843	0.179	0.005	9.52E-40	C8_CD4_Tres
chr18-26215275-26216137	3.42E-43	0.822532936	0.246	0.02	2.27E-38	C8_CD4_Tres
chr11-96105327-96105916	4.25E-43	0.831321507	0.216	0.013	2.81E-38	C8_CD4_Tres
chr2-2646398-2646936	2.67E-42	0.715100216	0.127	0.001	1.77E-37	C8_CD4_Tres
chr20-26208492-26209657	6.38E-42	0.773001513	0.175	0.006	4.23E-37	C8_CD4_Tres

chr1-54287235-54287619	1.67E-41	0.812106474	0.205	0.011	1.11E-36	C8_CD4_Tres
chr14-68968023-68968395	9.72E-41	0.779409388	0.179	0.007	6.44E-36	C8_CD4_Tres
chr20-26252603-26252961	1.97E-40	0.728439117	0.134	0.001	1.30E-35	C8_CD4_Tres
chr2-100653551-100654047	1.41E-39	0.736956436	0.138	0.002	9.34E-35	C8_CD4_Tres
chr4-140150254-140152549	2.28E-39	0.674753802	0.522	0.167	1.51E-34	C8_CD4_Tres
chr2-172496025-172496510	1.10E-38	0.756695816	0.16	0.005	7.30E-34	C8_CD4_Tres
chr14-49970870-49971818	3.08E-38	0.766625347	0.287	0.038	2.04E-33	C8_CD4_Tres
chr8-141265102-141265776	3.33E-38	0.764192436	0.299	0.045	2.20E-33	C8_CD4_Tres
chr1-234408552-234409644	3.98E-38	0.782991362	0.243	0.025	2.64E-33	C8_CD4_Tres
chr6-90218079-90219213	9.20E-38	0.755234136	0.343	0.058	6.10E-33	C8_CD4_Tres
chr7-139635508-139636238	9.22E-38	0.775776281	0.19	0.011	6.10E-33	C8_CD4_Tres
chr17-40602867-40603096	1.34E-37	0.770124926	0.228	0.02	8.86E-33	C8_CD4_Tres
chr17-19771878-19772442	2.71E-37	0.735753017	0.179	0.008	1.79E-32	C8_CD4_Tres
chr19-19817131-19817720	3.54E-37	0.756170651	0.25	0.026	2.35E-32	C8_CD4_Tres
chr8-20492942-20493637	9.44E-37	0.759294026	0.224	0.02	6.25E-32	C8_CD4_Tres
chr8-106269815-106270651	3.29E-36	0.755888387	0.228	0.02	2.18E-31	C8_CD4_Tres
chr18-76492241-76493159	1.12E-35	0.73341407	0.276	0.039	7.39E-31	C8_CD4_Tres
chr3-194396863-194397667	1.70E-35	0.726796145	0.19	0.015	1.13E-30	C8_CD4_Tres
chr11-62640420-62641044	2.77E-35	0.740872543	0.25	0.029	1.83E-30	C8_CD4_Tres
chr20-53070543-53071238	2.78E-35	0.708443758	0.153	0.005	1.84E-30	C8_CD4_Tres
chr20-52974097-52974482	3.24E-35	0.74631004	0.194	0.014	2.15E-30	C8_CD4_Tres
chr1-3032986-3033553	6.51E-35	0.699796893	0.157	0.007	4.31E-30	C8_CD4_Tres
chr21-14215863-14216542	6.54E-35	0.735332576	0.269	0.035	4.33E-30	C8_CD4_Tres
chr5-80152811-80153096	7.01E-35	0.705246602	0.146	0.004	4.64E-30	C8_CD4_Tres
chr20-62892700-62893159	8.76E-35	0.697550753	0.138	0.003	5.80E-30	C8_CD4_Tres
chr1-54356629-54357085	1.21E-34	0.67274439	0.134	0.003	7.99E-30	C8_CD4_Tres
chr21-38244078-38245338	1.98E-34	0.738827445	0.276	0.041	1.31E-29	C8_CD4_Tres

chr2-59887859-59888340	2.32E-34	0.671149662	0.123	0.002	1.54E-29	C8_CD4_Tres
chr4-153152856-153153871	4.16E-34	0.714462263	0.295	0.053	2.75E-29	C8_CD4_Tres
chr9-76023655-76024232	8.06E-34	0.699619407	0.149	0.006	5.34E-29	C8_CD4_Tres
chr3-98901024-98902157	1.34E-33	0.677796172	0.418	0.098	8.85E-29	C8_CD4_Tres
chr3-194396078-194396527	2.02E-33	0.665673543	0.142	0.006	1.34E-28	C8_CD4_Tres
chr20-53388110-53388617	2.95E-33	0.6661891	0.138	0.005	1.95E-28	C8_CD4_Tres
chr4-108080697-108081175	3.41E-33	0.681324189	0.142	0.005	2.26E-28	C8_CD4_Tres
chr14-68970124-68970568	3.81E-33	0.656539946	0.123	0.003	2.52E-28	C8_CD4_Tres
chr1-186288782-186289108	1.45E-32	0.624943928	0.108	0.002	9.59E-28	C8_CD4_Tres
chrX-56780889-56781254	1.98E-32	0.613181724	0.093	0	1.31E-27	C8_CD4_Tres
chr9-122846309-122846796	2.32E-32	0.624682027	0.112	0.002	1.54E-27	C8_CD4_Tres
chr20-44068738-44070678	5.08E-32	0.595201441	0.537	0.188	3.36E-27	C8_CD4_Tres
chr1-116345528-116346187	8.09E-32	0.669676648	0.142	0.005	5.36E-27	C8_CD4_Tres
chr4-38328756-38329303	9.20E-32	0.621138417	0.101	0.001	6.09E-27	C8_CD4_Tres
chr7-157881091-157881453	1.18E-31	0.620255444	0.097	0.001	7.81E-27	C8_CD4_Tres
chr2-2613210-2613940	2.82E-31	0.712607491	0.246	0.035	1.87E-26	C8_CD4_Tres
chr2-23452777-23453429	3.33E-31	0.655239894	0.134	0.005	2.21E-26	C8_CD4_Tres
chr12-95334343-95334722	1.12E-30	0.687137056	0.153	0.008	7.44E-26	C8_CD4_Tres
chr5-142104439-142104837	1.13E-30	0.683096442	0.287	0.053	7.45E-26	C8_CD4_Tres
chr12-51009077-51009364	2.44E-30	0.663274407	0.153	0.009	1.62E-25	C8_CD4_Tres
chr6-79536665-79537700	2.45E-30	0.681282562	0.299	0.055	1.63E-25	C8_CD4_Tres
chr6-90266909-90267552	4.01E-30	0.678312443	0.164	0.012	2.65E-25	C8_CD4_Tres
chr8-78444831-78445513	5.16E-30	0.682008204	0.168	0.013	3.42E-25	C8_CD4_Tres
chr2-105934515-105935382	6.69E-30	0.681501173	0.205	0.025	4.43E-25	C8_CD4_Tres
chr17-56592505-56594228	7.23E-30	0.680520251	0.228	0.033	4.79E-25	C8_CD4_Tres
chr10-35011505-35012193	7.50E-30	0.637882663	0.127	0.004	4.97E-25	C8_CD4_Tres
chr2-109030933-109032525	8.09E-30	0.606217765	0.437	0.142	5.36E-25	C8_CD4_Tres

chr13-73702210-73703076	1.41E-29	0.667409046	0.272	0.051	9.31E-25	C8_CD4_Tres
chr1-54285240-54285439	2.45E-29	0.591356824	0.097	0.001	1.62E-24	C8_CD4_Tres
chr18-70672090-70673009	3.09E-29	0.674720054	0.175	0.016	2.04E-24	C8_CD4_Tres
chr2-1509194-1509969	1.30E-28	0.637447414	0.127	0.005	8.63E-24	C8_CD4_Tres
chr17-56653942-56654206	2.10E-28	0.585677459	0.108	0.003	1.39E-23	C8_CD4_Tres
chr8-141000867-141002536	3.32E-28	0.615752036	0.388	0.099	2.20E-23	C8_CD4_Tres
chr14-106228511-106228830	3.73E-28	0.626807393	0.134	0.007	2.47E-23	C8_CD4_Tres
chr8-89243459-89243877	3.98E-28	0.635861712	0.119	0.004	2.64E-23	C8_CD4_Tres
chr17-75689432-75689866	4.21E-28	0.657237375	0.157	0.013	2.79E-23	C8_CD4_Tres
chr1-44305917-44306387	8.66E-28	0.535729493	0.082	0	5.73E-23	C8_CD4_Tres
chr7-158812659-158813019	8.92E-28	0.650493389	0.168	0.015	5.91E-23	C8_CD4_Tres
chrX-120697012-120697340	1.01E-27	0.555746095	0.09	0.001	6.67E-23	C8_CD4_Tres
chr10-1939091-1939370	1.07E-27	0.535850717	0.082	0	7.11E-23	C8_CD4_Tres
chr7-50321423-50322068	1.76E-27	0.644378753	0.25	0.047	1.17E-22	C8_CD4_Tres
chr16-85306206-85306512	2.22E-27	0.640021174	0.153	0.011	1.47E-22	C8_CD4_Tres
chr10-102611995-102613224	2.62E-27	0.6453413	0.213	0.028	1.73E-22	C8_CD4_Tres
chr4-69760457-69760838	4.42E-27	0.608740981	0.112	0.004	2.93E-22	C8_CD4_Tres
chr1-117049058-117049663	4.63E-27	0.609978731	0.131	0.006	3.06E-22	C8_CD4_Tres
chr21-45477623-45478816	5.04E-27	0.624254615	0.287	0.074	3.33E-22	C8_CD4_Tres
chr16-89534202-89534762	6.02E-27	0.623618105	0.146	0.011	3.98E-22	C8_CD4_Tres
chr15-98647186-98648976	6.78E-27	0.582564587	0.422	0.133	4.49E-22	C8_CD4_Tres
chr6-157755520-157756326	7.21E-27	0.647893622	0.194	0.026	4.77E-22	C8_CD4_Tres
chr12-52603354-52603639	8.54E-27	0.525015966	0.086	0.001	5.66E-22	C8_CD4_Tres
chr13-98994910-98995189	9.92E-27	0.60493673	0.119	0.005	6.57E-22	C8_CD4_Tres
chr12-55929153-55929738	1.19E-26	0.59195771	0.358	0.116	7.88E-22	C8_CD4_Tres
chr21-38323627-38324089	1.45E-26	0.584914664	0.104	0.003	9.59E-22	C8_CD4_Tres
chr1-239501490-239502121	1.61E-26	0.62152235	0.153	0.012	1.06E-21	C8_CD4_Tres

chr2-176826238-176826718	2.11E-26	0.604721725	0.131	0.008	1.40E-21	C8_CD4_Tres
chr3-68057468-68058182	2.26E-26	0.638235268	0.231	0.041	1.50E-21	C8_CD4_Tres
chr1-154386201-154387219	2.51E-26	0.632613344	0.291	0.063	1.66E-21	C8_CD4_Tres
chr21-43361645-43362764	2.64E-26	0.570005592	0.444	0.152	1.75E-21	C8_CD4_Tres
chr12-113103594-113104378	3.17E-26	0.64533539	0.224	0.037	2.10E-21	C8_CD4_Tres
chr17-78258573-78258863	3.53E-26	0.620145063	0.153	0.016	2.34E-21	C8_CD4_Tres
chr3-190301602-190301975	3.59E-26	0.569230298	0.093	0.002	2.38E-21	C8_CD4_Tres
chr1-213670261-213670648	4.53E-26	0.549455518	0.09	0.001	3.00E-21	C8_CD4_Tres
chr11-119306378-119306919	4.69E-26	0.612080136	0.119	0.006	3.10E-21	C8_CD4_Tres
chr12-120041315-120041732	5.06E-26	0.570617308	0.104	0.003	3.35E-21	C8_CD4_Tres
chr1-241677133-241677611	9.36E-26	0.632720343	0.198	0.024	6.20E-21	C8_CD4_Tres
chr12-6786545-6787142	1.28E-25	0.630930531	0.16	0.017	8.48E-21	C8_CD4_Tres
chr16-57266467-57267184	2.17E-25	0.63031156	0.175	0.022	1.44E-20	C8_CD4_Tres
chr1-226656648-226656889	2.81E-25	0.616163292	0.153	0.014	1.86E-20	C8_CD4_Tres
chr21-45766897-45767511	3.50E-25	0.597799369	0.146	0.01	2.32E-20	C8_CD4_Tres
chr20-41428044-41428663	4.75E-25	0.601475016	0.138	0.009	3.15E-20	C8_CD4_Tres
chr21-45614909-45615788	7.50E-25	0.584201865	0.343	0.102	4.97E-20	C8_CD4_Tres
chr4-108029853-108030668	7.85E-25	0.611651761	0.164	0.018	5.20E-20	C8_CD4_Tres
chr2-205077054-205077512	8.40E-25	0.555269262	0.097	0.003	5.57E-20	C8_CD4_Tres
chr22-37218585-37219771	1.06E-24	0.54872928	0.418	0.143	7.00E-20	C8_CD4_Tres
chr11-88843442-88844111	2.14E-24	0.599562496	0.149	0.014	1.42E-19	C8_CD4_Tres
chr19-18023805-18024127	2.63E-24	0.612479139	0.149	0.013	1.74E-19	C8_CD4_Tres
chr1-101111621-101112701	3.17E-24	0.571909421	0.366	0.11	2.10E-19	C8_CD4_Tres
chr3-45497838-45498287	4.27E-24	0.540485773	0.093	0.003	2.83E-19	C8_CD4_Tres
chr3-128316344-128316848	5.17E-24	0.589652047	0.149	0.014	3.42E-19	C8_CD4_Tres
chr17-22521254-22521613	5.28E-24	0.29628459	0.892	0.678	3.50E-19	C8_CD4_Tres
chr5-56148091-56148922	8.36E-24	0.590960374	0.142	0.012	5.54E-19	C8_CD4_Tres

chr15-33047089-33047383	9.87E-24	0.516749865	0.078	0.001	6.53E-19	C8_CD4_Tres
chr1-59814144-59815420	1.52E-23	0.488151768	0.537	0.235	1.00E-18	C8_CD4_Tres
chr2-112996556-112997022	1.56E-23	0.598752597	0.157	0.02	1.04E-18	C8_CD4_Tres
chr1-19074093-19075025	1.66E-23	0.591347113	0.299	0.07	1.10E-18	C8_CD4_Tres
chr21-42061903-42063035	1.96E-23	0.468945782	0.556	0.278	1.30E-18	C8_CD4_Tres
chr2-95074565-95074889	2.83E-23	0.593538487	0.134	0.012	1.87E-18	C8_CD4_Tres
chr15-90829567-90830386	3.04E-23	0.569389903	0.108	0.006	2.01E-18	C8_CD4_Tres
chr1-87045769-87046147	3.55E-23	0.578504844	0.138	0.011	2.35E-18	C8_CD4_Tres
chr7-1926900-1927206	3.88E-23	0.582298559	0.127	0.009	2.57E-18	C8_CD4_Tres
chr8-100809255-100810633	5.04E-23	0.506444712	0.478	0.193	3.34E-18	C8_CD4_Tres
chr7-134864897-134865560	5.61E-23	0.578121193	0.116	0.007	3.71E-18	C8_CD4_Tres
chr10-1938338-1938636	6.29E-23	0.490450351	0.071	0.001	4.17E-18	C8_CD4_Tres
chr4-89337776-89337987	6.69E-23	0.486544652	0.075	0.001	4.43E-18	C8_CD4_Tres
chr13-74290351-74290695	7.28E-23	0.589001141	0.272	0.062	4.82E-18	C8_CD4_Tres
chr22-39993961-39994372	8.02E-23	0.576855162	0.149	0.014	5.31E-18	C8_CD4_Tres
chr4-108031558-108031761	8.73E-23	0.503235091	0.09	0.002	5.78E-18	C8_CD4_Tres
chrX-57286220-57287198	1.10E-22	0.593092354	0.231	0.045	7.29E-18	C8_CD4_Tres
chr17-64348003-64348484	1.11E-22	0.580920867	0.157	0.019	7.36E-18	C8_CD4_Tres
chr3-196604586-196604872	1.20E-22	0.583379317	0.142	0.012	7.94E-18	C8_CD4_Tres
chr4-42627735-42628638	1.44E-22	0.573177767	0.291	0.08	9.53E-18	C8_CD4_Tres
chr1-203324153-203324359	1.53E-22	0.575502615	0.183	0.027	1.01E-17	C8_CD4_Tres
chr3-125357004-125357572	1.65E-22	0.577891489	0.142	0.013	1.09E-17	C8_CD4_Tres
chr19-16369616-16370355	2.07E-22	0.539576442	0.362	0.124	1.37E-17	C8_CD4_Tres
chr17-78272130-78272919	2.35E-22	0.582565892	0.168	0.023	1.55E-17	C8_CD4_Tres
chr10-14382040-14382418	2.61E-22	0.507667231	0.078	0.002	1.73E-17	C8_CD4_Tres
chr17-76191451-76192342	3.30E-22	0.568983873	0.284	0.072	2.19E-17	C8_CD4_Tres
chr15-38685964-38686245	3.91E-22	0.577252997	0.142	0.015	2.59E-17	C8_CD4_Tres

chr4-40301412-40302013	4.24E-22	0.562494652	0.291	0.074	2.81E-17	C8_CD4_Tres
chr8-127387001-127387283	4.45E-22	0.488182312	0.078	0.002	2.95E-17	C8_CD4_Tres
chr22-29701248-29701761	5.78E-22	0.5227444	0.097	0.004	3.83E-17	C8_CD4_Tres
chr14-58637081-58638156	6.11E-22	0.546087876	0.112	0.007	4.05E-17	C8_CD4_Tres
chr8-61013946-61014515	6.19E-22	0.575837734	0.168	0.024	4.10E-17	C8_CD4_Tres
chr19-40969729-40970887	6.60E-22	0.577255998	0.175	0.027	4.37E-17	C8_CD4_Tres
chr13-43057564-43058414	8.61E-22	0.584054191	0.157	0.02	5.70E-17	C8_CD4_Tres
chr2-69499986-69500268	1.17E-21	0.514463123	0.093	0.003	7.74E-17	C8_CD4_Tres
chr10-119501615-119502938	1.20E-21	0.46265014	0.556	0.249	7.94E-17	C8_CD4_Tres
chr3-72657700-72658017	1.25E-21	0.520549914	0.09	0.004	8.31E-17	C8_CD4_Tres
chr6-118719454-118719800	1.29E-21	0.503462991	0.09	0.003	8.52E-17	C8_CD4_Tres
chr8-6718011-6718559	1.37E-21	0.59061909	0.194	0.032	9.05E-17	C8_CD4_Tres
chr19-50724391-50725457	1.50E-21	0.551833328	0.313	0.09	9.94E-17	C8_CD4_Tres
chr12-92378780-92379353	1.73E-21	0.507377708	0.097	0.004	1.15E-16	C8_CD4_Tres
chr15-90539696-90540219	1.87E-21	0.550128749	0.134	0.013	1.24E-16	C8_CD4_Tres
chr12-123131669-123132365	2.08E-21	0.551528461	0.123	0.012	1.38E-16	C8_CD4_Tres
chr6-17589539-17590014	2.27E-21	0.498118936	0.086	0.003	1.50E-16	C8_CD4_Tres
chr19-29837346-29837770	2.35E-21	0.549875691	0.119	0.01	1.56E-16	C8_CD4_Tres
chr7-157851415-157852054	2.60E-21	0.529273944	0.108	0.006	1.72E-16	C8_CD4_Tres
chr4-99845754-99845961	2.61E-21	0.477644423	0.078	0.002	1.73E-16	C8_CD4_Tres
chr3-18443988-18445871	3.10E-21	0.469496215	0.507	0.223	2.05E-16	C8_CD4_Tres
chr1-154265567-154265909	3.29E-21	0.534658443	0.108	0.007	2.18E-16	C8_CD4_Tres
chr6-3455129-3455395	3.64E-21	0.527929963	0.104	0.006	2.41E-16	C8_CD4_Tres
chr16-29154993-29155525	4.49E-21	0.486079451	0.075	0.001	2.97E-16	C8_CD4_Tres
chr4-108171457-108171805	5.49E-21	0.564420776	0.175	0.028	3.64E-16	C8_CD4_Tres
chr1-186303877-186304254	5.80E-21	0.486852435	0.078	0.002	3.84E-16	C8_CD4_Tres
chr12-21676788-21677045	6.06E-21	0.504076949	0.09	0.003	4.01E-16	C8_CD4_Tres

chr20-43964340-43964695	6.47E-21	0.540280756	0.108	0.007	4.29E-16	C8_CD4_Tres
chr2-95076518-95077201	6.67E-21	0.557057044	0.257	0.065	4.42E-16	C8_CD4_Tres
chr10-3186194-3186680	6.75E-21	0.56007815	0.134	0.014	4.47E-16	C8_CD4_Tres
chr1-64469985-64470671	8.02E-21	0.501033375	0.097	0.004	5.31E-16	C8_CD4_Tres
chr19-12332303-12333893	9.36E-21	0.441902348	0.549	0.266	6.20E-16	C8_CD4_Tres
chr12-67590247-67590726	1.02E-20	0.509719474	0.104	0.006	6.76E-16	C8_CD4_Tres
chr1-151131118-151131599	1.08E-20	0.559370894	0.187	0.033	7.17E-16	C8_CD4_Tres
chr11-102421323-102421570	1.18E-20	0.532927801	0.108	0.008	7.81E-16	C8_CD4_Tres
chr12-107319650-107321616	1.44E-20	0.520450558	0.354	0.11	9.51E-16	C8_CD4_Tres
chr17-35285870-35286441	1.53E-20	0.517687641	0.101	0.006	1.01E-15	C8_CD4_Tres
chr4-48138579-48139318	1.66E-20	0.543984042	0.146	0.018	1.10E-15	C8_CD4_Tres
chr1-205313533-205314044	1.84E-20	0.538272013	0.123	0.011	1.22E-15	C8_CD4_Tres
chr15-52235099-52235447	1.90E-20	0.496382333	0.086	0.003	1.26E-15	C8_CD4_Tres
chr14-98223550-98224139	2.19E-20	0.564455593	0.22	0.047	1.45E-15	C8_CD4_Tres
chr13-46593347-46593825	2.73E-20	0.56320903	0.16	0.023	1.81E-15	C8_CD4_Tres
chr13-28492534-28492859	2.88E-20	0.525223392	0.097	0.006	1.91E-15	C8_CD4_Tres
chr7-3461773-3462269	3.49E-20	0.54649723	0.179	0.03	2.31E-15	C8_CD4_Tres
chr17-78343243-78344199	3.53E-20	0.537528253	0.269	0.077	2.34E-15	C8_CD4_Tres
chr10-28367842-28368938	3.86E-20	0.543427309	0.235	0.053	2.56E-15	C8_CD4_Tres
chr6-142395316-142396212	4.74E-20	0.539052452	0.269	0.071	3.14E-15	C8_CD4_Tres
chr9-114989570-114990132	5.19E-20	0.529313128	0.112	0.011	3.44E-15	C8_CD4_Tres
chr1-76074221-76075023	7.12E-20	0.552112608	0.239	0.055	4.71E-15	C8_CD4_Tres
chr17-76151814-76152175	8.58E-20	0.502800172	0.093	0.005	5.68E-15	C8_CD4_Tres
chr12-52610272-52610760	9.82E-20	0.519980632	0.108	0.008	6.50E-15	C8_CD4_Tres
chr5-142223267-142224134	1.10E-19	0.481794906	0.418	0.168	7.31E-15	C8_CD4_Tres
chr20-32971845-32972681	1.21E-19	0.503063814	0.358	0.124	8.04E-15	C8_CD4_Tres
chr9-89743241-89744283	1.27E-19	0.546000491	0.149	0.021	8.44E-15	C8_CD4_Tres

chr12-66964321-66964965	1.35E-19	0.546909603	0.153	0.02	8.94E-15	C8_CD4_Tres
chr17-78250773-78252846	1.39E-19	0.453366953	0.481	0.218	9.24E-15	C8_CD4_Tres
chr5-80958042-80958369	1.56E-19	0.541048632	0.146	0.017	1.03E-14	C8_CD4_Tres
chr10-43426969-43427732	1.75E-19	0.544133646	0.146	0.017	1.16E-14	C8_CD4_Tres
chr22-39612824-39613192	1.84E-19	0.499463436	0.086	0.004	1.22E-14	C8_CD4_Tres
chr9-96014068-96014465	2.06E-19	0.550636613	0.175	0.031	1.36E-14	C8_CD4_Tres
chr2-190947171-190948142	2.14E-19	0.519770389	0.149	0.02	1.42E-14	C8_CD4_Tres
chr1-206909120-206909783	2.42E-19	0.541404117	0.164	0.026	1.60E-14	C8_CD4_Tres
chr1-48776518-48777164	2.74E-19	0.540344191	0.157	0.023	1.82E-14	C8_CD4_Tres
chr22-45172734-45173508	2.85E-19	0.538636635	0.164	0.029	1.89E-14	C8_CD4_Tres
chr19-7705419-7706178	3.37E-19	0.531316772	0.168	0.029	2.23E-14	C8_CD4_Tres
chr6-6810880-6811959	3.47E-19	0.507323582	0.112	0.009	2.30E-14	C8_CD4_Tres
chr1-226690374-226691613	3.68E-19	0.52069634	0.291	0.087	2.44E-14	C8_CD4_Tres
chr18-76490779-76491831	4.91E-19	0.52466959	0.164	0.024	3.25E-14	C8_CD4_Tres
chr2-84845862-84846637	5.11E-19	0.528860373	0.216	0.048	3.39E-14	C8_CD4_Tres
chr3-128803856-128804503	5.28E-19	0.520614175	0.291	0.083	3.50E-14	C8_CD4_Tres
chr1-48471647-48472478	5.57E-19	0.516727994	0.302	0.092	3.69E-14	C8_CD4_Tres
chr8-106725861-106726144	5.76E-19	0.469672182	0.078	0.003	3.81E-14	C8_CD4_Tres
chr8-100826461-100827293	7.49E-19	0.517527416	0.254	0.069	4.96E-14	C8_CD4_Tres
chr14-51419576-51420179	9.53E-19	0.529086006	0.127	0.014	6.31E-14	C8_CD4_Tres
chr10-71756885-71757188	1.02E-18	0.518307582	0.116	0.01	6.73E-14	C8_CD4_Tres
chr22-19607227-19607690	1.13E-18	0.452830353	0.071	0.002	7.50E-14	C8_CD4_Tres
chr19-14506723-14507610	1.15E-18	0.513733421	0.287	0.085	7.62E-14	C8_CD4_Tres
chr15-85424071-85424455	1.20E-18	0.459466131	0.078	0.003	7.92E-14	C8_CD4_Tres
chr21-42527244-42528283	1.30E-18	0.51933764	0.254	0.063	8.58E-14	C8_CD4_Tres
chr9-4297374-4298823	1.34E-18	0.512765053	0.146	0.018	8.89E-14	C8_CD4_Tres
chr3-18438279-18438968	1.47E-18	0.533891228	0.183	0.035	9.75E-14	C8_CD4_Tres

chr3-190306611-190307013	1.47E-18	0.403679886	0.056	0	9.77E-14	C8_CD4_Tres
chr15-44194346-44195619	1.52E-18	0.513440762	0.284	0.084	1.01E-13	C8_CD4_Tres
chr8-80499486-80499722	1.56E-18	0.445057368	0.071	0.002	1.04E-13	C8_CD4_Tres
chr1-226001992-226002576	1.69E-18	0.52151433	0.254	0.067	1.12E-13	C8_CD4_Tres
chr14-98444073-98444446	1.88E-18	0.465779106	0.086	0.004	1.25E-13	C8_CD4_Tres
chr3-139677254-139678151	1.94E-18	0.530744773	0.235	0.058	1.29E-13	C8_CD4_Tres
chr12-106227575-106227887	1.98E-18	0.500688604	0.108	0.009	1.31E-13	C8_CD4_Tres
chr16-84819456-84820117	1.98E-18	0.531858598	0.19	0.037	1.31E-13	C8_CD4_Tres
chr1-41897886-41898707	2.21E-18	0.509196857	0.276	0.081	1.46E-13	C8_CD4_Tres
chr2-88754065-88754508	2.30E-18	0.463720936	0.078	0.003	1.52E-13	C8_CD4_Tres
chr18-70658921-70659305	2.31E-18	0.492330853	0.101	0.007	1.53E-13	C8_CD4_Tres
chr10-88064983-88065337	2.40E-18	0.472783862	0.078	0.003	1.59E-13	C8_CD4_Tres
chr10-6381982-6382418	2.51E-18	0.497744542	0.104	0.008	1.66E-13	C8_CD4_Tres
chr6-90233436-90234440	2.66E-18	0.517598924	0.205	0.05	1.76E-13	C8_CD4_Tres
chr19-58097520-58098745	2.90E-18	0.447119034	0.433	0.19	1.92E-13	C8_CD4_Tres
chr1-160628816-160630078	2.99E-18	0.485574579	0.328	0.118	1.98E-13	C8_CD4_Tres
chr10-104308366-104308866	3.66E-18	0.515418558	0.138	0.02	2.42E-13	C8_CD4_Tres
chr8-1759920-1760414	4.06E-18	0.521913953	0.239	0.057	2.69E-13	C8_CD4_Tres
chr6-16474724-16475354	4.30E-18	0.524542417	0.172	0.03	2.85E-13	C8_CD4_Tres
chr3-67947709-67948495	4.63E-18	0.526534269	0.194	0.041	3.07E-13	C8_CD4_Tres
chr13-73765278-73766386	4.68E-18	0.506683893	0.265	0.077	3.10E-13	C8_CD4_Tres
chr9-111937710-111938695	4.87E-18	0.513861912	0.146	0.021	3.22E-13	C8_CD4_Tres
chr3-183515954-183516702	5.15E-18	0.498680061	0.317	0.103	3.41E-13	C8_CD4_Tres
chr2-48448074-48449276	5.56E-18	0.517698668	0.149	0.026	3.68E-13	C8_CD4_Tres
chr7-111024983-111025302	5.59E-18	0.495181324	0.104	0.009	3.70E-13	C8_CD4_Tres
chr20-52973082-52973444	5.85E-18	0.483020003	0.101	0.007	3.88E-13	C8_CD4_Tres
chr11-65553175-65553682	5.91E-18	0.513262913	0.172	0.033	3.91E-13	C8_CD4_Tres

chr13-49371910-49372238	6.66E-18	0.452427353	0.071	0.002	4.41E-13	C8_CD4_Tres
chr20-44153574-44154024	7.45E-18	0.449300679	0.078	0.004	4.93E-13	C8_CD4_Tres
chr13-41286507-41286772	9.08E-18	0.431881229	0.067	0.002	6.01E-13	C8_CD4_Tres
chrX-48938643-48939055	9.12E-18	0.517625279	0.157	0.027	6.04E-13	C8_CD4_Tres
chr19-18014269-18014565	9.20E-18	0.496222158	0.104	0.011	6.09E-13	C8_CD4_Tres
chr1-25774449-25775787	1.02E-17	0.44573965	0.429	0.189	6.76E-13	C8_CD4_Tres
chr8-80485247-80487463	1.03E-17	0.394301024	0.556	0.301	6.80E-13	C8_CD4_Tres
chr2-105755812-105756277	1.10E-17	0.512738596	0.164	0.032	7.30E-13	C8_CD4_Tres
chr7-111083250-111083802	1.15E-17	0.51394889	0.25	0.068	7.58E-13	C8_CD4_Tres
chr1-109667768-109668231	1.22E-17	0.512849955	0.157	0.026	8.05E-13	C8_CD4_Tres
chr8-127733954-127736389	1.22E-17	0.349671619	0.694	0.41	8.05E-13	C8_CD4_Tres
chr3-102106355-102106754	1.40E-17	0.493257054	0.101	0.007	9.25E-13	C8_CD4_Tres
chr8-106271134-106271361	1.49E-17	0.451602089	0.078	0.003	9.88E-13	C8_CD4_Tres
chr5-131640445-131640819	1.63E-17	0.500266017	0.116	0.011	1.08E-12	C8_CD4_Tres
chr8-60865260-60865561	1.78E-17	0.451892286	0.082	0.004	1.18E-12	C8_CD4_Tres
chr1-213852119-213852522	1.82E-17	0.476968556	0.086	0.005	1.21E-12	C8_CD4_Tres
chr1-178724771-178726503	2.31E-17	0.510560984	0.198	0.039	1.53E-12	C8_CD4_Tres
chr17-80743162-80743444	2.43E-17	0.4411584	0.071	0.002	1.61E-12	C8_CD4_Tres
chr6-33668929-33669278	2.52E-17	0.476984933	0.097	0.008	1.67E-12	C8_CD4_Tres
chr6-33532859-33533845	2.58E-17	0.502625073	0.239	0.064	1.71E-12	C8_CD4_Tres
chr19-43468453-43469356	3.03E-17	0.510786037	0.16	0.026	2.01E-12	C8_CD4_Tres
chr19-47186306-47187379	3.13E-17	0.409991975	0.515	0.259	2.07E-12	C8_CD4_Tres
chr3-119218933-119219427	3.29E-17	0.486692369	0.112	0.011	2.18E-12	C8_CD4_Tres
chr2-168489921-168491103	3.46E-17	0.506753897	0.16	0.025	2.29E-12	C8_CD4_Tres
chr17-1473834-1474123	3.59E-17	0.458315072	0.086	0.005	2.37E-12	C8_CD4_Tres
chr12-92398366-92399172	3.71E-17	0.498339484	0.25	0.073	2.46E-12	C8_CD4_Tres
chr7-134995346-134995669	3.92E-17	0.450104247	0.075	0.003	2.60E-12	C8_CD4_Tres

chr18-13538919-13539374	5.04E-17	0.440868158	0.067	0.002	3.34E-12	C8_CD4_Tres
chr1-9728190-9729189	5.10E-17	0.500060593	0.183	0.039	3.38E-12	C8_CD4_Tres
chr7-6990644-6991633	6.26E-17	0.507761344	0.213	0.05	4.15E-12	C8_CD4_Tres
chr12-113232936-113233657	6.70E-17	0.508071495	0.209	0.047	4.44E-12	C8_CD4_Tres
chr8-141073044-141073534	6.81E-17	0.457315581	0.082	0.005	4.51E-12	C8_CD4_Tres
chr16-31695487-31696032	7.04E-17	0.497467943	0.231	0.057	4.67E-12	C8_CD4_Tres
chr9-79544130-79544747	7.07E-17	0.498412776	0.134	0.019	4.68E-12	C8_CD4_Tres
chr12-50517589-50518080	8.05E-17	0.479887259	0.116	0.011	5.33E-12	C8_CD4_Tres
chr12-95547953-95549269	8.27E-17	0.493411204	0.243	0.067	5.48E-12	C8_CD4_Tres
chr15-28890640-28891074	8.36E-17	0.47986779	0.123	0.014	5.54E-12	C8_CD4_Tres
chr14-92748351-92748817	9.53E-17	0.49949509	0.153	0.028	6.31E-12	C8_CD4_Tres
chr12-538680-539689	9.59E-17	0.492741769	0.272	0.077	6.35E-12	C8_CD4_Tres
chr4-112309170-112309475	1.01E-16	0.432316844	0.071	0.003	6.71E-12	C8_CD4_Tres
chr3-60010366-60011232	1.03E-16	0.500581992	0.19	0.042	6.84E-12	C8_CD4_Tres
chr1-15696759-15697561	1.04E-16	0.479916874	0.112	0.013	6.90E-12	C8_CD4_Tres
chr8-120706155-120707167	1.14E-16	0.495033934	0.239	0.072	7.57E-12	C8_CD4_Tres
chr16-89548782-89549304	1.21E-16	0.441953649	0.078	0.004	8.02E-12	C8_CD4_Tres
chr14-23352119-23353037	1.33E-16	0.490955806	0.146	0.021	8.78E-12	C8_CD4_Tres
chr8-143461255-143462999	1.39E-16	0.446128523	0.381	0.152	9.19E-12	C8_CD4_Tres
chr5-56102052-56102560	1.41E-16	0.46023979	0.097	0.008	9.36E-12	C8_CD4_Tres
chr10-9096282-9096612	1.47E-16	0.445048381	0.082	0.005	9.75E-12	C8_CD4_Tres
chr7-50148070-50148628	1.49E-16	0.462049319	0.097	0.008	9.88E-12	C8_CD4_Tres
chr9-133708987-133709724	1.60E-16	0.500983858	0.179	0.038	1.06E-11	C8_CD4_Tres
chr13-20475484-20476627	1.71E-16	0.478747107	0.276	0.094	1.13E-11	C8_CD4_Tres
chr17-40596301-40596704	1.88E-16	0.480046387	0.112	0.012	1.25E-11	C8_CD4_Tres
chr3-143751642-143751993	1.99E-16	0.438913917	0.078	0.004	1.31E-11	C8_CD4_Tres
chr2-23384932-23385535	1.99E-16	0.482821519	0.116	0.012	1.32E-11	C8_CD4_Tres

chr2-101570603-101571189	2.01E-16	0.461969677	0.097	0.008	1.33E-11	C8_CD4_Tres
chr6-41298832-41299460	2.19E-16	0.499609986	0.175	0.033	1.45E-11	C8_CD4_Tres
chr9-4595901-4596947	2.21E-16	0.49289622	0.16	0.032	1.46E-11	C8_CD4_Tres
chr1-62271888-62272482	2.23E-16	0.435800935	0.075	0.004	1.47E-11	C8_CD4_Tres
chr17-78257959-78258246	2.31E-16	0.436429077	0.078	0.005	1.53E-11	C8_CD4_Tres
chr18-77106301-77106718	2.35E-16	0.49155566	0.213	0.057	1.55E-11	C8_CD4_Tres
chr1-167460896-167461125	2.36E-16	0.461836511	0.097	0.007	1.57E-11	C8_CD4_Tres
chr3-15271567-15272227	2.51E-16	0.450459642	0.377	0.158	1.66E-11	C8_CD4_Tres
chr5-180085356-180086178	2.94E-16	0.48008255	0.134	0.019	1.95E-11	C8_CD4_Tres
chr17-67398709-67399075	3.07E-16	0.490422973	0.146	0.023	2.03E-11	C8_CD4_Tres
chr2-237603439-237604001	3.14E-16	0.486535069	0.164	0.035	2.08E-11	C8_CD4_Tres
chr17-40582034-40582847	3.43E-16	0.484797914	0.149	0.025	2.27E-11	C8_CD4_Tres
chr15-31360879-31361816	3.68E-16	0.415933585	0.459	0.214	2.44E-11	C8_CD4_Tres
chr4-165255390-165255853	3.77E-16	0.458042548	0.097	0.007	2.50E-11	C8_CD4_Tres
chr8-13132752-13133557	3.79E-16	0.480957839	0.123	0.018	2.51E-11	C8_CD4_Tres
chr2-203705036-203705569	4.80E-16	0.493122216	0.194	0.05	3.18E-11	C8_CD4_Tres
chr4-74632677-74632935	5.08E-16	0.396255403	0.06	0.002	3.36E-11	C8_CD4_Tres
chr13-51824965-51825545	5.57E-16	0.481068819	0.194	0.049	3.69E-11	C8_CD4_Tres
chr11-122675079-122675626	5.63E-16	0.485759484	0.138	0.024	3.73E-11	C8_CD4_Tres
chr9-31259851-31260139	6.20E-16	0.361425107	0.052	0.001	4.10E-11	C8_CD4_Tres
chr4-153114731-153116644	6.30E-16	0.419239789	0.425	0.197	4.17E-11	C8_CD4_Tres
chr10-7192776-7193777	6.63E-16	0.48463192	0.157	0.029	4.39E-11	C8_CD4_Tres
chrX-13051318-13052060	6.89E-16	0.483885925	0.157	0.03	4.56E-11	C8_CD4_Tres
chr3-43296487-43297080	7.87E-16	0.488327548	0.205	0.051	5.21E-11	C8_CD4_Tres
chr11-73034310-73035090	8.08E-16	0.469801673	0.116	0.013	5.35E-11	C8_CD4_Tres
chr7-50317103-50318637	8.25E-16	0.418342624	0.422	0.199	5.46E-11	C8_CD4_Tres
chr3-194306144-194307445	9.00E-16	0.466271676	0.116	0.015	5.96E-11	C8_CD4_Tres

chr17-77435199-77435543	9.77E-16	0.479671241	0.134	0.019	6.47E-11	C8_CD4_Tres
chr3-60048422-60048815	1.00E-15	0.396500009	0.06	0.002	6.64E-11	C8_CD4_Tres
chr8-80377118-80378180	1.01E-15	0.486272718	0.187	0.041	6.67E-11	C8_CD4_Tres
chr4-38856065-38857011	1.10E-15	0.484730982	0.172	0.038	7.27E-11	C8_CD4_Tres
chr4-108172562-108173451	1.12E-15	0.48472457	0.239	0.06	7.40E-11	C8_CD4_Tres
chr21-46550233-46551065	1.28E-15	0.473874526	0.216	0.067	8.51E-11	C8_CD4_Tres
chr4-108113233-108113704	1.32E-15	0.480451636	0.146	0.023	8.73E-11	C8_CD4_Tres
chr21-29449150-29449853	1.41E-15	0.450290853	0.097	0.009	9.36E-11	C8_CD4_Tres
chr21-31159153-31159465	1.42E-15	0.423328731	0.078	0.005	9.40E-11	C8_CD4_Tres
chr7-157874974-157875471	1.48E-15	0.411859182	0.067	0.003	9.83E-11	C8_CD4_Tres
chr15-99424968-99425526	1.64E-15	0.454129191	0.097	0.009	1.08E-10	C8_CD4_Tres
chr12-113152461-113153622	1.79E-15	0.475275166	0.239	0.069	1.19E-10	C8_CD4_Tres
chr11-12717834-12718289	1.86E-15	0.450057882	0.097	0.01	1.23E-10	C8_CD4_Tres
chr16-3503545-3503876	2.08E-15	0.463840735	0.131	0.02	1.38E-10	C8_CD4_Tres
chr9-128007349-128008353	2.17E-15	0.446370455	0.325	0.122	1.43E-10	C8_CD4_Tres
chr22-39951571-39952174	2.40E-15	0.469650461	0.123	0.017	1.59E-10	C8_CD4_Tres
chr21-34128944-34129864	2.43E-15	0.482892232	0.183	0.042	1.61E-10	C8_CD4_Tres
chr2-6880337-6880705	2.51E-15	0.443860051	0.097	0.011	1.66E-10	C8_CD4_Tres
chr14-76929788-76930339	2.68E-15	0.476271061	0.179	0.043	1.77E-10	C8_CD4_Tres
chr14-68977549-68979662	2.73E-15	0.472198925	0.198	0.047	1.81E-10	C8_CD4_Tres
chr16-89114598-89115866	2.85E-15	0.371562104	0.556	0.298	1.89E-10	C8_CD4_Tres
chr21-46626519-46626848	2.90E-15	0.422864209	0.086	0.006	1.92E-10	C8_CD4_Tres
chr1-203512644-203513343	2.91E-15	0.41299969	0.071	0.004	1.93E-10	C8_CD4_Tres
chr21-43921567-43922448	3.03E-15	0.480045105	0.168	0.037	2.01E-10	C8_CD4_Tres
chr1-25622589-25622851	3.19E-15	0.461024866	0.112	0.014	2.11E-10	C8_CD4_Tres
chr3-128448465-128448734	3.31E-15	0.395213995	0.067	0.003	2.19E-10	C8_CD4_Tres
chr9-5627070-5627577	3.34E-15	0.47327631	0.213	0.06	2.21E-10	C8_CD4_Tres

chr13-75760215-75760726	3.65E-15	0.430446522	0.09	0.006	2.42E-10	C8_CD4_Tres
chrX-101485045-101485531	3.68E-15	0.442476542	0.09	0.007	2.43E-10	C8_CD4_Tres
chr8-23243223-23243744	3.82E-15	0.4685377	0.22	0.067	2.53E-10	C8_CD4_Tres
chr13-99375056-99375719	3.95E-15	0.46810837	0.194	0.048	2.61E-10	C8_CD4_Tres
chr5-1315443-1316599	4.02E-15	0.463077628	0.224	0.066	2.66E-10	C8_CD4_Tres
chr18-46155660-46156716	4.12E-15	0.468426133	0.138	0.026	2.73E-10	C8_CD4_Tres
chr2-197847545-197848031	4.13E-15	0.42354818	0.086	0.006	2.74E-10	C8_CD4_Tres
chr6-162727061-162728294	4.89E-15	0.414973672	0.399	0.18	3.24E-10	C8_CD4_Tres
chr1-197391251-197391763	4.98E-15	0.446554993	0.097	0.009	3.30E-10	C8_CD4_Tres
chr17-17182047-17182344	5.15E-15	0.430657676	0.082	0.006	3.41E-10	C8_CD4_Tres
chr5-35853576-35854712	5.21E-15	0.368927681	0.541	0.288	3.45E-10	C8_CD4_Tres
chr5-16629145-16629776	5.34E-15	0.451091233	0.104	0.012	3.53E-10	C8_CD4_Tres
chr10-47264863-47265227	6.84E-15	0.438859171	0.104	0.01	4.53E-10	C8_CD4_Tres
chr18-23977791-23978389	7.00E-15	0.409023754	0.075	0.005	4.63E-10	C8_CD4_Tres
chr8-121348523-121349106	7.01E-15	0.376264347	0.063	0.002	4.65E-10	C8_CD4_Tres
chr1-36366168-36366682	7.49E-15	0.4726906	0.179	0.035	4.96E-10	C8_CD4_Tres
chr3-72100562-72101123	7.49E-15	0.456547826	0.116	0.016	4.96E-10	C8_CD4_Tres
chr5-180832544-180832897	7.66E-15	0.46888081	0.16	0.036	5.07E-10	C8_CD4_Tres
chr8-23250393-23250597	7.68E-15	0.43689454	0.09	0.008	5.09E-10	C8_CD4_Tres
chr11-6402036-6403092	7.71E-15	0.436814164	0.313	0.12	5.11E-10	C8_CD4_Tres
chr12-106301510-106303684	7.87E-15	0.302151748	0.664	0.467	5.21E-10	C8_CD4_Tres
chr15-88627297-88627807	9.00E-15	0.45022428	0.295	0.103	5.96E-10	C8_CD4_Tres
chr6-141153523-141154099	9.07E-15	0.419390092	0.082	0.006	6.01E-10	C8_CD4_Tres
chr9-114777851-114778143	9.09E-15	0.437982234	0.078	0.006	6.02E-10	C8_CD4_Tres
chr1-87044643-87045220	9.26E-15	0.470603639	0.16	0.033	6.13E-10	C8_CD4_Tres
chr1-171434779-171435134	9.67E-15	0.416512449	0.075	0.005	6.41E-10	C8_CD4_Tres
chr20-52972038-52972614	1.06E-14	0.436725548	0.101	0.012	7.04E-10	C8_CD4_Tres

chr5-131547859-131548573	1.21E-14	0.462927148	0.164	0.036	8.04E-10	C8_CD4_Tres
chrX-69615716-69616753	1.23E-14	0.46290417	0.131	0.021	8.13E-10	C8_CD4_Tres
chr3-152218122-152218468	1.24E-14	0.404052424	0.075	0.005	8.18E-10	C8_CD4_Tres
chr13-75762075-75762888	1.24E-14	0.441293383	0.112	0.016	8.24E-10	C8_CD4_Tres
chr16-57023403-57024065	1.26E-14	0.463193162	0.146	0.029	8.35E-10	C8_CD4_Tres
chr11-61021304-61022550	1.27E-14	0.404327255	0.422	0.191	8.38E-10	C8_CD4_Tres
chr19-7348549-7349090	1.38E-14	0.466191926	0.149	0.028	9.13E-10	C8_CD4_Tres
chr2-71226363-71227641	1.40E-14	0.326011124	0.649	0.389	9.29E-10	C8_CD4_Tres
chr8-60967199-60967905	1.45E-14	0.458322942	0.127	0.019	9.58E-10	C8_CD4_Tres
chr9-109107416-109107945	1.45E-14	0.446857375	0.108	0.013	9.60E-10	C8_CD4_Tres
chr22-37696574-37697098	1.45E-14	0.473381642	0.187	0.044	9.63E-10	C8_CD4_Tres
chr2-11757151-11757433	1.52E-14	0.459430942	0.149	0.032	1.01E-09	C8_CD4_Tres
chr5-134090561-134090829	1.58E-14	0.423932954	0.086	0.007	1.04E-09	C8_CD4_Tres
chr15-99421155-99421410	1.60E-14	0.399492213	0.067	0.003	1.06E-09	C8_CD4_Tres
chr6-111113657-111114733	1.81E-14	0.430797721	0.101	0.01	1.20E-09	C8_CD4_Tres
chr1-207320715-207322037	1.88E-14	0.281764382	0.754	0.507	1.25E-09	C8_CD4_Tres
chr5-180688407-180689690	1.96E-14	0.463051564	0.172	0.037	1.30E-09	C8_CD4_Tres
chr6-6614974-6615197	2.18E-14	0.412910135	0.067	0.004	1.44E-09	C8_CD4_Tres
chr6-149261373-149261623	2.22E-14	0.378792156	0.063	0.003	1.47E-09	C8_CD4_Tres
chr21-42460832-42461058	2.27E-14	0.41890278	0.078	0.006	1.51E-09	C8_CD4_Tres
chr12-92297504-92298232	2.34E-14	0.416233943	0.082	0.006	1.55E-09	C8_CD4_Tres
chr2-237581641-237581949	2.34E-14	0.426366817	0.078	0.006	1.55E-09	C8_CD4_Tres
chr8-99884955-99885238	2.59E-14	0.389805977	0.063	0.003	1.72E-09	C8_CD4_Tres
chr2-105753984-105754376	2.63E-14	0.454223751	0.16	0.04	1.74E-09	C8_CD4_Tres
chr2-100813453-100813778	2.65E-14	0.415013579	0.075	0.005	1.75E-09	C8_CD4_Tres
chr6-169959084-169959504	2.72E-14	0.435102904	0.093	0.01	1.80E-09	C8_CD4_Tres
chr1-213762074-213762346	2.80E-14	0.387113945	0.06	0.002	1.85E-09	C8_CD4_Tres

chr10-97454639-97454972	2.83E-14	0.384982727	0.063	0.003	1.88E-09	C8_CD4_Tres
chr11-129279072-129280040	3.16E-14	0.451735918	0.146	0.028	2.09E-09	C8_CD4_Tres
chr3-42501370-42502957	3.31E-14	0.401493149	0.429	0.184	2.19E-09	C8_CD4_Tres
chr3-13544459-13544774	3.45E-14	0.385746234	0.06	0.003	2.29E-09	C8_CD4_Tres
chr17-14404536-14404788	3.47E-14	0.37704643	0.056	0.002	2.30E-09	C8_CD4_Tres
chr22-40009710-40010132	3.51E-14	0.38408586	0.063	0.003	2.32E-09	C8_CD4_Tres
chr6-31260013-31260288	3.55E-14	0.38915063	0.063	0.003	2.35E-09	C8_CD4_Tres
chr2-197888974-197889360	3.56E-14	0.426217066	0.093	0.01	2.36E-09	C8_CD4_Tres
chr4-108138795-108139355	3.62E-14	0.398638463	0.067	0.004	2.40E-09	C8_CD4_Tres
chr1-154464803-154465156	3.79E-14	0.430166587	0.086	0.009	2.51E-09	C8_CD4_Tres
chr1-64505069-64506622	3.83E-14	0.450574631	0.119	0.018	2.54E-09	C8_CD4_Tres
chr6-11710200-11710502	3.84E-14	0.401139407	0.078	0.006	2.54E-09	C8_CD4_Tres
chr8-22531742-22532042	3.84E-14	0.403294702	0.075	0.005	2.54E-09	C8_CD4_Tres
chr5-44875576-44876230	3.86E-14	0.433107823	0.104	0.012	2.56E-09	C8_CD4_Tres
chr5-175796555-175797314	4.07E-14	0.452535373	0.205	0.052	2.70E-09	C8_CD4_Tres
chr15-98547819-98548572	4.17E-14	0.402300955	0.078	0.005	2.76E-09	C8_CD4_Tres
chr17-76256331-76257027	4.17E-14	0.462154896	0.157	0.033	2.76E-09	C8_CD4_Tres
chr11-33893440-33894248	4.37E-14	0.460922075	0.194	0.052	2.90E-09	C8_CD4_Tres
chr12-93245586-93245792	4.59E-14	0.378262137	0.056	0.002	3.04E-09	C8_CD4_Tres
chr19-12194575-12195247	4.74E-14	0.426072826	0.101	0.011	3.14E-09	C8_CD4_Tres
chr6-142331174-142331490	4.82E-14	0.350995257	0.056	0.002	3.19E-09	C8_CD4_Tres
chr2-7567514-7568019	4.97E-14	0.418113871	0.082	0.008	3.29E-09	C8_CD4_Tres
chr11-67416830-67417250	5.64E-14	0.443485085	0.243	0.079	3.74E-09	C8_CD4_Tres
chr6-39724762-39725612	5.66E-14	0.435590622	0.112	0.017	3.75E-09	C8_CD4_Tres
chr16-89201079-89202074	5.76E-14	0.4386539	0.272	0.096	3.81E-09	C8_CD4_Tres
chr3-13865998-13866900	6.24E-14	0.431300062	0.097	0.012	4.14E-09	C8_CD4_Tres
chr3-196111829-196112387	6.58E-14	0.405579324	0.075	0.006	4.36E-09	C8_CD4_Tres

chr5-58459578-58461045	6.73E-14	0.393096654	0.422	0.19	4.46E-09	C8_CD4_Tres
chr20-62891110-62891384	7.03E-14	0.426511797	0.09	0.009	4.66E-09	C8_CD4_Tres
chr17-18955508-18955901	7.10E-14	0.42136257	0.09	0.01	4.70E-09	C8_CD4_Tres
chr9-14993958-14994216	7.48E-14	0.374027637	0.063	0.003	4.95E-09	C8_CD4_Tres
chr5-126988002-126988439	7.52E-14	0.386243713	0.063	0.004	4.98E-09	C8_CD4_Tres
chr11-14078974-14079229	7.53E-14	0.414483029	0.082	0.008	4.98E-09	C8_CD4_Tres
chr10-11710691-11711020	7.79E-14	0.394625426	0.071	0.005	5.16E-09	C8_CD4_Tres
chr6-156953585-156953883	8.00E-14	0.420398936	0.09	0.008	5.30E-09	C8_CD4_Tres
chr2-101651646-101652205	8.92E-14	0.45038251	0.153	0.03	5.91E-09	C8_CD4_Tres
chr14-102020346-102020843	9.10E-14	0.394839817	0.075	0.005	6.03E-09	C8_CD4_Tres
chr19-22633794-22634686	1.02E-13	0.443092582	0.25	0.076	6.74E-09	C8_CD4_Tres
chr10-102673903-102674490	1.08E-13	0.447848918	0.168	0.04	7.14E-09	C8_CD4_Tres
chr1-101130048-101130248	1.08E-13	0.38640751	0.067	0.003	7.17E-09	C8_CD4_Tres
chr7-28328234-28328444	1.12E-13	0.374685606	0.06	0.003	7.43E-09	C8_CD4_Tres
chr1-38558924-38559993	1.22E-13	0.419547607	0.321	0.134	8.11E-09	C8_CD4_Tres
chr1-167489352-167489861	1.41E-13	0.446105727	0.16	0.037	9.33E-09	C8_CD4_Tres
chr10-22029358-22029829	1.41E-13	0.412707391	0.086	0.008	9.35E-09	C8_CD4_Tres
chr16-89122267-89122811	1.47E-13	0.437787336	0.123	0.021	9.75E-09	C8_CD4_Tres
chr4-108359348-108359631	1.55E-13	0.398646555	0.067	0.004	1.03E-08	C8_CD4_Tres
chr5-62299587-62299927	1.65E-13	0.416080812	0.09	0.009	1.09E-08	C8_CD4_Tres
chr7-105924847-105925186	1.85E-13	0.385794809	0.06	0.004	1.23E-08	C8_CD4_Tres
chr21-20997461-20998532	1.90E-13	0.435629421	0.116	0.02	1.26E-08	C8_CD4_Tres
chrX-357378-357978	1.96E-13	0.42847315	0.127	0.02	1.30E-08	C8_CD4_Tres
chr5-80135831-80137154	2.04E-13	0.441359557	0.175	0.041	1.35E-08	C8_CD4_Tres
chr19-8591726-8592924	2.34E-13	0.370360328	0.463	0.226	1.55E-08	C8_CD4_Tres
chr11-128544697-128544987	2.47E-13	0.384999646	0.071	0.005	1.63E-08	C8_CD4_Tres
chr4-88737500-88737919	2.50E-13	0.354077044	0.052	0.002	1.65E-08	C8_CD4_Tres

chr18-5214956-5215251	2.55E-13	0.374484106	0.06	0.003	1.69E-08	C8_CD4_Tres
chr9-90102270-90102634	2.57E-13	0.445964615	0.168	0.038	1.70E-08	C8_CD4_Tres
chr6-11728180-11728899	2.61E-13	0.396560569	0.075	0.006	1.73E-08	C8_CD4_Tres
chr6-135102750-135103608	2.66E-13	0.446183284	0.168	0.041	1.76E-08	C8_CD4_Tres
chr22-39412291-39413039	2.71E-13	0.439987638	0.157	0.036	1.80E-08	C8_CD4_Tres
chr1-98870973-98871909	2.75E-13	0.412666665	0.332	0.127	1.82E-08	C8_CD4_Tres
chr14-96126238-96126754	2.78E-13	0.400789074	0.082	0.008	1.84E-08	C8_CD4_Tres
chr14-32200889-32203910	3.05E-13	0.37469686	0.433	0.205	2.02E-08	C8_CD4_Tres
chr1-54460259-54460673	3.07E-13	0.383251264	0.067	0.004	2.03E-08	C8_CD4_Tres
chrX-108018411-108019284	3.09E-13	0.445444914	0.187	0.048	2.05E-08	C8_CD4_Tres
chr4-108148368-108148644	3.11E-13	0.389971509	0.078	0.006	2.06E-08	C8_CD4_Tres
chr1-83860408-83861157	3.25E-13	0.427799433	0.116	0.018	2.15E-08	C8_CD4_Tres
chr15-63859942-63860845	3.25E-13	0.416065297	0.097	0.011	2.15E-08	C8_CD4_Tres
chr5-75028163-75028381	3.28E-13	0.351593667	0.052	0.002	2.17E-08	C8_CD4_Tres
chr1-8970648-8971391	3.51E-13	0.434662979	0.146	0.03	2.33E-08	C8_CD4_Tres
chr9-133700751-133703054	3.71E-13	0.392723621	0.354	0.162	2.46E-08	C8_CD4_Tres
chr1-27161621-27162000	3.79E-13	0.391007916	0.071	0.005	2.51E-08	C8_CD4_Tres
chr10-14571730-14572503	3.82E-13	0.393827319	0.392	0.168	2.53E-08	C8_CD4_Tres
chr2-25273301-25273885	3.85E-13	0.435587217	0.216	0.068	2.55E-08	C8_CD4_Tres
chr6-149065962-149066492	3.96E-13	0.437164833	0.134	0.027	2.62E-08	C8_CD4_Tres
chr7-4739373-4740008	3.99E-13	0.418691987	0.104	0.013	2.64E-08	C8_CD4_Tres
chr19-5293115-5293643	4.23E-13	0.416479054	0.097	0.01	2.80E-08	C8_CD4_Tres
chr5-128196809-128197034	4.38E-13	0.36729671	0.06	0.003	2.90E-08	C8_CD4_Tres
chr20-53216564-53217358	4.38E-13	0.425235237	0.112	0.018	2.90E-08	C8_CD4_Tres
chr7-151768807-151769594	4.53E-13	0.437798927	0.235	0.073	3.00E-08	C8_CD4_Tres
chr17-73171790-73172182	4.66E-13	0.422561868	0.101	0.013	3.08E-08	C8_CD4_Tres
chr3-71128964-71131034	4.68E-13	0.397045328	0.328	0.151	3.10E-08	C8_CD4_Tres

chr3-141306926-141307575	4.69E-13	0.431690069	0.168	0.04	3.10E-08	C8_CD4_Tres
chr17-73722238-73722595	4.94E-13	0.400014833	0.082	0.008	3.27E-08	C8_CD4_Tres
chr6-90273842-90275042	5.00E-13	0.330647389	0.56	0.328	3.31E-08	C8_CD4_Tres
chr3-129481700-129482240	5.19E-13	0.409756268	0.09	0.01	3.44E-08	C8_CD4_Tres
chr12-15788419-15789835	5.27E-13	0.421115834	0.269	0.092	3.49E-08	C8_CD4_Tres
chr5-53031601-53032126	5.28E-13	0.41605245	0.097	0.013	3.50E-08	C8_CD4_Tres
chr10-129035381-129035855	5.47E-13	0.433243792	0.116	0.021	3.62E-08	C8_CD4_Tres
chr22-38266975-38267469	5.74E-13	0.426691794	0.119	0.023	3.80E-08	C8_CD4_Tres
chr1-172764423-172764711	5.95E-13	0.357679194	0.052	0.002	3.94E-08	C8_CD4_Tres
chr8-41971435-41971745	6.12E-13	0.348855556	0.056	0.002	4.05E-08	C8_CD4_Tres
chr2-1518360-1518815	6.20E-13	0.366305859	0.067	0.004	4.10E-08	C8_CD4_Tres
chr22-41873085-41873734	6.34E-13	0.42682249	0.16	0.038	4.20E-08	C8_CD4_Tres
chr4-37453000-37453993	6.36E-13	0.40410833	0.086	0.009	4.21E-08	C8_CD4_Tres
chr12-122393868-122394118	6.68E-13	0.366800713	0.06	0.003	4.42E-08	C8_CD4_Tres
chr8-17697171-17697779	6.73E-13	0.438343853	0.146	0.032	4.46E-08	C8_CD4_Tres
chr6-24359694-24360320	7.15E-13	0.429270772	0.187	0.051	4.74E-08	C8_CD4_Tres
chr4-37780561-37781098	7.83E-13	0.391334558	0.071	0.006	5.19E-08	C8_CD4_Tres
chr3-196218869-196219747	8.01E-13	0.414371462	0.28	0.104	5.30E-08	C8_CD4_Tres
chr15-28831788-28832603	8.11E-13	0.42753234	0.22	0.077	5.37E-08	C8_CD4_Tres
chr1-154784830-154785294	8.18E-13	0.40757222	0.09	0.011	5.42E-08	C8_CD4_Tres
chr5-1294844-1295423	8.24E-13	0.413469263	0.101	0.013	5.46E-08	C8_CD4_Tres
chr15-90561966-90562253	8.29E-13	0.394005278	0.075	0.007	5.49E-08	C8_CD4_Tres
chr22-19886884-19887126	8.57E-13	0.401352283	0.09	0.012	5.68E-08	C8_CD4_Tres
chr15-33194301-33195062	8.84E-13	0.427231201	0.246	0.079	5.86E-08	C8_CD4_Tres
chrX-48936837-48937796	8.98E-13	0.412200807	0.284	0.112	5.94E-08	C8_CD4_Tres
chr3-60017217-60018058	8.98E-13	0.423435755	0.134	0.028	5.95E-08	C8_CD4_Tres
chr13-26012076-26012788	9.29E-13	0.434651608	0.16	0.041	6.15E-08	C8_CD4_Tres

chr4-74444592-74445637	9.34E-13	0.375547565	0.407	0.192	6.18E-08	C8_CD4_Tres
chr22-39661990-39662637	9.36E-13	0.426537876	0.172	0.043	6.20E-08	C8_CD4_Tres
chr19-17605695-17606766	9.53E-13	0.402607337	0.31	0.119	6.31E-08	C8_CD4_Tres
chr18-70683273-70683640	9.55E-13	0.389547885	0.075	0.006	6.33E-08	C8_CD4_Tres
chr17-51096108-51096509	9.99E-13	0.409248349	0.09	0.013	6.61E-08	C8_CD4_Tres
chr17-16583836-16584640	1.00E-12	0.396871847	0.325	0.139	6.62E-08	C8_CD4_Tres
chr7-39633426-39634483	1.01E-12	0.424855863	0.104	0.018	6.70E-08	C8_CD4_Tres
chr15-65887491-65887918	1.14E-12	0.422578709	0.131	0.028	7.52E-08	C8_CD4_Tres
chr3-71299228-71299440	1.15E-12	0.344150046	0.052	0.002	7.65E-08	C8_CD4_Tres
chr2-158191023-158191491	1.17E-12	0.368459833	0.063	0.004	7.73E-08	C8_CD4_Tres
chr7-1925432-1926377	1.20E-12	0.424169464	0.134	0.027	7.92E-08	C8_CD4_Tres
chr1-173444712-173445091	1.22E-12	0.363139309	0.063	0.004	8.09E-08	C8_CD4_Tres
chr6-42045619-42046048	1.23E-12	0.417862277	0.243	0.084	8.12E-08	C8_CD4_Tres
chr9-135978776-135979455	1.23E-12	0.41817824	0.119	0.022	8.16E-08	C8_CD4_Tres
chr9-133682884-133684437	1.29E-12	0.384445181	0.377	0.169	8.53E-08	C8_CD4_Tres
chr15-70558469-70559600	1.29E-12	0.429912926	0.201	0.06	8.57E-08	C8_CD4_Tres
chr11-61041793-61043399	1.32E-12	0.392848754	0.317	0.141	8.71E-08	C8_CD4_Tres
chr4-40124645-40125207	1.33E-12	0.407349374	0.104	0.016	8.80E-08	C8_CD4_Tres
chr11-66317488-66318193	1.37E-12	0.433084386	0.175	0.047	9.07E-08	C8_CD4_Tres
chr15-67758465-67758909	1.40E-12	0.394486313	0.082	0.009	9.25E-08	C8_CD4_Tres
chr17-41786018-41787098	1.41E-12	0.430946028	0.216	0.068	9.36E-08	C8_CD4_Tres
chr7-21945360-21946244	1.47E-12	0.423759396	0.123	0.025	9.72E-08	C8_CD4_Tres
chr19-29830956-29831925	1.49E-12	0.421985478	0.119	0.021	9.83E-08	C8_CD4_Tres
chr9-125195063-125195364	1.49E-12	0.410769796	0.104	0.018	9.84E-08	C8_CD4_Tres
chr15-69782900-69783138	1.49E-12	0.366243895	0.06	0.004	9.87E-08	C8_CD4_Tres
chr8-24914285-24915131	1.52E-12	0.42566063	0.16	0.036	1.01E-07	C8_CD4_Tres
chr11-68471674-68472426	1.53E-12	0.421202062	0.19	0.066	1.02E-07	C8_CD4_Tres

chr6-105781918-105782269	1.55E-12	0.387634415	0.078	0.008	1.02E-07	C8_CD4_Tres
chrX-4240048-4241265	1.55E-12	0.418820529	0.104	0.017	1.03E-07	C8_CD4_Tres
chr6-11714291-11714827	1.57E-12	0.378872869	0.063	0.005	1.04E-07	C8_CD4_Tres
chr1-179709797-179710157	1.65E-12	0.391173391	0.071	0.007	1.09E-07	C8_CD4_Tres
chr8-119982109-119982764	1.69E-12	0.402072162	0.093	0.012	1.12E-07	C8_CD4_Tres
chr17-31310327-31310551	1.77E-12	0.423545743	0.157	0.038	1.17E-07	C8_CD4_Tres
chr17-57295178-57295461	1.83E-12	0.379902879	0.075	0.006	1.21E-07	C8_CD4_Tres
chr1-40385443-40386241	1.87E-12	0.327153788	0.515	0.306	1.24E-07	C8_CD4_Tres
chr19-45558192-45558736	1.93E-12	0.420861043	0.205	0.063	1.28E-07	C8_CD4_Tres
chrX-153582906-153583225	2.07E-12	0.415672324	0.104	0.018	1.37E-07	C8_CD4_Tres
chr14-102948969-102949652	2.08E-12	0.394920092	0.082	0.01	1.37E-07	C8_CD4_Tres
chr6-6609858-6610564	2.19E-12	0.416288526	0.127	0.028	1.45E-07	C8_CD4_Tres
chr11-114159059-114160400	2.19E-12	0.356863503	0.444	0.227	1.45E-07	C8_CD4_Tres
chr14-74922627-74923500	2.21E-12	0.42319175	0.153	0.035	1.46E-07	C8_CD4_Tres
chr17-418361-418609	2.22E-12	0.423846635	0.127	0.025	1.47E-07	C8_CD4_Tres
chr10-47257533-47258095	2.35E-12	0.410624714	0.112	0.018	1.56E-07	C8_CD4_Tres
chr14-51093870-51095661	2.36E-12	0.386789656	0.34	0.146	1.56E-07	C8_CD4_Tres
chr9-104984619-104984820	2.39E-12	0.342764936	0.056	0.002	1.58E-07	C8_CD4_Tres
chr14-68506586-68508247	2.49E-12	0.407732068	0.25	0.095	1.65E-07	C8_CD4_Tres
chr13-25888053-25888786	2.52E-12	0.389918384	0.09	0.011	1.67E-07	C8_CD4_Tres
chr14-70660997-70661751	2.66E-12	0.400570496	0.09	0.013	1.76E-07	C8_CD4_Tres
chr19-49473836-49474645	2.78E-12	0.345629709	0.44	0.24	1.84E-07	C8_CD4_Tres
chr22-39937944-39938146	3.00E-12	0.412924616	0.112	0.019	1.99E-07	C8_CD4_Tres
chr7-142508201-142508632	3.30E-12	0.389448183	0.075	0.007	2.18E-07	C8_CD4_Tres
chr7-157626395-157626679	3.33E-12	0.325789763	0.052	0.002	2.21E-07	C8_CD4_Tres
chr12-11727264-11727491	3.41E-12	0.367012251	0.06	0.004	2.26E-07	C8_CD4_Tres
chr1-19462870-19463619	3.53E-12	0.416295282	0.205	0.067	2.34E-07	C8_CD4_Tres

chr18-8755328-8755976	3.53E-12	0.421689233	0.172	0.049	2.34E-07	C8_CD4_Tres
chr11-129861062-129861355	3.71E-12	0.369217101	0.063	0.004	2.45E-07	C8_CD4_Tres
chr9-33081168-33081585	3.81E-12	0.41773223	0.187	0.058	2.53E-07	C8_CD4_Tres
chr1-23601107-23601311	3.88E-12	0.360077549	0.063	0.004	2.57E-07	C8_CD4_Tres
chr10-4825731-4826761	4.03E-12	0.421898423	0.175	0.051	2.67E-07	C8_CD4_Tres
chr19-47190133-47190450	4.18E-12	0.417110167	0.205	0.061	2.77E-07	C8_CD4_Tres
chr3-10603070-10603869	4.18E-12	0.419308124	0.131	0.026	2.77E-07	C8_CD4_Tres
chr3-142028337-142028705	4.24E-12	0.385658276	0.078	0.009	2.81E-07	C8_CD4_Tres
chr7-150973670-150975198	4.25E-12	0.375233099	0.351	0.176	2.81E-07	C8_CD4_Tres
chr6-1527140-1527358	4.29E-12	0.349159795	0.056	0.003	2.84E-07	C8_CD4_Tres
chr1-234522517-234523111	4.40E-12	0.390134299	0.078	0.009	2.91E-07	C8_CD4_Tres
chr4-2786175-2786471	4.53E-12	0.391619783	0.086	0.011	3.00E-07	C8_CD4_Tres
chr2-202642658-202643784	4.55E-12	0.407472046	0.108	0.019	3.01E-07	C8_CD4_Tres
chr8-70631357-70631636	4.56E-12	0.376608229	0.067	0.005	3.02E-07	C8_CD4_Tres
chr3-112357705-112358689	4.62E-12	0.399277463	0.101	0.015	3.06E-07	C8_CD4_Tres
chr2-96531850-96532475	4.86E-12	0.348039783	0.429	0.236	3.22E-07	C8_CD4_Tres
chr4-152183573-152183809	4.96E-12	0.337480324	0.052	0.002	3.28E-07	C8_CD4_Tres
chr3-72756760-72757362	5.02E-12	0.412384587	0.131	0.027	3.32E-07	C8_CD4_Tres
chr9-96491495-96491795	5.03E-12	0.357646462	0.06	0.004	3.33E-07	C8_CD4_Tres
chr19-56477290-56478164	5.06E-12	0.374784184	0.063	0.005	3.35E-07	C8_CD4_Tres
chr8-2000724-2002065	5.18E-12	0.406931901	0.131	0.027	3.43E-07	C8_CD4_Tres
chr7-150484128-150484446	5.24E-12	0.39617005	0.104	0.016	3.47E-07	C8_CD4_Tres
chr17-66201796-66202059	5.35E-12	0.398086107	0.086	0.011	3.54E-07	C8_CD4_Tres
chr17-39016815-39017089	5.56E-12	0.364216216	0.06	0.004	3.68E-07	C8_CD4_Tres
chr2-196914866-196915774	5.73E-12	0.412946662	0.153	0.035	3.79E-07	C8_CD4_Tres
chr5-131536468-131536758	5.86E-12	0.401361165	0.093	0.012	3.88E-07	C8_CD4_Tres
chr1-220689916-220690702	5.88E-12	0.414708628	0.168	0.043	3.89E-07	C8_CD4_Tres

chr21-38277432-38277709	5.92E-12	0.340860232	0.056	0.003	3.92E-07	C8_CD4_Tres
chr10-12605506-12606321	6.00E-12	0.400516557	0.112	0.017	3.98E-07	C8_CD4_Tres
chr22-31345113-31347731	6.02E-12	0.27785922	0.627	0.434	3.99E-07	C8_CD4_Tres
chr1-194056262-194056629	6.12E-12	0.359452577	0.067	0.005	4.06E-07	C8_CD4_Tres
chr20-53022034-53022260	6.39E-12	0.346909552	0.06	0.003	4.23E-07	C8_CD4_Tres
chr12-44863866-44864129	6.46E-12	0.360953163	0.06	0.004	4.27E-07	C8_CD4_Tres
chr12-1277840-1278243	6.50E-12	0.387529799	0.082	0.01	4.30E-07	C8_CD4_Tres
chr6-142902323-142903046	6.50E-12	0.405797693	0.213	0.071	4.31E-07	C8_CD4_Tres
chr19-32793912-32794828	6.62E-12	0.394476423	0.097	0.014	4.38E-07	C8_CD4_Tres
chr18-58687252-58688390	6.79E-12	0.402210079	0.25	0.095	4.49E-07	C8_CD4_Tres
chr4-98662779-98663780	7.05E-12	0.371362352	0.369	0.173	4.67E-07	C8_CD4_Tres
chr11-47523159-47524256	7.10E-12	0.414767628	0.164	0.044	4.70E-07	C8_CD4_Tres
chr1-159945367-159946057	7.16E-12	0.413512295	0.209	0.064	4.74E-07	C8_CD4_Tres
chr12-124608564-124609482	7.30E-12	0.407580581	0.194	0.06	4.84E-07	C8_CD4_Tres
chr1-8525085-8526207	7.46E-12	0.382992218	0.343	0.147	4.94E-07	C8_CD4_Tres
chr12-110349725-110350113	7.51E-12	0.368548469	0.067	0.006	4.97E-07	C8_CD4_Tres
chr16-19696293-19696702	7.84E-12	0.360057179	0.067	0.005	5.19E-07	C8_CD4_Tres
chr6-3456460-3457372	7.86E-12	0.412637336	0.198	0.058	5.20E-07	C8_CD4_Tres
chr18-26225894-26227146	7.91E-12	0.366312218	0.366	0.173	5.24E-07	C8_CD4_Tres
chr11-73995018-73996131	8.02E-12	0.391435189	0.284	0.11	5.31E-07	C8_CD4_Tres
chr11-60985594-60986875	8.47E-12	0.296189073	0.601	0.379	5.61E-07	C8_CD4_Tres
chr7-17118432-17118715	8.54E-12	0.368107818	0.067	0.006	5.66E-07	C8_CD4_Tres
chr3-32371484-32371802	8.89E-12	0.362950618	0.067	0.006	5.89E-07	C8_CD4_Tres
chr6-41199481-41201253	9.21E-12	0.409160751	0.168	0.052	6.10E-07	C8_CD4_Tres
chr1-77281695-77282470	9.54E-12	0.38606549	0.093	0.012	6.31E-07	C8_CD4_Tres
chr10-110010089-110010450	1.05E-11	0.386585133	0.313	0.133	6.97E-07	C8_CD4_Tres
chr9-127387926-127388843	1.08E-11	0.376624425	0.34	0.156	7.16E-07	C8_CD4_Tres

chr2-171247964-171248702	1.10E-11	0.394030193	0.104	0.019	7.28E-07	C8_CD4_Tres
chr10-22655049-22655957	1.10E-11	0.402781991	0.224	0.075	7.29E-07	C8_CD4_Tres
chr14-104393705-104394366	1.11E-11	0.401276422	0.112	0.02	7.35E-07	C8_CD4_Tres
chr10-104170658-104171154	1.11E-11	0.367719643	0.075	0.007	7.38E-07	C8_CD4_Tres
chr2-70767071-70768359	1.19E-11	0.386987727	0.09	0.012	7.87E-07	C8_CD4_Tres
chr2-101706724-101707056	1.22E-11	0.403382211	0.123	0.025	8.07E-07	C8_CD4_Tres
chr2-12081486-12081862	1.23E-11	0.349529175	0.06	0.004	8.14E-07	C8_CD4_Tres
chr9-93028081-93029070	1.24E-11	0.39310455	0.265	0.097	8.23E-07	C8_CD4_Tres
chr6-127918294-127919070	1.27E-11	0.403109713	0.19	0.055	8.39E-07	C8_CD4_Tres
chr7-98202429-98202861	1.30E-11	0.405035242	0.112	0.02	8.60E-07	C8_CD4_Tres
chr5-43110555-43111096	1.31E-11	0.380370665	0.078	0.008	8.71E-07	C8_CD4_Tres
chr1-25675673-25676760	1.34E-11	0.395056384	0.254	0.098	8.86E-07	C8_CD4_Tres
chr6-142551138-142551692	1.35E-11	0.346015117	0.056	0.004	8.97E-07	C8_CD4_Tres
chr8-127980149-127981716	1.38E-11	0.345306707	0.444	0.225	9.13E-07	C8_CD4_Tres
chr3-32459616-32460665	1.38E-11	0.366277602	0.362	0.17	9.17E-07	C8_CD4_Tres
chr8-70616554-70617291	1.45E-11	0.36834224	0.075	0.008	9.59E-07	C8_CD4_Tres
chr4-112296933-112297456	1.55E-11	0.38204202	0.078	0.01	1.02E-06	C8_CD4_Tres
chr15-78072560-78072809	1.58E-11	0.396133058	0.112	0.023	1.04E-06	C8_CD4_Tres
chr1-167491811-167492884	1.59E-11	0.395816364	0.22	0.08	1.05E-06	C8_CD4_Tres
chr5-157303587-157304308	1.64E-11	0.383554712	0.09	0.012	1.09E-06	C8_CD4_Tres
chr14-34930363-34931589	1.66E-11	0.393370964	0.272	0.106	1.10E-06	C8_CD4_Tres
chr18-13216112-13218047	1.67E-11	0.395823173	0.25	0.092	1.11E-06	C8_CD4_Tres
chr5-110043007-110043350	1.75E-11	0.341044401	0.06	0.004	1.16E-06	C8_CD4_Tres
chr4-153248607-153249600	1.79E-11	0.397086762	0.243	0.091	1.19E-06	C8_CD4_Tres
chr4-112183307-112183641	1.84E-11	0.344962088	0.056	0.003	1.22E-06	C8_CD4_Tres
chr11-61101814-61103611	1.88E-11	0.365106239	0.336	0.158	1.25E-06	C8_CD4_Tres
chr4-108141289-108141542	1.93E-11	0.344267314	0.06	0.004	1.28E-06	C8_CD4_Tres

chr16-324648-325396	1.94E-11	0.395254483	0.22	0.081	1.28E-06	C8_CD4_Tres
chr8-26440270-26441115	1.95E-11	0.357369812	0.067	0.005	1.29E-06	C8_CD4_Tres
chr2-171086490-171087104	1.98E-11	0.377422313	0.086	0.012	1.31E-06	C8_CD4_Tres
chr15-26081659-26082831	2.03E-11	0.374317409	0.321	0.142	1.35E-06	C8_CD4_Tres
chr19-40398745-40399080	2.07E-11	0.381368336	0.093	0.016	1.37E-06	C8_CD4_Tres
chr1-207363091-207363859	2.15E-11	0.39243421	0.112	0.018	1.42E-06	C8_CD4_Tres
chr22-28822875-28823407	2.17E-11	0.372658753	0.071	0.008	1.44E-06	C8_CD4_Tres
chr1-101308995-101310257	2.28E-11	0.340124808	0.429	0.224	1.51E-06	C8_CD4_Tres
chr14-61670928-61671527	2.30E-11	0.400265913	0.164	0.046	1.52E-06	C8_CD4_Tres
chr12-122452797-122454117	2.40E-11	0.394371556	0.224	0.082	1.59E-06	C8_CD4_Tres
chr4-165378697-165379476	2.46E-11	0.3854507	0.09	0.014	1.63E-06	C8_CD4_Tres
chr10-5446393-5446718	2.47E-11	0.392032927	0.104	0.017	1.63E-06	C8_CD4_Tres
chr1-167090160-167090929	2.47E-11	0.355372606	0.075	0.008	1.63E-06	C8_CD4_Tres
chr17-80663679-80664212	2.52E-11	0.364400423	0.067	0.006	1.67E-06	C8_CD4_Tres
chr12-132185814-132186088	2.56E-11	0.387631213	0.097	0.015	1.69E-06	C8_CD4_Tres
chr12-11555745-11556176	2.67E-11	0.323899983	0.052	0.003	1.77E-06	C8_CD4_Tres
chr1-109501891-109502870	2.67E-11	0.398720469	0.216	0.071	1.77E-06	C8_CD4_Tres
chr15-64514409-64514781	2.84E-11	0.343713079	0.056	0.003	1.88E-06	C8_CD4_Tres
chr6-15365294-15365525	2.86E-11	0.327754507	0.052	0.003	1.89E-06	C8_CD4_Tres
chrY-21180928-21181223	2.98E-11	0.362075376	0.071	0.007	1.98E-06	C8_CD4_Tres
chr20-53077566-53078140	3.01E-11	0.331230731	0.056	0.003	2.00E-06	C8_CD4_Tres
chr18-32531172-32531537	3.04E-11	0.320584819	0.052	0.002	2.01E-06	C8_CD4_Tres
chr2-127641876-127642449	3.16E-11	0.371335345	0.086	0.013	2.09E-06	C8_CD4_Tres
chr1-89781647-89782067	3.18E-11	0.357870358	0.067	0.007	2.11E-06	C8_CD4_Tres
chr4-100093210-100093831	3.19E-11	0.374591633	0.31	0.141	2.12E-06	C8_CD4_Tres
chr7-87216777-87217284	3.23E-11	0.401692326	0.16	0.047	2.14E-06	C8_CD4_Tres
chr22-33920577-33921368	3.35E-11	0.401360904	0.153	0.039	2.22E-06	C8_CD4_Tres

chr5-80192546-80193054	3.47E-11	0.374594747	0.086	0.012	2.30E-06	C8_CD4_Tres
chr12-92403421-92404233	3.61E-11	0.384358794	0.25	0.092	2.39E-06	C8_CD4_Tres
chr7-42887930-42888988	3.71E-11	0.381532005	0.097	0.016	2.46E-06	C8_CD4_Tres
chr1-160711561-160713027	3.74E-11	0.365245099	0.325	0.146	2.47E-06	C8_CD4_Tres
chr17-65556229-65556886	3.74E-11	0.398419857	0.168	0.048	2.47E-06	C8_CD4_Tres
chr1-111723260-111723743	3.84E-11	0.384788372	0.09	0.014	2.55E-06	C8_CD4_Tres
chr8-1823259-1824173	4.09E-11	0.389149446	0.112	0.019	2.71E-06	C8_CD4_Tres
chr6-24838942-24839263	4.13E-11	0.332250639	0.056	0.003	2.73E-06	C8_CD4_Tres
chr6-43223830-43224783	4.24E-11	0.367131777	0.34	0.155	2.81E-06	C8_CD4_Tres
chr9-133676693-133677641	4.24E-11	0.372827165	0.321	0.142	2.81E-06	C8_CD4_Tres
chr15-65981872-65982567	4.26E-11	0.401936257	0.153	0.038	2.82E-06	C8_CD4_Tres
chr7-50739678-50740449	4.32E-11	0.388134372	0.269	0.105	2.86E-06	C8_CD4_Tres
chr1-85200332-85202171	4.36E-11	0.331945775	0.459	0.24	2.89E-06	C8_CD4_Tres
chr10-80465244-80466204	4.51E-11	0.385735657	0.246	0.099	2.98E-06	C8_CD4_Tres
chr19-1081870-1082759	4.51E-11	0.346956901	0.407	0.196	2.98E-06	C8_CD4_Tres
chr16-50602316-50602849	4.52E-11	0.389664591	0.108	0.019	2.99E-06	C8_CD4_Tres
chr14-98188857-98190141	4.55E-11	0.392401406	0.175	0.053	3.02E-06	C8_CD4_Tres
chrX-9456144-9457013	4.56E-11	0.399143019	0.19	0.057	3.02E-06	C8_CD4_Tres
chr19-34997036-34997412	4.62E-11	0.386180839	0.108	0.022	3.06E-06	C8_CD4_Tres
chr7-906776-907415	4.65E-11	0.370742483	0.093	0.014	3.08E-06	C8_CD4_Tres
chr20-21548084-21549045	4.83E-11	0.395421669	0.164	0.043	3.20E-06	C8_CD4_Tres
chr2-197145428-197146035	4.87E-11	0.390031835	0.104	0.022	3.23E-06	C8_CD4_Tres
chr2-23532362-23532924	4.88E-11	0.343931813	0.063	0.006	3.23E-06	C8_CD4_Tres
chr10-105838031-105838269	4.95E-11	0.332384303	0.052	0.003	3.28E-06	C8_CD4_Tres
chr15-82680039-82681005	4.98E-11	0.371695502	0.31	0.132	3.30E-06	C8_CD4_Tres
chr5-96962480-96963507	5.13E-11	0.39809641	0.146	0.036	3.40E-06	C8_CD4_Tres
chr1-150281288-150282645	5.47E-11	0.36200169	0.325	0.145	3.62E-06	C8_CD4_Tres

chr12-117968732-117969440	5.48E-11	0.370139459	0.082	0.011	3.63E-06	C8_CD4_Tres
chr4-40213881-40214437	5.68E-11	0.393096442	0.194	0.067	3.76E-06	C8_CD4_Tres
chr19-6890286-6891246	5.69E-11	0.3647167	0.078	0.011	3.77E-06	C8_CD4_Tres
chr12-54983414-54985312	5.70E-11	0.353373044	0.377	0.182	3.77E-06	C8_CD4_Tres
chr15-90677895-90678739	5.77E-11	0.357231068	0.075	0.009	3.82E-06	C8_CD4_Tres
chr5-56155019-56155352	6.04E-11	0.350484787	0.071	0.007	4.00E-06	C8_CD4_Tres
chr13-114067370-114068148	6.22E-11	0.392262745	0.149	0.043	4.12E-06	C8_CD4_Tres
chr10-91200803-91202016	6.43E-11	0.384847362	0.134	0.031	4.26E-06	C8_CD4_Tres
chr12-121240101-121240961	6.53E-11	0.368984713	0.317	0.139	4.33E-06	C8_CD4_Tres
chr17-1870196-1870964	6.62E-11	0.390505254	0.138	0.033	4.38E-06	C8_CD4_Tres
chr22-45179215-45179727	6.64E-11	0.398027008	0.146	0.035	4.40E-06	C8_CD4_Tres
chr17-40598608-40599586	6.66E-11	0.374474685	0.284	0.12	4.41E-06	C8_CD4_Tres
chr12-92912103-92912475	7.04E-11	0.349888217	0.063	0.006	4.66E-06	C8_CD4_Tres
chr7-2508217-2509237	7.25E-11	0.344285487	0.396	0.2	4.80E-06	C8_CD4_Tres
chr1-222654388-222655336	7.31E-11	0.381623478	0.257	0.099	4.84E-06	C8_CD4_Tres
chr10-114499612-114499898	7.42E-11	0.37405546	0.097	0.014	4.92E-06	C8_CD4_Tres
chr2-54897516-54898153	7.61E-11	0.341339963	0.06	0.005	5.04E-06	C8_CD4_Tres
chr3-32334530-32334970	7.99E-11	0.35294906	0.071	0.008	5.29E-06	C8_CD4_Tres
chr15-25859700-25860183	8.33E-11	0.396169916	0.157	0.041	5.52E-06	C8_CD4_Tres
chr22-49945290-49945804	8.67E-11	0.392955177	0.134	0.033	5.74E-06	C8_CD4_Tres
chr5-157327866-157328727	8.68E-11	0.385893405	0.175	0.054	5.75E-06	C8_CD4_Tres
chr3-36892776-36893364	8.95E-11	0.391154042	0.164	0.048	5.93E-06	C8_CD4_Tres
chr3-119352167-119352495	8.97E-11	0.360934135	0.075	0.01	5.94E-06	C8_CD4_Tres
chr13-114148040-114148970	8.97E-11	0.300540969	0.515	0.318	5.94E-06	C8_CD4_Tres
chrX-16484307-16484638	9.17E-11	0.345886312	0.063	0.006	6.07E-06	C8_CD4_Tres
chr17-32503104-32504258	9.54E-11	0.39371259	0.179	0.053	6.31E-06	C8_CD4_Tres
chr8-144685265-144686815	9.55E-11	0.353645053	0.34	0.157	6.32E-06	C8_CD4_Tres

chr17-66399056-66399763	9.65E-11	0.370022043	0.09	0.013	6.39E-06	C8_CD4_Tres
chr7-5487835-5488047	9.66E-11	0.327987526	0.056	0.004	6.40E-06	C8_CD4_Tres
chr1-66214756-66215294	9.68E-11	0.385882589	0.119	0.025	6.41E-06	C8_CD4_Tres
chr21-36985829-36986041	1.01E-10	0.365524127	0.082	0.01	6.66E-06	C8_CD4_Tres
chr5-16616150-16617914	1.02E-10	0.359283521	0.321	0.148	6.74E-06	C8_CD4_Tres
chr12-67068411-67069811	1.04E-10	0.368182188	0.097	0.016	6.86E-06	C8_CD4_Tres
chr19-56476619-56477061	1.06E-10	0.33351873	0.056	0.004	7.01E-06	C8_CD4_Tres
chr19-4326855-4328981	1.17E-10	0.273295575	0.604	0.398	7.75E-06	C8_CD4_Tres
chr12-133129544-133131007	1.20E-10	0.275306749	0.601	0.393	7.97E-06	C8_CD4_Tres
chr17-65560758-65561933	1.20E-10	0.333390886	0.418	0.213	7.98E-06	C8_CD4_Tres
chr6-6515618-6516015	1.22E-10	0.345952041	0.056	0.005	8.08E-06	C8_CD4_Tres
chr19-35206384-35206844	1.24E-10	0.381974683	0.179	0.058	8.24E-06	C8_CD4_Tres
chr10-29681277-29682324	1.28E-10	0.387453451	0.146	0.041	8.48E-06	C8_CD4_Tres
chr9-36492794-36493240	1.30E-10	0.366678209	0.101	0.018	8.60E-06	C8_CD4_Tres
chr2-204967158-204968010	1.31E-10	0.362888396	0.086	0.013	8.70E-06	C8_CD4_Tres
chr13-36345038-36346947	1.32E-10	0.358790723	0.336	0.148	8.72E-06	C8_CD4_Tres
chr1-223813232-223813511	1.32E-10	0.314127004	0.052	0.003	8.72E-06	C8_CD4_Tres
chr3-42526619-42526984	1.32E-10	0.344501205	0.071	0.007	8.74E-06	C8_CD4_Tres
chr12-104720082-104721014	1.37E-10	0.369567134	0.269	0.112	9.09E-06	C8_CD4_Tres
chr1-8510083-8510753	1.43E-10	0.372091844	0.101	0.02	9.47E-06	C8_CD4_Tres
chr2-216142546-216142850	1.44E-10	0.325909545	0.06	0.004	9.52E-06	C8_CD4_Tres
chr4-153426261-153426826	1.46E-10	0.333859089	0.067	0.006	9.69E-06	C8_CD4_Tres
chr3-71304755-71304959	1.49E-10	0.330161126	0.06	0.005	9.84E-06	C8_CD4_Tres
chr10-42932711-42933021	1.49E-10	0.335878546	0.056	0.005	9.85E-06	C8_CD4_Tres
chr19-47145818-47146354	1.55E-10	0.350675511	0.075	0.01	1.03E-05	C8_CD4_Tres
chr1-6364892-6365461	1.65E-10	0.379413166	0.123	0.027	1.09E-05	C8_CD4_Tres
chr17-21550977-21551933	1.71E-10	0.363288925	0.276	0.12	1.13E-05	C8_CD4_Tres

chr3-187020348-187020582	1.72E-10	0.330629925	0.056	0.005	1.14E-05	C8_CD4_Tres
chr15-63436643-63437150	1.74E-10	0.355578519	0.078	0.01	1.15E-05	C8_CD4_Tres
chr7-139731259-139731566	1.80E-10	0.314975946	0.052	0.003	1.19E-05	C8_CD4_Tres
chr13-99384002-99384307	1.84E-10	0.378144205	0.116	0.022	1.22E-05	C8_CD4_Tres
chr3-61250801-61251671	1.85E-10	0.382718386	0.194	0.062	1.23E-05	C8_CD4_Tres
chr13-24159964-24161449	1.89E-10	0.341433159	0.392	0.194	1.25E-05	C8_CD4_Tres
chr16-3508930-3509980	1.91E-10	0.383487924	0.138	0.032	1.26E-05	C8_CD4_Tres
chr22-40289306-40290585	1.92E-10	0.363280107	0.299	0.127	1.27E-05	C8_CD4_Tres
chr6-130513406-130514207	2.04E-10	0.369916572	0.239	0.094	1.35E-05	C8_CD4_Tres
chr17-29722473-29723104	2.08E-10	0.378783232	0.119	0.026	1.37E-05	C8_CD4_Tres
chr1-172867272-172867710	2.08E-10	0.340846156	0.067	0.007	1.38E-05	C8_CD4_Tres
chr20-1225233-1226686	2.11E-10	0.353681933	0.325	0.145	1.40E-05	C8_CD4_Tres
chr17-39800325-39800862	2.17E-10	0.381366122	0.142	0.038	1.44E-05	C8_CD4_Tres
chr16-28148993-28149703	2.19E-10	0.343337961	0.071	0.008	1.45E-05	C8_CD4_Tres
chr4-83083666-83084456	2.25E-10	0.385351806	0.146	0.038	1.49E-05	C8_CD4_Tres
chr9-15354870-15355085	2.26E-10	0.308861413	0.052	0.003	1.50E-05	C8_CD4_Tres
chr3-13650247-13650775	2.26E-10	0.379608701	0.138	0.036	1.50E-05	C8_CD4_Tres
chr2-197152406-197152777	2.27E-10	0.353140829	0.071	0.009	1.50E-05	C8_CD4_Tres
chr7-2510808-2511146	2.28E-10	0.376834352	0.116	0.023	1.51E-05	C8_CD4_Tres
chr14-49977057-49977627	2.31E-10	0.381928182	0.123	0.029	1.53E-05	C8_CD4_Tres
chr9-36765625-36766346	2.33E-10	0.354266518	0.325	0.151	1.54E-05	C8_CD4_Tres
chr17-78758770-78759834	2.36E-10	0.354804651	0.302	0.134	1.56E-05	C8_CD4_Tres
chr2-108988785-108989405	2.45E-10	0.327630041	0.056	0.004	1.62E-05	C8_CD4_Tres
chr8-127169702-127170329	2.45E-10	0.363107367	0.097	0.018	1.62E-05	C8_CD4_Tres
chr4-48133787-48134385	2.48E-10	0.362676048	0.093	0.015	1.64E-05	C8_CD4_Tres
chr4-2713961-2714541	2.62E-10	0.351375164	0.078	0.01	1.74E-05	C8_CD4_Tres
chr3-37860357-37860945	2.66E-10	0.375823258	0.119	0.028	1.76E-05	C8_CD4_Tres

chr16-84765952-84766257	2.67E-10	0.309161364	0.052	0.003	1.77E-05	C8_CD4_Tres
chr9-129320538-129321322	2.69E-10	0.379201845	0.153	0.044	1.78E-05	C8_CD4_Tres
chr6-152233970-152234229	2.87E-10	0.332112036	0.06	0.005	1.90E-05	C8_CD4_Tres
chr13-23743251-23743566	2.87E-10	0.333529973	0.052	0.004	1.90E-05	C8_CD4_Tres
chr2-230261941-230262667	2.88E-10	0.368010027	0.101	0.02	1.91E-05	C8_CD4_Tres
chr12-96377521-96378290	2.90E-10	0.373861026	0.243	0.091	1.92E-05	C8_CD4_Tres
chr6-29708374-29709366	3.02E-10	0.317645566	0.433	0.245	2.00E-05	C8_CD4_Tres
chr6-6658897-6659163	3.08E-10	0.314480411	0.052	0.003	2.04E-05	C8_CD4_Tres
chr18-74373109-74373960	3.21E-10	0.356693969	0.306	0.143	2.13E-05	C8_CD4_Tres
chr6-134246555-134247783	3.26E-10	0.366717694	0.265	0.11	2.16E-05	C8_CD4_Tres
chr18-32470099-32471460	3.30E-10	0.372128873	0.254	0.1	2.19E-05	C8_CD4_Tres
chr21-31397876-31398305	3.33E-10	0.320650815	0.052	0.003	2.21E-05	C8_CD4_Tres
chr5-80270056-80270789	3.44E-10	0.333930557	0.063	0.006	2.28E-05	C8_CD4_Tres
chr9-124965606-124966248	3.46E-10	0.373905662	0.116	0.027	2.29E-05	C8_CD4_Tres
chr11-78291910-78292423	3.48E-10	0.312783083	0.052	0.004	2.31E-05	C8_CD4_Tres
chr3-32457886-32458428	3.71E-10	0.368498153	0.228	0.089	2.46E-05	C8_CD4_Tres
chr16-29148369-29149328	3.75E-10	0.37446695	0.194	0.067	2.48E-05	C8_CD4_Tres
chr19-41457144-41457549	3.89E-10	0.326659186	0.06	0.006	2.58E-05	C8_CD4_Tres
chr18-58657828-58658640	3.93E-10	0.378365746	0.198	0.068	2.60E-05	C8_CD4_Tres
chr10-45470013-45470492	3.98E-10	0.327039781	0.06	0.005	2.64E-05	C8_CD4_Tres
chr6-90294597-90297201	3.99E-10	0.296503205	0.493	0.298	2.64E-05	C8_CD4_Tres
chr11-121451268-121453120	4.06E-10	0.336540945	0.384	0.188	2.69E-05	C8_CD4_Tres
chr3-188170183-188170918	4.15E-10	0.385373996	0.149	0.041	2.75E-05	C8_CD4_Tres
chr5-81018330-81018878	4.26E-10	0.317865525	0.056	0.004	2.82E-05	C8_CD4_Tres
chr13-26482270-26482854	4.32E-10	0.375322024	0.119	0.03	2.86E-05	C8_CD4_Tres
chr6-111972838-111973716	4.34E-10	0.374869799	0.194	0.056	2.88E-05	C8_CD4_Tres
chr16-68210898-68211340	4.45E-10	0.319190125	0.052	0.004	2.95E-05	C8_CD4_Tres

chr3-177109792-177110040	4.50E-10	0.318267121	0.056	0.005	2.98E-05	C8_CD4_Tres
chr7-127404389-127404899	4.58E-10	0.319459376	0.06	0.005	3.03E-05	C8_CD4_Tres
chr1-9425784-9426833	4.61E-10	0.343167363	0.336	0.164	3.05E-05	C8_CD4_Tres
chr6-56843519-56844357	4.64E-10	0.32842496	0.063	0.007	3.08E-05	C8_CD4_Tres
chr8-80347644-80348270	4.67E-10	0.380827813	0.179	0.06	3.09E-05	C8_CD4_Tres
chr5-56588666-56589174	4.80E-10	0.342315111	0.067	0.007	3.18E-05	C8_CD4_Tres
chr8-20435319-20435984	5.33E-10	0.375983844	0.187	0.062	3.53E-05	C8_CD4_Tres
chr2-27965657-27966195	5.47E-10	0.373844132	0.127	0.029	3.62E-05	C8_CD4_Tres
chr9-111926299-111926951	5.48E-10	0.36573872	0.231	0.093	3.63E-05	C8_CD4_Tres
chr4-108168511-108169013	5.53E-10	0.372880509	0.146	0.041	3.67E-05	C8_CD4_Tres
chr19-51690314-51690996	5.58E-10	0.367683079	0.108	0.023	3.70E-05	C8_CD4_Tres
chr5-131209164-131210417	5.61E-10	0.348662059	0.313	0.139	3.72E-05	C8_CD4_Tres
chr4-36385545-36386606	5.64E-10	0.368716328	0.231	0.087	3.73E-05	C8_CD4_Tres
chr12-56020724-56021943	5.92E-10	0.365996409	0.112	0.028	3.92E-05	C8_CD4_Tres
chr1-22031140-22031735	6.10E-10	0.322510518	0.052	0.005	4.04E-05	C8_CD4_Tres
chr2-242031358-242032125	6.12E-10	0.373323329	0.19	0.074	4.05E-05	C8_CD4_Tres
chrX-103460026-103460299	6.24E-10	0.328814737	0.063	0.006	4.13E-05	C8_CD4_Tres
chr3-58007796-58009340	6.26E-10	0.343589242	0.321	0.151	4.15E-05	C8_CD4_Tres
chr3-15356694-15356996	6.32E-10	0.315046335	0.056	0.005	4.19E-05	C8_CD4_Tres
chr10-80378031-80378552	6.37E-10	0.364439498	0.108	0.022	4.22E-05	C8_CD4_Tres
chr13-48186081-48186613	6.44E-10	0.368415754	0.131	0.031	4.27E-05	C8_CD4_Tres
chr16-3214848-3215890	6.48E-10	0.367017943	0.198	0.075	4.29E-05	C8_CD4_Tres
chr8-41514454-41515547	6.50E-10	0.369939277	0.194	0.068	4.31E-05	C8_CD4_Tres
chr12-3869844-3870216	6.57E-10	0.371060816	0.123	0.032	4.35E-05	C8_CD4_Tres
chr2-240831720-240832790	6.60E-10	0.287862217	0.534	0.32	4.37E-05	C8_CD4_Tres
chr13-114124864-114125348	6.81E-10	0.37285646	0.205	0.07	4.51E-05	C8_CD4_Tres
chr12-110780310-110780733	6.88E-10	0.316651543	0.052	0.005	4.56E-05	C8_CD4_Tres

chr5-151612508-151613341	6.92E-10	0.34125741	0.075	0.012	4.58E-05	C8_CD4_Tres
chr5-134079721-134080091	6.95E-10	0.359732312	0.09	0.015	4.60E-05	C8_CD4_Tres
chr21-36112628-36114387	6.95E-10	0.347103122	0.291	0.134	4.60E-05	C8_CD4_Tres
chr6-14276360-14277613	7.06E-10	0.369908637	0.16	0.046	4.67E-05	C8_CD4_Tres
chr9-124894504-124895179	7.19E-10	0.36198845	0.108	0.021	4.76E-05	C8_CD4_Tres
chr19-54377894-54378576	7.26E-10	0.354768154	0.104	0.021	4.81E-05	C8_CD4_Tres
chr6-79671553-79672105	7.31E-10	0.337980557	0.071	0.009	4.84E-05	C8_CD4_Tres
chr19-34317385-34317892	7.34E-10	0.332809181	0.063	0.006	4.86E-05	C8_CD4_Tres
chr22-36384706-36385321	7.71E-10	0.358745596	0.25	0.1	5.10E-05	C8_CD4_Tres
chr9-99134813-99135758	7.85E-10	0.329380271	0.063	0.008	5.20E-05	C8_CD4_Tres
chr1-225897866-225898196	7.95E-10	0.351215538	0.093	0.015	5.27E-05	C8_CD4_Tres
chr6-16473999-16474291	8.34E-10	0.342840578	0.078	0.011	5.52E-05	C8_CD4_Tres
chr2-162343370-162344574	8.74E-10	0.265102183	0.601	0.386	5.79E-05	C8_CD4_Tres
chr7-67128731-67129510	8.79E-10	0.373532223	0.172	0.058	5.82E-05	C8_CD4_Tres
chr7-5695061-5695784	8.82E-10	0.366382771	0.224	0.077	5.84E-05	C8_CD4_Tres
chr5-179146225-179146603	8.87E-10	0.311652375	0.056	0.004	5.88E-05	C8_CD4_Tres
chr15-40129107-40129553	8.92E-10	0.308130781	0.052	0.005	5.91E-05	C8_CD4_Tres
chr7-63899747-63901071	9.26E-10	0.348927587	0.306	0.137	6.13E-05	C8_CD4_Tres
chr22-40116450-40117167	9.56E-10	0.36431772	0.127	0.031	6.33E-05	C8_CD4_Tres
chr1-25503860-25504083	9.63E-10	0.320582974	0.06	0.006	6.38E-05	C8_CD4_Tres
chr7-5836265-5836474	9.66E-10	0.31189774	0.056	0.004	6.40E-05	C8_CD4_Tres
chrX-74924195-74925417	9.72E-10	0.333580947	0.078	0.01	6.44E-05	C8_CD4_Tres
chr17-77609941-77610742	9.80E-10	0.361969612	0.116	0.028	6.49E-05	C8_CD4_Tres
chr1-84862578-84863254	9.82E-10	0.369398697	0.175	0.06	6.50E-05	C8_CD4_Tres
chr17-43906401-43907892	1.03E-09	0.36549592	0.194	0.069	6.85E-05	C8_CD4_Tres
chr16-3364661-3365303	1.09E-09	0.3636707	0.183	0.062	7.20E-05	C8_CD4_Tres
chr20-53706495-53707021	1.11E-09	0.332860301	0.063	0.009	7.38E-05	C8_CD4_Tres

chr8-120749099-120749998	1.13E-09	0.35791325	0.22	0.086	7.47E-05	C8_CD4_Tres
chr3-142723599-142725021	1.14E-09	0.331567173	0.366	0.182	7.57E-05	C8_CD4_Tres
chr20-58306124-58307329	1.14E-09	0.286029655	0.519	0.307	7.58E-05	C8_CD4_Tres
chr5-131068879-131069618	1.19E-09	0.368056205	0.157	0.04	7.85E-05	C8_CD4_Tres
chr6-35600596-35602220	1.19E-09	0.327291655	0.358	0.175	7.87E-05	C8_CD4_Tres
chr17-30808563-30809247	1.20E-09	0.368555075	0.164	0.051	7.94E-05	C8_CD4_Tres
chr3-48987692-48988112	1.20E-09	0.366178776	0.168	0.058	7.96E-05	C8_CD4_Tres
chr4-75654918-75655909	1.22E-09	0.36560971	0.172	0.053	8.07E-05	C8_CD4_Tres
chr19-43474881-43475638	1.23E-09	0.355458177	0.224	0.088	8.16E-05	C8_CD4_Tres
chr12-3988417-3988893	1.28E-09	0.326750836	0.06	0.006	8.51E-05	C8_CD4_Tres
chr12-68175137-68176200	1.29E-09	0.358712219	0.216	0.088	8.57E-05	C8_CD4_Tres
chr12-57063888-57064630	1.30E-09	0.360521672	0.201	0.079	8.59E-05	C8_CD4_Tres
chr19-37778627-37780020	1.35E-09	0.272209502	0.571	0.36	8.93E-05	C8_CD4_Tres
chr20-50569937-50570727	1.37E-09	0.357589569	0.101	0.02	9.09E-05	C8_CD4_Tres
chr8-41542691-41542965	1.39E-09	0.326055804	0.067	0.009	9.20E-05	C8_CD4_Tres
chr1-28965323-28965558	1.40E-09	0.30743158	0.052	0.004	9.28E-05	C8_CD4_Tres
chr7-5699962-5700343	1.46E-09	0.364379058	0.179	0.064	9.64E-05	C8_CD4_Tres
chr6-90186735-90187325	1.47E-09	0.302607139	0.052	0.005	9.72E-05	C8_CD4_Tres
chr15-59976129-59976881	1.49E-09	0.355313083	0.131	0.034	9.89E-05	C8_CD4_Tres
chr22-20979312-20979632	1.50E-09	0.335554353	0.075	0.012	9.91E-05	C8_CD4_Tres
chr21-45973176-45974319	1.53E-09	0.361045539	0.201	0.076	0.000100994	C8_CD4_Tres
chr19-50658910-50659246	1.55E-09	0.31857045	0.063	0.006	0.000102388	C8_CD4_Tres
chr3-46065049-46065762	1.55E-09	0.339793432	0.299	0.145	0.000102743	C8_CD4_Tres
chr20-3117953-3118205	1.56E-09	0.325979211	0.063	0.007	0.000103185	C8_CD4_Tres
chr1-21956652-21957477	1.59E-09	0.350278391	0.09	0.016	0.000105621	C8_CD4_Tres
chr4-185587347-185587769	1.63E-09	0.365911665	0.119	0.025	0.000107923	C8_CD4_Tres
chr11-95699289-95700729	1.66E-09	0.360186454	0.175	0.058	0.000110241	C8_CD4_Tres

chr1-23626986-23627593	1.68E-09	0.338070498	0.075	0.012	0.000111532	C8_CD4_Tres
chr12-122021376-122022717	1.73E-09	0.355302258	0.131	0.033	0.000114338	C8_CD4_Tres
chr1-39690899-39692125	1.83E-09	0.329563517	0.347	0.169	0.000121022	C8_CD4_Tres
chr5-14992314-14993715	1.84E-09	0.346230701	0.302	0.127	0.000121843	C8_CD4_Tres
chr13-67229919-67231188	1.97E-09	0.36576986	0.142	0.041	0.000130263	C8_CD4_Tres
chr2-74840370-74840636	1.99E-09	0.340231564	0.082	0.013	0.000131729	C8_CD4_Tres
chr15-41620912-41622135	2.02E-09	0.360258972	0.198	0.07	0.00013394	C8_CD4_Tres
chr17-2101395-2101847	2.03E-09	0.321941465	0.063	0.008	0.000134117	C8_CD4_Tres
chr11-126062236-126063536	2.06E-09	0.309778399	0.429	0.226	0.000136309	C8_CD4_Tres
chr5-176042097-176042560	2.06E-09	0.328231634	0.071	0.009	0.000136316	C8_CD4_Tres
chr8-23162430-23164119	2.06E-09	0.34587987	0.261	0.113	0.000136567	C8_CD4_Tres
chr15-84761427-84762135	2.07E-09	0.352815368	0.108	0.023	0.000137361	C8_CD4_Tres
chr5-137695589-137696764	2.13E-09	0.320587899	0.063	0.007	0.000141143	C8_CD4_Tres
chr19-7868417-7870845	2.19E-09	0.260282706	0.549	0.379	0.000144943	C8_CD4_Tres
chr6-135162718-135163673	2.21E-09	0.355236771	0.104	0.024	0.000146535	C8_CD4_Tres
chr1-23424005-23424892	2.31E-09	0.3363826	0.082	0.014	0.000152685	C8_CD4_Tres
chr2-168750814-168751567	2.38E-09	0.356241718	0.116	0.026	0.000157501	C8_CD4_Tres
chr5-76855781-76856341	2.39E-09	0.322678269	0.067	0.008	0.000158332	C8_CD4_Tres
chr10-6068483-6068703	2.40E-09	0.314690303	0.056	0.006	0.000158992	C8_CD4_Tres
chr2-98770366-98772519	2.46E-09	0.294698456	0.466	0.276	0.00016304	C8_CD4_Tres
chr8-19445167-19445761	2.51E-09	0.326123127	0.067	0.008	0.000166358	C8_CD4_Tres
chr15-88894284-88895829	2.51E-09	0.35820793	0.179	0.063	0.000166363	C8_CD4_Tres
chr14-21964874-21965416	2.60E-09	0.341082798	0.075	0.014	0.000171894	C8_CD4_Tres
chr11-14554576-14555398	2.64E-09	0.354616178	0.172	0.061	0.00017452	C8_CD4_Tres
chr14-88791740-88793188	2.65E-09	0.297105555	0.44	0.253	0.000175489	C8_CD4_Tres
chr4-153809018-153809665	2.66E-09	0.327727392	0.071	0.01	0.000176122	C8_CD4_Tres
chr4-108342612-108342894	2.68E-09	0.318362288	0.056	0.007	0.000177276	C8_CD4_Tres

chr11-108592771-108594036	2.68E-09	0.344494014	0.276	0.121	0.000177612	C8_CD4_Tres
chr4-153573887-153574646	2.69E-09	0.358358688	0.134	0.037	0.000177843	C8_CD4_Tres
chr18-2635965-2637576	2.71E-09	0.312397654	0.377	0.212	0.000179381	C8_CD4_Tres
chr2-43636589-43637671	2.73E-09	0.352877734	0.224	0.088	0.000181053	C8_CD4_Tres
chr7-150676283-150677222	2.77E-09	0.345349929	0.101	0.021	0.000183238	C8_CD4_Tres
chr14-68911611-68911901	2.79E-09	0.347481904	0.097	0.02	0.000184665	C8_CD4_Tres
chr10-450519-451497	2.89E-09	0.358479784	0.179	0.064	0.000191055	C8_CD4_Tres
chr3-32303171-32303658	2.89E-09	0.333928872	0.086	0.013	0.000191249	C8_CD4_Tres
chr1-52702806-52703581	2.95E-09	0.329526686	0.321	0.157	0.000195669	C8_CD4_Tres
chr10-62528315-62528964	2.99E-09	0.353690497	0.123	0.029	0.000197891	C8_CD4_Tres
chr3-187012575-187013084	3.00E-09	0.330005108	0.078	0.011	0.00019883	C8_CD4_Tres
chr6-149366046-149366592	3.09E-09	0.343925323	0.093	0.02	0.000204692	C8_CD4_Tres
chr5-96895950-96897156	3.13E-09	0.356047331	0.146	0.042	0.000207124	C8_CD4_Tres
chr2-230318633-230319735	3.19E-09	0.34281471	0.291	0.133	0.00021145	C8_CD4_Tres
chr16-87806817-87807074	3.41E-09	0.340927922	0.086	0.014	0.000225946	C8_CD4_Tres
chr1-110619126-110620321	3.43E-09	0.324286358	0.306	0.171	0.000227245	C8_CD4_Tres
chr5-139565445-139565753	3.62E-09	0.357214374	0.153	0.047	0.000239581	C8_CD4_Tres
chr4-142565459-142567574	3.64E-09	0.334010688	0.287	0.132	0.000241181	C8_CD4_Tres
chr2-106137806-106139198	3.83E-09	0.349526029	0.116	0.027	0.000253315	C8_CD4_Tres
chr10-6347325-6348596	4.03E-09	0.293611115	0.444	0.266	0.000267168	C8_CD4_Tres
chr2-109213462-109214436	4.11E-09	0.354956981	0.179	0.063	0.000272157	C8_CD4_Tres
chr17-5583706-5584867	4.24E-09	0.296610355	0.444	0.255	0.000280817	C8_CD4_Tres
chr4-151407888-151409396	4.24E-09	0.347482014	0.235	0.101	0.000280899	C8_CD4_Tres
chr11-118869781-118871007	4.30E-09	0.308031895	0.41	0.22	0.000284428	C8_CD4_Tres
chr3-187769898-187770606	4.32E-09	0.329633319	0.075	0.012	0.000286216	C8_CD4_Tres
chr15-91469028-91470405	4.35E-09	0.350291947	0.209	0.078	0.000288125	C8_CD4_Tres
chr6-3355286-3356545	4.41E-09	0.348516738	0.209	0.074	0.000292148	C8_CD4_Tres

chr4-108049409-108050020	4.52E-09	0.333480551	0.086	0.016	0.00029951	C8_CD4_Tres
chr10-80529561-80529880	4.53E-09	0.321186467	0.071	0.01	0.000299709	C8_CD4_Tres
chr16-53545412-53545721	4.62E-09	0.303100349	0.056	0.006	0.000306096	C8_CD4_Tres
chr22-20463087-20464540	4.64E-09	0.352291383	0.19	0.072	0.000307345	C8_CD4_Tres
chr9-129206943-129207150	4.65E-09	0.310241808	0.06	0.007	0.000308204	C8_CD4_Tres
chr11-129695394-129696343	4.70E-09	0.35254714	0.119	0.028	0.000311436	C8_CD4_Tres
chr9-93020770-93021193	4.83E-09	0.339294086	0.078	0.013	0.000319595	C8_CD4_Tres
chr20-32945720-32946053	4.84E-09	0.322576741	0.071	0.011	0.000320294	C8_CD4_Tres
chr8-128659491-128659921	4.84E-09	0.334484512	0.09	0.017	0.000320599	C8_CD4_Tres
chrX-134186480-134187124	4.97E-09	0.325148335	0.075	0.014	0.000328993	C8_CD4_Tres
chr13-40315242-40315766	5.01E-09	0.348174756	0.108	0.026	0.000331973	C8_CD4_Tres
chr2-230602472-230603588	5.04E-09	0.350307439	0.142	0.046	0.000334015	C8_CD4_Tres
chr17-49817826-49818462	5.22E-09	0.348724631	0.131	0.038	0.000345462	C8_CD4_Tres
chr6-53302359-53302981	5.22E-09	0.35434341	0.19	0.074	0.000345725	C8_CD4_Tres
chr10-132185413-132185905	5.25E-09	0.349560312	0.119	0.031	0.00034765	C8_CD4_Tres
chr11-45843046-45843534	5.27E-09	0.330756855	0.291	0.138	0.000348797	C8_CD4_Tres
chr17-419157-419647	5.35E-09	0.340241978	0.09	0.019	0.000354041	C8_CD4_Tres
chr8-121822496-121823104	5.35E-09	0.309960017	0.06	0.007	0.000354118	C8_CD4_Tres
chr4-183098172-183099464	5.45E-09	0.34881762	0.149	0.045	0.000360979	C8_CD4_Tres
chr18-830095-831228	5.47E-09	0.351670171	0.127	0.042	0.000362254	C8_CD4_Tres
chr1-169711439-169712316	5.53E-09	0.349624287	0.168	0.057	0.000366114	C8_CD4_Tres
chr1-31848141-31848940	5.54E-09	0.33941803	0.097	0.021	0.000366747	C8_CD4_Tres
chr11-3641851-3642617	5.56E-09	0.355939801	0.142	0.039	0.000368397	C8_CD4_Tres
chr1-25561794-25562997	5.63E-09	0.337687983	0.254	0.105	0.000373109	C8_CD4_Tres
chr3-50194041-50195219	5.69E-09	0.334452459	0.272	0.13	0.000376806	C8_CD4_Tres
chr8-61713853-61714897	5.75E-09	0.292045386	0.448	0.262	0.00038093	C8_CD4_Tres
chr10-102410555-102410754	5.89E-09	0.329893357	0.075	0.012	0.000389955	C8_CD4_Tres

chr3-161395027-161395483	5.94E-09	0.303841033	0.052	0.006	0.000393265	C8_CD4_Tres
chr20-25868078-25868654	5.98E-09	0.337801925	0.097	0.019	0.000396204	C8_CD4_Tres
chr13-102393889-102394903	6.02E-09	0.33021038	0.291	0.131	0.000398764	C8_CD4_Tres
chr2-237441532-237441819	6.05E-09	0.298985593	0.056	0.006	0.000400791	C8_CD4_Tres
chr5-98770390-98771779	6.06E-09	0.34371129	0.198	0.072	0.000401435	C8_CD4_Tres
chr3-171494426-171494801	6.11E-09	0.321615041	0.067	0.011	0.00040433	C8_CD4_Tres
chr22-40726566-40726831	6.14E-09	0.309281917	0.06	0.007	0.000406698	C8_CD4_Tres
chr6-28542510-28543278	6.15E-09	0.33956355	0.108	0.023	0.000407008	C8_CD4_Tres
chr11-96061836-96062462	6.27E-09	0.34078948	0.093	0.017	0.000415151	C8_CD4_Tres
chr4-3301814-3303402	6.30E-09	0.310118235	0.358	0.201	0.000416876	C8_CD4_Tres
chr5-80153520-80153836	6.34E-09	0.299736448	0.052	0.005	0.00041982	C8_CD4_Tres
chr6-142232230-142232808	6.43E-09	0.311919748	0.063	0.008	0.00042614	C8_CD4_Tres
chr8-2101236-2102257	6.44E-09	0.352756817	0.146	0.039	0.000426378	C8_CD4_Tres
chr19-8050146-8050978	6.52E-09	0.349280107	0.183	0.072	0.000431806	C8_CD4_Tres
chr6-28865798-28867028	6.57E-09	0.291015574	0.422	0.255	0.000435005	C8_CD4_Tres
chr19-11848308-11849324	6.58E-09	0.347815065	0.209	0.077	0.000435715	C8_CD4_Tres
chr16-30089993-30090192	6.60E-09	0.329010502	0.086	0.015	0.000437174	C8_CD4_Tres
chr15-99706659-99707172	6.64E-09	0.315367689	0.06	0.008	0.000439443	C8_CD4_Tres
chr19-2702211-2702989	6.72E-09	0.329090591	0.295	0.134	0.000444986	C8_CD4_Tres
chr19-18932044-18932793	6.78E-09	0.350421924	0.201	0.073	0.000449177	C8_CD4_Tres
chr11-134033824-134034272	6.83E-09	0.352065935	0.116	0.03	0.000452343	C8_CD4_Tres
chr14-106845476-106845675	6.86E-09	0.317980014	0.067	0.009	0.000454121	C8_CD4_Tres
chr14-64928345-64929195	7.00E-09	0.343639055	0.123	0.03	0.000463718	C8_CD4_Tres
chr1-2894464-2894963	7.02E-09	0.305307597	0.056	0.006	0.000464831	C8_CD4_Tres
chr15-81293594-81294384	7.12E-09	0.317905561	0.351	0.173	0.000471614	C8_CD4_Tres
chr9-20314872-20315317	7.17E-09	0.341624953	0.093	0.02	0.000474851	C8_CD4_Tres
chr2-215680929-215681513	7.20E-09	0.305198518	0.056	0.007	0.000476811	C8_CD4_Tres

chr8-22642831-22643458	7.21E-09	0.332937141	0.082	0.017	0.000477174	C8_CD4_Tres
chr13-112066311-112066913	7.26E-09	0.296375792	0.06	0.006	0.000480554	C8_CD4_Tres
chrX-38801080-38801672	7.26E-09	0.350232154	0.172	0.059	0.000480634	C8_CD4_Tres
chr14-56117661-56119506	7.74E-09	0.308362956	0.351	0.196	0.000512794	C8_CD4_Tres
chr3-195549821-195550581	7.80E-09	0.321243035	0.302	0.151	0.000516533	C8_CD4_Tres
chr7-107659665-107659981	7.90E-09	0.300303678	0.052	0.005	0.000523088	C8_CD4_Tres
chr18-76922152-76922411	7.93E-09	0.309900109	0.056	0.007	0.000525318	C8_CD4_Tres
chr2-7031503-7032044	7.99E-09	0.336678884	0.097	0.02	0.000528937	C8_CD4_Tres
chr20-49823177-49823827	8.27E-09	0.324858506	0.09	0.017	0.00054734	C8_CD4_Tres
chr16-11782958-11784091	8.33E-09	0.326829774	0.291	0.14	0.000551423	C8_CD4_Tres
chr18-46343891-46344255	8.41E-09	0.3123132	0.067	0.008	0.000556708	C8_CD4_Tres
chr14-37594904-37596254	8.42E-09	0.335403409	0.104	0.021	0.000557537	C8_CD4_Tres
chr3-37861225-37862515	8.43E-09	0.327686277	0.082	0.017	0.000558464	C8_CD4_Tres
chr9-20321539-20322047	8.49E-09	0.35014363	0.138	0.045	0.000562141	C8_CD4_Tres
chr3-63967612-63968856	8.71E-09	0.29441843	0.41	0.234	0.000576947	C8_CD4_Tres
chr17-66255061-66255362	8.73E-09	0.330508285	0.086	0.017	0.000578057	C8_CD4_Tres
chr9-112074216-112075051	9.05E-09	0.331041064	0.086	0.018	0.000599111	C8_CD4_Tres
chr22-24134065-24134388	9.21E-09	0.290959924	0.052	0.004	0.000610243	C8_CD4_Tres
chr19-2976818-2977673	9.29E-09	0.322643864	0.291	0.141	0.000615068	C8_CD4_Tres
chr7-151756000-151756419	9.67E-09	0.344875468	0.119	0.03	0.000640359	C8_CD4_Tres
chr22-25062044-25062947	9.73E-09	0.320594958	0.306	0.154	0.000644321	C8_CD4_Tres
chr15-38651029-38652094	9.85E-09	0.336157863	0.228	0.106	0.000652197	C8_CD4_Tres
chr12-52690855-52691542	1.02E-08	0.328805912	0.082	0.016	0.000675273	C8_CD4_Tres
chr6-2936707-2937265	1.05E-08	0.347146448	0.172	0.063	0.000693191	C8_CD4_Tres
chr10-14583215-14583699	1.06E-08	0.322138848	0.291	0.14	0.000702218	C8_CD4_Tres
chr11-77473258-77474673	1.08E-08	0.314485994	0.336	0.167	0.000712642	C8_CD4_Tres
chr1-100037520-100039291	1.12E-08	0.253322573	0.537	0.374	0.000740635	C8_CD4_Tres

chr3-119353010-119353578	1.17E-08	0.32163529	0.071	0.011	0.000776129	C8_CD4_Tres
chr8-9149994-9151906	1.19E-08	0.296256562	0.407	0.229	0.000787221	C8_CD4_Tres
chr12-75478423-75478752	1.20E-08	0.34456916	0.146	0.043	0.000793786	C8_CD4_Tres
chr10-22611430-22612645	1.23E-08	0.312708115	0.325	0.177	0.000815519	C8_CD4_Tres
chr5-134132105-134133318	1.23E-08	0.304081287	0.381	0.205	0.000816153	C8_CD4_Tres
chr6-11729134-11729420	1.25E-08	0.296869214	0.052	0.005	0.000826925	C8_CD4_Tres
chr5-177429254-177429569	1.27E-08	0.341983426	0.205	0.087	0.000839667	C8_CD4_Tres
chr6-29641397-29642045	1.28E-08	0.315285147	0.075	0.011	0.000847696	C8_CD4_Tres
chr6-35311302-35312028	1.31E-08	0.33959636	0.104	0.029	0.000867542	C8_CD4_Tres
chr1-207644649-207645408	1.36E-08	0.33265077	0.104	0.023	0.000900594	C8_CD4_Tres
chr22-19108908-19110012	1.41E-08	0.335576733	0.228	0.093	0.000934627	C8_CD4_Tres
chr21-41895863-41896133	1.42E-08	0.298189683	0.056	0.007	0.000941706	C8_CD4_Tres
chr9-129383299-129384135	1.43E-08	0.337094706	0.194	0.081	0.000947462	C8_CD4_Tres
chr11-128716319-128717284	1.47E-08	0.341344728	0.149	0.049	0.000976058	C8_CD4_Tres
chr12-51482714-51483444	1.49E-08	0.301342662	0.063	0.008	0.000989325	C8_CD4_Tres
chr15-52481501-52482318	1.50E-08	0.326470388	0.269	0.123	0.000992923	C8_CD4_Tres
chr9-137722026-137722864	1.50E-08	0.32135581	0.078	0.014	0.00099369	C8_CD4_Tres
chr19-17873725-17874449	1.50E-08	0.337161447	0.194	0.077	0.000995216	C8_CD4_Tres
chr7-100211261-100211792	1.51E-08	0.32654644	0.082	0.018	0.000997975	C8_CD4_Tres
chr14-92650743-92652022	1.51E-08	0.330901663	0.28	0.122	0.001001572	C8_CD4_Tres
chr1-111345981-111346794	1.51E-08	0.325424314	0.28	0.122	0.001001629	C8_CD4_Tres
chr5-80191507-80192278	1.55E-08	0.329398124	0.093	0.023	0.001027419	C8_CD4_Tres
chr16-70018279-70018690	1.58E-08	0.350438989	0.138	0.038	0.001045293	C8_CD4_Tres
chr17-20234205-20234631	1.61E-08	0.302654011	0.063	0.008	0.001066635	C8_CD4_Tres
chr2-234454230-234454770	1.61E-08	0.338526878	0.112	0.03	0.001068707	C8_CD4_Tres
chr17-76816273-76816820	1.64E-08	0.317315391	0.075	0.013	0.001084957	C8_CD4_Tres
chr4-176065489-176066137	1.66E-08	0.318842629	0.075	0.012	0.0010988	C8_CD4_Tres

chr6-20319136-20320385	1.68E-08	0.313406836	0.31	0.156	0.001115026	C8_CD4_Tres
chr2-105896000-105896396	1.69E-08	0.303441984	0.063	0.008	0.001115947	C8_CD4_Tres
chr21-33402721-33404158	1.70E-08	0.330802703	0.112	0.028	0.001128757	C8_CD4_Tres
chr1-111441263-111441865	1.71E-08	0.313650376	0.067	0.011	0.001132857	C8_CD4_Tres
chr11-33015631-33016185	1.71E-08	0.339400477	0.138	0.045	0.001133649	C8_CD4_Tres
chr11-78202511-78203649	1.73E-08	0.292724598	0.403	0.227	0.001145407	C8_CD4_Tres
chr4-154332725-154333386	1.73E-08	0.304081623	0.06	0.008	0.001146315	C8_CD4_Tres
chr7-66413112-66414056	1.75E-08	0.340999159	0.19	0.073	0.001157884	C8_CD4_Tres
chr10-61902368-61903891	1.76E-08	0.273754854	0.463	0.283	0.001162914	C8_CD4_Tres
chr2-234452014-234452718	1.76E-08	0.334759115	0.119	0.031	0.001165335	C8_CD4_Tres
chr1-247936711-247937420	1.77E-08	0.339507608	0.16	0.053	0.001170227	C8_CD4_Tres
chr22-28442062-28442561	1.78E-08	0.292223608	0.056	0.006	0.00117574	C8_CD4_Tres
chr6-107736157-107737204	1.81E-08	0.33786467	0.205	0.079	0.001195482	C8_CD4_Tres
chr19-14366514-14366901	1.87E-08	0.323694322	0.265	0.119	0.001237594	C8_CD4_Tres
chr3-108124430-108126092	1.89E-08	0.297608213	0.377	0.216	0.001250985	C8_CD4_Tres
chr8-124563657-124565073	2.01E-08	0.323249895	0.261	0.12	0.001331123	C8_CD4_Tres
chr13-23578522-23579929	2.07E-08	0.307663021	0.362	0.177	0.00136783	C8_CD4_Tres
chr10-14184677-14186034	2.08E-08	0.326361561	0.239	0.099	0.001375374	C8_CD4_Tres
chr4-119370317-119370756	2.08E-08	0.293800346	0.056	0.006	0.001377059	C8_CD4_Tres
chr13-45847416-45847966	2.12E-08	0.321173759	0.086	0.016	0.00140283	C8_CD4_Tres
chrX-48939729-48939998	2.12E-08	0.308894757	0.067	0.011	0.001405305	C8_CD4_Tres
chr3-128075252-128077313	2.14E-08	0.320196416	0.261	0.126	0.001416533	C8_CD4_Tres
chr1-144534108-144534632	2.14E-08	0.333144911	0.097	0.025	0.001418021	C8_CD4_Tres
chr21-46558996-46560053	2.23E-08	0.33067878	0.224	0.099	0.001476401	C8_CD4_Tres
chr6-131281200-131281893	2.28E-08	0.330746229	0.112	0.028	0.001509279	C8_CD4_Tres
chr19-51676031-51676989	2.30E-08	0.313470792	0.075	0.014	0.001521084	C8_CD4_Tres
chr19-50657797-50658465	2.32E-08	0.342471057	0.153	0.055	0.001535741	C8_CD4_Tres

chr16-28936149-28936898	2.36E-08	0.328212253	0.243	0.104	0.001562894	C8_CD4_Tres
chr10-79047893-79048586	2.37E-08	0.317443959	0.078	0.016	0.001570585	C8_CD4_Tres
chr1-167510766-167511126	2.42E-08	0.306591611	0.067	0.01	0.001604412	C8_CD4_Tres
chr19-1192187-1192890	2.45E-08	0.331820316	0.142	0.044	0.001624105	C8_CD4_Tres
chr5-173917409-173918774	2.47E-08	0.311524969	0.31	0.161	0.001634523	C8_CD4_Tres
chr7-149414691-149415308	2.49E-08	0.298307575	0.06	0.007	0.001646414	C8_CD4_Tres
chr4-89029539-89029966	2.54E-08	0.283243191	0.052	0.005	0.001684862	C8_CD4_Tres
chr8-60653087-60654042	2.55E-08	0.329175448	0.224	0.093	0.001688978	C8_CD4_Tres
chr15-55497888-55498624	2.59E-08	0.337877844	0.16	0.05	0.00171395	C8_CD4_Tres
chr6-1523968-1524660	2.62E-08	0.325430964	0.097	0.022	0.001735478	C8_CD4_Tres
chr17-76076895-76077210	2.63E-08	0.307139933	0.075	0.014	0.001739277	C8_CD4_Tres
chr19-39953253-39953782	2.63E-08	0.305057244	0.075	0.013	0.001743655	C8_CD4_Tres
chr16-27169333-27170139	2.66E-08	0.315143553	0.299	0.152	0.001761656	C8_CD4_Tres
chr19-1507526-1508472	2.67E-08	0.293839902	0.373	0.214	0.001765311	C8_CD4_Tres
chr15-38486909-38487408	2.67E-08	0.336859576	0.131	0.036	0.001769247	C8_CD4_Tres
chr20-36930337-36931332	2.68E-08	0.336698967	0.149	0.046	0.001773953	C8_CD4_Tres
chr22-30305501-30306854	2.74E-08	0.325215492	0.243	0.108	0.001811468	C8_CD4_Tres
chr3-179037253-179037722	2.74E-08	0.298945361	0.06	0.009	0.0018126	C8_CD4_Tres
chr3-170033457-170033781	2.74E-08	0.314982145	0.078	0.014	0.001813666	C8_CD4_Tres
chr19-11797439-11798913	2.81E-08	0.324730363	0.265	0.118	0.00186182	C8_CD4_Tres
chr9-105405161-105406022	2.86E-08	0.335242594	0.172	0.058	0.001895476	C8_CD4_Tres
chr11-128312044-128312312	2.95E-08	0.28792222	0.052	0.006	0.001955728	C8_CD4_Tres
chr4-83219344-83219940	2.96E-08	0.324888563	0.097	0.023	0.001962881	C8_CD4_Tres
chr6-28884116-28885677	3.18E-08	0.29770879	0.354	0.197	0.002105174	C8_CD4_Tres
chr16-50747150-50747928	3.19E-08	0.32492347	0.231	0.112	0.002110525	C8_CD4_Tres
chr21-37434963-37435466	3.20E-08	0.336704923	0.164	0.06	0.0021167	C8_CD4_Tres
chr4-122584363-122584854	3.22E-08	0.307626709	0.071	0.013	0.002135269	C8_CD4_Tres

chr7-1939095-1940953	3.24E-08	0.256162393	0.511	0.34	0.002146561	C8_CD4_Tres
chr12-12622168-12622812	3.26E-08	0.314101262	0.075	0.014	0.002156571	C8_CD4_Tres
chr6-90350556-90351059	3.33E-08	0.325121047	0.097	0.023	0.002207681	C8_CD4_Tres
chr7-23373112-23373479	3.38E-08	0.314799701	0.082	0.016	0.002236127	C8_CD4_Tres
chr6-90156690-90157232	3.38E-08	0.319408593	0.082	0.017	0.002239586	C8_CD4_Tres
chr11-8168515-8169244	3.42E-08	0.321540678	0.09	0.019	0.002262168	C8_CD4_Tres
chr1-226696615-226697454	3.42E-08	0.32944864	0.101	0.026	0.00226396	C8_CD4_Tres
chr16-4487821-4488972	3.48E-08	0.331455128	0.149	0.054	0.002301776	C8_CD4_Tres
chrX-78333534-78333906	3.59E-08	0.298783576	0.06	0.008	0.002378516	C8_CD4_Tres
chr1-84944521-84945566	3.60E-08	0.269340472	0.478	0.283	0.002382711	C8_CD4_Tres
chr19-17001273-17002247	3.63E-08	0.336435972	0.168	0.063	0.002404544	C8_CD4_Tres
chr9-20356366-20356591	3.63E-08	0.28149407	0.056	0.006	0.00240513	C8_CD4_Tres
chr22-23225873-23226540	3.68E-08	0.301980419	0.067	0.011	0.002438328	C8_CD4_Tres
chr8-19459825-19460225	3.71E-08	0.293275702	0.06	0.008	0.002456751	C8_CD4_Tres
chr13-74286961-74288638	3.74E-08	0.295657154	0.336	0.192	0.002476984	C8_CD4_Tres
chr1-241627384-241628238	3.80E-08	0.3186806	0.086	0.017	0.002514327	C8_CD4_Tres
chr7-142482901-142483604	4.07E-08	0.312677714	0.078	0.015	0.00269724	C8_CD4_Tres
chr1-203277845-203278652	4.08E-08	0.3214388	0.101	0.024	0.002700852	C8_CD4_Tres
chr17-63611367-63611676	4.09E-08	0.289986997	0.052	0.006	0.002707228	C8_CD4_Tres
chr3-10263609-10264389	4.17E-08	0.333833315	0.19	0.07	0.002760253	C8_CD4_Tres
chr8-1972996-1974359	4.26E-08	0.318207317	0.086	0.018	0.002821559	C8_CD4_Tres
chr8-80893653-80894070	4.29E-08	0.306781806	0.067	0.011	0.002844122	C8_CD4_Tres
chr1-111647753-111648664	4.34E-08	0.318428599	0.082	0.017	0.002872009	C8_CD4_Tres
chr11-62598951-62599496	4.35E-08	0.267717007	0.455	0.289	0.00288117	C8_CD4_Tres
chr22-45296530-45297075	4.45E-08	0.31393636	0.078	0.017	0.002948803	C8_CD4_Tres
chr5-168291365-168291956	4.52E-08	0.330401144	0.116	0.028	0.002994998	C8_CD4_Tres
chr12-14417708-14418050	4.52E-08	0.330815719	0.142	0.047	0.002996236	C8_CD4_Tres

chr7-155739260-155739940	4.54E-08	0.287127192	0.06	0.009	0.003004157	C8_CD4_Tres
chr11-102451619-102453572	4.66E-08	0.259825986	0.474	0.311	0.003085825	C8_CD4_Tres
chr9-22079362-22080370	4.69E-08	0.3300827	0.116	0.032	0.003106618	C8_CD4_Tres
chr3-71220276-71220570	4.80E-08	0.278876095	0.052	0.006	0.003180421	C8_CD4_Tres
chr19-16283563-16284148	4.83E-08	0.292300476	0.06	0.009	0.003197128	C8_CD4_Tres
chr5-124649550-124650020	4.98E-08	0.283475037	0.052	0.007	0.003297689	C8_CD4_Tres
chr11-73647058-73647984	5.00E-08	0.317657479	0.246	0.108	0.003314447	C8_CD4_Tres
chr9-34171773-34172085	5.16E-08	0.293090649	0.06	0.008	0.003419708	C8_CD4_Tres
chr2-189658403-189658728	5.36E-08	0.3207139	0.086	0.023	0.003549784	C8_CD4_Tres
chr8-2145110-2145682	5.39E-08	0.309947693	0.075	0.013	0.003569356	C8_CD4_Tres
chr3-52317318-52317978	5.42E-08	0.330779858	0.142	0.044	0.003587227	C8_CD4_Tres
chr4-88818658-88818930	5.49E-08	0.298964413	0.071	0.012	0.003637174	C8_CD4_Tres
chr20-36974607-36975637	5.51E-08	0.330777073	0.149	0.052	0.003646685	C8_CD4_Tres
chr12-26113885-26114716	5.57E-08	0.321337598	0.116	0.034	0.003686821	C8_CD4_Tres
chr3-99875674-99876384	5.77E-08	0.329546246	0.149	0.047	0.003820569	C8_CD4_Tres
chr1-13730172-13731444	5.89E-08	0.282626098	0.392	0.227	0.003901753	C8_CD4_Tres
chr6-106097372-106099189	5.92E-08	0.253765821	0.478	0.322	0.003923312	C8_CD4_Tres
chr3-58115966-58116538	6.06E-08	0.310146999	0.082	0.016	0.004011307	C8_CD4_Tres
chr17-20226788-20227070	6.11E-08	0.286816897	0.06	0.008	0.004049157	C8_CD4_Tres
chr16-67998766-67999884	6.20E-08	0.329269425	0.138	0.045	0.004107743	C8_CD4_Tres
chr3-71432479-71433038	6.30E-08	0.299280461	0.071	0.012	0.004169015	C8_CD4_Tres
chr3-169748605-169749000	6.39E-08	0.285170316	0.056	0.007	0.004228834	C8_CD4_Tres
chr14-64879442-64880758	6.43E-08	0.324768504	0.179	0.074	0.004260664	C8_CD4_Tres
chr7-44145418-44146555	6.43E-08	0.326613784	0.16	0.055	0.004261239	C8_CD4_Tres
chr20-52409915-52410930	6.54E-08	0.314504134	0.265	0.125	0.004328465	C8_CD4_Tres
chr10-100510527-100511388	6.77E-08	0.294311499	0.354	0.185	0.004482708	C8_CD4_Tres
chr15-28873754-28874055	6.78E-08	0.28843235	0.06	0.009	0.00449136	C8_CD4_Tres

chr15-70535012-70535978	6.86E-08	0.326838676	0.142	0.052	0.004542853	C8_CD4_Tres
chr14-75541972-75544034	6.90E-08	0.28736427	0.347	0.21	0.004566796	C8_CD4_Tres
chr17-66426023-66426689	6.98E-08	0.312691133	0.082	0.018	0.004621112	C8_CD4_Tres
chr4-83114583-83114901	7.01E-08	0.329355986	0.149	0.057	0.004640545	C8_CD4_Tres
chr16-89192765-89193395	7.05E-08	0.318069113	0.216	0.095	0.004665955	C8_CD4_Tres
chr14-92750191-92750751	7.21E-08	0.306409261	0.071	0.014	0.004777428	C8_CD4_Tres
chr3-194333402-194334063	7.39E-08	0.324220781	0.153	0.053	0.004892622	C8_CD4_Tres
chr16-28168877-28169369	7.40E-08	0.316357428	0.093	0.023	0.004903513	C8_CD4_Tres
chr3-49790069-49790408	7.49E-08	0.312156089	0.093	0.022	0.004960661	C8_CD4_Tres
chr1-27527920-27528351	7.51E-08	0.315430644	0.104	0.026	0.004971315	C8_CD4_Tres
chr3-146497768-146498465	7.55E-08	0.294329685	0.056	0.009	0.005001802	C8_CD4_Tres
chr5-157204401-157205071	7.70E-08	0.317277083	0.097	0.027	0.005096575	C8_CD4_Tres
chr4-88613140-88613853	7.78E-08	0.326067086	0.19	0.075	0.005155307	C8_CD4_Tres
chr10-113006884-113007652	7.87E-08	0.319909211	0.104	0.027	0.005213347	C8_CD4_Tres
chr4-48129386-48129835	7.93E-08	0.28375278	0.06	0.008	0.005252153	C8_CD4_Tres
chr16-71483901-71484825	8.13E-08	0.283568811	0.366	0.217	0.005386298	C8_CD4_Tres
chrX-47623581-47623950	8.35E-08	0.285740247	0.056	0.008	0.005529726	C8_CD4_Tres
chr5-75052504-75054047	8.44E-08	0.314059561	0.231	0.098	0.005589779	C8_CD4_Tres
chr17-77127541-77128387	8.62E-08	0.304693247	0.291	0.153	0.005710289	C8_CD4_Tres
chr13-26051410-26052194	9.06E-08	0.292720743	0.067	0.011	0.005999267	C8_CD4_Tres
chr2-172427037-172428427	9.08E-08	0.295243023	0.336	0.167	0.00601113	C8_CD4_Tres
chr14-56002757-56003529	9.42E-08	0.313419885	0.104	0.025	0.006239947	C8_CD4_Tres
chr3-52268059-52268325	9.50E-08	0.314676679	0.093	0.023	0.006290103	C8_CD4_Tres
chr9-95724120-95725286	1.02E-07	0.297420398	0.313	0.158	0.006776094	C8_CD4_Tres
chr16-14301362-14303097	1.03E-07	0.31748284	0.108	0.03	0.006791154	C8_CD4_Tres
chr13-99464395-99464867	1.03E-07	0.274714827	0.052	0.007	0.006804945	C8_CD4_Tres
chr11-111378628-111379509	1.03E-07	0.30656827	0.093	0.022	0.006817588	C8_CD4_Tres

chr19-55647923-55648529	1.04E-07	0.302530003	0.082	0.017	0.006863121	C8_CD4_Tres
chr19-16382650-16383571	1.07E-07	0.320152947	0.119	0.039	0.007082107	C8_CD4_Tres
chr17-28549672-28550055	1.07E-07	0.322976131	0.131	0.045	0.007108114	C8_CD4_Tres
chr10-80530209-80530530	1.11E-07	0.295884858	0.075	0.012	0.007338296	C8_CD4_Tres
chr14-65846455-65847646	1.11E-07	0.291062576	0.336	0.183	0.007365703	C8_CD4_Tres
chr7-140722991-140723303	1.13E-07	0.272586016	0.052	0.006	0.007465561	C8_CD4_Tres
chr13-112968220-112969617	1.15E-07	0.301411407	0.28	0.138	0.007588791	C8_CD4_Tres
chr20-3157101-3157715	1.16E-07	0.324846323	0.168	0.064	0.00767163	C8_CD4_Tres
chr2-172442043-172442337	1.18E-07	0.317564575	0.104	0.025	0.007837995	C8_CD4_Tres
chr16-15013573-15014795	1.19E-07	0.302574255	0.276	0.14	0.007902642	C8_CD4_Tres
chr11-62923741-62924336	1.20E-07	0.284743168	0.06	0.01	0.007968786	C8_CD4_Tres
chr14-71399002-71399345	1.21E-07	0.280436808	0.06	0.009	0.008005478	C8_CD4_Tres
chr5-74919393-74919814	1.23E-07	0.286582505	0.063	0.011	0.008171555	C8_CD4_Tres
chr8-129899852-129900496	1.24E-07	0.300446825	0.078	0.015	0.008213086	C8_CD4_Tres
chr11-46670637-46671731	1.27E-07	0.314191246	0.209	0.089	0.008400139	C8_CD4_Tres
chr20-25718977-25719472	1.28E-07	0.278297809	0.056	0.008	0.008506418	C8_CD4_Tres
chr11-118398631-118399385	1.32E-07	0.309859142	0.254	0.116	0.008719228	C8_CD4_Tres
chr9-137326395-137327034	1.32E-07	0.289120794	0.06	0.01	0.008748048	C8_CD4_Tres
chr1-6127623-6128120	1.33E-07	0.304287306	0.09	0.022	0.008826328	C8_CD4_Tres
chr20-47725331-47725668	1.33E-07	0.316657912	0.097	0.026	0.008830938	C8_CD4_Tres
chr10-6173225-6173539	1.37E-07	0.296861485	0.075	0.013	0.009092227	C8_CD4_Tres
chr20-44055995-44056344	1.41E-07	0.280431987	0.06	0.009	0.009367848	C8_CD4_Tres
chr1-169694286-169694730	1.48E-07	0.316887936	0.142	0.05	0.009786599	C8_CD4_Tres
chr13-34941883-34943256	1.51E-07	0.297042666	0.071	0.014	0.009967808	C8_CD4_Tres
chr11-16605599-16606604	1.51E-07	0.317142838	0.123	0.041	0.010032404	C8_CD4_Tres
chr18-596162-597241	1.57E-07	0.319322361	0.16	0.062	0.010387018	C8_CD4_Tres
chr11-134068604-134069377	1.57E-07	0.310727738	0.216	0.098	0.010393593	C8_CD4_Tres

chr11-35342918-35343560	1.58E-07	0.298125583	0.078	0.016	0.010462725	C8_CD4_Tres
chr6-152232861-152233475	1.65E-07	0.275727906	0.056	0.007	0.010947451	C8_CD4_Tres
chr3-154120945-154121830	1.70E-07	0.283390505	0.071	0.012	0.011256163	C8_CD4_Tres
chr2-109128021-109128523	1.72E-07	0.268615071	0.052	0.006	0.011400574	C8_CD4_Tres
chr1-154433135-154433466	1.74E-07	0.296308993	0.075	0.016	0.011498326	C8_CD4_Tres
chr4-108144518-108144833	1.75E-07	0.275282809	0.052	0.007	0.011590287	C8_CD4_Tres
chr15-88897310-88897605	1.77E-07	0.292333644	0.067	0.013	0.011711499	C8_CD4_Tres
chr22-31291874-31292654	1.81E-07	0.300366003	0.246	0.122	0.012014356	C8_CD4_Tres
chr21-45470858-45471189	1.82E-07	0.274872426	0.056	0.008	0.01204434	C8_CD4_Tres
chr6-32148627-32149036	1.86E-07	0.312673158	0.101	0.025	0.012317842	C8_CD4_Tres
chr12-113056548-113057002	1.87E-07	0.28542279	0.063	0.011	0.012353953	C8_CD4_Tres
chr15-70528049-70528642	1.89E-07	0.316104032	0.164	0.063	0.012521229	C8_CD4_Tres
chr5-131281028-131281751	1.90E-07	0.285347926	0.332	0.178	0.012586884	C8_CD4_Tres
chr21-44454685-44456039	1.94E-07	0.3007659	0.25	0.121	0.01283222	C8_CD4_Tres
chr3-84958545-84959238	1.96E-07	0.27409868	0.052	0.008	0.012961438	C8_CD4_Tres
chr17-29639047-29639812	1.96E-07	0.315469891	0.127	0.039	0.012971838	C8_CD4_Tres
chr1-12448952-12450012	1.98E-07	0.314275896	0.187	0.072	0.013119045	C8_CD4_Tres
chr14-99262140-99262856	2.00E-07	0.267394131	0.399	0.247	0.013229757	C8_CD4_Tres
chr2-236967862-236968381	2.00E-07	0.277222231	0.067	0.011	0.013276967	C8_CD4_Tres
chr2-234373093-234373344	2.07E-07	0.27290004	0.052	0.007	0.013701538	C8_CD4_Tres
chr11-46332562-46333529	2.07E-07	0.310085481	0.101	0.026	0.013707705	C8_CD4_Tres
chr12-132771207-132771772	2.08E-07	0.307556757	0.09	0.021	0.013758133	C8_CD4_Tres
chr12-66287435-66288552	2.12E-07	0.302246932	0.265	0.125	0.014060594	C8_CD4_Tres
chr7-140375766-140376698	2.15E-07	0.305200064	0.097	0.024	0.014260595	C8_CD4_Tres
chr22-40384985-40385742	2.16E-07	0.303754558	0.093	0.022	0.014286237	C8_CD4_Tres
chr14-98182303-98182999	2.19E-07	0.287044929	0.063	0.012	0.014495353	C8_CD4_Tres
chr20-62918231-62918442	2.19E-07	0.282092916	0.067	0.012	0.014501535	C8_CD4_Tres

chr11-59549908-59551377	2.20E-07	0.266282981	0.403	0.242	0.014561456	C8_CD4_Tres
chr19-3604865-3605418	2.21E-07	0.285101501	0.063	0.011	0.01463852	C8_CD4_Tres
chr19-4179952-4180449	2.21E-07	0.313641788	0.157	0.059	0.014643629	C8_CD4_Tres
chr17-65557303-65557533	2.22E-07	0.304570813	0.078	0.02	0.014721794	C8_CD4_Tres
chr15-100595895-100597210	2.24E-07	0.27810681	0.343	0.203	0.014812473	C8_CD4_Tres
chr8-38184371-38184758	2.26E-07	0.311547931	0.112	0.034	0.014948989	C8_CD4_Tres
chr16-70430601-70431251	2.27E-07	0.304306673	0.09	0.024	0.015024415	C8_CD4_Tres
chr11-66567885-66568767	2.30E-07	0.304449811	0.097	0.024	0.015214398	C8_CD4_Tres
chr3-15320417-15320867	2.32E-07	0.278635909	0.056	0.01	0.015379721	C8_CD4_Tres
chr20-32482700-32485561	2.39E-07	0.259200941	0.44	0.264	0.015827356	C8_CD4_Tres
chr12-71839203-71840800	2.40E-07	0.260629446	0.433	0.26	0.015861308	C8_CD4_Tres
chr6-134240598-134241047	2.40E-07	0.311440195	0.116	0.035	0.015889421	C8_CD4_Tres
chr16-12080021-12080518	2.42E-07	0.315958997	0.16	0.06	0.016050609	C8_CD4_Tres
chr10-119516192-119518135	2.43E-07	0.302405463	0.235	0.119	0.016069924	C8_CD4_Tres
chr2-172095256-172095806	2.43E-07	0.311657047	0.153	0.054	0.016104215	C8_CD4_Tres
chr5-173283535-173284078	2.43E-07	0.287475145	0.067	0.011	0.016122562	C8_CD4_Tres
chr4-83235369-83236028	2.50E-07	0.288691949	0.067	0.013	0.016528184	C8_CD4_Tres
chr17-19306153-19306735	2.51E-07	0.299676441	0.093	0.024	0.016588896	C8_CD4_Tres
chr16-57260266-57260698	2.52E-07	0.270948695	0.052	0.006	0.016703828	C8_CD4_Tres
chr15-70762874-70763726	2.57E-07	0.304872818	0.09	0.024	0.017047492	C8_CD4_Tres
chr1-76270326-76270694	2.59E-07	0.278349574	0.067	0.011	0.017132946	C8_CD4_Tres
chr3-197612961-197613571	2.59E-07	0.304375009	0.09	0.023	0.017133554	C8_CD4_Tres
chr10-13786642-13787482	2.63E-07	0.290563434	0.28	0.149	0.017415428	C8_CD4_Tres
chr20-1212748-1213241	2.68E-07	0.306506752	0.104	0.028	0.017721474	C8_CD4_Tres
chr3-179315766-179316376	2.69E-07	0.310705825	0.153	0.053	0.017843729	C8_CD4_Tres
chr2-96923968-96925045	2.71E-07	0.312054466	0.123	0.045	0.017938902	C8_CD4_Tres
chr5-95808149-95809098	2.76E-07	0.294407136	0.28	0.142	0.018261298	C8_CD4_Tres

chr11-4057708-4058466	2.79E-07	0.311439637	0.104	0.03	0.018499686	C8_CD4_Tres
chr11-61028371-61028662	2.83E-07	0.309766921	0.116	0.034	0.01870967	C8_CD4_Tres
chr2-6909378-6910289	2.87E-07	0.310288878	0.164	0.064	0.018997745	C8_CD4_Tres
chr12-124391371-124391873	2.93E-07	0.295650424	0.082	0.017	0.019421257	C8_CD4_Tres
chr2-84693919-84694622	2.97E-07	0.275317153	0.06	0.01	0.019672145	C8_CD4_Tres
chrX-124736533-124737214	2.99E-07	0.278562964	0.056	0.009	0.01979898	C8_CD4_Tres
chr2-43133957-43134649	2.99E-07	0.273022189	0.328	0.203	0.019819392	C8_CD4_Tres
chr10-103910787-103911469	3.04E-07	0.304640193	0.175	0.068	0.020124796	C8_CD4_Tres
chr1-154759096-154759592	3.04E-07	0.288293601	0.063	0.014	0.020151549	C8_CD4_Tres
chr5-159207566-159208624	3.05E-07	0.276734492	0.343	0.199	0.020213891	C8_CD4_Tres
chrX-73977236-73977876	3.13E-07	0.293566786	0.082	0.019	0.020739408	C8_CD4_Tres
chr8-127975532-127976036	3.24E-07	0.308490853	0.157	0.057	0.021462757	C8_CD4_Tres
chr6-33592610-33593414	3.25E-07	0.300659272	0.09	0.026	0.02154581	C8_CD4_Tres
chr2-42493364-42495245	3.25E-07	0.306865357	0.179	0.073	0.021553669	C8_CD4_Tres
chrX-56800754-56801160	3.40E-07	0.282686976	0.071	0.013	0.022488384	C8_CD4_Tres
chr5-40622939-40623837	3.40E-07	0.291660876	0.082	0.017	0.02250846	C8_CD4_Tres
chr8-132978752-132979545	3.57E-07	0.284022582	0.067	0.013	0.023618923	C8_CD4_Tres
chr20-36943921-36944602	3.63E-07	0.308273707	0.179	0.073	0.02403158	C8_CD4_Tres
chr2-42105326-42106336	3.63E-07	0.283067333	0.313	0.165	0.024057721	C8_CD4_Tres
chr19-9862545-9863117	3.68E-07	0.278316199	0.063	0.011	0.024400817	C8_CD4_Tres
chr19-3554248-3554604	3.70E-07	0.312083897	0.146	0.051	0.024490886	C8_CD4_Tres
chr22-17303559-17304598	3.70E-07	0.292071886	0.075	0.016	0.024491127	C8_CD4_Tres
chr3-194070541-194071198	3.72E-07	0.28807542	0.071	0.014	0.024644151	C8_CD4_Tres
chr10-80187074-80188348	3.74E-07	0.287576246	0.302	0.154	0.024761174	C8_CD4_Tres
chr2-239264545-239265697	3.83E-07	0.284525189	0.295	0.162	0.025341592	C8_CD4_Tres
chr3-58034811-58035307	3.87E-07	0.293146986	0.09	0.021	0.025649812	C8_CD4_Tres
chr2-240232910-240234054	3.88E-07	0.303517546	0.194	0.086	0.025724137	C8_CD4_Tres

chr12-117189576-117190721	3.90E-07	0.251802165	0.455	0.281	0.025832516	C8_CD4_Tres
chr12-6745636-6746009	3.90E-07	0.27490592	0.06	0.009	0.025848669	C8_CD4_Tres
chr15-81292589-81292788	3.92E-07	0.297234419	0.082	0.019	0.025947663	C8_CD4_Tres
chr17-21456808-21457846	3.95E-07	0.280589945	0.332	0.18	0.026171149	C8_CD4_Tres
chr7-128108672-128109290	4.01E-07	0.28997052	0.075	0.017	0.026576283	C8_CD4_Tres
chr1-40404356-40405120	4.05E-07	0.306096128	0.123	0.044	0.02684658	C8_CD4_Tres
chr9-92969004-92969313	4.10E-07	0.308833785	0.123	0.037	0.027125266	C8_CD4_Tres
chr12-132872556-132873377	4.10E-07	0.273315653	0.056	0.01	0.027133959	C8_CD4_Tres
chr15-28738456-28739180	4.16E-07	0.27380274	0.06	0.009	0.027515943	C8_CD4_Tres
chr10-100561965-100562598	4.20E-07	0.306408799	0.19	0.081	0.027786683	C8_CD4_Tres
chr1-36350652-36351036	4.28E-07	0.270760471	0.056	0.008	0.028372405	C8_CD4_Tres
chr2-7059158-7059556	4.31E-07	0.285147071	0.071	0.015	0.028536602	C8_CD4_Tres
chr7-121438014-121438727	4.32E-07	0.30466755	0.101	0.032	0.028633417	C8_CD4_Tres
chr7-30726551-30727749	4.35E-07	0.305362386	0.183	0.079	0.028810062	C8_CD4_Tres
chr4-80492489-80493267	4.39E-07	0.304875709	0.157	0.06	0.029064584	C8_CD4_Tres
chrX-75522755-75523631	4.47E-07	0.290860705	0.075	0.016	0.029617057	C8_CD4_Tres
chr11-68120214-68121965	4.57E-07	0.286179775	0.272	0.151	0.030244639	C8_CD4_Tres
chr2-143161914-143162569	4.58E-07	0.306508736	0.127	0.041	0.030297483	C8_CD4_Tres
chr15-81286391-81287078	4.59E-07	0.300813267	0.097	0.03	0.030407038	C8_CD4_Tres
chr5-140244310-140244991	4.66E-07	0.282568692	0.075	0.015	0.030865121	C8_CD4_Tres
chr19-33430011-33430934	4.71E-07	0.30195282	0.108	0.037	0.031182916	C8_CD4_Tres
chr12-64683968-64685368	4.74E-07	0.284028611	0.31	0.158	0.031415891	C8_CD4_Tres
chr13-25874732-25875243	4.86E-07	0.294815018	0.086	0.022	0.032198001	C8_CD4_Tres
chr7-148628367-148629224	4.87E-07	0.301699847	0.138	0.049	0.032282164	C8_CD4_Tres
chr2-29188904-29189409	4.95E-07	0.293083801	0.093	0.025	0.032794161	C8_CD4_Tres
chr9-127776231-127776683	5.06E-07	0.301367553	0.183	0.075	0.033536196	C8_CD4_Tres
chr16-84013850-84014615	5.10E-07	0.302722012	0.09	0.028	0.033790721	C8_CD4_Tres

chr7-76260123-76260653	5.11E-07	0.307335516	0.149	0.055	0.033838536	C8_CD4_Tres
chr3-167379643-167380510	5.13E-07	0.304076048	0.127	0.041	0.033985396	C8_CD4_Tres
chr3-18712806-18713585	5.14E-07	0.27311616	0.06	0.011	0.034012319	C8_CD4_Tres
chr5-140547120-140548193	5.16E-07	0.280990339	0.306	0.168	0.034196968	C8_CD4_Tres
chr21-34933642-34934318	5.36E-07	0.302050677	0.198	0.086	0.035463674	C8_CD4_Tres
chr19-48501415-48502530	5.36E-07	0.299466458	0.183	0.081	0.035466594	C8_CD4_Tres
chr8-141035889-141036347	5.37E-07	0.283260211	0.078	0.018	0.035543502	C8_CD4_Tres
chr7-151429487-151430243	5.38E-07	0.307891499	0.142	0.05	0.035623891	C8_CD4_Tres
chr17-67400229-67400508	5.40E-07	0.259833594	0.052	0.008	0.035754013	C8_CD4_Tres
chr17-80789597-80790422	5.41E-07	0.287007162	0.078	0.017	0.035838635	C8_CD4_Tres
chr2-109128854-109129673	5.51E-07	0.269925979	0.067	0.011	0.036467461	C8_CD4_Tres
chr22-50756513-50757373	5.59E-07	0.298384996	0.097	0.026	0.037044649	C8_CD4_Tres
chr10-22228869-22229745	5.67E-07	0.274584386	0.336	0.187	0.037536575	C8_CD4_Tres
chr5-74865580-74866791	5.70E-07	0.298021647	0.172	0.063	0.037768748	C8_CD4_Tres
chr4-40193321-40193566	5.73E-07	0.297900175	0.108	0.029	0.037917363	C8_CD4_Tres
chr12-7639566-7640916	5.75E-07	0.29639266	0.239	0.114	0.038097468	C8_CD4_Tres
chr4-89336791-89337191	5.78E-07	0.265032844	0.052	0.008	0.03824505	C8_CD4_Tres
chr1-22039304-22040301	6.01E-07	0.25014721	0.433	0.274	0.039779933	C8_CD4_Tres
chr4-76993858-76994597	6.07E-07	0.302377424	0.164	0.061	0.0402265	C8_CD4_Tres
chr15-92845155-92845424	6.09E-07	0.266620836	0.063	0.011	0.040298453	C8_CD4_Tres
chr4-88382307-88382999	6.14E-07	0.299626576	0.179	0.077	0.04063123	C8_CD4_Tres
chr17-2056661-2056898	6.25E-07	0.30045659	0.116	0.039	0.041383443	C8_CD4_Tres
chr11-128291863-128292145	6.27E-07	0.286878129	0.078	0.018	0.041533587	C8_CD4_Tres
chr10-15370362-15371603	6.29E-07	0.301248199	0.153	0.052	0.041649033	C8_CD4_Tres
chrX-41009899-41010507	6.36E-07	0.294123878	0.086	0.023	0.042102599	C8_CD4_Tres
chr3-61249946-61250327	6.38E-07	0.289369893	0.101	0.025	0.042277278	C8_CD4_Tres
chrX-9462749-9463476	6.55E-07	0.289251134	0.272	0.134	0.043371441	C8_CD4_Tres

chrY-20990849-20991238	6.62E-07	0.267048119	0.056	0.009	0.043850146	C8_CD4_Tres
chr19-57583523-57584423	6.65E-07	0.297946149	0.216	0.092	0.044037442	C8_CD4_Tres
chr7-56033630-56034605	6.78E-07	0.301839538	0.183	0.071	0.044915319	C8_CD4_Tres
chr11-128868707-128869583	6.84E-07	0.264855448	0.056	0.01	0.045270111	C8_CD4_Tres
chr5-32531301-32532622	6.87E-07	0.279472148	0.306	0.16	0.045526811	C8_CD4_Tres
chr10-47311281-47312421	7.01E-07	0.293273535	0.108	0.032	0.046409151	C8_CD4_Tres
chr14-70640771-70643189	7.05E-07	0.277024715	0.31	0.168	0.046662385	C8_CD4_Tres
chr7-142313449-142313926	7.07E-07	0.288114483	0.086	0.02	0.046809122	C8_CD4_Tres
chr12-50282857-50283898	7.15E-07	0.262725597	0.369	0.223	0.047341339	C8_CD4_Tres
chr5-59036317-59036622	7.21E-07	0.303566985	0.16	0.061	0.047771912	C8_CD4_Tres
chr1-94926661-94927456	7.24E-07	0.274474384	0.071	0.015	0.047955038	C8_CD4_Tres
chr4-153683714-153685052	7.27E-07	0.275613957	0.071	0.014	0.048153525	C8_CD4_Tres
chr3-23202421-23203630	7.29E-07	0.265632529	0.06	0.01	0.04829027	C8_CD4_Tres
chr19-56507410-56508156	7.44E-07	0.298891718	0.112	0.035	0.049256272	C8_CD4_Tres
chr5-149264286-149264983	7.45E-07	0.28781077	0.086	0.022	0.049369075	C8_CD4_Tres
chr5-67206478-67207348	7.53E-07	0.271898661	0.34	0.188	0.049875192	C8_CD4_Tres
chr17-78413035-78413614	7.61E-07	0.288195013	0.082	0.021	0.050415636	C8_CD4_Tres
chr8-128653305-128653612	7.64E-07	0.284981803	0.082	0.019	0.050563586	C8_CD4_Tres
chr12-53543338-53543787	7.66E-07	0.277999425	0.067	0.014	0.050734573	C8_CD4_Tres
chr14-97993653-97994741	7.68E-07	0.293500239	0.239	0.114	0.050826518	C8_CD4_Tres
chr4-39353169-39354321	7.73E-07	0.285485931	0.261	0.134	0.051162781	C8_CD4_Tres
chr1-231628255-231628738	7.73E-07	0.289264	0.093	0.027	0.051177156	C8_CD4_Tres
chr2-26298713-26299982	7.78E-07	0.295666893	0.101	0.029	0.051490206	C8_CD4_Tres
chr11-74004846-74005623	7.79E-07	0.283263412	0.284	0.144	0.051593264	C8_CD4_Tres
chr9-20504955-20505303	7.94E-07	0.266310719	0.063	0.01	0.052591158	C8_CD4_Tres
chr1-160069810-160070752	7.99E-07	0.299155013	0.138	0.053	0.052934658	C8_CD4_Tres
chr2-43136779-43137330	8.15E-07	0.260023997	0.384	0.229	0.053960669	C8_CD4_Tres

chr17-8630403-8631523	8.40E-07	0.287579905	0.261	0.123	0.055594297	C8_CD4_Tres
chr11-121367159-121367812	8.56E-07	0.25929489	0.052	0.008	0.056667398	C8_CD4_Tres
chr14-76926377-76927097	8.64E-07	0.299587757	0.153	0.058	0.05718454	C8_CD4_Tres
chr6-142884411-142885797	8.72E-07	0.267135418	0.351	0.203	0.057772587	C8_CD4_Tres
chr17-81481335-81481634	8.78E-07	0.282043198	0.075	0.017	0.058165091	C8_CD4_Tres
chr5-157172052-157172856	9.11E-07	0.289773789	0.231	0.113	0.060348427	C8_CD4_Tres
chr6-12051769-12052284	9.37E-07	0.297119778	0.101	0.031	0.062052007	C8_CD4_Tres
chr21-30718215-30718726	9.42E-07	0.258113847	0.052	0.008	0.06241516	C8_CD4_Tres
chr7-142636504-142637076	9.48E-07	0.288751842	0.09	0.024	0.062793886	C8_CD4_Tres
chr20-59121844-59123456	9.71E-07	0.294056671	0.168	0.07	0.06431417	C8_CD4_Tres
chr19-48414711-48415698	9.72E-07	0.297369819	0.179	0.072	0.064361692	C8_CD4_Tres
chr11-119115142-119115751	9.78E-07	0.266995935	0.063	0.011	0.06479492	C8_CD4_Tres
chr3-126654600-126655257	9.80E-07	0.284082171	0.082	0.019	0.064928884	C8_CD4_Tres
chr16-87635312-87636684	9.84E-07	0.297053188	0.179	0.08	0.065140036	C8_CD4_Tres
chr9-133238355-133239402	9.94E-07	0.286175338	0.093	0.023	0.065838885	C8_CD4_Tres
chr16-19885779-19886567	9.99E-07	0.292392514	0.108	0.036	0.066132157	C8_CD4_Tres
chr11-75328562-75329284	1.00E-06	0.294800605	0.138	0.047	0.066391659	C8_CD4_Tres
chr2-84919440-84920083	1.00E-06	0.269319903	0.063	0.011	0.066414397	C8_CD4_Tres
chr14-63649977-63650836	1.01E-06	0.295377271	0.116	0.041	0.067067951	C8_CD4_Tres
chr18-23070939-23071743	1.03E-06	0.283463508	0.243	0.123	0.06804758	C8_CD4_Tres
chr3-71478396-71478983	1.03E-06	0.298379487	0.153	0.059	0.068063169	C8_CD4_Tres
chr18-3096876-3097427	1.04E-06	0.289040067	0.086	0.023	0.068707216	C8_CD4_Tres
chr6-31094331-31094930	1.04E-06	0.260512728	0.06	0.011	0.068795261	C8_CD4_Tres
chr19-20999523-21000246	1.04E-06	0.271433223	0.328	0.179	0.069045363	C8_CD4_Tres
chr9-120903730-120904387	1.05E-06	0.291893247	0.142	0.05	0.06927498	C8_CD4_Tres
chr3-129067803-129068802	1.05E-06	0.281913546	0.078	0.019	0.069469961	C8_CD4_Tres
chr2-86041016-86041232	1.05E-06	0.292831069	0.116	0.042	0.069505023	C8_CD4_Tres

chr8-27404934-27405825	1.05E-06	0.293115122	0.183	0.085	0.069685353	C8_CD4_Tres
chr8-97774881-97776514	1.06E-06	0.289184407	0.243	0.113	0.070036925	C8_CD4_Tres
chr1-211228690-211229689	1.06E-06	0.293123446	0.119	0.039	0.070267085	C8_CD4_Tres
chr1-25565319-25565580	1.08E-06	0.276657742	0.078	0.019	0.071434336	C8_CD4_Tres
chr1-111225745-111226266	1.09E-06	0.284517589	0.086	0.021	0.071892577	C8_CD4_Tres
chr18-61892803-61894765	1.09E-06	0.298356542	0.142	0.053	0.071900211	C8_CD4_Tres
chr3-30657759-30659008	1.10E-06	0.267287914	0.347	0.19	0.072898966	C8_CD4_Tres
chr2-74848947-74849987	1.11E-06	0.298013111	0.16	0.064	0.073248361	C8_CD4_Tres
chr21-33360851-33362547	1.12E-06	0.268485969	0.325	0.182	0.074237422	C8_CD4_Tres
chr1-52685790-52686575	1.13E-06	0.295402022	0.153	0.057	0.074540933	C8_CD4_Tres
chr8-144583209-144583660	1.13E-06	0.289439909	0.093	0.027	0.074544157	C8_CD4_Tres
chr9-7935611-7936185	1.13E-06	0.295273765	0.108	0.036	0.075027611	C8_CD4_Tres
chr10-6583419-6583722	1.14E-06	0.290699652	0.116	0.041	0.07566377	C8_CD4_Tres
chr11-73177566-73178375	1.14E-06	0.290087613	0.108	0.034	0.075705421	C8_CD4_Tres
chr7-351210-352241	1.14E-06	0.292217298	0.127	0.047	0.075799476	C8_CD4_Tres
chr18-13436668-13436907	1.15E-06	0.260155215	0.052	0.009	0.07632755	C8_CD4_Tres
chr1-116325284-116326373	1.16E-06	0.288271703	0.101	0.03	0.076529631	C8_CD4_Tres
chr7-99579820-99580711	1.17E-06	0.295633911	0.127	0.043	0.077215812	C8_CD4_Tres
chr15-40453963-40454326	1.20E-06	0.270100959	0.056	0.011	0.079399579	C8_CD4_Tres
chr6-36539939-36540569	1.21E-06	0.292318687	0.112	0.035	0.080174028	C8_CD4_Tres
chr16-28622876-28623771	1.22E-06	0.294177884	0.142	0.052	0.080699775	C8_CD4_Tres
chr12-67630003-67631086	1.22E-06	0.275372646	0.291	0.152	0.081004609	C8_CD4_Tres
chr10-100968997-100970379	1.23E-06	0.295142398	0.134	0.046	0.08174887	C8_CD4_Tres
chr1-205593412-205593817	1.25E-06	0.272020813	0.071	0.015	0.0825155	C8_CD4_Tres
chr10-13371875-13372216	1.26E-06	0.280624055	0.082	0.02	0.083764562	C8_CD4_Tres
chr2-98656735-98657123	1.30E-06	0.298325669	0.142	0.054	0.085844257	C8_CD4_Tres
chr22-39557244-39557721	1.36E-06	0.265394802	0.06	0.01	0.089744428	C8_CD4_Tres

chr11-118530343-118531695	1.36E-06	0.281981243	0.254	0.125	0.090073994	C8_CD4_Tres
chr1-151539545-151540709	1.37E-06	0.256545746	0.354	0.22	0.090611122	C8_CD4_Tres
chr5-1104415-1104942	1.37E-06	0.292372554	0.116	0.04	0.090763379	C8_CD4_Tres
chr2-74833585-74834250	1.39E-06	0.288390884	0.224	0.105	0.091998227	C8_CD4_Tres
chr1-181159371-181160187	1.40E-06	0.28923133	0.112	0.038	0.092567588	C8_CD4_Tres
chr3-194404266-194404900	1.40E-06	0.262402797	0.06	0.011	0.092622353	C8_CD4_Tres
chr2-161992459-161992805	1.41E-06	0.263115289	0.056	0.01	0.093060785	C8_CD4_Tres
chr13-94419232-94419723	1.41E-06	0.251513163	0.052	0.007	0.093316497	C8_CD4_Tres
chr16-11297220-11298015	1.41E-06	0.284925923	0.112	0.033	0.093323703	C8_CD4_Tres
chrX-136646596-136646887	1.41E-06	0.262163163	0.06	0.013	0.093642502	C8_CD4_Tres
chr19-16001666-16002290	1.43E-06	0.272835975	0.063	0.015	0.094737023	C8_CD4_Tres
chr6-27332675-27333437	1.44E-06	0.280992456	0.086	0.024	0.095639951	C8_CD4_Tres
chr3-106176419-106177119	1.45E-06	0.288680303	0.205	0.097	0.096024628	C8_CD4_Tres
chr1-15578850-15579535	1.46E-06	0.268734292	0.071	0.015	0.096368746	C8_CD4_Tres
chr3-183115985-183116397	1.46E-06	0.264811466	0.056	0.011	0.096455414	C8_CD4_Tres
chr14-58330746-58331484	1.46E-06	0.285704068	0.216	0.107	0.096553403	C8_CD4_Tres
chr19-5949764-5950227	1.46E-06	0.27624034	0.078	0.018	0.096757109	C8_CD4_Tres
chr17-43900218-43900865	1.47E-06	0.290426288	0.131	0.047	0.09715644	C8_CD4_Tres
chr18-58043379-58044988	1.47E-06	0.288656853	0.198	0.083	0.097368597	C8_CD4_Tres
chr7-5701084-5701744	1.47E-06	0.287904992	0.123	0.044	0.097579961	C8_CD4_Tres
chr17-17848744-17850355	1.50E-06	0.265787302	0.313	0.181	0.099138492	C8_CD4_Tres
chr10-47206504-47207584	1.56E-06	0.271054646	0.075	0.017	0.103308983	C8_CD4_Tres
chr9-114928932-114929730	1.56E-06	0.289593433	0.187	0.076	0.1035197	C8_CD4_Tres
chr9-21443796-21444730	1.57E-06	0.28230838	0.086	0.026	0.104081194	C8_CD4_Tres
chr5-98796259-98796643	1.59E-06	0.253156912	0.052	0.008	0.105263953	C8_CD4_Tres
chr8-65951101-65952222	1.60E-06	0.262587315	0.317	0.19	0.105857219	C8_CD4_Tres
chr5-178120767-178121272	1.60E-06	0.290551357	0.146	0.052	0.106190058	C8_CD4_Tres

chr19-2507160-2507506	1.66E-06	0.277688099	0.082	0.02	0.109806712	C8_CD4_Tres
chr1-8918081-8918388	1.67E-06	0.273359136	0.071	0.015	0.110775764	C8_CD4_Tres
chr18-79516645-79517047	1.70E-06	0.260002176	0.06	0.012	0.112718222	C8_CD4_Tres
chr1-172393922-172394555	1.71E-06	0.286761897	0.116	0.039	0.11293176	C8_CD4_Tres
chr22-50267643-50267910	1.71E-06	0.282314806	0.086	0.023	0.113081296	C8_CD4_Tres
chr20-25148260-25148985	1.72E-06	0.281063555	0.093	0.025	0.113845892	C8_CD4_Tres
chr17-81967577-81968267	1.73E-06	0.289287816	0.172	0.071	0.114722118	C8_CD4_Tres
chr2-229835654-229836259	1.81E-06	0.289264205	0.112	0.037	0.120049978	C8_CD4_Tres
chr13-25973475-25973851	1.88E-06	0.270603657	0.075	0.017	0.124753432	C8_CD4_Tres
chr16-2486864-2487478	1.92E-06	0.290425983	0.16	0.067	0.126857904	C8_CD4_Tres
chr8-81034609-81035248	1.93E-06	0.287287247	0.112	0.035	0.127664557	C8_CD4_Tres
chr9-104967768-104968711	1.99E-06	0.26840855	0.299	0.162	0.13169644	C8_CD4_Tres
chr1-226444634-226445366	2.01E-06	0.287901342	0.146	0.053	0.133013015	C8_CD4_Tres
chr7-44104195-44105338	2.01E-06	0.259674998	0.063	0.01	0.133325787	C8_CD4_Tres
chr13-74136244-74136573	2.02E-06	0.288581815	0.149	0.064	0.133813589	C8_CD4_Tres
chrX-129769272-129769893	2.02E-06	0.283011844	0.205	0.097	0.134061837	C8_CD4_Tres
chr20-32967029-32967552	2.03E-06	0.291305417	0.138	0.053	0.13418545	C8_CD4_Tres
chr1-206922986-206923403	2.03E-06	0.288202553	0.183	0.078	0.134291165	C8_CD4_Tres
chrX-136694155-136695057	2.03E-06	0.288093637	0.138	0.051	0.134366623	C8_CD4_Tres
chr3-45005688-45006142	2.04E-06	0.250915955	0.052	0.01	0.134871185	C8_CD4_Tres
chr7-152144545-152145175	2.05E-06	0.278551792	0.078	0.02	0.135526568	C8_CD4_Tres
chr6-7139001-7139256	2.09E-06	0.270422835	0.075	0.017	0.138625865	C8_CD4_Tres
chr17-3754431-3755318	2.12E-06	0.277825502	0.243	0.12	0.14007889	C8_CD4_Tres
chr8-124972870-124973756	2.12E-06	0.290416668	0.146	0.061	0.140583064	C8_CD4_Tres
chr8-94119577-94120574	2.13E-06	0.285852523	0.164	0.069	0.141032623	C8_CD4_Tres
chr7-1919963-1920675	2.13E-06	0.285263619	0.093	0.03	0.141114628	C8_CD4_Tres
chr15-75349684-75350363	2.13E-06	0.287762872	0.168	0.07	0.141205124	C8_CD4_Tres

chr15-75347123-75348018	2.15E-06	0.282363797	0.119	0.043	0.142069231	C8_CD4_Tres
chr16-68272854-68273055	2.18E-06	0.264183142	0.063	0.014	0.14453015	C8_CD4_Tres
chr7-2715676-2716266	2.20E-06	0.291825882	0.138	0.055	0.145539171	C8_CD4_Tres
chr8-128543251-128543824	2.21E-06	0.285295478	0.123	0.043	0.146026905	C8_CD4_Tres
chr19-7871938-7872322	2.22E-06	0.28688417	0.153	0.067	0.14716726	C8_CD4_Tres
chr22-40203515-40203958	2.28E-06	0.286942499	0.119	0.037	0.15085426	C8_CD4_Tres
chr14-100083697-100084010	2.29E-06	0.284363192	0.097	0.028	0.151419705	C8_CD4_Tres
chr14-38064845-38065701	2.29E-06	0.273141285	0.28	0.143	0.151491135	C8_CD4_Tres
chr12-132904613-132905029	2.30E-06	0.287250649	0.149	0.065	0.152492518	C8_CD4_Tres
chr2-172461872-172463487	2.36E-06	0.267878179	0.295	0.153	0.155981037	C8_CD4_Tres
chr3-124584189-124585050	2.38E-06	0.251780507	0.052	0.009	0.157841046	C8_CD4_Tres
chr6-151413295-151414069	2.38E-06	0.285772436	0.134	0.049	0.157929301	C8_CD4_Tres
chr8-41544820-41545440	2.39E-06	0.283211255	0.101	0.033	0.158568351	C8_CD4_Tres
chr18-13438086-13438457	2.40E-06	0.264557753	0.063	0.013	0.15924953	C8_CD4_Tres
chr12-110887589-110888313	2.41E-06	0.286272981	0.123	0.043	0.159593043	C8_CD4_Tres
chr17-78484382-78484976	2.47E-06	0.269146112	0.071	0.018	0.163728389	C8_CD4_Tres
chr10-59864528-59865401	2.60E-06	0.257823817	0.063	0.012	0.172076381	C8_CD4_Tres
chr3-32240051-32240337	2.61E-06	0.264402525	0.067	0.015	0.172628237	C8_CD4_Tres
chr11-60775354-60775794	2.63E-06	0.277774581	0.086	0.024	0.173865267	C8_CD4_Tres
chr19-991348-991997	2.63E-06	0.274906358	0.265	0.13	0.174417462	C8_CD4_Tres
chr9-131744079-131744699	2.67E-06	0.277288391	0.09	0.024	0.176545153	C8_CD4_Tres
chr17-65042227-65042515	2.69E-06	0.276426311	0.086	0.025	0.178262136	C8_CD4_Tres
chr3-195458165-195458771	2.71E-06	0.268180137	0.067	0.017	0.179643302	C8_CD4_Tres
chr1-85179374-85179841	2.72E-06	0.256458107	0.063	0.012	0.180154565	C8_CD4_Tres
chr19-45971865-45972754	2.73E-06	0.283107102	0.104	0.034	0.180866082	C8_CD4_Tres
chr18-76709630-76710045	2.74E-06	0.255988941	0.052	0.01	0.181545525	C8_CD4_Tres
chr1-240611287-240612336	2.76E-06	0.275010356	0.082	0.02	0.182856851	C8_CD4_Tres

chr1-154352285-154353852	2.77E-06	0.28387858	0.138	0.05	0.183244369	C8_CD4_Tres
chr16-30055065-30055559	2.77E-06	0.270482664	0.086	0.023	0.183298565	C8_CD4_Tres
chr7-100866001-100866683	2.77E-06	0.25980714	0.063	0.014	0.183762921	C8_CD4_Tres
chr12-123931466-123932214	2.79E-06	0.264363978	0.071	0.017	0.18473641	C8_CD4_Tres
chr7-26097125-26097772	2.79E-06	0.250066977	0.052	0.008	0.184970005	C8_CD4_Tres
chr9-20208981-20210313	2.80E-06	0.277009265	0.093	0.032	0.185342382	C8_CD4_Tres
chr20-50146581-50147532	2.83E-06	0.274724631	0.246	0.126	0.187249998	C8_CD4_Tres
chr5-119302904-119303337	2.90E-06	0.255408039	0.06	0.011	0.192372903	C8_CD4_Tres
chr12-6792698-6793179	2.94E-06	0.264026613	0.067	0.015	0.194649486	C8_CD4_Tres
chr8-1780804-1781592	2.95E-06	0.285552903	0.142	0.062	0.195431388	C8_CD4_Tres
chr2-118855383-118855989	2.98E-06	0.275958728	0.097	0.028	0.197493089	C8_CD4_Tres
chr7-4883008-4884026	2.99E-06	0.275989012	0.216	0.102	0.19795189	C8_CD4_Tres
chr8-117439345-117440215	2.99E-06	0.275084793	0.086	0.022	0.198065361	C8_CD4_Tres
chr12-69329766-69331508	3.02E-06	0.281487546	0.194	0.082	0.200081528	C8_CD4_Tres
chr2-241094974-241095903	3.06E-06	0.277194503	0.097	0.029	0.202420773	C8_CD4_Tres
chr14-31124050-31124549	3.08E-06	0.279705033	0.116	0.042	0.204205098	C8_CD4_Tres
chrX-78516860-78517570	3.09E-06	0.279209996	0.235	0.111	0.204449968	C8_CD4_Tres
chr7-150568740-150569060	3.12E-06	0.269936511	0.078	0.02	0.206493637	C8_CD4_Tres
chr8-19013446-19014206	3.13E-06	0.264381981	0.067	0.017	0.207551404	C8_CD4_Tres
chr11-111944123-111945042	3.15E-06	0.266193684	0.075	0.016	0.208834479	C8_CD4_Tres
chr2-74834511-74835397	3.16E-06	0.283169307	0.164	0.067	0.209110059	C8_CD4_Tres
chr15-100339031-100339273	3.16E-06	0.280790543	0.127	0.045	0.209134102	C8_CD4_Tres
chr1-154760256-154760721	3.19E-06	0.281205342	0.127	0.045	0.211035359	C8_CD4_Tres
chr1-244074471-244075379	3.19E-06	0.278538171	0.198	0.092	0.211142789	C8_CD4_Tres
chr2-171210625-171211400	3.20E-06	0.274325686	0.093	0.028	0.211740969	C8_CD4_Tres
chr19-12603449-12604174	3.20E-06	0.271190591	0.086	0.02	0.211819429	C8_CD4_Tres
chr17-38718588-38718896	3.23E-06	0.267541896	0.071	0.017	0.213655121	C8_CD4_Tres

chr15-45117719-45118429	3.35E-06	0.281157609	0.16	0.069	0.221681378	C8_CD4_Tres
chr20-41231931-41232668	3.37E-06	0.280709022	0.123	0.042	0.223405457	C8_CD4_Tres
chr8-8942579-8942924	3.38E-06	0.263028094	0.067	0.016	0.223585337	C8_CD4_Tres
chr19-6161062-6161723	3.44E-06	0.268556635	0.09	0.026	0.227907853	C8_CD4_Tres
chr4-2570607-2571279	3.46E-06	0.281400853	0.123	0.046	0.228821179	C8_CD4_Tres
chr8-28493662-28494800	3.46E-06	0.26252236	0.295	0.163	0.229410205	C8_CD4_Tres
chr14-70654703-70655834	3.52E-06	0.277038873	0.209	0.102	0.23329114	C8_CD4_Tres
chr1-76267670-76268008	3.52E-06	0.2570625	0.063	0.014	0.233324217	C8_CD4_Tres
chrX-103214441-103215755	3.62E-06	0.279271313	0.194	0.089	0.239645196	C8_CD4_Tres
chr10-28594432-28594704	3.72E-06	0.25149349	0.056	0.008	0.246128441	C8_CD4_Tres
chr20-58228169-58228917	3.72E-06	0.273996716	0.104	0.033	0.246597919	C8_CD4_Tres
chr3-108100833-108101265	3.88E-06	0.259185693	0.067	0.015	0.256640873	C8_CD4_Tres
chr7-96837367-96838021	3.89E-06	0.264179156	0.071	0.017	0.257491054	C8_CD4_Tres
chr11-304970-305197	3.90E-06	0.277892841	0.108	0.034	0.258102993	C8_CD4_Tres
chr5-126852049-126852549	3.92E-06	0.269333735	0.082	0.022	0.259833759	C8_CD4_Tres
chr9-69121084-69122373	3.96E-06	0.271557789	0.09	0.026	0.262553336	C8_CD4_Tres
chr17-40107348-40108515	4.02E-06	0.263575273	0.28	0.157	0.266276391	C8_CD4_Tres
chr14-97719794-97720261	4.09E-06	0.272568775	0.101	0.031	0.270787033	C8_CD4_Tres
chr19-49690524-49691221	4.09E-06	0.269305145	0.09	0.025	0.27115617	C8_CD4_Tres
chr12-55930186-55930503	4.16E-06	0.280660514	0.183	0.079	0.275475437	C8_CD4_Tres
chr5-134586658-134587545	4.20E-06	0.278133472	0.198	0.1	0.278171175	C8_CD4_Tres
chr2-106085192-106086352	4.25E-06	0.273143569	0.228	0.115	0.281236983	C8_CD4_Tres
chr12-92569873-92570650	4.26E-06	0.27776818	0.153	0.061	0.281811465	C8_CD4_Tres
chr2-96527196-96528583	4.34E-06	0.273093709	0.205	0.107	0.287085504	C8_CD4_Tres
chr10-17428189-17429128	4.34E-06	0.278895004	0.183	0.084	0.28768918	C8_CD4_Tres
chr19-17830745-17831200	4.43E-06	0.26776054	0.078	0.022	0.293325242	C8_CD4_Tres
chr16-56610112-56610588	4.46E-06	0.2792088	0.146	0.059	0.295352	C8_CD4_Tres

chr10-32459154-32459693	4.50E-06	0.272568552	0.101	0.031	0.298245895	C8_CD4_Tres
chr5-80091594-80092154	4.58E-06	0.251535756	0.067	0.014	0.303374182	C8_CD4_Tres
chr6-31491696-31492193	4.62E-06	0.268117197	0.243	0.131	0.305874791	C8_CD4_Tres
chrX-1554452-1555247	4.65E-06	0.272303871	0.093	0.03	0.307650181	C8_CD4_Tres
chr9-96686502-96687193	4.66E-06	0.275821568	0.19	0.083	0.308710429	C8_CD4_Tres
chr4-7854483-7855485	4.66E-06	0.252863351	0.328	0.191	0.308793948	C8_CD4_Tres
chr22-41524824-41525099	4.68E-06	0.25596003	0.06	0.014	0.309728747	C8_CD4_Tres
chr5-111278513-111279163	4.72E-06	0.280264472	0.149	0.057	0.312301653	C8_CD4_Tres
chr11-61076548-61076826	4.86E-06	0.260672443	0.071	0.016	0.321679021	C8_CD4_Tres
chr17-46137542-46138093	4.86E-06	0.277728466	0.108	0.038	0.32181947	C8_CD4_Tres
chr8-85176750-85178060	4.89E-06	0.259569043	0.287	0.158	0.324135176	C8_CD4_Tres
chr3-56928752-56928999	4.95E-06	0.259908039	0.067	0.016	0.327490651	C8_CD4_Tres
chr3-141266546-141267459	4.97E-06	0.273175085	0.116	0.037	0.328912495	C8_CD4_Tres
chr12-25322376-25323215	4.97E-06	0.264709763	0.075	0.021	0.329024548	C8_CD4_Tres
chr20-25865370-25865959	5.02E-06	0.256240452	0.071	0.017	0.332148412	C8_CD4_Tres
chr17-7132693-7133801	5.08E-06	0.264101269	0.082	0.021	0.336689945	C8_CD4_Tres
chr14-24279266-24279898	5.18E-06	0.263906634	0.075	0.02	0.34318183	C8_CD4_Tres
chr17-20320541-20321115	5.19E-06	0.254742695	0.063	0.014	0.343423382	C8_CD4_Tres
chr21-45154725-45155132	5.20E-06	0.262454311	0.071	0.019	0.344358536	C8_CD4_Tres
chr20-3071691-3072476	5.28E-06	0.272604298	0.101	0.035	0.349589763	C8_CD4_Tres
chr11-76232136-76232414	5.32E-06	0.264398979	0.082	0.023	0.352356772	C8_CD4_Tres
chr2-98487763-98488231	5.34E-06	0.270564307	0.086	0.025	0.35353679	C8_CD4_Tres
chr1-52842361-52843347	5.62E-06	0.27426464	0.131	0.051	0.371867419	C8_CD4_Tres
chr7-138161530-138161893	5.72E-06	0.267612506	0.09	0.027	0.378898475	C8_CD4_Tres
chr4-140152828-140153182	5.81E-06	0.266363298	0.086	0.024	0.384979976	C8_CD4_Tres
chr4-48127559-48128275	5.91E-06	0.264268399	0.082	0.023	0.391118092	C8_CD4_Tres
chr17-82551654-82552091	5.93E-06	0.27750663	0.131	0.05	0.392661606	C8_CD4_Tres

chr6-7051362-7052599	6.02E-06	0.273660337	0.108	0.037	0.398335158	C8_CD4_Tres
chr12-44835569-44836267	6.03E-06	0.276081407	0.127	0.044	0.399245644	C8_CD4_Tres
chr5-157233063-157234120	6.06E-06	0.274421964	0.164	0.068	0.40110279	C8_CD4_Tres
chr3-13496307-13496585	6.16E-06	0.263075915	0.075	0.02	0.40762143	C8_CD4_Tres
chr2-84888289-84889541	6.23E-06	0.275785385	0.16	0.062	0.412396376	C8_CD4_Tres
chr12-10981744-10982780	6.43E-06	0.27231686	0.123	0.045	0.425897212	C8_CD4_Tres
chr10-80535319-80536337	6.60E-06	0.269152225	0.205	0.105	0.437171661	C8_CD4_Tres
chr2-38542361-38543327	6.60E-06	0.277822169	0.131	0.048	0.437399159	C8_CD4_Tres
chr11-3839030-3839697	6.64E-06	0.258473845	0.276	0.158	0.440015265	C8_CD4_Tres
chr3-71398153-71398751	6.70E-06	0.263188666	0.086	0.023	0.443689358	C8_CD4_Tres
chr2-234335408-234336249	6.76E-06	0.274939213	0.119	0.041	0.447578324	C8_CD4_Tres
chr7-76166077-76167090	6.78E-06	0.260407092	0.261	0.142	0.448842451	C8_CD4_Tres
chr20-59222107-59222840	6.83E-06	0.272996202	0.164	0.077	0.45199298	C8_CD4_Tres
chr16-23948645-23950307	6.84E-06	0.260089784	0.269	0.149	0.453024223	C8_CD4_Tres
chr9-109320242-109321291	6.88E-06	0.270821416	0.123	0.043	0.455639668	C8_CD4_Tres
chr5-131747211-131748217	7.04E-06	0.262795893	0.078	0.021	0.466522233	C8_CD4_Tres
chr17-74862179-74862484	7.05E-06	0.256640767	0.071	0.019	0.467126167	C8_CD4_Tres
chr11-121418514-121419009	7.17E-06	0.263922568	0.078	0.024	0.474966858	C8_CD4_Tres
chr16-68287346-68287875	7.17E-06	0.264941061	0.086	0.026	0.475046788	C8_CD4_Tres
chr13-99204670-99205058	7.22E-06	0.271284151	0.149	0.066	0.478048246	C8_CD4_Tres
chr2-157468873-157469748	7.32E-06	0.264645709	0.228	0.117	0.484793798	C8_CD4_Tres
chr1-101238024-101238405	7.44E-06	0.266504569	0.112	0.036	0.492383745	C8_CD4_Tres
chr19-10731038-10732050	7.46E-06	0.26040901	0.269	0.14	0.494198453	C8_CD4_Tres
chr5-39187062-39187580	7.50E-06	0.26969112	0.108	0.041	0.49659244	C8_CD4_Tres
chr2-71071626-71072129	7.78E-06	0.271879253	0.116	0.036	0.514959846	C8_CD4_Tres
chr2-64049375-64050009	7.81E-06	0.267585547	0.093	0.028	0.517135952	C8_CD4_Tres
chr15-84598264-84598642	8.07E-06	0.267047932	0.104	0.035	0.534301302	C8_CD4_Tres

chr13-114155938-114156532	8.07E-06	0.25008634	0.067	0.016	0.534724558	C8_CD4_Tres
chr1-146472182-146472711	8.13E-06	0.260839793	0.086	0.025	0.538268395	C8_CD4_Tres
chr4-113682783-113683554	8.16E-06	0.272124286	0.198	0.083	0.540066654	C8_CD4_Tres
chr9-89603057-89603430	8.16E-06	0.268587316	0.119	0.042	0.540637558	C8_CD4_Tres
chr11-96016159-96016481	8.17E-06	0.262598833	0.078	0.022	0.541010484	C8_CD4_Tres
chr7-32904934-32906040	8.18E-06	0.255217083	0.276	0.15	0.54152576	C8_CD4_Tres
chr5-173331670-173332261	8.26E-06	0.250205898	0.06	0.014	0.547023005	C8_CD4_Tres
chr4-124711932-124713188	8.69E-06	0.255006261	0.078	0.021	0.575555939	C8_CD4_Tres
chr16-10949351-10949646	8.72E-06	0.256211682	0.071	0.018	0.577330531	C8_CD4_Tres
chr17-77963501-77963776	8.84E-06	0.266389336	0.101	0.035	0.58548378	C8_CD4_Tres
chr17-78120942-78121917	9.10E-06	0.267909053	0.187	0.085	0.60234512	C8_CD4_Tres
chr19-1074353-1075077	9.22E-06	0.255703362	0.261	0.137	0.61024575	C8_CD4_Tres
chr6-112027394-112028499	9.22E-06	0.266027931	0.194	0.095	0.610714909	C8_CD4_Tres
chr1-235093249-235094158	9.34E-06	0.269039423	0.131	0.056	0.618723718	C8_CD4_Tres
chr13-99051605-99052040	9.42E-06	0.256306618	0.078	0.019	0.623844025	C8_CD4_Tres
chr5-65719394-65720210	9.49E-06	0.269914774	0.153	0.062	0.628366972	C8_CD4_Tres
chr5-177812192-177812598	9.53E-06	0.253478638	0.067	0.016	0.631361395	C8_CD4_Tres
chr1-6465361-6466378	9.55E-06	0.269685512	0.172	0.07	0.632627148	C8_CD4_Tres
chr2-236569187-236569917	9.70E-06	0.269726147	0.142	0.061	0.642124597	C8_CD4_Tres
chr10-30704160-30705722	9.70E-06	0.265830826	0.183	0.083	0.642213898	C8_CD4_Tres
chr6-155417959-155418805	9.86E-06	0.266760825	0.116	0.043	0.652849217	C8_CD4_Tres
chr20-50817814-50818749	9.87E-06	0.268842973	0.157	0.073	0.653809778	C8_CD4_Tres
chr14-91880594-91881425	9.98E-06	0.268934799	0.134	0.053	0.660733825	C8_CD4_Tres
chr18-76721135-76721810	1.00E-05	0.252158404	0.071	0.017	0.663348456	C8_CD4_Tres
chr4-113899253-113900043	1.01E-05	0.265673898	0.112	0.041	0.669046815	C8_CD4_Tres
chr15-45187432-45188756	1.02E-05	0.267513459	0.175	0.078	0.672210789	C8_CD4_Tres
chr2-219551629-219552845	1.02E-05	0.265759794	0.19	0.099	0.674492975	C8_CD4_Tres

chr11-313845-314589	1.02E-05	0.2557052	0.254	0.141	0.675528059	C8_CD4_Tres
chr22-31247898-31248420	1.04E-05	0.263220392	0.097	0.027	0.686341751	C8_CD4_Tres
chr17-47066809-47067682	1.06E-05	0.256439333	0.246	0.142	0.699469501	C8_CD4_Tres
chr1-144460773-144461736	1.08E-05	0.264725865	0.108	0.039	0.716744502	C8_CD4_Tres
chr4-71004246-71004883	1.11E-05	0.257351684	0.082	0.023	0.734339327	C8_CD4_Tres
chr4-9603659-9605107	1.11E-05	0.263422353	0.183	0.091	0.736416921	C8_CD4_Tres
chr14-61568401-61568931	1.12E-05	0.256228711	0.082	0.024	0.744995484	C8_CD4_Tres
chr11-2987866-2988719	1.13E-05	0.268969881	0.164	0.07	0.750930037	C8_CD4_Tres
chr1-6486305-6486768	1.16E-05	0.265377611	0.116	0.043	0.767264873	C8_CD4_Tres
chr16-74600780-74601487	1.16E-05	0.268490666	0.131	0.056	0.770181117	C8_CD4_Tres
chr8-27319914-27320198	1.16E-05	0.250615464	0.071	0.02	0.770454714	C8_CD4_Tres
chr16-68269321-68269586	1.17E-05	0.256092372	0.086	0.026	0.775349819	C8_CD4_Tres
chr17-78665713-78666425	1.17E-05	0.256050207	0.082	0.024	0.777552413	C8_CD4_Tres
chr8-124643865-124644425	1.20E-05	0.264796956	0.187	0.086	0.791388069	C8_CD4_Tres
chr3-45869558-45870568	1.21E-05	0.256631407	0.239	0.131	0.80148527	C8_CD4_Tres
chr11-96013827-96014532	1.22E-05	0.26765063	0.164	0.064	0.808715678	C8_CD4_Tres
chr3-72965598-72966263	1.26E-05	0.262504444	0.09	0.028	0.836751938	C8_CD4_Tres
chr3-30664494-30665301	1.28E-05	0.259130811	0.093	0.032	0.849679331	C8_CD4_Tres
chr9-125125431-125126577	1.28E-05	0.259154994	0.239	0.123	0.850041236	C8_CD4_Tres
chr3-184560832-184561872	1.29E-05	0.257668534	0.25	0.125	0.855282067	C8_CD4_Tres
chr12-92030053-92031090	1.30E-05	0.254083794	0.243	0.125	0.861116173	C8_CD4_Tres
chr1-2839967-2840722	1.31E-05	0.250203932	0.078	0.022	0.86436503	C8_CD4_Tres
chrX-107118073-107119013	1.32E-05	0.265388085	0.172	0.081	0.872607919	C8_CD4_Tres
chr5-77086615-77087495	1.32E-05	0.26223556	0.108	0.034	0.873766956	C8_CD4_Tres
chr14-105059106-105059912	1.33E-05	0.25676869	0.246	0.128	0.880486961	C8_CD4_Tres
chr2-112626585-112626914	1.34E-05	0.263907807	0.112	0.044	0.885647261	C8_CD4_Tres
chr13-102399689-102401174	1.40E-05	0.253578706	0.082	0.022	0.927467674	C8_CD4_Tres

chr11-96258056-96258777	1.40E-05	0.264245397	0.138	0.054	0.928927864	C8_CD4_Tres
chr12-132506455-132507103	1.41E-05	0.250251656	0.071	0.019	0.93110409	C8_CD4_Tres
chr6-149139815-149141152	1.49E-05	0.26292116	0.172	0.074	0.983690517	C8_CD4_Tres
chr18-109154-110106	1.50E-05	0.266255137	0.149	0.068	0.992737558	C8_CD4_Tres
chr14-75333653-75335042	1.50E-05	0.256893118	0.101	0.033	0.992819047	C8_CD4_Tres
chr14-22070214-22071365	1.50E-05	0.264485469	0.127	0.048	0.994745244	C8_CD4_Tres
chr12-10749737-10750506	1.55E-05	0.264297825	0.123	0.051	1	C8_CD4_Tres
chr10-22022164-22023093	1.55E-05	0.260820984	0.123	0.046	1	C8_CD4_Tres
chr12-50824838-50825632	1.56E-05	0.259613939	0.116	0.04	1	C8_CD4_Tres
chr11-59537249-59537938	1.57E-05	0.255557376	0.082	0.024	1	C8_CD4_Tres
chr5-14142798-14144272	1.58E-05	0.260389576	0.127	0.047	1	C8_CD4_Tres
chr19-47572945-47573549	1.61E-05	0.259488536	0.201	0.101	1	C8_CD4_Tres
chr9-89455434-89455726	1.62E-05	0.256591969	0.082	0.026	1	C8_CD4_Tres
chr2-55009577-55010439	1.64E-05	0.264957367	0.131	0.049	1	C8_CD4_Tres
chr3-56494477-56495006	1.65E-05	0.257042826	0.093	0.035	1	C8_CD4_Tres
chr21-17512118-17513532	1.72E-05	0.260739861	0.19	0.09	1	C8_CD4_Tres
chr1-167442066-167442456	1.73E-05	0.263484344	0.146	0.061	1	C8_CD4_Tres
chr1-181166240-181166874	1.73E-05	0.262921035	0.134	0.053	1	C8_CD4_Tres
chr16-70025946-70026896	1.74E-05	0.255806544	0.239	0.129	1	C8_CD4_Tres
chr5-126845503-126846043	1.74E-05	0.253526133	0.082	0.026	1	C8_CD4_Tres
chr12-47276228-47277218	1.76E-05	0.252476215	0.243	0.129	1	C8_CD4_Tres
chr1-100927047-100927772	1.76E-05	0.256916195	0.123	0.043	1	C8_CD4_Tres
chr13-46125554-46126897	1.76E-05	0.26297662	0.157	0.063	1	C8_CD4_Tres
chr12-53189972-53190504	1.76E-05	0.253002852	0.086	0.026	1	C8_CD4_Tres
chr2-233421892-233422408	1.77E-05	0.257681775	0.112	0.043	1	C8_CD4_Tres
chr18-34860224-34860894	1.78E-05	0.261234371	0.127	0.047	1	C8_CD4_Tres
chr17-77389163-77390433	1.81E-05	0.26062296	0.168	0.072	1	C8_CD4_Tres

chr10-100508571-100509478	1.82E-05	0.258690196	0.175	0.092	1	C8_CD4_Tres
chr19-32746727-32747203	1.91E-05	0.261712404	0.142	0.061	1	C8_CD4_Tres
chr6-38571743-38572542	1.91E-05	0.256803102	0.097	0.032	1	C8_CD4_Tres
chr3-47420741-47421489	1.94E-05	0.258941026	0.201	0.099	1	C8_CD4_Tres
chr11-35046697-35047817	1.95E-05	0.254232882	0.205	0.11	1	C8_CD4_Tres
chr3-45037491-45038046	1.97E-05	0.257220725	0.209	0.103	1	C8_CD4_Tres
chr3-33645123-33645806	1.98E-05	0.250037036	0.086	0.024	1	C8_CD4_Tres
chr3-120719156-120719892	2.00E-05	0.259326211	0.108	0.039	1	C8_CD4_Tres
chr21-43916714-43918296	2.00E-05	0.254917576	0.224	0.11	1	C8_CD4_Tres
chr9-131578371-131579083	2.03E-05	0.250194029	0.082	0.026	1	C8_CD4_Tres
chr14-76913127-76913896	2.03E-05	0.25884004	0.119	0.044	1	C8_CD4_Tres
chr5-73160041-73160990	2.05E-05	0.259800447	0.119	0.044	1	C8_CD4_Tres
chr8-141386583-141387072	2.06E-05	0.258414426	0.127	0.047	1	C8_CD4_Tres
chr6-7137166-7137885	2.10E-05	0.256377615	0.101	0.041	1	C8_CD4_Tres
chr6-31307952-31308774	2.10E-05	0.257627918	0.112	0.041	1	C8_CD4_Tres
chr17-8954327-8954691	2.16E-05	0.25522806	0.213	0.11	1	C8_CD4_Tres
chr2-98761391-98761743	2.18E-05	0.252462108	0.09	0.028	1	C8_CD4_Tres
chrX-79133736-79134266	2.19E-05	0.251338839	0.09	0.03	1	C8_CD4_Tres
chr9-97803058-97804095	2.21E-05	0.252410184	0.228	0.121	1	C8_CD4_Tres
chr4-54091181-54092298	2.22E-05	0.260215723	0.142	0.061	1	C8_CD4_Tres
chr21-45596615-45596968	2.22E-05	0.253942512	0.104	0.035	1	C8_CD4_Tres
chr4-184229719-184230545	2.28E-05	0.256429053	0.123	0.049	1	C8_CD4_Tres
chr20-33689996-33690343	2.36E-05	0.25899804	0.108	0.036	1	C8_CD4_Tres
chr2-64644338-64645312	2.46E-05	0.254943607	0.097	0.032	1	C8_CD4_Tres
chr6-26086221-26086705	2.48E-05	0.251177064	0.093	0.029	1	C8_CD4_Tres
chr12-119901985-119902360	2.49E-05	0.250031576	0.093	0.029	1	C8_CD4_Tres
chr19-21474320-21475022	2.49E-05	0.257218411	0.101	0.035	1	C8_CD4_Tres

chr19-7568238-7568727	2.51E-05	0.257192669	0.142	0.059	1	C8_CD4_Tres
chr9-133656312-133657664	2.54E-05	0.255161389	0.101	0.033	1	C8_CD4_Tres
chr4-83260281-83260865	2.55E-05	0.250930015	0.097	0.031	1	C8_CD4_Tres
chr21-44653950-44654450	2.59E-05	0.255862732	0.127	0.056	1	C8_CD4_Tres
chr19-16366924-16367513	2.65E-05	0.254604761	0.164	0.079	1	C8_CD4_Tres
chr11-114182689-114183518	2.66E-05	0.256319446	0.123	0.054	1	C8_CD4_Tres
chr14-68483693-68484328	2.79E-05	0.25174836	0.093	0.032	1	C8_CD4_Tres
chr20-36595397-36596128	2.83E-05	0.251184783	0.213	0.111	1	C8_CD4_Tres
chr17-43251577-43251794	3.10E-05	0.253610144	0.153	0.069	1	C8_CD4_Tres
chr11-27471707-27473063	3.10E-05	0.252320413	0.131	0.052	1	C8_CD4_Tres
chr1-89274089-89274710	3.20E-05	0.255128929	0.168	0.085	1	C8_CD4_Tres
chr21-45818488-45819285	3.28E-05	0.252117651	0.101	0.04	1	C8_CD4_Tres
chr22-37498188-37498720	3.37E-05	0.253284389	0.104	0.041	1	C8_CD4_Tres
chr12-111056500-111057484	3.38E-05	0.250759184	0.149	0.067	1	C8_CD4_Tres
chr19-8515352-8515723	3.43E-05	0.253298341	0.142	0.066	1	C8_CD4_Tres
chr4-3045328-3045783	3.48E-05	0.254412242	0.16	0.069	1	C8_CD4_Tres
chr14-100146547-100147647	3.52E-05	0.252660615	0.131	0.059	1	C8_CD4_Tres
chr9-129235749-129236394	3.55E-05	0.252904308	0.16	0.07	1	C8_CD4_Tres
chr8-128657071-128657789	3.58E-05	0.254087562	0.19	0.09	1	C8_CD4_Tres
chr10-88385394-88386231	3.61E-05	0.251080723	0.116	0.047	1	C8_CD4_Tres
chr13-99295988-99296823	3.66E-05	0.251792324	0.146	0.069	1	C8_CD4_Tres
chr8-100807069-100807404	3.86E-05	0.252045199	0.112	0.044	1	C8_CD4_Tres
chr3-59425875-59426305	4.02E-05	0.250573347	0.112	0.04	1	C8_CD4_Tres
chrX-51395670-51396818	4.53E-05	0.250451979	0.146	0.057	1	C8_CD4_Tres
chr17-77127541-77128387	8.62E-08	0.304693247	0.291	0.153	0.005710289	C8_CD4_Tres
chr6-90218079-90219213	9.20E-38	0.755234136	0.343	0.058	6.10E-33	C8_CD4_Tres
chr10-119501615-119502938	1.20E-21	0.46265014	0.556	0.249	7.94E-17	C8_CD4_Tres

chr11-134068604-134069377	1.57E-07	0.310727738	0.216	0.098	0.010393593	C8_CD4_Tres
chr4-3301814-3303402	6.30E-09	0.310118235	0.358	0.201	0.000416876	C8_CD4_Tres
chr21-38244078-38245338	1.98E-34	0.738827445	0.276	0.041	1.31E-29	C8_CD4_Tres
chr8-100809255-100810633	5.04E-23	0.506444712	0.478	0.193	3.34E-18	C8_CD4_Tres
chr15-25859700-25860183	8.33E-11	0.396169916	0.157	0.041	5.52E-06	C8_CD4_Tres
chr4-140150254-140152549	2.28E-39	0.674753802	0.522	0.167	1.51E-34	C8_CD4_Tres
chr2-157468873-157469748	7.32E-06	0.264645709	0.228	0.117	0.484793798	C8_CD4_Tres
chr19-8515352-8515723	3.43E-05	0.253298341	0.142	0.066	1	C8_CD4_Tres
chr12-44835569-44836267	6.03E-06	0.276081407	0.127	0.044	0.399245644	C8_CD4_Tres
chr11-66317488-66318193	1.37E-12	0.433084386	0.175	0.047	9.07E-08	C8_CD4_Tres
chr5-16616150-16617914	1.02E-10	0.359283521	0.321	0.148	6.74E-06	C8_CD4_Tres
chr17-418361-418609	2.22E-12	0.423846635	0.127	0.025	1.47E-07	C8_CD4_Tres
chr7-151756000-151756419	9.67E-09	0.344875468	0.119	0.03	0.000640359	C8_CD4_Tres
chr4-153114731-153116644	6.30E-16	0.419239789	0.425	0.197	4.17E-11	C8_CD4_Tres
chr17-40596301-40596704	1.88E-16	0.480046387	0.112	0.012	1.25E-11	C8_CD4_Tres
chr13-99375056-99375719	3.95E-15	0.46810837	0.194	0.048	2.61E-10	C8_CD4_Tres
chr3-52317318-52317978	5.42E-08	0.330779858	0.142	0.044	0.003587227	C8_CD4_Tres
chr11-59549908-59551377	2.20E-07	0.266282981	0.403	0.242	0.014561456	C8_CD4_Tres
chr22-37696574-37697098	1.45E-14	0.473381642	0.187	0.044	9.63E-10	C8_CD4_Tres
chr14-92650743-92652022	1.51E-08	0.330901663	0.28	0.122	0.001001572	C8_CD4_Tres
chr19-8591726-8592924	2.34E-13	0.370360328	0.463	0.226	1.55E-08	C8_CD4_Tres
chr15-65887491-65887918	1.14E-12	0.422578709	0.131	0.028	7.52E-08	C8_CD4_Tres
chr16-27169333-27170139	2.66E-08	0.315143553	0.299	0.152	0.001761656	C8_CD4_Tres
chr12-7639566-7640916	5.75E-07	0.29639266	0.239	0.114	0.038097468	C8_CD4_Tres
chr4-88382307-88382999	6.14E-07	0.299626576	0.179	0.077	0.04063123	C8_CD4_Tres
chr12-124391371-124391873	2.93E-07	0.295650424	0.082	0.017	0.019421257	C8_CD4_Tres
chr7-151768807-151769594	4.53E-13	0.437798927	0.235	0.073	3.00E-08	C8_CD4_Tres

chrX-78516860-78517570	3.09E-06	0.279209996	0.235	0.111	0.204449968	C8_CD4_Tres
chr8-100807069-100807404	3.86E-05	0.252045199	0.112	0.044	1	C8_CD4_Tres
chr11-96013827-96014532	1.22E-05	0.26765063	0.164	0.064	0.808715678	C8_CD4_Tres
chr15-26081659-26082831	2.03E-11	0.374317409	0.321	0.142	1.35E-06	C8_CD4_Tres
chr12-68175137-68176200	1.29E-09	0.358712219	0.216	0.088	8.57E-05	C8_CD4_Tres
chr5-111278513-111279163	4.72E-06	0.280264472	0.149	0.057	0.312301653	C8_CD4_Tres
chr13-112968220-112969617	1.15E-07	0.301411407	0.28	0.138	0.007588791	C8_CD4_Tres
chr1-12448952-12450012	1.98E-07	0.314275896	0.187	0.072	0.013119045	C8_CD4_Tres
chr15-91469028-91470405	4.35E-09	0.350291947	0.209	0.078	0.000288125	C8_CD4_Tres
chr1-167489352-167489861	1.41E-13	0.446105727	0.16	0.037	9.33E-09	C8_CD4_Tres
chr17-77127541-77128387	8.62E-08	0.304693247	0.291	0.153	0.005710289	C8_CD4_Tres
chr10-100510527-100511388	6.77E-08	0.294311499	0.354	0.185	0.004482708	C8_CD4_Tres
chr2-98656735-98657123	1.30E-06	0.298325669	0.142	0.054	0.085844257	C8_CD4_Tres
chr11-114182689-114183518	2.66E-05	0.256319446	0.123	0.054	1	C8_CD4_Tres
chr5-131209164-131210417	5.61E-10	0.348662059	0.313	0.139	3.72E-05	C8_CD4_Tres
chr20-33689996-33690343	2.36E-05	0.25899804	0.108	0.036	1	C8_CD4_Tres
chr19-47186306-47187379	3.13E-17	0.409991975	0.515	0.259	2.07E-12	C8_CD4_Tres
chr17-78413035-78413614	7.61E-07	0.288195013	0.082	0.021	0.050415636	C8_CD4_Tres
chr5-180688407-180689690	1.96E-14	0.463051564	0.172	0.037	1.30E-09	C8_CD4_Tres
chr8-85176750-85178060	4.89E-06	0.259569043	0.287	0.158	0.324135176	C8_CD4_Tres
chr7-151429487-151430243	5.38E-07	0.307891499	0.142	0.05	0.035623891	C8_CD4_Tres
chr1-23626986-23627593	1.68E-09	0.338070498	0.075	0.012	0.000111532	C8_CD4_Tres
chr15-40129107-40129553	8.92E-10	0.308130781	0.052	0.005	5.91E-05	C8_CD4_Tres
chr3-18443988-18445871	3.10E-21	0.469496215	0.507	0.223	2.05E-16	C8_CD4_Tres
chr17-77963501-77963776	8.84E-06	0.266389336	0.101	0.035	0.58548378	C8_CD4_Tres
chr3-183515954-183516702	5.15E-18	0.498680061	0.317	0.103	3.41E-13	C8_CD4_Tres
chr22-39951571-39952174	2.40E-15	0.469650461	0.123	0.017	1.59E-10	C8_CD4_Tres

chr18-23070939-23071743	1.03E-06	0.283463508	0.243	0.123	0.06804758	C8_CD4_Tres
chr3-119218933-119219427	3.29E-17	0.486692369	0.112	0.011	2.18E-12	C8_CD4_Tres
chr20-50817814-50818749	9.87E-06	0.268842973	0.157	0.073	0.653809778	C8_CD4_Tres
chr17-67398709-67399075	3.07E-16	0.490422973	0.146	0.023	2.03E-11	C8_CD4_Tres
chr4-2786175-2786471	4.53E-12	0.391619783	0.086	0.011	3.00E-07	C8_CD4_Tres
chr13-99375056-99375719	3.95E-15	0.46810837	0.194	0.048	2.61E-10	C8_CD4_Tres
chr7-1939095-1940953	3.24E-08	0.256162393	0.511	0.34	0.002146561	C8_CD4_Tres
chr4-3301814-3303402	6.30E-09	0.310118235	0.358	0.201	0.000416876	C8_CD4_Tres
chr14-68506586-68508247	2.49E-12	0.407732068	0.25	0.095	1.65E-07	C8_CD4_Tres
chr11-14554576-14555398	2.64E-09	0.354616178	0.172	0.061	0.00017452	C8_CD4_Tres
chr7-4883008-4884026	2.99E-06	0.275989012	0.216	0.102	0.19795189	C8_CD4_Tres
chr1-25561794-25562997	5.63E-09	0.337687983	0.254	0.105	0.000373109	C8_CD4_Tres
chr15-100339031-100339273	3.16E-06	0.280790543	0.127	0.045	0.209134102	C8_CD4_Tres
chr20-32971845-32972681	1.21E-19	0.503063814	0.358	0.124	8.04E-15	C8_CD4_Tres
chr5-911844-913044	3.32E-41	0.502711597	0.625	0.242	2.20E-36	C9_CD4_Tem
chr16-78894700-78896655	2.61E-38	0.590638313	0.416	0.106	1.73E-33	C9_CD4_Tem
chr8-9087912-9089120	3.53E-36	0.590423604	0.402	0.098	2.34E-31	C9_CD4_Tem
chr7-77830727-77831925	3.09E-31	0.568321155	0.256	0.049	2.05E-26	C9_CD4_Tem
chr16-78897103-78897904	3.35E-31	0.570252339	0.283	0.058	2.22E-26	C9_CD4_Tem
chr16-963615-965065	5.44E-29	0.533769092	0.348	0.095	3.60E-24	C9_CD4_Tem
chr10-5291424-5292650	1.10E-28	0.517295926	0.375	0.111	7.27E-24	C9_CD4_Tem
chr8-8843985-8844590	5.11E-28	0.55069547	0.245	0.046	3.39E-23	C9_CD4_Tem
chr7-37310382-37311803	6.28E-28	0.528139529	0.362	0.099	4.16E-23	C9_CD4_Tem
chr16-78777910-78778705	1.15E-27	0.538104166	0.191	0.028	7.61E-23	C9_CD4_Tem
chr3-196563586-196564238	6.43E-27	0.543691474	0.261	0.053	4.26E-22	C9_CD4_Tem
chr16-79639698-79640550	3.90E-26	0.524525545	0.209	0.037	2.58E-21	C9_CD4_Tem
chr3-141386319-141387061	5.32E-26	0.509894581	0.151	0.018	3.52E-21	C9_CD4_Tem

chr10-5317444-5318188	1.91E-25	0.513263093	0.29	0.067	1.27E-20	C9_CD4_Tem
chr8-8796896-8798212	3.76E-24	0.511406476	0.207	0.039	2.49E-19	C9_CD4_Tem
chr10-103757592-103758403	4.82E-24	0.497486808	0.306	0.08	3.19E-19	C9_CD4_Tem
chr16-79007492-79008321	1.69E-23	0.485873434	0.144	0.019	1.12E-18	C9_CD4_Tem
chr6-73695202-73696438	1.94E-23	0.495308558	0.261	0.062	1.28E-18	C9_CD4_Tem
chr20-63873332-63873812	1.95E-23	0.47975114	0.13	0.016	1.29E-18	C9_CD4_Tem
chr8-8868529-8869756	5.20E-23	0.489982524	0.294	0.079	3.44E-18	C9_CD4_Tem
chr9-87713197-87713846	5.73E-23	0.470745538	0.137	0.016	3.80E-18	C9_CD4_Tem
chr14-72451037-72451981	5.77E-23	0.468201018	0.355	0.115	3.82E-18	C9_CD4_Tem
chr9-115006571-115007084	6.03E-23	0.50322987	0.18	0.028	4.00E-18	C9_CD4_Tem
chr3-66472113-66473972	8.21E-23	0.426838413	0.519	0.207	5.44E-18	C9_CD4_Tem
chr1-236533710-236534731	3.42E-22	0.434624532	0.438	0.162	2.27E-17	C9_CD4_Tem
chr10-3754995-3755391	4.37E-22	0.495325195	0.245	0.058	2.89E-17	C9_CD4_Tem
chr13-98484347-98485269	6.96E-22	0.439936797	0.429	0.151	4.61E-17	C9_CD4_Tem
chr2-38663414-38664114	8.25E-22	0.475872988	0.27	0.071	5.47E-17	C9_CD4_Tem
chr3-9912046-9912875	1.07E-21	0.479608313	0.299	0.081	7.06E-17	C9_CD4_Tem
chr5-76790211-76791350	2.00E-21	0.468297237	0.281	0.078	1.32E-16	C9_CD4_Tem
chr9-129895290-129895783	2.71E-21	0.480761977	0.2	0.042	1.79E-16	C9_CD4_Tem
chr5-157551729-157552372	1.32E-20	0.460177684	0.146	0.023	8.76E-16	C9_CD4_Tem
chr5-139443778-139444510	3.13E-20	0.4777152	0.211	0.047	2.07E-15	C9_CD4_Tem
chrX-1208026-1208776	5.43E-20	0.46110096	0.231	0.056	3.59E-15	C9_CD4_Tem
chr11-123453839-123454698	1.28E-19	0.440681518	0.337	0.113	8.50E-15	C9_CD4_Tem
chr10-5293593-5294359	2.23E-19	0.463256539	0.236	0.059	1.48E-14	C9_CD4_Tem
chr5-149356409-149356633	2.30E-19	0.429128311	0.112	0.013	1.52E-14	C9_CD4_Tem
chr12-6789149-6789608	3.61E-19	0.455489424	0.144	0.022	2.39E-14	C9_CD4_Tem
chr1-160623049-160624086	8.67E-19	0.430113329	0.348	0.122	5.74E-14	C9_CD4_Tem
chr8-8860146-8861020	9.55E-19	0.436229329	0.13	0.019	6.33E-14	C9_CD4_Tem

chr3-71702063-71702983	2.13E-18	0.45045071	0.258	0.071	1.41E-13	C9_CD4_Tem
chr16-78956916-78957995	2.17E-18	0.420800243	0.13	0.017	1.43E-13	C9_CD4_Tem
chr7-140059213-140059829	2.36E-18	0.446828423	0.178	0.039	1.56E-13	C9_CD4_Tem
chr7-70787363-70788626	3.91E-18	0.433624306	0.151	0.028	2.59E-13	C9_CD4_Tem
chr13-98480746-98481317	4.45E-18	0.426137824	0.342	0.113	2.95E-13	C9_CD4_Tem
chr17-78381190-78382191	4.80E-18	0.351324841	0.571	0.278	3.18E-13	C9_CD4_Tem
chr8-58595097-58596320	6.75E-18	0.408199047	0.344	0.132	4.47E-13	C9_CD4_Tem
chr12-12456117-12457043	7.19E-18	0.443628003	0.225	0.06	4.76E-13	C9_CD4_Tem
chr19-39430360-39431582	9.18E-18	0.3571373	0.571	0.267	6.08E-13	C9_CD4_Tem
chr8-123148856-123149662	9.53E-18	0.442585514	0.258	0.073	6.31E-13	C9_CD4_Tem
chr12-109967381-109968397	1.48E-17	0.428848931	0.148	0.029	9.81E-13	C9_CD4_Tem
chr17-55089250-55090549	1.71E-17	0.422826511	0.279	0.086	1.14E-12	C9_CD4_Tem
chr8-8296057-8296572	1.76E-17	0.403038003	0.094	0.011	1.17E-12	C9_CD4_Tem
chr17-75123963-75124643	1.77E-17	0.44395013	0.204	0.049	1.17E-12	C9_CD4_Tem
chr2-203870881-203871836	2.10E-17	0.424401013	0.279	0.086	1.39E-12	C9_CD4_Tem
chr3-4906100-4906640	2.15E-17	0.428926616	0.169	0.035	1.42E-12	C9_CD4_Tem
chr2-218315799-218316938	2.25E-17	0.436166977	0.209	0.055	1.49E-12	C9_CD4_Tem
chr6-157760803-157761733	4.19E-17	0.349075161	0.524	0.244	2.77E-12	C9_CD4_Tem
chr2-238439071-238440133	4.73E-17	0.408541936	0.321	0.11	3.13E-12	C9_CD4_Tem
chr5-134436287-134437225	4.83E-17	0.419151998	0.267	0.088	3.20E-12	C9_CD4_Tem
chr18-13290640-13291397	4.91E-17	0.406019544	0.265	0.083	3.25E-12	C9_CD4_Tem
chrX-1582773-1583405	5.00E-17	0.435483002	0.252	0.072	3.31E-12	C9_CD4_Tem
chr2-96616905-96617951	5.95E-17	0.436005175	0.169	0.036	3.94E-12	C9_CD4_Tem
chr10-103747952-103748345	6.27E-17	0.380998995	0.088	0.01	4.15E-12	C9_CD4_Tem
chr20-50440479-50441792	7.21E-17	0.432583873	0.256	0.076	4.77E-12	C9_CD4_Tem
chr13-98509104-98509860	7.75E-17	0.413400612	0.315	0.107	5.13E-12	C9_CD4_Tem
chr17-80761178-80761807	8.60E-17	0.428727684	0.274	0.087	5.70E-12	C9_CD4_Tem

chr19-4840536-4841612	9.46E-17	0.427247865	0.184	0.042	6.27E-12	C9_CD4_Tem
chr15-64882205-64884501	1.17E-16	0.380838105	0.465	0.209	7.75E-12	C9_CD4_Tem
chr5-149348450-149348794	1.22E-16	0.429244404	0.182	0.041	8.08E-12	C9_CD4_Tem
chr10-3095161-3096047	2.13E-16	0.3943666	0.378	0.148	1.41E-11	C9_CD4_Tem
chr20-41002925-41004052	2.32E-16	0.308415151	0.649	0.342	1.53E-11	C9_CD4_Tem
chr10-119655529-119656093	2.34E-16	0.417022676	0.254	0.079	1.55E-11	C9_CD4_Tem
chr4-40672496-40673460	3.34E-16	0.389610186	0.306	0.11	2.21E-11	C9_CD4_Tem
chr20-63902379-63903354	3.67E-16	0.381758079	0.364	0.145	2.43E-11	C9_CD4_Tem
chr2-151423049-151424255	3.74E-16	0.389745438	0.33	0.124	2.48E-11	C9_CD4_Tem
chr2-233247436-233248149	4.20E-16	0.367188382	0.47	0.2	2.78E-11	C9_CD4_Tem
chr7-44617182-44617939	4.38E-16	0.406751273	0.261	0.081	2.90E-11	C9_CD4_Tem
chr3-20057204-20057566	5.72E-16	0.42308887	0.236	0.067	3.78E-11	C9_CD4_Tem
chr19-15466742-15467649	6.95E-16	0.396087769	0.375	0.142	4.60E-11	C9_CD4_Tem
chr2-197159068-197160249	1.12E-15	0.366858922	0.436	0.188	7.40E-11	C9_CD4_Tem
chr15-84681782-84682236	1.13E-15	0.401201048	0.146	0.027	7.50E-11	C9_CD4_Tem
chr3-71086871-71088054	1.70E-15	0.369208958	0.404	0.176	1.12E-10	C9_CD4_Tem
chr2-108618793-108619170	1.71E-15	0.411480275	0.148	0.032	1.13E-10	C9_CD4_Tem
chr19-47106997-47107896	1.71E-15	0.3887615	0.391	0.165	1.13E-10	C9_CD4_Tem
chr5-149355797-149356077	1.77E-15	0.378415495	0.097	0.014	1.17E-10	C9_CD4_Tem
chr1-117612735-117613313	1.78E-15	0.396420336	0.124	0.021	1.18E-10	C9_CD4_Tem
chr8-19473047-19473747	2.28E-15	0.376890841	0.373	0.148	1.51E-10	C9_CD4_Tem
chr8-47600277-47601292	2.33E-15	0.389455802	0.31	0.121	1.54E-10	C9_CD4_Tem
chr5-80253614-80254145	2.60E-15	0.409087248	0.187	0.047	1.72E-10	C9_CD4_Tem
chr14-76767656-76768871	2.62E-15	0.344594057	0.474	0.223	1.74E-10	C9_CD4_Tem
chr5-142014934-142016888	2.65E-15	0.353724558	0.485	0.227	1.76E-10	C9_CD4_Tem
chr8-22139011-22139338	3.24E-15	0.403784285	0.139	0.029	2.14E-10	C9_CD4_Tem
chr15-60787735-60788742	3.88E-15	0.401599303	0.207	0.062	2.57E-10	C9_CD4_Tem

chr2-234308137-234309280	4.10E-15	0.386890504	0.384	0.149	2.71E-10	C9_CD4_Tem
chr3-15269076-15269877	4.25E-15	0.346457692	0.483	0.226	2.81E-10	C9_CD4_Tem
chr5-157602392-157603773	4.56E-15	0.376200258	0.434	0.182	3.02E-10	C9_CD4_Tem
chr2-48343612-48344540	5.17E-15	0.396174456	0.306	0.107	3.42E-10	C9_CD4_Tem
chr1-243595599-243596987	6.58E-15	0.341654744	0.47	0.218	4.35E-10	C9_CD4_Tem
chr3-45166910-45167911	6.94E-15	0.396798756	0.29	0.101	4.60E-10	C9_CD4_Tem
chr6-45923690-45924372	7.19E-15	0.382076493	0.121	0.022	4.76E-10	C9_CD4_Tem
chr9-128094022-128094725	7.52E-15	0.398620855	0.198	0.055	4.98E-10	C9_CD4_Tem
chr16-50338338-50339423	7.83E-15	0.368224018	0.378	0.154	5.19E-10	C9_CD4_Tem
chr3-32296559-32298058	8.70E-15	0.388067044	0.267	0.091	5.76E-10	C9_CD4_Tem
chr3-191328414-191329941	1.04E-14	0.387080514	0.29	0.106	6.91E-10	C9_CD4_Tem
chr14-64755533-64756287	1.07E-14	0.363150776	0.373	0.147	7.12E-10	C9_CD4_Tem
chr17-80764767-80765901	1.09E-14	0.408018424	0.189	0.048	7.23E-10	C9_CD4_Tem
chr17-80605163-80606101	1.41E-14	0.384286038	0.2	0.059	9.32E-10	C9_CD4_Tem
chr10-110353290-110354533	1.48E-14	0.331490565	0.488	0.233	9.83E-10	C9_CD4_Tem
chr15-85620395-85620911	1.57E-14	0.399767697	0.164	0.037	1.04E-09	C9_CD4_Tem
chr6-46013854-46014734	1.95E-14	0.406322708	0.198	0.053	1.29E-09	C9_CD4_Tem
chr2-241862902-241863921	2.05E-14	0.381114741	0.335	0.133	1.36E-09	C9_CD4_Tem
chr7-131111162-131111414	2.05E-14	0.396785651	0.175	0.041	1.36E-09	C9_CD4_Tem
chr6-90411120-90411851	2.29E-14	0.345652914	0.447	0.196	1.52E-09	C9_CD4_Tem
chr5-126811000-126812526	2.89E-14	0.364099567	0.357	0.147	1.91E-09	C9_CD4_Tem
chr6-149232909-149234903	3.00E-14	0.356595435	0.44	0.19	1.98E-09	C9_CD4_Tem
chrX-2707275-2707747	3.19E-14	0.389426731	0.319	0.119	2.12E-09	C9_CD4_Tem
chr5-56011879-56012999	3.48E-14	0.392983541	0.213	0.066	2.31E-09	C9_CD4_Tem
chr9-135905096-135905931	3.50E-14	0.402083638	0.256	0.084	2.32E-09	C9_CD4_Tem
chr7-37354615-37355101	3.63E-14	0.405125435	0.184	0.046	2.40E-09	C9_CD4_Tem
chr12-122887370-122889055	3.70E-14	0.386449248	0.335	0.131	2.45E-09	C9_CD4_Tem

chr3-172520417-172520867	3.87E-14	0.378249034	0.303	0.111	2.56E-09	C9_CD4_Tem
chr16-80026863-80027654	3.98E-14	0.379146176	0.184	0.052	2.63E-09	C9_CD4_Tem
chr2-9655145-9656958	4.44E-14	0.329090985	0.481	0.223	2.94E-09	C9_CD4_Tem
chr7-149209160-149210278	4.55E-14	0.382821295	0.261	0.091	3.01E-09	C9_CD4_Tem
chr8-763527-764677	4.72E-14	0.354948874	0.384	0.156	3.12E-09	C9_CD4_Tem
chr7-44633252-44634170	4.81E-14	0.309293493	0.546	0.265	3.19E-09	C9_CD4_Tem
chr18-13376299-13377630	5.09E-14	0.399631285	0.231	0.071	3.37E-09	C9_CD4_Tem
chr1-193458475-193459060	6.04E-14	0.382406587	0.256	0.087	4.00E-09	C9_CD4_Tem
chr10-5284383-5284748	6.43E-14	0.370547676	0.11	0.02	4.26E-09	C9_CD4_Tem
chr5-142471988-142472953	6.43E-14	0.379480725	0.281	0.096	4.26E-09	C9_CD4_Tem
chr5-119287098-119288138	6.92E-14	0.354180235	0.418	0.186	4.58E-09	C9_CD4_Tem
chr19-6065663-6066219	9.73E-14	0.388325508	0.231	0.076	6.44E-09	C9_CD4_Tem
chr15-63841719-63842387	1.06E-13	0.385048849	0.211	0.064	7.02E-09	C9_CD4_Tem
chr2-43183820-43184763	1.26E-13	0.385951715	0.258	0.089	8.33E-09	C9_CD4_Tem
chr2-64325332-64326671	1.32E-13	0.311349955	0.503	0.25	8.74E-09	C9_CD4_Tem
chr10-12333018-12334141	1.33E-13	0.388367658	0.182	0.05	8.83E-09	C9_CD4_Tem
chr18-12839004-12840019	1.46E-13	0.344231805	0.436	0.196	9.66E-09	C9_CD4_Tem
chr9-131709602-131710571	1.48E-13	0.364960726	0.362	0.147	9.80E-09	C9_CD4_Tem
chr1-236168709-236169327	1.54E-13	0.336734616	0.07	0.007	1.02E-08	C9_CD4_Tem
chr1-40875530-40876133	1.73E-13	0.395247976	0.238	0.075	1.15E-08	C9_CD4_Tem
chr5-671624-673152	1.88E-13	0.343404806	0.389	0.177	1.25E-08	C9_CD4_Tem
chr16-72474275-72474958	1.90E-13	0.362693937	0.11	0.019	1.26E-08	C9_CD4_Tem
chr2-231068222-231068820	1.90E-13	0.377313833	0.146	0.034	1.26E-08	C9_CD4_Tem
chr19-3132728-3133839	2.11E-13	0.345405791	0.411	0.186	1.40E-08	C9_CD4_Tem
chr1-21022049-21023074	2.26E-13	0.349201679	0.387	0.168	1.49E-08	C9_CD4_Tem
chr11-60994311-60995082	2.40E-13	0.383818028	0.139	0.03	1.59E-08	C9_CD4_Tem
chr17-31318169-31318773	2.64E-13	0.361008329	0.355	0.152	1.75E-08	C9_CD4_Tem

chr6-166950318-166951589	2.69E-13	0.344152959	0.407	0.176	1.78E-08	C9_CD4_Tem
chr18-2889646-2890237	3.31E-13	0.375793469	0.198	0.06	2.19E-08	C9_CD4_Tem
chr16-30567958-30568566	3.36E-13	0.374856332	0.301	0.119	2.22E-08	C9_CD4_Tem
chr12-107027112-107028011	3.68E-13	0.357380402	0.375	0.152	2.44E-08	C9_CD4_Tem
chr3-172476397-172477640	3.81E-13	0.3607006	0.339	0.135	2.52E-08	C9_CD4_Tem
chr3-15796863-15798271	4.24E-13	0.371738347	0.308	0.116	2.81E-08	C9_CD4_Tem
chr8-8871973-8872681	4.39E-13	0.386162363	0.198	0.057	2.91E-08	C9_CD4_Tem
chr11-62024948-62026149	4.56E-13	0.290715814	0.548	0.296	3.02E-08	C9_CD4_Tem
chr17-55162182-55162841	4.61E-13	0.339517516	0.088	0.015	3.06E-08	C9_CD4_Tem
chr7-77834162-77834851	4.91E-13	0.365780425	0.121	0.024	3.25E-08	C9_CD4_Tem
chr17-78804683-78805729	5.02E-13	0.35199257	0.355	0.149	3.32E-08	C9_CD4_Tem
chr4-80016587-80017874	5.22E-13	0.381920909	0.182	0.048	3.46E-08	C9_CD4_Tem
chr15-40743298-40744759	5.49E-13	0.279705109	0.616	0.331	3.64E-08	C9_CD4_Tem
chr12-121214138-121214703	5.55E-13	0.378446193	0.162	0.047	3.67E-08	C9_CD4_Tem
chr17-80856148-80856910	5.70E-13	0.374932031	0.299	0.114	3.77E-08	C9_CD4_Tem
chr11-3511294-3512626	5.93E-13	0.370829488	0.234	0.081	3.92E-08	C9_CD4_Tem
chrX-136619777-136620391	5.93E-13	0.374943286	0.157	0.039	3.93E-08	C9_CD4_Tem
chr4-26075463-26076554	6.00E-13	0.353567803	0.326	0.133	3.97E-08	C9_CD4_Tem
chr2-102226947-102228524	6.39E-13	0.295576785	0.501	0.256	4.23E-08	C9_CD4_Tem
chr14-64723525-64724394	6.54E-13	0.376250909	0.187	0.053	4.33E-08	C9_CD4_Tem
chr4-122539514-122540452	6.87E-13	0.377627294	0.227	0.073	4.55E-08	C9_CD4_Tem
chr2-136238208-136239288	6.90E-13	0.337711316	0.409	0.196	4.57E-08	C9_CD4_Tem
chr10-103745191-103745983	7.75E-13	0.382150749	0.151	0.038	5.13E-08	C9_CD4_Tem
chr1-236510700-236511271	7.95E-13	0.35523476	0.103	0.018	5.26E-08	C9_CD4_Tem
chr16-67857443-67858863	8.94E-13	0.374298365	0.213	0.065	5.92E-08	C9_CD4_Tem
chr11-86182850-86184017	9.24E-13	0.362960732	0.243	0.085	6.12E-08	C9_CD4_Tem
chr10-3851288-3852848	1.00E-12	0.276070565	0.618	0.344	6.63E-08	C9_CD4_Tem

chr5-76468880-76469121	1.03E-12	0.297653021	0.052	0.004	6.84E-08	C9_CD4_Tem
chr5-52987434-52988237	1.13E-12	0.386174918	0.222	0.069	7.50E-08	C9_CD4_Tem
chr5-139458504-139459677	1.17E-12	0.303033862	0.506	0.263	7.72E-08	C9_CD4_Tem
chr8-141131689-141132139	1.18E-12	0.359216001	0.342	0.128	7.82E-08	C9_CD4_Tem
chr18-3304866-3306168	1.19E-12	0.324654441	0.393	0.174	7.90E-08	C9_CD4_Tem
chr10-17215994-17216841	1.20E-12	0.337438643	0.434	0.199	7.95E-08	C9_CD4_Tem
chr20-33369167-33370781	1.27E-12	0.296422359	0.546	0.285	8.41E-08	C9_CD4_Tem
chr1-58929483-58930098	1.31E-12	0.363847742	0.142	0.032	8.69E-08	C9_CD4_Tem
chr17-64081788-64082512	1.45E-12	0.375037882	0.247	0.086	9.59E-08	C9_CD4_Tem
chr2-38578721-38579215	1.54E-12	0.36382943	0.128	0.029	1.02E-07	C9_CD4_Tem
chr8-11853521-11854451	1.61E-12	0.362328903	0.335	0.139	1.07E-07	C9_CD4_Tem
chr8-19686699-19687530	1.75E-12	0.373921764	0.175	0.049	1.16E-07	C9_CD4_Tem
chr3-46369598-46370214	1.85E-12	0.362662449	0.317	0.127	1.23E-07	C9_CD4_Tem
chr1-116508136-116509326	2.01E-12	0.354079674	0.346	0.141	1.33E-07	C9_CD4_Tem
chr1-174964190-174965399	2.09E-12	0.316884652	0.465	0.223	1.38E-07	C9_CD4_Tem
chr1-167500396-167500800	2.11E-12	0.36301747	0.146	0.039	1.40E-07	C9_CD4_Tem
chr10-127990189-127991574	2.11E-12	0.350717207	0.364	0.157	1.40E-07	C9_CD4_Tem
chr1-204520820-204521625	2.20E-12	0.360124053	0.328	0.138	1.46E-07	C9_CD4_Tem
chr22-40651448-40652410	2.22E-12	0.334210002	0.431	0.191	1.47E-07	C9_CD4_Tem
chr8-47513301-47514143	2.29E-12	0.361534142	0.216	0.071	1.52E-07	C9_CD4_Tem
chr10-3867188-3868292	2.35E-12	0.368583895	0.258	0.09	1.56E-07	C9_CD4_Tem
chr6-37553397-37554076	2.53E-12	0.328674679	0.42	0.198	1.67E-07	C9_CD4_Tem
chr2-239380549-239381319	2.69E-12	0.360897734	0.292	0.115	1.78E-07	C9_CD4_Tem
chr7-131231107-131231912	2.72E-12	0.339813804	0.409	0.181	1.80E-07	C9_CD4_Tem
chr5-154197444-154198623	3.03E-12	0.34733258	0.357	0.151	2.00E-07	C9_CD4_Tem
chr3-152256823-152258002	3.04E-12	0.33951008	0.387	0.173	2.02E-07	C9_CD4_Tem
chr8-11865462-11865735	3.09E-12	0.343367877	0.09	0.014	2.05E-07	C9_CD4_Tem

chr10-103759005-103759372	3.15E-12	0.352917614	0.103	0.02	2.08E-07	C9_CD4_Tem
chr11-9565650-9566468	3.30E-12	0.300981988	0.481	0.239	2.19E-07	C9_CD4_Tem
chr6-24973935-24975042	3.52E-12	0.314487384	0.447	0.218	2.33E-07	C9_CD4_Tem
chr12-7989534-7990540	3.64E-12	0.303752349	0.449	0.231	2.41E-07	C9_CD4_Tem
chr10-103800571-103801159	3.65E-12	0.353357623	0.137	0.034	2.42E-07	C9_CD4_Tem
chr1-151311007-151312140	3.86E-12	0.314698314	0.429	0.199	2.55E-07	C9_CD4_Tem
chr9-69180395-69181037	4.44E-12	0.355523963	0.144	0.036	2.94E-07	C9_CD4_Tem
chr6-130452810-130453731	4.48E-12	0.350568287	0.29	0.111	2.97E-07	C9_CD4_Tem
chr15-67146917-67147212	4.67E-12	0.314803984	0.07	0.009	3.09E-07	C9_CD4_Tem
chr12-96484966-96485587	4.87E-12	0.339335014	0.351	0.153	3.23E-07	C9_CD4_Tem
chr8-28371357-28371763	5.06E-12	0.327283671	0.079	0.012	3.35E-07	C9_CD4_Tem
chr6-45709768-45710798	5.50E-12	0.353875399	0.139	0.037	3.64E-07	C9_CD4_Tem
chr22-46660420-46661825	5.89E-12	0.355191973	0.261	0.102	3.90E-07	C9_CD4_Tem
chr10-110081940-110082291	6.26E-12	0.362570404	0.133	0.029	4.14E-07	C9_CD4_Tem
chr10-30695364-30696271	6.28E-12	0.34791389	0.324	0.133	4.16E-07	C9_CD4_Tem
chr1-8181870-8182991	6.56E-12	0.341849522	0.317	0.134	4.34E-07	C9_CD4_Tem
chr1-234730640-234731942	6.64E-12	0.362604726	0.281	0.111	4.39E-07	C9_CD4_Tem
chr10-30446945-30447711	7.11E-12	0.356196957	0.175	0.051	4.71E-07	C9_CD4_Tem
chr3-71050282-71051242	7.27E-12	0.337024491	0.348	0.149	4.81E-07	C9_CD4_Tem
chr8-41986545-41987581	7.79E-12	0.358029814	0.236	0.079	5.16E-07	C9_CD4_Tem
chr6-389521-389984	7.83E-12	0.363116526	0.171	0.05	5.19E-07	C9_CD4_Tem
chr15-44545790-44546739	7.86E-12	0.357107969	0.308	0.118	5.20E-07	C9_CD4_Tem
chr12-51393879-51394537	8.15E-12	0.350001085	0.326	0.133	5.39E-07	C9_CD4_Tem
chr1-116555808-116556962	8.26E-12	0.302832777	0.492	0.238	5.47E-07	C9_CD4_Tem
chr13-98524294-98524743	8.36E-12	0.317564443	0.081	0.013	5.54E-07	C9_CD4_Tem
chr15-39641191-39642292	9.46E-12	0.341247357	0.324	0.135	6.26E-07	C9_CD4_Tem
chr19-4812769-4813304	9.54E-12	0.345014701	0.099	0.019	6.32E-07	C9_CD4_Tem

chr9-131677481-131678908	9.74E-12	0.304786066	0.503	0.243	6.45E-07	C9_CD4_Tem
chr14-73723186-73723723	1.01E-11	0.341534554	0.112	0.024	6.68E-07	C9_CD4_Tem
chr12-106288377-106288938	1.04E-11	0.337931513	0.11	0.024	6.89E-07	C9_CD4_Tem
chr1-200880056-200880647	1.08E-11	0.35398885	0.191	0.061	7.16E-07	C9_CD4_Tem
chr20-57697970-57699159	1.13E-11	0.323605452	0.409	0.185	7.49E-07	C9_CD4_Tem
chr11-96012030-96012570	1.14E-11	0.345673373	0.306	0.121	7.55E-07	C9_CD4_Tem
chr20-36733151-36734112	1.15E-11	0.370563251	0.196	0.063	7.59E-07	C9_CD4_Tem
chr22-23704649-23705733	1.29E-11	0.360548877	0.198	0.063	8.54E-07	C9_CD4_Tem
chr4-26092937-26093921	1.35E-11	0.343099528	0.148	0.041	8.95E-07	C9_CD4_Tem
chr3-4907946-4909045	1.40E-11	0.36371344	0.148	0.04	9.30E-07	C9_CD4_Tem
chr4-80007696-80008230	1.46E-11	0.364655071	0.198	0.063	9.65E-07	C9_CD4_Tem
chr8-115446402-115447129	1.55E-11	0.352935991	0.142	0.036	1.03E-06	C9_CD4_Tem
chr3-71066873-71067205	1.65E-11	0.35786458	0.231	0.083	1.09E-06	C9_CD4_Tem
chr18-58614917-58616188	1.75E-11	0.297242172	0.474	0.225	1.16E-06	C9_CD4_Tem
chrX-136658947-136659495	1.78E-11	0.325667359	0.085	0.014	1.18E-06	C9_CD4_Tem
chr8-129971239-129972733	1.88E-11	0.320517488	0.436	0.208	1.25E-06	C9_CD4_Tem
chr1-1216741-1217323	1.96E-11	0.357469663	0.144	0.036	1.30E-06	C9_CD4_Tem
chrX-136768405-136768900	2.07E-11	0.361351411	0.16	0.043	1.37E-06	C9_CD4_Tem
chr17-47705355-47706949	2.14E-11	0.342881016	0.373	0.16	1.42E-06	C9_CD4_Tem
chr5-43315324-43315674	2.19E-11	0.358468864	0.198	0.061	1.45E-06	C9_CD4_Tem
chr8-104664861-104666545	2.27E-11	0.329563891	0.213	0.077	1.50E-06	C9_CD4_Tem
chr9-5375766-5376258	2.28E-11	0.305428991	0.067	0.01	1.51E-06	C9_CD4_Tem
chr14-70893119-70894127	2.38E-11	0.336885486	0.11	0.024	1.57E-06	C9_CD4_Tem
chr4-40290704-40291215	2.51E-11	0.350752843	0.112	0.023	1.66E-06	C9_CD4_Tem
chr3-27533385-27534635	2.52E-11	0.357100053	0.276	0.102	1.67E-06	C9_CD4_Tem
chr17-59782839-59783542	2.61E-11	0.363760939	0.189	0.059	1.73E-06	C9_CD4_Tem
chr3-172129658-172130490	2.71E-11	0.354183611	0.265	0.096	1.79E-06	C9_CD4_Tem

chr6-36755265-36756275	2.79E-11	0.284140648	0.512	0.268	1.85E-06	C9_CD4_Tem
chr5-157613414-157613925	2.89E-11	0.297964859	0.065	0.009	1.92E-06	C9_CD4_Tem
chr9-19161181-19162137	2.93E-11	0.320212957	0.404	0.186	1.94E-06	C9_CD4_Tem
chr16-15590374-15591350	2.98E-11	0.288123556	0.526	0.29	1.98E-06	C9_CD4_Tem
chr12-108691012-108692242	3.11E-11	0.350345213	0.306	0.123	2.06E-06	C9_CD4_Tem
chr14-105294055-105294573	3.28E-11	0.339200101	0.124	0.031	2.17E-06	C9_CD4_Tem
chr4-122632689-122633566	3.47E-11	0.341534509	0.169	0.048	2.30E-06	C9_CD4_Tem
chrX-71636531-71637534	3.60E-11	0.340370347	0.254	0.095	2.38E-06	C9_CD4_Tem
chr20-4082237-4083080	3.66E-11	0.350844101	0.267	0.106	2.42E-06	C9_CD4_Tem
chr12-50220372-50220979	3.69E-11	0.349805919	0.135	0.035	2.44E-06	C9_CD4_Tem
chr16-31868659-31869914	3.76E-11	0.299339096	0.501	0.253	2.49E-06	C9_CD4_Tem
chr3-52216805-52217645	3.80E-11	0.348552518	0.263	0.101	2.52E-06	C9_CD4_Tem
chr10-3942601-3943086	3.86E-11	0.345131308	0.121	0.028	2.56E-06	C9_CD4_Tem
chr15-22263740-22265794	4.13E-11	0.316198746	0.384	0.181	2.74E-06	C9_CD4_Tem
chr5-758898-759475	4.34E-11	0.340411479	0.146	0.041	2.87E-06	C9_CD4_Tem
chr2-234258203-234258893	4.36E-11	0.335881548	0.101	0.018	2.89E-06	C9_CD4_Tem
chr20-47610192-47611272	4.42E-11	0.336075658	0.301	0.126	2.92E-06	C9_CD4_Tem
chr3-52892521-52893307	4.51E-11	0.360586292	0.236	0.091	2.99E-06	C9_CD4_Tem
chr3-20054241-20055644	4.57E-11	0.267824685	0.564	0.31	3.02E-06	C9_CD4_Tem
chr9-131719113-131720615	4.74E-11	0.354460508	0.162	0.045	3.14E-06	C9_CD4_Tem
chr22-42285911-42286587	4.91E-11	0.358151789	0.182	0.054	3.25E-06	C9_CD4_Tem
chr17-59800278-59800898	4.92E-11	0.31745879	0.09	0.015	3.26E-06	C9_CD4_Tem
chr2-232838127-232838895	4.97E-11	0.34885086	0.236	0.088	3.29E-06	C9_CD4_Tem
chr20-3989070-3989852	5.04E-11	0.359056329	0.227	0.076	3.34E-06	C9_CD4_Tem
chr2-28621184-28622118	5.40E-11	0.300746683	0.463	0.223	3.58E-06	C9_CD4_Tem
chr12-12505892-12506211	5.50E-11	0.32952347	0.094	0.016	3.64E-06	C9_CD4_Tem
chr2-111698639-111699488	5.66E-11	0.338443439	0.254	0.096	3.75E-06	C9_CD4_Tem

chr14-50038157-50039127	5.74E-11	0.339203214	0.301	0.12	3.80E-06	C9_CD4_Tem
chr14-69643291-69643925	5.91E-11	0.348432376	0.276	0.102	3.91E-06	C9_CD4_Tem
chr1-175004976-175005433	6.22E-11	0.323497353	0.097	0.021	4.12E-06	C9_CD4_Tem
chr21-42423100-42423710	6.53E-11	0.352058487	0.274	0.108	4.32E-06	C9_CD4_Tem
chr17-55093166-55093609	6.59E-11	0.283261015	0.058	0.006	4.37E-06	C9_CD4_Tem
chr2-204014980-204015880	6.63E-11	0.343455105	0.189	0.058	4.39E-06	C9_CD4_Tem
chr15-59919059-59920093	6.90E-11	0.330945197	0.274	0.11	4.57E-06	C9_CD4_Tem
chr14-91260813-91261599	8.01E-11	0.344830172	0.213	0.074	5.30E-06	C9_CD4_Tem
chr5-35773736-35774385	8.07E-11	0.352280846	0.178	0.054	5.35E-06	C9_CD4_Tem
chr7-44637763-44639509	8.13E-11	0.261653988	0.643	0.364	5.38E-06	C9_CD4_Tem
chr4-142406711-142407517	8.81E-11	0.338775491	0.29	0.117	5.83E-06	C9_CD4_Tem
chr1-160797274-160798259	8.87E-11	0.324588712	0.362	0.17	5.87E-06	C9_CD4_Tem
chr18-9090970-9091665	8.97E-11	0.323898556	0.252	0.098	5.94E-06	C9_CD4_Tem
chr4-7827981-7828987	9.15E-11	0.315841118	0.362	0.164	6.06E-06	C9_CD4_Tem
chr8-123083860-123084646	9.16E-11	0.335504162	0.191	0.066	6.06E-06	C9_CD4_Tem
chr12-50532171-50533321	9.21E-11	0.315310394	0.407	0.191	6.10E-06	C9_CD4_Tem
chr19-2627277-2628210	9.53E-11	0.341123753	0.216	0.076	6.31E-06	C9_CD4_Tem
chr5-157158945-157160332	9.57E-11	0.293009079	0.485	0.24	6.34E-06	C9_CD4_Tem
chr5-76608817-76609221	9.80E-11	0.301208875	0.074	0.012	6.49E-06	C9_CD4_Tem
chr1-161115296-161115684	9.95E-11	0.351700806	0.175	0.055	6.59E-06	C9_CD4_Tem
chr5-141691913-141693785	1.00E-10	0.313777255	0.427	0.209	6.66E-06	C9_CD4_Tem
chr6-36682464-36683091	1.05E-10	0.347468048	0.193	0.064	6.93E-06	C9_CD4_Tem
chr11-48018958-48020479	1.09E-10	0.330286201	0.315	0.131	7.21E-06	C9_CD4_Tem
chr15-92921387-92922141	1.10E-10	0.349852669	0.229	0.083	7.28E-06	C9_CD4_Tem
chr19-43701012-43701653	1.14E-10	0.342568851	0.231	0.087	7.53E-06	C9_CD4_Tem
chr10-110355991-110357698	1.17E-10	0.29067398	0.497	0.256	7.76E-06	C9_CD4_Tem
chr10-17205827-17206776	1.18E-10	0.27452957	0.553	0.285	7.78E-06	C9_CD4_Tem

chr8-99705382-99706622	1.19E-10	0.339365701	0.133	0.035	7.88E-06	C9_CD4_Tem
chr1-151945371-151946779	1.23E-10	0.266055789	0.555	0.315	8.15E-06	C9_CD4_Tem
chr9-131658309-131658634	1.25E-10	0.339330365	0.124	0.03	8.30E-06	C9_CD4_Tem
chr4-1329968-1331391	1.29E-10	0.335662172	0.281	0.114	8.52E-06	C9_CD4_Tem
chr19-17079770-17080390	1.31E-10	0.296852242	0.396	0.187	8.68E-06	C9_CD4_Tem
chr1-23118457-23119533	1.35E-10	0.333984027	0.321	0.138	8.96E-06	C9_CD4_Tem
chr15-38560391-38561241	1.42E-10	0.306502826	0.418	0.194	9.42E-06	C9_CD4_Tem
chr3-15805341-15806149	1.43E-10	0.347968876	0.139	0.035	9.46E-06	C9_CD4_Tem
chr1-22018821-22019429	1.43E-10	0.33917036	0.258	0.107	9.49E-06	C9_CD4_Tem
chr2-174600435-174600753	1.46E-10	0.346301635	0.157	0.046	9.64E-06	C9_CD4_Tem
chr5-119397446-119398002	1.51E-10	0.346222916	0.13	0.032	9.98E-06	C9_CD4_Tem
chr4-140103422-140104105	1.60E-10	0.34376879	0.164	0.048	1.06E-05	C9_CD4_Tem
chr11-85823641-85824333	1.61E-10	0.2967206	0.427	0.215	1.06E-05	C9_CD4_Tem
chr8-8288533-8289740	1.61E-10	0.336214752	0.139	0.04	1.07E-05	C9_CD4_Tem
chr12-8065975-8066697	1.67E-10	0.316134835	0.351	0.16	1.11E-05	C9_CD4_Tem
chr2-196259643-196260831	1.70E-10	0.334601161	0.299	0.127	1.12E-05	C9_CD4_Tem
chr9-97703617-97704354	1.71E-10	0.335715714	0.151	0.044	1.13E-05	C9_CD4_Tem
chr9-91071028-91071984	1.78E-10	0.334865611	0.151	0.045	1.18E-05	C9_CD4_Tem
chr6-142845079-142846023	1.93E-10	0.309803654	0.369	0.17	1.28E-05	C9_CD4_Tem
chr6-45830080-45831694	1.95E-10	0.328386161	0.139	0.044	1.29E-05	C9_CD4_Tem
chr12-91778427-91779340	2.03E-10	0.330708033	0.283	0.118	1.34E-05	C9_CD4_Tem
chr16-78790356-78791026	2.06E-10	0.319658957	0.106	0.024	1.37E-05	C9_CD4_Tem
chr2-108652269-108652961	2.09E-10	0.336797541	0.178	0.054	1.38E-05	C9_CD4_Tem
chr11-128342576-128343237	2.22E-10	0.344298546	0.238	0.085	1.47E-05	C9_CD4_Tem
chr19-54361712-54362971	2.23E-10	0.348550813	0.22	0.08	1.48E-05	C9_CD4_Tem
chr3-156482993-156484874	2.25E-10	0.28548624	0.454	0.24	1.49E-05	C9_CD4_Tem
chr3-20047339-20047600	2.43E-10	0.299138885	0.072	0.011	1.61E-05	C9_CD4_Tem

chr2-100580174-100581452	2.54E-10	0.281524451	0.479	0.246	1.68E-05	C9_CD4_Tem
chr15-92981098-92981897	2.55E-10	0.335176617	0.146	0.043	1.69E-05	C9_CD4_Tem
chr8-70136889-70137157	2.60E-10	0.298925001	0.079	0.014	1.72E-05	C9_CD4_Tem
chr11-85750466-85750991	2.70E-10	0.326273697	0.234	0.093	1.79E-05	C9_CD4_Tem
chr7-134669269-134670346	2.73E-10	0.289196831	0.452	0.223	1.81E-05	C9_CD4_Tem
chr14-106769851-106770454	2.94E-10	0.319739808	0.097	0.02	1.95E-05	C9_CD4_Tem
chr12-12465878-12466513	2.96E-10	0.329402217	0.225	0.085	1.96E-05	C9_CD4_Tem
chr8-143549050-143549719	3.10E-10	0.327718544	0.265	0.108	2.05E-05	C9_CD4_Tem
chr3-46353635-46354407	3.14E-10	0.33785893	0.148	0.042	2.08E-05	C9_CD4_Tem
chr10-110076483-110078021	3.18E-10	0.323772674	0.294	0.119	2.11E-05	C9_CD4_Tem
chr17-48505483-48506242	3.20E-10	0.284747916	0.425	0.213	2.12E-05	C9_CD4_Tem
chr2-111705671-111706269	3.21E-10	0.321849367	0.115	0.029	2.12E-05	C9_CD4_Tem
chr22-17074992-17075999	3.21E-10	0.348906492	0.193	0.062	2.13E-05	C9_CD4_Tem
chr12-12008999-12009782	3.24E-10	0.337307942	0.288	0.112	2.15E-05	C9_CD4_Tem
chr18-9082536-9084042	3.26E-10	0.345490024	0.182	0.062	2.16E-05	C9_CD4_Tem
chr20-45998370-45999078	3.33E-10	0.289104735	0.067	0.011	2.20E-05	C9_CD4_Tem
chr4-80101366-80102059	3.33E-10	0.332262398	0.119	0.029	2.21E-05	C9_CD4_Tem
chr20-4726673-4727448	3.54E-10	0.349786314	0.2	0.065	2.34E-05	C9_CD4_Tem
chr8-100381874-100382691	3.57E-10	0.328246631	0.245	0.099	2.36E-05	C9_CD4_Tem
chr9-98074971-98076183	3.59E-10	0.341355669	0.225	0.083	2.37E-05	C9_CD4_Tem
chr11-74735249-74735943	3.60E-10	0.322266579	0.13	0.038	2.38E-05	C9_CD4_Tem
chr3-5196676-5198041	3.76E-10	0.329082195	0.353	0.158	2.49E-05	C9_CD4_Tem
chr2-100587578-100588697	3.79E-10	0.306498382	0.382	0.182	2.51E-05	C9_CD4_Tem
chr7-130909824-130910532	3.86E-10	0.338701256	0.213	0.074	2.55E-05	C9_CD4_Tem
chr2-171415751-171416354	3.95E-10	0.325684165	0.169	0.056	2.62E-05	C9_CD4_Tem
chr4-80005707-80006193	3.95E-10	0.337697503	0.187	0.065	2.62E-05	C9_CD4_Tem
chr17-47762645-47763151	3.97E-10	0.329304118	0.139	0.043	2.63E-05	C9_CD4_Tem

chr11-35077333-35078468	4.02E-10	0.27682764	0.494	0.271	2.66E-05	C9_CD4_Tem
chr1-184870043-184870702	4.05E-10	0.337392104	0.238	0.084	2.68E-05	C9_CD4_Tem
chr7-149202812-149203252	4.10E-10	0.294004668	0.074	0.012	2.72E-05	C9_CD4_Tem
chr2-39099601-39100566	4.21E-10	0.332762828	0.267	0.1	2.79E-05	C9_CD4_Tem
chr1-234925693-234927241	4.41E-10	0.343831979	0.222	0.074	2.92E-05	C9_CD4_Tem
chr1-6459540-6460762	4.44E-10	0.297455001	0.413	0.193	2.94E-05	C9_CD4_Tem
chr14-20400614-20401434	4.46E-10	0.340690216	0.151	0.043	2.96E-05	C9_CD4_Tem
chr1-21779729-21780061	4.47E-10	0.335088155	0.144	0.044	2.96E-05	C9_CD4_Tem
chr5-40429275-40430259	4.72E-10	0.333542661	0.144	0.041	3.13E-05	C9_CD4_Tem
chr11-117078004-117078564	4.84E-10	0.321217831	0.306	0.127	3.21E-05	C9_CD4_Tem
chr3-187986308-187986927	4.85E-10	0.334124209	0.187	0.062	3.21E-05	C9_CD4_Tem
chr14-91245898-91246346	4.91E-10	0.279229539	0.479	0.249	3.25E-05	C9_CD4_Tem
chr17-65027149-65028700	5.00E-10	0.317019102	0.398	0.194	3.31E-05	C9_CD4_Tem
chr11-105099684-105100644	5.05E-10	0.331768376	0.166	0.056	3.35E-05	C9_CD4_Tem
chr2-161077026-161078356	5.08E-10	0.298576188	0.398	0.188	3.37E-05	C9_CD4_Tem
chr7-142440941-142441320	5.10E-10	0.286678147	0.067	0.01	3.38E-05	C9_CD4_Tem
chr13-49569683-49570585	5.14E-10	0.333757538	0.178	0.06	3.40E-05	C9_CD4_Tem
chr7-30347507-30348626	5.14E-10	0.28047198	0.519	0.282	3.41E-05	C9_CD4_Tem
chrX-2703270-2703599	5.19E-10	0.327356046	0.101	0.021	3.44E-05	C9_CD4_Tem
chr21-33229529-33231881	5.48E-10	0.29951537	0.472	0.246	3.63E-05	C9_CD4_Tem
chr4-139511175-139511622	5.64E-10	0.27283625	0.056	0.007	3.73E-05	C9_CD4_Tem
chr6-35709901-35710747	5.77E-10	0.317044637	0.308	0.135	3.82E-05	C9_CD4_Tem
chr2-102082410-102083466	5.84E-10	0.324761472	0.265	0.11	3.87E-05	C9_CD4_Tem
chr7-129530639-129531338	5.90E-10	0.331263432	0.213	0.076	3.91E-05	C9_CD4_Tem
chr4-122712864-122713083	5.93E-10	0.279628511	0.056	0.006	3.93E-05	C9_CD4_Tem
chrX-136673394-136674998	6.13E-10	0.333427795	0.146	0.041	4.06E-05	C9_CD4_Tem
chr2-85003735-85004185	6.52E-10	0.342662064	0.13	0.031	4.32E-05	C9_CD4_Tem

chr1-168386685-168387591	6.54E-10	0.33804064	0.227	0.085	4.33E-05	C9_CD4_Tem
chr1-184745673-184746317	6.72E-10	0.333062352	0.166	0.056	4.45E-05	C9_CD4_Tem
chr14-45089581-45090225	6.79E-10	0.344198838	0.227	0.078	4.50E-05	C9_CD4_Tem
chr6-152184680-152185716	6.96E-10	0.313054037	0.328	0.153	4.61E-05	C9_CD4_Tem
chr9-127954965-127956136	6.99E-10	0.294672252	0.431	0.21	4.63E-05	C9_CD4_Tem
chr9-89604248-89606232	7.08E-10	0.293888542	0.463	0.234	4.69E-05	C9_CD4_Tem
chr16-50780978-50781798	7.27E-10	0.308662539	0.099	0.023	4.81E-05	C9_CD4_Tem
chr2-9781835-9782724	7.29E-10	0.319207218	0.333	0.143	4.82E-05	C9_CD4_Tem
chr5-169649315-169649960	7.40E-10	0.33099874	0.157	0.047	4.90E-05	C9_CD4_Tem
chr19-49458005-49458729	7.66E-10	0.315392651	0.121	0.029	5.07E-05	C9_CD4_Tem
chr1-236531684-236531999	7.77E-10	0.313086577	0.106	0.023	5.14E-05	C9_CD4_Tem
chr8-29655469-29656763	7.79E-10	0.336666761	0.193	0.065	5.16E-05	C9_CD4_Tem
chr8-9079860-9080508	7.88E-10	0.2972894	0.088	0.019	5.22E-05	C9_CD4_Tem
chr5-40361348-40361764	7.90E-10	0.333610922	0.148	0.044	5.23E-05	C9_CD4_Tem
chr1-92583620-92585291	8.10E-10	0.301067398	0.416	0.2	5.36E-05	C9_CD4_Tem
chr6-155185969-155186428	8.17E-10	0.307267309	0.094	0.021	5.41E-05	C9_CD4_Tem
chr17-30331547-30332523	8.32E-10	0.33008583	0.288	0.128	5.51E-05	C9_CD4_Tem
chr2-88599576-88600516	9.26E-10	0.271446006	0.521	0.276	6.13E-05	C9_CD4_Tem
chr4-89316305-89317434	9.35E-10	0.315706721	0.31	0.139	6.19E-05	C9_CD4_Tem
chr15-73924294-73924559	9.37E-10	0.326120745	0.112	0.024	6.21E-05	C9_CD4_Tem
chr9-97906114-97907245	9.79E-10	0.316821621	0.33	0.142	6.48E-05	C9_CD4_Tem
chr11-128339693-128340101	1.01E-09	0.310430971	0.094	0.021	6.70E-05	C9_CD4_Tem
chr7-73850821-73852010	1.02E-09	0.320630961	0.234	0.09	6.75E-05	C9_CD4_Tem
chr15-101205906-101206664	1.05E-09	0.309244331	0.357	0.16	6.94E-05	C9_CD4_Tem
chr1-193591899-193593026	1.05E-09	0.322428171	0.139	0.038	6.97E-05	C9_CD4_Tem
chr10-87083621-87084926	1.12E-09	0.309287156	0.373	0.174	7.42E-05	C9_CD4_Tem
chr6-25025724-25026231	1.17E-09	0.335081159	0.135	0.036	7.74E-05	C9_CD4_Tem

chr20-33624306-33624906	1.17E-09	0.302250881	0.085	0.018	7.78E-05	C9_CD4_Tem
chr10-17025489-17025981	1.21E-09	0.333141694	0.272	0.11	8.02E-05	C9_CD4_Tem
chr5-140261334-140262059	1.26E-09	0.305919946	0.092	0.021	8.35E-05	C9_CD4_Tem
chr11-117010913-117011703	1.26E-09	0.344895971	0.169	0.049	8.37E-05	C9_CD4_Tem
chr12-25518432-25518859	1.27E-09	0.263106606	0.054	0.008	8.38E-05	C9_CD4_Tem
chr4-1300932-1301395	1.27E-09	0.267462231	0.058	0.009	8.39E-05	C9_CD4_Tem
chr14-91858278-91858884	1.35E-09	0.32998463	0.162	0.05	8.96E-05	C9_CD4_Tem
chr11-62427650-62428107	1.39E-09	0.316691717	0.11	0.026	9.20E-05	C9_CD4_Tem
chr15-50249088-50249434	1.40E-09	0.260905898	0.054	0.007	9.27E-05	C9_CD4_Tem
chr11-35029908-35031183	1.41E-09	0.32386435	0.312	0.132	9.33E-05	C9_CD4_Tem
chr7-2214547-2214969	1.45E-09	0.275696245	0.061	0.01	9.60E-05	C9_CD4_Tem
chr15-92906610-92907164	1.45E-09	0.288045222	0.44	0.232	9.63E-05	C9_CD4_Tem
chr6-135114334-135114792	1.52E-09	0.315323796	0.106	0.026	0.000100802	C9_CD4_Tem
chr1-89635903-89636505	1.56E-09	0.332990376	0.274	0.107	0.000103286	C9_CD4_Tem
chr16-31877064-31877797	1.57E-09	0.312512794	0.366	0.172	0.000104112	C9_CD4_Tem
chr2-200702511-200703859	1.59E-09	0.330539	0.202	0.073	0.000105022	C9_CD4_Tem
chr5-53041016-53041695	1.60E-09	0.328260044	0.157	0.048	0.000105856	C9_CD4_Tem
chr4-152553782-152554647	1.64E-09	0.32815227	0.227	0.086	0.000108283	C9_CD4_Tem
chr11-107840241-107841119	1.64E-09	0.314264461	0.281	0.118	0.000108776	C9_CD4_Tem
chr20-59165595-59166725	1.66E-09	0.289562105	0.449	0.225	0.000110216	C9_CD4_Tem
chr10-24466176-24466829	1.69E-09	0.294323136	0.081	0.017	0.000111685	C9_CD4_Tem
chr8-38362481-38363283	1.69E-09	0.328609325	0.2	0.072	0.000112161	C9_CD4_Tem
chr1-26123193-26123705	1.71E-09	0.320669272	0.164	0.051	0.000113341	C9_CD4_Tem
chr19-49553623-49554350	1.74E-09	0.331221679	0.182	0.059	0.000114963	C9_CD4_Tem
chr8-19510718-19511498	1.81E-09	0.329629911	0.198	0.069	0.000119931	C9_CD4_Tem
chr11-122748932-122749326	1.81E-09	0.323645399	0.216	0.08	0.000120045	C9_CD4_Tem
chr20-4037663-4038434	1.83E-09	0.30108817	0.315	0.139	0.000121159	C9_CD4_Tem

chr8-100313310-100313956	1.91E-09	0.329720294	0.274	0.109	0.000126488	C9_CD4_Tem
chr20-4160763-4161675	1.92E-09	0.302792095	0.103	0.026	0.000127331	C9_CD4_Tem
chr13-99050220-99050457	2.02E-09	0.279240416	0.067	0.011	0.000133864	C9_CD4_Tem
chr13-24253650-24254876	2.04E-09	0.25987888	0.53	0.288	0.000135116	C9_CD4_Tem
chr12-104603734-104604890	2.10E-09	0.328079497	0.207	0.072	0.000139071	C9_CD4_Tem
chr4-142416416-142417303	2.15E-09	0.315225523	0.285	0.118	0.000142506	C9_CD4_Tem
chr7-66859453-66860586	2.17E-09	0.276096193	0.07	0.014	0.000143675	C9_CD4_Tem
chr15-63479336-63480062	2.21E-09	0.30878166	0.272	0.111	0.000146039	C9_CD4_Tem
chr22-22210316-22210973	2.27E-09	0.323664346	0.124	0.032	0.000150336	C9_CD4_Tem
chr12-107759437-107759763	2.40E-09	0.322594476	0.155	0.051	0.000159073	C9_CD4_Tem
chr3-119637745-119639270	2.43E-09	0.294935104	0.128	0.037	0.000161217	C9_CD4_Tem
chr10-17001358-17003528	2.45E-09	0.256291252	0.528	0.293	0.000162363	C9_CD4_Tem
chr13-99310286-99310636	2.63E-09	0.331419461	0.146	0.043	0.000174082	C9_CD4_Tem
chr15-85690491-85691076	2.72E-09	0.323321045	0.263	0.112	0.000179891	C9_CD4_Tem
chr1-154404555-154406327	2.85E-09	0.318875494	0.335	0.155	0.000188461	C9_CD4_Tem
chr15-67076131-67076470	2.92E-09	0.283257663	0.065	0.01	0.000193473	C9_CD4_Tem
chr16-10957500-10958307	2.94E-09	0.324436374	0.137	0.036	0.000194683	C9_CD4_Tem
chr18-9094490-9095605	2.95E-09	0.321757251	0.249	0.098	0.00019514	C9_CD4_Tem
chr2-181106468-181107188	3.02E-09	0.315792105	0.238	0.098	0.000200142	C9_CD4_Tem
chr15-88623106-88623404	3.03E-09	0.331561107	0.144	0.041	0.000200639	C9_CD4_Tem
chr15-73920358-73920896	3.05E-09	0.322952749	0.151	0.046	0.000201956	C9_CD4_Tem
chr5-157198163-157199049	3.09E-09	0.273336211	0.438	0.236	0.000204592	C9_CD4_Tem
chr19-43438368-43439720	3.12E-09	0.313202051	0.285	0.124	0.000206494	C9_CD4_Tem
chr8-29458982-29459687	3.14E-09	0.320694261	0.178	0.064	0.000207793	C9_CD4_Tem
chr1-160637828-160638442	3.22E-09	0.311989671	0.299	0.132	0.00021305	C9_CD4_Tem
chr5-94574858-94575549	3.38E-09	0.302064483	0.148	0.048	0.000224132	C9_CD4_Tem
chr11-67884136-67885363	3.38E-09	0.312891696	0.108	0.029	0.00022416	C9_CD4_Tem

chr17-16027610-16028503	3.40E-09	0.317877649	0.299	0.129	0.00022536	C9_CD4_Tem
chr4-36394180-36394970	3.48E-09	0.287283523	0.378	0.187	0.000230783	C9_CD4_Tem
chr18-62420089-62421122	3.49E-09	0.26410813	0.483	0.261	0.00023104	C9_CD4_Tem
chr5-143605224-143606724	3.83E-09	0.321470192	0.162	0.054	0.000253428	C9_CD4_Tem
chr9-35084916-35085401	3.92E-09	0.276621715	0.074	0.014	0.000259375	C9_CD4_Tem
chr13-73714625-73716372	4.16E-09	0.268789847	0.488	0.26	0.000275282	C9_CD4_Tem
chr10-25061781-25062986	4.20E-09	0.296623754	0.155	0.053	0.000278014	C9_CD4_Tem
chr13-42354068-42355455	4.28E-09	0.312181963	0.261	0.105	0.000283356	C9_CD4_Tem
chr12-12502497-12503926	4.31E-09	0.321125453	0.157	0.048	0.000285125	C9_CD4_Tem
chr18-79246284-79246881	4.32E-09	0.284659379	0.344	0.169	0.000286212	C9_CD4_Tem
chr6-138592788-138593684	4.34E-09	0.324580149	0.191	0.064	0.000287561	C9_CD4_Tem
chr13-31044415-31046134	4.42E-09	0.296776586	0.369	0.183	0.00029271	C9_CD4_Tem
chr21-14481520-14482548	4.54E-09	0.310388168	0.11	0.027	0.000300828	C9_CD4_Tem
chr9-87709020-87709653	4.65E-09	0.264987613	0.065	0.01	0.000307731	C9_CD4_Tem
chr5-141598475-141599296	4.65E-09	0.315250275	0.128	0.036	0.000308234	C9_CD4_Tem
chr13-40205647-40207281	4.69E-09	0.319253097	0.238	0.092	0.000310784	C9_CD4_Tem
chr1-89669185-89670488	4.90E-09	0.313563822	0.272	0.117	0.00032429	C9_CD4_Tem
chr2-197343024-197343498	5.05E-09	0.260032628	0.058	0.011	0.000334117	C9_CD4_Tem
chr17-78769222-78769683	5.09E-09	0.273184623	0.07	0.012	0.000336985	C9_CD4_Tem
chr15-52559049-52559840	5.18E-09	0.320541237	0.16	0.053	0.000343007	C9_CD4_Tem
chr12-64665425-64665902	5.21E-09	0.317424171	0.225	0.094	0.000345013	C9_CD4_Tem
chr20-23147751-23148580	5.22E-09	0.279134806	0.065	0.01	0.000345905	C9_CD4_Tem
chr15-101205093-101205413	5.35E-09	0.296212676	0.094	0.023	0.000354465	C9_CD4_Tem
chr10-72305254-72305739	5.41E-09	0.301338646	0.085	0.018	0.000358326	C9_CD4_Tem
chr6-87731457-87732623	5.49E-09	0.317841817	0.155	0.052	0.000363406	C9_CD4_Tem
chr9-68974163-68974821	5.59E-09	0.267922239	0.07	0.015	0.00036988	C9_CD4_Tem
chr20-4665558-4666457	5.64E-09	0.325999613	0.258	0.101	0.000373758	C9_CD4_Tem

chr10-5283234-5283810	5.72E-09	0.303926714	0.085	0.016	0.000379072	C9_CD4_Tem
chr10-33263906-33264748	5.74E-09	0.309640894	0.22	0.085	0.000379818	C9_CD4_Tem
chr12-50542134-50542831	5.78E-09	0.305593722	0.112	0.03	0.000382655	C9_CD4_Tem
chr20-43750868-43751707	5.96E-09	0.313622595	0.207	0.082	0.000395018	C9_CD4_Tem
chr7-44630926-44631775	6.22E-09	0.297759058	0.324	0.15	0.000411843	C9_CD4_Tem
chr11-59857475-59858407	6.23E-09	0.312262414	0.121	0.032	0.000412736	C9_CD4_Tem
chr1-183915646-183916509	6.30E-09	0.280794988	0.396	0.194	0.000417467	C9_CD4_Tem
chr17-74513831-74514980	6.41E-09	0.316294008	0.236	0.099	0.000424808	C9_CD4_Tem
chr11-57791789-57792649	6.44E-09	0.293768168	0.36	0.173	0.000426756	C9_CD4_Tem
chr1-234771867-234773060	6.81E-09	0.282741082	0.443	0.229	0.000450868	C9_CD4_Tem
chr14-70998757-70999589	6.95E-09	0.318059206	0.148	0.049	0.000460027	C9_CD4_Tem
chr2-201262771-201263034	6.97E-09	0.32083983	0.146	0.046	0.000461743	C9_CD4_Tem
chr13-41355897-41357152	7.00E-09	0.321071345	0.207	0.076	0.000463454	C9_CD4_Tem
chr2-54572292-54573398	7.15E-09	0.289617889	0.429	0.213	0.000473803	C9_CD4_Tem
chr10-103504005-103504738	7.16E-09	0.331665669	0.178	0.063	0.000473908	C9_CD4_Tem
chr12-108696348-108696803	7.39E-09	0.313669003	0.11	0.028	0.000489082	C9_CD4_Tem
chr1-89603756-89603974	7.45E-09	0.291957719	0.083	0.018	0.000493256	C9_CD4_Tem
chr10-32307910-32308559	7.49E-09	0.295542945	0.106	0.026	0.000496045	C9_CD4_Tem
chr2-37149389-37149960	7.59E-09	0.310219542	0.135	0.042	0.000502405	C9_CD4_Tem
chr19-38427079-38427610	7.80E-09	0.274996831	0.079	0.017	0.000516566	C9_CD4_Tem
chr21-44078304-44078684	7.82E-09	0.279299386	0.07	0.012	0.000517747	C9_CD4_Tem
chr15-55388963-55389595	7.83E-09	0.289023189	0.081	0.017	0.000518201	C9_CD4_Tem
chr16-30469437-30470378	7.87E-09	0.325860994	0.211	0.08	0.000520986	C9_CD4_Tem
chr6-116386543-116387614	8.15E-09	0.297548724	0.09	0.021	0.000539675	C9_CD4_Tem
chr2-112181425-112183042	8.59E-09	0.319780984	0.171	0.054	0.000568907	C9_CD4_Tem
chr8-141238372-141239679	8.79E-09	0.303079191	0.33	0.151	0.000582108	C9_CD4_Tem
chr12-25495799-25496701	8.84E-09	0.26744309	0.076	0.015	0.000585264	C9_CD4_Tem

chr11-118001563-118003762	8.93E-09	0.287032645	0.422	0.208	0.000591698	C9_CD4_Tem
chr3-45972398-45973002	9.38E-09	0.330782521	0.166	0.056	0.00062111	C9_CD4_Tem
chr1-41811260-41811738	9.62E-09	0.301387916	0.162	0.062	0.000636823	C9_CD4_Tem
chr4-26325115-26325430	1.00E-08	0.308726156	0.146	0.047	0.000663515	C9_CD4_Tem
chr7-5641663-5642698	1.03E-08	0.278760152	0.387	0.189	0.000681254	C9_CD4_Tem
chr2-207163495-207163750	1.03E-08	0.293028287	0.09	0.018	0.000683548	C9_CD4_Tem
chr11-128316598-128316918	1.04E-08	0.285333145	0.076	0.014	0.000690331	C9_CD4_Tem
chr1-8394577-8395886	1.05E-08	0.322541878	0.169	0.055	0.00069235	C9_CD4_Tem
chr2-233067024-233067643	1.09E-08	0.317541122	0.16	0.05	0.000719204	C9_CD4_Tem
chr11-86176148-86176847	1.10E-08	0.30494753	0.137	0.043	0.000726761	C9_CD4_Tem
chr19-6062189-6062534	1.15E-08	0.30619187	0.099	0.023	0.000759201	C9_CD4_Tem
chr15-52729947-52730520	1.15E-08	0.311333238	0.169	0.058	0.000763316	C9_CD4_Tem
chr10-31353345-31353946	1.18E-08	0.317097048	0.204	0.076	0.000779216	C9_CD4_Tem
chr8-8285777-8286133	1.22E-08	0.281839471	0.079	0.018	0.000806681	C9_CD4_Tem
chr19-246602-247455	1.23E-08	0.313509399	0.169	0.058	0.000812913	C9_CD4_Tem
chr8-102107535-102109028	1.26E-08	0.297360544	0.173	0.063	0.000831242	C9_CD4_Tem
chr12-122399278-122400778	1.28E-08	0.32646784	0.166	0.052	0.000844925	C9_CD4_Tem
chr5-14925373-14926423	1.29E-08	0.314061541	0.222	0.082	0.000851539	C9_CD4_Tem
chr4-80006664-80007228	1.31E-08	0.299621058	0.103	0.027	0.000866565	C9_CD4_Tem
chr7-131235252-131236059	1.35E-08	0.289769353	0.366	0.176	0.000897305	C9_CD4_Tem
chr1-116538333-116539064	1.37E-08	0.293308622	0.285	0.124	0.000904835	C9_CD4_Tem
chr6-52392597-52393204	1.38E-08	0.307335919	0.227	0.091	0.000913377	C9_CD4_Tem
chr5-14809224-14810319	1.38E-08	0.251834079	0.479	0.275	0.000915054	C9_CD4_Tem
chr1-58812674-58813629	1.38E-08	0.289994958	0.274	0.125	0.000915153	C9_CD4_Tem
chr20-50165546-50166389	1.38E-08	0.311731291	0.249	0.1	0.00091707	C9_CD4_Tem
chr1-193457511-193458066	1.39E-08	0.300065864	0.126	0.038	0.000919796	C9_CD4_Tem
chr1-234980422-234981267	1.39E-08	0.309179599	0.285	0.121	0.000920646	C9_CD4_Tem

chr14-65206124-65207002	1.40E-08	0.315431601	0.126	0.035	0.000925593	C9_CD4_Tem
chr2-100924137-100925468	1.40E-08	0.297890828	0.22	0.087	0.000928718	C9_CD4_Tem
chr6-38713620-38715736	1.41E-08	0.303569619	0.249	0.107	0.000932141	C9_CD4_Tem
chr13-99307083-99308260	1.41E-08	0.273362761	0.447	0.238	0.000932605	C9_CD4_Tem
chr17-78386177-78386667	1.41E-08	0.313978125	0.124	0.034	0.000934257	C9_CD4_Tem
chr2-112251289-112251698	1.41E-08	0.279412574	0.074	0.015	0.000934663	C9_CD4_Tem
chr6-139125955-139126600	1.42E-08	0.307908593	0.137	0.046	0.00093713	C9_CD4_Tem
chr12-12481602-12482788	1.46E-08	0.304122375	0.308	0.138	0.000964237	C9_CD4_Tem
chr19-10408493-10409067	1.48E-08	0.293867672	0.344	0.158	0.000979795	C9_CD4_Tem
chr8-19756298-19758064	1.52E-08	0.312229579	0.184	0.063	0.001004142	C9_CD4_Tem
chr8-140588626-140589463	1.54E-08	0.282943451	0.431	0.217	0.001021642	C9_CD4_Tem
chr7-77802849-77803339	1.54E-08	0.313018316	0.148	0.047	0.001021757	C9_CD4_Tem
chr4-1710268-1710475	1.55E-08	0.29503026	0.094	0.024	0.001026747	C9_CD4_Tem
chr17-80678456-80679463	1.56E-08	0.287989483	0.094	0.022	0.001033767	C9_CD4_Tem
chr2-64212330-64213310	1.58E-08	0.311798069	0.213	0.081	0.001047458	C9_CD4_Tem
chr7-130925339-130925588	1.61E-08	0.292459659	0.083	0.017	0.001069048	C9_CD4_Tem
chr3-69178557-69179810	1.63E-08	0.297360998	0.106	0.027	0.001081096	C9_CD4_Tem
chr1-36373522-36375006	1.64E-08	0.295525147	0.369	0.177	0.001088097	C9_CD4_Tem
chr8-18957182-18957952	1.65E-08	0.30029886	0.137	0.043	0.001089633	C9_CD4_Tem
chr1-66345930-66346753	1.71E-08	0.314791887	0.225	0.087	0.00112983	C9_CD4_Tem
chr1-41862286-41863201	1.76E-08	0.267051113	0.067	0.014	0.001163702	C9_CD4_Tem
chr2-127389752-127389996	1.78E-08	0.304642214	0.13	0.042	0.001177274	C9_CD4_Tem
chr17-61806005-61806513	1.84E-08	0.282672593	0.092	0.023	0.001220875	C9_CD4_Tem
chr8-47951737-47952484	1.88E-08	0.284024045	0.085	0.019	0.00124515	C9_CD4_Tem
chr6-13302396-13303339	1.90E-08	0.281183334	0.297	0.138	0.001260034	C9_CD4_Tem
chr20-64285522-64287037	1.93E-08	0.284385528	0.292	0.139	0.001279368	C9_CD4_Tem
chr17-80720552-80721593	2.02E-08	0.280629514	0.389	0.188	0.001340269	C9_CD4_Tem

chr17-63597022-63597708	2.08E-08	0.315241168	0.245	0.098	0.001378246	C9_CD4_Tem
chr1-235074458-235075575	2.14E-08	0.298575092	0.297	0.141	0.001413896	C9_CD4_Tem
chr1-168421116-168421860	2.14E-08	0.293538659	0.319	0.153	0.001419814	C9_CD4_Tem
chr14-51827645-51828710	2.15E-08	0.307140619	0.211	0.081	0.001421857	C9_CD4_Tem
chr4-83125500-83126299	2.18E-08	0.316165156	0.193	0.069	0.001444165	C9_CD4_Tem
chr2-224927518-224928259	2.21E-08	0.257811595	0.465	0.249	0.001465114	C9_CD4_Tem
chr16-75108756-75109282	2.24E-08	0.313145334	0.211	0.079	0.001482087	C9_CD4_Tem
chr7-130988315-130988900	2.25E-08	0.30514551	0.144	0.043	0.0014911	C9_CD4_Tem
chr13-114268403-114269455	2.35E-08	0.30548888	0.139	0.044	0.001557549	C9_CD4_Tem
chr5-169655987-169656527	2.38E-08	0.293752008	0.112	0.035	0.001575824	C9_CD4_Tem
chr3-45693535-45694601	2.46E-08	0.291431673	0.387	0.191	0.001625974	C9_CD4_Tem
chr19-3141954-3142533	2.47E-08	0.302033202	0.106	0.025	0.001637949	C9_CD4_Tem
chr2-239247350-239247849	2.49E-08	0.274578017	0.079	0.017	0.001646096	C9_CD4_Tem
chr17-59842551-59843437	2.52E-08	0.291582731	0.342	0.164	0.001671131	C9_CD4_Tem
chr11-18383085-18384518	2.52E-08	0.258148123	0.431	0.232	0.001671525	C9_CD4_Tem
chr13-25901551-25902240	2.57E-08	0.282679368	0.303	0.143	0.001705008	C9_CD4_Tem
chr5-6829074-6829668	2.59E-08	0.294239748	0.103	0.027	0.001716693	C9_CD4_Tem
chr19-6057129-6057544	2.61E-08	0.295940514	0.088	0.019	0.001725354	C9_CD4_Tem
chr3-46360946-46361664	2.61E-08	0.307446979	0.157	0.052	0.001729199	C9_CD4_Tem
chr3-112676440-112677192	2.61E-08	0.272465872	0.369	0.182	0.001730504	C9_CD4_Tem
chr11-48093444-48094135	2.62E-08	0.30162473	0.101	0.026	0.0017358	C9_CD4_Tem
chr15-60695467-60696216	2.63E-08	0.308676765	0.166	0.056	0.001743617	C9_CD4_Tem
chr12-11978967-11979278	2.68E-08	0.300887407	0.108	0.027	0.001777841	C9_CD4_Tem
chr3-101936162-101937025	2.75E-08	0.285601543	0.328	0.156	0.001819119	C9_CD4_Tem
chr3-46286962-46287304	2.84E-08	0.291217402	0.119	0.036	0.001881413	C9_CD4_Tem
chr10-60714009-60714520	2.86E-08	0.258098861	0.065	0.012	0.001896782	C9_CD4_Tem
chr13-30739857-30740577	2.94E-08	0.254348535	0.472	0.256	0.001944399	C9_CD4_Tem

chr6-36383428-36383950	3.04E-08	0.268582564	0.081	0.021	0.002009972	C9_CD4_Tem
chr1-185439427-185440494	3.11E-08	0.273131674	0.362	0.175	0.00206031	C9_CD4_Tem
chr9-5446455-5447136	3.14E-08	0.304264413	0.155	0.055	0.002076518	C9_CD4_Tem
chr6-158283346-158284994	3.17E-08	0.293459964	0.155	0.057	0.002096199	C9_CD4_Tem
chr22-50542429-50542650	3.18E-08	0.287602381	0.09	0.022	0.002108019	C9_CD4_Tem
chr14-90263504-90264445	3.20E-08	0.296869073	0.169	0.063	0.002116094	C9_CD4_Tem
chr16-89388004-89388346	3.25E-08	0.297250385	0.099	0.024	0.00215361	C9_CD4_Tem
chr9-131656226-131657298	3.30E-08	0.280735096	0.337	0.162	0.002183548	C9_CD4_Tem
chr16-72482283-72483046	3.37E-08	0.269322336	0.076	0.017	0.002228838	C9_CD4_Tem
chr1-39816445-39817638	3.60E-08	0.296583903	0.261	0.11	0.002384736	C9_CD4_Tem
chr2-224925526-224926533	3.63E-08	0.293192893	0.333	0.154	0.002401511	C9_CD4_Tem
chr12-75943836-75945028	3.63E-08	0.291214575	0.355	0.17	0.002402147	C9_CD4_Tem
chr12-6790633-6791092	3.68E-08	0.279610418	0.092	0.022	0.00243415	C9_CD4_Tem
chr10-62050971-62051383	3.71E-08	0.264863989	0.427	0.219	0.002454057	C9_CD4_Tem
chr5-157343804-157344803	3.72E-08	0.292302223	0.238	0.104	0.002463873	C9_CD4_Tem
chrX-40278348-40279261	3.84E-08	0.288708094	0.279	0.121	0.002541374	C9_CD4_Tem
chr19-1213792-1214644	3.90E-08	0.293436509	0.106	0.026	0.002580767	C9_CD4_Tem
chr7-128933152-128933478	3.90E-08	0.270096136	0.072	0.014	0.002583101	C9_CD4_Tem
chr14-101876118-101877070	3.95E-08	0.282672797	0.328	0.166	0.002614074	C9_CD4_Tem
chr14-55303716-55304254	3.99E-08	0.260559606	0.07	0.015	0.002642785	C9_CD4_Tem
chr7-2757678-2758565	3.99E-08	0.289323753	0.279	0.126	0.00264466	C9_CD4_Tem
chr1-160626143-160626423	4.16E-08	0.321505772	0.162	0.053	0.002756678	C9_CD4_Tem
chr15-60362476-60363105	4.33E-08	0.290204549	0.124	0.039	0.002869742	C9_CD4_Tem
chr1-152034383-152037130	4.43E-08	0.274205576	0.425	0.227	0.002933914	C9_CD4_Tem
chr12-54935002-54935821	4.48E-08	0.30736597	0.285	0.122	0.002965662	C9_CD4_Tem
chr7-106717852-106718819	4.61E-08	0.288146797	0.315	0.147	0.00304994	C9_CD4_Tem
chr21-14629951-14631144	4.90E-08	0.314674926	0.196	0.071	0.003241919	C9_CD4_Tem

chr8-8848682-8849212	4.99E-08	0.290187012	0.088	0.019	0.003302057	C9_CD4_Tem
chr4-1337230-1337925	5.08E-08	0.288366048	0.148	0.05	0.003363331	C9_CD4_Tem
chr2-108620475-108621553	5.16E-08	0.275167776	0.404	0.203	0.00341942	C9_CD4_Tem
chr8-80918018-80919007	5.19E-08	0.30192623	0.142	0.048	0.003435354	C9_CD4_Tem
chr8-133216576-133218344	5.22E-08	0.253449529	0.515	0.275	0.003454224	C9_CD4_Tem
chrX-136652913-136653570	5.25E-08	0.271850944	0.072	0.013	0.003477973	C9_CD4_Tem
chr15-90414487-90414983	5.28E-08	0.293438544	0.117	0.033	0.003495401	C9_CD4_Tem
chr8-42893435-42894491	5.28E-08	0.306912674	0.229	0.092	0.003496707	C9_CD4_Tem
chr6-446306-447925	5.37E-08	0.278090729	0.369	0.177	0.003557428	C9_CD4_Tem
chr5-150780020-150781167	5.46E-08	0.298873279	0.164	0.058	0.003615718	C9_CD4_Tem
chr8-29994997-29995440	5.46E-08	0.286017189	0.13	0.044	0.003617055	C9_CD4_Tem
chr12-111505376-111506090	5.49E-08	0.297111715	0.121	0.037	0.003638473	C9_CD4_Tem
chrX-136598088-136599023	5.50E-08	0.263695177	0.072	0.013	0.003639201	C9_CD4_Tem
chr15-101199358-101200162	5.61E-08	0.289694654	0.321	0.144	0.003713182	C9_CD4_Tem
chrX-41382908-41384432	5.81E-08	0.291946524	0.187	0.072	0.003850092	C9_CD4_Tem
chr6-14732448-14733422	5.98E-08	0.278509599	0.321	0.151	0.00395761	C9_CD4_Tem
chr11-117026625-117027354	6.20E-08	0.305234999	0.227	0.089	0.004103593	C9_CD4_Tem
chr6-16659260-16660134	6.28E-08	0.313109369	0.13	0.039	0.004158406	C9_CD4_Tem
chr11-64885457-64885944	6.42E-08	0.292660169	0.133	0.044	0.004250562	C9_CD4_Tem
chr10-61991980-61994393	6.60E-08	0.261144295	0.425	0.223	0.00437304	C9_CD4_Tem
chr17-3048497-3049286	6.62E-08	0.275228474	0.353	0.174	0.00438259	C9_CD4_Tem
chr11-47539958-47541381	6.67E-08	0.307166891	0.139	0.044	0.00441997	C9_CD4_Tem
chr1-206757377-206758253	6.70E-08	0.309010702	0.207	0.081	0.004433788	C9_CD4_Tem
chr4-26311491-26312722	6.70E-08	0.294261001	0.204	0.08	0.004437893	C9_CD4_Tem
chr5-119333439-119333866	6.90E-08	0.301826651	0.22	0.088	0.004571521	C9_CD4_Tem
chr12-29390223-29390793	7.01E-08	0.289392419	0.112	0.032	0.004641672	C9_CD4_Tem
chr1-816763-817436	7.03E-08	0.257572838	0.07	0.016	0.004657534	C9_CD4_Tem

chr2-200944222-200945169	7.07E-08	0.279433904	0.119	0.036	0.004683266	C9_CD4_Tem
chr17-77391924-77392239	7.30E-08	0.269421638	0.076	0.016	0.004833439	C9_CD4_Tem
chr1-207823720-207824221	7.33E-08	0.28459556	0.319	0.152	0.004855097	C9_CD4_Tem
chr1-193558309-193559033	7.36E-08	0.275223996	0.117	0.035	0.004872021	C9_CD4_Tem
chr2-203711099-203711782	7.50E-08	0.292150744	0.276	0.122	0.004964319	C9_CD4_Tem
chr3-43388944-43390123	7.64E-08	0.283951294	0.312	0.15	0.005061424	C9_CD4_Tem
chr8-102097118-102098160	7.81E-08	0.275944042	0.303	0.144	0.005174511	C9_CD4_Tem
chr12-12439435-12439968	7.83E-08	0.291669053	0.283	0.122	0.005188185	C9_CD4_Tem
chr12-110590783-110591241	7.96E-08	0.273471648	0.081	0.018	0.005268992	C9_CD4_Tem
chr14-69685143-69686044	8.03E-08	0.303037152	0.142	0.044	0.005317682	C9_CD4_Tem
chr8-29974897-29975568	8.06E-08	0.278783364	0.101	0.029	0.005335243	C9_CD4_Tem
chr17-59361301-59362740	8.17E-08	0.293230451	0.204	0.082	0.005411259	C9_CD4_Tem
chr17-65022809-65023434	8.31E-08	0.289958069	0.198	0.075	0.005503287	C9_CD4_Tem
chr17-80575156-80575662	8.41E-08	0.310505037	0.182	0.067	0.005569297	C9_CD4_Tem
chr5-143409895-143410306	8.44E-08	0.273910214	0.074	0.015	0.005589565	C9_CD4_Tem
chr1-229252974-229253914	8.50E-08	0.302937977	0.189	0.073	0.005629308	C9_CD4_Tem
chr3-30623008-30623665	8.59E-08	0.286925038	0.121	0.039	0.005688393	C9_CD4_Tem
chr5-39164058-39165217	8.77E-08	0.285808512	0.357	0.174	0.005808786	C9_CD4_Tem
chr12-12486741-12487229	8.90E-08	0.286365638	0.092	0.023	0.005891071	C9_CD4_Tem
chr1-66243833-66244071	8.93E-08	0.28443547	0.099	0.027	0.005915038	C9_CD4_Tem
chr13-113493156-113493649	9.03E-08	0.303467273	0.207	0.078	0.005982652	C9_CD4_Tem
chr12-754851-755221	9.22E-08	0.289334611	0.285	0.138	0.006103964	C9_CD4_Tem
chr5-143311422-143312021	9.25E-08	0.269650371	0.074	0.016	0.00612582	C9_CD4_Tem
chr16-66521884-66522606	9.38E-08	0.257707641	0.4	0.206	0.006209956	C9_CD4_Tem
chr6-33694315-33694936	9.39E-08	0.284915968	0.133	0.044	0.006220639	C9_CD4_Tem
chr1-156222650-156223352	9.40E-08	0.286118291	0.256	0.117	0.006226176	C9_CD4_Tem
chr1-214439157-214440058	9.58E-08	0.280012211	0.142	0.049	0.006341843	C9_CD4_Tem

chr22-40240516-40241795	9.69E-08	0.26896248	0.389	0.202	0.006419186	C9_CD4_Tem
chr7-130930059-130930652	9.73E-08	0.289651603	0.204	0.082	0.006443711	C9_CD4_Tem
chr22-40247656-40248434	9.78E-08	0.293934103	0.243	0.106	0.006475636	C9_CD4_Tem
chr6-45420319-45420917	9.80E-08	0.297217647	0.182	0.067	0.006487745	C9_CD4_Tem
chr13-50236523-50237269	9.80E-08	0.297032154	0.238	0.105	0.006490887	C9_CD4_Tem
chr18-2891749-2892163	9.85E-08	0.282580057	0.09	0.02	0.006523188	C9_CD4_Tem
chr19-20922850-20923758	9.98E-08	0.263253801	0.387	0.211	0.006607514	C9_CD4_Tem
chr16-10963888-10965181	9.99E-08	0.277182642	0.382	0.191	0.006613149	C9_CD4_Tem
chr11-59106333-59108010	1.01E-07	0.268578577	0.4	0.202	0.006686044	C9_CD4_Tem
chr2-38681532-38682957	1.03E-07	0.27509785	0.375	0.193	0.006838757	C9_CD4_Tem
chr12-12474066-12475427	1.04E-07	0.263802333	0.393	0.212	0.006915563	C9_CD4_Tem
chr8-128539990-128541355	1.06E-07	0.282543198	0.303	0.137	0.007028118	C9_CD4_Tem
chr3-196641324-196642171	1.07E-07	0.273421608	0.366	0.188	0.007069037	C9_CD4_Tem
chr14-105025988-105026375	1.10E-07	0.308064584	0.178	0.063	0.007266069	C9_CD4_Tem
chr8-29654137-29654657	1.13E-07	0.274666247	0.088	0.021	0.007508565	C9_CD4_Tem
chr10-103696249-103696786	1.15E-07	0.30302308	0.225	0.093	0.007641914	C9_CD4_Tem
chr19-43430081-43430650	1.17E-07	0.268700925	0.088	0.023	0.007721509	C9_CD4_Tem
chr22-42443249-42444859	1.17E-07	0.306417798	0.218	0.089	0.007761331	C9_CD4_Tem
chr12-26298721-26299357	1.18E-07	0.286195351	0.133	0.044	0.007804871	C9_CD4_Tem
chr1-147494802-147495320	1.19E-07	0.272263146	0.088	0.024	0.007885078	C9_CD4_Tem
chr13-42392696-42393598	1.20E-07	0.292924002	0.263	0.114	0.007924277	C9_CD4_Tem
chr11-35145261-35146974	1.21E-07	0.26446563	0.447	0.233	0.007987581	C9_CD4_Tem
chr3-30654957-30655669	1.21E-07	0.288849675	0.24	0.108	0.008045143	C9_CD4_Tem
chr2-157420470-157421307	1.23E-07	0.294465994	0.27	0.121	0.00811716	C9_CD4_Tem
chr13-99655313-99657122	1.23E-07	0.273911997	0.335	0.17	0.008137458	C9_CD4_Tem
chr6-16730974-16732056	1.24E-07	0.296105348	0.258	0.112	0.008232441	C9_CD4_Tem
chr8-125434077-125434904	1.25E-07	0.287170542	0.263	0.117	0.008306475	C9_CD4_Tem

chr2-112793419-112794456	1.26E-07	0.253089073	0.07	0.015	0.008353837	C9_CD4_Tem
chr17-56786778-56787637	1.26E-07	0.287305267	0.151	0.053	0.00835978	C9_CD4_Tem
chr10-31745239-31746004	1.27E-07	0.290279701	0.218	0.087	0.008418985	C9_CD4_Tem
chr5-113020400-113021134	1.29E-07	0.274845042	0.097	0.027	0.008539877	C9_CD4_Tem
chr6-3176570-3177079	1.29E-07	0.274561808	0.094	0.025	0.00855424	C9_CD4_Tem
chr2-148643442-148643734	1.31E-07	0.280318661	0.119	0.037	0.008693354	C9_CD4_Tem
chr3-49387556-49388870	1.32E-07	0.290211377	0.137	0.044	0.008734751	C9_CD4_Tem
chr11-85827342-85828528	1.34E-07	0.295740095	0.285	0.13	0.008896015	C9_CD4_Tem
chr3-196726859-196727622	1.37E-07	0.293338533	0.133	0.044	0.009082025	C9_CD4_Tem
chr4-48699522-48700370	1.40E-07	0.286754824	0.207	0.087	0.009258695	C9_CD4_Tem
chr7-74061755-74062491	1.40E-07	0.28158176	0.283	0.133	0.009300163	C9_CD4_Tem
chr15-85639473-85640339	1.41E-07	0.270609216	0.076	0.018	0.009317128	C9_CD4_Tem
chr11-35235410-35236404	1.42E-07	0.286773998	0.27	0.121	0.009413363	C9_CD4_Tem
chr18-9116709-9117674	1.42E-07	0.285751935	0.375	0.186	0.009429239	C9_CD4_Tem
chr9-34185718-34186610	1.47E-07	0.305171422	0.256	0.107	0.009727462	C9_CD4_Tem
chr20-45884838-45885282	1.50E-07	0.284021473	0.261	0.112	0.009938424	C9_CD4_Tem
chr7-155763550-155764748	1.50E-07	0.291771802	0.213	0.086	0.009957218	C9_CD4_Tem
chr19-45614183-45614593	1.51E-07	0.273186618	0.092	0.025	0.009999389	C9_CD4_Tem
chr5-55977602-55978470	1.53E-07	0.294851416	0.173	0.067	0.010113826	C9_CD4_Tem
chr19-19605807-19606451	1.55E-07	0.267898391	0.387	0.206	0.010290766	C9_CD4_Tem
chr16-89483955-89484408	1.56E-07	0.29731486	0.169	0.067	0.010334306	C9_CD4_Tem
chr13-110676140-110677459	1.58E-07	0.287959804	0.142	0.046	0.010444268	C9_CD4_Tem
chr5-139414470-139415185	1.59E-07	0.257319595	0.067	0.013	0.010507705	C9_CD4_Tem
chr15-38612185-38612473	1.60E-07	0.255307177	0.065	0.012	0.010614988	C9_CD4_Tem
chr17-63598167-63598932	1.61E-07	0.29632111	0.227	0.095	0.010689407	C9_CD4_Tem
chr7-116859349-116859607	1.69E-07	0.269642286	0.076	0.015	0.011168122	C9_CD4_Tem
chr8-65750497-65751081	1.71E-07	0.274967387	0.09	0.022	0.011294398	C9_CD4_Tem

chr4-152680832-152681334	1.71E-07	0.286896518	0.126	0.042	0.011317591	C9_CD4_Tem
chr9-71766521-71766911	1.73E-07	0.295738929	0.178	0.065	0.011467126	C9_CD4_Tem
chr1-36299893-36300822	1.75E-07	0.283375195	0.142	0.05	0.011565565	C9_CD4_Tem
chr1-1219328-1219665	1.75E-07	0.273554909	0.079	0.016	0.011608237	C9_CD4_Tem
chr8-27413150-27413875	1.77E-07	0.301203303	0.193	0.077	0.011693762	C9_CD4_Tem
chr4-6898371-6899885	1.77E-07	0.28710539	0.216	0.089	0.011744124	C9_CD4_Tem
chr1-147617887-147618539	1.81E-07	0.274807146	0.097	0.025	0.011954988	C9_CD4_Tem
chr2-54046879-54047411	1.82E-07	0.280886145	0.119	0.039	0.01202378	C9_CD4_Tem
chr7-130893397-130893884	1.84E-07	0.298748963	0.117	0.035	0.012178347	C9_CD4_Tem
chr3-71777921-71778665	1.84E-07	0.252160701	0.065	0.013	0.012215869	C9_CD4_Tem
chr17-39621913-39622674	1.86E-07	0.266033667	0.38	0.2	0.012299688	C9_CD4_Tem
chr2-69774368-69775424	1.90E-07	0.275667457	0.173	0.066	0.012589825	C9_CD4_Tem
chr6-45895170-45895980	1.91E-07	0.277693241	0.209	0.088	0.012674742	C9_CD4_Tem
chr13-40764517-40765143	1.94E-07	0.28698554	0.146	0.05	0.012833894	C9_CD4_Tem
chr2-64034991-64035814	1.95E-07	0.28651621	0.124	0.038	0.012912178	C9_CD4_Tem
chr6-25014869-25015545	2.00E-07	0.287214748	0.133	0.043	0.01325969	C9_CD4_Tem
chr2-10653525-10654388	2.02E-07	0.292016172	0.261	0.117	0.013407335	C9_CD4_Tem
chr20-32737294-32737999	2.08E-07	0.293303029	0.249	0.11	0.013745061	C9_CD4_Tem
chr6-87742046-87742517	2.16E-07	0.279751602	0.211	0.087	0.014319063	C9_CD4_Tem
chr8-126505903-126506723	2.18E-07	0.279374763	0.128	0.045	0.014432702	C9_CD4_Tem
chr10-43140168-43140773	2.21E-07	0.290941978	0.279	0.119	0.014614752	C9_CD4_Tem
chr10-131903651-131904012	2.23E-07	0.297061859	0.171	0.063	0.014766477	C9_CD4_Tem
chr6-45495574-45496208	2.23E-07	0.260677829	0.079	0.019	0.014769892	C9_CD4_Tem
chr3-187170099-187170441	2.24E-07	0.25135252	0.056	0.009	0.014825739	C9_CD4_Tem
chr3-39146862-39147406	2.25E-07	0.283297186	0.267	0.115	0.014912027	C9_CD4_Tem
chr17-42342317-42343172	2.28E-07	0.28532589	0.202	0.084	0.015105769	C9_CD4_Tem
chr7-17304802-17305626	2.30E-07	0.297675705	0.243	0.1	0.015232666	C9_CD4_Tem

chr11-61023326-61023918	2.32E-07	0.289670542	0.191	0.076	0.01534599	C9_CD4_Tem
chr8-133249155-133250018	2.32E-07	0.271375922	0.085	0.022	0.015364733	C9_CD4_Tem
chr15-90157888-90158436	2.37E-07	0.289057965	0.112	0.035	0.01571149	C9_CD4_Tem
chr3-152309736-152310333	2.38E-07	0.286465313	0.24	0.104	0.015783412	C9_CD4_Tem
chr3-5194413-5195095	2.42E-07	0.286569589	0.301	0.135	0.016034754	C9_CD4_Tem
chr6-14270772-14271426	2.47E-07	0.264107945	0.092	0.026	0.016341885	C9_CD4_Tem
chr14-100063346-100063812	2.47E-07	0.291161976	0.234	0.104	0.01635107	C9_CD4_Tem
chr17-35072276-35073745	2.48E-07	0.299636519	0.238	0.096	0.016429869	C9_CD4_Tem
chr4-4032292-4033521	2.50E-07	0.272840785	0.115	0.036	0.016554717	C9_CD4_Tem
chr7-130971297-130971671	2.52E-07	0.293272188	0.207	0.085	0.016690486	C9_CD4_Tem
chr9-2055311-2055618	2.52E-07	0.259258343	0.067	0.012	0.016698355	C9_CD4_Tem
chr10-61987577-61988287	2.52E-07	0.290773805	0.169	0.061	0.016706747	C9_CD4_Tem
chr14-61562357-61563185	2.53E-07	0.296390695	0.184	0.065	0.016734328	C9_CD4_Tem
chr1-89611737-89612000	2.57E-07	0.290729633	0.175	0.067	0.016997816	C9_CD4_Tem
chr22-42283450-42284200	2.57E-07	0.28506121	0.126	0.044	0.017002635	C9_CD4_Tem
chr17-65018284-65018501	2.61E-07	0.251016339	0.07	0.015	0.017257185	C9_CD4_Tem
chr13-40096304-40096697	2.64E-07	0.277357139	0.081	0.019	0.017467723	C9_CD4_Tem
chr4-8414149-8414744	2.69E-07	0.299694082	0.16	0.05	0.017843883	C9_CD4_Tem
chr5-67221349-67222682	2.73E-07	0.281175929	0.182	0.073	0.018066684	C9_CD4_Tem
chr18-7950517-7951113	2.73E-07	0.274581736	0.142	0.05	0.018111553	C9_CD4_Tem
chr1-51569246-51570089	2.74E-07	0.29322615	0.173	0.063	0.018121176	C9_CD4_Tem
chr6-36911727-36913108	2.75E-07	0.280657796	0.258	0.12	0.018208625	C9_CD4_Tem
chr15-41150491-41151266	2.78E-07	0.278340761	0.106	0.03	0.018415609	C9_CD4_Tem
chr12-121222836-121223913	2.86E-07	0.2806847	0.121	0.041	0.018914832	C9_CD4_Tem
chr9-130034586-130034825	2.90E-07	0.305108248	0.166	0.062	0.01918812	C9_CD4_Tem
chr21-45550634-45551673	2.90E-07	0.301132606	0.18	0.068	0.019222837	C9_CD4_Tem
chr12-89225012-89226115	2.95E-07	0.267127948	0.285	0.136	0.019535132	C9_CD4_Tem

chr20-51636026-51636360	3.01E-07	0.261519762	0.074	0.016	0.019901922	C9_CD4_Tem
chr7-43572253-43572850	3.03E-07	0.256171437	0.088	0.026	0.020032949	C9_CD4_Tem
chr2-234289001-234289361	3.06E-07	0.291752702	0.171	0.066	0.020284552	C9_CD4_Tem
chr7-129493631-129494415	3.06E-07	0.269187821	0.108	0.032	0.020293234	C9_CD4_Tem
chr1-39394385-39394975	3.11E-07	0.282644328	0.236	0.1	0.0206048	C9_CD4_Tem
chr9-97904676-97905518	3.11E-07	0.297424721	0.231	0.093	0.020619781	C9_CD4_Tem
chr15-52331309-52332079	3.11E-07	0.284025931	0.178	0.07	0.020627073	C9_CD4_Tem
chr11-123429883-123431909	3.14E-07	0.283664001	0.274	0.125	0.020798693	C9_CD4_Tem
chr3-186589739-186590295	3.17E-07	0.27682697	0.108	0.031	0.021017686	C9_CD4_Tem
chr16-30472159-30472861	3.17E-07	0.254333581	0.463	0.242	0.021023226	C9_CD4_Tem
chr4-169659466-169660333	3.19E-07	0.26911904	0.267	0.127	0.021134878	C9_CD4_Tem
chr17-65181164-65181535	3.28E-07	0.276480168	0.103	0.03	0.021728754	C9_CD4_Tem
chr9-98007185-98007810	3.32E-07	0.278880883	0.103	0.029	0.021972165	C9_CD4_Tem
chr7-99379134-99379501	3.36E-07	0.286130975	0.252	0.112	0.022225025	C9_CD4_Tem
chr5-87408268-87408482	3.37E-07	0.267228648	0.099	0.03	0.022341921	C9_CD4_Tem
chr9-135468706-135469230	3.49E-07	0.250752658	0.067	0.016	0.023094269	C9_CD4_Tem
chr6-155049980-155050788	3.51E-07	0.283068395	0.202	0.084	0.023211465	C9_CD4_Tem
chr8-22672984-22673623	3.57E-07	0.294770953	0.187	0.074	0.023620804	C9_CD4_Tem
chr17-48510904-48511570	3.65E-07	0.28806857	0.245	0.112	0.024152587	C9_CD4_Tem
chr16-47141707-47142043	3.69E-07	0.287408514	0.213	0.084	0.024423037	C9_CD4_Tem
chr10-110522365-110522858	3.79E-07	0.26925595	0.085	0.021	0.025083351	C9_CD4_Tem
chr10-14573622-14573907	3.79E-07	0.280031401	0.092	0.022	0.025113357	C9_CD4_Tem
chr1-151944505-151944754	3.83E-07	0.261277911	0.085	0.023	0.025330716	C9_CD4_Tem
chr6-157833493-157834396	3.83E-07	0.276315604	0.106	0.032	0.025391237	C9_CD4_Tem
chr4-140095635-140096748	3.85E-07	0.26359492	0.317	0.156	0.025504608	C9_CD4_Tem
chr9-125521473-125522208	3.85E-07	0.278648515	0.153	0.055	0.025504612	C9_CD4_Tem
chr17-37089413-37090520	3.87E-07	0.268851863	0.366	0.192	0.02560194	C9_CD4_Tem

chr10-21740597-21741353	3.88E-07	0.276614762	0.162	0.064	0.025700083	C9_CD4_Tem
chr11-61082734-61084076	3.88E-07	0.280401591	0.252	0.115	0.025712674	C9_CD4_Tem
chr12-19125269-19125749	3.95E-07	0.269278688	0.085	0.021	0.026172755	C9_CD4_Tem
chr5-50595175-50595563	4.12E-07	0.286749007	0.135	0.049	0.027312844	C9_CD4_Tem
chr11-86292869-86293630	4.15E-07	0.281475172	0.126	0.043	0.02746295	C9_CD4_Tem
chr2-65300567-65301530	4.18E-07	0.27284007	0.312	0.147	0.027685794	C9_CD4_Tem
chr6-53306769-53307370	4.21E-07	0.292707634	0.238	0.1	0.027902379	C9_CD4_Tem
chr8-96327643-96328320	4.22E-07	0.28222017	0.133	0.043	0.027974873	C9_CD4_Tem
chr21-28831720-28832658	4.22E-07	0.282094802	0.2	0.082	0.027978126	C9_CD4_Tem
chr22-23216134-23216876	4.24E-07	0.283110235	0.139	0.048	0.028063838	C9_CD4_Tem
chr4-147781981-147782581	4.30E-07	0.256614466	0.079	0.019	0.028461714	C9_CD4_Tem
chr17-78338852-78339134	4.34E-07	0.265504491	0.088	0.023	0.028713776	C9_CD4_Tem
chr3-179608866-179609590	4.40E-07	0.266529504	0.11	0.034	0.029105226	C9_CD4_Tem
chr5-132103195-132104109	4.43E-07	0.256828165	0.407	0.219	0.029314586	C9_CD4_Tem
chr5-157607280-157608223	4.48E-07	0.283281444	0.279	0.125	0.029671165	C9_CD4_Tem
chr16-11253334-11253830	4.53E-07	0.290318162	0.276	0.119	0.030010468	C9_CD4_Tem
chr12-24838485-24839770	4.57E-07	0.250059443	0.182	0.082	0.030269008	C9_CD4_Tem
chr8-8917273-8918050	4.62E-07	0.284887423	0.292	0.133	0.030597306	C9_CD4_Tem
chr1-224623198-224624417	4.63E-07	0.25098029	0.4	0.206	0.030659656	C9_CD4_Tem
chr1-206577831-206578616	4.69E-07	0.254898094	0.411	0.223	0.031028443	C9_CD4_Tem
chr19-1415259-1415748	4.71E-07	0.273416478	0.108	0.032	0.031178341	C9_CD4_Tem
chr8-19474844-19475909	4.76E-07	0.252797415	0.362	0.181	0.031549898	C9_CD4_Tem
chr15-101167659-101168648	4.84E-07	0.282991876	0.126	0.04	0.032044162	C9_CD4_Tem
chr4-80070059-80070499	4.92E-07	0.298691542	0.139	0.042	0.032609138	C9_CD4_Tem
chr1-169363114-169363632	4.96E-07	0.277355525	0.202	0.084	0.032870345	C9_CD4_Tem
chr8-81153794-81154520	5.03E-07	0.282062339	0.184	0.073	0.033278199	C9_CD4_Tem
chr3-69197577-69198622	5.05E-07	0.256519364	0.382	0.199	0.033415781	C9_CD4_Tem

chr2-232781689-232782204	5.09E-07	0.274016936	0.142	0.051	0.033733953	C9_CD4_Tem
chr19-14353243-14353746	5.11E-07	0.281119381	0.218	0.091	0.033869419	C9_CD4_Tem
chr3-52670349-52671954	5.13E-07	0.298390613	0.211	0.083	0.033953384	C9_CD4_Tem
chr1-66436324-66436969	5.16E-07	0.27558973	0.182	0.076	0.034139049	C9_CD4_Tem
chr2-33263-33967	5.21E-07	0.280238134	0.187	0.075	0.034509548	C9_CD4_Tem
chr21-14581882-14582319	5.28E-07	0.275540597	0.108	0.033	0.034949147	C9_CD4_Tem
chr7-104956630-104957892	5.29E-07	0.280753375	0.218	0.099	0.035022335	C9_CD4_Tem
chr7-130903464-130904489	5.30E-07	0.289997671	0.209	0.084	0.035086248	C9_CD4_Tem
chr17-58417073-58417900	5.31E-07	0.252627217	0.355	0.191	0.03515461	C9_CD4_Tem
chr13-40220055-40220672	5.45E-07	0.269290187	0.209	0.086	0.036121917	C9_CD4_Tem
chr10-22605124-22605429	5.53E-07	0.287076172	0.126	0.039	0.036589243	C9_CD4_Tem
chr17-42337333-42337808	5.58E-07	0.285440091	0.191	0.073	0.03692309	C9_CD4_Tem
chr1-183956039-183957784	5.58E-07	0.252117064	0.337	0.173	0.036947464	C9_CD4_Tem
chr10-3948547-3949242	5.63E-07	0.276953549	0.308	0.145	0.037251163	C9_CD4_Tem
chr8-19385482-19386169	5.78E-07	0.276048493	0.094	0.026	0.038302365	C9_CD4_Tem
chr17-64050814-64051614	5.91E-07	0.269701359	0.155	0.061	0.039114919	C9_CD4_Tem
chr5-95842841-95843512	5.91E-07	0.27106069	0.173	0.066	0.039167821	C9_CD4_Tem
chr6-24927192-24928577	6.01E-07	0.26939896	0.288	0.133	0.039782235	C9_CD4_Tem
chr4-184237232-184237948	6.02E-07	0.27744212	0.187	0.078	0.039836895	C9_CD4_Tem
chr8-1437996-1438827	6.02E-07	0.25473448	0.088	0.023	0.039897253	C9_CD4_Tem
chr9-112327721-112328295	6.07E-07	0.294672815	0.155	0.054	0.040174417	C9_CD4_Tem
chr2-134258210-134259049	6.13E-07	0.266825037	0.225	0.101	0.040626434	C9_CD4_Tem
chr1-244322678-244323335	6.20E-07	0.281487285	0.261	0.123	0.041079906	C9_CD4_Tem
chr14-68682803-68683115	6.24E-07	0.288817519	0.121	0.036	0.041298574	C9_CD4_Tem
chr11-128341316-128341610	6.36E-07	0.270807212	0.117	0.038	0.042150342	C9_CD4_Tem
chr10-74589097-74589651	6.49E-07	0.270151058	0.281	0.134	0.042961187	C9_CD4_Tem
chr10-6299787-6300557	6.51E-07	0.275160663	0.133	0.045	0.043132844	C9_CD4_Tem

chr16-78705687-78706198	6.55E-07	0.279824075	0.148	0.055	0.04335707	C9_CD4_Tem
chr1-154463170-154464019	6.56E-07	0.287075661	0.157	0.057	0.043445203	C9_CD4_Tem
chr12-9667395-9667772	6.71E-07	0.289610233	0.135	0.044	0.044406096	C9_CD4_Tem
chr17-40485997-40486440	6.72E-07	0.263900866	0.11	0.036	0.044498577	C9_CD4_Tem
chr10-62082312-62083179	6.76E-07	0.274842333	0.175	0.07	0.044781783	C9_CD4_Tem
chr5-39197571-39198357	6.80E-07	0.282335007	0.243	0.103	0.045039302	C9_CD4_Tem
chr3-192916860-192918360	6.88E-07	0.272707071	0.155	0.057	0.045574699	C9_CD4_Tem
chr22-21816219-21817513	6.92E-07	0.294071896	0.234	0.1	0.045835586	C9_CD4_Tem
chr3-128787317-128788663	7.03E-07	0.281496857	0.283	0.135	0.04655749	C9_CD4_Tem
chr6-118844873-118845384	7.08E-07	0.26267275	0.103	0.031	0.046863733	C9_CD4_Tem
chr17-78777947-78778211	7.18E-07	0.269451268	0.117	0.036	0.047569236	C9_CD4_Tem
chr4-73222516-73223284	7.38E-07	0.266324243	0.294	0.143	0.048902504	C9_CD4_Tem
chr3-153623879-153624925	7.43E-07	0.257010501	0.202	0.091	0.049236212	C9_CD4_Tem
chr5-32583684-32583894	7.44E-07	0.256213784	0.076	0.017	0.049244373	C9_CD4_Tem
chr12-49753673-49754131	7.54E-07	0.273020633	0.124	0.042	0.049955572	C9_CD4_Tem
chr20-51388264-51388514	7.55E-07	0.251930876	0.067	0.014	0.049975249	C9_CD4_Tem
chr15-41940247-41940845	7.56E-07	0.274242189	0.225	0.093	0.050046414	C9_CD4_Tem
chr1-206784128-206784848	7.57E-07	0.281423173	0.187	0.075	0.05012789	C9_CD4_Tem
chr3-172517529-172518264	7.69E-07	0.282111524	0.18	0.073	0.0509082	C9_CD4_Tem
chr14-75562018-75562740	7.71E-07	0.270383922	0.153	0.058	0.05103636	C9_CD4_Tem
chr19-36132471-36132828	7.86E-07	0.276411105	0.121	0.042	0.052081805	C9_CD4_Tem
chr19-51272220-51273178	7.99E-07	0.282373832	0.139	0.048	0.052935923	C9_CD4_Tem
chr9-91130813-91131935	8.08E-07	0.275551935	0.276	0.129	0.05350413	C9_CD4_Tem
chr4-86939722-86939984	8.19E-07	0.260091619	0.092	0.029	0.054231073	C9_CD4_Tem
chr15-70196426-70197229	8.19E-07	0.269215292	0.117	0.038	0.054257577	C9_CD4_Tem
chr17-47763831-47764281	8.45E-07	0.279573725	0.103	0.028	0.055962266	C9_CD4_Tem
chr19-19614804-19615420	8.55E-07	0.262284103	0.382	0.194	0.056620917	C9_CD4_Tem

chr7-73715641-73715883	8.56E-07	0.250359145	0.07	0.016	0.056710134	C9_CD4_Tem
chr20-62623349-62624245	8.83E-07	0.276990995	0.281	0.128	0.058473079	C9_CD4_Tem
chr4-39031693-39033080	9.11E-07	0.266279446	0.391	0.197	0.060306186	C9_CD4_Tem
chr14-93962689-93963638	9.22E-07	0.258717127	0.389	0.201	0.061060553	C9_CD4_Tem
chr4-80062227-80062862	9.28E-07	0.271553913	0.094	0.028	0.061468753	C9_CD4_Tem
chr3-71010494-71011430	9.46E-07	0.279588443	0.263	0.118	0.062666709	C9_CD4_Tem
chr21-42192942-42193602	9.53E-07	0.286597198	0.209	0.088	0.063107028	C9_CD4_Tem
chr1-174204583-174205261	9.58E-07	0.279515402	0.184	0.076	0.063417656	C9_CD4_Tem
chr19-35239863-35240702	9.67E-07	0.276031238	0.155	0.06	0.064058547	C9_CD4_Tem
chr22-21876376-21877100	9.83E-07	0.293480677	0.144	0.047	0.065086221	C9_CD4_Tem
chr17-29138271-29138885	9.84E-07	0.279494734	0.274	0.128	0.065182109	C9_CD4_Tem
chr16-31880219-31880789	9.86E-07	0.291183405	0.193	0.081	0.065274787	C9_CD4_Tem
chr4-80077595-80078076	9.86E-07	0.280356278	0.218	0.095	0.065303798	C9_CD4_Tem
chr8-29523882-29524582	9.87E-07	0.278178932	0.173	0.067	0.065360673	C9_CD4_Tem
chr5-154214266-154214853	9.91E-07	0.270273377	0.106	0.031	0.065604074	C9_CD4_Tem
chr14-22566788-22567272	9.97E-07	0.288476052	0.24	0.104	0.066003572	C9_CD4_Tem
chr5-53069561-53070033	1.00E-06	0.256104586	0.09	0.025	0.06639585	C9_CD4_Tem
chr16-81716537-81716764	1.00E-06	0.271804722	0.115	0.036	0.066445371	C9_CD4_Tem
chr17-65173018-65173919	1.02E-06	0.264942505	0.272	0.128	0.067760445	C9_CD4_Tem
chr8-11860631-11861557	1.03E-06	0.260412605	0.074	0.016	0.068017955	C9_CD4_Tem
chr8-94959899-94960988	1.03E-06	0.278659043	0.148	0.058	0.068208598	C9_CD4_Tem
chr1-9294240-9295204	1.03E-06	0.294827292	0.182	0.071	0.068250778	C9_CD4_Tem
chr19-45615747-45616401	1.04E-06	0.266341556	0.216	0.092	0.069091186	C9_CD4_Tem
chr3-32963641-32964287	1.05E-06	0.264364896	0.142	0.052	0.069313047	C9_CD4_Tem
chr12-11998194-11999166	1.05E-06	0.274929518	0.216	0.095	0.069416388	C9_CD4_Tem
chr9-113214785-113215504	1.05E-06	0.280700597	0.171	0.063	0.069799535	C9_CD4_Tem
chr8-70172538-70173138	1.07E-06	0.266390959	0.144	0.056	0.070598428	C9_CD4_Tem

chr16-17364067-17364670	1.07E-06	0.264862018	0.312	0.156	0.070876879	C9_CD4_Tem
chr13-75325753-75327029	1.07E-06	0.253216382	0.312	0.16	0.071141601	C9_CD4_Tem
chr14-99243342-99243725	1.08E-06	0.281865252	0.22	0.091	0.07123542	C9_CD4_Tem
chr17-59753121-59753773	1.08E-06	0.285929268	0.151	0.054	0.071818101	C9_CD4_Tem
chr3-186882781-186883279	1.09E-06	0.256954208	0.106	0.032	0.072003159	C9_CD4_Tem
chr10-124693764-124694151	1.09E-06	0.274313467	0.137	0.048	0.072149606	C9_CD4_Tem
chr16-22357153-22358391	1.09E-06	0.271067729	0.2	0.091	0.072330102	C9_CD4_Tem
chr2-223881805-223882274	1.09E-06	0.265634839	0.108	0.035	0.072406737	C9_CD4_Tem
chr1-25329501-25330439	1.09E-06	0.284978747	0.18	0.071	0.072476815	C9_CD4_Tem
chr1-206744678-206745677	1.11E-06	0.266504781	0.153	0.061	0.073746784	C9_CD4_Tem
chr12-13179234-13180371	1.12E-06	0.281401491	0.137	0.046	0.074363466	C9_CD4_Tem
chr14-21229166-21230205	1.13E-06	0.262358866	0.346	0.169	0.07454944	C9_CD4_Tem
chr5-35816593-35817551	1.16E-06	0.272778266	0.256	0.123	0.076907761	C9_CD4_Tem
chr3-141552366-141553463	1.17E-06	0.277108521	0.234	0.105	0.077418578	C9_CD4_Tem
chr16-67055628-67056025	1.18E-06	0.282242614	0.126	0.039	0.078147151	C9_CD4_Tem
chrY-7326223-7327226	1.20E-06	0.27195331	0.153	0.058	0.079700792	C9_CD4_Tem
chr13-79457938-79458328	1.24E-06	0.27085901	0.112	0.033	0.082444258	C9_CD4_Tem
chr12-11958447-11959085	1.25E-06	0.250400949	0.083	0.023	0.082700756	C9_CD4_Tem
chr8-27613948-27614927	1.27E-06	0.266706627	0.265	0.121	0.084371033	C9_CD4_Tem
chr4-142264840-142265886	1.28E-06	0.273317087	0.112	0.035	0.084799485	C9_CD4_Tem
chr4-37954100-37954950	1.31E-06	0.266480853	0.137	0.048	0.086968751	C9_CD4_Tem
chr15-38688191-38689191	1.32E-06	0.26211525	0.324	0.158	0.087272098	C9_CD4_Tem
chr9-70563210-70564249	1.32E-06	0.270999811	0.151	0.059	0.087565893	C9_CD4_Tem
chr5-40390114-40390694	1.33E-06	0.278871555	0.162	0.059	0.088150571	C9_CD4_Tem
chr11-61111463-61112104	1.34E-06	0.272974559	0.18	0.079	0.08869345	C9_CD4_Tem
chr14-75535827-75536163	1.34E-06	0.258395307	0.076	0.019	0.088921804	C9_CD4_Tem
chr2-197209470-197210109	1.35E-06	0.281766473	0.196	0.083	0.089349089	C9_CD4_Tem

chr15-60392600-60392941	1.37E-06	0.289583804	0.16	0.059	0.090818646	C9_CD4_Tem
chr8-66490381-66491367	1.38E-06	0.275420885	0.27	0.126	0.091142888	C9_CD4_Tem
chr2-96485433-96486528	1.39E-06	0.266696862	0.335	0.174	0.09200078	C9_CD4_Tem
chr1-203671103-203671813	1.42E-06	0.272818419	0.128	0.043	0.093737866	C9_CD4_Tem
chr1-89719232-89719906	1.43E-06	0.269455377	0.209	0.093	0.094784531	C9_CD4_Tem
chr4-122503102-122503615	1.45E-06	0.250950757	0.09	0.025	0.09589765	C9_CD4_Tem
chr7-148772347-148773656	1.47E-06	0.275563792	0.189	0.074	0.09742671	C9_CD4_Tem
chr2-134118251-134118637	1.49E-06	0.289715912	0.211	0.087	0.098665776	C9_CD4_Tem
chr3-39260897-39261269	1.50E-06	0.266421721	0.117	0.039	0.099193614	C9_CD4_Tem
chr21-34967151-34968026	1.50E-06	0.281514635	0.211	0.083	0.09931001	C9_CD4_Tem
chr11-11171332-11172232	1.51E-06	0.264169962	0.18	0.07	0.099823695	C9_CD4_Tem
chr1-23103217-23103987	1.56E-06	0.277949604	0.265	0.117	0.103241622	C9_CD4_Tem
chr3-49602386-49603513	1.57E-06	0.278151493	0.191	0.08	0.103762245	C9_CD4_Tem
chr9-131500277-131501370	1.57E-06	0.260381109	0.366	0.186	0.10409867	C9_CD4_Tem
chrX-150482602-150483132	1.60E-06	0.251090478	0.092	0.025	0.106111161	C9_CD4_Tem
chr12-22395794-22396753	1.62E-06	0.259025345	0.285	0.134	0.10712443	C9_CD4_Tem
chr8-73990970-73992293	1.63E-06	0.263405834	0.115	0.036	0.107747479	C9_CD4_Tem
chr7-139100111-139100618	1.63E-06	0.282757037	0.209	0.085	0.108251138	C9_CD4_Tem
chr1-192530221-192531427	1.64E-06	0.261671046	0.276	0.14	0.108868447	C9_CD4_Tem
chr1-151941019-151941717	1.65E-06	0.267118863	0.247	0.111	0.109414827	C9_CD4_Tem
chr10-4130705-4132460	1.66E-06	0.270560828	0.288	0.136	0.109625505	C9_CD4_Tem
chr1-184882186-184883369	1.68E-06	0.269908493	0.182	0.076	0.111433286	C9_CD4_Tem
chr7-831634-832970	1.74E-06	0.269713958	0.306	0.144	0.115168364	C9_CD4_Tem
chr11-61004107-61005047	1.74E-06	0.251986584	0.079	0.021	0.115274393	C9_CD4_Tem
chr12-122412036-122413101	1.75E-06	0.283781706	0.128	0.041	0.115594533	C9_CD4_Tem
chr5-148620035-148621076	1.76E-06	0.259351114	0.117	0.039	0.116811715	C9_CD4_Tem
chr11-75536814-75538379	1.77E-06	0.281887543	0.227	0.096	0.117514013	C9_CD4_Tem

chr2-28594125-28595132	1.78E-06	0.294309576	0.182	0.071	0.118186687	C9_CD4_Tem
chr16-4957618-4958544	1.79E-06	0.255991598	0.337	0.168	0.118339771	C9_CD4_Tem
chr3-70989892-70991043	1.80E-06	0.262592896	0.112	0.036	0.119415175	C9_CD4_Tem
chr9-91099852-91100585	1.81E-06	0.256771179	0.364	0.182	0.119702551	C9_CD4_Tem
chr10-33005247-33005873	1.81E-06	0.269343678	0.124	0.044	0.119915862	C9_CD4_Tem
chr12-75722297-75723124	1.85E-06	0.266847509	0.146	0.054	0.122728373	C9_CD4_Tem
chr18-2658205-2658405	1.87E-06	0.275042807	0.135	0.048	0.123750103	C9_CD4_Tem
chr5-68274891-68275719	1.88E-06	0.271881816	0.281	0.133	0.12417845	C9_CD4_Tem
chr18-2998379-2999332	1.88E-06	0.251237545	0.094	0.028	0.124570982	C9_CD4_Tem
chr12-89019257-89019904	1.88E-06	0.277389411	0.162	0.061	0.124770763	C9_CD4_Tem
chr2-181305089-181306012	1.90E-06	0.259717609	0.299	0.151	0.125771822	C9_CD4_Tem
chr8-128542261-128542573	1.90E-06	0.267920953	0.121	0.039	0.125799546	C9_CD4_Tem
chr11-60987884-60988177	1.95E-06	0.271307085	0.11	0.035	0.129396612	C9_CD4_Tem
chr12-54411288-54411888	2.01E-06	0.27045168	0.187	0.079	0.132979904	C9_CD4_Tem
chr8-70141124-70142656	2.01E-06	0.262895216	0.319	0.159	0.133084018	C9_CD4_Tem
chr2-203869230-203870143	2.01E-06	0.267071936	0.216	0.098	0.133305953	C9_CD4_Tem
chrX-45803577-45803896	2.03E-06	0.272978751	0.106	0.032	0.134208695	C9_CD4_Tem
chr10-119314972-119315950	2.12E-06	0.258966982	0.292	0.141	0.140080792	C9_CD4_Tem
chr13-112688269-112690441	2.20E-06	0.250511092	0.36	0.186	0.145708845	C9_CD4_Tem
chr5-126758220-126759124	2.22E-06	0.261814293	0.169	0.069	0.146711664	C9_CD4_Tem
chr19-46828234-46828908	2.24E-06	0.251312856	0.09	0.026	0.148328339	C9_CD4_Tem
chr4-173332142-173332488	2.25E-06	0.254217631	0.092	0.027	0.148766086	C9_CD4_Tem
chr1-175174425-175175182	2.27E-06	0.257587952	0.117	0.041	0.150103988	C9_CD4_Tem
chr3-152193188-152194443	2.28E-06	0.259479998	0.324	0.163	0.150991335	C9_CD4_Tem
chr9-91150618-91151572	2.30E-06	0.250133723	0.344	0.178	0.152591831	C9_CD4_Tem
chr9-63337864-63338561	2.31E-06	0.253972251	0.088	0.024	0.15311863	C9_CD4_Tem
chr15-60408269-60408889	2.35E-06	0.273090494	0.178	0.071	0.155579514	C9_CD4_Tem

chr2-181467216-181467466	2.38E-06	0.263165219	0.117	0.044	0.157287778	C9_CD4_Tem
chr5-39200711-39201247	2.43E-06	0.264738142	0.133	0.047	0.161123874	C9_CD4_Tem
chr22-20323896-20324434	2.43E-06	0.272242571	0.137	0.05	0.161174152	C9_CD4_Tem
chr10-3761343-3761619	2.43E-06	0.2719197	0.115	0.035	0.161250002	C9_CD4_Tem
chr22-23695863-23696752	2.45E-06	0.263291825	0.297	0.149	0.162074964	C9_CD4_Tem
chr5-111227243-111227505	2.46E-06	0.274998828	0.157	0.058	0.162958756	C9_CD4_Tem
chr17-80254523-80255114	2.48E-06	0.270305433	0.267	0.128	0.163926047	C9_CD4_Tem
chr15-85792817-85793425	2.48E-06	0.276688563	0.13	0.044	0.164261085	C9_CD4_Tem
chr9-37528506-37529518	2.49E-06	0.261906531	0.222	0.106	0.164667007	C9_CD4_Tem
chr1-160640338-160640727	2.51E-06	0.271786238	0.211	0.091	0.166503375	C9_CD4_Tem
chr11-96155887-96157525	2.52E-06	0.272993698	0.198	0.083	0.166885972	C9_CD4_Tem
chr2-96806412-96807178	2.53E-06	0.263500451	0.157	0.062	0.167618548	C9_CD4_Tem
chr12-4287509-4288226	2.54E-06	0.265207502	0.099	0.029	0.168478389	C9_CD4_Tem
chr22-36781643-36782408	2.54E-06	0.258923763	0.308	0.156	0.168530523	C9_CD4_Tem
chr8-29680422-29681269	2.56E-06	0.255551714	0.108	0.035	0.169713555	C9_CD4_Tem
chr10-110221594-110222149	2.57E-06	0.253507564	0.36	0.18	0.170284342	C9_CD4_Tem
chr15-72554358-72555267	2.64E-06	0.253266024	0.103	0.034	0.174718459	C9_CD4_Tem
chr17-40612576-40613325	2.67E-06	0.259992326	0.33	0.166	0.17650686	C9_CD4_Tem
chr1-67193741-67195192	2.70E-06	0.259713335	0.254	0.128	0.178753917	C9_CD4_Tem
chr3-32960387-32961529	2.75E-06	0.266760499	0.115	0.036	0.181958834	C9_CD4_Tem
chr3-71503466-71504603	2.76E-06	0.267927795	0.128	0.046	0.183089226	C9_CD4_Tem
chr6-136787946-136788793	2.77E-06	0.261954004	0.308	0.154	0.183515595	C9_CD4_Tem
chr1-19390601-19391894	2.79E-06	0.27122666	0.283	0.136	0.184857164	C9_CD4_Tem
chr10-62433097-62433653	2.84E-06	0.256581655	0.101	0.029	0.188288341	C9_CD4_Tem
chr6-37239740-37240569	2.89E-06	0.270067906	0.218	0.087	0.191380552	C9_CD4_Tem
chr3-33054325-33055588	2.91E-06	0.267437806	0.173	0.07	0.192712492	C9_CD4_Tem
chr12-96237370-96238109	2.93E-06	0.271837386	0.18	0.071	0.193711488	C9_CD4_Tem

chr13-111176803-111177593	2.93E-06	0.254269811	0.119	0.04	0.194069842	C9_CD4_Tem
chr5-40428086-40428489	2.95E-06	0.285196704	0.164	0.06	0.19560266	C9_CD4_Tem
chr2-61762264-61762639	3.02E-06	0.255251623	0.092	0.028	0.199805656	C9_CD4_Tem
chr3-10241691-10242985	3.03E-06	0.259950865	0.281	0.138	0.20054682	C9_CD4_Tem
chr4-38523660-38524385	3.08E-06	0.257374418	0.166	0.069	0.203905966	C9_CD4_Tem
chr11-61026042-61026628	3.09E-06	0.266874456	0.153	0.056	0.204430207	C9_CD4_Tem
chr6-149031799-149033013	3.14E-06	0.257756252	0.22	0.102	0.208188837	C9_CD4_Tem
chr13-25971165-25971891	3.15E-06	0.260639918	0.173	0.07	0.208323792	C9_CD4_Tem
chr6-130027816-130028390	3.22E-06	0.262980416	0.155	0.062	0.213542887	C9_CD4_Tem
chr11-126284704-126285165	3.24E-06	0.272038382	0.222	0.099	0.214510277	C9_CD4_Tem
chr20-53899293-53900035	3.24E-06	0.273374236	0.216	0.092	0.214547907	C9_CD4_Tem
chr1-200889333-200889886	3.25E-06	0.276080303	0.157	0.062	0.215236204	C9_CD4_Tem
chr9-131651297-131651894	3.25E-06	0.250508256	0.081	0.021	0.215362875	C9_CD4_Tem
chr3-9354253-9354954	3.26E-06	0.252746373	0.11	0.037	0.216116371	C9_CD4_Tem
chr3-167691090-167691791	3.28E-06	0.258426561	0.178	0.073	0.217179297	C9_CD4_Tem
chr3-69204017-69204579	3.28E-06	0.253982882	0.106	0.033	0.217527342	C9_CD4_Tem
chr14-61335805-61336213	3.33E-06	0.253406338	0.285	0.138	0.220471586	C9_CD4_Tem
chr17-78753836-78754141	3.34E-06	0.266904674	0.106	0.032	0.221176825	C9_CD4_Tem
chr12-31748912-31749658	3.34E-06	0.26752302	0.267	0.124	0.221360659	C9_CD4_Tem
chr7-44046496-44046933	3.37E-06	0.258674995	0.13	0.048	0.223103662	C9_CD4_Tem
chr6-33692475-33693146	3.38E-06	0.275953222	0.178	0.07	0.223816018	C9_CD4_Tem
chr10-6239032-6239606	3.41E-06	0.2723277	0.191	0.081	0.225502163	C9_CD4_Tem
chr17-45311776-45312402	3.42E-06	0.265287903	0.326	0.162	0.226415676	C9_CD4_Tem
chr1-203606072-203606419	3.50E-06	0.261825818	0.115	0.037	0.231644209	C9_CD4_Tem
chr14-49631469-49631682	3.52E-06	0.273645733	0.135	0.049	0.233257618	C9_CD4_Tem
chr14-24274151-24274474	3.52E-06	0.257169239	0.101	0.03	0.233284421	C9_CD4_Tem
chr1-113878905-113879427	3.60E-06	0.280899391	0.133	0.044	0.238596274	C9_CD4_Tem

chr8-95099999-95101586	3.62E-06	0.267421157	0.13	0.046	0.240003195	C9_CD4_Tem
chr2-202266608-202267159	3.70E-06	0.267705466	0.22	0.102	0.245316969	C9_CD4_Tem
chr5-80125699-80126474	3.76E-06	0.256556852	0.126	0.046	0.249144062	C9_CD4_Tem
chr8-108242901-108243379	3.77E-06	0.261811556	0.178	0.069	0.249688286	C9_CD4_Tem
chr18-54263259-54264234	3.78E-06	0.260383177	0.126	0.045	0.250320442	C9_CD4_Tem
chr6-25049765-25050715	3.84E-06	0.27975095	0.254	0.115	0.254179973	C9_CD4_Tem
chr5-67215359-67216555	3.93E-06	0.260966884	0.173	0.075	0.260198717	C9_CD4_Tem
chr3-112773661-112774632	3.93E-06	0.263249209	0.202	0.083	0.260411887	C9_CD4_Tem
chr5-80246969-80247836	3.95E-06	0.270792138	0.139	0.047	0.261406677	C9_CD4_Tem
chr12-122946608-122947242	3.97E-06	0.262046017	0.184	0.078	0.263038331	C9_CD4_Tem
chr6-119315690-119316040	4.06E-06	0.253722107	0.126	0.047	0.268679057	C9_CD4_Tem
chr2-134288480-134289561	4.07E-06	0.2600005	0.173	0.074	0.269578692	C9_CD4_Tem
chr16-89440469-89441108	4.08E-06	0.281597165	0.13	0.041	0.27051997	C9_CD4_Tem
chr14-95518115-95518648	4.11E-06	0.26078225	0.112	0.037	0.272075237	C9_CD4_Tem
chr14-75124005-75124311	4.16E-06	0.256270506	0.081	0.022	0.275533491	C9_CD4_Tem
chr12-122898307-122898984	4.17E-06	0.264854035	0.27	0.128	0.276146961	C9_CD4_Tem
chr16-4700246-4700755	4.22E-06	0.265613655	0.155	0.061	0.279232969	C9_CD4_Tem
chr5-175480108-175480834	4.25E-06	0.273015829	0.178	0.072	0.281715569	C9_CD4_Tem
chr1-234978304-234979014	4.33E-06	0.271058333	0.204	0.085	0.286769051	C9_CD4_Tem
chr5-96537117-96537765	4.35E-06	0.257830828	0.213	0.095	0.287982424	C9_CD4_Tem
chr13-50244570-50245457	4.38E-06	0.269624561	0.218	0.092	0.289752789	C9_CD4_Tem
chr10-97648620-97649389	4.38E-06	0.25581736	0.218	0.098	0.290017371	C9_CD4_Tem
chr5-39187820-39188596	4.39E-06	0.260992088	0.276	0.131	0.291026622	C9_CD4_Tem
chr2-203729909-203730689	4.40E-06	0.26576249	0.148	0.057	0.291104489	C9_CD4_Tem
chr7-99375996-99376482	4.42E-06	0.273548061	0.22	0.098	0.293008227	C9_CD4_Tem
chr16-30458378-30459279	4.43E-06	0.261669446	0.27	0.132	0.293454523	C9_CD4_Tem
chr10-73956286-73957629	4.43E-06	0.26100253	0.166	0.068	0.293506077	C9_CD4_Tem

chr2-86583657-86584667	4.44E-06	0.256946361	0.119	0.039	0.294333323	C9_CD4_Tem
chr8-29339888-29340706	4.47E-06	0.269133929	0.169	0.07	0.296327238	C9_CD4_Tem
chr19-21028086-21028767	4.52E-06	0.2598046	0.155	0.065	0.299274053	C9_CD4_Tem
chr1-67162728-67163382	4.53E-06	0.256762484	0.148	0.055	0.300219637	C9_CD4_Tem
chr13-97257635-97258449	4.54E-06	0.269916439	0.162	0.065	0.300525055	C9_CD4_Tem
chr6-142838763-142839298	4.63E-06	0.262715451	0.207	0.09	0.306474218	C9_CD4_Tem
chr15-60572563-60572927	4.82E-06	0.253346475	0.085	0.023	0.319003126	C9_CD4_Tem
chr15-33104022-33104674	4.83E-06	0.268247331	0.148	0.053	0.319612683	C9_CD4_Tem
chr6-16836435-16837902	4.94E-06	0.256069037	0.108	0.036	0.327405208	C9_CD4_Tem
chr10-103854527-103855807	5.00E-06	0.256830969	0.094	0.024	0.330802168	C9_CD4_Tem
chr1-151898031-151899047	5.01E-06	0.266619203	0.178	0.076	0.33190049	C9_CD4_Tem
chr2-118309822-118310719	5.04E-06	0.252145588	0.119	0.045	0.333980568	C9_CD4_Tem
chr12-14255907-14256826	5.09E-06	0.255990676	0.276	0.135	0.336927336	C9_CD4_Tem
chr11-103035358-103036442	5.11E-06	0.260175001	0.189	0.08	0.338521052	C9_CD4_Tem
chr15-52463638-52464944	5.14E-06	0.25710394	0.157	0.063	0.340323586	C9_CD4_Tem
chr6-150491010-150491760	5.23E-06	0.2578327	0.139	0.054	0.346368285	C9_CD4_Tem
chr15-44632912-44633910	5.28E-06	0.266810042	0.162	0.062	0.349776939	C9_CD4_Tem
chr18-13135960-13136464	5.30E-06	0.258796567	0.106	0.034	0.351120036	C9_CD4_Tem
chr8-29229066-29230362	5.37E-06	0.265972121	0.234	0.106	0.355759841	C9_CD4_Tem
chr17-80649411-80650239	5.37E-06	0.254612139	0.094	0.027	0.355853406	C9_CD4_Tem
chr3-152204269-152204591	5.39E-06	0.251763786	0.083	0.023	0.357120686	C9_CD4_Tem
chr14-70980987-70981941	5.39E-06	0.255424558	0.124	0.045	0.357228618	C9_CD4_Tem
chr5-170312467-170313742	5.42E-06	0.258443782	0.272	0.133	0.358980951	C9_CD4_Tem
chr12-92134401-92135149	5.50E-06	0.26650402	0.294	0.14	0.364516446	C9_CD4_Tem
chr2-48420245-48421048	5.54E-06	0.260229814	0.294	0.151	0.367005835	C9_CD4_Tem
chr17-55237212-55239072	5.64E-06	0.258080089	0.249	0.112	0.373565811	C9_CD4_Tem
chr5-175652680-175653588	5.65E-06	0.268641373	0.151	0.055	0.374125981	C9_CD4_Tem

chr6-87718595-87719407	5.69E-06	0.262636718	0.166	0.064	0.37697696	C9_CD4_Tem
chr5-39176818-39178512	5.72E-06	0.252087223	0.362	0.194	0.378842941	C9_CD4_Tem
chr15-101641903-101642248	5.89E-06	0.255108778	0.106	0.033	0.389819713	C9_CD4_Tem
chrX-39196193-39197114	6.00E-06	0.256352614	0.13	0.045	0.397159101	C9_CD4_Tem
chr10-110013643-110014010	6.07E-06	0.257933047	0.117	0.038	0.402217317	C9_CD4_Tem
chr10-92594137-92594446	6.09E-06	0.256708898	0.101	0.032	0.403602133	C9_CD4_Tem
chr7-33053841-33054948	6.16E-06	0.269535221	0.216	0.095	0.40769478	C9_CD4_Tem
chr3-45963994-45964485	6.24E-06	0.26822128	0.16	0.059	0.413131278	C9_CD4_Tem
chr16-10738285-10739107	6.30E-06	0.264111594	0.207	0.09	0.416933615	C9_CD4_Tem
chr19-39398531-39398824	6.36E-06	0.272214494	0.187	0.08	0.421434068	C9_CD4_Tem
chr4-76989284-76990264	6.42E-06	0.259468763	0.281	0.133	0.425017841	C9_CD4_Tem
chr12-13165226-13166007	6.51E-06	0.260482612	0.153	0.063	0.431247601	C9_CD4_Tem
chr1-154431440-154432190	6.56E-06	0.251110467	0.18	0.078	0.434101827	C9_CD4_Tem
chr1-39193273-39193905	6.71E-06	0.264353841	0.283	0.137	0.444165887	C9_CD4_Tem
chr12-92603314-92604435	6.71E-06	0.25785883	0.128	0.047	0.444188746	C9_CD4_Tem
chr10-69045222-69045948	6.76E-06	0.259927279	0.245	0.118	0.447931852	C9_CD4_Tem
chr5-151059345-151060210	6.77E-06	0.253076208	0.099	0.031	0.448133923	C9_CD4_Tem
chr3-108834519-108835617	6.91E-06	0.250125163	0.315	0.157	0.457392303	C9_CD4_Tem
chrX-2709740-2710920	7.01E-06	0.262042595	0.279	0.133	0.46448172	C9_CD4_Tem
chr2-234491251-234491583	7.10E-06	0.27911205	0.164	0.064	0.470384614	C9_CD4_Tem
chr1-184742891-184743448	7.17E-06	0.252765448	0.213	0.096	0.474618047	C9_CD4_Tem
chr9-99056882-99058482	7.36E-06	0.252556309	0.312	0.156	0.487349265	C9_CD4_Tem
chr4-122666860-122667267	7.60E-06	0.268411398	0.097	0.025	0.503138256	C9_CD4_Tem
chr19-18486116-18486883	7.65E-06	0.25665203	0.18	0.075	0.506788475	C9_CD4_Tem
chr2-203948872-203949574	7.67E-06	0.252005534	0.162	0.065	0.508017995	C9_CD4_Tem
chr2-27920287-27920894	7.77E-06	0.262381074	0.144	0.054	0.51456216	C9_CD4_Tem
chr4-2484704-2485429	7.86E-06	0.252083937	0.139	0.057	0.520799676	C9_CD4_Tem

chr1-145984109-145984789	7.91E-06	0.251642985	0.119	0.042	0.523976787	C9_CD4_Tem
chr7-17144016-17144802	7.96E-06	0.259936603	0.229	0.099	0.527379861	C9_CD4_Tem
chr8-1744992-1746505	7.97E-06	0.256045834	0.252	0.121	0.527539752	C9_CD4_Tem
chr1-153565588-153566883	8.03E-06	0.255958158	0.285	0.136	0.531962603	C9_CD4_Tem
chr10-6140565-6141993	8.10E-06	0.268318683	0.231	0.106	0.53647471	C9_CD4_Tem
chrX-136634855-136635682	8.28E-06	0.258047916	0.133	0.048	0.548186245	C9_CD4_Tem
chr10-33115634-33116005	8.35E-06	0.254717157	0.112	0.04	0.55311247	C9_CD4_Tem
chr17-78725415-78726191	8.41E-06	0.259135347	0.124	0.043	0.556705318	C9_CD4_Tem
chr7-21338494-21339310	8.52E-06	0.251218725	0.202	0.092	0.56389506	C9_CD4_Tem
chr17-4696006-4697023	8.66E-06	0.262156649	0.204	0.092	0.573670458	C9_CD4_Tem
chr10-32352517-32352914	8.78E-06	0.252064311	0.099	0.032	0.581132779	C9_CD4_Tem
chr4-40183602-40184276	8.79E-06	0.255635205	0.097	0.029	0.582021309	C9_CD4_Tem
chr10-14554153-14555104	8.80E-06	0.25463468	0.312	0.154	0.582767649	C9_CD4_Tem
chr16-47136122-47136709	8.82E-06	0.250159073	0.099	0.031	0.583782729	C9_CD4_Tem
chr5-119274427-119275391	8.83E-06	0.275551431	0.231	0.104	0.584962534	C9_CD4_Tem
chr2-241309543-241310094	8.91E-06	0.254934488	0.182	0.079	0.59024075	C9_CD4_Tem
chr10-32356874-32357860	8.95E-06	0.254905065	0.18	0.081	0.592862439	C9_CD4_Tem
chr12-104218955-104220318	9.08E-06	0.258817104	0.204	0.089	0.601441966	C9_CD4_Tem
chr7-44735204-44735796	9.11E-06	0.257878545	0.13	0.046	0.60350286	C9_CD4_Tem
chr4-1693214-1694047	9.28E-06	0.267662058	0.169	0.064	0.614525861	C9_CD4_Tem
chr16-79627991-79628965	9.36E-06	0.25499884	0.157	0.062	0.619711963	C9_CD4_Tem
chr19-48649234-48649578	9.40E-06	0.261417387	0.209	0.094	0.622767588	C9_CD4_Tem
chr2-160279582-160280252	9.49E-06	0.263667772	0.166	0.064	0.628130483	C9_CD4_Tem
chr7-39589302-39590128	9.57E-06	0.256920975	0.267	0.129	0.633502805	C9_CD4_Tem
chr16-50277159-50278179	9.73E-06	0.259753526	0.24	0.117	0.644231347	C9_CD4_Tem
chr20-50561187-50561945	9.79E-06	0.268581567	0.142	0.049	0.648188512	C9_CD4_Tem
chr20-53608762-53610142	9.86E-06	0.253776956	0.227	0.107	0.653100151	C9_CD4_Tem

chr7-150448473-150449022	9.89E-06	0.266227035	0.236	0.112	0.654938274	C9_CD4_Tem
chr15-39579677-39581317	9.90E-06	0.262370781	0.171	0.069	0.655414126	C9_CD4_Tem
chr17-17957677-17958806	9.98E-06	0.261178081	0.178	0.074	0.660817862	C9_CD4_Tem
chr2-224885889-224886465	1.00E-05	0.25516071	0.112	0.037	0.662965061	C9_CD4_Tem
chr2-174603136-174603592	1.01E-05	0.268159542	0.155	0.061	0.669305292	C9_CD4_Tem
chr1-207861454-207862168	1.02E-05	0.259555922	0.155	0.063	0.673143096	C9_CD4_Tem
chr3-122609567-122610450	1.03E-05	0.260957614	0.18	0.073	0.682413826	C9_CD4_Tem
chr15-40492974-40493873	1.05E-05	0.263350027	0.202	0.089	0.692359428	C9_CD4_Tem
chr10-80498359-80499205	1.06E-05	0.267118127	0.124	0.041	0.701165965	C9_CD4_Tem
chr11-66115534-66116387	1.06E-05	0.250516026	0.121	0.046	0.704833103	C9_CD4_Tem
chr12-49750059-49750766	1.07E-05	0.254981888	0.22	0.099	0.710583255	C9_CD4_Tem
chr2-68721329-68721991	1.11E-05	0.254896362	0.133	0.049	0.734037776	C9_CD4_Tem
chr11-60830576-60831326	1.13E-05	0.258339818	0.117	0.04	0.746357981	C9_CD4_Tem
chr6-108895371-108896499	1.16E-05	0.257143969	0.24	0.114	0.771073504	C9_CD4_Tem
chr5-10635257-10635627	1.17E-05	0.253630515	0.175	0.074	0.772698713	C9_CD4_Tem
chr11-48055029-48056231	1.17E-05	0.25073634	0.213	0.092	0.772945968	C9_CD4_Tem
chr21-17794708-17796320	1.18E-05	0.25092155	0.265	0.132	0.778395142	C9_CD4_Tem
chr1-39398334-39398907	1.18E-05	0.250494501	0.117	0.042	0.779624446	C9_CD4_Tem
chr5-96823694-96824144	1.18E-05	0.264025877	0.135	0.05	0.779907883	C9_CD4_Tem
chr2-201431882-201432382	1.18E-05	0.254299288	0.128	0.049	0.781439256	C9_CD4_Tem
chr11-128345266-128345650	1.20E-05	0.259021581	0.142	0.05	0.791493778	C9_CD4_Tem
chr3-36988265-36988621	1.21E-05	0.256096014	0.119	0.041	0.802194777	C9_CD4_Tem
chr1-66283627-66284053	1.25E-05	0.253373317	0.157	0.065	0.825303173	C9_CD4_Tem
chr22-37303797-37304134	1.25E-05	0.259168252	0.139	0.051	0.826470399	C9_CD4_Tem
chr1-111470297-111471372	1.25E-05	0.254620477	0.243	0.117	0.830062577	C9_CD4_Tem
chr17-35171142-35172261	1.27E-05	0.277418104	0.207	0.089	0.841720359	C9_CD4_Tem
chr18-23573663-23574502	1.29E-05	0.259498362	0.166	0.07	0.856414352	C9_CD4_Tem

chr17-35177292-35178309	1.29E-05	0.257286712	0.193	0.083	0.856990154	C9_CD4_Tem
chr10-119270835-119271760	1.33E-05	0.254289593	0.189	0.083	0.879223141	C9_CD4_Tem
chr17-78735906-78737656	1.34E-05	0.260652652	0.312	0.156	0.885880757	C9_CD4_Tem
chr1-224430539-224430792	1.38E-05	0.255690158	0.133	0.054	0.916424425	C9_CD4_Tem
chr14-95495372-95496406	1.39E-05	0.261581576	0.173	0.07	0.918999455	C9_CD4_Tem
chr1-184838507-184839002	1.43E-05	0.251922676	0.139	0.05	0.947717781	C9_CD4_Tem
chr22-49869906-49870647	1.43E-05	0.252631552	0.204	0.092	0.948034433	C9_CD4_Tem
chr7-75427305-75428294	1.45E-05	0.257349958	0.187	0.083	0.958349828	C9_CD4_Tem
chr1-204544052-204544960	1.46E-05	0.260846916	0.204	0.086	0.969759168	C9_CD4_Tem
chr2-26085936-26086632	1.51E-05	0.252490599	0.204	0.092	1	C9_CD4_Tem
chr1-24539976-24540591	1.52E-05	0.2576738	0.153	0.057	1	C9_CD4_Tem
chr14-72476471-72477232	1.52E-05	0.260168758	0.146	0.054	1	C9_CD4_Tem
chr6-111605131-111606352	1.54E-05	0.256926022	0.236	0.116	1	C9_CD4_Tem
chr2-197265976-197266788	1.56E-05	0.251091338	0.124	0.045	1	C9_CD4_Tem
chr2-46229316-46230424	1.57E-05	0.253302288	0.204	0.089	1	C9_CD4_Tem
chr1-12157323-12157667	1.62E-05	0.258747408	0.157	0.062	1	C9_CD4_Tem
chr1-66349953-66350559	1.64E-05	0.250054849	0.276	0.137	1	C9_CD4_Tem
chr19-14195718-14196448	1.66E-05	0.251977988	0.124	0.046	1	C9_CD4_Tem
chr10-14562123-14562531	1.73E-05	0.255533688	0.155	0.06	1	C9_CD4_Tem
chr3-172562425-172563688	1.81E-05	0.258947547	0.124	0.042	1	C9_CD4_Tem
chr12-111993351-111994923	1.84E-05	0.256465402	0.227	0.105	1	C9_CD4_Tem
chr22-41618778-41619223	1.88E-05	0.264996876	0.191	0.082	1	C9_CD4_Tem
chr4-153496814-153497585	1.90E-05	0.250235181	0.169	0.069	1	C9_CD4_Tem
chr16-89314316-89314694	1.91E-05	0.261450112	0.126	0.046	1	C9_CD4_Tem
chr20-48823887-48824210	1.92E-05	0.257648109	0.285	0.139	1	C9_CD4_Tem
chr2-112690686-112691416	1.93E-05	0.257235808	0.162	0.068	1	C9_CD4_Tem
chr17-43720195-43721200	1.95E-05	0.257253337	0.213	0.101	1	C9_CD4_Tem

chr17-35245178-35245565	1.98E-05	0.254164571	0.243	0.119	1	C9_CD4_Tem
chr17-59845571-59846531	2.00E-05	0.263189602	0.211	0.094	1	C9_CD4_Tem
chr10-89003151-89003657	2.02E-05	0.265581749	0.153	0.057	1	C9_CD4_Tem
chr20-53948566-53949823	2.03E-05	0.252568212	0.258	0.128	1	C9_CD4_Tem
chr10-62048941-62049441	2.07E-05	0.252663022	0.252	0.117	1	C9_CD4_Tem
chr14-77124525-77125262	2.10E-05	0.252000438	0.263	0.128	1	C9_CD4_Tem
chr15-22262390-22263023	2.11E-05	0.252142429	0.126	0.049	1	C9_CD4_Tem
chr15-90594019-90594836	2.12E-05	0.253211647	0.187	0.079	1	C9_CD4_Tem
chr5-40386222-40386698	2.13E-05	0.251261752	0.169	0.072	1	C9_CD4_Tem
chr8-127982247-127982514	2.16E-05	0.251166701	0.139	0.053	1	C9_CD4_Tem
chr1-39185592-39186149	2.23E-05	0.255651628	0.148	0.059	1	C9_CD4_Tem
chr8-8919555-8920598	2.31E-05	0.258927025	0.218	0.097	1	C9_CD4_Tem
chr19-10406858-10407320	2.32E-05	0.254758441	0.135	0.053	1	C9_CD4_Tem
chr22-17767229-17768361	2.40E-05	0.251375207	0.204	0.092	1	C9_CD4_Tem
chr14-70888544-70888980	2.44E-05	0.257302734	0.162	0.066	1	C9_CD4_Tem
chr12-54560053-54560722	2.46E-05	0.26140752	0.2	0.085	1	C9_CD4_Tem
chr6-20201558-20202568	2.47E-05	0.25745099	0.166	0.068	1	C9_CD4_Tem
chr9-109101188-109102238	2.52E-05	0.251673162	0.184	0.083	1	C9_CD4_Tem
chr4-25859496-25860069	2.56E-05	0.252356912	0.202	0.093	1	C9_CD4_Tem
chr19-5953020-5954180	2.60E-05	0.256486527	0.117	0.04	1	C9_CD4_Tem
chr12-14390737-14391259	2.67E-05	0.253599129	0.171	0.075	1	C9_CD4_Tem
chr21-35043573-35043802	2.75E-05	0.250597724	0.137	0.053	1	C9_CD4_Tem
chr14-64707494-64708921	2.89E-05	0.254322332	0.182	0.072	1	C9_CD4_Tem
chr15-60520034-60521091	2.90E-05	0.251074971	0.139	0.056	1	C9_CD4_Tem
chr18-3250729-3250966	2.92E-05	0.257684132	0.121	0.043	1	C9_CD4_Tem
chr17-55264287-55266301	2.96E-05	0.252629561	0.252	0.116	1	C9_CD4_Tem
chr5-132102099-132102565	2.98E-05	0.253979725	0.187	0.082	1	C9_CD4_Tem

chr4-3106244-3106939	3.05E-05	0.257040662	0.211	0.094	1	C9_CD4_Tem
chr19-51926517-51927662	3.05E-05	0.253660679	0.29	0.152	1	C9_CD4_Tem
chr1-32980869-32981670	3.21E-05	0.252730526	0.189	0.087	1	C9_CD4_Tem
chr10-11232101-11232551	3.21E-05	0.254872487	0.155	0.066	1	C9_CD4_Tem
chr1-113931379-113931824	3.27E-05	0.253759749	0.164	0.073	1	C9_CD4_Tem
chr5-119343656-119344066	3.61E-05	0.257481268	0.166	0.069	1	C9_CD4_Tem
chr12-109887998-109888992	3.76E-05	0.253969254	0.175	0.075	1	C9_CD4_Tem
chr15-60568248-60570011	3.84E-05	0.253658789	0.2	0.093	1	C9_CD4_Tem
chr14-50233599-50234150	4.14E-05	0.251328865	0.166	0.066	1	C9_CD4_Tem
chr11-66125927-66127480	4.58E-05	0.251393889	0.133	0.053	1	C9_CD4_Tem
chr14-58281110-58281800	4.61E-05	0.250532885	0.126	0.046	1	C9_CD4_Tem
chr1-203662867-203664143	4.62E-05	0.258659199	0.225	0.101	1	C9_CD4_Tem
chr10-33143193-33143620	5.23E-05	0.250981043	0.135	0.052	1	C9_CD4_Tem
chr18-77069871-77070267	8.75E-05	0.251911375	0.198	0.091	1	C9_CD4_Tem
chr17-77324774-77325650	0.000111491	0.253384116	0.222	0.097	1	C9_CD4_Tem
chr17-83048299-83048927	0.000120231	0.255127759	0.191	0.086	1	C9_CD4_Tem
chr8-104664861-104666545	2.27E-11	0.329563891	0.213	0.077	1.50E-06	C9_CD4_Tem
chr1-6459540-6460762	4.44E-10	0.297455001	0.413	0.193	2.94E-05	C9_CD4_Tem
chr7-44633252-44634170	4.81E-14	0.309293493	0.546	0.265	3.19E-09	C9_CD4_Tem
chr1-193458475-193459060	6.04E-14	0.382406587	0.256	0.087	4.00E-09	C9_CD4_Tem
chr20-33369167-33370781	1.27E-12	0.296422359	0.546	0.285	8.41E-08	C9_CD4_Tem
chr13-98484347-98485269	6.96E-22	0.439936797	0.429	0.151	4.61E-17	C9_CD4_Tem
chr1-67162728-67163382	4.53E-06	0.256762484	0.148	0.055	0.300219637	C9_CD4_Tem
chr14-64755533-64756287	1.07E-14	0.363150776	0.373	0.147	7.12E-10	C9_CD4_Tem
chr4-1337230-1337925	5.08E-08	0.288366048	0.148	0.05	0.003363331	C9_CD4_Tem
chr4-1329968-1331391	1.29E-10	0.335662172	0.281	0.114	8.52E-06	C9_CD4_Tem
chr14-61562357-61563185	2.53E-07	0.296390695	0.184	0.065	0.016734328	C9_CD4_Tem

chr8-123083860-123084646	9.16E-11	0.335504162	0.191	0.066	6.06E-06	C9_CD4_Tem
chr6-14732448-14733422	5.98E-08	0.278509599	0.321	0.151	0.00395761	C9_CD4_Tem
chr1-193558309-193559033	7.36E-08	0.275223996	0.117	0.035	0.004872021	C9_CD4_Tem
chr11-128345266-128345650	1.20E-05	0.259021581	0.142	0.05	0.791493778	C9_CD4_Tem
chr6-90411120-90411851	2.29E-14	0.345652914	0.447	0.196	1.52E-09	C9_CD4_Tem
chr17-55089250-55090549	1.71E-17	0.422826511	0.279	0.086	1.14E-12	C9_CD4_Tem
chr13-111176803-111177593	2.93E-06	0.254269811	0.119	0.04	0.194069842	C9_CD4_Tem
chr8-47600277-47601292	2.33E-15	0.389455802	0.31	0.121	1.54E-10	C9_CD4_Tem
chr1-160623049-160624086	8.67E-19	0.430113329	0.348	0.122	5.74E-14	C9_CD4_Tem
chr13-98480746-98481317	4.45E-18	0.426137824	0.342	0.113	2.95E-13	C9_CD4_Tem
chr5-154214266-154214853	9.91E-07	0.270273377	0.106	0.031	0.065604074	C9_CD4_Tem
chr5-911844-913044	3.32E-41	0.502711597	0.625	0.242	2.20E-36	C9_CD4_Tem
chr9-97703617-97704354	1.71E-10	0.335715714	0.151	0.044	1.13E-05	C9_CD4_Tem
chr3-9912046-9912875	1.07E-21	0.479608313	0.299	0.081	7.06E-17	C9_CD4_Tem
chr7-44046496-44046933	3.37E-06	0.258674995	0.13	0.048	0.223103662	C9_CD4_Tem
chr2-174600435-174600753	1.46E-10	0.346301635	0.157	0.046	9.64E-06	C9_CD4_Tem
chr12-121214138-121214703	5.55E-13	0.378446193	0.162	0.047	3.67E-08	C9_CD4_Tem
chr1-193457511-193458066	1.39E-08	0.300065864	0.126	0.038	0.000919796	C9_CD4_Tem
chr20-4160763-4161675	1.92E-09	0.302792095	0.103	0.026	0.000127331	C9_CD4_Tem
chr19-49458005-49458729	7.66E-10	0.315392651	0.121	0.029	5.07E-05	C9_CD4_Tem
chr13-50244570-50245457	4.38E-06	0.269624561	0.218	0.092	0.289752789	C9_CD4_Tem
chr17-65022809-65023434	8.31E-08	0.289958069	0.198	0.075	0.005503287	C9_CD4_Tem
chr2-102226947-102228524	6.39E-13	0.295576785	0.501	0.256	4.23E-08	C9_CD4_Tem
chr8-66490381-66491367	1.38E-06	0.275420885	0.27	0.126	0.091142888	C9_CD4_Tem
chr16-72474275-72474958	1.90E-13	0.362693937	0.11	0.019	1.26E-08	C9_CD4_Tem
chr13-98509104-98509860	7.75E-17	0.413400612	0.315	0.107	5.13E-12	C9_CD4_Tem
chr9-115006571-115007084	6.03E-23	0.50322987	0.18	0.028	4.00E-18	C9_CD4_Tem

chr11-85750466-85750991	2.70E-10	0.326273697	0.234	0.093	1.79E-05	C9_CD4_Tem
chr5-132102099-132102565	2.98E-05	0.253979725	0.187	0.082	1	C9_CD4_Tem
chr4-80101366-80102059	3.33E-10	0.332262398	0.119	0.029	2.21E-05	C9_CD4_Tem
chr5-142471988-142472953	6.43E-14	0.379480725	0.281	0.096	4.26E-09	C9_CD4_Tem
chr9-87713197-87713846	5.73E-23	0.470745538	0.137	0.016	3.80E-18	C9_CD4_Tem
chr5-154197444-154198623	3.03E-12	0.34733258	0.357	0.151	2.00E-07	C9_CD4_Tem
chr9-131656226-131657298	3.30E-08	0.280735096	0.337	0.162	0.002183548	C9_CD4_Tem
chr7-70787363-70788626	3.91E-18	0.433624306	0.151	0.028	2.59E-13	C9_CD4_Tem
chr15-73920358-73920896	3.05E-09	0.322952749	0.151	0.046	0.000201956	C9_CD4_Tem
chr16-72482283-72483046	3.37E-08	0.269322336	0.076	0.017	0.002228838	C9_CD4_Tem
chr10-73956286-73957629	4.43E-06	0.26100253	0.166	0.068	0.293506077	C9_CD4_Tem
chr3-32296559-32298058	8.70E-15	0.388067044	0.267	0.091	5.76E-10	C9_CD4_Tem
chr13-97257635-97258449	4.54E-06	0.269916439	0.162	0.065	0.300525055	C9_CD4_Tem
chr13-98524294-98524743	8.36E-12	0.317564443	0.081	0.013	5.54E-07	C9_CD4_Tem
chr8-47513301-47514143	2.29E-12	0.361534142	0.216	0.071	1.52E-07	C9_CD4_Tem
chr1-116508136-116509326	2.01E-12	0.354079674	0.346	0.141	1.33E-07	C9_CD4_Tem
chr18-3304866-3306168	1.19E-12	0.324654441	0.393	0.174	7.90E-08	C9_CD4_Tem
chr5-157607280-157608223	4.48E-07	0.283281444	0.279	0.125	0.029671165	C9_CD4_Tem
chr6-35709901-35710747	5.77E-10	0.317044637	0.308	0.135	3.82E-05	C9_CD4_Tem
chr4-1300932-1301395	1.27E-09	0.267462231	0.058	0.009	8.39E-05	C9_CD4_Tem
chr2-43183820-43184763	1.26E-13	0.385951715	0.258	0.089	8.33E-09	C9_CD4_Tem
chr12-12465878-12466513	2.96E-10	0.329402217	0.225	0.085	1.96E-05	C9_CD4_Tem
chr17-64081788-64082512	1.45E-12	0.375037882	0.247	0.086	9.59E-08	C9_CD4_Tem
chr8-41986545-41987581	7.79E-12	0.358029814	0.236	0.079	5.16E-07	C9_CD4_Tem
chr5-35816593-35817551	1.16E-06	0.272778266	0.256	0.123	0.076907761	C9_CD4_Tem
chr10-5317444-5318188	1.91E-25	0.513263093	0.29	0.067	1.27E-20	C9_CD4_Tem
chr3-119637745-119639270	2.43E-09	0.294935104	0.128	0.037	0.000161217	C9_CD4_Tem

chr2-9781835-9782724	7.29E-10	0.319207218	0.333	0.143	4.82E-05	C9_CD4_Tem
chr10-127990189-127991574	2.11E-12	0.350717207	0.364	0.157	1.40E-07	C9_CD4_Tem
chr15-52463638-52464944	5.14E-06	0.25710394	0.157	0.063	0.340323586	C9_CD4_Tem
chr10-119655529-119656093	2.34E-16	0.417022676	0.254	0.079	1.55E-11	C9_CD4_Tem
chr6-87731457-87732623	5.49E-09	0.317841817	0.155	0.052	0.000363406	C9_CD4_Tem
chr16-75108756-75109282	2.24E-08	0.313145334	0.211	0.079	0.001482087	C9_CD4_Tem
chr6-38713620-38715736	1.41E-08	0.303569619	0.249	0.107	0.000932141	C9_CD4_Tem
chr5-80253614-80254145	2.60E-15	0.409087248	0.187	0.047	1.72E-10	C9_CD4_Tem
chr10-110353290-110354533	1.48E-14	0.331490565	0.488	0.233	9.83E-10	C9_CD4_Tem
chr9-19161181-19162137	2.93E-11	0.320212957	0.404	0.186	1.94E-06	C9_CD4_Tem
chr17-55237212-55239072	5.64E-06	0.258080089	0.249	0.112	0.373565811	C9_CD4_Tem
chr17-65173018-65173919	1.02E-06	0.264942505	0.272	0.128	0.067760445	C9_CD4_Tem
chr3-15796863-15798271	4.24E-13	0.371738347	0.308	0.116	2.81E-08	C9_CD4_Tem
chr14-64723525-64724394	6.54E-13	0.376250909	0.187	0.053	4.33E-08	C9_CD4_Tem
chr7-130903464-130904489	5.30E-07	0.289997671	0.209	0.084	0.035086248	C9_CD4_Tem
chr9-131500277-131501370	1.57E-06	0.260381109	0.366	0.186	0.10409867	C9_CD4_Tem
chr14-50038157-50039127	5.74E-11	0.339203214	0.301	0.12	3.80E-06	C9_CD4_Tem
chr4-153496814-153497585	1.90E-05	0.250235181	0.169	0.069	1	C9_CD4_Tem
chr6-116386543-116387614	8.15E-09	0.297548724	0.09	0.021	0.000539675	C9_CD4_Tem
chr2-234289001-234289361	3.06E-07	0.291752702	0.171	0.066	0.020284552	C9_CD4_Tem
chr17-80605163-80606101	1.41E-14	0.384286038	0.2	0.059	9.32E-10	C9_CD4_Tem
chr9-5446455-5447136	3.14E-08	0.304264413	0.155	0.055	0.002076518	C9_CD4_Tem
chr1-193591899-193593026	1.05E-09	0.322428171	0.139	0.038	6.97E-05	C9_CD4_Tem
chr17-55264287-55266301	2.96E-05	0.252629561	0.252	0.116	1	C9_CD4_Tem
chr22-22210316-22210973	2.27E-09	0.323664346	0.124	0.032	0.000150336	C9_CD4_Tem
chr1-184838507-184839002	1.43E-05	0.251922676	0.139	0.05	0.947717781	C9_CD4_Tem
chr1-203606072-203606419	3.50E-06	0.261825818	0.115	0.037	0.231644209	C9_CD4_Tem

chr12-12439435-12439968	7.83E-08	0.291669053	0.283	0.122	0.005188185	C9_CD4_Tem
chr17-78804683-78805729	5.02E-13	0.35199257	0.355	0.149	3.32E-08	C9_CD4_Tem
chr14-64707494-64708921	2.89E-05	0.254322332	0.182	0.072	1	C9_CD4_Tem
chr11-96155887-96157525	2.52E-06	0.272993698	0.198	0.083	0.166885972	C9_CD4_Tem
chr18-9090970-9091665	8.97E-11	0.323898556	0.252	0.098	5.94E-06	C9_CD4_Tem
chr12-12502497-12503926	4.31E-09	0.321125453	0.157	0.048	0.000285125	C9_CD4_Tem
chrX-1582773-1583405	5.00E-17	0.435483002	0.252	0.072	3.31E-12	C9_CD4_Tem
chr1-236510700-236511271	7.95E-13	0.35523476	0.103	0.018	5.26E-08	C9_CD4_Tem
chr3-52216805-52217645	3.80E-11	0.348552518	0.263	0.101	2.52E-06	C9_CD4_Tem
chr4-26092937-26093921	1.35E-11	0.343099528	0.148	0.041	8.95E-07	C9_CD4_Tem
chr16-67857443-67858863	8.94E-13	0.374298365	0.213	0.065	5.92E-08	C9_CD4_Tem
chr14-51827645-51828710	2.15E-08	0.307140619	0.211	0.081	0.001421857	C9_CD4_Tem
chr7-77830727-77831925	3.09E-31	0.568321155	0.256	0.049	2.05E-26	C9_CD4_Tem
chr11-75536814-75538379	1.77E-06	0.281887543	0.227	0.096	0.117514013	C9_CD4_Tem
chr13-114268403-114269455	2.35E-08	0.30548888	0.139	0.044	0.001557549	C9_CD4_Tem
chr8-102107535-102109028	1.26E-08	0.297360544	0.173	0.063	0.000831242	C9_CD4_Tem
chr8-102097118-102098160	7.81E-08	0.275944042	0.303	0.144	0.005174511	C9_CD4_Tem
chr1-8181870-8182991	6.56E-12	0.341849522	0.317	0.134	4.34E-07	C9_CD4_Tem
chr1-154431440-154432190	6.56E-06	0.251110467	0.18	0.078	0.434101827	C9_CD4_Tem
chr12-122946608-122947242	3.97E-06	0.262046017	0.184	0.078	0.263038331	C9_CD4_Tem
chr7-131231107-131231912	2.72E-12	0.339813804	0.409	0.181	1.80E-07	C9_CD4_Tem
chr9-91130813-91131935	8.08E-07	0.275551935	0.276	0.129	0.05350413	C9_CD4_Tem
chr3-122609567-122610450	1.03E-05	0.260957614	0.18	0.073	0.682413826	C9_CD4_Tem
chr3-39260897-39261269	1.50E-06	0.266421721	0.117	0.039	0.099193614	C9_CD4_Tem
chr2-96806412-96807178	2.53E-06	0.263500451	0.157	0.062	0.167618548	C9_CD4_Tem
chr3-32963641-32964287	1.05E-06	0.264364896	0.142	0.052	0.069313047	C9_CD4_Tem
chr18-9090970-9091665	8.97E-11	0.323898556	0.252	0.098	5.94E-06	C9_CD4_Tem

chr8-18957182-18957952	1.65E-08	0.30029886	0.137	0.043	0.001089633	C9_CD4_Tem
chr15-63479336-63480062	2.21E-09	0.30878166	0.272	0.111	0.000146039	C9_CD4_Tem
chr5-80125699-80126474	3.76E-06	0.256556852	0.126	0.046	0.249144062	C9_CD4_Tem
chr16-10963888-10965181	9.99E-08	0.277182642	0.382	0.191	0.006613149	C9_CD4_Tem
chr15-33104022-33104674	4.83E-06	0.268247331	0.148	0.053	0.319612683	C9_CD4_Tem
chr3-32296559-32298058	8.70E-15	0.388067044	0.267	0.091	5.76E-10	C9_CD4_Tem
chr5-143409895-143410306	8.44E-08	0.273910214	0.074	0.015	0.005589565	C9_CD4_Tem
chr18-2889646-2890237	3.31E-13	0.375793469	0.198	0.06	2.19E-08	C9_CD4_Tem
chr8-8868529-8869756	5.20E-23	0.489982524	0.294	0.079	3.44E-18	C9_CD4_Tem
chr5-157158945-157160332	9.57E-11	0.293009079	0.485	0.24	6.34E-06	C9_CD4_Tem
chr12-104218955-104220318	9.08E-06	0.258817104	0.204	0.089	0.601441966	C9_CD4_Tem
chr11-128339693-128340101	1.01E-09	0.310430971	0.094	0.021	6.70E-05	C9_CD4_Tem
chr4-36394180-36394970	3.48E-09	0.287283523	0.378	0.187	0.000230783	C9_CD4_Tem
chr9-131500277-131501370	1.57E-06	0.260381109	0.366	0.186	0.10409867	C9_CD4_Tem
chr18-9094490-9095605	2.95E-09	0.321757251	0.249	0.098	0.00019514	C9_CD4_Tem
chr5-52987434-52988237	1.13E-12	0.386174918	0.222	0.069	7.50E-08	C9_CD4_Tem
chr6-149232909-149234903	3.00E-14	0.356595435	0.44	0.19	1.98E-09	C9_CD4_Tem
chr16-10957500-10958307	2.94E-09	0.324436374	0.137	0.036	0.000194683	C9_CD4_Tem
chr2-39099601-39100566	4.21E-10	0.332762828	0.267	0.1	2.79E-05	C9_CD4_Tem
chr1-26123193-26123705	1.71E-09	0.320669272	0.164	0.051	0.000113341	C9_CD4_Tem
chr5-56011879-56012999	3.48E-14	0.392983541	0.213	0.066	2.31E-09	C9_CD4_Tem
chr10-5291424-5292650	1.10E-28	0.517295926	0.375	0.111	7.27E-24	C9_CD4_Tem
chr1-39185592-39186149	2.23E-05	0.255651628	0.148	0.059	1	C9_CD4_Tem
chr7-44633252-44634170	4.81E-14	0.309293493	0.546	0.265	3.19E-09	C9_CD4_Tem
chr14-70893119-70894127	2.38E-11	0.336885486	0.11	0.024	1.57E-06	C9_CD4_Tem
chr15-84681782-84682236	1.13E-15	0.401201048	0.146	0.027	7.50E-11	C9_CD4_Tem
chr7-831634-832970	1.74E-06	0.269713958	0.306	0.144	0.115168364	C9_CD4_Tem

chr12-11998194-11999166	1.05E-06	0.274929518	0.216	0.095	0.069416388	C9_CD4_Tem
chr17-77324774-77325650	0.000111491	0.253384116	0.222	0.097	1	C9_CD4_Tem
chr3-192916860-192918360	6.88E-07	0.272707071	0.155	0.057	0.045574699	C9_CD4_Tem
chr1-154463170-154464019	6.56E-07	0.287075661	0.157	0.057	0.043445203	C9_CD4_Tem
chrX-41382908-41384432	5.81E-08	0.291946524	0.187	0.072	0.003850092	C9_CD4_Tem
chr12-12008999-12009782	3.24E-10	0.337307942	0.288	0.112	2.15E-05	C9_CD4_Tem
chr1-6459540-6460762	4.44E-10	0.297455001	0.413	0.193	2.94E-05	C9_CD4_Tem
chrX-136619777-136620391	5.93E-13	0.374943286	0.157	0.039	3.93E-08	C9_CD4_Tem
chrX-1208026-1208776	5.43E-20	0.46110096	0.231	0.056	3.59E-15	C9_CD4_Tem
chr15-40492974-40493873	1.05E-05	0.263350027	0.202	0.089	0.692359428	C9_CD4_Tem
chr8-99705382-99706622	1.19E-10	0.339365701	0.133	0.035	7.88E-06	C9_CD4_Tem
chr16-89388004-89388346	3.25E-08	0.297250385	0.099	0.024	0.00215361	C9_CD4_Tem
chr15-63841719-63842387	1.06E-13	0.385048849	0.211	0.064	7.02E-09	C9_CD4_Tem
chr1-234978304-234979014	4.33E-06	0.271058333	0.204	0.085	0.286769051	C9_CD4_Tem
chr17-42337333-42337808	5.58E-07	0.285440091	0.191	0.073	0.03692309	C9_CD4_Tem
chr3-9912046-9912875	1.07E-21	0.479608313	0.299	0.081	7.06E-17	C9_CD4_Tem
chr9-89604248-89606232	7.08E-10	0.293888542	0.463	0.234	4.69E-05	C9_CD4_Tem
chr11-96012030-96012570	1.14E-11	0.345673373	0.306	0.121	7.55E-07	C9_CD4_Tem
chr8-8843985-8844590	5.11E-28	0.55069547	0.245	0.046	3.39E-23	C9_CD4_Tem
chr5-175652680-175653588	5.65E-06	0.268641373	0.151	0.055	0.374125981	C9_CD4_Tem
chr21-14629951-14631144	4.90E-08	0.314674926	0.196	0.071	0.003241919	C9_CD4_Tem
chr2-196259643-196260831	1.70E-10	0.334601161	0.299	0.127	1.12E-05	C9_CD4_Tem
chr3-52216805-52217645	3.80E-11	0.348552518	0.263	0.101	2.52E-06	C9_CD4_Tem
chr11-128345266-128345650	1.20E-05	0.259021581	0.142	0.05	0.791493778	C9_CD4_Tem
chr8-8871973-8872681	4.39E-13	0.386162363	0.198	0.057	2.91E-08	C9_CD4_Tem
chr14-64755533-64756287	1.07E-14	0.363150776	0.373	0.147	7.12E-10	C9_CD4_Tem
chr7-140059213-140059829	2.36E-18	0.446828423	0.178	0.039	1.56E-13	C9_CD4_Tem

chr4-140095635-140096748	3.85E-07	0.26359492	0.317	0.156	0.025504608	C9_CD4_Tem
chr5-80253614-80254145	2.60E-15	0.409087248	0.187	0.047	1.72E-10	C9_CD4_Tem
chr5-55977602-55978470	1.53E-07	0.294851416	0.173	0.067	0.010113826	C9_CD4_Tem
chr17-80605163-80606101	1.41E-14	0.384286038	0.2	0.059	9.32E-10	C9_CD4_Tem
chr6-45420319-45420917	9.80E-08	0.297217647	0.182	0.067	0.006487745	C9_CD4_Tem
chr16-17364067-17364670	1.07E-06	0.264862018	0.312	0.156	0.070876879	C9_CD4_Tem
chr10-74589097-74589651	6.49E-07	0.270151058	0.281	0.134	0.042961187	C9_CD4_Tem
chr10-5317444-5318188	1.91E-25	0.513263093	0.29	0.067	1.27E-20	C9_CD4_Tem
chr10-119655529-119656093	2.34E-16	0.417022676	0.254	0.079	1.55E-11	C9_CD4_Tem
chr1-111470297-111471372	1.25E-05	0.254620477	0.243	0.117	0.830062577	C9_CD4_Tem
chr10-3867188-3868292	2.35E-12	0.368583895	0.258	0.09	1.56E-07	C9_CD4_Tem
chr3-186589739-186590295	3.17E-07	0.27682697	0.108	0.031	0.021017686	C9_CD4_Tem
chr3-4906100-4906640	2.15E-17	0.428926616	0.169	0.035	1.42E-12	C9_CD4_Tem
chr8-8917273-8918050	4.62E-07	0.284887423	0.292	0.133	0.030597306	C9_CD4_Tem
chr1-234730640-234731942	6.64E-12	0.362604726	0.281	0.111	4.39E-07	C9_CD4_Tem
chr13-75325753-75327029	1.07E-06	0.253216382	0.312	0.16	0.071141601	C9_CD4_Tem
chr13-42354068-42355455	4.28E-09	0.312181963	0.261	0.105	0.000283356	C9_CD4_Tem
chr10-33115634-33116005	8.35E-06	0.254717157	0.112	0.04	0.55311247	C9_CD4_Tem
chr3-4907946-4909045	1.40E-11	0.36371344	0.148	0.04	9.30E-07	C9_CD4_Tem
chr9-125521473-125522208	3.85E-07	0.278648515	0.153	0.055	0.025504612	C9_CD4_Tem
chr15-60787735-60788742	3.88E-15	0.401599303	0.207	0.062	2.57E-10	C9_CD4_Tem
chr12-12465878-12466513	2.96E-10	0.329402217	0.225	0.085	1.96E-05	C9_CD4_Tem
chr4-37954100-37954950	1.31E-06	0.266480853	0.137	0.048	0.086968751	C9_CD4_Tem
chr15-59919059-59920093	6.90E-11	0.330945197	0.274	0.11	4.57E-06	C9_CD4_Tem
chr4-40290704-40291215	2.51E-11	0.350752843	0.112	0.023	1.66E-06	C9_CD4_Tem
chr1-89603756-89603974	7.45E-09	0.291957719	0.083	0.018	0.000493256	C9_CD4_Tem
chr8-29523882-29524582	9.87E-07	0.278178932	0.173	0.067	0.065360673	C9_CD4_Tem

chr17-80649411-80650239	5.37E-06	0.254612139	0.094	0.027	0.355853406	C9_CD4_Tem
chr10-33263906-33264748	5.74E-09	0.309640894	0.22	0.085	0.000379818	C9_CD4_Tem
chr22-23704649-23705733	1.29E-11	0.360548877	0.198	0.063	8.54E-07	C9_CD4_Tem
chr9-128094022-128094725	7.52E-15	0.398620855	0.198	0.055	4.98E-10	C9_CD4_Tem
chr13-41355897-41357152	7.00E-09	0.321071345	0.207	0.076	0.000463454	C9_CD4_Tem
chr14-24274151-24274474	3.52E-06	0.257169239	0.101	0.03	0.233284421	C9_CD4_Tem
chr5-175480108-175480834	4.25E-06	0.273015829	0.178	0.072	0.281715569	C9_CD4_Tem
chr12-110590783-110591241	7.96E-08	0.273471648	0.081	0.018	0.005268992	C9_CD4_Tem
chr17-59361301-59362740	8.17E-08	0.293230451	0.204	0.082	0.005411259	C9_CD4_Tem
chr9-69180395-69181037	4.44E-12	0.355523963	0.144	0.036	2.94E-07	C9_CD4_Tem
chr11-48055029-48056231	1.17E-05	0.25073634	0.213	0.092	0.772945968	C9_CD4_Tem
chr17-17957677-17958806	9.98E-06	0.261178081	0.178	0.074	0.660817862	C9_CD4_Tem
chr20-4037663-4038434	1.83E-09	0.30108817	0.315	0.139	0.000121159	C9_CD4_Tem
chr4-122503102-122503615	1.45E-06	0.250950757	0.09	0.025	0.09589765	C9_CD4_Tem
chr2-38578721-38579215	1.54E-12	0.36382943	0.128	0.029	1.02E-07	C9_CD4_Tem
chr7-149209160-149210278	4.55E-14	0.382821295	0.261	0.091	3.01E-09	C9_CD4_Tem
chrX-1582773-1583405	5.00E-17	0.435483002	0.252	0.072	3.31E-12	C9_CD4_Tem
chr7-37354615-37355101	3.63E-14	0.405125435	0.184	0.046	2.40E-09	C9_CD4_Tem
chr2-232781689-232782204	5.09E-07	0.274016936	0.142	0.051	0.033733953	C9_CD4_Tem
chr7-44617182-44617939	4.38E-16	0.406751273	0.261	0.081	2.90E-11	C9_CD4_Tem
chr2-203870881-203871836	2.10E-17	0.424401013	0.279	0.086	1.39E-12	C9_CD4_Tem
chr15-92981098-92981897	2.55E-10	0.335176617	0.146	0.043	1.69E-05	C9_CD4_Tem
chr14-75562018-75562740	7.71E-07	0.270383922	0.153	0.058	0.05103636	C9_CD4_Tem
chr7-77830727-77831925	3.09E-31	0.568321155	0.256	0.049	2.05E-26	C9_CD4_Tem
chr15-70196426-70197229	8.19E-07	0.269215292	0.117	0.038	0.054257577	C9_CD4_Tem
chr5-55977602-55978470	1.53E-07	0.294851416	0.173	0.067	0.010113826	C9_CD4_Tem
chr11-86176148-86176847	1.10E-08	0.30494753	0.137	0.043	0.000726761	C9_CD4_Tem

chr7-130930059-130930652	9.73E-08	0.289651603	0.204	0.082	0.006443711	C9_CD4_Tem
chr1-207823720-207824221	7.33E-08	0.28459556	0.319	0.152	0.004855097	C9_CD4_Tem
chr7-73850821-73852010	1.02E-09	0.320630961	0.234	0.09	6.75E-05	C9_CD4_Tem
chr15-60787735-60788742	3.88E-15	0.401599303	0.207	0.062	2.57E-10	C9_CD4_Tem
chr1-167500396-167500800	2.11E-12	0.36301747	0.146	0.039	1.40E-07	C9_CD4_Tem
chr18-54263259-54264234	3.78E-06	0.260383177	0.126	0.045	0.250320442	C9_CD4_Tem
chr19-38427079-38427610	7.80E-09	0.274996831	0.079	0.017	0.000516566	C9_CD4_Tem
chr9-68974163-68974821	5.59E-09	0.267922239	0.07	0.015	0.00036988	C9_CD4_Tem
chr22-36781643-36782408	2.54E-06	0.258923763	0.308	0.156	0.168530523	C9_CD4_Tem
chr17-16027610-16028503	3.40E-09	0.317877649	0.299	0.129	0.00022536	C9_CD4_Tem
chr13-49569683-49570585	5.14E-10	0.333757538	0.178	0.06	3.40E-05	C9_CD4_Tem
chr2-111705671-111706269	3.21E-10	0.321849367	0.115	0.029	2.12E-05	C9_CD4_Tem
chr12-31748912-31749658	3.34E-06	0.26752302	0.267	0.124	0.221360659	C9_CD4_Tem
chr8-1437996-1438827	6.02E-07	0.25473448	0.088	0.023	0.039897253	C9_CD4_Tem
chr7-30347507-30348626	5.14E-10	0.28047198	0.519	0.282	3.41E-05	C9_CD4_Tem
chr9-89604248-89606232	7.08E-10	0.293888542	0.463	0.234	4.69E-05	C9_CD4_Tem
chr16-10963888-10965181	9.99E-08	0.277182642	0.382	0.191	0.006613149	C9_CD4_Tem
chr20-4726673-4727448	3.54E-10	0.349786314	0.2	0.065	2.34E-05	C9_CD4_Tem
chr15-22263740-22265794	4.13E-11	0.316198746	0.384	0.181	2.74E-06	C9_CD4_Tem
chr8-99705382-99706622	1.19E-10	0.339365701	0.133	0.035	7.88E-06	C9_CD4_Tem
chr15-22262390-22263023	2.11E-05	0.252142429	0.126	0.049	1	C9_CD4_Tem
chr17-80605163-80606101	1.41E-14	0.384286038	0.2	0.059	9.32E-10	C9_CD4_Tem
chr1-193457511-193458066	1.39E-08	0.300065864	0.126	0.038	0.000919796	C9_CD4_Tem
chr13-75325753-75327029	1.07E-06	0.253216382	0.312	0.16	0.071141601	C9_CD4_Tem
chr10-43140168-43140773	2.21E-07	0.290941978	0.279	0.119	0.014614752	C9_CD4_Tem
chr2-112181425-112183042	8.59E-09	0.319780984	0.171	0.054	0.000568907	C9_CD4_Tem
chr15-59919059-59920093	6.90E-11	0.330945197	0.274	0.11	4.57E-06	C9_CD4_Tem

chr12-12481602-12482788	1.46E-08	0.304122375	0.308	0.138	0.000964237	C9_CD4_Tem
chr5-139414470-139415185	1.59E-07	0.257319595	0.067	0.013	0.010507705	C9_CD4_Tem
chr18-13376299-13377630	5.09E-14	0.399631285	0.231	0.071	3.37E-09	C9_CD4_Tem
chr5-134436287-134437225	4.83E-17	0.419151998	0.267	0.088	3.20E-12	C9_CD4_Tem
chr2-102226947-102228524	6.39E-13	0.295576785	0.501	0.256	4.23E-08	C9_CD4_Tem
chr6-20201558-20202568	2.47E-05	0.25745099	0.166	0.068	1	C9_CD4_Tem
chr2-200702511-200703859	1.59E-09	0.330539	0.202	0.073	0.000105022	C9_CD4_Tem
chr13-40205647-40207281	4.69E-09	0.319253097	0.238	0.092	0.000310784	C9_CD4_Tem
chr17-59361301-59362740	8.17E-08	0.293230451	0.204	0.082	0.005411259	C9_CD4_Tem
chr12-12502497-12503926	4.31E-09	0.321125453	0.157	0.048	0.000285125	C9_CD4_Tem
chr1-193458475-193459060	6.04E-14	0.382406587	0.256	0.087	4.00E-09	C9_CD4_Tem
chr7-5641663-5642698	1.03E-08	0.278760152	0.387	0.189	0.000681254	C9_CD4_Tem
chr15-85690491-85691076	2.72E-09	0.323321045	0.263	0.112	0.000179891	C9_CD4_Tem
chr4-169659466-169660333	3.19E-07	0.26911904	0.267	0.127	0.021134878	C9_CD4_Tem
chr3-196563586-196564238	6.43E-27	0.543691474	0.261	0.053	4.26E-22	C9_CD4_Tem
chr1-6459540-6460762	4.44E-10	0.297455001	0.413	0.193	2.94E-05	C9_CD4_Tem
chr2-203729909-203730689	4.40E-06	0.26576249	0.148	0.057	0.291104489	C9_CD4_Tem
chr10-103745191-103745983	7.75E-13	0.382150749	0.151	0.038	5.13E-08	C9_CD4_Tem
chr17-55089250-55090549	1.71E-17	0.422826511	0.279	0.086	1.14E-12	C9_CD4_Tem
chr17-78381190-78382191	4.80E-18	0.351324841	0.571	0.278	3.18E-13	C9_CD4_Tem
chr6-25025724-25026231	1.17E-09	0.335081159	0.135	0.036	7.74E-05	C9_CD4_Tem
chr20-43750868-43751707	5.96E-09	0.313622595	0.207	0.082	0.000395018	C9_CD4_Tem
chr8-70172538-70173138	1.07E-06	0.266390959	0.144	0.056	0.070598428	C9_CD4_Tem
chr19-1213792-1214644	3.90E-08	0.293436509	0.106	0.026	0.002580767	C9_CD4_Tem
chr19-46828234-46828908	2.24E-06	0.251312856	0.09	0.026	0.148328339	C9_CD4_Tem
chr8-9079860-9080508	7.88E-10	0.2972894	0.088	0.019	5.22E-05	C9_CD4_Tem
chr14-73723186-73723723	1.01E-11	0.341534554	0.112	0.024	6.68E-07	C9_CD4_Tem

chr7-106717852-106718819	4.61E-08	0.288146797	0.315	0.147	0.00304994	C9_CD4_Tem
chr7-66859453-66860586	2.17E-09	0.276096193	0.07	0.014	0.000143675	C9_CD4_Tem
chr18-9090970-9091665	8.97E-11	0.323898556	0.252	0.098	5.94E-06	C9_CD4_Tem
chr10-30446945-30447711	7.11E-12	0.356196957	0.175	0.051	4.71E-07	C9_CD4_Tem
chr17-80856148-80856910	5.70E-13	0.374932031	0.299	0.114	3.77E-08	C9_CD4_Tem
chr1-26123193-26123705	1.71E-09	0.320669272	0.164	0.051	0.000113341	C9_CD4_Tem
chr17-48510904-48511570	3.65E-07	0.28806857	0.245	0.112	0.024152587	C9_CD4_Tem
chr8-8796896-8798212	3.76E-24	0.511406476	0.207	0.039	2.49E-19	C9_CD4_Tem
chr2-64212330-64213310	1.58E-08	0.311798069	0.213	0.081	0.001047458	C9_CD4_Tem
chr17-17957677-17958806	9.98E-06	0.261178081	0.178	0.074	0.660817862	C9_CD4_Tem
chr1-236533710-236534731	3.42E-22	0.434624532	0.438	0.162	2.27E-17	C9_CD4_Tem
chr18-9094490-9095605	2.95E-09	0.321757251	0.249	0.098	0.00019514	C9_CD4_Tem
chr9-98074971-98076183	3.59E-10	0.341355669	0.225	0.083	2.37E-05	C9_CD4_Tem
chr16-50780978-50781798	7.27E-10	0.308662539	0.099	0.023	4.81E-05	C9_CD4_Tem
chr10-62082312-62083179	6.76E-07	0.274842333	0.175	0.07	0.044781783	C9_CD4_Tem
chr5-10635257-10635627	1.17E-05	0.253630515	0.175	0.074	0.772698713	C9_CD4_Tem
chr12-6790633-6791092	3.68E-08	0.279610418	0.092	0.022	0.00243415	C9_CD4_Tem
chr2-38681532-38682957	1.03E-07	0.27509785	0.375	0.193	0.006838757	C9_CD4_Tem
chr10-103759005-103759372	3.15E-12	0.352917614	0.103	0.02	2.08E-07	C9_CD4_Tem
chr4-140095635-140096748	3.85E-07	0.26359492	0.317	0.156	0.025504608	C9_CD4_Tem
chr1-116538333-116539064	1.37E-08	0.293308622	0.285	0.124	0.000904835	C9_CD4_Tem
chr12-122412036-122413101	1.75E-06	0.283781706	0.128	0.041	0.115594533	C9_CD4_Tem
chr14-22566788-22567272	9.97E-07	0.288476052	0.24	0.104	0.066003572	C9_CD4_Tem
chr1-21779729-21780061	4.47E-10	0.335088155	0.144	0.044	2.96E-05	C9_CD4_Tem
chr15-41150491-41151266	2.78E-07	0.278340761	0.106	0.03	0.018415609	C9_CD4_Tem
chr1-89635903-89636505	1.56E-09	0.332990376	0.274	0.107	0.000103286	C9_CD4_Tem
chr20-50561187-50561945	9.79E-06	0.268581567	0.142	0.049	0.648188512	C9_CD4_Tem

chr19-35239863-35240702	9.67E-07	0.276031238	0.155	0.06	0.064058547	C9_CD4_Tem
chr14-75535827-75536163	1.34E-06	0.258395307	0.076	0.019	0.088921804	C9_CD4_Tem
chr16-30567958-30568566	3.36E-13	0.374856332	0.301	0.119	2.22E-08	C9_CD4_Tem
chr8-38362481-38363283	1.69E-09	0.328609325	0.2	0.072	0.000112161	C9_CD4_Tem
chr2-174600435-174600753	1.46E-10	0.346301635	0.157	0.046	9.64E-06	C9_CD4_Tem
chr5-151059345-151060210	6.77E-06	0.253076208	0.099	0.031	0.448133923	C9_CD4_Tem
chr8-42893435-42894491	5.28E-08	0.306912674	0.229	0.092	0.003496707	C9_CD4_Tem
chr4-37954100-37954950	1.31E-06	0.266480853	0.137	0.048	0.086968751	C9_CD4_Tem
chr15-72554358-72555267	2.64E-06	0.253266024	0.103	0.034	0.174718459	C9_CD4_Tem
chr15-63479336-63480062	2.21E-09	0.30878166	0.272	0.111	0.000146039	C9_CD4_Tem
chr5-55977602-55978470	1.53E-07	0.294851416	0.173	0.067	0.010113826	C9_CD4_Tem
chr15-90594019-90594836	2.12E-05	0.253211647	0.187	0.079	1	C9_CD4_Tem
chr14-58281110-58281800	4.61E-05	0.250532885	0.126	0.046	1	C9_CD4_Tem
chr17-40612576-40613325	2.67E-06	0.259992326	0.33	0.166	0.17650686	C9_CD4_Tem
chr11-96012030-96012570	1.14E-11	0.345673373	0.306	0.121	7.55E-07	C9_CD4_Tem
chr10-21740597-21741353	3.88E-07	0.276614762	0.162	0.064	0.025700083	C9_CD4_Tem
chr18-9090970-9091665	8.97E-11	0.323898556	0.252	0.098	5.94E-06	C9_CD4_Tem
chr3-71010494-71011430	9.46E-07	0.279588443	0.263	0.118	0.062666709	C9_CD4_Tem
chr17-77324774-77325650	0.000111491	0.253384116	0.222	0.097	1	C9_CD4_Tem
chr1-6459540-6460762	4.44E-10	0.297455001	0.413	0.193	2.94E-05	C9_CD4_Tem
chr2-112181425-112183042	8.59E-09	0.319780984	0.171	0.054	0.000568907	C9_CD4_Tem
chr16-17364067-17364670	1.07E-06	0.264862018	0.312	0.156	0.070876879	C9_CD4_Tem
chr16-4957618-4958544	1.79E-06	0.255991598	0.337	0.168	0.118339771	C9_CD4_Tem
chr11-61026042-61026628	3.09E-06	0.266874456	0.153	0.056	0.204430207	C9_CD4_Tem
chr10-74589097-74589651	6.49E-07	0.270151058	0.281	0.134	0.042961187	C9_CD4_Tem
chr3-32296559-32298058	8.70E-15	0.388067044	0.267	0.091	5.76E-10	C9_CD4_Tem
chr8-19474844-19475909	4.76E-07	0.252797415	0.362	0.181	0.031549898	C9_CD4_Tem

chr17-42337333-42337808	5.58E-07	0.285440091	0.191	0.073	0.03692309	C9_CD4_Tem
chr11-61004107-61005047	1.74E-06	0.251986584	0.079	0.021	0.115274393	C9_CD4_Tem
chr9-70563210-70564249	1.32E-06	0.270999811	0.151	0.059	0.087565893	C9_CD4_Tem
chr8-81153794-81154520	5.03E-07	0.282062339	0.184	0.073	0.033278199	C9_CD4_Tem
chrX-41382908-41384432	5.81E-08	0.291946524	0.187	0.072	0.003850092	C9_CD4_Tem
chr12-12481602-12482788	1.46E-08	0.304122375	0.308	0.138	0.000964237	C9_CD4_Tem
chr8-73990970-73992293	1.63E-06	0.263405834	0.115	0.036	0.107747479	C9_CD4_Tem
chr7-831634-832970	1.74E-06	0.269713958	0.306	0.144	0.115168364	C9_CD4_Tem
chr2-151423049-151424255	3.74E-16	0.389745438	0.33	0.124	2.48E-11	C9_CD4_Tem
chr2-239380549-239381319	2.69E-12	0.360897734	0.292	0.115	1.78E-07	C9_CD4_Tem
chr11-85823641-85824333	1.61E-10	0.2967206	0.427	0.215	1.06E-05	C9_CD4_Tem
chr2-207163495-207163750	1.03E-08	0.293028287	0.09	0.018	0.000683548	C9_CD4_Tem
chr3-32963641-32964287	1.05E-06	0.264364896	0.142	0.052	0.069313047	C9_CD4_Tem
chr9-97904676-97905518	3.11E-07	0.297424721	0.231	0.093	0.020619781	C9_CD4_Tem
chr4-140095635-140096748	3.85E-07	0.26359492	0.317	0.156	0.025504608	C9_CD4_Tem
chr18-13376299-13377630	5.09E-14	0.399631285	0.231	0.071	3.37E-09	C9_CD4_Tem
chr16-89483955-89484408	1.56E-07	0.29731486	0.169	0.067	0.010334306	C9_CD4_Tem
chr12-110590783-110591241	7.96E-08	0.273471648	0.081	0.018	0.005268992	C9_CD4_Tem
chr8-18957182-18957952	1.65E-08	0.30029886	0.137	0.043	0.001089633	C9_CD4_Tem
chr19-43438368-43439720	3.12E-09	0.313202051	0.285	0.124	0.000206494	C9_CD4_Tem
chr9-98074971-98076183	3.59E-10	0.341355669	0.225	0.083	2.37E-05	C9_CD4_Tem
chr15-33104022-33104674	4.83E-06	0.268247331	0.148	0.053	0.319612683	C9_CD4_Tem
chr4-37954100-37954950	1.31E-06	0.266480853	0.137	0.048	0.086968751	C9_CD4_Tem
chr12-50542134-50542831	5.78E-09	0.305593722	0.112	0.03	0.000382655	C9_CD4_Tem
chr10-5291424-5292650	1.10E-28	0.517295926	0.375	0.111	7.27E-24	C9_CD4_Tem
chr4-153496814-153497585	1.90E-05	0.250235181	0.169	0.069	1	C9_CD4_Tem
chr8-19756298-19758064	1.52E-08	0.312229579	0.184	0.063	0.001004142	C9_CD4_Tem

chr5-175652680-175653588	5.65E-06	0.268641373	0.151	0.055	0.374125981	C9_CD4_Tem
chr17-59361301-59362740	8.17E-08	0.293230451	0.204	0.082	0.005411259	C9_CD4_Tem
chr5-76790211-76791350	2.00E-21	0.468297237	0.281	0.078	1.32E-16	C9_CD4_Tem
chr13-110676140-110677459	1.58E-07	0.287959804	0.142	0.046	0.010444268	C9_CD4_Tem
chr5-150780020-150781167	5.46E-08	0.298873279	0.164	0.058	0.003615718	C9_CD4_Tem
chr5-911844-913044	3.32E-41	0.502711597	0.625	0.242	2.20E-36	C9_CD4_Tem
chr17-80678456-80679463	1.56E-08	0.287989483	0.094	0.022	0.001033767	C9_CD4_Tem
chr13-98484347-98485269	6.96E-22	0.439936797	0.429	0.151	4.61E-17	C9_CD4_Tem
chr12-108696348-108696803	7.39E-09	0.313669003	0.11	0.028	0.000489082	C9_CD4_Tem
chr20-50440479-50441792	7.21E-17	0.432583873	0.256	0.076	4.77E-12	C9_CD4_Tem
chr9-97703617-97704354	1.71E-10	0.335715714	0.151	0.044	1.13E-05	C9_CD4_Tem
chr16-4700246-4700755	4.22E-06	0.265613655	0.155	0.061	0.279232969	C9_CD4_Tem
chr2-69774368-69775424	1.90E-07	0.275667457	0.173	0.066	0.012589825	C9_CD4_Tem
chr1-153565588-153566883	8.03E-06	0.255958158	0.285	0.136	0.531962603	C9_CD4_Tem
chr19-17079770-17080390	1.31E-10	0.296852242	0.396	0.187	8.68E-06	C9_CD4_Tem
chr17-80720552-80721593	2.02E-08	0.280629514	0.389	0.188	0.001340269	C9_CD4_Tem
chr20-33369167-33370781	1.27E-12	0.296422359	0.546	0.285	8.41E-08	C9_CD4_Tem
chr12-13179234-13180371	1.12E-06	0.281401491	0.137	0.046	0.074363466	C9_CD4_Tem
chr17-59361301-59362740	8.17E-08	0.293230451	0.204	0.082	0.005411259	C9_CD4_Tem
chr1-116508136-116509326	2.01E-12	0.354079674	0.346	0.141	1.33E-07	C9_CD4_Tem
chr15-101205906-101206664	1.05E-09	0.309244331	0.357	0.16	6.94E-05	C9_CD4_Tem
chr10-119314972-119315950	2.12E-06	0.258966982	0.292	0.141	0.140080792	C9_CD4_Tem
chr1-51569246-51570089	2.74E-07	0.29322615	0.173	0.063	0.018121176	C9_CD4_Tem
chr1-175004976-175005433	6.22E-11	0.323497353	0.097	0.021	4.12E-06	C9_CD4_Tem
chr17-42342317-42343172	2.28E-07	0.28532589	0.202	0.084	0.015105769	C9_CD4_Tem
chr6-38713620-38715736	1.41E-08	0.303569619	0.249	0.107	0.000932141	C9_CD4_Tem
chr8-1744992-1746505	7.97E-06	0.256045834	0.252	0.121	0.527539752	C9_CD4_Tem

chr7-104956630-104957892	5.29E-07	0.280753375	0.218	0.099	0.035022335	C9_CD4_Tem
chr2-43183820-43184763	1.26E-13	0.385951715	0.258	0.089	8.33E-09	C9_CD4_Tem
chr10-12333018-12334141	1.33E-13	0.388367658	0.182	0.05	8.83E-09	C9_CD4_Tem
chr12-51393879-51394537	8.15E-12	0.350001085	0.326	0.133	5.39E-07	C9_CD4_Tem
chr20-57697970-57699159	1.13E-11	0.323605452	0.409	0.185	7.49E-07	C9_CD4_Tem
chr13-99655313-99657122	1.23E-07	0.273911997	0.335	0.17	0.008137458	C9_CD4_Tem
chr6-90411120-90411851	2.29E-14	0.345652914	0.447	0.196	1.52E-09	C9_CD4_Tem
chr9-131500277-131501370	1.57E-06	0.260381109	0.366	0.186	0.10409867	C9_CD4_Tem
chr10-3942601-3943086	3.86E-11	0.345131308	0.121	0.028	2.56E-06	C9_CD4_Tem
chr10-110353290-110354533	1.48E-14	0.331490565	0.488	0.233	9.83E-10	C9_CD4_Tem
chr10-17215994-17216841	1.20E-12	0.337438643	0.434	0.199	7.95E-08	C9_CD4_Tem
chr22-23704649-23705733	1.29E-11	0.360548877	0.198	0.063	8.54E-07	C9_CD4_Tem
chr8-133216576-133218344	5.22E-08	0.253449529	0.515	0.275	0.003454224	C9_CD4_Tem
chr16-66521884-66522606	9.38E-08	0.257707641	0.4	0.206	0.006209956	C9_CD4_Tem
chr3-71503466-71504603	2.76E-06	0.267927795	0.128	0.046	0.183089226	C9_CD4_Tem
chr15-90414487-90414983	5.28E-08	0.293438544	0.117	0.033	0.003495401	C9_CD4_Tem
chr14-91260813-91261599	8.01E-11	0.344830172	0.213	0.074	5.30E-06	C9_CD4_Tem
chr15-60362476-60363105	4.33E-08	0.290204549	0.124	0.039	0.002869742	C9_CD4_Tem
chr6-149232909-149234903	3.00E-14	0.356595435	0.44	0.19	1.98E-09	C9_CD4_Tem
chr4-140103422-140104105	1.60E-10	0.34376879	0.164	0.048	1.06E-05	C9_CD4_Tem
chr13-98480746-98481317	4.45E-18	0.426137824	0.342	0.113	2.95E-13	C9_CD4_Tem
chr15-101167659-101168648	4.84E-07	0.282991876	0.126	0.04	0.032044162	C9_CD4_Tem
chr2-234308137-234309280	4.10E-15	0.386890504	0.384	0.149	2.71E-10	C9_CD4_Tem
chr4-140095635-140096748	3.85E-07	0.26359492	0.317	0.156	0.025504608	C9_CD4_Tem
chr6-130452810-130453731	4.48E-12	0.350568287	0.29	0.111	2.97E-07	C9_CD4_Tem
chr10-17205827-17206776	1.18E-10	0.27452957	0.553	0.285	7.78E-06	C9_CD4_Tem
chr7-149209160-149210278	4.55E-14	0.382821295	0.261	0.091	3.01E-09	C9_CD4_Tem

chr15-40743298-40744759	5.49E-13	0.279705109	0.616	0.331	3.64E-08	C9_CD4_Tem
chr1-160626143-160626423	4.16E-08	0.321505772	0.162	0.053	0.002756678	C9_CD4_Tem
chr2-233247436-233248149	4.20E-16	0.367188382	0.47	0.2	2.78E-11	C9_CD4_Tem
chr8-123083860-123084646	9.16E-11	0.335504162	0.191	0.066	6.06E-06	C9_CD4_Tem
chr19-2627277-2628210	9.53E-11	0.341123753	0.216	0.076	6.31E-06	C9_CD4_Tem
chr22-37303797-37304134	1.25E-05	0.259168252	0.139	0.051	0.826470399	C9_CD4_Tem
chr1-24539976-24540591	1.52E-05	0.2576738	0.153	0.057	1	C9_CD4_Tem
chr15-38688191-38689191	1.32E-06	0.26211525	0.324	0.158	0.087272098	C9_CD4_Tem
chr5-154197444-154198623	3.03E-12	0.34733258	0.357	0.151	2.00E-07	C9_CD4_Tem
chr3-52216805-52217645	3.80E-11	0.348552518	0.263	0.101	2.52E-06	C9_CD4_Tem
chr11-9565650-9566468	3.30E-12	0.300981988	0.481	0.239	2.19E-07	C9_CD4_Tem
chr11-128342576-128343237	2.22E-10	0.344298546	0.238	0.085	1.47E-05	C9_CD4_Tem
chr3-172129658-172130490	2.71E-11	0.354183611	0.265	0.096	1.79E-06	C9_CD4_Tem
chr17-80761178-80761807	8.60E-17	0.428727684	0.274	0.087	5.70E-12	C9_CD4_Tem
chr1-200880056-200880647	1.08E-11	0.35398885	0.191	0.061	7.16E-07	C9_CD4_Tem
chr6-149031799-149033013	3.14E-06	0.257756252	0.22	0.102	0.208188837	C9_CD4_Tem
chr2-231068222-231068820	1.90E-13	0.377313833	0.146	0.034	1.26E-08	C9_CD4_Tem
chr21-42192942-42193602	9.53E-07	0.286597198	0.209	0.088	0.063107028	C9_CD4_Tem
chr11-18383085-18384518	2.52E-08	0.258148123	0.431	0.232	0.001671525	C9_CD4_Tem
chr5-134436287-134437225	4.83E-17	0.419151998	0.267	0.088	3.20E-12	C9_CD4_Tem
chr2-224885889-224886465	1.00E-05	0.25516071	0.112	0.037	0.662965061	C9_CD4_Tem
chr19-39430360-39431582	9.18E-18	0.3571373	0.571	0.267	6.08E-13	C9_CD4_Tem
chr19-4840536-4841612	9.46E-17	0.427247865	0.184	0.042	6.27E-12	C9_CD4_Tem
chr1-206744678-206745677	1.11E-06	0.266504781	0.153	0.061	0.073746784	C9_CD4_Tem
chr6-157760803-157761733	4.19E-17	0.349075161	0.524	0.244	2.77E-12	C9_CD4_Tem
chr12-108691012-108692242	3.11E-11	0.350345213	0.306	0.123	2.06E-06	C9_CD4_Tem
chr12-104218955-104220318	9.08E-06	0.258817104	0.204	0.089	0.601441966	C9_CD4_Tem

chr4-80016587-80017874	5.22E-13	0.381920909	0.182	0.048	3.46E-08	C9_CD4_Tem
chr19-6065663-6066219	9.73E-14	0.388325508	0.231	0.076	6.44E-09	C9_CD4_Tem
chr3-71066873-71067205	1.65E-11	0.35786458	0.231	0.083	1.09E-06	C9_CD4_Tem
chr19-15466742-15467649	6.95E-16	0.396087769	0.375	0.142	4.60E-11	C9_CD4_Tem
chr21-14581882-14582319	5.28E-07	0.275540597	0.108	0.033	0.034949147	C9_CD4_Tem
chr19-19605807-19606451	1.55E-07	0.267898391	0.387	0.206	0.010290766	C9_CD4_Tem
chr1-116538333-116539064	1.37E-08	0.293308622	0.285	0.124	0.000904835	C9_CD4_Tem
chr1-234980422-234981267	1.39E-08	0.309179599	0.285	0.121	0.000920646	C9_CD4_Tem
chr4-8414149-8414744	2.69E-07	0.299694082	0.16	0.05	0.017843883	C9_CD4_Tem
chr5-35816593-35817551	1.16E-06	0.272778266	0.256	0.123	0.076907761	C9_CD4_Tem
chr8-19473047-19473747	2.28E-15	0.376890841	0.373	0.148	1.51E-10	C9_CD4_Tem
chr12-12486741-12487229	8.90E-08	0.286365638	0.092	0.023	0.005891071	C9_CD4_Tem
chr5-50595175-50595563	4.12E-07	0.286749007	0.135	0.049	0.027312844	C9_CD4_Tem
chr13-99307083-99308260	1.41E-08	0.273362761	0.447	0.238	0.000932605	C9_CD4_Tem
chr7-129530639-129531338	5.90E-10	0.331263432	0.213	0.076	3.91E-05	C9_CD4_Tem
chr10-17025489-17025981	1.21E-09	0.333141694	0.272	0.11	8.02E-05	C9_CD4_Tem
chr2-10653525-10654388	2.02E-07	0.292016172	0.261	0.117	0.013407335	C9_CD4_Tem
chrX-40278348-40279261	3.84E-08	0.288708094	0.279	0.121	0.002541374	C9_CD4_Tem
chr2-64212330-64213310	1.58E-08	0.311798069	0.213	0.081	0.001047458	C9_CD4_Tem
chr8-108242901-108243379	3.77E-06	0.261811556	0.178	0.069	0.249688286	C9_CD4_Tem
chr1-200889333-200889886	3.25E-06	0.276080303	0.157	0.062	0.215236204	C9_CD4_Tem
chr11-123453839-123454698	1.28E-19	0.440681518	0.337	0.113	8.50E-15	C9_CD4_Tem
chr20-41002925-41004052	2.32E-16	0.308415151	0.649	0.342	1.53E-11	C9_CD4_Tem
chr15-41940247-41940845	7.56E-07	0.274242189	0.225	0.093	0.050046414	C9_CD4_Tem
chr4-169659466-169660333	3.19E-07	0.26911904	0.267	0.127	0.021134878	C9_CD4_Tem
chr20-50165546-50166389	1.38E-08	0.311731291	0.249	0.1	0.00091707	C9_CD4_Tem
chr13-111176803-111177593	2.93E-06	0.254269811	0.119	0.04	0.194069842	C9_CD4_Tem

chr12-121214138-121214703	5.55E-13	0.378446193	0.162	0.047	3.67E-08	C9_CD4_Tem
chr2-9781835-9782724	7.29E-10	0.319207218	0.333	0.143	4.82E-05	C9_CD4_Tem
chr11-86182850-86184017	9.24E-13	0.362960732	0.243	0.085	6.12E-08	C9_CD4_Tem
chr2-196259643-196260831	1.70E-10	0.334601161	0.299	0.127	1.12E-05	C9_CD4_Tem
chr8-763527-764677	4.72E-14	0.354948874	0.384	0.156	3.12E-09	C9_CD4_Tem
chr2-54046879-54047411	1.82E-07	0.280886145	0.119	0.039	0.01202378	C9_CD4_Tem
chr8-141131689-141132139	1.18E-12	0.359216001	0.342	0.128	7.82E-08	C9_CD4_Tem
chr4-83125500-83126299	2.18E-08	0.316165156	0.193	0.069	0.001444165	C9_CD4_Tem
chr1-184882186-184883369	1.68E-06	0.269908493	0.182	0.076	0.111433286	C9_CD4_Tem
chr12-12474066-12475427	1.04E-07	0.263802333	0.393	0.212	0.006915563	C9_CD4_Tem
chr19-4812769-4813304	9.54E-12	0.345014701	0.099	0.019	6.32E-07	C9_CD4_Tem
chr17-42337333-42337808	5.58E-07	0.285440091	0.191	0.073	0.03692309	C9_CD4_Tem
chr15-60695467-60696216	2.63E-08	0.308676765	0.166	0.056	0.001743617	C9_CD4_Tem
chr11-35029908-35031183	1.41E-09	0.32386435	0.312	0.132	9.33E-05	C9_CD4_Tem
chr13-31044415-31046134	4.42E-09	0.296776586	0.369	0.183	0.00029271	C9_CD4_Tem
chr8-8296057-8296572	1.76E-17	0.403038003	0.094	0.011	1.17E-12	C9_CD4_Tem
chr5-53041016-53041695	1.60E-09	0.328260044	0.157	0.048	0.000105856	C9_CD4_Tem
chr22-40651448-40652410	2.22E-12	0.334210002	0.431	0.191	1.47E-07	C9_CD4_Tem
chr20-45884838-45885282	1.50E-07	0.284021473	0.261	0.112	0.009938424	C9_CD4_Tem
chr2-102226947-102228524	6.39E-13	0.295576785	0.501	0.256	4.23E-08	C9_CD4_Tem
chr12-122946608-122947242	3.97E-06	0.262046017	0.184	0.078	0.263038331	C9_CD4_Tem
chr2-224927518-224928259	2.21E-08	0.257811595	0.465	0.249	0.001465114	C9_CD4_Tem
chr7-37310382-37311803	6.28E-28	0.528139529	0.362	0.099	4.16E-23	C9_CD4_Tem
chr9-91071028-91071984	1.78E-10	0.334865611	0.151	0.045	1.18E-05	C9_CD4_Tem
chr5-80253614-80254145	2.60E-15	0.409087248	0.187	0.047	1.72E-10	C9_CD4_Tem
chr19-19614804-19615420	8.55E-07	0.262284103	0.382	0.194	0.056620917	C9_CD4_Tem
chr3-20047339-20047600	2.43E-10	0.299138885	0.072	0.011	1.61E-05	C9_CD4_Tem

chr4-26092937-26093921	1.35E-11	0.343099528	0.148	0.041	8.95E-07	C9_CD4_Tem
chr3-9354253-9354954	3.26E-06	0.252746373	0.11	0.037	0.216116371	C9_CD4_Tem
chr5-10635257-10635627	1.17E-05	0.253630515	0.175	0.074	0.772698713	C9_CD4_Tem
chr2-102082410-102083466	5.84E-10	0.324761472	0.265	0.11	3.87E-05	C9_CD4_Tem
chr5-53069561-53070033	1.00E-06	0.256104586	0.09	0.025	0.06639585	C9_CD4_Tem
chr12-75943836-75945028	3.63E-08	0.291214575	0.355	0.17	0.002402147	C9_CD4_Tem
chr1-183915646-183916509	6.30E-09	0.280794988	0.396	0.194	0.000417467	C9_CD4_Tem
chr2-224925526-224926533	3.63E-08	0.293192893	0.333	0.154	0.002401511	C9_CD4_Tem
chr1-67162728-67163382	4.53E-06	0.256762484	0.148	0.055	0.300219637	C9_CD4_Tem
chr17-30331547-30332523	8.32E-10	0.33008583	0.288	0.128	5.51E-05	C9_CD4_Tem
chr5-157198163-157199049	3.09E-09	0.273336211	0.438	0.236	0.000204592	C9_CD4_Tem
chr3-49602386-49603513	1.57E-06	0.278151493	0.191	0.08	0.103762245	C9_CD4_Tem
chr11-85750466-85750991	2.70E-10	0.326273697	0.234	0.093	1.79E-05	C9_CD4_Tem
chr15-92921387-92922141	1.10E-10	0.349852669	0.229	0.083	7.28E-06	C9_CD4_Tem
chr14-51827645-51828710	2.15E-08	0.307140619	0.211	0.081	0.001421857	C9_CD4_Tem
chr3-46369598-46370214	1.85E-12	0.362662449	0.317	0.127	1.23E-07	C9_CD4_Tem
chr5-154214266-154214853	9.91E-07	0.270273377	0.106	0.031	0.065604074	C9_CD4_Tem
chr14-61562357-61563185	2.53E-07	0.296390695	0.184	0.065	0.016734328	C9_CD4_Tem
chr10-33263906-33264748	5.74E-09	0.309640894	0.22	0.085	0.000379818	C9_CD4_Tem
chr2-64325332-64326671	1.32E-13	0.311349955	0.503	0.25	8.74E-09	C9_CD4_Tem
chr15-59919059-59920093	6.90E-11	0.330945197	0.274	0.11	4.57E-06	C9_CD4_Tem
chr11-57791789-57792649	6.44E-09	0.293768168	0.36	0.173	0.000426756	C9_CD4_Tem
chr12-107027112-107028011	3.68E-13	0.357380402	0.375	0.152	2.44E-08	C9_CD4_Tem
chr10-3754995-3755391	4.37E-22	0.495325195	0.245	0.058	2.89E-17	C9_CD4_Tem
chr3-15796863-15798271	4.24E-13	0.371738347	0.308	0.116	2.81E-08	C9_CD4_Tem
chr14-50038157-50039127	5.74E-11	0.339203214	0.301	0.12	3.80E-06	C9_CD4_Tem
chr17-65181164-65181535	3.28E-07	0.276480168	0.103	0.03	0.021728754	C9_CD4_Tem

chr15-92906610-92907164	1.45E-09	0.288045222	0.44	0.232	9.63E-05	C9_CD4_Tem
chr12-12439435-12439968	7.83E-08	0.291669053	0.283	0.122	0.005188185	C9_CD4_Tem
chr20-4665558-4666457	5.64E-09	0.325999613	0.258	0.101	0.000373758	C9_CD4_Tem
chr8-42893435-42894491	5.28E-08	0.306912674	0.229	0.092	0.003496707	C9_CD4_Tem
chr5-671624-673152	1.88E-13	0.343404806	0.389	0.177	1.25E-08	C9_CD4_Tem
chr8-29229066-29230362	5.37E-06	0.265972121	0.234	0.106	0.355759841	C9_CD4_Tem
chr5-35773736-35774385	8.07E-11	0.352280846	0.178	0.054	5.35E-06	C9_CD4_Tem
chr9-91130813-91131935	8.08E-07	0.275551935	0.276	0.129	0.05350413	C9_CD4_Tem
chr1-236168709-236169327	1.54E-13	0.336734616	0.07	0.007	1.02E-08	C9_CD4_Tem
chr1-161115296-161115684	9.95E-11	0.351700806	0.175	0.055	6.59E-06	C9_CD4_Tem
chr2-197159068-197160249	1.12E-15	0.366858922	0.436	0.188	7.40E-11	C9_CD4_Tem
chrX-2707275-2707747	3.19E-14	0.389426731	0.319	0.119	2.12E-09	C9_CD4_Tem
chr8-18957182-18957952	1.65E-08	0.30029886	0.137	0.043	0.001089633	C9_CD4_Tem
chr8-47600277-47601292	2.33E-15	0.389455802	0.31	0.121	1.54E-10	C9_CD4_Tem
chr8-9087912-9089120	3.53E-36	0.590423604	0.402	0.098	2.34E-31	C9_CD4_Tem
chr9-127954965-127956136	6.99E-10	0.294672252	0.431	0.21	4.63E-05	C9_CD4_Tem
chr7-149202812-149203252	4.10E-10	0.294004668	0.074	0.012	2.72E-05	C9_CD4_Tem
chr12-13165226-13166007	6.51E-06	0.260482612	0.153	0.063	0.431247601	C9_CD4_Tem
chr8-28371357-28371763	5.06E-12	0.327283671	0.079	0.012	3.35E-07	C9_CD4_Tem
chr3-20057204-20057566	5.72E-16	0.42308887	0.236	0.067	3.78E-11	C9_CD4_Tem
chr4-89316305-89317434	9.35E-10	0.315706721	0.31	0.139	6.19E-05	C9_CD4_Tem
chr1-151311007-151312140	3.86E-12	0.314698314	0.429	0.199	2.55E-07	C9_CD4_Tem
chr22-40240516-40241795	9.69E-08	0.26896248	0.389	0.202	0.006419186	C9_CD4_Tem
chr3-52670349-52671954	5.13E-07	0.298390613	0.211	0.083	0.033953384	C9_CD4_Tem
chr18-58614917-58616188	1.75E-11	0.297242172	0.474	0.225	1.16E-06	C9_CD4_Tem
chr2-100580174-100581452	2.54E-10	0.281524451	0.479	0.246	1.68E-05	C9_CD4_Tem
chr21-14629951-14631144	4.90E-08	0.314674926	0.196	0.071	0.003241919	C9_CD4_Tem

chr17-83048299-83048927	0.000120231	0.255127759	0.191	0.086	1	C9_CD4_Tem
chr17-80605163-80606101	1.41E-14	0.384286038	0.2	0.059	9.32E-10	C9_CD4_Tem
chr1-156222650-156223352	9.40E-08	0.286118291	0.256	0.117	0.006226176	C9_CD4_Tem
chr1-169363114-169363632	4.96E-07	0.277355525	0.202	0.084	0.032870345	C9_CD4_Tem
chr4-80062227-80062862	9.28E-07	0.271553913	0.094	0.028	0.061468753	C9_CD4_Tem
chr1-816763-817436	7.03E-08	0.257572838	0.07	0.016	0.004657534	C9_CD4_Tem
chr8-27613948-27614927	1.27E-06	0.266706627	0.265	0.121	0.084371033	C9_CD4_Tem
chr6-33692475-33693146	3.38E-06	0.275953222	0.178	0.07	0.223816018	C9_CD4_Tem
chr4-80077595-80078076	9.86E-07	0.280356278	0.218	0.095	0.065303798	C9_CD4_Tem
chr1-151898031-151899047	5.01E-06	0.266619203	0.178	0.076	0.33190049	C9_CD4_Tem
chr7-129493631-129494415	3.06E-07	0.269187821	0.108	0.032	0.020293234	C9_CD4_Tem
chr13-98509104-98509860	7.75E-17	0.413400612	0.315	0.107	5.13E-12	C9_CD4_Tem
chr5-96823694-96824144	1.18E-05	0.264025877	0.135	0.05	0.779907883	C9_CD4_Tem
chr3-27533385-27534635	2.52E-11	0.357100053	0.276	0.102	1.67E-06	C9_CD4_Tem
chr6-35709901-35710747	5.77E-10	0.317044637	0.308	0.135	3.82E-05	C9_CD4_Tem
chr5-52987434-52988237	1.13E-12	0.386174918	0.222	0.069	7.50E-08	C9_CD4_Tem
chr3-15805341-15806149	1.43E-10	0.347968876	0.139	0.035	9.46E-06	C9_CD4_Tem
chr6-52392597-52393204	1.38E-08	0.307335919	0.227	0.091	0.000913377	C9_CD4_Tem
chr2-241862902-241863921	2.05E-14	0.381114741	0.335	0.133	1.36E-09	C9_CD4_Tem
chr7-131235252-131236059	1.35E-08	0.289769353	0.366	0.176	0.000897305	C9_CD4_Tem
chr5-80125699-80126474	3.76E-06	0.256556852	0.126	0.046	0.249144062	C9_CD4_Tem
chr16-30458378-30459279	4.43E-06	0.261669446	0.27	0.132	0.293454523	C9_CD4_Tem
chr4-40183602-40184276	8.79E-06	0.255635205	0.097	0.029	0.582021309	C9_CD4_Tem
chr12-111505376-111506090	5.49E-08	0.297111715	0.121	0.037	0.003638473	C9_CD4_Tem
chr5-157602392-157603773	4.56E-15	0.376200258	0.434	0.182	3.02E-10	C9_CD4_Tem
chr4-7827981-7828987	9.15E-11	0.315841118	0.362	0.164	6.06E-06	C9_CD4_Tem
chr3-71050282-71051242	7.27E-12	0.337024491	0.348	0.149	4.81E-07	C9_CD4_Tem

chr14-64723525-64724394	6.54E-13	0.376250909	0.187	0.053	4.33E-08	C9_CD4_Tem
chr6-33694315-33694936	9.39E-08	0.284915968	0.133	0.044	0.006220639	C9_CD4_Tem
chr22-21816219-21817513	6.92E-07	0.294071896	0.234	0.1	0.045835586	C9_CD4_Tem
chr10-25061781-25062986	4.20E-09	0.296623754	0.155	0.053	0.000278014	C9_CD4_Tem
chr14-72476471-72477232	1.52E-05	0.260168758	0.146	0.054	1	C9_CD4_Tem
chr8-70141124-70142656	2.01E-06	0.262895216	0.319	0.159	0.133084018	C9_CD4_Tem
chr1-36373522-36375006	1.64E-08	0.295525147	0.369	0.177	0.001088097	C9_CD4_Tem
chr8-41986545-41987581	7.79E-12	0.358029814	0.236	0.079	5.16E-07	C9_CD4_Tem
chr12-89019257-89019904	1.88E-06	0.277389411	0.162	0.061	0.124770763	C9_CD4_Tem
chr12-96237370-96238109	2.93E-06	0.271837386	0.18	0.071	0.193711488	C9_CD4_Tem
chr11-75536814-75538379	1.77E-06	0.281887543	0.227	0.096	0.117514013	C9_CD4_Tem
chr6-45895170-45895980	1.91E-07	0.277693241	0.209	0.088	0.012674742	C9_CD4_Tem
chr17-59845571-59846531	2.00E-05	0.263189602	0.211	0.094	1	C9_CD4_Tem
chr3-69204017-69204579	3.28E-06	0.253982882	0.106	0.033	0.217527342	C9_CD4_Tem
chr17-29138271-29138885	9.84E-07	0.279494734	0.274	0.128	0.065182109	C9_CD4_Tem
chr8-115446402-115447129	1.55E-11	0.352935991	0.142	0.036	1.03E-06	C9_CD4_Tem
chr17-40612576-40613325	2.67E-06	0.259992326	0.33	0.166	0.17650686	C9_CD4_Tem
chr5-67215359-67216555	3.93E-06	0.260966884	0.173	0.075	0.260198717	C9_CD4_Tem
chr10-110221594-110222149	2.57E-06	0.253507564	0.36	0.18	0.170284342	C9_CD4_Tem
chr1-39185592-39186149	2.23E-05	0.255651628	0.148	0.059	1	C9_CD4_Tem
chr18-77069871-77070267	8.75E-05	0.251911375	0.198	0.091	1	C9_CD4_Tem
chr1-235074458-235075575	2.14E-08	0.298575092	0.297	0.141	0.001413896	C9_CD4_Tem
chr1-39394385-39394975	3.11E-07	0.282644328	0.236	0.1	0.0206048	C9_CD4_Tem
chr22-17074992-17075999	3.21E-10	0.348906492	0.193	0.062	2.13E-05	C9_CD4_Tem
chr15-101199358-101200162	5.61E-08	0.289694654	0.321	0.144	0.003713182	C9_CD4_Tem
chr15-101205093-101205413	5.35E-09	0.296212676	0.094	0.023	0.000354465	C9_CD4_Tem
chr7-44617182-44617939	4.38E-16	0.406751273	0.261	0.081	2.90E-11	C9_CD4_Tem

chr7-831634-832970	1.74E-06	0.269713958	0.306	0.144	0.115168364	C9_CD4_Tem
chr5-40386222-40386698	2.13E-05	0.251261752	0.169	0.072	1	C9_CD4_Tem
chr12-122887370-122889055	3.70E-14	0.386449248	0.335	0.131	2.45E-09	C9_CD4_Tem
chr1-207861454-207862168	1.02E-05	0.259555922	0.155	0.063	0.673143096	C9_CD4_Tem
chr1-40875530-40876133	1.73E-13	0.395247976	0.238	0.075	1.15E-08	C9_CD4_Tem
chr8-19385482-19386169	5.78E-07	0.276048493	0.094	0.026	0.038302365	C9_CD4_Tem
chr4-122539514-122540452	6.87E-13	0.377627294	0.227	0.073	4.55E-08	C9_CD4_Tem
chr13-50244570-50245457	4.38E-06	0.269624561	0.218	0.092	0.289752789	C9_CD4_Tem
chr7-17304802-17305626	2.30E-07	0.297675705	0.243	0.1	0.015232666	C9_CD4_Tem
chr14-61335805-61336213	3.33E-06	0.253406338	0.285	0.138	0.220471586	C9_CD4_Tem
chr1-168386685-168387591	6.54E-10	0.33804064	0.227	0.085	4.33E-05	C9_CD4_Tem
chr16-11253334-11253830	4.53E-07	0.290318162	0.276	0.119	0.030010468	C9_CD4_Tem
chr13-49569683-49570585	5.14E-10	0.333757538	0.178	0.06	3.40E-05	C9_CD4_Tem
chr4-80005707-80006193	3.95E-10	0.337697503	0.187	0.065	2.62E-05	C9_CD4_Tem
chr11-128345266-128345650	1.20E-05	0.259021581	0.142	0.05	0.791493778	C9_CD4_Tem
chr19-21028086-21028767	4.52E-06	0.2598046	0.155	0.065	0.299274053	C9_CD4_Tem
chr12-12465878-12466513	2.96E-10	0.329402217	0.225	0.085	1.96E-05	C9_CD4_Tem
chr6-142838763-142839298	4.63E-06	0.262715451	0.207	0.09	0.306474218	C9_CD4_Tem
chr10-119270835-119271760	1.33E-05	0.254289593	0.189	0.083	0.879223141	C9_CD4_Tem
chr1-184838507-184839002	1.43E-05	0.251922676	0.139	0.05	0.947717781	C9_CD4_Tem
chr8-126505903-126506723	2.18E-07	0.279374763	0.128	0.045	0.014432702	C9_CD4_Tem
chr8-29523882-29524582	9.87E-07	0.278178932	0.173	0.067	0.065360673	C9_CD4_Tem
chr10-32356874-32357860	8.95E-06	0.254905065	0.18	0.081	0.592862439	C9_CD4_Tem
chr20-50561187-50561945	9.79E-06	0.268581567	0.142	0.049	0.648188512	C9_CD4_Tem
chr22-20323896-20324434	2.43E-06	0.272242571	0.137	0.05	0.161174152	C9_CD4_Tem
chr10-131903651-131904012	2.23E-07	0.297061859	0.171	0.063	0.014766477	C9_CD4_Tem
chr19-39398531-39398824	6.36E-06	0.272214494	0.187	0.08	0.421434068	C9_CD4_Tem

chr8-8917273-8918050	4.62E-07	0.284887423	0.292	0.133	0.030597306	C9_CD4_Tem
chr8-8871973-8872681	4.39E-13	0.386162363	0.198	0.057	2.91E-08	C9_CD4_Tem
chr1-184870043-184870702	4.05E-10	0.337392104	0.238	0.084	2.68E-05	C9_CD4_Tem
chr10-103696249-103696786	1.15E-07	0.30302308	0.225	0.093	0.007641914	C9_CD4_Tem
chr16-89440469-89441108	4.08E-06	0.281597165	0.13	0.041	0.27051997	C9_CD4_Tem
chr8-123148856-123149662	9.53E-18	0.442585514	0.258	0.073	6.31E-13	C9_CD4_Tem
chr5-14925373-14926423	1.29E-08	0.314061541	0.222	0.082	0.000851539	C9_CD4_Tem
chr7-129530639-129531338	5.90E-10	0.331263432	0.213	0.076	3.91E-05	C9_CD4_Tem
chr10-119314972-119315950	2.12E-06	0.258966982	0.292	0.141	0.140080792	C9_CD4_Tem
chr5-132102099-132102565	2.98E-05	0.253979725	0.187	0.082	1	C9_CD4_Tem
chr20-33369167-33370781	1.27E-12	0.296422359	0.546	0.285	8.41E-08	C9_CD4_Tem
chr22-40247656-40248434	9.78E-08	0.293934103	0.243	0.106	0.006475636	C9_CD4_Tem
chr6-87731457-87732623	5.49E-09	0.317841817	0.155	0.052	0.000363406	C9_CD4_Tem
chr1-116508136-116509326	2.01E-12	0.354079674	0.346	0.141	1.33E-07	C9_CD4_Tem
chr1-23103217-23103987	1.56E-06	0.277949604	0.265	0.117	0.103241622	C9_CD4_Tem
chr10-31745239-31746004	1.27E-07	0.290279701	0.218	0.087	0.008418985	C9_CD4_Tem
chr8-47600277-47601292	2.33E-15	0.389455802	0.31	0.121	1.54E-10	C9_CD4_Tem
chr8-102097118-102098160	7.81E-08	0.275944042	0.303	0.144	0.005174511	C9_CD4_Tem
chr15-41940247-41940845	7.56E-07	0.274242189	0.225	0.093	0.050046414	C9_CD4_Tem
chr20-4665558-4666457	5.64E-09	0.325999613	0.258	0.101	0.000373758	C9_CD4_Tem
chr3-191328414-191329941	1.04E-14	0.387080514	0.29	0.106	6.91E-10	C9_CD4_Tem
chr19-51272220-51273178	7.99E-07	0.282373832	0.139	0.048	0.052935923	C9_CD4_Tem
chr17-64081788-64082512	1.45E-12	0.375037882	0.247	0.086	9.59E-08	C9_CD4_Tem
chr19-10408493-10409067	1.48E-08	0.293867672	0.344	0.158	0.000979795	C9_CD4_Tem
chr16-47136122-47136709	8.82E-06	0.250159073	0.099	0.031	0.583782729	C9_CD4_Tem
chr5-126811000-126812526	2.89E-14	0.364099567	0.357	0.147	1.91E-09	C9_CD4_Tem
chr9-131656226-131657298	3.30E-08	0.280735096	0.337	0.162	0.002183548	C9_CD4_Tem

chr4-83125500-83126299	2.18E-08	0.316165156	0.193	0.069	0.001444165	C9_CD4_Tem
chr10-6239032-6239606	3.41E-06	0.2723277	0.191	0.081	0.225502163	C9_CD4_Tem
chr14-72476471-72477232	1.52E-05	0.260168758	0.146	0.054	1	C9_CD4_Tem
chr10-110353290-110354533	1.48E-14	0.331490565	0.488	0.233	9.83E-10	C9_CD4_Tem
chr8-58595097-58596320	6.75E-18	0.408199047	0.344	0.132	4.47E-13	C9_CD4_Tem
chr9-5446455-5447136	3.14E-08	0.304264413	0.155	0.055	0.002076518	C9_CD4_Tem
chr10-80498359-80499205	1.06E-05	0.267118127	0.124	0.041	0.701165965	C9_CD4_Tem
chr8-123083860-123084646	9.16E-11	0.335504162	0.191	0.066	6.06E-06	C9_CD4_Tem
chr11-123453839-123454698	1.28E-19	0.440681518	0.337	0.113	8.50E-15	C9_CD4_Tem
chr15-40743298-40744759	5.49E-13	0.279705109	0.616	0.331	3.64E-08	C9_CD4_Tem
chr12-96237370-96238109	2.93E-06	0.271837386	0.18	0.071	0.193711488	C9_CD4_Tem
chr14-51827645-51828710	2.15E-08	0.307140619	0.211	0.081	0.001421857	C9_CD4_Tem
chr17-65022809-65023434	8.31E-08	0.289958069	0.198	0.075	0.005503287	C9_CD4_Tem
chr17-77391924-77392239	7.30E-08	0.269421638	0.076	0.016	0.004833439	C9_CD4_Tem
chr19-18486116-18486883	7.65E-06	0.25665203	0.18	0.075	0.506788475	C9_CD4_Tem
chr2-136238208-136239288	6.90E-13	0.337711316	0.409	0.196	4.57E-08	C9_CD4_Tem
chrX-40278348-40279261	3.84E-08	0.288708094	0.279	0.121	0.002541374	C9_CD4_Tem
chr9-99056882-99058482	7.36E-06	0.252556309	0.312	0.156	0.487349265	C9_CD4_Tem
chr3-46369598-46370214	1.85E-12	0.362662449	0.317	0.127	1.23E-07	C9_CD4_Tem
chr6-90411120-90411851	2.29E-14	0.345652914	0.447	0.196	1.52E-09	C9_CD4_Tem
chr3-196641324-196642171	1.07E-07	0.273421608	0.366	0.188	0.007069037	C9_CD4_Tem
chr6-35709901-35710747	5.77E-10	0.317044637	0.308	0.135	3.82E-05	C9_CD4_Tem
chr2-102082410-102083466	5.84E-10	0.324761472	0.265	0.11	3.87E-05	C9_CD4_Tem
chr21-35043573-35043802	2.75E-05	0.250597724	0.137	0.053	1	C9_CD4_Tem
chr2-223881805-223882274	1.09E-06	0.265634839	0.108	0.035	0.072406737	C9_CD4_Tem
chr2-203711099-203711782	7.50E-08	0.292150744	0.276	0.122	0.004964319	C9_CD4_Tem
chr8-133216576-133218344	5.22E-08	0.253449529	0.515	0.275	0.003454224	C9_CD4_Tem

chr14-61562357-61563185	2.53E-07	0.296390695	0.184	0.065	0.016734328	C9_CD4_Tem
chr16-4700246-4700755	4.22E-06	0.265613655	0.155	0.061	0.279232969	C9_CD4_Tem
chr22-40240516-40241795	9.69E-08	0.26896248	0.389	0.202	0.006419186	C9_CD4_Tem
chr12-75943836-75945028	3.63E-08	0.291214575	0.355	0.17	0.002402147	C9_CD4_Tem
chr3-39146862-39147406	2.25E-07	0.283297186	0.267	0.115	0.014912027	C9_CD4_Tem
chr9-19161181-19162137	2.93E-11	0.320212957	0.404	0.186	1.94E-06	C9_CD4_Tem
chr3-128787317-128788663	7.03E-07	0.281496857	0.283	0.135	0.04655749	C9_CD4_Tem
chr19-39430360-39431582	9.18E-18	0.3571373	0.571	0.267	6.08E-13	C9_CD4_Tem
chr7-43572253-43572850	3.03E-07	0.256171437	0.088	0.026	0.020032949	C9_CD4_Tem
chr16-66521884-66522606	9.38E-08	0.257707641	0.4	0.206	0.006209956	C9_CD4_Tem
chr2-38681532-38682957	1.03E-07	0.27509785	0.375	0.193	0.006838757	C9_CD4_Tem
chr5-157198163-157199049	3.09E-09	0.273336211	0.438	0.236	0.000204592	C9_CD4_Tem
chr11-123429883-123431909	3.14E-07	0.283664001	0.274	0.125	0.020798693	C9_CD4_Tem
chr14-93962689-93963638	9.22E-07	0.258717127	0.389	0.201	0.061060553	C9_CD4_Tem
chr9-91099852-91100585	1.81E-06	0.256771179	0.364	0.182	0.119702551	C9_CD4_Tem
chr19-2627277-2628210	9.53E-11	0.341123753	0.216	0.076	6.31E-06	C9_CD4_Tem
chr5-14809224-14810319	1.38E-08	0.251834079	0.479	0.275	0.000915054	C9_CD4_Tem
chr6-37553397-37554076	2.53E-12	0.328674679	0.42	0.198	1.67E-07	C9_CD4_Tem
chr21-42192942-42193602	9.53E-07	0.286597198	0.209	0.088	0.063107028	C9_CD4_Tem
chr22-23704649-23705733	1.29E-11	0.360548877	0.198	0.063	8.54E-07	C9_CD4_Tem
chr12-107027112-107028011	3.68E-13	0.357380402	0.375	0.152	2.44E-08	C9_CD4_Tem
chr2-102226947-102228524	6.39E-13	0.295576785	0.501	0.256	4.23E-08	C9_CD4_Tem
chr3-49602386-49603513	1.57E-06	0.278151493	0.191	0.08	0.103762245	C9_CD4_Tem
chr10-32356874-32357860	8.95E-06	0.254905065	0.18	0.081	0.592862439	C9_CD4_Tem
chrX-2707275-2707747	3.19E-14	0.389426731	0.319	0.119	2.12E-09	C9_CD4_Tem
chr2-33263-33967	5.21E-07	0.280238134	0.187	0.075	0.034509548	C9_CD4_Tem
chr1-156222650-156223352	9.40E-08	0.286118291	0.256	0.117	0.006226176	C9_CD4_Tem

chr2-197159068-197160249	1.12E-15	0.366858922	0.436	0.188	7.40E-11	C9_CD4_Tem
chr17-35245178-35245565	1.98E-05	0.254164571	0.243	0.119	1	C9_CD4_Tem
chr12-12439435-12439968	7.83E-08	0.291669053	0.283	0.122	0.005188185	C9_CD4_Tem
chr8-70141124-70142656	2.01E-06	0.262895216	0.319	0.159	0.133084018	C9_CD4_Tem
chr3-45963994-45964485	6.24E-06	0.26822128	0.16	0.059	0.413131278	C9_CD4_Tem
chr15-60520034-60521091	2.90E-05	0.251074971	0.139	0.056	1	C9_CD4_Tem
chr9-131500277-131501370	1.57E-06	0.260381109	0.366	0.186	0.10409867	C9_CD4_Tem
chr5-154197444-154198623	3.03E-12	0.34733258	0.357	0.151	2.00E-07	C9_CD4_Tem
chr17-80254523-80255114	2.48E-06	0.270305433	0.267	0.128	0.163926047	C9_CD4_Tem
chr15-60408269-60408889	2.35E-06	0.273090494	0.178	0.071	0.155579514	C9_CD4_Tem
chr20-53948566-53949823	2.03E-05	0.252568212	0.258	0.128	1	C9_CD4_Tem
chr2-227817822-227818927	7.30E-130	1.078387167	0.491	0.053	4.83E-125	C10_CD4_Th17
chr7-36207633-36208476	2.47E-107	1.015201546	0.316	0.018	1.64E-102	C10_CD4_Th17
chr12-68225520-68225959	5.21E-97	0.976831763	0.262	0.008	3.45E-92	C10_CD4_Th17
chr6-167113865-167114853	1.77E-95	0.953354986	0.406	0.048	1.17E-90	C10_CD4_Th17
chr6-161079586-161080584	2.52E-89	0.928023827	0.387	0.051	1.67E-84	C10_CD4_Th17
chr2-227848352-227849017	1.45E-81	0.877363375	0.257	0.015	9.61E-77	C10_CD4_Th17
chr5-127785079-127786007	9.07E-80	0.887883556	0.363	0.046	6.01E-75	C10_CD4_Th17
chr6-161076139-161076710	1.29E-79	0.873906621	0.283	0.022	8.53E-75	C10_CD4_Th17
chr2-119704467-119705063	1.47E-77	0.854622731	0.222	0.009	9.75E-73	C10_CD4_Th17
chr1-150596150-150597076	6.37E-76	0.82050782	0.2	0.006	4.22E-71	C10_CD4_Th17
chr10-32413228-32413966	7.07E-75	0.863131583	0.288	0.027	4.68E-70	C10_CD4_Th17
chr9-81688609-81690098	1.41E-71	0.848734467	0.302	0.031	9.31E-67	C10_CD4_Th17
chr12-95805402-95806330	2.56E-71	0.851550679	0.295	0.031	1.70E-66	C10_CD4_Th17
chr12-109062683-109063090	1.83E-70	0.775169763	0.16	0.002	1.21E-65	C10_CD4_Th17
chr8-92965226-92966277	4.65E-70	0.820930616	0.222	0.013	3.08E-65	C10_CD4_Th17
chr4-26130895-26132202	1.53E-66	0.804371691	0.295	0.036	1.01E-61	C10_CD4_Th17

chr14-49982189-49982981	1.85E-66	0.808463293	0.233	0.017	1.23E-61	C10_CD4_Th17
chr9-134648658-134649514	2.53E-66	0.77373059	0.203	0.01	1.67E-61	C10_CD4_Th17
chr15-90856526-90857139	8.02E-66	0.792590709	0.288	0.035	5.31E-61	C10_CD4_Th17
chr15-62122236-62122615	5.29E-62	0.752510086	0.179	0.007	3.50E-57	C10_CD4_Th17
chr17-82909342-82909983	7.16E-61	0.72562617	0.149	0.003	4.74E-56	C10_CD4_Th17
chr15-41515256-41515610	6.19E-60	0.722985607	0.167	0.006	4.10E-55	C10_CD4_Th17
chr6-125904531-125905320	5.42E-57	0.763655193	0.314	0.051	3.59E-52	C10_CD4_Th17
chr9-37503459-37504182	3.26E-56	0.737260534	0.252	0.03	2.16E-51	C10_CD4_Th17
chr6-137981912-137983144	3.40E-56	0.718017835	0.356	0.08	2.25E-51	C10_CD4_Th17
chr1-67166208-67166540	6.41E-56	0.715547983	0.184	0.013	4.25E-51	C10_CD4_Th17
chr11-114065813-114066852	7.59E-55	0.726499904	0.231	0.024	5.03E-50	C10_CD4_Th17
chr8-85495198-85495771	1.28E-54	0.699769268	0.144	0.003	8.44E-50	C10_CD4_Th17
chr18-45962379-45963321	2.05E-54	0.690201752	0.335	0.076	1.36E-49	C10_CD4_Th17
chr15-58209007-58209494	8.25E-54	0.720565814	0.177	0.01	5.47E-49	C10_CD4_Th17
chr2-227805051-227806081	2.21E-53	0.726087886	0.278	0.04	1.46E-48	C10_CD4_Th17
chr13-98511467-98511799	9.73E-53	0.739235412	0.189	0.013	6.44E-48	C10_CD4_Th17
chr22-31957999-31959012	2.92E-51	0.686099726	0.186	0.015	1.93E-46	C10_CD4_Th17
chr6-13725300-13725980	5.89E-51	0.688203143	0.208	0.022	3.90E-46	C10_CD4_Th17
chr2-161905344-161906009	1.71E-50	0.669697221	0.151	0.006	1.13E-45	C10_CD4_Th17
chr13-51644807-51645667	4.21E-50	0.685905004	0.248	0.034	2.79E-45	C10_CD4_Th17
chr7-36209034-36209548	4.95E-50	0.717987147	0.219	0.023	3.28E-45	C10_CD4_Th17
chr17-17119221-17120015	2.34E-49	0.662220566	0.363	0.095	1.55E-44	C10_CD4_Th17
chr8-37871015-37871537	4.19E-49	0.703447976	0.198	0.017	2.77E-44	C10_CD4_Th17
chr9-134641257-134642360	6.61E-49	0.672732979	0.186	0.015	4.38E-44	C10_CD4_Th17
chr5-133467064-133467755	2.13E-48	0.63350545	0.139	0.006	1.41E-43	C10_CD4_Th17
chrX-20252189-20253296	2.97E-48	0.637786318	0.349	0.09	1.97E-43	C10_CD4_Th17
chr3-56667937-56668459	7.50E-48	0.6664064	0.144	0.007	4.97E-43	C10_CD4_Th17

chr6-167350183-167351772	6.04E-47	0.628691959	0.342	0.086	4.00E-42	C10_CD4_Th17
chr4-86593771-86595286	1.52E-46	0.673212442	0.193	0.021	1.01E-41	C10_CD4_Th17
chr16-17590831-17592322	1.18E-45	0.637998164	0.368	0.106	7.80E-41	C10_CD4_Th17
chr3-171813902-171814427	1.91E-45	0.671874284	0.179	0.017	1.27E-40	C10_CD4_Th17
chr13-86757046-86758112	1.04E-44	0.672282563	0.191	0.019	6.91E-40	C10_CD4_Th17
chr2-148880632-148880921	1.89E-44	0.587246889	0.104	0.002	1.25E-39	C10_CD4_Th17
chr8-66467428-66468210	1.09E-43	0.653263548	0.252	0.044	7.24E-39	C10_CD4_Th17
chr14-67739060-67739535	1.10E-43	0.605627777	0.123	0.004	7.31E-39	C10_CD4_Th17
chr3-151226183-151226453	2.28E-43	0.614503906	0.127	0.006	1.51E-38	C10_CD4_Th17
chr15-62119321-62119669	2.91E-43	0.614072112	0.151	0.012	1.93E-38	C10_CD4_Th17
chr2-25305221-25305473	9.63E-43	0.627807795	0.144	0.009	6.38E-38	C10_CD4_Th17
chr20-44495271-44496462	2.45E-42	0.626285885	0.33	0.093	1.62E-37	C10_CD4_Th17
chr15-90837686-90838896	2.63E-42	0.619918571	0.318	0.08	1.74E-37	C10_CD4_Th17
chr8-144262121-144262803	1.19E-40	0.629682761	0.182	0.021	7.90E-36	C10_CD4_Th17
chr2-9801021-9801585	1.39E-40	0.621802541	0.219	0.036	9.19E-36	C10_CD4_Th17
chr3-9901857-9903161	1.49E-40	0.615052389	0.16	0.015	9.88E-36	C10_CD4_Th17
chr10-119807940-119808556	7.00E-40	0.56551597	0.108	0.004	4.64E-35	C10_CD4_Th17
chr11-132718804-132719896	3.31E-39	0.614223644	0.226	0.041	2.19E-34	C10_CD4_Th17
chr4-55866196-55866746	4.81E-39	0.603384331	0.21	0.037	3.18E-34	C10_CD4_Th17
chr9-72526824-72527815	4.99E-39	0.589463239	0.323	0.094	3.30E-34	C10_CD4_Th17
chr17-48294048-48295769	1.34E-38	0.559678072	0.264	0.073	8.91E-34	C10_CD4_Th17
chr20-35251409-35252730	1.54E-38	0.577964309	0.328	0.094	1.02E-33	C10_CD4_Th17
chr19-49896720-49898048	1.55E-38	0.500281598	0.46	0.193	1.03E-33	C10_CD4_Th17
chr17-66673230-66674233	8.55E-38	0.581928916	0.274	0.069	5.66E-33	C10_CD4_Th17
chr15-60704044-60705101	1.25E-37	0.598233894	0.245	0.054	8.31E-33	C10_CD4_Th17
chr3-33062904-33064000	1.49E-37	0.575066959	0.304	0.085	9.87E-33	C10_CD4_Th17
chr9-72525173-72525531	1.84E-37	0.581932764	0.123	0.007	1.22E-32	C10_CD4_Th17

chr5-126503445-126504007	3.48E-37	0.570640916	0.113	0.005	2.30E-32	C10_CD4_Th17
chr14-68210395-68211363	4.58E-37	0.574579808	0.318	0.091	3.03E-32	C10_CD4_Th17
chr6-167119521-167120297	1.52E-36	0.573595257	0.269	0.063	1.01E-31	C10_CD4_Th17
chr21-32569141-32570111	1.67E-36	0.53237944	0.373	0.144	1.11E-31	C10_CD4_Th17
chr2-100640494-100640836	1.90E-36	0.55096612	0.104	0.003	1.26E-31	C10_CD4_Th17
chr9-69485451-69485804	2.88E-36	0.513539713	0.087	0.002	1.91E-31	C10_CD4_Th17
chr2-178513847-178515083	3.52E-36	0.590651127	0.274	0.067	2.33E-31	C10_CD4_Th17
chr2-110804645-110805248	4.90E-36	0.594474081	0.198	0.033	3.25E-31	C10_CD4_Th17
chr4-114005622-114006382	5.25E-36	0.569023234	0.149	0.014	3.48E-31	C10_CD4_Th17
chr5-142425761-142426571	1.28E-35	0.570417724	0.137	0.012	8.44E-31	C10_CD4_Th17
chr5-177379339-177379698	1.45E-35	0.534906278	0.116	0.008	9.60E-31	C10_CD4_Th17
chr8-41477078-41477677	1.56E-35	0.594959489	0.16	0.018	1.03E-30	C10_CD4_Th17
chr6-161128247-161129161	3.34E-35	0.54685721	0.13	0.01	2.21E-30	C10_CD4_Th17
chr1-67170672-67172385	2.39E-34	0.532729234	0.278	0.081	1.58E-29	C10_CD4_Th17
chr7-139680676-139681239	2.73E-34	0.589282377	0.229	0.049	1.81E-29	C10_CD4_Th17
chr5-154058916-154059988	3.82E-34	0.566122405	0.127	0.011	2.53E-29	C10_CD4_Th17
chr20-35144828-35145367	4.60E-34	0.54362032	0.156	0.022	3.04E-29	C10_CD4_Th17
chr9-81814765-81815383	4.62E-34	0.502464692	0.09	0.003	3.06E-29	C10_CD4_Th17
chr9-129073371-129073779	5.78E-34	0.513491319	0.101	0.005	3.83E-29	C10_CD4_Th17
chr2-65319575-65320709	5.96E-34	0.569915044	0.274	0.071	3.95E-29	C10_CD4_Th17
chr9-2655258-2655979	6.48E-34	0.562331129	0.241	0.056	4.29E-29	C10_CD4_Th17
chr11-73907195-73907619	2.26E-33	0.51624819	0.094	0.004	1.50E-28	C10_CD4_Th17
chr10-96073599-96074055	2.85E-33	0.537645566	0.12	0.01	1.89E-28	C10_CD4_Th17
chr9-91694121-91694796	7.76E-33	0.49897062	0.085	0.002	5.14E-28	C10_CD4_Th17
chr1-57307131-57307908	1.21E-32	0.558033815	0.264	0.068	8.03E-28	C10_CD4_Th17
chr12-54428357-54429067	1.43E-32	0.545070184	0.16	0.024	9.45E-28	C10_CD4_Th17
chr8-60911538-60912419	2.41E-32	0.532876063	0.203	0.043	1.60E-27	C10_CD4_Th17

chr11-14074835-14075632	1.07E-31	0.541569943	0.245	0.058	7.11E-27	C10_CD4_Th17
chr7-151408489-151409399	8.43E-31	0.479733644	0.427	0.182	5.58E-26	C10_CD4_Th17
chr10-29635691-29636561	1.13E-30	0.533756916	0.203	0.044	7.50E-26	C10_CD4_Th17
chr16-79372426-79372948	2.12E-30	0.528367996	0.134	0.016	1.40E-25	C10_CD4_Th17
chr20-59144248-59144825	2.27E-30	0.521524894	0.255	0.07	1.50E-25	C10_CD4_Th17
chr10-126433311-126433678	2.35E-30	0.458850408	0.075	0.002	1.55E-25	C10_CD4_Th17
chr10-6409826-6410656	2.70E-30	0.521353309	0.231	0.064	1.79E-25	C10_CD4_Th17
chr10-6110211-6110788	2.97E-30	0.451722147	0.078	0.002	1.97E-25	C10_CD4_Th17
chr2-100218548-100218858	3.66E-30	0.465102199	0.068	0.001	2.42E-25	C10_CD4_Th17
chr15-64029278-64030445	4.89E-30	0.531249873	0.137	0.017	3.24E-25	C10_CD4_Th17
chr16-21565822-21566225	1.82E-29	0.475075469	0.099	0.007	1.21E-24	C10_CD4_Th17
chr2-41896061-41897028	5.63E-29	0.515379055	0.167	0.029	3.73E-24	C10_CD4_Th17
chr6-140809379-140810129	7.38E-29	0.50674317	0.106	0.008	4.89E-24	C10_CD4_Th17
chr18-23920063-23920381	8.44E-29	0.497216768	0.111	0.01	5.59E-24	C10_CD4_Th17
chr9-33426586-33427344	9.27E-29	0.501498082	0.111	0.01	6.14E-24	C10_CD4_Th17
chr18-54284614-54285581	1.42E-28	0.511475311	0.236	0.065	9.38E-24	C10_CD4_Th17
chr2-8455681-8456469	1.48E-28	0.510883163	0.189	0.045	9.80E-24	C10_CD4_Th17
chr2-213118936-213119809	2.64E-28	0.523057667	0.198	0.05	1.75E-23	C10_CD4_Th17
chr20-33350633-33350965	2.99E-28	0.460732253	0.087	0.005	1.98E-23	C10_CD4_Th17
chr19-18514456-18515253	3.25E-28	0.502635568	0.193	0.042	2.15E-23	C10_CD4_Th17
chr16-17396143-17397864	3.97E-28	0.504796885	0.179	0.038	2.63E-23	C10_CD4_Th17
chr2-227846490-227846703	1.02E-27	0.417511183	0.064	0.001	6.73E-23	C10_CD4_Th17
chr17-33233839-33234106	1.21E-27	0.456302248	0.073	0.002	8.03E-23	C10_CD4_Th17
chr17-4969337-4969558	1.47E-27	0.492816522	0.163	0.031	9.72E-23	C10_CD4_Th17
chr10-6912159-6913209	1.62E-27	0.505152812	0.142	0.021	1.07E-22	C10_CD4_Th17
chr17-48459089-48459298	1.79E-27	0.445776716	0.08	0.003	1.18E-22	C10_CD4_Th17
chr6-3850521-3850997	3.19E-27	0.498250277	0.125	0.016	2.12E-22	C10_CD4_Th17

chr2-100234974-100235684	4.54E-27	0.454118101	0.085	0.004	3.01E-22	C10_CD4_Th17
chr1-193448752-193449502	5.16E-27	0.5266301	0.175	0.035	3.42E-22	C10_CD4_Th17
chr17-55072867-55073264	6.00E-27	0.493552964	0.12	0.012	3.97E-22	C10_CD4_Th17
chr2-148813506-148814147	7.34E-27	0.511822171	0.215	0.06	4.86E-22	C10_CD4_Th17
chr6-79964535-79965015	9.66E-27	0.424093415	0.073	0.003	6.40E-22	C10_CD4_Th17
chr4-4461437-4462256	1.04E-26	0.493598203	0.139	0.02	6.89E-22	C10_CD4_Th17
chr12-27821235-27822714	1.09E-26	0.425963036	0.498	0.254	7.23E-22	C10_CD4_Th17
chr15-60573803-60574631	1.24E-26	0.483398865	0.224	0.063	8.21E-22	C10_CD4_Th17
chr2-9752414-9754775	1.56E-26	0.420403999	0.429	0.211	1.04E-21	C10_CD4_Th17
chr2-161912057-161912415	2.76E-26	0.419345077	0.068	0.002	1.83E-21	C10_CD4_Th17
chr9-69549144-69549701	3.22E-26	0.451631012	0.087	0.005	2.14E-21	C10_CD4_Th17
chr19-7651769-7652521	3.93E-26	0.440322814	0.281	0.108	2.60E-21	C10_CD4_Th17
chr15-90851021-90851484	4.02E-26	0.476367804	0.118	0.014	2.66E-21	C10_CD4_Th17
chr7-139681887-139682681	5.77E-26	0.49494323	0.139	0.023	3.82E-21	C10_CD4_Th17
chr3-188189591-188190116	7.98E-26	0.46555741	0.097	0.009	5.28E-21	C10_CD4_Th17
chr8-47643958-47644536	8.15E-26	0.476371934	0.108	0.011	5.40E-21	C10_CD4_Th17
chr6-109059312-109060140	9.64E-26	0.494030853	0.189	0.044	6.38E-21	C10_CD4_Th17
chr7-140252632-140253466	1.00E-25	0.473313118	0.25	0.079	6.65E-21	C10_CD4_Th17
chr16-69530264-69531572	1.03E-25	0.424835616	0.394	0.185	6.82E-21	C10_CD4_Th17
chr6-45486542-45487098	1.23E-25	0.488561249	0.125	0.017	8.15E-21	C10_CD4_Th17
chr1-32984227-32984776	1.35E-25	0.475103299	0.111	0.012	8.93E-21	C10_CD4_Th17
chr10-126468995-126469650	1.47E-25	0.457015561	0.092	0.006	9.74E-21	C10_CD4_Th17
chr10-102711614-102711895	1.49E-25	0.479232784	0.151	0.031	9.85E-21	C10_CD4_Th17
chr2-86139954-86140280	1.66E-25	0.417962073	0.073	0.003	1.10E-20	C10_CD4_Th17
chr5-126373443-126374288	1.81E-25	0.498565585	0.165	0.031	1.20E-20	C10_CD4_Th17
chr6-80453563-80454609	2.24E-25	0.48078952	0.104	0.01	1.48E-20	C10_CD4_Th17
chr2-161708368-161708991	2.47E-25	0.463459012	0.099	0.009	1.64E-20	C10_CD4_Th17

chr3-133882022-133882565	2.58E-25	0.473528407	0.12	0.018	1.71E-20	C10_CD4_Th17
chr1-116877538-116879084	3.39E-25	0.466850284	0.224	0.065	2.24E-20	C10_CD4_Th17
chr1-193185910-193186733	4.74E-25	0.471058409	0.111	0.013	3.14E-20	C10_CD4_Th17
chr6-14754405-14755441	1.10E-24	0.476139132	0.208	0.055	7.27E-20	C10_CD4_Th17
chr10-77801166-77802052	1.11E-24	0.473763475	0.208	0.059	7.34E-20	C10_CD4_Th17
chr19-8486986-8487803	1.13E-24	0.462256237	0.153	0.033	7.47E-20	C10_CD4_Th17
chr6-157884687-157886211	1.37E-24	0.445804727	0.292	0.108	9.07E-20	C10_CD4_Th17
chr14-35203648-35204531	1.64E-24	0.468145026	0.222	0.069	1.09E-19	C10_CD4_Th17
chr8-100514611-100515752	3.50E-24	0.474901199	0.189	0.05	2.32E-19	C10_CD4_Th17
chr7-139774376-139774627	3.59E-24	0.461191994	0.12	0.019	2.37E-19	C10_CD4_Th17
chr22-46734977-46735397	3.72E-24	0.450532823	0.191	0.046	2.47E-19	C10_CD4_Th17
chr8-60926284-60927881	4.02E-24	0.444107637	0.297	0.117	2.66E-19	C10_CD4_Th17
chr2-227826233-227826923	5.55E-24	0.440614492	0.092	0.008	3.68E-19	C10_CD4_Th17
chr15-90852005-90853142	6.03E-24	0.462014505	0.186	0.049	3.99E-19	C10_CD4_Th17
chr19-19473950-19474762	7.26E-24	0.464660809	0.156	0.031	4.81E-19	C10_CD4_Th17
chr6-83430510-83431262	1.08E-23	0.41559675	0.085	0.006	7.16E-19	C10_CD4_Th17
chr7-106604478-106604890	1.25E-23	0.458025217	0.104	0.012	8.28E-19	C10_CD4_Th17
chr2-161913774-161914080	1.26E-23	0.396163157	0.061	0.002	8.37E-19	C10_CD4_Th17
chr21-44868319-44868616	1.60E-23	0.451292041	0.137	0.027	1.06E-18	C10_CD4_Th17
chr10-126663287-126663754	1.91E-23	0.38260938	0.059	0.001	1.26E-18	C10_CD4_Th17
chr8-66513849-66514263	2.08E-23	0.409310262	0.073	0.004	1.38E-18	C10_CD4_Th17
chr16-78576960-78577960	2.27E-23	0.451979172	0.101	0.012	1.50E-18	C10_CD4_Th17
chr2-38653009-38654335	2.90E-23	0.408702259	0.356	0.164	1.92E-18	C10_CD4_Th17
chr11-126332215-126332648	3.24E-23	0.48245537	0.156	0.029	2.14E-18	C10_CD4_Th17
chr11-85780809-85781911	3.25E-23	0.469030806	0.137	0.025	2.15E-18	C10_CD4_Th17
chr15-72785349-72785762	3.61E-23	0.461144901	0.184	0.049	2.39E-18	C10_CD4_Th17
chr2-9662967-9663752	4.03E-23	0.426578426	0.094	0.01	2.67E-18	C10_CD4_Th17

chr16-2846458-2847278	4.03E-23	0.447409517	0.226	0.075	2.67E-18	C10_CD4_Th17
chr13-111031402-111031886	4.61E-23	0.396163135	0.066	0.003	3.05E-18	C10_CD4_Th17
chr2-241305108-241305678	6.55E-23	0.401432366	0.078	0.005	4.33E-18	C10_CD4_Th17
chr12-64400044-64400292	6.65E-23	0.441898878	0.113	0.018	4.40E-18	C10_CD4_Th17
chr8-85463167-85464357	7.03E-23	0.442188557	0.177	0.049	4.65E-18	C10_CD4_Th17
chr1-240576042-240576673	8.69E-23	0.456827813	0.13	0.023	5.75E-18	C10_CD4_Th17
chr11-35276432-35276662	1.04E-22	0.416395828	0.083	0.006	6.88E-18	C10_CD4_Th17
chr16-75110715-75111531	1.12E-22	0.430709886	0.25	0.096	7.39E-18	C10_CD4_Th17
chr6-14743459-14744292	1.14E-22	0.451152694	0.193	0.053	7.53E-18	C10_CD4_Th17
chr2-148875987-148877033	1.24E-22	0.455952003	0.16	0.036	8.24E-18	C10_CD4_Th17
chr6-45445613-45446115	1.27E-22	0.438882483	0.118	0.018	8.40E-18	C10_CD4_Th17
chr3-12345032-12345532	1.33E-22	0.420212087	0.087	0.009	8.80E-18	C10_CD4_Th17
chr3-183435609-183436073	1.34E-22	0.390347494	0.068	0.004	8.88E-18	C10_CD4_Th17
chr2-9779545-9780603	1.35E-22	0.428542387	0.165	0.042	8.93E-18	C10_CD4_Th17
chr9-33445631-33447874	1.37E-22	0.355337426	0.5	0.302	9.09E-18	C10_CD4_Th17
chr3-171827737-171828145	1.59E-22	0.364751237	0.057	0.002	1.06E-17	C10_CD4_Th17
chr9-72586340-72587114	1.69E-22	0.465606806	0.16	0.035	1.12E-17	C10_CD4_Th17
chr2-12686499-12686940	4.09E-22	0.330078329	0.05	0.001	2.71E-17	C10_CD4_Th17
chr11-11016105-11016472	4.29E-22	0.365677235	0.054	0.001	2.84E-17	C10_CD4_Th17
chr2-190352972-190353625	4.33E-22	0.385978399	0.068	0.004	2.87E-17	C10_CD4_Th17
chr6-36314226-36314607	5.96E-22	0.388590079	0.066	0.003	3.94E-17	C10_CD4_Th17
chr10-6267172-6267557	6.18E-22	0.430689182	0.097	0.012	4.09E-17	C10_CD4_Th17
chr1-46581171-46582211	6.25E-22	0.426977052	0.151	0.035	4.14E-17	C10_CD4_Th17
chr19-4153247-4153676	7.76E-22	0.437899567	0.118	0.019	5.14E-17	C10_CD4_Th17
chr2-204842686-204843391	7.89E-22	0.419614537	0.08	0.006	5.23E-17	C10_CD4_Th17
chr16-10333306-10333601	1.36E-21	0.400964795	0.075	0.005	9.01E-17	C10_CD4_Th17
chr16-79269224-79269811	1.50E-21	0.437441891	0.134	0.027	9.94E-17	C10_CD4_Th17

chr1-232580015-232580725	1.57E-21	0.432924678	0.153	0.036	1.04E-16	C10_CD4_Th17
chr19-14672179-14672585	1.88E-21	0.365391178	0.059	0.002	1.25E-16	C10_CD4_Th17
chrX-154718889-154719827	1.97E-21	0.420970046	0.257	0.093	1.31E-16	C10_CD4_Th17
chr12-89494272-89495220	2.09E-21	0.452317898	0.226	0.076	1.38E-16	C10_CD4_Th17
chr2-148524150-148525059	2.30E-21	0.43313594	0.212	0.066	1.52E-16	C10_CD4_Th17
chr11-9138977-9139270	2.47E-21	0.397646709	0.078	0.006	1.63E-16	C10_CD4_Th17
chr7-17022562-17023399	2.65E-21	0.436829788	0.17	0.043	1.75E-16	C10_CD4_Th17
chr1-87403957-87404678	3.42E-21	0.453977717	0.132	0.027	2.26E-16	C10_CD4_Th17
chr14-55107835-55108566	3.85E-21	0.434842085	0.127	0.027	2.55E-16	C10_CD4_Th17
chr7-74569487-74570050	3.97E-21	0.401406277	0.075	0.006	2.63E-16	C10_CD4_Th17
chr10-133475056-133476222	4.13E-21	0.430474562	0.108	0.016	2.73E-16	C10_CD4_Th17
chr13-97261653-97262370	5.30E-21	0.424897249	0.106	0.015	3.51E-16	C10_CD4_Th17
chr8-57000152-57000767	5.98E-21	0.420362824	0.252	0.098	3.96E-16	C10_CD4_Th17
chr8-42292107-42293538	6.63E-21	0.419923234	0.257	0.096	4.39E-16	C10_CD4_Th17
chr10-110535725-110536609	6.83E-21	0.419143025	0.276	0.115	4.52E-16	C10_CD4_Th17
chr2-69469407-69469683	8.96E-21	0.359540235	0.05	0.001	5.94E-16	C10_CD4_Th17
chr8-140790509-140791760	1.01E-20	0.406835106	0.29	0.132	6.70E-16	C10_CD4_Th17
chr11-114075849-114076084	1.25E-20	0.423188598	0.111	0.017	8.30E-16	C10_CD4_Th17
chr10-127945311-127945732	1.37E-20	0.389377908	0.08	0.007	9.06E-16	C10_CD4_Th17
chrX-19548455-19549140	1.92E-20	0.349161679	0.064	0.003	1.27E-15	C10_CD4_Th17
chr6-138944409-138944667	2.12E-20	0.401668454	0.08	0.008	1.41E-15	C10_CD4_Th17
chr19-4298298-4298743	2.25E-20	0.350319497	0.052	0.002	1.49E-15	C10_CD4_Th17
chr13-40653124-40654006	2.82E-20	0.42405736	0.139	0.032	1.86E-15	C10_CD4_Th17
chr17-62724320-62725192	2.91E-20	0.420648427	0.191	0.056	1.92E-15	C10_CD4_Th17
chr18-63223870-63224886	3.12E-20	0.419151349	0.146	0.036	2.06E-15	C10_CD4_Th17
chr10-72083641-72084362	3.43E-20	0.40558181	0.222	0.083	2.27E-15	C10_CD4_Th17
chr19-47182300-47182765	4.18E-20	0.42775386	0.151	0.039	2.77E-15	C10_CD4_Th17

chr12-117105531-117106254	6.28E-20	0.426954987	0.13	0.026	4.16E-15	C10_CD4_Th17
chr10-11156277-11156991	6.59E-20	0.416422713	0.142	0.035	4.36E-15	C10_CD4_Th17
chr17-58387528-58388196	6.82E-20	0.416261831	0.099	0.016	4.51E-15	C10_CD4_Th17
chr8-60908981-60910155	6.83E-20	0.409642034	0.149	0.034	4.52E-15	C10_CD4_Th17
chr11-114099393-114100021	7.19E-20	0.405788389	0.087	0.009	4.76E-15	C10_CD4_Th17
chr3-109806435-109807498	7.33E-20	0.382189471	0.337	0.16	4.85E-15	C10_CD4_Th17
chr1-151826072-151826408	8.32E-20	0.38128174	0.085	0.009	5.51E-15	C10_CD4_Th17
chr3-10232066-10232526	9.27E-20	0.345508555	0.052	0.002	6.14E-15	C10_CD4_Th17
chr11-13332626-13333039	1.00E-19	0.427553463	0.118	0.023	6.65E-15	C10_CD4_Th17
chr6-90078895-90081514	1.02E-19	0.359295661	0.394	0.208	6.75E-15	C10_CD4_Th17
chr12-92210105-92210495	1.08E-19	0.412841895	0.144	0.036	7.14E-15	C10_CD4_Th17
chr6-20266825-20267407	1.13E-19	0.378274775	0.061	0.003	7.49E-15	C10_CD4_Th17
chr19-7678958-7679757	1.15E-19	0.395576963	0.271	0.112	7.62E-15	C10_CD4_Th17
chr1-193478916-193479653	1.40E-19	0.398017092	0.276	0.115	9.26E-15	C10_CD4_Th17
chr10-69359169-69359953	1.49E-19	0.396798448	0.09	0.012	9.89E-15	C10_CD4_Th17
chr8-27333049-27333558	1.51E-19	0.416856926	0.118	0.024	1.00E-14	C10_CD4_Th17
chr17-69602834-69603373	1.67E-19	0.379417456	0.083	0.008	1.11E-14	C10_CD4_Th17
chr8-104588404-104589550	1.75E-19	0.412836997	0.175	0.046	1.16E-14	C10_CD4_Th17
chr3-138327695-138328096	1.78E-19	0.415955331	0.111	0.02	1.18E-14	C10_CD4_Th17
chr8-47737262-47739144	1.80E-19	0.403493839	0.215	0.074	1.19E-14	C10_CD4_Th17
chr15-68209730-68210150	1.83E-19	0.384724195	0.08	0.008	1.21E-14	C10_CD4_Th17
chr6-13747282-13747966	1.90E-19	0.40329107	0.245	0.089	1.26E-14	C10_CD4_Th17
chr8-11421601-11422174	2.01E-19	0.407859063	0.101	0.016	1.33E-14	C10_CD4_Th17
chr16-74888078-74888607	2.02E-19	0.354607012	0.061	0.004	1.34E-14	C10_CD4_Th17
chr1-50450889-50451613	2.31E-19	0.354540233	0.068	0.005	1.53E-14	C10_CD4_Th17
chr11-114073591-114074289	2.67E-19	0.430462904	0.172	0.045	1.77E-14	C10_CD4_Th17
chr5-154311383-154311735	3.61E-19	0.343530071	0.054	0.002	2.39E-14	C10_CD4_Th17

chr12-68289781-68290569	3.66E-19	0.400555877	0.241	0.095	2.43E-14	C10_CD4_Th17
chr10-110857274-110857907	3.77E-19	0.406188055	0.146	0.038	2.49E-14	C10_CD4_Th17
chr7-150579983-150580555	3.89E-19	0.382716216	0.066	0.006	2.58E-14	C10_CD4_Th17
chr18-3624044-3625432	4.17E-19	0.406830084	0.179	0.056	2.76E-14	C10_CD4_Th17
chr3-45993101-45994130	4.42E-19	0.346744984	0.396	0.217	2.93E-14	C10_CD4_Th17
chr14-71346394-71347012	5.23E-19	0.396659021	0.106	0.019	3.46E-14	C10_CD4_Th17
chr4-54657579-54658803	6.73E-19	0.357742015	0.066	0.005	4.46E-14	C10_CD4_Th17
chr18-23972303-23972734	7.09E-19	0.403750346	0.189	0.06	4.70E-14	C10_CD4_Th17
chr6-161029020-161029776	7.58E-19	0.370608671	0.083	0.01	5.02E-14	C10_CD4_Th17
chr20-53563774-53564141	7.64E-19	0.334912736	0.052	0.002	5.06E-14	C10_CD4_Th17
chr15-62098970-62099403	8.50E-19	0.335859465	0.054	0.002	5.63E-14	C10_CD4_Th17
chr9-69474785-69475156	8.71E-19	0.332865366	0.057	0.003	5.77E-14	C10_CD4_Th17
chr3-43769461-43771027	1.07E-18	0.417391766	0.219	0.075	7.11E-14	C10_CD4_Th17
chr10-8182810-8183774	1.28E-18	0.395847969	0.205	0.074	8.48E-14	C10_CD4_Th17
chr6-167091210-167091613	1.54E-18	0.390376656	0.094	0.014	1.02E-13	C10_CD4_Th17
chr10-110273509-110273786	1.54E-18	0.349805943	0.071	0.007	1.02E-13	C10_CD4_Th17
chr7-139771020-139771353	1.63E-18	0.371163278	0.075	0.008	1.08E-13	C10_CD4_Th17
chr12-12467236-12468134	1.90E-18	0.393318938	0.137	0.033	1.26E-13	C10_CD4_Th17
chr14-88044776-88045319	1.93E-18	0.36300437	0.066	0.005	1.28E-13	C10_CD4_Th17
chr22-42179959-42180790	1.95E-18	0.389776103	0.203	0.07	1.29E-13	C10_CD4_Th17
chr6-37134353-37134959	2.79E-18	0.401945001	0.17	0.05	1.85E-13	C10_CD4_Th17
chr10-3221985-3222470	2.79E-18	0.340205165	0.057	0.004	1.85E-13	C10_CD4_Th17
chr3-12335775-12336469	3.39E-18	0.385585797	0.09	0.013	2.24E-13	C10_CD4_Th17
chr14-68404523-68405009	4.67E-18	0.368921062	0.073	0.007	3.09E-13	C10_CD4_Th17
chr8-130022048-130022781	4.89E-18	0.376736965	0.073	0.007	3.24E-13	C10_CD4_Th17
chr5-177388823-177390851	5.37E-18	0.315714494	0.436	0.269	3.55E-13	C10_CD4_Th17
chr5-151745041-151745697	5.73E-18	0.392729088	0.132	0.03	3.80E-13	C10_CD4_Th17

chr2-136320293-136320773	6.52E-18	0.347009791	0.068	0.007	4.32E-13	C10_CD4_Th17
chr7-151206774-151207717	6.76E-18	0.383813861	0.108	0.023	4.48E-13	C10_CD4_Th17
chr6-90256690-90257351	6.81E-18	0.389316746	0.104	0.019	4.51E-13	C10_CD4_Th17
chr12-92065017-92066011	6.94E-18	0.389364089	0.137	0.034	4.60E-13	C10_CD4_Th17
chr10-6136172-6136752	9.07E-18	0.394537079	0.132	0.033	6.01E-13	C10_CD4_Th17
chr20-59242622-59243095	9.70E-18	0.394489289	0.158	0.044	6.42E-13	C10_CD4_Th17
chr7-87574464-87574973	1.08E-17	0.338310053	0.066	0.006	7.17E-13	C10_CD4_Th17
chr3-10191851-10192457	1.68E-17	0.373478851	0.177	0.058	1.11E-12	C10_CD4_Th17
chr16-85999262-85999600	1.74E-17	0.321531025	0.054	0.003	1.15E-12	C10_CD4_Th17
chr11-12189498-12190192	1.95E-17	0.39644782	0.12	0.027	1.29E-12	C10_CD4_Th17
chr4-54709370-54709817	2.54E-17	0.326322844	0.052	0.002	1.68E-12	C10_CD4_Th17
chr1-15416747-15417980	2.88E-17	0.334117744	0.34	0.183	1.91E-12	C10_CD4_Th17
chrX-118974150-118976707	2.88E-17	0.363882048	0.233	0.096	1.91E-12	C10_CD4_Th17
chr9-104927208-104928364	3.04E-17	0.366256895	0.179	0.07	2.01E-12	C10_CD4_Th17
chr3-183287204-183287477	3.05E-17	0.361850384	0.085	0.014	2.02E-12	C10_CD4_Th17
chr21-17799296-17799732	3.17E-17	0.383673326	0.108	0.023	2.10E-12	C10_CD4_Th17
chr12-107515226-107515892	3.36E-17	0.37982313	0.092	0.015	2.23E-12	C10_CD4_Th17
chr9-72557302-72557881	3.44E-17	0.370288188	0.087	0.012	2.28E-12	C10_CD4_Th17
chr6-167129175-167130547	4.14E-17	0.386693893	0.13	0.03	2.74E-12	C10_CD4_Th17
chr20-37990033-37990949	4.20E-17	0.399517063	0.196	0.067	2.78E-12	C10_CD4_Th17
chr12-95789734-95791401	4.28E-17	0.377076476	0.108	0.022	2.83E-12	C10_CD4_Th17
chr3-5002037-5002968	5.10E-17	0.346413798	0.382	0.194	3.38E-12	C10_CD4_Th17
chr19-34308731-34309315	5.28E-17	0.373932998	0.104	0.02	3.49E-12	C10_CD4_Th17
chr20-32967882-32968455	5.70E-17	0.377977604	0.193	0.066	3.77E-12	C10_CD4_Th17
chr21-46515022-46515535	5.78E-17	0.370885776	0.106	0.023	3.83E-12	C10_CD4_Th17
chr8-128076511-128077372	5.97E-17	0.381569156	0.259	0.106	3.95E-12	C10_CD4_Th17
chr11-121464061-121464975	6.05E-17	0.319107681	0.467	0.28	4.01E-12	C10_CD4_Th17

chr18-13276569-13276849	6.26E-17	0.347547742	0.059	0.005	4.14E-12	C10_CD4_Th17
chr7-131122216-131122728	6.33E-17	0.342077863	0.064	0.005	4.19E-12	C10_CD4_Th17
chr2-100060676-100061238	6.58E-17	0.338307279	0.061	0.006	4.36E-12	C10_CD4_Th17
chr17-4110216-4110523	6.64E-17	0.324062888	0.057	0.004	4.40E-12	C10_CD4_Th17
chr11-9158776-9159377	6.66E-17	0.36824066	0.085	0.012	4.41E-12	C10_CD4_Th17
chr16-53098482-53100073	7.83E-17	0.336735995	0.351	0.189	5.18E-12	C10_CD4_Th17
chr12-8492747-8493489	9.22E-17	0.394285374	0.149	0.04	6.11E-12	C10_CD4_Th17
chr6-5517794-5518190	9.24E-17	0.377938344	0.099	0.02	6.12E-12	C10_CD4_Th17
chr1-84853880-84854757	9.59E-17	0.378607313	0.248	0.106	6.35E-12	C10_CD4_Th17
chr5-131538061-131538535	1.07E-16	0.373372232	0.099	0.02	7.08E-12	C10_CD4_Th17
chr19-14683395-14683743	1.09E-16	0.323982892	0.05	0.003	7.19E-12	C10_CD4_Th17
chr12-12489029-12489492	1.11E-16	0.365598194	0.085	0.012	7.37E-12	C10_CD4_Th17
chr22-40257903-40258874	1.15E-16	0.374575783	0.17	0.054	7.62E-12	C10_CD4_Th17
chr15-56042896-56043976	1.19E-16	0.360619457	0.25	0.103	7.91E-12	C10_CD4_Th17
chr17-18563385-18564364	1.23E-16	0.370061409	0.101	0.019	8.12E-12	C10_CD4_Th17
chr2-10544065-10544785	1.25E-16	0.39312038	0.158	0.049	8.27E-12	C10_CD4_Th17
chr12-94180453-94181290	1.40E-16	0.384575637	0.153	0.046	9.24E-12	C10_CD4_Th17
chr22-49838544-49839669	1.45E-16	0.285652427	0.512	0.352	9.58E-12	C10_CD4_Th17
chr6-45434049-45434510	1.56E-16	0.346355868	0.08	0.012	1.03E-11	C10_CD4_Th17
chr17-76659451-76660554	1.58E-16	0.374441137	0.165	0.049	1.05E-11	C10_CD4_Th17
chr5-131513843-131515241	1.68E-16	0.361671982	0.226	0.099	1.11E-11	C10_CD4_Th17
chr5-57117109-57118076	1.72E-16	0.385566619	0.134	0.038	1.14E-11	C10_CD4_Th17
chr15-85024625-85025243	1.97E-16	0.315199234	0.054	0.004	1.30E-11	C10_CD4_Th17
chr22-42184905-42185202	2.26E-16	0.339775049	0.066	0.007	1.50E-11	C10_CD4_Th17
chr4-10106029-10106991	2.32E-16	0.329500744	0.38	0.204	1.54E-11	C10_CD4_Th17
chr9-126470334-126471491	2.33E-16	0.332098319	0.33	0.177	1.54E-11	C10_CD4_Th17
chr5-144177169-144177936	2.36E-16	0.356257625	0.097	0.019	1.56E-11	C10_CD4_Th17

chr1-185280844-185281643	2.90E-16	0.364758273	0.083	0.013	1.92E-11	C10_CD4_Th17
chr6-137967768-137969282	2.96E-16	0.373843852	0.13	0.034	1.96E-11	C10_CD4_Th17
chr7-130938262-130939034	3.06E-16	0.340680863	0.311	0.16	2.02E-11	C10_CD4_Th17
chr16-75098154-75098993	3.64E-16	0.357585765	0.262	0.118	2.41E-11	C10_CD4_Th17
chr11-123302728-123303680	3.82E-16	0.365457844	0.116	0.026	2.53E-11	C10_CD4_Th17
chr12-92624234-92624536	4.02E-16	0.321609309	0.064	0.005	2.66E-11	C10_CD4_Th17
chr22-47494984-47495456	4.99E-16	0.334494414	0.064	0.006	3.30E-11	C10_CD4_Th17
chr10-124657611-124658408	5.28E-16	0.365087671	0.163	0.054	3.49E-11	C10_CD4_Th17
chr6-37137037-37137801	5.49E-16	0.371951612	0.149	0.043	3.64E-11	C10_CD4_Th17
chr14-88029364-88030417	5.92E-16	0.370917587	0.172	0.055	3.92E-11	C10_CD4_Th17
chr1-194289384-194290132	7.99E-16	0.361544666	0.127	0.033	5.29E-11	C10_CD4_Th17
chr10-44576700-44577211	9.08E-16	0.343704196	0.061	0.006	6.01E-11	C10_CD4_Th17
chr8-27408908-27409173	9.83E-16	0.328514279	0.066	0.007	6.51E-11	C10_CD4_Th17
chr3-45946402-45947147	1.03E-15	0.359352006	0.196	0.076	6.83E-11	C10_CD4_Th17
chr14-73829509-73830403	1.07E-15	0.363865517	0.156	0.055	7.09E-11	C10_CD4_Th17
chr16-30567147-30567507	1.07E-15	0.355667734	0.215	0.084	7.11E-11	C10_CD4_Th17
chr15-60539991-60540712	1.09E-15	0.35262747	0.231	0.095	7.19E-11	C10_CD4_Th17
chr15-60563335-60564014	1.16E-15	0.356599575	0.144	0.042	7.71E-11	C10_CD4_Th17
chr2-119782296-119782616	1.19E-15	0.302227763	0.05	0.003	7.88E-11	C10_CD4_Th17
chr12-52243794-52244465	1.21E-15	0.358401057	0.104	0.02	8.02E-11	C10_CD4_Th17
chr22-38305504-38306477	1.40E-15	0.363258337	0.205	0.083	9.30E-11	C10_CD4_Th17
chr1-116389550-116390529	1.45E-15	0.339224069	0.231	0.107	9.58E-11	C10_CD4_Th17
chr10-62696639-62697146	1.45E-15	0.359643402	0.116	0.029	9.58E-11	C10_CD4_Th17
chr17-66691489-66691992	1.54E-15	0.340769017	0.068	0.008	1.02E-10	C10_CD4_Th17
chr8-118563808-118564448	1.62E-15	0.357796793	0.09	0.016	1.07E-10	C10_CD4_Th17
chr4-15725641-15726090	1.98E-15	0.316436771	0.05	0.003	1.31E-10	C10_CD4_Th17
chr2-234474955-234475474	2.09E-15	0.339194067	0.087	0.014	1.38E-10	C10_CD4_Th17

chr17-49748257-49749271	2.09E-15	0.315117029	0.347	0.19	1.38E-10	C10_CD4_Th17
chr15-78939119-78939535	2.17E-15	0.312621456	0.064	0.007	1.44E-10	C10_CD4_Th17
chr7-152344194-152344665	2.29E-15	0.339725233	0.064	0.008	1.52E-10	C10_CD4_Th17
chr6-44976789-44977679	2.54E-15	0.35507767	0.099	0.022	1.68E-10	C10_CD4_Th17
chr12-53236475-53236752	2.55E-15	0.343942943	0.094	0.018	1.69E-10	C10_CD4_Th17
chr1-32992407-32992873	2.65E-15	0.323920548	0.061	0.006	1.76E-10	C10_CD4_Th17
chr3-10198637-10199412	3.25E-15	0.354917238	0.153	0.053	2.16E-10	C10_CD4_Th17
chr11-128343864-128344192	3.43E-15	0.35602416	0.113	0.027	2.27E-10	C10_CD4_Th17
chr2-191037975-191038812	3.65E-15	0.358679793	0.198	0.077	2.42E-10	C10_CD4_Th17
chr21-44870488-44870976	3.85E-15	0.354121101	0.146	0.048	2.55E-10	C10_CD4_Th17
chr5-177372197-177373250	4.11E-15	0.343760893	0.198	0.079	2.72E-10	C10_CD4_Th17
chr1-198174311-198174649	4.46E-15	0.339652748	0.085	0.016	2.95E-10	C10_CD4_Th17
chr7-50396787-50397617	4.71E-15	0.354294989	0.156	0.055	3.12E-10	C10_CD4_Th17
chr6-167056170-167056709	5.33E-15	0.359041931	0.106	0.025	3.53E-10	C10_CD4_Th17
chr14-91193828-91194842	5.57E-15	0.350466794	0.151	0.05	3.69E-10	C10_CD4_Th17
chr13-31806431-31807534	6.55E-15	0.355083226	0.113	0.029	4.34E-10	C10_CD4_Th17
chr17-29154774-29155846	6.87E-15	0.352423263	0.153	0.056	4.55E-10	C10_CD4_Th17
chr10-6129689-6130528	7.82E-15	0.341933539	0.125	0.035	5.18E-10	C10_CD4_Th17
chr2-161952554-161953235	7.88E-15	0.355485311	0.127	0.033	5.22E-10	C10_CD4_Th17
chr5-132115179-132115701	8.70E-15	0.310048739	0.064	0.008	5.76E-10	C10_CD4_Th17
chr1-23108896-23109274	9.18E-15	0.327202402	0.073	0.011	6.08E-10	C10_CD4_Th17
chr15-62128880-62129222	9.39E-15	0.326989889	0.059	0.006	6.22E-10	C10_CD4_Th17
chr14-87959829-87960695	9.46E-15	0.343120027	0.09	0.016	6.27E-10	C10_CD4_Th17
chr16-24721591-24722189	9.77E-15	0.358316927	0.13	0.04	6.47E-10	C10_CD4_Th17
chr14-95480032-95480905	1.03E-14	0.352485195	0.177	0.068	6.83E-10	C10_CD4_Th17
chr2-12556641-12557091	1.13E-14	0.345673391	0.085	0.016	7.47E-10	C10_CD4_Th17
chr13-98556538-98557679	1.18E-14	0.360871475	0.123	0.034	7.83E-10	C10_CD4_Th17

chr8-47573303-47574822	1.20E-14	0.331349814	0.123	0.035	7.93E-10	C10_CD4_Th17
chr15-60932224-60932934	1.21E-14	0.355523145	0.172	0.066	8.02E-10	C10_CD4_Th17
chr10-70616681-70617364	1.27E-14	0.349263141	0.12	0.033	8.41E-10	C10_CD4_Th17
chr1-151836502-151837667	1.41E-14	0.316168297	0.264	0.13	9.35E-10	C10_CD4_Th17
chr15-39629099-39629839	1.47E-14	0.321376293	0.342	0.189	9.72E-10	C10_CD4_Th17
chr9-73869242-73869651	1.51E-14	0.359526453	0.113	0.029	9.98E-10	C10_CD4_Th17
chr1-212488422-212488879	1.51E-14	0.316755478	0.054	0.005	1.00E-09	C10_CD4_Th17
chr5-95631738-95632499	1.54E-14	0.355042199	0.193	0.08	1.02E-09	C10_CD4_Th17
chr6-138946733-138947227	1.60E-14	0.329249285	0.087	0.017	1.06E-09	C10_CD4_Th17
chr6-156396046-156397051	1.70E-14	0.335547412	0.087	0.017	1.13E-09	C10_CD4_Th17
chr6-167079773-167080194	1.76E-14	0.3402556	0.083	0.016	1.16E-09	C10_CD4_Th17
chr1-30191132-30191709	2.11E-14	0.337195065	0.101	0.022	1.40E-09	C10_CD4_Th17
chr21-32515402-32516067	2.18E-14	0.293501928	0.05	0.004	1.44E-09	C10_CD4_Th17
chr1-151830429-151831038	2.50E-14	0.337710714	0.193	0.076	1.66E-09	C10_CD4_Th17
chr10-100020029-100020861	3.07E-14	0.333038069	0.068	0.009	2.03E-09	C10_CD4_Th17
chr19-45455498-45456572	3.09E-14	0.31343777	0.278	0.141	2.04E-09	C10_CD4_Th17
chr1-210331995-210333134	3.10E-14	0.334066588	0.288	0.135	2.05E-09	C10_CD4_Th17
chr8-97979993-97980496	3.22E-14	0.332440706	0.087	0.017	2.13E-09	C10_CD4_Th17
chr11-3904661-3905532	3.33E-14	0.344611105	0.241	0.109	2.21E-09	C10_CD4_Th17
chr5-59132216-59132428	3.70E-14	0.32555263	0.066	0.009	2.45E-09	C10_CD4_Th17
chr11-118201941-118202904	3.74E-14	0.327861946	0.165	0.063	2.48E-09	C10_CD4_Th17
chr8-89728858-89729660	4.29E-14	0.334411763	0.236	0.111	2.84E-09	C10_CD4_Th17
chr6-140043297-140043746	4.29E-14	0.303048824	0.05	0.004	2.84E-09	C10_CD4_Th17
chr2-73896226-73897070	4.43E-14	0.330899733	0.099	0.023	2.94E-09	C10_CD4_Th17
chr6-161038172-161038783	5.04E-14	0.325789691	0.08	0.015	3.34E-09	C10_CD4_Th17
chr10-11183040-11183323	5.71E-14	0.318954926	0.087	0.02	3.78E-09	C10_CD4_Th17
chr16-81523422-81524781	5.99E-14	0.273190414	0.486	0.312	3.97E-09	C10_CD4_Th17

chr2-100643486-100644472	6.04E-14	0.33785735	0.146	0.046	4.00E-09	C10_CD4_Th17
chr15-73225304-73225745	6.20E-14	0.335881928	0.083	0.016	4.11E-09	C10_CD4_Th17
chr3-45915397-45916300	6.27E-14	0.318237306	0.212	0.101	4.15E-09	C10_CD4_Th17
chr9-128060897-128061424	6.32E-14	0.299888913	0.057	0.006	4.18E-09	C10_CD4_Th17
chr1-203755930-203757037	7.03E-14	0.330302865	0.267	0.128	4.66E-09	C10_CD4_Th17
chr5-56828983-56829541	7.70E-14	0.335271653	0.149	0.051	5.10E-09	C10_CD4_Th17
chr14-52918251-52918924	7.87E-14	0.329123451	0.215	0.095	5.21E-09	C10_CD4_Th17
chr6-158025904-158027160	7.99E-14	0.290912741	0.38	0.236	5.29E-09	C10_CD4_Th17
chr13-99045442-99046840	8.42E-14	0.309607394	0.333	0.192	5.57E-09	C10_CD4_Th17
chr12-48933563-48934303	8.99E-14	0.335270329	0.189	0.069	5.96E-09	C10_CD4_Th17
chr9-72443043-72444225	9.16E-14	0.325976625	0.241	0.113	6.07E-09	C10_CD4_Th17
chr3-133880266-133880595	9.43E-14	0.298528228	0.054	0.005	6.24E-09	C10_CD4_Th17
chr15-60899034-60899799	1.02E-13	0.32900783	0.111	0.03	6.74E-09	C10_CD4_Th17
chr14-69716206-69717035	1.28E-13	0.338077528	0.186	0.076	8.48E-09	C10_CD4_Th17
chr14-106395021-106395874	1.33E-13	0.323149698	0.092	0.021	8.83E-09	C10_CD4_Th17
chr9-109886576-109887420	1.49E-13	0.330936714	0.071	0.012	9.86E-09	C10_CD4_Th17
chr6-37173463-37173917	1.50E-13	0.31529977	0.262	0.131	9.90E-09	C10_CD4_Th17
chr10-27541185-27542063	1.58E-13	0.303020914	0.066	0.01	1.05E-08	C10_CD4_Th17
chr1-66351559-66352284	1.88E-13	0.319474098	0.17	0.072	1.24E-08	C10_CD4_Th17
chr8-100258271-100259260	1.94E-13	0.296886431	0.309	0.168	1.28E-08	C10_CD4_Th17
chr10-100506555-100506767	2.01E-13	0.319713201	0.068	0.01	1.33E-08	C10_CD4_Th17
chr15-70486282-70487163	2.09E-13	0.314956521	0.281	0.137	1.38E-08	C10_CD4_Th17
chr19-10754900-10755528	2.09E-13	0.325961988	0.219	0.095	1.39E-08	C10_CD4_Th17
chr6-53361405-53362042	2.24E-13	0.302688446	0.307	0.175	1.48E-08	C10_CD4_Th17
chr17-62703165-62704296	2.43E-13	0.337393212	0.142	0.048	1.61E-08	C10_CD4_Th17
chr6-111487675-111488000	2.61E-13	0.334296199	0.175	0.067	1.73E-08	C10_CD4_Th17
chr12-92207915-92208995	2.62E-13	0.30232851	0.267	0.141	1.73E-08	C10_CD4_Th17

chr8-11782647-11783521	2.73E-13	0.351337466	0.12	0.034	1.81E-08	C10_CD4_Th17
chr17-32903678-32904554	2.81E-13	0.288477772	0.054	0.005	1.86E-08	C10_CD4_Th17
chr15-90845159-90845531	2.97E-13	0.28749042	0.052	0.005	1.97E-08	C10_CD4_Th17
chr4-77007018-77007870	3.06E-13	0.317071014	0.099	0.026	2.02E-08	C10_CD4_Th17
chr10-6148976-6149188	3.07E-13	0.325395655	0.08	0.015	2.03E-08	C10_CD4_Th17
chr17-76997855-76998634	3.07E-13	0.332621691	0.196	0.08	2.03E-08	C10_CD4_Th17
chr17-55067689-55068009	3.09E-13	0.299338639	0.057	0.007	2.05E-08	C10_CD4_Th17
chr15-41513716-41514184	3.12E-13	0.312162682	0.066	0.009	2.07E-08	C10_CD4_Th17
chr12-56338147-56339151	3.20E-13	0.29954576	0.302	0.169	2.12E-08	C10_CD4_Th17
chr16-27464716-27465226	3.25E-13	0.324047963	0.118	0.037	2.16E-08	C10_CD4_Th17
chr20-43959087-43959778	3.48E-13	0.330992173	0.158	0.06	2.31E-08	C10_CD4_Th17
chrX-15246724-15247330	3.54E-13	0.299989548	0.057	0.007	2.35E-08	C10_CD4_Th17
chr11-18669178-18669580	3.67E-13	0.323038207	0.087	0.018	2.43E-08	C10_CD4_Th17
chr9-81596744-81597481	3.71E-13	0.286371767	0.05	0.004	2.46E-08	C10_CD4_Th17
chr15-64651283-64652050	4.20E-13	0.337586272	0.111	0.031	2.78E-08	C10_CD4_Th17
chr19-49564572-49564981	4.34E-13	0.318468842	0.094	0.024	2.87E-08	C10_CD4_Th17
chr12-120194189-120195457	4.39E-13	0.264021503	0.349	0.238	2.91E-08	C10_CD4_Th17
chr14-52960609-52961424	4.62E-13	0.3167907	0.097	0.025	3.06E-08	C10_CD4_Th17
chr4-114004445-114004920	4.78E-13	0.304259928	0.068	0.011	3.16E-08	C10_CD4_Th17
chr14-68685054-68686144	4.83E-13	0.250350584	0.519	0.362	3.20E-08	C10_CD4_Th17
chr6-157535151-157536392	5.66E-13	0.317121235	0.248	0.122	3.75E-08	C10_CD4_Th17
chr11-1619308-1619814	5.94E-13	0.307748164	0.066	0.011	3.93E-08	C10_CD4_Th17
chr5-93622061-93622827	5.96E-13	0.312193094	0.248	0.124	3.95E-08	C10_CD4_Th17
chr2-37587660-37588544	6.03E-13	0.321016267	0.17	0.068	3.99E-08	C10_CD4_Th17
chr12-47426670-47427356	6.05E-13	0.338633627	0.149	0.05	4.00E-08	C10_CD4_Th17
chr2-37459235-37460604	6.36E-13	0.336587749	0.104	0.025	4.21E-08	C10_CD4_Th17
chr4-98642912-98643439	6.54E-13	0.329405506	0.108	0.029	4.33E-08	C10_CD4_Th17

chr3-4750341-4750748	6.55E-13	0.30709542	0.068	0.011	4.34E-08	C10_CD4_Th17
chr11-18587521-18588998	7.22E-13	0.302106524	0.219	0.109	4.78E-08	C10_CD4_Th17
chr1-206714437-206715025	7.53E-13	0.320415944	0.215	0.098	4.99E-08	C10_CD4_Th17
chr13-21017087-21017687	7.55E-13	0.316802953	0.083	0.019	5.00E-08	C10_CD4_Th17
chr3-42080404-42081014	8.55E-13	0.321633544	0.08	0.019	5.66E-08	C10_CD4_Th17
chr11-128488077-128488837	9.29E-13	0.305083455	0.177	0.07	6.15E-08	C10_CD4_Th17
chr14-63712943-63713432	9.91E-13	0.281642822	0.057	0.007	6.57E-08	C10_CD4_Th17
chr2-10329826-10332253	1.01E-12	0.275826994	0.356	0.216	6.69E-08	C10_CD4_Th17
chr17-77692327-77692893	1.05E-12	0.318393406	0.09	0.021	6.96E-08	C10_CD4_Th17
chr15-60546764-60547094	1.11E-12	0.309697681	0.094	0.024	7.34E-08	C10_CD4_Th17
chr8-118379569-118380150	1.18E-12	0.283644415	0.052	0.006	7.81E-08	C10_CD4_Th17
chr20-62159715-62160218	1.20E-12	0.307946218	0.083	0.019	7.95E-08	C10_CD4_Th17
chr20-3076845-3077946	1.33E-12	0.315458329	0.085	0.02	8.78E-08	C10_CD4_Th17
chr2-162055259-162055750	1.36E-12	0.336595388	0.137	0.049	9.01E-08	C10_CD4_Th17
chr4-26878104-26879318	1.40E-12	0.293500877	0.217	0.111	9.25E-08	C10_CD4_Th17
chr12-13195937-13196934	1.49E-12	0.303215848	0.281	0.155	9.89E-08	C10_CD4_Th17
chr15-75836455-75837385	1.55E-12	0.323766136	0.156	0.059	1.03E-07	C10_CD4_Th17
chr3-101753197-101754015	1.60E-12	0.312251724	0.085	0.017	1.06E-07	C10_CD4_Th17
chr15-67133848-67135019	1.63E-12	0.300278355	0.229	0.122	1.08E-07	C10_CD4_Th17
chr6-89375368-89376080	1.81E-12	0.312421561	0.25	0.131	1.20E-07	C10_CD4_Th17
chr1-67322804-67323396	1.81E-12	0.286318828	0.052	0.005	1.20E-07	C10_CD4_Th17
chr2-227871409-227871799	1.95E-12	0.278942823	0.052	0.006	1.29E-07	C10_CD4_Th17
chr1-225865587-225866457	1.97E-12	0.315167217	0.139	0.051	1.30E-07	C10_CD4_Th17
chr16-87496370-87496731	2.06E-12	0.304487291	0.08	0.016	1.36E-07	C10_CD4_Th17
chr5-127936456-127937005	2.09E-12	0.312956992	0.071	0.012	1.38E-07	C10_CD4_Th17
chr19-41563193-41564195	2.09E-12	0.31429333	0.149	0.058	1.39E-07	C10_CD4_Th17
chr6-116392367-116393029	2.11E-12	0.310302132	0.101	0.03	1.40E-07	C10_CD4_Th17

chr11-65565723-65566380	2.27E-12	0.288918317	0.283	0.157	1.50E-07	C10_CD4_Th17
chr2-98505422-98505777	2.28E-12	0.302862881	0.075	0.015	1.51E-07	C10_CD4_Th17
chr1-153532011-153532446	2.37E-12	0.308560755	0.182	0.084	1.57E-07	C10_CD4_Th17
chr10-69450915-69451952	2.40E-12	0.310569203	0.184	0.085	1.59E-07	C10_CD4_Th17
chr1-110630630-110631786	2.55E-12	0.263319497	0.368	0.24	1.69E-07	C10_CD4_Th17
chr11-114077540-114078153	2.57E-12	0.310708158	0.094	0.025	1.70E-07	C10_CD4_Th17
chr4-55358434-55358962	2.62E-12	0.285076762	0.057	0.008	1.74E-07	C10_CD4_Th17
chr18-45874302-45875212	2.74E-12	0.318545568	0.163	0.067	1.82E-07	C10_CD4_Th17
chr16-4016646-4017009	2.83E-12	0.291031073	0.059	0.009	1.87E-07	C10_CD4_Th17
chr2-227783439-227783780	2.84E-12	0.297173831	0.064	0.009	1.88E-07	C10_CD4_Th17
chr15-73051280-73053417	2.88E-12	0.258065316	0.377	0.234	1.91E-07	C10_CD4_Th17
chr1-172845273-172845751	2.89E-12	0.299083604	0.066	0.01	1.91E-07	C10_CD4_Th17
chr15-84992505-84992877	2.97E-12	0.311684241	0.083	0.017	1.97E-07	C10_CD4_Th17
chr9-104926185-104926887	3.21E-12	0.304517508	0.175	0.074	2.13E-07	C10_CD4_Th17
chr2-161956486-161957047	3.35E-12	0.30396991	0.085	0.018	2.22E-07	C10_CD4_Th17
chr17-62892798-62893313	3.46E-12	0.278394632	0.05	0.005	2.29E-07	C10_CD4_Th17
chr6-37133719-37133978	3.60E-12	0.306361836	0.071	0.014	2.38E-07	C10_CD4_Th17
chr12-94172069-94172335	3.95E-12	0.27910494	0.05	0.005	2.62E-07	C10_CD4_Th17
chr14-64726412-64726865	4.14E-12	0.304812832	0.083	0.019	2.74E-07	C10_CD4_Th17
chr8-125968555-125969298	4.55E-12	0.317121485	0.13	0.046	3.01E-07	C10_CD4_Th17
chr3-119562278-119562977	4.79E-12	0.322501005	0.158	0.065	3.17E-07	C10_CD4_Th17
chr2-8487695-8488598	5.01E-12	0.297678269	0.248	0.133	3.32E-07	C10_CD4_Th17
chr11-13288399-13288949	5.07E-12	0.297084527	0.08	0.02	3.35E-07	C10_CD4_Th17
chr10-69507151-69508165	5.21E-12	0.295969839	0.205	0.097	3.45E-07	C10_CD4_Th17
chr4-75686208-75686780	5.44E-12	0.308265491	0.137	0.051	3.60E-07	C10_CD4_Th17
chr8-125697949-125699113	5.49E-12	0.300507929	0.233	0.118	3.63E-07	C10_CD4_Th17
chrX-136690913-136691409	6.42E-12	0.305741936	0.13	0.044	4.25E-07	C10_CD4_Th17

chr1-92315420-92316390	6.62E-12	0.306064701	0.212	0.108	4.38E-07	C10_CD4_Th17
chr6-45441519-45442249	6.72E-12	0.307972212	0.108	0.034	4.45E-07	C10_CD4_Th17
chr11-123300950-123301973	6.78E-12	0.301739069	0.12	0.046	4.49E-07	C10_CD4_Th17
chr4-113355340-113356394	7.47E-12	0.325336732	0.189	0.079	4.95E-07	C10_CD4_Th17
chr16-67244167-67245037	7.66E-12	0.25836293	0.382	0.256	5.08E-07	C10_CD4_Th17
chr19-44691671-44692560	8.70E-12	0.305169793	0.132	0.047	5.76E-07	C10_CD4_Th17
chr21-32493044-32493747	8.90E-12	0.305803695	0.087	0.024	5.89E-07	C10_CD4_Th17
chr6-137710500-137710766	8.91E-12	0.310106644	0.108	0.032	5.90E-07	C10_CD4_Th17
chrX-19625409-19625942	8.91E-12	0.306634206	0.146	0.054	5.90E-07	C10_CD4_Th17
chr14-92547392-92547850	9.69E-12	0.313803666	0.12	0.038	6.42E-07	C10_CD4_Th17
chr7-87590396-87590858	1.03E-11	0.276203294	0.05	0.005	6.85E-07	C10_CD4_Th17
chr1-147716927-147717501	1.07E-11	0.291368815	0.061	0.01	7.06E-07	C10_CD4_Th17
chr20-33372390-33372681	1.13E-11	0.295719658	0.116	0.037	7.51E-07	C10_CD4_Th17
chr9-129069083-129069745	1.23E-11	0.289539695	0.068	0.012	8.16E-07	C10_CD4_Th17
chr3-146550819-146551415	1.28E-11	0.285974751	0.068	0.013	8.49E-07	C10_CD4_Th17
chr6-167094817-167095391	1.41E-11	0.30895181	0.123	0.042	9.35E-07	C10_CD4_Th17
chr2-119827772-119828490	1.47E-11	0.319083523	0.127	0.045	9.74E-07	C10_CD4_Th17
chr1-193569550-193570281	1.51E-11	0.303672637	0.101	0.026	9.99E-07	C10_CD4_Th17
chr10-48633830-48634864	1.59E-11	0.288944567	0.061	0.011	1.05E-06	C10_CD4_Th17
chr4-139528391-139528994	1.60E-11	0.292602304	0.097	0.031	1.06E-06	C10_CD4_Th17
chr2-8408347-8409368	1.61E-11	0.300291558	0.226	0.106	1.07E-06	C10_CD4_Th17
chr9-130166843-130167273	1.65E-11	0.284872692	0.075	0.018	1.09E-06	C10_CD4_Th17
chr20-49620110-49621052	1.65E-11	0.29673141	0.078	0.018	1.09E-06	C10_CD4_Th17
chr4-147799864-147800522	2.14E-11	0.298783681	0.153	0.061	1.41E-06	C10_CD4_Th17
chr19-7807091-7808065	2.19E-11	0.287015658	0.182	0.082	1.45E-06	C10_CD4_Th17
chr1-151568621-151569180	2.46E-11	0.315418226	0.127	0.042	1.63E-06	C10_CD4_Th17
chr12-94207678-94208917	2.48E-11	0.284655626	0.217	0.11	1.64E-06	C10_CD4_Th17

chr3-51902167-51902700	2.54E-11	0.27265552	0.061	0.012	1.68E-06	C10_CD4_Th17
chr5-35832621-35833484	2.56E-11	0.287520764	0.101	0.033	1.69E-06	C10_CD4_Th17
chr2-86067922-86068639	2.75E-11	0.294213806	0.184	0.087	1.82E-06	C10_CD4_Th17
chr5-133423449-133424069	2.91E-11	0.277153946	0.061	0.012	1.93E-06	C10_CD4_Th17
chr9-85646059-85646549	3.00E-11	0.27401396	0.064	0.011	1.99E-06	C10_CD4_Th17
chr12-96176706-96177424	3.02E-11	0.293945658	0.092	0.025	2.00E-06	C10_CD4_Th17
chr19-6678504-6679035	3.02E-11	0.305382571	0.172	0.072	2.00E-06	C10_CD4_Th17
chr5-129114404-129115103	3.16E-11	0.299562449	0.075	0.017	2.09E-06	C10_CD4_Th17
chr17-1672879-1673546	3.24E-11	0.291827971	0.108	0.035	2.15E-06	C10_CD4_Th17
chrX-13387684-13388072	3.26E-11	0.279495998	0.054	0.008	2.16E-06	C10_CD4_Th17
chr14-71290877-71291357	3.36E-11	0.267840701	0.059	0.012	2.22E-06	C10_CD4_Th17
chr17-66655675-66656260	3.36E-11	0.278154686	0.054	0.008	2.22E-06	C10_CD4_Th17
chr14-100640014-100641033	3.67E-11	0.290690666	0.184	0.085	2.43E-06	C10_CD4_Th17
chr12-93846970-93847700	3.71E-11	0.302762121	0.083	0.019	2.45E-06	C10_CD4_Th17
chr22-30196160-30196870	4.04E-11	0.302397262	0.085	0.023	2.67E-06	C10_CD4_Th17
chr4-112167342-112168102	4.05E-11	0.287849702	0.075	0.018	2.68E-06	C10_CD4_Th17
chr20-31684382-31685375	4.20E-11	0.286175486	0.064	0.013	2.78E-06	C10_CD4_Th17
chr15-73140964-73141545	4.23E-11	0.258171795	0.05	0.007	2.80E-06	C10_CD4_Th17
chr11-3049433-3050260	4.28E-11	0.265622566	0.311	0.192	2.84E-06	C10_CD4_Th17
chr16-29290424-29291850	4.37E-11	0.293390112	0.144	0.057	2.89E-06	C10_CD4_Th17
chr8-60928971-60929558	4.40E-11	0.295743376	0.189	0.086	2.91E-06	C10_CD4_Th17
chr3-50316352-50317145	4.44E-11	0.276695407	0.271	0.148	2.94E-06	C10_CD4_Th17
chr5-56849291-56850026	4.52E-11	0.280785052	0.08	0.018	2.99E-06	C10_CD4_Th17
chr12-107317894-107318515	4.56E-11	0.282022387	0.25	0.134	3.02E-06	C10_CD4_Th17
chr11-119318537-119319008	4.57E-11	0.29224068	0.092	0.025	3.03E-06	C10_CD4_Th17
chr2-98911425-98912127	4.59E-11	0.28342318	0.075	0.016	3.04E-06	C10_CD4_Th17
chr3-126911097-126912196	4.91E-11	0.287596317	0.2	0.094	3.25E-06	C10_CD4_Th17

chr11-34233901-34234552	5.32E-11	0.292283944	0.184	0.077	3.52E-06	C10_CD4_Th17
chr13-109785441-109787258	5.33E-11	0.270759698	0.337	0.195	3.53E-06	C10_CD4_Th17
chr3-33029753-33031190	5.33E-11	0.293318461	0.146	0.056	3.53E-06	C10_CD4_Th17
chr7-156612703-156613280	5.40E-11	0.302893851	0.099	0.028	3.58E-06	C10_CD4_Th17
chr2-240624500-240625519	5.43E-11	0.257465058	0.33	0.197	3.60E-06	C10_CD4_Th17
chr9-113519909-113520623	5.50E-11	0.286480119	0.186	0.082	3.65E-06	C10_CD4_Th17
chr17-48264671-48265788	5.99E-11	0.290204783	0.134	0.051	3.97E-06	C10_CD4_Th17
chr17-55116324-55117300	6.05E-11	0.27965003	0.059	0.011	4.01E-06	C10_CD4_Th17
chr2-134232246-134233946	6.27E-11	0.279642541	0.224	0.111	4.15E-06	C10_CD4_Th17
chr7-104993172-104993738	6.41E-11	0.252429772	0.05	0.007	4.24E-06	C10_CD4_Th17
chr16-72787221-72788263	6.59E-11	0.308598248	0.132	0.044	4.36E-06	C10_CD4_Th17
chr22-36166068-36167237	6.63E-11	0.273372236	0.215	0.112	4.39E-06	C10_CD4_Th17
chr10-49177021-49177858	7.07E-11	0.295599353	0.149	0.064	4.68E-06	C10_CD4_Th17
chr6-79946341-79948027	7.10E-11	0.284209549	0.231	0.117	4.70E-06	C10_CD4_Th17
chr3-192892815-192893754	7.22E-11	0.292364504	0.137	0.052	4.78E-06	C10_CD4_Th17
chr16-82660246-82660521	7.41E-11	0.26808619	0.066	0.012	4.91E-06	C10_CD4_Th17
chr21-44297707-44298198	7.45E-11	0.288395304	0.158	0.069	4.93E-06	C10_CD4_Th17
chr16-53094019-53094366	8.10E-11	0.254154702	0.052	0.008	5.37E-06	C10_CD4_Th17
chr20-49676459-49677381	8.13E-11	0.27643968	0.25	0.144	5.38E-06	C10_CD4_Th17
chr10-8415943-8417132	8.55E-11	0.279551832	0.264	0.15	5.66E-06	C10_CD4_Th17
chrX-19627540-19627876	9.00E-11	0.287389322	0.09	0.026	5.96E-06	C10_CD4_Th17
chr13-74090377-74091556	9.15E-11	0.292527793	0.116	0.041	6.06E-06	C10_CD4_Th17
chr19-38693287-38694297	9.15E-11	0.27434462	0.203	0.111	6.06E-06	C10_CD4_Th17
chr14-71141928-71143729	9.33E-11	0.281764759	0.21	0.1	6.18E-06	C10_CD4_Th17
chr11-13286961-13287334	9.57E-11	0.282717938	0.075	0.019	6.34E-06	C10_CD4_Th17
chr1-66287844-66288414	1.01E-10	0.291893662	0.156	0.065	6.67E-06	C10_CD4_Th17
chr19-6639243-6639831	1.01E-10	0.294696095	0.125	0.047	6.67E-06	C10_CD4_Th17

chr15-90872836-90873160	1.01E-10	0.281603821	0.073	0.017	6.69E-06	C10_CD4_Th17
chr17-3740775-3741095	1.01E-10	0.276435078	0.078	0.018	6.71E-06	C10_CD4_Th17
chr8-47368994-47369760	1.06E-10	0.260356434	0.054	0.008	7.04E-06	C10_CD4_Th17
chr14-61594151-61595092	1.07E-10	0.292052076	0.144	0.052	7.07E-06	C10_CD4_Th17
chr2-224401023-224402243	1.08E-10	0.275742011	0.068	0.015	7.17E-06	C10_CD4_Th17
chr9-135887273-135887966	1.10E-10	0.270685186	0.066	0.015	7.26E-06	C10_CD4_Th17
chr13-29758427-29759203	1.10E-10	0.293482291	0.198	0.094	7.28E-06	C10_CD4_Th17
chr4-54942019-54943056	1.22E-10	0.286298747	0.092	0.025	8.09E-06	C10_CD4_Th17
chr5-35902161-35902620	1.30E-10	0.286677829	0.09	0.026	8.59E-06	C10_CD4_Th17
chr9-127976577-127976785	1.33E-10	0.284805494	0.094	0.029	8.79E-06	C10_CD4_Th17
chr3-192850360-192851020	1.37E-10	0.286504081	0.068	0.014	9.04E-06	C10_CD4_Th17
chr1-199172429-199173790	1.42E-10	0.292862382	0.172	0.076	9.37E-06	C10_CD4_Th17
chr8-37891644-37892174	1.46E-10	0.275042033	0.203	0.1	9.70E-06	C10_CD4_Th17
chr1-169908367-169909148	1.51E-10	0.274691634	0.099	0.032	9.97E-06	C10_CD4_Th17
chr11-125513944-125514931	1.55E-10	0.275389129	0.2	0.105	1.02E-05	C10_CD4_Th17
chr6-137978282-137978645	1.71E-10	0.276501174	0.083	0.023	1.13E-05	C10_CD4_Th17
chr3-8356603-8357740	1.76E-10	0.280230427	0.269	0.15	1.16E-05	C10_CD4_Th17
chr1-193417405-193417796	1.79E-10	0.265462032	0.066	0.014	1.18E-05	C10_CD4_Th17
chr6-167130813-167131317	1.79E-10	0.274543612	0.066	0.014	1.19E-05	C10_CD4_Th17
chr2-196229933-196230390	1.84E-10	0.282037055	0.106	0.036	1.22E-05	C10_CD4_Th17
chr17-2087584-2088039	1.90E-10	0.276004531	0.12	0.044	1.26E-05	C10_CD4_Th17
chr12-3727890-3729074	2.15E-10	0.277811146	0.222	0.107	1.42E-05	C10_CD4_Th17
chr3-27345438-27345822	2.16E-10	0.288005519	0.064	0.013	1.43E-05	C10_CD4_Th17
chr6-14635897-14636505	2.17E-10	0.281923874	0.083	0.022	1.44E-05	C10_CD4_Th17
chr6-157893183-157893573	2.17E-10	0.27130333	0.071	0.017	1.44E-05	C10_CD4_Th17
chr16-53445331-53445895	2.18E-10	0.27731994	0.094	0.027	1.44E-05	C10_CD4_Th17
chr11-114058953-114060800	2.28E-10	0.27148324	0.2	0.101	1.51E-05	C10_CD4_Th17

chr6-149077101-149077533	2.42E-10	0.275677324	0.068	0.015	1.60E-05	C10_CD4_Th17
chr3-50251272-50251540	2.45E-10	0.262683158	0.059	0.012	1.62E-05	C10_CD4_Th17
chr20-3665344-3666479	2.53E-10	0.292422884	0.189	0.093	1.67E-05	C10_CD4_Th17
chr11-74086206-74086771	2.88E-10	0.266094982	0.05	0.007	1.91E-05	C10_CD4_Th17
chr2-136362145-136363117	2.97E-10	0.282512351	0.172	0.086	1.96E-05	C10_CD4_Th17
chr14-61528509-61529053	3.37E-10	0.28785038	0.167	0.07	2.23E-05	C10_CD4_Th17
chr6-105810379-105810906	3.60E-10	0.280892578	0.08	0.025	2.38E-05	C10_CD4_Th17
chr3-58334925-58335368	3.61E-10	0.259217781	0.068	0.015	2.39E-05	C10_CD4_Th17
chr2-23891542-23892340	3.64E-10	0.282864859	0.165	0.074	2.41E-05	C10_CD4_Th17
chr14-35196384-35197321	3.65E-10	0.283035817	0.13	0.049	2.42E-05	C10_CD4_Th17
chr14-61525034-61525695	3.71E-10	0.262616448	0.241	0.142	2.45E-05	C10_CD4_Th17
chr17-66373973-66374748	3.77E-10	0.297300289	0.106	0.034	2.49E-05	C10_CD4_Th17
chr19-53851784-53852105	3.83E-10	0.260208946	0.066	0.014	2.54E-05	C10_CD4_Th17
chr10-45421154-45421545	3.86E-10	0.255496215	0.066	0.014	2.56E-05	C10_CD4_Th17
chr19-14237446-14237947	3.95E-10	0.258389234	0.054	0.009	2.62E-05	C10_CD4_Th17
chr15-92069190-92069971	4.05E-10	0.258586875	0.078	0.024	2.68E-05	C10_CD4_Th17
chr11-85742860-85743259	4.11E-10	0.268464935	0.064	0.013	2.72E-05	C10_CD4_Th17
chr14-99396004-99396540	4.18E-10	0.266220149	0.099	0.034	2.77E-05	C10_CD4_Th17
chr3-4394088-4394647	4.28E-10	0.278372069	0.087	0.027	2.84E-05	C10_CD4_Th17
chr3-15519975-15520491	4.84E-10	0.266012451	0.08	0.023	3.21E-05	C10_CD4_Th17
chr8-47703766-47704652	4.88E-10	0.274367248	0.087	0.026	3.23E-05	C10_CD4_Th17
chr1-159091528-159092334	5.14E-10	0.272288507	0.075	0.021	3.40E-05	C10_CD4_Th17
chr2-98479473-98480580	5.75E-10	0.27254677	0.212	0.108	3.81E-05	C10_CD4_Th17
chr17-63993469-63994017	6.25E-10	0.270586168	0.085	0.025	4.14E-05	C10_CD4_Th17
chr1-20187187-20187844	6.73E-10	0.259557118	0.078	0.024	4.46E-05	C10_CD4_Th17
chr1-20322131-20323405	7.32E-10	0.26884586	0.113	0.042	4.85E-05	C10_CD4_Th17
chr19-18021112-18022423	7.37E-10	0.26275753	0.144	0.063	4.88E-05	C10_CD4_Th17

chr12-121215049-121215261	7.81E-10	0.260319111	0.075	0.021	5.17E-05	C10_CD4_Th17
chr12-91870813-91871458	7.94E-10	0.267424617	0.139	0.058	5.26E-05	C10_CD4_Th17
chr6-119147106-119147713	7.96E-10	0.276122683	0.085	0.024	5.27E-05	C10_CD4_Th17
chr3-42555953-42556723	8.06E-10	0.267636391	0.182	0.093	5.34E-05	C10_CD4_Th17
chr2-148874497-148875694	8.18E-10	0.270641684	0.219	0.117	5.42E-05	C10_CD4_Th17
chr7-86978465-86979389	8.22E-10	0.270437179	0.184	0.093	5.44E-05	C10_CD4_Th17
chr1-234844749-234845682	8.33E-10	0.270092759	0.16	0.07	5.52E-05	C10_CD4_Th17
chr3-45988394-45989136	8.38E-10	0.269480704	0.118	0.048	5.55E-05	C10_CD4_Th17
chr17-66246229-66246701	8.92E-10	0.266951313	0.087	0.026	5.91E-05	C10_CD4_Th17
chr16-10987646-10989038	9.30E-10	0.26692652	0.127	0.052	6.16E-05	C10_CD4_Th17
chr10-126041608-126042736	9.49E-10	0.275202433	0.12	0.046	6.28E-05	C10_CD4_Th17
chr9-75083211-75083544	9.53E-10	0.28637077	0.12	0.047	6.31E-05	C10_CD4_Th17
chr9-72476745-72477354	9.69E-10	0.27103069	0.094	0.032	6.42E-05	C10_CD4_Th17
chr10-6406289-6406834	9.78E-10	0.26431071	0.075	0.02	6.48E-05	C10_CD4_Th17
chr6-16316586-16317072	9.93E-10	0.261748322	0.137	0.058	6.58E-05	C10_CD4_Th17
chr17-78154892-78155404	9.98E-10	0.267765477	0.061	0.013	6.61E-05	C10_CD4_Th17
chr2-65299224-65299583	1.02E-09	0.271305764	0.097	0.031	6.75E-05	C10_CD4_Th17
chr4-147793927-147795018	1.04E-09	0.276535534	0.113	0.038	6.86E-05	C10_CD4_Th17
chr6-167150847-167151405	1.13E-09	0.256498189	0.054	0.01	7.47E-05	C10_CD4_Th17
chr14-87994963-87995338	1.15E-09	0.270202225	0.113	0.039	7.63E-05	C10_CD4_Th17
chr4-75695851-75696388	1.18E-09	0.252496116	0.061	0.012	7.84E-05	C10_CD4_Th17
chr9-131504058-131504423	1.23E-09	0.262327358	0.149	0.063	8.13E-05	C10_CD4_Th17
chr12-93772937-93773510	1.24E-09	0.286880815	0.125	0.044	8.23E-05	C10_CD4_Th17
chr15-60526103-60526651	1.27E-09	0.26817472	0.137	0.058	8.40E-05	C10_CD4_Th17
chr17-65163803-65164484	1.28E-09	0.2669574	0.101	0.035	8.46E-05	C10_CD4_Th17
chr7-3062863-3063425	1.28E-09	0.269405334	0.182	0.093	8.48E-05	C10_CD4_Th17
chr1-110003587-110004366	1.36E-09	0.260508341	0.083	0.027	8.98E-05	C10_CD4_Th17

chr9-114484722-114485381	1.48E-09	0.262016359	0.182	0.089	9.81E-05	C10_CD4_Th17
chr3-149016678-149017726	1.49E-09	0.268342368	0.12	0.049	9.89E-05	C10_CD4_Th17
chr7-65798276-65798769	1.51E-09	0.274583273	0.099	0.034	9.99E-05	C10_CD4_Th17
chr22-40731502-40731884	1.57E-09	0.254446027	0.071	0.019	0.000103663	C10_CD4_Th17
chr2-162011620-162012049	1.58E-09	0.263776242	0.149	0.072	0.000104442	C10_CD4_Th17
chr17-66633280-66633639	1.59E-09	0.263108346	0.064	0.013	0.000105489	C10_CD4_Th17
chr11-121476267-121476689	1.59E-09	0.255151397	0.057	0.011	0.000105515	C10_CD4_Th17
chr11-3037313-3038053	1.66E-09	0.265818971	0.085	0.025	0.000109985	C10_CD4_Th17
chr2-33271956-33272421	1.71E-09	0.259844712	0.073	0.02	0.000112942	C10_CD4_Th17
chr19-49555937-49556672	1.75E-09	0.277322378	0.104	0.037	0.000116107	C10_CD4_Th17
chr8-2130857-2131533	1.83E-09	0.271833678	0.13	0.052	0.000121282	C10_CD4_Th17
chr6-167041203-167041953	1.84E-09	0.27319118	0.099	0.033	0.000121542	C10_CD4_Th17
chr6-53303451-53303697	1.87E-09	0.269735763	0.08	0.024	0.000123778	C10_CD4_Th17
chr2-12708586-12709707	1.91E-09	0.260890359	0.191	0.1	0.000126758	C10_CD4_Th17
chr4-174521723-174523253	2.12E-09	0.260547052	0.17	0.078	0.00014057	C10_CD4_Th17
chr13-99214321-99215835	2.18E-09	0.250623401	0.236	0.14	0.000144654	C10_CD4_Th17
chr4-148056547-148057231	2.36E-09	0.269604131	0.193	0.095	0.000156335	C10_CD4_Th17
chr7-87600029-87601050	2.53E-09	0.265381682	0.106	0.038	0.000167231	C10_CD4_Th17
chr2-12442483-12442877	2.53E-09	0.250905507	0.05	0.009	0.000167693	C10_CD4_Th17
chr15-62067498-62069032	2.79E-09	0.262001488	0.132	0.058	0.000184527	C10_CD4_Th17
chr5-59562745-59563294	2.90E-09	0.260802993	0.061	0.015	0.000192235	C10_CD4_Th17
chr3-13002327-13002932	2.95E-09	0.267822822	0.116	0.047	0.000195163	C10_CD4_Th17
chr17-66713231-66714033	3.04E-09	0.275250302	0.118	0.045	0.000201309	C10_CD4_Th17
chr22-40682751-40683431	3.11E-09	0.257450801	0.17	0.086	0.000206206	C10_CD4_Th17
chr2-28583595-28584569	3.12E-09	0.273434196	0.17	0.077	0.000206551	C10_CD4_Th17
chr6-45477827-45478595	3.15E-09	0.273492884	0.085	0.026	0.000208723	C10_CD4_Th17
chr6-87756776-87757261	3.18E-09	0.265155886	0.101	0.037	0.000210295	C10_CD4_Th17

chr17-64066512-64067113	3.22E-09	0.25837446	0.087	0.029	0.000213125	C10_CD4_Th17
chr4-174358650-174359167	3.36E-09	0.263040416	0.066	0.016	0.000222815	C10_CD4_Th17
chr12-93783792-93785545	3.50E-09	0.25271386	0.259	0.145	0.000231943	C10_CD4_Th17
chr15-78944408-78945573	3.58E-09	0.259616241	0.153	0.068	0.000236938	C10_CD4_Th17
chr1-46549395-46550267	3.59E-09	0.258663938	0.212	0.117	0.00023769	C10_CD4_Th17
chr2-33275472-33276071	3.90E-09	0.270432171	0.125	0.052	0.00025798	C10_CD4_Th17
chr12-93851159-93851691	4.22E-09	0.266511772	0.085	0.025	0.000279214	C10_CD4_Th17
chr9-2621194-2622965	4.51E-09	0.26120194	0.12	0.049	0.00029857	C10_CD4_Th17
chr12-68259227-68259799	4.89E-09	0.261488998	0.085	0.026	0.000323784	C10_CD4_Th17
chr6-45436654-45437106	5.23E-09	0.251080416	0.097	0.034	0.000346196	C10_CD4_Th17
chr4-122458393-122459006	5.24E-09	0.263006628	0.165	0.081	0.000346846	C10_CD4_Th17
chr14-21308205-21309096	5.25E-09	0.256538116	0.215	0.111	0.000347941	C10_CD4_Th17
chr4-153493611-153493964	5.44E-09	0.261339333	0.068	0.02	0.000360092	C10_CD4_Th17
chr2-174039493-174040227	5.95E-09	0.251336096	0.2	0.107	0.000393951	C10_CD4_Th17
chr5-35923950-35925289	5.99E-09	0.255778666	0.172	0.092	0.000396976	C10_CD4_Th17
chr4-141307171-141307735	6.43E-09	0.262872479	0.097	0.036	0.00042555	C10_CD4_Th17
chr16-57277352-57277998	7.07E-09	0.260290702	0.092	0.031	0.000468234	C10_CD4_Th17
chr1-40628211-40628877	7.82E-09	0.268022083	0.198	0.102	0.000518098	C10_CD4_Th17
chr3-10223517-10224076	7.95E-09	0.258607594	0.123	0.051	0.000526633	C10_CD4_Th17
chr10-73932104-73932872	7.98E-09	0.265829363	0.125	0.055	0.000528469	C10_CD4_Th17
chr16-10329387-10329866	8.59E-09	0.253007611	0.09	0.033	0.000569123	C10_CD4_Th17
chr4-87093377-87094067	8.70E-09	0.262099191	0.092	0.037	0.000576372	C10_CD4_Th17
chr2-65382460-65383326	9.13E-09	0.254512387	0.146	0.069	0.000604484	C10_CD4_Th17
chr4-83548014-83548830	9.38E-09	0.254669783	0.177	0.089	0.000621264	C10_CD4_Th17
chr9-71685347-71685809	9.77E-09	0.2545621	0.116	0.05	0.000646762	C10_CD4_Th17
chr17-77409462-77410211	1.06E-08	0.25936852	0.149	0.069	0.000704977	C10_CD4_Th17
chr4-112273289-112274142	1.19E-08	0.251722477	0.078	0.024	0.0007867	C10_CD4_Th17

chr9-112037436-112038280	1.23E-08	0.25376282	0.146	0.064	0.00081506	C10_CD4_Th17
chr14-97592306-97592928	1.23E-08	0.250422383	0.073	0.021	0.000815283	C10_CD4_Th17
chr2-160481840-160483052	1.30E-08	0.258047022	0.118	0.054	0.000859107	C10_CD4_Th17
chr1-193539802-193540816	1.30E-08	0.252162719	0.108	0.042	0.000859745	C10_CD4_Th17
chr10-77832147-77832594	1.33E-08	0.251965477	0.083	0.026	0.000882614	C10_CD4_Th17
chr20-47274406-47274862	1.36E-08	0.256783512	0.085	0.029	0.000903597	C10_CD4_Th17
chr13-24187009-24187839	1.51E-08	0.252626498	0.087	0.033	0.000998189	C10_CD4_Th17
chr10-91194457-91195121	1.56E-08	0.260272002	0.106	0.044	0.001034438	C10_CD4_Th17
chr20-24929998-24930763	1.60E-08	0.262485365	0.13	0.053	0.001057665	C10_CD4_Th17
chr2-160403047-160403559	1.99E-08	0.250771375	0.118	0.05	0.001319267	C10_CD4_Th17
chr6-139172678-139173531	2.82E-08	0.253564888	0.142	0.07	0.001866015	C10_CD4_Th17
chr7-115230796-115231520	3.63E-08	0.251331093	0.116	0.05	0.002403143	C10_CD4_Th17
chr13-31806431-31807534	6.55E-15	0.355083226	0.113	0.029	4.34E-10	C10_CD4_Th17
chr11-126332215-126332648	3.24E-23	0.48245537	0.156	0.029	2.14E-18	C10_CD4_Th17
chr1-30191132-30191709	2.11E-14	0.337195065	0.101	0.022	1.40E-09	C10_CD4_Th17
chr1-203755930-203757037	7.03E-14	0.330302865	0.267	0.128	4.66E-09	C10_CD4_Th17
chr6-80453563-80454609	2.24E-25	0.48078952	0.104	0.01	1.48E-20	C10_CD4_Th17
chr2-119827772-119828490	1.47E-11	0.319083523	0.127	0.045	9.74E-07	C10_CD4_Th17
chr5-151745041-151745697	5.73E-18	0.392729088	0.132	0.03	3.80E-13	C10_CD4_Th17
chr20-35251409-35252730	1.54E-38	0.577964309	0.328	0.094	1.02E-33	C10_CD4_Th17
chr12-3727890-3729074	2.15E-10	0.277811146	0.222	0.107	1.42E-05	C10_CD4_Th17
chr16-4016646-4017009	2.83E-12	0.291031073	0.059	0.009	1.87E-07	C10_CD4_Th17
chr14-61528509-61529053	3.37E-10	0.28785038	0.167	0.07	2.23E-05	C10_CD4_Th17
chr16-87496370-87496731	2.06E-12	0.304487291	0.08	0.016	1.36E-07	C10_CD4_Th17
chr16-81523422-81524781	5.99E-14	0.273190414	0.486	0.312	3.97E-09	C10_CD4_Th17
chr6-37137037-37137801	5.49E-16	0.371951612	0.149	0.043	3.64E-11	C10_CD4_Th17
chr1-92315420-92316390	6.62E-12	0.306064701	0.212	0.108	4.38E-07	C10_CD4_Th17

chr6-89375368-89376080	1.81E-12	0.312421561	0.25	0.131	1.20E-07	C10_CD4_Th17
chr1-199172429-199173790	1.42E-10	0.292862382	0.172	0.076	9.37E-06	C10_CD4_Th17
chr16-27464716-27465226	3.25E-13	0.324047963	0.118	0.037	2.16E-08	C10_CD4_Th17
chr8-89728858-89729660	4.29E-14	0.334411763	0.236	0.111	2.84E-09	C10_CD4_Th17
chr8-60926284-60927881	4.02E-24	0.444107637	0.297	0.117	2.66E-19	C10_CD4_Th17
chr15-70486282-70487163	2.09E-13	0.314956521	0.281	0.137	1.38E-08	C10_CD4_Th17
chr8-57000152-57000767	5.98E-21	0.420362824	0.252	0.098	3.96E-16	C10_CD4_Th17
chr11-114073591-114074289	2.67E-19	0.430462904	0.172	0.045	1.77E-14	C10_CD4_Th17
chr17-48264671-48265788	5.99E-11	0.290204783	0.134	0.051	3.97E-06	C10_CD4_Th17
chr3-15519975-15520491	4.84E-10	0.266012451	0.08	0.023	3.21E-05	C10_CD4_Th17
chr14-97592306-97592928	1.23E-08	0.250422383	0.073	0.021	0.000815283	C10_CD4_Th17
chr2-33275472-33276071	3.90E-09	0.270432171	0.125	0.052	0.00025798	C10_CD4_Th17
chr3-171813902-171814427	1.91E-45	0.671874284	0.179	0.017	1.27E-40	C10_CD4_Th17
chr10-70616681-70617364	1.27E-14	0.349263141	0.12	0.033	8.41E-10	C10_CD4_Th17
chr6-5517794-5518190	9.24E-17	0.377938344	0.099	0.02	6.12E-12	C10_CD4_Th17
chr14-69716206-69717035	1.28E-13	0.338077528	0.186	0.076	8.48E-09	C10_CD4_Th17
chr17-66373973-66374748	3.77E-10	0.297300289	0.106	0.034	2.49E-05	C10_CD4_Th17
chr13-109785441-109787258	5.33E-11	0.270759698	0.337	0.195	3.53E-06	C10_CD4_Th17
chr1-87403957-87404678	3.42E-21	0.453977717	0.132	0.027	2.26E-16	C10_CD4_Th17
chr5-57117109-57118076	1.72E-16	0.385566619	0.134	0.038	1.14E-11	C10_CD4_Th17
chr7-115230796-115231520	3.63E-08	0.251331093	0.116	0.05	0.002403143	C10_CD4_Th17
chr9-72526824-72527815	4.99E-39	0.589463239	0.323	0.094	3.30E-34	C10_CD4_Th17
chr2-148524150-148525059	2.30E-21	0.43313594	0.212	0.066	1.52E-16	C10_CD4_Th17
chr14-91193828-91194842	5.57E-15	0.350466794	0.151	0.05	3.69E-10	C10_CD4_Th17
chr7-86978465-86979389	8.22E-10	0.270437179	0.184	0.093	5.44E-05	C10_CD4_Th17
chr2-162011620-162012049	1.58E-09	0.263776242	0.149	0.072	0.000104442	C10_CD4_Th17
chr12-13195937-13196934	1.49E-12	0.303215848	0.281	0.155	9.89E-08	C10_CD4_Th17

chr21-44870488-44870976	3.85E-15	0.354121101	0.146	0.048	2.55E-10	C10_CD4_Th17
chr3-33029753-33031190	5.33E-11	0.293318461	0.146	0.056	3.53E-06	C10_CD4_Th17
chr11-121464061-121464975	6.05E-17	0.319107681	0.467	0.28	4.01E-12	C10_CD4_Th17
chr7-151408489-151409399	8.43E-31	0.479733644	0.427	0.182	5.58E-26	C10_CD4_Th17
chr9-72476745-72477354	9.69E-10	0.27103069	0.094	0.032	6.42E-05	C10_CD4_Th17
chr1-147716927-147717501	1.07E-11	0.291368815	0.061	0.01	7.06E-07	C10_CD4_Th17
chr8-140790509-140791760	1.01E-20	0.406835106	0.29	0.132	6.70E-16	C10_CD4_Th17
chr1-225865587-225866457	1.97E-12	0.315167217	0.139	0.051	1.30E-07	C10_CD4_Th17
chr2-98479473-98480580	5.75E-10	0.27254677	0.212	0.108	3.81E-05	C10_CD4_Th17
chr19-10754900-10755528	2.09E-13	0.325961988	0.219	0.095	1.39E-08	C10_CD4_Th17
chr8-2130857-2131533	1.83E-09	0.271833678	0.13	0.052	0.000121282	C10_CD4_Th17
chr7-156612703-156613280	5.40E-11	0.302893851	0.099	0.028	3.58E-06	C10_CD4_Th17
chr6-45441519-45442249	6.72E-12	0.307972212	0.108	0.034	4.45E-07	C10_CD4_Th17
chr6-45477827-45478595	3.15E-09	0.273492884	0.085	0.026	0.000208723	C10_CD4_Th17
chr10-73932104-73932872	7.98E-09	0.265829363	0.125	0.055	0.000528469	C10_CD4_Th17
chr13-31806431-31807534	6.55E-15	0.355083226	0.113	0.029	4.34E-10	C10_CD4_Th17
chr3-42555953-42556723	8.06E-10	0.267636391	0.182	0.093	5.34E-05	C10_CD4_Th17
chr5-95631738-95632499	1.54E-14	0.355042199	0.193	0.08	1.02E-09	C10_CD4_Th17
chr22-42179959-42180790	1.95E-18	0.389776103	0.203	0.07	1.29E-13	C10_CD4_Th17
chr19-47182300-47182765	4.18E-20	0.42775386	0.151	0.039	2.77E-15	C10_CD4_Th17
chr1-234844749-234845682	8.33E-10	0.270092759	0.16	0.07	5.52E-05	C10_CD4_Th17
chr2-100643486-100644472	6.04E-14	0.33785735	0.146	0.046	4.00E-09	C10_CD4_Th17
chr12-96176706-96177424	3.02E-11	0.293945658	0.092	0.025	2.00E-06	C10_CD4_Th17
chr8-11782647-11783521	2.73E-13	0.351337466	0.12	0.034	1.81E-08	C10_CD4_Th17
chr10-126041608-126042736	9.49E-10	0.275202433	0.12	0.046	6.28E-05	C10_CD4_Th17
chr13-109785441-109787258	5.33E-11	0.270759698	0.337	0.195	3.53E-06	C10_CD4_Th17
chr12-93783792-93785545	3.50E-09	0.25271386	0.259	0.145	0.000231943	C10_CD4_Th17

chr6-167130813-167131317	1.79E-10	0.274543612	0.066	0.014	1.19E-05	C10_CD4_Th17
chr2-213118936-213119809	2.64E-28	0.523057667	0.198	0.05	1.75E-23	C10_CD4_Th17
chr5-129114404-129115103	3.16E-11	0.299562449	0.075	0.017	2.09E-06	C10_CD4_Th17
chr6-157884687-157886211	1.37E-24	0.445804727	0.292	0.108	9.07E-20	C10_CD4_Th17
chr2-224401023-224402243	1.08E-10	0.275742011	0.068	0.015	7.17E-06	C10_CD4_Th17
chr6-167113865-167114853	1.77E-95	0.953354986	0.406	0.048	1.17E-90	C10_CD4_Th17
chr16-75110715-75111531	1.12E-22	0.430709886	0.25	0.096	7.39E-18	C10_CD4_Th17
chr7-130938262-130939034	3.06E-16	0.340680863	0.311	0.16	2.02E-11	C10_CD4_Th17
chr1-194289384-194290132	7.99E-16	0.361544666	0.127	0.033	5.29E-11	C10_CD4_Th17
chr3-119562278-119562977	4.79E-12	0.322501005	0.158	0.065	3.17E-07	C10_CD4_Th17
chr12-93772937-93773510	1.24E-09	0.286880815	0.125	0.044	8.23E-05	C10_CD4_Th17
chr7-36209034-36209548	4.95E-50	0.717987147	0.219	0.023	3.28E-45	C10_CD4_Th17
chr12-93846970-93847700	3.71E-11	0.302762121	0.083	0.019	2.45E-06	C10_CD4_Th17
chr6-167119521-167120297	1.52E-36	0.573595257	0.269	0.063	1.01E-31	C10_CD4_Th17
chr11-119318537-119319008	4.57E-11	0.29224068	0.092	0.025	3.03E-06	C10_CD4_Th17
chr8-118379569-118380150	1.18E-12	0.283644415	0.052	0.006	7.81E-08	C10_CD4_Th17
chr6-167094817-167095391	1.41E-11	0.30895181	0.123	0.042	9.35E-07	C10_CD4_Th17
chr3-192850360-192851020	1.37E-10	0.286504081	0.068	0.014	9.04E-06	C10_CD4_Th17
chr10-69507151-69508165	5.21E-12	0.295969839	0.205	0.097	3.45E-07	C10_CD4_Th17
chr12-12489029-12489492	1.11E-16	0.365598194	0.085	0.012	7.37E-12	C10_CD4_Th17
chr16-75098154-75098993	3.64E-16	0.357585765	0.262	0.118	2.41E-11	C10_CD4_Th17
chr5-56828983-56829541	7.70E-14	0.335271653	0.149	0.051	5.10E-09	C10_CD4_Th17
chr1-193185910-193186733	4.74E-25	0.471058409	0.111	0.013	3.14E-20	C10_CD4_Th17
chr14-21308205-21309096	5.25E-09	0.256538116	0.215	0.111	0.000347941	C10_CD4_Th17
chr7-17022562-17023399	2.65E-21	0.436829788	0.17	0.043	1.75E-16	C10_CD4_Th17
chr4-54942019-54943056	1.22E-10	0.286298747	0.092	0.025	8.09E-06	C10_CD4_Th17
chr19-49555937-49556672	1.75E-09	0.277322378	0.104	0.037	0.000116107	C10_CD4_Th17

chr2-148524150-148525059	2.30E-21	0.43313594	0.212	0.066	1.52E-16	C10_CD4_Th17
chr19-10754900-10755528	2.09E-13	0.325961988	0.219	0.095	1.39E-08	C10_CD4_Th17
chr8-60928971-60929558	4.40E-11	0.295743376	0.189	0.086	2.91E-06	C10_CD4_Th17
chr2-162011620-162012049	1.58E-09	0.263776242	0.149	0.072	0.000104442	C10_CD4_Th17
chr11-14074835-14075632	1.07E-31	0.541569943	0.245	0.058	7.11E-27	C10_CD4_Th17
chr17-66673230-66674233	8.55E-38	0.581928916	0.274	0.069	5.66E-33	C10_CD4_Th17
chr6-157884687-157886211	1.37E-24	0.445804727	0.292	0.108	9.07E-20	C10_CD4_Th17
chr13-109785441-109787258	5.33E-11	0.270759698	0.337	0.195	3.53E-06	C10_CD4_Th17
chr9-72526824-72527815	4.99E-39	0.589463239	0.323	0.094	3.30E-34	C10_CD4_Th17
chr6-156396046-156397051	1.70E-14	0.335547412	0.087	0.017	1.13E-09	C10_CD4_Th17
chr20-3076845-3077946	1.33E-12	0.315458329	0.085	0.02	8.78E-08	C10_CD4_Th17
chr7-86978465-86979389	8.22E-10	0.270437179	0.184	0.093	5.44E-05	C10_CD4_Th17
chr5-95631738-95632499	1.54E-14	0.355042199	0.193	0.08	1.02E-09	C10_CD4_Th17
chr12-92210105-92210495	1.08E-19	0.412841895	0.144	0.036	7.14E-15	C10_CD4_Th17
chr8-60908981-60910155	6.83E-20	0.409642034	0.149	0.034	4.52E-15	C10_CD4_Th17
chr17-66246229-66246701	8.92E-10	0.266951313	0.087	0.026	5.91E-05	C10_CD4_Th17
chr15-90837686-90838896	2.63E-42	0.619918571	0.318	0.08	1.74E-37	C10_CD4_Th17
chr6-157893183-157893573	2.17E-10	0.27130333	0.071	0.017	1.44E-05	C10_CD4_Th17
chr1-225865587-225866457	1.97E-12	0.315167217	0.139	0.051	1.30E-07	C10_CD4_Th17
chr19-49564572-49564981	4.34E-13	0.318468842	0.094	0.024	2.87E-08	C10_CD4_Th17
chr2-100643486-100644472	6.04E-14	0.33785735	0.146	0.046	4.00E-09	C10_CD4_Th17
chr19-18021112-18022423	7.37E-10	0.26275753	0.144	0.063	4.88E-05	C10_CD4_Th17
chr22-42179959-42180790	1.95E-18	0.389776103	0.203	0.07	1.29E-13	C10_CD4_Th17
chr20-32967882-32968455	5.70E-17	0.377977604	0.193	0.066	3.77E-12	C10_CD4_Th17
chr12-107317894-107318515	4.56E-11	0.282022387	0.25	0.134	3.02E-06	C10_CD4_Th17
chr3-192892815-192893754	7.22E-11	0.292364504	0.137	0.052	4.78E-06	C10_CD4_Th17
chr9-104927208-104928364	3.04E-17	0.366256895	0.179	0.07	2.01E-12	C10_CD4_Th17

chr14-106395021-106395874	1.33E-13	0.323149698	0.092	0.021	8.83E-09	C10_CD4_Th17
chr12-56338147-56339151	3.20E-13	0.29954576	0.302	0.169	2.12E-08	C10_CD4_Th17
chr9-135887273-135887966	1.10E-10	0.270685186	0.066	0.015	7.26E-06	C10_CD4_Th17
chr4-87093377-87094067	8.70E-09	0.262099191	0.092	0.037	0.000576372	C10_CD4_Th17
chr9-112037436-112038280	1.23E-08	0.25376282	0.146	0.064	0.00081506	C10_CD4_Th17
chr11-3904661-3905532	3.33E-14	0.344611105	0.241	0.109	2.21E-09	C10_CD4_Th17
chr3-42555953-42556723	8.06E-10	0.267636391	0.182	0.093	5.34E-05	C10_CD4_Th17
chr17-1672879-1673546	3.24E-11	0.291827971	0.108	0.035	2.15E-06	C10_CD4_Th17
chr16-17590831-17592322	1.18E-45	0.637998164	0.368	0.106	7.80E-41	C10_CD4_Th17
chr3-5002037-5002968	5.10E-17	0.346413798	0.382	0.194	3.38E-12	C10_CD4_Th17
chr19-45455498-45456572	3.09E-14	0.31343777	0.278	0.141	2.04E-09	C10_CD4_Th17
chr20-37990033-37990949	4.20E-17	0.399517063	0.196	0.067	2.78E-12	C10_CD4_Th17
chr1-66287844-66288414	1.01E-10	0.291893662	0.156	0.065	6.67E-06	C10_CD4_Th17
chr2-148813506-148814147	7.34E-27	0.511822171	0.215	0.06	4.86E-22	C10_CD4_Th17
chr20-3665344-3666479	2.53E-10	0.292422884	0.189	0.093	1.67E-05	C10_CD4_Th17
chr9-75083211-75083544	9.53E-10	0.28637077	0.12	0.047	6.31E-05	C10_CD4_Th17
chr11-121464061-121464975	6.05E-17	0.319107681	0.467	0.28	4.01E-12	C10_CD4_Th17
chr15-70486282-70487163	2.09E-13	0.314956521	0.281	0.137	1.38E-08	C10_CD4_Th17
chr12-13195937-13196934	1.49E-12	0.303215848	0.281	0.155	9.89E-08	C10_CD4_Th17
chr16-81523422-81524781	5.99E-14	0.273190414	0.486	0.312	3.97E-09	C10_CD4_Th17
chr10-11156277-11156991	6.59E-20	0.416422713	0.142	0.035	4.36E-15	C10_CD4_Th17
chr19-47182300-47182765	4.18E-20	0.42775386	0.151	0.039	2.77E-15	C10_CD4_Th17
chr14-61528509-61529053	3.37E-10	0.28785038	0.167	0.07	2.23E-05	C10_CD4_Th17
chr15-90852005-90853142	6.03E-24	0.462014505	0.186	0.049	3.99E-19	C10_CD4_Th17
chr14-91193828-91194842	5.57E-15	0.350466794	0.151	0.05	3.69E-10	C10_CD4_Th17
chr16-24721591-24722189	9.77E-15	0.358316927	0.13	0.04	6.47E-10	C10_CD4_Th17
chr2-9752414-9754775	1.56E-26	0.420403999	0.429	0.211	1.04E-21	C10_CD4_Th17

chr9-126470334-126471491	2.33E-16	0.332098319	0.33	0.177	1.54E-11	C10_CD4_Th17
chr20-59242622-59243095	9.70E-18	0.394489289	0.158	0.044	6.42E-13	C10_CD4_Th17
chr8-125697949-125699113	5.49E-12	0.300507929	0.233	0.118	3.63E-07	C10_CD4_Th17
chr19-38693287-38694297	9.15E-11	0.27434462	0.203	0.111	6.06E-06	C10_CD4_Th17
chr13-29758427-29759203	1.10E-10	0.293482291	0.198	0.094	7.28E-06	C10_CD4_Th17
chr20-62159715-62160218	1.20E-12	0.307946218	0.083	0.019	7.95E-08	C10_CD4_Th17
chr18-63223870-63224886	3.12E-20	0.419151349	0.146	0.036	2.06E-15	C10_CD4_Th17
chr16-67244167-67245037	7.66E-12	0.25836293	0.382	0.256	5.08E-07	C10_CD4_Th17
chr1-46549395-46550267	3.59E-09	0.258663938	0.212	0.117	0.00023769	C10_CD4_Th17
chr3-10223517-10224076	7.95E-09	0.258607594	0.123	0.051	0.000526633	C10_CD4_Th17
chr7-151408489-151409399	8.43E-31	0.479733644	0.427	0.182	5.58E-26	C10_CD4_Th17
chr16-53098482-53100073	7.83E-17	0.336735995	0.351	0.189	5.18E-12	C10_CD4_Th17
chr2-10544065-10544785	1.25E-16	0.39312038	0.158	0.049	8.27E-12	C10_CD4_Th17
chr2-136362145-136363117	2.97E-10	0.282512351	0.172	0.086	1.96E-05	C10_CD4_Th17
chr20-44495271-44496462	2.45E-42	0.626285885	0.33	0.093	1.62E-37	C10_CD4_Th17
chr9-114484722-114485381	1.48E-09	0.262016359	0.182	0.089	9.81E-05	C10_CD4_Th17
chr10-77801166-77802052	1.11E-24	0.473763475	0.208	0.059	7.34E-20	C10_CD4_Th17
chr16-2846458-2847278	4.03E-23	0.447409517	0.226	0.075	2.67E-18	C10_CD4_Th17
chr17-48264671-48265788	5.99E-11	0.290204783	0.134	0.051	3.97E-06	C10_CD4_Th17
chr11-128343864-128344192	3.43E-15	0.35602416	0.113	0.027	2.27E-10	C10_CD4_Th17
chr4-10106029-10106991	2.32E-16	0.329500744	0.38	0.204	1.54E-11	C10_CD4_Th17
chr8-128076511-128077372	5.97E-17	0.381569156	0.259	0.106	3.95E-12	C10_CD4_Th17
chr10-91194457-91195121	1.56E-08	0.260272002	0.106	0.044	0.001034438	C10_CD4_Th17
chr10-49177021-49177858	7.07E-11	0.295599353	0.149	0.064	4.68E-06	C10_CD4_Th17
chr11-85742860-85743259	4.11E-10	0.268464935	0.064	0.013	2.72E-05	C10_CD4_Th17
chr20-59144248-59144825	2.27E-30	0.521524894	0.255	0.07	1.50E-25	C10_CD4_Th17
chr14-71141928-71143729	9.33E-11	0.281764759	0.21	0.1	6.18E-06	C10_CD4_Th17

chr12-27821235-27822714	1.09E-26	0.425963036	0.498	0.254	7.23E-22	C10_CD4_Th17
chr12-68259227-68259799	4.89E-09	0.261488998	0.085	0.026	0.000323784	C10_CD4_Th17
chr11-118201941-118202904	3.74E-14	0.327861946	0.165	0.063	2.48E-09	C10_CD4_Th17
chr4-148056547-148057231	2.36E-09	0.269604131	0.193	0.095	0.000156335	C10_CD4_Th17
chr3-149016678-149017726	1.49E-09	0.268342368	0.12	0.049	9.89E-05	C10_CD4_Th17
chr3-45993101-45994130	4.42E-19	0.346744984	0.396	0.217	2.93E-14	C10_CD4_Th17
chr7-17022562-17023399	2.65E-21	0.436829788	0.17	0.043	1.75E-16	C10_CD4_Th17
chr10-110535725-110536609	6.83E-21	0.419143025	0.276	0.115	4.52E-16	C10_CD4_Th17
chr14-68685054-68686144	4.83E-13	0.250350584	0.519	0.362	3.20E-08	C10_CD4_Th17
chr2-119827772-119828490	1.47E-11	0.319083523	0.127	0.045	9.74E-07	C10_CD4_Th17
chr6-90078895-90081514	1.02E-19	0.359295661	0.394	0.208	6.75E-15	C10_CD4_Th17
chr14-99396004-99396540	4.18E-10	0.266220149	0.099	0.034	2.77E-05	C10_CD4_Th17
chr2-73896226-73897070	4.43E-14	0.330899733	0.099	0.023	2.94E-09	C10_CD4_Th17
chr2-65382460-65383326	9.13E-09	0.254512387	0.146	0.069	0.000604484	C10_CD4_Th17
chr3-50316352-50317145	4.44E-11	0.276695407	0.271	0.148	2.94E-06	C10_CD4_Th17
chr11-14074835-14075632	1.07E-31	0.541569943	0.245	0.058	7.11E-27	C10_CD4_Th17
chr4-83548014-83548830	9.38E-09	0.254669783	0.177	0.089	0.000621264	C10_CD4_Th17
chr22-36166068-36167237	6.63E-11	0.273372236	0.215	0.112	4.39E-06	C10_CD4_Th17
chr4-139528391-139528994	1.60E-11	0.292602304	0.097	0.031	1.06E-06	C10_CD4_Th17
chr2-9779545-9780603	1.35E-22	0.428542387	0.165	0.042	8.93E-18	C10_CD4_Th17
chr2-65299224-65299583	1.02E-09	0.271305764	0.097	0.031	6.75E-05	C10_CD4_Th17
chr10-8415943-8417132	8.55E-11	0.279551832	0.264	0.15	5.66E-06	C10_CD4_Th17
chr6-89375368-89376080	1.81E-12	0.312421561	0.25	0.131	1.20E-07	C10_CD4_Th17
chr1-40628211-40628877	7.82E-09	0.268022083	0.198	0.102	0.000518098	C10_CD4_Th17
chr6-158025904-158027160	7.99E-14	0.290912741	0.38	0.236	5.29E-09	C10_CD4_Th17
chr15-60932224-60932934	1.21E-14	0.355523145	0.172	0.066	8.02E-10	C10_CD4_Th17
chr10-70616681-70617364	1.27E-14	0.349263141	0.12	0.033	8.41E-10	C10_CD4_Th17

chr15-72785349-72785762	3.61E-23	0.461144901	0.184	0.049	2.39E-18	C10_CD4_Th17
chr6-37173463-37173917	1.50E-13	0.31529977	0.262	0.131	9.90E-09	C10_CD4_Th17
chr14-61525034-61525695	3.71E-10	0.262616448	0.241	0.142	2.45E-05	C10_CD4_Th17
chr12-93783792-93785545	3.50E-09	0.25271386	0.259	0.145	0.000231943	C10_CD4_Th17
chr10-45421154-45421545	3.86E-10	0.255496215	0.066	0.014	2.56E-05	C10_CD4_Th17
chr1-84853880-84854757	9.59E-17	0.378607313	0.248	0.106	6.35E-12	C10_CD4_Th17
chr17-4969337-4969558	1.47E-27	0.492816522	0.163	0.031	9.72E-23	C10_CD4_Th17
chr1-151836502-151837667	1.41E-14	0.316168297	0.264	0.13	9.35E-10	C10_CD4_Th17
chr8-11782647-11783521	2.73E-13	0.351337466	0.12	0.034	1.81E-08	C10_CD4_Th17
chr1-210331995-210333134	3.10E-14	0.334066588	0.288	0.135	2.05E-09	C10_CD4_Th17
chr2-98479473-98480580	5.75E-10	0.27254677	0.212	0.108	3.81E-05	C10_CD4_Th17
chr10-32413228-32413966	7.07E-75	0.863131583	0.288	0.027	4.68E-70	C10_CD4_Th17
chr9-130166843-130167273	1.65E-11	0.284872692	0.075	0.018	1.09E-06	C10_CD4_Th17
chr2-240624500-240625519	5.43E-11	0.257465058	0.33	0.197	3.60E-06	C10_CD4_Th17
chr6-139172678-139173531	2.82E-08	0.253564888	0.142	0.07	0.001866015	C10_CD4_Th17
chr3-45988394-45989136	8.38E-10	0.269480704	0.118	0.048	5.55E-05	C10_CD4_Th17
chr14-69716206-69717035	1.28E-13	0.338077528	0.186	0.076	8.48E-09	C10_CD4_Th17
chr12-89494272-89495220	2.09E-21	0.452317898	0.226	0.076	1.38E-16	C10_CD4_Th17
chr2-37587660-37588544	6.03E-13	0.321016267	0.17	0.068	3.99E-08	C10_CD4_Th17
chr2-178513847-178515083	3.52E-36	0.590651127	0.274	0.067	2.33E-31	C10_CD4_Th17
chr10-6267172-6267557	6.18E-22	0.430689182	0.097	0.012	4.09E-17	C10_CD4_Th17
chr15-39629099-39629839	1.47E-14	0.321376293	0.342	0.189	9.72E-10	C10_CD4_Th17
chr15-90837686-90838896	2.63E-42	0.619918571	0.318	0.08	1.74E-37	C10_CD4_Th17
chrX-20252189-20253296	2.97E-48	0.637786318	0.349	0.09	1.97E-43	C10_CD4_Th17
chr18-45874302-45875212	2.74E-12	0.318545568	0.163	0.067	1.82E-07	C10_CD4_Th17
chr22-40682751-40683431	3.11E-09	0.257450801	0.17	0.086	0.000206206	C10_CD4_Th17
chr2-10329826-10332253	1.01E-12	0.275826994	0.356	0.216	6.69E-08	C10_CD4_Th17

chr20-47274406-47274862	1.36E-08	0.256783512	0.085	0.029	0.000903597	C10_CD4_Th17
chr2-28583595-28584569	3.12E-09	0.273434196	0.17	0.077	0.000206551	C10_CD4_Th17
chr1-169908367-169909148	1.51E-10	0.274691634	0.099	0.032	9.97E-06	C10_CD4_Th17
chrX-19627540-19627876	9.00E-11	0.287389322	0.09	0.026	5.96E-06	C10_CD4_Th17
chr13-99214321-99215835	2.18E-09	0.250623401	0.236	0.14	0.000144654	C10_CD4_Th17
chr19-6639243-6639831	1.01E-10	0.294696095	0.125	0.047	6.67E-06	C10_CD4_Th17
chr9-131504058-131504423	1.23E-09	0.262327358	0.149	0.063	8.13E-05	C10_CD4_Th17
chr4-75686208-75686780	5.44E-12	0.308265491	0.137	0.051	3.60E-07	C10_CD4_Th17
chr6-137978282-137978645	1.71E-10	0.276501174	0.083	0.023	1.13E-05	C10_CD4_Th17
chr10-73932104-73932872	7.98E-09	0.265829363	0.125	0.055	0.000528469	C10_CD4_Th17
chr15-60539991-60540712	1.09E-15	0.35262747	0.231	0.095	7.19E-11	C10_CD4_Th17
chr11-65565723-65566380	2.27E-12	0.288918317	0.283	0.157	1.50E-07	C10_CD4_Th17
chr12-56338147-56339151	3.20E-13	0.29954576	0.302	0.169	2.12E-08	C10_CD4_Th17
chr17-2087584-2088039	1.90E-10	0.276004531	0.12	0.044	1.26E-05	C10_CD4_Th17
chr4-55866196-55866746	4.81E-39	0.603384331	0.21	0.037	3.18E-34	C10_CD4_Th17
chr1-67170672-67172385	2.39E-34	0.532729234	0.278	0.081	1.58E-29	C10_CD4_Th17
chr2-162055259-162055750	1.36E-12	0.336595388	0.137	0.049	9.01E-08	C10_CD4_Th17
chr17-77409462-77410211	1.06E-08	0.25936852	0.149	0.069	0.000704977	C10_CD4_Th17
chr1-116389550-116390529	1.45E-15	0.339224069	0.231	0.107	9.58E-11	C10_CD4_Th17
chr12-48933563-48934303	8.99E-14	0.335270329	0.189	0.069	5.96E-09	C10_CD4_Th17
chr15-60526103-60526651	1.27E-09	0.26817472	0.137	0.058	8.40E-05	C10_CD4_Th17
chr11-114065813-114066852	7.59E-55	0.726499904	0.231	0.024	5.03E-50	C10_CD4_Th17
chr11-114099393-114100021	7.19E-20	0.405788389	0.087	0.009	4.76E-15	C10_CD4_Th17
chr16-2846458-2847278	4.03E-23	0.447409517	0.226	0.075	2.67E-18	C10_CD4_Th17
chr11-114073591-114074289	2.67E-19	0.430462904	0.172	0.045	1.77E-14	C10_CD4_Th17
chr8-37871015-37871537	4.19E-49	0.703447976	0.198	0.017	2.77E-44	C10_CD4_Th17
chr11-114075849-114076084	1.25E-20	0.423188598	0.111	0.017	8.30E-16	C10_CD4_Th17

chr20-35251409-35252730	1.54E-38	0.577964309	0.328	0.094	1.02E-33	C10_CD4_Th17
chr6-83430510-83431262	1.08E-23	0.41559675	0.085	0.006	7.16E-19	C10_CD4_Th17
chr15-60526103-60526651	1.27E-09	0.26817472	0.137	0.058	8.40E-05	C10_CD4_Th17
chr11-126332215-126332648	3.24E-23	0.48245537	0.156	0.029	2.14E-18	C10_CD4_Th17
chr9-81688609-81690098	1.41E-71	0.848734467	0.302	0.031	9.31E-67	C10_CD4_Th17
chr3-5002037-5002968	5.10E-17	0.346413798	0.382	0.194	3.38E-12	C10_CD4_Th17
chr2-10544065-10544785	1.25E-16	0.39312038	0.158	0.049	8.27E-12	C10_CD4_Th17
chr2-25305221-25305473	9.63E-43	0.627807795	0.144	0.009	6.38E-38	C10_CD4_Th17
chr11-114058953-114060800	2.28E-10	0.27148324	0.2	0.101	1.51E-05	C10_CD4_Th17
chr9-81596744-81597481	3.71E-13	0.286371767	0.05	0.004	2.46E-08	C10_CD4_Th17
chr4-174521723-174523253	2.12E-09	0.260547052	0.17	0.078	0.00014057	C10_CD4_Th17
chr14-87994963-87995338	1.15E-09	0.270202225	0.113	0.039	7.63E-05	C10_CD4_Th17
chr14-92547392-92547850	9.69E-12	0.313803666	0.12	0.038	6.42E-07	C10_CD4_Th17
chr1-15416747-15417980	2.88E-17	0.334117744	0.34	0.183	1.91E-12	C10_CD4_Th17
chr6-111487675-111488000	2.61E-13	0.334296199	0.175	0.067	1.73E-08	C10_CD4_Th17
chr11-125513944-125514931	1.55E-10	0.275389129	0.2	0.105	1.02E-05	C10_CD4_Th17
chr19-45455498-45456572	3.09E-14	0.31343777	0.278	0.141	2.04E-09	C10_CD4_Th17
chr1-67322804-67323396	1.81E-12	0.286318828	0.052	0.005	1.20E-07	C10_CD4_Th17
chr14-71141928-71143729	9.33E-11	0.281764759	0.21	0.1	6.18E-06	C10_CD4_Th17
chr15-90852005-90853142	6.03E-24	0.462014505	0.186	0.049	3.99E-19	C10_CD4_Th17
chr8-27333049-27333558	1.51E-19	0.416856926	0.118	0.024	1.00E-14	C10_CD4_Th17
chr8-41477078-41477677	1.56E-35	0.594959489	0.16	0.018	1.03E-30	C10_CD4_Th17
chr3-10191851-10192457	1.68E-17	0.373478851	0.177	0.058	1.11E-12	C10_CD4_Th17
chr10-110535725-110536609	6.83E-21	0.419143025	0.276	0.115	4.52E-16	C10_CD4_Th17
chr7-151408489-151409399	8.43E-31	0.479733644	0.427	0.182	5.58E-26	C10_CD4_Th17
chr8-27408908-27409173	9.83E-16	0.328514279	0.066	0.007	6.51E-11	C10_CD4_Th17
chr22-38305504-38306477	1.40E-15	0.363258337	0.205	0.083	9.30E-11	C10_CD4_Th17

chr11-13332626-13333039	1.00E-19	0.427553463	0.118	0.023	6.65E-15	C10_CD4_Th17
chr7-140252632-140253466	1.00E-25	0.473313118	0.25	0.079	6.65E-21	C10_CD4_Th17
chr1-66287844-66288414	1.01E-10	0.291893662	0.156	0.065	6.67E-06	C10_CD4_Th17
chr10-11156277-11156991	6.59E-20	0.416422713	0.142	0.035	4.36E-15	C10_CD4_Th17
chr17-64066512-64067113	3.22E-09	0.25837446	0.087	0.029	0.000213125	C10_CD4_Th17
chr8-128076511-128077372	5.97E-17	0.381569156	0.259	0.106	3.95E-12	C10_CD4_Th17
chr15-70486282-70487163	2.09E-13	0.314956521	0.281	0.137	1.38E-08	C10_CD4_Th17
chr8-66467428-66468210	1.09E-43	0.653263548	0.252	0.044	7.24E-39	C10_CD4_Th17
chr19-38693287-38694297	9.15E-11	0.27434462	0.203	0.111	6.06E-06	C10_CD4_Th17
chr16-81523422-81524781	5.99E-14	0.273190414	0.486	0.312	3.97E-09	C10_CD4_Th17
chr15-60932224-60932934	1.21E-14	0.355523145	0.172	0.066	8.02E-10	C10_CD4_Th17
chr5-59562745-59563294	2.90E-09	0.260802993	0.061	0.015	0.000192235	C10_CD4_Th17
chr8-100258271-100259260	1.94E-13	0.296886431	0.309	0.168	1.28E-08	C10_CD4_Th17
chr3-149016678-149017726	1.49E-09	0.268342368	0.12	0.049	9.89E-05	C10_CD4_Th17
chr11-114077540-114078153	2.57E-12	0.310708158	0.094	0.025	1.70E-07	C10_CD4_Th17
chr21-46515022-46515535	5.78E-17	0.370885776	0.106	0.023	3.83E-12	C10_CD4_Th17
chr1-40628211-40628877	7.82E-09	0.268022083	0.198	0.102	0.000518098	C10_CD4_Th17
chr20-44495271-44496462	2.45E-42	0.626285885	0.33	0.093	1.62E-37	C10_CD4_Th17
chr8-60926284-60927881	4.02E-24	0.444107637	0.297	0.117	2.66E-19	C10_CD4_Th17
chr17-3740775-3741095	1.01E-10	0.276435078	0.078	0.018	6.71E-06	C10_CD4_Th17
chr17-62724320-62725192	2.91E-20	0.420648427	0.191	0.056	1.92E-15	C10_CD4_Th17
chr14-61525034-61525695	3.71E-10	0.262616448	0.241	0.142	2.45E-05	C10_CD4_Th17
chr6-137978282-137978645	1.71E-10	0.276501174	0.083	0.023	1.13E-05	C10_CD4_Th17
chr8-47643958-47644536	8.15E-26	0.476371934	0.108	0.011	5.40E-21	C10_CD4_Th17
chr2-73896226-73897070	4.43E-14	0.330899733	0.099	0.023	2.94E-09	C10_CD4_Th17
chr19-6678504-6679035	3.02E-11	0.305382571	0.172	0.072	2.00E-06	C10_CD4_Th17
chr8-37891644-37892174	1.46E-10	0.275042033	0.203	0.1	9.70E-06	C10_CD4_Th17

chr6-137967768-137969282	2.96E-16	0.373843852	0.13	0.034	1.96E-11	C10_CD4_Th17
chr3-188189591-188190116	7.98E-26	0.46555741	0.097	0.009	5.28E-21	C10_CD4_Th17
chr5-127785079-127786007	9.07E-80	0.887883556	0.363	0.046	6.01E-75	C10_CD4_Th17
chr16-69530264-69531572	1.03E-25	0.424835616	0.394	0.185	6.82E-21	C10_CD4_Th17
chr1-193448752-193449502	5.16E-27	0.5266301	0.175	0.035	3.42E-22	C10_CD4_Th17
chr11-132718804-132719896	3.31E-39	0.614223644	0.226	0.041	2.19E-34	C10_CD4_Th17
chr19-49896720-49898048	1.55E-38	0.500281598	0.46	0.193	1.03E-33	C10_CD4_Th17
chr8-60928971-60929558	4.40E-11	0.295743376	0.189	0.086	2.91E-06	C10_CD4_Th17
chr15-60573803-60574631	1.24E-26	0.483398865	0.224	0.063	8.21E-22	C10_CD4_Th17
chr19-8486986-8487803	1.13E-24	0.462256237	0.153	0.033	7.47E-20	C10_CD4_Th17
chr14-73829509-73830403	1.07E-15	0.363865517	0.156	0.055	7.09E-11	C10_CD4_Th17
chr6-167129175-167130547	4.14E-17	0.386693893	0.13	0.03	2.74E-12	C10_CD4_Th17
chr2-240624500-240625519	5.43E-11	0.257465058	0.33	0.197	3.60E-06	C10_CD4_Th17
chr19-18514456-18515253	3.25E-28	0.502635568	0.193	0.042	2.15E-23	C10_CD4_Th17
chr8-47737262-47739144	1.80E-19	0.403493839	0.215	0.074	1.19E-14	C10_CD4_Th17
chr12-3727890-3729074	2.15E-10	0.277811146	0.222	0.107	1.42E-05	C10_CD4_Th17
chr6-37173463-37173917	1.50E-13	0.31529977	0.262	0.131	9.90E-09	C10_CD4_Th17
chr12-48933563-48934303	8.99E-14	0.335270329	0.189	0.069	5.96E-09	C10_CD4_Th17
chr11-65565723-65566380	2.27E-12	0.288918317	0.283	0.157	1.50E-07	C10_CD4_Th17
chr1-67166208-67166540	6.41E-56	0.715547983	0.184	0.013	4.25E-51	C10_CD4_Th17
chr1-116389550-116390529	1.45E-15	0.339224069	0.231	0.107	9.58E-11	C10_CD4_Th17
chr3-45993101-45994130	4.42E-19	0.346744984	0.396	0.217	2.93E-14	C10_CD4_Th17
chr1-232580015-232580725	1.57E-21	0.432924678	0.153	0.036	1.04E-16	C10_CD4_Th17
chr6-53361405-53362042	2.24E-13	0.302688446	0.307	0.175	1.48E-08	C10_CD4_Th17
chr8-47368994-47369760	1.06E-10	0.260356434	0.054	0.008	7.04E-06	C10_CD4_Th17
chr10-110857274-110857907	3.77E-19	0.406188055	0.146	0.038	2.49E-14	C10_CD4_Th17
chr6-109059312-109060140	9.64E-26	0.494030853	0.189	0.044	6.38E-21	C10_CD4_Th17

chr2-8487695-8488598	5.01E-12	0.297678269	0.248	0.133	3.32E-07	C10_CD4_Th17
chr4-10106029-10106991	2.32E-16	0.329500744	0.38	0.204	1.54E-11	C10_CD4_Th17
chr15-90851021-90851484	4.02E-26	0.476367804	0.118	0.014	2.66E-21	C10_CD4_Th17
chr5-127936456-127937005	2.09E-12	0.312956992	0.071	0.012	1.38E-07	C10_CD4_Th17
chr20-24929998-24930763	1.60E-08	0.262485365	0.13	0.053	0.001057665	C10_CD4_Th17
chr3-15519975-15520491	4.84E-10	0.266012451	0.08	0.023	3.21E-05	C10_CD4_Th17
chr1-20187187-20187844	6.73E-10	0.259557118	0.078	0.024	4.46E-05	C10_CD4_Th17
chr20-37990033-37990949	4.20E-17	0.399517063	0.196	0.067	2.78E-12	C10_CD4_Th17
chr1-46549395-46550267	3.59E-09	0.258663938	0.212	0.117	0.00023769	C10_CD4_Th17
chr1-67170672-67172385	2.39E-34	0.532729234	0.278	0.081	1.58E-29	C10_CD4_Th17
chr11-13288399-13288949	5.07E-12	0.297084527	0.08	0.02	3.35E-07	C10_CD4_Th17
chr15-90837686-90838896	2.63E-42	0.619918571	0.318	0.08	1.74E-37	C10_CD4_Th17
chr10-70616681-70617364	1.27E-14	0.349263141	0.12	0.033	8.41E-10	C10_CD4_Th17
chr20-33372390-33372681	1.13E-11	0.295719658	0.116	0.037	7.51E-07	C10_CD4_Th17
chr10-8415943-8417132	8.55E-11	0.279551832	0.264	0.15	5.66E-06	C10_CD4_Th17
chr7-139680676-139681239	2.73E-34	0.589282377	0.229	0.049	1.81E-29	C10_CD4_Th17
chr14-61528509-61529053	3.37E-10	0.28785038	0.167	0.07	2.23E-05	C10_CD4_Th17
chr17-66633280-66633639	1.59E-09	0.263108346	0.064	0.013	0.000105489	C10_CD4_Th17
chr15-60704044-60705101	1.25E-37	0.598233894	0.245	0.054	8.31E-33	C10_CD4_Th17
chr2-161956486-161957047	3.35E-12	0.30396991	0.085	0.018	2.22E-07	C10_CD4_Th17
chr6-167094817-167095391	1.41E-11	0.30895181	0.123	0.042	9.35E-07	C10_CD4_Th17
chr9-113519909-113520623	5.50E-11	0.286480119	0.186	0.082	3.65E-06	C10_CD4_Th17
chr9-104926185-104926887	3.21E-12	0.304517508	0.175	0.074	2.13E-07	C10_CD4_Th17
chr17-29154774-29155846	6.87E-15	0.352423263	0.153	0.056	4.55E-10	C10_CD4_Th17
chr22-49838544-49839669	1.45E-16	0.285652427	0.512	0.352	9.58E-12	C10_CD4_Th17
chr12-92207915-92208995	2.62E-13	0.30232851	0.267	0.141	1.73E-08	C10_CD4_Th17
chr1-203755930-203757037	7.03E-14	0.330302865	0.267	0.128	4.66E-09	C10_CD4_Th17

chr12-68259227-68259799	4.89E-09	0.261488998	0.085	0.026	0.000323784	C10_CD4_Th17
chr5-177388823-177390851	5.37E-18	0.315714494	0.436	0.269	3.55E-13	C10_CD4_Th17
chr4-114005622-114006382	5.25E-36	0.569023234	0.149	0.014	3.48E-31	C10_CD4_Th17
chr2-161952554-161953235	7.88E-15	0.355485311	0.127	0.033	5.22E-10	C10_CD4_Th17
chr8-125697949-125699113	5.49E-12	0.300507929	0.233	0.118	3.63E-07	C10_CD4_Th17
chr17-66673230-66674233	8.55E-38	0.581928916	0.274	0.069	5.66E-33	C10_CD4_Th17
chr4-147799864-147800522	2.14E-11	0.298783681	0.153	0.061	1.41E-06	C10_CD4_Th17
chr2-234474955-234475474	2.09E-15	0.339194067	0.087	0.014	1.38E-10	C10_CD4_Th17
chr10-77832147-77832594	1.33E-08	0.251965477	0.083	0.026	0.000882614	C10_CD4_Th17
chr6-87756776-87757261	3.18E-09	0.265155886	0.101	0.037	0.000210295	C10_CD4_Th17
chr15-75836455-75837385	1.55E-12	0.323766136	0.156	0.059	1.03E-07	C10_CD4_Th17
chr12-94180453-94181290	1.40E-16	0.384575637	0.153	0.046	9.24E-12	C10_CD4_Th17
chr7-87600029-87601050	2.53E-09	0.265381682	0.106	0.038	0.000167231	C10_CD4_Th17
chr6-167350183-167351772	6.04E-47	0.628691959	0.342	0.086	4.00E-42	C10_CD4_Th17
chr1-199172429-199173790	1.42E-10	0.292862382	0.172	0.076	9.37E-06	C10_CD4_Th17
chr21-44870488-44870976	3.85E-15	0.354121101	0.146	0.048	2.55E-10	C10_CD4_Th17
chrX-15246724-15247330	3.54E-13	0.299989548	0.057	0.007	2.35E-08	C10_CD4_Th17
chr11-3049433-3050260	4.28E-11	0.265622566	0.311	0.192	2.84E-06	C10_CD4_Th17
chr11-128488077-128488837	9.29E-13	0.305083455	0.177	0.07	6.15E-08	C10_CD4_Th17
chr13-99214321-99215835	2.18E-09	0.250623401	0.236	0.14	0.000144654	C10_CD4_Th17
chr12-89494272-89495220	2.09E-21	0.452317898	0.226	0.076	1.38E-16	C10_CD4_Th17
chr2-191037975-191038812	3.65E-15	0.358679793	0.198	0.077	2.42E-10	C10_CD4_Th17
chr2-100060676-100061238	6.58E-17	0.338307279	0.061	0.006	4.36E-12	C10_CD4_Th17
chr17-66713231-66714033	3.04E-09	0.275250302	0.118	0.045	0.000201309	C10_CD4_Th17
chr9-104927208-104928364	3.04E-17	0.366256895	0.179	0.07	2.01E-12	C10_CD4_Th17
chr10-133475056-133476222	4.13E-21	0.430474562	0.108	0.016	2.73E-16	C10_CD4_Th17
chr19-10754900-10755528	2.09E-13	0.325961988	0.219	0.095	1.39E-08	C10_CD4_Th17

chr16-10333306-10333601	1.36E-21	0.400964795	0.075	0.005	9.01E-17	C10_CD4_Th17
chr17-66246229-66246701	8.92E-10	0.266951313	0.087	0.026	5.91E-05	C10_CD4_Th17
chr2-110804645-110805248	4.90E-36	0.594474081	0.198	0.033	3.25E-31	C10_CD4_Th17
chr20-3665344-3666479	2.53E-10	0.292422884	0.189	0.093	1.67E-05	C10_CD4_Th17
chr3-10198637-10199412	3.25E-15	0.354917238	0.153	0.053	2.16E-10	C10_CD4_Th17
chr14-69716206-69717035	1.28E-13	0.338077528	0.186	0.076	8.48E-09	C10_CD4_Th17
chr9-33445631-33447874	1.37E-22	0.355337426	0.5	0.302	9.09E-18	C10_CD4_Th17
chr1-6962118-6963668	9.46E-85	1.055424718	0.42	0.037	6.26E-80	C11_CD4_Treg
chr11-111531693-111532434	1.39E-77	1.032669669	0.295	0.011	9.23E-73	C11_CD4_Treg
chr12-49946158-49946404	4.16E-67	0.939381635	0.224	0.005	2.75E-62	C11_CD4_Treg
chr2-213219582-213220321	6.76E-56	0.851221509	0.186	0.004	4.47E-51	C11_CD4_Treg
chr3-39333899-39334524	7.19E-54	0.818082288	0.173	0.003	4.76E-49	C11_CD4_Treg
chr5-139437205-139437732	8.54E-54	0.881382548	0.275	0.025	5.65E-49	C11_CD4_Treg
chr2-213015457-213016118	9.98E-48	0.816980472	0.207	0.011	6.61E-43	C11_CD4_Treg
chr2-213232792-213233431	1.15E-43	0.805015461	0.22	0.015	7.62E-39	C11_CD4_Treg
chr3-133447911-133449082	2.26E-43	0.803154156	0.237	0.021	1.50E-38	C11_CD4_Treg
chr2-213151185-213152375	5.54E-43	0.736657702	0.407	0.083	3.67E-38	C11_CD4_Treg
chr11-111539312-111539744	2.07E-42	0.753362782	0.166	0.005	1.37E-37	C11_CD4_Treg
chr10-74853842-74854353	8.35E-42	0.762284217	0.183	0.01	5.53E-37	C11_CD4_Treg
chr1-193525872-193526271	2.31E-40	0.71624405	0.149	0.004	1.53E-35	C11_CD4_Treg
chr10-73366386-73367075	5.72E-40	0.700101161	0.153	0.006	3.79E-35	C11_CD4_Treg
chr3-183370787-183371819	4.96E-38	0.737340189	0.254	0.038	3.29E-33	C11_CD4_Treg
chr10-17648260-17648838	2.44E-37	0.713702976	0.312	0.06	1.62E-32	C11_CD4_Treg
chr8-94241589-94242663	3.29E-37	0.742084719	0.207	0.016	2.18E-32	C11_CD4_Treg
chr6-158322958-158323832	3.08E-36	0.721186126	0.251	0.033	2.04E-31	C11_CD4_Treg
chr3-16370836-16371582	1.32E-35	0.671365207	0.139	0.005	8.72E-31	C11_CD4_Treg
chr3-8501053-8501967	4.45E-34	0.668192842	0.363	0.088	2.95E-29	C11_CD4_Treg

chr5-96565652-96566586	1.08E-33	0.669377995	0.169	0.013	7.12E-29	C11_CD4_Treg
chr13-40102126-40103235	1.33E-33	0.68945061	0.22	0.028	8.83E-29	C11_CD4_Treg
chr10-26738270-26738613	7.23E-33	0.679569863	0.156	0.01	4.79E-28	C11_CD4_Treg
chr8-125227192-125227489	7.29E-33	0.602583412	0.105	0.002	4.82E-28	C11_CD4_Treg
chr6-143594615-143594988	1.04E-32	0.681381285	0.156	0.01	6.90E-28	C11_CD4_Treg
chr2-197934117-197935225	1.13E-31	0.668007102	0.203	0.024	7.50E-27	C11_CD4_Treg
chr3-40971188-40972064	1.82E-31	0.65543094	0.166	0.013	1.20E-26	C11_CD4_Treg
chr11-111540518-111540955	2.05E-31	0.60884604	0.129	0.005	1.35E-26	C11_CD4_Treg
chr2-108580858-108581371	3.05E-31	0.625979171	0.136	0.006	2.02E-26	C11_CD4_Treg
chr2-212929134-212929470	9.39E-31	0.614989015	0.125	0.005	6.22E-26	C11_CD4_Treg
chr10-17655435-17655972	1.23E-30	0.629467474	0.136	0.007	8.16E-26	C11_CD4_Treg
chr9-127911169-127911471	3.13E-30	0.628545285	0.136	0.008	2.07E-25	C11_CD4_Treg
chr12-93089978-93090926	3.39E-30	0.629999296	0.156	0.015	2.24E-25	C11_CD4_Treg
chr10-119705117-119705802	4.22E-30	0.643989001	0.159	0.015	2.79E-25	C11_CD4_Treg
chr10-119704003-119704793	4.40E-30	0.640319611	0.166	0.016	2.92E-25	C11_CD4_Treg
chr10-123287714-123288269	5.33E-30	0.620736456	0.129	0.007	3.53E-25	C11_CD4_Treg
chr4-105305965-105306841	1.05E-29	0.651833254	0.271	0.05	6.94E-25	C11_CD4_Treg
chr6-143441405-143442063	1.31E-29	0.651445511	0.176	0.02	8.68E-25	C11_CD4_Treg
chr9-6433385-6433878	1.34E-29	0.654630871	0.19	0.024	8.85E-25	C11_CD4_Treg
chr2-32189333-32190229	1.54E-29	0.630212859	0.159	0.015	1.02E-24	C11_CD4_Treg
chr21-29298338-29300857	2.83E-29	0.414221938	0.688	0.444	1.87E-24	C11_CD4_Treg
chr11-111538786-111539041	6.75E-29	0.558689703	0.102	0.002	4.47E-24	C11_CD4_Treg
chr17-55314380-55315152	8.39E-29	0.571806765	0.108	0.003	5.55E-24	C11_CD4_Treg
chr2-43569377-43570293	1.15E-28	0.628606767	0.163	0.016	7.62E-24	C11_CD4_Treg
chr5-81211881-81212589	1.52E-28	0.600588322	0.125	0.008	1.00E-23	C11_CD4_Treg
chr1-24203514-24203785	1.68E-27	0.562591631	0.105	0.004	1.11E-22	C11_CD4_Treg
chr10-95832061-95832722	2.15E-27	0.590397782	0.342	0.101	1.42E-22	C11_CD4_Treg

chr18-57017021-57017640	2.60E-27	0.586696578	0.132	0.008	1.72E-22	C11_CD4_Treg
chr14-51331651-51332192	3.53E-27	0.525930132	0.088	0.002	2.34E-22	C11_CD4_Treg
chr22-36790463-36790937	4.01E-27	0.538083069	0.095	0.003	2.66E-22	C11_CD4_Treg
chr12-121536158-121538195	1.01E-26	0.520755199	0.458	0.185	6.72E-22	C11_CD4_Treg
chr22-45064365-45064941	1.05E-26	0.587669274	0.136	0.011	6.96E-22	C11_CD4_Treg
chr12-93095435-93095742	1.42E-26	0.553242472	0.108	0.005	9.39E-22	C11_CD4_Treg
chr2-213157304-213157640	1.74E-26	0.611149437	0.139	0.009	1.15E-21	C11_CD4_Treg
chr19-50300687-50301160	4.52E-26	0.56331004	0.108	0.005	2.99E-21	C11_CD4_Treg
chr2-130823955-130824921	5.40E-26	0.53233374	0.454	0.175	3.58E-21	C11_CD4_Treg
chr15-63135514-63136173	6.00E-26	0.577456485	0.139	0.012	3.97E-21	C11_CD4_Treg
chr10-22308327-22308717	6.14E-26	0.591491603	0.156	0.017	4.07E-21	C11_CD4_Treg
chr22-30841271-30841751	6.31E-26	0.553692957	0.102	0.004	4.18E-21	C11_CD4_Treg
chr2-64179841-64180124	1.08E-25	0.519729545	0.085	0.002	7.19E-21	C11_CD4_Treg
chr8-64756570-64757235	1.28E-25	0.569417904	0.108	0.005	8.48E-21	C11_CD4_Treg
chr2-229821762-229822606	3.00E-25	0.577423263	0.254	0.064	1.99E-20	C11_CD4_Treg
chr8-29847895-29848543	1.76E-24	0.546746086	0.108	0.006	1.17E-19	C11_CD4_Treg
chr7-25967831-25968834	3.43E-24	0.57116034	0.169	0.025	2.27E-19	C11_CD4_Treg
chr1-67723609-67724007	4.32E-24	0.558389968	0.125	0.01	2.86E-19	C11_CD4_Treg
chr1-23624092-23624707	5.01E-24	0.583902192	0.22	0.046	3.32E-19	C11_CD4_Treg
chr3-56959894-56960572	5.43E-24	0.500786693	0.081	0.002	3.60E-19	C11_CD4_Treg
chr10-1036199-1036764	5.82E-24	0.594636362	0.156	0.018	3.85E-19	C11_CD4_Treg
chr11-113587293-113587733	6.64E-24	0.48271349	0.075	0.001	4.40E-19	C11_CD4_Treg
chr2-203987081-203987466	9.07E-24	0.553857422	0.132	0.012	6.01E-19	C11_CD4_Treg
chr6-106247362-106248095	9.31E-24	0.53864043	0.112	0.008	6.16E-19	C11_CD4_Treg
chr9-72396603-72396962	1.07E-23	0.470916273	0.075	0.001	7.11E-19	C11_CD4_Treg
chr20-49958450-49958927	1.55E-23	0.482775371	0.078	0.002	1.03E-18	C11_CD4_Treg
chr22-42030982-42031652	1.55E-23	0.516124808	0.098	0.005	1.03E-18	C11_CD4_Treg

chr1-207822694-207822987	1.94E-23	0.567367258	0.214	0.041	1.28E-18	C11_CD4_Treg
chr19-33235260-33236309	2.30E-23	0.552044823	0.169	0.027	1.52E-18	C11_CD4_Treg
chr1-193614739-193615144	2.34E-23	0.561196426	0.136	0.013	1.55E-18	C11_CD4_Treg
chr17-14118928-14119256	2.35E-23	0.491931107	0.085	0.002	1.56E-18	C11_CD4_Treg
chr6-5279718-5280346	2.75E-23	0.54918385	0.125	0.011	1.82E-18	C11_CD4_Treg
chr16-88588606-88589845	3.61E-23	0.564885766	0.176	0.028	2.39E-18	C11_CD4_Treg
chr17-57463157-57463534	4.75E-23	0.546794433	0.125	0.01	3.15E-18	C11_CD4_Treg
chr8-25467260-25467626	6.22E-23	0.489073519	0.081	0.002	4.12E-18	C11_CD4_Treg
chr3-39639183-39639639	6.64E-23	0.463539078	0.075	0.001	4.40E-18	C11_CD4_Treg
chr6-87705135-87705438	6.78E-23	0.56884935	0.234	0.049	4.49E-18	C11_CD4_Treg
chr8-127307185-127307982	8.21E-23	0.529437086	0.125	0.011	5.43E-18	C11_CD4_Treg
chr20-5865014-5866032	9.78E-23	0.52608995	0.122	0.011	6.47E-18	C11_CD4_Treg
chr3-195129411-195129892	1.27E-22	0.524695959	0.115	0.008	8.43E-18	C11_CD4_Treg
chr1-211304904-211305388	1.35E-22	0.535068855	0.112	0.008	8.93E-18	C11_CD4_Treg
chr12-108572300-108572608	1.39E-22	0.484679134	0.088	0.004	9.18E-18	C11_CD4_Treg
chr20-2993230-2993508	1.45E-22	0.440977792	0.068	0.001	9.60E-18	C11_CD4_Treg
chr2-159895264-159895725	1.69E-22	0.521537177	0.098	0.005	1.12E-17	C11_CD4_Treg
chr8-97798004-97799005	1.93E-22	0.484154181	0.092	0.004	1.28E-17	C11_CD4_Treg
chr8-29750724-29751112	2.87E-22	0.512643106	0.088	0.004	1.90E-17	C11_CD4_Treg
chr18-80202204-80202517	4.27E-22	0.513674582	0.095	0.005	2.83E-17	C11_CD4_Treg
chr1-193700697-193701352	5.32E-22	0.529139953	0.132	0.013	3.53E-17	C11_CD4_Treg
chr2-179041145-179041505	5.52E-22	0.483582835	0.085	0.003	3.65E-17	C11_CD4_Treg
chr9-89740725-89741002	6.62E-22	0.486908822	0.095	0.005	4.38E-17	C11_CD4_Treg
chr10-26739054-26739667	7.69E-22	0.541025539	0.125	0.012	5.09E-17	C11_CD4_Treg
chr2-64213948-64215295	8.37E-22	0.519959612	0.315	0.098	5.54E-17	C11_CD4_Treg
chr12-104494195-104495095	8.94E-22	0.538938788	0.183	0.035	5.92E-17	C11_CD4_Treg
chr2-190721956-190722795	9.34E-22	0.514490321	0.115	0.01	6.18E-17	C11_CD4_Treg

chr1-193516831-193517122	1.26E-21	0.419933813	0.064	0.001	8.33E-17	C11_CD4_Treg
chr11-12174228-12174840	1.38E-21	0.546040094	0.19	0.036	9.14E-17	C11_CD4_Treg
chr2-213021843-213022754	1.94E-21	0.544889732	0.156	0.022	1.29E-16	C11_CD4_Treg
chr1-226990169-226990434	2.79E-21	0.48921452	0.098	0.006	1.85E-16	C11_CD4_Treg
chr17-37147188-37147714	2.90E-21	0.46987011	0.085	0.003	1.92E-16	C11_CD4_Treg
chr6-143646812-143647448	6.84E-21	0.455666663	0.075	0.002	4.53E-16	C11_CD4_Treg
chr5-66828246-66829328	6.89E-21	0.510641659	0.315	0.11	4.56E-16	C11_CD4_Treg
chr18-13375048-13375693	8.06E-21	0.51432459	0.264	0.073	5.34E-16	C11_CD4_Treg
chr2-200454767-200455737	1.04E-20	0.518992554	0.146	0.02	6.86E-16	C11_CD4_Treg
chr9-68793696-68794284	1.04E-20	0.499649564	0.092	0.006	6.88E-16	C11_CD4_Treg
chr2-159879011-159879457	1.08E-20	0.460862609	0.081	0.003	7.14E-16	C11_CD4_Treg
chrX-30659306-30659767	1.21E-20	0.523480025	0.129	0.017	8.00E-16	C11_CD4_Treg
chr5-66840435-66841059	1.49E-20	0.531214281	0.142	0.019	9.84E-16	C11_CD4_Treg
chr1-117346244-117346729	2.32E-20	0.492237018	0.098	0.006	1.54E-15	C11_CD4_Treg
chr3-16378444-16379238	2.54E-20	0.539623588	0.186	0.037	1.68E-15	C11_CD4_Treg
chr4-77146387-77146799	2.96E-20	0.477538646	0.085	0.004	1.96E-15	C11_CD4_Treg
chr2-64541169-64541913	3.43E-20	0.501500745	0.108	0.01	2.27E-15	C11_CD4_Treg
chr3-47140878-47141508	4.48E-20	0.520247969	0.125	0.014	2.97E-15	C11_CD4_Treg
chr7-117110345-117111015	7.37E-20	0.492685145	0.125	0.014	4.88E-15	C11_CD4_Treg
chr1-27542371-27543065	8.76E-20	0.477821549	0.102	0.009	5.80E-15	C11_CD4_Treg
chr13-108915655-108916073	8.89E-20	0.493091846	0.122	0.015	5.89E-15	C11_CD4_Treg
chr2-101998849-101999254	1.07E-19	0.433515547	0.071	0.002	7.12E-15	C11_CD4_Treg
chr16-24774040-24774386	1.37E-19	0.496693746	0.102	0.009	9.08E-15	C11_CD4_Treg
chr3-133475232-133475860	2.14E-19	0.44978521	0.081	0.005	1.42E-14	C11_CD4_Treg
chr8-70322872-70323090	2.42E-19	0.41367284	0.061	0.001	1.60E-14	C11_CD4_Treg
chr13-46650871-46651334	2.53E-19	0.452606532	0.075	0.003	1.67E-14	C11_CD4_Treg
chr7-158603320-158603802	2.53E-19	0.425029505	0.071	0.002	1.68E-14	C11_CD4_Treg

chr2-203886637-203887207	2.57E-19	0.490672971	0.115	0.013	1.70E-14	C11_CD4_Treg
chr14-68728774-68729138	2.61E-19	0.42957602	0.068	0.002	1.73E-14	C11_CD4_Treg
chr17-51146990-51147391	2.77E-19	0.440759117	0.075	0.003	1.84E-14	C11_CD4_Treg
chr10-17668755-17669923	2.98E-19	0.511456322	0.163	0.029	1.97E-14	C11_CD4_Treg
chr2-204004937-204005219	3.06E-19	0.449209613	0.081	0.004	2.03E-14	C11_CD4_Treg
chr6-11317604-11318777	4.75E-19	0.414531028	0.492	0.244	3.15E-14	C11_CD4_Treg
chr19-1668546-1669388	4.96E-19	0.500513309	0.153	0.027	3.29E-14	C11_CD4_Treg
chr6-111086996-111088653	6.02E-19	0.493297591	0.261	0.076	3.99E-14	C11_CD4_Treg
chr11-110077505-110078170	6.18E-19	0.457133227	0.088	0.005	4.09E-14	C11_CD4_Treg
chr8-29815480-29815820	6.55E-19	0.424219721	0.064	0.002	4.34E-14	C11_CD4_Treg
chr13-40115661-40116412	6.79E-19	0.491744196	0.176	0.041	4.49E-14	C11_CD4_Treg
chr7-34991878-34992694	7.23E-19	0.472407594	0.346	0.138	4.79E-14	C11_CD4_Treg
chr11-115925027-115925392	7.65E-19	0.408401394	0.064	0.002	5.06E-14	C11_CD4_Treg
chr10-126043052-126043297	9.22E-19	0.440287655	0.078	0.004	6.11E-14	C11_CD4_Treg
chr1-65811918-65812403	1.07E-18	0.513299715	0.139	0.02	7.08E-14	C11_CD4_Treg
chr7-90595783-90596685	2.14E-18	0.45169209	0.085	0.006	1.42E-13	C11_CD4_Treg
chr10-50321884-50322311	2.49E-18	0.450095249	0.081	0.005	1.65E-13	C11_CD4_Treg
chr6-9520846-9521305	2.49E-18	0.386232589	0.054	0.001	1.65E-13	C11_CD4_Treg
chr8-125110513-125111202	2.74E-18	0.496538923	0.146	0.025	1.82E-13	C11_CD4_Treg
chr18-59952800-59953991	2.86E-18	0.49787984	0.241	0.065	1.90E-13	C11_CD4_Treg
chr6-156794874-156795532	2.89E-18	0.460677504	0.105	0.01	1.92E-13	C11_CD4_Treg
chr10-9294493-9295083	3.93E-18	0.404256598	0.061	0.002	2.60E-13	C11_CD4_Treg
chr4-8172143-8172547	3.98E-18	0.43062539	0.081	0.005	2.63E-13	C11_CD4_Treg
chr11-1325763-1326920	4.02E-18	0.457318616	0.095	0.01	2.66E-13	C11_CD4_Treg
chr17-44159368-44160086	4.71E-18	0.491625065	0.112	0.012	3.12E-13	C11_CD4_Treg
chr5-99004645-99005274	5.90E-18	0.476010906	0.108	0.012	3.90E-13	C11_CD4_Treg
chr10-50431180-50432803	6.01E-18	0.485219935	0.169	0.032	3.98E-13	C11_CD4_Treg

chr5-126446468-126446774	6.55E-18	0.489310267	0.112	0.013	4.34E-13	C11_CD4_Treg
chr6-157822693-157824394	7.21E-18	0.417307784	0.403	0.174	4.78E-13	C11_CD4_Treg
chr6-110944444-110945188	8.28E-18	0.471054137	0.332	0.126	5.48E-13	C11_CD4_Treg
chr10-88883272-88883788	8.72E-18	0.487830077	0.156	0.03	5.78E-13	C11_CD4_Treg
chr8-125151671-125152054	9.46E-18	0.479275821	0.112	0.012	6.27E-13	C11_CD4_Treg
chr6-12060507-12061408	9.88E-18	0.466269005	0.292	0.106	6.54E-13	C11_CD4_Treg
chr7-18639071-18639438	1.06E-17	0.442659199	0.078	0.006	7.02E-13	C11_CD4_Treg
chr3-126034476-126035032	1.14E-17	0.462846594	0.098	0.01	7.55E-13	C11_CD4_Treg
chr9-130239571-130240162	1.21E-17	0.471497161	0.115	0.015	7.98E-13	C11_CD4_Treg
chr16-26597839-26598554	1.27E-17	0.434152273	0.403	0.177	8.42E-13	C11_CD4_Treg
chr10-65501206-65501458	1.27E-17	0.416117975	0.064	0.002	8.42E-13	C11_CD4_Treg
chr15-59268094-59268728	1.30E-17	0.438097694	0.075	0.005	8.64E-13	C11_CD4_Treg
chr11-129960194-129960575	1.31E-17	0.418037788	0.068	0.002	8.68E-13	C11_CD4_Treg
chr8-125607684-125608560	1.75E-17	0.47573733	0.142	0.027	1.16E-12	C11_CD4_Treg
chr10-31321399-31321710	2.03E-17	0.477744206	0.18	0.046	1.34E-12	C11_CD4_Treg
chr19-41638604-41639067	2.04E-17	0.418414133	0.071	0.003	1.35E-12	C11_CD4_Treg
chr12-92172418-92173086	2.33E-17	0.47610755	0.129	0.024	1.54E-12	C11_CD4_Treg
chr1-246569353-246569929	2.51E-17	0.486414475	0.2	0.048	1.66E-12	C11_CD4_Treg
chr8-125340108-125340324	2.60E-17	0.41642647	0.071	0.003	1.72E-12	C11_CD4_Treg
chr7-128963196-128963662	2.89E-17	0.440613893	0.088	0.007	1.92E-12	C11_CD4_Treg
chr3-66469190-66469508	3.00E-17	0.479536578	0.142	0.025	1.99E-12	C11_CD4_Treg
chr1-193472130-193472632	3.05E-17	0.406339362	0.068	0.003	2.02E-12	C11_CD4_Treg
chr13-110964901-110965636	3.09E-17	0.48091423	0.142	0.027	2.05E-12	C11_CD4_Treg
chr19-11567730-11568616	4.11E-17	0.463187794	0.119	0.018	2.72E-12	C11_CD4_Treg
chrX-30657704-30658043	5.35E-17	0.462416885	0.112	0.014	3.54E-12	C11_CD4_Treg
chr8-9011534-9012382	6.29E-17	0.470829286	0.203	0.053	4.17E-12	C11_CD4_Treg
chr13-39732828-39733239	6.32E-17	0.402428279	0.064	0.003	4.19E-12	C11_CD4_Treg

chr19-7387194-7387916	6.51E-17	0.471078589	0.159	0.036	4.31E-12	C11_CD4_Treg
chr2-44713173-44713446	7.62E-17	0.414641555	0.068	0.003	5.04E-12	C11_CD4_Treg
chr11-113262239-113262946	8.41E-17	0.417750033	0.075	0.004	5.57E-12	C11_CD4_Treg
chr9-136766510-136767167	9.12E-17	0.46888211	0.173	0.041	6.04E-12	C11_CD4_Treg
chr17-72422382-72423143	9.50E-17	0.444119158	0.095	0.009	6.29E-12	C11_CD4_Treg
chr20-21557169-21558003	1.07E-16	0.473279223	0.153	0.027	7.06E-12	C11_CD4_Treg
chr8-78665332-78666780	1.27E-16	0.450889912	0.264	0.086	8.38E-12	C11_CD4_Treg
chr18-58639847-58640362	1.48E-16	0.386328444	0.061	0.002	9.77E-12	C11_CD4_Treg
chr13-75312818-75313186	1.78E-16	0.460551014	0.112	0.015	1.18E-11	C11_CD4_Treg
chr11-20374664-20375042	1.79E-16	0.424500995	0.078	0.006	1.19E-11	C11_CD4_Treg
chr1-246117052-246117844	1.80E-16	0.469895277	0.129	0.02	1.19E-11	C11_CD4_Treg
chr7-43076307-43076657	2.14E-16	0.386970891	0.064	0.002	1.42E-11	C11_CD4_Treg
chr19-5130283-5131108	2.20E-16	0.468055566	0.169	0.038	1.46E-11	C11_CD4_Treg
chr19-46633689-46634603	2.61E-16	0.357915451	0.522	0.301	1.73E-11	C11_CD4_Treg
chr3-16462409-16463082	3.13E-16	0.476196277	0.153	0.031	2.07E-11	C11_CD4_Treg
chr10-110773712-110774861	4.72E-16	0.422476161	0.369	0.156	3.12E-11	C11_CD4_Treg
chr3-33060661-33060935	5.36E-16	0.464905253	0.139	0.023	3.55E-11	C11_CD4_Treg
chr1-231387949-231388223	5.73E-16	0.378660502	0.058	0.002	3.79E-11	C11_CD4_Treg
chr6-137806942-137807583	6.12E-16	0.457855743	0.136	0.022	4.05E-11	C11_CD4_Treg
chr22-47090614-47091616	6.27E-16	0.465688043	0.193	0.049	4.15E-11	C11_CD4_Treg
chr10-95761981-95762255	8.22E-16	0.415348976	0.075	0.005	5.44E-11	C11_CD4_Treg
chr3-45553075-45553928	1.02E-15	0.400944089	0.075	0.005	6.75E-11	C11_CD4_Treg
chr3-156816066-156817881	1.04E-15	0.357089099	0.492	0.28	6.88E-11	C11_CD4_Treg
chr6-87182301-87182640	1.10E-15	0.435404594	0.102	0.014	7.26E-11	C11_CD4_Treg
chr12-123119847-123120166	1.14E-15	0.414727949	0.085	0.008	7.57E-11	C11_CD4_Treg
chr2-102001849-102002489	1.38E-15	0.402779904	0.071	0.004	9.16E-11	C11_CD4_Treg
chr8-27500108-27500573	1.47E-15	0.398115446	0.068	0.004	9.70E-11	C11_CD4_Treg

chr6-403311-404204	1.53E-15	0.45984045	0.163	0.039	1.01E-10	C11_CD4_Treg
chr1-202565905-202566224	1.57E-15	0.379583498	0.054	0.002	1.04E-10	C11_CD4_Treg
chr17-79804152-79804754	1.71E-15	0.446491335	0.132	0.027	1.13E-10	C11_CD4_Treg
chr8-29737096-29737752	1.72E-15	0.449180141	0.169	0.04	1.14E-10	C11_CD4_Treg
chr6-143607207-143607543	1.77E-15	0.388762777	0.061	0.002	1.17E-10	C11_CD4_Treg
chr2-102062297-102062860	1.81E-15	0.410710749	0.081	0.006	1.20E-10	C11_CD4_Treg
chr7-156777247-156777963	1.90E-15	0.446174602	0.122	0.019	1.26E-10	C11_CD4_Treg
chr14-92760013-92760310	2.78E-15	0.361244011	0.054	0.002	1.84E-10	C11_CD4_Treg
chr1-153597091-153597477	3.00E-15	0.415882459	0.081	0.008	1.98E-10	C11_CD4_Treg
chr13-110954982-110955413	3.02E-15	0.379504462	0.061	0.003	2.00E-10	C11_CD4_Treg
chr11-111526467-111526868	3.09E-15	0.399664448	0.071	0.004	2.04E-10	C11_CD4_Treg
chr6-2862631-2863246	3.68E-15	0.439949643	0.254	0.088	2.44E-10	C11_CD4_Treg
chr1-117093088-117093447	3.74E-15	0.416120432	0.075	0.007	2.47E-10	C11_CD4_Treg
chr1-107791420-107791711	3.98E-15	0.411056864	0.081	0.008	2.64E-10	C11_CD4_Treg
chr1-13702483-13703165	4.31E-15	0.409711734	0.342	0.15	2.86E-10	C11_CD4_Treg
chr1-1207954-1209089	4.54E-15	0.361257661	0.478	0.256	3.01E-10	C11_CD4_Treg
chr10-95789733-95790227	4.67E-15	0.417852122	0.088	0.01	3.09E-10	C11_CD4_Treg
chr6-36108987-36109444	4.69E-15	0.433913631	0.115	0.021	3.11E-10	C11_CD4_Treg
chr20-62651035-62652604	4.89E-15	0.368971878	0.427	0.223	3.24E-10	C11_CD4_Treg
chr13-40494317-40495333	5.06E-15	0.444906563	0.166	0.044	3.35E-10	C11_CD4_Treg
chr12-104505285-104505649	5.09E-15	0.397587258	0.078	0.006	3.37E-10	C11_CD4_Treg
chr21-45590378-45592664	5.24E-15	0.367113305	0.444	0.232	3.47E-10	C11_CD4_Treg
chr17-7951486-7951703	5.32E-15	0.352407022	0.054	0.002	3.52E-10	C11_CD4_Treg
chr10-74898132-74898590	6.22E-15	0.421189757	0.095	0.012	4.12E-10	C11_CD4_Treg
chr18-62869474-62870017	7.15E-15	0.415298999	0.085	0.008	4.73E-10	C11_CD4_Treg
chr2-213104005-213104437	7.99E-15	0.411530622	0.095	0.011	5.29E-10	C11_CD4_Treg
chrX-68508621-68509581	9.08E-15	0.445164972	0.173	0.047	6.01E-10	C11_CD4_Treg

chr10-50410005-50410729	9.36E-15	0.42996224	0.105	0.016	6.20E-10	C11_CD4_Treg
chr19-17776274-17776867	9.63E-15	0.413620324	0.088	0.009	6.38E-10	C11_CD4_Treg
chr13-27147492-27147994	9.77E-15	0.424248529	0.105	0.017	6.47E-10	C11_CD4_Treg
chr12-113135304-113136419	1.00E-14	0.42275353	0.268	0.101	6.62E-10	C11_CD4_Treg
chr4-10671147-10671744	1.06E-14	0.452843075	0.142	0.029	7.03E-10	C11_CD4_Treg
chr22-37100612-37100869	1.10E-14	0.368203688	0.061	0.004	7.28E-10	C11_CD4_Treg
chr3-71982627-71982905	1.12E-14	0.399215654	0.071	0.005	7.39E-10	C11_CD4_Treg
chr2-222451288-222451541	1.15E-14	0.34728781	0.054	0.002	7.60E-10	C11_CD4_Treg
chr7-34727473-34727694	1.29E-14	0.344423691	0.051	0.002	8.53E-10	C11_CD4_Treg
chr4-14856090-14856770	1.46E-14	0.415793764	0.085	0.009	9.69E-10	C11_CD4_Treg
chr7-90816666-90817055	1.56E-14	0.375794951	0.058	0.003	1.03E-09	C11_CD4_Treg
chr8-18755730-18755979	1.56E-14	0.341881433	0.051	0.002	1.03E-09	C11_CD4_Treg
chr10-17645796-17646273	1.84E-14	0.437453192	0.159	0.042	1.22E-09	C11_CD4_Treg
chr11-20367426-20367928	2.05E-14	0.426360415	0.231	0.087	1.36E-09	C11_CD4_Treg
chr16-23335753-23336340	2.19E-14	0.409238179	0.081	0.008	1.45E-09	C11_CD4_Treg
chr14-31927274-31927725	2.20E-14	0.38255741	0.068	0.005	1.46E-09	C11_CD4_Treg
chr11-105046876-105047083	2.70E-14	0.390730227	0.075	0.006	1.79E-09	C11_CD4_Treg
chr10-95839996-95840304	2.89E-14	0.387821502	0.064	0.004	1.91E-09	C11_CD4_Treg
chr18-58714828-58715082	3.35E-14	0.385324007	0.071	0.005	2.22E-09	C11_CD4_Treg
chr15-38069006-38069782	3.38E-14	0.423825556	0.139	0.033	2.24E-09	C11_CD4_Treg
chr1-8097254-8097773	3.40E-14	0.434490316	0.193	0.058	2.25E-09	C11_CD4_Treg
chr6-4059043-4060201	3.95E-14	0.397866274	0.339	0.144	2.61E-09	C11_CD4_Treg
chr10-119836783-119837132	4.00E-14	0.393057777	0.075	0.008	2.65E-09	C11_CD4_Treg
chr12-112751907-112752606	4.08E-14	0.396816466	0.081	0.008	2.70E-09	C11_CD4_Treg
chr14-49992839-49993233	4.13E-14	0.391099835	0.078	0.006	2.73E-09	C11_CD4_Treg
chr18-23228715-23229053	4.55E-14	0.424450239	0.132	0.027	3.01E-09	C11_CD4_Treg
chr2-232300853-232301179	4.59E-14	0.368065098	0.058	0.003	3.04E-09	C11_CD4_Treg

chr3-195133458-195133841	4.63E-14	0.439355463	0.122	0.024	3.07E-09	C11_CD4_Treg
chr17-75297288-75297880	4.66E-14	0.40984119	0.112	0.021	3.09E-09	C11_CD4_Treg
chr2-68789570-68789907	5.38E-14	0.391273932	0.081	0.009	3.56E-09	C11_CD4_Treg
chr15-85312362-85312747	5.95E-14	0.395630515	0.081	0.009	3.94E-09	C11_CD4_Treg
chr17-77098715-77099248	6.30E-14	0.403758112	0.095	0.014	4.17E-09	C11_CD4_Treg
chr18-58721713-58722058	6.42E-14	0.334833184	0.051	0.002	4.25E-09	C11_CD4_Treg
chr12-47889034-47889303	6.74E-14	0.38199358	0.071	0.007	4.46E-09	C11_CD4_Treg
chr10-62243355-62243959	6.78E-14	0.389107367	0.078	0.008	4.49E-09	C11_CD4_Treg
chr17-50511069-50511422	7.40E-14	0.41449328	0.112	0.022	4.90E-09	C11_CD4_Treg
chr2-213147617-213147823	7.95E-14	0.393883821	0.081	0.008	5.27E-09	C11_CD4_Treg
chr8-29774520-29775217	8.23E-14	0.372397817	0.064	0.004	5.45E-09	C11_CD4_Treg
chr2-241123839-241124156	8.46E-14	0.373410409	0.075	0.006	5.60E-09	C11_CD4_Treg
chr15-44882195-44883034	8.72E-14	0.416702859	0.095	0.014	5.78E-09	C11_CD4_Treg
chr3-65963911-65964536	8.93E-14	0.363035919	0.058	0.003	5.91E-09	C11_CD4_Treg
chr21-31173783-31174249	1.01E-13	0.370756611	0.068	0.005	6.67E-09	C11_CD4_Treg
chr10-86419104-86419428	1.04E-13	0.36387796	0.058	0.004	6.86E-09	C11_CD4_Treg
chr18-80194820-80195119	1.08E-13	0.345439555	0.051	0.002	7.18E-09	C11_CD4_Treg
chr17-57440085-57441355	1.12E-13	0.411705972	0.251	0.096	7.43E-09	C11_CD4_Treg
chr22-38389170-38389672	1.12E-13	0.349104566	0.054	0.003	7.45E-09	C11_CD4_Treg
chr3-195147567-195147999	1.19E-13	0.370679339	0.071	0.006	7.85E-09	C11_CD4_Treg
chr17-17179309-17179692	1.29E-13	0.419441048	0.139	0.031	8.54E-09	C11_CD4_Treg
chr1-166166214-166167260	1.34E-13	0.40820825	0.108	0.018	8.90E-09	C11_CD4_Treg
chr2-215979636-215979948	1.50E-13	0.398616953	0.088	0.012	9.96E-09	C11_CD4_Treg
chr3-188753311-188753568	1.68E-13	0.331720865	0.051	0.002	1.11E-08	C11_CD4_Treg
chr19-39258144-39258677	1.72E-13	0.418209715	0.125	0.028	1.14E-08	C11_CD4_Treg
chr11-19442082-19442601	1.73E-13	0.369861369	0.064	0.005	1.15E-08	C11_CD4_Treg
chr7-90708892-90709243	1.89E-13	0.339828934	0.051	0.002	1.25E-08	C11_CD4_Treg

chr6-2379329-2379557	1.89E-13	0.357800912	0.058	0.003	1.25E-08	C11_CD4_Treg
chr6-158808651-158809531	1.96E-13	0.408204431	0.258	0.1	1.30E-08	C11_CD4_Treg
chr11-76629466-76629988	2.01E-13	0.353905557	0.061	0.004	1.33E-08	C11_CD4_Treg
chr5-180873453-180874176	2.10E-13	0.422519736	0.166	0.046	1.39E-08	C11_CD4_Treg
chr4-158817736-158818308	2.26E-13	0.355690467	0.058	0.003	1.50E-08	C11_CD4_Treg
chr15-78756784-78757373	2.29E-13	0.404647115	0.098	0.015	1.51E-08	C11_CD4_Treg
chr11-117456452-117456912	2.63E-13	0.384233263	0.081	0.008	1.74E-08	C11_CD4_Treg
chr15-60855021-60855518	2.74E-13	0.378175808	0.075	0.009	1.81E-08	C11_CD4_Treg
chr21-42474391-42474854	2.81E-13	0.371874421	0.071	0.006	1.86E-08	C11_CD4_Treg
chr10-2949769-2950400	3.01E-13	0.398243611	0.095	0.014	1.99E-08	C11_CD4_Treg
chr3-32285284-32285783	3.72E-13	0.38436235	0.092	0.013	2.47E-08	C11_CD4_Treg
chr6-12077011-12077326	3.85E-13	0.342095863	0.051	0.003	2.55E-08	C11_CD4_Treg
chr10-98142286-98142702	3.99E-13	0.362497895	0.064	0.005	2.64E-08	C11_CD4_Treg
chr3-39327563-39328257	3.99E-13	0.352643717	0.068	0.006	2.64E-08	C11_CD4_Treg
chr9-37126144-37126492	4.09E-13	0.373276127	0.075	0.007	2.71E-08	C11_CD4_Treg
chr17-78359795-78361001	4.29E-13	0.291109476	0.59	0.372	2.84E-08	C11_CD4_Treg
chr2-159891011-159891278	4.35E-13	0.339291099	0.051	0.002	2.88E-08	C11_CD4_Treg
chr12-56034346-56034875	4.81E-13	0.387688875	0.088	0.01	3.19E-08	C11_CD4_Treg
chr14-67680011-67680449	5.42E-13	0.39581687	0.095	0.013	3.59E-08	C11_CD4_Treg
chr9-36342134-36342515	5.94E-13	0.378741015	0.078	0.009	3.94E-08	C11_CD4_Treg
chr3-72822065-72822680	6.05E-13	0.389256375	0.115	0.021	4.01E-08	C11_CD4_Treg
chr3-133490527-133491287	6.34E-13	0.397567028	0.098	0.016	4.20E-08	C11_CD4_Treg
chr8-66651688-66652342	6.68E-13	0.392732134	0.102	0.017	4.42E-08	C11_CD4_Treg
chr12-56032056-56032280	6.75E-13	0.350667986	0.061	0.004	4.47E-08	C11_CD4_Treg
chr9-136225144-136226416	6.80E-13	0.410143152	0.217	0.072	4.50E-08	C11_CD4_Treg
chr8-100451519-100453015	6.92E-13	0.337119886	0.468	0.261	4.58E-08	C11_CD4_Treg
chr11-8913976-8914611	7.64E-13	0.398988264	0.136	0.034	5.06E-08	C11_CD4_Treg

chr8-29349797-29351626	8.20E-13	0.289987392	0.569	0.37	5.43E-08	C11_CD4_Treg
chr2-60872460-60873008	1.02E-12	0.406601582	0.115	0.023	6.77E-08	C11_CD4_Treg
chr13-40377769-40378213	1.07E-12	0.3959863	0.108	0.02	7.09E-08	C11_CD4_Treg
chr13-75365657-75366252	1.25E-12	0.410728487	0.176	0.046	8.26E-08	C11_CD4_Treg
chrX-130179444-130179937	1.28E-12	0.364601858	0.071	0.007	8.50E-08	C11_CD4_Treg
chr2-119800379-119800837	1.35E-12	0.362366628	0.071	0.007	8.91E-08	C11_CD4_Treg
chr11-118808126-118808646	1.37E-12	0.334507309	0.054	0.003	9.04E-08	C11_CD4_Treg
chr10-17675620-17675937	1.46E-12	0.333146618	0.051	0.003	9.66E-08	C11_CD4_Treg
chr1-39798825-39799977	1.53E-12	0.352971475	0.38	0.198	1.01E-07	C11_CD4_Treg
chr11-110092518-110093745	1.74E-12	0.383819102	0.108	0.017	1.15E-07	C11_CD4_Treg
chr8-27490629-27491808	1.75E-12	0.396801743	0.166	0.05	1.16E-07	C11_CD4_Treg
chr19-39147168-39147777	1.81E-12	0.332789745	0.054	0.004	1.20E-07	C11_CD4_Treg
chr8-1474481-1474680	1.84E-12	0.333323863	0.051	0.003	1.22E-07	C11_CD4_Treg
chr7-8135456-8135705	2.19E-12	0.395773755	0.112	0.023	1.45E-07	C11_CD4_Treg
chr12-124493941-124494239	2.22E-12	0.340093982	0.058	0.004	1.47E-07	C11_CD4_Treg
chr5-179836341-179837238	2.32E-12	0.363949315	0.308	0.143	1.54E-07	C11_CD4_Treg
chr2-97688244-97688687	2.36E-12	0.339295107	0.064	0.006	1.57E-07	C11_CD4_Treg
chr15-59251066-59251516	2.38E-12	0.365515842	0.081	0.011	1.58E-07	C11_CD4_Treg
chr11-111454583-111454965	2.65E-12	0.346008505	0.058	0.004	1.76E-07	C11_CD4_Treg
chr4-102889771-102890227	2.73E-12	0.367734626	0.078	0.01	1.81E-07	C11_CD4_Treg
chr7-100242863-100243863	2.74E-12	0.398402191	0.166	0.051	1.81E-07	C11_CD4_Treg
chr8-29885801-29886229	3.01E-12	0.351412372	0.064	0.006	1.99E-07	C11_CD4_Treg
chr19-50330700-50331025	3.11E-12	0.391644693	0.19	0.063	2.06E-07	C11_CD4_Treg
chr5-160459044-160459508	3.78E-12	0.36986855	0.075	0.008	2.50E-07	C11_CD4_Treg
chr1-155535960-155536738	4.00E-12	0.387843857	0.176	0.054	2.65E-07	C11_CD4_Treg
chr2-213145636-213146045	4.16E-12	0.347036874	0.064	0.006	2.75E-07	C11_CD4_Treg
chr8-59630525-59631135	4.20E-12	0.327439954	0.054	0.004	2.78E-07	C11_CD4_Treg

chr2-190663558-190664477	4.23E-12	0.390872864	0.119	0.025	2.80E-07	C11_CD4_Treg
chr8-128063932-128064423	4.42E-12	0.38983011	0.098	0.018	2.92E-07	C11_CD4_Treg
chr10-26737604-26738015	4.59E-12	0.387778947	0.095	0.015	3.04E-07	C11_CD4_Treg
chr20-43736661-43737046	4.98E-12	0.333645635	0.058	0.004	3.30E-07	C11_CD4_Treg
chr12-49945303-49945885	5.58E-12	0.385373096	0.227	0.083	3.70E-07	C11_CD4_Treg
chr9-72473574-72473805	5.86E-12	0.344535154	0.061	0.005	3.88E-07	C11_CD4_Treg
chr7-102834933-102835314	5.97E-12	0.320291877	0.054	0.004	3.95E-07	C11_CD4_Treg
chr4-6191403-6191733	6.08E-12	0.35011367	0.061	0.006	4.03E-07	C11_CD4_Treg
chrX-30712998-30713488	6.23E-12	0.362371938	0.078	0.01	4.13E-07	C11_CD4_Treg
chr11-47663210-47663600	7.35E-12	0.360501029	0.078	0.01	4.87E-07	C11_CD4_Treg
chr10-95757949-95758227	7.45E-12	0.339712902	0.068	0.008	4.94E-07	C11_CD4_Treg
chr3-171561564-171562227	7.86E-12	0.34272501	0.061	0.007	5.20E-07	C11_CD4_Treg
chr10-6045076-6045577	8.23E-12	0.355049945	0.068	0.009	5.45E-07	C11_CD4_Treg
chr1-3921873-3922663	9.51E-12	0.386059641	0.149	0.044	6.30E-07	C11_CD4_Treg
chr2-10493121-10494006	1.00E-11	0.359838651	0.329	0.155	6.65E-07	C11_CD4_Treg
chr14-65425301-65426157	1.01E-11	0.348670272	0.081	0.009	6.71E-07	C11_CD4_Treg
chr3-70982577-70983145	1.03E-11	0.367380378	0.085	0.012	6.82E-07	C11_CD4_Treg
chr2-179031588-179032398	1.07E-11	0.386920659	0.139	0.036	7.10E-07	C11_CD4_Treg
chr3-194134185-194135283	1.16E-11	0.377576527	0.214	0.071	7.69E-07	C11_CD4_Treg
chr7-47291306-47291768	1.24E-11	0.324909302	0.051	0.003	8.22E-07	C11_CD4_Treg
chr5-67030302-67030575	1.25E-11	0.31607049	0.051	0.003	8.25E-07	C11_CD4_Treg
chr6-157800618-157801244	1.35E-11	0.383863027	0.139	0.037	8.96E-07	C11_CD4_Treg
chr14-75559550-75560067	1.36E-11	0.361373735	0.095	0.019	9.04E-07	C11_CD4_Treg
chr11-122696520-122697137	1.38E-11	0.383930198	0.159	0.046	9.14E-07	C11_CD4_Treg
chr5-143426468-143427421	1.42E-11	0.376936092	0.095	0.017	9.43E-07	C11_CD4_Treg
chr1-31009812-31010931	1.50E-11	0.383094176	0.214	0.08	9.93E-07	C11_CD4_Treg
chr5-156401908-156402946	1.62E-11	0.385985962	0.169	0.055	1.07E-06	C11_CD4_Treg

chr1-206466004-206466203	1.64E-11	0.346610847	0.071	0.008	1.09E-06	C11_CD4_Treg
chr13-40373536-40373897	1.65E-11	0.35474431	0.078	0.011	1.10E-06	C11_CD4_Treg
chr8-38943736-38944024	1.66E-11	0.377209333	0.105	0.023	1.10E-06	C11_CD4_Treg
chr5-96715613-96716384	1.67E-11	0.376041996	0.129	0.034	1.11E-06	C11_CD4_Treg
chr14-55071702-55072623	1.76E-11	0.372000304	0.227	0.096	1.17E-06	C11_CD4_Treg
chr13-19984719-19985364	1.81E-11	0.377236464	0.136	0.037	1.20E-06	C11_CD4_Treg
chr1-15290351-15290736	1.84E-11	0.362531166	0.088	0.014	1.22E-06	C11_CD4_Treg
chr11-110148716-110149111	1.84E-11	0.337295858	0.058	0.005	1.22E-06	C11_CD4_Treg
chr13-49098121-49098495	1.91E-11	0.33402269	0.061	0.005	1.27E-06	C11_CD4_Treg
chr8-125606112-125606853	1.92E-11	0.38568205	0.163	0.049	1.27E-06	C11_CD4_Treg
chrX-49264550-49264973	2.07E-11	0.375410176	0.115	0.024	1.37E-06	C11_CD4_Treg
chr12-50224208-50224793	2.17E-11	0.378488073	0.2	0.074	1.43E-06	C11_CD4_Treg
chr2-204026735-204027521	2.20E-11	0.374214101	0.224	0.086	1.46E-06	C11_CD4_Treg
chr1-160657221-160657559	2.29E-11	0.309152284	0.051	0.003	1.52E-06	C11_CD4_Treg
chr19-46522622-46523319	2.30E-11	0.376934744	0.129	0.031	1.53E-06	C11_CD4_Treg
chr1-107935394-107935783	2.37E-11	0.377394778	0.132	0.034	1.57E-06	C11_CD4_Treg
chr3-71489233-71489774	2.53E-11	0.377146831	0.173	0.061	1.68E-06	C11_CD4_Treg
chr7-126700963-126701509	2.66E-11	0.385827254	0.197	0.065	1.76E-06	C11_CD4_Treg
chr10-50396624-50397532	2.71E-11	0.370834204	0.105	0.021	1.79E-06	C11_CD4_Treg
chr5-35908165-35908604	2.83E-11	0.364576353	0.085	0.013	1.87E-06	C11_CD4_Treg
chr10-43160275-43161454	3.05E-11	0.374478636	0.105	0.024	2.02E-06	C11_CD4_Treg
chr10-32332140-32332707	3.25E-11	0.313361825	0.464	0.264	2.15E-06	C11_CD4_Treg
chr3-88141633-88142486	3.35E-11	0.36841427	0.119	0.029	2.22E-06	C11_CD4_Treg
chr4-152606832-152607183	3.61E-11	0.340265352	0.061	0.006	2.39E-06	C11_CD4_Treg
chr12-123474686-123475310	3.73E-11	0.336558925	0.068	0.009	2.47E-06	C11_CD4_Treg
chr3-18649512-18649987	3.90E-11	0.323952621	0.054	0.006	2.58E-06	C11_CD4_Treg
chr8-29926233-29927010	3.91E-11	0.346102776	0.075	0.01	2.59E-06	C11_CD4_Treg

chr7-105808732-105809620	4.13E-11	0.321274294	0.424	0.243	2.73E-06	C11_CD4_Treg
chr8-24387267-24387842	4.38E-11	0.321019142	0.058	0.005	2.90E-06	C11_CD4_Treg
chr8-1479952-1480233	4.39E-11	0.324683418	0.058	0.006	2.91E-06	C11_CD4_Treg
chr14-59274691-59276003	4.52E-11	0.373225361	0.186	0.061	2.99E-06	C11_CD4_Treg
chr10-124230729-124232196	4.54E-11	0.360110216	0.166	0.051	3.01E-06	C11_CD4_Treg
chr20-58660901-58661838	4.59E-11	0.373873744	0.146	0.042	3.04E-06	C11_CD4_Treg
chr2-105836856-105837638	5.37E-11	0.366513846	0.098	0.02	3.56E-06	C11_CD4_Treg
chr6-11653095-11653556	5.41E-11	0.33344862	0.064	0.006	3.58E-06	C11_CD4_Treg
chr2-43579185-43579556	5.53E-11	0.309378883	0.051	0.004	3.67E-06	C11_CD4_Treg
chr7-137923368-137923728	5.56E-11	0.333442353	0.064	0.007	3.68E-06	C11_CD4_Treg
chr2-161199714-161200428	5.61E-11	0.376832677	0.132	0.037	3.72E-06	C11_CD4_Treg
chr9-109974079-109974674	5.79E-11	0.314570685	0.058	0.005	3.84E-06	C11_CD4_Treg
chr11-122687051-122687462	5.87E-11	0.34645381	0.068	0.01	3.89E-06	C11_CD4_Treg
chr4-67713832-67714553	6.18E-11	0.32723026	0.054	0.005	4.09E-06	C11_CD4_Treg
chr2-28711708-28711983	6.43E-11	0.325727012	0.058	0.005	4.26E-06	C11_CD4_Treg
chr18-13381809-13382793	8.33E-11	0.366052467	0.19	0.064	5.52E-06	C11_CD4_Treg
chr16-74577562-74578351	8.43E-11	0.359981364	0.108	0.025	5.58E-06	C11_CD4_Treg
chr3-172142309-172142535	9.00E-11	0.314782848	0.054	0.005	5.96E-06	C11_CD4_Treg
chr1-229440967-229441374	9.10E-11	0.332422176	0.075	0.01	6.03E-06	C11_CD4_Treg
chr2-64606378-64607367	9.93E-11	0.333698136	0.329	0.177	6.58E-06	C11_CD4_Treg
chr10-62016804-62017117	9.99E-11	0.334581533	0.068	0.009	6.62E-06	C11_CD4_Treg
chr17-57460853-57461403	1.04E-10	0.353936418	0.095	0.018	6.90E-06	C11_CD4_Treg
chr5-145946402-145946895	1.06E-10	0.349623493	0.085	0.014	7.00E-06	C11_CD4_Treg
chr1-169572267-169572537	1.08E-10	0.315448399	0.051	0.005	7.18E-06	C11_CD4_Treg
chr2-28275826-28276457	1.11E-10	0.364272154	0.193	0.07	7.33E-06	C11_CD4_Treg
chr16-15960530-15961931	1.11E-10	0.330404537	0.295	0.142	7.33E-06	C11_CD4_Treg
chr4-2797784-2799125	1.15E-10	0.360388041	0.153	0.053	7.64E-06	C11_CD4_Treg

chr10-103677742-103678553	1.26E-10	0.355375216	0.227	0.094	8.31E-06	C11_CD4_Treg
chr3-58260176-58260977	1.26E-10	0.331766213	0.068	0.01	8.33E-06	C11_CD4_Treg
chr14-91108895-91109503	1.26E-10	0.359798572	0.098	0.019	8.34E-06	C11_CD4_Treg
chr18-23230837-23232105	1.37E-10	0.299222326	0.461	0.276	9.07E-06	C11_CD4_Treg
chr14-89305180-89305467	1.37E-10	0.314542773	0.054	0.004	9.09E-06	C11_CD4_Treg
chr12-68373775-68374805	1.56E-10	0.341906614	0.268	0.132	1.03E-05	C11_CD4_Treg
chr3-16467428-16467703	1.56E-10	0.305018152	0.054	0.005	1.04E-05	C11_CD4_Treg
chr6-11785437-11786547	1.58E-10	0.351278798	0.092	0.019	1.05E-05	C11_CD4_Treg
chrX-30649877-30650153	1.62E-10	0.356338962	0.108	0.023	1.07E-05	C11_CD4_Treg
chr6-158270820-158271475	1.71E-10	0.358670939	0.214	0.087	1.13E-05	C11_CD4_Treg
chr1-12079620-12079845	1.80E-10	0.30060392	0.051	0.004	1.19E-05	C11_CD4_Treg
chr19-47288386-47288664	2.09E-10	0.329764899	0.068	0.009	1.39E-05	C11_CD4_Treg
chr2-64101289-64101955	2.14E-10	0.321357348	0.071	0.009	1.41E-05	C11_CD4_Treg
chr10-33133765-33134651	2.14E-10	0.282141465	0.488	0.314	1.42E-05	C11_CD4_Treg
chr6-154606273-154606870	2.28E-10	0.357304617	0.112	0.025	1.51E-05	C11_CD4_Treg
chr9-137639150-137639935	2.33E-10	0.350432798	0.105	0.026	1.54E-05	C11_CD4_Treg
chr6-149500126-149500371	2.41E-10	0.319730579	0.058	0.006	1.60E-05	C11_CD4_Treg
chr12-12512077-12512565	2.53E-10	0.34719785	0.105	0.021	1.68E-05	C11_CD4_Treg
chr10-112530895-112531558	2.55E-10	0.344494624	0.105	0.023	1.69E-05	C11_CD4_Treg
chr1-89657688-89658322	2.59E-10	0.352271588	0.183	0.068	1.72E-05	C11_CD4_Treg
chr7-55556497-55556744	2.72E-10	0.311159402	0.054	0.005	1.80E-05	C11_CD4_Treg
chr20-49698681-49699604	2.72E-10	0.348540821	0.231	0.099	1.80E-05	C11_CD4_Treg
chr5-14685507-14686137	2.74E-10	0.337831225	0.092	0.019	1.82E-05	C11_CD4_Treg
chr1-12078887-12079387	2.82E-10	0.297646203	0.051	0.004	1.87E-05	C11_CD4_Treg
chr6-156866159-156866798	2.90E-10	0.335626856	0.085	0.015	1.92E-05	C11_CD4_Treg
chr13-23384098-23384447	2.98E-10	0.326579501	0.061	0.007	1.97E-05	C11_CD4_Treg
chr2-68426872-68427587	3.04E-10	0.361834222	0.166	0.056	2.01E-05	C11_CD4_Treg

chr17-31535595-31535937	3.11E-10	0.29362066	0.051	0.004	2.06E-05	C11_CD4_Treg
chr5-56638463-56639330	3.15E-10	0.342259887	0.081	0.015	2.08E-05	C11_CD4_Treg
chr9-112028338-112029099	3.21E-10	0.305199663	0.051	0.004	2.13E-05	C11_CD4_Treg
chr17-48915506-48916261	3.36E-10	0.350740284	0.153	0.051	2.22E-05	C11_CD4_Treg
chr14-102789199-102789642	3.45E-10	0.312040754	0.058	0.007	2.28E-05	C11_CD4_Treg
chr14-91388134-91388885	3.46E-10	0.351902726	0.203	0.076	2.29E-05	C11_CD4_Treg
chr17-57422574-57423022	3.47E-10	0.312003661	0.058	0.006	2.30E-05	C11_CD4_Treg
chr17-79810024-79810483	3.48E-10	0.333354564	0.268	0.126	2.30E-05	C11_CD4_Treg
chr6-149473393-149473788	3.54E-10	0.324600488	0.064	0.008	2.34E-05	C11_CD4_Treg
chr11-66221892-66223046	3.61E-10	0.316391744	0.332	0.181	2.39E-05	C11_CD4_Treg
chr2-46226640-46226849	3.63E-10	0.327752846	0.068	0.009	2.41E-05	C11_CD4_Treg
chr3-114344484-114344995	3.68E-10	0.314617442	0.061	0.008	2.44E-05	C11_CD4_Treg
chr22-20111025-20111737	3.97E-10	0.349518953	0.139	0.045	2.63E-05	C11_CD4_Treg
chr14-102822268-102822750	4.98E-10	0.330010284	0.088	0.015	3.30E-05	C11_CD4_Treg
chr2-102137082-102137424	5.14E-10	0.319530136	0.061	0.008	3.41E-05	C11_CD4_Treg
chr1-12052038-12052332	5.60E-10	0.329261588	0.075	0.013	3.71E-05	C11_CD4_Treg
chr1-116986501-116987514	5.92E-10	0.353308675	0.136	0.042	3.92E-05	C11_CD4_Treg
chr6-20877405-20877768	6.05E-10	0.315788201	0.061	0.006	4.00E-05	C11_CD4_Treg
chr10-6072489-6073024	6.13E-10	0.31646413	0.064	0.009	4.06E-05	C11_CD4_Treg
chr12-2867617-2868383	6.15E-10	0.347437201	0.18	0.064	4.08E-05	C11_CD4_Treg
chr9-89700866-89701738	6.37E-10	0.338224371	0.095	0.021	4.22E-05	C11_CD4_Treg
chr4-139657669-139658436	6.84E-10	0.353334912	0.139	0.042	4.53E-05	C11_CD4_Treg
chr11-47991364-47992512	7.04E-10	0.330125945	0.088	0.017	4.66E-05	C11_CD4_Treg
chr14-68368597-68369160	7.26E-10	0.310974269	0.064	0.008	4.81E-05	C11_CD4_Treg
chr1-198681336-198682329	7.35E-10	0.271529562	0.481	0.303	4.87E-05	C11_CD4_Treg
chr1-193537280-193538060	7.54E-10	0.345534233	0.136	0.038	4.99E-05	C11_CD4_Treg
chr7-16619744-16620380	7.63E-10	0.324456589	0.075	0.013	5.05E-05	C11_CD4_Treg

chr11-6319657-6321138	7.71E-10	0.324572546	0.078	0.014	5.11E-05	C11_CD4_Treg
chr1-244599427-244600257	8.93E-10	0.350734561	0.173	0.064	5.91E-05	C11_CD4_Treg
chr2-203909148-203909943	8.94E-10	0.334917515	0.098	0.023	5.92E-05	C11_CD4_Treg
chr2-106107586-106107874	9.76E-10	0.330718421	0.078	0.014	6.46E-05	C11_CD4_Treg
chr12-92096443-92097074	1.01E-09	0.332095406	0.081	0.017	6.67E-05	C11_CD4_Treg
chr1-24488101-24488495	1.05E-09	0.317721698	0.071	0.012	6.97E-05	C11_CD4_Treg
chr14-36315280-36315681	1.10E-09	0.30288675	0.061	0.007	7.28E-05	C11_CD4_Treg
chr6-53231452-53232021	1.18E-09	0.324570517	0.081	0.016	7.79E-05	C11_CD4_Treg
chr2-55590635-55591208	1.23E-09	0.342607314	0.176	0.073	8.17E-05	C11_CD4_Treg
chr16-75011600-75012683	1.25E-09	0.346380068	0.149	0.051	8.25E-05	C11_CD4_Treg
chr1-1692273-1693274	1.25E-09	0.287375989	0.403	0.239	8.30E-05	C11_CD4_Treg
chr14-75521543-75522754	1.25E-09	0.316242023	0.305	0.148	8.30E-05	C11_CD4_Treg
chr2-190670310-190670994	1.26E-09	0.345173963	0.129	0.041	8.32E-05	C11_CD4_Treg
chr3-190585667-190587087	1.26E-09	0.341434981	0.186	0.074	8.34E-05	C11_CD4_Treg
chr13-49454382-49455265	1.28E-09	0.338911851	0.122	0.035	8.47E-05	C11_CD4_Treg
chr5-75077913-75078334	1.33E-09	0.303529797	0.054	0.006	8.79E-05	C11_CD4_Treg
chr2-106144549-106145218	1.41E-09	0.338929466	0.197	0.071	9.36E-05	C11_CD4_Treg
chr18-74082201-74083383	1.48E-09	0.319761734	0.075	0.015	9.83E-05	C11_CD4_Treg
chr6-111776749-111777168	1.53E-09	0.348802033	0.139	0.054	0.000101246	C11_CD4_Treg
chr3-43755575-43755857	1.62E-09	0.284827644	0.051	0.005	0.000107232	C11_CD4_Treg
chr20-43914364-43916088	1.64E-09	0.311394163	0.315	0.162	0.000108723	C11_CD4_Treg
chr19-16371034-16372003	1.74E-09	0.266881542	0.475	0.306	0.000115084	C11_CD4_Treg
chr2-222426102-222426398	1.74E-09	0.307668145	0.068	0.011	0.000115511	C11_CD4_Treg
chr21-15064466-15066165	1.75E-09	0.317506365	0.275	0.131	0.000116049	C11_CD4_Treg
chr21-33955591-33956270	1.81E-09	0.312192658	0.075	0.013	0.0001198	C11_CD4_Treg
chr15-90749782-90750584	1.82E-09	0.307929305	0.064	0.009	0.000120803	C11_CD4_Treg
chr1-192575289-192575905	1.84E-09	0.270256061	0.444	0.292	0.00012212	C11_CD4_Treg

chr11-1330303-1330805	1.85E-09	0.34099896	0.098	0.023	0.000122392	C11_CD4_Treg
chr17-27339843-27340335	1.86E-09	0.336047013	0.098	0.022	0.00012339	C11_CD4_Treg
chr1-111624453-111624708	2.03E-09	0.334189695	0.108	0.03	0.00013465	C11_CD4_Treg
chr7-105448132-105448471	2.24E-09	0.296181711	0.054	0.006	0.000148186	C11_CD4_Treg
chr3-56593307-56593915	2.26E-09	0.338326387	0.163	0.058	0.000149943	C11_CD4_Treg
chr1-87061559-87062117	2.36E-09	0.320241887	0.068	0.013	0.00015612	C11_CD4_Treg
chr17-75189038-75189554	2.37E-09	0.337035266	0.108	0.028	0.000156994	C11_CD4_Treg
chr5-7876906-7877145	2.45E-09	0.309681113	0.064	0.009	0.000162279	C11_CD4_Treg
chr9-133115507-133116015	2.64E-09	0.334856814	0.2	0.085	0.000175006	C11_CD4_Treg
chr13-40484980-40486296	2.75E-09	0.278403598	0.454	0.277	0.000182232	C11_CD4_Treg
chrX-68503870-68504614	2.79E-09	0.340633241	0.186	0.075	0.000184751	C11_CD4_Treg
chr11-76560014-76560790	2.80E-09	0.327027853	0.085	0.018	0.000185174	C11_CD4_Treg
chr1-192536084-192536586	2.83E-09	0.321541742	0.085	0.017	0.000187396	C11_CD4_Treg
chr11-3013570-3014890	2.88E-09	0.327108704	0.227	0.102	0.000190777	C11_CD4_Treg
chr6-15776158-15776908	2.90E-09	0.311281939	0.061	0.009	0.000192007	C11_CD4_Treg
chr2-110858007-110859297	3.12E-09	0.300948473	0.353	0.195	0.000206288	C11_CD4_Treg
chr5-124583506-124584176	3.15E-09	0.291771351	0.058	0.007	0.000208669	C11_CD4_Treg
chr12-15087789-15088499	3.32E-09	0.324295473	0.088	0.021	0.000220062	C11_CD4_Treg
chr22-41862047-41862535	3.41E-09	0.32879701	0.207	0.09	0.000225544	C11_CD4_Treg
chr11-128552743-128553298	3.47E-09	0.333320643	0.132	0.038	0.000230116	C11_CD4_Treg
chr1-169699700-169700009	3.56E-09	0.284149466	0.051	0.005	0.000236015	C11_CD4_Treg
chr19-5961600-5962508	3.71E-09	0.311759532	0.305	0.159	0.000245995	C11_CD4_Treg
chr16-47142898-47144427	3.80E-09	0.299324155	0.298	0.162	0.000251504	C11_CD4_Treg
chr4-139560462-139561006	3.86E-09	0.337612544	0.146	0.048	0.000255619	C11_CD4_Treg
chr8-133045854-133047511	3.96E-09	0.331130007	0.183	0.074	0.00026197	C11_CD4_Treg
chr8-125636947-125637820	4.09E-09	0.324533512	0.088	0.021	0.000270747	C11_CD4_Treg
chr19-2168797-2169315	4.24E-09	0.329041802	0.153	0.054	0.000281094	C11_CD4_Treg

chr21-29436094-29436883	4.25E-09	0.293462152	0.054	0.008	0.000281308	C11_CD4_Treg
chr7-43668993-43670048	4.39E-09	0.312395108	0.302	0.146	0.000290489	C11_CD4_Treg
chr8-26306146-26306651	4.55E-09	0.310037567	0.068	0.012	0.000301523	C11_CD4_Treg
chr11-6351788-6352103	4.76E-09	0.291143572	0.054	0.007	0.000315494	C11_CD4_Treg
chr10-86412826-86413665	4.88E-09	0.331270534	0.146	0.05	0.000322852	C11_CD4_Treg
chr14-32702356-32702971	4.99E-09	0.291184062	0.054	0.006	0.000330742	C11_CD4_Treg
chr17-41717372-41717988	5.06E-09	0.307181294	0.058	0.009	0.000335136	C11_CD4_Treg
chr8-94261747-94262531	5.07E-09	0.323034083	0.105	0.028	0.000335963	C11_CD4_Treg
chr6-11368620-11369072	5.24E-09	0.33577733	0.149	0.053	0.000346939	C11_CD4_Treg
chr20-48788738-48789667	5.25E-09	0.335168737	0.166	0.069	0.000347655	C11_CD4_Treg
chr1-26305966-26307536	5.34E-09	0.259143464	0.464	0.303	0.000353506	C11_CD4_Treg
chr4-105060586-105061634	5.42E-09	0.291820929	0.058	0.007	0.000358696	C11_CD4_Treg
chrY-21239777-21240913	5.49E-09	0.329265636	0.146	0.05	0.000363861	C11_CD4_Treg
chr8-124267062-124267363	5.65E-09	0.322248925	0.081	0.016	0.000374385	C11_CD4_Treg
chr7-155166708-155167009	5.81E-09	0.286941423	0.051	0.007	0.000384544	C11_CD4_Treg
chr3-58111626-58112127	5.91E-09	0.279888833	0.051	0.006	0.000391166	C11_CD4_Treg
chr7-42869112-42869720	5.93E-09	0.29971697	0.058	0.008	0.000392568	C11_CD4_Treg
chr7-585260-585669	6.06E-09	0.318713674	0.098	0.025	0.000401127	C11_CD4_Treg
chr6-11459928-11461016	6.10E-09	0.296470298	0.064	0.011	0.000404195	C11_CD4_Treg
chr4-1081314-1081905	6.12E-09	0.312470632	0.078	0.016	0.000405341	C11_CD4_Treg
chr17-27299487-27299956	6.23E-09	0.301069078	0.064	0.011	0.00041287	C11_CD4_Treg
chr7-130366908-130367554	6.64E-09	0.279441782	0.051	0.005	0.000439401	C11_CD4_Treg
chr6-11414562-11414921	6.67E-09	0.324307084	0.129	0.042	0.000441718	C11_CD4_Treg
chr22-42217129-42217740	6.74E-09	0.321186881	0.092	0.023	0.000446066	C11_CD4_Treg
chr10-123241153-123242233	7.21E-09	0.316793646	0.261	0.12	0.000477769	C11_CD4_Treg
chr4-98603736-98604567	7.38E-09	0.320921848	0.095	0.023	0.000488662	C11_CD4_Treg
chr14-77119880-77120151	7.48E-09	0.295668024	0.068	0.012	0.000495238	C11_CD4_Treg

chr6-149494631-149495655	7.68E-09	0.298530906	0.302	0.165	0.000508707	C11_CD4_Treg
chr4-5758615-5759164	7.74E-09	0.294284176	0.058	0.007	0.000512593	C11_CD4_Treg
chr1-43836390-43836823	8.06E-09	0.29765588	0.064	0.01	0.000533802	C11_CD4_Treg
chr2-135948176-135949241	8.18E-09	0.291441378	0.319	0.171	0.000541998	C11_CD4_Treg
chr4-83503825-83504278	8.19E-09	0.283808109	0.054	0.007	0.000542391	C11_CD4_Treg
chr14-63872779-63873206	8.25E-09	0.303705214	0.064	0.012	0.000546546	C11_CD4_Treg
chr3-177057697-177058161	8.38E-09	0.320165446	0.115	0.034	0.000554856	C11_CD4_Treg
chr10-6291189-6292182	8.41E-09	0.320223444	0.19	0.079	0.000557168	C11_CD4_Treg
chr7-139118135-139119540	8.47E-09	0.313736178	0.227	0.103	0.000560859	C11_CD4_Treg
chr15-69060800-69061110	8.73E-09	0.284379957	0.054	0.007	0.000578112	C11_CD4_Treg
chr20-5835299-5836281	8.74E-09	0.327103138	0.142	0.048	0.000578835	C11_CD4_Treg
chr9-95852449-95852960	9.96E-09	0.302555565	0.064	0.011	0.000659283	C11_CD4_Treg
chr11-113242642-113243026	1.01E-08	0.2773775	0.051	0.005	0.00066598	C11_CD4_Treg
chr12-12582265-12583159	1.01E-08	0.281420791	0.054	0.008	0.000666994	C11_CD4_Treg
chr7-151745171-151745590	1.01E-08	0.296682062	0.061	0.009	0.000671359	C11_CD4_Treg
chr7-17029070-17029486	1.04E-08	0.30404765	0.071	0.014	0.000686244	C11_CD4_Treg
chr7-55533125-55534783	1.04E-08	0.300493905	0.288	0.148	0.00069004	C11_CD4_Treg
chr6-42339955-42340798	1.07E-08	0.3248166	0.115	0.037	0.000711499	C11_CD4_Treg
chr2-213125647-213126295	1.17E-08	0.298863752	0.075	0.013	0.000773956	C11_CD4_Treg
chr6-16639749-16640941	1.19E-08	0.325376551	0.105	0.029	0.000786675	C11_CD4_Treg
chr10-50394890-50395173	1.29E-08	0.292272077	0.058	0.009	0.000853644	C11_CD4_Treg
chr4-83561271-83561701	1.32E-08	0.306975483	0.078	0.019	0.000871415	C11_CD4_Treg
chr14-68822060-68822528	1.34E-08	0.300172009	0.085	0.019	0.000888746	C11_CD4_Treg
chr8-125220481-125220777	1.37E-08	0.285765334	0.061	0.009	0.000905692	C11_CD4_Treg
chr13-33678751-33679695	1.49E-08	0.310437079	0.227	0.113	0.00098646	C11_CD4_Treg
chr14-66507340-66509245	1.52E-08	0.259129987	0.461	0.293	0.00100933	C11_CD4_Treg
chr6-43275160-43276563	1.60E-08	0.314327019	0.125	0.04	0.001058274	C11_CD4_Treg

chr4-139554868-139557458	1.64E-08	0.26256585	0.403	0.258	0.001083531	C11_CD4_Treg
chr2-43559119-43560119	1.64E-08	0.274115636	0.054	0.006	0.001088884	C11_CD4_Treg
chr12-68745444-68746970	1.65E-08	0.284323566	0.339	0.191	0.001090027	C11_CD4_Treg
chr10-31100732-31101400	1.67E-08	0.314109849	0.095	0.023	0.001104523	C11_CD4_Treg
chr15-60798724-60799364	1.76E-08	0.320286937	0.115	0.038	0.00116269	C11_CD4_Treg
chr1-40185698-40186840	1.76E-08	0.316928374	0.142	0.05	0.001164651	C11_CD4_Treg
chr7-135147131-135148836	1.80E-08	0.286187429	0.336	0.186	0.00119428	C11_CD4_Treg
chr3-141529666-141530375	1.82E-08	0.300670958	0.081	0.018	0.001203859	C11_CD4_Treg
chr6-150632707-150633722	1.99E-08	0.316814044	0.115	0.034	0.001316737	C11_CD4_Treg
chr14-77302902-77304034	2.02E-08	0.321920289	0.166	0.06	0.001335551	C11_CD4_Treg
chr12-93771840-93772302	2.02E-08	0.301624686	0.088	0.021	0.001337366	C11_CD4_Treg
chr20-43721914-43722429	2.07E-08	0.297415624	0.068	0.014	0.001372315	C11_CD4_Treg
chr7-30321628-30323383	2.11E-08	0.295089251	0.268	0.14	0.001398585	C11_CD4_Treg
chr8-115494813-115495429	2.11E-08	0.301420598	0.088	0.021	0.001399536	C11_CD4_Treg
chr13-33639664-33640087	2.14E-08	0.280053949	0.051	0.007	0.001419025	C11_CD4_Treg
chr20-3419999-3420607	2.15E-08	0.318430864	0.156	0.058	0.001423203	C11_CD4_Treg
chr3-194103420-194103773	2.42E-08	0.284236596	0.061	0.009	0.001600427	C11_CD4_Treg
chr2-239321551-239323037	2.44E-08	0.309010119	0.169	0.068	0.001617524	C11_CD4_Treg
chr16-16025129-16026008	2.44E-08	0.314640318	0.095	0.024	0.001618445	C11_CD4_Treg
chr20-52975381-52975713	2.49E-08	0.292930452	0.064	0.011	0.001648896	C11_CD4_Treg
chr10-62199449-62199950	2.50E-08	0.299728464	0.078	0.017	0.001656533	C11_CD4_Treg
chr2-19971417-19972053	2.52E-08	0.297317518	0.068	0.013	0.001670033	C11_CD4_Treg
chr1-27418299-27418682	2.58E-08	0.307459781	0.102	0.029	0.001709727	C11_CD4_Treg
chr11-10657329-10657693	2.97E-08	0.281201587	0.054	0.009	0.001966522	C11_CD4_Treg
chr1-180507849-180508213	3.06E-08	0.312277195	0.092	0.023	0.002028394	C11_CD4_Treg
chr10-102408812-102409521	3.23E-08	0.307934178	0.139	0.049	0.002139523	C11_CD4_Treg
chr5-69234245-69235292	3.29E-08	0.250524171	0.431	0.274	0.002180312	C11_CD4_Treg

chr8-101003531-101004409	3.54E-08	0.29421994	0.071	0.015	0.002343577	C11_CD4_Treg
chr1-246329805-246330238	3.56E-08	0.299973476	0.068	0.013	0.002355028	C11_CD4_Treg
chr12-96223732-96224149	3.67E-08	0.266369106	0.051	0.007	0.002430601	C11_CD4_Treg
chr1-26838673-26839270	3.68E-08	0.307846562	0.105	0.03	0.002434694	C11_CD4_Treg
chr2-191624565-191625497	3.68E-08	0.304364956	0.18	0.075	0.002435573	C11_CD4_Treg
chr19-2171133-2171653	3.70E-08	0.310926065	0.156	0.058	0.002446944	C11_CD4_Treg
chr12-113962496-113962698	3.79E-08	0.27969288	0.058	0.009	0.00251179	C11_CD4_Treg
chr18-57434980-57435670	3.82E-08	0.292552535	0.075	0.014	0.002529569	C11_CD4_Treg
chr2-110811384-110812110	3.95E-08	0.300308652	0.092	0.024	0.002619025	C11_CD4_Treg
chr15-90574285-90574826	3.96E-08	0.294916797	0.085	0.019	0.002622235	C11_CD4_Treg
chr5-373385-374072	4.06E-08	0.305015617	0.102	0.033	0.00269059	C11_CD4_Treg
chr10-6037928-6038655	4.63E-08	0.308101731	0.166	0.07	0.003068654	C11_CD4_Treg
chr10-5775334-5776068	4.70E-08	0.312099482	0.159	0.063	0.003113884	C11_CD4_Treg
chr5-160479646-160480190	4.73E-08	0.299929505	0.085	0.018	0.003134033	C11_CD4_Treg
chr2-87476419-87477399	4.77E-08	0.309412827	0.132	0.047	0.003159383	C11_CD4_Treg
chr10-67753388-67753878	5.10E-08	0.276774209	0.061	0.011	0.003377021	C11_CD4_Treg
chr22-41217179-41218042	5.17E-08	0.294276199	0.085	0.019	0.003423987	C11_CD4_Treg
chr1-117076633-117077668	5.28E-08	0.3110947	0.115	0.039	0.003495621	C11_CD4_Treg
chr8-130067835-130068961	5.33E-08	0.257471548	0.424	0.266	0.003530268	C11_CD4_Treg
chr13-27130641-27131655	5.62E-08	0.2612703	0.414	0.261	0.003724353	C11_CD4_Treg
chr8-127625052-127625543	5.77E-08	0.295693067	0.075	0.017	0.003820143	C11_CD4_Treg
chr11-95715656-95716162	6.19E-08	0.290999135	0.075	0.016	0.004098746	C11_CD4_Treg
chr13-28357923-28358384	6.40E-08	0.267731028	0.051	0.008	0.004240095	C11_CD4_Treg
chr19-48876331-48876562	6.62E-08	0.298392988	0.166	0.066	0.004386786	C11_CD4_Treg
chr7-55469133-55469770	6.75E-08	0.303215406	0.098	0.026	0.004472307	C11_CD4_Treg
chr6-25053897-25054344	6.77E-08	0.301661569	0.125	0.039	0.004484755	C11_CD4_Treg
chr13-75481381-75482730	6.82E-08	0.295582589	0.115	0.037	0.004515719	C11_CD4_Treg

chr7-583060-584509	6.91E-08	0.268649132	0.339	0.206	0.004578308	C11_CD4_Treg
chr5-67031731-67032094	7.08E-08	0.270841804	0.054	0.009	0.004685305	C11_CD4_Treg
chr8-94928704-94929210	7.08E-08	0.266366826	0.051	0.007	0.00469053	C11_CD4_Treg
chr11-13439808-13440413	7.08E-08	0.264213577	0.051	0.007	0.00469109	C11_CD4_Treg
chrX-23732653-23733136	7.18E-08	0.267851138	0.054	0.009	0.004754295	C11_CD4_Treg
chr12-47884970-47885465	7.88E-08	0.268056138	0.061	0.01	0.005218865	C11_CD4_Treg
chr3-119628453-119629060	7.88E-08	0.274845219	0.061	0.009	0.005220756	C11_CD4_Treg
chr2-112154785-112155070	7.89E-08	0.270026284	0.058	0.01	0.005226323	C11_CD4_Treg
chr21-36309817-36310425	7.91E-08	0.26112244	0.051	0.008	0.005240735	C11_CD4_Treg
chr14-68711619-68712337	8.16E-08	0.275526426	0.068	0.013	0.005405635	C11_CD4_Treg
chr2-109348586-109349259	8.52E-08	0.280773678	0.071	0.015	0.00564286	C11_CD4_Treg
chr21-36909577-36910179	8.54E-08	0.267186661	0.054	0.008	0.005655001	C11_CD4_Treg
chr12-69161837-69162178	8.73E-08	0.300157577	0.088	0.022	0.005782712	C11_CD4_Treg
chr2-20097677-20098494	8.73E-08	0.288634131	0.092	0.026	0.005782782	C11_CD4_Treg
chr15-63668356-63668888	9.16E-08	0.260839139	0.054	0.009	0.006065111	C11_CD4_Treg
chr14-75539550-75539784	9.81E-08	0.293020336	0.095	0.027	0.006499776	C11_CD4_Treg
chr3-171225092-171225499	1.04E-07	0.28784941	0.081	0.018	0.006866577	C11_CD4_Treg
chr2-68792692-68793071	1.06E-07	0.275060108	0.061	0.012	0.007052248	C11_CD4_Treg
chr22-19576353-19577334	1.08E-07	0.295870798	0.115	0.036	0.007158912	C11_CD4_Treg
chr2-197803986-197805480	1.15E-07	0.264873894	0.332	0.197	0.007605322	C11_CD4_Treg
chr14-65879207-65879578	1.16E-07	0.274171378	0.058	0.01	0.007685306	C11_CD4_Treg
chr7-98131480-98132270	1.21E-07	0.297169604	0.102	0.034	0.00803536	C11_CD4_Treg
chr10-43166227-43166862	1.26E-07	0.289547751	0.081	0.018	0.008321273	C11_CD4_Treg
chr4-40908513-40909151	1.29E-07	0.273752908	0.071	0.015	0.008554704	C11_CD4_Treg
chr6-21438210-21439182	1.32E-07	0.262360673	0.064	0.011	0.008709536	C11_CD4_Treg
chr1-45550245-45551341	1.32E-07	0.252635576	0.38	0.234	0.008725378	C11_CD4_Treg
chr5-95817186-95817788	1.32E-07	0.298197969	0.136	0.05	0.008759018	C11_CD4_Treg

chr10-6036999-6037596	1.33E-07	0.280211181	0.075	0.017	0.008786842	C11_CD4_Treg
chr13-46177588-46177932	1.37E-07	0.296377742	0.108	0.039	0.009059556	C11_CD4_Treg
chr1-67313990-67315008	1.38E-07	0.278510414	0.075	0.018	0.009123824	C11_CD4_Treg
chr6-143545173-143546297	1.38E-07	0.299507442	0.176	0.071	0.009159473	C11_CD4_Treg
chr20-43942387-43943196	1.47E-07	0.29159419	0.132	0.051	0.009734637	C11_CD4_Treg
chr7-44995790-44996138	1.55E-07	0.29214852	0.136	0.051	0.010261609	C11_CD4_Treg
chr9-137095461-137096475	1.57E-07	0.250701025	0.373	0.234	0.010385282	C11_CD4_Treg
chr5-157350883-157351639	1.60E-07	0.267547185	0.061	0.012	0.010585247	C11_CD4_Treg
chr1-100046170-100047187	1.64E-07	0.286208481	0.102	0.032	0.010884265	C11_CD4_Treg
chr5-142095067-142096638	1.65E-07	0.262450392	0.342	0.204	0.010903345	C11_CD4_Treg
chr1-89626679-89627196	1.70E-07	0.289291033	0.122	0.041	0.011283453	C11_CD4_Treg
chr11-122703665-122704343	1.75E-07	0.286904373	0.085	0.023	0.011618914	C11_CD4_Treg
chr2-195477521-195477978	1.78E-07	0.278366427	0.068	0.013	0.011763245	C11_CD4_Treg
chr8-100463329-100464462	1.79E-07	0.296089646	0.173	0.079	0.011861927	C11_CD4_Treg
chrX-39174211-39175387	1.84E-07	0.290549761	0.149	0.06	0.012193836	C11_CD4_Treg
chr12-132178455-132179229	1.86E-07	0.277550212	0.071	0.018	0.012348892	C11_CD4_Treg
chr17-45307244-45307740	1.89E-07	0.294888998	0.136	0.056	0.012538507	C11_CD4_Treg
chrX-23745815-23746166	1.93E-07	0.280146077	0.081	0.02	0.012813802	C11_CD4_Treg
chr11-102300732-102301177	1.98E-07	0.281331411	0.068	0.015	0.013120769	C11_CD4_Treg
chr5-392379-392924	2.06E-07	0.293998027	0.132	0.05	0.013641475	C11_CD4_Treg
chr6-43246611-43247809	2.16E-07	0.275978123	0.22	0.121	0.014319014	C11_CD4_Treg
chr6-137810949-137811758	2.17E-07	0.279113131	0.254	0.132	0.014353463	C11_CD4_Treg
chr4-86977381-86978124	2.28E-07	0.281423354	0.071	0.018	0.015078846	C11_CD4_Treg
chr7-44915002-44915376	2.37E-07	0.27432354	0.064	0.013	0.015688713	C11_CD4_Treg
chr2-69648142-69648830	2.38E-07	0.278389809	0.227	0.117	0.015792381	C11_CD4_Treg
chr17-60151150-60151752	2.39E-07	0.283022144	0.078	0.02	0.015841433	C11_CD4_Treg
chr2-85017827-85018417	2.40E-07	0.27563515	0.081	0.021	0.015923657	C11_CD4_Treg

chr15-68795268-68795655	2.44E-07	0.281230881	0.092	0.025	0.016140709	C11_CD4_Treg
chr7-44038932-44039494	2.50E-07	0.295797005	0.159	0.065	0.016539689	C11_CD4_Treg
chr3-59450954-59452131	2.52E-07	0.282166806	0.231	0.113	0.016703307	C11_CD4_Treg
chr2-113390014-113390750	2.54E-07	0.277554361	0.166	0.076	0.016835722	C11_CD4_Treg
chrX-30652462-30654046	2.61E-07	0.251981234	0.366	0.223	0.017278521	C11_CD4_Treg
chr7-8175967-8177475	2.68E-07	0.276966487	0.264	0.144	0.017756747	C11_CD4_Treg
chr17-82604168-82604460	2.75E-07	0.265745846	0.054	0.01	0.018179171	C11_CD4_Treg
chr8-29992893-29993170	2.75E-07	0.283161404	0.078	0.022	0.018208507	C11_CD4_Treg
chr8-54134493-54135869	2.83E-07	0.274634917	0.271	0.147	0.018717016	C11_CD4_Treg
chr1-117013711-117014597	2.84E-07	0.283497044	0.092	0.029	0.01877543	C11_CD4_Treg
chr17-27480877-27481716	2.95E-07	0.286492261	0.119	0.042	0.019553938	C11_CD4_Treg
chr9-137757485-137757930	3.00E-07	0.261080485	0.058	0.01	0.019895775	C11_CD4_Treg
chr3-188165502-188165863	3.01E-07	0.263906855	0.054	0.009	0.019947893	C11_CD4_Treg
chr2-198004875-198005817	3.14E-07	0.2693941	0.075	0.019	0.020761853	C11_CD4_Treg
chr12-94007767-94008135	3.26E-07	0.26279322	0.051	0.008	0.021601138	C11_CD4_Treg
chr17-27466295-27467056	3.27E-07	0.281650283	0.183	0.087	0.021672734	C11_CD4_Treg
chr16-58732492-58732996	3.38E-07	0.278090972	0.075	0.021	0.022359541	C11_CD4_Treg
chr1-40388539-40388742	3.41E-07	0.275429166	0.085	0.025	0.02256303	C11_CD4_Treg
chr5-80687929-80688982	3.47E-07	0.273130934	0.068	0.016	0.022985306	C11_CD4_Treg
chr22-41842857-41843340	3.47E-07	0.280440108	0.092	0.027	0.022994873	C11_CD4_Treg
chr1-169586162-169586657	3.48E-07	0.280931668	0.085	0.023	0.023075148	C11_CD4_Treg
chr1-12386037-12386483	3.51E-07	0.285148744	0.095	0.026	0.023219629	C11_CD4_Treg
chr14-101635881-101636530	3.73E-07	0.266720872	0.068	0.014	0.024671527	C11_CD4_Treg
chr10-12122386-12123076	3.74E-07	0.289806782	0.136	0.05	0.024779308	C11_CD4_Treg
chr2-24827301-24827917	3.81E-07	0.280596487	0.102	0.035	0.025214721	C11_CD4_Treg
chr2-111534813-111535294	3.86E-07	0.253011755	0.054	0.009	0.025534338	C11_CD4_Treg
chr2-208013529-208014220	4.03E-07	0.270499912	0.064	0.015	0.026718781	C11_CD4_Treg

chr6-104789465-104790131	4.06E-07	0.287497093	0.125	0.043	0.026857137	C11_CD4_Treg
chr1-198175504-198176431	4.33E-07	0.288377162	0.146	0.059	0.028680789	C11_CD4_Treg
chr8-29805850-29806824	4.34E-07	0.282994339	0.183	0.088	0.028724246	C11_CD4_Treg
chr6-399602-400197	4.84E-07	0.282873356	0.149	0.064	0.03203965	C11_CD4_Treg
chr13-30408056-30408806	4.85E-07	0.260454833	0.298	0.164	0.032144728	C11_CD4_Treg
chr19-56537955-56539110	5.09E-07	0.257825413	0.288	0.174	0.033738975	C11_CD4_Treg
chr5-65742386-65742864	5.11E-07	0.252018995	0.058	0.012	0.033838201	C11_CD4_Treg
chr21-42456368-42456733	5.15E-07	0.288004331	0.115	0.038	0.034106852	C11_CD4_Treg
chr17-57662736-57663327	5.29E-07	0.260462248	0.058	0.011	0.035009039	C11_CD4_Treg
chr7-55517073-55517556	5.29E-07	0.284272791	0.098	0.032	0.03506126	C11_CD4_Treg
chr8-29569619-29570104	5.65E-07	0.252820949	0.051	0.008	0.037446643	C11_CD4_Treg
chr18-10530838-10531124	5.73E-07	0.25747809	0.061	0.013	0.03794263	C11_CD4_Treg
chr3-102114188-102114891	5.81E-07	0.26290091	0.061	0.013	0.038464742	C11_CD4_Treg
chr10-87761758-87762252	5.85E-07	0.270196599	0.071	0.018	0.038771621	C11_CD4_Treg
chr16-3625818-3626753	5.87E-07	0.282522693	0.115	0.044	0.038842141	C11_CD4_Treg
chr8-28375267-28375693	5.97E-07	0.251091795	0.061	0.012	0.039567467	C11_CD4_Treg
chr6-143539203-143540048	6.28E-07	0.275386005	0.129	0.051	0.041589918	C11_CD4_Treg
chr11-118883396-118883996	6.49E-07	0.273813712	0.085	0.021	0.042992794	C11_CD4_Treg
chr16-57133900-57134167	6.61E-07	0.250994197	0.051	0.011	0.043784305	C11_CD4_Treg
chr16-11379346-11379881	6.83E-07	0.260378967	0.068	0.017	0.045234251	C11_CD4_Treg
chr2-64036079-64036687	7.01E-07	0.274884972	0.217	0.118	0.046451146	C11_CD4_Treg
chr18-62784340-62785260	7.14E-07	0.265228972	0.081	0.022	0.047260022	C11_CD4_Treg
chr15-90686360-90686802	7.17E-07	0.251290017	0.058	0.012	0.04749097	C11_CD4_Treg
chr16-58126912-58127290	7.58E-07	0.277000539	0.18	0.083	0.050164211	C11_CD4_Treg
chr8-58845135-58845720	7.66E-07	0.264992494	0.064	0.014	0.050756519	C11_CD4_Treg
chr12-123024734-123025278	7.95E-07	0.25064032	0.064	0.015	0.052645152	C11_CD4_Treg
chr9-129992050-129992450	8.00E-07	0.276225816	0.125	0.045	0.052976163	C11_CD4_Treg

chr6-42813577-42814381	8.32E-07	0.258839821	0.075	0.02	0.055083285	C11_CD4_Treg
chr19-32642552-32643229	8.71E-07	0.27216447	0.098	0.032	0.057711337	C11_CD4_Treg
chr16-68005582-68006325	9.01E-07	0.271195935	0.095	0.029	0.059686052	C11_CD4_Treg
chr6-156791197-156791531	9.10E-07	0.261933303	0.075	0.019	0.060243165	C11_CD4_Treg
chr8-38359337-38360142	9.87E-07	0.273627773	0.149	0.062	0.065381076	C11_CD4_Treg
chr8-116708863-116709635	1.01E-06	0.280444599	0.139	0.056	0.066986881	C11_CD4_Treg
chr10-62183124-62184050	1.03E-06	0.270401804	0.197	0.095	0.068248068	C11_CD4_Treg
chr3-195181957-195182647	1.08E-06	0.26153439	0.075	0.017	0.071238227	C11_CD4_Treg
chr19-41595067-41595832	1.11E-06	0.270930244	0.136	0.059	0.07343418	C11_CD4_Treg
chr3-59388463-59389194	1.16E-06	0.25408077	0.058	0.012	0.076917838	C11_CD4_Treg
chr18-13435620-13436307	1.20E-06	0.265719454	0.078	0.018	0.079397515	C11_CD4_Treg
chr1-200483071-200483879	1.26E-06	0.276725049	0.166	0.077	0.083334707	C11_CD4_Treg
chr3-143118993-143121602	1.35E-06	0.263622595	0.217	0.105	0.089336741	C11_CD4_Treg
chr22-40898744-40899363	1.39E-06	0.250001264	0.064	0.017	0.092184682	C11_CD4_Treg
chr14-75514130-75515611	1.47E-06	0.264234745	0.193	0.093	0.097361086	C11_CD4_Treg
chr2-222423635-222425613	1.50E-06	0.263552368	0.153	0.06	0.099569901	C11_CD4_Treg
chr10-17616707-17617887	1.53E-06	0.268944011	0.139	0.054	0.101080058	C11_CD4_Treg
chr12-50059792-50060083	1.54E-06	0.257206396	0.075	0.02	0.101706937	C11_CD4_Treg
chr6-11495691-11496528	1.65E-06	0.266447042	0.125	0.048	0.108969137	C11_CD4_Treg
chr15-63707799-63708632	1.68E-06	0.268078998	0.095	0.034	0.111094902	C11_CD4_Treg
chr15-22222968-22223414	1.81E-06	0.267722159	0.095	0.034	0.119685228	C11_CD4_Treg
chr5-1792331-1793561	1.83E-06	0.25293947	0.261	0.149	0.120877133	C11_CD4_Treg
chr5-14706188-14706999	1.90E-06	0.265435403	0.183	0.086	0.125750582	C11_CD4_Treg
chr17-50903589-50904600	1.91E-06	0.265529085	0.169	0.079	0.126348241	C11_CD4_Treg
chr4-8199983-8200688	1.94E-06	0.265094061	0.105	0.037	0.128451105	C11_CD4_Treg
chrX-9340694-9342168	2.02E-06	0.260133638	0.203	0.107	0.133677216	C11_CD4_Treg
chr10-102246241-102246646	2.14E-06	0.259864776	0.095	0.034	0.141419813	C11_CD4_Treg

chr4-1511563-1512056	2.14E-06	0.259508526	0.085	0.025	0.142038919	C11_CD4_Treg
chr13-20174500-20175193	2.15E-06	0.265481056	0.108	0.038	0.142179438	C11_CD4_Treg
chr21-45606985-45607484	2.24E-06	0.266439111	0.088	0.027	0.148205389	C11_CD4_Treg
chr11-86115924-86116762	2.27E-06	0.261775883	0.139	0.059	0.150146641	C11_CD4_Treg
chr17-42458408-42459083	2.27E-06	0.263265757	0.183	0.084	0.150619474	C11_CD4_Treg
chr20-32248960-32249687	2.28E-06	0.264219175	0.2	0.105	0.150914463	C11_CD4_Treg
chr14-89616876-89617244	2.32E-06	0.257037334	0.217	0.113	0.153580754	C11_CD4_Treg
chr15-52658686-52659323	2.33E-06	0.26756043	0.112	0.044	0.154151193	C11_CD4_Treg
chr1-89357683-89358731	2.33E-06	0.262503189	0.112	0.041	0.154388759	C11_CD4_Treg
chr4-68438906-68439718	2.42E-06	0.255890459	0.088	0.027	0.160549257	C11_CD4_Treg
chr12-132153714-132154291	2.43E-06	0.264629041	0.156	0.074	0.160693404	C11_CD4_Treg
chr5-463802-464366	2.45E-06	0.26467522	0.132	0.055	0.162221862	C11_CD4_Treg
chr20-59163571-59164234	2.45E-06	0.257051956	0.203	0.106	0.162314011	C11_CD4_Treg
chr2-234469362-234469775	2.54E-06	0.251715516	0.068	0.019	0.167990752	C11_CD4_Treg
chr11-123069861-123070762	2.61E-06	0.26077868	0.132	0.061	0.172858708	C11_CD4_Treg
chr17-7047602-7047891	2.81E-06	0.26954465	0.115	0.046	0.186013768	C11_CD4_Treg
chr1-64948997-64949482	3.06E-06	0.264499481	0.129	0.058	0.202645647	C11_CD4_Treg
chr4-165215254-165216004	3.09E-06	0.262163478	0.078	0.026	0.204947998	C11_CD4_Treg
chr12-76456710-76457296	3.10E-06	0.257703038	0.098	0.036	0.204988317	C11_CD4_Treg
chr4-113564536-113565351	3.17E-06	0.260907129	0.163	0.077	0.2099561	C11_CD4_Treg
chr4-40197804-40198071	3.20E-06	0.255097921	0.203	0.103	0.211877416	C11_CD4_Treg
chr7-149439665-149440538	3.31E-06	0.250906345	0.064	0.017	0.219055229	C11_CD4_Treg
chr10-24622117-24623254	3.47E-06	0.256894014	0.085	0.026	0.229960291	C11_CD4_Treg
chr1-58573715-58574090	3.61E-06	0.253048666	0.078	0.022	0.238894899	C11_CD4_Treg
chr2-168233811-168234977	4.06E-06	0.252806946	0.085	0.027	0.268573941	C11_CD4_Treg
chr16-67667812-67668227	4.22E-06	0.257091265	0.207	0.104	0.279189287	C11_CD4_Treg
chr17-59329607-59330419	4.45E-06	0.259066021	0.173	0.083	0.294822092	C11_CD4_Treg

chr17-59853047-59853560	4.50E-06	0.254937911	0.095	0.036	0.297677833	C11_CD4_Treg
chr6-12044767-12045304	4.51E-06	0.25264352	0.075	0.022	0.298504873	C11_CD4_Treg
chrX-103463912-103465050	4.59E-06	0.262542787	0.112	0.045	0.303687165	C11_CD4_Treg
chr11-128579648-128580335	4.82E-06	0.254380114	0.098	0.036	0.319038982	C11_CD4_Treg
chr15-52651763-52652243	4.85E-06	0.254254496	0.102	0.036	0.321511498	C11_CD4_Treg
chr20-3092636-3094230	5.13E-06	0.252875272	0.112	0.043	0.339434748	C11_CD4_Treg
chr3-130093640-130094937	5.32E-06	0.250897608	0.088	0.03	0.352124373	C11_CD4_Treg
chr2-216388051-216388833	5.65E-06	0.257734815	0.092	0.036	0.374488722	C11_CD4_Treg
chr1-9950116-9950668	6.69E-06	0.253894431	0.119	0.05	0.443150928	C11_CD4_Treg
chr1-112390634-112391210	7.12E-06	0.253269855	0.149	0.067	0.471318232	C11_CD4_Treg
chr14-102819036-102820004	7.41E-06	0.250858964	0.122	0.048	0.491012513	C11_CD4_Treg
chr8-2146340-2146857	7.80E-06	0.256335025	0.146	0.068	0.516386099	C11_CD4_Treg
chr5-75049874-75050124	7.90E-06	0.251756253	0.098	0.036	0.522899321	C11_CD4_Treg
chr3-143847683-143848973	8.51E-06	0.252147236	0.159	0.08	0.563442241	C11_CD4_Treg
chr12-108568541-108569182	8.87E-06	0.250494234	0.139	0.067	0.587697225	C11_CD4_Treg
chr1-89200647-89201555	9.81E-06	0.250979455	0.132	0.059	0.649855223	C11_CD4_Treg
chr20-3092636-3094230	5.13E-06	0.252875272	0.112	0.043	0.339434748	C11_CD4_Treg
chr6-111086996-111088653	6.02E-19	0.493297591	0.261	0.076	3.99E-14	C11_CD4_Treg
chr11-3013570-3014890	2.88E-09	0.327108704	0.227	0.102	0.000190777	C11_CD4_Treg
chr20-43721914-43722429	2.07E-08	0.297415624	0.068	0.014	0.001372315	C11_CD4_Treg
chr3-102114188-102114891	5.81E-07	0.26290091	0.061	0.013	0.038464742	C11_CD4_Treg
chr3-194103420-194103773	2.42E-08	0.284236596	0.061	0.009	0.001600427	C11_CD4_Treg
chr1-246117052-246117844	1.80E-16	0.469895277	0.129	0.02	1.19E-11	C11_CD4_Treg
chr15-90686360-90686802	7.17E-07	0.251290017	0.058	0.012	0.04749097	C11_CD4_Treg
chr21-45590378-45592664	5.24E-15	0.367113305	0.444	0.232	3.47E-10	C11_CD4_Treg
chr7-135147131-135148836	1.80E-08	0.286187429	0.336	0.186	0.00119428	C11_CD4_Treg
chr16-15960530-15961931	1.11E-10	0.330404537	0.295	0.142	7.33E-06	C11_CD4_Treg

chr17-17179309-17179692	1.29E-13	0.419441048	0.139	0.031	8.54E-09	C11_CD4_Treg
chr20-58660901-58661838	4.59E-11	0.373873744	0.146	0.042	3.04E-06	C11_CD4_Treg
chr21-45606985-45607484	2.24E-06	0.266439111	0.088	0.027	0.148205389	C11_CD4_Treg
chr10-62183124-62184050	1.03E-06	0.270401804	0.197	0.095	0.068248068	C11_CD4_Treg
chr12-94007767-94008135	3.26E-07	0.26279322	0.051	0.008	0.021601138	C11_CD4_Treg
chr10-103677742-103678553	1.26E-10	0.355375216	0.227	0.094	8.31E-06	C11_CD4_Treg
chr11-102300732-102301177	1.98E-07	0.281331411	0.068	0.015	0.013120769	C11_CD4_Treg
chr10-24622117-24623254	3.47E-06	0.256894014	0.085	0.026	0.229960291	C11_CD4_Treg
chr8-27490629-27491808	1.75E-12	0.396801743	0.166	0.05	1.16E-07	C11_CD4_Treg
chr12-123474686-123475310	3.73E-11	0.336558925	0.068	0.009	2.47E-06	C11_CD4_Treg
chr10-31321399-31321710	2.03E-17	0.477744206	0.18	0.046	1.34E-12	C11_CD4_Treg
chr12-108568541-108569182	8.87E-06	0.250494234	0.139	0.067	0.587697225	C11_CD4_Treg
chr13-40484980-40486296	2.75E-09	0.278403598	0.454	0.277	0.000182232	C11_CD4_Treg
chr21-15064466-15066165	1.75E-09	0.317506365	0.275	0.131	0.000116049	C11_CD4_Treg
chr2-112154785-112155070	7.89E-08	0.270026284	0.058	0.01	0.005226323	C11_CD4_Treg
chr12-113135304-113136419	1.00E-14	0.42275353	0.268	0.101	6.62E-10	C11_CD4_Treg
chr5-142095067-142096638	1.65E-07	0.262450392	0.342	0.204	0.010903345	C11_CD4_Treg
chr5-56638463-56639330	3.15E-10	0.342259887	0.081	0.015	2.08E-05	C11_CD4_Treg
chr20-62651035-62652604	4.89E-15	0.368971878	0.427	0.223	3.24E-10	C11_CD4_Treg
chr1-207822694-207822987	1.94E-23	0.567367258	0.214	0.041	1.28E-18	C11_CD4_Treg
chr19-46633689-46634603	2.61E-16	0.357915451	0.522	0.301	1.73E-11	C11_CD4_Treg
chr20-52975381-52975713	2.49E-08	0.292930452	0.064	0.011	0.001648896	C11_CD4_Treg
chr22-47090614-47091616	6.27E-16	0.465688043	0.193	0.049	4.15E-11	C11_CD4_Treg
chr4-1511563-1512056	2.14E-06	0.259508526	0.085	0.025	0.142038919	C11_CD4_Treg
chr3-43755575-43755857	1.62E-09	0.284827644	0.051	0.005	0.000107232	C11_CD4_Treg
chr7-44915002-44915376	2.37E-07	0.27432354	0.064	0.013	0.015688713	C11_CD4_Treg
chr14-32702356-32702971	4.99E-09	0.291184062	0.054	0.006	0.000330742	C11_CD4_Treg

chr13-40494317-40495333	5.06E-15	0.444906563	0.166	0.044	3.35E-10	C11_CD4_Treg
chr3-143118993-143121602	1.35E-06	0.263622595	0.217	0.105	0.089336741	C11_CD4_Treg
chr10-6072489-6073024	6.13E-10	0.31646413	0.064	0.009	4.06E-05	C11_CD4_Treg
chrY-21239777-21240913	5.49E-09	0.329265636	0.146	0.05	0.000363861	C11_CD4_Treg
chr1-12386037-12386483	3.51E-07	0.285148744	0.095	0.026	0.023219629	C11_CD4_Treg
chr4-10671147-10671744	1.06E-14	0.452843075	0.142	0.029	7.03E-10	C11_CD4_Treg
chr12-123119847-123120166	1.14E-15	0.414727949	0.085	0.008	7.57E-11	C11_CD4_Treg
chr12-132153714-132154291	2.43E-06	0.264629041	0.156	0.074	0.160693404	C11_CD4_Treg
chr2-239321551-239323037	2.44E-08	0.309010119	0.169	0.068	0.001617524	C11_CD4_Treg
chr10-12122386-12123076	3.74E-07	0.289806782	0.136	0.05	0.024779308	C11_CD4_Treg
chr5-14706188-14706999	1.90E-06	0.265435403	0.183	0.086	0.125750582	C11_CD4_Treg
chr15-90574285-90574826	3.96E-08	0.294916797	0.085	0.019	0.002622235	C11_CD4_Treg
chr7-55469133-55469770	6.75E-08	0.303215406	0.098	0.026	0.004472307	C11_CD4_Treg
chr10-102408812-102409521	3.23E-08	0.307934178	0.139	0.049	0.002139523	C11_CD4_Treg
chr18-13381809-13382793	8.33E-11	0.366052467	0.19	0.064	5.52E-06	C11_CD4_Treg
chr1-116986501-116987514	5.92E-10	0.353308675	0.136	0.042	3.92E-05	C11_CD4_Treg
chr2-109348586-109349259	8.52E-08	0.280773678	0.071	0.015	0.00564286	C11_CD4_Treg
chrX-103463912-103465050	4.59E-06	0.262542787	0.112	0.045	0.303687165	C11_CD4_Treg
chr1-246569353-246569929	2.51E-17	0.486414475	0.2	0.048	1.66E-12	C11_CD4_Treg
chr16-67667812-67668227	4.22E-06	0.257091265	0.207	0.104	0.279189287	C11_CD4_Treg
chr6-36108987-36109444	4.69E-15	0.433913631	0.115	0.021	3.11E-10	C11_CD4_Treg
chr18-74082201-74083383	1.48E-09	0.319761734	0.075	0.015	9.83E-05	C11_CD4_Treg
chr10-123241153-123242233	7.21E-09	0.316793646	0.261	0.12	0.000477769	C11_CD4_Treg
chr6-154606273-154606870	2.28E-10	0.357304617	0.112	0.025	1.51E-05	C11_CD4_Treg
chr19-5130283-5131108	2.20E-16	0.468055566	0.169	0.038	1.46E-11	C11_CD4_Treg
chr19-46522622-46523319	2.30E-11	0.376934744	0.129	0.031	1.53E-06	C11_CD4_Treg
chr5-463802-464366	2.45E-06	0.26467522	0.132	0.055	0.162221862	C11_CD4_Treg

chr6-156791197-156791531	9.10E-07	0.261933303	0.075	0.019	0.060243165	C11_CD4_Treg
chr11-123069861-123070762	2.61E-06	0.26077868	0.132	0.061	0.172858708	C11_CD4_Treg
chr1-169586162-169586657	3.48E-07	0.280931668	0.085	0.023	0.023075148	C11_CD4_Treg
chr5-373385-374072	4.06E-08	0.305015617	0.102	0.033	0.00269059	C11_CD4_Treg
chr2-216388051-216388833	5.65E-06	0.257734815	0.092	0.036	0.374488722	C11_CD4_Treg
chr11-66221892-66223046	3.61E-10	0.316391744	0.332	0.181	2.39E-05	C11_CD4_Treg
chr8-29805850-29806824	4.34E-07	0.282994339	0.183	0.088	0.028724246	C11_CD4_Treg
chr10-5775334-5776068	4.70E-08	0.312099482	0.159	0.063	0.003113884	C11_CD4_Treg
chr14-75521543-75522754	1.25E-09	0.316242023	0.305	0.148	8.30E-05	C11_CD4_Treg
chr10-31100732-31101400	1.67E-08	0.314109849	0.095	0.023	0.001104523	C11_CD4_Treg
chrX-39174211-39175387	1.84E-07	0.290549761	0.149	0.06	0.012193836	C11_CD4_Treg
chr6-158270820-158271475	1.71E-10	0.358670939	0.214	0.087	1.13E-05	C11_CD4_Treg
chr3-56593307-56593915	2.26E-09	0.338326387	0.163	0.058	0.000149943	C11_CD4_Treg
chr5-75049874-75050124	7.90E-06	0.251756253	0.098	0.036	0.522899321	C11_CD4_Treg
chr3-71489233-71489774	2.53E-11	0.377146831	0.173	0.061	1.68E-06	C11_CD4_Treg
chr17-50903589-50904600	1.91E-06	0.265529085	0.169	0.079	0.126348241	C11_CD4_Treg
chr11-122703665-122704343	1.75E-07	0.286904373	0.085	0.023	0.011618914	C11_CD4_Treg
chr6-4059043-4060201	3.95E-14	0.397866274	0.339	0.144	2.61E-09	C11_CD4_Treg
chr16-26597839-26598554	1.27E-17	0.434152273	0.403	0.177	8.42E-13	C11_CD4_Treg
chr19-2168797-2169315	4.24E-09	0.329041802	0.153	0.054	0.000281094	C11_CD4_Treg
chr8-125110513-125111202	2.74E-18	0.496538923	0.146	0.025	1.82E-13	C11_CD4_Treg
chr20-48788738-48789667	5.25E-09	0.335168737	0.166	0.069	0.000347655	C11_CD4_Treg
chrX-68503870-68504614	2.79E-09	0.340633241	0.186	0.075	0.000184751	C11_CD4_Treg
chr10-32332140-32332707	3.25E-11	0.313361825	0.464	0.264	2.15E-06	C11_CD4_Treg
chr19-11567730-11568616	4.11E-17	0.463187794	0.119	0.018	2.72E-12	C11_CD4_Treg
chr5-80687929-80688982	3.47E-07	0.273130934	0.068	0.016	0.022985306	C11_CD4_Treg
chr1-117013711-117014597	2.84E-07	0.283497044	0.092	0.029	0.01877543	C11_CD4_Treg

chr7-139118135-139119540	8.47E-09	0.313736178	0.227	0.103	0.000560859	C11_CD4_Treg
chr5-35908165-35908604	2.83E-11	0.364576353	0.085	0.013	1.87E-06	C11_CD4_Treg
chr11-3013570-3014890	2.88E-09	0.327108704	0.227	0.102	0.000190777	C11_CD4_Treg
chr2-106144549-106145218	1.41E-09	0.338929466	0.197	0.071	9.36E-05	C11_CD4_Treg
chr15-52658686-52659323	2.33E-06	0.26756043	0.112	0.044	0.154151193	C11_CD4_Treg
chr5-392379-392924	2.06E-07	0.293998027	0.132	0.05	0.013641475	C11_CD4_Treg
chr7-17029070-17029486	1.04E-08	0.30404765	0.071	0.014	0.000686244	C11_CD4_Treg
chr7-98131480-98132270	1.21E-07	0.297169604	0.102	0.034	0.00803536	C11_CD4_Treg
chr14-77302902-77304034	2.02E-08	0.321920289	0.166	0.06	0.001335551	C11_CD4_Treg
chr6-2862631-2863246	3.68E-15	0.439949643	0.254	0.088	2.44E-10	C11_CD4_Treg
chr12-69161837-69162178	8.73E-08	0.300157577	0.088	0.022	0.005782712	C11_CD4_Treg
chr20-62651035-62652604	4.89E-15	0.368971878	0.427	0.223	3.24E-10	C11_CD4_Treg
chr17-75297288-75297880	4.66E-14	0.40984119	0.112	0.021	3.09E-09	C11_CD4_Treg
chr17-57440085-57441355	1.12E-13	0.411705972	0.251	0.096	7.43E-09	C11_CD4_Treg
chr20-32248960-32249687	2.28E-06	0.264219175	0.2	0.105	0.150914463	C11_CD4_Treg
chr5-373385-374072	4.06E-08	0.305015617	0.102	0.033	0.00269059	C11_CD4_Treg
chr10-17648260-17648838	2.44E-37	0.713702976	0.312	0.06	1.62E-32	C11_CD4_Treg
chr15-60798724-60799364	1.76E-08	0.320286937	0.115	0.038	0.00116269	C11_CD4_Treg
chr14-55071702-55072623	1.76E-11	0.372000304	0.227	0.096	1.17E-06	C11_CD4_Treg
chr17-27466295-27467056	3.27E-07	0.281650283	0.183	0.087	0.021672734	C11_CD4_Treg
chr20-49698681-49699604	2.72E-10	0.348540821	0.231	0.099	1.80E-05	C11_CD4_Treg
chr1-67313990-67315008	1.38E-07	0.278510414	0.075	0.018	0.009123824	C11_CD4_Treg
chr5-95817186-95817788	1.32E-07	0.298197969	0.136	0.05	0.008759018	C11_CD4_Treg
chr5-96715613-96716384	1.67E-11	0.376041996	0.129	0.034	1.11E-06	C11_CD4_Treg
chr6-4059043-4060201	3.95E-14	0.397866274	0.339	0.144	2.61E-09	C11_CD4_Treg
chr19-2171133-2171653	3.70E-08	0.310926065	0.156	0.058	0.002446944	C11_CD4_Treg
chr14-66507340-66509245	1.52E-08	0.259129987	0.461	0.293	0.00100933	C11_CD4_Treg

chr20-3419999-3420607	2.15E-08	0.318430864	0.156	0.058	0.001423203	C11_CD4_Treg
chr14-77302902-77304034	2.02E-08	0.321920289	0.166	0.06	0.001335551	C11_CD4_Treg
chr1-23624092-23624707	5.01E-24	0.583902192	0.22	0.046	3.32E-19	C11_CD4_Treg
chr8-127307185-127307982	8.21E-23	0.529437086	0.125	0.011	5.43E-18	C11_CD4_Treg
chr8-130067835-130068961	5.33E-08	0.257471548	0.424	0.266	0.003530268	C11_CD4_Treg
chr22-20111025-20111737	3.97E-10	0.349518953	0.139	0.045	2.63E-05	C11_CD4_Treg
chr3-188165502-188165863	3.01E-07	0.263906855	0.054	0.009	0.019947893	C11_CD4_Treg
chr7-100242863-100243863	2.74E-12	0.398402191	0.166	0.051	1.81E-07	C11_CD4_Treg
chr14-22460233-22460781	6.68E-26	1.361990349	0.357	0.005	4.43E-21	C12_gdT
chr14-22336747-22337125	8.42E-20	1.192461511	0.286	0.005	5.58E-15	C12_gdT
chr14-22466504-22466858	2.40E-18	1.123097448	0.232	0.004	1.59E-13	C12_gdT
chr14-22386608-22386958	9.84E-16	1.061139118	0.232	0.005	6.52E-11	C12_gdT
chr14-22333823-22334304	3.01E-15	1.04344326	0.232	0.006	1.99E-10	C12_gdT
chr14-22383348-22384016	6.19E-14	0.998689246	0.25	0.01	4.10E-09	C12_gdT
chr20-44609678-44610919	3.63E-12	0.82938605	0.536	0.112	2.40E-07	C12_gdT
chr11-114141906-114142137	1.58E-11	0.873973202	0.161	0.003	1.05E-06	C12_gdT
chr12-48932877-48933311	2.31E-11	0.902324775	0.304	0.026	1.53E-06	C12_gdT
chr12-128791870-128792180	1.34E-10	0.851504113	0.214	0.014	8.90E-06	C12_gdT
chr9-19155606-19156477	1.70E-10	0.847437438	0.214	0.012	1.12E-05	C12_gdT
chr14-22231033-22231456	1.96E-10	0.861225239	0.232	0.018	1.30E-05	C12_gdT
chr1-53411596-53411829	4.29E-10	0.846876096	0.286	0.026	2.84E-05	C12_gdT
chr7-38317371-38317880	8.10E-10	0.817360375	0.286	0.031	5.36E-05	C12_gdT
chr1-167473329-167474646	8.26E-10	0.804897648	0.357	0.054	5.47E-05	C12_gdT
chr15-91706099-91706801	1.53E-09	0.779117844	0.375	0.07	0.000101262	C12_gdT
chr11-114131540-114131901	5.57E-09	0.776249501	0.196	0.012	0.000369084	C12_gdT
chr1-23591397-23592492	8.72E-09	0.755845314	0.357	0.063	0.000577769	C12_gdT
chr2-195503214-195503568	1.19E-08	0.765740971	0.196	0.016	0.000785927	C12_gdT

chr15-91348820-91349503	1.45E-08	0.762437691	0.232	0.021	0.000962306	C12_gdT
chr3-122508049-122508471	1.66E-08	0.76524669	0.196	0.015	0.001099823	C12_gdT
chr16-85902567-85903082	3.04E-08	0.748917064	0.25	0.03	0.002015645	C12_gdT
chr19-659341-659906	3.60E-08	0.63671261	0.518	0.158	0.002386323	C12_gdT
chr7-142784718-142786248	1.65E-07	0.557265957	0.607	0.224	0.01094437	C12_gdT
chr7-132488959-132489202	1.73E-07	0.690930953	0.143	0.008	0.011449363	C12_gdT
chr21-46637994-46638606	1.90E-07	0.701199993	0.268	0.047	0.012580338	C12_gdT
chr14-55353687-55354948	1.98E-07	0.661496882	0.375	0.097	0.013118441	C12_gdT
chr9-129854475-129855048	2.00E-07	0.702627773	0.179	0.016	0.013212987	C12_gdT
chr6-37189628-37190270	2.04E-07	0.711797493	0.268	0.039	0.013507415	C12_gdT
chr2-105072084-105072364	2.28E-07	0.68081894	0.161	0.011	0.015100726	C12_gdT
chr3-151328403-151328976	2.29E-07	0.699799607	0.196	0.019	0.015157064	C12_gdT
chr8-99002249-99002627	3.23E-07	0.680704111	0.179	0.016	0.021393628	C12_gdT
chr2-212052222-212052797	4.07E-07	0.650028889	0.125	0.005	0.026980618	C12_gdT
chr2-46439221-46440197	4.90E-07	0.651634604	0.339	0.069	0.032417769	C12_gdT
chr17-17675991-17676511	5.25E-07	0.663419249	0.161	0.013	0.034794941	C12_gdT
chr1-167467438-167467670	5.94E-07	0.613893669	0.107	0.004	0.039313421	C12_gdT
chr5-10695806-10696382	6.14E-07	0.637463471	0.125	0.007	0.040685841	C12_gdT
chr2-231179831-231180269	6.37E-07	0.667564414	0.161	0.014	0.042166613	C12_gdT
chr6-125831744-125832093	6.82E-07	0.64427146	0.143	0.008	0.045160059	C12_gdT
chrX-136623449-136623648	1.24E-06	0.620153828	0.125	0.007	0.081866901	C12_gdT
chr17-49338793-49339221	1.27E-06	0.656537092	0.286	0.053	0.083919412	C12_gdT
chr19-6652556-6653061	1.31E-06	0.656706295	0.232	0.03	0.086422711	C12_gdT
chr16-29792426-29792673	1.36E-06	0.658215612	0.179	0.018	0.089754524	C12_gdT
chr16-27426016-27426826	1.42E-06	0.583854696	0.5	0.145	0.09430971	C12_gdT
chr3-196645146-196645347	1.89E-06	0.639663521	0.161	0.012	0.125463822	C12_gdT
chr7-140262553-140263614	1.95E-06	0.649677369	0.25	0.046	0.128874912	C12_gdT

chr17-40540363-40542001	2.06E-06	0.536064206	0.554	0.198	0.136209551	C12_gdT
chr22-49867574-49868421	2.15E-06	0.590535785	0.411	0.122	0.142109282	C12_gdT
chr1-205479917-205482045	2.45E-06	0.598535064	0.429	0.109	0.162067457	C12_gdT
chr9-19183545-19184782	2.57E-06	0.518977606	0.554	0.214	0.170469542	C12_gdT
chr12-8996631-8997098	3.85E-06	0.617684267	0.304	0.068	0.254989293	C12_gdT
chr1-67307039-67308443	4.53E-06	0.615930652	0.25	0.045	0.299876057	C12_gdT
chr1-200153142-200153701	4.76E-06	0.608921234	0.268	0.052	0.314896975	C12_gdT
chr6-111736117-111736526	4.96E-06	0.62723982	0.196	0.029	0.328488345	C12_gdT
chr5-142845232-142846686	5.33E-06	0.617293556	0.25	0.043	0.353163866	C12_gdT
chr6-41333631-41334623	5.35E-06	0.574505786	0.393	0.109	0.354344263	C12_gdT
chr1-67274467-67275119	5.39E-06	0.615053405	0.214	0.033	0.357198574	C12_gdT
chr15-85543578-85544124	6.13E-06	0.604995755	0.161	0.016	0.405990274	C12_gdT
chr12-113109841-113110461	6.41E-06	0.528431169	0.071	0.002	0.424409009	C12_gdT
chr8-120760131-120760347	6.91E-06	0.543830884	0.089	0.003	0.457455463	C12_gdT
chr5-14670021-14670244	7.29E-06	0.61563182	0.196	0.025	0.48261332	C12_gdT
chr12-103505200-103505705	8.12E-06	0.600689586	0.179	0.023	0.537769818	C12_gdT
chr19-51440683-51442204	8.14E-06	0.42094926	0.696	0.349	0.539079055	C12_gdT
chr14-71555214-71556263	8.23E-06	0.532593814	0.5	0.161	0.544749196	C12_gdT
chr19-18506164-18506898	8.33E-06	0.607445851	0.232	0.044	0.551361582	C12_gdT
chr7-100365442-100366078	8.48E-06	0.604449682	0.25	0.051	0.561772468	C12_gdT
chr11-134058092-134058651	9.13E-06	0.566702295	0.125	0.008	0.60472769	C12_gdT
chr9-134296152-134296471	9.59E-06	0.538050036	0.089	0.004	0.634923172	C12_gdT
chr19-41552622-41553241	9.69E-06	0.607601987	0.196	0.031	0.64165652	C12_gdT
chr2-233262272-233262763	1.03E-05	0.577655689	0.125	0.009	0.682015934	C12_gdT
chr16-47574094-47575038	1.08E-05	0.588664059	0.161	0.017	0.715329352	C12_gdT
chr4-26873014-26874048	1.10E-05	0.472071444	0.625	0.247	0.729318235	C12_gdT
chr12-120760382-120761097	1.11E-05	0.571712528	0.125	0.012	0.732569544	C12_gdT

chr8-22352361-22353147	1.13E-05	0.597386637	0.214	0.045	0.751580152	C12_gdT
chr16-11571884-11572994	1.18E-05	0.534251092	0.429	0.141	0.782179287	C12_gdT
chr6-87738725-87739077	1.35E-05	0.580396923	0.179	0.025	0.895727616	C12_gdT
chr3-46375800-46376062	1.38E-05	0.59337999	0.179	0.026	0.915192883	C12_gdT
chr15-50751157-50751770	1.42E-05	0.58851843	0.232	0.042	0.939258698	C12_gdT
chr7-2956465-2957000	1.50E-05	0.587443006	0.179	0.023	0.992615083	C12_gdT
chr9-7976019-7977225	1.57E-05	0.483291935	0.518	0.203	1	C12_gdT
chr7-70647532-70648779	1.59E-05	0.441680723	0.607	0.269	1	C12_gdT
chr9-76441189-76442171	1.60E-05	0.58048661	0.268	0.055	1	C12_gdT
chr2-29314625-29315567	1.65E-05	0.566781666	0.304	0.079	1	C12_gdT
chr2-201123817-201124500	1.67E-05	0.499442057	0.482	0.19	1	C12_gdT
chr3-188188807-188189323	1.71E-05	0.587066001	0.179	0.025	1	C12_gdT
chr6-37962614-37963073	1.71E-05	0.557947245	0.125	0.01	1	C12_gdT
chr1-67285881-67286493	2.13E-05	0.577571643	0.214	0.036	1	C12_gdT
chr19-6654211-6655291	2.30E-05	0.569706476	0.268	0.062	1	C12_gdT
chr5-151093444-151094730	2.31E-05	0.555645885	0.339	0.095	1	C12_gdT
chr19-45864035-45864395	2.38E-05	0.575849636	0.268	0.06	1	C12_gdT
chr20-8152876-8153675	2.44E-05	0.573046837	0.25	0.055	1	C12_gdT
chr12-9607470-9607951	2.46E-05	0.52419493	0.089	0.005	1	C12_gdT
chr8-47602305-47603195	2.50E-05	0.57316074	0.321	0.069	1	C12_gdT
chr12-92184750-92185479	2.50E-05	0.578242817	0.214	0.045	1	C12_gdT
chr5-177431202-177432128	2.53E-05	0.501180078	0.446	0.158	1	C12_gdT
chr19-19597343-19599475	2.64E-05	0.327532847	0.804	0.516	1	C12_gdT
chr22-30475020-30475303	2.68E-05	0.527346104	0.089	0.005	1	C12_gdT
chr2-102104121-102104811	2.74E-05	0.576344602	0.161	0.025	1	C12_gdT
chr15-76998760-76999035	2.84E-05	0.56543541	0.25	0.059	1	C12_gdT
chr12-104695184-104696080	3.03E-05	0.53785906	0.393	0.105	1	C12_gdT

chr5-176618946-176619534	3.09E-05	0.566560994	0.196	0.046	1	C12_gdT
chr11-126317563-126319327	3.09E-05	0.57313183	0.196	0.033	1	C12_gdT
chr2-195648650-195649108	3.32E-05	0.546700962	0.143	0.015	1	C12_gdT
chr10-91901490-91902766	3.54E-05	0.517927022	0.411	0.135	1	C12_gdT
chr17-78774580-78775296	3.60E-05	0.547354026	0.339	0.091	1	C12_gdT
chr8-120701911-120702512	3.65E-05	0.542732457	0.321	0.086	1	C12_gdT
chr14-22493224-22493428	3.68E-05	0.552062258	0.143	0.014	1	C12_gdT
chr14-65302754-65303253	3.78E-05	0.528052778	0.107	0.009	1	C12_gdT
chr10-79320988-79321466	3.79E-05	0.549744493	0.321	0.087	1	C12_gdT
chr11-43868541-43869348	3.81E-05	0.510177454	0.411	0.137	1	C12_gdT
chr17-40340819-40342040	3.86E-05	0.454308272	0.571	0.245	1	C12_gdT
chrX-100829494-100830107	3.95E-05	0.563678301	0.196	0.037	1	C12_gdT
chr19-10508610-10509056	4.03E-05	0.563964615	0.214	0.037	1	C12_gdT
chr14-22490569-22490935	4.04E-05	0.553958447	0.161	0.022	1	C12_gdT
chr13-30743263-30744731	4.14E-05	0.368590211	0.75	0.404	1	C12_gdT
chr14-22549641-22550456	4.16E-05	0.473552382	0.518	0.214	1	C12_gdT
chr19-18495529-18496851	4.21E-05	0.418850404	0.661	0.299	1	C12_gdT
chr7-129472354-129472649	4.31E-05	0.559813498	0.143	0.021	1	C12_gdT
chr2-105796788-105797775	4.33E-05	0.509922736	0.393	0.146	1	C12_gdT
chr1-227773874-227774764	4.37E-05	0.553069533	0.25	0.045	1	C12_gdT
chr1-23612919-23613923	4.40E-05	0.545138042	0.304	0.071	1	C12_gdT
chr15-65767840-65768161	4.49E-05	0.529812992	0.107	0.009	1	C12_gdT
chr8-80140552-80141193	4.68E-05	0.556735261	0.196	0.037	1	C12_gdT
chr10-124734092-124735011	4.77E-05	0.5293869	0.375	0.109	1	C12_gdT
chr2-190563037-190563452	4.94E-05	0.551264098	0.143	0.015	1	C12_gdT
chr11-114080069-114080617	5.24E-05	0.551103577	0.25	0.061	1	C12_gdT
chr5-142969726-142970112	5.29E-05	0.519125205	0.107	0.008	1	C12_gdT

chr5-142952236-142952623	5.41E-05	0.526525088	0.107	0.01	1	C12_gdT
chr20-34369172-34369614	5.48E-05	0.561364598	0.196	0.031	1	C12_gdT
chr19-35451253-35452114	5.53E-05	0.554468832	0.214	0.041	1	C12_gdT
chr16-19126775-19127535	5.74E-05	0.517944528	0.107	0.009	1	C12_gdT
chr13-99331868-99332312	5.85E-05	0.477369721	0.071	0.003	1	C12_gdT
chr6-111836647-111838431	5.88E-05	0.441092134	0.518	0.241	1	C12_gdT
chr7-2760260-2760895	5.92E-05	0.533855399	0.143	0.019	1	C12_gdT
chr22-26502518-26503530	6.03E-05	0.555625986	0.214	0.044	1	C12_gdT
chr5-172854270-172854650	6.05E-05	0.529741193	0.125	0.012	1	C12_gdT
chr8-79692759-79693641	6.09E-05	0.531970707	0.143	0.018	1	C12_gdT
chr9-89429240-89429535	6.41E-05	0.541441553	0.286	0.066	1	C12_gdT
chrX-66045891-66046281	6.48E-05	0.527874828	0.125	0.015	1	C12_gdT
chr15-91655609-91656225	6.49E-05	0.518949698	0.125	0.01	1	C12_gdT
chr22-38172640-38173190	6.61E-05	0.542318076	0.268	0.064	1	C12_gdT
chr3-122506465-122506791	7.03E-05	0.525750615	0.125	0.016	1	C12_gdT
chr11-118876224-118877225	7.09E-05	0.420635072	0.625	0.27	1	C12_gdT
chr14-22505745-22506198	7.14E-05	0.552434444	0.268	0.059	1	C12_gdT
chr4-82992467-82993104	7.16E-05	0.507566373	0.107	0.009	1	C12_gdT
chr18-45197402-45197754	7.30E-05	0.456124251	0.071	0.003	1	C12_gdT
chr19-19010383-19010932	7.47E-05	0.53542659	0.179	0.028	1	C12_gdT
chr5-143246101-143246326	7.64E-05	0.467905068	0.071	0.003	1	C12_gdT
chr7-38330039-38330430	7.68E-05	0.542581003	0.196	0.038	1	C12_gdT
chr20-8428580-8429320	7.73E-05	0.536417417	0.179	0.029	1	C12_gdT
chr20-34377039-34377460	7.94E-05	0.488411623	0.089	0.006	1	C12_gdT
chr9-79731448-79731764	8.03E-05	0.466667525	0.071	0.003	1	C12_gdT
chr4-184409454-184410304	8.22E-05	0.539949884	0.196	0.035	1	C12_gdT
chr1-32953534-32953739	8.28E-05	0.492814112	0.089	0.008	1	C12_gdT

chr19-52542666-52543433	8.55E-05	0.537292209	0.232	0.053	1	C12_gdT
chr17-8859170-8859387	8.64E-05	0.463474114	0.071	0.004	1	C12_gdT
chr21-42190068-42190339	9.01E-05	0.536091997	0.179	0.025	1	C12_gdT
chr19-10427839-10428546	9.11E-05	0.393113701	0.696	0.328	1	C12_gdT
chr1-101238754-101239821	9.53E-05	0.472505913	0.429	0.167	1	C12_gdT
chr1-84779982-84780278	9.77E-05	0.456833213	0.071	0.004	1	C12_gdT
chr12-49683691-49684376	9.77E-05	0.508459041	0.125	0.013	1	C12_gdT
chr2-101736320-101737299	9.95E-05	0.54823822	0.161	0.028	1	C12_gdT
chr2-86170445-86170685	0.000111267	0.494343241	0.089	0.005	1	C12_gdT
chr11-43830821-43831262	0.000111711	0.519593027	0.143	0.018	1	C12_gdT
chr17-1293537-1294629	0.000112599	0.439680516	0.536	0.218	1	C12_gdT
chrX-124238242-124238557	0.000112979	0.496771515	0.107	0.012	1	C12_gdT
chr4-14924192-14925342	0.000113367	0.496019858	0.107	0.011	1	C12_gdT
chr1-206579746-206580509	0.000114864	0.436276421	0.5	0.229	1	C12_gdT
chr2-191146024-191146339	0.00011748	0.53296132	0.179	0.029	1	C12_gdT
chr2-216606447-216607051	0.000117585	0.508189749	0.125	0.017	1	C12_gdT
chr12-75933913-75935024	0.000121515	0.510759642	0.321	0.099	1	C12_gdT
chr4-174538093-174538534	0.000126161	0.461148776	0.071	0.004	1	C12_gdT
chr2-102366662-102368134	0.000130913	0.463863411	0.429	0.156	1	C12_gdT
chr8-25250677-25251177	0.000131547	0.473894468	0.089	0.006	1	C12_gdT
chr20-8453892-8454778	0.000132694	0.516550551	0.161	0.025	1	C12_gdT
chr17-8868597-8868958	0.000132965	0.529682704	0.161	0.023	1	C12_gdT
chr14-55806678-55807685	0.000134204	0.476524643	0.393	0.13	1	C12_gdT
chr1-23720418-23720922	0.000135892	0.468003595	0.089	0.006	1	C12_gdT
chr10-11245507-11245804	0.000135923	0.502952966	0.125	0.014	1	C12_gdT
chr15-90361699-90363084	0.000136051	0.515027631	0.321	0.085	1	C12_gdT
chrX-48719963-48721208	0.000136447	0.514475272	0.268	0.066	1	C12_gdT

chr7-66124860-66126329	0.000141672	0.527801842	0.232	0.056	1	C12_gdT
chr3-113219673-113219997	0.000144683	0.409873635	0.054	0.001	1	C12_gdT
chr1-167439226-167439454	0.000146258	0.469833228	0.089	0.005	1	C12_gdT
chr10-35414158-35414918	0.000146724	0.518675917	0.143	0.023	1	C12_gdT
chr5-35899848-35900047	0.000147911	0.474121171	0.089	0.006	1	C12_gdT
chr13-80873600-80873916	0.000151896	0.450410225	0.071	0.005	1	C12_gdT
chr1-241706832-241707196	0.000152149	0.455022542	0.071	0.004	1	C12_gdT
chr15-91426345-91426776	0.00015303	0.473139587	0.089	0.007	1	C12_gdT
chr9-129656631-129657126	0.000153685	0.478394267	0.089	0.008	1	C12_gdT
chr2-190843881-190844683	0.000156079	0.512119435	0.143	0.024	1	C12_gdT
chr5-139651866-139652678	0.000156417	0.52359285	0.232	0.049	1	C12_gdT
chr1-145720593-145721452	0.000156462	0.527458534	0.196	0.035	1	C12_gdT
chr16-4526860-4527114	0.000156929	0.464264437	0.089	0.006	1	C12_gdT
chr7-29284955-29285803	0.000156943	0.514395191	0.232	0.054	1	C12_gdT
chr19-6661577-6662192	0.000158128	0.479647552	0.375	0.139	1	C12_gdT
chr8-55972878-55973349	0.000158279	0.489623527	0.107	0.012	1	C12_gdT
chr22-41603794-41604401	0.000160553	0.49906318	0.321	0.089	1	C12_gdT
chr10-32004822-32005264	0.000180666	0.476503625	0.089	0.008	1	C12_gdT
chr7-101099883-101100538	0.000191503	0.522085432	0.179	0.032	1	C12_gdT
chr15-83199281-83200411	0.00019594	0.468748989	0.089	0.008	1	C12_gdT
chr16-87860303-87860882	0.000199441	0.514876102	0.196	0.037	1	C12_gdT
chr17-32072069-32072303	0.00020031	0.442022775	0.071	0.004	1	C12_gdT
chr12-101795810-101796047	0.000204313	0.460622273	0.089	0.007	1	C12_gdT
chr22-30442148-30443165	0.000204623	0.511783863	0.25	0.064	1	C12_gdT
chr2-102094840-102095217	0.000204735	0.456819495	0.071	0.006	1	C12_gdT
chr20-53865883-53866489	0.000209824	0.47991643	0.107	0.011	1	C12_gdT
chr9-120924730-120926775	0.000210925	0.377470121	0.696	0.328	1	C12_gdT

chr1-168531516-168532245	0.000211518	0.513203154	0.196	0.05	1	C12_gdT
chr14-22483911-22484380	0.000212401	0.452327523	0.089	0.006	1	C12_gdT
chr10-110849609-110850576	0.000214116	0.504703669	0.232	0.057	1	C12_gdT
chr3-194584944-194585183	0.000215875	0.483454642	0.107	0.01	1	C12_gdT
chr10-104328762-104329932	0.000216893	0.333203912	0.75	0.402	1	C12_gdT
chr11-33722539-33723061	0.000221493	0.481413147	0.107	0.012	1	C12_gdT
chr13-98476074-98476882	0.000233945	0.3666594	0.643	0.33	1	C12_gdT
chr3-125821767-125822611	0.000237493	0.500893312	0.232	0.056	1	C12_gdT
chr7-112556680-112556922	0.00023915	0.410038901	0.054	0.002	1	C12_gdT
chr15-60649071-60650441	0.000241964	0.456130117	0.393	0.157	1	C12_gdT
chr2-240621247-240621949	0.000242326	0.491112293	0.339	0.104	1	C12_gdT
chr10-62680966-62681951	0.000243059	0.414269328	0.536	0.219	1	C12_gdT
chr10-17023184-17024161	0.00024587	0.393931237	0.607	0.276	1	C12_gdT
chr17-36206421-36206691	0.000249173	0.501615051	0.143	0.019	1	C12_gdT
chr3-44865368-44865902	0.000249938	0.490385484	0.143	0.019	1	C12_gdT
chr12-106747353-106748386	0.000252101	0.496335613	0.196	0.042	1	C12_gdT
chr22-22735108-22735543	0.000254284	0.480215398	0.107	0.012	1	C12_gdT
chr7-129535250-129535498	0.000258066	0.467238043	0.107	0.01	1	C12_gdT
chr12-121786315-121786824	0.000260651	0.488051669	0.321	0.109	1	C12_gdT
chr11-67434359-67434670	0.00026179	0.503417253	0.214	0.046	1	C12_gdT
chr19-8568732-8569769	0.000263417	0.425289183	0.518	0.209	1	C12_gdT
chr5-42960884-42961255	0.00026437	0.432338925	0.071	0.005	1	C12_gdT
chr1-156807576-156808011	0.000268036	0.510793941	0.196	0.045	1	C12_gdT
chr10-63210755-63211890	0.000268457	0.503490268	0.196	0.042	1	C12_gdT
chr5-139669668-139670814	0.000272586	0.503524448	0.196	0.041	1	C12_gdT
chr22-40323824-40325656	0.000274076	0.440967099	0.464	0.177	1	C12_gdT
chr6-130218012-130218662	0.000275202	0.50177974	0.232	0.059	1	C12_gdT

chr14-102057207-102058015	0.000276905	0.508522837	0.161	0.032	1	C12_gdT
chr7-44073866-44074372	0.00027941	0.479904678	0.125	0.017	1	C12_gdT
chr16-28473708-28474021	0.000281426	0.481012759	0.107	0.012	1	C12_gdT
chr17-8867499-8868032	0.00028197	0.482012895	0.107	0.012	1	C12_gdT
chr17-76561368-76561601	0.000283645	0.498723544	0.196	0.036	1	C12_gdT
chr3-122533515-122534313	0.000286709	0.473342345	0.304	0.102	1	C12_gdT
chrX-124341373-124341798	0.00028726	0.501317779	0.196	0.037	1	C12_gdT
chr1-41919702-41920973	0.000293737	0.404976923	0.518	0.253	1	C12_gdT
chr1-172662379-172663209	0.000303306	0.38110502	0.554	0.293	1	C12_gdT
chr6-138465011-138465272	0.000304396	0.436925845	0.071	0.005	1	C12_gdT
chr3-187987725-187988394	0.0003059	0.469475378	0.357	0.121	1	C12_gdT
chr16-75594517-75594751	0.000309006	0.443384855	0.071	0.004	1	C12_gdT
chr20-31666573-31667048	0.000314982	0.49439862	0.179	0.033	1	C12_gdT
chr13-94674966-94675190	0.000317439	0.393078156	0.054	0.002	1	C12_gdT
chr21-5101563-5102080	0.00031781	0.456700529	0.375	0.147	1	C12_gdT
chrX-9205494-9206106	0.000317889	0.460490089	0.107	0.012	1	C12_gdT
chr19-10759280-10760603	0.000320648	0.368661804	0.661	0.319	1	C12_gdT
chr15-31365574-31366244	0.000323666	0.433154085	0.429	0.18	1	C12_gdT
chr12-51368459-51369816	0.00032562	0.38617189	0.554	0.273	1	C12_gdT
chr2-160301190-160301740	0.00032914	0.485248526	0.125	0.015	1	C12_gdT
chr17-66191464-66192637	0.000330052	0.451761859	0.411	0.148	1	C12_gdT
chr15-40769412-40769903	0.000342661	0.504267255	0.196	0.042	1	C12_gdT
chr7-150432972-150433521	0.000347816	0.454204358	0.089	0.006	1	C12_gdT
chr5-159456663-159457136	0.000350228	0.493741619	0.196	0.038	1	C12_gdT
chr12-6074350-6075538	0.000352161	0.482334992	0.232	0.059	1	C12_gdT
chr7-132580315-132581031	0.000353287	0.468958848	0.107	0.013	1	C12_gdT
chr7-6234416-6234710	0.000358633	0.448521911	0.089	0.007	1	C12_gdT

chr8-130244112-130245260	0.000358709	0.469447221	0.304	0.099	1	C12_gdT
chr1-59071296-59071726	0.000366928	0.425860153	0.071	0.006	1	C12_gdT
chr7-70659280-70659528	0.000373937	0.454919805	0.107	0.01	1	C12_gdT
chr4-146159402-146160036	0.000394452	0.428701123	0.071	0.005	1	C12_gdT
chrX-41736880-41737441	0.000395229	0.466594505	0.107	0.013	1	C12_gdT
chr2-111115978-111116265	0.000404227	0.49531472	0.161	0.029	1	C12_gdT
chr2-37648015-37648964	0.000415364	0.484955806	0.232	0.07	1	C12_gdT
chr9-89521906-89522786	0.000418533	0.362400685	0.625	0.314	1	C12_gdT
chr6-37866579-37867379	0.000419779	0.494200366	0.143	0.024	1	C12_gdT
chrX-52346763-52347101	0.000421757	0.421328626	0.071	0.006	1	C12_gdT
chr2-9769163-9769612	0.000434164	0.472315327	0.107	0.012	1	C12_gdT
chr12-40146855-40147072	0.000434433	0.406207056	0.071	0.004	1	C12_gdT
chr12-128914848-128915410	0.000443563	0.48512664	0.179	0.039	1	C12_gdT
chr20-45052951-45053496	0.000445993	0.473925801	0.143	0.027	1	C12_gdT
chr9-123282388-123282836	0.000448359	0.406838498	0.071	0.004	1	C12_gdT
chr13-80385889-80386379	0.000459294	0.430379234	0.089	0.009	1	C12_gdT
chr10-7329224-7329606	0.000463817	0.4223874	0.071	0.004	1	C12_gdT
chr6-135087436-135089407	0.000474687	0.422536252	0.429	0.177	1	C12_gdT
chr11-14357827-14359757	0.000476513	0.440082149	0.411	0.147	1	C12_gdT
chr1-91555389-91556947	0.00047947	0.313803883	0.75	0.411	1	C12_gdT
chr2-27719900-27720532	0.000480507	0.488755184	0.196	0.052	1	C12_gdT
chr9-22452474-22452706	0.000484505	0.444439424	0.089	0.007	1	C12_gdT
chr2-216139930-216140201	0.000484961	0.440733678	0.089	0.007	1	C12_gdT
chr21-46540080-46540759	0.000485093	0.474216102	0.161	0.03	1	C12_gdT
chr2-74185527-74186302	0.000488079	0.464736251	0.143	0.021	1	C12_gdT
chr15-65303858-65305375	0.00048851	0.364187437	0.607	0.317	1	C12_gdT
chr13-86760706-86760940	0.000489208	0.421475402	0.071	0.006	1	C12_gdT

chr5-80837635-80838079	0.000490651	0.431664218	0.107	0.011	1	C12_gdT
chr4-2961910-2962492	0.000495173	0.486941834	0.232	0.061	1	C12_gdT
chr7-38258964-38259520	0.000502579	0.449256645	0.089	0.011	1	C12_gdT
chr10-70168878-70169329	0.000504239	0.475704778	0.214	0.053	1	C12_gdT
chr17-31008116-31009332	0.000516482	0.47883536	0.214	0.065	1	C12_gdT
chr14-22485136-22485398	0.000527243	0.408208837	0.071	0.005	1	C12_gdT
chr2-37656353-37656863	0.000527614	0.47809958	0.143	0.025	1	C12_gdT
chr3-124974691-124974988	0.000529114	0.417724152	0.071	0.005	1	C12_gdT
chr15-85649337-85649693	0.000538853	0.488664931	0.179	0.04	1	C12_gdT
chr19-19602171-19602464	0.000540828	0.480272621	0.268	0.074	1	C12_gdT
chr9-5851118-5851862	0.000541032	0.473650757	0.25	0.075	1	C12_gdT
chr16-81808839-81809374	0.00054876	0.467113879	0.143	0.024	1	C12_gdT
chr8-28411402-28412572	0.000553664	0.440680867	0.375	0.141	1	C12_gdT
chr3-170145270-170145578	0.000556778	0.446212396	0.089	0.011	1	C12_gdT
chr17-3693779-3694243	0.000563066	0.480890801	0.161	0.029	1	C12_gdT
chr3-5006515-5007056	0.000563424	0.465564751	0.286	0.083	1	C12_gdT
chr8-89992783-89993034	0.000563661	0.418532104	0.071	0.005	1	C12_gdT
chr3-114809564-114810216	0.000568455	0.468003455	0.161	0.028	1	C12_gdT
chr19-16588384-16588717	0.000574487	0.466769504	0.268	0.091	1	C12_gdT
chr10-14586783-14587224	0.000577102	0.449258853	0.304	0.119	1	C12_gdT
chr16-81810967-81811207	0.000586914	0.412434202	0.071	0.005	1	C12_gdT
chr2-161122832-161123886	0.000587659	0.482991149	0.179	0.033	1	C12_gdT
chr18-79191775-79192312	0.000589647	0.449412791	0.107	0.017	1	C12_gdT
chr1-7752513-7753133	0.000594186	0.436020858	0.089	0.008	1	C12_gdT
chrX-136586277-136586792	0.000596417	0.484159312	0.214	0.053	1	C12_gdT
chr1-167499216-167500049	0.00060088	0.472325258	0.161	0.028	1	C12_gdT
chr19-18843004-18843708	0.000603918	0.476298196	0.25	0.064	1	C12_gdT

chr2-46144201-46144849	0.000605211	0.462964849	0.143	0.022	1	C12_gdT
chr7-38244539-38244947	0.000612028	0.462930364	0.125	0.021	1	C12_gdT
chr10-71434298-71434874	0.000623036	0.447763505	0.107	0.013	1	C12_gdT
chr15-65785273-65785477	0.000631777	0.432884617	0.089	0.009	1	C12_gdT
chr11-118841072-118841881	0.000633745	0.47574462	0.214	0.052	1	C12_gdT
chr3-98460173-98460545	0.000634146	0.399150945	0.071	0.005	1	C12_gdT
chr1-38009293-38009629	0.000634871	0.4680994	0.214	0.048	1	C12_gdT
chr1-160874983-160875219	0.000638213	0.439493914	0.089	0.009	1	C12_gdT
chr6-107823021-107823382	0.000639137	0.44110122	0.339	0.123	1	C12_gdT
chr15-60799947-60800644	0.000641002	0.47227686	0.196	0.043	1	C12_gdT
chr10-43815677-43816850	0.000642699	0.467454332	0.268	0.072	1	C12_gdT
chr11-64874272-64874585	0.000649732	0.453899461	0.286	0.1	1	C12_gdT
chr12-67990863-67991524	0.000666457	0.467537932	0.179	0.04	1	C12_gdT
chr20-8131554-8132972	0.000668414	0.376442347	0.571	0.246	1	C12_gdT
chr13-23979525-23980561	0.00066916	0.462715828	0.25	0.075	1	C12_gdT
chr8-22704470-22705845	0.000674096	0.432906581	0.375	0.154	1	C12_gdT
chr12-67698503-67699393	0.000677313	0.417644723	0.411	0.165	1	C12_gdT
chr2-37635061-37636193	0.000679698	0.35729262	0.589	0.303	1	C12_gdT
chr15-60582275-60582734	0.000699364	0.43441999	0.393	0.149	1	C12_gdT
chr6-25872573-25873201	0.000708023	0.456480296	0.125	0.02	1	C12_gdT
chr12-89375509-89376216	0.000711842	0.472157579	0.179	0.042	1	C12_gdT
chr12-57065035-57065262	0.000713935	0.446157595	0.107	0.012	1	C12_gdT
chr10-11225823-11227785	0.000718898	0.395372907	0.464	0.225	1	C12_gdT
chr6-36357766-36358115	0.000721707	0.380787997	0.054	0.002	1	C12_gdT
chrX-48712653-48714151	0.000722146	0.43935089	0.321	0.118	1	C12_gdT
chr12-65171734-65172149	0.000725396	0.456249	0.232	0.066	1	C12_gdT
chr22-35900483-35901280	0.000726063	0.442801881	0.107	0.014	1	C12_gdT

chr2-25831180-25832151	0.000743698	0.474429392	0.179	0.04	1	C12_gdT
chr2-230757102-230757719	0.000744601	0.440544158	0.107	0.012	1	C12_gdT
chr15-65778319-65778912	0.000750243	0.460529279	0.143	0.025	1	C12_gdT
chr6-152631074-152631385	0.000767541	0.430413549	0.089	0.012	1	C12_gdT
chr17-49218801-49219711	0.000769943	0.430570903	0.357	0.134	1	C12_gdT
chr16-81798165-81799096	0.000785465	0.45556598	0.125	0.015	1	C12_gdT
chr2-7588336-7588626	0.000786592	0.410402375	0.071	0.006	1	C12_gdT
chr17-16285682-16286793	0.000791926	0.382943157	0.5	0.243	1	C12_gdT
chr18-54260719-54260939	0.000803981	0.417874001	0.089	0.008	1	C12_gdT
chr5-119357659-119358055	0.000825708	0.456916415	0.161	0.028	1	C12_gdT
chr17-17063461-17063848	0.000828302	0.403672532	0.071	0.006	1	C12_gdT
chr2-173370153-173370582	0.000831872	0.362363195	0.054	0.003	1	C12_gdT
chr17-57113916-57114365	0.000832207	0.440388594	0.107	0.013	1	C12_gdT
chr4-1055352-1056245	0.000836696	0.416830946	0.393	0.168	1	C12_gdT
chr8-133568409-133569161	0.000837967	0.379129724	0.554	0.25	1	C12_gdT
chr3-17022614-17023528	0.000850926	0.460493564	0.232	0.057	1	C12_gdT
chr3-196643223-196643491	0.000855023	0.464625174	0.179	0.038	1	C12_gdT
chr19-9583165-9584752	0.000855649	0.452906599	0.286	0.098	1	C12_gdT
chr1-231973820-231974224	0.000856838	0.41617407	0.089	0.008	1	C12_gdT
chr14-71395022-71395434	0.000863679	0.428803953	0.089	0.012	1	C12_gdT
chr15-43440708-43441197	0.000865538	0.459288074	0.125	0.023	1	C12_gdT
chr7-29189734-29190058	0.000870181	0.451459082	0.125	0.017	1	C12_gdT
chr6-75268098-75268591	0.000880974	0.428293996	0.089	0.011	1	C12_gdT
chr1-67226702-67226959	0.000883125	0.356067382	0.054	0.002	1	C12_gdT
chr8-129999910-130000245	0.000891189	0.455450992	0.161	0.03	1	C12_gdT
chr13-97238360-97239162	0.00090529	0.43943293	0.125	0.019	1	C12_gdT
chr10-7397048-7397343	0.000918155	0.427489461	0.089	0.011	1	C12_gdT

chr22-30705735-30706468	0.000919516	0.437391789	0.107	0.017	1	C12_gdT
chr5-172918391-172919326	0.000923417	0.433250175	0.125	0.023	1	C12_gdT
chr9-127746873-127747187	0.000931586	0.425449303	0.089	0.01	1	C12_gdT
chr5-88203873-88204795	0.000935662	0.450507658	0.232	0.068	1	C12_gdT
chr16-30895838-30896107	0.000938804	0.449954069	0.125	0.02	1	C12_gdT
chr3-151304982-151305677	0.000948982	0.442285018	0.107	0.015	1	C12_gdT
chr1-113875427-113875970	0.000970744	0.447762582	0.214	0.053	1	C12_gdT
chr2-102417600-102418727	0.000971337	0.457225057	0.25	0.081	1	C12_gdT
chr5-134131113-134131679	0.000981247	0.460758249	0.25	0.073	1	C12_gdT
chr4-26197042-26197840	0.000986999	0.45040151	0.304	0.095	1	C12_gdT
chr14-22226367-22226994	0.000995029	0.434839011	0.125	0.019	1	C12_gdT
chr10-104260182-104260973	0.000996601	0.457096196	0.161	0.034	1	C12_gdT
chr10-43417319-43418368	0.000996722	0.459398938	0.232	0.059	1	C12_gdT
chr17-64061214-64061682	0.001000523	0.448403932	0.143	0.026	1	C12_gdT
chr15-45456255-45458339	0.001001764	0.358100019	0.607	0.288	1	C12_gdT
chr19-54247601-54248489	0.001001962	0.427455847	0.089	0.011	1	C12_gdT
chr7-74566759-74567027	0.001004346	0.437138322	0.107	0.016	1	C12_gdT
chr14-61471720-61472745	0.001007859	0.414018539	0.411	0.174	1	C12_gdT
chr15-55250527-55251223	0.001020014	0.463384651	0.143	0.024	1	C12_gdT
chr8-127977585-127978166	0.001020366	0.398905883	0.429	0.187	1	C12_gdT
chr8-120740532-120741120	0.001033722	0.440940484	0.143	0.03	1	C12_gdT
chr14-73785348-73786389	0.001034525	0.438311568	0.339	0.118	1	C12_gdT
chr11-118395956-118396457	0.001037943	0.440535884	0.125	0.019	1	C12_gdT
chr1-92475860-92476631	0.001040923	0.441543919	0.25	0.081	1	C12_gdT
chr17-40605132-40605713	0.001050055	0.437101046	0.321	0.116	1	C12_gdT
chr16-30875994-30876725	0.001050839	0.452476774	0.25	0.078	1	C12_gdT
chr2-105776004-105776613	0.001053553	0.449955446	0.179	0.043	1	C12_gdT

chr10-133442430-133442744	0.001056102	0.422482158	0.089	0.008	1	C12_gdT
chr9-33161023-33161334	0.00105724	0.458346058	0.214	0.057	1	C12_gdT
chr20-4794857-4795746	0.001059081	0.446687554	0.125	0.02	1	C12_gdT
chr8-70160002-70160911	0.00106528	0.438886477	0.286	0.086	1	C12_gdT
chr16-21608682-21609240	0.001066567	0.417730755	0.089	0.01	1	C12_gdT
chr19-38514813-38515614	0.001068845	0.455161473	0.214	0.061	1	C12_gdT
chr7-100025789-100026459	0.001074913	0.461376775	0.179	0.043	1	C12_gdT
chr10-22660810-22661009	0.001088707	0.415323715	0.089	0.009	1	C12_gdT
chr7-142800100-142800383	0.001092757	0.456653806	0.25	0.072	1	C12_gdT
chr6-159973884-159974287	0.001099279	0.456122567	0.179	0.043	1	C12_gdT
chr19-52397237-52398306	0.001104256	0.447056652	0.286	0.095	1	C12_gdT
chr20-33681952-33682189	0.001108431	0.430902406	0.107	0.014	1	C12_gdT
chr5-40276891-40277803	0.001108659	0.450775196	0.125	0.019	1	C12_gdT
chr17-48814475-48814700	0.001110069	0.449037211	0.125	0.019	1	C12_gdT
chr14-22492060-22492325	0.001116984	0.443928768	0.143	0.025	1	C12_gdT
chr22-19955300-19956083	0.001126032	0.421992694	0.107	0.014	1	C12_gdT
chrY-12490607-12491168	0.001131666	0.412217308	0.089	0.011	1	C12_gdT
chr15-90306068-90306838	0.001133095	0.430792207	0.107	0.012	1	C12_gdT
chr20-8213754-8214311	0.001137526	0.430614458	0.107	0.017	1	C12_gdT
chr3-28250942-28251307	0.001139773	0.440107326	0.125	0.021	1	C12_gdT
chr1-161421835-161422272	0.001140742	0.424092601	0.107	0.013	1	C12_gdT
chr3-128758068-128758420	0.001155867	0.40040588	0.071	0.005	1	C12_gdT
chr3-98513314-98513520	0.0011578	0.401744407	0.071	0.005	1	C12_gdT
chr13-99431921-99433213	0.001159985	0.376768627	0.464	0.233	1	C12_gdT
chr6-139186021-139186633	0.001164791	0.461822478	0.196	0.052	1	C12_gdT
chr8-122920536-122920835	0.001167322	0.415072085	0.089	0.01	1	C12_gdT
chr18-22169007-22170092	0.001170348	0.44766637	0.161	0.034	1	C12_gdT

chr8-37881639-37882244	0.001183846	0.453088785	0.232	0.072	1	C12_gdT
chr6-90397638-90398606	0.001187212	0.344530205	0.607	0.293	1	C12_gdT
chr6-15296710-15296909	0.00119095	0.384780366	0.071	0.005	1	C12_gdT
chr22-46537014-46537868	0.001198027	0.440535382	0.125	0.02	1	C12_gdT
chr1-16078627-16079659	0.001199855	0.433904974	0.268	0.105	1	C12_gdT
chr6-46062057-46062579	0.001207947	0.416882596	0.089	0.012	1	C12_gdT
chr7-99897713-99898376	0.001219965	0.386601314	0.071	0.005	1	C12_gdT
chr9-93146601-93147045	0.001226487	0.436822986	0.125	0.022	1	C12_gdT
chr1-15347152-15347503	0.001227525	0.454565671	0.143	0.026	1	C12_gdT
chr3-119293419-119295316	0.00122802	0.321456405	0.625	0.355	1	C12_gdT
chr5-14817028-14817417	0.001229554	0.447474545	0.125	0.021	1	C12_gdT
chr17-66636083-66636332	0.001236797	0.362386135	0.054	0.003	1	C12_gdT
chr8-130278687-130279387	0.001240677	0.451937499	0.196	0.058	1	C12_gdT
chr19-18494173-18494550	0.001243294	0.454369222	0.214	0.052	1	C12_gdT
chr2-112630731-112631767	0.001248474	0.382742705	0.464	0.215	1	C12_gdT
chr12-68617547-68617845	0.001249866	0.450411222	0.179	0.035	1	C12_gdT
chr7-142711254-142712290	0.001252525	0.447459283	0.25	0.074	1	C12_gdT
chr5-68378254-68378468	0.001252682	0.398396657	0.071	0.006	1	C12_gdT
chr3-46187896-46188168	0.001253595	0.387834006	0.071	0.006	1	C12_gdT
chr20-36563866-36564414	0.00126517	0.420153223	0.107	0.019	1	C12_gdT
chr17-67244665-67247444	0.00126762	0.320070424	0.643	0.363	1	C12_gdT
chr13-46149216-46150177	0.001270762	0.454855512	0.143	0.031	1	C12_gdT
chr6-16694854-16695065	0.001272163	0.440415824	0.125	0.023	1	C12_gdT
chrX-56812524-56813906	0.001275606	0.427457795	0.232	0.094	1	C12_gdT
chr17-40542665-40543122	0.001279566	0.451348533	0.179	0.041	1	C12_gdT
chr15-85542752-85543106	0.001280824	0.431602392	0.107	0.013	1	C12_gdT
chr4-16884740-16885360	0.001292446	0.39801832	0.071	0.01	1	C12_gdT

chr12-96445383-96446719	0.001300899	0.333529472	0.607	0.32	1	C12_gdT
chr11-104907473-104908236	0.001301139	0.44541027	0.161	0.034	1	C12_gdT
chr14-24171285-24172216	0.001301295	0.425303324	0.321	0.106	1	C12_gdT
chr13-32314608-32316165	0.001315679	0.32258339	0.607	0.341	1	C12_gdT
chr7-150668046-150668980	0.001374358	0.361562721	0.536	0.248	1	C12_gdT
chr1-114458205-114458558	0.001378847	0.399234611	0.071	0.006	1	C12_gdT
chr2-162022621-162022859	0.001385274	0.397148171	0.071	0.005	1	C12_gdT
chr13-26930143-26930579	0.001397102	0.388851438	0.071	0.007	1	C12_gdT
chr19-51380699-51381214	0.001397262	0.452650688	0.161	0.031	1	C12_gdT
chr12-51323030-51324479	0.001426462	0.403409803	0.393	0.163	1	C12_gdT
chr13-100392631-100393083	0.001427412	0.426157098	0.107	0.021	1	C12_gdT
chr10-68406394-68407546	0.001454416	0.39273625	0.464	0.188	1	C12_gdT
chr10-132615857-132616861	0.001478435	0.42505998	0.268	0.093	1	C12_gdT
chr8-98011595-98011948	0.001486844	0.354490969	0.054	0.003	1	C12_gdT
chrX-19797458-19798969	0.00149014	0.443940737	0.268	0.086	1	C12_gdT
chr19-4914033-4914289	0.001502409	0.44595925	0.196	0.049	1	C12_gdT
chr17-4043473-4044265	0.001502655	0.442051437	0.232	0.066	1	C12_gdT
chr20-43925156-43925490	0.00150869	0.380760477	0.071	0.006	1	C12_gdT
chr14-52531476-52531769	0.001510232	0.385103856	0.071	0.007	1	C12_gdT
chr17-47850649-47851512	0.001512447	0.450621506	0.214	0.054	1	C12_gdT
chr6-143555040-143555913	0.001525727	0.436811494	0.143	0.027	1	C12_gdT
chr1-45749923-45751077	0.00153544	0.35330999	0.518	0.275	1	C12_gdT
chr16-89330215-89330835	0.001561888	0.44125338	0.161	0.041	1	C12_gdT
chr13-79194162-79194689	0.001571991	0.433335858	0.179	0.046	1	C12_gdT
chr20-28602102-28603285	0.001590088	0.381937111	0.446	0.184	1	C12_gdT
chr9-89436970-89437696	0.001612025	0.424098216	0.286	0.105	1	C12_gdT
chr2-233084385-233084790	0.001618572	0.437000807	0.232	0.073	1	C12_gdT

chrX-19624678-19625056	0.001648255	0.444066863	0.125	0.026	1	C12_gdT
chr10-79244837-79245183	0.001651444	0.437519804	0.161	0.038	1	C12_gdT
chr2-240686139-240686655	0.001655327	0.379216469	0.071	0.005	1	C12_gdT
chr22-43288577-43289321	0.001666545	0.406215013	0.089	0.01	1	C12_gdT
chrX-106481347-106481550	0.001669866	0.36495834	0.071	0.005	1	C12_gdT
chr19-3811991-3812804	0.001670078	0.408779466	0.339	0.137	1	C12_gdT
chr17-58328085-58329156	0.001672341	0.379273217	0.446	0.2	1	C12_gdT
chr15-58979334-58979571	0.001690929	0.388142776	0.071	0.007	1	C12_gdT
chr17-35529916-35530180	0.001693709	0.434357738	0.161	0.042	1	C12_gdT
chr19-4638832-4639658	0.001695746	0.292335448	0.714	0.414	1	C12_gdT
chr11-35282388-35282844	0.001700366	0.419845812	0.089	0.013	1	C12_gdT
chr11-63535740-63535985	0.001721555	0.438383583	0.161	0.036	1	C12_gdT
chr9-7879413-7880123	0.001728774	0.412190127	0.107	0.016	1	C12_gdT
chr5-131530778-131531117	0.001728823	0.426428642	0.107	0.016	1	C12_gdT
chr8-130444438-130445389	0.001740676	0.437306791	0.214	0.064	1	C12_gdT
chr22-23182502-23183005	0.001746953	0.436599844	0.214	0.055	1	C12_gdT
chr19-18303414-18305621	0.001758132	0.304843687	0.696	0.385	1	C12_gdT
chr3-122271399-122271857	0.001764383	0.380287358	0.071	0.008	1	C12_gdT
chr6-36033899-36034235	0.001770605	0.410755722	0.107	0.014	1	C12_gdT
chr5-87097721-87097993	0.001776057	0.402264357	0.089	0.011	1	C12_gdT
chr8-126421087-126421366	0.001776278	0.346498858	0.054	0.003	1	C12_gdT
chr1-31891314-31891567	0.001776979	0.410593543	0.089	0.011	1	C12_gdT
chr5-148832053-148832442	0.001777175	0.432526994	0.143	0.034	1	C12_gdT
chr17-48478011-48478796	0.001786594	0.435725657	0.179	0.05	1	C12_gdT
chr7-74660749-74661226	0.001788048	0.433455705	0.214	0.068	1	C12_gdT
chr21-46094004-46094262	0.001804185	0.369795033	0.071	0.006	1	C12_gdT
chr1-12018812-12020009	0.001809808	0.301675311	0.661	0.394	1	C12_gdT

chr17-74463106-74464291	0.001813927	0.394422829	0.429	0.173	1	C12_gdT
chr22-42201650-42202997	0.001821315	0.439978076	0.179	0.051	1	C12_gdT
chr2-230668081-230668614	0.001821699	0.439565588	0.179	0.051	1	C12_gdT
chr14-77738107-77738924	0.001822399	0.434577077	0.268	0.083	1	C12_gdT
chr2-177251782-177252365	0.001823504	0.429175282	0.214	0.075	1	C12_gdT
chr15-40873579-40874406	0.001831977	0.4409422	0.196	0.057	1	C12_gdT
chr19-38551270-38551800	0.001838232	0.419147861	0.125	0.025	1	C12_gdT
chr3-125018709-125019577	0.001842559	0.392207286	0.089	0.009	1	C12_gdT
chr2-160411215-160411471	0.001842909	0.433107939	0.161	0.032	1	C12_gdT
chr13-51861686-51862120	0.001847995	0.406913513	0.089	0.012	1	C12_gdT
chr12-120199930-120200241	0.001852119	0.446264061	0.179	0.042	1	C12_gdT
chr6-137217815-137219653	0.001856158	0.315914095	0.625	0.347	1	C12_gdT
chr16-2065231-2065621	0.00186573	0.439621374	0.161	0.038	1	C12_gdT
chr4-26787791-26788583	0.001867957	0.413597942	0.107	0.019	1	C12_gdT
chr13-99380817-99381487	0.001878985	0.419294693	0.286	0.089	1	C12_gdT
chr2-190792601-190793200	0.001883511	0.393012373	0.089	0.012	1	C12_gdT
chr19-10423996-10424554	0.001895651	0.431632829	0.286	0.096	1	C12_gdT
chr13-79480488-79482048	0.001896724	0.335395139	0.571	0.285	1	C12_gdT
chr12-122901581-122901940	0.001906127	0.410648226	0.125	0.019	1	C12_gdT
chr17-32126847-32127883	0.001908277	0.400222372	0.339	0.145	1	C12_gdT
chr1-160741200-160741501	0.001917266	0.423752098	0.161	0.035	1	C12_gdT
chr11-3824292-3825056	0.001936902	0.428385189	0.25	0.077	1	C12_gdT
chr1-12595053-12596115	0.001940212	0.412492621	0.107	0.016	1	C12_gdT
chr15-39716497-39716827	0.001941921	0.375058256	0.071	0.007	1	C12_gdT
chr20-1696561-1697073	0.001946724	0.401979075	0.089	0.009	1	C12_gdT
chr8-30094713-30095589	0.001947109	0.412327004	0.321	0.137	1	C12_gdT
chrX-1661077-1662086	0.001950912	0.36632082	0.429	0.208	1	C12_gdT

chr5-68405067-68405353	0.001951754	0.381365182	0.071	0.006	1	C12_gdT
chr9-33141372-33141731	0.001954269	0.400727389	0.107	0.017	1	C12_gdT
chr3-119149421-119149882	0.001954997	0.411941597	0.107	0.018	1	C12_gdT
chr15-84734780-84736757	0.00195919	0.36944563	0.518	0.224	1	C12_gdT
chr17-58152919-58153368	0.001962777	0.420250168	0.143	0.031	1	C12_gdT
chr7-127745393-127745836	0.001968536	0.413233519	0.107	0.016	1	C12_gdT
chr5-131252464-131253214	0.001972945	0.356126938	0.536	0.245	1	C12_gdT
chr11-44609176-44609719	0.001975067	0.42674105	0.179	0.047	1	C12_gdT
chr19-18328035-18328828	0.001982311	0.402798411	0.393	0.155	1	C12_gdT
chr5-143775339-143775596	0.002002475	0.359953824	0.054	0.005	1	C12_gdT
chr2-239399495-239399715	0.00202618	0.417682246	0.107	0.021	1	C12_gdT
chr1-42661118-42661317	0.002027232	0.393803023	0.089	0.012	1	C12_gdT
chr19-36572322-36573801	0.002048637	0.329908761	0.571	0.305	1	C12_gdT
chr1-67332642-67333587	0.002085078	0.386809614	0.357	0.15	1	C12_gdT
chr2-196291609-196292654	0.002094664	0.394738458	0.089	0.013	1	C12_gdT
chr21-33931016-33932182	0.00211654	0.348338825	0.536	0.261	1	C12_gdT
chr19-4264440-4265088	0.002121045	0.416332633	0.125	0.024	1	C12_gdT
chr8-141286861-141287442	0.002140557	0.381568721	0.089	0.013	1	C12_gdT
chr1-23634631-23635668	0.002148605	0.434694301	0.214	0.058	1	C12_gdT
chr9-125700786-125701665	0.002164048	0.427434344	0.125	0.022	1	C12_gdT
chr3-46538435-46539588	0.002165169	0.299644344	0.643	0.374	1	C12_gdT
chr18-23994252-23994859	0.002180955	0.435178571	0.214	0.064	1	C12_gdT
chr14-92576844-92577715	0.002181447	0.428812605	0.143	0.03	1	C12_gdT
chr14-21230581-21231398	0.002183063	0.416177905	0.268	0.092	1	C12_gdT
chr2-98454539-98455897	0.002183731	0.41405891	0.357	0.124	1	C12_gdT
chr3-138744245-138744573	0.002197704	0.362356852	0.054	0.005	1	C12_gdT
chr4-80182137-80182480	0.002200896	0.427283229	0.161	0.036	1	C12_gdT

chr12-13067135-13067603	0.002213497	0.372308572	0.071	0.008	1	C12_gdT
chr6-161393324-161394382	0.002213926	0.412113087	0.232	0.074	1	C12_gdT
chr6-41961638-41961951	0.002219127	0.390106133	0.089	0.015	1	C12_gdT
chr9-105127415-105128277	0.002221389	0.377580569	0.411	0.174	1	C12_gdT
chr17-32550768-32551648	0.002222685	0.410717	0.107	0.019	1	C12_gdT
chr9-132908300-132909051	0.002228723	0.422366715	0.143	0.034	1	C12_gdT
chrX-9355775-9356065	0.002233337	0.423281825	0.125	0.027	1	C12_gdT
chr19-42563403-42563636	0.002252497	0.372749557	0.071	0.007	1	C12_gdT
chr8-28077011-28077297	0.002255205	0.42413281	0.125	0.021	1	C12_gdT
chr11-102547000-102547393	0.002260244	0.330997934	0.054	0.004	1	C12_gdT
chr6-108966795-108967505	0.002265333	0.405613721	0.107	0.017	1	C12_gdT
chr10-1062892-1063806	0.002276854	0.424033026	0.179	0.045	1	C12_gdT
chr14-60151344-60152134	0.00228988	0.393074655	0.375	0.15	1	C12_gdT
chr3-98536479-98536900	0.002293693	0.413982436	0.125	0.023	1	C12_gdT
chr6-134435891-134436747	0.002295776	0.411254842	0.321	0.106	1	C12_gdT
chr2-28544631-28545400	0.002304915	0.423863957	0.125	0.023	1	C12_gdT
chr15-31265683-31266778	0.002308391	0.407824483	0.286	0.1	1	C12_gdT
chrX-136935287-136935969	0.002319273	0.399732355	0.089	0.014	1	C12_gdT
chr12-96427318-96427635	0.002324626	0.3766667	0.071	0.007	1	C12_gdT
chr16-89097944-89098443	0.002325957	0.418298341	0.143	0.033	1	C12_gdT
chr10-3093075-3093392	0.002326845	0.399165995	0.107	0.018	1	C12_gdT
chr21-29079025-29079769	0.002338062	0.421580114	0.143	0.036	1	C12_gdT
chr9-130871274-130871623	0.002338071	0.359618898	0.054	0.005	1	C12_gdT
chr1-23790740-23792067	0.002347805	0.401244341	0.357	0.143	1	C12_gdT
chr1-221727296-221727691	0.002351001	0.418610263	0.125	0.022	1	C12_gdT
chr1-46131747-46133558	0.002360243	0.329412855	0.571	0.296	1	C12_gdT
chrX-74290486-74290725	0.00238406	0.408271862	0.125	0.026	1	C12_gdT

chr16-31265035-31265724	0.002400748	0.418329178	0.161	0.036	1	C12_gdT
chr7-104206906-104208441	0.002405022	0.389308333	0.375	0.163	1	C12_gdT
chr19-14248546-14249625	0.002406759	0.405981071	0.107	0.017	1	C12_gdT
chr6-148760150-148761881	0.002424777	0.366192725	0.446	0.189	1	C12_gdT
chr5-142935676-142936193	0.002434741	0.399745894	0.107	0.018	1	C12_gdT
chr7-2521473-2522638	0.002442851	0.372131167	0.482	0.211	1	C12_gdT
chr17-64660951-64662663	0.002448691	0.351403494	0.536	0.26	1	C12_gdT
chr1-101307980-101308230	0.0024505	0.400737847	0.089	0.013	1	C12_gdT
chr11-122666827-122667071	0.002456478	0.36852855	0.071	0.007	1	C12_gdT
chr10-63225474-63226395	0.002457526	0.411648327	0.268	0.099	1	C12_gdT
chr6-137975059-137975926	0.002458193	0.401602088	0.339	0.119	1	C12_gdT
chr6-139764364-139765000	0.002459033	0.379963873	0.071	0.007	1	C12_gdT
chr1-200628126-200628946	0.002460112	0.410050374	0.143	0.028	1	C12_gdT
chr6-150417647-150418623	0.002466595	0.415664721	0.232	0.071	1	C12_gdT
chr16-17013190-17014181	0.002468776	0.37800491	0.411	0.172	1	C12_gdT
chr2-131528306-131529720	0.002470048	0.410888704	0.161	0.035	1	C12_gdT
chr17-27595229-27595586	0.002470083	0.38986947	0.089	0.016	1	C12_gdT
chr2-3222506-3223598	0.002475909	0.369160028	0.429	0.178	1	C12_gdT
chr13-88622262-88622860	0.002488432	0.372107571	0.071	0.008	1	C12_gdT
chr10-109892113-109892389	0.002489161	0.352085236	0.054	0.005	1	C12_gdT
chr12-103279474-103279785	0.002491692	0.365157264	0.071	0.008	1	C12_gdT
chrY-7339411-7339993	0.002492308	0.406766902	0.107	0.022	1	C12_gdT
chr3-184262462-184262813	0.002507956	0.413932744	0.125	0.024	1	C12_gdT
chr19-10104407-10106632	0.002512048	0.262636478	0.821	0.478	1	C12_gdT
chr12-47414538-47414852	0.002536949	0.368900979	0.071	0.007	1	C12_gdT
chr5-35904677-35905071	0.002559084	0.403704603	0.107	0.016	1	C12_gdT
chr22-36383075-36383603	0.002564411	0.363546229	0.464	0.215	1	C12_gdT

chr7-38220427-38220757	0.002596555	0.419494588	0.214	0.06	1	C12_gdT
chr1-149160062-149160460	0.002600285	0.394091969	0.089	0.014	1	C12_gdT
chr9-135926728-135927223	0.002620919	0.399741362	0.107	0.019	1	C12_gdT
chr7-43658620-43659527	0.002637492	0.407698781	0.125	0.026	1	C12_gdT
chr22-25060476-25061073	0.002656563	0.421864691	0.179	0.047	1	C12_gdT
chr2-96267723-96267966	0.002667165	0.408313985	0.125	0.027	1	C12_gdT
chr5-68464418-68465459	0.002668611	0.421167093	0.143	0.034	1	C12_gdT
chr10-62778942-62780013	0.002676747	0.399996325	0.321	0.123	1	C12_gdT
chr3-27521453-27521878	0.002679377	0.36884652	0.071	0.007	1	C12_gdT
chr2-38685245-38685493	0.00268115	0.396141531	0.107	0.014	1	C12_gdT
chr18-711374-712937	0.002692303	0.364286876	0.411	0.206	1	C12_gdT
chr1-8396776-8397663	0.00269565	0.415861744	0.25	0.085	1	C12_gdT
chr1-67342240-67342645	0.002699989	0.39265283	0.089	0.011	1	C12_gdT
chr5-40486325-40487137	0.002703672	0.403883068	0.286	0.104	1	C12_gdT
chr19-39897793-39898081	0.002703866	0.370964397	0.071	0.007	1	C12_gdT
chr14-52530891-52531233	0.002707306	0.363253553	0.071	0.008	1	C12_gdT
chr18-49949843-49950449	0.002717411	0.411124199	0.143	0.026	1	C12_gdT
chr10-27568278-27568943	0.00272794	0.391119369	0.107	0.019	1	C12_gdT
chr13-46340934-46341615	0.002734729	0.39076741	0.089	0.014	1	C12_gdT
chr19-6800735-6802738	0.002739808	0.355603635	0.482	0.228	1	C12_gdT
chr5-103138920-103139590	0.00275182	0.415748702	0.161	0.038	1	C12_gdT
chr10-6050501-6051537	0.002770857	0.299172996	0.643	0.359	1	C12_gdT
chr15-40405034-40406168	0.002776098	0.329809323	0.518	0.279	1	C12_gdT
chr4-48431255-48431601	0.002777679	0.337609469	0.054	0.004	1	C12_gdT
chr10-11205271-11205686	0.002785738	0.43039105	0.179	0.044	1	C12_gdT
chr2-98467031-98467345	0.002794104	0.415804332	0.125	0.025	1	C12_gdT
chr19-38680979-38681483	0.002796363	0.366099613	0.464	0.201	1	C12_gdT

chr11-12086986-12088226	0.002804576	0.418240647	0.196	0.064	1	C12_gdT
chr5-172721834-172722697	0.002814049	0.420267287	0.232	0.066	1	C12_gdT
chr15-81315087-81315885	0.002831916	0.413309754	0.143	0.034	1	C12_gdT
chr8-22540044-22540833	0.002834071	0.424982296	0.196	0.049	1	C12_gdT
chr12-9026990-9027546	0.002843907	0.427697973	0.161	0.037	1	C12_gdT
chr7-150439996-150440263	0.002858624	0.36186841	0.071	0.007	1	C12_gdT
chr2-100104654-100105376	0.002886243	0.379145041	0.089	0.012	1	C12_gdT
chr9-136901384-136903152	0.002901834	0.349952138	0.5	0.235	1	C12_gdT
chr2-30226404-30226998	0.002909622	0.412383127	0.179	0.047	1	C12_gdT
chr2-190382859-190383156	0.002924528	0.398460152	0.107	0.016	1	C12_gdT
chr9-112777722-112778221	0.002934734	0.392923595	0.089	0.012	1	C12_gdT
chr6-34486126-34486463	0.002973508	0.404906532	0.125	0.026	1	C12_gdT
chr10-11244628-11244980	0.002979214	0.371730223	0.071	0.007	1	C12_gdT
chr11-289977-290255	0.002997374	0.415585573	0.143	0.033	1	C12_gdT
chr1-25819271-25820956	0.003005779	0.308110806	0.661	0.351	1	C12_gdT
chrX-139738592-139739552	0.003087037	0.403809327	0.214	0.066	1	C12_gdT
chr1-235048127-235048399	0.003095655	0.339746088	0.054	0.004	1	C12_gdT
chr3-10194428-10195039	0.003127178	0.4204866	0.179	0.05	1	C12_gdT
chr3-183275450-183275782	0.003129325	0.354202035	0.071	0.006	1	C12_gdT
chr22-35894475-35894868	0.003130755	0.343304989	0.054	0.004	1	C12_gdT
chr2-102368385-102368872	0.003147386	0.393721396	0.107	0.017	1	C12_gdT
chr2-36333392-36333954	0.003149871	0.351465417	0.071	0.009	1	C12_gdT
chr8-133209100-133209736	0.003158503	0.406542771	0.125	0.025	1	C12_gdT
chr20-50913969-50914488	0.003164466	0.38868312	0.089	0.013	1	C12_gdT
chr15-67100938-67102603	0.003164992	0.329913477	0.536	0.277	1	C12_gdT
chr21-38831352-38831885	0.003188552	0.410536657	0.125	0.023	1	C12_gdT
chr8-125508191-125508960	0.003199199	0.409065266	0.125	0.029	1	C12_gdT

chr10-124636397-124637010	0.003211594	0.413240365	0.179	0.047	1	C12_gdT
chr13-86830969-86831449	0.003224792	0.332168017	0.054	0.003	1	C12_gdT
chr21-42424690-42426018	0.003235213	0.332962841	0.536	0.272	1	C12_gdT
chr7-100836978-100837642	0.003254528	0.413086608	0.161	0.042	1	C12_gdT
chr18-45212578-45213054	0.003282262	0.3820073	0.107	0.018	1	C12_gdT
chr8-63199887-63200359	0.003286263	0.368637995	0.071	0.007	1	C12_gdT
chr6-53244990-53245299	0.003291092	0.383793436	0.089	0.012	1	C12_gdT
chrX-1500828-1501514	0.003298584	0.406615388	0.125	0.029	1	C12_gdT
chr19-55207147-55207399	0.003300937	0.364229568	0.071	0.008	1	C12_gdT
chr3-155835725-155836011	0.003328227	0.336952125	0.054	0.005	1	C12_gdT
chr1-26781475-26781744	0.003338914	0.367265889	0.071	0.007	1	C12_gdT
chr1-36125025-36125424	0.003353375	0.368934664	0.071	0.006	1	C12_gdT
chr14-75274873-75275118	0.003362624	0.402034738	0.25	0.082	1	C12_gdT
chr7-140304221-140304802	0.0033627	0.406122089	0.125	0.024	1	C12_gdT
chr17-73204159-73204498	0.003372398	0.379273236	0.089	0.013	1	C12_gdT
chr11-73979775-73980709	0.003374122	0.371950875	0.375	0.165	1	C12_gdT
chr16-68064203-68065203	0.00339799	0.419375215	0.196	0.061	1	C12_gdT
chr7-151251226-151251964	0.003420708	0.402328591	0.107	0.017	1	C12_gdT
chr17-1374430-1375970	0.003422819	0.407227767	0.268	0.093	1	C12_gdT
chr7-41881388-41882181	0.003425171	0.379258694	0.304	0.118	1	C12_gdT
chr1-244665748-244666061	0.003428103	0.361807409	0.071	0.007	1	C12_gdT
chr12-119659937-119660416	0.003448592	0.394676168	0.107	0.023	1	C12_gdT
chr7-159112168-159112450	0.003455347	0.370554184	0.071	0.008	1	C12_gdT
chr5-150789782-150790618	0.003479373	0.415190588	0.196	0.061	1	C12_gdT
chr9-96382294-96382503	0.003489285	0.364090612	0.071	0.009	1	C12_gdT
chr5-139310626-139311211	0.003517928	0.408085899	0.143	0.029	1	C12_gdT
chr3-18501690-18502234	0.003521897	0.357119607	0.071	0.006	1	C12_gdT

chr12-10276778-10277095	0.003534374	0.394707973	0.089	0.014	1	C12_gdT
chr5-40333013-40333871	0.003595327	0.418068192	0.232	0.067	1	C12_gdT
chr15-72226693-72227473	0.003606783	0.324872921	0.536	0.268	1	C12_gdT
chr17-3916450-3916678	0.003652456	0.365499991	0.071	0.009	1	C12_gdT
chr11-69337124-69338166	0.003672293	0.404323991	0.196	0.057	1	C12_gdT
chr18-75216211-75216608	0.003689039	0.411313144	0.179	0.041	1	C12_gdT
chr3-14959042-14959823	0.003700728	0.361900393	0.071	0.008	1	C12_gdT
chr7-157302001-157302825	0.003710129	0.313305005	0.536	0.298	1	C12_gdT
chr17-39921260-39921730	0.003750466	0.407824316	0.125	0.023	1	C12_gdT
chr15-38453924-38454909	0.003753818	0.371598657	0.375	0.167	1	C12_gdT
chr2-113178931-113179200	0.003769629	0.418385112	0.179	0.044	1	C12_gdT
chr2-12856538-12857357	0.003776487	0.40133508	0.25	0.078	1	C12_gdT
chr13-50801034-50801682	0.003787967	0.40329715	0.107	0.025	1	C12_gdT
chr18-22559418-22560214	0.003806932	0.367903227	0.089	0.011	1	C12_gdT
chr17-82859428-82860382	0.003814744	0.404467405	0.232	0.078	1	C12_gdT
chr4-35525205-35525662	0.003815591	0.321331995	0.054	0.004	1	C12_gdT
chr1-156487857-156488242	0.003843976	0.413498787	0.143	0.03	1	C12_gdT
chr2-112634346-112634891	0.003850586	0.398008865	0.125	0.023	1	C12_gdT
chr15-70307789-70308709	0.003853714	0.405751872	0.196	0.057	1	C12_gdT
chr14-50725859-50727013	0.003891854	0.398924009	0.196	0.053	1	C12_gdT
chr10-96744044-96744243	0.003892085	0.317928421	0.054	0.003	1	C12_gdT
chr9-127778707-127779516	0.003894077	0.400034202	0.286	0.1	1	C12_gdT
chr3-111615665-111616907	0.003898883	0.30148019	0.571	0.315	1	C12_gdT
chr20-53729474-53729957	0.003914725	0.370567851	0.089	0.012	1	C12_gdT
chr20-62887400-62887801	0.003944043	0.388724148	0.107	0.024	1	C12_gdT
chr4-14994323-14994539	0.003947501	0.338169877	0.054	0.005	1	C12_gdT
chr20-34350181-34350593	0.003950239	0.367633935	0.071	0.009	1	C12_gdT

chr10-17075563-17075764	0.003951469	0.34438816	0.054	0.004	1	C12_gdT
chr12-107014082-107014393	0.003963299	0.383354864	0.089	0.016	1	C12_gdT
chr7-151935747-151936721	0.003968026	0.395433932	0.125	0.031	1	C12_gdT
chr1-113072255-113073741	0.003968742	0.263931084	0.714	0.444	1	C12_gdT
chr19-51273414-51273811	0.003979729	0.391879244	0.107	0.018	1	C12_gdT
chr1-206703547-206704074	0.00399462	0.406759081	0.196	0.054	1	C12_gdT
chr2-136007775-136008815	0.00399479	0.369200482	0.339	0.166	1	C12_gdT
chr4-101327816-101328712	0.00402471	0.393009105	0.286	0.11	1	C12_gdT
chr20-8399909-8400372	0.004070154	0.369501403	0.089	0.012	1	C12_gdT
chr20-63736123-63737678	0.004085863	0.317239635	0.554	0.296	1	C12_gdT
chr1-107842235-107842552	0.004090251	0.393251168	0.089	0.012	1	C12_gdT
chr7-44977127-44977649	0.004106034	0.368344976	0.339	0.149	1	C12_gdT
chr6-87737833-87738296	0.004113662	0.397039801	0.143	0.03	1	C12_gdT
chr5-132437488-132438255	0.004120064	0.402823088	0.196	0.068	1	C12_gdT
chr8-33512426-33514574	0.004122144	0.297794594	0.661	0.359	1	C12_gdT
chr17-78113526-78114140	0.004129963	0.403420451	0.232	0.063	1	C12_gdT
chr7-3027650-3028400	0.00414597	0.374976942	0.089	0.014	1	C12_gdT
chr3-12796378-12797141	0.00415235	0.396016594	0.125	0.026	1	C12_gdT
chr4-6688520-6690780	0.00416393	0.334470127	0.518	0.26	1	C12_gdT
chr15-63238893-63239174	0.004167929	0.376797983	0.071	0.01	1	C12_gdT
chr18-26710477-26710764	0.004193551	0.318516144	0.054	0.004	1	C12_gdT
chr5-143261649-143262167	0.004235668	0.382415608	0.125	0.023	1	C12_gdT
chr2-70133497-70134341	0.004237523	0.391493363	0.268	0.104	1	C12_gdT
chr3-46213071-46213654	0.004244003	0.406790275	0.179	0.048	1	C12_gdT
chr16-89564096-89564376	0.00425813	0.40141264	0.107	0.024	1	C12_gdT
chr2-173151696-173151987	0.004263667	0.357688202	0.071	0.011	1	C12_gdT
chr4-42670769-42671929	0.004272996	0.401348742	0.179	0.053	1	C12_gdT

chr15-68783375-68783983	0.004281357	0.389719599	0.107	0.023	1	C12_gdT
chr6-137949797-137950379	0.004289754	0.381555123	0.125	0.024	1	C12_gdT
chr17-46156732-46156996	0.004301816	0.378991979	0.089	0.016	1	C12_gdT
chr19-19617939-19619069	0.004307401	0.25762636	0.714	0.456	1	C12_gdT
chr1-234961869-234963686	0.004333413	0.368267014	0.357	0.145	1	C12_gdT
chr1-167430060-167430391	0.004359172	0.387363394	0.107	0.018	1	C12_gdT
chr10-7239029-7239648	0.004359247	0.388702718	0.125	0.023	1	C12_gdT
chr3-196629926-196630150	0.004383085	0.389115216	0.107	0.021	1	C12_gdT
chr9-133113477-133113691	0.004383451	0.378230094	0.089	0.015	1	C12_gdT
chr17-30363186-30364571	0.004393396	0.27078242	0.679	0.39	1	C12_gdT
chr20-20710850-20711779	0.004404734	0.397953401	0.179	0.054	1	C12_gdT
chr10-124732713-124733532	0.004406892	0.39031172	0.339	0.121	1	C12_gdT
chr17-50160938-50161851	0.004408232	0.36840741	0.411	0.178	1	C12_gdT
chr3-33046471-33046845	0.004446552	0.38458708	0.125	0.027	1	C12_gdT
chr6-125829362-125829724	0.004446717	0.391770211	0.107	0.02	1	C12_gdT
chr20-44520954-44522413	0.004447587	0.267641615	0.679	0.423	1	C12_gdT
chr2-234463647-234464682	0.004470654	0.337457274	0.482	0.234	1	C12_gdT
chr5-142952909-142953135	0.004472519	0.325439796	0.054	0.005	1	C12_gdT
chr2-8481556-8481984	0.004479543	0.40735463	0.143	0.032	1	C12_gdT
chr6-75268974-75269256	0.004481922	0.341558261	0.071	0.007	1	C12_gdT
chrX-48025688-48025906	0.004482535	0.379627343	0.089	0.015	1	C12_gdT
chr1-160578272-160578494	0.004485562	0.384338419	0.107	0.018	1	C12_gdT
chrX-130415143-130415515	0.004523395	0.34170339	0.071	0.009	1	C12_gdT
chrX-13486892-13487459	0.004528921	0.363979544	0.089	0.013	1	C12_gdT
chr4-17385101-17385746	0.004574721	0.330578407	0.054	0.005	1	C12_gdT
chr16-11206447-11207087	0.004586023	0.365055245	0.071	0.01	1	C12_gdT
chrX-41837253-41837552	0.004603101	0.322941675	0.054	0.004	1	C12_gdT

chr17-7042291-7043186	0.00460313	0.394973654	0.214	0.068	1	C12_gdT
chr3-14461772-14461997	0.004610337	0.347923696	0.071	0.008	1	C12_gdT
chr2-195563713-195564556	0.004622442	0.379873418	0.339	0.132	1	C12_gdT
chr14-61661424-61663027	0.004633331	0.354218727	0.464	0.208	1	C12_gdT
chr3-46186783-46187521	0.004663751	0.398441737	0.125	0.026	1	C12_gdT
chr12-67989196-67989503	0.004664985	0.380017033	0.107	0.02	1	C12_gdT
chr19-32700117-32700424	0.004678001	0.322044684	0.054	0.004	1	C12_gdT
chr18-59921297-59921922	0.004688361	0.383253094	0.143	0.032	1	C12_gdT
chr10-69237413-69237915	0.004690722	0.399490833	0.179	0.049	1	C12_gdT
chr17-58218313-58219577	0.004698259	0.313179599	0.589	0.31	1	C12_gdT
chr17-80454095-80455479	0.004704687	0.307954185	0.625	0.321	1	C12_gdT
chr5-134100056-134100788	0.00472471	0.367006359	0.411	0.161	1	C12_gdT
chr10-73781673-73782416	0.004726351	0.341396167	0.446	0.226	1	C12_gdT
chr11-118834441-118834720	0.004747981	0.319677507	0.054	0.004	1	C12_gdT
chr2-61763611-61764772	0.004750026	0.294098494	0.571	0.345	1	C12_gdT
chr1-58903137-58904239	0.004751155	0.364722976	0.339	0.167	1	C12_gdT
chr14-22268479-22269145	0.004787819	0.374841612	0.107	0.021	1	C12_gdT
chr17-45228471-45229581	0.00479027	0.311730232	0.571	0.294	1	C12_gdT
chr14-100106310-100106554	0.004810379	0.398708179	0.161	0.041	1	C12_gdT
chr18-46097502-46098720	0.004817481	0.35724544	0.429	0.193	1	C12_gdT
chr4-184250668-184251125	0.004828569	0.381883527	0.107	0.019	1	C12_gdT
chr1-16371776-16372062	0.004838599	0.401854371	0.125	0.027	1	C12_gdT
chr14-58198061-58198458	0.004845784	0.395090791	0.161	0.049	1	C12_gdT
chr6-137155466-137156249	0.004859722	0.3767084	0.339	0.13	1	C12_gdT
chr8-142623916-142624308	0.004875446	0.341888475	0.071	0.008	1	C12_gdT
chr19-42201372-42201843	0.00488203	0.385189669	0.143	0.044	1	C12_gdT
chr2-195202265-195202737	0.004888527	0.313432798	0.054	0.005	1	C12_gdT

chr2-197212625-197212904	0.004890452	0.389255002	0.125	0.024	1	C12_gdT
chr2-120287140-120287522	0.004917039	0.361894055	0.071	0.009	1	C12_gdT
chr4-54063294-54064977	0.004922564	0.301931626	0.625	0.337	1	C12_gdT
chr14-23539431-23539911	0.00496504	0.359380523	0.089	0.015	1	C12_gdT
chr2-134324847-134325306	0.004977617	0.386618073	0.143	0.035	1	C12_gdT
chr20-49951196-49951397	0.004982411	0.319148893	0.054	0.005	1	C12_gdT
chr3-128764013-128764328	0.004983606	0.389623885	0.107	0.019	1	C12_gdT
chr11-73249017-73249982	0.004994339	0.347652117	0.071	0.011	1	C12_gdT
chr6-155155741-155156415	0.005017412	0.385698106	0.214	0.076	1	C12_gdT
chr3-125835707-125837118	0.005020202	0.379081905	0.268	0.095	1	C12_gdT
chr10-35328410-35328633	0.005040167	0.337069228	0.054	0.005	1	C12_gdT
chr8-10838897-10840250	0.005054947	0.271124045	0.661	0.397	1	C12_gdT
chr2-27096282-27096507	0.005061396	0.338604052	0.071	0.008	1	C12_gdT
chr4-88319698-88321115	0.005086066	0.378936074	0.107	0.018	1	C12_gdT
chrX-78658930-78659597	0.00509001	0.328596455	0.054	0.006	1	C12_gdT
chr18-136384-136762	0.005111819	0.354052254	0.071	0.011	1	C12_gdT
chr5-65190375-65190794	0.00514344	0.320570196	0.054	0.005	1	C12_gdT
chr9-114898471-114899139	0.005155215	0.377596536	0.268	0.101	1	C12_gdT
chr19-48812206-48812571	0.005167571	0.381185947	0.107	0.021	1	C12_gdT
chr16-89657145-89658188	0.005196247	0.293474718	0.643	0.355	1	C12_gdT
chr6-87714200-87714832	0.005218779	0.398936591	0.196	0.061	1	C12_gdT
chr19-50363012-50363223	0.005221862	0.367624602	0.089	0.012	1	C12_gdT
chr5-112995699-112996337	0.005226949	0.39466322	0.125	0.027	1	C12_gdT
chrX-71195680-71196678	0.005235382	0.393000314	0.179	0.055	1	C12_gdT
chr7-38318292-38318523	0.005259246	0.361341809	0.089	0.014	1	C12_gdT
chr21-36173534-36174232	0.005263341	0.371079194	0.321	0.13	1	C12_gdT
chr21-42405793-42406623	0.005277836	0.39588904	0.214	0.071	1	C12_gdT

chr15-63226620-63226936	0.005283987	0.322322731	0.054	0.005	1	C12_gdT
chr2-237693175-237693472	0.005306575	0.390460024	0.321	0.106	1	C12_gdT
chr2-225056468-225056967	0.005309516	0.389817984	0.143	0.031	1	C12_gdT
chr14-75126153-75127416	0.005311069	0.367832414	0.393	0.164	1	C12_gdT
chr11-18267656-18268575	0.005320238	0.364253772	0.089	0.018	1	C12_gdT
chr1-167412007-167412418	0.005322719	0.320769243	0.054	0.003	1	C12_gdT
chr6-134250189-134250388	0.005331137	0.370710133	0.089	0.014	1	C12_gdT
chr3-105772623-105773593	0.005337913	0.397632267	0.161	0.041	1	C12_gdT
chr16-29273517-29273937	0.00534357	0.352529447	0.071	0.011	1	C12_gdT
chr14-91227149-91227478	0.00534904	0.398597125	0.179	0.049	1	C12_gdT
chr17-28981875-28982842	0.005353102	0.338396478	0.429	0.192	1	C12_gdT
chr5-139808456-139808841	0.005369547	0.330499751	0.054	0.006	1	C12_gdT
chr21-34946331-34947178	0.005376366	0.385771512	0.125	0.027	1	C12_gdT
chr17-63697481-63698188	0.00538396	0.350417426	0.393	0.186	1	C12_gdT
chr11-111601713-111603626	0.005430861	0.281549942	0.643	0.372	1	C12_gdT
chr9-33458237-33458907	0.005440539	0.391862388	0.286	0.095	1	C12_gdT
chr7-38334781-38335925	0.005450103	0.384991605	0.286	0.109	1	C12_gdT
chr3-17088691-17089324	0.005466453	0.375752097	0.143	0.038	1	C12_gdT
chr10-110992547-110993435	0.005489529	0.395320538	0.232	0.082	1	C12_gdT
chr19-13116263-13118454	0.005490241	0.284508188	0.661	0.379	1	C12_gdT
chr4-110055141-110055747	0.005539694	0.343660995	0.071	0.009	1	C12_gdT
chr7-29243867-29244683	0.005578785	0.371012891	0.125	0.031	1	C12_gdT
chr7-26194934-26195246	0.005602427	0.384998798	0.161	0.039	1	C12_gdT
chr9-6023808-6024457	0.005603772	0.366323158	0.089	0.016	1	C12_gdT
chr19-20558559-20558812	0.005613584	0.309730579	0.054	0.005	1	C12_gdT
chr1-206556123-206557520	0.00563161	0.316303685	0.518	0.279	1	C12_gdT
chr6-490179-491637	0.005645894	0.388954043	0.25	0.089	1	C12_gdT

chr1-165717659-165718134	0.005652113	0.316109246	0.054	0.004	1	C12_gdT
chr20-34390510-34391188	0.005664985	0.389979049	0.143	0.036	1	C12_gdT
chr10-16891059-16892239	0.005691048	0.381344278	0.286	0.104	1	C12_gdT
chr16-82456651-82456860	0.005691369	0.343835246	0.071	0.01	1	C12_gdT
chr1-64956132-64957373	0.005760627	0.392382413	0.179	0.049	1	C12_gdT
chr19-57465763-57466061	0.005780206	0.333861403	0.071	0.008	1	C12_gdT
chr2-105071438-105071694	0.005781562	0.343892191	0.071	0.007	1	C12_gdT
chr1-206713030-206713392	0.005784969	0.386961738	0.143	0.035	1	C12_gdT
chr17-714149-715489	0.005792828	0.359940352	0.375	0.183	1	C12_gdT
chr3-187041438-187042264	0.005797021	0.377255076	0.304	0.118	1	C12_gdT
chr3-11306743-11307448	0.005800812	0.389923618	0.179	0.05	1	C12_gdT
chr1-88985711-88985996	0.005801818	0.373630231	0.089	0.013	1	C12_gdT
chr5-172983242-172984778	0.005804013	0.309307721	0.589	0.294	1	C12_gdT
chr15-92821765-92822008	0.005818251	0.373741282	0.089	0.012	1	C12_gdT
chr10-6013878-6014870	0.005822921	0.373292399	0.286	0.109	1	C12_gdT
chr10-110503757-110504429	0.005831759	0.391293099	0.232	0.085	1	C12_gdT
chr19-19458984-19461274	0.005853666	0.30749491	0.5	0.293	1	C12_gdT
chr20-53915194-53916161	0.005858221	0.365662617	0.357	0.148	1	C12_gdT
chr1-156656911-156657749	0.005866388	0.404275893	0.232	0.069	1	C12_gdT
chr19-46878653-46878943	0.005873451	0.32052288	0.054	0.006	1	C12_gdT
chr13-48768884-48769457	0.005873524	0.38932279	0.25	0.086	1	C12_gdT
chr10-49254540-49254900	0.005884817	0.3176689	0.054	0.006	1	C12_gdT
chr15-41894292-41895489	0.005938511	0.315380952	0.5	0.268	1	C12_gdT
chr17-27332106-27333732	0.005959303	0.280763161	0.625	0.373	1	C12_gdT
chr5-96714482-96715221	0.005962257	0.388645239	0.214	0.07	1	C12_gdT
chr17-64792757-64793153	0.005970687	0.366575924	0.089	0.017	1	C12_gdT
chr2-25981896-25982950	0.005980025	0.35631951	0.357	0.173	1	C12_gdT

chr2-159124433-159124918	0.005984909	0.340746027	0.071	0.009	1	C12_gdT
chr9-134390469-134392266	0.005987861	0.276307334	0.661	0.367	1	C12_gdT
chr6-2911339-2912046	0.005990415	0.396578935	0.161	0.044	1	C12_gdT
chr2-144454539-144455211	0.005997942	0.38693043	0.179	0.056	1	C12_gdT
chr15-89068264-89068624	0.006012914	0.320867361	0.054	0.005	1	C12_gdT
chr6-37048546-37049063	0.006055744	0.37582388	0.321	0.126	1	C12_gdT
chr15-85704106-85704342	0.006082698	0.396130249	0.232	0.081	1	C12_gdT
chr15-78033868-78035707	0.006094529	0.316507297	0.464	0.259	1	C12_gdT
chr1-58391880-58392560	0.006094704	0.375963104	0.143	0.038	1	C12_gdT
chr12-10364189-10365469	0.006119491	0.379415796	0.268	0.102	1	C12_gdT
chr17-81663091-81663296	0.006129424	0.361276872	0.089	0.014	1	C12_gdT
chr3-56860850-56861191	0.006131426	0.31655929	0.054	0.004	1	C12_gdT
chr6-137883341-137883824	0.006132719	0.386589253	0.196	0.055	1	C12_gdT
chr11-19970803-19971362	0.00613679	0.356946984	0.089	0.015	1	C12_gdT
chr6-155093216-155093828	0.006143284	0.376192683	0.161	0.043	1	C12_gdT
chr1-28365957-28366177	0.006177255	0.340410681	0.071	0.009	1	C12_gdT
chr6-159053711-159054248	0.006184045	0.382138615	0.161	0.039	1	C12_gdT
chr3-132180278-132180723	0.006211245	0.316733762	0.054	0.004	1	C12_gdT
chr2-233142050-233142888	0.006221596	0.371012094	0.107	0.024	1	C12_gdT
chr15-43977058-43977354	0.006223222	0.313616761	0.054	0.006	1	C12_gdT
chr2-62222406-62223114	0.006225413	0.378955105	0.107	0.024	1	C12_gdT
chr4-101526809-101527577	0.006252768	0.381150285	0.268	0.096	1	C12_gdT
chr17-71921364-71922606	0.006252921	0.358431392	0.107	0.019	1	C12_gdT
chr5-140239912-140240123	0.006263292	0.347215663	0.071	0.008	1	C12_gdT
chr13-43905278-43905736	0.006265685	0.373644759	0.089	0.016	1	C12_gdT
chr8-143927925-143929685	0.006274379	0.344103725	0.429	0.198	1	C12_gdT
chrX-120302061-120302781	0.00628095	0.376727635	0.125	0.026	1	C12_gdT

chr2-180937450-180937895	0.006286307	0.341231875	0.071	0.009	1	C12_gdT
chr10-26479739-26480013	0.006291393	0.378666377	0.125	0.03	1	C12_gdT
chr16-69563866-69564644	0.006292807	0.377623239	0.304	0.125	1	C12_gdT
chr10-14601893-14602337	0.006377996	0.377476681	0.286	0.113	1	C12_gdT
chr5-80713436-80713965	0.006384141	0.339787722	0.071	0.01	1	C12_gdT
chr22-39013617-39014976	0.006405794	0.349935011	0.393	0.179	1	C12_gdT
chr11-63554498-63554704	0.006417901	0.378004965	0.125	0.027	1	C12_gdT
chr6-42028084-42028782	0.006420926	0.376583086	0.107	0.021	1	C12_gdT
chr1-212225137-212226187	0.006429018	0.379953014	0.214	0.06	1	C12_gdT
chr22-50541687-50541936	0.006430444	0.3865537	0.143	0.035	1	C12_gdT
chr9-129776951-129777617	0.006493076	0.369210338	0.107	0.025	1	C12_gdT
chr20-64034958-64035632	0.006498598	0.384785767	0.214	0.069	1	C12_gdT
chr11-3949085-3949420	0.00650335	0.385806312	0.143	0.039	1	C12_gdT
chr1-27152303-27152519	0.006504747	0.364259862	0.089	0.014	1	C12_gdT
chr9-124257232-124258634	0.006510842	0.382102844	0.232	0.078	1	C12_gdT
chr1-10494516-10495586	0.006515385	0.360813213	0.357	0.154	1	C12_gdT
chr22-36405030-36406318	0.006532809	0.372242917	0.25	0.097	1	C12_gdT
chr8-129984001-129984631	0.006544482	0.36689288	0.107	0.023	1	C12_gdT
chr4-56648308-56648624	0.00654736	0.338400313	0.071	0.01	1	C12_gdT
chr3-46107646-46108052	0.006556869	0.368803209	0.107	0.02	1	C12_gdT
chr2-64561955-64562596	0.006626572	0.353904027	0.089	0.015	1	C12_gdT
chr3-136932528-136932785	0.006631709	0.308471418	0.054	0.005	1	C12_gdT
chr2-144261941-144262325	0.0066494	0.369747994	0.107	0.02	1	C12_gdT
chr1-202341404-202342291	0.006656633	0.278585777	0.625	0.361	1	C12_gdT
chr17-2064638-2065319	0.006662488	0.384116368	0.125	0.031	1	C12_gdT
chr13-51814961-51815438	0.006670365	0.38086047	0.143	0.039	1	C12_gdT
chr5-172835399-172835624	0.00668044	0.363182058	0.089	0.012	1	C12_gdT

chr3-56754781-56755291	0.006745821	0.361522574	0.089	0.014	1	C12_gdT
chr16-57599905-57600108	0.006747841	0.343941121	0.071	0.011	1	C12_gdT
chr12-905301-906035	0.006748052	0.375750453	0.107	0.025	1	C12_gdT
chr5-138641635-138642152	0.006754563	0.362554501	0.089	0.015	1	C12_gdT
chr18-12744108-12744421	0.006757168	0.36085306	0.089	0.013	1	C12_gdT
chr16-27296395-27296620	0.006765173	0.303172125	0.054	0.005	1	C12_gdT
chr2-64643277-64643512	0.006773206	0.33663762	0.071	0.011	1	C12_gdT
chr1-181044223-181044803	0.006775723	0.370430855	0.107	0.023	1	C12_gdT
chr21-44143493-44143703	0.0067866	0.338219272	0.071	0.008	1	C12_gdT
chr19-48627112-48627452	0.00679731	0.379379729	0.161	0.043	1	C12_gdT
chr6-122393231-122393843	0.006827502	0.335532376	0.071	0.008	1	C12_gdT
chr2-7671680-7672899	0.006861426	0.367706647	0.107	0.027	1	C12_gdT
chr5-149860651-149861105	0.00687507	0.314456472	0.054	0.005	1	C12_gdT
chr6-7275624-7276244	0.0068819	0.350357184	0.089	0.015	1	C12_gdT
chr9-35790108-35790615	0.006906615	0.389154469	0.125	0.032	1	C12_gdT
chr2-174634260-174635429	0.006948235	0.36999077	0.268	0.12	1	C12_gdT
chr1-167428336-167428633	0.006976471	0.356276366	0.089	0.013	1	C12_gdT
chr8-81074282-81075008	0.007010983	0.386163157	0.161	0.045	1	C12_gdT
chr16-30713688-30714025	0.007013879	0.384707108	0.125	0.033	1	C12_gdT
chr12-68000962-68001797	0.00705528	0.325741332	0.054	0.006	1	C12_gdT
chr10-9302181-9302398	0.007064112	0.309711931	0.054	0.005	1	C12_gdT
chr15-43376111-43376383	0.007083598	0.357982974	0.089	0.016	1	C12_gdT
chr5-55420556-55421467	0.007105361	0.375774639	0.125	0.034	1	C12_gdT
chr8-95305256-95305621	0.007120495	0.304937633	0.054	0.004	1	C12_gdT
chr20-45036175-45036496	0.007151927	0.342001357	0.071	0.01	1	C12_gdT
chr7-50335686-50336484	0.007194716	0.335479796	0.411	0.197	1	C12_gdT
chr17-39769619-39770652	0.007213727	0.250059204	0.696	0.437	1	C12_gdT

chr2-201115569-201117047	0.007234611	0.316272086	0.446	0.235	1	C12_gdT
chr15-91472216-91472679	0.007259913	0.37535353	0.161	0.038	1	C12_gdT
chr19-18088434-18089440	0.007300649	0.341609555	0.375	0.192	1	C12_gdT
chr19-2561374-2562355	0.007315056	0.391949963	0.179	0.053	1	C12_gdT
chr9-85289616-85290443	0.007334901	0.342482759	0.089	0.013	1	C12_gdT
chr6-166221025-166221415	0.00735261	0.303203202	0.054	0.006	1	C12_gdT
chr15-92803681-92804192	0.007387583	0.369906278	0.125	0.031	1	C12_gdT
chr15-98953039-98953566	0.007391128	0.337145597	0.071	0.009	1	C12_gdT
chr8-67602390-67602895	0.007404663	0.346061544	0.089	0.013	1	C12_gdT
chr11-114215903-114216208	0.007426228	0.306136484	0.054	0.005	1	C12_gdT
chr3-184607926-184608654	0.00743741	0.372325484	0.179	0.051	1	C12_gdT
chr8-144082057-144082860	0.007447046	0.375263096	0.286	0.12	1	C12_gdT
chr9-4970875-4971713	0.007455943	0.375933158	0.161	0.047	1	C12_gdT
chr22-40200870-40201128	0.007458344	0.335981909	0.071	0.011	1	C12_gdT
chr17-17109287-17109555	0.00749509	0.330556859	0.071	0.009	1	C12_gdT
chr8-120758173-120758422	0.007522475	0.343265441	0.071	0.008	1	C12_gdT
chr19-15584329-15585037	0.007531244	0.368269294	0.107	0.024	1	C12_gdT
chr3-129593350-129594280	0.007541277	0.378568148	0.161	0.044	1	C12_gdT
chr17-47970319-47971553	0.007572965	0.256068187	0.696	0.43	1	C12_gdT
chr10-96695163-96695631	0.007587611	0.363067344	0.107	0.018	1	C12_gdT
chr10-97632839-97634223	0.007590147	0.339506644	0.411	0.195	1	C12_gdT
chr1-155167155-155168146	0.007596286	0.380032881	0.232	0.087	1	C12_gdT
chr7-136012463-136013011	0.007607517	0.302350028	0.054	0.004	1	C12_gdT
chr20-2651726-2653297	0.007620124	0.258101743	0.714	0.421	1	C12_gdT
chr11-65013169-65014784	0.007621555	0.280883542	0.589	0.359	1	C12_gdT
chr14-94056567-94057012	0.007623543	0.347168488	0.089	0.016	1	C12_gdT
chr3-71193691-71194104	0.007623649	0.322589823	0.054	0.005	1	C12_gdT

chr5-151098063-151098545	0.007659787	0.369959662	0.143	0.038	1	C12_gdT
chr1-221478037-221478405	0.007676277	0.352161813	0.089	0.016	1	C12_gdT
chr7-18665851-18666449	0.007688267	0.338228418	0.071	0.009	1	C12_gdT
chr2-105803347-105803967	0.007697943	0.372092227	0.232	0.089	1	C12_gdT
chr19-6862382-6863831	0.007727876	0.318232404	0.518	0.252	1	C12_gdT
chrX-63250093-63250526	0.007737186	0.327635769	0.071	0.011	1	C12_gdT
chr13-51802029-51802552	0.007738597	0.335549763	0.446	0.208	1	C12_gdT
chr5-138726689-138727172	0.007778137	0.307638076	0.054	0.005	1	C12_gdT
chr1-15248237-15248471	0.007788553	0.331030206	0.071	0.012	1	C12_gdT
chr2-234446150-234447016	0.007807895	0.344531655	0.375	0.164	1	C12_gdT
chr9-36165984-36166940	0.007826084	0.353913974	0.339	0.142	1	C12_gdT
chr3-46481731-46482277	0.00783179	0.353544213	0.089	0.019	1	C12_gdT
chr5-68730613-68731011	0.00789901	0.326152557	0.071	0.011	1	C12_gdT
chr3-122482909-122483366	0.007900418	0.321685381	0.054	0.006	1	C12_gdT
chr3-63937671-63937932	0.007900563	0.342385766	0.071	0.01	1	C12_gdT
chr6-143536305-143537612	0.007908736	0.362817449	0.286	0.121	1	C12_gdT
chr14-22509988-22510312	0.007924368	0.381769093	0.143	0.038	1	C12_gdT
chr10-6052168-6053200	0.007943723	0.309361457	0.571	0.272	1	C12_gdT
chr7-150586072-150587131	0.007949013	0.356723286	0.089	0.019	1	C12_gdT
chr17-9529124-9529730	0.007957806	0.301533937	0.054	0.006	1	C12_gdT
chr20-15155854-15156259	0.007985947	0.307831362	0.054	0.005	1	C12_gdT
chr1-224204631-224205148	0.0080019	0.374538561	0.161	0.053	1	C12_gdT
chr3-11764904-11765360	0.008030541	0.309777591	0.054	0.007	1	C12_gdT
chr17-8898595-8899604	0.00805248	0.308809247	0.518	0.259	1	C12_gdT
chr6-130215041-130215856	0.008083933	0.360866007	0.286	0.112	1	C12_gdT
chr6-137094322-137094918	0.008086653	0.357222543	0.107	0.024	1	C12_gdT
chr6-31465660-31465962	0.008112337	0.359655635	0.107	0.024	1	C12_gdT

chr7-4872342-4872939	0.008130657	0.319373886	0.054	0.006	1	C12_gdT
chr4-70992828-70994191	0.008132231	0.313803324	0.482	0.233	1	C12_gdT
chr12-6631524-6632573	0.00816338	0.380693535	0.214	0.072	1	C12_gdT
chr4-10098769-10099066	0.00816472	0.384333084	0.143	0.041	1	C12_gdT
chr22-38214363-38215019	0.008178578	0.367458154	0.232	0.092	1	C12_gdT
chr20-63863774-63865455	0.008179057	0.262992036	0.679	0.411	1	C12_gdT
chr20-57718377-57719052	0.008192632	0.318008768	0.482	0.238	1	C12_gdT
chr2-217988433-217988795	0.00821446	0.314568111	0.054	0.006	1	C12_gdT
chr11-108538007-108538349	0.008247146	0.294724046	0.054	0.005	1	C12_gdT
chr17-17363512-17364103	0.008281179	0.330584065	0.071	0.013	1	C12_gdT
chr10-73763325-73763693	0.008302141	0.375784287	0.143	0.033	1	C12_gdT
chr9-129220827-129221610	0.008315737	0.360144434	0.143	0.036	1	C12_gdT
chr3-46947133-46948865	0.008320311	0.250679303	0.679	0.43	1	C12_gdT
chr2-191152884-191153144	0.008324836	0.377757547	0.161	0.042	1	C12_gdT
chr10-17643388-17644469	0.008365986	0.286005315	0.607	0.33	1	C12_gdT
chr9-96241201-96241788	0.00838705	0.361718633	0.089	0.019	1	C12_gdT
chr12-9032666-9032869	0.008403126	0.305464814	0.054	0.005	1	C12_gdT
chr1-172426962-172427507	0.008431838	0.306937674	0.054	0.006	1	C12_gdT
chr3-48112078-48112978	0.008483107	0.360800323	0.107	0.022	1	C12_gdT
chrX-154515307-154516934	0.008485403	0.315806903	0.5	0.249	1	C12_gdT
chr16-75064435-75065416	0.008495419	0.362655405	0.107	0.021	1	C12_gdT
chrX-41733938-41734256	0.008519924	0.300141516	0.054	0.005	1	C12_gdT
chr21-29080446-29080731	0.008534547	0.367044712	0.125	0.027	1	C12_gdT
chr12-90091035-90091919	0.008554761	0.320222041	0.071	0.009	1	C12_gdT
chr13-20943462-20943818	0.008590137	0.321613381	0.071	0.008	1	C12_gdT
chr12-54502561-54502814	0.008595951	0.347071191	0.089	0.013	1	C12_gdT
chr11-45102362-45103340	0.008599578	0.349562216	0.321	0.147	1	C12_gdT

chr1-228185163-228186087	0.008614855	0.361563281	0.268	0.099	1	C12_gdT
chr7-38427311-38427918	0.008622388	0.331935053	0.071	0.01	1	C12_gdT
chr1-244353760-244354086	0.008628101	0.332725397	0.071	0.01	1	C12_gdT
chr14-91494404-91495716	0.008641241	0.374134745	0.196	0.06	1	C12_gdT
chr19-52095126-52096053	0.008650641	0.359494827	0.304	0.122	1	C12_gdT
chr17-64090968-64091451	0.008657889	0.347735232	0.089	0.014	1	C12_gdT
chr2-102252951-102253744	0.008685047	0.381116471	0.196	0.055	1	C12_gdT
chr4-174591263-174591977	0.00869291	0.360054187	0.089	0.013	1	C12_gdT
chr1-156488497-156489677	0.008732531	0.367195779	0.286	0.102	1	C12_gdT
chr1-31955938-31956381	0.008734165	0.377588468	0.196	0.059	1	C12_gdT
chr13-80340970-80341538	0.008745464	0.363220165	0.143	0.034	1	C12_gdT
chr11-43643479-43644683	0.008758858	0.360835623	0.286	0.116	1	C12_gdT
chr12-8989331-8989800	0.008762751	0.367718798	0.196	0.061	1	C12_gdT
chr3-109804567-109805094	0.008778664	0.368407506	0.268	0.097	1	C12_gdT
chr1-86774031-86774573	0.008792839	0.299455128	0.054	0.005	1	C12_gdT
chr7-27235458-27235964	0.008813594	0.315268587	0.054	0.005	1	C12_gdT
chr9-95506774-95508009	0.008815626	0.357443674	0.286	0.116	1	C12_gdT
chr12-132827637-132829362	0.008819322	0.286373656	0.607	0.339	1	C12_gdT
chr18-23259923-23260582	0.008875501	0.321914866	0.071	0.01	1	C12_gdT
chr20-51335362-51335815	0.008878496	0.318198429	0.054	0.007	1	C12_gdT
chr20-35740396-35740682	0.008899116	0.378743781	0.161	0.054	1	C12_gdT
chr3-149152383-149152797	0.008903974	0.343167311	0.089	0.018	1	C12_gdT
chr17-17823164-17824534	0.008905024	0.286161824	0.589	0.329	1	C12_gdT
chr6-43142184-43142470	0.008909154	0.339446997	0.071	0.011	1	C12_gdT
chr12-123483681-123484089	0.008937457	0.30661736	0.054	0.005	1	C12_gdT
chr10-84267399-84267837	0.00895151	0.3695386	0.125	0.034	1	C12_gdT
chr3-46980418-46980881	0.008965302	0.36063302	0.286	0.123	1	C12_gdT

chr12-46216212-46216480	0.008979699	0.377206439	0.196	0.062	1	C12_gdT
chr19-54850134-54850554	0.0089931	0.30802469	0.054	0.006	1	C12_gdT
chr10-88582151-88583476	0.008999701	0.350339969	0.25	0.104	1	C12_gdT
chr1-100745698-100746504	0.009024772	0.334576754	0.071	0.013	1	C12_gdT
chr16-10938322-10938583	0.009036977	0.359910768	0.107	0.023	1	C12_gdT
chr7-18509025-18509265	0.009052218	0.294988841	0.054	0.006	1	C12_gdT
chr19-57389346-57390368	0.009060685	0.259138419	0.679	0.407	1	C12_gdT
chr1-53553981-53554507	0.009088008	0.338152485	0.089	0.016	1	C12_gdT
chr2-196961875-196962181	0.009099299	0.288033081	0.054	0.004	1	C12_gdT
chr19-45508421-45508727	0.009104787	0.380284673	0.179	0.056	1	C12_gdT
chr22-50783023-50783905	0.009107833	0.350676684	0.339	0.147	1	C12_gdT
chr10-3934786-3935577	0.009121338	0.374471957	0.232	0.08	1	C12_gdT
chr15-77002869-77003809	0.009147872	0.369892008	0.25	0.095	1	C12_gdT
chr14-100103636-100103925	0.009194118	0.369707395	0.107	0.024	1	C12_gdT
chr5-68392636-68393018	0.009216804	0.296627176	0.054	0.005	1	C12_gdT
chr13-99370684-99371173	0.009216943	0.363831062	0.143	0.035	1	C12_gdT
chr13-99626579-99627034	0.009224039	0.313058405	0.054	0.005	1	C12_gdT
chr10-110455488-110455770	0.009225689	0.306535672	0.054	0.005	1	C12_gdT
chr10-6927092-6928985	0.009227912	0.323444085	0.429	0.205	1	C12_gdT
chr3-152325157-152325390	0.009232808	0.344710131	0.089	0.016	1	C12_gdT
chr3-45667961-45668228	0.009252478	0.354563201	0.089	0.015	1	C12_gdT
chr22-38982010-38982451	0.00925258	0.364970688	0.125	0.032	1	C12_gdT
chr10-11270221-11270881	0.009278492	0.362249772	0.125	0.032	1	C12_gdT
chr17-31548848-31550517	0.00928089	0.317317342	0.464	0.234	1	C12_gdT
chr7-102306831-102307323	0.00929773	0.348013549	0.071	0.01	1	C12_gdT
chrX-120549955-120550280	0.009329927	0.318791214	0.054	0.007	1	C12_gdT
chr1-185170085-185170711	0.009365015	0.353351924	0.107	0.019	1	C12_gdT

chr20-36645487-36646357	0.009390365	0.362154123	0.268	0.102	1	C12_gdT
chr9-127417972-127418491	0.009404825	0.370833806	0.125	0.031	1	C12_gdT
chr20-46010355-46011076	0.009411198	0.376029076	0.179	0.058	1	C12_gdT
chr11-88179142-88179533	0.009414846	0.339050187	0.089	0.016	1	C12_gdT
chr12-69284149-69284450	0.009437436	0.316344217	0.071	0.008	1	C12_gdT
chr17-35022429-35023134	0.009439784	0.360588826	0.268	0.102	1	C12_gdT
chr9-89556382-89556934	0.009445777	0.346813356	0.089	0.016	1	C12_gdT
chr1-31963835-31964434	0.009454407	0.371204741	0.125	0.036	1	C12_gdT
chr7-139249259-139249765	0.009456649	0.314874118	0.071	0.011	1	C12_gdT
chrX-47563827-47564068	0.009465825	0.350088688	0.089	0.02	1	C12_gdT
chr6-75611964-75612680	0.009469695	0.357151603	0.107	0.021	1	C12_gdT
chr1-235286623-235287142	0.009478753	0.330966829	0.071	0.009	1	C12_gdT
chr14-24692713-24693456	0.009517366	0.326109635	0.411	0.192	1	C12_gdT
chr9-114331181-114331606	0.009541943	0.323786732	0.071	0.011	1	C12_gdT
chr2-7438848-7439111	0.009542709	0.343390879	0.089	0.014	1	C12_gdT
chr6-12232421-12232682	0.009587821	0.30863282	0.054	0.006	1	C12_gdT
chrX-129798850-129799172	0.009613099	0.29706002	0.054	0.005	1	C12_gdT
chr2-161166427-161167334	0.009616702	0.338333961	0.339	0.154	1	C12_gdT
chr7-102404238-102404918	0.009637131	0.37535456	0.179	0.067	1	C12_gdT
chr17-67421166-67422670	0.009697181	0.288248903	0.554	0.316	1	C12_gdT
chr3-39101118-39101393	0.009712714	0.356289524	0.107	0.024	1	C12_gdT
chr7-121544667-121545144	0.009731366	0.357630266	0.107	0.02	1	C12_gdT
chr22-35256916-35258077	0.009735569	0.306112094	0.518	0.261	1	C12_gdT
chr4-100101109-100101413	0.009759457	0.294473437	0.054	0.005	1	C12_gdT
chr21-46183893-46185251	0.009771553	0.322525232	0.446	0.215	1	C12_gdT
chr11-35943805-35944436	0.009787935	0.291462206	0.054	0.005	1	C12_gdT
chr5-35810251-35811725	0.009788424	0.332425949	0.393	0.187	1	C12_gdT

chr19-1884000-1884685	0.009821723	0.373603587	0.196	0.066	1	C12_gdT
chr22-37162263-37164547	0.009832035	0.267778893	0.679	0.361	1	C12_gdT
chr6-4906471-4907178	0.009877314	0.31426469	0.054	0.007	1	C12_gdT
chr17-63434839-63435159	0.009886819	0.342196793	0.089	0.019	1	C12_gdT
chr17-48625735-48626892	0.009929179	0.37706988	0.214	0.067	1	C12_gdT
chr7-69651030-69651552	0.009944412	0.295974595	0.054	0.005	1	C12_gdT
chr22-40436455-40437256	0.009946226	0.351185163	0.107	0.021	1	C12_gdT
chr6-156828054-156828704	0.009963446	0.34498908	0.089	0.016	1	C12_gdT

**Differentially accessible peaks (DAPs) of T cell clusters in AS plaque and exhausted T cells.**						
**Peak**	***P* value**	**avg_logFC**	**pct.1**	**pct.2**	**adjusted *P* value**	**cluster**
chr17-22521254-22521613	3.80087E-69	0.745569079	0.772	0.278	2.51705E-64	C1_CD8_Tres
chrX-317539-319259	2.58242E-68	0.675579487	0.814	0.299	1.71015E-63	C1_CD8_Tres
chr1-633460-634698	2.63058E-61	0.684577475	0.769	0.293	1.74205E-56	C1_CD8_Tres
chr5-82356754-82357721	1.04741E-60	0.811227518	0.365	0.068	6.93626E-56	C1_CD8_Tres
chr3-32963641-32964287	3.99338E-59	0.844955276	0.269	0.026	2.64454E-54	C1_CD8_Tres
chr11-13868785-13869542	1.46732E-57	0.817486829	0.263	0.026	9.71703E-53	C1_CD8_Tres
chr17-82126395-82127458	3.57657E-52	0.70054836	0.449	0.129	2.36851E-47	C1_CD8_Tres
chr21-45642617-45643885	5.52663E-51	0.67568801	0.392	0.118	3.6599E-46	C1_CD8_Tres
chr11-129853443-129854439	4.39413E-50	0.750029984	0.18	0.01	2.90992E-45	C1_CD8_Tres
chr2-113876400-113877141	1.02008E-48	0.737727852	0.204	0.018	6.75529E-44	C1_CD8_Tres
chr3-32296559-32298058	7.04148E-48	0.716085073	0.287	0.055	4.66308E-43	C1_CD8_Tres
chr20-5736988-5737733	5.81466E-47	0.683078769	0.219	0.027	3.85064E-42	C1_CD8_Tres
chr17-2814110-2815366	1.2617E-45	0.653274964	0.497	0.175	8.35533E-41	C1_CD8_Tres
chr1-83508052-83508796	3.63921E-45	0.659821301	0.129	0.003	2.41E-40	C1_CD8_Tres
chr15-33104022-33104674	2.4215E-44	0.690465183	0.216	0.026	1.60359E-39	C1_CD8_Tres

chr2-206530475-206531411	1.19313E-43	0.620262611	0.129	0.005	7.90129E-39	C1_CD8_Tres
chr7-74451939-74452898	2.80838E-43	0.657824435	0.365	0.097	1.8598E-38	C1_CD8_Tres
chr19-43667974-43669476	9.67163E-40	0.522033166	0.602	0.282	6.40484E-35	C1_CD8_Tres
chr1-23958530-23960278	2.65789E-38	0.527470675	0.575	0.248	1.76013E-33	C1_CD8_Tres
chr22-28882669-28884423	9.25429E-38	0.529199688	0.488	0.22	6.12847E-33	C1_CD8_Tres
chr21-36288508-36289609	7.63958E-37	0.621435779	0.284	0.078	5.05916E-32	C1_CD8_Tres
chr19-50328720-50329660	2.24213E-36	0.552194172	0.395	0.157	1.48481E-31	C1_CD8_Tres
chr3-112074691-112075380	1.94249E-35	0.637549	0.132	0.006	1.28638E-30	C1_CD8_Tres
chr2-112187547-112188686	7.89414E-35	0.540559486	0.389	0.152	5.22774E-30	C1_CD8_Tres
chr12-109981321-109981669	8.27638E-35	0.5708341	0.114	0.006	5.48087E-30	C1_CD8_Tres
chr20-5714028-5715411	8.29342E-35	0.527268459	0.413	0.156	5.49215E-30	C1_CD8_Tres
chr12-133079608-133081270	3.11646E-34	0.51529982	0.569	0.241	2.06381E-29	C1_CD8_Tres
chr9-33415251-33415946	4.57764E-34	0.592112104	0.231	0.048	3.03145E-29	C1_CD8_Tres
chr11-46331145-46331748	4.90768E-34	0.626192784	0.153	0.014	3.25002E-29	C1_CD8_Tres
chr11-129846333-129846856	5.21613E-34	0.593206074	0.129	0.008	3.45428E-29	C1_CD8_Tres
chr16-27195818-27196446	9.43037E-34	0.625708625	0.192	0.032	6.24508E-29	C1_CD8_Tres
chr12-104218955-104220318	2.06991E-33	0.582720358	0.243	0.058	1.37075E-28	C1_CD8_Tres
chr19-49567775-49568531	2.1416E-33	0.61060134	0.24	0.051	1.41823E-28	C1_CD8_Tres
chr11-128840076-128840452	2.30596E-33	0.52726101	0.114	0.008	1.52708E-28	C1_CD8_Tres
chr7-86978465-86979389	4.45982E-33	0.588348445	0.234	0.049	2.95343E-28	C1_CD8_Tres
chr8-144146829-144148682	1.02688E-32	0.47427437	0.488	0.213	6.80028E-28	C1_CD8_Tres
chr5-77155202-77155772	2.62898E-32	0.468742466	0.081	0.002	1.74099E-27	C1_CD8_Tres
chr7-63925380-63926794	3.43633E-32	0.591528592	0.314	0.089	2.27564E-27	C1_CD8_Tres
chr1-33128184-33128619	5.487E-32	0.571484619	0.159	0.021	3.63365E-27	C1_CD8_Tres
chr18-2889646-2890237	7.18818E-32	0.602816413	0.219	0.04	4.76023E-27	C1_CD8_Tres
chr15-88904290-88905053	1.28201E-31	0.556587335	0.353	0.122	8.48982E-27	C1_CD8_Tres
chr3-187028971-187029250	1.87153E-31	0.548489634	0.105	0.006	1.23938E-26	C1_CD8_Tres

chr17-75036583-75037040	1.64037E-30	0.572139618	0.308	0.101	1.0863E-25	C1_CD8_Tres
chr10-131900106-131901808	1.66157E-30	0.503829364	0.446	0.179	1.10034E-25	C1_CD8_Tres
chr2-201448194-201448990	3.12648E-30	0.596182191	0.195	0.03	2.07045E-25	C1_CD8_Tres
chr5-35853576-35854712	1.36093E-29	0.465401258	0.44	0.208	9.01252E-25	C1_CD8_Tres
chr2-148524150-148525059	1.41956E-29	0.558295194	0.219	0.046	9.40073E-25	C1_CD8_Tres
chr15-97959959-97961285	2.2358E-29	0.582760621	0.326	0.101	1.48062E-24	C1_CD8_Tres
chr1-121184236-121185085	2.98094E-29	0.53496817	0.47	0.186	1.97407E-24	C1_CD8_Tres
chr12-56291386-56292107	5.87559E-29	0.526946718	0.135	0.016	3.89099E-24	C1_CD8_Tres
chr2-160417640-160418130	6.45285E-29	0.57914154	0.168	0.025	4.27327E-24	C1_CD8_Tres
chr3-112128704-112129279	1.11301E-28	0.566335937	0.168	0.025	7.3707E-24	C1_CD8_Tres
chr21-44870488-44870976	1.29624E-28	0.529051428	0.144	0.021	8.58412E-24	C1_CD8_Tres
chr21-36155689-36157441	1.40186E-28	0.339027665	0.76	0.56	9.28355E-24	C1_CD8_Tres
chr12-133036812-133038023	1.89258E-28	0.48786114	0.362	0.15	1.25332E-23	C1_CD8_Tres
chr5-98773002-98774550	2.61222E-28	0.502812344	0.296	0.111	1.72989E-23	C1_CD8_Tres
chr7-38433669-38434193	2.778E-28	0.531269642	0.102	0.005	1.83967E-23	C1_CD8_Tres
chr16-10963888-10965181	2.81992E-28	0.446105338	0.434	0.217	1.86744E-23	C1_CD8_Tres
chr15-92813428-92813750	3.70739E-28	0.514710868	0.141	0.02	2.45515E-23	C1_CD8_Tres
chr1-23979709-23980925	3.86076E-28	0.483689341	0.395	0.167	2.55671E-23	C1_CD8_Tres
chr4-122156762-122157172	4.92609E-28	0.53874323	0.141	0.018	3.26221E-23	C1_CD8_Tres
chr12-11998194-11999166	5.16212E-28	0.53357167	0.228	0.058	3.41851E-23	C1_CD8_Tres
chr12-64683968-64685368	5.64793E-28	0.522039815	0.275	0.081	3.74023E-23	C1_CD8_Tres
chr11-133944712-133945688	9.51792E-28	0.496429113	0.437	0.206	6.30305E-23	C1_CD8_Tres
chr19-46633689-46634603	1.2449E-27	0.443232722	0.404	0.188	8.24412E-23	C1_CD8_Tres
chr11-128339693-128340101	1.90685E-27	0.508354759	0.123	0.012	1.26277E-22	C1_CD8_Tres
chr2-39099601-39100566	2.11689E-27	0.531978444	0.249	0.074	1.40187E-22	C1_CD8_Tres
chr7-134710579-134711526	2.51477E-27	0.522748778	0.201	0.048	1.66536E-22	C1_CD8_Tres
chr22-31278702-31279429	2.53104E-27	0.541863188	0.165	0.029	1.67613E-22	C1_CD8_Tres

chr11-11149350-11150552	2.72354E-27	0.514979773	0.231	0.06	1.80361E-22	C1_CD8_Tres
chrX-8987543-8988639	4.61307E-27	0.520065508	0.114	0.01	3.05491E-22	C1_CD8_Tres
chr11-129813909-129814861	5.67441E-27	0.53121506	0.12	0.012	3.75776E-22	C1_CD8_Tres
chr10-5317444-5318188	7.08045E-27	0.517487735	0.18	0.04	4.68889E-22	C1_CD8_Tres
chr2-197869407-197870296	1.80334E-26	0.470312545	0.108	0.01	1.19422E-21	C1_CD8_Tres
chr19-8591726-8592924	1.85348E-26	0.477412579	0.344	0.138	1.22743E-21	C1_CD8_Tres
chr19-679697-681238	2.79859E-26	0.342468201	0.647	0.449	1.85331E-21	C1_CD8_Tres
chr11-68962496-68963150	2.82058E-26	0.484100372	0.081	0.003	1.86787E-21	C1_CD8_Tres
chr12-15546160-15546740	3.36978E-26	0.481700981	0.075	0.002	2.23157E-21	C1_CD8_Tres
chr11-121464061-121464975	3.91504E-26	0.463748041	0.455	0.218	2.59266E-21	C1_CD8_Tres
chr10-132536674-132537961	5.60645E-26	0.330815282	0.707	0.534	3.71276E-21	C1_CD8_Tres
chr2-96962262-96963250	5.91209E-26	0.438445048	0.302	0.122	3.91517E-21	C1_CD8_Tres
chr15-82286287-82287632	7.34749E-26	0.450926241	0.099	0.009	4.86573E-21	C1_CD8_Tres
chr3-52216805-52217645	7.48482E-26	0.491619564	0.225	0.067	4.95667E-21	C1_CD8_Tres
chr6-134529945-134530292	7.80928E-26	0.421875481	0.075	0.003	5.17154E-21	C1_CD8_Tres
chr3-13879587-13880852	1.08113E-25	0.502502221	0.168	0.033	7.15959E-21	C1_CD8_Tres
chr18-58605329-58605745	1.82744E-25	0.48961473	0.105	0.009	1.21019E-20	C1_CD8_Tres
chr3-31213468-31214195	4.41925E-25	0.481490566	0.135	0.02	2.92656E-20	C1_CD8_Tres
chr5-57117109-57118076	4.6624E-25	0.502966417	0.168	0.034	3.08758E-20	C1_CD8_Tres
chr1-120941840-120942777	5.79753E-25	0.494693148	0.302	0.112	3.8393E-20	C1_CD8_Tres
chr17-80605163-80606101	6.50264E-25	0.515226039	0.165	0.033	4.30624E-20	C1_CD8_Tres
chr2-162011620-162012049	6.92842E-25	0.485454971	0.189	0.047	4.58821E-20	C1_CD8_Tres
chr1-15523836-15525464	1.06113E-24	0.284260088	0.766	0.607	7.02711E-20	C1_CD8_Tres
chr10-9097450-9097902	1.39097E-24	0.473373637	0.081	0.004	9.21139E-20	C1_CD8_Tres
chr8-18957182-18957952	1.53075E-24	0.571110744	0.216	0.049	1.01371E-19	C1_CD8_Tres
chr1-30717614-30719544	1.9207E-24	0.27461632	0.787	0.648	1.27195E-19	C1_CD8_Tres
chr7-36152276-36153823	2.04189E-24	0.299457649	0.704	0.544	1.3522E-19	C1_CD8_Tres

chr10-5291424-5292650	2.21905E-24	0.501233344	0.278	0.097	1.46952E-19	C1_CD8_Tres
chr15-63446190-63446645	4.05359E-24	0.473315165	0.087	0.005	2.68441E-19	C1_CD8_Tres
chr19-19605807-19606451	4.12717E-24	0.440626722	0.308	0.127	2.73314E-19	C1_CD8_Tres
chr17-76198813-76199833	4.5193E-24	0.466128766	0.177	0.045	2.99282E-19	C1_CD8_Tres
chr5-142223267-142224134	5.57883E-24	0.444587947	0.254	0.096	3.69447E-19	C1_CD8_Tres
chr7-77393208-77393730	7.96235E-24	0.497998928	0.138	0.024	5.27291E-19	C1_CD8_Tres
chr5-134103456-134103722	1.3661E-23	0.43374811	0.084	0.006	9.04672E-19	C1_CD8_Tres
chr20-63552544-63554373	1.54318E-23	0.364703562	0.485	0.299	1.02194E-18	C1_CD8_Tres
chr19-3275065-3276762	1.56836E-23	0.387348192	0.494	0.289	1.03861E-18	C1_CD8_Tres
chr5-52987434-52988237	1.91292E-23	0.505813346	0.21	0.057	1.26679E-18	C1_CD8_Tres
chr8-19496478-19497608	3.63715E-23	0.425414229	0.404	0.199	2.40863E-18	C1_CD8_Tres
chr15-60580112-60580970	3.73532E-23	0.467031802	0.249	0.09	2.47364E-18	C1_CD8_Tres
chr16-352134-353545	3.74363E-23	0.331969925	0.584	0.391	2.47914E-18	C1_CD8_Tres
chr19-9791501-9793338	4.67648E-23	0.300767891	0.635	0.492	3.0969E-18	C1_CD8_Tres
chr8-144136968-144138476	4.84082E-23	0.432229091	0.377	0.162	3.20574E-18	C1_CD8_Tres
chr9-89456643-89457725	4.92121E-23	0.479408647	0.18	0.047	3.25897E-18	C1_CD8_Tres
chrX-1208026-1208776	5.3638E-23	0.487096744	0.168	0.038	3.55207E-18	C1_CD8_Tres
chr16-89114598-89115866	5.82418E-23	0.381789168	0.431	0.249	3.85695E-18	C1_CD8_Tres
chr2-230254210-230254912	8.46374E-23	0.450728938	0.111	0.016	5.60494E-18	C1_CD8_Tres
chr5-134927520-134928170	1.01018E-22	0.52776801	0.254	0.076	6.68969E-18	C1_CD8_Tres
chr16-4957618-4958544	1.03067E-22	0.448593458	0.254	0.095	6.82543E-18	C1_CD8_Tres
chr7-150478861-150479476	1.17116E-22	0.500323892	0.159	0.033	7.7558E-18	C1_CD8_Tres
chr18-9090970-9091665	1.82207E-22	0.463476366	0.344	0.155	1.20663E-17	C1_CD8_Tres
chr13-73984811-73986085	1.92196E-22	0.460303946	0.126	0.021	1.27278E-17	C1_CD8_Tres
chr4-84582300-84583857	2.45473E-22	0.486981832	0.228	0.066	1.62559E-17	C1_CD8_Tres
chr13-73058336-73059823	2.47708E-22	0.448098992	0.344	0.147	1.6404E-17	C1_CD8_Tres
chr19-18635842-18637151	3.17043E-22	0.319933812	0.656	0.485	2.09955E-17	C1_CD8_Tres

chr7-115230796-115231520	3.61823E-22	0.519769672	0.18	0.038	2.3961E-17	C1_CD8_Tres
chr4-108107558-108108443	4.38157E-22	0.463851997	0.246	0.089	2.90161E-17	C1_CD8_Tres
chr3-7876673-7877294	6.14285E-22	0.522709637	0.102	0.008	4.06798E-17	C1_CD8_Tres
chr1-3032986-3033553	6.95162E-22	0.350031776	0.057	0.002	4.60357E-17	C1_CD8_Tres
chr5-154829296-154830233	7.6524E-22	0.424963181	0.15	0.032	5.06765E-17	C1_CD8_Tres
chr5-81006557-81008275	7.88297E-22	0.444886545	0.189	0.051	5.22034E-17	C1_CD8_Tres
chr2-112181425-112183042	8.11328E-22	0.410582515	0.117	0.024	5.37285E-17	C1_CD8_Tres
chrX-1452437-1454445	9.5588E-22	0.294571942	0.713	0.553	6.33012E-17	C1_CD8_Tres
chr11-4090275-4090505	1.22747E-21	0.402406106	0.081	0.007	8.12866E-17	C1_CD8_Tres
chr9-79581567-79582252	1.33556E-21	0.476221886	0.105	0.012	8.84449E-17	C1_CD8_Tres
chr19-9818223-9819870	1.45016E-21	0.276063397	0.725	0.57	9.60338E-17	C1_CD8_Tres
chr7-63899747-63901071	1.50985E-21	0.472590611	0.219	0.066	9.99867E-17	C1_CD8_Tres
chr20-3122892-3123357	1.64824E-21	0.450590627	0.099	0.011	1.09152E-16	C1_CD8_Tres
chr19-35207312-35207769	1.65111E-21	0.47256939	0.174	0.041	1.09341E-16	C1_CD8_Tres
chr5-137737372-137738112	1.69135E-21	0.445042173	0.129	0.025	1.12006E-16	C1_CD8_Tres
chr4-102630512-102631095	1.74664E-21	0.466850494	0.132	0.024	1.15667E-16	C1_CD8_Tres
chr1-24506618-24507685	1.76849E-21	0.436619077	0.174	0.048	1.17115E-16	C1_CD8_Tres
chr1-1918791-1919873	1.8966E-21	0.333718434	0.536	0.366	1.25599E-16	C1_CD8_Tres
chr2-240232910-240234054	1.98185E-21	0.48303464	0.168	0.039	1.31244E-16	C1_CD8_Tres
chr19-2702211-2702989	2.13196E-21	0.410251654	0.216	0.079	1.41185E-16	C1_CD8_Tres
chr22-24305022-24305671	2.3274E-21	0.426572642	0.141	0.031	1.54128E-16	C1_CD8_Tres
chr1-235206595-235207509	2.80588E-21	0.445035563	0.237	0.091	1.85814E-16	C1_CD8_Tres
chr9-40991238-40992772	3.23214E-21	0.360534696	0.569	0.393	2.14042E-16	C1_CD8_Tres
chr10-3867188-3868292	3.26219E-21	0.443055402	0.198	0.066	2.16032E-16	C1_CD8_Tres
chr6-73391601-73391958	3.40432E-21	0.39296139	0.081	0.007	2.25444E-16	C1_CD8_Tres
chr10-43331916-43332565	3.98481E-21	0.480238485	0.141	0.025	2.63886E-16	C1_CD8_Tres
chr10-11684325-11685479	4.09722E-21	0.461931893	0.171	0.044	2.7133E-16	C1_CD8_Tres

chr1-147716927-147717501	4.36665E-21	0.400082492	0.069	0.004	2.89173E-16	C1_CD8_Tres
chr17-80791257-80792212	4.61558E-21	0.397367927	0.12	0.026	3.05658E-16	C1_CD8_Tres
chr8-8917273-8918050	5.14816E-21	0.453058911	0.249	0.091	3.40927E-16	C1_CD8_Tres
chr6-149488799-149489139	5.59634E-21	0.456757849	0.111	0.015	3.70606E-16	C1_CD8_Tres
chr1-87403957-87404678	5.99693E-21	0.514402785	0.147	0.026	3.97135E-16	C1_CD8_Tres
chr1-629778-630252	6.51468E-21	0.450485722	0.293	0.116	4.31422E-16	C1_CD8_Tres
chr17-78250773-78252846	6.86027E-21	0.396445266	0.32	0.16	4.54308E-16	C1_CD8_Tres
chr7-142535063-142535860	6.90644E-21	0.466574411	0.189	0.047	4.57365E-16	C1_CD8_Tres
chr3-112071979-112072769	7.13136E-21	0.4519781	0.096	0.01	4.7226E-16	C1_CD8_Tres
chr2-98479473-98480580	7.15881E-21	0.432692209	0.225	0.078	4.74078E-16	C1_CD8_Tres
chr4-98688335-98689318	8.53097E-21	0.445816598	0.105	0.013	5.64947E-16	C1_CD8_Tres
chr7-140059213-140059829	1.02969E-20	0.44535384	0.129	0.026	6.81892E-16	C1_CD8_Tres
chr4-122577849-122579037	1.35867E-20	0.41297179	0.24	0.096	8.99754E-16	C1_CD8_Tres
chr14-64755533-64756287	1.49086E-20	0.45616637	0.29	0.116	9.87289E-16	C1_CD8_Tres
chr1-154418172-154418597	1.57346E-20	0.344575543	0.06	0.004	1.04199E-15	C1_CD8_Tres
chr7-1046744-1046983	1.63613E-20	0.368814683	0.066	0.004	1.08349E-15	C1_CD8_Tres
chr15-33125871-33127048	1.63617E-20	0.413838907	0.102	0.013	1.08352E-15	C1_CD8_Tres
chr17-77324774-77325650	1.64585E-20	0.410666872	0.225	0.084	1.08993E-15	C1_CD8_Tres
chr19-16330210-16331296	1.83722E-20	0.387552326	0.461	0.273	1.21666E-15	C1_CD8_Tres
chr12-104498240-104498577	2.06254E-20	0.398726473	0.114	0.023	1.36587E-15	C1_CD8_Tres
chr3-187027208-187027918	2.08606E-20	0.442835224	0.186	0.057	1.38145E-15	C1_CD8_Tres
chr5-174454197-174454784	2.19337E-20	0.385440279	0.069	0.005	1.45252E-15	C1_CD8_Tres
chr7-130930059-130930652	2.22557E-20	0.462225548	0.165	0.041	1.47384E-15	C1_CD8_Tres
chr19-35140757-35141944	2.84099E-20	0.364285923	0.44	0.255	1.88139E-15	C1_CD8_Tres
chr8-8868529-8869756	3.9954E-20	0.451076603	0.257	0.09	2.64587E-15	C1_CD8_Tres
chr4-151407888-151409396	5.34941E-20	0.402257075	0.171	0.052	3.54254E-15	C1_CD8_Tres
chr19-1081870-1082759	5.93138E-20	0.419958072	0.311	0.138	3.92794E-15	C1_CD8_Tres

chr6-90456933-90457172	6.5754E-20	0.333476719	0.054	0.003	4.35442E-15	C1_CD8_Tres
chr4-109648192-109649022	6.84229E-20	0.44288329	0.183	0.054	4.53117E-15	C1_CD8_Tres
chr19-19602171-19602464	6.89305E-20	0.381149493	0.123	0.027	4.56479E-15	C1_CD8_Tres
chr14-64698882-64699230	7.01359E-20	0.378919625	0.066	0.004	4.64461E-15	C1_CD8_Tres
chr17-41403563-41404364	7.18867E-20	0.437789643	0.165	0.044	4.76055E-15	C1_CD8_Tres
chr3-112132950-112133478	7.93464E-20	0.403302098	0.108	0.019	5.25455E-15	C1_CD8_Tres
chr17-48194005-48194522	9.41219E-20	0.392723222	0.054	0.001	6.23304E-15	C1_CD8_Tres
chr11-95688151-95689305	9.76363E-20	0.460653042	0.159	0.038	6.46577E-15	C1_CD8_Tres
chr5-80125699-80126474	9.80436E-20	0.446005305	0.21	0.067	6.49274E-15	C1_CD8_Tres
chr1-63651066-63651653	1.2271E-19	0.368221565	0.066	0.003	8.1262E-15	C1_CD8_Tres
chr19-38263762-38264979	1.5212E-19	0.403452565	0.168	0.051	1.00739E-14	C1_CD8_Tres
chr3-187006868-187007491	1.67306E-19	0.411275212	0.084	0.009	1.10795E-14	C1_CD8_Tres
chr19-37778627-37780020	1.68525E-19	0.308407618	0.476	0.338	1.11602E-14	C1_CD8_Tres
chr9-99112799-99113629	2.49992E-19	0.437220579	0.123	0.025	1.65552E-14	C1_CD8_Tres
chr5-133170668-133171170	2.8614E-19	0.400246192	0.069	0.004	1.89491E-14	C1_CD8_Tres
chr14-31937565-31938397	4.0446E-19	0.496481565	0.201	0.059	2.67845E-14	C1_CD8_Tres
chr19-51354750-51355094	4.06605E-19	0.382263052	0.063	0.003	2.69266E-14	C1_CD8_Tres
chr11-35216770-35217513	4.10449E-19	0.405450713	0.21	0.074	2.71812E-14	C1_CD8_Tres
chr14-31944184-31945344	4.67141E-19	0.387558225	0.072	0.005	3.09355E-14	C1_CD8_Tres
chr5-95631738-95632499	4.90584E-19	0.430465115	0.177	0.051	3.24879E-14	C1_CD8_Tres
chr4-1511563-1512056	5.37151E-19	0.383028199	0.069	0.005	3.55718E-14	C1_CD8_Tres
chr8-60792613-60793213	5.63756E-19	0.424252324	0.171	0.049	3.73336E-14	C1_CD8_Tres
chrX-41382908-41384432	6.74087E-19	0.437035015	0.186	0.059	4.46401E-14	C1_CD8_Tres
chr17-22524315-22524530	8.05483E-19	0.47217194	0.177	0.044	5.33415E-14	C1_CD8_Tres
chr12-31736324-31736891	8.38247E-19	0.405094871	0.251	0.103	5.55112E-14	C1_CD8_Tres
chr22-40224483-40225060	9.46434E-19	0.435786663	0.087	0.009	6.26757E-14	C1_CD8_Tres
chr19-41774440-41775003	9.54529E-19	0.374420094	0.087	0.011	6.32118E-14	C1_CD8_Tres

chr6-161076139-161076710	9.55545E-19	0.41185918	0.102	0.016	6.3279E-14	C1_CD8_Tres
chr5-157158945-157160332	1.0407E-18	0.338335194	0.449	0.282	6.89182E-14	C1_CD8_Tres
chr7-5833513-5834769	1.06725E-18	0.373361061	0.072	0.007	7.06763E-14	C1_CD8_Tres
chr8-127196582-127197223	1.11121E-18	0.409382931	0.09	0.012	7.35876E-14	C1_CD8_Tres
chr3-42501370-42502957	1.19025E-18	0.391416952	0.305	0.139	7.8822E-14	C1_CD8_Tres
chr22-45172734-45173508	1.43227E-18	0.364145912	0.093	0.015	9.48494E-14	C1_CD8_Tres
chr2-9781835-9782724	1.4434E-18	0.393346852	0.263	0.108	9.55864E-14	C1_CD8_Tres
chr12-21615425-21615792	1.49997E-18	0.361488108	0.069	0.007	9.93322E-14	C1_CD8_Tres
chr11-96012030-96012570	1.52626E-18	0.42272032	0.26	0.106	1.01074E-13	C1_CD8_Tres
chr17-57879920-57880394	1.64702E-18	0.37300419	0.072	0.007	1.0907E-13	C1_CD8_Tres
chr5-35830501-35830963	1.6898E-18	0.38036937	0.072	0.006	1.11904E-13	C1_CD8_Tres
chr1-1098814-1099840	1.69621E-18	0.408288593	0.093	0.012	1.12328E-13	C1_CD8_Tres
chr4-108166096-108167509	1.83633E-18	0.40487048	0.228	0.082	1.21607E-13	C1_CD8_Tres
chr6-3370286-3371101	1.88292E-18	0.402765367	0.075	0.006	1.24692E-13	C1_CD8_Tres
chr10-101829579-101831176	2.17007E-18	0.2807712	0.629	0.469	1.43709E-13	C1_CD8_Tres
chr1-244047161-244049716	2.18278E-18	0.279101533	0.65	0.492	1.4455E-13	C1_CD8_Tres
chr19-10142429-10143140	2.21428E-18	0.411739395	0.156	0.049	1.46636E-13	C1_CD8_Tres
chr16-29827065-29827442	2.79119E-18	0.363465961	0.072	0.008	1.84841E-13	C1_CD8_Tres
chr19-7920040-7921116	2.82495E-18	0.274928525	0.611	0.463	1.87077E-13	C1_CD8_Tres
chr7-831634-832970	2.88879E-18	0.379161591	0.299	0.14	1.91305E-13	C1_CD8_Tres
chr11-133946903-133948357	3.05076E-18	0.432729341	0.186	0.056	2.02031E-13	C1_CD8_Tres
chr13-98998744-98999348	4.07606E-18	0.374790799	0.111	0.022	2.69929E-13	C1_CD8_Tres
chr3-93470260-93470829	4.37216E-18	0.366742856	0.674	0.293	2.89537E-13	C1_CD8_Tres
chr3-186589739-186590295	4.41615E-18	0.422886197	0.105	0.017	2.92451E-13	C1_CD8_Tres
chr11-133927455-133928846	4.6118E-18	0.437180477	0.183	0.057	3.05407E-13	C1_CD8_Tres
chr15-59803380-59804029	4.75877E-18	0.478096356	0.123	0.02	3.1514E-13	C1_CD8_Tres
chr10-470188-471014	4.83698E-18	0.43554951	0.138	0.032	3.2032E-13	C1_CD8_Tres

chr7-157502480-157503684	4.87158E-18	0.381660806	0.26	0.123	3.22611E-13	C1_CD8_Tres
chr14-104389873-104390222	4.92055E-18	0.33560665	0.06	0.004	3.25853E-13	C1_CD8_Tres
chr15-66911780-66912419	5.55781E-18	0.376926519	0.075	0.007	3.68055E-13	C1_CD8_Tres
chr1-223363515-223364829	5.58485E-18	0.36532791	0.105	0.021	3.69846E-13	C1_CD8_Tres
chrX-48714555-48715412	5.58802E-18	0.408263945	0.18	0.059	3.70055E-13	C1_CD8_Tres
chr17-19771878-19772442	5.62318E-18	0.326361824	0.06	0.006	3.72384E-13	C1_CD8_Tres
chr9-129895290-129895783	6.29692E-18	0.402013868	0.108	0.02	4.17001E-13	C1_CD8_Tres
chr2-109613514-109614698	6.39024E-18	0.389370425	0.153	0.043	4.23181E-13	C1_CD8_Tres
chr12-13195937-13196934	6.41713E-18	0.395444658	0.308	0.145	4.24962E-13	C1_CD8_Tres
chr1-101235126-101237022	6.47972E-18	0.370155822	0.338	0.183	4.29106E-13	C1_CD8_Tres
chr15-82539674-82541121	7.15086E-18	0.3290858	0.407	0.247	4.73552E-13	C1_CD8_Tres
chr4-185254344-185254839	7.87817E-18	0.405338137	0.135	0.033	5.21716E-13	C1_CD8_Tres
chr13-73702210-73703076	8.68743E-18	0.401511817	0.135	0.031	5.75307E-13	C1_CD8_Tres
chr5-758898-759475	9.27043E-18	0.384460798	0.114	0.023	6.13916E-13	C1_CD8_Tres
chr2-191739699-191740487	9.33449E-18	0.42611867	0.165	0.047	6.18158E-13	C1_CD8_Tres
chr9-120846384-120846831	1.03397E-17	0.429282411	0.171	0.049	6.84727E-13	C1_CD8_Tres
chrX-136619777-136620391	1.09259E-17	0.436349701	0.135	0.031	7.23547E-13	C1_CD8_Tres
chr13-109785441-109787258	1.13417E-17	0.314775219	0.425	0.254	7.5108E-13	C1_CD8_Tres
chr4-153205070-153206058	1.13936E-17	0.359398245	0.078	0.009	7.54521E-13	C1_CD8_Tres
chr11-129836518-129836864	1.2792E-17	0.36311407	0.06	0.003	8.47124E-13	C1_CD8_Tres
chr2-207143134-207144070	1.28959E-17	0.38470035	0.093	0.013	8.54004E-13	C1_CD8_Tres
chr8-8843985-8844590	1.30383E-17	0.449198745	0.153	0.037	8.63435E-13	C1_CD8_Tres
chr6-35331999-35332596	1.72418E-17	0.424259039	0.165	0.047	1.1418E-12	C1_CD8_Tres
chr5-56828983-56829541	1.84502E-17	0.405688128	0.111	0.022	1.22183E-12	C1_CD8_Tres
chr11-64899995-64900620	1.87118E-17	0.358287487	0.21	0.09	1.23915E-12	C1_CD8_Tres
chr1-61736160-61736819	2.07532E-17	0.336993367	0.072	0.008	1.37434E-12	C1_CD8_Tres
chr13-74069847-74070979	2.24882E-17	0.386432586	0.171	0.053	1.48923E-12	C1_CD8_Tres

chr2-73978805-73979404	2.33396E-17	0.408506101	0.195	0.071	1.54562E-12	C1_CD8_Tres
chr14-32200889-32203910	2.54105E-17	0.346156965	0.311	0.16	1.68276E-12	C1_CD8_Tres
chr4-87103173-87104125	2.88981E-17	0.411687158	0.159	0.05	1.91372E-12	C1_CD8_Tres
chr21-45590378-45592664	3.19096E-17	0.335141302	0.314	0.161	2.11315E-12	C1_CD8_Tres
chr1-234408552-234409644	3.28781E-17	0.393390482	0.09	0.012	2.17728E-12	C1_CD8_Tres
chr8-27897108-27897853	3.9435E-17	0.371912941	0.096	0.016	2.6115E-12	C1_CD8_Tres
chr3-188322747-188323755	4.00202E-17	0.390598236	0.192	0.068	2.65026E-12	C1_CD8_Tres
chr6-29845040-29845804	4.15097E-17	0.390361323	0.18	0.074	2.7489E-12	C1_CD8_Tres
chr1-37673680-37674333	4.22708E-17	0.424594798	0.096	0.015	2.7993E-12	C1_CD8_Tres
chr17-1871661-1872163	4.22737E-17	0.402524776	0.087	0.01	2.79949E-12	C1_CD8_Tres
chr5-35129908-35130508	4.63202E-17	0.370298047	0.069	0.006	3.06746E-12	C1_CD8_Tres
chr8-80864501-80865161	5.42237E-17	0.400201196	0.293	0.135	3.59086E-12	C1_CD8_Tres
chr15-88627297-88627807	5.58513E-17	0.363113924	0.177	0.067	3.69864E-12	C1_CD8_Tres
chr9-131500277-131501370	6.17837E-17	0.315308751	0.371	0.229	4.0915E-12	C1_CD8_Tres
chr18-77140699-77141089	6.36766E-17	0.333838565	0.063	0.005	4.21686E-12	C1_CD8_Tres
chr4-140150254-140152549	6.51865E-17	0.349429673	0.281	0.145	4.31684E-12	C1_CD8_Tres
chr19-47427236-47428586	6.562E-17	0.31395749	0.317	0.18	4.34556E-12	C1_CD8_Tres
chr2-218315799-218316938	7.05742E-17	0.33694187	0.117	0.031	4.67363E-12	C1_CD8_Tres
chr7-44633252-44634170	8.2448E-17	0.303120227	0.473	0.319	5.45996E-12	C1_CD8_Tres
chr7-94656122-94658687	8.42126E-17	0.332052375	0.545	0.35	5.57681E-12	C1_CD8_Tres
chrX-46320257-46320809	8.59182E-17	0.386982055	0.093	0.014	5.68976E-12	C1_CD8_Tres
chr1-234844749-234845682	8.66156E-17	0.355851565	0.156	0.051	5.73594E-12	C1_CD8_Tres
chr7-75920383-75920747	9.13811E-17	0.419490604	0.081	0.009	6.05153E-12	C1_CD8_Tres
chr10-6593172-6593523	9.88848E-17	0.346141829	0.057	0.004	6.54845E-12	C1_CD8_Tres
chr1-59814144-59815420	1.02231E-16	0.34693471	0.257	0.125	6.77007E-12	C1_CD8_Tres
chr5-1105490-1106169	1.0478E-16	0.423895232	0.162	0.045	6.93883E-12	C1_CD8_Tres
chr6-143549556-143549930	1.07154E-16	0.370661157	0.096	0.018	7.09608E-12	C1_CD8_Tres

chr10-73932104-73932872	1.26348E-16	0.400845067	0.144	0.034	8.36714E-12	C1_CD8_Tres
chr5-134086388-134087356	1.39174E-16	0.390749022	0.102	0.018	9.21653E-12	C1_CD8_Tres
chr4-36394180-36394970	1.69501E-16	0.318611892	0.374	0.237	1.12248E-11	C1_CD8_Tres
chr3-31377041-31377469	1.69905E-16	0.415455147	0.084	0.01	1.12516E-11	C1_CD8_Tres
chr13-36345038-36346947	1.72962E-16	0.37585861	0.21	0.091	1.14541E-11	C1_CD8_Tres
chr10-132158881-132159787	1.81985E-16	0.39288333	0.201	0.084	1.20516E-11	C1_CD8_Tres
chr14-69643291-69643925	2.10287E-16	0.359069533	0.189	0.08	1.39258E-11	C1_CD8_Tres
chr4-153496814-153497585	2.11454E-16	0.376762418	0.147	0.044	1.40031E-11	C1_CD8_Tres
chr11-6389570-6390881	2.32093E-16	0.25794573	0.563	0.444	1.53699E-11	C1_CD8_Tres
chr8-24914285-24915131	2.55551E-16	0.352039018	0.081	0.012	1.69234E-11	C1_CD8_Tres
chr12-110590783-110591241	2.6278E-16	0.325777508	0.072	0.011	1.74021E-11	C1_CD8_Tres
chr3-196563586-196564238	2.65685E-16	0.358776017	0.102	0.021	1.75945E-11	C1_CD8_Tres
chr17-65560758-65561933	2.8291E-16	0.320489987	0.335	0.192	1.87352E-11	C1_CD8_Tres
chr2-170651625-170652027	2.86047E-16	0.386452065	0.063	0.004	1.89429E-11	C1_CD8_Tres
chr11-118877972-118878403	2.9061E-16	0.366481603	0.081	0.011	1.92451E-11	C1_CD8_Tres
chr11-61021304-61022550	2.98065E-16	0.352152376	0.243	0.119	1.97388E-11	C1_CD8_Tres
chr22-40054988-40055943	3.13777E-16	0.33834903	0.305	0.16	2.07792E-11	C1_CD8_Tres
chr20-36988410-36988905	3.21218E-16	0.389702025	0.057	0.002	2.1272E-11	C1_CD8_Tres
chr10-69425550-69426156	3.38939E-16	0.361999616	0.072	0.008	2.24456E-11	C1_CD8_Tres
chr15-91469028-91470405	3.43343E-16	0.387344732	0.132	0.038	2.27372E-11	C1_CD8_Tres
chr3-161349586-161350276	3.43853E-16	0.401330828	0.132	0.034	2.2771E-11	C1_CD8_Tres
chr17-51820146-51820617	3.56115E-16	0.366163569	0.075	0.01	2.3583E-11	C1_CD8_Tres
chr3-32237980-32239111	3.71327E-16	0.38645166	0.171	0.058	2.45904E-11	C1_CD8_Tres
chr19-19817131-19817720	3.82903E-16	0.379935532	0.093	0.015	2.5357E-11	C1_CD8_Tres
chr3-33023056-33023996	4.10208E-16	0.390132082	0.153	0.044	2.71652E-11	C1_CD8_Tres
chr5-65796812-65797221	5.18206E-16	0.319136395	0.057	0.004	3.43172E-11	C1_CD8_Tres
chr7-23020655-23021404	5.45409E-16	0.413672839	0.171	0.056	3.61186E-11	C1_CD8_Tres

chr16-89388004-89388346	5.50277E-16	0.373289426	0.102	0.02	3.6441E-11	C1_CD8_Tres
chr16-17396143-17397864	5.69333E-16	0.368974231	0.105	0.022	3.77029E-11	C1_CD8_Tres
chr1-39183570-39184484	5.7493E-16	0.312451251	0.389	0.239	3.80736E-11	C1_CD8_Tres
chr5-56736925-56737623	6.12142E-16	0.329635931	0.066	0.007	4.05379E-11	C1_CD8_Tres
chr2-37243101-37244262	6.26205E-16	0.341241796	0.066	0.007	4.14691E-11	C1_CD8_Tres
chr12-120316891-120317993	7.52569E-16	0.287294197	0.488	0.337	4.98374E-11	C1_CD8_Tres
chr1-101295211-101295547	7.6982E-16	0.344416345	0.075	0.01	5.09798E-11	C1_CD8_Tres
chr19-16368879-16369329	8.68676E-16	0.385063389	0.165	0.055	5.75263E-11	C1_CD8_Tres
chr19-10754900-10755528	9.71061E-16	0.390673295	0.204	0.081	6.43066E-11	C1_CD8_Tres
chr21-36145297-36145547	1.00159E-15	0.327810381	0.06	0.006	6.63282E-11	C1_CD8_Tres
chr4-142845779-142847546	1.05298E-15	0.374659977	0.168	0.06	6.97314E-11	C1_CD8_Tres
chr7-50331555-50332145	1.07042E-15	0.376700635	0.21	0.083	7.08861E-11	C1_CD8_Tres
chr1-147540684-147541537	1.2471E-15	0.353993982	0.198	0.079	8.25868E-11	C1_CD8_Tres
chr11-121467993-121468350	1.25854E-15	0.39371439	0.126	0.03	8.3344E-11	C1_CD8_Tres
chr16-89170661-89171852	1.42051E-15	0.287368475	0.443	0.309	9.40701E-11	C1_CD8_Tres
chr5-55977602-55978470	1.52207E-15	0.408471985	0.165	0.054	1.00796E-10	C1_CD8_Tres
chr19-14506723-14507610	1.53733E-15	0.363663825	0.159	0.054	1.01807E-10	C1_CD8_Tres
chr11-121451268-121453120	1.62408E-15	0.351522715	0.284	0.14	1.07552E-10	C1_CD8_Tres
chr3-115791932-115792900	1.77297E-15	0.352446555	0.225	0.107	1.17411E-10	C1_CD8_Tres
chr5-134279596-134280290	1.81553E-15	0.369054344	0.078	0.01	1.2023E-10	C1_CD8_Tres
chr10-33263906-33264748	1.92612E-15	0.378275303	0.195	0.071	1.27553E-10	C1_CD8_Tres
chr2-12492984-12493465	2.00781E-15	0.329572531	0.057	0.005	1.32963E-10	C1_CD8_Tres
chr1-148371452-148373872	2.26161E-15	0.36870369	0.207	0.086	1.4977E-10	C1_CD8_Tres
chr1-156126095-156127656	2.37408E-15	0.276229646	0.497	0.337	1.57218E-10	C1_CD8_Tres
chr4-108433425-108434172	2.50825E-15	0.337836092	0.069	0.009	1.66104E-10	C1_CD8_Tres
chr1-225865587-225866457	2.64497E-15	0.388252061	0.138	0.041	1.75158E-10	C1_CD8_Tres
chr15-43151602-43152007	2.66381E-15	0.340873486	0.075	0.01	1.76406E-10	C1_CD8_Tres

chr5-75059857-75060218	2.73859E-15	0.367418719	0.057	0.004	1.81358E-10	C1_CD8_Tres
chr5-81749663-81751888	2.88849E-15	0.250769999	0.605	0.476	1.91284E-10	C1_CD8_Tres
chr2-7031503-7032044	2.90808E-15	0.353820721	0.069	0.007	1.92582E-10	C1_CD8_Tres
chr18-58321858-58322325	3.13182E-15	0.397390527	0.075	0.009	2.07398E-10	C1_CD8_Tres
chr5-157602392-157603773	3.15227E-15	0.314221213	0.299	0.166	2.08753E-10	C1_CD8_Tres
chr13-109779026-109779436	3.19622E-15	0.348667782	0.084	0.012	2.11663E-10	C1_CD8_Tres
chr22-40257903-40258874	3.21018E-15	0.350172368	0.126	0.037	2.12587E-10	C1_CD8_Tres
chr3-33022269-33022699	3.58574E-15	0.333709299	0.063	0.007	2.37459E-10	C1_CD8_Tres
chr1-64876955-64877577	3.73275E-15	0.357081693	0.168	0.059	2.47194E-10	C1_CD8_Tres
chr22-36887085-36887532	4.10619E-15	0.330818419	0.057	0.004	2.71924E-10	C1_CD8_Tres
chr1-25549312-25550465	4.13744E-15	0.341870631	0.293	0.151	2.73994E-10	C1_CD8_Tres
chr12-123447987-123448777	4.36144E-15	0.369987548	0.231	0.097	2.88828E-10	C1_CD8_Tres
chr3-32313450-32314223	4.37328E-15	0.337786581	0.09	0.019	2.89611E-10	C1_CD8_Tres
chr14-32102108-32102841	4.56886E-15	0.386507337	0.084	0.012	3.02563E-10	C1_CD8_Tres
chr11-60985594-60986875	4.61524E-15	0.273262392	0.506	0.374	3.05635E-10	C1_CD8_Tres
chr19-38387274-38388789	4.74388E-15	0.278841603	0.329	0.209	3.14154E-10	C1_CD8_Tres
chr17-76772497-76773639	4.91562E-15	0.310211688	0.332	0.198	3.25527E-10	C1_CD8_Tres
chr2-189580239-189581447	5.06398E-15	0.387981939	0.132	0.035	3.35352E-10	C1_CD8_Tres
chr1-8525085-8526207	5.08256E-15	0.355544817	0.225	0.099	3.36583E-10	C1_CD8_Tres
chr13-41355897-41357152	5.69211E-15	0.363578892	0.177	0.062	3.76949E-10	C1_CD8_Tres
chr2-2613210-2613940	5.78725E-15	0.380894027	0.105	0.021	3.83249E-10	C1_CD8_Tres
chr1-38977213-38978268	5.94822E-15	0.373257967	0.15	0.047	3.93909E-10	C1_CD8_Tres
chr1-26123193-26123705	6.26995E-15	0.399865371	0.174	0.057	4.15215E-10	C1_CD8_Tres
chr2-95022886-95023505	6.91052E-15	0.359248415	0.12	0.032	4.57635E-10	C1_CD8_Tres
chr17-75123963-75124643	7.47699E-15	0.330321399	0.108	0.029	4.95149E-10	C1_CD8_Tres
chr10-97851783-97851989	8.41357E-15	0.329754312	0.069	0.009	5.57172E-10	C1_CD8_Tres
chr15-63841719-63842387	8.43217E-15	0.42155146	0.174	0.052	5.58404E-10	C1_CD8_Tres

chr1-110630630-110631786	8.75355E-15	0.286485161	0.341	0.209	5.79686E-10	C1_CD8_Tres
chr12-3947462-3948089	9.78759E-15	0.315003273	0.066	0.009	6.48163E-10	C1_CD8_Tres
chr15-33091957-33092571	9.91715E-15	0.342164675	0.057	0.004	6.56743E-10	C1_CD8_Tres
chr6-166626350-166628465	1.0896E-14	0.251899499	0.635	0.517	7.21569E-10	C1_CD8_Tres
chr22-37218585-37219771	1.11414E-14	0.323901086	0.222	0.108	7.37816E-10	C1_CD8_Tres
chr1-92951077-92951575	1.12147E-14	0.36273642	0.081	0.013	7.42669E-10	C1_CD8_Tres
chr2-71226363-71227641	1.21743E-14	0.316168561	0.497	0.328	8.06218E-10	C1_CD8_Tres
chr12-66287435-66288552	1.23326E-14	0.332881079	0.213	0.096	8.167E-10	C1_CD8_Tres
chr2-143123982-143124199	1.27285E-14	0.295281784	0.054	0.005	8.42917E-10	C1_CD8_Tres
chr11-35082399-35084363	1.28546E-14	0.291891409	0.386	0.247	8.51272E-10	C1_CD8_Tres
chr10-17425918-17426234	1.42492E-14	0.38433883	0.075	0.008	9.43624E-10	C1_CD8_Tres
chr11-47234057-47235190	1.46022E-14	0.369850429	0.138	0.046	9.67004E-10	C1_CD8_Tres
chr1-61782618-61783124	1.5103E-14	0.360437419	0.108	0.029	1.00016E-09	C1_CD8_Tres
chr1-12386037-12386483	1.52966E-14	0.34422587	0.072	0.011	1.01299E-09	C1_CD8_Tres
chr4-40290704-40291215	1.55039E-14	0.344235454	0.087	0.017	1.02672E-09	C1_CD8_Tres
chr14-68968023-68968395	1.55452E-14	0.301871995	0.051	0.004	1.02945E-09	C1_CD8_Tres
chr9-78029850-78032610	1.6206E-14	0.289018709	0.332	0.181	1.07321E-09	C1_CD8_Tres
chr15-90061916-90062642	1.62636E-14	0.380198186	0.075	0.009	1.07703E-09	C1_CD8_Tres
chr16-89201079-89202074	1.8476E-14	0.342221571	0.144	0.05	1.22354E-09	C1_CD8_Tres
chr6-42045619-42046048	1.96191E-14	0.354515865	0.15	0.053	1.29924E-09	C1_CD8_Tres
chr5-77379400-77380032	1.96269E-14	0.330555629	0.075	0.012	1.29975E-09	C1_CD8_Tres
chr7-129906470-129907126	2.08355E-14	0.328609182	0.063	0.007	1.37979E-09	C1_CD8_Tres
chr1-151568621-151569180	2.15045E-14	0.377552231	0.114	0.024	1.42409E-09	C1_CD8_Tres
chr5-157233063-157234120	2.15781E-14	0.338319656	0.144	0.049	1.42896E-09	C1_CD8_Tres
chr3-112134742-112135071	2.15955E-14	0.359234798	0.078	0.011	1.43012E-09	C1_CD8_Tres
chr4-152099735-152101089	2.23457E-14	0.346851787	0.305	0.156	1.4798E-09	C1_CD8_Tres
chr1-40014173-40014678	2.36263E-14	0.3247375	0.096	0.023	1.56461E-09	C1_CD8_Tres

chr11-96105327-96105916	2.40392E-14	0.317234019	0.063	0.008	1.59195E-09	C1_CD8_Tres
chr1-144412046-144413315	2.40892E-14	0.315686143	0.305	0.172	1.59526E-09	C1_CD8_Tres
chr17-78139875-78140785	2.48962E-14	0.280498247	0.41	0.292	1.6487E-09	C1_CD8_Tres
chr11-102268345-102269043	2.55402E-14	0.331563553	0.189	0.085	1.69135E-09	C1_CD8_Tres
chr12-120248506-120248818	2.63007E-14	0.378447404	0.087	0.015	1.74171E-09	C1_CD8_Tres
chr10-3668289-3669209	2.93758E-14	0.327654803	0.195	0.084	1.94535E-09	C1_CD8_Tres
chr22-43103915-43104518	3.01126E-14	0.291617194	0.054	0.005	1.99414E-09	C1_CD8_Tres
chr19-50375967-50377486	3.29392E-14	0.25262031	0.479	0.354	2.18134E-09	C1_CD8_Tres
chr17-39952197-39953600	3.34179E-14	0.268520897	0.551	0.414	2.21303E-09	C1_CD8_Tres
chr1-45246430-45247084	3.84753E-14	0.330048579	0.069	0.009	2.54795E-09	C1_CD8_Tres
chr5-783299-785042	3.87641E-14	0.312612326	0.257	0.136	2.56707E-09	C1_CD8_Tres
chr19-40570079-40571126	4.04253E-14	0.273527332	0.449	0.311	2.67709E-09	C1_CD8_Tres
chr9-128094022-128094725	4.09359E-14	0.376176238	0.144	0.044	2.7109E-09	C1_CD8_Tres
chr14-95462999-95463674	4.12102E-14	0.329050648	0.096	0.021	2.72906E-09	C1_CD8_Tres
chr17-65522837-65523959	4.27831E-14	0.352810141	0.111	0.027	2.83322E-09	C1_CD8_Tres
chr13-48653150-48654064	4.45545E-14	0.371201799	0.171	0.061	2.95053E-09	C1_CD8_Tres
chr5-134964405-134964998	4.51479E-14	0.336798002	0.054	0.004	2.98983E-09	C1_CD8_Tres
chr20-62623349-62624245	5.25676E-14	0.349330674	0.165	0.061	3.48118E-09	C1_CD8_Tres
chr18-48455837-48456529	5.3482E-14	0.343236396	0.063	0.007	3.54174E-09	C1_CD8_Tres
chr10-114499612-114499898	6.05433E-14	0.32300028	0.057	0.005	4.00936E-09	C1_CD8_Tres
chr20-28602102-28603285	6.08925E-14	0.358619638	0.21	0.08	4.03248E-09	C1_CD8_Tres
chr19-908610-909828	6.27886E-14	0.278582335	0.443	0.305	4.15805E-09	C1_CD8_Tres
chr7-151381343-151382371	6.55253E-14	0.323059806	0.144	0.05	4.33928E-09	C1_CD8_Tres
chr8-2130857-2131533	6.82961E-14	0.346596796	0.147	0.045	4.52277E-09	C1_CD8_Tres
chr11-61026042-61026628	6.93993E-14	0.350080839	0.129	0.039	4.59583E-09	C1_CD8_Tres
chr6-36682464-36683091	7.71846E-14	0.350733417	0.126	0.037	5.11139E-09	C1_CD8_Tres
chr14-64707494-64708921	8.02786E-14	0.311430763	0.141	0.057	5.31629E-09	C1_CD8_Tres

chr1-145280570-145282226	8.15049E-14	0.331126486	0.249	0.117	5.3975E-09	C1_CD8_Tres
chr16-67236044-67236973	8.43003E-14	0.360063616	0.186	0.073	5.58262E-09	C1_CD8_Tres
chrX-1205702-1206264	8.69842E-14	0.332957729	0.084	0.017	5.76035E-09	C1_CD8_Tres
chr20-32534602-32535602	8.91098E-14	0.299504629	0.231	0.121	5.90112E-09	C1_CD8_Tres
chr5-67161007-67161300	8.94045E-14	0.363121426	0.093	0.017	5.92063E-09	C1_CD8_Tres
chr4-108071859-108072789	9.01666E-14	0.361614071	0.165	0.063	5.9711E-09	C1_CD8_Tres
chr1-98870973-98871909	9.17706E-14	0.32754336	0.222	0.11	6.07733E-09	C1_CD8_Tres
chr1-223248405-223249194	9.52447E-14	0.379100255	0.066	0.006	6.30739E-09	C1_CD8_Tres
chr11-62551598-62552120	9.98277E-14	0.269264989	0.329	0.204	6.61089E-09	C1_CD8_Tres
chr7-6990644-6991633	1.18776E-13	0.35211953	0.12	0.035	7.8657E-09	C1_CD8_Tres
chr8-20492942-20493637	1.28191E-13	0.317440276	0.075	0.014	8.48918E-09	C1_CD8_Tres
chr7-151408489-151409399	1.29158E-13	0.301626036	0.347	0.215	8.5532E-09	C1_CD8_Tres
chr21-5154334-5154970	1.31642E-13	0.349547233	0.228	0.094	8.7177E-09	C1_CD8_Tres
chr7-50206183-50206951	1.41334E-13	0.336108713	0.237	0.12	9.35953E-09	C1_CD8_Tres
chr16-28990309-28990992	1.41619E-13	0.337718553	0.183	0.077	9.37846E-09	C1_CD8_Tres
chr4-82284216-82285491	1.55078E-13	0.28098272	0.53	0.382	1.02697E-08	C1_CD8_Tres
chr8-141000867-141002536	1.577E-13	0.328878281	0.192	0.084	1.04434E-08	C1_CD8_Tres
chr5-132687864-132689114	1.6899E-13	0.321559611	0.147	0.051	1.1191E-08	C1_CD8_Tres
chr11-62853215-62853866	1.69863E-13	0.330416285	0.144	0.057	1.12488E-08	C1_CD8_Tres
chr14-91193828-91194842	1.7115E-13	0.337004368	0.171	0.069	1.13341E-08	C1_CD8_Tres
chr5-56011879-56012999	1.83266E-13	0.359038221	0.204	0.083	1.21364E-08	C1_CD8_Tres
chr19-7196940-7198555	2.07228E-13	0.283435541	0.401	0.261	1.37232E-08	C1_CD8_Tres
chr1-84853880-84854757	2.07734E-13	0.359899361	0.177	0.07	1.37568E-08	C1_CD8_Tres
chr15-63469133-63469588	2.09352E-13	0.35302072	0.081	0.014	1.38639E-08	C1_CD8_Tres
chr19-2503181-2504126	2.17271E-13	0.315658482	0.314	0.171	1.43883E-08	C1_CD8_Tres
chr5-132059009-132059400	2.26123E-13	0.354649051	0.108	0.024	1.49745E-08	C1_CD8_Tres
chr7-158811472-158811740	2.30294E-13	0.3211374	0.051	0.004	1.52508E-08	C1_CD8_Tres

chr12-89663546-89664413	2.56158E-13	0.330862227	0.126	0.039	1.69636E-08	C1_CD8_Tres
chr12-66964321-66964965	2.58815E-13	0.335344619	0.069	0.009	1.71395E-08	C1_CD8_Tres
chr8-42154163-42154463	2.66152E-13	0.363909928	0.12	0.033	1.76254E-08	C1_CD8_Tres
chr9-133708987-133709724	2.74155E-13	0.307528957	0.099	0.029	1.81554E-08	C1_CD8_Tres
chr4-1578981-1580867	2.84957E-13	0.250116203	0.497	0.366	1.88707E-08	C1_CD8_Tres
chrX-13151328-13152312	3.10665E-13	0.322870805	0.165	0.066	2.05731E-08	C1_CD8_Tres
chr18-67515877-67517322	3.1213E-13	0.301534689	0.392	0.255	2.06702E-08	C1_CD8_Tres
chr2-221517255-221519122	3.17043E-13	0.303510806	0.455	0.292	2.09955E-08	C1_CD8_Tres
chr10-3754995-3755391	3.21285E-13	0.334462525	0.111	0.031	2.12764E-08	C1_CD8_Tres
chr4-153471228-153472161	3.21382E-13	0.330259005	0.254	0.128	2.12829E-08	C1_CD8_Tres
chr12-124636086-124636573	3.3734E-13	0.352742283	0.093	0.021	2.23397E-08	C1_CD8_Tres
chr14-70660997-70661751	3.47398E-13	0.308626991	0.057	0.007	2.30057E-08	C1_CD8_Tres
chr12-124469301-124469836	3.53448E-13	0.347407057	0.132	0.039	2.34064E-08	C1_CD8_Tres
chr14-21087020-21087996	3.8222E-13	0.295591523	0.326	0.202	2.53118E-08	C1_CD8_Tres
chr7-130988315-130988900	3.9417E-13	0.326578978	0.102	0.029	2.61031E-08	C1_CD8_Tres
chr15-82680039-82681005	3.95897E-13	0.323836797	0.18	0.074	2.62175E-08	C1_CD8_Tres
chr5-111530075-111530703	4.08128E-13	0.332024336	0.201	0.094	2.70275E-08	C1_CD8_Tres
chrX-16889110-16889721	4.20192E-13	0.337598825	0.114	0.032	2.78264E-08	C1_CD8_Tres
chr10-14506919-14507504	4.27392E-13	0.339401199	0.078	0.015	2.83032E-08	C1_CD8_Tres
chr4-74444592-74445637	4.39675E-13	0.342344958	0.21	0.091	2.91166E-08	C1_CD8_Tres
chr20-3989070-3989852	4.62832E-13	0.334541595	0.162	0.063	3.06501E-08	C1_CD8_Tres
chr14-31947518-31948078	4.73367E-13	0.325884143	0.054	0.004	3.13478E-08	C1_CD8_Tres
chr14-91835529-91836905	4.95228E-13	0.272180666	0.395	0.262	3.27955E-08	C1_CD8_Tres
chr8-11820144-11820848	5.48372E-13	0.376848445	0.108	0.026	3.63148E-08	C1_CD8_Tres
chr13-108342454-108343194	5.49406E-13	0.292715412	0.054	0.005	3.63833E-08	C1_CD8_Tres
chr1-9389762-9390266	6.03952E-13	0.284355824	0.072	0.013	3.99955E-08	C1_CD8_Tres
chr12-55929153-55929738	6.17903E-13	0.316788328	0.234	0.107	4.09194E-08	C1_CD8_Tres

chr2-226423204-226423867	6.7094E-13	0.310756928	0.054	0.006	4.44317E-08	C1_CD8_Tres
chr5-178120767-178121272	6.87451E-13	0.30507469	0.12	0.039	4.55251E-08	C1_CD8_Tres
chr1-154463170-154464019	7.70867E-13	0.319082407	0.174	0.069	5.10492E-08	C1_CD8_Tres
chr3-52268059-52268325	8.19131E-13	0.289286355	0.06	0.008	5.42453E-08	C1_CD8_Tres
chrX-136658947-136659495	8.22521E-13	0.342063188	0.051	0.004	5.44698E-08	C1_CD8_Tres
chr11-121459423-121459742	8.61551E-13	0.317844792	0.057	0.006	5.70545E-08	C1_CD8_Tres
chr7-134669269-134670346	9.41334E-13	0.300033615	0.329	0.198	6.2338E-08	C1_CD8_Tres
chr9-75889731-75891611	9.65579E-13	0.351521246	0.153	0.057	6.39435E-08	C1_CD8_Tres
chr8-80485247-80487463	1.02763E-12	0.29053176	0.398	0.263	6.8053E-08	C1_CD8_Tres
chr1-26739289-26739664	1.02954E-12	0.275243075	0.06	0.01	6.81791E-08	C1_CD8_Tres
chr10-60727760-60728404	1.04542E-12	0.328075645	0.147	0.053	6.92311E-08	C1_CD8_Tres
chr11-128476613-128477067	1.12113E-12	0.302251887	0.249	0.138	7.42448E-08	C1_CD8_Tres
chr12-12465878-12466513	1.15302E-12	0.312287791	0.195	0.084	7.63564E-08	C1_CD8_Tres
chr19-12635499-12635851	1.16667E-12	0.347560768	0.081	0.014	7.72607E-08	C1_CD8_Tres
chr6-3355286-3356545	1.29973E-12	0.29788128	0.126	0.046	8.60721E-08	C1_CD8_Tres
chr9-107243581-107244128	1.35643E-12	0.35127904	0.102	0.026	8.98266E-08	C1_CD8_Tres
chr14-21308205-21309096	1.38008E-12	0.341188853	0.18	0.068	9.1393E-08	C1_CD8_Tres
chr5-175652680-175653588	1.40436E-12	0.343056527	0.153	0.054	9.30009E-08	C1_CD8_Tres
chr1-33430300-33431421	1.42051E-12	0.299224498	0.198	0.093	9.40706E-08	C1_CD8_Tres
chr6-35718831-35719415	1.48349E-12	0.306724771	0.069	0.013	9.82408E-08	C1_CD8_Tres
chr5-119330234-119330834	1.48449E-12	0.332587305	0.219	0.103	9.83072E-08	C1_CD8_Tres
chr2-234425673-234426466	1.49522E-12	0.281259711	0.234	0.126	9.90177E-08	C1_CD8_Tres
chr8-119638361-119639273	1.54401E-12	0.333668381	0.21	0.095	1.02249E-07	C1_CD8_Tres
chr14-56117661-56119506	1.56919E-12	0.290871261	0.254	0.142	1.03917E-07	C1_CD8_Tres
chr11-122580325-122581444	1.59096E-12	0.307311831	0.171	0.076	1.05358E-07	C1_CD8_Tres
chr17-66373973-66374748	1.61075E-12	0.369874458	0.171	0.066	1.06669E-07	C1_CD8_Tres
chr1-220689916-220690702	1.62511E-12	0.346298242	0.102	0.024	1.0762E-07	C1_CD8_Tres

chr1-149636033-149636990	1.67189E-12	0.321010816	0.216	0.108	1.10717E-07	C1_CD8_Tres
chr6-139160917-139162291	1.78699E-12	0.277609244	0.377	0.252	1.1834E-07	C1_CD8_Tres
chr12-8953797-8954571	1.80088E-12	0.328382055	0.192	0.084	1.19259E-07	C1_CD8_Tres
chr9-93028081-93029070	1.8087E-12	0.309368463	0.171	0.072	1.19777E-07	C1_CD8_Tres
chr6-3456460-3457372	1.90277E-12	0.315869843	0.123	0.037	1.26007E-07	C1_CD8_Tres
chr19-49555937-49556672	1.94749E-12	0.329017985	0.078	0.017	1.28968E-07	C1_CD8_Tres
chr4-113901802-113902396	2.01304E-12	0.357525339	0.102	0.026	1.3331E-07	C1_CD8_Tres
chr20-13230872-13231669	2.02981E-12	0.332782526	0.072	0.01	1.3442E-07	C1_CD8_Tres
chr4-99507514-99508118	2.1851E-12	0.326218696	0.06	0.007	1.44704E-07	C1_CD8_Tres
chr9-33458237-33458907	2.19736E-12	0.314677958	0.153	0.058	1.45516E-07	C1_CD8_Tres
chr3-15685900-15686174	2.29364E-12	0.355165732	0.057	0.004	1.51892E-07	C1_CD8_Tres
chr20-36943921-36944602	2.39817E-12	0.314756472	0.153	0.059	1.58814E-07	C1_CD8_Tres
chr1-67674051-67674973	2.40897E-12	0.311238804	0.102	0.027	1.59529E-07	C1_CD8_Tres
chr17-64348003-64348484	2.43808E-12	0.294124482	0.054	0.006	1.61457E-07	C1_CD8_Tres
chr18-62339492-62339920	2.49975E-12	0.327427925	0.057	0.006	1.65541E-07	C1_CD8_Tres
chr2-206442859-206444018	2.67404E-12	0.310970163	0.093	0.021	1.77083E-07	C1_CD8_Tres
chr20-13220538-13222122	2.67853E-12	0.339683022	0.144	0.049	1.7738E-07	C1_CD8_Tres
chr1-6459540-6460762	2.7501E-12	0.254274335	0.359	0.238	1.8212E-07	C1_CD8_Tres
chr10-29734990-29736750	2.80679E-12	0.300616696	0.171	0.08	1.85874E-07	C1_CD8_Tres
chr12-132985767-132986898	2.82645E-12	0.308257727	0.198	0.091	1.87176E-07	C1_CD8_Tres
chr1-101259667-101260103	2.88174E-12	0.323563838	0.132	0.045	1.90838E-07	C1_CD8_Tres
chr14-20400614-20401434	2.95617E-12	0.31339154	0.093	0.026	1.95766E-07	C1_CD8_Tres
chr17-81119210-81119977	3.17806E-12	0.300220881	0.159	0.067	2.1046E-07	C1_CD8_Tres
chr1-38558924-38559993	3.22188E-12	0.284681468	0.207	0.111	2.13363E-07	C1_CD8_Tres
chr19-41530765-41531866	3.247E-12	0.285172076	0.198	0.101	2.15026E-07	C1_CD8_Tres
chr12-7935321-7936575	3.34988E-12	0.263181444	0.386	0.283	2.21839E-07	C1_CD8_Tres
chr20-44552832-44553759	3.57038E-12	0.350021277	0.102	0.024	2.36441E-07	C1_CD8_Tres

chr17-39963810-39964169	3.58608E-12	0.295335451	0.06	0.009	2.37481E-07	C1_CD8_Tres
chr16-58755878-58756696	3.59105E-12	0.307233489	0.15	0.058	2.3781E-07	C1_CD8_Tres
chr17-72617949-72618607	3.73282E-12	0.279734557	0.054	0.008	2.47198E-07	C1_CD8_Tres
chr11-128479179-128479398	3.90347E-12	0.3164818	0.105	0.032	2.58499E-07	C1_CD8_Tres
chr2-240831720-240832790	3.9185E-12	0.292926316	0.374	0.244	2.59495E-07	C1_CD8_Tres
chr19-39073226-39074029	4.05978E-12	0.321045083	0.308	0.165	2.68851E-07	C1_CD8_Tres
chr7-138903069-138903853	4.16596E-12	0.311119508	0.123	0.04	2.75883E-07	C1_CD8_Tres
chr9-135905096-135905931	4.26032E-12	0.286918302	0.138	0.056	2.82131E-07	C1_CD8_Tres
chr1-9289939-9290271	4.54465E-12	0.305657204	0.099	0.03	3.00961E-07	C1_CD8_Tres
chr8-144685265-144686815	4.60578E-12	0.262623826	0.18	0.095	3.05009E-07	C1_CD8_Tres
chr11-128379351-128380164	4.70437E-12	0.305908955	0.171	0.075	3.11538E-07	C1_CD8_Tres
chr3-9912046-9912875	4.7736E-12	0.323126033	0.216	0.102	3.16122E-07	C1_CD8_Tres
chr5-151083343-151083684	4.82546E-12	0.306575991	0.153	0.064	3.19556E-07	C1_CD8_Tres
chr17-82200599-82201168	5.16485E-12	0.318657751	0.189	0.087	3.42032E-07	C1_CD8_Tres
chr1-85200332-85202171	5.24418E-12	0.265453267	0.323	0.2	3.47286E-07	C1_CD8_Tres
chr10-29635691-29636561	5.45754E-12	0.337042573	0.105	0.03	3.61415E-07	C1_CD8_Tres
chr2-74840370-74840636	5.52868E-12	0.274757208	0.054	0.007	3.66126E-07	C1_CD8_Tres
chr2-203711099-203711782	5.76398E-12	0.333187756	0.207	0.095	3.81708E-07	C1_CD8_Tres
chr1-31771868-31772831	5.85352E-12	0.301270359	0.189	0.083	3.87638E-07	C1_CD8_Tres
chr19-48492745-48493941	5.86203E-12	0.288063352	0.296	0.177	3.88201E-07	C1_CD8_Tres
chr7-5688922-5689525	6.05868E-12	0.315971983	0.12	0.039	4.01224E-07	C1_CD8_Tres
chr1-145464778-145465831	6.43739E-12	0.319342787	0.15	0.053	4.26303E-07	C1_CD8_Tres
chr1-181395947-181396693	6.50095E-12	0.322059063	0.108	0.03	4.30512E-07	C1_CD8_Tres
chr1-168419981-168420510	6.76537E-12	0.316207687	0.099	0.029	4.48023E-07	C1_CD8_Tres
chr4-108165590-108165834	6.8525E-12	0.30956005	0.081	0.017	4.53793E-07	C1_CD8_Tres
chr9-35910837-35911183	6.98461E-12	0.313379262	0.063	0.009	4.62542E-07	C1_CD8_Tres
chr12-95334343-95334722	7.11939E-12	0.298861206	0.051	0.006	4.71467E-07	C1_CD8_Tres

chr19-17538908-17540305	7.50075E-12	0.251232293	0.308	0.206	4.96722E-07	C1_CD8_Tres
chr22-45179215-45179727	7.8337E-12	0.319404328	0.087	0.017	5.18771E-07	C1_CD8_Tres
chr16-996722-998093	8.51708E-12	0.305426865	0.171	0.077	5.64027E-07	C1_CD8_Tres
chr10-62183124-62184050	8.51969E-12	0.281809916	0.177	0.083	5.642E-07	C1_CD8_Tres
chr11-114058953-114060800	8.86057E-12	0.327137306	0.168	0.066	5.86774E-07	C1_CD8_Tres
chr1-111354586-111354949	1.04659E-11	0.290584321	0.051	0.005	6.93085E-07	C1_CD8_Tres
chr1-144545929-144546983	1.04949E-11	0.305436231	0.177	0.075	6.95003E-07	C1_CD8_Tres
chr15-22757299-22758340	1.11261E-11	0.271281404	0.117	0.043	7.36806E-07	C1_CD8_Tres
chr19-47157786-47159607	1.15985E-11	0.277803381	0.222	0.113	7.68086E-07	C1_CD8_Tres
chr20-4037663-4038434	1.1824E-11	0.316533139	0.243	0.132	7.83022E-07	C1_CD8_Tres
chr11-13940393-13940985	1.25333E-11	0.316523243	0.06	0.009	8.29995E-07	C1_CD8_Tres
chr11-88843442-88844111	1.27663E-11	0.315562096	0.063	0.009	8.45424E-07	C1_CD8_Tres
chr4-153114731-153116644	1.33905E-11	0.256976818	0.305	0.2	8.86757E-07	C1_CD8_Tres
chr12-106247417-106249361	1.34947E-11	0.262339543	0.228	0.128	8.9366E-07	C1_CD8_Tres
chr6-37209460-37210717	1.35794E-11	0.29778398	0.159	0.076	8.99269E-07	C1_CD8_Tres
chr22-19022702-19023241	1.37085E-11	0.358894358	0.102	0.024	9.07819E-07	C1_CD8_Tres
chr3-31212741-31213107	1.37341E-11	0.312836224	0.075	0.015	9.09514E-07	C1_CD8_Tres
chr17-74852308-74852909	1.41543E-11	0.270139227	0.075	0.018	9.37339E-07	C1_CD8_Tres
chr1-39690899-39692125	1.42209E-11	0.259750693	0.263	0.159	9.4175E-07	C1_CD8_Tres
chr17-8898595-8899604	1.42218E-11	0.259653918	0.329	0.218	9.41813E-07	C1_CD8_Tres
chr21-5101563-5102080	1.58259E-11	0.303530481	0.162	0.071	1.04804E-06	C1_CD8_Tres
chr10-6013878-6014870	1.58862E-11	0.315032983	0.162	0.069	1.05203E-06	C1_CD8_Tres
chr13-30654090-30654855	1.62323E-11	0.296093573	0.111	0.04	1.07495E-06	C1_CD8_Tres
chr7-150484128-150484446	1.63167E-11	0.297756795	0.066	0.012	1.08054E-06	C1_CD8_Tres
chr2-172442043-172442337	1.75397E-11	0.337647532	0.075	0.013	1.16153E-06	C1_CD8_Tres
chr19-48501415-48502530	1.77322E-11	0.289792228	0.129	0.047	1.17428E-06	C1_CD8_Tres
chr5-175796555-175797314	1.90337E-11	0.30786734	0.099	0.027	1.26047E-06	C1_CD8_Tres

chr12-52024867-52025083	1.92647E-11	0.294996137	0.06	0.009	1.27576E-06	C1_CD8_Tres
chr11-62553211-62553659	2.08334E-11	0.258737415	0.165	0.08	1.37965E-06	C1_CD8_Tres
chr14-102774570-102775151	2.17257E-11	0.263236518	0.171	0.085	1.43874E-06	C1_CD8_Tres
chr2-105738026-105739249	2.27308E-11	0.276364448	0.237	0.133	1.5053E-06	C1_CD8_Tres
chr1-160628816-160630078	2.31544E-11	0.278201541	0.189	0.092	1.53335E-06	C1_CD8_Tres
chr1-240666520-240666960	2.36646E-11	0.279501965	0.051	0.007	1.56714E-06	C1_CD8_Tres
chr4-108355270-108355972	2.37433E-11	0.278977075	0.054	0.007	1.57236E-06	C1_CD8_Tres
chr20-34875858-34876868	2.47333E-11	0.250587487	0.314	0.205	1.63792E-06	C1_CD8_Tres
chr5-35832621-35833484	2.5025E-11	0.275518223	0.075	0.019	1.65723E-06	C1_CD8_Tres
chr7-45013349-45013764	2.55604E-11	0.289902756	0.051	0.006	1.69269E-06	C1_CD8_Tres
chr5-80487743-80488360	2.56396E-11	0.258393431	0.075	0.019	1.69793E-06	C1_CD8_Tres
chr8-27319914-27320198	2.57842E-11	0.274784245	0.063	0.012	1.7075E-06	C1_CD8_Tres
chr2-106057215-106057529	2.82013E-11	0.340635042	0.108	0.029	1.86758E-06	C1_CD8_Tres
chr19-49568852-49569348	2.92323E-11	0.301381892	0.093	0.024	1.93585E-06	C1_CD8_Tres
chr9-97904676-97905518	2.95575E-11	0.274402336	0.18	0.092	1.95739E-06	C1_CD8_Tres
chr6-53302359-53302981	2.97751E-11	0.311945567	0.138	0.051	1.9718E-06	C1_CD8_Tres
chr5-142104439-142104837	2.98332E-11	0.300932342	0.108	0.036	1.97564E-06	C1_CD8_Tres
chr8-78444831-78445513	3.08759E-11	0.299424436	0.054	0.006	2.0447E-06	C1_CD8_Tres
chr14-24274151-24274474	3.10467E-11	0.297206321	0.096	0.029	2.05601E-06	C1_CD8_Tres
chr22-42499281-42500799	3.11754E-11	0.269780308	0.314	0.209	2.06453E-06	C1_CD8_Tres
chr1-239501490-239502121	3.19852E-11	0.276748937	0.051	0.007	2.11816E-06	C1_CD8_Tres
chr17-76752882-76753875	3.54209E-11	0.290396138	0.135	0.054	2.34568E-06	C1_CD8_Tres
chr8-141402473-141403370	3.64706E-11	0.30936862	0.129	0.046	2.4152E-06	C1_CD8_Tres
chr19-12289014-12289628	3.66781E-11	0.302695534	0.174	0.084	2.42893E-06	C1_CD8_Tres
chr8-8871973-8872681	3.72287E-11	0.307881285	0.168	0.068	2.4654E-06	C1_CD8_Tres
chr7-106126279-106126712	3.77914E-11	0.27355111	0.072	0.016	2.50266E-06	C1_CD8_Tres
chr1-101308995-101310257	3.7829E-11	0.28197457	0.326	0.193	2.50515E-06	C1_CD8_Tres

chrX-108018411-108019284	3.86348E-11	0.315145802	0.096	0.025	2.55852E-06	C1_CD8_Tres
chr6-25865665-25866406	3.99378E-11	0.320769922	0.105	0.029	2.6448E-06	C1_CD8_Tres
chr16-17364067-17364670	4.10926E-11	0.279458004	0.29	0.172	2.72127E-06	C1_CD8_Tres
chr2-63855841-63856696	4.13432E-11	0.287004383	0.213	0.111	2.73787E-06	C1_CD8_Tres
chr10-8898769-8899165	4.19127E-11	0.335762329	0.099	0.029	2.77558E-06	C1_CD8_Tres
chr8-141403636-141404346	4.24701E-11	0.31060639	0.18	0.075	2.8125E-06	C1_CD8_Tres
chr15-92849056-92849741	4.50154E-11	0.316314549	0.138	0.053	2.98105E-06	C1_CD8_Tres
chr14-64673394-64674157	4.73032E-11	0.314163321	0.108	0.032	3.13256E-06	C1_CD8_Tres
chr17-78343243-78344199	4.79221E-11	0.297091969	0.159	0.069	3.17355E-06	C1_CD8_Tres
chr1-25356938-25357695	4.90007E-11	0.265959399	0.063	0.012	3.24498E-06	C1_CD8_Tres
chr17-3754431-3755318	5.42542E-11	0.303531253	0.168	0.078	3.59288E-06	C1_CD8_Tres
chr18-2891749-2892163	5.4632E-11	0.263208455	0.066	0.015	3.61789E-06	C1_CD8_Tres
chr17-78772479-78773053	5.61367E-11	0.316745943	0.12	0.043	3.71754E-06	C1_CD8_Tres
chr10-6036999-6037596	5.68694E-11	0.267182421	0.06	0.011	3.76606E-06	C1_CD8_Tres
chr13-40186805-40188189	5.82E-11	0.275207297	0.231	0.129	3.85418E-06	C1_CD8_Tres
chr14-102527467-102527755	5.82085E-11	0.294910101	0.063	0.011	3.85474E-06	C1_CD8_Tres
chr5-14413293-14413754	5.89253E-11	0.280192339	0.099	0.031	3.90221E-06	C1_CD8_Tres
chr10-49941284-49942406	6.09311E-11	0.286307699	0.162	0.069	4.03504E-06	C1_CD8_Tres
chr4-184792913-184793389	6.22558E-11	0.308943971	0.099	0.031	4.12276E-06	C1_CD8_Tres
chr12-102059274-102059663	6.3947E-11	0.314119597	0.15	0.059	4.23476E-06	C1_CD8_Tres
chr7-44046496-44046933	6.56419E-11	0.293312536	0.099	0.029	4.34701E-06	C1_CD8_Tres
chr7-100965668-100966641	6.63264E-11	0.275888041	0.093	0.029	4.39233E-06	C1_CD8_Tres
chr18-596162-597241	6.68811E-11	0.304835607	0.12	0.044	4.42907E-06	C1_CD8_Tres
chr1-39185592-39186149	6.70255E-11	0.292362484	0.171	0.082	4.43863E-06	C1_CD8_Tres
chr13-98484347-98485269	6.8264E-11	0.288192023	0.222	0.116	4.52065E-06	C1_CD8_Tres
chr1-57307131-57307908	6.8776E-11	0.343498585	0.108	0.03	4.55455E-06	C1_CD8_Tres
chr2-68829068-68830009	6.94748E-11	0.305909856	0.117	0.04	4.60083E-06	C1_CD8_Tres

chr1-241677133-241677611	7.01001E-11	0.294962519	0.075	0.017	4.64224E-06	C1_CD8_Tres
chr6-29708374-29709366	7.25535E-11	0.253192804	0.308	0.21	4.80471E-06	C1_CD8_Tres
chr2-197052280-197052589	7.85323E-11	0.282660145	0.054	0.006	5.20064E-06	C1_CD8_Tres
chr14-54773156-54774273	7.86136E-11	0.266195951	0.269	0.157	5.20603E-06	C1_CD8_Tres
chr19-49553623-49554350	7.95653E-11	0.296909174	0.126	0.045	5.26905E-06	C1_CD8_Tres
chr1-120850260-120851399	8.59246E-11	0.278036285	0.275	0.16	5.69018E-06	C1_CD8_Tres
chr3-45869558-45870568	8.5937E-11	0.26707616	0.213	0.119	5.691E-06	C1_CD8_Tres
chr19-7871938-7872322	8.80896E-11	0.301325906	0.123	0.048	5.83356E-06	C1_CD8_Tres
chr1-235074458-235075575	8.91623E-11	0.310783572	0.192	0.085	5.90459E-06	C1_CD8_Tres
chr3-31541040-31541436	9.01857E-11	0.280567356	0.069	0.016	5.97237E-06	C1_CD8_Tres
chr9-120890146-120890767	9.89662E-11	0.305522986	0.15	0.062	6.55384E-06	C1_CD8_Tres
chr2-28586895-28587213	9.9408E-11	0.270632262	0.06	0.011	6.5831E-06	C1_CD8_Tres
chr3-101518076-101518866	1.02747E-10	0.266303881	0.263	0.156	6.80419E-06	C1_CD8_Tres
chr20-53844774-53845489	1.03296E-10	0.264725997	0.132	0.061	6.84058E-06	C1_CD8_Tres
chr1-167446938-167448179	1.0515E-10	0.25619298	0.332	0.23	6.96336E-06	C1_CD8_Tres
chr3-192916860-192918360	1.07792E-10	0.313152822	0.168	0.072	7.13831E-06	C1_CD8_Tres
chr1-113389521-113391431	1.09961E-10	0.25679508	0.296	0.182	7.28195E-06	C1_CD8_Tres
chr9-79544130-79544747	1.11918E-10	0.259087925	0.06	0.013	7.41152E-06	C1_CD8_Tres
chr17-75872763-75872991	1.14555E-10	0.268243582	0.051	0.007	7.58616E-06	C1_CD8_Tres
chr12-51009077-51009364	1.15354E-10	0.262252753	0.06	0.011	7.63912E-06	C1_CD8_Tres
chr12-6789149-6789608	1.15686E-10	0.286862865	0.066	0.011	7.6611E-06	C1_CD8_Tres
chr14-98223550-98224139	1.26984E-10	0.318636221	0.114	0.038	8.40927E-06	C1_CD8_Tres
chr11-118919418-118920066	1.39527E-10	0.273014346	0.231	0.126	9.23991E-06	C1_CD8_Tres
chr8-142776583-142778001	1.40081E-10	0.251173218	0.302	0.192	9.27656E-06	C1_CD8_Tres
chr11-121418514-121419009	1.41571E-10	0.269548034	0.066	0.014	9.37528E-06	C1_CD8_Tres
chr12-117105531-117106254	1.43406E-10	0.27449368	0.081	0.024	9.49677E-06	C1_CD8_Tres
chrX-1582773-1583405	1.43424E-10	0.305282089	0.162	0.069	9.49799E-06	C1_CD8_Tres

chr3-153221387-153222517	1.55256E-10	0.26220581	0.347	0.215	1.02815E-05	C1_CD8_Tres
chr21-26169083-26171127	1.62964E-10	0.253410108	0.222	0.129	1.07919E-05	C1_CD8_Tres
chr1-226696615-226697454	1.63084E-10	0.277118832	0.063	0.014	1.07999E-05	C1_CD8_Tres
chr17-40602867-40603096	1.63396E-10	0.286939912	0.075	0.019	1.08205E-05	C1_CD8_Tres
chr6-166257734-166258201	1.69107E-10	0.293645116	0.15	0.07	1.11988E-05	C1_CD8_Tres
chr9-104960815-104961631	1.71052E-10	0.321795576	0.117	0.039	1.13276E-05	C1_CD8_Tres
chr20-1225233-1226686	1.7162E-10	0.273181884	0.195	0.1	1.13652E-05	C1_CD8_Tres
chr22-42285911-42286587	1.73387E-10	0.26422402	0.093	0.035	1.14822E-05	C1_CD8_Tres
chr3-56930666-56931064	1.73485E-10	0.281060876	0.075	0.018	1.14887E-05	C1_CD8_Tres
chr4-37954100-37954950	1.76624E-10	0.28242439	0.129	0.053	1.16966E-05	C1_CD8_Tres
chr10-110750898-110751288	1.78283E-10	0.254897849	0.06	0.013	1.18064E-05	C1_CD8_Tres
chr5-143434366-143435536	1.79195E-10	0.270726109	0.156	0.069	1.18668E-05	C1_CD8_Tres
chr22-40116450-40117167	1.82024E-10	0.259153197	0.072	0.019	1.20542E-05	C1_CD8_Tres
chr6-3869841-3870317	1.82737E-10	0.29026211	0.075	0.018	1.21014E-05	C1_CD8_Tres
chr3-5019459-5019917	1.84918E-10	0.264739928	0.156	0.077	1.22458E-05	C1_CD8_Tres
chr22-35551999-35552992	1.8945E-10	0.300636169	0.111	0.035	1.2546E-05	C1_CD8_Tres
chr12-12008999-12009782	1.93021E-10	0.253586614	0.254	0.151	1.27824E-05	C1_CD8_Tres
chr5-159337564-159338047	2.03885E-10	0.269456337	0.084	0.025	1.35018E-05	C1_CD8_Tres
chr18-58657828-58658640	2.07597E-10	0.298786991	0.135	0.054	1.37477E-05	C1_CD8_Tres
chr14-76926377-76927097	2.07734E-10	0.273648994	0.108	0.042	1.37567E-05	C1_CD8_Tres
chr9-93091520-93091803	2.14034E-10	0.322668434	0.09	0.023	1.4174E-05	C1_CD8_Tres
chr14-35222955-35223484	2.20188E-10	0.282542067	0.069	0.015	1.45815E-05	C1_CD8_Tres
chr16-29679437-29680044	2.20405E-10	0.300903963	0.135	0.052	1.45959E-05	C1_CD8_Tres
chr10-5293593-5294359	2.23289E-10	0.273613066	0.141	0.064	1.47869E-05	C1_CD8_Tres
chr22-24284770-24285679	2.2509E-10	0.289319616	0.099	0.034	1.49061E-05	C1_CD8_Tres
chr1-148458349-148459569	2.2714E-10	0.302530025	0.138	0.049	1.50419E-05	C1_CD8_Tres
chr8-140790509-140791760	2.34762E-10	0.251977735	0.266	0.167	1.55466E-05	C1_CD8_Tres

chr12-64665425-64665902	2.4521E-10	0.295754259	0.144	0.061	1.62385E-05	C1_CD8_Tres
chr1-232923575-232924398	2.45511E-10	0.312593698	0.12	0.045	1.62585E-05	C1_CD8_Tres
chr10-5566646-5566938	2.55305E-10	0.28291824	0.051	0.006	1.69071E-05	C1_CD8_Tres
chr15-63481072-63481443	2.57044E-10	0.304713126	0.093	0.027	1.70222E-05	C1_CD8_Tres
chr1-197391251-197391763	2.62653E-10	0.295686985	0.051	0.007	1.73937E-05	C1_CD8_Tres
chr1-109501891-109502870	2.65247E-10	0.279155258	0.141	0.058	1.75655E-05	C1_CD8_Tres
chr3-151282893-151283705	2.65586E-10	0.276383308	0.114	0.047	1.75879E-05	C1_CD8_Tres
chr9-127990721-127991610	2.69204E-10	0.270546491	0.06	0.012	1.78275E-05	C1_CD8_Tres
chr7-158812659-158813019	2.75102E-10	0.266746256	0.066	0.014	1.82181E-05	C1_CD8_Tres
chr10-43426969-43427732	2.75153E-10	0.268036408	0.054	0.007	1.82215E-05	C1_CD8_Tres
chr14-76844045-76844599	2.80744E-10	0.303899494	0.078	0.018	1.85917E-05	C1_CD8_Tres
chr18-58687252-58688390	2.84346E-10	0.304850313	0.18	0.083	1.88302E-05	C1_CD8_Tres
chr18-74373109-74373960	2.86778E-10	0.273167855	0.24	0.135	1.89913E-05	C1_CD8_Tres
chr5-173781685-173782434	3.12275E-10	0.281368284	0.135	0.055	2.06798E-05	C1_CD8_Tres
chr10-28367842-28368938	3.20632E-10	0.272182158	0.129	0.055	2.12332E-05	C1_CD8_Tres
chrX-150574856-150575791	3.21838E-10	0.279804474	0.129	0.058	2.13131E-05	C1_CD8_Tres
chr8-763527-764677	3.30006E-10	0.259508212	0.24	0.138	2.1854E-05	C1_CD8_Tres
chr9-104990822-104992139	3.31326E-10	0.264667621	0.311	0.2	2.19414E-05	C1_CD8_Tres
chr15-91472216-91472679	3.35097E-10	0.26016923	0.069	0.018	2.21911E-05	C1_CD8_Tres
chr3-150722776-150723110	3.37137E-10	0.270256549	0.075	0.021	2.23262E-05	C1_CD8_Tres
chr1-148388151-148389117	3.39071E-10	0.29384888	0.165	0.073	2.24543E-05	C1_CD8_Tres
chr11-62024948-62026149	3.40964E-10	0.265379884	0.41	0.277	2.25797E-05	C1_CD8_Tres
chr19-43468453-43469356	3.60816E-10	0.261836094	0.072	0.019	2.38943E-05	C1_CD8_Tres
chr12-91870813-91871458	3.76527E-10	0.278462193	0.105	0.036	2.49348E-05	C1_CD8_Tres
chr2-197145428-197146035	3.83177E-10	0.251344989	0.072	0.02	2.53751E-05	C1_CD8_Tres
chr11-2093101-2093449	4.08526E-10	0.256355943	0.057	0.01	2.70538E-05	C1_CD8_Tres
chr7-76153463-76153892	4.14438E-10	0.314952462	0.063	0.009	2.74453E-05	C1_CD8_Tres

chr7-45048620-45049166	4.21771E-10	0.274993306	0.084	0.022	2.79309E-05	C1_CD8_Tres
chr4-8221373-8221947	4.22834E-10	0.26458207	0.123	0.05	2.80014E-05	C1_CD8_Tres
chr18-62329615-62329886	4.26588E-10	0.270018036	0.054	0.009	2.82499E-05	C1_CD8_Tres
chr19-38841624-38842824	4.28536E-10	0.262770609	0.263	0.153	2.83789E-05	C1_CD8_Tres
chr2-172437422-172438372	4.33089E-10	0.264253657	0.183	0.091	2.86805E-05	C1_CD8_Tres
chr10-5563994-5565107	4.90022E-10	0.311886903	0.108	0.034	3.24507E-05	C1_CD8_Tres
chr10-14573622-14573907	5.24658E-10	0.255093049	0.075	0.02	3.47444E-05	C1_CD8_Tres
chr9-97903148-97903413	5.28334E-10	0.257879426	0.063	0.016	3.49879E-05	C1_CD8_Tres
chr22-42179959-42180790	5.71967E-10	0.261626748	0.162	0.076	3.78774E-05	C1_CD8_Tres
chr1-146376265-146377483	5.82881E-10	0.27400274	0.219	0.113	3.86002E-05	C1_CD8_Tres
chr5-80181195-80181968	5.84249E-10	0.259619133	0.063	0.015	3.86907E-05	C1_CD8_Tres
chr5-1294844-1295423	5.93682E-10	0.278737449	0.057	0.01	3.93154E-05	C1_CD8_Tres
chr2-43636589-43637671	5.99139E-10	0.268850735	0.156	0.074	3.96768E-05	C1_CD8_Tres
chr17-2786066-2786653	6.28671E-10	0.290749573	0.108	0.038	4.16325E-05	C1_CD8_Tres
chr2-201419966-201420780	6.66661E-10	0.272227009	0.075	0.019	4.41483E-05	C1_CD8_Tres
chr1-8181870-8182991	7.14602E-10	0.288527312	0.183	0.084	4.73231E-05	C1_CD8_Tres
chr2-43175939-43176179	7.44124E-10	0.28317294	0.078	0.019	4.92781E-05	C1_CD8_Tres
chr14-49911425-49911963	7.55466E-10	0.285198343	0.132	0.055	5.00292E-05	C1_CD8_Tres
chr13-112968220-112969617	7.84375E-10	0.252312824	0.189	0.094	5.19436E-05	C1_CD8_Tres
chr19-43438368-43439720	8.23751E-10	0.285538217	0.195	0.101	5.45512E-05	C1_CD8_Tres
chr4-38837308-38838234	9.0653E-10	0.279107348	0.078	0.019	6.00331E-05	C1_CD8_Tres
chr15-82530074-82531139	9.34808E-10	0.271096664	0.141	0.058	6.19058E-05	C1_CD8_Tres
chr12-89244467-89245416	9.39738E-10	0.2892774	0.189	0.086	6.22323E-05	C1_CD8_Tres
chr4-52588534-52589208	9.44089E-10	0.345258151	0.141	0.048	6.25204E-05	C1_CD8_Tres
chr12-3896836-3897918	1.02434E-09	0.291499348	0.108	0.038	6.78346E-05	C1_CD8_Tres
chr19-17001273-17002247	1.03414E-09	0.292358813	0.138	0.057	6.8484E-05	C1_CD8_Tres
chr4-122221248-122221713	1.03917E-09	0.272213272	0.069	0.016	6.88168E-05	C1_CD8_Tres

chr2-197803986-197805480	1.05042E-09	0.266988352	0.24	0.143	6.95623E-05	C1_CD8_Tres
chr20-50165546-50166389	1.06306E-09	0.27236205	0.174	0.091	7.03993E-05	C1_CD8_Tres
chr12-50224208-50224793	1.09064E-09	0.26166963	0.129	0.058	7.22255E-05	C1_CD8_Tres
chr6-16723769-16724222	1.13853E-09	0.251736551	0.093	0.034	7.53967E-05	C1_CD8_Tres
chr1-28971976-28972791	1.14408E-09	0.25535403	0.111	0.047	7.57643E-05	C1_CD8_Tres
chrX-72105175-72105816	1.14474E-09	0.264324342	0.216	0.111	7.58078E-05	C1_CD8_Tres
chr10-92901631-92902311	1.15166E-09	0.273333012	0.084	0.024	7.62662E-05	C1_CD8_Tres
chr7-139100111-139100618	1.18664E-09	0.265342022	0.153	0.068	7.8583E-05	C1_CD8_Tres
chr14-98983929-98985162	1.19644E-09	0.26203035	0.243	0.131	7.92321E-05	C1_CD8_Tres
chr10-132615857-132616861	1.22343E-09	0.299435599	0.168	0.074	8.10189E-05	C1_CD8_Tres
chr17-40612576-40613325	1.23188E-09	0.257877806	0.201	0.111	8.15785E-05	C1_CD8_Tres
chr22-23704649-23705733	1.24728E-09	0.268413799	0.156	0.072	8.25986E-05	C1_CD8_Tres
chr4-102627761-102628377	1.27871E-09	0.260657148	0.117	0.051	8.46799E-05	C1_CD8_Tres
chr5-56638463-56639330	1.30004E-09	0.284430677	0.057	0.009	8.60923E-05	C1_CD8_Tres
chr5-168291365-168291956	1.35387E-09	0.295991176	0.069	0.013	8.9657E-05	C1_CD8_Tres
chr7-4883008-4884026	1.38522E-09	0.289628464	0.141	0.059	9.17335E-05	C1_CD8_Tres
chr5-149348450-149348794	1.43623E-09	0.277124821	0.096	0.026	9.51113E-05	C1_CD8_Tres
chr3-33007748-33008622	1.43668E-09	0.25722461	0.06	0.015	9.51411E-05	C1_CD8_Tres
chr17-43906401-43907892	1.47079E-09	0.265697785	0.132	0.052	9.74004E-05	C1_CD8_Tres
chr10-88193938-88194699	1.5147E-09	0.272891684	0.114	0.045	0.000100308	C1_CD8_Tres
chr19-49564572-49564981	1.54093E-09	0.277527703	0.057	0.01	0.000102045	C1_CD8_Tres
chr11-2442623-2442867	1.56804E-09	0.2625816	0.054	0.01	0.000103841	C1_CD8_Tres
chr2-234335408-234336249	1.57827E-09	0.313947146	0.099	0.029	0.000104518	C1_CD8_Tres
chr3-52982868-52983957	1.58291E-09	0.271775467	0.141	0.059	0.000104825	C1_CD8_Tres
chr1-39845279-39846062	1.62298E-09	0.259523461	0.102	0.041	0.000107479	C1_CD8_Tres
chr16-10957500-10958307	1.64842E-09	0.253764186	0.144	0.071	0.000109163	C1_CD8_Tres
chr9-35844908-35845230	1.67452E-09	0.259889416	0.075	0.019	0.000110892	C1_CD8_Tres

chr3-49602386-49603513	1.69161E-09	0.257190683	0.156	0.073	0.000112024	C1_CD8_Tres
chr19-7345315-7345860	1.76772E-09	0.27477187	0.081	0.025	0.000117064	C1_CD8_Tres
chr19-35346361-35347695	1.81984E-09	0.27851249	0.144	0.065	0.000120515	C1_CD8_Tres
chr2-149069078-149069733	1.87914E-09	0.297136747	0.108	0.035	0.000124442	C1_CD8_Tres
chr17-78758770-78759834	1.90862E-09	0.268103494	0.249	0.131	0.000126394	C1_CD8_Tres
chr16-324648-325396	1.99155E-09	0.259556465	0.135	0.062	0.000131887	C1_CD8_Tres
chr17-41338688-41339538	2.019E-09	0.25382083	0.249	0.152	0.000133704	C1_CD8_Tres
chr16-15672337-15673212	2.25559E-09	0.264827102	0.165	0.078	0.000149372	C1_CD8_Tres
chr8-99705382-99706622	2.42908E-09	0.26796151	0.126	0.054	0.000160861	C1_CD8_Tres
chrX-72094718-72095441	2.52736E-09	0.265471884	0.09	0.029	0.000167369	C1_CD8_Tres
chr20-47727109-47727940	2.6344E-09	0.303384119	0.153	0.067	0.000174458	C1_CD8_Tres
chr6-41298832-41299460	2.75384E-09	0.272916345	0.087	0.028	0.000182368	C1_CD8_Tres
chr5-80140182-80140705	2.75867E-09	0.250908648	0.057	0.011	0.000182688	C1_CD8_Tres
chr12-50597329-50597819	2.76479E-09	0.289989448	0.102	0.037	0.000183093	C1_CD8_Tres
chr21-33402721-33404158	2.77378E-09	0.255943045	0.063	0.016	0.000183688	C1_CD8_Tres
chr6-90411120-90411851	2.92076E-09	0.253693676	0.237	0.153	0.000193422	C1_CD8_Tres
chr19-40803732-40804155	2.97034E-09	0.265020864	0.099	0.035	0.000196705	C1_CD8_Tres
chr11-93737922-93738486	3.00952E-09	0.250139834	0.159	0.084	0.0001993	C1_CD8_Tres
chr14-104876193-104876651	3.07286E-09	0.30748015	0.099	0.033	0.000203494	C1_CD8_Tres
chr13-99290592-99291195	3.37343E-09	0.258036272	0.066	0.018	0.000223398	C1_CD8_Tres
chr3-124639579-124640359	3.57214E-09	0.263845823	0.096	0.033	0.000236558	C1_CD8_Tres
chr8-127982247-127982514	3.67386E-09	0.256180758	0.108	0.044	0.000243294	C1_CD8_Tres
chr5-96344663-96345726	3.70545E-09	0.253353698	0.117	0.05	0.000245386	C1_CD8_Tres
chr15-40492974-40493873	3.76658E-09	0.251785476	0.216	0.12	0.000249434	C1_CD8_Tres
chr15-92981098-92981897	3.85227E-09	0.263206235	0.075	0.02	0.000255109	C1_CD8_Tres
chr7-77834162-77834851	4.17389E-09	0.270572459	0.078	0.022	0.000276408	C1_CD8_Tres
chr20-5835299-5836281	4.17437E-09	0.263564402	0.114	0.048	0.000276439	C1_CD8_Tres

chr22-47090614-47091616	4.24356E-09	0.293689107	0.105	0.035	0.000281021	C1_CD8_Tres
chr8-17026652-17027754	4.27378E-09	0.289205334	0.126	0.047	0.000283022	C1_CD8_Tres
chr22-40728755-40729037	4.37305E-09	0.281832228	0.06	0.011	0.000289596	C1_CD8_Tres
chr9-69180395-69181037	4.69871E-09	0.297060466	0.108	0.038	0.000311163	C1_CD8_Tres
chr13-40764517-40765143	4.76411E-09	0.252008951	0.117	0.053	0.000315494	C1_CD8_Tres
chr15-91706099-91706801	4.82342E-09	0.269281253	0.117	0.05	0.000319422	C1_CD8_Tres
chr13-113816631-113817278	4.94554E-09	0.275557289	0.099	0.034	0.000327508	C1_CD8_Tres
chr5-76790211-76791350	5.15538E-09	0.251417111	0.168	0.086	0.000341405	C1_CD8_Tres
chr1-28933212-28933711	5.24391E-09	0.2652973	0.087	0.031	0.000347268	C1_CD8_Tres
chr12-92055807-92056207	5.26719E-09	0.27060479	0.153	0.074	0.000348809	C1_CD8_Tres
chr1-247936711-247937420	5.55584E-09	0.256380184	0.084	0.027	0.000367925	C1_CD8_Tres
chr13-99339624-99340297	6.14923E-09	0.258381889	0.114	0.054	0.000407221	C1_CD8_Tres
chr3-196279167-196279688	7.01264E-09	0.25614851	0.072	0.022	0.000464398	C1_CD8_Tres
chr1-148037879-148039000	7.57692E-09	0.280199309	0.15	0.065	0.000501766	C1_CD8_Tres
chr11-59857475-59858407	7.92227E-09	0.279808648	0.099	0.031	0.000524636	C1_CD8_Tres
chr14-38064845-38065701	8.04613E-09	0.283244731	0.201	0.098	0.000532839	C1_CD8_Tres
chr2-106085192-106086352	8.18258E-09	0.260742928	0.201	0.111	0.000541875	C1_CD8_Tres
chr9-22079362-22080370	9.35364E-09	0.264849651	0.087	0.031	0.000619426	C1_CD8_Tres
chr2-207123347-207123803	9.6379E-09	0.261579557	0.066	0.015	0.000638251	C1_CD8_Tres
chr11-61028371-61028662	9.8139E-09	0.283238763	0.081	0.025	0.000649906	C1_CD8_Tres
chr3-33029753-33031190	1.00118E-08	0.256358038	0.162	0.08	0.000663013	C1_CD8_Tres
chr17-63899406-63899732	1.03166E-08	0.251784358	0.057	0.012	0.000683196	C1_CD8_Tres
chr6-111086996-111088653	1.07363E-08	0.256634007	0.144	0.07	0.000710988	C1_CD8_Tres
chr2-25273301-25273885	1.15222E-08	0.250406563	0.141	0.067	0.000763031	C1_CD8_Tres
chr16-28936149-28936898	1.27275E-08	0.257356911	0.15	0.073	0.000842856	C1_CD8_Tres
chr3-18438279-18438968	1.32631E-08	0.268599461	0.072	0.02	0.000878321	C1_CD8_Tres
chr6-149065962-149066492	1.33938E-08	0.255423116	0.072	0.021	0.000886977	C1_CD8_Tres

chr10-23340977-23341304	1.38356E-08	0.281253768	0.075	0.019	0.000916233	C1_CD8_Tres
chr13-51821533-51822768	1.46626E-08	0.250867909	0.138	0.067	0.000971001	C1_CD8_Tres
chr7-98869672-98870538	1.47626E-08	0.252589956	0.102	0.04	0.000977626	C1_CD8_Tres
chr16-31695487-31696032	1.51023E-08	0.282767993	0.123	0.049	0.001000117	C1_CD8_Tres
chr2-86583657-86584667	1.5167E-08	0.256536389	0.099	0.035	0.001004402	C1_CD8_Tres
chr12-96176706-96177424	1.54142E-08	0.26702524	0.084	0.025	0.001020774	C1_CD8_Tres
chr7-50339986-50340516	1.84672E-08	0.253227656	0.102	0.039	0.00122295	C1_CD8_Tres
chr4-8199983-8200688	1.89994E-08	0.258137327	0.087	0.032	0.001258196	C1_CD8_Tres
chr8-2101236-2102257	1.93849E-08	0.254955647	0.093	0.031	0.001283729	C1_CD8_Tres
chr19-7348549-7349090	2.00081E-08	0.259071035	0.072	0.021	0.001324997	C1_CD8_Tres
chr12-89039825-89040764	2.01136E-08	0.255193774	0.126	0.057	0.001331983	C1_CD8_Tres
chr2-38578721-38579215	2.20396E-08	0.251437387	0.096	0.034	0.001459526	C1_CD8_Tres
chr4-83114583-83114901	2.4831E-08	0.283414556	0.117	0.044	0.001644382	C1_CD8_Tres
chr6-125302788-125303291	2.51987E-08	0.273935404	0.099	0.036	0.001668731	C1_CD8_Tres
chr21-43733027-43734456	2.86729E-08	0.264264168	0.162	0.078	0.001898803	C1_CD8_Tres
chr11-3511294-3512626	2.97446E-08	0.252589748	0.138	0.07	0.001969775	C1_CD8_Tres
chr8-42893435-42894491	3.01109E-08	0.256300764	0.162	0.082	0.001994033	C1_CD8_Tres
chr11-76560014-76560790	3.15546E-08	0.250948222	0.06	0.015	0.002089639	C1_CD8_Tres
chr3-115790540-115791139	3.40509E-08	0.268857023	0.198	0.102	0.002254955	C1_CD8_Tres
chrX-123803310-123803865	3.64375E-08	0.268086383	0.099	0.033	0.002413003	C1_CD8_Tres
chr3-33060661-33060935	3.92584E-08	0.256067492	0.063	0.017	0.002599806	C1_CD8_Tres
chr11-6733322-6733997	4.09689E-08	0.269185498	0.144	0.066	0.002713085	C1_CD8_Tres
chr1-244074471-244075379	4.94571E-08	0.264715345	0.165	0.088	0.003275199	C1_CD8_Tres
chrX-44343421-44344907	5.26262E-08	0.251302007	0.123	0.055	0.003485066	C1_CD8_Tres
chr14-70893119-70894127	5.65197E-08	0.285907274	0.111	0.041	0.003742906	C1_CD8_Tres
chr13-67229919-67231188	5.71735E-08	0.267090702	0.093	0.035	0.003786198	C1_CD8_Tres
chr6-90222669-90222949	5.81797E-08	0.260007742	0.069	0.02	0.003852832	C1_CD8_Tres

chr5-175895298-175896056	5.98688E-08	0.254375332	0.096	0.033	0.00396469	C1_CD8_Tres
chr11-121328162-121329239	6.07625E-08	0.250958637	0.063	0.015	0.004023872	C1_CD8_Tres
chr1-101111621-101112701	6.09935E-08	0.26322676	0.162	0.08	0.004039175	C1_CD8_Tres
chr16-84013850-84014615	6.29335E-08	0.25906718	0.075	0.023	0.004167643	C1_CD8_Tres
chr18-70781711-70782229	6.97234E-08	0.257302216	0.066	0.018	0.004617291	C1_CD8_Tres
chr11-14074835-14075632	8.80044E-08	0.273711594	0.123	0.053	0.005827918	C1_CD8_Tres
chr8-118620736-118622436	1.07491E-07	0.26335111	0.138	0.06	0.007118389	C1_CD8_Tres
chr3-128316344-128316848	1.19958E-07	0.253773624	0.066	0.017	0.007943954	C1_CD8_Tres
chr3-67947709-67948495	1.26816E-07	0.255530082	0.084	0.027	0.008398106	C1_CD8_Tres
chr19-50657797-50658465	1.79348E-07	0.257682036	0.096	0.037	0.011876962	C1_CD8_Tres
chr16-31951775-31952529	2.07931E-07	0.263832107	0.072	0.025	0.013769782	C1_CD8_Tres
chr13-31806431-31807534	3.32152E-07	0.25527865	0.096	0.035	0.021996109	C1_CD8_Tres
chr7-107560896-107561246	3.88839E-07	0.262758566	0.096	0.033	0.025750055	C1_CD8_Tres
chr8-89765714-89766222	6.19506E-07	0.254856398	0.084	0.03	0.041025534	C1_CD8_Tres
chr3-93470260-93470829	4.37216E-18	0.366742856	0.674	0.293	2.89537E-13	C1_CD8_Tres
chr17-22521254-22521613	3.80087E-69	0.745569079	0.772	0.278	2.51705E-64	C1_CD8_Tres
chr12-52566301-52567390	2.46913E-79	0.874838695	0.424	0.062	1.63513E-74	C3_CD8_Tem
chr19-10515556-10516304	8.66568E-78	0.890608504	0.32	0.031	5.73868E-73	C3_CD8_Tem
chr1-633460-634698	2.63058E-61	0.684577475	0.769	0.293	1.74205E-56	C1_CD8_Tres
chr9-136315890-136316920	2.66125E-70	0.847677118	0.31	0.032	1.76236E-65	C3_CD8_Tem
chr19-14475801-14477045	1.79762E-69	0.776107046	0.419	0.088	1.19044E-64	C3_CD8_Tem
chr14-101712387-101713961	3.09958E-65	0.709255657	0.471	0.124	2.05264E-60	C3_CD8_Tem
chr7-33707213-33707881	7.12688E-63	0.757634627	0.18	0.005	4.71963E-58	C3_CD8_Tem
chr6-161241983-161242489	8.28052E-63	0.830088736	0.219	0.011	5.48361E-58	C3_CD8_Tem
chr4-15961711-15962460	1.08123E-62	0.788563507	0.219	0.014	7.16021E-58	C3_CD8_Tem
chrX-317539-319259	2.58242E-68	0.675579487	0.814	0.299	1.71015E-63	C1_CD8_Tres
chr20-20215934-20216617	3.84678E-62	0.762606835	0.188	0.007	2.54745E-57	C3_CD8_Tem

chr1-184386329-184388089	5.54197E-62	0.675188144	0.518	0.165	3.67006E-57	C3_CD8_Tem
chr22-46289074-46289906	1.81906E-57	0.73335262	0.195	0.011	1.20464E-52	C3_CD8_Tem
chr12-121557117-121558231	3.26604E-56	0.66964259	0.484	0.152	2.16287E-51	C3_CD8_Tem
chr9-126636966-126637873	6.12289E-56	0.745561807	0.331	0.051	4.05476E-51	C3_CD8_Tem
chr1-202197442-202198255	8.33705E-56	0.713658132	0.385	0.087	5.52104E-51	C3_CD8_Tem
chr16-87761941-87762604	1.58783E-53	0.702072339	0.37	0.076	1.05151E-48	C3_CD8_Tem
chr10-77877596-77878312	3.02819E-53	0.748593308	0.286	0.045	2.00536E-48	C3_CD8_Tem
chr17-83199175-83200859	6.06288E-53	0.640654781	0.396	0.109	4.01502E-48	C3_CD8_Tem
chr4-8408869-8410058	7.55219E-53	0.592720178	0.612	0.276	5.00129E-48	C3_CD8_Tem
chr10-124641962-124642445	2.54628E-51	0.697827797	0.167	0.008	1.68622E-46	C3_CD8_Tem
chr22-46280826-46281500	4.36501E-51	0.700425188	0.237	0.032	2.89064E-46	C3_CD8_Tem
chr3-196338092-196338771	1.04698E-50	0.618772621	0.133	0.004	6.93341E-46	C3_CD8_Tem
chr4-970184-971071	7.90883E-50	0.675961704	0.341	0.078	5.23746E-45	C3_CD8_Tem
chr17-36188180-36189273	3.18453E-49	0.58806807	0.503	0.165	2.10889E-44	C3_CD8_Tem
chr2-27572153-27573519	1.14258E-48	0.660719368	0.208	0.024	7.5665E-44	C3_CD8_Tem
chr1-10524905-10525628	2.02813E-47	0.690396415	0.279	0.05	1.34309E-42	C3_CD8_Tem
chr4-1087278-1087798	8.98463E-46	0.688732629	0.151	0.006	5.94989E-41	C3_CD8_Tem
chr15-58614423-58615291	1.57643E-45	0.679244992	0.161	0.01	1.04396E-40	C3_CD8_Tem
chr12-128795987-128796930	2.0251E-45	0.640937865	0.276	0.056	1.34108E-40	C3_CD8_Tem
chr11-104163407-104164411	2.11515E-44	0.650833916	0.201	0.022	1.40072E-39	C3_CD8_Tem
chr12-128782326-128783599	3.35657E-44	0.615965329	0.307	0.074	2.22282E-39	C3_CD8_Tem
chr12-52558738-52559814	2.09533E-43	0.634349612	0.292	0.06	1.38759E-38	C3_CD8_Tem
chr12-108332684-108333036	1.38806E-42	0.60070829	0.112	0.002	9.19214E-38	C3_CD8_Tem
chr4-15962971-15963912	1.48343E-42	0.624791981	0.276	0.058	9.82374E-38	C3_CD8_Tem
chr22-20122665-20123654	1.72888E-42	0.610928614	0.156	0.014	1.14492E-37	C3_CD8_Tem
chr2-36800224-36800983	4.74566E-42	0.643852154	0.258	0.046	3.14272E-37	C3_CD8_Tem
chr20-24156928-24157560	7.42764E-42	0.634697319	0.185	0.021	4.91881E-37	C3_CD8_Tem

chr19-12983457-12985835	2.66114E-41	0.579037719	0.372	0.128	1.76229E-36	C3_CD8_Tem
chr19-10517383-10518508	2.86577E-40	0.588369848	0.273	0.063	1.8978E-35	C3_CD8_Tem
chr3-39234573-39235058	4.00336E-40	0.627313163	0.177	0.018	2.65115E-35	C3_CD8_Tem
chr1-6294018-6295206	4.65747E-40	0.622103156	0.138	0.008	3.08432E-35	C3_CD8_Tem
chr16-82936709-82937497	5.94575E-40	0.620102607	0.146	0.009	3.93745E-35	C3_CD8_Tem
chr4-158895825-158897132	1.93837E-39	0.612221535	0.148	0.011	1.28364E-34	C3_CD8_Tem
chr1-1628692-1631735	3.98631E-39	0.38325495	0.701	0.469	2.63985E-34	C3_CD8_Tem
chr5-1518362-1519073	7.16787E-39	0.56881139	0.281	0.071	4.74678E-34	C3_CD8_Tem
chr6-163750735-163751431	8.41945E-39	0.540827635	0.102	0.003	5.57561E-34	C3_CD8_Tem
chr16-81816756-81818369	2.16306E-38	0.593449082	0.167	0.021	1.43244E-33	C3_CD8_Tem
chr1-23958530-23960278	2.65789E-38	0.527470675	0.575	0.248	1.76013E-33	C1_CD8_Tres
chr17-10114141-10115557	8.09413E-38	0.489782217	0.56	0.267	5.36017E-33	C3_CD8_Tem
chr14-23663092-23663505	1.15958E-37	0.558492493	0.094	0.001	7.67909E-33	C3_CD8_Tem
chr8-141152316-141153097	3.95915E-37	0.594412882	0.193	0.028	2.62187E-32	C3_CD8_Tem
chr12-133079608-133081270	3.11646E-34	0.51529982	0.569	0.241	2.06381E-29	C1_CD8_Tres
chr19-10523026-10523671	5.76932E-36	0.565651082	0.289	0.077	3.82061E-31	C3_CD8_Tem
chr4-4268731-4270272	1.92637E-35	0.542397708	0.372	0.132	1.2757E-30	C3_CD8_Tem
chr1-629778-630252	6.51468E-21	0.450485722	0.293	0.116	4.31422E-16	C1_CD8_Tres
chr16-89821523-89821914	1.07035E-34	0.557871605	0.18	0.03	7.08815E-30	C3_CD8_Tem
chr22-23263385-23264547	1.41352E-34	0.551089318	0.362	0.132	9.36074E-30	C3_CD8_Tem
chr12-52871343-52871596	2.33177E-34	0.563263878	0.117	0.006	1.54417E-29	C3_CD8_Tem
chr2-144598045-144598443	3.11893E-34	0.489934334	0.089	0.002	2.06545E-29	C3_CD8_Tem
chr8-144146829-144148682	1.02688E-32	0.47427437	0.488	0.213	6.80028E-28	C1_CD8_Tres
chr17-1890533-1891414	6.97062E-34	0.543681586	0.292	0.089	4.61615E-29	C3_CD8_Tem
chr3-3109424-3110729	8.21023E-34	0.560243291	0.224	0.048	5.43706E-29	C3_CD8_Tem
chr19-10870523-10871977	1.16606E-33	0.33839424	0.745	0.521	7.72201E-29	C3_CD8_Tem
chr7-134640813-134641492	1.47261E-33	0.586354536	0.19	0.028	9.75203E-29	C3_CD8_Tem

chr20-57604829-57606180	2.36162E-33	0.507442486	0.354	0.121	1.56394E-28	C3_CD8_Tem
chr1-167430678-167431750	3.60141E-33	0.5519712	0.135	0.013	2.38496E-28	C3_CD8_Tem
chr5-107474537-107475439	4.24597E-33	0.508538975	0.367	0.131	2.81181E-28	C3_CD8_Tem
chr19-40465090-40467143	8.6162E-33	0.291449452	0.836	0.695	5.70591E-28	C3_CD8_Tem
chr9-78238064-78238326	9.89765E-33	0.528175762	0.125	0.01	6.55452E-28	C3_CD8_Tem
chr20-18738034-18738493	1.3135E-32	0.572440991	0.138	0.011	8.69836E-28	C3_CD8_Tem
chr15-58517225-58518085	1.44868E-32	0.534629665	0.112	0.006	9.59357E-28	C3_CD8_Tem
chr2-195649750-195650636	1.87098E-32	0.550970588	0.188	0.033	1.23902E-27	C3_CD8_Tem
chr1-11335432-11335881	2.74984E-32	0.517499646	0.089	0.002	1.82103E-27	C3_CD8_Tem
chr11-115502847-115505075	3.48921E-32	0.492819631	0.37	0.137	2.31066E-27	C3_CD8_Tem
chr14-91377071-91377696	3.97322E-32	0.560237366	0.242	0.058	2.63119E-27	C3_CD8_Tem
chr7-98514235-98515138	5.70899E-32	0.517798224	0.141	0.016	3.78066E-27	C3_CD8_Tem
chr1-8181870-8182991	7.14602E-10	0.288527312	0.183	0.084	4.73231E-05	C1_CD8_Tres
chr19-40687552-40687751	1.66941E-31	0.531533838	0.109	0.006	1.10553E-26	C3_CD8_Tem
chr20-24148061-24148691	1.70093E-31	0.525735667	0.102	0.005	1.12641E-26	C3_CD8_Tem
chr19-38864390-38866046	2.7247E-31	0.4274194	0.542	0.271	1.80438E-26	C3_CD8_Tem
chr3-185582820-185583272	1.08711E-30	0.5353978	0.177	0.031	7.19914E-26	C3_CD8_Tem
chr19-48492745-48493941	5.86203E-12	0.288063352	0.296	0.177	3.88201E-07	C1_CD8_Tres
chr10-131900106-131901808	1.66157E-30	0.503829364	0.446	0.179	1.10034E-25	C1_CD8_Tres
chr1-53135352-53136106	2.67864E-30	0.549656237	0.135	0.014	1.77387E-25	C3_CD8_Tem
chr4-38677485-38677764	3.97361E-30	0.483486789	0.089	0.004	2.63145E-25	C3_CD8_Tem
chr21-46423897-46425970	7.20188E-30	0.41552081	0.414	0.213	4.7693E-25	C3_CD8_Tem
chr3-56732303-56733288	7.78181E-30	0.506368376	0.232	0.065	5.15335E-25	C3_CD8_Tem
chr5-155834127-155834990	1.07124E-29	0.523294737	0.112	0.008	7.09408E-25	C3_CD8_Tem
chr3-57671133-57671708	1.61774E-29	0.5093469	0.198	0.046	1.07132E-24	C3_CD8_Tem
chr2-159144828-159145758	2.9113E-29	0.532200869	0.151	0.021	1.92795E-24	C3_CD8_Tem
chr1-75784374-75785133	3.14862E-29	0.511336666	0.214	0.054	2.08511E-24	C3_CD8_Tem

chr5-139925797-139926640	3.34003E-29	0.533250622	0.203	0.046	2.21187E-24	C3_CD8_Tem
chr2-64880665-64881690	4.76594E-29	0.518748479	0.198	0.044	3.15615E-24	C3_CD8_Tem
chr18-12920744-12921812	5.59375E-29	0.491111609	0.299	0.103	3.70435E-24	C3_CD8_Tem
chr12-128763410-128763767	7.93343E-29	0.523174669	0.112	0.008	5.25376E-24	C3_CD8_Tem
chr18-22465727-22466370	1.07943E-28	0.518672049	0.148	0.023	7.1483E-24	C3_CD8_Tem
chr17-39952197-39953600	3.34179E-14	0.268520897	0.551	0.414	2.21303E-09	C1_CD8_Tres
chr8-89728858-89729660	1.85269E-28	0.502737603	0.237	0.064	1.22691E-23	C3_CD8_Tem
chr12-108334194-108334419	1.90305E-28	0.426404166	0.065	0	1.26026E-23	C3_CD8_Tem
chr5-155766982-155767882	2.00171E-28	0.512950298	0.206	0.049	1.3256E-23	C3_CD8_Tem
chr8-144136968-144138476	4.84082E-23	0.432229091	0.377	0.162	3.20574E-18	C1_CD8_Tres
chr7-50335686-50336484	3.94137E-28	0.490840708	0.276	0.093	2.61009E-23	C3_CD8_Tem
chr10-17853977-17854653	4.89495E-28	0.527711408	0.104	0.005	3.24159E-23	C3_CD8_Tem
chr8-60409105-60409515	7.48627E-28	0.504693399	0.089	0.003	4.95763E-23	C3_CD8_Tem
chr7-38242795-38243176	7.54563E-28	0.5240371	0.182	0.037	4.99694E-23	C3_CD8_Tem
chr3-50465936-50466510	9.54037E-28	0.519897375	0.112	0.01	6.31792E-23	C3_CD8_Tem
chrX-1452437-1454445	9.5588E-22	0.294571942	0.713	0.553	6.33012E-17	C1_CD8_Tres
chr1-53084389-53084876	2.10388E-27	0.47853472	0.083	0.003	1.39325E-22	C3_CD8_Tem
chr19-39828147-39828699	2.21041E-27	0.47603442	0.135	0.019	1.4638E-22	C3_CD8_Tem
chr18-44700560-44700996	2.21175E-27	0.51401199	0.221	0.056	1.46469E-22	C3_CD8_Tem
chr6-81751731-81754171	3.20255E-27	0.445503619	0.43	0.198	2.12083E-22	C3_CD8_Tem
chr19-29263284-29263735	3.6504E-27	0.470585607	0.112	0.013	2.4174E-22	C3_CD8_Tem
chr6-152634627-152635124	4.00852E-27	0.480072749	0.232	0.065	2.65456E-22	C3_CD8_Tem
chr6-168905458-168906167	4.96056E-27	0.513757517	0.141	0.02	3.28503E-22	C3_CD8_Tem
chr16-57889406-57890119	5.01465E-27	0.453975527	0.086	0.005	3.32085E-22	C3_CD8_Tem
chr3-43477309-43478624	7.72491E-27	0.462285859	0.31	0.117	5.11566E-22	C3_CD8_Tem
chr8-141156903-141157559	8.11922E-27	0.474017868	0.297	0.106	5.37679E-22	C3_CD8_Tem
chr9-37512116-37512807	1.06788E-26	0.489241616	0.167	0.032	7.07179E-22	C3_CD8_Tem

chr21-33227850-33228121	2.07048E-26	0.5062783	0.112	0.011	1.37114E-21	C3_CD8_Tem
chr20-61989751-61990109	2.32674E-26	0.444055486	0.076	0.002	1.54083E-21	C3_CD8_Tem
chr17-8925655-8926140	2.40887E-26	0.47175913	0.089	0.005	1.59522E-21	C3_CD8_Tem
chr21-36155689-36157441	1.40186E-28	0.339027665	0.76	0.56	9.28355E-24	C1_CD8_Tres
chr1-23979709-23980925	3.86076E-28	0.483689341	0.395	0.167	2.55671E-23	C1_CD8_Tres
chr15-65512623-65513362	3.80529E-26	0.444611257	0.328	0.127	2.51998E-21	C3_CD8_Tem
chr22-25054537-25055508	4.15633E-26	0.468325651	0.156	0.031	2.75244E-21	C3_CD8_Tem
chr19-52833277-52834042	4.30001E-26	0.485972142	0.13	0.018	2.8476E-21	C3_CD8_Tem
chr14-55337435-55338199	4.34578E-26	0.426042099	0.453	0.221	2.87791E-21	C3_CD8_Tem
chr2-231529810-231530789	4.74916E-26	0.481784075	0.24	0.083	3.14504E-21	C3_CD8_Tem
chr4-38666967-38667483	5.495E-26	0.468684908	0.271	0.086	3.63896E-21	C3_CD8_Tem
chr4-38663147-38664983	7.17285E-26	0.378673308	0.5	0.277	4.75008E-21	C3_CD8_Tem
chr17-2792476-2793160	8.25126E-26	0.509444529	0.214	0.054	5.46423E-21	C3_CD8_Tem
chr19-54803320-54803853	1.03234E-25	0.456459259	0.083	0.003	6.83646E-21	C3_CD8_Tem
chr7-28203798-28204143	1.49924E-25	0.485618664	0.099	0.007	9.9284E-21	C3_CD8_Tem
chr12-128794493-128795204	1.84793E-25	0.460251928	0.156	0.033	1.22375E-20	C3_CD8_Tem
chr17-5184249-5184741	1.86212E-25	0.472727476	0.117	0.014	1.23315E-20	C3_CD8_Tem
chr2-68378735-68379810	1.96629E-25	0.454572305	0.26	0.09	1.30214E-20	C3_CD8_Tem
chr4-169015460-169016498	1.98768E-25	0.464053206	0.146	0.029	1.3163E-20	C3_CD8_Tem
chr16-54928338-54929868	2.05784E-25	0.443364137	0.341	0.149	1.36276E-20	C3_CD8_Tem
chr6-161226089-161227237	2.44777E-25	0.463735328	0.117	0.015	1.62099E-20	C3_CD8_Tem
chr1-15523836-15525464	1.06113E-24	0.284260088	0.766	0.607	7.02711E-20	C1_CD8_Tres
chr19-13043761-13044879	3.87226E-25	0.436370202	0.346	0.146	2.56432E-20	C3_CD8_Tem
chr9-131621481-131622102	4.02386E-25	0.49459992	0.146	0.027	2.66472E-20	C3_CD8_Tem
chr7-73005202-73006292	5.89203E-25	0.433156199	0.302	0.12	3.90188E-20	C3_CD8_Tem
chr1-53090595-53091098	7.41569E-25	0.433852608	0.073	0.002	4.91089E-20	C3_CD8_Tem
chr8-55843559-55845375	8.60502E-25	0.45494669	0.289	0.105	5.6985E-20	C3_CD8_Tem

chr1-24856950-24857412	8.61356E-25	0.490016207	0.099	0.007	5.70416E-20	C3_CD8_Tem
chr1-25064358-25066301	1.06894E-24	0.388565188	0.401	0.204	7.07887E-20	C3_CD8_Tem
chr13-114108073-114108397	1.13847E-24	0.493894961	0.112	0.011	7.53929E-20	C3_CD8_Tem
chr15-58518658-58518888	1.20403E-24	0.44421483	0.07	0.001	7.97342E-20	C3_CD8_Tem
chr3-142269739-142270886	1.43137E-24	0.434573236	0.297	0.117	9.47896E-20	C3_CD8_Tem
chr6-168877798-168878184	1.70458E-24	0.369619267	0.055	0.001	1.12883E-19	C3_CD8_Tem
chr19-41327145-41328838	2.75384E-24	0.26927853	0.794	0.666	1.82367E-19	C3_CD8_Tem
chr13-113875597-113877300	3.55021E-24	0.441795829	0.266	0.093	2.35105E-19	C3_CD8_Tem
chr9-136790481-136792075	4.89578E-24	0.273118179	0.711	0.541	3.24213E-19	C3_CD8_Tem
chr19-7920040-7921116	2.82495E-18	0.274928525	0.611	0.463	1.87077E-13	C1_CD8_Tres
chr12-681735-682825	5.60221E-24	0.439803881	0.211	0.061	3.70995E-19	C3_CD8_Tem
chr5-112418813-112420570	6.05075E-24	0.457124964	0.13	0.022	4.00699E-19	C3_CD8_Tem
chr9-136977939-136978928	6.49847E-24	0.428114087	0.26	0.106	4.30348E-19	C3_CD8_Tem
chr5-102295715-102297223	1.10198E-23	0.458526675	0.195	0.055	7.29767E-19	C3_CD8_Tem
chr5-112417854-112418360	1.72241E-23	0.472949839	0.128	0.018	1.14063E-18	C3_CD8_Tem
chr11-130388522-130389781	1.9917E-23	0.458325855	0.12	0.018	1.31896E-18	C3_CD8_Tem
chr1-41916078-41916434	2.1349E-23	0.444317874	0.128	0.021	1.41379E-18	C3_CD8_Tem
chr13-113176236-113176601	2.3097E-23	0.457658926	0.125	0.021	1.52955E-18	C3_CD8_Tem
chr20-24108764-24109803	2.38813E-23	0.437389609	0.133	0.022	1.58149E-18	C3_CD8_Tem
chr5-32241013-32241763	2.52358E-23	0.437307228	0.115	0.017	1.67119E-18	C3_CD8_Tem
chr20-10350624-10351911	2.53735E-23	0.437654133	0.268	0.098	1.68031E-18	C3_CD8_Tem
chr16-89872601-89874669	2.85584E-23	0.268672153	0.648	0.517	1.89122E-18	C3_CD8_Tem
chr13-31806431-31807534	3.32152E-07	0.25527865	0.096	0.035	0.021996109	C1_CD8_Tres
chr5-14108596-14110068	3.32559E-23	0.469628955	0.117	0.014	2.20231E-18	C3_CD8_Tem
chr17-2795538-2796544	3.89466E-23	0.452543864	0.208	0.061	2.57916E-18	C3_CD8_Tem
chr7-37657812-37658119	3.96604E-23	0.385952896	0.06	0.001	2.62643E-18	C3_CD8_Tem
chr17-2814110-2815366	1.2617E-45	0.653274964	0.497	0.175	8.35533E-41	C1_CD8_Tres

chr6-132612264-132612817	4.92359E-23	0.410305417	0.073	0.004	3.26055E-18	C3_CD8_Tem
chr2-191206130-191207147	5.30231E-23	0.401227684	0.331	0.149	3.51135E-18	C3_CD8_Tem
chr7-150665882-150666234	6.09876E-23	0.440462186	0.224	0.075	4.03878E-18	C3_CD8_Tem
chr1-53106453-53106790	6.18283E-23	0.396945842	0.068	0.003	4.09446E-18	C3_CD8_Tem
chr4-83211996-83212861	6.66564E-23	0.443141412	0.253	0.09	4.41419E-18	C3_CD8_Tem
chr1-121184236-121185085	2.98094E-29	0.53496817	0.47	0.186	1.97407E-24	C1_CD8_Tres
chr12-124423792-124424175	6.98691E-23	0.466853874	0.099	0.008	4.62694E-18	C3_CD8_Tem
chr1-3809711-3810580	7.42422E-23	0.451066803	0.148	0.034	4.91654E-18	C3_CD8_Tem
chr6-117616136-117617545	8.111E-23	0.452030633	0.143	0.026	5.37135E-18	C3_CD8_Tem
chr2-36798760-36799012	8.5842E-23	0.423075935	0.078	0.005	5.68472E-18	C3_CD8_Tem
chr2-144659399-144660199	1.06491E-22	0.430371582	0.372	0.168	7.05214E-18	C3_CD8_Tem
chr8-125729895-125730351	1.39825E-22	0.425909185	0.091	0.008	9.2596E-18	C3_CD8_Tem
chr17-36196490-36197498	1.51618E-22	0.430208115	0.229	0.074	1.00406E-17	C3_CD8_Tem
chr2-233385348-233386049	1.81236E-22	0.463230108	0.159	0.037	1.2002E-17	C3_CD8_Tem
chr19-43667974-43669476	9.67163E-40	0.522033166	0.602	0.282	6.40484E-35	C1_CD8_Tres
chr6-157143009-157143795	2.22341E-22	0.443238013	0.312	0.118	1.47241E-17	C3_CD8_Tem
chr5-107476616-107477202	2.43201E-22	0.418501467	0.312	0.127	1.61055E-17	C3_CD8_Tem
chr3-50428075-50428409	3.20484E-22	0.455139297	0.094	0.008	2.12234E-17	C3_CD8_Tem
chr15-58313319-58313881	3.24285E-22	0.429606704	0.102	0.011	2.14751E-17	C3_CD8_Tem
chr14-91354247-91354655	3.61047E-22	0.460609447	0.206	0.064	2.39096E-17	C3_CD8_Tem
chr22-23185555-23186129	3.83068E-22	0.471319767	0.13	0.022	2.53679E-17	C3_CD8_Tem
chr18-22281989-22283234	4.44495E-22	0.428259554	0.242	0.084	2.94358E-17	C3_CD8_Tem
chr2-216349913-216350784	4.72924E-22	0.440638886	0.263	0.103	3.13185E-17	C3_CD8_Tem
chr7-94656122-94658687	8.42126E-17	0.332052375	0.545	0.35	5.57681E-12	C1_CD8_Tres
chr8-124649586-124650602	5.42407E-22	0.399680876	0.247	0.094	3.59199E-17	C3_CD8_Tem
chr16-57070100-57072258	5.51011E-22	0.382387073	0.315	0.145	3.64896E-17	C3_CD8_Tem
chr17-47634861-47635166	6.19798E-22	0.394520555	0.065	0.002	4.10449E-17	C3_CD8_Tem

chr17-10077500-10078331	6.73628E-22	0.433996017	0.125	0.02	4.46097E-17	C3_CD8_Tem
chr19-40826622-40827364	6.83207E-22	0.464681803	0.214	0.065	4.5244E-17	C3_CD8_Tem
chr2-24739351-24739990	6.94402E-22	0.458937234	0.159	0.032	4.59854E-17	C3_CD8_Tem
chr6-152565755-152566727	7.83554E-22	0.418681814	0.253	0.085	5.18893E-17	C3_CD8_Tem
chr1-158924181-158925048	9.33917E-22	0.445274958	0.154	0.035	6.18468E-17	C3_CD8_Tem
chrX-1507227-1508353	9.42355E-22	0.406291322	0.312	0.136	6.24056E-17	C3_CD8_Tem
chr16-87724547-87725360	9.62699E-22	0.445457138	0.148	0.033	6.37528E-17	C3_CD8_Tem
chr16-58264802-58265790	1.5338E-21	0.418263648	0.102	0.012	1.01573E-16	C3_CD8_Tem
chr7-70660047-70660932	1.58219E-21	0.452568874	0.216	0.065	1.04777E-16	C3_CD8_Tem
chr6-157146935-157147209	1.65576E-21	0.402997024	0.081	0.006	1.0965E-16	C3_CD8_Tem
chr16-11562843-11563505	1.79236E-21	0.421169253	0.156	0.039	1.18695E-16	C3_CD8_Tem
chr1-24915001-24915963	1.84425E-21	0.393070554	0.253	0.098	1.22132E-16	C3_CD8_Tem
chr13-30157033-30157717	1.84852E-21	0.439652788	0.143	0.034	1.22414E-16	C3_CD8_Tem
chr17-48472024-48472803	1.87873E-21	0.437449368	0.117	0.019	1.24415E-16	C3_CD8_Tem
chr2-144508210-144508577	2.47197E-21	0.436759871	0.201	0.061	1.63702E-16	C3_CD8_Tem
chr20-24959621-24961536	2.6311E-21	0.357805248	0.438	0.235	1.74239E-16	C3_CD8_Tem
chr10-132536674-132537961	5.60645E-26	0.330815282	0.707	0.534	3.71276E-21	C1_CD8_Tres
chr1-54807085-54807644	2.94643E-21	0.467257506	0.167	0.038	1.95122E-16	C3_CD8_Tem
chr16-85099230-85099696	3.06081E-21	0.437019264	0.128	0.025	2.02696E-16	C3_CD8_Tem
chr11-65637708-65640465	3.17295E-21	0.309727417	0.503	0.34	2.10122E-16	C3_CD8_Tem
chr6-40409221-40409430	3.20229E-21	0.370049164	0.057	0.002	2.12065E-16	C3_CD8_Tem
chr7-37657024-37657499	3.51992E-21	0.402369119	0.07	0.003	2.33099E-16	C3_CD8_Tem
chr17-1286281-1287032	4.05792E-21	0.381589818	0.065	0.003	2.68727E-16	C3_CD8_Tem
chr19-47229277-47231760	4.20534E-21	0.271597229	0.589	0.433	2.7849E-16	C3_CD8_Tem
chr11-1568276-1568696	4.23123E-21	0.413245336	0.193	0.061	2.80205E-16	C3_CD8_Tem
chr7-28200374-28200895	4.77008E-21	0.428056424	0.083	0.008	3.15889E-16	C3_CD8_Tem
chr6-151374675-151375151	4.90351E-21	0.419582192	0.135	0.03	3.24725E-16	C3_CD8_Tem

chr3-170276876-170278095	5.94624E-21	0.428171033	0.224	0.077	3.93778E-16	C3_CD8_Tem
chr16-87492259-87493349	6.4581E-21	0.344944041	0.456	0.251	4.27675E-16	C3_CD8_Tem
chr12-94649474-94650756	7.27654E-21	0.404214568	0.141	0.037	4.81874E-16	C3_CD8_Tem
chr19-41120609-41121507	7.6395E-21	0.389623995	0.391	0.197	5.05911E-16	C3_CD8_Tem
chr10-22912663-22913849	9.16177E-21	0.434410827	0.125	0.021	6.0672E-16	C3_CD8_Tem
chr7-159198281-159198619	9.56849E-21	0.417935802	0.109	0.016	6.33654E-16	C3_CD8_Tem
chr7-128422793-128423306	1.08814E-20	0.425855435	0.112	0.016	7.20598E-16	C3_CD8_Tem
chr8-59655615-59655962	1.37309E-20	0.355193598	0.057	0.002	9.09304E-16	C3_CD8_Tem
chr3-43012965-43014368	1.42404E-20	0.408145229	0.201	0.071	9.43045E-16	C3_CD8_Tem
chr9-137021568-137022588	1.44852E-20	0.370014119	0.32	0.147	9.59252E-16	C3_CD8_Tem
chr10-124640818-124641704	1.50115E-20	0.400769411	0.268	0.11	9.94103E-16	C3_CD8_Tem
chr22-20556585-20556784	1.56909E-20	0.456547722	0.12	0.019	1.0391E-15	C3_CD8_Tem
chr1-45393590-45394814	1.63552E-20	0.422927791	0.138	0.032	1.08309E-15	C3_CD8_Tem
chr17-64024859-64026046	1.77547E-20	0.337529473	0.487	0.297	1.17577E-15	C3_CD8_Tem
chr4-38654965-38655565	1.99331E-20	0.411629096	0.154	0.04	1.32003E-15	C3_CD8_Tem
chr4-71107300-71108096	2.09924E-20	0.393474139	0.117	0.022	1.39018E-15	C3_CD8_Tem
chr19-19419983-19421059	2.11386E-20	0.379792051	0.289	0.123	1.39986E-15	C3_CD8_Tem
chr3-50429657-50430497	2.16544E-20	0.406755803	0.081	0.006	1.43402E-15	C3_CD8_Tem
chr16-87764489-87766170	2.3089E-20	0.298208144	0.521	0.347	1.52902E-15	C3_CD8_Tem
chr16-57646598-57647057	2.45367E-20	0.440655314	0.177	0.046	1.62489E-15	C3_CD8_Tem
chr18-23570812-23571367	2.67479E-20	0.428759417	0.104	0.015	1.77133E-15	C3_CD8_Tem
chr16-23482470-23483873	2.95602E-20	0.377784242	0.396	0.198	1.95756E-15	C3_CD8_Tem
chr12-128569992-128570692	3.16053E-20	0.444932386	0.13	0.023	2.093E-15	C3_CD8_Tem
chr19-51131882-51132291	3.25496E-20	0.423926159	0.086	0.009	2.15553E-15	C3_CD8_Tem
chr22-19419104-19420340	3.64009E-20	0.399093915	0.26	0.106	2.41057E-15	C3_CD8_Tem
chr14-93036187-93036733	4.05321E-20	0.431575729	0.112	0.017	2.68416E-15	C3_CD8_Tem
chr19-16256510-16257138	4.08192E-20	0.435481009	0.112	0.018	2.70317E-15	C3_CD8_Tem

chr17-49387502-49388620	4.76474E-20	0.41153871	0.234	0.087	3.15535E-15	C3_CD8_Tem
chr4-71186263-71188267	4.79893E-20	0.399543878	0.224	0.079	3.17799E-15	C3_CD8_Tem
chr19-40570079-40571126	4.04253E-14	0.273527332	0.449	0.311	2.67709E-09	C1_CD8_Tres
chr17-47829370-47831773	5.60638E-20	0.276619679	0.638	0.455	3.71272E-15	C3_CD8_Tem
chr19-3788955-3789725	5.77766E-20	0.39338395	0.206	0.069	3.82614E-15	C3_CD8_Tem
chr19-14274356-14275171	6.42228E-20	0.411610311	0.169	0.046	4.25302E-15	C3_CD8_Tem
chr7-70693239-70694833	6.49712E-20	0.408922329	0.286	0.119	4.30258E-15	C3_CD8_Tem
chr2-233386461-233386779	7.58264E-20	0.35450462	0.062	0.004	5.02145E-15	C3_CD8_Tem
chr6-46174902-46175839	7.64716E-20	0.393147456	0.253	0.1	5.06418E-15	C3_CD8_Tem
chr14-54669599-54670304	7.71428E-20	0.417617043	0.099	0.014	5.10863E-15	C3_CD8_Tem
chr1-107785657-107786884	7.82373E-20	0.372589796	0.299	0.14	5.18111E-15	C3_CD8_Tem
chr19-38924134-38924793	7.96186E-20	0.435908187	0.138	0.031	5.27258E-15	C3_CD8_Tem
chr5-1503317-1503721	8.48892E-20	0.407808462	0.104	0.016	5.62162E-15	C3_CD8_Tem
chr11-6228448-6228797	9.66232E-20	0.333277121	0.06	0.004	6.39868E-15	C3_CD8_Tem
chr11-134582476-134582776	1.07001E-19	0.467095756	0.133	0.021	7.08593E-15	C3_CD8_Tem
chr5-74631355-74632512	1.08472E-19	0.414943833	0.247	0.098	7.18332E-15	C3_CD8_Tem
chr11-110565415-110566163	1.38241E-19	0.365427392	0.073	0.006	9.15474E-15	C3_CD8_Tem
chr5-139453681-139454599	1.40146E-19	0.42282445	0.141	0.03	9.28091E-15	C3_CD8_Tem
chr2-68385217-68385954	1.6138E-19	0.39968011	0.214	0.075	1.06871E-14	C3_CD8_Tem
chr4-82284216-82285491	1.55078E-13	0.28098272	0.53	0.382	1.02697E-08	C1_CD8_Tres
chr6-10751519-10752026	1.80739E-19	0.434812869	0.143	0.033	1.19691E-14	C3_CD8_Tem
chr1-53581990-53582744	2.15688E-19	0.409248655	0.188	0.061	1.42835E-14	C3_CD8_Tem
chr17-80708430-80709030	2.30409E-19	0.416683505	0.096	0.011	1.52584E-14	C3_CD8_Tem
chr17-12981952-12982730	2.37589E-19	0.406938454	0.185	0.054	1.57339E-14	C3_CD8_Tem
chr17-409666-411434	2.81603E-19	0.301369197	0.448	0.293	1.86486E-14	C3_CD8_Tem
chr17-38900699-38901837	3.06186E-19	0.335699536	0.359	0.2	2.02766E-14	C3_CD8_Tem
chr5-155773428-155774034	3.20147E-19	0.417767426	0.109	0.016	2.12011E-14	C3_CD8_Tem

chr3-39260897-39261269	3.25752E-19	0.389725653	0.112	0.019	2.15723E-14	C3_CD8_Tem
chr16-4465340-4466660	3.4496E-19	0.347904806	0.344	0.184	2.28443E-14	C3_CD8_Tem
chr19-7690692-7691941	3.4602E-19	0.36944322	0.195	0.069	2.29144E-14	C3_CD8_Tem
chr5-91389941-91390685	3.83644E-19	0.393805326	0.096	0.014	2.54061E-14	C3_CD8_Tem
chr13-48504671-48506020	4.19305E-19	0.371554452	0.333	0.161	2.77676E-14	C3_CD8_Tem
chr10-110085686-110086736	4.4647E-19	0.37143326	0.156	0.049	2.95666E-14	C3_CD8_Tem
chr20-24953770-24954170	4.7263E-19	0.413334876	0.164	0.047	3.1299E-14	C3_CD8_Tem
chr13-25737866-25738508	4.98305E-19	0.365882646	0.062	0.003	3.29992E-14	C3_CD8_Tem
chr22-39173749-39174989	5.56815E-19	0.342828171	0.326	0.163	3.68739E-14	C3_CD8_Tem
chr18-48813554-48815949	5.57112E-19	0.332170022	0.411	0.242	3.68937E-14	C3_CD8_Tem
chr14-24678582-24680022	5.74575E-19	0.386337748	0.266	0.118	3.80501E-14	C3_CD8_Tem
chr16-88636781-88637627	6.64603E-19	0.332599715	0.318	0.167	4.4012E-14	C3_CD8_Tem
chr6-158717301-158718256	6.65365E-19	0.398377584	0.24	0.096	4.40624E-14	C3_CD8_Tem
chr22-23278801-23279304	7.21303E-19	0.39227923	0.146	0.039	4.77668E-14	C3_CD8_Tem
chr22-36435230-36436318	8.3552E-19	0.349795208	0.367	0.188	5.53306E-14	C3_CD8_Tem
chr19-45087196-45087984	9.38316E-19	0.332268135	0.383	0.203	6.21381E-14	C3_CD8_Tem
chr12-124452954-124453904	1.04393E-18	0.275403762	0.646	0.463	6.91325E-14	C3_CD8_Tem
chr10-129517368-129518421	1.13042E-18	0.351916842	0.076	0.008	7.48595E-14	C3_CD8_Tem
chr9-114369205-114370071	1.1782E-18	0.323353843	0.479	0.279	7.80237E-14	C3_CD8_Tem
chr3-20080956-20081545	1.26406E-18	0.383923316	0.112	0.023	8.37099E-14	C3_CD8_Tem
chr21-34800971-34801962	1.26674E-18	0.410065402	0.195	0.069	8.38874E-14	C3_CD8_Tem
chr4-6825019-6825950	1.29245E-18	0.410310289	0.115	0.022	8.55899E-14	C3_CD8_Tem
chr19-3357402-3358618	1.35355E-18	0.37706807	0.208	0.079	8.96365E-14	C3_CD8_Tem
chr3-118851499-118852175	1.50236E-18	0.389878905	0.107	0.019	9.94907E-14	C3_CD8_Tem
chr13-80399322-80400669	1.59207E-18	0.394549044	0.133	0.03	1.05432E-13	C3_CD8_Tem
chr17-78781990-78782715	1.72902E-18	0.276589143	0.438	0.276	1.14501E-13	C3_CD8_Tem
chr12-111404641-111406934	1.73214E-18	0.262013792	0.576	0.424	1.14708E-13	C3_CD8_Tem

chr9-136572519-136572836	2.09732E-18	0.3467929	0.055	0.002	1.38891E-13	C3_CD8_Tem
chr17-43359949-43362035	2.17594E-18	0.317878477	0.583	0.388	1.44097E-13	C3_CD8_Tem
chr2-100278936-100279351	2.25533E-18	0.371402239	0.078	0.008	1.49354E-13	C3_CD8_Tem
chr1-25035751-25036614	2.28245E-18	0.382485486	0.271	0.115	1.51151E-13	C3_CD8_Tem
chr5-142977014-142978026	2.35514E-18	0.357277266	0.299	0.141	1.55964E-13	C3_CD8_Tem
chr11-13550683-13551063	2.41617E-18	0.350917937	0.057	0.003	1.60006E-13	C3_CD8_Tem
chr12-128388516-128388978	2.6694E-18	0.324971338	0.052	0.002	1.76776E-13	C3_CD8_Tem
chr6-10403687-10404781	2.7416E-18	0.350072052	0.276	0.124	1.81557E-13	C3_CD8_Tem
chr12-128797250-128797743	2.94786E-18	0.378479375	0.109	0.021	1.95216E-13	C3_CD8_Tem
chr7-1011025-1011836	3.45373E-18	0.410002751	0.099	0.013	2.28716E-13	C3_CD8_Tem
chr11-406564-407932	3.5122E-18	0.303140596	0.49	0.331	2.32588E-13	C3_CD8_Tem
chr8-56999227-56999847	3.56951E-18	0.374991406	0.099	0.017	2.36384E-13	C3_CD8_Tem
chr1-27624800-27625617	4.40355E-18	0.394606911	0.172	0.052	2.91616E-13	C3_CD8_Tem
chr19-34184824-34185469	4.65506E-18	0.386568863	0.122	0.029	3.08272E-13	C3_CD8_Tem
chr2-16663941-16664571	5.2461E-18	0.360061148	0.086	0.013	3.47412E-13	C3_CD8_Tem
chr12-132985767-132986898	2.82645E-12	0.308257727	0.198	0.091	1.87176E-07	C1_CD8_Tres
chr19-10516551-10517154	5.72155E-18	0.405856536	0.133	0.028	3.78898E-13	C3_CD8_Tem
chr16-391649-392432	5.87686E-18	0.32973607	0.375	0.209	3.89183E-13	C3_CD8_Tem
chr22-46373246-46373567	7.10244E-18	0.36889144	0.073	0.008	4.70345E-13	C3_CD8_Tem
chr11-114073591-114074289	8.0925E-18	0.411708404	0.133	0.032	5.35909E-13	C3_CD8_Tem
chr9-87276213-87277089	9.04173E-18	0.358244697	0.109	0.023	5.98771E-13	C3_CD8_Tem
chr5-74630330-74631054	9.19269E-18	0.379639428	0.198	0.071	6.08768E-13	C3_CD8_Tem
chr3-52459189-52459423	1.01382E-17	0.353039948	0.081	0.011	6.71383E-13	C3_CD8_Tem
chr17-5220631-5221894	1.02383E-17	0.37461876	0.224	0.085	6.7801E-13	C3_CD8_Tem
chr17-47740627-47742073	1.03287E-17	0.385217947	0.203	0.07	6.84E-13	C3_CD8_Tem
chr11-134402464-134402811	1.07421E-17	0.354622052	0.068	0.005	7.11372E-13	C3_CD8_Tem
chr15-89936034-89936904	1.30752E-17	0.375118236	0.099	0.019	8.65881E-13	C3_CD8_Tem

chr1-148401655-148403045	1.33869E-17	0.377978628	0.221	0.083	8.86518E-13	C3_CD8_Tem
chr22-37993882-37994570	1.38803E-17	0.376733936	0.089	0.012	9.19193E-13	C3_CD8_Tem
chr7-8152189-8153125	1.5299E-17	0.357738951	0.086	0.013	1.01314E-12	C3_CD8_Tem
chr8-144412646-144413811	1.5569E-17	0.289273289	0.417	0.262	1.03103E-12	C3_CD8_Tem
chr8-141172247-141172549	1.56138E-17	0.385784892	0.073	0.006	1.03399E-12	C3_CD8_Tem
chr13-114142248-114143533	1.71088E-17	0.320508713	0.367	0.199	1.133E-12	C3_CD8_Tem
chr14-91385208-91385523	1.88091E-17	0.38885366	0.109	0.023	1.2456E-12	C3_CD8_Tem
chr19-54550831-54551646	1.98704E-17	0.402129909	0.151	0.043	1.31588E-12	C3_CD8_Tem
chr14-101726547-101726808	1.99294E-17	0.408503001	0.138	0.038	1.31979E-12	C3_CD8_Tem
chr17-67449711-67450561	2.00038E-17	0.382836746	0.12	0.029	1.32471E-12	C3_CD8_Tem
chr6-168968129-168968581	2.03334E-17	0.392772019	0.091	0.012	1.34654E-12	C3_CD8_Tem
chr12-70049371-70050399	2.16969E-17	0.370298783	0.297	0.142	1.43683E-12	C3_CD8_Tem
chr9-131405174-131405600	2.18408E-17	0.405144837	0.115	0.024	1.44636E-12	C3_CD8_Tem
chr17-10198118-10199316	2.21119E-17	0.383501918	0.229	0.085	1.46431E-12	C3_CD8_Tem
chr22-50506952-50508750	2.23428E-17	0.250378072	0.591	0.444	1.47961E-12	C3_CD8_Tem
chr22-28882669-28884423	9.25429E-38	0.529199688	0.488	0.22	6.12847E-33	C1_CD8_Tres
chr2-231554181-231554979	2.65823E-17	0.36545537	0.07	0.006	1.76036E-12	C3_CD8_Tem
chr1-184388426-184388636	2.78717E-17	0.373346594	0.089	0.015	1.84575E-12	C3_CD8_Tem
chr20-24032404-24033610	2.84094E-17	0.386145365	0.112	0.02	1.88135E-12	C3_CD8_Tem
chr12-94675699-94676685	2.9392E-17	0.392068286	0.195	0.067	1.94643E-12	C3_CD8_Tem
chr8-28781891-28782316	2.93977E-17	0.320546471	0.055	0.003	1.9468E-12	C3_CD8_Tem
chr3-16789235-16789741	3.23747E-17	0.369233614	0.094	0.015	2.14395E-12	C3_CD8_Tem
chr5-1501091-1502379	3.49045E-17	0.389676935	0.169	0.05	2.31148E-12	C3_CD8_Tem
chr20-60058205-60058753	4.02568E-17	0.337856586	0.073	0.009	2.66593E-12	C3_CD8_Tem
chr10-89367247-89367952	4.36038E-17	0.377323261	0.141	0.036	2.88758E-12	C3_CD8_Tem
chr7-77428850-77429150	4.42658E-17	0.34136687	0.06	0.004	2.93141E-12	C3_CD8_Tem
chr10-79025064-79025989	4.51917E-17	0.353794914	0.284	0.143	2.99273E-12	C3_CD8_Tem

chr11-3099202-3099527	4.57103E-17	0.355156425	0.068	0.007	3.02707E-12	C3_CD8_Tem
chr12-128810142-128810932	5.43873E-17	0.396344087	0.146	0.04	3.60169E-12	C3_CD8_Tem
chr19-679697-681238	2.79859E-26	0.342468201	0.647	0.449	1.85331E-21	C1_CD8_Tres
chr11-115256133-115257162	5.93142E-17	0.355881847	0.177	0.06	3.92797E-12	C3_CD8_Tem
chr15-82539674-82541121	7.15086E-18	0.3290858	0.407	0.247	4.73552E-13	C1_CD8_Tres
chr1-94017157-94017843	8.26438E-17	0.385639261	0.112	0.025	5.47292E-12	C3_CD8_Tem
chr3-15519975-15520491	9.3691E-17	0.355711386	0.089	0.014	6.2045E-12	C3_CD8_Tem
chr20-32569246-32569647	1.00325E-16	0.317378937	0.057	0.005	6.64383E-12	C3_CD8_Tem
chr7-159195482-159196150	1.02499E-16	0.377090899	0.148	0.045	6.7878E-12	C3_CD8_Tem
chr2-201656079-201656495	1.06442E-16	0.373615066	0.096	0.017	7.0489E-12	C3_CD8_Tem
chr19-4104223-4105353	1.08966E-16	0.262346742	0.508	0.369	7.21606E-12	C3_CD8_Tem
chr19-3284533-3286006	1.1029E-16	0.36863174	0.182	0.073	7.30375E-12	C3_CD8_Tem
chr2-181393318-181395118	1.30227E-16	0.338698967	0.281	0.132	8.62403E-12	C3_CD8_Tem
chr19-10416274-10417062	1.41525E-16	0.277669529	0.414	0.269	9.37221E-12	C3_CD8_Tem
chrX-124641758-124642302	1.41968E-16	0.349149996	0.057	0.003	9.40156E-12	C3_CD8_Tem
chr6-40477130-40478225	1.4676E-16	0.352304449	0.292	0.142	9.71889E-12	C3_CD8_Tem
chr17-49714993-49716355	1.49736E-16	0.280506752	0.495	0.331	9.916E-12	C3_CD8_Tem
chr2-9680883-9681755	1.56438E-16	0.367581012	0.133	0.038	1.03598E-11	C3_CD8_Tem
chr17-3870861-3871325	1.75367E-16	0.288754373	0.052	0.005	1.16133E-11	C3_CD8_Tem
chr6-75781491-75782175	1.85092E-16	0.355280802	0.112	0.027	1.22573E-11	C3_CD8_Tem
chr4-25687707-25688167	1.92006E-16	0.343648895	0.062	0.005	1.27152E-11	C3_CD8_Tem
chr16-123924-124278	1.92241E-16	0.369252884	0.06	0.004	1.27308E-11	C3_CD8_Tem
chr8-66609955-66610470	1.97108E-16	0.367104858	0.146	0.041	1.30531E-11	C3_CD8_Tem
chr4-3879513-3880885	2.00702E-16	0.371709307	0.115	0.027	1.32911E-11	C3_CD8_Tem
chr16-53453967-53454220	2.05645E-16	0.385918071	0.115	0.024	1.36184E-11	C3_CD8_Tem
chr19-7692254-7693184	2.19912E-16	0.319946202	0.19	0.082	1.45632E-11	C3_CD8_Tem
chr4-9151875-9153538	2.32207E-16	0.261695621	0.602	0.455	1.53774E-11	C3_CD8_Tem

chr4-7539428-7540144	2.35718E-16	0.38211418	0.104	0.019	1.561E-11	C3_CD8_Tem
chr9-104990822-104992139	3.31326E-10	0.264667621	0.311	0.2	2.19414E-05	C1_CD8_Tres
chr17-1823106-1823993	2.48931E-16	0.33556185	0.344	0.194	1.6485E-11	C3_CD8_Tem
chr1-27990940-27991227	2.6448E-16	0.364830662	0.06	0.003	1.75147E-11	C3_CD8_Tem
chr15-58331692-58332623	2.71202E-16	0.333098137	0.318	0.173	1.79598E-11	C3_CD8_Tem
chr9-85743374-85743622	2.90713E-16	0.384716163	0.094	0.015	1.92519E-11	C3_CD8_Tem
chr7-158804277-158804969	3.02388E-16	0.337688804	0.141	0.043	2.00251E-11	C3_CD8_Tem
chr18-45106137-45106769	3.0253E-16	0.395573544	0.141	0.035	2.00344E-11	C3_CD8_Tem
chr4-168816422-168817018	3.03502E-16	0.35822803	0.073	0.009	2.00988E-11	C3_CD8_Tem
chr18-22385697-22386058	3.15522E-16	0.325637717	0.07	0.009	2.08948E-11	C3_CD8_Tem
chr1-2234180-2234998	3.22633E-16	0.332450825	0.286	0.15	2.13657E-11	C3_CD8_Tem
chr12-6613453-6614299	3.39478E-16	0.386290711	0.19	0.068	2.24813E-11	C3_CD8_Tem
chr20-28602102-28603285	6.08925E-14	0.358619638	0.21	0.08	4.03248E-09	C1_CD8_Tres
chr18-23546087-23546614	3.4448E-16	0.359792532	0.073	0.01	2.28125E-11	C3_CD8_Tem
chr10-120683857-120684576	3.53995E-16	0.34348906	0.06	0.005	2.34426E-11	C3_CD8_Tem
chr19-3275065-3276762	1.56836E-23	0.387348192	0.494	0.289	1.03861E-18	C1_CD8_Tres
chr5-142929335-142929995	3.88304E-16	0.358372195	0.138	0.042	2.57146E-11	C3_CD8_Tem
chr4-145623780-145624155	3.88332E-16	0.357429162	0.122	0.031	2.57165E-11	C3_CD8_Tem
chr21-5154334-5154970	1.31642E-13	0.349547233	0.228	0.094	8.7177E-09	C1_CD8_Tres
chr22-50561408-50562188	4.04784E-16	0.34203992	0.109	0.024	2.6806E-11	C3_CD8_Tem
chr12-128745688-128746405	4.59257E-16	0.304105337	0.06	0.007	3.04134E-11	C3_CD8_Tem
chr14-91390079-91390524	4.68373E-16	0.350066578	0.102	0.023	3.10171E-11	C3_CD8_Tem
chr19-14459391-14460097	4.79567E-16	0.348070102	0.195	0.078	3.17584E-11	C3_CD8_Tem
chr2-235669088-235669666	5.26691E-16	0.33411519	0.07	0.008	3.48791E-11	C3_CD8_Tem
chr19-49127628-49128613	5.56567E-16	0.258716174	0.469	0.325	3.68575E-11	C3_CD8_Tem
chr17-5237381-5238054	5.61587E-16	0.367789952	0.141	0.041	3.719E-11	C3_CD8_Tem
chr10-124225140-124226065	6.31956E-16	0.361381641	0.094	0.015	4.18501E-11	C3_CD8_Tem

chr6-145844988-145845928	6.61344E-16	0.34332974	0.096	0.021	4.37962E-11	C3_CD8_Tem
chr1-202203369-202204084	6.7511E-16	0.358565412	0.128	0.035	4.47078E-11	C3_CD8_Tem
chr3-15303830-15304820	8.65746E-16	0.371016536	0.112	0.023	5.73323E-11	C3_CD8_Tem
chr3-50588289-50588897	9.88203E-16	0.339502053	0.237	0.108	6.54418E-11	C3_CD8_Tem
chr3-39222454-39222817	1.02856E-15	0.331218209	0.073	0.01	6.81147E-11	C3_CD8_Tem
chr1-160480584-160481100	1.03406E-15	0.373497234	0.091	0.016	6.84784E-11	C3_CD8_Tem
chr8-143927925-143929685	1.10813E-15	0.322899144	0.284	0.145	7.33834E-11	C3_CD8_Tem
chr3-39249348-39249908	1.14233E-15	0.328733489	0.081	0.013	7.56484E-11	C3_CD8_Tem
chr1-198556590-198557308	1.15426E-15	0.354826655	0.185	0.068	7.64383E-11	C3_CD8_Tem
chr19-44760126-44761340	1.18492E-15	0.284595738	0.398	0.242	7.84693E-11	C3_CD8_Tem
chr17-62965173-62966688	1.18874E-15	0.310334529	0.349	0.182	7.87217E-11	C3_CD8_Tem
chr10-98408778-98409438	1.19326E-15	0.318536117	0.076	0.012	7.9021E-11	C3_CD8_Tem
chr7-2102032-2102670	1.23944E-15	0.352084065	0.078	0.011	8.20793E-11	C3_CD8_Tem
chr15-95864358-95864855	1.26752E-15	0.334744527	0.07	0.01	8.39391E-11	C3_CD8_Tem
chr15-58369239-58369703	1.37559E-15	0.336555701	0.057	0.004	9.10957E-11	C3_CD8_Tem
chr18-22237893-22238540	1.39682E-15	0.343645222	0.068	0.01	9.25014E-11	C3_CD8_Tem
chr5-107485956-107486918	1.44519E-15	0.34032355	0.237	0.101	9.57046E-11	C3_CD8_Tem
chr12-8987598-8988061	1.47991E-15	0.343085332	0.115	0.028	9.8004E-11	C3_CD8_Tem
chr1-147540684-147541537	1.2471E-15	0.353993982	0.198	0.079	8.25868E-11	C1_CD8_Tres
chr4-38654125-38654687	1.6119E-15	0.368478093	0.122	0.03	1.06745E-10	C3_CD8_Tem
chr6-80453563-80454609	1.62669E-15	0.398009287	0.091	0.013	1.07724E-10	C3_CD8_Tem
chr4-15970971-15971455	1.71478E-15	0.337830799	0.07	0.01	1.13558E-10	C3_CD8_Tem
chr17-67407593-67408095	1.74222E-15	0.338367473	0.086	0.015	1.15375E-10	C3_CD8_Tem
chr9-85715559-85716785	1.8138E-15	0.346648573	0.128	0.036	1.20115E-10	C3_CD8_Tem
chr7-133480899-133481198	2.24756E-15	0.348106764	0.057	0.004	1.4884E-10	C3_CD8_Tem
chr20-51553758-51554917	2.37026E-15	0.344300631	0.25	0.114	1.56966E-10	C3_CD8_Tem
chr10-73758806-73760041	2.47215E-15	0.318707877	0.247	0.123	1.63713E-10	C3_CD8_Tem

chr20-24027145-24027791	2.85605E-15	0.351157612	0.151	0.05	1.89136E-10	C3_CD8_Tem
chr19-16343147-16343471	2.9172E-15	0.314066162	0.083	0.015	1.93186E-10	C3_CD8_Tem
chr20-38776288-38776623	2.93887E-15	0.326135775	0.078	0.014	1.94621E-10	C3_CD8_Tem
chr19-38928395-38928678	2.99659E-15	0.367911966	0.104	0.022	1.98443E-10	C3_CD8_Tem
chr9-134024653-134026073	3.0321E-15	0.260029744	0.516	0.364	2.00795E-10	C3_CD8_Tem
chr19-51128884-51129150	3.3782E-15	0.318039805	0.062	0.008	2.23714E-10	C3_CD8_Tem
chr11-118699279-118700427	3.37858E-15	0.312956664	0.279	0.146	2.2374E-10	C3_CD8_Tem
chr6-152635971-152637143	3.496E-15	0.325194654	0.255	0.126	2.31516E-10	C3_CD8_Tem
chr18-44723355-44723585	3.65692E-15	0.313103378	0.065	0.009	2.42173E-10	C3_CD8_Tem
chr1-159799549-159800580	4.06132E-15	0.338411987	0.12	0.034	2.68953E-10	C3_CD8_Tem
chr12-49043678-49044518	4.12264E-15	0.348012104	0.185	0.071	2.73013E-10	C3_CD8_Tem
chr11-134228632-134229052	4.3296E-15	0.356477499	0.19	0.077	2.86719E-10	C3_CD8_Tem
chr11-121481647-121482948	5.04433E-15	0.315803905	0.359	0.204	3.34051E-10	C3_CD8_Tem
chr3-187828673-187829471	5.09431E-15	0.30558089	0.062	0.008	3.3736E-10	C3_CD8_Tem
chr17-78789249-78789852	5.3379E-15	0.364583213	0.078	0.011	3.53492E-10	C3_CD8_Tem
chr19-17538908-17540305	7.50075E-12	0.251232293	0.308	0.206	4.96722E-07	C1_CD8_Tres
chr1-101111621-101112701	6.09935E-08	0.26322676	0.162	0.08	0.004039175	C1_CD8_Tres
chr9-40991238-40992772	3.23214E-21	0.360534696	0.569	0.393	2.14042E-16	C1_CD8_Tres
chr5-152113146-152113770	5.76167E-15	0.351972852	0.083	0.013	3.81555E-10	C3_CD8_Tem
chr14-100408099-100408812	5.94827E-15	0.348460901	0.146	0.045	3.93912E-10	C3_CD8_Tem
chr19-543224-544491	5.9802E-15	0.2819524	0.357	0.209	3.96027E-10	C3_CD8_Tem
chr21-7775170-7776039	6.08216E-15	0.323449394	0.112	0.028	4.02779E-10	C3_CD8_Tem
chr6-40587005-40588094	6.12348E-15	0.342475287	0.151	0.052	4.05515E-10	C3_CD8_Tem
chr22-46370317-46371312	6.26933E-15	0.339534285	0.161	0.059	4.15174E-10	C3_CD8_Tem
chr5-177327484-177328163	6.32522E-15	0.364816516	0.138	0.038	4.18875E-10	C3_CD8_Tem
chr4-145626300-145626817	7.18659E-15	0.315849788	0.073	0.011	4.75917E-10	C3_CD8_Tem
chr15-40313542-40313961	7.19251E-15	0.333791992	0.115	0.03	4.76309E-10	C3_CD8_Tem

chr22-39353348-39353661	7.28807E-15	0.314167306	0.055	0.004	4.82638E-10	C3_CD8_Tem
chr16-85569565-85571266	7.29047E-15	0.318059979	0.255	0.129	4.82797E-10	C3_CD8_Tem
chr18-11818493-11818843	7.70031E-15	0.297058889	0.052	0.004	5.09938E-10	C3_CD8_Tem
chr18-48940224-48941255	7.98765E-15	0.277040919	0.471	0.318	5.28966E-10	C3_CD8_Tem
chr21-44427728-44428950	8.39367E-15	0.335325137	0.164	0.067	5.55854E-10	C3_CD8_Tem
chr10-131896262-131896784	9.54184E-15	0.352657415	0.177	0.064	6.31889E-10	C3_CD8_Tem
chr14-92615207-92615959	1.0615E-14	0.363547441	0.208	0.084	7.02958E-10	C3_CD8_Tem
chr10-17509096-17509486	1.1285E-14	0.354086683	0.117	0.031	7.47328E-10	C3_CD8_Tem
chr6-161242937-161243613	1.14297E-14	0.351565737	0.221	0.086	7.56908E-10	C3_CD8_Tem
chr6-193662-194430	1.35654E-14	0.33530034	0.221	0.101	8.98343E-10	C3_CD8_Tem
chr16-4064883-4065411	1.36122E-14	0.310682952	0.081	0.016	9.01441E-10	C3_CD8_Tem
chr12-132818116-132818950	1.36827E-14	0.354184933	0.125	0.036	9.06107E-10	C3_CD8_Tem
chr7-105346261-105347149	1.38625E-14	0.363565403	0.133	0.039	9.18015E-10	C3_CD8_Tem
chr6-46325308-46326080	1.53286E-14	0.333670848	0.167	0.061	1.01511E-09	C3_CD8_Tem
chr19-51122443-51122842	1.56008E-14	0.281785026	0.052	0.006	1.03313E-09	C3_CD8_Tem
chr5-88140701-88141866	1.59265E-14	0.32797038	0.263	0.129	1.0547E-09	C3_CD8_Tem
chr12-6337143-6337559	1.59553E-14	0.324466259	0.232	0.114	1.05661E-09	C3_CD8_Tem
chr1-25989958-25991332	1.5975E-14	0.345321181	0.154	0.055	1.05791E-09	C3_CD8_Tem
chr20-17612090-17612811	1.60685E-14	0.352601194	0.172	0.066	1.06411E-09	C3_CD8_Tem
chr7-70669706-70670633	1.66801E-14	0.348515634	0.133	0.037	1.10461E-09	C3_CD8_Tem
chr5-14208019-14208476	1.77643E-14	0.294586065	0.055	0.005	1.1764E-09	C3_CD8_Tem
chr2-208358913-208359863	2.03084E-14	0.325873833	0.076	0.011	1.34488E-09	C3_CD8_Tem
chr17-36212926-36213380	2.05367E-14	0.323490332	0.125	0.035	1.36E-09	C3_CD8_Tem
chr17-49403620-49404641	2.07583E-14	0.355642901	0.091	0.018	1.37468E-09	C3_CD8_Tem
chr19-10111953-10113270	2.24177E-14	0.26468045	0.406	0.277	1.48457E-09	C3_CD8_Tem
chr5-142786694-142787240	2.61447E-14	0.317866643	0.237	0.115	1.73138E-09	C3_CD8_Tem
chr2-96131228-96131850	2.64387E-14	0.328293737	0.143	0.049	1.75085E-09	C3_CD8_Tem

chr17-46019786-46020825	2.73547E-14	0.311678949	0.299	0.167	1.81151E-09	C3_CD8_Tem
chr12-120265007-120266070	2.94843E-14	0.279230768	0.268	0.147	1.95254E-09	C3_CD8_Tem
chr3-48931087-48931615	3.00676E-14	0.34125158	0.112	0.029	1.99117E-09	C3_CD8_Tem
chr7-70647532-70648779	3.10014E-14	0.311821097	0.393	0.222	2.053E-09	C3_CD8_Tem
chr13-80477733-80478369	3.25515E-14	0.332680053	0.081	0.012	2.15566E-09	C3_CD8_Tem
chr14-55806678-55807685	3.28921E-14	0.341951917	0.221	0.096	2.17821E-09	C3_CD8_Tem
chr17-80471993-80472202	3.34211E-14	0.30999807	0.055	0.005	2.21325E-09	C3_CD8_Tem
chr9-93624047-93625281	3.51627E-14	0.331564892	0.104	0.025	2.32858E-09	C3_CD8_Tem
chr17-82257065-82257733	3.57187E-14	0.347291557	0.172	0.068	2.3654E-09	C3_CD8_Tem
chr13-80338896-80339381	4.08507E-14	0.298502381	0.06	0.007	2.70525E-09	C3_CD8_Tem
chr4-3192121-3192568	4.50773E-14	0.279817396	0.055	0.008	2.98515E-09	C3_CD8_Tem
chr10-22596797-22597505	4.89431E-14	0.324064901	0.18	0.076	3.24116E-09	C3_CD8_Tem
chr3-135965264-135966458	4.9833E-14	0.364588532	0.135	0.041	3.30009E-09	C3_CD8_Tem
chr4-40264977-40266011	5.11887E-14	0.315068787	0.193	0.084	3.38987E-09	C3_CD8_Tem
chr9-38226982-38227814	5.18549E-14	0.313630806	0.062	0.008	3.43398E-09	C3_CD8_Tem
chr7-25861799-25862894	5.27125E-14	0.32405273	0.216	0.094	3.49078E-09	C3_CD8_Tem
chr1-146376265-146377483	5.82881E-10	0.27400274	0.219	0.113	3.86002E-05	C1_CD8_Tres
chr3-69281817-69282554	5.38001E-14	0.298798354	0.266	0.141	3.5628E-09	C3_CD8_Tem
chr18-44729308-44730171	5.42239E-14	0.299625722	0.328	0.189	3.59087E-09	C3_CD8_Tem
chr19-659341-659906	5.76111E-14	0.327988502	0.237	0.113	3.81518E-09	C3_CD8_Tem
chr10-44310373-44311620	6.41106E-14	0.339438291	0.164	0.062	4.24559E-09	C3_CD8_Tem
chr22-23137252-23138634	6.64212E-14	0.29864429	0.258	0.14	4.39861E-09	C3_CD8_Tem
chr1-158978573-158979257	6.91648E-14	0.322162703	0.148	0.053	4.5803E-09	C3_CD8_Tem
chr20-35069488-35070404	7.46273E-14	0.330952265	0.094	0.024	4.94205E-09	C3_CD8_Tem
chr13-55971544-55971783	7.77768E-14	0.350838013	0.13	0.035	5.15061E-09	C3_CD8_Tem
chr17-10046365-10047468	7.96171E-14	0.326779597	0.086	0.017	5.27249E-09	C3_CD8_Tem
chr5-147804838-147805626	8.17063E-14	0.325155779	0.146	0.05	5.41084E-09	C3_CD8_Tem

chr19-16329007-16329426	8.97058E-14	0.328113873	0.208	0.093	5.94059E-09	C3_CD8_Tem
chr16-3818357-3819464	9.21251E-14	0.326403643	0.104	0.026	6.1008E-09	C3_CD8_Tem
chr2-86079669-86080996	9.74864E-14	0.292991627	0.352	0.202	6.45584E-09	C3_CD8_Tem
chr19-7196940-7198555	2.07228E-13	0.283435541	0.401	0.261	1.37232E-08	C1_CD8_Tres
chr17-30975014-30975406	1.02821E-13	0.297278552	0.081	0.016	6.80913E-09	C3_CD8_Tem
chr7-36863792-36865390	1.0364E-13	0.291637612	0.268	0.145	6.86338E-09	C3_CD8_Tem
chr3-17075310-17075732	1.03756E-13	0.282204666	0.062	0.009	6.87105E-09	C3_CD8_Tem
chr19-16324026-16325430	1.05083E-13	0.254209086	0.406	0.276	6.9589E-09	C3_CD8_Tem
chr11-2270658-2271271	1.10945E-13	0.315608554	0.13	0.05	7.34712E-09	C3_CD8_Tem
chr1-243507985-243508809	1.18823E-13	0.333222606	0.143	0.049	7.86884E-09	C3_CD8_Tem
chr11-86800116-86800797	1.30318E-13	0.318713893	0.146	0.052	8.63003E-09	C3_CD8_Tem
chr19-8573198-8573976	1.43212E-13	0.272990204	0.318	0.19	9.48393E-09	C3_CD8_Tem
chr2-221517255-221519122	3.17043E-13	0.303510806	0.455	0.292	2.09955E-08	C1_CD8_Tres
chr22-21772845-21773238	1.47335E-13	0.322393194	0.068	0.01	9.75699E-09	C3_CD8_Tem
chr12-52645010-52646024	1.48679E-13	0.33899161	0.253	0.11	9.84597E-09	C3_CD8_Tem
chr19-49493630-49493854	1.55875E-13	0.323118875	0.133	0.045	1.03225E-08	C3_CD8_Tem
chr15-94324887-94325647	1.63808E-13	0.311413278	0.148	0.051	1.08479E-08	C3_CD8_Tem
chr8-101967543-101968103	1.73399E-13	0.322496418	0.078	0.013	1.1483E-08	C3_CD8_Tem
chr11-72743763-72744303	1.77827E-13	0.339143674	0.102	0.026	1.17762E-08	C3_CD8_Tem
chr20-45944148-45944437	1.84E-13	0.319552331	0.13	0.044	1.2185E-08	C3_CD8_Tem
chr1-64882580-64883097	1.94133E-13	0.300703665	0.055	0.006	1.2856E-08	C3_CD8_Tem
chr6-36123502-36124155	2.07913E-13	0.291909094	0.318	0.189	1.37686E-08	C3_CD8_Tem
chr3-153242772-153243234	2.22736E-13	0.330378173	0.104	0.026	1.47502E-08	C3_CD8_Tem
chr19-41875113-41875859	2.23148E-13	0.277536998	0.43	0.277	1.47775E-08	C3_CD8_Tem
chr19-35140757-35141944	2.84099E-20	0.364285923	0.44	0.255	1.88139E-15	C1_CD8_Tres
chr17-82350306-82350899	2.3457E-13	0.314232007	0.081	0.018	1.55339E-08	C3_CD8_Tem
chr16-82431989-82432632	2.35582E-13	0.315915265	0.096	0.021	1.5601E-08	C3_CD8_Tem

chr17-43251577-43251794	2.48706E-13	0.287034444	0.112	0.033	1.647E-08	C3_CD8_Tem
chr6-24948959-24949511	2.49557E-13	0.286885857	0.086	0.021	1.65264E-08	C3_CD8_Tem
chr10-22634033-22634531	2.54407E-13	0.319212681	0.156	0.064	1.68476E-08	C3_CD8_Tem
chr16-27491703-27492455	2.58062E-13	0.310442495	0.065	0.009	1.70896E-08	C3_CD8_Tem
chrY-7040598-7041260	2.59634E-13	0.352227222	0.096	0.021	1.71938E-08	C3_CD8_Tem
chr20-8131554-8132972	2.62397E-13	0.306232432	0.273	0.141	1.73767E-08	C3_CD8_Tem
chr9-86200569-86201022	2.89306E-13	0.301176839	0.057	0.006	1.91587E-08	C3_CD8_Tem
chr10-110441270-110442167	2.90911E-13	0.341724432	0.18	0.071	1.9265E-08	C3_CD8_Tem
chr16-85104759-85105382	2.99885E-13	0.332629316	0.107	0.029	1.98593E-08	C3_CD8_Tem
chr15-72805422-72805797	3.05699E-13	0.319613983	0.081	0.016	2.02443E-08	C3_CD8_Tem
chr9-124873648-124874001	3.12637E-13	0.309845356	0.13	0.044	2.07038E-08	C3_CD8_Tem
chr1-148522069-148523165	3.42945E-13	0.30178345	0.302	0.157	2.27109E-08	C3_CD8_Tem
chr6-101845117-101846042	3.76079E-13	0.33134781	0.086	0.017	2.49051E-08	C3_CD8_Tem
chr4-121253639-121254863	3.79628E-13	0.27041219	0.375	0.232	2.51401E-08	C3_CD8_Tem
chr10-123771922-123773586	3.82179E-13	0.313789301	0.089	0.018	2.53091E-08	C3_CD8_Tem
chr20-33369167-33370781	3.93941E-13	0.269654678	0.339	0.188	2.6088E-08	C3_CD8_Tem
chr20-23456455-23457109	3.9514E-13	0.3225922	0.099	0.026	2.61674E-08	C3_CD8_Tem
chr2-96806412-96807178	4.26284E-13	0.324224982	0.143	0.052	2.82298E-08	C3_CD8_Tem
chr17-1203077-1203834	4.42194E-13	0.301381995	0.143	0.06	2.92834E-08	C3_CD8_Tem
chr5-54646159-54647075	4.43002E-13	0.313398333	0.234	0.123	2.93369E-08	C3_CD8_Tem
chr20-62117493-62118052	4.44719E-13	0.298266955	0.276	0.15	2.94507E-08	C3_CD8_Tem
chr17-42015625-42015831	5.19495E-13	0.298218077	0.081	0.019	3.44025E-08	C3_CD8_Tem
chr6-137231582-137232242	5.19501E-13	0.321222988	0.135	0.048	3.44029E-08	C3_CD8_Tem
chr11-2217156-2217452	5.29096E-13	0.300122389	0.057	0.006	3.50383E-08	C3_CD8_Tem
chr12-122161588-122162025	5.35137E-13	0.324178936	0.094	0.022	3.54384E-08	C3_CD8_Tem
chr1-203755930-203757037	5.36993E-13	0.267231313	0.32	0.187	3.55613E-08	C3_CD8_Tem
chr16-82945391-82946046	5.56487E-13	0.30436076	0.073	0.012	3.68522E-08	C3_CD8_Tem

chr5-107501861-107502630	5.82855E-13	0.318947265	0.109	0.032	3.85984E-08	C3_CD8_Tem
chr5-155768313-155768661	5.93327E-13	0.317786667	0.104	0.028	3.92919E-08	C3_CD8_Tem
chr2-110674997-110676078	5.95705E-13	0.276010399	0.302	0.181	3.94494E-08	C3_CD8_Tem
chr1-101338011-101339141	6.04441E-13	0.337923542	0.172	0.063	4.00279E-08	C3_CD8_Tem
chr8-58790453-58791432	6.3278E-13	0.299387189	0.193	0.092	4.19046E-08	C3_CD8_Tem
chr18-23422625-23423422	6.4488E-13	0.343973102	0.12	0.04	4.27059E-08	C3_CD8_Tem
chr11-134348881-134349933	6.53154E-13	0.327430294	0.146	0.05	4.32538E-08	C3_CD8_Tem
chr3-14377448-14378683	6.54514E-13	0.304776821	0.193	0.085	4.33439E-08	C3_CD8_Tem
chr2-240831720-240832790	3.9185E-12	0.292926316	0.374	0.244	2.59495E-07	C1_CD8_Tres
chr16-326942-327829	7.00951E-13	0.253117023	0.406	0.278	4.64191E-08	C3_CD8_Tem
chr7-150999190-151000200	7.658E-13	0.305762817	0.081	0.018	5.07136E-08	C3_CD8_Tem
chr5-178113100-178115384	8.19057E-13	0.269133097	0.32	0.203	5.42404E-08	C3_CD8_Tem
chr1-53600208-53600946	8.50866E-13	0.312013543	0.154	0.058	5.63469E-08	C3_CD8_Tem
chr3-50502718-50503940	9.62132E-13	0.287540047	0.214	0.105	6.37153E-08	C3_CD8_Tem
chr17-31051892-31052728	9.89955E-13	0.313223109	0.081	0.017	6.55578E-08	C3_CD8_Tem
chr19-16369616-16370355	1.05869E-12	0.299462805	0.201	0.095	7.01094E-08	C3_CD8_Tem
chr1-92840417-92840927	1.08974E-12	0.296301885	0.07	0.013	7.21662E-08	C3_CD8_Tem
chr8-73167236-73167579	1.15851E-12	0.314896239	0.117	0.035	7.67198E-08	C3_CD8_Tem
chr1-161626522-161627073	1.20338E-12	0.296266862	0.117	0.038	7.96913E-08	C3_CD8_Tem
chr1-235074458-235075575	8.91623E-11	0.310783572	0.192	0.085	5.90459E-06	C1_CD8_Tres
chr19-1896931-1898310	1.22654E-12	0.278025981	0.354	0.215	8.12254E-08	C3_CD8_Tem
chr1-91730812-91731065	1.23542E-12	0.291960561	0.073	0.015	8.18131E-08	C3_CD8_Tem
chr7-158595018-158595361	1.23669E-12	0.318526117	0.159	0.064	8.18975E-08	C3_CD8_Tem
chr7-132450906-132451511	1.25278E-12	0.305918532	0.117	0.04	8.29627E-08	C3_CD8_Tem
chr7-25856468-25857283	1.28977E-12	0.299662975	0.148	0.06	8.54121E-08	C3_CD8_Tem
chr11-113867604-113868489	1.38363E-12	0.273565969	0.06	0.01	9.16283E-08	C3_CD8_Tem
chr5-155728080-155729632	1.40425E-12	0.295478129	0.07	0.012	9.29936E-08	C3_CD8_Tem

chr12-120253297-120253660	1.41284E-12	0.320610816	0.128	0.047	9.35624E-08	C3_CD8_Tem
chr13-114104456-114104877	1.41657E-12	0.304840784	0.062	0.01	9.38096E-08	C3_CD8_Tem
chr14-67875192-67875771	1.41791E-12	0.313971283	0.076	0.014	9.38985E-08	C3_CD8_Tem
chr6-37051112-37051757	1.51267E-12	0.287168746	0.263	0.149	1.00174E-07	C3_CD8_Tem
chr3-50450069-50451178	1.51631E-12	0.285128514	0.161	0.077	1.00415E-07	C3_CD8_Tem
chr15-20353048-20354546	1.5404E-12	0.291173786	0.221	0.119	1.0201E-07	C3_CD8_Tem
chr10-70610194-70611322	1.58622E-12	0.272078445	0.289	0.177	1.05044E-07	C3_CD8_Tem
chr14-23660023-23660690	1.64528E-12	0.345420611	0.096	0.023	1.08955E-07	C3_CD8_Tem
chr11-65902073-65902532	1.68796E-12	0.312998331	0.128	0.041	1.11782E-07	C3_CD8_Tem
chr18-74995694-74996447	1.69042E-12	0.308982613	0.125	0.048	1.11945E-07	C3_CD8_Tem
chr17-78474402-78475194	1.76478E-12	0.311220819	0.078	0.016	1.16869E-07	C3_CD8_Tem
chr12-94180453-94181290	1.77004E-12	0.302229254	0.117	0.038	1.17217E-07	C3_CD8_Tem
chr2-177251782-177252365	1.77849E-12	0.322270393	0.135	0.048	1.17777E-07	C3_CD8_Tem
chr1-16122578-16124085	1.8041E-12	0.320108177	0.208	0.091	1.19473E-07	C3_CD8_Tem
chr7-133483833-133484470	1.83107E-12	0.350907744	0.143	0.051	1.21259E-07	C3_CD8_Tem
chr6-30568132-30568616	1.84547E-12	0.296796729	0.182	0.079	1.22213E-07	C3_CD8_Tem
chr17-36182330-36184239	1.84827E-12	0.282950995	0.143	0.056	1.22398E-07	C3_CD8_Tem
chr3-20066969-20067533	1.87608E-12	0.280837569	0.078	0.016	1.2424E-07	C3_CD8_Tem
chr16-67542375-67542823	1.96682E-12	0.319875817	0.18	0.08	1.30249E-07	C3_CD8_Tem
chr1-167430060-167430391	2.0062E-12	0.286873814	0.062	0.01	1.32857E-07	C3_CD8_Tem
chr6-108837368-108838249	2.01587E-12	0.29313166	0.133	0.047	1.33497E-07	C3_CD8_Tem
chr11-134406573-134406855	2.02616E-12	0.312283889	0.07	0.011	1.34178E-07	C3_CD8_Tem
chr3-13233775-13234999	2.0461E-12	0.322529449	0.083	0.016	1.35499E-07	C3_CD8_Tem
chr1-150186466-150187633	2.05444E-12	0.2994733	0.167	0.067	1.36051E-07	C3_CD8_Tem
chr19-23378845-23379406	2.06329E-12	0.302326219	0.06	0.008	1.36637E-07	C3_CD8_Tem
chr17-48478011-48478796	2.25896E-12	0.318416794	0.109	0.034	1.49595E-07	C3_CD8_Tem
chr17-5226891-5227781	2.32976E-12	0.271653279	0.073	0.018	1.54284E-07	C3_CD8_Tem

chr19-5086165-5086940	2.34145E-12	0.31440928	0.094	0.026	1.55058E-07	C3_CD8_Tem
chr17-42483623-42484911	2.42871E-12	0.271139073	0.292	0.171	1.60836E-07	C3_CD8_Tem
chr6-36118475-36118945	2.58904E-12	0.30373336	0.25	0.127	1.71454E-07	C3_CD8_Tem
chr17-49968095-49969213	2.62372E-12	0.303488187	0.146	0.061	1.7375E-07	C3_CD8_Tem
chr5-1374037-1374398	2.66602E-12	0.300429189	0.055	0.007	1.76552E-07	C3_CD8_Tem
chr17-74730383-74731073	2.73083E-12	0.266861605	0.227	0.128	1.80844E-07	C3_CD8_Tem
chr1-144076968-144077758	2.73373E-12	0.27983098	0.068	0.012	1.81036E-07	C3_CD8_Tem
chr18-77080211-77081050	2.7976E-12	0.295836892	0.146	0.059	1.85266E-07	C3_CD8_Tem
chr1-111215714-111216491	2.87106E-12	0.262989866	0.438	0.287	1.9013E-07	C3_CD8_Tem
chr9-123304331-123305414	2.89258E-12	0.281890072	0.326	0.196	1.91555E-07	C3_CD8_Tem
chr12-10602523-10603301	3.01703E-12	0.291578974	0.128	0.047	1.99797E-07	C3_CD8_Tem
chr18-8601407-8602044	3.16852E-12	0.307164533	0.068	0.01	2.09829E-07	C3_CD8_Tem
chr19-42352176-42352961	3.19109E-12	0.326405184	0.078	0.017	2.11324E-07	C3_CD8_Tem
chr12-121750190-121751261	3.28189E-12	0.308114799	0.076	0.015	2.17337E-07	C3_CD8_Tem
chr7-3120260-3120830	3.63944E-12	0.287419557	0.089	0.024	2.41015E-07	C3_CD8_Tem
chr8-55864340-55864719	3.81183E-12	0.296089359	0.057	0.007	2.52431E-07	C3_CD8_Tem
chr5-142996190-142996868	3.81465E-12	0.3115215	0.096	0.028	2.52617E-07	C3_CD8_Tem
chr8-2133830-2134213	3.91617E-12	0.318821937	0.104	0.028	2.59341E-07	C3_CD8_Tem
chr11-115267533-115268247	4.2046E-12	0.301200347	0.107	0.03	2.78441E-07	C3_CD8_Tem
chr6-11260229-11260909	4.3267E-12	0.282300636	0.083	0.025	2.86527E-07	C3_CD8_Tem
chr1-8495849-8497059	4.37499E-12	0.297918377	0.242	0.126	2.89725E-07	C3_CD8_Tem
chr11-128189888-128190647	4.90265E-12	0.273433706	0.06	0.01	3.24668E-07	C3_CD8_Tem
chr5-42961865-42962585	5.30029E-12	0.312952622	0.083	0.018	3.51001E-07	C3_CD8_Tem
chr7-150556371-150557079	5.48474E-12	0.318432753	0.117	0.038	3.63216E-07	C3_CD8_Tem
chr1-171805282-171806053	5.52637E-12	0.303101927	0.086	0.021	3.65973E-07	C3_CD8_Tem
chr17-82548700-82548930	5.6357E-12	0.29752393	0.052	0.006	3.73213E-07	C3_CD8_Tem
chr12-133036812-133038023	1.89258E-28	0.48786114	0.362	0.15	1.25332E-23	C1_CD8_Tres

chrX-107449624-107451395	5.91649E-12	0.267019307	0.232	0.115	3.91808E-07	C3_CD8_Tem
chr1-55138744-55139160	6.05594E-12	0.316079215	0.091	0.023	4.01043E-07	C3_CD8_Tem
chr15-89136591-89137119	6.08276E-12	0.266823543	0.06	0.01	4.02819E-07	C3_CD8_Tem
chr12-94704331-94704803	6.42258E-12	0.298184448	0.065	0.009	4.25323E-07	C3_CD8_Tem
chr14-87959829-87960695	6.4552E-12	0.299274857	0.065	0.012	4.27482E-07	C3_CD8_Tem
chr7-105848750-105849993	6.47151E-12	0.25533245	0.393	0.246	4.28563E-07	C3_CD8_Tem
chr11-66421081-66422007	6.48974E-12	0.270286158	0.182	0.084	4.2977E-07	C3_CD8_Tem
chr6-4607340-4608186	6.54025E-12	0.288758984	0.055	0.007	4.33115E-07	C3_CD8_Tem
chr22-24951939-24952941	6.54513E-12	0.278461502	0.221	0.113	4.33438E-07	C3_CD8_Tem
chr16-85518417-85520080	6.90481E-12	0.287743472	0.109	0.038	4.57257E-07	C3_CD8_Tem
chr10-95290491-95291334	7.01186E-12	0.292706171	0.089	0.024	4.64346E-07	C3_CD8_Tem
chr10-128042170-128042547	7.10126E-12	0.318585041	0.128	0.044	4.70267E-07	C3_CD8_Tem
chr7-16346063-16346786	7.41205E-12	0.321590017	0.083	0.018	4.90848E-07	C3_CD8_Tem
chr16-31364431-31365532	7.51256E-12	0.301228026	0.203	0.093	4.97505E-07	C3_CD8_Tem
chr18-77621808-77622512	8.20717E-12	0.302391463	0.083	0.018	5.43503E-07	C3_CD8_Tem
chr1-148037879-148039000	7.57692E-09	0.280199309	0.15	0.065	0.000501766	C1_CD8_Tres
chr2-130726997-130729121	8.48556E-12	0.2673568	0.37	0.236	5.61939E-07	C3_CD8_Tem
chr18-79326345-79328199	8.59335E-12	0.291160088	0.117	0.037	5.69077E-07	C3_CD8_Tem
chr6-30963694-30964716	8.61885E-12	0.330556879	0.128	0.039	5.70766E-07	C3_CD8_Tem
chr12-1740319-1740894	8.92634E-12	0.284179435	0.055	0.006	5.91129E-07	C3_CD8_Tem
chr19-19039612-19040323	8.95742E-12	0.284085508	0.104	0.037	5.93187E-07	C3_CD8_Tem
chr11-11987445-11988281	9.09996E-12	0.309981017	0.143	0.06	6.02626E-07	C3_CD8_Tem
chr2-33275472-33276071	9.37276E-12	0.32255131	0.135	0.044	6.20692E-07	C3_CD8_Tem
chr7-69824019-69824874	9.56395E-12	0.286369776	0.068	0.011	6.33354E-07	C3_CD8_Tem
chr13-29529696-29530077	9.97745E-12	0.303464686	0.065	0.01	6.60737E-07	C3_CD8_Tem
chr16-57667384-57668198	1.00128E-11	0.281946287	0.073	0.016	6.63077E-07	C3_CD8_Tem
chr2-12064177-12064536	1.0148E-11	0.296123491	0.076	0.016	6.72029E-07	C3_CD8_Tem

chr3-4748585-4749193	1.02765E-11	0.28910881	0.065	0.011	6.80538E-07	C3_CD8_Tem
chr22-39351636-39352097	1.06242E-11	0.297844251	0.07	0.013	7.03568E-07	C3_CD8_Tem
chr6-36099343-36100456	1.06879E-11	0.286040022	0.208	0.101	7.07785E-07	C3_CD8_Tem
chr1-37822976-37823765	1.0719E-11	0.277416174	0.083	0.022	7.09842E-07	C3_CD8_Tem
chr17-78630966-78632304	1.13876E-11	0.317009443	0.219	0.103	7.54121E-07	C3_CD8_Tem
chr11-3216973-3217537	1.15025E-11	0.288127923	0.104	0.034	7.61728E-07	C3_CD8_Tem
chr7-130145661-130145954	1.16977E-11	0.272427361	0.055	0.009	7.74655E-07	C3_CD8_Tem
chr21-33937726-33938566	1.1753E-11	0.263840958	0.24	0.133	7.78318E-07	C3_CD8_Tem
chr7-3119492-3119792	1.20894E-11	0.2802874	0.07	0.016	8.00593E-07	C3_CD8_Tem
chr19-4059467-4060321	1.22236E-11	0.260372443	0.266	0.155	8.09485E-07	C3_CD8_Tem
chr12-64668656-64669921	1.2265E-11	0.263856063	0.305	0.187	8.12223E-07	C3_CD8_Tem
chr5-74326657-74327326	1.24575E-11	0.291478081	0.099	0.028	8.24976E-07	C3_CD8_Tem
chr10-11194958-11195311	1.27559E-11	0.265700855	0.055	0.008	8.44736E-07	C3_CD8_Tem
chr17-36192338-36192639	1.28857E-11	0.290327953	0.133	0.053	8.53328E-07	C3_CD8_Tem
chr7-50333272-50333646	1.2986E-11	0.295741441	0.112	0.034	8.59971E-07	C3_CD8_Tem
chrX-87899957-87900875	1.31843E-11	0.290934309	0.062	0.01	8.73107E-07	C3_CD8_Tem
chr11-36426147-36426816	1.3238E-11	0.311981092	0.091	0.023	8.76661E-07	C3_CD8_Tem
chr14-97393042-97394054	1.33344E-11	0.303450691	0.125	0.042	8.83044E-07	C3_CD8_Tem
chr6-10726322-10726976	1.41193E-11	0.319092728	0.141	0.049	9.35022E-07	C3_CD8_Tem
chr1-221521581-221522129	1.42399E-11	0.293769055	0.057	0.009	9.43008E-07	C3_CD8_Tem
chr6-125748748-125749927	1.44171E-11	0.253828633	0.182	0.1	9.54745E-07	C3_CD8_Tem
chr8-20251411-20252448	1.44733E-11	0.270499424	0.344	0.221	9.58462E-07	C3_CD8_Tem
chr5-139310626-139311211	1.48033E-11	0.26714431	0.07	0.016	9.80321E-07	C3_CD8_Tem
chr14-91402433-91402691	1.48393E-11	0.283097481	0.086	0.026	9.82704E-07	C3_CD8_Tem
chr14-52324842-52325579	1.57915E-11	0.303189109	0.167	0.066	1.04576E-06	C3_CD8_Tem
chr19-52778593-52779552	1.596E-11	0.281163328	0.102	0.034	1.05692E-06	C3_CD8_Tem
chr10-71698226-71699001	1.61909E-11	0.292454584	0.164	0.074	1.07221E-06	C3_CD8_Tem

chr1-41888593-41888878	1.62338E-11	0.284858453	0.062	0.01	1.07505E-06	C3_CD8_Tem
chr15-89129195-89130578	1.62638E-11	0.308200524	0.128	0.042	1.07704E-06	C3_CD8_Tem
chr1-8112997-8113548	1.63025E-11	0.265686742	0.055	0.009	1.0796E-06	C3_CD8_Tem
chr6-5506844-5507645	1.67641E-11	0.302405948	0.078	0.02	1.11017E-06	C3_CD8_Tem
chr19-45778988-45780048	1.75464E-11	0.254229346	0.273	0.158	1.16197E-06	C3_CD8_Tem
chr3-196144186-196145064	1.76397E-11	0.264499934	0.107	0.039	1.16815E-06	C3_CD8_Tem
chr10-4234389-4235123	1.80039E-11	0.297647664	0.201	0.095	1.19227E-06	C3_CD8_Tem
chr3-129633797-129634347	1.86003E-11	0.280818685	0.052	0.007	1.23177E-06	C3_CD8_Tem
chr10-79027120-79028053	1.9077E-11	0.292046776	0.273	0.146	1.26333E-06	C3_CD8_Tem
chr7-38240794-38241149	2.11439E-11	0.296546012	0.078	0.018	1.40021E-06	C3_CD8_Tem
chr19-2621692-2622070	2.12292E-11	0.288372072	0.143	0.056	1.40586E-06	C3_CD8_Tem
chr19-51386736-51387180	2.13936E-11	0.283617026	0.065	0.012	1.41675E-06	C3_CD8_Tem
chr1-101308995-101310257	3.7829E-11	0.28197457	0.326	0.193	2.50515E-06	C1_CD8_Tres
chr16-4059692-4060452	2.26668E-11	0.264498179	0.055	0.009	1.50107E-06	C3_CD8_Tem
chrX-1480086-1480445	2.28148E-11	0.28551132	0.138	0.053	1.51086E-06	C3_CD8_Tem
chr16-82451996-82452757	2.3019E-11	0.262332743	0.068	0.014	1.52439E-06	C3_CD8_Tem
chr2-16665304-16666092	2.32792E-11	0.283315549	0.12	0.042	1.54162E-06	C3_CD8_Tem
chr16-4016646-4017009	2.35473E-11	0.289225629	0.065	0.011	1.55937E-06	C3_CD8_Tem
chr16-2841950-2842989	2.35943E-11	0.293203129	0.141	0.056	1.56249E-06	C3_CD8_Tem
chr5-138565000-138565905	2.37853E-11	0.267267256	0.32	0.196	1.57514E-06	C3_CD8_Tem
chr7-29255737-29256426	2.41012E-11	0.293376344	0.102	0.032	1.59605E-06	C3_CD8_Tem
chr19-7185705-7186758	2.59598E-11	0.307751851	0.122	0.038	1.71913E-06	C3_CD8_Tem
chr19-991348-991997	2.61115E-11	0.296467807	0.182	0.08	1.72918E-06	C3_CD8_Tem
chr1-224205410-224205763	2.62429E-11	0.307493796	0.117	0.043	1.73788E-06	C3_CD8_Tem
chr17-36210420-36211235	2.68634E-11	0.289624807	0.12	0.042	1.77898E-06	C3_CD8_Tem
chr1-198788484-198788980	2.73046E-11	0.302132239	0.083	0.019	1.80819E-06	C3_CD8_Tem
chr19-703370-704434	2.80488E-11	0.279984998	0.122	0.047	1.85747E-06	C3_CD8_Tem

chr11-134411370-134413079	3.11309E-11	0.28587533	0.107	0.037	2.06158E-06	C3_CD8_Tem
chr5-32283680-32284051	3.11939E-11	0.319379408	0.096	0.026	2.06575E-06	C3_CD8_Tem
chr12-128811579-128812024	3.23093E-11	0.287818532	0.062	0.011	2.13962E-06	C3_CD8_Tem
chr3-13040011-13040691	3.24002E-11	0.300692621	0.164	0.067	2.14564E-06	C3_CD8_Tem
chr12-64657630-64658285	3.24522E-11	0.310863614	0.138	0.049	2.14908E-06	C3_CD8_Tem
chr7-1289898-1290656	3.26543E-11	0.263432374	0.06	0.014	2.16246E-06	C3_CD8_Tem
chr7-2849879-2850916	3.27035E-11	0.297619848	0.117	0.04	2.16572E-06	C3_CD8_Tem
chrX-13691803-13692928	3.27945E-11	0.276045502	0.135	0.059	2.17175E-06	C3_CD8_Tem
chr17-2786066-2786653	6.28671E-10	0.290749573	0.108	0.038	4.16325E-05	C1_CD8_Tres
chr1-151944505-151944754	3.49737E-11	0.283952649	0.068	0.016	2.31606E-06	C3_CD8_Tem
chr15-93047493-93048020	3.49755E-11	0.272431675	0.081	0.022	2.31618E-06	C3_CD8_Tem
chr15-40256386-40256899	3.52785E-11	0.286667658	0.086	0.024	2.33625E-06	C3_CD8_Tem
chr4-121354-121746	3.55032E-11	0.274018192	0.089	0.026	2.35113E-06	C3_CD8_Tem
chr3-15467103-15468158	3.62643E-11	0.264681409	0.057	0.011	2.40153E-06	C3_CD8_Tem
chr2-192672-193512	3.63727E-11	0.292418841	0.104	0.033	2.40871E-06	C3_CD8_Tem
chr7-136086708-136087097	3.67752E-11	0.296920215	0.062	0.01	2.43536E-06	C3_CD8_Tem
chr19-54162212-54162714	3.72803E-11	0.285320548	0.141	0.059	2.46881E-06	C3_CD8_Tem
chr8-124673311-124673644	4.16225E-11	0.293702293	0.057	0.007	2.75637E-06	C3_CD8_Tem
chrX-149829497-149830713	4.27001E-11	0.289804738	0.151	0.063	2.82773E-06	C3_CD8_Tem
chr20-45945960-45946761	4.32851E-11	0.300846825	0.203	0.094	2.86647E-06	C3_CD8_Tem
chr19-8591726-8592924	1.85348E-26	0.477412579	0.344	0.138	1.22743E-21	C1_CD8_Tres
chr11-68750938-68751783	4.34398E-11	0.254314769	0.224	0.127	2.87672E-06	C3_CD8_Tem
chr17-64392409-64393697	4.47564E-11	0.304861282	0.122	0.042	2.9639E-06	C3_CD8_Tem
chr3-153221387-153222517	1.55256E-10	0.26220581	0.347	0.215	1.02815E-05	C1_CD8_Tres
chr17-76681255-76681992	4.79329E-11	0.293265334	0.102	0.032	3.17426E-06	C3_CD8_Tem
chr12-96473063-96473740	4.884E-11	0.309673482	0.115	0.038	3.23433E-06	C3_CD8_Tem
chr12-48409863-48410272	4.88494E-11	0.292604132	0.065	0.011	3.23495E-06	C3_CD8_Tem

chr16-30767493-30768294	4.93269E-11	0.271316003	0.169	0.074	3.26657E-06	C3_CD8_Tem
chr2-69770122-69770552	5.27191E-11	0.30354054	0.12	0.039	3.49122E-06	C3_CD8_Tem
chr8-57000152-57000767	5.36953E-11	0.267571868	0.214	0.115	3.55586E-06	C3_CD8_Tem
chr1-224204631-224205148	5.44301E-11	0.292563754	0.115	0.042	3.60452E-06	C3_CD8_Tem
chr11-103682114-103682782	5.4626E-11	0.274145371	0.055	0.009	3.6175E-06	C3_CD8_Tem
chr14-105993495-105994314	5.46487E-11	0.277030207	0.156	0.068	3.619E-06	C3_CD8_Tem
chr6-151373020-151374384	5.48741E-11	0.266810849	0.273	0.168	3.63393E-06	C3_CD8_Tem
chr12-54017291-54018413	5.49048E-11	0.265440422	0.247	0.138	3.63596E-06	C3_CD8_Tem
chr15-94272057-94272936	5.72282E-11	0.282563568	0.214	0.111	3.78983E-06	C3_CD8_Tem
chr9-95462185-95463306	6.22224E-11	0.294123924	0.193	0.091	4.12056E-06	C3_CD8_Tem
chr2-221520381-221520677	6.27981E-11	0.284592527	0.083	0.019	4.15868E-06	C3_CD8_Tem
chr11-72821707-72822779	6.32168E-11	0.251787997	0.25	0.146	4.1864E-06	C3_CD8_Tem
chr3-66353737-66354383	6.50534E-11	0.279567185	0.07	0.015	4.30803E-06	C3_CD8_Tem
chr1-92029675-92030632	6.84545E-11	0.311262583	0.081	0.019	4.53327E-06	C3_CD8_Tem
chr1-33045281-33046369	6.88524E-11	0.265770493	0.193	0.098	4.55961E-06	C3_CD8_Tem
chr17-10081483-10082006	7.22676E-11	0.30357219	0.107	0.031	4.78577E-06	C3_CD8_Tem
chr4-168877670-168878512	7.36019E-11	0.294682313	0.07	0.016	4.87414E-06	C3_CD8_Tem
chr9-66269828-66270644	7.47077E-11	0.257805237	0.083	0.022	4.94737E-06	C3_CD8_Tem
chr3-43015524-43016642	7.78512E-11	0.283295024	0.125	0.046	5.15554E-06	C3_CD8_Tem
chr12-121614850-121615377	7.78623E-11	0.281461648	0.065	0.013	5.15628E-06	C3_CD8_Tem
chr3-196357886-196358280	8.32334E-11	0.308223464	0.096	0.031	5.51196E-06	C3_CD8_Tem
chr11-110524194-110524644	8.43072E-11	0.286554403	0.117	0.043	5.58307E-06	C3_CD8_Tem
chr19-603874-604745	8.67197E-11	0.283460717	0.104	0.035	5.74284E-06	C3_CD8_Tem
chr19-45615747-45616401	9.04026E-11	0.295011072	0.161	0.067	5.98673E-06	C3_CD8_Tem
chr22-22720135-22721253	9.05688E-11	0.257331621	0.135	0.058	5.99774E-06	C3_CD8_Tem
chr10-17453156-17454822	9.52085E-11	0.2673578	0.141	0.063	6.30499E-06	C3_CD8_Tem
chr10-131311491-131312586	9.58242E-11	0.260201368	0.062	0.014	6.34576E-06	C3_CD8_Tem

chr16-11556927-11557611	9.65431E-11	0.28225294	0.12	0.045	6.39338E-06	C3_CD8_Tem
chr19-10551638-10551902	9.71448E-11	0.272355331	0.068	0.016	6.43322E-06	C3_CD8_Tem
chr6-137588991-137589938	9.85609E-11	0.300720245	0.229	0.122	6.527E-06	C3_CD8_Tem
chr7-159208992-159209902	1.02032E-10	0.288898518	0.089	0.028	6.7569E-06	C3_CD8_Tem
chr7-129553822-129554510	1.02864E-10	0.280557047	0.12	0.042	6.81195E-06	C3_CD8_Tem
chr3-27880867-27881933	1.09588E-10	0.269535627	0.18	0.082	7.25725E-06	C3_CD8_Tem
chr10-104333173-104333562	1.15083E-10	0.280659141	0.161	0.076	7.62117E-06	C3_CD8_Tem
chr6-40788609-40789502	1.15368E-10	0.286577517	0.065	0.013	7.64005E-06	C3_CD8_Tem
chr2-239609499-239610074	1.15609E-10	0.278706176	0.065	0.014	7.65598E-06	C3_CD8_Tem
chr5-126816486-126816991	1.1623E-10	0.307775208	0.081	0.018	7.69711E-06	C3_CD8_Tem
chr5-1494704-1495740	1.16799E-10	0.290401295	0.104	0.034	7.73481E-06	C3_CD8_Tem
chr13-80340970-80341538	1.18312E-10	0.261044414	0.078	0.02	7.83495E-06	C3_CD8_Tem
chr12-94644054-94644939	1.18436E-10	0.268577471	0.081	0.023	7.84317E-06	C3_CD8_Tem
chr7-50331555-50332145	1.07042E-15	0.376700635	0.21	0.083	7.08861E-11	C1_CD8_Tres
chr13-114263501-114264004	1.21749E-10	0.267282667	0.076	0.02	8.0626E-06	C3_CD8_Tem
chr9-96435774-96436570	1.2344E-10	0.261318838	0.201	0.103	8.17457E-06	C3_CD8_Tem
chr1-12132673-12133385	1.24324E-10	0.274617856	0.109	0.04	8.23312E-06	C3_CD8_Tem
chr21-10271601-10272657	1.25809E-10	0.256398691	0.083	0.027	8.33146E-06	C3_CD8_Tem
chr10-69092930-69093615	1.26264E-10	0.256912184	0.219	0.121	8.36161E-06	C3_CD8_Tem
chr19-12784493-12785357	1.39919E-10	0.25417171	0.263	0.164	9.26584E-06	C3_CD8_Tem
chr4-7324150-7325121	1.5468E-10	0.270039252	0.094	0.03	1.02434E-05	C3_CD8_Tem
chr17-17109906-17110286	1.56906E-10	0.282038746	0.065	0.013	1.03908E-05	C3_CD8_Tem
chr7-123770266-123770758	1.61798E-10	0.269791421	0.065	0.014	1.07147E-05	C3_CD8_Tem
chr7-1961547-1962350	1.65646E-10	0.254604571	0.211	0.113	1.09696E-05	C3_CD8_Tem
chr20-23919665-23920428	1.69037E-10	0.269401993	0.076	0.022	1.11941E-05	C3_CD8_Tem
chr16-50335070-50335857	1.74063E-10	0.289104105	0.224	0.117	1.1527E-05	C3_CD8_Tem
chr5-154384422-154385470	1.75317E-10	0.266540912	0.148	0.065	1.161E-05	C3_CD8_Tem

chr4-158873881-158874451	1.76473E-10	0.304729319	0.102	0.03	1.16866E-05	C3_CD8_Tem
chr1-160738147-160738595	1.79426E-10	0.261351265	0.086	0.026	1.18821E-05	C3_CD8_Tem
chr7-29196076-29197235	1.8404E-10	0.26655971	0.227	0.126	1.21877E-05	C3_CD8_Tem
chr15-31341177-31342073	1.84345E-10	0.270268522	0.174	0.087	1.22079E-05	C3_CD8_Tem
chr2-68365906-68366543	1.91463E-10	0.277003638	0.096	0.027	1.26793E-05	C3_CD8_Tem
chr11-62557935-62558490	1.94105E-10	0.26627003	0.214	0.107	1.28542E-05	C3_CD8_Tem
chr16-11613566-11614218	1.98562E-10	0.282163271	0.096	0.028	1.31494E-05	C3_CD8_Tem
chr17-74733406-74733708	1.9984E-10	0.255400453	0.076	0.023	1.3234E-05	C3_CD8_Tem
chr15-72809259-72809634	2.0011E-10	0.258725473	0.057	0.011	1.32519E-05	C3_CD8_Tem
chr11-6200930-6201816	2.06873E-10	0.253409746	0.096	0.036	1.36997E-05	C3_CD8_Tem
chr16-57764361-57765161	2.0769E-10	0.278006816	0.086	0.024	1.37538E-05	C3_CD8_Tem
chr1-167450803-167451067	2.08011E-10	0.269118395	0.091	0.031	1.37751E-05	C3_CD8_Tem
chr16-21645118-21645754	2.26427E-10	0.26199278	0.172	0.086	1.49946E-05	C3_CD8_Tem
chr12-10307683-10307956	2.32593E-10	0.294532344	0.122	0.044	1.5403E-05	C3_CD8_Tem
chr1-3889848-3891382	2.5472E-10	0.270683744	0.154	0.071	1.68683E-05	C3_CD8_Tem
chr14-100406558-100406893	2.58482E-10	0.257803293	0.052	0.008	1.71174E-05	C3_CD8_Tem
chr2-233409095-233409966	2.68371E-10	0.277017613	0.117	0.046	1.77723E-05	C3_CD8_Tem
chr2-225027102-225027655	2.69182E-10	0.253287694	0.06	0.011	1.7826E-05	C3_CD8_Tem
chr11-86486452-86487087	2.7409E-10	0.273091976	0.169	0.078	1.81511E-05	C3_CD8_Tem
chr9-129410215-129410661	2.84384E-10	0.268167093	0.07	0.017	1.88328E-05	C3_CD8_Tem
chr10-29681277-29682324	2.85685E-10	0.253652583	0.089	0.027	1.89189E-05	C3_CD8_Tem
chr5-107570845-107572433	2.85738E-10	0.278378724	0.185	0.091	1.89224E-05	C3_CD8_Tem
chr19-43738375-43739147	3.14994E-10	0.250067089	0.065	0.017	2.08598E-05	C3_CD8_Tem
chr5-73693614-73694468	3.19126E-10	0.293708418	0.143	0.057	2.11335E-05	C3_CD8_Tem
chr16-81841425-81842485	3.23907E-10	0.26734942	0.133	0.056	2.14501E-05	C3_CD8_Tem
chr2-8315816-8316099	3.29097E-10	0.260476611	0.076	0.019	2.17938E-05	C3_CD8_Tem
chr19-29276950-29277477	3.34666E-10	0.268051565	0.102	0.035	2.21626E-05	C3_CD8_Tem

chr10-109933640-109934562	3.35138E-10	0.268042858	0.167	0.08	2.21938E-05	C3_CD8_Tem
chr7-50342425-50342974	3.46985E-10	0.25948274	0.151	0.067	2.29784E-05	C3_CD8_Tem
chr15-66471428-66472664	3.62681E-10	0.285206685	0.133	0.056	2.40178E-05	C3_CD8_Tem
chr14-89385981-89386791	3.84422E-10	0.271726681	0.065	0.015	2.54576E-05	C3_CD8_Tem
chr11-62538392-62538742	4.06096E-10	0.275855095	0.073	0.017	2.68929E-05	C3_CD8_Tem
chr1-148388151-148389117	3.39071E-10	0.29384888	0.165	0.073	2.24543E-05	C1_CD8_Tres
chr11-19280863-19281397	4.42401E-10	0.277816401	0.104	0.033	2.92971E-05	C3_CD8_Tem
chr22-46373864-46374642	4.59855E-10	0.272216172	0.091	0.029	3.0453E-05	C3_CD8_Tem
chr10-127293325-127294047	4.63167E-10	0.280902039	0.068	0.013	3.06723E-05	C3_CD8_Tem
chr14-105477586-105477889	4.67717E-10	0.2557345	0.143	0.069	3.09736E-05	C3_CD8_Tem
chr14-51930014-51930928	4.78456E-10	0.276947967	0.068	0.015	3.16848E-05	C3_CD8_Tem
chr1-53127345-53127787	4.81091E-10	0.285224021	0.076	0.016	3.18593E-05	C3_CD8_Tem
chr6-152185992-152186568	4.84535E-10	0.271446433	0.117	0.043	3.20873E-05	C3_CD8_Tem
chr7-66033509-66034026	4.90787E-10	0.274162073	0.06	0.011	3.25014E-05	C3_CD8_Tem
chr11-114058953-114060800	8.86057E-12	0.327137306	0.168	0.066	5.86774E-07	C1_CD8_Tres
chr6-10732633-10733726	5.1574E-10	0.280882917	0.115	0.041	3.41539E-05	C3_CD8_Tem
chrX-149513423-149514464	5.47356E-10	0.264679642	0.141	0.061	3.62476E-05	C3_CD8_Tem
chr20-32567524-32568306	5.57594E-10	0.25600128	0.052	0.009	3.69255E-05	C3_CD8_Tem
chr9-86106623-86107344	5.65084E-10	0.254079367	0.271	0.16	3.74215E-05	C3_CD8_Tem
chr14-94945532-94946593	5.69491E-10	0.318140432	0.164	0.066	3.77134E-05	C3_CD8_Tem
chr4-4270548-4270834	5.71298E-10	0.255737324	0.102	0.035	3.78331E-05	C3_CD8_Tem
chr16-89020070-89020713	5.78842E-10	0.268946927	0.117	0.045	3.83327E-05	C3_CD8_Tem
chr11-34650095-34650916	6.47119E-10	0.288037441	0.148	0.058	4.28541E-05	C3_CD8_Tem
chr16-89192765-89193395	6.49456E-10	0.261828991	0.141	0.069	4.30089E-05	C3_CD8_Tem
chr20-5996653-5997169	6.73351E-10	0.277702822	0.135	0.059	4.45913E-05	C3_CD8_Tem
chr15-39426779-39427607	6.83294E-10	0.282328418	0.208	0.102	4.52498E-05	C3_CD8_Tem
chr1-54474738-54475836	7.14767E-10	0.2587892	0.096	0.034	4.7334E-05	C3_CD8_Tem

chr2-136281055-136281929	7.28362E-10	0.250058148	0.169	0.092	4.82343E-05	C3_CD8_Tem
chr1-101285872-101286177	7.46762E-10	0.263586072	0.06	0.011	4.94528E-05	C3_CD8_Tem
chr2-70851627-70852078	7.70492E-10	0.258302524	0.086	0.026	5.10243E-05	C3_CD8_Tem
chr5-14144550-14145397	8.06118E-10	0.291712511	0.096	0.031	5.33836E-05	C3_CD8_Tem
chr17-83124835-83126264	9.53333E-10	0.261475503	0.128	0.052	6.31326E-05	C3_CD8_Tem
chr19-54532364-54532870	9.53524E-10	0.27039447	0.102	0.036	6.31452E-05	C3_CD8_Tem
chr13-48214433-48215171	9.72605E-10	0.25592448	0.138	0.063	6.44088E-05	C3_CD8_Tem
chr9-136960244-136960558	9.73215E-10	0.259246851	0.062	0.014	6.44492E-05	C3_CD8_Tem
chr4-158870854-158871126	9.74761E-10	0.280284586	0.062	0.011	6.45516E-05	C3_CD8_Tem
chr19-29293972-29294697	9.97326E-10	0.257288173	0.06	0.013	6.6046E-05	C3_CD8_Tem
chr21-44626134-44626560	1.22987E-09	0.280479104	0.078	0.02	8.14454E-05	C3_CD8_Tem
chr1-43581747-43582584	1.24019E-09	0.290724709	0.112	0.044	8.21289E-05	C3_CD8_Tem
chr3-31502345-31503474	1.24148E-09	0.266323167	0.146	0.062	8.22145E-05	C3_CD8_Tem
chr1-161545114-161545652	1.25632E-09	0.265446814	0.107	0.038	8.31974E-05	C3_CD8_Tem
chr2-170770081-170771488	1.25747E-09	0.252668212	0.156	0.077	8.32734E-05	C3_CD8_Tem
chr3-129017013-129017459	1.29429E-09	0.277493541	0.07	0.016	8.57119E-05	C3_CD8_Tem
chr16-3962590-3963461	1.29711E-09	0.26752342	0.203	0.103	8.58987E-05	C3_CD8_Tem
chr10-109945534-109946158	1.3572E-09	0.257768716	0.078	0.023	8.98776E-05	C3_CD8_Tem
chr2-234335408-234336249	1.57827E-09	0.313947146	0.099	0.029	0.000104518	C1_CD8_Tres
chr15-98647186-98648976	1.41006E-09	0.254606873	0.188	0.097	9.33787E-05	C3_CD8_Tem
chr18-45669987-45670872	1.56367E-09	0.272373594	0.201	0.103	0.000103551	C3_CD8_Tem
chr2-223881805-223882274	1.73518E-09	0.250519509	0.073	0.02	0.000114909	C3_CD8_Tem
chr2-60954055-60954421	1.75266E-09	0.267578875	0.128	0.055	0.000116067	C3_CD8_Tem
chr2-151669574-151670532	1.8596E-09	0.286486471	0.086	0.024	0.000123148	C3_CD8_Tem
chr10-124616109-124616310	1.95168E-09	0.27701388	0.068	0.016	0.000129246	C3_CD8_Tem
chr12-128829708-128830511	2.23873E-09	0.256352768	0.068	0.018	0.000148255	C3_CD8_Tem
chr17-78242332-78242988	2.31902E-09	0.252085502	0.057	0.014	0.000153572	C3_CD8_Tem

chr14-24680549-24681263	2.4131E-09	0.261324926	0.081	0.023	0.000159803	C3_CD8_Tem
chr5-14745675-14746143	2.41576E-09	0.273995436	0.078	0.021	0.000159979	C3_CD8_Tem
chr10-22505731-22506455	2.58931E-09	0.254622855	0.18	0.09	0.000171472	C3_CD8_Tem
chr1-120723413-120724378	2.66701E-09	0.258062148	0.094	0.031	0.000176617	C3_CD8_Tem
chr19-40143503-40144657	2.80989E-09	0.254221062	0.138	0.064	0.00018608	C3_CD8_Tem
chr7-24441740-24442645	2.90352E-09	0.254484535	0.055	0.01	0.00019228	C3_CD8_Tem
chr13-50427239-50427876	2.95627E-09	0.257727087	0.104	0.038	0.000195773	C3_CD8_Tem
chr1-149390092-149390941	2.99977E-09	0.251432468	0.081	0.023	0.000198654	C3_CD8_Tem
chr5-67514805-67516138	3.01486E-09	0.27176946	0.148	0.065	0.000199653	C3_CD8_Tem
chr9-99076203-99077051	3.11962E-09	0.255436352	0.141	0.06	0.000206591	C3_CD8_Tem
chr16-50313625-50313989	3.1532E-09	0.259458046	0.062	0.013	0.000208814	C3_CD8_Tem
chr16-355199-355431	3.6731E-09	0.250296764	0.06	0.014	0.000243244	C3_CD8_Tem
chr6-40436496-40437057	3.96839E-09	0.276807994	0.143	0.059	0.000262799	C3_CD8_Tem
chr2-143328872-143329683	4.17262E-09	0.257016679	0.055	0.01	0.000276323	C3_CD8_Tem
chr17-57982290-57982826	4.26788E-09	0.253128872	0.151	0.073	0.000282632	C3_CD8_Tem
chr3-67947709-67948495	1.26816E-07	0.255530082	0.084	0.027	0.008398106	C1_CD8_Tres
chr11-364121-364750	4.7597E-09	0.263765294	0.151	0.072	0.000315202	C3_CD8_Tem
chr1-36400534-36401245	4.91175E-09	0.279918267	0.156	0.071	0.000325271	C3_CD8_Tem
chr17-74473957-74474793	5.21358E-09	0.262796722	0.115	0.049	0.000345259	C3_CD8_Tem
chr17-5241943-5243263	5.82114E-09	0.261960884	0.109	0.045	0.000385493	C3_CD8_Tem
chr10-132427249-132428487	5.94439E-09	0.252750703	0.138	0.068	0.000393656	C3_CD8_Tem
chr16-50721688-50722453	6.00434E-09	0.252819889	0.094	0.033	0.000397626	C3_CD8_Tem
chr10-124341685-124342217	6.2063E-09	0.273052461	0.109	0.043	0.000411	C3_CD8_Tem
chr5-157292655-157292949	7.38247E-09	0.255401229	0.076	0.021	0.000488889	C3_CD8_Tem
chr7-135993911-135995065	7.61258E-09	0.251368359	0.078	0.026	0.000504128	C3_CD8_Tem
chr14-34856480-34857144	7.7377E-09	0.257684696	0.065	0.015	0.000512414	C3_CD8_Tem
chr5-148848399-148848913	8.1298E-09	0.253542605	0.138	0.066	0.00053838	C3_CD8_Tem

chr12-120480248-120481561	8.31469E-09	0.257789529	0.167	0.082	0.000550624	C3_CD8_Tem
chr1-184110280-184111219	8.31602E-09	0.256679431	0.052	0.01	0.000550712	C3_CD8_Tem
chr2-237898491-237899081	1.24549E-08	0.254573012	0.107	0.045	0.000824803	C3_CD8_Tem
chr3-50472751-50473378	1.2922E-08	0.253153025	0.107	0.045	0.000855733	C3_CD8_Tem
chr7-1042157-1042471	1.62897E-08	0.254166934	0.078	0.023	0.00107875	C3_CD8_Tem
chr15-58544853-58545667	1.64792E-08	0.262598604	0.073	0.018	0.001091303	C3_CD8_Tem
chr14-102500096-102500678	2.33801E-08	0.262334784	0.086	0.028	0.001548298	C3_CD8_Tem
chr9-71774703-71775317	2.94142E-08	0.263401848	0.109	0.042	0.001947896	C3_CD8_Tem
chr11-59728697-59729209	4.7573E-08	0.254131501	0.073	0.018	0.003150428	C3_CD8_Tem
chr12-95805402-95806330	5.06091E-08	0.278036984	0.109	0.036	0.003351486	C3_CD8_Tem
chr5-107670912-107671673	5.78369E-08	0.258155221	0.078	0.023	0.003830136	C3_CD8_Tem
chr15-63841719-63842387	8.43217E-15	0.42155146	0.174	0.052	5.58404E-10	C1_CD8_Tres
chrX-317539-319259	2.58242E-68	0.675579487	0.814	0.299	1.71015E-63	C1_CD8_Tres
chr3-93470260-93470829	4.37216E-18	0.366742856	0.674	0.293	2.89537E-13	C1_CD8_Tres
chr17-36188180-36189273	3.18453E-49	0.58806807	0.503	0.165	2.10889E-44	C3_CD8_Tem
chr1-23958530-23960278	2.65789E-38	0.527470675	0.575	0.248	1.76013E-33	C1_CD8_Tres
chr12-133079608-133081270	3.11646E-34	0.51529982	0.569	0.241	2.06381E-29	C1_CD8_Tres
chr8-144146829-144148682	1.02688E-32	0.47427437	0.488	0.213	6.80028E-28	C1_CD8_Tres
chr17-36198897-36199460	7.1471E-152	0.770173325	0.355	0.026	4.7331E-147	C4_CD8_Teff
chr19-1030247-1031629	1.3243E-151	0.72814457	0.508	0.089	8.7701E-147	C4_CD8_Teff
chr8-100409389-100410844	8.0641E-149	0.66571217	0.649	0.173	5.3403E-144	C4_CD8_Teff
chr1-121184236-121185085	2.98094E-29	0.53496817	0.47	0.186	1.97407E-24	C1_CD8_Tres
chr16-23482470-23483873	2.95602E-20	0.377784242	0.396	0.198	1.95756E-15	C3_CD8_Tem
chr6-152565755-152566727	7.83554E-22	0.418681814	0.253	0.085	5.18893E-17	C3_CD8_Tem
chr1-633460-634698	2.63058E-61	0.684577475	0.769	0.293	1.74205E-56	C1_CD8_Tres
chr1-3786925-3788055	8.7092E-133	0.688416408	0.513	0.107	5.7675E-128	C4_CD8_Teff
chr12-133036812-133038023	1.89258E-28	0.48786114	0.362	0.15	1.25332E-23	C1_CD8_Tres

chr19-13043761-13044879	3.87226E-25	0.436370202	0.346	0.146	2.56432E-20	C3_CD8_Tem
chr1-23979709-23980925	3.86076E-28	0.483689341	0.395	0.167	2.55671E-23	C1_CD8_Tres
chr14-101712387-101713961	3.09958E-65	0.709255657	0.471	0.124	2.05264E-60	C3_CD8_Tem
chr10-73758806-73760041	2.47215E-15	0.318707877	0.247	0.123	1.63713E-10	C3_CD8_Tem
chr10-131900106-131901808	1.66157E-30	0.503829364	0.446	0.179	1.10034E-25	C1_CD8_Tres
chr2-221517255-221519122	3.17043E-13	0.303510806	0.455	0.292	2.09955E-08	C1_CD8_Tres
chr1-91947948-91949312	2.4853E-123	0.679599195	0.443	0.077	1.6458E-118	C4_CD8_Teff
chr6-34484371-34485456	2.964E-123	0.699825929	0.403	0.063	1.9629E-118	C4_CD8_Teff
chr6-152635971-152637143	3.496E-15	0.325194654	0.255	0.126	2.31516E-10	C3_CD8_Tem
chr20-57604829-57606180	2.36162E-33	0.507442486	0.354	0.121	1.56394E-28	C3_CD8_Tem
chr17-22521254-22521613	3.80087E-69	0.745569079	0.772	0.278	2.51705E-64	C1_CD8_Tres
chr8-144136968-144138476	4.84082E-23	0.432229091	0.377	0.162	3.20574E-18	C1_CD8_Tres
chr19-38864390-38866046	2.7247E-31	0.4274194	0.542	0.271	1.80438E-26	C3_CD8_Tem
chr1-8495849-8497059	4.37499E-12	0.297918377	0.242	0.126	2.89725E-07	C3_CD8_Tem
chr15-82539674-82541121	7.15086E-18	0.3290858	0.407	0.247	4.73552E-13	C1_CD8_Tres
chr20-51499996-51501369	5.1356E-112	0.555862286	0.656	0.231	3.4009E-107	C4_CD8_Teff
chr6-215328-216209	1.0292E-111	0.616121037	0.509	0.128	6.8158E-107	C4_CD8_Teff
chr1-145280570-145282226	8.15049E-14	0.331126486	0.249	0.117	5.3975E-09	C1_CD8_Tres
chr1-15523836-15525464	1.06113E-24	0.284260088	0.766	0.607	7.02711E-20	C1_CD8_Tres
chr12-121557117-121558231	3.26604E-56	0.66964259	0.484	0.152	2.16287E-51	C3_CD8_Tem
chr11-35334234-35335006	2.7937E-109	0.678589184	0.297	0.029	1.8501E-104	C4_CD8_Teff
chr6-150310284-150311807	4.2001E-108	0.653541341	0.395	0.07	2.7815E-103	C4_CD8_Teff
chr16-75620163-75620836	3.2341E-105	0.635145821	0.457	0.098	2.1417E-100	C4_CD8_Teff
chr17-40479856-40481224	7.1493E-105	0.546971545	0.633	0.219	4.7345E-100	C4_CD8_Teff
chr1-202197442-202198255	8.33705E-56	0.713658132	0.385	0.087	5.52104E-51	C3_CD8_Tem
chr6-193662-194430	1.35654E-14	0.33530034	0.221	0.101	8.98343E-10	C3_CD8_Tem
chr14-103908297-103909068	3.5954E-100	0.645005173	0.224	0.014	2.38096E-95	C4_CD8_Teff

chr17-36196490-36197498	1.51618E-22	0.430208115	0.229	0.074	1.00406E-17	C3_CD8_Tem
chr16-57028997-57030129	3.4469E-99	0.656473769	0.248	0.022	2.28267E-94	C4_CD8_Teff
chr8-100415528-100417352	1.76153E-98	0.543293083	0.594	0.2	1.16654E-93	C4_CD8_Teff
chr22-39240764-39243606	2.94055E-98	0.604196885	0.442	0.1	1.94732E-93	C4_CD8_Teff
chr21-36155689-36157441	1.40186E-28	0.339027665	0.76	0.56	9.28355E-24	C1_CD8_Tres
chr1-120941840-120942777	5.79753E-25	0.494693148	0.302	0.112	3.8393E-20	C1_CD8_Tres
chr17-10114141-10115557	8.09413E-38	0.489782217	0.56	0.267	5.36017E-33	C3_CD8_Tem
chr9-40991238-40992772	3.23214E-21	0.360534696	0.569	0.393	2.14042E-16	C1_CD8_Tres
chr9-136790481-136792075	4.89578E-24	0.273118179	0.711	0.541	3.24213E-19	C3_CD8_Tem
chrX-1452437-1454445	9.5588E-22	0.294571942	0.713	0.553	6.33012E-17	C1_CD8_Tres
chr3-31502345-31503474	1.24148E-09	0.266323167	0.146	0.062	8.22145E-05	C3_CD8_Tem
chr16-352134-353545	3.74363E-23	0.331969925	0.584	0.391	2.47914E-18	C1_CD8_Tres
chr19-7920040-7921116	2.82495E-18	0.274928525	0.611	0.463	1.87077E-13	C1_CD8_Tres
chr13-114142248-114143533	1.71088E-17	0.320508713	0.367	0.199	1.133E-12	C3_CD8_Tem
chr8-29049783-29050546	3.79834E-92	0.61803097	0.222	0.017	2.51538E-87	C4_CD8_Teff
chr17-83199175-83200859	6.06288E-53	0.640654781	0.396	0.109	4.01502E-48	C3_CD8_Tem
chr20-51553758-51554917	2.37026E-15	0.344300631	0.25	0.114	1.56966E-10	C3_CD8_Tem
chr20-5714028-5715411	8.29342E-35	0.527268459	0.413	0.156	5.49215E-30	C1_CD8_Tres
chr2-238290100-238290499	2.09248E-89	0.619213987	0.332	0.057	1.3857E-84	C4_CD8_Teff
chr7-100182171-100183142	9.86371E-89	0.617437759	0.213	0.016	6.53204E-84	C4_CD8_Teff
chr19-3505376-3507649	1.03889E-88	0.3965237	0.807	0.438	6.87986E-84	C4_CD8_Teff
chr19-18635842-18637151	3.17043E-22	0.319933812	0.656	0.485	2.09955E-17	C1_CD8_Tres
chr1-144551201-144552431	1.31752E-87	0.606987583	0.286	0.038	8.72498E-83	C4_CD8_Teff
chr7-6240465-6242175	3.3406E-87	0.616187428	0.213	0.017	2.21224E-82	C4_CD8_Teff
chr19-45087196-45087984	9.38316E-19	0.332268135	0.383	0.203	6.21381E-14	C3_CD8_Tem
chr6-188656-189699	1.04694E-84	0.608709557	0.273	0.037	6.93312E-80	C4_CD8_Teff
chr8-125941480-125942739	2.27011E-84	0.473629021	0.65	0.263	1.50334E-79	C4_CD8_Teff

chr1-146376265-146377483	5.82881E-10	0.27400274	0.219	0.113	3.86002E-05	C1_CD8_Tres
chr4-970184-971071	7.90883E-50	0.675961704	0.341	0.078	5.23746E-45	C3_CD8_Tem
chr20-24959621-24961536	2.6311E-21	0.357805248	0.438	0.235	1.74239E-16	C3_CD8_Tem
chr10-132536674-132537961	5.60645E-26	0.330815282	0.707	0.534	3.71276E-21	C1_CD8_Tres
chr1-148522069-148523165	3.42945E-13	0.30178345	0.302	0.157	2.27109E-08	C3_CD8_Tem
chr3-31461768-31463020	1.78007E-83	0.596094477	0.286	0.048	1.17882E-78	C4_CD8_Teff
chr10-110225534-110227561	1.4863E-82	0.396012529	0.782	0.412	9.84271E-78	C4_CD8_Teff
chr11-118919418-118920066	1.39527E-10	0.273014346	0.231	0.126	9.23991E-06	C1_CD8_Tres
chr1-160812949-160813628	1.14815E-81	0.589286185	0.243	0.031	7.60342E-77	C4_CD8_Teff
chr9-97910196-97910809	1.27212E-81	0.586727195	0.217	0.021	8.42438E-77	C4_CD8_Teff
chr2-230877055-230878272	1.89204E-81	0.484136874	0.609	0.235	1.25296E-76	C4_CD8_Teff
chr17-64024859-64026046	1.77547E-20	0.337529473	0.487	0.297	1.17577E-15	C3_CD8_Tem
chr20-8131554-8132972	2.62397E-13	0.306232432	0.273	0.141	1.73767E-08	C3_CD8_Tem
chr19-19419983-19421059	2.11386E-20	0.379792051	0.289	0.123	1.39986E-15	C3_CD8_Tem
chr19-41935809-41936940	5.39033E-80	0.490953236	0.572	0.217	3.56964E-75	C4_CD8_Teff
chr3-183349015-183350695	1.11233E-79	0.542237169	0.444	0.131	7.3662E-75	C4_CD8_Teff
chr16-30767493-30768294	4.93269E-11	0.271316003	0.169	0.074	3.26657E-06	C3_CD8_Tem
chr14-101760063-101762546	2.02833E-78	0.348067516	0.837	0.5	1.34322E-73	C4_CD8_Teff
chr13-113930452-113931666	7.34572E-78	0.576766485	0.322	0.063	4.86456E-73	C4_CD8_Teff
chr19-47514207-47515664	8.73505E-78	0.345901718	0.849	0.499	5.78461E-73	C4_CD8_Teff
chr1-1628692-1631735	3.98631E-39	0.38325495	0.701	0.469	2.63985E-34	C3_CD8_Tem
chr10-131896262-131896784	9.54184E-15	0.352657415	0.177	0.064	6.31889E-10	C3_CD8_Tem
chr17-47829370-47831773	5.60638E-20	0.276619679	0.638	0.455	3.71272E-15	C3_CD8_Tem
chr1-38052567-38053460	4.85267E-76	0.577052134	0.237	0.033	3.21358E-71	C4_CD8_Teff
chr18-80033096-80034705	8.23453E-76	0.322565745	0.883	0.556	5.45315E-71	C4_CD8_Teff
chr3-101518076-101518866	1.02747E-10	0.266303881	0.263	0.156	6.80419E-06	C1_CD8_Tres
chr19-10652745-10654934	6.75081E-75	0.355761954	0.796	0.461	4.47059E-70	C4_CD8_Teff

chr21-42973696-42975706	1.77505E-74	0.317719798	0.871	0.553	1.17549E-69	C4_CD8_Teff
chr5-1518362-1519073	7.16787E-39	0.56881139	0.281	0.071	4.74678E-34	C3_CD8_Tem
chr14-106174117-106174932	2.56283E-73	0.535398567	0.403	0.104	1.69718E-68	C4_CD8_Teff
chr7-100428038-100430543	6.68601E-73	0.295048753	0.892	0.611	4.42767E-68	C4_CD8_Teff
chr1-156217552-156218363	8.09395E-73	0.536384806	0.382	0.099	5.36006E-68	C4_CD8_Teff
chr4-82284216-82285491	1.55078E-13	0.28098272	0.53	0.382	1.02697E-08	C1_CD8_Tres
chr13-113875597-113877300	3.55021E-24	0.441795829	0.266	0.093	2.35105E-19	C3_CD8_Tem
chr4-152099735-152101089	2.23457E-14	0.346851787	0.305	0.156	1.4798E-09	C1_CD8_Tres
chr20-63552544-63554373	1.54318E-23	0.364703562	0.485	0.299	1.02194E-18	C1_CD8_Tres
chr7-26166549-26168204	1.86329E-72	0.550129127	0.316	0.062	1.23393E-67	C4_CD8_Teff
chr9-123321919-123323358	3.37977E-72	0.465690401	0.562	0.23	2.23818E-67	C4_CD8_Teff
chr20-5719626-5720622	1.90509E-71	0.54256261	0.239	0.035	1.26161E-66	C4_CD8_Teff
chr19-53866992-53869754	1.5402E-70	0.320076198	0.858	0.538	1.01997E-65	C4_CD8_Teff
chr7-94656122-94658687	8.42126E-17	0.332052375	0.545	0.35	5.57681E-12	C1_CD8_Tres
chr17-36182330-36184239	1.84827E-12	0.282950995	0.143	0.056	1.22398E-07	C3_CD8_Tem
chr8-140463759-140465274	2.3166E-69	0.296152326	0.893	0.593	1.53412E-64	C4_CD8_Teff
chr14-91869738-91870325	3.54497E-69	0.544404379	0.303	0.062	2.34759E-64	C4_CD8_Teff
chr14-106506652-106507782	4.17652E-69	0.429527495	0.633	0.277	2.76582E-64	C4_CD8_Teff
chr6-2861395-2861985	4.71194E-69	0.524895288	0.373	0.101	3.12039E-64	C4_CD8_Teff
chr5-154384422-154385470	1.75317E-10	0.266540912	0.148	0.065	1.161E-05	C3_CD8_Tem
chr17-68182449-68183538	2.3077E-68	0.489275333	0.468	0.154	1.52823E-63	C4_CD8_Teff
chr13-48504671-48506020	4.19305E-19	0.371554452	0.333	0.161	2.77676E-14	C3_CD8_Tem
chr2-219386961-219388750	2.69351E-67	0.294504137	0.9	0.584	1.78373E-62	C4_CD8_Teff
chr12-132985767-132986898	2.82645E-12	0.308257727	0.198	0.091	1.87176E-07	C1_CD8_Tres
chr17-80720552-80721593	3.2612E-67	0.516808902	0.353	0.09	2.15966E-62	C4_CD8_Teff
chr2-105749545-105750333	3.66352E-67	0.538395752	0.277	0.052	2.42609E-62	C4_CD8_Teff
chr1-53945042-53946736	4.10817E-67	0.306183347	0.868	0.55	2.72055E-62	C4_CD8_Teff

chr19-10870523-10871977	1.16606E-33	0.33839424	0.745	0.521	7.72201E-29	C3_CD8_Tem
chr19-660252-660874	5.95391E-67	0.447524155	0.542	0.223	3.94286E-62	C4_CD8_Teff
chr1-24922243-24922632	1.01539E-66	0.527396153	0.353	0.087	6.72424E-62	C4_CD8_Teff
chr1-30717614-30719544	1.9207E-24	0.27461632	0.787	0.648	1.27195E-19	C1_CD8_Tres
chr16-9081917-9082880	2.83176E-66	0.532336547	0.188	0.021	1.87528E-61	C4_CD8_Teff
chr17-36192338-36192639	1.28857E-11	0.290327953	0.133	0.053	8.53328E-07	C3_CD8_Tem
chr6-30568132-30568616	1.84547E-12	0.296796729	0.182	0.079	1.22213E-07	C3_CD8_Tem
chr21-5101563-5102080	1.58259E-11	0.303530481	0.162	0.071	1.04804E-06	C1_CD8_Tres
chr9-41234540-41235024	7.18773E-66	0.539913493	0.199	0.024	4.75993E-61	C4_CD8_Teff
chr15-31341177-31342073	1.84345E-10	0.270268522	0.174	0.087	1.22079E-05	C3_CD8_Tem
chr7-50335686-50336484	3.94137E-28	0.490840708	0.276	0.093	2.61009E-23	C3_CD8_Tem
chr15-67106490-67107471	2.28682E-65	0.526609668	0.266	0.053	1.5144E-60	C4_CD8_Teff
chr1-144412046-144413315	2.40892E-14	0.315686143	0.305	0.172	1.59526E-09	C1_CD8_Tres
chr21-5154334-5154970	1.31642E-13	0.349547233	0.228	0.094	8.7177E-09	C1_CD8_Tres
chr12-132955395-132956898	7.53068E-65	0.530919317	0.223	0.032	4.98704E-60	C4_CD8_Teff
chr1-1918791-1919873	1.8966E-21	0.333718434	0.536	0.366	1.25599E-16	C1_CD8_Tres
chr6-143396717-143398041	9.31105E-65	0.529310091	0.271	0.053	6.16606E-60	C4_CD8_Teff
chr10-14586783-14587224	1.03011E-64	0.536417901	0.219	0.031	6.82172E-60	C4_CD8_Teff
chr7-106021478-106022922	1.5206E-64	0.440576234	0.56	0.232	1.00698E-59	C4_CD8_Teff
chr22-38873763-38874357	4.23253E-64	0.478681437	0.439	0.149	2.80291E-59	C4_CD8_Teff
chr2-234422145-234423481	4.96912E-64	0.477645383	0.458	0.15	3.2907E-59	C4_CD8_Teff
chr7-105675523-105676465	7.97291E-64	0.481065122	0.44	0.152	5.2799E-59	C4_CD8_Teff
chr1-148401655-148403045	1.33869E-17	0.377978628	0.221	0.083	8.86518E-13	C3_CD8_Tem
chr12-681735-682825	5.60221E-24	0.439803881	0.211	0.061	3.70995E-19	C3_CD8_Tem
chr19-40465090-40467143	8.6162E-33	0.291449452	0.836	0.695	5.70591E-28	C3_CD8_Tem
chr17-64392409-64393697	4.47564E-11	0.304861282	0.122	0.042	2.9639E-06	C3_CD8_Tem
chr16-87492259-87493349	6.4581E-21	0.344944041	0.456	0.251	4.27675E-16	C3_CD8_Tem

chr17-5220631-5221894	1.02383E-17	0.37461876	0.224	0.085	6.7801E-13	C3_CD8_Tem
chr5-142977014-142978026	2.35514E-18	0.357277266	0.299	0.141	1.55964E-13	C3_CD8_Tem
chr1-144545929-144546983	1.04949E-11	0.305436231	0.177	0.075	6.95003E-07	C1_CD8_Tres
chr15-65512623-65513362	3.80529E-26	0.444611257	0.328	0.127	2.51998E-21	C3_CD8_Tem
chr1-44806789-44808263	1.77304E-62	0.381351211	0.689	0.342	1.17416E-57	C4_CD8_Teff
chr4-74444592-74445637	4.39675E-13	0.342344958	0.21	0.091	2.91166E-08	C1_CD8_Tres
chr22-36435230-36436318	8.3552E-19	0.349795208	0.367	0.188	5.53306E-14	C3_CD8_Tem
chr22-50506952-50508750	2.23428E-17	0.250378072	0.591	0.444	1.47961E-12	C3_CD8_Tem
chr15-77021514-77022455	2.20799E-61	0.484913098	0.378	0.117	1.4622E-56	C4_CD8_Teff
chr9-20403156-20403890	2.73784E-61	0.532481253	0.219	0.034	1.81308E-56	C4_CD8_Teff
chr18-49958736-49959702	3.29313E-61	0.521575114	0.221	0.037	2.18081E-56	C4_CD8_Teff
chr19-4246517-4247768	3.29923E-61	0.342825622	0.738	0.415	2.18485E-56	C4_CD8_Teff
chr1-1658437-1659418	3.42463E-61	0.465044991	0.451	0.158	2.26789E-56	C4_CD8_Teff
chr2-144508210-144508577	2.47197E-21	0.436759871	0.201	0.061	1.63702E-16	C3_CD8_Tem
chr9-134301405-134302179	5.05287E-61	0.50176979	0.352	0.097	3.34616E-56	C4_CD8_Teff
chr19-41944151-41945945	1.0078E-60	0.336824509	0.759	0.436	6.67398E-56	C4_CD8_Teff
chr4-38663147-38664983	7.17285E-26	0.378673308	0.5	0.277	4.75008E-21	C3_CD8_Tem
chr1-120850260-120851399	8.59246E-11	0.278036285	0.275	0.16	5.69018E-06	C1_CD8_Tres
chr2-68769927-68770619	3.44006E-60	0.504773719	0.312	0.084	2.27811E-55	C4_CD8_Teff
chr19-47273133-47275176	4.09031E-60	0.294616897	0.837	0.545	2.70872E-55	C4_CD8_Teff
chr14-74617453-74618817	5.55964E-60	0.396181922	0.604	0.286	3.68176E-55	C4_CD8_Teff
chr5-151138721-151139887	6.1292E-60	0.500412493	0.319	0.081	4.05894E-55	C4_CD8_Teff
chrX-149539128-149541387	7.27555E-60	0.31062565	0.811	0.503	4.81808E-55	C4_CD8_Teff
chr10-14007906-14010051	7.70359E-60	0.460119739	0.44	0.162	5.10155E-55	C4_CD8_Teff
chr1-161529712-161531372	9.37659E-60	0.424041124	0.563	0.24	6.20946E-55	C4_CD8_Teff
chr19-18521056-18522749	9.88247E-60	0.273042443	0.891	0.597	6.54447E-55	C4_CD8_Teff
chr17-36212926-36213380	2.05367E-14	0.323490332	0.125	0.035	1.36E-09	C3_CD8_Tem

chr1-148388151-148389117	3.39071E-10	0.29384888	0.165	0.073	2.24543E-05	C1_CD8_Tres
chr19-16330210-16331296	1.83722E-20	0.387552326	0.461	0.273	1.21666E-15	C1_CD8_Tres
chr12-120316891-120317993	7.52569E-16	0.287294197	0.488	0.337	4.98374E-11	C1_CD8_Tres
chr19-16583861-16584718	2.23246E-58	0.375608503	0.66	0.329	1.4784E-53	C4_CD8_Teff
chr17-77440699-77442224	2.45758E-58	0.413549776	0.55	0.241	1.62748E-53	C4_CD8_Teff
chr22-18528018-18529368	2.89316E-58	0.468145754	0.404	0.133	1.91594E-53	C4_CD8_Teff
chr10-49941284-49942406	6.09311E-11	0.286307699	0.162	0.069	4.03504E-06	C1_CD8_Tres
chr8-143595790-143598462	4.5901E-58	0.346593053	0.701	0.386	3.0397E-53	C4_CD8_Teff
chr4-6855496-6856344	5.0904E-58	0.439209239	0.484	0.191	3.37102E-53	C4_CD8_Teff
chr19-9818223-9819870	1.45016E-21	0.276063397	0.725	0.57	9.60338E-17	C1_CD8_Tres
chr12-111404641-111406934	1.73214E-18	0.262013792	0.576	0.424	1.14708E-13	C3_CD8_Tem
chr7-50331555-50332145	1.07042E-15	0.376700635	0.21	0.083	7.08861E-11	C1_CD8_Tres
chr14-91835529-91836905	4.95228E-13	0.272180666	0.395	0.262	3.27955E-08	C1_CD8_Tres
chr5-74974290-74975239	5.04081E-57	0.500300428	0.212	0.036	3.33817E-52	C4_CD8_Teff
chr10-6491805-6492593	6.68955E-57	0.411695652	0.571	0.236	4.43002E-52	C4_CD8_Teff
chr17-75241875-75243049	6.69313E-57	0.490536986	0.303	0.081	4.43239E-52	C4_CD8_Teff
chr8-144465161-144467134	6.82024E-57	0.338948086	0.715	0.396	4.51657E-52	C4_CD8_Teff
chr3-46359594-46360492	8.13842E-57	0.463434748	0.395	0.128	5.3895E-52	C4_CD8_Teff
chr19-40570079-40571126	4.04253E-14	0.273527332	0.449	0.311	2.67709E-09	C1_CD8_Tres
chr11-35247140-35248293	1.85848E-56	0.427542028	0.519	0.211	1.23074E-51	C4_CD8_Teff
chr2-105863707-105864687	3.34484E-56	0.50230046	0.195	0.029	2.21505E-51	C4_CD8_Teff
chr2-221570958-221573882	4.11617E-56	0.408765418	0.562	0.235	2.72585E-51	C4_CD8_Teff
chr19-55598768-55600670	5.56459E-56	0.288630936	0.821	0.532	3.68504E-51	C4_CD8_Teff
chr1-52636599-52637735	6.75294E-56	0.418330165	0.506	0.212	4.472E-51	C4_CD8_Teff
chr7-100353226-100354065	1.13863E-55	0.494178067	0.179	0.023	7.54035E-51	C4_CD8_Teff
chr15-22263740-22265794	2.00486E-55	0.488077114	0.301	0.077	1.32768E-50	C4_CD8_Teff
chr13-41492087-41493279	2.82935E-55	0.473597686	0.354	0.11	1.87368E-50	C4_CD8_Teff

chr9-114369205-114370071	1.1782E-18	0.323353843	0.479	0.279	7.80237E-14	C3_CD8_Tem
chr4-2240986-2242588	3.4442E-55	0.315339622	0.771	0.449	2.28085E-50	C4_CD8_Teff
chr19-55420941-55421695	3.47939E-55	0.495085617	0.182	0.026	2.30416E-50	C4_CD8_Teff
chr17-38900699-38901837	3.06186E-19	0.335699536	0.359	0.2	2.02766E-14	C3_CD8_Tem
chr5-74630330-74631054	9.19269E-18	0.379639428	0.198	0.071	6.08768E-13	C3_CD8_Tem
chr9-123304331-123305414	2.89258E-12	0.281890072	0.326	0.196	1.91555E-07	C3_CD8_Tem
chr2-161233994-161234768	4.88647E-55	0.494140305	0.257	0.061	3.23597E-50	C4_CD8_Teff
chr20-5038646-5039755	5.69553E-55	0.471198218	0.356	0.112	3.77175E-50	C4_CD8_Teff
chr12-121671515-121673361	5.71129E-55	0.313644395	0.767	0.457	3.78219E-50	C4_CD8_Teff
chr15-38251201-38253230	6.56352E-55	0.478687278	0.295	0.079	4.34656E-50	C4_CD8_Teff
chr16-89784179-89785008	1.03883E-54	0.494505697	0.24	0.052	6.87947E-50	C4_CD8_Teff
chr15-20353048-20354546	1.5404E-12	0.291173786	0.221	0.119	1.0201E-07	C3_CD8_Tem
chr2-121745929-121746674	1.92504E-54	0.469652934	0.138	0.012	1.27482E-49	C4_CD8_Teff
chr17-40612576-40613325	1.23188E-09	0.257877806	0.201	0.111	8.15785E-05	C1_CD8_Tres
chr19-9791501-9793338	4.67648E-23	0.300767891	0.635	0.492	3.0969E-18	C1_CD8_Tres
chr19-679697-681238	2.79859E-26	0.342468201	0.647	0.449	1.85331E-21	C1_CD8_Tres
chr19-43667974-43669476	9.67163E-40	0.522033166	0.602	0.282	6.40484E-35	C1_CD8_Tres
chr19-14475801-14477045	1.79762E-69	0.776107046	0.419	0.088	1.19044E-64	C3_CD8_Tem
chr3-28049995-28051257	9.4499E-54	0.488973121	0.252	0.054	6.25801E-49	C4_CD8_Teff
chr1-156126095-156127656	2.37408E-15	0.276229646	0.497	0.337	1.57218E-10	C1_CD8_Tres
chr17-75864256-75865335	1.9099E-53	0.462484426	0.325	0.095	1.26479E-48	C4_CD8_Teff
chr11-118928062-118930467	2.40138E-53	0.279688841	0.824	0.546	1.59027E-48	C4_CD8_Teff
chr12-4806518-4808422	2.40227E-53	0.479145964	0.251	0.059	1.59086E-48	C4_CD8_Teff
chr3-27720978-27722529	3.21468E-53	0.432723976	0.436	0.163	2.12886E-48	C4_CD8_Teff
chr11-12215903-12216717	3.63052E-53	0.471416117	0.142	0.013	2.40424E-48	C4_CD8_Teff
chr19-6199031-6199946	3.67099E-53	0.395177271	0.541	0.244	2.43104E-48	C4_CD8_Teff
chr9-114366219-114366991	4.02264E-53	0.433715112	0.438	0.162	2.66391E-48	C4_CD8_Teff

chr14-105059106-105059912	4.07091E-53	0.478009306	0.241	0.053	2.69588E-48	C4_CD8_Teff
chr19-50375967-50377486	3.29392E-14	0.25262031	0.479	0.354	2.18134E-09	C1_CD8_Tres
chr19-14521961-14522806	5.67969E-53	0.427799809	0.472	0.185	3.76126E-48	C4_CD8_Teff
chr1-25795882-25797196	7.60953E-53	0.449623838	0.375	0.133	5.03926E-48	C4_CD8_Teff
chr17-12981952-12982730	2.37589E-19	0.406938454	0.185	0.054	1.57339E-14	C3_CD8_Tem
chr19-1862291-1864961	1.41208E-52	0.305418577	0.767	0.46	9.35122E-48	C4_CD8_Teff
chr17-36210420-36211235	2.68634E-11	0.289624807	0.12	0.042	1.77898E-06	C3_CD8_Tem
chr17-5241943-5243263	5.82114E-09	0.261960884	0.109	0.045	0.000385493	C3_CD8_Tem
chr2-105738026-105739249	2.27308E-11	0.276364448	0.237	0.133	1.5053E-06	C1_CD8_Tres
chr11-11987445-11988281	9.09996E-12	0.309981017	0.143	0.06	6.02626E-07	C3_CD8_Tem
chr12-52558738-52559814	2.09533E-43	0.634349612	0.292	0.06	1.38759E-38	C3_CD8_Tem
chr1-235074458-235075575	8.91623E-11	0.310783572	0.192	0.085	5.90459E-06	C1_CD8_Tres
chr19-40797961-40799552	4.5498E-52	0.293010275	0.794	0.486	3.01302E-47	C4_CD8_Teff
chr19-41327145-41328838	2.75384E-24	0.26927853	0.794	0.666	1.82367E-19	C3_CD8_Tem
chr7-35722248-35723372	6.58922E-52	0.401271485	0.51	0.228	4.36358E-47	C4_CD8_Teff
chr12-112012161-112014427	7.94329E-52	0.2987482	0.788	0.474	5.26028E-47	C4_CD8_Teff
chr10-100370276-100371692	8.54992E-52	0.387641052	0.549	0.261	5.66201E-47	C4_CD8_Teff
chr10-45971741-45972790	8.56577E-52	0.47619277	0.208	0.036	5.67251E-47	C4_CD8_Teff
chr1-154558046-154559783	9.40036E-52	0.298720168	0.791	0.476	6.2252E-47	C4_CD8_Teff
chr9-128032997-128033511	9.9236E-52	0.435392165	0.407	0.144	6.57171E-47	C4_CD8_Teff
chr2-169682683-169683766	1.07529E-51	0.463272507	0.315	0.091	7.12087E-47	C4_CD8_Teff
chr9-136482112-136484128	1.24E-51	0.291622906	0.781	0.482	8.21166E-47	C4_CD8_Teff
chr20-28602102-28603285	6.08925E-14	0.358619638	0.21	0.08	4.03248E-09	C1_CD8_Tres
chr9-134324251-134324675	1.71818E-51	0.478159407	0.217	0.041	1.13783E-46	C4_CD8_Teff
chr5-172132817-172133989	2.00185E-51	0.446793741	0.371	0.122	1.32568E-46	C4_CD8_Teff
chr11-61110341-61111058	2.52582E-51	0.486734619	0.218	0.043	1.67267E-46	C4_CD8_Teff
chr1-3889848-3891382	2.5472E-10	0.270683744	0.154	0.071	1.68683E-05	C3_CD8_Tem

chr5-74631355-74632512	1.08472E-19	0.414943833	0.247	0.098	7.18332E-15	C3_CD8_Tem
chr19-3275065-3276762	1.56836E-23	0.387348192	0.494	0.289	1.03861E-18	C1_CD8_Tres
chr18-13611875-13612700	6.01339E-51	0.385301123	0.562	0.258	3.98225E-46	C4_CD8_Teff
chr11-62557935-62558490	1.94105E-10	0.26627003	0.214	0.107	1.28542E-05	C3_CD8_Tem
chr4-83211996-83212861	6.66564E-23	0.443141412	0.253	0.09	4.41419E-18	C3_CD8_Tem
chr17-64384876-64385574	8.13828E-51	0.471297952	0.145	0.015	5.38941E-46	C4_CD8_Teff
chr3-43477309-43478624	7.72491E-27	0.462285859	0.31	0.117	5.11566E-22	C3_CD8_Tem
chr19-7829011-7830465	1.26705E-50	0.272108351	0.821	0.549	8.39079E-46	C4_CD8_Teff
chr22-22720135-22721253	9.05688E-11	0.257331621	0.135	0.058	5.99774E-06	C3_CD8_Tem
chr14-106004855-106006329	1.61373E-50	0.397134281	0.512	0.223	1.06866E-45	C4_CD8_Teff
chr1-148371452-148373872	2.26161E-15	0.36870369	0.207	0.086	1.4977E-10	C1_CD8_Tres
chr17-74872443-74873722	2.09589E-50	0.304871548	0.76	0.442	1.38796E-45	C4_CD8_Teff
chr17-409666-411434	2.81603E-19	0.301369197	0.448	0.293	1.86486E-14	C3_CD8_Tem
chr1-25549312-25550465	4.13744E-15	0.341870631	0.293	0.151	2.73994E-10	C1_CD8_Tres
chr8-126556354-126558700	3.6175E-50	0.383875023	0.561	0.252	2.39562E-45	C4_CD8_Teff
chr9-134325400-134326553	3.76444E-50	0.442131054	0.329	0.102	2.49293E-45	C4_CD8_Teff
chr12-57751778-57752833	6.8499E-50	0.326096287	0.701	0.388	4.53621E-45	C4_CD8_Teff
chr19-6766690-6768366	8.4248E-50	0.3267103	0.684	0.377	5.57916E-45	C4_CD8_Teff
chr19-10523026-10523671	5.76932E-36	0.565651082	0.289	0.077	3.82061E-31	C3_CD8_Tem
chr4-4268731-4270272	1.92637E-35	0.542397708	0.372	0.132	1.2757E-30	C3_CD8_Tem
chr7-36776701-36777574	1.2612E-49	0.456064751	0.301	0.087	8.35204E-45	C4_CD8_Teff
chr16-56261625-56262745	1.59968E-49	0.417100909	0.437	0.172	1.05936E-44	C4_CD8_Teff
chr8-22443319-22444348	1.67572E-49	0.378750945	0.554	0.257	1.10971E-44	C4_CD8_Teff
chr11-10307208-10308280	1.69199E-49	0.425991537	0.414	0.149	1.12049E-44	C4_CD8_Teff
chr18-58541077-58542576	1.69906E-49	0.448042763	0.323	0.097	1.12517E-44	C4_CD8_Teff
chr9-128025757-128027532	2.16898E-49	0.367601321	0.58	0.276	1.43636E-44	C4_CD8_Teff
chr5-150376673-150377594	2.45574E-49	0.451726667	0.147	0.019	1.62626E-44	C4_CD8_Teff

chr6-32971500-32972515	3.33873E-49	0.281973647	0.777	0.496	2.21101E-44	C4_CD8_Teff
chr9-97921744-97923970	3.51445E-49	0.287275186	0.782	0.497	2.32737E-44	C4_CD8_Teff
chr3-138780519-138781487	5.18803E-49	0.4233454	0.399	0.144	3.43567E-44	C4_CD8_Teff
chr14-106013425-106014366	6.08299E-49	0.462677207	0.254	0.064	4.02834E-44	C4_CD8_Teff
chr19-908610-909828	6.27886E-14	0.278582335	0.443	0.305	4.15805E-09	C1_CD8_Tres
chr16-87761941-87762604	1.58783E-53	0.702072339	0.37	0.076	1.05151E-48	C3_CD8_Tem
chr8-144412646-144413811	1.5569E-17	0.289273289	0.417	0.262	1.03103E-12	C3_CD8_Tem
chr1-154790841-154792148	1.11408E-48	0.410146688	0.438	0.173	7.3778E-44	C4_CD8_Teff
chr18-22281989-22283234	4.44495E-22	0.428259554	0.242	0.084	2.94358E-17	C3_CD8_Tem
chr19-996309-996912	1.32569E-48	0.438504064	0.345	0.114	8.77913E-44	C4_CD8_Teff
chr17-39952197-39953600	3.34179E-14	0.268520897	0.551	0.414	2.21303E-09	C1_CD8_Tres
chr19-16586712-16587917	2.26386E-48	0.411522953	0.43	0.172	1.4992E-43	C4_CD8_Teff
chr19-50383560-50384765	2.50319E-48	0.339875549	0.631	0.328	1.65769E-43	C4_CD8_Teff
chr8-29035430-29036887	2.62524E-48	0.469274078	0.185	0.032	1.73852E-43	C4_CD8_Teff
chr14-96391046-96392362	3.89202E-48	0.303933181	0.743	0.431	2.57741E-43	C4_CD8_Teff
chr2-27489058-27490129	4.1443E-48	0.305590164	0.723	0.424	2.74448E-43	C4_CD8_Teff
chr1-51520040-51521153	7.45327E-48	0.413238594	0.428	0.165	4.93578E-43	C4_CD8_Teff
chr20-59159150-59160047	7.46971E-48	0.393195629	0.487	0.209	4.94667E-43	C4_CD8_Teff
chr11-118051199-118052134	8.87929E-48	0.401627889	0.468	0.2	5.88014E-43	C4_CD8_Teff
chr5-134927520-134928170	1.01018E-22	0.52776801	0.254	0.076	6.68969E-18	C1_CD8_Tres
chr6-107715618-107717134	1.04945E-47	0.421860345	0.406	0.153	6.9498E-43	C4_CD8_Teff
chr8-58790453-58791432	6.3278E-13	0.299387189	0.193	0.092	4.19046E-08	C3_CD8_Tem
chr16-75622547-75623599	1.66608E-47	0.335788512	0.656	0.346	1.10333E-42	C4_CD8_Teff
chr3-66298568-66298893	1.73176E-47	0.455236838	0.139	0.017	1.14683E-42	C4_CD8_Teff
chr21-44064889-44066026	1.78586E-47	0.361037638	0.578	0.283	1.18265E-42	C4_CD8_Teff
chr20-57397146-57398815	1.89311E-47	0.338291412	0.639	0.347	1.25367E-42	C4_CD8_Teff
chr2-68907317-68908830	2.06908E-47	0.390476046	0.492	0.212	1.37021E-42	C4_CD8_Teff

chr5-32537250-32538363	2.29537E-47	0.440549564	0.292	0.09	1.52006E-42	C4_CD8_Teff
chr1-221839379-221840266	2.63174E-47	0.373306106	0.549	0.258	1.74282E-42	C4_CD8_Teff
chr2-60818936-60820092	2.70482E-47	0.386418838	0.502	0.221	1.79121E-42	C4_CD8_Teff
chr15-82530074-82531139	9.34808E-10	0.271096664	0.141	0.058	6.19058E-05	C1_CD8_Tres
chr1-26275597-26276590	3.57072E-47	0.36022029	0.586	0.287	2.36464E-42	C4_CD8_Teff
chr1-149636033-149636990	1.67189E-12	0.321010816	0.216	0.108	1.10717E-07	C1_CD8_Tres
chr17-83124835-83126264	9.53333E-10	0.261475503	0.128	0.052	6.31326E-05	C3_CD8_Tem
chr11-1568276-1568696	4.23123E-21	0.413245336	0.193	0.061	2.80205E-16	C3_CD8_Tem
chr20-8156300-8156927	5.02406E-47	0.448324575	0.175	0.028	3.32709E-42	C4_CD8_Teff
chr9-135906639-135908149	1.11079E-46	0.357145526	0.56	0.269	7.35599E-42	C4_CD8_Teff
chr6-143544221-143544640	1.54784E-46	0.444335511	0.128	0.014	1.02502E-41	C4_CD8_Teff
chr10-114808541-114809335	1.55865E-46	0.445498879	0.136	0.014	1.03219E-41	C4_CD8_Teff
chr7-102431570-102432147	3.21354E-46	0.456312423	0.216	0.049	2.1281E-41	C4_CD8_Teff
chr5-55023801-55024381	3.54535E-46	0.45181084	0.16	0.023	2.34784E-41	C4_CD8_Teff
chr22-22737509-22738033	4.44716E-46	0.440378428	0.147	0.019	2.94504E-41	C4_CD8_Teff
chr1-226682729-226683203	4.98414E-46	0.450677002	0.193	0.038	3.30065E-41	C4_CD8_Teff
chr14-22928676-22930651	5.0852E-46	0.289987762	0.765	0.454	3.36757E-41	C4_CD8_Teff
chr6-137155466-137156249	6.18965E-46	0.444660429	0.227	0.05	4.09897E-41	C4_CD8_Teff
chr19-7903057-7904640	6.27678E-46	0.314857806	0.661	0.367	4.15667E-41	C4_CD8_Teff
chr1-148458349-148459569	2.2714E-10	0.302530025	0.138	0.049	1.50419E-05	C1_CD8_Tres
chr21-43733027-43734456	2.86729E-08	0.264264168	0.162	0.078	0.001898803	C1_CD8_Tres
chr18-12920744-12921812	5.59375E-29	0.491111609	0.299	0.103	3.70435E-24	C3_CD8_Tem
chr20-37189590-37190679	1.02182E-45	0.440769257	0.149	0.02	6.76678E-41	C4_CD8_Teff
chr1-91116594-91117319	1.03089E-45	0.462735525	0.226	0.053	6.82687E-41	C4_CD8_Teff
chr7-150665882-150666234	6.09876E-23	0.440462186	0.224	0.075	4.03878E-18	C3_CD8_Tem
chr14-75229715-75230721	1.43782E-45	0.422858185	0.338	0.115	9.52166E-41	C4_CD8_Teff
chr9-271865-273241	1.56846E-45	0.323778946	0.655	0.366	1.03868E-40	C4_CD8_Teff

chr12-25333537-25334249	2.08312E-45	0.433639852	0.316	0.101	1.3795E-40	C4_CD8_Teff
chr17-78781990-78782715	1.72902E-18	0.276589143	0.438	0.276	1.14501E-13	C3_CD8_Tem
chr11-36393948-36394445	2.83051E-45	0.422743081	0.129	0.014	1.87445E-40	C4_CD8_Teff
chr20-51530978-51531758	3.01089E-45	0.337876317	0.62	0.313	1.9939E-40	C4_CD8_Teff
chr12-49043678-49044518	4.12264E-15	0.348012104	0.185	0.071	2.73013E-10	C3_CD8_Tem
chr16-11571884-11572994	4.15447E-45	0.443553606	0.25	0.06	2.75121E-40	C4_CD8_Teff
chr14-103909899-103910613	5.05742E-45	0.454107441	0.237	0.058	3.34917E-40	C4_CD8_Teff
chr6-10732633-10733726	5.1574E-10	0.280882917	0.115	0.041	3.41539E-05	C3_CD8_Tem
chr6-41533293-41533722	6.10778E-45	0.444758635	0.154	0.022	4.04476E-40	C4_CD8_Teff
chr4-2290508-2291625	7.06123E-45	0.386590771	0.464	0.202	4.67616E-40	C4_CD8_Teff
chr2-68378735-68379810	1.96629E-25	0.454572305	0.26	0.09	1.30214E-20	C3_CD8_Tem
chr7-143407229-143409343	9.34437E-45	0.429674633	0.299	0.091	6.18812E-40	C4_CD8_Teff
chr14-101730892-101732010	1.0404E-44	0.392822089	0.443	0.182	6.88982E-40	C4_CD8_Teff
chr18-23872449-23873091	1.56593E-44	0.438753315	0.174	0.029	1.037E-39	C4_CD8_Teff
chr5-142924201-142924891	1.66242E-44	0.448237106	0.275	0.077	1.10091E-39	C4_CD8_Teff
chrX-13691803-13692928	3.27945E-11	0.276045502	0.135	0.059	2.17175E-06	C3_CD8_Tem
chr19-543224-544491	5.9802E-15	0.2819524	0.357	0.209	3.96027E-10	C3_CD8_Tem
chr12-128795987-128796930	2.0251E-45	0.640937865	0.276	0.056	1.34108E-40	C3_CD8_Tem
chr21-44817155-44818526	2.46877E-44	0.329574208	0.603	0.306	1.6349E-39	C4_CD8_Teff
chr6-16487460-16488540	2.60899E-44	0.421202741	0.348	0.125	1.72775E-39	C4_CD8_Teff
chr1-26799587-26800842	2.71352E-44	0.4353539	0.273	0.08	1.79697E-39	C4_CD8_Teff
chr10-3874847-3875904	3.59026E-44	0.42538345	0.306	0.1	2.37758E-39	C4_CD8_Teff
chr4-10108841-10109483	3.59846E-44	0.427914464	0.306	0.097	2.38301E-39	C4_CD8_Teff
chr2-106094047-106094928	4.76033E-44	0.424634161	0.336	0.113	3.15243E-39	C4_CD8_Teff
chr20-62623349-62624245	5.25676E-14	0.349330674	0.165	0.061	3.48118E-09	C1_CD8_Tres
chr19-19605807-19606451	4.12717E-24	0.440626722	0.308	0.127	2.73314E-19	C1_CD8_Tres
chr5-178113100-178115384	8.19057E-13	0.269133097	0.32	0.203	5.42404E-08	C3_CD8_Tem

chr22-46035390-46037034	6.6154E-44	0.265690939	0.793	0.51	4.38091E-39	C4_CD8_Teff
chr1-101235126-101237022	6.47972E-18	0.370155822	0.338	0.183	4.29106E-13	C1_CD8_Tres
chr9-137021568-137022588	1.44852E-20	0.370014119	0.32	0.147	9.59252E-16	C3_CD8_Tem
chr18-46536388-46536937	7.49448E-44	0.42027875	0.113	0.009	4.96307E-39	C4_CD8_Teff
chr17-83050968-83052208	9.04287E-44	0.252468681	0.834	0.547	5.98846E-39	C4_CD8_Teff
chr16-29805070-29806620	9.20536E-44	0.289267457	0.713	0.435	6.09607E-39	C4_CD8_Teff
chr19-1248095-1249938	1.06746E-43	0.266248519	0.795	0.499	7.06902E-39	C4_CD8_Teff
chr22-28882669-28884423	9.25429E-38	0.529199688	0.488	0.22	6.12847E-33	C1_CD8_Tres
chr2-61662962-61663425	1.29594E-43	0.418880873	0.117	0.011	8.58211E-39	C4_CD8_Teff
chr6-26568122-26569395	1.3167E-43	0.416690644	0.343	0.12	8.71959E-39	C4_CD8_Teff
chr14-50789772-50790133	1.42935E-43	0.413459588	0.103	0.007	9.46556E-39	C4_CD8_Teff
chr1-107785657-107786884	7.82373E-20	0.372589796	0.299	0.14	5.18111E-15	C3_CD8_Tem
chr7-70647532-70648779	3.10014E-14	0.311821097	0.393	0.222	2.053E-09	C3_CD8_Tem
chr1-1908440-1909710	2.13765E-43	0.32721079	0.622	0.324	1.41562E-38	C4_CD8_Teff
chr21-33937726-33938566	1.1753E-11	0.263840958	0.24	0.133	7.78318E-07	C3_CD8_Tem
chr6-30720833-30722128	2.52584E-43	0.355582704	0.535	0.254	1.67268E-38	C4_CD8_Teff
chr17-1890533-1891414	6.97062E-34	0.543681586	0.292	0.089	4.61615E-29	C3_CD8_Tem
chr17-65038931-65039998	2.99628E-43	0.403246131	0.394	0.149	1.98423E-38	C4_CD8_Teff
chr3-27880867-27881933	1.09588E-10	0.269535627	0.18	0.082	7.25725E-06	C3_CD8_Tem
chr3-15269076-15269877	3.19223E-43	0.406646604	0.363	0.132	2.11399E-38	C4_CD8_Teff
chr11-60912103-60912668	3.62625E-43	0.427255015	0.143	0.022	2.40141E-38	C4_CD8_Teff
chr19-37778627-37780020	1.68525E-19	0.308407618	0.476	0.338	1.11602E-14	C1_CD8_Tres
chr1-12617052-12619578	3.88977E-43	0.382999307	0.436	0.183	2.57592E-38	C4_CD8_Teff
chr14-91871983-91873936	3.9724E-43	0.357340808	0.525	0.258	2.63064E-38	C4_CD8_Teff
chr1-150186466-150187633	2.05444E-12	0.2994733	0.167	0.067	1.36051E-07	C3_CD8_Tem
chr1-146036485-146038363	4.31296E-43	0.353984138	0.55	0.257	2.85617E-38	C4_CD8_Teff
chr2-99140602-99141892	5.18652E-43	0.304124445	0.671	0.382	3.43467E-38	C4_CD8_Teff

chr17-78250773-78252846	6.86027E-21	0.396445266	0.32	0.16	4.54308E-16	C1_CD8_Tres
chr9-136245215-136246169	5.44826E-43	0.389136168	0.399	0.164	3.608E-38	C4_CD8_Teff
chr20-62977422-62978333	6.25224E-43	0.413052868	0.354	0.13	4.14042E-38	C4_CD8_Teff
chr22-50018577-50019498	6.52726E-43	0.440497266	0.213	0.051	4.32255E-38	C4_CD8_Teff
chr8-144019212-144020607	7.13216E-43	0.439404297	0.156	0.024	4.72313E-38	C4_CD8_Teff
chr11-118911875-118912806	7.71351E-43	0.406821417	0.353	0.134	5.10812E-38	C4_CD8_Teff
chr7-116210224-116211665	8.06093E-43	0.354399175	0.534	0.25	5.33819E-38	C4_CD8_Teff
chr1-8520336-8520883	1.13679E-42	0.415392112	0.102	0.007	7.52818E-38	C4_CD8_Teff
chr11-64877938-64879151	1.21204E-42	0.2740678	0.754	0.47	8.02649E-38	C4_CD8_Teff
chr17-49762920-49764623	1.37835E-42	0.271028603	0.759	0.486	9.12784E-38	C4_CD8_Teff
chr2-230864386-230865285	1.55999E-42	0.4067617	0.363	0.132	1.03307E-37	C4_CD8_Teff
chr7-45001112-45001624	1.58736E-42	0.406426137	0.351	0.122	1.05119E-37	C4_CD8_Teff
chr19-659341-659906	5.76111E-14	0.327988502	0.237	0.113	3.81518E-09	C3_CD8_Tem
chr17-5048792-5049765	1.83902E-42	0.42009025	0.306	0.101	1.21786E-37	C4_CD8_Teff
chr14-97393042-97394054	1.33344E-11	0.303450691	0.125	0.042	8.83044E-07	C3_CD8_Tem
chr19-1940031-1941273	2.27254E-42	0.318699843	0.611	0.33	1.50495E-37	C4_CD8_Teff
chr9-96874301-96876019	2.55019E-42	0.410914861	0.332	0.118	1.68881E-37	C4_CD8_Teff
chr1-44801637-44802432	2.84914E-42	0.429403904	0.271	0.084	1.88679E-37	C4_CD8_Teff
chr17-44185951-44187838	2.89669E-42	0.276244005	0.743	0.453	1.91827E-37	C4_CD8_Teff
chr22-46370317-46371312	6.26933E-15	0.339534285	0.161	0.059	4.15174E-10	C3_CD8_Tem
chr1-59814144-59815420	1.02231E-16	0.34693471	0.257	0.125	6.77007E-12	C1_CD8_Tres
chr11-118607485-118609622	3.38673E-42	0.352759394	0.527	0.255	2.2428E-37	C4_CD8_Teff
chr9-124873648-124874001	3.12637E-13	0.309845356	0.13	0.044	2.07038E-08	C3_CD8_Tem
chr13-46295121-46296637	4.02915E-42	0.332234488	0.583	0.313	2.66822E-37	C4_CD8_Teff
chr3-56732303-56733288	7.78181E-30	0.506368376	0.232	0.065	5.15335E-25	C3_CD8_Tem
chr1-2305042-2305594	4.0922E-42	0.427490831	0.124	0.014	2.70998E-37	C4_CD8_Teff
chr20-10350624-10351911	2.53735E-23	0.437654133	0.268	0.098	1.68031E-18	C3_CD8_Tem

chr17-74473957-74474793	5.21358E-09	0.262796722	0.115	0.049	0.000345259	C3_CD8_Tem
chr2-241880944-241882639	1.11986E-41	0.285921969	0.705	0.433	7.41603E-37	C4_CD8_Teff
chr5-155766982-155767882	2.00171E-28	0.512950298	0.206	0.049	1.3256E-23	C3_CD8_Tem
chr2-181393318-181395118	1.30227E-16	0.338698967	0.281	0.132	8.62403E-12	C3_CD8_Tem
chr5-119287098-119288138	1.80032E-41	0.402329338	0.347	0.128	1.19223E-36	C4_CD8_Teff
chr7-50342425-50342974	3.46985E-10	0.25948274	0.151	0.067	2.29784E-05	C3_CD8_Tem
chr1-26377507-26378830	2.04054E-41	0.310380127	0.638	0.352	1.3513E-36	C4_CD8_Teff
chr1-8525085-8526207	5.08256E-15	0.355544817	0.225	0.099	3.36583E-10	C1_CD8_Tres
chr10-6493437-6493865	2.45545E-41	0.421125954	0.282	0.089	1.62607E-36	C4_CD8_Teff
chr19-6459601-6460944	2.47722E-41	0.308586051	0.639	0.359	1.64049E-36	C4_CD8_Teff
chr1-144560117-144561057	2.52861E-41	0.419071371	0.135	0.017	1.67452E-36	C4_CD8_Teff
chr17-49714993-49716355	1.49736E-16	0.280506752	0.495	0.331	9.916E-12	C3_CD8_Tem
chr12-52566301-52567390	2.46913E-79	0.874838695	0.424	0.062	1.63513E-74	C3_CD8_Tem
chr22-39115709-39118155	2.84403E-41	0.262378382	0.77	0.506	1.8834E-36	C4_CD8_Teff
chr1-150615613-150616066	3.04474E-41	0.434279726	0.194	0.041	2.01632E-36	C4_CD8_Teff
chr19-41875113-41875859	2.23148E-13	0.277536998	0.43	0.277	1.47775E-08	C3_CD8_Tem
chr5-134111599-134112485	3.71401E-41	0.405131371	0.342	0.125	2.45953E-36	C4_CD8_Teff
chr9-134024653-134026073	3.0321E-15	0.260029744	0.516	0.364	2.00795E-10	C3_CD8_Tem
chr14-106184342-106186119	4.87264E-41	0.361474867	0.48	0.219	3.22681E-36	C4_CD8_Teff
chr6-33417143-33418678	5.53694E-41	0.254739788	0.787	0.518	3.66673E-36	C4_CD8_Teff
chr17-27471322-27472502	5.72192E-41	0.353836584	0.49	0.24	3.78923E-36	C4_CD8_Teff
chr10-7268834-7269976	6.22052E-41	0.401611372	0.344	0.124	4.11942E-36	C4_CD8_Teff
chr19-919449-921116	7.41449E-41	0.414837896	0.293	0.095	4.9101E-36	C4_CD8_Teff
chr10-104333173-104333562	1.15083E-10	0.280659141	0.161	0.076	7.62117E-06	C3_CD8_Tem
chr1-629778-630252	6.51468E-21	0.450485722	0.293	0.116	4.31422E-16	C1_CD8_Tres
chr2-98835548-98836629	8.57051E-41	0.378778164	0.436	0.185	5.67565E-36	C4_CD8_Teff
chr11-19280863-19281397	4.42401E-10	0.277816401	0.104	0.033	2.92971E-05	C3_CD8_Tem

chr2-239297658-239298926	9.1088E-41	0.34687689	0.536	0.257	6.03212E-36	C4_CD8_Teff
chr19-510466-511962	9.15917E-41	0.27045681	0.759	0.48	6.06548E-36	C4_CD8_Teff
chr20-50730432-50732135	1.24716E-40	0.289304582	0.691	0.407	8.25907E-36	C4_CD8_Teff
chr2-128381991-128382821	1.34895E-40	0.411815758	0.122	0.014	8.93318E-36	C4_CD8_Teff
chr20-3810788-3812090	1.39475E-40	0.316902972	0.62	0.332	9.23647E-36	C4_CD8_Teff
chr1-25064358-25066301	1.06894E-24	0.388565188	0.401	0.204	7.07887E-20	C3_CD8_Tem
chr1-10032332-10033663	1.99698E-40	0.26973473	0.746	0.456	1.32246E-35	C4_CD8_Teff
chr7-73005202-73006292	5.89203E-25	0.433156199	0.302	0.12	3.90188E-20	C3_CD8_Tem
chr13-99366761-99367637	2.24601E-40	0.420336899	0.146	0.024	1.48737E-35	C4_CD8_Teff
chr14-20612877-20614023	2.32258E-40	0.343443943	0.548	0.26	1.53808E-35	C4_CD8_Teff
chr16-56278591-56279579	2.4272E-40	0.417715264	0.261	0.075	1.60736E-35	C4_CD8_Teff
chr8-27386227-27386923	2.94634E-40	0.417153791	0.139	0.022	1.95115E-35	C4_CD8_Teff
chr3-66297408-66298303	3.39608E-40	0.423087013	0.186	0.04	2.24899E-35	C4_CD8_Teff
chr2-37851387-37852354	3.43853E-40	0.427734047	0.152	0.026	2.2771E-35	C4_CD8_Teff
chr22-22787701-22788489	3.82523E-40	0.422056274	0.237	0.065	2.53319E-35	C4_CD8_Teff
chr17-7241805-7243214	4.08041E-40	0.334250912	0.534	0.267	2.70217E-35	C4_CD8_Teff
chr19-39073226-39074029	4.05978E-12	0.321045083	0.308	0.165	2.68851E-07	C1_CD8_Tres
chr10-14118784-14119354	4.10059E-40	0.400109685	0.105	0.008	2.71553E-35	C4_CD8_Teff
chr19-55623806-55625040	4.88483E-40	0.354422408	0.463	0.212	3.23488E-35	C4_CD8_Teff
chr14-55337435-55338199	4.34578E-26	0.426042099	0.453	0.221	2.87791E-21	C3_CD8_Tem
chr7-73578051-73579177	6.31202E-40	0.259597209	0.782	0.488	4.18001E-35	C4_CD8_Teff
chr14-106024756-106025729	6.83634E-40	0.391785954	0.099	0.007	4.52723E-35	C4_CD8_Teff
chr1-8873543-8873917	7.32628E-40	0.404335181	0.321	0.113	4.85168E-35	C4_CD8_Teff
chr8-123415937-123417564	7.74181E-40	0.265230459	0.733	0.467	5.12686E-35	C4_CD8_Teff
chr3-123491916-123492608	8.63534E-40	0.428226538	0.173	0.033	5.71858E-35	C4_CD8_Teff
chr19-1650675-1653262	9.88399E-40	0.271053896	0.716	0.451	6.54547E-35	C4_CD8_Teff
chr21-44343509-44343886	1.03275E-39	0.407108196	0.118	0.013	6.83917E-35	C4_CD8_Teff

chr2-110657494-110658231	1.09307E-39	0.408656173	0.153	0.025	7.2386E-35	C4_CD8_Teff
chr20-63902379-63903354	1.16271E-39	0.40689162	0.287	0.095	7.6998E-35	C4_CD8_Teff
chr14-101816009-101816233	1.24404E-39	0.39377003	0.105	0.01	8.2384E-35	C4_CD8_Teff
chr19-12782008-12783206	1.6332E-39	0.319749748	0.583	0.318	1.08156E-34	C4_CD8_Teff
chr20-62141216-62142022	2.07494E-39	0.424349432	0.218	0.055	1.37408E-34	C4_CD8_Teff
chr16-89994428-89996219	2.18508E-39	0.371836743	0.406	0.18	1.44703E-34	C4_CD8_Teff
chr18-38940334-38941537	2.27778E-39	0.391506607	0.336	0.117	1.50841E-34	C4_CD8_Teff
chr19-40756246-40757119	2.36258E-39	0.404550617	0.306	0.108	1.56457E-34	C4_CD8_Teff
chr1-203320345-203321303	2.59594E-39	0.397777821	0.337	0.122	1.71911E-34	C4_CD8_Teff
chr18-13453621-13454061	2.64087E-39	0.377681636	0.084	0.004	1.74887E-34	C4_CD8_Teff
chr13-111182833-111183595	3.06564E-39	0.424473088	0.155	0.027	2.03016E-34	C4_CD8_Teff
chr6-26025668-26027497	3.26156E-39	0.304457988	0.626	0.355	2.1599E-34	C4_CD8_Teff
chr8-123727616-123728227	3.62153E-39	0.393992342	0.106	0.01	2.39829E-34	C4_CD8_Teff
chr15-66115293-66116264	4.06232E-39	0.402055795	0.124	0.017	2.69019E-34	C4_CD8_Teff
chr2-218879617-218880954	5.42949E-39	0.345755897	0.504	0.243	3.59557E-34	C4_CD8_Teff
chr20-62860925-62862275	5.60288E-39	0.341974619	0.487	0.234	3.7104E-34	C4_CD8_Teff
chr13-30373890-30374880	6.65152E-39	0.367210963	0.427	0.188	4.40484E-34	C4_CD8_Teff
chr3-45957881-45958880	7.07305E-39	0.420747772	0.219	0.058	4.68399E-34	C4_CD8_Teff
chr17-7213785-7215240	7.69651E-39	0.281708891	0.686	0.414	5.09686E-34	C4_CD8_Teff
chr6-132812796-132815022	7.79731E-39	0.267114152	0.758	0.465	5.16361E-34	C4_CD8_Teff
chr17-39979878-39981285	9.36642E-39	0.259091162	0.748	0.479	6.20273E-34	C4_CD8_Teff
chr2-105855655-105857360	1.0282E-38	0.370551709	0.413	0.179	6.80902E-34	C4_CD8_Teff
chr11-121481647-121482948	5.04433E-15	0.315803905	0.359	0.204	3.34051E-10	C3_CD8_Tem
chr19-19661392-19662269	1.54376E-38	0.408241512	0.167	0.034	1.02232E-33	C4_CD8_Teff
chr19-5077800-5078647	1.63047E-38	0.408842697	0.144	0.025	1.07975E-33	C4_CD8_Teff
chr20-48759795-48761687	1.68316E-38	0.303168816	0.629	0.351	1.11464E-33	C4_CD8_Teff
chr7-143385779-143386618	1.87991E-38	0.396768922	0.322	0.116	1.24493E-33	C4_CD8_Teff

chr17-10198118-10199316	2.21119E-17	0.383501918	0.229	0.085	1.46431E-12	C3_CD8_Tem
chrX-107479597-107480657	2.38803E-38	0.410826559	0.15	0.027	1.58143E-33	C4_CD8_Teff
chr6-137588991-137589938	9.85609E-11	0.300720245	0.229	0.122	6.527E-06	C3_CD8_Tem
chrX-2629003-2631181	2.66594E-38	0.31238837	0.622	0.328	1.76547E-33	C4_CD8_Teff
chr22-44180249-44182890	3.08435E-38	0.325876947	0.527	0.281	2.04255E-33	C4_CD8_Teff
chr10-132331335-132332628	3.1973E-38	0.310396329	0.592	0.313	2.11735E-33	C4_CD8_Teff
chr4-40254631-40255993	3.24068E-38	0.404852319	0.231	0.066	2.14608E-33	C4_CD8_Teff
chr2-218395882-218397084	3.38111E-38	0.300418034	0.63	0.345	2.23907E-33	C4_CD8_Teff
chr17-28727642-28729137	3.39274E-38	0.260672706	0.736	0.466	2.24678E-33	C4_CD8_Teff
chr3-46980418-46980881	3.78899E-38	0.402390303	0.22	0.061	2.50918E-33	C4_CD8_Teff
chr16-50160451-50161037	4.08382E-38	0.411576243	0.177	0.039	2.70443E-33	C4_CD8_Teff
chr3-184248456-184250546	4.13231E-38	0.283748755	0.672	0.398	2.73654E-33	C4_CD8_Teff
chr19-1406701-1408982	4.33496E-38	0.267493884	0.715	0.444	2.87074E-33	C4_CD8_Teff
chr17-82775027-82775870	4.36546E-38	0.415127346	0.173	0.038	2.89094E-33	C4_CD8_Teff
chr13-23374861-23375945	4.52102E-38	0.268028163	0.727	0.447	2.99395E-33	C4_CD8_Teff
chr17-78139875-78140785	2.48962E-14	0.280498247	0.41	0.292	1.6487E-09	C1_CD8_Tres
chr6-195719-196150	4.71343E-38	0.399309562	0.119	0.015	3.12137E-33	C4_CD8_Teff
chr9-124868416-124870556	5.07578E-38	0.263400181	0.743	0.466	3.36133E-33	C4_CD8_Teff
chr16-4253220-4254275	5.32287E-38	0.271060754	0.71	0.436	3.52497E-33	C4_CD8_Teff
chr1-146386707-146387682	5.57285E-38	0.405550772	0.142	0.022	3.69051E-33	C4_CD8_Teff
chr16-56202633-56203391	6.38333E-38	0.416139189	0.171	0.036	4.22723E-33	C4_CD8_Teff
chr19-58543588-58545118	7.5114E-38	0.265950444	0.727	0.44	4.97427E-33	C4_CD8_Teff
chr2-96148311-96148944	8.06675E-38	0.282385515	0.681	0.406	5.34205E-33	C4_CD8_Teff
chr15-70299789-70301094	8.74078E-38	0.306605446	0.607	0.339	5.78841E-33	C4_CD8_Teff
chr8-30137089-30137602	9.18984E-38	0.394769332	0.317	0.111	6.08579E-33	C4_CD8_Teff
chr10-133307582-133309430	1.00764E-37	0.270036539	0.717	0.432	6.6729E-33	C4_CD8_Teff
chr22-49970261-49971724	1.03612E-37	0.290225415	0.658	0.375	6.86148E-33	C4_CD8_Teff

chr10-119305549-119307371	1.14188E-37	0.268571253	0.717	0.447	7.56185E-33	C4_CD8_Teff
chr1-40058897-40060781	1.33118E-37	0.380838278	0.344	0.132	8.81544E-33	C4_CD8_Teff
chr19-8412535-8413465	1.35708E-37	0.313995094	0.576	0.297	8.98699E-33	C4_CD8_Teff
chr19-3357402-3358618	1.35355E-18	0.37706807	0.208	0.079	8.96365E-14	C3_CD8_Tem
chr9-131732826-131734540	1.42558E-37	0.321723269	0.552	0.285	9.44063E-33	C4_CD8_Teff
chr11-505922-507628	1.42895E-37	0.265250319	0.722	0.455	9.46292E-33	C4_CD8_Teff
chr19-570851-572919	1.47677E-37	0.310958793	0.571	0.304	9.77958E-33	C4_CD8_Teff
chr5-143218472-143219003	1.52029E-37	0.405483457	0.129	0.018	1.00678E-32	C4_CD8_Teff
chr2-121735676-121737371	1.67324E-37	0.26518088	0.729	0.446	1.10807E-32	C4_CD8_Teff
chr1-160751298-160751835	1.68431E-37	0.395596827	0.305	0.103	1.1154E-32	C4_CD8_Teff
chr10-124650100-124651550	2.07235E-37	0.306071555	0.616	0.341	1.37237E-32	C4_CD8_Teff
chr9-128723479-128725129	2.15887E-37	0.265252044	0.726	0.439	1.42967E-32	C4_CD8_Teff
chr1-144523576-144524419	2.16804E-37	0.391850457	0.121	0.017	1.43574E-32	C4_CD8_Teff
chr19-2150869-2152142	2.18201E-37	0.264688453	0.718	0.442	1.44499E-32	C4_CD8_Teff
chr11-66421081-66422007	6.48974E-12	0.270286158	0.182	0.084	4.2977E-07	C3_CD8_Tem
chr20-58980863-58983206	2.30631E-37	0.323455369	0.536	0.277	1.52731E-32	C4_CD8_Teff
chr5-142799721-142801418	2.65852E-37	0.346211905	0.449	0.216	1.76055E-32	C4_CD8_Teff
chr7-100220756-100222056	2.75524E-37	0.355929516	0.436	0.196	1.8246E-32	C4_CD8_Teff
chr2-191206130-191207147	5.30231E-23	0.401227684	0.331	0.149	3.51135E-18	C3_CD8_Tem
chr2-181162826-181163908	3.02189E-37	0.386578684	0.304	0.11	2.00119E-32	C4_CD8_Teff
chr17-4899075-4899877	3.17995E-37	0.319390106	0.543	0.282	2.10586E-32	C4_CD8_Teff
chr1-201170280-201171791	3.29827E-37	0.326755005	0.525	0.284	2.18421E-32	C4_CD8_Teff
chr19-54200205-54201433	3.62128E-37	0.320900401	0.539	0.281	2.39812E-32	C4_CD8_Teff
chr20-53835364-53835846	4.03035E-37	0.406950288	0.233	0.066	2.66902E-32	C4_CD8_Teff
chr22-37228513-37231037	4.67117E-37	0.273103464	0.683	0.421	3.09339E-32	C4_CD8_Teff
chr17-78133905-78134571	5.42981E-37	0.38609495	0.332	0.122	3.59578E-32	C4_CD8_Teff
chr1-148037879-148039000	7.57692E-09	0.280199309	0.15	0.065	0.000501766	C1_CD8_Tres

chr6-33409564-33411481	5.97171E-37	0.25252566	0.763	0.482	3.95464E-32	C4_CD8_Teff
chr18-45669987-45670872	1.56367E-09	0.272373594	0.201	0.103	0.000103551	C3_CD8_Tem
chr1-161611874-161612938	6.4743E-37	0.354786809	0.441	0.195	4.28748E-32	C4_CD8_Teff
chr2-117814139-117815490	6.50138E-37	0.284434465	0.657	0.393	4.30541E-32	C4_CD8_Teff
chr16-70522891-70525051	6.62432E-37	0.3203025	0.543	0.283	4.38682E-32	C4_CD8_Teff
chr16-84593536-84595332	6.85911E-37	0.315083059	0.561	0.305	4.54231E-32	C4_CD8_Teff
chr2-203706055-203706728	7.78305E-37	0.377964001	0.351	0.136	5.15417E-32	C4_CD8_Teff
chr15-69721380-69722147	7.95068E-37	0.407554943	0.207	0.055	5.26518E-32	C4_CD8_Teff
chr3-45975843-45977120	7.95215E-37	0.366621334	0.4	0.17	5.26615E-32	C4_CD8_Teff
chr22-30434891-30436225	7.97357E-37	0.374937917	0.363	0.141	5.28034E-32	C4_CD8_Teff
chr16-3020156-3022540	8.22231E-37	0.289219126	0.624	0.369	5.44506E-32	C4_CD8_Teff
chr2-24739351-24739990	6.94402E-22	0.458937234	0.159	0.032	4.59854E-17	C3_CD8_Tem
chr9-131310396-131311231	8.54203E-37	0.404350502	0.243	0.075	5.65679E-32	C4_CD8_Teff
chr17-82448888-82451040	1.19386E-36	0.303492026	0.584	0.316	7.90612E-32	C4_CD8_Teff
chr4-40213881-40214437	1.22277E-36	0.401200319	0.13	0.019	8.09754E-32	C4_CD8_Teff
chr7-36784623-36785346	1.35394E-36	0.363163101	0.413	0.174	8.96621E-32	C4_CD8_Teff
chr10-118753904-118755525	1.44619E-36	0.280407049	0.652	0.385	9.57712E-32	C4_CD8_Teff
chr3-12967159-12968208	1.552E-36	0.330869712	0.509	0.248	1.02778E-31	C4_CD8_Teff
chr17-74470999-74471668	1.71283E-36	0.407961808	0.191	0.047	1.13429E-31	C4_CD8_Teff
chr10-11225823-11227785	2.06034E-36	0.36722806	0.373	0.153	1.36442E-31	C4_CD8_Teff
chr2-218567748-218569450	2.11791E-36	0.258498193	0.73	0.458	1.40254E-31	C4_CD8_Teff
chr19-14405683-14406428	2.22879E-36	0.373475037	0.36	0.141	1.47597E-31	C4_CD8_Teff
chr6-13453743-13455250	2.51424E-36	0.288972556	0.635	0.376	1.66501E-31	C4_CD8_Teff
chr17-8118072-8120871	2.66079E-36	0.295847523	0.622	0.352	1.76205E-31	C4_CD8_Teff
chr17-3910962-3911726	2.77984E-36	0.402655191	0.149	0.028	1.84089E-31	C4_CD8_Teff
chr2-169107254-169108739	3.04723E-36	0.387061974	0.311	0.114	2.01797E-31	C4_CD8_Teff
chr16-2204857-2206164	3.08149E-36	0.317442436	0.541	0.271	2.04065E-31	C4_CD8_Teff

chr19-41886761-41888221	4.08134E-36	0.292049681	0.619	0.35	2.70278E-31	C4_CD8_Teff
chr12-4808668-4809383	4.83991E-36	0.39490639	0.136	0.021	3.20513E-31	C4_CD8_Teff
chr18-59902107-59902595	4.90661E-36	0.38893363	0.298	0.106	3.2493E-31	C4_CD8_Teff
chr11-575175-577019	4.94306E-36	0.278954671	0.655	0.391	3.27344E-31	C4_CD8_Teff
chr8-27394817-27395510	5.04527E-36	0.37004813	0.387	0.155	3.34113E-31	C4_CD8_Teff
chr20-64285522-64287037	5.27449E-36	0.401714591	0.249	0.073	3.49293E-31	C4_CD8_Teff
chr16-30956779-30958447	5.41582E-36	0.281491975	0.649	0.371	3.58652E-31	C4_CD8_Teff
chr2-201257290-201258754	5.45782E-36	0.277736602	0.657	0.397	3.61433E-31	C4_CD8_Teff
chr6-130218012-130218662	5.66438E-36	0.37980113	0.122	0.019	3.75112E-31	C4_CD8_Teff
chr2-203719655-203720451	5.86386E-36	0.400995616	0.155	0.031	3.88322E-31	C4_CD8_Teff
chr22-44213017-44214167	7.12817E-36	0.387926964	0.123	0.017	4.72049E-31	C4_CD8_Teff
chr20-35454274-35455884	7.4998E-36	0.304134791	0.58	0.309	4.96659E-31	C4_CD8_Teff
chr19-45667368-45669445	7.56334E-36	0.31810094	0.53	0.283	5.00867E-31	C4_CD8_Teff
chr1-110879494-110881374	8.85428E-36	0.26212564	0.696	0.444	5.86357E-31	C4_CD8_Teff
chr1-161020499-161021600	9.08297E-36	0.34596873	0.432	0.193	6.01502E-31	C4_CD8_Teff
chr17-2049509-2050893	1.08552E-35	0.303914221	0.547	0.3	7.18862E-31	C4_CD8_Teff
chr4-38666967-38667483	5.495E-26	0.468684908	0.271	0.086	3.63896E-21	C3_CD8_Tem
chr7-1458812-1460149	1.09429E-35	0.288537625	0.64	0.355	7.2467E-31	C4_CD8_Teff
chr19-53196418-53197426	1.15905E-35	0.358191138	0.392	0.166	7.6756E-31	C4_CD8_Teff
chr11-65637708-65640465	3.17295E-21	0.309727417	0.503	0.34	2.10122E-16	C3_CD8_Tem
chr1-8396776-8397663	1.4033E-35	0.398877198	0.163	0.035	9.2931E-31	C4_CD8_Teff
chr17-2053611-2055340	1.76813E-35	0.307014709	0.557	0.298	1.17091E-30	C4_CD8_Teff
chr2-29010912-29012188	1.87784E-35	0.294328352	0.611	0.348	1.24356E-30	C4_CD8_Teff
chr16-68756090-68757132	2.01633E-35	0.39342914	0.27	0.092	1.33528E-30	C4_CD8_Teff
chr17-31487350-31488871	2.06152E-35	0.269806134	0.673	0.414	1.3652E-30	C4_CD8_Teff
chr3-14424240-14425081	2.21091E-35	0.39283003	0.234	0.073	1.46413E-30	C4_CD8_Teff
chr19-35931035-35931937	2.29194E-35	0.263488251	0.715	0.432	1.51779E-30	C4_CD8_Teff

chr19-2607565-2608631	2.4452E-35	0.329441374	0.468	0.234	1.61929E-30	C4_CD8_Teff
chr5-181216913-181218442	2.72684E-35	0.359365922	0.383	0.164	1.80579E-30	C4_CD8_Teff
chr14-106275951-106277107	4.57035E-35	0.396589661	0.162	0.035	3.02662E-30	C4_CD8_Teff
chr19-10516551-10517154	5.72155E-18	0.405856536	0.133	0.028	3.78898E-13	C3_CD8_Tem
chr5-155768313-155768661	5.93327E-13	0.317786667	0.104	0.028	3.92919E-08	C3_CD8_Tem
chr13-30371414-30372280	5.30763E-35	0.325299813	0.487	0.25	3.51487E-30	C4_CD8_Teff
chr11-57546909-57548228	5.42914E-35	0.399131303	0.169	0.039	3.59534E-30	C4_CD8_Teff
chr18-78642705-78643359	5.77418E-35	0.372953087	0.091	0.008	3.82384E-30	C4_CD8_Teff
chr3-161310358-161311501	5.86008E-35	0.393490019	0.211	0.058	3.88072E-30	C4_CD8_Teff
chr11-33777769-33778167	7.07304E-35	0.349844219	0.085	0.007	4.68398E-30	C4_CD8_Teff
chr16-85027306-85029738	7.23725E-35	0.266057927	0.686	0.424	4.79272E-30	C4_CD8_Teff
chr19-40143503-40144657	2.80989E-09	0.254221062	0.138	0.064	0.00018608	C3_CD8_Tem
chr10-110764818-110765711	8.41351E-35	0.391491066	0.108	0.013	5.57168E-30	C4_CD8_Teff
chr10-12041807-12043633	8.51202E-35	0.27156239	0.674	0.399	5.63692E-30	C4_CD8_Teff
chr1-205284427-205285142	9.4007E-35	0.371912685	0.322	0.121	6.22542E-30	C4_CD8_Teff
chr4-9151875-9153538	2.32207E-16	0.261695621	0.602	0.455	1.53774E-11	C3_CD8_Tem
chr21-46423897-46425970	7.20188E-30	0.41552081	0.414	0.213	4.7693E-25	C3_CD8_Tem
chr9-128275116-128276616	1.26484E-34	0.262640521	0.681	0.42	8.37613E-30	C4_CD8_Teff
chr2-38587698-38588105	1.30047E-34	0.388441449	0.11	0.014	8.6121E-30	C4_CD8_Teff
chr17-8246766-8249446	1.38051E-34	0.258290873	0.696	0.443	9.14215E-30	C4_CD8_Teff
chr3-14142106-14143151	1.40929E-34	0.357084835	0.392	0.169	9.33272E-30	C4_CD8_Teff
chr16-11562843-11563505	1.79236E-21	0.421169253	0.156	0.039	1.18695E-16	C3_CD8_Tem
chr16-3157478-3159307	1.5587E-34	0.313310319	0.516	0.269	1.03222E-29	C4_CD8_Teff
chr14-106163482-106164277	2.02171E-34	0.370807413	0.099	0.011	1.33883E-29	C4_CD8_Teff
chr13-99289490-99290186	2.30072E-34	0.375880257	0.296	0.11	1.5236E-29	C4_CD8_Teff
chr21-42227509-42228757	2.33425E-34	0.253178301	0.723	0.458	1.54581E-29	C4_CD8_Teff
chr2-64880665-64881690	4.76594E-29	0.518748479	0.198	0.044	3.15615E-24	C3_CD8_Tem

chr16-27235027-27235999	2.68228E-34	0.278940312	0.619	0.368	1.77629E-29	C4_CD8_Teff
chr6-137233676-137234822	2.82779E-34	0.392449016	0.156	0.033	1.87265E-29	C4_CD8_Teff
chr16-75617604-75617832	3.15204E-34	0.350304919	0.08	0.006	2.08737E-29	C4_CD8_Teff
chr17-63842103-63843710	3.22646E-34	0.258560226	0.707	0.43	2.13666E-29	C4_CD8_Teff
chr14-99259303-99259653	3.2888E-34	0.382680741	0.293	0.103	2.17794E-29	C4_CD8_Teff
chr11-406564-407932	3.5122E-18	0.303140596	0.49	0.331	2.32588E-13	C3_CD8_Tem
chr10-100535269-100536296	3.88415E-34	0.323653062	0.496	0.242	2.5722E-29	C4_CD8_Teff
chr14-99248859-99249767	4.19689E-34	0.377168274	0.281	0.101	2.7793E-29	C4_CD8_Teff
chr19-10963238-10963842	4.62319E-34	0.386673401	0.248	0.082	3.06161E-29	C4_CD8_Teff
chr19-7690692-7691941	3.4602E-19	0.36944322	0.195	0.069	2.29144E-14	C3_CD8_Tem
chr2-8551423-8552687	4.82088E-34	0.377641981	0.311	0.122	3.19253E-29	C4_CD8_Teff
chr3-98557230-98557783	4.91614E-34	0.389835712	0.24	0.077	3.25562E-29	C4_CD8_Teff
chr20-33369167-33370781	3.93941E-13	0.269654678	0.339	0.188	2.6088E-08	C3_CD8_Tem
chr19-7196940-7198555	2.07228E-13	0.283435541	0.401	0.261	1.37232E-08	C1_CD8_Tres
chr11-121464061-121464975	3.91504E-26	0.463748041	0.455	0.218	2.59266E-21	C1_CD8_Tres
chr13-50427239-50427876	2.95627E-09	0.257727087	0.104	0.038	0.000195773	C3_CD8_Tem
chr8-66774355-66775798	5.699E-34	0.26717267	0.696	0.406	3.77405E-29	C4_CD8_Teff
chr6-152634627-152635124	4.00852E-27	0.480072749	0.232	0.065	2.65456E-22	C3_CD8_Tem
chr4-89929326-89930097	6.43152E-34	0.383723345	0.122	0.019	4.25914E-29	C4_CD8_Teff
chr19-48169220-48170972	6.47216E-34	0.256528847	0.693	0.433	4.28606E-29	C4_CD8_Teff
chr16-4415461-4417126	7.00117E-34	0.261410115	0.688	0.423	4.63639E-29	C4_CD8_Teff
chr19-19039612-19040323	8.95742E-12	0.284085508	0.104	0.037	5.93187E-07	C3_CD8_Tem
chr22-39919971-39921390	7.89862E-34	0.328879564	0.438	0.217	5.2307E-29	C4_CD8_Teff
chr8-70217016-70218279	8.10569E-34	0.387850704	0.23	0.072	5.36783E-29	C4_CD8_Teff
chr19-47348669-47349678	8.81136E-34	0.293528515	0.581	0.32	5.83515E-29	C4_CD8_Teff
chr20-51410666-51411119	1.00345E-33	0.387197123	0.139	0.025	6.64517E-29	C4_CD8_Teff
chr7-123533959-123535436	1.09153E-33	0.279665315	0.622	0.359	7.22841E-29	C4_CD8_Teff

chr1-18955298-18957153	1.21202E-33	0.260790603	0.685	0.419	8.02634E-29	C4_CD8_Teff
chr2-208100304-208101691	1.2406E-33	0.383495143	0.133	0.023	8.21563E-29	C4_CD8_Teff
chr9-127954965-127956136	1.35699E-33	0.355154043	0.355	0.15	8.98638E-29	C4_CD8_Teff
chr9-137604700-137606259	1.36339E-33	0.257039596	0.696	0.423	9.02875E-29	C4_CD8_Teff
chr6-130222738-130223910	1.37309E-33	0.373479383	0.315	0.113	9.093E-29	C4_CD8_Teff
chr10-29658562-29659853	1.44412E-33	0.344070109	0.418	0.181	9.56341E-29	C4_CD8_Teff
chr17-7851097-7852081	1.55313E-33	0.328707538	0.465	0.221	1.02853E-28	C4_CD8_Teff
chr6-4139347-4139677	1.58867E-33	0.373864367	0.114	0.016	1.05207E-28	C4_CD8_Teff
chr9-97906114-97907245	1.70146E-33	0.377476365	0.259	0.087	1.12676E-28	C4_CD8_Teff
chr17-8898595-8899604	1.42218E-11	0.259653918	0.329	0.218	9.41813E-07	C1_CD8_Tres
chr10-17001358-17003528	1.77452E-33	0.315814907	0.506	0.254	1.17514E-28	C4_CD8_Teff
chr17-35519463-35520065	2.00776E-33	0.395555029	0.186	0.048	1.3296E-28	C4_CD8_Teff
chr4-112636423-112637651	2.20422E-33	0.252446054	0.724	0.452	1.4597E-28	C4_CD8_Teff
chrX-79169280-79170245	2.25996E-33	0.385916359	0.174	0.043	1.49661E-28	C4_CD8_Teff
chr11-126210929-126212113	2.32534E-33	0.297176691	0.562	0.297	1.53991E-28	C4_CD8_Teff
chrX-107449624-107451395	5.91649E-12	0.267019307	0.232	0.115	3.91808E-07	C3_CD8_Tem
chr22-27853335-27853831	2.61507E-33	0.342216938	0.077	0.005	1.73178E-28	C4_CD8_Teff
chr3-47037543-47038612	2.68776E-33	0.275521564	0.644	0.375	1.77991E-28	C4_CD8_Teff
chr21-45224281-45224986	3.70826E-33	0.331162278	0.429	0.205	2.45572E-28	C4_CD8_Teff
chr9-85940253-85941881	3.72753E-33	0.258089584	0.693	0.422	2.46848E-28	C4_CD8_Teff
chr1-53600208-53600946	8.50866E-13	0.312013543	0.154	0.058	5.63469E-08	C3_CD8_Tem
chr2-135953947-135954581	4.18068E-33	0.380842346	0.143	0.03	2.76857E-28	C4_CD8_Teff
chr3-46538435-46539588	4.39103E-33	0.28511103	0.593	0.341	2.90787E-28	C4_CD8_Teff
chr1-91803412-91805154	5.10406E-33	0.264854078	0.671	0.404	3.38006E-28	C4_CD8_Teff
chr19-2268991-2269926	5.10493E-33	0.29160444	0.576	0.319	3.38064E-28	C4_CD8_Teff
chr1-46246610-46247912	6.89016E-33	0.28978111	0.581	0.316	4.56287E-28	C4_CD8_Teff
chr19-19191544-19193186	7.10119E-33	0.255320852	0.695	0.427	4.70262E-28	C4_CD8_Teff

chr19-42216635-42218121	7.27519E-33	0.256163025	0.689	0.427	4.81785E-28	C4_CD8_Teff
chr1-9425784-9426833	7.55582E-33	0.372155332	0.279	0.1	5.00369E-28	C4_CD8_Teff
chr3-141369867-141370534	7.73973E-33	0.376429302	0.294	0.105	5.12548E-28	C4_CD8_Teff
chr2-96304672-96306236	8.15303E-33	0.271163215	0.63	0.381	5.39918E-28	C4_CD8_Teff
chr19-46600304-46601499	8.39769E-33	0.259940899	0.674	0.409	5.5612E-28	C4_CD8_Teff
chr19-53431315-53432525	8.52338E-33	0.305420888	0.516	0.271	5.64444E-28	C4_CD8_Teff
chr16-84603038-84603523	9.52771E-33	0.383152122	0.235	0.077	6.30954E-28	C4_CD8_Teff
chr10-89332036-89334371	9.92563E-33	0.308323071	0.52	0.272	6.57305E-28	C4_CD8_Teff
chr4-6640018-6641968	9.96691E-33	0.285229188	0.58	0.33	6.60038E-28	C4_CD8_Teff
chr16-57070100-57072258	5.51011E-22	0.382387073	0.315	0.145	3.64896E-17	C3_CD8_Tem
chr6-33343596-33345571	1.20912E-32	0.309769931	0.513	0.264	8.00715E-28	C4_CD8_Teff
chr14-100067927-100069030	1.22226E-32	0.260554797	0.674	0.411	8.09416E-28	C4_CD8_Teff
chr1-145464778-145465831	6.43739E-12	0.319342787	0.15	0.053	4.26303E-07	C1_CD8_Tres
chr2-85862138-85862826	1.28968E-32	0.367079998	0.311	0.125	8.54067E-28	C4_CD8_Teff
chr2-136212448-136213283	1.29531E-32	0.379962369	0.206	0.058	8.57792E-28	C4_CD8_Teff
chr17-8958263-8958907	1.30862E-32	0.37557075	0.27	0.093	8.66604E-28	C4_CD8_Teff
chr3-183377312-183378294	1.3213E-32	0.371070683	0.119	0.017	8.75005E-28	C4_CD8_Teff
chr8-143245590-143247447	1.49007E-32	0.254593474	0.683	0.421	9.8677E-28	C4_CD8_Teff
chr19-46633689-46634603	1.2449E-27	0.443232722	0.404	0.188	8.24412E-23	C1_CD8_Tres
chr9-33473245-33474252	1.51404E-32	0.282623997	0.601	0.34	1.00264E-27	C4_CD8_Teff
chr16-88469556-88470538	1.52543E-32	0.377816198	0.171	0.041	1.01018E-27	C4_CD8_Teff
chr12-8989331-8989800	1.5574E-32	0.36705	0.127	0.024	1.03135E-27	C4_CD8_Teff
chr1-161389340-161390723	1.74847E-32	0.284831739	0.584	0.317	1.15789E-27	C4_CD8_Teff
chr7-105848750-105849993	6.47151E-12	0.25533245	0.393	0.246	4.28563E-07	C3_CD8_Tem
chr6-29845040-29845804	4.15097E-17	0.390361323	0.18	0.074	2.7489E-12	C1_CD8_Tres
chr5-61407928-61409110	2.02761E-32	0.299665625	0.542	0.289	1.34274E-27	C4_CD8_Teff
chr19-4638832-4639658	2.27006E-32	0.286295594	0.559	0.314	1.5033E-27	C4_CD8_Teff

chr8-144826609-144828010	2.34871E-32	0.252202732	0.703	0.422	1.55539E-27	C4_CD8_Teff
chr1-160705280-160706149	2.49701E-32	0.382050574	0.173	0.043	1.6536E-27	C4_CD8_Teff
chr10-84330139-84330644	2.52635E-32	0.325174105	0.455	0.22	1.67303E-27	C4_CD8_Teff
chr10-47552472-47553609	2.76343E-32	0.367394664	0.127	0.02	1.83003E-27	C4_CD8_Teff
chr12-121786315-121786824	2.88061E-32	0.374372086	0.19	0.051	1.90762E-27	C4_CD8_Teff
chr1-226675790-226676289	3.16601E-32	0.378610432	0.189	0.05	2.09663E-27	C4_CD8_Teff
chr11-67292914-67293862	3.16757E-32	0.35096789	0.348	0.144	2.09766E-27	C4_CD8_Teff
chr19-49589548-49591889	3.22846E-32	0.264204352	0.636	0.392	2.13798E-27	C4_CD8_Teff
chr19-41363195-41364458	3.25149E-32	0.266907299	0.63	0.385	2.15323E-27	C4_CD8_Teff
chr4-121776309-121777398	3.31733E-32	0.38746558	0.15	0.033	2.19683E-27	C4_CD8_Teff
chr3-149003738-149004386	3.32654E-32	0.375313083	0.206	0.058	2.20294E-27	C4_CD8_Teff
chr1-24915001-24915963	1.84425E-21	0.393070554	0.253	0.098	1.22132E-16	C3_CD8_Tem
chr2-234463647-234464682	3.60535E-32	0.346006324	0.358	0.155	2.38757E-27	C4_CD8_Teff
chr17-143682-144224	3.66612E-32	0.36115489	0.1	0.012	2.42782E-27	C4_CD8_Teff
chr16-2846458-2847278	3.84891E-32	0.376358358	0.16	0.036	2.54887E-27	C4_CD8_Teff
chr6-33317159-33318236	3.93343E-32	0.275829875	0.614	0.358	2.60484E-27	C4_CD8_Teff
chr11-122842844-122844041	4.31549E-32	0.317880358	0.474	0.244	2.85785E-27	C4_CD8_Teff
chr12-49017824-49019215	4.38164E-32	0.257041689	0.679	0.415	2.90165E-27	C4_CD8_Teff
chr7-73154314-73155059	4.5855E-32	0.374177102	0.16	0.036	3.03666E-27	C4_CD8_Teff
chr6-29708374-29709366	7.25535E-11	0.253192804	0.308	0.21	4.80471E-06	C1_CD8_Tres
chr5-134370338-134372166	6.20358E-32	0.260989666	0.66	0.403	4.1082E-27	C4_CD8_Teff
chr4-2936723-2937154	6.3738E-32	0.348205169	0.345	0.149	4.22092E-27	C4_CD8_Teff
chr1-9626753-9627649	6.47246E-32	0.308113046	0.501	0.259	4.28626E-27	C4_CD8_Teff
chr21-8219415-8220192	7.58377E-32	0.35544119	0.097	0.011	5.0222E-27	C4_CD8_Teff
chr5-107485956-107486918	1.44519E-15	0.34032355	0.237	0.101	9.57046E-11	C3_CD8_Tem
chr6-33271096-33272222	9.64902E-32	0.285806525	0.578	0.322	6.38987E-27	C4_CD8_Teff
chr12-118371832-118373287	1.00747E-31	0.256504997	0.663	0.403	6.67175E-27	C4_CD8_Teff

chr12-47211956-47212706	1.04862E-31	0.348392856	0.333	0.143	6.9443E-27	C4_CD8_Teff
chr20-5056444-5057393	1.11831E-31	0.34298386	0.375	0.165	7.40581E-27	C4_CD8_Teff
chr22-23137252-23138634	6.64212E-14	0.29864429	0.258	0.14	4.39861E-09	C3_CD8_Tem
chr5-159099653-159100745	1.19357E-31	0.375405329	0.208	0.062	7.90416E-27	C4_CD8_Teff
chr17-57849359-57850657	1.2356E-31	0.279964355	0.592	0.338	8.18253E-27	C4_CD8_Teff
chr16-31179069-31180889	1.24067E-31	0.269471317	0.62	0.364	8.21608E-27	C4_CD8_Teff
chr7-148687-150085	1.28709E-31	0.250671772	0.684	0.432	8.5235E-27	C4_CD8_Teff
chr4-38654965-38655565	1.99331E-20	0.411629096	0.154	0.04	1.32003E-15	C3_CD8_Tem
chr16-88781069-88783155	2.02645E-31	0.296458631	0.529	0.283	1.34198E-26	C4_CD8_Teff
chr12-132488622-132490598	2.1098E-31	0.274812638	0.607	0.344	1.39717E-26	C4_CD8_Teff
chr16-88856236-88858015	2.33397E-31	0.287981381	0.538	0.296	1.54562E-26	C4_CD8_Teff
chr1-39394385-39394975	2.37981E-31	0.369928278	0.165	0.04	1.57598E-26	C4_CD8_Teff
chr16-75616348-75616998	2.49978E-31	0.37579756	0.149	0.033	1.65543E-26	C4_CD8_Teff
chr19-51272220-51273178	2.74138E-31	0.367083352	0.121	0.019	1.81543E-26	C4_CD8_Teff
chr22-22721908-22722289	3.24313E-31	0.367473868	0.121	0.021	2.1477E-26	C4_CD8_Teff
chr2-234425673-234426466	1.49522E-12	0.281259711	0.234	0.126	9.90177E-08	C1_CD8_Tres
chr1-156113857-156115386	3.43464E-31	0.255283141	0.657	0.402	2.27452E-26	C4_CD8_Teff
chr2-37599387-37600389	3.77386E-31	0.338534474	0.378	0.169	2.49916E-26	C4_CD8_Teff
chr7-150665070-150665525	3.88304E-31	0.3743735	0.16	0.036	2.57147E-26	C4_CD8_Teff
chr12-54358168-54359838	4.0421E-31	0.265047074	0.64	0.378	2.6768E-26	C4_CD8_Teff
chr14-24206945-24207575	4.06965E-31	0.362889147	0.103	0.014	2.69505E-26	C4_CD8_Teff
chr12-64683968-64685368	5.64793E-28	0.522039815	0.275	0.081	3.74023E-23	C1_CD8_Tres
chr22-40240516-40241795	4.3765E-31	0.342550325	0.35	0.15	2.89825E-26	C4_CD8_Teff
chr20-38961596-38962829	4.46667E-31	0.277999716	0.593	0.335	2.95796E-26	C4_CD8_Teff
chr1-8511474-8512048	4.54024E-31	0.335412382	0.075	0.006	3.00669E-26	C4_CD8_Teff
chr11-36420374-36421610	4.62567E-31	0.30317024	0.491	0.269	3.06326E-26	C4_CD8_Teff
chr10-22634033-22634531	2.54407E-13	0.319212681	0.156	0.064	1.68476E-08	C3_CD8_Tem

chr13-29934808-29936565	4.65119E-31	0.293981821	0.526	0.292	3.08016E-26	C4_CD8_Teff
chr6-34235273-34237756	4.65441E-31	0.262609282	0.617	0.378	3.08229E-26	C4_CD8_Teff
chr1-52403451-52405121	4.68587E-31	0.251986518	0.663	0.413	3.10312E-26	C4_CD8_Teff
chr20-38870531-38871035	4.89977E-31	0.373221517	0.206	0.061	3.24477E-26	C4_CD8_Teff
chr1-46603570-46604567	4.92787E-31	0.304917807	0.477	0.248	3.26338E-26	C4_CD8_Teff
chr3-98563422-98564975	5.11227E-31	0.30738207	0.471	0.255	3.3855E-26	C4_CD8_Teff
chr1-156214299-156214702	5.29481E-31	0.368096392	0.224	0.074	3.50638E-26	C4_CD8_Teff
chr14-106510787-106511672	6.77978E-31	0.344641279	0.332	0.145	4.48977E-26	C4_CD8_Teff
chr14-77039705-77041237	6.88044E-31	0.273997997	0.591	0.349	4.55643E-26	C4_CD8_Teff
chr16-67555402-67556295	7.0409E-31	0.361043646	0.294	0.114	4.66269E-26	C4_CD8_Teff
chr9-20358104-20358641	7.70051E-31	0.37197886	0.134	0.027	5.09951E-26	C4_CD8_Teff
chr3-101520965-101521766	7.91852E-31	0.36887557	0.162	0.04	5.24388E-26	C4_CD8_Teff
chr16-3170887-3172150	8.31079E-31	0.365808483	0.244	0.081	5.50366E-26	C4_CD8_Teff
chr19-3984616-3985631	9.21613E-31	0.309832036	0.459	0.229	6.1032E-26	C4_CD8_Teff
chr16-391649-392432	5.87686E-18	0.32973607	0.375	0.209	3.89183E-13	C3_CD8_Tem
chr5-142786694-142787240	2.61447E-14	0.317866643	0.237	0.115	1.73138E-09	C3_CD8_Tem
chr9-129868456-129869599	1.37391E-30	0.283093497	0.58	0.317	9.09846E-26	C4_CD8_Teff
chr1-21658983-21659718	1.43605E-30	0.365030031	0.238	0.077	9.50995E-26	C4_CD8_Teff
chr17-36206421-36206691	1.58985E-30	0.3092991	0.062	0.002	1.05285E-25	C4_CD8_Teff
chr3-46958859-46959827	1.61784E-30	0.325736956	0.406	0.201	1.07138E-25	C4_CD8_Teff
chr11-72736269-72737114	1.72923E-30	0.331651045	0.38	0.172	1.14515E-25	C4_CD8_Teff
chr19-12734198-12734993	1.83567E-30	0.315856906	0.443	0.214	1.21563E-25	C4_CD8_Teff
chr2-203763279-203764214	1.97481E-30	0.370136594	0.18	0.048	1.30778E-25	C4_CD8_Teff
chr22-30326189-30327456	1.98015E-30	0.283376318	0.56	0.301	1.31132E-25	C4_CD8_Teff
chr19-41947056-41947370	2.09125E-30	0.365014397	0.14	0.029	1.38489E-25	C4_CD8_Teff
chr1-228136881-228137236	2.11323E-30	0.365641595	0.132	0.026	1.39944E-25	C4_CD8_Teff
chr19-16329007-16329426	8.97058E-14	0.328113873	0.208	0.093	5.94059E-09	C3_CD8_Tem

chr16-89785283-89785996	2.32939E-30	0.367833205	0.152	0.038	1.54259E-25	C4_CD8_Teff
chrX-156000197-156000775	2.59681E-30	0.365512715	0.157	0.04	1.71969E-25	C4_CD8_Teff
chr1-198120924-198121694	2.70693E-30	0.347913079	0.095	0.011	1.79261E-25	C4_CD8_Teff
chr1-84156143-84156635	2.86735E-30	0.361897118	0.139	0.03	1.89885E-25	C4_CD8_Teff
chr13-99370684-99371173	3.00442E-30	0.340349749	0.081	0.008	1.98962E-25	C4_CD8_Teff
chr11-59549908-59551377	3.5782E-30	0.335611956	0.361	0.16	2.36959E-25	C4_CD8_Teff
chr19-10111953-10113270	2.24177E-14	0.26468045	0.406	0.277	1.48457E-09	C3_CD8_Tem
chr16-68752400-68753017	3.84952E-30	0.347287045	0.333	0.139	2.54927E-25	C4_CD8_Teff
chr14-74298870-74299288	4.18337E-30	0.351932642	0.114	0.02	2.77035E-25	C4_CD8_Teff
chr8-28621762-28623340	4.52594E-30	0.281920206	0.553	0.297	2.99721E-25	C4_CD8_Teff
chr10-109914741-109915379	4.65008E-30	0.356056609	0.271	0.1	3.07942E-25	C4_CD8_Teff
chr17-68180326-68181596	4.88992E-30	0.34252287	0.337	0.143	3.23825E-25	C4_CD8_Teff
chr19-5977392-5978573	4.99806E-30	0.267715749	0.604	0.349	3.30987E-25	C4_CD8_Teff
chr11-65545804-65547035	5.23719E-30	0.284734765	0.53	0.287	3.46823E-25	C4_CD8_Teff
chr12-121839091-121840027	5.48568E-30	0.264262088	0.619	0.363	3.63278E-25	C4_CD8_Teff
chr9-128249691-128250974	6.00246E-30	0.27476703	0.576	0.325	3.97501E-25	C4_CD8_Teff
chr12-120265007-120266070	2.94843E-14	0.279230768	0.268	0.147	1.95254E-09	C3_CD8_Tem
chr19-47229277-47231760	4.20534E-21	0.271597229	0.589	0.433	2.7849E-16	C3_CD8_Tem
chr5-172147762-172148141	6.9664E-30	0.350155731	0.105	0.016	4.61336E-25	C4_CD8_Teff
chr3-14402010-14403511	7.06643E-30	0.261220308	0.638	0.373	4.6796E-25	C4_CD8_Teff
chr6-159106451-159107921	7.20522E-30	0.272419657	0.593	0.343	4.77151E-25	C4_CD8_Teff
chr19-991348-991997	2.61115E-11	0.296467807	0.182	0.08	1.72918E-06	C3_CD8_Tem
chr11-45092887-45093822	7.47706E-30	0.344422917	0.3	0.122	4.95154E-25	C4_CD8_Teff
chr10-133334635-133337342	7.84631E-30	0.300175212	0.479	0.246	5.19606E-25	C4_CD8_Teff
chr19-1081870-1082759	5.93138E-20	0.419958072	0.311	0.138	3.92794E-15	C1_CD8_Tres
chr7-158804277-158804969	3.02388E-16	0.337688804	0.141	0.043	2.00251E-11	C3_CD8_Tem
chr9-92664259-92665110	9.07467E-30	0.36789911	0.168	0.044	6.00952E-25	C4_CD8_Teff

chr11-316938-317527	9.17875E-30	0.359260013	0.172	0.046	6.07844E-25	C4_CD8_Teff
chr12-121784424-121784732	9.34135E-30	0.365461703	0.153	0.037	6.18613E-25	C4_CD8_Teff
chr19-10418914-10420053	9.61825E-30	0.337681698	0.34	0.146	6.36949E-25	C4_CD8_Teff
chr1-198167012-198168407	9.94009E-30	0.349685388	0.296	0.123	6.58262E-25	C4_CD8_Teff
chr19-10517383-10518508	2.86577E-40	0.588369848	0.273	0.063	1.8978E-35	C3_CD8_Tem
chr10-129466266-129467794	1.0743E-29	0.288571228	0.523	0.279	7.11431E-25	C4_CD8_Teff
chr17-43359949-43362035	2.17594E-18	0.317878477	0.583	0.388	1.44097E-13	C3_CD8_Tem
chrX-107451771-107452071	1.11576E-29	0.356192939	0.105	0.015	7.38892E-25	C4_CD8_Teff
chr1-25035751-25036614	2.28245E-18	0.382485486	0.271	0.115	1.51151E-13	C3_CD8_Tem
chr1-158969126-158970202	1.33757E-29	0.361754219	0.172	0.045	8.85781E-25	C4_CD8_Teff
chr14-105993495-105994314	5.46487E-11	0.277030207	0.156	0.068	3.619E-06	C3_CD8_Tem
chr9-128159045-128160928	1.36816E-29	0.274609958	0.557	0.318	9.06034E-25	C4_CD8_Teff
chr19-39403386-39404168	1.41071E-29	0.294524725	0.485	0.26	9.34215E-25	C4_CD8_Teff
chr19-38647055-38647950	1.46757E-29	0.288389355	0.513	0.275	9.71869E-25	C4_CD8_Teff
chr7-157306434-157306782	1.62254E-29	0.346981628	0.112	0.019	1.07449E-24	C4_CD8_Teff
chr6-129686918-129687735	1.66664E-29	0.350699897	0.263	0.096	1.1037E-24	C4_CD8_Teff
chr19-1382956-1384238	1.81307E-29	0.297995859	0.497	0.251	1.20067E-24	C4_CD8_Teff
chr21-44083156-44084366	1.92356E-29	0.32893488	0.378	0.171	1.27384E-24	C4_CD8_Teff
chr10-69179181-69181157	2.3026E-29	0.281528826	0.531	0.298	1.52485E-24	C4_CD8_Teff
chr11-3421815-3423505	2.66032E-29	0.257032472	0.611	0.373	1.76174E-24	C4_CD8_Teff
chr17-82583520-82584286	2.74177E-29	0.368763196	0.187	0.052	1.81568E-24	C4_CD8_Teff
chr3-43012965-43014368	1.42404E-20	0.408145229	0.201	0.071	9.43045E-16	C3_CD8_Tem
chr19-10960162-10961495	2.89406E-29	0.281339481	0.531	0.295	1.91653E-24	C4_CD8_Teff
chr16-88619267-88620766	3.04628E-29	0.256141907	0.638	0.384	2.01734E-24	C4_CD8_Teff
chr1-2204389-2205690	3.64338E-29	0.295246114	0.48	0.243	2.41275E-24	C4_CD8_Teff
chr3-28243428-28244017	3.78522E-29	0.360523107	0.182	0.054	2.50668E-24	C4_CD8_Teff
chr17-488956-490300	3.80502E-29	0.349864391	0.111	0.019	2.5198E-24	C4_CD8_Teff

chr5-179811037-179811509	3.86514E-29	0.357863292	0.152	0.038	2.55961E-24	C4_CD8_Teff
chr1-181104486-181105943	3.89094E-29	0.273980763	0.559	0.317	2.5767E-24	C4_CD8_Teff
chr1-161621077-161622355	3.99257E-29	0.35847633	0.245	0.086	2.644E-24	C4_CD8_Teff
chr7-142535063-142535860	6.90644E-21	0.466574411	0.189	0.047	4.57365E-16	C1_CD8_Tres
chr5-181206641-181207910	4.43247E-29	0.346760629	0.285	0.113	2.93532E-24	C4_CD8_Teff
chr10-22626753-22627429	4.43305E-29	0.363003626	0.188	0.057	2.9357E-24	C4_CD8_Teff
chr10-123241153-123242233	4.81486E-29	0.357288496	0.211	0.071	3.18854E-24	C4_CD8_Teff
chr1-26692192-26693265	4.84373E-29	0.276592879	0.553	0.311	3.20766E-24	C4_CD8_Teff
chr21-42237622-42238947	5.28833E-29	0.300620034	0.466	0.243	3.50209E-24	C4_CD8_Teff
chr8-23230029-23231334	5.64042E-29	0.317803737	0.391	0.188	3.73526E-24	C4_CD8_Teff
chr1-167373983-167374849	5.91121E-29	0.365395087	0.194	0.058	3.91458E-24	C4_CD8_Teff
chr6-34486126-34486463	5.95769E-29	0.315787006	0.07	0.006	3.94536E-24	C4_CD8_Teff
chr9-96856664-96857311	6.03804E-29	0.354293352	0.267	0.1	3.99857E-24	C4_CD8_Teff
chr22-43085813-43086551	6.72752E-29	0.354197352	0.255	0.088	4.45516E-24	C4_CD8_Teff
chr17-32349453-32351007	7.31513E-29	0.285129763	0.519	0.282	4.8443E-24	C4_CD8_Teff
chr14-58198061-58198458	8.25658E-29	0.337840519	0.098	0.014	5.46776E-24	C4_CD8_Teff
chr1-1745564-1746898	8.8193E-29	0.342077413	0.315	0.128	5.8404E-24	C4_CD8_Teff
chr2-96531850-96532475	8.99346E-29	0.317841017	0.38	0.179	5.95574E-24	C4_CD8_Teff
chr16-15642364-15643748	9.79739E-29	0.251671584	0.648	0.385	6.48812E-24	C4_CD8_Teff
chr19-2620353-2621307	1.01661E-28	0.318526581	0.387	0.181	6.7323E-24	C4_CD8_Teff
chr17-58417073-58417900	1.09297E-28	0.334216034	0.333	0.149	7.23799E-24	C4_CD8_Teff
chr7-158704315-158705479	1.10091E-28	0.263548747	0.594	0.337	7.29057E-24	C4_CD8_Teff
chr8-70219352-70219826	1.12186E-28	0.353520051	0.135	0.03	7.42929E-24	C4_CD8_Teff
chr15-82680039-82681005	3.95897E-13	0.323836797	0.18	0.074	2.62175E-08	C1_CD8_Tres
chr20-36644017-36645047	1.22413E-28	0.318655688	0.38	0.182	8.10654E-24	C4_CD8_Teff
chr2-101328201-101329516	1.25322E-28	0.340782614	0.285	0.114	8.29917E-24	C4_CD8_Teff
chr11-65385266-65385954	1.2972E-28	0.361530147	0.158	0.04	8.59044E-24	C4_CD8_Teff

chr19-52569637-52570843	1.41881E-28	0.292459821	0.503	0.26	9.3958E-24	C4_CD8_Teff
chr17-39152269-39154624	1.48015E-28	0.262864178	0.584	0.346	9.80198E-24	C4_CD8_Teff
chr11-78202511-78203649	1.51831E-28	0.325046782	0.35	0.161	1.00547E-23	C4_CD8_Teff
chr19-2223703-2224311	1.71527E-28	0.368868684	0.143	0.034	1.1359E-23	C4_CD8_Teff
chr17-40528673-40529258	1.73977E-28	0.356740029	0.118	0.023	1.15213E-23	C4_CD8_Teff
chr6-147670-148976	1.73999E-28	0.336989629	0.105	0.017	1.15228E-23	C4_CD8_Teff
chr22-39318663-39320111	1.78513E-28	0.258635104	0.603	0.362	1.18216E-23	C4_CD8_Teff
chr16-787754-788876	1.8304E-28	0.282922554	0.519	0.279	1.21214E-23	C4_CD8_Teff
chr6-34181890-34183339	1.87673E-28	0.314561009	0.398	0.192	1.24282E-23	C4_CD8_Teff
chr15-30625249-30626515	2.10593E-28	0.310299601	0.427	0.204	1.39461E-23	C4_CD8_Teff
chrX-386166-387600	2.18458E-28	0.258259477	0.605	0.353	1.44669E-23	C4_CD8_Teff
chr3-66303282-66304068	2.28455E-28	0.306729677	0.427	0.213	1.5129E-23	C4_CD8_Teff
chr10-12412623-12413670	2.34518E-28	0.353227906	0.22	0.074	1.55305E-23	C4_CD8_Teff
chr17-17236209-17237838	2.58069E-28	0.291612982	0.473	0.247	1.70901E-23	C4_CD8_Teff
chr17-3737410-3739041	2.63653E-28	0.31676836	0.393	0.186	1.74599E-23	C4_CD8_Teff
chr16-56190646-56191876	2.89631E-28	0.327075607	0.336	0.147	1.91802E-23	C4_CD8_Teff
chr17-38727667-38728769	2.97242E-28	0.33810671	0.312	0.129	1.96843E-23	C4_CD8_Teff
chr20-59041986-59044082	3.02794E-28	0.280642353	0.509	0.284	2.0052E-23	C4_CD8_Teff
chr10-29657489-29658251	3.11866E-28	0.348941184	0.155	0.038	2.06527E-23	C4_CD8_Teff
chr11-128550976-128552304	3.17268E-28	0.313892411	0.402	0.201	2.10104E-23	C4_CD8_Teff
chr2-136052421-136053254	3.64794E-28	0.353037306	0.221	0.071	2.41578E-23	C4_CD8_Teff
chr7-134669269-134670346	9.41334E-13	0.300033615	0.329	0.198	6.2338E-08	C1_CD8_Tres
chr20-34875858-34876868	2.47333E-11	0.250587487	0.314	0.205	1.63792E-06	C1_CD8_Tres
chr19-6663130-6664168	4.96807E-28	0.318759675	0.356	0.163	3.29001E-23	C4_CD8_Teff
chr5-109857035-109858029	5.43024E-28	0.351796103	0.172	0.05	3.59607E-23	C4_CD8_Teff
chr19-19451245-19452586	5.47018E-28	0.35111035	0.195	0.061	3.62252E-23	C4_CD8_Teff
chr11-62551598-62552120	9.98277E-14	0.269264989	0.329	0.204	6.61089E-09	C1_CD8_Tres

chr10-124640818-124641704	1.50115E-20	0.400769411	0.268	0.11	9.94103E-16	C3_CD8_Tem
chr19-7692254-7693184	2.19912E-16	0.319946202	0.19	0.082	1.45632E-11	C3_CD8_Tem
chr20-63894439-63896589	6.4367E-28	0.290954651	0.464	0.24	4.26258E-23	C4_CD8_Teff
chr5-159411886-159412863	6.65316E-28	0.353242695	0.185	0.058	4.40592E-23	C4_CD8_Teff
chr1-161545114-161545652	1.25632E-09	0.265446814	0.107	0.038	8.31974E-05	C3_CD8_Tem
chr11-85755749-85756689	7.51831E-28	0.349441515	0.249	0.091	4.97885E-23	C4_CD8_Teff
chr10-109912877-109913260	8.28422E-28	0.332404508	0.089	0.012	5.48606E-23	C4_CD8_Teff
chr20-51409496-51409819	8.95885E-28	0.33617396	0.101	0.016	5.93282E-23	C4_CD8_Teff
chr9-128007349-128008353	9.69403E-28	0.348809608	0.206	0.07	6.41968E-23	C4_CD8_Teff
chr5-107476616-107477202	2.43201E-22	0.418501467	0.312	0.127	1.61055E-17	C3_CD8_Tem
chr11-62855064-62856388	9.9654E-28	0.262591499	0.574	0.336	6.59939E-23	C4_CD8_Teff
chr18-3610164-3610564	1.06097E-27	0.303893411	0.065	0.005	7.02609E-23	C4_CD8_Teff
chr17-7687091-7688540	1.10687E-27	0.251875293	0.619	0.37	7.33001E-23	C4_CD8_Teff
chr8-141156903-141157559	8.11922E-27	0.474017868	0.297	0.106	5.37679E-22	C3_CD8_Tem
chr11-2384117-2384734	1.17769E-27	0.346594613	0.124	0.026	7.79899E-23	C4_CD8_Teff
chr12-108561930-108562843	1.31203E-27	0.28030945	0.491	0.266	8.68866E-23	C4_CD8_Teff
chr6-3160099-3160760	1.4697E-27	0.323160967	0.336	0.149	9.73277E-23	C4_CD8_Teff
chr22-23786301-23787763	1.48053E-27	0.278454585	0.486	0.266	9.80454E-23	C4_CD8_Teff
chr19-53394535-53395728	1.49366E-27	0.29149003	0.466	0.237	9.89146E-23	C4_CD8_Teff
chr21-44074114-44074777	1.51256E-27	0.303289127	0.069	0.007	1.00166E-22	C4_CD8_Teff
chr14-90263504-90264445	1.52633E-27	0.348666497	0.146	0.035	1.01078E-22	C4_CD8_Teff
chr19-52637379-52638779	1.64301E-27	0.27149541	0.525	0.296	1.08805E-22	C4_CD8_Teff
chr19-2784904-2785677	1.65138E-27	0.266607319	0.557	0.32	1.09359E-22	C4_CD8_Teff
chr9-20041689-20043404	1.84485E-27	0.354950154	0.202	0.064	1.22172E-22	C4_CD8_Teff
chr18-74995694-74996447	1.69042E-12	0.308982613	0.125	0.048	1.11945E-07	C3_CD8_Tem
chr19-12983457-12985835	2.66114E-41	0.579037719	0.372	0.128	1.76229E-36	C3_CD8_Tem
chr14-99234956-99235814	1.88971E-27	0.321639388	0.35	0.162	1.25143E-22	C4_CD8_Teff

chr2-144323881-144325643	1.99094E-27	0.253282125	0.601	0.373	1.31846E-22	C4_CD8_Teff
chr6-32127594-32128546	2.032E-27	0.254264256	0.605	0.365	1.34565E-22	C4_CD8_Teff
chr19-44770040-44770977	2.25756E-27	0.264624857	0.567	0.323	1.49503E-22	C4_CD8_Teff
chr6-10403687-10404781	2.7416E-18	0.350072052	0.276	0.124	1.81557E-13	C3_CD8_Tem
chr22-46790637-46791845	2.61502E-27	0.267281907	0.568	0.318	1.73175E-22	C4_CD8_Teff
chr17-37607696-37609856	2.65778E-27	0.255622758	0.596	0.354	1.76006E-22	C4_CD8_Teff
chr21-34800971-34801962	1.26674E-18	0.410065402	0.195	0.069	8.38874E-14	C3_CD8_Tem
chr6-30527513-30528524	2.89661E-27	0.341134721	0.234	0.087	1.91822E-22	C4_CD8_Teff
chr14-105477586-105477889	4.67717E-10	0.2557345	0.143	0.069	3.09736E-05	C3_CD8_Tem
chr1-32272574-32274188	2.9579E-27	0.318941049	0.355	0.164	1.95881E-22	C4_CD8_Teff
chr20-62117493-62118052	4.44719E-13	0.298266955	0.276	0.15	2.94507E-08	C3_CD8_Tem
chr4-40237932-40239209	3.57167E-27	0.320816294	0.351	0.152	2.36526E-22	C4_CD8_Teff
chr22-31661678-31662700	3.82186E-27	0.272037463	0.528	0.292	2.53095E-22	C4_CD8_Teff
chr19-55062481-55063422	3.85208E-27	0.270309898	0.542	0.301	2.55096E-22	C4_CD8_Teff
chr19-17932114-17933318	3.93562E-27	0.290669356	0.463	0.238	2.60628E-22	C4_CD8_Teff
chr19-16627219-16629268	3.95154E-27	0.252307754	0.602	0.351	2.61683E-22	C4_CD8_Teff
chr11-35337134-35337825	4.04238E-27	0.345243535	0.132	0.03	2.67698E-22	C4_CD8_Teff
chr12-121033338-121034295	4.25215E-27	0.268779912	0.543	0.317	2.8159E-22	C4_CD8_Teff
chr5-159341175-159342321	4.51142E-27	0.345482427	0.212	0.071	2.9876E-22	C4_CD8_Teff
chr2-68365906-68366543	1.91463E-10	0.277003638	0.096	0.027	1.26793E-05	C3_CD8_Tem
chr15-74842541-74843534	4.57483E-27	0.277519476	0.502	0.268	3.02959E-22	C4_CD8_Teff
chr17-40607524-40608588	4.7856E-27	0.293406229	0.455	0.237	3.16917E-22	C4_CD8_Teff
chr7-2631190-2632270	4.91225E-27	0.255485578	0.592	0.343	3.25304E-22	C4_CD8_Teff
chr2-197159068-197160249	4.92683E-27	0.325160066	0.318	0.132	3.2627E-22	C4_CD8_Teff
chr16-48611489-48612178	5.12316E-27	0.291922908	0.459	0.239	3.39271E-22	C4_CD8_Teff
chr19-3987417-3988063	5.91938E-27	0.336581688	0.259	0.099	3.91999E-22	C4_CD8_Teff
chr9-97411367-97412913	7.24525E-27	0.294085102	0.436	0.223	4.79802E-22	C4_CD8_Teff

chr2-189660885-189661794	7.44497E-27	0.253014438	0.598	0.358	4.93028E-22	C4_CD8_Teff
chr17-74513831-74514980	7.45597E-27	0.345730151	0.166	0.049	4.93756E-22	C4_CD8_Teff
chr2-203711099-203711782	5.76398E-12	0.333187756	0.207	0.095	3.81708E-07	C1_CD8_Tres
chr17-82417968-82418991	8.72838E-27	0.270870956	0.51	0.285	5.7802E-22	C4_CD8_Teff
chr3-46360946-46361664	8.95721E-27	0.333885249	0.122	0.026	5.93173E-22	C4_CD8_Teff
chr19-639027-640295	9.05111E-27	0.271510624	0.509	0.283	5.99392E-22	C4_CD8_Teff
chr19-47240737-47241527	9.78832E-27	0.317450483	0.341	0.149	6.48212E-22	C4_CD8_Teff
chr9-124414790-124415743	1.09399E-26	0.300053641	0.415	0.207	7.24472E-22	C4_CD8_Teff
chr14-106736894-106737778	1.10335E-26	0.322218744	0.087	0.013	7.3067E-22	C4_CD8_Teff
chr1-17053199-17054446	1.24968E-26	0.251808006	0.601	0.355	8.27574E-22	C4_CD8_Teff
chr20-63641165-63642420	1.32541E-26	0.260345397	0.57	0.325	8.77726E-22	C4_CD8_Teff
chr5-139709652-139711649	1.33792E-26	0.30707048	0.381	0.183	8.86012E-22	C4_CD8_Teff
chr6-26321822-26323062	1.39293E-26	0.340527685	0.252	0.098	9.2244E-22	C4_CD8_Teff
chr1-52139610-52140503	1.47412E-26	0.326080981	0.304	0.129	9.76204E-22	C4_CD8_Teff
chr3-115164316-115166011	1.54595E-26	0.293167101	0.439	0.235	1.02377E-21	C4_CD8_Teff
chr5-138352085-138353694	1.59923E-26	0.258719356	0.559	0.321	1.05906E-21	C4_CD8_Teff
chr1-66246411-66248118	1.62194E-26	0.307996559	0.385	0.197	1.0741E-21	C4_CD8_Teff
chr12-56467905-56468748	1.66356E-26	0.282562942	0.471	0.248	1.10166E-21	C4_CD8_Teff
chr17-67421166-67422670	1.70466E-26	0.280654293	0.483	0.259	1.12888E-21	C4_CD8_Teff
chr17-57982290-57982826	4.26788E-09	0.253128872	0.151	0.073	0.000282632	C3_CD8_Tem
chr10-30529126-30530120	1.89363E-26	0.308155721	0.375	0.183	1.25402E-21	C4_CD8_Teff
chr11-67420523-67421562	1.92851E-26	0.258080481	0.56	0.327	1.27712E-21	C4_CD8_Teff
chr3-101685986-101687094	1.97747E-26	0.261792478	0.552	0.316	1.30954E-21	C4_CD8_Teff
chr16-3149642-3151097	1.99946E-26	0.282864102	0.479	0.258	1.3241E-21	C4_CD8_Teff
chr19-4456356-4458153	2.11705E-26	0.287662791	0.446	0.233	1.40197E-21	C4_CD8_Teff
chr1-120913695-120914354	2.55359E-26	0.335081824	0.102	0.017	1.69106E-21	C4_CD8_Teff
chr19-35140757-35141944	2.84099E-20	0.364285923	0.44	0.255	1.88139E-15	C1_CD8_Tres

chr7-99142052-99144411	2.82153E-26	0.260817529	0.552	0.318	1.8685E-21	C4_CD8_Teff
chr1-156096259-156098080	3.00384E-26	0.261009162	0.556	0.316	1.98923E-21	C4_CD8_Teff
chr1-228081698-228083569	3.41648E-26	0.261200828	0.543	0.314	2.2625E-21	C4_CD8_Teff
chr9-135905096-135905931	4.26032E-12	0.286918302	0.138	0.056	2.82131E-07	C1_CD8_Tres
chr22-44454921-44456171	3.43444E-26	0.262211248	0.556	0.32	2.27439E-21	C4_CD8_Teff
chr22-20566372-20567525	3.54528E-26	0.320797084	0.318	0.139	2.34779E-21	C4_CD8_Teff
chr2-68371111-68371926	3.56811E-26	0.341137683	0.147	0.038	2.36291E-21	C4_CD8_Teff
chr1-93817483-93818780	3.70565E-26	0.32704029	0.272	0.116	2.45399E-21	C4_CD8_Teff
chr4-71186263-71188267	4.79893E-20	0.399543878	0.224	0.079	3.17799E-15	C3_CD8_Tem
chr22-39173749-39174989	5.56815E-19	0.342828171	0.326	0.163	3.68739E-14	C3_CD8_Tem
chr20-57716215-57716565	4.07688E-26	0.322322662	0.083	0.01	2.69983E-21	C4_CD8_Teff
chr12-49827665-49828906	4.08762E-26	0.262771925	0.546	0.306	2.70695E-21	C4_CD8_Teff
chr2-86787734-86787992	4.14498E-26	0.347097121	0.176	0.053	2.74493E-21	C4_CD8_Teff
chr3-153221387-153222517	1.55256E-10	0.26220581	0.347	0.215	1.02815E-05	C1_CD8_Tres
chr1-121097113-121097847	4.52059E-26	0.327680458	0.102	0.017	2.99367E-21	C4_CD8_Teff
chr7-130069865-130070885	4.58141E-26	0.254338864	0.567	0.337	3.03395E-21	C4_CD8_Teff
chr10-14554153-14555104	5.04743E-26	0.324144704	0.286	0.119	3.34256E-21	C4_CD8_Teff
chr20-3744652-3745441	5.05776E-26	0.340246721	0.146	0.036	3.3494E-21	C4_CD8_Teff
chr15-38576418-38577719	5.16324E-26	0.3338885	0.272	0.113	3.41925E-21	C4_CD8_Teff
chr1-161539434-161540845	5.23301E-26	0.305839593	0.387	0.177	3.46545E-21	C4_CD8_Teff
chr7-1412343-1413186	5.75914E-26	0.337816827	0.21	0.07	3.81388E-21	C4_CD8_Teff
chr13-18211612-18212525	5.89585E-26	0.316856515	0.098	0.014	3.90441E-21	C4_CD8_Teff
chr6-96923430-96925286	6.11317E-26	0.272314642	0.503	0.274	4.04832E-21	C4_CD8_Teff
chr11-35216770-35217513	4.10449E-19	0.405450713	0.21	0.074	2.71812E-14	C1_CD8_Tres
chr4-40211314-40211712	6.30023E-26	0.328273205	0.098	0.017	4.1722E-21	C4_CD8_Teff
chr1-151069973-151071485	7.02486E-26	0.2547762	0.568	0.332	4.65207E-21	C4_CD8_Teff
chr22-39528942-39529487	7.27746E-26	0.328777832	0.268	0.11	4.81935E-21	C4_CD8_Teff

chr8-101080148-101081061	7.68402E-26	0.328798618	0.234	0.089	5.08859E-21	C4_CD8_Teff
chr19-16324026-16325430	1.05083E-13	0.254209086	0.406	0.276	6.9589E-09	C3_CD8_Tem
chr22-39080163-39080852	8.63476E-26	0.338564398	0.231	0.086	5.7182E-21	C4_CD8_Teff
chr18-48868097-48869427	8.70268E-26	0.287918787	0.431	0.237	5.76318E-21	C4_CD8_Teff
chr8-81106233-81107262	9.17207E-26	0.313663909	0.337	0.157	6.07402E-21	C4_CD8_Teff
chr11-35082399-35084363	1.28546E-14	0.291891409	0.386	0.247	8.51272E-10	C1_CD8_Tres
chr19-58181949-58183960	9.30465E-26	0.257957179	0.546	0.323	6.16182E-21	C4_CD8_Teff
chrX-72272557-72273155	9.98427E-26	0.324512386	0.099	0.016	6.61188E-21	C4_CD8_Teff
chr11-19723327-19724481	1.1422E-25	0.335541918	0.198	0.065	7.56399E-21	C4_CD8_Teff
chr11-10455699-10456707	1.15816E-25	0.287861606	0.438	0.221	7.66966E-21	C4_CD8_Teff
chr1-64876955-64877577	3.73275E-15	0.357081693	0.168	0.059	2.47194E-10	C1_CD8_Tres
chr17-77783640-77784842	1.30914E-25	0.298857763	0.385	0.19	8.66955E-21	C4_CD8_Teff
chr16-88636781-88637627	6.64603E-19	0.332599715	0.318	0.167	4.4012E-14	C3_CD8_Tem
chr11-133944712-133945688	9.51792E-28	0.496429113	0.437	0.206	6.30305E-23	C1_CD8_Tres
chr20-49805033-49805682	1.50131E-25	0.323394048	0.288	0.126	9.94213E-21	C4_CD8_Teff
chr17-19003801-19005037	1.50334E-25	0.299491645	0.382	0.185	9.9556E-21	C4_CD8_Teff
chr18-78641586-78642043	1.56431E-25	0.269458832	0.051	0.002	1.03593E-20	C4_CD8_Teff
chr3-71421672-71422664	1.57526E-25	0.325872305	0.285	0.118	1.04318E-20	C4_CD8_Teff
chr10-132427249-132428487	5.94439E-09	0.252750703	0.138	0.068	0.000393656	C3_CD8_Tem
chr21-45227809-45228640	1.64132E-25	0.263236307	0.539	0.306	1.08693E-20	C4_CD8_Teff
chr5-96702371-96703654	1.65045E-25	0.278284929	0.457	0.249	1.09297E-20	C4_CD8_Teff
chr6-224832-225792	1.80924E-25	0.28518241	0.437	0.231	1.19813E-20	C4_CD8_Teff
chr17-7023032-7024093	1.93185E-25	0.317095576	0.309	0.133	1.27933E-20	C4_CD8_Teff
chr2-234137366-234137708	2.0857E-25	0.283091588	0.057	0.004	1.38121E-20	C4_CD8_Teff
chr3-66306992-66307313	2.10078E-25	0.341308944	0.151	0.039	1.3912E-20	C4_CD8_Teff
chr11-117984168-117984752	2.14121E-25	0.339255315	0.161	0.046	1.41798E-20	C4_CD8_Teff
chr2-203733674-203733997	2.14638E-25	0.324662695	0.092	0.016	1.4214E-20	C4_CD8_Teff

chr3-51024430-51025677	2.15407E-25	0.310152317	0.34	0.157	1.42649E-20	C4_CD8_Teff
chr9-129027464-129028705	2.18829E-25	0.257632558	0.545	0.303	1.44915E-20	C4_CD8_Teff
chr18-13455552-13456423	2.30705E-25	0.335549068	0.141	0.038	1.5278E-20	C4_CD8_Teff
chr15-51727470-51727966	2.66629E-25	0.327444223	0.135	0.032	1.7657E-20	C4_CD8_Teff
chr2-241867876-241869439	2.77235E-25	0.281323625	0.458	0.249	1.83593E-20	C4_CD8_Teff
chr1-40801806-40803049	3.20858E-25	0.313146009	0.315	0.143	2.12482E-20	C4_CD8_Teff
chr19-49928559-49930247	3.32872E-25	0.260411054	0.532	0.309	2.20438E-20	C4_CD8_Teff
chr14-65791200-65792008	3.51682E-25	0.33240833	0.213	0.076	2.32894E-20	C4_CD8_Teff
chr11-43880208-43881191	3.67908E-25	0.308080474	0.35	0.158	2.4364E-20	C4_CD8_Teff
chr3-14175481-14175997	3.78335E-25	0.335397948	0.226	0.08	2.50545E-20	C4_CD8_Teff
chr17-22524315-22524530	8.05483E-19	0.47217194	0.177	0.044	5.33415E-14	C1_CD8_Tres
chr6-27600236-27601152	4.19683E-25	0.318315371	0.084	0.012	2.77927E-20	C4_CD8_Teff
chr4-40225534-40226673	4.83482E-25	0.332384648	0.215	0.076	3.20176E-20	C4_CD8_Teff
chr5-157233063-157234120	2.15781E-14	0.338319656	0.144	0.049	1.42896E-09	C1_CD8_Tres
chr19-14506723-14507610	1.53733E-15	0.363663825	0.159	0.054	1.01807E-10	C1_CD8_Tres
chr17-5041938-5042982	5.41E-25	0.321733214	0.107	0.021	3.58266E-20	C4_CD8_Teff
chr19-12944918-12946373	5.48886E-25	0.277148401	0.459	0.243	3.63489E-20	C4_CD8_Teff
chr21-46368226-46369182	5.62908E-25	0.33074771	0.204	0.071	3.72775E-20	C4_CD8_Teff
chr7-152027350-152028282	5.81715E-25	0.317455761	0.296	0.128	3.85229E-20	C4_CD8_Teff
chr19-7521925-7522930	6.22791E-25	0.304030657	0.342	0.162	4.12431E-20	C4_CD8_Teff
chr21-42198955-42200215	6.68355E-25	0.325253688	0.245	0.099	4.42605E-20	C4_CD8_Teff
chr2-68385217-68385954	1.6138E-19	0.39968011	0.214	0.075	1.06871E-14	C3_CD8_Tem
chr3-196641324-196642171	7.40051E-25	0.300873333	0.348	0.167	4.90084E-20	C4_CD8_Teff
chr1-167446938-167448179	1.0515E-10	0.25619298	0.332	0.23	6.96336E-06	C1_CD8_Tres
chr10-7483654-7485358	8.10817E-25	0.315954432	0.292	0.13	5.36947E-20	C4_CD8_Teff
chr21-7775170-7776039	6.08216E-15	0.323449394	0.112	0.028	4.02779E-10	C3_CD8_Tem
chr6-149053939-149054961	8.602E-25	0.325860844	0.108	0.023	5.6965E-20	C4_CD8_Teff

chr2-230781194-230782105	8.67398E-25	0.279121632	0.455	0.237	5.74417E-20	C4_CD8_Teff
chr1-160738839-160739406	8.72625E-25	0.311368268	0.311	0.137	5.77879E-20	C4_CD8_Teff
chr16-89246879-89247980	1.02044E-24	0.293889293	0.365	0.175	6.75764E-20	C4_CD8_Teff
chr11-35214696-35215174	1.03713E-24	0.32640309	0.237	0.089	6.86819E-20	C4_CD8_Teff
chr17-4902860-4903824	1.0546E-24	0.320439795	0.287	0.121	6.9839E-20	C4_CD8_Teff
chr7-70693239-70694833	6.49712E-20	0.408922329	0.286	0.119	4.30258E-15	C3_CD8_Tem
chr22-35371393-35372311	1.13917E-24	0.331331734	0.222	0.084	7.54391E-20	C4_CD8_Teff
chr19-1567791-1568653	1.23906E-24	0.315645082	0.276	0.114	8.20541E-20	C4_CD8_Teff
chr11-66465878-66467478	1.25628E-24	0.258740436	0.515	0.291	8.31947E-20	C4_CD8_Teff
chr14-20632462-20633362	1.30578E-24	0.336737899	0.142	0.04	8.64729E-20	C4_CD8_Teff
chr1-202203369-202204084	6.7511E-16	0.358565412	0.128	0.035	4.47078E-11	C3_CD8_Tem
chr16-57815530-57816711	1.43592E-24	0.323898821	0.243	0.097	9.50912E-20	C4_CD8_Teff
chr12-128782326-128783599	3.35657E-44	0.615965329	0.307	0.074	2.22282E-39	C3_CD8_Tem
chr4-36255271-36256001	1.5265E-24	0.30723997	0.312	0.138	1.01089E-19	C4_CD8_Teff
chr22-36383075-36383603	1.60058E-24	0.298307964	0.362	0.173	1.05995E-19	C4_CD8_Teff
chr7-925609-926009	1.65519E-24	0.310241896	0.289	0.131	1.09612E-19	C4_CD8_Teff
chr22-27875514-27876372	1.6999E-24	0.336443218	0.171	0.053	1.12572E-19	C4_CD8_Teff
chr17-67433918-67435509	1.70015E-24	0.261882917	0.517	0.288	1.12589E-19	C4_CD8_Teff
chr17-42483623-42484911	2.42871E-12	0.271139073	0.292	0.171	1.60836E-07	C3_CD8_Tem
chr18-22260170-22260899	2.00896E-24	0.312375506	0.08	0.012	1.33039E-19	C4_CD8_Teff
chr2-15038063-15038793	2.16188E-24	0.304238345	0.077	0.011	1.43166E-19	C4_CD8_Teff
chr4-152680832-152681334	2.21883E-24	0.314917058	0.108	0.022	1.46938E-19	C4_CD8_Teff
chr15-92907405-92907607	2.22327E-24	0.332163093	0.183	0.058	1.47231E-19	C4_CD8_Teff
chr17-77836360-77837135	2.22429E-24	0.324476352	0.227	0.089	1.47299E-19	C4_CD8_Teff
chr5-157198163-157199049	2.59864E-24	0.285696544	0.417	0.216	1.7209E-19	C4_CD8_Teff
chr15-77012654-77014551	2.70746E-24	0.259204241	0.518	0.297	1.79296E-19	C4_CD8_Teff
chr3-33047436-33048236	2.7652E-24	0.312486068	0.304	0.131	1.8312E-19	C4_CD8_Teff

chr11-118914246-118914785	2.79894E-24	0.313504294	0.301	0.131	1.85354E-19	C4_CD8_Teff
chr15-67839463-67840757	3.00905E-24	0.260732958	0.504	0.289	1.99269E-19	C4_CD8_Teff
chr3-63978845-63979787	3.13886E-24	0.317844759	0.26	0.104	2.07864E-19	C4_CD8_Teff
chr7-24907220-24907930	3.39451E-24	0.298767661	0.359	0.168	2.24795E-19	C4_CD8_Teff
chr19-17538908-17540305	7.50075E-12	0.251232293	0.308	0.206	4.96722E-07	C1_CD8_Tres
chr13-114148040-114148970	3.60844E-24	0.261717664	0.512	0.287	2.38962E-19	C4_CD8_Teff
chr16-2663483-2664349	3.66104E-24	0.32933787	0.227	0.081	2.42445E-19	C4_CD8_Teff
chr13-29925476-29925902	3.8023E-24	0.287795414	0.069	0.007	2.518E-19	C4_CD8_Teff
chr11-122838103-122838816	3.89923E-24	0.26911797	0.483	0.273	2.58219E-19	C4_CD8_Teff
chr8-133138417-133139840	4.05611E-24	0.269131098	0.492	0.272	2.68608E-19	C4_CD8_Teff
chr18-48940224-48941255	7.98765E-15	0.277040919	0.471	0.318	5.28966E-10	C3_CD8_Tem
chr19-10416274-10417062	1.41525E-16	0.277669529	0.414	0.269	9.37221E-12	C3_CD8_Tem
chr10-89337454-89337853	4.43677E-24	0.326598405	0.131	0.033	2.93816E-19	C4_CD8_Teff
chr9-104990822-104992139	3.31326E-10	0.264667621	0.311	0.2	2.19414E-05	C1_CD8_Tres
chrX-107741465-107742619	4.59355E-24	0.296386506	0.356	0.171	3.04199E-19	C4_CD8_Teff
chr11-116953413-116954230	4.70731E-24	0.331773121	0.163	0.047	3.11732E-19	C4_CD8_Teff
chr17-1823106-1823993	2.48931E-16	0.33556185	0.344	0.194	1.6485E-11	C3_CD8_Tem
chr6-107712642-107713482	4.87776E-24	0.322211422	0.166	0.053	3.2302E-19	C4_CD8_Teff
chr6-158852757-158853837	5.05227E-24	0.253812113	0.536	0.307	3.34576E-19	C4_CD8_Teff
chr9-21503901-21504597	5.18216E-24	0.279922431	0.057	0.004	3.43178E-19	C4_CD8_Teff
chr19-16078184-16079767	5.34028E-24	0.250305958	0.541	0.316	3.5365E-19	C4_CD8_Teff
chr10-124636397-124637010	5.51266E-24	0.318664414	0.11	0.022	3.65065E-19	C4_CD8_Teff
chr5-119277723-119278563	5.65385E-24	0.32596876	0.213	0.075	3.74415E-19	C4_CD8_Teff
chr1-107936381-107937456	6.039E-24	0.265110067	0.48	0.277	3.9992E-19	C4_CD8_Teff
chr1-34852454-34853221	6.47365E-24	0.302471437	0.341	0.157	4.28704E-19	C4_CD8_Teff
chr17-49387502-49388620	4.76474E-20	0.41153871	0.234	0.087	3.15535E-15	C3_CD8_Tem
chr9-134305425-134306484	6.8978E-24	0.300673346	0.086	0.014	4.56793E-19	C4_CD8_Teff

chr11-46929656-46930468	7.1618E-24	0.323109137	0.114	0.025	4.74276E-19	C4_CD8_Teff
chr11-119066931-119068016	7.22847E-24	0.257373291	0.513	0.3	4.78691E-19	C4_CD8_Teff
chr22-23263385-23264547	1.41352E-34	0.551089318	0.362	0.132	9.36074E-30	C3_CD8_Tem
chr14-103846725-103848246	7.45707E-24	0.279791896	0.411	0.206	4.93829E-19	C4_CD8_Teff
chr5-172178403-172179887	7.67028E-24	0.295636778	0.353	0.169	5.07949E-19	C4_CD8_Teff
chr22-37218585-37219771	1.11414E-14	0.323901086	0.222	0.108	7.37816E-10	C1_CD8_Tres
chr19-9933927-9934491	8.10402E-24	0.327757105	0.163	0.05	5.36672E-19	C4_CD8_Teff
chr7-55240414-55240993	8.55101E-24	0.293557854	0.064	0.007	5.66273E-19	C4_CD8_Teff
chr7-139084116-139085810	8.82991E-24	0.310629911	0.287	0.123	5.84743E-19	C4_CD8_Teff
chr14-68685054-68686144	9.5723E-24	0.255392433	0.498	0.29	6.33906E-19	C4_CD8_Teff
chr21-10271601-10272657	1.25809E-10	0.256398691	0.083	0.027	8.33146E-06	C3_CD8_Tem
chr22-30308503-30308820	9.63001E-24	0.318256665	0.109	0.023	6.37728E-19	C4_CD8_Teff
chr3-186107653-186109638	9.7815E-24	0.26355387	0.487	0.264	6.4776E-19	C4_CD8_Teff
chr10-71850493-71851558	1.00996E-23	0.263636711	0.483	0.27	6.68823E-19	C4_CD8_Teff
chr12-14771627-14772004	1.06892E-23	0.326828635	0.206	0.073	7.07868E-19	C4_CD8_Teff
chr17-7239248-7239916	1.07439E-23	0.25988694	0.491	0.28	7.11495E-19	C4_CD8_Teff
chr1-212558174-212559265	1.09236E-23	0.285780442	0.378	0.191	7.23396E-19	C4_CD8_Teff
chr17-40068520-40070664	1.10444E-23	0.315211785	0.255	0.101	7.3139E-19	C4_CD8_Teff
chr6-31805950-31807159	1.14458E-23	0.272798235	0.443	0.234	7.57977E-19	C4_CD8_Teff
chr5-134122346-134122925	1.23643E-23	0.323796241	0.154	0.044	8.188E-19	C4_CD8_Teff
chr2-317260-318382	1.24732E-23	0.326610832	0.204	0.068	8.2601E-19	C4_CD8_Teff
chr19-47157786-47159607	1.15985E-11	0.277803381	0.222	0.113	7.68086E-07	C1_CD8_Tres
chr21-42218518-42220679	1.34707E-23	0.257295299	0.488	0.279	8.92069E-19	C4_CD8_Teff
chr8-144462553-144463122	1.45587E-23	0.265691498	0.46	0.255	9.64124E-19	C4_CD8_Teff
chr1-3809711-3810580	7.42422E-23	0.451066803	0.148	0.034	4.91654E-18	C3_CD8_Tem
chr16-66933595-66934886	1.53196E-23	0.271488986	0.455	0.238	1.01451E-18	C4_CD8_Teff
chr11-9759348-9760157	1.64865E-23	0.27668011	0.417	0.217	1.09178E-18	C4_CD8_Teff

chr9-114363274-114363843	1.68684E-23	0.331261529	0.195	0.064	1.11708E-18	C4_CD8_Teff
chr1-200530457-200531292	1.75063E-23	0.312130147	0.099	0.02	1.15932E-18	C4_CD8_Teff
chr3-48685288-48686487	1.77101E-23	0.2563965	0.508	0.283	1.17282E-18	C4_CD8_Teff
chr19-53466942-53468224	1.79105E-23	0.276833022	0.409	0.216	1.18609E-18	C4_CD8_Teff
chr6-26571548-26572252	1.91082E-23	0.321981525	0.155	0.048	1.2654E-18	C4_CD8_Teff
chr4-123928-125323	1.93528E-23	0.261852571	0.481	0.265	1.2816E-18	C4_CD8_Teff
chr19-8573198-8573976	1.43212E-13	0.272990204	0.318	0.19	9.48393E-09	C3_CD8_Tem
chr12-64679444-64680046	2.25341E-23	0.319187227	0.234	0.087	1.49228E-18	C4_CD8_Teff
chr6-26360593-26361302	2.28617E-23	0.329636748	0.176	0.058	1.51397E-18	C4_CD8_Teff
chr1-53581990-53582744	2.15688E-19	0.409248655	0.188	0.061	1.42835E-14	C3_CD8_Tem
chr6-46325308-46326080	1.53286E-14	0.333670848	0.167	0.061	1.01511E-09	C3_CD8_Tem
chr1-42936414-42937495	2.4851E-23	0.278006807	0.415	0.218	1.64571E-18	C4_CD8_Teff
chr6-145844988-145845928	6.61344E-16	0.34332974	0.096	0.021	4.37962E-11	C3_CD8_Tem
chr13-114099862-114101465	2.59869E-23	0.314586509	0.106	0.023	1.72093E-18	C4_CD8_Teff
chr22-19419104-19420340	3.64009E-20	0.399093915	0.26	0.106	2.41057E-15	C3_CD8_Tem
chr19-53333050-53334257	2.67898E-23	0.263915917	0.463	0.259	1.7741E-18	C4_CD8_Teff
chr1-155062759-155064618	2.70182E-23	0.279920362	0.404	0.207	1.78923E-18	C4_CD8_Teff
chr12-22130321-22131038	2.71094E-23	0.300573951	0.074	0.01	1.79527E-18	C4_CD8_Teff
chr16-400830-402210	3.12243E-23	0.261742865	0.475	0.26	2.06777E-18	C4_CD8_Teff
chr1-10524905-10525628	2.02813E-47	0.690396415	0.279	0.05	1.34309E-42	C3_CD8_Tem
chr18-62420089-62421122	3.15873E-23	0.27903009	0.404	0.21	2.0918E-18	C4_CD8_Teff
chr10-6050501-6051537	3.16495E-23	0.252701279	0.513	0.298	2.09593E-18	C4_CD8_Teff
chr18-45686597-45689178	3.41885E-23	0.269265301	0.46	0.255	2.26406E-18	C4_CD8_Teff
chr6-166257734-166258201	1.69107E-10	0.293645116	0.15	0.07	1.11988E-05	C1_CD8_Tres
chr13-99274870-99275424	3.74351E-23	0.319843894	0.183	0.059	2.47906E-18	C4_CD8_Teff
chr17-43251577-43251794	2.48706E-13	0.287034444	0.112	0.033	1.647E-08	C3_CD8_Tem
chr16-89564096-89564376	3.9266E-23	0.284096655	0.067	0.008	2.60031E-18	C4_CD8_Teff

chr19-31339470-31340914	4.00556E-23	0.321372099	0.2	0.071	2.6526E-18	C4_CD8_Teff
chr17-8939402-8939794	4.03654E-23	0.321212093	0.194	0.069	2.67312E-18	C4_CD8_Teff
chr3-187744862-187745877	4.05958E-23	0.281425558	0.389	0.2	2.68838E-18	C4_CD8_Teff
chr14-106622028-106622629	4.09853E-23	0.304790297	0.088	0.015	2.71417E-18	C4_CD8_Teff
chr7-50339986-50340516	1.84672E-08	0.253227656	0.102	0.039	0.00122295	C1_CD8_Tres
chr12-54411288-54411888	4.48176E-23	0.318012565	0.142	0.04	2.96795E-18	C4_CD8_Teff
chr5-134115059-134115786	4.72119E-23	0.305537222	0.254	0.107	3.12651E-18	C4_CD8_Teff
chr4-101038995-101039746	4.95574E-23	0.311329157	0.22	0.084	3.28184E-18	C4_CD8_Teff
chr16-68417447-68418608	4.98294E-23	0.291660707	0.347	0.17	3.29985E-18	C4_CD8_Teff
chr6-31354130-31354531	5.00161E-23	0.322712659	0.197	0.069	3.31222E-18	C4_CD8_Teff
chr14-22549641-22550456	5.00797E-23	0.294616025	0.329	0.153	3.31643E-18	C4_CD8_Teff
chr13-20703044-20704721	5.32877E-23	0.289156324	0.369	0.179	3.52887E-18	C4_CD8_Teff
chr19-16371034-16372003	5.41251E-23	0.267524594	0.449	0.246	3.58432E-18	C4_CD8_Teff
chr19-7929244-7929703	5.4515E-23	0.326368413	0.152	0.044	3.61014E-18	C4_CD8_Teff
chr15-63203399-63204113	5.62847E-23	0.317481019	0.217	0.08	3.72734E-18	C4_CD8_Teff
chr19-46785922-46786919	5.67061E-23	0.257263845	0.486	0.279	3.75525E-18	C4_CD8_Teff
chr15-40753674-40755571	5.74089E-23	0.261278415	0.47	0.259	3.80179E-18	C4_CD8_Teff
chr10-69407868-69409188	6.30181E-23	0.254131539	0.504	0.291	4.17325E-18	C4_CD8_Teff
chr11-75524687-75526380	6.45772E-23	0.2690336	0.424	0.23	4.2765E-18	C4_CD8_Teff
chr6-27502428-27503966	6.52483E-23	0.319616304	0.201	0.071	4.32094E-18	C4_CD8_Teff
chrX-107711845-107713138	6.79456E-23	0.300565317	0.309	0.141	4.49956E-18	C4_CD8_Teff
chr1-202966456-202968208	6.86665E-23	0.269197306	0.433	0.231	4.5473E-18	C4_CD8_Teff
chr20-38839996-38841506	7.14143E-23	0.255421182	0.516	0.287	4.72927E-18	C4_CD8_Teff
chr19-7636645-7637782	7.37855E-23	0.275667661	0.399	0.206	4.88629E-18	C4_CD8_Teff
chr19-10142429-10143140	2.21428E-18	0.411739395	0.156	0.049	1.46636E-13	C1_CD8_Tres
chr2-231425680-231426544	7.93672E-23	0.319471121	0.197	0.074	5.25593E-18	C4_CD8_Teff
chr17-4795541-4796508	8.28948E-23	0.291971179	0.334	0.156	5.48954E-18	C4_CD8_Teff

chr2-234147113-234147957	8.75433E-23	0.306151888	0.118	0.027	5.79738E-18	C4_CD8_Teff
chr12-10602523-10603301	3.01703E-12	0.291578974	0.128	0.047	1.99797E-07	C3_CD8_Tem
chr17-75666694-75667675	9.12031E-23	0.273261138	0.417	0.223	6.03975E-18	C4_CD8_Teff
chr12-10595665-10596319	9.18694E-23	0.308131438	0.092	0.017	6.08387E-18	C4_CD8_Teff
chr20-51529421-51530098	9.66616E-23	0.275759683	0.413	0.218	6.40122E-18	C4_CD8_Teff
chr22-35660180-35661817	9.89816E-23	0.250889119	0.497	0.294	6.55486E-18	C4_CD8_Teff
chr3-170276876-170278095	5.94624E-21	0.428171033	0.224	0.077	3.93778E-16	C3_CD8_Tem
chr1-168531516-168532245	1.05957E-22	0.308383834	0.108	0.024	7.0168E-18	C4_CD8_Teff
chr6-32945173-32945747	1.07483E-22	0.313041903	0.262	0.108	7.11787E-18	C4_CD8_Teff
chr19-17419397-17420836	1.11147E-22	0.251860919	0.494	0.283	7.3605E-18	C4_CD8_Teff
chr2-219247439-219248340	1.16369E-22	0.255341766	0.495	0.285	7.70627E-18	C4_CD8_Teff
chr10-3776772-3777302	1.18017E-22	0.307469854	0.264	0.111	7.81543E-18	C4_CD8_Teff
chr1-205591456-205592373	1.21254E-22	0.289334678	0.349	0.165	8.02978E-18	C4_CD8_Teff
chr1-161626522-161627073	1.20338E-12	0.296266862	0.117	0.038	7.96913E-08	C3_CD8_Tem
chr6-37514251-37514771	1.31199E-22	0.304042922	0.278	0.122	8.68841E-18	C4_CD8_Teff
chr22-39316316-39316694	1.31427E-22	0.304075485	0.262	0.11	8.70348E-18	C4_CD8_Teff
chr20-33731448-33732841	1.36386E-22	0.261785009	0.471	0.259	9.0319E-18	C4_CD8_Teff
chr12-54412871-54413756	1.41962E-22	0.299936155	0.303	0.132	9.40112E-18	C4_CD8_Teff
chr4-148444349-148445940	1.46787E-22	0.292028937	0.314	0.147	9.72067E-18	C4_CD8_Teff
chr15-43776139-43777745	1.50832E-22	0.270728305	0.428	0.218	9.98852E-18	C4_CD8_Teff
chr19-49493630-49493854	1.55875E-13	0.323118875	0.133	0.045	1.03225E-08	C3_CD8_Tem
chr17-82882266-82883579	1.62467E-22	0.259619775	0.46	0.262	1.07591E-17	C4_CD8_Teff
chr2-203740503-203741793	1.62477E-22	0.309135574	0.27	0.114	1.07597E-17	C4_CD8_Teff
chr6-125299184-125299677	1.7148E-22	0.315961496	0.191	0.068	1.13559E-17	C4_CD8_Teff
chr7-99375996-99376482	1.73174E-22	0.31516275	0.16	0.05	1.14681E-17	C4_CD8_Teff
chr10-124734092-124735011	1.73274E-22	0.310835001	0.19	0.068	1.14747E-17	C4_CD8_Teff
chr6-34696098-34697326	1.86646E-22	0.257334383	0.459	0.257	1.23603E-17	C4_CD8_Teff

chr8-66609955-66610470	1.97108E-16	0.367104858	0.146	0.041	1.30531E-11	C3_CD8_Tem
chr19-53536173-53536491	2.08511E-22	0.308685899	0.102	0.02	1.38082E-17	C4_CD8_Teff
chr3-27719890-27720151	2.17462E-22	0.311948077	0.113	0.025	1.4401E-17	C4_CD8_Teff
chr1-120723413-120724378	2.66701E-09	0.258062148	0.094	0.031	0.000176617	C3_CD8_Tem
chr16-50335070-50335857	1.74063E-10	0.289104105	0.224	0.117	1.1527E-05	C3_CD8_Tem
chr17-38657220-38657677	2.64355E-22	0.266322042	0.055	0.004	1.75064E-17	C4_CD8_Teff
chr1-148151263-148152408	2.65745E-22	0.309342725	0.125	0.032	1.75984E-17	C4_CD8_Teff
chr3-66304302-66304635	2.72757E-22	0.310704551	0.106	0.023	1.80628E-17	C4_CD8_Teff
chr7-48084289-48084571	2.75602E-22	0.305420217	0.109	0.023	1.82512E-17	C4_CD8_Teff
chr2-61763611-61764772	2.76381E-22	0.252180427	0.496	0.28	1.83028E-17	C4_CD8_Teff
chr22-25054537-25055508	4.15633E-26	0.468325651	0.156	0.031	2.75244E-21	C3_CD8_Tem
chr11-117078004-117078564	2.80108E-22	0.311644194	0.242	0.1	1.85496E-17	C4_CD8_Teff
chr12-6069234-6069891	2.87037E-22	0.313354121	0.205	0.077	1.90084E-17	C4_CD8_Teff
chr11-59559807-59561140	2.97878E-22	0.303010821	0.271	0.115	1.97264E-17	C4_CD8_Teff
chr7-140229060-140231925	3.22551E-22	0.253211254	0.487	0.273	2.13603E-17	C4_CD8_Teff
chr16-81841425-81842485	3.23907E-10	0.26734942	0.133	0.056	2.14501E-05	C3_CD8_Tem
chr6-106194641-106195955	3.26414E-22	0.30845963	0.223	0.086	2.16161E-17	C4_CD8_Teff
chr17-63597022-63597708	3.28508E-22	0.314143646	0.194	0.066	2.17548E-17	C4_CD8_Teff
chr8-144685265-144686815	4.60578E-12	0.262623826	0.18	0.095	3.05009E-07	C1_CD8_Tres
chr1-24984496-24985576	3.46408E-22	0.310066865	0.237	0.098	2.29402E-17	C4_CD8_Teff
chr11-118565419-118566266	3.80414E-22	0.271607626	0.42	0.223	2.51922E-17	C4_CD8_Teff
chr9-21504865-21505616	3.89547E-22	0.274053243	0.061	0.006	2.5797E-17	C4_CD8_Teff
chr14-91880594-91881425	3.90182E-22	0.305114171	0.102	0.022	2.5839E-17	C4_CD8_Teff
chr2-240831720-240832790	3.9185E-12	0.292926316	0.374	0.244	2.59495E-07	C1_CD8_Tres
chr21-33936393-33936926	4.04788E-22	0.316459543	0.149	0.046	2.68063E-17	C4_CD8_Teff
chr2-86820019-86820947	4.47184E-22	0.317381543	0.149	0.044	2.96139E-17	C4_CD8_Teff
chr22-39094669-39094965	4.528E-22	0.309199599	0.217	0.083	2.99857E-17	C4_CD8_Teff

chr21-33931016-33932182	5.20035E-22	0.267085552	0.418	0.222	3.44383E-17	C4_CD8_Teff
chr17-67431290-67431893	5.72781E-22	0.305546899	0.251	0.102	3.79313E-17	C4_CD8_Teff
chr21-42233095-42233934	5.86308E-22	0.281679549	0.342	0.171	3.88271E-17	C4_CD8_Teff
chr2-105803347-105803967	6.10167E-22	0.30773956	0.179	0.062	4.04071E-17	C4_CD8_Teff
chr1-25642076-25643133	7.18652E-22	0.26904602	0.409	0.225	4.75913E-17	C4_CD8_Teff
chr19-14274356-14275171	6.42228E-20	0.411610311	0.169	0.046	4.25302E-15	C3_CD8_Tem
chr2-33263-33967	7.42774E-22	0.309767207	0.153	0.045	4.91887E-17	C4_CD8_Teff
chr10-22228869-22229745	7.61002E-22	0.288766377	0.315	0.147	5.03958E-17	C4_CD8_Teff
chr2-86815456-86815995	8.20998E-22	0.316798695	0.149	0.045	5.4369E-17	C4_CD8_Teff
chr14-61351428-61351787	8.54805E-22	0.30475715	0.111	0.026	5.66078E-17	C4_CD8_Teff
chr5-148844559-148845169	8.5753E-22	0.279228099	0.37	0.191	5.67882E-17	C4_CD8_Teff
chr16-71483901-71484825	9.3869E-22	0.286820521	0.325	0.164	6.21628E-17	C4_CD8_Teff
chr10-22912663-22913849	9.16177E-21	0.434410827	0.125	0.021	6.0672E-16	C3_CD8_Tem
chr1-24924873-24925461	9.75561E-22	0.270660435	0.387	0.2	6.46046E-17	C4_CD8_Teff
chr19-6521764-6522766	9.95086E-22	0.278267023	0.391	0.201	6.58976E-17	C4_CD8_Teff
chr5-139453681-139454599	1.40146E-19	0.42282445	0.141	0.03	9.28091E-15	C3_CD8_Tem
chr12-124988451-124989340	1.04896E-21	0.289449775	0.314	0.147	6.9465E-17	C4_CD8_Teff
chr1-173864200-173864584	1.13389E-21	0.308047403	0.169	0.058	7.50897E-17	C4_CD8_Teff
chr7-102671226-102671950	1.14584E-21	0.308914539	0.118	0.027	7.5881E-17	C4_CD8_Teff
chr5-109852127-109852711	1.16812E-21	0.306112092	0.123	0.033	7.73564E-17	C4_CD8_Teff
chr2-98466340-98466719	1.17184E-21	0.281069318	0.072	0.01	7.76029E-17	C4_CD8_Teff
chr19-289957-291073	1.22186E-21	0.313346128	0.172	0.059	8.09154E-17	C4_CD8_Teff
chr22-22753064-22753864	1.26353E-21	0.314743421	0.164	0.055	8.36747E-17	C4_CD8_Teff
chr17-36214896-36215196	1.27347E-21	0.277659478	0.065	0.007	8.43329E-17	C4_CD8_Teff
chr20-49935906-49937155	1.31987E-21	0.271473335	0.374	0.194	8.74057E-17	C4_CD8_Teff
chr2-230893993-230894713	1.32686E-21	0.293598432	0.08	0.014	8.78686E-17	C4_CD8_Teff
chr1-156746533-156747458	1.72365E-21	0.310067004	0.152	0.048	1.14145E-16	C4_CD8_Teff

chr2-38696807-38697971	1.79396E-21	0.274274026	0.371	0.193	1.18801E-16	C4_CD8_Teff
chr1-66349953-66350559	1.83252E-21	0.297641424	0.256	0.114	1.21355E-16	C4_CD8_Teff
chr17-36211820-36212128	2.026E-21	0.260529533	0.054	0.004	1.34168E-16	C4_CD8_Teff
chr10-71742093-71742966	2.02905E-21	0.303788058	0.178	0.068	1.3437E-16	C4_CD8_Teff
chr11-57649096-57650233	2.04197E-21	0.260880513	0.425	0.235	1.35226E-16	C4_CD8_Teff
chr8-133614979-133615492	2.20411E-21	0.303546196	0.112	0.027	1.45963E-16	C4_CD8_Teff
chr19-11091497-11092486	2.21535E-21	0.278772627	0.339	0.17	1.46707E-16	C4_CD8_Teff
chr1-2314402-2315380	2.22487E-21	0.306504717	0.206	0.078	1.47338E-16	C4_CD8_Teff
chr20-38471932-38473324	2.25325E-21	0.250038832	0.47	0.265	1.49217E-16	C4_CD8_Teff
chr8-126505903-126506723	2.436E-21	0.288996531	0.096	0.021	1.61319E-16	C4_CD8_Teff
chr6-35709901-35710747	2.71672E-21	0.294824883	0.254	0.108	1.7991E-16	C4_CD8_Teff
chr14-75247053-75247940	2.75771E-21	0.294065686	0.256	0.111	1.82624E-16	C4_CD8_Teff
chr17-35160382-35161809	2.77264E-21	0.294029802	0.285	0.131	1.83612E-16	C4_CD8_Teff
chr10-69182661-69182916	2.84924E-21	0.296786378	0.088	0.017	1.88685E-16	C4_CD8_Teff
chr21-44927273-44927898	3.03116E-21	0.27288913	0.362	0.187	2.00733E-16	C4_CD8_Teff
chr10-112949661-112950427	3.06024E-21	0.307136631	0.147	0.046	2.02659E-16	C4_CD8_Teff
chr19-4914033-4914289	3.07822E-21	0.30638462	0.113	0.028	2.03849E-16	C4_CD8_Teff
chr22-20549056-20549841	3.09517E-21	0.308311546	0.189	0.068	2.04972E-16	C4_CD8_Teff
chr21-42200888-42201253	3.40442E-21	0.279704085	0.072	0.011	2.25451E-16	C4_CD8_Teff
chr12-94243950-94245220	3.40785E-21	0.3081484	0.201	0.078	2.25678E-16	C4_CD8_Teff
chr8-130286360-130287025	3.66064E-21	0.28845884	0.083	0.015	2.42419E-16	C4_CD8_Teff
chr19-16369616-16370355	1.05869E-12	0.299462805	0.201	0.095	7.01094E-08	C3_CD8_Tem
chr1-65001188-65003206	3.70704E-21	0.268301312	0.395	0.204	2.45491E-16	C4_CD8_Teff
chr11-36357657-36358119	4.24197E-21	0.288460131	0.081	0.013	2.80916E-16	C4_CD8_Teff
chr19-35958669-35959893	4.57093E-21	0.286050186	0.299	0.142	3.02701E-16	C4_CD8_Teff
chr1-114340814-114342699	4.72086E-21	0.260582053	0.425	0.232	3.1263E-16	C4_CD8_Teff
chr4-153471228-153472161	3.21382E-13	0.330259005	0.254	0.128	2.12829E-08	C1_CD8_Tres

chr9-281823-282760	4.91135E-21	0.298518071	0.248	0.107	3.25244E-16	C4_CD8_Teff
chr17-47809886-47810847	5.18333E-21	0.290583754	0.268	0.123	3.43255E-16	C4_CD8_Teff
chr10-133258920-133259596	5.32783E-21	0.281602161	0.305	0.143	3.52825E-16	C4_CD8_Teff
chr2-195532256-195533283	5.41136E-21	0.306340019	0.157	0.052	3.58356E-16	C4_CD8_Teff
chr1-198187635-198188597	5.47785E-21	0.300882273	0.23	0.093	3.6276E-16	C4_CD8_Teff
chr6-26188117-26189363	5.64118E-21	0.259634746	0.426	0.232	3.73576E-16	C4_CD8_Teff
chr17-5281510-5282931	5.68045E-21	0.2780153	0.308	0.154	3.76176E-16	C4_CD8_Teff
chrX-153966946-153967202	5.71727E-21	0.302279371	0.1	0.022	3.78615E-16	C4_CD8_Teff
chr6-137231582-137232242	5.19501E-13	0.321222988	0.135	0.048	3.44029E-08	C3_CD8_Tem
chr1-24506618-24507685	1.76849E-21	0.436619077	0.174	0.048	1.17115E-16	C1_CD8_Tres
chr15-62856936-62857867	6.6515E-21	0.287587349	0.084	0.016	4.40482E-16	C4_CD8_Teff
chr5-107467115-107467752	6.82408E-21	0.273198602	0.059	0.007	4.51911E-16	C4_CD8_Teff
chr5-132673906-132674406	7.02159E-21	0.260651722	0.059	0.006	4.64991E-16	C4_CD8_Teff
chr12-92178279-92178728	7.03667E-21	0.25931899	0.051	0.004	4.65989E-16	C4_CD8_Teff
chr3-177652522-177653269	7.78967E-21	0.273679271	0.076	0.012	5.15855E-16	C4_CD8_Teff
chr19-58325564-58326172	8.32199E-21	0.301579181	0.116	0.032	5.51107E-16	C4_CD8_Teff
chr5-102295715-102297223	1.10198E-23	0.458526675	0.195	0.055	7.29767E-19	C3_CD8_Tem
chr19-662933-663534	8.66002E-21	0.275354057	0.332	0.165	5.73493E-16	C4_CD8_Teff
chr19-1939203-1939466	8.66196E-21	0.30307462	0.138	0.043	5.73621E-16	C4_CD8_Teff
chr10-12418885-12419856	8.76997E-21	0.304526394	0.157	0.052	5.80774E-16	C4_CD8_Teff
chr15-66471428-66472664	3.62681E-10	0.285206685	0.133	0.056	2.40178E-05	C3_CD8_Tem
chr17-40605132-40605713	9.09198E-21	0.298118383	0.213	0.085	6.02098E-16	C4_CD8_Teff
chr3-28053526-28054012	9.70961E-21	0.295817635	0.087	0.016	6.42999E-16	C4_CD8_Teff
chr4-40192310-40192559	1.02583E-20	0.268748511	0.064	0.008	6.79335E-16	C4_CD8_Teff
chr1-146411794-146412500	1.0418E-20	0.292867425	0.095	0.019	6.89908E-16	C4_CD8_Teff
chr17-58352950-58353766	1.10251E-20	0.302043194	0.194	0.075	7.30117E-16	C4_CD8_Teff
chr7-100351741-100352788	1.12738E-20	0.293924521	0.099	0.022	7.46582E-16	C4_CD8_Teff

chr14-101821570-101821803	1.13189E-20	0.303784699	0.198	0.074	7.49571E-16	C4_CD8_Teff
chr16-2911291-2912589	1.14586E-20	0.256190591	0.421	0.23	7.58823E-16	C4_CD8_Teff
chr20-10034653-10035439	1.17605E-20	0.293282311	0.228	0.09	7.78814E-16	C4_CD8_Teff
chr19-34269563-34270597	1.21506E-20	0.292542928	0.254	0.112	8.04648E-16	C4_CD8_Teff
chr19-43434182-43434701	1.24452E-20	0.263865772	0.055	0.005	8.2416E-16	C4_CD8_Teff
chr21-43739541-43741123	1.2887E-20	0.290859975	0.091	0.019	8.53413E-16	C4_CD8_Teff
chr1-113783097-113784687	1.39647E-20	0.254358028	0.44	0.252	9.24781E-16	C4_CD8_Teff
chr17-2792476-2793160	8.25126E-26	0.509444529	0.214	0.054	5.46423E-21	C3_CD8_Tem
chr8-143840519-143842057	1.45614E-20	0.255847193	0.417	0.233	9.64302E-16	C4_CD8_Teff
chr1-37553565-37554486	1.4705E-20	0.290903276	0.272	0.122	9.73812E-16	C4_CD8_Teff
chr4-121777655-121778050	1.52446E-20	0.278668918	0.072	0.011	1.00954E-15	C4_CD8_Teff
chr4-6182446-6182666	1.56095E-20	0.295365349	0.087	0.017	1.03371E-15	C4_CD8_Teff
chr6-24980377-24980744	1.56414E-20	0.250896344	0.05	0.004	1.03582E-15	C4_CD8_Teff
chr18-34978305-34979493	1.66307E-20	0.288462703	0.278	0.125	1.10133E-15	C4_CD8_Teff
chr5-157221925-157223223	1.66536E-20	0.26553843	0.374	0.198	1.10285E-15	C4_CD8_Teff
chr7-100884591-100885134	1.70745E-20	0.295277469	0.272	0.122	1.13073E-15	C4_CD8_Teff
chr1-31826680-31827733	1.74055E-20	0.292674697	0.257	0.112	1.15264E-15	C4_CD8_Teff
chr14-21230581-21231398	1.77714E-20	0.30882865	0.153	0.051	1.17688E-15	C4_CD8_Teff
chr22-38512741-38513371	1.79304E-20	0.285365145	0.078	0.014	1.1874E-15	C4_CD8_Teff
chr17-44141309-44142522	1.8485E-20	0.262371202	0.399	0.217	1.22413E-15	C4_CD8_Teff
chr19-38656537-38656999	2.04809E-20	0.299449225	0.149	0.049	1.35631E-15	C4_CD8_Teff
chr13-46202059-46202834	2.09241E-20	0.302156612	0.133	0.04	1.38566E-15	C4_CD8_Teff
chr1-160738147-160738595	1.79426E-10	0.261351265	0.086	0.026	1.18821E-05	C3_CD8_Tem
chrX-51395670-51396818	2.16068E-20	0.300988736	0.124	0.035	1.43087E-15	C4_CD8_Teff
chr17-7233953-7234751	2.37642E-20	0.261588775	0.374	0.2	1.57373E-15	C4_CD8_Teff
chr10-3739840-3740818	2.40218E-20	0.293649702	0.255	0.11	1.59079E-15	C4_CD8_Teff
chr14-55330264-55330678	2.50848E-20	0.291394908	0.116	0.029	1.66119E-15	C4_CD8_Teff

chr5-139925797-139926640	3.34003E-29	0.533250622	0.203	0.046	2.21187E-24	C3_CD8_Tem
chr1-248853194-248854037	2.56751E-20	0.305290124	0.176	0.061	1.70028E-15	C4_CD8_Teff
chr13-20668764-20669575	2.61067E-20	0.258628921	0.056	0.006	1.72886E-15	C4_CD8_Teff
chr14-21087020-21087996	3.8222E-13	0.295591523	0.326	0.202	2.53118E-08	C1_CD8_Tres
chr10-7817489-7819337	2.952E-20	0.254068752	0.442	0.237	1.9549E-15	C4_CD8_Teff
chr11-35160155-35160602	3.05938E-20	0.304192569	0.14	0.043	2.02601E-15	C4_CD8_Teff
chr1-156257213-156258026	3.10433E-20	0.272083075	0.07	0.012	2.05578E-15	C4_CD8_Teff
chr19-19010383-19010932	3.21669E-20	0.276537501	0.073	0.011	2.13019E-15	C4_CD8_Teff
chr19-14409541-14410411	3.31093E-20	0.297925173	0.186	0.068	2.1926E-15	C4_CD8_Teff
chr1-75784374-75785133	3.14862E-29	0.511336666	0.214	0.054	2.08511E-24	C3_CD8_Tem
chr14-106417041-106418015	3.57154E-20	0.30205673	0.153	0.049	2.36518E-15	C4_CD8_Teff
chr2-38740614-38741591	3.74246E-20	0.30091867	0.12	0.032	2.47837E-15	C4_CD8_Teff
chr16-48612737-48613497	3.76085E-20	0.259579958	0.404	0.215	2.49055E-15	C4_CD8_Teff
chr1-161523391-161524567	3.78504E-20	0.288385157	0.268	0.122	2.50657E-15	C4_CD8_Teff
chr3-189120952-189121619	3.95976E-20	0.301590347	0.132	0.039	2.62227E-15	C4_CD8_Teff
chr19-20660864-20661836	4.07249E-20	0.276589635	0.316	0.15	2.69693E-15	C4_CD8_Teff
chr7-812579-813519	4.31001E-20	0.297394073	0.187	0.07	2.85422E-15	C4_CD8_Teff
chr9-126636966-126637873	6.12289E-56	0.745561807	0.331	0.051	4.05476E-51	C3_CD8_Tem
chr10-131903651-131904012	4.36328E-20	0.287882337	0.105	0.023	2.88949E-15	C4_CD8_Teff
chr20-31612508-31613201	4.56587E-20	0.281483489	0.292	0.138	3.02366E-15	C4_CD8_Teff
chr5-115295809-115297010	4.72491E-20	0.293010371	0.206	0.08	3.12898E-15	C4_CD8_Teff
chr7-50333272-50333646	1.2986E-11	0.295741441	0.112	0.034	8.59971E-07	C3_CD8_Tem
chrX-71619070-71619528	4.84959E-20	0.283744861	0.284	0.132	3.21154E-15	C4_CD8_Teff
chr6-26553161-26555402	4.86831E-20	0.2690595	0.362	0.18	3.22394E-15	C4_CD8_Teff
chr17-40533110-40533839	5.25898E-20	0.269514799	0.342	0.174	3.48265E-15	C4_CD8_Teff
chr2-230865592-230867477	5.37672E-20	0.291370996	0.224	0.09	3.56063E-15	C4_CD8_Teff
chr4-185470292-185471891	5.44343E-20	0.256747279	0.37	0.195	3.6048E-15	C4_CD8_Teff

chr17-74463106-74464291	5.46666E-20	0.282638892	0.288	0.129	3.62019E-15	C4_CD8_Teff
chr1-168408758-168409328	5.56062E-20	0.261884108	0.353	0.19	3.68241E-15	C4_CD8_Teff
chr3-50335510-50335801	5.96771E-20	0.294873047	0.12	0.032	3.95199E-15	C4_CD8_Teff
chr7-75156628-75157437	6.22015E-20	0.298657816	0.196	0.076	4.11917E-15	C4_CD8_Teff
chr5-142223267-142224134	5.57883E-24	0.444587947	0.254	0.096	3.69447E-19	C1_CD8_Tres
chr3-188974768-188975646	6.88506E-20	0.29893897	0.184	0.07	4.5595E-15	C4_CD8_Teff
chr2-38742812-38743107	6.92457E-20	0.280441704	0.079	0.015	4.58565E-15	C4_CD8_Teff
chr9-137458755-137459579	7.01465E-20	0.253547183	0.392	0.211	4.64531E-15	C4_CD8_Teff
chr19-35462181-35463328	7.34613E-20	0.25236624	0.419	0.234	4.86483E-15	C4_CD8_Teff
chr10-114495192-114495890	8.17788E-20	0.300156489	0.144	0.044	5.41564E-15	C4_CD8_Teff
chr1-37689665-37690791	8.19151E-20	0.250683687	0.43	0.232	5.42466E-15	C4_CD8_Teff
chr9-114348407-114349465	8.30975E-20	0.262280461	0.381	0.197	5.50297E-15	C4_CD8_Teff
chr19-13833263-13833967	8.38245E-20	0.272052042	0.323	0.165	5.55111E-15	C4_CD8_Teff
chr11-72821707-72822779	6.32168E-11	0.251787997	0.25	0.146	4.1864E-06	C3_CD8_Tem
chr5-107474537-107475439	4.24597E-33	0.508538975	0.367	0.131	2.81181E-28	C3_CD8_Tem
chr1-30749570-30750173	9.71126E-20	0.27897918	0.271	0.128	6.43108E-15	C4_CD8_Teff
chr16-85518417-85520080	6.90481E-12	0.287743472	0.109	0.038	4.57257E-07	C3_CD8_Tem
chr14-99240565-99241669	1.00375E-19	0.271489894	0.345	0.177	6.64714E-15	C4_CD8_Teff
chr16-88705900-88706900	1.02322E-19	0.251375561	0.409	0.225	6.77606E-15	C4_CD8_Teff
chr2-8836573-8837825	1.03255E-19	0.260401404	0.369	0.196	6.83785E-15	C4_CD8_Teff
chr7-74451939-74452898	2.80838E-43	0.657824435	0.365	0.097	1.8598E-38	C1_CD8_Tres
chr3-46284941-46285553	1.11117E-19	0.283906613	0.266	0.121	7.35851E-15	C4_CD8_Teff
chr3-52454685-52456006	1.15576E-19	0.256455445	0.387	0.208	7.65381E-15	C4_CD8_Teff
chr17-42107246-42108066	1.21391E-19	0.277184041	0.285	0.131	8.03889E-15	C4_CD8_Teff
chr10-6498392-6499278	1.22448E-19	0.272472266	0.321	0.156	8.10885E-15	C4_CD8_Teff
chr16-49463107-49464038	1.32871E-19	0.292392029	0.138	0.041	8.79908E-15	C4_CD8_Teff
chr13-55971544-55971783	7.77768E-14	0.350838013	0.13	0.035	5.15061E-09	C3_CD8_Tem

chr3-39150944-39151194	1.40882E-19	0.291978936	0.169	0.059	9.32962E-15	C4_CD8_Teff
chr2-144659399-144660199	1.06491E-22	0.430371582	0.372	0.168	7.05214E-18	C3_CD8_Tem
chr6-26312475-26313784	1.44925E-19	0.289603487	0.243	0.104	9.59737E-15	C4_CD8_Teff
chr11-67391142-67392398	1.45721E-19	0.254043549	0.392	0.213	9.65008E-15	C4_CD8_Teff
chr11-65506356-65507822	1.47457E-19	0.282274755	0.248	0.113	9.76505E-15	C4_CD8_Teff
chr19-18117400-18118360	1.48841E-19	0.253178184	0.385	0.212	9.85672E-15	C4_CD8_Teff
chr2-112187547-112188686	7.89414E-35	0.540559486	0.389	0.152	5.22774E-30	C1_CD8_Tres
chr11-72743763-72744303	1.77827E-13	0.339143674	0.102	0.026	1.17762E-08	C3_CD8_Tem
chr3-46953178-46953995	1.61758E-19	0.295422173	0.195	0.074	1.07121E-14	C4_CD8_Teff
chr6-29707139-29707420	1.62881E-19	0.27110389	0.077	0.013	1.07864E-14	C4_CD8_Teff
chr15-101242180-101243023	1.62962E-19	0.279252604	0.299	0.139	1.07918E-14	C4_CD8_Teff
chr17-979239-980290	1.63704E-19	0.268341017	0.325	0.163	1.0841E-14	C4_CD8_Teff
chr1-101111621-101112701	6.09935E-08	0.26322676	0.162	0.08	0.004039175	C1_CD8_Tres
chrX-13093411-13093961	1.8992E-19	0.287208607	0.223	0.096	1.25771E-14	C4_CD8_Teff
chr19-4769073-4769865	1.97586E-19	0.292510937	0.112	0.03	1.30847E-14	C4_CD8_Teff
chr14-98704378-98704827	2.02143E-19	0.274805773	0.081	0.016	1.33865E-14	C4_CD8_Teff
chr1-156813778-156814456	2.12409E-19	0.258273853	0.356	0.194	1.40664E-14	C4_CD8_Teff
chr2-216114326-216115111	2.1355E-19	0.273001969	0.316	0.152	1.41419E-14	C4_CD8_Teff
chr7-100965668-100966641	6.63264E-11	0.275888041	0.093	0.029	4.39233E-06	C1_CD8_Tres
chr13-112968220-112969617	7.84375E-10	0.252312824	0.189	0.094	5.19436E-05	C1_CD8_Tres
chr6-27687183-27688548	2.22462E-19	0.272694951	0.318	0.155	1.47321E-14	C4_CD8_Teff
chr10-50503586-50504280	2.28011E-19	0.289141852	0.101	0.025	1.50995E-14	C4_CD8_Teff
chr5-140699623-140700794	2.32016E-19	0.256005597	0.406	0.212	1.53648E-14	C4_CD8_Teff
chr14-105981229-105981844	2.40254E-19	0.287568401	0.101	0.026	1.59103E-14	C4_CD8_Teff
chr7-128506664-128507252	2.41449E-19	0.263219857	0.068	0.009	1.59895E-14	C4_CD8_Teff
chr3-189142162-189142896	2.7509E-19	0.290506647	0.187	0.067	1.82173E-14	C4_CD8_Teff
chr3-108862366-108863843	2.82442E-19	0.292032488	0.164	0.058	1.87042E-14	C4_CD8_Teff

chrX-107454096-107454662	2.88572E-19	0.294487742	0.179	0.065	1.91101E-14	C4_CD8_Teff
chr3-46536702-46537036	2.88995E-19	0.288539393	0.107	0.027	1.91381E-14	C4_CD8_Teff
chr12-128794493-128795204	1.84793E-25	0.460251928	0.156	0.033	1.22375E-20	C3_CD8_Tem
chr6-26596487-26597466	2.97464E-19	0.250772575	0.415	0.223	1.9699E-14	C4_CD8_Teff
chr9-130007296-130008059	3.03397E-19	0.292733885	0.206	0.085	2.00918E-14	C4_CD8_Teff
chr1-147540684-147541537	1.2471E-15	0.353993982	0.198	0.079	8.25868E-11	C1_CD8_Tres
chr7-50367163-50367968	3.2128E-19	0.285991007	0.248	0.11	2.12762E-14	C4_CD8_Teff
chr19-1045175-1045527	3.35997E-19	0.295567311	0.131	0.04	2.22507E-14	C4_CD8_Teff
chr1-160766869-160768605	3.42706E-19	0.256347371	0.392	0.205	2.2695E-14	C4_CD8_Teff
chr15-40313542-40313961	7.19251E-15	0.333791992	0.115	0.03	4.76309E-10	C3_CD8_Tem
chr15-29057582-29058217	3.55734E-19	0.267661562	0.074	0.013	2.35577E-14	C4_CD8_Teff
chr6-148760150-148761881	3.57238E-19	0.276134092	0.288	0.136	2.36574E-14	C4_CD8_Teff
chr20-35740396-35740682	3.7742E-19	0.287905194	0.103	0.027	2.49939E-14	C4_CD8_Teff
chr20-32534602-32535602	8.91098E-14	0.299504629	0.231	0.121	5.90112E-09	C1_CD8_Tres
chr11-116931463-116931982	4.39582E-19	0.290802208	0.164	0.057	2.91105E-14	C4_CD8_Teff
chr20-53844774-53845489	1.03296E-10	0.264725997	0.132	0.061	6.84058E-06	C1_CD8_Tres
chr5-14745675-14746143	2.41576E-09	0.273995436	0.078	0.021	0.000159979	C3_CD8_Tem
chr14-61337588-61338216	4.45712E-19	0.262254943	0.351	0.181	2.95164E-14	C4_CD8_Teff
chr18-49491671-49492685	4.46538E-19	0.259845346	0.349	0.181	2.95711E-14	C4_CD8_Teff
chr22-38176976-38177222	4.46738E-19	0.265718197	0.068	0.011	2.95843E-14	C4_CD8_Teff
chr19-16328414-16328703	4.60394E-19	0.292720687	0.168	0.062	3.04887E-14	C4_CD8_Teff
chr11-60455396-60456242	4.84868E-19	0.255429154	0.063	0.009	3.21094E-14	C4_CD8_Teff
chr2-233076629-233077079	5.10914E-19	0.288310097	0.172	0.063	3.38342E-14	C4_CD8_Teff
chr20-51532933-51533471	5.15671E-19	0.286478085	0.122	0.037	3.41493E-14	C4_CD8_Teff
chrX-1480086-1480445	2.28148E-11	0.28551132	0.138	0.053	1.51086E-06	C3_CD8_Tem
chr4-169015460-169016498	1.98768E-25	0.464053206	0.146	0.029	1.3163E-20	C3_CD8_Tem
chr20-8152876-8153675	5.49403E-19	0.281684328	0.1	0.024	3.63831E-14	C4_CD8_Teff

chr4-102612376-102613463	5.544E-19	0.271288671	0.303	0.149	3.67141E-14	C4_CD8_Teff
chr1-23612919-23613923	5.61952E-19	0.289881978	0.14	0.045	3.72141E-14	C4_CD8_Teff
chr4-40309008-40309781	5.74671E-19	0.265362147	0.323	0.165	3.80564E-14	C4_CD8_Teff
chr19-45496368-45497325	5.9671E-19	0.255226499	0.363	0.192	3.95159E-14	C4_CD8_Teff
chr1-110619126-110620321	6.19182E-19	0.276261445	0.259	0.121	4.10041E-14	C4_CD8_Teff
chr1-168519863-168520828	6.43483E-19	0.274001746	0.297	0.144	4.26134E-14	C4_CD8_Teff
chr17-47220233-47221044	6.54376E-19	0.281138118	0.267	0.123	4.33347E-14	C4_CD8_Teff
chr6-28980855-28982825	6.55354E-19	0.279383161	0.255	0.113	4.33995E-14	C4_CD8_Teff
chr15-28985929-28986480	6.65801E-19	0.292190452	0.144	0.047	4.40913E-14	C4_CD8_Teff
chr1-40628211-40628877	6.72609E-19	0.279700487	0.199	0.082	4.45422E-14	C4_CD8_Teff
chrX-53088273-53088954	6.86478E-19	0.286033958	0.241	0.103	4.54606E-14	C4_CD8_Teff
chr4-139554868-139557458	6.87392E-19	0.262462083	0.316	0.15	4.55211E-14	C4_CD8_Teff
chr2-232309525-232310661	7.14836E-19	0.280853543	0.243	0.121	4.73386E-14	C4_CD8_Teff
chr19-3176839-3177481	7.28371E-19	0.276340081	0.268	0.123	4.82349E-14	C4_CD8_Teff
chr13-33743565-33744407	7.52317E-19	0.295632462	0.141	0.045	4.98207E-14	C4_CD8_Teff
chr9-105244100-105245734	7.62851E-19	0.250600679	0.393	0.203	5.05183E-14	C4_CD8_Teff
chr15-70424784-70425447	7.75099E-19	0.260128702	0.063	0.01	5.13293E-14	C4_CD8_Teff
chr16-89319915-89320538	7.83677E-19	0.286278021	0.191	0.072	5.18974E-14	C4_CD8_Teff
chr6-13375080-13375836	9.53492E-19	0.293883313	0.15	0.048	6.31431E-14	C4_CD8_Teff
chr19-35142645-35143303	9.64719E-19	0.286540604	0.138	0.043	6.38866E-14	C4_CD8_Teff
chr9-129235749-129236394	9.82026E-19	0.291765823	0.141	0.047	6.50327E-14	C4_CD8_Teff
chr14-68786002-68786376	9.92483E-19	0.285486747	0.171	0.064	6.57252E-14	C4_CD8_Teff
chr2-96204056-96204309	1.01296E-18	0.272750632	0.085	0.018	6.7081E-14	C4_CD8_Teff
chr3-141403417-141403956	1.03418E-18	0.291765975	0.172	0.063	6.84868E-14	C4_CD8_Teff
chr5-177434384-177434710	1.13796E-18	0.277005475	0.094	0.021	7.53591E-14	C4_CD8_Teff
chr12-123916635-123917094	1.15315E-18	0.288912684	0.151	0.052	7.63649E-14	C4_CD8_Teff
chr22-35306256-35306831	1.17716E-18	0.285427883	0.094	0.021	7.79548E-14	C4_CD8_Teff

chr12-57240019-57240804	1.23998E-18	0.277063779	0.24	0.107	8.21153E-14	C4_CD8_Teff
chr2-105861294-105861522	1.34012E-18	0.267844439	0.074	0.014	8.87468E-14	C4_CD8_Teff
chr3-141413259-141414298	1.44402E-18	0.27509712	0.252	0.117	9.56274E-14	C4_CD8_Teff
chr12-1660083-1660586	1.48404E-18	0.286158682	0.191	0.076	9.82775E-14	C4_CD8_Teff
chr12-101279312-101280435	1.51455E-18	0.25104339	0.361	0.187	1.00298E-13	C4_CD8_Teff
chr14-106145715-106146042	1.56547E-18	0.266500574	0.064	0.01	1.0367E-13	C4_CD8_Teff
chr8-22573948-22574670	1.59118E-18	0.277146915	0.261	0.121	1.05373E-13	C4_CD8_Teff
chr11-122849806-122850635	1.61692E-18	0.271117648	0.288	0.145	1.07078E-13	C4_CD8_Teff
chr19-57476928-57477893	1.63826E-18	0.264242088	0.294	0.149	1.08491E-13	C4_CD8_Teff
chr8-89728858-89729660	1.85269E-28	0.502737603	0.237	0.064	1.22691E-23	C3_CD8_Tem
chr17-35588392-35588820	1.85916E-18	0.288841715	0.14	0.047	1.23119E-13	C4_CD8_Teff
chr16-57984357-57985564	1.86704E-18	0.250156652	0.367	0.197	1.23641E-13	C4_CD8_Teff
chr7-24913108-24913356	1.87716E-18	0.258027463	0.061	0.008	1.24311E-13	C4_CD8_Teff
chr1-53603881-53604633	1.8819E-18	0.268536653	0.078	0.016	1.24625E-13	C4_CD8_Teff
chr17-8885118-8886166	1.92867E-18	0.285503452	0.151	0.05	1.27722E-13	C4_CD8_Teff
chr11-68750938-68751783	4.34398E-11	0.254314769	0.224	0.127	2.87672E-06	C3_CD8_Tem
chr10-109919978-109920280	2.40917E-18	0.275548362	0.088	0.019	1.59543E-13	C4_CD8_Teff
chr20-20710850-20711779	2.48845E-18	0.28608955	0.11	0.03	1.64793E-13	C4_CD8_Teff
chr12-538680-539689	2.49318E-18	0.279428524	0.133	0.042	1.65106E-13	C4_CD8_Teff
chr19-47427236-47428586	6.562E-17	0.31395749	0.317	0.18	4.34556E-12	C1_CD8_Tres
chrX-71622257-71623068	2.63783E-18	0.269696095	0.283	0.138	1.74685E-13	C4_CD8_Teff
chr2-238287604-238287946	2.89424E-18	0.28820424	0.154	0.053	1.91665E-13	C4_CD8_Teff
chr21-42234498-42235063	2.89487E-18	0.256262506	0.351	0.179	1.91707E-13	C4_CD8_Teff
chr16-81872605-81874152	3.06131E-18	0.286377795	0.147	0.048	2.02729E-13	C4_CD8_Teff
chr14-105537601-105538656	3.1423E-18	0.28136685	0.166	0.062	2.08092E-13	C4_CD8_Teff
chr20-31951211-31952284	3.15171E-18	0.250409955	0.377	0.2	2.08716E-13	C4_CD8_Teff
chr2-181465701-181466268	3.15267E-18	0.280397787	0.143	0.049	2.08779E-13	C4_CD8_Teff

chr8-21938183-21938667	3.27454E-18	0.276289894	0.095	0.023	2.1685E-13	C4_CD8_Teff
chr16-50721688-50722453	6.00434E-09	0.252819889	0.094	0.033	0.000397626	C3_CD8_Tem
chr10-6013878-6014870	1.58862E-11	0.315032983	0.162	0.069	1.05203E-06	C1_CD8_Tres
chr17-35548957-35549368	3.64728E-18	0.257861069	0.064	0.011	2.41534E-13	C4_CD8_Teff
chr16-79370347-79371115	3.65345E-18	0.27184582	0.091	0.022	2.41943E-13	C4_CD8_Teff
chr1-146035399-146035994	3.67582E-18	0.284164825	0.165	0.061	2.43424E-13	C4_CD8_Teff
chr9-136977939-136978928	6.49847E-24	0.428114087	0.26	0.106	4.30348E-19	C3_CD8_Tem
chr16-56201875-56202402	3.69902E-18	0.280714513	0.124	0.04	2.4496E-13	C4_CD8_Teff
chr8-66533041-66534234	3.84679E-18	0.252040707	0.378	0.198	2.54746E-13	C4_CD8_Teff
chr14-22547452-22547707	3.88313E-18	0.277311806	0.105	0.028	2.57152E-13	C4_CD8_Teff
chr12-42094064-42094682	4.03568E-18	0.28139955	0.19	0.077	2.67255E-13	C4_CD8_Teff
chr4-40316170-40317474	4.08211E-18	0.255037357	0.337	0.18	2.70329E-13	C4_CD8_Teff
chr2-156335439-156335792	4.32283E-18	0.265410393	0.3	0.149	2.86271E-13	C4_CD8_Teff
chr5-671624-673152	4.38596E-18	0.269690471	0.261	0.12	2.90451E-13	C4_CD8_Teff
chr17-16418759-16419440	4.42352E-18	0.272729731	0.244	0.109	2.92939E-13	C4_CD8_Teff
chr1-10307758-10308315	4.43755E-18	0.277691615	0.098	0.026	2.93868E-13	C4_CD8_Teff
chr19-57192947-57193349	4.49493E-18	0.288630012	0.128	0.04	2.97667E-13	C4_CD8_Teff
chr14-38073289-38075046	4.52184E-18	0.28754451	0.162	0.055	2.9945E-13	C4_CD8_Teff
chr18-22342578-22343028	4.53527E-18	0.281199853	0.151	0.053	3.00339E-13	C4_CD8_Teff
chr3-57671133-57671708	1.61774E-29	0.5093469	0.198	0.046	1.07132E-24	C3_CD8_Tem
chr19-861889-863063	4.57742E-18	0.26981995	0.257	0.121	3.03131E-13	C4_CD8_Teff
chr8-143927925-143929685	1.10813E-15	0.322899144	0.284	0.145	7.33834E-11	C3_CD8_Tem
chr4-169626903-169627554	5.02681E-18	0.268202948	0.08	0.018	3.3289E-13	C4_CD8_Teff
chr2-221546601-221547166	5.16931E-18	0.25376199	0.067	0.013	3.42327E-13	C4_CD8_Teff
chr6-152624869-152625572	5.1789E-18	0.254879825	0.064	0.011	3.42962E-13	C4_CD8_Teff
chr5-65021146-65022063	5.28516E-18	0.286230546	0.163	0.057	3.49999E-13	C4_CD8_Teff
chr10-73763325-73763693	5.31816E-18	0.254337558	0.065	0.011	3.52184E-13	C4_CD8_Teff

chr7-134640813-134641492	1.47261E-33	0.586354536	0.19	0.028	9.75203E-29	C3_CD8_Tem
chr2-68771057-68771646	5.419E-18	0.268378011	0.276	0.14	3.58863E-13	C4_CD8_Teff
chrX-72105175-72105816	1.14474E-09	0.264324342	0.216	0.111	7.58078E-05	C1_CD8_Tres
chr19-50475797-50476961	5.56445E-18	0.256541648	0.328	0.166	3.68495E-13	C4_CD8_Teff
chr19-19403375-19403706	5.59196E-18	0.278764099	0.119	0.036	3.70316E-13	C4_CD8_Teff
chr18-3602484-3603755	5.70328E-18	0.266769928	0.275	0.138	3.77688E-13	C4_CD8_Teff
chr20-36564814-36565280	5.75273E-18	0.269608181	0.095	0.023	3.80963E-13	C4_CD8_Teff
chr16-5117210-5117771	5.81426E-18	0.273852507	0.094	0.022	3.85038E-13	C4_CD8_Teff
chr5-111230124-111230769	6.07657E-18	0.28676686	0.191	0.072	4.02409E-13	C4_CD8_Teff
chr5-1516162-1516472	6.12131E-18	0.254828544	0.066	0.011	4.05371E-13	C4_CD8_Teff
chr3-46279866-46280683	6.21196E-18	0.267201487	0.265	0.127	4.11375E-13	C4_CD8_Teff
chr14-106176911-106177756	6.30951E-18	0.251492068	0.361	0.191	4.17835E-13	C4_CD8_Teff
chr4-109648192-109649022	6.84229E-20	0.44288329	0.183	0.054	4.53117E-15	C1_CD8_Tres
chr7-152675421-152676417	6.56002E-18	0.252836688	0.344	0.182	4.34424E-13	C4_CD8_Teff
chr22-36385999-36386762	6.69176E-18	0.26709804	0.261	0.122	4.43148E-13	C4_CD8_Teff
chr6-31575316-31575761	6.7391E-18	0.277465693	0.179	0.068	4.46283E-13	C4_CD8_Teff
chr10-6958100-6959108	6.79445E-18	0.272227766	0.099	0.026	4.49949E-13	C4_CD8_Teff
chr1-9825060-9825916	6.85242E-18	0.277062037	0.187	0.079	4.53788E-13	C4_CD8_Teff
chr11-63554498-63554704	7.24594E-18	0.26663198	0.074	0.015	4.79848E-13	C4_CD8_Teff
chr21-5128232-5128589	7.30473E-18	0.252360673	0.065	0.011	4.83741E-13	C4_CD8_Teff
chr1-198519185-198521065	7.52488E-18	0.262429511	0.29	0.145	4.9832E-13	C4_CD8_Teff
chr14-91377071-91377696	3.97322E-32	0.560237366	0.242	0.058	2.63119E-27	C3_CD8_Tem
chr11-46352141-46352659	8.05043E-18	0.281734618	0.187	0.071	5.33124E-13	C4_CD8_Teff
chr11-34650095-34650916	6.47119E-10	0.288037441	0.148	0.058	4.28541E-05	C3_CD8_Tem
chr5-68343379-68343949	8.17781E-18	0.276896388	0.15	0.051	5.41559E-13	C4_CD8_Teff
chr16-2519846-2521453	8.3548E-18	0.253518202	0.33	0.169	5.5328E-13	C4_CD8_Teff
chr7-43808245-43808852	8.37392E-18	0.256816806	0.064	0.01	5.54546E-13	C4_CD8_Teff

chr13-50421534-50422621	8.37586E-18	0.279565394	0.125	0.039	5.54674E-13	C4_CD8_Teff
chr6-108837368-108838249	2.01587E-12	0.29313166	0.133	0.047	1.33497E-07	C3_CD8_Tem
chr17-10149804-10150604	8.75248E-18	0.286395488	0.135	0.043	5.79616E-13	C4_CD8_Teff
chr19-492061-492334	8.82124E-18	0.267249083	0.08	0.018	5.84169E-13	C4_CD8_Teff
chr9-5841326-5842455	9.23077E-18	0.274332303	0.235	0.107	6.11289E-13	C4_CD8_Teff
chr2-219249317-219249833	9.23577E-18	0.271952778	0.228	0.104	6.1162E-13	C4_CD8_Teff
chr1-192546054-192547091	9.59189E-18	0.27823531	0.223	0.096	6.35204E-13	C4_CD8_Teff
chr1-206770437-206770829	1.00967E-17	0.277992184	0.106	0.031	6.68631E-13	C4_CD8_Teff
chr1-16167474-16168282	1.04629E-17	0.282595896	0.164	0.062	6.92885E-13	C4_CD8_Teff
chr15-38578506-38578944	1.09661E-17	0.285031282	0.128	0.041	7.26207E-13	C4_CD8_Teff
chr7-1961547-1962350	1.65646E-10	0.254604571	0.211	0.113	1.09696E-05	C3_CD8_Tem
chr16-79380583-79382128	1.21973E-17	0.279349435	0.188	0.075	8.07739E-13	C4_CD8_Teff
chr19-1884948-1885693	1.26553E-17	0.252104232	0.347	0.179	8.38074E-13	C4_CD8_Teff
chr17-78630966-78632304	1.13876E-11	0.317009443	0.219	0.103	7.54121E-07	C3_CD8_Tem
chr19-45778988-45780048	1.75464E-11	0.254229346	0.273	0.158	1.16197E-06	C3_CD8_Tem
chr19-41120609-41121507	7.6395E-21	0.389623995	0.391	0.197	5.05911E-16	C3_CD8_Tem
chr13-29923888-29924434	1.34198E-17	0.276354265	0.152	0.056	8.88696E-13	C4_CD8_Teff
chr14-75283363-75284214	1.35098E-17	0.275127025	0.144	0.049	8.94657E-13	C4_CD8_Teff
chr4-855743-856942	1.36574E-17	0.265358457	0.092	0.022	9.04432E-13	C4_CD8_Teff
chr12-64665425-64665902	2.4521E-10	0.295754259	0.144	0.061	1.62385E-05	C1_CD8_Tres
chr10-89367247-89367952	4.36038E-17	0.377323261	0.141	0.036	2.88758E-12	C3_CD8_Tem
chr7-74788462-74788932	1.44646E-17	0.279801504	0.182	0.071	9.57886E-13	C4_CD8_Teff
chr16-81715201-81715577	1.46135E-17	0.280262296	0.151	0.054	9.6775E-13	C4_CD8_Teff
chr8-125125285-125126199	1.61262E-17	0.277894139	0.191	0.077	1.06793E-12	C4_CD8_Teff
chr10-26470831-26471211	1.6217E-17	0.264741158	0.09	0.022	1.07394E-12	C4_CD8_Teff
chr5-171957368-171958385	1.64088E-17	0.270769546	0.233	0.115	1.08664E-12	C4_CD8_Teff
chr4-80127300-80127706	1.72567E-17	0.278142946	0.15	0.05	1.14279E-12	C4_CD8_Teff

chr22-40054988-40055943	3.13777E-16	0.33834903	0.305	0.16	2.07792E-11	C1_CD8_Tres
chr19-38506681-38507584	1.76716E-17	0.275278276	0.18	0.071	1.17027E-12	C4_CD8_Teff
chr19-43438368-43439720	8.23751E-10	0.285538217	0.195	0.101	5.45512E-05	C1_CD8_Tres
chr22-23984433-23984996	1.83989E-17	0.255849079	0.073	0.014	1.21843E-12	C4_CD8_Teff
chr9-89489522-89490441	1.86652E-17	0.261068827	0.283	0.142	1.23606E-12	C4_CD8_Teff
chr13-25971165-25971891	1.96976E-17	0.279339639	0.146	0.051	1.30443E-12	C4_CD8_Teff
chr8-29445670-29446425	1.97424E-17	0.278289423	0.091	0.023	1.3074E-12	C4_CD8_Teff
chr1-88993689-88994252	2.02772E-17	0.27930917	0.134	0.046	1.34282E-12	C4_CD8_Teff
chr1-1677363-1679152	2.10662E-17	0.276035154	0.185	0.075	1.39506E-12	C4_CD8_Teff
chr6-33312467-33313076	2.1102E-17	0.26486268	0.256	0.118	1.39744E-12	C4_CD8_Teff
chr14-38078091-38079039	2.16517E-17	0.280893349	0.123	0.04	1.43384E-12	C4_CD8_Teff
chr21-45266014-45267006	2.19384E-17	0.272360789	0.215	0.093	1.45283E-12	C4_CD8_Teff
chr1-202201227-202201590	2.22322E-17	0.285003185	0.123	0.037	1.47229E-12	C4_CD8_Teff
chr19-35445789-35446564	2.26106E-17	0.282464233	0.131	0.041	1.49734E-12	C4_CD8_Teff
chr17-2814110-2815366	1.2617E-45	0.653274964	0.497	0.175	8.35533E-41	C1_CD8_Tres
chr10-119686136-119687030	2.31331E-17	0.266176344	0.256	0.119	1.53195E-12	C4_CD8_Teff
chr1-146938154-146939156	2.35006E-17	0.260714009	0.088	0.019	1.55628E-12	C4_CD8_Teff
chr19-34184824-34185469	4.65506E-18	0.386568863	0.122	0.029	3.08272E-13	C3_CD8_Tem
chr19-55251198-55251985	2.43124E-17	0.269353849	0.212	0.092	1.61004E-12	C4_CD8_Teff
chr1-168410353-168410783	2.44974E-17	0.271663315	0.111	0.031	1.62229E-12	C4_CD8_Teff
chr9-70421870-70422103	2.48871E-17	0.275529727	0.117	0.034	1.6481E-12	C4_CD8_Teff
chr6-151373020-151374384	5.48741E-11	0.266810849	0.273	0.168	3.63393E-06	C3_CD8_Tem
chr15-28831788-28832603	2.51612E-17	0.26950501	0.119	0.036	1.66625E-12	C4_CD8_Teff
chr4-2470849-2471049	2.52456E-17	0.26211953	0.08	0.019	1.67184E-12	C4_CD8_Teff
chr1-8167096-8167570	2.569E-17	0.26952139	0.088	0.022	1.70127E-12	C4_CD8_Teff
chr9-33458237-33458907	2.19736E-12	0.314677958	0.153	0.058	1.45516E-07	C1_CD8_Tres
chr22-36370538-36371599	2.61992E-17	0.269374673	0.218	0.095	1.73499E-12	C4_CD8_Teff

chr3-128787317-128788663	2.66513E-17	0.266659937	0.257	0.12	1.76493E-12	C4_CD8_Teff
chr1-224204631-224205148	5.44301E-11	0.292563754	0.115	0.042	3.60452E-06	C3_CD8_Tem
chr8-127977585-127978166	2.86135E-17	0.256647271	0.306	0.155	1.89487E-12	C4_CD8_Teff
chr20-51494211-51494803	2.87603E-17	0.269561778	0.085	0.02	1.90459E-12	C4_CD8_Teff
chr2-144320147-144320851	3.01119E-17	0.269496733	0.24	0.107	1.9941E-12	C4_CD8_Teff
chr6-155155741-155156415	3.16511E-17	0.274167914	0.156	0.055	2.09603E-12	C4_CD8_Teff
chr2-112624045-112624907	3.31679E-17	0.263488903	0.256	0.122	2.19648E-12	C4_CD8_Teff
chrX-124342178-124343394	3.39665E-17	0.258029664	0.279	0.142	2.24936E-12	C4_CD8_Teff
chr2-10311625-10313028	3.65139E-17	0.250997553	0.069	0.013	2.41806E-12	C4_CD8_Teff
chr17-3754431-3755318	5.42542E-11	0.303531253	0.168	0.078	3.59288E-06	C1_CD8_Tres
chr14-51901507-51901923	3.91716E-17	0.257360316	0.072	0.014	2.59406E-12	C4_CD8_Teff
chr19-10508610-10509056	3.96907E-17	0.254274513	0.068	0.013	2.62844E-12	C4_CD8_Teff
chr1-112344441-112345311	4.15156E-17	0.266215723	0.08	0.019	2.74929E-12	C4_CD8_Teff
chrX-48712653-48714151	4.3253E-17	0.271661865	0.211	0.09	2.86434E-12	C4_CD8_Teff
chr22-50027654-50028185	4.48935E-17	0.277298001	0.163	0.06	2.97298E-12	C4_CD8_Teff
chr14-38064845-38065701	8.04613E-09	0.283244731	0.201	0.098	0.000532839	C1_CD8_Tres
chr3-45880671-45881550	4.63521E-17	0.266618594	0.242	0.111	3.06958E-12	C4_CD8_Teff
chr10-17453156-17454822	9.52085E-11	0.2673578	0.141	0.063	6.30499E-06	C3_CD8_Tem
chr14-20624672-20625774	5.91976E-17	0.265816149	0.11	0.032	3.92024E-12	C4_CD8_Teff
chr8-66615249-66615541	5.94611E-17	0.274601494	0.136	0.048	3.93769E-12	C4_CD8_Teff
chr16-4957618-4958544	1.03067E-22	0.448593458	0.254	0.095	6.82543E-18	C1_CD8_Tres
chr1-25099271-25100697	6.17406E-17	0.254997765	0.286	0.145	4.08865E-12	C4_CD8_Teff
chr13-29901673-29902832	6.1749E-17	0.262564192	0.262	0.124	4.08921E-12	C4_CD8_Teff
chr4-1581157-1581420	6.39489E-17	0.270102274	0.19	0.079	4.23489E-12	C4_CD8_Teff
chr13-30157033-30157717	1.84852E-21	0.439652788	0.143	0.034	1.22414E-16	C3_CD8_Tem
chr20-50929317-50929673	6.93044E-17	0.262083794	0.251	0.123	4.58955E-12	C4_CD8_Teff
chr2-96131228-96131850	2.64387E-14	0.328293737	0.143	0.049	1.75085E-09	C3_CD8_Tem

chr12-49342301-49343661	7.57715E-17	0.26391863	0.223	0.099	5.01782E-12	C4_CD8_Teff
chr15-60584079-60584976	8.25496E-17	0.256460557	0.289	0.139	5.46668E-12	C4_CD8_Teff
chr9-87276213-87277089	9.04173E-18	0.358244697	0.109	0.023	5.98771E-13	C3_CD8_Tem
chr12-757930-758385	9.20255E-17	0.278357837	0.127	0.04	6.09421E-12	C4_CD8_Teff
chr6-135102750-135103608	9.55383E-17	0.254366779	0.073	0.016	6.32684E-12	C4_CD8_Teff
chr1-192580320-192581740	1.02932E-16	0.268253348	0.216	0.094	6.81649E-12	C4_CD8_Teff
chr3-28294695-28295255	1.03393E-16	0.250842502	0.066	0.011	6.847E-12	C4_CD8_Teff
chr6-32760929-32761739	1.03808E-16	0.257378631	0.067	0.013	6.87447E-12	C4_CD8_Teff
chr17-2353063-2354351	1.06626E-16	0.252954225	0.312	0.162	7.06111E-12	C4_CD8_Teff
chr4-6201622-6201889	1.08537E-16	0.271393234	0.123	0.041	7.18762E-12	C4_CD8_Teff
chr9-132268767-132270525	1.14184E-16	0.26831571	0.133	0.045	7.5616E-12	C4_CD8_Teff
chr16-87979501-87980612	1.16662E-16	0.257527919	0.288	0.149	7.72569E-12	C4_CD8_Teff
chr7-4883008-4884026	1.38522E-09	0.289628464	0.141	0.059	9.17335E-05	C1_CD8_Tres
chr8-22455099-22455535	1.19952E-16	0.270720396	0.116	0.036	7.94357E-12	C4_CD8_Teff
chr22-25062044-25062947	1.27237E-16	0.257333965	0.277	0.138	8.426E-12	C4_CD8_Teff
chr20-62087952-62088847	1.27577E-16	0.268651662	0.172	0.072	8.44852E-12	C4_CD8_Teff
chr7-150478861-150479476	1.17116E-22	0.500323892	0.159	0.033	7.7558E-18	C1_CD8_Tres
chr2-216348988-216349622	1.31139E-16	0.271211479	0.143	0.052	8.6844E-12	C4_CD8_Teff
chr16-86563511-86564169	1.35281E-16	0.267298344	0.177	0.07	8.95873E-12	C4_CD8_Teff
chr1-8181870-8182991	7.14602E-10	0.288527312	0.183	0.084	4.73231E-05	C1_CD8_Tres
chr5-134131113-134131679	1.4008E-16	0.268837384	0.139	0.049	9.27651E-12	C4_CD8_Teff
chrX-7083577-7084536	1.45557E-16	0.270185817	0.139	0.05	9.63921E-12	C4_CD8_Teff
chr19-45816627-45817502	1.64646E-16	0.26138434	0.232	0.106	1.09033E-11	C4_CD8_Teff
chr19-3989895-3990482	1.65976E-16	0.254672337	0.267	0.135	1.09914E-11	C4_CD8_Teff
chr7-100365442-100366078	1.70411E-16	0.271125486	0.118	0.038	1.12851E-11	C4_CD8_Teff
chr2-161237113-161238201	1.72679E-16	0.264910577	0.233	0.109	1.14353E-11	C4_CD8_Teff
chr19-38924134-38924793	7.96186E-20	0.435908187	0.138	0.031	5.27258E-15	C3_CD8_Tem

chr18-49325808-49326540	1.77698E-16	0.259447983	0.079	0.018	1.17677E-11	C4_CD8_Teff
chr14-105986190-105987143	1.81714E-16	0.256591167	0.267	0.133	1.20336E-11	C4_CD8_Teff
chr7-134667777-134668181	1.91812E-16	0.251877419	0.083	0.019	1.27024E-11	C4_CD8_Teff
chr6-142935354-142936324	1.92991E-16	0.273596146	0.138	0.048	1.27804E-11	C4_CD8_Teff
chr1-64744269-64746053	1.9546E-16	0.267020562	0.153	0.054	1.2944E-11	C4_CD8_Teff
chr1-28232381-28233153	2.04415E-16	0.254918608	0.256	0.128	1.3537E-11	C4_CD8_Teff
chr1-145424557-145425819	2.07418E-16	0.253122844	0.077	0.016	1.37358E-11	C4_CD8_Teff
chr6-130215041-130215856	2.08607E-16	0.264249867	0.212	0.096	1.38146E-11	C4_CD8_Teff
chr10-124693764-124694151	2.09979E-16	0.268882392	0.114	0.035	1.39054E-11	C4_CD8_Teff
chr6-127879604-127880774	2.26834E-16	0.268227383	0.179	0.071	1.50217E-11	C4_CD8_Teff
chr6-16676897-16677812	2.34404E-16	0.262615418	0.095	0.026	1.55229E-11	C4_CD8_Teff
chr1-179317299-179318027	2.64139E-16	0.255768819	0.084	0.021	1.74921E-11	C4_CD8_Teff
chr4-38654125-38654687	1.6119E-15	0.368478093	0.122	0.03	1.06745E-10	C3_CD8_Tem
chr7-36780816-36781372	2.83832E-16	0.267662298	0.177	0.075	1.87962E-11	C4_CD8_Teff
chr3-27900129-27900802	2.87838E-16	0.255220374	0.285	0.141	1.90615E-11	C4_CD8_Teff
chr1-40698858-40699420	2.9115E-16	0.263624119	0.098	0.027	1.92809E-11	C4_CD8_Teff
chr13-99273061-99273690	2.95906E-16	0.266316186	0.182	0.074	1.95958E-11	C4_CD8_Teff
chr19-246602-247455	3.02521E-16	0.26378826	0.118	0.037	2.00339E-11	C4_CD8_Teff
chr18-75037048-75037596	3.06341E-16	0.254339386	0.076	0.017	2.02868E-11	C4_CD8_Teff
chr1-86714200-86714969	3.15621E-16	0.257574111	0.12	0.038	2.09014E-11	C4_CD8_Teff
chr7-5858314-5858888	3.22198E-16	0.252097219	0.076	0.016	2.13369E-11	C4_CD8_Teff
chr5-131266428-131266993	3.24585E-16	0.266026321	0.212	0.093	2.1495E-11	C4_CD8_Teff
chr22-42316743-42317483	3.31162E-16	0.265235379	0.204	0.091	2.19305E-11	C4_CD8_Teff
chr5-157210524-157210938	3.36718E-16	0.257289681	0.094	0.025	2.22985E-11	C4_CD8_Teff
chr6-31351263-31351901	3.39468E-16	0.267532692	0.178	0.075	2.24806E-11	C4_CD8_Teff
chr7-159196994-159197895	3.49376E-16	0.265398687	0.143	0.052	2.31367E-11	C4_CD8_Teff
chr14-63524317-63525177	3.50021E-16	0.267417344	0.117	0.038	2.31794E-11	C4_CD8_Teff

chr10-14583215-14583699	3.5348E-16	0.260980834	0.218	0.101	2.34085E-11	C4_CD8_Teff
chr22-38305504-38306477	3.63446E-16	0.26280278	0.138	0.05	2.40685E-11	C4_CD8_Teff
chr1-149390092-149390941	2.99977E-09	0.251432468	0.081	0.023	0.000198654	C3_CD8_Tem
chr3-136142213-136142946	3.68307E-16	0.271059786	0.136	0.048	2.43904E-11	C4_CD8_Teff
chr6-106513750-106514529	3.8105E-16	0.264706316	0.139	0.052	2.52343E-11	C4_CD8_Teff
chr15-91469028-91470405	3.43343E-16	0.387344732	0.132	0.038	2.27372E-11	C1_CD8_Tres
chr1-66367445-66367920	4.23734E-16	0.258990089	0.106	0.031	2.8061E-11	C4_CD8_Teff
chr6-111736886-111737612	4.64858E-16	0.257251224	0.228	0.105	3.07843E-11	C4_CD8_Teff
chr6-158645662-158645997	4.89148E-16	0.265451626	0.182	0.073	3.23929E-11	C4_CD8_Teff
chr12-11998194-11999166	5.16212E-28	0.53357167	0.228	0.058	3.41851E-23	C1_CD8_Tres
chr8-130235971-130236839	5.0381E-16	0.265613515	0.163	0.065	3.33638E-11	C4_CD8_Teff
chr6-127902865-127903304	5.31544E-16	0.266586789	0.194	0.081	3.52004E-11	C4_CD8_Teff
chr21-33199587-33200143	5.4494E-16	0.257927653	0.238	0.113	3.60876E-11	C4_CD8_Teff
chr20-53750872-53751930	5.51361E-16	0.26463639	0.221	0.098	3.65128E-11	C4_CD8_Teff
chr6-111767632-111768163	5.58928E-16	0.268982361	0.138	0.049	3.70139E-11	C4_CD8_Teff
chr17-7855969-7856180	5.69397E-16	0.259284771	0.1	0.029	3.77072E-11	C4_CD8_Teff
chr1-192559117-192559888	5.72541E-16	0.26786001	0.176	0.069	3.79154E-11	C4_CD8_Teff
chr22-46373864-46374642	4.59855E-10	0.272216172	0.091	0.029	3.0453E-05	C3_CD8_Tem
chr4-80077595-80078076	5.94878E-16	0.263065089	0.167	0.064	3.93946E-11	C4_CD8_Teff
chr19-10515556-10516304	8.66568E-78	0.890608504	0.32	0.031	5.73868E-73	C3_CD8_Tem
chr16-3151987-3152943	6.69883E-16	0.262875891	0.134	0.047	4.43616E-11	C4_CD8_Teff
chr14-77043496-77043949	6.8419E-16	0.25793685	0.097	0.027	4.53091E-11	C4_CD8_Teff
chr15-22757299-22758340	1.11261E-11	0.271281404	0.117	0.043	7.36806E-07	C1_CD8_Tres
chr9-89523816-89524260	7.54661E-16	0.26553887	0.141	0.05	4.99759E-11	C4_CD8_Teff
chr5-39271889-39272647	7.61614E-16	0.263767855	0.112	0.035	5.04364E-11	C4_CD8_Teff
chr1-156119599-156119848	7.639E-16	0.259547573	0.1	0.029	5.05878E-11	C4_CD8_Teff
chr4-1203540-1204028	7.7967E-16	0.261895287	0.196	0.085	5.16321E-11	C4_CD8_Teff

chrX-19333914-19334628	7.98189E-16	0.257785314	0.098	0.026	5.28584E-11	C4_CD8_Teff
chr2-197803986-197805480	1.05042E-09	0.266988352	0.24	0.143	6.95623E-05	C1_CD8_Tres
chr1-12438275-12438835	8.57913E-16	0.259190095	0.208	0.095	5.68135E-11	C4_CD8_Teff
chr6-142838763-142839298	9.41957E-16	0.263037618	0.168	0.069	6.23792E-11	C4_CD8_Teff
chr3-46440680-46441475	9.43221E-16	0.255668063	0.253	0.123	6.24629E-11	C4_CD8_Teff
chr22-44190923-44191455	9.49208E-16	0.255848322	0.105	0.029	6.28594E-11	C4_CD8_Teff
chr11-35330558-35331318	9.49533E-16	0.265199777	0.167	0.067	6.28809E-11	C4_CD8_Teff
chr10-84177301-84178035	9.55616E-16	0.265984259	0.136	0.05	6.32838E-11	C4_CD8_Teff
chr15-40903778-40904276	1.00191E-15	0.25830656	0.215	0.097	6.63492E-11	C4_CD8_Teff
chr15-67067971-67068344	1.01231E-15	0.26763844	0.124	0.042	6.70381E-11	C4_CD8_Teff
chr17-3788051-3788536	1.03106E-15	0.265560832	0.176	0.071	6.82797E-11	C4_CD8_Teff
chr16-78705687-78706198	1.06018E-15	0.264997337	0.118	0.037	7.02085E-11	C4_CD8_Teff
chr1-38558924-38559993	3.22188E-12	0.284681468	0.207	0.111	2.13363E-07	C1_CD8_Tres
chr22-20509324-20509708	1.30467E-15	0.262269315	0.131	0.045	8.63995E-11	C4_CD8_Teff
chr10-29734990-29736750	2.80679E-12	0.300616696	0.171	0.08	1.85874E-07	C1_CD8_Tres
chr1-156124587-156125379	1.37916E-15	0.259703058	0.147	0.051	9.13321E-11	C4_CD8_Teff
chr9-41358509-41359426	1.44093E-15	0.250112783	0.086	0.021	9.54224E-11	C4_CD8_Teff
chr1-55217513-55217826	1.48724E-15	0.262849567	0.142	0.051	9.84897E-11	C4_CD8_Teff
chr11-61021304-61022550	2.98065E-16	0.352152376	0.243	0.119	1.97388E-11	C1_CD8_Tres
chr2-46439221-46440197	1.59845E-15	0.251547272	0.105	0.03	1.05854E-10	C4_CD8_Teff
chr6-16316586-16317072	1.62512E-15	0.258618703	0.105	0.031	1.07621E-10	C4_CD8_Teff
chr16-78794405-78795284	1.81568E-15	0.261912123	0.15	0.058	1.2024E-10	C4_CD8_Teff
chr2-239630043-239630854	1.83793E-15	0.258197579	0.107	0.035	1.21714E-10	C4_CD8_Teff
chr11-62853215-62853866	1.69863E-13	0.330416285	0.144	0.057	1.12488E-08	C1_CD8_Tres
chr2-218230831-218231380	2.09649E-15	0.252151704	0.229	0.11	1.38836E-10	C4_CD8_Teff
chr10-110860118-110861209	2.22725E-15	0.256637137	0.217	0.097	1.47495E-10	C4_CD8_Teff
chr12-89039825-89040764	2.01136E-08	0.255193774	0.126	0.057	0.001331983	C1_CD8_Tres

chr2-161356884-161357449	2.38051E-15	0.256399408	0.122	0.041	1.57644E-10	C4_CD8_Teff
chr1-223843917-223844264	2.55826E-15	0.257456804	0.124	0.041	1.69416E-10	C4_CD8_Teff
chr7-122143423-122144686	2.5672E-15	0.259770393	0.129	0.045	1.70008E-10	C4_CD8_Teff
chr3-17179520-17180186	2.588E-15	0.255004319	0.099	0.028	1.71385E-10	C4_CD8_Teff
chrX-149513423-149514464	5.47356E-10	0.264679642	0.141	0.061	3.62476E-05	C3_CD8_Tem
chr6-37489657-37490288	2.77111E-15	0.259972014	0.13	0.046	1.83511E-10	C4_CD8_Teff
chr19-2257763-2258367	2.90016E-15	0.260314143	0.178	0.076	1.92057E-10	C4_CD8_Teff
chr8-141403636-141404346	4.24701E-11	0.31060639	0.18	0.075	2.8125E-06	C1_CD8_Tres
chr14-106061374-106062230	3.00279E-15	0.256839248	0.133	0.045	1.98854E-10	C4_CD8_Teff
chr14-106308753-106309349	3.27575E-15	0.251950994	0.089	0.026	2.1693E-10	C4_CD8_Teff
chr10-3867188-3868292	3.26219E-21	0.443055402	0.198	0.066	2.16032E-16	C1_CD8_Tres
chr5-50699466-50700075	3.5507E-15	0.256662413	0.114	0.037	2.35138E-10	C4_CD8_Teff
chr15-92907860-92908224	3.64229E-15	0.25200474	0.187	0.084	2.41204E-10	C4_CD8_Teff
chr7-70669706-70670633	1.66801E-14	0.348515634	0.133	0.037	1.10461E-09	C3_CD8_Tem
chrX-129786960-129787957	3.89025E-15	0.254618165	0.125	0.041	2.57624E-10	C4_CD8_Teff
chr1-9714260-9714637	3.97741E-15	0.256097539	0.1	0.031	2.63396E-10	C4_CD8_Teff
chr2-173145274-173145781	4.11831E-15	0.254732522	0.102	0.031	2.72727E-10	C4_CD8_Teff
chr11-11995396-11996007	4.24833E-15	0.260182133	0.109	0.033	2.81337E-10	C4_CD8_Teff
chr19-40528890-40529575	4.48871E-15	0.255389653	0.176	0.076	2.97256E-10	C4_CD8_Teff
chr2-106085192-106086352	8.18258E-09	0.260742928	0.201	0.111	0.000541875	C1_CD8_Tres
chr16-30068855-30069058	4.65461E-15	0.253996861	0.099	0.03	3.08242E-10	C4_CD8_Teff
chr5-150398547-150399063	4.66096E-15	0.25544359	0.096	0.026	3.08663E-10	C4_CD8_Teff
chr5-76473460-76474051	4.75565E-15	0.251666286	0.172	0.074	3.14934E-10	C4_CD8_Teff
chr10-3805891-3806195	4.82555E-15	0.250318317	0.195	0.087	3.19563E-10	C4_CD8_Teff
chr16-57083692-57084747	5.20988E-15	0.2582183	0.178	0.077	3.45014E-10	C4_CD8_Teff
chr3-50588289-50588897	9.88203E-16	0.339502053	0.237	0.108	6.54418E-11	C3_CD8_Tem
chr1-12159777-12160066	5.63432E-15	0.251051533	0.097	0.028	3.73122E-10	C4_CD8_Teff

chr6-139262193-139263168	5.98917E-15	0.254300243	0.123	0.044	3.96621E-10	C4_CD8_Teff
chr6-152185992-152186568	4.84535E-10	0.271446433	0.117	0.043	3.20873E-05	C3_CD8_Tem
chr1-6599293-6599844	6.536E-15	0.255232647	0.15	0.057	4.32834E-10	C4_CD8_Teff
chr16-78897103-78897904	6.78557E-15	0.258685999	0.129	0.044	4.49361E-10	C4_CD8_Teff
chr1-158978573-158979257	6.91648E-14	0.322162703	0.148	0.053	4.5803E-09	C3_CD8_Tem
chr14-73771549-73772628	7.5563E-15	0.250963556	0.175	0.078	5.00401E-10	C4_CD8_Teff
chr19-13215486-13216341	7.62038E-15	0.257651251	0.145	0.057	5.04645E-10	C4_CD8_Teff
chr2-105747999-105748419	8.42341E-15	0.253739669	0.162	0.064	5.57824E-10	C4_CD8_Teff
chr8-143974875-143975161	8.49599E-15	0.253370548	0.169	0.07	5.6263E-10	C4_CD8_Teff
chr10-3960681-3961472	8.58354E-15	0.254765658	0.157	0.066	5.68428E-10	C4_CD8_Teff
chr15-28984650-28985528	8.71381E-15	0.255864052	0.186	0.079	5.77055E-10	C4_CD8_Teff
chr15-78750624-78751398	9.00689E-15	0.258204989	0.15	0.058	5.96463E-10	C4_CD8_Teff
chr10-22599050-22599468	9.50463E-15	0.254000505	0.129	0.044	6.29425E-10	C4_CD8_Teff
chr16-30873288-30873655	1.01609E-14	0.256161818	0.157	0.062	6.72883E-10	C4_CD8_Teff
chr3-119437547-119438330	1.13337E-14	0.250738152	0.086	0.023	7.5055E-10	C4_CD8_Teff
chr1-27608555-27610024	1.19713E-14	0.252098209	0.176	0.077	7.92779E-10	C4_CD8_Teff
chr12-8088462-8088942	1.49839E-14	0.251205053	0.129	0.045	9.92281E-10	C4_CD8_Teff
chr3-111392379-111393380	1.55344E-14	0.255813263	0.139	0.052	1.02874E-09	C4_CD8_Teff
chr3-97910188-97911295	1.56623E-14	0.25037867	0.12	0.044	1.03721E-09	C4_CD8_Teff
chr5-75052504-75054047	1.73032E-14	0.250370875	0.176	0.073	1.14587E-09	C4_CD8_Teff
chr4-4260137-4260549	1.84888E-14	0.254319323	0.149	0.058	1.22438E-09	C4_CD8_Teff
chr19-49987087-49987666	1.87206E-14	0.250903231	0.116	0.041	1.23973E-09	C4_CD8_Teff
chr1-43581747-43582584	1.24019E-09	0.290724709	0.112	0.044	8.21289E-05	C3_CD8_Tem
chr1-25561794-25562997	2.09487E-14	0.252107966	0.164	0.069	1.38728E-09	C4_CD8_Teff
chr5-111231007-111231380	2.83463E-14	0.251775745	0.157	0.064	1.87718E-09	C4_CD8_Teff
chr5-107570845-107572433	2.85738E-10	0.278378724	0.185	0.091	1.89224E-05	C3_CD8_Tem
chr13-110676140-110677459	5.34894E-86	1.298777767	0.464	0.016	3.54223E-81	C5_CD8_Tem

chr5-911844-913044	1.7074E-52	0.807328372	0.781	0.236	1.13069E-47	C5_CD8_Tem
chr13-98484347-98485269	6.8264E-11	0.288192023	0.222	0.116	4.52065E-06	C1_CD8_Tres
chr5-150780020-150781167	1.15394E-40	0.868905669	0.481	0.088	7.64172E-36	C5_CD8_Tem
chr20-33369167-33370781	3.93941E-13	0.269654678	0.339	0.188	2.6088E-08	C3_CD8_Tem
chr17-80678456-80679463	2.24081E-37	0.890563008	0.246	0.013	1.48393E-32	C5_CD8_Tem
chr9-97703617-97704354	2.24922E-32	0.807870336	0.251	0.023	1.4895E-27	C5_CD8_Tem
chr17-80720552-80721593	3.2612E-67	0.516808902	0.353	0.09	2.15966E-62	C4_CD8_Teff
chr13-29885997-29887364	6.18268E-30	0.738437443	0.448	0.113	4.09436E-25	C5_CD8_Tem
chrX-317539-319259	2.58242E-68	0.675579487	0.814	0.299	1.71015E-63	C1_CD8_Tres
chr1-156126095-156127656	2.37408E-15	0.276229646	0.497	0.337	1.57218E-10	C1_CD8_Tres
chr11-121464061-121464975	3.91504E-26	0.463748041	0.455	0.218	2.59266E-21	C1_CD8_Tres
chr1-66287844-66288414	6.07496E-28	0.73520665	0.311	0.052	4.02302E-23	C5_CD8_Tem
chr16-4700246-4700755	1.06881E-27	0.737551845	0.273	0.038	7.078E-23	C5_CD8_Tem
chr12-133079608-133081270	3.11646E-34	0.51529982	0.569	0.241	2.06381E-29	C1_CD8_Tres
chr6-90411120-90411851	2.92076E-09	0.253693676	0.237	0.153	0.000193422	C1_CD8_Tres
chr3-93470260-93470829	4.37216E-18	0.366742856	0.674	0.293	2.89537E-13	C1_CD8_Tres
chr17-1672879-1673546	1.39765E-25	0.741994205	0.29	0.045	9.25566E-21	C5_CD8_Tem
chr4-1928299-1928994	2.59094E-25	0.734130852	0.202	0.015	1.7158E-20	C5_CD8_Tem
chr19-19605807-19606451	4.12717E-24	0.440626722	0.308	0.127	2.73314E-19	C1_CD8_Tres
chr16-89319915-89320538	7.83677E-19	0.286278021	0.191	0.072	5.18974E-14	C4_CD8_Teff
chr19-2627277-2628210	1.50417E-22	0.653789351	0.257	0.039	9.96108E-18	C5_CD8_Tem
chr19-45455498-45456572	1.54009E-22	0.583168551	0.475	0.166	1.0199E-17	C5_CD8_Tem
chr10-3942601-3943086	2.29708E-22	0.653821697	0.169	0.014	1.5212E-17	C5_CD8_Tem
chr12-13179234-13180371	2.50897E-22	0.662479472	0.24	0.032	1.66151E-17	C5_CD8_Tem
chr9-30075103-30075874	1.43471E-21	0.673255929	0.18	0.015	9.5011E-17	C5_CD8_Tem
chr1-148371452-148373872	2.26161E-15	0.36870369	0.207	0.086	1.4977E-10	C1_CD8_Tres
chr3-115791932-115792900	1.77297E-15	0.352446555	0.225	0.107	1.17411E-10	C1_CD8_Tres

chr9-40991238-40992772	3.23214E-21	0.360534696	0.569	0.393	2.14042E-16	C1_CD8_Tres
chr17-2239905-2240417	3.47511E-21	0.641713777	0.148	0.008	2.30132E-16	C5_CD8_Tem
chr9-75083211-75083544	5.39998E-21	0.666418161	0.24	0.033	3.57603E-16	C5_CD8_Tem
chr1-23958530-23960278	2.65789E-38	0.527470675	0.575	0.248	1.76013E-33	C1_CD8_Tres
chr8-144146829-144148682	1.02688E-32	0.47427437	0.488	0.213	6.80028E-28	C1_CD8_Tres
chr9-91795771-91796794	6.94187E-21	0.642466668	0.257	0.046	4.59711E-16	C5_CD8_Tem
chr10-11156277-11156991	2.4254E-20	0.633232439	0.197	0.024	1.60617E-15	C5_CD8_Tem
chr1-175004976-175005433	2.98372E-20	0.638443089	0.153	0.011	1.97591E-15	C5_CD8_Tem
chr19-43667974-43669476	9.67163E-40	0.522033166	0.602	0.282	6.40484E-35	C1_CD8_Tres
chr1-16727130-16727940	5.18086E-20	0.604258073	0.301	0.064	3.43092E-15	C5_CD8_Tem
chr1-207335669-207337433	1.39886E-19	0.553889166	0.421	0.142	9.26369E-15	C5_CD8_Tem
chr10-12333018-12334141	1.56782E-19	0.607879145	0.235	0.04	1.03826E-14	C5_CD8_Tem
chr12-51393879-51394537	2.41833E-19	0.568739928	0.388	0.114	1.60149E-14	C5_CD8_Tem
chr1-148522069-148523165	3.42945E-13	0.30178345	0.302	0.157	2.27109E-08	C3_CD8_Tem
chr6-31792847-31793216	3.0632E-19	0.601108647	0.137	0.01	2.02854E-14	C5_CD8_Tem
chr4-82284216-82285491	1.55078E-13	0.28098272	0.53	0.382	1.02697E-08	C1_CD8_Tres
chr2-43183820-43184763	4.24157E-19	0.600118873	0.311	0.074	2.8089E-14	C5_CD8_Tem
chr3-52216805-52217645	7.48482E-26	0.491619564	0.225	0.067	4.95667E-21	C1_CD8_Tres
chr20-5719626-5720622	1.90509E-71	0.54256261	0.239	0.035	1.26161E-66	C4_CD8_Teff
chr22-37082324-37082607	7.93389E-19	0.560092567	0.109	0.004	5.25406E-14	C5_CD8_Tem
chr12-13195937-13196934	6.41713E-18	0.395444658	0.308	0.145	4.24962E-13	C1_CD8_Tres
chr16-87457196-87458130	1.87293E-18	0.613409291	0.235	0.042	1.24031E-13	C5_CD8_Tem
chr1-146376265-146377483	5.82881E-10	0.27400274	0.219	0.113	3.86002E-05	C1_CD8_Tres
chr20-8131554-8132972	2.62397E-13	0.306232432	0.273	0.141	1.73767E-08	C3_CD8_Tem
chr1-59814144-59815420	1.02231E-16	0.34693471	0.257	0.125	6.77007E-12	C1_CD8_Tres
chr3-20047339-20047600	2.82794E-18	0.494498984	0.082	0.001	1.87275E-13	C5_CD8_Tem
chr20-44495271-44496462	3.23087E-18	0.583617761	0.306	0.073	2.13958E-13	C5_CD8_Tem

chr16-2846458-2847278	3.84891E-32	0.376358358	0.16	0.036	2.54887E-27	C4_CD8_Teff
chr2-43130912-43131861	5.94313E-18	0.369728277	0.76	0.461	3.93572E-13	C5_CD8_Tem
chr19-8591726-8592924	1.85348E-26	0.477412579	0.344	0.138	1.22743E-21	C1_CD8_Tres
chr1-116508136-116509326	7.43199E-18	0.520365544	0.432	0.149	4.92169E-13	C5_CD8_Tem
chr13-98480746-98481317	1.83931E-17	0.565080779	0.339	0.097	1.21805E-12	C5_CD8_Tem
chr16-3020156-3022540	8.22231E-37	0.289219126	0.624	0.369	5.44506E-32	C4_CD8_Teff
chr22-18151993-18152258	3.00671E-17	0.544179922	0.104	0.005	1.99113E-12	C5_CD8_Tem
chr8-30655795-30656170	3.13798E-17	0.571060151	0.202	0.032	2.07807E-12	C5_CD8_Tem
chr14-91260813-91261599	3.20341E-17	0.573919129	0.268	0.061	2.1214E-12	C5_CD8_Tem
chr14-68685054-68686144	9.5723E-24	0.255392433	0.498	0.29	6.33906E-19	C4_CD8_Teff
chr19-13850407-13851612	4.9456E-17	0.468930393	0.503	0.217	3.27512E-12	C5_CD8_Tem
chr20-5714028-5715411	8.29342E-35	0.527268459	0.413	0.156	5.49215E-30	C1_CD8_Tres
chr1-15523836-15525464	1.06113E-24	0.284260088	0.766	0.607	7.02711E-20	C1_CD8_Tres
chr16-352134-353545	3.74363E-23	0.331969925	0.584	0.391	2.47914E-18	C1_CD8_Tres
chr13-110677706-110677951	8.70334E-17	0.546427048	0.104	0.006	5.76361E-12	C5_CD8_Tem
chr20-3665344-3666479	1.20505E-16	0.5447042	0.344	0.099	7.9802E-12	C5_CD8_Tem
chr10-17238002-17239250	1.65842E-16	0.544925506	0.202	0.033	1.09826E-11	C5_CD8_Tem
chr12-104218955-104220318	2.06991E-33	0.582720358	0.243	0.058	1.37075E-28	C1_CD8_Tres
chr17-83199175-83200859	6.06288E-53	0.640654781	0.396	0.109	4.01502E-48	C3_CD8_Tem
chr6-159984234-159984684	5.95009E-16	0.563388798	0.164	0.019	3.94033E-11	C5_CD8_Tem
chr5-80759910-80760514	6.82085E-16	0.574286143	0.169	0.02	4.51697E-11	C5_CD8_Tem
chr10-3754995-3755391	3.21285E-13	0.334462525	0.111	0.031	2.12764E-08	C1_CD8_Tres
chr19-38838538-38838914	7.64211E-16	0.55590227	0.191	0.03	5.06084E-11	C5_CD8_Tem
chr1-52636599-52637735	6.75294E-56	0.418330165	0.506	0.212	4.472E-51	C4_CD8_Teff
chr1-633460-634698	2.63058E-61	0.684577475	0.769	0.293	1.74205E-56	C1_CD8_Tres
chr4-74444592-74445637	4.39675E-13	0.342344958	0.21	0.091	2.91166E-08	C1_CD8_Tres
chr8-144136968-144138476	4.84082E-23	0.432229091	0.377	0.162	3.20574E-18	C1_CD8_Tres

chr16-81493560-81493983	1.59946E-15	0.554760217	0.142	0.014	1.05921E-10	C5_CD8_Tem
chr10-45420434-45420818	1.69529E-15	0.546377665	0.131	0.013	1.12267E-10	C5_CD8_Tem
chr2-9781835-9782724	1.4434E-18	0.393346852	0.263	0.108	9.55864E-14	C1_CD8_Tres
chr10-29658562-29659853	1.44412E-33	0.344070109	0.418	0.181	9.56341E-29	C4_CD8_Teff
chr19-4812769-4813304	2.47345E-15	0.488036056	0.115	0.009	1.63799E-10	C5_CD8_Tem
chr6-193662-194430	1.35654E-14	0.33530034	0.221	0.101	8.98343E-10	C3_CD8_Tem
chr1-145280570-145282226	8.15049E-14	0.331126486	0.249	0.117	5.3975E-09	C1_CD8_Tres
chr14-91835529-91836905	4.95228E-13	0.272180666	0.395	0.262	3.27955E-08	C1_CD8_Tres
chr19-35247585-35249208	3.06785E-15	0.488876056	0.366	0.127	2.03162E-10	C5_CD8_Tem
chr10-71730365-71730636	3.08459E-15	0.560599124	0.23	0.049	2.04271E-10	C5_CD8_Tem
chr19-38841624-38842824	4.28536E-10	0.262770609	0.263	0.153	2.83789E-05	C1_CD8_Tres
chr12-31732958-31733460	3.99506E-15	0.538811075	0.24	0.056	2.64565E-10	C5_CD8_Tem
chr9-248582-249420	4.1139E-15	0.557396061	0.164	0.023	2.72435E-10	C5_CD8_Tem
chr1-121184236-121185085	2.98094E-29	0.53496817	0.47	0.186	1.97407E-24	C1_CD8_Tres
chr4-9151875-9153538	2.32207E-16	0.261695621	0.602	0.455	1.53774E-11	C3_CD8_Tem
chr6-224832-225792	1.80924E-25	0.28518241	0.437	0.231	1.19813E-20	C4_CD8_Teff
chr14-49902642-49902931	5.48177E-15	0.509961444	0.093	0.005	3.63019E-10	C5_CD8_Tem
chr10-22681345-22683701	6.71596E-15	0.3124979	0.76	0.506	4.44751E-10	C5_CD8_Tem
chr10-131900106-131901808	1.66157E-30	0.503829364	0.446	0.179	1.10034E-25	C1_CD8_Tres
chr12-89039825-89040764	2.01136E-08	0.255193774	0.126	0.057	0.001331983	C1_CD8_Tres
chr1-244341376-244342138	7.8234E-15	0.538837898	0.175	0.029	5.18089E-10	C5_CD8_Tem
chrX-1452437-1454445	9.5588E-22	0.294571942	0.713	0.553	6.33012E-17	C1_CD8_Tres
chr14-52324842-52325579	1.57915E-11	0.303189109	0.167	0.066	1.04576E-06	C3_CD8_Tem
chr2-102226947-102228524	1.18958E-14	0.442555945	0.503	0.214	7.87776E-10	C5_CD8_Tem
chr18-23872449-23873091	1.56593E-44	0.438753315	0.174	0.029	1.037E-39	C4_CD8_Teff
chr3-196246689-196247325	1.2692E-14	0.529809102	0.12	0.01	8.405E-10	C5_CD8_Tem
chr11-587475-588457	1.5028E-14	0.394355231	0.612	0.318	9.95202E-10	C5_CD8_Tem

chr4-140150254-140152549	6.51865E-17	0.349429673	0.281	0.145	4.31684E-12	C1_CD8_Tres
chr14-106174117-106174932	2.56283E-73	0.535398567	0.403	0.104	1.69718E-68	C4_CD8_Teff
chr19-908610-909828	6.27886E-14	0.278582335	0.443	0.305	4.15805E-09	C1_CD8_Tres
chr11-316938-317527	9.17875E-30	0.359260013	0.172	0.046	6.07844E-25	C4_CD8_Teff
chr11-10307208-10308280	1.69199E-49	0.425991537	0.414	0.149	1.12049E-44	C4_CD8_Teff
chr6-154296-154947	1.85162E-14	0.497107155	0.131	0.013	1.2262E-09	C5_CD8_Tem
chr2-24075797-24077419	2.01948E-14	0.256754104	0.88	0.674	1.33736E-09	C5_CD8_Tem
chr14-73567787-73568552	2.6395E-14	0.522846682	0.191	0.037	1.74796E-09	C5_CD8_Tem
chr17-40612576-40613325	1.23188E-09	0.257877806	0.201	0.111	8.15785E-05	C1_CD8_Tres
chr22-28882669-28884423	9.25429E-38	0.529199688	0.488	0.22	6.12847E-33	C1_CD8_Tres
chr17-2218522-2218736	4.32753E-14	0.480680017	0.087	0.005	2.86582E-09	C5_CD8_Tem
chr1-156113857-156115386	3.43464E-31	0.255283141	0.657	0.402	2.27452E-26	C4_CD8_Teff
chr10-132536674-132537961	5.60645E-26	0.330815282	0.707	0.534	3.71276E-21	C1_CD8_Tres
chr1-235074458-235075575	8.91623E-11	0.310783572	0.192	0.085	5.90459E-06	C1_CD8_Tres
chr1-1918791-1919873	1.8966E-21	0.333718434	0.536	0.366	1.25599E-16	C1_CD8_Tres
chr5-783299-785042	3.87641E-14	0.312612326	0.257	0.136	2.56707E-09	C1_CD8_Tres
chr3-71503466-71504603	6.72843E-14	0.516327203	0.208	0.045	4.45577E-09	C5_CD8_Tem
chr1-30717614-30719544	1.9207E-24	0.27461632	0.787	0.648	1.27195E-19	C1_CD8_Tres
chr21-36155689-36157441	1.40186E-28	0.339027665	0.76	0.56	9.28355E-24	C1_CD8_Tres
chr11-121481647-121482948	5.04433E-15	0.315803905	0.359	0.204	3.34051E-10	C3_CD8_Tem
chr17-27471322-27472502	5.72192E-41	0.353836584	0.49	0.24	3.78923E-36	C4_CD8_Teff
chr3-5016068-5016933	1.29572E-13	0.450937001	0.377	0.148	8.58067E-09	C5_CD8_Tem
chr8-763527-764677	3.30006E-10	0.259508212	0.24	0.138	2.1854E-05	C1_CD8_Tres
chr19-53866992-53869754	1.5402E-70	0.320076198	0.858	0.538	1.01997E-65	C4_CD8_Teff
chr1-120941840-120942777	5.79753E-25	0.494693148	0.302	0.112	3.8393E-20	C1_CD8_Tres
chr3-5002037-5002968	1.89235E-13	0.386919284	0.585	0.301	1.25317E-08	C5_CD8_Tem
chr18-63223870-63224886	2.1259E-13	0.514565001	0.18	0.032	1.40783E-08	C5_CD8_Tem

chr18-69895209-69896744	2.1715E-13	0.434687173	0.421	0.189	1.43803E-08	C5_CD8_Tem
chr10-133334635-133337342	7.84631E-30	0.300175212	0.479	0.246	5.19606E-25	C4_CD8_Teff
chr17-22521254-22521613	3.80087E-69	0.745569079	0.772	0.278	2.51705E-64	C1_CD8_Tres
chr1-84853880-84854757	2.07734E-13	0.359899361	0.177	0.07	1.37568E-08	C1_CD8_Tres
chr20-53835364-53835846	4.03035E-37	0.406950288	0.233	0.066	2.66902E-32	C4_CD8_Teff
chr7-73005202-73006292	5.89203E-25	0.433156199	0.302	0.12	3.90188E-20	C3_CD8_Tem
chr21-45224281-45224986	3.70826E-33	0.331162278	0.429	0.205	2.45572E-28	C4_CD8_Teff
chr19-7920040-7921116	2.82495E-18	0.274928525	0.611	0.463	1.87077E-13	C1_CD8_Tres
chr19-19403375-19403706	5.59196E-18	0.278764099	0.119	0.036	3.70316E-13	C4_CD8_Teff
chr15-101205906-101206664	4.41992E-13	0.41920664	0.437	0.202	2.927E-08	C5_CD8_Tem
chr11-9759348-9760157	1.64865E-23	0.27668011	0.417	0.217	1.09178E-18	C4_CD8_Teff
chr1-39394385-39394975	2.37981E-31	0.369928278	0.165	0.04	1.57598E-26	C4_CD8_Teff
chr2-234286306-234286606	5.15063E-13	0.488647088	0.169	0.027	3.4109E-08	C5_CD8_Tem
chr16-24721591-24722189	5.71272E-13	0.511691994	0.191	0.041	3.78313E-08	C5_CD8_Tem
chr11-59549908-59551377	3.5782E-30	0.335611956	0.361	0.16	2.36959E-25	C4_CD8_Teff
chr4-10108841-10109483	3.59846E-44	0.427914464	0.306	0.097	2.38301E-39	C4_CD8_Teff
chr19-13886722-13887896	9.89185E-13	0.413192394	0.486	0.204	6.55068E-08	C5_CD8_Tem
chr5-671624-673152	4.38596E-18	0.269690471	0.261	0.12	2.90451E-13	C4_CD8_Teff
chr13-111166524-111167378	1.04519E-12	0.485356752	0.169	0.032	6.92155E-08	C5_CD8_Tem
chr19-3275065-3276762	1.56836E-23	0.387348192	0.494	0.289	1.03861E-18	C1_CD8_Tres
chr11-118919418-118920066	1.39527E-10	0.273014346	0.231	0.126	9.23991E-06	C1_CD8_Tres
chr15-64697750-64698345	1.3354E-12	0.467696342	0.131	0.019	8.84345E-08	C5_CD8_Tem
chr11-121467993-121468350	1.25854E-15	0.39371439	0.126	0.03	8.3344E-11	C1_CD8_Tres
chr19-39430360-39431582	1.50251E-12	0.371452409	0.541	0.278	9.95007E-08	C5_CD8_Tem
chr4-83125500-83126299	1.54169E-12	0.489684037	0.213	0.052	1.02095E-07	C5_CD8_Tem
chr21-42267024-42268470	1.83593E-12	0.468030366	0.148	0.026	1.21581E-07	C5_CD8_Tem
chr5-35853576-35854712	1.36093E-29	0.465401258	0.44	0.208	9.01252E-25	C1_CD8_Tres

chr7-70634427-70635183	1.96332E-12	0.494204484	0.175	0.032	1.30017E-07	C5_CD8_Tem
chr1-1344800-1345081	2.03605E-12	0.481448369	0.131	0.02	1.34833E-07	C5_CD8_Tem
chr15-82539674-82541121	7.15086E-18	0.3290858	0.407	0.247	4.73552E-13	C1_CD8_Tres
chr5-35816593-35817551	2.14461E-12	0.455447505	0.317	0.109	1.42022E-07	C5_CD8_Tem
chr12-111404641-111406934	1.73214E-18	0.262013792	0.576	0.424	1.14708E-13	C3_CD8_Tem
chr16-67244167-67245037	2.51584E-12	0.376299204	0.525	0.266	1.66607E-07	C5_CD8_Tem
chr21-42227509-42228757	2.33425E-34	0.253178301	0.723	0.458	1.54581E-29	C4_CD8_Teff
chr2-196278382-196278703	2.6836E-12	0.432924413	0.082	0.005	1.77716E-07	C5_CD8_Tem
chr11-121465916-121466273	2.8042E-12	0.47206494	0.158	0.029	1.85702E-07	C5_CD8_Tem
chr22-23704649-23705733	1.24728E-09	0.268413799	0.156	0.072	8.25986E-05	C1_CD8_Tres
chr3-46359594-46360492	8.13842E-57	0.463434748	0.395	0.128	5.3895E-52	C4_CD8_Teff
chr2-218879617-218880954	5.42949E-39	0.345755897	0.504	0.243	3.59557E-34	C4_CD8_Teff
chr6-150310284-150311807	4.2001E-108	0.653541341	0.395	0.07	2.7815E-103	C4_CD8_Teff
chr12-56581086-56582251	5.4298E-12	0.429139036	0.388	0.153	3.59578E-07	C5_CD8_Tem
chr20-50165546-50166389	1.06306E-09	0.27236205	0.174	0.091	7.03993E-05	C1_CD8_Tres
chr16-1342296-1343583	6.35873E-12	0.321439239	0.661	0.374	4.21094E-07	C5_CD8_Tem
chr10-16948102-16948793	6.7592E-12	0.464677248	0.295	0.096	4.47614E-07	C5_CD8_Tem
chr1-9719099-9719404	7.08248E-12	0.459837528	0.262	0.077	4.69023E-07	C5_CD8_Tem
chr19-2089816-2090730	7.12886E-12	0.413437183	0.388	0.167	4.72094E-07	C5_CD8_Tem
chr2-197159068-197160249	4.92683E-27	0.325160066	0.318	0.132	3.2627E-22	C4_CD8_Teff
chr17-36188180-36189273	3.18453E-49	0.58806807	0.503	0.165	2.10889E-44	C3_CD8_Tem
chr1-120850260-120851399	8.59246E-11	0.278036285	0.275	0.16	5.69018E-06	C1_CD8_Tres
chr1-9128564-9129983	1.06043E-11	0.375114559	0.448	0.228	7.02245E-07	C5_CD8_Tem
chr6-34484371-34485456	2.964E-123	0.699825929	0.403	0.063	1.9629E-118	C4_CD8_Teff
chr6-130452810-130453731	1.17282E-11	0.435394251	0.328	0.121	7.76679E-07	C5_CD8_Tem
chr4-38663147-38664983	7.17285E-26	0.378673308	0.5	0.277	4.75008E-21	C3_CD8_Tem
chr1-51569246-51570089	1.351E-11	0.459369638	0.257	0.081	8.94674E-07	C5_CD8_Tem

chr9-127954965-127956136	1.35699E-33	0.355154043	0.355	0.15	8.98638E-29	C4_CD8_Teff
chr2-10329826-10332253	1.36126E-11	0.387569076	0.399	0.188	9.01464E-07	C5_CD8_Tem
chr16-11571884-11572994	4.15447E-45	0.443553606	0.25	0.06	2.75121E-40	C4_CD8_Teff
chr6-106107268-106107521	1.42596E-11	0.46563252	0.137	0.021	9.44312E-07	C5_CD8_Tem
chr7-22179415-22179871	1.56752E-11	0.416388398	0.077	0.004	1.03806E-06	C5_CD8_Tem
chr1-247406912-247407731	1.5751E-11	0.435768364	0.284	0.092	1.04308E-06	C5_CD8_Tem
chr19-9791501-9793338	4.67648E-23	0.300767891	0.635	0.492	3.0969E-18	C1_CD8_Tres
chr17-80605163-80606101	6.50264E-25	0.515226039	0.165	0.033	4.30624E-20	C1_CD8_Tres
chr7-99273963-99274662	1.7511E-11	0.457872927	0.104	0.012	1.15963E-06	C5_CD8_Tem
chr1-9686695-9687821	1.76574E-11	0.398275877	0.366	0.162	1.16932E-06	C5_CD8_Tem
chr22-18528018-18529368	2.89316E-58	0.468145754	0.404	0.133	1.91594E-53	C4_CD8_Teff
chr2-98835548-98836629	8.57051E-41	0.378778164	0.436	0.185	5.67565E-36	C4_CD8_Teff
chr16-66521884-66522606	2.18185E-11	0.37029897	0.481	0.244	1.44489E-06	C5_CD8_Tem
chr1-167446938-167448179	1.0515E-10	0.25619298	0.332	0.23	6.96336E-06	C1_CD8_Tres
chr8-143939083-143940463	2.33195E-11	0.341019418	0.552	0.32	1.54429E-06	C5_CD8_Tem
chr20-47543122-47543458	2.34093E-11	0.452242532	0.098	0.009	1.55023E-06	C5_CD8_Tem
chr13-29758427-29759203	2.52399E-11	0.436647414	0.295	0.102	1.67146E-06	C5_CD8_Tem
chr19-39073226-39074029	4.05978E-12	0.321045083	0.308	0.165	2.68851E-07	C1_CD8_Tres
chr19-9818223-9819870	1.45016E-21	0.276063397	0.725	0.57	9.60338E-17	C1_CD8_Tres
chr12-111438406-111438781	2.64843E-11	0.448560905	0.131	0.023	1.75387E-06	C5_CD8_Tem
chr6-156588-157425	2.66106E-11	0.422530546	0.109	0.014	1.76224E-06	C5_CD8_Tem
chr8-133302840-133303865	2.86492E-11	0.380007995	0.448	0.209	1.89724E-06	C5_CD8_Tem
chr3-15269076-15269877	3.19223E-43	0.406646604	0.363	0.132	2.11399E-38	C4_CD8_Teff
chr12-6332545-6332809	2.9264E-11	0.454765092	0.169	0.036	1.93795E-06	C5_CD8_Tem
chr20-62623349-62624245	5.25676E-14	0.349330674	0.165	0.061	3.48118E-09	C1_CD8_Tres
chr9-131500277-131501370	6.17837E-17	0.315308751	0.371	0.229	4.0915E-12	C1_CD8_Tres
chr11-11149350-11150552	2.72354E-27	0.514979773	0.231	0.06	1.80361E-22	C1_CD8_Tres

chr13-111176803-111177593	3.59844E-11	0.472174013	0.158	0.028	2.383E-06	C5_CD8_Tem
chr20-58312816-58313696	3.61345E-11	0.442610164	0.268	0.09	2.39294E-06	C5_CD8_Tem
chr13-112968220-112969617	7.84375E-10	0.252312824	0.189	0.094	5.19436E-05	C1_CD8_Tres
chr8-30059433-30060536	3.76259E-11	0.430266728	0.295	0.103	2.4917E-06	C5_CD8_Tem
chr22-36166068-36167237	4.03124E-11	0.425362709	0.279	0.101	2.66961E-06	C5_CD8_Tem
chr10-131903651-131904012	4.36328E-20	0.287882337	0.105	0.023	2.88949E-15	C4_CD8_Teff
chr10-11157328-11157601	4.40993E-11	0.409570424	0.077	0.005	2.92039E-06	C5_CD8_Tem
chr12-122887370-122889055	4.43245E-11	0.42005639	0.279	0.102	2.9353E-06	C5_CD8_Tem
chr4-185470292-185471891	5.44343E-20	0.256747279	0.37	0.195	3.6048E-15	C4_CD8_Teff
chr8-123415937-123417564	7.74181E-40	0.265230459	0.733	0.467	5.12686E-35	C4_CD8_Teff
chr13-29961481-29962153	5.88288E-11	0.455109317	0.219	0.065	3.89582E-06	C5_CD8_Tem
chr2-98479473-98480580	7.15881E-21	0.432692209	0.225	0.078	4.74078E-16	C1_CD8_Tres
chr13-29923888-29924434	1.34198E-17	0.276354265	0.152	0.056	8.88696E-13	C4_CD8_Teff
chr6-90218079-90219213	6.92078E-11	0.448780127	0.268	0.092	4.58315E-06	C5_CD8_Tem
chr19-2503181-2504126	2.17271E-13	0.315658482	0.314	0.171	1.43883E-08	C1_CD8_Tres
chr3-157127893-157129028	8.0591E-11	0.393351542	0.366	0.172	5.33698E-06	C5_CD8_Tem
chr16-57984357-57985564	1.86704E-18	0.250156652	0.367	0.197	1.23641E-13	C4_CD8_Teff
chr3-138327695-138328096	8.4261E-11	0.394984052	0.093	0.009	5.58001E-06	C5_CD8_Tem
chr8-100809255-100810633	8.99339E-11	0.386750376	0.421	0.192	5.9557E-06	C5_CD8_Tem
chr3-115790540-115791139	3.40509E-08	0.268857023	0.198	0.102	0.002254955	C1_CD8_Tres
chr12-101803950-101804293	1.09885E-10	0.413439541	0.093	0.009	7.27691E-06	C5_CD8_Tem
chr20-51499996-51501369	5.1356E-112	0.555862286	0.656	0.231	3.4009E-107	C4_CD8_Teff
chr16-81523422-81524781	1.10313E-10	0.29458591	0.656	0.413	7.30526E-06	C5_CD8_Tem
chr1-64876955-64877577	3.73275E-15	0.357081693	0.168	0.059	2.47194E-10	C1_CD8_Tres
chr5-96701055-96701439	1.21163E-10	0.436288994	0.153	0.031	8.02376E-06	C5_CD8_Tem
chr8-66534684-66536138	1.23699E-10	0.385131258	0.355	0.153	8.19169E-06	C5_CD8_Tem
chr1-25549312-25550465	4.13744E-15	0.341870631	0.293	0.151	2.73994E-10	C1_CD8_Tres

chr15-92921387-92922141	1.30228E-10	0.419839864	0.246	0.075	8.6241E-06	C5_CD8_Tem
chr1-91941494-91942178	1.35981E-10	0.437903234	0.126	0.023	9.00505E-06	C5_CD8_Tem
chr3-153221387-153222517	1.55256E-10	0.26220581	0.347	0.215	1.02815E-05	C1_CD8_Tres
chr2-96556544-96557329	1.46575E-10	0.395095893	0.366	0.157	9.70666E-06	C5_CD8_Tem
chr9-127555909-127556449	1.50652E-10	0.449153243	0.158	0.035	9.97663E-06	C5_CD8_Tem
chr8-22327004-22327768	1.54531E-10	0.437159729	0.104	0.013	1.02335E-05	C5_CD8_Tem
chr14-75276922-75279603	1.6313E-10	0.254361216	0.732	0.509	1.0803E-05	C5_CD8_Tem
chr5-52724672-52725892	1.63301E-10	0.360447949	0.454	0.238	1.08143E-05	C5_CD8_Tem
chr17-50909211-50909589	1.63603E-10	0.440264509	0.142	0.025	1.08343E-05	C5_CD8_Tem
chr19-58097520-58098745	1.66501E-10	0.391630908	0.328	0.135	1.10262E-05	C5_CD8_Tem
chr10-4651547-4652513	1.69993E-10	0.424118264	0.115	0.018	1.12574E-05	C5_CD8_Tem
chr10-29657489-29658251	3.11866E-28	0.348941184	0.155	0.038	2.06527E-23	C4_CD8_Teff
chr1-84862578-84863254	1.797E-10	0.432288955	0.153	0.03	1.19003E-05	C5_CD8_Tem
chr14-55330264-55330678	2.50848E-20	0.291394908	0.116	0.029	1.66119E-15	C4_CD8_Teff
chr19-2598750-2599757	1.85032E-10	0.327626018	0.536	0.299	1.22534E-05	C5_CD8_Tem
chr9-133406665-133407377	1.87421E-10	0.373086195	0.459	0.229	1.24116E-05	C5_CD8_Tem
chr8-20251411-20252448	1.44733E-11	0.270499424	0.344	0.221	9.58462E-07	C3_CD8_Tem
chr11-118232602-118233067	1.91013E-10	0.40647241	0.317	0.129	1.26494E-05	C5_CD8_Tem
chr11-14074835-14075632	8.80044E-08	0.273711594	0.123	0.053	0.005827918	C1_CD8_Tres
chr1-148037879-148039000	7.57692E-09	0.280199309	0.15	0.065	0.000501766	C1_CD8_Tres
chr8-8917273-8918050	5.14816E-21	0.453058911	0.249	0.091	3.40927E-16	C1_CD8_Tres
chr1-12617052-12619578	3.88977E-43	0.382999307	0.436	0.183	2.57592E-38	C4_CD8_Teff
chr2-11753459-11754635	2.15426E-10	0.295228751	0.612	0.399	1.42661E-05	C5_CD8_Tem
chr6-188656-189699	1.04694E-84	0.608709557	0.273	0.037	6.93312E-80	C4_CD8_Teff
chr3-10223517-10224076	2.24556E-10	0.445078217	0.202	0.052	1.48708E-05	C5_CD8_Tem
chr3-101518076-101518866	1.02747E-10	0.266303881	0.263	0.156	6.80419E-06	C1_CD8_Tres
chr17-36182330-36184239	1.84827E-12	0.282950995	0.143	0.056	1.22398E-07	C3_CD8_Tem

chr10-12412623-12413670	2.34518E-28	0.353227906	0.22	0.074	1.55305E-23	C4_CD8_Teff
chr7-17138775-17139518	2.72015E-10	0.408569232	0.24	0.079	1.80136E-05	C5_CD8_Tem
chr12-47211956-47212706	1.04862E-31	0.348392856	0.333	0.143	6.9443E-27	C4_CD8_Teff
chr7-77393208-77393730	7.96235E-24	0.497998928	0.138	0.024	5.27291E-19	C1_CD8_Tres
chr12-8242149-8244094	2.86143E-10	0.261717146	0.732	0.495	1.89492E-05	C5_CD8_Tem
chr16-87492259-87493349	6.4581E-21	0.344944041	0.456	0.251	4.27675E-16	C3_CD8_Tem
chr10-99993940-99994675	3.24323E-10	0.426640743	0.131	0.026	2.14776E-05	C5_CD8_Tem
chr1-148401655-148403045	1.33869E-17	0.377978628	0.221	0.083	8.86518E-13	C3_CD8_Tem
chr18-23870990-23871741	3.3441E-10	0.39865114	0.306	0.13	2.21456E-05	C5_CD8_Tem
chr5-81749663-81751888	2.88849E-15	0.250769999	0.605	0.476	1.91284E-10	C1_CD8_Tres
chr7-151408489-151409399	1.29158E-13	0.301626036	0.347	0.215	8.5532E-09	C1_CD8_Tres
chr22-40240516-40241795	4.3765E-31	0.342550325	0.35	0.15	2.89825E-26	C4_CD8_Teff
chr6-129686918-129687735	1.66664E-29	0.350699897	0.263	0.096	1.1037E-24	C4_CD8_Teff
chr1-146036485-146038363	4.31296E-43	0.353984138	0.55	0.257	2.85617E-38	C4_CD8_Teff
chr8-100807069-100807404	3.75696E-10	0.405389617	0.12	0.024	2.48797E-05	C5_CD8_Tem
chr14-71118875-71119413	3.92357E-10	0.429123243	0.12	0.022	2.59831E-05	C5_CD8_Tem
chr4-148444349-148445940	1.46787E-22	0.292028937	0.314	0.147	9.72067E-18	C4_CD8_Teff
chr5-157602392-157603773	3.15227E-15	0.314221213	0.299	0.166	2.08753E-10	C1_CD8_Tres
chr10-17205827-17206776	4.11261E-10	0.321398895	0.568	0.332	2.72349E-05	C5_CD8_Tem
chr10-73859195-73860037	4.33961E-10	0.429873385	0.169	0.04	2.87382E-05	C5_CD8_Tem
chr3-157081113-157082069	4.40698E-10	0.365639143	0.415	0.195	2.91844E-05	C5_CD8_Tem
chr11-121472323-121472641	4.52795E-10	0.424491722	0.109	0.015	2.99855E-05	C5_CD8_Tem
chr6-37202080-37203132	4.61383E-10	0.399396432	0.142	0.032	3.05542E-05	C5_CD8_Tem
chr14-61537146-61537848	4.95039E-10	0.41326424	0.169	0.042	3.2783E-05	C5_CD8_Tem
chr10-45421154-45421545	5.27105E-10	0.388663779	0.082	0.008	3.49065E-05	C5_CD8_Tem
chr17-75123963-75124643	7.47699E-15	0.330321399	0.108	0.029	4.95149E-10	C1_CD8_Tres
chr1-145464778-145465831	6.43739E-12	0.319342787	0.15	0.053	4.26303E-07	C1_CD8_Tres

chr1-23110298-23111268	5.66602E-10	0.34355936	0.475	0.241	3.75221E-05	C5_CD8_Tem
chr17-36192338-36192639	1.28857E-11	0.290327953	0.133	0.053	8.53328E-07	C3_CD8_Tem
chr5-56828983-56829541	1.84502E-17	0.405688128	0.111	0.022	1.22183E-12	C1_CD8_Tres
chr17-83124835-83126264	9.53333E-10	0.261475503	0.128	0.052	6.31326E-05	C3_CD8_Tem
chr10-6493437-6493865	2.45545E-41	0.421125954	0.282	0.089	1.62607E-36	C4_CD8_Teff
chrX-72107315-72107683	6.88411E-10	0.415893041	0.131	0.026	4.55886E-05	C5_CD8_Tem
chr12-133036812-133038023	1.89258E-28	0.48786114	0.362	0.15	1.25332E-23	C1_CD8_Tres
chr1-44806789-44808263	1.77304E-62	0.381351211	0.689	0.342	1.17416E-57	C4_CD8_Teff
chr2-172427037-172428427	7.25843E-10	0.385826548	0.295	0.127	4.80675E-05	C5_CD8_Tem
chr20-34875858-34876868	2.47333E-11	0.250587487	0.314	0.205	1.63792E-06	C1_CD8_Tres
chr10-33263906-33264748	1.92612E-15	0.378275303	0.195	0.071	1.27553E-10	C1_CD8_Tres
chr1-110630630-110631786	8.75355E-15	0.286485161	0.341	0.209	5.79686E-10	C1_CD8_Tres
chr1-148363409-148363721	7.74313E-10	0.377036551	0.082	0.008	5.12773E-05	C5_CD8_Tem
chr19-41935809-41936940	5.39033E-80	0.490953236	0.572	0.217	3.56964E-75	C4_CD8_Teff
chr2-233247436-233248149	8.60708E-10	0.338829103	0.47	0.245	5.69987E-05	C5_CD8_Tem
chr1-150569880-150570355	9.4607E-10	0.408464734	0.224	0.079	6.26516E-05	C5_CD8_Tem
chr12-121214138-121214703	9.52248E-10	0.41843989	0.18	0.048	6.30607E-05	C5_CD8_Tem
chr17-81094547-81095689	9.60839E-10	0.30597424	0.585	0.356	6.36296E-05	C5_CD8_Tem
chr5-108747592-108749807	1.00049E-09	0.279255042	0.689	0.431	6.62552E-05	C5_CD8_Tem
chr1-117006886-117007868	1.02477E-09	0.407299992	0.224	0.068	6.78634E-05	C5_CD8_Tem
chr1-23979709-23980925	3.86076E-28	0.483689341	0.395	0.167	2.55671E-23	C1_CD8_Tres
chr7-143385779-143386618	1.87991E-38	0.396768922	0.322	0.116	1.24493E-33	C4_CD8_Teff
chr1-38977213-38978268	5.94822E-15	0.373257967	0.15	0.047	3.93909E-10	C1_CD8_Tres
chr12-124469301-124469836	3.53448E-13	0.347407057	0.132	0.039	2.34064E-08	C1_CD8_Tres
chr17-62965173-62966688	1.18874E-15	0.310334529	0.349	0.182	7.87217E-11	C3_CD8_Tem
chr7-50335686-50336484	3.94137E-28	0.490840708	0.276	0.093	2.61009E-23	C3_CD8_Tem
chr10-73857235-73858101	1.3065E-09	0.403043019	0.202	0.065	8.65203E-05	C5_CD8_Tem

chr3-71066873-71067205	1.34085E-09	0.405015224	0.268	0.092	8.87954E-05	C5_CD8_Tem
chr7-37310382-37311803	1.43845E-09	0.394184035	0.273	0.106	9.52587E-05	C5_CD8_Tem
chr14-91193828-91194842	1.7115E-13	0.337004368	0.171	0.069	1.13341E-08	C1_CD8_Tres
chr2-240624500-240625519	1.53446E-09	0.356240655	0.404	0.186	0.000101616	C5_CD8_Tem
chr9-75151671-75152898	1.55205E-09	0.371348253	0.35	0.155	0.000102782	C5_CD8_Tem
chr13-99433974-99434864	1.56968E-09	0.263635366	0.678	0.457	0.000103949	C5_CD8_Tem
chr6-166257734-166258201	1.69107E-10	0.293645116	0.15	0.07	1.11988E-05	C1_CD8_Tres
chr13-99283464-99283788	1.66472E-09	0.410933038	0.148	0.032	0.000110243	C5_CD8_Tem
chr7-50331555-50332145	1.07042E-15	0.376700635	0.21	0.083	7.08861E-11	C1_CD8_Tres
chr9-128032997-128033511	9.9236E-52	0.435392165	0.407	0.144	6.57171E-47	C4_CD8_Teff
chr4-80077595-80078076	5.94878E-16	0.263065089	0.167	0.064	3.93946E-11	C4_CD8_Teff
chr19-1030247-1031629	1.3243E-151	0.72814457	0.508	0.089	8.7701E-147	C4_CD8_Teff
chr3-4983523-4984406	1.97988E-09	0.309824211	0.525	0.301	0.000131114	C5_CD8_Tem
chr8-8296057-8296572	2.01513E-09	0.394733322	0.082	0.01	0.000133448	C5_CD8_Tem
chr15-29012767-29013642	2.02927E-09	0.419717835	0.164	0.041	0.000134384	C5_CD8_Tem
chr10-12418885-12419856	8.76997E-21	0.304526394	0.157	0.052	5.80774E-16	C4_CD8_Teff
chr2-134216488-134216895	2.07386E-09	0.390871708	0.104	0.016	0.000137337	C5_CD8_Tem
chr5-80745473-80745863	2.1095E-09	0.397687711	0.093	0.012	0.000139697	C5_CD8_Tem
chr1-15352418-15352854	2.1951E-09	0.401774977	0.153	0.044	0.000145366	C5_CD8_Tem
chr14-56117661-56119506	1.56919E-12	0.290871261	0.254	0.142	1.03917E-07	C1_CD8_Tres
chr9-105244100-105245734	7.62851E-19	0.250600679	0.393	0.203	5.05183E-14	C4_CD8_Teff
chr16-391649-392432	5.87686E-18	0.32973607	0.375	0.209	3.89183E-13	C3_CD8_Tem
chr7-152027350-152028282	5.81715E-25	0.317455761	0.296	0.128	3.85229E-20	C4_CD8_Teff
chr3-194715029-194715816	2.42957E-09	0.389671016	0.126	0.027	0.000160893	C5_CD8_Tem
chr14-64723525-64724394	2.50304E-09	0.410091741	0.169	0.039	0.000165759	C5_CD8_Tem
chr1-40628211-40628877	6.72609E-19	0.279700487	0.199	0.082	4.45422E-14	C4_CD8_Teff
chr10-6513052-6513898	2.61186E-09	0.387580479	0.202	0.06	0.000172965	C5_CD8_Tem

chr16-89170661-89171852	1.42051E-15	0.287368475	0.443	0.309	9.40701E-11	C1_CD8_Tres
chr4-3301814-3303402	2.67334E-09	0.346448114	0.415	0.215	0.000177036	C5_CD8_Tem
chr12-8697074-8698685	2.7002E-09	0.348137064	0.377	0.188	0.000178815	C5_CD8_Tem
chr4-76200951-76201207	2.70738E-09	0.378497637	0.066	0.005	0.000179291	C5_CD8_Tem
chr2-69675442-69675785	3.0682E-09	0.372627494	0.087	0.01	0.000203185	C5_CD8_Tem
chr7-50342425-50342974	3.46985E-10	0.25948274	0.151	0.067	2.29784E-05	C3_CD8_Tem
chr1-161115296-161115684	3.36932E-09	0.397256176	0.175	0.049	0.000223126	C5_CD8_Tem
chr15-60507462-60508748	3.36991E-09	0.384380355	0.268	0.112	0.000223165	C5_CD8_Tem
chr1-206744678-206745677	3.4952E-09	0.402803776	0.191	0.068	0.000231462	C5_CD8_Tem
chr5-88140701-88141866	1.59265E-14	0.32797038	0.263	0.129	1.0547E-09	C3_CD8_Tem
chr6-106106396-106106752	3.56712E-09	0.390384883	0.104	0.017	0.000236225	C5_CD8_Tem
chr11-3421815-3423505	2.66032E-29	0.257032472	0.611	0.373	1.76174E-24	C4_CD8_Teff
chr10-73932104-73932872	1.26348E-16	0.400845067	0.144	0.034	8.36714E-12	C1_CD8_Tres
chr8-73356217-73356774	3.90191E-09	0.422302626	0.213	0.067	0.000258396	C5_CD8_Tem
chr22-36435230-36436318	8.3552E-19	0.349795208	0.367	0.188	5.53306E-14	C3_CD8_Tem
chr22-40054988-40055943	3.13777E-16	0.33834903	0.305	0.16	2.07792E-11	C1_CD8_Tres
chr7-94656122-94658687	8.42126E-17	0.332052375	0.545	0.35	5.57681E-12	C1_CD8_Tres
chr16-88469556-88470538	1.52543E-32	0.377816198	0.171	0.041	1.01018E-27	C4_CD8_Teff
chr7-142383916-142384929	4.1659E-09	0.381899336	0.087	0.012	0.000275878	C5_CD8_Tem
chr13-31044415-31046134	4.36343E-09	0.343180289	0.399	0.197	0.000288959	C5_CD8_Tem
chr21-31656217-31656445	4.5614E-09	0.388448229	0.109	0.022	0.00030207	C5_CD8_Tem
chr12-132955395-132956898	7.53068E-65	0.530919317	0.223	0.032	4.98704E-60	C4_CD8_Teff
chr5-50595175-50595563	4.7211E-09	0.403297069	0.18	0.051	0.000312645	C5_CD8_Tem
chr17-48816624-48817404	4.76453E-09	0.379130469	0.268	0.107	0.000315521	C5_CD8_Tem
chr19-16160548-16161222	4.7782E-09	0.398688701	0.109	0.018	0.000316427	C5_CD8_Tem
chr17-50199670-50201005	4.84605E-09	0.343942744	0.35	0.177	0.00032092	C5_CD8_Tem
chr1-151898031-151899047	5.0389E-09	0.38474675	0.202	0.064	0.000333691	C5_CD8_Tem

chr8-42893435-42894491	3.01109E-08	0.256300764	0.162	0.082	0.001994033	C1_CD8_Tres
chr17-68182449-68183538	2.3077E-68	0.489275333	0.468	0.154	1.52823E-63	C4_CD8_Teff
chr18-58541077-58542576	1.69906E-49	0.448042763	0.323	0.097	1.12517E-44	C4_CD8_Teff
chr12-108696348-108696803	5.2371E-09	0.395190871	0.235	0.077	0.000346816	C5_CD8_Tem
chr2-191114311-191116116	5.32886E-09	0.251040902	0.71	0.474	0.000352893	C5_CD8_Tem
chrX-72272557-72273155	9.98427E-26	0.324512386	0.099	0.016	6.61188E-21	C4_CD8_Teff
chr14-105069895-105071142	5.46482E-09	0.347811733	0.383	0.191	0.000361897	C5_CD8_Tem
chr6-2861395-2861985	4.71194E-69	0.524895288	0.373	0.101	3.12039E-64	C4_CD8_Teff
chr19-18514456-18515253	5.50988E-09	0.393544418	0.126	0.026	0.000364881	C5_CD8_Tem
chr3-49602386-49603513	1.69161E-09	0.257190683	0.156	0.073	0.000112024	C1_CD8_Tres
chr16-85615088-85615287	5.93148E-09	0.388994201	0.142	0.031	0.0003928	C5_CD8_Tem
chr17-77127541-77128387	6.04724E-09	0.330755426	0.448	0.228	0.000400467	C5_CD8_Tem
chr1-46549395-46550267	6.26649E-09	0.376451533	0.311	0.134	0.000414986	C5_CD8_Tem
chr8-126556354-126558700	3.6175E-50	0.383875023	0.561	0.252	2.39562E-45	C4_CD8_Teff
chr1-161020499-161021600	9.08297E-36	0.34596873	0.432	0.193	6.01502E-31	C4_CD8_Teff
chr18-13611875-13612700	6.01339E-51	0.385301123	0.562	0.258	3.98225E-46	C4_CD8_Teff
chr13-30576523-30577824	6.82275E-09	0.337110304	0.41	0.203	0.000451823	C5_CD8_Tem
chr19-38864390-38866046	2.7247E-31	0.4274194	0.542	0.271	1.80438E-26	C3_CD8_Tem
chr5-132100768-132101111	7.24137E-09	0.374587671	0.131	0.03	0.000479545	C5_CD8_Tem
chr11-121514259-121514960	7.48062E-09	0.393082037	0.104	0.015	0.000495389	C5_CD8_Tem
chr8-29251858-29252392	7.60131E-09	0.390426421	0.142	0.032	0.000503381	C5_CD8_Tem
chr1-144545929-144546983	1.04949E-11	0.305436231	0.177	0.075	6.95003E-07	C1_CD8_Tres
chr6-148746057-148748592	8.04162E-09	0.353897403	0.339	0.159	0.00053254	C5_CD8_Tem
chr6-137978282-137978645	8.13876E-09	0.369814864	0.104	0.015	0.000538973	C5_CD8_Tem
chr6-109461822-109462412	8.16742E-09	0.392968391	0.142	0.036	0.000540871	C5_CD8_Tem
chr12-120231020-120231923	8.18347E-09	0.342386181	0.35	0.165	0.000541934	C5_CD8_Tem
chr2-134217364-134218430	8.76365E-09	0.352942557	0.344	0.165	0.000580355	C5_CD8_Tem

chr8-20281983-20282492	8.80626E-09	0.381053697	0.087	0.012	0.000583177	C5_CD8_Tem
chr5-142786694-142787240	2.61447E-14	0.317866643	0.237	0.115	1.73138E-09	C3_CD8_Tem
chr4-80127300-80127706	1.72567E-17	0.278142946	0.15	0.05	1.14279E-12	C4_CD8_Teff
chr19-1746878-1747789	9.52661E-09	0.384049806	0.131	0.035	0.000630881	C5_CD8_Tem
chr11-1850299-1851392	1.01949E-08	0.294605068	0.525	0.329	0.000675136	C5_CD8_Tem
chr9-104927208-104928364	1.02216E-08	0.391785438	0.137	0.037	0.000676908	C5_CD8_Tem
chr1-156124587-156125379	1.37916E-15	0.259703058	0.147	0.051	9.13321E-11	C4_CD8_Teff
chr12-94644054-94644939	1.18436E-10	0.268577471	0.081	0.023	7.84317E-06	C3_CD8_Tem
chr9-91798086-91799208	1.05396E-08	0.368883869	0.279	0.103	0.000697966	C5_CD8_Tem
chr4-153114731-153116644	1.33905E-11	0.256976818	0.305	0.2	8.86757E-07	C1_CD8_Tres
chr1-148426851-148427257	1.12991E-08	0.350499381	0.082	0.011	0.000748257	C5_CD8_Tem
chr3-105792056-105792856	1.14669E-08	0.378617611	0.262	0.097	0.000759372	C5_CD8_Tem
chrX-18424617-18426200	1.17831E-08	0.306010409	0.53	0.294	0.000780315	C5_CD8_Tem
chr18-49690202-49690676	1.20404E-08	0.384724974	0.12	0.023	0.000797353	C5_CD8_Tem
chr16-996722-998093	8.51708E-12	0.305426865	0.171	0.077	5.64027E-07	C1_CD8_Tres
chr1-153565588-153566883	1.23218E-08	0.332017972	0.404	0.214	0.000815988	C5_CD8_Tem
chr11-119217855-119218824	1.28496E-08	0.39084788	0.131	0.031	0.000850941	C5_CD8_Tem
chr12-94618812-94619272	1.32236E-08	0.374103897	0.115	0.02	0.000875704	C5_CD8_Tem
chr7-158804277-158804969	3.02388E-16	0.337688804	0.141	0.043	2.00251E-11	C3_CD8_Tem
chr2-224909784-224910475	1.36295E-08	0.393633799	0.186	0.053	0.000902588	C5_CD8_Tem
chr10-6052168-6053200	1.44501E-08	0.305488896	0.448	0.251	0.000956931	C5_CD8_Tem
chr5-134122346-134122925	1.23643E-23	0.323796241	0.154	0.044	8.188E-19	C4_CD8_Teff
chr21-5154334-5154970	1.31642E-13	0.349547233	0.228	0.094	8.7177E-09	C1_CD8_Tres
chr2-69774368-69775424	1.51625E-08	0.382953793	0.29	0.112	0.001004109	C5_CD8_Tem
chr15-28831788-28832603	2.51612E-17	0.26950501	0.119	0.036	1.66625E-12	C4_CD8_Teff
chr15-45454650-45455247	1.52062E-08	0.366592977	0.251	0.098	0.001006998	C5_CD8_Tem
chr19-41886761-41888221	4.08134E-36	0.292049681	0.619	0.35	2.70278E-31	C4_CD8_Teff

chr1-52658711-52659613	1.57797E-08	0.393139995	0.164	0.044	0.001044976	C5_CD8_Tem
chr6-166258549-166259778	1.69323E-08	0.365764684	0.257	0.103	0.001121306	C5_CD8_Tem
chr16-30491852-30492398	1.72125E-08	0.378854678	0.126	0.03	0.001139867	C5_CD8_Tem
chr1-144551201-144552431	1.31752E-87	0.606987583	0.286	0.038	8.72498E-83	C4_CD8_Teff
chr12-106247417-106249361	1.34947E-11	0.262339543	0.228	0.128	8.9366E-07	C1_CD8_Tres
chr1-116514824-116516096	2.08403E-08	0.306243896	0.454	0.255	0.001380106	C5_CD8_Tem
chr21-42198955-42200215	6.68355E-25	0.325253688	0.245	0.099	4.42605E-20	C4_CD8_Teff
chr22-38873763-38874357	4.23253E-64	0.478681437	0.439	0.149	2.80291E-59	C4_CD8_Teff
chr5-96702371-96703654	1.65045E-25	0.278284929	0.457	0.249	1.09297E-20	C4_CD8_Teff
chr22-38984750-38985599	2.2988E-08	0.326763114	0.383	0.188	0.001522332	C5_CD8_Tem
chr1-629778-630252	6.51468E-21	0.450485722	0.293	0.116	4.31422E-16	C1_CD8_Tres
chr16-53098482-53100073	2.33201E-08	0.303485256	0.421	0.247	0.001544327	C5_CD8_Tem
chr1-185044541-185046123	2.34043E-08	0.301036258	0.464	0.276	0.001549904	C5_CD8_Tem
chr19-41875113-41875859	2.23148E-13	0.277536998	0.43	0.277	1.47775E-08	C3_CD8_Tem
chr7-123533959-123535436	1.09153E-33	0.279665315	0.622	0.359	7.22841E-29	C4_CD8_Teff
chr6-36691075-36691365	2.44186E-08	0.337764081	0.066	0.006	0.001617073	C5_CD8_Tem
chr6-139172678-139173531	2.52177E-08	0.377914584	0.18	0.06	0.001669994	C5_CD8_Tem
chr22-36328686-36329793	2.55501E-08	0.305018683	0.47	0.256	0.001692002	C5_CD8_Tem
chr2-144659399-144660199	1.06491E-22	0.430371582	0.372	0.168	7.05214E-18	C3_CD8_Tem
chr2-69770122-69770552	5.27191E-11	0.30354054	0.12	0.039	3.49122E-06	C3_CD8_Tem
chr6-35709901-35710747	2.71672E-21	0.294824883	0.254	0.108	1.7991E-16	C4_CD8_Teff
chr22-39919971-39921390	7.89862E-34	0.328879564	0.438	0.217	5.2307E-29	C4_CD8_Teff
chr1-153490519-153491082	2.63352E-08	0.37795037	0.093	0.018	0.001743996	C5_CD8_Tem
chr8-20302981-20304211	2.79612E-08	0.359285422	0.268	0.11	0.001851673	C5_CD8_Tem
chr2-102082410-102083466	2.79817E-08	0.361264315	0.279	0.119	0.001853034	C5_CD8_Tem
chr17-2053611-2055340	1.76813E-35	0.307014709	0.557	0.298	1.17091E-30	C4_CD8_Teff
chr1-210373706-210374813	2.86399E-08	0.377933652	0.175	0.055	0.001896623	C5_CD8_Tem

chr17-1707041-1707494	2.87275E-08	0.360593195	0.077	0.011	0.00190242	C5_CD8_Tem
chr16-72172180-72172725	2.91323E-08	0.340640758	0.066	0.007	0.001929227	C5_CD8_Tem
chr10-6013878-6014870	1.58862E-11	0.315032983	0.162	0.069	1.05203E-06	C1_CD8_Tres
chr9-224587-225420	3.07719E-08	0.383048328	0.158	0.041	0.002037808	C5_CD8_Tem
chr1-26799587-26800842	2.71352E-44	0.4353539	0.273	0.08	1.79697E-39	C4_CD8_Teff
chr5-67003640-67004819	3.17937E-08	0.2680993	0.568	0.367	0.002105477	C5_CD8_Tem
chr12-6345309-6346049	3.19011E-08	0.367616895	0.158	0.047	0.002112584	C5_CD8_Tem
chr11-225787-226040	3.20753E-08	0.344190433	0.055	0.003	0.002124122	C5_CD8_Tem
chr20-32534602-32535602	8.91098E-14	0.299504629	0.231	0.121	5.90112E-09	C1_CD8_Tres
chr15-34331685-34332149	3.2544E-08	0.374622336	0.186	0.061	0.002155162	C5_CD8_Tem
chr17-81097350-81097584	3.34496E-08	0.378976934	0.164	0.047	0.00221513	C5_CD8_Tem
chr7-143407229-143409343	9.34437E-45	0.429674633	0.299	0.091	6.18812E-40	C4_CD8_Teff
chr8-126505903-126506723	2.436E-21	0.288996531	0.096	0.021	1.61319E-16	C4_CD8_Teff
chr12-121786315-121786824	2.88061E-32	0.374372086	0.19	0.051	1.90762E-27	C4_CD8_Teff
chr2-197299946-197300457	3.72311E-08	0.372513221	0.142	0.036	0.002465558	C5_CD8_Tem
chr1-156119599-156119848	7.639E-16	0.259547573	0.1	0.029	5.05878E-11	C4_CD8_Teff
chr21-5101563-5102080	1.58259E-11	0.303530481	0.162	0.071	1.04804E-06	C1_CD8_Tres
chr3-183513151-183513641	4.08748E-08	0.377960081	0.169	0.049	0.002706854	C5_CD8_Tem
chr15-75201509-75202996	4.17454E-08	0.259095859	0.574	0.378	0.002764505	C5_CD8_Tem
chr8-93935396-93936515	4.18029E-08	0.366919729	0.186	0.059	0.002768314	C5_CD8_Tem
chr3-17774268-17775421	4.18424E-08	0.365224	0.262	0.115	0.002770929	C5_CD8_Tem
chr11-62553211-62553659	2.08334E-11	0.258737415	0.165	0.08	1.37965E-06	C1_CD8_Tres
chr19-50383560-50384765	2.50319E-48	0.339875549	0.631	0.328	1.65769E-43	C4_CD8_Teff
chr1-46246610-46247912	6.89016E-33	0.28978111	0.581	0.316	4.56287E-28	C4_CD8_Teff
chr16-2163039-2163533	4.58295E-08	0.33101565	0.06	0.005	0.003034965	C5_CD8_Tem
chr6-90458163-90458907	4.66081E-08	0.372174916	0.191	0.056	0.003086529	C5_CD8_Tem
chr5-157198163-157199049	2.59864E-24	0.285696544	0.417	0.216	1.7209E-19	C4_CD8_Teff

chr4-123616807-123617351	4.70551E-08	0.387482442	0.158	0.046	0.00311613	C5_CD8_Tem
chr9-6566384-6567492	4.77581E-08	0.35820997	0.093	0.015	0.003162685	C5_CD8_Tem
chr5-52987434-52988237	1.91292E-23	0.505813346	0.21	0.057	1.26679E-18	C1_CD8_Tres
chr15-92907860-92908224	3.64229E-15	0.25200474	0.187	0.084	2.41204E-10	C4_CD8_Teff
chr17-2192183-2193154	4.8622E-08	0.322966341	0.399	0.2	0.003219897	C5_CD8_Tem
chr9-129868456-129869599	1.37391E-30	0.283093497	0.58	0.317	9.09846E-26	C4_CD8_Teff
chr8-28621762-28623340	4.52594E-30	0.281920206	0.553	0.297	2.99721E-25	C4_CD8_Teff
chr6-30768952-30769914	5.07542E-08	0.35971121	0.202	0.073	0.003361096	C5_CD8_Tem
chr2-106159784-106161249	5.11763E-08	0.322282782	0.361	0.184	0.003389048	C5_CD8_Tem
chr5-61407928-61409110	2.02761E-32	0.299665625	0.542	0.289	1.34274E-27	C4_CD8_Teff
chr14-22549641-22550456	5.00797E-23	0.294616025	0.329	0.153	3.31643E-18	C4_CD8_Teff
chr4-170025921-170027730	5.15187E-08	0.336732209	0.306	0.136	0.00341172	C5_CD8_Tem
chr6-34242824-34243525	5.19491E-08	0.365668165	0.142	0.036	0.003440225	C5_CD8_Tem
chr1-58754179-58755866	5.31072E-08	0.318994719	0.377	0.179	0.003516918	C5_CD8_Tem
chr2-43129845-43130196	5.74933E-08	0.370525387	0.175	0.061	0.003807378	C5_CD8_Tem
chr9-134024653-134026073	3.0321E-15	0.260029744	0.516	0.364	2.00795E-10	C3_CD8_Tem
chr10-49941284-49942406	6.09311E-11	0.286307699	0.162	0.069	4.03504E-06	C1_CD8_Tres
chr19-10418914-10420053	9.61825E-30	0.337681698	0.34	0.146	6.36949E-25	C4_CD8_Teff
chr2-42353580-42354147	6.19423E-08	0.372267401	0.104	0.018	0.004102003	C5_CD8_Tem
chr5-35785590-35786080	6.20405E-08	0.315801934	0.055	0.004	0.00410851	C5_CD8_Tem
chr7-130909824-130910532	6.31926E-08	0.366582175	0.175	0.054	0.004184803	C5_CD8_Tem
chr14-69643291-69643925	2.10287E-16	0.359069533	0.189	0.08	1.39258E-11	C1_CD8_Tres
chr2-234422145-234423481	4.96912E-64	0.477645383	0.458	0.15	3.2907E-59	C4_CD8_Teff
chr4-80062227-80062862	6.5723E-08	0.355579918	0.109	0.024	0.004352373	C5_CD8_Tem
chr2-157463896-157464634	6.58013E-08	0.363611016	0.246	0.091	0.00435756	C5_CD8_Tem
chr21-43706359-43707102	6.76581E-08	0.370700852	0.18	0.059	0.004480522	C5_CD8_Tem
chr12-120316891-120317993	7.52569E-16	0.287294197	0.488	0.337	4.98374E-11	C1_CD8_Tres

chr9-37408810-37409703	7.02842E-08	0.325979304	0.339	0.162	0.00465443	C5_CD8_Tem
chr14-71004347-71005898	7.07221E-08	0.365056888	0.131	0.032	0.00468343	C5_CD8_Tem
chr12-11998194-11999166	5.16212E-28	0.53357167	0.228	0.058	3.41851E-23	C1_CD8_Tres
chr2-11743707-11744413	7.43902E-08	0.320052533	0.361	0.181	0.004926342	C5_CD8_Tem
chr10-119501615-119502938	7.72202E-08	0.288313155	0.514	0.296	0.005113751	C5_CD8_Tem
chr6-24645539-24646555	7.72519E-08	0.311585565	0.388	0.197	0.005115853	C5_CD8_Tem
chr19-46633689-46634603	1.2449E-27	0.443232722	0.404	0.188	8.24412E-23	C1_CD8_Tres
chr2-43194324-43195471	8.36844E-08	0.265544046	0.552	0.349	0.00554183	C5_CD8_Tem
chr2-9752414-9754775	8.41055E-08	0.277206704	0.503	0.304	0.00556972	C5_CD8_Tem
chr11-35029908-35031183	8.43165E-08	0.326251429	0.328	0.158	0.005583692	C5_CD8_Tem
chr7-139084116-139085810	8.82991E-24	0.310629911	0.287	0.123	5.84743E-19	C4_CD8_Teff
chr3-197393766-197395033	8.93519E-08	0.331065152	0.366	0.183	0.005917149	C5_CD8_Tem
chr19-35207312-35207769	1.65111E-21	0.47256939	0.174	0.041	1.09341E-16	C1_CD8_Tres
chr1-146938154-146939156	2.35006E-17	0.260714009	0.088	0.019	1.55628E-12	C4_CD8_Teff
chr20-59253037-59254373	1.02436E-07	0.328266788	0.311	0.156	0.00678365	C5_CD8_Tem
chr9-5446455-5447136	1.04592E-07	0.346493972	0.137	0.033	0.006926396	C5_CD8_Tem
chr20-8152876-8153675	5.49403E-19	0.281684328	0.1	0.024	3.63831E-14	C4_CD8_Teff
chr1-235077651-235077919	1.05741E-07	0.312377552	0.06	0.006	0.007002474	C5_CD8_Tem
chr9-114369205-114370071	1.1782E-18	0.323353843	0.479	0.279	7.80237E-14	C3_CD8_Tem
chr2-127401273-127402245	1.08919E-07	0.366533519	0.18	0.058	0.007212911	C5_CD8_Tem
chr2-70083311-70083646	1.09544E-07	0.351980822	0.169	0.054	0.007254344	C5_CD8_Tem
chr17-75864256-75865335	1.9099E-53	0.462484426	0.325	0.095	1.26479E-48	C4_CD8_Teff
chr21-42192942-42193602	1.10406E-07	0.33860847	0.268	0.115	0.007311406	C5_CD8_Tem
chr10-6274704-6275756	1.11653E-07	0.250708175	0.596	0.385	0.007394019	C5_CD8_Tem
chr19-16586712-16587917	2.26386E-48	0.411522953	0.43	0.172	1.4992E-43	C4_CD8_Teff
chr12-94621298-94622082	1.15707E-07	0.358244564	0.202	0.066	0.007662483	C5_CD8_Tem
chr1-42943071-42943442	1.21505E-07	0.360053348	0.158	0.049	0.008046393	C5_CD8_Tem

chr17-409666-411434	2.81603E-19	0.301369197	0.448	0.293	1.86486E-14	C3_CD8_Tem
chr12-13165226-13166007	1.26252E-07	0.357803328	0.18	0.059	0.008360766	C5_CD8_Tem
chr19-18477201-18477605	1.26612E-07	0.359824087	0.109	0.025	0.00838461	C5_CD8_Tem
chr16-84518295-84519273	1.28526E-07	0.316064939	0.361	0.18	0.008511382	C5_CD8_Tem
chrX-2707275-2707747	1.29795E-07	0.330404071	0.29	0.126	0.008595419	C5_CD8_Tem
chr20-63635056-63636218	1.30626E-07	0.264645804	0.546	0.339	0.008650468	C5_CD8_Tem
chr21-34807265-34808813	1.34598E-07	0.275269876	0.514	0.313	0.008913468	C5_CD8_Tem
chr19-2620353-2621307	1.01661E-28	0.318526581	0.387	0.181	6.7323E-24	C4_CD8_Teff
chr1-144412046-144413315	2.40892E-14	0.315686143	0.305	0.172	1.59526E-09	C1_CD8_Tres
chr10-133258920-133259596	5.32783E-21	0.281602161	0.305	0.143	3.52825E-16	C4_CD8_Teff
chr21-45255154-45256068	1.44414E-07	0.341945672	0.109	0.028	0.009563505	C5_CD8_Tem
chr1-156096259-156098080	3.00384E-26	0.261009162	0.556	0.316	1.98923E-21	C4_CD8_Teff
chr9-128249691-128250974	6.00246E-30	0.27476703	0.576	0.325	3.97501E-25	C4_CD8_Teff
chr13-98509104-98509860	1.48965E-07	0.346721369	0.251	0.106	0.009864937	C5_CD8_Tem
chr16-66933595-66934886	1.53196E-23	0.271488986	0.455	0.238	1.01451E-18	C4_CD8_Teff
chr1-30756163-30757917	1.60582E-07	0.258062909	0.546	0.34	0.010634215	C5_CD8_Tem
chr5-415603-416613	1.62839E-07	0.360004683	0.153	0.042	0.010783683	C5_CD8_Tem
chr13-73058336-73059823	2.47708E-22	0.448098992	0.344	0.147	1.6404E-17	C1_CD8_Tres
chr11-65545804-65547035	5.23719E-30	0.284734765	0.53	0.287	3.46823E-25	C4_CD8_Teff
chr13-114160955-114161300	1.66625E-07	0.339222653	0.082	0.013	0.011034405	C5_CD8_Tem
chr3-161310358-161311501	5.86008E-35	0.393490019	0.211	0.058	3.88072E-30	C4_CD8_Teff
chr3-11251301-11251791	1.70838E-07	0.333810938	0.066	0.008	0.011313427	C5_CD8_Tem
chr4-139554868-139557458	6.87392E-19	0.262462083	0.316	0.15	4.55211E-14	C4_CD8_Teff
chr6-137217815-137219653	1.71787E-07	0.258757532	0.563	0.352	0.011376268	C5_CD8_Tem
chr9-135905096-135905931	4.26032E-12	0.286918302	0.138	0.056	2.82131E-07	C1_CD8_Tres
chr1-64897939-64898683	1.73087E-07	0.345528778	0.164	0.049	0.011462337	C5_CD8_Tem
chr17-82126395-82127458	3.57657E-52	0.70054836	0.449	0.129	2.36851E-47	C1_CD8_Tres

chr1-61452542-61453693	1.81777E-07	0.323764588	0.306	0.157	0.012037813	C5_CD8_Tem
chr1-92475860-92476631	1.83918E-07	0.348609468	0.153	0.051	0.012179594	C5_CD8_Tem
chr3-98557230-98557783	4.91614E-34	0.389835712	0.24	0.077	3.25562E-29	C4_CD8_Teff
chr1-154790841-154792148	1.11408E-48	0.410146688	0.438	0.173	7.3778E-44	C4_CD8_Teff
chr19-41297646-41298585	1.89206E-07	0.342199495	0.191	0.077	0.012529799	C5_CD8_Tem
chr7-1412343-1413186	5.75914E-26	0.337816827	0.21	0.07	3.81388E-21	C4_CD8_Teff
chr6-38713620-38715736	1.91906E-07	0.315397303	0.339	0.175	0.012708584	C5_CD8_Tem
chr1-151836502-151837667	1.96342E-07	0.323891875	0.284	0.137	0.013002389	C5_CD8_Tem
chr20-3994987-3995699	1.98294E-07	0.349854328	0.131	0.038	0.013131642	C5_CD8_Tem
chr2-231397112-231397311	2.06122E-07	0.3434069	0.077	0.011	0.013650011	C5_CD8_Tem
chr2-172437422-172438372	4.33089E-10	0.264253657	0.183	0.091	2.86805E-05	C1_CD8_Tres
chr1-8394577-8395886	2.08269E-07	0.343857142	0.142	0.04	0.01379219	C5_CD8_Tem
chr1-86714200-86714969	3.15621E-16	0.257574111	0.12	0.038	2.09014E-11	C4_CD8_Teff
chr11-93737922-93738486	3.00952E-09	0.250139834	0.159	0.084	0.0001993	C1_CD8_Tres
chr14-105537601-105538656	3.1423E-18	0.28136685	0.166	0.062	2.08092E-13	C4_CD8_Teff
chr2-234425673-234426466	1.49522E-12	0.281259711	0.234	0.126	9.90177E-08	C1_CD8_Tres
chr11-35247140-35248293	1.85848E-56	0.427542028	0.519	0.211	1.23074E-51	C4_CD8_Teff
chr13-99321864-99323139	2.44025E-07	0.292739462	0.421	0.23	0.016160074	C5_CD8_Tem
chr13-30429999-30430725	2.45518E-07	0.331131125	0.257	0.104	0.016258913	C5_CD8_Tem
chr9-4725663-4726129	2.53153E-07	0.355811444	0.126	0.03	0.016764522	C5_CD8_Tem
chr2-317260-318382	1.24732E-23	0.326610832	0.204	0.068	8.2601E-19	C4_CD8_Teff
chr9-33458237-33458907	2.19736E-12	0.314677958	0.153	0.058	1.45516E-07	C1_CD8_Tres
chr3-98555296-98556764	2.64816E-07	0.281823269	0.475	0.279	0.01753689	C5_CD8_Tem
chr11-67391142-67392398	1.45721E-19	0.254043549	0.392	0.213	9.65008E-15	C4_CD8_Teff
chrX-72273746-72274052	2.69618E-07	0.347507934	0.104	0.025	0.017854898	C5_CD8_Tem
chr12-64683968-64685368	5.64793E-28	0.522039815	0.275	0.081	3.74023E-23	C1_CD8_Tres
chr6-130368653-130368926	2.72479E-07	0.358623774	0.131	0.038	0.018044356	C5_CD8_Tem

chr8-100415528-100417352	1.76153E-98	0.543293083	0.594	0.2	1.16654E-93	C4_CD8_Teff
chr4-153471228-153472161	3.21382E-13	0.330259005	0.254	0.128	2.12829E-08	C1_CD8_Tres
chr11-35214696-35215174	1.03713E-24	0.32640309	0.237	0.089	6.86819E-20	C4_CD8_Teff
chr12-53102878-53103402	2.82533E-07	0.333453847	0.273	0.109	0.018710196	C5_CD8_Tem
chr19-14206310-14207701	2.83742E-07	0.303626199	0.333	0.175	0.018790253	C5_CD8_Tem
chr6-90222669-90222949	5.81797E-08	0.260007742	0.069	0.02	0.003852832	C1_CD8_Tres
chr8-125941480-125942739	2.27011E-84	0.473629021	0.65	0.263	1.50334E-79	C4_CD8_Teff
chr14-22524050-22525004	2.90363E-07	0.347468518	0.197	0.073	0.019228721	C5_CD8_Tem
chr8-108242901-108243379	2.98843E-07	0.341720172	0.219	0.087	0.019790299	C5_CD8_Tem
chr19-19614804-19615420	3.12818E-07	0.288076061	0.393	0.23	0.020715715	C5_CD8_Tem
chr22-20323896-20324434	3.14403E-07	0.333487996	0.148	0.042	0.020820743	C5_CD8_Tem
chr2-143263101-143263523	3.16078E-07	0.342844302	0.109	0.022	0.020931642	C5_CD8_Tem
chr19-19419983-19421059	2.11386E-20	0.379792051	0.289	0.123	1.39986E-15	C3_CD8_Tem
chr6-137981912-137983144	3.19831E-07	0.345636196	0.208	0.074	0.021180172	C5_CD8_Tem
chr19-8573198-8573976	1.43212E-13	0.272990204	0.318	0.19	9.48393E-09	C3_CD8_Tem
chr9-134325400-134326553	3.76444E-50	0.442131054	0.329	0.102	2.49293E-45	C4_CD8_Teff
chr17-77420572-77421454	3.22328E-07	0.332850544	0.093	0.019	0.021345558	C5_CD8_Tem
chr3-141017374-141018166	3.23004E-07	0.343976906	0.12	0.034	0.021390304	C5_CD8_Tem
chr11-118911875-118912806	7.71351E-43	0.406821417	0.353	0.134	5.10812E-38	C4_CD8_Teff
chr2-70084915-70085393	3.37559E-07	0.340881347	0.224	0.094	0.02235416	C5_CD8_Tem
chr13-73702210-73703076	8.68743E-18	0.401511817	0.135	0.031	5.75307E-13	C1_CD8_Tres
chr1-235075902-235076130	3.46928E-07	0.313892458	0.066	0.011	0.022974615	C5_CD8_Tem
chr9-104926185-104926887	3.50194E-07	0.336328349	0.131	0.039	0.023190901	C5_CD8_Tem
chr13-79385738-79386850	3.59697E-07	0.3191454	0.273	0.116	0.023820208	C5_CD8_Tem
chr14-24670539-24670969	3.61447E-07	0.326843619	0.197	0.077	0.02393609	C5_CD8_Tem
chr2-68464737-68464983	3.61869E-07	0.319852953	0.077	0.013	0.023964064	C5_CD8_Tem
chr11-61021304-61022550	2.98065E-16	0.352152376	0.243	0.119	1.97388E-11	C1_CD8_Tres

chrX-135098023-135099222	3.64297E-07	0.295625306	0.383	0.199	0.024124828	C5_CD8_Tem
chr3-20057204-20057566	3.65177E-07	0.335973676	0.197	0.075	0.024183137	C5_CD8_Tem
chr12-54428357-54429067	3.68831E-07	0.336319701	0.093	0.017	0.024425104	C5_CD8_Tem
chr14-52328277-52328528	3.78408E-07	0.315055195	0.071	0.011	0.025059318	C5_CD8_Tem
chr1-168419981-168420510	6.76537E-12	0.316207687	0.099	0.029	4.48023E-07	C1_CD8_Tres
chr17-78139875-78140785	2.48962E-14	0.280498247	0.41	0.292	1.6487E-09	C1_CD8_Tres
chr5-151138721-151139887	6.1292E-60	0.500412493	0.319	0.081	4.05894E-55	C4_CD8_Teff
chr6-130602179-130602871	4.05171E-07	0.350366161	0.126	0.03	0.026831646	C5_CD8_Tem
chr8-66589185-66590031	4.10339E-07	0.337355459	0.115	0.028	0.027173861	C5_CD8_Tem
chr19-38506681-38507584	1.76716E-17	0.275278276	0.18	0.071	1.17027E-12	C4_CD8_Teff
chr14-68682803-68683115	4.26007E-07	0.329699986	0.104	0.025	0.028211429	C5_CD8_Tem
chr8-143955319-143955620	4.26157E-07	0.334392535	0.142	0.04	0.028221425	C5_CD8_Tem
chr14-75283363-75284214	1.35098E-17	0.275127025	0.144	0.049	8.94657E-13	C4_CD8_Teff
chr5-139709652-139711649	1.33792E-26	0.30707048	0.381	0.183	8.86012E-22	C4_CD8_Teff
chr2-234258203-234258893	4.39447E-07	0.295792792	0.071	0.01	0.029101528	C5_CD8_Tem
chr5-142977014-142978026	2.35514E-18	0.357277266	0.299	0.141	1.55964E-13	C3_CD8_Tem
chr22-40257903-40258874	3.21018E-15	0.350172368	0.126	0.037	2.12587E-10	C1_CD8_Tres
chrX-41695340-41695863	4.69056E-07	0.346456928	0.169	0.053	0.031062312	C5_CD8_Tem
chr9-130834161-130835912	4.69233E-07	0.281208169	0.426	0.247	0.031074005	C5_CD8_Tem
chr1-111515645-111516492	4.69974E-07	0.333835422	0.087	0.016	0.031123086	C5_CD8_Tem
chr1-41238448-41238971	4.7083E-07	0.319079805	0.077	0.014	0.031179788	C5_CD8_Tem
chr9-114516844-114517273	4.77488E-07	0.344364092	0.093	0.016	0.031620677	C5_CD8_Tem
chr6-139187398-139187789	4.81605E-07	0.330878724	0.104	0.023	0.031893355	C5_CD8_Tem
chr19-19602171-19602464	6.89305E-20	0.381149493	0.123	0.027	4.56479E-15	C1_CD8_Tres
chr4-101038995-101039746	4.95574E-23	0.311329157	0.22	0.084	3.28184E-18	C4_CD8_Teff
chr15-74383546-74385446	4.89705E-07	0.27296366	0.464	0.28	0.032429718	C5_CD8_Tem
chr2-181465701-181466268	3.15267E-18	0.280397787	0.143	0.049	2.08779E-13	C4_CD8_Teff

chr12-49342301-49343661	7.57715E-17	0.26391863	0.223	0.099	5.01782E-12	C4_CD8_Teff
chr19-49553623-49554350	7.95653E-11	0.296909174	0.126	0.045	5.26905E-06	C1_CD8_Tres
chr9-105127415-105128277	5.31995E-07	0.317509021	0.306	0.145	0.03523033	C5_CD8_Tem
chr19-17079770-17080390	5.45285E-07	0.274028418	0.492	0.297	0.036110428	C5_CD8_Tem
chr10-46022955-46023765	5.46831E-07	0.305972298	0.328	0.161	0.036212811	C5_CD8_Tem
chr9-41234540-41235024	7.18773E-66	0.539913493	0.199	0.024	4.75993E-61	C4_CD8_Teff
chr19-13833263-13833967	8.38245E-20	0.272052042	0.323	0.165	5.55111E-15	C4_CD8_Teff
chr6-46170486-46171256	5.96735E-07	0.333311484	0.175	0.06	0.039517571	C5_CD8_Tem
chr8-12194030-12195076	6.00868E-07	0.291677222	0.383	0.198	0.039791257	C5_CD8_Tem
chr3-32113428-32115253	6.04431E-07	0.261792361	0.519	0.316	0.040027234	C5_CD8_Tem
chr13-99307083-99308260	6.0698E-07	0.265271495	0.481	0.291	0.040196044	C5_CD8_Tem
chr14-75229715-75230721	1.43782E-45	0.422858185	0.338	0.115	9.52166E-41	C4_CD8_Teff
chr5-157216753-157217563	6.09414E-07	0.335868354	0.164	0.056	0.040357209	C5_CD8_Tem
chr5-40831877-40832183	6.10653E-07	0.332310812	0.109	0.024	0.040439288	C5_CD8_Tem
chr17-488956-490300	3.80502E-29	0.349864391	0.111	0.019	2.5198E-24	C4_CD8_Teff
chr7-50197180-50197973	6.34175E-07	0.339638453	0.142	0.046	0.041997004	C5_CD8_Tem
chr2-230924249-230925362	6.39092E-07	0.327612612	0.208	0.09	0.042322602	C5_CD8_Tem
chr12-121222836-121223913	6.44887E-07	0.330862803	0.115	0.028	0.042706327	C5_CD8_Tem
chr5-35904677-35905071	6.48876E-07	0.303874474	0.06	0.009	0.042970548	C5_CD8_Tem
chr1-25035751-25036614	2.28245E-18	0.382485486	0.271	0.115	1.51151E-13	C3_CD8_Tem
chr1-161611874-161612938	6.4743E-37	0.354786809	0.441	0.195	4.28748E-32	C4_CD8_Teff
chr15-92906610-92907164	6.66778E-07	0.266110125	0.432	0.264	0.044156021	C5_CD8_Tem
chr19-39893399-39894033	6.70822E-07	0.331828568	0.082	0.015	0.044423832	C5_CD8_Tem
chr3-14424240-14425081	2.21091E-35	0.39283003	0.234	0.073	1.46413E-30	C4_CD8_Teff
chr21-14581882-14582319	7.02829E-07	0.345061655	0.153	0.045	0.04654342	C5_CD8_Tem
chr10-5683052-5683534	7.30405E-07	0.33364877	0.142	0.044	0.048369598	C5_CD8_Tem
chr15-92813428-92813750	3.70739E-28	0.514710868	0.141	0.02	2.45515E-23	C1_CD8_Tres

chr2-216348988-216349622	1.31139E-16	0.271211479	0.143	0.052	8.6844E-12	C4_CD8_Teff
chr6-25026839-25027916	7.48496E-07	0.308310833	0.301	0.147	0.049567623	C5_CD8_Tem
chr9-87973935-87974992	7.55377E-07	0.268713913	0.443	0.248	0.050023311	C5_CD8_Tem
chr9-137611644-137612094	7.63965E-07	0.326230596	0.098	0.023	0.050592033	C5_CD8_Tem
chr19-8515352-8515723	7.65976E-07	0.337699699	0.197	0.074	0.05072522	C5_CD8_Tem
chr17-47809886-47810847	5.18333E-21	0.290583754	0.268	0.123	3.43255E-16	C4_CD8_Teff
chr17-50148438-50149449	7.78033E-07	0.316485658	0.235	0.109	0.051523664	C5_CD8_Tem
chr9-33445631-33447874	8.00532E-07	0.251225699	0.514	0.318	0.053013655	C5_CD8_Tem
chr3-51902167-51902700	8.15681E-07	0.286487458	0.06	0.008	0.054016854	C5_CD8_Tem
chr2-201419966-201420780	6.66661E-10	0.272227009	0.075	0.019	4.41483E-05	C1_CD8_Tres
chr4-4387607-4387952	8.55266E-07	0.309979156	0.066	0.01	0.056638299	C5_CD8_Tem
chr15-74396914-74397281	8.57968E-07	0.339296549	0.169	0.055	0.056817229	C5_CD8_Tem
chr7-74451939-74452898	2.80838E-43	0.657824435	0.365	0.097	1.8598E-38	C1_CD8_Tres
chr1-26335932-26336975	8.62014E-07	0.295605772	0.333	0.167	0.057085167	C5_CD8_Tem
chr1-149636033-149636990	1.67189E-12	0.321010816	0.216	0.108	1.10717E-07	C1_CD8_Tres
chr11-117984168-117984752	2.14121E-25	0.339255315	0.161	0.046	1.41798E-20	C4_CD8_Teff
chr11-68750938-68751783	4.34398E-11	0.254314769	0.224	0.127	2.87672E-06	C3_CD8_Tem
chr13-49499965-49500458	8.92011E-07	0.321705849	0.115	0.03	0.059071651	C5_CD8_Tem
chr3-56930666-56931064	1.73485E-10	0.281060876	0.075	0.018	1.14887E-05	C1_CD8_Tres
chr7-158855911-158857041	9.24701E-07	0.251942099	0.508	0.318	0.061236448	C5_CD8_Tem
chr1-198187635-198188597	5.47785E-21	0.300882273	0.23	0.093	3.6276E-16	C4_CD8_Teff
chr8-23064440-23064845	9.39779E-07	0.308539171	0.104	0.023	0.062235009	C5_CD8_Tem
chr4-40290704-40291215	1.55039E-14	0.344235454	0.087	0.017	1.02672E-09	C1_CD8_Tres
chr5-132406174-132406631	9.49233E-07	0.312050182	0.093	0.018	0.062861064	C5_CD8_Tem
chr15-85030033-85030519	9.54184E-07	0.297232331	0.06	0.008	0.06318894	C5_CD8_Tem
chr14-32200889-32203910	2.54105E-17	0.346156965	0.311	0.16	1.68276E-12	C1_CD8_Tres
chr2-239326744-239327330	9.69828E-07	0.330762259	0.219	0.09	0.064224944	C5_CD8_Tem

chr15-44394628-44395398	9.71116E-07	0.32130966	0.071	0.011	0.064310223	C5_CD8_Tem
chr9-126636966-126637873	6.12289E-56	0.745561807	0.331	0.051	4.05476E-51	C3_CD8_Tem
chr3-156160373-156161595	9.7814E-07	0.332747636	0.148	0.05	0.064775398	C5_CD8_Tem
chr9-66269828-66270644	7.47077E-11	0.257805237	0.083	0.022	4.94737E-06	C3_CD8_Tem
chr14-97712947-97713426	1.00978E-06	0.316213136	0.082	0.019	0.066870491	C5_CD8_Tem
chr8-8919555-8920598	1.01147E-06	0.317168168	0.208	0.08	0.06698227	C5_CD8_Tem
chr6-159989789-159990428	1.01452E-06	0.323464123	0.109	0.027	0.067184363	C5_CD8_Tem
chr17-42541185-42541749	1.03904E-06	0.297198321	0.071	0.013	0.068808461	C5_CD8_Tem
chr16-57815530-57816711	1.43592E-24	0.323898821	0.243	0.097	9.50912E-20	C4_CD8_Teff
chr10-17001358-17003528	1.77452E-33	0.315814907	0.506	0.254	1.17514E-28	C4_CD8_Teff
chr1-184838507-184839002	1.10785E-06	0.327331296	0.137	0.044	0.073365423	C5_CD8_Tem
chr3-98508509-98508910	1.11368E-06	0.313769198	0.06	0.009	0.07375152	C5_CD8_Tem
chr9-134301405-134302179	5.05287E-61	0.50176979	0.352	0.097	3.34616E-56	C4_CD8_Teff
chr13-99273061-99273690	2.95906E-16	0.266316186	0.182	0.074	1.95958E-11	C4_CD8_Teff
chr1-39183570-39184484	5.7493E-16	0.312451251	0.389	0.239	3.80736E-11	C1_CD8_Tres
chr1-16728179-16728666	1.12738E-06	0.309605731	0.093	0.02	0.074658247	C5_CD8_Tem
chr21-38804430-38806699	1.12906E-06	0.260230364	0.443	0.266	0.074769592	C5_CD8_Tem
chr1-52139610-52140503	1.47412E-26	0.326080981	0.304	0.129	9.76204E-22	C4_CD8_Teff
chr1-205284427-205285142	9.4007E-35	0.371912685	0.322	0.121	6.22542E-30	C4_CD8_Teff
chr14-64726412-64726865	1.17236E-06	0.298730679	0.066	0.009	0.077637235	C5_CD8_Tem
chr2-130370517-130370961	1.1851E-06	0.322117676	0.115	0.033	0.078480989	C5_CD8_Tem
chr12-10405331-10405627	1.22602E-06	0.329623552	0.137	0.042	0.081190885	C5_CD8_Tem
chr9-110015739-110016098	1.23592E-06	0.295661512	0.055	0.007	0.081846414	C5_CD8_Tem
chr17-77409462-77410211	1.25234E-06	0.32082878	0.169	0.061	0.082933986	C5_CD8_Tem
chr6-28079818-28081698	1.26307E-06	0.265138379	0.421	0.251	0.083644067	C5_CD8_Tem
chr1-145984109-145984789	1.26417E-06	0.318372611	0.098	0.024	0.08371707	C5_CD8_Tem
chr22-42300693-42301094	1.26432E-06	0.302454925	0.077	0.015	0.083726821	C5_CD8_Tem

chr7-45001112-45001624	1.58736E-42	0.406426137	0.351	0.122	1.05119E-37	C4_CD8_Teff
chr4-83535346-83536610	1.2924E-06	0.292232761	0.333	0.174	0.085586873	C5_CD8_Tem
chr6-107712642-107713482	4.87776E-24	0.322211422	0.166	0.053	3.2302E-19	C4_CD8_Teff
chr6-117677515-117677862	1.29774E-06	0.340945574	0.12	0.034	0.085940133	C5_CD8_Tem
chr12-101279312-101280435	1.51455E-18	0.25104339	0.361	0.187	1.00298E-13	C4_CD8_Teff
chr3-17151241-17152599	1.3199E-06	0.265296709	0.437	0.258	0.087407454	C5_CD8_Tem
chr7-831634-832970	2.88879E-18	0.379161591	0.299	0.14	1.91305E-13	C1_CD8_Tres
chr9-92097933-92098396	1.40261E-06	0.296940244	0.066	0.01	0.092884717	C5_CD8_Tem
chr6-195719-196150	4.71343E-38	0.399309562	0.119	0.015	3.12137E-33	C4_CD8_Teff
chr12-95547953-95549269	1.43485E-06	0.31982869	0.142	0.049	0.095020242	C5_CD8_Tem
chr11-85742860-85743259	1.44058E-06	0.32979645	0.093	0.022	0.095399358	C5_CD8_Tem
chr1-117013711-117014597	1.44935E-06	0.32306728	0.126	0.038	0.095979994	C5_CD8_Tem
chr5-40391649-40391964	1.45859E-06	0.319766162	0.087	0.019	0.096591955	C5_CD8_Tem
chr5-157221925-157223223	1.66536E-20	0.26553843	0.374	0.198	1.10285E-15	C4_CD8_Teff
chr9-123224905-123225788	1.49926E-06	0.317847662	0.191	0.073	0.099285231	C5_CD8_Tem
chr4-151407888-151409396	5.34941E-20	0.402257075	0.171	0.052	3.54254E-15	C1_CD8_Tres
chr5-35835883-35836351	1.55658E-06	0.303675022	0.077	0.013	0.103081339	C5_CD8_Tem
chr20-62523839-62524186	1.566E-06	0.283999746	0.055	0.006	0.103705209	C5_CD8_Tem
chr5-142826599-142827411	1.58308E-06	0.312657746	0.208	0.081	0.10483624	C5_CD8_Tem
chr20-8453892-8454778	1.61458E-06	0.301156732	0.077	0.014	0.106922145	C5_CD8_Tem
chr3-46542303-46542549	1.64775E-06	0.298553726	0.087	0.019	0.109119239	C5_CD8_Tem
chr10-110221594-110222149	1.66149E-06	0.291729346	0.355	0.18	0.110028825	C5_CD8_Tem
chr2-98757844-98758431	1.67454E-06	0.335868094	0.153	0.055	0.110893058	C5_CD8_Tem
chr5-119274427-119275391	1.67751E-06	0.311079937	0.23	0.094	0.111089721	C5_CD8_Tem
chr9-21559043-21560315	1.67981E-06	0.328133929	0.148	0.05	0.111242149	C5_CD8_Tem
chr7-134669269-134670346	9.41334E-13	0.300033615	0.329	0.198	6.2338E-08	C1_CD8_Tres
chr14-70640771-70643189	1.71508E-06	0.30159066	0.301	0.144	0.113577855	C5_CD8_Tem

chr9-137449890-137450919	1.72804E-06	0.314100593	0.104	0.029	0.114436265	C5_CD8_Tem
chr20-58309071-58310212	1.73701E-06	0.276695897	0.366	0.199	0.115029807	C5_CD8_Tem
chr4-101015825-101016276	1.74562E-06	0.326688841	0.23	0.094	0.115600194	C5_CD8_Tem
chrX-156000197-156000775	2.59681E-30	0.365512715	0.157	0.04	1.71969E-25	C4_CD8_Teff
chr16-89785283-89785996	2.32939E-30	0.367833205	0.152	0.038	1.54259E-25	C4_CD8_Teff
chr11-134068604-134069377	1.80732E-06	0.303975684	0.279	0.129	0.11968596	C5_CD8_Tem
chr2-234273944-234274588	1.81176E-06	0.309740933	0.23	0.101	0.119980405	C5_CD8_Tem
chr17-7354287-7354690	1.84644E-06	0.312303177	0.109	0.03	0.122277099	C5_CD8_Tem
chr1-168391987-168393210	1.85002E-06	0.311668923	0.131	0.042	0.122514088	C5_CD8_Tem
chr7-44046496-44046933	6.56419E-11	0.293312536	0.099	0.029	4.34701E-06	C1_CD8_Tres
chrX-72105175-72105816	1.14474E-09	0.264324342	0.216	0.111	7.58078E-05	C1_CD8_Tres
chr22-49934200-49934413	1.93219E-06	0.310791013	0.071	0.014	0.127955393	C5_CD8_Tem
chr7-36776701-36777574	1.2612E-49	0.456064751	0.301	0.087	8.35204E-45	C4_CD8_Teff
chr1-160689426-160689875	1.966E-06	0.30404041	0.077	0.015	0.130194688	C5_CD8_Tem
chr6-142838763-142839298	9.41957E-16	0.263037618	0.168	0.069	6.23792E-11	C4_CD8_Teff
chr22-39388036-39389139	2.03949E-06	0.300662281	0.251	0.116	0.135060876	C5_CD8_Tem
chr19-15466742-15467649	2.07902E-06	0.275977422	0.35	0.202	0.137678826	C5_CD8_Tem
chr14-105059106-105059912	4.07091E-53	0.478009306	0.241	0.053	2.69588E-48	C4_CD8_Teff
chr17-47763831-47764281	2.12861E-06	0.306445663	0.098	0.024	0.140963028	C5_CD8_Tem
chr5-163434342-163434752	2.18089E-06	0.327634362	0.12	0.03	0.14442527	C5_CD8_Tem
chr11-78202511-78203649	1.51831E-28	0.325046782	0.35	0.161	1.00547E-23	C4_CD8_Teff
chr18-34978305-34979493	1.66307E-20	0.288462703	0.278	0.125	1.10133E-15	C4_CD8_Teff
chr1-93878188-93879401	2.23507E-06	0.255003105	0.443	0.27	0.148012827	C5_CD8_Tem
chr11-47234057-47235190	1.46022E-14	0.369850429	0.138	0.046	9.67004E-10	C1_CD8_Tres
chr6-108781749-108782677	2.26819E-06	0.318564928	0.186	0.076	0.150206555	C5_CD8_Tem
chr5-139481044-139482930	2.30666E-06	0.258776252	0.415	0.25	0.152754127	C5_CD8_Tem
chr3-156535163-156535577	2.33143E-06	0.32658793	0.077	0.014	0.15439449	C5_CD8_Tem

chr16-67326404-67327082	2.34705E-06	0.316757132	0.12	0.038	0.155428612	C5_CD8_Tem
chr1-84998254-84999298	2.34712E-06	0.291663676	0.311	0.157	0.155433525	C5_CD8_Tem
chr3-111609809-111611067	2.35269E-06	0.255541345	0.426	0.267	0.155802324	C5_CD8_Tem
chr14-106004855-106006329	1.61373E-50	0.397134281	0.512	0.223	1.06866E-45	C4_CD8_Teff
chr2-102233971-102234562	2.46107E-06	0.318633768	0.126	0.034	0.162979542	C5_CD8_Tem
chr10-47552472-47553609	2.76343E-32	0.367394664	0.127	0.02	1.83003E-27	C4_CD8_Teff
chr14-64707494-64708921	8.02786E-14	0.311430763	0.141	0.057	5.31629E-09	C1_CD8_Tres
chr9-33452827-33453458	2.53813E-06	0.314816963	0.098	0.025	0.168082679	C5_CD8_Tem
chr2-10653525-10654388	2.5385E-06	0.296490976	0.306	0.154	0.168107299	C5_CD8_Tem
chr10-3761343-3761619	2.54214E-06	0.299775895	0.093	0.021	0.168348095	C5_CD8_Tem
chr6-137155466-137156249	6.18965E-46	0.444660429	0.227	0.05	4.09897E-41	C4_CD8_Teff
chr2-174023893-174024536	2.57485E-06	0.301253056	0.082	0.015	0.170514578	C5_CD8_Tem
chr21-45208406-45208978	2.61262E-06	0.303872897	0.093	0.021	0.173015658	C5_CD8_Tem
chr5-119287098-119288138	1.80032E-41	0.402329338	0.347	0.128	1.19223E-36	C4_CD8_Teff
chr6-29845040-29845804	4.15097E-17	0.390361323	0.18	0.074	2.7489E-12	C1_CD8_Tres
chr12-120959565-120960303	2.68235E-06	0.320355063	0.175	0.072	0.177633369	C5_CD8_Tem
chr8-418873-419406	2.68541E-06	0.290521093	0.066	0.012	0.177835935	C5_CD8_Tem
chr1-21658983-21659718	1.43605E-30	0.365030031	0.238	0.077	9.50995E-26	C4_CD8_Teff
chr15-91852811-91853905	2.69974E-06	0.282709932	0.333	0.17	0.178784578	C5_CD8_Tem
chr3-106185921-106186192	2.73672E-06	0.302726868	0.071	0.01	0.181233703	C5_CD8_Tem
chr2-28583595-28584569	2.75569E-06	0.316916536	0.186	0.077	0.182489758	C5_CD8_Tem
chr11-35082399-35084363	1.28546E-14	0.291891409	0.386	0.247	8.51272E-10	C1_CD8_Tres
chr9-134324251-134324675	1.71818E-51	0.478159407	0.217	0.041	1.13783E-46	C4_CD8_Teff
chr11-96020238-96020646	2.88397E-06	0.294617457	0.071	0.012	0.190985116	C5_CD8_Tem
chr17-39929217-39929496	2.90977E-06	0.31360094	0.137	0.044	0.19269344	C5_CD8_Tem
chr14-61419862-61420659	2.91881E-06	0.314196144	0.158	0.057	0.193292275	C5_CD8_Tem
chr17-74463106-74464291	5.46666E-20	0.282638892	0.288	0.129	3.62019E-15	C4_CD8_Teff

chr2-74919792-74920335	2.99773E-06	0.311944785	0.115	0.038	0.19851877	C5_CD8_Tem
chr11-6650892-6651177	3.00297E-06	0.287348386	0.071	0.013	0.198865402	C5_CD8_Tem
chr2-231068222-231068820	3.02015E-06	0.334657521	0.158	0.053	0.200003637	C5_CD8_Tem
chr3-45950340-45951392	3.02584E-06	0.276628424	0.35	0.195	0.200380162	C5_CD8_Tem
chr1-234980422-234981267	3.06869E-06	0.282113074	0.311	0.173	0.203217752	C5_CD8_Tem
chr7-130903464-130904489	3.0991E-06	0.307364559	0.191	0.076	0.205231419	C5_CD8_Tem
chr15-33104022-33104674	2.4215E-44	0.690465183	0.216	0.026	1.60359E-39	C1_CD8_Tres
chr12-56291386-56292107	5.87559E-29	0.526946718	0.135	0.016	3.89099E-24	C1_CD8_Tres
chr18-62420089-62421122	3.15873E-23	0.27903009	0.404	0.21	2.0918E-18	C4_CD8_Teff
chr5-379399-379715	3.27722E-06	0.300533246	0.071	0.012	0.217027343	C5_CD8_Tem
chr5-50699466-50700075	3.5507E-15	0.256662413	0.114	0.037	2.35138E-10	C4_CD8_Teff
chr18-67301515-67302327	3.33927E-06	0.28311188	0.06	0.009	0.221136543	C5_CD8_Tem
chr12-538680-539689	2.49318E-18	0.279428524	0.133	0.042	1.65106E-13	C4_CD8_Teff
chr6-159988907-159989533	3.38626E-06	0.321359455	0.115	0.03	0.224248396	C5_CD8_Tem
chr1-61782618-61783124	1.5103E-14	0.360437419	0.108	0.029	1.00016E-09	C1_CD8_Tres
chr2-216349913-216350784	4.72924E-22	0.440638886	0.263	0.103	3.13185E-17	C3_CD8_Tem
chr4-40183602-40184276	3.4908E-06	0.309291556	0.115	0.029	0.231171186	C5_CD8_Tem
chr5-95823670-95824747	3.59271E-06	0.278350867	0.306	0.163	0.23791987	C5_CD8_Tem
chr2-74329796-74331371	3.59623E-06	0.295804343	0.071	0.015	0.238152897	C5_CD8_Tem
chr13-99375056-99375719	3.63603E-06	0.325200983	0.142	0.048	0.240788952	C5_CD8_Tem
chr11-117078004-117078564	2.80108E-22	0.311644194	0.242	0.1	1.85496E-17	C4_CD8_Teff
chr19-6667304-6667600	3.70884E-06	0.307215793	0.153	0.056	0.245610708	C5_CD8_Tem
chr4-1337230-1337925	3.73986E-06	0.307581694	0.115	0.034	0.247664755	C5_CD8_Tem
chr8-144262121-144262803	3.78217E-06	0.295221148	0.06	0.008	0.250466817	C5_CD8_Tem
chr1-101235126-101237022	6.47972E-18	0.370155822	0.338	0.183	4.29106E-13	C1_CD8_Tres
chr22-40651448-40652410	3.82002E-06	0.264810439	0.41	0.236	0.252972904	C5_CD8_Tem
chr19-56537955-56539110	3.83693E-06	0.267959341	0.317	0.181	0.25409286	C5_CD8_Tem

chr6-136753350-136754042	3.86141E-06	0.294546629	0.219	0.094	0.255714475	C5_CD8_Tem
chr1-8525085-8526207	5.08256E-15	0.355544817	0.225	0.099	3.36583E-10	C1_CD8_Tres
chr17-979239-980290	1.63704E-19	0.268341017	0.325	0.163	1.0841E-14	C4_CD8_Teff
chr1-8495849-8497059	4.37499E-12	0.297918377	0.242	0.126	2.89725E-07	C3_CD8_Tem
chr6-36514876-36515410	4.14391E-06	0.284883098	0.055	0.007	0.274422115	C5_CD8_Tem
chr7-100220756-100222056	2.75524E-37	0.355929516	0.436	0.196	1.8246E-32	C4_CD8_Teff
chr15-51727470-51727966	2.66629E-25	0.327444223	0.135	0.032	1.7657E-20	C4_CD8_Teff
chr2-240831720-240832790	3.9185E-12	0.292926316	0.374	0.244	2.59495E-07	C1_CD8_Tres
chr9-89456643-89457725	4.92121E-23	0.479408647	0.18	0.047	3.25897E-18	C1_CD8_Tres
chr12-103840408-103842131	4.43371E-06	0.254367379	0.426	0.245	0.293613372	C5_CD8_Tem
chr8-30141130-30142087	4.45545E-06	0.275413743	0.311	0.167	0.295053557	C5_CD8_Tem
chr9-104990822-104992139	3.31326E-10	0.264667621	0.311	0.2	2.19414E-05	C1_CD8_Tres
chr7-1961547-1962350	1.65646E-10	0.254604571	0.211	0.113	1.09696E-05	C3_CD8_Tem
chr11-65385266-65385954	1.2972E-28	0.361530147	0.158	0.04	8.59044E-24	C4_CD8_Teff
chr19-35215991-35216331	4.51905E-06	0.295940355	0.082	0.017	0.299265329	C5_CD8_Tem
chr15-60526103-60526651	4.54121E-06	0.307023575	0.142	0.05	0.300732803	C5_CD8_Tem
chr17-40605132-40605713	9.09198E-21	0.298118383	0.213	0.085	6.02098E-16	C4_CD8_Teff
chr11-104907473-104908236	4.59368E-06	0.302415114	0.087	0.021	0.304207543	C5_CD8_Tem
chr11-286876-287442	4.64761E-06	0.304011292	0.158	0.059	0.307778624	C5_CD8_Tem
chr5-115295809-115297010	4.72491E-20	0.293010371	0.206	0.08	3.12898E-15	C4_CD8_Teff
chr14-61525034-61525695	4.66723E-06	0.27528548	0.322	0.168	0.309077765	C5_CD8_Tem
chr5-132055757-132056644	4.67207E-06	0.309331459	0.273	0.123	0.309398498	C5_CD8_Tem
chr1-64744269-64746053	1.9546E-16	0.267020562	0.153	0.054	1.2944E-11	C4_CD8_Teff
chr12-8065975-8066697	4.69938E-06	0.287255463	0.295	0.146	0.311207366	C5_CD8_Tem
chr19-7690692-7691941	3.4602E-19	0.36944322	0.195	0.069	2.29144E-14	C3_CD8_Tem
chr17-80761178-80761807	4.81524E-06	0.287658826	0.268	0.137	0.3188797	C5_CD8_Tem
chr19-14431799-14432186	4.84759E-06	0.301116097	0.142	0.05	0.321022087	C5_CD8_Tem

chr1-113389521-113391431	1.09961E-10	0.25679508	0.296	0.182	7.28195E-06	C1_CD8_Tres
chr9-131264301-131264515	4.88118E-06	0.294178517	0.071	0.011	0.32324643	C5_CD8_Tem
chr7-131013200-131013528	4.92826E-06	0.31252278	0.126	0.036	0.326363986	C5_CD8_Tem
chr20-45884838-45885282	4.93831E-06	0.29138239	0.284	0.141	0.327029936	C5_CD8_Tem
chr19-47572945-47573549	4.94413E-06	0.295742213	0.197	0.085	0.327415047	C5_CD8_Tem
chr11-45092887-45093822	7.47706E-30	0.344422917	0.3	0.122	4.95154E-25	C4_CD8_Teff
chr18-79681487-79682034	4.98546E-06	0.300659441	0.219	0.104	0.330152009	C5_CD8_Tem
chr16-89256421-89257469	4.99986E-06	0.277751319	0.317	0.168	0.331105629	C5_CD8_Tem
chr8-143927925-143929685	1.10813E-15	0.322899144	0.284	0.145	7.33834E-11	C3_CD8_Tem
chr11-62024948-62026149	3.40964E-10	0.265379884	0.41	0.277	2.25797E-05	C1_CD8_Tres
chr6-42031089-42031503	5.07527E-06	0.321522419	0.12	0.032	0.336099458	C5_CD8_Tem
chr7-23020655-23021404	5.45409E-16	0.413672839	0.171	0.056	3.61186E-11	C1_CD8_Tres
chr21-44870488-44870976	1.29624E-28	0.529051428	0.144	0.021	8.58412E-24	C1_CD8_Tres
chr4-140095635-140096748	5.22907E-06	0.267006591	0.421	0.227	0.346284726	C5_CD8_Tem
chr14-64755533-64756287	1.49086E-20	0.45616637	0.29	0.116	9.87289E-16	C1_CD8_Tres
chr19-38387274-38388789	4.74388E-15	0.278841603	0.329	0.209	3.14154E-10	C1_CD8_Tres
chr16-68417447-68418608	4.98294E-23	0.291660707	0.347	0.17	3.29985E-18	C4_CD8_Teff
chr3-161349586-161350276	3.43853E-16	0.401330828	0.132	0.034	2.2771E-11	C1_CD8_Tres
chr11-57457205-57459446	5.47433E-06	0.282597253	0.268	0.132	0.36252669	C5_CD8_Tem
chr2-241813396-241814135	5.54851E-06	0.303739654	0.109	0.033	0.36743884	C5_CD8_Tem
chr1-221855985-221856710	5.59683E-06	0.305611851	0.126	0.041	0.370638996	C5_CD8_Tem
chr1-41919702-41920973	5.60575E-06	0.257004938	0.399	0.241	0.371229258	C5_CD8_Tem
chr2-113167035-113167587	5.62459E-06	0.297095747	0.087	0.02	0.372477072	C5_CD8_Tem
chr8-133645062-133646171	5.65219E-06	0.297131895	0.142	0.048	0.374304755	C5_CD8_Tem
chr2-241619549-241620779	5.77549E-06	0.298795458	0.213	0.091	0.382470194	C5_CD8_Tem
chr8-101080148-101081061	7.68402E-26	0.328798618	0.234	0.089	5.08859E-21	C4_CD8_Teff
chr2-105747999-105748419	8.42341E-15	0.253739669	0.162	0.064	5.57824E-10	C4_CD8_Teff

chr3-170037534-170038972	5.99479E-06	0.291731944	0.224	0.105	0.396993132	C5_CD8_Tem
chr3-106215035-106215385	6.19091E-06	0.323409249	0.142	0.048	0.409980481	C5_CD8_Tem
chr18-38940334-38941537	2.27778E-39	0.391506607	0.336	0.117	1.50841E-34	C4_CD8_Teff
chr19-55372632-55373022	6.22629E-06	0.300799586	0.077	0.016	0.412323837	C5_CD8_Tem
chr5-1105490-1106169	1.0478E-16	0.423895232	0.162	0.045	6.93883E-12	C1_CD8_Tres
chr15-88627297-88627807	5.58513E-17	0.363113924	0.177	0.067	3.69864E-12	C1_CD8_Tres
chr1-148362433-148362934	6.30195E-06	0.266017734	0.055	0.008	0.417333982	C5_CD8_Tem
chr3-157129352-157130836	6.30857E-06	0.262607058	0.383	0.213	0.417772176	C5_CD8_Tem
chr4-80016587-80017874	6.31108E-06	0.302260816	0.18	0.08	0.417938771	C5_CD8_Tem
chr4-8408869-8410058	7.55219E-53	0.592720178	0.612	0.276	5.00129E-48	C3_CD8_Tem
chrX-1208026-1208776	5.3638E-23	0.487096744	0.168	0.038	3.55207E-18	C1_CD8_Tres
chr17-82985881-82986429	6.41716E-06	0.302808321	0.197	0.092	0.424963602	C5_CD8_Tem
chr6-142832768-142833393	6.58507E-06	0.305247344	0.169	0.064	0.436083043	C5_CD8_Tem
chr5-373385-374072	6.6757E-06	0.299940098	0.104	0.031	0.442084882	C5_CD8_Tem
chr13-99303876-99304151	6.72345E-06	0.285879663	0.087	0.025	0.445246874	C5_CD8_Tem
chr1-156813778-156814456	2.12409E-19	0.258273853	0.356	0.194	1.40664E-14	C4_CD8_Teff
chr7-925609-926009	1.65519E-24	0.310241896	0.289	0.131	1.09612E-19	C4_CD8_Teff
chr17-8934619-8935587	6.85969E-06	0.284771429	0.279	0.141	0.454269414	C5_CD8_Tem
chr9-33415251-33415946	4.57764E-34	0.592112104	0.231	0.048	3.03145E-29	C1_CD8_Tres
chr6-26568122-26569395	1.3167E-43	0.416690644	0.343	0.12	8.71959E-39	C4_CD8_Teff
chr15-50885239-50886167	7.08384E-06	0.278722972	0.301	0.158	0.469113036	C5_CD8_Tem
chr12-132985767-132986898	2.82645E-12	0.308257727	0.198	0.091	1.87176E-07	C1_CD8_Tres
chr1-228058759-228060157	7.31965E-06	0.287127824	0.186	0.08	0.484729476	C5_CD8_Tem
chr5-132059009-132059400	2.26123E-13	0.354649051	0.108	0.024	1.49745E-08	C1_CD8_Tres
chr15-60362476-60363105	7.61309E-06	0.309348245	0.191	0.074	0.504161775	C5_CD8_Tem
chr14-76767656-76768871	7.62445E-06	0.258940514	0.355	0.21	0.504914219	C5_CD8_Tem
chr1-84156143-84156635	2.86735E-30	0.361897118	0.139	0.03	1.89885E-25	C4_CD8_Teff

chr17-4714902-4715337	7.93846E-06	0.297715923	0.158	0.06	0.525708359	C5_CD8_Tem
chr1-40413722-40414567	7.94832E-06	0.292965462	0.082	0.02	0.526361655	C5_CD8_Tem
chr9-134305425-134306484	6.8978E-24	0.300673346	0.086	0.014	4.56793E-19	C4_CD8_Teff
chrX-40278348-40279261	8.02976E-06	0.277168028	0.301	0.162	0.531754964	C5_CD8_Tem
chr2-172084668-172085596	8.03301E-06	0.296023494	0.148	0.056	0.531970049	C5_CD8_Tem
chr17-4721449-4722079	8.12342E-06	0.300515891	0.098	0.028	0.537957413	C5_CD8_Tem
chr17-46108887-46109131	8.20597E-06	0.280174432	0.055	0.01	0.54342404	C5_CD8_Tem
chr7-150738216-150738966	8.32865E-06	0.293780104	0.202	0.083	0.551548104	C5_CD8_Tem
chr12-111443552-111443831	8.35373E-06	0.296947761	0.093	0.023	0.553208808	C5_CD8_Tem
chr6-30441110-30441527	8.42477E-06	0.290434012	0.087	0.02	0.557913275	C5_CD8_Tem
chr5-142432040-142433340	8.62954E-06	0.274229679	0.29	0.155	0.571474044	C5_CD8_Tem
chr1-116538333-116539064	8.63482E-06	0.276709044	0.317	0.173	0.571823359	C5_CD8_Tem
chr6-106528021-106528285	8.78975E-06	0.286724311	0.082	0.017	0.582083682	C5_CD8_Tem
chr5-159337564-159338047	2.03885E-10	0.269456337	0.084	0.025	1.35018E-05	C1_CD8_Tres
chr1-235206595-235207509	2.80588E-21	0.445035563	0.237	0.091	1.85814E-16	C1_CD8_Tres
chr13-36345038-36346947	1.72962E-16	0.37585861	0.21	0.091	1.14541E-11	C1_CD8_Tres
chr12-124391371-124391873	8.95076E-06	0.290054417	0.071	0.015	0.592746468	C5_CD8_Tem
chr16-89784179-89785008	1.03883E-54	0.494505697	0.24	0.052	6.87947E-50	C4_CD8_Teff
chr6-129678485-129678766	8.99703E-06	0.258953938	0.055	0.007	0.595810455	C5_CD8_Tem
chr12-31733746-31733978	9.18434E-06	0.298950326	0.115	0.03	0.608214597	C5_CD8_Tem
chr16-3039409-3039886	9.22023E-06	0.293226131	0.093	0.022	0.610591275	C5_CD8_Tem
chr2-127397933-127398589	9.25203E-06	0.299143389	0.175	0.076	0.612696884	C5_CD8_Tem
chr1-33430300-33431421	1.42051E-12	0.299224498	0.198	0.093	9.40706E-08	C1_CD8_Tres
chr19-10430776-10432295	9.32937E-06	0.251852037	0.383	0.215	0.617819163	C5_CD8_Tem
chr7-45048620-45049166	4.21771E-10	0.274993306	0.084	0.022	2.79309E-05	C1_CD8_Tres
chr3-50610261-50610470	9.38664E-06	0.295797024	0.104	0.03	0.621611454	C5_CD8_Tem
chr7-28119958-28120567	9.40533E-06	0.264205546	0.06	0.01	0.622848876	C5_CD8_Tem

chr15-52658686-52659323	9.55548E-06	0.300200391	0.142	0.046	0.632792653	C5_CD8_Tem
chr21-36141149-36141916	9.59201E-06	0.287855663	0.213	0.095	0.635211693	C5_CD8_Tem
chr5-177370637-177371098	9.61894E-06	0.301644227	0.175	0.071	0.636994787	C5_CD8_Tem
chr2-70133497-70134341	9.66093E-06	0.290758465	0.197	0.083	0.639775762	C5_CD8_Tem
chr15-91706099-91706801	4.82342E-09	0.269281253	0.117	0.05	0.000319422	C1_CD8_Tres
chr15-63203399-63204113	5.62847E-23	0.317481019	0.217	0.08	3.72734E-18	C4_CD8_Teff
chr14-55071702-55072623	9.85015E-06	0.288177865	0.213	0.094	0.65230672	C5_CD8_Tem
chr19-45087196-45087984	9.38316E-19	0.332268135	0.383	0.203	6.21381E-14	C3_CD8_Tem
chr11-47797435-47798033	9.93626E-06	0.313610271	0.142	0.053	0.658009009	C5_CD8_Tem
chr10-3873474-3874307	9.99785E-06	0.278133238	0.306	0.154	0.662087583	C5_CD8_Tem
chr10-110852269-110852489	1.00616E-05	0.265213843	0.06	0.012	0.666311667	C5_CD8_Tem
chr16-3170887-3172150	8.31079E-31	0.365808483	0.244	0.081	5.50366E-26	C4_CD8_Teff
chr22-36383075-36383603	1.60058E-24	0.298307964	0.362	0.173	1.05995E-19	C4_CD8_Teff
chr20-28602102-28603285	6.08925E-14	0.358619638	0.21	0.08	4.03248E-09	C1_CD8_Tres
chr17-77836360-77837135	2.22429E-24	0.324476352	0.227	0.089	1.47299E-19	C4_CD8_Teff
chr12-12006921-12007387	1.11761E-05	0.260543096	0.383	0.209	0.740115654	C5_CD8_Tem
chr12-56338147-56339151	1.1177E-05	0.257669377	0.328	0.182	0.740176764	C5_CD8_Tem
chr10-132615857-132616861	1.22343E-09	0.299435599	0.168	0.074	8.10189E-05	C1_CD8_Tres
chr1-160641440-160641922	1.13933E-05	0.292418606	0.186	0.085	0.754499675	C5_CD8_Tem
chr5-141681599-141682444	1.16198E-05	0.29965005	0.137	0.048	0.769499353	C5_CD8_Tem
chr12-53492330-53493553	1.17215E-05	0.262891134	0.301	0.157	0.776233937	C5_CD8_Tem
chr9-96149625-96150847	1.17886E-05	0.279976903	0.311	0.168	0.780676053	C5_CD8_Tem
chr9-136384932-136385369	1.19673E-05	0.299550747	0.131	0.05	0.792513194	C5_CD8_Tem
chr1-149635266-149635738	1.19758E-05	0.273533722	0.071	0.016	0.793075739	C5_CD8_Tem
chr4-113901802-113902396	2.01304E-12	0.357525339	0.102	0.026	1.3331E-07	C1_CD8_Tres
chr16-57475206-57476389	1.21397E-05	0.285156985	0.208	0.09	0.803929805	C5_CD8_Tem
chr9-114718278-114719263	1.21799E-05	0.271582055	0.262	0.138	0.806590347	C5_CD8_Tem

chr1-6599293-6599844	6.536E-15	0.255232647	0.15	0.057	4.32834E-10	C4_CD8_Teff
chr4-8414149-8414744	1.22238E-05	0.288073898	0.191	0.082	0.809497872	C5_CD8_Tem
chr17-48456624-48457598	1.22955E-05	0.285332376	0.235	0.109	0.814245931	C5_CD8_Tem
chr17-49337948-49338151	1.23004E-05	0.290368823	0.082	0.019	0.814572414	C5_CD8_Tem
chr6-106103371-106104183	1.23831E-05	0.288933452	0.186	0.078	0.820047417	C5_CD8_Tem
chr22-44709731-44711179	1.24042E-05	0.296457833	0.219	0.091	0.821445676	C5_CD8_Tem
chr17-8958263-8958907	1.30862E-32	0.37557075	0.27	0.093	8.66604E-28	C4_CD8_Teff
chr15-82680039-82681005	3.95897E-13	0.323836797	0.18	0.074	2.62175E-08	C1_CD8_Tres
chr11-77473258-77474673	1.25744E-05	0.274511054	0.279	0.145	0.83271515	C5_CD8_Tem
chr2-234071681-234072045	1.2734E-05	0.300118167	0.093	0.023	0.843280561	C5_CD8_Tem
chr17-75033617-75035232	1.28332E-05	0.259193157	0.295	0.159	0.849853825	C5_CD8_Tem
chr6-28988361-28989093	1.28758E-05	0.285786023	0.087	0.023	0.852674178	C5_CD8_Tem
chr15-60932224-60932934	1.30399E-05	0.28582472	0.191	0.087	0.863538886	C5_CD8_Tem
chr1-84782177-84782778	1.30703E-05	0.294252876	0.093	0.025	0.865557321	C5_CD8_Tem
chr15-92914300-92915477	1.30822E-05	0.280051769	0.23	0.107	0.866339805	C5_CD8_Tem
chr1-116389550-116390529	1.33096E-05	0.277774903	0.235	0.112	0.881400009	C5_CD8_Tem
chr2-233076629-233077079	5.10914E-19	0.288310097	0.172	0.063	3.38342E-14	C4_CD8_Teff
chr6-3160099-3160760	1.4697E-27	0.323160967	0.336	0.149	9.73277E-23	C4_CD8_Teff
chr4-76993858-76994597	1.40888E-05	0.285002829	0.115	0.038	0.933000509	C5_CD8_Tem
chr14-20626227-20626546	1.41628E-05	0.255503531	0.06	0.01	0.937901564	C5_CD8_Tem
chr6-162727061-162728294	1.44316E-05	0.26817511	0.306	0.162	0.955707	C5_CD8_Tem
chr17-40346908-40347142	1.44897E-05	0.258093366	0.066	0.013	0.959552257	C5_CD8_Tem
chr1-151989519-151989782	1.4541E-05	0.293498189	0.137	0.045	0.962945494	C5_CD8_Tem
chr2-7743584-7743968	1.46688E-05	0.281933837	0.087	0.024	0.971409558	C5_CD8_Tem
chr12-96237370-96238109	1.47032E-05	0.292240463	0.186	0.076	0.973688624	C5_CD8_Tem
chr2-9779545-9780603	1.49645E-05	0.30105228	0.153	0.06	0.990991108	C5_CD8_Tem
chr5-111278513-111279163	1.50318E-05	0.283762022	0.137	0.056	0.995453844	C5_CD8_Tem

chr10-133159871-133160517	1.50814E-05	0.295513374	0.109	0.034	0.998736787	C5_CD8_Tem
chr19-20660864-20661836	4.07249E-20	0.276589635	0.316	0.15	2.69693E-15	C4_CD8_Teff
chr3-71415466-71417239	1.5104E-05	0.278129049	0.202	0.089	1	C5_CD8_Tem
chr8-126488135-126488574	1.5593E-05	0.265917339	0.071	0.016	1	C5_CD8_Tem
chr17-77426314-77426584	1.56005E-05	0.271873039	0.066	0.011	1	C5_CD8_Tem
chr1-210394231-210394749	1.57253E-05	0.287005026	0.087	0.024	1	C5_CD8_Tem
chr16-38646-39563	1.58005E-05	0.288837575	0.137	0.048	1	C5_CD8_Tem
chr6-27250704-27252141	1.58372E-05	0.284604827	0.142	0.055	1	C5_CD8_Tem
chr2-197803986-197805480	1.05042E-09	0.266988352	0.24	0.143	6.95623E-05	C1_CD8_Tres
chr5-35856551-35857094	1.60527E-05	0.283084885	0.202	0.097	1	C5_CD8_Tem
chr12-120199930-120200241	1.6219E-05	0.284059932	0.109	0.034	1	C5_CD8_Tem
chr5-134131113-134131679	1.4008E-16	0.268837384	0.139	0.049	9.27651E-12	C4_CD8_Teff
chr1-206581612-206582024	1.64659E-05	0.280651703	0.23	0.106	1	C5_CD8_Tem
chr3-151209427-151210045	1.65323E-05	0.302797387	0.175	0.068	1	C5_CD8_Tem
chr12-120783056-120783602	1.65745E-05	0.275285585	0.066	0.013	1	C5_CD8_Tem
chr15-60580112-60580970	3.73532E-23	0.467031802	0.249	0.09	2.47364E-18	C1_CD8_Tres
chr4-139172282-139172515	1.68138E-05	0.270755519	0.071	0.014	1	C5_CD8_Tem
chr12-12465878-12466513	1.15302E-12	0.312287791	0.195	0.084	7.63564E-08	C1_CD8_Tres
chr11-3904661-3905532	1.71144E-05	0.279891892	0.235	0.11	1	C5_CD8_Tem
chr21-46368226-46369182	5.62908E-25	0.33074771	0.204	0.071	3.72775E-20	C4_CD8_Teff
chr7-50206183-50206951	1.41334E-13	0.336108713	0.237	0.12	9.35953E-09	C1_CD8_Tres
chr9-93091520-93091803	2.14034E-10	0.322668434	0.09	0.023	1.4174E-05	C1_CD8_Tres
chr3-11238758-11239264	1.77129E-05	0.28408963	0.087	0.023	1	C5_CD8_Tem
chr17-36196490-36197498	1.51618E-22	0.430208115	0.229	0.074	1.00406E-17	C3_CD8_Tem
chr15-67106490-67107471	2.28682E-65	0.526609668	0.266	0.053	1.5144E-60	C4_CD8_Teff
chr5-80757363-80757600	1.80639E-05	0.28017778	0.077	0.016	1	C5_CD8_Tem
chr6-37209460-37210717	1.35794E-11	0.29778398	0.159	0.076	8.99269E-07	C1_CD8_Tres

chr6-16316586-16317072	1.62512E-15	0.258618703	0.105	0.031	1.07621E-10	C4_CD8_Teff
chr11-123453839-123454698	1.88914E-05	0.262578469	0.317	0.169	1	C5_CD8_Tem
chr19-55586888-55587624	1.89112E-05	0.277630227	0.197	0.087	1	C5_CD8_Tem
chr9-132268767-132270525	1.14184E-16	0.26831571	0.133	0.045	7.5616E-12	C4_CD8_Teff
chr17-42107246-42108066	1.21391E-19	0.277184041	0.285	0.131	8.03889E-15	C4_CD8_Teff
chr1-160738839-160739406	8.72625E-25	0.311368268	0.311	0.137	5.77879E-20	C4_CD8_Teff
chr13-51821533-51822768	1.46626E-08	0.250867909	0.138	0.067	0.000971001	C1_CD8_Tres
chr3-50316352-50317145	2.01582E-05	0.257137402	0.339	0.189	1	C5_CD8_Tem
chr7-50339986-50340516	1.84672E-08	0.253227656	0.102	0.039	0.00122295	C1_CD8_Tres
chr2-11757151-11757433	2.02068E-05	0.274019427	0.082	0.021	1	C5_CD8_Tem
chr5-80761968-80762198	2.04293E-05	0.267925435	0.055	0.009	1	C5_CD8_Tem
chr3-188322747-188323755	4.00202E-17	0.390598236	0.192	0.068	2.65026E-12	C1_CD8_Tres
chr8-120701911-120702512	2.07424E-05	0.290095471	0.175	0.073	1	C5_CD8_Tem
chr6-108068389-108069053	2.07811E-05	0.286675381	0.109	0.039	1	C5_CD8_Tem
chr6-52392597-52393204	2.09247E-05	0.278575139	0.224	0.105	1	C5_CD8_Tem
chr1-232923575-232924398	2.45511E-10	0.312593698	0.12	0.045	1.62585E-05	C1_CD8_Tres
chr7-100217224-100217845	2.11601E-05	0.254298762	0.355	0.188	1	C5_CD8_Tem
chr22-23263385-23264547	1.41352E-34	0.551089318	0.362	0.132	9.36074E-30	C3_CD8_Tem
chr12-117105531-117106254	1.43406E-10	0.27449368	0.081	0.024	9.49677E-06	C1_CD8_Tres
chr12-107515226-107515892	2.18359E-05	0.273945735	0.077	0.018	1	C5_CD8_Tem
chr17-67431290-67431893	5.72781E-22	0.305546899	0.251	0.102	3.79313E-17	C4_CD8_Teff
chr5-72138841-72139361	2.26576E-05	0.282416814	0.104	0.031	1	C5_CD8_Tem
chr12-121784424-121784732	9.34135E-30	0.365461703	0.153	0.037	6.18613E-25	C4_CD8_Teff
chr14-74620541-74620955	2.28199E-05	0.270349459	0.257	0.129	1	C5_CD8_Tem
chr1-39690899-39692125	1.42209E-11	0.259750693	0.263	0.159	9.4175E-07	C1_CD8_Tres
chrX-129786960-129787957	3.89025E-15	0.254618165	0.125	0.041	2.57624E-10	C4_CD8_Teff
chr18-3339899-3340358	2.31796E-05	0.27707976	0.087	0.02	1	C5_CD8_Tem

chrX-2709740-2710920	2.32238E-05	0.265601141	0.257	0.123	1	C5_CD8_Tem
chr17-8154148-8154466	2.33804E-05	0.271188705	0.202	0.091	1	C5_CD8_Tem
chr17-36198897-36199460	7.1471E-152	0.770173325	0.355	0.026	4.7331E-147	C4_CD8_Teff
chr3-189142162-189142896	2.7509E-19	0.290506647	0.187	0.067	1.82173E-14	C4_CD8_Teff
chr9-127976577-127976785	2.36346E-05	0.260805435	0.06	0.011	1	C5_CD8_Tem
chr15-22262390-22263023	2.37312E-05	0.264907851	0.082	0.021	1	C5_CD8_Tem
chr5-55997774-55998031	2.38443E-05	0.287105103	0.12	0.042	1	C5_CD8_Tem
chr5-134435523-134435945	2.41508E-05	0.266925351	0.071	0.016	1	C5_CD8_Tem
chr9-33818659-33819261	2.42708E-05	0.275859974	0.213	0.096	1	C5_CD8_Tem
chr11-96013827-96014532	2.43629E-05	0.282760941	0.158	0.066	1	C5_CD8_Tem
chr14-106013425-106014366	6.08299E-49	0.462677207	0.254	0.064	4.02834E-44	C4_CD8_Teff
chr10-3900361-3900730	2.45271E-05	0.262538056	0.06	0.011	1	C5_CD8_Tem
chr2-218315799-218316938	7.05742E-17	0.33694187	0.117	0.031	4.67363E-12	C1_CD8_Tres
chr19-38213622-38214750	2.48369E-05	0.26590582	0.208	0.106	1	C5_CD8_Tem
chr16-30715390-30716124	2.50766E-05	0.256249704	0.35	0.188	1	C5_CD8_Tem
chr11-118200748-118201309	2.56141E-05	0.268290011	0.262	0.133	1	C5_CD8_Tem
chr1-89671786-89672473	2.56862E-05	0.28196895	0.12	0.043	1	C5_CD8_Tem
chr14-52318842-52319075	2.5752E-05	0.281554771	0.082	0.021	1	C5_CD8_Tem
chr2-234147113-234147957	8.75433E-23	0.306151888	0.118	0.027	5.79738E-18	C4_CD8_Teff
chr6-29632311-29633465	2.59019E-05	0.265888337	0.224	0.112	1	C5_CD8_Tem
chr11-114166610-114167398	2.60937E-05	0.275782139	0.18	0.08	1	C5_CD8_Tem
chr3-56800303-56800870	2.61074E-05	0.261916758	0.251	0.139	1	C5_CD8_Tem
chr1-224387248-224387840	2.61631E-05	0.292199332	0.087	0.024	1	C5_CD8_Tem
chr19-17317302-17317741	2.6769E-05	0.270152255	0.071	0.016	1	C5_CD8_Tem
chr6-28940401-28941744	2.72347E-05	0.2500504	0.322	0.189	1	C5_CD8_Tem
chr6-31737572-31738589	2.74269E-05	0.259854664	0.257	0.14	1	C5_CD8_Tem
chr4-49709368-49710893	2.78117E-05	0.260077465	0.066	0.014	1	C5_CD8_Tem

chr15-88623106-88623404	2.79334E-05	0.273074523	0.12	0.044	1	C5_CD8_Tem
chr15-40787129-40787874	2.79858E-05	0.277469831	0.137	0.049	1	C5_CD8_Tem
chr6-30813935-30814547	2.81218E-05	0.271784457	0.087	0.025	1	C5_CD8_Tem
chr5-154829296-154830233	7.6524E-22	0.424963181	0.15	0.032	5.06765E-17	C1_CD8_Tres
chr6-106545977-106547011	2.8749E-05	0.276224873	0.12	0.04	1	C5_CD8_Tem
chr12-124608564-124609482	2.89134E-05	0.274387838	0.131	0.046	1	C5_CD8_Tem
chr13-113875597-113877300	3.55021E-24	0.441795829	0.266	0.093	2.35105E-19	C3_CD8_Tem
chr2-169682683-169683766	1.07529E-51	0.463272507	0.315	0.091	7.12087E-47	C4_CD8_Teff
chr15-55282008-55282801	2.92414E-05	0.263019211	0.284	0.153	1	C5_CD8_Tem
chr7-1417277-1417829	2.97799E-05	0.267007564	0.246	0.119	1	C5_CD8_Tem
chr11-96012030-96012570	1.52626E-18	0.42272032	0.26	0.106	1.01074E-13	C1_CD8_Tres
chr3-43477309-43478624	7.72491E-27	0.462285859	0.31	0.117	5.11566E-22	C3_CD8_Tem
chr22-40116450-40117167	1.82024E-10	0.259153197	0.072	0.019	1.20542E-05	C1_CD8_Tres
chr5-134436287-134437225	3.01466E-05	0.266586208	0.273	0.149	1	C5_CD8_Tem
chr9-131656226-131657298	3.03997E-05	0.25839533	0.311	0.166	1	C5_CD8_Tem
chr10-72292208-72292480	3.08037E-05	0.276449926	0.082	0.02	1	C5_CD8_Tem
chr14-61460987-61462229	3.08698E-05	0.273081764	0.197	0.084	1	C5_CD8_Tem
chr10-4243638-4243991	3.12941E-05	0.267315676	0.071	0.018	1	C5_CD8_Tem
chr7-90178650-90179227	3.16704E-05	0.278202063	0.087	0.021	1	C5_CD8_Tem
chr16-89476470-89476922	3.18395E-05	0.262877553	0.055	0.008	1	C5_CD8_Tem
chr14-74495366-74495652	3.20963E-05	0.26733165	0.082	0.023	1	C5_CD8_Tem
chr20-48910236-48910874	3.25006E-05	0.26297905	0.066	0.015	1	C5_CD8_Tem
chr17-1258454-1260011	3.27315E-05	0.272907067	0.219	0.102	1	C5_CD8_Tem
chr5-177494324-177494861	3.41202E-05	0.285431865	0.142	0.05	1	C5_CD8_Tem
chr1-39398334-39398907	3.418E-05	0.271618148	0.126	0.041	1	C5_CD8_Tem
chr20-62159715-62160218	3.45595E-05	0.275864073	0.126	0.048	1	C5_CD8_Tem
chr18-58657828-58658640	2.07597E-10	0.298786991	0.135	0.054	1.37477E-05	C1_CD8_Tres

chr14-106605660-106606192	3.51317E-05	0.264830928	0.093	0.027	1	C5_CD8_Tem
chr3-50573508-50574737	3.53624E-05	0.264594866	0.23	0.116	1	C5_CD8_Tem
chr17-67503146-67503529	3.56157E-05	0.265846295	0.077	0.018	1	C5_CD8_Tem
chr1-160626143-160626423	3.63237E-05	0.272097415	0.219	0.101	1	C5_CD8_Tem
chr7-4791803-4792831	3.65498E-05	0.272054536	0.115	0.041	1	C5_CD8_Tem
chr11-76207223-76207876	3.65824E-05	0.263333651	0.202	0.097	1	C5_CD8_Tem
chr20-47316682-47317752	3.73402E-05	0.284778326	0.104	0.031	1	C5_CD8_Tem
chr2-162055259-162055750	3.75749E-05	0.278028414	0.137	0.053	1	C5_CD8_Tem
chr17-68462750-68463229	3.76566E-05	0.261480754	0.208	0.093	1	C5_CD8_Tem
chr17-81518937-81519781	3.78647E-05	0.25428525	0.273	0.143	1	C5_CD8_Tem
chr16-88470790-88471093	3.79583E-05	0.254258873	0.066	0.015	1	C5_CD8_Tem
chr20-63902379-63903354	1.16271E-39	0.40689162	0.287	0.095	7.6998E-35	C4_CD8_Teff
chr8-29230626-29231599	3.80837E-05	0.267168028	0.175	0.07	1	C5_CD8_Tem
chr1-120354590-120355111	3.81372E-05	0.260346153	0.066	0.014	1	C5_CD8_Tem
chr2-201261506-201261760	3.86851E-05	0.270817627	0.164	0.073	1	C5_CD8_Tem
chr13-45395720-45396421	3.93791E-05	0.28153594	0.153	0.065	1	C5_CD8_Tem
chr19-3721190-3721854	3.97294E-05	0.270055328	0.175	0.079	1	C5_CD8_Tem
chr6-116386543-116387614	4.01905E-05	0.258443326	0.071	0.016	1	C5_CD8_Tem
chr2-191680249-191680467	4.03548E-05	0.274907504	0.175	0.081	1	C5_CD8_Tem
chr14-105989699-105990239	4.0714E-05	0.271828048	0.208	0.092	1	C5_CD8_Tem
chr1-144523576-144524419	2.16804E-37	0.391850457	0.121	0.017	1.43574E-32	C4_CD8_Teff
chr12-91870813-91871458	3.76527E-10	0.278462193	0.105	0.036	2.49348E-05	C1_CD8_Tres
chr11-118914246-118914785	2.79894E-24	0.313504294	0.301	0.131	1.85354E-19	C4_CD8_Teff
chr10-45418849-45419677	4.21277E-05	0.279329508	0.098	0.028	1	C5_CD8_Tem
chr7-50367163-50367968	3.2128E-19	0.285991007	0.248	0.11	2.12762E-14	C4_CD8_Teff
chr22-37275602-37275923	4.31264E-05	0.266503581	0.082	0.02	1	C5_CD8_Tem
chr16-660447-661183	4.32945E-05	0.255911401	0.104	0.035	1	C5_CD8_Tem

chr15-26081659-26082831	4.37023E-05	0.251331708	0.284	0.155	1	C5_CD8_Tem
chr5-378613-378982	4.39828E-05	0.28028086	0.098	0.028	1	C5_CD8_Tem
chr6-158634578-158635224	4.39994E-05	0.264730988	0.071	0.018	1	C5_CD8_Tem
chr6-148760150-148761881	3.57238E-19	0.276134092	0.288	0.136	2.36574E-14	C4_CD8_Teff
chr9-70421870-70422103	2.48871E-17	0.275529727	0.117	0.034	1.6481E-12	C4_CD8_Teff
chr19-41774440-41775003	9.54529E-19	0.374420094	0.087	0.011	6.32118E-14	C1_CD8_Tres
chr11-128488077-128488837	4.59282E-05	0.264269162	0.158	0.061	1	C5_CD8_Tem
chr21-46550233-46551065	4.60483E-05	0.27551893	0.131	0.047	1	C5_CD8_Tem
chr11-14336812-14337472	4.62005E-05	0.275345765	0.153	0.064	1	C5_CD8_Tem
chr15-77005256-77005671	4.63183E-05	0.261033275	0.104	0.035	1	C5_CD8_Tem
chr8-128554733-128555434	4.63444E-05	0.274102199	0.131	0.05	1	C5_CD8_Tem
chr8-143974875-143975161	8.49599E-15	0.253370548	0.169	0.07	5.6263E-10	C4_CD8_Teff
chr7-156903350-156903857	4.66213E-05	0.254304551	0.06	0.013	1	C5_CD8_Tem
chr13-41355897-41357152	5.69211E-15	0.363578892	0.177	0.062	3.76949E-10	C1_CD8_Tres
chr16-21660924-21661602	4.6825E-05	0.273203983	0.148	0.058	1	C5_CD8_Tem
chr13-51793403-51793840	4.69245E-05	0.263696585	0.082	0.025	1	C5_CD8_Tem
chr1-24506618-24507685	1.76849E-21	0.436619077	0.174	0.048	1.17115E-16	C1_CD8_Tres
chr1-3889848-3891382	2.5472E-10	0.270683744	0.154	0.071	1.68683E-05	C3_CD8_Tem
chr10-71742093-71742966	2.02905E-21	0.303788058	0.178	0.068	1.3437E-16	C4_CD8_Teff
chr12-123931466-123932214	4.79944E-05	0.25653192	0.06	0.013	1	C5_CD8_Tem
chr1-234844749-234845682	8.66156E-17	0.355851565	0.156	0.051	5.73594E-12	C1_CD8_Tres
chr4-169621656-169622489	4.8109E-05	0.251100533	0.284	0.156	1	C5_CD8_Tem
chr10-132392019-132392738	4.92177E-05	0.271433019	0.098	0.028	1	C5_CD8_Tem
chr19-8451718-8452189	4.96278E-05	0.264473014	0.071	0.018	1	C5_CD8_Tem
chr3-52317318-52317978	5.00822E-05	0.275290081	0.131	0.052	1	C5_CD8_Tem
chr15-60539991-60540712	5.09684E-05	0.261391424	0.235	0.112	1	C5_CD8_Tem
chr4-122539514-122540452	5.12151E-05	0.266638004	0.186	0.086	1	C5_CD8_Tem

chr8-27333049-27333558	5.15225E-05	0.273688815	0.093	0.026	1	C5_CD8_Tem
chr20-36591907-36592374	5.19876E-05	0.271562518	0.142	0.054	1	C5_CD8_Tem
chr15-101167659-101168648	5.25963E-05	0.274653039	0.186	0.081	1	C5_CD8_Tem
chr6-144197790-144198476	5.30511E-05	0.27363849	0.131	0.047	1	C5_CD8_Tem
chr14-21308205-21309096	1.38008E-12	0.341188853	0.18	0.068	9.1393E-08	C1_CD8_Tres
chr20-8428580-8429320	5.31631E-05	0.263115627	0.077	0.017	1	C5_CD8_Tem
chr9-135939051-135939861	5.33194E-05	0.268891775	0.137	0.046	1	C5_CD8_Tem
chr10-45971741-45972790	8.56577E-52	0.47619277	0.208	0.036	5.67251E-47	C4_CD8_Teff
chr2-70553163-70554435	5.3651E-05	0.252043953	0.235	0.12	1	C5_CD8_Tem
chr6-3355286-3356545	1.29973E-12	0.29788128	0.126	0.046	8.60721E-08	C1_CD8_Tres
chr11-123456276-123457499	5.45408E-05	0.274829455	0.169	0.072	1	C5_CD8_Tem
chr1-23118457-23119533	5.48725E-05	0.256646665	0.246	0.13	1	C5_CD8_Tem
chr19-6652556-6653061	5.54045E-05	0.259841374	0.093	0.026	1	C5_CD8_Tem
chr16-28990309-28990992	1.41619E-13	0.337718553	0.183	0.077	9.37846E-09	C1_CD8_Tres
chr9-127990721-127991610	2.69204E-10	0.270546491	0.06	0.012	1.78275E-05	C1_CD8_Tres
chr7-99375996-99376482	1.73174E-22	0.31516275	0.16	0.05	1.14681E-17	C4_CD8_Teff
chr10-8365800-8366409	5.63888E-05	0.274335827	0.131	0.052	1	C5_CD8_Tem
chr6-149895809-149896871	5.66264E-05	0.263988275	0.077	0.02	1	C5_CD8_Tem
chr2-28406519-28407079	5.66639E-05	0.253071388	0.295	0.159	1	C5_CD8_Tem
chr22-38696516-38696880	5.68901E-05	0.266684402	0.191	0.084	1	C5_CD8_Tem
chr15-28885379-28886544	5.80631E-05	0.268292159	0.153	0.064	1	C5_CD8_Tem
chr12-67697399-67697745	5.82071E-05	0.265893473	0.071	0.016	1	C5_CD8_Tem
chr16-1002720-1003121	5.84227E-05	0.271509985	0.175	0.072	1	C5_CD8_Tem
chr19-55353370-55354516	5.95722E-05	0.2500098	0.23	0.123	1	C5_CD8_Tem
chr6-142850610-142850991	6.12625E-05	0.264600359	0.169	0.078	1	C5_CD8_Tem
chr5-142223267-142224134	5.57883E-24	0.444587947	0.254	0.096	3.69447E-19	C1_CD8_Tres
chr5-119277723-119278563	5.65385E-24	0.32596876	0.213	0.075	3.74415E-19	C4_CD8_Teff

chr7-156908716-156909284	6.20333E-05	0.265569821	0.137	0.052	1	C5_CD8_Tem
chr18-62585565-62586431	6.21351E-05	0.277558224	0.142	0.053	1	C5_CD8_Tem
chr3-71496056-71496530	6.25875E-05	0.268534735	0.158	0.067	1	C5_CD8_Tem
chr6-37535730-37536631	6.30006E-05	0.256495431	0.071	0.017	1	C5_CD8_Tem
chr11-108592771-108594036	6.38965E-05	0.258502128	0.23	0.117	1	C5_CD8_Tem
chr17-65557303-65557533	6.44522E-05	0.258706907	0.071	0.018	1	C5_CD8_Tem
chr2-233350704-233351049	6.52929E-05	0.261989853	0.12	0.041	1	C5_CD8_Tem
chr19-7929244-7929703	5.4515E-23	0.326368413	0.152	0.044	3.61014E-18	C4_CD8_Teff
chr3-46112278-46112563	6.55901E-05	0.269895453	0.109	0.031	1	C5_CD8_Tem
chr8-144402609-144402951	6.5992E-05	0.260506908	0.071	0.017	1	C5_CD8_Tem
chr4-26787791-26788583	6.60753E-05	0.254561895	0.066	0.015	1	C5_CD8_Tem
chr22-36374698-36374934	6.61287E-05	0.267959804	0.164	0.069	1	C5_CD8_Tem
chr4-1581157-1581420	6.39489E-17	0.270102274	0.19	0.079	4.23489E-12	C4_CD8_Teff
chr20-57350857-57351785	6.83937E-05	0.250724945	0.23	0.129	1	C5_CD8_Tem
chr8-28371357-28371763	6.89607E-05	0.260558427	0.077	0.019	1	C5_CD8_Tem
chr19-45442783-45443921	6.90767E-05	0.260518627	0.175	0.085	1	C5_CD8_Tem
chr10-3886692-3887255	6.95091E-05	0.257687809	0.082	0.023	1	C5_CD8_Tem
chr16-5117210-5117771	5.81426E-18	0.273852507	0.094	0.022	3.85038E-13	C4_CD8_Teff
chr3-18438279-18438968	1.32631E-08	0.268599461	0.072	0.02	0.000878321	C1_CD8_Tres
chr8-29289137-29289880	7.15908E-05	0.258033658	0.12	0.044	1	C5_CD8_Tem
chr6-34244522-34244801	7.28855E-05	0.268245904	0.137	0.048	1	C5_CD8_Tem
chr6-108565117-108565494	7.35495E-05	0.262609407	0.142	0.059	1	C5_CD8_Tem
chr7-121442756-121443205	7.45245E-05	0.272496549	0.131	0.054	1	C5_CD8_Tem
chr6-26321822-26323062	1.39293E-26	0.340527685	0.252	0.098	9.2244E-22	C4_CD8_Teff
chr12-122108513-122109047	7.68778E-05	0.262862737	0.082	0.022	1	C5_CD8_Tem
chr1-158969126-158970202	1.33757E-29	0.361754219	0.172	0.045	8.85781E-25	C4_CD8_Teff
chr18-69916359-69916954	7.81913E-05	0.268924527	0.109	0.039	1	C5_CD8_Tem

chr5-80958042-80958369	7.83133E-05	0.259167792	0.066	0.016	1	C5_CD8_Tem
chr18-12407443-12408557	7.96279E-05	0.25293671	0.235	0.118	1	C5_CD8_Tem
chr5-139453681-139454599	1.40146E-19	0.42282445	0.141	0.03	9.28091E-15	C3_CD8_Tem
chr2-105749545-105750333	3.66352E-67	0.538395752	0.277	0.052	2.42609E-62	C4_CD8_Teff
chr1-45393590-45394814	1.63552E-20	0.422927791	0.138	0.032	1.08309E-15	C3_CD8_Tem
chr7-23272869-23273592	8.2209E-05	0.256848369	0.208	0.094	1	C5_CD8_Tem
chr1-212260783-212261338	8.27397E-05	0.256331328	0.115	0.038	1	C5_CD8_Tem
chr13-111182833-111183595	3.06564E-39	0.424473088	0.155	0.027	2.03016E-34	C4_CD8_Teff
chr6-87731457-87732623	8.56389E-05	0.256717296	0.126	0.051	1	C5_CD8_Tem
chr7-129553822-129554510	1.02864E-10	0.280557047	0.12	0.042	6.81195E-06	C3_CD8_Tem
chr6-29015754-29017369	8.68137E-05	0.2610203	0.208	0.108	1	C5_CD8_Tem
chr9-92953944-92954541	8.68268E-05	0.251751576	0.208	0.106	1	C5_CD8_Tem
chr1-101259667-101260103	2.88174E-12	0.323563838	0.132	0.045	1.90838E-07	C1_CD8_Tres
chr17-4852260-4853626	8.8328E-05	0.262304509	0.104	0.036	1	C5_CD8_Tem
chrX-20252189-20253296	8.845E-05	0.252385839	0.24	0.123	1	C5_CD8_Tem
chr10-3867188-3868292	3.26219E-21	0.443055402	0.198	0.066	2.16032E-16	C1_CD8_Tres
chr10-50503586-50504280	2.28011E-19	0.289141852	0.101	0.025	1.50995E-14	C4_CD8_Teff
chr19-38263762-38264979	1.5212E-19	0.403452565	0.168	0.051	1.00739E-14	C1_CD8_Tres
chr16-67052469-67054173	8.95472E-05	0.252329749	0.219	0.108	1	C5_CD8_Tem
chr12-66287435-66288552	1.23326E-14	0.332881079	0.213	0.096	8.167E-10	C1_CD8_Tres
chr1-8899518-8900285	9.19192E-05	0.253850516	0.148	0.059	1	C5_CD8_Tem
chr4-139528391-139528994	9.35337E-05	0.27564863	0.131	0.046	1	C5_CD8_Tem
chr17-83048299-83048927	9.39011E-05	0.253616431	0.213	0.108	1	C5_CD8_Tem
chr19-7345315-7345860	1.76772E-09	0.27477187	0.081	0.025	0.000117064	C1_CD8_Tres
chr4-48016190-48017397	9.73372E-05	0.26664422	0.142	0.058	1	C5_CD8_Tem
chr10-132684279-132684760	9.84687E-05	0.261207967	0.093	0.026	1	C5_CD8_Tem
chr12-7639566-7640916	9.97906E-05	0.25699361	0.24	0.126	1	C5_CD8_Tem

chr12-64679444-64680046	2.25341E-23	0.319187227	0.234	0.087	1.49228E-18	C4_CD8_Teff
chr16-963615-965065	0.000102415	0.257304803	0.175	0.071	1	C5_CD8_Tem
chr3-189120952-189121619	3.95976E-20	0.301590347	0.132	0.039	2.62227E-15	C4_CD8_Teff
chr8-144019212-144020607	7.13216E-43	0.439404297	0.156	0.024	4.72313E-38	C4_CD8_Teff
chr1-24149753-24150805	0.000105824	0.252539096	0.191	0.093	1	C5_CD8_Tem
chr10-97422855-97423133	0.000106537	0.262412176	0.131	0.047	1	C5_CD8_Tem
chr20-47544264-47544561	0.000107089	0.263979361	0.093	0.024	1	C5_CD8_Tem
chr5-68343379-68343949	8.17781E-18	0.276896388	0.15	0.051	5.41559E-13	C4_CD8_Teff
chr12-101781478-101782561	0.000108494	0.272656187	0.175	0.075	1	C5_CD8_Tem
chr18-13578481-13579195	0.000108621	0.261267671	0.126	0.05	1	C5_CD8_Tem
chr1-24481076-24482289	0.000110318	0.255357764	0.175	0.083	1	C5_CD8_Tem
chr5-1111011-1112213	0.000110382	0.261044261	0.137	0.054	1	C5_CD8_Tem
chr5-80253614-80254145	0.000112143	0.263809761	0.169	0.074	1	C5_CD8_Tem
chr19-2610872-2611460	0.000114492	0.251327518	0.153	0.075	1	C5_CD8_Tem
chr19-10754900-10755528	9.71061E-16	0.390673295	0.204	0.081	6.43066E-11	C1_CD8_Tres
chr15-42056918-42057805	0.000117392	0.259239556	0.153	0.062	1	C5_CD8_Tem
chr5-91281465-91281787	0.000118966	0.253134321	0.082	0.024	1	C5_CD8_Tem
chr15-39774449-39775028	0.000120731	0.259374145	0.082	0.026	1	C5_CD8_Tem
chr13-40978146-40978401	0.000123037	0.251563282	0.093	0.031	1	C5_CD8_Tem
chr1-16676584-16677443	0.000124185	0.250411639	0.093	0.03	1	C5_CD8_Tem
chr1-161730056-161730515	0.000126982	0.255887113	0.153	0.061	1	C5_CD8_Tem
chr22-37696574-37697098	0.000127565	0.274319371	0.131	0.051	1	C5_CD8_Tem
chr21-46052450-46053732	0.000127615	0.265269375	0.142	0.057	1	C5_CD8_Tem
chr20-33372390-33372681	0.000128356	0.264032158	0.098	0.035	1	C5_CD8_Tem
chr13-46125554-46126897	0.000129778	0.259402053	0.126	0.053	1	C5_CD8_Tem
chr2-159225953-159226543	0.000130989	0.251972999	0.219	0.104	1	C5_CD8_Tem
chr2-224885889-224886465	0.000131319	0.26078943	0.164	0.068	1	C5_CD8_Tem

chr17-76565170-76565564	0.000132761	0.257103708	0.148	0.064	1	C5_CD8_Tem
chr2-160665844-160666780	0.000133293	0.257973757	0.109	0.042	1	C5_CD8_Tem
chr15-29050728-29051366	0.000136477	0.260698632	0.23	0.109	1	C5_CD8_Tem
chr1-148388151-148389117	3.39071E-10	0.29384888	0.165	0.073	2.24543E-05	C1_CD8_Tres
chr21-14550247-14550530	0.000137759	0.250030759	0.077	0.024	1	C5_CD8_Tem
chr19-13172174-13172899	0.000140318	0.25099944	0.169	0.082	1	C5_CD8_Tem
chr16-68270063-68270730	0.000140484	0.257124959	0.169	0.078	1	C5_CD8_Tem
chr4-83219344-83219940	0.000142384	0.250306636	0.071	0.019	1	C5_CD8_Tem
chr4-184792913-184793389	6.22558E-11	0.308943971	0.099	0.031	4.12276E-06	C1_CD8_Tres
chr9-114364129-114364520	0.000150639	0.250732533	0.18	0.078	1	C5_CD8_Tem
chr4-84582300-84583857	2.45473E-22	0.486981832	0.228	0.066	1.62559E-17	C1_CD8_Tres
chr10-119270835-119271760	0.000154136	0.255134399	0.191	0.093	1	C5_CD8_Tem
chr9-179019-179639	0.000158398	0.252508974	0.142	0.061	1	C5_CD8_Tem
chr4-56505124-56505906	0.000161954	0.254204187	0.126	0.053	1	C5_CD8_Tem
chr8-143966180-143966904	0.000177433	0.254961042	0.12	0.042	1	C5_CD8_Tem
chr14-60721309-60722140	0.000178772	0.252740341	0.109	0.046	1	C5_CD8_Tem
chr8-18957182-18957952	1.53075E-24	0.571110744	0.216	0.049	1.01371E-19	C1_CD8_Tres
chr1-166910240-166910820	0.000184511	0.252775816	0.202	0.095	1	C5_CD8_Tem
chr8-46917486-46918178	0.000199521	0.253773943	0.126	0.045	1	C5_CD8_Tem
chr5-82356754-82357721	1.04741E-60	0.811227518	0.365	0.068	6.93626E-56	C1_CD8_Tres
chr14-24631806-24632062	0.000207703	0.260224446	0.087	0.031	1	C5_CD8_Tem
chrX-38811629-38812393	0.000226407	0.250572615	0.12	0.047	1	C5_CD8_Tem
chr12-122081332-122081980	0.000241095	0.250291455	0.131	0.056	1	C5_CD8_Tem
chr6-30963694-30964716	8.61885E-12	0.330556879	0.128	0.039	5.70766E-07	C3_CD8_Tem
chr8-94241589-94242663	3.3573E-300	0.828327436	0.479	0.014	2.2233E-295	Tumor Tex
chr11-110077505-110078170	8.6175E-243	0.772610013	0.384	0.005	5.7068E-238	Tumor Tex
chr3-40971188-40972064	4.3091E-237	0.764681866	0.394	0.01	2.8536E-232	Tumor Tex

chr19-17776274-17776867	1.4888E-229	0.750479405	0.393	0.014	9.8591E-225	Tumor Tex
chr15-44882195-44883034	3.7359E-213	0.735481283	0.386	0.018	2.474E-208	Tumor Tex
chr1-148951598-148952687	7.2176E-212	0.687625563	0.482	0.085	4.7797E-207	Tumor Tex
chr11-111531693-111532434	1.9554E-211	0.735787603	0.373	0.014	1.2949E-206	Tumor Tex
chr15-50137275-50137951	1.4433E-204	0.705216519	0.457	0.061	9.5579E-200	Tumor Tex
chr15-64029278-64030445	2.7714E-203	0.720862181	0.409	0.033	1.8353E-198	Tumor Tex
chr3-168094718-168096447	4.3098E-201	0.688039876	0.471	0.066	2.8541E-196	Tumor Tex
chr20-2865582-2866467	1.5055E-196	0.713404347	0.385	0.028	9.9701E-192	Tumor Tex
chr1-15965254-15966633	2.4185E-196	0.68307187	0.463	0.075	1.6016E-191	Tumor Tex
chr11-111539312-111539744	1.1263E-195	0.713061997	0.332	0.007	7.4588E-191	Tumor Tex
chr12-12582265-12583159	6.9078E-195	0.713731787	0.336	0.01	4.5745E-190	Tumor Tex
chr5-157350883-157351639	2.8229E-191	0.711829323	0.357	0.02	1.8694E-186	Tumor Tex
chr6-45923690-45924372	1.2506E-190	0.70218113	0.382	0.029	8.2819E-186	Tumor Tex
chr7-151745171-151745590	6.5106E-187	0.704092567	0.344	0.016	4.3115E-182	Tumor Tex
chr5-66840435-66841059	4.7032E-185	0.702547903	0.365	0.027	3.1146E-180	Tumor Tex
chr6-105620790-105621255	6.4236E-185	0.697746266	0.364	0.027	4.2539E-180	Tumor Tex
chr14-49992839-49993233	2.7156E-178	0.698624485	0.311	0.008	1.7984E-173	Tumor Tex
chr8-124751393-124752265	2.4095E-175	0.665803671	0.424	0.065	1.5957E-170	Tumor Tex
chr2-190721956-190722795	3.8394E-174	0.678877371	0.332	0.017	2.5426E-169	Tumor Tex
chr2-222813226-222813776	2.0977E-170	0.679045134	0.29	0.005	1.3892E-165	Tumor Tex
chr12-75635603-75636274	5.874E-166	0.670369917	0.37	0.039	3.89E-161	Tumor Tex
chr5-57316837-57317137	3.8583E-165	0.678089403	0.285	0.006	2.5551E-160	Tumor Tex
chr8-132917934-132918561	9.1765E-164	0.668550932	0.32	0.018	6.0769E-159	Tumor Tex
chr14-88044776-88045319	9.8481E-164	0.662920748	0.287	0.006	6.5217E-159	Tumor Tex
chr15-50134784-50135559	1.0202E-163	0.652289246	0.403	0.056	6.7562E-159	Tumor Tex
chr5-66828246-66829328	5.7685E-163	0.603293729	0.507	0.133	3.82E-158	Tumor Tex
chr7-47291306-47291768	8.223E-163	0.664916024	0.271	0.003	5.4455E-158	Tumor Tex

chr15-48940397-48940885	1.3053E-161	0.671659148	0.327	0.024	8.6443E-157	Tumor Tex
chr6-11785437-11786547	2.5342E-161	0.65990574	0.324	0.021	1.6782E-156	Tumor Tex
chr8-132920726-132920979	2.3116E-159	0.665391389	0.279	0.007	1.5308E-154	Tumor Tex
chr14-77302902-77304034	8.1287E-159	0.625273553	0.445	0.085	5.3831E-154	Tumor Tex
chr11-110092518-110093745	8.0633E-157	0.653004157	0.311	0.018	5.3398E-152	Tumor Tex
chr18-54263259-54264234	2.8805E-155	0.648222413	0.366	0.046	1.9076E-150	Tumor Tex
chr7-149202812-149203252	8.8621E-153	0.663571413	0.286	0.014	5.8687E-148	Tumor Tex
chr6-16955947-16957024	2.8418E-151	0.649623416	0.296	0.017	1.8819E-146	Tumor Tex
chr1-230208534-230209331	3.5627E-151	0.596759535	0.476	0.118	2.3594E-146	Tumor Tex
chr17-57463157-57463534	1.4262E-150	0.646245422	0.294	0.015	9.4449E-146	Tumor Tex
chr8-8568934-8569330	1.3846E-149	0.653308876	0.293	0.018	9.1691E-145	Tumor Tex
chr8-11421601-11422174	1.0108E-148	0.643405019	0.314	0.025	6.694E-144	Tumor Tex
chr4-26178982-26179673	3.1953E-146	0.627323535	0.258	0.005	2.116E-141	Tumor Tex
chr8-94234377-94234980	3.4646E-145	0.631865653	0.251	0.004	2.2944E-140	Tumor Tex
chr16-74936891-74937281	1.7564E-143	0.638943108	0.287	0.016	1.1631E-138	Tumor Tex
chr2-7467817-7468707	7.3818E-142	0.601963902	0.427	0.094	4.8885E-137	Tumor Tex
chr3-58260176-58260977	1.0273E-141	0.639972289	0.285	0.018	6.8032E-137	Tumor Tex
chr6-45830080-45831694	6.0467E-141	0.61069309	0.369	0.061	4.0043E-136	Tumor Tex
chr14-65302754-65303253	3.6819E-140	0.630219054	0.27	0.013	2.4383E-135	Tumor Tex
chr2-227817822-227818927	4.1699E-140	0.620029932	0.338	0.041	2.7614E-135	Tumor Tex
chr4-105305965-105306841	1.8551E-139	0.612698471	0.379	0.065	1.2285E-134	Tumor Tex
chr5-137666157-137666487	3.1348E-139	0.632333328	0.257	0.008	2.076E-134	Tumor Tex
chr9-35848188-35848451	1.0809E-137	0.621238554	0.24	0.004	7.1583E-133	Tumor Tex
chr12-24838485-24839770	1.0521E-135	0.561055666	0.48	0.129	6.9673E-131	Tumor Tex
chr6-21438210-21439182	1.6498E-135	0.621201816	0.271	0.014	1.0925E-130	Tumor Tex
chr6-45801624-45802318	2.2687E-134	0.60816259	0.342	0.049	1.5024E-129	Tumor Tex
chr7-25967831-25968834	2.3735E-134	0.614853386	0.306	0.032	1.5718E-129	Tumor Tex

chr17-68381070-68381460	2.5992E-134	0.618264483	0.292	0.02	1.7213E-129	Tumor Tex
chr21-44133467-44133675	4.1101E-134	0.613548466	0.266	0.013	2.7218E-129	Tumor Tex
chr6-105599530-105600079	5.5481E-134	0.615426556	0.312	0.032	3.6741E-129	Tumor Tex
chr6-45709768-45710798	5.5447E-133	0.596335834	0.376	0.068	3.6719E-128	Tumor Tex
chr1-65811918-65812403	1.0091E-132	0.618538285	0.268	0.016	6.6823E-128	Tumor Tex
chr18-35577316-35577673	7.2351E-132	0.615747452	0.289	0.023	4.7913E-127	Tumor Tex
chr14-75562018-75562740	1.4526E-131	0.591749505	0.383	0.076	9.6195E-127	Tumor Tex
chr1-59477147-59477656	6.4892E-131	0.609873856	0.281	0.024	4.2974E-126	Tumor Tex
chr2-181036558-181037513	5.3858E-130	0.600786482	0.342	0.055	3.5666E-125	Tumor Tex
chr10-30736755-30737032	5.5654E-130	0.601715412	0.225	0.003	3.6856E-125	Tumor Tex
chr6-105560375-105560939	1.3861E-127	0.609321872	0.293	0.027	9.1793E-123	Tumor Tex
chr11-3013570-3014890	7.7795E-127	0.558414213	0.435	0.12	5.1518E-122	Tumor Tex
chr20-25223184-25223558	1.3946E-126	0.611702309	0.269	0.021	9.2354E-122	Tumor Tex
chrX-1661077-1662086	1.9595E-126	0.485361973	0.578	0.25	1.2977E-121	Tumor Tex
chr20-37990033-37990949	5.5559E-126	0.567334276	0.421	0.101	3.6793E-121	Tumor Tex
chr9-92631616-92632022	1.9236E-125	0.603643915	0.238	0.009	1.2739E-120	Tumor Tex
chr17-78154892-78155404	2.5909E-125	0.599946824	0.254	0.013	1.7158E-120	Tumor Tex
chr1-143729194-143729745	3.919E-125	0.600805654	0.297	0.034	2.5953E-120	Tumor Tex
chr20-49534831-49535313	1.972E-124	0.601104987	0.282	0.027	1.3059E-119	Tumor Tex
chr13-40102126-40103235	2.0646E-123	0.597692192	0.296	0.033	1.3672E-118	Tumor Tex
chr9-112028338-112029099	1.7281E-122	0.593862789	0.232	0.007	1.1444E-117	Tumor Tex
chr8-8227439-8229283	4.3572E-122	0.42449662	0.667	0.373	2.8855E-117	Tumor Tex
chr6-105484357-105485180	1.6575E-121	0.577294993	0.358	0.074	1.0976E-116	Tumor Tex
chr7-149439665-149440538	2.6257E-121	0.597696995	0.273	0.023	1.7388E-116	Tumor Tex
chr1-209751634-209752193	6.0428E-121	0.592251951	0.318	0.045	4.0017E-116	Tumor Tex
chr6-161029020-161029776	6.2286E-121	0.592681151	0.243	0.013	4.1247E-116	Tumor Tex
chr8-29926233-29927010	2.4036E-120	0.589894135	0.253	0.017	1.5917E-115	Tumor Tex

chr14-67680011-67680449	3.3438E-120	0.600231903	0.268	0.021	2.2144E-115	Tumor Tex
chr10-95832061-95832722	4.0251E-119	0.535191717	0.448	0.14	2.6655E-114	Tumor Tex
chr3-119562278-119562977	3.6911E-118	0.568039298	0.359	0.076	2.4443E-113	Tumor Tex
chr12-104494195-104495095	1.2779E-117	0.578276804	0.313	0.047	8.4624E-113	Tumor Tex
chr6-52227495-52227786	1.617E-117	0.580493058	0.218	0.006	1.0708E-112	Tumor Tex
chr2-200454767-200455737	1.65E-117	0.581018974	0.274	0.025	1.0927E-112	Tumor Tex
chr17-57422574-57423022	3.2105E-117	0.582336022	0.228	0.01	2.1261E-112	Tumor Tex
chr14-51331651-51332192	4.5133E-117	0.570754701	0.205	0.003	2.9888E-112	Tumor Tex
chr2-199228985-199229480	4.8163E-117	0.582525868	0.218	0.009	3.1895E-112	Tumor Tex
chr14-92556577-92557047	9.9195E-117	0.589250159	0.252	0.018	6.569E-112	Tumor Tex
chr3-110814048-110814875	3.143E-116	0.578848534	0.291	0.04	2.0814E-111	Tumor Tex
chr15-85318007-85318839	6.4428E-116	0.554031662	0.395	0.092	4.2666E-111	Tumor Tex
chr17-77106798-77107919	1.02E-115	0.575245417	0.315	0.05	6.7545E-111	Tumor Tex
chr3-16378444-16379238	2.1855E-115	0.574371647	0.324	0.057	1.4473E-110	Tumor Tex
chr13-75365657-75366252	2.4187E-115	0.570593419	0.335	0.061	1.6017E-110	Tumor Tex
chr8-125607684-125608560	1.2328E-114	0.577434923	0.276	0.031	8.1643E-110	Tumor Tex
chr8-19724702-19725267	2.281E-114	0.569885192	0.208	0.005	1.5105E-109	Tumor Tex
chr2-207165058-207167063	4.4888E-114	0.484497343	0.533	0.207	2.9726E-109	Tumor Tex
chr16-17590831-17592322	5.1274E-114	0.520113077	0.455	0.148	3.3955E-109	Tumor Tex
chr6-11324614-11324985	7.2036E-114	0.583023223	0.229	0.012	4.7704E-109	Tumor Tex
chr5-160459044-160459508	1.0928E-113	0.580328476	0.24	0.015	7.2368E-109	Tumor Tex
chr15-85407582-85408630	2.2446E-113	0.568878448	0.323	0.055	1.4865E-108	Tumor Tex
chr3-58357546-58358333	2.8182E-113	0.572476428	0.216	0.008	1.8663E-108	Tumor Tex
chr20-43750868-43751707	3.02E-113	0.533871883	0.419	0.127	1.9999E-108	Tumor Tex
chr22-35410030-35410595	6.4462E-113	0.577024112	0.246	0.022	4.2689E-108	Tumor Tex
chr8-19717007-19717914	7.3511E-113	0.556102189	0.354	0.073	4.8681E-108	Tumor Tex
chr2-241305108-241305678	1.8192E-112	0.570596178	0.223	0.01	1.2047E-107	Tumor Tex

chr17-68349235-68349445	3.3528E-112	0.579960326	0.257	0.023	2.2203E-107	Tumor Tex
chr19-11567730-11568616	1.1489E-111	0.579485808	0.254	0.025	7.6087E-107	Tumor Tex
chr12-50187161-50187769	1.7404E-111	0.571902457	0.219	0.008	1.1526E-106	Tumor Tex
chr15-90449349-90449802	4.2532E-111	0.560989386	0.207	0.006	2.8166E-106	Tumor Tex
chr18-13290640-13291397	4.9668E-111	0.521164204	0.436	0.127	3.2892E-106	Tumor Tex
chr3-133447911-133449082	6.5712E-111	0.571444746	0.249	0.019	4.3517E-106	Tumor Tex
chr4-26141939-26142244	6.4815E-110	0.550669463	0.201	0.003	4.2923E-105	Tumor Tex
chr8-125110513-125111202	6.8585E-110	0.570103247	0.276	0.033	4.5419E-105	Tumor Tex
chr4-10722943-10723244	4.276E-109	0.552181146	0.193	0.002	2.8317E-104	Tumor Tex
chr15-49286677-49286966	1.4159E-108	0.561277838	0.199	0.004	9.3768E-104	Tumor Tex
chr19-33235260-33236309	2.9738E-108	0.564419311	0.282	0.041	1.9693E-103	Tumor Tex
chr6-169530383-169530975	5.7623E-108	0.55818514	0.206	0.006	3.8159E-103	Tumor Tex
chr8-72000196-72000797	1.8385E-107	0.531969354	0.392	0.11	1.2175E-102	Tumor Tex
chr13-100393974-100394661	2.1924E-107	0.555928914	0.217	0.009	1.4519E-102	Tumor Tex
chr10-70656517-70656832	2.457E-107	0.555815436	0.208	0.006	1.6271E-102	Tumor Tex
chr8-118099324-118100030	3.7496E-107	0.559683981	0.213	0.009	2.4831E-102	Tumor Tex
chr2-203870881-203871836	5.0397E-107	0.522246827	0.402	0.119	3.3374E-102	Tumor Tex
chr1-229440967-229441374	3.5389E-106	0.565745863	0.221	0.013	2.3436E-101	Tumor Tex
chr12-30795178-30796649	4.8614E-105	0.546815424	0.317	0.065	3.2193E-100	Tumor Tex
chr2-203830101-203830716	8.525E-105	0.555200043	0.209	0.009	5.6455E-100	Tumor Tex
chr4-184513816-184514652	2.0306E-104	0.551097473	0.29	0.049	1.3447E-99	Tumor Tex
chr19-4840536-4841612	2.3733E-104	0.543531784	0.327	0.065	1.5717E-99	Tumor Tex
chr7-42869112-42869720	2.9136E-104	0.561288847	0.222	0.014	1.9295E-99	Tumor Tex
chr2-8608295-8608995	3.1284E-104	0.554257097	0.295	0.047	2.0717E-99	Tumor Tex
chr11-13286961-13287334	4.3641E-104	0.563980839	0.248	0.025	2.89E-99	Tumor Tex
chr7-149209160-149210278	7.0709E-104	0.514822045	0.411	0.138	4.6825E-99	Tumor Tex
chr8-115185164-115185850	7.7926E-104	0.553277669	0.217	0.012	5.1605E-99	Tumor Tex

chr11-111538786-111539041	8.8806E-104	0.53912104	0.183	0.002	5.881E-99	Tumor Tex
chr2-39492073-39492358	1.4756E-103	0.554567981	0.195	0.007	9.7717E-99	Tumor Tex
chr3-102092537-102093421	2.0122E-103	0.553932668	0.257	0.028	1.33255E-98	Tumor Tex
chr15-59268094-59268728	2.37E-103	0.553860382	0.204	0.008	1.56946E-98	Tumor Tex
chr11-111540518-111540955	2.4781E-103	0.543212798	0.194	0.006	1.6411E-98	Tumor Tex
chr9-135926728-135927223	2.531E-103	0.561003924	0.248	0.024	1.67612E-98	Tumor Tex
chr3-195129411-195129892	7.7702E-103	0.551347576	0.208	0.008	5.14567E-98	Tumor Tex
chr1-172857396-172857696	1.228E-102	0.547899097	0.199	0.007	8.13202E-98	Tumor Tex
chr6-14706163-14706786	1.7255E-101	0.552764611	0.236	0.022	1.14265E-96	Tumor Tex
chr1-175514328-175515104	2.1269E-101	0.554515503	0.255	0.03	1.40852E-96	Tumor Tex
chr2-7450068-7451142	2.6928E-101	0.543586993	0.315	0.065	1.78323E-96	Tumor Tex
chr2-148813506-148814147	1.6138E-100	0.526331132	0.356	0.092	1.06868E-95	Tumor Tex
chr8-132980959-132981312	2.5233E-100	0.555233189	0.237	0.022	1.671E-95	Tumor Tex
chr20-49958450-49958927	3.0818E-100	0.524690207	0.177	0.002	2.04085E-95	Tumor Tex
chr1-148844154-148844407	3.3564E-100	0.552050889	0.241	0.026	2.22271E-95	Tumor Tex
chr1-61332279-61332568	5.2846E-100	0.52561493	0.172	0.001	3.4996E-95	Tumor Tex
chr11-129960194-129960575	1.0068E-99	0.534936925	0.183	0.004	6.66753E-95	Tumor Tex
chr7-117124589-117125031	1.6598E-99	0.55418418	0.25	0.03	1.09917E-94	Tumor Tex
chr10-110614034-110614302	2.5758E-99	0.546419594	0.212	0.013	1.70576E-94	Tumor Tex
chr17-57440085-57441355	2.6052E-99	0.501076747	0.414	0.134	1.72522E-94	Tumor Tex
chr11-111378628-111379509	3.2916E-99	0.544550237	0.239	0.024	2.17979E-94	Tumor Tex
chr11-115194101-115194928	1.10422E-98	0.545614087	0.219	0.018	7.31247E-94	Tumor Tex
chr16-12646448-12647233	1.49994E-98	0.531351244	0.186	0.005	9.93302E-94	Tumor Tex
chr15-99324055-99324494	1.5143E-98	0.537374494	0.197	0.009	1.00281E-93	Tumor Tex
chr11-113587293-113587733	1.76106E-98	0.525974548	0.164	0.001	1.16622E-93	Tumor Tex
chr5-157343804-157344803	8.92962E-98	0.479183366	0.454	0.163	5.91347E-93	Tumor Tex
chr3-4776895-4777149	1.10026E-97	0.529969215	0.172	0.002	7.28628E-93	Tumor Tex

chr14-71475936-71476146	1.26771E-97	0.536456586	0.178	0.004	8.39513E-93	Tumor Tex
chr7-102712956-102713667	1.35647E-97	0.545055196	0.233	0.022	8.98293E-93	Tumor Tex
chr17-45329456-45329975	1.43369E-97	0.537766402	0.236	0.026	9.49434E-93	Tumor Tex
chr9-89700866-89701738	2.08363E-97	0.536156229	0.229	0.021	1.37985E-92	Tumor Tex
chr7-2757678-2758565	2.50739E-97	0.465203809	0.48	0.194	1.66047E-92	Tumor Tex
chr6-169589240-169590210	3.21904E-97	0.53409663	0.289	0.05	2.13175E-92	Tumor Tex
chr5-160467400-160468467	3.62888E-97	0.487466239	0.429	0.149	2.40315E-92	Tumor Tex
chr3-168024446-168024880	3.83181E-97	0.539135784	0.189	0.008	2.53754E-92	Tumor Tex
chr6-37033168-37033613	5.5812E-97	0.545085468	0.255	0.033	3.69604E-92	Tumor Tex
chr5-157389806-157391016	7.62845E-97	0.428625378	0.561	0.26	5.05179E-92	Tumor Tex
chr7-76008313-76009488	1.38198E-96	0.518327319	0.343	0.087	9.15189E-92	Tumor Tex
chr10-6267172-6267557	1.80924E-96	0.540710546	0.212	0.015	1.19813E-91	Tumor Tex
chr2-38653009-38654335	2.06469E-96	0.472665212	0.456	0.18	1.3673E-91	Tumor Tex
chr8-133152638-133153228	2.66889E-96	0.531262871	0.312	0.066	1.76742E-91	Tumor Tex
chr4-10671147-10671744	1.68376E-95	0.540177741	0.275	0.041	1.11504E-90	Tumor Tex
chr1-91526560-91526900	2.16956E-95	0.528781035	0.18	0.005	1.43675E-90	Tumor Tex
chr6-140809379-140810129	4.94721E-95	0.53888183	0.207	0.013	3.27619E-90	Tumor Tex
chr13-40145048-40145508	7.0744E-95	0.536532971	0.219	0.019	4.68488E-90	Tumor Tex
chr6-5302224-5303567	7.56156E-95	0.526263048	0.307	0.067	5.00749E-90	Tumor Tex
chr12-93846970-93847700	8.93952E-95	0.530236849	0.209	0.013	5.92002E-90	Tumor Tex
chr10-264729-265463	9.33442E-95	0.539803537	0.259	0.04	6.18154E-90	Tumor Tex
chr10-117653086-117653485	9.64628E-95	0.53897264	0.223	0.019	6.38805E-90	Tumor Tex
chr2-12554705-12555502	1.48874E-94	0.534555368	0.259	0.038	9.85886E-90	Tumor Tex
chr4-5758615-5759164	1.51215E-94	0.527066027	0.191	0.006	1.00139E-89	Tumor Tex
chr2-85693753-85694481	2.96006E-94	0.530982499	0.285	0.051	1.96024E-89	Tumor Tex
chr6-53231452-53232021	4.00926E-94	0.534139421	0.222	0.021	2.65505E-89	Tumor Tex
chr5-67030302-67030575	5.84309E-94	0.515534745	0.161	0.001	3.86947E-89	Tumor Tex

chr16-15509172-15509969	1.05855E-93	0.537161364	0.27	0.042	7.01002E-89	Tumor Tex
chr7-55533125-55534783	1.63729E-93	0.467235431	0.446	0.173	1.08426E-88	Tumor Tex
chr20-1357785-1358576	4.23439E-93	0.533778545	0.253	0.036	2.80414E-88	Tumor Tex
chr4-26175233-26175743	5.38413E-93	0.513595191	0.179	0.005	3.56554E-88	Tumor Tex
chr12-52308147-52308778	5.46087E-93	0.53266447	0.271	0.05	3.61635E-88	Tumor Tex
chr10-119704003-119704793	6.30142E-93	0.530715077	0.207	0.014	4.17299E-88	Tumor Tex
chr9-88303778-88304430	6.34622E-93	0.534366623	0.224	0.021	4.20266E-88	Tumor Tex
chr10-119705117-119705802	6.75875E-93	0.533755286	0.221	0.02	4.47584E-88	Tumor Tex
chr6-157822693-157824394	1.62221E-92	0.464784173	0.449	0.183	1.07428E-87	Tumor Tex
chr6-45486542-45487098	1.9422E-92	0.5306624	0.217	0.02	1.28618E-87	Tumor Tex
chr18-13566916-13568052	2.59855E-92	0.516651428	0.311	0.073	1.72084E-87	Tumor Tex
chr11-111526467-111526868	3.00108E-92	0.505210347	0.166	0.002	1.9874E-87	Tumor Tex
chr12-103505200-103505705	4.46914E-92	0.532058105	0.256	0.043	2.9596E-87	Tumor Tex
chr3-149657408-149658809	5.19371E-92	0.530838715	0.245	0.033	3.43943E-87	Tumor Tex
chr6-143441405-143442063	5.27032E-92	0.536235471	0.254	0.035	3.49016E-87	Tumor Tex
chr15-85312362-85312747	1.37881E-91	0.527949992	0.209	0.015	9.13088E-87	Tumor Tex
chr6-155185969-155186428	2.20086E-91	0.530460819	0.23	0.029	1.45748E-86	Tumor Tex
chr11-69055762-69056426	2.30833E-91	0.529181216	0.256	0.039	1.52864E-86	Tumor Tex
chr2-175154547-175154896	2.34187E-91	0.530229586	0.221	0.021	1.55086E-86	Tumor Tex
chr2-199977971-199978303	3.74989E-91	0.502911111	0.165	0.002	2.48329E-86	Tumor Tex
chr15-60787735-60788742	4.23611E-91	0.514182426	0.309	0.064	2.80528E-86	Tumor Tex
chr16-68319275-68319585	5.22697E-91	0.518804362	0.179	0.006	3.46146E-86	Tumor Tex
chr2-190718476-190719040	6.25633E-91	0.52324578	0.216	0.019	4.14313E-86	Tumor Tex
chr8-94261747-94262531	1.22485E-90	0.525454844	0.232	0.027	8.11134E-86	Tumor Tex
chr17-50903589-50904600	1.4428E-90	0.498468691	0.356	0.111	9.55465E-86	Tumor Tex
chr2-111624470-111625023	6.1269E-90	0.509885284	0.174	0.006	4.05741E-85	Tumor Tex
chr4-105060586-105061634	8.56257E-90	0.514943581	0.181	0.008	5.67039E-85	Tumor Tex

chr17-76997855-76998634	9.76036E-90	0.501737673	0.344	0.088	6.4636E-85	Tumor Tex
chr10-62811253-62811830	9.92207E-90	0.518038634	0.285	0.06	6.57069E-85	Tumor Tex
chr14-71141928-71143729	1.29098E-89	0.475328131	0.411	0.144	8.54926E-85	Tumor Tex
chr5-180909089-180909304	1.51468E-89	0.503845112	0.16	0.002	1.00307E-84	Tumor Tex
chr1-230123718-230124051	2.72744E-89	0.521491646	0.204	0.017	1.80619E-84	Tumor Tex
chr20-53077566-53078140	4.5141E-89	0.503988611	0.172	0.004	2.98937E-84	Tumor Tex
chr14-88029364-88030417	6.5063E-89	0.499225217	0.344	0.088	4.30866E-84	Tumor Tex
chr4-10954591-10955200	7.46398E-89	0.518811686	0.215	0.018	4.94287E-84	Tumor Tex
chr8-11860631-11861557	7.53612E-89	0.514555118	0.187	0.011	4.99065E-84	Tumor Tex
chr3-195203673-195204071	2.80624E-88	0.52365801	0.221	0.024	1.85838E-83	Tumor Tex
chr13-42405400-42406703	3.35236E-88	0.483935517	0.376	0.116	2.22003E-83	Tumor Tex
chrX-121308094-121308781	1.0423E-87	0.518100783	0.193	0.013	6.90243E-83	Tumor Tex
chr2-12556641-12557091	1.1252E-87	0.519082216	0.201	0.015	7.45143E-83	Tumor Tex
chr1-172845273-172845751	1.15004E-87	0.519830368	0.204	0.016	7.61594E-83	Tumor Tex
chr4-26130895-26132202	1.22818E-87	0.511518176	0.278	0.053	8.13335E-83	Tumor Tex
chr10-91944533-91945369	1.48589E-87	0.5069677	0.312	0.078	9.84E-83	Tumor Tex
chr13-49454382-49455265	2.79183E-87	0.51701437	0.274	0.052	1.84884E-82	Tumor Tex
chr6-135537548-135538735	3.07029E-87	0.439958095	0.477	0.194	2.03324E-82	Tumor Tex
chr3-72977065-72977731	4.80392E-87	0.518306356	0.217	0.024	3.1813E-82	Tumor Tex
chr3-12193461-12193917	5.51848E-87	0.50254819	0.173	0.006	3.6545E-82	Tumor Tex
chr15-100201415-100202261	9.58622E-87	0.516760038	0.219	0.025	6.34828E-82	Tumor Tex
chr15-75042673-75043371	1.3135E-86	0.512149309	0.278	0.059	8.69839E-82	Tumor Tex
chr11-3006477-3007219	2.36713E-86	0.50828937	0.183	0.011	1.56758E-81	Tumor Tex
chr7-13982849-13983216	2.38971E-86	0.510681646	0.192	0.013	1.58254E-81	Tumor Tex
chr8-125636947-125637820	3.61407E-86	0.512360373	0.231	0.031	2.39335E-81	Tumor Tex
chr10-110773712-110774861	3.9832E-86	0.419381612	0.518	0.248	2.63779E-81	Tumor Tex
chr2-47520774-47521779	6.40596E-86	0.480537383	0.371	0.12	4.24222E-81	Tumor Tex

chr21-43106851-43107642	6.8213E-86	0.510732092	0.232	0.03	4.51727E-81	Tumor Tex
chr3-12394872-12395933	7.25821E-86	0.506974914	0.181	0.01	4.8066E-81	Tumor Tex
chr1-243588091-243588424	9.9788E-86	0.488498286	0.15	0.002	6.60826E-81	Tumor Tex
chr1-58922254-58922886	1.20087E-85	0.510003687	0.277	0.055	7.95253E-81	Tumor Tex
chr17-59361301-59362740	1.60843E-85	0.481043617	0.373	0.113	1.06515E-80	Tumor Tex
chr14-31377974-31378365	2.15257E-85	0.513225692	0.204	0.018	1.42549E-80	Tumor Tex
chr1-93758660-93759371	4.41266E-85	0.510599161	0.202	0.02	2.92219E-80	Tumor Tex
chr1-61318800-61319149	1.0237E-84	0.506021595	0.186	0.013	6.77927E-80	Tumor Tex
chr6-12233518-12234307	1.15867E-84	0.50954667	0.233	0.034	7.67308E-80	Tumor Tex
chr5-53069561-53070033	1.28011E-84	0.515489953	0.243	0.039	8.47725E-80	Tumor Tex
chr6-30227452-30228309	1.28848E-84	0.497821488	0.314	0.082	8.53273E-80	Tumor Tex
chr9-127437097-127438230	1.37811E-84	0.483227282	0.345	0.099	9.12626E-80	Tumor Tex
chr22-43003950-43004637	1.9639E-84	0.51004717	0.232	0.033	1.30055E-79	Tumor Tex
chr3-196111829-196112387	2.19131E-84	0.496925472	0.166	0.005	1.45115E-79	Tumor Tex
chr10-73366386-73367075	2.66153E-84	0.493141039	0.162	0.006	1.76255E-79	Tumor Tex
chr11-77151854-77152477	2.72336E-84	0.505420204	0.277	0.057	1.80349E-79	Tumor Tex
chr7-151795018-151795782	3.66211E-84	0.50440094	0.285	0.058	2.42516E-79	Tumor Tex
chr1-184930291-184931068	5.90609E-84	0.499056265	0.303	0.072	3.91119E-79	Tumor Tex
chr18-54149045-54149336	1.13378E-83	0.503756133	0.182	0.01	7.50825E-79	Tumor Tex
chr1-173195242-173196174	1.60078E-83	0.495229562	0.305	0.073	1.06008E-78	Tumor Tex
chr6-21180108-21180507	2.06184E-83	0.483006878	0.148	0.002	1.36541E-78	Tumor Tex
chr18-9094490-9095605	2.3575E-83	0.465359045	0.393	0.136	1.5612E-78	Tumor Tex
chr6-21587217-21589522	2.48639E-83	0.488079927	0.314	0.087	1.64656E-78	Tumor Tex
chr3-4805634-4805844	3.22567E-83	0.483746248	0.153	0.002	2.13613E-78	Tumor Tex
chr2-203578923-203579475	3.4572E-83	0.492499959	0.174	0.008	2.28946E-78	Tumor Tex
chr10-86878974-86879286	5.32161E-83	0.50175256	0.174	0.01	3.52413E-78	Tumor Tex
chr8-60903495-60904370	5.47595E-83	0.502909542	0.208	0.023	3.62634E-78	Tumor Tex

chr20-23407822-23408391	6.20873E-83	0.509979523	0.203	0.019	4.11161E-78	Tumor Tex
chr11-126465407-126466100	6.2474E-83	0.492578043	0.171	0.007	4.13722E-78	Tumor Tex
chr18-80202204-80202517	9.29272E-83	0.496554599	0.17	0.008	6.15392E-78	Tumor Tex
chr1-109823025-109823495	1.37192E-82	0.486229162	0.15	0.002	9.08529E-78	Tumor Tex
chr6-45808958-45809286	2.03389E-82	0.499258779	0.176	0.009	1.3469E-77	Tumor Tex
chr8-19686699-19687530	2.31671E-82	0.488288548	0.32	0.083	1.53419E-77	Tumor Tex
chr10-8415943-8417132	2.34872E-82	0.431185736	0.463	0.208	1.55539E-77	Tumor Tex
chr3-126911097-126912196	2.45905E-82	0.471329675	0.371	0.123	1.62845E-77	Tumor Tex
chr21-31558627-31559975	2.66995E-82	0.454437445	0.419	0.145	1.76812E-77	Tumor Tex
chr6-105676120-105676404	2.67555E-82	0.490168737	0.162	0.007	1.77183E-77	Tumor Tex
chr12-47884970-47885465	2.71973E-82	0.500887616	0.189	0.013	1.80109E-77	Tumor Tex
chr13-76397260-76397754	2.97299E-82	0.504530667	0.18	0.012	1.9688E-77	Tumor Tex
chr2-178404369-178404670	3.20329E-82	0.483518473	0.15	0.003	2.12131E-77	Tumor Tex
chr14-65336052-65336479	4.14209E-82	0.50286697	0.181	0.012	2.74301E-77	Tumor Tex
chr13-44301100-44301622	4.89665E-82	0.5013343	0.203	0.018	3.24271E-77	Tumor Tex
chr14-93994905-93995321	5.12876E-82	0.502476801	0.258	0.052	3.39642E-77	Tumor Tex
chr16-57609271-57610284	5.23572E-82	0.431268262	0.466	0.212	3.46725E-77	Tumor Tex
chr7-66859453-66860586	6.92673E-82	0.491293035	0.167	0.007	4.58709E-77	Tumor Tex
chr6-87705135-87705438	7.29297E-82	0.495868613	0.291	0.07	4.82963E-77	Tumor Tex
chr6-166942461-166943155	8.29261E-82	0.506789582	0.198	0.017	5.49162E-77	Tumor Tex
chr10-95793510-95794013	8.3786E-82	0.495716787	0.181	0.013	5.54856E-77	Tumor Tex
chr11-93541977-93543693	8.86688E-82	0.499532796	0.243	0.039	5.87191E-77	Tumor Tex
chr3-8501053-8501967	1.4021E-81	0.474970999	0.351	0.109	9.28515E-77	Tumor Tex
chr12-106113029-106113339	1.8564E-81	0.479379552	0.148	0.003	1.22936E-76	Tumor Tex
chr5-139437205-139437732	1.99315E-81	0.504482826	0.22	0.028	1.31993E-76	Tumor Tex
chr1-200473407-200474191	2.03337E-81	0.501571877	0.243	0.039	1.34656E-76	Tumor Tex
chr1-3656719-3656999	3.52664E-81	0.487998207	0.152	0.004	2.33544E-76	Tumor Tex

chrX-9677166-9677687	3.72463E-81	0.504606422	0.196	0.019	2.46656E-76	Tumor Tex
chr14-65462346-65462791	5.05217E-81	0.498189268	0.187	0.015	3.3457E-76	Tumor Tex
chr1-67285881-67286493	5.90251E-81	0.499555504	0.262	0.051	3.90882E-76	Tumor Tex
chr5-132635970-132636193	6.32795E-81	0.477539554	0.147	0.002	4.19056E-76	Tumor Tex
chr9-133738065-133738627	6.36059E-81	0.49735987	0.192	0.017	4.21218E-76	Tumor Tex
chr14-73463544-73463786	6.483E-81	0.491788142	0.164	0.007	4.29323E-76	Tumor Tex
chr20-54064003-54064558	8.4264E-81	0.49572941	0.177	0.012	5.58021E-76	Tumor Tex
chr9-134648658-134649514	1.73991E-80	0.481870595	0.163	0.006	1.15222E-75	Tumor Tex
chr21-44107283-44107488	1.98498E-80	0.49138934	0.184	0.012	1.31451E-75	Tumor Tex
chr21-31354162-31355261	2.10816E-80	0.454659168	0.404	0.129	1.39609E-75	Tumor Tex
chr4-26058530-26058821	2.34789E-80	0.485367738	0.163	0.006	1.55484E-75	Tumor Tex
chr22-25749385-25750051	3.02125E-80	0.467366918	0.139	0.001	2.00077E-75	Tumor Tex
chr4-77586502-77587521	4.58605E-80	0.389662451	0.543	0.28	3.03702E-75	Tumor Tex
chr9-22147915-22148508	4.97479E-80	0.505491454	0.212	0.029	3.29446E-75	Tumor Tex
chr10-32244387-32245031	9.39507E-80	0.490141516	0.17	0.009	6.2217E-75	Tumor Tex
chr19-7387194-7387916	9.42877E-80	0.497435133	0.24	0.04	6.24401E-75	Tumor Tex
chr11-6319657-6321138	1.18802E-79	0.492363954	0.192	0.017	7.86745E-75	Tumor Tex
chr10-87761758-87762252	1.28768E-79	0.500103247	0.203	0.024	8.52742E-75	Tumor Tex
chr2-28347715-28348462	1.30655E-79	0.492144858	0.278	0.066	8.65238E-75	Tumor Tex
chr11-118448327-118448559	2.26005E-79	0.489010424	0.161	0.007	1.49668E-74	Tumor Tex
chr10-2949769-2950400	2.91129E-79	0.49919219	0.196	0.019	1.92794E-74	Tumor Tex
chr7-7944092-7945298	2.97882E-79	0.463519507	0.355	0.127	1.97266E-74	Tumor Tex
chr13-50244570-50245457	4.76422E-79	0.456103456	0.38	0.139	3.15501E-74	Tumor Tex
chr3-172142309-172142535	4.81416E-79	0.49210702	0.173	0.012	3.18808E-74	Tumor Tex
chr2-7430520-7431585	4.93776E-79	0.491914004	0.25	0.05	3.26993E-74	Tumor Tex
chr2-38806019-38806300	7.20187E-79	0.485481308	0.158	0.006	4.7693E-74	Tumor Tex
chr7-117110345-117111015	1.20581E-78	0.489140849	0.169	0.012	7.98523E-74	Tumor Tex

chr2-147449266-147450025	1.23255E-78	0.498187258	0.228	0.035	8.16231E-74	Tumor Tex
chr2-203891487-203891728	1.40015E-78	0.460861391	0.135	0.001	9.27224E-74	Tumor Tex
chr7-51123440-51123978	1.5515E-78	0.494179455	0.22	0.031	1.02745E-73	Tumor Tex
chr6-135114334-135114792	1.62317E-78	0.495776296	0.227	0.036	1.07491E-73	Tumor Tex
chrX-135213271-135213769	1.94385E-78	0.494683596	0.19	0.017	1.28728E-73	Tumor Tex
chr13-50236523-50237269	2.44001E-78	0.451941482	0.387	0.149	1.61585E-73	Tumor Tex
chr21-35088173-35088698	2.95868E-78	0.488064923	0.175	0.014	1.95933E-73	Tumor Tex
chr6-158207251-158207744	3.95205E-78	0.477238513	0.158	0.006	2.61716E-73	Tumor Tex
chr8-6690142-6690727	4.07399E-78	0.488675939	0.178	0.012	2.69792E-73	Tumor Tex
chr18-69885499-69886408	7.14578E-78	0.491949887	0.265	0.054	4.73215E-73	Tumor Tex
chr6-157884687-157886211	9.76404E-78	0.474077621	0.314	0.086	6.46604E-73	Tumor Tex
chr22-39065959-39066432	2.0562E-77	0.490969727	0.233	0.04	1.36168E-72	Tumor Tex
chr14-73696136-73696796	3.21283E-77	0.495441048	0.222	0.033	2.12763E-72	Tumor Tex
chr3-168061363-168061637	3.55439E-77	0.475172191	0.15	0.004	2.35382E-72	Tumor Tex
chr3-32992439-32993594	3.79775E-77	0.488657844	0.215	0.029	2.51498E-72	Tumor Tex
chr7-158655633-158656519	3.86469E-77	0.472068476	0.323	0.096	2.55932E-72	Tumor Tex
chr10-9294493-9295083	4.5757E-77	0.45492281	0.136	0.001	3.03017E-72	Tumor Tex
chr16-57610990-57611424	5.29746E-77	0.490814708	0.236	0.044	3.50814E-72	Tumor Tex
chr12-75738402-75738828	6.58489E-77	0.493739589	0.195	0.023	4.36071E-72	Tumor Tex
chr2-25561157-25561432	6.85352E-77	0.484704066	0.169	0.01	4.53861E-72	Tumor Tex
chr8-94259082-94259326	7.07983E-77	0.467967214	0.141	0.002	4.68848E-72	Tumor Tex
chr14-72963513-72963801	8.53871E-77	0.463611511	0.138	0.002	5.65459E-72	Tumor Tex
chr7-106424852-106425491	1.05683E-76	0.488759064	0.203	0.024	6.99864E-72	Tumor Tex
chr10-95757949-95758227	1.06467E-76	0.479493811	0.164	0.008	7.05057E-72	Tumor Tex
chr5-157100151-157100687	1.29402E-76	0.487154522	0.173	0.013	8.56941E-72	Tumor Tex
chr14-65286994-65288096	1.33904E-76	0.483088646	0.269	0.059	8.8675E-72	Tumor Tex
chr13-33610375-33611211	1.49816E-76	0.480081923	0.286	0.072	9.92124E-72	Tumor Tex

chr12-28252920-28253695	1.58043E-76	0.483154779	0.272	0.061	1.04661E-71	Tumor Tex
chr2-190670310-190670994	1.85714E-76	0.481731205	0.277	0.06	1.22985E-71	Tumor Tex
chr16-67311490-67312146	1.90126E-76	0.465964312	0.332	0.101	1.25907E-71	Tumor Tex
chr8-29773685-29774166	2.31229E-76	0.480953533	0.163	0.009	1.53127E-71	Tumor Tex
chr4-26088401-26088894	2.42244E-76	0.483327625	0.188	0.015	1.60422E-71	Tumor Tex
chr1-233112925-233113236	4.63946E-76	0.474129535	0.152	0.005	3.07239E-71	Tumor Tex
chr7-92664250-92665026	5.28097E-76	0.491351388	0.21	0.027	3.49721E-71	Tumor Tex
chr7-75842956-75843170	7.80635E-76	0.464052165	0.138	0.002	5.1696E-71	Tumor Tex
chr2-29314625-29315567	8.2857E-76	0.455799883	0.35	0.12	5.48704E-71	Tumor Tex
chr8-29990174-29991436	9.40526E-76	0.359771082	0.592	0.33	6.22845E-71	Tumor Tex
chr18-79962870-79963594	9.874E-76	0.441210181	0.396	0.16	6.53886E-71	Tumor Tex
chr12-75994191-75994621	9.91753E-76	0.480262662	0.16	0.007	6.56769E-71	Tumor Tex
chr15-76988064-76988885	1.17362E-75	0.484598658	0.252	0.052	7.77204E-71	Tumor Tex
chr6-21593392-21593945	1.31769E-75	0.486754162	0.243	0.047	8.72612E-71	Tumor Tex
chr4-98482850-98483548	1.44052E-75	0.485772994	0.204	0.026	9.53954E-71	Tumor Tex
chr3-189563890-189564171	1.47243E-75	0.476316581	0.15	0.006	9.7509E-71	Tumor Tex
chr6-20877405-20877768	2.22416E-75	0.478489079	0.162	0.008	1.47291E-70	Tumor Tex
chr17-55413904-55414432	2.30403E-75	0.480487525	0.162	0.012	1.5258E-70	Tumor Tex
chr20-2983448-2984502	2.94678E-75	0.486138908	0.242	0.045	1.95145E-70	Tumor Tex
chr21-35096959-35097256	4.51675E-75	0.454288555	0.131	0.001	2.99113E-70	Tumor Tex
chr7-16619744-16620380	4.58581E-75	0.485300257	0.189	0.019	3.03686E-70	Tumor Tex
chr5-40223844-40224404	5.33644E-75	0.482671731	0.204	0.029	3.53395E-70	Tumor Tex
chr4-98603736-98604567	5.54794E-75	0.484436301	0.22	0.033	3.67401E-70	Tumor Tex
chr3-126923457-126923724	6.53736E-75	0.453595076	0.13	0.001	4.32924E-70	Tumor Tex
chr11-1728861-1729725	6.6167E-75	0.486116024	0.206	0.03	4.38178E-70	Tumor Tex
chr1-172874854-172875271	6.69677E-75	0.472340647	0.164	0.009	4.4348E-70	Tumor Tex
chr12-56034346-56034875	9.67431E-75	0.482264424	0.189	0.017	6.40662E-70	Tumor Tex

chr16-88591597-88591821	1.36752E-74	0.461153455	0.143	0.003	9.0561E-70	Tumor Tex
chr22-24433931-24435044	1.71319E-74	0.343121262	0.615	0.392	1.13453E-69	Tumor Tex
chr11-85673210-85673769	2.02029E-74	0.483428432	0.185	0.018	1.3379E-69	Tumor Tex
chr15-50066174-50066563	2.27742E-74	0.48532796	0.206	0.029	1.50818E-69	Tumor Tex
chr8-59274821-59275303	2.38217E-74	0.469792412	0.156	0.008	1.57754E-69	Tumor Tex
chr22-45064365-45064941	2.42297E-74	0.479925446	0.178	0.015	1.60456E-69	Tumor Tex
chr3-12194260-12194852	2.66728E-74	0.468286329	0.149	0.006	1.76635E-69	Tumor Tex
chr6-45001563-45002699	2.92971E-74	0.482326562	0.227	0.039	1.94014E-69	Tumor Tex
chr16-72474275-72474958	3.05111E-74	0.47272369	0.167	0.01	2.02054E-69	Tumor Tex
chr6-5279718-5280346	4.50191E-74	0.470808627	0.166	0.01	2.9813E-69	Tumor Tex
chr2-176491158-176491662	5.43572E-74	0.483624542	0.249	0.048	3.59969E-69	Tumor Tex
chr7-65798276-65798769	6.04E-74	0.48347495	0.218	0.037	3.99987E-69	Tumor Tex
chr18-49161612-49162048	6.09101E-74	0.485893939	0.2	0.026	4.03365E-69	Tumor Tex
chr18-13284440-13284965	7.33289E-74	0.480781026	0.192	0.02	4.85606E-69	Tumor Tex
chr1-181062053-181063137	1.14786E-73	0.482005641	0.233	0.044	7.60148E-69	Tumor Tex
chr7-17022562-17023399	2.19027E-73	0.479206113	0.249	0.053	1.45046E-68	Tumor Tex
chrX-109774723-109775007	2.26709E-73	0.471503697	0.151	0.007	1.50133E-68	Tumor Tex
chr5-55420556-55421467	2.91576E-73	0.478777752	0.253	0.054	1.9309E-68	Tumor Tex
chr18-13376299-13377630	3.72851E-73	0.472795175	0.268	0.061	2.46913E-68	Tumor Tex
chr2-134683415-134683947	3.79691E-73	0.474825211	0.234	0.045	2.51443E-68	Tumor Tex
chr1-195195198-195195909	5.65642E-73	0.479435935	0.188	0.023	3.74585E-68	Tumor Tex
chr1-156524169-156524901	8.57085E-73	0.455986445	0.328	0.106	5.67587E-68	Tumor Tex
chr13-77345109-77346139	1.21417E-72	0.478562758	0.219	0.038	8.04063E-68	Tumor Tex
chr19-54905787-54906383	1.30345E-72	0.482486051	0.206	0.027	8.63183E-68	Tumor Tex
chr6-21263912-21264121	2.30035E-72	0.458245549	0.135	0.003	1.52336E-67	Tumor Tex
chr8-104664861-104666545	2.81554E-72	0.464096644	0.266	0.064	1.86453E-67	Tumor Tex
chr17-66633280-66633639	4.3602E-72	0.47307936	0.17	0.014	2.88746E-67	Tumor Tex

chr5-143827044-143827464	4.37601E-72	0.473573655	0.179	0.019	2.89793E-67	Tumor Tex
chr10-95789733-95790227	5.19177E-72	0.473027836	0.179	0.018	3.43815E-67	Tumor Tex
chr20-50561187-50561945	7.26588E-72	0.470817759	0.269	0.068	4.81168E-67	Tumor Tex
chr14-87994963-87995338	7.93792E-72	0.475589929	0.238	0.048	5.25673E-67	Tumor Tex
chr16-23669949-23670400	8.09128E-72	0.470864578	0.165	0.012	5.35829E-67	Tumor Tex
chr12-3743810-3744865	9.79773E-72	0.475215642	0.198	0.028	6.48835E-67	Tumor Tex
chr11-72668589-72668988	9.80111E-72	0.47788584	0.177	0.017	6.49059E-67	Tumor Tex
chr12-108822012-108822239	1.29166E-71	0.456314884	0.139	0.004	8.55378E-67	Tumor Tex
chr11-34161491-34162212	1.64217E-71	0.435452183	0.367	0.134	1.08749E-66	Tumor Tex
chr8-64756570-64757235	1.8649E-71	0.441369892	0.134	0.002	1.23499E-66	Tumor Tex
chr16-11379346-11379881	2.24122E-71	0.47039221	0.188	0.022	1.4842E-66	Tumor Tex
chr1-202565905-202566224	2.33585E-71	0.456331654	0.137	0.004	1.54687E-66	Tumor Tex
chr12-68225520-68225959	2.41283E-71	0.456442377	0.15	0.007	1.59785E-66	Tumor Tex
chr1-239718733-239720090	2.96384E-71	0.474364284	0.226	0.04	1.96275E-66	Tumor Tex
chr16-11099977-11100490	3.12141E-71	0.473529192	0.169	0.013	2.06709E-66	Tumor Tex
chr19-2168797-2169315	3.66087E-71	0.465603508	0.282	0.07	2.42434E-66	Tumor Tex
chr3-151268369-151269265	3.68996E-71	0.475889999	0.217	0.038	2.4436E-66	Tumor Tex
chr10-43781497-43782356	5.49662E-71	0.461101793	0.289	0.088	3.64003E-66	Tumor Tex
chrX-9131079-9131408	5.59786E-71	0.464138079	0.145	0.007	3.70707E-66	Tumor Tex
chr22-45254512-45255367	5.8535E-71	0.453965095	0.321	0.104	3.87636E-66	Tumor Tex
chr8-19719588-19719839	6.82622E-71	0.473244073	0.183	0.019	4.52053E-66	Tumor Tex
chrX-72621455-72621974	1.6275E-70	0.474976565	0.201	0.03	1.07778E-65	Tumor Tex
chr2-176508957-176509443	1.69015E-70	0.46553302	0.173	0.015	1.11927E-65	Tumor Tex
chr10-50431180-50432803	1.92773E-70	0.469764468	0.228	0.044	1.2766E-65	Tumor Tex
chr6-118680655-118681051	1.99866E-70	0.468780448	0.17	0.016	1.32358E-65	Tumor Tex
chr18-54261954-54262153	2.15329E-70	0.448582281	0.135	0.003	1.42597E-65	Tumor Tex
chr9-91071028-91071984	2.52483E-70	0.460825773	0.285	0.079	1.67202E-65	Tumor Tex

chr14-89507455-89508275	5.00235E-70	0.425930954	0.389	0.157	3.31271E-65	Tumor Tex
chr1-76294438-76294867	5.85194E-70	0.472474496	0.226	0.04	3.87533E-65	Tumor Tex
chr6-12044767-12045304	6.13176E-70	0.471982833	0.206	0.03	4.06063E-65	Tumor Tex
chr5-67031731-67032094	7.38287E-70	0.456137142	0.146	0.007	4.88916E-65	Tumor Tex
chr1-172870929-172871515	7.82952E-70	0.4681276	0.182	0.02	5.18494E-65	Tumor Tex
chr11-12174228-12174840	9.34495E-70	0.46628962	0.243	0.049	6.1885E-65	Tumor Tex
chr6-16524270-16524627	1.50802E-69	0.471162805	0.178	0.02	9.98656E-65	Tumor Tex
chr6-140774070-140774502	2.19031E-69	0.45833654	0.151	0.008	1.45049E-64	Tumor Tex
chr1-211304904-211305388	2.52452E-69	0.45883694	0.151	0.008	1.67181E-64	Tumor Tex
chr10-119253876-119254141	2.59826E-69	0.443603206	0.127	0.002	1.72065E-64	Tumor Tex
chr2-144233454-144234825	4.4368E-69	0.463256071	0.251	0.06	2.93818E-64	Tumor Tex
chr2-64338862-64339381	4.98895E-69	0.458813892	0.164	0.014	3.30383E-64	Tumor Tex
chr13-75312818-75313186	5.22785E-69	0.461815975	0.162	0.012	3.46204E-64	Tumor Tex
chr6-149444533-149445735	5.83017E-69	0.45976555	0.176	0.019	3.86091E-64	Tumor Tex
chr2-111208944-111209309	6.27742E-69	0.449487928	0.144	0.007	4.15709E-64	Tumor Tex
chr9-91341044-91341630	7.07111E-69	0.455707787	0.15	0.008	4.6827E-64	Tumor Tex
chr20-57786139-57786605	8.37844E-69	0.450061362	0.145	0.007	5.54845E-64	Tumor Tex
chr4-36348495-36348884	1.31688E-68	0.448066519	0.139	0.006	8.72074E-64	Tumor Tex
chr10-117656748-117657029	1.45637E-68	0.45090583	0.145	0.007	9.64449E-64	Tumor Tex
chr6-105591092-105591344	1.7532E-68	0.456842399	0.156	0.01	1.16102E-63	Tumor Tex
chr19-24054012-24054904	1.88038E-68	0.465983128	0.219	0.041	1.24525E-63	Tumor Tex
chr9-2655258-2655979	2.0076E-68	0.455184234	0.279	0.082	1.32949E-63	Tumor Tex
chr4-10729996-10730322	2.05297E-68	0.428579464	0.125	0.001	1.35954E-63	Tumor Tex
chr4-7649400-7650381	2.91818E-68	0.359478411	0.542	0.294	1.9325E-63	Tumor Tex
chr3-114231511-114232156	3.07638E-68	0.464927325	0.233	0.05	2.03727E-63	Tumor Tex
chr18-13216112-13218047	3.10789E-68	0.450822781	0.287	0.085	2.05814E-63	Tumor Tex
chr10-43120783-43121223	3.67539E-68	0.461074048	0.168	0.015	2.43395E-63	Tumor Tex

chr13-33678751-33679695	4.2783E-68	0.419338455	0.386	0.156	2.83322E-63	Tumor Tex
chr6-155126907-155127353	5.14893E-68	0.462515932	0.171	0.019	3.40978E-63	Tumor Tex
chr20-48767472-48767832	6.11558E-68	0.463450412	0.236	0.051	4.04992E-63	Tumor Tex
chr15-85329699-85331523	6.39244E-68	0.414790616	0.395	0.169	4.23327E-63	Tumor Tex
chr12-96425333-96425756	1.4284E-67	0.467431774	0.2	0.029	9.45931E-63	Tumor Tex
chr1-6962118-6963668	1.51101E-67	0.457557539	0.193	0.028	1.00064E-62	Tumor Tex
chr2-144374472-144374671	1.71317E-67	0.43905264	0.128	0.003	1.13451E-62	Tumor Tex
chr10-72218333-72218660	1.9849E-67	0.464877854	0.206	0.037	1.31446E-62	Tumor Tex
chr10-113681659-113682137	3.16577E-67	0.461840203	0.221	0.043	2.09647E-62	Tumor Tex
chr2-179031588-179032398	3.75579E-67	0.46015983	0.241	0.051	2.4872E-62	Tumor Tex
chr10-31100732-31101400	6.29065E-67	0.461155133	0.178	0.02	4.16586E-62	Tumor Tex
chr3-37430792-37431173	7.24157E-67	0.452128409	0.147	0.009	4.79559E-62	Tumor Tex
chr16-16025129-16026008	9.42656E-67	0.463347439	0.214	0.036	6.24255E-62	Tumor Tex
chr8-29885801-29886229	1.05993E-66	0.447653352	0.144	0.008	7.01916E-62	Tumor Tex
chr3-16462409-16463082	1.33251E-66	0.458526921	0.247	0.057	8.8243E-62	Tumor Tex
chr2-119827772-119828490	1.44463E-66	0.445666965	0.297	0.091	9.56677E-62	Tumor Tex
chr6-149139815-149141152	1.93773E-66	0.441903739	0.307	0.096	1.28323E-61	Tumor Tex
chr6-116136323-116137365	2.44587E-66	0.45586363	0.252	0.061	1.61973E-61	Tumor Tex
chr3-105876466-105877610	2.4899E-66	0.458336486	0.24	0.058	1.64889E-61	Tumor Tex
chr2-222451288-222451541	2.9208E-66	0.429013902	0.12	0.002	1.93424E-61	Tumor Tex
chr3-133595345-133595630	3.61222E-66	0.458826985	0.178	0.022	2.39212E-61	Tumor Tex
chr3-149579842-149580538	6.04789E-66	0.455651844	0.172	0.02	4.0051E-61	Tumor Tex
chrX-41518108-41518480	6.8934E-66	0.430418366	0.123	0.002	4.56501E-61	Tumor Tex
chr3-151135437-151136317	8.05029E-66	0.446725039	0.275	0.075	5.33114E-61	Tumor Tex
chr11-16749818-16750111	8.80028E-66	0.432889587	0.129	0.004	5.82781E-61	Tumor Tex
chr14-71218231-71218781	1.29076E-65	0.445009331	0.14	0.007	8.54779E-61	Tumor Tex
chr8-124757086-124757833	1.32083E-65	0.458019209	0.232	0.054	8.74692E-61	Tumor Tex

chr10-17648260-17648838	1.45924E-65	0.446252296	0.289	0.082	9.66352E-61	Tumor Tex
chr19-38675547-38675922	1.68533E-65	0.461919687	0.214	0.041	1.11608E-60	Tumor Tex
chr16-26597839-26598554	2.73047E-65	0.378772731	0.473	0.228	1.8082E-60	Tumor Tex
chr12-6167276-6168190	3.23437E-65	0.446470959	0.276	0.076	2.1419E-60	Tumor Tex
chr16-75011600-75012683	3.56277E-65	0.451333612	0.245	0.057	2.35937E-60	Tumor Tex
chr6-109490791-109492021	4.86232E-65	0.446248987	0.275	0.083	3.21998E-60	Tumor Tex
chr5-138790683-138790998	5.31189E-65	0.450756997	0.148	0.012	3.51769E-60	Tumor Tex
chr1-160807268-160807536	5.31327E-65	0.452747778	0.167	0.018	3.51861E-60	Tumor Tex
chr7-151747008-151747237	5.46489E-65	0.428875488	0.122	0.003	3.61901E-60	Tumor Tex
chr3-195147567-195147999	5.48868E-65	0.443518594	0.147	0.009	3.63477E-60	Tumor Tex
chr8-42292107-42293538	5.57904E-65	0.424404375	0.344	0.125	3.69461E-60	Tumor Tex
chr14-92650743-92652022	5.67373E-65	0.408823845	0.392	0.164	3.75731E-60	Tumor Tex
chr12-27185234-27185904	7.55562E-65	0.444335075	0.152	0.013	5.00356E-60	Tumor Tex
chr9-68793696-68794284	8.78806E-65	0.442573707	0.135	0.008	5.81971E-60	Tumor Tex
chr21-38937955-38938753	9.94727E-65	0.45256643	0.229	0.053	6.58738E-60	Tumor Tex
chr6-135624071-135624885	1.18498E-64	0.433439564	0.32	0.104	7.84732E-60	Tumor Tex
chr5-157172052-157172856	1.43646E-64	0.434473817	0.315	0.104	9.51265E-60	Tumor Tex
chr8-124629483-124630102	2.1825E-64	0.451546927	0.246	0.059	1.44531E-59	Tumor Tex
chr12-95102887-95103602	2.66826E-64	0.434976226	0.3	0.096	1.767E-59	Tumor Tex
chr5-56327606-56328201	2.72839E-64	0.450783703	0.168	0.018	1.80682E-59	Tumor Tex
chr8-97798004-97799005	5.52092E-64	0.436624894	0.134	0.006	3.65612E-59	Tumor Tex
chr7-98131480-98132270	6.83252E-64	0.452474267	0.223	0.049	4.5247E-59	Tumor Tex
chr3-195133458-195133841	7.70389E-64	0.452868153	0.197	0.031	5.10175E-59	Tumor Tex
chr6-44642405-44643221	8.19858E-64	0.451785386	0.186	0.029	5.42935E-59	Tumor Tex
chr13-42354068-42355455	8.23654E-64	0.400266905	0.395	0.175	5.45448E-59	Tumor Tex
chr7-157574258-157574468	9.41337E-64	0.429577748	0.13	0.005	6.23381E-59	Tumor Tex
chr14-75499046-75499320	1.09363E-63	0.44576429	0.156	0.014	7.24233E-59	Tumor Tex

chr10-70354478-70355657	1.70694E-63	0.446648283	0.17	0.019	1.13039E-58	Tumor Tex
chr3-46369598-46370214	2.64126E-63	0.392152474	0.415	0.196	1.74912E-58	Tumor Tex
chr1-67166208-67166540	2.77725E-63	0.437500955	0.146	0.009	1.83918E-58	Tumor Tex
chr10-122491022-122491535	2.81113E-63	0.450534167	0.166	0.019	1.86162E-58	Tumor Tex
chr1-204452780-204453566	2.91195E-63	0.418291091	0.348	0.135	1.92838E-58	Tumor Tex
chr6-11277458-11278552	3.00032E-63	0.401898598	0.385	0.171	1.9869E-58	Tumor Tex
chr6-90472687-90474013	3.20709E-63	0.447737699	0.237	0.055	2.12383E-58	Tumor Tex
chr15-50107185-50107937	3.56194E-63	0.444857513	0.16	0.015	2.35883E-58	Tumor Tex
chr1-169680617-169680944	3.9679E-63	0.432771479	0.133	0.007	2.62766E-58	Tumor Tex
chr1-7953678-7954908	4.62508E-63	0.411684592	0.366	0.154	3.06287E-58	Tumor Tex
chr2-168455852-168457138	4.85055E-63	0.44985514	0.209	0.038	3.21218E-58	Tumor Tex
chr20-50349300-50349619	5.1868E-63	0.435006613	0.136	0.009	3.43486E-58	Tumor Tex
chr10-26737604-26738015	5.18933E-63	0.436755811	0.149	0.01	3.43653E-58	Tumor Tex
chr13-30049589-30050211	5.28406E-63	0.448556884	0.169	0.02	3.49926E-58	Tumor Tex
chr6-106247362-106248095	5.31751E-63	0.437961022	0.146	0.011	3.52142E-58	Tumor Tex
chr12-108831501-108832025	5.97677E-63	0.447317003	0.167	0.019	3.95799E-58	Tumor Tex
chr16-10987646-10989038	6.09577E-63	0.445343595	0.238	0.057	4.0368E-58	Tumor Tex
chr14-72476471-72477232	6.48712E-63	0.442614909	0.258	0.07	4.29597E-58	Tumor Tex
chr2-101272198-101272723	7.05904E-63	0.447869102	0.164	0.018	4.67471E-58	Tumor Tex
chr3-72965598-72966263	9.23456E-63	0.44801527	0.186	0.028	6.11541E-58	Tumor Tex
chr7-151727523-151728027	1.59697E-62	0.444746144	0.165	0.02	1.05756E-57	Tumor Tex
chr11-129966324-129966527	3.2436E-62	0.414489498	0.113	0.002	2.14801E-57	Tumor Tex
chr4-123420121-123420439	3.569E-62	0.448071833	0.171	0.024	2.3635E-57	Tumor Tex
chr14-61528509-61529053	4.13703E-62	0.419156571	0.336	0.116	2.73966E-57	Tumor Tex
chr12-76030096-76032366	4.22254E-62	0.318432954	0.597	0.395	2.79629E-57	Tumor Tex
chr13-39688320-39689127	5.69152E-62	0.449820772	0.204	0.042	3.76909E-57	Tumor Tex
chr14-55128874-55130115	6.06158E-62	0.415762572	0.336	0.131	4.01416E-57	Tumor Tex

chr10-92101336-92102068	6.18185E-62	0.444745678	0.191	0.029	4.09381E-57	Tumor Tex
chr7-156777247-156777963	6.18717E-62	0.447259693	0.187	0.029	4.09733E-57	Tumor Tex
chr1-200866527-200866888	6.71402E-62	0.446733542	0.21	0.043	4.44623E-57	Tumor Tex
chr1-19045071-19045791	7.29072E-62	0.441830867	0.242	0.059	4.82813E-57	Tumor Tex
chr2-174844367-174844602	7.38665E-62	0.42497049	0.129	0.006	4.89166E-57	Tumor Tex
chr1-31912796-31913249	7.66449E-62	0.44587896	0.195	0.036	5.07566E-57	Tumor Tex
chr17-57454840-57455533	7.91912E-62	0.44528222	0.214	0.044	5.24428E-57	Tumor Tex
chr6-135586900-135587158	8.40433E-62	0.434620133	0.139	0.01	5.5656E-57	Tumor Tex
chr12-68932468-68933453	9.96687E-62	0.444539296	0.229	0.051	6.60036E-57	Tumor Tex
chr6-134437115-134437704	1.0046E-61	0.446934147	0.193	0.035	6.65278E-57	Tumor Tex
chr1-109687593-109687880	1.24973E-61	0.434429075	0.148	0.01	8.27611E-57	Tumor Tex
chr9-128558972-128559281	1.26939E-61	0.444424133	0.167	0.02	8.40627E-57	Tumor Tex
chr5-53041016-53041695	1.35848E-61	0.438496972	0.261	0.073	8.99625E-57	Tumor Tex
chr12-44827617-44828447	1.36294E-61	0.438727298	0.25	0.067	9.02582E-57	Tumor Tex
chr17-55089250-55090549	1.5336E-61	0.423104766	0.309	0.098	1.01559E-56	Tumor Tex
chr4-152553782-152554647	1.71203E-61	0.418924826	0.324	0.124	1.13376E-56	Tumor Tex
chr10-78578755-78579313	1.92771E-61	0.4339519	0.142	0.013	1.27659E-56	Tumor Tex
chr6-135115496-135116113	1.98446E-61	0.44012222	0.168	0.022	1.31417E-56	Tumor Tex
chr1-109161944-109162888	2.64419E-61	0.43879665	0.159	0.019	1.75106E-56	Tumor Tex
chr8-81061025-81061633	2.81842E-61	0.443047363	0.232	0.055	1.86644E-56	Tumor Tex
chr15-41798948-41799464	2.96399E-61	0.408921582	0.113	0.002	1.96284E-56	Tumor Tex
chr16-70737036-70738164	3.06861E-61	0.400172197	0.374	0.159	2.03212E-56	Tumor Tex
chr16-27254128-27254570	3.40032E-61	0.439530329	0.163	0.019	2.2518E-56	Tumor Tex
chr3-9778111-9778369	3.70737E-61	0.434326962	0.143	0.012	2.45513E-56	Tumor Tex
chr3-143118993-143121602	3.9337E-61	0.41385985	0.34	0.13	2.60501E-56	Tumor Tex
chr3-88141633-88142486	4.26489E-61	0.443747788	0.224	0.046	2.82434E-56	Tumor Tex
chr3-17044943-17045220	5.51118E-61	0.412532894	0.115	0.003	3.64967E-56	Tumor Tex

chr6-134435891-134436747	6.11848E-61	0.407615132	0.348	0.143	4.05184E-56	Tumor Tex
chr1-230542058-230542440	6.34812E-61	0.424167034	0.121	0.006	4.20392E-56	Tumor Tex
chr11-77147685-77148316	6.71508E-61	0.441011772	0.22	0.048	4.44693E-56	Tumor Tex
chr1-192936376-192937407	7.86543E-61	0.441682155	0.181	0.028	5.20872E-56	Tumor Tex
chr12-98745373-98746311	7.88367E-61	0.442974982	0.187	0.029	5.2208E-56	Tumor Tex
chr8-133045854-133047511	1.04742E-60	0.43230866	0.262	0.08	6.93635E-56	Tumor Tex
chr8-115451332-115452455	1.05441E-60	0.422943629	0.302	0.1	6.9826E-56	Tumor Tex
chr2-160516530-160517273	1.10981E-60	0.43564131	0.155	0.015	7.34949E-56	Tumor Tex
chr3-197338638-197339013	1.14784E-60	0.433381994	0.135	0.012	7.60134E-56	Tumor Tex
chr13-46340934-46341615	1.48849E-60	0.439885432	0.166	0.022	9.85721E-56	Tumor Tex
chr4-121227229-121227837	1.56129E-60	0.431931509	0.268	0.086	1.03394E-55	Tumor Tex
chr15-90829567-90830386	1.91962E-60	0.4167485	0.126	0.005	1.27123E-55	Tumor Tex
chr2-181275226-181276102	2.24269E-60	0.437505738	0.246	0.067	1.48517E-55	Tumor Tex
chr6-490179-491637	2.54043E-60	0.417991409	0.316	0.115	1.68235E-55	Tumor Tex
chr8-124953764-124954242	2.6852E-60	0.430250286	0.139	0.011	1.77822E-55	Tumor Tex
chr8-29402141-29402998	2.86384E-60	0.408604096	0.341	0.133	1.89652E-55	Tumor Tex
chr3-66469190-66469508	3.03877E-60	0.441490288	0.177	0.026	2.01236E-55	Tumor Tex
chr3-138234398-138235136	4.80559E-60	0.438486537	0.18	0.026	3.18241E-55	Tumor Tex
chr15-59919059-59920093	4.83433E-60	0.396411989	0.378	0.153	3.20144E-55	Tumor Tex
chr1-65830335-65830637	5.45669E-60	0.406837326	0.112	0.002	3.61358E-55	Tumor Tex
chr19-13121941-13122660	6.3246E-60	0.433116682	0.245	0.061	4.18834E-55	Tumor Tex
chr18-23283811-23284332	6.79749E-60	0.433938821	0.153	0.017	4.5015E-55	Tumor Tex
chrX-30712998-30713488	7.49434E-60	0.43125068	0.139	0.011	4.96297E-55	Tumor Tex
chr16-11076232-11076710	7.74176E-60	0.436114534	0.158	0.015	5.12682E-55	Tumor Tex
chr3-46097601-46098753	8.1365E-60	0.41715854	0.311	0.113	5.38824E-55	Tumor Tex
chr4-123477612-123478227	8.25143E-60	0.439566319	0.217	0.049	5.46435E-55	Tumor Tex
chr2-168167138-168167537	1.0156E-59	0.39765897	0.107	0.001	6.72558E-55	Tumor Tex

chr4-26145187-26145473	1.27436E-59	0.42272664	0.13	0.007	8.43922E-55	Tumor Tex
chr7-36115500-36115891	1.77087E-59	0.426145913	0.146	0.013	1.17272E-54	Tumor Tex
chr20-25321349-25321796	1.9006E-59	0.419890741	0.128	0.008	1.25863E-54	Tumor Tex
chr5-157539916-157541269	1.90315E-59	0.41518454	0.314	0.119	1.26032E-54	Tumor Tex
chr2-38907874-38908438	1.91339E-59	0.417487368	0.122	0.005	1.26711E-54	Tumor Tex
chr9-36342134-36342515	2.16069E-59	0.430581764	0.148	0.013	1.43087E-54	Tumor Tex
chr7-13778743-13779330	2.49578E-59	0.427391355	0.135	0.011	1.65278E-54	Tumor Tex
chr12-27179691-27180134	2.59655E-59	0.437353858	0.165	0.021	1.71952E-54	Tumor Tex
chr8-125606112-125606853	2.63923E-59	0.428767351	0.269	0.08	1.74778E-54	Tumor Tex
chr10-95761981-95762255	2.81901E-59	0.420385619	0.13	0.007	1.86683E-54	Tumor Tex
chr10-59880835-59881397	3.4075E-59	0.430523774	0.242	0.061	2.25655E-54	Tumor Tex
chr5-157024936-157025759	3.95339E-59	0.410086027	0.335	0.125	2.61805E-54	Tumor Tex
chr17-55162182-55162841	5.13792E-59	0.428586818	0.155	0.015	3.40248E-54	Tumor Tex
chr13-50366705-50367148	6.7605E-59	0.43403196	0.228	0.057	4.47701E-54	Tumor Tex
chr15-74857038-74858309	7.54566E-59	0.434244338	0.218	0.05	4.99696E-54	Tumor Tex
chr6-12051769-12052284	7.75099E-59	0.439390348	0.212	0.043	5.13294E-54	Tumor Tex
chr10-6291189-6292182	8.41239E-59	0.427254658	0.253	0.074	5.57094E-54	Tumor Tex
chr14-65302028-65302299	8.64903E-59	0.42123909	0.13	0.008	5.72764E-54	Tumor Tex
chr6-158031349-158031795	9.14396E-59	0.431699688	0.242	0.064	6.0554E-54	Tumor Tex
chr12-56032056-56032280	1.03859E-58	0.408012511	0.116	0.003	6.87782E-54	Tumor Tex
chr4-122632689-122633566	1.0922E-58	0.423726118	0.272	0.082	7.23286E-54	Tumor Tex
chr9-5509811-5510953	1.1085E-58	0.434435795	0.166	0.022	7.34083E-54	Tumor Tex
chr3-71086871-71088054	1.12107E-58	0.354539785	0.481	0.259	7.42406E-54	Tumor Tex
chr6-137827871-137828576	1.22975E-58	0.425597815	0.272	0.088	8.14378E-54	Tumor Tex
chr12-69161837-69162178	1.24412E-58	0.438768931	0.189	0.032	8.23895E-54	Tumor Tex
chr12-26298721-26299357	1.2554E-58	0.430092189	0.239	0.066	8.31366E-54	Tumor Tex
chr3-15635835-15636753	1.45316E-58	0.428635081	0.252	0.072	9.62324E-54	Tumor Tex

chr4-123421504-123422425	1.49529E-58	0.432639403	0.224	0.052	9.90225E-54	Tumor Tex
chr15-90815412-90815622	1.52237E-58	0.403573962	0.116	0.003	1.00816E-53	Tumor Tex
chr6-87718595-87719407	1.86885E-58	0.422239855	0.274	0.085	1.23761E-53	Tumor Tex
chr2-135819320-135820567	2.20431E-58	0.416592798	0.296	0.099	1.45976E-53	Tumor Tex
chr11-118443607-118443828	2.98233E-58	0.416729704	0.13	0.008	1.97499E-53	Tumor Tex
chr7-130649008-130649225	3.38316E-58	0.405700892	0.111	0.003	2.24043E-53	Tumor Tex
chr20-53146228-53146646	4.83379E-58	0.419043803	0.13	0.007	3.20108E-53	Tumor Tex
chr15-70141625-70142758	5.67684E-58	0.428298083	0.234	0.065	3.75938E-53	Tumor Tex
chr2-144430473-144431724	5.95136E-58	0.424823007	0.259	0.077	3.94117E-53	Tumor Tex
chr2-227410991-227411690	7.9886E-58	0.424783601	0.151	0.015	5.29029E-53	Tumor Tex
chr8-42196108-42196512	9.14838E-58	0.404099615	0.11	0.003	6.05833E-53	Tumor Tex
chr2-8376633-8377259	1.00914E-57	0.426789383	0.16	0.019	6.68281E-53	Tumor Tex
chr14-67641939-67642709	1.13183E-57	0.423448091	0.145	0.015	7.4953E-53	Tumor Tex
chr12-92096443-92097074	1.13311E-57	0.432075588	0.17	0.025	7.50377E-53	Tumor Tex
chr12-47356048-47356502	1.22407E-57	0.429758287	0.162	0.023	8.10618E-53	Tumor Tex
chr3-4421095-4421496	1.41857E-57	0.415508321	0.137	0.01	9.39417E-53	Tumor Tex
chr14-64447910-64448586	1.6829E-57	0.429928023	0.219	0.051	1.11447E-52	Tumor Tex
chr8-81285343-81285617	2.12361E-57	0.431555756	0.17	0.025	1.40632E-52	Tumor Tex
chr2-28465402-28465738	2.16286E-57	0.418370895	0.132	0.009	1.43231E-52	Tumor Tex
chr5-32138688-32139123	2.23426E-57	0.416681625	0.125	0.007	1.4796E-52	Tumor Tex
chr10-30746968-30747990	2.25776E-57	0.396322881	0.346	0.136	1.49515E-52	Tumor Tex
chr3-18556003-18556909	2.89423E-57	0.403239867	0.324	0.125	1.91665E-52	Tumor Tex
chr4-77594956-77595777	3.0813E-57	0.423490721	0.257	0.076	2.04053E-52	Tumor Tex
chr16-50684772-50685555	3.4248E-57	0.430259343	0.224	0.054	2.268E-52	Tumor Tex
chr16-17224454-17225255	3.67895E-57	0.432019797	0.194	0.038	2.43631E-52	Tumor Tex
chr10-110425756-110426445	3.68768E-57	0.424619622	0.259	0.073	2.44209E-52	Tumor Tex
chr2-20595436-20596528	4.25594E-57	0.426020128	0.148	0.017	2.81841E-52	Tumor Tex

chr16-75110715-75111531	4.4003E-57	0.416402373	0.289	0.096	2.91401E-52	Tumor Tex
chr8-28365720-28366140	4.75392E-57	0.429063501	0.222	0.056	3.14819E-52	Tumor Tex
chr12-89768901-89769578	4.87822E-57	0.429395578	0.194	0.038	3.2305E-52	Tumor Tex
chr16-10266853-10267075	4.91956E-57	0.400293839	0.106	0.003	3.25788E-52	Tumor Tex
chr2-233230821-233231330	6.22544E-57	0.427607028	0.161	0.022	4.12267E-52	Tumor Tex
chr2-171318734-171319646	7.52936E-57	0.42014949	0.26	0.078	4.98617E-52	Tumor Tex
chr22-45612408-45612989	8.23627E-57	0.423818761	0.146	0.015	5.45431E-52	Tumor Tex
chr4-7960578-7961131	9.25786E-57	0.424467833	0.152	0.019	6.13084E-52	Tumor Tex
chr14-65425301-65426157	1.13089E-56	0.413843686	0.131	0.009	7.48906E-52	Tumor Tex
chr6-12060507-12061408	1.15339E-56	0.393454632	0.352	0.147	7.63808E-52	Tumor Tex
chr1-19010784-19011423	1.43998E-56	0.427080128	0.225	0.055	9.53599E-52	Tumor Tex
chr7-17029070-17029486	1.72001E-56	0.423763213	0.146	0.017	1.13904E-51	Tumor Tex
chr8-102782757-102783458	1.8039E-56	0.427203307	0.172	0.028	1.19459E-51	Tumor Tex
chr11-110148716-110149111	1.84955E-56	0.406949036	0.118	0.005	1.22483E-51	Tumor Tex
chr9-84296203-84296569	1.99139E-56	0.397833987	0.115	0.003	1.31876E-51	Tumor Tex
chr13-102873677-102873934	2.06758E-56	0.421735108	0.145	0.015	1.36921E-51	Tumor Tex
chr3-4906100-4906640	2.15461E-56	0.425190744	0.217	0.048	1.42685E-51	Tumor Tex
chr6-2127301-2127987	2.65847E-56	0.426933077	0.189	0.037	1.76052E-51	Tumor Tex
chr2-97688244-97688687	3.10797E-56	0.411295652	0.13	0.008	2.05819E-51	Tumor Tex
chr1-40185698-40186840	4.35888E-56	0.419991091	0.26	0.082	2.88658E-51	Tumor Tex
chr14-68292233-68292599	4.92381E-56	0.392604272	0.106	0.002	3.26069E-51	Tumor Tex
chr1-230212485-230212818	5.83958E-56	0.424156242	0.153	0.019	3.86715E-51	Tumor Tex
chr8-125601766-125602204	6.52067E-56	0.424136927	0.162	0.025	4.31818E-51	Tumor Tex
chr22-42030982-42031652	6.60072E-56	0.403517821	0.122	0.006	4.3712E-51	Tumor Tex
chr3-16370836-16371582	9.29629E-56	0.397312477	0.116	0.004	6.15628E-51	Tumor Tex
chr18-54260719-54260939	9.59596E-56	0.414464072	0.132	0.009	6.35473E-51	Tumor Tex
chr16-27464716-27465226	1.18906E-55	0.419189564	0.255	0.076	7.87434E-51	Tumor Tex

chr15-90837686-90838896	1.2364E-55	0.409891322	0.29	0.098	8.18782E-51	Tumor Tex
chr10-6507024-6507707	1.48667E-55	0.408696033	0.293	0.105	9.84517E-51	Tumor Tex
chr1-235641570-235642981	1.94567E-55	0.376234577	0.391	0.186	1.28848E-50	Tumor Tex
chr9-125785154-125785689	2.07684E-55	0.401438108	0.115	0.006	1.37535E-50	Tumor Tex
chr10-63173798-63174140	2.13313E-55	0.421818931	0.147	0.017	1.41262E-50	Tumor Tex
chr1-231387949-231388223	2.25077E-55	0.383764258	0.1	0.001	1.49053E-50	Tumor Tex
chr9-109974079-109974674	2.2576E-55	0.390053024	0.104	0.002	1.49505E-50	Tumor Tex
chr11-126317563-126319327	2.26668E-55	0.422037623	0.202	0.048	1.50106E-50	Tumor Tex
chr16-88264744-88265942	2.30485E-55	0.398929393	0.323	0.123	1.52634E-50	Tumor Tex
chr16-12750138-12750809	2.62592E-55	0.414558706	0.139	0.012	1.73896E-50	Tumor Tex
chr7-92631405-92632241	2.739E-55	0.42416949	0.188	0.038	1.81385E-50	Tumor Tex
chr12-113962496-113962698	3.00141E-55	0.42039565	0.143	0.015	1.98762E-50	Tumor Tex
chr17-8056190-8056475	3.08852E-55	0.415396388	0.139	0.014	2.04531E-50	Tumor Tex
chr9-19999130-19999982	3.1204E-55	0.403499247	0.3	0.108	2.06642E-50	Tumor Tex
chr21-31195707-31196293	3.13223E-55	0.412213345	0.135	0.01	2.07426E-50	Tumor Tex
chr15-88918766-88919116	3.29323E-55	0.415500911	0.136	0.012	2.18087E-50	Tumor Tex
chr17-57645881-57646536	3.33123E-55	0.426440506	0.184	0.036	2.20604E-50	Tumor Tex
chrX-20515725-20516500	3.85956E-55	0.423550887	0.18	0.031	2.55592E-50	Tumor Tex
chr6-156794874-156795532	4.23453E-55	0.390614767	0.114	0.004	2.80423E-50	Tumor Tex
chr2-8239815-8240059	4.39229E-55	0.383386076	0.103	0.001	2.90871E-50	Tumor Tex
chr4-25854601-25855365	4.79691E-55	0.412386107	0.27	0.092	3.17666E-50	Tumor Tex
chr6-125904531-125905320	4.8594E-55	0.418851695	0.232	0.061	3.21804E-50	Tumor Tex
chr3-189622050-189622834	5.16403E-55	0.405670939	0.125	0.008	3.41978E-50	Tumor Tex
chr1-115073318-115073833	5.36717E-55	0.417629826	0.15	0.017	3.5543E-50	Tumor Tex
chr18-48801054-48801624	5.37347E-55	0.385848141	0.103	0.002	3.55847E-50	Tumor Tex
chr11-86176148-86176847	5.99935E-55	0.42327634	0.219	0.055	3.97295E-50	Tumor Tex
chr10-124728451-124728881	8.44573E-55	0.418400099	0.242	0.067	5.59301E-50	Tumor Tex

chr10-122301206-122301456	9.55256E-55	0.393797369	0.109	0.003	6.32599E-50	Tumor Tex
chr6-135851438-135851803	9.95705E-55	0.40872734	0.128	0.009	6.59386E-50	Tumor Tex
chr5-143595005-143595686	1.00462E-54	0.418567607	0.162	0.023	6.65293E-50	Tumor Tex
chr1-239586952-239587432	1.01059E-54	0.404745974	0.118	0.007	6.69244E-50	Tumor Tex
chr15-50046873-50047143	1.13113E-54	0.413096529	0.135	0.01	7.49071E-50	Tumor Tex
chr6-135583259-135583458	1.14802E-54	0.404274138	0.125	0.008	7.60254E-50	Tumor Tex
chr2-64212330-64213310	1.17035E-54	0.399439467	0.311	0.119	7.75039E-50	Tumor Tex
chr18-13562087-13562747	1.2965E-54	0.419324703	0.163	0.025	8.5858E-50	Tumor Tex
chr2-9708794-9709401	1.31433E-54	0.418560329	0.147	0.017	8.70385E-50	Tumor Tex
chr12-68042200-68042665	1.35152E-54	0.419265828	0.154	0.022	8.95018E-50	Tumor Tex
chr12-24719156-24719756	1.39642E-54	0.4214152	0.193	0.041	9.2475E-50	Tumor Tex
chr6-20978211-20978586	1.54052E-54	0.382266871	0.107	0.002	1.02018E-49	Tumor Tex
chr20-56414903-56415412	1.69594E-54	0.421590459	0.207	0.05	1.1231E-49	Tumor Tex
chr2-190678452-190679171	2.01437E-54	0.416683605	0.239	0.067	1.33398E-49	Tumor Tex
chr1-87449314-87449987	2.29671E-54	0.416081524	0.141	0.015	1.52095E-49	Tumor Tex
chr14-68695001-68695785	2.29923E-54	0.422595229	0.196	0.043	1.52262E-49	Tumor Tex
chr5-143105572-143105788	2.47563E-54	0.390257916	0.106	0.003	1.63944E-49	Tumor Tex
chr15-80711186-80711431	2.81128E-54	0.39051667	0.106	0.004	1.86171E-49	Tumor Tex
chr14-69716206-69717035	3.32628E-54	0.40028438	0.309	0.114	2.20276E-49	Tumor Tex
chr17-48915506-48916261	3.44887E-54	0.417894797	0.222	0.056	2.28395E-49	Tumor Tex
chr8-133016252-133017510	3.5232E-54	0.404000097	0.284	0.102	2.33317E-49	Tumor Tex
chr11-8963481-8965209	4.43108E-54	0.351164189	0.442	0.241	2.93439E-49	Tumor Tex
chr1-107935394-107935783	4.62673E-54	0.419133032	0.211	0.055	3.06396E-49	Tumor Tex
chr2-112984891-112985466	4.67347E-54	0.406956766	0.127	0.01	3.09491E-49	Tumor Tex
chr6-136593598-136594587	4.79594E-54	0.417289838	0.225	0.065	3.17602E-49	Tumor Tex
chr20-50440479-50441792	4.86864E-54	0.390790756	0.329	0.127	3.22416E-49	Tumor Tex
chr13-28492534-28492859	5.33001E-54	0.401211599	0.115	0.006	3.52969E-49	Tumor Tex

chr2-8281696-8283299	6.40654E-54	0.411140352	0.253	0.078	4.2426E-49	Tumor Tex
chr12-10497396-10498070	6.43899E-54	0.395053353	0.315	0.126	4.26409E-49	Tumor Tex
chr8-29750724-29751112	7.54438E-54	0.396695223	0.112	0.006	4.99611E-49	Tumor Tex
chr9-127911169-127911471	7.79947E-54	0.395537475	0.124	0.008	5.16504E-49	Tumor Tex
chr16-57614601-57614894	8.27297E-54	0.415213099	0.155	0.022	5.47861E-49	Tumor Tex
chr1-41698742-41699398	8.9684E-54	0.418754703	0.212	0.051	5.93914E-49	Tumor Tex
chr1-12067906-12068254	9.53661E-54	0.418913274	0.174	0.029	6.31543E-49	Tumor Tex
chr11-128610744-128611661	9.90769E-54	0.418769272	0.197	0.044	6.56117E-49	Tumor Tex
chr1-89465196-89465713	1.03412E-53	0.418146199	0.186	0.034	6.84826E-49	Tumor Tex
chr3-133490527-133491287	1.05648E-53	0.41471516	0.154	0.018	6.99635E-49	Tumor Tex
chr22-35352296-35352832	1.14986E-53	0.406498111	0.134	0.013	7.61471E-49	Tumor Tex
chr10-30759496-30760172	1.3027E-53	0.414508615	0.152	0.022	8.62687E-49	Tumor Tex
chrX-78073005-78073926	1.43808E-53	0.411783939	0.253	0.077	9.5234E-49	Tumor Tex
chr4-878889-880047	1.56441E-53	0.401324181	0.286	0.108	1.036E-48	Tumor Tex
chr11-105046876-105047083	1.74886E-53	0.393967104	0.113	0.006	1.15814E-48	Tumor Tex
chr6-159077084-159077549	2.08971E-53	0.416607021	0.17	0.03	1.38387E-48	Tumor Tex
chr2-227848352-227849017	2.48058E-53	0.397538731	0.127	0.009	1.64272E-48	Tumor Tex
chr6-157830471-157830682	2.49743E-53	0.398123873	0.118	0.007	1.65387E-48	Tumor Tex
chr2-204019581-204019876	2.61102E-53	0.416505097	0.167	0.026	1.7291E-48	Tumor Tex
chr8-128338556-128339018	2.62385E-53	0.380915291	0.1	0.002	1.73759E-48	Tumor Tex
chr1-155535960-155536738	2.67346E-53	0.410343144	0.259	0.082	1.77044E-48	Tumor Tex
chr8-29523882-29524582	3.7271E-53	0.3985691	0.302	0.114	2.4682E-48	Tumor Tex
chr12-75640883-75641454	3.80835E-53	0.40991751	0.251	0.076	2.522E-48	Tumor Tex
chr10-50325355-50325742	4.49834E-53	0.41432472	0.145	0.019	2.97893E-48	Tumor Tex
chr6-118843733-118844085	6.7483E-53	0.411349907	0.148	0.02	4.46892E-48	Tumor Tex
chr11-12150868-12151116	6.75676E-53	0.373451172	0.098	0.002	4.47453E-48	Tumor Tex
chr10-102611995-102613224	7.13895E-53	0.413740564	0.169	0.027	4.72763E-48	Tumor Tex

chr2-135244873-135245614	7.68494E-53	0.392087704	0.302	0.119	5.0892E-48	Tumor Tex
chr6-149031799-149033013	7.96293E-53	0.372619071	0.372	0.171	5.27329E-48	Tumor Tex
chr20-38997504-38998049	1.00668E-52	0.413141727	0.194	0.044	6.66651E-48	Tumor Tex
chr12-26274593-26275070	1.045E-52	0.393249394	0.113	0.006	6.9203E-48	Tumor Tex
chr16-24189516-24190101	1.23031E-52	0.403010566	0.127	0.01	8.14751E-48	Tumor Tex
chr15-59419922-59420462	1.46096E-52	0.406228838	0.13	0.012	9.67491E-48	Tumor Tex
chr12-743254-744217	1.66399E-52	0.412680365	0.225	0.062	1.10194E-47	Tumor Tex
chr2-37459235-37460604	1.7345E-52	0.412061713	0.174	0.029	1.14864E-47	Tumor Tex
chr2-53751710-53752005	1.78597E-52	0.381775542	0.102	0.002	1.18272E-47	Tumor Tex
chr7-111011574-111011801	1.84472E-52	0.385107558	0.107	0.004	1.22163E-47	Tumor Tex
chr7-104956630-104957892	2.07796E-52	0.371139594	0.368	0.177	1.37609E-47	Tumor Tex
chr15-59249293-59249651	2.20675E-52	0.403031667	0.13	0.011	1.46138E-47	Tumor Tex
chr10-102660760-102661885	2.29303E-52	0.414315564	0.208	0.05	1.51851E-47	Tumor Tex
chr10-62652086-62652419	2.88092E-52	0.404455072	0.14	0.015	1.90783E-47	Tumor Tex
chr6-169556719-169557128	3.11623E-52	0.409281291	0.143	0.017	2.06366E-47	Tumor Tex
chr11-115222115-115223260	3.15057E-52	0.373295872	0.366	0.165	2.0864E-47	Tumor Tex
chr8-125340108-125340324	3.24845E-52	0.381613785	0.103	0.003	2.15122E-47	Tumor Tex
chr17-79804152-79804754	4.02599E-52	0.413263443	0.176	0.035	2.66613E-47	Tumor Tex
chr7-3106217-3106891	4.0834E-52	0.404416157	0.268	0.085	2.70415E-47	Tumor Tex
chr14-101635881-101636530	4.39791E-52	0.408162078	0.151	0.022	2.91243E-47	Tumor Tex
chr7-98133075-98134208	4.47457E-52	0.413116311	0.177	0.036	2.96319E-47	Tumor Tex
chr6-110944444-110945188	4.61144E-52	0.366230315	0.374	0.176	3.05384E-47	Tumor Tex
chr22-40682751-40683431	4.91808E-52	0.394340972	0.303	0.114	3.2569E-47	Tumor Tex
chr11-13288399-13288949	6.0942E-52	0.410799387	0.164	0.028	4.03576E-47	Tumor Tex
chr15-65612487-65612843	6.26623E-52	0.410665183	0.21	0.054	4.14968E-47	Tumor Tex
chr3-9180060-9180677	6.45463E-52	0.389922465	0.111	0.006	4.27445E-47	Tumor Tex
chr7-55308550-55310016	6.53951E-52	0.356200308	0.412	0.208	4.33066E-47	Tumor Tex

chr19-4153247-4153676	7.02575E-52	0.412948873	0.161	0.025	4.65266E-47	Tumor Tex
chr2-8229524-8229921	7.15976E-52	0.415111165	0.166	0.029	4.74141E-47	Tumor Tex
chr8-25345238-25345791	9.02268E-52	0.412943005	0.215	0.054	5.97509E-47	Tumor Tex
chr6-135722663-135723055	9.97258E-52	0.397788686	0.126	0.011	6.60414E-47	Tumor Tex
chr7-4724682-4725747	1.11302E-51	0.410381698	0.209	0.051	7.37075E-47	Tumor Tex
chr11-13216334-13216796	1.15103E-51	0.396308106	0.124	0.01	7.62249E-47	Tumor Tex
chr22-25736275-25737002	1.18579E-51	0.382785969	0.105	0.003	7.85263E-47	Tumor Tex
chr1-223702996-223703441	1.25256E-51	0.40576932	0.137	0.016	8.29486E-47	Tumor Tex
chr14-59274691-59276003	1.31333E-51	0.400289602	0.254	0.082	8.69725E-47	Tumor Tex
chr8-73520678-73520970	1.40349E-51	0.372842154	0.096	0.002	9.29435E-47	Tumor Tex
chr1-207831756-207832144	1.47898E-51	0.40868415	0.16	0.026	9.79425E-47	Tumor Tex
chr15-90845159-90845531	1.69812E-51	0.38551648	0.112	0.006	1.12454E-46	Tumor Tex
chr1-230104630-230105094	1.76102E-51	0.410665794	0.21	0.054	1.1662E-46	Tumor Tex
chr6-135008662-135008934	1.83213E-51	0.374849002	0.097	0.003	1.21329E-46	Tumor Tex
chr14-90677576-90677984	2.6312E-51	0.398692055	0.124	0.01	1.74246E-46	Tumor Tex
chr11-110348823-110349985	2.7882E-51	0.405060702	0.147	0.019	1.84643E-46	Tumor Tex
chr16-26645110-26645901	3.08852E-51	0.407468459	0.16	0.027	2.04531E-46	Tumor Tex
chr12-124388728-124389574	4.10298E-51	0.402401656	0.245	0.074	2.71712E-46	Tumor Tex
chr7-139118135-139119540	4.5182E-51	0.372673414	0.348	0.159	2.99209E-46	Tumor Tex
chr8-115418134-115418664	4.71518E-51	0.405866703	0.162	0.027	3.12254E-46	Tumor Tex
chr15-60855021-60855518	6.02336E-51	0.38997642	0.122	0.01	3.98885E-46	Tumor Tex
chr1-200483071-200483879	6.52312E-51	0.391884809	0.288	0.108	4.31981E-46	Tumor Tex
chr3-37860357-37860945	6.75391E-51	0.408515871	0.176	0.034	4.47264E-46	Tumor Tex
chr1-61302249-61302537	7.71437E-51	0.382722692	0.108	0.004	5.10869E-46	Tumor Tex
chr3-133592821-133593486	8.17745E-51	0.406828531	0.219	0.058	5.41535E-46	Tumor Tex
chr10-79247786-79248035	8.45043E-51	0.408410601	0.155	0.025	5.59613E-46	Tumor Tex
chr17-3804270-3804876	8.8513E-51	0.411458021	0.182	0.04	5.8616E-46	Tumor Tex

chr17-66655675-66656260	1.18233E-50	0.396160814	0.13	0.012	7.82973E-46	Tumor Tex
chr5-157108783-157109413	1.20562E-50	0.395710833	0.271	0.094	7.98395E-46	Tumor Tex
chr20-32248960-32249687	1.34559E-50	0.381417746	0.32	0.133	8.91091E-46	Tumor Tex
chr22-31951715-31952201	1.39465E-50	0.402349878	0.143	0.019	9.23576E-46	Tumor Tex
chr5-123549289-123549904	1.44207E-50	0.400080914	0.256	0.085	9.54983E-46	Tumor Tex
chr15-64251173-64251438	1.67551E-50	0.408803617	0.164	0.026	1.10957E-45	Tumor Tex
chr8-60908981-60910155	1.82673E-50	0.405722051	0.183	0.037	1.20972E-45	Tumor Tex
chr12-68373775-68374805	1.85723E-50	0.364782727	0.365	0.172	1.22991E-45	Tumor Tex
chr13-44197982-44198280	2.04808E-50	0.366849433	0.099	0.002	1.3563E-45	Tumor Tex
chr5-79229487-79229970	2.94843E-50	0.401230298	0.149	0.018	1.95254E-45	Tumor Tex
chr12-9917415-9917897	3.00681E-50	0.379015078	0.107	0.006	1.9912E-45	Tumor Tex
chr1-193700697-193701352	3.02434E-50	0.399381315	0.136	0.016	2.00281E-45	Tumor Tex
chr8-81026210-81026826	3.13319E-50	0.391350566	0.129	0.013	2.07489E-45	Tumor Tex
chr14-68282805-68284054	3.13592E-50	0.397183771	0.263	0.088	2.0767E-45	Tumor Tex
chr12-124774146-124775146	3.2063E-50	0.37543899	0.329	0.144	2.1233E-45	Tumor Tex
chr9-84293238-84293645	3.9525E-50	0.372330633	0.098	0.003	2.61747E-45	Tumor Tex
chr15-86624254-86625061	4.22594E-50	0.378376539	0.317	0.133	2.79854E-45	Tumor Tex
chr3-197861047-197861548	4.43339E-50	0.391749134	0.121	0.011	2.93592E-45	Tumor Tex
chr22-25564179-25565355	5.17799E-50	0.403912213	0.169	0.03	3.42902E-45	Tumor Tex
chr1-172948471-172948722	5.49581E-50	0.390243482	0.121	0.011	3.63949E-45	Tumor Tex
chr20-36863257-36864301	5.51382E-50	0.393782864	0.265	0.088	3.65141E-45	Tumor Tex
chr10-59879309-59879701	5.715E-50	0.408559784	0.185	0.041	3.78464E-45	Tumor Tex
chr14-65566948-65567575	5.86013E-50	0.403801531	0.154	0.024	3.88075E-45	Tumor Tex
chr8-6417041-6418204	7.07874E-50	0.390809471	0.269	0.099	4.68776E-45	Tumor Tex
chr1-92315420-92316390	7.1225E-50	0.373983531	0.322	0.141	4.71673E-45	Tumor Tex
chr10-59886900-59887320	7.14921E-50	0.383382779	0.113	0.007	4.73442E-45	Tumor Tex
chr11-34235233-34235495	7.53088E-50	0.392918896	0.118	0.009	4.98718E-45	Tumor Tex

chr3-72951179-72951470	7.53113E-50	0.386646881	0.113	0.008	4.98734E-45	Tumor Tex
chr8-19461656-19462271	7.67779E-50	0.4017034	0.225	0.061	5.08446E-45	Tumor Tex
chr16-75098154-75098993	8.19063E-50	0.378422528	0.318	0.134	5.42408E-45	Tumor Tex
chr6-161038172-161038783	8.97089E-50	0.401141433	0.146	0.02	5.94079E-45	Tumor Tex
chr6-158046800-158047404	9.66318E-50	0.393064648	0.133	0.013	6.39925E-45	Tumor Tex
chr10-26446253-26446532	9.85585E-50	0.402136911	0.164	0.029	6.52684E-45	Tumor Tex
chr2-230750940-230751485	1.03305E-49	0.402687505	0.189	0.044	6.84119E-45	Tumor Tex
chr14-24289571-24289929	1.28607E-49	0.381224835	0.109	0.006	8.51675E-45	Tumor Tex
chrX-97398090-97398404	1.31392E-49	0.374540387	0.102	0.003	8.70117E-45	Tumor Tex
chr12-52835962-52836393	1.45698E-49	0.40416235	0.179	0.039	9.64854E-45	Tumor Tex
chr18-3458388-3459775	1.46004E-49	0.38464452	0.288	0.114	9.66882E-45	Tumor Tex
chr15-59251066-59251516	1.5069E-49	0.387886706	0.12	0.01	9.97914E-45	Tumor Tex
chr2-43569377-43570293	1.6472E-49	0.399944466	0.147	0.022	1.09082E-44	Tumor Tex
chr17-36089888-36090742	1.65314E-49	0.361150335	0.362	0.18	1.09476E-44	Tumor Tex
chr12-12505892-12506211	1.82189E-49	0.393821357	0.126	0.012	1.20651E-44	Tumor Tex
chr10-43160275-43161454	1.86698E-49	0.402824905	0.167	0.034	1.23637E-44	Tumor Tex
chr5-156920193-156920860	1.96916E-49	0.401595028	0.204	0.055	1.30404E-44	Tumor Tex
chr8-125151671-125152054	2.46277E-49	0.393746912	0.126	0.012	1.63092E-44	Tumor Tex
chr5-177471356-177472019	2.91659E-49	0.402045804	0.205	0.056	1.93146E-44	Tumor Tex
chr1-93715474-93715920	2.93092E-49	0.388247559	0.122	0.012	1.94094E-44	Tumor Tex
chr2-54680409-54680999	3.01296E-49	0.395330693	0.137	0.017	1.99527E-44	Tumor Tex
chr17-38485565-38486130	3.08584E-49	0.401700946	0.165	0.028	2.04354E-44	Tumor Tex
chrX-9285257-9285696	3.176E-49	0.389693183	0.12	0.01	2.10324E-44	Tumor Tex
chr16-13956943-13958085	3.36788E-49	0.389276187	0.123	0.014	2.23031E-44	Tumor Tex
chr16-69563866-69564644	3.53636E-49	0.371047436	0.344	0.155	2.34188E-44	Tumor Tex
chr1-168416248-168416455	4.1125E-49	0.361535295	0.091	0.002	2.72342E-44	Tumor Tex
chr11-93532094-93532584	4.25451E-49	0.365245843	0.095	0.003	2.81747E-44	Tumor Tex

chr1-172653194-172653539	4.67913E-49	0.394199318	0.129	0.013	3.09866E-44	Tumor Tex
chr6-45155883-45156420	4.84678E-49	0.391980256	0.131	0.013	3.20968E-44	Tumor Tex
chr1-184941728-184942008	4.98313E-49	0.367854665	0.101	0.003	3.29998E-44	Tumor Tex
chr20-50837478-50838094	5.43598E-49	0.376954449	0.109	0.007	3.59987E-44	Tumor Tex
chr3-13073074-13073862	6.35859E-49	0.392685967	0.25	0.085	4.21085E-44	Tumor Tex
chr4-26288265-26288700	6.36519E-49	0.396439315	0.137	0.017	4.21522E-44	Tumor Tex
chr8-100438004-100438459	6.36948E-49	0.396118132	0.146	0.02	4.21806E-44	Tumor Tex
chr22-50361213-50362026	6.47861E-49	0.398737199	0.215	0.063	4.29033E-44	Tumor Tex
chr14-75514130-75515611	8.30521E-49	0.379676713	0.304	0.124	5.49996E-44	Tumor Tex
chr2-28275826-28276457	8.51075E-49	0.385767363	0.288	0.106	5.63607E-44	Tumor Tex
chr7-26398027-26399177	1.07158E-48	0.391745776	0.138	0.018	7.09634E-44	Tumor Tex
chr15-50183422-50183745	1.07818E-48	0.397670755	0.15	0.023	7.14001E-44	Tumor Tex
chr10-95839996-95840304	1.14288E-48	0.362049002	0.096	0.002	7.56848E-44	Tumor Tex
chr19-18044590-18045578	1.43595E-48	0.38472809	0.288	0.112	9.50928E-44	Tumor Tex
chr15-90686360-90686802	1.44952E-48	0.378789969	0.11	0.007	9.59914E-44	Tumor Tex
chr2-178871519-178871775	1.50715E-48	0.381378243	0.114	0.009	9.98081E-44	Tumor Tex
chr11-107858026-107859827	1.60101E-48	0.369462771	0.325	0.146	1.06024E-43	Tumor Tex
chr11-78464339-78465918	1.61127E-48	0.402280691	0.205	0.051	1.06703E-43	Tumor Tex
chr10-133475056-133476222	1.82606E-48	0.391426701	0.132	0.017	1.20927E-43	Tumor Tex
chr3-195181957-195182647	1.89462E-48	0.397479696	0.161	0.028	1.25468E-43	Tumor Tex
chr3-196803851-196804533	2.03492E-48	0.366518265	0.102	0.004	1.34759E-43	Tumor Tex
chr12-69052805-69053336	2.25648E-48	0.380329588	0.114	0.007	1.49431E-43	Tumor Tex
chr3-4888936-4889853	2.34225E-48	0.397984451	0.168	0.033	1.55111E-43	Tumor Tex
chr11-72969659-72970333	2.40588E-48	0.389240785	0.262	0.093	1.59324E-43	Tumor Tex
chr19-50005530-50005768	2.40874E-48	0.385403042	0.12	0.01	1.59514E-43	Tumor Tex
chr20-50844400-50845079	2.62432E-48	0.396649156	0.204	0.055	1.7379E-43	Tumor Tex
chr11-16902077-16902450	2.83454E-48	0.352417406	0.088	0.001	1.87712E-43	Tumor Tex

chr11-44855247-44855711	3.0997E-48	0.387142403	0.122	0.012	2.05271E-43	Tumor Tex
chr10-5845882-5846444	3.25497E-48	0.365513182	0.095	0.004	2.15554E-43	Tumor Tex
chr9-134641257-134642360	3.7338E-48	0.372540659	0.112	0.008	2.47264E-43	Tumor Tex
chr5-142088214-142089105	4.16139E-48	0.398502822	0.164	0.031	2.7558E-43	Tumor Tex
chr21-32493044-32493747	4.63602E-48	0.399182568	0.164	0.031	3.07011E-43	Tumor Tex
chrX-135158689-135159028	4.94863E-48	0.380281769	0.113	0.007	3.27713E-43	Tumor Tex
chr20-50338443-50339316	5.03251E-48	0.396055538	0.199	0.054	3.33268E-43	Tumor Tex
chr6-73722525-73723016	5.11265E-48	0.394168134	0.14	0.023	3.38575E-43	Tumor Tex
chr18-54173929-54174398	5.3815E-48	0.380988582	0.116	0.009	3.56379E-43	Tumor Tex
chr17-28229957-28230321	7.5574E-48	0.382036901	0.119	0.01	5.00474E-43	Tumor Tex
chr10-43221123-43221669	7.6359E-48	0.396182227	0.172	0.034	5.05672E-43	Tumor Tex
chr12-10540546-10541263	8.16441E-48	0.39043822	0.25	0.081	5.40672E-43	Tumor Tex
chr7-102834933-102835314	9.91167E-48	0.367010793	0.099	0.004	6.5638E-43	Tumor Tex
chr19-50147508-50148431	1.08172E-47	0.396157656	0.18	0.043	7.16347E-43	Tumor Tex
chr21-46660601-46660910	1.09975E-47	0.389051763	0.124	0.015	7.28287E-43	Tumor Tex
chr9-97397206-97397605	1.17777E-47	0.393255742	0.146	0.021	7.79956E-43	Tumor Tex
chr12-12430729-12431533	1.18081E-47	0.386522768	0.252	0.09	7.81969E-43	Tumor Tex
chr9-70563210-70564249	1.19949E-47	0.392294608	0.224	0.071	7.94337E-43	Tumor Tex
chr4-121138041-121138342	1.28925E-47	0.38434613	0.126	0.014	8.53778E-43	Tumor Tex
chr9-95852449-95852960	1.31007E-47	0.391169324	0.132	0.018	8.67569E-43	Tumor Tex
chr12-122646630-122647161	1.34952E-47	0.396341232	0.217	0.059	8.93692E-43	Tumor Tex
chr3-41419153-41419716	1.40905E-47	0.39375683	0.151	0.025	9.33118E-43	Tumor Tex
chr11-33722539-33723061	1.43788E-47	0.388111728	0.13	0.015	9.52205E-43	Tumor Tex
chr11-18616054-18616357	1.54014E-47	0.376197869	0.112	0.008	1.01992E-42	Tumor Tex
chr2-227775286-227775619	1.86551E-47	0.360459198	0.094	0.003	1.2354E-42	Tumor Tex
chr10-93379195-93380076	2.0579E-47	0.391485207	0.202	0.057	1.3628E-42	Tumor Tex
chr3-111690233-111690550	2.07992E-47	0.393861725	0.144	0.022	1.37738E-42	Tumor Tex

chr16-26616136-26616458	2.09535E-47	0.360777248	0.099	0.003	1.3876E-42	Tumor Tex
chr13-30773890-30774682	2.46798E-47	0.385797552	0.257	0.092	1.63437E-42	Tumor Tex
chr9-4530194-4531201	2.53058E-47	0.394634643	0.204	0.056	1.67583E-42	Tumor Tex
chr3-149224735-149225554	2.66292E-47	0.393632932	0.151	0.022	1.76346E-42	Tumor Tex
chr11-13297797-13298248	3.20212E-47	0.380302486	0.117	0.011	2.12054E-42	Tumor Tex
chr3-66472113-66473972	3.2779E-47	0.310430066	0.486	0.294	2.17073E-42	Tumor Tex
chr6-157800618-157801244	3.50272E-47	0.395598771	0.188	0.044	2.31961E-42	Tumor Tex
chr1-212225137-212226187	3.51054E-47	0.385166939	0.254	0.088	2.32478E-42	Tumor Tex
chr2-101473964-101475254	3.65636E-47	0.393859217	0.167	0.033	2.42135E-42	Tumor Tex
chr19-48978835-48979196	3.70055E-47	0.3907365	0.143	0.02	2.45061E-42	Tumor Tex
chr2-171326034-171326539	4.06861E-47	0.392715245	0.145	0.022	2.69436E-42	Tumor Tex
chr9-127906366-127906797	4.23716E-47	0.397642753	0.199	0.051	2.80598E-42	Tumor Tex
chr7-128963196-128963662	4.48436E-47	0.372886614	0.107	0.007	2.96968E-42	Tumor Tex
chr21-34128944-34129864	4.63288E-47	0.393934327	0.191	0.05	3.06803E-42	Tumor Tex
chr2-12293880-12294273	4.88919E-47	0.381314819	0.118	0.01	3.23777E-42	Tumor Tex
chr16-81626171-81627260	5.52526E-47	0.390151715	0.201	0.049	3.65899E-42	Tumor Tex
chr1-181134438-181135613	6.86215E-47	0.391858249	0.221	0.069	4.54432E-42	Tumor Tex
chr15-63479336-63480062	7.9865E-47	0.371601026	0.307	0.121	5.2889E-42	Tumor Tex
chr1-185636723-185637094	8.00193E-47	0.362281955	0.097	0.004	5.29912E-42	Tumor Tex
chr10-33136326-33137128	8.68261E-47	0.339307315	0.397	0.208	5.74989E-42	Tumor Tex
chr2-8345685-8347031	9.06791E-47	0.39446915	0.193	0.046	6.00504E-42	Tumor Tex
chr1-235872805-235873637	1.01696E-46	0.370169534	0.301	0.126	6.73461E-42	Tumor Tex
chr10-74055758-74056625	1.03276E-46	0.392635358	0.148	0.023	6.83924E-42	Tumor Tex
chr12-92470514-92470928	1.04049E-46	0.37326491	0.109	0.008	6.89042E-42	Tumor Tex
chr1-76023699-76023960	1.04963E-46	0.353459359	0.087	0.002	6.95098E-42	Tumor Tex
chr14-94056567-94057012	1.13583E-46	0.392532501	0.146	0.024	7.52182E-42	Tumor Tex
chr15-55829567-55830226	1.22671E-46	0.366674622	0.103	0.006	8.12365E-42	Tumor Tex

chr6-131532506-131532942	1.31223E-46	0.378779739	0.122	0.013	8.69001E-42	Tumor Tex
chr3-172550412-172550892	1.58464E-46	0.38954954	0.196	0.052	1.0494E-41	Tumor Tex
chr12-25377052-25377801	1.60592E-46	0.392101967	0.168	0.036	1.06349E-41	Tumor Tex
chr11-128422224-128423073	1.64847E-46	0.352773616	0.357	0.172	1.09167E-41	Tumor Tex
chr5-115209163-115209734	1.65016E-46	0.386565216	0.24	0.08	1.09278E-41	Tumor Tex
chr4-184319392-184319648	1.65554E-46	0.346569234	0.086	0.001	1.09635E-41	Tumor Tex
chr12-10303912-10304558	1.91191E-46	0.376847098	0.278	0.108	1.26612E-41	Tumor Tex
chr16-79627991-79628965	2.01306E-46	0.379949327	0.251	0.091	1.33311E-41	Tumor Tex
chr9-36383011-36383639	2.07309E-46	0.392917617	0.208	0.06	1.37286E-41	Tumor Tex
chr2-12948190-12949287	2.12386E-46	0.385698286	0.229	0.072	1.40648E-41	Tumor Tex
chr6-167113865-167114853	2.22704E-46	0.386742257	0.163	0.031	1.47481E-41	Tumor Tex
chr5-81284800-81285091	2.23099E-46	0.385510885	0.124	0.013	1.47743E-41	Tumor Tex
chr16-31354297-31355200	2.26484E-46	0.392077027	0.185	0.044	1.49984E-41	Tumor Tex
chr15-60761129-60761445	2.31087E-46	0.378540702	0.122	0.01	1.53033E-41	Tumor Tex
chr2-37506437-37506700	2.57184E-46	0.371011001	0.105	0.007	1.70315E-41	Tumor Tex
chr10-30783278-30785406	2.6306E-46	0.348119404	0.376	0.19	1.74206E-41	Tumor Tex
chr3-114412378-114412667	2.65135E-46	0.375803145	0.116	0.01	1.75581E-41	Tumor Tex
chr4-26075463-26076554	3.0536E-46	0.339733437	0.395	0.2	2.02219E-41	Tumor Tex
chr16-30350358-30350838	3.45682E-46	0.37597473	0.28	0.109	2.28921E-41	Tumor Tex
chr14-64232144-64232766	4.13196E-46	0.379400014	0.123	0.014	2.73631E-41	Tumor Tex
chr8-102096158-102096610	4.33182E-46	0.389775773	0.155	0.027	2.86866E-41	Tumor Tex
chr13-23695695-23696435	4.65397E-46	0.387429132	0.139	0.023	3.082E-41	Tumor Tex
chr2-111705671-111706269	4.68862E-46	0.389104625	0.163	0.031	3.10495E-41	Tumor Tex
chr5-156401908-156402946	4.90989E-46	0.384083934	0.228	0.071	3.25148E-41	Tumor Tex
chr2-113886559-113886769	4.97619E-46	0.386389992	0.142	0.023	3.29539E-41	Tumor Tex
chr9-6893637-6894481	5.0154E-46	0.367840361	0.104	0.008	3.32135E-41	Tumor Tex
chr7-55557657-55558150	6.37595E-46	0.374022026	0.114	0.01	4.22234E-41	Tumor Tex

chr12-12481602-12482788	6.42633E-46	0.357714469	0.332	0.155	4.25571E-41	Tumor Tex
chr12-68240841-68241316	6.54701E-46	0.391092081	0.172	0.038	4.33563E-41	Tumor Tex
chr2-135112138-135112811	6.66233E-46	0.389201929	0.159	0.032	4.41199E-41	Tumor Tex
chr5-55533902-55535527	6.81584E-46	0.287045397	0.549	0.365	4.51365E-41	Tumor Tex
chr6-170394033-170394326	7.42591E-46	0.372630679	0.111	0.009	4.91766E-41	Tumor Tex
chr10-79331239-79332103	7.93015E-46	0.374627431	0.272	0.103	5.25159E-41	Tumor Tex
chr16-80656430-80657130	8.1155E-46	0.384809903	0.152	0.025	5.37432E-41	Tumor Tex
chr14-71346394-71347012	1.07698E-45	0.381650684	0.134	0.02	7.13212E-41	Tumor Tex
chr6-106163048-106164775	1.20001E-45	0.31407014	0.458	0.276	7.94684E-41	Tumor Tex
chr4-94214428-94215405	1.20386E-45	0.388925372	0.184	0.043	7.97235E-41	Tumor Tex
chr2-7437217-7437437	1.28286E-45	0.379843823	0.129	0.015	8.4955E-41	Tumor Tex
chr6-45441519-45442249	1.29831E-45	0.391112829	0.194	0.048	8.59782E-41	Tumor Tex
chr6-135560852-135561078	1.33107E-45	0.370312636	0.108	0.008	8.81472E-41	Tumor Tex
chr18-62845048-62845847	1.36861E-45	0.364009357	0.103	0.008	9.06337E-41	Tumor Tex
chr10-49177021-49177858	1.41378E-45	0.380999267	0.229	0.08	9.36246E-41	Tumor Tex
chr12-9364265-9365196	1.46189E-45	0.377317272	0.258	0.099	9.68106E-41	Tumor Tex
chr3-151183263-151183757	1.58595E-45	0.380509588	0.126	0.017	1.05026E-40	Tumor Tex
chr4-152458498-152459621	1.68323E-45	0.385945504	0.207	0.064	1.11469E-40	Tumor Tex
chr21-14452973-14453692	1.82161E-45	0.376386748	0.119	0.014	1.20632E-40	Tumor Tex
chr2-112821116-112822041	1.85612E-45	0.386428487	0.198	0.055	1.22918E-40	Tumor Tex
chr1-200153142-200153701	1.99731E-45	0.385682995	0.22	0.062	1.32268E-40	Tumor Tex
chr16-72787221-72788263	2.26102E-45	0.38590105	0.186	0.042	1.49731E-40	Tumor Tex
chr5-143605224-143606724	2.26842E-45	0.378534253	0.252	0.091	1.50222E-40	Tumor Tex
chr9-89743241-89744283	2.72556E-45	0.379970157	0.136	0.018	1.80495E-40	Tumor Tex
chr10-112519038-112519467	2.9081E-45	0.373834557	0.122	0.012	1.92583E-40	Tumor Tex
chr2-46229316-46230424	3.69763E-45	0.363726398	0.3	0.131	2.44868E-40	Tumor Tex
chr8-78515522-78516693	3.87387E-45	0.370965504	0.281	0.109	2.56539E-40	Tumor Tex

chr13-44768304-44768554	4.0761E-45	0.361138031	0.1	0.005	2.69931E-40	Tumor Tex
chr3-114809564-114810216	4.78024E-45	0.386227549	0.167	0.036	3.16562E-40	Tumor Tex
chr9-20562040-20562373	5.366E-45	0.387302842	0.15	0.027	3.55353E-40	Tumor Tex
chr6-75830887-75831273	5.41498E-45	0.378369217	0.122	0.014	3.58596E-40	Tumor Tex
chr2-156527116-156527571	5.92056E-45	0.382369846	0.131	0.018	3.92078E-40	Tumor Tex
chr7-2772892-2773203	6.01443E-45	0.340357509	0.086	0.001	3.98294E-40	Tumor Tex
chr3-41188213-41188598	6.26357E-45	0.357522603	0.094	0.005	4.14792E-40	Tumor Tex
chr17-57460853-57461403	6.28739E-45	0.385772164	0.156	0.031	4.1637E-40	Tumor Tex
chr17-17416110-17416870	6.47977E-45	0.38601595	0.166	0.036	4.2911E-40	Tumor Tex
chr1-230231264-230231620	6.97641E-45	0.34878013	0.085	0.002	4.61999E-40	Tumor Tex
chr3-183435609-183436073	7.24698E-45	0.355494016	0.094	0.004	4.79917E-40	Tumor Tex
chr10-86835958-86836402	7.3835E-45	0.382099678	0.135	0.022	4.88957E-40	Tumor Tex
chr20-50569937-50570727	7.61107E-45	0.377109613	0.132	0.017	5.04028E-40	Tumor Tex
chrX-150482602-150483132	7.80839E-45	0.386001302	0.172	0.039	5.17095E-40	Tumor Tex
chr14-65206124-65207002	8.65894E-45	0.386683412	0.195	0.054	5.73421E-40	Tumor Tex
chr21-31446237-31446820	9.24881E-45	0.382142861	0.137	0.019	6.12484E-40	Tumor Tex
chr2-173354023-173355770	9.74247E-45	0.379150727	0.225	0.069	6.45175E-40	Tumor Tex
chr6-24936581-24936913	1.02131E-44	0.377419312	0.246	0.089	6.76344E-40	Tumor Tex
chr18-2644540-2644846	1.02997E-44	0.373613022	0.116	0.012	6.82074E-40	Tumor Tex
chr4-112327906-112328680	1.11873E-44	0.381830743	0.137	0.022	7.40859E-40	Tumor Tex
chr6-105898368-105898649	1.23784E-44	0.35426401	0.097	0.004	8.19737E-40	Tumor Tex
chr3-143170714-143171541	1.33946E-44	0.384366375	0.154	0.03	8.87028E-40	Tumor Tex
chr17-3770352-3771391	1.38803E-44	0.3702675	0.271	0.107	9.19192E-40	Tumor Tex
chr6-42339955-42340798	1.51609E-44	0.380586614	0.214	0.063	1.004E-39	Tumor Tex
chr2-111537223-111537561	1.53776E-44	0.365686349	0.105	0.009	1.01835E-39	Tumor Tex
chr17-1473834-1474123	1.67025E-44	0.363937959	0.107	0.008	1.10609E-39	Tumor Tex
chrX-19566023-19566896	1.93396E-44	0.381265669	0.197	0.057	1.28073E-39	Tumor Tex

chr5-78953220-78953565	1.97203E-44	0.344796594	0.086	0.002	1.30594E-39	Tumor Tex
chr10-63707117-63707656	2.1961E-44	0.38196456	0.197	0.052	1.45432E-39	Tumor Tex
chr15-90414487-90414983	2.23625E-44	0.383205612	0.2	0.057	1.48091E-39	Tumor Tex
chr10-43128964-43129634	2.42673E-44	0.385935142	0.167	0.038	1.60705E-39	Tumor Tex
chr2-28680740-28681832	2.62718E-44	0.384457046	0.16	0.033	1.7398E-39	Tumor Tex
chr2-230582874-230583258	2.66927E-44	0.366097125	0.114	0.01	1.76767E-39	Tumor Tex
chr20-17895638-17895842	2.75551E-44	0.323573877	0.076	0	1.82478E-39	Tumor Tex
chr3-105836394-105836981	2.79524E-44	0.378797614	0.23	0.077	1.85109E-39	Tumor Tex
chr21-35036382-35037297	2.90633E-44	0.381631428	0.215	0.067	1.92466E-39	Tumor Tex
chr15-40061174-40061804	3.07791E-44	0.381923161	0.151	0.029	2.03828E-39	Tumor Tex
chr19-12899883-12900090	3.69147E-44	0.340936495	0.083	0.002	2.4446E-39	Tumor Tex
chr5-126446468-126446774	3.77623E-44	0.370535183	0.117	0.012	2.50073E-39	Tumor Tex
chr12-45415368-45415707	3.89026E-44	0.359400621	0.102	0.007	2.57625E-39	Tumor Tex
chr1-92333706-92333975	3.9336E-44	0.36235627	0.103	0.007	2.60495E-39	Tumor Tex
chr2-227783439-227783780	4.41502E-44	0.3539754	0.097	0.004	2.92376E-39	Tumor Tex
chr19-13794570-13795881	4.64367E-44	0.327725237	0.413	0.23	3.07517E-39	Tumor Tex
chr2-196200112-196200896	5.3397E-44	0.383783102	0.194	0.055	3.53611E-39	Tumor Tex
chr6-73695202-73696438	5.72571E-44	0.371190397	0.239	0.085	3.79174E-39	Tumor Tex
chr15-85649337-85649693	6.06885E-44	0.384827336	0.197	0.057	4.01897E-39	Tumor Tex
chr11-109946051-109946694	7.21191E-44	0.369545752	0.117	0.012	4.77595E-39	Tumor Tex
chr14-68210395-68211363	7.49041E-44	0.362598241	0.297	0.12	4.96037E-39	Tumor Tex
chr4-147793927-147795018	8.49282E-44	0.381176905	0.206	0.056	5.6242E-39	Tumor Tex
chr4-121278862-121279077	8.85326E-44	0.33081419	0.078	0.001	5.86289E-39	Tumor Tex
chr6-78867056-78868030	9.24408E-44	0.375877825	0.239	0.079	6.12171E-39	Tumor Tex
chr6-135212509-135212736	1.01143E-43	0.343388528	0.088	0.002	6.69801E-39	Tumor Tex
chr11-102360573-102362042	1.12234E-43	0.378581773	0.211	0.061	7.4325E-39	Tumor Tex
chr1-51504933-51505468	1.33584E-43	0.376636605	0.14	0.024	8.84632E-39	Tumor Tex

chr11-9510547-9511363	1.33997E-43	0.379825066	0.15	0.027	8.87366E-39	Tumor Tex
chr6-2161521-2162105	1.4864E-43	0.379232323	0.146	0.029	9.84337E-39	Tumor Tex
chr10-102704929-102705316	1.85983E-43	0.343541035	0.087	0.003	1.23164E-38	Tumor Tex
chr5-59380368-59380713	1.88933E-43	0.368650593	0.116	0.012	1.25117E-38	Tumor Tex
chr9-37503459-37504182	1.9962E-43	0.380669301	0.179	0.044	1.32195E-38	Tumor Tex
chr12-3727890-3729074	2.04995E-43	0.34152666	0.358	0.173	1.35754E-38	Tumor Tex
chr17-29154774-29155846	2.35843E-43	0.378200994	0.218	0.069	1.56182E-38	Tumor Tex
chr8-132978752-132979545	2.37894E-43	0.372240564	0.123	0.016	1.57541E-38	Tumor Tex
chr22-20111025-20111737	2.39386E-43	0.378962256	0.219	0.07	1.58528E-38	Tumor Tex
chr1-12052038-12052332	2.43474E-43	0.37868406	0.142	0.023	1.61236E-38	Tumor Tex
chr2-73896226-73897070	2.6366E-43	0.378730381	0.157	0.034	1.74604E-38	Tumor Tex
chr9-89604248-89606232	2.65992E-43	0.313345286	0.447	0.256	1.76148E-38	Tumor Tex
chr8-115222389-115223405	2.8897E-43	0.37181736	0.237	0.084	1.91365E-38	Tumor Tex
chr4-98601594-98602508	3.31061E-43	0.378096404	0.197	0.052	2.19238E-38	Tumor Tex
chr1-222653429-222653799	3.61747E-43	0.373509457	0.125	0.018	2.3956E-38	Tumor Tex
chr21-33078857-33079331	3.79933E-43	0.37730264	0.201	0.055	2.51603E-38	Tumor Tex
chr11-2940361-2940861	4.04959E-43	0.380506119	0.169	0.043	2.68176E-38	Tumor Tex
chr13-50224446-50224842	4.24728E-43	0.379137971	0.164	0.036	2.81268E-38	Tumor Tex
chr15-88912790-88913865	4.32649E-43	0.375294373	0.219	0.071	2.86513E-38	Tumor Tex
chr10-79249785-79250052	4.41738E-43	0.369693047	0.121	0.015	2.92532E-38	Tumor Tex
chr2-144454539-144455211	4.96197E-43	0.370807875	0.236	0.084	3.28597E-38	Tumor Tex
chr12-47889034-47889303	5.07412E-43	0.357940429	0.101	0.007	3.36024E-38	Tumor Tex
chr6-87182301-87182640	5.18907E-43	0.368867173	0.125	0.017	3.43636E-38	Tumor Tex
chr14-68368597-68369160	5.29459E-43	0.354282566	0.1	0.007	3.50624E-38	Tumor Tex
chr11-111436571-111437023	5.46648E-43	0.367211673	0.118	0.014	3.62007E-38	Tumor Tex
chr1-160623049-160624086	5.69157E-43	0.350628018	0.316	0.146	3.76913E-38	Tumor Tex
chr12-68010972-68011537	5.76114E-43	0.35967805	0.11	0.01	3.8152E-38	Tumor Tex

chr2-213118936-213119809	6.31783E-43	0.375189989	0.203	0.057	4.18385E-38	Tumor Tex
chr1-234668249-234668467	6.69252E-43	0.352947612	0.096	0.006	4.43199E-38	Tumor Tex
chr6-12163141-12163569	6.7249E-43	0.369131223	0.124	0.016	4.45343E-38	Tumor Tex
chrX-46628838-46629216	7.03295E-43	0.350259291	0.1	0.006	4.65743E-38	Tumor Tex
chr8-29169948-29170535	7.37615E-43	0.377428708	0.15	0.029	4.8847E-38	Tumor Tex
chr5-52709247-52709826	7.42184E-43	0.375174014	0.138	0.024	4.91496E-38	Tumor Tex
chr4-75939666-75941215	8.18624E-43	0.297162055	0.474	0.304	5.42117E-38	Tumor Tex
chr12-110349725-110350113	8.85877E-43	0.348536346	0.096	0.006	5.86654E-38	Tumor Tex
chrX-101429872-101430825	9.05276E-43	0.378278212	0.18	0.044	5.99501E-38	Tumor Tex
chr1-147647920-147648708	1.10919E-42	0.377517146	0.196	0.054	7.34536E-38	Tumor Tex
chr15-69509085-69509433	1.20728E-42	0.361408421	0.112	0.01	7.99495E-38	Tumor Tex
chr11-1701155-1701664	1.26417E-42	0.353820619	0.098	0.007	8.37172E-38	Tumor Tex
chr18-3209835-3210154	1.3323E-42	0.371988841	0.124	0.017	8.8229E-38	Tumor Tex
chr14-71371754-71372298	1.35622E-42	0.36659909	0.119	0.015	8.98131E-38	Tumor Tex
chr10-63629170-63631009	1.40877E-42	0.327112945	0.389	0.218	9.32931E-38	Tumor Tex
chr1-202488203-202488429	1.4092E-42	0.336112918	0.083	0.002	9.33215E-38	Tumor Tex
chr14-65637567-65638190	1.56712E-42	0.367250251	0.253	0.094	1.0378E-37	Tumor Tex
chr19-13261007-13261436	1.57853E-42	0.377494676	0.162	0.036	1.04535E-37	Tumor Tex
chr11-19442082-19442601	1.91385E-42	0.34610852	0.096	0.006	1.26741E-37	Tumor Tex
chr13-42720476-42720705	2.28918E-42	0.347958796	0.095	0.006	1.51597E-37	Tumor Tex
chr1-93680748-93681909	2.99939E-42	0.365722502	0.123	0.018	1.98629E-37	Tumor Tex
chr6-157944986-157945284	3.0052E-42	0.366338538	0.12	0.015	1.99013E-37	Tumor Tex
chr8-25385044-25385510	3.01762E-42	0.366095317	0.121	0.016	1.99836E-37	Tumor Tex
chr22-22110906-22111759	3.20308E-42	0.37327861	0.152	0.028	2.12117E-37	Tumor Tex
chr8-73756042-73756408	3.2249E-42	0.352197513	0.099	0.007	2.13563E-37	Tumor Tex
chr10-88117178-88117576	3.39333E-42	0.358955015	0.108	0.01	2.24716E-37	Tumor Tex
chr8-28077934-28079095	3.48763E-42	0.376277213	0.192	0.054	2.30961E-37	Tumor Tex

chr1-200877869-200878550	4.01672E-42	0.342820215	0.326	0.151	2.65999E-37	Tumor Tex
chr12-92065017-92066011	4.2657E-42	0.372681536	0.163	0.038	2.82487E-37	Tumor Tex
chr11-34214870-34215264	4.26799E-42	0.367747354	0.133	0.021	2.82639E-37	Tumor Tex
chr13-99228620-99229186	4.37239E-42	0.375123374	0.167	0.04	2.89553E-37	Tumor Tex
chr11-77141936-77142504	4.44566E-42	0.372828962	0.172	0.042	2.94405E-37	Tumor Tex
chr1-23720418-23720922	4.50458E-42	0.33968737	0.091	0.004	2.98307E-37	Tumor Tex
chr3-106068461-106069158	5.25877E-42	0.36759604	0.241	0.09	3.48251E-37	Tumor Tex
chr2-241443397-241444221	5.28932E-42	0.3658601	0.249	0.093	3.50275E-37	Tumor Tex
chr6-11459928-11461016	5.47644E-42	0.361468825	0.115	0.013	3.62666E-37	Tumor Tex
chr3-4406529-4407100	5.4877E-42	0.367037595	0.126	0.018	3.63412E-37	Tumor Tex
chr3-46102483-46103018	5.49705E-42	0.372063931	0.21	0.067	3.64031E-37	Tumor Tex
chr12-10558323-10558663	5.50122E-42	0.37315779	0.204	0.065	3.64308E-37	Tumor Tex
chr14-63510235-63510947	5.64163E-42	0.374518857	0.183	0.05	3.73606E-37	Tumor Tex
chr6-11330098-11330685	5.71531E-42	0.350566607	0.098	0.008	3.78485E-37	Tumor Tex
chr20-52972038-52972614	5.77561E-42	0.356319837	0.107	0.009	3.82478E-37	Tumor Tex
chr1-221800726-221801674	6.78395E-42	0.332027168	0.351	0.172	4.49254E-37	Tumor Tex
chr1-224497642-224498527	6.94846E-42	0.356554152	0.279	0.117	4.60148E-37	Tumor Tex
chr12-12407712-12408661	8.13249E-42	0.370561635	0.139	0.025	5.38558E-37	Tumor Tex
chr7-585260-585669	8.43727E-42	0.374240405	0.161	0.036	5.58741E-37	Tumor Tex
chr18-9082536-9084042	8.4746E-42	0.371106098	0.216	0.069	5.61214E-37	Tumor Tex
chr14-75466399-75466684	9.15529E-42	0.339453973	0.087	0.004	6.06291E-37	Tumor Tex
chr20-1150179-1150487	9.23262E-42	0.362789973	0.115	0.013	6.11412E-37	Tumor Tex
chr6-42402098-42402605	1.11341E-41	0.369591559	0.228	0.076	7.37333E-37	Tumor Tex
chrX-39874428-39874759	1.1451E-41	0.37274232	0.156	0.036	7.58319E-37	Tumor Tex
chr10-3720283-3720811	1.31283E-41	0.359706079	0.119	0.015	8.69393E-37	Tumor Tex
chr8-37267462-37267892	1.45399E-41	0.327466304	0.078	0.002	9.62873E-37	Tumor Tex
chr12-93783792-93785545	1.51882E-41	0.33239037	0.36	0.181	1.00581E-36	Tumor Tex

chr18-13381809-13382793	1.58274E-41	0.369442322	0.204	0.06	1.04814E-36	Tumor Tex
chr9-133682884-133684437	1.80591E-41	0.312082899	0.42	0.233	1.19593E-36	Tumor Tex
chr3-12287547-12288804	1.96321E-41	0.346533016	0.098	0.006	1.30009E-36	Tumor Tex
chr2-213219582-213220321	2.11171E-41	0.344846207	0.097	0.008	1.39844E-36	Tumor Tex
chr6-158040135-158040587	2.17689E-41	0.368480376	0.141	0.026	1.4416E-36	Tumor Tex
chr8-29774520-29775217	2.24437E-41	0.346144961	0.098	0.007	1.48629E-36	Tumor Tex
chr18-13375048-13375693	2.31712E-41	0.36621787	0.242	0.087	1.53447E-36	Tumor Tex
chr22-39099225-39100450	2.3283E-41	0.330345791	0.37	0.19	1.54187E-36	Tumor Tex
chr2-147182124-147182640	2.49645E-41	0.371370812	0.149	0.029	1.65322E-36	Tumor Tex
chr9-109101188-109102238	2.84477E-41	0.351281142	0.291	0.127	1.88389E-36	Tumor Tex
chr12-31746712-31747070	3.21943E-41	0.371061056	0.144	0.026	2.13201E-36	Tumor Tex
chr2-190695531-190696245	3.27554E-41	0.357878302	0.257	0.096	2.16916E-36	Tumor Tex
chr20-53154547-53154789	3.56609E-41	0.321566756	0.073	0.001	2.36157E-36	Tumor Tex
chr14-88039261-88039553	3.58169E-41	0.351902235	0.103	0.008	2.3719E-36	Tumor Tex
chr8-78665332-78666780	4.33064E-41	0.360997709	0.24	0.088	2.86788E-36	Tumor Tex
chr2-230658811-230659578	4.36916E-41	0.366950642	0.204	0.066	2.89339E-36	Tumor Tex
chr4-5863280-5863876	4.45427E-41	0.364046088	0.126	0.019	2.94975E-36	Tumor Tex
chr13-39936940-39937511	4.56306E-41	0.351190922	0.106	0.01	3.02179E-36	Tumor Tex
chr8-125513004-125513480	4.82061E-41	0.370012109	0.205	0.062	3.19235E-36	Tumor Tex
chr1-7940383-7941380	4.84136E-41	0.360658128	0.253	0.094	3.2061E-36	Tumor Tex
chr10-122541510-122542016	4.88886E-41	0.372122373	0.166	0.041	3.23755E-36	Tumor Tex
chr11-117456452-117456912	4.96541E-41	0.345229411	0.099	0.007	3.28824E-36	Tumor Tex
chr3-169811805-169814158	5.12234E-41	0.327188535	0.372	0.208	3.39216E-36	Tumor Tex
chr16-12536063-12536272	5.76195E-41	0.336918194	0.088	0.004	3.81574E-36	Tumor Tex
chr17-64572606-64573386	5.7628E-41	0.3700166	0.143	0.028	3.8163E-36	Tumor Tex
chr12-69534458-69534763	6.22045E-41	0.351108561	0.103	0.009	4.11937E-36	Tumor Tex
chr10-103759005-103759372	6.85363E-41	0.364820191	0.133	0.023	4.53868E-36	Tumor Tex

chr12-10453107-10453564	6.90851E-41	0.359582175	0.113	0.016	4.57503E-36	Tumor Tex
chr3-177448852-177450071	7.2015E-41	0.366423713	0.187	0.052	4.76905E-36	Tumor Tex
chr3-171813902-171814427	8.37987E-41	0.367807384	0.146	0.032	5.5494E-36	Tumor Tex
chr6-4059043-4060201	8.79365E-41	0.317429028	0.393	0.214	5.82342E-36	Tumor Tex
chr3-125110445-125111081	8.85068E-41	0.368068443	0.148	0.03	5.86119E-36	Tumor Tex
chr17-17179309-17179692	8.92143E-41	0.371695556	0.156	0.034	5.90804E-36	Tumor Tex
chr15-40163435-40163881	9.68949E-41	0.362357738	0.126	0.019	6.41667E-36	Tumor Tex
chr1-12087648-12088983	1.01782E-40	0.368029547	0.156	0.036	6.74031E-36	Tumor Tex
chr1-230270504-230270805	1.07606E-40	0.350798388	0.101	0.009	7.12602E-36	Tumor Tex
chr12-24900820-24901375	1.11472E-40	0.36509937	0.208	0.067	7.38199E-36	Tumor Tex
chr6-42198011-42198321	1.12633E-40	0.348767367	0.101	0.01	7.45888E-36	Tumor Tex
chr1-32992407-32992873	1.15108E-40	0.340755986	0.095	0.006	7.62277E-36	Tumor Tex
chr1-234842660-234843258	1.17595E-40	0.36550234	0.196	0.06	7.78749E-36	Tumor Tex
chr2-179049192-179050220	1.21074E-40	0.364339191	0.137	0.024	8.01787E-36	Tumor Tex
chr6-157460785-157461297	1.27634E-40	0.355413872	0.114	0.014	8.45228E-36	Tumor Tex
chr2-11203416-11203851	1.27839E-40	0.35557932	0.111	0.012	8.46585E-36	Tumor Tex
chr11-74011214-74012147	1.39373E-40	0.365471226	0.138	0.025	9.22967E-36	Tumor Tex
chr2-217223326-217223802	1.47161E-40	0.36042312	0.125	0.018	9.74541E-36	Tumor Tex
chr2-200305832-200307800	1.50334E-40	0.359569641	0.242	0.088	9.95558E-36	Tumor Tex
chr14-68379719-68379996	1.50419E-40	0.347892534	0.097	0.008	9.96121E-36	Tumor Tex
chr2-190843881-190844683	1.54924E-40	0.362120817	0.143	0.029	1.02596E-35	Tumor Tex
chr5-53004778-53005333	1.55934E-40	0.365388664	0.2	0.061	1.03264E-35	Tumor Tex
chr3-196183286-196183793	1.60028E-40	0.362191871	0.238	0.09	1.05975E-35	Tumor Tex
chr6-11711725-11711963	1.62726E-40	0.342543706	0.092	0.006	1.07762E-35	Tumor Tex
chr19-44691671-44692560	1.74958E-40	0.368088969	0.193	0.052	1.15863E-35	Tumor Tex
chr9-35618928-35620075	1.78388E-40	0.360235763	0.236	0.088	1.18134E-35	Tumor Tex
chr6-87714200-87714832	1.84921E-40	0.358321411	0.235	0.088	1.2246E-35	Tumor Tex

chr2-127025191-127026006	2.07002E-40	0.365296428	0.201	0.064	1.37083E-35	Tumor Tex
chr11-60307627-60308114	2.43029E-40	0.326984038	0.082	0.003	1.60941E-35	Tumor Tex
chr1-185559613-185560348	2.5098E-40	0.365025717	0.216	0.074	1.66206E-35	Tumor Tex
chr14-88165036-88165391	2.75075E-40	0.346402082	0.096	0.008	1.82163E-35	Tumor Tex
chr1-172826069-172826761	2.86208E-40	0.364777239	0.148	0.032	1.89535E-35	Tumor Tex
chr19-6069144-6069632	2.90524E-40	0.363187259	0.136	0.025	1.92394E-35	Tumor Tex
chr5-91162354-91163099	2.92177E-40	0.364999605	0.144	0.029	1.93489E-35	Tumor Tex
chr11-191583-192335	2.9836E-40	0.361443971	0.13	0.02	1.97583E-35	Tumor Tex
chr21-45818488-45819285	3.01286E-40	0.36463565	0.187	0.052	1.99521E-35	Tumor Tex
chr15-99424968-99425526	3.0929E-40	0.345671657	0.098	0.009	2.04821E-35	Tumor Tex
chr9-94878856-94879081	3.42238E-40	0.333798911	0.083	0.004	2.2664E-35	Tumor Tex
chr1-184882186-184883369	3.70963E-40	0.345337836	0.278	0.122	2.45663E-35	Tumor Tex
chr6-2081357-2081723	3.76428E-40	0.342029655	0.092	0.007	2.49282E-35	Tumor Tex
chr20-63567117-63568346	4.02336E-40	0.363851844	0.221	0.076	2.66439E-35	Tumor Tex
chr6-45619745-45620349	4.34207E-40	0.345869089	0.103	0.009	2.87545E-35	Tumor Tex
chr7-111083250-111083802	4.38022E-40	0.357648488	0.237	0.089	2.90071E-35	Tumor Tex
chr1-222048260-222048560	4.77052E-40	0.352139097	0.108	0.011	3.15918E-35	Tumor Tex
chr4-123415989-123416490	5.01945E-40	0.363470176	0.14	0.028	3.32403E-35	Tumor Tex
chr10-50396624-50397532	5.46017E-40	0.366069342	0.154	0.034	3.61589E-35	Tumor Tex
chr5-148708837-148709255	5.95277E-40	0.354594441	0.113	0.015	3.9421E-35	Tumor Tex
chr1-167500396-167500800	6.05908E-40	0.366292703	0.161	0.04	4.01251E-35	Tumor Tex
chr16-15998179-15998719	6.26319E-40	0.330643423	0.083	0.004	4.14767E-35	Tumor Tex
chr8-58922567-58923113	6.54169E-40	0.362217124	0.165	0.04	4.3321E-35	Tumor Tex
chr7-77266544-77266757	6.69475E-40	0.32662237	0.079	0.003	4.43347E-35	Tumor Tex
chr8-27817793-27818333	6.86792E-40	0.346255562	0.1	0.01	4.54814E-35	Tumor Tex
chr9-91416247-91416653	7.25192E-40	0.365210452	0.151	0.033	4.80244E-35	Tumor Tex
chr10-87504295-87505612	7.44701E-40	0.355326779	0.248	0.096	4.93163E-35	Tumor Tex

chr1-173297936-173298478	8.21447E-40	0.335563922	0.092	0.006	5.43987E-35	Tumor Tex
chr10-124722834-124723658	8.29382E-40	0.32264643	0.367	0.196	5.49242E-35	Tumor Tex
chr3-9248931-9249841	8.46725E-40	0.358727261	0.227	0.08	5.60727E-35	Tumor Tex
chr2-28649354-28650232	8.69716E-40	0.363166379	0.184	0.05	5.75952E-35	Tumor Tex
chr20-3419999-3420607	9.16527E-40	0.359556637	0.232	0.085	6.06952E-35	Tumor Tex
chr7-135147131-135148836	9.73768E-40	0.313145024	0.392	0.224	6.44858E-35	Tumor Tex
chr10-126041608-126042736	1.01268E-39	0.362959773	0.162	0.038	6.70628E-35	Tumor Tex
chr18-35573439-35573878	1.11281E-39	0.357646815	0.123	0.018	7.36936E-35	Tumor Tex
chr12-123742195-123742499	1.11795E-39	0.35584599	0.116	0.016	7.40341E-35	Tumor Tex
chr11-11856447-11856982	1.14654E-39	0.362713073	0.138	0.025	7.5927E-35	Tumor Tex
chr8-100514611-100515752	1.27604E-39	0.363378241	0.196	0.059	8.45034E-35	Tumor Tex
chr14-51328027-51328306	1.32138E-39	0.30988754	0.072	0.001	8.75058E-35	Tumor Tex
chr13-45398836-45399263	1.36803E-39	0.363583154	0.16	0.038	9.05948E-35	Tumor Tex
chr14-61594151-61595092	1.58093E-39	0.358454067	0.229	0.081	1.04694E-34	Tumor Tex
chr13-109785441-109787258	1.13417E-17	0.314775219	0.425	0.254	7.5108E-13	C1_CD8_Tres
chr1-28144486-28144765	1.70552E-39	0.341897441	0.097	0.008	1.12945E-34	Tumor Tex
chr17-45461841-45462250	1.72842E-39	0.360068942	0.128	0.023	1.14461E-34	Tumor Tex
chr9-2621194-2622965	1.80398E-39	0.361811801	0.184	0.054	1.19465E-34	Tumor Tex
chr7-66635991-66636294	1.85138E-39	0.362708912	0.138	0.024	1.22604E-34	Tumor Tex
chr13-40096304-40096697	1.96341E-39	0.357652997	0.121	0.018	1.30023E-34	Tumor Tex
chr6-42356959-42358089	1.97069E-39	0.325132026	0.362	0.186	1.30505E-34	Tumor Tex
chr9-112037436-112038280	1.9737E-39	0.358169724	0.236	0.084	1.30704E-34	Tumor Tex
chr10-70324444-70324838	2.31079E-39	0.348448558	0.107	0.012	1.53028E-34	Tumor Tex
chr17-49695220-49696615	2.51469E-39	0.34513561	0.284	0.124	1.6653E-34	Tumor Tex
chr1-15290351-15290736	2.61187E-39	0.360265814	0.135	0.025	1.72966E-34	Tumor Tex
chr19-10797000-10798246	2.63137E-39	0.293135527	0.467	0.272	1.74257E-34	Tumor Tex
chr2-174752335-174753609	2.73514E-39	0.30098077	0.422	0.238	1.81129E-34	Tumor Tex

chr17-17408458-17408829	2.90033E-39	0.35105847	0.112	0.015	1.92068E-34	Tumor Tex
chr2-7671680-7672899	2.94693E-39	0.3600883	0.143	0.029	1.95155E-34	Tumor Tex
chr8-97979993-97980496	3.09716E-39	0.361229792	0.145	0.03	2.05103E-34	Tumor Tex
chr3-192850360-192851020	3.13571E-39	0.345008982	0.1	0.01	2.07656E-34	Tumor Tex
chr1-184160160-184160807	3.13786E-39	0.361835231	0.168	0.038	2.07798E-34	Tumor Tex
chr22-36781643-36782408	3.25733E-39	0.310605279	0.392	0.23	2.1571E-34	Tumor Tex
chr5-131538061-131538535	3.39272E-39	0.358647824	0.137	0.028	2.24676E-34	Tumor Tex
chr8-29737096-29737752	3.43053E-39	0.362706437	0.191	0.058	2.2718E-34	Tumor Tex
chrX-65865923-65866527	3.55528E-39	0.361472118	0.152	0.032	2.35441E-34	Tumor Tex
chr10-119197882-119198304	3.58335E-39	0.358124249	0.128	0.021	2.373E-34	Tumor Tex
chr2-174833373-174833983	3.61437E-39	0.34202514	0.099	0.009	2.39355E-34	Tumor Tex
chr5-111254115-111255070	3.67716E-39	0.359670862	0.193	0.059	2.43512E-34	Tumor Tex
chr4-10655600-10656257	3.94313E-39	0.340634729	0.1	0.007	2.61126E-34	Tumor Tex
chr14-74867615-74868083	3.94527E-39	0.356885978	0.12	0.017	2.61268E-34	Tumor Tex
chr22-42964939-42965601	3.97708E-39	0.349377426	0.111	0.012	2.63374E-34	Tumor Tex
chr4-75555423-75556172	4.08275E-39	0.362183273	0.161	0.038	2.70372E-34	Tumor Tex
chr2-42299373-42299656	4.24275E-39	0.347170302	0.104	0.011	2.80968E-34	Tumor Tex
chr3-12187424-12187681	4.28903E-39	0.319801184	0.079	0.002	2.84032E-34	Tumor Tex
chr11-111512497-111513383	4.3403E-39	0.336358746	0.093	0.007	2.87428E-34	Tumor Tex
chr13-50222470-50222942	4.58549E-39	0.361025416	0.147	0.031	3.03665E-34	Tumor Tex
chr8-29525976-29526636	4.62274E-39	0.357567415	0.228	0.083	3.06132E-34	Tumor Tex
chr9-94909528-94909810	4.88905E-39	0.320112908	0.077	0.003	3.23767E-34	Tumor Tex
chr2-144470926-144471678	4.92219E-39	0.362250674	0.153	0.035	3.25962E-34	Tumor Tex
chr1-200887786-200888672	5.02443E-39	0.348186876	0.257	0.11	3.32733E-34	Tumor Tex
chr1-234866088-234866705	5.20607E-39	0.358696878	0.152	0.035	3.44762E-34	Tumor Tex
chr17-64808298-64808961	5.71965E-39	0.360691365	0.212	0.073	3.78773E-34	Tumor Tex
chr15-31458953-31459387	5.87974E-39	0.324402038	0.082	0.004	3.89374E-34	Tumor Tex

chr11-77136127-77136338	6.0539E-39	0.338879927	0.096	0.008	4.00907E-34	Tumor Tex
chr20-52047717-52048245	6.43815E-39	0.345772562	0.106	0.011	4.26354E-34	Tumor Tex
chr20-50450224-50451398	6.45116E-39	0.361775848	0.193	0.057	4.27215E-34	Tumor Tex
chr8-29538499-29538937	6.63095E-39	0.347741949	0.105	0.012	4.39122E-34	Tumor Tex
chr2-174847101-174847726	7.08606E-39	0.336611019	0.094	0.008	4.6926E-34	Tumor Tex
chr15-70196426-70197229	7.43826E-39	0.358649614	0.145	0.032	4.92584E-34	Tumor Tex
chr20-50563221-50563453	7.63648E-39	0.342929	0.101	0.01	5.05711E-34	Tumor Tex
chr11-69166554-69166824	7.89732E-39	0.332820647	0.091	0.006	5.22984E-34	Tumor Tex
chr4-26092937-26093921	8.06515E-39	0.359050178	0.181	0.05	5.34098E-34	Tumor Tex
chr5-133398771-133399035	8.44417E-39	0.32633736	0.085	0.004	5.59198E-34	Tumor Tex
chr12-93771840-93772302	8.67134E-39	0.355822584	0.125	0.022	5.74242E-34	Tumor Tex
chr21-14547899-14548328	8.82475E-39	0.35013395	0.109	0.014	5.84402E-34	Tumor Tex
chr16-15636329-15636725	8.92398E-39	0.34410618	0.101	0.011	5.90973E-34	Tumor Tex
chr11-86592479-86593274	9.00197E-39	0.361322193	0.172	0.048	5.96138E-34	Tumor Tex
chr9-68974163-68974821	9.9716E-39	0.34921072	0.11	0.015	6.60349E-34	Tumor Tex
chr4-40763180-40763648	1.17728E-38	0.336368408	0.094	0.008	7.79628E-34	Tumor Tex
chr3-106176419-106177119	1.24403E-38	0.332482232	0.309	0.149	8.23832E-34	Tumor Tex
chr20-52974097-52974482	1.26597E-38	0.350002825	0.116	0.013	8.38364E-34	Tumor Tex
chr12-124493941-124494239	1.28146E-38	0.330268026	0.085	0.006	8.48624E-34	Tumor Tex
chr2-222423635-222425613	1.31083E-38	0.357712882	0.208	0.066	8.68074E-34	Tumor Tex
chr9-39809273-39810110	1.32161E-38	0.360931548	0.172	0.049	8.75212E-34	Tumor Tex
chr1-192983373-192983900	1.35145E-38	0.350983192	0.118	0.017	8.94972E-34	Tumor Tex
chr18-80147172-80148063	1.51856E-38	0.359385921	0.17	0.049	1.00564E-33	Tumor Tex
chr16-31386203-31386954	1.71381E-38	0.359425819	0.166	0.041	1.13494E-33	Tumor Tex
chr4-184273747-184274096	1.87011E-38	0.337370907	0.096	0.009	1.23844E-33	Tumor Tex
chr15-74983841-74984190	1.88731E-38	0.334831199	0.092	0.009	1.24983E-33	Tumor Tex
chr2-64698661-64699284	1.98911E-38	0.356053058	0.129	0.024	1.31725E-33	Tumor Tex

chr2-73931832-73932494	2.08846E-38	0.357059111	0.198	0.064	1.38304E-33	Tumor Tex
chr20-5844048-5844689	2.16419E-38	0.354781633	0.205	0.064	1.43319E-33	Tumor Tex
chr2-214737161-214737598	2.18444E-38	0.336222598	0.094	0.008	1.4466E-33	Tumor Tex
chr1-204493553-204495289	2.23067E-38	0.30101774	0.418	0.252	1.47722E-33	Tumor Tex
chr13-34156897-34157365	2.37257E-38	0.344911534	0.112	0.014	1.57119E-33	Tumor Tex
chr2-32688575-32688815	2.39466E-38	0.312302745	0.074	0.002	1.58582E-33	Tumor Tex
chr12-52243794-52244465	2.41458E-38	0.357908635	0.144	0.03	1.59901E-33	Tumor Tex
chr1-172818339-172819563	2.45044E-38	0.290344503	0.449	0.276	1.62276E-33	Tumor Tex
chr1-24947013-24947515	2.51162E-38	0.351667487	0.118	0.019	1.66327E-33	Tumor Tex
chr5-37722715-37723206	2.54023E-38	0.358938974	0.166	0.045	1.68222E-33	Tumor Tex
chr7-601351-602853	2.62569E-38	0.320616143	0.36	0.196	1.73881E-33	Tumor Tex
chr17-68399908-68400342	2.78502E-38	0.335896001	0.096	0.009	1.84433E-33	Tumor Tex
chr10-74853842-74854353	2.84347E-38	0.342066395	0.101	0.011	1.88303E-33	Tumor Tex
chr12-22687372-22687995	3.15804E-38	0.354130294	0.12	0.02	2.09135E-33	Tumor Tex
chr2-3222506-3223598	3.20533E-38	0.30286584	0.406	0.215	2.12267E-33	Tumor Tex
chr1-234975106-234975352	3.37272E-38	0.354709756	0.134	0.028	2.23351E-33	Tumor Tex
chr1-234867253-234867551	3.77885E-38	0.337607215	0.095	0.008	2.50247E-33	Tumor Tex
chr1-21779729-21780061	3.97772E-38	0.359288061	0.187	0.059	2.63416E-33	Tumor Tex
chr12-92461338-92462768	4.07911E-38	0.353868789	0.208	0.073	2.70131E-33	Tumor Tex
chr11-72140775-72141464	4.12896E-38	0.359423521	0.166	0.042	2.73432E-33	Tumor Tex
chr1-193537280-193538060	4.21923E-38	0.358078476	0.16	0.039	2.7941E-33	Tumor Tex
chr2-147297569-147298175	4.43833E-38	0.358530457	0.144	0.031	2.9392E-33	Tumor Tex
chr6-42292026-42292365	4.55876E-38	0.358574075	0.153	0.033	3.01895E-33	Tumor Tex
chr22-20624224-20624423	4.67944E-38	0.313564163	0.077	0.002	3.09886E-33	Tumor Tex
chr14-75559550-75560067	4.74185E-38	0.354649388	0.132	0.024	3.14019E-33	Tumor Tex
chr7-141585876-141586198	4.79326E-38	0.314134503	0.075	0.002	3.17424E-33	Tumor Tex
chr15-50176892-50177164	5.20653E-38	0.349124568	0.112	0.017	3.44792E-33	Tumor Tex

chr2-238003661-238004001	5.32836E-38	0.332007343	0.09	0.007	3.5286E-33	Tumor Tex
chr17-75297288-75297880	5.42347E-38	0.359059175	0.154	0.036	3.59159E-33	Tumor Tex
chr11-14544480-14544958	5.47739E-38	0.358152415	0.175	0.048	3.62729E-33	Tumor Tex
chr7-92768893-92769153	5.48485E-38	0.3326684	0.091	0.008	3.63223E-33	Tumor Tex
chr3-12948893-12949899	5.61209E-38	0.355549045	0.19	0.059	3.71649E-33	Tumor Tex
chr7-3103636-3103889	5.62306E-38	0.355885499	0.141	0.03	3.72376E-33	Tumor Tex
chr13-75308264-75308463	5.72575E-38	0.310187143	0.075	0.002	3.79176E-33	Tumor Tex
chr13-40132169-40132611	5.73491E-38	0.339741996	0.1	0.01	3.79783E-33	Tumor Tex
chr4-146243041-146243754	5.76304E-38	0.356703809	0.16	0.041	3.81646E-33	Tumor Tex
chr7-106345417-106347384	5.85009E-38	0.278369699	0.476	0.317	3.87411E-33	Tumor Tex
chr13-40402310-40402673	6.18964E-38	0.345387679	0.106	0.013	4.09897E-33	Tumor Tex
chr22-38373999-38374465	6.67078E-38	0.344200532	0.107	0.013	4.41759E-33	Tumor Tex
chr9-111916197-111916599	6.77899E-38	0.341816985	0.103	0.012	4.48925E-33	Tumor Tex
chr1-206469931-206471688	6.90602E-38	0.327367984	0.324	0.164	4.57338E-33	Tumor Tex
chr2-28547802-28548208	6.92271E-38	0.359071017	0.174	0.05	4.58443E-33	Tumor Tex
chr17-36721540-36722977	6.96511E-38	0.341478789	0.277	0.12	4.6125E-33	Tumor Tex
chrX-100994465-100994686	7.09511E-38	0.31796018	0.077	0.003	4.6986E-33	Tumor Tex
chr13-112688269-112690441	7.1749E-38	0.320053632	0.345	0.186	4.75143E-33	Tumor Tex
chr1-76292323-76292686	7.61211E-38	0.350528378	0.125	0.022	5.04097E-33	Tumor Tex
chr8-124713950-124714215	7.80655E-38	0.331659026	0.089	0.007	5.16973E-33	Tumor Tex
chr6-17673423-17673829	8.06182E-38	0.359023024	0.154	0.036	5.33878E-33	Tumor Tex
chr7-13989740-13991801	9.14969E-38	0.342140018	0.262	0.115	6.0592E-33	Tumor Tex
chr12-24902207-24903167	1.02139E-37	0.354926688	0.159	0.039	6.76395E-33	Tumor Tex
chr5-145946402-145946895	1.04896E-37	0.352954968	0.143	0.03	6.94654E-33	Tumor Tex
chr6-149151075-149151294	1.10334E-37	0.347533856	0.121	0.02	7.30662E-33	Tumor Tex
chr3-37451288-37452322	1.23476E-37	0.346410493	0.12	0.02	8.17694E-33	Tumor Tex
chr2-64213948-64215295	1.51026E-37	0.329348755	0.305	0.138	1.00014E-32	Tumor Tex

chr6-137791858-137792929	1.56982E-37	0.355860868	0.171	0.05	1.03958E-32	Tumor Tex
chr2-25586688-25587055	1.59042E-37	0.29925492	0.069	0.001	1.05323E-32	Tumor Tex
chr14-92717665-92717972	1.61843E-37	0.336846382	0.098	0.009	1.07177E-32	Tumor Tex
chr15-90852005-90853142	1.63638E-37	0.353525706	0.209	0.066	1.08366E-32	Tumor Tex
chr11-48006136-48006998	1.66233E-37	0.305877636	0.391	0.228	1.10085E-32	Tumor Tex
chr12-108568541-108569182	1.78897E-37	0.351137302	0.227	0.081	1.18471E-32	Tumor Tex
chr12-10320746-10320992	1.968E-37	0.333089378	0.096	0.008	1.30327E-32	Tumor Tex
chr15-50262113-50262934	1.9943E-37	0.353608708	0.198	0.064	1.32069E-32	Tumor Tex
chr9-91415249-91415615	2.13118E-37	0.351111212	0.121	0.022	1.41133E-32	Tumor Tex
chr18-13178957-13179288	2.21734E-37	0.335926413	0.099	0.011	1.46839E-32	Tumor Tex
chr2-24577445-24577803	2.25633E-37	0.348962853	0.123	0.019	1.49421E-32	Tumor Tex
chr1-116128321-116129119	2.4027E-37	0.342755969	0.113	0.018	1.59114E-32	Tumor Tex
chr2-46226640-46226849	2.41495E-37	0.34028519	0.103	0.013	1.59926E-32	Tumor Tex
chr8-59972386-59972880	2.4988E-37	0.347583932	0.12	0.019	1.65478E-32	Tumor Tex
chr1-23577342-23577600	2.52433E-37	0.325356765	0.085	0.005	1.67169E-32	Tumor Tex
chr9-96478616-96479366	2.75841E-37	0.346839892	0.119	0.018	1.8267E-32	Tumor Tex
chr2-25557976-25558688	2.81991E-37	0.340701209	0.262	0.115	1.86743E-32	Tumor Tex
chr11-63518178-63518906	2.86482E-37	0.355850335	0.174	0.049	1.89717E-32	Tumor Tex
chr6-79536665-79537700	2.98912E-37	0.349563875	0.201	0.07	1.97949E-32	Tumor Tex
chr14-96497080-96497405	3.1207E-37	0.339414259	0.104	0.013	2.06662E-32	Tumor Tex
chr6-149153713-149154622	3.16868E-37	0.353778188	0.169	0.044	2.09839E-32	Tumor Tex
chr3-171225092-171225499	3.31936E-37	0.35148734	0.135	0.029	2.19818E-32	Tumor Tex
chr3-59388463-59389194	3.38789E-37	0.346292974	0.114	0.017	2.24356E-32	Tumor Tex
chr15-50181975-50182934	3.47612E-37	0.351860186	0.195	0.066	2.30199E-32	Tumor Tex
chr20-25310236-25310958	3.75983E-37	0.354081344	0.19	0.059	2.48987E-32	Tumor Tex
chr17-68312556-68312780	3.90549E-37	0.329085697	0.089	0.006	2.58633E-32	Tumor Tex
chr15-90856526-90857139	3.92816E-37	0.352870533	0.155	0.036	2.60135E-32	Tumor Tex

chr9-131233206-131234405	4.40417E-37	0.342296488	0.25	0.103	2.91657E-32	Tumor Tex
chr2-12506916-12507720	4.43408E-37	0.339650369	0.268	0.112	2.93638E-32	Tumor Tex
chr20-62501785-62502282	4.46964E-37	0.350211109	0.211	0.073	2.95993E-32	Tumor Tex
chr13-77429481-77429963	4.89751E-37	0.333607564	0.102	0.011	3.24328E-32	Tumor Tex
chr5-40384034-40384519	5.11178E-37	0.35306155	0.151	0.038	3.38517E-32	Tumor Tex
chr1-100712436-100713975	5.17717E-37	0.303852638	0.377	0.219	3.42848E-32	Tumor Tex
chr2-203673961-203674200	5.25834E-37	0.327264737	0.09	0.007	3.48223E-32	Tumor Tex
chr9-114696737-114697683	5.3524E-37	0.326143023	0.32	0.15	3.54452E-32	Tumor Tex
chr20-43736661-43737046	5.80371E-37	0.317212158	0.083	0.005	3.84339E-32	Tumor Tex
chr16-26666475-26666805	5.93197E-37	0.314557909	0.078	0.003	3.92833E-32	Tumor Tex
chr1-200869806-200870256	6.20572E-37	0.344548125	0.116	0.018	4.10961E-32	Tumor Tex
chr10-119807940-119808556	6.217E-37	0.309518393	0.073	0.003	4.11708E-32	Tumor Tex
chr17-50918950-50920059	6.37331E-37	0.333529214	0.279	0.121	4.2206E-32	Tumor Tex
chr17-19284194-19284800	6.73365E-37	0.329261166	0.091	0.008	4.45923E-32	Tumor Tex
chr13-51518711-51519202	6.87835E-37	0.33657988	0.1	0.013	4.55505E-32	Tumor Tex
chr12-75655246-75655494	6.9794E-37	0.323104334	0.085	0.006	4.62197E-32	Tumor Tex
chr2-203867549-203867895	7.12237E-37	0.35426657	0.154	0.038	4.71665E-32	Tumor Tex
chr2-238439071-238440133	7.34838E-37	0.313129056	0.348	0.177	4.86632E-32	Tumor Tex
chr12-68482486-68483093	7.43398E-37	0.349281661	0.19	0.066	4.923E-32	Tumor Tex
chr7-610497-611001	7.99841E-37	0.34343765	0.117	0.019	5.29678E-32	Tumor Tex
chr8-58850557-58851235	9.06154E-37	0.348212072	0.2	0.065	6.00082E-32	Tumor Tex
chr19-11581700-11582051	9.52941E-37	0.349687335	0.127	0.024	6.31066E-32	Tumor Tex
chr7-139886116-139886553	1.01605E-36	0.333527209	0.097	0.01	6.72858E-32	Tumor Tex
chr21-35138340-35139519	1.05919E-36	0.340289661	0.242	0.099	7.01424E-32	Tumor Tex
chr9-114484722-114485381	1.16902E-36	0.334228104	0.27	0.126	7.74163E-32	Tumor Tex
chr3-58420120-58420392	1.44551E-36	0.324249247	0.087	0.006	9.57258E-32	Tumor Tex
chr4-184282153-184282762	1.47123E-36	0.330047747	0.277	0.133	9.74291E-32	Tumor Tex

chr19-45614183-45614593	1.62029E-36	0.352566689	0.159	0.041	1.07301E-31	Tumor Tex
chr13-39016089-39017050	1.64825E-36	0.350720175	0.144	0.03	1.09152E-31	Tumor Tex
chr13-77729452-77730220	1.65364E-36	0.346089455	0.134	0.028	1.09509E-31	Tumor Tex
chr1-144460773-144461736	1.65752E-36	0.352067206	0.184	0.057	1.09766E-31	Tumor Tex
chr20-3045278-3046631	1.69607E-36	0.322503962	0.324	0.164	1.12319E-31	Tumor Tex
chr22-37273467-37273731	1.73987E-36	0.338642731	0.105	0.014	1.15219E-31	Tumor Tex
chr2-102301673-102302207	1.75168E-36	0.350991181	0.145	0.034	1.16002E-31	Tumor Tex
chr14-72800890-72801632	1.81859E-36	0.350797211	0.151	0.038	1.20433E-31	Tumor Tex
chr2-43508174-43508655	1.82657E-36	0.352067215	0.16	0.038	1.20961E-31	Tumor Tex
chr15-55244987-55245400	1.8484E-36	0.351221356	0.138	0.031	1.22406E-31	Tumor Tex
chr5-157072417-157072785	2.07965E-36	0.341208997	0.112	0.018	1.37721E-31	Tumor Tex
chr14-101927949-101928163	2.26E-36	0.29943376	0.068	0.001	1.49664E-31	Tumor Tex
chr6-3850521-3850997	2.26147E-36	0.338515471	0.113	0.018	1.49761E-31	Tumor Tex
chr6-90049249-90049639	2.34895E-36	0.337094084	0.103	0.013	1.55555E-31	Tumor Tex
chr21-25298504-25299400	2.43511E-36	0.346478346	0.135	0.026	1.6126E-31	Tumor Tex
chr14-69685143-69686044	2.51651E-36	0.348019742	0.164	0.043	1.66651E-31	Tumor Tex
chr6-127420356-127420962	2.61064E-36	0.337213879	0.252	0.109	1.72885E-31	Tumor Tex
chr6-89145460-89146826	2.6526E-36	0.313918805	0.345	0.19	1.75663E-31	Tumor Tex
chr19-38551270-38551800	2.66176E-36	0.350241813	0.157	0.039	1.7627E-31	Tumor Tex
chr13-30047559-30047761	2.79113E-36	0.329373203	0.09	0.007	1.84837E-31	Tumor Tex
chr20-49893536-49894387	2.79975E-36	0.348252005	0.134	0.028	1.85408E-31	Tumor Tex
chr1-193539802-193540816	2.82347E-36	0.350219008	0.172	0.05	1.86979E-31	Tumor Tex
chr2-208013529-208014220	2.97278E-36	0.336311702	0.109	0.018	1.96866E-31	Tumor Tex
chr8-19756298-19758064	3.0519E-36	0.345522418	0.22	0.08	2.02106E-31	Tumor Tex
chr6-4790049-4790786	3.17737E-36	0.34810371	0.141	0.03	2.10415E-31	Tumor Tex
chr16-46883506-46884647	3.37907E-36	0.345263616	0.214	0.081	2.23772E-31	Tumor Tex
chr4-165215254-165216004	3.41637E-36	0.349611517	0.149	0.039	2.26242E-31	Tumor Tex

chr1-114745276-114745704	3.5484E-36	0.339630825	0.113	0.017	2.34986E-31	Tumor Tex
chr11-66317488-66318193	3.56447E-36	0.342106441	0.214	0.08	2.3605E-31	Tumor Tex
chr20-23399477-23400073	3.61845E-36	0.3518167	0.154	0.038	2.39624E-31	Tumor Tex
chr11-120248427-120248993	3.62708E-36	0.334393722	0.102	0.013	2.40196E-31	Tumor Tex
chr16-11328304-11328880	3.64089E-36	0.338650468	0.109	0.017	2.41111E-31	Tumor Tex
chr15-48966117-48966974	3.65815E-36	0.337612651	0.244	0.104	2.42254E-31	Tumor Tex
chr1-76153980-76155041	3.67076E-36	0.348691234	0.197	0.069	2.43089E-31	Tumor Tex
chr2-74031888-74032866	3.69813E-36	0.346097145	0.22	0.076	2.44901E-31	Tumor Tex
chr18-79156679-79157477	4.03011E-36	0.346897516	0.205	0.07	2.66886E-31	Tumor Tex
chr4-174358650-174359167	4.0865E-36	0.344020144	0.118	0.019	2.7062E-31	Tumor Tex
chr11-6351788-6352103	4.38077E-36	0.314012462	0.079	0.004	2.90108E-31	Tumor Tex
chr1-23624092-23624707	4.42784E-36	0.345757515	0.203	0.065	2.93225E-31	Tumor Tex
chr1-227765833-227766694	4.46874E-36	0.298255793	0.386	0.222	2.95934E-31	Tumor Tex
chr2-65319575-65320709	4.58981E-36	0.33771014	0.233	0.092	3.03951E-31	Tumor Tex
chr14-71697800-71698396	4.67845E-36	0.342046233	0.118	0.021	3.09821E-31	Tumor Tex
chr2-161136421-161137297	4.69528E-36	0.326174115	0.306	0.152	3.10935E-31	Tumor Tex
chr18-3304866-3306168	5.2881E-36	0.292342724	0.392	0.23	3.50194E-31	Tumor Tex
chr17-55116324-55117300	5.35866E-36	0.323159196	0.093	0.008	3.54866E-31	Tumor Tex
chr7-20330291-20331687	5.68587E-36	0.339587869	0.12	0.022	3.76535E-31	Tumor Tex
chr10-11863185-11864069	5.69083E-36	0.345544379	0.19	0.061	3.76864E-31	Tumor Tex
chr6-105810379-105810906	5.90107E-36	0.346908218	0.151	0.038	3.90786E-31	Tumor Tex
chr10-102729103-102729514	6.11044E-36	0.33448704	0.1	0.013	4.04652E-31	Tumor Tex
chr15-60798724-60799364	6.22242E-36	0.350675084	0.169	0.048	4.12068E-31	Tumor Tex
chr9-99253629-99254311	6.23799E-36	0.348358648	0.169	0.047	4.13098E-31	Tumor Tex
chr15-90807686-90807933	6.4569E-36	0.304242793	0.073	0.002	4.27595E-31	Tumor Tex
chr9-2657395-2657704	6.73552E-36	0.334772695	0.105	0.012	4.46046E-31	Tumor Tex
chr5-54717445-54718241	6.82917E-36	0.345786385	0.137	0.03	4.52248E-31	Tumor Tex

chr12-27969875-27970750	7.11228E-36	0.339463205	0.121	0.021	4.70997E-31	Tumor Tex
chr4-140103422-140104105	7.38125E-36	0.343185183	0.21	0.076	4.88809E-31	Tumor Tex
chr11-1727091-1727419	7.58896E-36	0.342868421	0.12	0.022	5.02564E-31	Tumor Tex
chr17-45583380-45586189	7.67075E-36	0.251416343	0.548	0.403	5.0798E-31	Tumor Tex
chr6-155188845-155189374	7.74228E-36	0.348331026	0.146	0.036	5.12717E-31	Tumor Tex
chr21-34946331-34947178	8.26502E-36	0.347688655	0.164	0.045	5.47334E-31	Tumor Tex
chr12-12645334-12646201	8.35704E-36	0.350275639	0.165	0.048	5.53428E-31	Tumor Tex
chr1-235876399-235876880	8.59586E-36	0.347811591	0.187	0.061	5.69244E-31	Tumor Tex
chr9-91395649-91396081	8.76004E-36	0.320900498	0.087	0.007	5.80116E-31	Tumor Tex
chr5-147781983-147783016	8.8182E-36	0.324402844	0.291	0.138	5.83968E-31	Tumor Tex
chr6-52231513-52232101	9.00707E-36	0.345064279	0.136	0.029	5.96475E-31	Tumor Tex
chr2-9463325-9463947	9.13147E-36	0.344583786	0.147	0.035	6.04714E-31	Tumor Tex
chrX-20352521-20353116	9.73493E-36	0.327442303	0.095	0.01	6.44676E-31	Tumor Tex
chr16-84632-85149	1.09321E-35	0.342630811	0.133	0.03	7.23957E-31	Tumor Tex
chr6-138946733-138947227	1.1088E-35	0.339109053	0.121	0.024	7.34283E-31	Tumor Tex
chr15-59299651-59300706	1.16281E-35	0.333971212	0.255	0.112	7.7005E-31	Tumor Tex
chr2-156473398-156473710	1.19927E-35	0.329434986	0.099	0.012	7.94195E-31	Tumor Tex
chr17-66541521-66542609	1.21193E-35	0.345729703	0.146	0.033	8.02575E-31	Tumor Tex
chr8-42753698-42753916	1.31705E-35	0.305781716	0.073	0.003	8.72187E-31	Tumor Tex
chr1-160459809-160461171	1.38443E-35	0.338747878	0.119	0.022	9.1681E-31	Tumor Tex
chr5-40479909-40480810	1.4095E-35	0.337375526	0.243	0.099	9.33416E-31	Tumor Tex
chr3-193963989-193964426	1.43207E-35	0.293031564	0.068	0.001	9.48358E-31	Tumor Tex
chr16-75108756-75109282	1.44471E-35	0.337420259	0.242	0.098	9.5673E-31	Tumor Tex
chr6-166950318-166951589	1.45761E-35	0.304194563	0.354	0.194	9.65274E-31	Tumor Tex
chr1-109890174-109890515	1.54398E-35	0.319239318	0.084	0.007	1.02247E-30	Tumor Tex
chr12-10503277-10503827	1.55246E-35	0.34898001	0.19	0.065	1.02809E-30	Tumor Tex
chr5-132293184-132295501	1.60045E-35	0.302532299	0.368	0.208	1.05986E-30	Tumor Tex

chr2-19868580-19869240	1.61519E-35	0.344970891	0.169	0.05	1.06963E-30	Tumor Tex
chr9-88829276-88829583	1.65206E-35	0.324296903	0.089	0.01	1.09405E-30	Tumor Tex
chr20-47274406-47274862	1.66268E-35	0.347827241	0.152	0.038	1.10107E-30	Tumor Tex
chr10-283817-284346	1.67728E-35	0.337369152	0.112	0.018	1.11075E-30	Tumor Tex
chr6-44976789-44977679	1.73699E-35	0.341415909	0.121	0.022	1.15028E-30	Tumor Tex
chr2-238025045-238025346	1.78854E-35	0.32082618	0.086	0.008	1.18442E-30	Tumor Tex
chr6-119313756-119315180	1.79614E-35	0.310880634	0.329	0.174	1.18946E-30	Tumor Tex
chr1-59333031-59333336	1.80553E-35	0.310911498	0.08	0.004	1.19568E-30	Tumor Tex
chr9-91515014-91515510	1.8327E-35	0.323850656	0.094	0.01	1.21367E-30	Tumor Tex
chr13-26325838-26326177	2.02675E-35	0.325041116	0.093	0.009	1.34217E-30	Tumor Tex
chr14-71566296-71566575	2.03723E-35	0.324858099	0.091	0.009	1.34911E-30	Tumor Tex
chr11-95715656-95716162	2.09731E-35	0.336117746	0.11	0.016	1.3889E-30	Tumor Tex
chr21-44143493-44143703	2.10686E-35	0.330430035	0.103	0.014	1.39523E-30	Tumor Tex
chr13-27132581-27132869	2.16428E-35	0.312243105	0.082	0.005	1.43325E-30	Tumor Tex
chr3-45567787-45568011	2.25901E-35	0.296744854	0.068	0.001	1.49599E-30	Tumor Tex
chr2-237752741-237753094	2.33881E-35	0.334801554	0.105	0.015	1.54883E-30	Tumor Tex
chr1-179034661-179035093	2.37618E-35	0.341928232	0.133	0.028	1.57358E-30	Tumor Tex
chr12-47390904-47392148	2.67548E-35	0.304648338	0.367	0.203	1.77178E-30	Tumor Tex
chr1-154863651-154863943	2.84139E-35	0.338519516	0.116	0.022	1.88165E-30	Tumor Tex
chr3-66456579-66457138	2.92294E-35	0.337941977	0.112	0.018	1.93566E-30	Tumor Tex
chrX-9644332-9644649	2.93825E-35	0.315565405	0.084	0.006	1.9458E-30	Tumor Tex
chrX-30659306-30659767	3.07407E-35	0.338800174	0.118	0.018	2.03574E-30	Tumor Tex
chr6-139539917-139540647	3.63966E-35	0.334405294	0.236	0.097	2.41029E-30	Tumor Tex
chr14-92846338-92846836	3.69727E-35	0.34023612	0.121	0.022	2.44844E-30	Tumor Tex
chr7-96685414-96686566	3.7078E-35	0.342175782	0.135	0.03	2.45541E-30	Tumor Tex
chr7-17260841-17261797	3.78048E-35	0.288535746	0.397	0.236	2.50355E-30	Tumor Tex
chr5-37737660-37738457	4.00756E-35	0.342134392	0.128	0.027	2.65393E-30	Tumor Tex

chr22-24400655-24401563	4.02924E-35	0.337851734	0.119	0.02	2.66828E-30	Tumor Tex
chr1-25012381-25012676	4.03902E-35	0.303606744	0.074	0.003	2.67476E-30	Tumor Tex
chr8-71830785-71831599	4.1304E-35	0.338310952	0.127	0.026	2.73527E-30	Tumor Tex
chr8-29469060-29469536	4.13277E-35	0.340771646	0.21	0.076	2.73684E-30	Tumor Tex
chr5-143446885-143447154	4.33437E-35	0.326874789	0.097	0.01	2.87035E-30	Tumor Tex
chr6-3977989-3978630	4.35166E-35	0.343967831	0.159	0.046	2.8818E-30	Tumor Tex
chr6-104789465-104790131	4.89389E-35	0.342919027	0.166	0.049	3.24088E-30	Tumor Tex
chr7-128937694-128939367	4.95096E-35	0.337360823	0.124	0.025	3.27868E-30	Tumor Tex
chr10-99555587-99556279	5.25418E-35	0.343266065	0.149	0.039	3.47948E-30	Tumor Tex
chr6-111776749-111777168	5.37116E-35	0.340235069	0.224	0.087	3.55694E-30	Tumor Tex
chr1-193569550-193570281	6.11983E-35	0.335364957	0.121	0.024	4.05273E-30	Tumor Tex
chr2-147623196-147623683	6.35996E-35	0.329471859	0.097	0.012	4.21175E-30	Tumor Tex
chr6-4914655-4915129	6.45179E-35	0.330151767	0.1	0.015	4.27257E-30	Tumor Tex
chr6-16657468-16657670	6.54096E-35	0.32798117	0.099	0.012	4.33162E-30	Tumor Tex
chr1-236159739-236160337	6.80923E-35	0.346599701	0.174	0.052	4.50927E-30	Tumor Tex
chr6-2862631-2863246	6.84584E-35	0.321265416	0.293	0.14	4.53352E-30	Tumor Tex
chr1-153790567-153791281	6.92357E-35	0.331872521	0.105	0.015	4.585E-30	Tumor Tex
chr6-41027482-41029030	6.94415E-35	0.335605993	0.236	0.1	4.59862E-30	Tumor Tex
chr4-89221977-89222526	7.0733E-35	0.337802962	0.121	0.022	4.68415E-30	Tumor Tex
chr10-22308327-22308717	7.6489E-35	0.341560104	0.13	0.027	5.06533E-30	Tumor Tex
chr10-103053813-103054760	7.89608E-35	0.326985892	0.272	0.123	5.22902E-30	Tumor Tex
chr10-26739054-26739667	8.06049E-35	0.312648889	0.086	0.007	5.3379E-30	Tumor Tex
chr1-12203804-12204426	8.16323E-35	0.343759394	0.159	0.044	5.40594E-30	Tumor Tex
chr5-43304507-43304885	8.19843E-35	0.332268232	0.107	0.015	5.42925E-30	Tumor Tex
chr8-115774598-115774979	8.51542E-35	0.321202345	0.089	0.01	5.63917E-30	Tumor Tex
chr7-121530116-121531141	8.7917E-35	0.274578273	0.438	0.272	5.82213E-30	Tumor Tex
chr21-36134639-36136206	9.00086E-35	0.327342375	0.273	0.127	5.96064E-30	Tumor Tex

chr16-50708575-50709338	9.64435E-35	0.338917944	0.126	0.025	6.38678E-30	Tumor Tex
chr9-131853303-131853745	9.78619E-35	0.298600729	0.069	0.002	6.48071E-30	Tumor Tex
chr10-49280127-49280329	9.82848E-35	0.297553275	0.068	0.003	6.50872E-30	Tumor Tex
chr12-2867617-2868383	9.84869E-35	0.336418587	0.222	0.087	6.5221E-30	Tumor Tex
chr10-17668755-17669923	1.00369E-34	0.340573787	0.137	0.03	6.64671E-30	Tumor Tex
chr4-121199383-121200997	1.045E-34	0.341304105	0.201	0.071	6.9203E-30	Tumor Tex
chr1-230109865-230110443	1.05323E-34	0.341464301	0.147	0.036	6.97479E-30	Tumor Tex
chr10-12122386-12123076	1.09743E-34	0.342742462	0.181	0.053	7.26752E-30	Tumor Tex
chr7-111012848-111013677	1.10669E-34	0.339242223	0.141	0.033	7.32881E-30	Tumor Tex
chr2-227805051-227806081	1.12872E-34	0.333250363	0.122	0.022	7.47473E-30	Tumor Tex
chr4-6134247-6134978	1.175E-34	0.339215582	0.214	0.083	7.78119E-30	Tumor Tex
chr1-100745698-100746504	1.21927E-34	0.34155568	0.137	0.032	8.07434E-30	Tumor Tex
chr3-194070541-194071198	1.23546E-34	0.325807289	0.102	0.013	8.18161E-30	Tumor Tex
chr15-90412728-90413209	1.28512E-34	0.338743712	0.125	0.026	8.51046E-30	Tumor Tex
chr22-31582276-31582734	1.38412E-34	0.319939595	0.09	0.008	9.16604E-30	Tumor Tex
chr7-98930458-98931376	1.45546E-34	0.325924666	0.267	0.123	9.63853E-30	Tumor Tex
chr14-92768181-92768601	1.50889E-34	0.32917299	0.101	0.013	9.99231E-30	Tumor Tex
chr8-125514527-125515197	1.57023E-34	0.321512621	0.278	0.13	1.03986E-29	Tumor Tex
chr13-39095526-39095828	1.70989E-34	0.307029895	0.077	0.005	1.13234E-29	Tumor Tex
chr18-63936022-63936995	1.81653E-34	0.336518374	0.131	0.028	1.20296E-29	Tumor Tex
chr12-107368588-107369974	1.82549E-34	0.340280149	0.143	0.031	1.2089E-29	Tumor Tex
chr2-8154900-8155263	1.91355E-34	0.30839061	0.08	0.006	1.26721E-29	Tumor Tex
chr21-45766897-45767511	1.99848E-34	0.322668626	0.093	0.01	1.32345E-29	Tumor Tex
chr14-71711005-71711376	2.04922E-34	0.309076773	0.077	0.005	1.35706E-29	Tumor Tex
chr20-59144248-59144825	2.06949E-34	0.324231991	0.269	0.128	1.37048E-29	Tumor Tex
chr11-115282257-115282471	2.13975E-34	0.274340797	0.059	0	1.41701E-29	Tumor Tex
chr9-84751744-84752169	2.16724E-34	0.317214974	0.089	0.008	1.43521E-29	Tumor Tex

chr12-10305448-10306148	2.23751E-34	0.342292104	0.178	0.055	1.48174E-29	Tumor Tex
chr10-50410005-50410729	2.42552E-34	0.339969825	0.138	0.033	1.60625E-29	Tumor Tex
chr5-100840250-100841122	2.43144E-34	0.343097601	0.171	0.05	1.61017E-29	Tumor Tex
chr6-493971-494519	2.80955E-34	0.340384322	0.139	0.034	1.86057E-29	Tumor Tex
chr16-29111778-29112539	2.92532E-34	0.331781184	0.216	0.085	1.93724E-29	Tumor Tex
chr3-197905979-197906934	3.03169E-34	0.336231411	0.217	0.083	2.00768E-29	Tumor Tex
chr2-105873347-105874080	3.03797E-34	0.342472478	0.169	0.05	2.01184E-29	Tumor Tex
chr11-65418746-65419322	3.14828E-34	0.303054678	0.359	0.199	2.08489E-29	Tumor Tex
chr19-47093633-47094206	3.21151E-34	0.342817002	0.161	0.046	2.12676E-29	Tumor Tex
chr20-56411601-56412587	3.31062E-34	0.330540989	0.241	0.103	2.19239E-29	Tumor Tex
chr22-29821281-29821587	3.4378E-34	0.310202273	0.083	0.007	2.27661E-29	Tumor Tex
chr2-39379575-39380016	3.55571E-34	0.310456985	0.082	0.005	2.3547E-29	Tumor Tex
chr1-28055924-28056648	4.89789E-34	0.339710106	0.148	0.036	3.24353E-29	Tumor Tex
chr22-21689281-21690526	5.22924E-34	0.338498238	0.184	0.06	3.46296E-29	Tumor Tex
chr19-46963125-46963331	5.30652E-34	0.322700301	0.095	0.013	3.51414E-29	Tumor Tex
chr2-64113954-64114539	5.65126E-34	0.341132878	0.147	0.036	3.74243E-29	Tumor Tex
chr17-65228721-65229431	6.08871E-34	0.332248049	0.224	0.089	4.03213E-29	Tumor Tex
chr16-79269224-79269811	6.38133E-34	0.336937412	0.141	0.033	4.22591E-29	Tumor Tex
chr11-129695394-129696343	6.47472E-34	0.335517745	0.143	0.037	4.28775E-29	Tumor Tex
chr12-123075421-123076983	6.8044E-34	0.268862158	0.45	0.292	4.50607E-29	Tumor Tex
chr2-239303924-239304587	7.04973E-34	0.339480578	0.158	0.046	4.66854E-29	Tumor Tex
chr7-95501235-95501808	7.05752E-34	0.321495846	0.095	0.012	4.6737E-29	Tumor Tex
chr8-81289047-81289507	7.10174E-34	0.329148934	0.112	0.019	4.70299E-29	Tumor Tex
chr3-45946402-45947147	7.23367E-34	0.328893667	0.228	0.094	4.79035E-29	Tumor Tex
chr6-16950655-16951127	7.29061E-34	0.310005693	0.085	0.006	4.82806E-29	Tumor Tex
chr1-200437656-200438159	7.47872E-34	0.322441174	0.099	0.013	4.95264E-29	Tumor Tex
chr7-44257461-44258470	7.7714E-34	0.331477937	0.225	0.095	5.14645E-29	Tumor Tex

chr20-44028897-44029554	7.86678E-34	0.33333486	0.121	0.023	5.20962E-29	Tumor Tex
chr10-182506-183219	7.96826E-34	0.325480947	0.1	0.014	5.27682E-29	Tumor Tex
chr12-3962350-3963101	8.20002E-34	0.338800069	0.152	0.038	5.4303E-29	Tumor Tex
chr3-195468031-195468568	8.40974E-34	0.304561445	0.076	0.005	5.56918E-29	Tumor Tex
chr1-116986501-116987514	8.64554E-34	0.339061863	0.156	0.042	5.72534E-29	Tumor Tex
chr2-171317556-171317835	9.05719E-34	0.316286549	0.091	0.01	5.99795E-29	Tumor Tex
chr22-36860760-36861356	9.57739E-34	0.325239994	0.102	0.015	6.34244E-29	Tumor Tex
chr18-80194820-80195119	1.07199E-33	0.304510449	0.076	0.004	7.09904E-29	Tumor Tex
chr1-182259000-182259827	1.11223E-33	0.312796148	0.302	0.151	7.36554E-29	Tumor Tex
chr13-75481381-75482730	1.14797E-33	0.337753074	0.154	0.045	7.60218E-29	Tumor Tex
chr1-199218089-199218401	1.17877E-33	0.311087098	0.084	0.008	7.80615E-29	Tumor Tex
chr3-119628453-119629060	1.17969E-33	0.304871113	0.08	0.006	7.81225E-29	Tumor Tex
chr3-46089311-46090471	1.18748E-33	0.337436499	0.162	0.051	7.86383E-29	Tumor Tex
chr10-95864826-95865523	1.19521E-33	0.334606994	0.134	0.031	7.91502E-29	Tumor Tex
chr16-17238336-17238754	1.19783E-33	0.31679976	0.088	0.01	7.93238E-29	Tumor Tex
chr4-123599529-123600338	1.23422E-33	0.336888481	0.163	0.048	8.17334E-29	Tumor Tex
chr2-39377315-39377716	1.28564E-33	0.290617598	0.067	0.002	8.51391E-29	Tumor Tex
chr8-59968754-59969205	1.2963E-33	0.318532978	0.092	0.011	8.58448E-29	Tumor Tex
chr17-57442992-57443476	1.30091E-33	0.338993772	0.16	0.045	8.61502E-29	Tumor Tex
chr16-20865494-20866400	1.3017E-33	0.31471575	0.089	0.009	8.62025E-29	Tumor Tex
chr7-8134050-8134677	1.32556E-33	0.333116014	0.223	0.091	8.77828E-29	Tumor Tex
chr6-135506117-135506934	1.35977E-33	0.337596968	0.154	0.043	9.00481E-29	Tumor Tex
chr6-129875669-129876370	1.36353E-33	0.323003807	0.1	0.015	9.0297E-29	Tumor Tex
chr6-154606273-154606870	1.4178E-33	0.335193162	0.143	0.033	9.3891E-29	Tumor Tex
chr3-4850143-4851451	1.42055E-33	0.302234425	0.341	0.196	9.40733E-29	Tumor Tex
chr11-118820529-118821183	1.43092E-33	0.27830348	0.061	0.001	9.47598E-29	Tumor Tex
chr6-45436654-45437106	1.46076E-33	0.339414019	0.172	0.052	9.6736E-29	Tumor Tex

chr5-52771652-52771937	1.48058E-33	0.336289276	0.133	0.033	9.80483E-29	Tumor Tex
chr1-172154455-172154891	1.58098E-33	0.322506559	0.096	0.014	1.04697E-28	Tumor Tex
chr19-43780774-43781495	1.7721E-33	0.302740148	0.335	0.17	1.17354E-28	Tumor Tex
chr2-232781689-232782204	1.87706E-33	0.331987553	0.203	0.073	1.24305E-28	Tumor Tex
chr2-8477345-8478641	1.93097E-33	0.320036695	0.272	0.13	1.27875E-28	Tumor Tex
chr3-119579299-119580381	1.99939E-33	0.335373491	0.175	0.056	1.32406E-28	Tumor Tex
chr10-7538899-7539434	2.02678E-33	0.319949112	0.095	0.014	1.3422E-28	Tumor Tex
chr20-25346402-25346630	2.05468E-33	0.313658485	0.086	0.008	1.36067E-28	Tumor Tex
chr18-657249-658870	2.06744E-33	0.331375385	0.209	0.081	1.36912E-28	Tumor Tex
chr1-230084247-230084928	2.15074E-33	0.332484131	0.195	0.069	1.42428E-28	Tumor Tex
chr16-80580013-80580858	2.26964E-33	0.283595833	0.394	0.239	1.50303E-28	Tumor Tex
chr8-133092300-133093018	2.27258E-33	0.335049872	0.134	0.033	1.50497E-28	Tumor Tex
chr1-27356407-27357763	2.33103E-33	0.305749474	0.337	0.193	1.54368E-28	Tumor Tex
chr6-105652043-105652242	2.4145E-33	0.325644344	0.104	0.015	1.59895E-28	Tumor Tex
chr6-446306-447925	2.65325E-33	0.29067646	0.379	0.221	1.75706E-28	Tumor Tex
chr3-133506130-133506809	2.656E-33	0.334945232	0.135	0.033	1.75888E-28	Tumor Tex
chr14-75541972-75544034	2.83569E-33	0.275333379	0.431	0.282	1.87788E-28	Tumor Tex
chr16-72482283-72483046	2.9096E-33	0.314100286	0.09	0.01	1.92682E-28	Tumor Tex
chr8-58927168-58927820	2.98721E-33	0.329710614	0.12	0.025	1.97822E-28	Tumor Tex
chr22-24486377-24486816	3.01012E-33	0.323370014	0.108	0.019	1.99339E-28	Tumor Tex
chr15-50089391-50089684	3.11118E-33	0.28505834	0.065	0.002	2.06032E-28	Tumor Tex
chr2-64541169-64541913	3.14419E-33	0.31956332	0.098	0.012	2.08218E-28	Tumor Tex
chr6-137738140-137738550	3.20013E-33	0.335509632	0.147	0.039	2.11922E-28	Tumor Tex
chr3-58323913-58324554	3.21787E-33	0.299164488	0.073	0.004	2.13097E-28	Tumor Tex
chr6-71287711-71289632	3.21974E-33	0.271853738	0.432	0.283	2.13221E-28	Tumor Tex
chr7-92648055-92648438	3.48837E-33	0.313447039	0.089	0.009	2.3101E-28	Tumor Tex
chr8-67505784-67506344	3.53321E-33	0.334148671	0.135	0.032	2.3398E-28	Tumor Tex

chr8-41822631-41823042	3.84319E-33	0.31288369	0.085	0.009	2.54508E-28	Tumor Tex
chr6-11342644-11343476	4.00485E-33	0.313939537	0.087	0.009	2.65213E-28	Tumor Tex
[truncated: 140,418 more chars]
