# Supplementary material for: Targeting pro-inflammatory T cells as a novel therapeutic approach to potentially resolve atherosclerosis in humans
Source: Cell Res. 2024 Mar 15;34(6):407–27. doi: 10.1038/s41422-024-00945-0 (PMC11143203; doi:10.1038/s41422-024-00945-0)
Supplement: Supplementary file 18 — Supplementary information, Table S6 [file 41422_2024_945_MOESM18_ESM.pdf]

Supplementary information, Table S6. Summary of TCR clonotypes and distributions of TCR clonotypes across T cell clusters and tissue

Summmary information of TCR clonotypes of T cells

Cell ID	Tissue	Class ID	TCR Clonotype Name	Tissue Sharing	Clonal State	Clonotype Sequence (CDR3 amino acid)	TCR Clonotype Counts	Cluster
PB-1_AAACCTGAGGGTGTGT	PB	PB-1	P1-425	FALSE	1	TRA:CAVAPSNQFYF TRB:CSARGRGLAGGPAYEQYF	1	CD8-C5
PB-1_AAACCTGAGTGAAGAG	PB	PB-1	P1-1	TRUE	3	TRA:CAMSDRMTGGFKTIF TRB:CASSQVGRGGEETQYF	1047	CD8-C7
PB-1_AAACCTGAGTGTCCAT	PB	PB-1	P1-1	TRUE	3	TRA:CAMSDRMTGGFKTIF TRB:CASSQVGRGGEETQYF	1047	CD8-C7
PB-1_AAACCTGGTCAGCTAT	PB	PB-1	P1-4	TRUE	3	TRA:CAMKGRDDKIIF TRB:CASTKGVSREAFF	226	CD8-C7
PB-1_AAACCTGTCGCCTGTT	PB	PB-1	P1-5	TRUE	3	TRA:CALSGTPMNRDDKIIF TRB:CSARGRGLAGGPAYEQYF	223	CD8-C5
PB-1_AAACCTGTCGTTGCCT	PB	PB-1	P1-1	TRUE	3	TRA:CAMSDRMTGGFKTIF TRB:CASSQVGRGGEETQYF	1047	CD8-C7
PB-1_AAACGGGAGCTCCTCT	PB	PB-1	P1-1	TRUE	3	TRA:CAMSDRMTGGFKTIF TRB:CASSQVGRGGEETQYF	1047	CD8-C7
PB-1_AAACGGGAGGAGTTTA	PB	PB-1	P1-8	TRUE	3	TRA:CAVRPPGRNSGGYQKVTF TRB:CASSRRGVTDTQYF	204	CD8-C7
PB-1_AAACGGGCAGCCTGTG	PB	PB-1	P1-1	TRUE	3	TRA:CAMSDRMTGGFKTIF TRB:CASSQVGRGGEETQYF	1047	CD8-C7
PB-1_AAACGGGCATTTGCTT	PB	PB-1	P1-31	TRUE	3	TRA:CAVEEGADRLMF TRB:CASSLLGGGELFF	46	CD8-C7
PB-1_AAACGGGGTTGAACTC	PB	PB-1	P1-1	TRUE	3	TRA:CAMSDRMTGGFKTIF TRB:CASSQVGRGGEETQYF	1047	CD8-C7
PB-1_AAACGGGGTTGGAGGT	PB	PB-1	P1-5	TRUE	3	TRA:CALSGTPMNRDDKIIF TRB:CSARGRGLAGGPAYEQYF	223	CD8-C7
PB-1_AAACGGGTCGGTCCGA	PB	PB-1	P1-3	TRUE	3	TRA:CAFLSGTYKYIF TRB:CASSQSPGQRNYGYTF	308	CD8-C7
PB-1_AAAGATGAGACAGACC	PB	PB-1	P1-16	TRUE	3	TRA:CALSGTPMNRDDKIIF TRB:CASSQSWDLAVLNEQFF	79	CD8-C7
PB-1_AAAGATGAGAGGTAGA	PB	PB-1	P1-1	TRUE	3	TRA:CAMSDRMTGGFKTIF TRB:CASSQVGRGGEETQYF	1047	CD8-C7
PB-1_AAAGATGAGTCATGCT	PB	PB-1	P1-29	TRUE	3	TRA:CATVSFSGGYNKLIF TRB:CASSLDRRGLSYEQYF	50	CD8-C7
PB-1_AAAGATGAGTCGTACT	PB	PB-1	P1-1	TRUE	3	TRA:CAMSDRMTGGFKTIF TRB:CASSQVGRGGEETQYF	1047	CD8-C7
PB-1_AAAGATGCAGTACACT	PB	PB-1	P1-1	TRUE	3	TRA:CAMSDRMTGGFKTIF TRB:CASSQVGRGGEETQYF	1047	CD8-C7
PB-1_AAAGATGCATACGCCG	PB	PB-1	P1-1	TRUE	3	TRA:CAMSDRMTGGFKTIF TRB:CASSQVGRGGEETQYF	1047	CD8-C7
PB-1_AAAGATGGTCCCTACT	PB	PB-1	P1-1	TRUE	3	TRA:CAMSDRMTGGFKTIF TRB:CASSQVGRGGEETQYF	1047	CD8-C5
PB-1_AAAGATGTCCAATGGT	PB	PB-1	P1-1	TRUE	3	TRA:CAMSDRMTGGFKTIF TRB:CASSQVGRGGEETQYF	1047	CD8-C7

PB-1_AAAGATGTCGCTTAGA	PB	PB-1	P1-15	TRUE	3	TRA:CAVNSSPLGANSKLTF TRB:CASSDSGNFYNEQFF	92	CD8-C2
PB-1_AAAGCAAAGTCGATAA	PB	PB-1	P1-3	TRUE	3	TRA:CAFLSGTYKYIF TRB:CASSQSPGQRNYGYTF	308	CD8-C7
PB-1_AAAGCAAGTCCTCTTG	PB	PB-1	P1-1	TRUE	3	TRA:CAMSDRMTGGFKTIF TRB:CASSQVGRGGEETQYF	1047	CD8-C7
PB-1_AAAGCAAGTCTCTTTA	PB	PB-1	P1-11	TRUE	3	TRA:CAVEPLYGSGNTGKLIF TRB:CASSSHGQLNQPQHF	147	CD8-C7
PB-1_AAAGCAAGTTCGCGAC	PB	PB-1	P1-18	TRUE	3	TRA:CVADGNYGQNFVF TRB:CASSSPGLGGGFDTEAFF	75	CD8-C6
PB-1_AAAGCAATCTACCTGC	PB	PB-1	P1-5	TRUE	3	TRA:CALSGTPMNRDDKIIF TRB:CSARGRGLAGGPAYEQYF	223	CD8-C7
PB-1_AAAGTAGGTCCGTTAA	PB	PB-1	P1-22	TRUE	3	TRA:CASNSGYSTLTF TRB:CASSQVTGGARDEQYF	65	CD8-C5
PB-1_AAAGTAGGTCGCCATG	PB	PB-1	P1-40	TRUE	3	TRA:CAVRDVGNTGFQKLVF TRB:CASSLGQGSMKTQYF	27	CD8-C7
PB-1_AAAGTAGGTTCAGCGC	PB	PB-1	P1-1	TRUE	3	TRA:CAMSDRMTGGFKTIF TRB:CASSQVGRGGEETQYF	1047	CD8-C7
PB-1_AAAGTAGTCATGCATG	PB	PB-1	P1-1	TRUE	3	TRA:CAMSDRMTGGFKTIF TRB:CASSQVGRGGEETQYF	1047	CD8-C7
PB-1_AAAGTAGTCGGTCTAA	PB	PB-1	P1-3	TRUE	3	TRA:CAFLSGTYKYIF TRB:CASSQSPGQRNYGYTF	308	CD8-C7
PB-1_AAATGCCAGAGCCTAG	PB	PB-1	P1-1	TRUE	3	TRA:CAMSDRMTGGFKTIF TRB:CASSQVGRGGEETQYF	1047	CD8-C7
PB-1_AAATGCCAGATATGGT	PB	PB-1	P1-2	TRUE	3	TRA:CGQNFVF TRB:CASNQRGGPSLYNEQFF	323	CD8-C7
PB-1_AAATGCCAGTTCCACA	PB	PB-1	P1-14	TRUE	3	TRA:CAASVVNDMRF TRB:CASSHRGGAKNIQYF	109	CD8-C5
PB-1_AAATGCCTCAGATAAG	PB	PB-1	P1-4	TRUE	3	TRA:CAMKGRDDKIIF TRB:CASTKGVSREAFF	226	CD8-C7
PB-1_AAATGCCTCGACCAGC	PB	PB-1	P1-431	FALSE	1	TRA:CASFSGGSQGNLIF TRB:CASSLTDRVPQFELVYEQYF	1	CD8-C7
PB-1_AACACGTCAGGACGTA	PB	PB-1	P1-11	TRUE	3	TRA:CAVEPLYGSGNTGKLIF TRB:CASSSHGQLNQPQHF	147	CD8-C7
PB-1_AACACGTGTATTAGCC	PB	PB-1	P1-3	TRUE	3	TRA:CAFLSGTYKYIF TRB:CASSQSPGQRNYGYTF	308	CD8-C7
PB-1_AACACGTTCAACACAC	PB	PB-1	P1-14	TRUE	3	TRA:CAASVVNDMRF TRB:CASSHRGGAKNIQYF	109	CD8-C5
PB-1_AACACGTTCCAGAGGA	PB	PB-1	P1-13	TRUE	3	TRA:CVVMNYGGSQGNLIF TRB:CASSSWGDTDEAFF	112	CD8-C7
PB-1_AACCATGAGAAACGAG	PB	PB-1	P1-8	TRUE	3	TRA:CAVRPPGRNSGGYQKVTF TRB:CASSRRGVTDTQYF	204	CD8-C7
PB-1_AACCATGCAAGGACTG	PB	PB-1	P1-1	TRUE	3	TRA:CAMSDRMTGGFKTIF TRB:CASSQVGRGGEETQYF	1047	CD8-C7
PB-1_AACCATGCATTCTCAT	PB	PB-1	P1-5	TRUE	3	TRA:CALSGTPMNRDDKIIF TRB:CSARGRGLAGGPAYEQYF	223	CD8-C7
PB-1_AACCATGTCCACGTGG	PB	PB-1	P1-3	TRUE	3	TRA:CAFLSGTYKYIF TRB:CASSQSPGQRNYGYTF	308	CD8-C7
PB-1_AACCATGTCGACGGAA	PB	PB-1	P1-5	TRUE	3	TRA:CALSGTPMNRDDKIIF TRB:CSARGRGLAGGPAYEQYF	223	CD8-C7
PB-1_AACCGCGAGCTAGCCC	PB	PB-1	P1-3	TRUE	3	TRA:CAFLSGTYKYIF TRB:CASSQSPGQRNYGYTF	308	CD8-C7
PB-1_AACCGCGTCACGACTA	PB	PB-1	P1-41	TRUE	3	TRA:CVVSEPPGGSYIPTF TRB:CASSSRPEDTQYF	27	CD4-C6

PB-1_AACCGCGTCACGCGGT	PB	PB-1	P1-1	TRUE	3	TRA:CAMSDRMTGGFKTIF TRB:CASSQVGRGGEETQYF	1047	CD8-C5
PB-1_AACCGCGTCGCGCCAA	PB	PB-1	P1-11	TRUE	3	TRA:CAVEPLYGSGNTGKLIF TRB:CASSSHGQLNQPQHF	147	CD8-C7
PB-1_AACGTTGCAAATACAG	PB	PB-1	P1-3	TRUE	3	TRA:CAFLSGTYKYIF TRB:CASSQSPGQRNYGYTF	308	CD8-C7
PB-1_AACGTTGCACAAGCCC	PB	PB-1	P1-1	TRUE	3	TRA:CAMSDRMTGGFKTIF TRB:CASSQVGRGGEETQYF	1047	CD8-C7
PB-1_AACGTTGTCAAGGCTT	PB	PB-1	P1-14	TRUE	3	TRA:CAASVVNDMRF TRB:CASSHRGGAKNIQYF	109	CD8-C5
PB-1_AACGTTGTCATCGATG	PB	PB-1	P1-1	TRUE	3	TRA:CAMSDRMTGGFKTIF TRB:CASSQVGRGGEETQYF	1047	CD8-C7
PB-1_AACTCAGAGATGAGAG	PB	PB-1	P1-10	TRUE	3	TRA:CISNSPMDSSYKLIF TRB:CASSSWGDTDEAFF	162	CD8-C7
PB-1_AACTCAGCACTCGACG	PB	PB-1	P1-13	TRUE	3	TRA:CVVMNYGGSQGNLIF TRB:CASSSWGDTDEAFF	112	CD8-C7
PB-1_AACTCAGCATCAGTAC	PB	PB-1	P1-1	TRUE	3	TRA:CAMSDRMTGGFKTIF TRB:CASSQVGRGGEETQYF	1047	CD8-C7
PB-1_AACTCAGGTCATGCCG	PB	PB-1	P1-5	TRUE	3	TRA:CALSGTPMNRDDKIIF TRB:CSARGRGLAGGPAYEQYF	223	CD8-C7
PB-1_AACTCCCAGATATGGT	PB	PB-1	P1-1	TRUE	3	TRA:CAMSDRMTGGFKTIF TRB:CASSQVGRGGEETQYF	1047	CD8-C7
PB-1_AACTCCCCAAAGGTGC	PB	PB-1	P1-4	TRUE	3	TRA:CAMKGRDDKIIF TRB:CASTKGVSREAFF	226	CD8-C7
PB-1_AACTCCCTCTTTCCTC	PB	PB-1	P1-85	FALSE	3	TRA:CAPRSGGSYIPTF TRB:CASSQDTGLDYGYTF	6	CD8-C7
PB-1_AACTCTTAGAGTTGGC	PB	PB-1	P1-3	TRUE	3	TRA:CAFLSGTYKYIF TRB:CASSQSPGQRNYGYTF	308	CD8-C7
PB-1_AACTCTTAGGATGTAT	PB	PB-1	P1-14	TRUE	3	TRA:CAASVVNDMRF TRB:CASSHRGGAKNIQYF	109	CD8-C7
PB-1_AACTCTTCAATTGCTG	PB	PB-1	P1-3	TRUE	3	TRA:CAFLSGTYKYIF TRB:CASSQSPGQRNYGYTF	308	CD8-C7
PB-1_AACTCTTCACACTGCG	PB	PB-1	P1-14	TRUE	3	TRA:CAASVVNDMRF TRB:CASSHRGGAKNIQYF	109	CD8-C7
PB-1_AACTCTTGTGCGAAAC	PB	PB-1	P1-441	FALSE	1	TRA:CVVMNYGGSQGNLIF TRB:CSARGRGLAGGPAYEQYF	1	CD8-C7
PB-1_AACTCTTGTTTAGCTG	PB	PB-1	P1-11	TRUE	3	TRA:CAVEPLYGSGNTGKLIF TRB:CASSSHGQLNQPQHF	147	CD8-C5
PB-1_AACTGGTAGGCAAAGA	PB	PB-1	P1-22	TRUE	3	TRA:CASNSGYSTLTF TRB:CASSQVTGGARDEQYF	65	CD8-C7
PB-1_AACTGGTAGTGCCATT	PB	PB-1	P1-4	TRUE	3	TRA:CAMKGRDDKIIF TRB:CASTKGVSREAFF	226	CD8-C7
PB-1_AACTGGTCAACACCTA	PB	PB-1	P1-1	TRUE	3	TRA:CAMSDRMTGGFKTIF TRB:CASSQVGRGGEETQYF	1047	CD8-C7
PB-1_AACTGGTCAAGGACAC	PB	PB-1	P1-61	TRUE	3	TRA:CAARGGSQGNLIF TRB:CASRPRHTDTQYF	10	CD8-C7
PB-1_AACTGGTCAGCGTCCA	PB	PB-1	P1-4	TRUE	3	TRA:CAMKGRDDKIIF TRB:CASTKGVSREAFF	226	CD8-C7
PB-1_AACTGGTCAGGCTCAC	PB	PB-1	P1-254	FALSE	2	TRA:CALTVHSSASKIIF TRB:CASSLPGRYSGANVLTF	2	CD8-C7
PB-1_AACTTTCAGGGATCTG	PB	PB-1	P1-3	TRUE	3	TRA:CAFLSGTYKYIF TRB:CASSQSPGQRNYGYTF	308	CD8-C7
PB-1_AACTTTCAGGGTGTTG	PB	PB-1	P1-3	TRUE	3	TRA:CAFLSGTYKYIF TRB:CASSQSPGQRNYGYTF	308	CD8-C7

PB-1_AACTTTCTCAGAGCTT	PB	PB-1	P1-3	TRUE	3	TRA:CAFLSGTYKYIF TRB:CASSQSPGQRNYGYTF	308	CD8-C7
PB-1_AAGACCTAGAGTCGGT	PB	PB-1	P1-1	TRUE	3	TRA:CAMSDRMTGGFKTIF TRB:CASSQVGRGGEETQYF	1047	CD8-C7
PB-1_AAGACCTAGCGTGTCC	PB	PB-1	P1-1	TRUE	3	TRA:CAMSDRMTGGFKTIF TRB:CASSQVGRGGEETQYF	1047	CD8-C7
PB-1_AAGACCTAGTGGTAGC	PB	PB-1	P1-2	TRUE	3	TRA:CGQNFVF TRB:CASNQRGGPSLYNEQFF	323	CD8-C7
PB-1_AAGACCTCACTGCCAG	PB	PB-1	P1-8	TRUE	3	TRA:CAVRPPGRNSGGYQKVTF TRB:CASSRRGVTDTQYF	204	CD8-C5
PB-1_AAGACCTCAGATCGGA	PB	PB-1	P1-446	FALSE	1	TRA:CALAHSGGGADGLTF TRB:CASTGDSSYEQYF	1	CD8-C11
PB-1_AAGACCTCAGGGATTG	PB	PB-1	P1-1	TRUE	3	TRA:CAMSDRMTGGFKTIF TRB:CASSQVGRGGEETQYF	1047	CD8-C5
PB-1_AAGACCTGTCCAGTAT	PB	PB-1	P1-2	TRUE	3	TRA:CGQNFVF TRB:CASNQRGGPSLYNEQFF	323	CD8-C7
PB-1_AAGACCTGTGGCCCTA	PB	PB-1	P1-10	TRUE	3	TRA:CISNSPMDSSYKLIF TRB:CASSSWGDTDEAFF	162	CD8-C7
PB-1_AAGACCTTCGGGAGTA	PB	PB-1	P1-47	TRUE	3	TRA:CIVGGNFNKFYF TRB:CASSPEWDTDTQYF	18	CD8-C5
PB-1_AAGACCTTCTACCAGA	PB	PB-1	P1-1	TRUE	3	TRA:CAMSDRMTGGFKTIF TRB:CASSQVGRGGEETQYF	1047	CD8-C7
PB-1_AAGCCGCAGATCGGGT	PB	PB-1	P1-3	TRUE	3	TRA:CAFLSGTYKYIF TRB:CASSQSPGQRNYGYTF	308	CD8-C7
PB-1_AAGCCGCAGGGTCGAT	PB	PB-1	P1-447	FALSE	1	TRA:CAMKGRDDKIIF TRB:CATQREGREQYF	1	CD8-C7
PB-1_AAGCCGCCATTCTCAT	PB	PB-1	P1-16	TRUE	3	TRA:CALSGTPMNRDDKIIF TRB:CASSQSWDLAVLNEQFF	79	CD8-C7
PB-1_AAGCCGCGTCATTAGC	PB	PB-1	P1-8	TRUE	3	TRA:CAVRPPGRNSGGYQKVTF TRB:CASSRRGVTDTQYF	204	CD8-C7
PB-1_AAGCCGCTCAGCGACC	PB	PB-1	P1-1	TRUE	3	TRA:CAMSDRMTGGFKTIF TRB:CASSQVGRGGEETQYF	1047	CD8-C5
PB-1_AAGCCGCTCTCTAAGG	PB	PB-1	P1-50	TRUE	3	TRA:CARWLGSQGNLIF TRB:CASSLTDRVPQFELVYEQYF	15	CD8-C7
PB-1_AAGGAGCAGAGCCTAG	PB	PB-1	P1-10	TRUE	3	TRA:CISNSPMDSSYKLIF TRB:CASSSWGDTDEAFF	162	CD8-C7
PB-1_AAGGAGCAGGCTCAGA	PB	PB-1	P1-14	TRUE	3	TRA:CAASVVNDMRF TRB:CASSHRGGAKNIQYF	109	CD8-C5
PB-1_AAGGAGCCAAAGCGGT	PB	PB-1	P1-2	TRUE	3	TRA:CGQNFVF TRB:CASNQRGGPSLYNEQFF	323	CD8-C7
PB-1_AAGGAGCCATTGTGCA	PB	PB-1	P1-2	TRUE	3	TRA:CGQNFVF TRB:CASNQRGGPSLYNEQFF	323	CD8-C7
PB-1_AAGGAGCGTTGATTCG	PB	PB-1	P1-4	TRUE	3	TRA:CAMKGRDDKIIF TRB:CASTKGVSREAFF	226	CD8-C7
PB-1_AAGGAGCTCACTTATC	PB	PB-1	P1-1	TRUE	3	TRA:CAMSDRMTGGFKTIF TRB:CASSQVGRGGEETQYF	1047	CD8-C7
PB-1_AAGGAGCTCAGATAAG	PB	PB-1	P1-42	TRUE	3	TRA:CASFSGGSQGNLIF TRB:CSAGQGSNQPQHF	24	CD4-C6
PB-1_AAGGAGCTCCAGTATG	PB	PB-1	P1-5	TRUE	3	TRA:CALSGTPMNRDDKIIF TRB:CSARGRGLAGGPAYEQYF	223	CD8-C7
PB-1_AAGGAGCTCTGCGTAA	PB	PB-1	P1-1	TRUE	3	TRA:CAMSDRMTGGFKTIF TRB:CASSQVGRGGEETQYF	1047	CD8-C7
PB-1_AAGGCAGCATCACCCT	PB	PB-1	P1-3	TRUE	3	TRA:CAFLSGTYKYIF TRB:CASSQSPGQRNYGYTF	308	CD8-C7

PB-1_AAGGCAGTCGTCTGCT	PB	PB-1	P1-1	TRUE	3	TRA:CAMSDRMTGGFKTIF TRB:CASSQVGRGGEETQYF	1047	CD8-C7
PB-1_AAGGCAGTCGTTTAGG	PB	PB-1	P1-1	TRUE	3	TRA:CAMSDRMTGGFKTIF TRB:CASSQVGRGGEETQYF	1047	CD8-C7
PB-1_AAGGTTCAGACTCGGA	PB	PB-1	P1-5	TRUE	3	TRA:CALSGTPMNRDDKIIF TRB:CSARGRGLAGGPAYEQYF	223	CD8-C7
PB-1_AAGGTTCAGTCCGTAT	PB	PB-1	P1-1	TRUE	3	TRA:CAMSDRMTGGFKTIF TRB:CASSQVGRGGEETQYF	1047	CD8-C7
PB-1_AAGGTTCCAATGCCAT	PB	PB-1	P1-8	TRUE	3	TRA:CAVRPPGRNSGGYQKVTF TRB:CASSRRGVTDTQYF	204	CD8-C7
PB-1_AAGGTTCGTTATTCTC	PB	PB-1	P1-1	TRUE	3	TRA:CAMSDRMTGGFKTIF TRB:CASSQVGRGGEETQYF	1047	CD8-C7
PB-1_AAGGTTCTCCAGTATG	PB	PB-1	P1-11	TRUE	3	TRA:CAVEPLYGSGNTGKLIF TRB:CASSSHGQLNQPQHF	147	CD8-C7
PB-1_AAGGTTCTCGCCAAAT	PB	PB-1	P1-1	TRUE	3	TRA:CAMSDRMTGGFKTIF TRB:CASSQVGRGGEETQYF	1047	CD8-C7
PB-1_AAGGTTCTCGTATCAG	PB	PB-1	P1-1	TRUE	3	TRA:CAMSDRMTGGFKTIF TRB:CASSQVGRGGEETQYF	1047	CD8-C7
PB-1_AAGTCTGAGTACGTTC	PB	PB-1	P1-22	TRUE	3	TRA:CASNSGYSTLTF TRB:CASSQVTGGARDEQYF	65	CD8-C5
PB-1_AAGTCTGCACGGCTAC	PB	PB-1	P1-13	TRUE	3	TRA:CVVMNYGGSQGNLIF TRB:CASSSWGDTDEAFF	112	CD8-C7
PB-1_AAGTCTGTCACAACGT	PB	PB-1	P1-4	TRUE	3	TRA:CAMKGRDDKIIF TRB:CASTKGVSREAFF	226	CD8-C7
PB-1_AAGTCTGTCCGCAGTG	PB	PB-1	P1-8	TRUE	3	TRA:CAVRPPGRNSGGYQKVTF TRB:CASSRRGVTDTQYF	204	CD8-C7
PB-1_AAGTCTGTCCTCAATT	PB	PB-1	P1-10	TRUE	3	TRA:CISNSPMDSSYKLIF TRB:CASSSWGDTDEAFF	162	CD8-C7
PB-1_AAGTCTGTCCTGCCAT	PB	PB-1	P1-6	TRUE	3	TRA:CAMREGRERDDKIIF TRB:CASSLPNTEAFF	216	CD8-C5
PB-1_AAGTCTGTCTCGGACG	PB	PB-1	P1-6	TRUE	3	TRA:CAMREGRERDDKIIF TRB:CASSLPNTEAFF	216	CD8-C5
PB-1_AATCCAGGTGACAAAT	PB	PB-1	P1-1	TRUE	3	TRA:CAMSDRMTGGFKTIF TRB:CASSQVGRGGEETQYF	1047	CD8-C7
PB-1_AATCGGTCATCGATGT	PB	PB-1	P1-1	TRUE	3	TRA:CAMSDRMTGGFKTIF TRB:CASSQVGRGGEETQYF	1047	CD8-C7
PB-1_AATCGGTGTGCTCTTC	PB	PB-1	P1-3	TRUE	3	TRA:CAFLSGTYKYIF TRB:CASSQSPGQRNYGYTF	308	CD8-C7
PB-1_AATCGGTGTGTAATGA	PB	PB-1	P1-2	TRUE	3	TRA:CGQNFVF TRB:CASNQRGGPSLYNEQFF	323	CD8-C7
PB-1_AATCGGTTCAGGCAAG	PB	PB-1	P1-3	TRUE	3	TRA:CAFLSGTYKYIF TRB:CASSQSPGQRNYGYTF	308	CD8-C7
PB-1_AATCGGTTCCTTCAAT	PB	PB-1	P1-1	TRUE	3	TRA:CAMSDRMTGGFKTIF TRB:CASSQVGRGGEETQYF	1047	CD8-C7
PB-1_AATCGGTTCGTTGCCT	PB	PB-1	P1-47	TRUE	3	TRA:CIVGGNFNKFYF TRB:CASSPEWDTDTQYF	18	CD8-C7
PB-1_ACACCAAAGGCGACAT	PB	PB-1	P1-3	TRUE	3	TRA:CAFLSGTYKYIF TRB:CASSQSPGQRNYGYTF	308	CD8-C7
PB-1_ACACCAAAGTAGGCCA	PB	PB-1	P1-3	TRUE	3	TRA:CAFLSGTYKYIF TRB:CASSQSPGQRNYGYTF	308	CD8-C7
PB-1_ACACCAACATCCCACT	PB	PB-1	P1-13	TRUE	3	TRA:CVVMNYGGSQGNLIF TRB:CASSSWGDTDEAFF	112	CD8-C7
PB-1_ACACCAAGTCTAGTGT	PB	PB-1	P1-7	TRUE	3	TRA:CAVQAGNNDMRF TRB:CASSTTGDFNYGYTF	215	CD8-C5

PB-1_ACACCAATCAGCACAT	PB	PB-1	P1-2	TRUE	3	TRA:CGQNFVF TRB:CASNQRGGPSLYNEQFF	323	CD8-C7
PB-1_ACACCCTTCAGTCCCT	PB	PB-1	P1-1	TRUE	3	TRA:CAMSDRMTGGFKTIF TRB:CASSQVGRGGEETQYF	1047	CD8-C7
PB-1_ACACCCTTCCATTCTA	PB	PB-1	P1-2	TRUE	3	TRA:CGQNFVF TRB:CASNQRGGPSLYNEQFF	323	CD8-C7
PB-1_ACACCCTTCTGATACG	PB	PB-1	P1-13	TRUE	3	TRA:CVVMNYGGSQGNLIF TRB:CASSSWGDTDEAFF	112	CD8-C7
PB-1_ACACCGGAGCTTCGCG	PB	PB-1	P1-3	TRUE	3	TRA:CAFLSGTYKYIF TRB:CASSQSPGQRNYGYTF	308	CD8-C6
PB-1_ACACCGGCATAGGATA	PB	PB-1	P1-16	TRUE	3	TRA:CALSGTPMNRDDKIIF TRB:CASSQSWDLAVLNEQFF	79	CD8-C7
PB-1_ACACCGGCATGACATC	PB	PB-1	P1-10	TRUE	3	TRA:CISNSPMDSSYKLIF TRB:CASSSWGDTDEAFF	162	CD4-C6
PB-1_ACACCGGGTTTGACTG	PB	PB-1	P1-5	TRUE	3	TRA:CALSGTPMNRDDKIIF TRB:CSARGRGLAGGPAYEQYF	223	CD8-C7
PB-1_ACACCGGTCCACGTGG	PB	PB-1	P1-256	FALSE	2	TRA:CAVQAGNNDMRF TRB:CASSQSPGQRNYGYTF	2	CD8-C7
PB-1_ACACTGAAGCAACGGT	PB	PB-1	P1-3	TRUE	3	TRA:CAFLSGTYKYIF TRB:CASSQSPGQRNYGYTF	308	CD8-C2
PB-1_ACACTGACAAACTGTC	PB	PB-1	P1-5	TRUE	3	TRA:CALSGTPMNRDDKIIF TRB:CSARGRGLAGGPAYEQYF	223	CD8-C7
PB-1_ACACTGACAGACGCCT	PB	PB-1	P1-1	TRUE	3	TRA:CAMSDRMTGGFKTIF TRB:CASSQVGRGGEETQYF	1047	CD8-C7
PB-1_ACACTGAGTACCGAGA	PB	PB-1	P1-2	TRUE	3	TRA:CGQNFVF TRB:CASNQRGGPSLYNEQFF	323	CD8-C5
PB-1_ACACTGAGTAGCGATG	PB	PB-1	P1-3	TRUE	3	TRA:CAFLSGTYKYIF TRB:CASSQSPGQRNYGYTF	308	CD8-C7
PB-1_ACACTGATCCCACTTG	PB	PB-1	P1-26	TRUE	3	TRA:CAPRSGGSYIPTF TRB:CASSQDLGLDYGYTF	56	CD8-C7
PB-1_ACACTGATCGCAAACT	PB	PB-1	P1-13	TRUE	3	TRA:CVVMNYGGSQGNLIF TRB:CASSSWGDTDEAFF	112	CD8-C7
PB-1_ACACTGATCGGAGCAA	PB	PB-1	P1-40	TRUE	3	TRA:CAVRDVGNTGFQKLVF TRB:CASSLGQGSMKTQYF	27	CD8-C7
PB-1_ACACTGATCTCTAAGG	PB	PB-1	P1-5	TRUE	3	TRA:CALSGTPMNRDDKIIF TRB:CSARGRGLAGGPAYEQYF	223	CD8-C7
PB-1_ACACTGATCTTGTCAT	PB	PB-1	P1-7	TRUE	3	TRA:CAVQAGNNDMRF TRB:CASSTTGDFNYGYTF	215	CD8-C5
PB-1_ACAGCCGAGCCGATTT	PB	PB-1	P1-465	FALSE	1	TRA:CAFLSGTYKYIF TRB:CATSGGDNEQFF	1	CD8-C7
PB-1_ACAGCCGGTCGGCTCA	PB	PB-1	P1-1	TRUE	3	TRA:CAMSDRMTGGFKTIF TRB:CASSQVGRGGEETQYF	1047	CD8-C7
PB-1_ACAGCCGGTTCCACTC	PB	PB-1	P1-1	TRUE	3	TRA:CAMSDRMTGGFKTIF TRB:CASSQVGRGGEETQYF	1047	CD8-C7
PB-1_ACAGCCGTCGGAGGTA	PB	PB-1	P1-466	FALSE	1	TRA:CALRILRGSYIPTF TRB:CASSLPNTEAFF	1	CD8-C7
PB-1_ACAGCCGTCTATCCTA	PB	PB-1	P1-8	TRUE	3	TRA:CAVRPPGRNSGGYQKVTF TRB:CASSRRGVTDTQYF	204	CD8-C7
PB-1_ACAGCCGTCTTGGGTA	PB	PB-1	P1-3	TRUE	3	TRA:CAFLSGTYKYIF TRB:CASSQSPGQRNYGYTF	308	CD8-C7
PB-1_ACAGCTAAGGATGGAA	PB	PB-1	P1-8	TRUE	3	TRA:CAVRPPGRNSGGYQKVTF TRB:CASSRRGVTDTQYF	204	CD8-C7
PB-1_ACAGCTAAGTCCAGGA	PB	PB-1	P1-31	TRUE	3	TRA:CAVEEGADRLMF TRB:CASSLLGGGELFF	46	CD8-C7

PB-1_ACAGCTAGTTGAACTC	PB	PB-1	P1-61	TRUE	3	TRA:CAARGGSQGNLIF TRB:CASRPRHTDTQYF	10	CD8-C7
PB-1_ACATACGAGATCCCGC	PB	PB-1	P1-10	TRUE	3	TRA:CISNSPMDSSYKLIF TRB:CASSSWGDTDEAFF	162	CD8-C7
PB-1_ACATACGAGTAGCGGT	PB	PB-1	P1-1	TRUE	3	TRA:CAMSDRMTGGFKTIF TRB:CASSQVGRGGEETQYF	1047	CD8-C7
PB-1_ACATACGCAATCTGCA	PB	PB-1	P1-1	TRUE	3	TRA:CAMSDRMTGGFKTIF TRB:CASSQVGRGGEETQYF	1047	CD8-C7
PB-1_ACATACGCATCCCACT	PB	PB-1	P1-47	TRUE	3	TRA:CIVGGNFNKFYF TRB:CASSPEWDTDTQYF	18	CD8-C5
PB-1_ACATACGGTAACGTTC	PB	PB-1	P1-2	TRUE	3	TRA:CGQNFVF TRB:CASNQRGGPSLYNEQFF	323	CD8-C7
PB-1_ACATACGGTCAACATC	PB	PB-1	P1-1	TRUE	3	TRA:CAMSDRMTGGFKTIF TRB:CASSQVGRGGEETQYF	1047	CD8-C5
PB-1_ACATACGTCATCGGAT	PB	PB-1	P1-1	TRUE	3	TRA:CAMSDRMTGGFKTIF TRB:CASSQVGRGGEETQYF	1047	CD8-C5
PB-1_ACATCAGAGAGGGCTT	PB	PB-1	P1-4	TRUE	3	TRA:CAMKGRDDKIIF TRB:CASTKGVSREAFF	226	CD8-C7
PB-1_ACATCAGAGCTGTTCA	PB	PB-1	P1-8	TRUE	3	TRA:CAVRPPGRNSGGYQKVTF TRB:CASSRRGVTDTQYF	204	CD8-C7
PB-1_ACATCAGAGTACGCGA	PB	PB-1	P1-2	TRUE	3	TRA:CGQNFVF TRB:CASNQRGGPSLYNEQFF	323	CD8-C7
PB-1_ACATCAGCAGCGTTCG	PB	PB-1	P1-1	TRUE	3	TRA:CAMSDRMTGGFKTIF TRB:CASSQVGRGGEETQYF	1047	CD8-C5
PB-1_ACATCAGGTAAACGCG	PB	PB-1	P1-1	TRUE	3	TRA:CAMSDRMTGGFKTIF TRB:CASSQVGRGGEETQYF	1047	CD8-C7
PB-1_ACATCAGGTATAGGGC	PB	PB-1	P1-5	TRUE	3	TRA:CALSGTPMNRDDKIIF TRB:CSARGRGLAGGPAYEQYF	223	CD8-C7
PB-1_ACATCAGGTTATCACG	PB	PB-1	P1-3	TRUE	3	TRA:CAFLSGTYKYIF TRB:CASSQSPGQRNYGYTF	308	CD8-C7
PB-1_ACATCAGTCGGAGGTA	PB	PB-1	P1-1	TRUE	3	TRA:CAMSDRMTGGFKTIF TRB:CASSQVGRGGEETQYF	1047	CD8-C7
PB-1_ACATCAGTCTGTACGA	PB	PB-1	P1-8	TRUE	3	TRA:CAVRPPGRNSGGYQKVTF TRB:CASSRRGVTDTQYF	204	CD8-C7
PB-1_ACATGGTCACATTTCT	PB	PB-1	P1-29	TRUE	3	TRA:CATVSFSGGYNKLIF TRB:CASSLDRRGLSYEQYF	50	CD8-C7
PB-1_ACATGGTTCAGTGCAT	PB	PB-1	P1-5	TRUE	3	TRA:CALSGTPMNRDDKIIF TRB:CSARGRGLAGGPAYEQYF	223	CD8-C7
PB-1_ACATGGTTCATCGGAT	PB	PB-1	P1-1	TRUE	3	TRA:CAMSDRMTGGFKTIF TRB:CASSQVGRGGEETQYF	1047	CD8-C7
PB-1_ACATGGTTCGGTCTAA	PB	PB-1	P1-3	TRUE	3	TRA:CAFLSGTYKYIF TRB:CASSQSPGQRNYGYTF	308	CD8-C7
PB-1_ACCAGTACAACGATGG	PB	PB-1	P1-1	TRUE	3	TRA:CAMSDRMTGGFKTIF TRB:CASSQVGRGGEETQYF	1047	CD8-C6
PB-1_ACCAGTACATAGAAAC	PB	PB-1	P1-29	TRUE	3	TRA:CATVSFSGGYNKLIF TRB:CASSLDRRGLSYEQYF	50	CD8-C7
PB-1_ACCAGTAGTTATTCTC	PB	PB-1	P1-1	TRUE	3	TRA:CAMSDRMTGGFKTIF TRB:CASSQVGRGGEETQYF	1047	CD8-C7
PB-1_ACCAGTATCAGCTCTC	PB	PB-1	P1-14	TRUE	3	TRA:CAASVVNDMRF TRB:CASSHRGGAKNIQYF	109	CD8-C5
PB-1_ACCAGTATCGAGAACG	PB	PB-1	P1-140	TRUE	3	TRA:CAYRSGKGGKLIF TRB:CASSDTSSHINEQFF	4	CD8-C7
PB-1_ACCCACTAGAGATGAG	PB	PB-1	P1-35	TRUE	3	TRA:CAVSDRGYFGNEKLTF TRB:CSALDDVRPVIAVYNEQFF	40	CD8-C7

PB-1_ACCCACTAGCAGATCG	PB	PB-1	P1-1	TRUE	3	TRA:CAMSDRMTGGFKTIF TRB:CASSQVGRGGEETQYF	1047	CD8-C7
PB-1_ACCCACTAGGGCTTCC	PB	PB-1	P1-8	TRUE	3	TRA:CAVRPPGRNSGGYQKVTF TRB:CASSRRGVTDTQYF	204	CD8-C7
PB-1_ACCCACTTCAACGAAA	PB	PB-1	P1-1	TRUE	3	TRA:CAMSDRMTGGFKTIF TRB:CASSQVGRGGEETQYF	1047	CD8-C5
PB-1_ACCGTAACATCGATGT	PB	PB-1	P1-1	TRUE	3	TRA:CAMSDRMTGGFKTIF TRB:CASSQVGRGGEETQYF	1047	CD8-C7
PB-1_ACCGTAAGTAAACCTC	PB	PB-1	P1-1	TRUE	3	TRA:CAMSDRMTGGFKTIF TRB:CASSQVGRGGEETQYF	1047	CD8-C5
PB-1_ACCGTAAGTACAGTTC	PB	PB-1	P1-476	FALSE	1	TRA:CAASVVNDMRF TRB:CSARGRGLAGGPAYEQYF	1	CD8-C5
PB-1_ACCGTAAGTGGTCCGT	PB	PB-1	P1-11	TRUE	3	TRA:CAVEPLYGSGNTGKLIF TRB:CASSSHGQLNQPQHF	147	CD8-C7
PB-1_ACCGTAAGTTCCATGA	PB	PB-1	P1-13	TRUE	3	TRA:CVVMNYGGSQGNLIF TRB:CASSSWGDTDEAFF	112	CD8-C7
PB-1_ACCTTTAAGTGTTTGC	PB	PB-1	P1-1	TRUE	3	TRA:CAMSDRMTGGFKTIF TRB:CASSQVGRGGEETQYF	1047	CD8-C5
PB-1_ACCTTTACACCACGTG	PB	PB-1	P1-1	TRUE	3	TRA:CAMSDRMTGGFKTIF TRB:CASSQVGRGGEETQYF	1047	CD8-C7
PB-1_ACCTTTACAGGATCGA	PB	PB-1	P1-1	TRUE	3	TRA:CAMSDRMTGGFKTIF TRB:CASSQVGRGGEETQYF	1047	CD8-C7
PB-1_ACCTTTAGTAGCTAAA	PB	PB-1	P1-22	TRUE	3	TRA:CASNSGYSTLTF TRB:CASSQVTGGARDEQYF	65	CD8-C5
PB-1_ACCTTTAGTATGAAAC	PB	PB-1	P1-13	TRUE	3	TRA:CVVMNYGGSQGNLIF TRB:CASSSWGDTDEAFF	112	CD8-C7
PB-1_ACCTTTAGTCCATCCT	PB	PB-1	P1-1	TRUE	3	TRA:CAMSDRMTGGFKTIF TRB:CASSQVGRGGEETQYF	1047	CD8-C5
PB-1_ACCTTTAGTCGACTGC	PB	PB-1	P1-4	TRUE	3	TRA:CAMKGRDDKIIF TRB:CASTKGVSREAFF	226	CD8-C7
PB-1_ACCTTTATCTCGTATT	PB	PB-1	P1-11	TRUE	3	TRA:CAVEPLYGSGNTGKLIF TRB:CASSSHGQLNQPQHF	147	CD8-C7
PB-1_ACCTTTATCTTGTTTG	PB	PB-1	P1-2	TRUE	3	TRA:CGQNFVF TRB:CASNQRGGPSLYNEQFF	323	CD8-C7
PB-1_ACGAGCCAGCTGAACG	PB	PB-1	P1-478	FALSE	1	TRA:CALSGTPMNRDDKIIF TRB:CASSQVTGGARDEQYF	1	CD8-C7
PB-1_ACGAGCCAGGACAGAA	PB	PB-1	P1-13	TRUE	3	TRA:CVVMNYGGSQGNLIF TRB:CASSSWGDTDEAFF	112	CD4-C6
PB-1_ACGAGCCAGGAGTTTA	PB	PB-1	P1-11	TRUE	3	TRA:CAVEPLYGSGNTGKLIF TRB:CASSSHGQLNQPQHF	147	CD8-C7
PB-1_ACGAGCCCACGTTGGC	PB	PB-1	P1-1	TRUE	3	TRA:CAMSDRMTGGFKTIF TRB:CASSQVGRGGEETQYF	1047	CD8-C5
PB-1_ACGAGCCGTTACGGAG	PB	PB-1	P1-1	TRUE	3	TRA:CAMSDRMTGGFKTIF TRB:CASSQVGRGGEETQYF	1047	CD8-C7
PB-1_ACGAGCCGTTATCCGA	PB	PB-1	P1-480	FALSE	1	TRA:CALSGTPMNRDDKIIF TRB:CATSGGDNEQFF	1	CD8-C10
PB-1_ACGAGCCGTTTAGGAA	PB	PB-1	P1-1	TRUE	3	TRA:CAMSDRMTGGFKTIF TRB:CASSQVGRGGEETQYF	1047	CD8-C5
PB-1_ACGAGCCTCGTCCGTT	PB	PB-1	P1-22	TRUE	3	TRA:CASNSGYSTLTF TRB:CASSQVTGGARDEQYF	65	CD8-C5
PB-1_ACGAGCCTCTCAAGTG	PB	PB-1	P1-29	TRUE	3	TRA:CATVSFSGGYNKLIF TRB:CASSLDRRGLSYEQYF	50	CD8-C7
PB-1_ACGAGGAAGGCCGAAT	PB	PB-1	P1-1	TRUE	3	TRA:CAMSDRMTGGFKTIF TRB:CASSQVGRGGEETQYF	1047	CD8-C7

PB-1_ACGAGGACACCAGTTA	PB	PB-1	P1-47	TRUE	3	TRA:CIVGGNFNKFYF TRB:CASSPEWDTDTQYF	18	CD8-C7
PB-1_ACGAGGAGTACTTCTT	PB	PB-1	P1-260	FALSE	2	TRA:CAMSDRMTGGFKTIF TRB:CSARGRGLAGGPAYEQYF	2	CD8-C7
PB-1_ACGAGGAGTCAAAGCG	PB	PB-1	P1-1	TRUE	3	TRA:CAMSDRMTGGFKTIF TRB:CASSQVGRGGEETQYF	1047	CD8-C7
PB-1_ACGAGGATCAGAGACG	PB	PB-1	P1-1	TRUE	3	TRA:CAMSDRMTGGFKTIF TRB:CASSQVGRGGEETQYF	1047	CD8-C7
PB-1_ACGAGGATCTCCCTGA	PB	PB-1	P1-31	TRUE	3	TRA:CAVEEGADRLMF TRB:CASSLLGGGELFF	46	CD8-C7
PB-1_ACGATACAGTGTCTCA	PB	PB-1	P1-13	TRUE	3	TRA:CVVMNYGGSQGNLIF TRB:CASSSWGDTDEAFF	112	CD8-C7
PB-1_ACGATACCACGGTAGA	PB	PB-1	P1-13	TRUE	3	TRA:CVVMNYGGSQGNLIF TRB:CASSSWGDTDEAFF	112	CD8-C5
PB-1_ACGATACGTACCGCTG	PB	PB-1	P1-1	TRUE	3	TRA:CAMSDRMTGGFKTIF TRB:CASSQVGRGGEETQYF	1047	CD8-C7
PB-1_ACGATACGTCGGCATC	PB	PB-1	P1-1	TRUE	3	TRA:CAMSDRMTGGFKTIF TRB:CASSQVGRGGEETQYF	1047	CD8-C7
PB-1_ACGATACTCGCCTGAG	PB	PB-1	P1-3	TRUE	3	TRA:CAFLSGTYKYIF TRB:CASSQSPGQRNYGYTF	308	CD8-C7
PB-1_ACGATGTAGTTAGCGG	PB	PB-1	P1-1	TRUE	3	TRA:CAMSDRMTGGFKTIF TRB:CASSQVGRGGEETQYF	1047	CD8-C7
PB-1_ACGATGTCAGCGTTCG	PB	PB-1	P1-1	TRUE	3	TRA:CAMSDRMTGGFKTIF TRB:CASSQVGRGGEETQYF	1047	CD8-C7
PB-1_ACGATGTGTAGCACGA	PB	PB-1	P1-2	TRUE	3	TRA:CGQNFVF TRB:CASNQRGGPSLYNEQFF	323	CD8-C5
PB-1_ACGATGTGTCTGGAGA	PB	PB-1	P1-8	TRUE	3	TRA:CAVRPPGRNSGGYQKVTF TRB:CASSRRGVTDTQYF	204	CD8-C7
PB-1_ACGCAGCAGTACCGGA	PB	PB-1	P1-1	TRUE	3	TRA:CAMSDRMTGGFKTIF TRB:CASSQVGRGGEETQYF	1047	CD8-C7
PB-1_ACGCAGCCAAGGTGTG	PB	PB-1	P1-12	TRUE	3	TRA:CALSDERGDYKLSF TRB:CASSPGTGSYNEQFF	141	CD8-C5
PB-1_ACGCAGCGTGAAAGAG	PB	PB-1	P1-1	TRUE	3	TRA:CAMSDRMTGGFKTIF TRB:CASSQVGRGGEETQYF	1047	CD8-C7
PB-1_ACGCAGCTCCCTCTTT	PB	PB-1	P1-26	TRUE	3	TRA:CAPRSGGSYIPTF TRB:CASSQDLGLDYGYTF	56	CD8-C7
PB-1_ACGCAGCTCCCTTGCA	PB	PB-1	P1-2	TRUE	3	TRA:CGQNFVF TRB:CASNQRGGPSLYNEQFF	323	CD8-C7
PB-1_ACGCAGCTCCGGGTGT	PB	PB-1	P1-4	TRUE	3	TRA:CAMKGRDDKIIF TRB:CASTKGVSREAFF	226	CD8-C7
PB-1_ACGCCAGAGGAGTACC	PB	PB-1	P1-35	TRUE	3	TRA:CAVSDRGYFGNEKLTF TRB:CSALDDVRPVIAVYNEQFF	40	CD8-C6
PB-1_ACGCCAGGTCAGGACA	PB	PB-1	P1-1	TRUE	3	TRA:CAMSDRMTGGFKTIF TRB:CASSQVGRGGEETQYF	1047	CD8-C5
PB-1_ACGCCAGGTCATCCCT	PB	PB-1	P1-29	TRUE	3	TRA:CATVSFSGGYNKLIF TRB:CASSLDRRGLSYEQYF	50	CD8-C7
PB-1_ACGCCAGTCACCTCGT	PB	PB-1	P1-3	TRUE	3	TRA:CAFLSGTYKYIF TRB:CASSQSPGQRNYGYTF	308	CD8-C7
PB-1_ACGCCAGTCCGGGTGT	PB	PB-1	P1-488	FALSE	1	TRA:CVVNLMEYGNKLVF TRB:CASSQSPGQRNYGYTF	1	CD8-C2
PB-1_ACGCCGAAGATAGGAG	PB	PB-1	P1-2	TRUE	3	TRA:CGQNFVF TRB:CASNQRGGPSLYNEQFF	323	CD8-C7
PB-1_ACGCCGACAAGCGAGT	PB	PB-1	P1-489	FALSE	1	TRA:CAATGLVQGGSEKLVF TRB:CASSQEGLEQYF	1	CD4-C6

PB-1_ACGCCGACAGCTCCGA	PB	PB-1	P1-4	TRUE	3	TRA:CAMKGRDDKIIF TRB:CASTKGVSREAFF	226	CD8-C7
PB-1_ACGCCGACAGCTTCGG	PB	PB-1	P1-4	TRUE	3	TRA:CAMKGRDDKIIF TRB:CASTKGVSREAFF	226	CD8-C7
PB-1_ACGCCGACATACGCCG	PB	PB-1	P1-1	TRUE	3	TRA:CAMSDRMTGGFKTIF TRB:CASSQVGRGGEETQYF	1047	CD8-C7
PB-1_ACGCCGACATACGCTA	PB	PB-1	P1-1	TRUE	3	TRA:CAMSDRMTGGFKTIF TRB:CASSQVGRGGEETQYF	1047	CD8-C7
PB-1_ACGCCGAGTACATGTC	PB	PB-1	P1-11	TRUE	3	TRA:CAVEPLYGSGNTGKLIF TRB:CASSSHGQLNQPQHF	147	CD8-C7
PB-1_ACGCCGAGTTAGAACA	PB	PB-1	P1-5	TRUE	3	TRA:CALSGTPMNRDDKIIF TRB:CSARGRGLAGGPAYEQYF	223	CD8-C7
PB-1_ACGGAGAAGATGCCTT	PB	PB-1	P1-11	TRUE	3	TRA:CAVEPLYGSGNTGKLIF TRB:CASSSHGQLNQPQHF	147	CD8-C7
PB-1_ACGGAGAGTATAATGG	PB	PB-1	P1-1	TRUE	3	TRA:CAMSDRMTGGFKTIF TRB:CASSQVGRGGEETQYF	1047	CD8-C5
PB-1_ACGGAGAGTCTGCCAG	PB	PB-1	P1-173	FALSE	3	TRA:CAVVGYSSASKIIF TRB:CATSDLGYEQFF	3	CD8-C7
PB-1_ACGGAGAGTTAGATGA	PB	PB-1	P1-1	TRUE	3	TRA:CAMSDRMTGGFKTIF TRB:CASSQVGRGGEETQYF	1047	CD8-C5
PB-1_ACGGGCTAGATGAGAG	PB	PB-1	P1-3	TRUE	3	TRA:CAFLSGTYKYIF TRB:CASSQSPGQRNYGYTF	308	CD8-C7
PB-1_ACGGGCTAGGCTAGAC	PB	PB-1	P1-1	TRUE	3	TRA:CAMSDRMTGGFKTIF TRB:CASSQVGRGGEETQYF	1047	CD8-C7
PB-1_ACGGGCTCACGACGAA	PB	PB-1	P1-61	TRUE	3	TRA:CAARGGSQGNLIF TRB:CASRPRHTDTQYF	10	CD8-C7
PB-1_ACGGGCTCATAGACTC	PB	PB-1	P1-1	TRUE	3	TRA:CAMSDRMTGGFKTIF TRB:CASSQVGRGGEETQYF	1047	CD8-C7
PB-1_ACGGGCTGTCCGACGT	PB	PB-1	P1-2	TRUE	3	TRA:CGQNFVF TRB:CASNQRGGPSLYNEQFF	323	CD8-C7
PB-1_ACGGGCTGTCTGCCAG	PB	PB-1	P1-10	TRUE	3	TRA:CISNSPMDSSYKLIF TRB:CASSSWGDTDEAFF	162	CD8-C7
PB-1_ACGGGTCGTACAGTGG	PB	PB-1	P1-14	TRUE	3	TRA:CAASVVNDMRF TRB:CASSHRGGAKNIQYF	109	CD8-C7
PB-1_ACGGGTCTCCGCAGTG	PB	PB-1	P1-8	TRUE	3	TRA:CAVRPPGRNSGGYQKVTF TRB:CASSRRGVTDTQYF	204	CD8-C7
PB-1_ACGGGTCTCCTTCAAT	PB	PB-1	P1-1	TRUE	3	TRA:CAMSDRMTGGFKTIF TRB:CASSQVGRGGEETQYF	1047	CD8-C7
PB-1_ACGGGTCTCTTCGGTC	PB	PB-1	P1-18	TRUE	3	TRA:CVADGNYGQNFVF TRB:CASSSPGLGGGFDTEAFF	75	CD8-C6
PB-1_ACGTCAACAATGTAAG	PB	PB-1	P1-1	TRUE	3	TRA:CAMSDRMTGGFKTIF TRB:CASSQVGRGGEETQYF	1047	CD8-C5
PB-1_ACGTCAACAGCAGTTT	PB	PB-1	P1-11	TRUE	3	TRA:CAVEPLYGSGNTGKLIF TRB:CASSSHGQLNQPQHF	147	CD8-C7
PB-1_ACGTCAACAGCATACT	PB	PB-1	P1-1	TRUE	3	TRA:CAMSDRMTGGFKTIF TRB:CASSQVGRGGEETQYF	1047	CD8-C5
PB-1_ACGTCAACAGGATCGA	PB	PB-1	P1-39	TRUE	3	TRA:CAVRDLGGTSYDKVIF TRB:CSARVREVSYEQYF	31	CD8-C7
PB-1_ACGTCAAGTGTGAATA	PB	PB-1	P1-31	TRUE	3	TRA:CAVEEGADRLMF TRB:CASSLLGGGELFF	46	CD8-C7
PB-1_ACGTCAAGTTCCCGAG	PB	PB-1	P1-2	TRUE	3	TRA:CGQNFVF TRB:CASNQRGGPSLYNEQFF	323	CD8-C7
PB-1_ACGTCAATCTACCTGC	PB	PB-1	P1-22	TRUE	3	TRA:CASNSGYSTLTF TRB:CASSQVTGGARDEQYF	65	CD8-C7

PB-1_ACTATCTAGTACACCT	PB	PB-1	P1-496	FALSE	1	TRA:CAVVGAGSYQLTF TRB:CASSIGGNYEQYF	1	CD8-C7
PB-1_ACTATCTCATGGTAGG	PB	PB-1	P1-40	TRUE	3	TRA:CAVRDVGNTGFQKLVF TRB:CASSLGQGSMKTQYF	27	CD4-C6
PB-1_ACTATCTGTAGAAGGA	PB	PB-1	P1-11	TRUE	3	TRA:CAVEPLYGSGNTGKLIF TRB:CASSSHGQLNQPQHF	147	CD8-C7
PB-1_ACTATCTTCCGAAGAG	PB	PB-1	P1-10	TRUE	3	TRA:CISNSPMDSSYKLIF TRB:CASSSWGDTDEAFF	162	CD8-C7
PB-1_ACTATCTTCGGTCCGA	PB	PB-1	P1-22	TRUE	3	TRA:CASNSGYSTLTF TRB:CASSQVTGGARDEQYF	65	CD8-C7
PB-1_ACTGAACGTTGGACCC	PB	PB-1	P1-4	TRUE	3	TRA:CAMKGRDDKIIF TRB:CASTKGVSREAFF	226	CD8-C7
PB-1_ACTGAGTAGCGATCCC	PB	PB-1	P1-2	TRUE	3	TRA:CGQNFVF TRB:CASNQRGGPSLYNEQFF	323	CD8-C7
PB-1_ACTGAGTCAAGAGGCT	PB	PB-1	P1-13	TRUE	3	TRA:CVVMNYGGSQGNLIF TRB:CASSSWGDTDEAFF	112	CD8-C7
PB-1_ACTGAGTGTAGGCATG	PB	PB-1	P1-3	TRUE	3	TRA:CAFLSGTYKYIF TRB:CASSQSPGQRNYGYTF	308	CD8-C7
PB-1_ACTGATGAGCATCATC	PB	PB-1	P1-6	TRUE	3	TRA:CAMREGRERDDKIIF TRB:CASSLPNTEAFF	216	CD8-C5
PB-1_ACTGATGGTGGCGAAT	PB	PB-1	P1-1	TRUE	3	TRA:CAMSDRMTGGFKTIF TRB:CASSQVGRGGEETQYF	1047	CD8-C7
PB-1_ACTGATGTCGGCATCG	PB	PB-1	P1-35	TRUE	3	TRA:CAVSDRGYFGNEKLTF TRB:CSALDDVRPVIAVYNEQFF	40	CD8-C6
PB-1_ACTGATGTCTCCCTGA	PB	PB-1	P1-1	TRUE	3	TRA:CAMSDRMTGGFKTIF TRB:CASSQVGRGGEETQYF	1047	CD8-C7
PB-1_ACTGATGTCTGTCTCG	PB	PB-1	P1-17	TRUE	3	TRA:CGTGARGSSGSRLTF TRB:CASSRQGMGAFF	78	CD4-C6
PB-1_ACTGCTCAGAAACGCC	PB	PB-1	P1-1	TRUE	3	TRA:CAMSDRMTGGFKTIF TRB:CASSQVGRGGEETQYF	1047	CD8-C7
PB-1_ACTGCTCAGCGTCAAG	PB	PB-1	P1-2	TRUE	3	TRA:CGQNFVF TRB:CASNQRGGPSLYNEQFF	323	CD8-C7
PB-1_ACTGCTCAGGATCGCA	PB	PB-1	P1-11	TRUE	3	TRA:CAVEPLYGSGNTGKLIF TRB:CASSSHGQLNQPQHF	147	CD8-C7
PB-1_ACTGCTCAGTGTACTC	PB	PB-1	P1-13	TRUE	3	TRA:CVVMNYGGSQGNLIF TRB:CASSSWGDTDEAFF	112	CD8-C5
PB-1_ACTGCTCCAGTAAGAT	PB	PB-1	P1-2	TRUE	3	TRA:CGQNFVF TRB:CASNQRGGPSLYNEQFF	323	CD8-C7
PB-1_ACTGCTCCATGTCTCC	PB	PB-1	P1-19	TRUE	3	TRA:CALGAFGSARQLTF TRB:CASSKGQGPRETQYF	70	CD4-C6
PB-1_ACTGCTCTCGTGGACC	PB	PB-1	P1-13	TRUE	3	TRA:CVVMNYGGSQGNLIF TRB:CASSSWGDTDEAFF	112	CD8-C7
PB-1_ACTGCTCTCTGGTGTA	PB	PB-1	P1-2	TRUE	3	TRA:CGQNFVF TRB:CASNQRGGPSLYNEQFF	323	CD8-C7
PB-1_ACTGTCCAGAGGGATA	PB	PB-1	P1-2	TRUE	3	TRA:CGQNFVF TRB:CASNQRGGPSLYNEQFF	323	CD8-C7
PB-1_ACTGTCCAGCTCCCAG	PB	PB-1	P1-10	TRUE	3	TRA:CISNSPMDSSYKLIF TRB:CASSSWGDTDEAFF	162	CD8-C5
PB-1_ACTGTCCAGGGATCTG	PB	PB-1	P1-266	FALSE	2	TRA:CAFLSGTYKYIF TRB:CASSLPNTEAFF	2	CD8-C7
PB-1_ACTGTCCCACTGTGTA	PB	PB-1	P1-2	TRUE	3	TRA:CGQNFVF TRB:CASNQRGGPSLYNEQFF	323	CD8-C7
PB-1_ACTGTCCCAGACGCCT	PB	PB-1	P1-11	TRUE	3	TRA:CAVEPLYGSGNTGKLIF TRB:CASSSHGQLNQPQHF	147	CD8-C7

PB-1_ACTGTCCCAGCGAACA	PB	PB-1	P1-8	TRUE	3	TRA:CAVRPPGRNSGGYQKVTF TRB:CASSRRGVTDTQYF	204	CD8-C7
PB-1_ACTGTCCGTAGCGATG	PB	PB-1	P1-26	TRUE	3	TRA:CAPRSGGSYIPTF TRB:CASSQDLGLDYGYTF	56	CD8-C7
PB-1_ACTGTCCTCAGAGCTT	PB	PB-1	P1-3	TRUE	3	TRA:CAFLSGTYKYIF TRB:CASSQSPGQRNYGYTF	308	CD8-C7
PB-1_ACTGTCCTCATCATTC	PB	PB-1	P1-4	TRUE	3	TRA:CAMKGRDDKIIF TRB:CASTKGVSREAFF	226	CD8-C7
PB-1_ACTGTCCTCTCGTTTA	PB	PB-1	P1-11	TRUE	3	TRA:CAVEPLYGSGNTGKLIF TRB:CASSSHGQLNQPQHF	147	CD8-C7
PB-1_ACTTACTAGCCAGGAT	PB	PB-1	P1-10	TRUE	3	TRA:CISNSPMDSSYKLIF TRB:CASSSWGDTDEAFF	162	CD8-C7
PB-1_ACTTACTAGCTCCCAG	PB	PB-1	P1-26	TRUE	3	TRA:CAPRSGGSYIPTF TRB:CASSQDLGLDYGYTF	56	CD8-C5
PB-1_ACTTACTCATAGACTC	PB	PB-1	P1-54	TRUE	3	TRA:CAGWYSGGGADGLTF TRB:CATGAGDNEQFF	13	CD8-C7
PB-1_ACTTACTCATGGTAGG	PB	PB-1	P1-6	TRUE	3	TRA:CAMREGRERDDKIIF TRB:CASSLPNTEAFF	216	CD8-C5
PB-1_ACTTACTGTAAATGAC	PB	PB-1	P1-1	TRUE	3	TRA:CAMSDRMTGGFKTIF TRB:CASSQVGRGGEETQYF	1047	CD8-C7
PB-1_ACTTACTTCCCTTGCA	PB	PB-1	P1-1	TRUE	3	TRA:CAMSDRMTGGFKTIF TRB:CASSQVGRGGEETQYF	1047	CD8-C7
PB-1_ACTTGTTAGTAAGTAC	PB	PB-1	P1-11	TRUE	3	TRA:CAVEPLYGSGNTGKLIF TRB:CASSSHGQLNQPQHF	147	CD8-C7
PB-1_ACTTGTTCAACACCTA	PB	PB-1	P1-3	TRUE	3	TRA:CAFLSGTYKYIF TRB:CASSQSPGQRNYGYTF	308	CD8-C7
PB-1_ACTTGTTCACCTCGGA	PB	PB-1	P1-15	TRUE	3	TRA:CAVNSSPLGANSKLTF TRB:CASSDSGNFYNEQFF	92	CD8-C7
PB-1_ACTTGTTCACGTCTCT	PB	PB-1	P1-1	TRUE	3	TRA:CAMSDRMTGGFKTIF TRB:CASSQVGRGGEETQYF	1047	CD8-C7
PB-1_ACTTGTTGTACGAAAT	PB	PB-1	P1-3	TRUE	3	TRA:CAFLSGTYKYIF TRB:CASSQSPGQRNYGYTF	308	CD8-C7
PB-1_ACTTGTTTCCGTTGCT	PB	PB-1	P1-1	TRUE	3	TRA:CAMSDRMTGGFKTIF TRB:CASSQVGRGGEETQYF	1047	CD8-C5
PB-1_ACTTGTTTCGAACGGA	PB	PB-1	P1-10	TRUE	3	TRA:CISNSPMDSSYKLIF TRB:CASSSWGDTDEAFF	162	CD8-C7
PB-1_ACTTTCACAAATCCGT	PB	PB-1	P1-10	TRUE	3	TRA:CISNSPMDSSYKLIF TRB:CASSSWGDTDEAFF	162	CD8-C7
PB-1_ACTTTCACAGCGTCCA	PB	PB-1	P1-1	TRUE	3	TRA:CAMSDRMTGGFKTIF TRB:CASSQVGRGGEETQYF	1047	CD8-C7
PB-1_ACTTTCAGTCCTCCAT	PB	PB-1	P1-1	TRUE	3	TRA:CAMSDRMTGGFKTIF TRB:CASSQVGRGGEETQYF	1047	CD8-C7
PB-1_ACTTTCATCATTTGGG	PB	PB-1	P1-5	TRUE	3	TRA:CALSGTPMNRDDKIIF TRB:CSARGRGLAGGPAYEQYF	223	CD8-C7
PB-1_ACTTTCATCCGTCATC	PB	PB-1	P1-2	TRUE	3	TRA:CGQNFVF TRB:CASNQRGGPSLYNEQFF	323	CD8-C7
PB-1_AGACGTTCAATAGAGT	PB	PB-1	P1-1	TRUE	3	TRA:CAMSDRMTGGFKTIF TRB:CASSQVGRGGEETQYF	1047	CD8-C7
PB-1_AGACGTTCAATCTGCA	PB	PB-1	P1-5	TRUE	3	TRA:CALSGTPMNRDDKIIF TRB:CSARGRGLAGGPAYEQYF	223	CD8-C7
PB-1_AGACGTTCACCGATAT	PB	PB-1	P1-5	TRUE	3	TRA:CALSGTPMNRDDKIIF TRB:CSARGRGLAGGPAYEQYF	223	CD8-C7
PB-1_AGACGTTGTGAACCTT	PB	PB-1	P1-8	TRUE	3	TRA:CAVRPPGRNSGGYQKVTF TRB:CASSRRGVTDTQYF	204	CD8-C7

PB-1_AGACGTTGTTAAGAAC	PB	PB-1	P1-11	TRUE	3	TRA:CAVEPLYGSGNTGKLIF TRB:CASSSHGQLNQPQHF	147	CD8-C7
PB-1_AGACGTTTCCGTAGGC	PB	PB-1	P1-510	FALSE	1	TRA:CAVNYGGSQGNLIF TRB:CASATPIQFRGRGHEQYF	1	CD8-C5
PB-1_AGAGCGACATCCGCGA	PB	PB-1	P1-8	TRUE	3	TRA:CAVRPPGRNSGGYQKVTF TRB:CASSRRGVTDTQYF	204	CD8-C7
PB-1_AGAGCGAGTTCCGGCA	PB	PB-1	P1-3	TRUE	3	TRA:CAFLSGTYKYIF TRB:CASSQSPGQRNYGYTF	308	CD8-C7
PB-1_AGAGCGATCGGAAACG	PB	PB-1	P1-13	TRUE	3	TRA:CVVMNYGGSQGNLIF TRB:CASSSWGDTDEAFF	112	CD8-C5
PB-1_AGAGCTTAGAGCCTAG	PB	PB-1	P1-12	TRUE	3	TRA:CALSDERGDYKLSF TRB:CASSPGTGSYNEQFF	141	CD8-C7
PB-1_AGAGCTTCAAGTAATG	PB	PB-1	P1-22	TRUE	3	TRA:CASNSGYSTLTF TRB:CASSQVTGGARDEQYF	65	CD8-C7
PB-1_AGAGCTTGTCTGGTCG	PB	PB-1	P1-1	TRUE	3	TRA:CAMSDRMTGGFKTIF TRB:CASSQVGRGGEETQYF	1047	CD8-C7
PB-1_AGAGCTTGTGCTGTAT	PB	PB-1	P1-10	TRUE	3	TRA:CISNSPMDSSYKLIF TRB:CASSSWGDTDEAFF	162	CD8-C7
PB-1_AGAGCTTGTTCACCTC	PB	PB-1	P1-6	TRUE	3	TRA:CAMREGRERDDKIIF TRB:CASSLPNTEAFF	216	CD8-C7
PB-1_AGAGTGGAGGCTAGAC	PB	PB-1	P1-5	TRUE	3	TRA:CALSGTPMNRDDKIIF TRB:CSARGRGLAGGPAYEQYF	223	CD8-C7
PB-1_AGAGTGGCAGTAGAGC	PB	PB-1	P1-3	TRUE	3	TRA:CAFLSGTYKYIF TRB:CASSQSPGQRNYGYTF	308	CD8-C7
PB-1_AGAGTGGCAGTGACAG	PB	PB-1	P1-4	TRUE	3	TRA:CAMKGRDDKIIF TRB:CASTKGVSREAFF	226	CD8-C7
PB-1_AGAGTGGGTCTCTTAT	PB	PB-1	P1-5	TRUE	3	TRA:CALSGTPMNRDDKIIF TRB:CSARGRGLAGGPAYEQYF	223	CD8-C7
PB-1_AGAGTGGGTGGAAAGA	PB	PB-1	P1-1	TRUE	3	TRA:CAMSDRMTGGFKTIF TRB:CASSQVGRGGEETQYF	1047	CD8-C7
PB-1_AGAGTGGGTGGGTATG	PB	PB-1	P1-5	TRUE	3	TRA:CALSGTPMNRDDKIIF TRB:CSARGRGLAGGPAYEQYF	223	CD8-C7
PB-1_AGAGTGGGTGTTGGGA	PB	PB-1	P1-1	TRUE	3	TRA:CAMSDRMTGGFKTIF TRB:CASSQVGRGGEETQYF	1047	CD8-C7
PB-1_AGAGTGGGTTATCCGA	PB	PB-1	P1-40	TRUE	3	TRA:CAVRDVGNTGFQKLVF TRB:CASSLGQGSMKTQYF	27	CD8-C7
PB-1_AGAGTGGGTTGCGTTA	PB	PB-1	P1-29	TRUE	3	TRA:CATVSFSGGYNKLIF TRB:CASSLDRRGLSYEQYF	50	CD8-C7
PB-1_AGAGTGGGTTTGGGCC	PB	PB-1	P1-1	TRUE	3	TRA:CAMSDRMTGGFKTIF TRB:CASSQVGRGGEETQYF	1047	CD8-C5
PB-1_AGAGTGGTCTAAGCCA	PB	PB-1	P1-2	TRUE	3	TRA:CGQNFVF TRB:CASNQRGGPSLYNEQFF	323	CD8-C7
PB-1_AGAGTGGTCTGCGTAA	PB	PB-1	P1-1	TRUE	3	TRA:CAMSDRMTGGFKTIF TRB:CASSQVGRGGEETQYF	1047	CD8-C7
PB-1_AGATCTGAGCTAAGAT	PB	PB-1	P1-13	TRUE	3	TRA:CVVMNYGGSQGNLIF TRB:CASSSWGDTDEAFF	112	CD8-C7
PB-1_AGATCTGAGTGTTTGC	PB	PB-1	P1-3	TRUE	3	TRA:CAFLSGTYKYIF TRB:CASSQSPGQRNYGYTF	308	CD8-C7
PB-1_AGATCTGCAGTAAGAT	PB	PB-1	P1-2	TRUE	3	TRA:CGQNFVF TRB:CASNQRGGPSLYNEQFF	323	CD8-C7
PB-1_AGATCTGCATGATCCA	PB	PB-1	P1-72	FALSE	3	TRA:CALPDTNAGKSTF TRB:CSAAQGAYQPQHF	8	CD8-C7
PB-1_AGATCTGTCACGAAGG	PB	PB-1	P1-1	TRUE	3	TRA:CAMSDRMTGGFKTIF TRB:CASSQVGRGGEETQYF	1047	CD8-C5

PB-1_AGATCTGTCCGTAGGC	PB	PB-1	P1-13	TRUE	3	TRA:CVVMNYGGSQGNLIF TRB:CASSSWGDTDEAFF	112	CD8-C7
PB-1_AGATCTGTCGGAGGTA	PB	PB-1	P1-11	TRUE	3	TRA:CAVEPLYGSGNTGKLIF TRB:CASSSHGQLNQPQHF	147	CD8-C7
PB-1_AGATTGCCAAATCCGT	PB	PB-1	P1-1	TRUE	3	TRA:CAMSDRMTGGFKTIF TRB:CASSQVGRGGEETQYF	1047	CD8-C7
PB-1_AGATTGCGTGCACCAC	PB	PB-1	P1-5	TRUE	3	TRA:CALSGTPMNRDDKIIF TRB:CSARGRGLAGGPAYEQYF	223	CD8-C7
PB-1_AGATTGCTCGTCACGG	PB	PB-1	P1-1	TRUE	3	TRA:CAMSDRMTGGFKTIF TRB:CASSQVGRGGEETQYF	1047	CD8-C7
PB-1_AGCAGCCAGAACAATC	PB	PB-1	P1-1	TRUE	3	TRA:CAMSDRMTGGFKTIF TRB:CASSQVGRGGEETQYF	1047	CD8-C5
PB-1_AGCATACGTTGCGCAC	PB	PB-1	P1-14	TRUE	3	TRA:CAASVVNDMRF TRB:CASSHRGGAKNIQYF	109	CD8-C7
PB-1_AGCCTAAAGGATGCGT	PB	PB-1	P1-6	TRUE	3	TRA:CAMREGRERDDKIIF TRB:CASSLPNTEAFF	216	CD8-C5
PB-1_AGCCTAACACGGCCAT	PB	PB-1	P1-1	TRUE	3	TRA:CAMSDRMTGGFKTIF TRB:CASSQVGRGGEETQYF	1047	CD8-C7
PB-1_AGCCTAAGTACATCCA	PB	PB-1	P1-4	TRUE	3	TRA:CAMKGRDDKIIF TRB:CASTKGVSREAFF	226	CD8-C7
PB-1_AGCCTAAGTCCAGTAT	PB	PB-1	P1-5	TRUE	3	TRA:CALSGTPMNRDDKIIF TRB:CSARGRGLAGGPAYEQYF	223	CD8-C7
PB-1_AGCCTAATCAAGCCTA	PB	PB-1	P1-3	TRUE	3	TRA:CAFLSGTYKYIF TRB:CASSQSPGQRNYGYTF	308	CD8-C7
PB-1_AGCCTAATCCTCCTAG	PB	PB-1	P1-2	TRUE	3	TRA:CGQNFVF TRB:CASNQRGGPSLYNEQFF	323	CD8-C7
PB-1_AGCCTAATCTTATCTG	PB	PB-1	P1-11	TRUE	3	TRA:CAVEPLYGSGNTGKLIF TRB:CASSSHGQLNQPQHF	147	CD8-C7
PB-1_AGCGGTCAGCGGCTTC	PB	PB-1	P1-1	TRUE	3	TRA:CAMSDRMTGGFKTIF TRB:CASSQVGRGGEETQYF	1047	CD8-C5
PB-1_AGCGGTCAGCTCCTCT	PB	PB-1	P1-29	TRUE	3	TRA:CATVSFSGGYNKLIF TRB:CASSLDRRGLSYEQYF	50	CD8-C7
PB-1_AGCGGTCAGGCTACGA	PB	PB-1	P1-3	TRUE	3	TRA:CAFLSGTYKYIF TRB:CASSQSPGQRNYGYTF	308	CD8-C7
PB-1_AGCGGTCCAAGCTGAG	PB	PB-1	P1-1	TRUE	3	TRA:CAMSDRMTGGFKTIF TRB:CASSQVGRGGEETQYF	1047	CD8-C7
PB-1_AGCGGTCGTTGACGTT	PB	PB-1	P1-1	TRUE	3	TRA:CAMSDRMTGGFKTIF TRB:CASSQVGRGGEETQYF	1047	CD8-C7
PB-1_AGCGTATAGCGATCCC	PB	PB-1	P1-4	TRUE	3	TRA:CAMKGRDDKIIF TRB:CASTKGVSREAFF	226	CD8-C5
PB-1_AGCGTATAGGACAGAA	PB	PB-1	P1-9	TRUE	3	TRA:CAEAEGGGADGLTF TRB:CATSGGDNEQFF	174	CD8-C5
PB-1_AGCGTATAGGCAAAGA	PB	PB-1	P1-13	TRUE	3	TRA:CVVMNYGGSQGNLIF TRB:CASSSWGDTDEAFF	112	CD8-C7
PB-1_AGCGTATAGTTGAGAT	PB	PB-1	P1-10	TRUE	3	TRA:CISNSPMDSSYKLIF TRB:CASSSWGDTDEAFF	162	CD8-C7
PB-1_AGCGTATGTCACAAGG	PB	PB-1	P1-13	TRUE	3	TRA:CVVMNYGGSQGNLIF TRB:CASSSWGDTDEAFF	112	CD8-C7
PB-1_AGCGTATGTCTTCGTC	PB	PB-1	P1-1	TRUE	3	TRA:CAMSDRMTGGFKTIF TRB:CASSQVGRGGEETQYF	1047	CD8-C7
PB-1_AGCGTATGTGTGCCTG	PB	PB-1	P1-18	TRUE	3	TRA:CVADGNYGQNFVF TRB:CASSSPGLGGGFDTEAFF	75	CD8-C6
PB-1_AGCTCCTAGACCGGAT	PB	PB-1	P1-1	TRUE	3	TRA:CAMSDRMTGGFKTIF TRB:CASSQVGRGGEETQYF	1047	CD8-C7

PB-1_AGCTCCTAGATCGATA	PB	PB-1	P1-1	TRUE	3	TRA:CAMSDRMTGGFKTIF TRB:CASSQVGRGGEETQYF	1047	CD8-C7
PB-1_AGCTCCTAGGACACCA	PB	PB-1	P1-1	TRUE	3	TRA:CAMSDRMTGGFKTIF TRB:CASSQVGRGGEETQYF	1047	CD8-C7
PB-1_AGCTCCTCAGTGAGTG	PB	PB-1	P1-3	TRUE	3	TRA:CAFLSGTYKYIF TRB:CASSQSPGQRNYGYTF	308	CD8-C7
PB-1_AGCTCCTGTGTTCGAT	PB	PB-1	P1-11	TRUE	3	TRA:CAVEPLYGSGNTGKLIF TRB:CASSSHGQLNQPQHF	147	CD8-C7
PB-1_AGCTCCTTCGTTTATC	PB	PB-1	P1-3	TRUE	3	TRA:CAFLSGTYKYIF TRB:CASSQSPGQRNYGYTF	308	CD8-C7
PB-1_AGCTCTCCACACCGAC	PB	PB-1	P1-1	TRUE	3	TRA:CAMSDRMTGGFKTIF TRB:CASSQVGRGGEETQYF	1047	CD8-C7
PB-1_AGCTCTCCATGGTAGG	PB	PB-1	P1-145	FALSE	3	TRA:CAFMKHDFYTGANSKLTF TRB:CASSYMFLLGVNTEAFF	4	CD8-C6
PB-1_AGCTCTCGTCTGATCA	PB	PB-1	P1-1	TRUE	3	TRA:CAMSDRMTGGFKTIF TRB:CASSQVGRGGEETQYF	1047	CD8-C7
PB-1_AGCTCTCTCACCGGGT	PB	PB-1	P1-10	TRUE	3	TRA:CISNSPMDSSYKLIF TRB:CASSSWGDTDEAFF	162	CD8-C7
PB-1_AGCTTGAAGGATGCGT	PB	PB-1	P1-1	TRUE	3	TRA:CAMSDRMTGGFKTIF TRB:CASSQVGRGGEETQYF	1047	CD8-C7
PB-1_AGCTTGACACCTCGTT	PB	PB-1	P1-1	TRUE	3	TRA:CAMSDRMTGGFKTIF TRB:CASSQVGRGGEETQYF	1047	CD8-C7
PB-1_AGCTTGACACGAGGTA	PB	PB-1	P1-1	TRUE	3	TRA:CAMSDRMTGGFKTIF TRB:CASSQVGRGGEETQYF	1047	CD8-C7
PB-1_AGCTTGAGTAATCGTC	PB	PB-1	P1-1	TRUE	3	TRA:CAMSDRMTGGFKTIF TRB:CASSQVGRGGEETQYF	1047	CD8-C7
PB-1_AGCTTGAGTCCAGTAT	PB	PB-1	P1-8	TRUE	3	TRA:CAVRPPGRNSGGYQKVTF TRB:CASSRRGVTDTQYF	204	CD8-C7
PB-1_AGCTTGATCCCGGATG	PB	PB-1	P1-1	TRUE	3	TRA:CAMSDRMTGGFKTIF TRB:CASSQVGRGGEETQYF	1047	CD8-C7
PB-1_AGGCCACAGCAATCTC	PB	PB-1	P1-1	TRUE	3	TRA:CAMSDRMTGGFKTIF TRB:CASSQVGRGGEETQYF	1047	CD8-C7
PB-1_AGGCCACCATATGCTG	PB	PB-1	P1-35	TRUE	3	TRA:CAVSDRGYFGNEKLTF TRB:CSALDDVRPVIAVYNEQFF	40	CD8-C6
PB-1_AGGCCACGTACTCTCC	PB	PB-1	P1-8	TRUE	3	TRA:CAVRPPGRNSGGYQKVTF TRB:CASSRRGVTDTQYF	204	CD8-C7
PB-1_AGGCCACGTAGGGTAC	PB	PB-1	P1-8	TRUE	3	TRA:CAVRPPGRNSGGYQKVTF TRB:CASSRRGVTDTQYF	204	CD8-C5
PB-1_AGGCCACGTCCGACGT	PB	PB-1	P1-10	TRUE	3	TRA:CISNSPMDSSYKLIF TRB:CASSSWGDTDEAFF	162	CD8-C7
PB-1_AGGCCGTAGGCGATAC	PB	PB-1	P1-11	TRUE	3	TRA:CAVEPLYGSGNTGKLIF TRB:CASSSHGQLNQPQHF	147	CD8-C7
PB-1_AGGCCGTAGGCTCAGA	PB	PB-1	P1-26	TRUE	3	TRA:CAPRSGGSYIPTF TRB:CASSQDLGLDYGYTF	56	CD8-C7
PB-1_AGGCCGTAGTGTGAAT	PB	PB-1	P1-1	TRUE	3	TRA:CAMSDRMTGGFKTIF TRB:CASSQVGRGGEETQYF	1047	CD8-C7
PB-1_AGGCCGTCACGAGGTA	PB	PB-1	P1-1	TRUE	3	TRA:CAMSDRMTGGFKTIF TRB:CASSQVGRGGEETQYF	1047	CD8-C7
PB-1_AGGCCGTGTCGGGTCT	PB	PB-1	P1-3	TRUE	3	TRA:CAFLSGTYKYIF TRB:CASSQSPGQRNYGYTF	308	CD8-C6
PB-1_AGGCCGTTCATGTAGC	PB	PB-1	P1-53	TRUE	3	TRA:CALSERDTDKLIF TRB:CASSILQGVITEAFF	13	CD8-C7
PB-1_AGGCCGTTCTTTAGGG	PB	PB-1	P1-2	TRUE	3	TRA:CGQNFVF TRB:CASNQRGGPSLYNEQFF	323	CD8-C7

PB-1_AGGGAGTAGTGTGGCA	PB	PB-1	P1-1	TRUE	3	TRA:CAMSDRMTGGFKTIF TRB:CASSQVGRGGEETQYF	1047	CD8-C7
PB-1_AGGGAGTCACTTACGA	PB	PB-1	P1-10	TRUE	3	TRA:CISNSPMDSSYKLIF TRB:CASSSWGDTDEAFF	162	CD8-C7
PB-1_AGGGAGTCAGGGTTAG	PB	PB-1	P1-29	TRUE	3	TRA:CATVSFSGGYNKLIF TRB:CASSLDRRGLSYEQYF	50	CD8-C7
PB-1_AGGGAGTCATGCTGGC	PB	PB-1	P1-1	TRUE	3	TRA:CAMSDRMTGGFKTIF TRB:CASSQVGRGGEETQYF	1047	CD8-C7
PB-1_AGGGAGTGTCACTTCC	PB	PB-1	P1-1	TRUE	3	TRA:CAMSDRMTGGFKTIF TRB:CASSQVGRGGEETQYF	1047	CD8-C5
PB-1_AGGGAGTGTGAGTGAC	PB	PB-1	P1-1	TRUE	3	TRA:CAMSDRMTGGFKTIF TRB:CASSQVGRGGEETQYF	1047	CD8-C7
PB-1_AGGGAGTTCGTCACGG	PB	PB-1	P1-11	TRUE	3	TRA:CAVEPLYGSGNTGKLIF TRB:CASSSHGQLNQPQHF	147	CD8-C7
PB-1_AGGGAGTTCTATCCTA	PB	PB-1	P1-1	TRUE	3	TRA:CAMSDRMTGGFKTIF TRB:CASSQVGRGGEETQYF	1047	CD8-C7
PB-1_AGGGATGCACAGACAG	PB	PB-1	P1-11	TRUE	3	TRA:CAVEPLYGSGNTGKLIF TRB:CASSSHGQLNQPQHF	147	CD8-C7
PB-1_AGGGATGGTGACCAAG	PB	PB-1	P1-142	FALSE	3	TRA:CAVPYSSASKIIF TRB:CASSRTTGGSSYEQYF	4	CD8-C7
PB-1_AGGGATGTCCGGGTGT	PB	PB-1	P1-29	TRUE	3	TRA:CATVSFSGGYNKLIF TRB:CASSLDRRGLSYEQYF	50	CD8-C7
PB-1_AGGGATGTCTGTCCGT	PB	PB-1	P1-1	TRUE	3	TRA:CAMSDRMTGGFKTIF TRB:CASSQVGRGGEETQYF	1047	CD8-C5
PB-1_AGGGTGAAGATCCTGT	PB	PB-1	P1-8	TRUE	3	TRA:CAVRPPGRNSGGYQKVTF TRB:CASSRRGVTDTQYF	204	CD8-C7
PB-1_AGGGTGAAGGGATCTG	PB	PB-1	P1-1	TRUE	3	TRA:CAMSDRMTGGFKTIF TRB:CASSQVGRGGEETQYF	1047	CD8-C7
PB-1_AGGGTGACACGAGGTA	PB	PB-1	P1-1	TRUE	3	TRA:CAMSDRMTGGFKTIF TRB:CASSQVGRGGEETQYF	1047	CD8-C7
PB-1_AGGGTGAGTAGGGTAC	PB	PB-1	P1-1	TRUE	3	TRA:CAMSDRMTGGFKTIF TRB:CASSQVGRGGEETQYF	1047	CD8-C7
PB-1_AGGGTGAGTCGCGTGT	PB	PB-1	P1-8	TRUE	3	TRA:CAVRPPGRNSGGYQKVTF TRB:CASSRRGVTDTQYF	204	CD8-C7
PB-1_AGGGTGAGTCTGGAGA	PB	PB-1	P1-8	TRUE	3	TRA:CAVRPPGRNSGGYQKVTF TRB:CASSRRGVTDTQYF	204	CD8-C7
PB-1_AGGGTGATCAAGATCC	PB	PB-1	P1-11	TRUE	3	TRA:CAVEPLYGSGNTGKLIF TRB:CASSSHGQLNQPQHF	147	CD8-C7
PB-1_AGGGTGATCTAGCACA	PB	PB-1	P1-29	TRUE	3	TRA:CATVSFSGGYNKLIF TRB:CASSLDRRGLSYEQYF	50	CD8-C7
PB-1_AGGGTGATCTGGTGTA	PB	PB-1	P1-1	TRUE	3	TRA:CAMSDRMTGGFKTIF TRB:CASSQVGRGGEETQYF	1047	CD8-C5
PB-1_AGGTCATAGTGCCATT	PB	PB-1	P1-13	TRUE	3	TRA:CVVMNYGGSQGNLIF TRB:CASSSWGDTDEAFF	112	CD8-C7
PB-1_AGGTCATGTACCGTAT	PB	PB-1	P1-2	TRUE	3	TRA:CGQNFVF TRB:CASNQRGGPSLYNEQFF	323	CD8-C7
PB-1_AGGTCATGTGTGCGTC	PB	PB-1	P1-1	TRUE	3	TRA:CAMSDRMTGGFKTIF TRB:CASSQVGRGGEETQYF	1047	CD8-C7
PB-1_AGGTCCGCAGGACCCT	PB	PB-1	P1-12	TRUE	3	TRA:CALSDERGDYKLSF TRB:CASSPGTGSYNEQFF	141	CD8-C5
PB-1_AGGTCCGCAGTGGGAT	PB	PB-1	P1-3	TRUE	3	TRA:CAFLSGTYKYIF TRB:CASSQSPGQRNYGYTF	308	CD8-C7
PB-1_AGGTCCGGTACAGTGG	PB	PB-1	P1-5	TRUE	3	TRA:CALSGTPMNRDDKIIF TRB:CSARGRGLAGGPAYEQYF	223	CD8-C5

PB-1_AGGTCCGGTCCATGAT	PB	PB-1	P1-10	TRUE	3	TRA:CISNSPMDSSYKLIF TRB:CASSSWGDTDEAFF	162	CD8-C7
PB-1_AGGTCCGTCCTTTACA	PB	PB-1	P1-5	TRUE	3	TRA:CALSGTPMNRDDKIIF TRB:CSARGRGLAGGPAYEQYF	223	CD8-C5
PB-1_AGTAGTCAGACAGACC	PB	PB-1	P1-4	TRUE	3	TRA:CAMKGRDDKIIF TRB:CASTKGVSREAFF	226	CD8-C5
PB-1_AGTAGTCAGGGCTCTC	PB	PB-1	P1-7	TRUE	3	TRA:CAVQAGNNDMRF TRB:CASSTTGDFNYGYTF	215	CD8-C5
PB-1_AGTAGTCCAGCTCCGA	PB	PB-1	P1-1	TRUE	3	TRA:CAMSDRMTGGFKTIF TRB:CASSQVGRGGEETQYF	1047	CD8-C7
PB-1_AGTAGTCGTTCCGGCA	PB	PB-1	P1-1	TRUE	3	TRA:CAMSDRMTGGFKTIF TRB:CASSQVGRGGEETQYF	1047	CD8-C7
PB-1_AGTAGTCTCTCCGGTT	PB	PB-1	P1-5	TRUE	3	TRA:CALSGTPMNRDDKIIF TRB:CSARGRGLAGGPAYEQYF	223	CD8-C7
PB-1_AGTAGTCTCTTGTCAT	PB	PB-1	P1-1	TRUE	3	TRA:CAMSDRMTGGFKTIF TRB:CASSQVGRGGEETQYF	1047	CD8-C5
PB-1_AGTCTTTAGCGAGAAA	PB	PB-1	P1-1	TRUE	3	TRA:CAMSDRMTGGFKTIF TRB:CASSQVGRGGEETQYF	1047	CD8-C7
PB-1_AGTCTTTAGTCTTGCA	PB	PB-1	P1-1	TRUE	3	TRA:CAMSDRMTGGFKTIF TRB:CASSQVGRGGEETQYF	1047	CD8-C7
PB-1_AGTCTTTGTAGCCTAT	PB	PB-1	P1-1	TRUE	3	TRA:CAMSDRMTGGFKTIF TRB:CASSQVGRGGEETQYF	1047	CD8-C7
PB-1_AGTCTTTGTTGACGTT	PB	PB-1	P1-11	TRUE	3	TRA:CAVEPLYGSGNTGKLIF TRB:CASSSHGQLNQPQHF	147	CD8-C7
PB-1_AGTCTTTTCGGCTACG	PB	PB-1	P1-4	TRUE	3	TRA:CAMKGRDDKIIF TRB:CASTKGVSREAFF	226	CD8-C7
PB-1_AGTCTTTTCGTTTGCC	PB	PB-1	P1-10	TRUE	3	TRA:CISNSPMDSSYKLIF TRB:CASSSWGDTDEAFF	162	CD8-C7
PB-1_AGTGAGGAGAAGGTTT	PB	PB-1	P1-6	TRUE	3	TRA:CAMREGRERDDKIIF TRB:CASSLPNTEAFF	216	CD8-C5
PB-1_AGTGAGGCACCAGGTC	PB	PB-1	P1-1	TRUE	3	TRA:CAMSDRMTGGFKTIF TRB:CASSQVGRGGEETQYF	1047	CD8-C7
PB-1_AGTGAGGCACCTCGTT	PB	PB-1	P1-1	TRUE	3	TRA:CAMSDRMTGGFKTIF TRB:CASSQVGRGGEETQYF	1047	CD8-C7
PB-1_AGTGAGGTCACAAACC	PB	PB-1	P1-10	TRUE	3	TRA:CISNSPMDSSYKLIF TRB:CASSSWGDTDEAFF	162	CD8-C7
PB-1_AGTGAGGTCGCATGGC	PB	PB-1	P1-2	TRUE	3	TRA:CGQNFVF TRB:CASNQRGGPSLYNEQFF	323	CD8-C7
PB-1_AGTGGGAGTGGTGTAG	PB	PB-1	P1-530	FALSE	1	TRA:CGQNFVF TRB:CASGEGQGASSYEQYF	1	CD8-C2
PB-1_AGTGTCACATCCCACT	PB	PB-1	P1-17	TRUE	3	TRA:CGTGARGSSGSRLTF TRB:CASSRQGMGAFF	78	CD8-C7
PB-1_AGTGTCAGTAAACACA	PB	PB-1	P1-2	TRUE	3	TRA:CGQNFVF TRB:CASNQRGGPSLYNEQFF	323	CD8-C7
PB-1_AGTGTCATCACCCTCA	PB	PB-1	P1-1	TRUE	3	TRA:CAMSDRMTGGFKTIF TRB:CASSQVGRGGEETQYF	1047	CD8-C7
PB-1_AGTGTCATCGCATGAT	PB	PB-1	P1-11	TRUE	3	TRA:CAVEPLYGSGNTGKLIF TRB:CASSSHGQLNQPQHF	147	CD8-C7
PB-1_AGTTGGTAGGGAACGG	PB	PB-1	P1-266	FALSE	2	TRA:CAFLSGTYKYIF TRB:CASSLPNTEAFF	2	CD8-C7
PB-1_AGTTGGTAGTCAATAG	PB	PB-1	P1-183	TRUE	3	TRA:CASYYGGSQGNLIF TRB:CSAGQGSNQPQHF	3	CD8-C7
PB-1_AGTTGGTCATGTAGTC	PB	PB-1	P1-1	TRUE	3	TRA:CAMSDRMTGGFKTIF TRB:CASSQVGRGGEETQYF	1047	CD8-C7

PB-1_AGTTGGTTCAACGGGA	PB	PB-1	P1-26	TRUE	3	TRA:CAPRSGGSYIPTF TRB:CASSQDLGLDYGYTF	56	CD8-C7
PB-1_AGTTGGTTCCACGTTC	PB	PB-1	P1-31	TRUE	3	TRA:CAVEEGADRLMF TRB:CASSLLGGGELFF	46	CD8-C5
PB-1_AGTTGGTTCCGCGCAA	PB	PB-1	P1-2	TRUE	3	TRA:CGQNFVF TRB:CASNQRGGPSLYNEQFF	323	CD8-C7
PB-1_AGTTGGTTCTCTTGAT	PB	PB-1	P1-1	TRUE	3	TRA:CAMSDRMTGGFKTIF TRB:CASSQVGRGGEETQYF	1047	CD8-C7
PB-1_ATAACGCAGAGTCGGT	PB	PB-1	P1-2	TRUE	3	TRA:CGQNFVF TRB:CASNQRGGPSLYNEQFF	323	CD8-C7
PB-1_ATAACGCAGAGTCTGG	PB	PB-1	P1-1	TRUE	3	TRA:CAMSDRMTGGFKTIF TRB:CASSQVGRGGEETQYF	1047	CD8-C7
PB-1_ATAACGCAGGGCTTCC	PB	PB-1	P1-58	TRUE	3	TRA:CAVRPNRDDKIIF TRB:CASSSRLAGGTDTQYF	12	CD8-C7
PB-1_ATAACGCAGTTACCCA	PB	PB-1	P1-1	TRUE	3	TRA:CAMSDRMTGGFKTIF TRB:CASSQVGRGGEETQYF	1047	CD8-C7
PB-1_ATAACGCCAGCTGTAT	PB	PB-1	P1-31	TRUE	3	TRA:CAVEEGADRLMF TRB:CASSLLGGGELFF	46	CD8-C6
PB-1_ATAACGCGTTGCTCCT	PB	PB-1	P1-1	TRUE	3	TRA:CAMSDRMTGGFKTIF TRB:CASSQVGRGGEETQYF	1047	CD8-C7
PB-1_ATAACGCTCACCTTAT	PB	PB-1	P1-5	TRUE	3	TRA:CALSGTPMNRDDKIIF TRB:CSARGRGLAGGPAYEQYF	223	CD8-C7
PB-1_ATAACGCTCCGCGCAA	PB	PB-1	P1-7	TRUE	3	TRA:CAVQAGNNDMRF TRB:CASSTTGDFNYGYTF	215	CD8-C5
PB-1_ATAAGAGTCACATAGC	PB	PB-1	P1-3	TRUE	3	TRA:CAFLSGTYKYIF TRB:CASSQSPGQRNYGYTF	308	CD8-C7
PB-1_ATAAGAGTCCGTAGGC	PB	PB-1	P1-1	TRUE	3	TRA:CAMSDRMTGGFKTIF TRB:CASSQVGRGGEETQYF	1047	CD8-C7
PB-1_ATAAGAGTCGGTCCGA	PB	PB-1	P1-4	TRUE	3	TRA:CAMKGRDDKIIF TRB:CASTKGVSREAFF	226	CD8-C7
PB-1_ATAGACCAGAAACCGC	PB	PB-1	P1-10	TRUE	3	TRA:CISNSPMDSSYKLIF TRB:CASSSWGDTDEAFF	162	CD8-C7
PB-1_ATAGACCGTACGAAAT	PB	PB-1	P1-1	TRUE	3	TRA:CAMSDRMTGGFKTIF TRB:CASSQVGRGGEETQYF	1047	CD8-C5
PB-1_ATAGACCGTTCCGTCT	PB	PB-1	P1-6	TRUE	3	TRA:CAMREGRERDDKIIF TRB:CASSLPNTEAFF	216	CD8-C5
PB-1_ATAGACCTCCGCATCT	PB	PB-1	P1-11	TRUE	3	TRA:CAVEPLYGSGNTGKLIF TRB:CASSSHGQLNQPQHF	147	CD8-C7
PB-1_ATAGACCTCTATGTGG	PB	PB-1	P1-8	TRUE	3	TRA:CAVRPPGRNSGGYQKVTF TRB:CASSRRGVTDTQYF	204	CD8-C7
PB-1_ATCACGAAGCTCCTTC	PB	PB-1	P1-1	TRUE	3	TRA:CAMSDRMTGGFKTIF TRB:CASSQVGRGGEETQYF	1047	CD8-C7
PB-1_ATCACGACATGTAAGA	PB	PB-1	P1-3	TRUE	3	TRA:CAFLSGTYKYIF TRB:CASSQSPGQRNYGYTF	308	CD8-C7
PB-1_ATCACGAGTCCAAGTT	PB	PB-1	P1-2	TRUE	3	TRA:CGQNFVF TRB:CASNQRGGPSLYNEQFF	323	CD8-C7
PB-1_ATCACGATCCTCATTA	PB	PB-1	P1-1	TRUE	3	TRA:CAMSDRMTGGFKTIF TRB:CASSQVGRGGEETQYF	1047	CD8-C7
PB-1_ATCACGATCCTTGGTC	PB	PB-1	P1-10	TRUE	3	TRA:CISNSPMDSSYKLIF TRB:CASSSWGDTDEAFF	162	CD8-C7
PB-1_ATCATCTAGCGCCTCA	PB	PB-1	P1-2	TRUE	3	TRA:CGQNFVF TRB:CASNQRGGPSLYNEQFF	323	CD8-C7
PB-1_ATCATCTCAAGTCTGT	PB	PB-1	P1-4	TRUE	3	TRA:CAMKGRDDKIIF TRB:CASTKGVSREAFF	226	CD8-C7

PB-1_ATCATCTGTTGACGTT	PB	PB-1	P1-1	TRUE	3	TRA:CAMSDRMTGGFKTIF TRB:CASSQVGRGGEETQYF	1047	CD8-C7
PB-1_ATCATCTTCACAGGCC	PB	PB-1	P1-11	TRUE	3	TRA:CAVEPLYGSGNTGKLIF TRB:CASSSHGQLNQPQHF	147	CD8-C7
PB-1_ATCATGGCACATCCGG	PB	PB-1	P1-2	TRUE	3	TRA:CGQNFVF TRB:CASNQRGGPSLYNEQFF	323	CD8-C7
PB-1_ATCATGGCATGGATGG	PB	PB-1	P1-1	TRUE	3	TRA:CAMSDRMTGGFKTIF TRB:CASSQVGRGGEETQYF	1047	CD8-C7
PB-1_ATCATGGGTTTACTCT	PB	PB-1	P1-13	TRUE	3	TRA:CVVMNYGGSQGNLIF TRB:CASSSWGDTDEAFF	112	CD8-C7
PB-1_ATCATGGTCAAAGACA	PB	PB-1	P1-1	TRUE	3	TRA:CAMSDRMTGGFKTIF TRB:CASSQVGRGGEETQYF	1047	CD8-C5
PB-1_ATCATGGTCTACCTGC	PB	PB-1	P1-18	TRUE	3	TRA:CVADGNYGQNFVF TRB:CASSSPGLGGGFDTEAFF	75	CD8-C6
PB-1_ATCCACCAGGTAGCCA	PB	PB-1	P1-2	TRUE	3	TRA:CGQNFVF TRB:CASNQRGGPSLYNEQFF	323	CD8-C7
PB-1_ATCCACCAGTAACCCT	PB	PB-1	P1-10	TRUE	3	TRA:CISNSPMDSSYKLIF TRB:CASSSWGDTDEAFF	162	CD8-C7
PB-1_ATCCACCAGTACACCT	PB	PB-1	P1-10	TRUE	3	TRA:CISNSPMDSSYKLIF TRB:CASSSWGDTDEAFF	162	CD8-C7
PB-1_ATCCACCGTAAGTAGT	PB	PB-1	P1-10	TRUE	3	TRA:CISNSPMDSSYKLIF TRB:CASSSWGDTDEAFF	162	CD8-C7
PB-1_ATCCACCGTCAGTGGA	PB	PB-1	P1-2	TRUE	3	TRA:CGQNFVF TRB:CASNQRGGPSLYNEQFF	323	CD8-C5
PB-1_ATCCACCGTGCTCTTC	PB	PB-1	P1-3	TRUE	3	TRA:CAFLSGTYKYIF TRB:CASSQSPGQRNYGYTF	308	CD8-C7
PB-1_ATCCGAACAATAAGCA	PB	PB-1	P1-1	TRUE	3	TRA:CAMSDRMTGGFKTIF TRB:CASSQVGRGGEETQYF	1047	CD8-C7
PB-1_ATCCGAACACAGTCGC	PB	PB-1	P1-1	TRUE	3	TRA:CAMSDRMTGGFKTIF TRB:CASSQVGRGGEETQYF	1047	CD8-C7
PB-1_ATCCGAAGTAGATTAG	PB	PB-1	P1-29	TRUE	3	TRA:CATVSFSGGYNKLIF TRB:CASSLDRRGLSYEQYF	50	CD8-C7
PB-1_ATCCGAAGTCTGGAGA	PB	PB-1	P1-13	TRUE	3	TRA:CVVMNYGGSQGNLIF TRB:CASSSWGDTDEAFF	112	CD8-C7
PB-1_ATCCGAATCGGTCTAA	PB	PB-1	P1-8	TRUE	3	TRA:CAVRPPGRNSGGYQKVTF TRB:CASSRRGVTDTQYF	204	CD8-C7
PB-1_ATCCGAATCTTTCCTC	PB	PB-1	P1-3	TRUE	3	TRA:CAFLSGTYKYIF TRB:CASSQSPGQRNYGYTF	308	CD8-C7
PB-1_ATCGAGTAGGATCGCA	PB	PB-1	P1-7	TRUE	3	TRA:CAVQAGNNDMRF TRB:CASSTTGDFNYGYTF	215	CD8-C7
PB-1_ATCGAGTCACCCTATC	PB	PB-1	P1-31	TRUE	3	TRA:CAVEEGADRLMF TRB:CASSLLGGGELFF	46	CD8-C2
PB-1_ATCGAGTGTAGGGACT	PB	PB-1	P1-29	TRUE	3	TRA:CATVSFSGGYNKLIF TRB:CASSLDRRGLSYEQYF	50	CD8-C7
PB-1_ATCGAGTGTGCCTGTG	PB	PB-1	P1-8	TRUE	3	TRA:CAVRPPGRNSGGYQKVTF TRB:CASSRRGVTDTQYF	204	CD8-C7
PB-1_ATCGAGTTCAACTCTT	PB	PB-1	P1-1	TRUE	3	TRA:CAMSDRMTGGFKTIF TRB:CASSQVGRGGEETQYF	1047	CD8-C7
PB-1_ATCGAGTTCTTGTTTG	PB	PB-1	P1-547	FALSE	1	TRA:CAVAPSNQFYF TRB:CASNQRGGPSLYNEQFF	1	CD8-C7
PB-1_ATCTACTTCAAGGTAA	PB	PB-1	P1-5	TRUE	3	TRA:CALSGTPMNRDDKIIF TRB:CSARGRGLAGGPAYEQYF	223	CD8-C7
PB-1_ATCTACTTCCGTCAAA	PB	PB-1	P1-1	TRUE	3	TRA:CAMSDRMTGGFKTIF TRB:CASSQVGRGGEETQYF	1047	CD8-C7

PB-1_ATCTGCCAGACTACAA	PB	PB-1	P1-14	TRUE	3	TRA:CAASVVNDMRF TRB:CASSHRGGAKNIQYF	109	CD8-C7
PB-1_ATCTGCCAGATGTGTA	PB	PB-1	P1-1	TRUE	3	TRA:CAMSDRMTGGFKTIF TRB:CASSQVGRGGEETQYF	1047	CD8-C7
PB-1_ATCTGCCAGATTACCC	PB	PB-1	P1-2	TRUE	3	TRA:CGQNFVF TRB:CASNQRGGPSLYNEQFF	323	CD8-C7
PB-1_ATCTGCCCAGCTATTG	PB	PB-1	P1-1	TRUE	3	TRA:CAMSDRMTGGFKTIF TRB:CASSQVGRGGEETQYF	1047	CD8-C7
PB-1_ATCTGCCGTTCGGCAC	PB	PB-1	P1-3	TRUE	3	TRA:CAFLSGTYKYIF TRB:CASSQSPGQRNYGYTF	308	CD8-C7
PB-1_ATCTGCCGTTCGGGCT	PB	PB-1	P1-3	TRUE	3	TRA:CAFLSGTYKYIF TRB:CASSQSPGQRNYGYTF	308	CD8-C2
PB-1_ATGAGGGAGCCAGAAC	PB	PB-1	P1-4	TRUE	3	TRA:CAMKGRDDKIIF TRB:CASTKGVSREAFF	226	CD8-C7
PB-1_ATGCGATGTTACCAGT	PB	PB-1	P1-3	TRUE	3	TRA:CAFLSGTYKYIF TRB:CASSQSPGQRNYGYTF	308	CD8-C6
PB-1_ATGCGATTCTGCTGCT	PB	PB-1	P1-3	TRUE	3	TRA:CAFLSGTYKYIF TRB:CASSQSPGQRNYGYTF	308	CD8-C7
PB-1_ATGCGATTCTGGTGTA	PB	PB-1	P1-18	TRUE	3	TRA:CVADGNYGQNFVF TRB:CASSSPGLGGGFDTEAFF	75	CD8-C7
PB-1_ATGGGAGGTAAACGCG	PB	PB-1	P1-3	TRUE	3	TRA:CAFLSGTYKYIF TRB:CASSQSPGQRNYGYTF	308	CD8-C7
PB-1_ATGGGAGTCAAACGGG	PB	PB-1	P1-3	TRUE	3	TRA:CAFLSGTYKYIF TRB:CASSQSPGQRNYGYTF	308	CD8-C7
PB-1_ATGTGTGCAAAGTCAA	PB	PB-1	P1-10	TRUE	3	TRA:CISNSPMDSSYKLIF TRB:CASSSWGDTDEAFF	162	CD8-C7
PB-1_ATGTGTGCATCACAAC	PB	PB-1	P1-5	TRUE	3	TRA:CALSGTPMNRDDKIIF TRB:CSARGRGLAGGPAYEQYF	223	CD8-C5
PB-1_ATGTGTGCATTCACTT	PB	PB-1	P1-3	TRUE	3	TRA:CAFLSGTYKYIF TRB:CASSQSPGQRNYGYTF	308	CD8-C7
PB-1_ATGTGTGGTGTCTGAT	PB	PB-1	P1-2	TRUE	3	TRA:CGQNFVF TRB:CASNQRGGPSLYNEQFF	323	CD4-C6
PB-1_ATGTGTGGTTCCCGAG	PB	PB-1	P1-12	TRUE	3	TRA:CALSDERGDYKLSF TRB:CASSPGTGSYNEQFF	141	CD8-C7
PB-1_ATGTGTGTCTCGCATC	PB	PB-1	P1-11	TRUE	3	TRA:CAVEPLYGSGNTGKLIF TRB:CASSSHGQLNQPQHF	147	CD8-C5
PB-1_ATGTGTGTCTTAGCCC	PB	PB-1	P1-11	TRUE	3	TRA:CAVEPLYGSGNTGKLIF TRB:CASSSHGQLNQPQHF	147	CD8-C7
PB-1_ATTACTCCATAACCTG	PB	PB-1	P1-13	TRUE	3	TRA:CVVMNYGGSQGNLIF TRB:CASSSWGDTDEAFF	112	CD8-C7
PB-1_ATTACTCCATTTCACT	PB	PB-1	P1-11	TRUE	3	TRA:CAVEPLYGSGNTGKLIF TRB:CASSSHGQLNQPQHF	147	CD8-C7
PB-1_ATTACTCGTTCTCATT	PB	PB-1	P1-14	TRUE	3	TRA:CAASVVNDMRF TRB:CASSHRGGAKNIQYF	109	CD8-C7
PB-1_ATTATCCAGTATCGAA	PB	PB-1	P1-58	TRUE	3	TRA:CAVRPNRDDKIIF TRB:CASSSRLAGGTDTQYF	12	CD8-C7
PB-1_ATTATCCAGTTGAGTA	PB	PB-1	P1-1	TRUE	3	TRA:CAMSDRMTGGFKTIF TRB:CASSQVGRGGEETQYF	1047	CD8-C7
PB-1_ATTATCCCATCAGTAC	PB	PB-1	P1-1	TRUE	3	TRA:CAMSDRMTGGFKTIF TRB:CASSQVGRGGEETQYF	1047	CD8-C7
PB-1_ATTATCCGTGTATGGG	PB	PB-1	P1-8	TRUE	3	TRA:CAVRPPGRNSGGYQKVTF TRB:CASSRRGVTDTQYF	204	CD8-C7
PB-1_ATTATCCGTTACGTCA	PB	PB-1	P1-2	TRUE	3	TRA:CGQNFVF TRB:CASNQRGGPSLYNEQFF	323	CD8-C7

PB-1_ATTCTACCACGAAGCA	PB	PB-1	P1-3	TRUE	3	TRA:CAFLSGTYKYIF TRB:CASSQSPGQRNYGYTF	308	CD8-C7
PB-1_ATTCTACCACGGTAGA	PB	PB-1	P1-3	TRUE	3	TRA:CAFLSGTYKYIF TRB:CASSQSPGQRNYGYTF	308	CD8-C7
PB-1_ATTCTACGTACATCCA	PB	PB-1	P1-1	TRUE	3	TRA:CAMSDRMTGGFKTIF TRB:CASSQVGRGGEETQYF	1047	CD8-C7
PB-1_ATTCTACGTTGGTAAA	PB	PB-1	P1-16	TRUE	3	TRA:CALSGTPMNRDDKIIF TRB:CASSQSWDLAVLNEQFF	79	CD8-C7
PB-1_ATTGGACAGACGACGT	PB	PB-1	P1-3	TRUE	3	TRA:CAFLSGTYKYIF TRB:CASSQSPGQRNYGYTF	308	CD8-C7
PB-1_ATTGGACCAGTCAGAG	PB	PB-1	P1-1	TRUE	3	TRA:CAMSDRMTGGFKTIF TRB:CASSQVGRGGEETQYF	1047	CD8-C5
PB-1_ATTGGACGTAGAAGGA	PB	PB-1	P1-5	TRUE	3	TRA:CALSGTPMNRDDKIIF TRB:CSARGRGLAGGPAYEQYF	223	CD8-C7
PB-1_ATTGGACGTCAAAGCG	PB	PB-1	P1-271	FALSE	2	TRA:CADSGAGSYQLTF TRB:CASSFTLDSGSVDEQFF	2	CD8-C5
PB-1_ATTGGTGAGCGTCTAT	PB	PB-1	P1-16	TRUE	3	TRA:CALSGTPMNRDDKIIF TRB:CASSQSWDLAVLNEQFF	79	CD8-C7
PB-1_ATTGGTGCAGTAAGCG	PB	PB-1	P1-259	FALSE	2	TRA:CAVNEGTNAGKSTF TRB:CASSAGGDYNEQFF	2	CD8-C7
PB-1_ATTGGTGCATCATCCC	PB	PB-1	P1-3	TRUE	3	TRA:CAFLSGTYKYIF TRB:CASSQSPGQRNYGYTF	308	CD8-C7
PB-1_ATTGGTGGTCGAACAG	PB	PB-1	P1-2	TRUE	3	TRA:CGQNFVF TRB:CASNQRGGPSLYNEQFF	323	CD8-C7
PB-1_ATTGGTGGTCGGATCC	PB	PB-1	P1-1	TRUE	3	TRA:CAMSDRMTGGFKTIF TRB:CASSQVGRGGEETQYF	1047	CD8-C7
PB-1_ATTGGTGGTCTAGTCA	PB	PB-1	P1-3	TRUE	3	TRA:CAFLSGTYKYIF TRB:CASSQSPGQRNYGYTF	308	CD8-C7
PB-1_ATTGGTGGTGACAAAT	PB	PB-1	P1-10	TRUE	3	TRA:CISNSPMDSSYKLIF TRB:CASSSWGDTDEAFF	162	CD8-C7
PB-1_ATTTCTGAGCAGGCTA	PB	PB-1	P1-564	FALSE	1	TRA:CISNSPMDSSYKLIF TRB:CASSQVGRGGEETQYF	1	CD8-C7
PB-1_ATTTCTGAGCTCAACT	PB	PB-1	P1-58	TRUE	3	TRA:CAVRPNRDDKIIF TRB:CASSSRLAGGTDTQYF	12	CD8-C7
PB-1_ATTTCTGAGGGATGGG	PB	PB-1	P1-14	TRUE	3	TRA:CAASVVNDMRF TRB:CASSHRGGAKNIQYF	109	CD8-C7
PB-1_ATTTCTGCAGGACCCT	PB	PB-1	P1-22	TRUE	3	TRA:CASNSGYSTLTF TRB:CASSQVTGGARDEQYF	65	CD8-C5
PB-1_ATTTCTGGTAAGGGAA	PB	PB-1	P1-1	TRUE	3	TRA:CAMSDRMTGGFKTIF TRB:CASSQVGRGGEETQYF	1047	CD8-C7
PB-1_ATTTCTGGTCCATCCT	PB	PB-1	P1-11	TRUE	3	TRA:CAVEPLYGSGNTGKLIF TRB:CASSSHGQLNQPQHF	147	CD8-C5
PB-1_ATTTCTGGTCCGCTGA	PB	PB-1	P1-2	TRUE	3	TRA:CGQNFVF TRB:CASNQRGGPSLYNEQFF	323	CD8-C5
PB-1_ATTTCTGGTCGTGGCT	PB	PB-1	P1-4	TRUE	3	TRA:CAMKGRDDKIIF TRB:CASTKGVSREAFF	226	CD8-C7
PB-1_ATTTCTGGTGGACGAT	PB	PB-1	P1-11	TRUE	3	TRA:CAVEPLYGSGNTGKLIF TRB:CASSSHGQLNQPQHF	147	CD8-C7
PB-1_CAACCAAAGATCGATA	PB	PB-1	P1-3	TRUE	3	TRA:CAFLSGTYKYIF TRB:CASSQSPGQRNYGYTF	308	CD8-C7
PB-1_CAACCAACATGATCCA	PB	PB-1	P1-3	TRUE	3	TRA:CAFLSGTYKYIF TRB:CASSQSPGQRNYGYTF	308	CD8-C7
PB-1_CAACCAATCCCTAACC	PB	PB-1	P1-2	TRUE	3	TRA:CGQNFVF TRB:CASNQRGGPSLYNEQFF	323	CD8-C7

PB-1_CAACCTCAGTGGTAGC	PB	PB-1	P1-10	TRUE	3	TRA:CISNSPMDSSYKLIF TRB:CASSSWGDTDEAFF	162	CD8-C7
PB-1_CAACCTCCAATGTAAG	PB	PB-1	P1-5	TRUE	3	TRA:CALSGTPMNRDDKIIF TRB:CSARGRGLAGGPAYEQYF	223	CD8-C5
PB-1_CAACCTCCAGCGTTCG	PB	PB-1	P1-4	TRUE	3	TRA:CAMKGRDDKIIF TRB:CASTKGVSREAFF	226	CD8-C7
PB-1_CAACCTCCATTCTTAC	PB	PB-1	P1-8	TRUE	3	TRA:CAVRPPGRNSGGYQKVTF TRB:CASSRRGVTDTQYF	204	CD8-C7
PB-1_CAACCTCTCACGACTA	PB	PB-1	P1-1	TRUE	3	TRA:CAMSDRMTGGFKTIF TRB:CASSQVGRGGEETQYF	1047	CD8-C7
PB-1_CAACCTCTCCCTTGCA	PB	PB-1	P1-10	TRUE	3	TRA:CISNSPMDSSYKLIF TRB:CASSSWGDTDEAFF	162	CD4-C6
PB-1_CAACCTCTCTCCCTGA	PB	PB-1	P1-1	TRUE	3	TRA:CAMSDRMTGGFKTIF TRB:CASSQVGRGGEETQYF	1047	CD8-C7
PB-1_CAACTAGCATAGTAAG	PB	PB-1	P1-16	TRUE	3	TRA:CALSGTPMNRDDKIIF TRB:CASSQSWDLAVLNEQFF	79	CD8-C7
PB-1_CAACTAGCATGTTGAC	PB	PB-1	P1-11	TRUE	3	TRA:CAVEPLYGSGNTGKLIF TRB:CASSSHGQLNQPQHF	147	CD8-C7
PB-1_CAACTAGTCTACTTAC	PB	PB-1	P1-8	TRUE	3	TRA:CAVRPPGRNSGGYQKVTF TRB:CASSRRGVTDTQYF	204	CD8-C7
PB-1_CAAGAAAAGGTTACCT	PB	PB-1	P1-29	TRUE	3	TRA:CATVSFSGGYNKLIF TRB:CASSLDRRGLSYEQYF	50	CD8-C7
PB-1_CAAGAAACACTACAGT	PB	PB-1	P1-1	TRUE	3	TRA:CAMSDRMTGGFKTIF TRB:CASSQVGRGGEETQYF	1047	CD8-C6
PB-1_CAAGAAACATCACCCT	PB	PB-1	P1-1	TRUE	3	TRA:CAMSDRMTGGFKTIF TRB:CASSQVGRGGEETQYF	1047	CD8-C7
PB-1_CAAGAAAGTCATGCAT	PB	PB-1	P1-1	TRUE	3	TRA:CAMSDRMTGGFKTIF TRB:CASSQVGRGGEETQYF	1047	CD8-C7
PB-1_CAAGATCAGGAATCGC	PB	PB-1	P1-1	TRUE	3	TRA:CAMSDRMTGGFKTIF TRB:CASSQVGRGGEETQYF	1047	CD8-C7
PB-1_CAAGATCAGGGATGGG	PB	PB-1	P1-1	TRUE	3	TRA:CAMSDRMTGGFKTIF TRB:CASSQVGRGGEETQYF	1047	CD8-C7
PB-1_CAAGATCCACTTAACG	PB	PB-1	P1-2	TRUE	3	TRA:CGQNFVF TRB:CASNQRGGPSLYNEQFF	323	CD8-C7
PB-1_CAAGATCTCAGGATCT	PB	PB-1	P1-1	TRUE	3	TRA:CAMSDRMTGGFKTIF TRB:CASSQVGRGGEETQYF	1047	CD8-C7
PB-1_CAAGATCTCTTGAGGT	PB	PB-1	P1-9	TRUE	3	TRA:CAEAEGGGADGLTF TRB:CATSGGDNEQFF	174	CD8-C5
PB-1_CAAGGCCAGACTGGGT	PB	PB-1	P1-12	TRUE	3	TRA:CALSDERGDYKLSF TRB:CASSPGTGSYNEQFF	141	CD8-C7
PB-1_CAAGGCCAGATCCGAG	PB	PB-1	P1-1	TRUE	3	TRA:CAMSDRMTGGFKTIF TRB:CASSQVGRGGEETQYF	1047	CD8-C7
PB-1_CAAGGCCAGGCCCTCA	PB	PB-1	P1-1	TRUE	3	TRA:CAMSDRMTGGFKTIF TRB:CASSQVGRGGEETQYF	1047	CD8-C7
PB-1_CAAGGCCTCTGGAGCC	PB	PB-1	P1-11	TRUE	3	TRA:CAVEPLYGSGNTGKLIF TRB:CASSSHGQLNQPQHF	147	CD8-C5
PB-1_CAAGTTGAGGGTCGAT	PB	PB-1	P1-1	TRUE	3	TRA:CAMSDRMTGGFKTIF TRB:CASSQVGRGGEETQYF	1047	CD8-C7
PB-1_CAAGTTGGTACAGCAG	PB	PB-1	P1-1	TRUE	3	TRA:CAMSDRMTGGFKTIF TRB:CASSQVGRGGEETQYF	1047	CD8-C5
PB-1_CAAGTTGGTCACCTAA	PB	PB-1	P1-5	TRUE	3	TRA:CALSGTPMNRDDKIIF TRB:CSARGRGLAGGPAYEQYF	223	CD8-C7
PB-1_CAAGTTGGTCTCGTTC	PB	PB-1	P1-7	TRUE	3	TRA:CAVQAGNNDMRF TRB:CASSTTGDFNYGYTF	215	CD8-C5

PB-1_CAAGTTGTCAGTCCCT	PB	PB-1	P1-4	TRUE	3	TRA:CAMKGRDDKIIF TRB:CASTKGVSREAFF	226	CD8-C7
PB-1_CAAGTTGTCCTTTACA	PB	PB-1	P1-148	FALSE	3	TRA:CISNSPMDSSYKLIF TRB:CASSQSPGQRNYGYTF	4	CD8-C7
PB-1_CACAAACAGCACCGTC	PB	PB-1	P1-2	TRUE	3	TRA:CGQNFVF TRB:CASNQRGGPSLYNEQFF	323	CD8-C7
PB-1_CACAAACAGCCTTGAT	PB	PB-1	P1-2	TRUE	3	TRA:CGQNFVF TRB:CASNQRGGPSLYNEQFF	323	CD8-C7
PB-1_CACAAACCAAACGCGA	PB	PB-1	P1-575	FALSE	1	TRA:CAMSDRMTGGFKTIF TRB:CSAAQGAYQPQHF	1	CD8-C7
PB-1_CACAAACGTGATGCCC	PB	PB-1	P1-26	TRUE	3	TRA:CAPRSGGSYIPTF TRB:CASSQDLGLDYGYTF	56	CD8-C7
PB-1_CACAAACTCGCGTAGC	PB	PB-1	P1-13	TRUE	3	TRA:CVVMNYGGSQGNLIF TRB:CASSSWGDTDEAFF	112	CD8-C7
PB-1_CACACAACATTTCACT	PB	PB-1	P1-13	TRUE	3	TRA:CVVMNYGGSQGNLIF TRB:CASSSWGDTDEAFF	112	CD8-C7
PB-1_CACACAAGTCAACATC	PB	PB-1	P1-5	TRUE	3	TRA:CALSGTPMNRDDKIIF TRB:CSARGRGLAGGPAYEQYF	223	CD8-C7
PB-1_CACACAAGTTCGCTAA	PB	PB-1	P1-4	TRUE	3	TRA:CAMKGRDDKIIF TRB:CASTKGVSREAFF	226	CD8-C7
PB-1_CACACAATCACTTCAT	PB	PB-1	P1-1	TRUE	3	TRA:CAMSDRMTGGFKTIF TRB:CASSQVGRGGEETQYF	1047	CD8-C5
PB-1_CACACCTGTACCGTTA	PB	PB-1	P1-2	TRUE	3	TRA:CGQNFVF TRB:CASNQRGGPSLYNEQFF	323	CD8-C7
PB-1_CACACCTGTCCGAGTC	PB	PB-1	P1-3	TRUE	3	TRA:CAFLSGTYKYIF TRB:CASSQSPGQRNYGYTF	308	CD8-C7
PB-1_CACACTCAGAGTCTGG	PB	PB-1	P1-5	TRUE	3	TRA:CALSGTPMNRDDKIIF TRB:CSARGRGLAGGPAYEQYF	223	CD8-C7
PB-1_CACACTCCAAACCTAC	PB	PB-1	P1-2	TRUE	3	TRA:CGQNFVF TRB:CASNQRGGPSLYNEQFF	323	CD8-C7
PB-1_CACACTCCAAGTAATG	PB	PB-1	P1-1	TRUE	3	TRA:CAMSDRMTGGFKTIF TRB:CASSQVGRGGEETQYF	1047	CD8-C7
PB-1_CACACTCGTAAAGGAG	PB	PB-1	P1-14	TRUE	3	TRA:CAASVVNDMRF TRB:CASSHRGGAKNIQYF	109	CD8-C5
PB-1_CACACTCTCATCGATG	PB	PB-1	P1-11	TRUE	3	TRA:CAVEPLYGSGNTGKLIF TRB:CASSSHGQLNQPQHF	147	CD8-C5
PB-1_CACACTCTCCTAAGTG	PB	PB-1	P1-2	TRUE	3	TRA:CGQNFVF TRB:CASNQRGGPSLYNEQFF	323	CD8-C7
PB-1_CACAGGCAGAGTAAGG	PB	PB-1	P1-5	TRUE	3	TRA:CALSGTPMNRDDKIIF TRB:CSARGRGLAGGPAYEQYF	223	CD8-C7
PB-1_CACAGGCAGAGTCTGG	PB	PB-1	P1-85	FALSE	3	TRA:CAPRSGGSYIPTF TRB:CASSQDTGLDYGYTF	6	CD8-C5
PB-1_CACAGGCAGCTCCTCT	PB	PB-1	P1-1	TRUE	3	TRA:CAMSDRMTGGFKTIF TRB:CASSQVGRGGEETQYF	1047	CD8-C7
PB-1_CACAGGCCAAAGGAAG	PB	PB-1	P1-16	TRUE	3	TRA:CALSGTPMNRDDKIIF TRB:CASSQSWDLAVLNEQFF	79	CD8-C7
PB-1_CACAGGCGTAAATGTG	PB	PB-1	P1-16	TRUE	3	TRA:CALSGTPMNRDDKIIF TRB:CASSQSWDLAVLNEQFF	79	CD8-C6
PB-1_CACAGGCGTACCATCA	PB	PB-1	P1-1	TRUE	3	TRA:CAMSDRMTGGFKTIF TRB:CASSQVGRGGEETQYF	1047	CD8-C7
PB-1_CACAGGCGTGACCAAG	PB	PB-1	P1-4	TRUE	3	TRA:CAMKGRDDKIIF TRB:CASTKGVSREAFF	226	CD8-C7
PB-1_CACAGGCTCGGGAGTA	PB	PB-1	P1-1	TRUE	3	TRA:CAMSDRMTGGFKTIF TRB:CASSQVGRGGEETQYF	1047	CD8-C7

PB-1_CACAGTAAGATATGGT	PB	PB-1	P1-29	TRUE	3	TRA:CATVSFSGGYNKLIF TRB:CASSLDRRGLSYEQYF	50	CD8-C7
PB-1_CACAGTAAGCAGCCTC	PB	PB-1	P1-35	TRUE	3	TRA:CAVSDRGYFGNEKLTF TRB:CSALDDVRPVIAVYNEQFF	40	CD8-C7
PB-1_CACAGTAAGCCAGTTT	PB	PB-1	P1-1	TRUE	3	TRA:CAMSDRMTGGFKTIF TRB:CASSQVGRGGEETQYF	1047	CD8-C7
PB-1_CACAGTAAGGAGTACC	PB	PB-1	P1-582	FALSE	1	TRA:CAFLSGTYKYIF TRB:CASIEGQGPYNEQFF	1	CD8-C2
PB-1_CACAGTAAGGTGCAAC	PB	PB-1	P1-1	TRUE	3	TRA:CAMSDRMTGGFKTIF TRB:CASSQVGRGGEETQYF	1047	CD8-C7
PB-1_CACAGTAAGTTGTCGT	PB	PB-1	P1-10	TRUE	3	TRA:CISNSPMDSSYKLIF TRB:CASSSWGDTDEAFF	162	CD8-C7
PB-1_CACAGTAGTACCCAAT	PB	PB-1	P1-31	TRUE	3	TRA:CAVEEGADRLMF TRB:CASSLLGGGELFF	46	CD8-C7
PB-1_CACAGTAGTACTTAGC	PB	PB-1	P1-1	TRUE	3	TRA:CAMSDRMTGGFKTIF TRB:CASSQVGRGGEETQYF	1047	CD8-C5
PB-1_CACAGTAGTCTAGCCG	PB	PB-1	P1-1	TRUE	3	TRA:CAMSDRMTGGFKTIF TRB:CASSQVGRGGEETQYF	1047	CD8-C5
PB-1_CACAGTAGTCTTCAAG	PB	PB-1	P1-1	TRUE	3	TRA:CAMSDRMTGGFKTIF TRB:CASSQVGRGGEETQYF	1047	CD8-C7
PB-1_CACATAGAGTTACGGG	PB	PB-1	P1-1	TRUE	3	TRA:CAMSDRMTGGFKTIF TRB:CASSQVGRGGEETQYF	1047	CD8-C7
PB-1_CACATAGCAATGGAAT	PB	PB-1	P1-1	TRUE	3	TRA:CAMSDRMTGGFKTIF TRB:CASSQVGRGGEETQYF	1047	CD8-C7
PB-1_CACATAGGTACTCAAC	PB	PB-1	P1-14	TRUE	3	TRA:CAASVVNDMRF TRB:CASSHRGGAKNIQYF	109	CD8-C7
PB-1_CACATAGGTCGAACAG	PB	PB-1	P1-8	TRUE	3	TRA:CAVRPPGRNSGGYQKVTF TRB:CASSRRGVTDTQYF	204	CD8-C7
PB-1_CACATAGGTCTTGCGG	PB	PB-1	P1-18	TRUE	3	TRA:CVADGNYGQNFVF TRB:CASSSPGLGGGFDTEAFF	75	CD8-C6
PB-1_CACATAGGTTCCACGG	PB	PB-1	P1-10	TRUE	3	TRA:CISNSPMDSSYKLIF TRB:CASSSWGDTDEAFF	162	CD8-C7
PB-1_CACATAGTCAACCAAC	PB	PB-1	P1-2	TRUE	3	TRA:CGQNFVF TRB:CASNQRGGPSLYNEQFF	323	CD8-C7
PB-1_CACATAGTCATGTCTT	PB	PB-1	P1-3	TRUE	3	TRA:CAFLSGTYKYIF TRB:CASSQSPGQRNYGYTF	308	CD8-C7
PB-1_CACATAGTCCGCGCAA	PB	PB-1	P1-11	TRUE	3	TRA:CAVEPLYGSGNTGKLIF TRB:CASSSHGQLNQPQHF	147	CD8-C7
PB-1_CACATTTAGAGTAATC	PB	PB-1	P1-22	TRUE	3	TRA:CASNSGYSTLTF TRB:CASSQVTGGARDEQYF	65	CD8-C5
PB-1_CACATTTCATTTGCCC	PB	PB-1	P1-258	FALSE	2	TRA:CGQNFVF TRB:CASSSHGQLNQPQHF	2	CD8-C7
PB-1_CACATTTGTAAATGAC	PB	PB-1	P1-1	TRUE	3	TRA:CAMSDRMTGGFKTIF TRB:CASSQVGRGGEETQYF	1047	CD8-C7
PB-1_CACCACTAGAAACGCC	PB	PB-1	P1-47	TRUE	3	TRA:CIVGGNFNKFYF TRB:CASSPEWDTDTQYF	18	CD8-C7
PB-1_CACCACTCACAGACAG	PB	PB-1	P1-10	TRUE	3	TRA:CISNSPMDSSYKLIF TRB:CASSSWGDTDEAFF	162	CD8-C7
PB-1_CACCACTCATACAGCT	PB	PB-1	P1-1	TRUE	3	TRA:CAMSDRMTGGFKTIF TRB:CASSQVGRGGEETQYF	1047	CD8-C5
PB-1_CACCACTCATGTCCTC	PB	PB-1	P1-5	TRUE	3	TRA:CALSGTPMNRDDKIIF TRB:CSARGRGLAGGPAYEQYF	223	CD8-C7
PB-1_CACCACTGTCTTCTCG	PB	PB-1	P1-8	TRUE	3	TRA:CAVRPPGRNSGGYQKVTF TRB:CASSRRGVTDTQYF	204	CD8-C7

PB-1_CACCACTGTTTGACAC	PB	PB-1	P1-31	TRUE	3	TRA:CAVEEGADRLMF TRB:CASSLLGGGELFF	46	CD8-C7
PB-1_CACCAGGAGACTAGAT	PB	PB-1	P1-14	TRUE	3	TRA:CAASVVNDMRF TRB:CASSHRGGAKNIQYF	109	CD8-C7
PB-1_CACCAGGAGAGTTGGC	PB	PB-1	P1-12	TRUE	3	TRA:CALSDERGDYKLSF TRB:CASSPGTGSYNEQFF	141	CD8-C5
PB-1_CACCAGGAGCCAGTTT	PB	PB-1	P1-1	TRUE	3	TRA:CAMSDRMTGGFKTIF TRB:CASSQVGRGGEETQYF	1047	CD8-C7
PB-1_CACCAGGAGGACATTA	PB	PB-1	P1-2	TRUE	3	TRA:CGQNFVF TRB:CASNQRGGPSLYNEQFF	323	CD8-C7
PB-1_CACCAGGCACACTGCG	PB	PB-1	P1-22	TRUE	3	TRA:CASNSGYSTLTF TRB:CASSQVTGGARDEQYF	65	CD8-C7
PB-1_CACCAGGCATCCAACA	PB	PB-1	P1-16	TRUE	3	TRA:CALSGTPMNRDDKIIF TRB:CASSQSWDLAVLNEQFF	79	CD8-C7
PB-1_CACCAGGCATCCTAGA	PB	PB-1	P1-1	TRUE	3	TRA:CAMSDRMTGGFKTIF TRB:CASSQVGRGGEETQYF	1047	CD8-C7
PB-1_CACCAGGGTAAATGAC	PB	PB-1	P1-1	TRUE	3	TRA:CAMSDRMTGGFKTIF TRB:CASSQVGRGGEETQYF	1047	CD8-C7
PB-1_CACCAGGGTCACCTAA	PB	PB-1	P1-1	TRUE	3	TRA:CAMSDRMTGGFKTIF TRB:CASSQVGRGGEETQYF	1047	CD8-C7
PB-1_CACCAGGTCGCCCTTA	PB	PB-1	P1-8	TRUE	3	TRA:CAVRPPGRNSGGYQKVTF TRB:CASSRRGVTDTQYF	204	CD8-C7
PB-1_CACCAGGTCTACTATC	PB	PB-1	P1-588	FALSE	1	TRA:CAVEALIYGGATNKLIF TRB:CASSPLGQGDQPQHF	1	CD8-C2
PB-1_CACCAGGTCTGTCAAG	PB	PB-1	P1-3	TRUE	3	TRA:CAFLSGTYKYIF TRB:CASSQSPGQRNYGYTF	308	CD8-C7
PB-1_CACCAGGTCTTCAACT	PB	PB-1	P1-2	TRUE	3	TRA:CGQNFVF TRB:CASNQRGGPSLYNEQFF	323	CD8-C7
PB-1_CACCTTGAGGGAACGG	PB	PB-1	P1-22	TRUE	3	TRA:CASNSGYSTLTF TRB:CASSQVTGGARDEQYF	65	CD8-C7
PB-1_CACCTTGAGGGCATGT	PB	PB-1	P1-5	TRUE	3	TRA:CALSGTPMNRDDKIIF TRB:CSARGRGLAGGPAYEQYF	223	CD8-C7
PB-1_CACCTTGCATGCAACT	PB	PB-1	P1-35	TRUE	3	TRA:CAVSDRGYFGNEKLTF TRB:CSALDDVRPVIAVYNEQFF	40	CD8-C6
PB-1_CACCTTGGTTCACCTC	PB	PB-1	P1-4	TRUE	3	TRA:CAMKGRDDKIIF TRB:CASTKGVSREAFF	226	CD8-C7
PB-1_CACCTTGGTTTGGGCC	PB	PB-1	P1-3	TRUE	3	TRA:CAFLSGTYKYIF TRB:CASSQSPGQRNYGYTF	308	CD8-C7
PB-1_CACCTTGTCCAACCAA	PB	PB-1	P1-2	TRUE	3	TRA:CGQNFVF TRB:CASNQRGGPSLYNEQFF	323	CD8-C7
PB-1_CACCTTGTCCGAGCCA	PB	PB-1	P1-1	TRUE	3	TRA:CAMSDRMTGGFKTIF TRB:CASSQVGRGGEETQYF	1047	CD8-C7
PB-1_CACCTTGTCGTAGATC	PB	PB-1	P1-10	TRUE	3	TRA:CISNSPMDSSYKLIF TRB:CASSSWGDTDEAFF	162	CD8-C7
PB-1_CACCTTGTCTCCTATA	PB	PB-1	P1-35	TRUE	3	TRA:CAVSDRGYFGNEKLTF TRB:CSALDDVRPVIAVYNEQFF	40	CD8-C6
PB-1_CACTCCAAGAGTTGGC	PB	PB-1	P1-1	TRUE	3	TRA:CAMSDRMTGGFKTIF TRB:CASSQVGRGGEETQYF	1047	CD8-C7
PB-1_CACTCCAAGGTGCTTT	PB	PB-1	P1-3	TRUE	3	TRA:CAFLSGTYKYIF TRB:CASSQSPGQRNYGYTF	308	CD8-C7
PB-1_CACTCCACAATGAATG	PB	PB-1	P1-4	TRUE	3	TRA:CAMKGRDDKIIF TRB:CASTKGVSREAFF	226	CD8-C7
PB-1_CACTCCACAGCCAATT	PB	PB-1	P1-592	FALSE	1	TRA:CAFLSGTYKYTF TRB:CASSQSPGQRNYGYTF	1	CD8-C7

PB-1_CACTCCAGTACCATCA	PB	PB-1	P1-1	TRUE	3	TRA:CAMSDRMTGGFKTIF TRB:CASSQVGRGGEETQYF	1047	CD8-C7
PB-1_CACTCCAGTCACCCAG	PB	PB-1	P1-16	TRUE	3	TRA:CALSGTPMNRDDKIIF TRB:CASSQSWDLAVLNEQFF	79	CD8-C7
PB-1_CACTCCAGTCACTGGC	PB	PB-1	P1-4	TRUE	3	TRA:CAMKGRDDKIIF TRB:CASTKGVSREAFF	226	CD8-C7
PB-1_CACTCCATCACAACGT	PB	PB-1	P1-16	TRUE	3	TRA:CALSGTPMNRDDKIIF TRB:CASSQSWDLAVLNEQFF	79	CD8-C7
PB-1_CACTCCATCATGTGGT	PB	PB-1	P1-1	TRUE	3	TRA:CAMSDRMTGGFKTIF TRB:CASSQVGRGGEETQYF	1047	CD8-C7
PB-1_CACTCCATCTACTCAT	PB	PB-1	P1-273	TRUE	2	TRA:CAVPLNTGNQFYF TRB:CAISVGNEQFF	2	CD8-C7
PB-1_CAGAATCAGACTAAGT	PB	PB-1	P1-3	TRUE	3	TRA:CAFLSGTYKYIF TRB:CASSQSPGQRNYGYTF	308	CD8-C7
PB-1_CAGAATCAGCTAGTCT	PB	PB-1	P1-6	TRUE	3	TRA:CAMREGRERDDKIIF TRB:CASSLPNTEAFF	216	CD8-C5
PB-1_CAGAATCCAAGTAGTA	PB	PB-1	P1-11	TRUE	3	TRA:CAVEPLYGSGNTGKLIF TRB:CASSSHGQLNQPQHF	147	CD8-C7
PB-1_CAGAATCCACCAACCG	PB	PB-1	P1-40	TRUE	3	TRA:CAVRDVGNTGFQKLVF TRB:CASSLGQGSMKTQYF	27	CD8-C7
PB-1_CAGAATCCACTGTGTA	PB	PB-1	P1-1	TRUE	3	TRA:CAMSDRMTGGFKTIF TRB:CASSQVGRGGEETQYF	1047	CD8-C5
PB-1_CAGAATCGTACATCCA	PB	PB-1	P1-1	TRUE	3	TRA:CAMSDRMTGGFKTIF TRB:CASSQVGRGGEETQYF	1047	CD8-C7
PB-1_CAGAATCGTACGCTGC	PB	PB-1	P1-29	TRUE	3	TRA:CATVSFSGGYNKLIF TRB:CASSLDRRGLSYEQYF	50	CD8-C7
PB-1_CAGAATCGTCATCGGC	PB	PB-1	P1-5	TRUE	3	TRA:CALSGTPMNRDDKIIF TRB:CSARGRGLAGGPAYEQYF	223	CD8-C7
PB-1_CAGAATCGTCTTCTCG	PB	PB-1	P1-18	TRUE	3	TRA:CVADGNYGGQNFVF TRB:CASSSPGLGGGFDTEAFF	75	CD8-C6
PB-1_CAGAATCTCCGTTGCT	PB	PB-1	P1-35	TRUE	3	TRA:CAVSDRGYFGNEKLTF TRB:CSALDDVRPVIAVYNEQFF	40	CD8-C6
PB-1_CAGAGAGAGGAGTTTA	PB	PB-1	P1-3	TRUE	3	TRA:CAFLSGTYKYIF TRB:CASSQSPGQRNYGYTF	308	CD8-C7
PB-1_CAGAGAGGTCGCGTGT	PB	PB-1	P1-1	TRUE	3	TRA:CAMSDRMTGGFKTIF TRB:CASSQVGRGGEETQYF	1047	CD8-C7
PB-1_CAGATCAAGAATAGGG	PB	PB-1	P1-1	TRUE	3	TRA:CAMSDRMTGGFKTIF TRB:CASSQVGRGGEETQYF	1047	CD8-C7
PB-1_CAGATCACAGATAATG	PB	PB-1	P1-1	TRUE	3	TRA:CAMSDRMTGGFKTIF TRB:CASSQVGRGGEETQYF	1047	CD8-C7
PB-1_CAGATCAGTTCGGCAC	PB	PB-1	P1-11	TRUE	3	TRA:CAVEPLYGSGNTGKLIF TRB:CASSSHGQLNQPQHF	147	CD8-C7
PB-1_CAGATCATCATGCAAC	PB	PB-1	P1-1	TRUE	3	TRA:CAMSDRMTGGFKTIF TRB:CASSQVGRGGEETQYF	1047	CD8-C7
PB-1_CAGATCATCGCCTGTT	PB	PB-1	P1-1	TRUE	3	TRA:CAMSDRMTGGFKTIF TRB:CASSQVGRGGEETQYF	1047	CD8-C7
PB-1_CAGCAGCGTAATAGCA	PB	PB-1	P1-274	FALSE	2	TRA:CAVNFHDMRF TRB:CASSRTGTRANYGYTF	2	CD8-C5
PB-1_CAGCAGCGTTTACTCT	PB	PB-1	P1-14	TRUE	3	TRA:CAASVVNDMRF TRB:CASSHRGGAKNIQYF	109	CD8-C5
PB-1_CAGCATAAGCCCGAAA	PB	PB-1	P1-8	TRUE	3	TRA:CAVRPPGRNSGGYQKVTF TRB:CASSRRGVTDTQYF	204	CD8-C7
PB-1_CAGCATACACCTCGTT	PB	PB-1	P1-112	FALSE	3	TRA:CALTPLTGGGNKLTF TRB:CSAREGGSMDTQYF	5	CD8-C5

PB-1_CAGCATAGTTGGGACA	PB	PB-1	P1-11	TRUE	3	TRA:CAVEPLYGSGNTGKLIF TRB:CASSSHGQLNQPQHF	147	CD8-C7
PB-1_CAGCATATCGTAGATC	PB	PB-1	P1-3	TRUE	3	TRA:CAFLSGTYKYIF TRB:CASSQSPGQRNYGYTF	308	CD8-C7
PB-1_CAGCATATCTAACGGT	PB	PB-1	P1-5	TRUE	3	TRA:CALSGTPMNRDDKIIF TRB:CSARGRGLAGGPAYEQYF	223	CD8-C5
PB-1_CAGCCGAGTCAGAGGT	PB	PB-1	P1-1	TRUE	3	TRA:CAMSDRMTGGFKTIF TRB:CASSQVGRGGEETQYF	1047	CD8-C7
PB-1_CAGCGACGTCCATCCT	PB	PB-1	P1-14	TRUE	3	TRA:CAASVVNDMRF TRB:CASSHRGGAKNIQYF	109	CD8-C5
PB-1_CAGCGACTCGTAGGTT	PB	PB-1	P1-14	TRUE	3	TRA:CAASVVNDMRF TRB:CASSHRGGAKNIQYF	109	CD8-C5
PB-1_CAGCTAAAGCATCATC	PB	PB-1	P1-10	TRUE	3	TRA:CISNSPMDSSYKLIF TRB:CASSSWGDTDEAFF	162	CD8-C7
PB-1_CAGCTAAGTTAAGGGC	PB	PB-1	P1-16	TRUE	3	TRA:CALSGTPMNRDDKIIF TRB:CASSQSWDLAVLNEQFF	79	CD8-C7
PB-1_CAGCTAATCAGCGACC	PB	PB-1	P1-1	TRUE	3	TRA:CAMSDRMTGGFKTIF TRB:CASSQVGRGGEETQYF	1047	CD8-C5
PB-1_CAGCTGGAGAGACTTA	PB	PB-1	P1-8	TRUE	3	TRA:CAVRPPGRNSGGYQKVTF TRB:CASSRRGVTDTQYF	204	CD8-C7
PB-1_CAGCTGGAGAGTAAGG	PB	PB-1	P1-10	TRUE	3	TRA:CISNSPMDSSYKLIF TRB:CASSSWGDTDEAFF	162	CD8-C7
PB-1_CAGCTGGTCTGGCGAC	PB	PB-1	P1-26	TRUE	3	TRA:CAPRSGGSYIPTF TRB:CASSQDLGLDYGYTF	56	CD8-C7
PB-1_CAGGTGCAGATAGTCA	PB	PB-1	P1-5	TRUE	3	TRA:CALSGTPMNRDDKIIF TRB:CSARGRGLAGGPAYEQYF	223	CD8-C7
PB-1_CAGGTGCAGGCACATG	PB	PB-1	P1-3	TRUE	3	TRA:CAFLSGTYKYIF TRB:CASSQSPGQRNYGYTF	308	CD8-C7
PB-1_CAGGTGCAGGTGATAT	PB	PB-1	P1-1	TRUE	3	TRA:CAMSDRMTGGFKTIF TRB:CASSQVGRGGEETQYF	1047	CD8-C7
PB-1_CAGGTGCCAGCCACCA	PB	PB-1	P1-2	TRUE	3	TRA:CGQNFVF TRB:CASNQRGGPSLYNEQFF	323	CD8-C7
PB-1_CAGTAACAGATCCGAG	PB	PB-1	P1-3	TRUE	3	TRA:CAFLSGTYKYIF TRB:CASSQSPGQRNYGYTF	308	CD8-C7
PB-1_CAGTAACCACCTCGGA	PB	PB-1	P1-8	TRUE	3	TRA:CAVRPPGRNSGGYQKVTF TRB:CASSRRGVTDTQYF	204	CD8-C7
PB-1_CAGTAACGTCTCTTTA	PB	PB-1	P1-14	TRUE	3	TRA:CAASVVNDMRF TRB:CASSHRGGAKNIQYF	109	CD8-C5
PB-1_CAGTAACGTTAGAACA	PB	PB-1	P1-1	TRUE	3	TRA:CAMSDRMTGGFKTIF TRB:CASSQVGRGGEETQYF	1047	CD8-C7
PB-1_CAGTAACGTTATGCGT	PB	PB-1	P1-4	TRUE	3	TRA:CAMKGRDDKIIF TRB:CASTKGVSREAFF	226	CD8-C7
PB-1_CAGTAACTCAATCACG	PB	PB-1	P1-16	TRUE	3	TRA:CALSGTPMNRDDKIIF TRB:CASSQSWDLAVLNEQFF	79	CD8-C7
PB-1_CAGTAACTCTTTAGTC	PB	PB-1	P1-11	TRUE	3	TRA:CAVEPLYGSGNTGKLIF TRB:CASSSHGQLNQPQHF	147	CD8-C7
PB-1_CAGTCCTAGGCATGGT	PB	PB-1	P1-16	TRUE	3	TRA:CALSGTPMNRDDKIIF TRB:CASSQSWDLAVLNEQFF	79	CD8-C7
PB-1_CAGTCCTCACCTATCC	PB	PB-1	P1-10	TRUE	3	TRA:CISNSPMDSSYKLIF TRB:CASSSWGDTDEAFF	162	CD8-C7
PB-1_CAGTCCTGTGTAAGTA	PB	PB-1	P1-4	TRUE	3	TRA:CAMKGRDDKIIF TRB:CASTKGVSREAFF	226	CD8-C7
PB-1_CAGTCCTGTTCCGGCA	PB	PB-1	P1-254	FALSE	2	TRA:CALTVHSSASKIIF TRB:CASSLPGRYSGANVLTF	2	CD8-C7

PB-1_CAGTCCTTCCACTCCA	PB	PB-1	P1-4	TRUE	3	TRA:CAMKGRDDKIIF TRB:CASTKGVSREAFF	226	CD8-C7
PB-1_CAGTCCTTCTAACGGT	PB	PB-1	P1-13	TRUE	3	TRA:CVVMNYGGSQGNLIF TRB:CASSSWGDTDEAFF	112	CD8-C7
PB-1_CAGTCCTTCTTGACGA	PB	PB-1	P1-3	TRUE	3	TRA:CAFLSGTYKYIF TRB:CASSQSPGQRNYGYTF	308	CD8-C7
PB-1_CATATGGAGTCGCCGT	PB	PB-1	P1-12	TRUE	3	TRA:CALSDERGDYKLSF TRB:CASSPGTGSYNEQFF	141	CD8-C5
PB-1_CATATGGCACCGAATT	PB	PB-1	P1-39	TRUE	3	TRA:CAVRDLGGTSYDKVIF TRB:CSARVREVSYEQYF	31	CD8-C5
PB-1_CATATGGCACTTGGAT	PB	PB-1	P1-2	TRUE	3	TRA:CGQNFVF TRB:CASNQRGGPSLYNEQFF	323	CD8-C7
PB-1_CATATGGCATCCGGGT	PB	PB-1	P1-4	TRUE	3	TRA:CAMKGRDDKIIF TRB:CASTKGVSREAFF	226	CD8-C7
PB-1_CATATGGCATGTAGTC	PB	PB-1	P1-1	TRUE	3	TRA:CAMSDRMTGGFKTIF TRB:CASSQVGRGGEETQYF	1047	CD8-C7
PB-1_CATATGGGTTGTCGCG	PB	PB-1	P1-1	TRUE	3	TRA:CAMSDRMTGGFKTIF TRB:CASSQVGRGGEETQYF	1047	CD8-C7
PB-1_CATATGGTCAGGATCT	PB	PB-1	P1-1	TRUE	3	TRA:CAMSDRMTGGFKTIF TRB:CASSQVGRGGEETQYF	1047	CD8-C7
PB-1_CATATTCAGAGCCTAG	PB	PB-1	P1-1	TRUE	3	TRA:CAMSDRMTGGFKTIF TRB:CASSQVGRGGEETQYF	1047	CD8-C7
PB-1_CATATTCAGATAGGAG	PB	PB-1	P1-2	TRUE	3	TRA:CGQNFVF TRB:CASNQRGGPSLYNEQFF	323	CD8-C7
PB-1_CATATTCCACCAGCAC	PB	PB-1	P1-1	TRUE	3	TRA:CAMSDRMTGGFKTIF TRB:CASSQVGRGGEETQYF	1047	CD8-C7
PB-1_CATATTCCACGAGGTA	PB	PB-1	P1-1	TRUE	3	TRA:CAMSDRMTGGFKTIF TRB:CASSQVGRGGEETQYF	1047	CD8-C5
PB-1_CATATTCGTCGCATAT	PB	PB-1	P1-11	TRUE	3	TRA:CAVEPLYGSGNTGKLIF TRB:CASSSHGQLNQPQHF	147	CD8-C7
PB-1_CATATTCTCACCCTCA	PB	PB-1	P1-1	TRUE	3	TRA:CAMSDRMTGGFKTIF TRB:CASSQVGRGGEETQYF	1047	CD8-C7
PB-1_CATATTCTCCGCATAA	PB	PB-1	P1-8	TRUE	3	TRA:CAVRPPGRNSGGYQKVTF TRB:CASSRRGVTDTQYF	204	CD8-C7
PB-1_CATATTCTCTGATACG	PB	PB-1	P1-1	TRUE	3	TRA:CAMSDRMTGGFKTIF TRB:CASSQVGRGGEETQYF	1047	CD8-C7
PB-1_CATCAAGAGGTAAACT	PB	PB-1	P1-3	TRUE	3	TRA:CAFLSGTYKYIF TRB:CASSQSPGQRNYGYTF	308	CD8-C7
PB-1_CATCAAGAGTGCCATT	PB	PB-1	P1-1	TRUE	3	TRA:CAMSDRMTGGFKTIF TRB:CASSQVGRGGEETQYF	1047	CD8-C7
PB-1_CATCAAGCAAAGTGCG	PB	PB-1	P1-40	TRUE	3	TRA:CAVRDVGNTGFQKLVF TRB:CASSLGQGSMKTQYF	27	CD8-C7
PB-1_CATCAAGCACCGAATT	PB	PB-1	P1-149	TRUE	3	TRA:CAVGSSGGSYIPTF TRB:CASSLRTINEKLFF	4	CD8-C5
PB-1_CATCAAGGTCTCCACT	PB	PB-1	P1-3	TRUE	3	TRA:CAFLSGTYKYIF TRB:CASSQSPGQRNYGYTF	308	CD8-C7
PB-1_CATCAAGGTGTTTGTG	PB	PB-1	P1-8	TRUE	3	TRA:CAVRPPGRNSGGYQKVTF TRB:CASSRRGVTDTQYF	204	CD8-C7
PB-1_CATCAAGTCAACTCTT	PB	PB-1	P1-1	TRUE	3	TRA:CAMSDRMTGGFKTIF TRB:CASSQVGRGGEETQYF	1047	CD8-C7
PB-1_CATCAAGTCAGAGCTT	PB	PB-1	P1-35	TRUE	3	TRA:CAVSDRGYFGNEKLTF TRB:CSALDDVRPVIAVYNEQFF	40	CD8-C7
PB-1_CATCAAGTCAGGTTCA	PB	PB-1	P1-1	TRUE	3	TRA:CAMSDRMTGGFKTIF TRB:CASSQVGRGGEETQYF	1047	CD8-C7

PB-1_CATCAGAAGATCTGAA	PB	PB-1	P1-29	TRUE	3	TRA:CATVSFSGGYNKLIF TRB:CASSLDRRGLSYEQYF	50	CD8-C7
PB-1_CATCAGAAGGAGTACC	PB	PB-1	P1-10	TRUE	3	TRA:CISNSPMDSSYKLIF TRB:CASSSWGDTDEAFF	162	CD8-C7
PB-1_CATCAGACAGATCTGT	PB	PB-1	P1-1	TRUE	3	TRA:CAMSDRMTGGFKTIF TRB:CASSQVGRGGEETQYF	1047	CD8-C7
PB-1_CATCAGACAGTCAGCC	PB	PB-1	P1-4	TRUE	3	TRA:CAMKGRDDKIIF TRB:CASTKGVSREAFF	226	CD8-C7
PB-1_CATCAGAGTACGCACC	PB	PB-1	P1-22	TRUE	3	TRA:CASNSGYSTLTF TRB:CASSQVTGGARDEQYF	65	CD8-C7
PB-1_CATCAGAGTTTAGCTG	PB	PB-1	P1-22	TRUE	3	TRA:CASNSGYSTLTF TRB:CASSQVTGGARDEQYF	65	CD8-C5
PB-1_CATCAGATCTTGCAAG	PB	PB-1	P1-1	TRUE	3	TRA:CAMSDRMTGGFKTIF TRB:CASSQVGRGGEETQYF	1047	CD8-C7
PB-1_CATCCACAGAAGGTTT	PB	PB-1	P1-13	TRUE	3	TRA:CVVMNYGGSQGNLIF TRB:CASSSWGDTDEAFF	112	CD8-C7
PB-1_CATCCACAGTGACTCT	PB	PB-1	P1-1	TRUE	3	TRA:CAMSDRMTGGFKTIF TRB:CASSQVGRGGEETQYF	1047	CD8-C7
PB-1_CATCCACTCCTCGCAT	PB	PB-1	P1-11	TRUE	3	TRA:CAVEPLYGSGNTGKLIF TRB:CASSSHGQLNQPQHF	147	CD8-C7
PB-1_CATCCACTCTTGCCGT	PB	PB-1	P1-1	TRUE	3	TRA:CAMSDRMTGGFKTIF TRB:CASSQVGRGGEETQYF	1047	CD8-C7
PB-1_CATCGAACAAATTGCC	PB	PB-1	P1-1	TRUE	3	TRA:CAMSDRMTGGFKTIF TRB:CASSQVGRGGEETQYF	1047	CD8-C5
PB-1_CATCGAACAAGAAAGG	PB	PB-1	P1-31	TRUE	3	TRA:CAVEEGADRLMF TRB:CASSLLGGGELFF	46	CD8-C7
PB-1_CATCGAACAAGCCCAC	PB	PB-1	P1-2	TRUE	3	TRA:CGQNFVF TRB:CASNQRGGPSLYNEQFF	323	CD8-C7
PB-1_CATCGAACAAGCGATG	PB	PB-1	P1-1	TRUE	3	TRA:CAMSDRMTGGFKTIF TRB:CASSQVGRGGEETQYF	1047	CD8-C7
PB-1_CATCGAACAAGTTGTC	PB	PB-1	P1-31	TRUE	3	TRA:CAVEEGADRLMF TRB:CASSLLGGGELFF	46	CD8-C7
PB-1_CATCGAACACGTCAGC	PB	PB-1	P1-2	TRUE	3	TRA:CGQNFVF TRB:CASNQRGGPSLYNEQFF	323	CD8-C7
PB-1_CATCGAAGTCAGCTAT	PB	PB-1	P1-1	TRUE	3	TRA:CAMSDRMTGGFKTIF TRB:CASSQVGRGGEETQYF	1047	CD8-C7
PB-1_CATCGAAGTGCGAAAC	PB	PB-1	P1-39	TRUE	3	TRA:CAVRDLGGTSYDKVIF TRB:CSARVREVSYEQYF	31	CD8-C5
PB-1_CATCGAATCAAACCGT	PB	PB-1	P1-1	TRUE	3	TRA:CAMSDRMTGGFKTIF TRB:CASSQVGRGGEETQYF	1047	CD8-C7
PB-1_CATCGAATCACTTCAT	PB	PB-1	P1-1	TRUE	3	TRA:CAMSDRMTGGFKTIF TRB:CASSQVGRGGEETQYF	1047	CD8-C7
PB-1_CATCGGGGTACTTGAC	PB	PB-1	P1-1	TRUE	3	TRA:CAMSDRMTGGFKTIF TRB:CASSQVGRGGEETQYF	1047	CD8-C7
PB-1_CATCGGGTCCCGGATG	PB	PB-1	P1-10	TRUE	3	TRA:CISNSPMDSSYKLIF TRB:CASSSWGDTDEAFF	162	CD8-C7
PB-1_CATGACAAGGGAAACA	PB	PB-1	P1-11	TRUE	3	TRA:CAVEPLYGSGNTGKLIF TRB:CASSSHGQLNQPQHF	147	CD8-C7
PB-1_CATGACAAGGGATCTG	PB	PB-1	P1-1	TRUE	3	TRA:CAMSDRMTGGFKTIF TRB:CASSQVGRGGEETQYF	1047	CD8-C7
PB-1_CATGACACACGACTCG	PB	PB-1	P1-2	TRUE	3	TRA:CGQNFVF TRB:CASNQRGGPSLYNEQFF	323	CD8-C7
PB-1_CATGACACATCCGCGA	PB	PB-1	P1-14	TRUE	3	TRA:CAASVVNDMRF TRB:CASSHRGGAKNIQYF	109	CD8-C7

PB-1_CATGACACATTCTCAT	PB	PB-1	P1-4	TRUE	3	TRA:CAMKGRDDKIIF TRB:CASTKGVSREAFF	226	CD8-C7
PB-1_CATGCCTAGTACGACG	PB	PB-1	P1-1	TRUE	3	TRA:CAMSDRMTGGFKTIF TRB:CASSQVGRGGEETQYF	1047	CD8-C7
PB-1_CATGCCTCACTCGACG	PB	PB-1	P1-10	TRUE	3	TRA:CISNSPMDSSYKLIF TRB:CASSSWGDTDEAFF	162	CD8-C7
PB-1_CATGCCTTCTCCCTGA	PB	PB-1	P1-1	TRUE	3	TRA:CAMSDRMTGGFKTIF TRB:CASSQVGRGGEETQYF	1047	CD8-C7
PB-1_CATGGCGAGGGCTTGA	PB	PB-1	P1-3	TRUE	3	TRA:CAFLSGTYKYIF TRB:CASSQSPGQRNYGYTF	308	CD8-C7
PB-1_CATGGCGCAATCACAC	PB	PB-1	P1-1	TRUE	3	TRA:CAMSDRMTGGFKTIF TRB:CASSQVGRGGEETQYF	1047	CD8-C7
PB-1_CATGGCGCATCACAAC	PB	PB-1	P1-12	TRUE	3	TRA:CALSDERGDYKLSF TRB:CASSPGTGSYNEQFF	141	CD8-C5
PB-1_CATGGCGGTACGAAAT	PB	PB-1	P1-3	TRUE	3	TRA:CAFLSGTYKYIF TRB:CASSQSPGQRNYGYTF	308	CD8-C7
PB-1_CATGGCGGTTCAGGCC	PB	PB-1	P1-1	TRUE	3	TRA:CAMSDRMTGGFKTIF TRB:CASSQVGRGGEETQYF	1047	CD8-C7
PB-1_CATGGCGTCTGCAAGT	PB	PB-1	P1-1	TRUE	3	TRA:CAMSDRMTGGFKTIF TRB:CASSQVGRGGEETQYF	1047	CD8-C5
PB-1_CATTATCAGAGTACCG	PB	PB-1	P1-1	TRUE	3	TRA:CAMSDRMTGGFKTIF TRB:CASSQVGRGGEETQYF	1047	CD8-C7
PB-1_CATTATCAGGGTGTGT	PB	PB-1	P1-22	TRUE	3	TRA:CASNSGYSTLTF TRB:CASSQVTGGARDEQYF	65	CD8-C7
PB-1_CATTATCCAATGAATG	PB	PB-1	P1-9	TRUE	3	TRA:CAEAEGGGADGLTF TRB:CATSGGDNEQFF	174	CD8-C7
PB-1_CATTATCCATCTATGG	PB	PB-1	P1-629	FALSE	1	TRA:CALSGTPMNRDDKIIF TRB:CASSQVGRGGEETQYF	1	CD8-C7
PB-1_CATTATCGTGAAGGCT	PB	PB-1	P1-1	TRUE	3	TRA:CAMSDRMTGGFKTIF TRB:CASSQVGRGGEETQYF	1047	CD8-C5
PB-1_CATTATCGTTGATTGC	PB	PB-1	P1-1	TRUE	3	TRA:CAMSDRMTGGFKTIF TRB:CASSQVGRGGEETQYF	1047	CD8-C7
PB-1_CATTATCTCCAGAGGA	PB	PB-1	P1-14	TRUE	3	TRA:CAASVVNDMRF TRB:CASSHRGGAKNIQYF	109	CD8-C6
PB-1_CATTATCTCTGTCTAT	PB	PB-1	P1-1	TRUE	3	TRA:CAMSDRMTGGFKTIF TRB:CASSQVGRGGEETQYF	1047	CD8-C5
PB-1_CATTCGCAGATCCCGC	PB	PB-1	P1-2	TRUE	3	TRA:CGQNFVF TRB:CASNQRGGPSLYNEQFF	323	CD8-C7
PB-1_CATTCGCAGCACACAG	PB	PB-1	P1-11	TRUE	3	TRA:CAVEPLYGSGNTGKLIF TRB:CASSSHGQLNQPQHF	147	CD8-C7
PB-1_CATTCGCGTACATGTC	PB	PB-1	P1-5	TRUE	3	TRA:CALSGTPMNRDDKIIF TRB:CSARGRGLAGGPAYEQYF	223	CD8-C7
PB-1_CATTCGCTCTTAACCT	PB	PB-1	P1-11	TRUE	3	TRA:CAVEPLYGSGNTGKLIF TRB:CASSSHGQLNQPQHF	147	CD8-C5
PB-1_CCAATCCGTCCAACTA	PB	PB-1	P1-1	TRUE	3	TRA:CAMSDRMTGGFKTIF TRB:CASSQVGRGGEETQYF	1047	CD8-C7
PB-1_CCAATCCGTCTGATCA	PB	PB-1	P1-13	TRUE	3	TRA:CVVMNYGGSQGNLIF TRB:CASSSWGDTDEAFF	112	CD8-C7
PB-1_CCAATCCGTGCTAGCC	PB	PB-1	P1-5	TRUE	3	TRA:CALSGTPMNRDDKIIF TRB:CSARGRGLAGGPAYEQYF	223	CD8-C7
PB-1_CCACCTACAGATCTGT	PB	PB-1	P1-3	TRUE	3	TRA:CAFLSGTYKYIF TRB:CASSQSPGQRNYGYTF	308	CD8-C7
PB-1_CCACCTAGTCGGCACT	PB	PB-1	P1-1	TRUE	3	TRA:CAMSDRMTGGFKTIF TRB:CASSQVGRGGEETQYF	1047	CD8-C7

PB-1_CCACCTATCAGCATGT	PB	PB-1	P1-10	TRUE	3	TRA:CISNSPMDSSYKLIF TRB:CASSSWGDTDEAFF	162	CD8-C7
PB-1_CCACCTATCGTCTGCT	PB	PB-1	P1-1	TRUE	3	TRA:CAMSDRMTGGFKTIF TRB:CASSQVGRGGEETQYF	1047	CD8-C7
PB-1_CCACGGAAGTTGAGTA	PB	PB-1	P1-18	TRUE	3	TRA:CVADGNYGGQNFVF TRB:CASSSPGLGGGFDTEAFF	75	CD8-C6
PB-1_CCACGGAGTAAGCACG	PB	PB-1	P1-11	TRUE	3	TRA:CAVEPLYGSGNTGKLIF TRB:CASSSHGQLNQPQHF	147	CD8-C7
PB-1_CCACTACCAAACGCGA	PB	PB-1	P1-4	TRUE	3	TRA:CAMKGRDDKIIF TRB:CASTKGVSREAFF	226	CD8-C7
PB-1_CCACTACTCACTTATC	PB	PB-1	P1-26	TRUE	3	TRA:CAPRSGGSYIPTF TRB:CASSQDLGLDYGYTF	56	CD8-C7
PB-1_CCAGCGACAAAGTGCG	PB	PB-1	P1-2	TRUE	3	TRA:CGQNFVF TRB:CASNQRGGPSLYNEQFF	323	CD8-C7
PB-1_CCAGCGAGTAACGACG	PB	PB-1	P1-2	TRUE	3	TRA:CGQNFVF TRB:CASNQRGGPSLYNEQFF	323	CD8-C7
PB-1_CCAGCGAGTAGGAGTC	PB	PB-1	P1-58	TRUE	3	TRA:CAVRPNRDDKIIF TRB:CASSSRLAGGTDTQYF	12	CD8-C7
PB-1_CCAGCGAGTCTAGCGC	PB	PB-1	P1-17	TRUE	3	TRA:CGTGARGSSGSRLTF TRB:CASSRQGMGAFF	78	CD8-C7
PB-1_CCAGCGATCAAGGCTT	PB	PB-1	P1-12	TRUE	3	TRA:CALSDERGDYKLSF TRB:CASSPGTGSYNEQFF	141	CD8-C5
PB-1_CCAGCGATCAATAAGG	PB	PB-1	P1-3	TRUE	3	TRA:CAFLSGTYKYIF TRB:CASSQSPGQRNYGYTF	308	CD8-C7
PB-1_CCAGCGATCAGCGACC	PB	PB-1	P1-2	TRUE	3	TRA:CGQNFVF TRB:CASNQRGGPSLYNEQFF	323	CD8-C7
PB-1_CCAGCGATCTCAAACG	PB	PB-1	P1-1	TRUE	3	TRA:CAMSDRMTGGFKTIF TRB:CASSQVGRGGEETQYF	1047	CD8-C7
PB-1_CCATGTCAGCCCTAAT	PB	PB-1	P1-1	TRUE	3	TRA:CAMSDRMTGGFKTIF TRB:CASSQVGRGGEETQYF	1047	CD8-C5
PB-1_CCATGTCAGTTGTCGT	PB	PB-1	P1-1	TRUE	3	TRA:CAMSDRMTGGFKTIF TRB:CASSQVGRGGEETQYF	1047	CD8-C5
PB-1_CCATGTCCAACAACCT	PB	PB-1	P1-22	TRUE	3	TRA:CASNSGYSTLTF TRB:CASSQVTGGARDEQYF	65	CD8-C5
PB-1_CCATGTCGTTGAACTC	PB	PB-1	P1-8	TRUE	3	TRA:CAVRPPGRNSGGYQKVTF TRB:CASSRRGVTDTQYF	204	CD8-C7
PB-1_CCATTCGGTTCGGGCT	PB	PB-1	P1-4	TRUE	3	TRA:CAMKGRDDKIIF TRB:CASTKGVSREAFF	226	CD8-C7
PB-1_CCCAATCAGACTTTCG	PB	PB-1	P1-35	TRUE	3	TRA:CAVSDRGYFGNEKLTF TRB:CSALDDVRPVIAVYNEQFF	40	CD8-C7
PB-1_CCCAATCAGAGGGCTT	PB	PB-1	P1-3	TRUE	3	TRA:CAFLSGTYKYIF TRB:CASSQSPGQRNYGYTF	308	CD8-C7
PB-1_CCCAATCGTCCTGCTT	PB	PB-1	P1-9	TRUE	3	TRA:CAEAEGGGADGLTF TRB:CATSGGDNEQFF	174	CD8-C7
PB-1_CCCAATCTCCACTCCA	PB	PB-1	P1-71	FALSE	3	TRA:CAVAGGGGADGLTF TRB:CASSCPGLGGSTDTQYF	8	CD8-C6
PB-1_CCCAATCTCGCCTGAG	PB	PB-1	P1-2	TRUE	3	TRA:CGQNFVF TRB:CASNQRGGPSLYNEQFF	323	CD8-C5
PB-1_CCCAGTTAGGTGTTAA	PB	PB-1	P1-145	FALSE	3	TRA:CAFMKHDFYTGANSKLTF TRB:CASSYMFLLGVNTEAFF	4	CD8-C6
PB-1_CCCAGTTAGTGGAGTC	PB	PB-1	P1-647	FALSE	1	TRA:CGTGARGSSGSRLTF TRB:CASSSWGDTDEAFF	1	CD8-C7
PB-1_CCCAGTTCAAGCTGAG	PB	PB-1	P1-12	TRUE	3	TRA:CALSDERGDYKLSF TRB:CASSPGTGSYNEQFF	141	CD8-C5

PB-1_CCCAGTTCATTAGGCT	PB	PB-1	P1-11	TRUE	3	TRA:CAVEPLYGSGNTGKLIF TRB:CASSSHGQLNQPQHF	147	CD8-C7
PB-1_CCCAGTTGTTGTGGAG	PB	PB-1	P1-14	TRUE	3	TRA:CAASVVNDMRF TRB:CASSHRGGAKNIQYF	109	CD8-C5
PB-1_CCCAGTTTCAAAGACA	PB	PB-1	P1-1	TRUE	3	TRA:CAMSDRMTGGFKTIF TRB:CASSQVGRGGEETQYF	1047	CD8-C7
PB-1_CCCAGTTTCCCTTGCA	PB	PB-1	P1-2	TRUE	3	TRA:CGQNFVF TRB:CASNQRGGPSLYNEQFF	323	CD8-C7
PB-1_CCCATACGTGCGGTAA	PB	PB-1	P1-3	TRUE	3	TRA:CAFLSGTYKYIF TRB:CASSQSPGQRNYGYTF	308	CD8-C7
PB-1_CCCTCCTAGATCCGAG	PB	PB-1	P1-15	TRUE	3	TRA:CAVNSSPLGANSKLTF TRB:CASSDSGNFYNEQFF	92	CD8-C2
PB-1_CCCTCCTCAGGTGGAT	PB	PB-1	P1-54	TRUE	3	TRA:CAGWYSGGGADGLTF TRB:CATGAGDNEQFF	13	CD8-C7
PB-1_CCCTCCTTCAGAGCTT	PB	PB-1	P1-8	TRUE	3	TRA:CAVRPPGRNSGGYQKVTF TRB:CASSRRGVTDTQYF	204	CD8-C7
PB-1_CCCTCCTTCTCGATGA	PB	PB-1	P1-5	TRUE	3	TRA:CALSGTPMNRDDKIIF TRB:CSARGRGLAGGPAYEQYF	223	CD8-C7
PB-1_CCCTCCTTCTGCCAGG	PB	PB-1	P1-1	TRUE	3	TRA:CAMSDRMTGGFKTIF TRB:CASSQVGRGGEETQYF	1047	CD8-C7
PB-1_CCGGGATAGACCCACC	PB	PB-1	P1-1	TRUE	3	TRA:CAMSDRMTGGFKTIF TRB:CASSQVGRGGEETQYF	1047	CD8-C7
PB-1_CCGGGATAGACTTGAA	PB	PB-1	P1-1	TRUE	3	TRA:CAMSDRMTGGFKTIF TRB:CASSQVGRGGEETQYF	1047	CD8-C7
PB-1_CCGGGATAGGTCGGAT	PB	PB-1	P1-5	TRUE	3	TRA:CALSGTPMNRDDKIIF TRB:CSARGRGLAGGPAYEQYF	223	CD8-C7
PB-1_CCGGGATAGTTAGCGG	PB	PB-1	P1-1	TRUE	3	TRA:CAMSDRMTGGFKTIF TRB:CASSQVGRGGEETQYF	1047	CD8-C7
PB-1_CCGGGATAGTTAGGTA	PB	PB-1	P1-1	TRUE	3	TRA:CAMSDRMTGGFKTIF TRB:CASSQVGRGGEETQYF	1047	CD8-C7
PB-1_CCGGGATTCTTGTACT	PB	PB-1	P1-11	TRUE	3	TRA:CAVEPLYGSGNTGKLIF TRB:CASSSHGQLNQPQHF	147	CD8-C7
PB-1_CCGGTAGAGAGCCTAG	PB	PB-1	P1-1	TRUE	3	TRA:CAMSDRMTGGFKTIF TRB:CASSQVGRGGEETQYF	1047	CD8-C7
PB-1_CCGGTAGAGATGTTAG	PB	PB-1	P1-3	TRUE	3	TRA:CAFLSGTYKYIF TRB:CASSQSPGQRNYGYTF	308	CD8-C7
PB-1_CCGGTAGAGGGAGTAA	PB	PB-1	P1-14	TRUE	3	TRA:CAASVVNDMRF TRB:CASSHRGGAKNIQYF	109	CD8-C5
PB-1_CCGGTAGAGTGAACAT	PB	PB-1	P1-3	TRUE	3	TRA:CAFLSGTYKYIF TRB:CASSQSPGQRNYGYTF	308	CD8-C7
PB-1_CCGGTAGCACGACGAA	PB	PB-1	P1-1	TRUE	3	TRA:CAMSDRMTGGFKTIF TRB:CASSQVGRGGEETQYF	1047	CD8-C7
PB-1_CCGGTAGGTGAGTATA	PB	PB-1	P1-1	TRUE	3	TRA:CAMSDRMTGGFKTIF TRB:CASSQVGRGGEETQYF	1047	CD8-C7
PB-1_CCGTACTAGATGCGAC	PB	PB-1	P1-1	TRUE	3	TRA:CAMSDRMTGGFKTIF TRB:CASSQVGRGGEETQYF	1047	CD8-C7
PB-1_CCGTACTAGCAAATCA	PB	PB-1	P1-11	TRUE	3	TRA:CAVEPLYGSGNTGKLIF TRB:CASSSHGQLNQPQHF	147	CD8-C7
PB-1_CCGTACTAGTCAAGCG	PB	PB-1	P1-1	TRUE	3	TRA:CAMSDRMTGGFKTIF TRB:CASSQVGRGGEETQYF	1047	CD8-C7
PB-1_CCGTACTCAAAGGAAG	PB	PB-1	P1-9	TRUE	3	TRA:CAEAEGGGADGLTF TRB:CATSGGDNEQFF	174	CD8-C7
PB-1_CCGTACTCATCCGCGA	PB	PB-1	P1-11	TRUE	3	TRA:CAVEPLYGSGNTGKLIF TRB:CASSSHGQLNQPQHF	147	CD8-C7

PB-1_CCGTACTGTCTCATCC	PB	PB-1	P1-8	TRUE	3	TRA:CAVRPPGRNSGGYQKVTF TRB:CASSRRGVTDTQYF	204	CD8-C7
PB-1_CCGTACTTCAGCTTAG	PB	PB-1	P1-1	TRUE	3	TRA:CAMSDRMTGGFKTIF TRB:CASSQVGRGGEETQYF	1047	CD8-C7
PB-1_CCGTACTTCCGATATG	PB	PB-1	P1-5	TRUE	3	TRA:CALSGTPMNRDDKIIF TRB:CSARGRGLAGGPAYEQYF	223	CD8-C7
PB-1_CCGTACTTCTTGAGAC	PB	PB-1	P1-1	TRUE	3	TRA:CAMSDRMTGGFKTIF TRB:CASSQVGRGGEETQYF	1047	CD8-C5
PB-1_CCGTGGAAGCGATCCC	PB	PB-1	P1-14	TRUE	3	TRA:CAASVVNDMRF TRB:CASSHRGGAKNIQYF	109	CD8-C7
PB-1_CCGTGGAAGCTCCTCT	PB	PB-1	P1-3	TRUE	3	TRA:CAFLSGTYKYIF TRB:CASSQSPGQRNYGYTF	308	CD8-C7
PB-1_CCGTGGAAGTGGACGT	PB	PB-1	P1-16	TRUE	3	TRA:CALSGTPMNRDDKIIF TRB:CASSQSWDLAVLNEQFF	79	CD8-C7
PB-1_CCGTGGACAGACAAAT	PB	PB-1	P1-18	TRUE	3	TRA:CVADGNYGQNFVF TRB:CASSSPGLGGGFDTEAFF	75	CD8-C6
PB-1_CCGTGGACAGCTGCTG	PB	PB-1	P1-4	TRUE	3	TRA:CAMKGRDDKIIF TRB:CASTKGVSREAFF	226	CD8-C7
PB-1_CCGTGGACAGTTTACG	PB	PB-1	P1-22	TRUE	3	TRA:CASNSGYSTLTF TRB:CASSQVTGGARDEQYF	65	CD8-C7
PB-1_CCGTGGACATCCTTGC	PB	PB-1	P1-4	TRUE	3	TRA:CAMKGRDDKIIF TRB:CASTKGVSREAFF	226	CD8-C7
PB-1_CCGTGGAGTAACGCGA	PB	PB-1	P1-5	TRUE	3	TRA:CALSGTPMNRDDKIIF TRB:CSARGRGLAGGPAYEQYF	223	CD8-C7
PB-1_CCGTGGAGTAAGGGCT	PB	PB-1	P1-1	TRUE	3	TRA:CAMSDRMTGGFKTIF TRB:CASSQVGRGGEETQYF	1047	CD8-C7
PB-1_CCGTGGAGTATATGAG	PB	PB-1	P1-1	TRUE	3	TRA:CAMSDRMTGGFKTIF TRB:CASSQVGRGGEETQYF	1047	CD8-C7
PB-1_CCGTGGATCACCAGGC	PB	PB-1	P1-40	TRUE	3	TRA:CAVRDVGNTGFQKLVF TRB:CASSLGQGSMKTQYF	27	CD8-C7
PB-1_CCGTGGATCATCACCC	PB	PB-1	P1-1	TRUE	3	TRA:CAMSDRMTGGFKTIF TRB:CASSQVGRGGEETQYF	1047	CD8-C7
PB-1_CCGTGGATCGGCGCTA	PB	PB-1	P1-2	TRUE	3	TRA:CGQNFVF TRB:CASNQRGGPSLYNEQFF	323	CD8-C7
PB-1_CCGTGGATCTGTGCAA	PB	PB-1	P1-1	TRUE	3	TRA:CAMSDRMTGGFKTIF TRB:CASSQVGRGGEETQYF	1047	CD8-C7
PB-1_CCGTTCAAGTTTAGGA	PB	PB-1	P1-1	TRUE	3	TRA:CAMSDRMTGGFKTIF TRB:CASSQVGRGGEETQYF	1047	CD8-C7
PB-1_CCGTTCACAGCTGCTG	PB	PB-1	P1-31	TRUE	3	TRA:CAVEEGADRLMF TRB:CASSLLGGGELFF	46	CD8-C7
PB-1_CCGTTCACATGATCCA	PB	PB-1	P1-7	TRUE	3	TRA:CAVQAGNNDMRF TRB:CASSTTGDFNYGYTF	215	CD8-C5
PB-1_CCGTTCAGTAAGAGGA	PB	PB-1	P1-6	TRUE	3	TRA:CAMREGRERDDKIIF TRB:CASSLPNTEAFF	216	CD8-C7
PB-1_CCGTTCAGTAATCGTC	PB	PB-1	P1-29	TRUE	3	TRA:CATVSFSGGYNKLIF TRB:CASSLDRRGLSYEQYF	50	CD8-C7
PB-1_CCGTTCAGTCCATCCT	PB	PB-1	P1-3	TRUE	3	TRA:CAFLSGTYKYIF TRB:CASSQSPGQRNYGYTF	308	CD8-C7
PB-1_CCGTTCAGTTTGACAC	PB	PB-1	P1-2	TRUE	3	TRA:CGQNFVF TRB:CASNQRGGPSLYNEQFF	323	CD8-C7
PB-1_CCTAAAGAGACCCACC	PB	PB-1	P1-11	TRUE	3	TRA:CAVEPLYGSGNTGKLIF TRB:CASSSHGQLNQPQHF	147	CD8-C7
PB-1_CCTAAAGAGGCTCTTA	PB	PB-1	P1-14	TRUE	3	TRA:CAASVVNDMRF TRB:CASSHRGGAKNIQYF	109	CD8-C5

PB-1_CCTAAAGCATCGATTG	PB	PB-1	P1-1	TRUE	3	TRA:CAMSDRMTGGFKTIF TRB:CASSQVGRGGEETQYF	1047	CD8-C7
PB-1_CCTAAAGGTTCCGGCA	PB	PB-1	P1-1	TRUE	3	TRA:CAMSDRMTGGFKTIF TRB:CASSQVGRGGEETQYF	1047	CD8-C7
PB-1_CCTAAAGTCCAAGTAC	PB	PB-1	P1-3	TRUE	3	TRA:CAFLSGTYKYIF TRB:CASSQSPGQRNYGYTF	308	CD8-C7
PB-1_CCTAAAGTCGACCAGC	PB	PB-1	P1-3	TRUE	3	TRA:CAFLSGTYKYIF TRB:CASSQSPGQRNYGYTF	308	CD8-C7
PB-1_CCTACACAGATGCCAG	PB	PB-1	P1-10	TRUE	3	TRA:CISNSPMDSSYKLIF TRB:CASSSWGDTDEAFF	162	CD8-C7
PB-1_CCTACACCACGTCTCT	PB	PB-1	P1-8	TRUE	3	TRA:CAVRPPGRNSGGYQKVTF TRB:CASSRRGVTDTQYF	204	CD8-C7
PB-1_CCTACACCATATACGC	PB	PB-1	P1-26	TRUE	3	TRA:CAPRSGGSYIPTF TRB:CASSQDLGLDYGYTF	56	CD8-C5
PB-1_CCTACACGTGGTTTCA	PB	PB-1	P1-1	TRUE	3	TRA:CAMSDRMTGGFKTIF TRB:CASSQVGRGGEETQYF	1047	CD8-C7
PB-1_CCTACACGTGTGGTTT	PB	PB-1	P1-3	TRUE	3	TRA:CAFLSGTYKYIF TRB:CASSQSPGQRNYGYTF	308	CD8-C7
PB-1_CCTACACTCTGGAGCC	PB	PB-1	P1-4	TRUE	3	TRA:CAMKGRDDKIIF TRB:CASTKGVSREAFF	226	CD8-C7
PB-1_CCTACCAAGTCAATAG	PB	PB-1	P1-3	TRUE	3	TRA:CAFLSGTYKYIF TRB:CASSQSPGQRNYGYTF	308	CD8-C5
PB-1_CCTACCAAGTTACGGG	PB	PB-1	P1-8	TRUE	3	TRA:CAVRPPGRNSGGYQKVTF TRB:CASSRRGVTDTQYF	204	CD8-C7
PB-1_CCTACCAGTATTCGTG	PB	PB-1	P1-3	TRUE	3	TRA:CAFLSGTYKYIF TRB:CASSQSPGQRNYGYTF	308	CD8-C7
PB-1_CCTACCAGTCGCCATG	PB	PB-1	P1-29	TRUE	3	TRA:CATVSFSGGYNKLIF TRB:CASSLDRRGLSYEQYF	50	CD8-C7
PB-1_CCTACCAGTCTGGTCG	PB	PB-1	P1-10	TRUE	3	TRA:CISNSPMDSSYKLIF TRB:CASSSWGDTDEAFF	162	CD8-C7
PB-1_CCTACCAGTGCCTGCA	PB	PB-1	P1-1	TRUE	3	TRA:CAMSDRMTGGFKTIF TRB:CASSQVGRGGEETQYF	1047	CD8-C7
PB-1_CCTACCATCAGCTGGC	PB	PB-1	P1-5	TRUE	3	TRA:CALSGTPMNRDDKIIF TRB:CSARGRGLAGGPAYEQYF	223	CD8-C5
PB-1_CCTACCATCGGTCTAA	PB	PB-1	P1-1	TRUE	3	TRA:CAMSDRMTGGFKTIF TRB:CASSQVGRGGEETQYF	1047	CD8-C7
PB-1_CCTAGCTAGCCACGCT	PB	PB-1	P1-3	TRUE	3	TRA:CAFLSGTYKYIF TRB:CASSQSPGQRNYGYTF	308	CD8-C7
PB-1_CCTAGCTCACATCCAA	PB	PB-1	P1-8	TRUE	3	TRA:CAVRPPGRNSGGYQKVTF TRB:CASSRRGVTDTQYF	204	CD8-C7
PB-1_CCTAGCTGTCGAACAG	PB	PB-1	P1-29	TRUE	3	TRA:CATVSFSGGYNKLIF TRB:CASSLDRRGLSYEQYF	50	CD8-C7
PB-1_CCTAGCTTCGTTACGA	PB	PB-1	P1-1	TRUE	3	TRA:CAMSDRMTGGFKTIF TRB:CASSQVGRGGEETQYF	1047	CD8-C5
PB-1_CCTAGCTTCTTCAACT	PB	PB-1	P1-1	TRUE	3	TRA:CAMSDRMTGGFKTIF TRB:CASSQVGRGGEETQYF	1047	CD8-C7
PB-1_CCTATTAGTGGCAAAC	PB	PB-1	P1-4	TRUE	3	TRA:CAMKGRDDKIIF TRB:CASTKGVSREAFF	226	CD8-C5
PB-1_CCTATTAGTTTGACTG	PB	PB-1	P1-26	TRUE	3	TRA:CAPRSGGSYIPTF TRB:CASSQDLGLDYGYTF	56	CD8-C5
PB-1_CCTATTATCTAACCGA	PB	PB-1	P1-8	TRUE	3	TRA:CAVRPPGRNSGGYQKVTF TRB:CASSRRGVTDTQYF	204	CD8-C7
PB-1_CCTCAGTAGACAATAC	PB	PB-1	P1-1	TRUE	3	TRA:CAMSDRMTGGFKTIF TRB:CASSQVGRGGEETQYF	1047	CD8-C7

PB-1_CCTCAGTAGCCTTGAT	PB	PB-1	P1-1	TRUE	3	TRA:CAMSDRMTGGFKTIF TRB:CASSQVGRGGEETQYF	1047	CD8-C7
PB-1_CCTCAGTTCAACACTG	PB	PB-1	P1-114	TRUE	3	TRA:CAEPENTGNQFYF TRB:CAISLGNEQFF	5	CD8-C5
PB-1_CCTCAGTTCTACCTGC	PB	PB-1	P1-58	TRUE	3	TRA:CAVRPNRDDKIIF TRB:CASSSRLAGGTDTQYF	12	CD8-C5
PB-1_CCTCTGAAGGGCTCTC	PB	PB-1	P1-4	TRUE	3	TRA:CAMKGRDDKIIF TRB:CASTKGVSREAFF	226	CD8-C7
PB-1_CCTCTGAGTCTACCTC	PB	PB-1	P1-8	TRUE	3	TRA:CAVRPPGRNSGGYQKVTF TRB:CASSRRGVTDTQYF	204	CD8-C7
PB-1_CCTCTGAGTGGACGAT	PB	PB-1	P1-1	TRUE	3	TRA:CAMSDRMTGGFKTIF TRB:CASSQVGRGGEETQYF	1047	CD8-C7
PB-1_CCTCTGATCAACCATG	PB	PB-1	P1-8	TRUE	3	TRA:CAVRPPGRNSGGYQKVTF TRB:CASSRRGVTDTQYF	204	CD8-C7
PB-1_CCTCTGATCTAAGCCA	PB	PB-1	P1-2	TRUE	3	TRA:CGQNFVF TRB:CASNQRGGPSLYNEQFF	323	CD8-C7
PB-1_CCTCTGATCTCACATT	PB	PB-1	P1-14	TRUE	3	TRA:CAASVVNDMRF TRB:CASSHRGGAKNIQYF	109	CD8-C7
PB-1_CCTTACGAGAATGTTG	PB	PB-1	P1-1	TRUE	3	TRA:CAMSDRMTGGFKTIF TRB:CASSQVGRGGEETQYF	1047	CD8-C7
PB-1_CCTTACGCAGCTGTGC	PB	PB-1	P1-1	TRUE	3	TRA:CAMSDRMTGGFKTIF TRB:CASSQVGRGGEETQYF	1047	CD8-C7
PB-1_CCTTACGGTACCTACA	PB	PB-1	P1-4	TRUE	3	TRA:CAMKGRDDKIIF TRB:CASTKGVSREAFF	226	CD8-C7
PB-1_CCTTACGGTTAGTGGG	PB	PB-1	P1-256	FALSE	2	TRA:CAVQAGNNDMRF TRB:CASSQSPGQRNYGYTF	2	CD8-C7
PB-1_CCTTACGTCGGCTTGG	PB	PB-1	P1-8	TRUE	3	TRA:CAVRPPGRNSGGYQKVTF TRB:CASSRRGVTDTQYF	204	CD8-C7
PB-1_CCTTCCCAGTCCAGGA	PB	PB-1	P1-12	TRUE	3	TRA:CALSDERGDYKLSF TRB:CASSPGTGSYNEQFF	141	CD8-C5
PB-1_CCTTCCCCAGTCAGAG	PB	PB-1	P1-9	TRUE	3	TRA:CAEAEGGGADGLTF TRB:CATSGGDNEQFF	174	CD8-C7
PB-1_CCTTCCCCAGTGACAG	PB	PB-1	P1-10	TRUE	3	TRA:CISNSPMDSSYKLIF TRB:CASSSWGDTDEAFF	162	CD8-C7
PB-1_CCTTCCCTCAGTTTGG	PB	PB-1	P1-1	TRUE	3	TRA:CAMSDRMTGGFKTIF TRB:CASSQVGRGGEETQYF	1047	CD8-C7
PB-1_CCTTCCCTCTCTGCTG	PB	PB-1	P1-3	TRUE	3	TRA:CAFLSGTYKYIF TRB:CASSQSPGQRNYGYTF	308	CD8-C7
PB-1_CCTTCGAAGACAAGCC	PB	PB-1	P1-1	TRUE	3	TRA:CAMSDRMTGGFKTIF TRB:CASSQVGRGGEETQYF	1047	CD8-C7
PB-1_CCTTCGAAGGCAGTCA	PB	PB-1	P1-1	TRUE	3	TRA:CAMSDRMTGGFKTIF TRB:CASSQVGRGGEETQYF	1047	CD8-C7
PB-1_CCTTCGACACTTAACG	PB	PB-1	P1-2	TRUE	3	TRA:CGQNFVF TRB:CASNQRGGPSLYNEQFF	323	CD8-C7
PB-1_CCTTCGAGTACCGAGA	PB	PB-1	P1-2	TRUE	3	TRA:CGQNFVF TRB:CASNQRGGPSLYNEQFF	323	CD8-C7
PB-1_CCTTTCTAGCTAGGCA	PB	PB-1	P1-8	TRUE	3	TRA:CAVRPPGRNSGGYQKVTF TRB:CASSRRGVTDTQYF	204	CD8-C7
PB-1_CCTTTCTAGCTGGAAC	PB	PB-1	P1-14	TRUE	3	TRA:CAASVVNDMRF TRB:CASSHRGGAKNIQYF	109	CD8-C7
PB-1_CCTTTCTAGGGTGTTG	PB	PB-1	P1-3	TRUE	3	TRA:CAFLSGTYKYIF TRB:CASSQSPGQRNYGYTF	308	CD8-C7
PB-1_CCTTTCTTCAACGAAA	PB	PB-1	P1-2	TRUE	3	TRA:CGQNFVF TRB:CASNQRGGPSLYNEQFF	323	CD8-C7

PB-1_CGAACATGTCATATGC	PB	PB-1	P1-4	TRUE	3	TRA:CAMKGRDDKIIF TRB:CASTKGVSREAFF	226	CD8-C7
PB-1_CGAACATGTTAAGGGC	PB	PB-1	P1-4	TRUE	3	TRA:CAMKGRDDKIIF TRB:CASTKGVSREAFF	226	CD8-C5
PB-1_CGAACATGTTGGTTTG	PB	PB-1	P1-35	TRUE	3	TRA:CAVSDRGYFGNEKLTF TRB:CSALDDVRPVIAVYNEQFF	40	CD8-C6
PB-1_CGAACATTCTGGAGCC	PB	PB-1	P1-14	TRUE	3	TRA:CAASVVNDMRF TRB:CASSHRGGAKNIQYF	109	CD8-C7
PB-1_CGAATGTAGAGATGAG	PB	PB-1	P1-667	FALSE	1	TRA:CAFLSGTYKYIF TRB:CASTKGVSREAFF	1	CD8-C7
PB-1_CGAATGTAGTAGGCCA	PB	PB-1	P1-1	TRUE	3	TRA:CAMSDRMTGGFKTIF TRB:CASSQVGRGGEETQYF	1047	CD8-C7
PB-1_CGAATGTCAGCAGTTT	PB	PB-1	P1-8	TRUE	3	TRA:CAVRPPGRNSGGYQKVTF TRB:CASSRRGVTDTQYF	204	CD8-C7
PB-1_CGAATGTCATGACGGA	PB	PB-1	P1-63	TRUE	3	TRA:CAEPLNTGNQFYF TRB:CASSVGNEQFF	10	CD8-C7
PB-1_CGAATGTGTCTCGTTC	PB	PB-1	P1-11	TRUE	3	TRA:CAVEPLYGSGNTGKLIF TRB:CASSSHGQLNQPQHF	147	CD8-C7
PB-1_CGACCTTAGCGATGAC	PB	PB-1	P1-8	TRUE	3	TRA:CAVRPPGRNSGGYQKVTF TRB:CASSRRGVTDTQYF	204	CD8-C7
PB-1_CGACCTTAGTGTACCT	PB	PB-1	P1-3	TRUE	3	TRA:CAFLSGTYKYIF TRB:CASSQSPGQRNYGYTF	308	CD8-C7
PB-1_CGACCTTCACCCATGG	PB	PB-1	P1-66	TRUE	3	TRA:CVVSDPPDKLIF TRB:CASSASETGSVYEQYF	9	CD8-C7
PB-1_CGACCTTCACGTCAGC	PB	PB-1	P1-4	TRUE	3	TRA:CAMKGRDDKIIF TRB:CASTKGVSREAFF	226	CD8-C7
PB-1_CGACCTTCAGCCAATT	PB	PB-1	P1-39	TRUE	3	TRA:CAVRDLGGTSYDKVIF TRB:CSARVREVSYEQYF	31	CD8-C7
PB-1_CGACCTTGTCAATGTC	PB	PB-1	P1-42	TRUE	3	TRA:CASFSGGSQGNLIF TRB:CSAGQGSNQPQHF	24	CD4-C6
PB-1_CGACCTTGTGCCTTGG	PB	PB-1	P1-5	TRUE	3	TRA:CALSGTPMNRDDKIIF TRB:CSARGRGLAGGPAYEQYF	223	CD8-C7
PB-1_CGACCTTGTTACTGAC	PB	PB-1	P1-3	TRUE	3	TRA:CAFLSGTYKYIF TRB:CASSQSPGQRNYGYTF	308	CD8-C7
PB-1_CGACCTTTCCCTAATT	PB	PB-1	P1-11	TRUE	3	TRA:CAVEPLYGSGNTGKLIF TRB:CASSSHGQLNQPQHF	147	CD8-C7
PB-1_CGACCTTTCCTCCTAG	PB	PB-1	P1-1	TRUE	3	TRA:CAMSDRMTGGFKTIF TRB:CASSQVGRGGEETQYF	1047	CD8-C7
PB-1_CGACTTCAGATGGCGT	PB	PB-1	P1-1	TRUE	3	TRA:CAMSDRMTGGFKTIF TRB:CASSQVGRGGEETQYF	1047	CD8-C7
PB-1_CGACTTCTCACCGGGT	PB	PB-1	P1-11	TRUE	3	TRA:CAVEPLYGSGNTGKLIF TRB:CASSSHGQLNQPQHF	147	CD8-C7
PB-1_CGACTTCTCAGCAACT	PB	PB-1	P1-2	TRUE	3	TRA:CGQNFVF TRB:CASNQRGGPSLYNEQFF	323	CD8-C7
PB-1_CGAGAAGAGGGCTTCC	PB	PB-1	P1-2	TRUE	3	TRA:CGQNFVF TRB:CASNQRGGPSLYNEQFF	323	CD8-C2
PB-1_CGAGAAGTCACGACTA	PB	PB-1	P1-1	TRUE	3	TRA:CAMSDRMTGGFKTIF TRB:CASSQVGRGGEETQYF	1047	CD8-C7
PB-1_CGAGAAGTCTTGTCAT	PB	PB-1	P1-1	TRUE	3	TRA:CAMSDRMTGGFKTIF TRB:CASSQVGRGGEETQYF	1047	CD8-C7
PB-1_CGAGCACAGACAATAC	PB	PB-1	P1-26	TRUE	3	TRA:CAPRSGGSYIPTF TRB:CASSQDLGLDYGYTF	56	CD8-C7
PB-1_CGAGCACAGTGGTCCC	PB	PB-1	P1-3	TRUE	3	TRA:CAFLSGTYKYIF TRB:CASSQSPGQRNYGYTF	308	CD8-C7

PB-1_CGAGCACCAAGCGAGT	PB	PB-1	P1-1	TRUE	3	TRA:CAMSDRMTGGFKTIF TRB:CASSQVGRGGEETQYF	1047	CD8-C7
PB-1_CGAGCACTCGATAGAA	PB	PB-1	P1-85	FALSE	3	TRA:CAPRSGGSYIPTF TRB:CASSQDTGLDYGYTF	6	CD8-C7
PB-1_CGAGCCAAGCACCGTC	PB	PB-1	P1-3	TRUE	3	TRA:CAFLSGTYKYIF TRB:CASSQSPGQRNYGYTF	308	CD8-C2
PB-1_CGAGCCACAAAGGAAG	PB	PB-1	P1-10	TRUE	3	TRA:CISNSPMDSSYKLIF TRB:CASSSWGDTDEAFF	162	CD8-C7
PB-1_CGAGCCACACGGTAAG	PB	PB-1	P1-36	TRUE	3	TRA:CILRDNAGNNRKLIW TRB:CASSSRQGFPGGANVLTF	40	CD8-C6
PB-1_CGAGCCAGTTACCAGT	PB	PB-1	P1-1	TRUE	3	TRA:CAMSDRMTGGFKTIF TRB:CASSQVGRGGEETQYF	1047	CD8-C7
PB-1_CGAGCCATCATCGATG	PB	PB-1	P1-22	TRUE	3	TRA:CASNSGYSTLTF TRB:CASSQVTGGARDEQYF	65	CD8-C7
PB-1_CGAGCCATCCGCATAA	PB	PB-1	P1-11	TRUE	3	TRA:CAVEPLYGSGNTGKLIF TRB:CASSSHGQLNQPQHF	147	CD8-C7
PB-1_CGAGCCATCTGTGCAA	PB	PB-1	P1-1	TRUE	3	TRA:CAMSDRMTGGFKTIF TRB:CASSQVGRGGEETQYF	1047	CD8-C7
PB-1_CGATCGGGTCGAACAG	PB	PB-1	P1-35	TRUE	3	TRA:CAVSDRGYFGNEKLTF TRB:CSALDDVRPVIAVYNEQFF	40	CD8-C7
PB-1_CGATCGGGTTCCACGG	PB	PB-1	P1-7	TRUE	3	TRA:CAVQAGNNDMRF TRB:CASSTTGDFNYGYTF	215	CD8-C7
PB-1_CGATCGGTCTACGAGT	PB	PB-1	P1-18	TRUE	3	TRA:CVADGNYGQNFVF TRB:CASSSPGLGGGFDTEAFF	75	CD8-C7
PB-1_CGATGGCCAGTGGAGT	PB	PB-1	P1-13	TRUE	3	TRA:CVVMNYGGSQGNLIF TRB:CASSSWGDTDEAFF	112	CD8-C7
PB-1_CGATGGCTCGCAAACT	PB	PB-1	P1-22	TRUE	3	TRA:CASNSGYSTLTF TRB:CASSQVTGGARDEQYF	65	CD8-C7
PB-1_CGATGGCTCTATCCCG	PB	PB-1	P1-8	TRUE	3	TRA:CAVRPPGRNSGGYQKVTF TRB:CASSRRGVTDTQYF	204	CD8-C7
PB-1_CGATGTAAGACTCGGA	PB	PB-1	P1-3	TRUE	3	TRA:CAFLSGTYKYIF TRB:CASSQSPGQRNYGYTF	308	CD8-C7
PB-1_CGATGTACACACTGCG	PB	PB-1	P1-13	TRUE	3	TRA:CVVMNYGGSQGNLIF TRB:CASSSWGDTDEAFF	112	CD8-C7
PB-1_CGATGTATCTCGATGA	PB	PB-1	P1-1	TRUE	3	TRA:CAMSDRMTGGFKTIF TRB:CASSQVGRGGEETQYF	1047	CD8-C7
PB-1_CGATGTATCTCTGTCG	PB	PB-1	P1-2	TRUE	3	TRA:CGQNFVF TRB:CASNQRGGPSLYNEQFF	323	CD8-C7
PB-1_CGATGTATCTGGGCCA	PB	PB-1	P1-12	TRUE	3	TRA:CALSDERGDYKLSF TRB:CASSPGTGSYNEQFF	141	CD8-C7
PB-1_CGATTGAAGAGTACAT	PB	PB-1	P1-2	TRUE	3	TRA:CGQNFVF TRB:CASNQRGGPSLYNEQFF	323	CD8-C7
PB-1_CGATTGAAGGGCATGT	PB	PB-1	P1-3	TRUE	3	TRA:CAFLSGTYKYIF TRB:CASSQSPGQRNYGYTF	308	CD8-C7
PB-1_CGATTGAAGTTACCCA	PB	PB-1	P1-2	TRUE	3	TRA:CGQNFVF TRB:CASNQRGGPSLYNEQFF	323	CD8-C5
PB-1_CGATTGACAGACGCAA	PB	PB-1	P1-40	TRUE	3	TRA:CAVRDVGNTGFQKLVF TRB:CASSLGQGSMKTQYF	27	CD8-C7
PB-1_CGATTGACAGCTCGCA	PB	PB-1	P1-1	TRUE	3	TRA:CAMSDRMTGGFKTIF TRB:CASSQVGRGGEETQYF	1047	CD8-C7
PB-1_CGATTGAGTACTCAAC	PB	PB-1	P1-4	TRUE	3	TRA:CAMKGRDDKIIF TRB:CASTKGVSREAFF	226	CD8-C7
PB-1_CGATTGAGTTGGTTTG	PB	PB-1	P1-10	TRUE	3	TRA:CISNSPMDSSYKLIF TRB:CASSSWGDTDEAFF	162	CD8-C7

PB-1_CGCCAAGAGCGATATA	PB	PB-1	P1-8	TRUE	3	TRA:CAVRPPGRNSGGYQKVTF TRB:CASSRRGVTDTQYF	204	CD8-C7
PB-1_CGCCAAGAGTGAACGC	PB	PB-1	P1-115	FALSE	3	TRA:CAVKGSSNTGKLIF TRB:CASSYSYGRTGTGTYEQYF	5	CD8-C7
PB-1_CGCCAAGCAATCGGTT	PB	PB-1	P1-1	TRUE	3	TRA:CAMSDRMTGGFKTIF TRB:CASSQVGRGGEETQYF	1047	CD8-C5
PB-1_CGCCAAGCATATGAGA	PB	PB-1	P1-1	TRUE	3	TRA:CAMSDRMTGGFKTIF TRB:CASSQVGRGGEETQYF	1047	CD8-C7
PB-1_CGCCAAGGTAAACGCG	PB	PB-1	P1-29	TRUE	3	TRA:CATVSFSGGYNKLIF TRB:CASSLDRRGLSYEQYF	50	CD8-C7
PB-1_CGCCAAGGTCAAAGCG	PB	PB-1	P1-1	TRUE	3	TRA:CAMSDRMTGGFKTIF TRB:CASSQVGRGGEETQYF	1047	CD8-C7
PB-1_CGCCAAGGTGGCGAAT	PB	PB-1	P1-3	TRUE	3	TRA:CAFLSGTYKYIF TRB:CASSQSPGQRNYGYTF	308	CD8-C7
PB-1_CGCCAAGTCTCAACTT	PB	PB-1	P1-1	TRUE	3	TRA:CAMSDRMTGGFKTIF TRB:CASSQVGRGGEETQYF	1047	CD8-C7
PB-1_CGCCAAGTCTTGTATC	PB	PB-1	P1-1	TRUE	3	TRA:CAMSDRMTGGFKTIF TRB:CASSQVGRGGEETQYF	1047	CD8-C7
PB-1_CGCGGTACAACTGCGC	PB	PB-1	P1-3	TRUE	3	TRA:CAFLSGTYKYIF TRB:CASSQSPGQRNYGYTF	308	CD8-C7
PB-1_CGCGGTACAATAAGCA	PB	PB-1	P1-1	TRUE	3	TRA:CAMSDRMTGGFKTIF TRB:CASSQVGRGGEETQYF	1047	CD8-C7
PB-1_CGCGGTACACGAAAGC	PB	PB-1	P1-13	TRUE	3	TRA:CVVMNYGGSQGNLIF TRB:CASSSWGDTDEAFF	112	CD8-C7
PB-1_CGCGGTACAGGAATGC	PB	PB-1	P1-1	TRUE	3	TRA:CAMSDRMTGGFKTIF TRB:CASSQVGRGGEETQYF	1047	CD8-C7
PB-1_CGCGGTACATTGTGCA	PB	PB-1	P1-40	TRUE	3	TRA:CAVRDVGNTGFQKLVF TRB:CASSLGQGSMKTQYF	27	CD8-C7
PB-1_CGCGGTAGTCCGACGT	PB	PB-1	P1-14	TRUE	3	TRA:CAASVVNDMRF TRB:CASSHRGGAKNIQYF	109	CD8-C5
PB-1_CGCGGTAGTGACCAAG	PB	PB-1	P1-1	TRUE	3	TRA:CAMSDRMTGGFKTIF TRB:CASSQVGRGGEETQYF	1047	CD8-C7
PB-1_CGCGGTAGTTTAGCTG	PB	PB-1	P1-1	TRUE	3	TRA:CAMSDRMTGGFKTIF TRB:CASSQVGRGGEETQYF	1047	CD8-C7
PB-1_CGCGGTATCCACGACG	PB	PB-1	P1-3	TRUE	3	TRA:CAFLSGTYKYIF TRB:CASSQSPGQRNYGYTF	308	CD8-C7
PB-1_CGCGGTATCGTACCGG	PB	PB-1	P1-16	TRUE	3	TRA:CALSGTPMNRDDKIIF TRB:CASSQSWDLAVLNEQFF	79	CD8-C7
PB-1_CGCGGTATCTCCCTGA	PB	PB-1	P1-11	TRUE	3	TRA:CAVEPLYGSGNTGKLIF TRB:CASSSHGQLNQPQHF	147	CD8-C7
PB-1_CGCGTTTAGGCCCTCA	PB	PB-1	P1-3	TRUE	3	TRA:CAFLSGTYKYIF TRB:CASSQSPGQRNYGYTF	308	CD8-C7
PB-1_CGCGTTTAGTGTTTGC	PB	PB-1	P1-8	TRUE	3	TRA:CAVRPPGRNSGGYQKVTF TRB:CASSRRGVTDTQYF	204	CD8-C7
PB-1_CGCGTTTGTGGCAAAC	PB	PB-1	P1-5	TRUE	3	TRA:CALSGTPMNRDDKIIF TRB:CSARGRGLAGGPAYEQYF	223	CD8-C7
PB-1_CGCGTTTTCACTTACT	PB	PB-1	P1-11	TRUE	3	TRA:CAVEPLYGSGNTGKLIF TRB:CASSSHGQLNQPQHF	147	CD8-C7
PB-1_CGCGTTTTCATTATCC	PB	PB-1	P1-1	TRUE	3	TRA:CAMSDRMTGGFKTIF TRB:CASSQVGRGGEETQYF	1047	CD8-C5
PB-1_CGCTATCAGCCGATTT	PB	PB-1	P1-1	TRUE	3	TRA:CAMSDRMTGGFKTIF TRB:CASSQVGRGGEETQYF	1047	CD8-C5
PB-1_CGCTATCAGGTAAACT	PB	PB-1	P1-13	TRUE	3	TRA:CVVMNYGGSQGNLIF TRB:CASSSWGDTDEAFF	112	CD8-C7

PB-1_CGCTATCGTTGTGGCC	PB	PB-1	P1-1	TRUE	3	TRA:CAMSDRMTGGFKTIF TRB:CASSQVGRGGEETQYF	1047	CD8-C7
PB-1_CGCTATCTCTGCAGTA	PB	PB-1	P1-16	TRUE	3	TRA:CALSGTPMNRDDKIIF TRB:CASSQSWDLAVLNEQFF	79	CD8-C7
PB-1_CGCTGGAAGGCTCATT	PB	PB-1	P1-1	TRUE	3	TRA:CAMSDRMTGGFKTIF TRB:CASSQVGRGGEETQYF	1047	CD8-C7
PB-1_CGCTGGAAGTACGCGA	PB	PB-1	P1-177	FALSE	3	TRA:CAVLQAGTALIF TRB:CASSVNRDKRTDTQYF	3	CD8-C7
PB-1_CGCTGGACAGGTCGTC	PB	PB-1	P1-26	TRUE	3	TRA:CAPRSGGSYIPTF TRB:CASSQDLGLDYGYTF	56	CD8-C7
PB-1_CGCTGGAGTATGAATG	PB	PB-1	P1-26	TRUE	3	TRA:CAPRSGGSYIPTF TRB:CASSQDLGLDYGYTF	56	CD8-C5
PB-1_CGCTGGAGTCTCTTTA	PB	PB-1	P1-12	TRUE	3	TRA:CALSDERGDYKLSF TRB:CASSPGTGSYNEQFF	141	CD8-C5
PB-1_CGCTGGATCGGCGCTA	PB	PB-1	P1-2	TRUE	3	TRA:CGQNFVF TRB:CASNQRGGPSLYNEQFF	323	CD8-C7
PB-1_CGCTTCAAGCTGAACG	PB	PB-1	P1-1	TRUE	3	TRA:CAMSDRMTGGFKTIF TRB:CASSQVGRGGEETQYF	1047	CD8-C5
PB-1_CGCTTCAAGGCCCTTG	PB	PB-1	P1-6	TRUE	3	TRA:CAMREGRERDDKIIF TRB:CASSLPNTEAFF	216	CD8-C5
PB-1_CGCTTCAAGTACGATA	PB	PB-1	P1-5	TRUE	3	TRA:CALSGTPMNRDDKIIF TRB:CSARGRGLAGGPAYEQYF	223	CD8-C7
PB-1_CGCTTCAAGTAGGCCA	PB	PB-1	P1-13	TRUE	3	TRA:CVVMNYGGSQGNLIF TRB:CASSSWGDTDEAFF	112	CD8-C7
PB-1_CGCTTCACACATTCGA	PB	PB-1	P1-2	TRUE	3	TRA:CGQNFVF TRB:CASNQRGGPSLYNEQFF	323	CD8-C7
PB-1_CGCTTCAGTCTCTCTG	PB	PB-1	P1-1	TRUE	3	TRA:CAMSDRMTGGFKTIF TRB:CASSQVGRGGEETQYF	1047	CD8-C7
PB-1_CGCTTCATCTTAACCT	PB	PB-1	P1-5	TRUE	3	TRA:CALSGTPMNRDDKIIF TRB:CSARGRGLAGGPAYEQYF	223	CD8-C5
PB-1_CGGACACAGTGGGATC	PB	PB-1	P1-35	TRUE	3	TRA:CAVSDRGYFGNEKLTF TRB:CSALDDVRPVIAVYNEQFF	40	CD8-C6
PB-1_CGGACACGTCCTGCTT	PB	PB-1	P1-5	TRUE	3	TRA:CALSGTPMNRDDKIIF TRB:CSARGRGLAGGPAYEQYF	223	CD8-C7
PB-1_CGGACACTCCCGGATG	PB	PB-1	P1-2	TRUE	3	TRA:CGQNFVF TRB:CASNQRGGPSLYNEQFF	323	CD8-C7
PB-1_CGGACACTCTAACGGT	PB	PB-1	P1-11	TRUE	3	TRA:CAVEPLYGSGNTGKLIF TRB:CASSSHGQLNQPQHF	147	CD8-C7
PB-1_CGGACACTCTGGGCCA	PB	PB-1	P1-4	TRUE	3	TRA:CAMKGRDDKIIF TRB:CASTKGVSREAFF	226	CD8-C7
PB-1_CGGACGTAGATCCCGC	PB	PB-1	P1-10	TRUE	3	TRA:CISNSPMDSSYKLIF TRB:CASSSWGDTDEAFF	162	CD8-C7
PB-1_CGGACGTCACAACGCC	PB	PB-1	P1-18	TRUE	3	TRA:CVADGNYGQNFVF TRB:CASSSPGLGGGFDTEAFF	75	CD8-C6
PB-1_CGGACGTCACACTGCG	PB	PB-1	P1-14	TRUE	3	TRA:CAASVVNDMRF TRB:CASSHRGGAKNIQYF	109	CD8-C5
PB-1_CGGACGTTCGTCGTTC	PB	PB-1	P1-699	FALSE	1	TRA:CAMKGRDDKIIF TRB:CASSFPTGGRTGRTEAFF	1	CD8-C7
PB-1_CGGACGTTCTGATACG	PB	PB-1	P1-4	TRUE	3	TRA:CAMKGRDDKIIF TRB:CASTKGVSREAFF	226	CD8-C7
PB-1_CGGACTGAGGCGACAT	PB	PB-1	P1-3	TRUE	3	TRA:CAFLSGTYKYIF TRB:CASSQSPGQRNYGYTF	308	CD8-C7
PB-1_CGGACTGAGTGCCATT	PB	PB-1	P1-1	TRUE	3	TRA:CAMSDRMTGGFKTIF TRB:CASSQVGRGGEETQYF	1047	CD8-C7

PB-1_CGGACTGGTTCGTTGA	PB	PB-1	P1-40	TRUE	3	TRA:CAVRDVGNTGFQKLVF TRB:CASSLGQGSMKTQYF	27	CD8-C5
PB-1_CGGACTGTCATTTGGG	PB	PB-1	P1-1	TRUE	3	TRA:CAMSDRMTGGFKTIF TRB:CASSQVGRGGEETQYF	1047	CD8-C7
PB-1_CGGACTGTCTAGCACA	PB	PB-1	P1-39	TRUE	3	TRA:CAVRDLGGTSYDKVIF TRB:CSARVREVSYEQYF	31	CD8-C5
PB-1_CGGAGCTAGTAGCCGA	PB	PB-1	P1-1	TRUE	3	TRA:CAMSDRMTGGFKTIF TRB:CASSQVGRGGEETQYF	1047	CD8-C7
PB-1_CGGAGCTAGTGAACAT	PB	PB-1	P1-8	TRUE	3	TRA:CAVRPPGRNSGGYQKVTF TRB:CASSRRGVTDTQYF	204	CD8-C7
PB-1_CGGAGCTCATGGATGG	PB	PB-1	P1-6	TRUE	3	TRA:CAMREGRERDDKIIF TRB:CASSLPNTEAFF	216	CD8-C5
PB-1_CGGAGCTCATTCCTGC	PB	PB-1	P1-1	TRUE	3	TRA:CAMSDRMTGGFKTIF TRB:CASSQVGRGGEETQYF	1047	CD8-C7
PB-1_CGGAGCTTCGATCCCT	PB	PB-1	P1-1	TRUE	3	TRA:CAMSDRMTGGFKTIF TRB:CASSQVGRGGEETQYF	1047	CD8-C7
PB-1_CGGAGCTTCGTTACAG	PB	PB-1	P1-16	TRUE	3	TRA:CALSGTPMNRDDKIIF TRB:CASSQSWDLAVLNEQFF	79	CD8-C7
PB-1_CGGAGTCAGAAGAAGC	PB	PB-1	P1-1	TRUE	3	TRA:CAMSDRMTGGFKTIF TRB:CASSQVGRGGEETQYF	1047	CD8-C5
PB-1_CGGAGTCAGAATGTGT	PB	PB-1	P1-1	TRUE	3	TRA:CAMSDRMTGGFKTIF TRB:CASSQVGRGGEETQYF	1047	CD8-C5
PB-1_CGGAGTCAGGAGCGAG	PB	PB-1	P1-1	TRUE	3	TRA:CAMSDRMTGGFKTIF TRB:CASSQVGRGGEETQYF	1047	CD8-C7
PB-1_CGGAGTCCAGCTCGCA	PB	PB-1	P1-1	TRUE	3	TRA:CAMSDRMTGGFKTIF TRB:CASSQVGRGGEETQYF	1047	CD8-C7
PB-1_CGGCTAGAGACCTTTG	PB	PB-1	P1-1	TRUE	3	TRA:CAMSDRMTGGFKTIF TRB:CASSQVGRGGEETQYF	1047	CD8-C7
PB-1_CGGCTAGCAAGCGATG	PB	PB-1	P1-14	TRUE	3	TRA:CAASVVNDMRF TRB:CASSHRGGAKNIQYF	109	CD8-C7
PB-1_CGGCTAGCAGTGACAG	PB	PB-1	P1-1	TRUE	3	TRA:CAMSDRMTGGFKTIF TRB:CASSQVGRGGEETQYF	1047	CD8-C7
PB-1_CGGCTAGGTGAGGGTT	PB	PB-1	P1-53	TRUE	3	TRA:CALSERDTDKLIF TRB:CASSILQGVITEAFF	13	CD8-C7
PB-1_CGGCTAGGTTCAGTAC	PB	PB-1	P1-22	TRUE	3	TRA:CASNSGYSTLTF TRB:CASSQVTGGARDEQYF	65	CD8-C5
PB-1_CGGCTAGGTTGCCTCT	PB	PB-1	P1-11	TRUE	3	TRA:CAVEPLYGSGNTGKLIF TRB:CASSSHGQLNQPQHF	147	CD8-C7
PB-1_CGGGTCAAGTGAACGC	PB	PB-1	P1-6	TRUE	3	TRA:CAMREGRERDDKIIF TRB:CASSLPNTEAFF	216	CD8-C5
PB-1_CGGGTCACAAGCTGTT	PB	PB-1	P1-10	TRUE	3	TRA:CISNSPMDSSYKLIF TRB:CASSSWGDTDEAFF	162	CD8-C7
PB-1_CGGGTCACATAAGACA	PB	PB-1	P1-5	TRUE	3	TRA:CALSGTPMNRDDKIIF TRB:CSARGRGLAGGPAYEQYF	223	CD8-C7
PB-1_CGGGTCAGTGTTAAGA	PB	PB-1	P1-1	TRUE	3	TRA:CAMSDRMTGGFKTIF TRB:CASSQVGRGGEETQYF	1047	CD8-C5
PB-1_CGGGTCATCTGCAAGT	PB	PB-1	P1-2	TRUE	3	TRA:CGQNFVF TRB:CASNQRGGPSLYNEQFF	323	CD8-C7
PB-1_CGGTTAACAACGATGG	PB	PB-1	P1-1	TRUE	3	TRA:CAMSDRMTGGFKTIF TRB:CASSQVGRGGEETQYF	1047	CD8-C7
PB-1_CGGTTAACAGTGAGTG	PB	PB-1	P1-2	TRUE	3	TRA:CGQNFVF TRB:CASNQRGGPSLYNEQFF	323	CD8-C7
PB-1_CGGTTAATCACAAACC	PB	PB-1	P1-5	TRUE	3	TRA:CALSGTPMNRDDKIIF TRB:CSARGRGLAGGPAYEQYF	223	CD8-C7

PB-1_CGGTTAATCCGCGGTA	PB	PB-1	P1-1	TRUE	3	TRA:CAMSDRMTGGFKTIF TRB:CASSQVGRGGEETQYF	1047	CD8-C7
PB-1_CGGTTAATCCTAAGTG	PB	PB-1	P1-85	FALSE	3	TRA:CAPRSGGSYIPTF TRB:CASSQDTGLDYGYTF	6	CD8-C5
PB-1_CGGTTAATCCTTTCGG	PB	PB-1	P1-63	TRUE	3	TRA:CAEPLNTGNQFYF TRB:CASSVGNEQFF	10	CD8-C7
PB-1_CGGTTAATCTCGAGTA	PB	PB-1	P1-3	TRUE	3	TRA:CAFLSGTYKYIF TRB:CASSQSPGQRNYGYTF	308	CD8-C7
PB-1_CGTAGCGGTCGAACAG	PB	PB-1	P1-1	TRUE	3	TRA:CAMSDRMTGGFKTIF TRB:CASSQVGRGGEETQYF	1047	CD8-C7
PB-1_CGTAGCGGTCGGCTCA	PB	PB-1	P1-5	TRUE	3	TRA:CALSGTPMNRDDKIIF TRB:CSARGRGLAGGPAYEQYF	223	CD8-C7
PB-1_CGTAGCGTCAGCACAT	PB	PB-1	P1-4	TRUE	3	TRA:CAMKGRDDKIIF TRB:CASTKGVSREAFF	226	CD8-C7
PB-1_CGTAGGCAGAGGTTGC	PB	PB-1	P1-1	TRUE	3	TRA:CAMSDRMTGGFKTIF TRB:CASSQVGRGGEETQYF	1047	CD8-C7
PB-1_CGTAGGCAGATATGGT	PB	PB-1	P1-22	TRUE	3	TRA:CASNSGYSTLTF TRB:CASSQVTGGARDEQYF	65	CD8-C7
PB-1_CGTAGGCCAGGGTATG	PB	PB-1	P1-3	TRUE	3	TRA:CAFLSGTYKYIF TRB:CASSQSPGQRNYGYTF	308	CD8-C7
PB-1_CGTAGGCCATTGTGCA	PB	PB-1	P1-1	TRUE	3	TRA:CAMSDRMTGGFKTIF TRB:CASSQVGRGGEETQYF	1047	CD8-C5
PB-1_CGTCACTAGCCTTGAT	PB	PB-1	P1-8	TRUE	3	TRA:CAVRPPGRNSGGYQKVTF TRB:CASSRRGVTDTQYF	204	CD8-C7
PB-1_CGTCACTCAAAGGAAG	PB	PB-1	P1-1	TRUE	3	TRA:CAMSDRMTGGFKTIF TRB:CASSQVGRGGEETQYF	1047	CD8-C7
PB-1_CGTCACTCACCAGGTC	PB	PB-1	P1-16	TRUE	3	TRA:CALSGTPMNRDDKIIF TRB:CASSQSWDLAVLNEQFF	79	CD8-C7
PB-1_CGTCACTCAGGACGTA	PB	PB-1	P1-3	TRUE	3	TRA:CAFLSGTYKYIF TRB:CASSQSPGQRNYGYTF	308	CD8-C7
PB-1_CGTCACTGTAGGACAC	PB	PB-1	P1-1	TRUE	3	TRA:CAMSDRMTGGFKTIF TRB:CASSQVGRGGEETQYF	1047	CD8-C7
PB-1_CGTCACTGTGAGTGAC	PB	PB-1	P1-1	TRUE	3	TRA:CAMSDRMTGGFKTIF TRB:CASSQVGRGGEETQYF	1047	CD8-C7
PB-1_CGTCACTTCCGAATGT	PB	PB-1	P1-1	TRUE	3	TRA:CAMSDRMTGGFKTIF TRB:CASSQVGRGGEETQYF	1047	CD8-C7
PB-1_CGTCACTTCGCAGGCT	PB	PB-1	P1-10	TRUE	3	TRA:CISNSPMDSSYKLIF TRB:CASSSWGDTDEAFF	162	CD8-C7
PB-1_CGTCACTTCGGCATCG	PB	PB-1	P1-3	TRUE	3	TRA:CAFLSGTYKYIF TRB:CASSQSPGQRNYGYTF	308	CD8-C6
PB-1_CGTCACTTCTAACGGT	PB	PB-1	P1-9	TRUE	3	TRA:CAEAEGGGADGLTF TRB:CATSGGDNEQFF	174	CD8-C5
PB-1_CGTCAGGAGAACTCGG	PB	PB-1	P1-9	TRUE	3	TRA:CAEAEGGGADGLTF TRB:CATSGGDNEQFF	174	CD8-C5
PB-1_CGTCAGGAGAGGTACC	PB	PB-1	P1-1	TRUE	3	TRA:CAMSDRMTGGFKTIF TRB:CASSQVGRGGEETQYF	1047	CD8-C7
PB-1_CGTCAGGCAATTCCTT	PB	PB-1	P1-1	TRUE	3	TRA:CAMSDRMTGGFKTIF TRB:CASSQVGRGGEETQYF	1047	CD8-C7
PB-1_CGTCAGGCACGTCAGC	PB	PB-1	P1-10	TRUE	3	TRA:CISNSPMDSSYKLIF TRB:CASSSWGDTDEAFF	162	CD8-C7
PB-1_CGTCAGGCAGACTCGC	PB	PB-1	P1-1	TRUE	3	TRA:CAMSDRMTGGFKTIF TRB:CASSQVGRGGEETQYF	1047	CD8-C5
PB-1_CGTCAGGGTCTTTCAT	PB	PB-1	P1-1	TRUE	3	TRA:CAMSDRMTGGFKTIF TRB:CASSQVGRGGEETQYF	1047	CD8-C7

PB-1_CGTCAGGGTTCTGAAC	PB	PB-1	P1-9	TRUE	3	TRA:CAEAEGGGADGLTF TRB:CATSGGDNEQFF	174	CD8-C7
PB-1_CGTCAGGGTTGACGTT	PB	PB-1	P1-16	TRUE	3	TRA:CALSGTPMNRDDKIIF TRB:CASSQSWDLAVLNEQFF	79	CD8-C7
PB-1_CGTCAGGTCGTGGACC	PB	PB-1	P1-10	TRUE	3	TRA:CISNSPMDSSYKLIF TRB:CASSSWGDTDEAFF	162	CD8-C7
PB-1_CGTCCATGTTGAGGTG	PB	PB-1	P1-22	TRUE	3	TRA:CASNSGYSTLTF TRB:CASSQVTGGARDEQYF	65	CD8-C5
PB-1_CGTCCATTCAAGGCTT	PB	PB-1	P1-2	TRUE	3	TRA:CGQNFVF TRB:CASNQRGGPSLYNEQFF	323	CD8-C7
PB-1_CGTCTACAGTAATCCC	PB	PB-1	P1-22	TRUE	3	TRA:CASNSGYSTLTF TRB:CASSQVTGGARDEQYF	65	CD8-C7
PB-1_CGTCTACCAAATCCGT	PB	PB-1	P1-713	FALSE	1	TRA:CAVRDLGGTSYDKVIF TRB:CASSLGQRGLQYF	1	CD8-C5
PB-1_CGTCTACCAGTCTTCC	PB	PB-1	P1-2	TRUE	3	TRA:CGQNFVF TRB:CASNQRGGPSLYNEQFF	323	CD8-C7
PB-1_CGTCTACGTCAGAATA	PB	PB-1	P1-1	TRUE	3	TRA:CAMSDRMTGGFKTIF TRB:CASSQVGRGGEETQYF	1047	CD8-C7
PB-1_CGTCTACTCTTCGGTC	PB	PB-1	P1-1	TRUE	3	TRA:CAMSDRMTGGFKTIF TRB:CASSQVGRGGEETQYF	1047	CD8-C7
PB-1_CGTGAGCCAGGGAGAG	PB	PB-1	P1-1	TRUE	3	TRA:CAMSDRMTGGFKTIF TRB:CASSQVGRGGEETQYF	1047	CD8-C5
PB-1_CGTGAGCGTATATCCG	PB	PB-1	P1-2	TRUE	3	TRA:CGQNFVF TRB:CASNQRGGPSLYNEQFF	323	CD8-C7
PB-1_CGTGAGCTCAACCAAC	PB	PB-1	P1-35	TRUE	3	TRA:CAVSDRGYFGNEKLTF TRB:CSALDDVRPVIAVYNEQFF	40	CD8-C7
PB-1_CGTGTAACAGAGCCAA	PB	PB-1	P1-1	TRUE	3	TRA:CAMSDRMTGGFKTIF TRB:CASSQVGRGGEETQYF	1047	CD8-C7
PB-1_CGTGTAACAGTAAGCG	PB	PB-1	P1-3	TRUE	3	TRA:CAFLSGTYKYIF TRB:CASSQSPGQRNYGYTF	308	CD8-C7
PB-1_CGTGTAAGTCCTCTTG	PB	PB-1	P1-18	TRUE	3	TRA:CVADGNYGQNFVF TRB:CASSSPGLGGGFDTEAFF	75	CD8-C7
PB-1_CGTGTAAGTCGAATCT	PB	PB-1	P1-2	TRUE	3	TRA:CGQNFVF TRB:CASNQRGGPSLYNEQFF	323	CD8-C7
PB-1_CGTGTAAGTCTTCGTC	PB	PB-1	P1-2	TRUE	3	TRA:CGQNFVF TRB:CASNQRGGPSLYNEQFF	323	CD8-C7
PB-1_CGTGTAAGTGCGGTAA	PB	PB-1	P1-1	TRUE	3	TRA:CAMSDRMTGGFKTIF TRB:CASSQVGRGGEETQYF	1047	CD8-C7
PB-1_CGTGTAATCTACTTAC	PB	PB-1	P1-2	TRUE	3	TRA:CGQNFVF TRB:CASNQRGGPSLYNEQFF	323	CD8-C7
PB-1_CGTGTCTAGAACAATC	PB	PB-1	P1-35	TRUE	3	TRA:CAVSDRGYFGNEKLTF TRB:CSALDDVRPVIAVYNEQFF	40	CD8-C6
PB-1_CGTGTCTAGACGCAAC	PB	PB-1	P1-10	TRUE	3	TRA:CISNSPMDSSYKLIF TRB:CASSSWGDTDEAFF	162	CD8-C7
PB-1_CGTGTCTAGAGCTTCT	PB	PB-1	P1-63	TRUE	3	TRA:CAEPLNTGNQFYF TRB:CASSVGNEQFF	10	CD8-C7
PB-1_CGTGTCTAGGCCCGTT	PB	PB-1	P1-8	TRUE	3	TRA:CAVRPPGRNSGGYQKVTF TRB:CASSRRGVTDTQYF	204	CD8-C7
PB-1_CGTGTCTGTAAGTTCC	PB	PB-1	P1-1	TRUE	3	TRA:CAMSDRMTGGFKTIF TRB:CASSQVGRGGEETQYF	1047	CD8-C7
PB-1_CGTGTCTGTATTACCG	PB	PB-1	P1-7	TRUE	3	TRA:CAVQAGNNDMRF TRB:CASSTTGDFNYGYTF	215	CD8-C5
PB-1_CGTGTCTGTGTTGGGA	PB	PB-1	P1-1	TRUE	3	TRA:CAMSDRMTGGFKTIF TRB:CASSQVGRGGEETQYF	1047	CD8-C5

PB-1_CGTGTCTTCCTAGTGA	PB	PB-1	P1-8	TRUE	3	TRA:CAVRPPGRNSGGYQKVTF TRB:CASSRRGVTDTQYF	204	CD8-C7
PB-1_CGTTAGAAGCAGGTCA	PB	PB-1	P1-25	TRUE	3	TRA:CIVGRDSSYKLIF TRB:CASNPQRGAHRDTQYF	58	CD8-C7
PB-1_CGTTAGACAAACCTAC	PB	PB-1	P1-1	TRUE	3	TRA:CAMSDRMTGGFKTIF TRB:CASSQVGRGGEETQYF	1047	CD8-C5
PB-1_CGTTAGACAAGACGTG	PB	PB-1	P1-12	TRUE	3	TRA:CALSDERGDYKLSF TRB:CASSPGTGSYNEQFF	141	CD8-C7
PB-1_CGTTAGACAATGAATG	PB	PB-1	P1-35	TRUE	3	TRA:CAVSDRGYFGNEKLTF TRB:CSALDDVRPVIAVYNEQFF	40	CD8-C6
PB-1_CGTTAGACATGTCCTC	PB	PB-1	P1-4	TRUE	3	TRA:CAMKGRDDKIIF TRB:CASTKGVSREAFF	226	CD8-C7
PB-1_CGTTAGAGTAAGGATT	PB	PB-1	P1-3	TRUE	3	TRA:CAFLSGTYKYIF TRB:CASSQSPGQRNYGYTF	308	CD8-C7
PB-1_CGTTCTGAGGCACATG	PB	PB-1	P1-1	TRUE	3	TRA:CAMSDRMTGGFKTIF TRB:CASSQVGRGGEETQYF	1047	CD8-C7
PB-1_CGTTCTGAGGCTACGA	PB	PB-1	P1-3	TRUE	3	TRA:CAFLSGTYKYIF TRB:CASSQSPGQRNYGYTF	308	CD8-C7
PB-1_CGTTCTGAGTATGACA	PB	PB-1	P1-16	TRUE	3	TRA:CALSGTPMNRDDKIIF TRB:CASSQSWDLAVLNEQFF	79	CD8-C5
PB-1_CGTTCTGCAGACAGGT	PB	PB-1	P1-3	TRUE	3	TRA:CAFLSGTYKYIF TRB:CASSQSPGQRNYGYTF	308	CD8-C7
PB-1_CGTTCTGGTACACCGC	PB	PB-1	P1-16	TRUE	3	TRA:CALSGTPMNRDDKIIF TRB:CASSQSWDLAVLNEQFF	79	CD8-C5
PB-1_CGTTCTGGTATAGGGC	PB	PB-1	P1-4	TRUE	3	TRA:CAMKGRDDKIIF TRB:CASTKGVSREAFF	226	CD8-C7
PB-1_CGTTCTGGTTATTCTC	PB	PB-1	P1-1	TRUE	3	TRA:CAMSDRMTGGFKTIF TRB:CASSQVGRGGEETQYF	1047	CD8-C7
PB-1_CGTTCTGTCACCACCT	PB	PB-1	P1-9	TRUE	3	TRA:CAEAEGGGADGLTF TRB:CATSGGDNEQFF	174	CD8-C7
PB-1_CGTTCTGTCTTGTACT	PB	PB-1	P1-1	TRUE	3	TRA:CAMSDRMTGGFKTIF TRB:CASSQVGRGGEETQYF	1047	CD8-C7
PB-1_CGTTGGGCACAAGTAA	PB	PB-1	P1-1	TRUE	3	TRA:CAMSDRMTGGFKTIF TRB:CASSQVGRGGEETQYF	1047	CD8-C7
PB-1_CGTTGGGCATCATCCC	PB	PB-1	P1-5	TRUE	3	TRA:CALSGTPMNRDDKIIF TRB:CSARGRGLAGGPAYEQYF	223	CD8-C5
PB-1_CGTTGGGCATTCGACA	PB	PB-1	P1-1	TRUE	3	TRA:CAMSDRMTGGFKTIF TRB:CASSQVGRGGEETQYF	1047	CD8-C7
PB-1_CGTTGGGGTTAAGGGC	PB	PB-1	P1-2	TRUE	3	TRA:CGQNFVF TRB:CASNQRGGPSLYNEQFF	323	CD8-C7
PB-1_CGTTGGGTCAGCCTAA	PB	PB-1	P1-3	TRUE	3	TRA:CAFLSGTYKYIF TRB:CASSQSPGQRNYGYTF	308	CD8-C6
PB-1_CGTTGGGTCGTACCGG	PB	PB-1	P1-722	FALSE	1	TRA:CAMSDRMTGGFKTIF TRB:CASSLAVNYGYTF	1	CD8-C7
PB-1_CTAACTTAGGCAGTCA	PB	PB-1	P1-5	TRUE	3	TRA:CALSGTPMNRDDKIIF TRB:CSARGRGLAGGPAYEQYF	223	CD8-C7
PB-1_CTAACTTAGTACGATA	PB	PB-1	P1-8	TRUE	3	TRA:CAVRPPGRNSGGYQKVTF TRB:CASSRRGVTDTQYF	204	CD8-C5
PB-1_CTAACTTAGTCGAGTG	PB	PB-1	P1-1	TRUE	3	TRA:CAMSDRMTGGFKTIF TRB:CASSQVGRGGEETQYF	1047	CD8-C7
PB-1_CTAACTTCAATGGTCT	PB	PB-1	P1-3	TRUE	3	TRA:CAFLSGTYKYIF TRB:CASSQSPGQRNYGYTF	308	CD8-C6
PB-1_CTAACTTCACACCGAC	PB	PB-1	P1-2	TRUE	3	TRA:CGQNFVF TRB:CASNQRGGPSLYNEQFF	323	CD8-C7

PB-1_CTAACTTCAGTCACTA	PB	PB-1	P1-1	TRUE	3	TRA:CAMSDRMTGGFKTIF TRB:CASSQVGRGGEETQYF	1047	CD8-C7
PB-1_CTAACTTCATAAAGGT	PB	PB-1	P1-6	TRUE	3	TRA:CAMREGRERDDKIIF TRB:CASSLPNTEAFF	216	CD8-C5
PB-1_CTAACTTTCTGAGGGA	PB	PB-1	P1-3	TRUE	3	TRA:CAFLSGTYKYIF TRB:CASSQSPGQRNYGYTF	308	CD8-C7
PB-1_CTAAGACCAAGCTGGA	PB	PB-1	P1-50	TRUE	3	TRA:CARWLGSQGNLIF TRB:CASSLTDRVPQFELVYEQYF	15	CD8-C7
PB-1_CTAAGACCACATGACT	PB	PB-1	P1-3	TRUE	3	TRA:CAFLSGTYKYIF TRB:CASSQSPGQRNYGYTF	308	CD8-C6
PB-1_CTAAGACCAGGCTCAC	PB	PB-1	P1-4	TRUE	3	TRA:CAMKGRDDKIIF TRB:CASTKGVSREAFF	226	CD8-C7
PB-1_CTAAGACGTCCCGACA	PB	PB-1	P1-31	TRUE	3	TRA:CAVEEGADRLMF TRB:CASSLLGGGELFF	46	CD8-C7
PB-1_CTAAGACGTTATCGGT	PB	PB-1	P1-1	TRUE	3	TRA:CAMSDRMTGGFKTIF TRB:CASSQVGRGGEETQYF	1047	CD8-C5
PB-1_CTAAGACTCCTGTAGA	PB	PB-1	P1-22	TRUE	3	TRA:CASNSGYSTLTF TRB:CASSQVTGGARDEQYF	65	CD8-C5
PB-1_CTAATGGGTCGACTAT	PB	PB-1	P1-3	TRUE	3	TRA:CAFLSGTYKYIF TRB:CASSQSPGQRNYGYTF	308	CD8-C7
PB-1_CTACACCAGCTCAACT	PB	PB-1	P1-8	TRUE	3	TRA:CAVRPPGRNSGGYQKVTF TRB:CASSRRGVTDTQYF	204	CD8-C7
PB-1_CTACACCCAAGTAATG	PB	PB-1	P1-5	TRUE	3	TRA:CALSGTPMNRDDKIIF TRB:CSARGRGLAGGPAYEQYF	223	CD8-C7
PB-1_CTACACCCACGGTGTC	PB	PB-1	P1-1	TRUE	3	TRA:CAMSDRMTGGFKTIF TRB:CASSQVGRGGEETQYF	1047	CD8-C7
PB-1_CTACATTAGAGTAAGG	PB	PB-1	P1-4	TRUE	3	TRA:CAMKGRDDKIIF TRB:CASTKGVSREAFF	226	CD8-C7
PB-1_CTACATTAGCACCGCT	PB	PB-1	P1-8	TRUE	3	TRA:CAVRPPGRNSGGYQKVTF TRB:CASSRRGVTDTQYF	204	CD8-C7
PB-1_CTACATTAGTACGCGA	PB	PB-1	P1-3	TRUE	3	TRA:CAFLSGTYKYIF TRB:CASSQSPGQRNYGYTF	308	CD8-C7
PB-1_CTACATTCAATGGAAT	PB	PB-1	P1-284	FALSE	2	TRA:CALHSVSQAGTALIF TRB:CSVSTGITDTQYF	2	CD8-C5
PB-1_CTACATTCATCTATGG	PB	PB-1	P1-3	TRUE	3	TRA:CAFLSGTYKYIF TRB:CASSQSPGQRNYGYTF	308	CD8-C7
PB-1_CTACATTTCGTAGATC	PB	PB-1	P1-1	TRUE	3	TRA:CAMSDRMTGGFKTIF TRB:CASSQVGRGGEETQYF	1047	CD8-C7
PB-1_CTACCCAAGAAACGAG	PB	PB-1	P1-3	TRUE	3	TRA:CAFLSGTYKYIF TRB:CASSQSPGQRNYGYTF	308	CD8-C7
PB-1_CTACCCAGTACAGTGG	PB	PB-1	P1-93	TRUE	3	TRA:CAFTGGGGADGLTF TRB:CASSVAWGTGGWNTEAFF	6	CD8-C7
PB-1_CTACCCAGTCGTTGTA	PB	PB-1	P1-10	TRUE	3	TRA:CISNSPMDSSYKLIF TRB:CASSSWGDTDEAFF	162	CD8-C7
PB-1_CTACCCATCCACGTTC	PB	PB-1	P1-1	TRUE	3	TRA:CAMSDRMTGGFKTIF TRB:CASSQVGRGGEETQYF	1047	CD8-C5
PB-1_CTACCCATCCTGTAGA	PB	PB-1	P1-16	TRUE	3	TRA:CALSGTPMNRDDKIIF TRB:CASSQSWDLAVLNEQFF	79	CD8-C7
PB-1_CTACCCATCTGCTGCT	PB	PB-1	P1-31	TRUE	3	TRA:CAVEEGADRLMF TRB:CASSLLGGGELFF	46	CD8-C7
PB-1_CTACGTCAGCCGATTT	PB	PB-1	P1-4	TRUE	3	TRA:CAMKGRDDKIIF TRB:CASTKGVSREAFF	226	CD8-C7
PB-1_CTACGTCAGTTCGCGC	PB	PB-1	P1-10	TRUE	3	TRA:CISNSPMDSSYKLIF TRB:CASSSWGDTDEAFF	162	CD8-C7

PB-1_CTACGTCGTCTCTCGT	PB	PB-1	P1-1	TRUE	3	TRA:CAMSDRMTGGFKTIF TRB:CASSQVGRGGEETQYF	1047	CD8-C7
PB-1_CTACGTCTCAACCATG	PB	PB-1	P1-18	TRUE	3	TRA:CVADGNYGQNFVF TRB:CASSSPGLGGGFDTEAFF	75	CD8-C7
PB-1_CTACGTCTCCATGCTC	PB	PB-1	P1-1	TRUE	3	TRA:CAMSDRMTGGFKTIF TRB:CASSQVGRGGEETQYF	1047	CD8-C7
PB-1_CTACGTCTCGGAAATA	PB	PB-1	P1-12	TRUE	3	TRA:CALSDERGDYKLSF TRB:CASSPGTGSYNEQFF	141	CD8-C5
PB-1_CTAGAGTAGACAGACC	PB	PB-1	P1-29	TRUE	3	TRA:CATVSFSGGYNKLIF TRB:CASSLDRRGLSYEQYF	50	CD8-C7
PB-1_CTAGAGTAGAGCTGGT	PB	PB-1	P1-1	TRUE	3	TRA:CAMSDRMTGGFKTIF TRB:CASSQVGRGGEETQYF	1047	CD8-C5
PB-1_CTAGAGTAGGTGCACA	PB	PB-1	P1-14	TRUE	3	TRA:CAASVVNDMRF TRB:CASSHRGGAKNIQYF	109	CD8-C5
PB-1_CTAGAGTCATGAGCGA	PB	PB-1	P1-191	TRUE	3	TRA:CIVSGAYSGAGSYQLTF TRB:CASSLVRGGETQYF	3	CD8-C5
PB-1_CTAGAGTGTCCGCTGA	PB	PB-1	P1-2	TRUE	3	TRA:CGQNFVF TRB:CASNQRGGPSLYNEQFF	323	CD8-C7
PB-1_CTAGAGTTCAAAGTAG	PB	PB-1	P1-5	TRUE	3	TRA:CALSGTPMNRDDKIIF TRB:CSARGRGLAGGPAYEQYF	223	CD8-C7
PB-1_CTAGCCTAGTGAATTG	PB	PB-1	P1-2	TRUE	3	TRA:CGQNFVF TRB:CASNQRGGPSLYNEQFF	323	CD8-C7
PB-1_CTAGCCTCAGACAAGC	PB	PB-1	P1-14	TRUE	3	TRA:CAASVVNDMRF TRB:CASSHRGGAKNIQYF	109	CD8-C7
PB-1_CTAGCCTCATTATCTC	PB	PB-1	P1-1	TRUE	3	TRA:CAMSDRMTGGFKTIF TRB:CASSQVGRGGEETQYF	1047	CD8-C7
PB-1_CTAGCCTGTATATCCG	PB	PB-1	P1-2	TRUE	3	TRA:CGQNFVF TRB:CASNQRGGPSLYNEQFF	323	CD8-C7
PB-1_CTAGCCTTCTGGGCCA	PB	PB-1	P1-58	TRUE	3	TRA:CAVRPNRDDKIIF TRB:CASSSRLAGGTDTQYF	12	CD8-C7
PB-1_CTAGTGAAGCAGATCG	PB	PB-1	P1-2	TRUE	3	TRA:CGQNFVF TRB:CASNQRGGPSLYNEQFF	323	CD8-C7
PB-1_CTAGTGAAGGTGCACA	PB	PB-1	P1-2	TRUE	3	TRA:CGQNFVF TRB:CASNQRGGPSLYNEQFF	323	CD8-C5
PB-1_CTAGTGACAAGCCATT	PB	PB-1	P1-31	TRUE	3	TRA:CAVEEGADRLMF TRB:CASSLLGGGELFF	46	CD8-C5
PB-1_CTAGTGATCAGCTCGG	PB	PB-1	P1-1	TRUE	3	TRA:CAMSDRMTGGFKTIF TRB:CASSQVGRGGEETQYF	1047	CD8-C7
PB-1_CTCACACCATGCAACT	PB	PB-1	P1-1	TRUE	3	TRA:CAMSDRMTGGFKTIF TRB:CASSQVGRGGEETQYF	1047	CD8-C5
PB-1_CTCACACTCAGGCGAA	PB	PB-1	P1-2	TRUE	3	TRA:CGQNFVF TRB:CASNQRGGPSLYNEQFF	323	CD8-C5
PB-1_CTCAGAAAGATATGCA	PB	PB-1	P1-1	TRUE	3	TRA:CAMSDRMTGGFKTIF TRB:CASSQVGRGGEETQYF	1047	CD8-C7
PB-1_CTCAGAAAGCTGCAAG	PB	PB-1	P1-10	TRUE	3	TRA:CISNSPMDSSYKLIF TRB:CASSSWGDTDEAFF	162	CD8-C7
PB-1_CTCAGAAAGGCATGTG	PB	PB-1	P1-54	TRUE	3	TRA:CAGWYSGGGADGLTF TRB:CATGAGDNEQFF	13	CD8-C5
PB-1_CTCAGAACACCACGTG	PB	PB-1	P1-1	TRUE	3	TRA:CAMSDRMTGGFKTIF TRB:CASSQVGRGGEETQYF	1047	CD8-C7
PB-1_CTCAGAACATGCAACT	PB	PB-1	P1-1	TRUE	3	TRA:CAMSDRMTGGFKTIF TRB:CASSQVGRGGEETQYF	1047	CD8-C7
PB-1_CTCAGAAGTTATCCGA	PB	PB-1	P1-13	TRUE	3	TRA:CVVMNYGGSQGNLIF TRB:CASSSWGDTDEAFF	112	CD8-C7

PB-1_CTCAGAATCAAACAAG	PB	PB-1	P1-2	TRUE	3	TRA:CGQNFVF TRB:CASNQRGGPSLYNEQFF	323	CD4-C6
PB-1_CTCAGAATCAGTGCAT	PB	PB-1	P1-286	FALSE	2	TRA:CAMRGSYIPTF TRB:CASRWEGLGVNEQFF	2	CD8-C6
PB-1_CTCATTAAGACACGAC	PB	PB-1	P1-1	TRUE	3	TRA:CAMSDRMTGGFKTIF TRB:CASSQVGRGGEETQYF	1047	CD8-C7
PB-1_CTCCTAGGTAAGTGGC	PB	PB-1	P1-1	TRUE	3	TRA:CAMSDRMTGGFKTIF TRB:CASSQVGRGGEETQYF	1047	CD8-C7
PB-1_CTCCTAGGTTAAGAAC	PB	PB-1	P1-114	TRUE	3	TRA:CAEPENTGNQFYF TRB:CAISLGNEQFF	5	CD8-C7
PB-1_CTCCTAGTCAAACAAG	PB	PB-1	P1-16	TRUE	3	TRA:CALSGTPMNRDDKIIF TRB:CASSQSWDLAVLNEQFF	79	CD8-C5
PB-1_CTCCTAGTCGGATGGA	PB	PB-1	P1-2	TRUE	3	TRA:CGQNFVF TRB:CASNQRGGPSLYNEQFF	323	CD4-C6
PB-1_CTCCTAGTCGTATCAG	PB	PB-1	P1-3	TRUE	3	TRA:CAFLSGTYKYIF TRB:CASSQSPGQRNYGYTF	308	CD8-C5
PB-1_CTCGAAAAGACATAAC	PB	PB-1	P1-737	FALSE	1	TRA:CAMKGRDDKIIF TRB:CASSQSPGQRNYGYTF	1	CD8-C7
PB-1_CTCGAAAAGTTGAGTA	PB	PB-1	P1-3	TRUE	3	TRA:CAFLSGTYKYIF TRB:CASSQSPGQRNYGYTF	308	CD8-C7
PB-1_CTCGAAACAGCTATTG	PB	PB-1	P1-1	TRUE	3	TRA:CAMSDRMTGGFKTIF TRB:CASSQVGRGGEETQYF	1047	CD8-C7
PB-1_CTCGAAATCTGTTTGT	PB	PB-1	P1-6	TRUE	3	TRA:CAMREGRERDDKIIF TRB:CASSLPNTEAFF	216	CD8-C5
PB-1_CTCGAGGCATTGGCGC	PB	PB-1	P1-2	TRUE	3	TRA:CGQNFVF TRB:CASNQRGGPSLYNEQFF	323	CD8-C7
PB-1_CTCGAGGTCCACTGGG	PB	PB-1	P1-4	TRUE	3	TRA:CAMKGRDDKIIF TRB:CASTKGVSREAFF	226	CD8-C7
PB-1_CTCGAGGTCCTCGCAT	PB	PB-1	P1-287	FALSE	2	TRA:CVVMNYGGSQGNLIF TRB:CASSQVGRGGEETQYF	2	CD8-C7
PB-1_CTCGGAGAGCCTTGAT	PB	PB-1	P1-1	TRUE	3	TRA:CAMSDRMTGGFKTIF TRB:CASSQVGRGGEETQYF	1047	CD8-C5
PB-1_CTCGGAGAGTCGATAA	PB	PB-1	P1-26	TRUE	3	TRA:CAPRSGGSYIPTF TRB:CASSQDLGLDYGYTF	56	CD8-C5
PB-1_CTCGGAGCACTGTTAG	PB	PB-1	P1-18	TRUE	3	TRA:CVADGNYGQNFVF TRB:CASSSPGLGGGFDTEAFF	75	CD8-C7
PB-1_CTCGGAGCATGTAAGA	PB	PB-1	P1-739	FALSE	1	TRA:CAVPYSSASKIIF TRB:CASSSPGLGGGFDTEAFF	1	CD8-C7
PB-1_CTCGGAGTCACATGCA	PB	PB-1	P1-31	TRUE	3	TRA:CAVEEGADRLMF TRB:CASSLLGGGELFF	46	CD8-C7
PB-1_CTCGGAGTCAGGTAAA	PB	PB-1	P1-8	TRUE	3	TRA:CAVRPPGRNSGGYQKVTF TRB:CASSRRGVTDTQYF	204	CD8-C7
PB-1_CTCGGAGTCAGTGTTG	PB	PB-1	P1-1	TRUE	3	TRA:CAMSDRMTGGFKTIF TRB:CASSQVGRGGEETQYF	1047	CD8-C7
PB-1_CTCGGAGTCTTAACCT	PB	PB-1	P1-1	TRUE	3	TRA:CAMSDRMTGGFKTIF TRB:CASSQVGRGGEETQYF	1047	CD8-C7
PB-1_CTCGGGAAGAAACGAG	PB	PB-1	P1-11	TRUE	3	TRA:CAVEPLYGSGNTGKLIF TRB:CASSSHGQLNQPQHF	147	CD8-C7
PB-1_CTCGGGAAGCCCTAAT	PB	PB-1	P1-11	TRUE	3	TRA:CAVEPLYGSGNTGKLIF TRB:CASSSHGQLNQPQHF	147	CD8-C7
PB-1_CTCGGGAAGTGCGATG	PB	PB-1	P1-3	TRUE	3	TRA:CAFLSGTYKYIF TRB:CASSQSPGQRNYGYTF	308	CD8-C7
PB-1_CTCGGGACACTTCTGC	PB	PB-1	P1-11	TRUE	3	TRA:CAVEPLYGSGNTGKLIF TRB:CASSSHGQLNQPQHF	147	CD8-C7

PB-1_CTCGGGACAGACGCAA	PB	PB-1	P1-10	TRUE	3	TRA:CISNSPMDSSYKLIF TRB:CASSSWGDTDEAFF	162	CD8-C7
PB-1_CTCGGGAGTCTCCCTA	PB	PB-1	P1-1	TRUE	3	TRA:CAMSDRMTGGFKTIF TRB:CASSQVGRGGEETQYF	1047	CD8-C7
PB-1_CTCGGGAGTCTCGTTC	PB	PB-1	P1-1	TRUE	3	TRA:CAMSDRMTGGFKTIF TRB:CASSQVGRGGEETQYF	1047	CD8-C7
PB-1_CTCGGGAGTTTGTTGG	PB	PB-1	P1-3	TRUE	3	TRA:CAFLSGTYKYIF TRB:CASSQSPGQRNYGYTF	308	CD8-C7
PB-1_CTCGGGATCGTTACAG	PB	PB-1	P1-2	TRUE	3	TRA:CGQNFVF TRB:CASNQRGGPSLYNEQFF	323	CD8-C7
PB-1_CTCGTACCACAGAGGT	PB	PB-1	P1-5	TRUE	3	TRA:CALSGTPMNRDDKIIF TRB:CSARGRGLAGGPAYEQYF	223	CD8-C5
PB-1_CTCGTACGTAGCGTGA	PB	PB-1	P1-1	TRUE	3	TRA:CAMSDRMTGGFKTIF TRB:CASSQVGRGGEETQYF	1047	CD8-C7
PB-1_CTCGTCAAGATGGGTC	PB	PB-1	P1-5	TRUE	3	TRA:CALSGTPMNRDDKIIF TRB:CSARGRGLAGGPAYEQYF	223	CD8-C7
PB-1_CTCGTCACAAACCCAT	PB	PB-1	P1-13	TRUE	3	TRA:CVVMNYGGSQGNLIF TRB:CASSSWGDTDEAFF	112	CD8-C7
PB-1_CTCGTCACACCGAAAG	PB	PB-1	P1-2	TRUE	3	TRA:CGQNFVF TRB:CASNQRGGPSLYNEQFF	323	CD8-C7
PB-1_CTCGTCAGTAAATACG	PB	PB-1	P1-1	TRUE	3	TRA:CAMSDRMTGGFKTIF TRB:CASSQVGRGGEETQYF	1047	CD8-C7
PB-1_CTCGTCAGTACTTAGC	PB	PB-1	P1-4	TRUE	3	TRA:CAMKGRDDKIIF TRB:CASTKGVSREAFF	226	CD8-C7
PB-1_CTCGTCATCCTGCAGG	PB	PB-1	P1-35	TRUE	3	TRA:CAVSDRGYFGNEKLTF TRB:CSALDDVRPVIAVYNEQFF	40	CD8-C6
PB-1_CTCGTCATCGGCGCAT	PB	PB-1	P1-1	TRUE	3	TRA:CAMSDRMTGGFKTIF TRB:CASSQVGRGGEETQYF	1047	CD8-C7
PB-1_CTCTAATAGAAGGTTT	PB	PB-1	P1-3	TRUE	3	TRA:CAFLSGTYKYIF TRB:CASSQSPGQRNYGYTF	308	CD8-C7
PB-1_CTCTAATCAAGTTGTC	PB	PB-1	P1-1	TRUE	3	TRA:CAMSDRMTGGFKTIF TRB:CASSQVGRGGEETQYF	1047	CD8-C7
PB-1_CTCTAATCAATCCAAC	PB	PB-1	P1-73	TRUE	3	TRA:CAGI TRB:CASSRTTGGSSYEQYF	8	CD8-C7
PB-1_CTCTAATTCAATCACG	PB	PB-1	P1-1	TRUE	3	TRA:CAMSDRMTGGFKTIF TRB:CASSQVGRGGEETQYF	1047	CD8-C7
PB-1_CTCTAATTCCTTTCTC	PB	PB-1	P1-3	TRUE	3	TRA:CAFLSGTYKYIF TRB:CASSQSPGQRNYGYTF	308	CD8-C7
PB-1_CTCTACGAGCCGCCTA	PB	PB-1	P1-2	TRUE	3	TRA:CGQNFVF TRB:CASNQRGGPSLYNEQFF	323	CD8-C7
PB-1_CTCTACGCAATCCGAT	PB	PB-1	P1-1	TRUE	3	TRA:CAMSDRMTGGFKTIF TRB:CASSQVGRGGEETQYF	1047	CD8-C7
PB-1_CTCTACGCACAAGCCC	PB	PB-1	P1-746	FALSE	1	TRA:CAPRSGGSYIPTF TRB:CASTKGVSREAFF	1	CD8-C7
PB-1_CTCTACGTCCTCTAGC	PB	PB-1	P1-1	TRUE	3	TRA:CAMSDRMTGGFKTIF TRB:CASSQVGRGGEETQYF	1047	CD8-C7
PB-1_CTCTACGTCTGCCCTA	PB	PB-1	P1-10	TRUE	3	TRA:CISNSPMDSSYKLIF TRB:CASSSWGDTDEAFF	162	CD8-C7
PB-1_CTCTGGTTCAATAAGG	PB	PB-1	P1-1	TRUE	3	TRA:CAMSDRMTGGFKTIF TRB:CASSQVGRGGEETQYF	1047	CD8-C7
PB-1_CTGAAACAGAAGGACA	PB	PB-1	P1-6	TRUE	3	TRA:CAMREGRERDDKIIF TRB:CASSLPNTEAFF	216	CD8-C5
PB-1_CTGAAACAGAAGGTTT	PB	PB-1	P1-11	TRUE	3	TRA:CAVEPLYGSGNTGKLIF TRB:CASSSHGQLNQPQHF	147	CD8-C7

PB-1_CTGAAACCAACGCACC	PB	PB-1	P1-1	TRUE	3	TRA:CAMSDRMTGGFKTIF TRB:CASSQVGRGGEETQYF	1047	CD8-C7
PB-1_CTGAAACCACAACTGT	PB	PB-1	P1-1	TRUE	3	TRA:CAMSDRMTGGFKTIF TRB:CASSQVGRGGEETQYF	1047	CD8-C7
PB-1_CTGAAACGTAGCACGA	PB	PB-1	P1-5	TRUE	3	TRA:CALSGTPMNRDDKIIF TRB:CSARGRGLAGGPAYEQYF	223	CD8-C7
PB-1_CTGAAGTCAAGCCCAC	PB	PB-1	P1-16	TRUE	3	TRA:CALSGTPMNRDDKIIF TRB:CASSQSWDLAVLNEQFF	79	CD8-C7
PB-1_CTGAAGTCAGAAGCAC	PB	PB-1	P1-1	TRUE	3	TRA:CAMSDRMTGGFKTIF TRB:CASSQVGRGGEETQYF	1047	CD8-C7
PB-1_CTGAAGTGTATATGAG	PB	PB-1	P1-8	TRUE	3	TRA:CAVRPPGRNSGGYQKVTF TRB:CASSRRGVTDTQYF	204	CD8-C7
PB-1_CTGATAGAGGGCTTCC	PB	PB-1	P1-3	TRUE	3	TRA:CAFLSGTYKYIF TRB:CASSQSPGQRNYGYTF	308	CD8-C7
PB-1_CTGATAGCACTAGTAC	PB	PB-1	P1-14	TRUE	3	TRA:CAASVVNDMRF TRB:CASSHRGGAKNIQYF	109	CD8-C7
PB-1_CTGATAGCATAGAAAC	PB	PB-1	P1-1	TRUE	3	TRA:CAMSDRMTGGFKTIF TRB:CASSQVGRGGEETQYF	1047	CD8-C5
PB-1_CTGATAGGTCTCAACA	PB	PB-1	P1-22	TRUE	3	TRA:CASNSGYSTLTF TRB:CASSQVTGGARDEQYF	65	CD8-C5
PB-1_CTGATAGTCTGAAAGA	PB	PB-1	P1-1	TRUE	3	TRA:CAMSDRMTGGFKTIF TRB:CASSQVGRGGEETQYF	1047	CD8-C7
PB-1_CTGATCCAGACTAGAT	PB	PB-1	P1-4	TRUE	3	TRA:CAMKGRDDKIIF TRB:CASTKGVSREAFF	226	CD8-C7
PB-1_CTGATCCAGATATGGT	PB	PB-1	P1-194	FALSE	3	TRA:CAFIYNQGGKLIF TRB:CAWSVGRGTEQFF	3	CD8-C7
PB-1_CTGATCCGTATGCTTG	PB	PB-1	P1-11	TRUE	3	TRA:CAVEPLYGSGNTGKLIF TRB:CASSSHGQLNQPQHF	147	CD8-C7
PB-1_CTGATCCGTCGCGTGT	PB	PB-1	P1-29	TRUE	3	TRA:CATVSFSGGYNKLIF TRB:CASSLDRRGLSYEQYF	50	CD8-C7
PB-1_CTGATCCTCAGTACGT	PB	PB-1	P1-1	TRUE	3	TRA:CAMSDRMTGGFKTIF TRB:CASSQVGRGGEETQYF	1047	CD8-C6
PB-1_CTGATCCTCTAGCACA	PB	PB-1	P1-1	TRUE	3	TRA:CAMSDRMTGGFKTIF TRB:CASSQVGRGGEETQYF	1047	CD8-C7
PB-1_CTGCCTAAGTGTTAGA	PB	PB-1	P1-7	TRUE	3	TRA:CAVQAGNNDMRF TRB:CASSTTGDFNYGYTF	215	CD8-C5
PB-1_CTGCCTATCAACACGT	PB	PB-1	P1-13	TRUE	3	TRA:CVVMNYGGSQGNLIF TRB:CASSSWGDTDEAFF	112	CD8-C7
PB-1_CTGCCTATCTAACGGT	PB	PB-1	P1-1	TRUE	3	TRA:CAMSDRMTGGFKTIF TRB:CASSQVGRGGEETQYF	1047	CD8-C5
PB-1_CTGCCTATCTGCTTGC	PB	PB-1	P1-4	TRUE	3	TRA:CAMKGRDDKIIF TRB:CASTKGVSREAFF	226	CD8-C7
PB-1_CTGCGGAAGCGATGAC	PB	PB-1	P1-287	FALSE	2	TRA:CVVMNYGGSQGNLIF TRB:CASSQVGRGGEETQYF	2	CD8-C7
PB-1_CTGCGGAAGCGTCAAG	PB	PB-1	P1-31	TRUE	3	TRA:CAVEEGADRLMF TRB:CASSLLGGGELFF	46	CD8-C7
PB-1_CTGCGGAAGGGTCTCC	PB	PB-1	P1-1	TRUE	3	TRA:CAMSDRMTGGFKTIF TRB:CASSQVGRGGEETQYF	1047	CD8-C7
PB-1_CTGCGGACATAAAGGT	PB	PB-1	P1-7	TRUE	3	TRA:CAVQAGNNDMRF TRB:CASSTTGDFNYGYTF	215	CD8-C5
PB-1_CTGCGGACATACGCCG	PB	PB-1	P1-14	TRUE	3	TRA:CAASVVNDMRF TRB:CASSHRGGAKNIQYF	109	CD8-C7
PB-1_CTGCGGAGTAGAGGAA	PB	PB-1	P1-3	TRUE	3	TRA:CAFLSGTYKYIF TRB:CASSQSPGQRNYGYTF	308	CD8-C7

PB-1_CTGCGGATCATATCGG	PB	PB-1	P1-195	TRUE	3	TRA:CAANLYPHSGAGSYQLTF TRB:CASSSGTGGETQYF	3	CD8-C7
PB-1_CTGCGGATCTTCATGT	PB	PB-1	P1-25	TRUE	3	TRA:CIVGRDSSYKLIF TRB:CASNPQRGAHRDTQYF	58	CD8-C7
PB-1_CTGCTGTAGTCGATAA	PB	PB-1	P1-8	TRUE	3	TRA:CAVRPPGRNSGGYQKVTF TRB:CASSRRGVTDTQYF	204	CD8-C7
PB-1_CTGCTGTCAGCTGCTG	PB	PB-1	P1-1	TRUE	3	TRA:CAMSDRMTGGFKTIF TRB:CASSQVGRGGEETQYF	1047	CD8-C7
PB-1_CTGCTGTCATGAAGTA	PB	PB-1	P1-1	TRUE	3	TRA:CAMSDRMTGGFKTIF TRB:CASSQVGRGGEETQYF	1047	CD8-C7
PB-1_CTGCTGTGTAGCTAAA	PB	PB-1	P1-759	FALSE	1	TRA:CVVMNYGGSQGNLIF TRB:CASNQRGGPSLYNEQFF	1	CD8-C7
PB-1_CTGCTGTGTTGAACTC	PB	PB-1	P1-1	TRUE	3	TRA:CAMSDRMTGGFKTIF TRB:CASSQVGRGGEETQYF	1047	CD8-C7
PB-1_CTGCTGTGTTTGGCGC	PB	PB-1	P1-1	TRUE	3	TRA:CAMSDRMTGGFKTIF TRB:CASSQVGRGGEETQYF	1047	CD8-C7
PB-1_CTGCTGTTCCAAACTG	PB	PB-1	P1-1	TRUE	3	TRA:CAMSDRMTGGFKTIF TRB:CASSQVGRGGEETQYF	1047	CD8-C7
PB-1_CTGGTCTGTAGCGTCC	PB	PB-1	P1-761	FALSE	1	TRA:CAVKEGAQKLVF TRB:CASSEAFTGGGKTQYF	1	CD8-C2
PB-1_CTGGTCTTCATCGGAT	PB	PB-1	P1-8	TRUE	3	TRA:CAVRPPGRNSGGYQKVTF TRB:CASSRRGVTDTQYF	204	CD8-C7
PB-1_CTGGTCTTCCCAAGTA	PB	PB-1	P1-11	TRUE	3	TRA:CAVEPLYGSGNTGKLIF TRB:CASSSHGQLNQPQHF	147	CD8-C7
PB-1_CTGTGCTCATGTAGTC	PB	PB-1	P1-3	TRUE	3	TRA:CAFLSGTYKYIF TRB:CASSQSPGQRNYGYTF	308	CD8-C7
PB-1_CTGTGCTGTAAATACG	PB	PB-1	P1-10	TRUE	3	TRA:CISNSPMDSSYKLIF TRB:CASSSWGDTDEAFF	162	CD8-C7
PB-1_CTGTGCTGTGACGGTA	PB	PB-1	P1-2	TRUE	3	TRA:CGQNFVF TRB:CASNQRGGPSLYNEQFF	323	CD8-C7
PB-1_CTGTTTATCACTTACT	PB	PB-1	P1-1	TRUE	3	TRA:CAMSDRMTGGFKTIF TRB:CASSQVGRGGEETQYF	1047	CD8-C5
PB-1_CTGTTTATCGACCAGC	PB	PB-1	P1-194	FALSE	3	TRA:CAFIYNQGGKLIF TRB:CAWSVGRGTEQFF	3	CD8-C7
PB-1_CTTAACTAGGTGTTAA	PB	PB-1	P1-179	TRUE	3	TRA:CALAPTNTGNQFYF TRB:CASQRVGLSRYEQYF	3	CD8-C3
PB-1_CTTAACTGTCGCTTCT	PB	PB-1	P1-13	TRUE	3	TRA:CVVMNYGGSQGNLIF TRB:CASSSWGDTDEAFF	112	CD8-C7
PB-1_CTTACCGCATCTCGCT	PB	PB-1	P1-4	TRUE	3	TRA:CAMKGRDDKIIF TRB:CASTKGVSREAFF	226	CD8-C7
PB-1_CTTACCGGTCTTCAAG	PB	PB-1	P1-11	TRUE	3	TRA:CAVEPLYGSGNTGKLIF TRB:CASSSHGQLNQPQHF	147	CD8-C7
PB-1_CTTACCGGTGACTCAT	PB	PB-1	P1-2	TRUE	3	TRA:CGQNFVF TRB:CASNQRGGPSLYNEQFF	323	CD8-C7
PB-1_CTTACCGGTTCATGGT	PB	PB-1	P1-10	TRUE	3	TRA:CISNSPMDSSYKLIF TRB:CASSSWGDTDEAFF	162	CD4-C6
PB-1_CTTACCGTCACTCCTG	PB	PB-1	P1-1	TRUE	3	TRA:CAMSDRMTGGFKTIF TRB:CASSQVGRGGEETQYF	1047	CD8-C7
PB-1_CTTAGGAAGTGACATA	PB	PB-1	P1-1	TRUE	3	TRA:CAMSDRMTGGFKTIF TRB:CASSQVGRGGEETQYF	1047	CD8-C7
PB-1_CTTAGGAGTCTAGGTT	PB	PB-1	P1-771	FALSE	1	TRA:CAVRDMMDSNYQLIW TRB:CASSSWGDTDEAFF	1	CD8-C2
PB-1_CTTAGGAGTTAAGACA	PB	PB-1	P1-3	TRUE	3	TRA:CAFLSGTYKYIF TRB:CASSQSPGQRNYGYTF	308	CD8-C6

PB-1_CTTAGGAGTTCATGGT	PB	PB-1	P1-11	TRUE	3	TRA:CAVEPLYGSGNTGKLIF TRB:CASSSHGQLNQPQHF	147	CD8-C7
PB-1_CTTAGGATCCCGACTT	PB	PB-1	P1-4	TRUE	3	TRA:CAMKGRDDKIIF TRB:CASTKGVSREAFF	226	CD8-C7
PB-1_CTTCTCTCAAATCCGT	PB	PB-1	P1-1	TRUE	3	TRA:CAMSDRMTGGFKTIF TRB:CASSQVGRGGEETQYF	1047	CD8-C7
PB-1_CTTCTCTCAAGTCTGT	PB	PB-1	P1-114	TRUE	3	TRA:CAEPENTGNQFYF TRB:CAISLGNEQFF	5	CD8-C7
PB-1_CTTCTCTCAGACAAAT	PB	PB-1	P1-1	TRUE	3	TRA:CAMSDRMTGGFKTIF TRB:CASSQVGRGGEETQYF	1047	CD8-C7
PB-1_CTTGGCTCAGCTGGCT	PB	PB-1	P1-8	TRUE	3	TRA:CAVRPPGRNSGGYQKVTF TRB:CASSRRGVTDTQYF	204	CD8-C7
PB-1_CTTGGCTTCAGTACGT	PB	PB-1	P1-18	TRUE	3	TRA:CVADGNYGGQNFVF TRB:CASSSPGLGGGFDTEAFF	75	CD8-C6
PB-1_CTTGGCTTCCGTAGTA	PB	PB-1	P1-11	TRUE	3	TRA:CAVEPLYGSGNTGKLIF TRB:CASSSHGQLNQPQHF	147	CD8-C7
PB-1_CTTGGCTTCTTGACGA	PB	PB-1	P1-8	TRUE	3	TRA:CAVRPPGRNSGGYQKVTF TRB:CASSRRGVTDTQYF	204	CD8-C7
PB-1_CTTTGCGAGCAGCCTC	PB	PB-1	P1-3	TRUE	3	TRA:CAFLSGTYKYIF TRB:CASSQSPGQRNYGYTF	308	CD8-C7
PB-1_CTTTGCGAGTAAGTAC	PB	PB-1	P1-10	TRUE	3	TRA:CISNSPMDSSYKLIF TRB:CASSSWGDTDEAFF	162	CD8-C7
PB-1_CTTTGCGAGTCTTGCA	PB	PB-1	P1-267	FALSE	2	TRA:CAVGLMDSSYKLIF TRB:CASAPKRERGGTQYF	2	CD8-C7
PB-1_CTTTGCGCAAAGTCAA	PB	PB-1	P1-22	TRUE	3	TRA:CASNSGYSTLTF TRB:CASSQVTGGARDEQYF	65	CD8-C7
PB-1_CTTTGCGCAGGAACGT	PB	PB-1	P1-5	TRUE	3	TRA:CALSGTPMNRDDKIIF TRB:CSARGRGLAGGPAYEQYF	223	CD8-C7
PB-1_CTTTGCGGTCCGTGAC	PB	PB-1	P1-4	TRUE	3	TRA:CAMKGRDDKIIF TRB:CASTKGVSREAFF	226	CD8-C7
PB-1_CTTTGCGTCGAACTGT	PB	PB-1	P1-13	TRUE	3	TRA:CVVMNYGGSQGNLIF TRB:CASSSWGDTDEAFF	112	CD8-C7
PB-1_GAAATGAAGATTACCC	PB	PB-1	P1-4	TRUE	3	TRA:CAMKGRDDKIIF TRB:CASTKGVSREAFF	226	CD8-C7
PB-1_GAAATGAAGTTCGATC	PB	PB-1	P1-11	TRUE	3	TRA:CAVEPLYGSGNTGKLIF TRB:CASSSHGQLNQPQHF	147	CD8-C7
PB-1_GAAATGACAAAGTCAA	PB	PB-1	P1-1	TRUE	3	TRA:CAMSDRMTGGFKTIF TRB:CASSQVGRGGEETQYF	1047	CD8-C7
PB-1_GAAATGACACAGCCCA	PB	PB-1	P1-1	TRUE	3	TRA:CAMSDRMTGGFKTIF TRB:CASSQVGRGGEETQYF	1047	CD8-C7
PB-1_GAAATGATCGGGAGTA	PB	PB-1	P1-1	TRUE	3	TRA:CAMSDRMTGGFKTIF TRB:CASSQVGRGGEETQYF	1047	CD8-C7
PB-1_GAACATCGTGGGTCAA	PB	PB-1	P1-10	TRUE	3	TRA:CISNSPMDSSYKLIF TRB:CASSSWGDTDEAFF	162	CD8-C7
PB-1_GAACATCTCCTAAGTG	PB	PB-1	P1-1	TRUE	3	TRA:CAMSDRMTGGFKTIF TRB:CASSQVGRGGEETQYF	1047	CD8-C7
PB-1_GAACATCTCGCAGGCT	PB	PB-1	P1-2	TRUE	3	TRA:CGQNFVF TRB:CASNQRGGPSLYNEQFF	323	CD8-C7
PB-1_GAACCTAAGCTACCGC	PB	PB-1	P1-1	TRUE	3	TRA:CAMSDRMTGGFKTIF TRB:CASSQVGRGGEETQYF	1047	CD8-C7
PB-1_GAACCTAAGGAGTACC	PB	PB-1	P1-3	TRUE	3	TRA:CAFLSGTYKYIF TRB:CASSQSPGQRNYGYTF	308	CD8-C7
PB-1_GAACCTAGTGCCTGCA	PB	PB-1	P1-1	TRUE	3	TRA:CAMSDRMTGGFKTIF TRB:CASSQVGRGGEETQYF	1047	CD8-C7

PB-1_GAACCTATCAGATAAG	PB	PB-1	P1-2	TRUE	3	TRA:CGQNFVF TRB:CASNQRGGPSLYNEQFF	323	CD8-C7
PB-1_GAACCTATCCCAACGG	PB	PB-1	P1-54	TRUE	3	TRA:CAGWYSGGGADGLTF TRB:CATGAGDNEQFF	13	CD8-C5
PB-1_GAACGGAGTACACCGC	PB	PB-1	P1-12	TRUE	3	TRA:CALSDERGDYKLSF TRB:CASSPGTGSYNEQFF	141	CD8-C7
PB-1_GAACGGAGTCATTAGC	PB	PB-1	P1-3	TRUE	3	TRA:CAFLSGTYKYIF TRB:CASSQSPGQRNYGYTF	308	CD8-C7
PB-1_GAACGGAGTGTAAGTA	PB	PB-1	P1-9	TRUE	3	TRA:CAEAEGGGADGLTF TRB:CATSGGDNEQFF	174	CD8-C5
PB-1_GAACGGAGTGTCTGAT	PB	PB-1	P1-14	TRUE	3	TRA:CAASVVNDMRF TRB:CASSHRGGAKNIQYF	109	CD8-C7
PB-1_GAACGGATCGTTGACA	PB	PB-1	P1-1	TRUE	3	TRA:CAMSDRMTGGFKTIF TRB:CASSQVGRGGEETQYF	1047	CD8-C7
PB-1_GAAGCAGAGTACGCGA	PB	PB-1	P1-150	FALSE	3	TRA:CALGAQGGSEKLVF TRB:CASSCPGLGGSTDTQYF	4	CD8-C6
PB-1_GAAGCAGCAATCAGAA	PB	PB-1	P1-1	TRUE	3	TRA:CAMSDRMTGGFKTIF TRB:CASSQVGRGGEETQYF	1047	CD8-C7
PB-1_GAAGCAGCAATGGAAT	PB	PB-1	P1-22	TRUE	3	TRA:CASNSGYSTLTF TRB:CASSQVTGGARDEQYF	65	CD8-C7
PB-1_GAAGCAGGTATAGGTA	PB	PB-1	P1-1	TRUE	3	TRA:CAMSDRMTGGFKTIF TRB:CASSQVGRGGEETQYF	1047	CD8-C7
PB-1_GAAGCAGTCTTGTATC	PB	PB-1	P1-16	TRUE	3	TRA:CALSGTPMNRDDKIIF TRB:CASSQSWDLAVLNEQFF	79	CD8-C7
PB-1_GAATAAGAGAGTAATC	PB	PB-1	P1-1	TRUE	3	TRA:CAMSDRMTGGFKTIF TRB:CASSQVGRGGEETQYF	1047	CD8-C7
PB-1_GAATAAGAGCACCGTC	PB	PB-1	P1-1	TRUE	3	TRA:CAMSDRMTGGFKTIF TRB:CASSQVGRGGEETQYF	1047	CD8-C7
PB-1_GAATAAGAGTACTTGC	PB	PB-1	P1-5	TRUE	3	TRA:CALSGTPMNRDDKIIF TRB:CSARGRGLAGGPAYEQYF	223	CD8-C5
PB-1_GAATAAGCAAGGCTCC	PB	PB-1	P1-1	TRUE	3	TRA:CAMSDRMTGGFKTIF TRB:CASSQVGRGGEETQYF	1047	CD8-C7
PB-1_GAATAAGCATAAAGGT	PB	PB-1	P1-1	TRUE	3	TRA:CAMSDRMTGGFKTIF TRB:CASSQVGRGGEETQYF	1047	CD8-C7
PB-1_GAATAAGCATCCAACA	PB	PB-1	P1-16	TRUE	3	TRA:CALSGTPMNRDDKIIF TRB:CASSQSWDLAVLNEQFF	79	CD8-C7
PB-1_GAATAAGGTACCATCA	PB	PB-1	P1-3	TRUE	3	TRA:CAFLSGTYKYIF TRB:CASSQSPGQRNYGYTF	308	CD8-C7
PB-1_GAATAAGGTTGTGGAG	PB	PB-1	P1-4	TRUE	3	TRA:CAMKGRDDKIIF TRB:CASTKGVSREAFF	226	CD8-C5
PB-1_GAATGAACACGGTTTA	PB	PB-1	P1-1	TRUE	3	TRA:CAMSDRMTGGFKTIF TRB:CASSQVGRGGEETQYF	1047	CD8-C7
PB-1_GAATGAACAGGATCGA	PB	PB-1	P1-1	TRUE	3	TRA:CAMSDRMTGGFKTIF TRB:CASSQVGRGGEETQYF	1047	CD8-C7
PB-1_GAATGAATCTCTGAGA	PB	PB-1	P1-5	TRUE	3	TRA:CALSGTPMNRDDKIIF TRB:CSARGRGLAGGPAYEQYF	223	CD8-C7
PB-1_GACACGCCAAGCGAGT	PB	PB-1	P1-14	TRUE	3	TRA:CAASVVNDMRF TRB:CASSHRGGAKNIQYF	109	CD8-C7
PB-1_GACACGCGTAGCGATG	PB	PB-1	P1-2	TRUE	3	TRA:CGQNFVF TRB:CASNQRGGPSLYNEQFF	323	CD8-C7
PB-1_GACACGCGTCATACTG	PB	PB-1	P1-13	TRUE	3	TRA:CVVMNYGGSQGNLIF TRB:CASSSWGDTDEAFF	112	CD8-C7
PB-1_GACAGAGAGTGTGGCA	PB	PB-1	P1-790	FALSE	1	TRA:CALSGTPMNRDDKIIF TRB:CASNQRGGPSLYNEQFF	1	CD8-C7

PB-1_GACAGAGCAAAGGAAG	PB	PB-1	P1-31	TRUE	3	TRA:CAVEEGADRLMF TRB:CASSLLGGGELFF	46	CD8-C5
PB-1_GACAGAGCACGGCTAC	PB	PB-1	P1-1	TRUE	3	TRA:CAMSDRMTGGFKTIF TRB:CASSQVGRGGEETQYF	1047	CD8-C5
PB-1_GACAGAGTCTACTCAT	PB	PB-1	P1-50	TRUE	3	TRA:CARWLGSQGNLIF TRB:CASSLTDRVPQFELVYEQYF	15	CD8-C5
PB-1_GACCAATAGCTTCGCG	PB	PB-1	P1-6	TRUE	3	TRA:CAMREGRERDDKIIF TRB:CASSLPNTEAFF	216	CD8-C5
PB-1_GACCAATTCGCCTGAG	PB	PB-1	P1-1	TRUE	3	TRA:CAMSDRMTGGFKTIF TRB:CASSQVGRGGEETQYF	1047	CD8-C7
PB-1_GACCAATTCTGTTTGT	PB	PB-1	P1-3	TRUE	3	TRA:CAFLSGTYKYIF TRB:CASSQSPGQRNYGYTF	308	CD8-C7
PB-1_GACCTGGCACTGTCGG	PB	PB-1	P1-3	TRUE	3	TRA:CAFLSGTYKYIF TRB:CASSQSPGQRNYGYTF	308	CD8-C7
PB-1_GACCTGGCATGCCCGA	PB	PB-1	P1-5	TRUE	3	TRA:CALSGTPMNRDDKIIF TRB:CSARGRGLAGGPAYEQYF	223	CD8-C7
PB-1_GACCTGGGTACATCCA	PB	PB-1	P1-1	TRUE	3	TRA:CAMSDRMTGGFKTIF TRB:CASSQVGRGGEETQYF	1047	CD8-C5
PB-1_GACCTGGGTGGGTCAA	PB	PB-1	P1-1	TRUE	3	TRA:CAMSDRMTGGFKTIF TRB:CASSQVGRGGEETQYF	1047	CD8-C7
PB-1_GACCTGGTCCCAAGAT	PB	PB-1	P1-3	TRUE	3	TRA:CAFLSGTYKYIF TRB:CASSQSPGQRNYGYTF	308	CD8-C7
PB-1_GACCTGGTCGAGGTAG	PB	PB-1	P1-4	TRUE	3	TRA:CAMKGRDDKIIF TRB:CASTKGVSREAFF	226	CD8-C7
PB-1_GACGCGTCAAGAGGCT	PB	PB-1	P1-8	TRUE	3	TRA:CAVRPPGRNSGGYQKVTF TRB:CASSRRGVTDTQYF	204	CD8-C7
PB-1_GACGCGTTCCGCAGTG	PB	PB-1	P1-11	TRUE	3	TRA:CAVEPLYGSGNTGKLIF TRB:CASSSHGQLNQPQHF	147	CD8-C7
PB-1_GACGCGTTCCTAGGGC	PB	PB-1	P1-8	TRUE	3	TRA:CAVRPPGRNSGGYQKVTF TRB:CASSRRGVTDTQYF	204	CD8-C7
PB-1_GACGGCTCATGTCCTC	PB	PB-1	P1-8	TRUE	3	TRA:CAVRPPGRNSGGYQKVTF TRB:CASSRRGVTDTQYF	204	CD8-C7
PB-1_GACGGCTTCAAGGCTT	PB	PB-1	P1-14	TRUE	3	TRA:CAASVVNDMRF TRB:CASSHRGGAKNIQYF	109	CD8-C5
PB-1_GACGGCTTCAGCATGT	PB	PB-1	P1-6	TRUE	3	TRA:CAMREGRERDDKIIF TRB:CASSLPNTEAFF	216	CD8-C5
PB-1_GACGTGCAGCAGATCG	PB	PB-1	P1-22	TRUE	3	TRA:CASNSGYSTLTF TRB:CASSQVTGGARDEQYF	65	CD8-C5
PB-1_GACGTGCCAGTGGGAT	PB	PB-1	P1-3	TRUE	3	TRA:CAFLSGTYKYIF TRB:CASSQSPGQRNYGYTF	308	CD8-C7
PB-1_GACGTGCCATCACCCT	PB	PB-1	P1-9	TRUE	3	TRA:CAEAEGGGADGLTF TRB:CATSGGDNEQFF	174	CD8-C5
PB-1_GACGTGCGTATAGGGC	PB	PB-1	P1-16	TRUE	3	TRA:CALSGTPMNRDDKIIF TRB:CASSQSWDLAVLNEQFF	79	CD8-C5
PB-1_GACGTGCGTCAGATAA	PB	PB-1	P1-47	TRUE	3	TRA:CIVGGNFNKFYF TRB:CASSPEWDTDTQYF	18	CD8-C7
PB-1_GACGTTAAGAATGTGT	PB	PB-1	P1-31	TRUE	3	TRA:CAVEEGADRLMF TRB:CASSLLGGGELFF	46	CD8-C5
PB-1_GACGTTAAGATGTAAC	PB	PB-1	P1-35	TRUE	3	TRA:CAVSDRGYFGNEKLTF TRB:CSALDDVRPVIAVYNEQFF	40	CD8-C7
PB-1_GACGTTACATATACGC	PB	PB-1	P1-10	TRUE	3	TRA:CISNSPMDSSYKLIF TRB:CASSSWGDTDEAFF	162	CD8-C7
PB-1_GACGTTAGTCAATGTC	PB	PB-1	P1-12	TRUE	3	TRA:CALSDERGDYKLSF TRB:CASSPGTGSYNEQFF	141	CD8-C5

PB-1_GACGTTAGTCTAAACC	PB	PB-1	P1-4	TRUE	3	TRA:CAMKGRDDKIIF TRB:CASTKGVSREAFF	226	CD8-C7
PB-1_GACGTTAGTTAAAGTG	PB	PB-1	P1-1	TRUE	3	TRA:CAMSDRMTGGFKTIF TRB:CASSQVGRGGEETQYF	1047	CD8-C5
PB-1_GACGTTATCCTAAGTG	PB	PB-1	P1-1	TRUE	3	TRA:CAMSDRMTGGFKTIF TRB:CASSQVGRGGEETQYF	1047	CD8-C7
PB-1_GACTAACAGAGTACAT	PB	PB-1	P1-7	TRUE	3	TRA:CAVQAGNNDMRF TRB:CASSTTGDFNYGYTF	215	CD8-C7
PB-1_GACTAACAGCGATGAC	PB	PB-1	P1-1	TRUE	3	TRA:CAMSDRMTGGFKTIF TRB:CASSQVGRGGEETQYF	1047	CD8-C7
PB-1_GACTAACCAGGACGTA	PB	PB-1	P1-10	TRUE	3	TRA:CISNSPMDSSYKLIF TRB:CASSSWGDTDEAFF	162	CD8-C7
PB-1_GACTAACGTCCAGTGC	PB	PB-1	P1-54	TRUE	3	TRA:CAGWYSGGGADGLTF TRB:CATGAGDNEQFF	13	CD8-C7
PB-1_GACTAACGTTGGACCC	PB	PB-1	P1-26	TRUE	3	TRA:CAPRSGGSYIPTF TRB:CASSQDLGLDYGYTF	56	CD8-C7
PB-1_GACTACAAGACAGACC	PB	PB-1	P1-29	TRUE	3	TRA:CATVSFSGGYNKLIF TRB:CASSLDRRGLSYEQYF	50	CD8-C7
PB-1_GACTACAAGAGTCGGT	PB	PB-1	P1-16	TRUE	3	TRA:CALSGTPMNRDDKIIF TRB:CASSQSWDLAVLNEQFF	79	CD8-C7
PB-1_GACTACAAGCCTATGT	PB	PB-1	P1-3	TRUE	3	TRA:CAFLSGTYKYIF TRB:CASSQSPGQRNYGYTF	308	CD8-C7
PB-1_GACTGCGAGACAGGCT	PB	PB-1	P1-1	TRUE	3	TRA:CAMSDRMTGGFKTIF TRB:CASSQVGRGGEETQYF	1047	CD8-C5
PB-1_GACTGCGAGATCTGCT	PB	PB-1	P1-2	TRUE	3	TRA:CGQNFVF TRB:CASNQRGGPSLYNEQFF	323	CD4-C6
PB-1_GACTGCGCACAGACTT	PB	PB-1	P1-1	TRUE	3	TRA:CAMSDRMTGGFKTIF TRB:CASSQVGRGGEETQYF	1047	CD8-C7
PB-1_GACTGCGCATCGGTTA	PB	PB-1	P1-6	TRUE	3	TRA:CAMREGRERDDKIIF TRB:CASSLPNTEAFF	216	CD8-C5
PB-1_GACTGCGGTAGCCTAT	PB	PB-1	P1-1	TRUE	3	TRA:CAMSDRMTGGFKTIF TRB:CASSQVGRGGEETQYF	1047	CD8-C7
PB-1_GACTGCGGTCTACCTC	PB	PB-1	P1-1	TRUE	3	TRA:CAMSDRMTGGFKTIF TRB:CASSQVGRGGEETQYF	1047	CD8-C7
PB-1_GACTGCGGTGTTGGGA	PB	PB-1	P1-3	TRUE	3	TRA:CAFLSGTYKYIF TRB:CASSQSPGQRNYGYTF	308	CD8-C7
PB-1_GACTGCGTCCCTGACT	PB	PB-1	P1-4	TRUE	3	TRA:CAMKGRDDKIIF TRB:CASTKGVSREAFF	226	CD8-C7
PB-1_GACTGCGTCTCGGACG	PB	PB-1	P1-4	TRUE	3	TRA:CAMKGRDDKIIF TRB:CASTKGVSREAFF	226	CD8-C7
PB-1_GACTGCGTCTGTCCGT	PB	PB-1	P1-13	TRUE	3	TRA:CVVMNYGGSQGNLIF TRB:CASSSWGDTDEAFF	112	CD8-C7
PB-1_GAGCAGAAGTCGTACT	PB	PB-1	P1-1	TRUE	3	TRA:CAMSDRMTGGFKTIF TRB:CASSQVGRGGEETQYF	1047	CD8-C7
PB-1_GAGCAGATCATGTAGC	PB	PB-1	P1-7	TRUE	3	TRA:CAVQAGNNDMRF TRB:CASSTTGDFNYGYTF	215	CD8-C7
PB-1_GAGGTGAAGCGTAATA	PB	PB-1	P1-11	TRUE	3	TRA:CAVEPLYGSGNTGKLIF TRB:CASSSHGQLNQPQHF	147	CD8-C5
PB-1_GAGGTGACATGTAAGA	PB	PB-1	P1-8	TRUE	3	TRA:CAVRPPGRNSGGYQKVTF TRB:CASSRRGVTDTQYF	204	CD8-C7
PB-1_GAGGTGAGTCAATACC	PB	PB-1	P1-11	TRUE	3	TRA:CAVEPLYGSGNTGKLIF TRB:CASSSHGQLNQPQHF	147	CD8-C7
PB-1_GAGGTGAGTCTCACCT	PB	PB-1	P1-3	TRUE	3	TRA:CAFLSGTYKYIF TRB:CASSQSPGQRNYGYTF	308	CD8-C7

PB-1_GAGGTGAGTGCAGTAG	PB	PB-1	P1-10	TRUE	3	TRA:CISNSPMDSSYKLIF TRB:CASSSWGDTDEAFF	162	CD8-C7
PB-1_GAGTCCGAGCATGGCA	PB	PB-1	P1-22	TRUE	3	TRA:CASNSGYSTLTF TRB:CASSQVTGGARDEQYF	65	CD8-C7
PB-1_GAGTCCGGTCCGCTGA	PB	PB-1	P1-1	TRUE	3	TRA:CAMSDRMTGGFKTIF TRB:CASSQVGRGGEETQYF	1047	CD8-C7
PB-1_GAGTCCGTCCTAAGTG	PB	PB-1	P1-1	TRUE	3	TRA:CAMSDRMTGGFKTIF TRB:CASSQVGRGGEETQYF	1047	CD8-C7
PB-1_GAGTCCGTCGAGCCCA	PB	PB-1	P1-1	TRUE	3	TRA:CAMSDRMTGGFKTIF TRB:CASSQVGRGGEETQYF	1047	CD8-C7
PB-1_GATCAGTCAGCTGTTA	PB	PB-1	P1-7	TRUE	3	TRA:CAVQAGNNDMRF TRB:CASSTTGDFNYGYTF	215	CD8-C7
PB-1_GATCAGTGTTCGTGAT	PB	PB-1	P1-8	TRUE	3	TRA:CAVRPPGRNSGGYQKVTF TRB:CASSRRGVTDTQYF	204	CD8-C7
PB-1_GATCAGTTCAACGAAA	PB	PB-1	P1-5	TRUE	3	TRA:CALSGTPMNRDDKIIF TRB:CSARGRGLAGGPAYEQYF	223	CD8-C7
PB-1_GATCGATCATCGATGT	PB	PB-1	P1-9	TRUE	3	TRA:CAEAEGGGADGLTF TRB:CATSGGDNEQFF	174	CD8-C5
PB-1_GATCGATGTCTGCGGT	PB	PB-1	P1-14	TRUE	3	TRA:CAASVVNDMRF TRB:CASSHRGGAKNIQYF	109	CD8-C5
PB-1_GATCGATGTGTTCTTT	PB	PB-1	P1-13	TRUE	3	TRA:CVVMNYGGSQGNLIF TRB:CASSSWGDTDEAFF	112	CD8-C7
PB-1_GATCGATGTGTTTGGT	PB	PB-1	P1-1	TRUE	3	TRA:CAMSDRMTGGFKTIF TRB:CASSQVGRGGEETQYF	1047	CD8-C7
PB-1_GATCGATGTTACTGAC	PB	PB-1	P1-2	TRUE	3	TRA:CGQNFVF TRB:CASNQRGGPSLYNEQFF	323	CD8-C7
PB-1_GATCGATTCACTTCAT	PB	PB-1	P1-5	TRUE	3	TRA:CALSGTPMNRDDKIIF TRB:CSARGRGLAGGPAYEQYF	223	CD8-C7
PB-1_GATCGATTCTCAAGTG	PB	PB-1	P1-3	TRUE	3	TRA:CAFLSGTYKYIF TRB:CASSQSPGQRNYGYTF	308	CD8-C7
PB-1_GATCGCGAGACTTGAA	PB	PB-1	P1-3	TRUE	3	TRA:CAFLSGTYKYIF TRB:CASSQSPGQRNYGYTF	308	CD8-C7
PB-1_GATCGCGCAAGCGTAG	PB	PB-1	P1-3	TRUE	3	TRA:CAFLSGTYKYIF TRB:CASSQSPGQRNYGYTF	308	CD8-C7
PB-1_GATCGCGCACGAAAGC	PB	PB-1	P1-8	TRUE	3	TRA:CAVRPPGRNSGGYQKVTF TRB:CASSRRGVTDTQYF	204	CD8-C7
PB-1_GATCGCGCAGGGCATA	PB	PB-1	P1-5	TRUE	3	TRA:CALSGTPMNRDDKIIF TRB:CSARGRGLAGGPAYEQYF	223	CD8-C7
PB-1_GATCGCGGTTGTCGCG	PB	PB-1	P1-1	TRUE	3	TRA:CAMSDRMTGGFKTIF TRB:CASSQVGRGGEETQYF	1047	CD8-C7
PB-1_GATCGCGTCAGGCAAG	PB	PB-1	P1-1	TRUE	3	TRA:CAMSDRMTGGFKTIF TRB:CASSQVGRGGEETQYF	1047	CD8-C7
PB-1_GATCGCGTCCACGACG	PB	PB-1	P1-16	TRUE	3	TRA:CALSGTPMNRDDKIIF TRB:CASSQSWDLAVLNEQFF	79	CD8-C5
PB-1_GATCGTAAGACTTTCG	PB	PB-1	P1-1	TRUE	3	TRA:CAMSDRMTGGFKTIF TRB:CASSQVGRGGEETQYF	1047	CD8-C7
PB-1_GATCGTACACAGCCCA	PB	PB-1	P1-807	FALSE	1	TRA:CAVRPPGRNSGGYQKVTF TRB:CASSDSGNFYNEQFF	1	CD8-C2
PB-1_GATCGTAGTCTCATCC	PB	PB-1	P1-1	TRUE	3	TRA:CAMSDRMTGGFKTIF TRB:CASSQVGRGGEETQYF	1047	CD8-C7
PB-1_GATCTAGAGTACATGA	PB	PB-1	P1-14	TRUE	3	TRA:CAASVVNDMRF TRB:CASSHRGGAKNIQYF	109	CD8-C5
PB-1_GATCTAGCACTCAGGC	PB	PB-1	P1-5	TRUE	3	TRA:CALSGTPMNRDDKIIF TRB:CSARGRGLAGGPAYEQYF	223	CD8-C7

PB-1_GATCTAGCAGACACTT	PB	PB-1	P1-31	TRUE	3	TRA:CAVEEGADRLMF TRB:CASSLLGGGELFF	46	CD8-C5
PB-1_GATCTAGCAGGGCATA	PB	PB-1	P1-8	TRUE	3	TRA:CAVRPPGRNSGGYQKVTF TRB:CASSRRGVTDTQYF	204	CD8-C7
PB-1_GATCTAGTCAGCTGGC	PB	PB-1	P1-3	TRUE	3	TRA:CAFLSGTYKYIF TRB:CASSQSPGQRNYGYTF	308	CD8-C7
PB-1_GATCTAGTCAGGCAAG	PB	PB-1	P1-8	TRUE	3	TRA:CAVRPPGRNSGGYQKVTF TRB:CASSRRGVTDTQYF	204	CD8-C7
PB-1_GATGAAAAGGTACTCT	PB	PB-1	P1-18	TRUE	3	TRA:CVADGNYGQNFVF TRB:CASSSPGLGGGFDTEAFF	75	CD8-C7
PB-1_GATGAAACATTTCAGG	PB	PB-1	P1-1	TRUE	3	TRA:CAMSDRMTGGFKTIF TRB:CASSQVGRGGEETQYF	1047	CD8-C7
PB-1_GATGAAAGTCTGGAGA	PB	PB-1	P1-26	TRUE	3	TRA:CAPRSGGSYIPTF TRB:CASSQDLGLDYGYTF	56	CD8-C7
PB-1_GATGAAATCAGTCAGT	PB	PB-1	P1-4	TRUE	3	TRA:CAMKGRDDKIIF TRB:CASTKGVSREAFF	226	CD8-C7
PB-1_GATGAGGCAGTTAACC	PB	PB-1	P1-814	FALSE	1	TRA:CAMREGRERDDKIIF TRB:CASSLLGGGELFF	1	CD8-C7
PB-1_GATGAGGGTCCGAAGA	PB	PB-1	P1-1	TRUE	3	TRA:CAMSDRMTGGFKTIF TRB:CASSQVGRGGEETQYF	1047	CD8-C7
PB-1_GATGAGGGTTCCTCCA	PB	PB-1	P1-11	TRUE	3	TRA:CAVEPLYGSGNTGKLIF TRB:CASSSHGQLNQPQHF	147	CD8-C7
PB-1_GATGAGGGTTGCGTTA	PB	PB-1	P1-1	TRUE	3	TRA:CAMSDRMTGGFKTIF TRB:CASSQVGRGGEETQYF	1047	CD8-C7
PB-1_GATGAGGTCGTTGCCT	PB	PB-1	P1-3	TRUE	3	TRA:CAFLSGTYKYIF TRB:CASSQSPGQRNYGYTF	308	CD8-C7
PB-1_GATGCTAAGACATAAC	PB	PB-1	P1-1	TRUE	3	TRA:CAMSDRMTGGFKTIF TRB:CASSQVGRGGEETQYF	1047	CD8-C5
PB-1_GATGCTAAGACTACAA	PB	PB-1	P1-5	TRUE	3	TRA:CALSGTPMNRDDKIIF TRB:CSARGRGLAGGPAYEQYF	223	CD8-C7
PB-1_GATGCTACAAGCTGAG	PB	PB-1	P1-4	TRUE	3	TRA:CAMKGRDDKIIF TRB:CASTKGVSREAFF	226	CD8-C7
PB-1_GATGCTACACACAGAG	PB	PB-1	P1-1	TRUE	3	TRA:CAMSDRMTGGFKTIF TRB:CASSQVGRGGEETQYF	1047	CD8-C7
PB-1_GATGCTACACCCATTC	PB	PB-1	P1-1	TRUE	3	TRA:CAMSDRMTGGFKTIF TRB:CASSQVGRGGEETQYF	1047	CD8-C7
PB-1_GATGCTAGTGTTCGAT	PB	PB-1	P1-817	FALSE	1	TRA:CAMSDRMTGGFKTIF TRB:CASNQRGGPSLYNEQFF	1	CD8-C7
PB-1_GATGCTATCACCACCT	PB	PB-1	P1-1	TRUE	3	TRA:CAMSDRMTGGFKTIF TRB:CASSQVGRGGEETQYF	1047	CD8-C7
PB-1_GATGCTATCACCATAG	PB	PB-1	P1-5	TRUE	3	TRA:CALSGTPMNRDDKIIF TRB:CSARGRGLAGGPAYEQYF	223	CD8-C7
PB-1_GATTCAGCAAGGTTTC	PB	PB-1	P1-1	TRUE	3	TRA:CAMSDRMTGGFKTIF TRB:CASSQVGRGGEETQYF	1047	CD8-C7
PB-1_GATTCAGTCAAACGGG	PB	PB-1	P1-50	TRUE	3	TRA:CARWLGSQGNLIF TRB:CASSLTDRVPQFELVYEQYF	15	CD8-C7
PB-1_GATTCAGTCACAATGC	PB	PB-1	P1-3	TRUE	3	TRA:CAFLSGTYKYIF TRB:CASSQSPGQRNYGYTF	308	CD8-C7
PB-1_GCAAACTAGACAAAGG	PB	PB-1	P1-14	TRUE	3	TRA:CAASVVNDMRF TRB:CASSHRGGAKNIQYF	109	CD8-C5
PB-1_GCAAACTAGTATGACA	PB	PB-1	P1-1	TRUE	3	TRA:CAMSDRMTGGFKTIF TRB:CASSQVGRGGEETQYF	1047	CD8-C7
PB-1_GCAAACTCAGCCTTGG	PB	PB-1	P1-5	TRUE	3	TRA:CALSGTPMNRDDKIIF TRB:CSARGRGLAGGPAYEQYF	223	CD8-C7

PB-1_GCAAACTTCCCTCTTT	PB	PB-1	P1-14	TRUE	3	TRA:CAASVVNDMRF TRB:CASSHRGGAKNIQYF	109	CD8-C5
PB-1_GCAAACTTCTCTGTCG	PB	PB-1	P1-1	TRUE	3	TRA:CAMSDRMTGGFKTIF TRB:CASSQVGRGGEETQYF	1047	CD8-C7
PB-1_GCAAACTTCTGAGGGA	PB	PB-1	P1-2	TRUE	3	TRA:CGQNFVF TRB:CASNQRGGPSLYNEQFF	323	CD8-C7
PB-1_GCAAACTTCTGCCAGG	PB	PB-1	P1-31	TRUE	3	TRA:CAVEEGADRLMF TRB:CASSLLGGGELFF	46	CD8-C7
PB-1_GCAATCAAGGTGATAT	PB	PB-1	P1-1	TRUE	3	TRA:CAMSDRMTGGFKTIF TRB:CASSQVGRGGEETQYF	1047	CD8-C7
PB-1_GCAATCACAATGTAAG	PB	PB-1	P1-1	TRUE	3	TRA:CAMSDRMTGGFKTIF TRB:CASSQVGRGGEETQYF	1047	CD8-C7
PB-1_GCAATCACACCGCTAG	PB	PB-1	P1-5	TRUE	3	TRA:CALSGTPMNRDDKIIF TRB:CSARGRGLAGGPAYEQYF	223	CD8-C7
PB-1_GCACATACAATAGCGG	PB	PB-1	P1-26	TRUE	3	TRA:CAPRSGGSYIPTF TRB:CASSQDLGLDYGYTF	56	CD8-C7
PB-1_GCACATATCATGTCTT	PB	PB-1	P1-1	TRUE	3	TRA:CAMSDRMTGGFKTIF TRB:CASSQVGRGGEETQYF	1047	CD8-C7
PB-1_GCACATATCCTGTACC	PB	PB-1	P1-5	TRUE	3	TRA:CALSGTPMNRDDKIIF TRB:CSARGRGLAGGPAYEQYF	223	CD8-C5
PB-1_GCACTCTAGCTGCCCA	PB	PB-1	P1-2	TRUE	3	TRA:CGQNFVF TRB:CASNQRGGPSLYNEQFF	323	CD8-C7
PB-1_GCACTCTCACGGTAAG	PB	PB-1	P1-2	TRUE	3	TRA:CGQNFVF TRB:CASNQRGGPSLYNEQFF	323	CD8-C7
PB-1_GCACTCTGTGACAAAT	PB	PB-1	P1-115	FALSE	3	TRA:CAVKGSSNTGKLIF TRB:CASSYSYGRTGTGTYEQYF	5	CD8-C7
PB-1_GCACTCTTCAGAGCTT	PB	PB-1	P1-11	TRUE	3	TRA:CAVEPLYGSGNTGKLIF TRB:CASSSHGQLNQPQHF	147	CD8-C7
PB-1_GCACTCTTCCAATGGT	PB	PB-1	P1-5	TRUE	3	TRA:CALSGTPMNRDDKIIF TRB:CSARGRGLAGGPAYEQYF	223	CD8-C7
PB-1_GCAGCCAAGTAGTGCG	PB	PB-1	P1-2	TRUE	3	TRA:CGQNFVF TRB:CASNQRGGPSLYNEQFF	323	CD8-C5
PB-1_GCAGCCACAAGCGATG	PB	PB-1	P1-3	TRUE	3	TRA:CAFLSGTYKYIF TRB:CASSQSPGQRNYGYTF	308	CD8-C7
PB-1_GCAGCCACACAACTGT	PB	PB-1	P1-5	TRUE	3	TRA:CALSGTPMNRDDKIIF TRB:CSARGRGLAGGPAYEQYF	223	CD8-C7
PB-1_GCAGCCACATCGATGT	PB	PB-1	P1-2	TRUE	3	TRA:CGQNFVF TRB:CASNQRGGPSLYNEQFF	323	CD8-C7
PB-1_GCAGCCAGTCTGGAGA	PB	PB-1	P1-5	TRUE	3	TRA:CALSGTPMNRDDKIIF TRB:CSARGRGLAGGPAYEQYF	223	CD8-C7
PB-1_GCAGCCAGTGAAATCA	PB	PB-1	P1-13	TRUE	3	TRA:CVVMNYGGSQGNLIF TRB:CASSSWGDTDEAFF	112	CD8-C7
PB-1_GCAGCCATCACTCCTG	PB	PB-1	P1-11	TRUE	3	TRA:CAVEPLYGSGNTGKLIF TRB:CASSSHGQLNQPQHF	147	CD8-C7
PB-1_GCAGTTAAGCAACGGT	PB	PB-1	P1-8	TRUE	3	TRA:CAVRPPGRNSGGYQKVTF TRB:CASSRRGVTDTQYF	204	CD8-C5
PB-1_GCAGTTAAGCTACCTA	PB	PB-1	P1-2	TRUE	3	TRA:CGQNFVF TRB:CASNQRGGPSLYNEQFF	323	CD8-C7
PB-1_GCAGTTACAGATCCAT	PB	PB-1	P1-7	TRUE	3	TRA:CAVQAGNNDMRF TRB:CASSTTGDFNYGYTF	215	CD8-C5
PB-1_GCAGTTAGTGATGTCT	PB	PB-1	P1-1	TRUE	3	TRA:CAMSDRMTGGFKTIF TRB:CASSQVGRGGEETQYF	1047	CD8-C7
PB-1_GCAGTTAGTGTTTGTG	PB	PB-1	P1-1	TRUE	3	TRA:CAMSDRMTGGFKTIF TRB:CASSQVGRGGEETQYF	1047	CD8-C7

PB-1_GCATACAAGGCTACGA	PB	PB-1	P1-18	TRUE	3	TRA:CVADGNYGQNFVF TRB:CASSSPGLGGGFDTEAFF	75	CD8-C6
PB-1_GCATACACAGCTATTG	PB	PB-1	P1-1	TRUE	3	TRA:CAMSDRMTGGFKTIF TRB:CASSQVGRGGEETQYF	1047	CD8-C7
PB-1_GCATACACATCTCGCT	PB	PB-1	P1-13	TRUE	3	TRA:CVVMNYGGSQGNLIF TRB:CASSSWGDTDEAFF	112	CD8-C7
PB-1_GCATACAGTATATCCG	PB	PB-1	P1-3	TRUE	3	TRA:CAFLSGTYKYIF TRB:CASSQSPGQRNYGYTF	308	CD8-C7
PB-1_GCATACATCCAAGCCG	PB	PB-1	P1-13	TRUE	3	TRA:CVVMNYGGSQGNLIF TRB:CASSSWGDTDEAFF	112	CD8-C7
PB-1_GCATACATCTCTGAGA	PB	PB-1	P1-10	TRUE	3	TRA:CISNSPMDSSYKLIF TRB:CASSSWGDTDEAFF	162	CD8-C7
PB-1_GCATACATCTTCGGTC	PB	PB-1	P1-3	TRUE	3	TRA:CAFLSGTYKYIF TRB:CASSQSPGQRNYGYTF	308	CD8-C7
PB-1_GCATACATCTTTAGGG	PB	PB-1	P1-827	FALSE	1	TRA:CVVSEVNNQAGTALIF TRB:CASSYTGEGYTF	1	CD8-C7
PB-1_GCATGATAGTCGCCGT	PB	PB-1	P1-3	TRUE	3	TRA:CAFLSGTYKYIF TRB:CASSQSPGQRNYGYTF	308	CD8-C5
PB-1_GCATGATGTGAAAGAG	PB	PB-1	P1-26	TRUE	3	TRA:CAPRSGGSYIPTF TRB:CASSQDLGLDYGYTF	56	CD8-C7
PB-1_GCATGATTCACTATTC	PB	PB-1	P1-1	TRUE	3	TRA:CAMSDRMTGGFKTIF TRB:CASSQVGRGGEETQYF	1047	CD8-C7
PB-1_GCATGCGAGTGCGTGA	PB	PB-1	P1-1	TRUE	3	TRA:CAMSDRMTGGFKTIF TRB:CASSQVGRGGEETQYF	1047	CD8-C5
PB-1_GCATGCGTCAAACCAC	PB	PB-1	P1-10	TRUE	3	TRA:CISNSPMDSSYKLIF TRB:CASSSWGDTDEAFF	162	CD8-C7
PB-1_GCATGCGTCCTTAATC	PB	PB-1	P1-17	TRUE	3	TRA:CGTGARGSSGSRLTF TRB:CASSRQGMGAFF	78	CD8-C7
PB-1_GCATGTAAGGACAGCT	PB	PB-1	P1-10	TRUE	3	TRA:CISNSPMDSSYKLIF TRB:CASSSWGDTDEAFF	162	CD8-C7
PB-1_GCATGTACATCTCCCA	PB	PB-1	P1-144	FALSE	3	TRA:CAMKGRDDKIIF TRB:CASSQVGRGGEETQYF	4	CD8-C11
PB-1_GCATGTATCAACACCA	PB	PB-1	P1-26	TRUE	3	TRA:CAPRSGGSYIPTF TRB:CASSQDLGLDYGYTF	56	CD8-C7
PB-1_GCATGTATCATGCTCC	PB	PB-1	P1-1	TRUE	3	TRA:CAMSDRMTGGFKTIF TRB:CASSQVGRGGEETQYF	1047	CD8-C7
PB-1_GCATGTATCGCGCCAA	PB	PB-1	P1-1	TRUE	3	TRA:CAMSDRMTGGFKTIF TRB:CASSQVGRGGEETQYF	1047	CD8-C7
PB-1_GCATGTATCTCTAAGG	PB	PB-1	P1-1	TRUE	3	TRA:CAMSDRMTGGFKTIF TRB:CASSQVGRGGEETQYF	1047	CD8-C7
PB-1_GCATGTATCTGCCAGG	PB	PB-1	P1-1	TRUE	3	TRA:CAMSDRMTGGFKTIF TRB:CASSQVGRGGEETQYF	1047	CD8-C7
PB-1_GCCAAATAGTGCTGCC	PB	PB-1	P1-1	TRUE	3	TRA:CAMSDRMTGGFKTIF TRB:CASSQVGRGGEETQYF	1047	CD8-C7
PB-1_GCCAAATCAGCGTCCA	PB	PB-1	P1-16	TRUE	3	TRA:CALSGTPMNRDDKIIF TRB:CASSQSWDLAVLNEQFF	79	CD8-C7
PB-1_GCCAAATGTCGCGAAA	PB	PB-1	P1-8	TRUE	3	TRA:CAVRPPGRNSGGYQKVTF TRB:CASSRRGVTDTQYF	204	CD8-C7
PB-1_GCCAAATGTTCAGTAC	PB	PB-1	P1-1	TRUE	3	TRA:CAMSDRMTGGFKTIF TRB:CASSQVGRGGEETQYF	1047	CD8-C7
PB-1_GCCAAATTCCTAAGTG	PB	PB-1	P1-8	TRUE	3	TRA:CAVRPPGRNSGGYQKVTF TRB:CASSRRGVTDTQYF	204	CD8-C7
PB-1_GCCTCTAAGTGAACGC	PB	PB-1	P1-2	TRUE	3	TRA:CGQNFVF TRB:CASNQRGGPSLYNEQFF	323	CD8-C7

PB-1_GCCTCTACACACCGAC	PB	PB-1	P1-4	TRUE	3	TRA:CAMKGRDDKIIF TRB:CASTKGVSREAFF	226	CD8-C7
PB-1_GCCTCTACATGCAACT	PB	PB-1	P1-3	TRUE	3	TRA:CAFLSGTYKYIF TRB:CASSQSPGQRNYGYTF	308	CD8-C7
PB-1_GCCTCTAGTAGATTAG	PB	PB-1	P1-5	TRUE	3	TRA:CALSGTPMNRDDKIIF TRB:CSARGRGLAGGPAYEQYF	223	CD8-C7
PB-1_GCCTCTAGTTACCAGT	PB	PB-1	P1-8	TRUE	3	TRA:CAVRPPGRNSGGYQKVTF TRB:CASSRRGVTDTQYF	204	CD8-C7
PB-1_GCCTCTATCAATCACG	PB	PB-1	P1-1	TRUE	3	TRA:CAMSDRMTGGFKTIF TRB:CASSQVGRGGEETQYF	1047	CD8-C5
PB-1_GCCTCTATCAGTCCCT	PB	PB-1	P1-10	TRUE	3	TRA:CISNSPMDSSYKLIF TRB:CASSSWGDTDEAFF	162	CD8-C7
PB-1_GCGACCAAGCATCATC	PB	PB-1	P1-8	TRUE	3	TRA:CAVRPPGRNSGGYQKVTF TRB:CASSRRGVTDTQYF	204	CD8-C7
PB-1_GCGACCAAGTGAAGAG	PB	PB-1	P1-4	TRUE	3	TRA:CAMKGRDDKIIF TRB:CASTKGVSREAFF	226	CD8-C7
PB-1_GCGACCACATGCAATC	PB	PB-1	P1-8	TRUE	3	TRA:CAVRPPGRNSGGYQKVTF TRB:CASSRRGVTDTQYF	204	CD8-C7
PB-1_GCGACCAGTACCGCTG	PB	PB-1	P1-1	TRUE	3	TRA:CAMSDRMTGGFKTIF TRB:CASSQVGRGGEETQYF	1047	CD8-C7
PB-1_GCGAGAAAGATAGGAG	PB	PB-1	P1-35	TRUE	3	TRA:CAVSDRGYFGNEKLTF TRB:CSALDDVRPVIAVYNEQFF	40	CD8-C7
PB-1_GCGAGAAAGCGGCTTC	PB	PB-1	P1-43	TRUE	3	TRA:CAASIIQGAQKLVF TRB:CASSFPGQGILEQYF	23	CD8-C2
PB-1_GCGAGAAGTATTCGTG	PB	PB-1	P1-1	TRUE	3	TRA:CAMSDRMTGGFKTIF TRB:CASSQVGRGGEETQYF	1047	CD8-C7
PB-1_GCGAGAATCCTCGCAT	PB	PB-1	P1-1	TRUE	3	TRA:CAMSDRMTGGFKTIF TRB:CASSQVGRGGEETQYF	1047	CD8-C7
PB-1_GCGCAACCATCCGCGA	PB	PB-1	P1-8	TRUE	3	TRA:CAVRPPGRNSGGYQKVTF TRB:CASSRRGVTDTQYF	204	CD8-C5
PB-1_GCGCAACCATCGGGTC	PB	PB-1	P1-11	TRUE	3	TRA:CAVEPLYGSGNTGKLIF TRB:CASSSHGQLNQPQHF	147	CD8-C7
PB-1_GCGCAACGTGTTGGGA	PB	PB-1	P1-39	TRUE	3	TRA:CAVRDLGGTSYDKVIF TRB:CSARVREVSYEQYF	31	CD8-C7
PB-1_GCGCAACGTTGGTGGA	PB	PB-1	P1-290	TRUE	2	TRA:CATNTDKLIF TRB:CASSLEGDWNEQYF	2	CD8-C2
PB-1_GCGCAACTCTCCAACC	PB	PB-1	P1-31	TRUE	3	TRA:CAVEEGADRLMF TRB:CASSLLGGGELFF	46	CD8-C7
PB-1_GCGCAGTGTTCGTTGA	PB	PB-1	P1-6	TRUE	3	TRA:CAMREGRERDDKIIF TRB:CASSLPNTEAFF	216	CD8-C5
PB-1_GCGCAGTTCTTGACGA	PB	PB-1	P1-1	TRUE	3	TRA:CAMSDRMTGGFKTIF TRB:CASSQVGRGGEETQYF	1047	CD8-C7
PB-1_GCGCCAAAGAGGACGG	PB	PB-1	P1-10	TRUE	3	TRA:CISNSPMDSSYKLIF TRB:CASSSWGDTDEAFF	162	CD8-C7
PB-1_GCGCCAAAGGAATGGA	PB	PB-1	P1-1	TRUE	3	TRA:CAMSDRMTGGFKTIF TRB:CASSQVGRGGEETQYF	1047	CD8-C7
PB-1_GCGCCAAAGGCTAGCA	PB	PB-1	P1-10	TRUE	3	TRA:CISNSPMDSSYKLIF TRB:CASSSWGDTDEAFF	162	CD8-C7
PB-1_GCGCCAAGTCCAGTGC	PB	PB-1	P1-31	TRUE	3	TRA:CAVEEGADRLMF TRB:CASSLLGGGELFF	46	CD8-C7
PB-1_GCGCCAAGTCTTCGTC	PB	PB-1	P1-13	TRUE	3	TRA:CVVMNYGGSQGNLIF TRB:CASSSWGDTDEAFF	112	CD8-C7
PB-1_GCGCCAAGTGAGGGTT	PB	PB-1	P1-3	TRUE	3	TRA:CAFLSGTYKYIF TRB:CASSQSPGQRNYGYTF	308	CD8-C7

PB-1_GCGCCAATCGCCTGTT	PB	PB-1	P1-1	TRUE	3	TRA:CAMSDRMTGGFKTIF TRB:CASSQVGRGGEETQYF	1047	CD8-C7
PB-1_GCGCCAATCTAAGCCA	PB	PB-1	P1-1	TRUE	3	TRA:CAMSDRMTGGFKTIF TRB:CASSQVGRGGEETQYF	1047	CD8-C7
PB-1_GCGCCAATCTGCCAGG	PB	PB-1	P1-1	TRUE	3	TRA:CAMSDRMTGGFKTIF TRB:CASSQVGRGGEETQYF	1047	CD8-C7
PB-1_GCGCGATAGCTGCCCA	PB	PB-1	P1-8	TRUE	3	TRA:CAVRPPGRNSGGYQKVTF TRB:CASSRRGVTDTQYF	204	CD8-C7
PB-1_GCGCGATGTAAAGTCA	PB	PB-1	P1-11	TRUE	3	TRA:CAVEPLYGSGNTGKLIF TRB:CASSSHGQLNQPQHF	147	CD8-C5
PB-1_GCGCGATGTCTACCTC	PB	PB-1	P1-1	TRUE	3	TRA:CAMSDRMTGGFKTIF TRB:CASSQVGRGGEETQYF	1047	CD8-C7
PB-1_GCGCGATGTGTTGAGG	PB	PB-1	P1-11	TRUE	3	TRA:CAVEPLYGSGNTGKLIF TRB:CASSSHGQLNQPQHF	147	CD8-C7
PB-1_GCGCGATGTTGATTCG	PB	PB-1	P1-2	TRUE	3	TRA:CGQNFVF TRB:CASNQRGGPSLYNEQFF	323	CD8-C7
PB-1_GCGCGATGTTGCCTCT	PB	PB-1	P1-25	TRUE	3	TRA:CIVGRDSSYKLIF TRB:CASNPQRGAHRDTQYF	58	CD8-C6
PB-1_GCGGGTTAGCCTATGT	PB	PB-1	P1-3	TRUE	3	TRA:CAFLSGTYKYIF TRB:CASSQSPGQRNYGYTF	308	CD8-C7
PB-1_GCGGGTTCAGCCTGTG	PB	PB-1	P1-1	TRUE	3	TRA:CAMSDRMTGGFKTIF TRB:CASSQVGRGGEETQYF	1047	CD8-C7
PB-1_GCGGGTTCAGGAACGT	PB	PB-1	P1-1	TRUE	3	TRA:CAMSDRMTGGFKTIF TRB:CASSQVGRGGEETQYF	1047	CD8-C5
PB-1_GCGGGTTGTACTCGCG	PB	PB-1	P1-1	TRUE	3	TRA:CAMSDRMTGGFKTIF TRB:CASSQVGRGGEETQYF	1047	CD8-C7
PB-1_GCGGGTTTCAGCTTAG	PB	PB-1	P1-18	TRUE	3	TRA:CVADGNYGQNFVF TRB:CASSSPGLGGGFDTEAFF	75	CD8-C6
PB-1_GCGGGTTTCGTCCGTT	PB	PB-1	P1-2	TRUE	3	TRA:CGQNFVF TRB:CASNQRGGPSLYNEQFF	323	CD8-C7
PB-1_GCTCCTAAGGCGCTCT	PB	PB-1	P1-16	TRUE	3	TRA:CALSGTPMNRDDKIIF TRB:CASSQSWDLAVLNEQFF	79	CD8-C7
PB-1_GCTCCTAAGGGTCTCC	PB	PB-1	P1-113	TRUE	3	TRA:CAVNGGSQGNLIF TRB:CASSLAVNYGYTF	5	CD8-C7
PB-1_GCTCCTAAGTTAACGA	PB	PB-1	P1-2	TRUE	3	TRA:CGQNFVF TRB:CASNQRGGPSLYNEQFF	323	CD4-C6
PB-1_GCTCCTACAGGTCTCG	PB	PB-1	P1-1	TRUE	3	TRA:CAMSDRMTGGFKTIF TRB:CASSQVGRGGEETQYF	1047	CD8-C7
PB-1_GCTCCTACATATACCG	PB	PB-1	P1-10	TRUE	3	TRA:CISNSPMDSSYKLIF TRB:CASSSWGDTDEAFF	162	CD8-C7
PB-1_GCTCCTACATGACATC	PB	PB-1	P1-1	TRUE	3	TRA:CAMSDRMTGGFKTIF TRB:CASSQVGRGGEETQYF	1047	CD8-C7
PB-1_GCTCCTAGTCAGATAA	PB	PB-1	P1-13	TRUE	3	TRA:CVVMNYGGSQGNLIF TRB:CASSSWGDTDEAFF	112	CD8-C7
PB-1_GCTCTGTAGAGTAATC	PB	PB-1	P1-1	TRUE	3	TRA:CAMSDRMTGGFKTIF TRB:CASSQVGRGGEETQYF	1047	CD8-C7
PB-1_GCTCTGTAGCCGGTAA	PB	PB-1	P1-22	TRUE	3	TRA:CASNSGYSTLTF TRB:CASSQVTGGARDEQYF	65	CD8-C7
PB-1_GCTCTGTAGGGCTTGA	PB	PB-1	P1-13	TRUE	3	TRA:CVVMNYGGSQGNLIF TRB:CASSSWGDTDEAFF	112	CD8-C7
PB-1_GCTCTGTCAACACCTA	PB	PB-1	P1-16	TRUE	3	TRA:CALSGTPMNRDDKIIF TRB:CASSQSWDLAVLNEQFF	79	CD8-C7
PB-1_GCTCTGTCACTGTTAG	PB	PB-1	P1-1	TRUE	3	TRA:CAMSDRMTGGFKTIF TRB:CASSQVGRGGEETQYF	1047	CD8-C7

PB-1_GCTCTGTCATGCAACT	PB	PB-1	P1-13	TRUE	3	TRA:CVVMNYGGSQGNLIF TRB:CASSSWGDTDEAFF	112	CD4-C6
PB-1_GCTCTGTGTAGGACAC	PB	PB-1	P1-5	TRUE	3	TRA:CALSGTPMNRDDKIIF TRB:CSARGRGLAGGPAYEQYF	223	CD8-C7
PB-1_GCTCTGTGTCATCGGC	PB	PB-1	P1-1	TRUE	3	TRA:CAMSDRMTGGFKTIF TRB:CASSQVGRGGEETQYF	1047	CD8-C7
PB-1_GCTCTGTGTCCGAAGA	PB	PB-1	P1-5	TRUE	3	TRA:CALSGTPMNRDDKIIF TRB:CSARGRGLAGGPAYEQYF	223	CD8-C7
PB-1_GCTCTGTGTCTTCGTC	PB	PB-1	P1-29	TRUE	3	TRA:CATVSFSGGYNKLIF TRB:CASSLDRRGLSYEQYF	50	CD8-C7
PB-1_GCTGCAGAGATATGGT	PB	PB-1	P1-3	TRUE	3	TRA:CAFLSGTYKYIF TRB:CASSQSPGQRNYGYTF	308	CD8-C7
PB-1_GCTGCAGAGCAACGGT	PB	PB-1	P1-13	TRUE	3	TRA:CVVMNYGGSQGNLIF TRB:CASSSWGDTDEAFF	112	CD8-C7
PB-1_GCTGCAGCACGGCCAT	PB	PB-1	P1-26	TRUE	3	TRA:CAPRSGGSYIPTF TRB:CASSQDLGLDYGYTF	56	CD8-C7
PB-1_GCTGCAGCACTCAGGC	PB	PB-1	P1-10	TRUE	3	TRA:CISNSPMDSSYKLIF TRB:CASSSWGDTDEAFF	162	CD8-C7
PB-1_GCTGCAGTCCTTTACA	PB	PB-1	P1-13	TRUE	3	TRA:CVVMNYGGSQGNLIF TRB:CASSSWGDTDEAFF	112	CD8-C7
PB-1_GCTGCAGTCGCAGGCT	PB	PB-1	P1-4	TRUE	3	TRA:CAMKGRDDKIIF TRB:CASTKGVSREAFF	226	CD8-C7
PB-1_GCTGCAGTCGCTTAGA	PB	PB-1	P1-1	TRUE	3	TRA:CAMSDRMTGGFKTIF TRB:CASSQVGRGGEETQYF	1047	CD8-C6
PB-1_GCTGCAGTCTTAGAGC	PB	PB-1	P1-31	TRUE	3	TRA:CAVEEGADRLMF TRB:CASSLLGGGELFF	46	CD8-C7
PB-1_GCTGCGAAGCAGATCG	PB	PB-1	P1-1	TRUE	3	TRA:CAMSDRMTGGFKTIF TRB:CASSQVGRGGEETQYF	1047	CD8-C7
PB-1_GCTGCGACAAAGTCAA	PB	PB-1	P1-1	TRUE	3	TRA:CAMSDRMTGGFKTIF TRB:CASSQVGRGGEETQYF	1047	CD8-C5
PB-1_GCTGCGACAATCCGAT	PB	PB-1	P1-195	TRUE	3	TRA:CAANLYPHSGAGSYQLTF TRB:CASSSGTGGETQYF	3	CD8-C7
PB-1_GCTGCGACATCGATGT	PB	PB-1	P1-1	TRUE	3	TRA:CAMSDRMTGGFKTIF TRB:CASSQVGRGGEETQYF	1047	CD8-C7
PB-1_GCTGCGAGTCGGATCC	PB	PB-1	P1-18	TRUE	3	TRA:CVADGNYGQNFVF TRB:CASSSPGLGGGFDTEAFF	75	CD8-C7
PB-1_GCTGCGATCAGATAAG	PB	PB-1	P1-1	TRUE	3	TRA:CAMSDRMTGGFKTIF TRB:CASSQVGRGGEETQYF	1047	CD8-C5
PB-1_GCTGCGATCATGGTCA	PB	PB-1	P1-1	TRUE	3	TRA:CAMSDRMTGGFKTIF TRB:CASSQVGRGGEETQYF	1047	CD8-C5
PB-1_GCTGCGATCCGCGGTA	PB	PB-1	P1-1	TRUE	3	TRA:CAMSDRMTGGFKTIF TRB:CASSQVGRGGEETQYF	1047	CD8-C7
PB-1_GCTGCGATCGATCCCT	PB	PB-1	P1-8	TRUE	3	TRA:CAVRPPGRNSGGYQKVTF TRB:CASSRRGVTDTQYF	204	CD8-C7
PB-1_GCTGCTTCAATAGCAA	PB	PB-1	P1-1	TRUE	3	TRA:CAMSDRMTGGFKTIF TRB:CASSQVGRGGEETQYF	1047	CD8-C7
PB-1_GCTGGGTAGCGCTCCA	PB	PB-1	P1-1	TRUE	3	TRA:CAMSDRMTGGFKTIF TRB:CASSQVGRGGEETQYF	1047	CD8-C7
PB-1_GCTGGGTAGTGTACTC	PB	PB-1	P1-15	TRUE	3	TRA:CAVNSSPLGANSKLTF TRB:CASSDSGNFYNEQFF	92	CD8-C2
PB-1_GCTGGGTCAATGGAGC	PB	PB-1	P1-1	TRUE	3	TRA:CAMSDRMTGGFKTIF TRB:CASSQVGRGGEETQYF	1047	CD8-C7
PB-1_GCTGGGTCAGCATGAG	PB	PB-1	P1-18	TRUE	3	TRA:CVADGNYGQNFVF TRB:CASSSPGLGGGFDTEAFF	75	CD8-C6

PB-1_GCTTCCAAGGCCCTTG	PB	PB-1	P1-73	TRUE	3	TRA:CAGI TRB:CASSRTTGGSSYEQYF	8	CD8-C7
PB-1_GCTTCCACACCCTATC	PB	PB-1	P1-3	TRUE	3	TRA:CAFLSGTYKYIF TRB:CASSQSPGQRNYGYTF	308	CD8-C7
PB-1_GCTTCCACACGGCCAT	PB	PB-1	P1-5	TRUE	3	TRA:CALSGTPMNRDDKIIF TRB:CSARGRGLAGGPAYEQYF	223	CD8-C7
PB-1_GCTTCCAGTGAGCGAT	PB	PB-1	P1-67	TRUE	3	TRA:CALNTNQAGTALIF TRB:CASSLGTRYAYEQYF	9	CD8-C7
PB-1_GCTTCCAGTGTCAATC	PB	PB-1	P1-1	TRUE	3	TRA:CAMSDRMTGGFKTIF TRB:CASSQVGRGGEETQYF	1047	CD8-C5
PB-1_GCTTGAAAGAATTGTG	PB	PB-1	P1-3	TRUE	3	TRA:CAFLSGTYKYIF TRB:CASSQSPGQRNYGYTF	308	CD8-C7
PB-1_GCTTGAAAGGAATCGC	PB	PB-1	P1-39	TRUE	3	TRA:CAVRDLGGTSYDKVIF TRB:CSARVREVSYEQYF	31	CD8-C7
PB-1_GCTTGAAAGTGGTCCC	PB	PB-1	P1-13	TRUE	3	TRA:CVVMNYGGSQGNLIF TRB:CASSSWGDTDEAFF	112	CD8-C7
PB-1_GCTTGAACACCTGGTG	PB	PB-1	P1-3	TRUE	3	TRA:CAFLSGTYKYIF TRB:CASSQSPGQRNYGYTF	308	CD8-C7
PB-1_GCTTGAAGTAGGAGTC	PB	PB-1	P1-2	TRUE	3	TRA:CGQNFVF TRB:CASNQRGGPSLYNEQFF	323	CD8-C7
PB-1_GCTTGAATCATCTGTT	PB	PB-1	P1-10	TRUE	3	TRA:CISNSPMDSSYKLIF TRB:CASSSWGDTDEAFF	162	CD8-C7
PB-1_GGAAAGCAGAGTTGGC	PB	PB-1	P1-1	TRUE	3	TRA:CAMSDRMTGGFKTIF TRB:CASSQVGRGGEETQYF	1047	CD8-C5
PB-1_GGAAAGCAGGCCCTCA	PB	PB-1	P1-1	TRUE	3	TRA:CAMSDRMTGGFKTIF TRB:CASSQVGRGGEETQYF	1047	CD8-C7
PB-1_GGAAAGCCAGTGAGTG	PB	PB-1	P1-1	TRUE	3	TRA:CAMSDRMTGGFKTIF TRB:CASSQVGRGGEETQYF	1047	CD8-C7
PB-1_GGAAAGCGTGGTCTCG	PB	PB-1	P1-2	TRUE	3	TRA:CGQNFVF TRB:CASNQRGGPSLYNEQFF	323	CD8-C7
PB-1_GGAAAGCTCACTTCAT	PB	PB-1	P1-3	TRUE	3	TRA:CAFLSGTYKYIF TRB:CASSQSPGQRNYGYTF	308	CD8-C7
PB-1_GGAACTTAGGGATGGG	PB	PB-1	P1-9	TRUE	3	TRA:CAEAEGGGADGLTF TRB:CATSGGDNEQFF	174	CD8-C7
PB-1_GGAACTTCAATCGGTT	PB	PB-1	P1-1	TRUE	3	TRA:CAMSDRMTGGFKTIF TRB:CASSQVGRGGEETQYF	1047	CD8-C7
PB-1_GGAACTTGTCAAACTC	PB	PB-1	P1-7	TRUE	3	TRA:CAVQAGNNDMRF TRB:CASSTTGDFNYGYTF	215	CD8-C7
PB-1_GGAACTTGTGGTGTAG	PB	PB-1	P1-140	TRUE	3	TRA:CAYRSGKGGKLIF TRB:CASSDTSSHINEQFF	4	CD8-C7
PB-1_GGAACTTGTTGTGGCC	PB	PB-1	P1-8	TRUE	3	TRA:CAVRPPGRNSGGYQKVTF TRB:CASSRRGVTDTQYF	204	CD8-C7
PB-1_GGAACTTGTTTGTGTG	PB	PB-1	P1-8	TRUE	3	TRA:CAVRPPGRNSGGYQKVTF TRB:CASSRRGVTDTQYF	204	CD8-C7
PB-1_GGAACTTTCATAAAGG	PB	PB-1	P1-1	TRUE	3	TRA:CAMSDRMTGGFKTIF TRB:CASSQVGRGGEETQYF	1047	CD8-C7
PB-1_GGAATAAAGTCTCAAC	PB	PB-1	P1-2	TRUE	3	TRA:CGQNFVF TRB:CASNQRGGPSLYNEQFF	323	CD8-C7
PB-1_GGAATAACAACGATCT	PB	PB-1	P1-1	TRUE	3	TRA:CAMSDRMTGGFKTIF TRB:CASSQVGRGGEETQYF	1047	CD8-C7
PB-1_GGAATAACAAGAAAGG	PB	PB-1	P1-14	TRUE	3	TRA:CAASVVNDMRF TRB:CASSHRGGAKNIQYF	109	CD8-C5
PB-1_GGAATAACAGCTCGAC	PB	PB-1	P1-5	TRUE	3	TRA:CALSGTPMNRDDKIIF TRB:CSARGRGLAGGPAYEQYF	223	CD8-C7

PB-1_GGAATAAGTGGGTATG	PB	PB-1	P1-3	TRUE	3	TRA:CAFLSGTYKYIF TRB:CASSQSPGQRNYGYTF	308	CD8-C7
PB-1_GGAATAATCGGCTTGG	PB	PB-1	P1-3	TRUE	3	TRA:CAFLSGTYKYIF TRB:CASSQSPGQRNYGYTF	308	CD8-C7
PB-1_GGACAAGAGAGCTGCA	PB	PB-1	P1-39	TRUE	3	TRA:CAVRDLGGTSYDKVIF TRB:CSARVREVSYEQYF	31	CD8-C5
PB-1_GGACAAGCACACTGCG	PB	PB-1	P1-3	TRUE	3	TRA:CAFLSGTYKYIF TRB:CASSQSPGQRNYGYTF	308	CD8-C6
PB-1_GGACAAGCAGGAACGT	PB	PB-1	P1-36	TRUE	3	TRA:CILRDNAGNNRKLIW TRB:CASSSRQGFPGGANVLTF	40	CD4-C6
PB-1_GGACAAGGTTAAGGGC	PB	PB-1	P1-8	TRUE	3	TRA:CAVRPPGRNSGGYQKVTF TRB:CASSRRGVTDTQYF	204	CD8-C5
PB-1_GGACAAGGTTGAGTTC	PB	PB-1	P1-11	TRUE	3	TRA:CAVEPLYGSGNTGKLIF TRB:CASSSHGQLNQPQHF	147	CD8-C7
PB-1_GGACAAGTCTATCGCC	PB	PB-1	P1-5	TRUE	3	TRA:CALSGTPMNRDDKIIF TRB:CSARGRGLAGGPAYEQYF	223	CD8-C7
PB-1_GGACAGAAGCTAGGCA	PB	PB-1	P1-8	TRUE	3	TRA:CAVRPPGRNSGGYQKVTF TRB:CASSRRGVTDTQYF	204	CD8-C7
PB-1_GGACAGAGTGGTAACG	PB	PB-1	P1-8	TRUE	3	TRA:CAVRPPGRNSGGYQKVTF TRB:CASSRRGVTDTQYF	204	CD8-C7
PB-1_GGACAGATCACGCGGT	PB	PB-1	P1-7	TRUE	3	TRA:CAVQAGNNDMRF TRB:CASSTTGDFNYGYTF	215	CD8-C5
PB-1_GGACATTAGGCCCTCA	PB	PB-1	P1-1	TRUE	3	TRA:CAMSDRMTGGFKTIF TRB:CASSQVGRGGEETQYF	1047	CD8-C7
PB-1_GGACATTAGTTCGCGC	PB	PB-1	P1-1	TRUE	3	TRA:CAMSDRMTGGFKTIF TRB:CASSQVGRGGEETQYF	1047	CD8-C7
PB-1_GGACATTCAAAGGTGC	PB	PB-1	P1-2	TRUE	3	TRA:CGQNFVF TRB:CASNQRGGPSLYNEQFF	323	CD8-C7
PB-1_GGACATTCAGACGCAA	PB	PB-1	P1-2	TRUE	3	TRA:CGQNFVF TRB:CASNQRGGPSLYNEQFF	323	CD8-C7
PB-1_GGACATTGTCGGGTCT	PB	PB-1	P1-47	TRUE	3	TRA:CIVGGNFNKFYF TRB:CASSPEWDTDTQYF	18	CD8-C5
PB-1_GGACATTGTGCCTGGT	PB	PB-1	P1-8	TRUE	3	TRA:CAVRPPGRNSGGYQKVTF TRB:CASSRRGVTDTQYF	204	CD8-C7
PB-1_GGACATTGTTCCACAA	PB	PB-1	P1-2	TRUE	3	TRA:CGQNFVF TRB:CASNQRGGPSLYNEQFF	323	CD8-C7
PB-1_GGACATTTCCACGCAG	PB	PB-1	P1-3	TRUE	3	TRA:CAFLSGTYKYIF TRB:CASSQSPGQRNYGYTF	308	CD8-C7
PB-1_GGACGTCAGAGACTTA	PB	PB-1	P1-4	TRUE	3	TRA:CAMKGRDDKIIF TRB:CASTKGVSREAFF	226	CD4-C6
PB-1_GGACGTCGTGGGTATG	PB	PB-1	P1-2	TRUE	3	TRA:CGQNFVF TRB:CASNQRGGPSLYNEQFF	323	CD4-C6
PB-1_GGACGTCTCAGTGTTG	PB	PB-1	P1-9	TRUE	3	TRA:CAEAEGGGADGLTF TRB:CATSGGDNEQFF	174	CD8-C7
PB-1_GGACGTCTCGCCTGTT	PB	PB-1	P1-1	TRUE	3	TRA:CAMSDRMTGGFKTIF TRB:CASSQVGRGGEETQYF	1047	CD8-C5
PB-1_GGAGCAAAGCAGACTG	PB	PB-1	P1-10	TRUE	3	TRA:CISNSPMDSSYKLIF TRB:CASSSWGDTDEAFF	162	CD8-C7
PB-1_GGAGCAAAGCGTTCCG	PB	PB-1	P1-22	TRUE	3	TRA:CASNSGYSTLTF TRB:CASSQVTGGARDEQYF	65	CD8-C7
PB-1_GGAGCAAAGTGCGTGA	PB	PB-1	P1-2	TRUE	3	TRA:CGQNFVF TRB:CASNQRGGPSLYNEQFF	323	CD8-C7
PB-1_GGAGCAACACACGCTG	PB	PB-1	P1-2	TRUE	3	TRA:CGQNFVF TRB:CASNQRGGPSLYNEQFF	323	CD8-C7

PB-1_GGAGCAACACAGGAGT	PB	PB-1	P1-2	TRUE	3	TRA:CGQNFVF TRB:CASNQRGGPSLYNEQFF	323	CD8-C7
PB-1_GGAGCAACACCACCAG	PB	PB-1	P1-3	TRUE	3	TRA:CAFLSGTYKYIF TRB:CASSQSPGQRNYGYTF	308	CD8-C7
PB-1_GGAGCAAGTTCCCGAG	PB	PB-1	P1-3	TRUE	3	TRA:CAFLSGTYKYIF TRB:CASSQSPGQRNYGYTF	308	CD8-C2
PB-1_GGAGCAAGTTTGACAC	PB	PB-1	P1-1	TRUE	3	TRA:CAMSDRMTGGFKTIF TRB:CASSQVGRGGEETQYF	1047	CD8-C5
PB-1_GGAGCAATCATATCGG	PB	PB-1	P1-2	TRUE	3	TRA:CGQNFVF TRB:CASNQRGGPSLYNEQFF	323	CD8-C7
PB-1_GGATGTTAGAACTGTA	PB	PB-1	P1-1	TRUE	3	TRA:CAMSDRMTGGFKTIF TRB:CASSQVGRGGEETQYF	1047	CD8-C7
PB-1_GGATGTTCACAGACTT	PB	PB-1	P1-15	TRUE	3	TRA:CAVNSSPLGANSKLTF TRB:CASSDSGNFYNEQFF	92	CD8-C7
PB-1_GGATGTTGTATTACCG	PB	PB-1	P1-3	TRUE	3	TRA:CAFLSGTYKYIF TRB:CASSQSPGQRNYGYTF	308	CD8-C7
PB-1_GGATGTTTCTCTGTCG	PB	PB-1	P1-2	TRUE	3	TRA:CGQNFVF TRB:CASNQRGGPSLYNEQFF	323	CD8-C7
PB-1_GGATTACGTACCGTAT	PB	PB-1	P1-26	TRUE	3	TRA:CAPRSGGSYIPTF TRB:CASSQDLGLDYGYTF	56	CD8-C7
PB-1_GGATTACGTACTTAGC	PB	PB-1	P1-1	TRUE	3	TRA:CAMSDRMTGGFKTIF TRB:CASSQVGRGGEETQYF	1047	CD8-C5
PB-1_GGATTACGTGGTGTAG	PB	PB-1	P1-8	TRUE	3	TRA:CAVRPPGRNSGGYQKVTF TRB:CASSRRGVTDTQYF	204	CD8-C6
PB-1_GGCAATTAGCTCCTTC	PB	PB-1	P1-11	TRUE	3	TRA:CAVEPLYGSGNTGKLIF TRB:CASSSHGQLNQPQHF	147	CD8-C5
PB-1_GGCAATTGTCAACTGT	PB	PB-1	P1-1	TRUE	3	TRA:CAMSDRMTGGFKTIF TRB:CASSQVGRGGEETQYF	1047	CD8-C7
PB-1_GGCAATTGTTATTCTC	PB	PB-1	P1-1	TRUE	3	TRA:CAMSDRMTGGFKTIF TRB:CASSQVGRGGEETQYF	1047	CD8-C7
PB-1_GGCAATTTCCTTTACA	PB	PB-1	P1-2	TRUE	3	TRA:CGQNFVF TRB:CASNQRGGPSLYNEQFF	323	CD8-C7
PB-1_GGCAATTTCGACGGAA	PB	PB-1	P1-1	TRUE	3	TRA:CAMSDRMTGGFKTIF TRB:CASSQVGRGGEETQYF	1047	CD8-C7
PB-1_GGCAATTTCGCCAAAT	PB	PB-1	P1-9	TRUE	3	TRA:CAEAEGGGADGLTF TRB:CATSGGDNEQFF	174	CD8-C7
PB-1_GGCCGATCACCAGATT	PB	PB-1	P1-71	FALSE	3	TRA:CAVAGGGGADGLTF TRB:CASSCPGLGGSTDTQYF	8	CD8-C6
PB-1_GGCCGATCATCAGTCA	PB	PB-1	P1-1	TRUE	3	TRA:CAMSDRMTGGFKTIF TRB:CASSQVGRGGEETQYF	1047	CD8-C5
PB-1_GGCCGATTCAAACCAC	PB	PB-1	P1-10	TRUE	3	TRA:CISNSPMDSSYKLIF TRB:CASSSWGDTDEAFF	162	CD8-C7
PB-1_GGCCGATTCATGCAAC	PB	PB-1	P1-4	TRUE	3	TRA:CAMKGRDDKIIF TRB:CASTKGVSREAFF	226	CD8-C7
PB-1_GGCGACTAGACTAGAT	PB	PB-1	P1-1	TRUE	3	TRA:CAMSDRMTGGFKTIF TRB:CASSQVGRGGEETQYF	1047	CD8-C7
PB-1_GGCGACTAGTGTCCCG	PB	PB-1	P1-6	TRUE	3	TRA:CAMREGRERDDKIIF TRB:CASSLPNTEAFF	216	CD8-C5
PB-1_GGCGACTCAACAACCT	PB	PB-1	P1-9	TRUE	3	TRA:CAEAEGGGADGLTF TRB:CATSGGDNEQFF	174	CD8-C7
PB-1_GGCGACTCAAGCTGAG	PB	PB-1	P1-24	TRUE	3	TRA:CAVKGNTDKLIF TRB:CASSLRTGAYNEQFF	62	CD4-C6
PB-1_GGCGACTTCCTGCTTG	PB	PB-1	P1-5	TRUE	3	TRA:CALSGTPMNRDDKIIF TRB:CSARGRGLAGGPAYEQYF	223	CD8-C7

PB-1_GGCGACTTCCTTGACC	PB	PB-1	P1-1	TRUE	3	TRA:CAMSDRMTGGFKTIF TRB:CASSQVGRGGEETQYF	1047	CD8-C7
PB-1_GGCGTGTGTGTTTGTG	PB	PB-1	P1-12	TRUE	3	TRA:CALSDERGDYKLSF TRB:CASSPGTGSYNEQFF	141	CD8-C7
PB-1_GGCGTGTTCATCGCTC	PB	PB-1	P1-10	TRUE	3	TRA:CISNSPMDSSYKLIF TRB:CASSSWGDTDEAFF	162	CD8-C7
PB-1_GGCGTGTTCGTGACAT	PB	PB-1	P1-14	TRUE	3	TRA:CAASVVNDMRF TRB:CASSHRGGAKNIQYF	109	CD8-C5
PB-1_GGCTCGACAATGGAAT	PB	PB-1	P1-2	TRUE	3	TRA:CGQNFVF TRB:CASNQRGGPSLYNEQFF	323	CD8-C7
PB-1_GGCTCGAGTCCAGTTA	PB	PB-1	P1-293	TRUE	2	TRA:CAGWYSGGGADGLTF TRB:CATSAGDNEQFF	2	CD8-C7
PB-1_GGCTCGAGTGCTCTTC	PB	PB-1	P1-1	TRUE	3	TRA:CAMSDRMTGGFKTIF TRB:CASSQVGRGGEETQYF	1047	CD8-C5
PB-1_GGCTCGAGTTTAGGAA	PB	PB-1	P1-2	TRUE	3	TRA:CGQNFVF TRB:CASNQRGGPSLYNEQFF	323	CD8-C7
PB-1_GGCTCGAGTTTGCATG	PB	PB-1	P1-16	TRUE	3	TRA:CALSGTPMNRDDKIIF TRB:CASSQSWDLAVLNEQFF	79	CD8-C7
PB-1_GGCTCGATCATCATTC	PB	PB-1	P1-16	TRUE	3	TRA:CALSGTPMNRDDKIIF TRB:CASSQSWDLAVLNEQFF	79	CD8-C7
PB-1_GGCTCGATCTAACTGG	PB	PB-1	P1-16	TRUE	3	TRA:CALSGTPMNRDDKIIF TRB:CASSQSWDLAVLNEQFF	79	CD8-C7
PB-1_GGCTGGTCAAGCGATG	PB	PB-1	P1-1	TRUE	3	TRA:CAMSDRMTGGFKTIF TRB:CASSQVGRGGEETQYF	1047	CD8-C5
PB-1_GGCTGGTGTAAGAGGA	PB	PB-1	P1-869	FALSE	1	TRA:CALNTNQAGTALIF TRB:CSARGRGLAGGPAYEQYF	1	CD8-C7
PB-1_GGCTGGTGTAGCCTAT	PB	PB-1	P1-7	TRUE	3	TRA:CAVQAGNNDMRF TRB:CASSTTGDFNYGYTF	215	CD8-C5
PB-1_GGCTGGTTCGTTTATC	PB	PB-1	P1-4	TRUE	3	TRA:CAMKGRDDKIIF TRB:CASTKGVSREAFF	226	CD8-C7
PB-1_GGGAATGAGTGTTTGC	PB	PB-1	P1-3	TRUE	3	TRA:CAFLSGTYKYIF TRB:CASSQSPGQRNYGYTF	308	CD8-C5
PB-1_GGGAATGCAGCTCGAC	PB	PB-1	P1-1	TRUE	3	TRA:CAMSDRMTGGFKTIF TRB:CASSQVGRGGEETQYF	1047	CD8-C7
PB-1_GGGAATGCAGGTCGTC	PB	PB-1	P1-10	TRUE	3	TRA:CISNSPMDSSYKLIF TRB:CASSSWGDTDEAFF	162	CD8-C7
PB-1_GGGAATGGTCCTGCTT	PB	PB-1	P1-9	TRUE	3	TRA:CAEAEGGGADGLTF TRB:CATSGGDNEQFF	174	CD8-C5
PB-1_GGGAATGGTCTCCATC	PB	PB-1	P1-10	TRUE	3	TRA:CISNSPMDSSYKLIF TRB:CASSSWGDTDEAFF	162	CD8-C7
PB-1_GGGACCTGTAAGTAGT	PB	PB-1	P1-1	TRUE	3	TRA:CAMSDRMTGGFKTIF TRB:CASSQVGRGGEETQYF	1047	CD8-C7
PB-1_GGGACCTGTTAAGAAC	PB	PB-1	P1-14	TRUE	3	TRA:CAASVVNDMRF TRB:CASSHRGGAKNIQYF	109	CD8-C7
PB-1_GGGAGATAGCTACCGC	PB	PB-1	P1-10	TRUE	3	TRA:CISNSPMDSSYKLIF TRB:CASSSWGDTDEAFF	162	CD8-C7
PB-1_GGGAGATCATCGTCGG	PB	PB-1	P1-24	TRUE	3	TRA:CAVKGNTDKLIF TRB:CASSLRTGAYNEQFF	62	CD4-C6
PB-1_GGGAGATTCAAACCGT	PB	PB-1	P1-4	TRUE	3	TRA:CAMKGRDDKIIF TRB:CASTKGVSREAFF	226	CD8-C7
PB-1_GGGAGATTCCGATATG	PB	PB-1	P1-16	TRUE	3	TRA:CALSGTPMNRDDKIIF TRB:CASSQSWDLAVLNEQFF	79	CD8-C7
PB-1_GGGAGATTCGTCCAGG	PB	PB-1	P1-50	TRUE	3	TRA:CARWLGSQGNLIF TRB:CASSLTDRVPQFELVYEQYF	15	CD8-C7

PB-1_GGGATGAAGCCTTGAT	PB	PB-1	P1-4	TRUE	3	TRA:CAMKGRDDKIIF TRB:CASTKGVSREAFF	226	CD8-C7
PB-1_GGGATGAAGTACACCT	PB	PB-1	P1-11	TRUE	3	TRA:CAVEPLYGSGNTGKLIF TRB:CASSSHGQLNQPQHF	147	CD8-C7
PB-1_GGGATGAAGTCCAGGA	PB	PB-1	P1-1	TRUE	3	TRA:CAMSDRMTGGFKTIF TRB:CASSQVGRGGEETQYF	1047	CD8-C7
PB-1_GGGATGACATACAGCT	PB	PB-1	P1-5	TRUE	3	TRA:CALSGTPMNRDDKIIF TRB:CSARGRGLAGGPAYEQYF	223	CD8-C7
PB-1_GGGATGAGTAGAAAGG	PB	PB-1	P1-22	TRUE	3	TRA:CASNSGYSTLTF TRB:CASSQVTGGARDEQYF	65	CD8-C7
PB-1_GGGCACTAGGCCCGTT	PB	PB-1	P1-2	TRUE	3	TRA:CGQNFVF TRB:CASNQRGGPSLYNEQFF	323	CD8-C7
PB-1_GGGCACTCATCGGACC	PB	PB-1	P1-1	TRUE	3	TRA:CAMSDRMTGGFKTIF TRB:CASSQVGRGGEETQYF	1047	CD8-C7
PB-1_GGGCACTGTCCGTTAA	PB	PB-1	P1-1	TRUE	3	TRA:CAMSDRMTGGFKTIF TRB:CASSQVGRGGEETQYF	1047	CD8-C7
PB-1_GGGCACTTCTAACCGA	PB	PB-1	P1-3	TRUE	3	TRA:CAFLSGTYKYIF TRB:CASSQSPGQRNYGYTF	308	CD8-C7
PB-1_GGGCATCAGAAGAAGC	PB	PB-1	P1-11	TRUE	3	TRA:CAVEPLYGSGNTGKLIF TRB:CASSSHGQLNQPQHF	147	CD8-C7
PB-1_GGGCATCAGCTCCCAG	PB	PB-1	P1-2	TRUE	3	TRA:CGQNFVF TRB:CASNQRGGPSLYNEQFF	323	CD8-C7
PB-1_GGGCATCCACGCCAGT	PB	PB-1	P1-10	TRUE	3	TRA:CISNSPMDSSYKLIF TRB:CASSSWGDTDEAFF	162	CD8-C7
PB-1_GGGCATCGTACGCTGC	PB	PB-1	P1-1	TRUE	3	TRA:CAMSDRMTGGFKTIF TRB:CASSQVGRGGEETQYF	1047	CD8-C7
PB-1_GGGTCTGAGAGACTTA	PB	PB-1	P1-5	TRUE	3	TRA:CALSGTPMNRDDKIIF TRB:CSARGRGLAGGPAYEQYF	223	CD8-C5
PB-1_GGGTCTGAGAGCTGCA	PB	PB-1	P1-2	TRUE	3	TRA:CGQNFVF TRB:CASNQRGGPSLYNEQFF	323	CD8-C7
PB-1_GGGTCTGAGGACATTA	PB	PB-1	P1-39	TRUE	3	TRA:CAVRDLGGTSYDKVIF TRB:CSARVREVSYEQYF	31	CD8-C2
PB-1_GGGTCTGCAAGAGTCG	PB	PB-1	P1-14	TRUE	3	TRA:CAASVVNDMRF TRB:CASSHRGGAKNIQYF	109	CD8-C7
PB-1_GGGTCTGCATTGGGCC	PB	PB-1	P1-1	TRUE	3	TRA:CAMSDRMTGGFKTIF TRB:CASSQVGRGGEETQYF	1047	CD8-C5
PB-1_GGGTTGCAGCTCTCGG	PB	PB-1	P1-16	TRUE	3	TRA:CALSGTPMNRDDKIIF TRB:CASSQSWDLAVLNEQFF	79	CD8-C5
PB-1_GGGTTGCCAAGTCTGT	PB	PB-1	P1-3	TRUE	3	TRA:CAFLSGTYKYIF TRB:CASSQSPGQRNYGYTF	308	CD8-C7
PB-1_GGGTTGCGTCAATGTC	PB	PB-1	P1-1	TRUE	3	TRA:CAMSDRMTGGFKTIF TRB:CASSQVGRGGEETQYF	1047	CD8-C7
PB-1_GGGTTGCTCATGCAAC	PB	PB-1	P1-1	TRUE	3	TRA:CAMSDRMTGGFKTIF TRB:CASSQVGRGGEETQYF	1047	CD8-C7
PB-1_GGTATTGAGAAGGTTT	PB	PB-1	P1-1	TRUE	3	TRA:CAMSDRMTGGFKTIF TRB:CASSQVGRGGEETQYF	1047	CD8-C7
PB-1_GGTATTGCATGCCTAA	PB	PB-1	P1-13	TRUE	3	TRA:CVVMNYGGSQGNLIF TRB:CASSSWGDTDEAFF	112	CD4-C6
PB-1_GGTATTGGTCCATCCT	PB	PB-1	P1-13	TRUE	3	TRA:CVVMNYGGSQGNLIF TRB:CASSSWGDTDEAFF	112	CD8-C7
PB-1_GGTATTGTCCTAGAAC	PB	PB-1	P1-8	TRUE	3	TRA:CAVRPPGRNSGGYQKVTF TRB:CASSRRGVTDTQYF	204	CD8-C5
PB-1_GGTGAAGAGCTCTCGG	PB	PB-1	P1-4	TRUE	3	TRA:CAMKGRDDKIIF TRB:CASTKGVSREAFF	226	CD8-C7

PB-1_GGTGAAGCATAAGACA	PB	PB-1	P1-85	FALSE	3	TRA:CAPRSGGSYIPTF TRB:CASSQDTGLDYGYTF	6	CD8-C7
PB-1_GGTGAAGTCTTGTACT	PB	PB-1	P1-880	FALSE	1	TRA:CALRPLYGNNRLAF TRB:CASSLRAGLAGSFLNYEQYF	1	CD8-C5
PB-1_GGTGCGTCAAGTTAAG	PB	PB-1	P1-8	TRUE	3	TRA:CAVRPPGRNSGGYQKVTF TRB:CASSRRGVTDTQYF	204	CD8-C7
PB-1_GGTGCGTCACCATCCT	PB	PB-1	P1-40	TRUE	3	TRA:CAVRDVGNTGFQKLVF TRB:CASSLGQGSMKTQYF	27	CD4-C6
PB-1_GGTGCGTCATACTACG	PB	PB-1	P1-199	FALSE	3	TRA:CAMSDRMTGGFKTIF TRB:CASSRRGVTDTQYF	3	CD8-C7
PB-1_GGTGCGTGTTAAGACA	PB	PB-1	P1-13	TRUE	3	TRA:CVVMNYGGSQGNLIF TRB:CASSSWGDTDEAFF	112	CD8-C7
PB-1_GGTGCGTGTTGTCGCG	PB	PB-1	P1-5	TRUE	3	TRA:CALSGTPMNRDDKIIF TRB:CSARGRGLAGGPAYEQYF	223	CD8-C7
PB-1_GGTGTTAGTAAACACA	PB	PB-1	P1-1	TRUE	3	TRA:CAMSDRMTGGFKTIF TRB:CASSQVGRGGEETQYF	1047	CD8-C7
PB-1_GGTGTTAGTCGCCATG	PB	PB-1	P1-8	TRUE	3	TRA:CAVRPPGRNSGGYQKVTF TRB:CASSRRGVTDTQYF	204	CD8-C7
PB-1_GGTGTTAGTTCAGGCC	PB	PB-1	P1-24	TRUE	3	TRA:CAVKGNTDKLIF TRB:CASSLRTGAYNEQFF	62	CD4-C6
PB-1_GTAACGTAGACACGAC	PB	PB-1	P1-1	TRUE	3	TRA:CAMSDRMTGGFKTIF TRB:CASSQVGRGGEETQYF	1047	CD8-C7
PB-1_GTAACGTAGGCTCAGA	PB	PB-1	P1-16	TRUE	3	TRA:CALSGTPMNRDDKIIF TRB:CASSQSWDLAVLNEQFF	79	CD8-C5
PB-1_GTAACGTCATTACCTT	PB	PB-1	P1-1	TRUE	3	TRA:CAMSDRMTGGFKTIF TRB:CASSQVGRGGEETQYF	1047	CD8-C5
PB-1_GTAACGTGTACCGAGA	PB	PB-1	P1-13	TRUE	3	TRA:CVVMNYGGSQGNLIF TRB:CASSSWGDTDEAFF	112	CD8-C7
PB-1_GTAACGTGTTCAGGCC	PB	PB-1	P1-13	TRUE	3	TRA:CVVMNYGGSQGNLIF TRB:CASSSWGDTDEAFF	112	CD8-C7
PB-1_GTAACGTTCATAAAGG	PB	PB-1	P1-2	TRUE	3	TRA:CGQNFVF TRB:CASNQRGGPSLYNEQFF	323	CD8-C7
PB-1_GTAACTGAGGAGTTGC	PB	PB-1	P1-3	TRUE	3	TRA:CAFLSGTYKYIF TRB:CASSQSPGQRNYGYTF	308	CD8-C7
PB-1_GTAACTGAGGTGCTTT	PB	PB-1	P1-115	FALSE	3	TRA:CAVKGSSNTGKLIF TRB:CASSYSYGRTGTGTYEQYF	5	CD8-C7
PB-1_GTAACTGCAAGAAAGG	PB	PB-1	P1-4	TRUE	3	TRA:CAMKGRDDKIIF TRB:CASTKGVSREAFF	226	CD8-C7
PB-1_GTAACTGCACAAGTAA	PB	PB-1	P1-11	TRUE	3	TRA:CAVEPLYGSGNTGKLIF TRB:CASSSHGQLNQPQHF	147	CD8-C7
PB-1_GTAACTGGTCGCTTTC	PB	PB-1	P1-14	TRUE	3	TRA:CAASVVNDMRF TRB:CASSHRGGAKNIQYF	109	CD4-C6
PB-1_GTAACTGGTTGGACCC	PB	PB-1	P1-3	TRUE	3	TRA:CAFLSGTYKYIF TRB:CASSQSPGQRNYGYTF	308	CD8-C7
PB-1_GTAACTGTCATAGCAC	PB	PB-1	P1-10	TRUE	3	TRA:CISNSPMDSSYKLIF TRB:CASSSWGDTDEAFF	162	CD8-C7
PB-1_GTAACTGTCGAATCCA	PB	PB-1	P1-4	TRUE	3	TRA:CAMKGRDDKIIF TRB:CASTKGVSREAFF	226	CD8-C7
PB-1_GTAACTGTCTTAGAGC	PB	PB-1	P1-22	TRUE	3	TRA:CASNSGYSTLTF TRB:CASSQVTGGARDEQYF	65	CD8-C7
PB-1_GTACGTAGTCCTAGCG	PB	PB-1	P1-14	TRUE	3	TRA:CAASVVNDMRF TRB:CASSHRGGAKNIQYF	109	CD8-C5
PB-1_GTACGTAGTTTGCATG	PB	PB-1	P1-1	TRUE	3	TRA:CAMSDRMTGGFKTIF TRB:CASSQVGRGGEETQYF	1047	CD8-C5

PB-1_GTACGTATCGGAAACG	PB	PB-1	P1-1	TRUE	3	TRA:CAMSDRMTGGFKTIF	TRB:CASSQVGRGGEETQYF	1047	CD8-C5
PB-1_GTACTCCAGCTACCGC	PB	PB-1	P1-35	TRUE	3	TRA:CAVSDRGYFGNEKLTF	TRB:CSALDDVRPVIAVYNEQFF	40	CD8-C6
PB-1_GTACTCCCAAACTGTC	PB	PB-1	P1-889	FALSE	1	TRA:CALNTNQAGTALIF	TRB:CASSQSPGQRNYGYTF	1	CD8-C7
PB-1_GTACTCCGTCTAACGT	PB	PB-1	P1-63	TRUE	3	TRA:CAEPLNTGNQFYF	TRB:CASSVGNEQFF	10	CD8-C7
PB-1_GTACTCCGTTAGATGA	PB	PB-1	P1-890	FALSE	1	TRA:CAASDTGTASKLTF	TRB:CASSFVQGTDEAFF	1	CD8-C7
PB-1_GTACTCCGTTCAGTAC	PB	PB-1	P1-8	TRUE	3	TRA:CAVRPPGRNSGGYQKVTF	TRB:CASSRRGVTDTQYF	204	CD8-C7
PB-1_GTACTCCGTTCGCGAC	PB	PB-1	P1-26	TRUE	3	TRA:CAPRSGGSYIPTF	TRB:CASSQDLGLDYGYTF	56	CD8-C7
PB-1_GTACTCCGTTTGCATG	PB	PB-1	P1-47	TRUE	3	TRA:CIVGGNFNKFYF	TRB:CASSPEWDTDTQYF	18	CD8-C7
PB-1_GTACTCCTCCACGTTC	PB	PB-1	P1-1	TRUE	3	TRA:CAMSDRMTGGFKTIF	TRB:CASSQVGRGGEETQYF	1047	CD8-C7
PB-1_GTACTTTAGGCTACGA	PB	PB-1	P1-2	TRUE	3	TRA:CGQNFVF	TRB:CASNQRGGPSLYNEQFF	323	CD8-C7
PB-1_GTACTTTCAACACCTA	PB	PB-1	P1-1	TRUE	3	TRA:CAMSDRMTGGFKTIF	TRB:CASSQVGRGGEETQYF	1047	CD8-C7
PB-1_GTACTTTGTGTGAAAT	PB	PB-1	P1-892	FALSE	1	TRA:CAMSDRMTGGFKTIF	TRB:CASSSHGQLNQPQHF	1	CD8-C7
PB-1_GTACTTTTCAGTTTGG	PB	PB-1	P1-1	TRUE	3	TRA:CAMSDRMTGGFKTIF	TRB:CASSQVGRGGEETQYF	1047	CD8-C5
PB-1_GTACTTTTCCAACCAA	PB	PB-1	P1-5	TRUE	3	TRA:CALSGTPMNRDDKIIF	TRB:CSARGRGLAGGPAYEQYF	223	CD8-C7
PB-1_GTACTTTTCGGCTACG	PB	PB-1	P1-29	TRUE	3	TRA:CATVSFSGGYNKLIF	TRB:CASSLDRRGLSYEQYF	50	CD8-C7
PB-1_GTACTTTTCGTTGCCT	PB	PB-1	P1-4	TRUE	3	TRA:CAMKGRDDKIIF	TRB:CASTKGVSREAFF	226	CD8-C7
PB-1_GTACTTTTCTGTACGA	PB	PB-1	P1-1	TRUE	3	TRA:CAMSDRMTGGFKTIF	TRB:CASSQVGRGGEETQYF	1047	CD8-C7
PB-1_GTAGGCCAGACAATAC	PB	PB-1	P1-39	TRUE	3	TRA:CAVRDLGGTSYDKVIF	TRB:CSARVREVSYEQYF	31	CD8-C5
PB-1_GTAGGCCAGATCCCAT	PB	PB-1	P1-2	TRUE	3	TRA:CGQNFVF	TRB:CASNQRGGPSLYNEQFF	323	CD8-C7
PB-1_GTAGGCCAGCGAAGGG	PB	PB-1	P1-13	TRUE	3	TRA:CVVMNYGGSQGNLIF	TRB:CASSSWGDTDEAFF	112	CD8-C7
PB-1_GTAGGCCAGTGGTCCC	PB	PB-1	P1-12	TRUE	3	TRA:CALSDERGDYKLSF	TRB:CASSPGTGSYNEQFF	141	CD8-C7
PB-1_GTAGGCCCATTTGCTT	PB	PB-1	P1-35	TRUE	3	TRA:CAVSDRGYFGNEKLTF	TRB:CSALDDVRPVIAVYNEQFF	40	CD8-C6
PB-1_GTAGGCCGTGTAATGA	PB	PB-1	P1-22	TRUE	3	TRA:CASNSGYSTLTF	TRB:CASSQVTGGARDEQYF	65	CD8-C7
PB-1_GTAGGCCTCTTGCCGT	PB	PB-1	P1-1	TRUE	3	TRA:CAMSDRMTGGFKTIF	TRB:CASSQVGRGGEETQYF	1047	CD8-C7
PB-1_GTAGTCAAGACCACGA	PB	PB-1	P1-3	TRUE	3	TRA:CAFLSGTYKYIF	TRB:CASSQSPGQRNYGYTF	308	CD8-C7
PB-1_GTAGTCAAGGAACTGC	PB	PB-1	P1-1	TRUE	3	TRA:CAMSDRMTGGFKTIF	TRB:CASSQVGRGGEETQYF	1047	CD8-C5
PB-1_GTAGTCAGTACAGACG	PB	PB-1	P1-1	TRUE	3	TRA:CAMSDRMTGGFKTIF	TRB:CASSQVGRGGEETQYF	1047	CD8-C5

PB-1_GTAGTCATCACCCTCA	PB	PB-1	P1-50	TRUE	3	TRA:CARWLGSQGNLIF TRB:CASSLTDRVPQFELVYEQYF	15	CD8-C7
PB-1_GTAGTCATCATGCAAC	PB	PB-1	P1-142	FALSE	3	TRA:CAVPYSSASKIIF TRB:CASSRTTGGSSYEQYF	4	CD8-C7
PB-1_GTAGTCATCTGCTGTC	PB	PB-1	P1-1	TRUE	3	TRA:CAMSDRMTGGFKTIF TRB:CASSQVGRGGEETQYF	1047	CD8-C5
PB-1_GTATCTTCACCGCTAG	PB	PB-1	P1-3	TRUE	3	TRA:CAFLSGTYKYIF TRB:CASSQSPGQRNYGYTF	308	CD8-C7
PB-1_GTATCTTCATGGTCTA	PB	PB-1	P1-1	TRUE	3	TRA:CAMSDRMTGGFKTIF TRB:CASSQVGRGGEETQYF	1047	CD8-C7
PB-1_GTATCTTGTACAAGTA	PB	PB-1	P1-8	TRUE	3	TRA:CAVRPPGRNSGGYQKVTF TRB:CASSRRGVTDTQYF	204	CD8-C7
PB-1_GTATCTTGTCCCGACA	PB	PB-1	P1-1	TRUE	3	TRA:CAMSDRMTGGFKTIF TRB:CASSQVGRGGEETQYF	1047	CD8-C7
PB-1_GTATCTTGTCGCTTTC	PB	PB-1	P1-10	TRUE	3	TRA:CISNSPMDSSYKLIF TRB:CASSSWGDTDEAFF	162	CD8-C7
PB-1_GTATCTTTCAGTCCCT	PB	PB-1	P1-1	TRUE	3	TRA:CAMSDRMTGGFKTIF TRB:CASSQVGRGGEETQYF	1047	CD8-C7
PB-1_GTATTCTAGATATACG	PB	PB-1	P1-47	TRUE	3	TRA:CIVGGNFNKFYF TRB:CASSPEWDTDTQYF	18	CD4-C6
PB-1_GTATTCTGTTCAGACT	PB	PB-1	P1-10	TRUE	3	TRA:CISNSPMDSSYKLIF TRB:CASSSWGDTDEAFF	162	CD8-C7
PB-1_GTATTCTGTTTGACAC	PB	PB-1	P1-1	TRUE	3	TRA:CAMSDRMTGGFKTIF TRB:CASSQVGRGGEETQYF	1047	CD8-C7
PB-1_GTATTCTTCCTTGCCA	PB	PB-1	P1-1	TRUE	3	TRA:CAMSDRMTGGFKTIF TRB:CASSQVGRGGEETQYF	1047	CD8-C7
PB-1_GTATTCTTCGACGGAA	PB	PB-1	P1-4	TRUE	3	TRA:CAMKGRDDKIIF TRB:CASTKGVSREAFF	226	CD8-C7
PB-1_GTCAAGTCAAGCTGTT	PB	PB-1	P1-1	TRUE	3	TRA:CAMSDRMTGGFKTIF TRB:CASSQVGRGGEETQYF	1047	CD8-C7
PB-1_GTCAAGTCATGGAATA	PB	PB-1	P1-4	TRUE	3	TRA:CAMKGRDDKIIF TRB:CASTKGVSREAFF	226	CD8-C7
PB-1_GTCAAGTCATTGGGCC	PB	PB-1	P1-4	TRUE	3	TRA:CAMKGRDDKIIF TRB:CASTKGVSREAFF	226	CD8-C7
PB-1_GTCAAGTGTGAAGGCT	PB	PB-1	P1-16	TRUE	3	TRA:CALSGTPMNRDDKIIF TRB:CASSQSWDLAVLNEQFF	79	CD8-C7
PB-1_GTCAAGTGTGTTAAGA	PB	PB-1	P1-3	TRUE	3	TRA:CAFLSGTYKYIF TRB:CASSQSPGQRNYGYTF	308	CD8-C7
PB-1_GTCAAGTGTTCCATGA	PB	PB-1	P1-40	TRUE	3	TRA:CAVRDVGNTGFQKLVF TRB:CASSLGQGSMKTQYF	27	CD8-C7
PB-1_GTCAAGTTCAATCACG	PB	PB-1	P1-14	TRUE	3	TRA:CAASVVNDMRF TRB:CASSHRGGAKNIQYF	109	CD8-C7
PB-1_GTCAAGTTCACAATGC	PB	PB-1	P1-5	TRUE	3	TRA:CALSGTPMNRDDKIIF TRB:CSARGRGLAGGPAYEQYF	223	CD8-C7
PB-1_GTCAAGTTCGGCTTGG	PB	PB-1	P1-14	TRUE	3	TRA:CAASVVNDMRF TRB:CASSHRGGAKNIQYF	109	CD8-C5
PB-1_GTCACAACAGGGTATG	PB	PB-1	P1-5	TRUE	3	TRA:CALSGTPMNRDDKIIF TRB:CSARGRGLAGGPAYEQYF	223	CD8-C7
PB-1_GTCACAATCTCCCTGA	PB	PB-1	P1-3	TRUE	3	TRA:CAFLSGTYKYIF TRB:CASSQSPGQRNYGYTF	308	CD8-C7
PB-1_GTCACGGAGCTACCGC	PB	PB-1	P1-66	TRUE	3	TRA:CVVSDPPDKLIF TRB:CASSASETGSVYEQYF	9	CD8-C7
PB-1_GTCACGGGTGTGGCTC	PB	PB-1	P1-8	TRUE	3	TRA:CAVRPPGRNSGGYQKVTF TRB:CASSRRGVTDTQYF	204	CD8-C7

PB-1_GTCACGGGTTATGTGC	PB	PB-1	P1-8	TRUE	3	TRA:CAVRPPGRNSGGYQKVTF TRB:CASSRRGVTDTQYF	204	CD8-C7
PB-1_GTCACGGTCAACGGCC	PB	PB-1	P1-2	TRUE	3	TRA:CGQNFVF TRB:CASNQRGGPSLYNEQFF	323	CD8-C7
PB-1_GTCACGGTCATCGGAT	PB	PB-1	P1-1	TRUE	3	TRA:CAMSDRMTGGFKTIF TRB:CASSQVGRGGEETQYF	1047	CD8-C7
PB-1_GTCATTTAGAGACTAT	PB	PB-1	P1-1	TRUE	3	TRA:CAMSDRMTGGFKTIF TRB:CASSQVGRGGEETQYF	1047	CD8-C7
PB-1_GTCATTTAGCGATTCT	PB	PB-1	P1-144	FALSE	3	TRA:CAMKGRDDKIIF TRB:CASSQVGRGGEETQYF	4	CD8-C7
PB-1_GTCATTTAGCTAACTC	PB	PB-1	P1-1	TRUE	3	TRA:CAMSDRMTGGFKTIF TRB:CASSQVGRGGEETQYF	1047	CD8-C7
PB-1_GTCATTTAGGAATTAC	PB	PB-1	P1-7	TRUE	3	TRA:CAVQAGNNDMRF TRB:CASSTTGDFNYGYTF	215	CD8-C7
PB-1_GTCATTTAGTACACCT	PB	PB-1	P1-8	TRUE	3	TRA:CAVRPPGRNSGGYQKVTF TRB:CASSRRGVTDTQYF	204	CD8-C5
PB-1_GTCATTTCAGTAAGAT	PB	PB-1	P1-9	TRUE	3	TRA:CAEAEGGGADGLTF TRB:CATSGGDNEQFF	174	CD8-C7
PB-1_GTCATTTTCACGATGT	PB	PB-1	P1-13	TRUE	3	TRA:CVVMNYGGSQGNLIF TRB:CASSSWGDTDEAFF	112	CD8-C7
PB-1_GTCATTTTCTCTGTCG	PB	PB-1	P1-2	TRUE	3	TRA:CGQNFVF TRB:CASNQRGGPSLYNEQFF	323	CD8-C7
PB-1_GTCCTCAAGTTCGCAT	PB	PB-1	P1-4	TRUE	3	TRA:CAMKGRDDKIIF TRB:CASTKGVSREAFF	226	CD8-C7
PB-1_GTCCTCACAGTTCATG	PB	PB-1	P1-3	TRUE	3	TRA:CAFLSGTYKYIF TRB:CASSQSPGQRNYGYTF	308	CD8-C7
PB-1_GTCCTCATCATGCATG	PB	PB-1	P1-3	TRUE	3	TRA:CAFLSGTYKYIF TRB:CASSQSPGQRNYGYTF	308	CD8-C7
PB-1_GTCGGGTAGTTGAGAT	PB	PB-1	P1-18	TRUE	3	TRA:CVADGNYGQNFVF TRB:CASSSPGLGGGFDTEAFF	75	CD8-C6
PB-1_GTCGGGTCAATGGAAT	PB	PB-1	P1-16	TRUE	3	TRA:CALSGTPMNRDDKIIF TRB:CASSQSWDLAVLNEQFF	79	CD8-C7
PB-1_GTCGGGTGTAGGACAC	PB	PB-1	P1-5	TRUE	3	TRA:CALSGTPMNRDDKIIF TRB:CSARGRGLAGGPAYEQYF	223	CD8-C5
PB-1_GTCGGGTTCCGAAGAG	PB	PB-1	P1-1	TRUE	3	TRA:CAMSDRMTGGFKTIF TRB:CASSQVGRGGEETQYF	1047	CD8-C7
PB-1_GTCGTAAAGATAGGAG	PB	PB-1	P1-4	TRUE	3	TRA:CAMKGRDDKIIF TRB:CASTKGVSREAFF	226	CD8-C7
PB-1_GTCGTAAAGGGCTTCC	PB	PB-1	P1-3	TRUE	3	TRA:CAFLSGTYKYIF TRB:CASSQSPGQRNYGYTF	308	CD8-C7
PB-1_GTCGTAAAGTCCGTAT	PB	PB-1	P1-284	FALSE	2	TRA:CALHSVSQAGTALIF TRB:CSVSTGITDTQYF	2	CD8-C7
PB-1_GTCGTAACACCCATGG	PB	PB-1	P1-3	TRUE	3	TRA:CAFLSGTYKYIF TRB:CASSQSPGQRNYGYTF	308	CD8-C6
PB-1_GTCGTAACAGCGTTCG	PB	PB-1	P1-1	TRUE	3	TRA:CAMSDRMTGGFKTIF TRB:CASSQVGRGGEETQYF	1047	CD8-C7
PB-1_GTCGTAACATACCATG	PB	PB-1	P1-8	TRUE	3	TRA:CAVRPPGRNSGGYQKVTF TRB:CASSRRGVTDTQYF	204	CD8-C7
PB-1_GTCGTAAGTGCATCTA	PB	PB-1	P1-1	TRUE	3	TRA:CAMSDRMTGGFKTIF TRB:CASSQVGRGGEETQYF	1047	CD8-C5
PB-1_GTCGTAAGTGGCCCTA	PB	PB-1	P1-6	TRUE	3	TRA:CAMREGRERDDKIIF TRB:CASSLPNTEAFF	216	CD8-C5
PB-1_GTCGTAATCAACGCTA	PB	PB-1	P1-3	TRUE	3	TRA:CAFLSGTYKYIF TRB:CASSQSPGQRNYGYTF	308	CD8-C7

PB-1_GTCGTAATCTAAGCCA	PB	PB-1	P1-5	TRUE	3	TRA:CALSGTPMNRDDKIIF TRB:CSARGRGLAGGPAYEQYF	223	CD8-C5
PB-1_GTCTCGTAGCCACGCT	PB	PB-1	P1-5	TRUE	3	TRA:CALSGTPMNRDDKIIF TRB:CSARGRGLAGGPAYEQYF	223	CD8-C5
PB-1_GTCTCGTAGCTAGTCT	PB	PB-1	P1-14	TRUE	3	TRA:CAASVVNDMRF TRB:CASSHRGGAKNIQYF	109	CD8-C7
PB-1_GTCTCGTAGGAGTACC	PB	PB-1	P1-910	FALSE	1	TRA:CALSGTPMNRDDKIIF TRB:CASSKGQGPRETQYF	1	CD8-C5
PB-1_GTCTCGTAGTTTAGGA	PB	PB-1	P1-1	TRUE	3	TRA:CAMSDRMTGGFKTIF TRB:CASSQVGRGGEETQYF	1047	CD8-C7
PB-1_GTCTCGTCAATGGACG	PB	PB-1	P1-13	TRUE	3	TRA:CVVMNYGGSQGNLIF TRB:CASSSWGDTDEAFF	112	CD8-C7
PB-1_GTCTCGTCACGGTGTC	PB	PB-1	P1-9	TRUE	3	TRA:CAEAEGGGADGLTF TRB:CATSGGDNEQFF	174	CD8-C7
PB-1_GTCTCGTCAGCTCGCA	PB	PB-1	P1-2	TRUE	3	TRA:CGQNFVF TRB:CASNQRGGPSLYNEQFF	323	CD8-C7
PB-1_GTCTCGTCAGCTTAAC	PB	PB-1	P1-31	TRUE	3	TRA:CAVEEGADRLMF TRB:CASSLLGGGELFF	46	CD8-C7
PB-1_GTCTCGTCATCACGTA	PB	PB-1	P1-2	TRUE	3	TRA:CGQNFVF TRB:CASNQRGGPSLYNEQFF	323	CD8-C7
PB-1_GTCTCGTGTCAAACTC	PB	PB-1	P1-5	TRUE	3	TRA:CALSGTPMNRDDKIIF TRB:CSARGRGLAGGPAYEQYF	223	CD8-C7
PB-1_GTCTCGTTCCAGTAGT	PB	PB-1	P1-29	TRUE	3	TRA:CATVSFSGGYNKLIF TRB:CASSLDRRGLSYEQYF	50	CD8-C7
PB-1_GTCTCGTTCTAACGGT	PB	PB-1	P1-1	TRUE	3	TRA:CAMSDRMTGGFKTIF TRB:CASSQVGRGGEETQYF	1047	CD8-C7
PB-1_GTCTTCGAGACAAGCC	PB	PB-1	P1-14	TRUE	3	TRA:CAASVVNDMRF TRB:CASSHRGGAKNIQYF	109	CD8-C5
PB-1_GTCTTCGAGACACTAA	PB	PB-1	P1-35	TRUE	3	TRA:CAVSDRGYFGNEKLTF TRB:CSALDDVRPVIAVYNEQFF	40	CD8-C7
PB-1_GTCTTCGAGAGCTTCT	PB	PB-1	P1-2	TRUE	3	TRA:CGQNFVF TRB:CASNQRGGPSLYNEQFF	323	CD8-C7
PB-1_GTCTTCGAGGACAGAA	PB	PB-1	P1-1	TRUE	3	TRA:CAMSDRMTGGFKTIF TRB:CASSQVGRGGEETQYF	1047	CD8-C7
PB-1_GTCTTCGAGTAATCCC	PB	PB-1	P1-4	TRUE	3	TRA:CAMKGRDDKIIF TRB:CASTKGVSREAFF	226	CD8-C7
PB-1_GTCTTCGAGTGTTAGA	PB	PB-1	P1-2	TRUE	3	TRA:CGQNFVF TRB:CASNQRGGPSLYNEQFF	323	CD8-C7
PB-1_GTCTTCGCATGGGAAC	PB	PB-1	P1-8	TRUE	3	TRA:CAVRPPGRNSGGYQKVTF TRB:CASSRRGVTDTQYF	204	CD8-C7
PB-1_GTCTTCGGTCAATGTC	PB	PB-1	P1-2	TRUE	3	TRA:CGQNFVF TRB:CASNQRGGPSLYNEQFF	323	CD8-C7
PB-1_GTCTTCGTCTAACTTC	PB	PB-1	P1-1	TRUE	3	TRA:CAMSDRMTGGFKTIF TRB:CASSQVGRGGEETQYF	1047	CD8-C7
PB-1_GTGAAGGCAAGTAGTA	PB	PB-1	P1-2	TRUE	3	TRA:CGQNFVF TRB:CASNQRGGPSLYNEQFF	323	CD4-C6
PB-1_GTGAAGGGTCTTCGTC	PB	PB-1	P1-26	TRUE	3	TRA:CAPRSGGSYIPTF TRB:CASSQDLGLDYGYTF	56	CD8-C7
PB-1_GTGAAGGTCCTGCCAT	PB	PB-1	P1-4	TRUE	3	TRA:CAMKGRDDKIIF TRB:CASTKGVSREAFF	226	CD8-C7
PB-1_GTGCAGCAGTTATCGC	PB	PB-1	P1-13	TRUE	3	TRA:CVVMNYGGSQGNLIF TRB:CASSSWGDTDEAFF	112	CD8-C7
PB-1_GTGCAGCTCAATCTCT	PB	PB-1	P1-29	TRUE	3	TRA:CATVSFSGGYNKLIF TRB:CASSLDRRGLSYEQYF	50	CD8-C7

PB-1_GTGCAGCTCCACGAAT	PB	PB-1	P1-5	TRUE	3	TRA:CALSGTPMNRDDKIIF TRB:CSARGRGLAGGPAYEQYF	223	CD8-C7
PB-1_GTGCAGCTCTGGGCCA	PB	PB-1	P1-23	TRUE	3	TRA:CALRAITQGGSEKLVF TRB:CASSLTGAVYGYTF	65	CD8-C5
PB-1_GTGCATAGTAAAGGAG	PB	PB-1	P1-2	TRUE	3	TRA:CGQNFVF TRB:CASNQRGGPSLYNEQFF	323	CD8-C7
PB-1_GTGCATAGTAGGAGTC	PB	PB-1	P1-7	TRUE	3	TRA:CAVQAGNNDMRF TRB:CASSTTGDFNYGYTF	215	CD8-C5
PB-1_GTGCATAGTTAAAGTG	PB	PB-1	P1-1	TRUE	3	TRA:CAMSDRMTGGFKTIF TRB:CASSQVGRGGEETQYF	1047	CD8-C7
PB-1_GTGCATATCTCGCTTG	PB	PB-1	P1-5	TRUE	3	TRA:CALSGTPMNRDDKIIF TRB:CSARGRGLAGGPAYEQYF	223	CD8-C7
PB-1_GTGCGGTAGAGGACGG	PB	PB-1	P1-11	TRUE	3	TRA:CAVEPLYGSGNTGKLIF TRB:CASSSHGQLNQPQHF	147	CD8-C5
PB-1_GTGCGGTCATACGCCG	PB	PB-1	P1-35	TRUE	3	TRA:CAVSDRGYFGNEKLTF TRB:CSALDDVRPVIAVYNEQFF	40	CD8-C7
PB-1_GTGCGGTCATGTAGTC	PB	PB-1	P1-10	TRUE	3	TRA:CISNSPMDSSYKLIF TRB:CASSSWGDTDEAFF	162	CD8-C7
PB-1_GTGCGGTCATTGCGGC	PB	PB-1	P1-199	FALSE	3	TRA:CAMSDRMTGGFKTIF TRB:CASSRRGVTDTQYF	3	CD8-C5
PB-1_GTGCGGTGTTCCATGA	PB	PB-1	P1-27	TRUE	3	TRA:CALVDFSGYSTLTF TRB:CASSGRGTNEKLFF	55	CD8-C6
PB-1_GTGCTTCAGAGTGAGA	PB	PB-1	P1-1	TRUE	3	TRA:CAMSDRMTGGFKTIF TRB:CASSQVGRGGEETQYF	1047	CD8-C7
PB-1_GTGCTTCGTACTCAAC	PB	PB-1	P1-3	TRUE	3	TRA:CAFLSGTYKYIF TRB:CASSQSPGQRNYGYTF	308	CD8-C5
PB-1_GTGCTTCTCACGACTA	PB	PB-1	P1-1	TRUE	3	TRA:CAMSDRMTGGFKTIF TRB:CASSQVGRGGEETQYF	1047	CD8-C7
PB-1_GTGGGTCAGGATGGAA	PB	PB-1	P1-9	TRUE	3	TRA:CAEAEGGGADGLTF TRB:CATSGGDNEQFF	174	CD8-C7
PB-1_GTGGGTCAGTTCGCGC	PB	PB-1	P1-58	TRUE	3	TRA:CAVRPNRDDKIIF TRB:CASSSRLAGGTDTQYF	12	CD8-C7
PB-1_GTGGGTCCACGGTAAG	PB	PB-1	P1-22	TRUE	3	TRA:CASNSGYSTLTF TRB:CASSQVTGGARDEQYF	65	CD8-C7
PB-1_GTGGGTCCATGGGACA	PB	PB-1	P1-1	TRUE	3	TRA:CAMSDRMTGGFKTIF TRB:CASSQVGRGGEETQYF	1047	CD8-C7
PB-1_GTGGGTCGTATAGTAG	PB	PB-1	P1-14	TRUE	3	TRA:CAASVVNDMRF TRB:CASSHRGGAKNIQYF	109	CD8-C5
PB-1_GTGTGCGAGCATGGCA	PB	PB-1	P1-5	TRUE	3	TRA:CALSGTPMNRDDKIIF TRB:CSARGRGLAGGPAYEQYF	223	CD8-C7
PB-1_GTGTGCGCAAGACACG	PB	PB-1	P1-2	TRUE	3	TRA:CGQNFVF TRB:CASNQRGGPSLYNEQFF	323	CD8-C7
PB-1_GTGTTAGAGAGTAATC	PB	PB-1	P1-1	TRUE	3	TRA:CAMSDRMTGGFKTIF TRB:CASSQVGRGGEETQYF	1047	CD8-C7
PB-1_GTGTTAGGTCAGCTAT	PB	PB-1	P1-1	TRUE	3	TRA:CAMSDRMTGGFKTIF TRB:CASSQVGRGGEETQYF	1047	CD8-C7
PB-1_GTTAAGCAGTTTAGGA	PB	PB-1	P1-1	TRUE	3	TRA:CAMSDRMTGGFKTIF TRB:CASSQVGRGGEETQYF	1047	CD8-C7
PB-1_GTTAAGCCAAGGTTTC	PB	PB-1	P1-919	FALSE	1	TRA:CAEAEGGGADGLTF TRB:CASTKGVSREAFF	1	CD8-C7
PB-1_GTTAAGCCACACGCTG	PB	PB-1	P1-3	TRUE	3	TRA:CAFLSGTYKYIF TRB:CASSQSPGQRNYGYTF	308	CD8-C7
PB-1_GTTAAGCGTAAAGTCA	PB	PB-1	P1-11	TRUE	3	TRA:CAVEPLYGSGNTGKLIF TRB:CASSSHGQLNQPQHF	147	CD8-C7

PB-1_GTTAAGCGTATAAACG	PB	PB-1	P1-8	TRUE	3	TRA:CAVRPPGRNSGGYQKVTF TRB:CASSRRGVTDTQYF	204	CD8-C7
PB-1_GTTAAGCGTGCATCTA	PB	PB-1	P1-8	TRUE	3	TRA:CAVRPPGRNSGGYQKVTF TRB:CASSRRGVTDTQYF	204	CD8-C7
PB-1_GTTAAGCTCACGGTTA	PB	PB-1	P1-3	TRUE	3	TRA:CAFLSGTYKYIF TRB:CASSQSPGQRNYGYTF	308	CD8-C7
PB-1_GTTAAGCTCCTGCCAT	PB	PB-1	P1-1	TRUE	3	TRA:CAMSDRMTGGFKTIF TRB:CASSQVGRGGEETQYF	1047	CD8-C7
PB-1_GTTACAGAGAAACGAG	PB	PB-1	P1-18	TRUE	3	TRA:CVADGNYGQNFVF TRB:CASSSPGLGGGFDTEAFF	75	CD8-C6
PB-1_GTTACAGAGCTGTCTA	PB	PB-1	P1-2	TRUE	3	TRA:CGQNFVF TRB:CASNQRGGPSLYNEQFF	323	CD8-C7
PB-1_GTTACAGCATCGATGT	PB	PB-1	P1-1	TRUE	3	TRA:CAMSDRMTGGFKTIF TRB:CASSQVGRGGEETQYF	1047	CD8-C7
PB-1_GTTACAGGTAGGGACT	PB	PB-1	P1-5	TRUE	3	TRA:CALSGTPMNRDDKIIF TRB:CSARGRGLAGGPAYEQYF	223	CD8-C7
PB-1_GTTCATTAGATGTCGG	PB	PB-1	P1-1	TRUE	3	TRA:CAMSDRMTGGFKTIF TRB:CASSQVGRGGEETQYF	1047	CD8-C7
PB-1_GTTCATTAGGTGTGGT	PB	PB-1	P1-10	TRUE	3	TRA:CISNSPMDSSYKLIF TRB:CASSSWGDTDEAFF	162	CD8-C7
PB-1_GTTCATTGTAAAGGAG	PB	PB-1	P1-8	TRUE	3	TRA:CAVRPPGRNSGGYQKVTF TRB:CASSRRGVTDTQYF	204	CD8-C6
PB-1_GTTCATTGTAAGTTCC	PB	PB-1	P1-11	TRUE	3	TRA:CAVEPLYGSGNTGKLIF TRB:CASSSHGQLNQPQHF	147	CD8-C7
PB-1_GTTCATTTCATGTGGT	PB	PB-1	P1-1	TRUE	3	TRA:CAMSDRMTGGFKTIF TRB:CASSQVGRGGEETQYF	1047	CD8-C7
PB-1_GTTCGGGAGCAGACTG	PB	PB-1	P1-22	TRUE	3	TRA:CASNSGYSTLTF TRB:CASSQVTGGARDEQYF	65	CD8-C7
PB-1_GTTCGGGCATCGTCGG	PB	PB-1	P1-1	TRUE	3	TRA:CAMSDRMTGGFKTIF TRB:CASSQVGRGGEETQYF	1047	CD8-C5
PB-1_GTTCGGGGTCACAAGG	PB	PB-1	P1-4	TRUE	3	TRA:CAMKGRDDKIIF TRB:CASTKGVSREAFF	226	CD8-C7
PB-1_GTTCGGGTCGCAAACT	PB	PB-1	P1-5	TRUE	3	TRA:CALSGTPMNRDDKIIF TRB:CSARGRGLAGGPAYEQYF	223	CD8-C7
PB-1_GTTCTCGAGAGGACGG	PB	PB-1	P1-29	TRUE	3	TRA:CATVSFSGGYNKLIF TRB:CASSLDRRGLSYEQYF	50	CD8-C7
PB-1_GTTCTCGTCATTCACT	PB	PB-1	P1-5	TRUE	3	TRA:CALSGTPMNRDDKIIF TRB:CSARGRGLAGGPAYEQYF	223	CD8-C7
PB-1_GTTCTCGTCCGGCACA	PB	PB-1	P1-1	TRUE	3	TRA:CAMSDRMTGGFKTIF TRB:CASSQVGRGGEETQYF	1047	CD8-C7
PB-1_GTTTCTAAGGCATTGG	PB	PB-1	P1-4	TRUE	3	TRA:CAMKGRDDKIIF TRB:CASTKGVSREAFF	226	CD8-C7
PB-1_GTTTCTACACATTCGA	PB	PB-1	P1-26	TRUE	3	TRA:CAPRSGGSYIPTF TRB:CASSQDLGLDYGYTF	56	CD8-C7
PB-1_GTTTCTACATACCATG	PB	PB-1	P1-7	TRUE	3	TRA:CAVQAGNNDMRF TRB:CASSTTGDFNYGYTF	215	CD8-C7
PB-1_GTTTCTATCAGGCCCA	PB	PB-1	P1-1	TRUE	3	TRA:CAMSDRMTGGFKTIF TRB:CASSQVGRGGEETQYF	1047	CD8-C5
PB-1_TAAACCGAGTGTACTC	PB	PB-1	P1-2	TRUE	3	TRA:CGQNFVF TRB:CASNQRGGPSLYNEQFF	323	CD8-C7
PB-1_TAAACCGCATTTGCCC	PB	PB-1	P1-4	TRUE	3	TRA:CAMKGRDDKIIF TRB:CASTKGVSREAFF	226	CD8-C7
PB-1_TAAACCGTCAGAGGTG	PB	PB-1	P1-22	TRUE	3	TRA:CASNSGYSTLTF TRB:CASSQVTGGARDEQYF	65	CD8-C7

PB-1_TAAGAGAAGGGATGGG	PB	PB-1	P1-1	TRUE	3	TRA:CAMSDRMTGGFKTIF TRB:CASSQVGRGGEETQYF	1047	CD8-C5
PB-1_TAAGAGAAGTGAAGAG	PB	PB-1	P1-2	TRUE	3	TRA:CGQNFVF TRB:CASNQRGGPSLYNEQFF	323	CD8-C7
PB-1_TAAGAGAAGTGTCCAT	PB	PB-1	P1-8	TRUE	3	TRA:CAVRPPGRNSGGYQKVTF TRB:CASSRRGVTDTQYF	204	CD8-C7
PB-1_TAAGAGAGTCGAGTTT	PB	PB-1	P1-13	TRUE	3	TRA:CVVMNYGGSQGNLIF TRB:CASSSWGDTDEAFF	112	CD8-C7
PB-1_TAAGAGATCACCATAG	PB	PB-1	P1-1	TRUE	3	TRA:CAMSDRMTGGFKTIF TRB:CASSQVGRGGEETQYF	1047	CD8-C7
PB-1_TAAGAGATCTTCGGTC	PB	PB-1	P1-22	TRUE	3	TRA:CASNSGYSTLTF TRB:CASSQVTGGARDEQYF	65	CD8-C7
PB-1_TAAGCGTAGCTAGGCA	PB	PB-1	P1-4	TRUE	3	TRA:CAMKGRDDKIIF TRB:CASTKGVSREAFF	226	CD8-C7
PB-1_TAAGCGTAGGTGATTA	PB	PB-1	P1-934	FALSE	1	TRA:CAMSDRMTGGFKTIF TRB:CATSGGDNEQFF	1	CD8-C5
PB-1_TAAGCGTCATGTTCCC	PB	PB-1	P1-2	TRUE	3	TRA:CGQNFVF TRB:CASNQRGGPSLYNEQFF	323	CD8-C7
PB-1_TAAGCGTTCGCGCCAA	PB	PB-1	P1-935	FALSE	1	TRA:CAVADGNKLVF TRB:CASSLPGTIGNTIYF	1	CD8-C7
PB-1_TAAGTGCCAAGTCTAC	PB	PB-1	P1-286	FALSE	2	TRA:CAMRGSYIPTF TRB:CASRWEGLGVNEQFF	2	CD8-C6
PB-1_TAAGTGCGTTGCGCAC	PB	PB-1	P1-1	TRUE	3	TRA:CAMSDRMTGGFKTIF TRB:CASSQVGRGGEETQYF	1047	CD8-C7
PB-1_TAAGTGCTCCTAGGGC	PB	PB-1	P1-5	TRUE	3	TRA:CALSGTPMNRDDKIIF TRB:CSARGRGLAGGPAYEQYF	223	CD8-C7
PB-1_TAAGTGCTCCTATGTT	PB	PB-1	P1-1	TRUE	3	TRA:CAMSDRMTGGFKTIF TRB:CASSQVGRGGEETQYF	1047	CD8-C5
PB-1_TAAGTGCTCGGCTTGG	PB	PB-1	P1-1	TRUE	3	TRA:CAMSDRMTGGFKTIF TRB:CASSQVGRGGEETQYF	1047	CD8-C7
PB-1_TACACGAAGGCTAGAC	PB	PB-1	P1-3	TRUE	3	TRA:CAFLSGTYKYIF TRB:CASSQSPGQRNYGYTF	308	CD8-C7
PB-1_TACACGACAAGCCCAC	PB	PB-1	P1-50	TRUE	3	TRA:CARWLGSQGNLIF TRB:CASSLTDRVPQFELVYEQYF	15	CD8-C5
PB-1_TACACGACAGACGCTC	PB	PB-1	P1-1	TRUE	3	TRA:CAMSDRMTGGFKTIF TRB:CASSQVGRGGEETQYF	1047	CD8-C7
PB-1_TACACGAGTTATCACG	PB	PB-1	P1-8	TRUE	3	TRA:CAVRPPGRNSGGYQKVTF TRB:CASSRRGVTDTQYF	204	CD8-C7
PB-1_TACACGAGTTTCGCTC	PB	PB-1	P1-3	TRUE	3	TRA:CAFLSGTYKYIF TRB:CASSQSPGQRNYGYTF	308	CD8-C7
PB-1_TACACGAGTTTGTTTC	PB	PB-1	P1-1	TRUE	3	TRA:CAMSDRMTGGFKTIF TRB:CASSQVGRGGEETQYF	1047	CD8-C5
PB-1_TACAGTGAGTAGGCCA	PB	PB-1	P1-937	FALSE	1	TRA:CATVSFSGGYNKLIF TRB:CASNQRGGPSLYNEQFF	1	CD8-C7
PB-1_TACAGTGGTTTCGCTC	PB	PB-1	P1-1	TRUE	3	TRA:CAMSDRMTGGFKTIF TRB:CASSQVGRGGEETQYF	1047	CD8-C7
PB-1_TACAGTGTCACATGCA	PB	PB-1	P1-26	TRUE	3	TRA:CAPRSGGSYIPTF TRB:CASSQDLGLDYGYTF	56	CD8-C5
PB-1_TACAGTGTCGTTACGA	PB	PB-1	P1-22	TRUE	3	TRA:CASNSGYSTLTF TRB:CASSQVTGGARDEQYF	65	CD8-C7
PB-1_TACCTATAGCGATTCT	PB	PB-1	P1-3	TRUE	3	TRA:CAFLSGTYKYIF TRB:CASSQSPGQRNYGYTF	308	CD8-C5
PB-1_TACCTATAGGCATGGT	PB	PB-1	P1-4	TRUE	3	TRA:CAMKGRDDKIIF TRB:CASTKGVSREAFF	226	CD8-C7

PB-1_TACCTATCATCCGTGG	PB	PB-1	P1-53	TRUE	3	TRA:CALSERDTDKLIF TRB:CASSILQGVITEAFF	13	CD8-C7
PB-1_TACCTATGTTACGACT	PB	PB-1	P1-3	TRUE	3	TRA:CAFLSGTYKYIF TRB:CASSQSPGQRNYGYTF	308	CD8-C5
PB-1_TACCTTAAGGTAAACT	PB	PB-1	P1-2	TRUE	3	TRA:CGQNFVF TRB:CASNQRGGPSLYNEQFF	323	CD8-C7
PB-1_TACCTTACATCAGTCA	PB	PB-1	P1-1	TRUE	3	TRA:CAMSDRMTGGFKTIF TRB:CASSQVGRGGEETQYF	1047	CD8-C7
PB-1_TACCTTAGTCACTGGC	PB	PB-1	P1-1	TRUE	3	TRA:CAMSDRMTGGFKTIF TRB:CASSQVGRGGEETQYF	1047	CD8-C7
PB-1_TACCTTAGTCCCTACT	PB	PB-1	P1-16	TRUE	3	TRA:CALSGTPMNRDDKIIF TRB:CASSQSWDLAVLNEQFF	79	CD8-C7
PB-1_TACCTTAGTGTGCCTG	PB	PB-1	P1-31	TRUE	3	TRA:CAVEEGADRLMF TRB:CASSLLGGGELFF	46	CD8-C7
PB-1_TACCTTATCTGTGCAA	PB	PB-1	P1-10	TRUE	3	TRA:CISNSPMDSSYKLIF TRB:CASSSWGDTDEAFF	162	CD8-C7
PB-1_TACGGATAGACTAGGC	PB	PB-1	P1-1	TRUE	3	TRA:CAMSDRMTGGFKTIF TRB:CASSQVGRGGEETQYF	1047	CD8-C7
PB-1_TACGGATCACACAGAG	PB	PB-1	P1-8	TRUE	3	TRA:CAVRPPGRNSGGYQKVTF TRB:CASSRRGVTDTQYF	204	CD8-C7
PB-1_TACGGATGTACATGTC	PB	PB-1	P1-10	TRUE	3	TRA:CISNSPMDSSYKLIF TRB:CASSSWGDTDEAFF	162	CD8-C7
PB-1_TACGGGCAGAAGGGTA	PB	PB-1	P1-1	TRUE	3	TRA:CAMSDRMTGGFKTIF TRB:CASSQVGRGGEETQYF	1047	CD8-C7
PB-1_TACGGGCAGTGCCAGA	PB	PB-1	P1-112	FALSE	3	TRA:CALTPLTGGGNKLTF TRB:CSAREGGSMDTQYF	5	CD8-C7
PB-1_TACGGGCCAGGTGCCT	PB	PB-1	P1-8	TRUE	3	TRA:CAVRPPGRNSGGYQKVTF TRB:CASSRRGVTDTQYF	204	CD8-C7
PB-1_TACGGGCCATAGTAAG	PB	PB-1	P1-1	TRUE	3	TRA:CAMSDRMTGGFKTIF TRB:CASSQVGRGGEETQYF	1047	CD8-C7
PB-1_TACGGGCGTACGCACC	PB	PB-1	P1-26	TRUE	3	TRA:CAPRSGGSYIPTF TRB:CASSQDLGLDYGYTF	56	CD8-C5
PB-1_TACGGGCGTATAGGGC	PB	PB-1	P1-1	TRUE	3	TRA:CAMSDRMTGGFKTIF TRB:CASSQVGRGGEETQYF	1047	CD8-C7
PB-1_TACGGGCGTTACGGAG	PB	PB-1	P1-1	TRUE	3	TRA:CAMSDRMTGGFKTIF TRB:CASSQVGRGGEETQYF	1047	CD8-C7
PB-1_TACGGGCTCATCTGTT	PB	PB-1	P1-39	TRUE	3	TRA:CAVRDLGGTSYDKVIF TRB:CSARVREVSYEQYF	31	CD8-C5
PB-1_TACGGGCTCTCGCTTG	PB	PB-1	P1-18	TRUE	3	TRA:CVADGNYGQNFVF TRB:CASSSPGLGGGFDTEAFF	75	CD8-C6
PB-1_TACGGTAAGACTGGGT	PB	PB-1	P1-18	TRUE	3	TRA:CVADGNYGQNFVF TRB:CASSSPGLGGGFDTEAFF	75	CD8-C6
PB-1_TACGGTAAGCCTCGTG	PB	PB-1	P1-944	FALSE	1	TRA:CGQNFVF TRB:CASTKGVSREAFF	1	CD8-C7
PB-1_TACGGTAAGTTGAGAT	PB	PB-1	P1-8	TRUE	3	TRA:CAVRPPGRNSGGYQKVTF TRB:CASSRRGVTDTQYF	204	CD8-C7
PB-1_TACGGTACAGACTCGC	PB	PB-1	P1-26	TRUE	3	TRA:CAPRSGGSYIPTF TRB:CASSQDLGLDYGYTF	56	CD8-C7
PB-1_TACGGTATCACCAGGC	PB	PB-1	P1-1	TRUE	3	TRA:CAMSDRMTGGFKTIF TRB:CASSQVGRGGEETQYF	1047	CD8-C7
PB-1_TACGGTATCTGCGACG	PB	PB-1	P1-3	TRUE	3	TRA:CAFLSGTYKYIF TRB:CASSQSPGQRNYGYTF	308	CD8-C7
PB-1_TACTCATAGAGCTGGT	PB	PB-1	P1-112	FALSE	3	TRA:CALTPLTGGGNKLTF TRB:CSAREGGSMDTQYF	5	CD8-C7

PB-1_TACTCATCATGGTCAT	PB	PB-1	P1-22	TRUE	3	TRA:CASNSGYSTLTF TRB:CASSQVTGGARDEQYF	65	CD8-C7
PB-1_TACTCATCATTTGCCC	PB	PB-1	P1-12	TRUE	3	TRA:CALSDERGDYKLSF TRB:CASSPGTGSYNEQFF	141	CD8-C7
PB-1_TACTCATGTGTTCTTT	PB	PB-1	P1-2	TRUE	3	TRA:CGQNFVF TRB:CASNQRGGPSLYNEQFF	323	CD8-C7
PB-1_TACTCATTCGAACGGA	PB	PB-1	P1-26	TRUE	3	TRA:CAPRSGGSYIPTF TRB:CASSQDLGLDYGYTF	56	CD8-C7
PB-1_TACTCATTCTCCGGTT	PB	PB-1	P1-3	TRUE	3	TRA:CAFLSGTYKYIF TRB:CASSQSPGQRNYGYTF	308	CD8-C7
PB-1_TACTCGCCAGCCAGAA	PB	PB-1	P1-3	TRUE	3	TRA:CAFLSGTYKYIF TRB:CASSQSPGQRNYGYTF	308	CD8-C7
PB-1_TACTCGCCAGTGGGAT	PB	PB-1	P1-1	TRUE	3	TRA:CAMSDRMTGGFKTIF TRB:CASSQVGRGGEETQYF	1047	CD8-C7
PB-1_TACTCGCTCTAAGCCA	PB	PB-1	P1-31	TRUE	3	TRA:CAVEEGADRLMF TRB:CASSLLGGGELFF	46	CD8-C7
PB-1_TACTTACCACGGACAA	PB	PB-1	P1-4	TRUE	3	TRA:CAMKGRDDKIIF TRB:CASTKGVSREAFF	226	CD8-C7
PB-1_TACTTACCACTTCGAA	PB	PB-1	P1-10	TRUE	3	TRA:CISNSPMDSSYKLIF TRB:CASSSWGDTDEAFF	162	CD8-C7
PB-1_TACTTACCAGCTTAAC	PB	PB-1	P1-3	TRUE	3	TRA:CAFLSGTYKYIF TRB:CASSQSPGQRNYGYTF	308	CD8-C7
PB-1_TACTTACCATGGAATA	PB	PB-1	P1-12	TRUE	3	TRA:CALSDERGDYKLSF TRB:CASSPGTGSYNEQFF	141	CD8-C5
PB-1_TACTTACTCCGATATG	PB	PB-1	P1-71	FALSE	3	TRA:CAVAGGGGADGLTF TRB:CASSCPGLGGSTDTQYF	8	CD8-C6
PB-1_TACTTACTCGTTGACA	PB	PB-1	P1-13	TRUE	3	TRA:CVVMNYGGSQGNLIF TRB:CASSSWGDTDEAFF	112	CD8-C7
PB-1_TACTTGTAGCAATCTC	PB	PB-1	P1-14	TRUE	3	TRA:CAASVVNDMRF TRB:CASSHRGGAKNIQYF	109	CD8-C5
PB-1_TACTTGTAGGTGATTA	PB	PB-1	P1-10	TRUE	3	TRA:CISNSPMDSSYKLIF TRB:CASSSWGDTDEAFF	162	CD8-C7
PB-1_TACTTGTAGTCGTTTG	PB	PB-1	P1-4	TRUE	3	TRA:CAMKGRDDKIIF TRB:CASTKGVSREAFF	226	CD8-C7
PB-1_TACTTGTAGTTCCACA	PB	PB-1	P1-11	TRUE	3	TRA:CAVEPLYGSGNTGKLIF TRB:CASSSHGQLNQPQHF	147	CD8-C7
PB-1_TACTTGTCATGAAGTA	PB	PB-1	P1-8	TRUE	3	TRA:CAVRPPGRNSGGYQKVTF TRB:CASSRRGVTDTQYF	204	CD8-C7
PB-1_TACTTGTGTCGAATCT	PB	PB-1	P1-1	TRUE	3	TRA:CAMSDRMTGGFKTIF TRB:CASSQVGRGGEETQYF	1047	CD8-C7
PB-1_TACTTGTGTGCATCTA	PB	PB-1	P1-11	TRUE	3	TRA:CAVEPLYGSGNTGKLIF TRB:CASSSHGQLNQPQHF	147	CD8-C7
PB-1_TACTTGTTCAGTACGT	PB	PB-1	P1-2	TRUE	3	TRA:CGQNFVF TRB:CASNQRGGPSLYNEQFF	323	CD8-C7
PB-1_TACTTGTTCGTCGTTC	PB	PB-1	P1-951	FALSE	1	TRA:CAESGFTTSGTYKYIF TRB:CSARDPGARADTQYF	1	CD8-C7
PB-1_TACTTGTTCTTAGCCC	PB	PB-1	P1-5	TRUE	3	TRA:CALSGTPMNRDDKIIF TRB:CSARGRGLAGGPAYEQYF	223	CD8-C7
PB-1_TAGACCACAAGCGTAG	PB	PB-1	P1-29	TRUE	3	TRA:CATVSFSGGYNKLIF TRB:CASSLDRRGLSYEQYF	50	CD8-C7
PB-1_TAGACCACACACGCTG	PB	PB-1	P1-2	TRUE	3	TRA:CGQNFVF TRB:CASNQRGGPSLYNEQFF	323	CD8-C7
PB-1_TAGACCACACACTGCG	PB	PB-1	P1-1	TRUE	3	TRA:CAMSDRMTGGFKTIF TRB:CASSQVGRGGEETQYF	1047	CD8-C7

PB-1_TAGACCACAGGAATGC	PB	PB-1	P1-6	TRUE	3	TRA:CAMREGRERDDKIIF TRB:CASSLPNTEAFF	216	CD8-C5
PB-1_TAGACCAGTATAAACG	PB	PB-1	P1-4	TRUE	3	TRA:CAMKGRDDKIIF TRB:CASTKGVSREAFF	226	CD8-C2
PB-1_TAGAGCTAGGATGCGT	PB	PB-1	P1-4	TRUE	3	TRA:CAMKGRDDKIIF TRB:CASTKGVSREAFF	226	CD8-C7
PB-1_TAGAGCTGTGTGCGTC	PB	PB-1	P1-5	TRUE	3	TRA:CALSGTPMNRDDKIIF TRB:CSARGRGLAGGPAYEQYF	223	CD8-C7
PB-1_TAGAGCTTCCTTGCCA	PB	PB-1	P1-2	TRUE	3	TRA:CGQNFVF TRB:CASNQRGGPSLYNEQFF	323	CD8-C7
PB-1_TAGAGCTTCGGATGTT	PB	PB-1	P1-1	TRUE	3	TRA:CAMSDRMTGGFKTIF TRB:CASSQVGRGGEETQYF	1047	CD8-C7
PB-1_TAGCCGGCAAACTGCT	PB	PB-1	P1-8	TRUE	3	TRA:CAVRPPGRNSGGYQKVTF TRB:CASSRRGVTDTQYF	204	CD8-C5
PB-1_TAGCCGGGTTCGTTGA	PB	PB-1	P1-73	TRUE	3	TRA:CAGI TRB:CASSRTTGGSSYEQYF	8	CD8-C7
PB-1_TAGCCGGTCTCTGCTG	PB	PB-1	P1-956	FALSE	1	TRA:CVVSDPPDKLIF TRB:CSARGRGLAGGPAYEQYF	1	CD8-C7
PB-1_TAGGCATAGAGACGAA	PB	PB-1	P1-10	TRUE	3	TRA:CISNSPMDSSYKLIF TRB:CASSSWGDTDEAFF	162	CD8-C7
PB-1_TAGTGGTAGGACGAAA	PB	PB-1	P1-8	TRUE	3	TRA:CAVRPPGRNSGGYQKVTF TRB:CASSRRGVTDTQYF	204	CD8-C7
PB-1_TAGTGGTAGTACGCGA	PB	PB-1	P1-29	TRUE	3	TRA:CATVSFSGGYNKLIF TRB:CASSLDRRGLSYEQYF	50	CD8-C7
PB-1_TAGTGGTCACGCATCG	PB	PB-1	P1-8	TRUE	3	TRA:CAVRPPGRNSGGYQKVTF TRB:CASSRRGVTDTQYF	204	CD8-C7
PB-1_TAGTGGTCAGGGTACA	PB	PB-1	P1-1	TRUE	3	TRA:CAMSDRMTGGFKTIF TRB:CASSQVGRGGEETQYF	1047	CD8-C7
PB-1_TAGTGGTGTCGGCTCA	PB	PB-1	P1-3	TRUE	3	TRA:CAFLSGTYKYIF TRB:CASSQSPGQRNYGYTF	308	CD8-C7
PB-1_TAGTGGTTCCATGAAC	PB	PB-1	P1-1	TRUE	3	TRA:CAMSDRMTGGFKTIF TRB:CASSQVGRGGEETQYF	1047	CD8-C7
PB-1_TAGTTGGAGCAAATCA	PB	PB-1	P1-1	TRUE	3	TRA:CAMSDRMTGGFKTIF TRB:CASSQVGRGGEETQYF	1047	CD8-C7
PB-1_TAGTTGGCAAGGTTTC	PB	PB-1	P1-13	TRUE	3	TRA:CVVMNYGGSQGNLIF TRB:CASSSWGDTDEAFF	112	CD8-C7
PB-1_TAGTTGGCAATCTGCA	PB	PB-1	P1-11	TRUE	3	TRA:CAVEPLYGSGNTGKLIF TRB:CASSSHGQLNQPQHF	147	CD8-C7
PB-1_TAGTTGGCAGGGAGAG	PB	PB-1	P1-10	TRUE	3	TRA:CISNSPMDSSYKLIF TRB:CASSSWGDTDEAFF	162	CD8-C7
PB-1_TAGTTGGCATGTTGAC	PB	PB-1	P1-11	TRUE	3	TRA:CAVEPLYGSGNTGKLIF TRB:CASSSHGQLNQPQHF	147	CD8-C7
PB-1_TAGTTGGCATTTGCTT	PB	PB-1	P1-1	TRUE	3	TRA:CAMSDRMTGGFKTIF TRB:CASSQVGRGGEETQYF	1047	CD8-C7
PB-1_TATCAGGAGGTGTGGT	PB	PB-1	P1-3	TRUE	3	TRA:CAFLSGTYKYIF TRB:CASSQSPGQRNYGYTF	308	CD8-C7
PB-1_TATCAGGCAAGCGAGT	PB	PB-1	P1-4	TRUE	3	TRA:CAMKGRDDKIIF TRB:CASTKGVSREAFF	226	CD8-C7
PB-1_TATCAGGCAGATGAGC	PB	PB-1	P1-10	TRUE	3	TRA:CISNSPMDSSYKLIF TRB:CASSSWGDTDEAFF	162	CD8-C7
PB-1_TATCAGGGTCAGAAGC	PB	PB-1	P1-10	TRUE	3	TRA:CISNSPMDSSYKLIF TRB:CASSSWGDTDEAFF	162	CD8-C7
PB-1_TATCAGGGTTTACTCT	PB	PB-1	P1-29	TRUE	3	TRA:CATVSFSGGYNKLIF TRB:CASSLDRRGLSYEQYF	50	CD8-C7

PB-1_TATCTCAAGAATAGGG	PB	PB-1	P1-2	TRUE	3	TRA:CGQNFVF TRB:CASNQRGGPSLYNEQFF	323	CD8-C7
PB-1_TATCTCAAGGAATGGA	PB	PB-1	P1-4	TRUE	3	TRA:CAMKGRDDKIIF TRB:CASTKGVSREAFF	226	CD8-C7
PB-1_TATCTCAGTAGCTGCC	PB	PB-1	P1-12	TRUE	3	TRA:CALSDERGDYKLSF TRB:CASSPGTGSYNEQFF	141	CD8-C5
PB-1_TATCTCAGTCGTCTTC	PB	PB-1	P1-35	TRUE	3	TRA:CAVSDRGYFGNEKLTF TRB:CSALDDVRPVIAVYNEQFF	40	CD8-C6
PB-1_TATCTCATCGGTCCGA	PB	PB-1	P1-1	TRUE	3	TRA:CAMSDRMTGGFKTIF TRB:CASSQVGRGGEETQYF	1047	CD8-C5
PB-1_TATCTCATCTGAAAGA	PB	PB-1	P1-1	TRUE	3	TRA:CAMSDRMTGGFKTIF TRB:CASSQVGRGGEETQYF	1047	CD8-C7
PB-1_TATGCCCAGTACGATA	PB	PB-1	P1-5	TRUE	3	TRA:CALSGTPMNRDDKIIF TRB:CSARGRGLAGGPAYEQYF	223	CD8-C7
PB-1_TATGCCCGTATTAGCC	PB	PB-1	P1-22	TRUE	3	TRA:CASNSGYSTLTF TRB:CASSQVTGGARDEQYF	65	CD8-C7
PB-1_TATGCCCGTTGTCGCG	PB	PB-1	P1-3	TRUE	3	TRA:CAFLSGTYKYIF TRB:CASSQSPGQRNYGYTF	308	CD8-C7
PB-1_TATGCCCTCTCGTATT	PB	PB-1	P1-50	TRUE	3	TRA:CARWLGSQGNLIF TRB:CASSLTDRVPQFELVYEQYF	15	CD8-C7
PB-1_TATTACCAGGTGATAT	PB	PB-1	P1-13	TRUE	3	TRA:CVVMNYGGSQGNLIF TRB:CASSSWGDTDEAFF	112	CD8-C7
PB-1_TATTACCCATTGTGCA	PB	PB-1	P1-1	TRUE	3	TRA:CAMSDRMTGGFKTIF TRB:CASSQVGRGGEETQYF	1047	CD8-C7
PB-1_TATTACCGTCAGCTAT	PB	PB-1	P1-22	TRUE	3	TRA:CASNSGYSTLTF TRB:CASSQVTGGARDEQYF	65	CD8-C7
PB-1_TATTACCGTGTCGCTG	PB	PB-1	P1-964	FALSE	1	TRA:CAVEEGADRLMF TRB:CASSKGQGPRETQYF	1	CD8-C7
PB-1_TATTACCGTGTTTGGT	PB	PB-1	P1-10	TRUE	3	TRA:CISNSPMDSSYKLIF TRB:CASSSWGDTDEAFF	162	CD8-C7
PB-1_TATTACCTCTCTGTCG	PB	PB-1	P1-3	TRUE	3	TRA:CAFLSGTYKYIF TRB:CASSQSPGQRNYGYTF	308	CD8-C7
PB-1_TCAACGAAGAGAGCTC	PB	PB-1	P1-4	TRUE	3	TRA:CAMKGRDDKIIF TRB:CASTKGVSREAFF	226	CD8-C7
PB-1_TCAACGAAGCACAGGT	PB	PB-1	P1-4	TRUE	3	TRA:CAMKGRDDKIIF TRB:CASTKGVSREAFF	226	CD8-C7
PB-1_TCAACGACAGTGACAG	PB	PB-1	P1-10	TRUE	3	TRA:CISNSPMDSSYKLIF TRB:CASSSWGDTDEAFF	162	CD8-C7
PB-1_TCAACGACATGGATGG	PB	PB-1	P1-67	TRUE	3	TRA:CALNTNQAGTALIF TRB:CASSLGTRYAYEQYF	9	CD8-C7
PB-1_TCAACGATCGCTTGTC	PB	PB-1	P1-3	TRUE	3	TRA:CAFLSGTYKYIF TRB:CASSQSPGQRNYGYTF	308	CD8-C5
PB-1_TCAATCTCATGCTGGC	PB	PB-1	P1-1	TRUE	3	TRA:CAMSDRMTGGFKTIF TRB:CASSQVGRGGEETQYF	1047	CD8-C7
PB-1_TCAATCTCATGGTCAT	PB	PB-1	P1-22	TRUE	3	TRA:CASNSGYSTLTF TRB:CASSQVTGGARDEQYF	65	CD8-C5
PB-1_TCAATCTGTAATTGGA	PB	PB-1	P1-1	TRUE	3	TRA:CAMSDRMTGGFKTIF TRB:CASSQVGRGGEETQYF	1047	CD8-C7
PB-1_TCAATCTGTCCCTTGT	PB	PB-1	P1-5	TRUE	3	TRA:CALSGTPMNRDDKIIF TRB:CSARGRGLAGGPAYEQYF	223	CD8-C7
PB-1_TCAATCTTCCCGACTT	PB	PB-1	P1-2	TRUE	3	TRA:CGQNFVF TRB:CASNQRGGPSLYNEQFF	323	CD8-C7
PB-1_TCAATCTTCTTCGAGA	PB	PB-1	P1-8	TRUE	3	TRA:CAVRPPGRNSGGYQKVTF TRB:CASSRRGVTDTQYF	204	CD8-C7

PB-1_TCACAAGAGACGCAAC	PB	PB-1	P1-1	TRUE	3	TRA:CAMSDRMTGGFKTIF TRB:CASSQVGRGGEETQYF	1047	CD8-C7
PB-1_TCACAAGAGACGCTTT	PB	PB-1	P1-3	TRUE	3	TRA:CAFLSGTYKYIF TRB:CASSQSPGQRNYGYTF	308	CD8-C7
PB-1_TCACAAGAGACTACAA	PB	PB-1	P1-4	TRUE	3	TRA:CAMKGRDDKIIF TRB:CASTKGVSREAFF	226	CD8-C7
PB-1_TCACAAGAGATGTAAC	PB	PB-1	P1-1	TRUE	3	TRA:CAMSDRMTGGFKTIF TRB:CASSQVGRGGEETQYF	1047	CD8-C7
PB-1_TCACAAGAGTTTCCTT	PB	PB-1	P1-11	TRUE	3	TRA:CAVEPLYGSGNTGKLIF TRB:CASSSHGQLNQPQHF	147	CD8-C7
PB-1_TCACAAGCAAGCGCTC	PB	PB-1	P1-7	TRUE	3	TRA:CAVQAGNNDMRF TRB:CASSTTGDFNYGYTF	215	CD8-C5
PB-1_TCACAAGCAAGTTAAG	PB	PB-1	P1-1	TRUE	3	TRA:CAMSDRMTGGFKTIF TRB:CASSQVGRGGEETQYF	1047	CD8-C7
PB-1_TCACAAGCACAACTGT	PB	PB-1	P1-2	TRUE	3	TRA:CGQNFVF TRB:CASNQRGGPSLYNEQFF	323	CD8-C7
PB-1_TCACAAGCACCGTTGG	PB	PB-1	P1-13	TRUE	3	TRA:CVVMNYGGSQGNLIF TRB:CASSSWGDTDEAFF	112	CD8-C7
PB-1_TCACAAGCACGGTAGA	PB	PB-1	P1-1	TRUE	3	TRA:CAMSDRMTGGFKTIF TRB:CASSQVGRGGEETQYF	1047	CD8-C7
PB-1_TCACAAGCAGGATCGA	PB	PB-1	P1-1	TRUE	3	TRA:CAMSDRMTGGFKTIF TRB:CASSQVGRGGEETQYF	1047	CD8-C6
PB-1_TCACAAGGTCACCCAG	PB	PB-1	P1-18	TRUE	3	TRA:CVADGNYGQNFVF TRB:CASSSPGLGGGFDTEAFF	75	CD8-C7
PB-1_TCACAAGGTCCTCTTG	PB	PB-1	P1-1	TRUE	3	TRA:CAMSDRMTGGFKTIF TRB:CASSQVGRGGEETQYF	1047	CD8-C7
PB-1_TCACAAGTCGAGGTAG	PB	PB-1	P1-12	TRUE	3	TRA:CALSDERGDYKLSF TRB:CASSPGTGSYNEQFF	141	CD8-C6
PB-1_TCACAAGTCTGTCTCG	PB	PB-1	P1-5	TRUE	3	TRA:CALSGTPMNRDDKIIF TRB:CSARGRGLAGGPAYEQYF	223	CD8-C7
PB-1_TCACAAGTCTTAGAGC	PB	PB-1	P1-17	TRUE	3	TRA:CGTGARGSSGSRLTF TRB:CASSRQGMGAFF	78	CD8-C7
PB-1_TCACGAACACTTCTGC	PB	PB-1	P1-29	TRUE	3	TRA:CATVSFSGGYNKLIF TRB:CASSLDRRGLSYEQYF	50	CD8-C7
PB-1_TCACGAAGTATGGTTC	PB	PB-1	P1-3	TRUE	3	TRA:CAFLSGTYKYIF TRB:CASSQSPGQRNYGYTF	308	CD8-C7
PB-1_TCAGATGGTAATCACC	PB	PB-1	P1-15	TRUE	3	TRA:CAVNSSPLGANSKLTF TRB:CASSDSGNFYNEQFF	92	CD8-C6
PB-1_TCAGATGGTGGCGAAT	PB	PB-1	P1-14	TRUE	3	TRA:CAASVVNDMRF TRB:CASSHRGGAKNIQYF	109	CD8-C5
PB-1_TCAGATGTCTGCAAGT	PB	PB-1	P1-38	TRUE	3	TRA:CAFTAGGATNKLIF TRB:CASIEGQGPYNEQFF	38	CD8-C7
PB-1_TCAGCAAAGCCCTAAT	PB	PB-1	P1-10	TRUE	3	TRA:CISNSPMDSSYKLIF TRB:CASSSWGDTDEAFF	162	CD8-C7
PB-1_TCAGCAAAGGAGTAGA	PB	PB-1	P1-150	FALSE	3	TRA:CALGAQGGSEKLVF TRB:CASSCPGLGGSTDTQYF	4	CD8-C6
PB-1_TCAGCAAAGGTAAACT	PB	PB-1	P1-2	TRUE	3	TRA:CGQNFVF TRB:CASNQRGGPSLYNEQFF	323	CD4-C6
PB-1_TCAGCAAAGTGCGTGA	PB	PB-1	P1-1	TRUE	3	TRA:CAMSDRMTGGFKTIF TRB:CASSQVGRGGEETQYF	1047	CD8-C7
PB-1_TCAGCAAAGTTCGATC	PB	PB-1	P1-2	TRUE	3	TRA:CGQNFVF TRB:CASNQRGGPSLYNEQFF	323	CD8-C7
PB-1_TCAGCAATCGGGAGTA	PB	PB-1	P1-974	FALSE	1	TRA:CAYRSAPGGTSYGKLTF TRB:CASSTGIYGYTF	1	CD8-C7

PB-1_TCAGCAATCGTAGGTT	PB	PB-1	P1-1	TRUE	3	TRA:CAMSDRMTGGFKTIF TRB:CASSQVGRGGEETQYF	1047	CD8-C7
PB-1_TCAGCTCAGGAGTCTG	PB	PB-1	P1-199	FALSE	3	TRA:CAMSDRMTGGFKTIF TRB:CASSRRGVTDTQYF	3	CD8-C7
PB-1_TCAGCTCAGGATCGCA	PB	PB-1	P1-14	TRUE	3	TRA:CAASVVNDMRF TRB:CASSHRGGAKNIQYF	109	CD8-C7
PB-1_TCAGCTCAGTATCTCG	PB	PB-1	P1-1	TRUE	3	TRA:CAMSDRMTGGFKTIF TRB:CASSQVGRGGEETQYF	1047	CD8-C7
PB-1_TCAGCTCCAAGGTTTC	PB	PB-1	P1-1	TRUE	3	TRA:CAMSDRMTGGFKTIF TRB:CASSQVGRGGEETQYF	1047	CD8-C7
PB-1_TCAGCTCCACAACTGT	PB	PB-1	P1-1	TRUE	3	TRA:CAMSDRMTGGFKTIF TRB:CASSQVGRGGEETQYF	1047	CD8-C7
PB-1_TCAGCTCCACGCCAGT	PB	PB-1	P1-26	TRUE	3	TRA:CAPRSGGSYIPTF TRB:CASSQDLGLDYGYTF	56	CD8-C7
PB-1_TCAGCTCGTATCACCA	PB	PB-1	P1-1	TRUE	3	TRA:CAMSDRMTGGFKTIF TRB:CASSQVGRGGEETQYF	1047	CD8-C5
PB-1_TCAGGATAGCAGGCTA	PB	PB-1	P1-1	TRUE	3	TRA:CAMSDRMTGGFKTIF TRB:CASSQVGRGGEETQYF	1047	CD8-C7
PB-1_TCAGGATGTCGCCATG	PB	PB-1	P1-3	TRUE	3	TRA:CAFLSGTYKYIF TRB:CASSQSPGQRNYGYTF	308	CD8-C7
PB-1_TCAGGATGTGATAAGT	PB	PB-1	P1-2	TRUE	3	TRA:CGQNFVF TRB:CASNQRGGPSLYNEQFF	323	CD8-C7
PB-1_TCAGGATTCCAAGCCG	PB	PB-1	P1-6	TRUE	3	TRA:CAMREGRERDDKIIF TRB:CASSLPNTEAFF	216	CD8-C7
PB-1_TCAGGTACACATTAGC	PB	PB-1	P1-26	TRUE	3	TRA:CAPRSGGSYIPTF TRB:CASSQDLGLDYGYTF	56	CD8-C5
PB-1_TCATTACAGCCAGGAT	PB	PB-1	P1-3	TRUE	3	TRA:CAFLSGTYKYIF TRB:CASSQSPGQRNYGYTF	308	CD8-C7
PB-1_TCATTACCAAGAGGCT	PB	PB-1	P1-1	TRUE	3	TRA:CAMSDRMTGGFKTIF TRB:CASSQVGRGGEETQYF	1047	CD8-C7
PB-1_TCATTACCACCGCTAG	PB	PB-1	P1-3	TRUE	3	TRA:CAFLSGTYKYIF TRB:CASSQSPGQRNYGYTF	308	CD8-C7
PB-1_TCATTACGTAAAGTCA	PB	PB-1	P1-976	FALSE	1	TRA:CALSEGRSSNDYKLSF TRB:CASSAEGSEETQYF	1	CD8-C2
PB-1_TCATTACGTTCGCTAA	PB	PB-1	P1-4	TRUE	3	TRA:CAMKGRDDKIIF TRB:CASTKGVSREAFF	226	CD8-C7
PB-1_TCATTACTCAACACCA	PB	PB-1	P1-13	TRUE	3	TRA:CVVMNYGGSQGNLIF TRB:CASSSWGDTDEAFF	112	CD8-C7
PB-1_TCATTACTCCATGAAC	PB	PB-1	P1-3	TRUE	3	TRA:CAFLSGTYKYIF TRB:CASSQSPGQRNYGYTF	308	CD8-C7
PB-1_TCATTTGAGGCAGGTT	PB	PB-1	P1-8	TRUE	3	TRA:CAVRPPGRNSGGYQKVTF TRB:CASSRRGVTDTQYF	204	CD8-C7
PB-1_TCATTTGAGGCGTACA	PB	PB-1	P1-5	TRUE	3	TRA:CALSGTPMNRDDKIIF TRB:CSARGRGLAGGPAYEQYF	223	CD8-C7
PB-1_TCATTTGAGTCATGCT	PB	PB-1	P1-2	TRUE	3	TRA:CGQNFVF TRB:CASNQRGGPSLYNEQFF	323	CD8-C5
PB-1_TCATTTGCAATGAATG	PB	PB-1	P1-14	TRUE	3	TRA:CAASVVNDMRF TRB:CASSHRGGAKNIQYF	109	CD8-C7
PB-1_TCATTTGCATGAAGTA	PB	PB-1	P1-1	TRUE	3	TRA:CAMSDRMTGGFKTIF TRB:CASSQVGRGGEETQYF	1047	CD8-C7
PB-1_TCATTTGGTCTTCTCG	PB	PB-1	P1-2	TRUE	3	TRA:CGQNFVF TRB:CASNQRGGPSLYNEQFF	323	CD8-C7
PB-1_TCATTTGTCCAAAGTC	PB	PB-1	P1-3	TRUE	3	TRA:CAFLSGTYKYIF TRB:CASSQSPGQRNYGYTF	308	CD8-C7

PB-1_TCCACACCAAGCTGAG	PB	PB-1	P1-13	TRUE	3	TRA:CVVMNYGGSQGNLIF TRB:CASSSWGDTDEAFF	112	CD8-C7
PB-1_TCCACACCACCACCAG	PB	PB-1	P1-47	TRUE	3	TRA:CIVGGNFNKFYF TRB:CASSPEWDTDTQYF	18	CD8-C5
PB-1_TCCACACCAGCTTCGG	PB	PB-1	P1-9	TRUE	3	TRA:CAEAEGGGADGLTF TRB:CATSGGDNEQFF	174	CD8-C7
PB-1_TCCACACGTCGTGGCT	PB	PB-1	P1-1	TRUE	3	TRA:CAMSDRMTGGFKTIF TRB:CASSQVGRGGEETQYF	1047	CD8-C7
PB-1_TCCACACTCCAGAAGG	PB	PB-1	P1-3	TRUE	3	TRA:CAFLSGTYKYIF TRB:CASSQSPGQRNYGYTF	308	CD8-C7
PB-1_TCCACACTCTGTTGAG	PB	PB-1	P1-11	TRUE	3	TRA:CAVEPLYGSGNTGKLIF TRB:CASSSHGQLNQPQHF	147	CD8-C7
PB-1_TCCCGATAGGCATGGT	PB	PB-1	P1-3	TRUE	3	TRA:CAFLSGTYKYIF TRB:CASSQSPGQRNYGYTF	308	CD8-C7
PB-1_TCCCGATCAGGTTTCA	PB	PB-1	P1-10	TRUE	3	TRA:CISNSPMDSSYKLIF TRB:CASSSWGDTDEAFF	162	CD4-C6
PB-1_TCCCGATCATCTACGA	PB	PB-1	P1-14	TRUE	3	TRA:CAASVVNDMRF TRB:CASSHRGGAKNIQYF	109	CD8-C5
PB-1_TCCCGATTCCAAACAC	PB	PB-1	P1-2	TRUE	3	TRA:CGQNFVF TRB:CASNQRGGPSLYNEQFF	323	CD8-C7
PB-1_TCCCGATTCGGAAATA	PB	PB-1	P1-5	TRUE	3	TRA:CALSGTPMNRDDKIIF TRB:CSARGRGLAGGPAYEQYF	223	CD8-C7
PB-1_TCCCGATTCTGTCTCG	PB	PB-1	P1-1	TRUE	3	TRA:CAMSDRMTGGFKTIF TRB:CASSQVGRGGEETQYF	1047	CD8-C7
PB-1_TCCCGATTCTTCAACT	PB	PB-1	P1-4	TRUE	3	TRA:CAMKGRDDKIIF TRB:CASTKGVSREAFF	226	CD8-C7
PB-1_TCGAGGCCATTCGACA	PB	PB-1	P1-1	TRUE	3	TRA:CAMSDRMTGGFKTIF TRB:CASSQVGRGGEETQYF	1047	CD8-C7
PB-1_TCGAGGCGTGAACCTT	PB	PB-1	P1-1	TRUE	3	TRA:CAMSDRMTGGFKTIF TRB:CASSQVGRGGEETQYF	1047	CD8-C7
PB-1_TCGAGGCGTGCGAAAC	PB	PB-1	P1-26	TRUE	3	TRA:CAPRSGGSYIPTF TRB:CASSQDLGLDYGYTF	56	CD8-C7
PB-1_TCGAGGCTCCACTCCA	PB	PB-1	P1-1	TRUE	3	TRA:CAMSDRMTGGFKTIF TRB:CASSQVGRGGEETQYF	1047	CD8-C7
PB-1_TCGCGAGAGTGAACAT	PB	PB-1	P1-1	TRUE	3	TRA:CAMSDRMTGGFKTIF TRB:CASSQVGRGGEETQYF	1047	CD8-C7
PB-1_TCGCGAGAGTGCTGCC	PB	PB-1	P1-3	TRUE	3	TRA:CAFLSGTYKYIF TRB:CASSQSPGQRNYGYTF	308	CD8-C7
PB-1_TCGCGAGCAACGATCT	PB	PB-1	P1-983	FALSE	1	TRA:CAVVKGTGAQKLVF TRB:CASSSTLGTEAFF	1	CD8-C7
PB-1_TCGCGAGCATGTCCTC	PB	PB-1	P1-14	TRUE	3	TRA:CAASVVNDMRF TRB:CASSHRGGAKNIQYF	109	CD8-C5
PB-1_TCGCGAGGTCTCATCC	PB	PB-1	P1-2	TRUE	3	TRA:CGQNFVF TRB:CASNQRGGPSLYNEQFF	323	CD8-C7
PB-1_TCGCGTTAGAGCTATA	PB	PB-1	P1-11	TRUE	3	TRA:CAVEPLYGSGNTGKLIF TRB:CASSSHGQLNQPQHF	147	CD8-C7
PB-1_TCGCGTTCATGCGCAC	PB	PB-1	P1-115	FALSE	3	TRA:CAVKGSSNTGKLIF TRB:CASSYSYGRTGTGTYEQYF	5	CD8-C7
PB-1_TCGCGTTCATGCTGGC	PB	PB-1	P1-1	TRUE	3	TRA:CAMSDRMTGGFKTIF TRB:CASSQVGRGGEETQYF	1047	CD8-C7
PB-1_TCGCGTTTCATTGCGA	PB	PB-1	P1-29	TRUE	3	TRA:CATVSFSGGYNKLIF TRB:CASSLDRRGLSYEQYF	50	CD8-C7
PB-1_TCGCGTTTCCTTTCTC	PB	PB-1	P1-1	TRUE	3	TRA:CAMSDRMTGGFKTIF TRB:CASSQVGRGGEETQYF	1047	CD8-C7

PB-1_TCGGGACCACGCATCG	PB	PB-1	P1-10	TRUE	3	TRA:CISNSPMDSSYKLIF TRB:CASSSWGDTDEAFF	162	CD8-C7
PB-1_TCGGGACGTCGCGTGT	PB	PB-1	P1-10	TRUE	3	TRA:CISNSPMDSSYKLIF TRB:CASSSWGDTDEAFF	162	CD8-C7
PB-1_TCGGGACTCGGCGCAT	PB	PB-1	P1-3	TRUE	3	TRA:CAFLSGTYKYIF TRB:CASSQSPGQRNYGYTF	308	CD8-C7
PB-1_TCGGGACTCTCTTGAT	PB	PB-1	P1-989	FALSE	1	TRA:CLVGDGYGQNFVF TRB:CASSLRTINEKLFF	1	CD8-C7
PB-1_TCGGTAAAGTCGATAA	PB	PB-1	P1-3	TRUE	3	TRA:CAFLSGTYKYIF TRB:CASSQSPGQRNYGYTF	308	CD8-C7
PB-1_TCGGTAACAGTCAGCC	PB	PB-1	P1-3	TRUE	3	TRA:CAFLSGTYKYIF TRB:CASSQSPGQRNYGYTF	308	CD8-C7
PB-1_TCGGTAAGTCCGAACC	PB	PB-1	P1-1	TRUE	3	TRA:CAMSDRMTGGFKTIF TRB:CASSQVGRGGEETQYF	1047	CD8-C7
PB-1_TCGGTAATCGAGCCCA	PB	PB-1	P1-18	TRUE	3	TRA:CVADGNYGQNFVF TRB:CASSSPGLGGGFDTEAFF	75	CD8-C6
PB-1_TCGGTAATCTGGGCCA	PB	PB-1	P1-1	TRUE	3	TRA:CAMSDRMTGGFKTIF TRB:CASSQVGRGGEETQYF	1047	CD8-C5
PB-1_TCGTACCAGGGTCTCC	PB	PB-1	P1-35	TRUE	3	TRA:CAVSDRGYFGNEKLTF TRB:CSALDDVRPVIAVYNEQFF	40	CD8-C6
PB-1_TCGTACCCACACAGAG	PB	PB-1	P1-26	TRUE	3	TRA:CAPRSGGSYIPTF TRB:CASSQDLGLDYGYTF	56	CD8-C7
PB-1_TCGTACCCACAGACAG	PB	PB-1	P1-8	TRUE	3	TRA:CAVRPPGRNSGGYQKVTF TRB:CASSRRGVTDTQYF	204	CD8-C7
PB-1_TCGTACCTCTCTTGAT	PB	PB-1	P1-2	TRUE	3	TRA:CGQNFVF TRB:CASNQRGGPSLYNEQFF	323	CD8-C7
PB-1_TCGTACCTCTGTTGAG	PB	PB-1	P1-22	TRUE	3	TRA:CASNSGYSTLTF TRB:CASSQVTGGARDEQYF	65	CD8-C7
PB-1_TCGTAGAAGGCAGGTT	PB	PB-1	P1-26	TRUE	3	TRA:CAPRSGGSYIPTF TRB:CASSQDLGLDYGYTF	56	CD8-C7
PB-1_TCGTAGAAGGGTGTTG	PB	PB-1	P1-47	TRUE	3	TRA:CIVGGNFNKFYF TRB:CASSPEWDTDTQYF	18	CD8-C5
PB-1_TCGTAGAAGTGTCCCG	PB	PB-1	P1-13	TRUE	3	TRA:CVVMNYGGSQGNLIF TRB:CASSSWGDTDEAFF	112	CD8-C7
PB-1_TCGTAGAAGTTGAGAT	PB	PB-1	P1-26	TRUE	3	TRA:CAPRSGGSYIPTF TRB:CASSQDLGLDYGYTF	56	CD8-C5
PB-1_TCGTAGACAGCGTCCA	PB	PB-1	P1-1	TRUE	3	TRA:CAMSDRMTGGFKTIF TRB:CASSQVGRGGEETQYF	1047	CD8-C7
PB-1_TCGTAGACATTCGACA	PB	PB-1	P1-1	TRUE	3	TRA:CAMSDRMTGGFKTIF TRB:CASSQVGRGGEETQYF	1047	CD8-C7
PB-1_TCGTAGAGTCTAGTGT	PB	PB-1	P1-5	TRUE	3	TRA:CALSGTPMNRDDKIIF TRB:CSARGRGLAGGPAYEQYF	223	CD8-C7
PB-1_TCGTAGAGTGTAACGG	PB	PB-1	P1-1	TRUE	3	TRA:CAMSDRMTGGFKTIF TRB:CASSQVGRGGEETQYF	1047	CD8-C7
PB-1_TCTATTGAGGCATGTG	PB	PB-1	P1-3	TRUE	3	TRA:CAFLSGTYKYIF TRB:CASSQSPGQRNYGYTF	308	CD8-C7
PB-1_TCTATTGAGTGGAGTC	PB	PB-1	P1-2	TRUE	3	TRA:CGQNFVF TRB:CASNQRGGPSLYNEQFF	323	CD8-C7
PB-1_TCTATTGCATTAGGCT	PB	PB-1	P1-40	TRUE	3	TRA:CAVRDVGNTGFQKLVF TRB:CASSLGQGSMKTQYF	27	CD8-C7
PB-1_TCTATTGGTCCTAGCG	PB	PB-1	P1-12	TRUE	3	TRA:CALSDERGDYKLSF TRB:CASSPGTGSYNEQFF	141	CD8-C6
PB-1_TCTATTGGTGAAGGCT	PB	PB-1	P1-2	TRUE	3	TRA:CGQNFVF TRB:CASNQRGGPSLYNEQFF	323	CD8-C7

PB-1_TCTATTGTCGGTGTTA	PB	PB-1	P1-9	TRUE	3	TRA:CAEAEGGGADGLTF TRB:CATSGGDNEQFF	174	CD8-C7
PB-1_TCTCTAAAGAGTAAGG	PB	PB-1	P1-8	TRUE	3	TRA:CAVRPPGRNSGGYQKVTF TRB:CASSRRGVTDTQYF	204	CD8-C7
PB-1_TCTCTAAAGGGTTCCC	PB	PB-1	P1-1	TRUE	3	TRA:CAMSDRMTGGFKTIF TRB:CASSQVGRGGEETQYF	1047	CD8-C7
PB-1_TCTCTAACAGATCTGT	PB	PB-1	P1-2	TRUE	3	TRA:CGQNFVF TRB:CASNQRGGPSLYNEQFF	323	CD8-C7
PB-1_TCTCTAACATGAGCGA	PB	PB-1	P1-1	TRUE	3	TRA:CAMSDRMTGGFKTIF TRB:CASSQVGRGGEETQYF	1047	CD8-C7
PB-1_TCTCTAAGTCTGCGGT	PB	PB-1	P1-3	TRUE	3	TRA:CAFLSGTYKYIF TRB:CASSQSPGQRNYGYTF	308	CD8-C7
PB-1_TCTCTAATCATGCTCC	PB	PB-1	P1-5	TRUE	3	TRA:CALSGTPMNRDDKIIF TRB:CSARGRGLAGGPAYEQYF	223	CD8-C5
PB-1_TCTGAGACATATACGC	PB	PB-1	P1-15	TRUE	3	TRA:CAVNSSPLGANSKLTF TRB:CASSDSGNFYNEQFF	92	CD8-C2
PB-1_TCTGAGAGTGTGTGCC	PB	PB-1	P1-8	TRUE	3	TRA:CAVRPPGRNSGGYQKVTF TRB:CASSRRGVTDTQYF	204	CD8-C7
PB-1_TCTGAGATCATGTGGT	PB	PB-1	P1-22	TRUE	3	TRA:CASNSGYSTLTF TRB:CASSQVTGGARDEQYF	65	CD8-C5
PB-1_TCTGAGATCGACAGCC	PB	PB-1	P1-11	TRUE	3	TRA:CAVEPLYGSGNTGKLIF TRB:CASSSHGQLNQPQHF	147	CD8-C7
PB-1_TCTGAGATCTAACTCT	PB	PB-1	P1-2	TRUE	3	TRA:CGQNFVF TRB:CASNQRGGPSLYNEQFF	323	CD8-C7
PB-1_TCTGGAAAGAAGGTGA	PB	PB-1	P1-2	TRUE	3	TRA:CGQNFVF TRB:CASNQRGGPSLYNEQFF	323	CD8-C7
PB-1_TCTGGAACACTCTGTC	PB	PB-1	P1-8	TRUE	3	TRA:CAVRPPGRNSGGYQKVTF TRB:CASSRRGVTDTQYF	204	CD8-C5
PB-1_TCTGGAAGTCACTGGC	PB	PB-1	P1-31	TRUE	3	TRA:CAVEEGADRLMF TRB:CASSLLGGGELFF	46	CD8-C7
PB-1_TCTGGAAGTTGGTTTG	PB	PB-1	P1-11	TRUE	3	TRA:CAVEPLYGSGNTGKLIF TRB:CASSSHGQLNQPQHF	147	CD8-C5
PB-1_TCTTCGGAGGTAGCCA	PB	PB-1	P1-3	TRUE	3	TRA:CAFLSGTYKYIF TRB:CASSQSPGQRNYGYTF	308	CD8-C7
PB-1_TCTTCGGAGTGGGTTG	PB	PB-1	P1-10	TRUE	3	TRA:CISNSPMDSSYKLIF TRB:CASSSWGDTDEAFF	162	CD8-C7
PB-1_TCTTCGGCAGGGTTAG	PB	PB-1	P1-1	TRUE	3	TRA:CAMSDRMTGGFKTIF TRB:CASSQVGRGGEETQYF	1047	CD8-C7
PB-1_TCTTCGGGTCCTGCTT	PB	PB-1	P1-6	TRUE	3	TRA:CAMREGRERDDKIIF TRB:CASSLPNTEAFF	216	CD8-C5
PB-1_TCTTCGGGTGCGCTTG	PB	PB-1	P1-1	TRUE	3	TRA:CAMSDRMTGGFKTIF TRB:CASSQVGRGGEETQYF	1047	CD8-C5
PB-1_TCTTCGGGTTCGGCAC	PB	PB-1	P1-1	TRUE	3	TRA:CAMSDRMTGGFKTIF TRB:CASSQVGRGGEETQYF	1047	CD8-C7
PB-1_TCTTTCCCACGAAAGC	PB	PB-1	P1-3	TRUE	3	TRA:CAFLSGTYKYIF TRB:CASSQSPGQRNYGYTF	308	CD8-C7
PB-1_TCTTTCCGTCAGTGGA	PB	PB-1	P1-3	TRUE	3	TRA:CAFLSGTYKYIF TRB:CASSQSPGQRNYGYTF	308	CD8-C7
PB-1_TGAAAGAAGCCACTAT	PB	PB-1	P1-2	TRUE	3	TRA:CGQNFVF TRB:CASNQRGGPSLYNEQFF	323	CD8-C7
PB-1_TGAAAGAGTCTCCCTA	PB	PB-1	P1-2	TRUE	3	TRA:CGQNFVF TRB:CASNQRGGPSLYNEQFF	323	CD8-C7
PB-1_TGAAAGAGTGTGCCTG	PB	PB-1	P1-26	TRUE	3	TRA:CAPRSGGSYIPTF TRB:CASSQDLGLDYGYTF	56	CD8-C7

PB-1_TGACAACAGGCAGGTT	PB	PB-1	P1-1	TRUE	3	TRA:CAMSDRMTGGFKTIF TRB:CASSQVGRGGEETQYF	1047	CD8-C5
PB-1_TGACAACAGTAAGTAC	PB	PB-1	P1-11	TRUE	3	TRA:CAVEPLYGSGNTGKLIF TRB:CASSSHGQLNQPQHF	147	CD8-C7
PB-1_TGACAACCAACCGCCA	PB	PB-1	P1-15	TRUE	3	TRA:CAVNSSPLGANSKLTF TRB:CASSDSGNFYNEQFF	92	CD8-C2
PB-1_TGACAACCAGGGCATA	PB	PB-1	P1-47	TRUE	3	TRA:CIVGGNFNKFYF TRB:CASSPEWDTDTQYF	18	CD8-C5
PB-1_TGACAACGTAAGTAGT	PB	PB-1	P1-3	TRUE	3	TRA:CAFLSGTYKYIF TRB:CASSQSPGQRNYGYTF	308	CD8-C6
PB-1_TGACAACGTTGGTTTG	PB	PB-1	P1-29	TRUE	3	TRA:CATVSFSGGYNKLIF TRB:CASSLDRRGLSYEQYF	50	CD8-C7
PB-1_TGACAACTCCTTTCGG	PB	PB-1	P1-1	TRUE	3	TRA:CAMSDRMTGGFKTIF TRB:CASSQVGRGGEETQYF	1047	CD8-C7
PB-1_TGACGGCCACGAAGCA	PB	PB-1	P1-1	TRUE	3	TRA:CAMSDRMTGGFKTIF TRB:CASSQVGRGGEETQYF	1047	CD8-C7
PB-1_TGACGGCCAGCCTTGG	PB	PB-1	P1-3	TRUE	3	TRA:CAFLSGTYKYIF TRB:CASSQSPGQRNYGYTF	308	CD8-C7
PB-1_TGACGGCCATACGCCG	PB	PB-1	P1-35	TRUE	3	TRA:CAVSDRGYFGNEKLTF TRB:CSALDDVRPVIAVYNEQFF	40	CD8-C7
PB-1_TGACGGCGTTCTCATT	PB	PB-1	P1-14	TRUE	3	TRA:CAASVVNDMRF TRB:CASSHRGGAKNIQYF	109	CD8-C7
PB-1_TGACTAGGTATAGTAG	PB	PB-1	P1-29	TRUE	3	TRA:CATVSFSGGYNKLIF TRB:CASSLDRRGLSYEQYF	50	CD8-C7
PB-1_TGACTAGGTCGAGTTT	PB	PB-1	P1-3	TRUE	3	TRA:CAFLSGTYKYIF TRB:CASSQSPGQRNYGYTF	308	CD8-C7
PB-1_TGACTAGTCGTATCAG	PB	PB-1	P1-68	TRUE	3	TRA:CAVIPDILTGGGNKLTF TRB:CASSLQGFGNTIYF	9	CD8-C7
PB-1_TGACTAGTCTCGATGA	PB	PB-1	P1-3	TRUE	3	TRA:CAFLSGTYKYIF TRB:CASSQSPGQRNYGYTF	308	CD8-C7
PB-1_TGACTTTAGGGAAACA	PB	PB-1	P1-149	TRUE	3	TRA:CAVGSSGGSYIPTF TRB:CASSLRTINEKLFF	4	CD8-C7
PB-1_TGACTTTAGTTGTCGT	PB	PB-1	P1-6	TRUE	3	TRA:CAMREGRERDDKIIF TRB:CASSLPNTEAFF	216	CD8-C5
PB-1_TGACTTTCAGACAAGC	PB	PB-1	P1-10	TRUE	3	TRA:CISNSPMDSSYKLIF TRB:CASSSWGDTDEAFF	162	CD8-C7
PB-1_TGACTTTCAGGCTGAA	PB	PB-1	P1-3	TRUE	3	TRA:CAFLSGTYKYIF TRB:CASSQSPGQRNYGYTF	308	CD8-C7
PB-1_TGACTTTTCACGATGT	PB	PB-1	P1-10	TRUE	3	TRA:CISNSPMDSSYKLIF TRB:CASSSWGDTDEAFF	162	CD8-C7
PB-1_TGAGAGGAGAATAGGG	PB	PB-1	P1-5	TRUE	3	TRA:CALSGTPMNRDDKIIF TRB:CSARGRGLAGGPAYEQYF	223	CD8-C5
PB-1_TGAGAGGAGTTAACGA	PB	PB-1	P1-10	TRUE	3	TRA:CISNSPMDSSYKLIF TRB:CASSSWGDTDEAFF	162	CD8-C7
PB-1_TGAGAGGCAGTCTTCC	PB	PB-1	P1-1	TRUE	3	TRA:CAMSDRMTGGFKTIF TRB:CASSQVGRGGEETQYF	1047	CD8-C7
PB-1_TGAGAGGCATGCCTAA	PB	PB-1	P1-1	TRUE	3	TRA:CAMSDRMTGGFKTIF TRB:CASSQVGRGGEETQYF	1047	CD8-C7
PB-1_TGAGAGGGTCCGAAGA	PB	PB-1	P1-3	TRUE	3	TRA:CAFLSGTYKYIF TRB:CASSQSPGQRNYGYTF	308	CD8-C7
PB-1_TGAGAGGGTTAAAGAC	PB	PB-1	P1-2	TRUE	3	TRA:CGQNFVF TRB:CASNQRGGPSLYNEQFF	323	CD8-C7
PB-1_TGAGAGGTCATAAAGG	PB	PB-1	P1-5	TRUE	3	TRA:CALSGTPMNRDDKIIF TRB:CSARGRGLAGGPAYEQYF	223	CD8-C7

PB-1_TGAGAGGTCGCCTGAG	PB	PB-1	P1-1	TRUE	3	TRA:CAMSDRMTGGFKTIF TRB:CASSQVGRGGEETQYF	1047	CD8-C7
PB-1_TGAGCATAGACAGGCT	PB	PB-1	P1-1	TRUE	3	TRA:CAMSDRMTGGFKTIF TRB:CASSQVGRGGEETQYF	1047	CD8-C7
PB-1_TGAGCATCATTACCTT	PB	PB-1	P1-2	TRUE	3	TRA:CGQNFVF TRB:CASNQRGGPSLYNEQFF	323	CD8-C7
PB-1_TGAGCATCATTTCACT	PB	PB-1	P1-1010	FALSE	1	TRA:CALSEAGKPGRKAYLRT TRB:CASSQSWDLAVLNEQFF	1	CD8-C7
PB-1_TGAGCCGAGAAACCTA	PB	PB-1	P1-8	TRUE	3	TRA:CAVRPPGRNSGGYQKVTF TRB:CASSRRGVTDTQYF	204	CD8-C5
PB-1_TGAGCCGAGTTAGGTA	PB	PB-1	P1-6	TRUE	3	TRA:CAMREGRERDDKIIF TRB:CASSLPNTEAFF	216	CD8-C5
PB-1_TGAGCCGAGTTCGATC	PB	PB-1	P1-8	TRUE	3	TRA:CAVRPPGRNSGGYQKVTF TRB:CASSRRGVTDTQYF	204	CD8-C5
PB-1_TGAGCCGTCAAACCGT	PB	PB-1	P1-8	TRUE	3	TRA:CAVRPPGRNSGGYQKVTF TRB:CASSRRGVTDTQYF	204	CD8-C5
PB-1_TGAGCCGTCGTTACGA	PB	PB-1	P1-3	TRUE	3	TRA:CAFLSGTYKYIF TRB:CASSQSPGQRNYGYTF	308	CD8-C7
PB-1_TGAGGGAAGGGTATCG	PB	PB-1	P1-2	TRUE	3	TRA:CGQNFVF TRB:CASNQRGGPSLYNEQFF	323	CD8-C7
PB-1_TGAGGGAGTACCAGTT	PB	PB-1	P1-1	TRUE	3	TRA:CAMSDRMTGGFKTIF TRB:CASSQVGRGGEETQYF	1047	CD8-C5
PB-1_TGAGGGAGTACTCGCG	PB	PB-1	P1-3	TRUE	3	TRA:CAFLSGTYKYIF TRB:CASSQSPGQRNYGYTF	308	CD8-C7
PB-1_TGAGGGAGTCTGGAGA	PB	PB-1	P1-142	FALSE	3	TRA:CAVPYSSASKIIF TRB:CASSRTTGGSSYEQYF	4	CD8-C5
PB-1_TGAGGGATCATGGTCA	PB	PB-1	P1-1	TRUE	3	TRA:CAMSDRMTGGFKTIF TRB:CASSQVGRGGEETQYF	1047	CD8-C7
PB-1_TGAGGGATCCGCTGTT	PB	PB-1	P1-2	TRUE	3	TRA:CGQNFVF TRB:CASNQRGGPSLYNEQFF	323	CD8-C7
PB-1_TGATTTCGTCATACTG	PB	PB-1	P1-1	TRUE	3	TRA:CAMSDRMTGGFKTIF TRB:CASSQVGRGGEETQYF	1047	CD8-C5
PB-1_TGATTTCGTGCTGTAT	PB	PB-1	P1-8	TRUE	3	TRA:CAVRPPGRNSGGYQKVTF TRB:CASSRRGVTDTQYF	204	CD8-C7
PB-1_TGATTTCGTTGAACTC	PB	PB-1	P1-1	TRUE	3	TRA:CAMSDRMTGGFKTIF TRB:CASSQVGRGGEETQYF	1047	CD8-C5
PB-1_TGCACCTCAAGCCTAT	PB	PB-1	P1-10	TRUE	3	TRA:CISNSPMDSSYKLIF TRB:CASSSWGDTDEAFF	162	CD8-C7
PB-1_TGCACCTCACAGCCCA	PB	PB-1	P1-4	TRUE	3	TRA:CAMKGRDDKIIF TRB:CASTKGVSREAFF	226	CD8-C7
PB-1_TGCACCTGTTCCGGCA	PB	PB-1	P1-5	TRUE	3	TRA:CALSGTPMNRDDKIIF TRB:CSARGRGLAGGPAYEQYF	223	CD8-C7
PB-1_TGCACCTGTTGACGTT	PB	PB-1	P1-275	FALSE	2	TRA:CVADGNYGQNFVF TRB:CASSQSPGQRNYGYTF	2	CD8-C7
PB-1_TGCACCTGTTGTCGCG	PB	PB-1	P1-5	TRUE	3	TRA:CALSGTPMNRDDKIIF TRB:CSARGRGLAGGPAYEQYF	223	CD8-C7
PB-1_TGCACCTTCCAGATCA	PB	PB-1	P1-12	TRUE	3	TRA:CALSDERGDYKLSF TRB:CASSPGTGSYNEQFF	141	CD8-C5
PB-1_TGCACCTTCCCAAGTA	PB	PB-1	P1-3	TRUE	3	TRA:CAFLSGTYKYIF TRB:CASSQSPGQRNYGYTF	308	CD8-C7
PB-1_TGCCAAACAAGACGTG	PB	PB-1	P1-26	TRUE	3	TRA:CAPRSGGSYIPTF TRB:CASSQDLGLDYGYTF	56	CD8-C5
PB-1_TGCCAAAGTCGATTGT	PB	PB-1	P1-2	TRUE	3	TRA:CGQNFVF TRB:CASNQRGGPSLYNEQFF	323	CD8-C7

PB-1_TGCCAAATCATAAAGG	PB	PB-1	P1-1	TRUE	3	TRA:CAMSDRMTGGFKTIF TRB:CASSQVGRGGEETQYF	1047	CD8-C7
PB-1_TGCCAAATCCACTCCA	PB	PB-1	P1-1	TRUE	3	TRA:CAMSDRMTGGFKTIF TRB:CASSQVGRGGEETQYF	1047	CD8-C7
PB-1_TGCCAAATCCTAAGTG	PB	PB-1	P1-5	TRUE	3	TRA:CALSGTPMNRDDKIIF TRB:CSARGRGLAGGPAYEQYF	223	CD8-C7
PB-1_TGCCCATCAGACAGGT	PB	PB-1	P1-1	TRUE	3	TRA:CAMSDRMTGGFKTIF TRB:CASSQVGRGGEETQYF	1047	CD8-C7
PB-1_TGCCCATTCTCGATGA	PB	PB-1	P1-1	TRUE	3	TRA:CAMSDRMTGGFKTIF TRB:CASSQVGRGGEETQYF	1047	CD8-C7
PB-1_TGCCCTAAGCCATCGC	PB	PB-1	P1-2	TRUE	3	TRA:CGQNFVF TRB:CASNQRGGPSLYNEQFF	323	CD8-C7
PB-1_TGCCCTAAGTCGATAA	PB	PB-1	P1-2	TRUE	3	TRA:CGQNFVF TRB:CASNQRGGPSLYNEQFF	323	CD8-C7
PB-1_TGCCCTACAAGCCCAC	PB	PB-1	P1-1	TRUE	3	TRA:CAMSDRMTGGFKTIF TRB:CASSQVGRGGEETQYF	1047	CD8-C7
PB-1_TGCCCTACACAGACTT	PB	PB-1	P1-1	TRUE	3	TRA:CAMSDRMTGGFKTIF TRB:CASSQVGRGGEETQYF	1047	CD8-C7
PB-1_TGCCCTACAGTATCTG	PB	PB-1	P1-1	TRUE	3	TRA:CAMSDRMTGGFKTIF TRB:CASSQVGRGGEETQYF	1047	CD8-C7
PB-1_TGCCCTATCCAAACAC	PB	PB-1	P1-3	TRUE	3	TRA:CAFLSGTYKYIF TRB:CASSQSPGQRNYGYTF	308	CD8-C7
PB-1_TGCCCTATCTCTAGGA	PB	PB-1	P1-5	TRUE	3	TRA:CALSGTPMNRDDKIIF TRB:CSARGRGLAGGPAYEQYF	223	CD8-C7
PB-1_TGCCCTATCTTACCTA	PB	PB-1	P1-1	TRUE	3	TRA:CAMSDRMTGGFKTIF TRB:CASSQVGRGGEETQYF	1047	CD8-C7
PB-1_TGCGCAGAGACACGAC	PB	PB-1	P1-8	TRUE	3	TRA:CAVRPPGRNSGGYQKVTF TRB:CASSRRGVTDTQYF	204	CD8-C7
PB-1_TGCGCAGAGCTTTGGT	PB	PB-1	P1-4	TRUE	3	TRA:CAMKGRDDKIIF TRB:CASTKGVSREAFF	226	CD8-C7
PB-1_TGCGCAGAGGAATGGA	PB	PB-1	P1-5	TRUE	3	TRA:CALSGTPMNRDDKIIF TRB:CSARGRGLAGGPAYEQYF	223	CD8-C7
PB-1_TGCGCAGAGGGATGGG	PB	PB-1	P1-1	TRUE	3	TRA:CAMSDRMTGGFKTIF TRB:CASSQVGRGGEETQYF	1047	CD8-C7
PB-1_TGCGCAGAGTTATCGC	PB	PB-1	P1-3	TRUE	3	TRA:CAFLSGTYKYIF TRB:CASSQSPGQRNYGYTF	308	CD8-C7
PB-1_TGCGCAGGTCTTTCAT	PB	PB-1	P1-6	TRUE	3	TRA:CAMREGRERDDKIIF TRB:CASSLPNTEAFF	216	CD8-C5
PB-1_TGCGGGTCACGAAATA	PB	PB-1	P1-10	TRUE	3	TRA:CISNSPMDSSYKLIF TRB:CASSSWGDTDEAFF	162	CD8-C7
PB-1_TGCGGGTCACTAAGTC	PB	PB-1	P1-1	TRUE	3	TRA:CAMSDRMTGGFKTIF TRB:CASSQVGRGGEETQYF	1047	CD8-C7
PB-1_TGCGGGTGTTCGCTAA	PB	PB-1	P1-5	TRUE	3	TRA:CALSGTPMNRDDKIIF TRB:CSARGRGLAGGPAYEQYF	223	CD8-C7
PB-1_TGCGGGTTCGAGGTAG	PB	PB-1	P1-1	TRUE	3	TRA:CAMSDRMTGGFKTIF TRB:CASSQVGRGGEETQYF	1047	CD8-C7
PB-1_TGCGTGGAGTTTGCGT	PB	PB-1	P1-3	TRUE	3	TRA:CAFLSGTYKYIF TRB:CASSQSPGQRNYGYTF	308	CD8-C7
PB-1_TGCGTGGCACCAGATT	PB	PB-1	P1-5	TRUE	3	TRA:CALSGTPMNRDDKIIF TRB:CSARGRGLAGGPAYEQYF	223	CD8-C7
PB-1_TGCGTGGGTCGAATCT	PB	PB-1	P1-2	TRUE	3	TRA:CGQNFVF TRB:CASNQRGGPSLYNEQFF	323	CD8-C7
PB-1_TGCGTGGGTGTGAATA	PB	PB-1	P1-39	TRUE	3	TRA:CAVRDLGGTSYDKVIF TRB:CSARVREVSYEQYF	31	CD8-C7

PB-1_TGCTACCCAATAGCAA	PB	PB-1	P1-1026	FALSE	1	TRA:CAVQAGNNDMRF TRB:CASSQVTGGARDEQYF	1	CD8-C5
PB-1_TGCTACCCACAGACAG	PB	PB-1	P1-12	TRUE	3	TRA:CALSDERGDYKLSF TRB:CASSPGTGSYNEQFF	141	CD8-C7
PB-1_TGCTACCCAGAAGCAC	PB	PB-1	P1-14	TRUE	3	TRA:CAASVVNDMRF TRB:CASSHRGGAKNIQYF	109	CD8-C5
PB-1_TGCTACCGTAGCGCTC	PB	PB-1	P1-4	TRUE	3	TRA:CAMKGRDDKIIF TRB:CASTKGVSREAFF	226	CD8-C7
PB-1_TGCTGCTAGCTGCAAG	PB	PB-1	P1-1	TRUE	3	TRA:CAMSDRMTGGFKTIF TRB:CASSQVGRGGEETQYF	1047	CD8-C7
PB-1_TGCTGCTCAATAGCAA	PB	PB-1	P1-1	TRUE	3	TRA:CAMSDRMTGGFKTIF TRB:CASSQVGRGGEETQYF	1047	CD8-C7
PB-1_TGCTGCTTCAGCGATT	PB	PB-1	P1-7	TRUE	3	TRA:CAVQAGNNDMRF TRB:CASSTTGDFNYGYTF	215	CD8-C5
PB-1_TGCTGCTTCATATCGG	PB	PB-1	P1-1	TRUE	3	TRA:CAMSDRMTGGFKTIF TRB:CASSQVGRGGEETQYF	1047	CD8-C7
PB-1_TGCTGCTTCATGTGGT	PB	PB-1	P1-40	TRUE	3	TRA:CAVRDVGNTGFQKLVF TRB:CASSLGQGSMKTQYF	27	CD8-C7
PB-1_TGCTGCTTCTGCCCTA	PB	PB-1	P1-1	TRUE	3	TRA:CAMSDRMTGGFKTIF TRB:CASSQVGRGGEETQYF	1047	CD8-C7
PB-1_TGGACGCAGCGTCAAG	PB	PB-1	P1-29	TRUE	3	TRA:CATVSFSGGYNKLIF TRB:CASSLDRRGLSYEQYF	50	CD8-C7
PB-1_TGGACGCCAAGCGCTC	PB	PB-1	P1-1	TRUE	3	TRA:CAMSDRMTGGFKTIF TRB:CASSQVGRGGEETQYF	1047	CD8-C7
PB-1_TGGACGCGTCCGACGT	PB	PB-1	P1-4	TRUE	3	TRA:CAMKGRDDKIIF TRB:CASTKGVSREAFF	226	CD8-C5
PB-1_TGGCCAGGTCTAGTGT	PB	PB-1	P1-13	TRUE	3	TRA:CVVMNYGGSQGNLIF TRB:CASSSWGDTDEAFF	112	CD8-C7
PB-1_TGGCCAGTCGGCATCG	PB	PB-1	P1-18	TRUE	3	TRA:CVADGNYGQNFVF TRB:CASSSPGLGGGFDTEAFF	75	CD8-C6
PB-1_TGGCCAGTCTTCGAGA	PB	PB-1	P1-1	TRUE	3	TRA:CAMSDRMTGGFKTIF TRB:CASSQVGRGGEETQYF	1047	CD8-C7
PB-1_TGGCGCACACTTAACG	PB	PB-1	P1-4	TRUE	3	TRA:CAMKGRDDKIIF TRB:CASTKGVSREAFF	226	CD8-C5
PB-1_TGGCGCATCAACACGT	PB	PB-1	P1-29	TRUE	3	TRA:CATVSFSGGYNKLIF TRB:CASSLDRRGLSYEQYF	50	CD8-C7
PB-1_TGGCGCATCCGCGGTA	PB	PB-1	P1-9	TRUE	3	TRA:CAEAEGGGADGLTF TRB:CATSGGDNEQFF	174	CD8-C7
PB-1_TGGCGCATCGGCTACG	PB	PB-1	P1-58	TRUE	3	TRA:CAVRPNRDDKIIF TRB:CASSSRLAGGTDTQYF	12	CD8-C7
PB-1_TGGCGCATCTGGTTCC	PB	PB-1	P1-24	TRUE	3	TRA:CAVKGNTDKLIF TRB:CASSLRTGAYNEQFF	62	CD4-C6
PB-1_TGGCTGGAGGATGCGT	PB	PB-1	P1-16	TRUE	3	TRA:CALSGTPMNRDDKIIF TRB:CASSQSWDLAVLNEQFF	79	CD8-C7
PB-1_TGGCTGGCACCCATGG	PB	PB-1	P1-12	TRUE	3	TRA:CALSDERGDYKLSF TRB:CASSPGTGSYNEQFF	141	CD8-C5
PB-1_TGGCTGGCAGGTCCAC	PB	PB-1	P1-14	TRUE	3	TRA:CAASVVNDMRF TRB:CASSHRGGAKNIQYF	109	CD8-C7
PB-1_TGGCTGGCATGTCCTC	PB	PB-1	P1-5	TRUE	3	TRA:CALSGTPMNRDDKIIF TRB:CSARGRGLAGGPAYEQYF	223	CD8-C7
PB-1_TGGCTGGTCACAAACC	PB	PB-1	P1-14	TRUE	3	TRA:CAASVVNDMRF TRB:CASSHRGGAKNIQYF	109	CD8-C7
PB-1_TGGCTGGTCTTGGGTA	PB	PB-1	P1-3	TRUE	3	TRA:CAFLSGTYKYIF TRB:CASSQSPGQRNYGYTF	308	CD8-C7

PB-1_TGGGAAGAGATAGGAG	PB	PB-1	P1-1	TRUE	3	TRA:CAMSDRMTGGFKTIF TRB:CASSQVGRGGEETQYF	1047	CD8-C7
PB-1_TGGGAAGCAGATCCAT	PB	PB-1	P1-1	TRUE	3	TRA:CAMSDRMTGGFKTIF TRB:CASSQVGRGGEETQYF	1047	CD8-C7
PB-1_TGGGCGTCATACGCTA	PB	PB-1	P1-26	TRUE	3	TRA:CAPRSGGSYIPTF TRB:CASSQDLGLDYGYTF	56	CD8-C7
PB-1_TGGGCGTTCAAGATCC	PB	PB-1	P1-22	TRUE	3	TRA:CASNSGYSTLTF TRB:CASSQVTGGARDEQYF	65	CD8-C5
PB-1_TGGTTAGTCACCACCT	PB	PB-1	P1-1	TRUE	3	TRA:CAMSDRMTGGFKTIF TRB:CASSQVGRGGEETQYF	1047	CD8-C7
PB-1_TGGTTAGTCAGTGCAT	PB	PB-1	P1-3	TRUE	3	TRA:CAFLSGTYKYIF TRB:CASSQSPGQRNYGYTF	308	CD8-C7
PB-1_TGGTTCCGTACAAGTA	PB	PB-1	P1-1	TRUE	3	TRA:CAMSDRMTGGFKTIF TRB:CASSQVGRGGEETQYF	1047	CD8-C7
PB-1_TGGTTCCGTTCAGCGC	PB	PB-1	P1-13	TRUE	3	TRA:CVVMNYGGSQGNLIF TRB:CASSSWGDTDEAFF	112	CD8-C7
PB-1_TGGTTCCTCAGCTTAG	PB	PB-1	P1-4	TRUE	3	TRA:CAMKGRDDKIIF TRB:CASTKGVSREAFF	226	CD8-C7
PB-1_TGGTTCCTCGCCAAAT	PB	PB-1	P1-1	TRUE	3	TRA:CAMSDRMTGGFKTIF TRB:CASSQVGRGGEETQYF	1047	CD8-C5
PB-1_TGGTTCCTCTGCAAGT	PB	PB-1	P1-1	TRUE	3	TRA:CAMSDRMTGGFKTIF TRB:CASSQVGRGGEETQYF	1047	CD8-C7
PB-1_TGTATTCAGTCACGCC	PB	PB-1	P1-144	FALSE	3	TRA:CAMKGRDDKIIF TRB:CASSQVGRGGEETQYF	4	CD8-C5
PB-1_TGTATTCCAGTCCTTC	PB	PB-1	P1-1	TRUE	3	TRA:CAMSDRMTGGFKTIF TRB:CASSQVGRGGEETQYF	1047	CD8-C7
PB-1_TGTATTCGTACCGAGA	PB	PB-1	P1-148	FALSE	3	TRA:CISNSPMDSSYKLIF TRB:CASSQSPGQRNYGYTF	4	CD8-C7
PB-1_TGTATTCGTGATAAGT	PB	PB-1	P1-1	TRUE	3	TRA:CAMSDRMTGGFKTIF TRB:CASSQVGRGGEETQYF	1047	CD8-C7
PB-1_TGTATTCTCGAGGTAG	PB	PB-1	P1-1039	FALSE	1	TRA:CAMSDRMTGGFKTIF TRB:CASSPGTGSYNEQFF	1	CD8-C7
PB-1_TGTATTCTCGCCAAAT	PB	PB-1	P1-3	TRUE	3	TRA:CAFLSGTYKYIF TRB:CASSQSPGQRNYGYTF	308	CD8-C7
PB-1_TGTCCCAGTGGTTTCA	PB	PB-1	P1-1	TRUE	3	TRA:CAMSDRMTGGFKTIF TRB:CASSQVGRGGEETQYF	1047	CD8-C7
PB-1_TGTCCCAGTTGGGACA	PB	PB-1	P1-1	TRUE	3	TRA:CAMSDRMTGGFKTIF TRB:CASSQVGRGGEETQYF	1047	CD8-C7
PB-1_TGTCCCATCGCGATCG	PB	PB-1	P1-13	TRUE	3	TRA:CVVMNYGGSQGNLIF TRB:CASSSWGDTDEAFF	112	CD8-C7
PB-1_TGTGGTACAGGTTTCA	PB	PB-1	P1-3	TRUE	3	TRA:CAFLSGTYKYIF TRB:CASSQSPGQRNYGYTF	308	CD8-C6
PB-1_TGTGTTTCATGTCTCC	PB	PB-1	P1-1	TRUE	3	TRA:CAMSDRMTGGFKTIF TRB:CASSQVGRGGEETQYF	1047	CD8-C7
PB-1_TGTGTTTGTCCAGTAT	PB	PB-1	P1-6	TRUE	3	TRA:CAMREGRERDDKIIF TRB:CASSLPNTEAFF	216	CD8-C5
PB-1_TGTTCCGAGCTGCGAA	PB	PB-1	P1-1	TRUE	3	TRA:CAMSDRMTGGFKTIF TRB:CASSQVGRGGEETQYF	1047	CD8-C7
PB-1_TGTTCCGAGGGATCTG	PB	PB-1	P1-1047	FALSE	1	TRA:CALVDFSGYSTLTF TRB:CASSQVTGGARDEQYF	1	CD4-C6
PB-1_TGTTCCGAGTTGTCGT	PB	PB-1	P1-3	TRUE	3	TRA:CAFLSGTYKYIF TRB:CASSQSPGQRNYGYTF	308	CD8-C7
PB-1_TGTTCCGCACATGACT	PB	PB-1	P1-1	TRUE	3	TRA:CAMSDRMTGGFKTIF TRB:CASSQVGRGGEETQYF	1047	CD8-C7

PB-1_TGTTCCGCATTAGCCA	PB	PB-1	P1-1	TRUE	3	TRA:CAMSDRMTGGFKTIF TRB:CASSQVGRGGEETQYF	1047	CD8-C7
PB-1_TGTTCCGGTCAACATC	PB	PB-1	P1-1	TRUE	3	TRA:CAMSDRMTGGFKTIF TRB:CASSQVGRGGEETQYF	1047	CD8-C5
PB-1_TGTTCCGGTCTAACGT	PB	PB-1	P1-2	TRUE	3	TRA:CGQNFVF TRB:CASNQRGGPSLYNEQFF	323	CD8-C7
PB-1_TGTTCCGTCTATGTGG	PB	PB-1	P1-26	TRUE	3	TRA:CAPRSGGSYIPTF TRB:CASSQDLGLDYGYTF	56	CD8-C7
PB-1_TTAACTCCAAGACGTG	PB	PB-1	P1-10	TRUE	3	TRA:CISNSPMDSSYKLIF TRB:CASSSWGDTDEAFF	162	CD4-C6
PB-1_TTAACTCCATTCACTT	PB	PB-1	P1-1	TRUE	3	TRA:CAMSDRMTGGFKTIF TRB:CASSQVGRGGEETQYF	1047	CD8-C7
PB-1_TTAACTCTCCGTCAAA	PB	PB-1	P1-50	TRUE	3	TRA:CARWLGSQGNLIF TRB:CASSLTDRVPQFELVYEQYF	15	CD8-C5
PB-1_TTAACTCTCTCACATT	PB	PB-1	P1-1	TRUE	3	TRA:CAMSDRMTGGFKTIF TRB:CASSQVGRGGEETQYF	1047	CD8-C7
PB-1_TTAGGACCATAAAGGT	PB	PB-1	P1-2	TRUE	3	TRA:CGQNFVF TRB:CASNQRGGPSLYNEQFF	323	CD8-C7
PB-1_TTAGGACGTAAATGAC	PB	PB-1	P1-4	TRUE	3	TRA:CAMKGRDDKIIF TRB:CASTKGVSREAFF	226	CD8-C7
PB-1_TTAGGACTCTTAGAGC	PB	PB-1	P1-4	TRUE	3	TRA:CAMKGRDDKIIF TRB:CASTKGVSREAFF	226	CD8-C7
PB-1_TTAGGCAAGAAAGTGG	PB	PB-1	P1-4	TRUE	3	TRA:CAMKGRDDKIIF TRB:CASTKGVSREAFF	226	CD8-C7
PB-1_TTAGGCAAGATACACA	PB	PB-1	P1-1	TRUE	3	TRA:CAMSDRMTGGFKTIF TRB:CASSQVGRGGEETQYF	1047	CD8-C7
PB-1_TTAGGCAAGGTGTTAA	PB	PB-1	P1-18	TRUE	3	TRA:CVADGNYGQNFVF TRB:CASSSPGLGGGFDTEAFF	75	CD8-C6
PB-1_TTAGGCAAGTAGATGT	PB	PB-1	P1-1	TRUE	3	TRA:CAMSDRMTGGFKTIF TRB:CASSQVGRGGEETQYF	1047	CD8-C7
PB-1_TTAGGCACAGATAATG	PB	PB-1	P1-1	TRUE	3	TRA:CAMSDRMTGGFKTIF TRB:CASSQVGRGGEETQYF	1047	CD8-C7
PB-1_TTAGGCACAGGCTGAA	PB	PB-1	P1-2	TRUE	3	TRA:CGQNFVF TRB:CASNQRGGPSLYNEQFF	323	CD8-C7
PB-1_TTAGGCACATGTCTCC	PB	PB-1	P1-3	TRUE	3	TRA:CAFLSGTYKYIF TRB:CASSQSPGQRNYGYTF	308	CD8-C7
PB-1_TTAGGCAGTAAGTGGC	PB	PB-1	P1-5	TRUE	3	TRA:CALSGTPMNRDDKIIF TRB:CSARGRGLAGGPAYEQYF	223	CD8-C5
PB-1_TTAGGCAGTCTCTTTA	PB	PB-1	P1-16	TRUE	3	TRA:CALSGTPMNRDDKIIF TRB:CASSQSWDLAVLNEQFF	79	CD8-C5
PB-1_TTAGGCATCGAGCCCA	PB	PB-1	P1-1	TRUE	3	TRA:CAMSDRMTGGFKTIF TRB:CASSQVGRGGEETQYF	1047	CD8-C5
PB-1_TTAGGCATCGGCTTGG	PB	PB-1	P1-2	TRUE	3	TRA:CGQNFVF TRB:CASNQRGGPSLYNEQFF	323	CD8-C7
PB-1_TTAGGCATCTGTCTAT	PB	PB-1	P1-3	TRUE	3	TRA:CAFLSGTYKYIF TRB:CASSQSPGQRNYGYTF	308	CD8-C7
PB-1_TTAGTTCAGAGACTAT	PB	PB-1	P1-50	TRUE	3	TRA:CARWLGSQGNLIF TRB:CASSLTDRVPQFELVYEQYF	15	CD8-C5
PB-1_TTAGTTCTCTCTGCTG	PB	PB-1	P1-1052	FALSE	1	TRA:CAASRGTGANNLFF TRB:CASSQSWDLAVLNEQFF	1	CD4-C6
PB-1_TTAGTTCTCTTCAACT	PB	PB-1	P1-1	TRUE	3	TRA:CAMSDRMTGGFKTIF TRB:CASSQVGRGGEETQYF	1047	CD8-C7
PB-1_TTATGCTAGAGGTTAT	PB	PB-1	P1-10	TRUE	3	TRA:CISNSPMDSSYKLIF TRB:CASSSWGDTDEAFF	162	CD8-C7

PB-1_TTATGCTGTCTGCGGT	PB	PB-1	P1-288	FALSE	2	TRA:CVVMNYGGSQGNLIF TRB:CASSQSPGQRNYGYTF	2	CD8-C7
PB-1_TTATGCTGTTAGAACA	PB	PB-1	P1-22	TRUE	3	TRA:CASNSGYSTLTF TRB:CASSQVTGGARDEQYF	65	CD8-C7
PB-1_TTATGCTGTTGCGCAC	PB	PB-1	P1-6	TRUE	3	TRA:CAMREGRERDDKIIF TRB:CASSLPNTEAFF	216	CD8-C5
PB-1_TTATGCTTCGTTACGA	PB	PB-1	P1-29	TRUE	3	TRA:CATVSFSGGYNKLIF TRB:CASSLDRRGLSYEQYF	50	CD8-C7
PB-1_TTATGCTTCTAGCACA	PB	PB-1	P1-5	TRUE	3	TRA:CALSGTPMNRDDKIIF TRB:CSARGRGLAGGPAYEQYF	223	CD8-C7
PB-1_TTCCCAGAGCCAGTAG	PB	PB-1	P1-3	TRUE	3	TRA:CAFLSGTYKYIF TRB:CASSQSPGQRNYGYTF	308	CD8-C7
PB-1_TTCCCAGAGCCGATTT	PB	PB-1	P1-3	TRUE	3	TRA:CAFLSGTYKYIF TRB:CASSQSPGQRNYGYTF	308	CD8-C7
PB-1_TTCCCAGAGGAACTGC	PB	PB-1	P1-2	TRUE	3	TRA:CGQNFVF TRB:CASNQRGGPSLYNEQFF	323	CD8-C7
PB-1_TTCCCAGGTAAACACA	PB	PB-1	P1-7	TRUE	3	TRA:CAVQAGNNDMRF TRB:CASSTTGDFNYGYTF	215	CD8-C5
PB-1_TTCCCAGGTCTGATTG	PB	PB-1	P1-1	TRUE	3	TRA:CAMSDRMTGGFKTIF TRB:CASSQVGRGGEETQYF	1047	CD8-C7
PB-1_TTCGAAGAGCCAGGAT	PB	PB-1	P1-9	TRUE	3	TRA:CAEAEGGGADGLTF TRB:CATSGGDNEQFF	174	CD8-C7
PB-1_TTCGAAGCAACCGCCA	PB	PB-1	P1-3	TRUE	3	TRA:CAFLSGTYKYIF TRB:CASSQSPGQRNYGYTF	308	CD8-C2
PB-1_TTCGAAGCAAGCGATG	PB	PB-1	P1-1	TRUE	3	TRA:CAMSDRMTGGFKTIF TRB:CASSQVGRGGEETQYF	1047	CD8-C7
PB-1_TTCGAAGCAAGGGTCA	PB	PB-1	P1-58	TRUE	3	TRA:CAVRPNRDDKIIF TRB:CASSSRLAGGTDTQYF	12	CD8-C7
PB-1_TTCGAAGCAGGAACGT	PB	PB-1	P1-5	TRUE	3	TRA:CALSGTPMNRDDKIIF TRB:CSARGRGLAGGPAYEQYF	223	CD8-C7
PB-1_TTCGAAGCATGCCCGA	PB	PB-1	P1-146	TRUE	3	TRA:CAMSDRMTGGFKTIF TRB:CASSLGQRGLQYF	4	CD8-C7
PB-1_TTCGAAGTCTGGAGCC	PB	PB-1	P1-61	TRUE	3	TRA:CAARGGSQGNLIF TRB:CASRPRHTDTQYF	10	CD8-C7
PB-1_TTCGGTCAGGATCGCA	PB	PB-1	P1-2	TRUE	3	TRA:CGQNFVF TRB:CASNQRGGPSLYNEQFF	323	CD8-C7
PB-1_TTCGGTCCACATTCGA	PB	PB-1	P1-14	TRUE	3	TRA:CAASVVNDMRF TRB:CASSHRGGAKNIQYF	109	CD8-C7
PB-1_TTCGGTCCACTTAAGC	PB	PB-1	P1-10	TRUE	3	TRA:CISNSPMDSSYKLIF TRB:CASSSWGDTDEAFF	162	CD8-C7
PB-1_TTCGGTCGTCCAGTGC	PB	PB-1	P1-4	TRUE	3	TRA:CAMKGRDDKIIF TRB:CASTKGVSREAFF	226	CD8-C7
PB-1_TTCGGTCGTTTGTTTC	PB	PB-1	P1-3	TRUE	3	TRA:CAFLSGTYKYIF TRB:CASSQSPGQRNYGYTF	308	CD8-C7
PB-1_TTCGGTCTCCTTTCTC	PB	PB-1	P1-5	TRUE	3	TRA:CALSGTPMNRDDKIIF TRB:CSARGRGLAGGPAYEQYF	223	CD8-C7
PB-1_TTCTACAAGAAGCCCA	PB	PB-1	P1-14	TRUE	3	TRA:CAASVVNDMRF TRB:CASSHRGGAKNIQYF	109	CD8-C7
PB-1_TTCTACAAGAGCTGGT	PB	PB-1	P1-2	TRUE	3	TRA:CGQNFVF TRB:CASNQRGGPSLYNEQFF	323	CD8-C7
PB-1_TTCTACAAGCACGCCT	PB	PB-1	P1-13	TRUE	3	TRA:CVVMNYGGSQGNLIF TRB:CASSSWGDTDEAFF	112	CD8-C7
PB-1_TTCTACAAGCTAGTGG	PB	PB-1	P1-1	TRUE	3	TRA:CAMSDRMTGGFKTIF TRB:CASSQVGRGGEETQYF	1047	CD8-C7

PB-1_TTCTACAGTTCGCTAA	PB	PB-1	P1-1	TRUE	3	TRA:CAMSDRMTGGFKTIF TRB:CASSQVGRGGEETQYF	1047	CD8-C7
PB-1_TTCTACAGTTTGGCGC	PB	PB-1	P1-1	TRUE	3	TRA:CAMSDRMTGGFKTIF TRB:CASSQVGRGGEETQYF	1047	CD8-C7
PB-1_TTCTCAAGTCGACTAT	PB	PB-1	P1-2	TRUE	3	TRA:CGQNFVF TRB:CASNQRGGPSLYNEQFF	323	CD8-C7
PB-1_TTCTCAAGTGACCAAG	PB	PB-1	P1-1061	FALSE	1	TRA:CAASVVNDMRF TRB:CASSQSPGQRNYGYTF	1	CD8-C7
PB-1_TTCTCAATCTAAGCCA	PB	PB-1	P1-29	TRUE	3	TRA:CATVSFSGGYNKLIF TRB:CASSLDRRGLSYEQYF	50	CD8-C7
PB-1_TTCTCAATCTACCAGA	PB	PB-1	P1-3	TRUE	3	TRA:CAFLSGTYKYIF TRB:CASSQSPGQRNYGYTF	308	CD8-C7
PB-1_TTCTCAATCTCACATT	PB	PB-1	P1-3	TRUE	3	TRA:CAFLSGTYKYIF TRB:CASSQSPGQRNYGYTF	308	CD8-C7
PB-1_TTCTCAATCTTAACCT	PB	PB-1	P1-47	TRUE	3	TRA:CIVGGNFNKFYF TRB:CASSPEWDTDTQYF	18	CD8-C5
PB-1_TTCTCCTAGTTACCCA	PB	PB-1	P1-2	TRUE	3	TRA:CGQNFVF TRB:CASNQRGGPSLYNEQFF	323	CD8-C7
PB-1_TTCTCCTAGTTGTAGA	PB	PB-1	P1-12	TRUE	3	TRA:CALSDERGDYKLSF TRB:CASSPGTGSYNEQFF	141	CD8-C7
PB-1_TTCTCCTCATTATCTC	PB	PB-1	P1-1	TRUE	3	TRA:CAMSDRMTGGFKTIF TRB:CASSQVGRGGEETQYF	1047	CD8-C7
PB-1_TTCTCCTGTCACTGGC	PB	PB-1	P1-5	TRUE	3	TRA:CALSGTPMNRDDKIIF TRB:CSARGRGLAGGPAYEQYF	223	CD8-C7
PB-1_TTCTCCTTCCTACAGA	PB	PB-1	P1-112	FALSE	3	TRA:CALTPLTGGGNKLTF TRB:CSAREGGSMDTQYF	5	CD8-C7
PB-1_TTCTTAGAGGCAATTA	PB	PB-1	P1-1	TRUE	3	TRA:CAMSDRMTGGFKTIF TRB:CASSQVGRGGEETQYF	1047	CD8-C7
PB-1_TTCTTAGAGTGTACTC	PB	PB-1	P1-2	TRUE	3	TRA:CGQNFVF TRB:CASNQRGGPSLYNEQFF	323	CD8-C5
PB-1_TTCTTAGCAAGCGATG	PB	PB-1	P1-29	TRUE	3	TRA:CATVSFSGGYNKLIF TRB:CASSLDRRGLSYEQYF	50	CD8-C7
PB-1_TTCTTAGGTACATCCA	PB	PB-1	P1-1	TRUE	3	TRA:CAMSDRMTGGFKTIF TRB:CASSQVGRGGEETQYF	1047	CD8-C7
PB-1_TTCTTAGGTAGAAAGG	PB	PB-1	P1-1	TRUE	3	TRA:CAMSDRMTGGFKTIF TRB:CASSQVGRGGEETQYF	1047	CD8-C7
PB-1_TTCTTAGGTCATATCG	PB	PB-1	P1-5	TRUE	3	TRA:CALSGTPMNRDDKIIF TRB:CSARGRGLAGGPAYEQYF	223	CD8-C7
PB-1_TTCTTAGGTCTTCTCG	PB	PB-1	P1-1	TRUE	3	TRA:CAMSDRMTGGFKTIF TRB:CASSQVGRGGEETQYF	1047	CD8-C7
PB-1_TTGAACGAGATCGATA	PB	PB-1	P1-2	TRUE	3	TRA:CGQNFVF TRB:CASNQRGGPSLYNEQFF	323	CD8-C7
PB-1_TTGAACGCACAGACTT	PB	PB-1	P1-10	TRUE	3	TRA:CISNSPMDSSYKLIF TRB:CASSSWGDTDEAFF	162	CD8-C7
PB-1_TTGAACGCACGTCTCT	PB	PB-1	P1-53	TRUE	3	TRA:CALSERDTDKLIF TRB:CASSILQGVITEAFF	13	CD8-C7
PB-1_TTGAACGCAGTCCTTC	PB	PB-1	P1-3	TRUE	3	TRA:CAFLSGTYKYIF TRB:CASSQSPGQRNYGYTF	308	CD8-C7
PB-1_TTGAACGTCCGCGCAA	PB	PB-1	P1-1	TRUE	3	TRA:CAMSDRMTGGFKTIF TRB:CASSQVGRGGEETQYF	1047	CD8-C7
PB-1_TTGAACGTCGTCGTTC	PB	PB-1	P1-13	TRUE	3	TRA:CVVMNYGGSQGNLIF TRB:CASSSWGDTDEAFF	112	CD8-C7
PB-1_TTGACTTCACTGTTAG	PB	PB-1	P1-1	TRUE	3	TRA:CAMSDRMTGGFKTIF TRB:CASSQVGRGGEETQYF	1047	CD8-C5

PB-1_TTGACTTGTACGAAAT	PB	PB-1	P1-2	TRUE	3	TRA:CGQNFVF TRB:CASNQRGGPSLYNEQFF	323	CD4-C6
PB-1_TTGACTTGTAGTGAAT	PB	PB-1	P1-18	TRUE	3	TRA:CVADGNYGQNFVF TRB:CASSSPGLGGGFDTEAFF	75	CD8-C6
PB-1_TTGACTTGTCATATGC	PB	PB-1	P1-73	TRUE	3	TRA:CAGI TRB:CASSRTTGGSSYEQYF	8	CD8-C7
PB-1_TTGACTTGTCCGTTAA	PB	PB-1	P1-10	TRUE	3	TRA:CISNSPMDSSYKLIF TRB:CASSSWGDTDEAFF	162	CD8-C7
PB-1_TTGACTTTCCGTACAA	PB	PB-1	P1-12	TRUE	3	TRA:CALSDERGDYKLSF TRB:CASSPGTGSYNEQFF	141	CD8-C7
PB-1_TTGACTTTCGGAGGTA	PB	PB-1	P1-11	TRUE	3	TRA:CAVEPLYGSGNTGKLIF TRB:CASSSHGQLNQPQHF	147	CD8-C7
PB-1_TTGCCGTCACTTACGA	PB	PB-1	P1-2	TRUE	3	TRA:CGQNFVF TRB:CASNQRGGPSLYNEQFF	323	CD8-C7
PB-1_TTGCCGTCAGCTGTGC	PB	PB-1	P1-204	TRUE	3	TRA:CAVRDMMDSNYQLIW TRB:CASSLDGAGGETQYF	3	CD8-C7
PB-1_TTGCCGTTCTCAAACG	PB	PB-1	P1-5	TRUE	3	TRA:CALSGTPMNRDDKIIF TRB:CSARGRGLAGGPAYEQYF	223	CD8-C7
PB-1_TTGCGTCCACCGGAAA	PB	PB-1	P1-29	TRUE	3	TRA:CATVSFSGGYNKLIF TRB:CASSLDRRGLSYEQYF	50	CD8-C7
PB-1_TTGCGTCCATCTCGCT	PB	PB-1	P1-2	TRUE	3	TRA:CGQNFVF TRB:CASNQRGGPSLYNEQFF	323	CD8-C7
PB-1_TTGGAACAGACAGGCT	PB	PB-1	P1-1	TRUE	3	TRA:CAMSDRMTGGFKTIF TRB:CASSQVGRGGEETQYF	1047	CD8-C5
PB-1_TTGGAACAGCTGAACG	PB	PB-1	P1-1	TRUE	3	TRA:CAMSDRMTGGFKTIF TRB:CASSQVGRGGEETQYF	1047	CD8-C5
PB-1_TTGGAACAGTGCCATT	PB	PB-1	P1-2	TRUE	3	TRA:CGQNFVF TRB:CASNQRGGPSLYNEQFF	323	CD8-C7
PB-1_TTGGAACCAATGGATA	PB	PB-1	P1-66	TRUE	3	TRA:CVVSDPPDKLIF TRB:CASSASETGSVYEQYF	9	CD8-C7
PB-1_TTGGAACCAGCCTGTG	PB	PB-1	P1-5	TRUE	3	TRA:CALSGTPMNRDDKIIF TRB:CSARGRGLAGGPAYEQYF	223	CD8-C7
PB-1_TTGGAACCATGCGCAC	PB	PB-1	P1-10	TRUE	3	TRA:CISNSPMDSSYKLIF TRB:CASSSWGDTDEAFF	162	CD8-C7
PB-1_TTGGAACGTCTCATCC	PB	PB-1	P1-1	TRUE	3	TRA:CAMSDRMTGGFKTIF TRB:CASSQVGRGGEETQYF	1047	CD8-C7
PB-1_TTGGAACGTGTCAATC	PB	PB-1	P1-1	TRUE	3	TRA:CAMSDRMTGGFKTIF TRB:CASSQVGRGGEETQYF	1047	CD8-C5
PB-1_TTGGAACTCACCCGAG	PB	PB-1	P1-3	TRUE	3	TRA:CAFLSGTYKYIF TRB:CASSQSPGQRNYGYTF	308	CD8-C7
PB-1_TTGGAACTCTAACGGT	PB	PB-1	P1-1	TRUE	3	TRA:CAMSDRMTGGFKTIF TRB:CASSQVGRGGEETQYF	1047	CD8-C5
PB-1_TTGGAACTCTCGTTTA	PB	PB-1	P1-2	TRUE	3	TRA:CGQNFVF TRB:CASNQRGGPSLYNEQFF	323	CD8-C7
PB-1_TTGGCAACAACAACCT	PB	PB-1	P1-1	TRUE	3	TRA:CAMSDRMTGGFKTIF TRB:CASSQVGRGGEETQYF	1047	CD8-C7
PB-1_TTGGCAACAGGGTACA	PB	PB-1	P1-13	TRUE	3	TRA:CVVMNYGGSQGNLIF TRB:CASSSWGDTDEAFF	112	CD8-C7
PB-1_TTGGCAAGTAAATACG	PB	PB-1	P1-1	TRUE	3	TRA:CAMSDRMTGGFKTIF TRB:CASSQVGRGGEETQYF	1047	CD8-C7
PB-1_TTGGCAAGTCACTTCC	PB	PB-1	P1-4	TRUE	3	TRA:CAMKGRDDKIIF TRB:CASTKGVSREAFF	226	CD8-C7
PB-1_TTGGCAAGTCCGTCAG	PB	PB-1	P1-5	TRUE	3	TRA:CALSGTPMNRDDKIIF TRB:CSARGRGLAGGPAYEQYF	223	CD8-C7

PB-1_TTGGCAATCCATTCTA	PB	PB-1	P1-6	TRUE	3	TRA:CAMREGRERDDKIIF TRB:CASSLPNTEAFF	216	CD8-C7
PB-1_TTGTAGGAGGTGGGTT	PB	PB-1	P1-4	TRUE	3	TRA:CAMKGRDDKIIF TRB:CASTKGVSREAFF	226	CD8-C7
PB-1_TTGTAGGAGTACGACG	PB	PB-1	P1-2	TRUE	3	TRA:CGQNFVF TRB:CASNQRGGPSLYNEQFF	323	CD8-C7
PB-1_TTGTAGGGTATAATGG	PB	PB-1	P1-2	TRUE	3	TRA:CGQNFVF TRB:CASNQRGGPSLYNEQFF	323	CD8-C7
PB-1_TTGTAGGGTCGATTGT	PB	PB-1	P1-13	TRUE	3	TRA:CVVMNYGGSQGNLIF TRB:CASSSWGDTDEAFF	112	CD8-C7
PB-1_TTGTAGGTCGTGGGAA	PB	PB-1	P1-2	TRUE	3	TRA:CGQNFVF TRB:CASNQRGGPSLYNEQFF	323	CD8-C7
PB-1_TTGTAGGTCGTTGACA	PB	PB-1	P1-187	FALSE	3	TRA:CAASMWDTDKLIF TRB:CASSAPDEQETQYF	3	CD8-C5
PB-1_TTTACTGAGAGTTGGC	PB	PB-1	P1-13	TRUE	3	TRA:CVVMNYGGSQGNLIF TRB:CASSSWGDTDEAFF	112	CD8-C7
PB-1_TTTACTGCAATTCCTT	PB	PB-1	P1-3	TRUE	3	TRA:CAFLSGTYKYIF TRB:CASSQSPGQRNYGYTF	308	CD8-C7
PB-1_TTTACTGCATTCCTCG	PB	PB-1	P1-7	TRUE	3	TRA:CAVQAGNNDMRF TRB:CASSTTGDFNYGYTF	215	CD8-C5
PB-1_TTTATGCCACTCTGTC	PB	PB-1	P1-4	TRUE	3	TRA:CAMKGRDDKIIF TRB:CASTKGVSREAFF	226	CD8-C7
PB-1_TTTATGCCACTGTCGG	PB	PB-1	P1-11	TRUE	3	TRA:CAVEPLYGSGNTGKLIF TRB:CASSSHGQLNQPQHF	147	CD8-C7
PB-1_TTTATGCCAGCCTATA	PB	PB-1	P1-12	TRUE	3	TRA:CALSDERGDYKLSF TRB:CASSPGTGSYNEQFF	141	CD8-C5
PB-1_TTTCCTCAGAGAACAG	PB	PB-1	P1-1	TRUE	3	TRA:CAMSDRMTGGFKTIF TRB:CASSQVGRGGEETQYF	1047	CD8-C7
PB-1_TTTCCTCAGATAGTCA	PB	PB-1	P1-4	TRUE	3	TRA:CAMKGRDDKIIF TRB:CASTKGVSREAFF	226	CD8-C7
PB-1_TTTCCTCAGCTAACAA	PB	PB-1	P1-10	TRUE	3	TRA:CISNSPMDSSYKLIF TRB:CASSSWGDTDEAFF	162	CD8-C7
PB-1_TTTCCTCGTAATCACC	PB	PB-1	P1-11	TRUE	3	TRA:CAVEPLYGSGNTGKLIF TRB:CASSSHGQLNQPQHF	147	CD8-C7
PB-1_TTTCCTCTCTCGTTTA	PB	PB-1	P1-4	TRUE	3	TRA:CAMKGRDDKIIF TRB:CASTKGVSREAFF	226	CD8-C7
PB-1_TTTGCGCAGATAGCAT	PB	PB-1	P1-18	TRUE	3	TRA:CVADGNYGQNFVF TRB:CASSSPGLGGGFDTEAFF	75	CD8-C6
PB-1_TTTGCGCCAACTGCTA	PB	PB-1	P1-4	TRUE	3	TRA:CAMKGRDDKIIF TRB:CASTKGVSREAFF	226	CD8-C7
PB-1_TTTGCGCCATCAGTAC	PB	PB-1	P1-1	TRUE	3	TRA:CAMSDRMTGGFKTIF TRB:CASSQVGRGGEETQYF	1047	CD8-C7
PB-1_TTTGCGCCATCTATGG	PB	PB-1	P1-50	TRUE	3	TRA:CARWLGSQGNLIF TRB:CASSLTDRVPQFELVYEQYF	15	CD8-C7
PB-1_TTTGCGCGTTACGACT	PB	PB-1	P1-13	TRUE	3	TRA:CVVMNYGGSQGNLIF TRB:CASSSWGDTDEAFF	112	CD8-C7
PB-1_TTTGGTTAGCCAGTAG	PB	PB-1	P1-1	TRUE	3	TRA:CAMSDRMTGGFKTIF TRB:CASSQVGRGGEETQYF	1047	CD8-C5
PB-1_TTTGGTTAGGCAATTA	PB	PB-1	P1-2	TRUE	3	TRA:CGQNFVF TRB:CASNQRGGPSLYNEQFF	323	CD8-C5
PB-1_TTTGGTTAGTACGTTC	PB	PB-1	P1-4	TRUE	3	TRA:CAMKGRDDKIIF TRB:CASTKGVSREAFF	226	CD8-C7
PB-1_TTTGGTTAGTGGAGAA	PB	PB-1	P1-8	TRUE	3	TRA:CAVRPPGRNSGGYQKVTF TRB:CASSRRGVTDTQYF	204	CD8-C7

PB-1_TTTGGTTCAGCGTTCG	PB	PB-1	P1-1	TRUE	3	TRA:CAMSDRMTGGFKTIF TRB:CASSQVGRGGEETQYF	1047	CD8-C5
PB-1_TTTGGTTGTACTTAGC	PB	PB-1	P1-3	TRUE	3	TRA:CAFLSGTYKYIF TRB:CASSQSPGQRNYGYTF	308	CD8-C7
PB-1_TTTGGTTGTATCAGTC	PB	PB-1	P1-1	TRUE	3	TRA:CAMSDRMTGGFKTIF TRB:CASSQVGRGGEETQYF	1047	CD8-C5
PB-1_TTTGGTTTCAGTACGT	PB	PB-1	P1-10	TRUE	3	TRA:CISNSPMDSSYKLIF TRB:CASSSWGDTDEAFF	162	CD8-C7
PB-1_TTTGTCACAGTGGGAT	PB	PB-1	P1-8	TRUE	3	TRA:CAVRPPGRNSGGYQKVTF TRB:CASSRRGVTDTQYF	204	CD8-C7
PB-1_TTTGTCACATGATCCA	PB	PB-1	P1-3	TRUE	3	TRA:CAFLSGTYKYIF TRB:CASSQSPGQRNYGYTF	308	CD8-C5
PB-1_TTTGTCAGTGCTCTTC	PB	PB-1	P1-16	TRUE	3	TRA:CALSGTPMNRDDKIIF TRB:CASSQSWDLAVLNEQFF	79	CD8-C7
PB-1_TTTGTCAGTTCCATGA	PB	PB-1	P1-1	TRUE	3	TRA:CAMSDRMTGGFKTIF TRB:CASSQVGRGGEETQYF	1047	CD8-C5
PB-1_TTTGTCAGTTGGTAAA	PB	PB-1	P1-4	TRUE	3	TRA:CAMKGRDDKIIF TRB:CASTKGVSREAFF	226	CD8-C7
PB-1_TTTGTCATCACTCCTG	PB	PB-1	P1-29	TRUE	3	TRA:CATVSFSGGYNKLIF TRB:CASSLDRRGLSYEQYF	50	CD8-C7
PB-1_TTTGTCATCTACCTGC	PB	PB-1	P1-8	TRUE	3	TRA:CAVRPPGRNSGGYQKVTF TRB:CASSRRGVTDTQYF	204	CD8-C7
PB-1_AAACCTGAGTGTACGG	PB	PB-1	P1-6	TRUE	3	TRA:CAMREGRERDDKIIF TRB:CASSLPNTEAFF	216	CD8-C5
PB-1_AAACGGGCAGATGGGT	PB	PB-1	P1-9	TRUE	3	TRA:CAEAEGGGADGLTF TRB:CATSGGDNEQFF	174	CD8-C5
PB-1_AAAGATGCAAGGTGTG	PB	PB-1	P1-7	TRUE	3	TRA:CAVQAGNNDMRF TRB:CASSTTGDFNYGYTF	215	CD8-C5
PB-1_AAAGCAAAGAAGGACA	PB	PB-1	P1-6	TRUE	3	TRA:CAMREGRERDDKIIF TRB:CASSLPNTEAFF	216	CD8-C5
PB-1_AAAGCAACACTTCGAA	PB	PB-1	P1-52	TRUE	3	TRA:CAETPDYKLSF TRB:CASRDLYEQYF	13	CD8-C5
PB-1_AAAGTAGTCGCAAACT	PB	PB-1	P1-7	TRUE	3	TRA:CAVQAGNNDMRF TRB:CASSTTGDFNYGYTF	215	CD8-C5
PB-1_AAATGCCAGCTAAGAT	PB	PB-1	P1-22	TRUE	3	TRA:CASNSGYSTLTF TRB:CASSQVTGGARDEQYF	65	CD8-C5
PB-1_AACCATGGTTGTCGCG	PB	PB-1	P1-6	TRUE	3	TRA:CAMREGRERDDKIIF TRB:CASSLPNTEAFF	216	CD8-C5
PB-1_AACCATGGTTTGGGCC	PB	PB-1	P1-9	TRUE	3	TRA:CAEAEGGGADGLTF TRB:CATSGGDNEQFF	174	CD8-C5
PB-1_AACCATGTCCATGAAC	PB	PB-1	P1-23	TRUE	3	TRA:CALRAITQGGSEKLVF TRB:CASSLTGAVYGYTF	65	CD8-C4
PB-1_AACCGCGCAATGAATG	PB	PB-1	P1-6	TRUE	3	TRA:CAMREGRERDDKIIF TRB:CASSLPNTEAFF	216	CD8-C5
PB-1_AACGTTGAGCGTCTAT	PB	PB-1	P1-47	TRUE	3	TRA:CIVGGNFNKFYF TRB:CASSPEWDTDTQYF	18	CD8-C7
PB-1_AACGTTGTCGTTGCCT	PB	PB-1	P1-9	TRUE	3	TRA:CAEAEGGGADGLTF TRB:CATSGGDNEQFF	174	CD8-C5
PB-1_AACTCAGCAAACTGTC	PB	PB-1	P1-9	TRUE	3	TRA:CAEAEGGGADGLTF TRB:CATSGGDNEQFF	174	CD8-C5
PB-1_AACTCAGTCACAAACC	PB	PB-1	P1-84	FALSE	3	TRA:CAMREPPHRGSTLGRLYF TRB:CSATGLAGVPFTDTQYF	6	CD8-C5
PB-1_AACTCAGTCGATGAGG	PB	PB-1	P1-12	TRUE	3	TRA:CALSDERGDYKLSF TRB:CASSPGTGSYNEQFF	141	CD8-C5

PB-1_AACTCCCAGGAGTTTA	PB	PB-1	P1-12	TRUE	3	TRA:CALSDERGDYKLSF TRB:CASSPGTGSYNEQFF	141	CD8-C5
PB-1_AACTCCCGTGCATCTA	PB	PB-1	P1-15	TRUE	3	TRA:CAVNSSPLGANSKLTF TRB:CASSDSGNFYNEQFF	92	CD8-C2
PB-1_AACTCCCGTGTAAGTA	PB	PB-1	P1-7	TRUE	3	TRA:CAVQAGNNDMRF TRB:CASSTTGDFNYGYTF	215	CD8-C4
PB-1_AACTCTTCAGACGCAA	PB	PB-1	P1-14	TRUE	3	TRA:CAASVVNDMRF TRB:CASSHRGGAKNIQYF	109	CD8-C5
PB-1_AACTGGTAGCTTCGCG	PB	PB-1	P1-12	TRUE	3	TRA:CALSDERGDYKLSF TRB:CASSPGTGSYNEQFF	141	CD8-C5
PB-1_AACTGGTTCAGCGATT	PB	PB-1	P1-66	TRUE	3	TRA:CVVSDPPDKLIF TRB:CASSASETGSVYEQYF	9	CD8-C5
PB-1_AACTGGTTCTTACCTA	PB	PB-1	P1-6	TRUE	3	TRA:CAMREGRERDDKIIF TRB:CASSLPNTEAFF	216	CD8-C5
PB-1_AAGACCTAGCCTCGTG	PB	PB-1	P1-7	TRUE	3	TRA:CAVQAGNNDMRF TRB:CASSTTGDFNYGYTF	215	CD8-C5
PB-1_AAGACCTGTCTTCTCG	PB	PB-1	P1-9	TRUE	3	TRA:CAEAEGGGADGLTF TRB:CATSGGDNEQFF	174	CD8-C5
PB-1_AAGACCTGTTACGGAG	PB	PB-1	P1-12	TRUE	3	TRA:CALSDERGDYKLSF TRB:CASSPGTGSYNEQFF	141	CD8-C5
PB-1_AAGACCTTCCCTGACT	PB	PB-1	P1-4	TRUE	3	TRA:CAMKGRDDKIIF TRB:CASTKGVSREAFF	226	CD8-C5
PB-1_AAGACCTTCTGGTATG	PB	PB-1	P1-9	TRUE	3	TRA:CAEAEGGGADGLTF TRB:CATSGGDNEQFF	174	CD8-C5
PB-1_AAGCCGCAGCACACAG	PB	PB-1	P1-6	TRUE	3	TRA:CAMREGRERDDKIIF TRB:CASSLPNTEAFF	216	CD8-C5
PB-1_AAGCCGCCAGTCACTA	PB	PB-1	P1-86	TRUE	3	TRA:CAVGGRSNTGRRALTF TRB:CAISLTGDGKETQYF	6	CD8-C5
PB-1_AAGGAGCTCAAACAAG	PB	PB-1	P1-14	TRUE	3	TRA:CAASVVNDMRF TRB:CASSHRGGAKNIQYF	109	CD8-C5
PB-1_AAGGCAGGTTACGGAG	PB	PB-1	P1-9	TRUE	3	TRA:CAEAEGGGADGLTF TRB:CATSGGDNEQFF	174	CD8-C5
PB-1_AAGTCTGCAATGGAAT	PB	PB-1	P1-8	TRUE	3	TRA:CAVRPPGRNSGGYQKVTF TRB:CASSRRGVTDTQYF	204	CD8-C7
PB-1_AATCCAGAGCGTTCCG	PB	PB-1	P1-7	TRUE	3	TRA:CAVQAGNNDMRF TRB:CASSTTGDFNYGYTF	215	CD8-C5
PB-1_AATCCAGAGTGAACAT	PB	PB-1	P1-7	TRUE	3	TRA:CAVQAGNNDMRF TRB:CASSTTGDFNYGYTF	215	CD8-C5
PB-1_AATCGGTCAATTGCTG	PB	PB-1	P1-12	TRUE	3	TRA:CALSDERGDYKLSF TRB:CASSPGTGSYNEQFF	141	CD8-C5
PB-1_AATCGGTCACAGAGGT	PB	PB-1	P1-12	TRUE	3	TRA:CALSDERGDYKLSF TRB:CASSPGTGSYNEQFF	141	CD8-C5
PB-1_ACACCAAGTTGAACTC	PB	PB-1	P1-6	TRUE	3	TRA:CAMREGRERDDKIIF TRB:CASSLPNTEAFF	216	CD8-C5
PB-1_ACACCCTGTAGAGTGC	PB	PB-1	P1-87	TRUE	3	TRA:CGTVYNYGGQNFVF TRB:CASSRGRGAQPQHF	6	CD8-C5
PB-1_ACACTGAAGGCAAAGA	PB	PB-1	P1-6	TRUE	3	TRA:CAMREGRERDDKIIF TRB:CASSLPNTEAFF	216	CD8-C5
PB-1_ACACTGATCTTGACGA	PB	PB-1	P1-6	TRUE	3	TRA:CAMREGRERDDKIIF TRB:CASSLPNTEAFF	216	CD8-C5
PB-1_ACAGCCGAGACTGTAA	PB	PB-1	P1-7	TRUE	3	TRA:CAVQAGNNDMRF TRB:CASSTTGDFNYGYTF	215	CD8-C5
PB-1_ACAGCCGAGTAGGTGC	PB	PB-1	P1-7	TRUE	3	TRA:CAVQAGNNDMRF TRB:CASSTTGDFNYGYTF	215	CD8-C5

PB-1_ACAGCCGTCGTATCAG	PB	PB-1	P1-257	FALSE	2	TRA:CAVSARGGSGNTGKLIF TRB:CSARDRRLSYEQYF	2	CD8-C2
PB-1_ACAGCTAGTATGGTTC	PB	PB-1	P1-3	TRUE	3	TRA:CAFLSGTYKYIF TRB:CASSQSPGQRNYGYTF	308	CD8-C7
PB-1_ACATACGAGACTTGAA	PB	PB-1	P1-173	FALSE	3	TRA:CAVVGYSSASKIIF TRB:CATSDLGYEQFF	3	CD8-C7
PB-1_ACATACGCAGAGCCAA	PB	PB-1	P1-6	TRUE	3	TRA:CAMREGRERDDKIIF TRB:CASSLPNTEAFF	216	CD8-C5
PB-1_ACATCAGTCATAAAGG	PB	PB-1	P1-53	TRUE	3	TRA:CALSERDTDKLIF TRB:CASSILQGVITEAFF	13	CD8-C5
PB-1_ACATGGTGTCACCCAG	PB	PB-1	P1-9	TRUE	3	TRA:CAEAEGGGADGLTF TRB:CATSGGDNEQFF	174	CD8-C5
PB-1_ACCAGTAGTCTAGCGC	PB	PB-1	P1-9	TRUE	3	TRA:CAEAEGGGADGLTF TRB:CATSGGDNEQFF	174	CD8-C5
PB-1_ACCGTAACAGATGAGC	PB	PB-1	P1-474	FALSE	1	TRA:CAVKEYGNKLVF TRB:CASSWGSYTGELFF	1	CD8-C5
PB-1_ACCTTTACAATAGCGG	PB	PB-1	P1-12	TRUE	3	TRA:CALSDERGDYKLSF TRB:CASSPGTGSYNEQFF	141	CD8-C5
PB-1_ACCTTTATCACTCCTG	PB	PB-1	P1-9	TRUE	3	TRA:CAEAEGGGADGLTF TRB:CATSGGDNEQFF	174	CD8-C5
PB-1_ACGAGCCAGAGTGAGA	PB	PB-1	P1-4	TRUE	3	TRA:CAMKGRDDKIIF TRB:CASTKGVSREAFF	226	CD8-C5
PB-1_ACGAGCCTCGCCAGCA	PB	PB-1	P1-9	TRUE	3	TRA:CAEAEGGGADGLTF TRB:CATSGGDNEQFF	174	CD8-C7
PB-1_ACGAGGAAGTATTGGA	PB	PB-1	P1-12	TRUE	3	TRA:CALSDERGDYKLSF TRB:CASSPGTGSYNEQFF	141	CD8-C5
PB-1_ACGATACTCGCCAAAT	PB	PB-1	P1-1	TRUE	3	TRA:CAMSDRMTGGFKTIF TRB:CASSQVGRGGEETQYF	1047	CD8-C2
PB-1_ACGATGTTCACAATGC	PB	PB-1	P1-7	TRUE	3	TRA:CAVQAGNNDMRF TRB:CASSTTGDFNYGYTF	215	CD8-C5
PB-1_ACGCAGCAGACTGTAA	PB	PB-1	P1-6	TRUE	3	TRA:CAMREGRERDDKIIF TRB:CASSLPNTEAFF	216	CD8-C5
PB-1_ACGCAGCCAGATAATG	PB	PB-1	P1-6	TRUE	3	TRA:CAMREGRERDDKIIF TRB:CASSLPNTEAFF	216	CD8-C5
PB-1_ACGCAGCCAGCTCGCA	PB	PB-1	P1-485	FALSE	1	TRA:CAMREGRERDDKIIF TRB:CASSQSPGQRNYGYTF	1	CD8-C5
PB-1_ACGCAGCGTTTGCATG	PB	PB-1	P1-85	FALSE	3	TRA:CAPRSGGSYIPTF TRB:CASSQDTGLDYGYTF	6	CD8-C5
PB-1_ACGCCAGCACATGACT	PB	PB-1	P1-6	TRUE	3	TRA:CAMREGRERDDKIIF TRB:CASSLPNTEAFF	216	CD8-C5
PB-1_ACGCCAGGTGTTCTTT	PB	PB-1	P1-7	TRUE	3	TRA:CAVQAGNNDMRF TRB:CASSTTGDFNYGYTF	215	CD8-C5
PB-1_ACGCCGAGTTGTCTTT	PB	PB-1	P1-14	TRUE	3	TRA:CAASVVNDMRF TRB:CASSHRGGAKNIQYF	109	CD8-C5
PB-1_ACGGGCTGTCAACTGT	PB	PB-1	P1-39	TRUE	3	TRA:CAVRDLGGTSYDKVIF TRB:CSARVREVSYEQYF	31	CD8-C5
PB-1_ACGGGCTGTCGAGATG	PB	PB-1	P1-494	FALSE	1	TRA:CAEIDNYGQNFVF TRB:CASSLEGSSGNTIYF	1	CD8-C5
PB-1_ACGGGCTGTTGGACCC	PB	PB-1	P1-6	TRUE	3	TRA:CAMREGRERDDKIIF TRB:CASSLPNTEAFF	216	CD8-C5
PB-1_ACGGGCTTCGCATGAT	PB	PB-1	P1-143	TRUE	3	TRA:CAEGNQAGTALIF TRB:CASSTIGQGIPGANVLTF	4	CD8-C5
PB-1_ACGGGTCAGTGTCCCG	PB	PB-1	P1-176	TRUE	3	TRA:CATVRTGANNLFF TRB:CASSQGLYEQYF	3	CD8-C5

PB-1_ACGGGTCTCCACGTGG	PB	PB-1	P1-52	TRUE	3	TRA:CAETPDYKLSF TRB:CASRDLYEQYF	13	CD8-C7
PB-1_ACGGGTCTCCCGACTT	PB	PB-1	P1-37	TRUE	3	TRA:CAVNIVGNQFYF TRB:CASSPLGTEAFF	40	CD8-C5
PB-1_ACGTCAACAATCTGCA	PB	PB-1	P1-7	TRUE	3	TRA:CAVQAGNNDMRF TRB:CASSTTGDFNYGYTF	215	CD8-C5
PB-1_ACTATCTGTAGGCTGA	PB	PB-1	P1-12	TRUE	3	TRA:CALSDERGDYKLSF TRB:CASSPGTGSYNEQFF	141	CD8-C5
PB-1_ACTGAACAGCCAGGAT	PB	PB-1	P1-9	TRUE	3	TRA:CAEAEGGGADGLTF TRB:CATSGGDNEQFF	174	CD8-C5
PB-1_ACTGAACCAGATGGGT	PB	PB-1	P1-264	TRUE	2	TRA:CAVQAGNNDMRF TRB:CASSPGTGSYNEQFF	2	CD8-C5
PB-1_ACTGATGGTCTAGCCG	PB	PB-1	P1-7	TRUE	3	TRA:CAVQAGNNDMRF TRB:CASSTTGDFNYGYTF	215	CD8-C5
PB-1_ACTGCTCAGGATGTAT	PB	PB-1	P1-39	TRUE	3	TRA:CAVRDLGGTSYDKVIF TRB:CSARVREVSYEQYF	31	CD8-C5
PB-1_ACTGCTCTCGGCATCG	PB	PB-1	P1-265	TRUE	2	TRA:CATSPPQGAQKLVF TRB:CASSLDRAFTDTQYF	2	CD8-C4
PB-1_ACTGTCCTCCGATATG	PB	PB-1	P1-12	TRUE	3	TRA:CALSDERGDYKLSF TRB:CASSPGTGSYNEQFF	141	CD8-C5
PB-1_ACTTACTAGCGCCTTG	PB	PB-1	P1-177	FALSE	3	TRA:CAVLQAGTALIF TRB:CASSVNRDKRTDTQYF	3	CD8-C2
PB-1_ACTTACTAGTCCAGGA	PB	PB-1	P1-47	TRUE	3	TRA:CIVGGNFNKFYF TRB:CASSPEWDTDTQYF	18	CD8-C5
PB-1_ACTTACTCAGAGCCAA	PB	PB-1	P1-6	TRUE	3	TRA:CAMREGRERDDKIIF TRB:CASSLPNTEAFF	216	CD8-C5
PB-1_ACTTGTTGTCATGCCG	PB	PB-1	P1-7	TRUE	3	TRA:CAVQAGNNDMRF TRB:CASSTTGDFNYGYTF	215	CD8-C5
PB-1_ACTTGTTTCCGCGGTA	PB	PB-1	P1-6	TRUE	3	TRA:CAMREGRERDDKIIF TRB:CASSLPNTEAFF	216	CD8-C5
PB-1_AGAATAGAGCTACCTA	PB	PB-1	P1-9	TRUE	3	TRA:CAEAEGGGADGLTF TRB:CATSGGDNEQFF	174	CD8-C5
PB-1_AGAATAGAGTGTACTC	PB	PB-1	P1-12	TRUE	3	TRA:CALSDERGDYKLSF TRB:CASSPGTGSYNEQFF	141	CD8-C5
PB-1_AGAATAGGTTTGTGTG	PB	PB-1	P1-9	TRUE	3	TRA:CAEAEGGGADGLTF TRB:CATSGGDNEQFF	174	CD8-C5
PB-1_AGACGTTTCGGAATCT	PB	PB-1	P1-20	TRUE	3	TRA:CVVVGGRALTF TRB:CASSLATNPHETQYF	69	CD8-C4
PB-1_AGAGCGAAGAGCTGCA	PB	PB-1	P1-29	TRUE	3	TRA:CATVSFSGGYNKLIF TRB:CASSLDRRGLSYEQYF	50	CD8-C4
PB-1_AGAGCGAAGCATCATC	PB	PB-1	P1-12	TRUE	3	TRA:CALSDERGDYKLSF TRB:CASSPGTGSYNEQFF	141	CD8-C5
PB-1_AGAGCGACAATGGTCT	PB	PB-1	P1-14	TRUE	3	TRA:CAASVVNDMRF TRB:CASSHRGGAKNIQYF	109	CD8-C5
PB-1_AGAGCGAGTACTTAGC	PB	PB-1	P1-144	FALSE	3	TRA:CAMKGRDDKIIF TRB:CASSQVGRGGEETQYF	4	CD8-C7
PB-1_AGATCTGGTATCGCAT	PB	PB-1	P1-9	TRUE	3	TRA:CAEAEGGGADGLTF TRB:CATSGGDNEQFF	174	CD8-C5
PB-1_AGATCTGGTCATCGGC	PB	PB-1	P1-7	TRUE	3	TRA:CAVQAGNNDMRF TRB:CASSTTGDFNYGYTF	215	CD8-C5
PB-1_AGATTGCAGAAGGTTT	PB	PB-1	P1-7	TRUE	3	TRA:CAVQAGNNDMRF TRB:CASSTTGDFNYGYTF	215	CD8-C5
PB-1_AGATTGCCACATGACT	PB	PB-1	P1-9	TRUE	3	TRA:CAEAEGGGADGLTF TRB:CATSGGDNEQFF	174	CD8-C5

PB-1_AGATTGCTCTTGAGAC	PB	PB-1	P1-179	TRUE	3	TRA:CALAPTNTGNQFYF TRB:CASQRVGLSRYEQYF	3	CD8-C4
PB-1_AGCAGCCCAAGAGTCG	PB	PB-1	P1-52	TRUE	3	TRA:CAETPDYKLSF TRB:CASRDLYEQYF	13	CD8-C5
PB-1_AGCATACAGACACTAA	PB	PB-1	P1-12	TRUE	3	TRA:CALSDERGDYKLSF TRB:CASSPGTGSYNEQFF	141	CD8-C4
PB-1_AGCATACAGGAGTCTG	PB	PB-1	P1-4	TRUE	3	TRA:CAMKGRDDKIIF TRB:CASTKGVSREAFF	226	CD8-C5
PB-1_AGCATACTCCAATGGT	PB	PB-1	P1-11	TRUE	3	TRA:CAVEPLYGSGNTGKLIF TRB:CASSSHGQLNQPQHF	147	CD8-C5
PB-1_AGCATACTCGAGCCCA	PB	PB-1	P1-516	FALSE	1	TRA:CAEPTYNFNKFYF TRB:CAISEGNEQFF	1	CD8-C5
PB-1_AGCCTAACAACACCCG	PB	PB-1	P1-517	FALSE	1	TRA:CAVQAGNNDMRF TRB:CATSGGDNEQFF	1	CD8-C5
PB-1_AGCCTAAGTGTCCTCT	PB	PB-1	P1-12	TRUE	3	TRA:CALSDERGDYKLSF TRB:CASSPGTGSYNEQFF	141	CD8-C5
PB-1_AGCGGTCAGTGGCACA	PB	PB-1	P1-5	TRUE	3	TRA:CALSGTPMNRDDKIIF TRB:CSARGRGLAGGPAYEQYF	223	CD8-C5
PB-1_AGCGGTCGTAAGTGTA	PB	PB-1	P1-520	FALSE	1	TRA:CALSEDRDDKIIF TRB:CASSYGRMNTGELFF	1	CD8-C7
PB-1_AGCGGTCTCTGAAAGA	PB	PB-1	P1-12	TRUE	3	TRA:CALSDERGDYKLSF TRB:CASSPGTGSYNEQFF	141	CD8-C5
PB-1_AGCGTATGTCAAAGCG	PB	PB-1	P1-12	TRUE	3	TRA:CALSDERGDYKLSF TRB:CASSPGTGSYNEQFF	141	CD8-C5
PB-1_AGCGTATTCCAGAAGG	PB	PB-1	P1-37	TRUE	3	TRA:CAVNIVGNQFYF TRB:CASSPLGTEAFF	40	CD8-C4
PB-1_AGCGTATTCTCAACTT	PB	PB-1	P1-1	TRUE	3	TRA:CAMSDRMTGGFKTIF TRB:CASSQVGRGGEETQYF	1047	CD8-C5
PB-1_AGCGTCGAGCTAGTTC	PB	PB-1	P1-7	TRUE	3	TRA:CAVQAGNNDMRF TRB:CASSTTGDFNYGYTF	215	CD8-C5
PB-1_AGCGTCGCATTCACTT	PB	PB-1	P1-7	TRUE	3	TRA:CAVQAGNNDMRF TRB:CASSTTGDFNYGYTF	215	CD8-C5
PB-1_AGCTTGAAGATCCGAG	PB	PB-1	P1-9	TRUE	3	TRA:CAEAEGGGADGLTF TRB:CATSGGDNEQFF	174	CD8-C5
PB-1_AGGCCACAGGCCATAG	PB	PB-1	P1-6	TRUE	3	TRA:CAMREGRERDDKIIF TRB:CASSLPNTEAFF	216	CD8-C5
PB-1_AGGCCACCACCAGTTA	PB	PB-1	P1-6	TRUE	3	TRA:CAMREGRERDDKIIF TRB:CASSLPNTEAFF	216	CD8-C5
PB-1_AGGCCGTCATTGGCGC	PB	PB-1	P1-9	TRUE	3	TRA:CAEAEGGGADGLTF TRB:CATSGGDNEQFF	174	CD8-C5
PB-1_AGGGAGTTCTATCCCG	PB	PB-1	P1-12	TRUE	3	TRA:CALSDERGDYKLSF TRB:CASSPGTGSYNEQFF	141	CD8-C5
PB-1_AGGGATGCAGGAATGC	PB	PB-1	P1-12	TRUE	3	TRA:CALSDERGDYKLSF TRB:CASSPGTGSYNEQFF	141	CD8-C5
PB-1_AGGGTGATCGTAGGTT	PB	PB-1	P1-37	TRUE	3	TRA:CAVNIVGNQFYF TRB:CASSPLGTEAFF	40	CD8-C4
PB-1_AGGGTGATCTCCTATA	PB	PB-1	P1-9	TRUE	3	TRA:CAEAEGGGADGLTF TRB:CATSGGDNEQFF	174	CD8-C5
PB-1_AGGTCCGCAAGCTGAG	PB	PB-1	P1-15	TRUE	3	TRA:CAVNSSPLGANSKLTF TRB:CASSDSGNFYNEQFF	92	CD8-C7
PB-1_AGGTCCGGTAGTAGTA	PB	PB-1	P1-54	TRUE	3	TRA:CAGWYSGGGADGLTF TRB:CATGAGDNEQFF	13	CD8-C5
PB-1_AGGTCCGGTTCGTGAT	PB	PB-1	P1-7	TRUE	3	TRA:CAVQAGNNDMRF TRB:CASSTTGDFNYGYTF	215	CD8-C5

PB-1_AGGTCCGGTTGTACAC	PB	PB-1	P1-1	TRUE	3	TRA:CAMSDRMTGGFKTIF TRB:CASSQVGRGGEETQYF	1047	CD8-C5
PB-1_AGTAGTCAGCTGCGAA	PB	PB-1	P1-5	TRUE	3	TRA:CALSGTPMNRDDKIIF TRB:CSARGRGLAGGPAYEQYF	223	CD8-C5
PB-1_AGTAGTCCACAACTGT	PB	PB-1	P1-7	TRUE	3	TRA:CAVQAGNNDMRF TRB:CASSTTGDFNYGYTF	215	CD8-C5
PB-1_AGTAGTCGTCGCGGTT	PB	PB-1	P1-16	TRUE	3	TRA:CALSGTPMNRDDKIIF TRB:CASSQSWDLAVLNEQFF	79	CD8-C7
PB-1_AGTCTTTGTAATCGTC	PB	PB-1	P1-6	TRUE	3	TRA:CAMREGRERDDKIIF TRB:CASSLPNTEAFF	216	CD8-C5
PB-1_AGTCTTTGTAGGAGTC	PB	PB-1	P1-9	TRUE	3	TRA:CAEAEGGGADGLTF TRB:CATSGGDNEQFF	174	CD8-C7
PB-1_AGTCTTTGTCGCATAT	PB	PB-1	P1-5	TRUE	3	TRA:CALSGTPMNRDDKIIF TRB:CSARGRGLAGGPAYEQYF	223	CD8-C5
PB-1_AGTGAGGCAACAACCT	PB	PB-1	P1-4	TRUE	3	TRA:CAMKGRDDKIIF TRB:CASTKGVSREAFF	226	CD8-C7
PB-1_AGTGAGGTCTTGTTTG	PB	PB-1	P1-7	TRUE	3	TRA:CAVQAGNNDMRF TRB:CASSTTGDFNYGYTF	215	CD8-C5
PB-1_AGTGTCACAAATCCGT	PB	PB-1	P1-6	TRUE	3	TRA:CAMREGRERDDKIIF TRB:CASSLPNTEAFF	216	CD8-C5
PB-1_AGTTGGTTCATGTGGT	PB	PB-1	P1-8	TRUE	3	TRA:CAVRPPGRNSGGYQKVTF TRB:CASSRRGVTDTQYF	204	CD8-C5
PB-1_ATAACGCGTCAGATAA	PB	PB-1	P1-20	TRUE	3	TRA:CVVVGGRALTF TRB:CASSLATNPHETQYF	69	CD8-C4
PB-1_ATAACGCTCACTCCTG	PB	PB-1	P1-55	TRUE	3	TRA:CALSPQDSNYQLIW TRB:CASSFWTSANEQFF	13	CD8-C5
PB-1_ATAAGAGGTGCGGTAA	PB	PB-1	P1-535	FALSE	1	TRA:CAMKGRDDKIIF TRB:CASAGDLRRAQHF	1	CD4-C6
PB-1_ATAAGAGTCAGGCGAA	PB	PB-1	P1-5	TRUE	3	TRA:CALSGTPMNRDDKIIF TRB:CSARGRGLAGGPAYEQYF	223	CD8-C5
PB-1_ATAGACCGTGCTAGCC	PB	PB-1	P1-7	TRUE	3	TRA:CAVQAGNNDMRF TRB:CASSTTGDFNYGYTF	215	CD8-C5
PB-1_ATCACGAAGACCTTTG	PB	PB-1	P1-39	TRUE	3	TRA:CAVRDLGGTSYDKVIF TRB:CSARVREVSYEQYF	31	CD8-C5
PB-1_ATCACGAGTCGCATCG	PB	PB-1	P1-9	TRUE	3	TRA:CAEAEGGGADGLTF TRB:CATSGGDNEQFF	174	CD8-C5
PB-1_ATCACGATCGTTTAGG	PB	PB-1	P1-7	TRUE	3	TRA:CAVQAGNNDMRF TRB:CASSTTGDFNYGYTF	215	CD8-C5
PB-1_ATCATCTGTGTTCGAT	PB	PB-1	P1-9	TRUE	3	TRA:CAEAEGGGADGLTF TRB:CATSGGDNEQFF	174	CD8-C5
PB-1_ATCCACCTCACCATAG	PB	PB-1	P1-6	TRUE	3	TRA:CAMREGRERDDKIIF TRB:CASSLPNTEAFF	216	CD8-C5
PB-1_ATCCGAAAGAAGGCCT	PB	PB-1	P1-110	TRUE	3	TRA:CAVAPGTGRRALTF TRB:CSARDRTAQETQYF	5	CD8-C5
PB-1_ATCGAGTAGCGATGAC	PB	PB-1	P1-6	TRUE	3	TRA:CAMREGRERDDKIIF TRB:CASSLPNTEAFF	216	CD8-C5
PB-1_ATCGAGTTCACTGGGC	PB	PB-1	P1-6	TRUE	3	TRA:CAMREGRERDDKIIF TRB:CASSLPNTEAFF	216	CD8-C5
PB-1_ATCGAGTTCTGCCAGG	PB	PB-1	P1-12	TRUE	3	TRA:CALSDERGDYKLSF TRB:CASSPGTGSYNEQFF	141	CD8-C5
PB-1_ATGAGGGGTGTTAAGA	PB	PB-1	P1-4	TRUE	3	TRA:CAMKGRDDKIIF TRB:CASTKGVSREAFF	226	CD8-C7
PB-1_ATGAGGGTCACAGTAC	PB	PB-1	P1-9	TRUE	3	TRA:CAEAEGGGADGLTF TRB:CATSGGDNEQFF	174	CD8-C5

PB-1_ATGCGATGTACAGCAG	PB	PB-1	P1-7	TRUE	3	TRA:CAVQAGNNDMRF TRB:CASSTTGDFNYGYTF	215	CD8-C5
PB-1_ATGGGAGCACAGACTT	PB	PB-1	P1-16	TRUE	3	TRA:CALSGTPMNRDDKIIF TRB:CASSQSWDLAVLNEQFF	79	CD8-C2
PB-1_ATGGGAGCACGAAACG	PB	PB-1	P1-185	TRUE	3	TRA:CATDEDKFYF TRB:CASSESWTGGTYEQYF	3	CD8-C5
PB-1_ATGGGAGTCCCAAGTA	PB	PB-1	P1-554	FALSE	1	TRA:CAVPYSSASKIIF TRB:CASSEVGFGDTQYF	1	CD8-C5
PB-1_ATGTGTGAGGAGTTTA	PB	PB-1	P1-39	TRUE	3	TRA:CAVRDLGGTSYDKVIF TRB:CSARVREVSYEQYF	31	CD8-C5
PB-1_ATGTGTGGTGGTCCGT	PB	PB-1	P1-6	TRUE	3	TRA:CAMREGRERDDKIIF TRB:CASSLPNTEAFF	216	CD8-C5
PB-1_ATGTGTGTCTTAGAGC	PB	PB-1	P1-555	FALSE	1	TRA:CAATSTNFGNEKLTF TRB:CASSLVTNTGELFF	1	CD8-C5
PB-1_ATTACTCAGGCATGTG	PB	PB-1	P1-6	TRUE	3	TRA:CAMREGRERDDKIIF TRB:CASSLPNTEAFF	216	CD8-C5
PB-1_ATTATCCCATACTACG	PB	PB-1	P1-23	TRUE	3	TRA:CALRAITQGGSEKLVF TRB:CASSLTGAVYGYTF	65	CD8-C5
PB-1_ATTCTACTCTTGCAAG	PB	PB-1	P1-16	TRUE	3	TRA:CALSGTPMNRDDKIIF TRB:CASSQSWDLAVLNEQFF	79	CD8-C5
PB-1_ATTGGACGTTCAGCGC	PB	PB-1	P1-9	TRUE	3	TRA:CAEAEGGGADGLTF TRB:CATSGGDNEQFF	174	CD8-C5
PB-1_ATTGGTGCAGCGTAAG	PB	PB-1	P1-6	TRUE	3	TRA:CAMREGRERDDKIIF TRB:CASSLPNTEAFF	216	CD8-C5
PB-1_ATTGGTGTCACCCTCA	PB	PB-1	P1-6	TRUE	3	TRA:CAMREGRERDDKIIF TRB:CASSLPNTEAFF	216	CD8-C5
PB-1_ATTGGTGTCGTTGACA	PB	PB-1	P1-7	TRUE	3	TRA:CAVQAGNNDMRF TRB:CASSTTGDFNYGYTF	215	CD8-C5
PB-1_ATTTCTGAGTGGTAAT	PB	PB-1	P1-9	TRUE	3	TRA:CAEAEGGGADGLTF TRB:CATSGGDNEQFF	174	CD8-C5
PB-1_ATTTCTGGTTTGTGTG	PB	PB-1	P1-4	TRUE	3	TRA:CAMKGRDDKIIF TRB:CASTKGVSREAFF	226	CD8-C5
PB-1_CAACCAACAAAGGCGT	PB	PB-1	P1-7	TRUE	3	TRA:CAVQAGNNDMRF TRB:CASSTTGDFNYGYTF	215	CD8-C4
PB-1_CAACCAACAGCGTCCA	PB	PB-1	P1-9	TRUE	3	TRA:CAEAEGGGADGLTF TRB:CATSGGDNEQFF	174	CD8-C5
PB-1_CAACCAACAGGCGATA	PB	PB-1	P1-10	TRUE	3	TRA:CISNSPMDSSYKLIF TRB:CASSSWGDTDEAFF	162	CD8-C7
PB-1_CAACCAAGTACCATCA	PB	PB-1	P1-9	TRUE	3	TRA:CAEAEGGGADGLTF TRB:CATSGGDNEQFF	174	CD8-C5
PB-1_CAACCAATCGCATGAT	PB	PB-1	P1-1	TRUE	3	TRA:CAMSDRMTGGFKTIF TRB:CASSQVGRGGEETQYF	1047	CD8-C5
PB-1_CAACCTCTCCTTGACC	PB	PB-1	P1-187	FALSE	3	TRA:CAASMWDTDKLIF TRB:CASSAPDEQETQYF	3	CD8-C5
PB-1_CAACTAGAGTCAAGCG	PB	PB-1	P1-7	TRUE	3	TRA:CAVQAGNNDMRF TRB:CASSTTGDFNYGYTF	215	CD8-C4
PB-1_CAACTAGGTAGATTAG	PB	PB-1	P1-9	TRUE	3	TRA:CAEAEGGGADGLTF TRB:CATSGGDNEQFF	174	CD8-C5
PB-1_CAAGAAAAGGCTAGCA	PB	PB-1	P1-12	TRUE	3	TRA:CALSDERGDYKLSF TRB:CASSPGTGSYNEQFF	141	CD8-C5
PB-1_CAAGAAACAATGGAGC	PB	PB-1	P1-9	TRUE	3	TRA:CAEAEGGGADGLTF TRB:CATSGGDNEQFF	174	CD8-C5
PB-1_CAAGAAAGTTTGTTTC	PB	PB-1	P1-1	TRUE	3	TRA:CAMSDRMTGGFKTIF TRB:CASSQVGRGGEETQYF	1047	CD8-C7

PB-1_CAAGATCCACATAACC	PB	PB-1	P1-4	TRUE	3	TRA:CAMKGRDDKIIF TRB:CASTKGVSREAFF	226	CD8-C5
PB-1_CAAGTTGAGTAGATGT	PB	PB-1	P1-572	FALSE	1	TRA:CAVRPPGRNSGGYQKVTF TRB:CATSGGDNEQFF	1	CD8-C5
PB-1_CAAGTTGGTCAACATC	PB	PB-1	P1-23	TRUE	3	TRA:CALRAITQGGSEKLVF TRB:CASSLTGAVYGYTF	65	CD8-C4
PB-1_CAAGTTGTCTATCCTA	PB	PB-1	P1-573	FALSE	1	TRA:CALSEAGDRDDKIIF TRB:CASSIEEGIFTEAFF	1	CD8-C5
PB-1_CACAAACAGCTAGCCC	PB	PB-1	P1-45	TRUE	3	TRA:CAYRPLGAQKLVF TRB:CASSTLDGRESSYEQYF	20	CD8-C5
PB-1_CACACAAAGTCAATAG	PB	PB-1	P1-6	TRUE	3	TRA:CAMREGRERDDKIIF TRB:CASSLPNTEAFF	216	CD8-C5
PB-1_CACACAAGTGTGACCC	PB	PB-1	P1-9	TRUE	3	TRA:CAEAEGGGADGLTF TRB:CATSGGDNEQFF	174	CD8-C5
PB-1_CACACCTGTCATGCCG	PB	PB-1	P1-6	TRUE	3	TRA:CAMREGRERDDKIIF TRB:CASSLPNTEAFF	216	CD8-C5
PB-1_CACACCTTCGTTACGA	PB	PB-1	P1-6	TRUE	3	TRA:CAMREGRERDDKIIF TRB:CASSLPNTEAFF	216	CD8-C5
PB-1_CACACCTTCTATCCTA	PB	PB-1	P1-12	TRUE	3	TRA:CALSDERGDYKLSF TRB:CASSPGTGSYNEQFF	141	CD8-C4
PB-1_CACACTCCAAAGGTGC	PB	PB-1	P1-9	TRUE	3	TRA:CAEAEGGGADGLTF TRB:CATSGGDNEQFF	174	CD8-C5
PB-1_CACAGGCCAATCACAC	PB	PB-1	P1-7	TRUE	3	TRA:CAVQAGNNDMRF TRB:CASSTTGDFNYGYTF	215	CD8-C5
PB-1_CACAGGCTCATAAAGG	PB	PB-1	P1-9	TRUE	3	TRA:CAEAEGGGADGLTF TRB:CATSGGDNEQFF	174	CD8-C5
PB-1_CACAGTATCTTGGGTA	PB	PB-1	P1-9	TRUE	3	TRA:CAEAEGGGADGLTF TRB:CATSGGDNEQFF	174	CD8-C5
PB-1_CACATAGTCATGTCCC	PB	PB-1	P1-585	FALSE	1	TRA:CVVRVYRDDKIIF TRB:CASSSRLAGGITDTQYF	1	CD8-C5
PB-1_CACATTTGTCGATTGT	PB	PB-1	P1-4	TRUE	3	TRA:CAMKGRDDKIIF TRB:CASTKGVSREAFF	226	CD8-C7
PB-1_CACATTTTCAGGCCCA	PB	PB-1	P1-6	TRUE	3	TRA:CAMREGRERDDKIIF TRB:CASSLPNTEAFF	216	CD8-C5
PB-1_CACCACTAGGCTCATT	PB	PB-1	P1-9	TRUE	3	TRA:CAEAEGGGADGLTF TRB:CATSGGDNEQFF	174	CD8-C5
PB-1_CACCAGGAGAGAACAG	PB	PB-1	P1-7	TRUE	3	TRA:CAVQAGNNDMRF TRB:CASSTTGDFNYGYTF	215	CD8-C5
PB-1_CACCAGGCAAGGTGTG	PB	PB-1	P1-7	TRUE	3	TRA:CAVQAGNNDMRF TRB:CASSTTGDFNYGYTF	215	CD8-C5
PB-1_CACCAGGGTGCGCTTG	PB	PB-1	P1-4	TRUE	3	TRA:CAMKGRDDKIIF TRB:CASTKGVSREAFF	226	CD8-C5
PB-1_CACCTTGCACTGCCAG	PB	PB-1	P1-31	TRUE	3	TRA:CAVEEGADRLMF TRB:CASSLLGGGELFF	46	CD8-C5
PB-1_CACCTTGCAGACAAGC	PB	PB-1	P1-6	TRUE	3	TRA:CAMREGRERDDKIIF TRB:CASSLPNTEAFF	216	CD8-C5
PB-1_CAGAATCGTAGTACCT	PB	PB-1	P1-23	TRUE	3	TRA:CALRAITQGGSEKLVF TRB:CASSLTGAVYGYTF	65	CD8-C4
PB-1_CAGAGAGCACATTCGA	PB	PB-1	P1-9	TRUE	3	TRA:CAEAEGGGADGLTF TRB:CATSGGDNEQFF	174	CD8-C5
PB-1_CAGATCAAGTCCGGTC	PB	PB-1	P1-7	TRUE	3	TRA:CAVQAGNNDMRF TRB:CASSTTGDFNYGYTF	215	CD8-C5
PB-1_CAGCAGCGTTGCCTCT	PB	PB-1	P1-52	TRUE	3	TRA:CAETPDYKLSF TRB:CASRDLYEQYF	13	CD8-C4

PB-1_CAGCATAAGGAGCGAG	PB	PB-1	P1-6	TRUE	3	TRA:CAMREGRERDDKIIF TRB:CASSLPNTEAFF	216	CD8-C5
PB-1_CAGCATACATGCCTAA	PB	PB-1	P1-10	TRUE	3	TRA:CISNSPMDSSYKLIF TRB:CASSSWGDTDEAFF	162	CD8-C7
PB-1_CAGCATATCTTGGGTA	PB	PB-1	P1-597	FALSE	1	TRA:CIVRVEGYGGQNFVF TRB:CASSPGALAYEQYF	1	CD8-C5
PB-1_CAGCCGACAACGCACC	PB	PB-1	P1-12	TRUE	3	TRA:CALSDERGDYKLSF TRB:CASSPGTGSYNEQFF	141	CD8-C5
PB-1_CAGCCGATCTGCCAGG	PB	PB-1	P1-9	TRUE	3	TRA:CAEAEGGGADGLTF TRB:CATSGGDNEQFF	174	CD8-C5
PB-1_CAGCGACCAGCCTGTG	PB	PB-1	P1-7	TRUE	3	TRA:CAVQAGNNDMRF TRB:CASSTTGDFNYGYTF	215	CD8-C5
PB-1_CAGCGACCATTTCAGG	PB	PB-1	P1-601	FALSE	1	TRA:CALDPGDSNYQLIW TRB:CASSIDPAVVSTGELFF	1	CD8-C5
PB-1_CAGCTAACAACTGGCC	PB	PB-1	P1-6	TRUE	3	TRA:CAMREGRERDDKIIF TRB:CASSLPNTEAFF	216	CD8-C5
PB-1_CAGCTAACAAGAGGCT	PB	PB-1	P1-2	TRUE	3	TRA:CGQNFVF TRB:CASNQRGGPSLYNEQFF	323	CD8-C5
PB-1_CAGCTAACATACTCTT	PB	PB-1	P1-9	TRUE	3	TRA:CAEAEGGGADGLTF TRB:CATSGGDNEQFF	174	CD8-C5
PB-1_CAGCTAAGTAAAGGAG	PB	PB-1	P1-7	TRUE	3	TRA:CAVQAGNNDMRF TRB:CASSTTGDFNYGYTF	215	CD8-C5
PB-1_CAGCTGGCAGAAGCAC	PB	PB-1	P1-6	TRUE	3	TRA:CAMREGRERDDKIIF TRB:CASSLPNTEAFF	216	CD8-C5
PB-1_CAGCTGGCATCCCATC	PB	PB-1	P1-6	TRUE	3	TRA:CAMREGRERDDKIIF TRB:CASSLPNTEAFF	216	CD8-C5
PB-1_CAGCTGGGTACATGTC	PB	PB-1	P1-6	TRUE	3	TRA:CAMREGRERDDKIIF TRB:CASSLPNTEAFF	216	CD8-C5
PB-1_CAGCTGGTCGTGGTCG	PB	PB-1	P1-6	TRUE	3	TRA:CAMREGRERDDKIIF TRB:CASSLPNTEAFF	216	CD8-C5
PB-1_CAGGTGCGTGCACCAC	PB	PB-1	P1-9	TRUE	3	TRA:CAEAEGGGADGLTF TRB:CATSGGDNEQFF	174	CD8-C5
PB-1_CAGGTGCTCACCCTCA	PB	PB-1	P1-6	TRUE	3	TRA:CAMREGRERDDKIIF TRB:CASSLPNTEAFF	216	CD8-C5
PB-1_CAGGTGCTCGAATCCA	PB	PB-1	P1-143	TRUE	3	TRA:CAEGNQAGTALIF TRB:CASSTIGQGIPGANVLTF	4	CD8-C5
PB-1_CAGTAACAGTCCTCCT	PB	PB-1	P1-9	TRUE	3	TRA:CAEAEGGGADGLTF TRB:CATSGGDNEQFF	174	CD8-C5
PB-1_CAGTAACGTCCCTACT	PB	PB-1	P1-6	TRUE	3	TRA:CAMREGRERDDKIIF TRB:CASSLPNTEAFF	216	CD8-C4
PB-1_CAGTCCTAGAGTAATC	PB	PB-1	P1-14	TRUE	3	TRA:CAASVVNDMRF TRB:CASSHRGGAKNIQYF	109	CD8-C5
PB-1_CAGTCCTCAAGACGTG	PB	PB-1	P1-12	TRUE	3	TRA:CALSDERGDYKLSF TRB:CASSPGTGSYNEQFF	141	CD8-C5
PB-1_CAGTCCTGTCCGAACC	PB	PB-1	P1-7	TRUE	3	TRA:CAVQAGNNDMRF TRB:CASSTTGDFNYGYTF	215	CD8-C5
PB-1_CATATGGTCGAATGGG	PB	PB-1	P1-7	TRUE	3	TRA:CAVQAGNNDMRF TRB:CASSTTGDFNYGYTF	215	CD8-C5
PB-1_CATATGGTCGCGGATC	PB	PB-1	P1-7	TRUE	3	TRA:CAVQAGNNDMRF TRB:CASSTTGDFNYGYTF	215	CD8-C5
PB-1_CATCAAGAGCGTAATA	PB	PB-1	P1-12	TRUE	3	TRA:CALSDERGDYKLSF TRB:CASSPGTGSYNEQFF	141	CD8-C5
PB-1_CATCAAGGTCTAGAGG	PB	PB-1	P1-6	TRUE	3	TRA:CAMREGRERDDKIIF TRB:CASSLPNTEAFF	216	CD8-C5

PB-1_CATCAGAGTCGAAAGC	PB	PB-1	P1-7	TRUE	3	TRA:CAVQAGNNDMRF TRB:CASSTTGDFNYGYTF	215	CD8-C5
PB-1_CATCCACGTTCACCTC	PB	PB-1	P1-9	TRUE	3	TRA:CAEAEGGGADGLTF TRB:CATSGGDNEQFF	174	CD8-C5
PB-1_CATCGAACACCGATAT	PB	PB-1	P1-52	TRUE	3	TRA:CAETPDYKLSF TRB:CASRDLYEQYF	13	CD8-C4
PB-1_CATCGAAGTTTGTGTG	PB	PB-1	P1-7	TRUE	3	TRA:CAVQAGNNDMRF TRB:CASSTTGDFNYGYTF	215	CD8-C5
PB-1_CATCGGGCATAACCTG	PB	PB-1	P1-6	TRUE	3	TRA:CAMREGRERDDKIIF TRB:CASSLPNTEAFF	216	CD8-C5
PB-1_CATGACACACTGTTAG	PB	PB-1	P1-12	TRUE	3	TRA:CALSDERGDYKLSF TRB:CASSPGTGSYNEQFF	141	CD8-C5
PB-1_CATGACAGTCTAAACC	PB	PB-1	P1-7	TRUE	3	TRA:CAVQAGNNDMRF TRB:CASSTTGDFNYGYTF	215	CD8-C5
PB-1_CATGGCGCACTTCTGC	PB	PB-1	P1-6	TRUE	3	TRA:CAMREGRERDDKIIF TRB:CASSLPNTEAFF	216	CD8-C5
PB-1_CATTATCCACAGATTC	PB	PB-1	P1-628	FALSE	1	TRA:CAVSDGYQKVTF TRB:CASSLSKGATGNTIYF	1	CD8-C5
PB-1_CATTATCCAGACAAAT	PB	PB-1	P1-12	TRUE	3	TRA:CALSDERGDYKLSF TRB:CASSPGTGSYNEQFF	141	CD8-C5
PB-1_CATTATCCAGCTGGCT	PB	PB-1	P1-9	TRUE	3	TRA:CAEAEGGGADGLTF TRB:CATSGGDNEQFF	174	CD8-C5
PB-1_CATTATCTCGTCCGTT	PB	PB-1	P1-7	TRUE	3	TRA:CAVQAGNNDMRF TRB:CASSTTGDFNYGYTF	215	CD8-C5
PB-1_CATTATCTCTTTACGT	PB	PB-1	P1-5	TRUE	3	TRA:CALSGTPMNRDDKIIF TRB:CSARGRGLAGGPAYEQYF	223	CD8-C7
PB-1_CATTCGCAGGATATAC	PB	PB-1	P1-7	TRUE	3	TRA:CAVQAGNNDMRF TRB:CASSTTGDFNYGYTF	215	CD8-C5
PB-1_CCAATCCGTGAACCTT	PB	PB-1	P1-5	TRUE	3	TRA:CALSGTPMNRDDKIIF TRB:CSARGRGLAGGPAYEQYF	223	CD8-C5
PB-1_CCACGGAGTCCGTCAG	PB	PB-1	P1-14	TRUE	3	TRA:CAASVVNDMRF TRB:CASSHRGGAKNIQYF	109	CD8-C7
PB-1_CCACGGATCCGTCATC	PB	PB-1	P1-7	TRUE	3	TRA:CAVQAGNNDMRF TRB:CASSTTGDFNYGYTF	215	CD8-C5
PB-1_CCAGCGACAGCTCCGA	PB	PB-1	P1-9	TRUE	3	TRA:CAEAEGGGADGLTF TRB:CATSGGDNEQFF	174	CD8-C5
PB-1_CCAGCGACATATACCG	PB	PB-1	P1-642	FALSE	1	TRA:CAVRDVGVIW TRB:CASSYLGTYLYREQFF	1	CD8-C5
PB-1_CCAGCGATCAAACGGG	PB	PB-1	P1-7	TRUE	3	TRA:CAVQAGNNDMRF TRB:CASSTTGDFNYGYTF	215	CD8-C5
PB-1_CCAGCGATCTGTTTGT	PB	PB-1	P1-4	TRUE	3	TRA:CAMKGRDDKIIF TRB:CASTKGVSREAFF	226	CD8-C5
PB-1_CCATGTCAGTTAGGTA	PB	PB-1	P1-53	TRUE	3	TRA:CALSERDTDKLIF TRB:CASSILQGVITEAFF	13	CD8-C5
PB-1_CCCAATCGTAGCCTCG	PB	PB-1	P1-9	TRUE	3	TRA:CAEAEGGGADGLTF TRB:CATSGGDNEQFF	174	CD8-C5
PB-1_CCCAGTTAGAGAGCTC	PB	PB-1	P1-84	FALSE	3	TRA:CAMREPPHRGSTLGRLYF TRB:CSATGLAGVPFTDTQYF	6	CD8-C4
PB-1_CCCAGTTTCTGCAGTA	PB	PB-1	P1-6	TRUE	3	TRA:CAMREGRERDDKIIF TRB:CASSLPNTEAFF	216	CD8-C5
PB-1_CCCATACTCGTGGTCG	PB	PB-1	P1-7	TRUE	3	TRA:CAVQAGNNDMRF TRB:CASSTTGDFNYGYTF	215	CD8-C5
PB-1_CCCATACTCTTGTTTG	PB	PB-1	P1-7	TRUE	3	TRA:CAVQAGNNDMRF TRB:CASSTTGDFNYGYTF	215	CD8-C5

PB-1_CCCTCCTAGCGGCTTC	PB	PB-1	P1-6	TRUE	3	TRA:CAMREGRERDDKIIF TRB:CASSLPNTEAFF	216	CD8-C5
PB-1_CCCTCCTGTACAGTGG	PB	PB-1	P1-7	TRUE	3	TRA:CAVQAGNNDMRF TRB:CASSTTGDFNYGYTF	215	CD8-C5
PB-1_CCCTCCTGTCTCCATC	PB	PB-1	P1-9	TRUE	3	TRA:CAEAEGGGADGLTF TRB:CATSGGDNEQFF	174	CD8-C5
PB-1_CCCTCCTTCTTGTACT	PB	PB-1	P1-9	TRUE	3	TRA:CAEAEGGGADGLTF TRB:CATSGGDNEQFF	174	CD8-C5
PB-1_CCGGGATAGCACACAG	PB	PB-1	P1-6	TRUE	3	TRA:CAMREGRERDDKIIF TRB:CASSLPNTEAFF	216	CD8-C5
PB-1_CCGTACTAGAACTCGG	PB	PB-1	P1-2	TRUE	3	TRA:CGQNFVF TRB:CASNQRGGPSLYNEQFF	323	CD8-C5
PB-1_CCGTACTTCATCTGTT	PB	PB-1	P1-61	TRUE	3	TRA:CAARGGSQGNLIF TRB:CASRPRHTDTQYF	10	CD8-C5
PB-1_CCGTACTTCTGATTCT	PB	PB-1	P1-12	TRUE	3	TRA:CALSDERGDYKLSF TRB:CASSPGTGSYNEQFF	141	CD8-C5
PB-1_CCGTTCACATGCAACT	PB	PB-1	P1-7	TRUE	3	TRA:CAVQAGNNDMRF TRB:CASSTTGDFNYGYTF	215	CD8-C5
PB-1_CCGTTCAGTACATGTC	PB	PB-1	P1-7	TRUE	3	TRA:CAVQAGNNDMRF TRB:CASSTTGDFNYGYTF	215	CD8-C5
PB-1_CCTAAAGTCGAGGTAG	PB	PB-1	P1-6	TRUE	3	TRA:CAMREGRERDDKIIF TRB:CASSLPNTEAFF	216	CD8-C5
PB-1_CCTAGCTCATCCCATC	PB	PB-1	P1-9	TRUE	3	TRA:CAEAEGGGADGLTF TRB:CATSGGDNEQFF	174	CD8-C5
PB-1_CCTAGCTGTACCGAGA	PB	PB-1	P1-112	FALSE	3	TRA:CALTPLTGGGNKLTF TRB:CSAREGGSMDTQYF	5	CD8-C5
PB-1_CCTATTACAAGCCCAC	PB	PB-1	P1-658	FALSE	1	TRA:CAVTGGFKTIF TRB:CSARDSYQETQYF	1	CD8-C4
PB-1_CCTATTATCGGTTAAC	PB	PB-1	P1-9	TRUE	3	TRA:CAEAEGGGADGLTF TRB:CATSGGDNEQFF	174	CD8-C5
PB-1_CCTCAGTAGCCCTAAT	PB	PB-1	P1-22	TRUE	3	TRA:CASNSGYSTLTF TRB:CASSQVTGGARDEQYF	65	CD8-C5
PB-1_CCTCAGTGTTAAGGGC	PB	PB-1	P1-16	TRUE	3	TRA:CALSGTPMNRDDKIIF TRB:CASSQSWDLAVLNEQFF	79	CD8-C7
PB-1_CCTCAGTTCAGCCTAA	PB	PB-1	P1-7	TRUE	3	TRA:CAVQAGNNDMRF TRB:CASSTTGDFNYGYTF	215	CD8-C5
PB-1_CCTCTGACAGTAAGAT	PB	PB-1	P1-4	TRUE	3	TRA:CAMKGRDDKIIF TRB:CASTKGVSREAFF	226	CD8-C5
PB-1_CCTCTGAGTATCGCAT	PB	PB-1	P1-9	TRUE	3	TRA:CAEAEGGGADGLTF TRB:CATSGGDNEQFF	174	CD8-C7
PB-1_CCTTACGAGACAATAC	PB	PB-1	P1-12	TRUE	3	TRA:CALSDERGDYKLSF TRB:CASSPGTGSYNEQFF	141	CD8-C5
PB-1_CCTTACGAGCGATCCC	PB	PB-1	P1-5	TRUE	3	TRA:CALSGTPMNRDDKIIF TRB:CSARGRGLAGGPAYEQYF	223	CD8-C5
PB-1_CCTTACGAGCTAGTCT	PB	PB-1	P1-16	TRUE	3	TRA:CALSGTPMNRDDKIIF TRB:CASSQSWDLAVLNEQFF	79	CD8-C7
PB-1_CCTTCCCCAGCTGGCT	PB	PB-1	P1-7	TRUE	3	TRA:CAVQAGNNDMRF TRB:CASSTTGDFNYGYTF	215	CD8-C5
PB-1_CCTTTCTCAGGCTCAC	PB	PB-1	P1-12	TRUE	3	TRA:CALSDERGDYKLSF TRB:CASSPGTGSYNEQFF	141	CD8-C5
PB-1_CCTTTCTGTTCCCTTG	PB	PB-1	P1-20	TRUE	3	TRA:CVVVGGRALTF TRB:CASSLATNPHETQYF	69	CD8-C4
PB-1_CCTTTCTGTTGATTGC	PB	PB-1	P1-7	TRUE	3	TRA:CAVQAGNNDMRF TRB:CASSTTGDFNYGYTF	215	CD8-C5

PB-1_CGAACATAGTCCAGGA	PB	PB-1	P1-12	TRUE	3	TRA:CALSDERGDYKLSF TRB:CASSPGTGSYNEQFF	141	CD8-C5
PB-1_CGAATGTTCCCACTTG	PB	PB-1	P1-4	TRUE	3	TRA:CAMKGRDDKIIF TRB:CASTKGVSREAFF	226	CD8-C5
PB-1_CGACCTTAGCGGCTTC	PB	PB-1	P1-7	TRUE	3	TRA:CAVQAGNNDMRF TRB:CASSTTGDFNYGYTF	215	CD8-C5
PB-1_CGACCTTCAAGTTGTC	PB	PB-1	P1-9	TRUE	3	TRA:CAEAEGGGADGLTF TRB:CATSGGDNEQFF	174	CD8-C5
PB-1_CGACCTTCAATAGAGT	PB	PB-1	P1-1	TRUE	3	TRA:CAMSDRMTGGFKTIF TRB:CASSQVGRGGEETQYF	1047	CD8-C4
PB-1_CGACTTCGTCAAGCGA	PB	PB-1	P1-669	FALSE	1	TRA:CAVPSPGGGADGLTF TRB:CASSPHRANEQFF	1	CD8-C5
PB-1_CGACTTCGTGTGTGCC	PB	PB-1	P1-7	TRUE	3	TRA:CAVQAGNNDMRF TRB:CASSTTGDFNYGYTF	215	CD8-C5
PB-1_CGACTTCTCCATGCTC	PB	PB-1	P1-6	TRUE	3	TRA:CAMREGRERDDKIIF TRB:CASSLPNTEAFF	216	CD8-C5
PB-1_CGAGAAGAGTACCGGA	PB	PB-1	P1-84	FALSE	3	TRA:CAMREPPHRGSTLGRLYF TRB:CSATGLAGVPFTDTQYF	6	CD8-C5
PB-1_CGAGAAGCACCGCTAG	PB	PB-1	P1-7	TRUE	3	TRA:CAVQAGNNDMRF TRB:CASSTTGDFNYGYTF	215	CD8-C5
PB-1_CGAGAAGCACTGCCAG	PB	PB-1	P1-6	TRUE	3	TRA:CAMREGRERDDKIIF TRB:CASSLPNTEAFF	216	CD8-C5
PB-1_CGAGAAGTCCCATTTA	PB	PB-1	P1-140	TRUE	3	TRA:CAYRSGKGGKLIF TRB:CASSDTSSHINEQFF	4	CD8-C7
PB-1_CGAGAAGTCGTCCAGG	PB	PB-1	P1-671	FALSE	1	TRA:CAELYNFNKFYF TRB:CASSFRTGDHEQYF	1	CD8-C5
PB-1_CGAGCACAGGTCGGAT	PB	PB-1	P1-12	TRUE	3	TRA:CALSDERGDYKLSF TRB:CASSPGTGSYNEQFF	141	CD8-C5
PB-1_CGAGCCACATAGTAAG	PB	PB-1	P1-4	TRUE	3	TRA:CAMKGRDDKIIF TRB:CASTKGVSREAFF	226	CD8-C7
PB-1_CGATCGGTCAAAGTAG	PB	PB-1	P1-677	FALSE	1	TRA:CASLEGGGNKLTF TRB:CASSLSYEQYF	1	CD8-C5
PB-1_CGATGTAAGTGTTAGA	PB	PB-1	P1-6	TRUE	3	TRA:CAMREGRERDDKIIF TRB:CASSLPNTEAFF	216	CD8-C5
PB-1_CGATGTACAAAGAATC	PB	PB-1	P1-54	TRUE	3	TRA:CAGWYSGGGADGLTF TRB:CATGAGDNEQFF	13	CD8-C5
PB-1_CGATGTACATGCCTTC	PB	PB-1	P1-6	TRUE	3	TRA:CAMREGRERDDKIIF TRB:CASSLPNTEAFF	216	CD8-C5
PB-1_CGATTGAAGGGTATCG	PB	PB-1	P1-6	TRUE	3	TRA:CAMREGRERDDKIIF TRB:CASSLPNTEAFF	216	CD8-C5
PB-1_CGCCAAGAGTTTCCTT	PB	PB-1	P1-16	TRUE	3	TRA:CALSGTPMNRDDKIIF TRB:CASSQSWDLAVLNEQFF	79	CD8-C7
PB-1_CGCCAAGCAGGGATTG	PB	PB-1	P1-4	TRUE	3	TRA:CAMKGRDDKIIF TRB:CASTKGVSREAFF	226	CD8-C7
PB-1_CGCGGTACATTGGTAC	PB	PB-1	P1-37	TRUE	3	TRA:CAVNIVGNQFYF TRB:CASSPLGTEAFF	40	CD8-C4
PB-1_CGCGGTAGTGTTGGGA	PB	PB-1	P1-7	TRUE	3	TRA:CAVQAGNNDMRF TRB:CASSTTGDFNYGYTF	215	CD8-C5
PB-1_CGCGGTATCTATCCCG	PB	PB-1	P1-1	TRUE	3	TRA:CAMSDRMTGGFKTIF TRB:CASSQVGRGGEETQYF	1047	CD8-C7
PB-1_CGCGTTTAGATGTAAC	PB	PB-1	P1-7	TRUE	3	TRA:CAVQAGNNDMRF TRB:CASSTTGDFNYGYTF	215	CD8-C5
PB-1_CGCGTTTAGTGAATTG	PB	PB-1	P1-9	TRUE	3	TRA:CAEAEGGGADGLTF TRB:CATSGGDNEQFF	174	CD8-C5

PB-1_CGCTATCCACATGGGA	PB	PB-1	P1-2	TRUE	3	TRA:CGQNFVF TRB:CASNQRGGPSLYNEQFF	323	CD8-C2
PB-1_CGCTGGACACAGATTC	PB	PB-1	P1-6	TRUE	3	TRA:CAMREGRERDDKIIF TRB:CASSLPNTEAFF	216	CD8-C5
PB-1_CGCTGGAGTGAGCGAT	PB	PB-1	P1-9	TRUE	3	TRA:CAEAEGGGADGLTF TRB:CATSGGDNEQFF	174	CD8-C5
PB-1_CGCTTCAGTCATCGGC	PB	PB-1	P1-39	TRUE	3	TRA:CAVRDLGGTSYDKVIF TRB:CSARVREVSYEQYF	31	CD8-C2
PB-1_CGGACACGTAGATTAG	PB	PB-1	P1-6	TRUE	3	TRA:CAMREGRERDDKIIF TRB:CASSLPNTEAFF	216	CD8-C5
PB-1_CGGACTGAGCTACCGC	PB	PB-1	P1-9	TRUE	3	TRA:CAEAEGGGADGLTF TRB:CATSGGDNEQFF	174	CD8-C5
PB-1_CGGACTGCAGGGTTAG	PB	PB-1	P1-5	TRUE	3	TRA:CALSGTPMNRDDKIIF TRB:CSARGRGLAGGPAYEQYF	223	CD8-C5
PB-1_CGGACTGCATGCAACT	PB	PB-1	P1-37	TRUE	3	TRA:CAVNIVGNQFYF TRB:CASSPLGTEAFF	40	CD8-C4
PB-1_CGGAGCTCAGCTGCAC	PB	PB-1	P1-6	TRUE	3	TRA:CAMREGRERDDKIIF TRB:CASSLPNTEAFF	216	CD8-C7
PB-1_CGGAGCTGTTCACGGC	PB	PB-1	P1-9	TRUE	3	TRA:CAEAEGGGADGLTF TRB:CATSGGDNEQFF	174	CD8-C7
PB-1_CGGAGTCAGCATGGCA	PB	PB-1	P1-6	TRUE	3	TRA:CAMREGRERDDKIIF TRB:CASSLPNTEAFF	216	CD8-C5
PB-1_CGGCTAGCATACGCCG	PB	PB-1	P1-5	TRUE	3	TRA:CALSGTPMNRDDKIIF TRB:CSARGRGLAGGPAYEQYF	223	CD8-C5
PB-1_CGGCTAGGTCGCCATG	PB	PB-1	P1-9	TRUE	3	TRA:CAEAEGGGADGLTF TRB:CATSGGDNEQFF	174	CD8-C5
PB-1_CGGGTCATCGCCGTGA	PB	PB-1	P1-6	TRUE	3	TRA:CAMREGRERDDKIIF TRB:CASSLPNTEAFF	216	CD8-C5
PB-1_CGGTTAATCGACCAGC	PB	PB-1	P1-7	TRUE	3	TRA:CAVQAGNNDMRF TRB:CASSTTGDFNYGYTF	215	CD8-C5
PB-1_CGTAGCGGTTAAGAAC	PB	PB-1	P1-7	TRUE	3	TRA:CAVQAGNNDMRF TRB:CASSTTGDFNYGYTF	215	CD8-C5
PB-1_CGTAGGCGTGGCAAAC	PB	PB-1	P1-709	FALSE	1	TRA:CAVRDWGNARLMF TRB:CAWTAEPDRRNSPLHF	1	CD8-C5
PB-1_CGTCACTAGATCTGCT	PB	PB-1	P1-5	TRUE	3	TRA:CALSGTPMNRDDKIIF TRB:CSARGRGLAGGPAYEQYF	223	CD8-C7
PB-1_CGTCACTAGGCAGGTT	PB	PB-1	P1-7	TRUE	3	TRA:CAVQAGNNDMRF TRB:CASSTTGDFNYGYTF	215	CD8-C5
PB-1_CGTCACTTCTGCCCTA	PB	PB-1	P1-12	TRUE	3	TRA:CALSDERGDYKLSF TRB:CASSPGTGSYNEQFF	141	CD8-C4
PB-1_CGTCAGGTCAATCACG	PB	PB-1	P1-16	TRUE	3	TRA:CALSGTPMNRDDKIIF TRB:CASSQSWDLAVLNEQFF	79	CD8-C5
PB-1_CGTCCATGTTCCACTC	PB	PB-1	P1-44	TRUE	3	TRA:CAYRGLGAQKLVF TRB:CASSSLDGQDNHYGYTF	22	CD8-C4
PB-1_CGTCTACAGGATTCGG	PB	PB-1	P1-91	TRUE	3	TRA:CVVSDQYNTDKLIF TRB:CASGEGQGASSYEQYF	6	CD8-C5
PB-1_CGTCTACCACTCTGTC	PB	PB-1	P1-7	TRUE	3	TRA:CAVQAGNNDMRF TRB:CASSTTGDFNYGYTF	215	CD8-C5
PB-1_CGTGTAATCGTGGACC	PB	PB-1	P1-9	TRUE	3	TRA:CAEAEGGGADGLTF TRB:CATSGGDNEQFF	174	CD8-C5
PB-1_CGTGTCTAGGTGATTA	PB	PB-1	P1-12	TRUE	3	TRA:CALSDERGDYKLSF TRB:CASSPGTGSYNEQFF	141	CD8-C5
PB-1_CGTTAGACACATGACT	PB	PB-1	P1-4	TRUE	3	TRA:CAMKGRDDKIIF TRB:CASTKGVSREAFF	226	CD8-C7

PB-1_CGTTCTGAGACGACGT	PB	PB-1	P1-152	TRUE	3	TRA:CALSEAGKPGRKAYLRT TRB:CASSPGTGSYNEQFF	4	CD8-C5
PB-1_CGTTCTGTCATACGGT	PB	PB-1	P1-93	TRUE	3	TRA:CAFTGGGGADGLTF TRB:CASSVAWGTGGWNTEAFF	6	CD8-C2
PB-1_CTAACTTAGTGGTAGC	PB	PB-1	P1-9	TRUE	3	TRA:CAEAEGGGADGLTF TRB:CATSGGDNEQFF	174	CD8-C7
PB-1_CTAACTTGTTAAGTAG	PB	PB-1	P1-26	TRUE	3	TRA:CAPRSGGSYIPTF TRB:CASSQDLGLDYGYTF	56	CD8-C5
PB-1_CTAACTTGTTCTGGTA	PB	PB-1	P1-12	TRUE	3	TRA:CALSDERGDYKLSF TRB:CASSPGTGSYNEQFF	141	CD8-C5
PB-1_CTAACTTTCACAGGCC	PB	PB-1	P1-187	FALSE	3	TRA:CAASMWDTDKLIF TRB:CASSAPDEQETQYF	3	CD8-C5
PB-1_CTAAGACGTACAAGTA	PB	PB-1	P1-723	FALSE	1	TRA:CAFTQGGSEKLVF TRB:CASSQEEGAGGNQETQYF	1	CD8-C5
PB-1_CTAAGACGTTTACTCT	PB	PB-1	P1-84	FALSE	3	TRA:CAMREPPHRGSTLGRLYF TRB:CSATGLAGVPFTDTQYF	6	CD8-C5
PB-1_CTAAGACTCTGTACGA	PB	PB-1	P1-9	TRUE	3	TRA:CAEAEGGGADGLTF TRB:CATSGGDNEQFF	174	CD8-C7
PB-1_CTAATGGAGACTGGGT	PB	PB-1	P1-283	TRUE	2	TRA:CIVRVPNTGNQFYF TRB:CAIRTADMVIGTDTQYF	2	CD8-C5
PB-1_CTAATGGTCACAGTAC	PB	PB-1	P1-12	TRUE	3	TRA:CALSDERGDYKLSF TRB:CASSPGTGSYNEQFF	141	CD8-C5
PB-1_CTACACCCAGATGAGC	PB	PB-1	P1-7	TRUE	3	TRA:CAVQAGNNDMRF TRB:CASSTTGDFNYGYTF	215	CD8-C5
PB-1_CTACACCCAGCGTAAG	PB	PB-1	P1-9	TRUE	3	TRA:CAEAEGGGADGLTF TRB:CATSGGDNEQFF	174	CD8-C5
PB-1_CTACATTAGTGTACCT	PB	PB-1	P1-4	TRUE	3	TRA:CAMKGRDDKIIF TRB:CASTKGVSREAFF	226	CD8-C5
PB-1_CTACCCAAGGTGATAT	PB	PB-1	P1-7	TRUE	3	TRA:CAVQAGNNDMRF TRB:CASSTTGDFNYGYTF	215	CD8-C5
PB-1_CTACCCAGTCAAGCGA	PB	PB-1	P1-12	TRUE	3	TRA:CALSDERGDYKLSF TRB:CASSPGTGSYNEQFF	141	CD8-C7
PB-1_CTAGAGTAGCCAGTTT	PB	PB-1	P1-9	TRUE	3	TRA:CAEAEGGGADGLTF TRB:CATSGGDNEQFF	174	CD8-C5
PB-1_CTAGAGTAGGCTAGCA	PB	PB-1	P1-14	TRUE	3	TRA:CAASVVNDMRF TRB:CASSHRGGAKNIQYF	109	CD8-C5
PB-1_CTAGAGTCACCGCTAG	PB	PB-1	P1-12	TRUE	3	TRA:CALSDERGDYKLSF TRB:CASSPGTGSYNEQFF	141	CD8-C5
PB-1_CTAGAGTTCGACGGAA	PB	PB-1	P1-4	TRUE	3	TRA:CAMKGRDDKIIF TRB:CASTKGVSREAFF	226	CD8-C7
PB-1_CTAGCCTCATCGGTTA	PB	PB-1	P1-9	TRUE	3	TRA:CAEAEGGGADGLTF TRB:CATSGGDNEQFF	174	CD8-C5
PB-1_CTAGTGAAGGGCTTCC	PB	PB-1	P1-12	TRUE	3	TRA:CALSDERGDYKLSF TRB:CASSPGTGSYNEQFF	141	CD8-C5
PB-1_CTCACACCAGGTCGTC	PB	PB-1	P1-8	TRUE	3	TRA:CAVRPPGRNSGGYQKVTF TRB:CASSRRGVTDTQYF	204	CD8-C7
PB-1_CTCACACGTGTGACGA	PB	PB-1	P1-12	TRUE	3	TRA:CALSDERGDYKLSF TRB:CASSPGTGSYNEQFF	141	CD8-C5
PB-1_CTCACACGTTACGCGC	PB	PB-1	P1-9	TRUE	3	TRA:CAEAEGGGADGLTF TRB:CATSGGDNEQFF	174	CD8-C5
PB-1_CTCACACTCGGAGCAA	PB	PB-1	P1-6	TRUE	3	TRA:CAMREGRERDDKIIF TRB:CASSLPNTEAFF	216	CD8-C5
PB-1_CTCAGAAAGTACATGA	PB	PB-1	P1-9	TRUE	3	TRA:CAEAEGGGADGLTF TRB:CATSGGDNEQFF	174	CD8-C5

PB-1_CTCAGAACAGCGTAAG	PB	PB-1	P1-9	TRUE	3	TRA:CAEAEGGGADGLTF TRB:CATSGGDNEQFF	174	CD8-C5
PB-1_CTCAGAACAGGCTGAA	PB	PB-1	P1-7	TRUE	3	TRA:CAVQAGNNDMRF TRB:CASSTTGDFNYGYTF	215	CD8-C5
PB-1_CTCAGAATCGGCGGTT	PB	PB-1	P1-733	FALSE	1	TRA:CAYRGYGGATNKLIF TRB:CATSVGGLSNQPQHF	1	CD8-C7
PB-1_CTCATTACATCCTTGC	PB	PB-1	P1-12	TRUE	3	TRA:CALSDERGDYKLSF TRB:CASSPGTGSYNEQFF	141	CD8-C5
PB-1_CTCATTATCATTCACT	PB	PB-1	P1-12	TRUE	3	TRA:CALSDERGDYKLSF TRB:CASSPGTGSYNEQFF	141	CD8-C5
PB-1_CTCATTATCTGTCTAT	PB	PB-1	P1-9	TRUE	3	TRA:CAEAEGGGADGLTF TRB:CATSGGDNEQFF	174	CD8-C5
PB-1_CTCCTAGCACAGCCCA	PB	PB-1	P1-735	FALSE	1	TRA:CATEEVPNFGNEKLTF TRB:CASRGTF	1	CD8-C4
PB-1_CTCGAAAGTCTCAACA	PB	PB-1	P1-9	TRUE	3	TRA:CAEAEGGGADGLTF TRB:CATSGGDNEQFF	174	CD8-C5
PB-1_CTCGAAAGTTCAGTAC	PB	PB-1	P1-7	TRUE	3	TRA:CAVQAGNNDMRF TRB:CASSTTGDFNYGYTF	215	CD8-C5
PB-1_CTCGAAAGTTGAGTTC	PB	PB-1	P1-9	TRUE	3	TRA:CAEAEGGGADGLTF TRB:CATSGGDNEQFF	174	CD8-C5
PB-1_CTCGAGGAGCACGCCT	PB	PB-1	P1-7	TRUE	3	TRA:CAVQAGNNDMRF TRB:CASSTTGDFNYGYTF	215	CD8-C5
PB-1_CTCGAGGGTACAGTGG	PB	PB-1	P1-39	TRUE	3	TRA:CAVRDLGGTSYDKVIF TRB:CSARVREVSYEQYF	31	CD8-C5
PB-1_CTCGGAGGTGATGTCT	PB	PB-1	P1-7	TRUE	3	TRA:CAVQAGNNDMRF TRB:CASSTTGDFNYGYTF	215	CD8-C7
PB-1_CTCTAATCAAGCCGCT	PB	PB-1	P1-12	TRUE	3	TRA:CALSDERGDYKLSF TRB:CASSPGTGSYNEQFF	141	CD8-C5
PB-1_CTGAAACAGGATGGAA	PB	PB-1	P1-8	TRUE	3	TRA:CAVRPPGRNSGGYQKVTF TRB:CASSRRGVTDTQYF	204	CD8-C7
PB-1_CTGAAGTAGGGAACGG	PB	PB-1	P1-37	TRUE	3	TRA:CAVNIVGNQFYF TRB:CASSPLGTEAFF	40	CD8-C4
PB-1_CTGAAGTCATCGATTG	PB	PB-1	P1-12	TRUE	3	TRA:CALSDERGDYKLSF TRB:CASSPGTGSYNEQFF	141	CD8-C5
PB-1_CTGAAGTGTCAAAGCG	PB	PB-1	P1-7	TRUE	3	TRA:CAVQAGNNDMRF TRB:CASSTTGDFNYGYTF	215	CD8-C5
PB-1_CTGAAGTGTGTTGGGA	PB	PB-1	P1-9	TRUE	3	TRA:CAEAEGGGADGLTF TRB:CATSGGDNEQFF	174	CD8-C5
PB-1_CTGAAGTTCCTTCAAT	PB	PB-1	P1-7	TRUE	3	TRA:CAVQAGNNDMRF TRB:CASSTTGDFNYGYTF	215	CD8-C5
PB-1_CTGATAGAGGATATAC	PB	PB-1	P1-7	TRUE	3	TRA:CAVQAGNNDMRF TRB:CASSTTGDFNYGYTF	215	CD8-C5
PB-1_CTGATAGAGGTGCTTT	PB	PB-1	P1-14	TRUE	3	TRA:CAASVVNDMRF TRB:CASSHRGGAKNIQYF	109	CD8-C5
PB-1_CTGATCCAGCACGCCT	PB	PB-1	P1-14	TRUE	3	TRA:CAASVVNDMRF TRB:CASSHRGGAKNIQYF	109	CD8-C5
PB-1_CTGATCCAGTCGTTTG	PB	PB-1	P1-9	TRUE	3	TRA:CAEAEGGGADGLTF TRB:CATSGGDNEQFF	174	CD8-C5
PB-1_CTGATCCTCACAGGCC	PB	PB-1	P1-6	TRUE	3	TRA:CAMREGRERDDKIIF TRB:CASSLPNTEAFF	216	CD8-C5
PB-1_CTGCCTAAGTCCATAC	PB	PB-1	P1-6	TRUE	3	TRA:CAMREGRERDDKIIF TRB:CASSLPNTEAFF	216	CD8-C5
PB-1_CTGCCTAGTAAGGATT	PB	PB-1	P1-7	TRUE	3	TRA:CAVQAGNNDMRF TRB:CASSTTGDFNYGYTF	215	CD8-C5

PB-1_CTGCGGAAGTGGGCTA	PB	PB-1	P1-7	TRUE	3	TRA:CAVQAGNNDMRF TRB:CASSTTGDFNYGYTF	215	CD8-C5
PB-1_CTGCGGACAAAGCAAT	PB	PB-1	P1-39	TRUE	3	TRA:CAVRDLGGTSYDKVIF TRB:CSARVREVSYEQYF	31	CD8-C5
PB-1_CTGCGGATCCACTGGG	PB	PB-1	P1-7	TRUE	3	TRA:CAVQAGNNDMRF TRB:CASSTTGDFNYGYTF	215	CD8-C5
PB-1_CTGCTGTCACCAGATT	PB	PB-1	P1-12	TRUE	3	TRA:CALSDERGDYKLSF TRB:CASSPGTGSYNEQFF	141	CD8-C5
PB-1_CTGCTGTGTCATGCCG	PB	PB-1	P1-5	TRUE	3	TRA:CALSGTPMNRDDKIIF TRB:CSARGRGLAGGPAYEQYF	223	CD8-C5
PB-1_CTGCTGTTCCTCGCAT	PB	PB-1	P1-288	FALSE	2	TRA:CVVMNYGGSQGNLIF TRB:CASSQSPGQRNYGYTF	2	CD8-C4
PB-1_CTGGTCTAGGCTATCT	PB	PB-1	P1-7	TRUE	3	TRA:CAVQAGNNDMRF TRB:CASSTTGDFNYGYTF	215	CD8-C5
PB-1_CTGGTCTCATGGTCAT	PB	PB-1	P1-4	TRUE	3	TRA:CAMKGRDDKIIF TRB:CASTKGVSREAFF	226	CD8-C5
PB-1_CTGGTCTTCACCACCT	PB	PB-1	P1-87	TRUE	3	TRA:CGTVYNYGGQNFVF TRB:CASSRGRGAQPQHF	6	CD8-C5
PB-1_CTGTGCTCACGGACAA	PB	PB-1	P1-4	TRUE	3	TRA:CAMKGRDDKIIF TRB:CASTKGVSREAFF	226	CD8-C5
PB-1_CTGTTTAGTCTAGCCG	PB	PB-1	P1-6	TRUE	3	TRA:CAMREGRERDDKIIF TRB:CASSLPNTEAFF	216	CD8-C5
PB-1_CTTAACTAGGGATACC	PB	PB-1	P1-40	TRUE	3	TRA:CAVRDVGNTGFQKLVF TRB:CASSLGQGSMKTQYF	27	CD8-C4
PB-1_CTTAACTCAAACTGCT	PB	PB-1	P1-7	TRUE	3	TRA:CAVQAGNNDMRF TRB:CASSTTGDFNYGYTF	215	CD8-C5
PB-1_CTTAACTGTTGAGTTC	PB	PB-1	P1-6	TRUE	3	TRA:CAMREGRERDDKIIF TRB:CASSLPNTEAFF	216	CD8-C5
PB-1_CTTACCGCAACAACCT	PB	PB-1	P1-7	TRUE	3	TRA:CAVQAGNNDMRF TRB:CASSTTGDFNYGYTF	215	CD8-C5
PB-1_CTTACCGCATCCGGGT	PB	PB-1	P1-7	TRUE	3	TRA:CAVQAGNNDMRF TRB:CASSTTGDFNYGYTF	215	CD8-C4
PB-1_CTTACCGTCCAATGGT	PB	PB-1	P1-12	TRUE	3	TRA:CALSDERGDYKLSF TRB:CASSPGTGSYNEQFF	141	CD8-C5
PB-1_CTTCTCTAGTTAAGTG	PB	PB-1	P1-26	TRUE	3	TRA:CAPRSGGSYIPTF TRB:CASSQDLGLDYGYTF	56	CD8-C7
PB-1_CTTCTCTGTTCCACAA	PB	PB-1	P1-6	TRUE	3	TRA:CAMREGRERDDKIIF TRB:CASSLPNTEAFF	216	CD8-C5
PB-1_CTTGGCTCACCCTATC	PB	PB-1	P1-774	FALSE	1	TRA:CAEPRFTGNQFYF TRB:CAISGGNEQFF	1	CD8-C5
PB-1_CTTGGCTCACCTATCC	PB	PB-1	P1-37	TRUE	3	TRA:CAVNIVGNQFYF TRB:CASSPLGTEAFF	40	CD8-C4
PB-1_CTTGGCTCAGTATAAG	PB	PB-1	P1-9	TRUE	3	TRA:CAEAEGGGADGLTF TRB:CATSGGDNEQFF	174	CD8-C7
PB-1_CTTTGCGAGGAGCGTT	PB	PB-1	P1-3	TRUE	3	TRA:CAFLSGTYKYIF TRB:CASSQSPGQRNYGYTF	308	CD8-C7
PB-1_CTTTGCGCATAGGATA	PB	PB-1	P1-7	TRUE	3	TRA:CAVQAGNNDMRF TRB:CASSTTGDFNYGYTF	215	CD8-C7
PB-1_GAAACTCCAGGACCCT	PB	PB-1	P1-4	TRUE	3	TRA:CAMKGRDDKIIF TRB:CASTKGVSREAFF	226	CD8-C5
PB-1_GAAACTCGTACCGTAT	PB	PB-1	P1-7	TRUE	3	TRA:CAVQAGNNDMRF TRB:CASSTTGDFNYGYTF	215	CD8-C5
PB-1_GAAATGAAGGTGATTA	PB	PB-1	P1-52	TRUE	3	TRA:CAETPDYKLSF TRB:CASRDLYEQYF	13	CD8-C3

PB-1_GAAATGAGTTCGTGAT	PB	PB-1	P1-5	TRUE	3	TRA:CALSGTPMNRDDKIIF TRB:CSARGRGLAGGPAYEQYF	223	CD8-C7
PB-1_GAACATCAGAGTACAT	PB	PB-1	P1-7	TRUE	3	TRA:CAVQAGNNDMRF TRB:CASSTTGDFNYGYTF	215	CD8-C5
PB-1_GAACATCGTGCTTCTC	PB	PB-1	P1-31	TRUE	3	TRA:CAVEEGADRLMF TRB:CASSLLGGGELFF	46	CD8-C7
PB-1_GAACATCTCACCTTAT	PB	PB-1	P1-6	TRUE	3	TRA:CAMREGRERDDKIIF TRB:CASSLPNTEAFF	216	CD8-C5
PB-1_GAACCTAAGAAGAAGC	PB	PB-1	P1-4	TRUE	3	TRA:CAMKGRDDKIIF TRB:CASTKGVSREAFF	226	CD8-C5
PB-1_GAACCTATCACAACGT	PB	PB-1	P1-4	TRUE	3	TRA:CAMKGRDDKIIF TRB:CASTKGVSREAFF	226	CD8-C7
PB-1_GAACGGAAGCCGATTT	PB	PB-1	P1-14	TRUE	3	TRA:CAASVVNDMRF TRB:CASSHRGGAKNIQYF	109	CD8-C7
PB-1_GAACGGAGTCTACCTC	PB	PB-1	P1-5	TRUE	3	TRA:CALSGTPMNRDDKIIF TRB:CSARGRGLAGGPAYEQYF	223	CD8-C5
PB-1_GAACGGATCTTGTTTG	PB	PB-1	P1-4	TRUE	3	TRA:CAMKGRDDKIIF TRB:CASTKGVSREAFF	226	CD8-C7
PB-1_GAAGCAGAGGGATCTG	PB	PB-1	P1-16	TRUE	3	TRA:CALSGTPMNRDDKIIF TRB:CASSQSWDLAVLNEQFF	79	CD8-C5
PB-1_GAAGCAGGTCTGATTG	PB	PB-1	P1-6	TRUE	3	TRA:CAMREGRERDDKIIF TRB:CASSLPNTEAFF	216	CD8-C5
PB-1_GAATAAGAGATATGCA	PB	PB-1	P1-9	TRUE	3	TRA:CAEAEGGGADGLTF TRB:CATSGGDNEQFF	174	CD8-C5
PB-1_GAATAAGCAGTATCTG	PB	PB-1	P1-6	TRUE	3	TRA:CAMREGRERDDKIIF TRB:CASSLPNTEAFF	216	CD8-C5
PB-1_GAATGAACAACACCTA	PB	PB-1	P1-7	TRUE	3	TRA:CAVQAGNNDMRF TRB:CASSTTGDFNYGYTF	215	CD8-C5
PB-1_GACACGCCAAATCCGT	PB	PB-1	P1-7	TRUE	3	TRA:CAVQAGNNDMRF TRB:CASSTTGDFNYGYTF	215	CD8-C5
PB-1_GACCAATGTTCACCTC	PB	PB-1	P1-9	TRUE	3	TRA:CAEAEGGGADGLTF TRB:CATSGGDNEQFF	174	CD8-C5
PB-1_GACGGCTAGCACACAG	PB	PB-1	P1-7	TRUE	3	TRA:CAVQAGNNDMRF TRB:CASSTTGDFNYGYTF	215	CD8-C8
PB-1_GACGTTAAGATGGCGT	PB	PB-1	P1-9	TRUE	3	TRA:CAEAEGGGADGLTF TRB:CATSGGDNEQFF	174	CD8-C5
PB-1_GACGTTAAGCGTGAAC	PB	PB-1	P1-6	TRUE	3	TRA:CAMREGRERDDKIIF TRB:CASSLPNTEAFF	216	CD8-C5
PB-1_GACGTTAGTCAGAGGT	PB	PB-1	P1-4	TRUE	3	TRA:CAMKGRDDKIIF TRB:CASTKGVSREAFF	226	CD8-C7
PB-1_GACTAACAGTCCATAC	PB	PB-1	P1-9	TRUE	3	TRA:CAEAEGGGADGLTF TRB:CATSGGDNEQFF	174	CD8-C5
PB-1_GACTAACGTCACTGGC	PB	PB-1	P1-26	TRUE	3	TRA:CAPRSGGSYIPTF TRB:CASSQDLGLDYGYTF	56	CD8-C5
PB-1_GACTACAAGACACTAA	PB	PB-1	P1-143	TRUE	3	TRA:CAEGNQAGTALIF TRB:CASSTIGQGIPGANVLTF	4	CD8-C4
PB-1_GACTACATCACGATGT	PB	PB-1	P1-7	TRUE	3	TRA:CAVQAGNNDMRF TRB:CASSTTGDFNYGYTF	215	CD8-C5
PB-1_GACTGCGAGATGTGGC	PB	PB-1	P1-6	TRUE	3	TRA:CAMREGRERDDKIIF TRB:CASSLPNTEAFF	216	CD8-C5
PB-1_GACTGCGAGGGCTCTC	PB	PB-1	P1-800	FALSE	1	TRA:CALSEAHGGSQGNLIF TRB:CASSVEVFTDTQYF	1	CD8-C4
PB-1_GAGCAGAAGAGTAAGG	PB	PB-1	P1-39	TRUE	3	TRA:CAVRDLGGTSYDKVIF TRB:CSARVREVSYEQYF	31	CD8-C5

PB-1_GAGCAGAAGATAGCAT	PB	PB-1	P1-12	TRUE	3	TRA:CALSDERGDYKLSF TRB:CASSPGTGSYNEQFF	141	CD8-C4
PB-1_GAGCAGACAGCTCCGA	PB	PB-1	P1-12	TRUE	3	TRA:CALSDERGDYKLSF TRB:CASSPGTGSYNEQFF	141	CD8-C4
PB-1_GAGCAGACATGCGCAC	PB	PB-1	P1-53	TRUE	3	TRA:CALSERDTDKLIF TRB:CASSILQGVITEAFF	13	CD8-C5
PB-1_GAGCAGATCTGTTGAG	PB	PB-1	P1-12	TRUE	3	TRA:CALSDERGDYKLSF TRB:CASSPGTGSYNEQFF	141	CD8-C5
PB-1_GATCAGTCAATCGAAA	PB	PB-1	P1-5	TRUE	3	TRA:CALSGTPMNRDDKIIF TRB:CSARGRGLAGGPAYEQYF	223	CD8-C7
PB-1_GATCGATAGCCAGTTT	PB	PB-1	P1-93	TRUE	3	TRA:CAFTGGGGADGLTF TRB:CASSVAWGTGGWNTEAFF	6	CD8-C4
PB-1_GATCGATAGGCCCGTT	PB	PB-1	P1-197	TRUE	3	TRA:CAENFQGAQKLVF TRB:CSVDPSGNTGELFF	3	CD8-C5
PB-1_GATCGATCAAATACAG	PB	PB-1	P1-12	TRUE	3	TRA:CALSDERGDYKLSF TRB:CASSPGTGSYNEQFF	141	CD8-C5
PB-1_GATCGCGAGAGTCTGG	PB	PB-1	P1-4	TRUE	3	TRA:CAMKGRDDKIIF TRB:CASTKGVSREAFF	226	CD8-C7
PB-1_GATCGTAAGACGCACA	PB	PB-1	P1-7	TRUE	3	TRA:CAVQAGNNDMRF TRB:CASSTTGDFNYGYTF	215	CD8-C5
PB-1_GATCGTAAGGCCCGTT	PB	PB-1	P1-7	TRUE	3	TRA:CAVQAGNNDMRF TRB:CASSTTGDFNYGYTF	215	CD8-C4
PB-1_GATCTAGAGAAGCCCA	PB	PB-1	P1-7	TRUE	3	TRA:CAVQAGNNDMRF TRB:CASSTTGDFNYGYTF	215	CD8-C5
PB-1_GATCTAGAGGCGCTCT	PB	PB-1	P1-113	TRUE	3	TRA:CAVNGGSQGNLIF TRB:CASSLAVNYGYTF	5	CD8-C7
PB-1_GATCTAGAGGGTTTCT	PB	PB-1	P1-6	TRUE	3	TRA:CAMREGRERDDKIIF TRB:CASSLPNTEAFF	216	CD8-C4
PB-1_GATCTAGCACTCTGTC	PB	PB-1	P1-93	TRUE	3	TRA:CAFTGGGGADGLTF TRB:CASSVAWGTGGWNTEAFF	6	CD8-C4
PB-1_GATCTAGGTCAACTGT	PB	PB-1	P1-6	TRUE	3	TRA:CAMREGRERDDKIIF TRB:CASSLPNTEAFF	216	CD8-C5
PB-1_GATGAAAGTATTCTCT	PB	PB-1	P1-7	TRUE	3	TRA:CAVQAGNNDMRF TRB:CASSTTGDFNYGYTF	215	CD8-C5
PB-1_GATGAAATCGGCGCTA	PB	PB-1	P1-9	TRUE	3	TRA:CAEAEGGGADGLTF TRB:CATSGGDNEQFF	174	CD8-C7
PB-1_GATGCTACACTAAGTC	PB	PB-1	P1-12	TRUE	3	TRA:CALSDERGDYKLSF TRB:CASSPGTGSYNEQFF	141	CD8-C5
PB-1_GATGCTATCACCCGAG	PB	PB-1	P1-198	TRUE	3	TRA:CAVRDMDDKIIF TRB:CASRPQGAGGYEQYF	3	CD8-C5
PB-1_GATGCTATCAGTTGAC	PB	PB-1	P1-6	TRUE	3	TRA:CAMREGRERDDKIIF TRB:CASSLPNTEAFF	216	CD8-C5
PB-1_GATGCTATCTCCGGTT	PB	PB-1	P1-818	FALSE	1	TRA:CAAILTGGGNKLTF TRB:CASSLGQRGLQYF	1	CD8-C5
PB-1_GATTCAGAGATGTGGC	PB	PB-1	P1-9	TRUE	3	TRA:CAEAEGGGADGLTF TRB:CATSGGDNEQFF	174	CD8-C5
PB-1_GCAAACTAGACTTGAA	PB	PB-1	P1-6	TRUE	3	TRA:CAMREGRERDDKIIF TRB:CASSLPNTEAFF	216	CD8-C5
PB-1_GCAAACTTCGTAGGTT	PB	PB-1	P1-9	TRUE	3	TRA:CAEAEGGGADGLTF TRB:CATSGGDNEQFF	174	CD8-C5
PB-1_GCAATCACATGCGCAC	PB	PB-1	P1-1	TRUE	3	TRA:CAMSDRMTGGFKTIF TRB:CASSQVGRGGEETQYF	1047	CD8-C5
PB-1_GCAATCAGTTGATTGC	PB	PB-1	P1-1	TRUE	3	TRA:CAMSDRMTGGFKTIF TRB:CASSQVGRGGEETQYF	1047	CD8-C5

PB-1_GCAATCATCTCTGAGA	PB	PB-1	P1-50	TRUE	3	TRA:CARWLGSQGNLIF TRB:CASSLTDRVPQFELVYEQYF	15	CD8-C7
PB-1_GCACATACAATCACAC	PB	PB-1	P1-68	TRUE	3	TRA:CAVIPDILTGGGNKLTF TRB:CASSLQGFGNTIYF	9	CD8-C4
PB-1_GCACATAGTCGACTGC	PB	PB-1	P1-6	TRUE	3	TRA:CAMREGRERDDKIIF TRB:CASSLPNTEAFF	216	CD8-C5
PB-1_GCACTCTAGGTGTTAA	PB	PB-1	P1-84	FALSE	3	TRA:CAMREPPHRGSTLGRLYF TRB:CSATGLAGVPFTDTQYF	6	CD8-C5
PB-1_GCACTCTCACATTTCT	PB	PB-1	P1-6	TRUE	3	TRA:CAMREGRERDDKIIF TRB:CASSLPNTEAFF	216	CD8-C5
PB-1_GCACTCTCATCTACGA	PB	PB-1	P1-6	TRUE	3	TRA:CAMREGRERDDKIIF TRB:CASSLPNTEAFF	216	CD8-C5
PB-1_GCACTCTTCCGCATAA	PB	PB-1	P1-7	TRUE	3	TRA:CAVQAGNNDMRF TRB:CASSTTGDFNYGYTF	215	CD8-C5
PB-1_GCACTCTTCTCTGAGA	PB	PB-1	P1-6	TRUE	3	TRA:CAMREGRERDDKIIF TRB:CASSLPNTEAFF	216	CD8-C5
PB-1_GCAGTTAGTGAGGGAG	PB	PB-1	P1-12	TRUE	3	TRA:CALSDERGDYKLSF TRB:CASSPGTGSYNEQFF	141	CD8-C5
PB-1_GCAGTTAGTGTCGCTG	PB	PB-1	P1-825	FALSE	1	TRA:CAVGGADGLTF TRB:CASSPTSYEQYF	1	CD8-C4
PB-1_GCATACAAGTGCAAGC	PB	PB-1	P1-7	TRUE	3	TRA:CAVQAGNNDMRF TRB:CASSTTGDFNYGYTF	215	CD8-C5
PB-1_GCATACATCGCTGATA	PB	PB-1	P1-6	TRUE	3	TRA:CAMREGRERDDKIIF TRB:CASSLPNTEAFF	216	CD8-C5
PB-1_GCATGATCAGCGTCCA	PB	PB-1	P1-141	TRUE	3	TRA:CALRILRGSYIPTF TRB:CASSLPGRYSGANVLTF	4	CD8-C2
PB-1_GCATGATTCCCAAGAT	PB	PB-1	P1-12	TRUE	3	TRA:CALSDERGDYKLSF TRB:CASSPGTGSYNEQFF	141	CD8-C5
PB-1_GCATGCGGTTTAGCTG	PB	PB-1	P1-7	TRUE	3	TRA:CAVQAGNNDMRF TRB:CASSTTGDFNYGYTF	215	CD8-C5
PB-1_GCATGCGTCCGCAAGC	PB	PB-1	P1-6	TRUE	3	TRA:CAMREGRERDDKIIF TRB:CASSLPNTEAFF	216	CD8-C5
PB-1_GCATGTACACCGAAAG	PB	PB-1	P1-9	TRUE	3	TRA:CAEAEGGGADGLTF TRB:CATSGGDNEQFF	174	CD8-C5
PB-1_GCCAAATCATTAGGCT	PB	PB-1	P1-117	TRUE	3	TRA:CIVGETSGSRLTF TRB:CASSLVGGVGTDTQYF	5	CD8-C2
PB-1_GCCAAATGTAAGAGAG	PB	PB-1	P1-7	TRUE	3	TRA:CAVQAGNNDMRF TRB:CASSTTGDFNYGYTF	215	CD8-C5
PB-1_GCCAAATGTGCGGTAA	PB	PB-1	P1-8	TRUE	3	TRA:CAVRPPGRNSGGYQKVTF TRB:CASSRRGVTDTQYF	204	CD8-C5
PB-1_GCCTCTACACTGTCGG	PB	PB-1	P1-141	TRUE	3	TRA:CALRILRGSYIPTF TRB:CASSLPGRYSGANVLTF	4	CD8-C7
PB-1_GCGAGAAAGAGTAATC	PB	PB-1	P1-4	TRUE	3	TRA:CAMKGRDDKIIF TRB:CASTKGVSREAFF	226	CD8-C7
PB-1_GCGAGAAAGGCTAGCA	PB	PB-1	P1-6	TRUE	3	TRA:CAMREGRERDDKIIF TRB:CASSLPNTEAFF	216	CD8-C7
PB-1_GCGCAACGTACAAGTA	PB	PB-1	P1-6	TRUE	3	TRA:CAMREGRERDDKIIF TRB:CASSLPNTEAFF	216	CD8-C7
PB-1_GCGCAGTAGAGGTACC	PB	PB-1	P1-4	TRUE	3	TRA:CAMKGRDDKIIF TRB:CASTKGVSREAFF	226	CD8-C5
PB-1_GCGCAGTAGCACAGGT	PB	PB-1	P1-7	TRUE	3	TRA:CAVQAGNNDMRF TRB:CASSTTGDFNYGYTF	215	CD8-C5
PB-1_GCGCAGTCATCGATTG	PB	PB-1	P1-3	TRUE	3	TRA:CAFLSGTYKYIF TRB:CASSQSPGQRNYGYTF	308	CD8-C5

PB-1_GCGCAGTTCACCTCGT	PB	PB-1	P1-176	TRUE	3	TRA:CATVRTGANNLFF TRB:CASSQGLYEQYF	3	CD8-C5
PB-1_GCGCCAAAGAGCTTCT	PB	PB-1	P1-6	TRUE	3	TRA:CAMREGRERDDKIIF TRB:CASSLPNTEAFF	216	CD8-C5
PB-1_GCGCCAAAGTCATCCA	PB	PB-1	P1-7	TRUE	3	TRA:CAVQAGNNDMRF TRB:CASSTTGDFNYGYTF	215	CD8-C5
PB-1_GCGCGATAGCCCAGCT	PB	PB-1	P1-9	TRUE	3	TRA:CAEAEGGGADGLTF TRB:CATSGGDNEQFF	174	CD8-C5
PB-1_GCGCGATTCACCAGGC	PB	PB-1	P1-6	TRUE	3	TRA:CAMREGRERDDKIIF TRB:CASSLPNTEAFF	216	CD8-C5
PB-1_GCGGGTTAGCCCAACC	PB	PB-1	P1-12	TRUE	3	TRA:CALSDERGDYKLSF TRB:CASSPGTGSYNEQFF	141	CD8-C5
PB-1_GCGGGTTCAACACCTA	PB	PB-1	P1-1	TRUE	3	TRA:CAMSDRMTGGFKTIF TRB:CASSQVGRGGEETQYF	1047	CD8-C5
PB-1_GCGGGTTCAGCGTTCG	PB	PB-1	P1-22	TRUE	3	TRA:CASNSGYSTLTF TRB:CASSQVTGGARDEQYF	65	CD8-C7
PB-1_GCTCCTACAGTGGAGT	PB	PB-1	P1-47	TRUE	3	TRA:CIVGGNFNKFYF TRB:CASSPEWDTDTQYF	18	CD8-C5
PB-1_GCTCCTAGTGGTCCGT	PB	PB-1	P1-6	TRUE	3	TRA:CAMREGRERDDKIIF TRB:CASSLPNTEAFF	216	CD8-C5
PB-1_GCTCTGTTCCGGCACA	PB	PB-1	P1-1	TRUE	3	TRA:CAMSDRMTGGFKTIF TRB:CASSQVGRGGEETQYF	1047	CD8-C5
PB-1_GCTGCAGAGAGCTGCA	PB	PB-1	P1-842	FALSE	1	TRA:CAMREGPGAGSYQLTF TRB:CASSAGTSGNYEQYF	1	CD8-C4
PB-1_GCTGCAGCACGGTAGA	PB	PB-1	P1-6	TRUE	3	TRA:CAMREGRERDDKIIF TRB:CASSLPNTEAFF	216	CD8-C5
PB-1_GCTGCGAGTCTGGTCG	PB	PB-1	P1-23	TRUE	3	TRA:CALRAITQGGSEKLVF TRB:CASSLTGAVYGYTF	65	CD8-C5
PB-1_GCTGGGTCAAGCGTAG	PB	PB-1	P1-7	TRUE	3	TRA:CAVQAGNNDMRF TRB:CASSTTGDFNYGYTF	215	CD8-C5
PB-1_GCTGGGTTCGTTACAG	PB	PB-1	P1-91	TRUE	3	TRA:CVVSDQYNTDKLIF TRB:CASGEGQGASSYEQYF	6	CD8-C5
PB-1_GCTGGGTTCTCAACTT	PB	PB-1	P1-6	TRUE	3	TRA:CAMREGRERDDKIIF TRB:CASSLPNTEAFF	216	CD8-C5
PB-1_GCTTCCAAGATCCCAT	PB	PB-1	P1-7	TRUE	3	TRA:CAVQAGNNDMRF TRB:CASSTTGDFNYGYTF	215	CD8-C5
PB-1_GCTTCCACATACCATG	PB	PB-1	P1-6	TRUE	3	TRA:CAMREGRERDDKIIF TRB:CASSLPNTEAFF	216	CD8-C5
PB-1_GCTTCCATCTTACCGC	PB	PB-1	P1-9	TRUE	3	TRA:CAEAEGGGADGLTF TRB:CATSGGDNEQFF	174	CD8-C5
PB-1_GGAAAGCGTAATTGGA	PB	PB-1	P1-6	TRUE	3	TRA:CAMREGRERDDKIIF TRB:CASSLPNTEAFF	216	CD8-C5
PB-1_GGAAAGCGTTCGAATC	PB	PB-1	P1-7	TRUE	3	TRA:CAVQAGNNDMRF TRB:CASSTTGDFNYGYTF	215	CD8-C5
PB-1_GGAACTTAGTGTTAGA	PB	PB-1	P1-23	TRUE	3	TRA:CALRAITQGGSEKLVF TRB:CASSLTGAVYGYTF	65	CD8-C4
PB-1_GGAACTTTCGGCGCTA	PB	PB-1	P1-185	TRUE	3	TRA:CATDEDKFYF TRB:CASSESWTGGTYEQYF	3	CD8-C4
PB-1_GGAATAAAGACTGGGT	PB	PB-1	P1-6	TRUE	3	TRA:CAMREGRERDDKIIF TRB:CASSLPNTEAFF	216	CD8-C5
PB-1_GGACAAGTCGTAGGAG	PB	PB-1	P1-12	TRUE	3	TRA:CALSDERGDYKLSF TRB:CASSPGTGSYNEQFF	141	CD8-C5
PB-1_GGACAGATCCTTAATC	PB	PB-1	P1-12	TRUE	3	TRA:CALSDERGDYKLSF TRB:CASSPGTGSYNEQFF	141	CD8-C5

PB-1_GGACGTCTCACATGCA	PB	PB-1	P1-9	TRUE	3	TRA:CAEAEGGGADGLTF TRB:CATSGGDNEQFF	174	CD8-C5
PB-1_GGAGCAACAGCATGAG	PB	PB-1	P1-7	TRUE	3	TRA:CAVQAGNNDMRF TRB:CASSTTGDFNYGYTF	215	CD8-C5
PB-1_GGATGTTGTCTAGTGT	PB	PB-1	P1-9	TRUE	3	TRA:CAEAEGGGADGLTF TRB:CATSGGDNEQFF	174	CD8-C5
PB-1_GGATTACAGACAAGCC	PB	PB-1	P1-6	TRUE	3	TRA:CAMREGRERDDKIIF TRB:CASSLPNTEAFF	216	CD8-C5
PB-1_GGCAATTAGATCTGCT	PB	PB-1	P1-5	TRUE	3	TRA:CALSGTPMNRDDKIIF TRB:CSARGRGLAGGPAYEQYF	223	CD8-C7
PB-1_GGCAATTGTTCCGTCT	PB	PB-1	P1-9	TRUE	3	TRA:CAEAEGGGADGLTF TRB:CATSGGDNEQFF	174	CD8-C5
PB-1_GGCAATTTCTTTACAC	PB	PB-1	P1-7	TRUE	3	TRA:CAVQAGNNDMRF TRB:CASSTTGDFNYGYTF	215	CD8-C5
PB-1_GGCCGATCAACACCCG	PB	PB-1	P1-12	TRUE	3	TRA:CALSDERGDYKLSF TRB:CASSPGTGSYNEQFF	141	CD8-C5
PB-1_GGCCGATGTAAGGGCT	PB	PB-1	P1-6	TRUE	3	TRA:CAMREGRERDDKIIF TRB:CASSLPNTEAFF	216	CD8-C5
PB-1_GGCCGATTCCCAGGTG	PB	PB-1	P1-4	TRUE	3	TRA:CAMKGRDDKIIF TRB:CASTKGVSREAFF	226	CD8-C5
PB-1_GGCCGATTCCGCAGTG	PB	PB-1	P1-274	FALSE	2	TRA:CAVNFHDMRF TRB:CASSRTGTRANYGYTF	2	CD8-C5
PB-1_GGCGACTAGGTCATCT	PB	PB-1	P1-1	TRUE	3	TRA:CAMSDRMTGGFKTIF TRB:CASSQVGRGGEETQYF	1047	CD8-C5
PB-1_GGCGTGTCACTCAGGC	PB	PB-1	P1-7	TRUE	3	TRA:CAVQAGNNDMRF TRB:CASSTTGDFNYGYTF	215	CD8-C5
PB-1_GGCGTGTTCTATCCCG	PB	PB-1	P1-12	TRUE	3	TRA:CALSDERGDYKLSF TRB:CASSPGTGSYNEQFF	141	CD8-C5
PB-1_GGCTCGATCGGCGCTA	PB	PB-1	P1-4	TRUE	3	TRA:CAMKGRDDKIIF TRB:CASTKGVSREAFF	226	CD8-C5
PB-1_GGCTCGATCTTCGAGA	PB	PB-1	P1-6	TRUE	3	TRA:CAMREGRERDDKIIF TRB:CASSLPNTEAFF	216	CD8-C5
PB-1_GGCTGGTCACTCAGGC	PB	PB-1	P1-4	TRUE	3	TRA:CAMKGRDDKIIF TRB:CASTKGVSREAFF	226	CD8-C7
PB-1_GGGAATGAGATATGCA	PB	PB-1	P1-23	TRUE	3	TRA:CALRAITQGGSEKLVF TRB:CASSLTGAVYGYTF	65	CD8-C4
PB-1_GGGAATGAGCCCAGCT	PB	PB-1	P1-7	TRUE	3	TRA:CAVQAGNNDMRF TRB:CASSTTGDFNYGYTF	215	CD8-C5
PB-1_GGGAATGAGGGATACC	PB	PB-1	P1-31	TRUE	3	TRA:CAVEEGADRLMF TRB:CASSLLGGGELFF	46	CD8-C5
PB-1_GGGAATGCACTGCCAG	PB	PB-1	P1-42	TRUE	3	TRA:CASFSGGSQGNLIF TRB:CSAGQGSNQPQHF	24	CD8-C3
PB-1_GGGAATGCATACTCTT	PB	PB-1	P1-7	TRUE	3	TRA:CAVQAGNNDMRF TRB:CASSTTGDFNYGYTF	215	CD8-C5
PB-1_GGGACCTAGGGATACC	PB	PB-1	P1-4	TRUE	3	TRA:CAMKGRDDKIIF TRB:CASTKGVSREAFF	226	CD8-C7
PB-1_GGGACCTGTTGGTGGA	PB	PB-1	P1-118	TRUE	3	TRA:CAVVKAGGTSYGKLTF TRB:CASKSLAGKSSYEQYF	5	CD8-C4
PB-1_GGGATGAAGGCATTGG	PB	PB-1	P1-7	TRUE	3	TRA:CAVQAGNNDMRF TRB:CASSTTGDFNYGYTF	215	CD8-C5
PB-1_GGGCACTAGTCGAGTG	PB	PB-1	P1-7	TRUE	3	TRA:CAVQAGNNDMRF TRB:CASSTTGDFNYGYTF	215	CD8-C5
PB-1_GGGCACTAGTCTCAAC	PB	PB-1	P1-37	TRUE	3	TRA:CAVNIVGNQFYF TRB:CASSPLGTEAFF	40	CD8-C4

PB-1_GGGCACTCACCATGTA	PB	PB-1	P1-12	TRUE	3	TRA:CALSDERGDYKLSF TRB:CASSPGTGSYNEQFF	141	CD8-C5
PB-1_GGGCACTGTACCTACA	PB	PB-1	P1-295	TRUE	2	TRA:CALPFISGNTPLVF TRB:CASSLGPLENIQYF	2	CD8-C4
PB-1_GGGCATCAGACATAAC	PB	PB-1	P1-7	TRUE	3	TRA:CAVQAGNNDMRF TRB:CASSTTGDFNYGYTF	215	CD8-C5
PB-1_GGGTCTGCACAGAGGT	PB	PB-1	P1-9	TRUE	3	TRA:CAEAEGGGADGLTF TRB:CATSGGDNEQFF	174	CD8-C5
PB-1_GGGTCTGCACATTAGC	PB	PB-1	P1-173	FALSE	3	TRA:CAVVGYSSASKIIF TRB:CATSDLGYEQFF	3	CD8-C7
PB-1_GGGTCTGGTTGAGTTC	PB	PB-1	P1-6	TRUE	3	TRA:CAMREGRERDDKIIF TRB:CASSLPNTEAFF	216	CD8-C5
PB-1_GGGTCTGGTTGCGTTA	PB	PB-1	P1-9	TRUE	3	TRA:CAEAEGGGADGLTF TRB:CATSGGDNEQFF	174	CD8-C5
PB-1_GGGTCTGTCCAAACAC	PB	PB-1	P1-1	TRUE	3	TRA:CAMSDRMTGGFKTIF TRB:CASSQVGRGGEETQYF	1047	CD8-C5
PB-1_GGTGAAGAGCCACGTC	PB	PB-1	P1-296	TRUE	2	TRA:CALSGWDTDKLIF TRB:CASTADREGQETQYF	2	CD8-C5
PB-1_GGTGAAGAGCGATATA	PB	PB-1	P1-6	TRUE	3	TRA:CAMREGRERDDKIIF TRB:CASSLPNTEAFF	216	CD8-C5
PB-1_GGTGAAGTCCGTAGTA	PB	PB-1	P1-8	TRUE	3	TRA:CAVRPPGRNSGGYQKVTF TRB:CASSRRGVTDTQYF	204	CD8-C7
PB-1_GGTGCGTTCAGAAATG	PB	PB-1	P1-12	TRUE	3	TRA:CALSDERGDYKLSF TRB:CASSPGTGSYNEQFF	141	CD8-C5
PB-1_GGTGTTAAGGACCACA	PB	PB-1	P1-7	TRUE	3	TRA:CAVQAGNNDMRF TRB:CASSTTGDFNYGYTF	215	CD8-C5
PB-1_GGTGTTAAGTCCAGGA	PB	PB-1	P1-6	TRUE	3	TRA:CAMREGRERDDKIIF TRB:CASSLPNTEAFF	216	CD8-C5
PB-1_GTAACTGGTAGCACGA	PB	PB-1	P1-87	TRUE	3	TRA:CGTVYNYGQNFVF TRB:CASSRGRGAQPQHF	6	CD8-C5
PB-1_GTAACTGGTGATGCCC	PB	PB-1	P1-7	TRUE	3	TRA:CAVQAGNNDMRF TRB:CASSTTGDFNYGYTF	215	CD8-C5
PB-1_GTACGTAGTGTAACGG	PB	PB-1	P1-23	TRUE	3	TRA:CALRAITQGGSEKLVF TRB:CASSLTGAVYGYTF	65	CD8-C4
PB-1_GTACGTATCGAACTGT	PB	PB-1	P1-9	TRUE	3	TRA:CAEAEGGGADGLTF TRB:CATSGGDNEQFF	174	CD8-C5
PB-1_GTACTCCAGCCTTGAT	PB	PB-1	P1-68	TRUE	3	TRA:CAVIPDILTGGGNKLTF TRB:CASSLQGFGNTIYF	9	CD8-C4
PB-1_GTACTTTAGGCAAAGA	PB	PB-1	P1-7	TRUE	3	TRA:CAVQAGNNDMRF TRB:CASSTTGDFNYGYTF	215	CD8-C5
PB-1_GTACTTTAGTCCATAC	PB	PB-1	P1-4	TRUE	3	TRA:CAMKGRDDKIIF TRB:CASTKGVSREAFF	226	CD8-C5
PB-1_GTACTTTCATCACGAT	PB	PB-1	P1-54	TRUE	3	TRA:CAGWYSGGGADGLTF TRB:CATGAGDNEQFF	13	CD8-C7
PB-1_GTACTTTTCGTAGGTT	PB	PB-1	P1-4	TRUE	3	TRA:CAMKGRDDKIIF TRB:CASTKGVSREAFF	226	CD8-C5
PB-1_GTAGGCCTCATACGGT	PB	PB-1	P1-12	TRUE	3	TRA:CALSDERGDYKLSF TRB:CASSPGTGSYNEQFF	141	CD8-C5
PB-1_GTAGTCAAGAATGTTG	PB	PB-1	P1-4	TRUE	3	TRA:CAMKGRDDKIIF TRB:CASTKGVSREAFF	226	CD8-C5
PB-1_GTAGTCAAGACTGGGT	PB	PB-1	P1-54	TRUE	3	TRA:CAGWYSGGGADGLTF TRB:CATGAGDNEQFF	13	CD8-C5
PB-1_GTAGTCACAGTGGAGT	PB	PB-1	P1-6	TRUE	3	TRA:CAMREGRERDDKIIF TRB:CASSLPNTEAFF	216	CD8-C5

PB-1_GTAGTCATCGGAATCT	PB	PB-1	P1-6	TRUE	3	TRA:CAMREGRERDDKIIF TRB:CASSLPNTEAFF	216	CD8-C5
PB-1_GTATCTTAGACGCACA	PB	PB-1	P1-7	TRUE	3	TRA:CAVQAGNNDMRF TRB:CASSTTGDFNYGYTF	215	CD8-C5
PB-1_GTATCTTCACTGTGTA	PB	PB-1	P1-200	TRUE	3	TRA:CAVGGNNNDMRF TRB:CASSRQDLHEQYF	3	CD8-C4
PB-1_GTATCTTGTCTGGAGA	PB	PB-1	P1-9	TRUE	3	TRA:CAEAEGGGADGLTF TRB:CATSGGDNEQFF	174	CD8-C5
PB-1_GTATTCTAGGACACCA	PB	PB-1	P1-6	TRUE	3	TRA:CAMREGRERDDKIIF TRB:CASSLPNTEAFF	216	CD8-C5
PB-1_GTATTCTGTGTTGAGG	PB	PB-1	P1-4	TRUE	3	TRA:CAMKGRDDKIIF TRB:CASTKGVSREAFF	226	CD8-C5
PB-1_GTCAAGTCACGAAATA	PB	PB-1	P1-9	TRUE	3	TRA:CAEAEGGGADGLTF TRB:CATSGGDNEQFF	174	CD8-C5
PB-1_GTCAAGTGTACCAGTT	PB	PB-1	P1-6	TRUE	3	TRA:CAMREGRERDDKIIF TRB:CASSLPNTEAFF	216	CD8-C5
PB-1_GTCACAACAGTCTTCC	PB	PB-1	P1-5	TRUE	3	TRA:CALSGTPMNRDDKIIF TRB:CSARGRGLAGGPAYEQYF	223	CD8-C7
PB-1_GTCACAAGTCGCATAT	PB	PB-1	P1-9	TRUE	3	TRA:CAEAEGGGADGLTF TRB:CATSGGDNEQFF	174	CD8-C5
PB-1_GTCACAAGTTAGATGA	PB	PB-1	P1-12	TRUE	3	TRA:CALSDERGDYKLSF TRB:CASSPGTGSYNEQFF	141	CD8-C5
PB-1_GTCACAAGTTCCCTTG	PB	PB-1	P1-37	TRUE	3	TRA:CAVNIVGNQFYF TRB:CASSPLGTEAFF	40	CD8-C4
PB-1_GTCATTTAGCTGATAA	PB	PB-1	P1-12	TRUE	3	TRA:CALSDERGDYKLSF TRB:CASSPGTGSYNEQFF	141	CD8-C5
PB-1_GTCATTTTCACCTCGT	PB	PB-1	P1-6	TRUE	3	TRA:CAMREGRERDDKIIF TRB:CASSLPNTEAFF	216	CD8-C5
PB-1_GTCATTTTCGTACCGG	PB	PB-1	P1-902	FALSE	1	TRA:CALSEAEIQGAQKLVF TRB:CASRSGGGFNSPLHF	1	CD8-C5
PB-1_GTCCTCAGTCATATGC	PB	PB-1	P1-12	TRUE	3	TRA:CALSDERGDYKLSF TRB:CASSPGTGSYNEQFF	141	CD8-C5
PB-1_GTCCTCAGTGTCCTCT	PB	PB-1	P1-6	TRUE	3	TRA:CAMREGRERDDKIIF TRB:CASSLPNTEAFF	216	CD8-C5
PB-1_GTCCTCATCTCACATT	PB	PB-1	P1-14	TRUE	3	TRA:CAASVVNDMRF TRB:CASSHRGGAKNIQYF	109	CD8-C5
PB-1_GTCGGGTCATCCTTGC	PB	PB-1	P1-7	TRUE	3	TRA:CAVQAGNNDMRF TRB:CASSTTGDFNYGYTF	215	CD8-C5
PB-1_GTCGGGTGTCATATCG	PB	PB-1	P1-6	TRUE	3	TRA:CAMREGRERDDKIIF TRB:CASSLPNTEAFF	216	CD8-C5
PB-1_GTCGGGTTCGAATGGG	PB	PB-1	P1-93	TRUE	3	TRA:CAFTGGGGADGLTF TRB:CASSVAWGTGGWNTEAFF	6	CD8-C4
PB-1_GTCGTAAGTCAGAGGT	PB	PB-1	P1-12	TRUE	3	TRA:CALSDERGDYKLSF TRB:CASSPGTGSYNEQFF	141	CD8-C5
PB-1_GTCTCGTGTCCGAACC	PB	PB-1	P1-9	TRUE	3	TRA:CAEAEGGGADGLTF TRB:CATSGGDNEQFF	174	CD8-C5
PB-1_GTCTTCGTCAACGGGA	PB	PB-1	P1-15	TRUE	3	TRA:CAVNSSPLGANSKLTF TRB:CASSDSGNFYNEQFF	92	CD8-C4
PB-1_GTGCATAGTATAAACG	PB	PB-1	P1-6	TRUE	3	TRA:CAMREGRERDDKIIF TRB:CASSLPNTEAFF	216	CD8-C5
PB-1_GTGCATATCGGTTAAC	PB	PB-1	P1-31	TRUE	3	TRA:CAVEEGADRLMF TRB:CASSLLGGGELFF	46	CD8-C5
PB-1_GTGGGTCGTAAAGTCA	PB	PB-1	P1-12	TRUE	3	TRA:CALSDERGDYKLSF TRB:CASSPGTGSYNEQFF	141	CD8-C5

PB-1_GTGTGCGTCAGAAATG	PB	PB-1	P1-9	TRUE	3	TRA:CAEAEGGGADGLTF TRB:CATSGGDNEQFF	174	CD8-C5
PB-1_GTGTTAGAGGCATGGT	PB	PB-1	P1-6	TRUE	3	TRA:CAMREGRERDDKIIF TRB:CASSLPNTEAFF	216	CD8-C5
PB-1_GTTACAGAGCCAGGAT	PB	PB-1	P1-9	TRUE	3	TRA:CAEAEGGGADGLTF TRB:CATSGGDNEQFF	174	CD8-C5
PB-1_GTTACAGTCGTCGTTC	PB	PB-1	P1-7	TRUE	3	TRA:CAVQAGNNDMRF TRB:CASSTTGDFNYGYTF	215	CD8-C5
PB-1_GTTCATTAGCAAATCA	PB	PB-1	P1-45	TRUE	3	TRA:CAYRPLGAQKLVF TRB:CASSTLDGRESSYEQYF	20	CD8-C4
PB-1_GTTCATTCACCAACCG	PB	PB-1	P1-63	TRUE	3	TRA:CAEPLNTGNQFYF TRB:CASSVGNEQFF	10	CD8-C7
PB-1_GTTCATTTCGGAGGTA	PB	PB-1	P1-5	TRUE	3	TRA:CALSGTPMNRDDKIIF TRB:CSARGRGLAGGPAYEQYF	223	CD8-C5
PB-1_GTTCTCGCACTTGGAT	PB	PB-1	P1-4	TRUE	3	TRA:CAMKGRDDKIIF TRB:CASTKGVSREAFF	226	CD8-C5
PB-1_GTTCTCGGTCAATACC	PB	PB-1	P1-7	TRUE	3	TRA:CAVQAGNNDMRF TRB:CASSTTGDFNYGYTF	215	CD8-C5
PB-1_GTTTCTAAGACCGGAT	PB	PB-1	P1-22	TRUE	3	TRA:CASNSGYSTLTF TRB:CASSQVTGGARDEQYF	65	CD8-C7
PB-1_TAAACCGTCTTGGGTA	PB	PB-1	P1-56	TRUE	3	TRA:CVVNPWGSGTYKYIF TRB:CASSRDITDTQYF	13	CD8-C4
PB-1_TAAGAGACAGTCACTA	PB	PB-1	P1-12	TRUE	3	TRA:CALSDERGDYKLSF TRB:CASSPGTGSYNEQFF	141	CD8-C5
PB-1_TACAGTGCACCATGTA	PB	PB-1	P1-6	TRUE	3	TRA:CAMREGRERDDKIIF TRB:CASSLPNTEAFF	216	CD8-C5
PB-1_TACAGTGGTAAACACA	PB	PB-1	P1-23	TRUE	3	TRA:CALRAITQGGSEKLVF TRB:CASSLTGAVYGYTF	65	CD8-C5
PB-1_TACAGTGGTGGGTCAA	PB	PB-1	P1-6	TRUE	3	TRA:CAMREGRERDDKIIF TRB:CASSLPNTEAFF	216	CD8-C5
PB-1_TACAGTGTCAAAGACA	PB	PB-1	P1-5	TRUE	3	TRA:CALSGTPMNRDDKIIF TRB:CSARGRGLAGGPAYEQYF	223	CD8-C7
PB-1_TACCTATAGCTTCGCG	PB	PB-1	P1-7	TRUE	3	TRA:CAVQAGNNDMRF TRB:CASSTTGDFNYGYTF	215	CD8-C5
PB-1_TACCTATCACGGCTAC	PB	PB-1	P1-5	TRUE	3	TRA:CALSGTPMNRDDKIIF TRB:CSARGRGLAGGPAYEQYF	223	CD8-C5
PB-1_TACCTTAAGGTGACCA	PB	PB-1	P1-6	TRUE	3	TRA:CAMREGRERDDKIIF TRB:CASSLPNTEAFF	216	CD8-C5
PB-1_TACCTTAGTCTAAACC	PB	PB-1	P1-9	TRUE	3	TRA:CAEAEGGGADGLTF TRB:CATSGGDNEQFF	174	CD8-C5
PB-1_TACGGATCAATCAGAA	PB	PB-1	P1-7	TRUE	3	TRA:CAVQAGNNDMRF TRB:CASSTTGDFNYGYTF	215	CD8-C5
PB-1_TACGGGCCAAGTCTGT	PB	PB-1	P1-4	TRUE	3	TRA:CAMKGRDDKIIF TRB:CASTKGVSREAFF	226	CD8-C5
PB-1_TACGGGCTCTTGCCGT	PB	PB-1	P1-943	FALSE	1	TRA:CAVGVWAGTALIF TRB:CASSPRRAGENSPLHF	1	CD8-C4
PB-1_TACTCATAGATGCGAC	PB	PB-1	P1-6	TRUE	3	TRA:CAMREGRERDDKIIF TRB:CASSLPNTEAFF	216	CD8-C5
PB-1_TACTCATCAATCGGTT	PB	PB-1	P1-6	TRUE	3	TRA:CAMREGRERDDKIIF TRB:CASSLPNTEAFF	216	CD8-C5
PB-1_TACTCATGTGCCTGGT	PB	PB-1	P1-23	TRUE	3	TRA:CALRAITQGGSEKLVF TRB:CASSLTGAVYGYTF	65	CD8-C5
PB-1_TACTCATTCTATGTGG	PB	PB-1	P1-6	TRUE	3	TRA:CAMREGRERDDKIIF TRB:CASSLPNTEAFF	216	CD8-C5

PB-1_TACTCGCAGTGGACGT	PB	PB-1	P1-6	TRUE	3	TRA:CAMREGRERDDKIIF TRB:CASSLPNTEAFF	216	CD8-C5
PB-1_TACTCGCCAGTGGAGT	PB	PB-1	P1-6	TRUE	3	TRA:CAMREGRERDDKIIF TRB:CASSLPNTEAFF	216	CD8-C5
PB-1_TACTCGCGTTACGGAG	PB	PB-1	P1-12	TRUE	3	TRA:CALSDERGDYKLSF TRB:CASSPGTGSYNEQFF	141	CD8-C5
PB-1_TACTTACCACCCATGG	PB	PB-1	P1-154	TRUE	3	TRA:CAGEKSSGDKLTF TRB:CSARGTGLNYGYTF	4	CD8-C5
PB-1_TACTTACGTACCGCTG	PB	PB-1	P1-12	TRUE	3	TRA:CALSDERGDYKLSF TRB:CASSPGTGSYNEQFF	141	CD8-C5
PB-1_TACTTACGTTAAGTAG	PB	PB-1	P1-6	TRUE	3	TRA:CAMREGRERDDKIIF TRB:CASSLPNTEAFF	216	CD8-C5
PB-1_TAGCCGGAGCGTAGTG	PB	PB-1	P1-54	TRUE	3	TRA:CAGWYSGGGADGLTF TRB:CATGAGDNEQFF	13	CD8-C5
PB-1_TAGGCATCATTCACTT	PB	PB-1	P1-12	TRUE	3	TRA:CALSDERGDYKLSF TRB:CASSPGTGSYNEQFF	141	CD8-C5
PB-1_TAGTGGTTCACATGCA	PB	PB-1	P1-1	TRUE	3	TRA:CAMSDRMTGGFKTIF TRB:CASSQVGRGGEETQYF	1047	CD8-C7
PB-1_TAGTTGGAGTGAACGC	PB	PB-1	P1-12	TRUE	3	TRA:CALSDERGDYKLSF TRB:CASSPGTGSYNEQFF	141	CD8-C5
PB-1_TAGTTGGGTGCCTGTG	PB	PB-1	P1-14	TRUE	3	TRA:CAASVVNDMRF TRB:CASSHRGGAKNIQYF	109	CD8-C5
PB-1_TAGTTGGTCAGTACGT	PB	PB-1	P1-6	TRUE	3	TRA:CAMREGRERDDKIIF TRB:CASSLPNTEAFF	216	CD8-C5
PB-1_TATCAGGTCGTCCAGG	PB	PB-1	P1-37	TRUE	3	TRA:CAVNIVGNQFYF TRB:CASSPLGTEAFF	40	CD8-C4
PB-1_TATCTCACACGGTTTA	PB	PB-1	P1-7	TRUE	3	TRA:CAVQAGNNDMRF TRB:CASSTTGDFNYGYTF	215	CD8-C5
PB-1_TATGCCCAGGGCACTA	PB	PB-1	P1-14	TRUE	3	TRA:CAASVVNDMRF TRB:CASSHRGGAKNIQYF	109	CD8-C5
PB-1_TATGCCCGTACCCAAT	PB	PB-1	P1-6	TRUE	3	TRA:CAMREGRERDDKIIF TRB:CASSLPNTEAFF	216	CD8-C5
PB-1_TATGCCCGTCGGCATC	PB	PB-1	P1-9	TRUE	3	TRA:CAEAEGGGADGLTF TRB:CATSGGDNEQFF	174	CD8-C5
PB-1_TATGCCCGTTCGGGCT	PB	PB-1	P1-7	TRUE	3	TRA:CAVQAGNNDMRF TRB:CASSTTGDFNYGYTF	215	CD8-C5
PB-1_TCAACGAGTCACTTCC	PB	PB-1	P1-297	FALSE	2	TRA:CAGGNNNDMRF TRB:CASTRFGNEQFF	2	CD8-C4
PB-1_TCAACGAGTGGGTATG	PB	PB-1	P1-6	TRUE	3	TRA:CAMREGRERDDKIIF TRB:CASSLPNTEAFF	216	CD8-C5
PB-1_TCAATCTAGAAACGCC	PB	PB-1	P1-6	TRUE	3	TRA:CAMREGRERDDKIIF TRB:CASSLPNTEAFF	216	CD8-C5
PB-1_TCAATCTGTAAACACA	PB	PB-1	P1-7	TRUE	3	TRA:CAVQAGNNDMRF TRB:CASSTTGDFNYGYTF	215	CD8-C5
PB-1_TCAATCTGTTAAGAAC	PB	PB-1	P1-39	TRUE	3	TRA:CAVRDLGGTSYDKVIF TRB:CSARVREVSYEQYF	31	CD8-C2
PB-1_TCACAAGAGCGAAGGG	PB	PB-1	P1-6	TRUE	3	TRA:CAMREGRERDDKIIF TRB:CASSLPNTEAFF	216	CD8-C5
PB-1_TCACAAGCACGCGAAA	PB	PB-1	P1-4	TRUE	3	TRA:CAMKGRDDKIIF TRB:CASTKGVSREAFF	226	CD8-C5
PB-1_TCACAAGGTGCATCTA	PB	PB-1	P1-37	TRUE	3	TRA:CAVNIVGNQFYF TRB:CASSPLGTEAFF	40	CD8-C4
PB-1_TCACGAAAGATGTAAC	PB	PB-1	P1-12	TRUE	3	TRA:CALSDERGDYKLSF TRB:CASSPGTGSYNEQFF	141	CD8-C5

PB-1_TCACGAACATTGTGCA	PB	PB-1	P1-6	TRUE	3	TRA:CAMREGRERDDKIIF TRB:CASSLPNTEAFF	216	CD8-C5
PB-1_TCACGAAGTATCTGCA	PB	PB-1	P1-7	TRUE	3	TRA:CAVQAGNNDMRF TRB:CASSTTGDFNYGYTF	215	CD8-C5
PB-1_TCAGATGAGCGATTCT	PB	PB-1	P1-6	TRUE	3	TRA:CAMREGRERDDKIIF TRB:CASSLPNTEAFF	216	CD8-C5
PB-1_TCAGCAAAGTCCGTAT	PB	PB-1	P1-9	TRUE	3	TRA:CAEAEGGGADGLTF TRB:CATSGGDNEQFF	174	CD8-C5
PB-1_TCAGCAACAACACCTA	PB	PB-1	P1-9	TRUE	3	TRA:CAEAEGGGADGLTF TRB:CATSGGDNEQFF	174	CD8-C7
PB-1_TCAGCTCAGGTGATTA	PB	PB-1	P1-12	TRUE	3	TRA:CALSDERGDYKLSF TRB:CASSPGTGSYNEQFF	141	CD8-C5
PB-1_TCAGCTCCAGGCGATA	PB	PB-1	P1-6	TRUE	3	TRA:CAMREGRERDDKIIF TRB:CASSLPNTEAFF	216	CD8-C5
PB-1_TCAGCTCGTAATCGTC	PB	PB-1	P1-5	TRUE	3	TRA:CALSGTPMNRDDKIIF TRB:CSARGRGLAGGPAYEQYF	223	CD8-C5
PB-1_TCAGCTCGTACCCAAT	PB	PB-1	P1-45	TRUE	3	TRA:CAYRPLGAQKLVF TRB:CASSTLDGRESSYEQYF	20	CD8-C5
PB-1_TCAGGATCATACAGCT	PB	PB-1	P1-54	TRUE	3	TRA:CAGWYSGGGADGLTF TRB:CATGAGDNEQFF	13	CD8-C5
PB-1_TCAGGATGTAATCACC	PB	PB-1	P1-6	TRUE	3	TRA:CAMREGRERDDKIIF TRB:CASSLPNTEAFF	216	CD8-C5
PB-1_TCAGGATGTGCAGTAG	PB	PB-1	P1-6	TRUE	3	TRA:CAMREGRERDDKIIF TRB:CASSLPNTEAFF	216	CD8-C5
PB-1_TCATTACTCTCCAGGG	PB	PB-1	P1-1	TRUE	3	TRA:CAMSDRMTGGFKTIF TRB:CASSQVGRGGEETQYF	1047	CD8-C5
PB-1_TCATTTGGTACAAGTA	PB	PB-1	P1-63	TRUE	3	TRA:CAEPLNTGNQFYF TRB:CASSVGNEQFF	10	CD8-C7
PB-1_TCATTTGTCCTTTCTC	PB	PB-1	P1-14	TRUE	3	TRA:CAASVVNDMRF TRB:CASSHRGGAKNIQYF	109	CD8-C5
PB-1_TCCACACGTACTTGAC	PB	PB-1	P1-12	TRUE	3	TRA:CALSDERGDYKLSF TRB:CASSPGTGSYNEQFF	141	CD8-C5
PB-1_TCCACACGTATAGGGC	PB	PB-1	P1-16	TRUE	3	TRA:CALSGTPMNRDDKIIF TRB:CASSQSWDLAVLNEQFF	79	CD8-C5
PB-1_TCCACACGTCAACTGT	PB	PB-1	P1-979	FALSE	1	TRA:CAEILNSGNTPLVF TRB:CASSLYGQITDTQYF	1	CD8-C4
PB-1_TCCCGATGTCGACTAT	PB	PB-1	P1-12	TRUE	3	TRA:CALSDERGDYKLSF TRB:CASSPGTGSYNEQFF	141	CD8-C5
PB-1_TCGCGAGAGAAACCTA	PB	PB-1	P1-299	TRUE	2	TRA:CAFMKATGTASKLTF TRB:CASSSPGLAGVSYNEQFF	2	CD8-C5
PB-1_TCGCGAGAGTACGCGA	PB	PB-1	P1-9	TRUE	3	TRA:CAEAEGGGADGLTF TRB:CATSGGDNEQFF	174	CD8-C5
PB-1_TCGCGAGCAGACGTAG	PB	PB-1	P1-271	FALSE	2	TRA:CADSGAGSYQLTF TRB:CASSFTLDSGSVDEQFF	2	CD8-C5
PB-1_TCGCGAGCATATGAGA	PB	PB-1	P1-7	TRUE	3	TRA:CAVQAGNNDMRF TRB:CASSTTGDFNYGYTF	215	CD8-C5
PB-1_TCGGTAAGTTAAGTAG	PB	PB-1	P1-12	TRUE	3	TRA:CALSDERGDYKLSF TRB:CASSPGTGSYNEQFF	141	CD8-C5
PB-1_TCGTACCCAAGGGTCA	PB	PB-1	P1-6	TRUE	3	TRA:CAMREGRERDDKIIF TRB:CASSLPNTEAFF	216	CD8-C5
PB-1_TCGTAGAAGTGTCCAT	PB	PB-1	P1-6	TRUE	3	TRA:CAMREGRERDDKIIF TRB:CASSLPNTEAFF	216	CD4-C6
PB-1_TCGTAGACACATGTGT	PB	PB-1	P1-14	TRUE	3	TRA:CAASVVNDMRF TRB:CASSHRGGAKNIQYF	109	CD8-C5

PB-1_TCGTAGACAGGGAGAG	PB	PB-1	P1-7	TRUE	3	TRA:CAVQAGNNDMRF TRB:CASSTTGDFNYGYTF	215	CD8-C5
PB-1_TCTGAGACAAGGGTCA	PB	PB-1	P1-12	TRUE	3	TRA:CALSDERGDYKLSF TRB:CASSPGTGSYNEQFF	141	CD8-C5
PB-1_TCTGAGACACTTACGA	PB	PB-1	P1-6	TRUE	3	TRA:CAMREGRERDDKIIF TRB:CASSLPNTEAFF	216	CD8-C5
PB-1_TCTGGAAAGGTAGCCA	PB	PB-1	P1-2	TRUE	3	TRA:CGQNFVF TRB:CASNQRGGPSLYNEQFF	323	CD8-C7
PB-1_TCTTCGGAGAGTTGGC	PB	PB-1	P1-6	TRUE	3	TRA:CAMREGRERDDKIIF TRB:CASSLPNTEAFF	216	CD8-C5
PB-1_TCTTCGGAGGGTCTCC	PB	PB-1	P1-12	TRUE	3	TRA:CALSDERGDYKLSF TRB:CASSPGTGSYNEQFF	141	CD8-C4
PB-1_TCTTCGGCACTCGACG	PB	PB-1	P1-14	TRUE	3	TRA:CAASVVNDMRF TRB:CASSHRGGAKNIQYF	109	CD8-C5
PB-1_TCTTCGGTCCTTGGTC	PB	PB-1	P1-1002	FALSE	1	TRA:CAGGDNFNKFYF TRB:CASSLSWGYEQYF	1	CD8-C4
PB-1_TCTTTCCCATTATCTC	PB	PB-1	P1-5	TRUE	3	TRA:CALSGTPMNRDDKIIF TRB:CSARGRGLAGGPAYEQYF	223	CD8-C5
PB-1_TCTTTCCTCGACAGCC	PB	PB-1	P1-7	TRUE	3	TRA:CAVQAGNNDMRF TRB:CASSTTGDFNYGYTF	215	CD8-C5
PB-1_TGACAACCATCGGGTC	PB	PB-1	P1-3	TRUE	3	TRA:CAFLSGTYKYIF TRB:CASSQSPGQRNYGYTF	308	CD8-C5
PB-1_TGACGGCCATAGTAAG	PB	PB-1	P1-7	TRUE	3	TRA:CAVQAGNNDMRF TRB:CASSTTGDFNYGYTF	215	CD8-C5
PB-1_TGACGGCTCGGCATCG	PB	PB-1	P1-23	TRUE	3	TRA:CALRAITQGGSEKLVF TRB:CASSLTGAVYGYTF	65	CD8-C4
PB-1_TGACTAGCACAGGTTT	PB	PB-1	P1-6	TRUE	3	TRA:CAMREGRERDDKIIF TRB:CASSLPNTEAFF	216	CD8-C5
PB-1_TGACTAGGTGGAAAGA	PB	PB-1	P1-7	TRUE	3	TRA:CAVQAGNNDMRF TRB:CASSTTGDFNYGYTF	215	CD8-C5
PB-1_TGAGAGGAGGCATTGG	PB	PB-1	P1-14	TRUE	3	TRA:CAASVVNDMRF TRB:CASSHRGGAKNIQYF	109	CD8-C5
PB-1_TGAGAGGGTTATTCTC	PB	PB-1	P1-37	TRUE	3	TRA:CAVNIVGNQFYF TRB:CASSPLGTEAFF	40	CD8-C5
PB-1_TGAGCATCACGGATAG	PB	PB-1	P1-9	TRUE	3	TRA:CAEAEGGGADGLTF TRB:CATSGGDNEQFF	174	CD8-C5
PB-1_TGAGCATCACTACAGT	PB	PB-1	P1-7	TRUE	3	TRA:CAVQAGNNDMRF TRB:CASSTTGDFNYGYTF	215	CD8-C5
PB-1_TGAGCCGTCAAAGACA	PB	PB-1	P1-7	TRUE	3	TRA:CAVQAGNNDMRF TRB:CASSTTGDFNYGYTF	215	CD8-C5
PB-1_TGAGCCGTCATGCTCC	PB	PB-1	P1-9	TRUE	3	TRA:CAEAEGGGADGLTF TRB:CATSGGDNEQFF	174	CD8-C5
PB-1_TGAGGGAAGTTAGCGG	PB	PB-1	P1-1013	FALSE	1	TRA:CAEAEGGGADGLTF TRB:CASSLPNTEAFF	1	CD8-C5
PB-1_TGATTTCAGCGCCTCA	PB	PB-1	P1-9	TRUE	3	TRA:CAEAEGGGADGLTF TRB:CATSGGDNEQFF	174	CD8-C5
PB-1_TGATTTCTCTGCCCTA	PB	PB-1	P1-6	TRUE	3	TRA:CAMREGRERDDKIIF TRB:CASSLPNTEAFF	216	CD8-C5
PB-1_TGCACCTAGACCGGAT	PB	PB-1	P1-6	TRUE	3	TRA:CAMREGRERDDKIIF TRB:CASSLPNTEAFF	216	CD8-C5
PB-1_TGCACCTAGAGACGAA	PB	PB-1	P1-12	TRUE	3	TRA:CALSDERGDYKLSF TRB:CASSPGTGSYNEQFF	141	CD8-C5
PB-1_TGCACCTAGAGGTAGA	PB	PB-1	P1-1015	FALSE	1	TRA:CALRAITQGGSEKLVF TRB:CASSQVTGGARDEQYF	1	CD8-C4

PB-1_TGCCAAAAGAGGTTGC	PB	PB-1	P1-12	TRUE	3	TRA:CALSDERGDYKLSF TRB:CASSPGTGSYNEQFF	141	CD8-C5
PB-1_TGCCAAATCAAACAAG	PB	PB-1	P1-6	TRUE	3	TRA:CAMREGRERDDKIIF TRB:CASSLPNTEAFF	216	CD8-C5
PB-1_TGCCCATCACTAAGTC	PB	PB-1	P1-33	TRUE	3	TRA:CLVGTGGFKTIF TRB:CSARVPGQMKETQYF	41	CD8-C4
PB-1_TGCCCTAAGACTGTAA	PB	PB-1	P1-6	TRUE	3	TRA:CAMREGRERDDKIIF TRB:CASSLPNTEAFF	216	CD8-C5
PB-1_TGCCCTACACAAGACG	PB	PB-1	P1-12	TRUE	3	TRA:CALSDERGDYKLSF TRB:CASSPGTGSYNEQFF	141	CD8-C5
PB-1_TGCCCTATCGCTGATA	PB	PB-1	P1-6	TRUE	3	TRA:CAMREGRERDDKIIF TRB:CASSLPNTEAFF	216	CD8-C5
PB-1_TGCGCAGGTACGACCC	PB	PB-1	P1-9	TRUE	3	TRA:CAEAEGGGADGLTF TRB:CATSGGDNEQFF	174	CD8-C5
PB-1_TGCGCAGTCAACGGGA	PB	PB-1	P1-6	TRUE	3	TRA:CAMREGRERDDKIIF TRB:CASSLPNTEAFF	216	CD8-C5
PB-1_TGCGCAGTCCAGGGCT	PB	PB-1	P1-9	TRUE	3	TRA:CAEAEGGGADGLTF TRB:CATSGGDNEQFF	174	CD8-C7
PB-1_TGCGGGTCAAGCCTAT	PB	PB-1	P1-7	TRUE	3	TRA:CAVQAGNNDMRF TRB:CASSTTGDFNYGYTF	215	CD8-C5
PB-1_TGCGGGTTCCTTAATC	PB	PB-1	P1-9	TRUE	3	TRA:CAEAEGGGADGLTF TRB:CATSGGDNEQFF	174	CD8-C7
PB-1_TGCGTGGCACAAGCCC	PB	PB-1	P1-12	TRUE	3	TRA:CALSDERGDYKLSF TRB:CASSPGTGSYNEQFF	141	CD8-C5
PB-1_TGCGTGGCACGAAACG	PB	PB-1	P1-12	TRUE	3	TRA:CALSDERGDYKLSF TRB:CASSPGTGSYNEQFF	141	CD8-C5
PB-1_TGCGTGGGTCAGATAA	PB	PB-1	P1-7	TRUE	3	TRA:CAVQAGNNDMRF TRB:CASSTTGDFNYGYTF	215	CD8-C5
PB-1_TGGACGCAGCCAGAAC	PB	PB-1	P1-9	TRUE	3	TRA:CAEAEGGGADGLTF TRB:CATSGGDNEQFF	174	CD8-C7
PB-1_TGGACGCAGGGTGTGT	PB	PB-1	P1-91	TRUE	3	TRA:CVVSDQYNTDKLIF TRB:CASGEGQGASSYEQYF	6	CD8-C5
PB-1_TGGACGCGTCTAAACC	PB	PB-1	P1-55	TRUE	3	TRA:CALSPQDSNYQLIW TRB:CASSFWTSANEQFF	13	CD8-C5
PB-1_TGGCCAGCAAGAAAGG	PB	PB-1	P1-7	TRUE	3	TRA:CAVQAGNNDMRF TRB:CASSTTGDFNYGYTF	215	CD8-C5
PB-1_TGGCCAGGTCTGCGGT	PB	PB-1	P1-300	TRUE	2	TRA:CAASFNDYKLSF TRB:CASKSVRTPQFF	2	CD8-C5
PB-1_TGGCCAGTCTGTCTCG	PB	PB-1	P1-6	TRUE	3	TRA:CAMREGRERDDKIIF TRB:CASSLPNTEAFF	216	CD8-C7
PB-1_TGGCCAGTCTTGCATT	PB	PB-1	P1-6	TRUE	3	TRA:CAMREGRERDDKIIF TRB:CASSLPNTEAFF	216	CD8-C5
PB-1_TGGCGCAGTCTTCGTC	PB	PB-1	P1-6	TRUE	3	TRA:CAMREGRERDDKIIF TRB:CASSLPNTEAFF	216	CD8-C5
PB-1_TGGGAAGCATGCAACT	PB	PB-1	P1-7	TRUE	3	TRA:CAVQAGNNDMRF TRB:CASSTTGDFNYGYTF	215	CD8-C5
PB-1_TGGGAAGCATTCTCAT	PB	PB-1	P1-9	TRUE	3	TRA:CAEAEGGGADGLTF TRB:CATSGGDNEQFF	174	CD8-C7
PB-1_TGGTTAGAGTACCGGA	PB	PB-1	P1-14	TRUE	3	TRA:CAASVVNDMRF TRB:CASSHRGGAKNIQYF	109	CD8-C5
PB-1_TGTATTCCAGGCAGTA	PB	PB-1	P1-91	TRUE	3	TRA:CVVSDQYNTDKLIF TRB:CASGEGQGASSYEQYF	6	CD8-C5
PB-1_TGTATTCTCGTTACGA	PB	PB-1	P1-1040	FALSE	1	TRA:CALSERDTDKLIF TRB:CSARGRGLAGGPAYEQYF	1	CD8-C7

PB-1_TGTCCCATCCTTTACA	PB	PB-1	P1-7	TRUE	3	TRA:CAVQAGNNDMRF TRB:CASSTTGDFNYGYTF	215	CD8-C5
PB-1_TGTGGTAAGCGCCTTG	PB	PB-1	P1-9	TRUE	3	TRA:CAEAEGGGADGLTF TRB:CATSGGDNEQFF	174	CD8-C7
PB-1_TGTGGTATCGCTTGTC	PB	PB-1	P1-12	TRUE	3	TRA:CALSDERGDYKLSF TRB:CASSPGTGSYNEQFF	141	CD8-C5
PB-1_TGTGGTATCGTAGATC	PB	PB-1	P1-9	TRUE	3	TRA:CAEAEGGGADGLTF TRB:CATSGGDNEQFF	174	CD8-C7
PB-1_TGTGTTTCAGTTAACC	PB	PB-1	P1-18	TRUE	3	TRA:CVADGNYGQNFVF TRB:CASSSPGLGGGFDTEAFF	75	CD8-C6
PB-1_TGTGTTTGTGCGATAG	PB	PB-1	P1-6	TRUE	3	TRA:CAMREGRERDDKIIF TRB:CASSLPNTEAFF	216	CD8-C5
PB-1_TTAACTCAGAGCCTAG	PB	PB-1	P1-39	TRUE	3	TRA:CAVRDLGGTSYDKVIF TRB:CSARVREVSYEQYF	31	CD8-C7
PB-1_TTAACTCAGCGTTTAC	PB	PB-1	P1-9	TRUE	3	TRA:CAEAEGGGADGLTF TRB:CATSGGDNEQFF	174	CD8-C5
PB-1_TTAACTCCAAAGTGCG	PB	PB-1	P1-6	TRUE	3	TRA:CAMREGRERDDKIIF TRB:CASSLPNTEAFF	216	CD8-C5
PB-1_TTAACTCCAATAAGCA	PB	PB-1	P1-1	TRUE	3	TRA:CAMSDRMTGGFKTIF TRB:CASSQVGRGGEETQYF	1047	CD8-C5
PB-1_TTAACTCGTTGACGTT	PB	PB-1	P1-8	TRUE	3	TRA:CAVRPPGRNSGGYQKVTF TRB:CASSRRGVTDTQYF	204	CD8-C7
PB-1_TTAGGACTCAGTTAGC	PB	PB-1	P1-3	TRUE	3	TRA:CAFLSGTYKYIF TRB:CASSQSPGQRNYGYTF	308	CD8-C7
PB-1_TTAGTTCAGAGGACGG	PB	PB-1	P1-6	TRUE	3	TRA:CAMREGRERDDKIIF TRB:CASSLPNTEAFF	216	CD8-C5
PB-1_TTATGCTCACGTCAGC	PB	PB-1	P1-20	TRUE	3	TRA:CVVVGGRALTF TRB:CASSLATNPHETQYF	69	CD8-C4
PB-1_TTATGCTTCACTGGGC	PB	PB-1	P1-1	TRUE	3	TRA:CAMSDRMTGGFKTIF TRB:CASSQVGRGGEETQYF	1047	CD8-C5
PB-1_TTATGCTTCTCCAGGG	PB	PB-1	P1-66	TRUE	3	TRA:CVVSDPPDKLIF TRB:CASSASETGSVYEQYF	9	CD8-C5
PB-1_TTCCCAGCAGTATCTG	PB	PB-1	P1-7	TRUE	3	TRA:CAVQAGNNDMRF TRB:CASSTTGDFNYGYTF	215	CD8-C5
PB-1_TTCGGTCCAGCCAATT	PB	PB-1	P1-12	TRUE	3	TRA:CALSDERGDYKLSF TRB:CASSPGTGSYNEQFF	141	CD8-C4
PB-1_TTCTACACACTTAACG	PB	PB-1	P1-2	TRUE	3	TRA:CGQNFVF TRB:CASNQRGGPSLYNEQFF	323	CD8-C5
PB-1_TTCTCAACAATTGCTG	PB	PB-1	P1-177	FALSE	3	TRA:CAVLQAGTALIF TRB:CASSVNRDKRTDTQYF	3	CD8-C5
PB-1_TTCTCAACAGGACCCT	PB	PB-1	P1-12	TRUE	3	TRA:CALSDERGDYKLSF TRB:CASSPGTGSYNEQFF	141	CD8-C5
PB-1_TTCTCAATCGGTCTAA	PB	PB-1	P1-53	TRUE	3	TRA:CALSERDTDKLIF TRB:CASSILQGVITEAFF	13	CD8-C5
PB-1_TTCTCCTCAGTCGATT	PB	PB-1	P1-6	TRUE	3	TRA:CAMREGRERDDKIIF TRB:CASSLPNTEAFF	216	CD8-C5
PB-1_TTCTTAGTCCGAACGC	PB	PB-1	P1-1	TRUE	3	TRA:CAMSDRMTGGFKTIF TRB:CASSQVGRGGEETQYF	1047	CD8-C5
PB-1_TTGAACGAGAAACCGC	PB	PB-1	P1-5	TRUE	3	TRA:CALSGTPMNRDDKIIF TRB:CSARGRGLAGGPAYEQYF	223	CD8-C7
PB-1_TTGAACGAGAGACGAA	PB	PB-1	P1-5	TRUE	3	TRA:CALSGTPMNRDDKIIF TRB:CSARGRGLAGGPAYEQYF	223	CD8-C5
PB-1_TTGAACGTCACCAGGC	PB	PB-1	P1-84	FALSE	3	TRA:CAMREPPHRGSTLGRLYF TRB:CSATGLAGVPFTDTQYF	6	CD8-C5

PB-1_TTGACTTCAATCAGAA	PB	PB-1	P1-12	TRUE	3	TRA:CALSDERGDYKLSF TRB:CASSPGTGSYNEQFF	141	CD8-C5
PB-1_TTGACTTTCTCCTATA	PB	PB-1	P1-4	TRUE	3	TRA:CAMKGRDDKIIF TRB:CASTKGVSREAFF	226	CD8-C5
PB-1_TTGACTTTCTGGCGTG	PB	PB-1	P1-12	TRUE	3	TRA:CALSDERGDYKLSF TRB:CASSPGTGSYNEQFF	141	CD8-C5
PB-1_TTGCCGTAGGTGTTAA	PB	PB-1	P1-9	TRUE	3	TRA:CAEAEGGGADGLTF TRB:CATSGGDNEQFF	174	CD8-C5
PB-1_TTGCCGTGTCACTTCC	PB	PB-1	P1-8	TRUE	3	TRA:CAVRPPGRNSGGYQKVTF TRB:CASSRRGVTDTQYF	204	CD8-C5
PB-1_TTGGAACGTGTTTGGT	PB	PB-1	P1-9	TRUE	3	TRA:CAEAEGGGADGLTF TRB:CATSGGDNEQFF	174	CD8-C5
PB-1_TTGGCAAAGGATGGAA	PB	PB-1	P1-53	TRUE	3	TRA:CALSERDTDKLIF TRB:CASSILQGVITEAFF	13	CD8-C5
PB-1_TTGGCAAGTGACGGTA	PB	PB-1	P1-7	TRUE	3	TRA:CAVQAGNNDMRF TRB:CASSTTGDFNYGYTF	215	CD8-C5
PB-1_TTTATGCAGAGATGAG	PB	PB-1	P1-4	TRUE	3	TRA:CAMKGRDDKIIF TRB:CASTKGVSREAFF	226	CD8-C5
PB-1_TTTATGCCATTTCACT	PB	PB-1	P1-6	TRUE	3	TRA:CAMREGRERDDKIIF TRB:CASSLPNTEAFF	216	CD8-C5
PB-1_TTTATGCTCTGGTGTA	PB	PB-1	P1-12	TRUE	3	TRA:CALSDERGDYKLSF TRB:CASSPGTGSYNEQFF	141	CD8-C5
PB-1_TTTCCTCCACGCCAGT	PB	PB-1	P1-7	TRUE	3	TRA:CAVQAGNNDMRF TRB:CASSTTGDFNYGYTF	215	CD8-C5
PB-1_TTTCCTCTCTGCCAGG	PB	PB-1	P1-1076	FALSE	1	TRA:CTVSNDYKLSF TRB:CASSHSLGGGELFF	1	CD8-C4
PB-1_TTTGGTTAGGCCATAG	PB	PB-1	P1-12	TRUE	3	TRA:CALSDERGDYKLSF TRB:CASSPGTGSYNEQFF	141	CD8-C5
PB-1_TTTGGTTGTTCAGGCC	PB	PB-1	P1-14	TRUE	3	TRA:CAASVVNDMRF TRB:CASSHRGGAKNIQYF	109	CD8-C5
PB-1_TTTGGTTTCAGCTGGC	PB	PB-1	P1-9	TRUE	3	TRA:CAEAEGGGADGLTF TRB:CATSGGDNEQFF	174	CD8-C5
PB-1_TTTGTCAAGTCACGCC	PB	PB-1	P1-12	TRUE	3	TRA:CALSDERGDYKLSF TRB:CASSPGTGSYNEQFF	141	CD8-C5
PB-1_TTTGTCATCGAACGGA	PB	PB-1	P1-12	TRUE	3	TRA:CALSDERGDYKLSF TRB:CASSPGTGSYNEQFF	141	CD8-C5
PB-1_ACATACGCATGTAGTC	PB	PB-1	P1-470	FALSE	1	TRA:CAESWAGGSYIPTF TRB:CSARGLSTYEQYF	1	CD8-C3
PB-1_AGCATACTCTTGTATC	PB	PB-1	P1-6	TRUE	3	TRA:CAMREGRERDDKIIF TRB:CASSLPNTEAFF	216	CD8-C3
PB-1_CGTCACTTCCCTGACT	PB	PB-1	P1-3	TRUE	3	TRA:CAFLSGTYKYIF TRB:CASSQSPGQRNYGYTF	308	CD8-C7
PB-1_TGTATTCAGCGGCTTC	PB	PB-1	P1-23	TRUE	3	TRA:CALRAITQGGSEKLVF TRB:CASSLTGAVYGYTF	65	CD8-C4
PB-1_AACACGTAGCCCAGCT	PB	PB-1	P1-432	FALSE	1	TRA:CAAGGGADGLTF TRB:CAISGGGRDYGYTF	1	CD4-C4
PB-1_AAGGTTCCACCGAAAG	PB	PB-1	P1-453	FALSE	1	TRA:CAVNMGGADGLTF TRB:CSVASSYEQYF	1	CD4-C4
PB-1_AGCTCCTCACCAGGTC	PB	PB-1	P1-522	FALSE	1	TRA:CAYRRKSNYQLIW TRB:CASSHRGGAKNIQYF	1	CD4-C3
PB-1_CCCAATCTCAGTACGT	PB	PB-1	P1-188	TRUE	3	TRA:CPRNNNARLMF TRB:CASTSGGFNQPQHF	3	CD4-C3
PB-1_CGCTTCAGTATCACCA	PB	PB-1	P1-694	FALSE	1	TRA:CIVRVAQGGSEKLVF TRB:CASSSTGTGNQPQHF	1	CD4-C3

PB-1_CTCGAAACATACAGCT	PB	PB-1	P1-738	FALSE	1	TRA:CAAAYSGAGSYQLTF TRB:CASSGAVGVGEQFF	1	CD4-C2
PB-1_GCATGTACAAAGGTGC	PB	PB-1	P1-828	FALSE	1	TRA:CAASAIQAAGNKLTF TRB:CASGKRGGFTDTQYF	1	CD4-C4
PB-1_GGCGACTCATGCAACT	PB	PB-1	P1-292	TRUE	2	TRA:CAAPSNTNAGKSTF TRB:CASSLDVYQDNEQFF	2	CD4-C2
PB-1_AAACCTGGTGCCTGCA	PB	PB-1	P1-19	TRUE	3	TRA:CALGAFGSARQLTF TRB:CASSKGQGPRETQYF	70	CD4-C2
PB-1_AAATGCCGTTATTCTC	PB	PB-1	P1-429	FALSE	1	TRA:CAATYGGATNKLIF TRB:CSVQRGTATGELFF	1	CD4-C1
PB-1_AACCATGGTACCATCA	PB	PB-1	P1-435	FALSE	1	TRA:CAVGGQGAQKLVF TRB:CASSRGTDSAEAFF	1	CD4-C2
PB-1_AACTCAGGTAGCGCTC	PB	PB-1	P1-438	FALSE	1	TRA:CATDRGSARQLTF TRB:CASSMLAGGPEQFF	1	CD4-C2
PB-1_AAGGCAGGTTATGTGC	PB	PB-1	P1-450	FALSE	1	TRA:CAVSKRGSQGNLIF TRB:CASSQDRGTTYNEQFF	1	CD4-C2
PB-1_AAGTCTGCACTGTCGG	PB	PB-1	P1-456	FALSE	1	TRA:CALSDPYGGSQGNLIF TRB:CASSLGQNPLYEQYF	1	CD4-C2
PB-1_AATCCAGCACAAGCCC	PB	PB-1	P1-460	FALSE	1	TRA:CAAIGMDTGRRALTF TRB:CASSFRRGGSDTQYF	1	CD4-C1
PB-1_ACACCGGAGAACTCGG	PB	PB-1	P1-464	FALSE	1	TRA:CAVGITGANNLFF TRB:CASSVEPSGAGEQYF	1	CD4-C2
PB-1_ACAGCTAGTAGCTAAA	PB	PB-1	P1-467	FALSE	1	TRA:CAVSHAGGTSYGKLTF TRB:CATSVTGSETQYF	1	CD4-C2
PB-1_ACCGTAATCGTTACAG	PB	PB-1	P1-477	FALSE	1	TRA:CAVNTDKLIF TRB:CASSLGTSSFEQYF	1	CD4-C3
PB-1_ACGAGCCCAGTGAGTG	PB	PB-1	P1-479	FALSE	1	TRA:CAATRTDTDKLIF TRB:CASSTGTSDPNEQYF	1	CD4-C2
PB-1_ACGAGCCGTTGAGTTC	PB	PB-1	P1-481	FALSE	1	TRA:CAAGSGNTPLVF TRB:CSARDRQSNIKAFF	1	CD4-C2
PB-1_ACGATGTGTCAGTGGA	PB	PB-1	P1-484	FALSE	1	TRA:CALRHGNQFYF TRB:CASSLVGGMDYGYTF	1	CD4-C2
PB-1_ACGCAGCGTGCGGTAA	PB	PB-1	P1-487	FALSE	1	TRA:CVPSSSGGSYIPTF TRB:CASSANRNTEAFF	1	CD4-C1
PB-1_ACGGAGATCCCTTGTG	PB	PB-1	P1-491	FALSE	1	TRA:CAPAGGSNYKLTF TRB:CASTIGQKNQPQHF	1	CD4-C2
PB-1_ACGGCCAAGGAGTCTG	PB	PB-1	P1-493	FALSE	1	TRA:CAVNAGSGGYQKVTF TRB:CASSSTPGFNEKLFF	1	CD4-C2
PB-1_ACTATCTAGTGTACGG	PB	PB-1	P1-497	FALSE	1	TRA:CAASDGYSSASKIIF TRB:CASREQGGTSEQYF	1	CD4-C1
PB-1_ACTGAACTCCAAGTAC	PB	PB-1	P1-499	FALSE	1	TRA:CATRTQGGSEKLVF TRB:CASSLGGAGPDTQYF	1	CD4-C2
PB-1_ACTGATGTCAACTCTT	PB	PB-1	P1-501	FALSE	1	TRA:CARAGSYQLTF TRB:CASSLGLGNTGELFF	1	CD4-C2
PB-1_ACTGTCCGTGGCTCCA	PB	PB-1	P1-504	FALSE	1	TRA:CAVEIPSNYQLIW TRB:CASSPGERGHNEQFF	1	CD4-C1
PB-1_AGAATAGCAGTATCTG	PB	PB-1	P1-507	FALSE	1	TRA:CAVGGDNDMRF TRB:CASSLRPGTGVGYTF	1	CD4-C2
PB-1_AGAGCTTAGGGTGTGT	PB	PB-1	P1-88	TRUE	3	TRA:CAVRDRGSGNTPLVF TRB:CASSGDRVYEQYF	6	CD4-C2
PB-1_AGGGATGGTATCTGCA	PB	PB-1	P1-525	FALSE	1	TRA:CAAPDDKIIF TRB:CASSLVGRGVTGELFF	1	CD4-C2
PB-1_AGGTCATAGACAGACC	PB	PB-1	P1-527	FALSE	1	TRA:CVVSDISGGYNKLIF TRB:CASSPGDFSNQPQHF	1	CD4-C2

PB-1_AGTGGGATCACATACG	PB	PB-1	P1-531	FALSE	1	TRA:CAVGERFGNEKLTF TRB:CASSQDGARVTEAFF	1	CD4-C2
PB-1_ATAACGCCAAGAAGAG	PB	PB-1	P1-533	FALSE	1	TRA:CIVANSGYALNF TRB:CASSLATGTYEQYF	1	CD4-C1
PB-1_ATCATCTAGACGACGT	PB	PB-1	P1-538	FALSE	1	TRA:CAAILISGGYNKLIF TRB:CASQKGGQVYGGELFF	1	CD4-C2
PB-1_ATCATGGTCTTTACGT	PB	PB-1	P1-542	FALSE	1	TRA:CAETSRGSNASKIIF TRB:CASSLVDRSNSPLHF	1	CD4-C1
PB-1_ATCCGAACAAGTAATG	PB	PB-1	P1-545	FALSE	1	TRA:CAGLRIKAAGNKLTF TRB:CASSYVGGAAEAFF	1	CD4-C2
PB-1_ATCTGCCCATAAAGGT	PB	PB-1	P1-549	FALSE	1	TRA:CADVGRDSSYKLIF TRB:CASSLTRQETQYF	1	CD4-C2
PB-1_ATGAGGGTCGAGGTAG	PB	PB-1	P1-551	FALSE	1	TRA:CAPNGAMSGSRLTF TRB:CASSLGQGNYNEQFF	1	CD4-C2
PB-1_ATTACTCGTCTAGTGT	PB	PB-1	P1-557	FALSE	1	TRA:CATPTFSGGYQKVTF TRB:CASSQGAGRGRYEQYF	1	CD4-C2
PB-1_ATTCTACAGATCGATA	PB	PB-1	P1-560	FALSE	1	TRA:CAVRVLGSGNTGKLIF TRB:CASSLRREAFF	1	CD4-C2
PB-1_ATTGGTGCACGGCTAC	PB	PB-1	P1-561	FALSE	1	TRA:CAYRSSETSGSRLTF TRB:CASSTDSGGIDTQYF	1	CD4-C1
PB-1_ATTGGTGCATGAGCGA	PB	PB-1	P1-562	FALSE	1	TRA:CALSDSGGYQKVTF TRB:CASSRDRGGTTEAFF	1	CD4-C2
PB-1_ATTTCTGCATAGAAAC	PB	PB-1	P1-566	FALSE	1	TRA:CAASGGLNFGNEKLTF TRB:CATSKDIGSYNEQFF	1	CD4-C2
PB-1_CAACCTCTCGCCGTGA	PB	PB-1	P1-567	FALSE	1	TRA:CAFMKLNNARLMF TRB:CASSRSEGYEQYF	1	CD4-C2
PB-1_CAAGATCCAGGCTCAC	PB	PB-1	P1-570	FALSE	1	TRA:CAAAAAGGTSYGKLTF TRB:CASSRGPGSPGANVLTF	1	CD4-C2
PB-1_CAAGATCTCTCTGTCG	PB	PB-1	P1-571	FALSE	1	TRA:CAASGQAGANNLFF TRB:CASSQVSGVAEAFF	1	CD4-C2
PB-1_CACACAAAGCTCAACT	PB	PB-1	P1-577	FALSE	1	TRA:CAVQAGNNDMRF TRB:CASSLSTDTQYF	1	CD4-C3
PB-1_CACACAACAGATCCAT	PB	PB-1	P1-578	FALSE	1	TRA:CAVNRGGGNKLTF TRB:CASSFQRITRYNEQFF	1	CD4-C2
PB-1_CACACAACAGGTTTCA	PB	PB-1	P1-579	FALSE	1	TRA:CAVQAGLYNTDKLIF TRB:CSARASGNTIYF	1	CD4-C2
PB-1_CACCAGGGTCAGCTAT	PB	PB-1	P1-587	FALSE	1	TRA:CAVRDGGRGSTLGRLYF TRB:CAISGTGTSGNTIYF	1	CD4-C3
PB-1_CACCTTGGTTCGGCAC	PB	PB-1	P1-590	FALSE	1	TRA:CAATHNQGGKLIF TRB:CATNTGTPSYNEQFF	1	CD4-C2
PB-1_CAGCATAAGCCATCGC	PB	PB-1	P1-25	TRUE	3	TRA:CIVGRDSSYKLIF TRB:CASNPQRGAHRDTQYF	58	CD8-C2
PB-1_CAGCATAGTCTTCAAG	PB	PB-1	P1-596	FALSE	1	TRA:CATTQGNNRLAF TRB:CSVGQGEGEAFF	1	CD4-C2
PB-1_CAGGTGCGTAAGGGCT	PB	PB-1	P1-605	FALSE	1	TRA:CALRANFGNEKLTF TRB:CASSVLRDSSGNTIYF	1	CD4-C2
PB-1_CAGTAACAGACGACGT	PB	PB-1	P1-607	FALSE	1	TRA:CAAMEGSGSEKLVF TRB:CASSGFLAGGLNEQFF	1	CD4-C2
PB-1_CAGTAACGTGTCGCTG	PB	PB-1	P1-610	FALSE	1	TRA:CAVVAGSNYKLTF TRB:CASSPSGRSSDTQYF	1	CD4-C1
PB-1_CAGTCCTAGAAAGTGG	PB	PB-1	P1-612	FALSE	1	TRA:CIVRPPPSQGAQKLVF TRB:CSARPLLAAEQFF	1	CD4-C1
PB-1_CAGTCCTCACCTCGGA	PB	PB-1	P1-614	FALSE	1	TRA:CGTGARGSSGSRLTF TRB:CASSVAGTYSSYNEQFF	1	CD4-C2

PB-1_CAGTCCTCACGAAACG	PB	PB-1	P1-615	FALSE	1	TRA:CAVTGAGSYQLTF TRB:CASSLRTGRVDSEKLFF	1	CD4-C2
PB-1_CATATGGGTCGAACAG	PB	PB-1	P1-616	FALSE	1	TRA:CAVNGSSNTGKLIF TRB:CASSRWTQRGEQYF	1	CD4-C1
PB-1_CATATGGGTGCTAGCC	PB	PB-1	P1-276	FALSE	2	TRA:CAAPPLTTSGTYKYIF TRB:CASRRGLAGASNTGELFF	2	CD4-C3
PB-1_CATATGGTCACAACGT	PB	PB-1	P1-617	FALSE	1	TRA:CAGPTGANSKLTF TRB:CASSRGTYYGYTF	1	CD4-C1
PB-1_CATATGGTCTTAGCCC	PB	PB-1	P1-277	TRUE	2	TRA:CGTEWSGANNDMRF TRB:CATAEGDQPQHF	2	CD4-C2
PB-1_CATCAAGGTCAGAAGC	PB	PB-1	P1-621	FALSE	1	TRA:CAVGPAVFSGGYNKLIF TRB:CASGLVLAGAHNEQFF	1	CD4-C2
PB-1_CATCAAGGTTGGTGGA	PB	PB-1	P1-622	FALSE	1	TRA:CAVASTSYGKLTF TRB:CASSQDRRQGGTGELFF	1	CD4-C2
PB-1_CATCCACTCGAATGGG	PB	PB-1	P1-624	FALSE	1	TRA:CAVGAGDYGGSQGNLIF TRB:CASSRRERGLTGELFF	1	CD4-C2
PB-1_CCACCTATCACCTCGT	PB	PB-1	P1-634	FALSE	1	TRA:CAVGIYGNKLVF TRB:CASSLVGLNTEAFF	1	CD4-C2
PB-1_CCACTACGTCGAAAGC	PB	PB-1	P1-638	FALSE	1	TRA:CATDTGRRALTF TRB:CASSGGEGGTDTQYF	1	CD4-C2
PB-1_CCCATACCAGCTTCGG	PB	PB-1	P1-648	FALSE	1	TRA:CVVNLRGGADGLTF TRB:CASSIGTSPLNEQFF	1	CD4-C2
PB-1_CCCATACTCTTCAACT	PB	PB-1	P1-650	FALSE	1	TRA:CAMREYSGGSNYKLTF TRB:CASNLGQGADTQYF	1	CD4-C2
PB-1_CCGTACTCAAGTCTAC	PB	PB-1	P1-652	FALSE	1	TRA:CIAAQGSSNTGKLIF TRB:CASSLVSLMGELFF	1	CD4-C2
PB-1_CCTACCAGTATTACCG	PB	PB-1	P1-656	FALSE	1	TRA:CAVRDPNTNAGKSTF TRB:CASSEGADTQYF	1	CD4-C2
PB-1_CCTATTACATTGAGCT	PB	PB-1	P1-660	FALSE	1	TRA:CAVNAPASGGYNKLIF TRB:CASSLSGHSTDTQYF	1	CD4-C1
PB-1_CGAATGTAGATCCGAG	PB	PB-1	P1-668	FALSE	1	TRA:CAMREGQGSSNTGKLIF TRB:CASSVVPQAVSYNEQFF	1	CD4-C1
PB-1_CGATGTATCCGCGTTT	PB	PB-1	P1-680	FALSE	1	TRA:CAGALYNNNDMRF TRB:CASSPPGVAGGSYEQYF	1	CD4-C1
PB-1_CGCTATCTCGGCTACG	PB	PB-1	P1-689	FALSE	1	TRA:CVVNRGNSGYSTLTF TRB:CASSITVRNQPQHF	1	CD4-C2
PB-1_CGCTGGAAGAGACGAA	PB	PB-1	P1-690	FALSE	1	TRA:CIVGRDSSYKLIF TRB:CASSRTVAEAQYF	1	CD4-C3
PB-1_CGCTGGAAGTACGTTC	PB	PB-1	P1-691	FALSE	1	TRA:CAGDQAGTALIF TRB:CASKVGPAHTGELFF	1	CD4-C1
PB-1_CGGACGTCAATCGAAA	PB	PB-1	P1-698	FALSE	1	TRA:CVVSRSGGYNKLIF TRB:CASSPSQTNSPLHF	1	CD4-C2
PB-1_CGGACTGTCGTGGACC	PB	PB-1	P1-702	FALSE	1	TRA:CAVSLISSGSARQLTF TRB:CASQQTSGTQYF	1	CD4-C2
PB-1_CGTAGCGCACCGCTAG	PB	PB-1	P1-707	FALSE	1	TRA:CALSLDSGAGSYQLTF TRB:CASSYGLRGLEQYF	1	CD4-C2
PB-1_CGTCTACTCCGTCAAA	PB	PB-1	P1-714	FALSE	1	TRA:CAAASGEDDKIIF TRB:CATNPGQGSYNEQFF	1	CD4-C1
PB-1_CGTTCTGGTAGGAGTC	PB	PB-1	P1-720	FALSE	1	TRA:CAYREDYTGTASKLTF TRB:CSAVTGSSYNSPLHF	1	CD4-C1
PB-1_CTAGTGATCATCGATG	PB	PB-1	P1-728	FALSE	1	TRA:CAFMKTGYALNF TRB:CAWSVAGTSTDTQYF	1	CD4-C2
PB-1_CTAGTGATCGTGGACC	PB	PB-1	P1-729	FALSE	1	TRA:CAYRSRF TRB:CASSLTGGNTEAFF	1	CD4-C2

PB-1_CTCACACAGTTCGCAT	PB	PB-1	P1-730	FALSE	1	TRA:CAVGPWDDYKLSF TRB:CASGPGLAGVGEQFF	1	CD4-C2
PB-1_CTCGTACTCTCTAAGG	PB	PB-1	P1-742	FALSE	1	TRA:CALILTQGGSEKLVF TRB:CSARGGGRRPQHF	1	CD4-C7
PB-1_CTCGTCACAATAGAGT	PB	PB-1	P1-743	FALSE	1	TRA:CAVSHSGGYQKVTF TRB:CASSVGGTSGGAPSYNNEQFF	1	CD4-C2
PB-1_CTCTGGTCATTGAGCT	PB	PB-1	P1-747	FALSE	1	TRA:CAVDKSSGTYKYIF TRB:CASSDWSGGHTDTQYF	1	CD4-C1
PB-1_CTGCCTAAGATGTAAC	PB	PB-1	P1-753	FALSE	1	TRA:CALSDRDSGYSTLTF TRB:CASSVAGSGLSSYNEQFF	1	CD4-C2
PB-1_CTGCCTATCGGAAATA	PB	PB-1	P1-755	FALSE	1	TRA:CAVNTNAGKSTF TRB:CASSFETSGGTDTQYF	1	CD4-C1
PB-1_CTTAACTTCAGAGGTG	PB	PB-1	P1-768	FALSE	1	TRA:CATYCMDSNYQLIW TRB:CASSYGQGLDTQYF	1	CD4-C2
PB-1_CTTGGCTGTCCAGTGC	PB	PB-1	P1-776	FALSE	1	TRA:CAVDRGSTLGRLYF TRB:CASSPEQGDTGELFF	1	CD4-C2
PB-1_CTTTGCGTCTTACCTA	PB	PB-1	P1-777	FALSE	1	TRA:CAATPSGAGSYQLTF TRB:CASIRGGDLGETQYF	1	CD4-C2
PB-1_GAAACTCAGAGATGAG	PB	PB-1	P1-778	FALSE	1	TRA:CAVVRGGGADGLTF TRB:CASSPHKGLITEAFF	1	CD4-C2
PB-1_GAAACTCGTGTGAATA	PB	PB-1	P1-780	FALSE	1	TRA:CAVGVAGGGNKLTF TRB:CASRTTGLAKNIQYF	1	CD4-C2
PB-1_GAACCTATCCCATTAT	PB	PB-1	P1-784	FALSE	1	TRA:CALSAYNQGGKLIF TRB:CASIGTDEETQYF	1	CD4-C2
PB-1_GAAGCAGCAGCTGGCT	PB	PB-1	P1-276	FALSE	2	TRA:CAAPPLTTSGTYKYIF TRB:CASRRGLAGASNTGELFF	2	CD4-C2
PB-1_GACAGAGAGGAATCGC	PB	PB-1	P1-789	FALSE	1	TRA:CAMREGPGTGFQKLVF TRB:CASSLRKAGGNQPQHF	1	CD4-C2
PB-1_GACAGAGGTGTTCGAT	PB	PB-1	P1-791	FALSE	1	TRA:CSRYKASYDKVIF TRB:CASSPLRQAQLRDSPLHF	1	CD4-C2
PB-1_GACGTGCTCTTTCCTC	PB	PB-1	P1-795	FALSE	1	TRA:CAVSTWGNRDDKIIF TRB:CASSVRASGGGETQYF	1	CD4-C2
PB-1_GACTGCGGTTCGGCAC	PB	PB-1	P1-802	FALSE	1	TRA:CALGVPGGGNKLTF TRB:CASSLDDRTSSYEQYF	1	CD4-C2
PB-1_GATGCTAAGCCGATTT	PB	PB-1	P1-815	FALSE	1	TRA:CAGLGYSGSRLTF TRB:CASSQSSSWNTGELFF	1	CD4-C3
PB-1_GATTCAGCATGCCCGA	PB	PB-1	P1-820	FALSE	1	TRA:CAATTRTGRRALTF TRB:CASTGQSSYNSPLHF	1	CD4-C2
PB-1_GCAGCCACATCTACGA	PB	PB-1	P1-824	FALSE	1	TRA:CAVEVGNAGKSTF TRB:CASSFSGTRGETQYF	1	CD4-C3
PB-1_GCTCTGTGTAAATGAC	PB	PB-1	P1-841	FALSE	1	TRA:CATDAERGGADGLTF TRB:CASSVTGLLFSDTQYF	1	CD4-C2
PB-1_GCTGCAGAGTGTACCT	PB	PB-1	P1-843	FALSE	1	TRA:CAVGGRYSSASKIIF TRB:CASSPSGATDTQYF	1	CD4-C2
PB-1_GCTGCGATCTACTCAT	PB	PB-1	P1-291	FALSE	2	TRA:CIVAGGSEKLVF TRB:CASSSHRFAYNEQFF	2	CD4-C2
PB-1_GCTGGGTCATTTCAGG	PB	PB-1	P1-848	FALSE	1	TRA:CAYKVGRMNRDDKIIF TRB:CATSRTLNTEAFF	1	CD4-C3
PB-1_GCTTCCAAGCTAAACA	PB	PB-1	P1-849	FALSE	1	TRA:CAGLGWRGETSGSRLTF TRB:CASSGSSSYNSPLHF	1	CD4-C1
PB-1_GGAAAGCCAGCTGCTG	PB	PB-1	P1-853	FALSE	1	TRA:CALIWDNQFYF TRB:CASSHAGLDGYNEQFF	1	CD4-C2
PB-1_GGAATAACACCGTTGG	PB	PB-1	P1-855	FALSE	1	TRA:CAVRDAIRSGSARQLTF TRB:CASSSDGPRGELFF	1	CD4-C2

PB-1_GGATTACGTTCTCATT	PB	PB-1	P1-862	FALSE	1	TRA:CAAQALTGANNLFF TRB:CASSTRELPLHF	1	CD4-C2
PB-1_GGCTCGATCCGGGTGT	PB	PB-1	P1-867	FALSE	1	TRA:CAASGDRGGSEKLVF TRB:CASSQMRPTGNTIYF	1	CD4-C2
PB-1_GGGAATGAGAGACTAT	PB	PB-1	P1-870	FALSE	1	TRA:CGTELSNYQLIW TRB:CASSLGRLLAGVYNEQFF	1	CD4-C2
PB-1_GGGAGATAGAGAACAG	PB	PB-1	P1-872	FALSE	1	TRA:CAVSDLHGTSYGKLTF TRB:CASSSAYLAGGRETQYF	1	CD4-C1
PB-1_GGGTCTGTCGCAGGCT	PB	PB-1	P1-877	FALSE	1	TRA:CAASKAAGNKLTF TRB:CASSTDPLGNSPLHF	1	CD4-C2
PB-1_GGTGTTACATGGTAGG	PB	PB-1	P1-881	FALSE	1	TRA:CALSEDYNNNDMRF TRB:CSASGGTQYF	1	CD4-C2
PB-1_GTAACTGAGCATGGCA	PB	PB-1	P1-886	FALSE	1	TRA:CAASHDYKLSF TRB:CASTLGTERETQYF	1	CD4-C2
PB-1_GTAACTGTCAAACCAC	PB	PB-1	P1-888	FALSE	1	TRA:CAVYTGRRALTF TRB:CASSQGTGKNNEQFF	1	CD4-C1
PB-1_GTACTTTAGTCAATAG	PB	PB-1	P1-891	FALSE	1	TRA:CAVSIKAAGNKLTF TRB:CASSQVGNNSPLHF	1	CD4-C2
PB-1_GTAGTCATCCTTGCCA	PB	PB-1	P1-895	FALSE	1	TRA:CAVGTRF TRB:CASSSGSVEADTQYF	1	CD4-C2
PB-1_GTATTCTTCGCCATAA	PB	PB-1	P1-899	FALSE	1	TRA:CAMKGGNNAGNMLTF TRB:CASRFIRGLQPQHF	1	CD4-C1
PB-1_GTCACAACATCTCGCT	PB	PB-1	P1-901	FALSE	1	TRA:CAVGAQGGGADGLTF TRB:CSARDSGRAAEQFF	1	CD4-C2
PB-1_GTCCTCAGTAAGGATT	PB	PB-1	P1-904	FALSE	1	TRA:CAVALI TRB:CASSVEGHETQYF	1	CD4-C1
PB-1_GTCCTCAGTTCACGGC	PB	PB-1	P1-905	FALSE	1	TRA:CALKGDRGSTLGRLYF TRB:CAWSARSSGNTIYF	1	CD4-C2
PB-1_GTCTCGTAGATAGGAG	PB	PB-1	P1-909	FALSE	1	TRA:CAASKIGGAGSYQLTF TRB:CASRGTSGNTIYF	1	CD4-C2
PB-1_GTCTCGTAGTCGATAA	PB	PB-1	P1-911	FALSE	1	TRA:CAVYSNYQLIW TRB:CSARVGSDNEKLFF	1	CD4-C1
PB-1_GTGAAGGAGTATCGAA	PB	PB-1	P1-913	FALSE	1	TRA:CAYRSGDTDKLIF TRB:CASSDGQGYSNQPQHF	1	CD4-C2
PB-1_GTGCATACAGACAAGC	PB	PB-1	P1-914	FALSE	1	TRA:CAMRADFGNEKLTF TRB:CASSFIPTSGGDEQYF	1	CD4-C2
PB-1_GTGCGGTAGAGACTAT	PB	PB-1	P1-915	FALSE	1	TRA:CAVQASNTDKLIF TRB:CASSPNLGSETQYF	1	CD4-C2
PB-1_GTGCGGTGTATCGCAT	PB	PB-1	P1-916	FALSE	1	TRA:CAAKNAGNNRKLIW TRB:CASRSTSQGGYQPQHF	1	CD4-C2
PB-1_GTTCATTGTAGAGTGC	PB	PB-1	P1-921	FALSE	1	TRA:CAVDFNKFYF TRB:CASSPPLGAGGVSGELFF	1	CD4-C7
PB-1_GTTCATTGTCGCTTTC	PB	PB-1	P1-922	FALSE	1	TRA:CAENPEDDKIIF TRB:CASSEQGAGYNEQFF	1	CD4-C2
PB-1_GTTTCTATCATCATTC	PB	PB-1	P1-927	FALSE	1	TRA:CATDKYGGSQGNLIF TRB:CASSQRTVTAREPQHF	1	CD4-C3
PB-1_GTTTCTATCTTGCATT	PB	PB-1	P1-928	FALSE	1	TRA:CAARSKAAGNKLTF TRB:CATSREYSVEETQYF	1	CD4-C1
PB-1_TAAACCGCATTACGAC	PB	PB-1	P1-932	FALSE	1	TRA:CACGGGSQGNLIF TRB:CAASRDRGRDGYTF	1	CD4-C1
PB-1_TAAGAGACACAGGAGT	PB	PB-1	P1-933	FALSE	1	TRA:CATVNTGGFKTIF TRB:CSAQNLGQGVSNEKLFF	1	CD4-C2
PB-1_TAAGCGTTCTGTTGAG	PB	PB-1	P1-936	FALSE	1	TRA:CAETLSGYALNF TRB:CASSQLLATNEKLFF	1	CD4-C2

PB-1_TACAGTGTCCTACAGA	PB	PB-1	P1-938	FALSE	1	TRA:CALSEASNTGKLIF TRB:CASSIDIHRISGANVLTF	1	CD4-C2
PB-1_TACTCGCCACACGCTG	PB	PB-1	P1-946	FALSE	1	TRA:CAVKGAGSYQLTF TRB:CASSFGQTDNSPLHF	1	CD4-C2
PB-1_TACTTACCAGTCGTGC	PB	PB-1	P1-947	FALSE	1	TRA:CGTVTGGANNLFF TRB:CSARDWDSYGYTF	1	CD4-C2
PB-1_TACTTGTTCAGGTTCA	PB	PB-1	P1-950	FALSE	1	TRA:CAARVPNTGNQFYF TRB:CASSYSGGGNQPQHF	1	CD4-C1
PB-1_TATGCCCTCTTCAACT	PB	PB-1	P1-960	FALSE	1	TRA:CAASRGSGYALNF TRB:CASSLEQGVGSPLHF	1	CD4-C2
PB-1_TATTACCGTAGGGTAC	PB	PB-1	P1-963	FALSE	1	TRA:CATDAEYGNKLVF TRB:CASSLAQGSWNEQFF	1	CD4-C2
PB-1_TCACAAGGTAGCTAAA	PB	PB-1	P1-968	FALSE	1	TRA:CIVRSLNTGTASKLTF TRB:CSARGNRRAGAYNEQFF	1	CD4-C2
PB-1_TCACGAAAGTTATCGC	PB	PB-1	P1-970	FALSE	1	TRA:CAPLSGTYKYIF TRB:CASSPDRARNTEAFF	1	CD4-C2
PB-1_TCAGATGCAGGGTACA	PB	PB-1	P1-972	FALSE	1	TRA:CALSERGGSSNTGKLIF TRB:CASSAGGTLGEQYF	1	CD4-C2
PB-1_TCCACACGTGTTGGGA	PB	PB-1	P1-980	FALSE	1	TRA:CALRVGGNKLVF TRB:CASSDVWSNQPQHF	1	CD4-C2
PB-1_TCGGGACGTGTGACCC	PB	PB-1	P1-987	FALSE	1	TRA:CVVNGNTGFQKLVF TRB:CASRAAGVYEQYF	1	CD4-C1
PB-1_TCGGGACGTTAGATGA	PB	PB-1	P1-988	FALSE	1	TRA:CAVRVGNQFYF TRB:CASSFRRDSETQYF	1	CD4-C2
PB-1_TCGTAGATCGGTTCGG	PB	PB-1	P1-993	FALSE	1	TRA:CAVEEMEYGNKLVF TRB:CASSQSMGGYNSPLHF	1	CD4-C2
PB-1_TCTATTGTCGCTTGTC	PB	PB-1	P1-995	FALSE	1	TRA:CVVSLNTGFQKLVF TRB:CASRRDGVTGELFF	1	CD4-C3
PB-1_TGAAAGAGTCCGAATT	PB	PB-1	P1-1004	FALSE	1	TRA:CAGSYNTDKLIF TRB:CASSITLDRSHEQYF	1	CD4-C1
PB-1_TGACAACGTCGAATCT	PB	PB-1	P1-1006	FALSE	1	TRA:CALRPGGAQKLVF TRB:CASSLFLAGGSYEQYF	1	CD4-C2
PB-1_TGAGAGGCAATACGCT	PB	PB-1	P1-1008	FALSE	1	TRA:CASILTGGGNKLTF TRB:CASSLQGGPNEKLFF	1	CD4-C2
PB-1_TGAGCCGGTATAGGTA	PB	PB-1	P1-1011	FALSE	1	TRA:CAVSETGANNLFF TRB:CASSPQGAGGELFF	1	CD4-C1
PB-1_TGCCCTAAGTGTCCAT	PB	PB-1	P1-1021	FALSE	1	TRA:CALTGNQFYF TRB:CSARVRTGQVMNTEAFF	1	CD4-C2
PB-1_TGCGGGTTCGCAAACT	PB	PB-1	P1-1023	FALSE	1	TRA:CALASRTGYSTLTF TRB:CASSQRTSGRDDEQYF	1	CD4-C2
PB-1_TGCTGCTAGTGTCCCG	PB	PB-1	P1-1027	FALSE	1	TRA:CAVNPFSYNQGGKLIF TRB:CASRPIDRGRGTEAFF	1	CD4-C1
PB-1_TGCTGCTGTCGAATCT	PB	PB-1	P1-1028	FALSE	1	TRA:CASSGAGSYQLTF TRB:CASSLSSGVNQPQHF	1	CD4-C2
PB-1_TGGTTCCGTACTCGCG	PB	PB-1	P1-1036	FALSE	1	TRA:CAGRYSGAGSYQLTF TRB:CASSPTGFGEKLFF	1	CD4-C2
PB-1_TGTATTCGTCGGGTCT	PB	PB-1	P1-1037	FALSE	1	TRA:CAGSHYNQGGKLIF TRB:CASSLVPGDTTYEQYF	1	CD4-C3
PB-1_TGTATTCTCCTGCTTG	PB	PB-1	P1-1038	FALSE	1	TRA:CAENKNQAGTALIF TRB:CASQGQGNQPQHF	1	CD4-C2
PB-1_TGTGGTACACATGGGA	PB	PB-1	P1-1041	FALSE	1	TRA:CAVSGNNNARLMF TRB:CASSQEGQIKGSPLHF	1	CD4-C3
PB-1_TGTGTTTGTGGGTCAA	PB	PB-1	P1-1045	FALSE	1	TRA:CAVSKSGGGADGLTF TRB:CASSDGPYSNQPQHF	1	CD4-C2

PB-1_TGTGTTTGTGGTCTCG	PB	PB-1	P1-1046	FALSE	1	TRA:CAVTPSGGYQKVTF TRB:CASSLSRRLALSTDTQYF	1	CD4-C7
PB-1_TTCGAAGTCCTACAGA	PB	PB-1	P1-1056	FALSE	1	TRA:CAVSRKFTGTASKLTF TRB:CASSPLTTNEQFF	1	CD4-C1
PB-1_TTCGGTCAGCGTGAAC	PB	PB-1	P1-1057	FALSE	1	TRA:CAVEAR TRB:CATSSTSARGEQYF	1	CD4-C2
PB-1_TTCGGTCAGTCATCCA	PB	PB-1	P1-1058	FALSE	1	TRA:CAATTGYSTLTF TRB:CASSTIAGPYEQYF	1	CD4-C2
PB-1_TTGCCGTAGGATGGTC	PB	PB-1	P1-1066	FALSE	1	TRA:CATHNNARLMF TRB:CASSFHGQGAYEQYF	1	CD4-C1
PB-1_TTGCCGTTCTGGCGAC	PB	PB-1	P1-1067	FALSE	1	TRA:CALISYGGSQGNLIF TRB:CASSAPERGGRNEQFF	1	CD4-C2
PB-1_TTGCGTCCAACACCCG	PB	PB-1	P1-1068	FALSE	1	TRA:CAVERNTGGFKTIF TRB:CASSLEDRGRAFF	1	CD4-C2
PB-1_TTGCGTCGTCTTGCGG	PB	PB-1	P1-1069	FALSE	1	TRA:CAVFGNEKLTF TRB:CSVRGAVYGYTF	1	CD4-C2
PB-1_TTGGCAACAGGATTGG	PB	PB-1	P1-1071	FALSE	1	TRA:CAVSDRSSASKIIF TRB:CASNPDRDPYYGYTF	1	CD4-C2
PB-1_TTTATGCTCCTATGTT	PB	PB-1	P1-1073	FALSE	1	TRA:CALGGRGGADGLTF TRB:CASSPDRTGQPQHF	1	CD4-C2
PB-1_TTTCCTCGTTATCGGT	PB	PB-1	P1-1075	FALSE	1	TRA:CAVSYMDSNYQLIW TRB:CATSEDTNTGELFF	1	CD4-C3
PB-1_TTTGCGCCAAGGTGTG	PB	PB-1	P1-1077	FALSE	1	TRA:CIVRVPRGAQKLVF TRB:CASSRDRFSNQPQHF	1	CD4-C1
PB-1_TTTGCGCCACGGCCAT	PB	PB-1	P1-1078	FALSE	1	TRA:CAVSGGSGNQFYF TRB:CASSQSPGQRNYGYTF	1	CD4-C3
PB-1_TTTGGTTAGAACAACT	PB	PB-1	P1-1079	FALSE	1	TRA:CAMREGGTYNNNDMRF TRB:CASSQPSGTAGYTEAFF	1	CD4-C1
PB-1_AAAGTAGGTCCAAGTT	PB	PB-1	P1-43	TRUE	3	TRA:CAASIIQGAQKLVF TRB:CASSFPGQGILEQYF	23	CD8-C2
PB-1_AACCATGTCTACTATC	PB	PB-1	P1-139	TRUE	3	TRA:CGTERDQFYF TRB:CATSRELFGEKLFF	4	CD8-C2
PB-1_AACTTTCAGCCGATTT	PB	PB-1	P1-15	TRUE	3	TRA:CAVNSSPLGANSKLTF TRB:CASSDSGNFYNEQFF	92	CD8-C2
PB-1_AAGACCTTCTGGCGAC	PB	PB-1	P1-15	TRUE	3	TRA:CAVNSSPLGANSKLTF TRB:CASSDSGNFYNEQFF	92	CD8-C2
PB-1_ACACCGGGTTCCGTCT	PB	PB-1	P1-43	TRUE	3	TRA:CAASIIQGAQKLVF TRB:CASSFPGQGILEQYF	23	CD8-C2
PB-1_ACATACGTCCTTTCGG	PB	PB-1	P1-471	FALSE	1	TRA:CAVDSNYQLIW TRB:CASSDSGGAYEQYF	1	CD8-C2
PB-1_ACCGTAAAGATAGCAT	PB	PB-1	P1-259	FALSE	2	TRA:CAVNEGTNAGKSTF TRB:CASSAGGDYNEQFF	2	CD8-C2
PB-1_ACGAGGATCTTTACAC	PB	PB-1	P1-483	FALSE	1	TRA:CAFMKQRGGGSEKLVF TRB:CASSFGTSPLRGELFF	1	CD4-C7
PB-1_ACGATGTCAAGCGCTC	PB	PB-1	P1-43	TRUE	3	TRA:CAASIIQGAQKLVF TRB:CASSFPGQGILEQYF	23	CD8-C2
PB-1_ACGCAGCTCACCGTAA	PB	PB-1	P1-141	TRUE	3	TRA:CALRILRGSYIPTF TRB:CASSLPGRYSGANVLTF	4	CD8-C2
PB-1_ACGCCAGGTATATCCG	PB	PB-1	P1-1	TRUE	3	TRA:CAMSDRMTGGFKTIF TRB:CASSQVGRGGEETQYF	1047	CD8-C1
PB-1_ACGGAGAGTTTCGCTC	PB	PB-1	P1-3	TRUE	3	TRA:CAFLSGTYKYIF TRB:CASSQSPGQRNYGYTF	308	CD8-C2
PB-1_ACTGAACTCGATGAGG	PB	PB-1	P1-14	TRUE	3	TRA:CAASVVNDMRF TRB:CASSHRGGAKNIQYF	109	CD8-C2

PB-1_ACTGTCCTCGAATCCA	PB	PB-1	P1-267	FALSE	2	TRA:CAVGLMDSSYKLIF TRB:CASAPKRERGGTQYF	2	CD8-C2
PB-1_AGCCTAAAGTGTCTCA	PB	PB-1	P1-15	TRUE	3	TRA:CAVNSSPLGANSKLTF TRB:CASSDSGNFYNEQFF	92	CD8-C2
PB-1_AGCTTGATCAGGCAAG	PB	PB-1	P1-523	FALSE	1	TRA:CAYRYFYGGSQGNLIF TRB:CSARGRGLAGGPAYEQYF	1	CD8-C2
PB-1_AGGGTGAAGAGTTGGC	PB	PB-1	P1-88	TRUE	3	TRA:CAVRDRGSGNTPLVF TRB:CASSGDRVYEQYF	6	CD8-C2
PB-1_AGGGTGACATTACCTT	PB	PB-1	P1-15	TRUE	3	TRA:CAVNSSPLGANSKLTF TRB:CASSDSGNFYNEQFF	92	CD8-C2
PB-1_AGTAGTCCAGCTGGCT	PB	PB-1	P1-15	TRUE	3	TRA:CAVNSSPLGANSKLTF TRB:CASSDSGNFYNEQFF	92	CD8-C2
PB-1_AGTCTTTCACCCAGTG	PB	PB-1	P1-269	TRUE	2	TRA:CAFMLTYGGSQGNLIF TRB:CASSLTVLDEQFF	2	CD8-C2
PB-1_AGTTGGTAGAGTACAT	PB	PB-1	P1-15	TRUE	3	TRA:CAVNSSPLGANSKLTF TRB:CASSDSGNFYNEQFF	92	CD8-C2
PB-1_ATCGAGTAGTGTCCAT	PB	PB-1	P1-15	TRUE	3	TRA:CAVNSSPLGANSKLTF TRB:CASSDSGNFYNEQFF	92	CD8-C2
PB-1_ATGAGGGCAGTTTACG	PB	PB-1	P1-4	TRUE	3	TRA:CAMKGRDDKIIF TRB:CASTKGVSREAFF	226	CD8-C2
PB-1_ATGGGAGAGAGTGAGA	PB	PB-1	P1-553	FALSE	1	TRA:CALSDPHNNARLMF TRB:CASMDPVHYEQYF	1	CD8-C2
PB-1_ATGGGAGCAAGCGATG	PB	PB-1	P1-43	TRUE	3	TRA:CAASIIQGAQKLVF TRB:CASSFPGQGILEQYF	23	CD8-C2
PB-1_ATTCTACAGGCTATCT	PB	PB-1	P1-15	TRUE	3	TRA:CAVNSSPLGANSKLTF TRB:CASSDSGNFYNEQFF	92	CD8-C2
PB-1_ATTTCTGAGCCACGCT	PB	PB-1	P1-565	FALSE	1	TRA:CAVRSPPPFNYQLIW TRB:CASSLEVRDRVYGYTF	1	CD8-C2
PB-1_ATTTCTGAGTATGACA	PB	PB-1	P1-15	TRUE	3	TRA:CAVNSSPLGANSKLTF TRB:CASSDSGNFYNEQFF	92	CD8-C2
PB-1_CACACCTAGGTTCCTA	PB	PB-1	P1-43	TRUE	3	TRA:CAASIIQGAQKLVF TRB:CASSFPGQGILEQYF	23	CD8-C2
PB-1_CACATAGAGTGATCGG	PB	PB-1	P1-52	TRUE	3	TRA:CAETPDYKLSF TRB:CASRDLYEQYF	13	CD8-C2
PB-1_CACATTTAGCTCTCGG	PB	PB-1	P1-3	TRUE	3	TRA:CAFLSGTYKYIF TRB:CASSQSPGQRNYGYTF	308	CD8-C2
PB-1_CACATTTGTTAGATGA	PB	PB-1	P1-586	FALSE	1	TRA:CADLNQAGTALIF TRB:CASSRETGGLSGNTIYF	1	CD8-C4
PB-1_CACCTTGCACCCATTC	PB	PB-1	P1-4	TRUE	3	TRA:CAMKGRDDKIIF TRB:CASTKGVSREAFF	226	CD8-C5
PB-1_CAGCGACCATGCAACT	PB	PB-1	P1-600	FALSE	1	TRA:CVVNGDNFNKFYF TRB:CASSLVTGATYEQYF	1	CD8-C1
PB-1_CAGCTGGGTAGCGCTC	PB	PB-1	P1-15	TRUE	3	TRA:CAVNSSPLGANSKLTF TRB:CASSDSGNFYNEQFF	92	CD8-C2
PB-1_CAGGTGCCAGTCGTGC	PB	PB-1	P1-603	FALSE	1	TRA:CAESRNARLMF TRB:CSVVRDSSYNEQFF	1	CD8-C4
PB-1_CAGGTGCTCTCAAGTG	PB	PB-1	P1-606	FALSE	1	TRA:CAVLRGGFKTIF TRB:CASSPGTSGKSPYEQYF	1	CD8-C2
PB-1_CAGTAACAGTTTGCGT	PB	PB-1	P1-38	TRUE	3	TRA:CAFTAGGATNKLIF TRB:CASIEGQGPYNEQFF	38	CD8-C2
PB-1_CATCAGAGTCGCATCG	PB	PB-1	P1-1	TRUE	3	TRA:CAMSDRMTGGFKTIF TRB:CASSQVGRGGEETQYF	1047	CD8-C2
PB-1_CATGCCTAGGATGTAT	PB	PB-1	P1-43	TRUE	3	TRA:CAASIIQGAQKLVF TRB:CASSFPGQGILEQYF	23	CD8-C2

PB-1_CATGCCTAGTGCGATG	PB	PB-1	P1-66	TRUE	3	TRA:CVVSDPPDKLIF TRB:CASSASETGSVYEQYF	9	CD8-C5
PB-1_CATTATCTCATCGGAT	PB	PB-1	P1-630	FALSE	1	TRA:CAVRPPGRNSGGYQKVTF TRB:CSAPGGGLSSPLHF	1	CD8-C2
PB-1_CCAGCGACACAGACTT	PB	PB-1	P1-640	FALSE	1	TRA:CVVNHYGGSQGNLIF TRB:CASSQSPGQRNYGYTF	1	CD8-C2
PB-1_CCATGTCTCTGCCCTA	PB	PB-1	P1-15	TRUE	3	TRA:CAVNSSPLGANSKLTF TRB:CASSDSGNFYNEQFF	92	CD8-C2
PB-1_CCGTGGAGTCCTGCTT	PB	PB-1	P1-67	TRUE	3	TRA:CALNTNQAGTALIF TRB:CASSLGTRYAYEQYF	9	CD8-C2
PB-1_CCTTTCTGTTCTGAAC	PB	PB-1	P1-43	TRUE	3	TRA:CAASIIQGAQKLVF TRB:CASSFPGQGILEQYF	23	CD8-C2
PB-1_CGAGAAGGTAATTGGA	PB	PB-1	P1-62	TRUE	3	TRA:CVVNLMEYGNKLVF TRB:CASSGARQGQYEQYF	10	CD4-C3
PB-1_CGGACACTCATTATCC	PB	PB-1	P1-697	FALSE	1	TRA:CATPTNTGTASKLTF TRB:CASSYPQGAGTEAFF	1	CD8-C2
PB-1_CGGCTAGTCTGCTGCT	PB	PB-1	P1-706	FALSE	1	TRA:CAFMSTNFGNEKLTF TRB:CASSFDLGELGYGYTF	1	CD8-C2
PB-1_CGTCACTGTAGCTTGT	PB	PB-1	P1-282	TRUE	2	TRA:CAEPRNTGNQFYF TRB:CAISVGNEQFF	2	CD8-C2
PB-1_CGTCAGGGTCTGGAGA	PB	PB-1	P1-710	FALSE	1	TRA:CAGEETSGSRLTF TRB:CASSPGLILEGGPAAFF	1	CD8-C2
PB-1_CGTTCTGCAGGTCCAC	PB	PB-1	P1-15	TRUE	3	TRA:CAVNSSPLGANSKLTF TRB:CASSDSGNFYNEQFF	92	CD8-C2
PB-1_CTAACTTTCAGCTCGG	PB	PB-1	P1-190	TRUE	3	TRA:CAEGGVDGQKLLF TRB:CASSEDAGELFF	3	CD8-C2
PB-1_CTACCCAAGAGATGAG	PB	PB-1	P1-31	TRUE	3	TRA:CAVEEGADRLMF TRB:CASSLLGGGELFF	46	CD8-C10
PB-1_CTCACACAGCACGCCT	PB	PB-1	P1-15	TRUE	3	TRA:CAVNSSPLGANSKLTF TRB:CASSDSGNFYNEQFF	92	CD8-C2
PB-1_CTCATTACAAGCCTAT	PB	PB-1	P1-192	TRUE	3	TRA:CAASGNNAGNMLTF TRB:CASSLMAGGEREQYF	3	CD8-C2
PB-1_CTCCTAGAGTAATCCC	PB	PB-1	P1-116	TRUE	3	TRA:CALFAYSGAGSYQLTF TRB:CASRDDARTDTQYF	5	CD8-C2
PB-1_CTCCTAGTCGTGGTCG	PB	PB-1	P1-193	TRUE	3	TRA:CAVRFSGGYNKLIF TRB:CASLEGPYSNQPQHF	3	CD8-C4
PB-1_CTCGAGGTCGTACCGG	PB	PB-1	P1-6	TRUE	3	TRA:CAMREGRERDDKIIF TRB:CASSLPNTEAFF	216	CD8-C2
PB-1_CTCGTACTCTGCTTGC	PB	PB-1	P1-257	FALSE	2	TRA:CAVSARGGSGNTGKLIF TRB:CSARDRRLSYEQYF	2	CD8-C2
PB-1_CTGATCCGTAGTACCT	PB	PB-1	P1-15	TRUE	3	TRA:CAVNSSPLGANSKLTF TRB:CASSDSGNFYNEQFF	92	CD8-C2
PB-1_CTGCCTATCATTTGGG	PB	PB-1	P1-754	FALSE	1	TRA:CAPTHSGSARQLTF TRB:CSAPSGFTNQPQHF	1	CD8-C2
PB-1_CTTAACTAGGCATGGT	PB	PB-1	P1-766	FALSE	1	TRA:CAALGTGNQFYF TRB:CASRAPGTKGGYTF	1	CD8-C2
PB-1_CTTAGGATCAGAGCTT	PB	PB-1	P1-15	TRUE	3	TRA:CAVNSSPLGANSKLTF TRB:CASSDSGNFYNEQFF	92	CD8-C2
PB-1_GAGGTGAAGACGACGT	PB	PB-1	P1-3	TRUE	3	TRA:CAFLSGTYKYIF TRB:CASSQSPGQRNYGYTF	308	CD8-C5
PB-1_GCAAACTAGCCAGAAC	PB	PB-1	P1-15	TRUE	3	TRA:CAVNSSPLGANSKLTF TRB:CASSDSGNFYNEQFF	92	CD8-C2
PB-1_GCCTCTACATTGGCGC	PB	PB-1	P1-15	TRUE	3	TRA:CAVNSSPLGANSKLTF TRB:CASSDSGNFYNEQFF	92	CD8-C2

PB-1_GCGACCAAGACGCTTT	PB	PB-1	P1-832	FALSE	1	TRA:CLVGDGGYSSASKIIF TRB:CASSAIDRVTYEQYF	1	CD8-C2
PB-1_GCGACCAAGTCTCAAC	PB	PB-1	P1-15	TRUE	3	TRA:CAVNSSPLGANSKLTF TRB:CASSDSGNFYNEQFF	92	CD8-C2
PB-1_GCGAGAAAGTCGTACT	PB	PB-1	P1-3	TRUE	3	TRA:CAFLSGTYKYIF TRB:CASSQSPGQRNYGYTF	308	CD8-C2
PB-1_GCGCGATCAGAGTGTG	PB	PB-1	P1-4	TRUE	3	TRA:CAMKGRDDKIIF TRB:CASTKGVSREAFF	226	CD8-C2
PB-1_GCGCGATCAGCGAACA	PB	PB-1	P1-43	TRUE	3	TRA:CAASIIQGAQKLVF TRB:CASSFPGQGILEQYF	23	CD8-C2
PB-1_GCGGGTTAGTGTCCAT	PB	PB-1	P1-8	TRUE	3	TRA:CAVRPPGRNSGGYQKVTF TRB:CASSRRGVTDTQYF	204	CD8-C2
PB-1_GCTCTGTCACAGAGGT	PB	PB-1	P1-15	TRUE	3	TRA:CAVNSSPLGANSKLTF TRB:CASSDSGNFYNEQFF	92	CD8-C2
PB-1_GGACAAGTCGTCACGG	PB	PB-1	P1-88	TRUE	3	TRA:CAVRDRGSGNTPLVF TRB:CASSGDRVYEQYF	6	CD8-C2
PB-1_GGACATTGTTTGCATG	PB	PB-1	P1-858	FALSE	1	TRA:CAVQAGNNDMRF TRB:CASSPGTVSYEQYF	1	CD8-C2
PB-1_GGACGTCGTCGCATCG	PB	PB-1	P1-40	TRUE	3	TRA:CAVRDVGNTGFQKLVF TRB:CASSLGQGSMKTQYF	27	CD8-C2
PB-1_GGATGTTGTCCTCCAT	PB	PB-1	P1-15	TRUE	3	TRA:CAVNSSPLGANSKLTF TRB:CASSDSGNFYNEQFF	92	CD8-C2
PB-1_GGCCGATTCTGGAGCC	PB	PB-1	P1-115	FALSE	3	TRA:CAVKGSSNTGKLIF TRB:CASSYSYGRTGTGTYEQYF	5	CD4-C3
PB-1_GGGATGATCCTCGCAT	PB	PB-1	P1-15	TRUE	3	TRA:CAVNSSPLGANSKLTF TRB:CASSDSGNFYNEQFF	92	CD8-C2
PB-1_GGGATGATCTTGTTTG	PB	PB-1	P1-16	TRUE	3	TRA:CALSGTPMNRDDKIIF TRB:CASSQSWDLAVLNEQFF	79	CD8-C2
PB-1_GTACTCCGTGGGTATG	PB	PB-1	P1-12	TRUE	3	TRA:CALSDERGDYKLSF TRB:CASSPGTGSYNEQFF	141	CD8-C2
PB-1_GTACTCCTCCGAGCCA	PB	PB-1	P1-15	TRUE	3	TRA:CAVNSSPLGANSKLTF TRB:CASSDSGNFYNEQFF	92	CD8-C2
PB-1_GTACTTTGTAAGAGGA	PB	PB-1	P1-3	TRUE	3	TRA:CAFLSGTYKYIF TRB:CASSQSPGQRNYGYTF	308	CD8-C2
PB-1_GTAGTCAGTCGCGGTT	PB	PB-1	P1-13	TRUE	3	TRA:CVVMNYGGSQGNLIF TRB:CASSSWGDTDEAFF	112	CD8-C2
PB-1_GTATTCTTCCGAATGT	PB	PB-1	P1-15	TRUE	3	TRA:CAVNSSPLGANSKLTF TRB:CASSDSGNFYNEQFF	92	CD8-C2
PB-1_GTCACGGAGAATGTTG	PB	PB-1	P1-15	TRUE	3	TRA:CAVNSSPLGANSKLTF TRB:CASSDSGNFYNEQFF	92	CD8-C2
PB-1_GTCACGGGTCAGGACA	PB	PB-1	P1-43	TRUE	3	TRA:CAASIIQGAQKLVF TRB:CASSFPGQGILEQYF	23	CD8-C2
PB-1_GTGCATACACATCCGG	PB	PB-1	P1-201	TRUE	3	TRA:CAYVGGNTPLVF TRB:CASSFPPSGGARETQYF	3	CD8-C2
PB-1_GTTCGGGCATTGGGCC	PB	PB-1	P1-2	TRUE	3	TRA:CGQNFVF TRB:CASNQRGGPSLYNEQFF	323	CD8-C2
PB-1_TAAACCGCAACTTGAC	PB	PB-1	P1-52	TRUE	3	TRA:CAETPDYKLSF TRB:CASRDLYEQYF	13	CD8-C8
PB-1_TACGGGCAGCGTCAAG	PB	PB-1	P1-3	TRUE	3	TRA:CAFLSGTYKYIF TRB:CASSQSPGQRNYGYTF	308	CD8-C2
PB-1_TACTTACAGACACTAA	PB	PB-1	P1-4	TRUE	3	TRA:CAMKGRDDKIIF TRB:CASTKGVSREAFF	226	CD8-C10
PB-1_TACTTACCAGCCTTGG	PB	PB-1	P1-190	TRUE	3	TRA:CAEGGVDGQKLLF TRB:CASSEDAGELFF	3	CD8-C2

PB-1_TATTACCAGTGAATTG	PB	PB-1	P1-961	FALSE	1	TRA:CAGQNTGGFKTIF TRB:CASSLGLGRGRNIQYF	1	CD8-C10
PB-1_TCAACGACAGCCACCA	PB	PB-1	P1-15	TRUE	3	TRA:CAVNSSPLGANSKLTF TRB:CASSDSGNFYNEQFF	92	CD8-C2
PB-1_TCACAAGGTAAACACA	PB	PB-1	P1-67	TRUE	3	TRA:CALNTNQAGTALIF TRB:CASSLGTRYAYEQYF	9	CD8-C2
PB-1_TCACGAATCCGTAGTA	PB	PB-1	P1-67	TRUE	3	TRA:CALNTNQAGTALIF TRB:CASSLGTRYAYEQYF	9	CD8-C2
PB-1_TCCACACGTACAGTTC	PB	PB-1	P1-88	TRUE	3	TRA:CAVRDRGSGNTPLVF TRB:CASSGDRVYEQYF	6	CD8-C2
PB-1_TCCCGATAGTATCGAA	PB	PB-1	P1-298	TRUE	2	TRA:CVVNDNYGQNFVF TRB:CRPGGGFSYNEQFF	2	CD8-C2
PB-1_TCCCGATCAAGTCATC	PB	PB-1	P1-15	TRUE	3	TRA:CAVNSSPLGANSKLTF TRB:CASSDSGNFYNEQFF	92	CD8-C7
PB-1_TCGGTAAAGCTGCCCA	PB	PB-1	P1-15	TRUE	3	TRA:CAVNSSPLGANSKLTF TRB:CASSDSGNFYNEQFF	92	CD8-C2
PB-1_TCGTAGAAGGGCTCTC	PB	PB-1	P1-43	TRUE	3	TRA:CAASIIQGAQKLVF TRB:CASSFPGQGILEQYF	23	CD8-C2
PB-1_TCTCTAACAGACGCAA	PB	PB-1	P1-15	TRUE	3	TRA:CAVNSSPLGANSKLTF TRB:CASSDSGNFYNEQFF	92	CD8-C2
PB-1_TCTGAGAGTCTCCATC	PB	PB-1	P1-999	FALSE	1	TRA:CAALPQGAQKLVF TRB:CASGGDRGGGYGYTF	1	CD8-C2
PB-1_TGACTAGCACGTGAGA	PB	PB-1	P1-15	TRUE	3	TRA:CAVNSSPLGANSKLTF TRB:CASSDSGNFYNEQFF	92	CD8-C2
PB-1_TGCCAAAGTGTTCGAT	PB	PB-1	P1-1018	FALSE	1	TRA:CAASIIQGAQKLVF TRB:CASSQSPGQRNYGYTF	1	CD8-C2
PB-1_TGGACGCCAGCATGAG	PB	PB-1	P1-13	TRUE	3	TRA:CVVMNYGGSQGNLIF TRB:CASSSWGDTDEAFF	112	CD8-C2
PB-1_TGGCCAGAGATCGATA	PB	PB-1	P1-15	TRUE	3	TRA:CAVNSSPLGANSKLTF TRB:CASSDSGNFYNEQFF	92	CD8-C2
PB-1_TGGTTAGAGAAGCCCA	PB	PB-1	P1-10	TRUE	3	TRA:CISNSPMDSSYKLIF TRB:CASSSWGDTDEAFF	162	CD8-C2
PB-1_TGTCCCATCCTAAGTG	PB	PB-1	P1-12	TRUE	3	TRA:CALSDERGDYKLSF TRB:CASSPGTGSYNEQFF	141	CD8-C2
PB-1_TGTGTTTTCTTTAGGG	PB	PB-1	P1-74	TRUE	3	TRA:CAVGGGSTLGRLYF TRB:CASSIVAGADTEAFF	8	CD8-C2
PB-1_TTAACTCGTGATGTGG	PB	PB-1	P1-29	TRUE	3	TRA:CATVSFSGGYNKLIF TRB:CASSLDRRGLSYEQYF	50	CD8-C2
PB-1_TTCTACATCGTGACAT	PB	PB-1	P1-15	TRUE	3	TRA:CAVNSSPLGANSKLTF TRB:CASSDSGNFYNEQFF	92	CD8-C2
PB-1_TTCTCAACAGCCTGTG	PB	PB-1	P1-1060	FALSE	1	TRA:CAVRESGSARQLTF TRB:CASTGTSLGEQYF	1	CD8-C2
PB-1_TTCTCCTTCTGGCGAC	PB	PB-1	P1-5	TRUE	3	TRA:CALSGTPMNRDDKIIF TRB:CSARGRGLAGGPAYEQYF	223	CD8-C7
PB-1_TTGACTTTCTTCAACT	PB	PB-1	P1-15	TRUE	3	TRA:CAVNSSPLGANSKLTF TRB:CASSDSGNFYNEQFF	92	CD8-C2
PB-1_TTTGGTTGTGTAACGG	PB	PB-1	P1-15	TRUE	3	TRA:CAVNSSPLGANSKLTF TRB:CASSDSGNFYNEQFF	92	CD8-C2
PB-1_AAAGCAACAACACCTA	PB	PB-1	P1-25	TRUE	3	TRA:CIVGRDSSYKLIF TRB:CASNPQRGAHRDTQYF	58	CD4-C3
PB-1_AACACGTAGGGTCGAT	PB	PB-1	P1-106	TRUE	3	TRA:CAVIEGNQFYF TRB:CASSLTPGLNIQYF	5	CD4-C3
PB-1_AACTCTTCATCGATGT	PB	PB-1	P1-253	TRUE	2	TRA:CAMTRTGGGNKLTF TRB:CASSLGTGKGGPGSYEQYF	2	CD4-C3

PB-1_AAGGAGCAGTATGACA	PB	PB-1	P1-17	TRUE	3	TRA:CGTGARGSSGSRLTF TRB:CASSRQGMGAFF	78	CD4-C3
PB-1_AAGGAGCCAGTTCCCT	PB	PB-1	P1-255	FALSE	2	TRA:CAVSDRSGNTGKLIF TRB:CASSHRTRENEQFF	2	CD4-C2
PB-1_AAGGAGCTCCGGGTGT	PB	PB-1	P1-15	TRUE	3	TRA:CAVNSSPLGANSKLTF TRB:CASSDSGNFYNEQFF	92	CD8-C2
PB-1_AAGTCTGGTACGCACC	PB	PB-1	P1-15	TRUE	3	TRA:CAVNSSPLGANSKLTF TRB:CASSDSGNFYNEQFF	92	CD4-C3
PB-1_AATCCAGCAAGGTGTG	PB	PB-1	P1-459	FALSE	1	TRA:CAVSERNSNYQLIW TRB:CASSFSGQRSHEQYF	1	CD4-C3
PB-1_ACAGCTATCTCGCATC	PB	PB-1	P1-468	FALSE	1	TRA:CATDRAGGFKTIF TRB:CSARPLGTSNTGELFF	1	CD4-C3
PB-1_ACCGTAATCTCAAACG	PB	PB-1	P1-174	FALSE	3	TRA:CAAPENSNSGYALNF TRB:CSGGLANEQFF	3	CD4-C3
PB-1_ACCTTTAGTGAGTGAC	PB	PB-1	P1-109	TRUE	3	TRA:CAAQQGSGGSNYKLTF TRB:CASSLGDKQYF	5	CD4-C3
PB-1_ACGATACCACAGAGGT	PB	PB-1	P1-261	TRUE	2	TRA:CALSGAAGNKLTF TRB:CASSLLAGDPSYNEQFF	2	CD4-C3
PB-1_ACGGGTCCACCAGGTC	PB	PB-1	P1-263	TRUE	2	TRA:CAASKHSWGKLQF TRB:CSAGISSGGRYEQYF	2	CD4-C3
PB-1_ACTGCTCCACGTAAGG	PB	PB-1	P1-503	FALSE	1	TRA:CAERRNTGRRALTF TRB:CASSEESSGANVLTF	1	CD4-C3
PB-1_AGAGCTTCATGCATGT	PB	PB-1	P1-89	TRUE	3	TRA:CAVDVNYGGQNFVF TRB:CASSWGDTQYF	6	CD4-C3
PB-1_AGAGCTTTCCTTGCCA	PB	PB-1	P1-511	FALSE	1	TRA:CAPYTGGFKTIF TRB:CASRVFQGGVNTGELFF	1	CD8-C2
PB-1_AGATCTGTCCTTCAAT	PB	PB-1	P1-513	FALSE	1	TRA:CAFSDGQKLLF TRB:CASSLGTVSISGANVLTF	1	CD4-C3
PB-1_AGGGAGTCACAACGTT	PB	PB-1	P1-524	FALSE	1	TRA:CAMSHRANAGGTSYGKLTF TRB:CASRDTPNTEAFF	1	CD4-C3
PB-1_AGGTCATCAGTCGTGC	PB	PB-1	P1-181	TRUE	3	TRA:CAVTRVMGTYKYIF TRB:CASGTQNSPLHF	3	CD4-C3
PB-1_ATAACGCGTAACGACG	PB	PB-1	P1-62	TRUE	3	TRA:CVVNLMEYGNKLVF TRB:CASSGARQGQYEQYF	10	CD4-C3
PB-1_ATAACGCGTCGAGATG	PB	PB-1	P1-534	FALSE	1	TRA:CATGEAGRRALTF TRB:CASSYSQNTEAFF	1	CD4-C3
PB-1_ATCTACTCAGTAAGCG	PB	PB-1	P1-147	TRUE	3	TRA:CAGQLSGGSNYKLTF TRB:CASSERQGKAYGYTF	4	CD4-C3
PB-1_ATCTGCCCATTCCTGC	PB	PB-1	P1-184	TRUE	3	TRA:CILRDGLMGDYKLSF TRB:CASSRDSRNSPLHF	3	CD8-C2
PB-1_ATGAGGGAGGCAATTA	PB	PB-1	P1-270	TRUE	2	TRA:CAAKSNTGRRALTF TRB:CASRMGVDQPQHF	2	CD4-C3
PB-1_ATTACTCGTGAGGCTA	PB	PB-1	P1-558	FALSE	1	TRA:CAVSDNAGNMLTF TRB:CASSPDRGYTDTQYF	1	CD4-C4
PB-1_CAAGTTGGTCAGAGGT	PB	PB-1	P1-109	TRUE	3	TRA:CAAQQGSGGSNYKLTF TRB:CASSLGDKQYF	5	CD4-C3
PB-1_CACATAGTCTAACTTC	PB	PB-1	P1-78	TRUE	3	TRA:CAAAQGGSEKLVF TRB:CASTRGDWIYNEQFF	7	CD4-C3
PB-1_CACCTTGGTATTCGTG	PB	PB-1	P1-589	FALSE	1	TRA:CAVNDYKLSF TRB:CSARETAGGPWSTDTQYF	1	CD4-C3
PB-1_CAGCCGATCGATAGAA	PB	PB-1	P1-599	FALSE	1	TRA:CALDMRGNTPLVF TRB:CASSPTRTHLAEAFF	1	CD4-C3
PB-1_CAGCGACGTTAGGGTG	PB	PB-1	P1-602	FALSE	1	TRA:CAVGAFRTGFQKLVF TRB:CASSQDDQGNQPQHF	1	CD4-C3

PB-1_CATTATCAGCGACGTA	PB	PB-1	P1-627	FALSE	1	TRA:CALSEVKGAQKLVF TRB:CATSDLGTGITDTQYF	1	CD4-C3
PB-1_CCACGGAGTCAATACC	PB	PB-1	P1-635	FALSE	1	TRA:CATVLWGGGKLIF TRB:CSVEDRGTSGGRRDTQYF	1	CD4-C3
PB-1_CCACTACTCAGCAACT	PB	PB-1	P1-639	FALSE	1	TRA:CAGLTSGSRLTF TRB:CASSLTGIYTGELFF	1	CD4-C3
PB-1_CGATGGCCAGGGCATA	PB	PB-1	P1-678	FALSE	1	TRA:CALPLGTGGFKTIF TRB:CASSLGTGNQPQHF	1	CD4-C3
PB-1_CGCCAAGAGGTGCTTT	PB	PB-1	P1-682	FALSE	1	TRA:CVVSEVGGYQKVTF TRB:CAISEGSRTEAFF	1	CD4-C3
PB-1_CGCTTCAGTCGGATCC	PB	PB-1	P1-695	FALSE	1	TRA:CAETSNTGKLIF TRB:CASSSGSREAFF	1	CD8-C2
PB-1_CGGACTGCAGTAGAGC	PB	PB-1	P1-700	FALSE	1	TRA:CVVADRGSTLGRLYF TRB:CASSKHLQGIHEQYF	1	CD4-C3
PB-1_CGGACTGGTCTTCAAG	PB	PB-1	P1-189	TRUE	3	TRA:CAPGLLF TRB:CASSLSHQGSPLHF	3	CD4-C3
PB-1_CGTGAGCCATGGTCTA	PB	PB-1	P1-715	FALSE	1	TRA:CAGPLSRAAGNKLTF TRB:CASSPGGGGDTQYF	1	CD4-C3
PB-1_CGTGTAACATTACGAC	PB	PB-1	P1-92	TRUE	3	TRA:CIVQLTGTASKLTF TRB:CASSFKSGRTNEQFF	6	CD4-C3
PB-1_CTACACCTCCGCGGTA	PB	PB-1	P1-726	FALSE	1	TRA:CAATLRYNGGGSNYKLTF TRB:CASSQDYLATGKNIQYF	1	CD4-C3
PB-1_CTCACACTCTCATTCA	PB	PB-1	P1-731	FALSE	1	TRA:CALDTSGGYNKLIF TRB:CASSLSASGRGTEAFF	1	CD4-C3
PB-1_CTGAAGTCAGGTCCAC	PB	PB-1	P1-751	FALSE	1	TRA:CAVNTIATGKSTF TRB:CASSQQGPNTEAFF	1	CD4-C3
PB-1_CTGATAGGTACCGTTA	PB	PB-1	P1-28	TRUE	3	TRA:CAVKKGTGRRALTF TRB:CASETGQGTDTQYF	53	CD8-C10
PB-1_CTGTGCTGTGTTGAGG	PB	PB-1	P1-762	FALSE	1	TRA:CAGSSGYSTLTF TRB:CASSPSRETQYF	1	CD4-C3
PB-1_CTGTGCTTCAGAAATG	PB	PB-1	P1-763	FALSE	1	TRA:CAETLYSSASKIIF TRB:CASSEPNQETQYF	1	CD4-C3
PB-1_CTTCTCTTCCATTCTA	PB	PB-1	P1-773	FALSE	1	TRA:CALSPGGFERAGNQFYF TRB:CSARGRGQSHQPQHF	1	CD4-C3
PB-1_GATCTAGTCCGTCAAA	PB	PB-1	P1-811	FALSE	1	TRA:CAMRANSGGSNYKLTF TRB:CASSTRTGHQPQHF	1	CD4-C2
PB-1_GATGCTAGTCCGAAGA	PB	PB-1	P1-816	FALSE	1	TRA:CAVRGSWGKLQF TRB:CASNHPPAGTNTEAFF	1	CD8-C10
PB-1_GCAAACTGTCACAAGG	PB	PB-1	P1-822	FALSE	1	TRA:CATDAGYGNNRLAF TRB:CASTYTRQVSGQPQHF	1	CD4-C3
PB-1_GCACTCTTCGCATGGC	PB	PB-1	P1-106	TRUE	3	TRA:CAVIEGNQFYF TRB:CASSLTPGLNIQYF	5	CD4-C3
PB-1_GCATGTAGTCGAATCT	PB	PB-1	P1-192	TRUE	3	TRA:CAASGNNAGNMLTF TRB:CASSLMAGGEREQYF	3	CD8-C2
PB-1_GCCTCTAGTGCTTCTC	PB	PB-1	P1-181	TRUE	3	TRA:CAVTRVMGTYKYIF TRB:CASGTQNSPLHF	3	CD4-C3
PB-1_GCCTCTAGTGGTAACG	PB	PB-1	P1-174	FALSE	3	TRA:CAAPENSNSGYALNF TRB:CSGGLANEQFF	3	CD4-C3
PB-1_GCGACCAAGGAATGGA	PB	PB-1	P1-833	FALSE	1	TRA:CATDRNNNNDMRF TRB:CASSLTSGERGNTGELFF	1	CD4-C3
PB-1_GCGCAACGTACCGGCT	PB	PB-1	P1-289	TRUE	2	TRA:CAVNDGSSNTGKLIF TRB:CSVGGDPQETQYF	2	CD4-C3
PB-1_GCGCAGTAGCCGATTT	PB	PB-1	P1-109	TRUE	3	TRA:CAAQQGSGGSNYKLTF TRB:CASSLGDKQYF	5	CD4-C3

PB-1_GCTGGGTGTAAGTGTA	PB	PB-1	P1-147	TRUE	3	TRA:CAGQLSGGSNYKLTF TRB:CASSERQGKAYGYTF	4	CD4-C3
PB-1_GGAGCAACACTACAGT	PB	PB-1	P1-153	TRUE	3	TRA:CAVKPLDTGRRALTF TRB:CATSLRVTDTQYF	4	CD4-C3
PB-1_GGCTGGTAGCGGCTTC	PB	PB-1	P1-868	FALSE	1	TRA:CAASRNTGNQFYF TRB:CASSFNPLAGTQYF	1	CD4-C3
PB-1_GGCTGGTGTGTGACGA	PB	PB-1	P1-147	TRUE	3	TRA:CAGQLSGGSNYKLTF TRB:CASSERQGKAYGYTF	4	CD4-C3
PB-1_GGGAGATCATCTACGA	PB	PB-1	P1-873	FALSE	1	TRA:CATDAGDDKIIF TRB:CASSAATTSGQETQYF	1	CD4-C3
PB-1_GGGTCTGAGCACCGCT	PB	PB-1	P1-876	FALSE	1	TRA:CAASASGGTSYGKLTF TRB:CASSEAPPGGSTNEKLFF	1	CD4-C3
PB-1_GTAACGTAGTGCAAGC	PB	PB-1	P1-884	FALSE	1	TRA:CAMRGDTGFQKLVF TRB:CASSISGGYTEAFF	1	CD4-C3
PB-1_GTGAAGGAGGAATTAC	PB	PB-1	P1-912	FALSE	1	TRA:CAWAESGQNFVF TRB:CASSDRTEGRAFF	1	CD4-C3
PB-1_TAAACCGAGCAATATG	PB	PB-1	P1-929	FALSE	1	TRA:CAMIPSGNTGKLIF TRB:CASSDGLAGGEDTQYF	1	CD4-C2
PB-1_TACTCATGTTCTGGTA	PB	PB-1	P1-27	TRUE	3	TRA:CALVDFSGYSTLTF TRB:CASSGRGTNEKLFF	55	CD4-C3
PB-1_TACTCGCGTGCACCAC	PB	PB-1	P1-27	TRUE	3	TRA:CALVDFSGYSTLTF TRB:CASSGRGTNEKLFF	55	CD4-C3
PB-1_TACTTGTAGACGCACA	PB	PB-1	P1-948	FALSE	1	TRA:CAVTYSGGYQKVTF TRB:CASRVGGSGQLSGANVLTF	1	CD4-C3
PB-1_TAGACCAGTTTAGGAA	PB	PB-1	P1-953	FALSE	1	TRA:CAVQGTGGYQKVTF TRB:CASSRGTGSYEQYF	1	CD4-C3
PB-1_TAGGCATAGAGTAATC	PB	PB-1	P1-958	FALSE	1	TRA:CATDAADSWGKLQF TRB:CASTATPPRTGQETQYF	1	CD4-C3
PB-1_TCAGCAAAGCGGCTTC	PB	PB-1	P1-973	FALSE	1	TRA:CVVNIGGAQKLVF TRB:CASSEAGPDQFF	1	CD4-C3
PB-1_TCGAGGCAGCTATGCT	PB	PB-1	P1-982	FALSE	1	TRA:CIVRGGNNARLMF TRB:CASSPFRGGNEQFF	1	CD4-C3
PB-1_TCGTACCCAGACGCTC	PB	PB-1	P1-991	FALSE	1	TRA:CALGNFNKFYF TRB:CSAFSRNTGELFF	1	CD4-C3
PB-1_TCTCATAGTGGTCTCG	PB	PB-1	P1-996	FALSE	1	TRA:CALQGEYGNKLVF TRB:CASSGGSYQETQYF	1	CD4-C3
PB-1_TGAAAGAGTGTCGCTG	PB	PB-1	P1-1005	FALSE	1	TRA:CIVGRDSSYKLIF TRB:CASSKGQGPRETQYF	1	CD4-C2
PB-1_TGAGGGACACACCGAC	PB	PB-1	P1-1014	FALSE	1	TRA:CAVHGSGNTGKLIF TRB:CASSDSRVNEQFF	1	CD4-C3
PB-1_TGCGTGGAGCCCTAAT	PB	PB-1	P1-9	TRUE	3	TRA:CAEAEGGGADGLTF TRB:CATSGGDNEQFF	174	CD8-C2
PB-1_TGCTACCCACCGAAAG	PB	PB-1	P1-19	TRUE	3	TRA:CALGAFGSARQLTF TRB:CASSKGQGPRETQYF	70	CD4-C6
PB-1_TGCTACCTCTCATTCA	PB	PB-1	P1-106	TRUE	3	TRA:CAVIEGNQFYF TRB:CASSLTPGLNIQYF	5	CD4-C3
PB-1_TGGACGCTCCATGAAC	PB	PB-1	P1-95	TRUE	3	TRA:CIVRVARGNEKLTF TRB:CASSLYGEGHQPQHF	6	CD4-C6
PB-1_TGGGAAGGTTGTGGCC	PB	PB-1	P1-203	TRUE	3	TRA:CAGVLAGNMLTF TRB:CASRRDRDPNEQFF	3	CD4-C3
PB-1_TGGGCGTGTCAGAATA	PB	PB-1	P1-1033	FALSE	1	TRA:CALSDPHNNARLMF TRB:CASSLPNTEAFF	1	CD8-C2
PB-1_TGGTTCCAGCTAGGCA	PB	PB-1	P1-1035	FALSE	1	TRA:CAENVRAGTALIF TRB:CASSPPSSYEQYF	1	CD4-C3

PB-1_TTAACTCCAGCTATTG	PB	PB-1	P1-1048	FALSE	1	TRA:CIVRVLNTESQGNLIF TRB:CASSLEEGALYGYTF	1	CD4-C3
PB-1_TTCTTAGCAGGGATTG	PB	PB-1	P1-1063	FALSE	1	TRA:CAVKKGTGRRALTF TRB:CASRRTGTTGLAGGPRIYEQYF	1	CD4-C3
PB-1_TTGAACGCAATAGCAA	PB	PB-1	P1-17	TRUE	3	TRA:CGTGARGSSGSRLTF TRB:CASSRQGMGAFF	78	CD4-C3
PB-1_TTGAACGCAGCTGCTG	PB	PB-1	P1-27	TRUE	3	TRA:CALVDFSGYSTLTF TRB:CASSGRGTNEKLFF	55	CD4-C3
PB-1_AACCATGAGGTGATTA	PB	PB-1	P1-28	TRUE	3	TRA:CAVKKGTGRRALTF TRB:CASETGQGTDTQYF	53	CD8-C10
PB-1_AAGGAGCGTACAGTTC	PB	PB-1	P1-448	FALSE	1	TRA:CAVSGGNYQLIW TRB:CSARATRDYEQYF	1	CD8-C10
PB-1_AAGTCTGGTAACGCGA	PB	PB-1	P1-28	TRUE	3	TRA:CAVKKGTGRRALTF TRB:CASETGQGTDTQYF	53	CD8-C10
PB-1_AAGTCTGGTGCAGGTA	PB	PB-1	P1-172	TRUE	3	TRA:CAVRRDDKIIF TRB:CASSPIAGGSFDEQYF	3	CD8-C10
PB-1_ACACCCTCACCGCTAG	PB	PB-1	P1-15	TRUE	3	TRA:CAVNSSPLGANSKLTF TRB:CASSDSGNFYNEQFF	92	CD8-C2
PB-1_ACAGCTAAGGATATAC	PB	PB-1	P1-15	TRUE	3	TRA:CAVNSSPLGANSKLTF TRB:CASSDSGNFYNEQFF	92	CD8-C2
PB-1_ACTGTCCTCCTTCAAT	PB	PB-1	P1-28	TRUE	3	TRA:CAVKKGTGRRALTF TRB:CASETGQGTDTQYF	53	CD8-C10
PB-1_AGGTCCGCACCAGGCT	PB	PB-1	P1-172	TRUE	3	TRA:CAVRRDDKIIF TRB:CASSPIAGGSFDEQYF	3	CD8-C10
PB-1_AGTGGGACATCACAAC	PB	PB-1	P1-529	FALSE	1	TRA:CVVSDRGSTLGRLYF TRB:CASSEGPTGFNSPLHF	1	CD8-C2
PB-1_AGTGTCAAGAATGTGT	PB	PB-1	P1-28	TRUE	3	TRA:CAVKKGTGRRALTF TRB:CASETGQGTDTQYF	53	CD8-C10
PB-1_CACCACTCACCATCCT	PB	PB-1	P1-28	TRUE	3	TRA:CAVKKGTGRRALTF TRB:CASETGQGTDTQYF	53	CD8-C10
PB-1_CAGAGAGTCTGGAGCC	PB	PB-1	P1-28	TRUE	3	TRA:CAVKKGTGRRALTF TRB:CASETGQGTDTQYF	53	CD4-C3
PB-1_CATGCCTAGTTGTAGA	PB	PB-1	P1-28	TRUE	3	TRA:CAVKKGTGRRALTF TRB:CASETGQGTDTQYF	53	CD8-C10
PB-1_CCGGGATAGCATCATC	PB	PB-1	P1-28	TRUE	3	TRA:CAVKKGTGRRALTF TRB:CASETGQGTDTQYF	53	CD8-C10
PB-1_CGCGTTTTCACGCGGT	PB	PB-1	P1-28	TRUE	3	TRA:CAVKKGTGRRALTF TRB:CASETGQGTDTQYF	53	CD8-C10
PB-1_CGGACACTCAAGCCTA	PB	PB-1	P1-62	TRUE	3	TRA:CVVNLMEYGNKLVF TRB:CASSGARQGQYEQYF	10	CD8-C10
PB-1_CTACCCAGTGCAGGTA	PB	PB-1	P1-28	TRUE	3	TRA:CAVKKGTGRRALTF TRB:CASETGQGTDTQYF	53	CD8-C10
PB-1_CTACCCATCTGCGGCA	PB	PB-1	P1-28	TRUE	3	TRA:CAVKKGTGRRALTF TRB:CASETGQGTDTQYF	53	CD8-C10
PB-1_CTTCTCTTCGCCGTGA	PB	PB-1	P1-28	TRUE	3	TRA:CAVKKGTGRRALTF TRB:CASETGQGTDTQYF	53	CD8-C10
PB-1_GACCAATGTATAATGG	PB	PB-1	P1-68	TRUE	3	TRA:CAVIPDILTGGGNKLTF TRB:CASSLQGFGNTIYF	9	CD8-C4
PB-1_GACCTGGAGCAGGTCA	PB	PB-1	P1-28	TRUE	3	TRA:CAVKKGTGRRALTF TRB:CASETGQGTDTQYF	53	CD8-C10
PB-1_GATGAGGTCTCCCTGA	PB	PB-1	P1-28	TRUE	3	TRA:CAVKKGTGRRALTF TRB:CASETGQGTDTQYF	53	CD8-C10
PB-1_GCAATCACAACTGCGC	PB	PB-1	P1-15	TRUE	3	TRA:CAVNSSPLGANSKLTF TRB:CASSDSGNFYNEQFF	92	CD8-C10

PB-1_GCACTCTGTCGGCATC	PB	PB-1	P1-28	TRUE	3	TRA:CAVKKGTGRRALTF TRB:CASETGQGTDTQYF	53	CD8-C6
PB-1_GCGCAACAGTGTTGAA	PB	PB-1	P1-28	TRUE	3	TRA:CAVKKGTGRRALTF TRB:CASETGQGTDTQYF	53	CD8-C10
PB-1_GCGCGATCATGAACCT	PB	PB-1	P1-28	TRUE	3	TRA:CAVKKGTGRRALTF TRB:CASETGQGTDTQYF	53	CD8-C10
PB-1_GCTCTGTCACCAACCG	PB	PB-1	P1-28	TRUE	3	TRA:CAVKKGTGRRALTF TRB:CASETGQGTDTQYF	53	CD8-C10
PB-1_GGCGTGTTCATTGCGA	PB	PB-1	P1-864	FALSE	1	TRA:CAVKKGTGRRALTF TRB:CASSQVTGGARDEQYF	1	CD8-C10
PB-1_GTATTCTGTGTTCGAT	PB	PB-1	P1-898	FALSE	1	TRA:CVVSDRGSTLGRLYF TRB:CASSDRGPRYEQYF	1	CD8-C10
PB-1_GTCACGGTCTTGCCGT	PB	PB-1	P1-28	TRUE	3	TRA:CAVKKGTGRRALTF TRB:CASETGQGTDTQYF	53	CD8-C10
PB-1_GTTCGGGAGGTTCCTA	PB	PB-1	P1-28	TRUE	3	TRA:CAVKKGTGRRALTF TRB:CASETGQGTDTQYF	53	CD8-C10
PB-1_TAGTGGTGTACCGTTA	PB	PB-1	P1-15	TRUE	3	TRA:CAVNSSPLGANSKLTF TRB:CASSDSGNFYNEQFF	92	CD4-C3
PB-1_TCAACGAAGCGCCTCA	PB	PB-1	P1-965	FALSE	1	TRA:CAPYSNASKIIF TRB:CASSLVTGATYEQYF	1	CD8-C2
PB-1_TCAGCTCGTGGCGAAT	PB	PB-1	P1-975	FALSE	1	TRA:CAEGGGATNKLIF TRB:CSATPGTRSWSQYF	1	CD8-C2
PB-1_TCTCATACAGACGTAG	PB	PB-1	P1-28	TRUE	3	TRA:CAVKKGTGRRALTF TRB:CASETGQGTDTQYF	53	CD8-C10
PB-1_TGACGGCTCACCAGGC	PB	PB-1	P1-28	TRUE	3	TRA:CAVKKGTGRRALTF TRB:CASETGQGTDTQYF	53	CD8-C10
PB-1_TGCCCATGTTCGGGCT	PB	PB-1	P1-1019	FALSE	1	TRA:CVVSPYGGSQGNLIF TRB:CASSLLQGNTIYF	1	CD8-C10
PB-1_TTCGGTCTCACCGGGT	PB	PB-1	P1-28	TRUE	3	TRA:CAVKKGTGRRALTF TRB:CASETGQGTDTQYF	53	CD8-C10
PB-1_TTCTTAGGTTGGAGGT	PB	PB-1	P1-28	TRUE	3	TRA:CAVKKGTGRRALTF TRB:CASETGQGTDTQYF	53	CD4-C3
PB-1_TTTGCGCCACAGACTT	PB	PB-1	P1-28	TRUE	3	TRA:CAVKKGTGRRALTF TRB:CASETGQGTDTQYF	53	CD8-C10
PB-1_AAAGTAGGTCTCGTTC	PB	PB-1	P1-252	TRUE	2	TRA:CAASINGRGSTLGRLYF TRB:CASLPGHGVTDTQYF	2	CD4-C5
PB-1_ACGCCGATCCTCGCAT	PB	PB-1	P1-490	FALSE	1	TRA:CAVSFGNARLMF TRB:CASSFRAGGRITDTQYF	1	CD4-C6
PB-1_CACACCTCATGTAGTC	PB	PB-1	P1-272	TRUE	2	TRA:CAFMSNNNDMRF TRB:CASKPRDTEAFF	2	CD4-C5
PB-1_CTAGCCTTCATCGGAT	PB	PB-1	P1-151	TRUE	3	TRA:CAGTNSGYSTLTF TRB:CASSPKESGQTFQETQYF	4	CD4-C5
PB-1_CTCGTCAAGCCTCGTG	PB	PB-1	P1-27	TRUE	3	TRA:CALVDFSGYSTLTF TRB:CASSGRGTNEKLFF	55	CD4-C6
PB-1_GTGCGGTGTGATGTCT	PB	PB-1	P1-119	TRUE	3	TRA:CVVSQAGSGNTGKLIF TRB:CSAIPWRGYGYTF	5	CD4-C3
PB-1_TCTATTGGTGTTCTTT	PB	PB-1	P1-994	FALSE	1	TRA:CAVRSMDSNYQLIW TRB:CASSQVRVHSSHNEQFF	1	CD4-C2
PB-1_AACGTTGTCGAGAGCA	PB	PB-1	P1-437	FALSE	1	TRA:CAVPDGQKLLF TRB:CASSPPGADSYEQYF	1	CD4-C7
PB-1_AACTCAGTCTACCTGC	PB	PB-1	P1-439	FALSE	1	TRA:CAMSDRMTGGFKTIF TRB:CASSQDRDRNQETQYF	1	CD4-C7
PB-1_AACTTTCCAACGATGG	PB	PB-1	P1-443	FALSE	1	TRA:CALLRGGGNKLTF TRB:CASSFSLSSSYEQYF	1	CD4-C7

PB-1_AAGGCAGCAAACGCGA	PB	PB-1	P1-449	FALSE	1	TRA:CAMREPLLDGQKLLF TRB:CASSAIFSSGSINEQFF	1	CD4-C7
PB-1_AAGTCTGCACGGATAG	PB	PB-1	P1-455	FALSE	1	TRA:CAETGRSQGNLIF TRB:CASSFRPSDEKLFF	1	CD4-C7
PB-1_AAGTCTGGTGATGATA	PB	PB-1	P1-457	FALSE	1	TRA:CAYRSESGGGADGLTF TRB:CSARVPAAIYTEAFF	1	CD4-C7
PB-1_ACACCAACATAGTAAG	PB	PB-1	P1-462	FALSE	1	TRA:CAVSGGNTGKLIF TRB:CSARDARVDTQYF	1	CD4-C7
PB-1_ACACCCTTCGTGGGAA	PB	PB-1	P1-463	FALSE	1	TRA:CAVRSGTYKYIF TRB:CASSRLQGAEETQYF	1	CD4-C7
PB-1_ACCAGTATCCTTTACA	PB	PB-1	P1-472	FALSE	1	TRA:CALNTGNQFYF TRB:CSASPTRAGELFF	1	CD4-C7
PB-1_ACTTTCAGTCCATCCT	PB	PB-1	P1-506	FALSE	1	TRA:CAVNQGAQKLVF TRB:CASSFGTLNAYNEQFF	1	CD4-C7
PB-1_AGATTGCCAGAGCCAA	PB	PB-1	P1-514	FALSE	1	TRA:CALYLQGAQKLVF TRB:CAILPDSSSYEQYF	1	CD4-C2
PB-1_AGGGATGGTCCCGACA	PB	PB-1	P1-526	FALSE	1	TRA:CATAGRAGGYNKLIF TRB:CASSLLRGGEETQYF	1	CD4-C7
PB-1_AGGTCATGTCGGATCC	PB	PB-1	P1-182	TRUE	3	TRA:CAASRGGGADGLTF TRB:CASSLEVGGFGNTIYF	3	CD4-C7
PB-1_ATCCGAAAGGCTCATT	PB	PB-1	P1-544	FALSE	1	TRA:CALRSSGTASKLTF TRB:CATSDYTGGTGELFF	1	CD4-C7
PB-1_ATCTACTGTTCGCTAA	PB	PB-1	P1-548	FALSE	1	TRA:CALGAFGSARQLTF TRB:CASSLGGGRSTDTQYF	1	CD4-C7
PB-1_CACAGTAAGAGACTAT	PB	PB-1	P1-581	FALSE	1	TRA:CALSEAKSAMDTGRRALTF TRB:CASSELVEQYF	1	CD4-C7
PB-1_CACATAGAGGATGCGT	PB	PB-1	P1-584	FALSE	1	TRA:CIVRVGQGAQKLVF TRB:CSAIDRERTDTQYF	1	CD4-C7
PB-1_CAGAGAGGTCGTTGTA	PB	PB-1	P1-593	FALSE	1	TRA:CALRGNQAGTALIF TRB:CASSTAGQGGYEQYF	1	CD4-C7
PB-1_CATATTCCACACCGCA	PB	PB-1	P1-618	FALSE	1	TRA:CAVVNNQAGTALIF TRB:CASSIQPGQGADTQYF	1	CD4-C7
PB-1_CATTCGCCACATTCGA	PB	PB-1	P1-279	FALSE	2	TRA:CAVEDRDGQKLLF TRB:CASSLTDTQYF	2	CD4-C7
PB-1_CCCAGTTAGGGTCTCC	PB	PB-1	P1-646	FALSE	1	TRA:CAPTGGFKTIF TRB:CASRLIAGLNEQFF	1	CD4-C2
PB-1_CCGGGATCACGTAAGG	PB	PB-1	P1-651	FALSE	1	TRA:CALSDRGYKLSF TRB:CASSLVSEQPDTQYF	1	CD4-C7
PB-1_CCGTGGAGTCCAGTAT	PB	PB-1	P1-653	FALSE	1	TRA:CVVNAGGYNKLIF TRB:CASSLGQGRATDTQYF	1	CD4-C2
PB-1_CCTAGCTCAACCGCCA	PB	PB-1	P1-657	FALSE	1	TRA:CAASKSGSARQLTF TRB:CASSVGQQYF	1	CD4-C7
PB-1_CGCGGTAGTCACCTAA	PB	PB-1	P1-685	FALSE	1	TRA:CAAISRIGTASKLTF TRB:CASSPGTAPGGTQYF	1	CD4-C7
PB-1_CGCGTTTTCTGGTTCC	PB	PB-1	P1-687	FALSE	1	TRA:CAASAMYSGGGADGLTF TRB:CASRHRTTSGETQYF	1	CD4-C7
PB-1_CGCTGGACACTGCCAG	PB	PB-1	P1-692	FALSE	1	TRA:CAGQRTNFGNEKLTF TRB:CSARAGGGGNEQF	1	CD4-C2
PB-1_CGCTGGATCTGCAAGT	PB	PB-1	P1-693	FALSE	1	TRA:CGTVSNFGNEKLTF TRB:CSARGQGTRGTETQYF	1	CD4-C7
PB-1_CGTTCTGCAATGTTGC	PB	PB-1	P1-719	FALSE	1	TRA:CAVFQGAQKLVF TRB:CASSLGLSSYNEQFF	1	CD4-C7
PB-1_CTAGTGATCGAGAACG	PB	PB-1	P1-285	TRUE	2	TRA:CAMSDASGGYNKLIF TRB:CASGTHNRNTGELFF	2	CD4-C7

PB-1_CTCGGAGTCGTCTGAA	PB	PB-1	P1-741	FALSE	1	TRA:CVVKAQNAGNMLTF TRB:CASTTGGLTDTQYF	1	CD4-C7
PB-1_CTCTGGTGTCTCTCTG	PB	PB-1	P1-748	FALSE	1	TRA:CAMSFNNARLMF TRB:CASSPPSEREKLFF	1	CD4-C7
PB-1_CTGCTGTCAGATAATG	PB	PB-1	P1-757	FALSE	1	TRA:CAVRDISGSARQLTF TRB:CASSPGTAPGGTQYF	1	CD4-C7
PB-1_CTGTTTATCAACCATG	PB	PB-1	P1-765	FALSE	1	TRA:CAGLETSGSRLTF TRB:CASSGGLAKNIQYF	1	CD4-C7
PB-1_GAAACTCCAAGCGAGT	PB	PB-1	P1-779	FALSE	1	TRA:CAVGPGTGGFKTIF TRB:CASSLNRGSTDTQYF	1	CD4-C7
PB-1_GACGTGCGTACCGGCT	PB	PB-1	P1-794	FALSE	1	TRA:CVVNGESSNTGKLIF TRB:CSAEKGGGEETQYF	1	CD4-C7
PB-1_GACTAACCACGTAAGG	PB	PB-1	P1-797	FALSE	1	TRA:CAAIDGGSTLGRLYF TRB:CASSNRGGTEAFF	1	CD4-C7
PB-1_GACTACAGTGGGTCAA	PB	PB-1	P1-799	FALSE	1	TRA:CAMSAETGGFKTIF TRB:CASSARGPYGYTF	1	CD4-C7
PB-1_GAGCAGAAGGTAAACT	PB	PB-1	P1-803	FALSE	1	TRA:CAGQSSGTYKYIF TRB:CSVAQGGETQYF	1	CD4-C7
PB-1_GATCGATGTTCGTGAT	PB	PB-1	P1-805	FALSE	1	TRA:CAGRGGATNKLIF TRB:CASSIGGRGNQPQHF	1	CD4-C7
PB-1_GATCGCGTCCAACCAA	PB	PB-1	P1-806	FALSE	1	TRA:CAVQSGGSYIPTF TRB:CASSFRSEQFF	1	CD4-C7
PB-1_GATCGTACATTCACTT	PB	PB-1	P1-808	FALSE	1	TRA:CAASAYIYNTDKLIF TRB:CASSVGQGYNYGYTF	1	CD4-C7
PB-1_GATGAGGAGAGTCTGG	PB	PB-1	P1-813	FALSE	1	TRA:CILYTGANSKLTF TRB:CASNYGGTGSYEQYF	1	CD4-C7
PB-1_GATTCAGGTTGGTAAA	PB	PB-1	P1-821	FALSE	1	TRA:CAIRNTGTASKLTF TRB:CASSETGGNQPQHF	1	CD4-C7
PB-1_GCCTCTATCGGCGCAT	PB	PB-1	P1-831	FALSE	1	TRA:CAVRDMNGGSQGNLIF TRB:CASSLVSGSGNTIYF	1	CD4-C2
PB-1_GCGCAACAGCGACGTA	PB	PB-1	P1-837	FALSE	1	TRA:CAATEFSGGASKLTF TRB:CSARHGSYNSPLHF	1	CD4-C2
PB-1_GCTGCAGCAATCGAAA	PB	PB-1	P1-844	FALSE	1	TRA:CAASRREYGNKLVF TRB:CASSIGGANYGYTF	1	CD4-C7
PB-1_GCTGCGACAGTTAACC	PB	PB-1	P1-846	FALSE	1	TRA:CAFPRYSGGGADGLTF TRB:CASSLYTLGNEKLFF	1	CD4-C7
PB-1_GGACATTAGTGTCCCG	PB	PB-1	P1-857	FALSE	1	TRA:CALILRNTPLVF TRB:CSVPSGVGEQFF	1	CD4-C7
PB-1_GGACATTTCATAACCG	PB	PB-1	P1-859	FALSE	1	TRA:CAVSRSNDYKLSF TRB:CASSSTGSYGYTF	1	CD4-C7
PB-1_GGGAATGAGTCCATAC	PB	PB-1	P1-291	FALSE	2	TRA:CIVAGGSEKLVF TRB:CASSSHRFAYNEQFF	2	CD4-C7
PB-1_GGGAGATTCGACAGCC	PB	PB-1	P1-875	FALSE	1	TRA:CAESRNQGGKLIF TRB:CASSRSGSSIYEQYF	1	CD4-C2
PB-1_GGTGAAGTCCAATGGT	PB	PB-1	P1-879	FALSE	1	TRA:CAGGYWPAGNKLTF TRB:CASSQEGSGGGYTF	1	CD4-C7
PB-1_GTAACGTTCGCATGAT	PB	PB-1	P1-885	FALSE	1	TRA:CATDENQGAQKLVF TRB:CAISEGRGLYEQYF	1	CD4-C7
PB-1_GTATCTTGTAGAGCTG	PB	PB-1	P1-896	FALSE	1	TRA:CAETPSGNTPLVF TRB:CASRHGAGLWGEQYF	1	CD4-C7
PB-1_GTATTCTGTGGCGAAT	PB	PB-1	P1-897	FALSE	1	TRA:CALSDSNSGYALNF TRB:CASSLAGVQETQYF	1	CD4-C7
PB-1_GTCAAGTGTGCACGAA	PB	PB-1	P1-900	FALSE	1	TRA:CAVPMNRDDKIIF TRB:CASSLTRPYEQYF	1	CD4-C7

PB-1_GTCCTCATCTAACTTC	PB	PB-1	P1-908	FALSE	1	TRA:CALLSGGYNKLIF TRB:CASSHRKSRNTEAFF	1	CD4-C7
PB-1_GTTCTCGGTATCAGTC	PB	PB-1	P1-923	FALSE	1	TRA:CAVNAVGTGGFKTIF TRB:CASSPEVGVYSAQHF	1	CD4-C7
PB-1_GTTTCTAAGGATGTAT	PB	PB-1	P1-926	FALSE	1	TRA:CALARGSSASKIIF TRB:CSYPGREETQYF	1	CD4-C7
PB-1_TAAACCGAGGGAACGG	PB	PB-1	P1-930	FALSE	1	TRA:CAYRTGDATDKLIF TRB:CASRRGAGVGTQYF	1	CD4-C7
PB-1_TAAACCGAGTGCCAGA	PB	PB-1	P1-931	FALSE	1	TRA:CAVSGFNDYKLSF TRB:CAWSVKLEAFF	1	CD4-C7
PB-1_TATGCCCGTTAGATGA	PB	PB-1	P1-959	FALSE	1	TRA:CAASGPGTASKLTF TRB:CASSKGRGEGAQNIQYF	1	CD4-C7
PB-1_TCACAAGTCACGGTTA	PB	PB-1	P1-969	FALSE	1	TRA:CVVSLFNTGKLIF TRB:CASSYWQDSYEQYF	1	CD4-C7
PB-1_TCTTCGGCAGCTGTTA	PB	PB-1	P1-1000	FALSE	1	TRA:CAVSLSGSARQLTF TRB:CSARRTGTTNEQFF	1	CD4-C7
PB-1_TGACTAGAGACTACAA	PB	PB-1	P1-1007	FALSE	1	TRA:CAASVRGNQFYF TRB:CASSALGRPTTDTQYF	1	CD4-C7
PB-1_TGCGCAGGTACAAGTA	PB	PB-1	P1-1022	FALSE	1	TRA:CIASRMDSSYKLIF TRB:CASSVHRGRRETQYF	1	CD4-C7
PB-1_TGCGTGGGTAGAGTGC	PB	PB-1	P1-1024	FALSE	1	TRA:CAVKPGTALIF TRB:CASSLTSSGNTIYF	1	CD4-C7
PB-1_TGTGGTAGTCGGATCC	PB	PB-1	P1-1042	FALSE	1	TRA:CAVRDQNNAGNMLTF TRB:CSTGLAGTSTDTQYF	1	CD4-C7
PB-1_TGTGTTTGTGGCGAAT	PB	PB-1	P1-279	FALSE	2	TRA:CAVEDRDGQKLLF TRB:CASSLTDTQYF	2	CD4-C7
PB-1_TTTGGTTCAGGACGTA	PB	PB-1	P1-1081	FALSE	1	TRA:CVVTGEGNTGFQKLVF TRB:CASNSRDRVFNEQYF	1	CD4-C7
PB-1_AAAGTAGCACGAGGTA	PB	PB-1	P1-428	FALSE	1	TRA:CAVRPWAGGTSYGKLTF TRB:CSARGRGADHEQYF	1	CD4-C1
PB-1_AACTCCCAGTAACCCT	PB	PB-1	P1-440	FALSE	1	TRA:CATGISGAGSYQLTF TRB:CASSVGSSDRHPSYGYTF	1	CD4-C1
PB-1_AACTGGTTCTAACGGT	PB	PB-1	P1-442	FALSE	1	TRA:CAAGWGAQKLVF TRB:CASSIFSGGAGELFF	1	CD4-C1
PB-1_AAGACCTAGCGTGAGT	PB	PB-1	P1-445	FALSE	1	TRA:CIVGRWGGSNYKLTF TRB:CARSGAANEKLFF	1	CD4-C1
PB-1_AAGGCAGTCAGCATGT	PB	PB-1	P1-451	FALSE	1	TRA:CAATINTNAGKSTF TRB:CASSILGSPGRYGYTF	1	CD4-C1
PB-1_AAGGTTCAGGCCCTTG	PB	PB-1	P1-452	FALSE	1	TRA:CAVNRANQAGTALIF TRB:CSDMTVTSFDEQFF	1	CD4-C1
PB-1_AAGGTTCCAGCCTGTG	PB	PB-1	P1-454	FALSE	1	TRA:CVVSPDSWGKLQF TRB:CASTSPQTGELFF	1	CD4-C1
PB-1_ACATACGCATGGTCAT	PB	PB-1	P1-469	FALSE	1	TRA:CIVRVFGGATNKLIF TRB:CASSAGVETQYF	1	CD4-C1
PB-1_ACCCACTCAGACGCTC	PB	PB-1	P1-473	FALSE	1	TRA:CAVGETSGSRLTF TRB:CASSPGIDEKLFF	1	CD4-C1
PB-1_ACGGAGATCGCTTGTC	PB	PB-1	P1-492	FALSE	1	TRA:CVVMNYGGSQGNLIF TRB:CAWHRAYSYNSPLHF	1	CD4-C1
PB-1_ACGTCAACATGTCTCC	PB	PB-1	P1-495	FALSE	1	TRA:CVVSAYSSASKIIF TRB:CASSEQQGNIQYF	1	CD4-C1
PB-1_ACTGAACCAGTCACTA	PB	PB-1	P1-498	FALSE	1	TRA:CAVGGSSNTGKLIF TRB:CASSESTGGWNEQYF	1	CD4-C1
PB-1_ACTGAGTTCGCGCCAA	PB	PB-1	P1-500	FALSE	1	TRA:CALSDRNQGAQKLVF TRB:CASSDSYEQYF	1	CD4-C1

PB-1_AGACGTTCATCGGAAG	PB	PB-1	P1-509	FALSE	1	TRA:CIAYAGGTSYGKLTF TRB:CASSQDGRANYGYTF	1	CD4-C1
PB-1_AGGTCATTCGATAGAA	PB	PB-1	P1-528	FALSE	1	TRA:CAVTKGGSEKLVF TRB:CSVVGGSGLQEQYF	1	CD4-C1
PB-1_AGTGTCAGTTAGTGGG	PB	PB-1	P1-532	FALSE	1	TRA:CAVRTGFQKLVF TRB:CSARDANQETQYF	1	CD4-C1
PB-1_ATCATCTGTCTGATCA	PB	PB-1	P1-540	FALSE	1	TRA:CAVGSNYQLIW TRB:CASSLPEAGSYEQYF	1	CD4-C1
PB-1_ATCCACCAGAGGGCTT	PB	PB-1	P1-543	FALSE	1	TRA:CAGLSGNTPLVF TRB:CASSKSDRGMVRYNEQFF	1	CD4-C1
PB-1_ATCGAGTAGAAGGACA	PB	PB-1	P1-546	FALSE	1	TRA:CAMRRTTSGTYKYIF TRB:CASSFSQVVAKNIQYF	1	CD4-C1
PB-1_ATGAGGGGTAGCTAAA	PB	PB-1	P1-550	FALSE	1	TRA:CALTSGGSNYKLTF TRB:CASTPPGRGSYNEQFF	1	CD4-C1
PB-1_ATGCGATTCGGACAAG	PB	PB-1	P1-552	FALSE	1	TRA:CALSSSSGSARQLTF TRB:CSAFITGPGEQYF	1	CD4-C1
PB-1_ATTACTCCACATTAGC	PB	PB-1	P1-556	FALSE	1	TRA:CAMSVINQGGKLIF TRB:CSASDSLNTEAFF	1	CD4-C1
PB-1_ATTATCCGTAAGTAGT	PB	PB-1	P1-559	FALSE	1	TRA:CAASARSGAGKSTF TRB:CASSFLAGPDTQYF	1	CD4-C1
PB-1_CAACTAGGTGTCCTCT	PB	PB-1	P1-568	FALSE	1	TRA:CAAVPSGANNLFF TRB:CASSRDSHTEAFF	1	CD4-C1
PB-1_CAAGAAAGTCAGCTAT	PB	PB-1	P1-569	FALSE	1	TRA:CALSEAPGKGNLIF TRB:CAISPRGEGTDTQYF	1	CD4-C1
PB-1_CACACAAAGACAGGCT	PB	PB-1	P1-576	FALSE	1	TRA:CAVNAFGSWGKLQF TRB:CASSQEGGGVSYNEQFF	1	CD4-C1
PB-1_CACAGTAAGTCTCGGC	PB	PB-1	P1-583	FALSE	1	TRA:CAVSDRDARLMF TRB:CASRRTGGYEQYF	1	CD4-C1
PB-1_CACCTTGTCAGTCAGT	PB	PB-1	P1-591	FALSE	1	TRA:CAYRRNHGGSQGNLIF TRB:CASSGPRAYRRFF	1	CD4-C1
PB-1_CAGCAGCCATTCCTCG	PB	PB-1	P1-594	FALSE	1	TRA:CAVNSGNTPLVF TRB:CASSLARLAGVETQYF	1	CD4-C1
PB-1_CAGTAACCACACCGCA	PB	PB-1	P1-608	FALSE	1	TRA:CAFREWNTDKLIF TRB:CASSRTDRVINVLTF	1	CD4-C1
PB-1_CAGTAACTCACGCGGT	PB	PB-1	P1-611	FALSE	1	TRA:CAVYPPSGGSYIPTF TRB:CASSLGTGGDMEYF	1	CD4-C1
PB-1_CAGTCCTCAAGCTGGA	PB	PB-1	P1-613	FALSE	1	TRA:CAGDTGRRALTF TRB:CASSVAGTGGTDTQYF	1	CD4-C1
PB-1_CATCGGGAGTACACCT	PB	PB-1	P1-625	FALSE	1	TRA:CAENFVLFPSSGSARQLTF TRB:CASSPRDTRRASPLHF	1	CD4-C1
PB-1_CCACCTATCAACACCA	PB	PB-1	P1-633	FALSE	1	TRA:CIVRTSGTYKYIF TRB:CSAIEPGLAGVNEQFF	1	CD4-C1
PB-1_CCACGGATCCGCGTTT	PB	PB-1	P1-636	FALSE	1	TRA:CAVSFQGGKLIF TRB:CASSLDGQGKTYEQYF	1	CD4-C1
PB-1_CCACTACAGTAGTGCG	PB	PB-1	P1-637	FALSE	1	TRA:CIFYTNQAGTALIF TRB:CASSLGGANSPLHF	1	CD4-C1
PB-1_CCAGCGACACGGACAA	PB	PB-1	P1-641	FALSE	1	TRA:CAADGGATNKLIF TRB:CASSLDRGRTGELFF	1	CD4-C1
PB-1_CCATTCGCACAAGTAA	PB	PB-1	P1-645	FALSE	1	TRA:CVVNSNALNF TRB:CASRKDRVDGYTF	1	CD4-C1
PB-1_CCTATTACACCCAGTG	PB	PB-1	P1-659	FALSE	1	TRA:CAVKAGGTSYGKLTF TRB:CASSPGDSSITGELFF	1	CD4-C1
PB-1_CCTTCGAGTACTCAAC	PB	PB-1	P1-663	FALSE	1	TRA:CAACSFSGGYNKLIF TRB:CASSYSQTGLYSYEQYF	1	CD4-C1

PB-1_CGAACATCATGTTGAC	PB	PB-1	P1-666	FALSE	1	TRA:CAVEEYSGGGADGLTF TRB:CASTEGGGPRDTQYF	1	CD4-C1
PB-1_CGAGAAGAGTGTCCAT	PB	PB-1	P1-670	FALSE	1	TRA:CASQAGTALIF TRB:CASSQGQGDRGSEQYF	1	CD4-C2
PB-1_CGAGCACAGAGCTTCT	PB	PB-1	P1-672	FALSE	1	TRA:CAVERTTDSWGKLQF TRB:CASSLAAYEQYF	1	CD4-C1
PB-1_CGAGCACCAGGGTACA	PB	PB-1	P1-673	FALSE	1	TRA:CAASTGNQFYF TRB:CASSLGYNEQFF	1	CD4-C1
PB-1_CGAGCCAGTTCCGTCT	PB	PB-1	P1-675	FALSE	1	TRA:CAERSDGQKLLF TRB:CASSYDRISEAFF	1	CD4-C1
PB-1_CGATGTACATGCTGGC	PB	PB-1	P1-679	FALSE	1	TRA:CAVDPSGTYKYIF TRB:CASSMTGTQPSTDTQYF	1	CD4-C1
PB-1_CGCGGTAAGGCGATAC	PB	PB-1	P1-683	FALSE	1	TRA:CIVKWTAAGTASKLTF TRB:CASSVVPSGANVLTF	1	CD4-C1
PB-1_CGCGGTATCCCACTTG	PB	PB-1	P1-686	FALSE	1	TRA:CAMSPVLTGGGNKLTF TRB:CSARDYRGDQPQHF	1	CD4-C1
PB-1_CGGAGCTCATGGTTGT	PB	PB-1	P1-703	FALSE	1	TRA:CAASIGKAAGNKLTF TRB:CASSYSDRGYEQYF	1	CD4-C1
PB-1_CGTAGGCCAGACGCAA	PB	PB-1	P1-708	FALSE	1	TRA:CAATTWNNARLMF TRB:CASSLVSSGGSDTQYF	1	CD4-C1
PB-1_CGTGTAAAGGGATCTG	PB	PB-1	P1-717	FALSE	1	TRA:CALVPNNAGNMLTF TRB:CASNKGSSYEQYF	1	CD4-C1
PB-1_CGTGTCTCATTGGTAC	PB	PB-1	P1-718	FALSE	1	TRA:CAVEGDSNYQLIW TRB:CASSFDGGAFF	1	CD4-C1
PB-1_CGTTGGGGTTTAGGAA	PB	PB-1	P1-721	FALSE	1	TRA:CAVEVLGGQKLLF TRB:CASSDRGHPSGYEQYF	1	CD4-C1
PB-1_CTACACCGTGCCTGGT	PB	PB-1	P1-725	FALSE	1	TRA:CAAKNQAGTALIF TRB:CASSQRSPRILGTDTQYF	1	CD4-C1
PB-1_CTACATTGTCCGAATT	PB	PB-1	P1-727	FALSE	1	TRA:CAETDTGRRALTF TRB:CASIPGQGRGYTF	1	CD4-C1
PB-1_CTCAGAAGTACGACCC	PB	PB-1	P1-732	FALSE	1	TRA:CALNRGIYNQGGKLIF TRB:CASSPPRETQYF	1	CD4-C1
PB-1_CTCGGAGGTATATCCG	PB	PB-1	P1-740	FALSE	1	TRA:CILKFNFGNEKLTF TRB:CASSGLAGLTDTQYF	1	CD4-C1
PB-1_CTCGTCAGTTACGCGC	PB	PB-1	P1-744	FALSE	1	TRA:CAYRSARGGSEKLVF TRB:CASSLEYRSYGYTF	1	CD4-C1
PB-1_CTCTGGTTCTATCCTA	PB	PB-1	P1-749	FALSE	1	TRA:CAASSNQAGTALIF TRB:CASSGTAADTQYF	1	CD4-C1
PB-1_CTGCCTATCTCGAGTA	PB	PB-1	P1-756	FALSE	1	TRA:CALSGLSNAGNMLTF TRB:CASSPLKRGGMNTQYF	1	CD4-C1
PB-1_CTGTTTAAGATAGGAG	PB	PB-1	P1-764	FALSE	1	TRA:CAGLFGSSNTGKLIF TRB:CSGTGNTEAFF	1	CD4-C1
PB-1_CTTAACTCATTATCTC	PB	PB-1	P1-767	FALSE	1	TRA:CATNLQGGSEKLVF TRB:CASSRQGGFEAFF	1	CD4-C1
PB-1_CTTACCGGTACTCAAC	PB	PB-1	P1-770	FALSE	1	TRA:CAVVLISDGQKLLF TRB:CASSRLAPDYGYTF	1	CD4-C1
PB-1_CTTAGGAGTTGTACAC	PB	PB-1	P1-772	FALSE	1	TRA:CAVNGADKLIF TRB:CASSQRGPVGGEQYF	1	CD4-C1
PB-1_CTTGGCTCAGGGTACA	PB	PB-1	P1-775	FALSE	1	TRA:CAASMAGNTPLVF TRB:CASSLESYVVNGYTF	1	CD4-C1
PB-1_GAACATCTCTGCTGCT	PB	PB-1	P1-783	FALSE	1	TRA:CAAKRNTPLVF TRB:CASSLATVNTEAFF	1	CD4-C1
PB-1_GAATAAGAGCTCTCGG	PB	PB-1	P1-786	FALSE	1	TRA:CALLVTGGGNKLTF TRB:CSVGDRSLYEQYF	1	CD4-C1

PB-1_GAGTCCGCATCGGTTA	PB	PB-1	P1-804	FALSE	1	TRA:CAVGSRGGGADGLTF TRB:CASSLQGSRGTDTQYF	1	CD4-C1
PB-1_GATCGTAGTTATCCGA	PB	PB-1	P1-809	FALSE	1	TRA:CALTNAGNMLTF TRB:CASSQRGDPYEQYF	1	CD4-C1
PB-1_GATCGTAGTTCATGGT	PB	PB-1	P1-810	FALSE	1	TRA:CAVRARGGGNKLTF TRB:CASSPPSDRIGYEQFF	1	CD4-C1
PB-1_GATGAAATCTAACTTC	PB	PB-1	P1-812	FALSE	1	TRA:CAARGAGNQFYF TRB:CATSREKGGSYGYTF	1	CD4-C1
PB-1_GATTCAGAGGAATGGA	PB	PB-1	P1-819	FALSE	1	TRA:CAVGAAGNKLTF TRB:CAWMLVGAETQYF	1	CD4-C1
PB-1_GCATACAGTCTCCATC	PB	PB-1	P1-826	FALSE	1	TRA:CAVALIWGWELPTHF TRB:CASSFQGPGEQYF	1	CD4-C1
PB-1_GCGAGAAAGATGTTAG	PB	PB-1	P1-834	FALSE	1	TRA:CLLVSAVGDSNYQLIW TRB:CAGQGSLYEQYF	1	CD4-C1
PB-1_GCGAGAACATCCAACA	PB	PB-1	P1-835	FALSE	1	TRA:CIVRPPTSYGKLTF TRB:CASSLGDQGRETQYF	1	CD4-C1
PB-1_GCGAGAATCCTAGAAC	PB	PB-1	P1-836	FALSE	1	TRA:CAETLPSGNTPLVF TRB:CATSRDRADTQYF	1	CD4-C1
PB-1_GCGCAACCAGACAAAT	PB	PB-1	P1-838	FALSE	1	TRA:CAEMFNNDMRF TRB:CASSVSGDYTQYF	1	CD4-C1
PB-1_GCTGCGACAGGGTACA	PB	PB-1	P1-845	FALSE	1	TRA:CAMSLAQKLVF TRB:CASSLGTSEYYNEQFF	1	CD4-C1
PB-1_GCTGCGAGTTTAGGAA	PB	PB-1	P1-847	FALSE	1	TRA:CALGNDYKLSF TRB:CASSPSPPGVGYTF	1	CD4-C2
PB-1_GCTTCCATCGCTGATA	PB	PB-1	P1-850	FALSE	1	TRA:CAVKTTDSWGKLQF TRB:CASSQGPPDLTEYF	1	CD4-C1
PB-1_GCTTGAACATGATCCA	PB	PB-1	P1-851	FALSE	1	TRA:CALTGAGSYQLTF TRB:CSVAYRLAAVVGTDTQYF	1	CD4-C1
PB-1_GGAAAGCAGGCAAAGA	PB	PB-1	P1-852	FALSE	1	TRA:CAVYDRGSTLGRLYF TRB:CSATRLVGDTQYF	1	CD4-C1
PB-1_GGAACTTTCTGCTTGC	PB	PB-1	P1-854	FALSE	1	TRA:CVVSVAVRGNTGKLIF TRB:CASSRGDRSITGELFF	1	CD4-C1
PB-1_GGATTACAGGCGACAT	PB	PB-1	P1-860	FALSE	1	TRA:CAFINPSGGGADGLTF TRB:CASSPMTGWTGELFF	1	CD4-C1
PB-1_GGCCGATTCGTATCAG	PB	PB-1	P1-863	FALSE	1	TRA:CAATAKSNTGKLIF TRB:CASSVAGSSYNEQFF	1	CD4-C1
PB-1_GGCTCGAAGCTAGCCC	PB	PB-1	P1-865	FALSE	1	TRA:CATASGDYKLSF TRB:CASIRPGTDTQYF	1	CD4-C1
PB-1_GGGACCTTCCTCATTA	PB	PB-1	P1-871	FALSE	1	TRA:CAGYNNNDMRF TRB:CASRRGGGPFQPQHF	1	CD4-C1
PB-1_GTAACGTAGTGAACAT	PB	PB-1	P1-883	FALSE	1	TRA:CAMSQNSGYSTLTF TRB:CASSRRGGGLNTEAFF	1	CD4-C1
PB-1_GTAACTGCAAGGTTTC	PB	PB-1	P1-887	FALSE	1	TRA:CVVRFASNTGKLIF TRB:CASSAYGKQTDTQYF	1	CD4-C1
PB-1_GTAGTCAGTGTTAAGA	PB	PB-1	P1-894	FALSE	1	TRA:CALISGTYKYIF TRB:CASSSSGPGQPQHF	1	CD4-C1
PB-1_GTCCTCACATGGTAGG	PB	PB-1	P1-903	FALSE	1	TRA:CIVSRTDSWGKLQF TRB:CASSQGTSGRYEQYF	1	CD4-C1
PB-1_GTCCTCATCAGCAACT	PB	PB-1	P1-906	FALSE	1	TRA:CATALGIQGAQKLVF TRB:CASSSGTSGNTIYF	1	CD4-C1
PB-1_GTCCTCATCATCTGTT	PB	PB-1	P1-907	FALSE	1	TRA:CAARNPGNTPLVF TRB:CASSTTTGVNEKLFF	1	CD4-C1
PB-1_GTGGGTCAGGATATAC	PB	PB-1	P1-918	FALSE	1	TRA:CVVSGGGYQKVTF TRB:CAISTTSVDTDTQYF	1	CD4-C1

PB-1_GTTCTCGGTCTAGCGC	PB	PB-1	P1-924	FALSE	1	TRA:CAVSRGDSSYKLIF TRB:CASSLGQPYNSPLHF	1	CD4-C1
PB-1_TACGGATTCCAGGGCT	PB	PB-1	P1-942	FALSE	1	TRA:CIPSTTGGFKTIF TRB:CASSLEGGLSYGYTF	1	CD4-C1
PB-1_TACTTGTCAGTTTACG	PB	PB-1	P1-949	FALSE	1	TRA:CAGASNTGKLIF TRB:CASSQGLAGSSYEQYF	1	CD4-C1
PB-1_TAGACCAGTTAAGTAG	PB	PB-1	P1-952	FALSE	1	TRA:CAESIPNDYKLSF TRB:CATSAPRGGEETQYF	1	CD4-C1
PB-1_TAGACCATCGTACCGG	PB	PB-1	P1-954	FALSE	1	TRA:CAARDSGTYKYIF TRB:CASLAGTSGYEQYF	1	CD4-C1
PB-1_TAGCCGGCAGTATAAG	PB	PB-1	P1-955	FALSE	1	TRA:CILRAYSGGGADGLTF TRB:CASGTSGRKGNEQFF	1	CD4-C1
PB-1_TCAATCTAGTATCTCG	PB	PB-1	P1-966	FALSE	1	TRA:CIVRVAAAGNKLTF TRB:CASSYLGGGANSPLHF	1	CD4-C2
PB-1_TCCCGATGTGTAAGTA	PB	PB-1	P1-981	FALSE	1	TRA:CAVGAKNDMRF TRB:CASRRLMNTEAFF	1	CD4-C1
PB-1_TCGCGAGCACTAAGTC	PB	PB-1	P1-984	FALSE	1	TRA:CAVGSGAGSYQLTF TRB:CSARGYRETQYF	1	CD4-C1
PB-1_TCGCGAGTCTACTTAC	PB	PB-1	P1-985	FALSE	1	TRA:CAVGARQAGTALIF TRB:CASSFGPGGLAQGEQFF	1	CD4-C1
PB-1_TCGCGTTCAGTGGGAT	PB	PB-1	P1-986	FALSE	1	TRA:CAASKGGTSYGKLTF TRB:CATSDRQGVPGELFF	1	CD4-C1
PB-1_TCGGTAAAGTTATCGC	PB	PB-1	P1-990	FALSE	1	TRA:CAVKDNYGGQNFVF TRB:CASSLASLSYNSPLHF	1	CD4-C2
PB-1_TCTCTAAAGTCTCCTC	PB	PB-1	P1-997	FALSE	1	TRA:CETDKLIF TRB:CASSVQGGTEAFF	1	CD4-C1
PB-1_TCTTTCCTCGTACGGC	PB	PB-1	P1-1003	FALSE	1	TRA:CVVSENSGYSTLTF TRB:CASSGSGNYNEQFF	1	CD4-C1
PB-1_TGAGAGGCATGACGGA	PB	PB-1	P1-1009	FALSE	1	TRA:CAVKQASGSRLTF TRB:CASSQGPQQYF	1	CD4-C1
PB-1_TGCCAAAAGTATTGGA	PB	PB-1	P1-1017	FALSE	1	TRA:CAASANDYKLSF TRB:CATSPRGNEQFF	1	CD4-C1
PB-1_TGGGAAGTCGGCATCG	PB	PB-1	P1-1032	FALSE	1	TRA:CAGAGGGADGLTF TRB:CAISALGVNTEAFF	1	CD4-C1
PB-1_TGTGTTTCAGTGAGTG	PB	PB-1	P1-1043	FALSE	1	TRA:CAVSAFQNARLMF TRB:CASSLGTVPNEQFF	1	CD4-C1
PB-1_TGTGTTTGTCAACATC	PB	PB-1	P1-1044	FALSE	1	TRA:CAATDSWGKLQF TRB:CAWKSYPGQRLNSGNTIYF	1	CD4-C1
PB-1_TTAACTCTCGACCAGC	PB	PB-1	P1-1049	FALSE	1	TRA:CALKANDMRF TRB:CASSQNRYGTTYNEQFF	1	CD4-C1
PB-1_TTATGCTTCCGAATGT	PB	PB-1	P1-1053	FALSE	1	TRA:CVVTAGVGGYNKLIF TRB:CSVWGVANSGELFF	1	CD4-C1
PB-1_TTCGAAGCATGTCGAT	PB	PB-1	P1-1054	FALSE	1	TRA:CAASGDDYKLSF TRB:CASRTLAGVGNEQFF	1	CD4-C1
PB-1_TTCGAAGGTGTGAATA	PB	PB-1	P1-1055	FALSE	1	TRA:CAVRDIRNTGTASKLTF TRB:CASREGQLYGYTF	1	CD4-C1
PB-1_TTCTACAGTACCGGCT	PB	PB-1	P1-1059	FALSE	1	TRA:CAVRASGGSYIPTF TRB:CATTSQRSTDTQYF	1	CD4-C1
PB-1_TTCTCCTCAATCACAC	PB	PB-1	P1-1062	FALSE	1	TRA:CAVNTGGFKTIF TRB:CATSSVSGQGAYEQYF	1	CD4-C1
PB-1_TTCTTAGGTACCGTTA	PB	PB-1	P1-1064	FALSE	1	TRA:CIAAI TRB:CASSERDTTNTGELFF	1	CD4-C1
PB-1_TTGAACGAGAACTGTA	PB	PB-1	P1-1065	FALSE	1	TRA:CAMREAGTNTDKLIF TRB:CASSKGQGAGGELFF	1	CD4-C1

PB-1_TTGGCAAAGTACGCGA	PB	PB-1	P1-1070	FALSE	1	TRA:CAVGVNRDDKIIF TRB:CASRSQSILSGNTIYF	1	CD4-C2
PB-1_TTTCCTCAGCCAGTTT	PB	PB-1	P1-1074	FALSE	1	TRA:CAVQAANTGNQFYF TRB:CSVRRTSTDTQYF	1	CD4-C1
PB-1_TTTGGTTCAGACAAGC	PB	PB-1	P1-1080	FALSE	1	TRA:CAVQAWGEGAQKLVF TRB:CASSESIASYEQYF	1	CD4-C1
PB-1_AAACCTGCATCCGTGG	PB	PB-1	P1-27	TRUE	3	TRA:CALVDFSGYSTLTF TRB:CASSGRGTNEKLFF	55	CD4-C6
PB-1_AAACGGGGTCAGGACA	PB	PB-1	P1-10	TRUE	3	TRA:CISNSPMDSSYKLIF TRB:CASSSWGDTDEAFF	162	CD8-C7
PB-1_AAAGCAAGTACAAGTA	PB	PB-1	P1-426	FALSE	1	TRA:CAVRDGGGTSYGKLTF TRB:CASSPPGTSGRAKEKDEQY[illegible]	1	CD8-C4
PB-1_AAAGTAGCACGGTAGA	PB	PB-1	P1-21	TRUE	3	TRA:CAASRGTGANNLFF TRB:CATQREGREQYF	67	CD4-C6
PB-1_AAATGCCCATGGAATA	PB	PB-1	P1-19	TRUE	3	TRA:CALGAFGSARQLTF TRB:CASSKGQGPRETQYF	70	CD4-C6
PB-1_AACACGTGTCATATGC	PB	PB-1	P1-433	FALSE	1	TRA:CAVLSGGGADGLTF TRB:CASSLHKGQGSSEAFF	1	CD4-C3
PB-1_AACCATGGTACCGGCT	PB	PB-1	P1-19	TRUE	3	TRA:CALGAFGSARQLTF TRB:CASSKGQGPRETQYF	70	CD4-C6
PB-1_AACGTTGCAGACGCAA	PB	PB-1	P1-21	TRUE	3	TRA:CAASRGTGANNLFF TRB:CATQREGREQYF	67	CD4-C6
PB-1_AACTGGTAGTGGGCTA	PB	PB-1	P1-24	TRUE	3	TRA:CAVKGNTDKLIF TRB:CASSLRTGAYNEQFF	62	CD4-C6
PB-1_AACTGGTTCCTCAATT	PB	PB-1	P1-27	TRUE	3	TRA:CALVDFSGYSTLTF TRB:CASSGRGTNEKLFF	55	CD4-C6
PB-1_AACTTTCGTCATTAGC	PB	PB-1	P1-36	TRUE	3	TRA:CILRDNAGNNRKLIW TRB:CASSSRQGFPGGANVLTF	40	CD4-C6
PB-1_AAGACCTAGCGTTTAC	PB	PB-1	P1-30	TRUE	3	TRA:CALPPGGNNRKLIW TRB:CASSFGDRGGTEAFF	47	CD4-C6
PB-1_AAGACCTAGGCAAAGA	PB	PB-1	P1-24	TRUE	3	TRA:CAVKGNTDKLIF TRB:CASSLRTGAYNEQFF	62	CD4-C6
PB-1_AAGCCGCCAATGTAAG	PB	PB-1	P1-30	TRUE	3	TRA:CALPPGGNNRKLIW TRB:CASSFGDRGGTEAFF	47	CD4-C6
PB-1_AAGGAGCAGGGCTTCC	PB	PB-1	P1-42	TRUE	3	TRA:CASFSGGSQGNLIF TRB:CSAGQGSNQPQHF	24	CD4-C6
PB-1_AAGGAGCAGTTAGCGG	PB	PB-1	P1-17	TRUE	3	TRA:CGTGARGSSGSRLTF TRB:CASSRQGMGAFF	78	CD4-C6
PB-1_AAGGAGCGTTCCATGA	PB	PB-1	P1-21	TRUE	3	TRA:CAASRGTGANNLFF TRB:CATQREGREQYF	67	CD4-C6
PB-1_AAGGCAGAGAGATGAG	PB	PB-1	P1-24	TRUE	3	TRA:CAVKGNTDKLIF TRB:CASSLRTGAYNEQFF	62	CD4-C6
PB-1_AAGGTTCAGCTAAGAT	PB	PB-1	P1-17	TRUE	3	TRA:CGTGARGSSGSRLTF TRB:CASSRQGMGAFF	78	CD4-C6
PB-1_AAGGTTCTCTGTACGA	PB	PB-1	P1-19	TRUE	3	TRA:CALGAFGSARQLTF TRB:CASSKGQGPRETQYF	70	CD4-C6
PB-1_AAGTCTGAGTGTACGG	PB	PB-1	P1-30	TRUE	3	TRA:CALPPGGNNRKLIW TRB:CASSFGDRGGTEAFF	47	CD4-C6
PB-1_AATCCAGCAAGGTTCT	PB	PB-1	P1-19	TRUE	3	TRA:CALGAFGSARQLTF TRB:CASSKGQGPRETQYF	70	CD4-C6
PB-1_AATCGGTTCATTATCC	PB	PB-1	P1-41	TRUE	3	TRA:CVVSEPPGGSYIPTF TRB:CASSSRPEDTQYF	27	CD4-C6
PB-1_ACACCCTGTATAAACG	PB	PB-1	P1-25	TRUE	3	TRA:CIVGRDSSYKLIF TRB:CASNPQRGAHRDTQYF	58	CD4-C6

PB-1_ACAGCTAGTTCGTGAT	PB	PB-1	P1-107	TRUE	3	TRA:CALSDKEKLTF TRB:CAISEGGGVGYTF	5	CD4-C6
PB-1_ACATACGAGTCACGCC	PB	PB-1	P1-10	TRUE	3	TRA:CISNSPMDSSYKLIF TRB:CASSSWGDTDEAFF	162	CD4-C6
PB-1_ACATCAGGTCAGAATA	PB	PB-1	P1-21	TRUE	3	TRA:CAASRGTGANNLFF TRB:CATQREGREQYF	67	CD4-C6
PB-1_ACCAGTACACGACTCG	PB	PB-1	P1-19	TRUE	3	TRA:CALGAFGSARQLTF TRB:CASSKGQGPRETQYF	70	CD4-C6
PB-1_ACCCACTTCCGTAGTA	PB	PB-1	P1-108	TRUE	3	TRA:CAASNGDTGNQFYF TRB:CSASKQGRNQPQHF	5	CD4-C6
PB-1_ACCGTAAGTCTCTCGT	PB	PB-1	P1-25	TRUE	3	TRA:CIVGRDSSYKLIF TRB:CASNPQRGAHRDTQYF	58	CD4-C6
PB-1_ACGAGGAAGACTCGGA	PB	PB-1	P1-30	TRUE	3	TRA:CALPPGGNNRKLIW TRB:CASSFGDRGGTEAFF	47	CD4-C6
PB-1_ACGATACAGGTGTTAA	PB	PB-1	P1-25	TRUE	3	TRA:CIVGRDSSYKLIF TRB:CASNPQRGAHRDTQYF	58	CD4-C6
PB-1_ACGATACTCCTATTCA	PB	PB-1	P1-30	TRUE	3	TRA:CALPPGGNNRKLIW TRB:CASSFGDRGGTEAFF	47	CD4-C6
PB-1_ACGATGTTCAGCTCGG	PB	PB-1	P1-19	TRUE	3	TRA:CALGAFGSARQLTF TRB:CASSKGQGPRETQYF	70	CD4-C6
PB-1_ACGATGTTCATCGATG	PB	PB-1	P1-25	TRUE	3	TRA:CIVGRDSSYKLIF TRB:CASNPQRGAHRDTQYF	58	CD4-C6
PB-1_ACGATGTTCTCCGGTT	PB	PB-1	P1-24	TRUE	3	TRA:CAVKGNTDKLIF TRB:CASSLRTGAYNEQFF	62	CD4-C6
PB-1_ACGCAGCCAAAGAATC	PB	PB-1	P1-262	TRUE	2	TRA:CISNSPMDSSYKLIF TRB:CASSKGQGPRETQYF	2	CD4-C6
PB-1_ACGCCAGCAATCGAAA	PB	PB-1	P1-25	TRUE	3	TRA:CIVGRDSSYKLIF TRB:CASNPQRGAHRDTQYF	58	CD4-C6
PB-1_ACGCCAGGTCATATCG	PB	PB-1	P1-36	TRUE	3	TRA:CILRDNAGNNRKLIW TRB:CASSSRQGFPGGANVLTF	40	CD4-C6
PB-1_ACGCCAGTCCAATGGT	PB	PB-1	P1-27	TRUE	3	TRA:CALVDFSGYSTLTF TRB:CASSGRGTNEKLFF	55	CD4-C6
PB-1_ACGGAGAAGACAGACC	PB	PB-1	P1-17	TRUE	3	TRA:CGTGARGSSGSRLTF TRB:CASSRQGMGAFF	78	CD4-C6
PB-1_ACGGAGAAGATAGTCA	PB	PB-1	P1-17	TRUE	3	TRA:CGTGARGSSGSRLTF TRB:CASSRQGMGAFF	78	CD4-C6
PB-1_ACGGAGACATTACGAC	PB	PB-1	P1-175	TRUE	3	TRA:CAASVVDFGNEKLTF TRB:CASSSQGLSYNEQFF	3	CD4-C6
PB-1_ACGGAGATCAGTCCCT	PB	PB-1	P1-25	TRUE	3	TRA:CIVGRDSSYKLIF TRB:CASNPQRGAHRDTQYF	58	CD4-C6
PB-1_ACTATCTCACATTCGA	PB	PB-1	P1-27	TRUE	3	TRA:CALVDFSGYSTLTF TRB:CASSGRGTNEKLFF	55	CD4-C6
PB-1_ACTGATGAGGTGCTTT	PB	PB-1	P1-24	TRUE	3	TRA:CAVKGNTDKLIF TRB:CASSLRTGAYNEQFF	62	CD4-C6
PB-1_ACTGTCCGTTGAGTTC	PB	PB-1	P1-48	TRUE	3	TRA:CAEVSGYSTLTF TRB:CASSFPTGGRTGRTEAFF	16	CD4-C6
PB-1_ACTTGTTAGTCCGTAT	PB	PB-1	P1-21	TRUE	3	TRA:CAASRGTGANNLFF TRB:CATQREGREQYF	67	CD4-C6
PB-1_ACTTGTTCAAGTTCTG	PB	PB-1	P1-27	TRUE	3	TRA:CALVDFSGYSTLTF TRB:CASSGRGTNEKLFF	55	CD4-C6
PB-1_ACTTTCACACCACCAG	PB	PB-1	P1-1	TRUE	3	TRA:CAMSDRMTGGFKTIF TRB:CASSQVGRGGEETQYF	1047	CD4-C6
PB-1_ACTTTCACAGACAGGT	PB	PB-1	P1-178	TRUE	3	TRA:CAVFLWEARLMF TRB:CASSFGGQGGTEAFF	3	CD4-C6

PB-1_AGACGTTCAATAGCAA	PB	PB-1	P1-30	TRUE	3	TRA:CALPPGGNNRKLIW TRB:CASSFGDRGGTEAFF	47	CD4-C6
PB-1_AGAGCTTTCTAACGGT	PB	PB-1	P1-512	FALSE	1	TRA:CAEAEGGGADGLTF TRB:CSARGGAGTVRSETQYF	1	CD8-C2
PB-1_AGAGCTTTCTTACCGC	PB	PB-1	P1-13	TRUE	3	TRA:CVVMNYGGSQGNLIF TRB:CASSSWGDTDEAFF	112	CD8-C7
PB-1_AGAGTGGGTTAAGATG	PB	PB-1	P1-19	TRUE	3	TRA:CALGAFGSARQLTF TRB:CASSKGQGPRETQYF	70	CD4-C6
PB-1_AGAGTGGGTTTGTTTC	PB	PB-1	P1-36	TRUE	3	TRA:CILRDNAGNNRKLIW TRB:CASSSRQGFPGGANVLTF	40	CD4-C6
PB-1_AGAGTGGTCACAAACC	PB	PB-1	P1-17	TRUE	3	TRA:CGTGARGSSGSRLTF TRB:CASSRQGMGAFF	78	CD4-C6
PB-1_AGAGTGGTCGGATGGA	PB	PB-1	P1-41	TRUE	3	TRA:CVVSEPPGGSYIPTF TRB:CASSSRPEDTQYF	27	CD4-C6
PB-1_AGATCTGCAGGGTTAG	PB	PB-1	P1-25	TRUE	3	TRA:CIVGRDSSYKLIF TRB:CASNPQRGAHRDTQYF	58	CD4-C6
PB-1_AGATTGCCACAGAGGT	PB	PB-1	P1-48	TRUE	3	TRA:CAEVSGYSTLTF TRB:CASSFPTGGRTGRTEAFF	16	CD4-C6
PB-1_AGATTGCGTATATCCG	PB	PB-1	P1-19	TRUE	3	TRA:CALGAFGSARQLTF TRB:CASSKGQGPRETQYF	70	CD4-C6
PB-1_AGATTGCGTCCAGTAT	PB	PB-1	P1-25	TRUE	3	TRA:CIVGRDSSYKLIF TRB:CASNPQRGAHRDTQYF	58	CD4-C6
PB-1_AGCAGCCGTAATCGTC	PB	PB-1	P1-25	TRUE	3	TRA:CIVGRDSSYKLIF TRB:CASNPQRGAHRDTQYF	58	CD4-C6
PB-1_AGCAGCCTCAGAGCTT	PB	PB-1	P1-515	FALSE	1	TRA:CALVDFSGYSTLTF TRB:CASSTTGDFNYGYTF	1	CD4-C6
PB-1_AGCAGCCTCGTTTAGG	PB	PB-1	P1-19	TRUE	3	TRA:CALGAFGSARQLTF TRB:CASSKGQGPRETQYF	70	CD4-C6
PB-1_AGCAGCCTCTTTAGTC	PB	PB-1	P1-27	TRUE	3	TRA:CALVDFSGYSTLTF TRB:CASSGRGTNEKLFF	55	CD4-C6
PB-1_AGCCTAACAACTGGCC	PB	PB-1	P1-518	FALSE	1	TRA:CAESPTGGGNKLTF TRB:CASSVVAGGHNEQFF	1	CD4-C7
PB-1_AGCGGTCTCAACCATG	PB	PB-1	P1-24	TRUE	3	TRA:CAVKGNTDKLIF TRB:CASSLRTGAYNEQFF	62	CD4-C6
PB-1_AGCGGTCTCACCGGGT	PB	PB-1	P1-27	TRUE	3	TRA:CALVDFSGYSTLTF TRB:CASSGRGTNEKLFF	55	CD4-C6
PB-1_AGCTTGACAGGACGTA	PB	PB-1	P1-27	TRUE	3	TRA:CALVDFSGYSTLTF TRB:CASSGRGTNEKLFF	55	CD4-C6
PB-1_AGGCCGTTCATGCTCC	PB	PB-1	P1-25	TRUE	3	TRA:CIVGRDSSYKLIF TRB:CASNPQRGAHRDTQYF	58	CD4-C6
PB-1_AGGTCATGTGGAAAGA	PB	PB-1	P1-24	TRUE	3	TRA:CAVKGNTDKLIF TRB:CASSLRTGAYNEQFF	62	CD8-C7
PB-1_AGGTCCGGTTCAGACT	PB	PB-1	P1-25	TRUE	3	TRA:CIVGRDSSYKLIF TRB:CASNPQRGAHRDTQYF	58	CD4-C6
PB-1_AGTAGTCAGGATTCGG	PB	PB-1	P1-19	TRUE	3	TRA:CALGAFGSARQLTF TRB:CASSKGQGPRETQYF	70	CD4-C6
PB-1_AGTCTTTCAGATGGGT	PB	PB-1	P1-27	TRUE	3	TRA:CALVDFSGYSTLTF TRB:CASSGRGTNEKLFF	55	CD4-C6
PB-1_AGTGAGGCACTTAACG	PB	PB-1	P1-27	TRUE	3	TRA:CALVDFSGYSTLTF TRB:CASSGRGTNEKLFF	55	CD4-C6
PB-1_AGTGAGGTCATGTCTT	PB	PB-1	P1-72	FALSE	3	TRA:CALPDTNAGKSTF TRB:CSAAQGAYQPQHF	8	CD4-C6
PB-1_AGTGGGAGTCAGCTAT	PB	PB-1	P1-10	TRUE	3	TRA:CISNSPMDSSYKLIF TRB:CASSSWGDTDEAFF	162	CD4-C6

PB-1_AGTGTCAAGAATTGTG	PB	PB-1	P1-30	TRUE	3	TRA:CALPPGGNNRKLIW TRB:CASSFGDRGGTEAFF	47	CD4-C6
PB-1_ATAACGCGTCACAAGG	PB	PB-1	P1-10	TRUE	3	TRA:CISNSPMDSSYKLIF TRB:CASSSWGDTDEAFF	162	CD4-C6
PB-1_ATAAGAGGTAAACCTC	PB	PB-1	P1-17	TRUE	3	TRA:CGTGARGSSGSRLTF TRB:CASSRQGMGAFF	78	CD8-C7
PB-1_ATAAGAGTCTTCATGT	PB	PB-1	P1-27	TRUE	3	TRA:CALVDFSGYSTLTF TRB:CASSGRGTNEKLFF	55	CD4-C6
PB-1_ATAGACCAGAGACGAA	PB	PB-1	P1-536	FALSE	1	TRA:CAVRRNSGNTPLVF TRB:CASSQSPGQRNYGYTF	1	CD4-C7
PB-1_ATAGACCAGCTGCGAA	PB	PB-1	P1-537	FALSE	1	TRA:CVVMNYGGSQGNLIF TRB:CASSARGVTYNEQFF	1	CD4-C6
PB-1_ATCATCTGTGAAATCA	PB	PB-1	P1-146	TRUE	3	TRA:CAMSDRMTGGFKTIF TRB:CASSLGQRGLQYF	4	CD4-C6
PB-1_ATCATGGCACGGCCAT	PB	PB-1	P1-21	TRUE	3	TRA:CAASRGTGANNLFF TRB:CATQREGREQYF	67	CD4-C6
PB-1_ATCATGGGTGAAATCA	PB	PB-1	P1-17	TRUE	3	TRA:CGTGARGSSGSRLTF TRB:CASSRQGMGAFF	78	CD4-C6
PB-1_ATCTACTAGGGTTTCT	PB	PB-1	P1-5	TRUE	3	TRA:CALSGTPMNRDDKIIF TRB:CSARGRGLAGGPAYEQYF	223	CD8-C2
PB-1_ATCTGCCCAAGAGTCG	PB	PB-1	P1-21	TRUE	3	TRA:CAASRGTGANNLFF TRB:CATQREGREQYF	67	CD4-C6
PB-1_ATGAGGGCACGCATCG	PB	PB-1	P1-25	TRUE	3	TRA:CIVGRDSSYKLIF TRB:CASNPQRGAHRDTQYF	58	CD4-C6
PB-1_ATGTGTGAGAGCAATT	PB	PB-1	P1-30	TRUE	3	TRA:CALPPGGNNRKLIW TRB:CASSFGDRGGTEAFF	47	CD4-C6
PB-1_ATGTGTGAGCTAGGCA	PB	PB-1	P1-186	TRUE	3	TRA:CAVPPYMDSNYQLIW TRB:CASSLFGASPLHF	3	CD4-C6
PB-1_ATTACTCCAAGCCATT	PB	PB-1	P1-30	TRUE	3	TRA:CALPPGGNNRKLIW TRB:CASSFGDRGGTEAFF	47	CD4-C6
PB-1_ATTACTCTCGGAATCT	PB	PB-1	P1-30	TRUE	3	TRA:CALPPGGNNRKLIW TRB:CASSFGDRGGTEAFF	47	CD4-C6
PB-1_ATTATCCCACCGAATT	PB	PB-1	P1-19	TRUE	3	TRA:CALGAFGSARQLTF TRB:CASSKGQGPRETQYF	70	CD4-C6
PB-1_ATTCTACTCTATCCTA	PB	PB-1	P1-48	TRUE	3	TRA:CAEVSGYSTLTF TRB:CASSFPTGGRTGRTEAFF	16	CD4-C6
PB-1_ATTGGTGCAGCCTTGG	PB	PB-1	P1-36	TRUE	3	TRA:CILRDNAGNNRKLIW TRB:CASSSRQGFPGGANVLTF	40	CD4-C6
PB-1_ATTGGTGCAGTTCCCT	PB	PB-1	P1-21	TRUE	3	TRA:CAASRGTGANNLFF TRB:CATQREGREQYF	67	CD4-C6
PB-1_ATTGGTGCATTGCGGC	PB	PB-1	P1-563	FALSE	1	TRA:CALVDFSGYSTLTF TRB:CASSRQGMGAFF	1	CD4-C6
PB-1_ATTGGTGGTCATGCCG	PB	PB-1	P1-2	TRUE	3	TRA:CGQNFVF TRB:CASNQRGGPSLYNEQFF	323	CD8-C7
PB-1_ATTTCTGAGAAGGACA	PB	PB-1	P1-27	TRUE	3	TRA:CALVDFSGYSTLTF TRB:CASSGRGTNEKLFF	55	CD4-C6
PB-1_CAACCAACAGGGAGAG	PB	PB-1	P1-21	TRUE	3	TRA:CAASRGTGANNLFF TRB:CATQREGREQYF	67	CD8-C7
PB-1_CAACCTCTCAGGTTCA	PB	PB-1	P1-24	TRUE	3	TRA:CAVKGNTDKLIF TRB:CASSLRTGAYNEQFF	62	CD4-C6
PB-1_CAACCTCTCTCAAGTG	PB	PB-1	P1-25	TRUE	3	TRA:CIVGRDSSYKLIF TRB:CASNPQRGAHRDTQYF	58	CD4-C6
PB-1_CAACTAGGTCGTCTTC	PB	PB-1	P1-13	TRUE	3	TRA:CVVMNYGGSQGNLIF TRB:CASSSWGDTDEAFF	112	CD8-C7

PB-1_CAAGAAATCGGCGCTA	PB	PB-1	P1-24	TRUE	3	TRA:CAVKGNTDKLIF TRB:CASSLRTGAYNEQFF	62	CD4-C6
PB-1_CAAGGCCCACAGGTTT	PB	PB-1	P1-19	TRUE	3	TRA:CALGAFGSARQLTF TRB:CASSKGQGPRETQYF	70	CD4-C6
PB-1_CAAGGCCGTACTTGAC	PB	PB-1	P1-2	TRUE	3	TRA:CGQNFVF TRB:CASNQRGGPSLYNEQFF	323	CD4-C6
PB-1_CACAAACTCTCCCTGA	PB	PB-1	P1-19	TRUE	3	TRA:CALGAFGSARQLTF TRB:CASSKGQGPRETQYF	70	CD4-C6
PB-1_CACACAAAGTGGTAAT	PB	PB-1	P1-27	TRUE	3	TRA:CALVDFSGYSTLTF TRB:CASSGRGTNEKLFF	55	CD4-C6
PB-1_CACACCTTCGCGGATC	PB	PB-1	P1-580	FALSE	1	TRA:CLVVELTGGGNKLTF TRB:CASSRNPGVNTEAFF	1	CD8-C7
PB-1_CACAGGCAGTGTCCCG	PB	PB-1	P1-2	TRUE	3	TRA:CGQNFVF TRB:CASNQRGGPSLYNEQFF	323	CD8-C7
PB-1_CACAGGCGTCCATCCT	PB	PB-1	P1-108	TRUE	3	TRA:CAASNGDTGNQFYF TRB:CSASKQGRNQPQHF	5	CD4-C6
PB-1_CACAGGCTCTTTACAC	PB	PB-1	P1-30	TRUE	3	TRA:CALPPGGNNRKLIW TRB:CASSFGDRGGTEAFF	47	CD4-C6
PB-1_CACAGTAGTAGCGATG	PB	PB-1	P1-19	TRUE	3	TRA:CALGAFGSARQLTF TRB:CASSKGQGPRETQYF	70	CD4-C6
PB-1_CACAGTATCACCGTAA	PB	PB-1	P1-19	TRUE	3	TRA:CALGAFGSARQLTF TRB:CASSKGQGPRETQYF	70	CD4-C6
PB-1_CACATAGGTAGTAGTA	PB	PB-1	P1-27	TRUE	3	TRA:CALVDFSGYSTLTF TRB:CASSGRGTNEKLFF	55	CD4-C6
PB-1_CACCACTCAGCTCGCA	PB	PB-1	P1-36	TRUE	3	TRA:CILRDNAGNNRKLIW TRB:CASSSRQGFPGGANVLTF	40	CD4-C6
PB-1_CACCAGGCAAGAAGAG	PB	PB-1	P1-111	TRUE	3	TRA:CALSPYGGSQGNLIF TRB:CASSWNLGQAYEQYF	5	CD4-C6
PB-1_CACTCCATCCGCGCAA	PB	PB-1	P1-27	TRUE	3	TRA:CALVDFSGYSTLTF TRB:CASSGRGTNEKLFF	55	CD4-C6
PB-1_CAGAGAGCACGGCTAC	PB	PB-1	P1-30	TRUE	3	TRA:CALPPGGNNRKLIW TRB:CASSFGDRGGTEAFF	47	CD4-C6
PB-1_CAGATCAGTATATGGA	PB	PB-1	P1-36	TRUE	3	TRA:CILRDNAGNNRKLIW TRB:CASSSRQGFPGGANVLTF	40	CD4-C6
PB-1_CAGATCAGTTGGACCC	PB	PB-1	P1-25	TRUE	3	TRA:CIVGRDSSYKLIF TRB:CASNPQRGAHRDTQYF	58	CD4-C6
PB-1_CAGCAGCTCGCTAGCG	PB	PB-1	P1-19	TRUE	3	TRA:CALGAFGSARQLTF TRB:CASSKGQGPRETQYF	70	CD4-C6
PB-1_CAGCCGATCAGGTAAA	PB	PB-1	P1-598	FALSE	1	TRA:CALKHFGNEKLTF TRB:CASSEMRGRETQYF	1	CD8-C6
PB-1_CAGCCGATCCTCAATT	PB	PB-1	P1-41	TRUE	3	TRA:CVVSEPPGGSYIPTF TRB:CASSSRPEDTQYF	27	CD4-C6
PB-1_CAGTCCTAGAGCTGGT	PB	PB-1	P1-19	TRUE	3	TRA:CALGAFGSARQLTF TRB:CASSKGQGPRETQYF	70	CD4-C6
PB-1_CATATTCAGCAGATCG	PB	PB-1	P1-25	TRUE	3	TRA:CIVGRDSSYKLIF TRB:CASNPQRGAHRDTQYF	58	CD4-C6
PB-1_CATCAAGGTGGTTTCA	PB	PB-1	P1-36	TRUE	3	TRA:CILRDNAGNNRKLIW TRB:CASSSRQGFPGGANVLTF	40	CD4-C6
PB-1_CATCAGAGTCGGATCC	PB	PB-1	P1-24	TRUE	3	TRA:CAVKGNTDKLIF TRB:CASSLRTGAYNEQFF	62	CD4-C6
PB-1_CATCGAATCACAGGCC	PB	PB-1	P1-27	TRUE	3	TRA:CALVDFSGYSTLTF TRB:CASSGRGTNEKLFF	55	CD4-C6
PB-1_CATCGAATCATTGCCC	PB	PB-1	P1-72	FALSE	3	TRA:CALPDTNAGKSTF TRB:CSAAQGAYQPQHF	8	CD4-C6

PB-1_CATCGGGGTATGAATG	PB	PB-1	P1-19	TRUE	3	TRA:CALGAFGSARQLTF TRB:CASSKGQGPRETQYF	70	CD4-C6
PB-1_CATGACATCTTAGAGC	PB	PB-1	P1-10	TRUE	3	TRA:CISNSPMDSSYKLIF TRB:CASSSWGDTDEAFF	162	CD8-C7
PB-1_CATGCCTCATTTGCTT	PB	PB-1	P1-17	TRUE	3	TRA:CGTGARGSSGSRLTF TRB:CASSRQGMGAFF	78	CD4-C6
PB-1_CATGCCTTCGCCGTGA	PB	PB-1	P1-17	TRUE	3	TRA:CGTGARGSSGSRLTF TRB:CASSRQGMGAFF	78	CD4-C6
PB-1_CATGCCTTCTCGGACG	PB	PB-1	P1-17	TRUE	3	TRA:CGTGARGSSGSRLTF TRB:CASSRQGMGAFF	78	CD8-C7
PB-1_CATTCGCAGGAGTACC	PB	PB-1	P1-111	TRUE	3	TRA:CALSPYGGSQGNLIF TRB:CASSWNLGQAYEQYF	5	CD4-C3
PB-1_CCAATCCGTGCTCTTC	PB	PB-1	P1-24	TRUE	3	TRA:CAVKGNTDKLIF TRB:CASSLRTGAYNEQFF	62	CD4-C6
PB-1_CCAATCCTCTTATCTG	PB	PB-1	P1-41	TRUE	3	TRA:CVVSEPPGGSYIPTF TRB:CASSSRPEDTQYF	27	CD4-C6
PB-1_CCAATCCTCTTCGAGA	PB	PB-1	P1-280	FALSE	2	TRA:CIVGRDSSYKLIF TRB:CASSLGQRGLQYF	2	CD4-C6
PB-1_CCACCTACATCGTCGG	PB	PB-1	P1-42	TRUE	3	TRA:CASFSGGSQGNLIF TRB:CSAGQGSNQPQHF	24	CD4-C6
PB-1_CCACCTAGTGTCTGAT	PB	PB-1	P1-27	TRUE	3	TRA:CALVDFSGYSTLTF TRB:CASSGRGTNEKLFF	55	CD4-C6
PB-1_CCACGGAAGAATCTCC	PB	PB-1	P1-25	TRUE	3	TRA:CIVGRDSSYKLIF TRB:CASNPQRGAHRDTQYF	58	CD4-C6
PB-1_CCACGGAAGTGTCTCA	PB	PB-1	P1-21	TRUE	3	TRA:CAASRGTGANNLFF TRB:CATQREGREQYF	67	CD4-C6
PB-1_CCACGGACAAGGTTCT	PB	PB-1	P1-30	TRUE	3	TRA:CALPPGGNNRKLIW TRB:CASSFGDRGGTEAFF	47	CD4-C6
PB-1_CCACTACAGAGACGAA	PB	PB-1	P1-5	TRUE	3	TRA:CALSGTPMNRDDKIIF TRB:CSARGRGLAGGPAYEQYF	223	CD8-C7
PB-1_CCACTACCACAGCGTC	PB	PB-1	P1-21	TRUE	3	TRA:CAASRGTGANNLFF TRB:CATQREGREQYF	67	CD4-C6
PB-1_CCACTACTCCGGGTGT	PB	PB-1	P1-72	FALSE	3	TRA:CALPDTNAGKSTF TRB:CSAAQGAYQPQHF	8	CD4-C6
PB-1_CCAGCGAGTACAGTGG	PB	PB-1	P1-643	FALSE	1	TRA:CAVNNAGNMLTF TRB:CASSLRGAHQPQHF	1	CD8-C6
PB-1_CCAGCGAGTATATGAG	PB	PB-1	P1-19	TRUE	3	TRA:CALGAFGSARQLTF TRB:CASSKGQGPRETQYF	70	CD4-C6
PB-1_CCATTCGGTTCTGAAC	PB	PB-1	P1-30	TRUE	3	TRA:CALPPGGNNRKLIW TRB:CASSFGDRGGTEAFF	47	CD4-C6
PB-1_CCCTCCTGTTACGTCA	PB	PB-1	P1-10	TRUE	3	TRA:CISNSPMDSSYKLIF TRB:CASSSWGDTDEAFF	162	CD8-C7
PB-1_CCGGGATTCGTATCAG	PB	PB-1	P1-21	TRUE	3	TRA:CAASRGTGANNLFF TRB:CATQREGREQYF	67	CD4-C6
PB-1_CCGGTAGGTGTGACCC	PB	PB-1	P1-10	TRUE	3	TRA:CISNSPMDSSYKLIF TRB:CASSSWGDTDEAFF	162	CD4-C6
PB-1_CCGTACTGTAAACGCG	PB	PB-1	P1-30	TRUE	3	TRA:CALPPGGNNRKLIW TRB:CASSFGDRGGTEAFF	47	CD4-C6
PB-1_CCGTGGATCTCTGAGA	PB	PB-1	P1-19	TRUE	3	TRA:CALGAFGSARQLTF TRB:CASSKGQGPRETQYF	70	CD4-C6
PB-1_CCGTTCACAATGGAGC	PB	PB-1	P1-17	TRUE	3	TRA:CGTGARGSSGSRLTF TRB:CASSRQGMGAFF	78	CD4-C6
PB-1_CCGTTCACAGTTCATG	PB	PB-1	P1-654	FALSE	1	TRA:CGQNFVF TRB:CASSLGQRGLQYF	1	CD4-C6

PB-1_CCGTTCATCAAGGCTT	PB	PB-1	P1-2	TRUE	3	TRA:CGQNFVF TRB:CASNQRGGPSLYNEQFF	323	CD4-C6
PB-1_CCTAAAGTCTTCAACT	PB	PB-1	P1-24	TRUE	3	TRA:CAVKGNTDKLIF TRB:CASSLRTGAYNEQFF	62	CD4-C6
PB-1_CCTACACTCTGTCCGT	PB	PB-1	P1-655	FALSE	1	TRA:CAVSLQGGSEKLVF TRB:CASSLAGWGEYF	1	CD8-C6
PB-1_CCTACCAAGACATAAC	PB	PB-1	P1-25	TRUE	3	TRA:CIVGRDSSYKLIF TRB:CASNPQRGAHRDTQYF	58	CD4-C6
PB-1_CCTACCAGTAAGGGCT	PB	PB-1	P1-21	TRUE	3	TRA:CAASRGTGANNLFF TRB:CATQREGREQYF	67	CD4-C6
PB-1_CCTACCATCGTTTATC	PB	PB-1	P1-25	TRUE	3	TRA:CIVGRDSSYKLIF TRB:CASNPQRGAHRDTQYF	58	CD4-C6
PB-1_CCTATTACACCATGTA	PB	PB-1	P1-19	TRUE	3	TRA:CALGAFGSARQLTF TRB:CASSKGQGPRETQYF	70	CD4-C6
PB-1_CCTATTATCGCACTCT	PB	PB-1	P1-17	TRUE	3	TRA:CGTGARGSSGSRLTF TRB:CASSRQGMGAFF	78	CD4-C6
PB-1_CCTCTGATCAGCTGGC	PB	PB-1	P1-17	TRUE	3	TRA:CGTGARGSSGSRLTF TRB:CASSRQGMGAFF	78	CD8-C7
PB-1_CCTTCCCCATCCGGGT	PB	PB-1	P1-25	TRUE	3	TRA:CIVGRDSSYKLIF TRB:CASNPQRGAHRDTQYF	58	CD4-C6
PB-1_CCTTCCCCATCGGACC	PB	PB-1	P1-30	TRUE	3	TRA:CALPPGGNNRKLIW TRB:CASSFGDRGGTEAFF	47	CD4-C6
PB-1_CCTTCGACAATCGGTT	PB	PB-1	P1-30	TRUE	3	TRA:CALPPGGNNRKLIW TRB:CASSFGDRGGTEAFF	47	CD4-C6
PB-1_CCTTCGACATACTACG	PB	PB-1	P1-19	TRUE	3	TRA:CALGAFGSARQLTF TRB:CASSKGQGPRETQYF	70	CD4-C6
PB-1_CCTTTCTGTCAGGACA	PB	PB-1	P1-24	TRUE	3	TRA:CAVKGNTDKLIF TRB:CASSLRTGAYNEQFF	62	CD4-C6
PB-1_CCTTTCTTCCGAATGT	PB	PB-1	P1-30	TRUE	3	TRA:CALPPGGNNRKLIW TRB:CASSFGDRGGTEAFF	47	CD4-C6
PB-1_CGAACATTCTCAAGTG	PB	PB-1	P1-19	TRUE	3	TRA:CALGAFGSARQLTF TRB:CASSKGQGPRETQYF	70	CD4-C6
PB-1_CGAATGTCATCGATTG	PB	PB-1	P1-2	TRUE	3	TRA:CGQNFVF TRB:CASNQRGGPSLYNEQFF	323	CD4-C6
PB-1_CGAATGTTCTCGATGA	PB	PB-1	P1-21	TRUE	3	TRA:CAASRGTGANNLFF TRB:CATQREGREQYF	67	CD4-C6
PB-1_CGACCTTCAAGTTAAG	PB	PB-1	P1-25	TRUE	3	TRA:CIVGRDSSYKLIF TRB:CASNPQRGAHRDTQYF	58	CD4-C6
PB-1_CGACCTTTCCTTTACA	PB	PB-1	P1-281	TRUE	2	TRA:CVVNMRFTGGGNKLTF TRB:CASSPQGGTSTGELFF	2	CD4-C6
PB-1_CGACTTCGTCGGCACT	PB	PB-1	P1-42	TRUE	3	TRA:CASFSGGSQGNLIF TRB:CSAGQGSNQPQHF	24	CD4-C6
PB-1_CGACTTCGTGTATGGG	PB	PB-1	P1-30	TRUE	3	TRA:CALPPGGNNRKLIW TRB:CASSFGDRGGTEAFF	47	CD4-C6
PB-1_CGAGCCATCGCCATAA	PB	PB-1	P1-19	TRUE	3	TRA:CALGAFGSARQLTF TRB:CASSKGQGPRETQYF	70	CD4-C6
PB-1_CGATCGGCACCGAAAG	PB	PB-1	P1-676	FALSE	1	TRA:CAASCPVGFGNEKLTF TRB:CASSQTNTEAFF	1	CD8-C2
PB-1_CGATCGGCACGCGAAA	PB	PB-1	P1-17	TRUE	3	TRA:CGTGARGSSGSRLTF TRB:CASSRQGMGAFF	78	CD4-C6
PB-1_CGATCGGCAGTATAAG	PB	PB-1	P1-151	TRUE	3	TRA:CAGTNSGYSTLTF TRB:CASSPKESGQTFQETQYF	4	CD4-C6
PB-1_CGATCGGGTTTGGGCC	PB	PB-1	P1-2	TRUE	3	TRA:CGQNFVF TRB:CASNQRGGPSLYNEQFF	323	CD4-C6

PB-1_CGATGGCCATTAACCG	PB	PB-1	P1-30	TRUE	3	TRA:CALPPGGNNRKLIW TRB:CASSFGDRGGTEAFF	47	CD8-C7
PB-1_CGATGGCTCGCACTCT	PB	PB-1	P1-21	TRUE	3	TRA:CAASRGTGANNLFF TRB:CATQREGREQYF	67	CD4-C6
PB-1_CGATGTAGTCCGAACC	PB	PB-1	P1-41	TRUE	3	TRA:CVVSEPPGGSYIPTF TRB:CASSSRPEDTQYF	27	CD4-C6
PB-1_CGATTGAGTGAGTATA	PB	PB-1	P1-21	TRUE	3	TRA:CAASRGTGANNLFF TRB:CATQREGREQYF	67	CD4-C6
PB-1_CGCGGTACAAACGTGG	PB	PB-1	P1-19	TRUE	3	TRA:CALGAFGSARQLTF TRB:CASSKGQGPRETQYF	70	CD4-C6
PB-1_CGCGGTAGTCTAAAGA	PB	PB-1	P1-25	TRUE	3	TRA:CIVGRDSSYKLIF TRB:CASNPQRGAHRDTQYF	58	CD4-C6
PB-1_CGCTATCCATGGTCAT	PB	PB-1	P1-2	TRUE	3	TRA:CGQNFVF TRB:CASNQRGGPSLYNEQFF	323	CD4-C6
PB-1_CGCTATCGTGCTGTAT	PB	PB-1	P1-21	TRUE	3	TRA:CAASRGTGANNLFF TRB:CATQREGREQYF	67	CD4-C6
PB-1_CGCTATCTCCTGTAGA	PB	PB-1	P1-17	TRUE	3	TRA:CGTGARGSSGSRLTF TRB:CASSRQGMGAFF	78	CD8-C7
PB-1_CGCTGGACATGAAGTA	PB	PB-1	P1-27	TRUE	3	TRA:CALVDFSGYSTLTF TRB:CASSGRGTNEKLFF	55	CD4-C6
PB-1_CGCTTCATCGGTGTCG	PB	PB-1	P1-696	FALSE	1	TRA:CAMSDRMTGGFKTIF TRB:CSAMDMLNTEAFF	1	CD4-C7
PB-1_CGGACACAGCTGCCCA	PB	PB-1	P1-25	TRUE	3	TRA:CIVGRDSSYKLIF TRB:CASNPQRGAHRDTQYF	58	CD4-C6
PB-1_CGGACACAGTACGACG	PB	PB-1	P1-25	TRUE	3	TRA:CIVGRDSSYKLIF TRB:CASNPQRGAHRDTQYF	58	CD4-C6
PB-1_CGGACACTCGAATGCT	PB	PB-1	P1-36	TRUE	3	TRA:CILRDNAGNNRKLIW TRB:CASSSRQGFPGGANVLTF	40	CD4-C6
PB-1_CGGACGTCACGTAAGG	PB	PB-1	P1-19	TRUE	3	TRA:CALGAFGSARQLTF TRB:CASSKGQGPRETQYF	70	CD4-C6
PB-1_CGGACTGGTCAAACTC	PB	PB-1	P1-701	FALSE	1	TRA:CAVSANTGGFKTIF TRB:CASSRRGVTDTQYF	1	CD8-C2
PB-1_CGGAGCTCAGACGCAA	PB	PB-1	P1-21	TRUE	3	TRA:CAASRGTGANNLFF TRB:CATQREGREQYF	67	CD4-C6
PB-1_CGGAGTCCAGACAAGC	PB	PB-1	P1-19	TRUE	3	TRA:CALGAFGSARQLTF TRB:CASSKGQGPRETQYF	70	CD4-C6
PB-1_CGGTTAAGTCATATCG	PB	PB-1	P1-19	TRUE	3	TRA:CALGAFGSARQLTF TRB:CASSKGQGPRETQYF	70	CD4-C6
PB-1_CGGTTAAGTTATCGGT	PB	PB-1	P1-19	TRUE	3	TRA:CALGAFGSARQLTF TRB:CASSKGQGPRETQYF	70	CD4-C6
PB-1_CGGTTAATCAACGCTA	PB	PB-1	P1-17	TRUE	3	TRA:CGTGARGSSGSRLTF TRB:CASSRQGMGAFF	78	CD8-C7
PB-1_CGTAGCGAGGCCCTTG	PB	PB-1	P1-21	TRUE	3	TRA:CAASRGTGANNLFF TRB:CATQREGREQYF	67	CD4-C6
PB-1_CGTAGCGTCATTGCGA	PB	PB-1	P1-108	TRUE	3	TRA:CAASNGDTGNQFYF TRB:CSASKQGRNQPQHF	5	CD4-C6
PB-1_CGTAGCGTCCAAAGTC	PB	PB-1	P1-27	TRUE	3	TRA:CALVDFSGYSTLTF TRB:CASSGRGTNEKLFF	55	CD4-C6
PB-1_CGTAGGCCACGCTTTC	PB	PB-1	P1-19	TRUE	3	TRA:CALGAFGSARQLTF TRB:CASSKGQGPRETQYF	70	CD4-C6
PB-1_CGTAGGCTCGGAAACG	PB	PB-1	P1-19	TRUE	3	TRA:CALGAFGSARQLTF TRB:CASSKGQGPRETQYF	70	CD4-C6
PB-1_CGTCACTAGATGGGTC	PB	PB-1	P1-24	TRUE	3	TRA:CAVKGNTDKLIF TRB:CASSLRTGAYNEQFF	62	CD4-C6

PB-1_CGTCACTGTCGAGTTT	PB	PB-1	P1-111	TRUE	3	TRA:CALSPYGGSQGNLIF TRB:CASSWNLGQAYEQYF	5	CD4-C6
PB-1_CGTCAGGCAAAGCAAT	PB	PB-1	P1-27	TRUE	3	TRA:CALVDFSGYSTLTF TRB:CASSGRGTNEKLFF	55	CD4-C6
PB-1_CGTCCATTCCATGAGT	PB	PB-1	P1-712	FALSE	1	TRA:CALTMDSSYKLIF TRB:CASSLSHQGSPLHF	1	CD4-C6
PB-1_CGTGAGCTCGTTACAG	PB	PB-1	P1-716	FALSE	1	TRA:CAMREGRERDDKIIF TRB:CASSFPTGGRTGRTEAFF	1	CD4-C6
PB-1_CGTGTCTCACATCTTT	PB	PB-1	P1-24	TRUE	3	TRA:CAVKGNTDKLIF TRB:CASSLRTGAYNEQFF	62	CD4-C6
PB-1_CGTGTCTTCCGTTGTC	PB	PB-1	P1-3	TRUE	3	TRA:CAFLSGTYKYIF TRB:CASSQSPGQRNYGYTF	308	CD8-C7
PB-1_CGTTCTGTCGAGAACG	PB	PB-1	P1-25	TRUE	3	TRA:CIVGRDSSYKLIF TRB:CASNPQRGAHRDTQYF	58	CD4-C6
PB-1_CTAACTTAGCTCTCGG	PB	PB-1	P1-10	TRUE	3	TRA:CISNSPMDSSYKLIF TRB:CASSSWGDTDEAFF	162	CD8-C7
PB-1_CTACACCAGATATGGT	PB	PB-1	P1-724	FALSE	1	TRA:CAACGSNDYKLSF TRB:CAIKGQGAQGYTF	1	CD4-C6
PB-1_CTACACCCACCAACCG	PB	PB-1	P1-19	TRUE	3	TRA:CALGAFGSARQLTF TRB:CASSKGQGPRETQYF	70	CD4-C6
PB-1_CTACGTCAGACTCGGA	PB	PB-1	P1-24	TRUE	3	TRA:CAVKGNTDKLIF TRB:CASSLRTGAYNEQFF	62	CD4-C6
PB-1_CTAGAGTAGGAGCGAG	PB	PB-1	P1-27	TRUE	3	TRA:CALVDFSGYSTLTF TRB:CASSGRGTNEKLFF	55	CD4-C5
PB-1_CTAGCCTCATAAGACA	PB	PB-1	P1-25	TRUE	3	TRA:CIVGRDSSYKLIF TRB:CASNPQRGAHRDTQYF	58	CD4-C6
PB-1_CTCACACCAAACTGCT	PB	PB-1	P1-27	TRUE	3	TRA:CALVDFSGYSTLTF TRB:CASSGRGTNEKLFF	55	CD4-C6
PB-1_CTCAGAACAGACGTAG	PB	PB-1	P1-19	TRUE	3	TRA:CALGAFGSARQLTF TRB:CASSKGQGPRETQYF	70	CD4-C6
PB-1_CTCAGAATCTAACTCT	PB	PB-1	P1-24	TRUE	3	TRA:CAVKGNTDKLIF TRB:CASSLRTGAYNEQFF	62	CD4-C6
PB-1_CTCGAAACAAAGTCAA	PB	PB-1	P1-21	TRUE	3	TRA:CAASRGTGANNLFF TRB:CATQREGREQYF	67	CD4-C6
PB-1_CTCGAGGAGTACGTAA	PB	PB-1	P1-17	TRUE	3	TRA:CGTGARGSSGSRLTF TRB:CASSRQGMGAFF	78	CD4-C6
PB-1_CTCGAGGGTAGTACCT	PB	PB-1	P1-30	TRUE	3	TRA:CALPPGGNNRKLIW TRB:CASSFGDRGGTEAFF	47	CD4-C6
PB-1_CTCGTACTCCGTCATC	PB	PB-1	P1-27	TRUE	3	TRA:CALVDFSGYSTLTF TRB:CASSGRGTNEKLFF	55	CD4-C6
PB-1_CTCGTCATCCAAGTAC	PB	PB-1	P1-107	TRUE	3	TRA:CALSDKEKLTF TRB:CAISEGGGVGYTF	5	CD4-C6
PB-1_CTCTGGTCAAAGCAAT	PB	PB-1	P1-24	TRUE	3	TRA:CAVKGNTDKLIF TRB:CASSLRTGAYNEQFF	62	CD4-C6
PB-1_CTCTGGTCAATGCCAT	PB	PB-1	P1-19	TRUE	3	TRA:CALGAFGSARQLTF TRB:CASSKGQGPRETQYF	70	CD4-C6
PB-1_CTGAAACAGAACAACT	PB	PB-1	P1-21	TRUE	3	TRA:CAASRGTGANNLFF TRB:CATQREGREQYF	67	CD4-C6
PB-1_CTGAAACAGTACGTAA	PB	PB-1	P1-750	FALSE	1	TRA:CALVDFSGYSTLTF TRB:CASSQVGRGGEETQYF	1	CD4-C6
PB-1_CTGATCCGTGGTCCGT	PB	PB-1	P1-21	TRUE	3	TRA:CAASRGTGANNLFF TRB:CATQREGREQYF	67	CD8-C7
PB-1_CTGCCTAGTAGAGCTG	PB	PB-1	P1-42	TRUE	3	TRA:CASFSGGSQGNLIF TRB:CSAGQGSNQPQHF	24	CD4-C3

PB-1_CTGCGGAGTTCAACCA	PB	PB-1	P1-25	TRUE	3	TRA:CIVGRDSSYKLIF TRB:CASNPQRGAHRDTQYF	58	CD4-C6
PB-1_CTGCGGATCACTGGGC	PB	PB-1	P1-10	TRUE	3	TRA:CISNSPMDSSYKLIF TRB:CASSSWGDTDEAFF	162	CD8-C7
PB-1_CTGCGGATCTTGTTTG	PB	PB-1	P1-24	TRUE	3	TRA:CAVKGNTDKLIF TRB:CASSLRTGAYNEQFF	62	CD4-C6
PB-1_CTGCTGTGTAAACGCG	PB	PB-1	P1-758	FALSE	1	TRA:CIVRVTTGNQFYF TRB:CASSLVSTTDTQYF	1	CD4-C7
PB-1_CTGCTGTGTGTGGCTC	PB	PB-1	P1-25	TRUE	3	TRA:CIVGRDSSYKLIF TRB:CASNPQRGAHRDTQYF	58	CD4-C6
PB-1_CTGCTGTTCGGTCCGA	PB	PB-1	P1-2	TRUE	3	TRA:CGQNFVF TRB:CASNQRGGPSLYNEQFF	323	CD8-C7
PB-1_CTGTGCTGTCCTGCTT	PB	PB-1	P1-27	TRUE	3	TRA:CALVDFSGYSTLTF TRB:CASSGRGTNEKLFF	55	CD4-C6
PB-1_CTTAACTGTTCCGGCA	PB	PB-1	P1-17	TRUE	3	TRA:CGTGARGSSGSRLTF TRB:CASSRQGMGAFF	78	CD4-C6
PB-1_CTTCTCTAGACCCACC	PB	PB-1	P1-196	TRUE	3	TRA:CAVESPGRGGATNKLIF TRB:CASSARGVTYNEQFF	3	CD4-C6
PB-1_GAAACTCGTCTGCAAT	PB	PB-1	P1-27	TRUE	3	TRA:CALVDFSGYSTLTF TRB:CASSGRGTNEKLFF	55	CD4-C6
PB-1_GAAACTCTCAAACCGT	PB	PB-1	P1-48	TRUE	3	TRA:CAEVSGYSTLTF TRB:CASSFPTGGRTGRTEAFF	16	CD4-C4
PB-1_GAAACTCTCTGCCCTA	PB	PB-1	P1-19	TRUE	3	TRA:CALGAFGSARQLTF TRB:CASSKGQGPRETQYF	70	CD4-C6
PB-1_GAAATGAAGCGATCCC	PB	PB-1	P1-25	TRUE	3	TRA:CIVGRDSSYKLIF TRB:CASNPQRGAHRDTQYF	58	CD4-C6
PB-1_GAACATCAGGGAACGG	PB	PB-1	P1-21	TRUE	3	TRA:CAASRGTGANNLFF TRB:CATQREGREQYF	67	CD4-C6
PB-1_GAACATCAGTCTTGCA	PB	PB-1	P1-27	TRUE	3	TRA:CALVDFSGYSTLTF TRB:CASSGRGTNEKLFF	55	CD4-C6
PB-1_GAACGGACATGAACCT	PB	PB-1	P1-72	FALSE	3	TRA:CALPDTNAGKSTF TRB:CSAAQGAYQPQHF	8	CD4-C6
PB-1_GAACGGATCAATCACG	PB	PB-1	P1-24	TRUE	3	TRA:CAVKGNTDKLIF TRB:CASSLRTGAYNEQFF	62	CD4-C6
PB-1_GAAGCAGCATCCGTGG	PB	PB-1	P1-280	FALSE	2	TRA:CIVGRDSSYKLIF TRB:CASSLGQRGLQYF	2	CD4-C6
PB-1_GAATAAGCACACATGT	PB	PB-1	P1-19	TRUE	3	TRA:CALGAFGSARQLTF TRB:CASSKGQGPRETQYF	70	CD4-C6
PB-1_GAATAAGCAGTCTTCC	PB	PB-1	P1-24	TRUE	3	TRA:CAVKGNTDKLIF TRB:CASSLRTGAYNEQFF	62	CD4-C6
PB-1_GACACGCGTAATCACC	PB	PB-1	P1-19	TRUE	3	TRA:CALGAFGSARQLTF TRB:CASSKGQGPRETQYF	70	CD4-C6
PB-1_GACACGCGTTAAGAAC	PB	PB-1	P1-788	FALSE	1	TRA:CALAGGYNKLIF TRB:CSAQQGSGNQPQHF	1	CD4-C6
PB-1_GACCAATAGTTCGCAT	PB	PB-1	P1-10	TRUE	3	TRA:CISNSPMDSSYKLIF TRB:CASSSWGDTDEAFF	162	CD8-C7
PB-1_GACCAATTCCAGATCA	PB	PB-1	P1-30	TRUE	3	TRA:CALPPGGNNRKLIW TRB:CASSFGDRGGTEAFF	47	CD4-C6
PB-1_GACCTGGGTTCAACCA	PB	PB-1	P1-36	TRUE	3	TRA:CILRDNAGNNRKLIW TRB:CASSSRQGFPGGANVLTF	40	CD4-C6
PB-1_GACGCGTCATCCCACT	PB	PB-1	P1-17	TRUE	3	TRA:CGTGARGSSGSRLTF TRB:CASSRQGMGAFF	78	CD4-C6
PB-1_GACGCGTTCTCCAGGG	PB	PB-1	P1-24	TRUE	3	TRA:CAVKGNTDKLIF TRB:CASSLRTGAYNEQFF	62	CD4-C6

PB-1_GACGGCTAGTTCGCGC	PB	PB-1	P1-17	TRUE	3	TRA:CGTGARGSSGSRLTF TRB:CASSRQGMGAFF	78	CD4-C6
PB-1_GACGGCTGTTCAGTAC	PB	PB-1	P1-25	TRUE	3	TRA:CIVGRDSSYKLIF TRB:CASNPQRGAHRDTQYF	58	CD4-C6
PB-1_GACTACAGTAATCGTC	PB	PB-1	P1-798	FALSE	1	TRA:CAVKGNTDKLIF TRB:CASGGGLRILAKNIQYF	1	CD4-C3
PB-1_GACTGCGCATGGTAGG	PB	PB-1	P1-41	TRUE	3	TRA:CVVSEPPGGSYIPTF TRB:CASSSRPEDTQYF	27	CD4-C6
PB-1_GACTGCGTCAACGGGA	PB	PB-1	P1-17	TRUE	3	TRA:CGTGARGSSGSRLTF TRB:CASSRQGMGAFF	78	CD4-C6
PB-1_GAGCAGATCATCGGAT	PB	PB-1	P1-24	TRUE	3	TRA:CAVKGNTDKLIF TRB:CASSLRTGAYNEQFF	62	CD4-C6
PB-1_GAGGTGAGTCGAGTTT	PB	PB-1	P1-10	TRUE	3	TRA:CISNSPMDSSYKLIF TRB:CASSSWGDTDEAFF	162	CD4-C6
PB-1_GAGTCCGTCGATGAGG	PB	PB-1	P1-21	TRUE	3	TRA:CAASRGTGANNLFF TRB:CATQREGREQYF	67	CD4-C6
PB-1_GATCGATAGTTCGCAT	PB	PB-1	P1-19	TRUE	3	TRA:CALGAFGSARQLTF TRB:CASSKGQGPRETQYF	70	CD4-C6
PB-1_GATCGCGAGTGGGATC	PB	PB-1	P1-30	TRUE	3	TRA:CALPPGGNNRKLIW TRB:CASSFGDRGGTEAFF	47	CD4-C6
PB-1_GATCGCGTCTGGCGAC	PB	PB-1	P1-24	TRUE	3	TRA:CAVKGNTDKLIF TRB:CASSLRTGAYNEQFF	62	CD4-C6
PB-1_GATCTAGAGTAAGTAC	PB	PB-1	P1-30	TRUE	3	TRA:CALPPGGNNRKLIW TRB:CASSFGDRGGTEAFF	47	CD4-C6
PB-1_GATGAAATCCCATTAT	PB	PB-1	P1-17	TRUE	3	TRA:CGTGARGSSGSRLTF TRB:CASSRQGMGAFF	78	CD4-C6
PB-1_GATGAGGAGTATCGAA	PB	PB-1	P1-42	TRUE	3	TRA:CASFSGGSQGNLIF TRB:CSAGQGSNQPQHF	24	CD4-C6
PB-1_GATTCAGGTAGAGTGC	PB	PB-1	P1-25	TRUE	3	TRA:CIVGRDSSYKLIF TRB:CASNPQRGAHRDTQYF	58	CD4-C6
PB-1_GCAAACTAGAGCTGCA	PB	PB-1	P1-107	TRUE	3	TRA:CALSDKEKLTF TRB:CAISEGGGVGYTF	5	CD4-C6
PB-1_GCAATCAAGTTACCCA	PB	PB-1	P1-25	TRUE	3	TRA:CIVGRDSSYKLIF TRB:CASNPQRGAHRDTQYF	58	CD4-C6
PB-1_GCAATCATCTGATACG	PB	PB-1	P1-823	FALSE	1	TRA:CAEAEGGGADGLTF TRB:CASSTGGDPSNQPQHF	1	CD4-C6
PB-1_GCACATAGTTAGTGGG	PB	PB-1	P1-24	TRUE	3	TRA:CAVKGNTDKLIF TRB:CASSLRTGAYNEQFF	62	CD4-C6
PB-1_GCACATATCATGCAAC	PB	PB-1	P1-30	TRUE	3	TRA:CALPPGGNNRKLIW TRB:CASSFGDRGGTEAFF	47	CD4-C6
PB-1_GCACTCTAGTAATCCC	PB	PB-1	P1-27	TRUE	3	TRA:CALVDFSGYSTLTF TRB:CASSGRGTNEKLFF	55	CD4-C6
PB-1_GCACTCTTCTACTTAC	PB	PB-1	P1-30	TRUE	3	TRA:CALPPGGNNRKLIW TRB:CASSFGDRGGTEAFF	47	CD4-C3
PB-1_GCAGTTACACCTCGTT	PB	PB-1	P1-21	TRUE	3	TRA:CAASRGTGANNLFF TRB:CATQREGREQYF	67	CD4-C6
PB-1_GCCAAATTCCAAACAC	PB	PB-1	P1-19	TRUE	3	TRA:CALGAFGSARQLTF TRB:CASSKGQGPRETQYF	70	CD4-C6
PB-1_GCGAGAACACTGTCGG	PB	PB-1	P1-19	TRUE	3	TRA:CALGAFGSARQLTF TRB:CASSKGQGPRETQYF	70	CD4-C6
PB-1_GCGCAACAGCCGCCTA	PB	PB-1	P1-30	TRUE	3	TRA:CALPPGGNNRKLIW TRB:CASSFGDRGGTEAFF	47	CD4-C6
PB-1_GCGCAACTCATGTCCC	PB	PB-1	P1-17	TRUE	3	TRA:CGTGARGSSGSRLTF TRB:CASSRQGMGAFF	78	CD4-C6

PB-1_GCGCCAATCCCAAGAT	PB	PB-1	P1-839	FALSE	1	TRA:CAVRPPGRNSGGYQKVTF TRB:CASSLGQRGLQYF	1	CD4-C6
PB-1_GCGCGATAGTGCCATT	PB	PB-1	P1-17	TRUE	3	TRA:CGTGARGSSGSRLTF TRB:CASSRQGMGAFF	78	CD4-C6
PB-1_GCTCCTAAGTAACCCT	PB	PB-1	P1-36	TRUE	3	TRA:CILRDNAGNNRKLIW TRB:CASSSRQGFPGGANVLTF	40	CD4-C6
PB-1_GCTCTGTGTGTTGAGG	PB	PB-1	P1-2	TRUE	3	TRA:CGQNFVF TRB:CASNQRGGPSLYNEQFF	323	CD8-C7
PB-1_GCTGGGTGTAGGGACT	PB	PB-1	P1-24	TRUE	3	TRA:CAVKGNTDKLIF TRB:CASSLRTGAYNEQFF	62	CD4-C6
PB-1_GCTTCCAAGAGCCTAG	PB	PB-1	P1-25	TRUE	3	TRA:CIVGRDSSYKLIF TRB:CASNPQRGAHRDTQYF	58	CD4-C6
PB-1_GGAAAGCAGAGTAATC	PB	PB-1	P1-25	TRUE	3	TRA:CIVGRDSSYKLIF TRB:CASNPQRGAHRDTQYF	58	CD4-C6
PB-1_GGAAAGCGTTCAGACT	PB	PB-1	P1-27	TRUE	3	TRA:CALVDFSGYSTLTF TRB:CASSGRGTNEKLFF	55	CD8-C7
PB-1_GGAAAGCGTTCGCGAC	PB	PB-1	P1-24	TRUE	3	TRA:CAVKGNTDKLIF TRB:CASSLRTGAYNEQFF	62	CD4-C6
PB-1_GGAATAACACTATCTT	PB	PB-1	P1-17	TRUE	3	TRA:CGTGARGSSGSRLTF TRB:CASSRQGMGAFF	78	CD4-C6
PB-1_GGAATAACATACAGCT	PB	PB-1	P1-72	FALSE	3	TRA:CALPDTNAGKSTF TRB:CSAAQGAYQPQHF	8	CD4-C6
PB-1_GGAATAAGTAGCGATG	PB	PB-1	P1-856	FALSE	1	TRA:CATASLYNFNKFYF TRB:CASSQVGRGGEETQYF	1	CD8-C2
PB-1_GGACGTCAGTCGCCGT	PB	PB-1	P1-41	TRUE	3	TRA:CVVSEPPGGSYIPTF TRB:CASSSRPEDTQYF	27	CD4-C6
PB-1_GGATTACAGCGTAGTG	PB	PB-1	P1-2	TRUE	3	TRA:CGQNFVF TRB:CASNQRGGPSLYNEQFF	323	CD8-C7
PB-1_GGCGACTCACATGACT	PB	PB-1	P1-27	TRUE	3	TRA:CALVDFSGYSTLTF TRB:CASSGRGTNEKLFF	55	CD4-C6
PB-1_GGCGACTCATCGGTTA	PB	PB-1	P1-42	TRUE	3	TRA:CASFSGGSQGNLIF TRB:CSAGQGSNQPQHF	24	CD4-C3
PB-1_GGCTCGAAGGGTCTCC	PB	PB-1	P1-41	TRUE	3	TRA:CVVSEPPGGSYIPTF TRB:CASSSRPEDTQYF	27	CD4-C6
PB-1_GGGAATGGTAGCCTCG	PB	PB-1	P1-30	TRUE	3	TRA:CALPPGGNNRKLIW TRB:CASSFGDRGGTEAFF	47	CD4-C6
PB-1_GGGACCTGTAGCAAAT	PB	PB-1	P1-27	TRUE	3	TRA:CALVDFSGYSTLTF TRB:CASSGRGTNEKLFF	55	CD4-C6
PB-1_GGGCACTGTAGGGTAC	PB	PB-1	P1-21	TRUE	3	TRA:CAASRGTGANNLFF TRB:CATQREGREQYF	67	CD4-C6
PB-1_GGGTCTGGTACATCCA	PB	PB-1	P1-17	TRUE	3	TRA:CGTGARGSSGSRLTF TRB:CASSRQGMGAFF	78	CD4-C6
PB-1_GGGTCTGTCTGGGCCA	PB	PB-1	P1-27	TRUE	3	TRA:CALVDFSGYSTLTF TRB:CASSGRGTNEKLFF	55	CD4-C6
PB-1_GGTATTGTCTCTTATG	PB	PB-1	P1-24	TRUE	3	TRA:CAVKGNTDKLIF TRB:CASSLRTGAYNEQFF	62	CD4-C6
PB-1_GGTGTTAGTACCGGCT	PB	PB-1	P1-25	TRUE	3	TRA:CIVGRDSSYKLIF TRB:CASNPQRGAHRDTQYF	58	CD4-C6
PB-1_GGTGTTATCGTGTAGT	PB	PB-1	P1-25	TRUE	3	TRA:CIVGRDSSYKLIF TRB:CASNPQRGAHRDTQYF	58	CD4-C6
PB-1_GTAACTGAGAACAATC	PB	PB-1	P1-25	TRUE	3	TRA:CIVGRDSSYKLIF TRB:CASNPQRGAHRDTQYF	58	CD4-C6
PB-1_GTACTCCAGTAGCCGA	PB	PB-1	P1-30	TRUE	3	TRA:CALPPGGNNRKLIW TRB:CASSFGDRGGTEAFF	47	CD4-C6

PB-1_GTACTCCGTTATGTGC	PB	PB-1	P1-19	TRUE	3	TRA:CALGAFGSARQLTF TRB:CASSKGQGPRETQYF	70	CD4-C6
PB-1_GTACTTTAGCCCGAAA	PB	PB-1	P1-48	TRUE	3	TRA:CAEVSGYSTLTF TRB:CASSFPTGGRTGRTEAFF	16	CD4-C6
PB-1_GTACTTTCAAAGAATC	PB	PB-1	P1-19	TRUE	3	TRA:CALGAFGSARQLTF TRB:CASSKGQGPRETQYF	70	CD4-C6
PB-1_GTAGGCCCAGGACCCT	PB	PB-1	P1-36	TRUE	3	TRA:CILRDNAGNNRKLIW TRB:CASSSRQGFPGGANVLTF	40	CD4-C6
PB-1_GTAGTCATCTGGCGAC	PB	PB-1	P1-17	TRUE	3	TRA:CGTGARGSSGSRLTF TRB:CASSRQGMGAFF	78	CD4-C6
PB-1_GTATCTTGTGCAGTAG	PB	PB-1	P1-17	TRUE	3	TRA:CGTGARGSSGSRLTF TRB:CASSRQGMGAFF	78	CD4-C6
PB-1_GTATTCTAGCACAGGT	PB	PB-1	P1-13	TRUE	3	TRA:CVVMNYGGSQGNLIF TRB:CASSSWGDTDEAFF	112	CD4-C6
PB-1_GTATTCTAGGGTGTTG	PB	PB-1	P1-19	TRUE	3	TRA:CALGAFGSARQLTF TRB:CASSKGQGPRETQYF	70	CD4-C6
PB-1_GTATTCTTCTGAAAGA	PB	PB-1	P1-42	TRUE	3	TRA:CASFSGGSQGNLIF TRB:CSAGQGSNQPQHF	24	CD4-C6
PB-1_GTCACGGCAGCGTCCA	PB	PB-1	P1-17	TRUE	3	TRA:CGTGARGSSGSRLTF TRB:CASSRQGMGAFF	78	CD4-C6
PB-1_GTCACGGGTCGTGGCT	PB	PB-1	P1-24	TRUE	3	TRA:CAVKGNTDKLIF TRB:CASSLRTGAYNEQFF	62	CD4-C6
PB-1_GTCACGGTCAAGCCTA	PB	PB-1	P1-42	TRUE	3	TRA:CASFSGGSQGNLIF TRB:CSAGQGSNQPQHF	24	CD4-C6
PB-1_GTCCTCAGTAGCGATG	PB	PB-1	P1-30	TRUE	3	TRA:CALPPGGNNRKLIW TRB:CASSFGDRGGTEAFF	47	CD4-C6
PB-1_GTCGTAATCACAAACC	PB	PB-1	P1-94	TRUE	3	TRA:CAASIQDFGNEKLTF TRB:CASSLQVPPNTEAFF	6	CD4-C6
PB-1_GTCTCGTGTTCTGAAC	PB	PB-1	P1-30	TRUE	3	TRA:CALPPGGNNRKLIW TRB:CASSFGDRGGTEAFF	47	CD4-C6
PB-1_GTGCAGCCACCATCCT	PB	PB-1	P1-8	TRUE	3	TRA:CAVRPPGRNSGGYQKVTF TRB:CASSRRGVTDTQYF	204	CD8-C7
PB-1_GTGCATACAGCCTATA	PB	PB-1	P1-41	TRUE	3	TRA:CVVSEPPGGSYIPTF TRB:CASSSRPEDTQYF	27	CD4-C6
PB-1_GTGCTTCAGTTCGCGC	PB	PB-1	P1-30	TRUE	3	TRA:CALPPGGNNRKLIW TRB:CASSFGDRGGTEAFF	47	CD4-C6
PB-1_GTGCTTCTCAATACCG	PB	PB-1	P1-917	FALSE	1	TRA:CAENAGANSKLTF TRB:CASSYGGWSYNSPLHF	1	CD8-C2
PB-1_GTGTTAGAGCGTGAGT	PB	PB-1	P1-41	TRUE	3	TRA:CVVSEPPGGSYIPTF TRB:CASSSRPEDTQYF	27	CD4-C6
PB-1_GTTACAGCAGATGAGC	PB	PB-1	P1-189	TRUE	3	TRA:CAPGLLF TRB:CASSLSHQGSPLHF	3	CD4-C6
PB-1_GTTCGGGGTGCTGTAT	PB	PB-1	P1-30	TRUE	3	TRA:CALPPGGNNRKLIW TRB:CASSFGDRGGTEAFF	47	CD4-C6
PB-1_GTTCGGGTCACCAGGC	PB	PB-1	P1-17	TRUE	3	TRA:CGTGARGSSGSRLTF TRB:CASSRQGMGAFF	78	CD4-C6
PB-1_GTTCTCGGTACCGGCT	PB	PB-1	P1-17	TRUE	3	TRA:CGTGARGSSGSRLTF TRB:CASSRQGMGAFF	78	CD4-C6
PB-1_GTTTCTAAGATCCCAT	PB	PB-1	P1-925	FALSE	1	TRA:CAASNDKLVF TRB:CASSQLGGHTGELFF	1	CD8-C7
PB-1_GTTTCTACATGTTGAC	PB	PB-1	P1-22	TRUE	3	TRA:CASNSGYSTLTF TRB:CASSQVTGGARDEQYF	65	CD8-C7
PB-1_GTTTCTAGTCACTTCC	PB	PB-1	P1-21	TRUE	3	TRA:CAASRGTGANNLFF TRB:CATQREGREQYF	67	CD4-C6

PB-1_GTTTCTAGTCGTTGTA	PB	PB-1	P1-19	TRUE	3	TRA:CALGAFGSARQLTF TRB:CASSKGQGPRETQYF	70	CD4-C6
PB-1_TAAGAGAGTGTTCTTT	PB	PB-1	P1-30	TRUE	3	TRA:CALPPGGNNRKLIW TRB:CASSFGDRGGTEAFF	47	CD4-C6
PB-1_TAAGTGCCACGTAAGG	PB	PB-1	P1-27	TRUE	3	TRA:CALVDFSGYSTLTF TRB:CASSGRGTNEKLFF	55	CD4-C6
PB-1_TAAGTGCGTCTAGTGT	PB	PB-1	P1-17	TRUE	3	TRA:CGTGARGSSGSRLTF TRB:CASSRQGMGAFF	78	CD4-C6
PB-1_TACACGATCGGAAATA	PB	PB-1	P1-25	TRUE	3	TRA:CIVGRDSSYKLIF TRB:CASNPQRGAHRDTQYF	58	CD4-C6
PB-1_TACCTATCAGGGATTG	PB	PB-1	P1-27	TRUE	3	TRA:CALVDFSGYSTLTF TRB:CASSGRGTNEKLFF	55	CD4-C3
PB-1_TACCTATGTCCAGTTA	PB	PB-1	P1-30	TRUE	3	TRA:CALPPGGNNRKLIW TRB:CASSFGDRGGTEAFF	47	CD4-C3
PB-1_TACCTATGTCTCAACA	PB	PB-1	P1-27	TRUE	3	TRA:CALVDFSGYSTLTF TRB:CASSGRGTNEKLFF	55	CD4-C6
PB-1_TACCTTAGTTTAGGAA	PB	PB-1	P1-17	TRUE	3	TRA:CGTGARGSSGSRLTF TRB:CASSRQGMGAFF	78	CD8-C7
PB-1_TACGGGCCACACCGAC	PB	PB-1	P1-41	TRUE	3	TRA:CVVSEPPGGSYIPTF TRB:CASSSRPEDTQYF	27	CD4-C6
PB-1_TACTCATAGTGTGAAT	PB	PB-1	P1-21	TRUE	3	TRA:CAASRGTGANNLFF TRB:CATQREGREQYF	67	CD4-C6
PB-1_TACTCATAGTGTTGAA	PB	PB-1	P1-19	TRUE	3	TRA:CALGAFGSARQLTF TRB:CASSKGQGPRETQYF	70	CD4-C6
PB-1_TACTCATGTGCTGTAT	PB	PB-1	P1-945	FALSE	1	TRA:CAVVVSYGGATNKLIF TRB:CASNSGGSGANVLTF	1	CD8-C6
PB-1_TACTCGCAGAAGGGTA	PB	PB-1	P1-27	TRUE	3	TRA:CALVDFSGYSTLTF TRB:CASSGRGTNEKLFF	55	CD4-C6
PB-1_TACTCGCTCGGAGGTA	PB	PB-1	P1-36	TRUE	3	TRA:CILRDNAGNNRKLIW TRB:CASSSRQGFPGGANVLTF	40	CD4-C6
PB-1_TACTTACAGTATGACA	PB	PB-1	P1-27	TRUE	3	TRA:CALVDFSGYSTLTF TRB:CASSGRGTNEKLFF	55	CD4-C6
PB-1_TACTTACGTTGCGTTA	PB	PB-1	P1-42	TRUE	3	TRA:CASFSGGSQGNLIF TRB:CSAGQGSNQPQHF	24	CD4-C6
PB-1_TACTTACTCGTTACAG	PB	PB-1	P1-19	TRUE	3	TRA:CALGAFGSARQLTF TRB:CASSKGQGPRETQYF	70	CD4-C6
PB-1_TACTTGTAGAAACCAT	PB	PB-1	P1-17	TRUE	3	TRA:CGTGARGSSGSRLTF TRB:CASSRQGMGAFF	78	CD4-C6
PB-1_TACTTGTTCGGCTACG	PB	PB-1	P1-17	TRUE	3	TRA:CGTGARGSSGSRLTF TRB:CASSRQGMGAFF	78	CD4-C6
PB-1_TAGACCACAAGCCGCT	PB	PB-1	P1-21	TRUE	3	TRA:CAASRGTGANNLFF TRB:CATQREGREQYF	67	CD4-C6
PB-1_TAGCCGGGTAGGGACT	PB	PB-1	P1-17	TRUE	3	TRA:CGTGARGSSGSRLTF TRB:CASSRQGMGAFF	78	CD4-C6
PB-1_TAGCCGGTCACCGGGT	PB	PB-1	P1-21	TRUE	3	TRA:CAASRGTGANNLFF TRB:CATQREGREQYF	67	CD4-C6
PB-1_TAGGCATCATCTACGA	PB	PB-1	P1-19	TRUE	3	TRA:CALGAFGSARQLTF TRB:CASSKGQGPRETQYF	70	CD4-C6
PB-1_TAGTGGTTCCTTGGTC	PB	PB-1	P1-21	TRUE	3	TRA:CAASRGTGANNLFF TRB:CATQREGREQYF	67	CD4-C6
PB-1_TATCAGGGTCTTGCGG	PB	PB-1	P1-174	FALSE	3	TRA:CAAPENSNSGYALNF TRB:CSGGLANEQFF	3	CD4-C6
PB-1_TATCTCATCACAAACC	PB	PB-1	P1-27	TRUE	3	TRA:CALVDFSGYSTLTF TRB:CASSGRGTNEKLFF	55	CD4-C6

PB-1_TCAACGAGTGACTCAT	PB	PB-1	P1-30	TRUE	3	TRA:CALPPGGNNRKLIW TRB:CASSFGDRGGTEAFF	47	CD4-C6
PB-1_TCAATCTCAAGGACTG	PB	PB-1	P1-13	TRUE	3	TRA:CVVMNYGGSQGNLIF TRB:CASSSWGDTDEAFF	112	CD8-C7
PB-1_TCAATCTTCACTTATC	PB	PB-1	P1-967	FALSE	1	TRA:CAVQAGNNDMRF TRB:CASSLGQRGLQYF	1	CD4-C6
PB-1_TCACGAAAGTGGTCCC	PB	PB-1	P1-24	TRUE	3	TRA:CAVKGNTDKLIF TRB:CASSLRTGAYNEQFF	62	CD4-C3
PB-1_TCACGAAGTCTAAACC	PB	PB-1	P1-24	TRUE	3	TRA:CAVKGNTDKLIF TRB:CASSLRTGAYNEQFF	62	CD4-C6
PB-1_TCACGAAGTCTCTCTG	PB	PB-1	P1-27	TRUE	3	TRA:CALVDFSGYSTLTF TRB:CASSGRGTNEKLFF	55	CD4-C6
PB-1_TCAGCAATCAACCATG	PB	PB-1	P1-24	TRUE	3	TRA:CAVKGNTDKLIF TRB:CASSLRTGAYNEQFF	62	CD4-C6
PB-1_TCAGGATCATGTTGAC	PB	PB-1	P1-42	TRUE	3	TRA:CASFSGGSQGNLIF TRB:CSAGQGSNQPQHF	24	CD4-C6
PB-1_TCAGGTACAGGTGCCT	PB	PB-1	P1-79	TRUE	3	TRA:CAFMKLGGATNKLIF TRB:CASSLDRYSVDEQFF	7	CD4-C6
PB-1_TCCACACGTCTCTTAT	PB	PB-1	P1-21	TRUE	3	TRA:CAASRGTGANNLFF TRB:CATQREGREQYF	67	CD4-C6
PB-1_TCGCGAGGTAGCGTGA	PB	PB-1	P1-36	TRUE	3	TRA:CILRDNAGNNRKLIW TRB:CASSSRQGFPGGANVLTF	40	CD4-C6
PB-1_TCGCGAGTCGCCTGTT	PB	PB-1	P1-30	TRUE	3	TRA:CALPPGGNNRKLIW TRB:CASSFGDRGGTEAFF	47	CD4-C6
PB-1_TCGGGACGTCTTCAAG	PB	PB-1	P1-27	TRUE	3	TRA:CALVDFSGYSTLTF TRB:CASSGRGTNEKLFF	55	CD4-C5
PB-1_TCGGTAACAAACCTAC	PB	PB-1	P1-27	TRUE	3	TRA:CALVDFSGYSTLTF TRB:CASSGRGTNEKLFF	55	CD4-C6
PB-1_TCGTACCCAGACAAGC	PB	PB-1	P1-17	TRUE	3	TRA:CGTGARGSSGSRLTF TRB:CASSRQGMGAFF	78	CD4-C6
PB-1_TCGTAGACAGCGAACA	PB	PB-1	P1-21	TRUE	3	TRA:CAASRGTGANNLFF TRB:CATQREGREQYF	67	CD4-C6
PB-1_TCGTAGAGTGAACCTT	PB	PB-1	P1-2	TRUE	3	TRA:CGQNFVF TRB:CASNQRGGPSLYNEQFF	323	CD4-C6
PB-1_TCTCATAGTTTAAGCC	PB	PB-1	P1-183	TRUE	3	TRA:CASYYGGSQGNLIF TRB:CSAGQGSNQPQHF	3	CD4-C6
PB-1_TCTCTAACACAGACAG	PB	PB-1	P1-48	TRUE	3	TRA:CAEVSGYSTLTF TRB:CASSFPTGGRTGRTEAFF	16	CD4-C6
PB-1_TCTCTAATCTAGCACA	PB	PB-1	P1-998	FALSE	1	TRA:CAVRAYGQNFVF TRB:CASSLGQRGLQYF	1	CD4-C6
PB-1_TCTGAGATCACATACG	PB	PB-1	P1-8	TRUE	3	TRA:CAVRPPGRNSGGYQKVTF TRB:CASSRRGVTDTQYF	204	CD8-C2
PB-1_TCTGAGATCTACTTAC	PB	PB-1	P1-25	TRUE	3	TRA:CIVGRDSSYKLIF TRB:CASNPQRGAHRDTQYF	58	CD4-C6
PB-1_TCTGGAAAGTAGGTGC	PB	PB-1	P1-36	TRUE	3	TRA:CILRDNAGNNRKLIW TRB:CASSSRQGFPGGANVLTF	40	CD4-C6
PB-1_TCTTCGGAGCCACGTC	PB	PB-1	P1-25	TRUE	3	TRA:CIVGRDSSYKLIF TRB:CASNPQRGAHRDTQYF	58	CD4-C6
PB-1_TCTTCGGCATCCAACA	PB	PB-1	P1-36	TRUE	3	TRA:CILRDNAGNNRKLIW TRB:CASSSRQGFPGGANVLTF	40	CD4-C3
PB-1_TGAAAGATCTTATCTG	PB	PB-1	P1-19	TRUE	3	TRA:CALGAFGSARQLTF TRB:CASSKGQGPRETQYF	70	CD4-C6
PB-1_TGACAACAGCAATCTC	PB	PB-1	P1-24	TRUE	3	TRA:CAVKGNTDKLIF TRB:CASSLRTGAYNEQFF	62	CD4-C6

PB-1_TGACAACGTCCGACGT	PB	PB-1	P1-21	TRUE	3	TRA:CAASRGTGANNLFF TRB:CATQREGREQYF	67	CD4-C6
PB-1_TGACGGCGTCTAAAGA	PB	PB-1	P1-17	TRUE	3	TRA:CGTGARGSSGSRLTF TRB:CASSRQGMGAFF	78	CD4-C6
PB-1_TGACTAGAGGGCTTGA	PB	PB-1	P1-21	TRUE	3	TRA:CAASRGTGANNLFF TRB:CATQREGREQYF	67	CD4-C6
PB-1_TGAGCCGGTGTGAATA	PB	PB-1	P1-10	TRUE	3	TRA:CISNSPMDSSYKLIF TRB:CASSSWGDTDEAFF	162	CD8-C7
PB-1_TGAGGGAAGTTGCAGG	PB	PB-1	P1-30	TRUE	3	TRA:CALPPGGNNRKLIW TRB:CASSFGDRGGTEAFF	47	CD4-C6
PB-1_TGAGGGAGTAACGTTC	PB	PB-1	P1-10	TRUE	3	TRA:CISNSPMDSSYKLIF TRB:CASSSWGDTDEAFF	162	CD4-C6
PB-1_TGCACCTTCAATACCG	PB	PB-1	P1-27	TRUE	3	TRA:CALVDFSGYSTLTF TRB:CASSGRGTNEKLFF	55	CD4-C6
PB-1_TGCCCATAGTGAACAT	PB	PB-1	P1-17	TRUE	3	TRA:CGTGARGSSGSRLTF TRB:CASSRQGMGAFF	78	CD8-C7
PB-1_TGCGTGGCATGGGACA	PB	PB-1	P1-17	TRUE	3	TRA:CGTGARGSSGSRLTF TRB:CASSRQGMGAFF	78	CD4-C6
PB-1_TGCTACCAGATCTGAA	PB	PB-1	P1-17	TRUE	3	TRA:CGTGARGSSGSRLTF TRB:CASSRQGMGAFF	78	CD4-C6
PB-1_TGCTACCGTTCGGGCT	PB	PB-1	P1-30	TRUE	3	TRA:CALPPGGNNRKLIW TRB:CASSFGDRGGTEAFF	47	CD4-C6
PB-1_TGCTGCTAGGCTAGGT	PB	PB-1	P1-17	TRUE	3	TRA:CGTGARGSSGSRLTF TRB:CASSRQGMGAFF	78	CD4-C6
PB-1_TGGACGCAGCGGCTTC	PB	PB-1	P1-17	TRUE	3	TRA:CGTGARGSSGSRLTF TRB:CASSRQGMGAFF	78	CD4-C6
PB-1_TGGACGCAGTACGCCC	PB	PB-1	P1-21	TRUE	3	TRA:CAASRGTGANNLFF TRB:CATQREGREQYF	67	CD4-C6
PB-1_TGGACGCCAACACCCG	PB	PB-1	P1-30	TRUE	3	TRA:CALPPGGNNRKLIW TRB:CASSFGDRGGTEAFF	47	CD4-C6
PB-1_TGGCCAGCAAACTGCT	PB	PB-1	P1-27	TRUE	3	TRA:CALVDFSGYSTLTF TRB:CASSGRGTNEKLFF	55	CD4-C6
PB-1_TGGCCAGCACAGATTC	PB	PB-1	P1-1029	FALSE	1	TRA:CGQNFVF TRB:CSAAQGAYQPQHF	1	CD4-C6
PB-1_TGGCCAGCAGGAATCG	PB	PB-1	P1-17	TRUE	3	TRA:CGTGARGSSGSRLTF TRB:CASSRQGMGAFF	78	CD4-C6
PB-1_TGGCGCATCAACTCTT	PB	PB-1	P1-41	TRUE	3	TRA:CVVSEPPGGSYIPTF TRB:CASSSRPEDTQYF	27	CD4-C6
PB-1_TGGCTGGGTCTTGTCC	PB	PB-1	P1-21	TRUE	3	TRA:CAASRGTGANNLFF TRB:CATQREGREQYF	67	CD4-C6
PB-1_TGGCTGGTCATACGGT	PB	PB-1	P1-94	TRUE	3	TRA:CAASIQDFGNEKLTF TRB:CASSLQVPPNTEAFF	6	CD4-C6
PB-1_TGGCTGGTCTTACCGC	PB	PB-1	P1-25	TRUE	3	TRA:CIVGRDSSYKLIF TRB:CASNPQRGAHRDTQYF	58	CD4-C6
PB-1_TGGGAAGCACTCAGGC	PB	PB-1	P1-1030	FALSE	1	TRA:CGQNFVF TRB:CASSHRGGAKNIQYF	1	CD4-C6
PB-1_TGGGCGTAGGGATGGG	PB	PB-1	P1-21	TRUE	3	TRA:CAASRGTGANNLFF TRB:CATQREGREQYF	67	CD8-C7
PB-1_TGGGCGTCAGTAAGCG	PB	PB-1	P1-17	TRUE	3	TRA:CGTGARGSSGSRLTF TRB:CASSRQGMGAFF	78	CD8-C7
PB-1_TGTATTCGTCCTAGCG	PB	PB-1	P1-21	TRUE	3	TRA:CAASRGTGANNLFF TRB:CATQREGREQYF	67	CD4-C6
PB-1_TGTCCCAGTGGAAAGA	PB	PB-1	P1-21	TRUE	3	TRA:CAASRGTGANNLFF TRB:CATQREGREQYF	67	CD4-C6

PB-1_TGTCCCATCGACAGCC	PB	PB-1	P1-19	TRUE	3	TRA:CALGAFGSARQLTF TRB:CASSKGQGPRETQYF	70	CD4-C6
PB-1_TTAACTCCACCAGGCT	PB	PB-1	P1-41	TRUE	3	TRA:CVVSEPPGGSYIPTF TRB:CASSSRPEDTQYF	27	CD4-C3
PB-1_TTAACTCTCTCGTTTA	PB	PB-1	P1-17	TRUE	3	TRA:CGTGARGSSGSRLTF TRB:CASSRQGMGAFF	78	CD4-C6
PB-1_TTAACTCTCTTTAGGG	PB	PB-1	P1-24	TRUE	3	TRA:CAVKGNTDKLIF TRB:CASSLRTGAYNEQFF	62	CD4-C6
PB-1_TTAGGCATCACATACG	PB	PB-1	P1-13	TRUE	3	TRA:CVVMNYGGSQGNLIF TRB:CASSSWGDTDEAFF	112	CD8-C7
PB-1_TTATGCTCAGCCTTTC	PB	PB-1	P1-10	TRUE	3	TRA:CISNSPMDSSYKLIF TRB:CASSSWGDTDEAFF	162	CD8-C7
PB-1_TTATGCTTCATATCGG	PB	PB-1	P1-17	TRUE	3	TRA:CGTGARGSSGSRLTF TRB:CASSRQGMGAFF	78	CD4-C6
PB-1_TTCCCAGGTAGCTTGT	PB	PB-1	P1-2	TRUE	3	TRA:CGQNFVF TRB:CASNQRGGPSLYNEQFF	323	CD8-C7
PB-1_TTCGAAGGTCATGCCG	PB	PB-1	P1-24	TRUE	3	TRA:CAVKGNTDKLIF TRB:CASSLRTGAYNEQFF	62	CD4-C6
PB-1_TTCGAAGTCAATCACG	PB	PB-1	P1-19	TRUE	3	TRA:CALGAFGSARQLTF TRB:CASSKGQGPRETQYF	70	CD4-C6
PB-1_TTCTACAGTACAGCAG	PB	PB-1	P1-48	TRUE	3	TRA:CAEVSGYSTLTF TRB:CASSFPTGGRTGRTEAFF	16	CD4-C6
PB-1_TTCTACATCTACGAGT	PB	PB-1	P1-25	TRUE	3	TRA:CIVGRDSSYKLIF TRB:CASNPQRGAHRDTQYF	58	CD4-C6
PB-1_TTCTCAACATGACGGA	PB	PB-1	P1-27	TRUE	3	TRA:CALVDFSGYSTLTF TRB:CASSGRGTNEKLFF	55	CD4-C6
PB-1_TTCTCCTGTCCGTCAG	PB	PB-1	P1-79	TRUE	3	TRA:CAFMKLGGATNKLIF TRB:CASSLDRYSVDEQFF	7	CD4-C6
PB-1_TTGAACGGTATTAGCC	PB	PB-1	P1-41	TRUE	3	TRA:CVVSEPPGGSYIPTF TRB:CASSSRPEDTQYF	27	CD4-C6
PB-1_TTGAACGGTCATTAGC	PB	PB-1	P1-2	TRUE	3	TRA:CGQNFVF TRB:CASNQRGGPSLYNEQFF	323	CD8-C7
PB-1_TTGAACGGTCTGATCA	PB	PB-1	P1-25	TRUE	3	TRA:CIVGRDSSYKLIF TRB:CASNPQRGAHRDTQYF	58	CD4-C6
PB-1_TTGACTTAGTTATCGC	PB	PB-1	P1-19	TRUE	3	TRA:CALGAFGSARQLTF TRB:CASSKGQGPRETQYF	70	CD4-C6
PB-1_TTGGAACGTACGCTGC	PB	PB-1	P1-42	TRUE	3	TRA:CASFSGGSQGNLIF TRB:CSAGQGSNQPQHF	24	CD4-C6
PB-1_TTGGCAATCATAACCG	PB	PB-1	P1-17	TRUE	3	TRA:CGTGARGSSGSRLTF TRB:CASSRQGMGAFF	78	CD4-C6
PB-1_TTGTAGGCAGCCAATT	PB	PB-1	P1-21	TRUE	3	TRA:CAASRGTGANNLFF TRB:CATQREGREQYF	67	CD8-C2
PB-1_TTGTAGGTCAGAGGTG	PB	PB-1	P1-27	TRUE	3	TRA:CALVDFSGYSTLTF TRB:CASSGRGTNEKLFF	55	CD4-C6
PB-1_TTTACTGGTGAGTATA	PB	PB-1	P1-1072	FALSE	1	TRA:CAASLSGGYNKLIF TRB:CSAPTGAYNEQFF	1	CD8-C6
PB-1_TTTACTGGTTGCGCAC	PB	PB-1	P1-25	TRUE	3	TRA:CIVGRDSSYKLIF TRB:CASNPQRGAHRDTQYF	58	CD4-C6
PB-1_TTTATGCAGTCTCCTC	PB	PB-1	P1-2	TRUE	3	TRA:CGQNFVF TRB:CASNQRGGPSLYNEQFF	323	CD8-C5
PB-1_TTTATGCTCGATGAGG	PB	PB-1	P1-17	TRUE	3	TRA:CGTGARGSSGSRLTF TRB:CASSRQGMGAFF	78	CD4-C6
PB-1_TTTGCGCGTTGACGTT	PB	PB-1	P1-17	TRUE	3	TRA:CGTGARGSSGSRLTF TRB:CASSRQGMGAFF	78	CD4-C6

PB-1_TTTGTCAGTGAGTATA	PB	PB-1	P1-1	TRUE	3	TRA:CAMSDRMTGGFKTIF TRB:CASSQVGRGGEETQYF	1047	CD4-C6
PB-1_TTTGTCATCTCGTATT	PB	PB-1	P1-27	TRUE	3	TRA:CALVDFSGYSTLTF TRB:CASSGRGTNEKLFF	55	CD4-C6
PB-1_AACACGTGTCCAAGTT	PB	PB-1	P1-434	FALSE	1	TRA:CAVRDLQGQNFVF TRB:CASSEIGGSYEQYF	1	CD8-C1
PB-1_AATCGGTCAGTGAGTG	PB	PB-1	P1-461	FALSE	1	TRA:CAVGARVRGSYIPTF TRB:CANPWRELAKNIQYF	1	CD8-C1
PB-1_CATGCCTCAGCTTCGG	PB	PB-1	P1-626	FALSE	1	TRA:CAEKDTGGFKTIF TRB:CASSQDPLGETQYF	1	CD8-C1
PB-1_CCCATACTCCCTAATT	PB	PB-1	P1-649	FALSE	1	TRA:CALSWGAVNAGGTSYGKLTF TRB:CSARERHRSTDTQYF	1	CD8-C1
PB-1_CCTTACGAGTTGCAGG	PB	PB-1	P1-662	FALSE	1	TRA:CATDPALSGGSYIPTF TRB:CASRPTENSNQPQHF	1	CD4-C1
PB-1_CGAGCACGTTCGCGAC	PB	PB-1	P1-674	FALSE	1	TRA:CAVRNNNDMRF TRB:CASSGTGNTGELFF	1	CD8-C1
PB-1_CGATTGAGTTCCGGCA	PB	PB-1	P1-681	FALSE	1	TRA:CLVGDWTGANNLFF TRB:CASSPFEAGERILSGELFF	1	CD8-C1
PB-1_CGGAGTCCAAACTGTC	PB	PB-1	P1-704	FALSE	1	TRA:CAVGVETSGSRLTF TRB:CASSLTGGRNSPLHF	1	CD8-C1
PB-1_GAAGCAGAGTCAAGCG	PB	PB-1	P1-785	FALSE	1	TRA:CVVKMTNSGYALNF TRB:CATSDLDQGMVNEQFF	1	CD8-C1
PB-1_GCATGTACAAGCCGTC	PB	PB-1	P1-829	FALSE	1	TRA:CAASNNAGNMLTF TRB:CASSLGPLGAETQYF	1	CD8-C1
PB-1_GGCTCGACAGCTCGAC	PB	PB-1	P1-866	FALSE	1	TRA:CAVREKRGNNAGNMLTF TRB:CASSTGTGVDTQYF	1	CD8-C1
PB-1_GTACTTTTCAACACCA	PB	PB-1	P1-893	FALSE	1	TRA:CLVGYILTGGGNKLTF TRB:CASSGREAYGYTF	1	CD8-C1
PB-1_GTTACAGGTCGAGTTT	PB	PB-1	P1-920	FALSE	1	TRA:CVVTVTGTASKLTF TRB:CASSQSWDLAVLNEQFF	1	CD8-C2
PB-1_TACCTATTCCTGCAGG	PB	PB-1	P1-940	FALSE	1	TRA:CAVRLFTGGGNKLTF TRB:CASSIRGTGPFRPEKLFF	1	CD8-C1
PB-1_TATTACCCACCAGCAC	PB	PB-1	P1-962	FALSE	1	TRA:CAMREGTSGGYQKVTF TRB:CASSGLAGHPRTGELFF	1	CD8-C1
PB-1_TGCCAAAAGCATGGCA	PB	PB-1	P1-1016	FALSE	1	TRA:CAGINDYKLSF TRB:CASSEVRMNTEAFF	1	CD8-C1
PB-1_TGGGAAGTCCGTAGTA	PB	PB-1	P1-1031	FALSE	1	TRA:CLVGPVSGNTPLVF TRB:CASSNLMQKQETQYF	1	CD8-C1
PB-1_TGGGCGTGTCCAGTAT	PB	PB-1	P1-1034	FALSE	1	TRA:CAMPQGGYNKLIF TRB:CASSESPASTDTQYF	1	CD8-C1
PB-1_TTAGGACCAAGCCATT	PB	PB-1	P1-1050	FALSE	1	TRA:CAVRGSGAGSYQLTF TRB:CASSLDNRDEYEQYF	1	CD8-C1
PB-1_CAGCTGGCATCCTAGA	PB	PB-1	P1-2	TRUE	3	TRA:CGQNFVF TRB:CASNQRGGPSLYNEQFF	323	CD8-C11
PB-1_CCAATCCTCAGTGTTG	PB	PB-1	P1-632	FALSE	1	TRA:CALLTSGTYKYIF TRB:CAWSDWTGIQNTGELFF	1	CD8-C11
PB-1_CCGTACTCAAGTCTGT	PB	PB-1	P1-19	TRUE	3	TRA:CALGAFGSARQLTF TRB:CASSKGQGPRETQYF	70	CD8-C11
PB-1_CGGGTCATCGTACGGC	PB	PB-1	P1-4	TRUE	3	TRA:CAMKGRDDKIIF TRB:CASTKGVSREAFF	226	CD8-C11
PB-1_CGTCAGGGTACATGTC	PB	PB-1	P1-5	TRUE	3	TRA:CALSGTPMNRDDKIIF TRB:CSARGRGLAGGPAYEQYF	223	CD8-C11
PB-1_GAAACTCCACTTCTGC	PB	PB-1	P1-1	TRUE	3	TRA:CAMSDRMTGGFKTIF TRB:CASSQVGRGGEETQYF	1047	CD8-C11

PB-1_GGATGTTCAGGGTTAG	PB	PB-1	P1-1	TRUE	3	TRA:CAMSDRMTGGFKTIF TRB:CASSQVGRGGEETQYF	1047	CD8-C11
PB-1_TAAGTGCAGCTGGAAC	PB	PB-1	P1-1	TRUE	3	TRA:CAMSDRMTGGFKTIF TRB:CASSQVGRGGEETQYF	1047	CD8-C11
PB-1_TATGCCCAGTCCTCCT	PB	PB-1	P1-2	TRUE	3	TRA:CGQNFVF TRB:CASNQRGGPSLYNEQFF	323	CD8-C11
PB-1_TTTACTGAGCTCCTTC	PB	PB-1	P1-3	TRUE	3	TRA:CAFLSGTYKYIF TRB:CASSQSPGQRNYGYTF	308	CD8-C11
PB-2_AAACCTGAGTCAAGCG	PB	PB-2	P2-5	TRUE	3	TRA:CASSFSGGYNKLIF TRB:CASSYLGAGYQETQYF	127	CD8-C5
PB-2_AAACGGGAGACACGAC	PB	PB-2	P2-43	TRUE	3	TRA:CVWEADFNKFYF TRB:CATSAGNNQPQHF	11	CD8-C5
PB-2_AAACGGGTCATCGATG	PB	PB-2	P2-3	TRUE	3	TRA:CAVNPLESSYKLIF TRB:CASSPGTANTGELFF	177	CD8-C7
PB-2_AAACGGGTCGGAAATA	PB	PB-2	P2-1	TRUE	3	TRA:CVVNIIGYQKVTF TRB:CASTRQGKQPQHF	270	CD8-C7
PB-2_AAAGCAAGTTTACTCT	PB	PB-2	P2-3	TRUE	3	TRA:CAVNPLESSYKLIF TRB:CASSPGTANTGELFF	177	CD8-C5
PB-2_AAATGCCCAAGGACTG	PB	PB-2	P2-5	TRUE	3	TRA:CASSFSGGYNKLIF TRB:CASSYLGAGYQETQYF	127	CD8-C7
PB-2_AAATGCCCACCTATCC	PB	PB-2	P2-1	TRUE	3	TRA:CVVNIIGYQKVTF TRB:CASTRQGKQPQHF	270	CD8-C7
PB-2_AAATGCCGTCAAAGCG	PB	PB-2	P2-17	TRUE	3	TRA:CAVQASNAGNMLTF TRB:CASSVEQGNVETQYF	30	CD8-C5
PB-2_AACACGTAGATCACGG	PB	PB-2	P2-5	TRUE	3	TRA:CASSFSGGYNKLIF TRB:CASSYLGAGYQETQYF	127	CD8-C7
PB-2_AACACGTTCCACGCAG	PB	PB-2	P2-11	TRUE	3	TRA:CAVHNQGGKLIF TRB:CASSYLGAGYQETQYF	43	CD8-C7
PB-2_AACCGCGCAATGAATG	PB	PB-2	P2-11	TRUE	3	TRA:CAVHNQGGKLIF TRB:CASSYLGAGYQETQYF	43	CD8-C7
PB-2_AACTCTTTCTTGCATT	PB	PB-2	P2-5	TRUE	3	TRA:CASSFSGGYNKLIF TRB:CASSYLGAGYQETQYF	127	CD8-C5
PB-2_AACTGGTAGGCGACAT	PB	PB-2	P2-43	TRUE	3	TRA:CVWEADFNKFYF TRB:CATSAGNNQPQHF	11	CD8-C7
PB-2_AACTTTCAGCCTTGAT	PB	PB-2	P2-37	TRUE	3	TRA:CLVVIRHRWGSQGNLIF TRB:CASSFRTLTPLQETQYF	14	CD8-C7
PB-2_AACTTTCTCTCGCATC	PB	PB-2	P2-7	TRUE	3	TRA:CAESDSGNTPLVF TRB:CASSYSTPRGEAFF	84	CD8-C7
PB-2_AAGACCTAGCGCTTAT	PB	PB-2	P2-650	FALSE	1	TRA:CALSSSGGSNYKLTF TRB:CASSQALVEHTEAFF	1	CD8-C6
PB-2_AAGACCTCAATCCAAC	PB	PB-2	P2-12	TRUE	3	TRA:CGADAEYGNKLVF TRB:CASSEGQGAIMQPQHF	42	CD8-C7
PB-2_AAGACCTCATTTGCCC	PB	PB-2	P2-1	TRUE	3	TRA:CVVNIIGYQKVTF TRB:CASTRQGKQPQHF	270	CD8-C5
PB-2_AAGCCGCCACGCATCG	PB	PB-2	P2-170	TRUE	3	TRA:CALPAYSGAGSYQLTF TRB:CASSLYENTEAFF	3	CD8-C7
PB-2_AAGGAGCAGCTAAGAT	PB	PB-2	P2-17	TRUE	3	TRA:CAVQASNAGNMLTF TRB:CASSVEQGNVETQYF	30	CD8-C5
PB-2_AAGGCAGCACATCCAA	PB	PB-2	P2-676	FALSE	1	TRA:CASSFSGGYNKLIF TRB:CASSVEQGNVETQYF	1	CD8-C5
PB-2_AAGGCAGGTGAGCGAT	PB	PB-2	P2-4	TRUE	3	TRA:CAIRYQSFNFGNEKLTF TRB:CASYRRVTEQYF	148	CD8-C7
PB-2_AAGGTTCGTCGACTAT	PB	PB-2	P2-11	TRUE	3	TRA:CAVHNQGGKLIF TRB:CASSYLGAGYQETQYF	43	CD8-C5

PB-2_AAGGTTCTCCCATTAT	PB	PB-2	P2-11	TRUE	3	TRA:CAVHNQGGKLIF TRB:CASSYLGAGYQETQYF	43	CD8-C5
PB-2_AAGTCTGCAGTATAAG	PB	PB-2	P2-5	TRUE	3	TRA:CASSFSGGYNKLIF TRB:CASSYLGAGYQETQYF	127	CD8-C7
PB-2_AATCCAGGTGATAAAC	PB	PB-2	P2-4	TRUE	3	TRA:CAIRYQSFNFGNEKLTF TRB:CASYRRVTEQYF	148	CD8-C7
PB-2_AATCGGTGTTATCGGT	PB	PB-2	P2-17	TRUE	3	TRA:CAVQASNAGNMLTF TRB:CASSVEQGNVETQYF	30	CD8-C5
PB-2_ACACCCTTCGGCATCG	PB	PB-2	P2-5	TRUE	3	TRA:CASSFSGGYNKLIF TRB:CASSYLGAGYQETQYF	127	CD8-C7
PB-2_ACACCGGAGCAGCGTA	PB	PB-2	P2-7	TRUE	3	TRA:CAESDSGNTPLVF TRB:CASSYSTPRGEAFF	84	CD8-C7
PB-2_ACACCGGAGGGTCTCC	PB	PB-2	P2-5	TRUE	3	TRA:CASSFSGGYNKLIF TRB:CASSYLGAGYQETQYF	127	CD8-C6
PB-2_ACACCGGCAAAGGCGT	PB	PB-2	P2-1	TRUE	3	TRA:CVVNIIGYQKVTF TRB:CASTRQGKQPQHF	270	CD8-C7
PB-2_ACACTGAGTTTGGCGC	PB	PB-2	P2-12	TRUE	3	TRA:CGADAEYGNKLVF TRB:CASSEGQGAIMQPQHF	42	CD8-C5
PB-2_ACAGCCGCAGGGCATA	PB	PB-2	P2-4	TRUE	3	TRA:CAIRYQSFNFGNEKLTF TRB:CASYRRVTEQYF	148	CD8-C7
PB-2_ACAGCCGGTCAAAGCG	PB	PB-2	P2-54	FALSE	3	TRA:CALNFMNRDDKIIF TRB:CASSYSPGQAASYGYTF	10	CD8-C7
PB-2_ACAGCTAGTTCCATGA	PB	PB-2	P2-7	TRUE	3	TRA:CAESDSGNTPLVF TRB:CASSYSTPRGEAFF	84	CD8-C7
PB-2_ACATACGAGCCAACAG	PB	PB-2	P2-17	TRUE	3	TRA:CAVQASNAGNMLTF TRB:CASSVEQGNVETQYF	30	CD8-C5
PB-2_ACATACGTCAGCTCTC	PB	PB-2	P2-12	TRUE	3	TRA:CGADAEYGNKLVF TRB:CASSEGQGAIMQPQHF	42	CD8-C7
PB-2_ACATCAGCAGCTCGAC	PB	PB-2	P2-5	TRUE	3	TRA:CASSFSGGYNKLIF TRB:CASSYLGAGYQETQYF	127	CD8-C7
PB-2_ACCAGTATCGGAGCAA	PB	PB-2	P2-25	FALSE	3	TRA:CAMREAPNTDKLIF TRB:CASSGGGGGLETQYF	22	CD8-C5
PB-2_ACCAGTATCGTCCGTT	PB	PB-2	P2-3	TRUE	3	TRA:CAVNPLESSYKLIF TRB:CASSPGTANTGELFF	177	CD8-C5
PB-2_ACCCACTGTGAACCTT	PB	PB-2	P2-43	TRUE	3	TRA:CVVWEADFNKFYF TRB:CATSAGNNQPQHF	11	CD8-C7
PB-2_ACCCACTTCTTCTGGC	PB	PB-2	P2-1	TRUE	3	TRA:CVVNIIGYQKVTF TRB:CASTRQGKQPQHF	270	CD8-C7
PB-2_ACCGTAAGTACCGAGA	PB	PB-2	P2-3	TRUE	3	TRA:CAVNPLESSYKLIF TRB:CASSPGTANTGELFF	177	CD8-C7
PB-2_ACCTTTAGTCACTTCC	PB	PB-2	P2-3	TRUE	3	TRA:CAVNPLESSYKLIF TRB:CASSPGTANTGELFF	177	CD8-C5
PB-2_ACGAGCCAGTGAATTG	PB	PB-2	P2-25	FALSE	3	TRA:CAMREAPNTDKLIF TRB:CASSGGGGGLETQYF	22	CD8-C7
PB-2_ACGAGGAGTAGCACGA	PB	PB-2	P2-801	FALSE	1	TRA:CAMREGDNDMRF TRB:CASSEGGGAGETQYF	1	CD8-C6
PB-2_ACGATACTCACCCGAG	PB	PB-2	P2-11	TRUE	3	TRA:CAVHNQGGKLIF TRB:CASSYLGAGYQETQYF	43	CD8-C7
PB-2_ACGATGTAGCTCCTCT	PB	PB-2	P2-17	TRUE	3	TRA:CAVQASNAGNMLTF TRB:CASSVEQGNVETQYF	30	CD8-C7
PB-2_ACGCAGCCAGTCACTA	PB	PB-2	P2-43	TRUE	3	TRA:CVVWEADFNKFYF TRB:CATSAGNNQPQHF	11	CD8-C7
PB-2_ACGCCGATCTTACCGC	PB	PB-2	P2-1	TRUE	3	TRA:CVVNIIGYQKVTF TRB:CASTRQGKQPQHF	270	CD8-C7

PB-2_ACGGCCATCAGGTAAA	PB	PB-2	P2-4	TRUE	3	TRA:CAIRYQSFNFGNEKLTF TRB:CASYRRVTEQYF	148	CD8-C5
PB-2_ACGGGCTCAGCTCCGA	PB	PB-2	P2-3	TRUE	3	TRA:CAVNPLESSYKLIF TRB:CASSPGTANTGELFF	177	CD8-C5
PB-2_ACGGGCTTCATGGTCA	PB	PB-2	P2-7	TRUE	3	TRA:CAESDSGNTPLVF TRB:CASSYSTPRGEAFF	84	CD8-C6
PB-2_ACGTCAATCACGCATA	PB	PB-2	P2-7	TRUE	3	TRA:CAESDSGNTPLVF TRB:CASSYSTPRGEAFF	84	CD8-C7
PB-2_ACTATCTTCCCAAGAT	PB	PB-2	P2-1	TRUE	3	TRA:CVVNIIGYQKVTF TRB:CASTRQGKQPQHF	270	CD8-C7
PB-2_ACTGAACCAAGGCTCC	PB	PB-2	P2-12	TRUE	3	TRA:CGADAEYGNKLVF TRB:CASSEGQGAIMQPQHF	42	CD8-C5
PB-2_ACTGAGTAGAGCAATT	PB	PB-2	P2-5	TRUE	3	TRA:CASSFSGGYNKLIF TRB:CASSYLGAGYQETQYF	127	CD8-C7
PB-2_ACTGAGTAGTCGCCGT	PB	PB-2	P2-7	TRUE	3	TRA:CAESDSGNTPLVF TRB:CASSYSTPRGEAFF	84	CD8-C7
PB-2_ACTGAGTAGTGGTAGC	PB	PB-2	P2-65	TRUE	3	TRA:CAPNQAGTALIF TRB:CASKLSGTSGSYEQYF	8	CD8-C7
PB-2_ACTGATGGTAAGTAGT	PB	PB-2	P2-4	TRUE	3	TRA:CAIRYQSFNFGNEKLTF TRB:CASYRRVTEQYF	148	CD8-C7
PB-2_ACTGCTCAGAATAGGG	PB	PB-2	P2-8	TRUE	3	TRA:CAALEGDNYGQNFVF TRB:CASSPTWGGVRDTQYF	53	CD8-C5
PB-2_ACTTACTGTAAATGAC	PB	PB-2	P2-17	TRUE	3	TRA:CAVQASNAGNMLTF TRB:CASSVEQGNVETQYF	30	CD8-C7
PB-2_ACTTACTGTGATGTGG	PB	PB-2	P2-1	TRUE	3	TRA:CVVNIIGYQKVTF TRB:CASTRQGKQPQHF	270	CD4-C6
PB-2_ACTTACTTCGACAGCC	PB	PB-2	P2-27	FALSE	3	TRA:CAPKSYNTDKLIF TRB:CASSEGGGGAETQYF	22	CD8-C7
PB-2_ACTTTCAGTGAGTATA	PB	PB-2	P2-5	TRUE	3	TRA:CASSFSGGYNKLIF TRB:CASSYLGAGYQETQYF	127	CD8-C7
PB-2_AGAATAGAGTTGTCGT	PB	PB-2	P2-7	TRUE	3	TRA:CAESDSGNTPLVF TRB:CASSYSTPRGEAFF	84	CD8-C7
PB-2_AGAATAGCAATTCCTT	PB	PB-2	P2-7	TRUE	3	TRA:CAESDSGNTPLVF TRB:CASSYSTPRGEAFF	84	CD8-C7
PB-2_AGAATAGCACCACGTG	PB	PB-2	P2-7	TRUE	3	TRA:CAESDSGNTPLVF TRB:CASSYSTPRGEAFF	84	CD8-C7
PB-2_AGAATAGGTGCTGTAT	PB	PB-2	P2-1	TRUE	3	TRA:CVVNIIGYQKVTF TRB:CASTRQGKQPQHF	270	CD8-C7
PB-2_AGACGTTGTTTCGCTC	PB	PB-2	P2-7	TRUE	3	TRA:CAESDSGNTPLVF TRB:CASSYSTPRGEAFF	84	CD8-C7
PB-2_AGACGTTTCTGATACG	PB	PB-2	P2-7	TRUE	3	TRA:CAESDSGNTPLVF TRB:CASSYSTPRGEAFF	84	CD8-C7
PB-2_AGAGTGGGTTGGGACA	PB	PB-2	P2-12	TRUE	3	TRA:CGADAEYGNKLVF TRB:CASSEGQGAIMQPQHF	42	CD8-C7
PB-2_AGATTGCCACAGGTTT	PB	PB-2	P2-7	TRUE	3	TRA:CAESDSGNTPLVF TRB:CASSYSTPRGEAFF	84	CD8-C6
PB-2_AGCAGCCAGAAACCAT	PB	PB-2	P2-37	TRUE	3	TRA:CLVVIRHRWGSQGNLIF TRB:CASSFRTLTPLQETQYF	14	CD8-C7
PB-2_AGCAGCCAGTGGAGTC	PB	PB-2	P2-1	TRUE	3	TRA:CVVNIIGYQKVTF TRB:CASTRQGKQPQHF	270	CD8-C7
PB-2_AGCATACCACCATGTA	PB	PB-2	P2-4	TRUE	3	TRA:CAIRYQSFNFGNEKLTF TRB:CASYRRVTEQYF	148	CD8-C7
PB-2_AGCGGTCCACAGACAG	PB	PB-2	P2-12	TRUE	3	TRA:CGADAEYGNKLVF TRB:CASSEGQGAIMQPQHF	42	CD8-C7

PB-2_AGCGGTCGTGCTTCTC	PB	PB-2	P2-3	TRUE	3	TRA:CAVNPLESSYKLIF TRB:CASSPGTANTGELFF	177	CD8-C7
PB-2_AGCGTCGCATTATCTC	PB	PB-2	P2-12	TRUE	3	TRA:CGADAEYGNKLVF TRB:CASSEGQGAIMQPQHF	42	CD8-C5
PB-2_AGCTCCTGTTCGGCAC	PB	PB-2	P2-995	FALSE	1	TRA:CAVRGSYIPTF TRB:CASSQDRLGGGTYEQYF	1	CD8-C7
PB-2_AGCTCCTTCATTCACT	PB	PB-2	P2-29	TRUE	3	TRA:CAVGGDFNKFYF TRB:CSARSDRLGYQPQHF	21	CD8-C7
PB-2_AGCTCCTTCCCTGACT	PB	PB-2	P2-996	FALSE	1	TRA:CAVIRQGAQKLVF TRB:CASSQDPPRGYTGELFF	1	CD8-C7
PB-2_AGCTTGACAAGGTTCT	PB	PB-2	P2-3	TRUE	3	TRA:CAVNPLESSYKLIF TRB:CASSPGTANTGELFF	177	CD8-C7
PB-2_AGCTTGACATGCCTTC	PB	PB-2	P2-17	TRUE	3	TRA:CAVQASNAGNMLTF TRB:CASSVEQGNVETQYF	30	CD8-C7
PB-2_AGCTTGAGTCTGCGGT	PB	PB-2	P2-5	TRUE	3	TRA:CASSFSGGYNKLIF TRB:CASSYLGAGYQETQYF	127	CD8-C7
PB-2_AGCTTGATCCCAAGTA	PB	PB-2	P2-11	TRUE	3	TRA:CAVHNQGGKLIF TRB:CASSYLGAGYQETQYF	43	CD8-C7
PB-2_AGGCCGTAGCATGGCA	PB	PB-2	P2-1013	FALSE	1	TRA:CARVDTGRRALTF TRB:CAWSLGGAIQETQYF	1	CD8-C7
PB-2_AGGCCGTTCAGGCAAG	PB	PB-2	P2-4	TRUE	3	TRA:CAIRYQSFNFGNEKLTF TRB:CASYRRVTEQYF	148	CD8-C7
PB-2_AGGGAGTCATTTCACT	PB	PB-2	P2-1	TRUE	3	TRA:CVVNIIGYQKVTF TRB:CASTRQGKQPQHF	270	CD8-C7
PB-2_AGGTCATTCCACGACG	PB	PB-2	P2-3	TRUE	3	TRA:CAVNPLESSYKLIF TRB:CASSPGTANTGELFF	177	CD8-C7
PB-2_AGTCTTTCATAGACTC	PB	PB-2	P2-3	TRUE	3	TRA:CAVNPLESSYKLIF TRB:CASSPGTANTGELFF	177	CD8-C7
PB-2_AGTGAGGCACCAGATT	PB	PB-2	P2-3	TRUE	3	TRA:CAVNPLESSYKLIF TRB:CASSPGTANTGELFF	177	CD8-C7
PB-2_AGTGGGAGTCATCCCT	PB	PB-2	P2-4	TRUE	3	TRA:CAIRYQSFNFGNEKLTF TRB:CASYRRVTEQYF	148	CD8-C7
PB-2_AGTGTCATCAGGCAAG	PB	PB-2	P2-5	TRUE	3	TRA:CASSFSGGYNKLIF TRB:CASSYLGAGYQETQYF	127	CD8-C7
PB-2_AGTTGGTCAATCACAC	PB	PB-2	P2-3	TRUE	3	TRA:CAVNPLESSYKLIF TRB:CASSPGTANTGELFF	177	CD8-C7
PB-2_ATAAGAGTCCAAGTAC	PB	PB-2	P2-1	TRUE	3	TRA:CVVNIIGYQKVTF TRB:CASTRQGKQPQHF	270	CD8-C7
PB-2_ATAGACCAGGGAGTAA	PB	PB-2	P2-1	TRUE	3	TRA:CVVNIIGYQKVTF TRB:CASTRQGKQPQHF	270	CD8-C7
PB-2_ATCACGAGTGAAATCA	PB	PB-2	P2-5	TRUE	3	TRA:CASSFSGGYNKLIF TRB:CASSYLGAGYQETQYF	127	CD8-C6
PB-2_ATCATCTGTAACGTTC	PB	PB-2	P2-5	TRUE	3	TRA:CASSFSGGYNKLIF TRB:CASSYLGAGYQETQYF	127	CD8-C7
PB-2_ATCATGGGTAAACCTC	PB	PB-2	P2-5	TRUE	3	TRA:CASSFSGGYNKLIF TRB:CASSYLGAGYQETQYF	127	CD8-C7
PB-2_ATCATGGTCGGATGGA	PB	PB-2	P2-97	TRUE	3	TRA:CALLQGGSYIPTF TRB:CASSKTGGTDTQYF	5	CD4-C6
PB-2_ATCCACCAGAACAATC	PB	PB-2	P2-5	TRUE	3	TRA:CASSFSGGYNKLIF TRB:CASSYLGAGYQETQYF	127	CD8-C5
PB-2_ATCCACCTCCACGAAT	PB	PB-2	P2-7	TRUE	3	TRA:CAESDSGNTPLVF TRB:CASSYSTPRGEAFF	84	CD8-C7
PB-2_ATCCGAAAGACCGGAT	PB	PB-2	P2-37	TRUE	3	TRA:CLVVIRHRWGSQGNLIF TRB:CASSFRTLTPLQETQYF	14	CD8-C7

PB-2_ATCCGAACAATCCGAT	PB	PB-2	P2-1	TRUE	3	TRA:CVVNIIGYQKVTF TRB:CASTRQGKQPQHF	270	CD4-C6
PB-2_ATCGAGTCACAACGCC	PB	PB-2	P2-5	TRUE	3	TRA:CASSFSGGYNKLIF TRB:CASSYLGAGYQETQYF	127	CD8-C7
PB-2_ATCGAGTCAGATGGGT	PB	PB-2	P2-1	TRUE	3	TRA:CVVNIIGYQKVTF TRB:CASTRQGKQPQHF	270	CD8-C7
PB-2_ATCTACTAGTCACGCC	PB	PB-2	P2-1	TRUE	3	TRA:CVVNIIGYQKVTF TRB:CASTRQGKQPQHF	270	CD4-C6
PB-2_ATCTGCCCATCCGTGG	PB	PB-2	P2-4	TRUE	3	TRA:CAIRYQSFNFGNEKLTF TRB:CASYRRVTEQYF	148	CD8-C6
PB-2_ATGAGGGGTTACGCGC	PB	PB-2	P2-5	TRUE	3	TRA:CASSFSGGYNKLIF TRB:CASSYLGAGYQETQYF	127	CD8-C7
PB-2_ATGTGTGCATTACCTT	PB	PB-2	P2-103	FALSE	3	TRA:CAVGALSRDDKIIF TRB:CASSQSPGQIYGYTF	5	CD8-C5
PB-2_ATTACTCCAACTGCGC	PB	PB-2	P2-3	TRUE	3	TRA:CAVNPLESSYKLIF TRB:CASSPGTANTGELFF	177	CD8-C7
PB-2_ATTACTCGTGTATGGG	PB	PB-2	P2-12	TRUE	3	TRA:CGADAEYGNKLVF TRB:CASSEGQGAIMQPQHF	42	CD8-C7
PB-2_ATTATCCAGGGTGTTG	PB	PB-2	P2-5	TRUE	3	TRA:CASSFSGGYNKLIF TRB:CASSYLGAGYQETQYF	127	CD8-C7
PB-2_ATTATCCGTGCAGTAG	PB	PB-2	P2-17	TRUE	3	TRA:CAVQASNAGNMLTF TRB:CASSVEQGNVETQYF	30	CD8-C5
PB-2_ATTATCCTCAAACAAG	PB	PB-2	P2-5	TRUE	3	TRA:CASSFSGGYNKLIF TRB:CASSYLGAGYQETQYF	127	CD8-C5
PB-2_ATTATCCTCACTCTTA	PB	PB-2	P2-37	TRUE	3	TRA:CLVVIRHRWGSQGNLIF TRB:CASSFRTLTPLQETQYF	14	CD8-C7
PB-2_ATTATCCTCCGTACAA	PB	PB-2	P2-5	TRUE	3	TRA:CASSFSGGYNKLIF TRB:CASSYLGAGYQETQYF	127	CD8-C7
PB-2_ATTATCCTCTTGTCAT	PB	PB-2	P2-1	TRUE	3	TRA:CVVNIIGYQKVTF TRB:CASTRQGKQPQHF	270	CD4-C6
PB-2_ATTCTACGTACATCCA	PB	PB-2	P2-3	TRUE	3	TRA:CAVNPLESSYKLIF TRB:CASSPGTANTGELFF	177	CD8-C7
PB-2_ATTTCTGAGGCAATTA	PB	PB-2	P2-17	TRUE	3	TRA:CAVQASNAGNMLTF TRB:CASSVEQGNVETQYF	30	CD8-C7
PB-2_CAACCTCTCCAAAGTC	PB	PB-2	P2-4	TRUE	3	TRA:CAIRYQSFNFGNEKLTF TRB:CASYRRVTEQYF	148	CD8-C5
PB-2_CAAGATCCAACCGCCA	PB	PB-2	P2-1219	FALSE	1	TRA:CALSEDSQAGTALIF TRB:CASSLSASYNEQFF	1	CD8-C7
PB-2_CAAGGCCAGTGTCTCA	PB	PB-2	P2-1	TRUE	3	TRA:CVVNIIGYQKVTF TRB:CASTRQGKQPQHF	270	CD8-C7
PB-2_CACAAACCAGTAACGG	PB	PB-2	P2-55	TRUE	3	TRA:CIVRGIQGAQKLVF TRB:CASSLQGGGSGGLTQYF	10	CD8-C6
PB-2_CACAAACTCGTAGGAG	PB	PB-2	P2-2	TRUE	3	TRA:CVVPNTNAGKSTF TRB:CASSLQGSTYNEQFF	220	CD8-C5
PB-2_CACACCTAGTGTACGG	PB	PB-2	P2-11	TRUE	3	TRA:CAVHNQGGKLIF TRB:CASSYLGAGYQETQYF	43	CD8-C7
PB-2_CACACCTAGTGTCCCG	PB	PB-2	P2-5	TRUE	3	TRA:CASSFSGGYNKLIF TRB:CASSYLGAGYQETQYF	127	CD8-C7
PB-2_CACACCTTCTACCTGC	PB	PB-2	P2-7	TRUE	3	TRA:CAESDSGNTPLVF TRB:CASSYSTPRGEAFF	84	CD8-C7
PB-2_CACACCTTCTCGCTTG	PB	PB-2	P2-1	TRUE	3	TRA:CVVNIIGYQKVTF TRB:CASTRQGKQPQHF	270	CD4-C6
PB-2_CACAGGCCAGCCAGAA	PB	PB-2	P2-1	TRUE	3	TRA:CVVNIIGYQKVTF TRB:CASTRQGKQPQHF	270	CD8-C7

PB-2_CACAGTAAGCTGAAAT	PB	PB-2	P2-5	TRUE	3	TRA:CASSFSGGYNKLIF TRB:CASSYLGAGYQETQYF	127	CD8-C7
PB-2_CACATAGTCGTACCGG	PB	PB-2	P2-11	TRUE	3	TRA:CAVHNQGGKLIF TRB:CASSYLGAGYQETQYF	43	CD8-C5
PB-2_CACCACTTCTCGGACG	PB	PB-2	P2-8	TRUE	3	TRA:CAALEGDNYGGQNFVF TRB:CASSPTWGGVRDTQYF	53	CD8-C7
PB-2_CACTCCACAGCTGTTA	PB	PB-2	P2-1	TRUE	3	TRA:CVVNIIGYQKVTF TRB:CASTRQGKQPQHF	270	CD8-C7
PB-2_CACTCCATCACTGGGC	PB	PB-2	P2-12	TRUE	3	TRA:CGADAEYGNKLVF TRB:CASSEGQGAIMQPQHF	42	CD8-C7
PB-2_CAGAATCGTCCTAGCG	PB	PB-2	P2-5	TRUE	3	TRA:CASSFSGGYNKLIF TRB:CASSYLGAGYQETQYF	127	CD8-C7
PB-2_CAGATCATCCTACAGA	PB	PB-2	P2-5	TRUE	3	TRA:CASSFSGGYNKLIF TRB:CASSYLGAGYQETQYF	127	CD8-C7
PB-2_CAGCAGCTCGCCTGTT	PB	PB-2	P2-1	TRUE	3	TRA:CVVNIIGYQKVTF TRB:CASTRQGKQPQHF	270	CD8-C7
PB-2_CAGCAGCTCTAACTGG	PB	PB-2	P2-4	TRUE	3	TRA:CAIRYQSFNFGNEKLTF TRB:CASYRRVTEQYF	148	CD8-C7
PB-2_CAGCATACATACGCTA	PB	PB-2	P2-4	TRUE	3	TRA:CAIRYQSFNFGNEKLTF TRB:CASYRRVTEQYF	148	CD8-C7
PB-2_CAGCCGATCATTTGGG	PB	PB-2	P2-12	TRUE	3	TRA:CGADAEYGNKLVF TRB:CASSEGQGAIMQPQHF	42	CD8-C5
PB-2_CAGCTAAAGATAGTCA	PB	PB-2	P2-1	TRUE	3	TRA:CVVNIIGYQKVTF TRB:CASTRQGKQPQHF	270	CD8-C7
PB-2_CAGCTGGCAGACGCCT	PB	PB-2	P2-5	TRUE	3	TRA:CASSFSGGYNKLIF TRB:CASSYLGAGYQETQYF	127	CD8-C7
PB-2_CAGCTGGTCGACAGCC	PB	PB-2	P2-5	TRUE	3	TRA:CASSFSGGYNKLIF TRB:CASSYLGAGYQETQYF	127	CD8-C5
PB-2_CAGGTGCTCCTTGACC	PB	PB-2	P2-37	TRUE	3	TRA:CLVVIRHRWGSQGNLIF TRB:CASSFRTLTPLQETQYF	14	CD8-C7
PB-2_CAGTAACCAACGCACC	PB	PB-2	P2-4	TRUE	3	TRA:CAIRYQSFNFGNEKLTF TRB:CASYRRVTEQYF	148	CD8-C7
PB-2_CAGTAACCAAGCCGTC	PB	PB-2	P2-1351	FALSE	1	TRA:CASSFSGGYNKLIF TRB:CASSPPSAGGGNEQYF	1	CD8-C5
PB-2_CATCAAGAGGGTCTCC	PB	PB-2	P2-54	FALSE	3	TRA:CALNFMNRDDKIIF TRB:CASSYSPGQAASYGYTF	10	CD8-C7
PB-2_CATCAGACAAGTTGTC	PB	PB-2	P2-12	TRUE	3	TRA:CGADAEYGNKLVF TRB:CASSEGQGAIMQPQHF	42	CD8-C7
PB-2_CATCGAAAGACACGAC	PB	PB-2	P2-3	TRUE	3	TRA:CAVNPLESSYKLIF TRB:CASSPGTANTGELFF	177	CD8-C7
PB-2_CATCGGGAGACTAGGC	PB	PB-2	P2-5	TRUE	3	TRA:CASSFSGGYNKLIF TRB:CASSYLGAGYQETQYF	127	CD8-C7
PB-2_CATGCCTGTGGTCTCG	PB	PB-2	P2-7	TRUE	3	TRA:CAESDSGNTPLVF TRB:CASSYSTPRGEAFF	84	CD8-C7
PB-2_CATGGCGAGATGTTAG	PB	PB-2	P2-32	TRUE	3	TRA:CAMREANVGADGLTF TRB:CASSTRIAGGPFPETQYF	17	CD8-C6
PB-2_CATTATCAGCTAAGAT	PB	PB-2	P2-17	TRUE	3	TRA:CAVQASNAGNMLTF TRB:CASSVEQGNVETQYF	30	CD8-C5
PB-2_CATTATCCATCCTAGA	PB	PB-2	P2-1	TRUE	3	TRA:CVVNIIGYQKVTF TRB:CASTRQGKQPQHF	270	CD8-C7
PB-2_CATTCGCTCCACTCCA	PB	PB-2	P2-11	TRUE	3	TRA:CAVHNQGGKLIF TRB:CASSYLGAGYQETQYF	43	CD8-C7
PB-2_CCAATCCAGTACGTTC	PB	PB-2	P2-11	TRUE	3	TRA:CAVHNQGGKLIF TRB:CASSYLGAGYQETQYF	43	CD8-C7

PB-2_CCAATCCCATTCCTCG	PB	PB-2	P2-1	TRUE	3	TRA:CVVNIIGYQKVTF TRB:CASTRQGKQPQHF	270	CD8-C7
PB-2_CCACCTACATTGTGCA	PB	PB-2	P2-307	FALSE	2	TRA:CVVNIIGYQKVTF TRB:CASYRRVTEQYF	2	CD8-C7
PB-2_CCACGGAAGACTAGGC	PB	PB-2	P2-4	TRUE	3	TRA:CAIRYQSFNFGNEKLTF TRB:CASYRRVTEQYF	148	CD8-C5
PB-2_CCACGGATCACATGCA	PB	PB-2	P2-4	TRUE	3	TRA:CAIRYQSFNFGNEKLTF TRB:CASYRRVTEQYF	148	CD8-C7
PB-2_CCACTACAGTCCCACG	PB	PB-2	P2-4	TRUE	3	TRA:CAIRYQSFNFGNEKLTF TRB:CASYRRVTEQYF	148	CD8-C7
PB-2_CCACTACGTCCGAAGA	PB	PB-2	P2-4	TRUE	3	TRA:CAIRYQSFNFGNEKLTF TRB:CASYRRVTEQYF	148	CD8-C7
PB-2_CCATGTCGTTACGTCA	PB	PB-2	P2-3	TRUE	3	TRA:CAVNPLESSYKLIF TRB:CASSPGTANTGELFF	177	CD8-C7
PB-2_CCATGTCTCACCCTCA	PB	PB-2	P2-4	TRUE	3	TRA:CAIRYQSFNFGNEKLTF TRB:CASYRRVTEQYF	148	CD8-C7
PB-2_CCATGTCTCTGTTGAG	PB	PB-2	P2-17	TRUE	3	TRA:CAVQASNAGNMLTF TRB:CASSVEQGNVETQYF	30	CD8-C7
PB-2_CCATTCGAGATGTCGG	PB	PB-2	P2-17	TRUE	3	TRA:CAVQASNAGNMLTF TRB:CASSVEQGNVETQYF	30	CD8-C6
PB-2_CCATTCGAGCCTATGT	PB	PB-2	P2-67	FALSE	3	TRA:CVVSDPLWDNYGQNFVF TRB:CASSPSGGLLHYNEQFF	8	CD8-C6
PB-2_CCCAATCAGAGATGAG	PB	PB-2	P2-5	TRUE	3	TRA:CASSFSGGYNKLIF TRB:CASSYLGAGYQETQYF	127	CD8-C5
PB-2_CCCAGTTGTCTAACGT	PB	PB-2	P2-3	TRUE	3	TRA:CAVNPLESSYKLIF TRB:CASSPGTANTGELFF	177	CD8-C5
PB-2_CCCTCCTAGCCGGTAA	PB	PB-2	P2-5	TRUE	3	TRA:CASSFSGGYNKLIF TRB:CASSYLGAGYQETQYF	127	CD8-C5
PB-2_CCGGGATCAGACAAGC	PB	PB-2	P2-3	TRUE	3	TRA:CAVNPLESSYKLIF TRB:CASSPGTANTGELFF	177	CD8-C5
PB-2_CCGGTAGAGTACGCCC	PB	PB-2	P2-4	TRUE	3	TRA:CAIRYQSFNFGNEKLTF TRB:CASYRRVTEQYF	148	CD8-C7
PB-2_CCGTACTAGAAGGGTA	PB	PB-2	P2-1	TRUE	3	TRA:CVVNIIGYQKVTF TRB:CASTRQGKQPQHF	270	CD8-C7
PB-2_CCGTACTTCACCGGGT	PB	PB-2	P2-12	TRUE	3	TRA:CGADAEYGNKLVF TRB:CASSEGQGAIMQPQHF	42	CD8-C5
PB-2_CCTAAAGCACTCTGTC	PB	PB-2	P2-27	FALSE	3	TRA:CAPKSYNTDKLIF TRB:CASSEGGGGAETQYF	22	CD8-C6
PB-2_CCTAAAGCATGTCCTC	PB	PB-2	P2-12	TRUE	3	TRA:CGADAEYGNKLVF TRB:CASSEGQGAIMQPQHF	42	CD8-C5
PB-2_CCTAGCTAGCTGATAA	PB	PB-2	P2-7	TRUE	3	TRA:CAESDSGNTPLVF TRB:CASSYSTPRGEAFF	84	CD8-C7
PB-2_CCTAGCTCAGTAGAGC	PB	PB-2	P2-1	TRUE	3	TRA:CVVNIIGYQKVTF TRB:CASTRQGKQPQHF	270	CD8-C7
PB-2_CCTATTAAGATCACGG	PB	PB-2	P2-5	TRUE	3	TRA:CASSFSGGYNKLIF TRB:CASSYLGAGYQETQYF	127	CD8-C7
PB-2_CCTATTAAGCCATCGC	PB	PB-2	P2-103	FALSE	3	TRA:CAVGALSRDDKIIF TRB:CASSQSPGQIYGYTF	5	CD8-C7
PB-2_CCTCAGTGTCGACTGC	PB	PB-2	P2-1	TRUE	3	TRA:CVVNIIGYQKVTF TRB:CASTRQGKQPQHF	270	CD8-C7
PB-2_CCTCTGAAGACGCAAC	PB	PB-2	P2-1	TRUE	3	TRA:CVVNIIGYQKVTF TRB:CASTRQGKQPQHF	270	CD8-C7
PB-2_CCTCTGATCGGCGCAT	PB	PB-2	P2-3	TRUE	3	TRA:CAVNPLESSYKLIF TRB:CASSPGTANTGELFF	177	CD8-C5

PB-2_CCTTACGCATAGACTC	PB	PB-2	P2-4	TRUE	3	TRA:CAIRYQSFNFGNEKLTF TRB:CASYRRVTEQYF	148	CD8-C5
PB-2_CCTTACGGTTCACGGC	PB	PB-2	P2-7	TRUE	3	TRA:CAESDSGNTPLVF TRB:CASSYSTPRGEAFF	84	CD8-C7
PB-2_CCTTACGGTTGATTCG	PB	PB-2	P2-309	FALSE	2	TRA:CAVNMFQSDYKLSF TRB:CSARASSAREQFF	2	CD8-C7
PB-2_CCTTCGAAGGCCCTCA	PB	PB-2	P2-11	TRUE	3	TRA:CAVHNQGGKLIF TRB:CASSYLGAGYQETQYF	43	CD8-C7
PB-2_CCTTCGAGTTTGTTTC	PB	PB-2	P2-5	TRUE	3	TRA:CASSFSGGYNKLIF TRB:CASSYLGAGYQETQYF	127	CD8-C5
PB-2_CCTTTCTCACCTATCC	PB	PB-2	P2-3	TRUE	3	TRA:CAVNPLESSYKLIF TRB:CASSPGTANTGELFF	177	CD8-C7
PB-2_CGAACATCACCCATGG	PB	PB-2	P2-12	TRUE	3	TRA:CGADAEYGNKLVF TRB:CASSEGQGAIMQPQHF	42	CD8-C7
PB-2_CGACCTTAGACAAAGG	PB	PB-2	P2-86	TRUE	3	TRA:CAVTPLRNTDKLIF TRB:CSASPSGRGRGEQFF	6	CD8-C7
PB-2_CGACCTTGTCGAACAG	PB	PB-2	P2-1	TRUE	3	TRA:CVVNIIGYQKVTF TRB:CASTRQGKQPQHF	270	CD8-C6
PB-2_CGACTTCTCTGCAAGT	PB	PB-2	P2-7	TRUE	3	TRA:CAESDSGNTPLVF TRB:CASSYSTPRGEAFF	84	CD8-C7
PB-2_CGAGCACGTCCGTGAC	PB	PB-2	P2-1649	FALSE	1	TRA:CAIRYQSFNFGNEKLTF TRB:CASSEGQGAIMQPQHF	1	CD8-C7
PB-2_CGATCGGAGCGTTCCG	PB	PB-2	P2-4	TRUE	3	TRA:CAIRYQSFNFGNEKLTF TRB:CASYRRVTEQYF	148	CD8-C7
PB-2_CGATCGGCACACTGCG	PB	PB-2	P2-3	TRUE	3	TRA:CAVNPLESSYKLIF TRB:CASSPGTANTGELFF	177	CD8-C7
PB-2_CGATGGCGTTCTGGTA	PB	PB-2	P2-1	TRUE	3	TRA:CVVNIIGYQKVTF TRB:CASTRQGKQPQHF	270	CD8-C7
PB-2_CGCCAAGCAACACGCC	PB	PB-2	P2-3	TRUE	3	TRA:CAVNPLESSYKLIF TRB:CASSPGTANTGELFF	177	CD8-C5
PB-2_CGCGTTTAGCGTAGTG	PB	PB-2	P2-7	TRUE	3	TRA:CAESDSGNTPLVF TRB:CASSYSTPRGEAFF	84	CD8-C7
PB-2_CGCGTTTCAGTCTTCC	PB	PB-2	P2-1	TRUE	3	TRA:CVVNIIGYQKVTF TRB:CASTRQGKQPQHF	270	CD8-C7
PB-2_CGCTATCAGACTGGGT	PB	PB-2	P2-3	TRUE	3	TRA:CAVNPLESSYKLIF TRB:CASSPGTANTGELFF	177	CD8-C7
PB-2_CGCTTCACAGGCGATA	PB	PB-2	P2-1	TRUE	3	TRA:CVVNIIGYQKVTF TRB:CASTRQGKQPQHF	270	CD8-C7
PB-2_CGCTTCAGTGTAACGG	PB	PB-2	P2-1709	FALSE	1	TRA:CAVNDYKLSF TRB:CSVYGETGYGYTF	1	CD8-C7
PB-2_CGGACACAGCGATTCT	PB	PB-2	P2-1	TRUE	3	TRA:CVVNIIGYQKVTF TRB:CASTRQGKQPQHF	270	CD8-C7
PB-2_CGGACACCAATCGAAA	PB	PB-2	P2-168	FALSE	3	TRA:CIVETSYDKVIF TRB:CATARDSNQPQHF	3	CD8-C5
PB-2_CGGACACGTCTCACCT	PB	PB-2	P2-3	TRUE	3	TRA:CAVNPLESSYKLIF TRB:CASSPGTANTGELFF	177	CD8-C7
PB-2_CGGACGTTCTCCAACC	PB	PB-2	P2-3	TRUE	3	TRA:CAVNPLESSYKLIF TRB:CASSPGTANTGELFF	177	CD8-C7
PB-2_CGGACTGAGATCCCGC	PB	PB-2	P2-96	FALSE	3	TRA:CAMRGANFGNEKLTF TRB:CASSRDFGGPGQETQYF	5	CD8-C7
PB-2_CGGACTGCACACAGAG	PB	PB-2	P2-7	TRUE	3	TRA:CAESDSGNTPLVF TRB:CASSYSTPRGEAFF	84	CD8-C7
PB-2_CGGACTGGTATCAGTC	PB	PB-2	P2-3	TRUE	3	TRA:CAVNPLESSYKLIF TRB:CASSPGTANTGELFF	177	CD8-C5

PB-2_CGGAGCTAGGACATTA	PB	PB-2	P2-12	TRUE	3	TRA:CGADAEYGNKLVF TRB:CASSEGQGAIMQPQHF	42	CD8-C7
PB-2_CGGAGCTCATACCATG	PB	PB-2	P2-4	TRUE	3	TRA:CAIRYQSFNFGNEKLTF TRB:CASYRRVTEQYF	148	CD8-C7
PB-2_CGGAGCTGTACACCGC	PB	PB-2	P2-46	TRUE	3	TRA:CAFSTWGNQGGKLIF TRB:CASSQDRLGGGTYEQYF	11	CD8-C7
PB-2_CGGAGTCCACCTCGGA	PB	PB-2	P2-5	TRUE	3	TRA:CASSFSGGYNKLIF TRB:CASSYLGAGYQETQYF	127	CD8-C7
PB-2_CGGCTAGCATAACCTG	PB	PB-2	P2-5	TRUE	3	TRA:CASSFSGGYNKLIF TRB:CASSYLGAGYQETQYF	127	CD8-C7
PB-2_CGGCTAGGTACTTGAC	PB	PB-2	P2-7	TRUE	3	TRA:CAESDSGNTPLVF TRB:CASSYSTPRGEAFF	84	CD8-C7
PB-2_CGGGTCAAGCCAACAG	PB	PB-2	P2-1	TRUE	3	TRA:CVVNIIGYQKVTF TRB:CASTRQGKQPQHF	270	CD8-C7
PB-2_CGGGTCACACGAAGCA	PB	PB-2	P2-3	TRUE	3	TRA:CAVNPLESSYKLIF TRB:CASSPGTANTGELFF	177	CD8-C7
PB-2_CGGGTCATCGCCCTTA	PB	PB-2	P2-1	TRUE	3	TRA:CVVNIIGYQKVTF TRB:CASTRQGKQPQHF	270	CD8-C7
PB-2_CGGTTAAAGGTAAACT	PB	PB-2	P2-7	TRUE	3	TRA:CAESDSGNTPLVF TRB:CASSYSTPRGEAFF	84	CD8-C7
PB-2_CGTCACTGTTCCGTCT	PB	PB-2	P2-17	TRUE	3	TRA:CAVQASNAGNMLTF TRB:CASSVEQGNVETQYF	30	CD8-C5
PB-2_CGTCAGGAGCAGATCG	PB	PB-2	P2-1	TRUE	3	TRA:CVVNIIGYQKVTF TRB:CASTRQGKQPQHF	270	CD8-C7
PB-2_CGTCAGGTCAGTTCGA	PB	PB-2	P2-17	TRUE	3	TRA:CAVQASNAGNMLTF TRB:CASSVEQGNVETQYF	30	CD8-C7
PB-2_CGTCCATTCGAATGGG	PB	PB-2	P2-7	TRUE	3	TRA:CAESDSGNTPLVF TRB:CASSYSTPRGEAFF	84	CD8-C6
PB-2_CGTGTAAAGATCCCGC	PB	PB-2	P2-7	TRUE	3	TRA:CAESDSGNTPLVF TRB:CASSYSTPRGEAFF	84	CD8-C7
PB-2_CGTGTAAGTGGCCCTA	PB	PB-2	P2-3	TRUE	3	TRA:CAVNPLESSYKLIF TRB:CASSPGTANTGELFF	177	CD8-C5
PB-2_CGTGTCTTCTTGCCGT	PB	PB-2	P2-1	TRUE	3	TRA:CVVNIIGYQKVTF TRB:CASTRQGKQPQHF	270	CD4-C6
PB-2_CGTTAGATCTCGCATC	PB	PB-2	P2-11	TRUE	3	TRA:CAVHNQGGKLIF TRB:CASSYLGAGYQETQYF	43	CD8-C7
PB-2_CGTTGGGGTCGCATCG	PB	PB-2	P2-5	TRUE	3	TRA:CASSFSGGYNKLIF TRB:CASSYLGAGYQETQYF	127	CD8-C5
PB-2_CTAACTTCAAGGTTTC	PB	PB-2	P2-317	TRUE	2	TRA:CAGYQIKAAGNKLTF TRB:CASSWRQKDTQYF	2	CD8-C7
PB-2_CTAAGACGTGTGCCTG	PB	PB-2	P2-77	FALSE	3	TRA:CAVDSGWGRSGGSNYKLTF TRB:CASSADRPYSYEQYF	7	CD8-C6
PB-2_CTAAGACTCGAGGTAG	PB	PB-2	P2-189	TRUE	3	TRA:CAVRDWGRDDKIIF TRB:CASSSKPRSGANVLTF	3	CD8-C5
PB-2_CTAAGACTCTCATTCA	PB	PB-2	P2-3	TRUE	3	TRA:CAVNPLESSYKLIF TRB:CASSPGTANTGELFF	177	CD8-C7
PB-2_CTACATTTCAGCGACC	PB	PB-2	P2-12	TRUE	3	TRA:CGADAEYGNKLVF TRB:CASSEGQGAIMQPQHF	42	CD8-C7
PB-2_CTACCCAGTTTGACAC	PB	PB-2	P2-12	TRUE	3	TRA:CGADAEYGNKLVF TRB:CASSEGQGAIMQPQHF	42	CD8-C7
PB-2_CTACCCATCTTCGAGA	PB	PB-2	P2-1	TRUE	3	TRA:CVVNIIGYQKVTF TRB:CASTRQGKQPQHF	270	CD8-C7
PB-2_CTACGTCGTTTCGCTC	PB	PB-2	P2-1	TRUE	3	TRA:CVVNIIGYQKVTF TRB:CASTRQGKQPQHF	270	CD8-C7

PB-2_CTCACACCACCAGATT	PB	PB-2	P2-5	TRUE	3	TRA:CASSFSGGYNKLIF TRB:CASSYLGAGYQETQYF	127	CD8-C5
PB-2_CTCAGAACAAGGACAC	PB	PB-2	P2-1	TRUE	3	TRA:CVVNIIGYQKVTF TRB:CASTRQGKQPQHF	270	CD8-C5
PB-2_CTCATTATCTTGGGTA	PB	PB-2	P2-1	TRUE	3	TRA:CVVNIIGYQKVTF TRB:CASTRQGKQPQHF	270	CD8-C7
PB-2_CTCCTAGGTTGAGTTC	PB	PB-2	P2-65	TRUE	3	TRA:CAPNQAGTALIF TRB:CASKLSGTSGSYEQYF	8	CD8-C7
PB-2_CTCCTAGTCCGTAGGC	PB	PB-2	P2-1	TRUE	3	TRA:CVVNIIGYQKVTF TRB:CASTRQGKQPQHF	270	CD8-C7
PB-2_CTCGAAACAGGGTATG	PB	PB-2	P2-7	TRUE	3	TRA:CAESDSGNTPLVF TRB:CASSYSTPRGEAFF	84	CD8-C7
PB-2_CTCGAAATCTTATCTG	PB	PB-2	P2-11	TRUE	3	TRA:CAVHNQGGKLIF TRB:CASSYLGAGYQETQYF	43	CD8-C7
PB-2_CTCGAGGCAGACGCCT	PB	PB-2	P2-37	TRUE	3	TRA:CLVVIRHRWGSQGNLIF TRB:CASSFRTLTPLQETQYF	14	CD8-C7
PB-2_CTCGAGGGTGTGAATA	PB	PB-2	P2-1933	FALSE	1	TRA:CASSFSGGYNKLIF TRB:CASSLDLAPSTEAFF	1	CD8-C5
PB-2_CTCGGAGAGCTAGCCC	PB	PB-2	P2-12	TRUE	3	TRA:CGADAEYGNKLVF TRB:CASSEGQGAIMQPQHF	42	CD8-C7
PB-2_CTCGGGACAGGTGCCT	PB	PB-2	P2-3	TRUE	3	TRA:CAVNPLESSYKLIF TRB:CASSPGTANTGELFF	177	CD8-C5
PB-2_CTCGGGAGTGTAATGA	PB	PB-2	P2-1	TRUE	3	TRA:CVVNIIGYQKVTF TRB:CASTRQGKQPQHF	270	CD8-C7
PB-2_CTCGGGATCTATCGCC	PB	PB-2	P2-263	FALSE	2	TRA:CALTGNQFYF TRB:CASSFPGQDAEQFF	2	CD8-C7
PB-2_CTCGTACGTGAAAGAG	PB	PB-2	P2-25	FALSE	3	TRA:CAMREAPNTDKLIF TRB:CASSGGGGGLETQYF	22	CD8-C5
PB-2_CTCGTACTCTAACGGT	PB	PB-2	P2-7	TRUE	3	TRA:CAESDSGNTPLVF TRB:CASSYSTPRGEAFF	84	CD8-C7
PB-2_CTCGTACTCTCTGAGA	PB	PB-2	P2-4	TRUE	3	TRA:CAIRYQSFNFGNEKLTF TRB:CASYRRVTEQYF	148	CD8-C5
PB-2_CTCGTACTCTGAGTGT	PB	PB-2	P2-37	TRUE	3	TRA:CLVVIRHRWGSQGNLIF TRB:CASSFRTLTPLQETQYF	14	CD8-C7
PB-2_CTCGTCACATCATCCC	PB	PB-2	P2-5	TRUE	3	TRA:CASSFSGGYNKLIF TRB:CASSYLGAGYQETQYF	127	CD8-C7
PB-2_CTCTGGTAGTTAACGA	PB	PB-2	P2-4	TRUE	3	TRA:CAIRYQSFNFGNEKLTF TRB:CASYRRVTEQYF	148	CD8-C7
PB-2_CTCTGGTCAAAGGAAG	PB	PB-2	P2-5	TRUE	3	TRA:CASSFSGGYNKLIF TRB:CASSYLGAGYQETQYF	127	CD8-C7
PB-2_CTCTGGTTCCTTTACA	PB	PB-2	P2-1	TRUE	3	TRA:CVVNIIGYQKVTF TRB:CASTRQGKQPQHF	270	CD4-C6
PB-2_CTGAAACTCCGCATCT	PB	PB-2	P2-1975	FALSE	1	TRA:CAVEDDYKLSF TRB:CASRDRGVGDTQYF	1	CD8-C2
PB-2_CTGAAGTAGTCCTCCT	PB	PB-2	P2-37	TRUE	3	TRA:CLVVIRHRWGSQGNLIF TRB:CASSFRTLTPLQETQYF	14	CD8-C7
PB-2_CTGAAGTCAGTCGATT	PB	PB-2	P2-1980	FALSE	1	TRA:CGTDHNQGGKLIF TRB:CASKPVVNTGELFF	1	CD8-C5
PB-2_CTGAAGTGTTCAGTAC	PB	PB-2	P2-1981	FALSE	1	TRA:CAGILTGGGNKLTF TRB:CATSRGEVQETQYF	1	CD8-C7
PB-2_CTGAAGTTCCAGTATG	PB	PB-2	P2-3	TRUE	3	TRA:CAVNPLESSYKLIF TRB:CASSPGTANTGELFF	177	CD8-C7
PB-2_CTGATAGAGAGGTACC	PB	PB-2	P2-1	TRUE	3	TRA:CVVNIIGYQKVTF TRB:CASTRQGKQPQHF	270	CD4-C6

PB-2_CTGATAGCAATGAAAC	PB	PB-2	P2-3	TRUE	3	TRA:CAVNPLESSYKLIF TRB:CASSPGTANTGELFF	177	CD8-C5
PB-2_CTGATCCCAACTGCGC	PB	PB-2	P2-7	TRUE	3	TRA:CAESDSGNTPLVF TRB:CASSYSTPRGEAFF	84	CD8-C7
PB-2_CTGATCCTCCACTCCA	PB	PB-2	P2-5	TRUE	3	TRA:CASSFSGGYNKLIF TRB:CASSYLGAGYQETQYF	127	CD8-C7
PB-2_CTGCCTAAGTGTTGAA	PB	PB-2	P2-5	TRUE	3	TRA:CASSFSGGYNKLIF TRB:CASSYLGAGYQETQYF	127	CD8-C7
PB-2_CTGCCTACATTGGCGC	PB	PB-2	P2-1999	FALSE	1	TRA:CAVRRPGNFNKFYF TRB:CASSSSSGTGSLDTRTGELFF	1	CD8-C6
PB-2_CTGCCTATCCTGCCAT	PB	PB-2	P2-11	TRUE	3	TRA:CAVHNQGGKLIF TRB:CASSYLGAGYQETQYF	43	CD8-C7
PB-2_CTGCGGAGTCTAGTCA	PB	PB-2	P2-7	TRUE	3	TRA:CAESDSGNTPLVF TRB:CASSYSTPRGEAFF	84	CD8-C7
PB-2_CTGCTGTAGAGCCTAG	PB	PB-2	P2-65	TRUE	3	TRA:CAPNQAGTALIF TRB:CASKLSGTSGSYEQYF	8	CD8-C7
PB-2_CTGCTGTGTTCGCGAC	PB	PB-2	P2-5	TRUE	3	TRA:CASSFSGGYNKLIF TRB:CASSYLGAGYQETQYF	127	CD8-C7
PB-2_CTGGTCTTCGCAAGCC	PB	PB-2	P2-5	TRUE	3	TRA:CASSFSGGYNKLIF TRB:CASSYLGAGYQETQYF	127	CD8-C5
PB-2_CTGTTTATCCAGTATG	PB	PB-2	P2-12	TRUE	3	TRA:CGADAEYGNKLVF TRB:CASSEGQGAIMQPQHF	42	CD8-C7
PB-2_CTTAACTGTATCACCA	PB	PB-2	P2-3	TRUE	3	TRA:CAVNPLESSYKLIF TRB:CASSPGTANTGELFF	177	CD8-C5
PB-2_CTTAACTTCTTCGAGA	PB	PB-2	P2-1	TRUE	3	TRA:CVVNIIGYQKVTF TRB:CASTRQGKQPQHF	270	CD8-C7
PB-2_CTTTGCGCACTAGTAC	PB	PB-2	P2-1	TRUE	3	TRA:CVVNIIGYQKVTF TRB:CASTRQGKQPQHF	270	CD8-C7
PB-2_GAAACTCAGTGGGCTA	PB	PB-2	P2-11	TRUE	3	TRA:CAVHNQGGKLIF TRB:CASSYLGAGYQETQYF	43	CD8-C7
PB-2_GAAATGACACGCTTTC	PB	PB-2	P2-5	TRUE	3	TRA:CASSFSGGYNKLIF TRB:CASSYLGAGYQETQYF	127	CD8-C5
PB-2_GAACCTAGTGATAAGT	PB	PB-2	P2-4	TRUE	3	TRA:CAIRYQSFNFGNEKLTF TRB:CASYRRVTEQYF	148	CD8-C5
PB-2_GAATAAGAGAGCTATA	PB	PB-2	P2-1	TRUE	3	TRA:CVVNIIGYQKVTF TRB:CASTRQGKQPQHF	270	CD4-C6
PB-2_GAATAAGCAAAGGTGC	PB	PB-2	P2-3	TRUE	3	TRA:CAVNPLESSYKLIF TRB:CASSPGTANTGELFF	177	CD8-C5
PB-2_GAATGAAGTACAGCAG	PB	PB-2	P2-4	TRUE	3	TRA:CAIRYQSFNFGNEKLTF TRB:CASYRRVTEQYF	148	CD8-C7
PB-2_GACACGCAGCTCAACT	PB	PB-2	P2-11	TRUE	3	TRA:CAVHNQGGKLIF TRB:CASSYLGAGYQETQYF	43	CD8-C7
PB-2_GACAGAGAGTAGCGGT	PB	PB-2	P2-3	TRUE	3	TRA:CAVNPLESSYKLIF TRB:CASSPGTANTGELFF	177	CD8-C5
PB-2_GACCAATTCCACGACG	PB	PB-2	P2-1	TRUE	3	TRA:CVVNIIGYQKVTF TRB:CASTRQGKQPQHF	270	CD8-C7
PB-2_GACGCGTTCCGAAGAG	PB	PB-2	P2-5	TRUE	3	TRA:CASSFSGGYNKLIF TRB:CASSYLGAGYQETQYF	127	CD8-C7
PB-2_GACGTGCAGCTAACTC	PB	PB-2	P2-1	TRUE	3	TRA:CVVNIIGYQKVTF TRB:CASTRQGKQPQHF	270	CD8-C7
PB-2_GACTACAGTGAGGCTA	PB	PB-2	P2-5	TRUE	3	TRA:CASSFSGGYNKLIF TRB:CASSYLGAGYQETQYF	127	CD8-C5
PB-2_GACTGCGCATTGTGCA	PB	PB-2	P2-27	FALSE	3	TRA:CAPKSYNTDKLIF TRB:CASSEGGGGAETQYF	22	CD8-C7

PB-2_GACTGCGGTTTGACAC	PB	PB-2	P2-12	TRUE	3	TRA:CGADAEYGNKLVF TRB:CASSEGQGAIMQPQHF	42	CD8-C7
PB-2_GACTGCGTCTCCAGGG	PB	PB-2	P2-1	TRUE	3	TRA:CVVNIIGYQKVTF TRB:CASTRQGKQPQHF	270	CD8-C7
PB-2_GAGGTGAAGTGATCGG	PB	PB-2	P2-4	TRUE	3	TRA:CAIRYQSFNFGNEKLTF TRB:CASYRRVTEQYF	148	CD8-C7
PB-2_GAGGTGACAGATTGCT	PB	PB-2	P2-4	TRUE	3	TRA:CAIRYQSFNFGNEKLTF TRB:CASYRRVTEQYF	148	CD8-C7
PB-2_GAGTCCGTCCCGGATG	PB	PB-2	P2-1	TRUE	3	TRA:CVVNIIGYQKVTF TRB:CASTRQGKQPQHF	270	CD8-C7
PB-2_GAGTCCGTCTTGCAAG	PB	PB-2	P2-5	TRUE	3	TRA:CASSFSGGYNKLIF TRB:CASSYLGAGYQETQYF	127	CD8-C7
PB-2_GATCGCGGTTAGGGTG	PB	PB-2	P2-65	TRUE	3	TRA:CAPNQAGTALIF TRB:CASKLSGTSGSYEQYF	8	CD8-C7
PB-2_GATCGTACACCTTGTC	PB	PB-2	P2-17	TRUE	3	TRA:CAVQASNAGNMLTF TRB:CASSVEQGNVETQYF	30	CD8-C5
PB-2_GATCGTAGTTCGCTAA	PB	PB-2	P2-3	TRUE	3	TRA:CAVNPLESSYKLIF TRB:CASSPGTANTGELFF	177	CD8-C5
PB-2_GATGAGGCATACCATG	PB	PB-2	P2-1	TRUE	3	TRA:CVVNIIGYQKVTF TRB:CASTRQGKQPQHF	270	CD8-C7
PB-2_GATGAGGCATTGCGGC	PB	PB-2	P2-11	TRUE	3	TRA:CAVHNQGGKLIF TRB:CASSYLGAGYQETQYF	43	CD8-C7
PB-2_GATGAGGCATTTCAGG	PB	PB-2	P2-3	TRUE	3	TRA:CAVNPLESSYKLIF TRB:CASSPGTANTGELFF	177	CD8-C5
PB-2_GATGCTAAGCGATTCT	PB	PB-2	P2-1	TRUE	3	TRA:CVVNIIGYQKVTF TRB:CASTRQGKQPQHF	270	CD8-C7
PB-2_GCACATAAGGAGTAGA	PB	PB-2	P2-43	TRUE	3	TRA:CVWEADFNKFYF TRB:CATSAGNNQPQHF	11	CD8-C7
PB-2_GCACATATCATCTGTT	PB	PB-2	P2-46	TRUE	3	TRA:CAFSTWGNQGGKLIF TRB:CASSQDRLGGGTYEQYF	11	CD8-C5
PB-2_GCACTCTAGTGAAGTT	PB	PB-2	P2-5	TRUE	3	TRA:CASSFSGGYNKLIF TRB:CASSYLGAGYQETQYF	127	CD8-C5
PB-2_GCAGCCAAGGGATGGG	PB	PB-2	P2-5	TRUE	3	TRA:CASSFSGGYNKLIF TRB:CASSYLGAGYQETQYF	127	CD8-C7
PB-2_GCAGCCACACCAGGCT	PB	PB-2	P2-37	TRUE	3	TRA:CLVVIRHRWGSQGNLIF TRB:CASSFRTLTPLQETQYF	14	CD8-C7
PB-2_GCAGTTATCCGTTGCT	PB	PB-2	P2-4	TRUE	3	TRA:CAIRYQSFNFGNEKLTF TRB:CASYRRVTEQYF	148	CD8-C7
PB-2_GCATACAAGTACGCCC	PB	PB-2	P2-1	TRUE	3	TRA:CVVNIIGYQKVTF TRB:CASTRQGKQPQHF	270	CD8-C7
PB-2_GCATACAGTCTCTTTA	PB	PB-2	P2-17	TRUE	3	TRA:CAVQASNAGNMLTF TRB:CASSVEQGNVETQYF	30	CD8-C7
PB-2_GCATACAGTGCCTGCA	PB	PB-2	P2-3	TRUE	3	TRA:CAVNPLESSYKLIF TRB:CASSPGTANTGELFF	177	CD8-C7
PB-2_GCATACATCAACCATG	PB	PB-2	P2-96	FALSE	3	TRA:CAMRGANFGNEKLTF TRB:CASSRDFGGPGQETQYF	5	CD8-C7
PB-2_GCATGATGTCCATGAT	PB	PB-2	P2-3	TRUE	3	TRA:CAVNPLESSYKLIF TRB:CASSPGTANTGELFF	177	CD8-C7
PB-2_GCATGATGTGCCTGGT	PB	PB-2	P2-1	TRUE	3	TRA:CVVNIIGYQKVTF TRB:CASTRQGKQPQHF	270	CD8-C7
PB-2_GCATGTAAGACTGGGT	PB	PB-2	P2-12	TRUE	3	TRA:CGADAEYGNKLVF TRB:CASSEGQGAIMQPQHF	42	CD8-C5
PB-2_GCATGTACAGCGTCCA	PB	PB-2	P2-5	TRUE	3	TRA:CASSFSGGYNKLIF TRB:CASSYLGAGYQETQYF	127	CD8-C5

PB-2_GCGACCATCACATAGC	PB	PB-2	P2-5	TRUE	3	TRA:CASSFSGGYNKLIF TRB:CASSYLGAGYQETQYF	127	CD8-C5
PB-2_GCGAGAAAGCAGGCTA	PB	PB-2	P2-2355	FALSE	1	TRA:CVVNIIGYQKVTF TRB:CASSFEGGPNEQFF	1	CD8-C7
PB-2_GCGAGAACACACATGT	PB	PB-2	P2-17	TRUE	3	TRA:CAVQASNAGNMLTF TRB:CASSVEQGNVETQYF	30	CD8-C5
PB-2_GCGAGAAGTTCCGGCA	PB	PB-2	P2-1	TRUE	3	TRA:CVVNIIGYQKVTF TRB:CASTRQGKQPQHF	270	CD8-C7
PB-2_GCGAGAATCTCAACTT	PB	PB-2	P2-1	TRUE	3	TRA:CVVNIIGYQKVTF TRB:CASTRQGKQPQHF	270	CD8-C7
PB-2_GCGCAACCACGCATCG	PB	PB-2	P2-43	TRUE	3	TRA:CVWEADFNKFYF TRB:CATSAGNNQPQHF	11	CD8-C7
PB-2_GCGCAGTTCCTTTCTC	PB	PB-2	P2-3	TRUE	3	TRA:CAVNPLESSYKLIF TRB:CASSPGTANTGELFF	177	CD8-C5
PB-2_GCGCCAACAATCAGAA	PB	PB-2	P2-37	TRUE	3	TRA:CLVVIRHRWGSQGNLIF TRB:CASSFRTLTPLQETQYF	14	CD8-C7
PB-2_GCTCCTACAAGGACAC	PB	PB-2	P2-1	TRUE	3	TRA:CVVNIIGYQKVTF TRB:CASTRQGKQPQHF	270	CD8-C7
PB-2_GCTCTGTAGTACGTAA	PB	PB-2	P2-3	TRUE	3	TRA:CAVNPLESSYKLIF TRB:CASSPGTANTGELFF	177	CD8-C5
PB-2_GCTCTGTGTGCACGAA	PB	PB-2	P2-1	TRUE	3	TRA:CVVNIIGYQKVTF TRB:CASTRQGKQPQHF	270	CD8-C7
PB-2_GCTCTGTTCCTGCAGG	PB	PB-2	P2-7	TRUE	3	TRA:CAESDSGNTPLVF TRB:CASSYSTPRGEAFF	84	CD8-C7
PB-2_GCTGCAGCATCACCCT	PB	PB-2	P2-3	TRUE	3	TRA:CAVNPLESSYKLIF TRB:CASSPGTANTGELFF	177	CD8-C5
PB-2_GCTGCGAAGTTGTAGA	PB	PB-2	P2-27	FALSE	3	TRA:CAPKSYNTDKLIF TRB:CASSEGGGGAETQYF	22	CD8-C7
PB-2_GCTGCTTAGCTAAGAT	PB	PB-2	P2-54	FALSE	3	TRA:CALNFMNRDDKIIF TRB:CASSYSPGQAASYGYTF	10	CD8-C7
PB-2_GCTGCTTAGTCGCCGT	PB	PB-2	P2-1	TRUE	3	TRA:CVVNIIGYQKVTF TRB:CASTRQGKQPQHF	270	CD4-C6
PB-2_GCTGCTTAGTGTTGAA	PB	PB-2	P2-2418	FALSE	1	TRA:CAMREGRQGGSEKLVF TRB:CASSVEQGNVETQYF	1	CD8-C5
PB-2_GCTGGGTTCGCCTGTT	PB	PB-2	P2-1	TRUE	3	TRA:CVVNIIGYQKVTF TRB:CASTRQGKQPQHF	270	CD8-C7
PB-2_GCTTCCAAGGACAGAA	PB	PB-2	P2-3	TRUE	3	TRA:CAVNPLESSYKLIF TRB:CASSPGTANTGELFF	177	CD8-C5
PB-2_GCTTCCATCTGTCTCG	PB	PB-2	P2-27	FALSE	3	TRA:CAPKSYNTDKLIF TRB:CASSEGGGGAETQYF	22	CD8-C5
PB-2_GGAACTTGTCTAAACC	PB	PB-2	P2-2443	FALSE	1	TRA:CAVRKETGGSTLGRLYF TRB:CASSLGRREQYF	1	CD8-C7
PB-2_GGAATAATCCGGGTGT	PB	PB-2	P2-25	FALSE	3	TRA:CAMREAPNTDKLIF TRB:CASSGGGGGLETQYF	22	CD8-C5
PB-2_GGACAGATCCGCGTTT	PB	PB-2	P2-37	TRUE	3	TRA:CLVVIRHRWGSQGNLIF TRB:CASSFRTLTPLQETQYF	14	CD8-C7
PB-2_GGACATTTCCTAGTGA	PB	PB-2	P2-4	TRUE	3	TRA:CAIRYQSFNFGNEKLTF TRB:CASYRRVTEQYF	148	CD8-C7
PB-2_GGAGCAACACCGCTAG	PB	PB-2	P2-1	TRUE	3	TRA:CVVNIIGYQKVTF TRB:CASTRQGKQPQHF	270	CD8-C7
PB-2_GGATGTTCACAGAGGT	PB	PB-2	P2-7	TRUE	3	TRA:CAESDSGNTPLVF TRB:CASSYSTPRGEAFF	84	CD8-C7
PB-2_GGATGTTCAGGAATGC	PB	PB-2	P2-12	TRUE	3	TRA:CGADAEYGNKLVF TRB:CASSEGQGAIMQPQHF	42	CD8-C7

PB-2_GGATGTTTCCAAGTAC	PB	PB-2	P2-17	TRUE	3	TRA:CAVQASNAGNMLTF TRB:CASSVEQGNVETQYF	30	CD8-C5
PB-2_GGATTACGTTCCCGAG	PB	PB-2	P2-11	TRUE	3	TRA:CAVHNQGGKLIF TRB:CASSYLGAGYQETQYF	43	CD8-C5
PB-2_GGCGTGTGTACTCAAC	PB	PB-2	P2-43	TRUE	3	TRA:CVWEADFNKFYF TRB:CATSAGNNQPQHF	11	CD8-C7
PB-2_GGCGTGTGTTAAGATG	PB	PB-2	P2-43	TRUE	3	TRA:CVWEADFNKFYF TRB:CATSAGNNQPQHF	11	CD8-C7
PB-2_GGCTCGATCCTTGGTC	PB	PB-2	P2-1	TRUE	3	TRA:CVVNIIGYQKVTF TRB:CASTRQGKQPQHF	270	CD8-C7
PB-2_GGCTCGATCTGTACGA	PB	PB-2	P2-7	TRUE	3	TRA:CAESDSGNTPLVF TRB:CASSYSTPRGEAFF	84	CD8-C7
PB-2_GGGAATGGTTCCAACA	PB	PB-2	P2-5	TRUE	3	TRA:CASSFSGGYNKLIF TRB:CASSYLGAGYQETQYF	127	CD8-C5
PB-2_GGGACCTAGGTGGGTT	PB	PB-2	P2-2	TRUE	3	TRA:CVVPNTNAGKSTF TRB:CASSLQGSTYNEQFF	220	CD8-C7
PB-2_GGGAGATGTTGTACAC	PB	PB-2	P2-2547	FALSE	1	TRA:CVVSVDLNTDKLIF TRB:CASSLGGNTGELFF	1	CD8-C6
PB-2_GGGATGAAGTACGACG	PB	PB-2	P2-54	FALSE	3	TRA:CALNFMNRDDKIIF TRB:CASSYSPGQAASYGYTF	10	CD8-C7
PB-2_GGGCATCCAACGATGG	PB	PB-2	P2-3	TRUE	3	TRA:CAVNPLESSYKLIF TRB:CASSPGTANTGELFF	177	CD8-C7
PB-2_GGGCATCGTCCGAAGA	PB	PB-2	P2-3	TRUE	3	TRA:CAVNPLESSYKLIF TRB:CASSPGTANTGELFF	177	CD8-C7
PB-2_GGGTCTGAGTGTGAAT	PB	PB-2	P2-122	TRUE	3	TRA:CAYAPSGSRLTF TRB:CASSQGGWGRRDEQFF	4	CD8-C7
PB-2_GGGTTGCAGATGGCGT	PB	PB-2	P2-30	TRUE	3	TRA:CAVGGDTGNQFYF TRB:CASSLFPTGVAYGYTF	20	CD8-C6
PB-2_GGGTTGCAGCCGATTT	PB	PB-2	P2-1	TRUE	3	TRA:CVVNIIGYQKVTF TRB:CASTRQGKQPQHF	270	CD8-C7
PB-2_GGGTTGCCAAGCTGGA	PB	PB-2	P2-4	TRUE	3	TRA:CAIRYQSFNFGNEKLTF TRB:CASYRRVTEQYF	148	CD8-C7
PB-2_GGTATTGAGACGCACA	PB	PB-2	P2-3	TRUE	3	TRA:CAVNPLESSYKLIF TRB:CASSPGTANTGELFF	177	CD8-C5
PB-2_GGTGCGTCAAGTAATG	PB	PB-2	P2-1	TRUE	3	TRA:CVVNIIGYQKVTF TRB:CASTRQGKQPQHF	270	CD8-C7
PB-2_GGTGCGTCAGATCGGA	PB	PB-2	P2-1	TRUE	3	TRA:CVVNIIGYQKVTF TRB:CASTRQGKQPQHF	270	CD8-C7
PB-2_GTAACGTTCCGCGTTT	PB	PB-2	P2-96	FALSE	3	TRA:CAMRGANFGNEKLTF TRB:CASSRDFGGPGQETQYF	5	CD8-C7
PB-2_GTAACGTTCGGATGGA	PB	PB-2	P2-3	TRUE	3	TRA:CAVNPLESSYKLIF TRB:CASSPGTANTGELFF	177	CD8-C7
PB-2_GTAACTGGTACTCTCC	PB	PB-2	P2-1	TRUE	3	TRA:CVVNIIGYQKVTF TRB:CASTRQGKQPQHF	270	CD8-C7
PB-2_GTACGTACAAGCGATG	PB	PB-2	P2-307	FALSE	2	TRA:CVVNIIGYQKVTF TRB:CASYRRVTEQYF	2	CD8-C7
PB-2_GTACGTAGTAAGAGAG	PB	PB-2	P2-1	TRUE	3	TRA:CVVNIIGYQKVTF TRB:CASTRQGKQPQHF	270	CD8-C7
PB-2_GTACGTAGTGTCCTCT	PB	PB-2	P2-5	TRUE	3	TRA:CASSFSGGYNKLIF TRB:CASSYLGAGYQETQYF	127	CD8-C5
PB-2_GTACTTTCAAGCGATG	PB	PB-2	P2-3	TRUE	3	TRA:CAVNPLESSYKLIF TRB:CASSPGTANTGELFF	177	CD8-C7
PB-2_GTACTTTGTCTACCTC	PB	PB-2	P2-4	TRUE	3	TRA:CAIRYQSFNFGNEKLTF TRB:CASYRRVTEQYF	148	CD8-C7

PB-2_GTAGTCAAGTCCGTAT	PB	PB-2	P2-5	TRUE	3	TRA:CASSFSGGYNKLIF TRB:CASSYLGAGYQETQYF	127	CD8-C7
PB-2_GTATCTTGTCTGCCAG	PB	PB-2	P2-1	TRUE	3	TRA:CVVNIIGYQKVTF TRB:CASTRQGKQPQHF	270	CD8-C7
PB-2_GTCAAGTGTAGAGTGC	PB	PB-2	P2-2635	FALSE	1	TRA:CALSETFSGANSKLTF TRB:CASRLTGFGEAFF	1	CD8-C6
PB-2_GTCACAACACGTTGGC	PB	PB-2	P2-7	TRUE	3	TRA:CAESDSGNTPLVF TRB:CASSYSTPRGEAFF	84	CD8-C7
PB-2_GTCATTTAGGCTAGGT	PB	PB-2	P2-77	FALSE	3	TRA:CAVDSGWGRSGGSNYKLTF TRB:CASSADRPYSYEQYF	7	CD8-C6
PB-2_GTCATTTAGTTGTCGT	PB	PB-2	P2-54	FALSE	3	TRA:CALNFMNRDDKIIF TRB:CASSYSPGQAASYGYTF	10	CD8-C7
PB-2_GTCATTTCAAGAGTCG	PB	PB-2	P2-17	TRUE	3	TRA:CAVQASNAGNMLTF TRB:CASSVEQGNVETQYF	30	CD8-C7
PB-2_GTCATTTCATTCCTCG	PB	PB-2	P2-1	TRUE	3	TRA:CVVNIIGYQKVTF TRB:CASTRQGKQPQHF	270	CD8-C7
PB-2_GTCCTCAAGAGTAAGG	PB	PB-2	P2-3	TRUE	3	TRA:CAVNPLESSYKLIF TRB:CASSPGTANTGELFF	177	CD8-C7
PB-2_GTCCTCACATGACATC	PB	PB-2	P2-46	TRUE	3	TRA:CAFSTWGNQGGKLIF TRB:CASSQDRLGGGTYEQYF	11	CD8-C7
PB-2_GTCGTAAAGCCTTGAT	PB	PB-2	P2-7	TRUE	3	TRA:CAESDSGNTPLVF TRB:CASSYSTPRGEAFF	84	CD8-C7
PB-2_GTCGTAAAGTCCTCCT	PB	PB-2	P2-3	TRUE	3	TRA:CAVNPLESSYKLIF TRB:CASSPGTANTGELFF	177	CD8-C7
PB-2_GTCTCGTTCGGCCGAT	PB	PB-2	P2-12	TRUE	3	TRA:CGADAEYGNKLVF TRB:CASSEGQGAIMQPQHF	42	CD8-C7
PB-2_GTGCATAAGGCGTACA	PB	PB-2	P2-3	TRUE	3	TRA:CAVNPLESSYKLIF TRB:CASSPGTANTGELFF	177	CD8-C5
PB-2_GTGCATAGTAAGCACG	PB	PB-2	P2-4	TRUE	3	TRA:CAIRYQSFNFGNEKLTF TRB:CASYRRVTEQYF	148	CD8-C7
PB-2_GTGCGGTTCACCGGGT	PB	PB-2	P2-1	TRUE	3	TRA:CVVNIIGYQKVTF TRB:CASTRQGKQPQHF	270	CD8-C7
PB-2_GTGCTTCCAGCCAGAA	PB	PB-2	P2-7	TRUE	3	TRA:CAESDSGNTPLVF TRB:CASSYSTPRGEAFF	84	CD8-C7
PB-2_GTGGGTCAGTGTCCCG	PB	PB-2	P2-1	TRUE	3	TRA:CVVNIIGYQKVTF TRB:CASTRQGKQPQHF	270	CD8-C7
PB-2_GTGTGCGAGCGGATCA	PB	PB-2	P2-7	TRUE	3	TRA:CAESDSGNTPLVF TRB:CASSYSTPRGEAFF	84	CD8-C7
PB-2_GTGTGCGGTCAATGTC	PB	PB-2	P2-3	TRUE	3	TRA:CAVNPLESSYKLIF TRB:CASSPGTANTGELFF	177	CD8-C7
PB-2_GTGTGCGGTCGCTTCT	PB	PB-2	P2-1	TRUE	3	TRA:CVVNIIGYQKVTF TRB:CASTRQGKQPQHF	270	CD4-C6
PB-2_GTGTGCGTCACCTCGT	PB	PB-2	P2-3	TRUE	3	TRA:CAVNPLESSYKLIF TRB:CASSPGTANTGELFF	177	CD8-C7
PB-2_GTTACAGAGACCGGAT	PB	PB-2	P2-4	TRUE	3	TRA:CAIRYQSFNFGNEKLTF TRB:CASYRRVTEQYF	148	CD8-C5
PB-2_GTTACAGCACAAGACG	PB	PB-2	P2-5	TRUE	3	TRA:CASSFSGGYNKLIF TRB:CASSYLGAGYQETQYF	127	CD8-C7
PB-2_GTTACAGCAGCGTTCG	PB	PB-2	P2-46	TRUE	3	TRA:CAFSTWGNQGGKLIF TRB:CASSQDRLGGGTYEQYF	11	CD8-C7
PB-2_GTTACAGGTGCTAGCC	PB	PB-2	P2-12	TRUE	3	TRA:CGADAEYGNKLVF TRB:CASSEGQGAIMQPQHF	42	CD8-C7
PB-2_GTTACAGGTTACTGAC	PB	PB-2	P2-1	TRUE	3	TRA:CVVNIIGYQKVTF TRB:CASTRQGKQPQHF	270	CD8-C7

PB-2_GTTACAGTCGCATGAT	PB	PB-2	P2-5	TRUE	3	TRA:CASSFSGGYNKLIF TRB:CASSYLGAGYQETQYF	127	CD8-C7
PB-2_GTTCGGGAGGTTCCTA	PB	PB-2	P2-3	TRUE	3	TRA:CAVNPLESSYKLIF TRB:CASSPGTANTGELFF	177	CD8-C5
PB-2_GTTCTCGTCATAACCG	PB	PB-2	P2-1	TRUE	3	TRA:CVVNIIGYQKVTF TRB:CASTRQGKQPQHF	270	CD8-C7
PB-2_GTTTCTAAGAGGTTAT	PB	PB-2	P2-43	TRUE	3	TRA:CVWEADFNKFYF TRB:CATSAGNNQPQHF	11	CD8-C7
PB-2_TAAACCGAGACAATAC	PB	PB-2	P2-1	TRUE	3	TRA:CVVNIIGYQKVTF TRB:CASTRQGKQPQHF	270	CD4-C6
PB-2_TAAACCGAGACAGAGA	PB	PB-2	P2-1	TRUE	3	TRA:CVVNIIGYQKVTF TRB:CASTRQGKQPQHF	270	CD8-C7
PB-2_TAAACCGGTAGGACAC	PB	PB-2	P2-5	TRUE	3	TRA:CASSFSGGYNKLIF TRB:CASSYLGAGYQETQYF	127	CD8-C7
PB-2_TAAGAGAAGGTGACCA	PB	PB-2	P2-8	TRUE	3	TRA:CAALEGDNYGGQNFVF TRB:CASSPTWGGVRDTQYF	53	CD8-C5
PB-2_TAAGAGAGTATCACCA	PB	PB-2	P2-3	TRUE	3	TRA:CAVNPLESSYKLIF TRB:CASSPGTANTGELFF	177	CD8-C5
PB-2_TAAGAGAGTGAAAGAG	PB	PB-2	P2-8	TRUE	3	TRA:CAALEGDNYGGQNFVF TRB:CASSPTWGGVRDTQYF	53	CD8-C6
PB-2_TAAGCGTGTCCAGTTA	PB	PB-2	P2-7	TRUE	3	TRA:CAESDSGNTPLVF TRB:CASSYSTPRGEAFF	84	CD8-C7
PB-2_TAAGTGCGTCTAGAGG	PB	PB-2	P2-4	TRUE	3	TRA:CAIRYQSFNFGNEKLTF TRB:CASYRRVTEQYF	148	CD8-C7
PB-2_TAAGTGCTCATATCGG	PB	PB-2	P2-5	TRUE	3	TRA:CASSFSGGYNKLIF TRB:CASSYLGAGYQETQYF	127	CD8-C7
PB-2_TACCTATGTTGATTGC	PB	PB-2	P2-3	TRUE	3	TRA:CAVNPLESSYKLIF TRB:CASSPGTANTGELFF	177	CD8-C5
PB-2_TACCTATTCAACTCTT	PB	PB-2	P2-27	FALSE	3	TRA:CAPKSYNTDKLIF TRB:CASSEGGGGAETQYF	22	CD8-C7
PB-2_TACCTTATCACGCGGT	PB	PB-2	P2-5	TRUE	3	TRA:CASSFSGGYNKLIF TRB:CASSYLGAGYQETQYF	127	CD8-C7
PB-2_TACTCGCAGTTGAGAT	PB	PB-2	P2-200	TRUE	3	TRA:CAFNSKEVGANNLFF TRB:CATSRGRGREGYTF	3	CD8-C5
PB-2_TACTTACCAGATTGCT	PB	PB-2	P2-5	TRUE	3	TRA:CASSFSGGYNKLIF TRB:CASSYLGAGYQETQYF	127	CD8-C5
PB-2_TACTTACCAGTCGTGC	PB	PB-2	P2-1	TRUE	3	TRA:CVVNIIGYQKVTF TRB:CASTRQGKQPQHF	270	CD8-C7
PB-2_TACTTACGTTCCGGCA	PB	PB-2	P2-5	TRUE	3	TRA:CASSFSGGYNKLIF TRB:CASSYLGAGYQETQYF	127	CD8-C5
PB-2_TACTTACTCCCTCAGT	PB	PB-2	P2-4	TRUE	3	TRA:CAIRYQSFNFGNEKLTF TRB:CASYRRVTEQYF	148	CD8-C7
PB-2_TACTTGTCAGCGTTCG	PB	PB-2	P2-2867	FALSE	1	TRA:CAIRYQSFNFGNEKLTF TRB:CASSPGTANTGELFF	1	CD8-C7
PB-2_TAGAGCTAGCTGCCCA	PB	PB-2	P2-12	TRUE	3	TRA:CGADAEYGNKLVF TRB:CASSEGQGAIMQPQHF	42	CD8-C5
PB-2_TAGAGCTGTCTCTCGT	PB	PB-2	P2-4	TRUE	3	TRA:CAIRYQSFNFGNEKLTF TRB:CASYRRVTEQYF	148	CD8-C5
PB-2_TAGCCGGCACATGACT	PB	PB-2	P2-7	TRUE	3	TRA:CAESDSGNTPLVF TRB:CASSYSTPRGEAFF	84	CD8-C7
PB-2_TAGGCATCAATGTTGC	PB	PB-2	P2-4	TRUE	3	TRA:CAIRYQSFNFGNEKLTF TRB:CASYRRVTEQYF	148	CD8-C7
PB-2_TAGGCATGTACCAGTT	PB	PB-2	P2-5	TRUE	3	TRA:CASSFSGGYNKLIF TRB:CASSYLGAGYQETQYF	127	CD8-C7

PB-2_TAGTGGTAGGATGTAT	PB	PB-2	P2-4	TRUE	3	TRA:CAIRYQSFNFGNEKLTF TRB:CASYRRVTEQYF	148	CD8-C6
PB-2_TAGTTGGAGGTGCACA	PB	PB-2	P2-4	TRUE	3	TRA:CAIRYQSFNFGNEKLTF TRB:CASYRRVTEQYF	148	CD8-C5
PB-2_TAGTTGGGTCCTGCTT	PB	PB-2	P2-7	TRUE	3	TRA:CAESDSGNTPLVF TRB:CASSYSTPRGEAFF	84	CD8-C7
PB-2_TATCAGGAGCGGCTTC	PB	PB-2	P2-1	TRUE	3	TRA:CVVNIIGYQKVTF TRB:CASTRQGKQPQHF	270	CD8-C7
PB-2_TATCTCATCCTCTAGC	PB	PB-2	P2-11	TRUE	3	TRA:CAVHNQGGKLIF TRB:CASSYLGAGYQETQYF	43	CD8-C5
PB-2_TATTACCCACAGCCCA	PB	PB-2	P2-43	TRUE	3	TRA:CVVEADFNKFYF TRB:CATSAGNNQPQHF	11	CD8-C5
PB-2_TCAACGATCTCAAGTG	PB	PB-2	P2-7	TRUE	3	TRA:CAESDSGNTPLVF TRB:CASSYSTPRGEAFF	84	CD8-C6
PB-2_TCAATCTTCCTATGTT	PB	PB-2	P2-5	TRUE	3	TRA:CASSFSGGYNKLIF TRB:CASSYLGAGYQETQYF	127	CD8-C7
PB-2_TCACAAGTCAGTCAGT	PB	PB-2	P2-3	TRUE	3	TRA:CAVNPLESSYKLIF TRB:CASSPGTANTGELFF	177	CD8-C7
PB-2_TCACGAATCGCGGATC	PB	PB-2	P2-4	TRUE	3	TRA:CAIRYQSFNFGNEKLTF TRB:CASYRRVTEQYF	148	CD8-C7
PB-2_TCAGATGCAAGCCCAC	PB	PB-2	P2-7	TRUE	3	TRA:CAESDSGNTPLVF TRB:CASSYSTPRGEAFF	84	CD8-C6
PB-2_TCAGCAAGTAAGAGAG	PB	PB-2	P2-11	TRUE	3	TRA:CAVHNQGGKLIF TRB:CASSYLGAGYQETQYF	43	CD8-C7
PB-2_TCAGCAAGTCATTAGC	PB	PB-2	P2-5	TRUE	3	TRA:CASSFSGGYNKLIF TRB:CASSYLGAGYQETQYF	127	CD8-C7
PB-2_TCAGCTCTCTGTTGAG	PB	PB-2	P2-122	TRUE	3	TRA:CAYAPSGSRLTF TRB:CASSQGGWGRRDEQFF	4	CD8-C5
PB-2_TCAGGATGTAAGCACG	PB	PB-2	P2-1	TRUE	3	TRA:CVVNIIGYQKVTF TRB:CASTRQGKQPQHF	270	CD8-C7
PB-2_TCAGGTATCCGCGTTT	PB	PB-2	P2-12	TRUE	3	TRA:CGADAEYGNKLVF TRB:CASSEGQGAIMQPQHF	42	CD8-C7
PB-2_TCATTACTCCGAGCCA	PB	PB-2	P2-2995	FALSE	1	TRA:CAASDNYGQNFVF TRB:CASSFRGDRGYNEQFF	1	CD8-C6
PB-2_TCCACACTCCTTCAAT	PB	PB-2	P2-4	TRUE	3	TRA:CAIRYQSFNFGNEKLTF TRB:CASYRRVTEQYF	148	CD8-C7
PB-2_TCCCGATAGGAGTCTG	PB	PB-2	P2-5	TRUE	3	TRA:CASSFSGGYNKLIF TRB:CASSYLGAGYQETQYF	127	CD8-C5
PB-2_TCCCGATCATCAGTCA	PB	PB-2	P2-7	TRUE	3	TRA:CAESDSGNTPLVF TRB:CASSYSTPRGEAFF	84	CD8-C7
PB-2_TCGAGGCAGTACGTAA	PB	PB-2	P2-202	TRUE	3	TRA:CALTTAGGTSYGKLTF TRB:CASKVSGTSGRNEQFF	3	CD8-C7
PB-2_TCGCGAGGTGCGAAAC	PB	PB-2	P2-1	TRUE	3	TRA:CVVNIIGYQKVTF TRB:CASTRQGKQPQHF	270	CD8-C7
PB-2_TCGTACCCATCCGCGA	PB	PB-2	P2-5	TRUE	3	TRA:CASSFSGGYNKLIF TRB:CASSYLGAGYQETQYF	127	CD8-C7
PB-2_TCTATTGGTTCTGTTT	PB	PB-2	P2-1	TRUE	3	TRA:CVVNIIGYQKVTF TRB:CASTRQGKQPQHF	270	CD4-C6
PB-2_TCTCTAAAGAGTGACC	PB	PB-2	P2-103	FALSE	3	TRA:CAVGALSRDDKIIF TRB:CASSQSPGQIYGYTF	5	CD8-C5
PB-2_TCTCTAATCCTAGAAC	PB	PB-2	P2-4	TRUE	3	TRA:CAIRYQSFNFGNEKLTF TRB:CASYRRVTEQYF	148	CD8-C7
PB-2_TCTCTAATCGGCGCTA	PB	PB-2	P2-7	TRUE	3	TRA:CAESDSGNTPLVF TRB:CASSYSTPRGEAFF	84	CD8-C7

PB-2_TCTGAGAGTAAGGGAA	PB	PB-2	P2-1	TRUE	3	TRA:CVVNIIGYQKVTF TRB:CASTRQGKQPQHF	270	CD8-C7
PB-2_TCTGAGAGTGAAGGCT	PB	PB-2	P2-7	TRUE	3	TRA:CAESDSGNTPLVF TRB:CASSYSTPRGEAFF	84	CD8-C7
PB-2_TCTGGAACAGGATCGA	PB	PB-2	P2-17	TRUE	3	TRA:CAVQASNAGNMLTF TRB:CASSVEQGNVETQYF	30	CD8-C7
PB-2_TCTGGAATCTATGTGG	PB	PB-2	P2-295	FALSE	2	TRA:CAYRTTQTGANNLFF TRB:CASHVFKNIQYF	2	CD8-C5
PB-2_TCTTCGGCACCCATTC	PB	PB-2	P2-17	TRUE	3	TRA:CAVQASNAGNMLTF TRB:CASSVEQGNVETQYF	30	CD8-C5
PB-2_TCTTCGGCATATGGTC	PB	PB-2	P2-17	TRUE	3	TRA:CAVQASNAGNMLTF TRB:CASSVEQGNVETQYF	30	CD8-C5
PB-2_TGAAAGATCTTTAGTC	PB	PB-2	P2-4	TRUE	3	TRA:CAIRYQSFNFGNEKLTF TRB:CASYRRVTEQYF	148	CD8-C7
PB-2_TGACAACCAAGCGTAG	PB	PB-2	P2-3	TRUE	3	TRA:CAVNPLESSYKLIF TRB:CASSPGTANTGELFF	177	CD8-C7
PB-2_TGACAACGTTGGTAAA	PB	PB-2	P2-1	TRUE	3	TRA:CVVNIIGYQKVTF TRB:CASTRQGKQPQHF	270	CD8-C7
PB-2_TGACGGCAGAAACCTA	PB	PB-2	P2-12	TRUE	3	TRA:CGADAEYGNKLVF TRB:CASSEGQGAIMQPQHF	42	CD8-C7
PB-2_TGACGGCTCTCTAGGA	PB	PB-2	P2-11	TRUE	3	TRA:CAVHNQGGKLIF TRB:CASSYLGAGYQETQYF	43	CD8-C7
PB-2_TGACTAGAGGTGCAAC	PB	PB-2	P2-3100	FALSE	1	TRA:CAANSGYSTLTF TRB:CSAAAGAGTDTQYF	1	CD8-C6
PB-2_TGACTAGCAGTTCATG	PB	PB-2	P2-3	TRUE	3	TRA:CAVNPLESSYKLIF TRB:CASSPGTANTGELFF	177	CD8-C7
PB-2_TGACTAGTCACCATAG	PB	PB-2	P2-3	TRUE	3	TRA:CAVNPLESSYKLIF TRB:CASSPGTANTGELFF	177	CD8-C5
PB-2_TGACTTTTCCTCGCAT	PB	PB-2	P2-3	TRUE	3	TRA:CAVNPLESSYKLIF TRB:CASSPGTANTGELFF	177	CD8-C5
PB-2_TGAGAGGAGAGCTATA	PB	PB-2	P2-5	TRUE	3	TRA:CASSFSGGYNKLIF TRB:CASSYLGAGYQETQYF	127	CD8-C7
PB-2_TGAGAGGTCTTGTCAT	PB	PB-2	P2-3	TRUE	3	TRA:CAVNPLESSYKLIF TRB:CASSPGTANTGELFF	177	CD8-C7
PB-2_TGAGCATAGTCGTTTG	PB	PB-2	P2-3	TRUE	3	TRA:CAVNPLESSYKLIF TRB:CASSPGTANTGELFF	177	CD8-C7
PB-2_TGAGCATGTTCCGGCA	PB	PB-2	P2-17	TRUE	3	TRA:CAVQASNAGNMLTF TRB:CASSVEQGNVETQYF	30	CD8-C5
PB-2_TGAGGGAAGGTGCTAG	PB	PB-2	P2-3	TRUE	3	TRA:CAVNPLESSYKLIF TRB:CASSPGTANTGELFF	177	CD8-C7
PB-2_TGAGGGAGTCTAGCGC	PB	PB-2	P2-1	TRUE	3	TRA:CVVNIIGYQKVTF TRB:CASTRQGKQPQHF	270	CD8-C7
PB-2_TGCACCTTCTCCAACC	PB	PB-2	P2-3	TRUE	3	TRA:CAVNPLESSYKLIF TRB:CASSPGTANTGELFF	177	CD8-C7
PB-2_TGCCCATAGGGTATCG	PB	PB-2	P2-3152	FALSE	1	TRA:CAAEGGSQGNLIF TRB:CASSISRGKTNTGELFF	1	CD8-C6
PB-2_TGCCCTACATCGATTG	PB	PB-2	P2-27	FALSE	3	TRA:CAPKSYNTDKLIF TRB:CASSEGGGGAETQYF	22	CD8-C7
PB-2_TGCCCTATCCAACCAA	PB	PB-2	P2-4	TRUE	3	TRA:CAIRYQSFNFGNEKLTF TRB:CASYRRVTEQYF	148	CD8-C5
PB-2_TGCGGGTGTCCTCCAT	PB	PB-2	P2-4	TRUE	3	TRA:CAIRYQSFNFGNEKLTF TRB:CASYRRVTEQYF	148	CD8-C7
PB-2_TGCGGGTTCGGTCTAA	PB	PB-2	P2-17	TRUE	3	TRA:CAVQASNAGNMLTF TRB:CASSVEQGNVETQYF	30	CD8-C7

PB-2_TGCGTGGCAAGCTGGA	PB	PB-2	P2-1	TRUE	3	TRA:CVVNIIGYQKVTF TRB:CASTRQGKQPQHF	270	CD8-C7
PB-2_TGCGTGGCAGTAACGG	PB	PB-2	P2-67	FALSE	3	TRA:CVVSDPLWDNYGQNFVF TRB:CASSPSGGLLHYNEQFF	8	CD8-C5
PB-2_TGCGTGGGTTGCGTTA	PB	PB-2	P2-5	TRUE	3	TRA:CASSFSGGYNKLIF TRB:CASSYLGAGYQETQYF	127	CD8-C7
PB-2_TGGACGCAGCTTCGCG	PB	PB-2	P2-7	TRUE	3	TRA:CAESDSGNTPLVF TRB:CASSYSTPRGEAFF	84	CD8-C7
PB-2_TGGACGCGTAAGAGGA	PB	PB-2	P2-11	TRUE	3	TRA:CAVHNQGGKLIF TRB:CASSYLGAGYQETQYF	43	CD8-C7
PB-2_TGGCCAGAGCTTATCG	PB	PB-2	P2-5	TRUE	3	TRA:CASSFSGGYNKLIF TRB:CASSYLGAGYQETQYF	127	CD8-C7
PB-2_TGGCCAGTCCATGAGT	PB	PB-2	P2-1	TRUE	3	TRA:CVVNIIGYQKVTF TRB:CASTRQGKQPQHF	270	CD4-C6
PB-2_TGGCCAGTCTACTATC	PB	PB-2	P2-7	TRUE	3	TRA:CAESDSGNTPLVF TRB:CASSYSTPRGEAFF	84	CD8-C6
PB-2_TGGCGCATCGTCACGG	PB	PB-2	P2-3	TRUE	3	TRA:CAVNPLESSYKLIF TRB:CASSPGTANTGELFF	177	CD8-C7
PB-2_TGGCTGGGTTCATGGT	PB	PB-2	P2-37	TRUE	3	TRA:CLVVIRHRWGSQGNLIF TRB:CASSFRTLTPLQETQYF	14	CD8-C5
PB-2_TGGGAAGCAAATTGCC	PB	PB-2	P2-3217	FALSE	1	TRA:CAVRVPVLLYGGSQGNLIF TRB:CASSEGQGAIMQPQHF	1	CD8-C7
PB-2_TGGGCGTAGGCTAGGT	PB	PB-2	P2-1	TRUE	3	TRA:CVVNIIGYQKVTF TRB:CASTRQGKQPQHF	270	CD8-C7
PB-2_TGGTTAGTCATTTGGG	PB	PB-2	P2-1	TRUE	3	TRA:CVVNIIGYQKVTF TRB:CASTRQGKQPQHF	270	CD8-C7
PB-2_TGGTTCCCATCTATGG	PB	PB-2	P2-4	TRUE	3	TRA:CAIRYQSFNFGNEKLTF TRB:CASYRRVTEQYF	148	CD8-C5
PB-2_TGTATTCTCCGATATG	PB	PB-2	P2-3	TRUE	3	TRA:CAVNPLESSYKLIF TRB:CASSPGTANTGELFF	177	CD8-C5
PB-2_TGTCCCAAGTCAAGGC	PB	PB-2	P2-1	TRUE	3	TRA:CVVNIIGYQKVTF TRB:CASTRQGKQPQHF	270	CD8-C7
PB-2_TGTCCCACAGGAATCG	PB	PB-2	P2-4	TRUE	3	TRA:CAIRYQSFNFGNEKLTF TRB:CASYRRVTEQYF	148	CD8-C7
PB-2_TGTCCCATCAGTGTTG	PB	PB-2	P2-54	FALSE	3	TRA:CALNFMNRDDKIIF TRB:CASSYSPGQAASYGYTF	10	CD8-C7
PB-2_TGTGGTAGTTAAGACA	PB	PB-2	P2-11	TRUE	3	TRA:CAVHNQGGKLIF TRB:CASSYLGAGYQETQYF	43	CD8-C7
PB-2_TGTTCCGCATCATCCC	PB	PB-2	P2-5	TRUE	3	TRA:CASSFSGGYNKLIF TRB:CASSYLGAGYQETQYF	127	CD8-C5
PB-2_TTAGGACCAAATTGCC	PB	PB-2	P2-1	TRUE	3	TRA:CVVNIIGYQKVTF TRB:CASTRQGKQPQHF	270	CD8-C7
PB-2_TTAGTTCTCAGAGACG	PB	PB-2	P2-5	TRUE	3	TRA:CASSFSGGYNKLIF TRB:CASSYLGAGYQETQYF	127	CD8-C7
PB-2_TTCCCAGTCAGCAACT	PB	PB-2	P2-3295	FALSE	1	TRA:CAGGPGTYKYIF TRB:CASKVSGTSGRNEQFF	1	CD8-C7
PB-2_TTCCCAGTCTCACATT	PB	PB-2	P2-1	TRUE	3	TRA:CVVNIIGYQKVTF TRB:CASTRQGKQPQHF	270	CD8-C7
PB-2_TTCGGTCTCCTCAATT	PB	PB-2	P2-4	TRUE	3	TRA:CAIRYQSFNFGNEKLTF TRB:CASYRRVTEQYF	148	CD8-C7
PB-2_TTCTTAGAGCCACTAT	PB	PB-2	P2-7	TRUE	3	TRA:CAESDSGNTPLVF TRB:CASSYSTPRGEAFF	84	CD8-C7
PB-2_TTGAACGCATGAAGTA	PB	PB-2	P2-4	TRUE	3	TRA:CAIRYQSFNFGNEKLTF TRB:CASYRRVTEQYF	148	CD8-C7

PB-2_TTGAACGCATTACCTT	PB	PB-2	P2-1	TRUE	3	TRA:CVVNIIGYQKVTF TRB:CASTRQGKQPQHF	270	CD4-C6
PB-2_TTGACTTCACAACTGT	PB	PB-2	P2-1	TRUE	3	TRA:CVVNIIGYQKVTF TRB:CASTRQGKQPQHF	270	CD8-C7
PB-2_TTGCGTCCAAAGTGCG	PB	PB-2	P2-4	TRUE	3	TRA:CAIRYQSFNFGNEKLTF TRB:CASYRRVTEQYF	148	CD8-C7
PB-2_TTGCGTCGTCTCACCT	PB	PB-2	P2-7	TRUE	3	TRA:CAESDSGNTPLVF TRB:CASSYSTPRGEAFF	84	CD8-C7
PB-2_TTGGAACGTAAGCACG	PB	PB-2	P2-67	FALSE	3	TRA:CVVSDPLWDNYGQNFVF TRB:CASSPSGGLLHYNEQFF	8	CD8-C5
PB-2_TTGGCAAGTCTTCTCG	PB	PB-2	P2-3368	FALSE	1	TRA:CALYSGNTPLVF TRB:CASSRGQIEAFF	1	CD8-C6
PB-2_TTGGCAATCTGTCAAG	PB	PB-2	P2-7	TRUE	3	TRA:CAESDSGNTPLVF TRB:CASSYSTPRGEAFF	84	CD8-C6
PB-2_TTTACTGCATCGGTTA	PB	PB-2	P2-3	TRUE	3	TRA:CAVNPLESSYKLIF TRB:CASSPGTANTGELFF	177	CD8-C7
PB-2_TTTGGTTAGACAAGCC	PB	PB-2	P2-4	TRUE	3	TRA:CAIRYQSFNFGNEKLTF TRB:CASYRRVTEQYF	148	CD8-C7
PB-2_TTTGGTTCAAGTCTGT	PB	PB-2	P2-3402	FALSE	1	TRA:CAMREGDNDMRF TRB:CASSEGGGASETQYF	1	CD8-C7
PB-2_TTTGGTTGTAAAGTCA	PB	PB-2	P2-202	TRUE	3	TRA:CALTTAGGTSYGKLTF TRB:CASKVSGTSGRNEQFF	3	CD8-C7
PB-2_TTTGTCACACCAGATT	PB	PB-2	P2-1	TRUE	3	TRA:CVVNIIGYQKVTF TRB:CASTRQGKQPQHF	270	CD8-C7
PB-2_TTTGTCATCCAAGCCG	PB	PB-2	P2-11	TRUE	3	TRA:CAVHNQGGKLIF TRB:CASSYLGAGYQETQYF	43	CD8-C7
PB-2_AAACCTGAGAACAACT	PB	PB-2	P2-263	FALSE	2	TRA:CALTGNQFYF TRB:CASSFPGQDAEQFF	2	CD8-C5
PB-2_AAACGGGAGATGTAAC	PB	PB-2	P2-20	TRUE	3	TRA:CAYDSNYQLIW TRB:CASSLSIDRADTGELFF	25	CD8-C4
PB-2_AAACGGGAGGTAGCCA	PB	PB-2	P2-4	TRUE	3	TRA:CAIRYQSFNFGNEKLTF TRB:CASYRRVTEQYF	148	CD8-C5
PB-2_AACACGTAGACAGACC	PB	PB-2	P2-25	FALSE	3	TRA:CAMREAPNTDKLIF TRB:CASSGGGGGLETQYF	22	CD8-C5
PB-2_AACCATGCACCGTTGG	PB	PB-2	P2-19	TRUE	3	TRA:CAVRDMENNRLAF TRB:CASSLGGGLTYEQYF	27	CD8-C4
PB-2_AACCATGGTGTGTGCC	PB	PB-2	P2-10	TRUE	3	TRA:CAPLTPNDMRF TRB:CASLNGAGGSYEQYF	45	CD8-C4
PB-2_AACCATGTCAGCGACC	PB	PB-2	P2-2	TRUE	3	TRA:CVVPNTNAGKSTF TRB:CASSLQGSTYNEQFF	220	CD8-C5
PB-2_AACCATGTCTAACGGT	PB	PB-2	P2-12	TRUE	3	TRA:CGADAEYGNKLVF TRB:CASSEGQGAIMQPQHF	42	CD8-C5
PB-2_AACCGCGTCGCCATAA	PB	PB-2	P2-4	TRUE	3	TRA:CAIRYQSFNFGNEKLTF TRB:CASYRRVTEQYF	148	CD8-C5
PB-2_AACTCTTTCTCGTTTA	PB	PB-2	P2-11	TRUE	3	TRA:CAVHNQGGKLIF TRB:CASSYLGAGYQETQYF	43	CD8-C5
PB-2_AAGACCTAGGTGGGTT	PB	PB-2	P2-25	FALSE	3	TRA:CAMREAPNTDKLIF TRB:CASSGGGGGLETQYF	22	CD8-C5
PB-2_AAGACCTGTAAGGGAA	PB	PB-2	P2-2	TRUE	3	TRA:CVVPNTNAGKSTF TRB:CASSLQGSTYNEQFF	220	CD8-C4
PB-2_AAGCCGCGTATCGCAT	PB	PB-2	P2-10	TRUE	3	TRA:CAPLTPNDMRF TRB:CASLNGAGGSYEQYF	45	CD8-C4
PB-2_AAGGAGCAGGAACTGC	PB	PB-2	P2-45	TRUE	3	TRA:CAVSGDSNYQLIW TRB:CASSLGGNGNTIYF	11	CD8-C4

PB-2_AAGGCAGAGCGATGAC	PB	PB-2	P2-20	TRUE	3	TRA:CAYDSNYQLIW TRB:CASSLSIDRADTGELFF	25	CD8-C5
PB-2_AAGGTTCCATCTCCCA	PB	PB-2	P2-54	FALSE	3	TRA:CALNFMNRDDKIIF TRB:CASSYSPGQAASYGYTF	10	CD8-C5
PB-2_AATCCAGAGAGTCTGG	PB	PB-2	P2-699	FALSE	1	TRA:CAVSEFDIGGGTSYGKLTF TRB:CASSLDGTEYNEQFF	1	CD8-C5
PB-2_AATCCAGGTTCAGGCC	PB	PB-2	P2-4	TRUE	3	TRA:CAIRYQSFNFGNEKLTF TRB:CASYRRVTEQYF	148	CD8-C5
PB-2_ACACCAAAGTGTTGAA	PB	PB-2	P2-6	TRUE	3	TRA:CVVSVAAGNKLTF TRB:CASSPGTDLYEQYF	85	CD8-C4
PB-2_ACACCAACAGGGTACA	PB	PB-2	P2-4	TRUE	3	TRA:CAIRYQSFNFGNEKLTF TRB:CASYRRVTEQYF	148	CD8-C5
PB-2_ACACCCTGTTGAACTC	PB	PB-2	P2-269	FALSE	2	TRA:CATDGLNNAGNMLTF TRB:CASRTSGRFRTDTQYF	2	CD8-C4
PB-2_ACATACGGTATATGAG	PB	PB-2	P2-27	FALSE	3	TRA:CAPKSYNTDKLIF TRB:CASSEGGGGAETQYF	22	CD8-C5
PB-2_ACATACGGTCATATCG	PB	PB-2	P2-4	TRUE	3	TRA:CAIRYQSFNFGNEKLTF TRB:CASYRRVTEQYF	148	CD8-C5
PB-2_ACATACGTCCTAGTGA	PB	PB-2	P2-5	TRUE	3	TRA:CASSFSGGYNKLIF TRB:CASSYLGAGYQETQYF	127	CD8-C5
PB-2_ACATACGTCGGCGGTT	PB	PB-2	P2-5	TRUE	3	TRA:CASSFSGGYNKLIF TRB:CASSYLGAGYQETQYF	127	CD8-C7
PB-2_ACATGGTAGAGTCTGG	PB	PB-2	P2-30	TRUE	3	TRA:CAVGGDTGNQFYF TRB:CASSLFPTGVAYGYTF	20	CD8-C5
PB-2_ACATGGTTCCTAGGGC	PB	PB-2	P2-25	FALSE	3	TRA:CAMREAPNTDKLIF TRB:CASSGGGGGLETQYF	22	CD8-C5
PB-2_ACCTTTAAGGCCCTCA	PB	PB-2	P2-2	TRUE	3	TRA:CVVPNTNAGKSTF TRB:CASSLQGSTYNEQFF	220	CD8-C4
PB-2_ACCTTTAAGGCTAGGT	PB	PB-2	P2-2	TRUE	3	TRA:CVVPNTNAGKSTF TRB:CASSLQGSTYNEQFF	220	CD8-C5
PB-2_ACGAGGACACATGTGT	PB	PB-2	P2-5	TRUE	3	TRA:CASSFSGGYNKLIF TRB:CASSYLGAGYQETQYF	127	CD8-C5
PB-2_ACGCCGACATATACCG	PB	PB-2	P2-21	TRUE	3	TRA:CALSSYNNNDMRF TRB:CASSLDLAPSTEAFF	25	CD8-C5
PB-2_ACGCCGATCACCAGGC	PB	PB-2	P2-823	FALSE	1	TRA:CAPPPRLWGWELPTHF TRB:CASSQNRPQGMSNQPQHF	1	CD8-C4
PB-2_ACGGGCTCATCTATGG	PB	PB-2	P2-2	TRUE	3	TRA:CVVPNTNAGKSTF TRB:CASSLQGSTYNEQFF	220	CD8-C5
PB-2_ACGGGCTGTTCAACCA	PB	PB-2	P2-4	TRUE	3	TRA:CAIRYQSFNFGNEKLTF TRB:CASYRRVTEQYF	148	CD8-C5
PB-2_ACGGGTCCAAAGCAAT	PB	PB-2	P2-275	TRUE	2	TRA:CATGGGADGLTF TRB:CASSYFGENTEAFF	2	CD8-C5
PB-2_ACGGGTCTCATCTGCC	PB	PB-2	P2-15	TRUE	3	TRA:CAVGDNFNKFYF TRB:CASSRTSAAGELFF	35	CD8-C4
PB-2_ACTATCTGTTATCACG	PB	PB-2	P2-856	FALSE	1	TRA:CAHNDMRF TRB:CATSRDGLVMGEQYF	1	CD8-C5
PB-2_ACTGAACAGACATAAC	PB	PB-2	P2-2	TRUE	3	TRA:CVVPNTNAGKSTF TRB:CASSLQGSTYNEQFF	220	CD8-C5
PB-2_ACTGAACAGCGATAGC	PB	PB-2	P2-11	TRUE	3	TRA:CAVHNQGGKLIF TRB:CASSYLGAGYQETQYF	43	CD8-C5
PB-2_ACTGAACAGTTCGCGC	PB	PB-2	P2-860	FALSE	1	TRA:CAVVDQLTF TRB:CATSRDFPGQGGNGYTF	1	CD8-C4
PB-2_ACTGATGCACATAACC	PB	PB-2	P2-99	TRUE	3	TRA:CLVGDYAGGYQKVTF TRB:CASSEQGPTEAFF	5	CD8-C5

PB-2_ACTGATGGTCTGCCAG	PB	PB-2	P2-5	TRUE	3	TRA:CASSFSGGYNKLIF TRB:CASSYLGAGYQETQYF	127	CD8-C5
PB-2_ACTGATGGTTACCAGT	PB	PB-2	P2-12	TRUE	3	TRA:CGADAEYGNKLVF TRB:CASSEGQGAIMQPQHF	42	CD8-C5
PB-2_ACTGATGTCTGATACG	PB	PB-2	P2-11	TRUE	3	TRA:CAVHNQGGKLIF TRB:CASSYLGAGYQETQYF	43	CD8-C5
PB-2_ACTGTCCAGGCATTGG	PB	PB-2	P2-892	FALSE	1	TRA:CAFMNPLNFGNEKLTF TRB:CASSYSQDSSSGYTF	1	CD8-C5
PB-2_ACTTTCAAGTGAATTG	PB	PB-2	P2-908	FALSE	1	TRA:CASPYGGQNFVF TRB:CASRSGQGSYEQYF	1	CD8-C4
PB-2_ACTTTCAGTACTCAAC	PB	PB-2	P2-66	TRUE	3	TRA:CAVCRNRDDKIIF TRB:CASSTPQGHYGYTF	8	CD8-C4
PB-2_ACTTTCAGTCCATGAT	PB	PB-2	P2-4	TRUE	3	TRA:CAIRYQSFNFGNEKLTF TRB:CASYRRVTEQYF	148	CD8-C5
PB-2_AGAGCTTTCTACTTAC	PB	PB-2	P2-4	TRUE	3	TRA:CAIRYQSFNFGNEKLTF TRB:CASYRRVTEQYF	148	CD8-C5
PB-2_AGAGTGGGTCGAACAG	PB	PB-2	P2-2	TRUE	3	TRA:CVVPNTNAGKSTF TRB:CASSLQGSTYNEQFF	220	CD8-C5
PB-2_AGATCTGAGAAGAAGC	PB	PB-2	P2-100	TRUE	3	TRA:CAVGPRLLGNEKLTF TRB:CASSQDSTSVYNEQFF	5	CD8-C4
PB-2_AGATTGCCAGGGTATG	PB	PB-2	P2-5	TRUE	3	TRA:CASSFSGGYNKLIF TRB:CASSYLGAGYQETQYF	127	CD8-C5
PB-2_AGCCTAAAGGTAAACT	PB	PB-2	P2-11	TRUE	3	TRA:CAVHNQGGKLIF TRB:CASSYLGAGYQETQYF	43	CD8-C7
PB-2_AGCGGTCCATAGACTC	PB	PB-2	P2-27	FALSE	3	TRA:CAPKSYNTDKLIF TRB:CASSEGGGGAETQYF	22	CD8-C5
PB-2_AGCGGTCGTAGCGTGA	PB	PB-2	P2-57	TRUE	3	TRA:CAVRDMSSGAGSYQLTF TRB:CASSELEDQETQYF	9	CD8-C5
PB-2_AGCGTATTCAATAAGG	PB	PB-2	P2-4	TRUE	3	TRA:CAIRYQSFNFGNEKLTF TRB:CASYRRVTEQYF	148	CD8-C5
PB-2_AGCGTCGTCAGTTAGC	PB	PB-2	P2-25	FALSE	3	TRA:CAMREAPNTDKLIF TRB:CASSGGGGGLETQYF	22	CD8-C4
PB-2_AGCTCTCCAATCCAAC	PB	PB-2	P2-58	TRUE	3	TRA:CAVNERSNDYKLSF TRB:CAISGGDSWGNQPQHF	9	CD8-C5
PB-2_AGCTTGACAATCAGAA	PB	PB-2	P2-1	TRUE	3	TRA:CVVNIIGYQKVTF TRB:CASTRQGKQPQHF	270	CD4-C6
PB-2_AGCTTGAGTTGTGGCC	PB	PB-2	P2-3	TRUE	3	TRA:CAVNPLESSYKLIF TRB:CASSPGTANTGELFF	177	CD8-C5
PB-2_AGCTTGATCCGTAGGC	PB	PB-2	P2-3	TRUE	3	TRA:CAVNPLESSYKLIF TRB:CASSPGTANTGELFF	177	CD8-C5
PB-2_AGCTTGATCCTTTCTC	PB	PB-2	P2-4	TRUE	3	TRA:CAIRYQSFNFGNEKLTF TRB:CASYRRVTEQYF	148	CD8-C5
PB-2_AGGGATGCAAGGTTCT	PB	PB-2	P2-27	FALSE	3	TRA:CAPKSYNTDKLIF TRB:CASSEGGGGAETQYF	22	CD8-C5
PB-2_AGGGATGTCGTGGTCG	PB	PB-2	P2-66	TRUE	3	TRA:CAVCRNRDDKIIF TRB:CASSTPQGHYGYTF	8	CD8-C4
PB-2_AGGTCCGTCTCCGGTT	PB	PB-2	P2-5	TRUE	3	TRA:CASSFSGGYNKLIF TRB:CASSYLGAGYQETQYF	127	CD8-C5
PB-2_AGTAGTCAGAATCTCC	PB	PB-2	P2-4	TRUE	3	TRA:CAIRYQSFNFGNEKLTF TRB:CASYRRVTEQYF	148	CD8-C5
PB-2_AGTAGTCGTTGAGTTC	PB	PB-2	P2-8	TRUE	3	TRA:CAALEGDNYGGQNFVF TRB:CASSPTWGGVRDTQYF	53	CD8-C5
PB-2_AGTGAGGAGTCATGCT	PB	PB-2	P2-4	TRUE	3	TRA:CAIRYQSFNFGNEKLTF TRB:CASYRRVTEQYF	148	CD8-C5

PB-2_AGTGAGGTCAGCGATT	PB	PB-2	P2-1061	FALSE	1	TRA:CALLPPLGGSEKLVF TRB:CASSQDWGLQETQYF	1	CD8-C7
PB-2_AGTGAGGTCATCATTC	PB	PB-2	P2-25	FALSE	3	TRA:CAMREAPNTDKLIF TRB:CASSGGGGGLETQYF	22	CD8-C5
PB-2_AGTGAGGTCGCGGATC	PB	PB-2	P2-57	TRUE	3	TRA:CAVRDMSSGAGSYQLTF TRB:CASSELEDQETQYF	9	CD8-C5
PB-2_ATAAGAGCACAGGTTT	PB	PB-2	P2-59	TRUE	3	TRA:CAVDEGNTPLVF TRB:CAISESVDNTEAFF	9	CD8-C4
PB-2_ATAAGAGCATTTGCCC	PB	PB-2	P2-4	TRUE	3	TRA:CAIRYQSFNFGNEKLTF TRB:CASYRRVTEQYF	148	CD8-C7
PB-2_ATAGACCGTAGTGAAT	PB	PB-2	P2-8	TRUE	3	TRA:CAALEGDNYGQNFVF TRB:CASSPTWGGVRDTQYF	53	CD8-C4
PB-2_ATCATCTAGAAACCTA	PB	PB-2	P2-2	TRUE	3	TRA:CVVPNTNAGKSTF TRB:CASSLQGSTYNEQFF	220	CD8-C5
PB-2_ATCATGGTCTGAGGGA	PB	PB-2	P2-21	TRUE	3	TRA:CALSSYNNNDMRF TRB:CASSLDLAPSTEAFF	25	CD8-C5
PB-2_ATCCACCAGAGACGAA	PB	PB-2	P2-8	TRUE	3	TRA:CAALEGDNYGQNFVF TRB:CASSPTWGGVRDTQYF	53	CD8-C5
PB-2_ATCCACCCAAGTCATC	PB	PB-2	P2-176	TRUE	3	TRA:CAMREPGANNLFF TRB:CASSLGGDGNTIYF	3	CD8-C5
PB-2_ATCTACTGTGCCTGTG	PB	PB-2	P2-5	TRUE	3	TRA:CASSFSGGYNKLIF TRB:CASSYLGAGYQETQYF	127	CD8-C5
PB-2_ATGAGGGTCGAATGGG	PB	PB-2	P2-8	TRUE	3	TRA:CAALEGDNYGQNFVF TRB:CASSPTWGGVRDTQYF	53	CD8-C5
PB-2_ATGCGATCAGATGGGT	PB	PB-2	P2-4	TRUE	3	TRA:CAIRYQSFNFGNEKLTF TRB:CASYRRVTEQYF	148	CD8-C4
PB-2_ATGTGTGGTTACGACT	PB	PB-2	P2-295	FALSE	2	TRA:CAYRTTQTGANNLFF TRB:CASHVFKNIQYF	2	CD8-C5
PB-2_ATTATCCAGCGCCTCA	PB	PB-2	P2-296	TRUE	2	TRA:CALKKSSGDKLTF TRB:CASTSGGGGQETQYF	2	CD8-C5
PB-2_ATTTCTGAGGAGCGTT	PB	PB-2	P2-2	TRUE	3	TRA:CVVPNTNAGKSTF TRB:CASSLQGSTYNEQFF	220	CD8-C5
PB-2_ATTTCTGGTTGAGGTG	PB	PB-2	P2-25	FALSE	3	TRA:CAMREAPNTDKLIF TRB:CASSGGGGGLETQYF	22	CD8-C5
PB-2_CAACCAAAGTGGAGTC	PB	PB-2	P2-4	TRUE	3	TRA:CAIRYQSFNFGNEKLTF TRB:CASYRRVTEQYF	148	CD8-C5
PB-2_CAACCTCAGTTTGCGT	PB	PB-2	P2-2	TRUE	3	TRA:CVVPNTNAGKSTF TRB:CASSLQGSTYNEQFF	220	CD8-C5
PB-2_CAACCTCGTAGCTCCG	PB	PB-2	P2-3	TRUE	3	TRA:CAVNPLESSYKLIF TRB:CASSPGTANTGELFF	177	CD8-C5
PB-2_CAAGTTGAGTGTACGG	PB	PB-2	P2-4	TRUE	3	TRA:CAIRYQSFNFGNEKLTF TRB:CASYRRVTEQYF	148	CD8-C5
PB-2_CAAGTTGCACTAAGTC	PB	PB-2	P2-4	TRUE	3	TRA:CAIRYQSFNFGNEKLTF TRB:CASYRRVTEQYF	148	CD8-C5
PB-2_CACAAACGTGCAGTAG	PB	PB-2	P2-2	TRUE	3	TRA:CVVPNTNAGKSTF TRB:CASSLQGSTYNEQFF	220	CD8-C5
PB-2_CACACAAGTACTTGAC	PB	PB-2	P2-99	TRUE	3	TRA:CLVGDYAGGYQKVTF TRB:CASSEQGPTEAFF	5	CD8-C5
PB-2_CACAGGCTCTAACGGT	PB	PB-2	P2-5	TRUE	3	TRA:CASSFSGGYNKLIF TRB:CASSYLGAGYQETQYF	127	CD8-C5
PB-2_CACAGTAGTTCCATGA	PB	PB-2	P2-54	FALSE	3	TRA:CALNFMNRDDKIIF TRB:CASSYSPGQAASYGYTF	10	CD8-C5
PB-2_CACAGTATCAGATAAG	PB	PB-2	P2-4	TRUE	3	TRA:CAIRYQSFNFGNEKLTF TRB:CASYRRVTEQYF	148	CD8-C5

PB-2_CACATTTAGCCTTGAT	PB	PB-2	P2-27	FALSE	3	TRA:CAPKSYNTDKLIF TRB:CASSEGGGGAETQYF	22	CD8-C4
PB-2_CACCACTCAAACGTGG	PB	PB-2	P2-3	TRUE	3	TRA:CAVNPLESSYKLIF TRB:CASSPGTANTGELFF	177	CD8-C5
PB-2_CACCAGGTCAGTGTTG	PB	PB-2	P2-15	TRUE	3	TRA:CAVGDNFNKFYF TRB:CASSRTSAAGELFF	35	CD8-C5
PB-2_CACCAGGTCGCAAACT	PB	PB-2	P2-15	TRUE	3	TRA:CAVGDNFNKFYF TRB:CASSRTSAAGELFF	35	CD8-C5
PB-2_CACCAGGTCTGACCTC	PB	PB-2	P2-4	TRUE	3	TRA:CAIRYQSFNFGNEKLTF TRB:CASYRRVTEQYF	148	CD8-C7
PB-2_CACCTTGAGCTATGCT	PB	PB-2	P2-4	TRUE	3	TRA:CAIRYQSFNFGNEKLTF TRB:CASYRRVTEQYF	148	CD8-C5
PB-2_CACCTTGGTAGCCTAT	PB	PB-2	P2-66	TRUE	3	TRA:CAVCRNRDDKIIF TRB:CASSTPQGHYGYTF	8	CD8-C4
PB-2_CACCTTGGTCCGTCAG	PB	PB-2	P2-27	FALSE	3	TRA:CAPKSYNTDKLIF TRB:CASSEGGGGAETQYF	22	CD8-C5
PB-2_CACTCCAGTAGAAGGA	PB	PB-2	P2-2	TRUE	3	TRA:CVVPNTNAGKSTF TRB:CASSLQGSTYNEQFF	220	CD8-C5
PB-2_CAGAATCAGCAGCGTA	PB	PB-2	P2-1295	FALSE	1	TRA:CAVTTGIQGAQKLVF TRB:CASSQPQHF	1	CD8-C4
PB-2_CAGAATCAGCGCTCCA	PB	PB-2	P2-1296	FALSE	1	TRA:CAVSEASSYKLIF TRB:CASSLQGSTYNEQFF	1	CD8-C5
PB-2_CAGAGAGGTAAAGGAG	PB	PB-2	P2-2	TRUE	3	TRA:CVVPNTNAGKSTF TRB:CASSLQGSTYNEQFF	220	CD8-C4
PB-2_CAGATCAGTCTAGAGG	PB	PB-2	P2-8	TRUE	3	TRA:CAALEGDNYGQNFVF TRB:CASSPTWGGVRDTQYF	53	CD8-C4
PB-2_CAGCAGCCACGCATCG	PB	PB-2	P2-15	TRUE	3	TRA:CAVGDNFNKFYF TRB:CASSRTSAAGELFF	35	CD8-C4
PB-2_CAGCCGAAGGCATTGG	PB	PB-2	P2-25	FALSE	3	TRA:CAMREAPNTDKLIF TRB:CASSGGGGGLETQYF	22	CD8-C5
PB-2_CAGCGACGTGTCGCTG	PB	PB-2	P2-27	FALSE	3	TRA:CAPKSYNTDKLIF TRB:CASSEGGGGAETQYF	22	CD8-C2
PB-2_CAGCTGGTCGCTGATA	PB	PB-2	P2-4	TRUE	3	TRA:CAIRYQSFNFGNEKLTF TRB:CASYRRVTEQYF	148	CD8-C5
PB-2_CAGTAACAGTATCTCG	PB	PB-2	P2-21	TRUE	3	TRA:CALSSYNNNDMRF TRB:CASSLDLAPSTEAFF	25	CD8-C5
PB-2_CATATGGAGTATTGGA	PB	PB-2	P2-11	TRUE	3	TRA:CAVHNQGGKLIF TRB:CASSYLGAGYQETQYF	43	CD8-C5
PB-2_CATATGGCACGCATCG	PB	PB-2	P2-1365	FALSE	1	TRA:CAVNRDSNYQLIW TRB:CASSLGGGGAYEQYF	1	CD8-C5
PB-2_CATCAAGTCCATTCTA	PB	PB-2	P2-104	TRUE	3	TRA:CAMREAGGSGAGSYQLTF TRB:CASSLGSGNTIYF	5	CD8-C5
PB-2_CATCAGACATAGTAAG	PB	PB-2	P2-19	TRUE	3	TRA:CAVRDMENNRLAF TRB:CASSLGGGLTYEQYF	27	CD8-C4
PB-2_CATCAGAGTCCGAAGA	PB	PB-2	P2-2	TRUE	3	TRA:CVVPNTNAGKSTF TRB:CASSLQGSTYNEQFF	220	CD8-C5
PB-2_CATCCACTCACGACTA	PB	PB-2	P2-27	FALSE	3	TRA:CAPKSYNTDKLIF TRB:CASSEGGGGAETQYF	22	CD8-C5
PB-2_CATCGAATCCCTAATT	PB	PB-2	P2-6	TRUE	3	TRA:CVVSVAAGNKLTF TRB:CASSPGTDLYEQYF	85	CD8-C4
PB-2_CATCGGGCACATTAGC	PB	PB-2	P2-2	TRUE	3	TRA:CVVPNTNAGKSTF TRB:CASSLQGSTYNEQFF	220	CD8-C5
PB-2_CATCGGGCATGCAACT	PB	PB-2	P2-1403	FALSE	1	TRA:CAVHNQGGKLIF TRB:CASSLDLAPSTEAFF	1	CD8-C5

PB-2_CATCGGGGTGACGCCT	PB	PB-2	P2-1405	FALSE	1	TRA:CAVKNTNAGKSTF TRB:CASSPGPLPPYGYTF	1	CD8-C4
PB-2_CATGCCTCACCATCCT	PB	PB-2	P2-2	TRUE	3	TRA:CVVPNTNAGKSTF TRB:CASSLQGSTYNEQFF	220	CD8-C5
PB-2_CATGCCTTCTAACCGA	PB	PB-2	P2-2	TRUE	3	TRA:CVVPNTNAGKSTF TRB:CASSLQGSTYNEQFF	220	CD8-C5
PB-2_CATTCGCGTTATGTGC	PB	PB-2	P2-67	FALSE	3	TRA:CVVSDPLWDNYGQNFVF TRB:CASSPSGGLLHYNEQFF	8	CD8-C5
PB-2_CCAATCCAGAGAACAG	PB	PB-2	P2-5	TRUE	3	TRA:CASSFSGGYNKLIF TRB:CASSYLGAGYQETQYF	127	CD8-C5
PB-2_CCAATCCCAGGACGTA	PB	PB-2	P2-21	TRUE	3	TRA:CALSSYNNNDMRF TRB:CASSLDLAPSTEAFF	25	CD8-C5
PB-2_CCACGGAGTCCGAATT	PB	PB-2	P2-2	TRUE	3	TRA:CVVPNTNAGKSTF TRB:CASSLQGSTYNEQFF	220	CD8-C5
PB-2_CCACTACTCACTTCAT	PB	PB-2	P2-4	TRUE	3	TRA:CAIRYQSFNFGNEKLTF TRB:CASYRRVTEQYF	148	CD8-C5
PB-2_CCATGTCCATCGGAAG	PB	PB-2	P2-1483	FALSE	1	TRA:CALSSYNNNDMRF TRB:CASTPPRVSSYNEQFF	1	CD8-C5
PB-2_CCATGTCCATTCTCAT	PB	PB-2	P2-125	TRUE	3	TRA:CAASEGNFGNEKLTF TRB:CASSQSGGPFREQYF	4	CD8-C4
PB-2_CCATTCGCAGACGCAA	PB	PB-2	P2-1490	FALSE	1	TRA:CGADAEYGNKLVF TRB:CASSPRYGTGGTSSTDTQYF	1	CD8-C5
PB-2_CCCAATCGTCCAGTAT	PB	PB-2	P2-57	TRUE	3	TRA:CAVRDMSSGAGSYQLTF TRB:CASSELEDQETQYF	9	CD8-C5
PB-2_CCCAATCTCTAACTTC	PB	PB-2	P2-21	TRUE	3	TRA:CALSSYNNNDMRF TRB:CASSLDLAPSTEAFF	25	CD8-C5
PB-2_CCCAGTTAGACTTGAA	PB	PB-2	P2-1	TRUE	3	TRA:CVVNIIGYQKVTF TRB:CASTRQGKQPQHF	270	CD8-C5
PB-2_CCCATACTCAGTACGT	PB	PB-2	P2-45	TRUE	3	TRA:CAVSGDSNYQLIW TRB:CASSLGGNGNTIYF	11	CD8-C5
PB-2_CCCTCCTGTCCGTTAA	PB	PB-2	P2-5	TRUE	3	TRA:CASSFSGGYNKLIF TRB:CASSYLGAGYQETQYF	127	CD8-C5
PB-2_CCGGGATCACGAAACG	PB	PB-2	P2-4	TRUE	3	TRA:CAIRYQSFNFGNEKLTF TRB:CASYRRVTEQYF	148	CD8-C5
PB-2_CCGTACTAGATAGGAG	PB	PB-2	P2-126	TRUE	3	TRA:CATDGDFGNEKLTF TRB:CASSLTGETQYF	4	CD8-C4
PB-2_CCGTGGAGTCAGATAA	PB	PB-2	P2-2	TRUE	3	TRA:CVVPNTNAGKSTF TRB:CASSLQGSTYNEQFF	220	CD8-C5
PB-2_CCGTTCATCCATTCTA	PB	PB-2	P2-19	TRUE	3	TRA:CAVRDMENNRLAF TRB:CASSLGGGLTYEQYF	27	CD8-C4
PB-2_CCTACACAGATCCGAG	PB	PB-2	P2-2	TRUE	3	TRA:CVVPNTNAGKSTF TRB:CASSLQGSTYNEQFF	220	CD8-C5
PB-2_CCTAGCTCATGTCTCC	PB	PB-2	P2-21	TRUE	3	TRA:CALSSYNNNDMRF TRB:CASSLDLAPSTEAFF	25	CD8-C5
PB-2_CCTATTAGTGGTCTCG	PB	PB-2	P2-3	TRUE	3	TRA:CAVNPLESSYKLIF TRB:CASSPGTANTGELFF	177	CD8-C5
PB-2_CCTCTGAGTGTGACGA	PB	PB-2	P2-25	FALSE	3	TRA:CAMREAPNTDKLIF TRB:CASSGGGGGLETQYF	22	CD8-C5
PB-2_CCTTACGCAATAAGCA	PB	PB-2	P2-2	TRUE	3	TRA:CVVPNTNAGKSTF TRB:CASSLQGSTYNEQFF	220	CD8-C5
PB-2_CGACCTTAGAGTGAGA	PB	PB-2	P2-1623	FALSE	1	TRA:CAALAEYGNKLVF TRB:CSGDSSYNEQFF	1	CD8-C5
PB-2_CGACCTTCAGGGTATG	PB	PB-2	P2-176	TRUE	3	TRA:CAMREPGANNLFF TRB:CASSLGGDGNTIYF	3	CD8-C4

PB-2_CGACTTCAGCTCCTTC	PB	PB-2	P2-15	TRUE	3	TRA:CAVGDNFNKFYF TRB:CASSRTSAAGELFF	35	CD8-C4
PB-2_CGACTTCCAAGGTTTC	PB	PB-2	P2-2	TRUE	3	TRA:CVVPNTNAGKSTF TRB:CASSLQGSTYNEQFF	220	CD8-C5
PB-2_CGACTTCGTTGGTAAA	PB	PB-2	P2-25	FALSE	3	TRA:CAMREAPNTDKLIF TRB:CASSGGGGGLETQYF	22	CD8-C5
PB-2_CGAGAAGAGCTAGTGG	PB	PB-2	P2-30	TRUE	3	TRA:CAVGGDTGNQFYF TRB:CASSLFPTGVAYGYTF	20	CD8-C5
PB-2_CGAGCCAGTGTGGTTT	PB	PB-2	P2-66	TRUE	3	TRA:CAVCRNRDDKIIF TRB:CASSTPQGHYGYTF	8	CD8-C4
PB-2_CGAGCCATCCGCATCT	PB	PB-2	P2-4	TRUE	3	TRA:CAIRYQSFNFGNEKLTF TRB:CASYRRVTEQYF	148	CD8-C5
PB-2_CGATCGGAGTAGGTGC	PB	PB-2	P2-5	TRUE	3	TRA:CASSFSGGYNKLIF TRB:CASSYLGAGYQETQYF	127	CD8-C7
PB-2_CGATGGCAGGCATGGT	PB	PB-2	P2-2	TRUE	3	TRA:CVVPNTNAGKSTF TRB:CASSLQGSTYNEQFF	220	CD8-C5
PB-2_CGATGTATCCGGCACA	PB	PB-2	P2-4	TRUE	3	TRA:CAIRYQSFNFGNEKLTF TRB:CASYRRVTEQYF	148	CD8-C5
PB-2_CGCCAAGAGCTAAGAT	PB	PB-2	P2-4	TRUE	3	TRA:CAIRYQSFNFGNEKLTF TRB:CASYRRVTEQYF	148	CD8-C7
PB-2_CGCCAAGCAATTCCTT	PB	PB-2	P2-29	TRUE	3	TRA:CAVGGDFNKFYF TRB:CSARSDRLGYQPQHF	21	CD8-C5
PB-2_CGCGGTACAAGTCTAC	PB	PB-2	P2-5	TRUE	3	TRA:CASSFSGGYNKLIF TRB:CASSYLGAGYQETQYF	127	CD8-C5
PB-2_CGCGGTACACAGGTTT	PB	PB-2	P2-127	TRUE	3	TRA:CAARRSGYSTLTF TRB:CASSLFPTGVAYGYTF	4	CD8-C5
PB-2_CGCGGTACATACGCTA	PB	PB-2	P2-5	TRUE	3	TRA:CASSFSGGYNKLIF TRB:CASSYLGAGYQETQYF	127	CD8-C5
PB-2_CGCGTTTAGAAGGTGA	PB	PB-2	P2-127	TRUE	3	TRA:CAARRSGYSTLTF TRB:CASSLFPTGVAYGYTF	4	CD8-C6
PB-2_CGCGTTTAGAATTCCC	PB	PB-2	P2-29	TRUE	3	TRA:CAVGGDFNKFYF TRB:CSARSDRLGYQPQHF	21	CD8-C5
PB-2_CGCGTTTAGGCATGTG	PB	PB-2	P2-1684	FALSE	1	TRA:CALSETLGSGGGADGLTF TRB:CASSPGTSGVSSYEQYF	1	CD8-C4
PB-2_CGCGTTTGTGCTTCTC	PB	PB-2	P2-4	TRUE	3	TRA:CAIRYQSFNFGNEKLTF TRB:CASYRRVTEQYF	148	CD8-C5
PB-2_CGCTGGAGTTACGCGC	PB	PB-2	P2-2	TRUE	3	TRA:CVVPNTNAGKSTF TRB:CASSLQGSTYNEQFF	220	CD8-C5
PB-2_CGCTTCAGTTCGGCAC	PB	PB-2	P2-3	TRUE	3	TRA:CAVNPLESSYKLIF TRB:CASSPGTANTGELFF	177	CD8-C5
PB-2_CGGGTCACATGGGACA	PB	PB-2	P2-5	TRUE	3	TRA:CASSFSGGYNKLIF TRB:CASSYLGAGYQETQYF	127	CD8-C5
PB-2_CGGTTAAAGGAGTTGC	PB	PB-2	P2-10	TRUE	3	TRA:CAPLTPNDMRF TRB:CASLNGAGGSYEQYF	45	CD8-C4
PB-2_CGGTTAAAGTTTGCGT	PB	PB-2	P2-15	TRUE	3	TRA:CAVGDNFNKFYF TRB:CASSRTSAAGELFF	35	CD8-C5
PB-2_CGTAGCGGTTAAGAAC	PB	PB-2	P2-2	TRUE	3	TRA:CVVPNTNAGKSTF TRB:CASSLQGSTYNEQFF	220	CD8-C5
PB-2_CGTAGGCAGCTAGTCT	PB	PB-2	P2-25	FALSE	3	TRA:CAMREAPNTDKLIF TRB:CASSGGGGGLETQYF	22	CD8-C5
PB-2_CGTAGGCTCTGGAGCC	PB	PB-2	P2-54	FALSE	3	TRA:CALNFMNRDDKIIF TRB:CASSYSPGQAASYGYTF	10	CD8-C5
PB-2_CGTCAGGGTCATATCG	PB	PB-2	P2-129	TRUE	3	TRA:CARGGTGNQFYF TRB:CAWSPMPNEQFF	4	CD8-C5

PB-2_CGTCTACCACATTAGC	PB	PB-2	P2-5	TRUE	3	TRA:CASSFSGGYNKLIF TRB:CASSYLGAGYQETQYF	127	CD8-C7
PB-2_CGTGTCTAGTGGTAGC	PB	PB-2	P2-4	TRUE	3	TRA:CAIRYQSFNFGNEKLTF TRB:CASYRRVTEQYF	148	CD8-C5
PB-2_CGTTAGATCAGCTCTC	PB	PB-2	P2-189	TRUE	3	TRA:CAVRDWGRDDKIIF TRB:CASSSKPRSGANVLTF	3	CD8-C5
PB-2_CGTTCTGCACAACTGT	PB	PB-2	P2-1825	FALSE	1	TRA:CAMRNTGFQKLVF TRB:CSASPGGRDEQYF	1	CD8-C4
PB-2_CTAATGGCAGGTTTCA	PB	PB-2	P2-8	TRUE	3	TRA:CAALEGDNYGQNFVF TRB:CASSPTWGGVRDTQYF	53	CD8-C4
PB-2_CTACATTAGTCATGCT	PB	PB-2	P2-21	TRUE	3	TRA:CALSSYNNNDMRF TRB:CASSLDLAPSTEAFF	25	CD8-C5
PB-2_CTACATTAGTTTGCGT	PB	PB-2	P2-27	FALSE	3	TRA:CAPKSYNTDKLIF TRB:CASSEGGGGAETQYF	22	CD8-C5
PB-2_CTACCCATCCGCAAGC	PB	PB-2	P2-4	TRUE	3	TRA:CAIRYQSFNFGNEKLTF TRB:CASYRRVTEQYF	148	CD8-C5
PB-2_CTACCCATCCTTCAAT	PB	PB-2	P2-318	TRUE	2	TRA:CAVEEGVRLMF TRB:CATSAGQGAFTDTQYF	2	CD8-C4
PB-2_CTAGCCTTCGTCTGAA	PB	PB-2	P2-2	TRUE	3	TRA:CVVPNTNAGKSTF TRB:CASSLQGSTYNEQFF	220	CD8-C4
PB-2_CTAGTGATCCGCGTTT	PB	PB-2	P2-4	TRUE	3	TRA:CAIRYQSFNFGNEKLTF TRB:CASYRRVTEQYF	148	CD8-C5
PB-2_CTCCTAGCACACATGT	PB	PB-2	P2-30	TRUE	3	TRA:CAVGGDTGNQFYF TRB:CASSLFPTGVAYGYTF	20	CD8-C4
PB-2_CTCGAAAAGCGATTCT	PB	PB-2	P2-320	TRUE	2	TRA:CAGEADKLIF TRB:CASSQETAGGDTQYF	2	CD8-C4
PB-2_CTCGAAACACAGCCCA	PB	PB-2	P2-30	TRUE	3	TRA:CAVGGDTGNQFYF TRB:CASSLFPTGVAYGYTF	20	CD8-C4
PB-2_CTCGAAACATCGGGTC	PB	PB-2	P2-8	TRUE	3	TRA:CAALEGDNYGQNFVF TRB:CASSPTWGGVRDTQYF	53	CD8-C5
PB-2_CTCGAAACATGGTCTA	PB	PB-2	P2-4	TRUE	3	TRA:CAIRYQSFNFGNEKLTF TRB:CASYRRVTEQYF	148	CD8-C5
PB-2_CTCGAAATCAACGGCC	PB	PB-2	P2-4	TRUE	3	TRA:CAIRYQSFNFGNEKLTF TRB:CASYRRVTEQYF	148	CD8-C7
PB-2_CTCGGGAAGAAACCGC	PB	PB-2	P2-6	TRUE	3	TRA:CVVSVAAGNKLTF TRB:CASSPGTDLYEQYF	85	CD8-C4
PB-2_CTCTACGAGAGCTTCT	PB	PB-2	P2-27	FALSE	3	TRA:CAPKSYNTDKLIF TRB:CASSEGGGGAETQYF	22	CD8-C5
PB-2_CTCTGGTGTGCAACTT	PB	PB-2	P2-4	TRUE	3	TRA:CAIRYQSFNFGNEKLTF TRB:CASYRRVTEQYF	148	CD8-C5
PB-2_CTGAAACAGATAGGAG	PB	PB-2	P2-3	TRUE	3	TRA:CAVNPLESSYKLIF TRB:CASSPGTANTGELFF	177	CD8-C7
PB-2_CTGATAGTCATGCTCC	PB	PB-2	P2-4	TRUE	3	TRA:CAIRYQSFNFGNEKLTF TRB:CASYRRVTEQYF	148	CD8-C5
PB-2_CTGCCTAGTAGCAAAT	PB	PB-2	P2-2	TRUE	3	TRA:CVVPNTNAGKSTF TRB:CASSLQGSTYNEQFF	220	CD8-C5
PB-2_CTGCGGACAAACTGTC	PB	PB-2	P2-15	TRUE	3	TRA:CAVGDNFNKFYF TRB:CASSRTSAAGELFF	35	CD8-C4
PB-2_CTGCGGACATCAGTAC	PB	PB-2	P2-4	TRUE	3	TRA:CAIRYQSFNFGNEKLTF TRB:CASYRRVTEQYF	148	CD8-C5
PB-2_CTGCTGTAGGCTACGA	PB	PB-2	P2-2	TRUE	3	TRA:CVVPNTNAGKSTF TRB:CASSLQGSTYNEQFF	220	CD8-C4
PB-2_CTTACCGCATGCGCAC	PB	PB-2	P2-66	TRUE	3	TRA:CAVCRNRDDKIIF TRB:CASSTPQGHYGYTF	8	CD8-C4

PB-2_CTTCTCTGTTCAGGCC	PB	PB-2	P2-2	TRUE	3	TRA:CVVPNTNAGKSTF TRB:CASSLQGSTYNEQFF	220	CD8-C5
PB-2_CTTGGCTAGTACGATA	PB	PB-2	P2-3	TRUE	3	TRA:CAVNPLESSYKLIF TRB:CASSPGTANTGELFF	177	CD8-C5
PB-2_GAAATGAGTATAAACG	PB	PB-2	P2-25	FALSE	3	TRA:CAMREAPNTDKLIF TRB:CASSGGGGGLETQYF	22	CD8-C5
PB-2_GAAATGATCTACTTAC	PB	PB-2	P2-2	TRUE	3	TRA:CVVPNTNAGKSTF TRB:CASSLQGSTYNEQFF	220	CD8-C5
PB-2_GAACATCCATAGTAAG	PB	PB-2	P2-57	TRUE	3	TRA:CAVRDMSSGAGSYQLTF TRB:CASSELEDQETQYF	9	CD8-C5
PB-2_GAACATCTCCACGACG	PB	PB-2	P2-65	TRUE	3	TRA:CAPNQAGTALIF TRB:CASKLSGTSGSYEQYF	8	CD8-C4
PB-2_GAACGGATCCCATTAT	PB	PB-2	P2-309	FALSE	2	TRA:CAVNMFQSDYKLSF TRB:CSARASSAREQFF	2	CD8-C5
PB-2_GAAGCAGAGGACAGCT	PB	PB-2	P2-2	TRUE	3	TRA:CVVPNTNAGKSTF TRB:CASSLQGSTYNEQFF	220	CD8-C5
PB-2_GAATAAGCACCTTGTC	PB	PB-2	P2-99	TRUE	3	TRA:CLVGDYAGGYQKVTF TRB:CASSEQGPTEAFF	5	CD8-C5
PB-2_GAATAAGCATGCCACG	PB	PB-2	P2-3	TRUE	3	TRA:CAVNPLESSYKLIF TRB:CASSPGTANTGELFF	177	CD8-C5
PB-2_GACACGCAGGAGCGTT	PB	PB-2	P2-4	TRUE	3	TRA:CAIRYQSFNFGNEKLTF TRB:CASYRRVTEQYF	148	CD8-C5
PB-2_GACACGCGTCACCCAG	PB	PB-2	P2-2113	FALSE	1	TRA:CAATLNDYKLSF TRB:CASSLQGSTYNEQFF	1	CD8-C5
PB-2_GACAGAGTCTGGCGAC	PB	PB-2	P2-15	TRUE	3	TRA:CAVGDNFNKFYF TRB:CASSRTSAAGELFF	35	CD8-C4
PB-2_GACCAATTCATTGCCC	PB	PB-2	P2-2	TRUE	3	TRA:CVVPNTNAGKSTF TRB:CASSLQGSTYNEQFF	220	CD8-C5
PB-2_GACCTGGGTCTTTCAT	PB	PB-2	P2-168	FALSE	3	TRA:CIVETSYDKVIF TRB:CATARDSNQPQHF	3	CD8-C5
PB-2_GACCTGGGTGTTCGAT	PB	PB-2	P2-65	TRUE	3	TRA:CAPNQAGTALIF TRB:CASKLSGTSGSYEQYF	8	CD8-C5
PB-2_GACGCGTAGGTAGCCA	PB	PB-2	P2-8	TRUE	3	TRA:CAALEGDNYGQNFVF TRB:CASSPTWGGVRDTQYF	53	CD8-C5
PB-2_GACGCGTGTCAGGACA	PB	PB-2	P2-21	TRUE	3	TRA:CALSSYNNNDMRF TRB:CASSLDLAPSTEAFF	25	CD8-C5
PB-2_GACGCGTTCTATGTGG	PB	PB-2	P2-21	TRUE	3	TRA:CALSSYNNNDMRF TRB:CASSLDLAPSTEAFF	25	CD8-C5
PB-2_GACGGCTAGTCGATAA	PB	PB-2	P2-4	TRUE	3	TRA:CAIRYQSFNFGNEKLTF TRB:CASYRRVTEQYF	148	CD8-C7
PB-2_GACGGCTCATATACGC	PB	PB-2	P2-8	TRUE	3	TRA:CAALEGDNYGQNFVF TRB:CASSPTWGGVRDTQYF	53	CD8-C5
PB-2_GACGGCTGTGAGGCTA	PB	PB-2	P2-57	TRUE	3	TRA:CAVRDMSSGAGSYQLTF TRB:CASSELEDQETQYF	9	CD8-C4
PB-2_GACGTGCGTGACGCCT	PB	PB-2	P2-130	TRUE	3	TRA:CAFMTPNGGATNKLIF TRB:CASSQENTAGSYF	4	CD8-C5
PB-2_GACGTTAGTCTCCACT	PB	PB-2	P2-22	TRUE	3	TRA:CAMRERSWVRQKLVF TRB:CASSRGTSGTDTQYF	24	CD8-C4
PB-2_GAGCAGAGTAAGTGTA	PB	PB-2	P2-60	TRUE	3	TRA:CALSEGSGANSKLTF TRB:CASSSHSGLAGGFF	9	CD8-C4
PB-2_GAGCAGATCGCTAGCG	PB	PB-2	P2-19	TRUE	3	TRA:CAVRDMENNRLAF TRB:CASSLGGGLTYEQYF	27	CD8-C4
PB-2_GATCGATTCGGAGGTA	PB	PB-2	P2-2	TRUE	3	TRA:CVVPNTNAGKSTF TRB:CASSLQGSTYNEQFF	220	CD8-C5

PB-2_GATCGCGGTCCGAATT	PB	PB-2	P2-5	TRUE	3	TRA:CASSFSGGYNKLIF TRB:CASSYLGAGYQETQYF	127	CD8-C5
PB-2_GATCGTACAAAGTCAA	PB	PB-2	P2-65	TRUE	3	TRA:CAPNQAGTALIF TRB:CASKLSGTSGSYEQYF	8	CD8-C5
PB-2_GATCGTATCCAACCAA	PB	PB-2	P2-2239	FALSE	1	TRA:CAALAEYGNKLVF TRB:CASSPRLESGYEQYF	1	CD8-C5
PB-2_GATCTAGGTGTGCCTG	PB	PB-2	P2-47	TRUE	3	TRA:CAASAGGNKLTF TRB:CASSTEDEQYF	11	CD8-C5
PB-2_GATGCTAAGCGTCTAT	PB	PB-2	P2-4	TRUE	3	TRA:CAIRYQSFNFGNEKLTF TRB:CASYRRVTEQYF	148	CD8-C5
PB-2_GATTCAGAGATCGGGT	PB	PB-2	P2-4	TRUE	3	TRA:CAIRYQSFNFGNEKLTF TRB:CASYRRVTEQYF	148	CD8-C7
PB-2_GATTCAGCAAAGGCGT	PB	PB-2	P2-4	TRUE	3	TRA:CAIRYQSFNFGNEKLTF TRB:CASYRRVTEQYF	148	CD8-C5
PB-2_GATTCAGCAGCTCGCA	PB	PB-2	P2-2	TRUE	3	TRA:CVVPNTNAGKSTF TRB:CASSLQGSTYNEQFF	220	CD8-C4
PB-2_GCAAACTGTGTAACGG	PB	PB-2	P2-3	TRUE	3	TRA:CAVNPLESSYKLIF TRB:CASSPGTANTGELFF	177	CD8-C5
PB-2_GCAAACTTCTGCTGCT	PB	PB-2	P2-4	TRUE	3	TRA:CAIRYQSFNFGNEKLTF TRB:CASYRRVTEQYF	148	CD8-C7
PB-2_GCAATCACAGCGAACA	PB	PB-2	P2-109	TRUE	3	TRA:CAPPFGGSEKLVF TRB:CASSKTGSSYEQYF	5	CD8-C4
PB-2_GCACATATCAGTTGAC	PB	PB-2	P2-269	FALSE	2	TRA:CATDGLNNAGNMLTF TRB:CASRTSGRFRTDTQYF	2	CD8-C4
PB-2_GCACTCTGTATTCTCT	PB	PB-2	P2-5	TRUE	3	TRA:CASSFSGGYNKLIF TRB:CASSYLGAGYQETQYF	127	CD8-C7
PB-2_GCACTCTGTCGCGGTT	PB	PB-2	P2-25	FALSE	3	TRA:CAMREAPNTDKLIF TRB:CASSGGGGGLETQYF	22	CD8-C5
PB-2_GCAGTTATCCGGCACA	PB	PB-2	P2-2	TRUE	3	TRA:CVVPNTNAGKSTF TRB:CASSLQGSTYNEQFF	220	CD8-C4
PB-2_GCATACAAGCGACGTA	PB	PB-2	P2-45	TRUE	3	TRA:CAVSGDSNYQLIW TRB:CASSLGGNGNTIYF	11	CD8-C4
PB-2_GCATACAAGCGATCCC	PB	PB-2	P2-5	TRUE	3	TRA:CASSFSGGYNKLIF TRB:CASSYLGAGYQETQYF	127	CD8-C5
PB-2_GCATGCGAGCTGGAAC	PB	PB-2	P2-2	TRUE	3	TRA:CVVPNTNAGKSTF TRB:CASSLQGSTYNEQFF	220	CD8-C5
PB-2_GCCAAATTCACTTATC	PB	PB-2	P2-2334	FALSE	1	TRA:CAEGGGSYIPTF TRB:CASSGGQGEETQYF	1	CD8-C5
PB-2_GCGCAACAGCAGATCG	PB	PB-2	P2-2363	FALSE	1	TRA:CAHNDMRF TRB:CATSRDGVIMNEQFF	1	CD8-C4
PB-2_GCGCAGTAGATATGGT	PB	PB-2	P2-46	TRUE	3	TRA:CAFSTWGNQGGKLIF TRB:CASSQDRLGGGTYEQYF	11	CD8-C5
PB-2_GCGCCAAAGGCATGGT	PB	PB-2	P2-8	TRUE	3	TRA:CAALEGDNYGQNFVF TRB:CASSPTWGGVRDTQYF	53	CD8-C4
PB-2_GCGGGTTCATGACGGA	PB	PB-2	P2-2	TRUE	3	TRA:CVVPNTNAGKSTF TRB:CASSLQGSTYNEQFF	220	CD8-C3
PB-2_GCTCCTACATTACGAC	PB	PB-2	P2-2398	FALSE	1	TRA:CITYQGGSEKLVF TRB:CASSHRGSGEQYF	1	CD8-C5
PB-2_GCTCTGTAGCGACGTA	PB	PB-2	P2-2	TRUE	3	TRA:CVVPNTNAGKSTF TRB:CASSLQGSTYNEQFF	220	CD8-C3
PB-2_GCTCTGTGTCAGAGGT	PB	PB-2	P2-8	TRUE	3	TRA:CAALEGDNYGQNFVF TRB:CASSPTWGGVRDTQYF	53	CD8-C7
PB-2_GCTGCAGAGAAACGAG	PB	PB-2	P2-5	TRUE	3	TRA:CASSFSGGYNKLIF TRB:CASSYLGAGYQETQYF	127	CD8-C5

PB-2_GCTGCAGAGATCCCGC	PB	PB-2	P2-8	TRUE	3	TRA:CAALEGDNYGQNFVF TRB:CASSPTWGGVRDTQYF	53	CD8-C5
PB-2_GCTGCGAAGCGTAATA	PB	PB-2	P2-25	FALSE	3	TRA:CAMREAPNTDKLIF TRB:CASSGGGGGLETQYF	22	CD8-C5
PB-2_GCTGCTTCATCGGAAG	PB	PB-2	P2-27	FALSE	3	TRA:CAPKSYNTDKLIF TRB:CASSEGGGGAETQYF	22	CD8-C5
PB-2_GCTGCTTCATGCCACG	PB	PB-2	P2-19	TRUE	3	TRA:CAVRDMENNRLAF TRB:CASSLGGGLTYEQYF	27	CD8-C4
PB-2_GCTTCCAAGGTTACCT	PB	PB-2	P2-4	TRUE	3	TRA:CAIRYQSFNFGNEKLTF TRB:CASYRRVTEQYF	148	CD8-C5
PB-2_GCTTGAACACATCCAA	PB	PB-2	P2-4	TRUE	3	TRA:CAIRYQSFNFGNEKLTF TRB:CASYRRVTEQYF	148	CD8-C5
PB-2_GCTTGAAGTGGAAAGA	PB	PB-2	P2-4	TRUE	3	TRA:CAIRYQSFNFGNEKLTF TRB:CASYRRVTEQYF	148	CD8-C7
PB-2_GGAAAGCCAATGACCT	PB	PB-2	P2-125	TRUE	3	TRA:CAASEGNFGNEKLTF TRB:CASSQSGGPFREQYF	4	CD8-C4
PB-2_GGACAAGAGCCTTGAT	PB	PB-2	P2-4	TRUE	3	TRA:CAIRYQSFNFGNEKLTF TRB:CASYRRVTEQYF	148	CD8-C7
PB-2_GGACAGACATGGTCAT	PB	PB-2	P2-5	TRUE	3	TRA:CASSFSGGYNKLIF TRB:CASSYLGAGYQETQYF	127	CD8-C7
PB-2_GGACATTGTATGAATG	PB	PB-2	P2-30	TRUE	3	TRA:CAVGGDTGNQFYF TRB:CASSLFPTGVAYGYTF	20	CD8-C5
PB-2_GGACGTCGTGATAAGT	PB	PB-2	P2-196	TRUE	3	TRA:CAVYNAGNMLTF TRB:CASSSRQVYF	3	CD8-C5
PB-2_GGATGTTCACCTGGTG	PB	PB-2	P2-10	TRUE	3	TRA:CAPLTPNDMRF TRB:CASLNGAGGSYEQYF	45	CD8-C4
PB-2_GGATTACGTAAACACA	PB	PB-2	P2-19	TRUE	3	TRA:CAVRDMENNRLAF TRB:CASSLGGGLTYEQYF	27	CD8-C4
PB-2_GGATTACGTTTACTCT	PB	PB-2	P2-2	TRUE	3	TRA:CVVPNTNAGKSTF TRB:CASSLQGSTYNEQFF	220	CD8-C5
PB-2_GGCAATTCACGGTTTA	PB	PB-2	P2-2500	FALSE	1	TRA:CAVNDNYGQNFVF TRB:CASSRAGGLDTQYF	1	CD8-C4
PB-2_GGCAATTGTCTAGCGC	PB	PB-2	P2-5	TRUE	3	TRA:CASSFSGGYNKLIF TRB:CASSYLGAGYQETQYF	127	CD8-C5
PB-2_GGCGACTAGAGTCTGG	PB	PB-2	P2-2514	FALSE	1	TRA:CALDYSGGGADGLTF TRB:CASSDNRGGETQYF	1	CD8-C4
PB-2_GGCGACTCATGTTGAC	PB	PB-2	P2-59	TRUE	3	TRA:CAVDEGNTPLVF TRB:CAISESVDNTEAFF	9	CD8-C4
PB-2_GGCTGGTGTGCCTTGG	PB	PB-2	P2-21	TRUE	3	TRA:CALSSYNNNDMRF TRB:CASSLDLAPSTEAFF	25	CD8-C5
PB-2_GGCTGGTTCATGCAAC	PB	PB-2	P2-8	TRUE	3	TRA:CAALEGDNYGQNFVF TRB:CASSPTWGGVRDTQYF	53	CD8-C5
PB-2_GGGAATGTCATTCACT	PB	PB-2	P2-19	TRUE	3	TRA:CAVRDMENNRLAF TRB:CASSLGGGLTYEQYF	27	CD8-C5
PB-2_GGTATTGTCAGGTTCA	PB	PB-2	P2-2577	FALSE	1	TRA:CAVGAKTSYDKVIF TRB:CASSPTGTPNNEQFF	1	CD8-C5
PB-2_GGTGCGTCATTAACCG	PB	PB-2	P2-5	TRUE	3	TRA:CASSFSGGYNKLIF TRB:CASSYLGAGYQETQYF	127	CD8-C5
PB-2_GTAACGTTCTTGAGAC	PB	PB-2	P2-4	TRUE	3	TRA:CAIRYQSFNFGNEKLTF TRB:CASYRRVTEQYF	148	CD8-C5
PB-2_GTAACTGGTGCATCTA	PB	PB-2	P2-8	TRUE	3	TRA:CAALEGDNYGQNFVF TRB:CASSPTWGGVRDTQYF	53	CD8-C5
PB-2_GTACTCCAGCAGACTG	PB	PB-2	P2-5	TRUE	3	TRA:CASSFSGGYNKLIF TRB:CASSYLGAGYQETQYF	127	CD8-C5

PB-2_GTACTCCAGGAGTACC	PB	PB-2	P2-4	TRUE	3	TRA:CAIRYQSFNFGNEKLTF TRB:CASYRRVTEQYF	148	CD8-C7
PB-2_GTACTTTAGTGTCTCA	PB	PB-2	P2-2	TRUE	3	TRA:CVVPNTNAGKSTF TRB:CASSLQGSTYNEQFF	220	CD8-C5
PB-2_GTAGGCCCACGAGGTA	PB	PB-2	P2-353	TRUE	2	TRA:CAVSTDKLIF TRB:CASSLAYLGGGANVLTF	2	CD4-C6
PB-2_GTAGTCATCATGGTCA	PB	PB-2	P2-11	TRUE	3	TRA:CAVHNQGGKLIF TRB:CASSYLGAGYQETQYF	43	CD8-C5
PB-2_GTATCTTCAATACGCT	PB	PB-2	P2-4	TRUE	3	TRA:CAIRYQSFNFGNEKLTF TRB:CASYRRVTEQYF	148	CD8-C5
PB-2_GTATTCTCAATGGATA	PB	PB-2	P2-4	TRUE	3	TRA:CAIRYQSFNFGNEKLTF TRB:CASYRRVTEQYF	148	CD8-C5
PB-2_GTCACAACAACTGGCC	PB	PB-2	P2-2640	FALSE	1	TRA:CAASPGPGGKLTF TRB:CASSLDAEKLFF	1	CD8-C5
PB-2_GTCATTTTCGTACCGG	PB	PB-2	P2-8	TRUE	3	TRA:CAALEGDNYGQNFVF TRB:CASSPTWGGVRDTQYF	53	CD8-C5
PB-2_GTCCTCACACAACGCC	PB	PB-2	P2-354	TRUE	2	TRA:CALRQGARQLTF TRB:CALVGSSLETQYF	2	CD8-C5
PB-2_GTCTCGTAGGTGCTTT	PB	PB-2	P2-2	TRUE	3	TRA:CVVPNTNAGKSTF TRB:CASSLQGSTYNEQFF	220	CD8-C4
PB-2_GTCTTCGCACCATCCT	PB	PB-2	P2-8	TRUE	3	TRA:CAALEGDNYGQNFVF TRB:CASSPTWGGVRDTQYF	53	CD8-C5
PB-2_GTCTTCGGTGATAAGT	PB	PB-2	P2-5	TRUE	3	TRA:CASSFSGGYNKLIF TRB:CASSYLGAGYQETQYF	127	CD8-C5
PB-2_GTGCATAAGATAGGAG	PB	PB-2	P2-8	TRUE	3	TRA:CAALEGDNYGQNFVF TRB:CASSPTWGGVRDTQYF	53	CD8-C4
PB-2_GTGCATACACTTCTGC	PB	PB-2	P2-8	TRUE	3	TRA:CAALEGDNYGQNFVF TRB:CASSPTWGGVRDTQYF	53	CD8-C4
PB-2_GTGCATAGTCAGAAGC	PB	PB-2	P2-8	TRUE	3	TRA:CAALEGDNYGQNFVF TRB:CASSPTWGGVRDTQYF	53	CD8-C4
PB-2_GTGCGGTAGACGCTTT	PB	PB-2	P2-4	TRUE	3	TRA:CAIRYQSFNFGNEKLTF TRB:CASYRRVTEQYF	148	CD8-C5
PB-2_GTGTGCGGTTGGGACA	PB	PB-2	P2-4	TRUE	3	TRA:CAIRYQSFNFGNEKLTF TRB:CASYRRVTEQYF	148	CD8-C5
PB-2_GTGTGCGTCTCAAACG	PB	PB-2	P2-2	TRUE	3	TRA:CVVPNTNAGKSTF TRB:CASSLQGSTYNEQFF	220	CD8-C5
PB-2_GTTACAGCAGCGAACA	PB	PB-2	P2-6	TRUE	3	TRA:CVVSVAAGNKLTF TRB:CASSPGTDLYEQYF	85	CD8-C4
PB-2_GTTCATTTCGCGTTTC	PB	PB-2	P2-11	TRUE	3	TRA:CAVHNQGGKLIF TRB:CASSYLGAGYQETQYF	43	CD8-C7
PB-2_GTTCGGGAGGCAGGTT	PB	PB-2	P2-4	TRUE	3	TRA:CAIRYQSFNFGNEKLTF TRB:CASYRRVTEQYF	148	CD8-C7
PB-2_GTTCGGGCACAGACAG	PB	PB-2	P2-25	FALSE	3	TRA:CAMREAPNTDKLIF TRB:CASSGGGGGLETQYF	22	CD8-C5
PB-2_GTTTCTACAAACCCAT	PB	PB-2	P2-15	TRUE	3	TRA:CAVGDNFNKFYF TRB:CASSRTSAAGELFF	35	CD8-C4
PB-2_TAAGAGAGTACGCACC	PB	PB-2	P2-27	FALSE	3	TRA:CAPKSYNTDKLIF TRB:CASSEGGGGAETQYF	22	CD8-C5
PB-2_TAAGCGTTCGCCCTTA	PB	PB-2	P2-21	TRUE	3	TRA:CALSSYNNNDMRF TRB:CASSLDLAPSTEAFF	25	CD8-C5
PB-2_TACACGATCACCGGGT	PB	PB-2	P2-5	TRUE	3	TRA:CASSFSGGYNKLIF TRB:CASSYLGAGYQETQYF	127	CD8-C5
PB-2_TACAGTGCACATTTCT	PB	PB-2	P2-4	TRUE	3	TRA:CAIRYQSFNFGNEKLTF TRB:CASYRRVTEQYF	148	CD8-C5

PB-2_TACAGTGCATGCATGT	PB	PB-2	P2-27	FALSE	3	TRA:CAPKSYNTDKLIF TRB:CASSEGGGGAETQYF	22	CD8-C3
PB-2_TACAGTGGTCCGCTGA	PB	PB-2	P2-21	TRUE	3	TRA:CALSSYNNNDMRF TRB:CASSLDLAPSTEAFF	25	CD8-C5
PB-2_TACCTATGTTGGTAAA	PB	PB-2	P2-2829	FALSE	1	TRA:CVVGTISGGYNKLIF TRB:CASSQLGQGSNEKLFF	1	CD8-C5
PB-2_TACCTTACAGACAAAT	PB	PB-2	P2-2	TRUE	3	TRA:CVVPNTNAGKSTF TRB:CASSLQGSTYNEQFF	220	CD8-C5
PB-2_TACCTTAGTGATGTGG	PB	PB-2	P2-20	TRUE	3	TRA:CAYDSNYQLIW TRB:CASSLSIDRADTGELFF	25	CD8-C4
PB-2_TACGGGCCATGCTGGC	PB	PB-2	P2-2840	FALSE	1	TRA:CAGGSQPEKLVF TRB:CASRWTEFHTQYF	1	CD8-C10
PB-2_TACGGGCGTCGCCATG	PB	PB-2	P2-19	TRUE	3	TRA:CAVRDMENNRLAF TRB:CASSLGGGLTYEQYF	27	CD8-C4
PB-2_TACGGTAGTAATCACC	PB	PB-2	P2-25	FALSE	3	TRA:CAMREAPNTDKLIF TRB:CASSGGGGGLETQYF	22	CD8-C5
PB-2_TACGGTAGTCCGTTAA	PB	PB-2	P2-2	TRUE	3	TRA:CVVPNTNAGKSTF TRB:CASSLQGSTYNEQFF	220	CD8-C5
PB-2_TACTCATAGCTAAGAT	PB	PB-2	P2-19	TRUE	3	TRA:CAVRDMENNRLAF TRB:CASSLGGGLTYEQYF	27	CD8-C4
PB-2_TACTCGCCAATCCAAC	PB	PB-2	P2-2	TRUE	3	TRA:CVVPNTNAGKSTF TRB:CASSLQGSTYNEQFF	220	CD8-C5
PB-2_TACTCGCTCCTCTAGC	PB	PB-2	P2-11	TRUE	3	TRA:CAVHNQGGKLIF TRB:CASSYLGAGYQETQYF	43	CD8-C5
PB-2_TACTTGTAGTGGCACA	PB	PB-2	P2-2	TRUE	3	TRA:CVVPNTNAGKSTF TRB:CASSLQGSTYNEQFF	220	CD8-C5
PB-2_TACTTGTCAGTAAGCG	PB	PB-2	P2-4	TRUE	3	TRA:CAIRYQSFNFGNEKLTF TRB:CASYRRVTEQYF	148	CD8-C7
PB-2_TAGCCGGGTGACCAAG	PB	PB-2	P2-2	TRUE	3	TRA:CVVPNTNAGKSTF TRB:CASSLQGSTYNEQFF	220	CD8-C5
PB-2_TAGCCGGTCACGCATA	PB	PB-2	P2-6	TRUE	3	TRA:CVVSVAAGNKLTF TRB:CASSPGTDLYEQYF	85	CD8-C4
PB-2_TAGTGGTCAAGTAATG	PB	PB-2	P2-67	FALSE	3	TRA:CVVSDPLWDNYGQNFVF TRB:CASSPSGGLLHYNEQFF	8	CD8-C5
PB-2_TAGTGGTTCATACGGT	PB	PB-2	P2-69	TRUE	3	TRA:CAVRAGSARQLTF TRB:CATTGTSLDEQYF	8	CD8-C5
PB-2_TAGTGGTTCCCTCTTT	PB	PB-2	P2-5	TRUE	3	TRA:CASSFSGGYNKLIF TRB:CASSYLGAGYQETQYF	127	CD8-C5
PB-2_TAGTGGTTCGAATGCT	PB	PB-2	P2-87	TRUE	3	TRA:CAFMKPIGTDKLIF TRB:CASRLPGTGSRETQYF	6	CD8-C4
PB-2_TATCAGGGTCCGAGTC	PB	PB-2	P2-2	TRUE	3	TRA:CVVPNTNAGKSTF TRB:CASSLQGSTYNEQFF	220	CD8-C5
PB-2_TCACGAAAGTTGTCGT	PB	PB-2	P2-19	TRUE	3	TRA:CAVRDMENNRLAF TRB:CASSLGGGLTYEQYF	27	CD8-C4
PB-2_TCAGATGAGGCTCATT	PB	PB-2	P2-27	FALSE	3	TRA:CAPKSYNTDKLIF TRB:CASSEGGGGAETQYF	22	CD8-C5
PB-2_TCAGATGTCGAACGGA	PB	PB-2	P2-2	TRUE	3	TRA:CVVPNTNAGKSTF TRB:CASSLQGSTYNEQFF	220	CD8-C5
PB-2_TCAGCTCCAAGTTCTG	PB	PB-2	P2-5	TRUE	3	TRA:CASSFSGGYNKLIF TRB:CASSYLGAGYQETQYF	127	CD8-C5
PB-2_TCAGGTACATCCGCGA	PB	PB-2	P2-2982	FALSE	1	TRA:CAFNPLDDYKLSF TRB:CSVDTGGDYEQYF	1	CD8-C2
PB-2_TCAGGTAGTGAAGGCT	PB	PB-2	P2-21	TRUE	3	TRA:CALSSYNNNDMRF TRB:CASSLDLAPSTEAFF	25	CD8-C5

PB-2_TCATTACCAACACCTA	PB	PB-2	P2-47	TRUE	3	TRA:CAASAGGNKLTF TRB:CASSTEDEQYF	11	CD8-C5
PB-2_TCCCGATGTCATATGC	PB	PB-2	P2-130	TRUE	3	TRA:CAFMTPNGGATNKLIF TRB:CASSQENTAGSYF	4	CD8-C4
PB-2_TCGCGTTTCAATCACG	PB	PB-2	P2-5	TRUE	3	TRA:CASSFSGGYNKLIF TRB:CASSYLGAGYQETQYF	127	CD8-C7
PB-2_TCGGTAAAGTTGCAGG	PB	PB-2	P2-4	TRUE	3	TRA:CAIRYQSFNFGNEKLTF TRB:CASYRRVTEQYF	148	CD8-C5
PB-2_TCGGTAACAGATCTGT	PB	PB-2	P2-4	TRUE	3	TRA:CAIRYQSFNFGNEKLTF TRB:CASYRRVTEQYF	148	CD8-C5
PB-2_TCGGTAATCCCTCTTT	PB	PB-2	P2-365	TRUE	2	TRA:CAMREASLSGGYNKLIF TRB:CASRQDRQETQYF	2	CD8-C4
PB-2_TCTATTGAGGGTATCG	PB	PB-2	P2-59	TRUE	3	TRA:CAVDEGNTPLVF TRB:CAISESVDNTEAFF	9	CD8-C4
PB-2_TCTATTGGTAGGCTGA	PB	PB-2	P2-57	TRUE	3	TRA:CAVRDMSSGAGSYQLTF TRB:CASSELEDQETQYF	9	CD8-C5
PB-2_TCTATTGTCACGCGGT	PB	PB-2	P2-3045	FALSE	1	TRA:CALSEADTGNQFYF TRB:CASSHGAWETQYF	1	CD8-C5
PB-2_TCTCATAAGCCGGTAA	PB	PB-2	P2-27	FALSE	3	TRA:CAPKSYNTDKLIF TRB:CASSEGGGGAETQYF	22	CD8-C5
PB-2_TCTGGAAGTAGAGCTG	PB	PB-2	P2-2	TRUE	3	TRA:CVVPNTNAGKSTF TRB:CASSLQGSTYNEQFF	220	CD8-C5
PB-2_TCTTTCCAGATGCCAG	PB	PB-2	P2-25	FALSE	3	TRA:CAMREAPNTDKLIF TRB:CASSGGGGGLETQYF	22	CD8-C5
PB-2_TGAAAGATCACATGCA	PB	PB-2	P2-2	TRUE	3	TRA:CVVPNTNAGKSTF TRB:CASSLQGSTYNEQFF	220	CD8-C5
PB-2_TGACAACCAGTGGGAT	PB	PB-2	P2-67	FALSE	3	TRA:CVVSDPLWDNYGQNFVF TRB:CASSPSGGLLHYNEQFF	8	CD8-C4
PB-2_TGACTAGAGACAAAGG	PB	PB-2	P2-5	TRUE	3	TRA:CASSFSGGYNKLIF TRB:CASSYLGAGYQETQYF	127	CD8-C5
PB-2_TGACTAGTCTGGTATG	PB	PB-2	P2-5	TRUE	3	TRA:CASSFSGGYNKLIF TRB:CASSYLGAGYQETQYF	127	CD8-C5
PB-2_TGAGCATCACCGCTAG	PB	PB-2	P2-76	TRUE	3	TRA:CAGQEQGGSEKLVF TRB:CASSQMGLYGYTF	7	CD8-C5
PB-2_TGAGCCGGTGACGCCT	PB	PB-2	P2-10	TRUE	3	TRA:CAPLTPNDMRF TRB:CASLNGAGGSYEQYF	45	CD8-C4
PB-2_TGATTTCAGTCCGGTC	PB	PB-2	P2-67	FALSE	3	TRA:CVVSDPLWDNYGQNFVF TRB:CASSPSGGLLHYNEQFF	8	CD8-C5
PB-2_TGCCCTAGTACGAAAT	PB	PB-2	P2-111	TRUE	3	TRA:CAVYSGGSNYKLTF TRB:CASSLQGGGSGGLTQYF	5	CD8-C4
PB-2_TGCCCTATCCGGCACA	PB	PB-2	P2-2	TRUE	3	TRA:CVVPNTNAGKSTF TRB:CASSLQGSTYNEQFF	220	CD8-C5
PB-2_TGCGGGTTCGCCATAA	PB	PB-2	P2-2	TRUE	3	TRA:CVVPNTNAGKSTF TRB:CASSLQGSTYNEQFF	220	CD8-C5
PB-2_TGCGGGTTCGGTCCGA	PB	PB-2	P2-5	TRUE	3	TRA:CASSFSGGYNKLIF TRB:CASSYLGAGYQETQYF	127	CD8-C5
PB-2_TGGCCAGAGGTCGGAT	PB	PB-2	P2-59	TRUE	3	TRA:CAVDEGNTPLVF TRB:CAISESVDNTEAFF	9	CD8-C4
PB-2_TGGCGCACATCGTCGG	PB	PB-2	P2-4	TRUE	3	TRA:CAIRYQSFNFGNEKLTF TRB:CASYRRVTEQYF	148	CD8-C5
PB-2_TGGCTGGCACGGACAA	PB	PB-2	P2-30	TRUE	3	TRA:CAVGGDTGNQFYF TRB:CASSLFPTGVAYGYTF	20	CD8-C5
PB-2_TGTATTCGTGTCGCTG	PB	PB-2	P2-103	FALSE	3	TRA:CAVGALSRDDKIIF TRB:CASSQSPGQIYGYTF	5	CD8-C5

PB-2_TGTGGTAGTTATGTGC	PB	PB-2	P2-2	TRUE	3	TRA:CVVPNTNAGKSTF TRB:CASSLQGSTYNEQFF	220	CD8-C5
PB-2_TTAACTCCAAGGACAC	PB	PB-2	P2-3	TRUE	3	TRA:CAVNPLESSYKLIF TRB:CASSPGTANTGELFF	177	CD8-C5
PB-2_TTAGGCACATCTACGA	PB	PB-2	P2-203	TRUE	3	TRA:CAFIGGQNFVF TRB:CASSQNRPQGMSNQPQHF	3	CD8-C4
PB-2_TTAGTTCCACGCATCG	PB	PB-2	P2-15	TRUE	3	TRA:CAVGDNFNKFYF TRB:CASSRTSAAGELFF	35	CD8-C4
PB-2_TTCGAAGGTAAATGAC	PB	PB-2	P2-2	TRUE	3	TRA:CVVPNTNAGKSTF TRB:CASSLQGSTYNEQFF	220	CD8-C5
PB-2_TTCGGTCGTCTCTTTA	PB	PB-2	P2-78	TRUE	3	TRA:CAATTDSWGKFQF TRB:CASSSSASDYNEQFF	7	CD8-C4
PB-2_TTCTACACAGTCTTCC	PB	PB-2	P2-3313	FALSE	1	TRA:CALGGHGSGNTGKLIF TRB:CSANNPAGNTEAFF	1	CD8-C4
PB-2_TTCTCAATCGGCCGAT	PB	PB-2	P2-4	TRUE	3	TRA:CAIRYQSFNFGNEKLTF TRB:CASYRRVTEQYF	148	CD8-C4
PB-2_TTCTCCTCAGTAGAGC	PB	PB-2	P2-15	TRUE	3	TRA:CAVGDNFNKFYF TRB:CASSRTSAAGELFF	35	CD8-C4
PB-2_TTCTCCTGTAAACACA	PB	PB-2	P2-11	TRUE	3	TRA:CAVHNQGGKLIF TRB:CASSYLGAGYQETQYF	43	CD8-C5
PB-2_TTCTCCTGTCAATGTC	PB	PB-2	P2-57	TRUE	3	TRA:CAVRDMSSGAGSYQLTF TRB:CASSELEDQETQYF	9	CD8-C5
PB-2_TTCTTAGCACTATCTT	PB	PB-2	P2-3	TRUE	3	TRA:CAVNPLESSYKLIF TRB:CASSPGTANTGELFF	177	CD8-C5
PB-2_TTGAACGGTAGCCTCG	PB	PB-2	P2-67	FALSE	3	TRA:CVVSDPLWDNYGQNFVF TRB:CASSPSGGLLHYNEQFF	8	CD8-C5
PB-2_TTGACTTAGCGATCCC	PB	PB-2	P2-3	TRUE	3	TRA:CAVNPLESSYKLIF TRB:CASSPGTANTGELFF	177	CD8-C5
PB-2_TTGCGTCAGAACAACT	PB	PB-2	P2-2	TRUE	3	TRA:CVVPNTNAGKSTF TRB:CASSLQGSTYNEQFF	220	CD8-C5
PB-2_TTGCGTCAGCACCGCT	PB	PB-2	P2-3349	FALSE	1	TRA:CAVAHNTNAGKSTF TRB:CASSLADSSYNEQFF	1	CD8-C4
PB-2_TTGTAGGAGCAGCCTC	PB	PB-2	P2-57	TRUE	3	TRA:CAVRDMSSGAGSYQLTF TRB:CASSELEDQETQYF	9	CD8-C5
PB-2_TTTACTGCAGTATGCT	PB	PB-2	P2-4	TRUE	3	TRA:CAIRYQSFNFGNEKLTF TRB:CASYRRVTEQYF	148	CD8-C5
PB-2_TTTACTGTCACAATGC	PB	PB-2	P2-15	TRUE	3	TRA:CAVGDNFNKFYF TRB:CASSRTSAAGELFF	35	CD8-C4
PB-2_TTTCCTCTCTGTCTAT	PB	PB-2	P2-11	TRUE	3	TRA:CAVHNQGGKLIF TRB:CASSYLGAGYQETQYF	43	CD8-C5
PB-2_TTTGGTTAGTACGACG	PB	PB-2	P2-6	TRUE	3	TRA:CVVSVAAGNKLTF TRB:CASSPGTDLYEQYF	85	CD8-C4
PB-2_TTTGGTTTCACAAACC	PB	PB-2	P2-21	TRUE	3	TRA:CALSSYNNNDMRF TRB:CASSLDLAPSTEAFF	25	CD8-C5
PB-2_TTTGTCATCATTGCCC	PB	PB-2	P2-170	TRUE	3	TRA:CALPAYSGAGSYQLTF TRB:CASSLYENTEAFF	3	CD8-C5
PB-2_AAGGAGCTCTGCTGCT	PB	PB-2	P2-4	TRUE	3	TRA:CAIRYQSFNFGNEKLTF TRB:CASYRRVTEQYF	148	CD8-C3
PB-2_AGGCCGTCACCCAGTG	PB	PB-2	P2-21	TRUE	3	TRA:CALSSYNNNDMRF TRB:CASSLDLAPSTEAFF	25	CD8-C3
PB-2_ATAACGCAGAAGCCCA	PB	PB-2	P2-1083	FALSE	1	TRA:CIVRDGNTDKLIF TRB:CASSIAGLAGVGWFF	1	CD8-C3
PB-2_CGGCTAGTCCGCAGTG	PB	PB-2	P2-15	TRUE	3	TRA:CAVGDNFNKFYF TRB:CASSRTSAAGELFF	35	CD8-C3

PB-2_CTACACCTCGGTGTTA	PB	PB-2	P2-68	TRUE	3	TRA:CAVYSGYSTLTF TRB:CSVEDVGGKSSYNEQFF	8	CD8-C3
PB-2_CTCGTACCACGAAATA	PB	PB-2	P2-2	TRUE	3	TRA:CVVPNTNAGKSTF TRB:CASSLQGSTYNEQFF	220	CD8-C3
PB-2_CTTAGGAAGTGTTGAA	PB	PB-2	P2-2044	FALSE	1	TRA:CAVDDSGANNLFF TRB:CASRHGANEKLFF	1	CD8-C3
PB-2_GCCAAATAGATCGATA	PB	PB-2	P2-2	TRUE	3	TRA:CVVPNTNAGKSTF TRB:CASSLQGSTYNEQFF	220	CD8-C3
PB-2_GTCCTCACAGCCAATT	PB	PB-2	P2-2	TRUE	3	TRA:CVVPNTNAGKSTF TRB:CASSLQGSTYNEQFF	220	CD8-C5
PB-2_TTAGGCAGTACCGTTA	PB	PB-2	P2-2	TRUE	3	TRA:CVVPNTNAGKSTF TRB:CASSLQGSTYNEQFF	220	CD8-C5
PB-2_AACCGCGAGACCACGA	PB	PB-2	P2-616	FALSE	1	TRA:CIVRVSNNARLMF TRB:CASSPSSDSSPLHF	1	CD4-C5
PB-2_AAGCCGCAGCTCAACT	PB	PB-2	P2-659	FALSE	1	TRA:CALSVLGGSEKLVF TRB:CASKGSTLNQPQHF	1	CD4-C4
PB-2_AAGGCAGAGACTACAA	PB	PB-2	P2-674	FALSE	1	TRA:CAVNRNTGTASKLTF TRB:CASSPTVNQPQHF	1	CD4-C2
PB-2_AAGGTTCGTATTACCG	PB	PB-2	P2-687	FALSE	1	TRA:CAAFQTGANNLFF TRB:CASSQGLNRGDTQYF	1	CD4-C3
PB-2_ACCGTAATCAACCAAC	PB	PB-2	P2-784	FALSE	1	TRA:CAGPRSDGLTF TRB:CASSFWVATKGGYGYTF	1	CD4-C2
PB-2_ACGCCGAAGGGCATGT	PB	PB-2	P2-819	FALSE	1	TRA:CAFMPPYNNNDMRF TRB:CASSQDRQTQYF	1	CD4-C3
PB-2_ACGCCGAGTTCCACGG	PB	PB-2	P2-38	TRUE	3	TRA:CAVRDGSDYKLSF TRB:CASKADLNNQPQHF	14	CD4-C4
PB-2_ACGGGCTCATGTCCTC	PB	PB-2	P2-840	FALSE	1	TRA:CVVNPGNTGKLIF TRB:CASSSGQTYEQYF	1	CD4-C3
PB-2_ACTATCTCATCGATTG	PB	PB-2	P2-855	FALSE	1	TRA:CAVEASGTYKYIF TRB:CASSDPNTGLEQYF	1	CD8-C8
PB-2_ACTGTCCTCATGTAGC	PB	PB-2	P2-896	FALSE	1	TRA:CAVASGGGNKLTF TRB:CASSLMITDTQYF	1	CD4-C3
PB-2_ACTTACTTCACCGGGT	PB	PB-2	P2-278	TRUE	2	TRA:CAAGQGAQKLVF TRB:CASSLDGLLNTGELFF	2	CD4-C3
PB-2_ACTTTCATCGGAATCT	PB	PB-2	P2-911	FALSE	1	TRA:CAPMDSNYQLIW TRB:CASSTDRGEQYF	1	CD4-C3
PB-2_AGAGTGGTCTTAACCT	PB	PB-2	P2-941	FALSE	1	TRA:CAVGPEGYSSASKIIF TRB:CASSRETNTGELFF	1	CD4-C3
PB-2_AGCGTATTCTTTACGT	PB	PB-2	P2-985	FALSE	1	TRA:CGADSGSARQLTF TRB:CASSRDSYEQYF	1	CD4-C3
PB-2_AGCTCTCCAAACAACA	PB	PB-2	P2-998	FALSE	1	TRA:CAVQNNAGNMLTF TRB:CASRTGTSSYEQYF	1	CD4-C3
PB-2_AGTGGGAAGATGTGTA	PB	PB-2	P2-1064	FALSE	1	TRA:CAVGYSSASKIIF TRB:CSARAGTSGGNEQFF	1	CD4-C2
PB-2_ATAAGAGGTCATATGC	PB	PB-2	P2-1092	FALSE	1	TRA:CAVTSNSGYALNF TRB:CATNREANTGELFF	1	CD4-C4
PB-2_ATCACGACACATAACC	PB	PB-2	P2-1100	FALSE	1	TRA:CAVQGLYNFNKFYF TRB:CASSLSRGLSILRHEQYF	1	CD4-C2
PB-2_ATCCACCTCACTTACT	PB	PB-2	P2-1122	FALSE	1	TRA:CVVSRNNDMRF TRB:CASSYRAGDTDTQYF	1	CD4-C3
PB-2_ATTCTACTCGAATGGG	PB	PB-2	P2-179	TRUE	3	TRA:CAVEDTDKLIF TRB:CASSRQGSDTQYF	3	CD4-C3
PB-2_ATTGGTGAGCTAAGAT	PB	PB-2	P2-1	TRUE	3	TRA:CVVNIIGYQKVTF TRB:CASTRQGKQPQHF	270	CD4-C5

PB-2_ATTGGTGAGCTAGTTC	PB	PB-2	P2-1184	FALSE	1	TRA:CVVKFTGGGNKLTF TRB:CASSLTGELAFF	1	CD4-C5
PB-2_CACAGTAGTTGGTGGA	PB	PB-2	P2-1269	FALSE	1	TRA:CAAQRGTSGTYKYIF TRB:CASSVRGGAYEQYF	1	CD4-C4
PB-2_CACCTTGGTGGACGAT	PB	PB-2	P2-1290	FALSE	1	TRA:CIVRVSTDSWGKFQF TRB:CASSGYTVTNEKLFF	1	CD4-C4
PB-2_CAGCCGAAGCTGAACG	PB	PB-2	P2-302	TRUE	2	TRA:CADNNAGNMLTF TRB:CATSEFLFEQFF	2	CD4-C5
PB-2_CATCAGAGTCCCGACA	PB	PB-2	P2-1385	FALSE	1	TRA:CALSDNQGAQKLVF TRB:CASSQQGLGTQPQHF	1	CD4-C4
PB-2_CATGCCTAGAAGGTTT	PB	PB-2	P2-1417	FALSE	1	TRA:CAVNRDGGATNKLIF TRB:CASSIVGAGDETQYF	1	CD4-C3
PB-2_CCGTACTAGGTAAACT	PB	PB-2	P2-105	TRUE	3	TRA:CAYRRREGGSEKLVF TRB:CASSTSGAIANYGYTF	5	CD4-C5
PB-2_CCTAAAGCATTTCACT	PB	PB-2	P2-1552	FALSE	1	TRA:CAVAPRVGFGNVLHC TRB:CASRTAQGINTEAFF	1	CD4-C4
PB-2_CCTAGCTGTAGGGACT	PB	PB-2	P2-1569	FALSE	1	TRA:CAVGSPNTGGFKTIF TRB:CAWSTSGGYGEQFF	1	CD4-C4
PB-2_CCTAGCTTCACCATAG	PB	PB-2	P2-1570	FALSE	1	TRA:CAFSESGGYNKLIF TRB:CASSPDRGGNIQYF	1	CD4-C3
PB-2_CCTATTATCCGAGCCA	PB	PB-2	P2-1581	FALSE	1	TRA:CALSDRGRDMRF TRB:CASSLDQGGYMGYTF	1	CD4-C2
PB-2_CCTTCCCCAGTTAACC	PB	PB-2	P2-1600	FALSE	1	TRA:CAASAGGNQFYF TRB:CASIRRDTQYF	1	CD4-C3
PB-2_CCTTCGAAGCTAAACA	PB	PB-2	P2-1608	FALSE	1	TRA:CAALMNYGGSQGNLIF TRB:CASSFVADTGELFF	1	CD4-C3
PB-2_CGACCTTTCAGAGACG	PB	PB-2	P2-1628	FALSE	1	TRA:CAVNRNAGNMLTF TRB:CASSIAEEQTQYF	1	CD4-C2
PB-2_CGAGCACAGGGCTCTC	PB	PB-2	P2-1645	FALSE	1	TRA:CAMSGGGTSYGKLTF TRB:CAWNRGHGTEQFF	1	CD4-C3
PB-2_CGGCTAGAGCAGACTG	PB	PB-2	P2-1745	FALSE	1	TRA:CIVRFTQGGSEKLVF TRB:CSAPSANYGYTF	1	CD4-C3
PB-2_CGTAGGCGTTATGTGC	PB	PB-2	P2-312	FALSE	2	TRA:CLVALNRDDKIIF TRB:CSARGEVAKNIQYF	2	CD4-C4
PB-2_CGTCACTCAAGAGGCT	PB	PB-2	P2-1774	FALSE	1	TRA:CAAGFAQGAQKLVF TRB:CASSPRPGGSGNTIYF	1	CD4-C2
PB-2_CGTCCATCATTACGAC	PB	PB-2	P2-1789	FALSE	1	TRA:CAFISNNAGNMLTF TRB:CASSATGGGAEAFF	1	CD4-C3
PB-2_CTACACCAGGTGCAAC	PB	PB-2	P2-1858	FALSE	1	TRA:CATDGGADDMRF TRB:CASNRGPQPQHF	1	CD4-C4
PB-2_CTCCTAGCACAGGCCT	PB	PB-2	P2-1919	FALSE	1	TRA:CAMREAQGGKLIF TRB:CASSLFPGGLAPTDTQYF	1	CD4-C3
PB-2_CTGAAACCAGCAGTTT	PB	PB-2	P2-1972	FALSE	1	TRA:CIVRVDPGNTPLVF TRB:CASSLRQGSYNEQFF	1	CD4-C3
PB-2_CTGCTGTCATCTACGA	PB	PB-2	P2-2009	FALSE	1	TRA:CAVRNPGNEKLTF TRB:CASRDRGASGGNYGELFF	1	CD4-C4
PB-2_GAACCTACAATGGTCT	PB	PB-2	P2-2081	FALSE	1	TRA:CAPWGGYQKVTF TRB:CASRATGNANQPQHF	1	CD4-C4
PB-2_GAGGTGATCAGTTTGG	PB	PB-2	P2-326	FALSE	2	TRA:CIVRHLNTDKLIF TRB:CASSRRETQYF	2	CD4-C4
PB-2_GATCGTAAGATCGGGT	PB	PB-2	P2-328	FALSE	2	TRA:CAFMKPYNNNDMRF TRB:CASSQDRAGGQYF	2	CD4-C3
PB-2_GCAAACTAGGTAAACT	PB	PB-2	P2-123	TRUE	3	TRA:CAASNLGTYKYIF TRB:CASSPPIGSPLHF	4	CD4-C3

PB-2_GCATGTAGTGAACCTT	PB	PB-2	P2-2326	FALSE	1	TRA:CAVRGGGETSGSRLTF TRB:CASSRGLPYEQYF	1	CD4-C4
PB-2_GCGACCACAATCTACG	PB	PB-2	P2-2346	FALSE	1	TRA:CALRTGGGADGLTF TRB:CASSLEGRGTEAFF	1	CD4-C2
PB-2_GGGCATCGTGGGTATG	PB	PB-2	P2-2558	FALSE	1	TRA:CALSDGSGNTGKLIF TRB:CASSHRLATSDETQYF	1	CD4-C4
PB-2_GTAACTGGTCGACTAT	PB	PB-2	P2-2595	FALSE	1	TRA:CAVNRAGGTSYGKLTF TRB:CASSPRAGDTQYF	1	CD4-C3
PB-2_GTACTTTTCCAAACTG	PB	PB-2	P2-2612	FALSE	1	TRA:CTYRGSTLGRLYF TRB:CASSPQGQSEKLFF	1	CD4-C5
PB-2_GTCCTCAGTGGCGAAT	PB	PB-2	P2-2667	FALSE	1	TRA:CAVNTGNQFYF TRB:CASSKVATHNEQFF	1	CD4-C3
PB-2_GTCGTAAGTTCGTTGA	PB	PB-2	P2-2682	FALSE	1	TRA:CIVRVAGSGYALNF TRB:CASSQGVAVTDTQYF	1	CD4-C3
PB-2_GTGAAGGCAGATGGGT	PB	PB-2	P2-2702	FALSE	1	TRA:CAESLGGGADGLTF TRB:CASSFDRGSKEKLFF	1	CD4-C3
PB-2_TACGGATTCTGTCTCG	PB	PB-2	P2-2838	FALSE	1	TRA:CAESRSSASKIIF TRB:CASSLVELAGGGNEQFF	1	CD8-C8
PB-2_TACTCATTCAGTCAGT	PB	PB-2	P2-2852	FALSE	1	TRA:CALSVVTGFQKLVF TRB:CASSLRLAGDRGTQYF	1	CD4-C2
PB-2_TACTTACAGCCTATGT	PB	PB-2	P2-201	TRUE	3	TRA:CAFRTDSNYQLIW TRB:CASSLASGNQETQYF	3	CD4-C3
PB-2_TAGTGGTAGGAGTTTA	PB	PB-2	P2-360	TRUE	2	TRA:CAASAPGTYKYIF TRB:CASSLLRPSADTQYF	2	CD4-C3
PB-2_TCACAAGAGACTTGAA	PB	PB-2	P2-2944	FALSE	1	TRA:CATPNQAGTALIF TRB:CASSSKDYYEQYF	1	CD4-C3
PB-2_TCACGAACACTTAACG	PB	PB-2	P2-362	FALSE	2	TRA:CAPRNTGFQKLVF TRB:CASSFEGISYEQYF	2	CD4-C4
PB-2_TCTGGAAAGTGTACTC	PB	PB-2	P2-28	TRUE	3	TRA:CATDANTGNQFYF TRB:CASSLGQWVEQYF	22	CD4-C3
PB-2_TGACGGCGTCTCTCTG	PB	PB-2	P2-3095	FALSE	1	TRA:CALANQAGTALIF TRB:CASSPLLQSFNSPLHF	1	CD4-C4
PB-2_TGCGTGGAGCTCCTCT	PB	PB-2	P2-3178	FALSE	1	TRA:CAVRENTGGFKTIF TRB:CASSPLGGSGELFF	1	CD4-C3
PB-2_TGCTGCTGTGAGTGAC	PB	PB-2	P2-343	FALSE	2	TRA:CAASALGSGNTGKLIF TRB:CASSLGANEQYF	2	CD4-C4
PB-2_TTCTCCTTCGGATGTT	PB	PB-2	P2-3323	FALSE	1	TRA:CALSLSNSGNTPLVF TRB:CASSIGTSGKDTQYF	1	CD4-C4
PB-2_AAACCTGAGACTGGGT	PB	PB-2	P2-566	FALSE	1	TRA:CAPGQQTDSWGKFQF TRB:CASSRSGQGQGYTF	1	CD4-C1
PB-2_AAACCTGAGCCGGTAA	PB	PB-2	P2-568	FALSE	1	TRA:CAEIGGANSKLTF TRB:CASSLYLAGYSYNEQFF	1	CD4-C1
PB-2_AAACCTGGTGTTAAGA	PB	PB-2	P2-572	FALSE	1	TRA:CALSVSTGNQFYF TRB:CASSEGQVRSGANVLTF	1	CD4-C2
PB-2_AAACCTGTCCGCGTTT	PB	PB-2	P2-574	FALSE	1	TRA:CLVGDTSGTYKYIF TRB:CASTTGLASTDTQYF	1	CD4-C1
PB-2_AAAGATGAGGAATGGA	PB	PB-2	P2-580	FALSE	1	TRA:CAARNDYKLSF TRB:CSAQAGFGNQPQHF	1	CD4-C1
PB-2_AAAGATGAGGAGTACC	PB	PB-2	P2-581	FALSE	1	TRA:CALSDQETGGFKTIF TRB:CASSKDRSNQPQHF	1	CD4-C2
PB-2_AAAGATGGTTCCTCCA	PB	PB-2	P2-582	FALSE	1	TRA:CAVNAGGGADGLTF TRB:CASSVQPTYGYTF	1	CD4-C1
PB-2_AAAGATGTCTGCTGCT	PB	PB-2	P2-584	FALSE	1	TRA:CALDSGNTGKLIF TRB:CASSSSSGSFAYEQYF	1	CD4-C3

PB-2_AAAGCAAAGAGCAATT	PB	PB-2	P2-585	FALSE	1	TRA:CAVRSDGSGNTGKLIF TRB:CASSLIVNSEQYF	1	CD4-C2
PB-2_AAAGCAAAGTAGTGCG	PB	PB-2	P2-586	FALSE	1	TRA:CAVVYNFNKFYF TRB:CASSQVAGDTQYF	1	CD4-C2
PB-2_AAAGCAACAGACAGGT	PB	PB-2	P2-587	FALSE	1	TRA:CAFMKSTGEYGNKLVF TRB:CSAGGQGRGYTF	1	CD4-C2
PB-2_AAAGCAAGTAGCTTGT	PB	PB-2	P2-588	FALSE	1	TRA:CGTEQGDNFNKFYF TRB:CSALPAGRRLSYGYTF	1	CD4-C2
PB-2_AAAGCAATCGGAAACG	PB	PB-2	P2-591	FALSE	1	TRA:CAGAHSGGGADGLTF TRB:CASSLGTHKGTDTQYF	1	CD4-C3
PB-2_AAAGTAGAGAGTAAGG	PB	PB-2	P2-592	FALSE	1	TRA:CAVPVYGGSQGNLIF TRB:CASSLGPHPTEAFF	1	CD4-C2
PB-2_AAAGTAGAGCGAAGGG	PB	PB-2	P2-593	FALSE	1	TRA:CAGTNTGNQFYF TRB:CSARRGDRGETQYF	1	CD4-C1
PB-2_AAAGTAGTCCTACAGA	PB	PB-2	P2-599	FALSE	1	TRA:CVNAGKSTF TRB:CASSSTLLTAELFF	1	CD4-C2
PB-2_AACACGTTCGGCGCAT	PB	PB-2	P2-607	FALSE	1	TRA:CALSVSFGNEKLTF TRB:CAWSAGQGGNTQYF	1	CD4-C2
PB-2_AACCATGAGGAATTAC	PB	PB-2	P2-608	FALSE	1	TRA:CALSVFSGNTGKLIF TRB:CASTPRRAATDTQYF	1	CD4-C2
PB-2_AACCATGCACTGCCAG	PB	PB-2	P2-611	FALSE	1	TRA:CAEIPPPTGANNLFF TRB:CAWSTAWRGLSTDTQYF	1	CD4-C2
PB-2_AACCATGGTTTGGGCC	PB	PB-2	P2-612	FALSE	1	TRA:CAVRPVSGNTGKLIF TRB:CAWSGASGVYNEQFF	1	CD4-C2
PB-2_AACCATGTCACAGTAC	PB	PB-2	P2-613	FALSE	1	TRA:CALSVPGSGYSTLTF TRB:CASSAGGDTQYF	1	CD4-C2
PB-2_AACCATGTCGGCGGTT	PB	PB-2	P2-615	FALSE	1	TRA:CAVRDYGGATNKLIF TRB:CSVGYSSYEQYF	1	CD4-C3
PB-2_AACCGCGAGATCTGAA	PB	PB-2	P2-617	FALSE	1	TRA:CALDRGSTLGRLYF TRB:CASRGEDRNYGYTF	1	CD4-C2
PB-2_AACTCAGGTCGCGAAA	PB	PB-2	P2-628	FALSE	1	TRA:CALSEAGVNKLVF TRB:CASSPDREGYEQYF	1	CD4-C2
PB-2_AACTCAGTCCTGCAGG	PB	PB-2	P2-630	FALSE	1	TRA:CALRKTGANNLFF TRB:CAISESRTANTEAFF	1	CD4-C2
PB-2_AACTCCCAGACTAGAT	PB	PB-2	P2-633	FALSE	1	TRA:CAVRDLGDYKLSF TRB:CASSLVGLASLSNTGELFF	1	CD4-C2
PB-2_AACTCCCGTTACCGAT	PB	PB-2	P2-636	FALSE	1	TRA:CAGRTGGGADGLTF TRB:CASTAATGGWYTEAFF	1	CD4-C2
PB-2_AACTCTTGTTCTGGTA	PB	PB-2	P2-639	FALSE	1	TRA:CVHNDMRF TRB:CASSSTGRTDTQYF	1	CD4-C2
PB-2_AACTGGTAGATGTCGG	PB	PB-2	P2-642	FALSE	1	TRA:CAVRVSRPF TRB:CASSLQGQGTGEQYF	1	CD4-C2
PB-2_AACTGGTAGGAGCGAG	PB	PB-2	P2-268	TRUE	2	TRA:CAEKNGANSKLTF TRB:CASSLVGTGLGEQFF	2	CD4-C2
PB-2_AACTGGTCACTTCGAA	PB	PB-2	P2-643	FALSE	1	TRA:CAVRDIDSGAGSYQLTF TRB:CASRSLGSGNQPQHF	1	CD4-C2
PB-2_AACTTTCGTCATCCCT	PB	PB-2	P2-647	FALSE	1	TRA:CAVNKKRGGATNKLIF TRB:CASSLAAGRGYTF	1	CD4-C2
PB-2_AAGACCTCAACTGCTA	PB	PB-2	P2-651	FALSE	1	TRA:CAVSQAGTALIF TRB:CASRLGGYNEQFF	1	CD4-C3
PB-2_AAGACCTGTCTGGAGA	PB	PB-2	P2-653	FALSE	1	TRA:CGTTTPSSGNTGKLIF TRB:CASSLFFGLFQDTQYF	1	CD4-C2
PB-2_AAGACCTTCAAACAAG	PB	PB-2	P2-655	FALSE	1	TRA:CALSDTRSGGGADGLTF TRB:CASSQGPINQPQHF	1	CD4-C2

PB-2_AAGACCTTCAACCAAC	PB	PB-2	P2-656	FALSE	1	TRA:CAVVATDSWGKFQF TRB:CASNRGDVGSPLHF	1	CD4-C2
PB-2_AAGCCGCCAAGTACCT	PB	PB-2	P2-661	FALSE	1	TRA:CIVRVAPKGGYQKVTF TRB:CASSRATGKGELFF	1	CD4-C7
PB-2_AAGCCGCCATGTCGAT	PB	PB-2	P2-664	FALSE	1	TRA:CAVSGTASKLTF TRB:CAWRTAGLYEQYF	1	CD4-C2
PB-2_AAGCCGCGTACCTACA	PB	PB-2	P2-665	FALSE	1	TRA:CAVKVPNRDDKIIF TRB:CASTAGVVATEAFF	1	CD4-C2
PB-2_AAGCCGCGTAGCTGCC	PB	PB-2	P2-666	FALSE	1	TRA:CAVRSNSGNTPLVF TRB:CSAQEGLQPQHF	1	CD4-C2
PB-2_AAGCCGCTCTGCAAGT	PB	PB-2	P2-667	FALSE	1	TRA:CALRGVRLMF TRB:CASSEDRANTEAFF	1	CD4-C2
PB-2_AAGGAGCAGGCATTGG	PB	PB-2	P2-670	FALSE	1	TRA:CAVRGNQAGTALIF TRB:CASSLVARVETQYF	1	CD4-C2
PB-2_AAGGAGCAGGGAGTAA	PB	PB-2	P2-671	FALSE	1	TRA:CAASNNARLMF TRB:CSARVGQETQYF	1	CD8-C2
PB-2_AAGGCAGCATTGGGCC	PB	PB-2	P2-677	FALSE	1	TRA:CIVRVAENRDDKIIF TRB:CASSRTGAGANVLTF	1	CD4-C2
PB-2_AAGGCAGCATTTCACT	PB	PB-2	P2-678	FALSE	1	TRA:CAECFHTGGFKTIF TRB:CASSEGRAFF	1	CD4-C2
PB-2_AAGGCAGTCGGAAATA	PB	PB-2	P2-683	FALSE	1	TRA:CAMREGDTDKLIF TRB:CASSPPLRLAGVNEQFF	1	CD4-C1
PB-2_AAGGTTCCAAACGTGG	PB	PB-2	P2-685	FALSE	1	TRA:CAVSSNTGNQFYF TRB:CASSPIGRDEQYF	1	CD4-C3
PB-2_AAGGTTCCAATGTTGC	PB	PB-2	P2-686	FALSE	1	TRA:CGADIRDNYGQNFVF TRB:CASSYTGGNYNSPLHF	1	CD4-C3
PB-2_AAGTCTGAGATCCGAG	PB	PB-2	P2-689	FALSE	1	TRA:CAVKDVLHC TRB:CASSPAEGNTQYF	1	CD4-C5
PB-2_AAGTCTGAGTAGTGCG	PB	PB-2	P2-690	FALSE	1	TRA:CAVEKNQYGNKLVF TRB:CAISRTDQETQYF	1	CD4-C1
PB-2_AAGTCTGGTCTAGTCA	PB	PB-2	P2-693	FALSE	1	TRA:CAMSAPGGSYIPTF TRB:CSARESSEETQYF	1	CD4-C1
PB-2_AAGTCTGTCATTGCCC	PB	PB-2	P2-695	FALSE	1	TRA:CAFMKHSGNMLTF TRB:CASLGGANVLTF	1	CD4-C2
PB-2_AAGTCTGTCGGTCCGA	PB	PB-2	P2-696	FALSE	1	TRA:CAERGSYGKLTF TRB:CASTRQGKQPQHF	1	CD4-C2
PB-2_AAGTCTGTCGGTCTAA	PB	PB-2	P2-697	FALSE	1	TRA:CAVRDLPRDGSGGSYIPTF TRB:CATSRTLATNEKLFF	1	CD4-C2
PB-2_AATCCAGAGCTCCCAG	PB	PB-2	P2-700	FALSE	1	TRA:CAEGAAGNKLTF TRB:CASSESGTVRYTF	1	CD4-C2
PB-2_AATCCAGCACTCGACG	PB	PB-2	P2-702	FALSE	1	TRA:CAATPFGNEKLTF TRB:CSARDGQGGGGKLFF	1	CD4-C2
PB-2_AATCCAGGTGTATGGG	PB	PB-2	P2-703	FALSE	1	TRA:CAASFNQAGTALIF TRB:CASSPSPGQGRKGSYEQYF	1	CD4-C2
PB-2_AATCGGTAGCCAGTTT	PB	PB-2	P2-707	FALSE	1	TRA:CALSVVQGAQKLVF TRB:CASSPAMGSGANVLTF	1	CD4-C2
PB-2_AATCGGTAGCGTCAAG	PB	PB-2	P2-708	FALSE	1	TRA:CAVTGFQKLVF TRB:CASSDGTPPSGTQYF	1	CD4-C2
PB-2_AATCGGTGTAACGTTC	PB	PB-2	P2-711	FALSE	1	TRA:CATAITGGGNKLTF TRB:CASGLGSYNEQFF	1	CD4-C1
PB-2_ACACCAAAGTGGGCTA	PB	PB-2	P2-714	FALSE	1	TRA:CAEINGGFQKLVF TRB:CASSYSRVDTGELFF	1	CD4-C2
PB-2_ACACCAACAAGCCTAT	PB	PB-2	P2-715	FALSE	1	TRA:CAVGSSGSARQLTF TRB:CASSRSGADTQYF	1	CD4-C2

PB-2_ACACCAACAGTCCTTC	PB	PB-2	P2-717	FALSE	1	TRA:CAVSETNTGNQFYF TRB:CASSIRGTSYEQYF	1	CD4-C7
PB-2_ACACCAATCCTGCAGG	PB	PB-2	P2-722	FALSE	1	TRA:CAASAKTSYDKVIF TRB:CASSSRVYGYTF	1	CD4-C2
PB-2_ACACCAATCGACCAGC	PB	PB-2	P2-723	FALSE	1	TRA:CALSSGGYQKVTF TRB:CSAHRGEKLFF	1	CD4-C1
PB-2_ACACCAATCTCTTGAT	PB	PB-2	P2-725	FALSE	1	TRA:CILRGTQGGSEKLVF TRB:CASSAGTSLPYEQYF	1	CD4-C2
PB-2_ACACCCTCAAACCCAT	PB	PB-2	P2-728	FALSE	1	TRA:CAMSANFGNEKLTF TRB:CASSLDLDRRAFF	1	CD4-C3
PB-2_ACACCCTGTATTAGCC	PB	PB-2	P2-730	FALSE	1	TRA:CALSVQSGGSNYKLTF TRB:CASSLSGSFPNEQFF	1	CD4-C2
PB-2_ACACTGACATTATCTC	PB	PB-2	P2-739	FALSE	1	TRA:CAMREGRPSGSARQLTF TRB:CSAINPGPREGSNQPQHF	1	CD4-C2
PB-2_ACACTGAGTCGAGATG	PB	PB-2	P2-740	FALSE	1	TRA:CATDSGYQKVTF TRB:CASTIGRGGLGSEQFF	1	CD4-C1
PB-2_ACACTGATCAAAGTAG	PB	PB-2	P2-741	FALSE	1	TRA:CAPGSDGQKLLF TRB:CSARQGASTEAFF	1	CD4-C1
PB-2_ACAGCTACAGAAGCAC	PB	PB-2	P2-744	FALSE	1	TRA:CIVRVAHQAGTALIF TRB:CSAAPGIGTQYF	1	CD4-C1
PB-2_ACAGCTAGTAAGAGGA	PB	PB-2	P2-745	FALSE	1	TRA:CVVNPKGSQGNLIF TRB:CSVAASGGVDTQYF	1	CD4-C1
PB-2_ACATACGAGAACAACT	PB	PB-2	P2-748	FALSE	1	TRA:CAASSSGNTGKLIF TRB:CASSRGDYTF	1	CD4-C2
PB-2_ACATACGCAGTGACAG	PB	PB-2	P2-751	FALSE	1	TRA:CALSATDSNYQLIW TRB:CASSPGQVGSQPQHF	1	CD4-C2
PB-2_ACATCAGCAGGGAGAG	PB	PB-2	P2-758	FALSE	1	TRA:CAASARVVYGGSQGNLIF TRB:CASSRGIRYEQYF	1	CD4-C2
PB-2_ACATCAGGTCATACTG	PB	PB-2	P2-759	FALSE	1	TRA:CVVSPNQAGTALIF TRB:CASSLVGQGGEKLFF	1	CD4-C1
PB-2_ACATGGTAGCTAGTGG	PB	PB-2	P2-762	FALSE	1	TRA:CAAPKDPEGGGADGLTF TRB:CASSYGQGITDEQFF	1	CD4-C1
PB-2_ACCAGTAAGTTAACGA	PB	PB-2	P2-767	FALSE	1	TRA:CAAGGNKFYF TRB:CSARGDRGRSYEQYF	1	CD4-C1
PB-2_ACCAGTACACATTAGC	PB	PB-2	P2-768	FALSE	1	TRA:CAVQGDISNSGYALNF TRB:CASRGPLQPQHF	1	CD4-C2
PB-2_ACCAGTACACTTACGA	PB	PB-2	P2-769	FALSE	1	TRA:CALLTGANNLFF TRB:CASSITPGQAFYEQFF	1	CD4-C2
PB-2_ACCAGTATCGTGTAGT	PB	PB-2	P2-772	FALSE	1	TRA:CVVGGSARQLTF TRB:CATTGLNSYEQFF	1	CD4-C2
PB-2_ACCAGTATCTGCCCTA	PB	PB-2	P2-773	FALSE	1	TRA:CAVGGGTNAGKSTF TRB:CASSPGPYYEQYF	1	CD4-C7
PB-2_ACCCACTAGCATGGCA	PB	PB-2	P2-774	FALSE	1	TRA:CAGENSGTYKYIF TRB:CASSWDRATGELFF	1	CD4-C1
PB-2_ACCCACTCAATAGCGG	PB	PB-2	P2-776	FALSE	1	TRA:CAVRDPNNNARLMF TRB:CASSTRTGTGGLRETQYF	1	CD4-C2
PB-2_ACCCACTTCTAACGGT	PB	PB-2	P2-780	FALSE	1	TRA:CALSEDTGRRALTF TRB:CSARRPGTGGETQYF	1	CD4-C1
PB-2_ACCGTAAGTCTAGAGG	PB	PB-2	P2-783	FALSE	1	TRA:CALNKFTGANSKLTF TRB:CASSPGGADLYEQYF	1	CD4-C1
PB-2_ACCGTAATCAGATAAG	PB	PB-2	P2-785	FALSE	1	TRA:CLVRDNYGQNFVF TRB:CASSSGLAGVWAPDTQYF	1	CD4-C2
PB-2_ACCTTTACAGTAACGG	PB	PB-2	P2-789	FALSE	1	TRA:CAVRAMYNNNDMRF TRB:CASSSGTGRGSIQYF	1	CD4-C2

PB-2_ACCTTTATCAACTCTT	PB	PB-2	P2-790	FALSE	1	TRA:CAVGLTGGGNKLTF TRB:CASSALLGGTQYF	1	CD4-C3
PB-2_ACCTTTATCTGCTTGC	PB	PB-2	P2-792	FALSE	1	TRA:CALDITGGGNKLTF TRB:CATSSRDIHEQYF	1	CD4-C2
PB-2_ACGAGCCCACTAGTAC	PB	PB-2	P2-796	FALSE	1	TRA:CAAGGGTSYGKLTF TRB:CASSPRGDTQYF	1	CD4-C1
PB-2_ACGATACAGCTCCTTC	PB	PB-2	P2-803	FALSE	1	TRA:CAGAGGSNYKLTF TRB:CASSLDGGGTDTQYF	1	CD4-C3
PB-2_ACGATACGTCCGAGTC	PB	PB-2	P2-806	FALSE	1	TRA:CIVRPWNTGNQFYF TRB:CASSVRAGGQSEQFF	1	CD4-C2
PB-2_ACGATACTCAGCCTAA	PB	PB-2	P2-807	FALSE	1	TRA:CAVRERYGGATNKLIF TRB:CASSYQLAKKETQYF	1	CD4-C2
PB-2_ACGATGTCAGATCCAT	PB	PB-2	P2-812	FALSE	1	TRA:CAVSEGEYGNKLVF TRB:CASSLSPGVAYEQYF	1	CD4-C2
PB-2_ACGATGTCATTCTCAT	PB	PB-2	P2-813	FALSE	1	TRA:CAELSPRDQAGTALIF TRB:CAWTSGYNEQFF	1	CD4-C1
PB-2_ACGCAGCGTCTTGCGG	PB	PB-2	P2-815	FALSE	1	TRA:CASAGTASKLTF TRB:CASSPETGFLETQYF	1	CD4-C1
PB-2_ACGCAGCTCTTACCTA	PB	PB-2	P2-172	TRUE	3	TRA:CAVSAKTSYDKVIF TRB:CASSQGGGTGTDTQYF	3	CD4-C2
PB-2_ACGCCAGCACGCATCG	PB	PB-2	P2-173	TRUE	3	TRA:CAENNGHGGSTLGRLYF TRB:CSLRGDSGYTF	3	CD4-C3
PB-2_ACGCCAGTCTTATCTG	PB	PB-2	P2-817	FALSE	1	TRA:CAVSGRGSYIPTF TRB:CATATGEGTGELFF	1	CD4-C1
PB-2_ACGCCGAAGCTGTTCA	PB	PB-2	P2-818	FALSE	1	TRA:CAGLRRAGSYQLTF TRB:CAISESDGGGQYF	1	CD4-C1
PB-2_ACGCCGACATGAAGTA	PB	PB-2	P2-821	FALSE	1	TRA:CAEGFFF TRB:CSVRQGNEQFF	1	CD4-C1
PB-2_ACGGAGAAGACTCGGA	PB	PB-2	P2-825	FALSE	1	TRA:CIGGGTASKLTF TRB:CSTSKGGAEAFF	1	CD4-C1
PB-2_ACGGAGATCGTTGCCT	PB	PB-2	P2-829	FALSE	1	TRA:CAENSGGSNYKLTF TRB:CSARDVLRASTDTQYF	1	CD4-C2
PB-2_ACGGAGATCTCGCTTG	PB	PB-2	P2-830	FALSE	1	TRA:CAMRELFSGGYQKVTF TRB:CASSTSPGQGYNEQFF	1	CD4-C1
PB-2_ACGGCCAGTGATAAGT	PB	PB-2	P2-832	FALSE	1	TRA:CALSKPPSGGSNYKLTF TRB:CASSLRGRLGGNTIYF	1	CD4-C1
PB-2_ACGGGCTAGGGTCTCC	PB	PB-2	P2-835	FALSE	1	TRA:CVVNQAGTALIF TRB:CASRRIGGGQGTPSYEQYF	1	CD4-C1
PB-2_ACGGGCTCAGGGTACA	PB	PB-2	P2-837	FALSE	1	TRA:CAVQAGNNARLMF TRB:CASSLLAGIDTQYF	1	CD4-C1
PB-2_ACGGGCTCATATACGC	PB	PB-2	P2-839	FALSE	1	TRA:CAVGTDKLIF TRB:CASSWTGHQPQHF	1	CD4-C2
PB-2_ACGGGCTGTCACCCAG	PB	PB-2	P2-841	FALSE	1	TRA:CAETVSGYSTLTF TRB:CASSVREAVTGELFF	1	CD4-C2
PB-2_ACGGGTCGTGGGTCAA	PB	PB-2	P2-845	FALSE	1	TRA:CAMRVKYSGNTPLVF TRB:CASSQDYNEQFF	1	CD4-C2
PB-2_ACGTCAAAGCAGATCG	PB	PB-2	P2-847	FALSE	1	TRA:CAVGAPNFGNEKLTF TRB:CASSGALTGELFF	1	CD4-C2
PB-2_ACGTCAAAGGGTGTTG	PB	PB-2	P2-848	FALSE	1	TRA:CALSDPAGSEKLVF TRB:CASSLTGVRADTQYF	1	CD4-C2
PB-2_ACGTCAAAGGTGGGTT	PB	PB-2	P2-849	FALSE	1	TRA:CAVGGRLNNNDMRF TRB:CASSLDRAYEQYF	1	CD4-C1
PB-2_ACGTCAATCTGCTTGC	PB	PB-2	P2-852	FALSE	1	TRA:CAEGATNKLIF TRB:CAISETGQGEYNEQFF	1	CD4-C2

PB-2_ACTATCTCAAGACGTG	PB	PB-2	P2-854	FALSE	1	TRA:CALSGPGTGNQFYF TRB:CSATRGSYNEQFF	1	CD4-C3
PB-2_ACTGAACAGGATGTAT	PB	PB-2	P2-858	FALSE	1	TRA:CAAGWFGNEKLTF TRB:CASSFAGAGELFF	1	CD4-C2
PB-2_ACTGAACAGTTAAGTG	PB	PB-2	P2-859	FALSE	1	TRA:CAASVGGSNYKLTF TRB:CASSLAQGDTGELFF	1	CD4-C7
PB-2_ACTGAACCAAGGTTTC	PB	PB-2	P2-861	FALSE	1	TRA:CAVANQAGTALIF TRB:CSARASLEEQFF	1	CD4-C1
PB-2_ACTGAACTCACAAACC	PB	PB-2	P2-862	FALSE	1	TRA:CAASTGNQFYF TRB:CASSLVIGAGELFF	1	CD4-C1
PB-2_ACTGAGTAGTGGAGAA	PB	PB-2	P2-864	FALSE	1	TRA:CAVSNSVSGNTPLVF TRB:CASTLKVGTQYF	1	CD4-C1
PB-2_ACTGAGTCACATGTGT	PB	PB-2	P2-865	FALSE	1	TRA:CIVKTSYDKVIF TRB:CASSTNRGGDTQYF	1	CD4-C1
PB-2_ACTGAGTCATCAGTAC	PB	PB-2	P2-866	FALSE	1	TRA:CVVSVTYKYIF TRB:CSAILTRAGASSDEQYF	1	CD4-C2
PB-2_ACTGAGTTCTTATCTG	PB	PB-2	P2-869	FALSE	1	TRA:CAASRGDMRF TRB:CASSLDSSYNEQFF	1	CD4-C1
PB-2_ACTGATGAGAAACGCC	PB	PB-2	P2-870	FALSE	1	TRA:CATDVGGATNKLIF TRB:CASSPTNSGNTIYF	1	CD4-C1
PB-2_ACTGATGCAAGCCCAC	PB	PB-2	P2-873	FALSE	1	TRA:CALLDSGAGSYQLTF TRB:CASSATGPREKLFF	1	CD4-C2
PB-2_ACTGATGCAAGCGATG	PB	PB-2	P2-874	FALSE	1	TRA:CAVSYGNNRLAF TRB:CSARSSSPGELFF	1	CD4-C1
PB-2_ACTGATGTCCACGAAT	PB	PB-2	P2-877	FALSE	1	TRA:CAARSVDSNYQLIW TRB:CASSGGRGVGTGELFF	1	CD4-C2
PB-2_ACTGCTCAGCGAAGGG	PB	PB-2	P2-882	FALSE	1	TRA:CAASVGGNKLVF TRB:CASSLVVGQGEQYF	1	CD4-C1
PB-2_ACTGCTCAGGTGATAT	PB	PB-2	P2-883	FALSE	1	TRA:CALSASGAGSYQLTF TRB:CAWSGGPDTQYF	1	CD4-C2
PB-2_ACTGCTCCAGTAGAGC	PB	PB-2	P2-885	FALSE	1	TRA:CAVSPVNKLIF TRB:CASSLPATSGRVTDTQYF	1	CD4-C2
PB-2_ACTGCTCTCAACGGCC	PB	PB-2	P2-887	FALSE	1	TRA:CAVSEEGGSEKLVF TRB:CASRRTGVREQYF	1	CD4-C1
PB-2_ACTGCTCTCAAGATCC	PB	PB-2	P2-888	FALSE	1	TRA:CAGGRNAGGTSYGKLTF TRB:CASSKSGQGGLNTEAFF	1	CD4-C2
PB-2_ACTGTCCCAAGTACCT	PB	PB-2	P2-893	FALSE	1	TRA:CAASNQGNKLVF TRB:CATSRVLGASPLHF	1	CD4-C1
PB-2_ACTGTCCGTACAGACG	PB	PB-2	P2-894	FALSE	1	TRA:CAGPSGTYKYIF TRB:CASSYSGWRGVYNEQFF	1	CD4-C3
PB-2_ACTTACTAGAAACCAT	PB	PB-2	P2-897	FALSE	1	TRA:CLHTDKLIF TRB:CASSQTRNSNQPQHF	1	CD4-C2
PB-2_ACTTACTCACAGACTT	PB	PB-2	P2-898	FALSE	1	TRA:CLVGDNDMRF TRB:CSTTGVTYYEQYF	1	CD4-C2
PB-2_ACTTACTGTGCACCAC	PB	PB-2	P2-899	FALSE	1	TRA:CAGQTAGGYQKVTF TRB:CASSSTRLARTDTQYF	1	CD4-C2
PB-2_ACTTACTGTGTGACGA	PB	PB-2	P2-900	FALSE	1	TRA:CAGTNAGGTSYGKLTF TRB:CASSLGSHLNEQFF	1	CD4-C2
PB-2_ACTTACTTCTTTCCTC	PB	PB-2	P2-902	FALSE	1	TRA:CAVSAGRAGNTPLVF TRB:CASSEGGQFF	1	CD4-C2
PB-2_ACTTGTTAGAAGGTTT	PB	PB-2	P2-904	FALSE	1	TRA:CAVRLTDSWGKFQF TRB:CASTTMGNLITEAFF	1	CD4-C1
PB-2_ACTTGTTTCTTCGAGA	PB	PB-2	P2-906	FALSE	1	TRA:CATDDSLRETSGSRLTF TRB:CASSSSFWGRQPQHF	1	CD4-C2

PB-2_ACTTTCACACCGGAAA	PB	PB-2	P2-909	FALSE	1	TRA:CAFMKGRAGNTPLVF TRB:CASRLATNEKLFF	1	CD4-C1
PB-2_AGAATAGAGGAGTCTG	PB	PB-2	P2-912	FALSE	1	TRA:CAVRVEYGNKLVF TRB:CASSRIAGGETQYF	1	CD4-C2
PB-2_AGACGTTAGGCATTGG	PB	PB-2	P2-919	FALSE	1	TRA:CAASDGGGADGLTF TRB:CAWNQGSGNTIYF	1	CD4-C2
PB-2_AGACGTTAGTGTTTGC	PB	PB-2	P2-920	FALSE	1	TRA:CAGLRYSGGGADGLTF TRB:CAWRRYEKLFF	1	CD4-C1
PB-2_AGACGTTGTAAGGATT	PB	PB-2	P2-922	FALSE	1	TRA:CILRDVHNQGGKLIF TRB:CASSENTGGTQYF	1	CD4-C2
PB-2_AGACGTTGTCCTAGCG	PB	PB-2	P2-923	FALSE	1	TRA:CAASRGTSYGKLTF TRB:CASKRGTTLGETQYF	1	CD4-C2
PB-2_AGAGCGAAGAGACTTA	PB	PB-2	P2-924	FALSE	1	TRA:CAPLRDNYGQNFVF TRB:CASSLLIAKNIQYF	1	CD4-C2
PB-2_AGAGCGAAGCACAGGT	PB	PB-2	P2-925	FALSE	1	TRA:CASPNTGGFKTIF TRB:CASSYGGRGEQYF	1	CD4-C2
PB-2_AGAGCGAAGCTAGTTC	PB	PB-2	P2-926	FALSE	1	TRA:CAYRSARRALTF TRB:CASSWPTVGLSGNTIYF	1	CD4-C2
PB-2_AGAGCGAAGTGCGATG	PB	PB-2	P2-927	FALSE	1	TRA:CAMRRNNAGNNRKLIW TRB:CASSQVRGSLNEKLFF	1	CD4-C2
PB-2_AGAGCGAGTCATCCCT	PB	PB-2	P2-930	FALSE	1	TRA:CAARNNARLMF TRB:CASSPGTSIPDTQYF	1	CD4-C1
PB-2_AGAGCGAGTCTCTCTG	PB	PB-2	P2-931	FALSE	1	TRA:CAENPSRSSASKIIF TRB:CSAGGSTQGSWQFF	1	CD4-C2
PB-2_AGAGTGGTCCCTTGTG	PB	PB-2	P2-940	FALSE	1	TRA:CAARGNAGGTSYGKLTF TRB:CSASQTSYGYTF	1	CD4-C2
PB-2_AGATCTGCAGAGCCAA	PB	PB-2	P2-944	FALSE	1	TRA:CAASSYGNEKLTF TRB:CASSPAVEQYF	1	CD4-C2
PB-2_AGATCTGCATCAGTAC	PB	PB-2	P2-946	FALSE	1	TRA:CAVGGDFGNEKLTF TRB:CASSLGTGFSTQYF	1	CD4-C2
PB-2_AGATCTGGTCCCTACT	PB	PB-2	P2-948	FALSE	1	TRA:CAASKNGNQFYF TRB:CATRPSGSRGYEQFF	1	CD4-C2
PB-2_AGATCTGGTTACAGAA	PB	PB-2	P2-950	FALSE	1	TRA:CAASEGSSNYKLTF TRB:CASSEGGRGYTF	1	CD4-C2
PB-2_AGATCTGTCTACTATC	PB	PB-2	P2-951	FALSE	1	TRA:CAAHTGGGNKLTF TRB:CASSYSPYSNQPQHF	1	CD4-C1
PB-2_AGATTGCCAACTGCTA	PB	PB-2	P2-953	FALSE	1	TRA:CAVEDHNTDKLIF TRB:CASSQGQGASVRGEKLFF	1	CD4-C2
PB-2_AGATTGCCACTTGGAT	PB	PB-2	P2-955	FALSE	1	TRA:CATDKKLSF TRB:CASSTSTSGSVHGELFF	1	CD4-C1
PB-2_AGATTGCTCAAGATCC	PB	PB-2	P2-956	FALSE	1	TRA:CAVGARYGGSQGNLIF TRB:CASRRDRGPAYEQYF	1	CD4-C2
PB-2_AGCAGCCTCACCCTCA	PB	PB-2	P2-962	FALSE	1	TRA:CAKKTGGTSYGKLTF TRB:CAISDPSSGNTIYF	1	CD4-C2
PB-2_AGCAGCCTCACGGTTA	PB	PB-2	P2-963	FALSE	1	TRA:CAASIRGQKLLF TRB:CASRAPPGEQYF	1	CD4-C2
PB-2_AGCATACCAGATCGGA	PB	PB-2	P2-966	FALSE	1	TRA:CAARGNAGNMLTF TRB:CSALDPGLRVGTEAFF	1	CD4-C2
PB-2_AGCCTAAAGGGCATGT	PB	PB-2	P2-970	FALSE	1	TRA:CAPVGSGNTGKLIF TRB:CASSHYRDRVGEQFF	1	CD4-C2
PB-2_AGCGGTCCACCATGTA	PB	PB-2	P2-974	FALSE	1	TRA:CAASFGNEKLTF TRB:CSAAPGTGTYEQYF	1	CD4-C2
PB-2_AGCGGTCGTGTGACGA	PB	PB-2	P2-977	FALSE	1	TRA:CAVSNAGNMLTF TRB:CASSPGTGNSPLHF	1	CD4-C2

PB-2_AGCGTATTCTTCCTTC	PB	PB-2	P2-984	FALSE	1	TRA:CALSSTGANSKLTF TRB:CSASTGTGNTGELFF	1	CD4-C2
PB-2_AGCTCCTAGGAGTAGA	PB	PB-2	P2-990	FALSE	1	TRA:CASRQGGSEKLVF TRB:CSASGLTNTGELFF	1	CD4-C1
PB-2_AGCTCCTCAGCTCCGA	PB	PB-2	P2-992	FALSE	1	TRA:CAVSEKSGYSTLTF TRB:CASSGGLESSYNSPLHF	1	CD4-C2
PB-2_AGCTCCTGTCGCTTTC	PB	PB-2	P2-994	FALSE	1	TRA:CAVSERGSYIPTF TRB:CASTLGGVQETQYF	1	CD4-C2
PB-2_AGCTCCTGTTGTACAC	PB	PB-2	P2-284	TRUE	2	TRA:CAASMAGSQGNLIF TRB:CASSDPPTYEQYF	2	CD4-C3
PB-2_AGCTCCTTCTAACTGG	PB	PB-2	P2-997	FALSE	1	TRA:CAASMDYGQNFVF TRB:CSALGGTGGSEQYF	1	CD4-C2
PB-2_AGCTCTCTCCTCGCAT	PB	PB-2	P2-1001	FALSE	1	TRA:CGADNLGNEKLTF TRB:CASSRNGQNYGYTF	1	CD4-C2
PB-2_AGCTTGAAGATGGGTC	PB	PB-2	P2-1002	FALSE	1	TRA:CASRDGGSQGNLIF TRB:CAWSVTPSSTDTQYF	1	CD4-C2
PB-2_AGCTTGATCAGTGTTG	PB	PB-2	P2-1006	FALSE	1	TRA:CAASIGTYKYIF TRB:CASSAGGTEAFF	1	CD4-C1
PB-2_AGGCCACCACCCATGG	PB	PB-2	P2-1009	FALSE	1	TRA:CANRRKDAGGTSYGKLTF TRB:CASSTTFTGELFF	1	CD4-C2
PB-2_AGGCCACTCAGGCAAG	PB	PB-2	P2-1012	FALSE	1	TRA:CAVRGAGGTSYGKLTF TRB:CATSRNQGAYEQYF	1	CD4-C1
PB-2_AGGCCGTCACGCGAAA	PB	PB-2	P2-1015	FALSE	1	TRA:CAASRAPSWEKLTF TRB:CASSQGRTSGRAWGSSYNEQF	1	CD4-C2
PB-2_AGGCCGTCATGGTAGG	PB	PB-2	P2-1016	FALSE	1	TRA:CAASMITPGNMLTF TRB:CATSRGGAGDTEAFF	1	CD4-C2
PB-2_AGGGAGTCATGACATC	PB	PB-2	P2-1022	FALSE	1	TRA:CAVIPIAGGTSYGKLTF TRB:CASSLGLAGSTDTQYF	1	CD4-C1
PB-2_AGGGAGTGTCCGTTAA	PB	PB-2	P2-1023	FALSE	1	TRA:CAETYNYGQNFVF TRB:CASSSTQGVHEQYF	1	CD4-C2
PB-2_AGGGATGAGCAATATG	PB	PB-2	P2-1025	FALSE	1	TRA:CAVVNYGGSQGNLIF TRB:CASRKGTPPVYGYTF	1	CD4-C2
PB-2_AGGGATGGTATGGTTC	PB	PB-2	P2-1028	FALSE	1	TRA:CALVWGNTGKLIF TRB:CASSEDGFSYEQYF	1	CD4-C2
PB-2_AGGGTGAGTCTGCAAT	PB	PB-2	P2-1034	FALSE	1	TRA:CAMSPPGFKTIF TRB:CSARGTPSRETQYF	1	CD4-C2
PB-2_AGGGTGAGTGTTTGTG	PB	PB-2	P2-1035	FALSE	1	TRA:CAVGSGRRALTF TRB:CASSLAPDSSYEQYF	1	CD4-C2
PB-2_AGGTCATAGAGATGAG	PB	PB-2	P2-1038	FALSE	1	TRA:CATGTYGNNRLAF TRB:CASSLGGRDYEQYF	1	CD4-C2
PB-2_AGGTCATAGAGGACGG	PB	PB-2	P2-1039	FALSE	1	TRA:CATSSTGTYKYIF TRB:CSVPDDRRADTQYF	1	CD4-C1
PB-2_AGGTCATCAACTGCTA	PB	PB-2	P2-1044	FALSE	1	TRA:CAVFMDTGRRALTF TRB:CASSYSDRVWYF	1	CD4-C2
PB-2_AGGTCATCAAGAGGCT	PB	PB-2	P2-1045	FALSE	1	TRA:CAVVSFTGANSKLTF TRB:CATSRERGGGMNEQYF	1	CD4-C2
PB-2_AGGTCCGTCAGCACAT	PB	PB-2	P2-1052	FALSE	1	TRA:CAASPNNNARLMF TRB:CASSRFSSYNEQFF	1	CD4-C1
PB-2_AGTAGTCCATCCTAGA	PB	PB-2	P2-1054	FALSE	1	TRA:CAMRELQGGGGNKLTF TRB:CASSRYDQGDGYTF	1	CD4-C2
PB-2_AGTCTTTAGCACAGGT	PB	PB-2	P2-1055	FALSE	1	TRA:CALKRNQFYF TRB:CASSLGSSSYNEQFF	1	CD4-C1
PB-2_AGTCTTTTCCCAACGG	PB	PB-2	P2-1056	FALSE	1	TRA:CAGPLNSGGSNYKLTF TRB:CASSLVGATGNTIYF	1	CD4-C1

PB-2_AGTGAGGAGTGCTGCC	PB	PB-2	P2-1057	FALSE	1	TRA:CAVEDGNAGNMLTF TRB:CASSPQGQGTEQYF	1	CD4-C1
PB-2_AGTGAGGCATACTCTT	PB	PB-2	P2-1058	FALSE	1	TRA:CALKMDSGYSTLTF TRB:CASSQVGELFF	1	CD4-C2
PB-2_AGTGAGGGTGTATGGG	PB	PB-2	P2-1059	FALSE	1	TRA:CALSQGDSSYKLIF TRB:CATQRTLGAGNTIYF	1	CD4-C2
PB-2_AGTGAGGTCCTCTAGC	PB	PB-2	P2-1062	FALSE	1	TRA:CAVKDAGNMLTF TRB:CASRSPGQGAGGELFF	1	CD4-C1
PB-2_AGTGGGACACCCAGTG	PB	PB-2	P2-1066	FALSE	1	TRA:CATVLGEYGNKLVF TRB:CASSQARGGGGYTF	1	CD4-C1
PB-2_AGTGGGAGTTCACGGC	PB	PB-2	P2-1067	FALSE	1	TRA:CAASAEYGGSQGNLIF TRB:CASADRAPNTIYF	1	CD4-C2
PB-2_AGTGGGATCACCGGGT	PB	PB-2	P2-1068	FALSE	1	TRA:CAANNPNNAGNMLTF TRB:CASSPRTGRSQVEQYF	1	CD4-C2
PB-2_AGTGGGATCGCGCCAA	PB	PB-2	P2-1069	FALSE	1	TRA:CAVRGSASKIIF TRB:CATSTTGEETQYF	1	CD4-C2
PB-2_AGTGTCAAGACAGACC	PB	PB-2	P2-1072	FALSE	1	TRA:CAVRPGGYQKVTF TRB:CARQGLDNSPLHF	1	CD4-C2
PB-2_AGTGTCAAGAGCCCAA	PB	PB-2	P2-1073	FALSE	1	TRA:CALRENNARLMF TRB:CASSSPTYEQYF	1	CD4-C2
PB-2_AGTGTCAAGTGTGAAT	PB	PB-2	P2-1075	FALSE	1	TRA:CAVNMLGRDDKIIF TRB:CASSSGPPGPQHF	1	CD4-C1
PB-2_AGTGTCACAGAGTGTG	PB	PB-2	P2-1076	FALSE	1	TRA:CAGQLIPGAQKLVF TRB:CASSQETAWRTQYF	1	CD4-C3
PB-2_AGTGTCATCTGTACGA	PB	PB-2	P2-1079	FALSE	1	TRA:CAVNTGTASKLTF TRB:CASTTDFSSYEQYF	1	CD4-C2
PB-2_AGTTGGTAGCTAGTGG	PB	PB-2	P2-1082	FALSE	1	TRA:CAVNKKAGTALIF TRB:CASSLNQETQYF	1	CD4-C2
PB-2_ATAAGAGGTAAGGGAA	PB	PB-2	P2-1091	FALSE	1	TRA:CAASSGAQKLVF TRB:CSANSILSPGGASAFF	1	CD4-C2
PB-2_ATAGACCGTGACGGTA	PB	PB-2	P2-1098	FALSE	1	TRA:CAVNWISGGYNKLIF TRB:CASSETSGRATGNIQYF	1	CD4-C3
PB-2_ATAGACCTCTCCAGGG	PB	PB-2	P2-1099	FALSE	1	TRA:CAASKDFNKFYF TRB:CASSEVGGVSPLHF	1	CD4-C1
PB-2_ATCACGAGTGACGGTA	PB	PB-2	P2-1102	FALSE	1	TRA:CAAISQGAQKLVF TRB:CASSPGTGGPNSPLHF	1	CD4-C2
PB-2_ATCACGATCTGATTCT	PB	PB-2	P2-1105	FALSE	1	TRA:CAVTPFPFVRF TRB:CAWTLKGGAYEQYF	1	CD4-C2
PB-2_ATCATCTAGTAGATGT	PB	PB-2	P2-1106	FALSE	1	TRA:CAMTRGDSSYKLIF TRB:CASSQAVRGQEQYF	1	CD4-C2
PB-2_ATCATGGCACGTGAGA	PB	PB-2	P2-1112	FALSE	1	TRA:CAGRVNAGNMLTF TRB:CASSIGRAGEQFF	1	CD4-C1
PB-2_ATCATGGTCAACCATG	PB	PB-2	P2-1116	FALSE	1	TRA:CAVDSEYNNNDMRF TRB:CASSQDSGSQPQHF	1	CD4-C5
PB-2_ATCCACCAGCCGATTT	PB	PB-2	P2-1119	FALSE	1	TRA:CIVRVVNSGYALNF TRB:CASSLKGGEDYGYTF	1	CD4-C2
PB-2_ATCCACCAGTGCAAGC	PB	PB-2	P2-1120	FALSE	1	TRA:CVVRPDMEYGNKLVF TRB:CASSSSYEQYF	1	CD4-C2
PB-2_ATCCACCTCAATACCG	PB	PB-2	P2-1121	FALSE	1	TRA:CAVHTGANSKLTF TRB:CASRTGQFYTDTQYF	1	CD4-C1
PB-2_ATCGAGTAGGGAACGG	PB	PB-2	P2-1130	FALSE	1	TRA:CAMSEGNYGGSQGNLIF TRB:CASTTRREYF	1	CD4-C2
PB-2_ATCGAGTCAAGTTGTC	PB	PB-2	P2-1131	FALSE	1	TRA:CAMRVLNQAGTALIF TRB:CASSDGSNQPQHF	1	CD4-C1

PB-2_ATCGAGTGTTCTGTTT	PB	PB-2	P2-1133	FALSE	1	TRA:CAVEGNFGNEKLTF TRB:CATKATTGVYNEQFF	1	CD4-C7
PB-2_ATCGAGTTCCAGAGGA	PB	PB-2	P2-1134	FALSE	1	TRA:CAGSGGYGGATNKLIF TRB:CASSITGGSGNQPQHF	1	CD4-C2
PB-2_ATCTGCCCAAGTAGTA	PB	PB-2	P2-1140	FALSE	1	TRA:CAGRNTGNQFYF TRB:CSAPPGTTTNYGYTF	1	CD4-C2
PB-2_ATCTGCCCACGCATCG	PB	PB-2	P2-1141	FALSE	1	TRA:CAATGANSKLTF TRB:CASTFGTSDYTGELFF	1	CD4-C7
PB-2_ATCTGCCGTCTCCACT	PB	PB-2	P2-1143	FALSE	1	TRA:CAVHTGGFKTIF TRB:CASSMDGYSYEQYF	1	CD4-C1
PB-2_ATGAGGGAGCCTTGAT	PB	PB-2	P2-1144	FALSE	1	TRA:CAEEADYKLSF TRB:CATSDFVSGANVLTF	1	CD4-C2
PB-2_ATGAGGGAGCGTAGTG	PB	PB-2	P2-1145	FALSE	1	TRA:CAVVRPHGSGNTGKLIF TRB:CATSRLGRLNTEAFF	1	CD4-C2
PB-2_ATGAGGGCACCTCGTT	PB	PB-2	P2-1146	FALSE	1	TRA:CAMREGLSAGKSTF TRB:CATAGVINSGANVLTF	1	CD4-C3
PB-2_ATGAGGGGTCTCCATC	PB	PB-2	P2-1147	FALSE	1	TRA:CVVSGRGSQGNLIF TRB:CASSRGAGTDTQYF	1	CD4-C2
PB-2_ATGCGATAGCCAGGAT	PB	PB-2	P2-1151	FALSE	1	TRA:CAAHTSGTYKYIF TRB:CASSLQGSTEEQYF	1	CD4-C2
PB-2_ATGGGAGCAAGGTTCT	PB	PB-2	P2-1155	FALSE	1	TRA:CAISNTGNQFYF TRB:CSGSPPGTGRNEQFF	1	CD4-C2
PB-2_ATGGGAGGTGTTTGGT	PB	PB-2	P2-294	FALSE	2	TRA:CAASNYGGTSYGKLTF TRB:CASSRPERPLEQYF	2	CD4-C2
PB-2_ATGGGAGGTTTCGCTC	PB	PB-2	P2-1158	FALSE	1	TRA:CAVEETNFGNEKLTF TRB:CASSQGTSITQFSHGVYEQYF	1	CD4-C2
PB-2_ATGTGTGGTTCAGCGC	PB	PB-2	P2-1162	FALSE	1	TRA:CAVDRRTSGSRLTF TRB:CSVTPQHRFNEQFF	1	CD4-C2
PB-2_ATTACTCAGAAGGCCT	PB	PB-2	P2-1163	FALSE	1	TRA:CAVIDTGTASKLTF TRB:CASGGNMNTEAFF	1	CD4-C3
PB-2_ATTCTACGTTCAGCGC	PB	PB-2	P2-297	FALSE	2	TRA:CAVALSNNNDMRF TRB:CASRGDNEKLFF	2	CD4-C2
PB-2_ATTGGACAGTCCTCCT	PB	PB-2	P2-1179	FALSE	1	TRA:CALTWRGFKTIF TRB:CASSLEPPGHQNSPLHF	1	CD4-C1
PB-2_ATTGGACAGTTCGCAT	PB	PB-2	P2-1180	FALSE	1	TRA:CVVSNFNTDKLIF TRB:CASSPRTGLGEQYF	1	CD4-C1
PB-2_ATTGGACTCCGTCATC	PB	PB-2	P2-1182	FALSE	1	TRA:CAENEGGSYIPTF TRB:CASSRDSPGNQPQHF	1	CD4-C1
PB-2_ATTGGTGCATGGTAGG	PB	PB-2	P2-1186	FALSE	1	TRA:CAVRLNNAGNMLTF TRB:CASSAQRTDTQYF	1	CD4-C1
PB-2_ATTTCTGAGTCATGCT	PB	PB-2	P2-1194	FALSE	1	TRA:CIVRAYSGGGADGLTF TRB:CASSLTGPRVGETQYF	1	CD4-C1
PB-2_ATTTCTGTCTCCCTGA	PB	PB-2	P2-1197	FALSE	1	TRA:CAVNRGATNKLIF TRB:CASSTGQATYDGYTF	1	CD4-C2
PB-2_CAACCAAAGTTCGCGC	PB	PB-2	P2-1199	FALSE	1	TRA:CLPAGYSTLTF TRB:CASSRRLAGEDNEQFF	1	CD4-C1
PB-2_CAACCAATCGGAGGTA	PB	PB-2	P2-1203	FALSE	1	TRA:CASTGNQFYF TRB:CSASSFTGSYEQYF	1	CD4-C2
PB-2_CAACCTCAGAGGACGG	PB	PB-2	P2-1204	FALSE	1	TRA:CVAGVYGGSQGNLIF TRB:CASSLGITNTGELFF	1	CD4-C2
PB-2_CAACCTCGTGATGATA	PB	PB-2	P2-1207	FALSE	1	TRA:CAASRTDGAGSYQLTF TRB:CASSQDRLAGLFGTYEQYF	1	CD4-C1
PB-2_CAACTAGGTGTCCTCT	PB	PB-2	P2-299	FALSE	2	TRA:CAATDDNAGNMLTF TRB:CASSLAGLAGPPGTQYF	2	CD4-C2

PB-2_CAACTAGTCTATCCCG	PB	PB-2	P2-1213	FALSE	1	TRA:CAPTPGAGSYQLTF TRB:CASSSGTSRDTQYF	1	CD4-C2
PB-2_CAAGAAAAGCACCGCT	PB	PB-2	P2-1214	FALSE	1	TRA:CAVKNSGGYQKVTF TRB:CASSISRATEAFF	1	CD4-C1
PB-2_CAAGAAATCGGATGTT	PB	PB-2	P2-1216	FALSE	1	TRA:CGCGSEKLVF TRB:CSVGQGRGNTIYF	1	CD4-C1
PB-2_CAAGATCAGACAAGCC	PB	PB-2	P2-1217	FALSE	1	TRA:CAVRPPRGNEKLTF TRB:CASSPGTVNTGELFF	1	CD4-C7
PB-2_CAAGATCAGGTCATCT	PB	PB-2	P2-1218	FALSE	1	TRA:CAVNIWGGGYNKLIF TRB:CASSQGRGTGTEAFF	1	CD4-C2
PB-2_CAAGATCTCCCTCAGT	PB	PB-2	P2-1220	FALSE	1	TRA:CVVNYSGTYKYIF TRB:CASSEPLGAYEQYF	1	CD4-C2
PB-2_CAAGATCTCTGCCAGG	PB	PB-2	P2-1222	FALSE	1	TRA:CAVGAPGGANNLFF TRB:CASSSGLAGGQDTQYF	1	CD4-C7
PB-2_CAAGTTGAGTTCCACA	PB	PB-2	P2-1228	FALSE	1	TRA:CAASHFGNEKLTF TRB:CASSLEVGEQFF	1	CD4-C2
PB-2_CACAAACCACCTATCC	PB	PB-2	P2-1234	FALSE	1	TRA:CAETLPAYSSASKIIF TRB:CAGGGGSGSMNEQFF	1	CD4-C1
PB-2_CACACAAAGATGTCGG	PB	PB-2	P2-1236	FALSE	1	TRA:CAVRDITNNARLMF TRB:CASSPRQTGELFF	1	CD4-C3
PB-2_CACACAACATGAAGTA	PB	PB-2	P2-1237	FALSE	1	TRA:CALFDGGTSYGKLTF TRB:CASSYGEPGGASPLHF	1	CD4-C1
PB-2_CACACAACATTGGTAC	PB	PB-2	P2-1238	FALSE	1	TRA:CGTVSIAGGTSYGKLTF TRB:CASSPAQGSSYNEQFF	1	CD4-C1
PB-2_CACACAAGTATCACCA	PB	PB-2	P2-1240	FALSE	1	TRA:CATDRYTGTASKLTF TRB:CASSSGSRGEQFF	1	CD4-C2
PB-2_CACACAATCCCATTAT	PB	PB-2	P2-1243	FALSE	1	TRA:CAEKNRDDKIIF TRB:CASTKSGSSYNEQFF	1	CD4-C2
PB-2_CACACCTAGCGCTCCA	PB	PB-2	P2-1245	FALSE	1	TRA:CATDGPTSGSRLTF TRB:CASSLRGSGEAFF	1	CD4-C2
PB-2_CACACCTGTGCACGAA	PB	PB-2	P2-1247	FALSE	1	TRA:CAVSFTGGGNKLTF TRB:CASSLRTDGELFF	1	CD4-C1
PB-2_CACACCTTCGTGGGAA	PB	PB-2	P2-1249	FALSE	1	TRA:CVVLEGYSTLTF TRB:CSATGTARSSGNTIYF	1	CD4-C2
PB-2_CACACTCCAGCTGTGC	PB	PB-2	P2-1251	FALSE	1	TRA:CAVEDGTGFQKLVF TRB:CAISSGTETNEQFF	1	CD4-C1
PB-2_CACACTCCATGCCTAA	PB	PB-2	P2-1253	FALSE	1	TRA:CAVNLRDNYGQNFVF TRB:CASSLDRGSRTEAFF	1	CD8-C1
PB-2_CACACTCGTACCAGTT	PB	PB-2	P2-1254	FALSE	1	TRA:CAGLVARTSGSRLTF TRB:CASSVGTVSSYEQYF	1	CD4-C1
PB-2_CACACTCGTCAGATAA	PB	PB-2	P2-1255	FALSE	1	TRA:CAENDSNYKLTF TRB:CASSGTGVQQYF	1	CD4-C3
PB-2_CACAGGCAGATGGGTC	PB	PB-2	P2-1256	FALSE	1	TRA:CAVQAMGTSGSRLTF TRB:CASSSGTANSPLHF	1	CD4-C2
PB-2_CACAGGCAGGGTATCG	PB	PB-2	P2-1257	FALSE	1	TRA:CVVTRSGGSYIPTF TRB:CASSLGGTSPYEQYF	1	CD4-C1
PB-2_CACAGTAAGCGGATCA	PB	PB-2	P2-1261	FALSE	1	TRA:CAVEKSNSGYALNF TRB:CASSVSSILTDLSGANVLTF	1	CD4-C2
PB-2_CACAGTACAATCGAAA	PB	PB-2	P2-1264	FALSE	1	TRA:CASRRDTGRRALTF TRB:CASSEAGYDLFF	1	CD4-C2
PB-2_CACAGTACATGCATGT	PB	PB-2	P2-1265	FALSE	1	TRA:CSRYGNKLVF TRB:CASGGGALSKAFF	1	CD4-C3
PB-2_CACAGTATCACTGGGC	PB	PB-2	P2-1270	FALSE	1	TRA:CAVSGISGGYNKLIF TRB:CASSSNTDTQYF	1	CD4-C1

PB-2_CACATAGAGAGACTTA	PB	PB-2	P2-1272	FALSE	1	TRA:CAVKTNAGGTSYGKLTF TRB:CASSLIRDQETQYF	1	CD4-C1
PB-2_CACATTTAGAAGGCCT	PB	PB-2	P2-1274	FALSE	1	TRA:CAGLTF TRB:CSADHGSRDTQYF	1	CD4-C2
PB-2_CACCTTGCACCAGGTC	PB	PB-2	P2-1289	FALSE	1	TRA:CAASARTGNQFYF TRB:CASSPRDRGEQFF	1	CD4-C1
PB-2_CACCTTGTCCCAAGAT	PB	PB-2	P2-1291	FALSE	1	TRA:CAVGTSGSRLTF TRB:CASSEGLAGYNEQFF	1	CD4-C1
PB-2_CACTCCAAGTCTCGGC	PB	PB-2	P2-1292	FALSE	1	TRA:CAIQAAGNKLTF TRB:CATTQDRVNTEAFF	1	CD4-C1
PB-2_CACTCCAGTCTTGTCC	PB	PB-2	P2-1293	FALSE	1	TRA:CATISNFGNEKLTF TRB:CASSLLGRLAGVEQYF	1	CD4-C2
PB-2_CAGAATCAGGGAGTAA	PB	PB-2	P2-1297	FALSE	1	TRA:CAVRGNYGQNFVF TRB:CASNTDRGANTGELFF	1	CD4-C2
PB-2_CAGAATCCATCCAACA	PB	PB-2	P2-1299	FALSE	1	TRA:CAADDSNYQLIW TRB:CSASGGYGYTF	1	CD4-C2
PB-2_CAGAGAGAGGATGTAT	PB	PB-2	P2-1303	FALSE	1	TRA:CAARGGVDKLIF TRB:CASMSGLASYNEQFF	1	CD4-C2
PB-2_CAGAGAGCAGCTCCGA	PB	PB-2	P2-1304	FALSE	1	TRA:CALSKANSKLTF TRB:CASSLGRTWVWETQYF	1	CD4-C2
PB-2_CAGAGAGTCACATAGC	PB	PB-2	P2-1306	FALSE	1	TRA:CAESSGGTYKYIF TRB:CASSLRGLNTIYF	1	CD4-C2
PB-2_CAGAGAGTCTTGTCAT	PB	PB-2	P2-1309	FALSE	1	TRA:CALGGSQGNLIF TRB:CASSVAWDRSYEQYF	1	CD4-C1
PB-2_CAGATCAAGCCAACAG	PB	PB-2	P2-1310	FALSE	1	TRA:CAVQAEALNF TRB:CASSERLVGIFTYEQYF	1	CD4-C3
PB-2_CAGATCACACCGTTGG	PB	PB-2	P2-1311	FALSE	1	TRA:CAVRPPSNTGKLIF TRB:CASSSSGSGANVLTF	1	CD4-C2
PB-2_CAGATCAGTTCAGACT	PB	PB-2	P2-1313	FALSE	1	TRA:CAVEYNFNKFYF TRB:CASSLRNRETQYF	1	CD4-C2
PB-2_CAGATCATCTGGTGTA	PB	PB-2	P2-1316	FALSE	1	TRA:CAVDGGGGADGLTF TRB:CASSPTPFETQYF	1	CD4-C1
PB-2_CAGCAGCGTGACAAAT	PB	PB-2	P2-1320	FALSE	1	TRA:CAPDRFGYALNF TRB:CASRTSWDTGELFF	1	CD4-C2
PB-2_CAGCATAAGCCATCGC	PB	PB-2	P2-1322	FALSE	1	TRA:CAVRTGGGNKLTF TRB:CASSLWENEQFF	1	CD4-C2
PB-2_CAGCATATCTGAGTGT	PB	PB-2	P2-1328	FALSE	1	TRA:CAGRGNNARLMF TRB:CSASAVNYGYTF	1	CD4-C1
PB-2_CAGCCGAGTCATACTG	PB	PB-2	P2-1331	FALSE	1	TRA:CAMRGKNSGYSTLTF TRB:CASRETDLNTEAFF	1	CD4-C3
PB-2_CAGCGACAGGAGTTTA	PB	PB-2	P2-1334	FALSE	1	TRA:CIVPNTNAGKSTF TRB:CASFLAGAGETQYF	1	CD4-C2
PB-2_CAGCTGGCAGACAGGT	PB	PB-2	P2-1340	FALSE	1	TRA:CAVRGRDTGGFKTIF TRB:CASSMGLAGYEQFF	1	CD4-C2
PB-2_CAGCTGGGTTGCTCCT	PB	PB-2	P2-1344	FALSE	1	TRA:CVVSPAGNTGKLIF TRB:CAWSPQGWGYTF	1	CD4-C1
PB-2_CAGGTGCCAGACACTT	PB	PB-2	P2-1345	FALSE	1	TRA:CAYRSLNSNSGYALNF TRB:CASSPGTPGNQPQHF	1	CD4-C1
PB-2_CAGGTGCTCACGACTA	PB	PB-2	P2-1347	FALSE	1	TRA:CATLRNSGNTPLVF TRB:CASSWEQGGADTQYF	1	CD4-C1
PB-2_CAGGTGCTCCTGTACC	PB	PB-2	P2-1348	FALSE	1	TRA:CAVRAGSYQLTF TRB:CASSLSRDRHQPQHF	1	CD4-C2
PB-2_CAGTAACAGTAGTGCG	PB	PB-2	P2-1350	FALSE	1	TRA:CAAAKETSGSRLTF TRB:CASSPLYTNYGYTF	1	CD4-C2

PB-2_CAGTAACCACTCTGTC	PB	PB-2	P2-1352	FALSE	1	TRA:CAVRRTSGGGADGLTF TRB:CASSFTGISYEQYF	1	CD4-C2
PB-2_CAGTCCTAGCCGTCGT	PB	PB-2	P2-1357	FALSE	1	TRA:CAVSDFLTGGGNKLTF TRB:CASSAPGIGEQYF	1	CD4-C3
PB-2_CAGTCCTAGGCATGGT	PB	PB-2	P2-1358	FALSE	1	TRA:CAGQGTGGTSYGKLTF TRB:CASSLGAGTGELFF	1	CD4-C1
PB-2_CAGTCCTCACCAACCG	PB	PB-2	P2-1359	FALSE	1	TRA:CAASLYNNNDMRF TRB:CASSYSAGGPLSSYEQYF	1	CD4-C2
PB-2_CAGTCCTCATTTCACT	PB	PB-2	P2-1360	FALSE	1	TRA:CAVGERDYGGSQGNLIF TRB:CASSWVTGTEAFF	1	CD4-C1
PB-2_CAGTCCTGTCTAGGTT	PB	PB-2	P2-1361	FALSE	1	TRA:CAEYTGANSKLTF TRB:CASRTGGNQPQHF	1	CD4-C2
PB-2_CATATGGCAAGGTTCT	PB	PB-2	P2-1364	FALSE	1	TRA:CAYRSAASGSRLTF TRB:CASTRTGITYEQYF	1	CD4-C2
PB-2_CATATGGCACTGAAGG	PB	PB-2	P2-1366	FALSE	1	TRA:CATDAISGNKLVF TRB:CASSERRGQETQYF	1	CD4-C2
PB-2_CATATTCAGAAACCTA	PB	PB-2	P2-1367	FALSE	1	TRA:CATTLGEYGNKLVF TRB:CASSPLGTGELFF	1	CD4-C2
PB-2_CATATTCAGGAATTAC	PB	PB-2	P2-1368	FALSE	1	TRA:CVVKGRISNFGNEKLTF TRB:CAWSVRVSYEQYF	1	CD4-C2
PB-2_CATATTCCAACTGCGC	PB	PB-2	P2-1371	FALSE	1	TRA:CAVNEAAGNKLTF TRB:CASSPYVETGNSPLHF	1	CD4-C2
PB-2_CATATTCGTAACGCGA	PB	PB-2	P2-1373	FALSE	1	TRA:CAENGAAGNKLTF TRB:CASSDLPRGDGELFF	1	CD4-C3
PB-2_CATATTCGTGCTGTAT	PB	PB-2	P2-1375	FALSE	1	TRA:CALNKAAGNKLTF TRB:CATTGQSSYNEQFF	1	CD4-C2
PB-2_CATATTCTCGCGTTTC	PB	PB-2	P2-1376	FALSE	1	TRA:CAVLNQGGKLIF TRB:CASSSTGTGYEQYF	1	CD4-C2
PB-2_CATCAAGAGCGTGAAC	PB	PB-2	P2-1377	FALSE	1	TRA:CAAGSGNTGKLIF TRB:CASSERPGANQPQHF	1	CD4-C2
PB-2_CATCAGACAACGCACC	PB	PB-2	P2-1382	FALSE	1	TRA:CAVIPFSGNTPLVF TRB:CASSFSGGEQYF	1	CD4-C1
PB-2_CATCAGACATCGGAAG	PB	PB-2	P2-1384	FALSE	1	TRA:CAENEYGGATNKLIF TRB:CAISESPSTDTQYF	1	CD4-C2
PB-2_CATCCACAGCGTTCCG	PB	PB-2	P2-1386	FALSE	1	TRA:CAVRPQGGSEKLVF TRB:CASRSGGTEAFF	1	CD4-C3
PB-2_CATCCACAGTGCAAGC	PB	PB-2	P2-1387	FALSE	1	TRA:CAVTNTGNQFYF TRB:CASSLSGSMGEQYF	1	CD4-C3
PB-2_CATCCACCACTGTGTA	PB	PB-2	P2-1388	FALSE	1	TRA:CATVAYGNNRLAF TRB:CASSQGFGGNTGELFF	1	CD4-C2
PB-2_CATCCACGTCAATGTC	PB	PB-2	P2-1391	FALSE	1	TRA:CATDEGSNYKLTF TRB:CSAKFGELFF	1	CD4-C2
PB-2_CATCGAACACCGAAAG	PB	PB-2	P2-1395	FALSE	1	TRA:CAGHPSGTYKYIF TRB:CASSLGQGSGGYTF	1	CD4-C2
PB-2_CATCGAACATACGCCG	PB	PB-2	P2-1397	FALSE	1	TRA:CAAAGLVF TRB:CASSSGRRQETQYF	1	CD4-C2
PB-2_CATCGAACATGCAATC	PB	PB-2	P2-1398	FALSE	1	TRA:CATDSGGSNYKLTF TRB:CASSKDGSSYNEQFF	1	CD4-C2
PB-2_CATCGGGAGCTAACTC	PB	PB-2	P2-1399	FALSE	1	TRA:CALILLGGATNKLIF TRB:CASRKAGGPLEQFF	1	CD4-C2
PB-2_CATCGGGAGTTCGCAT	PB	PB-2	P2-1401	FALSE	1	TRA:CAVSELDNNDMRF TRB:CASSRGLASEQYF	1	CD4-C2
PB-2_CATCGGGCACTTACGA	PB	PB-2	P2-1402	FALSE	1	TRA:CAFAKYNFNKFYF TRB:CASSLRGEETQYF	1	CD4-C3

PB-2_CATCGGGGTCAAAGCG	PB	PB-2	P2-1404	FALSE	1	TRA:CATDALPSNFNKFYF TRB:CASSLWQNTGELFF	1	CD4-C1
PB-2_CATCGGGGTTATCGGT	PB	PB-2	P2-304	TRUE	2	TRA:CVVSLKPGGGNKLTF TRB:CASSLQGAKNTGELFF	2	CD4-C2
PB-2_CATCGGGTCAGAGCTT	PB	PB-2	P2-1407	FALSE	1	TRA:CVVSEAGANNLFF TRB:CASSLSRTGEKRYNEQFF	1	CD4-C2
PB-2_CATGACACACATTCGA	PB	PB-2	P2-1409	FALSE	1	TRA:CATGSGGYNKLIF TRB:CASSLVRTSGKLYNEQFF	1	CD4-C2
PB-2_CATGACAGTCCCTTGT	PB	PB-2	P2-1413	FALSE	1	TRA:CAVNAGGTSYGKLTF TRB:CASSLWFTDTQYF	1	CD4-C2
PB-2_CATGACATCACTTACT	PB	PB-2	P2-305	FALSE	2	TRA:CAVSKPNFGNEKLTF TRB:CASSFWEEAFF	2	CD4-C2
PB-2_CATGCCTTCAATAAGG	PB	PB-2	P2-1421	FALSE	1	TRA:CAENMKTGGFKTIF TRB:CASSPSGGASNEQFF	1	CD4-C3
PB-2_CATGGCGAGTGACATA	PB	PB-2	P2-1422	FALSE	1	TRA:CAVGSNYQLIW TRB:CASSLSATTLWRGYNEQFF	1	CD4-C2
PB-2_CATGGCGGTCTCTTTA	PB	PB-2	P2-1424	FALSE	1	TRA:CAPGYSSASKIIF TRB:CATSRDYGPGISYEQYF	1	CD4-C1
PB-2_CATGGCGTCTGGCGAC	PB	PB-2	P2-1429	FALSE	1	TRA:CAGRLFDNARLMF TRB:CASSLHPQDRPLAKNIQYF	1	CD4-C2
PB-2_CATTATCAGTGGTAGC	PB	PB-2	P2-1430	FALSE	1	TRA:CAVGDTGTASKLTF TRB:CASSQGLPTQGFYEQYF	1	CD4-C1
PB-2_CATTATCAGTTGAGAT	PB	PB-2	P2-1431	FALSE	1	TRA:CAASIIGSARQLTF TRB:CASRPAGTGHLYNEQFF	1	CD4-C1
PB-2_CATTATCTCGGCGGTT	PB	PB-2	P2-1436	FALSE	1	TRA:CAGQTIEETSGSRLTF TRB:CAISARRTDTQYF	1	CD4-C2
PB-2_CATTCGCAGCCCTAAT	PB	PB-2	P2-1438	FALSE	1	TRA:CAATARSGNTGKLIF TRB:CASSQEPTVGGEFF	1	CD4-C1
PB-2_CATTCGCTCAACCAAC	PB	PB-2	P2-1441	FALSE	1	TRA:CALSGHSGNTGKLIF TRB:CASSLKRINSPLHF	1	CD4-C2
PB-2_CATTCGCTCCTAGAAC	PB	PB-2	P2-1442	FALSE	1	TRA:CALMTGYNQGGKLIF TRB:CASSLNPRLGSPLHF	1	CD4-C2
PB-2_CATTCGCTCTATCCTA	PB	PB-2	P2-1444	FALSE	1	TRA:CAVKGSGGYQKVTF TRB:CASSMRGGPTDTQYF	1	CD4-C2
PB-2_CCAATCCAGAAACGAG	PB	PB-2	P2-306	TRUE	2	TRA:CAVVGNTDKLIF TRB:CASSLKGSGANVLTF	2	CD4-C3
PB-2_CCAATCCCAATACGCT	PB	PB-2	P2-1446	FALSE	1	TRA:CADQAGTALIF TRB:CASSPSRGAGGYTF	1	CD4-C1
PB-2_CCAATCCGTAGGCATG	PB	PB-2	P2-1447	FALSE	1	TRA:CIVSPHQAGTALIF TRB:CASRGQGSNEQFF	1	CD4-C3
PB-2_CCAATCCGTGCAGTAG	PB	PB-2	P2-1448	FALSE	1	TRA:CAASLSGAGSYQLTF TRB:CGSSGDPQETQYF	1	CD4-C2
PB-2_CCACCTAAGATCTGAA	PB	PB-2	P2-1451	FALSE	1	TRA:CALSSTGTASKLTF TRB:CASSSSGIRQETQYF	1	CD4-C2
PB-2_CCACCTAGTTCCGTCT	PB	PB-2	P2-1455	FALSE	1	TRA:CVVSDNRQNAGNNRKLIW TRB:CASSFVSGTRRYEQYF	1	CD4-C2
PB-2_CCACCTATCAAGCCTA	PB	PB-2	P2-1456	FALSE	1	TRA:CALSYNFNKFYF TRB:CASSHPRNPYEQYF	1	CD4-C2
PB-2_CCACCTATCCAAACTG	PB	PB-2	P2-1457	FALSE	1	TRA:CAVVTHQFYF TRB:CASSPRGAGTDTQYF	1	CD4-C2
PB-2_CCACCTATCCACTCCA	PB	PB-2	P2-1458	FALSE	1	TRA:CAVYRSGGYNKLIF TRB:CASSPGAMNTEAFF	1	CD4-C2
PB-2_CCACCTATCGGCATCG	PB	PB-2	P2-1459	FALSE	1	TRA:CVVRDIAGTYKYIF TRB:CASSLEGRVGTEAFF	1	CD4-C1

PB-2_CCACGGAAGTGTCCAT	PB	PB-2	P2-1462	FALSE	1	TRA:CVVSDQGGYQKVTF TRB:CASSQAGLAKNIQYF	1	CD4-C1
PB-2_CCACGGAGTGCAACTT	PB	PB-2	P2-1464	FALSE	1	TRA:CAVSDRGKSGGGADGLTF TRB:CASSFTENTEAFF	1	CD4-C2
PB-2_CCACGGAGTTTGGCGC	PB	PB-2	P2-1466	FALSE	1	TRA:CAVVTSGTYKYIF TRB:CASSRGTDPGANVLTF	1	CD4-C2
PB-2_CCACGGATCAGATAAG	PB	PB-2	P2-1467	FALSE	1	TRA:CALRGYSSASKIIF TRB:CASSLEPETQYF	1	CD4-C1
PB-2_CCACTACAGTTACCCA	PB	PB-2	P2-1472	FALSE	1	TRA:CAASKGGGTSYGKLTF TRB:CASSASTDTQYF	1	CD4-C2
PB-2_CCAGCGACACACAGAG	PB	PB-2	P2-1478	FALSE	1	TRA:CAAESVGNTGKLIF TRB:CASSYTRGQYF	1	CD4-C2
PB-2_CCAGCGAGTCTCTCTG	PB	PB-2	P2-1479	FALSE	1	TRA:CAESNPTGFQKLVF TRB:CASSARQGGNEQFF	1	CD4-C2
PB-2_CCAGCGAGTTACGACT	PB	PB-2	P2-1480	FALSE	1	TRA:CATDGEEYGNKLVF TRB:CASSMGQGGYEQYF	1	CD4-C2
PB-2_CCATGTCCATGGTCTA	PB	PB-2	P2-1485	FALSE	1	TRA:CAESTLKSGYNKLIF TRB:CASSEGQQGTDTQYF	1	CD4-C2
PB-2_CCATGTCTCGTTACAG	PB	PB-2	P2-1486	FALSE	1	TRA:CAVRDTGGFKTIF TRB:CASSQSLGERGKNIQYF	1	CD4-C2
PB-2_CCATTCGAGTGCCAGA	PB	PB-2	P2-1487	FALSE	1	TRA:CAMRDPAYSGAGSYQLTF TRB:CASSLGQGISYNEQFF	1	CD4-C3
PB-2_CCATTCGCACCGGAAA	PB	PB-2	P2-1488	FALSE	1	TRA:CAATDSGGSYIPTF TRB:CASSWGTGVNQPQHF	1	CD4-C2
PB-2_CCATTCGCACCGTTGG	PB	PB-2	P2-1489	FALSE	1	TRA:CAVGGTSYGKLTF TRB:CASSWTENTEAFF	1	CD4-C1
PB-2_CCCAATCAGCAGGTCA	PB	PB-2	P2-1494	FALSE	1	TRA:CATFNFGNEKLTF TRB:CASTNGQGRSPLHF	1	CD4-C2
PB-2_CCCAATCAGCCTCGTG	PB	PB-2	P2-1495	FALSE	1	TRA:CAMDNFNKFYF TRB:CASTAGAGNENTIYF	1	CD4-C1
PB-2_CCCAATCCAATTCCTT	PB	PB-2	P2-1497	FALSE	1	TRA:CAAKDTDKLIF TRB:CASSLVPDRGGEETQYF	1	CD4-C2
PB-2_CCCAATCCACAGTCGC	PB	PB-2	P2-1499	FALSE	1	TRA:CAFMEYGNKLVF TRB:CASSLGSGSYNEQFF	1	CD4-C1
PB-2_CCCAATCGTACATGTC	PB	PB-2	P2-1501	FALSE	1	TRA:CAVKGNTGFQKLVF TRB:CASSQDIQGEQYF	1	CD4-C2
PB-2_CCCAATCGTCGGCATC	PB	PB-2	P2-1502	FALSE	1	TRA:CAVQDTGTASKLTF TRB:CASSQGGNTGELFF	1	CD4-C2
PB-2_CCCAATCGTTGTTTGG	PB	PB-2	P2-1503	FALSE	1	TRA:CIVRMNRDDKIIF TRB:CASSGRRQETQYF	1	CD4-C1
PB-2_CCCAGTTCATGTCCTC	PB	PB-2	P2-1506	FALSE	1	TRA:CAVRSGANSKLTF TRB:CASSLDGNTEAFF	1	CD4-C2
PB-2_CCCAGTTCATGTCTCC	PB	PB-2	P2-1507	FALSE	1	TRA:CAVSGRGSYIPTF TRB:CSVWGQGSYEAFF	1	CD4-C2
PB-2_CCCAGTTGTCGGGTCT	PB	PB-2	P2-1508	FALSE	1	TRA:CAARGTGANSKLTF TRB:CASSVGGSYNSPLHF	1	CD4-C2
PB-2_CCCAGTTGTGTGTGCC	PB	PB-2	P2-1509	FALSE	1	TRA:CAHPGSGGSNYKLTF TRB:CASTKRGLVIGELFF	1	CD4-C2
PB-2_CCCTCCTAGAAGCCCA	PB	PB-2	P2-1515	FALSE	1	TRA:CAVTGTASKLTF TRB:CSAARGHFGTGELFF	1	CD4-C2
PB-2_CCCTCCTCACTAAGTC	PB	PB-2	P2-1516	FALSE	1	TRA:CAAPRSGGSNYKLTF TRB:CASSFPSGRGYNEQFF	1	CD4-C2
PB-2_CCCTCCTCAGAAGCAC	PB	PB-2	P2-1517	FALSE	1	TRA:CAVDPGGNNNARLMF TRB:CASSSSRLGYTF	1	CD4-C3

PB-2_CCCTCCTCAGATCCAT	PB	PB-2	P2-1518	FALSE	1	TRA:CALTLSYNTDKLIF TRB:CASSQQETQYF	1	CD4-C2
PB-2_CCCTCCTCATTGTGCA	PB	PB-2	P2-1519	FALSE	1	TRA:CAYRRGNYGQNFVF TRB:CASSFLQGAGGYTF	1	CD4-C1
PB-2_CCGGGATTCCTGTACC	PB	PB-2	P2-1522	FALSE	1	TRA:CAASIRGAQKLVF TRB:CASSLASGTGELFF	1	CD4-C2
PB-2_CCGGTAGAGCCAGTAG	PB	PB-2	P2-1525	FALSE	1	TRA:CAVQIIQGAQKLVF TRB:CASSAIGQGQSPLHF	1	CD4-C1
PB-2_CCGGTAGCAGCTGTTA	PB	PB-2	P2-1527	FALSE	1	TRA:CATNSGGSNYKLTF TRB:CSVAVLDRAGLAGYEQYF	1	CD4-C1
PB-2_CCGGTAGCATCTCCCA	PB	PB-2	P2-1529	FALSE	1	TRA:CAEKEGFGNEKLTF TRB:CASFLPAGTIYF	1	CD4-C1
PB-2_CCGGTAGTCCGTTGTC	PB	PB-2	P2-1530	FALSE	1	TRA:CALRNNAGNNRKLIW TRB:CASSLQGYEQYF	1	CD4-C2
PB-2_CCGTACTCACTGAAGG	PB	PB-2	P2-1532	FALSE	1	TRA:CALKSKVGGAQKLVF TRB:CASSLMGAGNGYTF	1	CD4-C1
PB-2_CCGTACTCATTACGAC	PB	PB-2	P2-1533	FALSE	1	TRA:CAATNAGGTSYGKLTF TRB:CASRSGTLYNEQFF	1	CD8-C1
PB-2_CCGTACTGTGTGAATA	PB	PB-2	P2-1534	FALSE	1	TRA:CAVGTHAGKSTF TRB:CASSLGTGGPGELFF	1	CD4-C1
PB-2_CCGTGGACACTAGTAC	PB	PB-2	P2-1537	FALSE	1	TRA:CAVSLTGTASKLTF TRB:CSAEGTGGAYGYTF	1	CD4-C2
PB-2_CCGTTCAGTCATCCCT	PB	PB-2	P2-1544	FALSE	1	TRA:CADKGGSNYKLTF TRB:CASSLKFSGIIYEQYF	1	CD4-C2
PB-2_CCTAAAGAGGTGCTAG	PB	PB-2	P2-1549	FALSE	1	TRA:CATEPRGGTASKLTF TRB:CASNPPPRHHLYTF	1	CD4-C2
PB-2_CCTAAAGGTGTCTGAT	PB	PB-2	P2-1553	FALSE	1	TRA:CALSADTNAGKSTF TRB:CASSDPGAGYTF	1	CD4-C2
PB-2_CCTACACAGTTAGCGG	PB	PB-2	P2-1556	FALSE	1	TRA:CAASLYSSGTYKYIF TRB:CASSPARRGQPQHF	1	CD4-C2
PB-2_CCTACACGTAGAGCTG	PB	PB-2	P2-1558	FALSE	1	TRA:CAVGAGGNNRLAF TRB:CASRSSTTGWETQYF	1	CD4-C1
PB-2_CCTACACTCATAAAGG	PB	PB-2	P2-1560	FALSE	1	TRA:CALSDTPPPQGGSEKLVF TRB:CASTRPPDGELFF	1	CD4-C1
PB-2_CCTACCACAGATGGGT	PB	PB-2	P2-1563	FALSE	1	TRA:CAVRRSGNTPLVF TRB:CSVSETQYF	1	CD4-C2
PB-2_CCTACCAGTGATAAGT	PB	PB-2	P2-1564	FALSE	1	TRA:CAVNLPDSWGKFQF TRB:CASSRLASVAGEQFF	1	CD4-C7
PB-2_CCTACCAGTGCGAAAC	PB	PB-2	P2-1565	FALSE	1	TRA:CAENIEGNNNDMRF TRB:CASSLAGQNGYTF	1	CD4-C2
PB-2_CCTACCATCGGCATCG	PB	PB-2	P2-1566	FALSE	1	TRA:CASGYGNNRLAF TRB:CASSHNRGQTTGELFF	1	CD4-C2
PB-2_CCTACCATCGTAGGTT	PB	PB-2	P2-1567	FALSE	1	TRA:CALSRNSGGSNYKLTF TRB:CATSDEGSGEQFF	1	CD4-C2
PB-2_CCTATTAGTTAAGTAG	PB	PB-2	P2-1575	FALSE	1	TRA:CAVNTGTASKLTF TRB:CASSLTVSGANVLTF	1	CD4-C2
PB-2_CCTATTAGTTCCACTC	PB	PB-2	P2-1577	FALSE	1	TRA:CAVHNTGNQFYF TRB:CASSTYQTGELFF	1	CD4-C2
PB-2_CCTCTGAGTAGCCTAT	PB	PB-2	P2-1587	FALSE	1	TRA:CAVSPLAGGGNKLTF TRB:CASSEGLAGLSKTGELFF	1	CD4-C2
PB-2_CCTCTGATCAAGATCC	PB	PB-2	P2-1591	FALSE	1	TRA:CATDGGGSGNTGKLIF TRB:CASSEGWSGPGNTIYF	1	CD4-C2
PB-2_CCTTCCCCAGTCCTTC	PB	PB-2	P2-1599	FALSE	1	TRA:CAASKAAGNKLTF TRB:CASSYALSAVYEQYF	1	CD4-C2

PB-2_CCTTCCCGTACCGCTG	PB	PB-2	P2-1601	FALSE	1	TRA:CAYRSGRSRDDKIIF TRB:CSASRGQRYEQYF	1	CD4-C2
PB-2_CCTTCCCTCACATACG	PB	PB-2	P2-1602	FALSE	1	TRA:CAVSPSGGYQKVTF TRB:CASRPTGTGHQPQHF	1	CD4-C1
PB-2_CCTTCCCTCTCGTATT	PB	PB-2	P2-1605	FALSE	1	TRA:CAVNERGSQGNLIF TRB:CASSSDPGTSVYEQYF	1	CD4-C2
PB-2_CCTTCCCTCTGCAGTA	PB	PB-2	P2-1607	FALSE	1	TRA:CAVFNTNAGKSTF TRB:CASSQVQGAGRGGEQYF	1	CD4-C2
PB-2_CCTTCGAGTTGTACAC	PB	PB-2	P2-1611	FALSE	1	TRA:CAVLKGGSYIPTF TRB:CASSSGQVAYEQYF	1	CD4-C2
PB-2_CCTTTCTAGGCTCAGA	PB	PB-2	P2-1612	FALSE	1	TRA:CVVSAMNYGQNFVF TRB:CASSYRDGADTQYF	1	CD4-C1
PB-2_CCTTTCTCACAGGCCT	PB	PB-2	P2-1613	FALSE	1	TRA:CAVKDSNYQLIW TRB:CASNRDRAGELFF	1	CD4-C2
PB-2_CCTTTCTTCAACACAC	PB	PB-2	P2-1615	FALSE	1	TRA:CAVGAGAAGNKLTF TRB:CSARFGLDQPQHF	1	CD4-C2
PB-2_CGAACATAGTACGTAA	PB	PB-2	P2-1617	FALSE	1	TRA:CAVGASGSGNTGKLIF TRB:CAIRSAGGSQETQYF	1	CD4-C2
PB-2_CGAATGTCAAAGCAAT	PB	PB-2	P2-1621	FALSE	1	TRA:CAASIARGSARQLTF TRB:CASSNQAHNEQFF	1	CD4-C2
PB-2_CGACCTTTCATTGCGA	PB	PB-2	P2-1629	FALSE	1	TRA:CAASDHNFNKFYF TRB:CASTRNTEAFF	1	CD4-C2
PB-2_CGACTTCAGACAATAC	PB	PB-2	P2-1630	FALSE	1	TRA:CAMREGPRYSSASKIIF TRB:CAWSGKSQGGYTF	1	CD4-C1
PB-2_CGACTTCAGCTTCGCG	PB	PB-2	P2-1631	FALSE	1	TRA:CVVSDRRNAGNMLTF TRB:CASSKMGQNQETQYF	1	CD4-C2
PB-2_CGACTTCAGTGAAGAG	PB	PB-2	P2-1632	FALSE	1	TRA:CVPSFSGNTGKLIF TRB:CSASLRGFEQYF	1	CD4-C2
PB-2_CGACTTCCAAAGAATC	PB	PB-2	P2-1633	FALSE	1	TRA:CALSVKAGKSTF TRB:CSASTGQIRETQYF	1	CD4-C2
PB-2_CGAGAAGAGGGATCTG	PB	PB-2	P2-1637	FALSE	1	TRA:CAMSLPDFGNEKLTF TRB:CASSFGGGGTEAFF	1	CD4-C2
PB-2_CGAGAAGCAAGCGCTC	PB	PB-2	P2-1638	FALSE	1	TRA:CAVRDGGSYIPTF TRB:CASSQDSAGGPHNEQFF	1	CD4-C2
PB-2_CGAGAAGCAAGGTGTG	PB	PB-2	P2-1639	FALSE	1	TRA:CAVRDTLVGGSASKIIF TRB:CASRGTGLSGNTIYF	1	CD4-C2
PB-2_CGAGCACAGCTGCAAG	PB	PB-2	P2-1643	FALSE	1	TRA:CALSQYSGGSNYKLTF TRB:CSAIEGSTDTQYF	1	CD4-C1
PB-2_CGAGCACAGGCTAGCA	PB	PB-2	P2-1644	FALSE	1	TRA:CAAERGGSNYKLTF TRB:CASRTPQGRWWNEQYF	1	CD4-C1
PB-2_CGAGCACCAGATTGCT	PB	PB-2	P2-1646	FALSE	1	TRA:CAVRDDQAGTALIF TRB:CASSYFLGPTGELFF	1	CD4-C7
PB-2_CGATCGGCACCATGTA	PB	PB-2	P2-1657	FALSE	1	TRA:CAGPSVFGNEKLTF TRB:CASLTNRNTEAFF	1	CD4-C2
PB-2_CGATCGGCATACTACG	PB	PB-2	P2-1658	FALSE	1	TRA:CAVENFGNEKLTF TRB:CSVEGGLNYGYTF	1	CD4-C2
PB-2_CGATGGCTCTGCTGTC	PB	PB-2	P2-1663	FALSE	1	TRA:CALSTGGGNKLTF TRB:CSAGMYGSGANVLTF	1	CD4-C3
PB-2_CGATGTATCTACGAGT	PB	PB-2	P2-1667	FALSE	1	TRA:CAVIPSGTYKYIF TRB:CASSQVGLEETQYF	1	CD4-C1
PB-2_CGATTGAAGCGAGAAA	PB	PB-2	P2-1668	FALSE	1	TRA:CAGNNNARLMF TRB:CASSQAPAGRRSF	1	CD4-C2
PB-2_CGATTGACAGGCAGTA	PB	PB-2	P2-1669	FALSE	1	TRA:CAVRPYSGAGSYQLTF TRB:CSVETNPHGELFF	1	CD4-C2

PB-2_CGATTGAGTCTAAACC	PB	PB-2	P2-1670	FALSE	1	TRA:CAASRHGATNKLIF TRB:CASSPTGVALYEQYF	1	CD4-C1
PB-2_CGATTGATCCAGTATG	PB	PB-2	P2-1671	FALSE	1	TRA:CATDGNQFYF TRB:CASSLGLLGYEQYF	1	CD4-C2
PB-2_CGATTGATCGGAGGTA	PB	PB-2	P2-1673	FALSE	1	TRA:CAVRSGGGADGLTF TRB:CATSRAYSFTDTQYF	1	CD4-C2
PB-2_CGCCAAGCACCCTATC	PB	PB-2	P2-1674	FALSE	1	TRA:CADHGNTGNQFYF TRB:CASSFQGGPYEQYF	1	CD4-C2
PB-2_CGCGGTAGTTACGGAG	PB	PB-2	P2-1680	FALSE	1	TRA:CAVSSLSSGSARQLTF TRB:CASSFGGGTSYEQYF	1	CD4-C1
PB-2_CGCGTTTTCTTGCATT	PB	PB-2	P2-1691	FALSE	1	TRA:CAATSYGQNFVF TRB:CSASPVNQPQHF	1	CD4-C2
PB-2_CGCTATCCACGGTAGA	PB	PB-2	P2-1693	FALSE	1	TRA:CIVRVAETSYDKVIF TRB:CASSLDSATPLHF	1	CD4-C1
PB-2_CGCTATCTCAATCACG	PB	PB-2	P2-1697	FALSE	1	TRA:CAVSDVGKDTGRRALTF TRB:CASSRSGGSVYNEQFF	1	CD4-C1
PB-2_CGCTATCTCGCACTCT	PB	PB-2	P2-1698	FALSE	1	TRA:CAVVSGYSTLTF TRB:CATSKQENNSPLHF	1	CD4-C2
PB-2_CGCTGGACACAAGTAA	PB	PB-2	P2-1702	FALSE	1	TRA:CAVPDTSGTYKYIF TRB:CASSGARGGGANVLTF	1	CD4-C2
PB-2_CGCTGGAGTTCTGTTT	PB	PB-2	P2-1704	FALSE	1	TRA:CLALPGAGSYQLTF TRB:CASSLLDPPDTQYF	1	CD4-C2
PB-2_CGGACACAGTGCAAGC	PB	PB-2	P2-1713	FALSE	1	TRA:CTPKSNNAGNMLTF TRB:CAIAQGMNTEAFF	1	CD4-C2
PB-2_CGGACACCAGCTGGCT	PB	PB-2	P2-1714	FALSE	1	TRA:CAVKETGGFKTIF TRB:CASSSQGANTEAFF	1	CD4-C2
PB-2_CGGACACGTGTGACGA	PB	PB-2	P2-1715	FALSE	1	TRA:CAVFNAGNMLTF TRB:CSATRGGGADTQYF	1	CD4-C1
PB-2_CGGACGTAGATGTCGG	PB	PB-2	P2-1717	FALSE	1	TRA:CAGHQSNDYKLSF TRB:CASSLTGTYTEAFF	1	CD4-C2
PB-2_CGGACGTAGGATGCGT	PB	PB-2	P2-1718	FALSE	1	TRA:CAVSEGTGGSYIPTF TRB:CATSRDMGNEQFF	1	CD4-C3
PB-2_CGGACGTCACGAAACG	PB	PB-2	P2-1723	FALSE	1	TRA:CIVRVGRDNQGGKLIF TRB:CASSQGLGRTGELFF	1	CD4-C2
PB-2_CGGACGTGTCAGAGGT	PB	PB-2	P2-1725	FALSE	1	TRA:CAGAPGGYQKVTF TRB:CASSLQGYEQYF	1	CD4-C2
PB-2_CGGACTGGTCGCGGTT	PB	PB-2	P2-1728	FALSE	1	TRA:CAVESNFGNEKLTF TRB:CASNDGSMNTEAFF	1	CD4-C2
PB-2_CGGAGCTAGCTCAACT	PB	PB-2	P2-1730	FALSE	1	TRA:CALDQSGGYQKVTF TRB:CASRSGTGSYGYTF	1	CD4-C1
PB-2_CGGAGCTAGGGTCGAT	PB	PB-2	P2-1731	FALSE	1	TRA:CIVRVADPGNTGKLIF TRB:CAISEVVGNTGELFF	1	CD4-C1
PB-2_CGGAGCTAGTGGAGAA	PB	PB-2	P2-1733	FALSE	1	TRA:CAMSDSGGSNYKLTF TRB:CASSLYEGGETQYF	1	CD4-C2
PB-2_CGGAGCTCACGGCCAT	PB	PB-2	P2-1734	FALSE	1	TRA:CAVKGNNDMRF TRB:CASSQELGPNYEQYF	1	CD4-C2
PB-2_CGGAGCTTCAAACAAG	PB	PB-2	P2-1739	FALSE	1	TRA:CAARAGTGGFKTIF TRB:CASGAPGSCSF	1	CD4-C2
PB-2_CGGCTAGTCGCAAACT	PB	PB-2	P2-1751	FALSE	1	TRA:CAGQVSWGKFQF TRB:CASSPRLAGLNTGELFF	1	CD4-C1
PB-2_CGGCTAGTCTGATACG	PB	PB-2	P2-1753	FALSE	1	TRA:CAASNGGTSYGKLTF TRB:CASSLGLGGSEQYF	1	CD4-C2
PB-2_CGGGTCACACTGTGTA	PB	PB-2	P2-1756	FALSE	1	TRA:CAYTRNSGGSNYKLTF TRB:CASSVMGRGGTDTQYF	1	CD4-C3

PB-2_CGGTTAAAGAATTGTG	PB	PB-2	P2-1760	FALSE	1	TRA:CAASDKSSGNTGKLIF TRB:CASSLGGSSKTQYF	1	CD4-C2
PB-2_CGGTTAATCAGGTTCA	PB	PB-2	P2-1765	FALSE	1	TRA:CALPNYGGSQGNLIF TRB:CASSEVIGSLGDTQYF	1	CD4-C2
PB-2_CGTAGGCGTATGCTTG	PB	PB-2	P2-1771	FALSE	1	TRA:CAMRRNRDDKIIF TRB:CASSQAGAPEETQYF	1	CD4-C2
PB-2_CGTCACTAGTCCGTAT	PB	PB-2	P2-1773	FALSE	1	TRA:CAASDPSTNAGKSTF TRB:CASSSPHRAEYNEQFF	1	CD4-C1
PB-2_CGTCACTAGTCTCAAC	PB	PB-2	P2-313	FALSE	2	TRA:CAVNVYSGAGSYQLTF TRB:CASSVSGQGRQPQHF	2	CD4-C4
PB-2_CGTCACTCAGTCGTGC	PB	PB-2	P2-1776	FALSE	1	TRA:CALLYNFNKFYF TRB:CASSQLKTGGLARTYEQYF	1	CD4-C3
PB-2_CGTCAGGAGGTCGGAT	PB	PB-2	P2-1778	FALSE	1	TRA:CATDAGSSYKLIF TRB:CASSQGGPTGELFF	1	CD4-C2
PB-2_CGTCAGGCAGATCTGT	PB	PB-2	P2-1779	FALSE	1	TRA:CAENSGNTGKLIF TRB:CASSPGGENYGYTF	1	CD4-C2
PB-2_CGTCAGGTCGGATGGA	PB	PB-2	P2-1784	FALSE	1	TRA:CAVIGNSGNTPLVF TRB:CASSLSQSRGYTF	1	CD4-C2
PB-2_CGTCAGGTCTTGAGGT	PB	PB-2	P2-1785	FALSE	1	TRA:CAVEPRMNSGYSTLTF TRB:CASSQYRNSPLHF	1	CD4-C2
PB-2_CGTCCATAGAAGGTGA	PB	PB-2	P2-314	FALSE	2	TRA:CAMLYDKIIF TRB:CASSLAVGSGNTIYF	2	CD4-C2
PB-2_CGTCCATAGTAGCCGA	PB	PB-2	P2-1788	FALSE	1	TRA:CAVSRDSNYQLIW TRB:CASSVGWRYNEQFF	1	CD4-C2
PB-2_CGTCCATTCGGTTAAC	PB	PB-2	P2-1792	FALSE	1	TRA:CAYRAGSYQLTF TRB:CASSLGQVYNEQFF	1	CD4-C1
PB-2_CGTCTACAGCCAGTAG	PB	PB-2	P2-1793	FALSE	1	TRA:CALDMRNYGGSQGNLIF TRB:CSASAGTGTYEQYF	1	CD4-C3
PB-2_CGTCTACAGTAGTGCG	PB	PB-2	P2-1794	FALSE	1	TRA:CALSDRSYQKVTF TRB:CASSSADRGNTGELFF	1	CD4-C2
PB-2_CGTCTACCAAGACGTG	PB	PB-2	P2-1797	FALSE	1	TRA:CAVRAGSNAGNNRKLIW TRB:CASSTGAGDTGELFF	1	CD4-C2
PB-2_CGTCTACCACGTTGGC	PB	PB-2	P2-1798	FALSE	1	TRA:CAFVYNNNDMRF TRB:CASRGQGSSGNTIYF	1	CD4-C2
PB-2_CGTCTACGTATGCTTG	PB	PB-2	P2-1800	FALSE	1	TRA:CAASADNYGQNFVF TRB:CASSLPRGASDTQYF	1	CD4-C1
PB-2_CGTGAGCAGATGTCGG	PB	PB-2	P2-1802	FALSE	1	TRA:CAESMVRASKIIF TRB:CSLEDSRDSYEQYF	1	CD4-C2
PB-2_CGTGAGCAGGAGCGAG	PB	PB-2	P2-1803	FALSE	1	TRA:CAMSAVGFGNVLHC TRB:CSASPRLAQYNEQFF	1	CD4-C2
PB-2_CGTGAGCGTCATATCG	PB	PB-2	P2-1805	FALSE	1	TRA:CASTDSNYQLIW TRB:CAWSPVQGPYGYTF	1	CD4-C2
PB-2_CGTGAGCTCATGGTCA	PB	PB-2	P2-1806	FALSE	1	TRA:CAARVDGTASKLTF TRB:CASGTSSRNEQFF	1	CD4-C2
PB-2_CGTGTAATCGGCCGAT	PB	PB-2	P2-1809	FALSE	1	TRA:CEGGGATNKLIF TRB:CASRTDQKSNQETQYF	1	CD4-C2
PB-2_CGTGTCTAGCAGACTG	PB	PB-2	P2-1812	FALSE	1	TRA:CAEQGGANNLFF TRB:CSARDGSGLLNTEAFF	1	CD4-C2
PB-2_CGTGTCTAGGGCTTGA	PB	PB-2	P2-1814	FALSE	1	TRA:CAVVGNQGGKLIF TRB:CASSGTGQAKYTGELFF	1	CD4-C1
PB-2_CGTGTCTGTGAGGGTT	PB	PB-2	P2-1816	FALSE	1	TRA:CAVGGDNNDMRF TRB:CASSLGTGKFYEQYF	1	CD4-C1
PB-2_CGTTAGAGTGAGTATA	PB	PB-2	P2-1818	FALSE	1	TRA:CAASSNNNNDMRF TRB:CASSPFSSGVAGGDTQYF	1	CD4-C2

PB-2_CGTTCTGAGCAGATCG	PB	PB-2	P2-1823	FALSE	1	TRA:CAGRGAQKLVF TRB:CASSRPSGYRHQPQHF	1	CD4-C2
PB-2_CGTTCTGCAGACACTT	PB	PB-2	P2-1827	FALSE	1	TRA:CAPIGGYQKVTF TRB:CASSRTSGVTDTQYF	1	CD4-C3
PB-2_CGTTCTGCATTCTCAT	PB	PB-2	P2-1828	FALSE	1	TRA:CAVGFSGGYQKVTF TRB:CASSLVSDTQYF	1	CD4-C1
PB-2_CGTTCTGGTGCAGGTA	PB	PB-2	P2-1829	FALSE	1	TRA:CAVDNQAGTALIF TRB:CASSPGQAYEQYF	1	CD4-C1
PB-2_CGTTCTGTCGAGAACG	PB	PB-2	P2-1831	FALSE	1	TRA:CAENTLGNYGQNFVF TRB:CASSLVGAKNIQYF	1	CD4-C2
PB-2_CGTTGGGCATGGGAAC	PB	PB-2	P2-315	FALSE	2	TRA:CAVSKTAGTYKYIF TRB:CSVKSGLSTDTQYF	2	CD4-C2
PB-2_CGTTGGGTCTTACCGC	PB	PB-2	P2-1835	FALSE	1	TRA:CAGNYGGSQGNLIF TRB:CASSESGQSSTEAFF	1	CD4-C3
PB-2_CTAACTTGTCTCATCC	PB	PB-2	P2-1838	FALSE	1	TRA:CALSGGNDMRF TRB:CASRGSLDTQYF	1	CD4-C2
PB-2_CTAACTTTCATATCGG	PB	PB-2	P2-1839	FALSE	1	TRA:CALKTGTASKLTF TRB:CASSGTGGFYEQYF	1	CD4-C2
PB-2_CTAACTTTCATCGATG	PB	PB-2	P2-1840	FALSE	1	TRA:CAVRGSGTYKYIF TRB:CASSMTGANTEAFF	1	CD4-C7
PB-2_CTAACTTTCCAGATCA	PB	PB-2	P2-1841	FALSE	1	TRA:CAASPNNQGGKLIF TRB:CASSSGTTNEKLFF	1	CD4-C2
PB-2_CTAACTTTCCGCATCT	PB	PB-2	P2-1842	FALSE	1	TRA:CNNAGNMLTF TRB:CASSSTSGSVEQYF	1	CD4-C1
PB-2_CTAACTTTCTTTAGGG	PB	PB-2	P2-1843	FALSE	1	TRA:CAVTRNNNARLMF TRB:CASSEEGTRSRYEQYF	1	CD4-C2
PB-2_CTAAGACAGGGTGTGT	PB	PB-2	P2-1845	FALSE	1	TRA:CAFVGSKLTF TRB:CASRLAGPKNIQYF	1	CD4-C2
PB-2_CTAAGACCACAGTCGC	PB	PB-2	P2-1846	FALSE	1	TRA:CAASAGYGGSQGNLIF TRB:CASSLALAGEELFF	1	CD4-C1
PB-2_CTAAGACGTAAACCTC	PB	PB-2	P2-1848	FALSE	1	TRA:CAVNRAGNNRKLIW TRB:CASSSGLAIPEQYF	1	CD4-C2
PB-2_CTAAGACGTATTCGTG	PB	PB-2	P2-1849	FALSE	1	TRA:CAVGPNFGNEKLTF TRB:CASSQGLTASTDTQYF	1	CD4-C1
PB-2_CTAATGGGTGGTTTCA	PB	PB-2	P2-1854	FALSE	1	TRA:CAVTNAGNMLTF TRB:CSVEGLAGGPVTTDTQYF	1	CD4-C2
PB-2_CTAATGGTCAACCATG	PB	PB-2	P2-1855	FALSE	1	TRA:CAVNPWGAGNMLTF TRB:CASSSQGSSYEQYF	1	CD4-C2
PB-2_CTACACCCATTTGCCC	PB	PB-2	P2-1860	FALSE	1	TRA:CATVLDQGAQKLVF TRB:CASSDRSSYNEQFF	1	CD4-C2
PB-2_CTACATTCAACTGCGC	PB	PB-2	P2-1863	FALSE	1	TRA:CAYRGGSEKLVF TRB:CASSVSAYNEQFF	1	CD4-C2
PB-2_CTACATTGTCGCTTTC	PB	PB-2	P2-1865	FALSE	1	TRA:CAASKYSGGGADGLTF TRB:CASSSRTYTDTQYF	1	CD4-C2
PB-2_CTACATTGTGTGGCTC	PB	PB-2	P2-1866	FALSE	1	TRA:CAVSRTDKLIF TRB:CASSLTWDRELYEQYF	1	CD4-C2
PB-2_CTACATTTCACGCGGT	PB	PB-2	P2-1868	FALSE	1	TRA:CAYRSAMQGGSEKLVF TRB:CASAIGVVSDEQFF	1	CD4-C2
PB-2_CTACCCAAGGGCTTGA	PB	PB-2	P2-1870	FALSE	1	TRA:CATTVGGAQKLVF TRB:CAVQVTKWETQYF	1	CD4-C2
PB-2_CTACCCAGTACATGTC	PB	PB-2	P2-1871	FALSE	1	TRA:CASQSWGKFQF TRB:CASSYRVGEADTQYF	1	CD4-C2
PB-2_CTACCCAGTTATCACG	PB	PB-2	P2-1874	FALSE	1	TRA:CAVYDNAGNNRKLIW TRB:CASRDRGSSYEQYF	1	CD4-C2

PB-2_CTACCCAGTTGGTGGA	PB	PB-2	P2-1875	FALSE	1	TRA:CAVNGGFGNVLHC TRB:CASSQEYASGRETQYF	1	CD4-C3
PB-2_CTACGTCGTAGGAGTC	PB	PB-2	P2-1876	FALSE	1	TRA:CAVGADRGSTLGRLYF TRB:CASSQDIWREGTYEQYF	1	CD4-C2
PB-2_CTACGTCTCAGCACAT	PB	PB-2	P2-1879	FALSE	1	TRA:CAVTEGGGAQKLVF TRB:CASSARLGGNEQFF	1	CD4-C3
PB-2_CTACGTCTCCTCGCAT	PB	PB-2	P2-1880	FALSE	1	TRA:CAAINTGANSKLTF TRB:CATSRAGVSYNEQFF	1	CD4-C2
PB-2_CTAGAGTGTCTAGGTT	PB	PB-2	P2-1888	FALSE	1	TRA:CAVNPFYGGATNKLIF TRB:CASLAGSYEQYF	1	CD4-C2
PB-2_CTAGCCTCAACACCTA	PB	PB-2	P2-1891	FALSE	1	TRA:CAEKAGTSYDKVIF TRB:CASSPTPYEQYF	1	CD4-C2
PB-2_CTAGCCTGTGACAAAT	PB	PB-2	P2-1893	FALSE	1	TRA:CAMREGDTGRRALTF TRB:CSAPKRGGVNTEAFF	1	CD4-C2
PB-2_CTAGTGACACCAGTTA	PB	PB-2	P2-1894	FALSE	1	TRA:CAETRGKSGGSYIPTF TRB:CSAKGATGLAGGRGETQYF	1	CD4-C1
PB-2_CTAGTGACAGTGGAGT	PB	PB-2	P2-1896	FALSE	1	TRA:CAASSSGGSNYKLTF TRB:CASSFVGGRTGGEQFF	1	CD4-C2
PB-2_CTAGTGAGTACGACCC	PB	PB-2	P2-1898	FALSE	1	TRA:CAVTAYSGAGSYQLTF TRB:CASSNRGRDEQYF	1	CD4-C2
PB-2_CTAGTGAGTTATCACG	PB	PB-2	P2-1899	FALSE	1	TRA:CAPPYGSGNTGKLIF TRB:CASSAPPVSTGELFF	1	CD4-C1
PB-2_CTCACACAGGCTCATT	PB	PB-2	P2-1900	FALSE	1	TRA:CAASGRGGSNYKLTF TRB:CASSFSGGAGDTQYF	1	CD4-C1
PB-2_CTCACACAGTAAGTAC	PB	PB-2	P2-1901	FALSE	1	TRA:CAASRSGGSNYKLTF TRB:CSVHFSGANVLTF	1	CD4-C1
PB-2_CTCACACCAAGGACAC	PB	PB-2	P2-1902	FALSE	1	TRA:CAVEDLGNNARLMF TRB:CASSSNGLAGLGGYNEQFF	1	CD4-C2
PB-2_CTCACACGTGAGTATA	PB	PB-2	P2-1905	FALSE	1	TRA:CAVRAPTDRGSTLGRLYF TRB:CASSLTAGGYTF	1	CD4-C2
PB-2_CTCACACGTGGTCCGT	PB	PB-2	P2-1906	FALSE	1	TRA:CIVFNRANAGKSTF TRB:CSVFQTGGNEQFF	1	CD4-C2
PB-2_CTCACACTCATGTGGT	PB	PB-2	P2-288	FALSE	2	TRA:CGTVLGSSYGKLTF TRB:CSARDPNRDYSYNEQFF	2	CD4-C2
PB-2_CTCAGAACAAACTGCT	PB	PB-2	P2-1910	FALSE	1	TRA:CALLQGGKLIF TRB:CASSPGHSGANVLTF	1	CD4-C1
PB-2_CTCATTAAGCTGCAAG	PB	PB-2	P2-1915	FALSE	1	TRA:CAVVTNTNAGKSTF TRB:CASTSPGSRNIQYF	1	CD4-C2
PB-2_CTCATTATCCACTCCA	PB	PB-2	P2-1916	FALSE	1	TRA:CALRGTMDSSYKLIF TRB:CASRLAGDNEQFF	1	CD4-C1
PB-2_CTCCTAGAGGCCGAAT	PB	PB-2	P2-1917	FALSE	1	TRA:CAGFKAAGNKLTF TRB:CATSRAIGTSGGYGPVDTQYF	1	CD4-C2
PB-2_CTCCTAGTCCTATTCA	PB	PB-2	P2-1921	FALSE	1	TRA:CAYTGTASKLTF TRB:CASSYSQDRGSGYTF	1	CD4-C3
PB-2_CTCGAAAAGCCAGGAT	PB	PB-2	P2-1923	FALSE	1	TRA:CAASALSGGSNYKLTF TRB:CASKEGRGYNEQFF	1	CD4-C2
PB-2_CTCGAAAAGCCTATGT	PB	PB-2	P2-1924	FALSE	1	TRA:CVVSFTGGGNKLTF TRB:CASSLDRASTDTQYF	1	CD4-C2
PB-2_CTCGAAAAGTACGACG	PB	PB-2	P2-1926	FALSE	1	TRA:CAVNEGAGRRALTF TRB:CASSRAGSEITGELFF	1	CD8-C6
PB-2_CTCGAAACAGGATCGA	PB	PB-2	P2-1928	FALSE	1	TRA:CAVNSRRGSYIPTF TRB:CASSELLGADGELFF	1	CD4-C2
PB-2_CTCGAAAGTTCCCGAG	PB	PB-2	P2-1929	FALSE	1	TRA:CVVSEGNTPLVF TRB:CASSKSAGTGANVLTF	1	CD4-C2

PB-2_CTCGAGGAGAGGTTGC	PB	PB-2	P2-1931	FALSE	1	TRA:CAVYQAGTALIF TRB:CSARDLASEAFF	1	CD4-C1
PB-2_CTCGAGGTCGGTTAAC	PB	PB-2	P2-1935	FALSE	1	TRA:CALSGANSKLTF TRB:CASTRAGFGTGFTGELFF	1	CD4-C1
PB-2_CTCGGAGAGATCACGG	PB	PB-2	P2-1936	FALSE	1	TRA:CAVRATSGGSYIPTF TRB:CSARGGTHGYTF	1	CD4-C2
PB-2_CTCGGAGCAGAGTGTG	PB	PB-2	P2-1938	FALSE	1	TRA:CAVRGGTSYGKLTF TRB:CASSPLTSGGRETQYF	1	CD4-C1
PB-2_CTCGGGAGTCCTCTTG	PB	PB-2	P2-1943	FALSE	1	TRA:CIVRVGGTGTASKLTF TRB:CASSLMMRTYEQYF	1	CD4-C2
PB-2_CTCGTACTCCTTTCGG	PB	PB-2	P2-1947	FALSE	1	TRA:CAAPSNYGGSQGNLIF TRB:CASSLQVTGWQETQYF	1	CD4-C1
PB-2_CTCGTCAGTCAACATC	PB	PB-2	P2-1952	FALSE	1	TRA:CATQVSWGSARQLTF TRB:CASGQGLYNEQFF	1	CD4-C2
PB-2_CTCGTCATCCCAAGAT	PB	PB-2	P2-1953	FALSE	1	TRA:CATVGNAGKSTF TRB:CASSPGTGFETQYF	1	CD4-C2
PB-2_CTCGTCATCTTAGCCC	PB	PB-2	P2-1954	FALSE	1	TRA:CAGPIFDGGSQGNLIF TRB:CSVVRIAWDTQYF	1	CD4-C2
PB-2_CTCTAATCATGAAGTA	PB	PB-2	P2-1955	FALSE	1	TRA:CAVRDSGTNAGKSTF TRB:CASSYGGNTGELFF	1	CD4-C2
PB-2_CTCTACGCATTTGCCC	PB	PB-2	P2-1959	FALSE	1	TRA:CLVGLPRGQKLLF TRB:CASSPSGSTDTQYF	1	CD4-C3
PB-2_CTCTACGGTCTGATTG	PB	PB-2	P2-1961	FALSE	1	TRA:CAYTSGTYKYIF TRB:CASSRTGGGSINEQYF	1	CD4-C2
PB-2_CTCTACGTCGACGGAA	PB	PB-2	P2-1962	FALSE	1	TRA:CALLKTTDSWGKFQF TRB:CAISTSQETQYF	1	CD4-C1
PB-2_CTCTGGTAGTACGTTC	PB	PB-2	P2-290	FALSE	2	TRA:CAVIRAGGSYIPTF TRB:CAWNIRDTNTGELFF	2	CD4-C2
PB-2_CTCTGGTCATTCCTCG	PB	PB-2	P2-1964	FALSE	1	TRA:CAVVESYGGATNKLIF TRB:CASSYDLFSYEQYF	1	CD4-C1
PB-2_CTGAAACAGCGTGTCC	PB	PB-2	P2-1970	FALSE	1	TRA:CAMILNQGGKLIF TRB:CSAPMNTGELFF	1	CD4-C1
PB-2_CTGAAGTCACCCTATC	PB	PB-2	P2-1978	FALSE	1	TRA:CAVGKGGADGLTF TRB:CASSSQAIEQFF	1	CD4-C2
PB-2_CTGAAGTCAGCTGTGC	PB	PB-2	P2-1979	FALSE	1	TRA:CAVAFYGGATNKLIF TRB:CASKVDEGTYEQYF	1	CD4-C1
PB-2_CTGAAGTGTTGGGACA	PB	PB-2	P2-1982	FALSE	1	TRA:CAVAADGQKLLF TRB:CASRTSDGNTQYF	1	CD4-C2
PB-2_CTGAAGTTCGCGTAGC	PB	PB-2	P2-1983	FALSE	1	TRA:CAVGARGGGADGLTF TRB:CASSLLSGTSNTGELFF	1	CD4-C2
PB-2_CTGATAGAGACTAAGT	PB	PB-2	P2-1985	FALSE	1	TRA:CAGPHSGAGSYQLTF TRB:CASSPPGVPGTQYF	1	CD4-C2
PB-2_CTGATAGAGAGGTTAT	PB	PB-2	P2-315	FALSE	2	TRA:CAVSKTAGTYKYIF TRB:CSVKSGLSTDTQYF	2	CD4-C2
PB-2_CTGATAGGTTTGTTGG	PB	PB-2	P2-1988	FALSE	1	TRA:CAYTAPGANNLFF TRB:CASSRRETGGKTATGELFF	1	CD4-C1
PB-2_CTGATCCCACTGCCAG	PB	PB-2	P2-1993	FALSE	1	TRA:CAESWDTGNQFYF TRB:CASSLEGLPNSGNTIYF	1	CD4-C2
PB-2_CTGATCCTCCGTTGTC	PB	PB-2	P2-1996	FALSE	1	TRA:CAASRMDSSYKLIF TRB:CASSSPQREGGNEQYF	1	CD4-C2
PB-2_CTGCCTAAGGCCCTCA	PB	PB-2	P2-1998	FALSE	1	TRA:CAMNSGGYQKVTF TRB:CASRSSGVGTDTQYF	1	CD4-C2
PB-2_CTGCCTAGTCGCGGTT	PB	PB-2	P2-2000	FALSE	1	TRA:CATLFSGGYNKLIF TRB:CASSVDRGGAGELFF	1	CD4-C2

PB-2_CTGCCTATCGAGAACG	PB	PB-2	P2-2002	FALSE	1	TRA:CALTPSGGSYIPTF TRB:CASSSPGLAGTSTYEQYF	1	CD4-C1
PB-2_CTGCTGTAGTCAAGGC	PB	PB-2	P2-2006	FALSE	1	TRA:CAVVVDSNSNSGYALNF TRB:CASSSLGNTEAFF	1	CD4-C2
PB-2_CTGCTGTCATCCAACA	PB	PB-2	P2-2008	FALSE	1	TRA:CAENIDWNTGFQKLVF TRB:CASSPDRSNSNQPQHF	1	CD4-C2
PB-2_CTGCTGTTCACATACG	PB	PB-2	P2-2011	FALSE	1	TRA:CALRTGTASKLTF TRB:CASIPWWGSTDTQYF	1	CD4-C2
PB-2_CTGCTGTTCCGCGGTA	PB	PB-2	P2-2013	FALSE	1	TRA:CALKTSYDKVIF TRB:CASSPAGTLSGANVLTF	1	CD4-C1
PB-2_CTGGTCTGTCATGCAT	PB	PB-2	P2-2016	FALSE	1	TRA:CAVRDGNYGGSQGNLIF TRB:CASALAYERVLYEQYF	1	CD4-C2
PB-2_CTGGTCTGTCGGCATC	PB	PB-2	P2-2017	FALSE	1	TRA:CAVEGAGSYQLTF TRB:CASSRRTGLKNIQYF	1	CD4-C2
PB-2_CTGGTCTTCAAGATCC	PB	PB-2	P2-314	FALSE	2	TRA:CAMLYDKIIF TRB:CASSLAVGSGNTIYF	2	CD4-C2
PB-2_CTGGTCTTCGCCAAAT	PB	PB-2	P2-2018	FALSE	1	TRA:CAPGSGNTGKLIF TRB:CATSRDILAGDTGELFF	1	CD4-C2
PB-2_CTGTGCTAGAATAGGG	PB	PB-2	P2-2019	FALSE	1	TRA:CAVDDEAAGNKLTF TRB:CSVEGGGVTYNEQFF	1	CD4-C2
PB-2_CTGTGCTCAATGTAAG	PB	PB-2	P2-2021	FALSE	1	TRA:CVVSRTGNQFYF TRB:CASSLARETQYF	1	CD4-C1
PB-2_CTGTGCTGTCCAAGTT	PB	PB-2	P2-2023	FALSE	1	TRA:CAVNVNNDMRF TRB:CASTSAPGGRDYGYTF	1	CD4-C1
PB-2_CTGTTTATCACCACCT	PB	PB-2	P2-2030	FALSE	1	TRA:CAASSTGAGGTSYGKLTF TRB:CASQGTVVDNEQFF	1	CD4-C2
PB-2_CTTAACTCACTAGTAC	PB	PB-2	P2-2032	FALSE	1	TRA:CLVVLEGIYNQGGKLIF TRB:CASSPRASRKNTQYF	1	CD4-C2
PB-2_CTTAACTCATAGAAAC	PB	PB-2	P2-2033	FALSE	1	TRA:CAENRGTYKYIF TRB:CASSGYNEQFF	1	CD4-C2
PB-2_CTTAACTCATGTCCTC	PB	PB-2	P2-2034	FALSE	1	TRA:CAYRSAYTGANSKLTF TRB:CASRLGTSPRQHLAKNIQYF	1	CD4-C2
PB-2_CTTAACTTCACCCGAG	PB	PB-2	P2-2036	FALSE	1	TRA:CAETLTGTASKLTF TRB:CSARPAGALLYF	1	CD4-C2
PB-2_CTTAACTTCGGTGTCG	PB	PB-2	P2-2037	FALSE	1	TRA:CAVRVSGNTGKLIF TRB:CASSPGRGYTF	1	CD4-C2
PB-2_CTTACCGCACTTAACG	PB	PB-2	P2-2039	FALSE	1	TRA:CAVSDRGTGGFKTIF TRB:CASRPHREGNTEAFF	1	CD4-C2
PB-2_CTTAGGAAGACAGAGA	PB	PB-2	P2-2042	FALSE	1	TRA:CGTEPRGSARQLTF TRB:CAWSVRGGLTYEQYF	1	CD4-C2
PB-2_CTTAGGAAGTAACCCT	PB	PB-2	P2-2043	FALSE	1	TRA:CALLGAGTALIF TRB:CASSVGTALASGELFF	1	CD4-C2
PB-2_CTTCTCTAGGGTCGAT	PB	PB-2	P2-2049	FALSE	1	TRA:CALSSGGSNYKLTF TRB:CASREDSTFADEQFF	1	CD4-C2
PB-2_CTTCTCTAGGTGATAT	PB	PB-2	P2-2050	FALSE	1	TRA:CAGRAGSARQLTF TRB:CASEGQGARNTEAFF	1	CD4-C2
PB-2_CTTCTCTCACGTCAGC	PB	PB-2	P2-2052	FALSE	1	TRA:CAVRDYGGRRALTF TRB:CASSSMGDTGELFF	1	CD4-C7
PB-2_CTTGGCTGTTTCGCTC	PB	PB-2	P2-2055	FALSE	1	TRA:CEIPGGTSYGKLTF TRB:CASSEMALYNSPLHF	1	CD4-C1
PB-2_CTTGGCTTCAAGGTAA	PB	PB-2	P2-2056	FALSE	1	TRA:CAASPFQGAQKLVF TRB:CASSFGGATGELFF	1	CD4-C2
PB-2_CTTGGCTTCTTTACAC	PB	PB-2	P2-2057	FALSE	1	TRA:CAGSMNTGFQKLVF TRB:CASSLRGLGSGAYNEQFF	1	CD4-C2

PB-2_CTTTGCGCAGGATCGA	PB	PB-2	P2-2059	FALSE	1	TRA:CAASIMNNNDMRF TRB:CASSGTAQTPLHF	1	CD4-C2
PB-2_CTTTGCGTCTGCCCTA	PB	PB-2	P2-2064	FALSE	1	TRA:CALSPREYGNKLVF TRB:CASSLKSPTNEKLFF	1	CD4-C2
PB-2_GAAACTCAGCTACCGC	PB	PB-2	P2-2065	FALSE	1	TRA:CATVNFGNEKLTF TRB:CSVQQGFLYNEQFF	1	CD4-C2
PB-2_GAAATGAAGCCCAATT	PB	PB-2	P2-2068	FALSE	1	TRA:CAVRERAGGGNKLTF TRB:CASSEYTGVDEQYF	1	CD4-C2
PB-2_GAAATGAGTATAGGGC	PB	PB-2	P2-2070	FALSE	1	TRA:CAFMKQSTGANNLFF TRB:CASSLQLAGGEEQFF	1	CD4-C2
PB-2_GAAATGATCGCGTAGC	PB	PB-2	P2-2071	FALSE	1	TRA:CAADDYIPTF TRB:CASSYSVGGQGVSEQYF	1	CD4-C1
PB-2_GAAATGATCGTACCGG	PB	PB-2	P2-2072	FALSE	1	TRA:CAVLYNFNKFYF TRB:CATSDWRNTEAFF	1	CD4-C2
PB-2_GAACATCAGGCCATAG	PB	PB-2	P2-2074	FALSE	1	TRA:CAVRDNYGQNFVF TRB:CAISEDLTGPAYEQYF	1	CD4-C2
PB-2_GAACATCCACATTAGC	PB	PB-2	P2-2075	FALSE	1	TRA:CAMMWNAGNMLTF TRB:CSVVTGGRGREQYF	1	CD4-C2
PB-2_GAACATCCAGCTGGCT	PB	PB-2	P2-2076	FALSE	1	TRA:CAVRGTGNQFYF TRB:CASRSGTGTETQYF	1	CD4-C2
PB-2_GAACATCTCCAAAGTC	PB	PB-2	P2-2077	FALSE	1	TRA:CVVSNPPGGGNKLTF TRB:CASSERRDRNIQYF	1	CD4-C2
PB-2_GAACATCTCCTATGTT	PB	PB-2	P2-2078	FALSE	1	TRA:CVVSEASKLVF TRB:CSATSGGGYEQYF	1	CD4-C2
PB-2_GAACATCTCGCTTAGA	PB	PB-2	P2-2079	FALSE	1	TRA:CAMSGSFGNEKLTF TRB:CASSSPPMNTEAFF	1	CD4-C2
PB-2_GAACCTAGTAATAGCA	PB	PB-2	P2-2082	FALSE	1	TRA:CIVRAFSTSGGSNYKLTF TRB:CASSLFENQETQYF	1	CD4-C3
PB-2_GAACGGAAGCGAGAAA	PB	PB-2	P2-2085	FALSE	1	TRA:CAVNSPNNNDMRF TRB:CASSSRAGKETQYF	1	CD4-C7
PB-2_GAACGGAGTGCAGTAG	PB	PB-2	P2-2086	FALSE	1	TRA:CVVSEGAGNNRKLIW TRB:CSVVELAGFRADTQYF	1	CD4-C1
PB-2_GAACGGAGTGTATGGG	PB	PB-2	P2-2087	FALSE	1	TRA:CAVAIAGGTSYGKLTF TRB:CASSPTELRSSYNEQFF	1	CD4-C2
PB-2_GAAGCAGCAATGCCAT	PB	PB-2	P2-2093	FALSE	1	TRA:CAGRLTGANSKLTF TRB:CASSGQSNEKLFF	1	CD4-C1
PB-2_GAATAAGAGCAGACTG	PB	PB-2	P2-2097	FALSE	1	TRA:CAIGIVGSARQLTF TRB:CAGNSQNTEAFF	1	CD4-C1
PB-2_GAATAAGGTGACGCCT	PB	PB-2	P2-2102	FALSE	1	TRA:CALIPQGGSEKLVF TRB:CASSLSYAYEQYF	1	CD4-C1
PB-2_GAATAAGGTGACGGTA	PB	PB-2	P2-2103	FALSE	1	TRA:CAMSPMDSSYKLIF TRB:CASSPKDWVTGELFF	1	CD4-C1
PB-2_GAATGAAGTTGTGGCC	PB	PB-2	P2-2107	FALSE	1	TRA:CAVSDFRGGGADGLTF TRB:CASSENGGLNTGELFF	1	CD4-C2
PB-2_GAATGAAGTTTCCACC	PB	PB-2	P2-2108	FALSE	1	TRA:CVVALNYQLIW TRB:CASSLTGSEQYF	1	CD4-C1
PB-2_GAATGAATCTATCCCG	PB	PB-2	P2-2110	FALSE	1	TRA:CAANNNDMRF TRB:CASSTQGAQVGELFF	1	CD4-C2
PB-2_GACACGCGTGAGGCTA	PB	PB-2	P2-192	TRUE	3	TRA:CAMKHDYKLSF TRB:CASSQDPNQPQHF	3	CD4-C2
PB-2_GACACGCTCAGTGTTG	PB	PB-2	P2-2114	FALSE	1	TRA:CAVTIYNFNKFYF TRB:CSANLGGNQPQHF	1	CD4-C1
PB-2_GACAGAGCAAACAACA	PB	PB-2	P2-2117	FALSE	1	TRA:CAGDRDGYNKLIF TRB:CASGRSSYEQYF	1	CD4-C2

PB-2_GACAGAGGTAAGGGCT	PB	PB-2	P2-2119	FALSE	1	TRA:CIVRVRNTDKLIF TRB:CASNVRSRVANTGELFF	1	CD4-C2
PB-2_GACCAATAGATGTGTA	PB	PB-2	P2-2121	FALSE	1	TRA:CLVGDMRITGGGNKLTF TRB:CSARGALADNYEQYF	1	CD4-C2
PB-2_GACCAATAGCCTATGT	PB	PB-2	P2-2122	FALSE	1	TRA:CAASGSGGSNYKLTF TRB:CASSVAGHQAEAFF	1	CD4-C2
PB-2_GACCAATCACTGAAGG	PB	PB-2	P2-2125	FALSE	1	TRA:CAVQVERAGNMLTF TRB:CASSPPNGEQYF	1	CD4-C2
PB-2_GACCTGGAGAAGAAGC	PB	PB-2	P2-2131	FALSE	1	TRA:CALSEAGDTDKLIF TRB:CASSQILDSRLTDTQYF	1	CD4-C2
PB-2_GACCTGGAGCCCAACC	PB	PB-2	P2-2133	FALSE	1	TRA:CAVRGGADGLTF TRB:CASSKDGVSYEQYF	1	CD4-C1
PB-2_GACCTGGAGCCGTCGT	PB	PB-2	P2-2134	FALSE	1	TRA:CAASQDTGRRALTF TRB:CSARDDINTGELFF	1	CD4-C2
PB-2_GACCTGGTCACAACGT	PB	PB-2	P2-2138	FALSE	1	TRA:CAVRADRGSTLGRLYF TRB:CSVERGTGRYNEQFF	1	CD4-C2
PB-2_GACGCGTAGATGGGTC	PB	PB-2	P2-2139	FALSE	1	TRA:CIVPGGYQKVTF TRB:CSARDQGRGLLNYEQYF	1	CD4-C2
PB-2_GACGCGTAGTACACCT	PB	PB-2	P2-2141	FALSE	1	TRA:CAMSANNAGNMLTF TRB:CASSFVGNTDTQYF	1	CD4-C1
PB-2_GACGGCTAGCTAGTCT	PB	PB-2	P2-2145	FALSE	1	TRA:CLPRPLGGSEKLVF TRB:CASSADRESGYTF	1	CD4-C2
PB-2_GACGGCTCAAGCTGTT	PB	PB-2	P2-2147	FALSE	1	TRA:CALSFIQGAQKLVF TRB:CASSQDFPGLAGDSVTQYF	1	CD4-C3
PB-2_GACGGCTTCTAACTCT	PB	PB-2	P2-2152	FALSE	1	TRA:CAGARSGGYQKVTF TRB:CASRSGSGVIQETQYF	1	CD4-C1
PB-2_GACGTTAAGACCCACC	PB	PB-2	P2-2162	FALSE	1	TRA:CAVPNSGYSTLTF TRB:CAWTGGDRGTDTQYF	1	CD4-C2
PB-2_GACGTTAAGGACTGGT	PB	PB-2	P2-2163	FALSE	1	TRA:CAEITSGNTGKLIF TRB:CASSLVGTSNQPQHF	1	CD4-C1
PB-2_GACGTTAAGGCAAAGA	PB	PB-2	P2-2164	FALSE	1	TRA:CAVRTSGGSNYKLTF TRB:CASSPRGRGVLAGELFF	1	CD4-C2
PB-2_GACGTTAAGGGCTCTC	PB	PB-2	P2-2165	FALSE	1	TRA:CALSGGTSGSRLTF TRB:CSARDQETQYF	1	CD4-C3
PB-2_GACGTTACATGCCTAA	PB	PB-2	P2-2166	FALSE	1	TRA:CAPIRGSGGYNKLIF TRB:CSAKSTENSPLHF	1	CD4-C2
PB-2_GACGTTAGTTAAGAAC	PB	PB-2	P2-2167	FALSE	1	TRA:CAMRGRLSMFSGGYNKLIF TRB:CSAKTGTGNGGYTF	1	CD4-C1
PB-2_GACGTTATCTGGTGTA	PB	PB-2	P2-2168	FALSE	1	TRA:CAPSQAYSGAGSYQLTF TRB:CASSLWGAQETQYF	1	CD4-C2
PB-2_GACTAACCAGCGTAAG	PB	PB-2	P2-2170	FALSE	1	TRA:CAVRARSGYSTLTF TRB:CASSQAPPIFQETQYF	1	CD4-C1
PB-2_GACTAACGTTGCTCCT	PB	PB-2	P2-2171	FALSE	1	TRA:CGADSTGANSKLTF TRB:CASSLAKTQYF	1	CD4-C1
PB-2_GACTAACTCTGTGCAA	PB	PB-2	P2-2172	FALSE	1	TRA:CAVTRPSGTYKYIF TRB:CSAKGMVAGANEQFF	1	CD4-C1
PB-2_GACTACAAGTGGGCTA	PB	PB-2	P2-2173	FALSE	1	TRA:CAARGKEGGKLIF TRB:CASSQVTWGLAVPNEQFF	1	CD4-C2
PB-2_GACTACAGTCCAAGTT	PB	PB-2	P2-2175	FALSE	1	TRA:CAVRGGQGGGATNKLIF TRB:CASSTAGVSNEQFF	1	CD4-C2
PB-2_GACTACAGTGAGGGAG	PB	PB-2	P2-2176	FALSE	1	TRA:CAASIPGYGGSQGNLIF TRB:CASSQVPSWGRAREQYF	1	CD4-C2
PB-2_GACTGCGAGTGTGAAT	PB	PB-2	P2-2182	FALSE	1	TRA:CAVRDRIYNFNKFYF TRB:CASTLGDRMNTEAFF	1	CD4-C2

PB-2_GACTGCGCAACTGGCC	PB	PB-2	P2-2183	FALSE	1	TRA:CAGSARQLTF TRB:CASGMRYNEQFF	1	CD4-C2
PB-2_GACTGCGCACCGAAAG	PB	PB-2	P2-2184	FALSE	1	TRA:CAASRRGNKLTF TRB:CASSLRTGNVGELFF	1	CD4-C2
PB-2_GACTGCGTCCAAAGTC	PB	PB-2	P2-325	FALSE	2	TRA:CADTYNNNDMRF TRB:CASSPSGFEQYF	2	CD4-C2
PB-2_GACTGCGTCTCGATGA	PB	PB-2	P2-2187	FALSE	1	TRA:CVVSGGGSQGNLIF TRB:CASSLAGSWNEKLFF	1	CD4-C1
PB-2_GACTGCGTCTGCGTAA	PB	PB-2	P2-2188	FALSE	1	TRA:CAVGSSTNAGKSTF TRB:CASSLGTGGNTQYF	1	CD4-C2
PB-2_GAGCAGAAGGAGCGTT	PB	PB-2	P2-2190	FALSE	1	TRA:CAVASNTNAGKSTF TRB:CASSLGTGSNQPQHF	1	CD4-C1
PB-2_GAGCAGACATCGATTG	PB	PB-2	P2-2194	FALSE	1	TRA:CAENAFGGGSNYKLTF TRB:CASSLGATVNTEAFF	1	CD4-C2
PB-2_GAGCAGACATGCAACT	PB	PB-2	P2-2195	FALSE	1	TRA:CGTEKRDSNYQLIW TRB:CASSDRTGEHGYTF	1	CD4-C1
PB-2_GAGCAGATCATTGCGA	PB	PB-2	P2-2196	FALSE	1	TRA:CAVVGLGNTDKLIF TRB:CASSRTSGGYEQYF	1	CD4-C1
PB-2_GAGGTGACACGGCTAC	PB	PB-2	P2-2198	FALSE	1	TRA:CAGPPTSGTYKYIF TRB:CASSLPANLNEKLFF	1	CD4-C2
PB-2_GAGGTGAGTTGCGCAC	PB	PB-2	P2-2201	FALSE	1	TRA:CAMSRGRSYQLTF TRB:CASSSSDRFSNEQYF	1	CD4-C2
PB-2_GAGGTGATCCTCAATT	PB	PB-2	P2-2202	FALSE	1	TRA:CAASPSAGGSYIPTF TRB:CASSLGSTPGHYTF	1	CD4-C2
PB-2_GAGTCCGCAGATGAGC	PB	PB-2	P2-2205	FALSE	1	TRA:CAELGASKIIF TRB:CASSYSGGATDTQYF	1	CD4-C1
PB-2_GAGTCCGCATACGCCG	PB	PB-2	P2-2206	FALSE	1	TRA:CIVRVADQFYF TRB:CASSLIWGNRFNEQFF	1	CD4-C7
PB-2_GAGTCCGTCGTCACGG	PB	PB-2	P2-2213	FALSE	1	TRA:CAVNAQGNTGKLIF TRB:CASSLNHLSGNTIYF	1	CD4-C2
PB-2_GATCAGTGTACGCACC	PB	PB-2	P2-2216	FALSE	1	TRA:CAVRVGAAGGSYIPTF TRB:CASRAMASGPYEQYF	1	CD4-C1
PB-2_GATCAGTTCTTTCCTC	PB	PB-2	P2-2219	FALSE	1	TRA:CALRGMDTGRRALTF TRB:CASSSGTNTDTQYF	1	CD4-C2
PB-2_GATCGATCACTAGTAC	PB	PB-2	P2-2221	FALSE	1	TRA:CAVGGLTGGGNKLTF TRB:CASRNGGSKNIQYF	1	CD4-C2
PB-2_GATCGATGTACCGTTA	PB	PB-2	P2-2223	FALSE	1	TRA:CAPCLYNTDKLIF TRB:CASSQARGYEQYF	1	CD4-C1
PB-2_GATCGCGCACGAAATA	PB	PB-2	P2-2228	FALSE	1	TRA:CAVINYGGSQGNLIF TRB:CASSEYGGWEQYF	1	CD4-C2
PB-2_GATCGCGCATACGCCG	PB	PB-2	P2-2230	FALSE	1	TRA:CAGQRGAGSYQLTF TRB:CASSFASLGTAQYF	1	CD4-C2
PB-2_GATCGCGGTACTCTCC	PB	PB-2	P2-2231	FALSE	1	TRA:CAESIAGTASKLTF TRB:CASSTAFETQYF	1	CD4-C2
PB-2_GATCGCGTCATGTGGT	PB	PB-2	P2-2233	FALSE	1	TRA:CVVNPGNTGKLIF TRB:CASSSGSTYEQYF	1	CD4-C2
PB-2_GATCGCGTCTGTCAAG	PB	PB-2	P2-2235	FALSE	1	TRA:CASNGGSQGNLIF TRB:CASSSGWDRRAYEQYF	1	CD4-C2
PB-2_GATCGTAGTCGCCATG	PB	PB-2	P2-2237	FALSE	1	TRA:CVAPSGNTGKLIF TRB:CSARDSARRLYSNQPQHF	1	CD4-C1
PB-2_GATCTAGAGGCCATAG	PB	PB-2	P2-2241	FALSE	1	TRA:CAVSPTTNAGKSTF TRB:CASSLIGGRTEAFF	1	CD4-C2
PB-2_GATCTAGAGGCCGAAT	PB	PB-2	P2-2242	FALSE	1	TRA:CALDYARLMF TRB:CSARTGGEDQPQHF	1	CD4-C1

PB-2_GATCTAGAGGCTACGA	PB	PB-2	P2-2243	FALSE	1	TRA:CIVRVAGNQGGKLIF TRB:CASSLGGRGEKLFF	1	CD4-C1
PB-2_GATCTAGCAGATGAGC	PB	PB-2	P2-330	FALSE	2	TRA:CATDASYSGAGSYQLTF TRB:CASSLASGFQETQYF	2	CD4-C2
PB-2_GATCTAGTCAGCTGGC	PB	PB-2	P2-2247	FALSE	1	TRA:CAVISGGGSQGNLIF TRB:CRAAGANVLTF	1	CD4-C2
PB-2_GATCTAGTCGGATGTT	PB	PB-2	P2-2248	FALSE	1	TRA:CAAGVAYSGAGSYQLTF TRB:CASSPTGVIYNEQFF	1	CD4-C3
PB-2_GATCTAGTCGTACCGG	PB	PB-2	P2-2249	FALSE	1	TRA:CALSGGGNQFYF TRB:CASSLYARPQHF	1	CD4-C2
PB-2_GATGAAACATGCCCGA	PB	PB-2	P2-2254	FALSE	1	TRA:CAASDNAGNMLTF TRB:CASSDRGYGYTF	1	CD4-C1
PB-2_GATGAAAGTCCTCTTG	PB	PB-2	P2-2257	FALSE	1	TRA:CVVSGNNDMRF TRB:CASSPSRAETQYF	1	CD4-C2
PB-2_GATGAAAGTGATAAAC	PB	PB-2	P2-2258	FALSE	1	TRA:CAVSGPGNTGKLIF TRB:CASSQVNKETQYF	1	CD4-C2
PB-2_GATGAAATCAATAAGG	PB	PB-2	P2-2259	FALSE	1	TRA:CAVRDGGSQGNLIF TRB:CASSLRGVGPSYEQYF	1	CD4-C2
PB-2_GATGAAATCACTTACT	PB	PB-2	P2-2260	FALSE	1	TRA:CAVSDRDTGGFKTIF TRB:CASSLDTSYGYTF	1	CD4-C1
PB-2_GATGAGGAGGCATTGG	PB	PB-2	P2-2262	FALSE	1	TRA:CAVSASKIIF TRB:CASSERDLYNEQFF	1	CD4-C2
PB-2_GATGAGGTCACGAAGG	PB	PB-2	P2-2264	FALSE	1	TRA:CAAGMGGSQGNLIF TRB:CASSLAEGHNYGYTF	1	CD4-C2
PB-2_GATGAGGTCGGTCCGA	PB	PB-2	P2-2265	FALSE	1	TRA:CAARKAAGNKLTF TRB:CATSERSQGGSQYF	1	CD4-C2
PB-2_GATGCTAGTTCTGTTT	PB	PB-2	P2-2269	FALSE	1	TRA:CAASMHNFNKFYF TRB:CASSQGPGPSTDTQYF	1	CD4-C2
PB-2_GATTCAGCATTGGCGC	PB	PB-2	P2-2270	FALSE	1	TRA:CAARSHSGGSNYKLTF TRB:CASSSGGGGLNEQYF	1	CD4-C2
PB-2_GCAAACTAGCAGATCG	PB	PB-2	P2-2273	FALSE	1	TRA:CAARGGGADGLTF TRB:CASTGTRSDTQYF	1	CD4-C2
PB-2_GCAAACTCAGACGCCT	PB	PB-2	P2-2276	FALSE	1	TRA:CAVTSTNAGKSTF TRB:CASSEEGTSRGETQYF	1	CD4-C2
PB-2_GCAAACTCAGGACGTA	PB	PB-2	P2-2277	FALSE	1	TRA:CAVILTGGGNKLTF TRB:CASRGDRGGHTEAFF	1	CD4-C2
PB-2_GCAATCACAGCCTGTG	PB	PB-2	P2-2281	FALSE	1	TRA:CAFSAAGTASKLTF TRB:CASSLVQGRDYEQYF	1	CD4-C2
PB-2_GCAATCAGTAACGACG	PB	PB-2	P2-2282	FALSE	1	TRA:CAVRDPGGANNLFF TRB:CASGGRATGELFF	1	CD4-C2
PB-2_GCACATACAACACGCC	PB	PB-2	P2-2287	FALSE	1	TRA:CAVGAYSGAGSYQLTF TRB:CASSLETRGSYEQYF	1	CD4-C2
PB-2_GCACATACAGCGTTCG	PB	PB-2	P2-2288	FALSE	1	TRA:CAGREAGNNRKLIW TRB:CASSNEEKLFF	1	CD4-C2
PB-2_GCACTCTCATGGTAGG	PB	PB-2	P2-2293	FALSE	1	TRA:CAVGVYGGATNKLIF TRB:CAWSSGGGPTGELFF	1	CD4-C3
PB-2_GCACTCTGTCCAGTGC	PB	PB-2	P2-2294	FALSE	1	TRA:CAVNRATNKLIF TRB:CASSISGGDTEAFF	1	CD4-C3
PB-2_GCACTCTGTTGTCGCG	PB	PB-2	P2-2296	FALSE	1	TRA:CAVRDIAGAGSYQLTF TRB:CASSSQGSDTGELFF	1	CD4-C2
PB-2_GCACTCTTCTGCTTGC	PB	PB-2	P2-2298	FALSE	1	TRA:CEGSGTYKYIF TRB:CASSRDLRVGYTF	1	CD4-C1
PB-2_GCAGCCAAGGAATCGC	PB	PB-2	P2-2299	FALSE	1	TRA:CAANRNQAGTALIF TRB:CASSLNRGGRETQYF	1	CD4-C2

PB-2_GCAGCCACAAACGCGA	PB	PB-2	P2-2300	FALSE	1	TRA:CALTREGYQKVTF TRB:CASSQDRGQRGTDTQYF	1	CD4-C2
PB-2_GCAGTTACAACTGCGC	PB	PB-2	P2-2305	FALSE	1	TRA:CAEPYSGAGSYQLTF TRB:CASAQGLRTDTQYF	1	CD4-C2
PB-2_GCAGTTAGTAGCCTCG	PB	PB-2	P2-2306	FALSE	1	TRA:CGTADGQKLLF TRB:CASSSLPYVGQGEEEKLFF	1	CD4-C2
PB-2_GCATACAGTAGCGTAG	PB	PB-2	P2-2310	FALSE	1	TRA:CAVRDNDNDMRF TRB:CASSSTGYNQPQHF	1	CD4-C2
PB-2_GCATACAGTCCGTTAA	PB	PB-2	P2-2311	FALSE	1	TRA:CAMSYIQGAQKLVF TRB:CASSQGLGQRQETQYF	1	CD4-C2
PB-2_GCATGATCAAACGCGA	PB	PB-2	P2-2313	FALSE	1	TRA:CAVGAARNGNKLVF TRB:CSAITGGAFSDEQFF	1	CD4-C2
PB-2_GCATGATGTTTGTGTG	PB	PB-2	P2-2317	FALSE	1	TRA:CIVRSHFGNEKLTF TRB:CSARDVRANEQFF	1	CD4-C1
PB-2_GCATGCGAGTCGTACT	PB	PB-2	P2-2319	FALSE	1	TRA:CAASDLRKGAQKLVF TRB:CASSLVSSAYNEQFF	1	CD4-C2
PB-2_GCCTCTACACAGACTT	PB	PB-2	P2-2340	FALSE	1	TRA:CAVMRGTSYGKLTF TRB:CASSRAGTEAFF	1	CD4-C2
PB-2_GCCTCTACATACTACG	PB	PB-2	P2-2341	FALSE	1	TRA:CAVGIQGAQKLVF TRB:CASGKPGLTYNEQFF	1	CD4-C2
PB-2_GCCTCTATCCGTACAA	PB	PB-2	P2-2343	FALSE	1	TRA:CALGGTNAGGTSYGKLTF TRB:CASSQGGFRTEAFF	1	CD4-C1
PB-2_GCCTCTATCTTCCTTC	PB	PB-2	P2-2344	FALSE	1	TRA:CALSEGYYGGSQGNLIF TRB:CAISTGGTDTQYF	1	CD4-C1
PB-2_GCGACCACAATGCCAT	PB	PB-2	P2-2347	FALSE	1	TRA:CAGYPSGTYKYIF TRB:CSADEGAQRETQYF	1	CD4-C2
PB-2_GCGACCACAATGGAAT	PB	PB-2	P2-2348	FALSE	1	TRA:CAVNTPLVF TRB:CASSLAVALAGGPTGRQQFF	1	CD4-C2
PB-2_GCGACCATCCGTTGCT	PB	PB-2	P2-336	FALSE	2	TRA:CAVPGRGFNKFYF TRB:CASSLGGYQQFF	2	CD4-C2
PB-2_GCGACCATCTGCAAGT	PB	PB-2	P2-2351	FALSE	1	TRA:CAVRTDGSGNTGKLIF TRB:CASSLVLTGADTQYF	1	CD4-C2
PB-2_GCGAGAAAGATGTGTA	PB	PB-2	P2-2354	FALSE	1	TRA:CAVNTVGNAGNMLTF TRB:CASSVGNSGNTIYF	1	CD4-C1
PB-2_GCGAGAAGTCACTGGC	PB	PB-2	P2-2359	FALSE	1	TRA:CALSEAGGSQGNLIF TRB:CASSLGSKLTGNTIYF	1	CD4-C2
PB-2_GCGAGAAGTGCCTGCA	PB	PB-2	P2-2360	FALSE	1	TRA:CVVRTTSGTYKYIF TRB:CASSDTGGTEQFF	1	CD4-C1
PB-2_GCGCAACGTAAAGGAG	PB	PB-2	P2-2364	FALSE	1	TRA:CAASSTGANNLFF TRB:CASSSVGEAAFYEQYF	1	CD4-C2
PB-2_GCGCAACGTGCAGTAG	PB	PB-2	P2-2366	FALSE	1	TRA:CAGEGAGGTSYGKLTF TRB:CASSIGMGYNTGELFF	1	CD4-C2
PB-2_GCGCAACGTGCCTGCA	PB	PB-2	P2-2367	FALSE	1	TRA:CAVGNNAGNMLTF TRB:CASTHRGANEQYF	1	CD4-C2
PB-2_GCGCAACGTGCCTGGT	PB	PB-2	P2-2368	FALSE	1	TRA:CAASTNHGNKLVF TRB:CASTVTELNTEAFF	1	CD8-C1
PB-2_GCGCAGTCATACGCTA	PB	PB-2	P2-2373	FALSE	1	TRA:CAVVQNSGAGSYQLTF TRB:CASSRGSGETQYF	1	CD4-C2
PB-2_GCGCAGTCATCTCCCA	PB	PB-2	P2-2374	FALSE	1	TRA:CAANSNSGYALNF TRB:CASRLTSGGEQFF	1	CD4-C1
PB-2_GCGCCAAAGCCACGCT	PB	PB-2	P2-2378	FALSE	1	TRA:CALPATRGYSSASKIIF TRB:CASSDTTSGRNTDTQYF	1	CD4-C2
PB-2_GCGCCAAAGTACGTTC	PB	PB-2	P2-2379	FALSE	1	TRA:CAVGASLTGNQFYF TRB:CASSGRDSKDEKLFF	1	CD4-C1

PB-2_GCGCCAACAGGAACGT	PB	PB-2	P2-2382	FALSE	1	TRA:CASYNFNKFYF TRB:CASSRGGAIDEQYF	1	CD4-C2
PB-2_GCGCCAATCTCATTCA	PB	PB-2	P2-2385	FALSE	1	TRA:CAVWGSGGSYIPTF TRB:CASSRATGNTIYF	1	CD4-C2
PB-2_GCGCGATGTATGAAAC	PB	PB-2	P2-2386	FALSE	1	TRA:CIVRAPRGRGSARQLTF TRB:CASSIGTGVIDTQYF	1	CD4-C3
PB-2_GCGGGTTAGCAGCCTC	PB	PB-2	P2-2389	FALSE	1	TRA:CAVGATGTASKLTF TRB:CATSDSSLAGVSAEQFF	1	CD4-C1
PB-2_GCGGGTTCAGTCTTCC	PB	PB-2	P2-2390	FALSE	1	TRA:CAAGDYKLSF TRB:CASRSGTGRTEAFF	1	CD4-C2
PB-2_GCGGGTTGTTCGTTGA	PB	PB-2	P2-2393	FALSE	1	TRA:CAASDTGANNLFF TRB:CASSRSGGAPGELFF	1	CD4-C2
PB-2_GCGGGTTTCCTTTCGG	PB	PB-2	P2-2395	FALSE	1	TRA:CATSEYGNKLVF TRB:CATSEGNQPQHF	1	CD4-C1
PB-2_GCTCCTAAGCGCTTAT	PB	PB-2	P2-2396	FALSE	1	TRA:CAVTRAGGTSYGKLTF TRB:CASSPRTSGSQETQYF	1	CD4-C2
PB-2_GCTCCTAAGGTCATCT	PB	PB-2	P2-2397	FALSE	1	TRA:CAVTARGNNRLAF TRB:CASKGLAGTLYEQYF	1	CD4-C1
PB-2_GCTCCTACACATTAGC	PB	PB-2	P2-337	FALSE	2	TRA:CAPQNQAGTALIF TRB:CASSIVGPNSQPQHF	2	CD4-C2
PB-2_GCTCCTAGTACTTCTT	PB	PB-2	P2-2399	FALSE	1	TRA:CAPIPPNNQFYF TRB:CASSLTGQGGTGELFF	1	CD4-C2
PB-2_GCTCCTAGTCTCTTTA	PB	PB-2	P2-2400	FALSE	1	TRA:CAVRPTGANNLFF TRB:CASSAGIQREQYF	1	CD4-C2
PB-2_GCTCCTATCAACGGCC	PB	PB-2	P2-2401	FALSE	1	TRA:CASSGAGSYQLTF TRB:CASSLFPGPNTGELFF	1	CD4-C2
PB-2_GCTCCTATCAGCTTAG	PB	PB-2	P2-2402	FALSE	1	TRA:CAVSDGRVFSGGYNKLIF TRB:CASSIMRSGGQSNYGYTF	1	CD4-C2
PB-2_GCTCTGTAGTAGGCCA	PB	PB-2	P2-2404	FALSE	1	TRA:CIPFTSGTYKYIF TRB:CSVEFYNEQFF	1	CD4-C1
PB-2_GCTCTGTGTAGCCTCG	PB	PB-2	P2-2405	FALSE	1	TRA:CATLYSGGGADGLTF TRB:CASAQAYSNQPQHF	1	CD4-C1
PB-2_GCTCTGTGTAGCGTAG	PB	PB-2	P2-2406	FALSE	1	TRA:CAGFSGNTPLVF TRB:CASSPGLAVYNEQFF	1	CD4-C7
PB-2_GCTGCAGGTAGCGTAG	PB	PB-2	P2-2409	FALSE	1	TRA:CAVGRGGADGLTF TRB:CASSKRRLASLEGTGELFF	1	CD4-C2
PB-2_GCTGCAGGTCCGAATT	PB	PB-2	P2-2411	FALSE	1	TRA:CAASMNTGFQKLVF TRB:CASLGQGSGEQYF	1	CD4-C2
PB-2_GCTGCAGGTGCGAAAC	PB	PB-2	P2-2412	FALSE	1	TRA:CALKSTGGGNKLTF TRB:CASSYSSGTSYEQYF	1	CD4-C1
PB-2_GCTGCAGGTTGAACTC	PB	PB-2	P2-2414	FALSE	1	TRA:CAENNAGNMLTF TRB:CAWSYRDRAGYGYTF	1	CD4-C1
PB-2_GCTGCAGTCCTGCTTG	PB	PB-2	P2-2415	FALSE	1	TRA:CAVPSWGKFQF TRB:CASSLAGSPTDTQYF	1	CD4-C2
PB-2_GCTGCGAAGGATGCGT	PB	PB-2	P2-2416	FALSE	1	TRA:CVVSSPDIPTF TRB:CASSLGTVGEQYF	1	CD4-C1
PB-2_GCTGCTTGTTTGTTGG	PB	PB-2	P2-2420	FALSE	1	TRA:CADPGGTSYGKLTF TRB:CASSGDHSSGFF	1	CD4-C1
PB-2_GCTGGGTAGAGTCTGG	PB	PB-2	P2-2421	FALSE	1	TRA:CGNSGGSNYKLTF TRB:CASSRDPSYEQYF	1	CD4-C2
PB-2_GCTGGGTGTAGCGCTC	PB	PB-2	P2-2423	FALSE	1	TRA:CAWPRFNKFYF TRB:CAWSAGQGSEQYF	1	CD4-C2
PB-2_GCTTGAACATTTCACT	PB	PB-2	P2-2433	FALSE	1	TRA:CAGEGAQKLVF TRB:CASSITGSLGEKLFF	1	CD4-C2

PB-2_GGAACTTGTCACTTCC	PB	PB-2	P2-2441	FALSE	1	TRA:CAARTGRYGSGNTGKLIF TRB:CASSPGQNQPQHF	1	CD4-C3
PB-2_GGAACTTTCAGTCAGT	PB	PB-2	P2-2444	FALSE	1	TRA:CADFSGNTPLVF TRB:CATSRGGGGETQYF	1	CD4-C2
PB-2_GGAACTTTCAGTTTGG	PB	PB-2	P2-2445	FALSE	1	TRA:CILRDETQGGKLIF TRB:CASSQGDFYNEQFF	1	CD4-C1
PB-2_GGAATAAAGGTGATAT	PB	PB-2	P2-2446	FALSE	1	TRA:CALSPVGTYKYIF TRB:CASSRPRGDGETQYF	1	CD4-C2
PB-2_GGAATAACAAGTAATG	PB	PB-2	P2-2447	FALSE	1	TRA:CAVSEPSGNTGKLIF TRB:CASSLAQVTDTQYF	1	CD4-C1
PB-2_GGAATAACAGACGCCT	PB	PB-2	P2-2449	FALSE	1	TRA:CALDVNYGQNFVF TRB:CASSVDRTDEQYF	1	CD4-C2
PB-2_GGAATAACATGCAACT	PB	PB-2	P2-2451	FALSE	1	TRA:CVVSDPEGSYIPTF TRB:CASSLTDTQYF	1	CD4-C3
PB-2_GGAATAAGTAAGAGAG	PB	PB-2	P2-2453	FALSE	1	TRA:CAEKSLNTGGFKTIF TRB:CASSDTNGPKRPQHF	1	CD4-C2
PB-2_GGAATAAGTGTGCCTG	PB	PB-2	P2-2454	FALSE	1	TRA:CAYLMNRDDKIIF TRB:CASSPGQGPSGANVLTF	1	CD4-C2
PB-2_GGAATAATCTGTCTAT	PB	PB-2	P2-2456	FALSE	1	TRA:CVVRKGGGSQGNLIF TRB:CASGLGEGFPAEAFF	1	CD4-C2
PB-2_GGAATAATCTTTAGGG	PB	PB-2	P2-2457	FALSE	1	TRA:CALSEANAGGTSYGKLTF TRB:CSAITVQGLQETQYF	1	CD4-C2
PB-2_GGACAAGAGGGCTTCC	PB	PB-2	P2-2460	FALSE	1	TRA:CALELSGGTSYGKLTF TRB:CASSPNTDTQYF	1	CD4-C1
PB-2_GGACAAGCAGCTGTTA	PB	PB-2	P2-344	TRUE	2	TRA:CAASNRNYGQNFVF TRB:CASSPRDGYGYTF	2	CD4-C2
PB-2_GGACAAGGTTAGATGA	PB	PB-2	P2-2466	FALSE	1	TRA:CAGGDTGGFKTIF TRB:CASSLGGVWTEAFF	1	CD4-C2
PB-2_GGACAAGGTTTAGCTG	PB	PB-2	P2-2467	FALSE	1	TRA:CIVRARGTSYGKLTF TRB:CASSLAGQGGNEKLFF	1	CD4-C2
PB-2_GGACAAGTCAAAGACA	PB	PB-2	P2-2468	FALSE	1	TRA:CAVNAPRAGNMLTF TRB:CASSQTSGREGAETQYF	1	CD4-C2
PB-2_GGACAAGTCCGCGCAA	PB	PB-2	P2-2469	FALSE	1	TRA:CALQGAQKLVF TRB:CASRGTGPVAQYF	1	CD4-C2
PB-2_GGACAGAAGAGACGAA	PB	PB-2	P2-2470	FALSE	1	TRA:CALSVETGGFKTIF TRB:CAWRMTDLNNSPLHF	1	CD4-C1
PB-2_GGACAGAGTAACGACG	PB	PB-2	P2-2473	FALSE	1	TRA:CAASRTGTASKLTF TRB:CASSRAGVWQPQHF	1	CD4-C2
PB-2_GGACAGATCATGCATG	PB	PB-2	P2-2475	FALSE	1	TRA:CGTGGGGTSYGKLTF TRB:CASSRTTQLYNSPLHF	1	CD4-C2
PB-2_GGACAGATCTCGATGA	PB	PB-2	P2-2476	FALSE	1	TRA:CAESHSFSGAGSYQLTF TRB:CASSVAADNEQFF	1	CD4-C2
PB-2_GGACATTCATCCTAGA	PB	PB-2	P2-2478	FALSE	1	TRA:CAATGAGGFKTIF TRB:CSARAGSLERGIQYF	1	CD4-C1
PB-2_GGACGTCAGTGAAGAG	PB	PB-2	P2-2480	FALSE	1	TRA:CAVSSSGTYKYIF TRB:CSAMKTGGTTYEQYF	1	CD4-C2
PB-2_GGACGTCTCATTGCCC	PB	PB-2	P2-2484	FALSE	1	TRA:CAVALNQFYF TRB:CASSFNLYSYEQYF	1	CD4-C1
PB-2_GGATGTTTCATCACCC	PB	PB-2	P2-2492	FALSE	1	TRA:CAPSGSARQLTF TRB:CASSGGGADTQYF	1	CD4-C2
PB-2_GGATGTTTCGATGAGG	PB	PB-2	P2-2493	FALSE	1	TRA:CAASMSAAGANNLFF TRB:CSARRGGTDTQYF	1	CD4-C3
PB-2_GGATTACGTCCCGACA	PB	PB-2	P2-299	FALSE	2	TRA:CAATDDNAGNMLTF TRB:CASSLAGLAGPPGTQYF	2	CD4-C2

PB-2_GGCAATTAGATTACCC	PB	PB-2	P2-2497	FALSE	1	TRA:CAVNNNARLMF TRB:CASNLAGTETQYF	1	CD4-C1
PB-2_GGCAATTCATGCCTTC	PB	PB-2	P2-2502	FALSE	1	TRA:CAVNTGGFKTIF TRB:CASSLPAATGGNTIYF	1	CD4-C2
PB-2_GGCAATTGTTCTGAAC	PB	PB-2	P2-2504	FALSE	1	TRA:CATLGGATNKLIF TRB:CASSIDRGGQPQHF	1	CD4-C1
PB-2_GGCCGATAGTAGTGCG	PB	PB-2	P2-2508	FALSE	1	TRA:CALSDRDDKIIF TRB:CASRQRGETQYF	1	CD4-C3
PB-2_GGCGACTAGGAGCGTT	PB	PB-2	P2-2515	FALSE	1	TRA:CALSERNFGNEKLTF TRB:CAIREGGGSSYEQYF	1	CD4-C1
PB-2_GGCGACTGTAAAGTCA	PB	PB-2	P2-2516	FALSE	1	TRA:CAATYGQNFVF TRB:CATSRELGSYNEQFF	1	CD4-C2
PB-2_GGCGACTGTCAGCTAT	PB	PB-2	P2-2518	FALSE	1	TRA:CALGGNQFYF TRB:CASSLGGGLGSYNEQFF	1	CD4-C1
PB-2_GGCGACTGTCTAGAGG	PB	PB-2	P2-2519	FALSE	1	TRA:CAVRYSGGFGNVLHC TRB:CASSQASGTTDTQYF	1	CD4-C2
PB-2_GGCGTGTCAAGACACG	PB	PB-2	P2-2521	FALSE	1	TRA:CAVRSESGYSTLTF TRB:CASSPGSSQPQHF	1	CD4-C2
PB-2_GGCGTGTTCATAACCG	PB	PB-2	P2-2522	FALSE	1	TRA:CALSDTGGFKTIF TRB:CASTRTQGTGELFF	1	CD4-C7
PB-2_GGCTCGAAGGACGAAA	PB	PB-2	P2-2525	FALSE	1	TRA:CAGLVGAAGNKLTF TRB:CASSTGTGGWEYF	1	CD4-C2
PB-2_GGCTCGACACGTTGGC	PB	PB-2	P2-2528	FALSE	1	TRA:CVVSEGGSARQLTF TRB:CASSLGVTSKNNEQFF	1	CD4-C2
PB-2_GGCTCGATCGCGCCAA	PB	PB-2	P2-2530	FALSE	1	TRA:CATVSGSARQLTF TRB:CASSLADRAYEQYF	1	CD4-C1
PB-2_GGCTCGATCTCTTGAT	PB	PB-2	P2-2531	FALSE	1	TRA:CAMREVSDNFNKFYF TRB:CASSLTSGGVGATQYF	1	CD4-C1
PB-2_GGCTGGTAGGCACATG	PB	PB-2	P2-2533	FALSE	1	TRA:CAERDTGRRALTF TRB:CASMGGSTDTQYF	1	CD4-C3
PB-2_GGCTGGTCATGTCTCC	PB	PB-2	P2-2534	FALSE	1	TRA:CAANSNSGYALNF TRB:CASSRSGASSYNEQFF	1	CD4-C2
PB-2_GGGAATGCACCATGTA	PB	PB-2	P2-2538	FALSE	1	TRA:CAVRDPRDFGNNRKLIW TRB:CASSYSGSGGSETQYF	1	CD4-C1
PB-2_GGGACCTAGGCACATG	PB	PB-2	P2-2540	FALSE	1	TRA:CGTEIVGGTSYGKLTF TRB:CASSSQGLGTDTQYF	1	CD4-C1
PB-2_GGGACCTAGTTACCCA	PB	PB-2	P2-2542	FALSE	1	TRA:CIVNTNAGKSTF TRB:CASSLNRGTDTQYF	1	CD4-C1
PB-2_GGGACCTGTACCGGCT	PB	PB-2	P2-324	FALSE	2	TRA:CAVSGTKAGTALIF TRB:CSARGLNAYNSPLHF	2	CD4-C3
PB-2_GGGAGATGTTCGGGCT	PB	PB-2	P2-325	FALSE	2	TRA:CADTYNNNDMRF TRB:CASSPSGFEQYF	2	CD4-C2
PB-2_GGGCACTCAGTATAAG	PB	PB-2	P2-2553	FALSE	1	TRA:CATGSDGTSYGKLTF TRB:CASSQTSVVEQYF	1	CD4-C3
PB-2_GGGCATCTCGAACTGT	PB	PB-2	P2-2559	FALSE	1	TRA:CATEETSGSRLTF TRB:CASSQGTGLSYEQYF	1	CD4-C2
PB-2_GGGTCTGCAAGCGCTC	PB	PB-2	P2-2563	FALSE	1	TRA:CAASARGFGNVLHC TRB:CATSGSGTGGTEAFF	1	CD4-C1
PB-2_GGGTCTGCATGTTCCC	PB	PB-2	P2-2565	FALSE	1	TRA:CAMSGGSYIPTF TRB:CASGSSGTLANNEQFF	1	CD4-C1
PB-2_GGGTCTGTCCGAAGAG	PB	PB-2	P2-2566	FALSE	1	TRA:CAVTTSGTYKYIF TRB:CAWSRGDGQPQHF	1	CD4-C3
PB-2_GGGTTGCTCCATGCTC	PB	PB-2	P2-2573	FALSE	1	TRA:CAMFGNEKLTF TRB:CASSQEAGRVVGLGKKGGYTF	1	CD4-C2

PB-2_GGTATTGAGATCACGG	PB	PB-2	P2-2574	FALSE	1	TRA:CAPSNNAGNMLTF TRB:CASRRTENTEAFF	1	CD4-C2
PB-2_GGTATTGAGTTGTAGA	PB	PB-2	P2-2575	FALSE	1	TRA:CALDSSHSGGYQKVTF TRB:CASSHTTGTGSFQAFF	1	CD4-C2
PB-2_GGTATTGGTAAGTGTA	PB	PB-2	P2-2576	FALSE	1	TRA:CAIKQGGKLIF TRB:CSVGTGQGELFF	1	CD4-C1
PB-2_GGTGCGTAGGTGATAT	PB	PB-2	P2-2578	FALSE	1	TRA:CAFVNSNSGYALNF TRB:CASSLGVGGGNQPQHF	1	CD4-C2
PB-2_GGTGCGTGTCCAGTTA	PB	PB-2	P2-2579	FALSE	1	TRA:CAVGAQGAQKLVF TRB:CASSPRGGGYNEQFF	1	CD4-C2
PB-2_GTAACGTAGGGTGTTG	PB	PB-2	P2-2587	FALSE	1	TRA:CAATAAGGTSYGKLTF TRB:CASSDFGLIPTDTQYF	1	CD4-C2
PB-2_GTAACGTCAAGCCTAT	PB	PB-2	P2-2589	FALSE	1	TRA:CAVLGGGSYIPTF TRB:CAWSVGLAGGPSNEQFF	1	CD4-C3
PB-2_GTAACGTGTATTCGTG	PB	PB-2	P2-2590	FALSE	1	TRA:CAVMNYGGSQGNLIF TRB:CAGGRTDTEAFF	1	CD4-C1
PB-2_GTAACTGAGCGATATA	PB	PB-2	P2-2593	FALSE	1	TRA:CAMRAYGGATNKLIF TRB:CASSPAGGGTEAFF	1	CD4-C2
PB-2_GTAACTGCACCAGGTC	PB	PB-2	P2-352	FALSE	2	TRA:CAMIRTQGGSEKLVF TRB:CASSAIGRSWQFF	2	CD4-C2
PB-2_GTAACTGTCCCTCTTT	PB	PB-2	P2-2596	FALSE	1	TRA:CVVRSASGGSYIPTF TRB:CSVEAGDPNTEAFF	1	CD4-C2
PB-2_GTACGTAAGCTATGCT	PB	PB-2	P2-2598	FALSE	1	TRA:CAAFPNTGNQFYF TRB:CSARIPGRSTDTQYF	1	CD4-C2
PB-2_GTACGTACAATCACAC	PB	PB-2	P2-2600	FALSE	1	TRA:CAAVQQTGANNLFF TRB:CASSLGMGTDTQYF	1	CD4-C2
PB-2_GTACGTAGTGCCTTGG	PB	PB-2	P2-2601	FALSE	1	TRA:CAFRADNQGGKLIF TRB:CASSLGAKISNQPQHF	1	CD4-C1
PB-2_GTACTTTCACAGGAGT	PB	PB-2	P2-2611	FALSE	1	TRA:CIVRPREGAGGTSYGKLTF TRB:CASSTGRGVDTQYF	1	CD4-C2
PB-2_GTAGGCCAGGCCATAG	PB	PB-2	P2-2613	FALSE	1	TRA:CAENDGYGGSQGNLIF TRB:CASSEAQAGLRPQHF	1	CD4-C1
PB-2_GTAGGCCCACACGCTG	PB	PB-2	P2-2614	FALSE	1	TRA:CAPSNTGNQFYF TRB:CASSLPQGANEQFF	1	CD4-C2
PB-2_GTAGGCCGTACTTCTT	PB	PB-2	P2-2616	FALSE	1	TRA:CAVSVTGGFKTIF TRB:CASSPGATNEKLFF	1	CD4-C1
PB-2_GTAGGCCGTTCCTCCA	PB	PB-2	P2-2618	FALSE	1	TRA:CAVRLGKSGNQFYF TRB:CASSQGLLSSYNSPLHF	1	CD4-C2
PB-2_GTAGTCACAAAGGAAG	PB	PB-2	P2-2621	FALSE	1	TRA:CAVFLYSSASKIIF TRB:CASSLRGGDSGFGGEQYF	1	CD4-C2
PB-2_GTAGTCAGTAAGGGAA	PB	PB-2	P2-2622	FALSE	1	TRA:CAVEDLDYGGSQGNLIF TRB:CASSLTGANVLTF	1	CD4-C2
PB-2_GTAGTCATCTGAAAGA	PB	PB-2	P2-2623	FALSE	1	TRA:CAVTRSGGSNYKLTF TRB:CASSPTGVEQYF	1	CD4-C1
PB-2_GTATCTTAGGCAGGTT	PB	PB-2	P2-2625	FALSE	1	TRA:CAENVKGGSEKLVF TRB:CSAFRDRGYEQYF	1	CD4-C1
PB-2_GTATCTTTCAAGATCC	PB	PB-2	P2-2626	FALSE	1	TRA:CAMSDYGNKLVF TRB:CASSPTRGGVETQYF	1	CD4-C2
PB-2_GTATTCTGTCTACCTC	PB	PB-2	P2-2629	FALSE	1	TRA:CAVKNTDKLIF TRB:CSARDLHESSGPSDTQYF	1	CD4-C1
PB-2_GTCAAGTAGCCGGTAA	PB	PB-2	P2-2634	FALSE	1	TRA:CVVLSPLGYQLIW TRB:CASSTGQGGSYEQYF	1	CD4-C2
PB-2_GTCAAGTCAGCCAATT	PB	PB-2	P2-352	FALSE	2	TRA:CAMIRTQGGSEKLVF TRB:CASSAIGRSWQFF	2	CD4-C2

PB-2_GTCAAGTTCAATAAGG	PB	PB-2	P2-2638	FALSE	1	TRA:CAMREAFGNQFYF TRB:CASSLRAGEQYF	1	CD4-C1
PB-2_GTCACAAGTAGATTAG	PB	PB-2	P2-2641	FALSE	1	TRA:CAMRGKNTDKLIF TRB:CASARGGGEETQYF	1	CD4-C2
PB-2_GTCACAAGTTATGCGT	PB	PB-2	P2-2642	FALSE	1	TRA:CATGGSGNTPLVF TRB:CASSPDGGSGNTIYF	1	CD4-C2
PB-2_GTCACAAGTTCTGAAC	PB	PB-2	P2-2643	FALSE	1	TRA:CAVRKATSGSARQLTF TRB:CASSLAGGLVTEAFF	1	CD4-C2
PB-2_GTCACGGAGTATTGGA	PB	PB-2	P2-2649	FALSE	1	TRA:CAENTSPRNQFYF TRB:CASSRDRGYAGANVLTF	1	CD4-C2
PB-2_GTCATTTAGGATTCGG	PB	PB-2	P2-2652	FALSE	1	TRA:CALGVYNQGGKLIF TRB:CASSLDSASTYQPQHF	1	CD4-C2
PB-2_GTCATTTCAGCTTAAC	PB	PB-2	P2-2656	FALSE	1	TRA:CAASPKGNDYKLSF TRB:CASRTGTLSYEQYF	1	CD4-C2
PB-2_GTCCTCAAGCTCTCGG	PB	PB-2	P2-2660	FALSE	1	TRA:CAASGDSGGSNYKLTF TRB:CASRIPGQGAGEQFF	1	CD4-C1
PB-2_GTCCTCAAGGCCGAAT	PB	PB-2	P2-2661	FALSE	1	TRA:CAGAGGSEKLVF TRB:CASSLGFGTARSGANVLTF	1	CD4-C1
PB-2_GTCCTCACACGGTGTC	PB	PB-2	P2-2663	FALSE	1	TRA:CIVRVLSSARQLTF TRB:CSASTSNTDTQYF	1	CD4-C1
PB-2_GTCCTCACATGGATGG	PB	PB-2	P2-2665	FALSE	1	TRA:CALSEVGYSSASKIIF TRB:CASSPLAGTGGGELFF	1	CD4-C2
PB-2_GTCGGGTCATGCAATC	PB	PB-2	P2-2672	FALSE	1	TRA:CAVTVSSGTYKYIF TRB:CASSLGLASTDTQYF	1	CD4-C2
PB-2_GTCGGGTTCGACAGCC	PB	PB-2	P2-2674	FALSE	1	TRA:CAVGARGTYKYIF TRB:CASSPGAYLSKNEQFF	1	CD4-C2
PB-2_GTCGGGTTCTGTGCAA	PB	PB-2	P2-2675	FALSE	1	TRA:CARPKTSYDKVIF TRB:CASSIRENEQFF	1	CD4-C2
PB-2_GTCGTAAAGGGTTTCT	PB	PB-2	P2-2679	FALSE	1	TRA:CAVRTNNAGNMLTF TRB:CADPRLEGRLATEAFF	1	CD4-C1
PB-2_GTCTCGTCATAACCTG	PB	PB-2	P2-2684	FALSE	1	TRA:CAASSGGGADGLTF TRB:CASSNRGYEQYF	1	CD4-C1
PB-2_GTCTCGTGTCCGCTGA	PB	PB-2	P2-2687	FALSE	1	TRA:CAVKPGNTGKLIF TRB:CASSLDGVNTEAFF	1	CD4-C2
PB-2_GTCTTCGTCGAGCCCA	PB	PB-2	P2-2697	FALSE	1	TRA:CALSDPGNTGKLIF TRB:CASSQRGADGYTF	1	CD4-C2
PB-2_GTGAAGGAGTGTTAGA	PB	PB-2	P2-2701	FALSE	1	TRA:CAAISTGGGNKLTF TRB:CASSPPGLMETQYF	1	CD4-C2
PB-2_GTGCAGCTCTGAGTGT	PB	PB-2	P2-2706	FALSE	1	TRA:CVVSGGSNYKLTF TRB:CASSYSWGRPQHF	1	CD4-C1
PB-2_GTGCATAGTTGCGTTA	PB	PB-2	P2-2709	FALSE	1	TRA:CAVSHPGNKLTF TRB:CASSQGPGNTEAFF	1	CD4-C1
PB-2_GTGCGGTCAAACTGCT	PB	PB-2	P2-2713	FALSE	1	TRA:CIVRAPAGGTSYGKLTF TRB:CASSYSQREITDTQYF	1	CD4-C1
PB-2_GTGCGGTCATGGTAGG	PB	PB-2	P2-2715	FALSE	1	TRA:CGAGPNSGYSTLTF TRB:CASSLGQGKQYF	1	CD4-C2
PB-2_GTGCTTCCACAGACTT	PB	PB-2	P2-2722	FALSE	1	TRA:CAASKDTNAGKSTF TRB:CASSRSSASHEQYF	1	CD4-C2
PB-2_GTGCTTCGTAAGGGAA	PB	PB-2	P2-2724	FALSE	1	TRA:CAAIGVYNQGGKLIF TRB:CASSQVLRGHQETQYF	1	CD4-C2
PB-2_GTGCTTCGTGCTCTTC	PB	PB-2	P2-2725	FALSE	1	TRA:CASNDYKLSF TRB:CASKLRGGDRSTDTQYF	1	CD4-C2
PB-2_GTGCTTCTCTGCAGTA	PB	PB-2	P2-2726	FALSE	1	TRA:CAAGEEYGNKLVF TRB:CASSPRLAGTNTGELFF	1	CD4-C2

PB-2_GTGGGTCGTCAAAGAT	PB	PB-2	P2-2730	FALSE	1	TRA:CIVRVGYTGNQFYF TRB:CSVEEGGKGGNTIYF	1	CD4-C2
PB-2_GTGGGTCGTCCGTCAG	PB	PB-2	P2-2731	FALSE	1	TRA:CAEKLVF TRB:CASSLAVAGGLGGELFF	1	CD4-C1
PB-2_GTGGGTCTCATCTGTT	PB	PB-2	P2-2732	FALSE	1	TRA:CVVSNPNAGNNRKLIW TRB:CASSFRTDTEAFF	1	CD4-C1
PB-2_GTGGGTCTCGTCTGAA	PB	PB-2	P2-2733	FALSE	1	TRA:CAGVTSGSRLTF TRB:CASSPKSGGSYEQYF	1	CD4-C2
PB-2_GTGTGCGCAAGTTAAG	PB	PB-2	P2-2734	FALSE	1	TRA:CAVGAQEGTYKYIF TRB:CASSQESDSFYGYTF	1	CD4-C2
PB-2_GTGTTAGCAAGCGAGT	PB	PB-2	P2-2744	FALSE	1	TRA:CAGSGNSGNTPLVF TRB:CASSYLGGNQPQHF	1	CD4-C2
PB-2_GTGTTAGGTGGTGTAG	PB	PB-2	P2-2748	FALSE	1	TRA:CALSLSGGGSNYKLTF TRB:CASRDNTGELFF	1	CD4-C7
PB-2_GTGTTAGTCATGCTCC	PB	PB-2	P2-2749	FALSE	1	TRA:CAMREEILYGGSQGNLIF TRB:CASSSLPGRTDTQYF	1	CD4-C2
PB-2_GTGTTAGTCCAGAAGG	PB	PB-2	P2-2750	FALSE	1	TRA:CAMRPLIQGAQKLVF TRB:CASSLSEFGGQYF	1	CD4-C2
PB-2_GTTAAGCAGTCTCCTC	PB	PB-2	P2-2752	FALSE	1	TRA:CAASRGFGNEKLTF TRB:CSFGQGTNEQFF	1	CD4-C2
PB-2_GTTAAGCCACACATGT	PB	PB-2	P2-2753	FALSE	1	TRA:CAVSPNSGYSTLTF TRB:CASSQGAGVGEQYF	1	CD4-C2
PB-2_GTTAAGCGTCTCCATC	PB	PB-2	P2-2756	FALSE	1	TRA:CAAKYQGAQKLVF TRB:CASSHQQGEAFF	1	CD4-C2
PB-2_GTTAAGCTCAGAGGTG	PB	PB-2	P2-2757	FALSE	1	TRA:CAVRPGDNFNKFYF TRB:CAISEGLAYEQYF	1	CD4-C2
PB-2_GTTAAGCTCTGCGTAA	PB	PB-2	P2-2760	FALSE	1	TRA:CAMREGVGAQKLVF TRB:CAITRGSGGPTDTQYF	1	CD4-C2
PB-2_GTTACAGAGTAGGCCA	PB	PB-2	P2-294	FALSE	2	TRA:CAASNYGGTSYGKLTF TRB:CASSRPERPLEQYF	2	CD4-C2
PB-2_GTTACAGGTACCGTAT	PB	PB-2	P2-2762	FALSE	1	TRA:CAVEDRGSGGGADGLTF TRB:CASSLLPQTEAFF	1	CD4-C2
PB-2_GTTCATTAGAGACTAT	PB	PB-2	P2-198	TRUE	3	TRA:CAVRDGDYKLSF TRB:CASSLEGVETQYF	3	CD4-C2
PB-2_GTTCATTAGCGGCTTC	PB	PB-2	P2-2765	FALSE	1	TRA:CAVETNYGGSQGNLIF TRB:CASSLARGPGNEQFF	1	CD4-C2
PB-2_GTTCATTCAACACGCC	PB	PB-2	P2-2766	FALSE	1	TRA:CVVSDHTNAGKSTF TRB:CASSMGQASYEQYF	1	CD4-C2
PB-2_GTTCGGGTCATAACCG	PB	PB-2	P2-2775	FALSE	1	TRA:CALSDNNAGNMLTF TRB:CASSQDRRNSGNTIYF	1	CD4-C2
PB-2_GTTCTCGAGGCTCAGA	PB	PB-2	P2-2776	FALSE	1	TRA:CIVGRYSGGGADGLTF TRB:CASSLVFDRTSGNTIYF	1	CD4-C1
PB-2_GTTTCTAAGCGTCTAT	PB	PB-2	P2-2780	FALSE	1	TRA:CAVSFADGLTF TRB:CASSLAARQSFNEQFF	1	CD4-C2
PB-2_TAAACCGGTTACAGAA	PB	PB-2	P2-2787	FALSE	1	TRA:CAASMDNDMRF TRB:CSARPGDTQYF	1	CD4-C2
PB-2_TAAGAGACATATGCTG	PB	PB-2	P2-356	FALSE	2	TRA:CALTLNNARLMF TRB:CASSQGPFSNQPQHF	2	CD4-C2
PB-2_TAAGAGATCCGGCACA	PB	PB-2	P2-2794	FALSE	1	TRA:CALRPNQGGKLIF TRB:CSAKENRGNSPLHF	1	CD4-C2
PB-2_TAAGCGTAGATGGGTC	PB	PB-2	P2-2796	FALSE	1	TRA:CAVNMRTGGFKTIF TRB:CATSDPVGVYF	1	CD4-C2
PB-2_TAAGCGTAGTTCCACA	PB	PB-2	P2-2799	FALSE	1	TRA:CAGGLSGNTPLVF TRB:CASSRGLGSPLHF	1	CD4-C3

PB-2_TAAGCGTCAGTCAGAG	PB	PB-2	P2-2800	FALSE	1	TRA:CADSPYSGAGSYQLTF TRB:CASSQGTGRETQYF	1	CD4-C2
PB-2_TAAGCGTGTCCGTCAG	PB	PB-2	P2-2801	FALSE	1	TRA:CAVRRGGADGLTF TRB:CSASNMRGANSPLHF	1	CD4-C2
PB-2_TAAGCGTTCTCCAGGG	PB	PB-2	P2-2802	FALSE	1	TRA:CALTTPTGNQFYF TRB:CASSLPQGINYGYTF	1	CD4-C2
PB-2_TACACGACAGAAGCAC	PB	PB-2	P2-2810	FALSE	1	TRA:CAMRGTGNQFYF TRB:CASSKQVGDYGYTF	1	CD4-C1
PB-2_TACACGATCCCGGATG	PB	PB-2	P2-2812	FALSE	1	TRA:CAFISTTSGTYKYIF TRB:CASSDRPGTDTQYF	1	CD4-C2
PB-2_TACAGTGCAAGCTGAG	PB	PB-2	P2-2813	FALSE	1	TRA:CAMRSGNTPLVF TRB:CSGAPGNSPLHF	1	CD4-C2
PB-2_TACAGTGCACGAGGTA	PB	PB-2	P2-2814	FALSE	1	TRA:CAASLNSGGSNYKLTF TRB:CASSSGQGYGYTF	1	CD4-C1
PB-2_TACAGTGCATAGTAAG	PB	PB-2	P2-2817	FALSE	1	TRA:CAVPIGLTGGGNKLTF TRB:CASSGVGQGRKGYTF	1	CD4-C2
PB-2_TACAGTGGTTAGTGGG	PB	PB-2	P2-2818	FALSE	1	TRA:CALAGGTSYGKLTF TRB:CASSPIRTGGTYGYTF	1	CD4-C3
PB-2_TACAGTGGTTCCTCCA	PB	PB-2	P2-2819	FALSE	1	TRA:CATARGGATNKLIF TRB:CSAEGLNSPLHF	1	CD4-C2
PB-2_TACCTATAGATCCCAT	PB	PB-2	P2-2821	FALSE	1	TRA:CAVRPLWGYQKVTF TRB:CASSYLGTGTDTQYF	1	CD4-C1
PB-2_TACCTATAGCCAGGAT	PB	PB-2	P2-2822	FALSE	1	TRA:CAFMKHVGARLMF TRB:CASSKEGGGAYEQYF	1	CD4-C2
PB-2_TACCTATTCGGCGGTT	PB	PB-2	P2-2830	FALSE	1	TRA:CALSGGTSGSRLTF TRB:CAIRDRDRGFEAFF	1	CD4-C2
PB-2_TACCTTACAAAGGCGT	PB	PB-2	P2-2832	FALSE	1	TRA:CALSRSTSYGKLTF TRB:CASSGAGTGEANEQFF	1	CD4-C2
PB-2_TACGGATCAGTAAGAT	PB	PB-2	P2-2836	FALSE	1	TRA:CAERWDNAGNMLTF TRB:CASSLGGTDTQYF	1	CD4-C2
PB-2_TACGGATTCGGACAAG	PB	PB-2	P2-2837	FALSE	1	TRA:CAVRDMASYQKVTF TRB:CATSDSVGANTGELFF	1	CD4-C7
PB-2_TACGGGCTCGTAGGAG	PB	PB-2	P2-2842	FALSE	1	TRA:CAMSVTGGFKTIF TRB:CASSPTVSTDTQYF	1	CD4-C1
PB-2_TACGGTACATGGATGG	PB	PB-2	P2-2846	FALSE	1	TRA:CAVGGNRDDKIIF TRB:CATGAGRQGKNTGELFF	1	CD4-C2
PB-2_TACGGTATCGATAGAA	PB	PB-2	P2-2847	FALSE	1	TRA:CLVGDGGGGFKTIF TRB:CASSTGGGRGKNIQYF	1	CD4-C3
PB-2_TACTCGCAGGAATCGC	PB	PB-2	P2-2855	FALSE	1	TRA:CAASNNAGNMLTF TRB:CASTITFGDGEQFF	1	CD4-C2
PB-2_TACTCGCCAAAGGCGT	PB	PB-2	P2-2857	FALSE	1	TRA:CAYRSRAGNTPLVF TRB:CASSFVGIMNTEAFF	1	CD4-C2
PB-2_TACTCGCGTAAGTGTA	PB	PB-2	P2-2858	FALSE	1	TRA:CAVSSQAGTALIF TRB:CSARDLAKPTDTQYF	1	CD4-C2
PB-2_TACTCGCGTCGAATCT	PB	PB-2	P2-2859	FALSE	1	TRA:CAEGRGGQKLLF TRB:CASSLVGQGTDTQYF	1	CD4-C2
PB-2_TACTCGCGTTCCCTTG	PB	PB-2	P2-2860	FALSE	1	TRA:CASPPPMSGGGGADGLTF TRB:CASSTRQGANTGELFF	1	CD4-C2
PB-2_TACTTACCACCACGTG	PB	PB-2	P2-2862	FALSE	1	TRA:CAVNAPTSYDKVIF TRB:CASSFPASHIYNEQFF	1	CD4-C2
PB-2_TACTTACTCAATCACG	PB	PB-2	P2-2863	FALSE	1	TRA:CAMREGGGGNQFYF TRB:CASSFGAQETQYF	1	CD4-C1
PB-2_TACTTGTAGATTACCC	PB	PB-2	P2-2864	FALSE	1	TRA:CVVVGSGAGSYQLTF TRB:CASSLLSATPLGQYF	1	CD4-C2

PB-2_TACTTGTAGCTTATCG	PB	PB-2	P2-2865	FALSE	1	TRA:CAVRQGGSEKLVF TRB:CAWSKHNEKLFF	1	CD4-C1
PB-2_TACTTGTCATGCAACT	PB	PB-2	P2-2868	FALSE	1	TRA:CGTENQTILNFGNEKLTF TRB:CASSLGLIGAQPQHF	1	CD4-C1
PB-2_TAGACCAAGACAAGCC	PB	PB-2	P2-2870	FALSE	1	TRA:CVGQVTGTASKLTF TRB:CASSLGQGFHNSPLHF	1	CD4-C1
PB-2_TAGACCAGTGGCTCCA	PB	PB-2	P2-2873	FALSE	1	TRA:CLVAPASNYQLIW TRB:CASSLAGMNNEQFF	1	CD4-C2
PB-2_TAGACCATCTGCGTAA	PB	PB-2	P2-2876	FALSE	1	TRA:CALSEGGGGADGLTF TRB:CATSDFTHSSAGGADTQYF	1	CD4-C2
PB-2_TAGAGCTCATCCGCGA	PB	PB-2	P2-2879	FALSE	1	TRA:CAVLNSGNTPLVF TRB:CSAMGLAGVEQFF	1	CD8-C6
PB-2_TAGCCGGGTACCCAAT	PB	PB-2	P2-2885	FALSE	1	TRA:CAGPSGGSYIPTF TRB:CASSEGGTVTGNTIYF	1	CD4-C2
PB-2_TAGGCATAGCCAGTAG	PB	PB-2	P2-2887	FALSE	1	TRA:CAVTPPGGGNKLTF TRB:CASSSHSWGGYTF	1	CD4-C2
PB-2_TAGGCATAGGATATAC	PB	PB-2	P2-2888	FALSE	1	TRA:CALELGGGADGLTF TRB:CASSLGGRGRPGYTF	1	CD4-C2
PB-2_TAGTGGTAGATGGGTC	PB	PB-2	P2-2890	FALSE	1	TRA:CAVQANYGQNFVF TRB:CASSLTPSPSDTQYF	1	CD4-C2
PB-2_TAGTGGTCACAGACAG	PB	PB-2	P2-2891	FALSE	1	TRA:CALDTFGNDMRF TRB:CASSSAGHINEKLFF	1	CD4-C2
PB-2_TAGTGGTGTCGGATCC	PB	PB-2	P2-2893	FALSE	1	TRA:CAVAGGFKTIF TRB:CSAESREGRDTDTQYF	1	CD4-C3
PB-2_TAGTGGTTCTTGAGGT	PB	PB-2	P2-2897	FALSE	1	TRA:CATLHSGNTPLVF TRB:CASSLVSGSTNEQFF	1	CD4-C1
PB-2_TAGTTGGAGCTACCGC	PB	PB-2	P2-2899	FALSE	1	TRA:CAGRGSNFGNEKLTF TRB:CASSLGIANTGELFF	1	CD4-C2
PB-2_TAGTTGGAGGAGTACC	PB	PB-2	P2-2900	FALSE	1	TRA:CAFMKDNARLMF TRB:CASSPAGGDDNEQFF	1	CD4-C2
PB-2_TAGTTGGCATACTACG	PB	PB-2	P2-2902	FALSE	1	TRA:CALPIQGGAQKLVF TRB:CASRTSGTGEQYF	1	CD4-C2
PB-2_TAGTTGGGTACCGCTG	PB	PB-2	P2-2904	FALSE	1	TRA:CAVDRNNDMRF TRB:CSARTGGYEQYF	1	CD4-C2
PB-2_TATCAGGAGTCTCCTC	PB	PB-2	P2-2907	FALSE	1	TRA:CAASFLGNQFYF TRB:CASRRAPLAPNEKLFF	1	CD4-C2
PB-2_TATCAGGCACAGATTC	PB	PB-2	P2-2908	FALSE	1	TRA:CGADGRNEKLTF TRB:CASSPPGLATQYF	1	CD4-C2
PB-2_TATCAGGCATCTCGCT	PB	PB-2	P2-2909	FALSE	1	TRA:CAVIQRGGNEKLTF TRB:CSAARGSYNEQFF	1	CD4-C2
PB-2_TATCAGGGTTTGTTGG	PB	PB-2	P2-2912	FALSE	1	TRA:CAYRTRLSQGAQKLVF TRB:CASSTYNQPQHF	1	CD4-C3
PB-2_TATCAGGTCATGTCCC	PB	PB-2	P2-2913	FALSE	1	TRA:CAVVNQAGTALIF TRB:CASSQAVGGGWKQFF	1	CD4-C2
PB-2_TATCAGGTCGTTACAG	PB	PB-2	P2-361	FALSE	2	TRA:CAVRDMRGSTLGRLYF TRB:CASEEYKAGGDTGELFF	2	CD4-C2
PB-2_TATCTCACAAGCCGCT	PB	PB-2	P2-2917	FALSE	1	TRA:CAGPETSGSRLTF TRB:CASSYNGGYYGYTF	1	CD4-C2
PB-2_TATCTCATCTTGAGGT	PB	PB-2	P2-2922	FALSE	1	TRA:CASTGTASKLTF TRB:CASSPRWGGTDTQYF	1	CD4-C1
PB-2_TATGCCCGTCCGTCAG	PB	PB-2	P2-2927	FALSE	1	TRA:CALSTDTGFQKLVF TRB:CASSEARGGGYTF	1	CD4-C2
PB-2_TATGCCCGTTTGTGTG	PB	PB-2	P2-2930	FALSE	1	TRA:CAVRPSFGNEKLTF TRB:CASASGQTDTQYF	1	CD4-C2

PB-2_TATGCCCTCAGTGTTG	PB	PB-2	P2-2931	FALSE	1	TRA:CAELYSSASKIIF TRB:CSASLASETQYF	1	CD4-C1
PB-2_TATTACCTCATCACCC	PB	PB-2	P2-2936	FALSE	1	TRA:CLLVLNTDKLIF TRB:CASSPNRRGAGGELFF	1	CD4-C2
PB-2_TCACAAGCATCACGTA	PB	PB-2	P2-2946	FALSE	1	TRA:CALSGGNTPLVF TRB:CASSRPGGGYTDTQYF	1	CD4-C2
PB-2_TCACAAGGTAAATGTG	PB	PB-2	P2-2947	FALSE	1	TRA:CAATGSSGYALNF TRB:CASSPTGAGKEEQYF	1	CD4-C2
PB-2_TCACAAGGTCGAGTTT	PB	PB-2	P2-2948	FALSE	1	TRA:CAVQEETSGSRLTF TRB:CASSGTSGTFTQYF	1	CD4-C1
PB-2_TCACGAACAGTACACT	PB	PB-2	P2-2951	FALSE	1	TRA:CGTEGDKAAGNKLTF TRB:CASSRMGLAGSTQETQYF	1	CD4-C3
PB-2_TCACGAATCACTTATC	PB	PB-2	P2-2954	FALSE	1	TRA:CVSQAGTALIF TRB:CSARVWGQETQYF	1	CD4-C1
PB-2_TCAGATGAGTGATCGG	PB	PB-2	P2-2956	FALSE	1	TRA:CAGLIQGAQKLVF TRB:CASSLILGPVDSPLHF	1	CD4-C2
PB-2_TCAGATGCATTGCGGC	PB	PB-2	P2-2958	FALSE	1	TRA:CAVNPYGQKLLF TRB:CASSQEVTDTQYF	1	CD4-C3
PB-2_TCAGCAAAGATCCCAT	PB	PB-2	P2-2960	FALSE	1	TRA:CAVSENTGNQFYF TRB:CASSETPRGPLETQYF	1	CD4-C2
PB-2_TCAGCAACAAACGCGA	PB	PB-2	P2-2962	FALSE	1	TRA:CALGAGSEKLVF TRB:CASTLEGSPNQPQHF	1	CD4-C2
PB-2_TCAGCAACACAGGAGT	PB	PB-2	P2-2964	FALSE	1	TRA:CAMGFGNEKLTF TRB:CSVDGDNTDTQYF	1	CD4-C2
PB-2_TCAGCAACACATTCGA	PB	PB-2	P2-2965	FALSE	1	TRA:CATPEGGAQKLVF TRB:CAISVGTGLRSEQYF	1	CD4-C1
PB-2_TCAGCAACATCTGGTA	PB	PB-2	P2-2966	FALSE	1	TRA:CAENNAGNMLTF TRB:CASSEYNSIYGYTF	1	CD4-C1
PB-2_TCAGCAAGTAGTACCT	PB	PB-2	P2-2967	FALSE	1	TRA:CALSTARLMF TRB:CASSIAWASGTYNEQFF	1	CD4-C2
PB-2_TCAGCAAGTGTGAATA	PB	PB-2	P2-2968	FALSE	1	TRA:CALRITGANSKLTF TRB:CSACPGLGDTYEQYF	1	CD4-C2
PB-2_TCAGCTCCACCCTATC	PB	PB-2	P2-2973	FALSE	1	TRA:CIVRVGGGSEKLVF TRB:CASSQVQHLGQPQHF	1	CD4-C2
PB-2_TCAGCTCGTTCCTCCA	PB	PB-2	P2-2974	FALSE	1	TRA:CAVEPSKRGADKLIF TRB:CASSFRTGSTDTQYF	1	CD4-C2
PB-2_TCAGCTCTCGAACGGA	PB	PB-2	P2-2975	FALSE	1	TRA:CAPFSSGSARQLTF TRB:CAWSLTAGAEQFF	1	CD4-C1
PB-2_TCAGGATTCCCAAGTA	PB	PB-2	P2-2977	FALSE	1	TRA:CAVKETSGSRLTF TRB:CASSPAVTGELFF	1	CD4-C2
PB-2_TCAGGTACAATGGTCT	PB	PB-2	P2-2981	FALSE	1	TRA:CAVSRAEYGNKLVF TRB:CSVTSGSSNTGELFF	1	CD4-C7
PB-2_TCAGGTAGTAGCGCTC	PB	PB-2	P2-2983	FALSE	1	TRA:CAENRVTF TRB:CASSSTGTSKDGEQFF	1	CD4-C2
PB-2_TCAGGTAGTATCGCAT	PB	PB-2	P2-2984	FALSE	1	TRA:CAVSFKGGATNKLIF TRB:CASSLSGMHYEQYF	1	CD4-C2
PB-2_TCAGGTAGTTACAGAA	PB	PB-2	P2-2985	FALSE	1	TRA:CAMRGVYNQGGKLIF TRB:CASSSPGSGYNEQFF	1	CD4-C2
PB-2_TCAGGTAGTTAGGGTG	PB	PB-2	P2-2986	FALSE	1	TRA:CAVTPGRGLSF TRB:CASSSLSGSDEQFF	1	CD4-C3
PB-2_TCATTACCAAGGGTCA	PB	PB-2	P2-2989	FALSE	1	TRA:CAMREAVL TRB:CASSFGGRNQPQHF	1	CD4-C2
PB-2_TCATTACGTTAAAGTG	PB	PB-2	P2-2993	FALSE	1	TRA:CAVGSSGNTPLVF TRB:CASSYGQFTDTQYF	1	CD4-C2

PB-2_TCATTACTCGCAAGCC	PB	PB-2	P2-2997	FALSE	1	TRA:CAVSDHTGGFKTIF TRB:CASSLEPTDYGYTF	1	CD4-C1
PB-2_TCATTTGCAGCTCCGA	PB	PB-2	P2-2998	FALSE	1	TRA:CAASRSGLTSGTYKYIF TRB:CASSGTGMSNQPQHF	1	CD4-C2
PB-2_TCATTTGCAGTCAGAG	PB	PB-2	P2-2999	FALSE	1	TRA:CVVKGRDGQKLLF TRB:CSGIGTGTYEQYF	1	CD4-C5
PB-2_TCATTTGTCTTGCCGT	PB	PB-2	P2-3001	FALSE	1	TRA:CLVGSTGFQKLVF TRB:CAISESAGGSYEQYF	1	CD4-C1
PB-2_TCCCGATCAGAGTGTG	PB	PB-2	P2-3008	FALSE	1	TRA:CAVNARSGAGSYQLTF TRB:CASSLVGVGNQPQHF	1	CD4-C2
PB-2_TCCCGATGTAACGACG	PB	PB-2	P2-3009	FALSE	1	TRA:CADYPNTGNQFYF TRB:CASSQEAAGAQYF	1	CD4-C2
PB-2_TCCCGATGTAAGGATT	PB	PB-2	P2-3010	FALSE	1	TRA:CALDNAGNMLTF TRB:CASSPLSASTDTQYF	1	CD4-C2
PB-2_TCCCGATGTCAGGACA	PB	PB-2	P2-3011	FALSE	1	TRA:CAASNPHGSSASKIIF TRB:CASTQSNQPQHF	1	CD4-C2
PB-2_TCGCGAGGTTGCGTTA	PB	PB-2	P2-3016	FALSE	1	TRA:CAANSITGGGNKLTF TRB:CASSLGAVETEAFF	1	CD4-C2
PB-2_TCGCGTTAGGCATGTG	PB	PB-2	P2-3018	FALSE	1	TRA:CAAFHGSGNTGKLIF TRB:CASSLGQGTETQYF	1	CD4-C1
PB-2_TCGCGTTCAGGGATTG	PB	PB-2	P2-3020	FALSE	1	TRA:CAVMNRNNNARLMF TRB:CASSHPPTNEKLFF	1	CD4-C1
PB-2_TCGCGTTCAGTAACGG	PB	PB-2	P2-363	TRUE	2	TRA:CAVTSTGGFKTIF TRB:CASSLGASGSLYEQYF	2	CD4-C2
PB-2_TCGGGACAGCACCGTC	PB	PB-2	P2-336	FALSE	2	TRA:CAVPGRGFNKFYF TRB:CASSLGGYQQFF	2	CD4-C2
PB-2_TCGGTAACAAACCCAT	PB	PB-2	P2-3027	FALSE	1	TRA:CAVEAGTASKLTF TRB:CATASGEANYGYTF	1	CD4-C2
PB-2_TCGGTAATCCACTGGG	PB	PB-2	P2-3029	FALSE	1	TRA:CALTENTGNQFYF TRB:CASSLVQGSTYEQYF	1	CD4-C2
PB-2_TCGTAGAAGCCGTCGT	PB	PB-2	P2-3033	FALSE	1	TRA:CAVGLAGGFKTIF TRB:CSARDVLAGGLSSYNEQFF	1	CD4-C3
PB-2_TCGTAGATCCAACCAA	PB	PB-2	P2-3037	FALSE	1	TRA:CAMRDAFSGGYQKVTF TRB:CASSQQQYF	1	CD4-C2
PB-2_TCTATTGCACGGACAA	PB	PB-2	P2-3039	FALSE	1	TRA:CAVPTTDQGGSEKLVF TRB:CARGPSSGTGELFF	1	CD4-C1
PB-2_TCTATTGCACGTCAGC	PB	PB-2	P2-3040	FALSE	1	TRA:CAVQAGSGNQFYF TRB:CSAYSTTVGYTF	1	CD4-C2
PB-2_TCTATTGGTCTTGCGG	PB	PB-2	P2-3043	FALSE	1	TRA:CAVSATGGKLIF TRB:CASLAGAVADTQYF	1	CD4-C1
PB-2_TCTATTGTCAAGGCTT	PB	PB-2	P2-3044	FALSE	1	TRA:CAGRRGGYQKVTF TRB:CASSHNTGAPQETQYF	1	CD4-C2
PB-2_TCTCATATCGAATCCA	PB	PB-2	P2-3049	FALSE	1	TRA:CALSEAGTNSGYALNF TRB:CASSVGGTSTDTQYF	1	CD4-C2
PB-2_TCTCTAAAGGTAAACT	PB	PB-2	P2-3050	FALSE	1	TRA:CAATGSNSGYALNF TRB:CASSRDRGVTGELFF	1	CD4-C1
PB-2_TCTCTAAGTCACAAGG	PB	PB-2	P2-3051	FALSE	1	TRA:CAVLYTGGFKTIF TRB:CASSHQLKKTGYNSPLHF	1	CD4-C2
PB-2_TCTCTAAGTCGGATCC	PB	PB-2	P2-3053	FALSE	1	TRA:CLVARNTGGFKTIF TRB:CSVVGGTNTGELFF	1	CD4-C2
PB-2_TCTCTAATCCTGCCAT	PB	PB-2	P2-3056	FALSE	1	TRA:CATEMNNNARLMF TRB:CASGLVVGQGNEQFF	1	CD4-C2
PB-2_TCTGAGAAGCACACAG	PB	PB-2	P2-3058	FALSE	1	TRA:CAVRRNQAGTALIF TRB:CSVTPGTGGRETQYF	1	CD4-C2

PB-2_TCTGAGAGTTCAACCA	PB	PB-2	P2-3062	FALSE	1	TRA:CAYRSGEGNTGTASKLTF TRB:CASSPLYGNTIYF	1	CD4-C2
PB-2_TCTGGAAGTGTAATGA	PB	PB-2	P2-3063	FALSE	1	TRA:CAVTLAGAYKYIF TRB:CASTPVRTQNEQYF	1	CD4-C2
PB-2_TCTTCGGAGTACTTGC	PB	PB-2	P2-3066	FALSE	1	TRA:CAASNSGGGADGLTF TRB:CASSPSDRGREQYF	1	CD4-C1
PB-2_TCTTCGGGTCCGAATT	PB	PB-2	P2-366	FALSE	2	TRA:CIVRVPPNNAGNMLTF TRB:CASSYESSGRTDTQYF	2	CD4-C2
PB-2_TCTTTCCAGCTGCCCA	PB	PB-2	P2-3074	FALSE	1	TRA:CAENTNAGGTSYGKLTF TRB:CASSWTDQPQHF	1	CD4-C2
PB-2_TCTTTCCCATTTCACT	PB	PB-2	P2-3077	FALSE	1	TRA:CAVARRNQGGKLIF TRB:CASFEGGTQYF	1	CD4-C1
PB-2_TGAAAGAAGATTACCC	PB	PB-2	P2-3079	FALSE	1	TRA:CATVPNAGGTSYGKLTF TRB:CASSLAFGTGNTIYF	1	CD4-C2
PB-2_TGAAAGAGTCGTGGCT	PB	PB-2	P2-3081	FALSE	1	TRA:CAVGFGNVLHC TRB:CATSYYYEQYF	1	CD4-C2
PB-2_TGAAAGATCCGCATAA	PB	PB-2	P2-3084	FALSE	1	TRA:CVVDQFYF TRB:CASSAYDGSPLHF	1	CD4-C2
PB-2_TGACAACAGTCACGCC	PB	PB-2	P2-3085	FALSE	1	TRA:CAVLFSGGYNKLIF TRB:CASSLRTGTGVYEKLFF	1	CD4-C1
PB-2_TGACAACAGTTGTAGA	PB	PB-2	P2-3087	FALSE	1	TRA:CAGARNNDMRF TRB:CSARDSRQGYMGELFF	1	CD4-C1
PB-2_TGACAACCAGTGACAG	PB	PB-2	P2-3089	FALSE	1	TRA:CAYRSASNARLMF TRB:CASSPGWAEQYF	1	CD4-C2
PB-2_TGACGGCCATGCTGGC	PB	PB-2	P2-3093	FALSE	1	TRA:CIVRHQGAQKLVF TRB:CASSLDGRSSYEQYF	1	CD4-C2
PB-2_TGACGGCCATTGGGCC	PB	PB-2	P2-3094	FALSE	1	TRA:CAPDSSASKIIF TRB:CSARGRDSNNSPLHF	1	CD4-C2
PB-2_TGACGGCTCAGCTCGG	PB	PB-2	P2-3096	FALSE	1	TRA:CAETYTGTASKLTF TRB:CASSMIRQPQHF	1	CD4-C1
PB-2_TGACGGCTCTACTATC	PB	PB-2	P2-3099	FALSE	1	TRA:CAGPASYGKLTF TRB:CASRPSAGNYGYTF	1	CD4-C1
PB-2_TGACTAGCAAGTCATC	PB	PB-2	P2-3101	FALSE	1	TRA:CAVGSDRGSTLGRLYF TRB:CASSSQSNTGELFF	1	CD4-C1
PB-2_TGACTAGCAAGTTAAG	PB	PB-2	P2-3102	FALSE	1	TRA:CAASMRNNNARLMF TRB:CASTSSFGTGEKLFF	1	CD4-C2
PB-2_TGACTAGCACAACGTT	PB	PB-2	P2-3103	FALSE	1	TRA:CAADLTGKLIF TRB:CSARSPGTPAGETQYF	1	CD4-C2
PB-2_TGACTAGCACTGTTAG	PB	PB-2	P2-3104	FALSE	1	TRA:CAVVTGGGNKLTF TRB:CAGKPQPLSQETQYF	1	CD4-C1
PB-2_TGACTAGCAGACAAAT	PB	PB-2	P2-3105	FALSE	1	TRA:CAVNSRGGSNYKLTF TRB:CASSSMGGSSYEQYF	1	CD4-C1
PB-2_TGACTAGGTTGGACCC	PB	PB-2	P2-3109	FALSE	1	TRA:CAVKGRGRTSGSRLTF TRB:CASSRHLGGERNEQYF	1	CD4-C2
PB-2_TGAGAGGGTAAACGCG	PB	PB-2	P2-3117	FALSE	1	TRA:CAENIYSGGSNYKLTF TRB:CASKSPTNEKLFF	1	CD4-C3
PB-2_TGAGCATAGAGTAAGG	PB	PB-2	P2-3119	FALSE	1	TRA:CAGGGSNYKLTF TRB:CASSLYVLELFF	1	CD4-C2
PB-2_TGAGCATAGTAGCCGA	PB	PB-2	P2-3120	FALSE	1	TRA:CAVKRGSTLGRLYF TRB:CASSPPGQAGNEQFF	1	CD4-C1
PB-2_TGAGCCGAGTTTAGGA	PB	PB-2	P2-3123	FALSE	1	TRA:CAGRPYGPASKLTF TRB:CASSPPGTGPVREQFF	1	CD4-C1
PB-2_TGAGCCGGTAAAGGAG	PB	PB-2	P2-3125	FALSE	1	TRA:CAVGVQGAQKLVF TRB:CASSENTGYNEQFF	1	CD4-C1

PB-2_TGAGGGAGTATGAAAC	PB	PB-2	P2-3132	FALSE	1	TRA:CALSEGSGANSKLTF TRB:CASSTGGNQETQYF	1	CD4-C2
PB-2_TGAGGGATCTGTTGAG	PB	PB-2	P2-355	FALSE	2	TRA:CAARRSNYQLIW TRB:CASSLGGFGYTF	2	CD4-C2
PB-2_TGCACCTGTCAAAGAT	PB	PB-2	P2-3142	FALSE	1	TRA:CATVSVSGSARQLTF TRB:CASSQGLNGQFGKKFF	1	CD4-C2
PB-2_TGCACCTGTCTGGAGA	PB	PB-2	P2-3143	FALSE	1	TRA:CAVTRSGGYQKVTF TRB:CASSLNPGSTGANVLTF	1	CD4-C2
PB-2_TGCACCTTCAACACAC	PB	PB-2	P2-3145	FALSE	1	TRA:CAVGIFGNEKLTF TRB:CASNRGTDTQYF	1	CD4-C2
PB-2_TGCCAAAAGTACGCCC	PB	PB-2	P2-366	FALSE	2	TRA:CIVRVPPNNAGNMLTF TRB:CASSYESSGRTDTQYF	2	CD4-C2
PB-2_TGCCAAACACGGTTTA	PB	PB-2	P2-3149	FALSE	1	TRA:CALSQWGQNFVF TRB:CSGTGTNEETQYF	1	CD4-C1
PB-2_TGCCAAACAGACTCGC	PB	PB-2	P2-3150	FALSE	1	TRA:CAVRGGGGFKTIF TRB:CASSFVTDTQYF	1	CD4-C2
PB-2_TGCCCATGTTACGACT	PB	PB-2	P2-337	FALSE	2	TRA:CAPQNQAGTALIF TRB:CASSIVGPNSQPQHF	2	CD4-C2
PB-2_TGCCCTAAGTTGTAGA	PB	PB-2	P2-3158	FALSE	1	TRA:CASQKTGANNLFF TRB:CASSFSGGAYTQYF	1	CD4-C2
PB-2_TGCCCTACATGGATGG	PB	PB-2	P2-3159	FALSE	1	TRA:CAVGDNSGYSTLTF TRB:CAWARPVDNSPLHF	1	CD4-C2
PB-2_TGCCCTACATGTCTCC	PB	PB-2	P2-362	FALSE	2	TRA:CAPRNTGFQKLVF TRB:CASSFEGISYEQYF	2	CD4-C2
PB-2_TGCCCTAGTACAGACG	PB	PB-2	P2-3161	FALSE	1	TRA:CALSEGNDKIIF TRB:CASSIATGSNQPQHF	1	CD4-C1
PB-2_TGCCCTATCACATACG	PB	PB-2	P2-3163	FALSE	1	TRA:CAASAQGTDKLIF TRB:CASSFRQGSFENEQFF	1	CD4-C2
PB-2_TGCCCTATCTAAGCCA	PB	PB-2	P2-3164	FALSE	1	TRA:CAAPIKTSYDKVIF TRB:CASSLSGTGANEQFF	1	CD4-C1
PB-2_TGCGCAGCAAGTCTAC	PB	PB-2	P2-3166	FALSE	1	TRA:CAVSGNQGGKLIF TRB:CSARNTLATNEKLFF	1	CD4-C2
PB-2_TGCGCAGCATATGCTG	PB	PB-2	P2-3168	FALSE	1	TRA:CATAPNSGGYQKVTF TRB:CASSATGPQHF	1	CD4-C2
PB-2_TGCGCAGCATCGGACC	PB	PB-2	P2-3169	FALSE	1	TRA:CAVQANYGQNFVF TRB:CASGSPGVGKLFF	1	CD4-C2
PB-2_TGCGCAGTCCGTACAA	PB	PB-2	P2-3172	FALSE	1	TRA:CAVKTQGGSEKLVF TRB:CSAISYTDTQYF	1	CD4-C1
PB-2_TGCGGGTCAAGAAAGG	PB	PB-2	P2-3174	FALSE	1	TRA:CAAPADSGYALNF TRB:CASSFQQGAGETQYF	1	CD4-C3
PB-2_TGCGGGTGTACCGTAT	PB	PB-2	P2-3175	FALSE	1	TRA:CAGMSGGSYIPTF TRB:CASSQGFGTGGRTQHF	1	CD4-C1
PB-2_TGCGGGTTCGGACAAG	PB	PB-2	P2-3176	FALSE	1	TRA:CATVPNTGTASKLTF TRB:CATSGGRQASTDTQYF	1	CD4-C2
PB-2_TGCGTGGAGCACGCCT	PB	PB-2	P2-3177	FALSE	1	TRA:CAEKEGTGANNLFF TRB:CASSPATNLNEQFF	1	CD4-C3
PB-2_TGCGTGGAGTCACGCC	PB	PB-2	P2-3179	FALSE	1	TRA:CAGLNTGTASKLTF TRB:CASSPARLGPTQYF	1	CD4-C2
PB-2_TGCGTGGCAATGACCT	PB	PB-2	P2-3182	FALSE	1	TRA:CAEKFAGYSTLTF TRB:CASSQAFDTGELFF	1	CD4-C2
PB-2_TGCGTGGGTCACAAGG	PB	PB-2	P2-3184	FALSE	1	TRA:CAVATPGAQKLVF TRB:CASATGFTSTDTQYF	1	CD4-C2
PB-2_TGCGTGGGTGGCTCCA	PB	PB-2	P2-3185	FALSE	1	TRA:CAISQGGKLIF TRB:CSAIAGGPEETQYF	1	CD4-C1

PB-2_TGCGTGGGTGTCGCTG	PB	PB-2	P2-3186	FALSE	1	TRA:CALRWENTPLVF TRB:CASRRDSLNTEAFF	1	CD4-C2
PB-2_TGCTACCAGCTCCCAG	PB	PB-2	P2-3189	FALSE	1	TRA:CAMREGRWYSSASKIIF TRB:CASSPEATGGGRLLSNQPQ	1	CD4-C2
PB-2_TGCTGCTGTTCCCTTG	PB	PB-2	P2-3195	FALSE	1	TRA:CAVRHMEYGNKLVF TRB:CASSEGQLNSPLHF	1	CD4-C1
PB-2_TGGACGCCATCTCCCA	PB	PB-2	P2-3199	FALSE	1	TRA:CAAFGGSNYKLTF TRB:CASSPSLAGYNEQFF	1	CD4-C2
PB-2_TGGCCAGGTGCTGTAT	PB	PB-2	P2-3201	FALSE	1	TRA:CVVSGNTGRRALTF TRB:CASSQDLLRAQETQYF	1	CD4-C1
PB-2_TGGCGCATCGAGAGCA	PB	PB-2	P2-3206	FALSE	1	TRA:CAMSKGTYKYIF TRB:CATSDRGPSYEQYF	1	CD4-C1
PB-2_TGGCTGGAGTTGAGAT	PB	PB-2	P2-3208	FALSE	1	TRA:CAASNSGGSGNTGKLIF TRB:CASSTRDSNEKLFF	1	CD4-C1
PB-2_TGGCTGGGTACATCCA	PB	PB-2	P2-3211	FALSE	1	TRA:CAYRSGIQGAQKLVF TRB:CASRVSMNTEAFF	1	CD4-C1
PB-2_TGGCTGGGTTTGACAC	PB	PB-2	P2-3212	FALSE	1	TRA:CAARGTSGSRLTF TRB:CASSSTGGSNSNQPQHF	1	CD4-C2
PB-2_TGGCTGGTCAAAGTAG	PB	PB-2	P2-3213	FALSE	1	TRA:CAYYREYNFNKFYF TRB:CASSRSQGANYGYTF	1	CD4-C2
PB-2_TGGGAAGGTGGCAAAC	PB	PB-2	P2-3221	FALSE	1	TRA:CALSQYTGGFKTIF TRB:CAIRAGETQYF	1	CD4-C1
PB-2_TGGGAAGTCTTCATGT	PB	PB-2	P2-3224	FALSE	1	TRA:CVVSGDLSGTYKYIF TRB:CASSSRTGVNNQPQHF	1	CD4-C1
PB-2_TGGGCGTCAGTCAGCC	PB	PB-2	P2-3225	FALSE	1	TRA:CAMRGNNNARLMF TRB:CASTTTGHYEQYF	1	CD4-C2
PB-2_TGGGCGTTCTCAAGTG	PB	PB-2	P2-3227	FALSE	1	TRA:CVSGSRLTF TRB:CSARESGPPAADTQYF	1	CD4-C2
PB-2_TGGTTAGAGCCGGTAA	PB	PB-2	P2-3230	FALSE	1	TRA:CAVQNQAGTALIF TRB:CAIIGQGALTYEQYF	1	CD4-C3
PB-2_TGGTTAGAGTGAACGC	PB	PB-2	P2-356	FALSE	2	TRA:CALTLNNARLMF TRB:CASSQGPFSNQPQHF	2	CD4-C2
PB-2_TGGTTAGGTGCCTGGT	PB	PB-2	P2-3231	FALSE	1	TRA:CALSDQGGSQGNLIF TRB:CASRYGDLNTEAFF	1	CD4-C3
PB-2_TGGTTAGTCTCGCATC	PB	PB-2	P2-330	FALSE	2	TRA:CATDASYSGAGSYQLTF TRB:CASSLASGFQETQYF	2	CD4-C2
PB-2_TGGTTCCTCTCTTATG	PB	PB-2	P2-3236	FALSE	1	TRA:CAGYNTNAGKSTF TRB:CASSVGTGAAQETQYF	1	CD4-C7
PB-2_TGTATTCTCGGGAGTA	PB	PB-2	P2-3240	FALSE	1	TRA:CAYRSVYSGGGADGLTF TRB:CASSQPGTAWQETQYF	1	CD4-C2
PB-2_TGTATTCTCTCGAGTA	PB	PB-2	P2-3241	FALSE	1	TRA:CVVTLYYGGATNKLIF TRB:CSAKTTGQPQHF	1	CD4-C1
PB-2_TGTCCCAAGGGTGTGT	PB	PB-2	P2-3243	FALSE	1	TRA:CAAGNNDMRF TRB:CASSEGLLAGVNEQFF	1	CD4-C2
PB-2_TGTCCCACACTTGGAT	PB	PB-2	P2-3245	FALSE	1	TRA:CAVGEDSNYQLIW TRB:CASKGQGDNQPQHF	1	CD4-C1
PB-2_TGTCCCAGTAAGTTCC	PB	PB-2	P2-3247	FALSE	1	TRA:CAMSLAPYGNNRLAF TRB:CASKGSGGVTDTQYF	1	CD4-C1
PB-2_TGTCCCAGTTCCACTC	PB	PB-2	P2-3248	FALSE	1	TRA:CALNSFGNEKLTF TRB:CASSPRGGDEDTIYF	1	CD4-C2
PB-2_TGTGGTATCGCTAGCG	PB	PB-2	P2-3252	FALSE	1	TRA:CAVRGTGRRALTF TRB:CASSLSHQETQYF	1	CD4-C1
PB-2_TGTGTTTAGTCCAGGA	PB	PB-2	P2-3253	FALSE	1	TRA:CAVKGLRAAGNKLTF TRB:CASSPGGTSGTYEQYF	1	CD4-C1

PB-2_TGTGTTTCAATAGAGT	PB	PB-2	P2-3254	FALSE	1	TRA:CAVGAGNMLTF TRB:CSARDGQRVETQYF	1	CD4-C2
PB-2_TGTGTTTCACGGCCAT	PB	PB-2	P2-3256	FALSE	1	TRA:CIVRAEHAGNNRKLIW TRB:CAIPPRFSNQPQHF	1	CD4-C2
PB-2_TGTGTTTGTGCGATAG	PB	PB-2	P2-3257	FALSE	1	TRA:CASLSGRGGLGVTNSLS TRB:CAWGPGVGELFF	1	CD4-C1
PB-2_TGTTCCGAGTTCGATC	PB	PB-2	P2-3261	FALSE	1	TRA:CAAVAGSARQLTF TRB:CASSVVPGRAGNTIYF	1	CD4-C2
PB-2_TGTTCCGCAGATCGGA	PB	PB-2	P2-3263	FALSE	1	TRA:CAVRGGITGGFKTIF TRB:CASSDGQGSTQYF	1	CD4-C2
PB-2_TGTTCCGGTTTGCATG	PB	PB-2	P2-3265	FALSE	1	TRA:CALRGANAGNMLTF TRB:CSARSSAPSTDTQYF	1	CD4-C1
PB-2_TTAACTCCACAGCGTC	PB	PB-2	P2-3269	FALSE	1	TRA:CAALPTGGGNKLTF TRB:CASGPHTNTGELFF	1	CD4-C1
PB-2_TTAGGACCAAAGGTGC	PB	PB-2	P2-3272	FALSE	1	TRA:CAPRLDARLMF TRB:CASSQPGQGLEQYF	1	CD4-C1
PB-2_TTAGGACCAAGTCATC	PB	PB-2	P2-3273	FALSE	1	TRA:CASANTDKLIF TRB:CASASGELYGYTF	1	CD4-C3
PB-2_TTAGGACGTCGTCTTC	PB	PB-2	P2-3276	FALSE	1	TRA:CVVRRDYKLSF TRB:CASSSQGRDYGYTF	1	CD4-C2
PB-2_TTAGGCAAGTGCCATT	PB	PB-2	P2-3279	FALSE	1	TRA:CAVPTWSGGSNYKLTF TRB:CASGGALTGELFF	1	CD4-C2
PB-2_TTAGTTCAGATCGGGT	PB	PB-2	P2-3283	FALSE	1	TRA:CAENRSGGSNYKLTF TRB:CASSQGYAGELFF	1	CD4-C1
PB-2_TTATGCTCAGATCGGA	PB	PB-2	P2-3290	FALSE	1	TRA:CALHPNFGNEKLTF TRB:CASRRDGAMGSEQYF	1	CD4-C1
PB-2_TTCCCAGAGATGCCAG	PB	PB-2	P2-373	TRUE	2	TRA:CAEHNARLMF TRB:CASSYLDGTEAFF	2	CD4-C2
PB-2_TTCCCAGAGCTGCGAA	PB	PB-2	P2-3291	FALSE	1	TRA:CAGGGSNYKLTF TRB:CASSESQGSRVFF	1	CD4-C3
PB-2_TTCCCAGCAAGGTTTC	PB	PB-2	P2-3292	FALSE	1	TRA:CAVVFTGGGNKLTF TRB:CARTRGCEQFF	1	CD4-C1
PB-2_TTCGAAGTCAGTTGAC	PB	PB-2	P2-3300	FALSE	1	TRA:CATDVSGGYNKLIF TRB:CAQGRETQYF	1	CD4-C1
PB-2_TTCGAAGTCCTAGTGA	PB	PB-2	P2-3302	FALSE	1	TRA:CAVSWRGNEKLTF TRB:CASSLVRSADTQYF	1	CD4-C1
PB-2_TTCGGTCTCACGGTTA	PB	PB-2	P2-3307	FALSE	1	TRA:CAFMKIKSGANNLFF TRB:CASSLGWTDNSPLHF	1	CD4-C1
PB-2_TTCGGTCTCGTGGACC	PB	PB-2	P2-3308	FALSE	1	TRA:CAASKAGANSKLTF TRB:CASSYSAGARTEAFF	1	CD4-C1
PB-2_TTCTACAAGAACTGTA	PB	PB-2	P2-3309	FALSE	1	TRA:CASLSGNTPLVF TRB:CASSPRVTANTEAFF	1	CD4-C2
PB-2_TTCTACACATCACCCT	PB	PB-2	P2-3314	FALSE	1	TRA:CAMTKGGATNKLIF TRB:CASSRLAGPNNEQFF	1	CD4-C4
PB-2_TTCTCAAAGCTACCGC	PB	PB-2	P2-3317	FALSE	1	TRA:CLVGGSNYQLIW TRB:CASRIDSVRNEQFF	1	CD4-C7
PB-2_TTCTCCTCATGATCCA	PB	PB-2	P2-3322	FALSE	1	TRA:CAASGSGSARQLTF TRB:CASRVKGNRHEQYF	1	CD4-C1
PB-2_TTCTTAGTCACATACG	PB	PB-2	P2-3328	FALSE	1	TRA:CAAGATGTASKLTF TRB:CASVPTGGARAFF	1	CD4-C2
PB-2_TTGAACGCACGCCAGT	PB	PB-2	P2-3329	FALSE	1	TRA:CAASIRGRDDKIIF TRB:CASSDTPGVGDQPQHF	1	CD4-C1
PB-2_TTGAACGCATCACGTA	PB	PB-2	P2-3331	FALSE	1	TRA:CVVSDQRGAQKLVF TRB:CASSRDSSGNTIYF	1	CD4-C2

PB-2_TTGAACGGTGAGGGTT	PB	PB-2	P2-3333	FALSE	1	TRA:CAVPDFKAAGNKLTF TRB:CASSLTGTGFFYNEQFF	1	CD4-C2
PB-2_TTGAACGTCGCTGATA	PB	PB-2	P2-3334	FALSE	1	TRA:CAVGANSGYALNF TRB:CAWSVLMAYNEQFF	1	CD4-C2
PB-2_TTGACTTAGACCTTTG	PB	PB-2	P2-3338	FALSE	1	TRA:CAVRDMGSSSASKIIF TRB:CASSGLTEAFF	1	CD4-C2
PB-2_TTGCCGTAGATACACA	PB	PB-2	P2-3340	FALSE	1	TRA:CAAWDNFNKFYF TRB:CASSLVTGSEAFF	1	CD4-C1
PB-2_TTGCCGTCAATGGAAT	PB	PB-2	P2-3343	FALSE	1	TRA:CAVLPLGQKLLF TRB:CASSVVGVPNQPQHF	1	CD4-C2
PB-2_TTGCCGTCACGAAAGC	PB	PB-2	P2-3344	FALSE	1	TRA:CASSFSGGYNKLIF TRB:CASRIVNEQFF	1	CD4-C2
PB-2_TTGCCGTGTATATCCG	PB	PB-2	P2-3345	FALSE	1	TRA:CAVRDPYSSASKIIF TRB:CASSTGQGAYGYTF	1	CD4-C2
PB-2_TTGCCGTTCTTGTACT	PB	PB-2	P2-3348	FALSE	1	TRA:CAASITGNQFYF TRB:CASHTGQGAYEQYF	1	CD4-C2
PB-2_TTGCGTCCAACAACCT	PB	PB-2	P2-3351	FALSE	1	TRA:CAVGPMNRDDKIIF TRB:CASSLGVLAGALHTDTQYF	1	CD4-C2
PB-2_TTGCGTCTCCTGTACC	PB	PB-2	P2-3354	FALSE	1	TRA:CIVRGNAGGTSYGKLTF TRB:CSASDYEQYF	1	CD4-C1
PB-2_TTGGAACCACGCTTTC	PB	PB-2	P2-3356	FALSE	1	TRA:CAASTPNTGNQFYF TRB:CAWKTGVNTGELFF	1	CD4-C2
PB-2_TTGGAACTCACCGGGT	PB	PB-2	P2-3359	FALSE	1	TRA:CAVNNAGGTSYGKLTF TRB:CASSPRPAETQYF	1	CD4-C3
PB-2_TTGGAACTCGATGAGG	PB	PB-2	P2-3360	FALSE	1	TRA:CATALNDYKLSF TRB:CASETGGTDTQYF	1	CD4-C4
PB-2_TTGGAACTCTGATACG	PB	PB-2	P2-3361	FALSE	1	TRA:CVVSARPQGNNAGNMLTF TRB:CASREAGGHNEQFF	1	CD4-C1
PB-2_TTGGCAAAGCTACCGC	PB	PB-2	P2-3363	FALSE	1	TRA:CLVFSGNTGKLIF TRB:CASSYFTPGNTNYGYTF	1	CD4-C2
PB-2_TTGGCAAAGTTACGGG	PB	PB-2	P2-3366	FALSE	1	TRA:CAEKSGGSYIPTF TRB:CAWSVTWTDQETQYF	1	CD4-C2
PB-2_TTGGCAATCATGCAAC	PB	PB-2	P2-3369	FALSE	1	TRA:CIVRGLYNNNDMRF TRB:CSAYRSGALGYTF	1	CD4-C3
PB-2_TTGGCAATCCTCATTA	PB	PB-2	P2-3370	FALSE	1	TRA:CAESETGANNLFF TRB:CASRSAGGLSYNEQFF	1	CD4-C2
PB-2_TTGTAGGGTCCGACGT	PB	PB-2	P2-3373	FALSE	1	TRA:CAVEDDEGSQGNLIF TRB:CASSPLYEQYF	1	CD4-C3
PB-2_TTGTAGGGTCTTTCAT	PB	PB-2	P2-3374	FALSE	1	TRA:CAGQGGSEKLVF TRB:CASRGYNEQFF	1	CD4-C1
PB-2_TTTACTGAGAGGTTAT	PB	PB-2	P2-3378	FALSE	1	TRA:CALSTSGYSTLTF TRB:CASSQAGQYYGYTF	1	CD4-C3
PB-2_TTTACTGAGGAGTTGC	PB	PB-2	P2-3379	FALSE	1	TRA:CAGGGKNQGEKLIF TRB:CASSPGQGLAAYEQYF	1	CD4-C2
PB-2_TTTACTGTCACGATGT	PB	PB-2	P2-3384	FALSE	1	TRA:CAVRNYGGSQGNLIF TRB:CASSHRGPDEKLFF	1	CD4-C2
PB-2_TTTATGCGTAAATACG	PB	PB-2	P2-3387	FALSE	1	TRA:CAVRDTSGGSYIPTF TRB:CSVVDTPGEKLFF	1	CD4-C1
PB-2_TTTATGCTCTGATACG	PB	PB-2	P2-3392	FALSE	1	TRA:CAVVVTGGGNKLTF TRB:CAWSVRLAGVFLDEQFF	1	CD4-C2
PB-2_TTTCCTCCATTTCACT	PB	PB-2	P2-3395	FALSE	1	TRA:CILLNTGNQFYF TRB:CASSLSFATNTGELFF	1	CD4-C1
PB-2_TTTCCTCTCGATAGAA	PB	PB-2	P2-3396	FALSE	1	TRA:CAENKGPGANNLFF TRB:CASSMNSYGYTF	1	CD4-C2

PB-2_TTTGCGCGTAGCCTCG	PB	PB-2	P2-3400	FALSE	1	TRA:CAMEEPSGSARQLTF TRB:CASKVGGGETQYF	1	CD4-C3
PB-2_TTTGGTTAGAGTCGGT	PB	PB-2	P2-361	FALSE	2	TRA:CAVRDMRGSTLGRLYF TRB:CASEEYKAGGDTGELFF	2	CD4-C2
PB-2_TTTGGTTTCACATAGC	PB	PB-2	P2-3404	FALSE	1	TRA:CAVRGPYSGAGSYQLTF TRB:CASSEQGERYNEQFF	1	CD4-C2
PB-2_TTTGTCAAGTGACTCT	PB	PB-2	P2-3406	FALSE	1	TRA:CILRPQNTGFQKLVF TRB:CASSPGPARDNTIYF	1	CD4-C1
PB-2_TTTGTCAGTAGCTAAA	PB	PB-2	P2-3407	FALSE	1	TRA:CAVRDDTNAGKSTF TRB:CASSWQDISYEQYF	1	CD4-C1
PB-2_AAACCTGGTACTCGCG	PB	PB-2	P2-570	FALSE	1	TRA:CAVWGGGADGLTF TRB:CASSSLAASSYNEQFF	1	CD8-C5
PB-2_AACCGCGCAATGTTGC	PB	PB-2	P2-620	FALSE	1	TRA:CAMNPI TRB:CASSRRTSGRTYEQYF	1	CD8-C2
PB-2_AACTCAGAGAAGAAGC	PB	PB-2	P2-626	FALSE	1	TRA:CAESTGYSTLTF TRB:CAWSAGMETQYF	1	CD8-C2
PB-2_AACTCCCTCAGCTTAG	PB	PB-2	P2-168	FALSE	3	TRA:CIVETSYDKVIF TRB:CATARDSNQPQHF	3	CD8-C2
PB-2_ACACCCTCATGGGAAC	PB	PB-2	P2-729	FALSE	1	TRA:CLVGDIWGAGNMLTF TRB:CASSNPSGQGSYEQYF	1	CD8-C2
PB-2_ACGATGTCACAAGCCC	PB	PB-2	P2-811	FALSE	1	TRA:CATPGGNNRLAF TRB:CASSGAIYEQYF	1	CD8-C2
PB-2_ACTGATGAGCTGATAA	PB	PB-2	P2-871	FALSE	1	TRA:CAPGGATNKLIF TRB:CASSTDSHSTDTQYF	1	CD8-C2
PB-2_ACTTTCAGTGGTGTAG	PB	PB-2	P2-279	FALSE	2	TRA:CAASMGNYGQNFVF TRB:CASSYDSYTGELFF	2	CD8-C2
PB-2_AGCTTGAAGTCAATAG	PB	PB-2	P2-1004	FALSE	1	TRA:CALLESGSRLTF TRB:CASSFRADSYEQYF	1	CD8-C2
PB-2_AGGTCCGGTGCAGGTA	PB	PB-2	P2-1050	FALSE	1	TRA:CAGGLNYGGQNFVF TRB:CASSLRGGGPSGNTIYF	1	CD8-C2
PB-2_ATCATGGAGTAGGTGC	PB	PB-2	P2-1110	FALSE	1	TRA:CAVRDIPSGTYKYIF TRB:CSVDPEDGYTF	1	CD8-C1
PB-2_ATCCGAACATGGGACA	PB	PB-2	P2-1126	FALSE	1	TRA:CAAIRVEKLVF TRB:CAISSGRGETQYF	1	CD8-C2
PB-2_ATCTGCCGTCGACTAT	PB	PB-2	P2-293	TRUE	2	TRA:CAVNMGTYKYIF TRB:CASSRTYEQYF	2	CD8-C5
PB-2_ATGCGATCAAGCCGTC	PB	PB-2	P2-178	FALSE	3	TRA:CAATYYGNKLVF TRB:CSAKVGHSGNTIYF	3	CD8-C2
PB-2_CACAAACCATCCGGGT	PB	PB-2	P2-60	TRUE	3	TRA:CALSEGSGANSKLTF TRB:CASSSHSGLAGGFF	9	CD8-C2
PB-2_CACAAACCATCTACGA	PB	PB-2	P2-46	TRUE	3	TRA:CAFSTWGNQGGKLIF TRB:CASSQDRLGGGTYEQYF	11	CD8-C2
PB-2_CACACAAGTAAATGTG	PB	PB-2	P2-1239	FALSE	1	TRA:CLVGMYNTDKLIF TRB:CASSVQDRTFSGANVLTF	1	CD4-C3
PB-2_CAGAATCAGATTACCC	PB	PB-2	P2-279	FALSE	2	TRA:CAASMGNYGQNFVF TRB:CASSYDSYTGELFF	2	CD8-C2
PB-2_CAGCCGATCACATGCA	PB	PB-2	P2-1332	FALSE	1	TRA:CAVEDYSGAGSYQLTF TRB:CASSPYRGNTIYF	1	CD8-C2
PB-2_CATATGGAGGACGAAA	PB	PB-2	P2-1363	FALSE	1	TRA:CAMREGGDSWGKFQF TRB:CASSLPGTTMYQETQYF	1	CD8-C2
PB-2_CATCAAGCAGCTCGCA	PB	PB-2	P2-13	TRUE	3	TRA:CAYDLLTGNQFYF TRB:CASVRQLNTEAFF	38	CD8-C2
PB-2_CATGACACATTCTTAC	PB	PB-2	P2-1411	FALSE	1	TRA:CAVGPPGADGLTF TRB:CASSSWTGEYEQYF	1	CD4-C2

PB-2_CATTATCCAGGACGTA	PB	PB-2	P2-1432	FALSE	1	TRA:CAFRTSGSRLTF TRB:CSVELDGGGYTF		1	CD8-C2
PB-2_CCTTCCCAGCGACGTA	PB	PB-2	P2-1597	FALSE	1	TRA:CAVVFTGTASKLTF TRB:CASTRDKRETQYF		1	CD8-C2
PB-2_CGAACATAGGCTACGA	PB	PB-2	P2-1616	FALSE	1	TRA:CAVTGVTGFQKLVF TRB:CASSLAGTEAFF		1	CD8-C2
PB-2_CGAACATAGTTACGGG	PB	PB-2	P2-1618	FALSE	1	TRA:CAVSPQGYNKLIF TRB:CASTPGGLVAQHF		1	CD8-C2
PB-2_CGAGCACGTAGGGACT	PB	PB-2	P2-1648	FALSE	1	TRA:CATDQYYNTDKLIF TRB:CASRRTGSRNEQFF		1	CD8-C2
PB-2_CGAGCCACAGCTCGCA	PB	PB-2	P2-1655	FALSE	1	TRA:CAEWRSGGSNYKLTF TRB:CASSYGAGVKNIQYF		1	CD8-C2
PB-2_CGCCAAGTCCAAACTG	PB	PB-2	P2-1677	FALSE	1	TRA:CVVSVQTGANNLFF TRB:CATSRGGTGPYNEQFF		1	CD8-C6
PB-2_CGCTGGAAGTCGCCGT	PB	PB-2	P2-1700	FALSE	1	TRA:CAVKEDSNYQLIW TRB:CASSGEANQEKLFF		1	CD8-C2
PB-2_CGCTTCAAGGCAGTCA	PB	PB-2	P2-1706	FALSE	1	TRA:CAGQETSGSRLTF TRB:CASSQNRPQGMSNQPQHF		1	CD8-C2
PB-2_CGGACGTCAACTGCGC	PB	PB-2	P2-1722	FALSE	1	TRA:CALGGSSGNTGKLIF TRB:CASTLGPGPTDTQYF		1	CD8-C2
PB-2_CGTAGCGGTCTAGCCG	PB	PB-2	P2-187	TRUE	3	TRA:CAVLTGTASKLTF TRB:CASSGSTDTQYF		3	CD8-C2
PB-2_CGTGAGCTCAGCGACC	PB	PB-2	P2-4	TRUE	3	TRA:CAIRYQSFNFGNEKLTF TRB:CASYRRVTEQYF		148	CD8-C2
PB-2_CGTTCTGTCAGCTTAG	PB	PB-2	P2-4	TRUE	3	TRA:CAIRYQSFNFGNEKLTF TRB:CASYRRVTEQYF		148	CD8-C2
PB-2_CGTTGGGGTCTGGTCG	PB	PB-2	P2-316	FALSE	2	TRA:CAASWTGGFKTIF TRB:CASSRGGAGNTEAFF		2	CD4-C4
PB-2_CTCGGGACAGAAGCAC	PB	PB-2	P2-1941	FALSE	1	TRA:CAMSKPGSARQLTF TRB:CSVDRAGEQYF		1	CD8-C2
PB-2_CTCGTCACAAATTGCC	PB	PB-2	P2-1949	FALSE	1	TRA:CAFMNRGSTLGRLYF TRB:CASSFLSGTDTQYF		1	CD8-C2
PB-2_CTGATCCAGTTTGCGT	PB	PB-2	P2-1991	FALSE	1	TRA:CAVNFNNAGNMLTF TRB:CASSSGTPPRF		1	CD8-C2
PB-2_CTGCGGATCCGAATGT	PB	PB-2	P2-2005	FALSE	1	TRA:CLVGDRWGYGNNRLAF TRB:CATSDPRTSDTGELFF		1	CD8-C2
PB-2_GAACCTAGTCTGCCAG	PB	PB-2	P2-2083	FALSE	1	TRA:CALTGANSKLTF TRB:CATSDPMGPSGEQYF		1	CD4-C6
PB-2_GAACGGAAGCAGACTG	PB	PB-2	P2-2084	FALSE	1	TRA:CAVRDVNQLIW TRB:CATQDGRRSRPLDEQYF		1	CD8-C2
PB-2_GACCAATTCCTTGCCA	PB	PB-2	P2-2129	FALSE	1	TRA:CLAHSKEYIF TRB:CAITGDGYTQYF		1	CD8-C2
PB-2_GACGTTACATTGTGCA	PB	PB-2	P2-13	TRUE	3	TRA:CAYDLLTGNQFYF TRB:CASVRQLNTEAFF		38	CD8-C2
PB-2_GACTGCGGTCTAGTCA	PB	PB-2	P2-108	TRUE	3	TRA:CAMFPIKAAGNKLTF TRB:CASNTEANYGYTF		5	CD8-C2
PB-2_GATCGATAGCTACCGC	PB	PB-2	P2-178	FALSE	3	TRA:CAATYYGNKLVF TRB:CSAKVGHSGNTIYF		3	CD8-C2
PB-2_GATGAAACACCGAATT	PB	PB-2	P2-2253	FALSE	1	TRA:CVVPNTNAGKSTF TRB:CASSLDGLSLGQPQHF		1	CD8-C2
PB-2_GATGCTAGTGCCTGGT	PB	PB-2	P2-2267	FALSE	1	TRA:CAASRSGGSNYKLTF TRB:CASSPWGDGEAFF		1	CD8-C6
PB-2_GCACTCTAGGCTAGCA	PB	PB-2	P2-2290	FALSE	1	TRA:CATDGAGSYQLTF TRB:CASSPSSVEQFF		1	CD8-C2

PB-2_GCGCAACCAACTTGAC	PB	PB-2	P2-178	FALSE	3	TRA:CAATYYGNKLVF TRB:CSAKVGHSGNTIYF	3	CD8-C2
PB-2_GCGCAACGTATTAGCC	PB	PB-2	P2-108	TRUE	3	TRA:CAMFPIKAAGNKLTF TRB:CASNTEANYGYTF	5	CD8-C2
PB-2_GCGCAGTCAAGCCCAC	PB	PB-2	P2-2372	FALSE	1	TRA:CAMRGSMMDTGRRALTF TRB:CSARSDRLGYQPQHF	1	CD8-C2
PB-2_GCGCCAACAGGACCCT	PB	PB-2	P2-3	TRUE	3	TRA:CAVNPLESSYKLIF TRB:CASSPGTANTGELFF	177	CD8-C2
PB-2_GCTCTGTTCAGCTCGG	PB	PB-2	P2-2407	FALSE	1	TRA:CASDGGSQGNLIF TRB:CATSRGGPGETQYF	1	CD8-C2
PB-2_GGAAAGCAGTCGTTTG	PB	PB-2	P2-2434	FALSE	1	TRA:CAATSYGQNFVF TRB:CASSEFASNTGELFF	1	CD8-C5
PB-2_GGACAAGCAAATTGCC	PB	PB-2	P2-2461	FALSE	1	TRA:CAATSTYKYIF TRB:CASSPDGRYEQYF	1	CD8-C2
PB-2_GGACAAGGTATTCGTG	PB	PB-2	P2-2464	FALSE	1	TRA:CVVNIIGYQKVTF TRB:CAWSSDGGNTIYF	1	CD8-C2
PB-2_GGCTGGTAGACATAAC	PB	PB-2	P2-2532	FALSE	1	TRA:CAVRLEYNFNKFYF TRB:CASSVGQARDYTF	1	CD8-C2
PB-2_GGGAGATTCACAGTAC	PB	PB-2	P2-350	TRUE	2	TRA:CAVKVDSNYQLIW TRB:CASSPEGAGGGTGELFF	2	CD8-C2
PB-2_GTACGTAAGATACACA	PB	PB-2	P2-2597	FALSE	1	TRA:CAPAHFGNEKLTF TRB:CASSDHTGELFF	1	CD8-C2
PB-2_GTACTTTAGGATGGAA	PB	PB-2	P2-2610	FALSE	1	TRA:CAMMGVQGGSEKLVF TRB:CASSHRKGGRQYF	1	CD8-C2
PB-2_GTATCTTGTAGCTAAA	PB	PB-2	P2-2	TRUE	3	TRA:CVVPNTNAGKSTF TRB:CASSLQGSTYNEQFF	220	CD8-C5
PB-2_TACAGTGGTTCGGGCT	PB	PB-2	P2-2820	FALSE	1	TRA:CALSEVGTDSWGKFQF TRB:CASSKWDSTSYGYTF	1	CD8-C2
PB-2_TCAACGAGTGTGACGA	PB	PB-2	P2-2939	FALSE	1	TRA:CAAHGGYQKVTF TRB:CASSLGTTYNEQFF	1	CD4-C1
PB-2_TCACGAACAATGACCT	PB	PB-2	P2-5	TRUE	3	TRA:CASSFSGGYNKLIF TRB:CASSYLGAGYQETQYF	127	CD8-C5
PB-2_TCAGGTACAGCCTGTG	PB	PB-2	P2-316	FALSE	2	TRA:CAASWTGGFKTIF TRB:CASSRGGAGNTEAFF	2	CD8-C2
PB-2_TCTCTAATCCTGTACC	PB	PB-2	P2-329	FALSE	2	TRA:CALADGQKLLF TRB:CAWSSDGGNTIYF	2	CD8-C2
PB-2_TCTTCGGAGGTGCTAG	PB	PB-2	P2-3065	FALSE	1	TRA:CAVNKYNFNKFYF TRB:CASSFGTGSGEQYF	1	CD8-C2
PB-2_TGACAACTCGTTACGA	PB	PB-2	P2-367	TRUE	2	TRA:CALSDPYNTDKLIF TRB:CASSFYKGELFF	2	CD8-C2
PB-2_TGACTAGGTCACAAGG	PB	PB-2	P2-3107	FALSE	1	TRA:CAVEDGNDMRF TRB:CASTPRTSSNTGELFF	1	CD8-C2
PB-2_TGCGGGTTCCGAATGT	PB	PB-2	P2-369	TRUE	2	TRA:CVVSDDANDTDKLIF TRB:CASSVPGLAGGLLVFNEQFF	2	CD8-C5
PB-2_TGCTGCTGTCCCTACT	PB	PB-2	P2-3193	FALSE	1	TRA:CAESTNAGKSTF TRB:CASSVGQGAHADTQYF	1	CD8-C2
PB-2_TGGACGCAGCGTGAGT	PB	PB-2	P2-3197	FALSE	1	TRA:CVVNLADRGSTLGRLYF TRB:CASSSQRTSGGYPYEQYF	1	CD8-C2
PB-2_TGTTCCGTCGTCCGTT	PB	PB-2	P2-5	TRUE	3	TRA:CASSFSGGYNKLIF TRB:CASSYLGAGYQETQYF	127	CD8-C2
PB-2_TTCCCAGAGCGAAGGG	PB	PB-2	P2-374	TRUE	2	TRA:CALSEAFNTNAGKSTF TRB:CASSLKWTGGAYQPQHF	2	CD8-C2
PB-2_TTCGAAGAGCAGGTCA	PB	PB-2	P2-3298	FALSE	1	TRA:CATDVRMDSSYKLIF TRB:CATSRRTPPGNTIYF	1	CD8-C2

PB-2_TTTGCGCTCATCATTC	PB	PB-2	P2-104	TRUE	3	TRA:CAMREAGGSGAGSYQLTF TRB:CASSLGSGNTIYF	5	CD8-C2
PB-2_AAACCTGGTCTAGTCA	PB	PB-2	P2-571	FALSE	1	TRA:CVAYQTGGGNKLTF TRB:CASSEVWTGANVLTF	1	CD4-C3
PB-2_AAACCTGGTCTCTTAT	PB	PB-2	P2-264	FALSE	2	TRA:CAGLRGQKLLF TRB:CASSRRGGTRSGANVLTF	2	CD4-C3
PB-2_AAACGGGAGTACGACG	PB	PB-2	P2-576	FALSE	1	TRA:CIVRMNSGGSNYKLTF TRB:CASSPRLTGNEQFF	1	CD4-C3
PB-2_AAACGGGGTGGTACAG	PB	PB-2	P2-578	FALSE	1	TRA:CAASSMDSNYQLIW TRB:CASSGVGRNEKLFF	1	CD4-C3
PB-2_AAAGATGTCAATACCG	PB	PB-2	P2-265	FALSE	2	TRA:CAVLGRGSGNTGKLIF TRB:CSARSAQGVTEAFF	2	CD4-C3
PB-2_AAAGCAAAGAGAGCTC	PB	PB-2	P2-167	TRUE	3	TRA:CGTRLMRRSSGSARQLTF TRB:CASSRGLALTQGETQYF	3	CD4-C3
PB-2_AAAGTAGGTAGAGGAA	PB	PB-2	P2-598	FALSE	1	TRA:CAVGSQAGTALIF TRB:CATSDYSDRGSGELFF	1	CD4-C3
PB-2_AAAGTAGTCCGCAAGC	PB	PB-2	P2-266	FALSE	2	TRA:CAVGAMLTGGGNKLTF TRB:CASRGSGGGYEQYF	2	CD4-C3
PB-2_AAATGCCAGAGTTGGC	PB	PB-2	P2-601	FALSE	1	TRA:CAAVTQGGSEKLVF TRB:CASSLDLRRTQYF	1	CD4-C3
PB-2_AAATGCCCAGGTCTCG	PB	PB-2	P2-603	FALSE	1	TRA:CAVNDNARLMF TRB:CATSGGHKNTEAFF	1	CD4-C3
PB-2_AACCATGCACACCGCA	PB	PB-2	P2-610	FALSE	1	TRA:CALSDRGGTGNQFYF TRB:CASPKREGWEQFF	1	CD4-C3
PB-2_AACGTTGCAGTTCATG	PB	PB-2	P2-40	TRUE	3	TRA:CALRETSGSRLTF TRB:CASSPGRYEQYF	12	CD4-C3
PB-2_AACGTTGGTCATCGGC	PB	PB-2	P2-267	TRUE	2	TRA:CIVRVNYGNNRLAF TRB:CASSFSGAWNEQFF	2	CD4-C6
PB-2_AACTCTTAGGCCCTCA	PB	PB-2	P2-121	TRUE	3	TRA:CAGASRDTDKLIF TRB:CSVESFLRGASLMTEAFF	4	CD4-C3
PB-2_AACTTTCTCCTCAATT	PB	PB-2	P2-648	FALSE	1	TRA:CAVGGSGGFKTIF TRB:CASTGLAGENTGELFF	1	CD4-C3
PB-2_AAGACCTAGTGCCATT	PB	PB-2	P2-40	TRUE	3	TRA:CALRETSGSRLTF TRB:CASSPGRYEQYF	12	CD4-C3
PB-2_AAGCCGCCACTCGACG	PB	PB-2	P2-662	FALSE	1	TRA:CAESFAGAQKLVF TRB:CASSPPIGTGTYDEQFF	1	CD4-C3
PB-2_AAGTCTGCATGGTAGG	PB	PB-2	P2-692	FALSE	1	TRA:CALMINRDDKIIF TRB:CASSYGTAYEQYF	1	CD4-C2
PB-2_AAGTCTGGTTATGTGC	PB	PB-2	P2-694	FALSE	1	TRA:CAVNIYSGAGSYQLTF TRB:CASSLGKGLGRSPLHF	1	CD4-C3
PB-2_AATCCAGGTTGTTTGG	PB	PB-2	P2-704	FALSE	1	TRA:CAYRSNDRLMF TRB:CASSLDLAAFYEQYF	1	CD4-C3
PB-2_AATCCAGTCAGTCCCT	PB	PB-2	P2-705	FALSE	1	TRA:CATDGKEYGNKLVF TRB:CASSQHRDTGELFF	1	CD8-C6
PB-2_AATCGGTAGGACTGGT	PB	PB-2	P2-709	FALSE	1	TRA:CAVQGAQKLVF TRB:CASSLVPRSRGGNEQFF	1	CD4-C3
PB-2_AATCGGTGTTGGTGGA	PB	PB-2	P2-712	FALSE	1	TRA:CAALRAGNNRKLIW TRB:CASSPGRTNTGELFF	1	CD4-C3
PB-2_AATCGGTTCCAGAAGG	PB	PB-2	P2-98	TRUE	3	TRA:CATDAIRGNTPLVF TRB:CASSDPGGPLHF	5	CD4-C3
PB-2_ACACCAAGTGGTGTAG	PB	PB-2	P2-719	FALSE	1	TRA:CALLSGSASKIIF TRB:CASSFSVGTGVSSGANVLTF	1	CD4-C5
PB-2_ACACCCTGTCGCGGTT	PB	PB-2	P2-731	FALSE	1	TRA:CAVNQAGTALIF TRB:CASSPPGGIAGQETQYF	1	CD4-C3

PB-2_ACACCCTGTCTAGTGT	PB	PB-2	P2-732	FALSE	1	TRA:CALSDNTGTASKLTF TRB:CASSPQVGSRDEHIDTQYF	1	CD4-C3
PB-2_ACCAGTAAGGATGTAT	PB	PB-2	P2-765	FALSE	1	TRA:CAYRSGTTGNQFYF TRB:CASRDIRGASNSPLHF	1	CD4-C3
PB-2_ACCAGTATCACCGGGT	PB	PB-2	P2-771	FALSE	1	TRA:CAVSDDDKIIF TRB:CASSSTGKYGYTF	1	CD4-C3
PB-2_ACCCACTCACGCATCG	PB	PB-2	P2-270	FALSE	2	TRA:CVVWGSGGGADGLTF TRB:CASRDRGRMNTEAFF	2	CD4-C3
PB-2_ACCGTAACACCAGTTA	PB	PB-2	P2-782	FALSE	1	TRA:CALGGGFNQGGKLIF TRB:CATTRDSAVYEQYF	1	CD4-C3
PB-2_ACCTTTATCCGTCATC	PB	PB-2	P2-791	FALSE	1	TRA:CIVRSGGSNYKLTF TRB:CASRPEQKNIQYF	1	CD4-C3
PB-2_ACGAGCCGTTCCTCCA	PB	PB-2	P2-271	TRUE	2	TRA:CALSTPNTDKLIF TRB:CASSRGQGLTDTQYF	2	CD4-C3
PB-2_ACGAGGAGTGTGCCTG	PB	PB-2	P2-802	FALSE	1	TRA:CAMREGFQLNTDKLIF TRB:CASSRGLAGDKEQYF	1	CD4-C3
PB-2_ACGATACCACTACAGT	PB	PB-2	P2-272	TRUE	2	TRA:CAMKGDDKIIF TRB:CSARDASFASYEQYF	2	CD4-C2
PB-2_ACGATACCACTGTTAG	PB	PB-2	P2-273	TRUE	2	TRA:CAVFDRGSTLGRLYF TRB:CASSASQSARDSPTQYF	2	CD4-C3
PB-2_ACGCCAGCACTGTTAG	PB	PB-2	P2-816	FALSE	1	TRA:CAVTVNQAGTALIF TRB:CASSLAARPFMSTDTQYF	1	CD4-C3
PB-2_ACGCCGATCACGAAGG	PB	PB-2	P2-824	FALSE	1	TRA:CAVGGRETSGSRLTF TRB:CASSEGLGTDTQYF	1	CD4-C3
PB-2_ACGGAGATCACTTACT	PB	PB-2	P2-828	FALSE	1	TRA:CEEGGANSKLTF TRB:CASRLQGGASPLHF	1	CD4-C3
PB-2_ACGGCCATCTTTAGGG	PB	PB-2	P2-833	FALSE	1	TRA:CALPSGGYNKLIF TRB:CASSPMGHRGMNTEAFF	1	CD4-C3
PB-2_ACGGGCTCACACAGAG	PB	PB-2	P2-836	FALSE	1	TRA:CLVGASGAGSYQLTF TRB:CASSLLAGTDTQYF	1	CD4-C3
PB-2_ACGGGTCAGCGTTTAC	PB	PB-2	P2-843	FALSE	1	TRA:CAVGTPGTYKYIF TRB:CASSLTREQYF	1	CD8-C2
PB-2_ACGTCAATCATTTGGG	PB	PB-2	P2-276	TRUE	2	TRA:CALSEAYTGANSKLTF TRB:CASSLLGTSYEQYF	2	CD4-C3
PB-2_ACTATCTAGTGAACAT	PB	PB-2	P2-853	FALSE	1	TRA:CALSVGSNFGNEKLTF TRB:CASSRLATEQFF	1	CD4-C3
PB-2_ACTGAGTAGGAGTAGA	PB	PB-2	P2-863	FALSE	1	TRA:CAVRSNAGNNRKLIW TRB:CASSSRDTGLVGEQFF	1	CD4-C3
PB-2_ACTGAGTAGTGTCCCG	PB	PB-2	P2-277	TRUE	2	TRA:CATALYNFNKFYF TRB:CASGSTGTSNQPQHF	2	CD4-C3
PB-2_ACTGATGGTCAAAGAT	PB	PB-2	P2-876	FALSE	1	TRA:CAADTTGGFKTIF TRB:CASSLGAGGPYEQYF	1	CD4-C5
PB-2_ACTGCTCAGCCACTAT	PB	PB-2	P2-881	FALSE	1	TRA:CAVEDGNKYIF TRB:CASRGDISYEQYF	1	CD4-C3
PB-2_ACTGCTCTCTCTGCTG	PB	PB-2	P2-890	FALSE	1	TRA:CALRGGGFQKLVF TRB:CASSGQLNSPLHF	1	CD4-C3
PB-2_ACTGTCCGTTTAGGAA	PB	PB-2	P2-895	FALSE	1	TRA:CAVNRRGSQGNLIF TRB:CASSPRTGGTNTIYF	1	CD4-C3
PB-2_ACTTGTTAGAACAATC	PB	PB-2	P2-903	FALSE	1	TRA:CAMSNSGGYQKVTF TRB:CASSLSTSAGVNDEQFF	1	CD4-C3
PB-2_ACTTGTTGTCTCTTAT	PB	PB-2	P2-905	FALSE	1	TRA:CILRKQGGSEKLVF TRB:CASKQDTTSSGYTF	1	CD4-C3
PB-2_AGAATAGCAAATCCGT	PB	PB-2	P2-914	FALSE	1	TRA:CAVRDMRGDGQKLLF TRB:CASSFERNQPQHF	1	CD4-C3

PB-2_AGAATAGCAAGGCTCC	PB	PB-2	P2-28	TRUE	3	TRA:CATDANTGNQFYF TRB:CASSLGQWVEQYF	22	CD4-C3
PB-2_AGAATAGGTTAAGACA	PB	PB-2	P2-915	FALSE	1	TRA:CATDPGGNEKLTF TRB:CASSGLSADTQYF	1	CD4-C3
PB-2_AGACGTTAGAGTAAGG	PB	PB-2	P2-916	FALSE	1	TRA:CAVRANNDMRF TRB:CASSDRGRTEAFF	1	CD4-C3
PB-2_AGACGTTAGGACTGGT	PB	PB-2	P2-917	FALSE	1	TRA:CVVSAQTGGFKTIF TRB:CASSPFGGQGSQYF	1	CD4-C3
PB-2_AGATCTGGTATAGGTA	PB	PB-2	P2-947	FALSE	1	TRA:CAATGSARQLTF TRB:CASSLGGSFDTQYF	1	CD4-C3
PB-2_AGATTGCTCAACACCA	PB	PB-2	P2-281	FALSE	2	TRA:CAVYSGGSNYKLTF TRB:CASTSGTDHTDTQYF	2	CD4-C3
PB-2_AGCAGCCCAGTCTTCC	PB	PB-2	P2-959	FALSE	1	TRA:CASFNNRKLIW TRB:CASSLGQATGDTQYF	1	CD4-C3
PB-2_AGCATACAGCACCGTC	PB	PB-2	P2-965	FALSE	1	TRA:CAYAGNNRKLIW TRB:CASSASGYQYNEQFF	1	CD4-C2
PB-2_AGCCTAAAGGAATCGC	PB	PB-2	P2-39	TRUE	3	TRA:CAARAVTGGGNKLTF TRB:CASSGRSSAWNEQFF	13	CD4-C3
PB-2_AGCCTAATCCGGCACA	PB	PB-2	P2-282	TRUE	2	TRA:CAMRELILTGANSKLTF TRB:CASSLGVGQGFHNEQFF	2	CD4-C3
PB-2_AGCGTATAGACTTTCG	PB	PB-2	P2-980	FALSE	1	TRA:CAVKQKNQGGKLIF TRB:CSVASGSSYNEQFF	1	CD4-C3
PB-2_AGGCCACCATGACATC	PB	PB-2	P2-1010	FALSE	1	TRA:CAASLDNNNDMRF TRB:CASSRYSGTSYEQYF	1	CD4-C3
PB-2_AGGCCACGTGGTACAG	PB	PB-2	P2-1011	FALSE	1	TRA:CALSGGSNYKLTF TRB:CASSTQKGPTDTQYF	1	CD4-C3
PB-2_AGGGAGTAGGGCTCTC	PB	PB-2	P2-1019	FALSE	1	TRA:CALPDDNAGNMLTF TRB:CASSSWTSGGNTQYF	1	CD4-C3
PB-2_AGGGAGTGTCACTTCC	PB	PB-2	P2-287	FALSE	2	TRA:CAGYLNAGGTSYGKLTF TRB:CASSFRGATGELFF	2	CD4-C3
PB-2_AGGGATGTCAAGCCTA	PB	PB-2	P2-1029	FALSE	1	TRA:CAGPTLNNFNKFYF TRB:CASSTRPGQPQHF	1	CD4-C3
PB-2_AGGTCATAGATAGTCA	PB	PB-2	P2-1040	FALSE	1	TRA:CAVNSGYQKVTF TRB:CASSSGLSYEQYF	1	CD4-C3
PB-2_AGGTCATAGTGGTAAT	PB	PB-2	P2-1043	FALSE	1	TRA:CVVTYFGNEKLTF TRB:CASSLAAGGLYGYTF	1	CD4-C3
PB-2_AGTAGTCAGCTTCGCG	PB	PB-2	P2-1053	FALSE	1	TRA:CAVKGNTDKLIF TRB:CASSFGTGFVGNQPQHF	1	CD4-C2
PB-2_AGTGTCACACACCGCA	PB	PB-2	P2-101	TRUE	3	TRA:CATDASGNEKLTF TRB:CASSLGLADTQYF	5	CD4-C3
PB-2_AGTTGGTAGATGCGAC	PB	PB-2	P2-1081	FALSE	1	TRA:CGAASPIQGAQKLVF TRB:CASSLRTFPYGYTF	1	CD4-C3
PB-2_ATAACGCAGACTAGAT	PB	PB-2	P2-102	TRUE	3	TRA:CAVIYGSGNTGKLIF TRB:CASSLVNTIYF	5	CD4-C3
PB-2_ATAAGAGAGAGGTACC	PB	PB-2	P2-289	TRUE	2	TRA:CAASYRGAGNNRKLIW TRB:CSARTSGGGNNEQFF	2	CD4-C3
PB-2_ATAAGAGAGGGATGGG	PB	PB-2	P2-1090	FALSE	1	TRA:CAFMKHGNTGTASKLTF TRB:CASSVGTTGGNTIYF	1	CD4-C3
PB-2_ATCATCTCACATCCGG	PB	PB-2	P2-290	FALSE	2	TRA:CAVIRAGGSYIPTF TRB:CAWNIRDTNTGELFF	2	CD4-C3
PB-2_ATCATGGGTTCTCATT	PB	PB-2	P2-1115	FALSE	1	TRA:CVVSEGGSQGNLIF TRB:CASSMGVNRATNEKLFF	1	CD4-C3
PB-2_ATCCACCCAGATGGGT	PB	PB-2	P2-291	FALSE	2	TRA:CALGNTPLVF TRB:CASSLDLNQPQHF	2	CD4-C3

PB-2_ATCCGAACAGCAGTTT	PB	PB-2	P2-1125	FALSE	1	TRA:CAASKNTGNQFYF TRB:CASRRTGGSGQPQHF	1	CD4-C3
PB-2_ATCCGAAGTCCTCTTG	PB	PB-2	P2-292	TRUE	2	TRA:CAAREGASKIIF TRB:CASSLVGQGAFF	2	CD4-C3
PB-2_ATCGAGTGTCGGCTCA	PB	PB-2	P2-1132	FALSE	1	TRA:CILRDVDFGNEKLTF TRB:CASRTGSGANVLTF	1	CD4-C3
PB-2_ATGCGATGTACGCTGC	PB	PB-2	P2-1152	FALSE	1	TRA:CAASGSGGSNYKLTF TRB:CISAPAVSRMDKRKQGADSPL	1	CD4-C3
PB-2_ATGGGAGAGTACGCGA	PB	PB-2	P2-38	TRUE	3	TRA:CAVRDGSDYKLSF TRB:CASKADLNNQPQHF	14	CD4-C6
PB-2_ATGGGAGGTACGAAAT	PB	PB-2	P2-1157	FALSE	1	TRA:CAVRQIGGATNKLIF TRB:CASSLATGGRETQYF	1	CD4-C3
PB-2_ATTATCCGTGACGCCT	PB	PB-2	P2-1172	FALSE	1	TRA:CAASNAGNNRKLIW TRB:CASSFGSGSNQPQHF	1	CD4-C3
PB-2_ATTGGACAGAGCTATA	PB	PB-2	P2-298	TRUE	2	TRA:CALDAVRSASKIIF TRB:CSARLGTSQETQYF	2	CD4-C3
PB-2_ATTGGACTCTAAGCCA	PB	PB-2	P2-1183	FALSE	1	TRA:CVVSDQWGDDMRF TRB:CASSRGDSSTGELFF	1	CD4-C3
PB-2_ATTGGTGAGGAGTTTA	PB	PB-2	P2-1185	FALSE	1	TRA:CIAPFINDYKLSF TRB:CSARTGLRYEQYF	1	CD4-C1
PB-2_ATTGGTGGTGATAAGT	PB	PB-2	P2-1188	FALSE	1	TRA:CAVRARISGNTPLVF TRB:CSARAGGPGETQYF	1	CD4-C3
PB-2_ATTTCTGTCGTGGTCG	PB	PB-2	P2-1196	FALSE	1	TRA:CAGRDSGYALNF TRB:CASSAVDRGSSPLHF	1	CD4-C2
PB-2_CAACCTCCAAGGTGTG	PB	PB-2	P2-1205	FALSE	1	TRA:CAVIGSGNTGKLIF TRB:CSARYGGSGANVLTF	1	CD4-C2
PB-2_CAACCTCTCGAGCCCA	PB	PB-2	P2-1208	FALSE	1	TRA:CAMRGGNNTDKLIF TRB:CASSKRAGTGNNEQFF	1	CD4-C4
PB-2_CAACTAGTCATTTGGG	PB	PB-2	P2-1212	FALSE	1	TRA:CAVINTGANSKLTF TRB:CASSLAGGSGTGELFF	1	CD4-C3
PB-2_CAAGGCCCACGACTCG	PB	PB-2	P2-1225	FALSE	1	TRA:CGAYLSAGNKLTF TRB:CASRPGGGGQYNEQFF	1	CD4-C3
PB-2_CAAGGCCTCATAAAGG	PB	PB-2	P2-300	FALSE	2	TRA:CALTPSSGTYKYIF TRB:CASNERGPVSEQYF	2	CD4-C3
PB-2_CACACCTCACAGATTC	PB	PB-2	P2-1246	FALSE	1	TRA:CAVNSHSGGYQKVTF TRB:CASTANPGVTEAFF	1	CD4-C6
PB-2_CACACTCCAATACGCT	PB	PB-2	P2-1250	FALSE	1	TRA:CAARVQNAGNMLTF TRB:CASSESPGNYEQYF	1	CD4-C3
PB-2_CACACTCCAGGTCGTC	PB	PB-2	P2-1252	FALSE	1	TRA:CALHAGNNRKLIW TRB:CASSFGQGKDYGYTF	1	CD4-C3
PB-2_CACAGTAAGTTAAGTG	PB	PB-2	P2-1262	FALSE	1	TRA:CALSPGGTSYGKLTF TRB:CSASDRALTGSGNQPQHF	1	CD4-C3
PB-2_CACATAGAGAAGGTTT	PB	PB-2	P2-1271	FALSE	1	TRA:CAVSDRGATNKLIF TRB:CASSGGTDTEAFF	1	CD4-C3
PB-2_CACATTTCAAGTACCT	PB	PB-2	P2-1275	FALSE	1	TRA:CADANSGGSNYKLTF TRB:CASSPQGLAGGEQYF	1	CD4-C3
PB-2_CACCACTAGCTAGTCT	PB	PB-2	P2-1278	FALSE	1	TRA:CAASTSGSARQLTF TRB:CASSVGHWGSYGYTF	1	CD4-C3
PB-2_CAGAGAGGTTGTTTGG	PB	PB-2	P2-1305	FALSE	1	TRA:CAVRDSGAGSYQLTF TRB:CASSLEAGAPYNEQFF	1	CD4-C3
PB-2_CAGAGAGTCCAGTATG	PB	PB-2	P2-1307	FALSE	1	TRA:CAVSGLRGSTLGRLYF TRB:CASSLGLYNEQFF	1	CD4-C3
PB-2_CAGAGAGTCCTTAATC	PB	PB-2	P2-1308	FALSE	1	TRA:CLVGDSRDDKIIF TRB:CASSLKGRDSPLHF	1	CD4-C3

PB-2_CAGATCACAGCCTGTG	PB	PB-2	P2-180	TRUE	3	TRA:CAVNGNNFNKFYF TRB:CASSSGQDYEQYF	3	CD4-C3
PB-2_CAGATCACAGGCGATA	PB	PB-2	P2-1312	FALSE	1	TRA:CAEIEKYGNKLVF TRB:CSAQILGGGETQYF	1	CD4-C3
PB-2_CAGATCATCTGAAAGA	PB	PB-2	P2-1315	FALSE	1	TRA:CAVNNAGNMLTF TRB:CSARDNRINEKLFF	1	CD4-C3
PB-2_CAGCAGCTCGGCTTGG	PB	PB-2	P2-181	TRUE	3	TRA:CAVEWTDSSYKLIF TRB:CASRYGTTYEQYF	3	CD4-C6
PB-2_CAGCATAGTGTCTGAT	PB	PB-2	P2-1327	FALSE	1	TRA:CAGLPAGGNFNKFYF TRB:CASSVETGVETQYF	1	CD4-C3
PB-2_CAGCATATCTCCTATA	PB	PB-2	P2-124	TRUE	3	TRA:CAFFNTDKLIF TRB:CASSPRPNYGYTF	4	CD4-C3
PB-2_CAGCGACGTAGCGTGA	PB	PB-2	P2-1335	FALSE	1	TRA:CAVIERNAGNMLTF TRB:CASSYGTGGYTDTQYF	1	CD4-C3
PB-2_CAGCTGGGTCCCGACA	PB	PB-2	P2-1343	FALSE	1	TRA:CALSPYNNNDMRF TRB:CASSSQGLSGANVLTF	1	CD4-C3
PB-2_CAGCTGGTCCTAAGTG	PB	PB-2	P2-264	FALSE	2	TRA:CAGLRGQKLLF TRB:CASSRRGGTRSGANVLTF	2	CD4-C3
PB-2_CAGTAACTCATCTGTT	PB	PB-2	P2-1353	FALSE	1	TRA:CAMTLESSYKLIF TRB:CASRIRGTNTGELFF	1	CD4-C3
PB-2_CAGTAACTCCTATGTT	PB	PB-2	P2-1354	FALSE	1	TRA:CAASAGGRTGANNLFF TRB:CASSAGGSGSTDTQYF	1	CD4-C3
PB-2_CATATGGAGACCACGA	PB	PB-2	P2-1362	FALSE	1	TRA:CAASSAGGGADGLTF TRB:CATRESYEQYF	1	CD4-C3
PB-2_CATCAAGGTTCAGCGC	PB	PB-2	P2-1381	FALSE	1	TRA:CLVVNRDDKIIF TRB:CASSPFGNGGLDTEAFF	1	CD4-C3
PB-2_CATCGAAAGATCCTGT	PB	PB-2	P2-1394	FALSE	1	TRA:CAVVGSGNTGKLIF TRB:CAWRKQGVETQYF	1	CD4-C3
PB-2_CCAATCCAGAAACGCC	PB	PB-2	P2-1445	FALSE	1	TRA:CVVSDPGFQKLVF TRB:CASRRDGKGNQPQHF	1	CD4-C3
PB-2_CCACCTAAGAAGATTC	PB	PB-2	P2-1450	FALSE	1	TRA:CAVRRETSGSRLTF TRB:CASSLLGGLGGGSLYNEQFF	1	CD4-C6
PB-2_CCACCTATCGGGAGTA	PB	PB-2	P2-1460	FALSE	1	TRA:CALRERGSTLGRLYF TRB:CASSYSEGKGVNTEAFF	1	CD4-C6
PB-2_CCACGGATCGGCGCAT	PB	PB-2	P2-1468	FALSE	1	TRA:CAAKGAGGGNKLTF TRB:CASSELITSYNEQFF	1	CD4-C3
PB-2_CCACTACAGTACGCCC	PB	PB-2	P2-1470	FALSE	1	TRA:CATDARGNNRLAF TRB:CAISGGQGNEKLFF	1	CD4-C3
PB-2_CCACTACAGTGGTAAT	PB	PB-2	P2-1471	FALSE	1	TRA:CAVNNQGGKLIF TRB:CASSQVRDNYGYTF	1	CD4-C3
PB-2_CCACTACCACTTGGAT	PB	PB-2	P2-28	TRUE	3	TRA:CATDANTGNQFYF TRB:CASSLGQWVEQYF	22	CD4-C3
PB-2_CCATTCGCAAGCTGTT	PB	PB-2	P2-123	TRUE	3	TRA:CAASNLGTYKYIF TRB:CASSPPIGSPLHF	4	CD4-C3
PB-2_CCATTCGGTAGTAGTA	PB	PB-2	P2-1493	FALSE	1	TRA:CAANGVHGSGNTGKLIF TRB:CASSLTSGGTDTQYF	1	CD4-C3
PB-2_CCGGTAGCACCCATGG	PB	PB-2	P2-1526	FALSE	1	TRA:CAVEDPGGAARQLTF TRB:CASSLQSGGTEAFF	1	CD4-C3
PB-2_CCGTGGAGTACCAGTT	PB	PB-2	P2-1538	FALSE	1	TRA:CAVDDNQGGKLIF TRB:CSARAPTVNYEQYF	1	CD4-C3
PB-2_CCGTTCAAGTCAAGCG	PB	PB-2	P2-1541	FALSE	1	TRA:CALVLYNTDKLIF TRB:CASRSSGGEKPTQYF	1	CD4-C6
PB-2_CCTAAAGAGCTGCCCA	PB	PB-2	P2-1547	FALSE	1	TRA:CAVKGFARAGNKLTF TRB:CASSLNPPGAGETQYF	1	CD4-C3

PB-2_CCTAAAGAGGATGGAA	PB	PB-2	P2-1548	FALSE	1	TRA:CAGTLRGSQGNLIF TRB:CASNRLGEAFF	1	CD4-C3
PB-2_CCTACACCATACGCCG	PB	PB-2	P2-1557	FALSE	1	TRA:CAEKPPNTDKLIF TRB:CASSLYTGELFF	1	CD4-C3
PB-2_CCTACCAAGGAATGGA	PB	PB-2	P2-1561	FALSE	1	TRA:CAVRAPGGNFNKFYF TRB:CASSLGGPVYEQYF	1	CD4-C3
PB-2_CCTAGCTAGTGCGATG	PB	PB-2	P2-1568	FALSE	1	TRA:CAAKESNFGNEKLTF TRB:CASGVWQPRDQETQYF	1	CD4-C3
PB-2_CCTAGCTAGTGTTGAA	PB	PB-2	P2-98	TRUE	3	TRA:CATDAIRGNTPLVF TRB:CASSDPGGPLHF	5	CD4-C3
PB-2_CCTATTATCGTCCAGG	PB	PB-2	P2-182	TRUE	3	TRA:CAPNFGNEKLTF TRB:CASSFSSAYEQYF	3	CD4-C3
PB-2_CCTCAGTCATCGTCGG	PB	PB-2	P2-1582	FALSE	1	TRA:CAASNQFYF TRB:CASIRTHQGNSGNTIYF	1	CD4-C3
PB-2_CCTCTGACATGGGAAC	PB	PB-2	P2-1586	FALSE	1	TRA:CLVVTRGAGSGGYNKLIF TRB:CASSLSGPSSYNSPLHF	1	CD4-C3
PB-2_CCTCTGAGTGATAAAC	PB	PB-2	P2-1590	FALSE	1	TRA:CAVRTGTSGTYKYIF TRB:CASSQLSGNEQYF	1	CD4-C3
PB-2_CCTTCCCAGTCGTTTG	PB	PB-2	P2-1598	FALSE	1	TRA:CAGDLASGSRLTF TRB:CASRRRATTGYNEQFF	1	CD4-C3
PB-2_CCTTCCCTCTGAAAGA	PB	PB-2	P2-1606	FALSE	1	TRA:CAMSGRGGYQKVTF TRB:CASSLSGAQGRTEAFF	1	CD4-C4
PB-2_CCTTCGATCACCTTAT	PB	PB-2	P2-28	TRUE	3	TRA:CATDANTGNQFYF TRB:CASSLGQWVEQYF	22	CD4-C3
PB-2_CGAATGTCACGAAATA	PB	PB-2	P2-1622	FALSE	1	TRA:CAATGFKTIF TRB:CASSEQGALNNEQFF	1	CD4-C3
PB-2_CGAATGTTCCTCGCAT	PB	PB-2	P2-28	TRUE	3	TRA:CATDANTGNQFYF TRB:CASSLGQWVEQYF	22	CD4-C3
PB-2_CGACTTCGTGATAAGT	PB	PB-2	P2-1636	FALSE	1	TRA:CAFMAVQGAQKLVF TRB:CASSLDRADEQYF	1	CD4-C3
PB-2_CGAGCACCATAAAGGT	PB	PB-2	P2-1647	FALSE	1	TRA:CAAVPTSSSL TRB:CASRSPPGDEQYF	1	CD4-C3
PB-2_CGAGCACGTGAGTATA	PB	PB-2	P2-1650	FALSE	1	TRA:CALRGLKTSYDKVIF TRB:CASSLGVMGGSGNTIYF	1	CD4-C3
PB-2_CGAGCACGTGCGAAAC	PB	PB-2	P2-1651	FALSE	1	TRA:CAVGQSSGSARQLTF TRB:CASSQPVGGKAFF	1	CD4-C3
PB-2_CGAGCCAAGCACGCCT	PB	PB-2	P2-1654	FALSE	1	TRA:CLVASNQAGTALIF TRB:CASSPGQVGNTGELFF	1	CD4-C3
PB-2_CGATGGCAGTTACGGG	PB	PB-2	P2-1662	FALSE	1	TRA:CAASKNAGKSTF TRB:CASSRAGTGFGANVLTF	1	CD4-C3
PB-2_CGATGTACAGATCTGT	PB	PB-2	P2-1665	FALSE	1	TRA:CVVSPGIQF TRB:CASSVARAYNEQFF	1	CD4-C3
PB-2_CGATTGATCGCTTGTC	PB	PB-2	P2-1672	FALSE	1	TRA:CAVSEGSGNQFYF TRB:CSARLAGVTGELFF	1	CD4-C3
PB-2_CGCGGTAAGATATGCA	PB	PB-2	P2-1678	FALSE	1	TRA:CVVRFTGGGNKLTF TRB:CASTATGPPPEAFF	1	CD4-C3
PB-2_CGCGTTTAGGACCACA	PB	PB-2	P2-1683	FALSE	1	TRA:CAVRDAQGGKLIF TRB:CASSPSSGARGTEAFF	1	CD4-C3
PB-2_CGCGTTTCAAGAAGAG	PB	PB-2	P2-1685	FALSE	1	TRA:CAMREVYNQGGKLIF TRB:CATSRDKGDREKLFF	1	CD4-C3
PB-2_CGCGTTTCAGACAAAT	PB	PB-2	P2-1686	FALSE	1	TRA:CALRGRNNFNKFYF TRB:CASRPSTSGIGTDTQYF	1	CD4-C2
PB-2_CGCGTTTCAGATAATG	PB	PB-2	P2-183	TRUE	3	TRA:CAASTGTGGFKTIF TRB:CASKPTSGTYEQYF	3	CD4-C3

PB-2_CGCGTTTCAGGCTCAC	PB	PB-2	P2-175	FALSE	3	TRA:CAVSDLPLEGYQKVTF TRB:CASSITGGSLSSYEQYF	3	CD4-C3
PB-2_CGCGTTTCATGATCCA	PB	PB-2	P2-184	TRUE	3	TRA:CARYQGGKLIF TRB:CASSPRWGLGSYNEQFF	3	CD4-C3
PB-2_CGCTATCCACGACGAA	PB	PB-2	P2-1692	FALSE	1	TRA:CALSDNYGQNFVF TRB:CSAKVAGTNNEQFF	1	CD4-C3
PB-2_CGCTGGACAATCTACG	PB	PB-2	P2-1701	FALSE	1	TRA:CLVDKIIF TRB:CASTERGDSYNEQFF	1	CD4-C3
PB-2_CGCTGGATCATTGCGA	PB	PB-2	P2-1705	FALSE	1	TRA:CLVGDRQSSASKIIF TRB:CASSSPLRGIQETQYF	1	CD4-C3
PB-2_CGCTTCATCCAAAGTC	PB	PB-2	P2-1710	FALSE	1	TRA:CSQGAQKLVF TRB:CASKAGNHQETQYF	1	CD4-C3
PB-2_CGCTTCATCCTGCAGG	PB	PB-2	P2-1711	FALSE	1	TRA:CAAIRAGNNRKLIW TRB:CASRPTGVNTGELFF	1	CD4-C3
PB-2_CGGACGTGTCTAGAGG	PB	PB-2	P2-185	TRUE	3	TRA:CAPSGNTPLVF TRB:CSARKTTSGNTIYF	3	CD4-C3
PB-2_CGGAGCTCAGATGGCA	PB	PB-2	P2-1735	FALSE	1	TRA:CAVSGYSTLTF TRB:CASSIDLRPQGQINEQFF	1	CD4-C3
PB-2_CGGAGTCGTAAAGTCA	PB	PB-2	P2-1740	FALSE	1	TRA:CAATVYGGATNKLIF TRB:CASTGEGRRTQYF	1	CD4-C3
PB-2_CGGCTAGGTTTGGCGC	PB	PB-2	P2-1750	FALSE	1	TRA:CALSPGGNTGKLIF TRB:CASSPSGTGVTGELFF	1	CD4-C3
PB-2_CGGGTCATCATACGGT	PB	PB-2	P2-128	TRUE	3	TRA:CAYKKTSYDKVIF TRB:CASSPTSQPQHF	4	CD4-C3
PB-2_CGGGTCATCCGAACGC	PB	PB-2	P2-311	FALSE	2	TRA:CAGRNQGAQKLVF TRB:CASGGSNQPQHF	2	CD4-C3
PB-2_CGGTTAAAGTCGTTTG	PB	PB-2	P2-28	TRUE	3	TRA:CATDANTGNQFYF TRB:CASSLGQWVEQYF	22	CD4-C3
PB-2_CGTAGGCCATGTAGTC	PB	PB-2	P2-188	TRUE	3	TRA:CAVRRQGGNAGNMLTF TRB:CASSEQGSGSPYEQYF	3	CD4-C3
PB-2_CGTCTACAGTGGTCCC	PB	PB-2	P2-1795	FALSE	1	TRA:CVVSDQSGTASKLTF TRB:CATSRGTANQPQHF	1	CD4-C3
PB-2_CGTGAGCTCCTTTCTC	PB	PB-2	P2-1807	FALSE	1	TRA:CAVGFLSDGQKLLF TRB:CSARDLAGTTDTQYF	1	CD4-C2
PB-2_CGTGTAATCTTGAGAC	PB	PB-2	P2-1810	FALSE	1	TRA:CATLNDYKLSF TRB:CSARALVFSTDTQYF	1	CD4-C3
PB-2_CGTTCTGAGCTGAAAT	PB	PB-2	P2-1824	FALSE	1	TRA:CIVRVGGNAGNMLTF TRB:CSARDPRTSGSNEQFF	1	CD4-C3
PB-2_CTAACTTTCCTGCCAT	PB	PB-2	P2-190	TRUE	3	TRA:CAVSHSGGYQKVTF TRB:CASRRAAHEQYF	3	CD4-C3
PB-2_CTAAGACAGGCCGAAT	PB	PB-2	P2-4	TRUE	3	TRA:CAIRYQSFNFGNEKLTF TRB:CASYRRVTEQYF	148	CD8-C2
PB-2_CTAATGGGTTATCGGT	PB	PB-2	P2-191	TRUE	3	TRA:CAPLTGSSGDKLTF TRB:CASSLTGNYGYTF	3	CD4-C4
PB-2_CTACATTGTTCGAATC	PB	PB-2	P2-1867	FALSE	1	TRA:CAERPVAAGNKLTF TRB:CAWSVKGNQPQHF	1	CD4-C3
PB-2_CTACGTCGTCCAACTA	PB	PB-2	P2-319	TRUE	2	TRA:CALKGSSGYSTLTF TRB:CASSYSIVRGQQPQHF	2	CD8-C10
PB-2_CTAGTGACAGTGACAG	PB	PB-2	P2-1895	FALSE	1	TRA:CAVKGTYKYIF TRB:CAISDGAAETQYF	1	CD4-C3
PB-2_CTCACACCACGGTGTC	PB	PB-2	P2-1903	FALSE	1	TRA:CAMKAGTASKLTF TRB:CASSAGGGYEQYF	1	CD4-C3
PB-2_CTCAGAATCACGCATA	PB	PB-2	P2-1913	FALSE	1	TRA:CAVINTGFQKLVF TRB:CASSLGGTHDQPQHF	1	CD4-C3

PB-2_CTCGAAAAGGGTTCCC	PB	PB-2	P2-1925	FALSE	1	TRA:CIVTEYGNKLVF TRB:CASSFSGSNQPQHF	1	CD4-C3
PB-2_CTCGAGGCAAAGGAAG	PB	PB-2	P2-281	FALSE	2	TRA:CAVYSGGSNYKLTF TRB:CASTSGTDHTDTQYF	2	CD4-C3
PB-2_CTCTACGGTCAAGCGA	PB	PB-2	P2-1960	FALSE	1	TRA:CAVRDKKLSF TRB:CSARFQSGGLRRDEQYF	1	CD4-C3
PB-2_CTCTACGTCTACCTGC	PB	PB-2	P2-313	FALSE	2	TRA:CAVNVYSGAGSYQLTF TRB:CASSVSGQGRQPQHF	2	CD4-C3
PB-2_CTCTGGTCACCATGTA	PB	PB-2	P2-1963	FALSE	1	TRA:CAVRSPGTMRF TRB:CASSTYRELSSTDTQYF	1	CD4-C3
PB-2_CTCTGGTGTGGTAACG	PB	PB-2	P2-1967	FALSE	1	TRA:CAVSYSGGGADGLTF TRB:CSAQGGLKPQHF	1	CD4-C3
PB-2_CTCTGGTTCAACACCA	PB	PB-2	P2-1969	FALSE	1	TRA:CAASIDGQKLLF TRB:CASSIGGSGNEKLFF	1	CD4-C3
PB-2_CTCTGGTTCCCATTAT	PB	PB-2	P2-321	FALSE	2	TRA:CVVSILNQAGTALIF TRB:CASSLSGRGTDTQYF	2	CD4-C3
PB-2_CTGAAACCACGAAGCA	PB	PB-2	P2-1971	FALSE	1	TRA:CAVRGGGGNTGKLIF TRB:CASRRTGTTDTQYF	1	CD4-C3
PB-2_CTGAAACGTCTTCTCG	PB	PB-2	P2-311	FALSE	2	TRA:CAGRNQGAQKLVF TRB:CASGGSNQPQHF	2	CD4-C3
PB-2_CTGAAGTGTTGATTCG	PB	PB-2	P2-270	FALSE	2	TRA:CVVWGSGGGADGLTF TRB:CASRDRGRMNTEAFF	2	CD4-C3
PB-2_CTGATAGTCACATACG	PB	PB-2	P2-1989	FALSE	1	TRA:CAMREGRGSARQLTF TRB:CASGTVNTEAFF	1	CD4-C1
PB-2_CTGATCCAGTGAACGC	PB	PB-2	P2-1990	FALSE	1	TRA:CAASGGSSYKLIF TRB:CASSPRLAGTFYNEQFF	1	CD4-C3
PB-2_CTGATCCGTGGCCCTA	PB	PB-2	P2-1994	FALSE	1	TRA:CAVSAQEYGNKLVF TRB:CASSVAGNTDTQYF	1	CD4-C3
PB-2_CTGCGGACAATGAATG	PB	PB-2	P2-322	FALSE	2	TRA:CAASAKARLMF TRB:CASSWRTGAQVAETQYF	2	CD4-C3
PB-2_CTGGTCTCACGAGGTA	PB	PB-2	P2-2015	FALSE	1	TRA:CAAVNYGQNFVF TRB:CASSTVRASQQETQYF	1	CD4-C3
PB-2_CTGTTTATCAGAGACG	PB	PB-2	P2-28	TRUE	3	TRA:CATDANTGNQFYF TRB:CASSLGQWVEQYF	22	CD4-C3
PB-2_CTTACCGCACGAGGTA	PB	PB-2	P2-40	TRUE	3	TRA:CALRETSGSRLTF TRB:CASSPGRYEQYF	12	CD4-C3
PB-2_CTTACCGCAGCTCGCA	PB	PB-2	P2-2040	FALSE	1	TRA:CALQGAQKLVF TRB:CASSVGQGEGQPQHF	1	CD4-C3
PB-2_CTTAGGAGTTCGGGCT	PB	PB-2	P2-2046	FALSE	1	TRA:CAGRGSNYKLTF TRB:CASTAAGLETQYF	1	CD4-C3
PB-2_CTTGGCTGTAGAGCTG	PB	PB-2	P2-2054	FALSE	1	TRA:CAVEDQGTYKYIF TRB:CASSMQITDTQYF	1	CD4-C3
PB-2_GAAACTCCAACTGCTA	PB	PB-2	P2-40	TRUE	3	TRA:CALRETSGSRLTF TRB:CASSPGRYEQYF	12	CD4-C3
PB-2_GAACGGATCGATGAGG	PB	PB-2	P2-2088	FALSE	1	TRA:CAVLFWDSNYQLIW TRB:CASSLKGATGEQFF	1	CD4-C2
PB-2_GAACGGATCTGCTGCT	PB	PB-2	P2-2089	FALSE	1	TRA:CALSDRGGTGNQFYF TRB:CASSSGQGMGNQPQHF	1	CD4-C3
PB-2_GAAGCAGAGGTTCCTA	PB	PB-2	P2-2090	FALSE	1	TRA:CLTFTGGGNKLTF TRB:CASSPEAGQRTEAFF	1	CD4-C3
PB-2_GAAGCAGCAAACCTAC	PB	PB-2	P2-2091	FALSE	1	TRA:CAGDSGNTGKLIF TRB:CASSLGYDNEQFF	1	CD4-C3
PB-2_GAAGCAGCAAGCTGGA	PB	PB-2	P2-2092	FALSE	1	TRA:CAVRDRSGGSYIPTF TRB:CASSLSLSGANVLTF	1	CD8-C2

PB-2_GAAGCAGCATCACAAC	PB	PB-2	P2-2095	FALSE	1	TRA:CAASRIVSWGWELPTHF TRB:CASSYSEGKGVNTEAFF	1	CD4-C3
PB-2_GAATAAGTCCTCGCAT	PB	PB-2	P2-2105	FALSE	1	TRA:CAVNQYNQGGKLIF TRB:CSVVKGIGTEAFF	1	CD4-C3
PB-2_GAATGAAAGCACCGCT	PB	PB-2	P2-323	TRUE	2	TRA:CLVGPAYNQGGKLIF TRB:CASSSSTANSGNTIYF	2	CD4-C3
PB-2_GAATGAATCGTCTGAA	PB	PB-2	P2-2109	FALSE	1	TRA:CALYGGSQGNLIF TRB:CASSFRGTGGASSYNSPLHF	1	CD4-C3
PB-2_GACCTGGGTGGCCCTA	PB	PB-2	P2-2137	FALSE	1	TRA:CAFLGAGSYQLTF TRB:CASSLGTVKEQPQHF	1	CD4-C2
PB-2_GACGCGTAGACATAAC	PB	PB-2	P2-40	TRUE	3	TRA:CALRETSGSRLTF TRB:CASSPGRYEQYF	12	CD4-C3
PB-2_GACGCGTAGGATCGCA	PB	PB-2	P2-2140	FALSE	1	TRA:CAEKKDNFNKFYF TRB:CASRGQGDNSPLHF	1	CD4-C3
PB-2_GACGGCTAGTAGCCGA	PB	PB-2	P2-2146	FALSE	1	TRA:CALSGGTSYGKLTF TRB:CASSPRQGGLGMNTEAFF	1	CD4-C3
PB-2_GACGGCTTCATAACCG	PB	PB-2	P2-2151	FALSE	1	TRA:CAALGRSDKYIF TRB:CSAIGGGTDTQYF	1	CD4-C2
PB-2_GACGGCTTCTGAAAGA	PB	PB-2	P2-2153	FALSE	1	TRA:CVVNEGGGGADGLTF TRB:CASSPAGQNYGYTF	1	CD4-C3
PB-2_GACGTGCAGATATACG	PB	PB-2	P2-2154	FALSE	1	TRA:CAVGETSGSRLTF TRB:CASSLGKRFQPQHF	1	CD4-C2
PB-2_GACGTGCCAGTGGAGT	PB	PB-2	P2-193	TRUE	3	TRA:CAPGGSGGSNYKLTF TRB:CASSPGTGGNEQFF	3	CD4-C3
PB-2_GACTACAGTGCACCAC	PB	PB-2	P2-2177	FALSE	1	TRA:CAYRSRGMGQKDNDMRF TRB:CASSQDWGWPLHF	1	CD4-C5
PB-2_GACTGCGAGGTGCTTT	PB	PB-2	P2-2180	FALSE	1	TRA:CAFMKRSGANSKLTF TRB:CASTKTESWLFF	1	CD4-C3
PB-2_GACTGCGTCGCACTCT	PB	PB-2	P2-2186	FALSE	1	TRA:CIVVESGANSKLTF TRB:CASSPTRLAGSYNEQFF	1	CD4-C3
PB-2_GAGGTGACAAAGCAAT	PB	PB-2	P2-2197	FALSE	1	TRA:CAVSRARGGANNLFF TRB:CASSARDKIETQYF	1	CD4-C3
PB-2_GAGTCCGTCAGAGGTG	PB	PB-2	P2-2212	FALSE	1	TRA:CIVRALTDSWGKFQF TRB:CASSRTGSKETQYF	1	CD4-C3
PB-2_GATCGATAGATCCTGT	PB	PB-2	P2-2220	FALSE	1	TRA:CLVGEGAGTALIF TRB:CAWSRGTGSYNEQFF	1	CD4-C3
PB-2_GATCGATGTTTGGCGC	PB	PB-2	P2-327	TRUE	2	TRA:CAVHSNYQLIW TRB:CASSTMGQAQETQYF	2	CD4-C3
PB-2_GATCGTACAAAGCAAT	PB	PB-2	P2-194	TRUE	3	TRA:CAGRLQGAQKLVF TRB:CAWSQSQGYYGYTF	3	CD4-C3
PB-2_GATCGTAGTCAATACC	PB	PB-2	P2-2236	FALSE	1	TRA:CAVSRAGGATNKLIF TRB:CASTLGGQGNNEQFF	1	CD4-C3
PB-2_GATCTAGAGCTCCCAG	PB	PB-2	P2-2240	FALSE	1	TRA:CAVRNAGNNRKLIW TRB:CAWSNRGEWTEAFF	1	CD4-C3
PB-2_GATCTAGCAAGGACAC	PB	PB-2	P2-2246	FALSE	1	TRA:CAVNMGNNARLMF TRB:CASSIRPSSGKETQYF	1	CD4-C3
PB-2_GATGAAAAGGGTGTTG	PB	PB-2	P2-2251	FALSE	1	TRA:CAVRISSGSARQLTF TRB:CASSTPLGSSYEQYF	1	CD4-C3
PB-2_GATGAAACAAACCTAC	PB	PB-2	P2-2252	FALSE	1	TRA:CAERGQRGNTGTASKLTF TRB:CSASTGAGSTQYF	1	CD4-C3
PB-2_GCAAACTTCTTATCTG	PB	PB-2	P2-2278	FALSE	1	TRA:CAVTTNNNAGNMLTF TRB:CASGGDPGGNTQYF	1	CD4-C3
PB-2_GCAATCACAATCGGTT	PB	PB-2	P2-2280	FALSE	1	TRA:CAARNNNAGNMLTF TRB:CSVTGQGYTF	1	CD4-C3

PB-2_GCAATCAGTCCGTTAA	PB	PB-2	P2-2284	FALSE	1	TRA:CAGVYGGSQGNLIF TRB:CASRPPGLPGNEQFF	1	CD4-C2
PB-2_GCAATCAGTTGCCTCT	PB	PB-2	P2-326	FALSE	2	TRA:CIVRHLNTDKLIF TRB:CASSRRETQYF	2	CD4-C3
PB-2_GCAATCATCACTCTTA	PB	PB-2	P2-2286	FALSE	1	TRA:CAVKRSNTGNQFYF TRB:CASRLRLAQDEQYF	1	CD4-C3
PB-2_GCAGTTAAGGCGTACA	PB	PB-2	P2-305	FALSE	2	TRA:CAVSKPNFGNEKLTF TRB:CASSFWEEAFF	2	CD4-C3
PB-2_GCAGTTAGTCACAAGG	PB	PB-2	P2-331	TRUE	2	TRA:CAAHWGNTGFQKLVF TRB:CSASAGLINQPQHF	2	CD4-C3
PB-2_GCATGATCACGCCAGT	PB	PB-2	P2-2316	FALSE	1	TRA:CATGGGETSGSRLTF TRB:CASSPYVGAAEAFF	1	CD4-C3
PB-2_GCATGATGTGGTCCGT	PB	PB-2	P2-333	TRUE	2	TRA:CAVSVSGGSYIPTF TRB:CASSVGAQSPGANVLTF	2	CD4-C4
PB-2_GCATGCGGTAGCACGA	PB	PB-2	P2-28	TRUE	3	TRA:CATDANTGNQFYF TRB:CASSLGQWVEQYF	22	CD4-C3
PB-2_GCATGTAGTCATCCCT	PB	PB-2	P2-2324	FALSE	1	TRA:CAVKSNNNAGNMLTF TRB:CASSLFGTLNSPLHF	1	CD4-C3
PB-2_GCATGTAGTCTCAACA	PB	PB-2	P2-2325	FALSE	1	TRA:CALSAQNDYKLSF TRB:CASTPLYGRTFNEQFF	1	CD4-C3
PB-2_GCCAAATGTGCATCTA	PB	PB-2	P2-2332	FALSE	1	TRA:CAVGGSQGNLIF TRB:CASSTAWLRATQYF	1	CD4-C3
PB-2_GCCAAATGTGTCTGAT	PB	PB-2	P2-2333	FALSE	1	TRA:CAVSVVRGSRLTF TRB:CASSQDSMRVQETQYF	1	CD4-C3
PB-2_GCCAAATTCGATGAGG	PB	PB-2	P2-2335	FALSE	1	TRA:CAFMSRETSGSRLTF TRB:CASSFSYTGELFF	1	CD4-C3
PB-2_GCCTCTAAGGCCCTCA	PB	PB-2	P2-335	TRUE	2	TRA:CIVRTGEYGNKLVF TRB:CAWRGQGTSTQYF	2	CD4-C3
PB-2_GCGACCATCTGCCAGG	PB	PB-2	P2-2352	FALSE	1	TRA:CAMREAGGSGAGSYQLTF TRB:CSARYRVGETQYF	1	CD8-C2
PB-2_GCGAGAACACAGCCCA	PB	PB-2	P2-2357	FALSE	1	TRA:CVVSDQGSGYSTLTF TRB:CASSSLAGGPRELFF	1	CD4-C3
PB-2_GCGAGAATCGACGGAA	PB	PB-2	P2-2361	FALSE	1	TRA:CAVNAYSGAGSYQLTF TRB:CASSYPGQGRQPQHF	1	CD4-C3
PB-2_GCGCAGTTCGCAGGCT	PB	PB-2	P2-2375	FALSE	1	TRA:CAVKGSNFGNEKLTF TRB:CASSFFPLSVTDTQYF	1	CD4-C2
PB-2_GCGCCAAAGACGCACA	PB	PB-2	P2-2377	FALSE	1	TRA:CAVEMDSSYKLIF TRB:CASSPLAGGTDTQYF	1	CD4-C3
PB-2_GCGCGATGTCGCTTTC	PB	PB-2	P2-195	TRUE	3	TRA:CAVSKGGSYIPTF TRB:CASRSGSSTDTQYF	3	CD4-C3
PB-2_GCGCGATTCATGTCCC	PB	PB-2	P2-2387	FALSE	1	TRA:CAVGTGTASKLTF TRB:CASSTTSGVSYEQYF	1	CD4-C3
PB-2_GCTCCTATCCTCGCAT	PB	PB-2	P2-2403	FALSE	1	TRA:CAEMDSGYSTLTF TRB:CASSSGGQGFGSEGYF	1	CD4-C3
PB-2_GCTGCAGGTAGCTAAA	PB	PB-2	P2-2410	FALSE	1	TRA:CALSGGSNYKLTF TRB:CASSSSGGDNSPLHF	1	CD4-C3
PB-2_GCTGCGATCTGGCGAC	PB	PB-2	P2-2417	FALSE	1	TRA:CAYRRGNSGGSNYKLTF TRB:CASSLGPSTDTQYF	1	CD4-C3
PB-2_GCTGGGTGTTATCCGA	PB	PB-2	P2-2424	FALSE	1	TRA:CAPNFGNEKLTF TRB:CSVEGMRNGYTF	1	CD4-C3
PB-2_GCTTCCAAGCGATGAC	PB	PB-2	P2-2427	FALSE	1	TRA:CAVSSFTGNQFYF TRB:CASSSLEGGLGKLFF	1	CD4-C2
PB-2_GCTTCCACACCCTATC	PB	PB-2	P2-339	TRUE	2	TRA:CAESNPSNFGNEKLTF TRB:CASSSAGDGYTF	2	CD4-C3

PB-2_GGAACTTAGGCTAGAC	PB	PB-2	P2-342	TRUE	2	TRA:CAVSEGTGRRALTF TRB:CASSLGTQRSTDTQYF	2	CD4-C3
PB-2_GGAATAAAGCAGCCTC	PB	PB-2	P2-343	FALSE	2	TRA:CAASALGSGNTGKLIF TRB:CASSLGANEQYF	2	CD4-C3
PB-2_GGAATAACACAGGAGT	PB	PB-2	P2-2448	FALSE	1	TRA:CAVSGLTGANSKLTF TRB:CASSLGLTANSPLHF	1	CD4-C3
PB-2_GGAATAACATCCTAGA	PB	PB-2	P2-2450	FALSE	1	TRA:CAANRYNSGYALNF TRB:CASYHSGSTGELFF	1	CD4-C3
PB-2_GGACAAGCAAGCTGTT	PB	PB-2	P2-2462	FALSE	1	TRA:CAVEDRAGGGADGLTF TRB:CASSVDSLVGNTIYF	1	CD4-C3
PB-2_GGACAAGCATATGCTG	PB	PB-2	P2-2463	FALSE	1	TRA:CAVAPREAGGTSYGKLTF TRB:CASSRGGDTDTQYF	1	CD4-C3
PB-2_GGACAGATCTCTAGGA	PB	PB-2	P2-345	TRUE	2	TRA:CAVSSGSRLTF TRB:CASSPNVGGFTYEQYF	2	CD4-C3
PB-2_GGACATTCAATGTTGC	PB	PB-2	P2-2477	FALSE	1	TRA:CAFVLNNNDMRF TRB:CAISGGQGVYGYTF	1	CD4-C3
PB-2_GGACGTCCACAACGTT	PB	PB-2	P2-2481	FALSE	1	TRA:CAVNLVYNAGNMLTF TRB:CSVGRDRVNEQFF	1	CD4-C2
PB-2_GGAGCAACACTAAGTC	PB	PB-2	P2-2486	FALSE	1	TRA:CATDVTNSGYALNF TRB:CASSLAQGDTGELFF	1	CD4-C3
PB-2_GGATGTTTCGGTTCGG	PB	PB-2	P2-2494	FALSE	1	TRA:CALRVRGSGNTGKLIF TRB:CASSSTGDTVQETQYF	1	CD4-C2
PB-2_GGCAATTCAATGAAAC	PB	PB-2	P2-2499	FALSE	1	TRA:CAVSQIQGAQKLVF TRB:CASEDRGRGKKLFF	1	CD4-C3
PB-2_GGCAATTTCGTTTGCC	PB	PB-2	P2-2506	FALSE	1	TRA:CAVETTNAGKSTF TRB:CASSERDSYGYTF	1	CD4-C3
PB-2_GGCCGATCACAGGAGT	PB	PB-2	P2-2509	FALSE	1	TRA:CALSNNARLMF TRB:CSADLRDYGYTF	1	CD4-C3
PB-2_GGCCGATGTCAATGTC	PB	PB-2	P2-2510	FALSE	1	TRA:CAVSASPLTGANSKLTF TRB:CASSVDSPSGANNEQFF	1	CD4-C3
PB-2_GGCGACTGTATTACCG	PB	PB-2	P2-2517	FALSE	1	TRA:CAQIRSGGSNYKLTF TRB:CASRQASGYSNQPQHF	1	CD4-C3
PB-2_GGCTCGAGTAATCGTC	PB	PB-2	P2-2529	FALSE	1	TRA:CAAFSGSARQLTF TRB:CASSSLGDQYF	1	CD4-C3
PB-2_GGGAATGCAACCGCCA	PB	PB-2	P2-39	TRUE	3	TRA:CAARAVTGGGNKLTF TRB:CASSGRSSAWNEQFF	13	CD4-C3
PB-2_GGGCACTAGTAGGCCA	PB	PB-2	P2-2551	FALSE	1	TRA:CAVRASGSARQLTF TRB:CASSLRQGPGELFF	1	CD4-C3
PB-2_GGGCACTAGTGGTAAT	PB	PB-2	P2-2552	FALSE	1	TRA:CAMSHPNTGGFKTIF TRB:CASSPDRTVSDTQYF	1	CD4-C3
PB-2_GGGCATCCAAAGGAAG	PB	PB-2	P2-2557	FALSE	1	TRA:CAVSGATQTGANNLFF TRB:CASNRQGRRNTEAFF	1	CD4-C3
PB-2_GGTGAAGGTCGGCACT	PB	PB-2	P2-197	TRUE	3	TRA:CVVSARAGGFKTIF TRB:CASSEAFGGSYNEQFF	3	CD4-C3
PB-2_GGTGTTACAAGCCCAC	PB	PB-2	P2-2581	FALSE	1	TRA:CAVIAAGNKLTF TRB:CASSKGLGSQETQYF	1	CD4-C2
PB-2_GGTGTTAGTCTCTCGT	PB	PB-2	P2-2584	FALSE	1	TRA:CAVRVGTGANNLFF TRB:CASAVTQGLEKLFF	1	CD4-C3
PB-2_GTACGTAAGGTGTTAA	PB	PB-2	P2-2599	FALSE	1	TRA:CATLSGNTPLVF TRB:CASSFQGSSSYGYTF	1	CD4-C3
PB-2_GTAGGCCCAGGACGTA	PB	PB-2	P2-2615	FALSE	1	TRA:CAVNNKADSNYQLIW TRB:CASSETGGSYNEQFF	1	CD8-C10
PB-2_GTAGTCAAGGGAACGG	PB	PB-2	P2-2620	FALSE	1	TRA:CAVNPRKGTASKLTF TRB:CASSQQTYEQYF	1	CD4-C3

PB-2_GTATCTTGTCCGAATT	PB	PB-2	P2-193	TRUE	3	TRA:CAPGGSGGSNYKLTF TRB:CASSPGTGGNEQFF	3	CD4-C3
PB-2_GTATTCTTCACGCGGT	PB	PB-2	P2-2631	FALSE	1	TRA:CAASDGGSQGNLIF TRB:CASSQDPGRPSLGEQYF	1	CD4-C3
PB-2_GTATTCTTCCCTTGCA	PB	PB-2	P2-2633	FALSE	1	TRA:CIVRVATGGTSYGKLTF TRB:CASSFRGGTGELFF	1	CD4-C3
PB-2_GTATTCTTCGTTTAGG	PB	PB-2	P2-38	TRUE	3	TRA:CAVRDGSDYKLSF TRB:CASKADLNNQPQHF	14	CD4-C3
PB-2_GTCAAGTTCAACACTG	PB	PB-2	P2-2637	FALSE	1	TRA:CAMSAAYGNNRLAF TRB:CATAPSGGEQFF	1	CD4-C3
PB-2_GTCACAAAGTGTCCAT	PB	PB-2	P2-2639	FALSE	1	TRA:CAPSKGGQNFVF TRB:CATSRDGLYNSPLHF	1	CD4-C3
PB-2_GTCACAAGTTTAGGAA	PB	PB-2	P2-2644	FALSE	1	TRA:CIVRGYTGNQFYF TRB:CASSFERASYEQYF	1	CD4-C3
PB-2_GTCGGGTCAAGAGTCG	PB	PB-2	P2-2671	FALSE	1	TRA:CAEKDDKIIF TRB:CASSLLHSYEQYF	1	CD4-C3
PB-2_GTCGGGTCATATGGTC	PB	PB-2	P2-40	TRUE	3	TRA:CALRETSGSRLTF TRB:CASSPGRYEQYF	12	CD4-C3
PB-2_GTCTCGTTCGCCCTTA	PB	PB-2	P2-297	FALSE	2	TRA:CAVALSNNNDMRF TRB:CASRGDNEKLFF	2	CD4-C3
PB-2_GTCTTCGAGAAAGTGG	PB	PB-2	P2-2692	FALSE	1	TRA:CAVRGAGGTSYGKLTF TRB:CASTRQGKQPQHF	1	CD4-C3
PB-2_GTCTTCGGTCATTAGC	PB	PB-2	P2-2695	FALSE	1	TRA:CAVSDSIAGGTSYGKLTF TRB:CASSLDPSGRNTIYF	1	CD4-C3
PB-2_GTGAAGGAGATCACGG	PB	PB-2	P2-2699	FALSE	1	TRA:CAVSDTGRRALTF TRB:CASSLREYNEQFF	1	CD4-C3
PB-2_GTGCATAAGGACTGGT	PB	PB-2	P2-2707	FALSE	1	TRA:CAASRGGSNYKLTF TRB:CAISNLAADTQYF	1	CD4-C3
PB-2_GTGCGGTAGCGCTCCA	PB	PB-2	P2-2712	FALSE	1	TRA:CAETTDKLIF TRB:CASRAGEWSETQYF	1	CD4-C3
PB-2_GTGCGGTGTGCTTCTC	PB	PB-2	P2-2717	FALSE	1	TRA:CAASITGGSYIPTF TRB:CASSLGGTLMNTEAFF	1	CD4-C3
PB-2_GTGCTTCAGAGACGAA	PB	PB-2	P2-2718	FALSE	1	TRA:CAVTSSGSARQLTF TRB:CASSQDLAGGPVVLFF	1	CD4-C2
PB-2_GTGCTTCAGCATGGCA	PB	PB-2	P2-2719	FALSE	1	TRA:CAVNAYSGAGSYQLTF TRB:CASSLVKGTGRQPQHF	1	CD4-C3
PB-2_GTGGGTCCATGAAGTA	PB	PB-2	P2-2727	FALSE	1	TRA:CAMSGNNNARLMF TRB:CASSPLEDITGETQYF	1	CD4-C3
PB-2_GTGGGTCGTAGAAAGG	PB	PB-2	P2-2728	FALSE	1	TRA:CGTDPDNNAGNMLTF TRB:CASRVDRGYEQYF	1	CD4-C3
PB-2_GTGTTAGAGAGCTGGT	PB	PB-2	P2-2741	FALSE	1	TRA:CAMDGTSYGKLTF TRB:CASSLGARGEQFF	1	CD4-C3
PB-2_GTGTTAGCATGGTAGG	PB	PB-2	P2-265	FALSE	2	TRA:CAVLGRGSGNTGKLIF TRB:CSARSAQGVTEAFF	2	CD4-C3
PB-2_GTTAAGCCAGTCTTCC	PB	PB-2	P2-2754	FALSE	1	TRA:CAVSANSGYSTLTF TRB:CSARGQTGNTEAFF	1	CD4-C3
PB-2_GTTCATTGTAAATACG	PB	PB-2	P2-2768	FALSE	1	TRA:CAVNAQGGRRALTF TRB:CASSLGETKGTYEQYF	1	CD4-C3
PB-2_GTTCATTGTCCATCCT	PB	PB-2	P2-2770	FALSE	1	TRA:CAFGTNAGKSTF TRB:CASSFVHEQYF	1	CD8-C10
PB-2_GTTTCTAAGTAGCCGA	PB	PB-2	P2-195	TRUE	3	TRA:CAVSKGGSYIPTF TRB:CASRSGSSTDTQYF	3	CD4-C3
PB-2_TAAACCGTCCAAAGTC	PB	PB-2	P2-334	FALSE	2	TRA:CAALQNTGKLIF TRB:CASSRDGYEQYF	2	CD4-C3

PB-2_TAAACCGTCGGTTCGG	PB	PB-2	P2-40	TRUE	3	TRA:CALRETSGSRLTF TRB:CASSPGRYEQYF	12	CD4-C3
PB-2_TAAGAGAAGGTGTTAA	PB	PB-2	P2-2789	FALSE	1	TRA:CAASARTGNQFYF TRB:CASSPQPGTDVGEEQYF	1	CD4-C3
PB-2_TACACGAAGTGGGATC	PB	PB-2	P2-2809	FALSE	1	TRA:CAVSRGNEKLTF TRB:CSVKSGTPQTQYF	1	CD4-C3
PB-2_TACCTATCAGACAAGC	PB	PB-2	P2-357	FALSE	2	TRA:CAVRSRGSGATNKLIF TRB:CASSAPGLSIYEQYF	2	CD4-C3
PB-2_TACCTATCAGTTTACG	PB	PB-2	P2-2826	FALSE	1	TRA:CAVRDNSGTYKYIF TRB:CSPRNRDRAADTQYF	1	CD4-C3
PB-2_TACCTATGTACACCGC	PB	PB-2	P2-2827	FALSE	1	TRA:CAMSSGYGGSQGNLIF TRB:CSADRASYEQYF	1	CD4-C3
PB-2_TACCTTACACTGAAGG	PB	PB-2	P2-358	TRUE	2	TRA:CGTEETSGSRLTF TRB:CASSVQRGVEQYF	2	CD4-C3
PB-2_TACGGATAGGCTCTTA	PB	PB-2	P2-2835	FALSE	1	TRA:CVVIFSGGYQKVTF TRB:CAWSVGQLAKNIQYF	1	CD4-C3
PB-2_TACGGTAAGCGGATCA	PB	PB-2	P2-2845	FALSE	1	TRA:CAVEGVPMDSNYQLIW TRB:CASSRGTSSPLHF	1	CD4-C3
PB-2_TACTTGTCATTATCTC	PB	PB-2	P2-2869	FALSE	1	TRA:CATTPYSGAGSYQLTF TRB:CASDRIGLAGDEQFF	1	CD4-C3
PB-2_TAGAGCTCACAGGTTT	PB	PB-2	P2-2878	FALSE	1	TRA:CAMWAGKSTF TRB:CSAPVSGGRRSTDTQYF	1	CD4-C3
PB-2_TAGAGCTGTACCGCTG	PB	PB-2	P2-2881	FALSE	1	TRA:CVVNIIGYQKVTF TRB:CAWNSGLNTEAFF	1	CD4-C6
PB-2_TAGTGGTTCTTTAGTC	PB	PB-2	P2-2898	FALSE	1	TRA:CAVRPFGNEKLTF TRB:CASSSKYRDRGMEQPQHF	1	CD4-C3
PB-2_TAGTTGGCAAGTTGTC	PB	PB-2	P2-291	FALSE	2	TRA:CALGNTPLVF TRB:CASSLDLNQPQHF	2	CD4-C3
PB-2_TAGTTGGTCGGAGCAA	PB	PB-2	P2-2906	FALSE	1	TRA:CALSGQGAQKLVF TRB:CASAITDRIRDVSGNTIYF	1	CD4-C3
PB-2_TATCAGGTCTCGGACG	PB	PB-2	P2-2915	FALSE	1	TRA:CAANPRSSGSARQLTF TRB:CASSTTGGDQPQHF	1	CD4-C3
PB-2_TATCTCATCATGTCTT	PB	PB-2	P2-2921	FALSE	1	TRA:CAVSEGGGYNKLIF TRB:CASSLFGNQPQHF	1	CD4-C6
PB-2_TCACAAGGTCGCATCG	PB	PB-2	P2-2949	FALSE	1	TRA:CAARRSGKLIF TRB:CSALAGGWPYNEQFF	1	CD4-C3
PB-2_TCACGAATCTCGTTTA	PB	PB-2	P2-2955	FALSE	1	TRA:CAASAVARDMRF TRB:CASSRQGTSSGYTF	1	CD4-C3
PB-2_TCAGATGGTCTCCATC	PB	PB-2	P2-312	FALSE	2	TRA:CLVALNRDDKIIF TRB:CSARGEVAKNIQYF	2	CD4-C3
PB-2_TCAGATGTCCCTGACT	PB	PB-2	P2-2959	FALSE	1	TRA:CAVEEGSGANNLFF TRB:CASSRSTGELFF	1	CD4-C3
PB-2_TCAGCAACAACACCTA	PB	PB-2	P2-2963	FALSE	1	TRA:CAGGNEKLTF TRB:CASLQTGGSYNEQFF	1	CD4-C3
PB-2_TCCACACAGATCCGAG	PB	PB-2	P2-132	TRUE	3	TRA:CAANRAGNNRKLIW TRB:CASSFKKAGGPVGELFF	4	CD4-C3
PB-2_TCCACACTCCCAAGAT	PB	PB-2	P2-3004	FALSE	1	TRA:CAVNSGGGADGLTF TRB:CAWRLNGPSQFF	1	CD4-C3
PB-2_TCCACACTCTGTCAAG	PB	PB-2	P2-3005	FALSE	1	TRA:CAMAPTSGTYKYIF TRB:CQQQSSRGSQPQHF	1	CD4-C3
PB-2_TCGAGGCTCAACTCTT	PB	PB-2	P2-40	TRUE	3	TRA:CALRETSGSRLTF TRB:CASSPGRYEQYF	12	CD4-C3
PB-2_TCGAGGCTCTCCCTGA	PB	PB-2	P2-3014	FALSE	1	TRA:CAVATGNQFYF TRB:CASSYQGTAYEQYF	1	CD4-C6

PB-2_TCGGTAACACAAGCCC	PB	PB-2	P2-28	TRUE	3	TRA:CATDANTGNQFYF TRB:CASSLGQWVEQYF	22	CD4-C3
PB-2_TCGTAGAAGGCCCGTT	PB	PB-2	P2-3034	FALSE	1	TRA:CAVSSSGGSYIPTF TRB:CATRVGSGNQPQHF	1	CD4-C3
PB-2_TCTCTAAGTCCCGACA	PB	PB-2	P2-3052	FALSE	1	TRA:CAMSGRSGGSNYKLTF TRB:CASSLSDSGNTIYF	1	CD4-C3
PB-2_TCTTCGGAGCGAAGGG	PB	PB-2	P2-322	FALSE	2	TRA:CAASAKARLMF TRB:CASSWRTGAQVAETQYF	2	CD4-C3
PB-2_TCTTTCCAGGATGCGT	PB	PB-2	P2-3075	FALSE	1	TRA:CAVRDQSNYQLIW TRB:CASSVRHRDTEAFF	1	CD4-C3
PB-2_TGACGGCTCTGTCTCG	PB	PB-2	P2-28	TRUE	3	TRA:CATDANTGNQFYF TRB:CASSLGQWVEQYF	22	CD4-C3
PB-2_TGACTAGGTAGCACGA	PB	PB-2	P2-3106	FALSE	1	TRA:CAVQASSGGYNKLIF TRB:CASSTTGETQYF	1	CD4-C3
PB-2_TGACTAGTCGAACGGA	PB	PB-2	P2-3111	FALSE	1	TRA:CAASAITNAGKSTF TRB:CASSRTADVGTYEQYF	1	CD4-C3
PB-2_TGACTTTTCCGCATCT	PB	PB-2	P2-3114	FALSE	1	TRA:CATDRGSTLGRLYF TRB:CATSDPTSGRSGTQYF	1	CD4-C3
PB-2_TGAGCCGCACGCCAGT	PB	PB-2	P2-3124	FALSE	1	TRA:CALDEDDKIIF TRB:CSANIRDRGLHEQFF	1	CD4-C3
PB-2_TGAGCCGGTTCACCTC	PB	PB-2	P2-3128	FALSE	1	TRA:CAGGYSGGSNYKLTF TRB:CASSPTGGRWNEQFF	1	CD4-C3
PB-2_TGATTTCTCATGCATG	PB	PB-2	P2-40	TRUE	3	TRA:CALRETSGSRLTF TRB:CASSPGRYEQYF	12	CD4-C3
PB-2_TGATTTCTCCGGCACA	PB	PB-2	P2-368	TRUE	2	TRA:CAVNAPTGGFKTIF TRB:CASSAWTGIGDEQYF	2	CD4-C3
PB-2_TGCACCTAGTGGAGTC	PB	PB-2	P2-3140	FALSE	1	TRA:CAASRRGNTPLVF TRB:CASSLEQGDTQYF	1	CD4-C3
PB-2_TGCACCTCAAGAAGAG	PB	PB-2	P2-110	TRUE	3	TRA:CAVSTSGTYKYIF TRB:CASSSSGGDNSPLHF	5	CD4-C3
PB-2_TGCACCTTCTTATCTG	PB	PB-2	P2-3147	FALSE	1	TRA:CALQGSARQLTF TRB:CASSLGQGDFGNTIYF	1	CD4-C3
PB-2_TGCCCATCAGGCTCAC	PB	PB-2	P2-3153	FALSE	1	TRA:CAVRDEPGGYQKVTF TRB:CASTESPGNEQFF	1	CD4-C3
PB-2_TGCCCTAGTGCAGACA	PB	PB-2	P2-175	FALSE	3	TRA:CAVSDLPLEGYQKVTF TRB:CASSITGGSLSSYEQYF	3	CD4-C3
PB-2_TGCGTGGTCAAGATCC	PB	PB-2	P2-3187	FALSE	1	TRA:CAANSELFGGSQGNLIF TRB:CASRESGHEQYF	1	CD4-C3
PB-2_TGCGTGGTCGCTGATA	PB	PB-2	P2-370	TRUE	2	TRA:CAVGAGGGSQGNLIF TRB:CSARTGLGGYTF	2	CD4-C3
PB-2_TGCTACCAGAATGTGT	PB	PB-2	P2-3188	FALSE	1	TRA:CAQETSGSRLTF TRB:CASSPGYRGYEQYF	1	CD4-C3
PB-2_TGCTGCTGTAAGTTCC	PB	PB-2	P2-3192	FALSE	1	TRA:CAASINTGFQKLVF TRB:CASSLRTSGVRGGEQYF	1	CD4-C3
PB-2_TGGCCAGAGGACATTA	PB	PB-2	P2-3200	FALSE	1	TRA:CAVSNTDKLIF TRB:CASSPQRGDQETQYF	1	CD4-C3
PB-2_TGGCCAGTCTTGAGAC	PB	PB-2	P2-3203	FALSE	1	TRA:CALIGGTSYGKLTF TRB:CASSGSGAEQFF	1	CD4-C3
PB-2_TGGCTGGAGGTCATCT	PB	PB-2	P2-3207	FALSE	1	TRA:CAVSRSGGSYIPTF TRB:CASSQDLQGPYEQYF	1	CD4-C3
PB-2_TGGGAAGCACGTAAGG	PB	PB-2	P2-3218	FALSE	1	TRA:CAMLRFGNEKLTF TRB:CASSSPRDTYEQYF	1	CD4-C3
PB-2_TGGGAAGGTATAAACG	PB	PB-2	P2-3220	FALSE	1	TRA:CAMKAPGSGNTGKLIF TRB:CSADHGSRDTQYF	1	CD4-C5

PB-2_TGGGCGTTCTCGTTTA	PB	PB-2	P2-3228	FALSE	1	TRA:CAGITGNQFYF TRB:CASSLVPGGNQPQHF	1	CD4-C3
PB-2_TGGTTAGAGATCTGAA	PB	PB-2	P2-328	FALSE	2	TRA:CAFMKPYNNNDMRF TRB:CASSQDRAGGQYF	2	CD4-C3
PB-2_TGGTTCCTCTCAACTT	PB	PB-2	P2-3235	FALSE	1	TRA:CATDAYNQGGKLIF TRB:CASSPQIYSSYNEQFF	1	CD4-C3
PB-2_TGTCCCACACCGAATT	PB	PB-2	P2-3244	FALSE	1	TRA:CATDEKGAQKLVF TRB:CASSQDPRGRETQYF	1	CD4-C3
PB-2_TGTTCCGCAATAGCGG	PB	PB-2	P2-105	TRUE	3	TRA:CAYRRREGGSEKLVF TRB:CASSTSGAIANYGYTF	5	CD4-C3
PB-2_TTAACTCTCATGCAAC	PB	PB-2	P2-3271	FALSE	1	TRA:CAMREMIRTGANSKLTF TRB:CAIGPGQGASGANVLTF	1	CD4-C3
PB-2_TTAGGACAGCGTAGTG	PB	PB-2	P2-372	FALSE	2	TRA:CAVSESTTGFQKLVF TRB:CSARPGTSRADTQYF	2	CD4-C3
PB-2_TTATGCTAGATCGGGT	PB	PB-2	P2-38	TRUE	3	TRA:CAVRDGSDYKLSF TRB:CASKADLNNQPQHF	14	CD4-C3
PB-2_TTCGAAGCAGCCAATT	PB	PB-2	P2-3299	FALSE	1	TRA:CAVQGVYTGRRALTF TRB:CSATHTGDSGYTF	1	CD4-C3
PB-2_TTCGAAGCAGGCGATA	PB	PB-2	P2-357	FALSE	2	TRA:CAVRSRGSGATNKLIF TRB:CASSAPGLSIYEQYF	2	CD4-C4
PB-2_TTCTCAATCACAAACC	PB	PB-2	P2-3320	FALSE	1	TRA:CAGGTGTASKLTF TRB:CATSDAASSYEQYF	1	CD4-C2
PB-2_TTCTTAGCAGCCACCA	PB	PB-2	P2-3325	FALSE	1	TRA:CAMSGVGNEKLTF TRB:CASSPASTDTQYF	1	CD4-C3
PB-2_TTGAACGTCGGCTTGG	PB	PB-2	P2-3335	FALSE	1	TRA:CLVEGTGFQKLVF TRB:CSAAGQGGNTIYF	1	CD4-C3
PB-2_TTGACTTTCACCCTCA	PB	PB-2	P2-3339	FALSE	1	TRA:CATDAGSGGGADGLTF TRB:CASSEFGSGSSYEQYF	1	CD4-C3
PB-2_TTGCGTCAGGAATTAC	PB	PB-2	P2-28	TRUE	3	TRA:CATDANTGNQFYF TRB:CASSLGQWVEQYF	22	CD4-C3
PB-2_TTGGCAAAGTCCAGGA	PB	PB-2	P2-372	FALSE	2	TRA:CAVSESTTGFQKLVF TRB:CSARPGTSRADTQYF	2	CD4-C3
PB-2_TTGGCAAGTATGCTTG	PB	PB-2	P2-321	FALSE	2	TRA:CVVSILNQAGTALIF TRB:CASSLSGRGTDTQYF	2	CD4-C3
PB-2_TTTACTGGTAAATGTG	PB	PB-2	P2-3382	FALSE	1	TRA:CAVRRGSTLGRLYF TRB:CASRTGTDTGELFF	1	CD4-C3
PB-2_TTTATGCTCTGAAAGA	PB	PB-2	P2-3391	FALSE	1	TRA:CAMYTGGYNKLIF TRB:CASSLRASGLYNEQFF	1	CD4-C2
PB-2_TTTCCTCTCTCGTTTA	PB	PB-2	P2-3398	FALSE	1	TRA:CAVSAGGSYIPTF TRB:CASSQPTSGAHNEQFF	1	CD4-C3
PB-2_TTTGCGCTCGTACGGC	PB	PB-2	P2-3401	FALSE	1	TRA:CAVTAGANSKLTF TRB:CASSERMGFSNQPQHF	1	CD4-C3
PB-2_TTTGTCACAGGTGGAT	PB	PB-2	P2-287	FALSE	2	TRA:CAGYLNAGGTSYGKLTF TRB:CASSFRGATGELFF	2	CD4-C3
PB-2_AACTCCCGTGAGGCTA	PB	PB-2	P2-34	TRUE	3	TRA:CAVRDSNYQLIW TRB:CASSLYPSGGGTDTQYF	15	CD8-C10
PB-2_AACTTTCCATTACGAC	PB	PB-2	P2-646	FALSE	1	TRA:CAVERLSDGQKLLF TRB:CASSLEGTGIYGYTF	1	CD8-C10
PB-2_ACACCAATCGGAGCAA	PB	PB-2	P2-724	FALSE	1	TRA:CAVYSGGGADGLTF TRB:CATSVLKDHEQYF	1	CD8-C8
PB-2_ACGGAGAGTAGATTAG	PB	PB-2	P2-826	FALSE	1	TRA:CAVMDSSYKLIF TRB:CSARDGGPQETQYF	1	CD8-C10
PB-2_ACTGATGAGACGCACA	PB	PB-2	P2-174	TRUE	3	TRA:CAVRDGDYKLSF TRB:CSAKDGGQDYEQYF	3	CD8-C10

PB-2_ACTTTCAGTTACGTCA	PB	PB-2	P2-280	TRUE	2	TRA:CASMDSNYQLIW TRB:CASSVLAGEEGEQFF	2	CD8-C10
PB-2_AGCGGTCAGTTGAGAT	PB	PB-2	P2-973	FALSE	1	TRA:CAVNETGGSYIPTF TRB:CAWKSAGRDTGELFF	1	CD8-C10
PB-2_AGGGATGTCGCCGTGA	PB	PB-2	P2-1031	FALSE	1	TRA:CAVVDSNYQLIW TRB:CAWSRGDTGELFF	1	CD8-C10
PB-2_AGGTCATGTAAGAGGA	PB	PB-2	P2-1046	FALSE	1	TRA:CAVVDSNYQLIW TRB:CASSYTQEGGEQFF	1	CD8-C10
PB-2_AGGTCCGGTCTGATCA	PB	PB-2	P2-1049	FALSE	1	TRA:CAALTNDYKLSF TRB:CASSEEAGGRQEQFF	1	CD8-C10
PB-2_ATTTCTGAGGTCATCT	PB	PB-2	P2-1193	FALSE	1	TRA:CAVNDGSYQLTF TRB:CASNPGTGYNEQFF	1	CD8-C10
PB-2_CAACCTCCACTGTCGG	PB	PB-2	P2-1206	FALSE	1	TRA:CAVIDSNYQLIW TRB:CASSPDIYNEQFF	1	CD8-C10
PB-2_CAAGAAAGTCACAAGG	PB	PB-2	P2-1215	FALSE	1	TRA:CAVTDSNYQLIW TRB:CSARDGGQGDSFPQYF	1	CD8-C10
PB-2_CAAGGCCGTTACCGAT	PB	PB-2	P2-34	TRUE	3	TRA:CAVRDSNYQLIW TRB:CASSLYPSGGGTDTQYF	15	CD8-C10
PB-2_CACATTTGTCGGCACT	PB	PB-2	P2-301	TRUE	2	TRA:CAVMDSNYQLIW TRB:CASSRTGGSYNEQFF	2	CD8-C10
PB-2_CATCGGGAGTAAGTAC	PB	PB-2	P2-1400	FALSE	1	TRA:CAAMDSNYQLIW TRB:CASSDSGDTGGYTF	1	CD8-C10
PB-2_CATTATCGTACAGTTC	PB	PB-2	P2-1433	FALSE	1	TRA:CAVMDSNYQLIW TRB:CASSEQGARDEQFF	1	CD8-C10
PB-2_CCACCTACATGCTAGT	PB	PB-2	P2-1454	FALSE	1	TRA:CAVLDSNYQLIW TRB:CASSDPAGGDIDEQFF	1	CD8-C10
PB-2_CCACTACTCTACTTAC	PB	PB-2	P2-1474	FALSE	1	TRA:CAAMDSNYQLIW TRB:CASSEDPEATGELFF	1	CD8-C10
PB-2_CCCAGTTGTTCGGGCT	PB	PB-2	P2-1510	FALSE	1	TRA:CAGLDSNYQLIW TRB:CASSEYLAGGDEQFF	1	CD8-C10
PB-2_CCGTTCAAGCGATCCC	PB	PB-2	P2-1539	FALSE	1	TRA:CAGHGNNRLAF TRB:CASSYSGTGSYEQYF	1	CD8-C10
PB-2_CCTAAAGCACGGTAGA	PB	PB-2	P2-1551	FALSE	1	TRA:CAAMDSNYQLIW TRB:CASSEQAGEVDEQYF	1	CD8-C10
PB-2_CCTATTAGTCACAAGG	PB	PB-2	P2-308	FALSE	2	TRA:CACMDSNYQLIW TRB:CASSYGVSGANVLTF	2	CD8-C10
PB-2_CCTTACGCAGATGGCA	PB	PB-2	P2-1594	FALSE	1	TRA:CAVMDSNYQLIW TRB:CASSGLFDTDTQYF	1	CD8-C10
PB-2_CGCGTTTAGACGCACA	PB	PB-2	P2-1682	FALSE	1	TRA:CAVGALGNNFNKFYF TRB:CASSEAPGGQETQYF	1	CD8-C10
PB-2_CGCTGGACAGTCGATT	PB	PB-2	P2-310	TRUE	2	TRA:CAVMDSNYQLIW TRB:CASSAQGTDTGELFF	2	CD8-C10
PB-2_CGGACGTGTAGCTTGT	PB	PB-2	P2-1724	FALSE	1	TRA:CAVTDNDYKLSF TRB:CASSEGAEGPNNEQFF	1	CD8-C10
PB-2_CGGCTAGGTGGTGTAG	PB	PB-2	P2-1748	FALSE	1	TRA:CASLDSSYKLIF TRB:CASSLRLGTGELFF	1	CD8-C10
PB-2_CGGCTAGGTTTGACTG	PB	PB-2	P2-34	TRUE	3	TRA:CAVRDSNYQLIW TRB:CASSLYPSGGGTDTQYF	15	CD8-C10
PB-2_CTGAAGTAGGACGAAA	PB	PB-2	P2-1977	FALSE	1	TRA:CVIMDSNYQLIW TRB:CASSATDYSYNEQFF	1	CD8-C10
PB-2_CTGCCTAAGATGCCAG	PB	PB-2	P2-1997	FALSE	1	TRA:CASMDSNYQLIW TRB:CASSYSQEGGHEQYF	1	CD8-C10
PB-2_CTTAACTCACAACTGT	PB	PB-2	P2-308	FALSE	2	TRA:CACMDSNYQLIW TRB:CASSYGVSGANVLTF	2	CD8-C10

PB-2_GACACGCCACGGCTAC	PB	PB-2	P2-2112	FALSE	1	TRA:CAVMDSNYQLIW TRB:CSARGLAGNEQFF	1	CD8-C10
PB-2_GACGGCTAGATCTGAA	PB	PB-2	P2-2143	FALSE	1	TRA:CAVMDSNYQLIW TRB:CASSGRETGELFF	1	CD8-C10
PB-2_GACGTGCTCAACCAAC	PB	PB-2	P2-2160	FALSE	1	TRA:CAPMDSNYQLIW TRB:CSARTGAHQETQYF	1	CD8-C10
PB-2_GATCGTACATAGGATA	PB	PB-2	P2-329	FALSE	2	TRA:CALADGGQKLLF TRB:CAWSSDGGNTIYF	2	CD4-C3
PB-2_GGAAAGCAGCTGTCTA	PB	PB-2	P2-341	TRUE	2	TRA:CAGMDSNYQLIW TRB:CSARDLSEPYEQYF	2	CD8-C10
PB-2_GGAAAGCCAGGGTTAG	PB	PB-2	P2-2435	FALSE	1	TRA:CAVVDSSYKLIF TRB:CSARGGAEYEQYF	1	CD8-C10
PB-2_GGACAAGAGACACTAA	PB	PB-2	P2-2458	FALSE	1	TRA:CASMDSSYKLIF TRB:CASSTSETSTDTQYF	1	CD8-C10
PB-2_GGAGCAAGTACTTCTT	PB	PB-2	P2-2487	FALSE	1	TRA:CAVKDSNYQLIW TRB:CASSYSGGADTQYF	1	CD8-C10
PB-2_GGCCGATCAATTCCTT	PB	PB-2	P2-346	TRUE	2	TRA:CAVSDGYTNAGKSTF TRB:CASSQDSRQGLNTEAFF	2	CD8-C10
PB-2_GGCGACTTCGGTCTAA	PB	PB-2	P2-348	TRUE	2	TRA:CAVSDSNYQLIW TRB:CASGEGTGSDTQYF	2	CD8-C10
PB-2_GTAACTGGTAGCTGCC	PB	PB-2	P2-2594	FALSE	1	TRA:CAVADSNYQLIW TRB:CASSSGTGDANVLTF	1	CD8-C10
PB-2_GTACTTTAGAAGGTGA	PB	PB-2	P2-2609	FALSE	1	TRA:CAVMDSNYQLIW TRB:CASSLTPNPEETQYF	1	CD8-C10
PB-2_GTCTTCGAGGCTAGGT	PB	PB-2	P2-2693	FALSE	1	TRA:CAVINSNYQLIW TRB:CASSVGGAGNEQFF	1	CD8-C10
PB-2_TAAACCGAGCGATGAC	PB	PB-2	P2-2786	FALSE	1	TRA:CAMGEDSNYQLIW TRB:CAADGDYSANEQFF	1	CD8-C10
PB-2_TACACGATCTAAGCCA	PB	PB-2	P2-199	TRUE	3	TRA:CAVMDSSYKLIF TRB:CSVPGQGDYEQYF	3	CD8-C10
PB-2_TAGACCATCGTAGGTT	PB	PB-2	P2-34	TRUE	3	TRA:CAVRDSNYQLIW TRB:CASSLYPSGGGTDTQYF	15	CD8-C10
PB-2_TAGTGGTTCTGCAGTA	PB	PB-2	P2-2896	FALSE	1	TRA:CAVKDGDYQLIW TRB:CASTRLAGGPDEQFF	1	CD8-C10
PB-2_TCGCGTTTCGGATGGA	PB	PB-2	P2-3023	FALSE	1	TRA:CAVRDSNYQLIW TRB:CASSQGRGDQETQYF	1	CD8-C10
PB-2_TCGGGACCATTGCGGC	PB	PB-2	P2-364	TRUE	2	TRA:CAAMDSNYQLIW TRB:CASSEEGGSYDEQFF	2	CD8-C10
PB-2_TGACGGCAGTTACGGG	PB	PB-2	P2-41	TRUE	3	TRA:CAVRDSDYKLSF TRB:CASSQGESSYEQYF	12	CD8-C10
PB-2_TGACGGCCACGTCAGC	PB	PB-2	P2-3092	FALSE	1	TRA:CALSGGSWGKFQF TRB:CASSAGATNEQFF	1	CD8-C10
PB-2_TGACTAGGTAGGGACT	PB	PB-2	P2-41	TRUE	3	TRA:CAVRDSDYKLSF TRB:CASSQGESSYEQYF	12	CD8-C10
PB-2_TGAGGGAAGAATGTTG	PB	PB-2	P2-3131	FALSE	1	TRA:CAVTDSNYQLIW TRB:CSARRGDTEAFF	1	CD8-C10
PB-2_TGATTTCCACCCAGTG	PB	PB-2	P2-88	TRUE	3	TRA:CAVPGAGNKLTF TRB:CASSRSLAGVDTGELFF	6	CD8-C10
PB-2_TGCACCTAGTCCGTAT	PB	PB-2	P2-3139	FALSE	1	TRA:CAGPSTSGTYKYIF TRB:CAWGRGDQETQYF	1	CD8-C10
PB-2_TGCCCATGTGCAACTT	PB	PB-2	P2-41	TRUE	3	TRA:CAVRDSDYKLSF TRB:CASSQGESSYEQYF	12	CD8-C10
PB-2_TGCCCATTCCTACAGA	PB	PB-2	P2-3157	FALSE	1	TRA:CAGVAGAGSYQLTF TRB:CSARDRGDTEAFF	1	CD8-C10

PB-2_TGCGTGGCAATGAATG	PB	PB-2	P2-3181	FALSE	1	TRA:CVVSDRGSTLGRLYF TRB:CASSRDRVDDQPQHF	1	CD8-C10
PB-2_TTAGGCAAGAGGACGG	PB	PB-2	P2-3278	FALSE	1	TRA:CAVLDSNYQLIW TRB:CASSVSQGSGGETQYF	1	CD8-C10
PB-2_TTAGTTCGTAGGGACT	PB	PB-2	P2-3284	FALSE	1	TRA:CAVLDSSYKLIF TRB:CASSEASGGATDTQYF	1	CD8-C10
PB-2_TTGCCGTTCCAGTATG	PB	PB-2	P2-3347	FALSE	1	TRA:CAVRDSNYQLIW TRB:CASTPGTSGADTQYF	1	CD8-C10
PB-2_TTTGTCAAGGATGGAA	PB	PB-2	P2-375	TRUE	2	TRA:CAVGDSNYQLIW TRB:CASSDSGGATDTQYF	2	CD8-C10
PB-2_AACTCCCCAACTGGCC	PB	PB-2	P2-634	FALSE	1	TRA:CAEGGGGADGLTF TRB:CASSLQGSTYNEQFF	1	CD4-C6
PB-2_AACTCTTAGAGGGATA	PB	PB-2	P2-169	TRUE	3	TRA:CGARNNARLMF TRB:CASSRLGAANTGELFF	3	CD4-C3
PB-2_AACTTTCTCTACTATC	PB	PB-2	P2-649	FALSE	1	TRA:CAMREGSRGGNQFYF TRB:CASSLREAKEQYF	1	CD4-C7
PB-2_AAGCCGCTCTTGTTTG	PB	PB-2	P2-44	FALSE	3	TRA:CALVNTGGFKTIF TRB:CASSPPSAGGGNEQYF	11	CD4-C6
PB-2_ACACCAAGTTCAGACT	PB	PB-2	P2-720	FALSE	1	TRA:CADNPGAAAGNKLTF TRB:CASSWGGQNGYTF	1	CD4-C2
PB-2_ACGATGTCAATGCCAT	PB	PB-2	P2-810	FALSE	1	TRA:CAPVRARTF TRB:CASSTRPGGTDTQYF	1	CD4-C3
PB-2_ACGCCGAGTCGCATAT	PB	PB-2	P2-822	FALSE	1	TRA:CAVGAQGSYIPTF TRB:CASSLVGTRTIAETQYF	1	CD4-C5
PB-2_ACGGGCTCATACTACG	PB	PB-2	P2-838	FALSE	1	TRA:CAVRTTGNQFYF TRB:CASSLVGSRRASTDTQYF	1	CD4-C5
PB-2_AGCATACTCAGTTAGC	PB	PB-2	P2-968	FALSE	1	TRA:CAMRERVDRGSTLGRLYF TRB:CASSLGQPGANVLTF	1	CD4-C5
PB-2_AGCTCCTAGACAGGCT	PB	PB-2	P2-989	FALSE	1	TRA:CAGRPPDNTDKLIF TRB:CASSLGVNTIYF	1	CD4-C3
PB-2_AGGCCGTCAAGACGTG	PB	PB-2	P2-1014	FALSE	1	TRA:CAVGGLLAASKLTF TRB:CASSPVGGGVGTEAFF	1	CD8-C6
PB-2_AGGTCCGCAATGGATA	PB	PB-2	P2-175	FALSE	3	TRA:CAVSDLPLEGYQKVTF TRB:CASSITGGSLSSYEQYF	3	CD4-C5
PB-2_ATCATCTCAAATACAG	PB	PB-2	P2-1107	FALSE	1	TRA:CALSEETGNQFYF TRB:CAISESLGMGTDTQYF	1	CD8-C11
PB-2_ATCCGAACAATGTAAG	PB	PB-2	P2-1124	FALSE	1	TRA:CAASVGAGSYQLTF TRB:CASSSGQGAFGYTF	1	CD4-C2
PB-2_CAAGATCTCTTGCAAG	PB	PB-2	P2-1223	FALSE	1	TRA:CALWGQGGKLIF TRB:CASSGGQGGGNTIYF	1	CD4-C5
PB-2_CAGCATACAACTTGAC	PB	PB-2	P2-1323	FALSE	1	TRA:CAVNMQNSGGSNYKLTF TRB:CATSREIQPNEKLFF	1	CD4-C5
PB-2_CAGCCGAAGTACTTGC	PB	PB-2	P2-1329	FALSE	1	TRA:CALSGVRLMF TRB:CASSIDGGYYEQYF	1	CD4-C5
PB-2_CATTCGCTCGATAGAA	PB	PB-2	P2-1443	FALSE	1	TRA:CATVSSGYALNF TRB:CAWSSIQGQETQYF	1	CD4-C2
PB-2_CCTATTACACCGCTAG	PB	PB-2	P2-1573	FALSE	1	TRA:CAVRDRAGTASKLTF TRB:CASSQTLSAVDTQYF	1	CD4-C6
PB-2_CCTTACGTCTTGCATT	PB	PB-2	P2-1596	FALSE	1	TRA:CALSVGGGADGLTF TRB:CASSQISGGLYEQYF	1	CD4-C3
PB-2_CGATCGGTCCAGATCA	PB	PB-2	P2-1659	FALSE	1	TRA:CGTEILRSNDYKLSF TRB:CASSHPGGGTYGYTF	1	CD4-C2
PB-2_CGGCTAGAGATGGGTC	PB	PB-2	P2-186	TRUE	3	TRA:CAVRADTNAGKSTF TRB:CASSKVGTEAFF	3	CD4-C7

PB-2_CGGGTCACAGTCCTTC	PB	PB-2	P2-1757	FALSE	1	TRA:CAVVSRGYNNNDMRF TRB:CASAFGMSINSNQPQHF	1	CD4-C4
PB-2_CGTCAGGTCCCGGATG	PB	PB-2	P2-1782	FALSE	1	TRA:CAVPGANSKLTF TRB:CASSNLRGMSGNTIYF	1	CD4-C2
PB-2_CTCGTACCAAATCCGT	PB	PB-2	P2-1944	FALSE	1	TRA:CVVSAYGGATNKLIF TRB:CASSPRGTSGGRTDTQYF	1	CD4-C3
PB-2_GAATAAGCAGGATCGA	PB	PB-2	P2-2099	FALSE	1	TRA:CAGPRGGSQGNLIF TRB:CASSLGTSLSTDTQYF	1	CD4-C3
PB-2_GAATAAGGTTCATGGT	PB	PB-2	P2-2104	FALSE	1	TRA:CAGAGSGAGSYQLTF TRB:CASSYGIVRRTGRRPEAFF	1	CD4-C3
PB-2_GACGTGCCATAGTAAG	PB	PB-2	P2-2157	FALSE	1	TRA:CAVTLNYGGSQGNLIF TRB:CASFQQLGTEAFF	1	CD4-C5
PB-2_GCATGATCAAGCGCTC	PB	PB-2	P2-2315	FALSE	1	TRA:CAVGDSGAGSYQLTF TRB:CASSVGSLLGTSPLHF	1	CD4-C5
PB-2_GCATGTATCGGTCCGA	PB	PB-2	P2-334	FALSE	2	TRA:CAALQNTGKLIF TRB:CASSRDGYEQYF	2	CD4-C5
PB-2_GCATGTATCGTAGGTT	PB	PB-2	P2-2329	FALSE	1	TRA:CALSGTQGGSEKLVF TRB:CASSGGMANEQFF	1	CD4-C3
PB-2_GCTGCAGTCTGCCCTA	PB	PB-2	P2-338	TRUE	2	TRA:CAVAGRGYSTLTF TRB:CAWSVTDRGSYNEQFF	2	CD4-C5
PB-2_GCTTGAACATCTATGG	PB	PB-2	P2-340	FALSE	2	TRA:CGAAPNSGGSNYKLTF TRB:CASSLGGRSGEGETQYF	2	CD4-C5
PB-2_GGAACTTGTCATATCG	PB	PB-2	P2-2442	FALSE	1	TRA:CALRQSGGYQKVTF TRB:CASGLTSTGLAYQETQYF	1	CD4-C5
PB-2_GGACATTTCAGTTAGC	PB	PB-2	P2-2479	FALSE	1	TRA:CAVSGNSGYSTLTF TRB:CASSASSDLPQYF	1	CD4-C4
PB-2_GGCTCGAAGCTGCGAA	PB	PB-2	P2-2524	FALSE	1	TRA:CAVSDRNAGGTSYGKLTF TRB:CASSAVLSNQPQHF	1	CD4-C5
PB-2_GGGAATGGTATCGCAT	PB	PB-2	P2-349	TRUE	2	TRA:CAMRDRGANSKLTF TRB:CSASSSSGGANYIGELFF	2	CD4-C3
PB-2_GTAACGTTCAATAAGG	PB	PB-2	P2-2591	FALSE	1	TRA:CVVSDRGAGSYQLTF TRB:CSARPVGIGELFF	1	CD4-C5
PB-2_GTCACAATCATCGATG	PB	PB-2	P2-2645	FALSE	1	TRA:CIVIGGANSKLTF TRB:CASSYSKGGTGNTIYF	1	CD4-C2
PB-2_GTCCTCAGTCAACATC	PB	PB-2	P2-2666	FALSE	1	TRA:CATEGTGGFKTIF TRB:CASSLGLAGGPDTQYF	1	CD4-C5
PB-2_GTGTGCGTCGGATGTT	PB	PB-2	P2-2740	FALSE	1	TRA:CAVNEKDARLMF TRB:CASSYLGAGYQETQYF	1	CD4-C6
PB-2_GTTCGGGGTTCCACGG	PB	PB-2	P2-2774	FALSE	1	TRA:CALPSYKLSF TRB:CASYRRVTEQYF	1	CD4-C6
PB-2_TAAGTGCGTCATGCAT	PB	PB-2	P2-2804	FALSE	1	TRA:CAAAPDSNYQLIW TRB:CAISEIGANSGNTIYF	1	CD4-C2
PB-2_TACACGAGTTAGAACA	PB	PB-2	P2-2811	FALSE	1	TRA:CALSESLTGANNLFF TRB:CASSRLRTGAGYEQYF	1	CD4-C5
PB-2_TCGGGACCAGTATAAG	PB	PB-2	P2-3025	FALSE	1	TRA:CAAVRGSGGGADGLTF TRB:CASSLHVTGELFF	1	CD4-C3
PB-2_TCTTCGGCATGATCCA	PB	PB-2	P2-3068	FALSE	1	TRA:CILRDGGSQGNLIF TRB:CASSLYGGKETQYF	1	CD4-C6
PB-2_TGAGCCGGTGTGTGCC	PB	PB-2	P2-3127	FALSE	1	TRA:CAVGGDKLTF TRB:CASSPSGSYTQYF	1	CD4-C7
PB-2_TGAGCCGTCTTGTACT	PB	PB-2	P2-3129	FALSE	1	TRA:CAEPPGGSYIPTF TRB:CASSQHHGSYEQYF	1	CD4-C2
PB-2_TGCTGCTAGTTGTCGT	PB	PB-2	P2-371	TRUE	2	TRA:CLVGNQGGKLIF TRB:CASTPGRESDIQYF	2	CD4-C5

PB-2_TGGCTGGTCAACGCTA	PB	PB-2	P2-3214	FALSE	1	TRA:CAERDNNARLMF TRB:CASGRAENTEAFF	1	CD8-C2
PB-2_TTCCCAGGTACATCCA	PB	PB-2	P2-204	TRUE	3	TRA:CAVGRRGSGGSNYKLTF TRB:CASQQGYSGNTIYF	3	CD4-C5
PB-2_TTCCCAGTCTGATTCT	PB	PB-2	P2-3296	FALSE	1	TRA:CAVRPGLKTSYDKVIF TRB:CASSMDSATPTGELFF	1	CD4-C5
PB-2_TTCGGTCAGGCCCTTG	PB	PB-2	P2-3303	FALSE	1	TRA:CALSDLAGGGADGLTF TRB:CASSLTGASGNEQFF	1	CD4-C2
PB-2_TTTACTGTCAGCTCGG	PB	PB-2	P2-3385	FALSE	1	TRA:CAVPLGAQKLVF TRB:CASSLNSGGFLSYNEQFF	1	CD4-C2
PB-2_TTTATGCAGGGTATCG	PB	PB-2	P2-340	FALSE	2	TRA:CGAAPNSGGSNYKLTF TRB:CASSLGGRSGEGETQYF	2	CD4-C5
PB-2_AACCATGCAACAACCT	PB	PB-2	P2-609	FALSE	1	TRA:CAVVNGGSYIPTF TRB:CASILGGLAALNNEQFF	1	CD4-C7
PB-2_AACCATGTCCTGTAGA	PB	PB-2	P2-614	FALSE	1	TRA:CAASTDKLIF TRB:CAISLAASTDTQYF	1	CD4-C7
PB-2_AACCGCGAGCACACAG	PB	PB-2	P2-618	FALSE	1	TRA:CAVRFLSNAGGTSYGKLTF TRB:CASMMSLGGEQYF	1	CD4-C7
PB-2_AACCGCGCACACTGCG	PB	PB-2	P2-621	FALSE	1	TRA:CAMYHSGTYKYIF TRB:CASSSQPGQNYEQYF	1	CD4-C7
PB-2_AACTCCCCAATTGCTG	PB	PB-2	P2-635	FALSE	1	TRA:CAVRGPNNNDMRF TRB:CAWAQGARQPQHF	1	CD4-C7
PB-2_AACTCTTGTGTAACGG	PB	PB-2	P2-638	FALSE	1	TRA:CAMGRGNNNDMRF TRB:CASSQDRMMETQYF	1	CD4-C7
PB-2_AAGACCTGTGCCTGTG	PB	PB-2	P2-654	FALSE	1	TRA:CAGEGNQGGKLIF TRB:CASRKVDRLSSPLHF	1	CD4-C7
PB-2_AAGGAGCAGCACCGTC	PB	PB-2	P2-669	FALSE	1	TRA:CAFMIRNNNARLMF TRB:CSASVAGGRETQYF	1	CD4-C7
PB-2_AAGTCTGCAATCTACG	PB	PB-2	P2-691	FALSE	1	TRA:CAYGGATNKLIF TRB:CASSLFGGRNSPLHF	1	CD4-C7
PB-2_AATCGGTAGGCTCATT	PB	PB-2	P2-710	FALSE	1	TRA:CAAIRNTGFQKLVF TRB:CASSLPLYEQYF	1	CD4-C7
PB-2_ACACCAAGTAAGGATT	PB	PB-2	P2-718	FALSE	1	TRA:CALRLYSGGSNYKLTF TRB:CASSDPAGGIGYEQYF	1	CD4-C2
PB-2_ACACCGGTCACCTCGT	PB	PB-2	P2-737	FALSE	1	TRA:CAALADQFYF TRB:CASSAGTAYGYTF	1	CD4-C2
PB-2_ACACCGGTCTTCTGGC	PB	PB-2	P2-738	FALSE	1	TRA:CAFIRGTGFQKLVF TRB:CASSETNGELFF	1	CD4-C7
PB-2_ACATCAGAGGGCTCTC	PB	PB-2	P2-754	FALSE	1	TRA:CAVENSGGSNYKLTF TRB:CASSLGLATGELFF	1	CD4-C7
PB-2_ACATCAGCAGCTTCGG	PB	PB-2	P2-757	FALSE	1	TRA:CAVRAGGTYKYIF TRB:CASSLSPPRGWDYEQFF	1	CD4-C2
PB-2_ACCCACTCAGCTGCAC	PB	PB-2	P2-777	FALSE	1	TRA:CARAGKLIF TRB:CAISTVNGQQYF	1	CD4-C7
PB-2_ACCTTTACACAGACAG	PB	PB-2	P2-788	FALSE	1	TRA:CAMREGPGGYNKLIF TRB:CSAHRTVSSYNEQFF	1	CD4-C7
PB-2_ACGATGTAGGTCATCT	PB	PB-2	P2-809	FALSE	1	TRA:CAASGSGNTGKLIF TRB:CASSVGHAGNGYTF	1	CD4-C7
PB-2_ACGCAGCCAGGTCGTC	PB	PB-2	P2-171	TRUE	3	TRA:CAVNILDGNKLVF TRB:CAHASGTSVYEQYF	3	CD4-C7
PB-2_AGACGTTCACCACGTG	PB	PB-2	P2-921	FALSE	1	TRA:CAVQRSAGNKLTF TRB:CASSWTGAVNYGYTF	1	CD4-C7
PB-2_AGAGCTTTCTTCGGTC	PB	PB-2	P2-938	FALSE	1	TRA:CAVSMDSSYKLIF TRB:CSAIDLSSSSYEQYF	1	CD4-C2

PB-2_AGATCTGCAAGACACG	PB	PB-2	P2-942	FALSE	1	TRA:CAVSDQGYRDDKIIF TRB:CASSRGVSYEQYF	1	CD4-C7
PB-2_AGCAGCCCAGGAACGT	PB	PB-2	P2-958	FALSE	1	TRA:CAERRSGGYQKVTF TRB:CASSPARGRSSTDTQYF	1	CD4-C7
PB-2_AGCGTATTCTCGCATC	PB	PB-2	P2-983	FALSE	1	TRA:CAGAGNFNKFYF TRB:CASSLIPGLGGLATDTQYF	1	CD4-C7
PB-2_AGCTCCTAGTCTCGGC	PB	PB-2	P2-991	FALSE	1	TRA:CARYRGSQGNLIF TRB:CASSPRGLKDQPQHF	1	CD4-C7
PB-2_AGGCCGTAGTATTGGA	PB	PB-2	P2-286	FALSE	2	TRA:CALFEGTYKYIF TRB:CASSSGQGSSPLHF	2	CD4-C7
PB-2_AGGGATGAGGTGACCA	PB	PB-2	P2-1026	FALSE	1	TRA:CALTPLIYNQGGKLIF TRB:CASGGQGSTGELFF	1	CD4-C7
PB-2_AGGGTGATCTACTTAC	PB	PB-2	P2-1037	FALSE	1	TRA:CAVTTNSGYALNF TRB:CASSFVGGELFF	1	CD4-C7
PB-2_AGGTCATGTTGTGGAG	PB	PB-2	P2-1047	FALSE	1	TRA:CATYSNSGYALNF TRB:CACSQTSLRWGNTIYF	1	CD4-C7
PB-2_ATCTGCCAGGCGATAC	PB	PB-2	P2-1139	FALSE	1	TRA:CAMRGVYSSASKIIF TRB:CASREGNEKLFF	1	CD4-C7
PB-2_ATGAGGGAGTGGAGAA	PB	PB-2	P2-286	FALSE	2	TRA:CALFEGTYKYIF TRB:CASSSGQGSSPLHF	2	CD4-C7
PB-2_ATGCGATGTTCAGACT	PB	PB-2	P2-1153	FALSE	1	TRA:CAYRSGGGSQGNLIF TRB:CASSYRFSDYEQYF	1	CD4-C7
PB-2_ATGTGTGCAGTGGAGT	PB	PB-2	P2-1160	FALSE	1	TRA:CAARLTGGGNKLTF TRB:CSAADFRETQYF	1	CD4-C7
PB-2_ATGTGTGCATTCGACA	PB	PB-2	P2-1161	FALSE	1	TRA:CIVRVNQAGTALIF TRB:CASSSLLERGPNEQFF	1	CD4-C7
PB-2_ATTATCCTCATGTCTT	PB	PB-2	P2-1174	FALSE	1	TRA:CVVMATGRRALTF TRB:CASSDPGGVETQYF	1	CD4-C2
PB-2_ATTCTACCACTAGTAC	PB	PB-2	P2-1176	FALSE	1	TRA:CFHTGANSKLTF TRB:CASRRRQESYEQYF	1	CD4-C7
PB-2_ATTGGACCACAGGTTT	PB	PB-2	P2-1181	FALSE	1	TRA:CAASRGDSSYKLIF TRB:CASSPSIGEQYF	1	CD4-C7
PB-2_ATTGGTGGTCTCACCT	PB	PB-2	P2-1187	FALSE	1	TRA:CAVGGGDNYGQNFVF TRB:CASSQAQGTGFWTNEKLFF	1	CD4-C7
PB-2_CAACCAAGTAAGTGGC	PB	PB-2	P2-1200	FALSE	1	TRA:CAVSANFGNEKLTF TRB:CSAREPLWGTIQYF	1	CD4-C2
PB-2_CACACCTGTGTGACCC	PB	PB-2	P2-1248	FALSE	1	TRA:CVVGSGYSTLTF TRB:CASSIAGLAGGSTGELFF	1	CD4-C7
PB-2_CACAGTAAGAGCTATA	PB	PB-2	P2-1260	FALSE	1	TRA:CAVKFAGGTSYGKLTF TRB:CASSLGQETQYF	1	CD4-C7
PB-2_CACAGTAGTGAACCTT	PB	PB-2	P2-1268	FALSE	1	TRA:CAVNANGGATNKLIF TRB:CASSSGGSRVNEQFF	1	CD4-C7
PB-2_CAGCAGCAGACCCACC	PB	PB-2	P2-1317	FALSE	1	TRA:CAGNSGTYKYIF TRB:CASSQTGLGETQYF	1	CD4-C7
PB-2_CATATTCAGGCATGTG	PB	PB-2	P2-1369	FALSE	1	TRA:CAGRGGATNKLIF TRB:CASSLDSYNEQFF	1	CD4-C7
PB-2_CATCAAGGTCTCAACA	PB	PB-2	P2-1379	FALSE	1	TRA:CIVLYGNNRLAF TRB:CASSLEGIMNTEAFF	1	CD4-C7
PB-2_CATCCACTCAACTCTT	PB	PB-2	P2-1392	FALSE	1	TRA:CAVIIFGNEKLTF TRB:CASSALQGRNTEAFF	1	CD4-C7
PB-2_CATGCCTAGCGTGAGT	PB	PB-2	P2-1420	FALSE	1	TRA:CIVRPSSARQLTF TRB:CASSRTSGMKNEQFF	1	CD4-C7
PB-2_CCAATCCTCCCTTGTG	PB	PB-2	P2-1449	FALSE	1	TRA:CGTDYNTDKLIF TRB:CASSYSAGELFF	1	CD4-C7

PB-2_CCATTCGCAGCTCCGA	PB	PB-2	P2-1491	FALSE	1	TRA:CAVRTLYQGGKLIF TRB:CSASSGGAIGTGELFF	1	CD4-C7
PB-2_CCCAGTTTCACATGCA	PB	PB-2	P2-1511	FALSE	1	TRA:CAVMNTGFQKLVF TRB:CASSLENNQPQHF	1	CD4-C7
PB-2_CCGTTCAGTGGACGAT	PB	PB-2	P2-1545	FALSE	1	TRA:CIVRVDPSDGQKLLF TRB:CAIRARSYEQYF	1	CD4-C2
PB-2_CCGTTCATCTACGAGT	PB	PB-2	P2-1546	FALSE	1	TRA:CAVVIKAAGNKLTF TRB:CASSQAWGQRDTGELFF	1	CD4-C2
PB-2_CCTATTATCCACGTTC	PB	PB-2	P2-1580	FALSE	1	TRA:CGADAEYGNKLVF TRB:CASSADFGQETQYF	1	CD4-C7
PB-2_CCTCTGAAGAGCCCAA	PB	PB-2	P2-1584	FALSE	1	TRA:CAAFMFSGGYNKLIF TRB:CSANRDRGAWETQYF	1	CD4-C2
PB-2_CGAGCACAGATATGCA	PB	PB-2	P2-1642	FALSE	1	TRA:CAVVNAGNTGKLIF TRB:CASSLNIVDNEQFF	1	CD4-C7
PB-2_CGATGTACACAGTCGC	PB	PB-2	P2-1664	FALSE	1	TRA:CAASVAGGSQGNLIF TRB:CASRLTGVGTQYF	1	CD4-C7
PB-2_CGATGTACATTCGACA	PB	PB-2	P2-1666	FALSE	1	TRA:CAMRGMTTDSWGKFQF TRB:CAWSVVRGVISPLHF	1	CD4-C7
PB-2_CGCTGGAAGCCCGAAA	PB	PB-2	P2-1699	FALSE	1	TRA:CIPMNNAGNMLTF TRB:CATTRELAASSYNEQFF	1	CD4-C7
PB-2_CGGACTGAGCGAGAAA	PB	PB-2	P2-1726	FALSE	1	TRA:CALDMNTGFQKLVF TRB:CASSVSSNTEAFF	1	CD4-C7
PB-2_CGGAGTCGTTCACGGC	PB	PB-2	P2-1742	FALSE	1	TRA:CALERTSYDKVIF TRB:CSAGYRGKQETQYF	1	CD4-C7
PB-2_CGGCTAGTCGTCCGTT	PB	PB-2	P2-1752	FALSE	1	TRA:CAVYLGNEKLTF TRB:CASRATSETYEQYF	1	CD4-C7
PB-2_CGTCCATAGCTCCTCT	PB	PB-2	P2-1787	FALSE	1	TRA:CAVRDTKGGSYIPTF TRB:CASSLAGENTEAFF	1	CD4-C7
PB-2_CGTGAGCCAGTCGTGC	PB	PB-2	P2-1804	FALSE	1	TRA:CAVSDLRDYKLSF TRB:CAWGAGTGASPLHF	1	CD4-C7
PB-2_CGTTAGACAAGTTGTC	PB	PB-2	P2-1817	FALSE	1	TRA:CAVEERGSYIPTF TRB:CASRPLAGGQYNEQFF	1	CD4-C7
PB-2_CGTTCTGTCCCATTTA	PB	PB-2	P2-1830	FALSE	1	TRA:CAGHPGGYQKVTF TRB:CASSLSAGSSSPLHF	1	CD4-C7
PB-2_CTAACTTAGCAGACTG	PB	PB-2	P2-1836	FALSE	1	TRA:CALSYGNQFYF TRB:CASRPQQGVLDTQYF	1	CD4-C7
PB-2_CTAATGGTCCGTCATC	PB	PB-2	P2-1856	FALSE	1	TRA:CAVRDSNTGGFKTIF TRB:CASSLYRGATDSTGELFF	1	CD4-C7
PB-2_CTACACCCAGCTGCAC	PB	PB-2	P2-1859	FALSE	1	TRA:CALNRDDKIIF TRB:CASTSSLGSGRAAGNEQYF	1	CD4-C7
PB-2_CTCTGGTGTATCACCA	PB	PB-2	P2-1965	FALSE	1	TRA:CATDAAQGAQKLVF TRB:CASVKPSTLTGELFF	1	CD4-C7
PB-2_CTGCTGTCAGGATCGA	PB	PB-2	P2-2007	FALSE	1	TRA:CALSDGGATNKLIF TRB:CASSQQDLGGDTQYF	1	CD4-C7
PB-2_CTGCTGTGTTCATGGT	PB	PB-2	P2-2010	FALSE	1	TRA:CALFAGNNRKLIW TRB:CAWTGTSGYGYTF	1	CD4-C2
PB-2_CTTACCGGTCTCAACA	PB	PB-2	P2-2041	FALSE	1	TRA:CATAPDTGRRALTF TRB:CASSAQGPNTGELFF	1	CD4-C2
PB-2_CTTCTCTTCCTGTAGA	PB	PB-2	P2-2053	FALSE	1	TRA:CAVRDRGGYQKVTF TRB:CAWSRGAGGVDEQFF	1	CD4-C7
PB-2_GAATGAAGTCGAGATG	PB	PB-2	P2-2106	FALSE	1	TRA:CAVTPPWGRRALTF TRB:CASSSEGNTEAFF	1	CD4-C7
PB-2_GACAGAGAGCCAGTTT	PB	PB-2	P2-2115	FALSE	1	TRA:CAVNLNNAGNMLTF TRB:CASSPMGGSYNEQFF	1	CD4-C7

PB-2_GACAGAGAGTACACCT	PB	PB-2	P2-2116	FALSE	1	TRA:CLVGSDSNYQLIW TRB:CASSSRPGQGLGTQYF	1	CD4-C7
PB-2_GACCAATGTAGGGACT	PB	PB-2	P2-2128	FALSE	1	TRA:CAASLPNSGNTPLVF TRB:CASSLLAVFSNQPQHF	1	CD4-C7
PB-2_GACCTGGAGGCAATTA	PB	PB-2	P2-2135	FALSE	1	TRA:CAASKGDYKLSF TRB:CASSIIGGNGLGRGNQPQHF	1	CD4-C7
PB-2_GATCAGTCACTAAGTC	PB	PB-2	P2-2214	FALSE	1	TRA:CAVSPLKGAQKLVF TRB:CASSPTGGHTDTQYF	1	CD4-C7
PB-2_GATCGTATCATGGTCA	PB	PB-2	P2-2238	FALSE	1	TRA:CAVNAGGTSYGKLTF TRB:CASSSRDRGQAFF	1	CD4-C7
PB-2_GATGAGGGTAAGGGAA	PB	PB-2	P2-2263	FALSE	1	TRA:CAFMKHGRGGSEKLVF TRB:CASRQQTKEKLFF	1	CD4-C2
PB-2_GCAATCAAGACAAGCC	PB	PB-2	P2-2279	FALSE	1	TRA:CAFMKHDNNARLMF TRB:CAWSTPGFFAFF	1	CD4-C7
PB-2_GCACATACATCTCCCA	PB	PB-2	P2-2289	FALSE	1	TRA:CVVNIIGYQKVTF TRB:CSASLGINEKLFF	1	CD4-C7
PB-2_GCACTCTCATCTCGCT	PB	PB-2	P2-2292	FALSE	1	TRA:CAANSGGGADGLTF TRB:CASHVTRNTEAFF	1	CD4-C7
PB-2_GCACTCTGTTACGTCA	PB	PB-2	P2-2295	FALSE	1	TRA:CVVSKGGSTLGRLYF TRB:CATSDPGTSGINEQFF	1	CD4-C2
PB-2_GCATGATCAATAACGA	PB	PB-2	P2-332	TRUE	2	TRA:CAVPGDQAGTALIF TRB:CASSLANAGPVEKLFF	2	CD4-C7
PB-2_GCATGCGAGGCCATAG	PB	PB-2	P2-2318	FALSE	1	TRA:CAVNLYNFNKFYF TRB:CASSALEKTGTQYF	1	CD4-C2
PB-2_GCCAAATCATGTAGTC	PB	PB-2	P2-2331	FALSE	1	TRA:CALSEAQAGFQKLVF TRB:CASSHYRGRDEQYF	1	CD4-C7
PB-2_GCGCAACAGATCCCAT	PB	PB-2	P2-2362	FALSE	1	TRA:CALEARRSNGNEKLTF TRB:CASGKAPNTGELFF	1	CD4-C2
PB-2_GCTTCCAGTTATCACG	PB	PB-2	P2-2429	FALSE	1	TRA:CAGIYDKLIF TRB:CASSRQGLSSTQYF	1	CD4-C7
PB-2_GGACGTCTCATGTGGT	PB	PB-2	P2-2483	FALSE	1	TRA:CAVGPDPFKAAGNKLTF TRB:CSARDRGAGANVLTF	1	CD4-C7
PB-2_GGATGTTAGAGGTTGC	PB	PB-2	P2-2489	FALSE	1	TRA:CALLNFGNEKLTF TRB:CASSPGTYEQYF	1	CD4-C7
PB-2_GGATTACAGGATGTAT	PB	PB-2	P2-2495	FALSE	1	TRA:CAVRPDTGFQKLVF TRB:CASSQETGRGNQPQHF	1	CD4-C2
PB-2_GGCTCGACAAACTGCT	PB	PB-2	P2-2526	FALSE	1	TRA:CAVKGSQGNLIF TRB:CASSEQGLARETQYF	1	CD4-C7
PB-2_GGGAATGAGCGTAGTG	PB	PB-2	P2-2535	FALSE	1	TRA:CALPRGGTSYGKLTF TRB:CASSLVAGPSNTEAFF	1	CD4-C7
PB-2_GGGAATGCAGGTTTCA	PB	PB-2	P2-2539	FALSE	1	TRA:CAMVKAAGNKLTF TRB:CASSYDRINTEAFF	1	CD4-C7
PB-2_GGGAGATAGGGTCGAT	PB	PB-2	P2-2544	FALSE	1	TRA:CIVGGGTSYGKLTF TRB:CASSFVSSTEAFF	1	CD4-C7
PB-2_GGGAGATTCTATCGCC	PB	PB-2	P2-2549	FALSE	1	TRA:CAVSFETSYDKVIF TRB:CAISATTSGVYEQYF	1	CD4-C2
PB-2_GGGCATCTCGGTTAAC	PB	PB-2	P2-2560	FALSE	1	TRA:CIVLFGNEKLTF TRB:CSALALAGKSSYEQYF	1	CD4-C7
PB-2_GGGTTGCGTCCGTCAG	PB	PB-2	P2-2572	FALSE	1	TRA:CATDARSDGQKLLF TRB:CASSVTPRDKTYEQYF	1	CD4-C7
PB-2_GTAACGTAGTGGACGT	PB	PB-2	P2-2588	FALSE	1	TRA:CAFMAPTSGTYKYIF TRB:CASSGIDRDFADTQYF	1	CD4-C7
PB-2_GTCGGGTAGTTCGATC	PB	PB-2	P2-2670	FALSE	1	TRA:CAMRELISNFGNEKLTF TRB:CASSYGYILHNEQFF	1	CD4-C7

PB-2_GTCGGGTTCCATGAAC	PB	PB-2	P2-2673	FALSE	1	TRA:CAVRRSYIPTF TRB:CSAQDAATNEKLFF	1	CD4-C7
PB-2_GTCTTCGCAAACCCAT	PB	PB-2	P2-2694	FALSE	1	TRA:CAVNTNAGKSTF TRB:CASSQVAGANTGELFF	1	CD4-C7
PB-2_GTCTTCGGTCCGACGT	PB	PB-2	P2-2696	FALSE	1	TRA:CILKDNTDKLIF TRB:CATSRGTGAIGELFF	1	CD4-C7
PB-2_GTTTCTACACCCATGG	PB	PB-2	P2-2783	FALSE	1	TRA:CAQFMNSGGYQKVTF TRB:CASSLMAAGELFF	1	CD4-C7
PB-2_TAAGCGTAGACAAGCC	PB	PB-2	P2-2795	FALSE	1	TRA:CVVSAGGNNRLAF TRB:CASSYHRTDDYGYTF	1	CD4-C7
PB-2_TAAGCGTAGATGTTAG	PB	PB-2	P2-2797	FALSE	1	TRA:CAVHRGSTLGRLYF TRB:CSGIAGTSGTRAKNIQYF	1	CD4-C7
PB-2_TAAGCGTAGCGTGAGT	PB	PB-2	P2-2798	FALSE	1	TRA:CAVNMLKTGANNLFF TRB:CASTLEVQGLYGYTF	1	CD4-C7
PB-2_TAAGTGCGTCCGTCAG	PB	PB-2	P2-2805	FALSE	1	TRA:CIVGAGGTSYGKLTF TRB:CASRQASHRVSNQPQHF	1	CD4-C7
PB-2_TACAGTGCAGATGGCA	PB	PB-2	P2-2815	FALSE	1	TRA:CAASDDHGQNFVF TRB:CASSPAQETQYF	1	CD4-C7
PB-2_TACCTTAAGCGTGAAC	PB	PB-2	P2-2831	FALSE	1	TRA:CAVAHSGTYKYIF TRB:CASSLSQGETQYF	1	CD4-C7
PB-2_TACTCGCAGTACGCGA	PB	PB-2	P2-2856	FALSE	1	TRA:CAVGPPYNTDKLIF TRB:CASSPKGQSDNEQFF	1	CD4-C7
PB-2_TAGCCGGGTGTCCTCT	PB	PB-2	P2-359	FALSE	2	TRA:CIVRPIYNQGGKLIF TRB:CASRQGSYEQYF	2	CD4-C7
PB-2_TATCTCACAGGCAGTA	PB	PB-2	P2-2919	FALSE	1	TRA:CAFTGANSKLTF TRB:CASSQVTGRRDEQFF	1	CD4-C7
PB-2_TATGCCCGTAAGTAGT	PB	PB-2	P2-2926	FALSE	1	TRA:CAGPREIGTASKLTF TRB:CASSFRLDWGTDTQYF	1	CD4-C7
PB-2_TATGCCCGTTAGATGA	PB	PB-2	P2-2929	FALSE	1	TRA:CAVRPFSGGYNKLIF TRB:CAWRERGSGAYEQYF	1	CD4-C7
PB-2_TATTACCTCAGCTTAG	PB	PB-2	P2-2935	FALSE	1	TRA:CAVNSGGYNKLIF TRB:CSASSPGRTSGVDTQYF	1	CD4-C7
PB-2_TCACAAGTCAAGATCC	PB	PB-2	P2-2950	FALSE	1	TRA:CALSEDPESSASKIIF TRB:CASSLAAGASYNEQFF	1	CD4-C7
PB-2_TCATTACAGTACTTGC	PB	PB-2	P2-2988	FALSE	1	TRA:CAVSGGGADGLTF TRB:CSARAWDRGNTEAFF	1	CD4-C7
PB-2_TCGCGTTGTAGCGCTC	PB	PB-2	P2-3021	FALSE	1	TRA:CAVRDSSGGSYIPTF TRB:CASSWGERPDTQYF	1	CD4-C2
PB-2_TCGGGACAGGGCTCTC	PB	PB-2	P2-3024	FALSE	1	TRA:CAVSRARRAGSYQLTF TRB:CASATSGVGETQYF	1	CD4-C7
PB-2_TCGTAGAGTCCGAAGA	PB	PB-2	P2-3036	FALSE	1	TRA:CAVRGYAPLGNDKIIF TRB:CASSIGDSGNEQFF	1	CD4-C7
PB-2_TCTCATAAGTTAGGTA	PB	PB-2	P2-3048	FALSE	1	TRA:CALSEALVTGNQFYF TRB:CASSSFVGVTGELFF	1	CD4-C7
PB-2_TCTGAGACATACGCCG	PB	PB-2	P2-3060	FALSE	1	TRA:CAARQAGTALIF TRB:CASSFWDVPSTDTQYF	1	CD4-C7
PB-2_TGAGAGGCACTGCCAG	PB	PB-2	P2-3116	FALSE	1	TRA:CAAPNAGNMLTF TRB:CAGGEIQGSLAKNIQYF	1	CD4-C2
PB-2_TGGCGCAAGAAGGTGA	PB	PB-2	P2-359	FALSE	2	TRA:CIVRPIYNQGGKLIF TRB:CASRQGSYEQYF	2	CD4-C7
PB-2_TGGGCGTTCTTGAGAC	PB	PB-2	P2-3229	FALSE	1	TRA:CALSGGETSGSRLTF TRB:CASSDRAFSTQYF	1	CD4-C7
PB-2_TGGTTCCTCGCATGGC	PB	PB-2	P2-3234	FALSE	1	TRA:CALLRGEFSGGSYIPTF TRB:CASFGQVNTGELFF	1	CD4-C7

PB-2_TGTCCCAGTAAACCTC	PB	PB-2	P2-3246	FALSE	1	TRA:CAVLPGGYQKVTF TRB:CASRQGASNQPQHF	1	CD4-C7
PB-2_TGTGTTTCACCACGTG	PB	PB-2	P2-3255	FALSE	1	TRA:CAVLSGSARQLTF TRB:CASSQEQGSDGYTF	1	CD4-C7
PB-2_TGTGTTTTCCCAGGTG	PB	PB-2	P2-3258	FALSE	1	TRA:CIVPRAGSARQLTF TRB:CSARDGTDPRINSPLHF	1	CD4-C7
PB-2_TGTTCCGGTCCTGCTT	PB	PB-2	P2-3264	FALSE	1	TRA:CAVKRAAGNKLTF TRB:CASNQGLAGGHGETQYF	1	CD4-C7
PB-2_TTATGCTCACCCATTC	PB	PB-2	P2-3289	FALSE	1	TRA:CLVAPYNQGGKLIF TRB:CASSQGQVLSSYEQYF	1	CD4-C7
PB-2_TTCGGTCCACGAAACG	PB	PB-2	P2-3306	FALSE	1	TRA:CAVYTGTASKLTF TRB:CASSPPPPSYEQYF	1	CD4-C7
PB-2_TTGCCGTCATCACGAT	PB	PB-2	P2-205	TRUE	3	TRA:CALSESTNAGKSTF TRB:CAWSYADTQYF	3	CD4-C7
PB-2_TTGCCGTTCCAGGGCT	PB	PB-2	P2-3346	FALSE	1	TRA:CLVVGGGADGLTF TRB:CSGLSGSLSYEQYF	1	CD4-C7
PB-2_TTGGCAACACCCTATC	PB	PB-2	P2-3367	FALSE	1	TRA:CIVRVAGTASKLTF TRB:CASSSANTGELFF	1	CD4-C7
PB-2_TTTACTGAGAATTGTG	PB	PB-2	P2-3376	FALSE	1	TRA:CADPFQGAQKLVF TRB:CAYSGGFTDTQYF	1	CD4-C7
PB-2_TTTGTCAGTTGCTCCT	PB	PB-2	P2-3408	FALSE	1	TRA:CAMREGGTGRRALTF TRB:CASGLPQHF	1	CD4-C7
PB-2_GCGCAGTTCTTAGAGC	PB	PB-2	P2-2376	FALSE	1	TRA:CALSSGGSNYKLTF TRB:CASHTGDSRPYEQYF	1	CD4-C2
PB-2_AAACCTGAGAGTTGGC	PB	PB-2	P2-567	FALSE	1	TRA:CVVSESAGNNRKLIW TRB:CASSPGLAGVSDTQYF	1	CD4-C1
PB-2_AAACCTGTCATGGTCA	PB	PB-2	P2-573	FALSE	1	TRA:CAENNDYKLSF TRB:CASSQVMGGTEAFF	1	CD4-C1
PB-2_AAACGGGGTATCGCAT	PB	PB-2	P2-577	FALSE	1	TRA:CAASASQGGSEKLVF TRB:CASSPGTGYGYTF	1	CD4-C1
PB-2_AAACGGGTCATGTAGC	PB	PB-2	P2-579	FALSE	1	TRA:CGTENPTPLVF TRB:CASGYSRLFNFQPQHF	1	CD4-C1
PB-2_AAAGATGTCAGTACGT	PB	PB-2	P2-583	FALSE	1	TRA:CAGAESNQFYF TRB:CASSFLRGVSGYTF	1	CD4-C1
PB-2_AAAGCAATCAGAGACG	PB	PB-2	P2-590	FALSE	1	TRA:CALSAQGAQKLVF TRB:CASSPGTAGTQYF	1	CD4-C1
PB-2_AAAGTAGAGTATCGAA	PB	PB-2	P2-594	FALSE	1	TRA:CASPLSNSGYALNF TRB:CASRGHTEAFF	1	CD4-C1
PB-2_AAAGTAGGTAAATACG	PB	PB-2	P2-597	FALSE	1	TRA:CAVSFYTDKLIF TRB:CASSLGDRGYGYTF	1	CD4-C1
PB-2_AAATGCCAGACTGTAA	PB	PB-2	P2-600	FALSE	1	TRA:CAETGGGGADGLTF TRB:CAWGKTVRNTIYF	1	CD4-C1
PB-2_AAATGCCGTCCGACGT	PB	PB-2	P2-604	FALSE	1	TRA:CVVSARNDYKLSF TRB:CASGSGALNTEAFF	1	CD4-C1
PB-2_AACACGTAGGCAATTA	PB	PB-2	P2-606	FALSE	1	TRA:CALDPRGRNRDDKIIF TRB:CSAILAGDGTDTQYF	1	CD4-C1
PB-2_AACCGCGCAGACGCTC	PB	PB-2	P2-622	FALSE	1	TRA:CAVRATTSGTYKYIF TRB:CASSPPPSGTDTQYF	1	CD4-C1
PB-2_AACCGCGTCAAAGACA	PB	PB-2	P2-623	FALSE	1	TRA:CALSDYGGSQGNLIF TRB:CASSSRTALNSGNTIYF	1	CD4-C1
PB-2_AACGTTGCACGCATCG	PB	PB-2	P2-625	FALSE	1	TRA:CAASMDGTASKLTF TRB:CASSILPGHHEQYF	1	CD4-C1
PB-2_AACTCAGTCTACTATC	PB	PB-2	P2-631	FALSE	1	TRA:CALPTYHSGNTPLVF TRB:CASSQSDRGSYEQYF	1	CD4-C1

PB-2_AACTCAGTCTTAGAGC	PB	PB-2	P2-632	FALSE	1	TRA:CGGATNKLIF TRB:CACQRDSNEKLFF	1	CD4-C1
PB-2_AACTCCCTCAGTCAGT	PB	PB-2	P2-637	FALSE	1	TRA:CAVRTSRRALTF TRB:CASSFESNQPQHF	1	CD4-C1
PB-2_AACTCTTTCCTCAACC	PB	PB-2	P2-640	FALSE	1	TRA:CATDAWAAGNKLTF TRB:CAWTLPLFGQFF	1	CD4-C1
PB-2_AACTGGTAGACGCAAC	PB	PB-2	P2-641	FALSE	1	TRA:CAASRGSGGYNKLIF TRB:CASSLGLAGGEQFF	1	CD4-C1
PB-2_AACTTTCAGCTAGTGG	PB	PB-2	P2-645	FALSE	1	TRA:CAGRRDSGAGSYQLTF TRB:CSVYRGWDYGYTF	1	CD4-C1
PB-2_AAGACCTTCATCGGAT	PB	PB-2	P2-657	FALSE	1	TRA:CAHHSGSARQLTF TRB:CASSLDLGGYTF	1	CD4-C1
PB-2_AAGCCGCAGCTTCGCG	PB	PB-2	P2-660	FALSE	1	TRA:CAGNTGNQFYF TRB:CASSAHRTAYEQYF	1	CD4-C1
PB-2_AAGCCGCCATGCTGGC	PB	PB-2	P2-663	FALSE	1	TRA:CAAKGNTGKLIF TRB:CATSDFQGGYTF	1	CD4-C1
PB-2_AAGGCAGCAAAGCGGT	PB	PB-2	P2-675	FALSE	1	TRA:CAVETGGSYIPTF TRB:CASSLAGGGTGELFF	1	CD4-C1
PB-2_AAGGCAGGTGCAGGTA	PB	PB-2	P2-681	FALSE	1	TRA:CAVRPGDYKLSF TRB:CASSRYTNQETQYF	1	CD4-C1
PB-2_AAGGCAGTCCGTCATC	PB	PB-2	P2-682	FALSE	1	TRA:CAVYNYGQNFVF TRB:CASSRAGGVYEQYF	1	CD4-C2
PB-2_AAGGTTCAGGGCACTA	PB	PB-2	P2-684	FALSE	1	TRA:CAVQAGNSGAGSYQLTF TRB:CASSRIGGHSYEQYF	1	CD4-C1
PB-2_AAGTCTGTCTCTTATG	PB	PB-2	P2-698	FALSE	1	TRA:CASKINAGGTSYGKLTF TRB:CASSSGIYNSPLHF	1	CD4-C1
PB-2_ACACCAATCACTGGGC	PB	PB-2	P2-721	FALSE	1	TRA:CAVISSYGGATNKLIF TRB:CASSSSFGGAYEQYF	1	CD4-C1
PB-2_ACACCCTAGACACGAC	PB	PB-2	P2-726	FALSE	1	TRA:CAMRGTYSGGGADGLTF TRB:CASSQPERGDPNYNEQFI	1	CD4-C1
PB-2_ACACCCTAGTGTTGAA	PB	PB-2	P2-727	FALSE	1	TRA:CAVGAR TRB:CASNLKTTEAFF	1	CD4-C1
PB-2_ACACCGGAGTTACCCA	PB	PB-2	P2-733	FALSE	1	TRA:CAVEGRTGGYNKLIF TRB:CAWIRDSYEQYF	1	CD4-C1
PB-2_ACACCGGCAAAGGAAG	PB	PB-2	P2-734	FALSE	1	TRA:CAASARGNTGKLIF TRB:CASSLIEESWARNEQFF	1	CD4-C1
PB-2_ACACCGGCAGTATCTG	PB	PB-2	P2-735	FALSE	1	TRA:CAMREGLNTGNQFYF TRB:CASSKEDRGRYQPQHF	1	CD4-C1
PB-2_ACAGCCGTCCTGCAGG	PB	PB-2	P2-742	FALSE	1	TRA:CAPNNKLIF TRB:CASSLGLQATEAFF	1	CD4-C2
PB-2_ACAGCTAAGTTGCAGG	PB	PB-2	P2-743	FALSE	1	TRA:CAGAGGYSTLTF TRB:CASSLARGGNTEAFF	1	CD4-C1
PB-2_ACAGCTATCGTTACGA	PB	PB-2	P2-746	FALSE	1	TRA:CAQWGGANSKLTF TRB:CASAGWGEQFF	1	CD4-C1
PB-2_ACATACGCAACTTGAC	PB	PB-2	P2-749	FALSE	1	TRA:CAGPTNAGKSTF TRB:CASRMGFLNTEAFF	1	CD4-C1
PB-2_ACATACGCACGAGGTA	PB	PB-2	P2-750	FALSE	1	TRA:CAASNTGGFKTIF TRB:CASSIERFEKLFF	1	CD4-C1
PB-2_ACATCAGAGCTCCTTC	PB	PB-2	P2-753	FALSE	1	TRA:CAVNKGNAGKSTF TRB:CASSPKPWDTEAFF	1	CD4-C1
PB-2_ACATCAGCACAGAGGT	PB	PB-2	P2-756	FALSE	1	TRA:CAVLKGGGADGLTF TRB:CASSPRQELNTEAFF	1	CD4-C1
PB-2_ACATCAGTCAGTACGT	PB	PB-2	P2-760	FALSE	1	TRA:CAATDSWGKFQF TRB:CASSPDSGGTGELFF	1	CD4-C1

PB-2_ACATGGTAGGCGTACA	PB	PB-2	P2-763	FALSE	1	TRA:CVVNGAGSNDYKLSF TRB:CASSSSYEQYF	1	CD4-C1
PB-2_ACCAGTAAGCTAGTTC	PB	PB-2	P2-764	FALSE	1	TRA:CAFQETSGSRLTF TRB:CASSPVQRYTQYF	1	CD4-C1
PB-2_ACCAGTATCAACGAAA	PB	PB-2	P2-770	FALSE	1	TRA:CLVGDGRNTPLVF TRB:CASSFPSEPLHF	1	CD4-C1
PB-2_ACCCACTAGGAATGGA	PB	PB-2	P2-775	FALSE	1	TRA:CIVRVQIYNQGGKLIF TRB:CASRTGQNTEAFF	1	CD4-C1
PB-2_ACCCACTTCCTAGTGA	PB	PB-2	P2-778	FALSE	1	TRA:CAASGYGTASKLTF TRB:CSAREVTSGWNSYNEQFF	1	CD4-C1
PB-2_ACCCACTTCGAATGGG	PB	PB-2	P2-779	FALSE	1	TRA:CILRDLRSNFGNEKLTF TRB:CASSLRGSVEQYF	1	CD4-C1
PB-2_ACCGTAACACAGACAG	PB	PB-2	P2-781	FALSE	1	TRA:CALRGSGNTGKLIF TRB:CASSIGPDYEQYF	1	CD4-C1
PB-2_ACCTTTAAGAGACGAA	PB	PB-2	P2-786	FALSE	1	TRA:CAGQRRSSASKIIF TRB:CSARSPSGKETQYF	1	CD4-C1
PB-2_ACCTTTAAGAGCCCAA	PB	PB-2	P2-787	FALSE	1	TRA:CAVPQGGKLIF TRB:CATSDGGGGSPLHF	1	CD4-C1
PB-2_ACGAGCCAGGAATCGC	PB	PB-2	P2-793	FALSE	1	TRA:CLVVSSYKLIF TRB:CASSENSNNQPQHF	1	CD4-C1
PB-2_ACGAGCCAGTTTCCTT	PB	PB-2	P2-794	FALSE	1	TRA:CATDKAGGTSYGKLTF TRB:CASSTTGGKGYTF	1	CD4-C1
PB-2_ACGAGCCGTTGTCTTT	PB	PB-2	P2-797	FALSE	1	TRA:CAARRTGTASKLTF TRB:CASSPPAGTGDGYTF	1	CD4-C1
PB-2_ACGAGGAAGCGAGAAA	PB	PB-2	P2-799	FALSE	1	TRA:CAESGAGSYQLTF TRB:CASSELDRPKGGTQYF	1	CD4-C1
PB-2_ACGAGGACAAATACAG	PB	PB-2	P2-800	FALSE	1	TRA:CAARNFGNEKLTF TRB:CASSSGLALVDTQYF	1	CD4-C1
PB-2_ACGATACCAACTGCGC	PB	PB-2	P2-804	FALSE	1	TRA:CAASRAGGTSYGKLTF TRB:CASSPSSNQPQHF	1	CD4-C1
PB-2_ACGATACCATTACGAC	PB	PB-2	P2-805	FALSE	1	TRA:CAVSDGGFKTIF TRB:CASSSQGAEADTQYF	1	CD4-C1
PB-2_ACGATGTAGATCCTGT	PB	PB-2	P2-808	FALSE	1	TRA:CAMREGGFKTIF TRB:CAWSATGGQETQYF	1	CD4-C2
PB-2_ACGCAGCAGCTGATAA	PB	PB-2	P2-814	FALSE	1	TRA:CAVGLDTGRRALTF TRB:CASSSQGNTEAFF	1	CD4-C1
PB-2_ACGCCGACAGGTCTCG	PB	PB-2	P2-820	FALSE	1	TRA:CAASGSGSARQLTF TRB:CSARKSWDTQYF	1	CD4-C1
PB-2_ACGGAGAGTTAAGTAG	PB	PB-2	P2-827	FALSE	1	TRA:CAMRERSGSTLGRLYF TRB:CASSLQKVGTGVSETQYF	1	CD4-C1
PB-2_ACGGCCAAGGCTATCT	PB	PB-2	P2-831	FALSE	1	TRA:CALRDPGAAGNKLTF TRB:CASSLKTGAATGELFF	1	CD4-C1
PB-2_ACGGGCTAGCTGTCTA	PB	PB-2	P2-834	FALSE	1	TRA:CALINQGGSEKLVF TRB:CASSLSGSYEQYF	1	CD4-C1
PB-2_ACGGGTCGTAGCTTGT	PB	PB-2	P2-844	FALSE	1	TRA:CAGANAGGTSYGKLTF TRB:CASSIQGALNTEAFF	1	CD4-C1
PB-2_ACGTCAAAGACAATAC	PB	PB-2	P2-846	FALSE	1	TRA:CAPNTDKLIF TRB:CASSPTTWTGGGEQYF	1	CD4-C1
PB-2_ACGTCAACATTGCGGC	PB	PB-2	P2-850	FALSE	1	TRA:CAVYRAAQKLVF TRB:CASRSPSGRAVDTQYF	1	CD4-C1
PB-2_ACTGAGTGTCACAAGG	PB	PB-2	P2-868	FALSE	1	TRA:CAYRVDNAGNMLTF TRB:CASSPHRNNSPLHF	1	CD4-C2
PB-2_ACTGATGTCCCTCAGT	PB	PB-2	P2-878	FALSE	1	TRA:CVLDSNYQLIW TRB:CASSLDGSRNSGANVLTF	1	CD4-C1

PB-2_ACTGCTCCAAGTCTAC	PB	PB-2	P2-884	FALSE	1	TRA:CAVKAYNTDKLIF TRB:CASSTGDYEQYF	1	CD4-C1
PB-2_ACTGCTCTCGAGCCCA	PB	PB-2	P2-889	FALSE	1	TRA:CAVRDGYSGGGADGLTF TRB:CASSRGSSGANVLTF	1	CD4-C1
PB-2_ACTTACTTCTGCGTAA	PB	PB-2	P2-901	FALSE	1	TRA:CAVESPLTGANNLFF TRB:CAIAPAQGNDYGYTF	1	CD4-C1
PB-2_ACTTTCAAGCTCTCGG	PB	PB-2	P2-907	FALSE	1	TRA:CAVNKGGSEKLVF TRB:CSARGSSYEQYF	1	CD4-C1
PB-2_ACTTTCACATCCCACT	PB	PB-2	P2-910	FALSE	1	TRA:CAVFPKAAGNKLTF TRB:CASSSTGLNYGYTF	1	CD4-C1
PB-2_AGACGTTAGGATGGTC	PB	PB-2	P2-918	FALSE	1	TRA:CAASREDYGGSQGNLIF TRB:CATSRDNRVYTEAFF	1	CD4-C1
PB-2_AGAGCGATCCTCATTA	PB	PB-2	P2-933	FALSE	1	TRA:CALTSGTYKYIF TRB:CASSRTTGSYNSPLHF	1	CD4-C1
PB-2_AGATCTGCACTTAAGC	PB	PB-2	P2-943	FALSE	1	TRA:CAESPFSGGYNKLIF TRB:CASSAAGGLSTDTQYF	1	CD4-C1
PB-2_AGATCTGGTCTTGTCC	PB	PB-2	P2-949	FALSE	1	TRA:CASMAGGTSYGKLTF TRB:CASSSRGNRNEQFF	1	CD4-C1
PB-2_AGATTGCAGCTGTCTA	PB	PB-2	P2-952	FALSE	1	TRA:CAVRDFGNEKLTF TRB:CASSLGGQGYNEQFF	1	CD4-C1
PB-2_AGATTGCCACATGTGT	PB	PB-2	P2-954	FALSE	1	TRA:CAVGVPWKPPGGGADGLTF TRB:CASSLDRTDTQYF	1	CD4-C1
PB-2_AGCAGCCGTAGGAGTC	PB	PB-2	P2-960	FALSE	1	TRA:CAARGTGTASKLTF TRB:CASSIDRDTEAFF	1	CD4-C1
PB-2_AGCAGCCTCAGCGATT	PB	PB-2	P2-964	FALSE	1	TRA:CAENQGDSNYQLIW TRB:CASGGETQYF	1	CD4-C1
PB-2_AGCCTAATCTGGAGCC	PB	PB-2	P2-972	FALSE	1	TRA:CAMRETMDSSYKLIF TRB:CASTPEYNSPLHF	1	CD4-C1
PB-2_AGCGGTCGTTAAGTAG	PB	PB-2	P2-978	FALSE	1	TRA:CAVAPPYGNNRLAF TRB:CASSQDNSGQTQYF	1	CD4-C1
PB-2_AGCGTATGTTGCGTTA	PB	PB-2	P2-981	FALSE	1	TRA:CVVSGRSNYKLTF TRB:CATRGSWTDTQYF	1	CD4-C1
PB-2_AGCGTATTCTCCAGGG	PB	PB-2	P2-982	FALSE	1	TRA:CAEKGDFGNEKLTF TRB:CASSWPTSGALGNEQFF	1	CD4-C1
PB-2_AGCTCCTCAGGATCGA	PB	PB-2	P2-993	FALSE	1	TRA:CALLSGNTGKLIF TRB:CSARNLFRAAQETQYF	1	CD4-C1
PB-2_AGCTCTCCAAGCTGAG	PB	PB-2	P2-999	FALSE	1	TRA:CAAHATYYGQNFVF TRB:CASSPPRGGALTGELFF	1	CD4-C1
PB-2_AGCTCTCTCACATGCA	PB	PB-2	P2-1000	FALSE	1	TRA:CAASSGGSNYKLTF TRB:CASSLGGVRETQYF	1	CD4-C1
PB-2_AGCTTGATCCGCGTTT	PB	PB-2	P2-1007	FALSE	1	TRA:CAENYLRSGGYNKLIF TRB:CASRPGLHTDTQYF	1	CD4-C1
PB-2_AGGCCGTGTGGCAAAC	PB	PB-2	P2-1017	FALSE	1	TRA:CIVITGTSGTYKYIF TRB:CASSLHLAGDYEQYF	1	CD4-C1
PB-2_AGGGAGTAGATCCGAG	PB	PB-2	P2-1018	FALSE	1	TRA:CILTPGGSQGNLIF TRB:CSGLTPNTEAFF	1	CD4-C1
PB-2_AGGGAGTCAATAGAGT	PB	PB-2	P2-1020	FALSE	1	TRA:CAASARVNAGGTSYGKLTF TRB:CASSIRPGSNEQFF	1	CD4-C1
PB-2_AGGGAGTCACAAGACG	PB	PB-2	P2-1021	FALSE	1	TRA:CAASDYGGATNKLIF TRB:CASSQEGASVTGELFF	1	CD4-C1
PB-2_AGGGATGTCAGCCTAA	PB	PB-2	P2-1030	FALSE	1	TRA:CAVTFGYQLIW TRB:CASRPGPQYNEQFF	1	CD4-C1
PB-2_AGGGATGTCTATCGCC	PB	PB-2	P2-1032	FALSE	1	TRA:CAGRNGGSQGNLIF TRB:CASSEGAYEQYF	1	CD4-C1

PB-2_AGGGTGACACCGAATT	PB	PB-2	P2-1033	FALSE	1	TRA:CAYRRKYNNNDMRF TRB:CSARVGLAGLAKNIQYF	1	CD4-C1
PB-2_AGGTCATAGCATGGCA	PB	PB-2	P2-1041	FALSE	1	TRA:CAVNRPLASGGYQKVTF TRB:CASSESKNSGIRGNTIYF	1	CD4-C1
PB-2_AGGTCATAGCTTATCG	PB	PB-2	P2-1042	FALSE	1	TRA:CAVGAAGNTDKLIF TRB:CASSQGIGVLETQYF	1	CD4-C1
PB-2_AGGTCATTCCAGTAGT	PB	PB-2	P2-1048	FALSE	1	TRA:CAVEKGNTPLVF TRB:CASSRQGRETQYF	1	CD4-C1
PB-2_AGTGAGGTCAGATAAG	PB	PB-2	P2-1060	FALSE	1	TRA:CAVRDTGANSKLTF TRB:CASAIGPGSWNTIYF	1	CD4-C1
PB-2_AGTGTCAAGAGTGAGA	PB	PB-2	P2-1074	FALSE	1	TRA:CAYSLTPNAGNMLTF TRB:CASSPSFTSAGELFF	1	CD4-C1
PB-2_AGTGTCAGTAATTGGA	PB	PB-2	P2-1078	FALSE	1	TRA:CAASRNNNARLMF TRB:CASRTFAKQKAFF	1	CD4-C1
PB-2_AGTTGGTAGAGTGACC	PB	PB-2	P2-1080	FALSE	1	TRA:CAGLKWGGGADGLTF TRB:CASSEDWNTEAFF	1	CD4-C2
PB-2_ATAACGCAGGCGATAC	PB	PB-2	P2-1084	FALSE	1	TRA:CATAQQGGSEKLVF TRB:CASSPAGVSANQPQHF	1	CD4-C1
PB-2_ATAAGAGAGCTGAACG	PB	PB-2	P2-1088	FALSE	1	TRA:CAVRDSNYQLIW TRB:CASSSQPGAKTYEQYF	1	CD4-C1
PB-2_ATAAGAGTCAGAGGTG	PB	PB-2	P2-1093	FALSE	1	TRA:CAFFDTDKLIF TRB:CASSQILDRSYEQYF	1	CD4-C1
PB-2_ATAAGAGTCATGCTCC	PB	PB-2	P2-1094	FALSE	1	TRA:CAMREVNSGYSTLTF TRB:CASSLRTGTNTEAFF	1	CD4-C1
PB-2_ATAGACCAGGAGTTGC	PB	PB-2	P2-1097	FALSE	1	TRA:CIIQGAQKLVF TRB:CASRGQLHGQPQHF	1	CD4-C1
PB-2_ATCACGATCACCGGGT	PB	PB-2	P2-1103	FALSE	1	TRA:CAPIGGTASKLTF TRB:CASNLFAGANTGELFF	1	CD4-C1
PB-2_ATCACGATCGTACCGG	PB	PB-2	P2-1104	FALSE	1	TRA:CASDQGGKLIF TRB:CASSLVSGPGEKLFF	1	CD4-C2
PB-2_ATCATCTGTACCCAAT	PB	PB-2	P2-1108	FALSE	1	TRA:CAVKNTGFQKLVF TRB:CASSPPGGMNTEAFF	1	CD4-C2
PB-2_ATCATGGTCAGTGTTG	PB	PB-2	P2-1117	FALSE	1	TRA:CVVIYYNTDKLIF TRB:CASSLEARTTLYEQYF	1	CD4-C1
PB-2_ATCATGGTCATCATTC	PB	PB-2	P2-1118	FALSE	1	TRA:CAVGAKGTGFQKLVF TRB:CASSTDRLTGELFF	1	CD4-C1
PB-2_ATCGAGTAGATGTGGC	PB	PB-2	P2-1127	FALSE	1	TRA:CAHYNNAGNMLTF TRB:CASSPGSGGSYEQYF	1	CD4-C1
PB-2_ATCGAGTAGCGTAATA	PB	PB-2	P2-1128	FALSE	1	TRA:CAASLLNQNFVF TRB:CSARDSRGTGSTQYF	1	CD4-C1
PB-2_ATCGAGTAGGATATAC	PB	PB-2	P2-1129	FALSE	1	TRA:CAVNTGANSKLTF TRB:CASSSTPLNTEAFF	1	CD4-C1
PB-2_ATCTACTAGCAGGCTA	PB	PB-2	P2-1135	FALSE	1	TRA:CAVTGGSNYKLTF TRB:CASSQFAMYIAGADTQYF	1	CD4-C2
PB-2_ATCTACTCAGACGCAA	PB	PB-2	P2-1137	FALSE	1	TRA:CALSRRGSGNQFYF TRB:CASSQDITGGQPQHF	1	CD4-C1
PB-2_ATCTGCCGTACACCGC	PB	PB-2	P2-1142	FALSE	1	TRA:CAVIPNTGGFKTIF TRB:CASSLRRAASTDTQYF	1	CD4-C1
PB-2_ATGAGGGGTTGGTTTG	PB	PB-2	P2-1148	FALSE	1	TRA:CAASADSNYQLIW TRB:CASSEVGVYEQYF	1	CD4-C1
PB-2_ATGAGGGTCCGAACGC	PB	PB-2	P2-1149	FALSE	1	TRA:CVVSGNWGSQGNLIF TRB:CASRNRQGPQETQYF	1	CD4-C1
PB-2_ATGCGATAGAGCAATT	PB	PB-2	P2-1150	FALSE	1	TRA:CAGEGTGGFKTIF TRB:CASSSDTYNEQFF	1	CD4-C1

PB-2_ATGGGAGAGGGAGTAA	PB	PB-2	P2-1154	FALSE	1	TRA:CILSKSGNTGKLIF TRB:CASSLEGSSYNEQFF	1	CD4-C1
PB-2_ATGTGTGAGCGACGTA	PB	PB-2	P2-1159	FALSE	1	TRA:CVVDFTSGTYKYIF TRB:CASSFYSSYEQYF	1	CD4-C2
PB-2_ATTACTCAGATGTAAC	PB	PB-2	P2-1165	FALSE	1	TRA:CAEMWGGKLIF TRB:CASSVAGGGNGYTF	1	CD8-C1
PB-2_ATTACTCAGGTGATTA	PB	PB-2	P2-1166	FALSE	1	TRA:CAAREGRGNQFYF TRB:CASSSAGTLRHTF	1	CD4-C1
PB-2_ATTACTCCAAGTCTGT	PB	PB-2	P2-1167	FALSE	1	TRA:CVVTVYSSASKIIF TRB:CASSLYGGGSHESSGANVLTF	1	CD4-C1
PB-2_ATTACTCTCTGCTGTC	PB	PB-2	P2-1168	FALSE	1	TRA:CPVTRDDKIIF TRB:CASSSDSGELFF	1	CD4-C1
PB-2_ATTATCCAGATCTGCT	PB	PB-2	P2-1169	FALSE	1	TRA:CAVTMAGGFKTIF TRB:CAVPDNTGELFF	1	CD4-C1
PB-2_ATTATCCGTCTTCTCG	PB	PB-2	P2-1171	FALSE	1	TRA:CAVRNFGNEKLTF TRB:CASSYDYTGELFF	1	CD4-C1
PB-2_ATTATCCTCATCTGTT	PB	PB-2	P2-1173	FALSE	1	TRA:CAGAEGDSGYALNF TRB:CASSPLSGQGVEKLFF	1	CD4-C1
PB-2_ATTGGTGTCAGGCGAA	PB	PB-2	P2-1189	FALSE	1	TRA:CAASTSGGSNYKLTF TRB:CATSQLRGLGEAFF	1	CD4-C1
PB-2_ATTGGTGTCTTCGGTC	PB	PB-2	P2-1190	FALSE	1	TRA:CALSGGSNYKLTF TRB:CASSMGTSGGAGGTDTQYF	1	CD4-C1
PB-2_ATTTCTGAGACTAGAT	PB	PB-2	P2-1191	FALSE	1	TRA:CAVRGNQAGTALIF TRB:CASSQATQTQYF	1	CD4-C1
PB-2_ATTTCTGAGGAGTTGC	PB	PB-2	P2-1192	FALSE	1	TRA:CAVNSNSGYALNF TRB:CASRGQGATGELFF	1	CD4-C1
PB-2_ATTTCTGTCAGTTTGG	PB	PB-2	P2-1195	FALSE	1	TRA:CLLGEGGDNNDMRF TRB:CASSAMGKLFF	1	CD4-C1
PB-2_CAACCAAAGAGTACCG	PB	PB-2	P2-1198	FALSE	1	TRA:CAMGVNTGFQKLVF TRB:CASNTGISYEQYF	1	CD4-C1
PB-2_CAACCAAGTACCCAAT	PB	PB-2	P2-1201	FALSE	1	TRA:CAVALNRNQAGTALIF TRB:CASSATGTGGQPQHF	1	CD4-C1
PB-2_CAACCAATCATAGCAC	PB	PB-2	P2-1202	FALSE	1	TRA:CAERVEGFGNVLHC TRB:CASEKGVGEQYF	1	CD4-C1
PB-2_CAACTAGTCATAAAGG	PB	PB-2	P2-1211	FALSE	1	TRA:CAVEGGGSYIPTF TRB:CASSMGGPNSPLHF	1	CD4-C1
PB-2_CAAGGCCCAAGCCATT	PB	PB-2	P2-1224	FALSE	1	TRA:CAASIWGAQKLVF TRB:CSARDGTASYGYTF	1	CD4-C1
PB-2_CAAGTTGGTCGAGATG	PB	PB-2	P2-1230	FALSE	1	TRA:CAASTGGNQFYF TRB:CASSGDRNFYEQYF	1	CD4-C1
PB-2_CAAGTTGGTCTCCCTA	PB	PB-2	P2-1231	FALSE	1	TRA:CAQSTGNQFYF TRB:CASSLGQGASPLHF	1	CD4-C1
PB-2_CACAAACAGGCTAGAC	PB	PB-2	P2-1232	FALSE	1	TRA:CAYRPNFGNEKLTF TRB:CASIKQPGRGDQPQHF	1	CD4-C1
PB-2_CACAAACCAAGGGTCA	PB	PB-2	P2-1233	FALSE	1	TRA:CAVSRASGTYKYIF TRB:CASSRTGSYEQYF	1	CD4-C1
PB-2_CACACCTAGACAAAGG	PB	PB-2	P2-1244	FALSE	1	TRA:CAYHGSGNTGKLIF TRB:CASSMVAGGPTANEQFF	1	CD4-C1
PB-2_CACATTTTCCTCAATT	PB	PB-2	P2-1277	FALSE	1	TRA:CAAKGAGSYQLTF TRB:CASSWAGYAATGELFF	1	CD4-C1
PB-2_CACCACTAGTACATGA	PB	PB-2	P2-1279	FALSE	1	TRA:CVVSDHPNTGGFKTIF TRB:CASPERSGNTIYF	1	CD4-C1
PB-2_CACCACTTCTTGTACT	PB	PB-2	P2-1283	FALSE	1	TRA:CYMNRDDKIIF TRB:CASSLGGSTGTNIQYF	1	CD4-C1

PB-2_CACCAGGTCAGCTCGG	PB	PB-2	P2-1286	FALSE	1	TRA:CAVNGGSGNTGKLIF TRB:CASGALATYEQYF	1	CD4-C1
PB-2_CACCAGGTCCTAGGGC	PB	PB-2	P2-1288	FALSE	1	TRA:CAVGWGKFQF TRB:CASSMDSGGREQFF	1	CD4-C1
PB-2_CACTCCAGTTGAACTC	PB	PB-2	P2-1294	FALSE	1	TRA:CIVRKGIYNQGGKLIF TRB:CASSSETIYDEQFF	1	CD4-C1
PB-2_CAGAATCTCAACGGGA	PB	PB-2	P2-1301	FALSE	1	TRA:CAYLKGGADGLTF TRB:CASSLVGATQYF	1	CD4-C1
PB-2_CAGATCATCGAACGGA	PB	PB-2	P2-1314	FALSE	1	TRA:CALSASGSARQLTF TRB:CASSADRGYSGNTIYF	1	CD4-C1
PB-2_CAGCAGCTCGGTCCGA	PB	PB-2	P2-1321	FALSE	1	TRA:CAVDPLSGNTPLVF TRB:CASSTGQGTGNTIYF	1	CD4-C1
PB-2_CAGCATACAGACGCCT	PB	PB-2	P2-1324	FALSE	1	TRA:CALTTGNQFYF TRB:CASSQDGLAGTGELFF	1	CD4-C1
PB-2_CAGCCGAGTAACGACG	PB	PB-2	P2-1330	FALSE	1	TRA:CAENIYGNNRLAF TRB:CASSEFRGDYSGANVLTF	1	CD4-C2
PB-2_CAGCGACTCCTAAGTG	PB	PB-2	P2-1336	FALSE	1	TRA:CAVGARDSSYKLIF TRB:CASSEGGGPNEQFF	1	CD4-C1
PB-2_CAGCTAAGTCATTAGC	PB	PB-2	P2-1337	FALSE	1	TRA:CVVSAMKTSYDKVIF TRB:CASSTSSGTGELFF	1	CD4-C1
PB-2_CAGCTAATCCGTAGTA	PB	PB-2	P2-1339	FALSE	1	TRA:CILSVAAGNKLTF TRB:CASSFSGGQGRIPLHF	1	CD4-C1
PB-2_CAGCTGGGTACGCTGC	PB	PB-2	P2-1341	FALSE	1	TRA:CARDKARGAQKLVF TRB:CSARDFGGPQETQYF	1	CD4-C1
PB-2_CAGCTGGGTAGGCATG	PB	PB-2	P2-1342	FALSE	1	TRA:CAGPGAGGTSYGKLTF TRB:CASSRRVLEKLFF	1	CD4-C1
PB-2_CAGGTGCCAGACGTAG	PB	PB-2	P2-1346	FALSE	1	TRA:CAVPLIF TRB:CASSLVTTGSGELFF	1	CD4-C1
PB-2_CAGGTGCTCTTGTCAT	PB	PB-2	P2-1349	FALSE	1	TRA:CAVEGPGNTPLVF TRB:CSATDRVPNEQYF	1	CD4-C1
PB-2_CAGTAACTCGTCGTTC	PB	PB-2	P2-1355	FALSE	1	TRA:CVVTPRGSGNTGKLIF TRB:CASSSLSTGAPRGYTF	1	CD4-C1
PB-2_CATATTCAGTGCCATT	PB	PB-2	P2-1370	FALSE	1	TRA:CAFGGSGGYQKVTF TRB:CASSYRGPPNEKLFF	1	CD4-C1
PB-2_CATATTCCAAGTACCT	PB	PB-2	P2-1372	FALSE	1	TRA:CAVGGYTGNQFYF TRB:CASSRTAQETQYF	1	CD4-C1
PB-2_CATATTCGTCTAGCCG	PB	PB-2	P2-1374	FALSE	1	TRA:CAARRGNTPLVF TRB:CASSQPGYQETQYF	1	CD4-C2
PB-2_CATCAAGGTAATCACC	PB	PB-2	P2-1378	FALSE	1	TRA:CIVRVAERNAGGTSYGKLTF TRB:CASSLNGYQNNEQFF	1	CD4-C1
PB-2_CATCAAGGTGGCAAAC	PB	PB-2	P2-1380	FALSE	1	TRA:CAVLNTGNQFYF TRB:CAISEERAQVYGYTF	1	CD4-C1
PB-2_CATCCACTCCGGCACA	PB	PB-2	P2-1393	FALSE	1	TRA:CAVGSTSGPGNTGKLIF TRB:CAWSYRRTGLASTYEQYF	1	CD4-C1
PB-2_CATCGAACAGGGTATG	PB	PB-2	P2-1396	FALSE	1	TRA:CGADITGANSKLTF TRB:CSARDPLISGNTIYF	1	CD4-C1
PB-2_CATCGGGGTTAGGGTG	PB	PB-2	P2-1406	FALSE	1	TRA:CALSGESGNTPLVF TRB:CASSSQNGYTF	1	CD4-C1
PB-2_CATGACACAGTTCATG	PB	PB-2	P2-1410	FALSE	1	TRA:CAVIISGNTGKLIF TRB:CASSGLAGGFTGELFF	1	CD4-C1
PB-2_CATGACATCGTTACAG	PB	PB-2	P2-1415	FALSE	1	TRA:CAETVYSGGGADGLTF TRB:CATSRVKTSRPPQETQYF	1	CD4-C1
PB-2_CATGACATCGTTTGCC	PB	PB-2	P2-1416	FALSE	1	TRA:CAAKNNDYKLSF TRB:CASSPLPGTSRNEQFF	1	CD4-C1

PB-2_CATGCCTAGAGACTTA	PB	PB-2	P2-1418	FALSE	1	TRA:CAASSSYKLIF TRB:CASSALGATGNTIYF	1	CD4-C1
PB-2_CATGCCTAGCACACAG	PB	PB-2	P2-1419	FALSE	1	TRA:CALISYKLIF TRB:CSALAGGNTGELFF	1	CD4-C1
PB-2_CATGGCGTCACCAGGC	PB	PB-2	P2-1425	FALSE	1	TRA:CVPTGAGSYQLTF TRB:CAWSVVGGNQPQHF	1	CD4-C1
PB-2_CATTATCTCTCGCTTG	PB	PB-2	P2-1437	FALSE	1	TRA:CAMSTNAGKSTF TRB:CASSLWGAYNEQFF	1	CD4-C1
PB-2_CATTCGCGTAATAGCA	PB	PB-2	P2-1440	FALSE	1	TRA:CAEKEGGSQGNLIF TRB:CSARGTGFTDTQYF	1	CD4-C1
PB-2_CCACCTACAAGCTGTT	PB	PB-2	P2-1452	FALSE	1	TRA:CVVNGGNNNARLMF TRB:CASSPAPIGQPQHF	1	CD4-C1
PB-2_CCACGGACACTGTCGG	PB	PB-2	P2-1463	FALSE	1	TRA:CAVSDVGGSQGNLIF TRB:CASRQLAGLQETQYF	1	CD4-C1
PB-2_CCACGGAGTTACGACT	PB	PB-2	P2-1465	FALSE	1	TRA:CALIGGGSYIPTF TRB:CASSQTWAGELFF	1	CD4-C1
PB-2_CCACTACAGACTTGAA	PB	PB-2	P2-1469	FALSE	1	TRA:CAEPVSGGYNKLIF TRB:CSARRLAGETQYF	1	CD4-C1
PB-2_CCACTACTCTCTTATG	PB	PB-2	P2-1475	FALSE	1	TRA:CAASRGGTASKLTF TRB:CARNSGTGLSGELFF	1	CD4-C1
PB-2_CCAGCGAAGCCTCGTG	PB	PB-2	P2-1476	FALSE	1	TRA:CAVTTDSWGKFQF TRB:CASSPTANSPLHF	1	CD4-C1
PB-2_CCAGCGACACAAGTAA	PB	PB-2	P2-1477	FALSE	1	TRA:CAGPVHQAGTALIF TRB:CSAGQGTYEQYF	1	CD4-C1
PB-2_CCATGTCCACTAAGTC	PB	PB-2	P2-1482	FALSE	1	TRA:CAGRNNNARLMF TRB:CASSERGPETQYF	1	CD4-C1
PB-2_CCATTCGGTACAAGTA	PB	PB-2	P2-1492	FALSE	1	TRA:CAGLSPQGAQKLVF TRB:CASREGSNQPQHF	1	CD4-C1
PB-2_CCCAATCAGCGATTCT	PB	PB-2	P2-1496	FALSE	1	TRA:CAANFFSGGYNKLIF TRB:CASSTGYEQYF	1	CD4-C1
PB-2_CCCTCCTGTGGCCCTA	PB	PB-2	P2-1520	FALSE	1	TRA:CALSSTGTASKLTF TRB:CASSIAAGGSQPQHF	1	CD4-C1
PB-2_CCGGGATAGAACTCGG	PB	PB-2	P2-1521	FALSE	1	TRA:CAVNADRGSTLGRLYF TRB:CASSQGFTGRHYGYTF	1	CD4-C2
PB-2_CCGGGATTCTATCCTA	PB	PB-2	P2-1523	FALSE	1	TRA:CAASTRGSQGNLIF TRB:CASSSSGTGYNEQFF	1	CD4-C1
PB-2_CCGGTAGCATAAAGGT	PB	PB-2	P2-1528	FALSE	1	TRA:CAANNNARLMF TRB:CASRTGFSTDTQYF	1	CD4-C1
PB-2_CCGTTCAAGGCTAGAC	PB	PB-2	P2-1540	FALSE	1	TRA:CALRTGNQFYF TRB:CATSDGTASYEQYF	1	CD4-C2
PB-2_CCGTTCACATCACGTA	PB	PB-2	P2-1543	FALSE	1	TRA:CAGSTSGTYKYIF TRB:CASSLPGGFYEQYF	1	CD4-C2
PB-2_CCTAAAGTCGTCTGCT	PB	PB-2	P2-1555	FALSE	1	TRA:CAASKDDKIIF TRB:CASSYGTFNQPQHF	1	CD4-C1
PB-2_CCTACCAAGTCAATAG	PB	PB-2	P2-1562	FALSE	1	TRA:CAVRDPNSGYALNF TRB:CASSRQGGTEAFF	1	CD4-C1
PB-2_CCTATTAAGGCGCTCT	PB	PB-2	P2-1571	FALSE	1	TRA:CAASLWGWELPTHF TRB:CASSRTSGAGELFF	1	CD4-C1
PB-2_CCTATTAAGTCTCGGC	PB	PB-2	P2-1572	FALSE	1	TRA:CALNSGTYKYIF TRB:CAWNRGTGAPLGTEAFF	1	CD4-C1
PB-2_CCTATTAGTATAGGGC	PB	PB-2	P2-1574	FALSE	1	TRA:CAVAASGTYKYIF TRB:CATSRGVTGELFF	1	CD4-C1
PB-2_CCTATTAGTTACCGAT	PB	PB-2	P2-1576	FALSE	1	TRA:CATDRSTNAGKSTF TRB:CASSPGQGGTYEQYF	1	CD4-C1

PB-2_CCTCTGAAGTCATGCT	PB	PB-2	P2-1585	FALSE	1	TRA:CAGSYGGSQGNLIF TRB:CAWGTGYEQYF	1	CD4-C1
PB-2_CCTCTGAGTAGCGCAA	PB	PB-2	P2-1588	FALSE	1	TRA:CAARLGALNF TRB:CAWWNLGTGELFF	1	CD4-C1
PB-2_CCTCTGAGTCAATGTC	PB	PB-2	P2-1589	FALSE	1	TRA:CAVPIQGGSEKLVF TRB:CSAQRTSGVHEQFF	1	CD4-C1
PB-2_CCTTACGGTGCCTTGG	PB	PB-2	P2-1595	FALSE	1	TRA:CAVTRSGAGSYQLTF TRB:CASSSRVGSDEQYF	1	CD4-C1
PB-2_CCTTCCCTCGCACTCT	PB	PB-2	P2-1604	FALSE	1	TRA:CALKEYSGAGSYQLTF TRB:CASSVGQEVWDGYTF	1	CD4-C1
PB-2_CCTTCGAGTAGCTTGT	PB	PB-2	P2-1609	FALSE	1	TRA:CAMSANNNDMRF TRB:CASKLRGYEQYF	1	CD4-C1
PB-2_CGACCTTAGCACAGGT	PB	PB-2	P2-1624	FALSE	1	TRA:CAYSLDSNYQLIW TRB:CASSPRPDPRGEQFF	1	CD4-C1
PB-2_CGACCTTAGGCACATG	PB	PB-2	P2-1625	FALSE	1	TRA:CAASVDSSASKIIF TRB:CASSDNLVSYEQYF	1	CD4-C1
PB-2_CGACCTTCAAGGTTCT	PB	PB-2	P2-1626	FALSE	1	TRA:CAGYSNDYKLSF TRB:CAWRFVGRGYTF	1	CD4-C1
PB-2_CGACTTCCACATCCAA	PB	PB-2	P2-1634	FALSE	1	TRA:CAVYFMNNNAGNMLTF TRB:CASSLSGYEQYF	1	CD4-C1
PB-2_CGACTTCGTCAAACTC	PB	PB-2	P2-1635	FALSE	1	TRA:CAVQANTNAGKSTF TRB:CAISAVSQGSGELFF	1	CD4-C1
PB-2_CGAGAAGCAGCTTAAC	PB	PB-2	P2-1640	FALSE	1	TRA:CVVSDQYDMRF TRB:CASSFRDRVGLKNTGELFF	1	CD4-C1
PB-2_CGAGAAGGTACACCGC	PB	PB-2	P2-1641	FALSE	1	TRA:CAVSVISNFGNEKLTF TRB:CASSLGRWEQYF	1	CD4-C1
PB-2_CGAGCACGTTTGACAC	PB	PB-2	P2-1652	FALSE	1	TRA:CVCHFGNEKLTF TRB:CSVAWLAGGNEQFF	1	CD4-C1
PB-2_CGATGGCAGTCGATAA	PB	PB-2	P2-1661	FALSE	1	TRA:CAVSENNARLMF TRB:CASSVDRDESQYF	1	CD4-C1
PB-2_CGCCAAGGTACCCAAT	PB	PB-2	P2-1676	FALSE	1	TRA:CAVKISNTGNQFYF TRB:CASSYETLTGEQYF	1	CD4-C1
PB-2_CGCGTTTTCCATGAAC	PB	PB-2	P2-1690	FALSE	1	TRA:CAGGAWGKLIF TRB:CASTARGVTDTQYF	1	CD4-C1
PB-2_CGCTATCCATTCTTAC	PB	PB-2	P2-1694	FALSE	1	TRA:CAENGGGGGGNKLTF TRB:CAISESGGGVGGYTF	1	CD4-C1
PB-2_CGCTATCGTTATCGGT	PB	PB-2	P2-1695	FALSE	1	TRA:CAASRAGNNRKLIW TRB:CASSWDRAGGEQYF	1	CD4-C1
PB-2_CGCTATCGTTCCGTCT	PB	PB-2	P2-1696	FALSE	1	TRA:CAGTGAGSYQLTF TRB:CASSFMGSKGYTF	1	CD4-C1
PB-2_CGCTTCACACCTATCC	PB	PB-2	P2-1708	FALSE	1	TRA:CALTQGGSEKLVF TRB:CASSFLAGTTGELFF	1	CD4-C1
PB-2_CGCTTCATCGCATGAT	PB	PB-2	P2-1712	FALSE	1	TRA:CAVDLNQAGTALIF TRB:CATSSTQGLNTEAFF	1	CD4-C1
PB-2_CGGACGTAGGCGCTCT	PB	PB-2	P2-1719	FALSE	1	TRA:CAARRDSGAGSYQLTF TRB:CASAGASVGTQYF	1	CD4-C1
PB-2_CGGACGTAGTCCATAC	PB	PB-2	P2-1720	FALSE	1	TRA:CAVLTSGTYKYIF TRB:CSVDRGQGETQYF	1	CD4-C1
PB-2_CGGACTGTCGTGGACC	PB	PB-2	P2-1729	FALSE	1	TRA:CAVRLISNFGNEKLTF TRB:CASSLYGETQYF	1	CD4-C1
PB-2_CGGAGCTAGGTTACCT	PB	PB-2	P2-1732	FALSE	1	TRA:CAFSGNKLVF TRB:CASSQIGEDTEAFF	1	CD4-C1
PB-2_CGGAGCTGTGACAAAT	PB	PB-2	P2-1738	FALSE	1	TRA:CAVNTYSGGYQKVTF TRB:CASSVDRTGSGYTF	1	CD4-C1

PB-2_CGGAGTCTCACATGCA	PB	PB-2	P2-1743	FALSE	1	TRA:CAANTGNQFYF TRB:CASSQDQGSGVYNEQFF	1	CD4-C1
PB-2_CGGCTAGAGCGCTTAT	PB	PB-2	P2-1746	FALSE	1	TRA:CAVRDANYGGSQGNLIF TRB:CASRPTLAGGSEQYF	1	CD4-C1
PB-2_CGGCTAGGTCATGCAT	PB	PB-2	P2-1747	FALSE	1	TRA:CAVNTDKLIF TRB:CSARRGGAASGFNYNEQFF	1	CD4-C2
PB-2_CGGCTAGGTTCCACAA	PB	PB-2	P2-1749	FALSE	1	TRA:CALSGSGGSNYKLTF TRB:CASSLGTSGRWNEQFF	1	CD4-C1
PB-2_CGGCTAGTCTGATTCT	PB	PB-2	P2-1754	FALSE	1	TRA:CAGPKNTGTASKLTF TRB:CASSRPGHNNEQFF	1	CD4-C2
PB-2_CGGGTCATCACCATAG	PB	PB-2	P2-1759	FALSE	1	TRA:CAATPPGANSKLTF TRB:CASREGTGGYNEQFF	1	CD4-C1
PB-2_CGGTTAAAGAGTCTGG	PB	PB-2	P2-1762	FALSE	1	TRA:CAVQSGGSYIPTF TRB:CSVSLAGVNEQYF	1	CD4-C1
PB-2_CGGTTAAAGCAACGGT	PB	PB-2	P2-1763	FALSE	1	TRA:CAVVGRGSQGNLIF TRB:CASSILLGNTEAFF	1	CD4-C1
PB-2_CGGTTAACAGGATCGA	PB	PB-2	P2-1764	FALSE	1	TRA:CAVTSKTSYDKVIF TRB:CATNRGGADTQYF	1	CD4-C1
PB-2_CGTAGCGCACAACGCC	PB	PB-2	P2-1766	FALSE	1	TRA:CATGPPSIISSGSARQLTF TRB:CASSEGMGGANYGYTF	1	CD4-C1
PB-2_CGTAGGCAGGCTCTTA	PB	PB-2	P2-1768	FALSE	1	TRA:CAETSRLDSSYKLIF TRB:CASSPQGTEAFF	1	CD4-C1
PB-2_CGTAGGCAGTAGCCGA	PB	PB-2	P2-1769	FALSE	1	TRA:CAMRVGTSYGKLTF TRB:CASSKDRVQYF	1	CD4-C1
PB-2_CGTAGGCCAGACAAAT	PB	PB-2	P2-1770	FALSE	1	TRA:CAVSAPVGNARLMF TRB:CASSSDRGNYNEQFF	1	CD4-C1
PB-2_CGTCACTCAGTATCTG	PB	PB-2	P2-1775	FALSE	1	TRA:CALSEARGFGNVLHC TRB:CATRRESYNEQFF	1	CD4-C1
PB-2_CGTCAGGGTAGCTAAA	PB	PB-2	P2-1781	FALSE	1	TRA:CIVRVAPLGGAGNKLTF TRB:CASSVASGTGFETGELFF	1	CD4-C1
PB-2_CGTCCATGTCAAAGCG	PB	PB-2	P2-1790	FALSE	1	TRA:CALYMGYSSASKIIF TRB:CSGTGGSSYNSPLHF	1	CD4-C1
PB-2_CGTCCATTCAGCAACT	PB	PB-2	P2-1791	FALSE	1	TRA:CAMKAGSNYKLTF TRB:CASSPGPTNEKLFF	1	CD4-C1
PB-2_CGTCTACAGTTTGCGT	PB	PB-2	P2-1796	FALSE	1	TRA:CAVSFLSGGYNKLIF TRB:CASSQGSGAYEQYF	1	CD4-C1
PB-2_CGTGTAAAGTGTCCAT	PB	PB-2	P2-1808	FALSE	1	TRA:CAMSSTIAGGTSYGKLTF TRB:CASSPQGGSGANVLTF	1	CD4-C1
PB-2_CGTGTCTAGCTCAACT	PB	PB-2	P2-1813	FALSE	1	TRA:CAVGGGATNKLIF TRB:CASSQVRNEQYF	1	CD4-C1
PB-2_CGTGTCTCATAACCTG	PB	PB-2	P2-1815	FALSE	1	TRA:CALSGGNQFYF TRB:CASRSSTANTEAFF	1	CD4-C1
PB-2_CGTTAGATCGCACTCT	PB	PB-2	P2-1820	FALSE	1	TRA:CAVSKTSGSRLTF TRB:CASSPDNMNTEAFF	1	CD4-C1
PB-2_CGTTCTGAGAAGGGTA	PB	PB-2	P2-1821	FALSE	1	TRA:CAVRDGGFQKLVF TRB:CASSLGQGTNEKLFF	1	CD4-C1
PB-2_CGTTCTGAGAGGGATA	PB	PB-2	P2-1822	FALSE	1	TRA:CALLMDSSYKLIF TRB:CASSLTTGLGADTQYF	1	CD4-C1
PB-2_CGTTGGGAGTTTGCGT	PB	PB-2	P2-1832	FALSE	1	TRA:CASPTNAGGTSYGKLTF TRB:CASSRPGSSSEQYF	1	CD4-C1
PB-2_CGTTGGGCACGCCAGT	PB	PB-2	P2-1833	FALSE	1	TRA:CAALNTGFQKLVF TRB:CASRLPGQGRNEQFF	1	CD4-C1
PB-2_CGTTGGGTCGCAGGCT	PB	PB-2	P2-1834	FALSE	1	TRA:CAATRYSSASKIIF TRB:CASSPGEGSEAFF	1	CD4-C1

PB-2_CTAACTTCAGTCGTGC	PB	PB-2	P2-1837	FALSE	1	TRA:CAEIDIQGAQKLVF TRB:CASSLDVGEQFF	1	CD4-C1
PB-2_CTAAGACAGCTGTCTA	PB	PB-2	P2-1844	FALSE	1	TRA:CATENSGGSNYKLTF TRB:CASSPGQGVRSPLHF	1	CD4-C1
PB-2_CTAAGACCATTGGCGC	PB	PB-2	P2-1847	FALSE	1	TRA:CAVNGWGAAGNKLTF TRB:CASSGPGGANVLTF	1	CD4-C1
PB-2_CTAAGACTCCGAGCCA	PB	PB-2	P2-1850	FALSE	1	TRA:CATEKIGNYGQNFVF TRB:CASGGRALYGYTF	1	CD4-C1
PB-2_CTAATGGAGGGTTCCC	PB	PB-2	P2-1851	FALSE	1	TRA:CAGPGGARLMF TRB:CASSFVDAGDTQYF	1	CD4-C1
PB-2_CTAATGGAGTACGCCC	PB	PB-2	P2-1852	FALSE	1	TRA:CAYRSARFGGATNKLIF TRB:CASSLPGTKNIQYF	1	CD4-C1
PB-2_CTAATGGCAATGACCT	PB	PB-2	P2-1853	FALSE	1	TRA:CVVSAPLAGGTSYGKLTF TRB:CAITGTGTGELFF	1	CD4-C1
PB-2_CTACACCAGATGTGTA	PB	PB-2	P2-1857	FALSE	1	TRA:CAGMASGGSNYKLTF TRB:CASTPWRLAGNNEQFF	1	CD4-C1
PB-2_CTACACCTCTGTGCAA	PB	PB-2	P2-1862	FALSE	1	TRA:CAARNPGGYNKLIF TRB:CATSRDDRVADTQYF	1	CD4-C1
PB-2_CTACCCAGTAGCTAAA	PB	PB-2	P2-1872	FALSE	1	TRA:CALSSNTGFQKLVF TRB:CASSLVKGSGELFF	1	CD4-C2
PB-2_CTAGAGTCACAGCGTC	PB	PB-2	P2-1883	FALSE	1	TRA:CAASETYGGSQGNLIF TRB:CAWSYKTLSRGVSTGELFF	1	CD4-C1
PB-2_CTAGCCTAGCCCAGCT	PB	PB-2	P2-1890	FALSE	1	TRA:CALLMDSSYKLIF TRB:CASSFSTETTEAFF	1	CD4-C1
PB-2_CTAGCCTCATGACGGA	PB	PB-2	P2-1892	FALSE	1	TRA:CAVKAGGTSYGKLTF TRB:CASRQGDSPLHF	1	CD4-C1
PB-2_CTAGTGACATGCATGT	PB	PB-2	P2-1897	FALSE	1	TRA:CAFWGFGNEKLTF TRB:CASSFLQGYEQYF	1	CD4-C1
PB-2_CTCACACCAGGTGCCT	PB	PB-2	P2-1904	FALSE	1	TRA:CAMRPSGGYQKVTF TRB:CSARVDTGEAFF	1	CD4-C1
PB-2_CTCACACTCCATGCTC	PB	PB-2	P2-1907	FALSE	1	TRA:CAMRAPPRGGKLIF TRB:CASSRTGTRETQYF	1	CD4-C1
PB-2_CTCAGAAAGACACTAA	PB	PB-2	P2-1908	FALSE	1	TRA:CAAVLSNDYKLSF TRB:CASSRSTLNTEAFF	1	CD4-C1
PB-2_CTCAGAACAAACTGTC	PB	PB-2	P2-1911	FALSE	1	TRA:CLVTKGNYGQNFVF TRB:CASSKIAGNSQETQYF	1	CD4-C1
PB-2_CTCAGAACAGATGGCA	PB	PB-2	P2-1912	FALSE	1	TRA:CALNTGGFKTIF TRB:CSATESLTGGVGELFF	1	CD4-C1
PB-2_CTCAGAATCGTGGACC	PB	PB-2	P2-1914	FALSE	1	TRA:CVTGANSKLTF TRB:CASSPASAGPYEQYF	1	CD4-C1
PB-2_CTCCTAGAGTGCCAGA	PB	PB-2	P2-1918	FALSE	1	TRA:CADRPNTGNQFYF TRB:CASRREGLGEKLFF	1	CD4-C1
PB-2_CTCCTAGGTACGCACC	PB	PB-2	P2-1920	FALSE	1	TRA:CAASAKGQGGSEKLVF TRB:CASSPGPETQYF	1	CD4-C1
PB-2_CTCGAAAAGAAGGTGA	PB	PB-2	P2-1922	FALSE	1	TRA:CASSYGQNFVF TRB:CASSPGQGSDTQYF	1	CD4-C1
PB-2_CTCGAAACACTACAGT	PB	PB-2	P2-1927	FALSE	1	TRA:CAVNIGYNNNDMRF TRB:CASSFKETQYF	1	CD4-C1
PB-2_CTCGAGGAGGGTGTGT	PB	PB-2	P2-1932	FALSE	1	TRA:CAVGF TRB:CASSQADRPVEAFF	1	CD4-C1
PB-2_CTCGGAGAGGTACTCT	PB	PB-2	P2-1937	FALSE	1	TRA:CAVGDYKLSF TRB:CASSVGQGGTDTQYF	1	CD4-C1
PB-2_CTCGGGAAGGGTTCCC	PB	PB-2	P2-1939	FALSE	1	TRA:CAVQAKDTNAGKSTF TRB:CASSLLAGNEQFF	1	CD4-C1

PB-2_CTCGGGACACCGTTGG	PB	PB-2	P2-1940	FALSE	1	TRA:CAATLTSGSRLTF TRB:CSARDPVISGNTIYF	1	CD4-C1
PB-2_CTCGTACGTGTGAATA	PB	PB-2	P2-1946	FALSE	1	TRA:CAASIGNDYKLSF TRB:CASSPPTFGEKLFF	1	CD4-C1
PB-2_CTCGTCAAGGTAGCTG	PB	PB-2	P2-1948	FALSE	1	TRA:CAVEERRGNTPLVF TRB:CARGPGKGDTEAFF	1	CD4-C1
PB-2_CTCGTCACACACAGAG	PB	PB-2	P2-1950	FALSE	1	TRA:CAVGARGGKLIF TRB:CASSSDIYEQYF	1	CD4-C1
PB-2_CTCTGGTGTGTGCCTG	PB	PB-2	P2-1968	FALSE	1	TRA:CALTSGGSNYKLTF TRB:CASSETTGTPKETQYF	1	CD4-C1
PB-2_CTGAAGTAGAGAACAG	PB	PB-2	P2-1976	FALSE	1	TRA:CAVKSKLIF TRB:CASSQESAGGLGEQYF	1	CD4-C1
PB-2_CTGATAGAGAGTAATC	PB	PB-2	P2-1986	FALSE	1	TRA:CAVLTGANNLFF TRB:CASSHGQGGDTQYF	1	CD4-C1
PB-2_CTGATAGCAGTTCCCT	PB	PB-2	P2-1987	FALSE	1	TRA:CAEGASGTYKYIF TRB:CASSQVPGGAFF	1	CD4-C2
PB-2_CTGATCCCAATCTACG	PB	PB-2	P2-1992	FALSE	1	TRA:CAENAGGTSYGKLTF TRB:CASSYSEGRYGYTF	1	CD4-C1
PB-2_CTGCGGACAGGGCATA	PB	PB-2	P2-2003	FALSE	1	TRA:CAVPPSGGYNKLIF TRB:CASSPGTGNNEQFF	1	CD4-C1
PB-2_CTGTGCTCACCGAAAG	PB	PB-2	P2-2022	FALSE	1	TRA:CATDARNRDDKIIF TRB:CASRGDGYSYEQYF	1	CD4-C1
PB-2_CTGTTTAAGCTCTCGG	PB	PB-2	P2-2026	FALSE	1	TRA:CAVADNYGQNFVF TRB:CASLTFVQETQYF	1	CD4-C1
PB-2_CTGTTTACAAAGTGCG	PB	PB-2	P2-2027	FALSE	1	TRA:CAASAQSTGTASKLTF TRB:CASSESWGRPQHF	1	CD4-C1
PB-2_CTGTTTACATCACCCT	PB	PB-2	P2-2028	FALSE	1	TRA:CAATHGSGNTGKLIF TRB:CASSRDSLNHNEQFF	1	CD4-C1
PB-2_CTGTTTAGTTCTGTTT	PB	PB-2	P2-2029	FALSE	1	TRA:CIVRGLRGDDKIIF TRB:CASSIRGRAPTEAFF	1	CD4-C1
PB-2_CTTAACTAGCTAGTGG	PB	PB-2	P2-2031	FALSE	1	TRA:CAVSPREGTSYGKLTF TRB:CASSVDGVYLRNEQFF	1	CD4-C1
PB-2_CTTAACTGTAGCGTCC	PB	PB-2	P2-2035	FALSE	1	TRA:CAYRSLGGATNKLIF TRB:CSASVAGSYEQYF	1	CD4-C1
PB-2_CTTAACTTCTAACTGG	PB	PB-2	P2-2038	FALSE	1	TRA:CALSKETSGSRLTF TRB:CASSPRQNYEQYF	1	CD4-C1
PB-2_CTTAGGAGTCTCTCGT	PB	PB-2	P2-2045	FALSE	1	TRA:CAVSQGSSASKIIF TRB:CASTFPAEAFF	1	CD4-C1
PB-2_CTTAGGATCTCTTGAT	PB	PB-2	P2-2047	FALSE	1	TRA:CAVAAIQGAQKLVF TRB:CASSARGGNTEAFF	1	CD4-C1
PB-2_CTTCTCTAGGATATAC	PB	PB-2	P2-2048	FALSE	1	TRA:CAPRSGNTGKLIF TRB:CASSLQHGTGWNEQFF	1	CD4-C1
PB-2_CTTTGCGAGAATCTCC	PB	PB-2	P2-2058	FALSE	1	TRA:CAPAVARSNYQLIW TRB:CASRATATGIAGELFF	1	CD4-C1
PB-2_CTTTGCGTCACGCGGT	PB	PB-2	P2-2063	FALSE	1	TRA:CAATFTRQLTF TRB:CAWRLAGGSKETQYF	1	CD4-C1
PB-2_GAAATGAAGAGACTTA	PB	PB-2	P2-2066	FALSE	1	TRA:CALSNGNQFYF TRB:CASSIGDSYEQYF	1	CD4-C1
PB-2_GAACCTAAGTGGTCCC	PB	PB-2	P2-2080	FALSE	1	TRA:CAVGARGADGLTF TRB:CAWTRQGRETQYF	1	CD8-C1
PB-2_GAATAAGAGGGCATGT	PB	PB-2	P2-2098	FALSE	1	TRA:CAVGSGGYQKVTF TRB:CASAFDSDRTIYF	1	CD4-C1
PB-2_GAATAAGCAGGATTGG	PB	PB-2	P2-2100	FALSE	1	TRA:CASVGGSQGNLIF TRB:CASSYGQRNTIYF	1	CD4-C1

PB-2_GAATAAGGTAAGAGGA	PB	PB-2	P2-2101	FALSE	1	TRA:CASSGVGGTSYGKLTF TRB:CASSLGSTSYNEQFF	1	CD4-C1
PB-2_GACACGCAGTTTAGGA	PB	PB-2	P2-2111	FALSE	1	TRA:CAVDNNNDMRF TRB:CASSYAKWETQYF	1	CD4-C1
PB-2_GACCAATCACCCTATC	PB	PB-2	P2-2124	FALSE	1	TRA:CGTDIETSGSRLTF TRB:CASSVIVGATEAFF	1	CD4-C1
PB-2_GACCAATCAGGACGTA	PB	PB-2	P2-2126	FALSE	1	TRA:CAQNTGGFKTIF TRB:CASSFVGNTGELFF	1	CD4-C1
PB-2_GACCTGGAGCACACAG	PB	PB-2	P2-2132	FALSE	1	TRA:CAVNANQAGTALIF TRB:CSARDSRPPLHF	1	CD4-C1
PB-2_GACGGCTAGCCACTAT	PB	PB-2	P2-2144	FALSE	1	TRA:CAMSDRGAQKLVF TRB:CAWSVGSHLDEQYF	1	CD4-C1
PB-2_GACGGCTGTACCCAAT	PB	PB-2	P2-2149	FALSE	1	TRA:CAMRGPIKAAGNKLTF TRB:CASRNSGPLGETQYF	1	CD4-C1
PB-2_GACGGCTGTTTAGCTG	PB	PB-2	P2-2150	FALSE	1	TRA:CSNAGGTSYGKLTF TRB:CASSLPTGANVLTF	1	CD4-C1
PB-2_GACGTGCTCGTTACAG	PB	PB-2	P2-2161	FALSE	1	TRA:CAFTNSGYALNF TRB:CASSPLGGATDTQYF	1	CD4-C1
PB-2_GACTACAGTTCCCTTG	PB	PB-2	P2-2178	FALSE	1	TRA:CAVREGSYIPTF TRB:CASSQVMDRANNEQFF	1	CD4-C1
PB-2_GACTGCGTCAGCGATT	PB	PB-2	P2-2185	FALSE	1	TRA:CIVRVRSNTGNQFYF TRB:CSARGPGTGEAFF	1	CD4-C1
PB-2_GAGCAGAAGTAGCGGT	PB	PB-2	P2-2191	FALSE	1	TRA:CIVREGKNGGSQGNLIF TRB:CASSVAPSSGDTQYF	1	CD4-C1
PB-2_GAGCAGACACTTCGAA	PB	PB-2	P2-2192	FALSE	1	TRA:CAGVNGQNFVF TRB:CASSPQGASNQPQHF	1	CD4-C1
PB-2_GAGGTGACATGCCACG	PB	PB-2	P2-2200	FALSE	1	TRA:CAASTHAGNMLTF TRB:CASSRGGSFMNTEAFF	1	CD4-C1
PB-2_GAGTCCGAGACTAGGC	PB	PB-2	P2-2203	FALSE	1	TRA:CAVSGGSGYALNF TRB:CASSLSGGTEGTQYF	1	CD4-C1
PB-2_GAGTCCGGTTCAGACT	PB	PB-2	P2-2211	FALSE	1	TRA:CAASRRGTGNTPLVF TRB:CASSLAGPNEKLFF	1	CD4-C1
PB-2_GATCAGTGTAAAGTCA	PB	PB-2	P2-2215	FALSE	1	TRA:CAASEKAAGNKLTF TRB:CASSQLAGGSYEQYF	1	CD4-C1
PB-2_GATCAGTGTTCAGTAC	PB	PB-2	P2-2217	FALSE	1	TRA:CVVTNTGGFKTIF TRB:CASSVGQGGASGSSYEQYF	1	CD4-C1
PB-2_GATCGATCATGGTAGG	PB	PB-2	P2-2222	FALSE	1	TRA:CAVKAGINAGNMLTF TRB:CASSQEDGSNTGELFF	1	CD4-C1
PB-2_GATCGATGTCCAGTAT	PB	PB-2	P2-2224	FALSE	1	TRA:CALSENTGNQFYF TRB:CSAPTGALSNQPQHF	1	CD4-C1
PB-2_GATCGATTCACCAGGC	PB	PB-2	P2-2226	FALSE	1	TRA:CAGQETSGSRLTF TRB:CASSLRVNQPQHF	1	CD4-C1
PB-2_GATCGATTCATCGATG	PB	PB-2	P2-2227	FALSE	1	TRA:CAPGRGGSQGNLIF TRB:CASSLPTGDYGYTF	1	CD4-C1
PB-2_GATCGCGTCTGATACG	PB	PB-2	P2-2234	FALSE	1	TRA:CAVQASNYGQNFVF TRB:CSARARGASNQPQHF	1	CD4-C1
PB-2_GATCTAGAGTCCTCCT	PB	PB-2	P2-2244	FALSE	1	TRA:CAMREGAGTGGFKTIF TRB:CASRPPYTGYTQYF	1	CD4-C1
PB-2_GATGAAAAGGAGTAGA	PB	PB-2	P2-2250	FALSE	1	TRA:CAVRLTGGGNKLTF TRB:CASSFGPGFRELFF	1	CD4-C1
PB-2_GATGAAAGTAGCGATG	PB	PB-2	P2-2256	FALSE	1	TRA:CLVGDRRQLTF TRB:CATSESGLNGETQYF	1	CD4-C1
PB-2_GATGAAATCGACCAGC	PB	PB-2	P2-2261	FALSE	1	TRA:CIATDQGAQKLVF TRB:CATSRGQGGSGNTIYF	1	CD4-C1

PB-2_GATGCTACAATGTTGC	PB	PB-2	P2-2266	FALSE	1	TRA:CAGLDSNTGNQFYF TRB:CASRALYEQYF	1	CD4-C1
PB-2_GATGCTAGTTCTGAAC	PB	PB-2	P2-2268	FALSE	1	TRA:CAVRDDTGRRALTF TRB:CASSAGTSESYEQYF	1	CD4-C2
PB-2_GATTCAGGTTCAGGCC	PB	PB-2	P2-2271	FALSE	1	TRA:CAVAYMNTGFQKLVF TRB:CASSPDSGETQYF	1	CD4-C1
PB-2_GATTCAGTCTTAGCCC	PB	PB-2	P2-2272	FALSE	1	TRA:CAMRDEDNDMRF TRB:CATSDPSVGKTQYF	1	CD4-C1
PB-2_GCAATCAGTTTGCATG	PB	PB-2	P2-2285	FALSE	1	TRA:CAVGAVASYDKVIF TRB:CASRASGQSGTGELFF	1	CD4-C1
PB-2_GCACTCTTCTGAAAGA	PB	PB-2	P2-2297	FALSE	1	TRA:CAVGETSGSRLTF TRB:CASSRVTDTQYF	1	CD4-C1
PB-2_GCAGCCACATCCGCGA	PB	PB-2	P2-2301	FALSE	1	TRA:CAVGAGGATNKLIF TRB:CASGIAGVETQYF	1	CD4-C1
PB-2_GCAGCCACATGCCACG	PB	PB-2	P2-2302	FALSE	1	TRA:CAGQLREIGGSEKLVF TRB:CASSYPRGKNTEAFF	1	CD4-C1
PB-2_GCAGTTATCTCAAGTG	PB	PB-2	P2-2307	FALSE	1	TRA:CAVSDPNTGNQFYF TRB:CASGGSIRGYEQYF	1	CD4-C2
PB-2_GCATGATCAACACCTA	PB	PB-2	P2-2314	FALSE	1	TRA:CAATFGNQFYF TRB:CSARGGTNTGELFF	1	CD4-C1
PB-2_GCATGCGGTTCTGAAC	PB	PB-2	P2-2322	FALSE	1	TRA:CVVSEGGYNKLIF TRB:CSADRTDNTEAFF	1	CD4-C2
PB-2_GCATGCGTCAAGCCTA	PB	PB-2	P2-2323	FALSE	1	TRA:CAENPLTTYGQNFVF TRB:CASSLMKGTGELFF	1	CD4-C2
PB-2_GCCTCTAAGACTAGAT	PB	PB-2	P2-2338	FALSE	1	TRA:CAVGANNNDMRF TRB:CASSVGPGFYNEQFF	1	CD4-C2
PB-2_GCCTCTAAGATGCCAG	PB	PB-2	P2-2339	FALSE	1	TRA:CVVSERSGYSTLTF TRB:CASSLAASGTGGYGYTF	1	CD4-C1
PB-2_GCGACCAAGACAATAC	PB	PB-2	P2-2345	FALSE	1	TRA:CAVLNTGGFKTIF TRB:CASTPGQMNTEAFF	1	CD4-C1
PB-2_GCGCAACGTATGAAAC	PB	PB-2	P2-2365	FALSE	1	TRA:CALSDPKTSYDKVIF TRB:CASSGLGSGTDTQYF	1	CD4-C1
PB-2_GCGCCAAAGTGAAGAG	PB	PB-2	P2-2380	FALSE	1	TRA:CALTIILWGWELPTHF TRB:CASSVTVYEQYF	1	CD4-C1
PB-2_GCGCCAAAGTTTAGGA	PB	PB-2	P2-2381	FALSE	1	TRA:CAASSGTYKYIF TRB:CASSLTVVERGRYEQYF	1	CD4-C1
PB-2_GCGCGATTCGTCCAGG	PB	PB-2	P2-2388	FALSE	1	TRA:CAMRERSGNTGKLIF TRB:CSANRDVGNTIYF	1	CD4-C1
PB-2_GCGGGTTTCCTGCCAT	PB	PB-2	P2-2394	FALSE	1	TRA:CVLLNTGGGNKLTF TRB:CSVAIGRVNTEAFF	1	CD4-C1
PB-2_GCTGCAGAGATAGTCA	PB	PB-2	P2-2408	FALSE	1	TRA:CAENAGGTSYGKLTF TRB:CASSERAKETQYF	1	CD4-C1
PB-2_GCTGGGTTCGCCAAAT	PB	PB-2	P2-2426	FALSE	1	TRA:CAISQAGTALIF TRB:CASSLDGGRARDTQYF	1	CD4-C1
PB-2_GCTTCCAGTCTTGATG	PB	PB-2	P2-2428	FALSE	1	TRA:CAASTGSNYQLIW TRB:CASRTDVYGYTF	1	CD4-C1
PB-2_GCTTCCATCGCTGATA	PB	PB-2	P2-2431	FALSE	1	TRA:CAAPSRGHIKAAGNKLTF TRB:CASASRTEAFF	1	CD4-C1
PB-2_GCTTGAAAGCAACGGT	PB	PB-2	P2-2432	FALSE	1	TRA:CGTEGPFRANNLFF TRB:CASSRRTGGRKETEAFF	1	CD4-C1
PB-2_GGAACTTAGTTATCGC	PB	PB-2	P2-2438	FALSE	1	TRA:CAWAIDYKLSF TRB:CASSLTYEQYF	1	CD4-C1
PB-2_GGAACTTCACGCATCG	PB	PB-2	P2-2439	FALSE	1	TRA:CALTTSGTYKYIF TRB:CSAGDGQAGTDEQYF	1	CD4-C1

PB-2_GGAATAACATTTGCCC	PB	PB-2	P2-2452	FALSE	1	TRA:CVVSTSGGSYIPTF TRB:CASSLGLAGGVNTGELFF	1	CD4-C1
PB-2_GGAATAATCCGTACAA	PB	PB-2	P2-2455	FALSE	1	TRA:CIVRVASSNSGGSNYKLTF TRB:CASSPELGSGRYNEQFF	1	CD4-C1
PB-2_GGACAAGAGCCAGTTT	PB	PB-2	P2-2459	FALSE	1	TRA:CAASGGSGTYKYIF TRB:CSATHYRGLGYTF	1	CD4-C1
PB-2_GGACAAGGTCAACATC	PB	PB-2	P2-2465	FALSE	1	TRA:CAVNINSGYALNF TRB:CASSQESGQAREQYF	1	CD4-C1
PB-2_GGACAGAAGTCGATAA	PB	PB-2	P2-2471	FALSE	1	TRA:CALDMSSGTYKYIF TRB:CASSHNRASSYEQYF	1	CD4-C1
PB-2_GGACAGACAATCAGAA	PB	PB-2	P2-2472	FALSE	1	TRA:CAALIDQGGKLIF TRB:CSVQEGRETQYF	1	CD4-C1
PB-2_GGACAGAGTTCCATGA	PB	PB-2	P2-2474	FALSE	1	TRA:CIVRAYSGAGSYQLTF TRB:CASSSGTVDGYTF	1	CD4-C1
PB-2_GGACGTCGTGGGTCAA	PB	PB-2	P2-2482	FALSE	1	TRA:CAASIQGGAQKLVF TRB:CAISPVLDRYNEQFF	1	CD4-C2
PB-2_GGAGCAAAGATAGTCA	PB	PB-2	P2-2485	FALSE	1	TRA:CATDVSAGGTSYGKLTF TRB:CASSVAPDRGYQTQYF	1	CD4-C1
PB-2_GGATGTTCAGCTCGCA	PB	PB-2	P2-2491	FALSE	1	TRA:CAMSAVGQGGSEKLVF TRB:CASSADRVSGNTIYF	1	CD4-C1
PB-2_GGCAATTAGGGTATCG	PB	PB-2	P2-2498	FALSE	1	TRA:CALRIQGGAQKLVF TRB:CASSWGDNEKLFF	1	CD4-C1
PB-2_GGCAATTCAGTCAGAG	PB	PB-2	P2-2501	FALSE	1	TRA:CAVPPLYNTDKLIF TRB:CASSTPLTATNEKLFF	1	CD4-C1
PB-2_GGCAATTGTTGCGCAC	PB	PB-2	P2-2505	FALSE	1	TRA:CAMHSGGSNYKLTF TRB:CSLQGPNTDTQYF	1	CD4-C1
PB-2_GGCCGATTCCCAACGG	PB	PB-2	P2-2511	FALSE	1	TRA:CAVIGTGNQFYF TRB:CASSLAIGEQFF	1	CD4-C1
PB-2_GGCGACTAGAAGCCCA	PB	PB-2	P2-2513	FALSE	1	TRA:CALNGGSEKLVF TRB:CASGFGESYEQFF	1	CD4-C1
PB-2_GGCGACTGTGCTCTTC	PB	PB-2	P2-2520	FALSE	1	TRA:CAVQGFGNVLHC TRB:CASSFQDSGTQYF	1	CD4-C1
PB-2_GGCTCGACACCGATAT	PB	PB-2	P2-2527	FALSE	1	TRA:CADRLSGGSYIPTF TRB:CAWSHSSRINNEQFF	1	CD4-C1
PB-2_GGGAATGCAATCCAAC	PB	PB-2	P2-2537	FALSE	1	TRA:CAGRIGQFYF TRB:CASRNQGGGGSTEAFF	1	CD4-C1
PB-2_GGGACCTAGTCCCACG	PB	PB-2	P2-2541	FALSE	1	TRA:CLVGDEGKEGFQKLVF TRB:CASSFWVATNEKLFF	1	CD4-C1
PB-2_GGGATGAGTGCAGACA	PB	PB-2	P2-2550	FALSE	1	TRA:CVVSPGGNKLVF TRB:CASSPPGGSYGYTF	1	CD4-C1
PB-2_GGGCATCCAAACGCGA	PB	PB-2	P2-2556	FALSE	1	TRA:CAVRPFLLNSGGGADGLTF TRB:CASILTLNAYNSPLHF	1	CD4-C1
PB-2_GGGCATCTCTGCCAGG	PB	PB-2	P2-2562	FALSE	1	TRA:CALSGSARQLTF TRB:CSARADRGDTGELFF	1	CD4-C1
PB-2_GGGTTGCAGATACACA	PB	PB-2	P2-2568	FALSE	1	TRA:CAAIYNNNDMRF TRB:CASSAGTPALNTEAFF	1	CD4-C1
PB-2_GGGTTGCCACCTATCC	PB	PB-2	P2-2570	FALSE	1	TRA:CAVEDRRGRRALTF TRB:CASSEGSVYEQYF	1	CD4-C1
PB-2_GGGTTGCCACCTCGTT	PB	PB-2	P2-2571	FALSE	1	TRA:CARRLFQGGSEKLVF TRB:CASSLTKSGVTYNEQFF	1	CD4-C2
PB-2_GGTGTTAAGGCATTGG	PB	PB-2	P2-2580	FALSE	1	TRA:CAARGDAGGTSYGKLTF TRB:CAQRGRPPFSYEQYF	1	CD4-C1
PB-2_GGTGTTACACAGACTT	PB	PB-2	P2-2582	FALSE	1	TRA:CAPSGNTPLVF TRB:CASSLDRERGYTF	1	CD4-C1

PB-2_GGTGTTAGTACCGAGA	PB	PB-2	P2-2583	FALSE	1	TRA:CAEGGFGNVLHC TRB:CASSLLGDSNQPQHF	1	CD4-C1
PB-2_GGTGTTAGTGAGGGAG	PB	PB-2	P2-2585	FALSE	1	TRA:CAVEATDSWGKFQF TRB:CASSSGTNNYGYTF	1	CD4-C1
PB-2_GGTGTTATCAGCTCTC	PB	PB-2	P2-2586	FALSE	1	TRA:CALGDNTGNQFYF TRB:CAISESLTGGYGYTF	1	CD4-C1
PB-2_GTACGTAGTTTGACAC	PB	PB-2	P2-2602	FALSE	1	TRA:CVVRSVSNDYKLSF TRB:CASSDQWGQDGELFF	1	CD4-C1
PB-2_GTACTCCCAAACCTAC	PB	PB-2	P2-2604	FALSE	1	TRA:CAVTSGGGADGLTF TRB:CSARDTRGTEQFF	1	CD4-C1
PB-2_GTACTCCGTAGCACGA	PB	PB-2	P2-2607	FALSE	1	TRA:CALSGGNTPLVF TRB:CASSVAPGQGTFF	1	CD4-C1
PB-2_GTAGGCCGTTATCACG	PB	PB-2	P2-2617	FALSE	1	TRA:CAVSGNNRLAF TRB:CAWSNLQTGTEAFF	1	CD4-C1
PB-2_GTATCTTAGATCCGAG	PB	PB-2	P2-2624	FALSE	1	TRA:CILRGLPYTGTASKLTF TRB:CAWSTYPRQDYGYTF	1	CD4-C1
PB-2_GTATTCTAGGCAGTCA	PB	PB-2	P2-2627	FALSE	1	TRA:CALSDRAGGTSYGKLTF TRB:CASSQGPPGGPYEQYF	1	CD4-C1
PB-2_GTATTCTCAGCTGCTG	PB	PB-2	P2-2628	FALSE	1	TRA:CAVGSEAAGNKLTF TRB:CASSALAGNTDTQYF	1	CD4-C1
PB-2_GTCAAGTGTTTACTCT	PB	PB-2	P2-2636	FALSE	1	TRA:CAVRLQGGTSYGKLTF TRB:CASRNSGEYGYTF	1	CD4-C1
PB-2_GTCACGGAGAGTAATC	PB	PB-2	P2-2646	FALSE	1	TRA:CAAISRSGYALNF TRB:CASSLGSASTDTQYF	1	CD4-C2
PB-2_GTCACGGAGGCTAGAC	PB	PB-2	P2-2647	FALSE	1	TRA:CAVERGGGNKLTF TRB:CASSSQRGGPQETQYF	1	CD4-C1
PB-2_GTCACGGAGTACGTTC	PB	PB-2	P2-2648	FALSE	1	TRA:CVVSDRTSYDKVIF TRB:CASSISGSHTGELFF	1	CD4-C1
PB-2_GTCACGGCAAGGTTTC	PB	PB-2	P2-2650	FALSE	1	TRA:CAAGGTSYGKLTF TRB:CASSGVSGNTIYF	1	CD4-C1
PB-2_GTCACGGTCCGTCATC	PB	PB-2	P2-2651	FALSE	1	TRA:CAASANYGGSQGNLIF TRB:CASSRSSPYGATNEKLFF	1	CD4-C2
PB-2_GTCATTTAGTACATGA	PB	PB-2	P2-2653	FALSE	1	TRA:CAAPQGAQKLVF TRB:CASSQVLSGLNEQFF	1	CD4-C1
PB-2_GTCATTTCAGATCTGT	PB	PB-2	P2-2655	FALSE	1	TRA:CAVKYSGGGADGLTF TRB:CASSQFDRAASEQYF	1	CD4-C1
PB-2_GTCCTCACAGACGCAA	PB	PB-2	P2-2664	FALSE	1	TRA:CILRDEYALNF TRB:CASSHGLAGGYDTQYF	1	CD4-C1
PB-2_GTCCTCAGTTGTCGCG	PB	PB-2	P2-2668	FALSE	1	TRA:CAYRSAPRYTGTASKLTF TRB:CASSPPSGASGWSQYF	1	CD4-C1
PB-2_GTCGGGTTCTTACCGC	PB	PB-2	P2-2676	FALSE	1	TRA:CAASRYSGGYQKVTF TRB:CAISEAAEETQYF	1	CD4-C2
PB-2_GTCGTAAAGCTCCTTC	PB	PB-2	P2-2677	FALSE	1	TRA:CARDTGRRALTF TRB:CASSLALLTGGGFGYTF	1	CD4-C1
PB-2_GTCGTAAAGGCTACGA	PB	PB-2	P2-2678	FALSE	1	TRA:CAGGTQGGSEKLVF TRB:CASSPNPWRNTGELFF	1	CD4-C1
PB-2_GTCGTAACATGCATGT	PB	PB-2	P2-2681	FALSE	1	TRA:CAVQALIGGFKTIF TRB:CASSSQGEETQYF	1	CD4-C1
PB-2_GTCTCGTCAGCCTATA	PB	PB-2	P2-2683	FALSE	1	TRA:CAAPFYF TRB:CSAQSGQGGGETQYF	1	CD4-C1
PB-2_GTCTCGTCATCCTAGA	PB	PB-2	P2-2685	FALSE	1	TRA:CAVLSASKLTF TRB:CASSQGSGGTYEQYF	1	CD4-C1
PB-2_GTCTCGTGTACCGCTG	PB	PB-2	P2-2686	FALSE	1	TRA:CAASIGNTGKLIF TRB:CASSVWAGVGETQYF	1	CD4-C1

PB-2_GTCTCGTGTTAAAGTG	PB	PB-2	P2-2688	FALSE	1	TRA:CAIGSNQFYF TRB:CASSLTGGAVVKDYGYTF	1	CD4-C2
PB-2_GTCTCGTTCAACTCTT	PB	PB-2	P2-2689	FALSE	1	TRA:CAVLRNNNARLMF TRB:CASSQVGGGVADTQYF	1	CD4-C1
PB-2_GTCTTCGTCGGCGGTT	PB	PB-2	P2-2698	FALSE	1	TRA:CAENDYSGSARQLTF TRB:CASSRSGAQNQPQHF	1	CD4-C1
PB-2_GTGAAGGAGCGACGTA	PB	PB-2	P2-2700	FALSE	1	TRA:CAVARNYGQNFVF TRB:CASSQAGEKLFF	1	CD4-C1
PB-2_GTGCAGCCACTGAAGG	PB	PB-2	P2-2704	FALSE	1	TRA:CAASYSGGGADGLTF TRB:CASSLATSGPYEQYF	1	CD4-C1
PB-2_GTGCAGCGTGGGTATG	PB	PB-2	P2-2705	FALSE	1	TRA:CAVIKNTGNQFYF TRB:CAISARGVSYNEQFF	1	CD4-C1
PB-2_GTGCATATCACCGGGT	PB	PB-2	P2-2710	FALSE	1	TRA:CAVETDKLIF TRB:CASRQNRGPLYF	1	CD4-C1
PB-2_GTGCATATCTCTGCTG	PB	PB-2	P2-2711	FALSE	1	TRA:CAVEDRHSGAGSYQLTF TRB:CASSLFGSDTQYF	1	CD4-C1
PB-2_GTGCTTCAGCCAGTAG	PB	PB-2	P2-2720	FALSE	1	TRA:CAVRDMNSGATNKLIF TRB:CSVVPSWQTSYNEQFF	1	CD4-C1
PB-2_GTGTGCGGTTACTGAC	PB	PB-2	P2-2736	FALSE	1	TRA:CAVGRGSNYKLTF TRB:CAWRSGVSNTDTQYF	1	CD4-C1
PB-2_GTGTTAGAGCTTATCG	PB	PB-2	P2-2743	FALSE	1	TRA:CAVGNNDMRF TRB:CASSQAFAGNYEQYF	1	CD4-C1
PB-2_GTGTTAGCACTTAAGC	PB	PB-2	P2-2745	FALSE	1	TRA:CAVPSSGSARQLTF TRB:CASSSKQRGGSYEQYF	1	CD4-C1
PB-2_GTGTTAGGTGATGTGG	PB	PB-2	P2-2746	FALSE	1	TRA:CIVRVVNVLRGSQGNLIF TRB:CASSTRASNYGYTF	1	CD4-C1
PB-2_GTGTTAGGTGCCTGTG	PB	PB-2	P2-2747	FALSE	1	TRA:CIVSRGSNYQLIW TRB:CSASSGYEETQYF	1	CD4-C1
PB-2_GTTAAGCAGATCCCAT	PB	PB-2	P2-2751	FALSE	1	TRA:CAVKGTGGSYIPTF TRB:CASSIGLAGGSYEQYF	1	CD4-C1
PB-2_GTTAAGCGTACCTACA	PB	PB-2	P2-2755	FALSE	1	TRA:CAVSENTGNQFYF TRB:CAWSGTSFGWNQPQHF	1	CD4-C1
PB-2_GTTAAGCTCTGCCAGG	PB	PB-2	P2-2759	FALSE	1	TRA:CILRDAGNQFYF TRB:CAIRDTGGSTDTQYF	1	CD4-C1
PB-2_GTTCATTCAGGATTGG	PB	PB-2	P2-2767	FALSE	1	TRA:CGTENSGGSNYKLTF TRB:CASSPDLSGGEAFF	1	CD4-C1
PB-2_GTTCATTGTCAGTGGA	PB	PB-2	P2-2769	FALSE	1	TRA:CATDRSFGNVLHC TRB:CASSLQGPDSYNEQFF	1	CD4-C1
PB-2_GTTCGGGCACAAGACG	PB	PB-2	P2-2772	FALSE	1	TRA:CAMREKGTNAGKSTF TRB:CASSASDFQETQYF	1	CD4-C1
PB-2_GTTCGGGCATATACGC	PB	PB-2	P2-2773	FALSE	1	TRA:CAVGPREGQKLLF TRB:CASSYIDRGGGYTF	1	CD4-C1
PB-2_GTTCTCGTCCAAGCCG	PB	PB-2	P2-2778	FALSE	1	TRA:CAVGGFTGGGNKLTF TRB:CASSVGVGGPTDTQYF	1	CD4-C1
PB-2_GTTTCTAAGCTGAACG	PB	PB-2	P2-2781	FALSE	1	TRA:CAVTAGGTSYGKLTF TRB:CASSQGDRGYEQYF	1	CD4-C1
PB-2_GTTTCTACAGATGGCA	PB	PB-2	P2-2784	FALSE	1	TRA:CAPSGYSSASKIIF TRB:CASRSRDYNEQFF	1	CD4-C1
PB-2_TAAGAGAAGAAGGCCT	PB	PB-2	P2-2788	FALSE	1	TRA:CASSVSGKLSF TRB:CAYSGQANSPLHF	1	CD4-C1
PB-2_TAAGAGAGTGACGGTA	PB	PB-2	P2-2793	FALSE	1	TRA:CAGPRYRSGNTGKLIF TRB:CAITGQADQPQHF	1	CD4-C1
PB-2_TACACGAAGAAACGAG	PB	PB-2	P2-2806	FALSE	1	TRA:CVVSRPGTASKLTF TRB:CSASSVRGTDTQYF	1	CD4-C1

PB-2_TACACGAAGAATGTGT	PB	PB-2	P2-2808	FALSE	1	TRA:CAVDSWVGGSQGNLIF TRB:CASSSGTGLYQPQHF	1	CD4-C1
PB-2_TACGGGCCACTTAACG	PB	PB-2	P2-2839	FALSE	1	TRA:CAVRPKDSNYQLIW TRB:CAWSSGTGLTNEQFF	1	CD4-C1
PB-2_TACGGGCGTGGCTCCA	PB	PB-2	P2-2841	FALSE	1	TRA:CAATITSGTYKYIF TRB:CASTPDISYNSPLHF	1	CD4-C1
PB-2_TACGGTAAGATCACGG	PB	PB-2	P2-2843	FALSE	1	TRA:CAVRDRVDSNYQLIW TRB:CASSQVGPVNEQFF	1	CD4-C1
PB-2_TACGGTATCGTTTAGG	PB	PB-2	P2-2848	FALSE	1	TRA:CAMREGRDGFNKFYF TRB:CASSLAQSGAKAFF	1	CD4-C1
PB-2_TACTCATCAGCTGCAC	PB	PB-2	P2-2849	FALSE	1	TRA:CAVRGDNAGNMLTF TRB:CASSKGYQPQHF	1	CD4-C1
PB-2_TACTCATGTCAAAGAT	PB	PB-2	P2-2850	FALSE	1	TRA:CANGGYALNF TRB:CATSDPGQGAYEQYF	1	CD4-C1
PB-2_TACTCATTCCTGCCAT	PB	PB-2	P2-2853	FALSE	1	TRA:CVVKLSSGYALNF TRB:CASRPLGGGDTEAFF	1	CD4-C1
PB-2_TAGACCATCAACCATG	PB	PB-2	P2-2874	FALSE	1	TRA:CAGGGDTDKLIF TRB:CASSRSRGNTYNEQFF	1	CD4-C1
PB-2_TAGACCATCCCAACGG	PB	PB-2	P2-2875	FALSE	1	TRA:CIVRVGGSGNTGKLIF TRB:CASGTTNNSPLHF	1	CD4-C1
PB-2_TAGAGCTGTTACCAGT	PB	PB-2	P2-2883	FALSE	1	TRA:CAATGGSGGGADGLTF TRB:CASSTFSGIRDNEQFF	1	CD4-C1
PB-2_TAGCCGGCAGCGATCC	PB	PB-2	P2-2884	FALSE	1	TRA:CAPLDNTDKLIF TRB:CASSLGSGNTIYF	1	CD4-C1
PB-2_TAGCCGGTCCTATTCA	PB	PB-2	P2-2886	FALSE	1	TRA:CAAATSTSGTYKYIF TRB:CAWSVQGALHF	1	CD4-C1
PB-2_TAGTGGTCACAGGCCT	PB	PB-2	P2-2892	FALSE	1	TRA:CAVEDRRVFNTGGFKTIF TRB:CATAQGANSPLHF	1	CD4-C1
PB-2_TAGTGGTTCACCACCT	PB	PB-2	P2-2894	FALSE	1	TRA:CAVSGGNKLVF TRB:CSARPIPGQGREQYF	1	CD4-C2
PB-2_TAGTTGGGTAAATGAC	PB	PB-2	P2-2903	FALSE	1	TRA:CAAPSFQGAQKLVF TRB:CASSEAREQYF	1	CD4-C1
PB-2_TAGTTGGGTGATGTGG	PB	PB-2	P2-2905	FALSE	1	TRA:CVVSEDSWGKFQF TRB:CSARRGGNPYNEQFF	1	CD4-C1
PB-2_TATCAGGGTGCGCTTG	PB	PB-2	P2-2911	FALSE	1	TRA:CAALNQAGTALIF TRB:CARGRQETQYF	1	CD4-C1
PB-2_TATCAGGTCTGCGGCA	PB	PB-2	P2-2916	FALSE	1	TRA:CAVRNQAGTALIF TRB:CASSPATGLNSPLHF	1	CD4-C1
PB-2_TATCTCACAGCTCCGA	PB	PB-2	P2-2918	FALSE	1	TRA:CAAPRNKLVF TRB:CASSYSNRPSSSYNSPLHF	1	CD4-C1
PB-2_TATGCCCAGCTGTTCA	PB	PB-2	P2-2923	FALSE	1	TRA:CAVRATYQSGSARQLTF TRB:CASSEALGGGHGELFF	1	CD4-C1
PB-2_TATGCCCAGGGTGTTG	PB	PB-2	P2-2924	FALSE	1	TRA:CAVITWGAQKLVF TRB:CASSFGNEQFF	1	CD4-C1
PB-2_TATGCCCGTGTGTGCC	PB	PB-2	P2-2928	FALSE	1	TRA:CAVTGTASKLTF TRB:CASSLRQGSSYNEQFF	1	CD4-C1
PB-2_TATTACCGTCGAGATG	PB	PB-2	P2-2934	FALSE	1	TRA:CIVRVGYNNAGNMLTF TRB:CSALSGNSGNTIYF	1	CD4-C1
PB-2_TCAACGATCCCACTTG	PB	PB-2	P2-2940	FALSE	1	TRA:CAASMNNDMRF TRB:CASSLGRAITEAFF	1	CD4-C1
PB-2_TCAATCTCATTCTTAC	PB	PB-2	P2-2941	FALSE	1	TRA:CAAPSSASKIIF TRB:CASSLTALTDNEQFF	1	CD4-C1
PB-2_TCAATCTTCGCTGATA	PB	PB-2	P2-2942	FALSE	1	TRA:CAGRLPGAQKLVF TRB:CASQPGRLTYEQYF	1	CD4-C1

PB-2_TCACAAGAGACCTAGG	PB	PB-2	P2-2943	FALSE	1	TRA:CAARSFQKLLF TRB:CSADSKTGSGGYTF	1	CD4-C1
PB-2_TCAGCAAAGTAGGCCA	PB	PB-2	P2-2961	FALSE	1	TRA:CAVNSGGYQKVTF TRB:CAWSVDRGSYGYTF	1	CD4-C1
PB-2_TCAGCTCAGATGTGGC	PB	PB-2	P2-2970	FALSE	1	TRA:CILQNNDMRF TRB:CASSQGDRGYGYTF	1	CD4-C1
PB-2_TCAGCTCAGCTGCCCA	PB	PB-2	P2-2971	FALSE	1	TRA:CAPGRYNFNKFYF TRB:CASYRDLNTEAFF	1	CD4-C1
PB-2_TCAGGATCATGAGCGA	PB	PB-2	P2-2976	FALSE	1	TRA:CAVRPEAGNMLTF TRB:CASRSHSGIGYTF	1	CD4-C1
PB-2_TCAGGTAAGAATGTGT	PB	PB-2	P2-2979	FALSE	1	TRA:CIVRVANFGNEKLTF TRB:CADANPGQGSGNTIYF	1	CD4-C1
PB-2_TCAGGTAAGCCCTAAT	PB	PB-2	P2-2980	FALSE	1	TRA:CAASGLGGGADGLTF TRB:CASSKGLLDYEQYF	1	CD4-C1
PB-2_TCATTACAGGCCCTTG	PB	PB-2	P2-2987	FALSE	1	TRA:CAFTLYNFNKFYF TRB:CASSLPPHEQYF	1	CD4-C1
PB-2_TCATTACGTACTCTCC	PB	PB-2	P2-2991	FALSE	1	TRA:CAVEGAMNTGFQKLVF TRB:CASSGTSGMSTDTQYF	1	CD4-C1
PB-2_TCATTACGTAGGACAC	PB	PB-2	P2-2992	FALSE	1	TRA:CAVQARHTDKLIF TRB:CASSLPSGGASYNEQFF	1	CD8-C1
PB-2_TCATTACTCCAAACTG	PB	PB-2	P2-2994	FALSE	1	TRA:CAASPPLKTSYDKVIF TRB:CASSISTGVTGELFF	1	CD4-C1
PB-2_TCATTACTCCTCAATT	PB	PB-2	P2-2996	FALSE	1	TRA:CADAGGTSYGKLTF TRB:CASSEIGYEQYF	1	CD4-C1
PB-2_TCCACACCACCATCCT	PB	PB-2	P2-3003	FALSE	1	TRA:CAASSYNTNAGKSTF TRB:CASSGWGRGSGELFF	1	CD4-C1
PB-2_TCCCGATAGGCACATG	PB	PB-2	P2-3006	FALSE	1	TRA:CAANFYNNNDMRF TRB:CASTSQGPEQFF	1	CD4-C1
PB-2_TCCCGATTCAACACGT	PB	PB-2	P2-3012	FALSE	1	TRA:CIVRSSNFGNEKLTF TRB:CASSVGPEPSETQYF	1	CD4-C2
PB-2_TCGAGGCGTCATCGGC	PB	PB-2	P2-3013	FALSE	1	TRA:CAAGNTGANNLFF TRB:CAISKDRDYEQYF	1	CD4-C1
PB-2_TCGGTAACATTGGCGC	PB	PB-2	P2-3028	FALSE	1	TRA:CALRGRDDKIIF TRB:CASSMSDRGLYEQYF	1	CD4-C1
PB-2_TCGTACCCACACATGT	PB	PB-2	P2-3030	FALSE	1	TRA:CAENIRSYIPTF TRB:CASSFNLGGETQYF	1	CD4-C1
PB-2_TCGTAGAGTCCATGAT	PB	PB-2	P2-3035	FALSE	1	TRA:CAVIPYNQGGKLIF TRB:CASREDRGQGPQHF	1	CD4-C1
PB-2_TCGTAGATCCCACTTG	PB	PB-2	P2-3038	FALSE	1	TRA:CAASNIVDGNKLVF TRB:CASSFGSGYEQYF	1	CD4-C1
PB-2_TCTATTGCACTAAGTC	PB	PB-2	P2-3041	FALSE	1	TRA:CAVSQTGANNLFF TRB:CASSLDNSYEQYF	1	CD4-C1
PB-2_TCTATTGTCAGTGTTG	PB	PB-2	P2-3046	FALSE	1	TRA:CAENGSGNTGKLIF TRB:CASSEMRSAVASNYGYTF	1	CD4-C1
PB-2_TCTCTAAGTGTGAATA	PB	PB-2	P2-3054	FALSE	1	TRA:CATGSIQGAQKLVF TRB:CASSQGPGVTEAFF	1	CD4-C1
PB-2_TCTCTAATCCGCAGTG	PB	PB-2	P2-3055	FALSE	1	TRA:CAPQGAGGFKTIF TRB:CASSPERQPQHF	1	CD4-C1
PB-2_TCTGAGAAGAGGTAGA	PB	PB-2	P2-3057	FALSE	1	TRA:CALFSGTSYGKLTF TRB:CASSSDRGQYF	1	CD4-C1
PB-2_TCTGAGACAGGAATCG	PB	PB-2	P2-3059	FALSE	1	TRA:CALRGRGNKLVF TRB:CASSSDRGLYGYTF	1	CD4-C1
PB-2_TCTGAGACATGAGCGA	PB	PB-2	P2-3061	FALSE	1	TRA:CAVNDRVATNKLIF TRB:CASSLDQGPPYEQYF	1	CD4-C1

PB-2_TCTTCGGCATCCGTGG	PB	PB-2	P2-3067	FALSE	1	TRA:CAMSGYTGNQFYF TRB:CASSTVPRSSRNTIYF	1	CD4-C1
PB-2_TCTTCGGGTATATGAG	PB	PB-2	P2-3069	FALSE	1	TRA:CALSDPGGGYNKLIF TRB:CASSPYALSSGANVLTF	1	CD4-C1
PB-2_TCTTCGGTCACATGCA	PB	PB-2	P2-3071	FALSE	1	TRA:CAASGGAQKLVF TRB:CASSLGAGHYGYTF	1	CD4-C1
PB-2_TCTTCGGTCGGTGTTA	PB	PB-2	P2-3072	FALSE	1	TRA:CAVSLLYGNNRLAF TRB:CAISEDTSNQPQHF	1	CD4-C1
PB-2_TCTTTCCAGGTAAACT	PB	PB-2	P2-3076	FALSE	1	TRA:CAVNGPGNTGKLIF TRB:CAISEREGLDTQYF	1	CD4-C1
PB-2_TCTTTCCTCACAGTAC	PB	PB-2	P2-3078	FALSE	1	TRA:CAAGGDTGTASKLTF TRB:CASSDPTPSRYF	1	CD4-C1
PB-2_TGACAACTCAACGCTA	PB	PB-2	P2-3090	FALSE	1	TRA:CAAGAGSYQLTF TRB:CASSLAGSGEQYF	1	CD4-C1
PB-2_TGACGGCTCATGTCCC	PB	PB-2	P2-3097	FALSE	1	TRA:CAVRAMDSSYKLIF TRB:CASSRAGQADTQYF	1	CD4-C2
PB-2_TGACGGCTCCTAGGGC	PB	PB-2	P2-3098	FALSE	1	TRA:CAFRANAGKSTF TRB:CASSQDVAAGKQYF	1	CD4-C1
PB-2_TGACTTTAGTCTCGGC	PB	PB-2	P2-3113	FALSE	1	TRA:CAMREGRGRLTGGGNKLTF TRB:CASRGLAGGSDTQYF	1	CD4-C1
PB-2_TGAGAGGCAAGCCGCT	PB	PB-2	P2-3115	FALSE	1	TRA:CALSATNSGYALNF TRB:CASRLEATNEKLFF	1	CD4-C1
PB-2_TGAGCATGTACTCAAC	PB	PB-2	P2-3121	FALSE	1	TRA:CAARSGGSNYKLTF TRB:CASRTTGWGQPQHF	1	CD4-C1
PB-2_TGAGCATTCTCACATT	PB	PB-2	P2-3122	FALSE	1	TRA:CIVRVATGTYKYIF TRB:CASSYGQGRDTQYF	1	CD4-C1
PB-2_TGAGCCGGTAGTGAAT	PB	PB-2	P2-3126	FALSE	1	TRA:CAAGYGNKLVF TRB:CSAFERGGIKETQYF	1	CD4-C1
PB-2_TGAGGGAAGAACAATC	PB	PB-2	P2-3130	FALSE	1	TRA:CAGQLAGSQGNLIF TRB:CASSQAAGQFSEAFF	1	CD4-C1
PB-2_TGATTTCGTACTCGCG	PB	PB-2	P2-3135	FALSE	1	TRA:CALSGGSNFGNEKLTF TRB:CAISPGSSRKGSYEQYF	1	CD4-C1
PB-2_TGATTTCGTATAATGG	PB	PB-2	P2-3136	FALSE	1	TRA:CALKSTGGGADGLTF TRB:CSAGTGNTEAFF	1	CD4-C1
PB-2_TGATTTCTCCAGAGGA	PB	PB-2	P2-3138	FALSE	1	TRA:CAMREGAGGTSYGKLTF TRB:CASSLQSSDRSPLHF	1	CD4-C2
PB-2_TGCACCTGTTCCACTC	PB	PB-2	P2-3144	FALSE	1	TRA:CATAAQGGGGYNKLIF TRB:CASGQPQHF	1	CD4-C1
PB-2_TGCACCTTCACAGTAC	PB	PB-2	P2-3146	FALSE	1	TRA:CALFGASNYQLIW TRB:CASSSSGVLSGNTIYF	1	CD4-C1
PB-2_TGCCAAACAAGGTTCT	PB	PB-2	P2-3148	FALSE	1	TRA:CAASIPLRPGGSEKLVF TRB:CASFGTGGPNEQYF	1	CD4-C1
PB-2_TGCCAAAGTCTTGCGG	PB	PB-2	P2-3151	FALSE	1	TRA:CAVSPTGGYNKLIF TRB:CASGKSGANVLTF	1	CD4-C1
PB-2_TGCCCTAGTATTAGCC	PB	PB-2	P2-3162	FALSE	1	TRA:CPHFGNEKLTF TRB:CASSPREAWSYEQYF	1	CD4-C1
PB-2_TGCCCTATCTGCGGCA	PB	PB-2	P2-3165	FALSE	1	TRA:CARLILWGWELPTHF TRB:CASSPGLAAFQETQYF	1	CD4-C1
PB-2_TGCGCAGCAGCTGCAC	PB	PB-2	P2-3167	FALSE	1	TRA:CAYRSPTSGTYKYIF TRB:CASSRPLWGGASGSTDTQYF	1	CD4-C1
PB-2_TGCGCAGGTAGCTCCG	PB	PB-2	P2-3170	FALSE	1	TRA:CAVERTGGTSYGKLTF TRB:CASSQVGVGYTF	1	CD4-C1
PB-2_TGCGCAGGTTCACCTC	PB	PB-2	P2-3171	FALSE	1	TRA:CATDHTGTASKLTL TRB:CASKLAVSTDTQYF	1	CD4-C1

PB-2_TGCGCAGTCCTGCAGG	PB	PB-2	P2-3173	FALSE	1	TRA:CAVGARPGGSQGNLIF TRB:CASSPDRSSSYNEQFF	1	CD4-C1
PB-2_TGCGTGGAGTCCTCCT	PB	PB-2	P2-3180	FALSE	1	TRA:CAPKPSGYSTLTF TRB:CASGRGTESQHF	1	CD4-C1
PB-2_TGCGTGGCATTTCACT	PB	PB-2	P2-3183	FALSE	1	TRA:CAVTSGYSTLTF TRB:CASSDGTNSYEQYF	1	CD4-C1
PB-2_TGGCCAGTCACAACGT	PB	PB-2	P2-3202	FALSE	1	TRA:CATDPSGGANSKLTF TRB:CASSLVRDDQPQHF	1	CD4-C1
PB-2_TGGCGCAGTACCGGCT	PB	PB-2	P2-3205	FALSE	1	TRA:CAVGGGNTDKLIF TRB:CASSLDQGGADTQYF	1	CD4-C1
PB-2_TGGCTGGAGTTGTCGT	PB	PB-2	P2-3209	FALSE	1	TRA:CALSDSGNTPLVF TRB:CASSPLPGSKNIQYF	1	CD4-C1
PB-2_TGGGAAGAGGTCATCT	PB	PB-2	P2-3216	FALSE	1	TRA:CAVSVAGGTSYGKLTF TRB:CATSRDQAYEQYF	1	CD4-C1
PB-2_TGGGAAGCATGCAATC	PB	PB-2	P2-3219	FALSE	1	TRA:CVVSEGGTYKYIF TRB:CASSLWQKETQYF	1	CD4-C1
PB-2_TGGGAAGTCTAACTTC	PB	PB-2	P2-3223	FALSE	1	TRA:CIVRGRDYTGGFKTIF TRB:CASSFQETQYF	1	CD4-C1
PB-2_TGGGCGTTCGGAATCT	PB	PB-2	P2-3226	FALSE	1	TRA:CALSDGGTSYGKLTF TRB:CASRPRQGAFEAFF	1	CD4-C1
PB-2_TGGTTCCCAGCGTTCG	PB	PB-2	P2-3233	FALSE	1	TRA:CILRSEGGSNYKLTF TRB:CSARPRGQPLAEAFF	1	CD4-C1
PB-2_TGTATTCGTGTCCTCT	PB	PB-2	P2-3239	FALSE	1	TRA:CATSTKGAQKLVF TRB:CASSLEGGGETQYF	1	CD4-C1
PB-2_TGTGGTATCCATTCTA	PB	PB-2	P2-3251	FALSE	1	TRA:CAASYQGGSEKLVF TRB:CASSQANSNEQFF	1	CD4-C1
PB-2_TGTTCCGAGTTAACGA	PB	PB-2	P2-3260	FALSE	1	TRA:CVVSDGGTSGTYKYIF TRB:CASSRQRGTGAYNEQFF	1	CD4-C1
PB-2_TGTTCCGCAAGCTGGA	PB	PB-2	P2-3262	FALSE	1	TRA:CIVRRPQTGGGNKLTF TRB:CSVFDRLTSYNEQFF	1	CD4-C1
PB-2_TTAACTCAGCCAGTAG	PB	PB-2	P2-3266	FALSE	1	TRA:CAVSGGYSGGSNYKLTF TRB:CASSLGGLQSPLHF	1	CD4-C1
PB-2_TTAACTCCAAGCTGGA	PB	PB-2	P2-3267	FALSE	1	TRA:CAVQAAGGNAGNMLTF TRB:CASSLAGSSYEQYF	1	CD4-C1
PB-2_TTAGGACTCTAACTGG	PB	PB-2	P2-3277	FALSE	1	TRA:CAVRDAGNTGKLIF TRB:CASLEQGASYEQYF	1	CD4-C1
PB-2_TTAGTTCGTATGAATG	PB	PB-2	P2-3285	FALSE	1	TRA:CVVNDNQGGKLIF TRB:CASSYSRSYEQYF	1	CD4-C1
PB-2_TTAGTTCTCGGTTAAC	PB	PB-2	P2-3286	FALSE	1	TRA:CAVSDLGNTGGFKTIF TRB:CASSFGGPGQGEKLFF	1	CD4-C1
PB-2_TTATGCTAGCCAGGAT	PB	PB-2	P2-3288	FALSE	1	TRA:CAASAPSGTYKYIF TRB:CASSLDLRAGGYF	1	CD4-C1
PB-2_TTCCCAGCAGGATTGG	PB	PB-2	P2-3293	FALSE	1	TRA:CAVTPSNTDKLIF TRB:CASSFSAVSGNTIYF	1	CD4-C1
PB-2_TTCCCAGCAGTCAGCC	PB	PB-2	P2-3294	FALSE	1	TRA:CATGGSNYKLTF TRB:CASSLGLGLGTEAFF	1	CD4-C1
PB-2_TTCTACAAGAAGCCCA	PB	PB-2	P2-3310	FALSE	1	TRA:CALPEDSGGYQKVTF TRB:CASSYSRNTGELFF	1	CD4-C1
PB-2_TTCTACAAGGAATCGC	PB	PB-2	P2-3311	FALSE	1	TRA:CAAKRGGSQGNLIF TRB:CATSPRGFGVNQPQHF	1	CD4-C1
PB-2_TTCTACATCGCCCTTA	PB	PB-2	P2-3315	FALSE	1	TRA:CALTTGNQFYF TRB:CASSPPGVGEQFF	1	CD4-C1
PB-2_TTCTCAACACAGGCCT	PB	PB-2	P2-3318	FALSE	1	TRA:CLVGDIAGGYQKVTF TRB:CASWDSSFNEQFF	1	CD4-C1

PB-2_TTCTCCTAGGCTCAGA	PB	PB-2	P2-3321	FALSE	1	TRA:CATVAGEYGNKLVF TRB:CASSLRQGGDTQYF	1	CD4-C1
PB-2_TTCTTAGCACGCGAAA	PB	PB-2	P2-3324	FALSE	1	TRA:CAVSRSGGGADGLTF TRB:CASSVEGWNTGELFF	1	CD4-C1
PB-2_TTCTTAGCATGCCTAA	PB	PB-2	P2-3326	FALSE	1	TRA:CIRYNTDKLIF TRB:CASRRGGTGELFF	1	CD4-C1
PB-2_TTCTTAGGTACTTCTT	PB	PB-2	P2-3327	FALSE	1	TRA:CAASHLNDYKLSF TRB:CASRRRGRSNEKLFF	1	CD4-C1
PB-2_TTGAACGCAGGAATCG	PB	PB-2	P2-3330	FALSE	1	TRA:CAVSESGGSYIPTF TRB:CASERGLGEKLFF	1	CD4-C1
PB-2_TTGAACGTCTGGGCCA	PB	PB-2	P2-3336	FALSE	1	TRA:CALSGGGSNYKLTF TRB:CASSGGGYGYTF	1	CD4-C1
PB-2_TTGAACGTCTTTACGT	PB	PB-2	P2-3337	FALSE	1	TRA:CAMSGGSQGNLIF TRB:CASSPTPGTGLDGYTF	1	CD4-C1
PB-2_TTGCGTCAGCCACTAT	PB	PB-2	P2-3350	FALSE	1	TRA:CAVEDPSGGSYIPTF TRB:CASSPAVNNQPQHF	1	CD4-C1
PB-2_TTGGCAAAGTGTCCCG	PB	PB-2	P2-3365	FALSE	1	TRA:CAVAYSGGSNYKLTF TRB:CASSLGQGARDYGYTF	1	CD4-C1
PB-2_TTGTAGGAGGCGATAC	PB	PB-2	P2-3372	FALSE	1	TRA:CAATRNTNTGRRALTF TRB:CASSLSPGLAGYEQYF	1	CD4-C1
PB-2_TTTACTGAGACAATAC	PB	PB-2	P2-3377	FALSE	1	TRA:CASGSGNTPLVF TRB:CASSQAGNGIGELFF	1	CD4-C1
PB-2_TTTACTGAGGGATCTG	PB	PB-2	P2-3380	FALSE	1	TRA:CAASKTTGGTSYGKLTF TRB:CSASAPTYNEQFF	1	CD4-C1
PB-2_TTTACTGCAGGCTCAC	PB	PB-2	P2-3381	FALSE	1	TRA:CVVSDRRGSGNTGKLIF TRB:CASSSRMMVSNTEAFF	1	CD4-C1
PB-2_TTTACTGGTTCATGGT	PB	PB-2	P2-3383	FALSE	1	TRA:CALSGGSSGSARQLTF TRB:CASSLRAGTGVGNEQFF	1	CD4-C1
PB-2_TTTATGCCAACCGCCA	PB	PB-2	P2-3386	FALSE	1	TRA:CGTVAGNTPLVF TRB:CASSSASSGSGNQPQHF	1	CD4-C1
PB-2_TTTATGCGTTCGTGAT	PB	PB-2	P2-3388	FALSE	1	TRA:CAGHNFQGAQKLVF TRB:CSALRQGEVYGYTF	1	CD4-C1
PB-2_TTTATGCTCAAAGTAG	PB	PB-2	P2-3389	FALSE	1	TRA:CAVPTQGGSEKLVF TRB:CASSYSGTNEKLFF	1	CD4-C1
PB-2_TTTATGCTCGAATGCT	PB	PB-2	P2-3390	FALSE	1	TRA:CAARGNTNAGKSTF TRB:CASSYSSGTDTQYF	1	CD4-C1
PB-2_TTTCCTCAGCGACGTA	PB	PB-2	P2-3393	FALSE	1	TRA:CAVGGRGQKLLF TRB:CASSPGTGAKSSYEQYF	1	CD4-C1
PB-2_TTTCCTCAGCTAGTGG	PB	PB-2	P2-3394	FALSE	1	TRA:CAVEARGATNKLIF TRB:CASSYGGLPQHF	1	CD4-C1
PB-2_AAACCTGGTGGTGTAG	PB	PB-2	P2-1	TRUE	3	TRA:CVVNIIGYQKVTF TRB:CASTRQGKQPQHF	270	CD4-C6
PB-2_AAACCTGTCTCGTTTA	PB	PB-2	P2-575	FALSE	1	TRA:CAVTFTGGGNKLTF TRB:CASSLYPQIGSTGELFF	1	CD8-C6
PB-2_AAAGATGGTAGCTCCG	PB	PB-2	P2-1	TRUE	3	TRA:CVVNIIGYQKVTF TRB:CASTRQGKQPQHF	270	CD4-C6
PB-2_AAAGTAGAGTCCCACG	PB	PB-2	P2-595	FALSE	1	TRA:CAVGNTNAGKSTF TRB:CASSQEGQGNYGYTF	1	CD8-C6
PB-2_AACACGTAGAATGTTG	PB	PB-2	P2-1	TRUE	3	TRA:CVVNIIGYQKVTF TRB:CASTRQGKQPQHF	270	CD8-C7
PB-2_AACCGCGCAAACGTGG	PB	PB-2	P2-619	FALSE	1	TRA:CAMSATPSGNTGKLIF TRB:CASSYSMWTEETQYF	1	CD8-C6
PB-2_AACGTTGAGGCAAAGA	PB	PB-2	P2-624	FALSE	1	TRA:CAARSEGNEKLTF TRB:CASSLAYQQDFSGNTIYF	1	CD8-C6

PB-2_AACTCAGTCACCGGGT	PB	PB-2	P2-629	FALSE	1	TRA:CAASGGAGTASKLTF TRB:CASSSTGLAGTDTQYF	1	CD8-C2
PB-2_AACTGGTTCTGTGCAA	PB	PB-2	P2-644	FALSE	1	TRA:CAMRPTGANSKLTF TRB:CASDRYNEQFF	1	CD8-C6
PB-2_AAGACCTGTCAGATAA	PB	PB-2	P2-652	FALSE	1	TRA:CAASAYSGAGSYQLTF TRB:CASTLGIANEKLFF	1	CD8-C7
PB-2_AAGACCTTCATCGATG	PB	PB-2	P2-26	TRUE	3	TRA:CALVNTGGFKTIF TRB:CASSPPSSGGANEQYF	22	CD4-C5
PB-2_AAGGCAGGTATATGGA	PB	PB-2	P2-679	FALSE	1	TRA:CAVLWDDYKLSF TRB:CSASTRGQETQYF	1	CD8-C6
PB-2_AATCCAGCACAGGAGT	PB	PB-2	P2-97	TRUE	3	TRA:CALLQGGSYIPTF TRB:CASSKTGGTDTQYF	5	CD4-C6
PB-2_AATCGGTTCTCAACTT	PB	PB-2	P2-44	FALSE	3	TRA:CALVNTGGFKTIF TRB:CASSPPSAGGGNEQYF	11	CD4-C6
PB-2_ACAGCTAAGAGTCGGT	PB	PB-2	P2-1	TRUE	3	TRA:CVVNIIGYQKVTF TRB:CASTRQGKQPQHF	270	CD8-C7
PB-2_ACATCAGAGTCACGCC	PB	PB-2	P2-755	FALSE	1	TRA:CAASVFGNEKLTF TRB:CASSWDPGEQYF	1	CD8-C6
PB-2_ACATCAGTCTCGTTTA	PB	PB-2	P2-761	FALSE	1	TRA:CVVSASERGSQGNLIF TRB:CASSRGRGLYEQYF	1	CD8-C6
PB-2_ACATGGTGTAGCCTAT	PB	PB-2	P2-85	TRUE	3	TRA:CAVTAYTNAGKSTF TRB:CASRGRSSGNTIYF	6	CD8-C6
PB-2_ACGATACAGTGAACGC	PB	PB-2	P2-1	TRUE	3	TRA:CVVNIIGYQKVTF TRB:CASTRQGKQPQHF	270	CD4-C6
PB-2_ACGATACCAATAGCGG	PB	PB-2	P2-1	TRUE	3	TRA:CVVNIIGYQKVTF TRB:CASTRQGKQPQHF	270	CD4-C6
PB-2_ACGCCGAGTTCGTTGA	PB	PB-2	P2-1	TRUE	3	TRA:CVVNIIGYQKVTF TRB:CASTRQGKQPQHF	270	CD4-C6
PB-2_ACGTCAAAGGCTACGA	PB	PB-2	P2-1	TRUE	3	TRA:CVVNIIGYQKVTF TRB:CASTRQGKQPQHF	270	CD8-C7
PB-2_ACTATCTAGCTCCTTC	PB	PB-2	P2-1	TRUE	3	TRA:CVVNIIGYQKVTF TRB:CASTRQGKQPQHF	270	CD8-C7
PB-2_ACTGAGTCATGGATGG	PB	PB-2	P2-867	FALSE	1	TRA:CAVNRNARLMF TRB:CASSDGGNPSTDTQYF	1	CD8-C6
PB-2_ACTGAGTGTTGAGGTG	PB	PB-2	P2-1	TRUE	3	TRA:CVVNIIGYQKVTF TRB:CASTRQGKQPQHF	270	CD8-C7
PB-2_ACTGATGTCAACGCTA	PB	PB-2	P2-1	TRUE	3	TRA:CVVNIIGYQKVTF TRB:CASTRQGKQPQHF	270	CD8-C7
PB-2_ACTGATGTCCGTTGCT	PB	PB-2	P2-880	FALSE	1	TRA:CARRGNAGGTSYGKLTF TRB:CSAQTGDNSPLHF	1	CD8-C6
PB-2_ACTGCTCCACATGTGT	PB	PB-2	P2-1	TRUE	3	TRA:CVVNIIGYQKVTF TRB:CASTRQGKQPQHF	270	CD4-C6
PB-2_ACTGCTCGTAAGAGGA	PB	PB-2	P2-886	FALSE	1	TRA:CAVRATVSGTYKYIF TRB:CASRIPGSVDTQYF	1	CD8-C6
PB-2_ACTGCTCGTGTTAAGA	PB	PB-2	P2-18	TRUE	3	TRA:CAATSSSNFGNEKLTF TRB:CAWSRAALTEAFF	28	CD4-C6
PB-2_ACTTACTGTGTTCGAT	PB	PB-2	P2-1	TRUE	3	TRA:CVVNIIGYQKVTF TRB:CASTRQGKQPQHF	270	CD4-C6
PB-2_ACTTGTTCACAGAGGT	PB	PB-2	P2-44	FALSE	3	TRA:CALVNTGGFKTIF TRB:CASSPPSAGGGNEQYF	11	CD4-C6
PB-2_AGAATAGCACCCAGTG	PB	PB-2	P2-1	TRUE	3	TRA:CVVNIIGYQKVTF TRB:CASTRQGKQPQHF	270	CD4-C6
PB-2_AGAGCGACACCGTTGG	PB	PB-2	P2-929	FALSE	1	TRA:CALRVGAQKLVF TRB:CASTRQGKQPQHF	1	CD4-C6

PB-2_AGAGCTTGTTTAAGCC	PB	PB-2	P2-936	FALSE	1	TRA:CAVRRGNEKLTF TRB:CASSKSALTDTQYF	1	CD8-C6
PB-2_AGATCTGCAGATGAGC	PB	PB-2	P2-945	FALSE	1	TRA:CAPNTGGFKTIF TRB:CASSHRTSGTDTQYF	1	CD8-C6
PB-2_AGCGGTCCATCCGCGA	PB	PB-2	P2-976	FALSE	1	TRA:CVVRRRGSTLGRLYF TRB:CASSYGGPDETQYF	1	CD8-C6
PB-2_AGCTCCTCAATGGATA	PB	PB-2	P2-26	TRUE	3	TRA:CALVNTGGFKTIF TRB:CASSPPSSGGANEQYF	22	CD4-C6
PB-2_AGCTTGACACAGAGGT	PB	PB-2	P2-1005	FALSE	1	TRA:CALLTGTASKLTF TRB:CSAGAGLAYSYEQYF	1	CD8-C6
PB-2_AGGCCGTTCTTAGCCC	PB	PB-2	P2-1	TRUE	3	TRA:CVVNIIGYQKVTF TRB:CASTRQGKQPQHF	270	CD8-C7
PB-2_AGGGAGTCACCCATGG	PB	PB-2	P2-1	TRUE	3	TRA:CVVNIIGYQKVTF TRB:CASTRQGKQPQHF	270	CD4-C6
PB-2_AGGGAGTTCCAGAAGG	PB	PB-2	P2-44	FALSE	3	TRA:CALVNTGGFKTIF TRB:CASSPPSAGGGNEQYF	11	CD4-C6
PB-2_AGGTCCGAGACCCACC	PB	PB-2	P2-123	TRUE	3	TRA:CAASNLGTYKYIF TRB:CASSPPIGSPLHF	4	CD8-C6
PB-2_AGTAGTCAGGTGCTTT	PB	PB-2	P2-1	TRUE	3	TRA:CVVNIIGYQKVTF TRB:CASTRQGKQPQHF	270	CD4-C6
PB-2_AGTGGGAAGGCATGTG	PB	PB-2	P2-1065	FALSE	1	TRA:CIVRVPTPNTDKLIF TRB:CASRDTSTDTQYF	1	CD8-C6
PB-2_AGTGGGATCTATCGCC	PB	PB-2	P2-1070	FALSE	1	TRA:CALSDRGGGFKTIF TRB:CASSGTGTAGANVLTF	1	CD8-C7
PB-2_AGTTGGTTCAAACCAC	PB	PB-2	P2-1	TRUE	3	TRA:CVVNIIGYQKVTF TRB:CASTRQGKQPQHF	270	CD4-C6
PB-2_ATAACGCCATTGTGCA	PB	PB-2	P2-1085	FALSE	1	TRA:CAFRRNNNARLMF TRB:CASSQTGTGSNQPQHF	1	CD8-C6
PB-2_ATAACGCTCATCGCTC	PB	PB-2	P2-1086	FALSE	1	TRA:CALSFGAAGNKLTF TRB:CASSIGSGRTYEQYF	1	CD8-C6
PB-2_ATAAGAGAGACTCGGA	PB	PB-2	P2-1	TRUE	3	TRA:CVVNIIGYQKVTF TRB:CASTRQGKQPQHF	270	CD4-C6
PB-2_ATAAGAGAGATCGATA	PB	PB-2	P2-1087	FALSE	1	TRA:CAPTEGGADGLTF TRB:CASQVQGFFNEQFF	1	CD8-C6
PB-2_ATAAGAGAGGCTACGA	PB	PB-2	P2-1089	FALSE	1	TRA:CAVSWTSYDKVIF TRB:CASRHESGYEQYF	1	CD8-C6
PB-2_ATAAGAGTCGTATCAG	PB	PB-2	P2-1096	FALSE	1	TRA:CALIAAAAGNKLTF TRB:CASSLDPGTSYEQYF	1	CD8-C6
PB-2_ATAGACCGTAAATGAC	PB	PB-2	P2-1	TRUE	3	TRA:CVVNIIGYQKVTF TRB:CASTRQGKQPQHF	270	CD8-C7
PB-2_ATCATCTTCACCCGAG	PB	PB-2	P2-1109	FALSE	1	TRA:CAASSRGDYKLSF TRB:CASSYVGDTYEQYF	1	CD8-C6
PB-2_ATCATGGCACAGCGTC	PB	PB-2	P2-1111	FALSE	1	TRA:CAVSDVFTGTASKLTF TRB:CSARDWAGANVLTF	1	CD8-C6
PB-2_ATCATGGGTATAAACG	PB	PB-2	P2-1113	FALSE	1	TRA:CAYTIGGFKTIF TRB:CASSFPRGRAEHEQYF	1	CD8-C2
PB-2_ATCCGAAAGTTGCAGG	PB	PB-2	P2-1123	FALSE	1	TRA:CAMRVLNTGGFKTIF TRB:CASTRQGKQPQHF	1	CD4-C6
PB-2_ATCGAGTAGTGTCTCA	PB	PB-2	P2-1	TRUE	3	TRA:CVVNIIGYQKVTF TRB:CASTRQGKQPQHF	270	CD4-C6
PB-2_ATCTACTCACTGTCGG	PB	PB-2	P2-1136	FALSE	1	TRA:CAFNAGNNRKLIW TRB:CASYRRVTEQYF	1	CD8-C7
PB-2_ATGAGGGAGTATTGGA	PB	PB-2	P2-26	TRUE	3	TRA:CALVNTGGFKTIF TRB:CASSPPSSGGANEQYF	22	CD4-C6

PB-2_ATGAGGGCACCCTATC	PB	PB-2	P2-26	TRUE	3	TRA:CALVNTGGFKTIF TRB:CASSPPSSGGANEQYF	22	CD4-C6
PB-2_ATGAGGGCAGATGAGC	PB	PB-2	P2-18	TRUE	3	TRA:CAATSSSNFGNEKLTF TRB:CAWSRAALTEAFF	28	CD4-C6
PB-2_ATTACTCAGTGTACGG	PB	PB-2	P2-1	TRUE	3	TRA:CVVNIIGYQKVTF TRB:CASTRQGKQPQHF	270	CD8-C5
PB-2_ATTCTACAGACATAAC	PB	PB-2	P2-1	TRUE	3	TRA:CVVNIIGYQKVTF TRB:CASTRQGKQPQHF	270	CD4-C6
PB-2_ATTCTACTCACGCATA	PB	PB-2	P2-1178	FALSE	1	TRA:CAASASGTSYGKLTF TRB:CASNPRDRGSWSNQPQHF	1	CD8-C6
PB-2_CAAGGCCTCTGGCGAC	PB	PB-2	P2-1	TRUE	3	TRA:CVVNIIGYQKVTF TRB:CASTRQGKQPQHF	270	CD4-C6
PB-2_CAAGTTGAGATAGCAT	PB	PB-2	P2-1226	FALSE	1	TRA:CVVNKAAAGNKLTF TRB:CATSRDTQARGQYF	1	CD8-C7
PB-2_CACACCTTCTTATCTG	PB	PB-2	P2-1	TRUE	3	TRA:CVVNIIGYQKVTF TRB:CASTRQGKQPQHF	270	CD8-C7
PB-2_CACAGGCGTTTGGCGC	PB	PB-2	P2-1259	FALSE	1	TRA:CAETPIMYSGGGADGLTF TRB:CASSGSGGGLYNEQFF	1	CD8-C6
PB-2_CACATTTGTATGAAAC	PB	PB-2	P2-26	TRUE	3	TRA:CALVNTGGFKTIF TRB:CASSPPSSGGANEQYF	22	CD4-C6
PB-2_CACCACTTCTAAGCCA	PB	PB-2	P2-1282	FALSE	1	TRA:CAASAGNTGKLIF TRB:CSATTGAANEKLFF	1	CD8-C6
PB-2_CACCAGGTCAACGAAA	PB	PB-2	P2-1285	FALSE	1	TRA:CVVNIIGYQKVTF TRB:CASSLGGEMNQPQHF	1	CD8-C7
PB-2_CACCAGGTCGAATCCA	PB	PB-2	P2-1	TRUE	3	TRA:CVVNIIGYQKVTF TRB:CASTRQGKQPQHF	270	CD8-C7
PB-2_CAGAATCCATCTCCCA	PB	PB-2	P2-26	TRUE	3	TRA:CALVNTGGFKTIF TRB:CASSPPSSGGANEQYF	22	CD4-C6
PB-2_CAGATCACATCCGCGA	PB	PB-2	P2-1	TRUE	3	TRA:CVVNIIGYQKVTF TRB:CASTRQGKQPQHF	270	CD8-C7
PB-2_CAGCCGATCTGGTATG	PB	PB-2	P2-1333	FALSE	1	TRA:CAVNYKLTF TRB:CSVEGGGVTYNEQFF	1	CD8-C2
PB-2_CAGTAACAGCAATCTC	PB	PB-2	P2-303	FALSE	2	TRA:CALKDDYKLSF TRB:CASSPGQPGKLFF	2	CD4-C6
PB-2_CAGTCCTAGCAATATG	PB	PB-2	P2-303	FALSE	2	TRA:CALKDDYKLSF TRB:CASSPGQPGKLFF	2	CD4-C6
PB-2_CATATTCTCCAGAGGA	PB	PB-2	P2-26	TRUE	3	TRA:CALVNTGGFKTIF TRB:CASSPPSSGGANEQYF	22	CD4-C6
PB-2_CATCAGACAAGTTCTG	PB	PB-2	P2-1	TRUE	3	TRA:CVVNIIGYQKVTF TRB:CASTRQGKQPQHF	270	CD4-C6
PB-2_CATCCACCATGAGCGA	PB	PB-2	P2-1389	FALSE	1	TRA:CAVNIFKDSSYKLIF TRB:CASSVGGLLQETQYF	1	CD8-C6
PB-2_CATCCACCATTGGCGC	PB	PB-2	P2-1390	FALSE	1	TRA:CALEVAGNMLTF TRB:CSARDLGGGVPETQYF	1	CD8-C6
PB-2_CATGGCGTCCTAGAAC	PB	PB-2	P2-1427	FALSE	1	TRA:CAESDSGNTPLVF TRB:CASTELPTGTEAFF	1	CD8-C7
PB-2_CATTATCGTTCCTCCA	PB	PB-2	P2-121	TRUE	3	TRA:CAGASRDTDKLIF TRB:CSVESFLRGASLMTEAFF	4	CD8-C6
PB-2_CATTCGCAGGACAGAA	PB	PB-2	P2-1	TRUE	3	TRA:CVVNIIGYQKVTF TRB:CASTRQGKQPQHF	270	CD8-C7
PB-2_CCACCTACACTGTTAG	PB	PB-2	P2-1453	FALSE	1	TRA:CAVTTRGGGGSQGNLIF TRB:CASRGSSLTNEQFF	1	CD8-C7
PB-2_CCACGGAAGAAGGACA	PB	PB-2	P2-1461	FALSE	1	TRA:CAVKLTGGYQKVTF TRB:CASSQDRRLSTDTQYF	1	CD8-C6

PB-2_CCATGTCTCTGATTCT	PB	PB-2	P2-44	FALSE	3	TRA:CALVNTGGFKTIF TRB:CASSPPSAGGGNEQYF	11	CD4-C6
PB-2_CCCATACGTAAGGGCT	PB	PB-2	P2-1	TRUE	3	TRA:CVVNIIGYQKVTF TRB:CASTRQGKQPQHF	270	CD8-C7
PB-2_CCGGGATTCTGTACGA	PB	PB-2	P2-1524	FALSE	1	TRA:CALLQGGSYIPTF TRB:CAWSRAALTEAFF	1	CD4-C6
PB-2_CCGTGGATCAGGCAAG	PB	PB-2	P2-26	TRUE	3	TRA:CALVNTGGFKTIF TRB:CASSPPSSGGANEQYF	22	CD4-C6
PB-2_CCGTTCACACAGCCCA	PB	PB-2	P2-1542	FALSE	1	TRA:CAMNVQGAQKLVF TRB:CASSLGTGTNEKLFF	1	CD8-C6
PB-2_CCGTTCAGTCCCTTGT	PB	PB-2	P2-1	TRUE	3	TRA:CVVNIIGYQKVTF TRB:CASTRQGKQPQHF	270	CD4-C6
PB-2_CCTAAAGAGTGTGGCA	PB	PB-2	P2-1	TRUE	3	TRA:CVVNIIGYQKVTF TRB:CASTRQGKQPQHF	270	CD4-C6
PB-2_CCTAAAGTCGCTTGTC	PB	PB-2	P2-1	TRUE	3	TRA:CVVNIIGYQKVTF TRB:CASTRQGKQPQHF	270	CD4-C6
PB-2_CCTAGCTTCATGTCTT	PB	PB-2	P2-1	TRUE	3	TRA:CVVNIIGYQKVTF TRB:CASTRQGKQPQHF	270	CD4-C6
PB-2_CCTATTATCACTTCAT	PB	PB-2	P2-1578	FALSE	1	TRA:CATDSGGGADGLTF TRB:CASSSGTSGTLEQYF	1	CD8-C6
PB-2_CCTCAGTGTTCAGACT	PB	PB-2	P2-1583	FALSE	1	TRA:CAALSLGYNNNDMRF TRB:CASRGGGAYEQYF	1	CD8-C6
PB-2_CGAACATCAACTGGCC	PB	PB-2	P2-1619	FALSE	1	TRA:CALSKKGNTPLVF TRB:CATSRDSSQGNEQFF	1	CD8-C6
PB-2_CGAACATCACAAGCCC	PB	PB-2	P2-18	TRUE	3	TRA:CAATSSSNFGNEKLTF TRB:CAWSRAALTEAFF	28	CD4-C6
PB-2_CGAGAAGCAGTATGCT	PB	PB-2	P2-1	TRUE	3	TRA:CVVNIIGYQKVTF TRB:CASTRQGKQPQHF	270	CD4-C6
PB-2_CGAGAAGGTAGAGGAA	PB	PB-2	P2-97	TRUE	3	TRA:CALLQGGSYIPTF TRB:CASSKTGGTDTQYF	5	CD4-C6
PB-2_CGCTGGACAGCGTTCG	PB	PB-2	P2-1703	FALSE	1	TRA:CVVSARTGNTGKLIF TRB:CASSYQTVYEQYF	1	CD8-C6
PB-2_CGGACACTCGGGAGTA	PB	PB-2	P2-1	TRUE	3	TRA:CVVNIIGYQKVTF TRB:CASTRQGKQPQHF	270	CD8-C7
PB-2_CGGACTGGTATAATGG	PB	PB-2	P2-1727	FALSE	1	TRA:CLPWTGSARQLTF TRB:CASSLAGELFF	1	CD8-C7
PB-2_CGGAGCTAGTCCATAC	PB	PB-2	P2-76	TRUE	3	TRA:CAGQEQGGSEKLVF TRB:CASSQMGLYGYTF	7	CD4-C6
PB-2_CGGAGCTCAGCTCCGA	PB	PB-2	P2-1736	FALSE	1	TRA:CLVAPGGDRDDKIIF TRB:CASRPTMSEGNTIYF	1	CD8-C6
PB-2_CGTAGGCCATGGTCTA	PB	PB-2	P2-26	TRUE	3	TRA:CALVNTGGFKTIF TRB:CASSPPSSGGANEQYF	22	CD4-C6
PB-2_CGTCACTTCTCTGAGA	PB	PB-2	P2-1	TRUE	3	TRA:CVVNIIGYQKVTF TRB:CASTRQGKQPQHF	270	CD4-C6
PB-2_CGTCACTTCTTGTACT	PB	PB-2	P2-1777	FALSE	1	TRA:CAASYSGGGADGLTF TRB:CASSPVGLNTGELFF	1	CD8-C6
PB-2_CGTCAGGTCGAGGTAG	PB	PB-2	P2-1783	FALSE	1	TRA:CAVRDLMTDKLIF TRB:CASSYSTPRGEAFF	1	CD8-C7
PB-2_CGTCTACTCCTTGGTC	PB	PB-2	P2-26	TRUE	3	TRA:CALVNTGGFKTIF TRB:CASSPPSSGGANEQYF	22	CD4-C6
PB-2_CGTGTAAAGTTATCGC	PB	PB-2	P2-1	TRUE	3	TRA:CVVNIIGYQKVTF TRB:CASTRQGKQPQHF	270	CD8-C7
PB-2_CGTGTCTGTAGCGCTC	PB	PB-2	P2-1	TRUE	3	TRA:CVVNIIGYQKVTF TRB:CASTRQGKQPQHF	270	CD4-C6

PB-2_CGTTAGAAGACAATAC	PB	PB-2	P2-1	TRUE	3	TRA:CVVNIIGYQKVTF TRB:CASTRQGKQPQHF	270	CD4-C6
PB-2_CTAATGGTCCGTAGTA	PB	PB-2	P2-26	TRUE	3	TRA:CALVNTGGFKTIF TRB:CASSPPSSGGANEQYF	22	CD4-C6
PB-2_CTACACCTCCGAGCCA	PB	PB-2	P2-1861	FALSE	1	TRA:CAIRYQSFNFGNEKLTF TRB:CASSQMGLYGYTF	1	CD4-C6
PB-2_CTACGTCAGGATGGAA	PB	PB-2	P2-1	TRUE	3	TRA:CVVNIIGYQKVTF TRB:CASTRQGKQPQHF	270	CD4-C6
PB-2_CTAGAGTCACCAACCG	PB	PB-2	P2-1884	FALSE	1	TRA:CAVLGRTDSWGKFQF TRB:CASSQDPRGYNSPLHF	1	CD4-C6
PB-2_CTAGTGAGTAGGACAC	PB	PB-2	P2-1	TRUE	3	TRA:CVVNIIGYQKVTF TRB:CASTRQGKQPQHF	270	CD8-C7
PB-2_CTCACACCAGCTGTAT	PB	PB-2	P2-1	TRUE	3	TRA:CVVNIIGYQKVTF TRB:CASTRQGKQPQHF	270	CD4-C6
PB-2_CTCAGAACACGAAAGC	PB	PB-2	P2-1	TRUE	3	TRA:CVVNIIGYQKVTF TRB:CASTRQGKQPQHF	270	CD8-C7
PB-2_CTCATTATCTGGAGCC	PB	PB-2	P2-1	TRUE	3	TRA:CVVNIIGYQKVTF TRB:CASTRQGKQPQHF	270	CD4-C6
PB-2_CTCGAGGCAGCTGGCT	PB	PB-2	P2-26	TRUE	3	TRA:CALVNTGGFKTIF TRB:CASSPPSSGGANEQYF	22	CD4-C6
PB-2_CTCGGGATCCGCTGTT	PB	PB-2	P2-18	TRUE	3	TRA:CAATSSSNFGNEKLTF TRB:CAWSRAALTEAFF	28	CD4-C6
PB-2_CTCGTACGTGTTTGGT	PB	PB-2	P2-1	TRUE	3	TRA:CVVNIIGYQKVTF TRB:CASTRQGKQPQHF	270	CD4-C6
PB-2_CTCTAATTCTCGATGA	PB	PB-2	P2-1958	FALSE	1	TRA:CAVNRATGGFKTIF TRB:CATSDHGAGKLFF	1	CD8-C6
PB-2_CTGAAACGTCATGCAT	PB	PB-2	P2-1974	FALSE	1	TRA:CAENRDNAGNMLTF TRB:CASSGGHSYEQYF	1	CD8-C6
PB-2_CTGATCCTCACTCCTG	PB	PB-2	P2-1995	FALSE	1	TRA:CAFMKHEGGAQKLVF TRB:CASSHIPGEGAEQYF	1	CD8-C6
PB-2_CTGCCTATCCTCGCAT	PB	PB-2	P2-2001	FALSE	1	TRA:CAVTQGGSYIPTF TRB:CASSYGTDPSYEQYF	1	CD8-C6
PB-2_CTGCTGTCACCCATGG	PB	PB-2	P2-1	TRUE	3	TRA:CVVNIIGYQKVTF TRB:CASTRQGKQPQHF	270	CD8-C7
PB-2_CTGCTGTTCACTCCTG	PB	PB-2	P2-2012	FALSE	1	TRA:CAVPRLRRALTF TRB:CASSPPRPGTTDTQYF	1	CD8-C7
PB-2_CTGTGCTGTCGGCATC	PB	PB-2	P2-2024	FALSE	1	TRA:CAVNYFGNEKLTF TRB:CASSLEGRSLQETQYF	1	CD8-C6
PB-2_CTGTTTAAGCCGGTAA	PB	PB-2	P2-2025	FALSE	1	TRA:CLVSGGSYIPTF TRB:CASSVTGVLGQETQYF	1	CD8-C6
PB-2_CTTAGGAAGTGAAGTT	PB	PB-2	P2-1	TRUE	3	TRA:CVVNIIGYQKVTF TRB:CASTRQGKQPQHF	270	CD4-C6
PB-2_CTTGGCTGTTATGCGT	PB	PB-2	P2-1	TRUE	3	TRA:CVVNIIGYQKVTF TRB:CASTRQGKQPQHF	270	CD8-C7
PB-2_CTTGGCTTCCCTGACT	PB	PB-2	P2-26	TRUE	3	TRA:CALVNTGGFKTIF TRB:CASSPPSSGGANEQYF	22	CD4-C6
PB-2_CTTTGCGGTACAAGTA	PB	PB-2	P2-2062	FALSE	1	TRA:CVVYILTGGGNKLTF TRB:CASSVPPGTGSNEQFF	1	CD8-C7
PB-2_GAAACTCGTGTGGCTC	PB	PB-2	P2-1	TRUE	3	TRA:CVVNIIGYQKVTF TRB:CASTRQGKQPQHF	270	CD4-C6
PB-2_GAAATGACACTTCGAA	PB	PB-2	P2-2069	FALSE	1	TRA:CVVNIIGYQKVTF TRB:CASSGGRESVSTDTQYF	1	CD4-C6
PB-2_GACAGAGTCGTAGATC	PB	PB-2	P2-2120	FALSE	1	TRA:CVVSDRSRGSYIPTF TRB:CASSPGGDTKNTEAFF	1	CD8-C6

PB-2_GACCAATTCGCCAAAT	PB	PB-2	P2-2130	FALSE	1	TRA:CATDQAGTALIF TRB:CAWSATWGGRETQYF	1	CD8-C6
PB-2_GACGGCTCACATGTGT	PB	PB-2	P2-2148	FALSE	1	TRA:CAVNNAGGTSYGKLTF TRB:CASSPPDRGYNEQFF	1	CD8-C6
PB-2_GACGTTATCTTCAACT	PB	PB-2	P2-2169	FALSE	1	TRA:CVVSGSTGRRALTF TRB:CASSDSYEQYF	1	CD8-C7
PB-2_GACTGCGAGTGAAGTT	PB	PB-2	P2-2181	FALSE	1	TRA:CLVGDNRDDKIIF TRB:CASSLSPRMSSYEQYF	1	CD8-C2
PB-2_GAGCAGAAGACCTAGG	PB	PB-2	P2-2189	FALSE	1	TRA:CAPSNSGGSNYKLTF TRB:CASSLGVFPYNEQFF	1	CD8-C6
PB-2_GAGGTGACAATGGAAT	PB	PB-2	P2-1	TRUE	3	TRA:CVVNIIGYQKVTF TRB:CASTRQGKQPQHF	270	CD4-C6
PB-2_GAGTCCGAGTGGTAGC	PB	PB-2	P2-2204	FALSE	1	TRA:CAVIIGSSASKIIF TRB:CASREVGGSQPQHF	1	CD8-C6
PB-2_GAGTCCGCATTCCTGC	PB	PB-2	P2-2208	FALSE	1	TRA:CAEKGNSGYALNF TRB:CASRPSGNTIYF	1	CD8-C6
PB-2_GAGTCCGGTGTGTGCC	PB	PB-2	P2-2210	FALSE	1	TRA:CAGAKVVTGGGNKLTF TRB:CASTRQGKQPQHF	1	CD4-C6
PB-2_GAGTCCGTCATGGTCA	PB	PB-2	P2-1	TRUE	3	TRA:CVVNIIGYQKVTF TRB:CASTRQGKQPQHF	270	CD4-C6
PB-2_GATCGCGAGACATAAC	PB	PB-2	P2-1	TRUE	3	TRA:CVVNIIGYQKVTF TRB:CASTRQGKQPQHF	270	CD4-C6
PB-2_GATCGCGCAGTTTACG	PB	PB-2	P2-2229	FALSE	1	TRA:CAAPRSGNTPLVF TRB:CASSPGAGANVLTF	1	CD8-C10
PB-2_GATCTAGCAATTGCTG	PB	PB-2	P2-1	TRUE	3	TRA:CVVNIIGYQKVTF TRB:CASTRQGKQPQHF	270	CD4-C6
PB-2_GATTCAGTCCAATGGT	PB	PB-2	P2-61	TRUE	3	TRA:CAVSFITQGGSEKLVF TRB:CASSRDRGTYNEQFF	9	CD4-C6
PB-2_GCAATCACACGGATAG	PB	PB-2	P2-1	TRUE	3	TRA:CVVNIIGYQKVTF TRB:CASTRQGKQPQHF	270	CD8-C7
PB-2_GCACTCTAGTGCCAGA	PB	PB-2	P2-2291	FALSE	1	TRA:CAATTSGTYKYIF TRB:CSARAGTGGHEQYF	1	CD8-C6
PB-2_GCATACAGTGCATCTA	PB	PB-2	P2-76	TRUE	3	TRA:CAGQEQGGSEKLVF TRB:CASSQMGLYGYTF	7	CD4-C6
PB-2_GCATGTAAGACCTTTG	PB	PB-2	P2-18	TRUE	3	TRA:CAATSSSNFGNEKLTF TRB:CAWSRAALTEAFF	28	CD4-C6
PB-2_GCATGTAGTTCGTGAT	PB	PB-2	P2-2327	FALSE	1	TRA:CAVEDPGANNLFF TRB:CASRVGTGVHEQYF	1	CD8-C6
PB-2_GCCAAATAGAAGGCCT	PB	PB-2	P2-1	TRUE	3	TRA:CVVNIIGYQKVTF TRB:CASTRQGKQPQHF	270	CD4-C6
PB-2_GCCTCTAGTTGATTCG	PB	PB-2	P2-1	TRUE	3	TRA:CVVNIIGYQKVTF TRB:CASTRQGKQPQHF	270	CD8-C7
PB-2_GCGACCAGTGCGATAG	PB	PB-2	P2-131	TRUE	3	TRA:CATVGQIQGAQKLVF TRB:CASRTGTSSYEQYF	4	CD8-C6
PB-2_GCGCCAAGTAGCCTCG	PB	PB-2	P2-2383	FALSE	1	TRA:CAMRPNNNARLMF TRB:CSARDPGQGSWTQYF	1	CD8-C6
PB-2_GCGCCAAGTAGCGTCC	PB	PB-2	P2-18	TRUE	3	TRA:CAATSSSNFGNEKLTF TRB:CAWSRAALTEAFF	28	CD4-C6
PB-2_GCGCCAATCAGAGGTG	PB	PB-2	P2-1	TRUE	3	TRA:CVVNIIGYQKVTF TRB:CASTRQGKQPQHF	270	CD4-C6
PB-2_GCGCCAATCTTCATGT	PB	PB-2	P2-44	FALSE	3	TRA:CALVNTGGFKTIF TRB:CASSPPSAGGGNEQYF	11	CD4-C6
PB-2_GCGCGATCAATGCCAT	PB	PB-2	P2-1	TRUE	3	TRA:CVVNIIGYQKVTF TRB:CASTRQGKQPQHF	270	CD4-C6

PB-2_GCGGGTTTCATGTCTT	PB	PB-2	P2-1	TRUE	3	TRA:CVVNIIGYQKVTF TRB:CASTRQGKQPQHF	270	CD8-C7
PB-2_GCTGCAGGTTATGTGC	PB	PB-2	P2-2413	FALSE	1	TRA:CVVNIIGYQKVTF TRB:CATTENSYNEQFF	1	CD8-C7
PB-2_GGAACTTCACCAGTTA	PB	PB-2	P2-1	TRUE	3	TRA:CVVNIIGYQKVTF TRB:CASTRQGKQPQHF	270	CD4-C6
PB-2_GGACATTAGCTATGCT	PB	PB-2	P2-266	FALSE	2	TRA:CAVGAMLTGGGNKLTF TRB:CASRGSGGGYEQYF	2	CD8-C6
PB-2_GGAGCAACAGCGTTCG	PB	PB-2	P2-1	TRUE	3	TRA:CVVNIIGYQKVTF TRB:CASTRQGKQPQHF	270	CD4-C6
PB-2_GGATGTTCATGTTCCC	PB	PB-2	P2-26	TRUE	3	TRA:CALVNTGGFKTIF TRB:CASSPPSSGGANEQYF	22	CD4-C6
PB-2_GGCAATTCAAGGCTCC	PB	PB-2	P2-5	TRUE	3	TRA:CASSFSGGYNKLIF TRB:CASSYLGAGYQETQYF	127	CD8-C2
PB-2_GGCCGATAGAACTGTA	PB	PB-2	P2-1	TRUE	3	TRA:CVVNIIGYQKVTF TRB:CASTRQGKQPQHF	270	CD4-C6
PB-2_GGCCGATAGAATAGGG	PB	PB-2	P2-1	TRUE	3	TRA:CVVNIIGYQKVTF TRB:CASTRQGKQPQHF	270	CD4-C6
PB-2_GGCCGATTCTGAAAGA	PB	PB-2	P2-1	TRUE	3	TRA:CVVNIIGYQKVTF TRB:CASTRQGKQPQHF	270	CD4-C6
PB-2_GGCTGGTTCCTCGCAT	PB	PB-2	P2-96	FALSE	3	TRA:CAMRGANFGNEKLTF TRB:CASSRDFGGPGQETQYF	5	CD8-C6
PB-2_GGGAATGCAAGCCTAT	PB	PB-2	P2-2536	FALSE	1	TRA:CATDSGGSNYKLTF TRB:CASSLPVTNEQFF	1	CD8-C2
PB-2_GGGAGATAGCTCCCAG	PB	PB-2	P2-2543	FALSE	1	TRA:CALSEARNTGKLIF TRB:CASSPGTANTGELFF	1	CD8-C5
PB-2_GGGCACTGTGTTGGGA	PB	PB-2	P2-2554	FALSE	1	TRA:CAVRWDYKLSF TRB:CASSRGQLETQYF	1	CD8-C6
PB-2_GGGTCTGCAGGTGCCT	PB	PB-2	P2-2564	FALSE	1	TRA:CLVGGAYNTDKLIF TRB:CASSDRDQNTGELFF	1	CD8-C6
PB-2_GGGTCTGCATTCTTAC	PB	PB-2	P2-1	TRUE	3	TRA:CVVNIIGYQKVTF TRB:CASTRQGKQPQHF	270	CD4-C6
PB-2_GGGTTGCAGGTGATTA	PB	PB-2	P2-2569	FALSE	1	TRA:CAVRDKNAGNNRKLIW TRB:CASSPSGQSATEKLFF	1	CD8-C6
PB-2_GGTATTGTCACTATTC	PB	PB-2	P2-351	FALSE	2	TRA:CAANDNAGNMLTF TRB:CAWRGQGVGSPLHF	2	CD8-C6
PB-2_GGTGAAGTCGCCTGAG	PB	PB-2	P2-1	TRUE	3	TRA:CVVNIIGYQKVTF TRB:CASTRQGKQPQHF	270	CD4-C6
PB-2_GGTGTTATCTCGCTTG	PB	PB-2	P2-1	TRUE	3	TRA:CVVNIIGYQKVTF TRB:CASTRQGKQPQHF	270	CD4-C6
PB-2_GTAGGCCTCCTGCAGG	PB	PB-2	P2-2619	FALSE	1	TRA:CAVKEYGNKLVF TRB:CSASSGNSGNTIYF	1	CD8-C7
PB-2_GTATCTTAGGCGCTCT	PB	PB-2	P2-1	TRUE	3	TRA:CVVNIIGYQKVTF TRB:CASTRQGKQPQHF	270	CD8-C7
PB-2_GTCACGGCACATCCGG	PB	PB-2	P2-1	TRUE	3	TRA:CVVNIIGYQKVTF TRB:CASTRQGKQPQHF	270	CD4-C6
PB-2_GTCATTTGTGATGCCC	PB	PB-2	P2-2658	FALSE	1	TRA:CVVSVLTGGFKTIF TRB:CASSSWTSGYEQYF	1	CD4-C6
PB-2_GTCGGGTGTCGCTTTC	PB	PB-2	P2-33	TRUE	3	TRA:CAFKLSDGQKLLF TRB:CAWSRAALTEAFF	17	CD4-C6
PB-2_GTCGTAACAGGACCCT	PB	PB-2	P2-2680	FALSE	1	TRA:CALRANFGNEKLTF TRB:CASSLEQGGTQYF	1	CD8-C6
PB-2_GTCTCGTTCGGAAATA	PB	PB-2	P2-2691	FALSE	1	TRA:CAASTAGTYKYIF TRB:CASSVGTGYTGELFF	1	CD8-C6

PB-2_GTGCAGCTCTTTACGT	PB	PB-2	P2-1	TRUE	3	TRA:CVVNIIGYQKVTF TRB:CASTRQGKQPQHF	270	CD8-C7
PB-2_GTGCTTCCAGTGACAG	PB	PB-2	P2-2723	FALSE	1	TRA:CAYRSSSGSRLTF TRB:CASSLTSGQGTDTQYF	1	CD8-C6
PB-2_GTTAAGCTCCACGAAT	PB	PB-2	P2-2758	FALSE	1	TRA:CAIRYQSFNFGNEKLTF TRB:CSATKAEVTDTQYF	1	CD8-C5
PB-2_GTTCATTAGCGTGAGT	PB	PB-2	P2-44	FALSE	3	TRA:CALVNTGGFKTIF TRB:CASSPPSAGGGNEQYF	11	CD4-C6
PB-2_TAAACCGAGACCACGA	PB	PB-2	P2-1	TRUE	3	TRA:CVVNIIGYQKVTF TRB:CASTRQGKQPQHF	270	CD8-C7
PB-2_TAAGTGCAGACCTTTG	PB	PB-2	P2-1	TRUE	3	TRA:CVVNIIGYQKVTF TRB:CASTRQGKQPQHF	270	CD8-C7
PB-2_TACACGAAGAAACGCC	PB	PB-2	P2-2807	FALSE	1	TRA:CIVRVGTNDYKLSF TRB:CASSFDTSRAFF	1	CD8-C6
PB-2_TACGGATAGAGCTGCA	PB	PB-2	P2-2834	FALSE	1	TRA:CAYSPLVQGGKLIF TRB:CASSPDLGTYEQYF	1	CD8-C6
PB-2_TACGGTAAGCGAAGGG	PB	PB-2	P2-2844	FALSE	1	TRA:CAVGAYGGSQGNLIF TRB:CATSDRNRVSGELFF	1	CD8-C6
PB-2_TACTTACAGTTACGGG	PB	PB-2	P2-2861	FALSE	1	TRA:CAVVPNSGGSNYKLTF TRB:CASSPVRDRVSGANVLTF	1	CD8-C6
PB-2_TAGACCAAGTTACCCA	PB	PB-2	P2-2871	FALSE	1	TRA:CAVKQTGANNLFF TRB:CASSEGFGNTIYF	1	CD8-C6
PB-2_TAGCCGGCATACGCTA	PB	PB-2	P2-44	FALSE	3	TRA:CALVNTGGFKTIF TRB:CASSPPSAGGGNEQYF	11	CD4-C6
PB-2_TAGTGGTTCATGTGGT	PB	PB-2	P2-26	TRUE	3	TRA:CALVNTGGFKTIF TRB:CASSPPSSGGANEQYF	22	CD4-C6
PB-2_TATCAGGTCCTTTCTC	PB	PB-2	P2-2914	FALSE	1	TRA:CAAPRGNTDKLIF TRB:CASSSYNEQFF	1	CD8-C6
PB-2_TATCTCATCTAAGCCA	PB	PB-2	P2-1	TRUE	3	TRA:CVVNIIGYQKVTF TRB:CASTRQGKQPQHF	270	CD8-C7
PB-2_TCAATCTCACAAGCCC	PB	PB-2	P2-1	TRUE	3	TRA:CVVNIIGYQKVTF TRB:CASTRQGKQPQHF	270	CD4-C6
PB-2_TCACAAGAGGTTACCT	PB	PB-2	P2-2945	FALSE	1	TRA:CALPPGGYNKLIF TRB:CASSRDRGTEAFF	1	CD4-C6
PB-2_TCAGATGCACAACGCC	PB	PB-2	P2-44	FALSE	3	TRA:CALVNTGGFKTIF TRB:CASSPPSAGGGNEQYF	11	CD4-C6
PB-2_TCAGATGCACAAGTAA	PB	PB-2	P2-2957	FALSE	1	TRA:CAVLINKFYF TRB:CASSLDWQTEAFF	1	CD8-C7
PB-2_TCAGCTCAGTATTGGA	PB	PB-2	P2-2972	FALSE	1	TRA:CAASYNQGGKLIF TRB:CASSPTPGVVGANVLTF	1	CD8-C6
PB-2_TCAGGATGTTGTCGCG	PB	PB-2	P2-104	TRUE	3	TRA:CAMREAGGSGAGSYQLTF TRB:CASSLGSGNTIYF	5	CD8-C2
PB-2_TCAGGATTCGCGTAGC	PB	PB-2	P2-2978	FALSE	1	TRA:CAVGGGGATNKLIF TRB:CSARKTGGLDEQYF	1	CD8-C7
PB-2_TCAGGTAGTCCATGAT	PB	PB-2	P2-11	TRUE	3	TRA:CAVHNQGGKLIF TRB:CASSYLGAGYQETQYF	43	CD8-C6
PB-2_TCCCGATTCGAATCCA	PB	PB-2	P2-1	TRUE	3	TRA:CVVNIIGYQKVTF TRB:CASTRQGKQPQHF	270	CD8-C7
PB-2_TCGCGTTTCCGCGTTT	PB	PB-2	P2-3022	FALSE	1	TRA:CVVSAPHNTDKLIF TRB:CSASGVSGSLEQYF	1	CD8-C6
PB-2_TCGTACCCACGCCAGT	PB	PB-2	P2-3031	FALSE	1	TRA:CALSRGGAQKLVF TRB:CASSYSTPRGEAFF	1	CD8-C7
PB-2_TCTATTGGTCTCACCT	PB	PB-2	P2-3042	FALSE	1	TRA:CAASYLNRDDKIIF TRB:CASNRARGYNEQFF	1	CD8-C7

PB-2_TCTTCGGAGCACACAG	PB	PB-2	P2-1	TRUE	3	TRA:CVVNIIGYQKVTF TRB:CASTRQGKQPQHF	270	CD8-C7
PB-2_TGACAACTCAAACGGG	PB	PB-2	P2-1	TRUE	3	TRA:CVVNIIGYQKVTF TRB:CASTRQGKQPQHF	270	CD4-C6
PB-2_TGACGGCTCCATGAAC	PB	PB-2	P2-1	TRUE	3	TRA:CVVNIIGYQKVTF TRB:CASTRQGKQPQHF	270	CD4-C6
PB-2_TGACTAGGTTTGTGTG	PB	PB-2	P2-3110	FALSE	1	TRA:CAVGSYNTDKLIF TRB:CASSYPRGTQYF	1	CD8-C6
PB-2_TGACTAGTCGCCAGCA	PB	PB-2	P2-54	FALSE	3	TRA:CALNFMNRDDKIIF TRB:CASSYSPGQAASYGYTF	10	CD8-C6
PB-2_TGAGGGAAGGACTGGT	PB	PB-2	P2-26	TRUE	3	TRA:CALVNTGGFKTIF TRB:CASSPPSSGGANEQYF	22	CD4-C6
PB-2_TGCACCTTCAGTGTTG	PB	PB-2	P2-1	TRUE	3	TRA:CVVNIIGYQKVTF TRB:CASTRQGKQPQHF	270	CD4-C6
PB-2_TGCCAAATCACTGGGC	PB	PB-2	P2-1	TRUE	3	TRA:CVVNIIGYQKVTF TRB:CASTRQGKQPQHF	270	CD4-C6
PB-2_TGCCCTAAGCGTAATA	PB	PB-2	P2-133	TRUE	3	TRA:CAVNKPAGNKLTF TRB:CASSTRPGGANVLTF	4	CD4-C6
PB-2_TGCCCTAGTAAGAGAG	PB	PB-2	P2-3160	FALSE	1	TRA:CALSDRGAQKLVF TRB:CASSPAGIIGEQFF	1	CD8-C6
PB-2_TGCGCAGAGTGGGATC	PB	PB-2	P2-1	TRUE	3	TRA:CVVNIIGYQKVTF TRB:CASTRQGKQPQHF	270	CD4-C6
PB-2_TGGACGCAGACTGGGT	PB	PB-2	P2-3196	FALSE	1	TRA:CVVNNAGKSTF TRB:CAWSPPGHYGQPQHF	1	CD8-C10
PB-2_TGGACGCCAAGCTGTT	PB	PB-2	P2-3198	FALSE	1	TRA:CAVSNLEYGNKLVF TRB:CASSQDLSGTGRGTQYF	1	CD8-C6
PB-2_TGGACGCCAGACGTAG	PB	PB-2	P2-1	TRUE	3	TRA:CVVNIIGYQKVTF TRB:CASTRQGKQPQHF	270	CD4-C6
PB-2_TGGACGCTCTGTCTAT	PB	PB-2	P2-1	TRUE	3	TRA:CVVNIIGYQKVTF TRB:CASTRQGKQPQHF	270	CD4-C6
PB-2_TGGCGCAGTCTACCTC	PB	PB-2	P2-1	TRUE	3	TRA:CVVNIIGYQKVTF TRB:CASTRQGKQPQHF	270	CD8-C7
PB-2_TGGCGCATCCTCGCAT	PB	PB-2	P2-1	TRUE	3	TRA:CVVNIIGYQKVTF TRB:CASTRQGKQPQHF	270	CD4-C6
PB-2_TGGGAAGGTCATCGGC	PB	PB-2	P2-1	TRUE	3	TRA:CVVNIIGYQKVTF TRB:CASTRQGKQPQHF	270	CD4-C6
PB-2_TGGGAAGTCCAAACTG	PB	PB-2	P2-1	TRUE	3	TRA:CVVNIIGYQKVTF TRB:CASTRQGKQPQHF	270	CD8-C7
PB-2_TGGTTAGCATATACGC	PB	PB-2	P2-1	TRUE	3	TRA:CVVNIIGYQKVTF TRB:CASTRQGKQPQHF	270	CD8-C7
PB-2_TGTATTCCACCACGTG	PB	PB-2	P2-1	TRUE	3	TRA:CVVNIIGYQKVTF TRB:CASTRQGKQPQHF	270	CD4-C6
PB-2_TGTCCCAAGCTAGTGG	PB	PB-2	P2-18	TRUE	3	TRA:CAATSSSNFGNEKLTF TRB:CAWSRAALTEAFF	28	CD4-C6
PB-2_TGTGGTACAAGGTTCT	PB	PB-2	P2-1	TRUE	3	TRA:CVVNIIGYQKVTF TRB:CASTRQGKQPQHF	270	CD8-C7
PB-2_TGTGTTTCATCAGTCA	PB	PB-2	P2-351	FALSE	2	TRA:CAANDNAGNMLTF TRB:CAWRGQGVGSPLHF	2	CD8-C6
PB-2_TGTTCCGAGACTGTAA	PB	PB-2	P2-1	TRUE	3	TRA:CVVNIIGYQKVTF TRB:CASTRQGKQPQHF	270	CD4-C6
PB-2_TGTTCCGAGCACACAG	PB	PB-2	P2-1	TRUE	3	TRA:CVVNIIGYQKVTF TRB:CASTRQGKQPQHF	270	CD4-C6
PB-2_TGTTCCGAGGATTCGG	PB	PB-2	P2-3259	FALSE	1	TRA:CAASTSSNYKLTF TRB:CASAGTGRYNEQFF	1	CD4-C7

PB-2_TTAGGACCACAGGAGT	PB	PB-2	P2-3274	FALSE	1	TRA:CAAIGGWTAGNMLTF TRB:CASSSLGGTYEQYF	1	CD4-C7
PB-2_TTAGGACCAGCTATTG	PB	PB-2	P2-3275	FALSE	1	TRA:CALSEAGSGYSTLTF TRB:CSGELADENNEQFF	1	CD8-C6
PB-2_TTAGGCACACTTCTGC	PB	PB-2	P2-44	FALSE	3	TRA:CALVNTGGFKTIF TRB:CASSPPSAGGGNEQYF	11	CD4-C6
PB-2_TTAGGCATCCGTTGCT	PB	PB-2	P2-3281	FALSE	1	TRA:CALFGGSNYKLTF TRB:CASSLGSNQPQHF	1	CD8-C7
PB-2_TTATGCTCATGTAAGA	PB	PB-2	P2-26	TRUE	3	TRA:CALVNTGGFKTIF TRB:CASSPPSSGGANEQYF	22	CD4-C6
PB-2_TTCCCAGAGTGCGTGA	PB	PB-2	P2-1	TRUE	3	TRA:CVVNIIGYQKVTF TRB:CASTRQGKQPQHF	270	CD4-C6
PB-2_TTCGAAGTCCTAGGGC	PB	PB-2	P2-3301	FALSE	1	TRA:CAYFRVDYKLSF TRB:CSATDREAYEQYF	1	CD8-C6
PB-2_TTCGGTCAGTTAAGTG	PB	PB-2	P2-3304	FALSE	1	TRA:CAVRGFNDYKLSF TRB:CASSPSETGVNEQFF	1	CD8-C6
PB-2_TTCTACAAGTTAAGTG	PB	PB-2	P2-3312	FALSE	1	TRA:CGTGSNFGNEKLTF TRB:CASSLLGGPGEQYF	1	CD8-C6
PB-2_TTCTCAAAGGACATTA	PB	PB-2	P2-1	TRUE	3	TRA:CVVNIIGYQKVTF TRB:CASTRQGKQPQHF	270	CD8-C7
PB-2_TTGAACGGTTCCACTC	PB	PB-2	P2-1	TRUE	3	TRA:CVVNIIGYQKVTF TRB:CASTRQGKQPQHF	270	CD4-C6
PB-2_TTGGCAATCGCATGAT	PB	PB-2	P2-3371	FALSE	1	TRA:CALSDGNFNKFYF TRB:CASSLRTSGSGETQYF	1	CD4-C7
PB-2_TTTATGCGTAGAGTGC	PB	PB-2	P2-1	TRUE	3	TRA:CVVNIIGYQKVTF TRB:CASTRQGKQPQHF	270	CD4-C6
PB-2_TTTATGCTCGTTTATC	PB	PB-2	P2-1	TRUE	3	TRA:CVVNIIGYQKVTF TRB:CASTRQGKQPQHF	270	CD8-C7
PB-2_TTTATGCTCTCGAGTA	PB	PB-2	P2-1	TRUE	3	TRA:CVVNIIGYQKVTF TRB:CASTRQGKQPQHF	270	CD4-C6
PB-2_TTTGGTTGTTGTTTGG	PB	PB-2	P2-3403	FALSE	1	TRA:CTRNNAGNMLTF TRB:CAWSGQGGPDIQYF	1	CD8-C6
PB-2_TTTGGTTTCTTAGCCC	PB	PB-2	P2-3405	FALSE	1	TRA:CLVGDRGNTPLVF TRB:CASSSPGGDTEAFF	1	CD8-C6
PB-2_TTTGTCACATAGTAAG	PB	PB-2	P2-1	TRUE	3	TRA:CVVNIIGYQKVTF TRB:CASTRQGKQPQHF	270	CD4-C6
PB-2_TTTGTCATCTTGTTTG	PB	PB-2	P2-3410	FALSE	1	TRA:CVVNIIGYQKVTF TRB:CAWIPFGTAGELFF	1	CD4-C6
PB-2_AAAGCAAGTCTACCTC	PB	PB-2	P2-589	FALSE	1	TRA:CLVGDKTGRRALTF TRB:CASSPRPAGDQETQYF	1	CD8-C1
PB-2_AAAGTAGCACAAGACG	PB	PB-2	P2-596	FALSE	1	TRA:CAVNRNSGYSTLTF TRB:CASSEGVGAGELFF	1	CD8-C1
PB-2_AAATGCCAGGATCGCA	PB	PB-2	P2-602	FALSE	1	TRA:CIVINDYKLSF TRB:CASSAFGTANEKLFF	1	CD8-C1
PB-2_AACACGTAGAGGTACC	PB	PB-2	P2-605	FALSE	1	TRA:CALRGANNLFF TRB:CASSEGQGSFYEQYF	1	CD4-C2
PB-2_AACTCAGAGCTTATCG	PB	PB-2	P2-627	FALSE	1	TRA:CAVSDPGNQFYF TRB:CASSLGAGGGTDTQYF	1	CD8-C1
PB-2_AAGGAGCAGAAACCTA	PB	PB-2	P2-668	FALSE	1	TRA:CAAPYSGGGADGLTF TRB:CASSLAIWGYGETQYF	1	CD8-C1
PB-2_AAGGAGCTCCTTGCCA	PB	PB-2	P2-673	FALSE	1	TRA:CAMREGDSGGSNYKLTF TRB:CASSLGTDYGYTF	1	CD8-C1
PB-2_AAGGTTCGTGCAACTT	PB	PB-2	P2-688	FALSE	1	TRA:CAPLYNNNDMRF TRB:CASSSSSYNEQFF	1	CD8-C1

PB-2_AATCCAGAGGTGACCA	PB	PB-2	P2-701	FALSE	1	TRA:CAYGNEDSSYKLIF TRB:CASSLGRYTDTQYF	1	CD8-C1
PB-2_AATCCAGTCCGCAGTG	PB	PB-2	P2-706	FALSE	1	TRA:CAVEDGTNAGKSTF TRB:CASSLGGLAAGNEQFF	1	CD8-C1
PB-2_AATCGGTTCGCGTTTC	PB	PB-2	P2-713	FALSE	1	TRA:CASRGGTYKYIF TRB:CASSVGFGNTIYF	1	CD8-C1
PB-2_ACACCGGGTCACTGGC	PB	PB-2	P2-736	FALSE	1	TRA:CAVGESGGGADGLTF TRB:CASTYQGVAQETQYF	1	CD8-C1
PB-2_ACAGCTATCTTCATGT	PB	PB-2	P2-747	FALSE	1	TRA:CAVQAETGGGADGLTF TRB:CASSLTTDKDTQYF	1	CD8-C1
PB-2_ACGAGCCTCTCGGACG	PB	PB-2	P2-798	FALSE	1	TRA:CAVNDRSRDDKIIF TRB:CASSLDWRERTGELFF	1	CD8-C1
PB-2_ACGGGCTTCGCGATCG	PB	PB-2	P2-842	FALSE	1	TRA:CAMREEGYSTLTF TRB:CATSREGMGGYTF	1	CD8-C1
PB-2_ACTGAACAGCCACCTG	PB	PB-2	P2-857	FALSE	1	TRA:CALSEADTGGFKTIF TRB:CSVEVDSGGNLDTQYF	1	CD8-C1
PB-2_ACTGATGTCCCTTGTG	PB	PB-2	P2-879	FALSE	1	TRA:CALSDLTGANSKLTF TRB:CASSDGTYPYEQYF	1	CD8-C1
PB-2_ACTGCTCTCTTGTACT	PB	PB-2	P2-891	FALSE	1	TRA:CVVRSGNTGKLIF TRB:CASSYLPGQLTTYTGELFF	1	CD8-C1
PB-2_AGAATAGAGTACCGGA	PB	PB-2	P2-913	FALSE	1	TRA:CAALSDGQKLLF TRB:CASSFRGIYEQFF	1	CD8-C1
PB-2_AGAGCGACAATCGGTT	PB	PB-2	P2-928	FALSE	1	TRA:CAFTDGQNFVF TRB:CASSLGLASTGELFF	1	CD8-C1
PB-2_AGAGCTTTCGCCTGAG	PB	PB-2	P2-937	FALSE	1	TRA:CAEANSNSGYALNF TRB:CASSQDALANYGYTF	1	CD8-C1
PB-2_AGAGTGGCAGCTCGAC	PB	PB-2	P2-939	FALSE	1	TRA:CAASVPPRGNTPLVF TRB:CASSRTSGFYEQYF	1	CD8-C1
PB-2_AGATTGCTCGTGGTCG	PB	PB-2	P2-957	FALSE	1	TRA:CAGHTVTGNQFYF TRB:CASSEGTSGTYEQYF	1	CD8-C1
PB-2_AGCAGCCGTCCAACTA	PB	PB-2	P2-961	FALSE	1	TRA:CAVSSDGQKLLF TRB:CASSFPSGAGDSYEQYF	1	CD8-C1
PB-2_AGCGGTCCAGAGTGTG	PB	PB-2	P2-975	FALSE	1	TRA:CAVASSSASKIIF TRB:CASSLASSGPYNEQFF	1	CD4-C2
PB-2_AGCTTGAAGCTGAACG	PB	PB-2	P2-1003	FALSE	1	TRA:CAVEFEGGATNKLIF TRB:CASSLGDLNEKLFF	1	CD8-C1
PB-2_AGGCCACCACACATGT	PB	PB-2	P2-1008	FALSE	1	TRA:CAPLESSASKIIF TRB:CASSLTRGSNQPQHF	1	CD8-C1
PB-2_AGGGAGTTCAATACCG	PB	PB-2	P2-1024	FALSE	1	TRA:CAASGARLMF TRB:CASSLDPGDLEKLFF	1	CD8-C1
PB-2_AGGGATGCAGACAGGT	PB	PB-2	P2-1027	FALSE	1	TRA:CAASVDWGGATNKLIF TRB:CASSLAVTDYGYTF	1	CD8-C1
PB-2_AGGTCCGTCACCACCT	PB	PB-2	P2-1051	FALSE	1	TRA:CAFLTSGTYKYIF TRB:CASSARSSGDFNEQYF	1	CD8-C1
PB-2_AGTGGGATCTTGAGGT	PB	PB-2	P2-1071	FALSE	1	TRA:CAASKSRGGSQGNLIF TRB:CASSLKGTSGRTSQYNEQFF	1	CD4-C2
PB-2_ATAAGAGTCGCCCTTA	PB	PB-2	P2-1095	FALSE	1	TRA:CAMRANTGGFKTIF TRB:CASSQNRDSKNSPLHF	1	CD8-C1
PB-2_ATCTACTTCCAGTAGT	PB	PB-2	P2-1138	FALSE	1	TRA:CALSEAGNNDMRF TRB:CAWRADGSRQPQHF	1	CD8-C1
PB-2_ATGGGAGCATAGTAAG	PB	PB-2	P2-1156	FALSE	1	TRA:CAVISGGYQKVTF TRB:CASSEALGELFF	1	CD8-C1
PB-2_ATTACTCAGATGCCAG	PB	PB-2	P2-1164	FALSE	1	TRA:CAVIFSGNTGKLIF TRB:CSARAPSGTGELFF	1	CD8-C1

PB-2_ATTATCCCACGGACAA	PB	PB-2	P2-1170	FALSE	1	TRA:CLVGDSNYQLIW TRB:CASSPQTEVTEAFF	1	CD8-C1
PB-2_ATTCTACAGATGTGTA	PB	PB-2	P2-1175	FALSE	1	TRA:CAVKNSGYALNF TRB:CASSLGGYEQYF	1	CD8-C1
PB-2_ATTCTACCATCAGTCA	PB	PB-2	P2-1177	FALSE	1	TRA:CAVEGFQKLVF TRB:CASSFPRAPAYNEQFF	1	CD8-C1
PB-2_CAACTAGAGATATGCA	PB	PB-2	P2-1209	FALSE	1	TRA:CALAGDNYGQNFVF TRB:CASSQGAGSYEQYF	1	CD8-C1
PB-2_CAACTAGCATTGGGCC	PB	PB-2	P2-1210	FALSE	1	TRA:CAVYDRYGGSQGNLIF TRB:CSASTPGTGAGYTF	1	CD8-C1
PB-2_CAAGATCTCTAGCACA	PB	PB-2	P2-1221	FALSE	1	TRA:CALSEAGAPYGGSNYKLTF TRB:CASSWEEKLFF	1	CD8-C1
PB-2_CAAGTTGAGTGCCAGA	PB	PB-2	P2-1227	FALSE	1	TRA:CGTVDTYSGAGSYQLTF TRB:CASSDGRGVYGYTF	1	CD8-C1
PB-2_CAAGTTGCAGGTGCCT	PB	PB-2	P2-1229	FALSE	1	TRA:CAMKGDGNTDKLIF TRB:CASIQESSHPEKLFF	1	CD8-C1
PB-2_CACAAACCAGACGCAA	PB	PB-2	P2-1235	FALSE	1	TRA:CGTDNYGQNFVF TRB:CASSFGLGPLHF	1	CD8-C1
PB-2_CACACAAGTGCTAGCC	PB	PB-2	P2-1241	FALSE	1	TRA:CAVDSTSGTYKYIF TRB:CAWSLYSDGYTF	1	CD8-C1
PB-2_CACAGGCCATATACGC	PB	PB-2	P2-1258	FALSE	1	TRA:CLVGSHASGGSYIPTF TRB:CASSLYVAGNTGELFF	1	CD8-C1
PB-2_CACAGTACATTCTCAT	PB	PB-2	P2-1266	FALSE	1	TRA:CALGAGNMLTF TRB:CASSRGGTEAFF	1	CD8-C1
PB-2_CACAGTAGTCCAGTGC	PB	PB-2	P2-1267	FALSE	1	TRA:CALKEGGNKLVF TRB:CASSQTGANYGYTF	1	CD8-C1
PB-2_CACATAGTCTCGTTTA	PB	PB-2	P2-1273	FALSE	1	TRA:CAGAWRAQKLVF TRB:CASSQHHRQGFFNQPQHF	1	CD8-C1
PB-2_CACATTTCATTAGCCA	PB	PB-2	P2-1276	FALSE	1	TRA:CATDDNAGNMLTF TRB:CASGVESSGSREQFF	1	CD8-C1
PB-2_CACCACTGTCCTGCTT	PB	PB-2	P2-1281	FALSE	1	TRA:CAATDSWGKFQF TRB:CASSHVKGDTKNIQYF	1	CD8-C1
PB-2_CACCAGGAGATCGGGT	PB	PB-2	P2-1284	FALSE	1	TRA:CAVRVLYGNNRLAF TRB:CASSPPGNRENTEAFF	1	CD8-C1
PB-2_CAGAATCCAACACCCG	PB	PB-2	P2-1298	FALSE	1	TRA:CVVNIAGGSQGNLIF TRB:CASSESMDYGYTF	1	CD8-C1
PB-2_CAGAATCGTCTGCAAT	PB	PB-2	P2-1300	FALSE	1	TRA:CAVLDGQKLLF TRB:CATEGGTTLYNEQFF	1	CD8-C1
PB-2_CAGAGAGAGCCATCGC	PB	PB-2	P2-1302	FALSE	1	TRA:CATALPVGGSQGNLIF TRB:CASSKSFQSSYEQYF	1	CD8-C1
PB-2_CAGCAGCCACATCCAA	PB	PB-2	P2-1318	FALSE	1	TRA:CAVRAQTGANNLFF TRB:CASSVEPGLSYEQYF	1	CD8-C1
PB-2_CAGCATACAGGAATCG	PB	PB-2	P2-1325	FALSE	1	TRA:CALSGRFDTGFQKLVF TRB:CASSHTGAEQYF	1	CD8-C1
PB-2_CAGCTAATCCAGTAGT	PB	PB-2	P2-1338	FALSE	1	TRA:CAVRDNNNNDMRF TRB:CASILGTGNYGYTF	1	CD8-C1
PB-2_CATGACAGTCGCGGTT	PB	PB-2	P2-1414	FALSE	1	TRA:CALDRYSSASKIIF TRB:CASSTYRDREYNEQFF	1	CD8-C1
PB-2_CATGGCGGTATAGGTA	PB	PB-2	P2-1423	FALSE	1	TRA:CAVDPMDSSYKLIF TRB:CASSPERESTYEQYF	1	CD8-C1
PB-2_CATGGCGTCCCTCAGT	PB	PB-2	P2-1426	FALSE	1	TRA:CILRDGTGANNLFF TRB:CASSKGLAGVETQYF	1	CD8-C1
PB-2_CATTCGCCAAAGCGGT	PB	PB-2	P2-1439	FALSE	1	TRA:CINDYKLSF TRB:CASSLGGQGDGELFF	1	CD8-C1

PB-2_CCACTACGTCGCGGTT	PB	PB-2	P2-1473	FALSE	1	TRA:CAVRYYGGSQGNLIF TRB:CAWRTGSSYEQYF	1	CD8-C1
PB-2_CCATGTCCATGGTCAT	PB	PB-2	P2-1484	FALSE	1	TRA:CAMRATTSGTYKYIF TRB:CATLTDSTDTQYF	1	CD8-C1
PB-2_CCCAATCCACACGCTG	PB	PB-2	P2-1498	FALSE	1	TRA:CVRANFGNEKLTF TRB:CASLLSGGTDTQYF	1	CD8-C1
PB-2_CCCAATCCAGTCCTTC	PB	PB-2	P2-1500	FALSE	1	TRA:CAVADNDMRF TRB:CASSLEAGLEQFF	1	CD8-C1
PB-2_CCCAGTTAGATATGGT	PB	PB-2	P2-1504	FALSE	1	TRA:CAVRDNYGQNFVF TRB:CSARGPTGYNEKLFF	1	CD8-C1
PB-2_CCCAGTTTCTGGAGCC	PB	PB-2	P2-1512	FALSE	1	TRA:CAVRSDYKLSF TRB:CASSGGLAENTGELFF	1	CD8-C1
PB-2_CCGTACTAGCTGTTCA	PB	PB-2	P2-1531	FALSE	1	TRA:CALSEAGNTPLVF TRB:CASSLGTSGYEQYF	1	CD8-C1
PB-2_CCGTGGAAGGTAGCTG	PB	PB-2	P2-1536	FALSE	1	TRA:CAGQGSVGYQKVTF TRB:CASSIGGNMQPQHF	1	CD8-C1
PB-2_CCTAAAGAGTGCCAGA	PB	PB-2	P2-1550	FALSE	1	TRA:CALRQGGSYIPTF TRB:CASRRNQGFGETQYF	1	CD8-C1
PB-2_CCTACACGTTGCTCCT	PB	PB-2	P2-1559	FALSE	1	TRA:CAVSPPSGYALNF TRB:CASSLEGPSYEQYF	1	CD8-C1
PB-2_CCTATTATCCACGACG	PB	PB-2	P2-1579	FALSE	1	TRA:CAPPVGAGNMLTF TRB:CASSTHGYYEQYF	1	CD8-C1
PB-2_CCTTCCCTCCGGCACA	PB	PB-2	P2-1603	FALSE	1	TRA:CAGGGSQGNLIF TRB:CASSFGGGGGEKLFF	1	CD8-C1
PB-2_CCTTTCTGTATTAGCC	PB	PB-2	P2-1614	FALSE	1	TRA:CAVSLGTASKLTF TRB:CASSLGWYEQYF	1	CD8-C1
PB-2_CGAACATTCAAGGCTT	PB	PB-2	P2-1620	FALSE	1	TRA:CALSNDYKLSF TRB:CASSLLAGGYNEQFF	1	CD4-C2
PB-2_CGACCTTCATCAGTAC	PB	PB-2	P2-1627	FALSE	1	TRA:CAEYPGGSYIPTF TRB:CASSLHSRTGYQETQYF	1	CD8-C1
PB-2_CGAGCACTCTATCCCG	PB	PB-2	P2-1653	FALSE	1	TRA:CALDNAGNMLTF TRB:CASSGTSFTSGGSDTQYF	1	CD8-C2
PB-2_CGATGGCAGGGTGTTG	PB	PB-2	P2-1660	FALSE	1	TRA:CALSATDSGNTPLVF TRB:CASSEGGGEYNEQFF	1	CD8-C1
PB-2_CGCCAAGCATTCACTT	PB	PB-2	P2-1675	FALSE	1	TRA:CVVIDSGGYNKLIF TRB:CASSPATHEQYF	1	CD8-C1
PB-2_CGCGGTACAAAGTGCG	PB	PB-2	P2-1679	FALSE	1	TRA:CAEKGDQKLVF TRB:CASSLSSGSYTGELFF	1	CD8-C1
PB-2_CGCGTTTGTTTGGGCC	PB	PB-2	P2-1689	FALSE	1	TRA:CAMREGGGFKTIF TRB:CASSLGASQPQHF	1	CD8-C1
PB-2_CGCTTCACAATAGCAA	PB	PB-2	P2-1707	FALSE	1	TRA:CAVRAAPSRDTDKLIF TRB:CATSVSGGAGNEQFF	1	CD8-C1
PB-2_CGGACACTCTTCGAGA	PB	PB-2	P2-1716	FALSE	1	TRA:CAVTTRDNYGQNFVF TRB:CASRTLTGDTEAFF	1	CD8-C1
PB-2_CGGACGTAGTGTCTCA	PB	PB-2	P2-1721	FALSE	1	TRA:CAARSGYALNF TRB:CASSFIPRADTEAFF	1	CD8-C1
PB-2_CGGAGCTCATAGAAAC	PB	PB-2	P2-1737	FALSE	1	TRA:CAVTTLLNTDKLIF TRB:CASSLLGPSGTDEQYF	1	CD8-C1
PB-2_CGGAGTCGTCAAGCGA	PB	PB-2	P2-1741	FALSE	1	TRA:CAASGDSGAGSYQLTF TRB:CASSVTTTDTQYF	1	CD8-C1
PB-2_CGGAGTCTCATGCATG	PB	PB-2	P2-1744	FALSE	1	TRA:CAYYGGATNKLIF TRB:CASSWDFLPRVRYNEQFF	1	CD8-C1
PB-2_CGGGTCAAGTGCCATT	PB	PB-2	P2-1755	FALSE	1	TRA:CVVSGGTYKYIF TRB:CASSLTETGFYEQYF	1	CD8-C1

PB-2_CGGTTAAAGACTGGGT	PB	PB-2	P2-1761	FALSE	1	TRA:CALSEGNNAGNMLTF TRB:CASKSGGGTYTQYF	1	CD8-C1
PB-2_CGTAGCGTCCTTGGTC	PB	PB-2	P2-1767	FALSE	1	TRA:CAVDRPLGAAGNKLTF TRB:CSARDEGGSWYEQYF	1	CD8-C1
PB-2_CGTAGGCTCCTTGGTC	PB	PB-2	P2-1772	FALSE	1	TRA:CAMKGASGTYKYIF TRB:CASSHPAGEGTDTQYF	1	CD8-C1
PB-2_CGTCAGGCATCGTCGG	PB	PB-2	P2-1780	FALSE	1	TRA:CAMRASLWMRF TRB:CASSKGGGELYEQYF	1	CD8-C1
PB-2_CGTCCATAGCGATTCT	PB	PB-2	P2-1786	FALSE	1	TRA:CAAINYGGATNKLIF TRB:CASSPPLPLAKNIQYF	1	CD8-C1
PB-2_CGTCTACCAGCTGTGC	PB	PB-2	P2-1799	FALSE	1	TRA:CAASINAGKSTF TRB:CASSIAHAGYEQYF	1	CD8-C1
PB-2_CGTCTACTCGCGTTTC	PB	PB-2	P2-1801	FALSE	1	TRA:CAVEDYGGSQGNLIF TRB:CASSVGGGRVSTDTQYF	1	CD8-C1
PB-2_CGTGTCTAGACAGGCT	PB	PB-2	P2-1811	FALSE	1	TRA:CAGFAGNKLTF TRB:CASSLVTGGHYNEQFF	1	CD8-C1
PB-2_CGTTAGAGTGTGAAAT	PB	PB-2	P2-1819	FALSE	1	TRA:CIVREAGNMLTF TRB:CASDSGAYEQYF	1	CD8-C1
PB-2_CGTTCTGCACGAAATA	PB	PB-2	P2-1826	FALSE	1	TRA:CAMNSDNNDMRF TRB:CASSFITGNEQFF	1	CD8-C1
PB-2_CTACATTGTCAAGCGA	PB	PB-2	P2-1864	FALSE	1	TRA:CAGGSQGNLIF TRB:CSAPDRGLHEQYF	1	CD8-C1
PB-2_CTACCCAAGAACTCGG	PB	PB-2	P2-1869	FALSE	1	TRA:CAVISPSDGQKLLF TRB:CSVIRDLGDYEQYF	1	CD8-C1
PB-2_CTACGTCTCGCATGAT	PB	PB-2	P2-1881	FALSE	1	TRA:CAVRDTGTQAGTALIF TRB:CASSQGTGHGETQYF	1	CD8-C1
PB-2_CTAGAGTCAAGCCCAC	PB	PB-2	P2-1882	FALSE	1	TRA:CATDREDDMRF TRB:CASSQVLAQGEAFF	1	CD8-C1
PB-2_CTAGAGTCATGTCTCC	PB	PB-2	P2-1885	FALSE	1	TRA:CAVASGAGSYQLTF TRB:CASSFRGPNTEAFF	1	CD8-C1
PB-2_CTAGAGTGTAATTGGA	PB	PB-2	P2-1886	FALSE	1	TRA:CAEWGTGDYKLSF TRB:CASSMAGGQETQYF	1	CD8-C1
PB-2_CTCGAAAGTTGGTGGA	PB	PB-2	P2-1930	FALSE	1	TRA:CAENMVSNSGYALNF TRB:CASSQYSGGNYEQYF	1	CD8-C1
PB-2_CTCGAGGGTTTGACAC	PB	PB-2	P2-1934	FALSE	1	TRA:CAVNLPTGTASKLTF TRB:CASSAGTGELFF	1	CD8-C1
PB-2_CTCGTCACATTTCACT	PB	PB-2	P2-1951	FALSE	1	TRA:CAVMGGNYKLTF TRB:CASSPGQMATEQYF	1	CD8-C1
PB-2_CTCTAATGTGCAACGA	PB	PB-2	P2-1956	FALSE	1	TRA:CAMREGGGDYKLSF TRB:CASGRSGGAYNEQFF	1	CD8-C1
PB-2_CTCTGGTGTGCACTTA	PB	PB-2	P2-1966	FALSE	1	TRA:CALSEEDYTGNQFYF TRB:CASSSDRVYRTDTQYF	1	CD8-C1
PB-2_CTGAAACCATGGGACA	PB	PB-2	P2-1973	FALSE	1	TRA:CAVNARDNNNDMRF TRB:CASSLGLAAYNEQFF	1	CD8-C1
PB-2_CTGATAGAGAATGTTG	PB	PB-2	P2-1984	FALSE	1	TRA:CASDSNSGYALNF TRB:CASSTSGAQSGTQYF	1	CD8-C1
PB-2_CTGCGGACATGCGCAC	PB	PB-2	P2-2004	FALSE	1	TRA:CAVQLGVTSYGKLTF TRB:CASSISGHEQYF	1	CD8-C1
PB-2_CTGCTGTTCTATCCCG	PB	PB-2	P2-2014	FALSE	1	TRA:CAMREAGSARQLTF TRB:CASSPRQGSEKLFF	1	CD8-C2
PB-2_CTTCTCTCAAGCGATG	PB	PB-2	P2-2051	FALSE	1	TRA:CAMREGSNSGYALNF TRB:CASSPGTAPSTNEKLFF	1	CD8-C1
PB-2_CTTTGCGCAGTTTACG	PB	PB-2	P2-2060	FALSE	1	TRA:CATERDLYNEKLTF TRB:CASSLGRDNEKLFF	1	CD8-C1

PB-2_GAAATGAAGATCCTGT	PB	PB-2	P2-2067	FALSE	1	TRA:CAVGRAGNNRKLIW TRB:CSAKEELSTDTQYF	1	CD8-C1
PB-2_GAAGCAGCACCAGGTC	PB	PB-2	P2-2094	FALSE	1	TRA:CATDAGGNNRLAF TRB:CASSYGAGGEETQYF	1	CD8-C1
PB-2_GAAGCAGTCCAGGGCT	PB	PB-2	P2-2096	FALSE	1	TRA:CAMREGGSYIPTF TRB:CASSLALTGADSPLHF	1	CD8-C1
PB-2_GACAGAGCAAACTGTC	PB	PB-2	P2-2118	FALSE	1	TRA:CAYRIPYNNNDMRF TRB:CASRGSGYNEQFF	1	CD8-C1
PB-2_GACCTGGCAAATACAG	PB	PB-2	P2-2136	FALSE	1	TRA:CAVASGTYKYIF TRB:CASSPDREGETQYF	1	CD4-C1
PB-2_GACGTGCAGGTGATAT	PB	PB-2	P2-2155	FALSE	1	TRA:CAGAHSGAGSYQLTF TRB:CSARSGPWDRYGYTF	1	CD8-C1
PB-2_GACGTGCGTCATACTG	PB	PB-2	P2-2158	FALSE	1	TRA:CAENSNSGYALNF TRB:CASSLVGDDEQFF	1	CD8-C1
PB-2_GACGTGCGTTGATTGC	PB	PB-2	P2-2159	FALSE	1	TRA:CATAPRDDKIIF TRB:CASHGATYEQYF	1	CD8-C1
PB-2_GACTACATCTCGTTTA	PB	PB-2	P2-2179	FALSE	1	TRA:CAVRDLQGAGSYQLTF TRB:CASSLAGGAYEQYF	1	CD8-C1
PB-2_GAGCAGACATCGATGT	PB	PB-2	P2-2193	FALSE	1	TRA:CAVRDRGSQGNLIF TRB:CASSEIWQEDGEAFF	1	CD8-C1
PB-2_GAGGTGACAGGGTATG	PB	PB-2	P2-2199	FALSE	1	TRA:CALRLPEDSSYKLIF TRB:CASSYSRSGEQYF	1	CD4-C2
PB-2_GAGTCCGCATTAGGCT	PB	PB-2	P2-2207	FALSE	1	TRA:CAESLDYKLSF TRB:CASSSRKDLNIQYF	1	CD8-C1
PB-2_GATCAGTGTTCGCGAC	PB	PB-2	P2-2218	FALSE	1	TRA:CAVVSGYSTLTF TRB:CASSEVGELFF	1	CD8-C1
PB-2_GATCGATGTTCACGGC	PB	PB-2	P2-2225	FALSE	1	TRA:CAVNGNARLMF TRB:CASSIGGGDEQFF	1	CD8-C1
PB-2_GATCTAGAGTTAAGTG	PB	PB-2	P2-2245	FALSE	1	TRA:CAVKNNNDMRF TRB:CASVGDTQYF	1	CD8-C1
PB-2_GATGAAAGTACTTAGC	PB	PB-2	P2-2255	FALSE	1	TRA:CLVGFSGNTGKLIF TRB:CASSGGTGWSGELFF	1	CD8-C1
PB-2_GCAAACTCAAGGCTCC	PB	PB-2	P2-2274	FALSE	1	TRA:CVVNHNQGGKLIF TRB:CASSRTYASNQPQHF	1	CD8-C1
PB-2_GCAAACTCACACTGCG	PB	PB-2	P2-2275	FALSE	1	TRA:CALSIVSGAGSYQLTF TRB:CASKSRGVGNIQYF	1	CD8-C1
PB-2_GCAATCAGTACGACCC	PB	PB-2	P2-2283	FALSE	1	TRA:CAVRDDPTSGTYKYIF TRB:CASSEGTGLYEQYF	1	CD4-C2
PB-2_GCAGTTAAGAAGATTC	PB	PB-2	P2-2303	FALSE	1	TRA:CAVKGPKGGSYIPTF TRB:CASSLGQQETQYF	1	CD8-C1
PB-2_GCAGTTAAGTCATGCT	PB	PB-2	P2-2304	FALSE	1	TRA:CAVRGADSNYQLIW TRB:CASSLKEGTDTQYF	1	CD8-C1
PB-2_GCATACAAGCACCGTC	PB	PB-2	P2-2308	FALSE	1	TRA:CAASRGGAGSYQLTF TRB:CSARDDSADVNSPLHF	1	CD8-C1
PB-2_GCATACACACGACGAA	PB	PB-2	P2-2309	FALSE	1	TRA:CAMREGGAGNNRKLIW TRB:CASSPQGGSTPDTQYF	1	CD8-C1
PB-2_GCATGCGCAATGTAAG	PB	PB-2	P2-2320	FALSE	1	TRA:CAVPSSNDYKLSF TRB:CASSTDRVETQYF	1	CD8-C1
PB-2_GCATGCGGTCAGTGGA	PB	PB-2	P2-2321	FALSE	1	TRA:CAVSQAGTALIF TRB:CASGTDIHEQFF	1	CD4-C1
PB-2_GCATGTATCATGTGGT	PB	PB-2	P2-2328	FALSE	1	TRA:CAFMTPTPGQAGTALIF TRB:CASSLIDRGDTEAFF	1	CD8-C1
PB-2_GCCAAATAGATCACGG	PB	PB-2	P2-2330	FALSE	1	TRA:CAVNSGTYKYIF TRB:CSAEQETQYF	1	CD8-C1

PB-2_GCCAAATTCTTAGAGC	PB	PB-2	P2-2337	FALSE	1	TRA:CAMRDHSGTYKYIF TRB:CASSPSGTGYTDTQYF	1	CD4-C1
PB-2_GCGAGAAAGCGATCCC	PB	PB-2	P2-2356	FALSE	1	TRA:CAPTGFGNEKLTF TRB:CASSLDRGSGNTIYF	1	CD8-C1
PB-2_GCGAGAACAGCGTAAG	PB	PB-2	P2-2358	FALSE	1	TRA:CAFLSYNQGGKLIF TRB:CSAREGGTTRRETQYF	1	CD8-C1
PB-2_GCGCAACTCAGAGCTT	PB	PB-2	P2-2370	FALSE	1	TRA:CALDPVSGGYNKLIF TRB:CASSIRTGSLTEAFF	1	CD8-C1
PB-2_GCTGCTTGTGCCTGGT	PB	PB-2	P2-2419	FALSE	1	TRA:CATDDNWDDYKLSF TRB:CASSLVGLGGDEQYF	1	CD8-C1
PB-2_GCTGGGTGTTTGACTG	PB	PB-2	P2-2425	FALSE	1	TRA:CAVRTL TRB:CSARDLGEVLNEQFF	1	CD8-C1
PB-2_GCTTCCATCCCGACTT	PB	PB-2	P2-2430	FALSE	1	TRA:CAVASHRDDKIIF TRB:CATNHGHSYNSPLHF	1	CD8-C1
PB-2_GGAAAGCTCTTTAGTC	PB	PB-2	P2-2436	FALSE	1	TRA:CALSEAPSGTYKYIF TRB:CASSSPRGPHQETQYF	1	CD8-C1
PB-2_GGAGCAAGTTCGCGAC	PB	PB-2	P2-2488	FALSE	1	TRA:CAVHPVKAGTALIF TRB:CASSPWVNQETQYF	1	CD4-C2
PB-2_GGATGTTAGATCCCAT	PB	PB-2	P2-2490	FALSE	1	TRA:CALSGLYNFNKFYF TRB:CASSPPGQGAGGANVLTF	1	CD8-C1
PB-2_GGCAATTGTGGTAACG	PB	PB-2	P2-2503	FALSE	1	TRA:CALSEARHDFGNEKLTF TRB:CASSQDRDRGIRSSYEQYF	1	CD8-C1
PB-2_GGCCGATTCCTAGAAC	PB	PB-2	P2-2512	FALSE	1	TRA:CAYRSAIGNTGKLIF TRB:CASSQEDPERNSGANVLTF	1	CD4-C1
PB-2_GGGAGATGTGATAAGT	PB	PB-2	P2-2545	FALSE	1	TRA:CAPHGGSARQLTF TRB:CASSLGQSAQQYF	1	CD8-C1
PB-2_GGGAGATTCCATGCTC	PB	PB-2	P2-2548	FALSE	1	TRA:CALSESSSGNTGKLIF TRB:CASSAGTGEAFF	1	CD8-C1
PB-2_GGGCACTTCACCCTCA	PB	PB-2	P2-2555	FALSE	1	TRA:CAVGVIGNTPLVF TRB:CASSSPDRYHRAFF	1	CD8-C1
PB-2_GGGCATCTCGTGGACC	PB	PB-2	P2-2561	FALSE	1	TRA:CAMREGRAAMNRDDKIIF TRB:CASSFPQNNRNTEAFF	1	CD8-C1
PB-2_GGGTTGCAGAGTCGGT	PB	PB-2	P2-2567	FALSE	1	TRA:CAVTTGANNLFF TRB:CASSVTGTGNTIYF	1	CD8-C1
PB-2_GTACTCCAGTTCGATC	PB	PB-2	P2-2603	FALSE	1	TRA:CVVTSYQTGANNLFF TRB:CASSLAGGADTQYF	1	CD8-C1
PB-2_GTACTCCCACCGGAAA	PB	PB-2	P2-2605	FALSE	1	TRA:CLVVDDNNDMRF TRB:CASSAGLGASYNEQFF	1	CD8-C1
PB-2_GTACTCCCATTTCACT	PB	PB-2	P2-2606	FALSE	1	TRA:CAASTGNQFYF TRB:CASSQEWERETQYF	1	CD8-C1
PB-2_GTACTCCGTCTTCGTC	PB	PB-2	P2-2608	FALSE	1	TRA:CADKGSGGYQKVTF TRB:CATSRHPTDQNTEAFF	1	CD8-C1
PB-2_GTATTCTTCAAAGTAG	PB	PB-2	P2-2630	FALSE	1	TRA:CAASNSGGFKTIF TRB:CSARIKTRDSDNYGYTF	1	CD8-C1
PB-2_GTCATTTCACTGTTAG	PB	PB-2	P2-2654	FALSE	1	TRA:CAYRSPQGGSEKLVF TRB:CASSVGGQGTGELFF	1	CD8-C1
PB-2_GTCATTTGTCAGATAA	PB	PB-2	P2-2657	FALSE	1	TRA:CAVDIVEGTNAGKSTF TRB:CASSVEDAGPGYNEQFF	1	CD8-C1
PB-2_GTCATTTTCTTGCCGT	PB	PB-2	P2-2659	FALSE	1	TRA:CALRNQGGKLIF TRB:CSAKESGSFYEQYF	1	CD8-C1
PB-2_GTCCTCAAGTGTACCT	PB	PB-2	P2-2662	FALSE	1	TRA:CARPGGVRNTPLVF TRB:CASSPSGGRGETQYF	1	CD8-C1
PB-2_GTCTCGTTCCTTGCCA	PB	PB-2	P2-2690	FALSE	1	TRA:CIGYNNNDMRF TRB:CASSEAGVQPQHF	1	CD8-C1

PB-2_GTGCATACAGTTTACG	PB	PB-2	P2-2708	FALSE	1	TRA:CAVRDLNTDKLIF TRB:CASSQGGWGAYEQYF	1	CD8-C1
PB-2_GTGGGTCGTAGCGCAA	PB	PB-2	P2-2729	FALSE	1	TRA:CAVEDQIRGDKIIF TRB:CASSLAGASTDTQYF	1	CD8-C1
PB-2_GTGTGCGCACCTCGTT	PB	PB-2	P2-2735	FALSE	1	TRA:CALSEASTGTASKLTF TRB:CASSLRSAAPYEQYF	1	CD8-C1
PB-2_GTGTGCGGTTTACTCT	PB	PB-2	P2-2737	FALSE	1	TRA:CAMRGVNYGQNFVF TRB:CAWSVALAGANQETQYF	1	CD4-C2
PB-2_GTGTGCGTCACAGGCC	PB	PB-2	P2-2738	FALSE	1	TRA:CARGGSQGNLIF TRB:CASLGGSSYNEQFF	1	CD4-C1
PB-2_GTGTTAGAGATGCCTT	PB	PB-2	P2-2742	FALSE	1	TRA:CAETSYDKVIF TRB:CASSEQGSGETQYF	1	CD4-C1
PB-2_GTTACAGGTTATCACG	PB	PB-2	P2-2763	FALSE	1	TRA:CAASIGGAQKLVF TRB:CASSVGGANYEQYF	1	CD8-C1
PB-2_GTTACAGTCATGCATG	PB	PB-2	P2-2764	FALSE	1	TRA:CASRASNYGQNFVF TRB:CASSLGWTAENKQYF	1	CD8-C1
PB-2_GTTTCTAAGCCATCGC	PB	PB-2	P2-2779	FALSE	1	TRA:CAVSAPGAGSYQLTF TRB:CSVEGLGEGYEQYF	1	CD8-C1
PB-2_TAAGAGAAGTATGACA	PB	PB-2	P2-2790	FALSE	1	TRA:CAGRVNRDDKIIF TRB:CASSLPGEVDTQYF	1	CD4-C1
PB-2_TAAGAGAAGTCACGCC	PB	PB-2	P2-2791	FALSE	1	TRA:CAVGLNNDMRF TRB:CASREDRGHAPLHF	1	CD8-C1
PB-2_TAAGAGAGTCCAACTA	PB	PB-2	P2-2792	FALSE	1	TRA:CALSEERGKLIF TRB:CASRLGQGSNQPQHF	1	CD8-C1
PB-2_TACAGTGCAGCCAGAA	PB	PB-2	P2-2816	FALSE	1	TRA:CAVSMDTGRRALTF TRB:CASSLGEGGYEQYF	1	CD8-C1
PB-2_TACCTATAGGTGATTA	PB	PB-2	P2-2823	FALSE	1	TRA:CAESADNAGNMLTF TRB:CASSYLSTEGDTQYF	1	CD8-C1
PB-2_TACCTATAGTTACCCA	PB	PB-2	P2-2824	FALSE	1	TRA:CALGFGNEKLTF TRB:CASSQELGAVTYNEQFF	1	CD8-C1
PB-2_TACTCATGTGGAAAGA	PB	PB-2	P2-2851	FALSE	1	TRA:CAVKIGARALTF TRB:CASSVQVTSRADANTGELFF	1	CD8-C1
PB-2_TACTCATTCGGCGCTA	PB	PB-2	P2-2854	FALSE	1	TRA:CAEILTDSWGKFQF TRB:CASSVGTDLPSQETQYF	1	CD8-C1
PB-2_TACTTGTAGGCAATTA	PB	PB-2	P2-2866	FALSE	1	TRA:CAENYGQNFVF TRB:CASSLFGTGGDYGYTF	1	CD8-C1
PB-2_TAGACCACATTTGCCC	PB	PB-2	P2-2872	FALSE	1	TRA:CASEVGGTSYGKLTF TRB:CASSPIPDGPYEQYF	1	CD8-C1
PB-2_TAGAGCTGTCAGGACA	PB	PB-2	P2-2882	FALSE	1	TRA:CALGGGRRALTF TRB:CASSIGAGGSYEQYF	1	CD4-C1
PB-2_TAGTGGTTCACCATAG	PB	PB-2	P2-2895	FALSE	1	TRA:CAVNSPSGGSYIPTF TRB:CASSPSTGVTIAFF	1	CD8-C1
PB-2_TAGTTGGCAGACAAGC	PB	PB-2	P2-2901	FALSE	1	TRA:CGPGGATNKLIF TRB:CASSVGEGLETQYF	1	CD8-C1
PB-2_TATCAGGCATGCGCAC	PB	PB-2	P2-2910	FALSE	1	TRA:CAVNTGFQKLVF TRB:CRVGKVEVVQGENEQFF	1	CD8-C1
PB-2_TATGCCCAGTACTTGC	PB	PB-2	P2-2925	FALSE	1	TRA:CASPGVSSASKIIF TRB:CSARTTSGRLTDTQYF	1	CD8-C1
PB-2_TATGCCCTCGCACTCT	PB	PB-2	P2-2932	FALSE	1	TRA:CAVRDIRSQTGANNLFF TRB:CASSPDRGTGELFF	1	CD8-C1
PB-2_TCACGAAGTGTTTGTG	PB	PB-2	P2-2952	FALSE	1	TRA:CAFMKEFYNNNDMRF TRB:CASSLDSSGAWEQFF	1	CD8-C1
PB-2_TCAGCTCAGATCCTGT	PB	PB-2	P2-2969	FALSE	1	TRA:CAVRGLNRDDKIIF TRB:CASSLKDLAVYEQYF	1	CD4-C1

PB-2_TCATTACCACAACTGT	PB	PB-2	P2-2990	FALSE	1	TRA:CGWSGGGADGLTF TRB:CASTPQSGWAAEAFF	1	CD8-C1
PB-2_TCCCGATAGGGTTTCT	PB	PB-2	P2-3007	FALSE	1	TRA:CAGQDEWGGSEKLVF TRB:CAISSTGGEGSPLHF	1	CD8-C1
PB-2_TCGCGAGAGATAGGAG	PB	PB-2	P2-3015	FALSE	1	TRA:CAMRVRRRDNYGQNFVF TRB:CASSFASTDTQYF	1	CD8-C1
PB-2_TCGCGAGTCATCTGTT	PB	PB-2	P2-3017	FALSE	1	TRA:CAVRYYGNNRLAF TRB:CATSAPGQGVYEQYF	1	CD8-C1
PB-2_TCGCGTTCAGCGTTCG	PB	PB-2	P2-3019	FALSE	1	TRA:CAMSLITGNQFYF TRB:CASSPWALISGANVLTF	1	CD4-C1
PB-2_TCTCATAAGGGCACTA	PB	PB-2	P2-3047	FALSE	1	TRA:CATDAMRGGSEKLVF TRB:CASSSRGVDTQYF	1	CD8-C1
PB-2_TCTTCGGAGCCGGTAA	PB	PB-2	P2-3064	FALSE	1	TRA:CAFIWAGSYQLTF TRB:CASSPDSRTEAFF	1	CD8-C1
PB-2_TCTTCGGTCTGTACGA	PB	PB-2	P2-3073	FALSE	1	TRA:CALKEAAAGNKLTF TRB:CASSPSGTGDGYTF	1	CD8-C1
PB-2_TGAAAGACATTTGCCC	PB	PB-2	P2-3080	FALSE	1	TRA:CAVSEGYGNNRLAF TRB:CASSAPGSPLHF	1	CD8-C1
PB-2_TGAAAGAGTGGCAAAC	PB	PB-2	P2-3082	FALSE	1	TRA:CAVRFHASGGSYIPTF TRB:CASSQVVEDNQPQHF	1	CD8-C1
PB-2_TGAAAGATCCAGAAGG	PB	PB-2	P2-3083	FALSE	1	TRA:CLVGEGTSGSARQLTF TRB:CASSPLAGGRETQYF	1	CD8-C1
PB-2_TGACAACAGTCATGCT	PB	PB-2	P2-3086	FALSE	1	TRA:CAADNYGQNFVF TRB:CASGGQGIYEQYF	1	CD8-C1
PB-2_TGACAACCAATTGCTG	PB	PB-2	P2-3088	FALSE	1	TRA:CAVSSNDYKLSF TRB:CASSPQGDDSGANVLTF	1	CD8-C1
PB-2_TGACGGCCAATGTTGC	PB	PB-2	P2-3091	FALSE	1	TRA:CAVGGSNYKLTF TRB:CASSYSGQGLETQYF	1	CD8-C1
PB-2_TGACTAGGTTCGCTAA	PB	PB-2	P2-3108	FALSE	1	TRA:CAYRGETSGSRLTF TRB:CASSETGGGNTEAFF	1	CD8-C1
PB-2_TGACTAGTCGACAGCC	PB	PB-2	P2-3112	FALSE	1	TRA:CAVGARGNLIF TRB:CASSQVPGGRKETQYF	1	CD8-C1
PB-2_TGAGAGGGTGACGGTA	PB	PB-2	P2-3118	FALSE	1	TRA:CALNYGNNRLAF TRB:CASKGTYNEQFF	1	CD8-C1
PB-2_TGAGGGATCCGGCACA	PB	PB-2	P2-3134	FALSE	1	TRA:CAVPGYNFNKFYF TRB:CASSVGQGEYSGANVLTF	1	CD8-C1
PB-2_TGATTTCGTCTAAACC	PB	PB-2	P2-3137	FALSE	1	TRA:CAVNQAGTALIF TRB:CASSRTGDNYGYTF	1	CD8-C1
PB-2_TGCACCTCAGATGGCA	PB	PB-2	P2-3141	FALSE	1	TRA:CAMRENTGGFKTIF TRB:CASSQGTSGTGPGYTF	1	CD8-C1
PB-2_TGCCCATGTCTCACCT	PB	PB-2	P2-3155	FALSE	1	TRA:CAVFNSNFGNEKLTF TRB:CASNAVGTDTQYF	1	CD8-C1
PB-2_TGCCCATGTGACTCAT	PB	PB-2	P2-3156	FALSE	1	TRA:CAVSQTGNQFYF TRB:CASSLARDVTGELFF	1	CD8-C1
PB-2_TGCTGCTCAGACAAAT	PB	PB-2	P2-3191	FALSE	1	TRA:CAVDGSTLTF TRB:CASSRQGAIQETQYF	1	CD8-C1
PB-2_TGGCGCACAAACGCGA	PB	PB-2	P2-3204	FALSE	1	TRA:CILRQGNAGNMLTF TRB:CASSAWDRGEETQYF	1	CD4-C1
PB-2_TGGCTGGCATGGATGG	PB	PB-2	P2-3210	FALSE	1	TRA:CAVRELSGYSTLTF TRB:CASSFLAGGRETQYF	1	CD8-C1
PB-2_TGGCTGGTCTGAAAGA	PB	PB-2	P2-3215	FALSE	1	TRA:CAVPSPPHTGTASKLTF TRB:CASSTTGTGREYF	1	CD8-C1
PB-2_TGGGAAGTCAACACCA	PB	PB-2	P2-3222	FALSE	1	TRA:CATPTTSGTYKYIF TRB:CACGSTSYNEQFF	1	CD8-C1

PB-2_TGTATTCCAGTTTACG	PB	PB-2	P2-3238	FALSE	1	TRA:CGTVQGAQKLVF TRB:CASSRRQGEQYF	1	CD8-C2
PB-2_TGTATTCTCTGCGTAA	PB	PB-2	P2-3242	FALSE	1	TRA:CAVNEGYGGATNKLIF TRB:CASSLATSPLHF	1	CD8-C1
PB-2_TTAACTCCAATTGCTG	PB	PB-2	P2-3268	FALSE	1	TRA:CAVSGSYNTDKLIF TRB:CAISDEGDGYTF	1	CD8-C1
PB-2_TTAGGCACATTGCGGC	PB	PB-2	P2-3280	FALSE	1	TRA:CAVRDTSGFQKLVF TRB:CASGGDTDTQYF	1	CD8-C1
PB-2_TTAGTTCAGAAACCGC	PB	PB-2	P2-3282	FALSE	1	TRA:CAVLNAGNMLTF TRB:CASSQEGRDTQYF	1	CD8-C1
PB-2_TTCTCAACATCACGTA	PB	PB-2	P2-3319	FALSE	1	TRA:CVVIADSNYQLIW TRB:CASSVGSDYSYEQYF	1	CD8-C1
PB-2_TTGAACGCATGTAGTC	PB	PB-2	P2-3332	FALSE	1	TRA:CSNDYKLSF TRB:CASRLLLAGGPSTDTQYF	1	CD8-C1
PB-2_TTGCCGTAGTACGCGA	PB	PB-2	P2-3341	FALSE	1	TRA:CAGPNFGNEKLTF TRB:CASSVGDRAETQYF	1	CD8-C1
PB-2_TTGCCGTAGTGTTGAA	PB	PB-2	P2-3342	FALSE	1	TRA:CAVPGAGSYQLTF TRB:CATSRDHQADTQYF	1	CD8-C1
PB-2_TTGCGTCCATAGTAAG	PB	PB-2	P2-3352	FALSE	1	TRA:CVVSDDFNQAGTALIF TRB:CASSPSGFSSYEQYF	1	CD8-C1
PB-2_TTGCGTCTCATTGCCC	PB	PB-2	P2-3353	FALSE	1	TRA:CATDADSEPIMRF TRB:CASSISSYYRELFF	1	CD8-C1
PB-2_TTGGAACCAATTGCTG	PB	PB-2	P2-3355	FALSE	1	TRA:CAVGLNYGQNFVF TRB:CASRLQGSGELFF	1	CD8-C1
PB-2_TTGGCAAAGAACAACT	PB	PB-2	P2-3362	FALSE	1	TRA:CAGGSGNTGKLIF TRB:CARKGLAGSYEQYF	1	CD4-C1
PB-2_TTGGCAAAGTAATCCC	PB	PB-2	P2-3364	FALSE	1	TRA:CAPNSGNTPLVF TRB:CASSYSIQEYSTDTQYF	1	CD8-C1
PB-2_TTGTAGGTCCTCTAGC	PB	PB-2	P2-3375	FALSE	1	TRA:CAVRDQNAGNMLTF TRB:CSVAGQGREQYF	1	CD8-C1
PB-2_TTTGCGCAGTACCGGA	PB	PB-2	P2-3399	FALSE	1	TRA:CALSAYSGAGSYQLTF TRB:CASSLQERGEQYF	1	CD8-C1
PB-2_TTTGTCATCAATCACG	PB	PB-2	P2-3409	FALSE	1	TRA:CALSEDDYKLSF TRB:CASSFARGGTDTQYF	1	CD8-C1
PB-2_ACCGTAATCAGGCCCA	PB	PB-2	P2-5	TRUE	3	TRA:CASSFSGGYNKLIF TRB:CASSYLGAGYQETQYF	127	CD8-C11
PB-2_AGAGCTTCATGCTGGC	PB	PB-2	P2-4	TRUE	3	TRA:CAIRYQSFNFGNEKLTF TRB:CASYRRVTEQYF	148	CD8-C11
PB-2_AGTGTCACAGCTCCGA	PB	PB-2	P2-1077	FALSE	1	TRA:CGTESNAGGTSYGKLTF TRB:CASSLFLPPGYTIYF	1	CD8-C11
PB-2_CGGTTAAAGAAGCCCA	PB	PB-2	P2-5	TRUE	3	TRA:CASSFSGGYNKLIF TRB:CASSYLGAGYQETQYF	127	CD8-C11
PB-2_CTGCCTATCAGGCAAG	PB	PB-2	P2-1	TRUE	3	TRA:CVVNIIGYQKVTF TRB:CASTRQGKQPQHF	270	CD8-C11
PB-2_GATCGCGTCCTCATTA	PB	PB-2	P2-21	TRUE	3	TRA:CALSSYNNNDMRF TRB:CASSLDLAPSTEAFF	25	CD8-C5
PB-2_GCATACATCTTCAACT	PB	PB-2	P2-2312	FALSE	1	TRA:CAVSPMDTGRRALTF TRB:CASSRRTGGGYGYTF	1	CD8-C11
PB-3_AACTCTTCATATACGC	PB	PB-3	P3-505	FALSE	1	TRA:CAENGWGGSQGNLIF TRB:CASSLGGGEQYF	1	CD8-C6
PB-3_AAGGAGCAGATGCGAC	PB	PB-3	P3-74	TRUE	3	TRA:CALSQGGKLIF TRB:CASSLGTGRDDTQYF	5	CD8-C7
PB-3_ACATACGTCGAACTGT	PB	PB-3	P3-148	TRUE	3	TRA:CAVTPISSGSARQLTF TRB:CASSPGGGGYGYTF	3	CD8-C2

PB-3_ACGAGGAGTCATCGGC	PB	PB-3	P3-585	FALSE	1	TRA:CAESGGGANNLFF TRB:CASSYSIERAAFF	1	CD8-C6
PB-3_ACGATACTCTGGAGCC	PB	PB-3	P3-13	TRUE	3	TRA:CAASARGVRGAQKLVF TRB:CASSRTGFQPQHF	15	CD8-C5
PB-3_ACGCAGCGTAATCACC	PB	PB-3	P3-106	TRUE	3	TRA:CAVQTWNSGNTPLVF TRB:CASSPRLAGDNTGELFF	4	CD8-C5
PB-3_ACGGGCTTCACTGGGC	PB	PB-3	P3-19	FALSE	3	TRA:CALTSGGGADGLTF TRB:CASSQRGGTGLSGANVLTF	12	CD8-C7
PB-3_ACGGGTCAGAAACCGC	PB	PB-3	P3-241	FALSE	2	TRA:CAAKDYQLIW TRB:CASSEPGQETQYF	2	CD8-C7
PB-3_ACTGAACCAACTGGCC	PB	PB-3	P3-1	TRUE	3	TRA:CAVAPISSGSARQLTF TRB:CASSQGMSEAFF	83	CD8-C5
PB-3_ACTGAGTGTATGCTTG	PB	PB-3	P3-106	TRUE	3	TRA:CAVQTWNSGNTPLVF TRB:CASSPRLAGDNTGELFF	4	CD8-C7
PB-3_ACTGCTCAGTTAAGTG	PB	PB-3	P3-10	TRUE	3	TRA:CAGHQFSGGYNKLIF TRB:CASRASGTANEQFF	21	CD8-C7
PB-3_ACTTACTGTTTGGCGC	PB	PB-3	P3-618	FALSE	1	TRA:CAMRESYSGGGADGLTF TRB:CASRPSYQPQHF	1	CD8-C6
PB-3_AGAGCGACAACACCCG	PB	PB-3	P3-2	TRUE	3	TRA:CAASAPNSGGGADGLTF TRB:CSARRGSSNQPQHF	63	CD4-C6
PB-3_AGCATACGTACACCGC	PB	PB-3	P3-644	FALSE	1	TRA:CVVSEDNYGQNFVF TRB:CASSLSIDRNGNTIYF	1	CD8-C6
PB-3_AGCTCTCTCCTCAATT	PB	PB-3	P3-1	TRUE	3	TRA:CAVAPISSGSARQLTF TRB:CASSQGMSEAFF	83	CD8-C5
PB-3_AGCTTGAGTGAAGGCT	PB	PB-3	P3-59	FALSE	3	TRA:CLVGGNFNKFYF TRB:CSAYLGDRGITPLHF	6	CD8-C5
PB-3_AGGTCCGAGGTGGGTT	PB	PB-3	P3-30	TRUE	3	TRA:CVVSEASGTYKYIF TRB:CASSLNLRKNTEAFF	10	CD8-C5
PB-3_AGTCTTTGTGATGATA	PB	PB-3	P3-38	FALSE	3	TRA:CALNSGGSNYKLTF TRB:CASSRTGFQPQHF	8	CD8-C7
PB-3_ATGAGGGCAGTCTTCC	PB	PB-3	P3-13	TRUE	3	TRA:CAASARGVRGAQKLVF TRB:CASSRTGFQPQHF	15	CD8-C7
PB-3_ATGCGATCAGGAATGC	PB	PB-3	P3-13	TRUE	3	TRA:CAASARGVRGAQKLVF TRB:CASSRTGFQPQHF	15	CD8-C7
PB-3_ATGGGAGCATGGTCTA	PB	PB-3	P3-729	FALSE	1	TRA:CVVNMGTGNQFYF TRB:CATLGGAETQYF	1	CD8-C6
PB-3_ATTTCTGAGCCCGAAA	PB	PB-3	P3-19	FALSE	3	TRA:CALTSGGGADGLTF TRB:CASSQRGGTGLSGANVLTF	12	CD8-C5
PB-3_CAACCTCAGTGCGTGA	PB	PB-3	P3-750	FALSE	1	TRA:CAFMKPVGGAQKLVF TRB:CASRSRRDRNTDTQYF	1	CD8-C7
PB-3_CAACTAGGTCATACTG	PB	PB-3	P3-753	FALSE	1	TRA:CAEGNQGGKLIF TRB:CASSLAGLGAKDTQYF	1	CD8-C6
PB-3_CCACGGACACCAGTTA	PB	PB-3	P3-867	FALSE	1	TRA:CVAPRGTGKLIF TRB:CASSAGAAEAFF	1	CD8-C5
PB-3_CCGGGATCAGGGAGAG	PB	PB-3	P3-17	TRUE	3	TRA:CAVRVRNARLMF TRB:CSVEGPRGDSGNTIYF	14	CD8-C7
PB-3_CCGGGATGTTTAGGAA	PB	PB-3	P3-883	FALSE	1	TRA:CALERNARLMF TRB:CASTPMSGGITDTQYF	1	CD8-C7
PB-3_CCGTGGATCACTTACT	PB	PB-3	P3-85	FALSE	3	TRA:CAVGDQAGTALIF TRB:CASSQNTEAFF	5	CD8-C7
PB-3_CCTACCATCACTATTC	PB	PB-3	P3-38	FALSE	3	TRA:CALNSGGSNYKLTF TRB:CASSRTGFQPQHF	8	CD8-C5
PB-3_CCTCTGAAGAAGATTC	PB	PB-3	P3-1	TRUE	3	TRA:CAVAPISSGSARQLTF TRB:CASSQGMSEAFF	83	CD8-C7

PB-3_CGAACATCACCTTGTC	PB	PB-3	P3-167	FALSE	3	TRA:CAAGFSNSNSGYALNF TRB:CASSQNTEAFF	3	CD8-C7
PB-3_CGAGCACAGAGCCCAA	PB	PB-3	P3-13	TRUE	3	TRA:CAASARGVRGAQKLVF TRB:CASSRTGFQPQHF	15	CD8-C7
PB-3_CGCGGTAAGCCTTGAT	PB	PB-3	P3-59	FALSE	3	TRA:CLVGGNFNKFYF TRB:CSAYLGDRGITPLHF	6	CD8-C7
PB-3_CGCGGTATCGGAAATA	PB	PB-3	P3-951	FALSE	1	TRA:CAASVGTYKYIF TRB:CASSLEGSAYEQYF	1	CD8-C7
PB-3_CGCGGTATCTCAACTT	PB	PB-3	P3-1	TRUE	3	TRA:CAVAPISSGSARQLTF TRB:CASSQGMSEAFF	83	CD8-C7
PB-3_CGCTTCAGTGGCCCTA	PB	PB-3	P3-1	TRUE	3	TRA:CAVAPISSGSARQLTF TRB:CASSQGMSEAFF	83	CD8-C5
PB-3_CGGACACAGTCGTACT	PB	PB-3	P3-59	FALSE	3	TRA:CLVGGNFNKFYF TRB:CSAYLGDRGITPLHF	6	CD8-C5
PB-3_CGTCTACAGAAACGAG	PB	PB-3	P3-994	FALSE	1	TRA:CAVNRGSGTYKYIF TRB:CASSLAGGPFYEQYF	1	CD8-C5
PB-3_CGTGTAACACTTCGAA	PB	PB-3	P3-1004	FALSE	1	TRA:CAVRDPNSGNTPLVF TRB:CASNGPGTNEKLFF	1	CD8-C6
PB-3_CGTGTCTTCTTGAGAC	PB	PB-3	P3-1008	FALSE	1	TRA:CAESSRDSSYKLIF TRB:CSARRGSSNQPQHF	1	CD4-C6
PB-3_CTACACCAGGGTCTCC	PB	PB-3	P3-124	TRUE	3	TRA:CALRLYRGGFKTIF TRB:CASRGGYEQYF	4	CD8-C5
PB-3_CTAGCCTAGTTAACGA	PB	PB-3	P3-280	FALSE	2	TRA:CAVISNFGNEKLTF TRB:CASSFFDNSNQPQHF	2	CD8-C7
PB-3_CTCGAAAAGCCCAATT	PB	PB-3	P3-58	FALSE	3	TRA:CALTRRLGDTDKLIF TRB:CASSKGVLEKLFF	6	CD8-C7
PB-3_CTCGAAAAGGCCATAG	PB	PB-3	P3-85	FALSE	3	TRA:CAVGDQAGTALIF TRB:CASSQNTEAFF	5	CD8-C7
PB-3_CTCGAGGAGTGGGTTG	PB	PB-3	P3-85	FALSE	3	TRA:CAVGDQAGTALIF TRB:CASSQNTEAFF	5	CD8-C7
PB-3_CTCGGAGAGTGGGATC	PB	PB-3	P3-19	FALSE	3	TRA:CALTSGGGADGLTF TRB:CASSQRGGTGLSGANVLTF	12	CD8-C5
PB-3_CTCGTACGTCTTGATG	PB	PB-3	P3-124	TRUE	3	TRA:CALRLYRGGFKTIF TRB:CASRGGYEQYF	4	CD8-C5
PB-3_CTGATAGCACGCTTTC	PB	PB-3	P3-1087	FALSE	1	TRA:CAVERGAGNMLTF TRB:CASSRGGASNTIYF	1	CD8-C7
PB-3_CTTTGCGTCGGATGGA	PB	PB-3	P3-288	FALSE	2	TRA:CAVGIQGAQKLVF TRB:CASSQDIGGEQFF	2	CD8-C7
PB-3_GAATGAAAGGCGTACA	PB	PB-3	P3-17	TRUE	3	TRA:CAVRVRNARLMF TRB:CSVEGPRGDSGNTIYF	14	CD8-C7
PB-3_GACAGAGAGTGTTTGC	PB	PB-3	P3-1	TRUE	3	TRA:CAVAPISSGSARQLTF TRB:CASSQGMSEAFF	83	CD8-C7
PB-3_GACCAATTCCTCGCAT	PB	PB-3	P3-19	FALSE	3	TRA:CALTSGGGADGLTF TRB:CASSQRGGTGLSGANVLTF	12	CD8-C5
PB-3_GACCTGGGTAAATGTG	PB	PB-3	P3-1143	FALSE	1	TRA:CAMRGLVDTNAGKSTF TRB:CASSNTGLETQYF	1	CD8-C7
PB-3_GAGGTGAAGTCTCGGC	PB	PB-3	P3-18	TRUE	3	TRA:CLVGVGNNDMRF TRB:CASSPGTGKYEQYF	12	CD8-C5
PB-3_GATTCAGAGACAGGCT	PB	PB-3	P3-17	TRUE	3	TRA:CAVRVRNARLMF TRB:CSVEGPRGDSGNTIYF	14	CD8-C2
PB-3_GATTCAGCAAACTGCT	PB	PB-3	P3-1184	FALSE	1	TRA:CAVRDSNYQLIW TRB:CSARGVGLNEKLFF	1	CD8-C6
PB-3_GCACATACAGTCGATT	PB	PB-3	P3-1189	FALSE	1	TRA:CIVRVANSNSGYALNF TRB:CSARPLNPGNGYTF	1	CD8-C7

PB-3_GCACTCTGTTTAAGCC	PB	PB-3	P3-19	FALSE	3	TRA:CALTSGGGADGLTF TRB:CASSQRGGTGLSGANVLTF	12	CD8-C7
PB-3_GCAGCCACATTGCGGC	PB	PB-3	P3-10	TRUE	3	TRA:CAGHQFSGGYNKLIF TRB:CASRASGTANEQFF	21	CD8-C5
PB-3_GCGACCAGTGTGCGTC	PB	PB-3	P3-1212	FALSE	1	TRA:CAVSDRLSNMLTF TRB:CASSEWEETQYF	1	CD8-C7
PB-3_GCTGGGTGTCGGCACT	PB	PB-3	P3-10	TRUE	3	TRA:CAGHQFSGGYNKLIF TRB:CASRASGTANEQFF	21	CD8-C5
PB-3_GGAAAGCAGCCCTAAT	PB	PB-3	P3-59	FALSE	3	TRA:CLVGGNFNKFYF TRB:CSAYLGDRGITPLHF	6	CD8-C7
PB-3_GGACAAGAGATAGGAG	PB	PB-3	P3-1249	FALSE	1	TRA:CAYSGYGGSQGNLIF TRB:CARPRSGGADTQYF	1	CD8-C6
PB-3_GGACAAGCATCCTTGC	PB	PB-3	P3-167	FALSE	3	TRA:CAAGFSNSNSGYALNF TRB:CASSQNTEAFF	3	CD8-C7
PB-3_GGACGTCTCTTTAGGG	PB	PB-3	P3-19	FALSE	3	TRA:CALTSGGGADGLTF TRB:CASSQRGGTGLSGANVLTF	12	CD8-C7
PB-3_GGGAATGAGAGGGCTT	PB	PB-3	P3-1279	FALSE	1	TRA:CAVGFPDSNYQLIW TRB:CSVEVGGSAYEQYF	1	CD8-C7
PB-3_GGGTCTGGTGAGGCTA	PB	PB-3	P3-13	TRUE	3	TRA:CAASARGVRGAQKLVF TRB:CASSRTGFQPQHF	15	CD8-C7
PB-3_GGTGTTAAGCCGTCGT	PB	PB-3	P3-285	FALSE	2	TRA:CAGKPAGYNKLIF TRB:CASSLGGYTEAFF	2	CD8-C7
PB-3_GTAACGTGTTAAGGGC	PB	PB-3	P3-1309	FALSE	1	TRA:CALSDLEAAGNKLTF TRB:CASSPWDTGELFF	1	CD8-C6
PB-3_GTAACTGCATTTGCCC	PB	PB-3	P3-1310	FALSE	1	TRA:CAPNTGNQFYF TRB:CASSLVGEFNGYTF	1	CD8-C7
PB-3_GTATTCTAGTACGTTC	PB	PB-3	P3-18	TRUE	3	TRA:CLVGVGNNDMRF TRB:CASSPGTGKYEQYF	12	CD8-C5
PB-3_GTGCGGTTCTCTAAGG	PB	PB-3	P3-1371	FALSE	1	TRA:CAVRSASSNTGKLIF TRB:CASSGTGGQPQHF	1	CD8-C6
PB-3_GTTCATTGTGATAAAC	PB	PB-3	P3-185	TRUE	3	TRA:CVVNMDEGSQGNLIF TRB:CASSWGVPPRGYTF	3	CD8-C5
PB-3_TAAGAGAGTCTGCAAT	PB	PB-3	P3-13	TRUE	3	TRA:CAASARGVRGAQKLVF TRB:CASSRTGFQPQHF	15	CD8-C5
PB-3_TAAGCGTAGGCTAGGT	PB	PB-3	P3-1405	FALSE	1	TRA:CAVNGGANNLFF TRB:CASSDRGIRSGANVLTF	1	CD8-C5
PB-3_TACCTTAGTCTAACGT	PB	PB-3	P3-74	TRUE	3	TRA:CALSQGGKLIF TRB:CASSLGTGRDDTQYF	5	CD8-C7
PB-3_TACTCGCGTCATGCAT	PB	PB-3	P3-250	FALSE	2	TRA:CVVNKLGGTYKYIF TRB:CASSPQGASSYNEQFF	2	CD8-C2
PB-3_TAGCCGGAGTGGTAGC	PB	PB-3	P3-74	TRUE	3	TRA:CALSQGGKLIF TRB:CASSLGTGRDDTQYF	5	CD8-C7
PB-3_TCGCGTTCAGGTGGAT	PB	PB-3	P3-169	FALSE	3	TRA:CAGTKGAQKLVF TRB:CASSPMNTEAFF	3	CD8-C7
PB-3_TCTCTAAGTTCTCATT	PB	PB-3	P3-19	FALSE	3	TRA:CALTSGGGADGLTF TRB:CASSQRGGTGLSGANVLTF	12	CD8-C7
PB-3_TCTGAGAAGAGGACGG	PB	PB-3	P3-318	TRUE	2	TRA:CATLDNTNAGKSTF TRB:CASSLSSWGAAGYTF	2	CD8-C2
PB-3_TCTTTCCGTGTTTGGT	PB	PB-3	P3-317	FALSE	2	TRA:CARPGAGGYQKVTF TRB:CASSSRPNTEAFF	2	CD8-C6
PB-3_TGAAAGATCCTCAACC	PB	PB-3	P3-38	FALSE	3	TRA:CALNSGGSNYKLTF TRB:CASSRTGFQPQHF	8	CD8-C7
PB-3_TGATTTCTCGGGAGTA	PB	PB-3	P3-1	TRUE	3	TRA:CAVAPISSGSARQLTF TRB:CASSQGMSEAFF	83	CD8-C7

PB-3_TGCGGGTCAAGCTGGA	PB	PB-3	P3-167	FALSE	3	TRA:CAAGFSNSNSGYALNF TRB:CASSQNTEAFF	3	CD8-C7
PB-3_TGCGGGTGTGACGGTA	PB	PB-3	P3-58	FALSE	3	TRA:CALTRRLGDTDKLIF TRB:CASSKGVLEKLFF	6	CD8-C5
PB-3_TGGCCAGAGCATGGCA	PB	PB-3	P3-19	FALSE	3	TRA:CALTSGGGADGLTF TRB:CASSQRGGTGLSGANVLTF	12	CD8-C7
PB-3_TGTTCCGCACAGAGGT	PB	PB-3	P3-280	FALSE	2	TRA:CAVISNFGNEKLTF TRB:CASSFFDNSNQPQHF	2	CD8-C7
PB-3_TTCGGTCAGTTAGCGG	PB	PB-3	P3-59	FALSE	3	TRA:CLVGGNFNKFYF TRB:CSAYLGDRGITPLHF	6	CD8-C7
PB-3_TTCTCCTGTTTACTCT	PB	PB-3	P3-74	TRUE	3	TRA:CALSQGGKLIF TRB:CASSLGTGRDDTQYF	5	CD8-C7
PB-3_AAAGTAGCAGCGTTCG	PB	PB-3	P3-1	TRUE	3	TRA:CAVAPISSGSARQLTF TRB:CASSQGMSEAFF	83	CD8-C5
PB-3_AACACGTAGCTGTCTA	PB	PB-3	P3-230	FALSE	2	TRA:CAASAPNFGNEKLTF TRB:CASSYRGGQGTEAFF	2	CD8-C4
PB-3_AACCATGGTACCGTAT	PB	PB-3	P3-1	TRUE	3	TRA:CAVAPISSGSARQLTF TRB:CASSQGMSEAFF	83	CD8-C5
PB-3_AACTCTTAGGTTACCT	PB	PB-3	P3-18	TRUE	3	TRA:CLVGVGNNDMRF TRB:CASSPGTGKYEQYF	12	CD8-C5
PB-3_AAGCCGCAGCGATCCC	PB	PB-3	P3-520	FALSE	1	TRA:CAVFGPENNNAGNMLTF TRB:CASSDDRGPALAKNIQYF	1	CD8-C2
PB-3_AAGCCGCAGTCGTTTG	PB	PB-3	P3-1	TRUE	3	TRA:CAVAPISSGSARQLTF TRB:CASSQGMSEAFF	83	CD8-C5
PB-3_AAGCCGCCACATTCGA	PB	PB-3	P3-19	FALSE	3	TRA:CALTSGGGADGLTF TRB:CASSQRGGTGLSGANVLTF	12	CD8-C5
PB-3_AAGCCGCGTGAGTGAC	PB	PB-3	P3-232	FALSE	2	TRA:CALSFSGYSTLTF TRB:CASSLGGRELFF	2	CD8-C4
PB-3_AAGGTTCTCTCTGTCG	PB	PB-3	P3-13	TRUE	3	TRA:CAASARGVRGAQKLVF TRB:CASSRTGFQPQHF	15	CD8-C5
PB-3_AAGTCTGTCGTAGGAG	PB	PB-3	P3-16	TRUE	3	TRA:CAASRNNNDMRF TRB:CASTNYQETQYF	14	CD8-C4
PB-3_AATCGGTCACGCCAGT	PB	PB-3	P3-542	FALSE	1	TRA:CAGAFNDYKLSF TRB:CASSSGTSSYNEQFF	1	CD8-C4
PB-3_AATCGGTTCAAGGTAA	PB	PB-3	P3-103	TRUE	3	TRA:CARDNTGKLIF TRB:CASSPGQGFTEAFF	4	CD8-C5
PB-3_ACACTGAAGACGACGT	PB	PB-3	P3-47	TRUE	3	TRA:CAVRDSVRVGSARQLTF TRB:CASSASTGANSPLHF	7	CD8-C5
PB-3_ACACTGATCGCTTGTC	PB	PB-3	P3-38	FALSE	3	TRA:CALNSGGSNYKLTF TRB:CASSRTGFQPQHF	8	CD8-C5
PB-3_ACAGCCGTCCGCGCAA	PB	PB-3	P3-16	TRUE	3	TRA:CAASRNNNDMRF TRB:CASTNYQETQYF	14	CD8-C4
PB-3_ACAGCTAAGCCAGAAC	PB	PB-3	P3-235	TRUE	2	TRA:CVALNTGNQFYF TRB:CATQGQGGLSGELFF	2	CD8-C4
PB-3_ACATGGTAGGCGATAC	PB	PB-3	P3-150	TRUE	3	TRA:CVVNRGYGGSQGNLIF TRB:CASSLRGNNQPQHF	3	CD8-C5
PB-3_ACCGTAAGTTTGTGTG	PB	PB-3	P3-1	TRUE	3	TRA:CAVAPISSGSARQLTF TRB:CASSQGMSEAFF	83	CD8-C4
PB-3_ACGAGCCTCATACGGT	PB	PB-3	P3-1	TRUE	3	TRA:CAVAPISSGSARQLTF TRB:CASSQGMSEAFF	83	CD8-C5
PB-3_ACGCCAGGTCTTTCAT	PB	PB-3	P3-240	TRUE	2	TRA:CALYYGNNRLAF TRB:CASSVGQGQGYEQYF	2	CD8-C4
PB-3_ACGGAGAAGCTAGTCT	PB	PB-3	P3-5	TRUE	3	TRA:CALRNSNYQLIW TRB:CASSLGDRAYNEQFF	38	CD8-C5

PB-3_ACGGAGAAGTACGATA	PB	PB-3	P3-1	TRUE	3	TRA:CAVAPISSGSARQLTF TRB:CASSQGMSEAFF	83	CD8-C4
PB-3_ACGGAGATCGATGAGG	PB	PB-3	P3-8	TRUE	3	TRA:CAFDRGSTLGRLYF TRB:CASNPRQGTSTYEQYF	26	CD8-C4
PB-3_ACGGCCATCTCTAGGA	PB	PB-3	P3-108	TRUE	3	TRA:CILRDPLKDGQKLLF TRB:CASSPSDSGANVLTF	4	CD8-C5
PB-3_ACGTCAAAGACGCTTT	PB	PB-3	P3-58	FALSE	3	TRA:CALTRRLGDTDKLIF TRB:CASSKGVLEKLFF	6	CD8-C5
PB-3_ACGTCAAAGTCAAGGC	PB	PB-3	P3-10	TRUE	3	TRA:CAGHQFSGGYNKLIF TRB:CASRASGTANEQFF	21	CD8-C5
PB-3_ACTATCTCACTACAGT	PB	PB-3	P3-10	TRUE	3	TRA:CAGHQFSGGYNKLIF TRB:CASRASGTANEQFF	21	CD8-C5
PB-3_ACTATCTCAGGTGGAT	PB	PB-3	P3-8	TRUE	3	TRA:CAFDRGSTLGRLYF TRB:CASNPRQGTSTYEQYF	26	CD8-C5
PB-3_ACTATCTGTACTCTCC	PB	PB-3	P3-39	TRUE	3	TRA:CAVRDIGGSYIPTF TRB:CASSLGGPEETQYF	8	CD8-C4
PB-3_ACTGAACAGGCAAAGA	PB	PB-3	P3-8	TRUE	3	TRA:CAFDRGSTLGRLYF TRB:CASNPRQGTSTYEQYF	26	CD8-C4
PB-3_ACTGAGTAGCATCATC	PB	PB-3	P3-606	FALSE	1	TRA:CAVRALYNQGGKLIF TRB:CASSQGVFAGELFF	1	CD8-C5
PB-3_ACTGATGGTTACCAGT	PB	PB-3	P3-609	FALSE	1	TRA:CVVSHLFSGGYNKLIF TRB:CASSLDLTGGMEAFF	1	CD8-C5
PB-3_ACTGTCCCATCCGTGG	PB	PB-3	P3-8	TRUE	3	TRA:CAFDRGSTLGRLYF TRB:CASNPRQGTSTYEQYF	26	CD8-C4
PB-3_ACTGTCCTCTGCCAGG	PB	PB-3	P3-1	TRUE	3	TRA:CAVAPISSGSARQLTF TRB:CASSQGMSEAFF	83	CD8-C5
PB-3_ACTTACTTCCCTCAGT	PB	PB-3	P3-1	TRUE	3	TRA:CAVAPISSGSARQLTF TRB:CASSQGMSEAFF	83	CD8-C5
PB-3_ACTTACTTCCGAACGC	PB	PB-3	P3-619	FALSE	1	TRA:CAVSDQGSSNTGKLIF TRB:CASSPARSGAFF	1	CD8-C5
PB-3_AGAGTGGGTTTGTTTC	PB	PB-3	P3-1	TRUE	3	TRA:CAVAPISSGSARQLTF TRB:CASSQGMSEAFF	83	CD8-C5
PB-3_AGATTGCCAAGTTGTC	PB	PB-3	P3-636	FALSE	1	TRA:CAASYFGNEKLTF TRB:CASAASSGGNEQFF	1	CD8-C5
PB-3_AGCAGCCCACACCGCA	PB	PB-3	P3-16	TRUE	3	TRA:CAASRNNNDMRF TRB:CASTNYQETQYF	14	CD8-C4
PB-3_AGCGTATCATAGAAAC	PB	PB-3	P3-1	TRUE	3	TRA:CAVAPISSGSARQLTF TRB:CASSQGMSEAFF	83	CD8-C5
PB-3_AGCGTATGTACTTCTT	PB	PB-3	P3-654	FALSE	1	TRA:CAFMKHVASGGSNYKLTF TRB:CAWKQAGNTIYF	1	CD4-C3
PB-3_AGCTTGACATTATCTC	PB	PB-3	P3-111	TRUE	3	TRA:CALSEARTGNQFYF TRB:CASSYGPGHATQYF	4	CD8-C5
PB-3_AGGGATGGTGAAATCA	PB	PB-3	P3-82	TRUE	3	TRA:CAVYLDRGSTLGRLYF TRB:CASSFGSGPNYGYTF	5	CD8-C4
PB-3_AGTGAGGTCTCCGGTT	PB	PB-3	P3-692	FALSE	1	TRA:CAELKGNNRLAF TRB:CASSDPTGEDETQYF	1	CD8-C5
PB-3_AGTTGGTGTAAATACG	PB	PB-3	P3-48	TRUE	3	TRA:CAYDGATNKLIF TRB:CASSLYGHQPQHF	7	CD8-C5
PB-3_ATAAGAGGTGGACGAT	PB	PB-3	P3-48	TRUE	3	TRA:CAYDGATNKLIF TRB:CASSLYGHQPQHF	7	CD8-C5
PB-3_ATCATCTCACGTGAGA	PB	PB-3	P3-12	TRUE	3	TRA:CAVGDLGNEKLTF TRB:CASSAEGYYNEQFF	16	CD8-C5
PB-3_ATCATCTGTTATTCTC	PB	PB-3	P3-5	TRUE	3	TRA:CALRNSNYQLIW TRB:CASSLGDRAYNEQFF	38	CD8-C5

PB-3_ATCATCTTCTTGACGA	PB	PB-3	P3-1	TRUE	3	TRA:CAVAPISSGSARQLTF TRB:CASSQGMSEAFF	83	CD8-C5
PB-3_ATCTACTAGTTACGGG	PB	PB-3	P3-250	FALSE	2	TRA:CVVNKLGGTYKYIF TRB:CASSPQGASSYNEQFF	2	CD8-C2
PB-3_ATGTGTGAGGAATGGA	PB	PB-3	P3-113	TRUE	3	TRA:CAVSDYAGGTSYGKLTF TRB:CASSVGGGVREQYF	4	CD8-C4
PB-3_ATTACTCCAAGCCGCT	PB	PB-3	P3-3	TRUE	3	TRA:CVVPWMDSSYKLIF TRB:CASSQRAGDGNYGYTF	42	CD8-C5
PB-3_ATTGGACGTCGGCTCA	PB	PB-3	P3-745	FALSE	1	TRA:CAAPRVGARLMF TRB:CASSLSWVEGGGAKGDTQYF	1	CD8-C5
PB-3_ATTTCTGAGACGCACA	PB	PB-3	P3-1	TRUE	3	TRA:CAVAPISSGSARQLTF TRB:CASSQGMSEAFF	83	CD8-C5
PB-3_CAAGATCGTATATGGA	PB	PB-3	P3-161	TRUE	3	TRA:CAFMKPGNTGNQFYF TRB:CASSPGPIGTVNYGYTF	3	CD8-C5
PB-3_CACAAACAGATCTGCT	PB	PB-3	P3-10	TRUE	3	TRA:CAGHQFSGGYNKLIF TRB:CASRASGTANEQFF	21	CD8-C5
PB-3_CACACAATCTGCAAGT	PB	PB-3	P3-6	TRUE	3	TRA:CAVRGANNLFF TRB:CASSPGTGYETQYF	29	CD8-C4
PB-3_CACACTCCATATACGC	PB	PB-3	P3-9	TRUE	3	TRA:CAVRDMDTGRRALTF TRB:CASSWDTRQAEAFF	24	CD8-C4
PB-3_CACAGTACACGTGAGA	PB	PB-3	P3-10	TRUE	3	TRA:CAGHQFSGGYNKLIF TRB:CASRASGTANEQFF	21	CD8-C5
PB-3_CACCAGGCAGGAATGC	PB	PB-3	P3-790	FALSE	1	TRA:CAVSRAGNNRKLIW TRB:CASSLRAGEPDTQYF	1	CD8-C5
PB-3_CACTCCAAGGAATTAC	PB	PB-3	P3-14	TRUE	3	TRA:CALPFSGGYNKLIF TRB:CSARKTEPRSYEQYF	15	CD8-C5
PB-3_CAGATCAGTACATGTC	PB	PB-3	P3-258	TRUE	2	TRA:CAVSGDSNYQLIW TRB:CASSLATGPYNEQFF	2	CD8-C5
PB-3_CAGATCAGTTATCCGA	PB	PB-3	P3-10	TRUE	3	TRA:CAGHQFSGGYNKLIF TRB:CASRASGTANEQFF	21	CD8-C4
PB-3_CAGCGACAGCTGCGAA	PB	PB-3	P3-29	TRUE	3	TRA:CAVSGGNQGAQKLVF TRB:CASSLGDPRGKKLFF	10	CD8-C2
PB-3_CAGCGACCACCCAGTG	PB	PB-3	P3-259	TRUE	2	TRA:CAPGGSNYKLTF TRB:CASSLAQQAALNEQFF	2	CD8-C4
PB-3_CAGCGACCAGCTGTTA	PB	PB-3	P3-809	FALSE	1	TRA:CAASGNGGNQFYF TRB:CASSASSYEQYF	1	CD8-C5
PB-3_CAGCGACGTCATATGC	PB	PB-3	P3-61	TRUE	3	TRA:CALIRVDSSYKLIF TRB:CASRLGGGYTF	6	CD8-C7
PB-3_CAGCTAACAGCGTAAG	PB	PB-3	P3-20	TRUE	3	TRA:CAVSSLQMDSSYKLIF TRB:CASSHRTENSPLHF	12	CD8-C5
PB-3_CAGGTGCGTCTAGTGT	PB	PB-3	P3-1	TRUE	3	TRA:CAVAPISSGSARQLTF TRB:CASSQGMSEAFF	83	CD8-C5
PB-3_CATATGGAGAGGGATA	PB	PB-3	P3-1	TRUE	3	TRA:CAVAPISSGSARQLTF TRB:CASSQGMSEAFF	83	CD8-C5
PB-3_CATATTCTCATGCATG	PB	PB-3	P3-164	TRUE	3	TRA:CALILTGGGNKLTF TRB:CASSWDTRQAEAFF	3	CD8-C4
PB-3_CATCAAGGTAGCTAAA	PB	PB-3	P3-12	TRUE	3	TRA:CAVGDLGNEKLTF TRB:CASSAEGYYNEQFF	16	CD8-C5
PB-3_CATCGAAGTTAAAGTG	PB	PB-3	P3-83	TRUE	3	TRA:CALSDNAGNNRKLIW TRB:CSVGGGLNQPQHF	5	CD8-C5
PB-3_CATCGGGTCCAGGGCT	PB	PB-3	P3-42	TRUE	3	TRA:CALGGSTLGRLYF TRB:CASSASTGANSPLHF	8	CD8-C5
PB-3_CATGCCTAGCAGGTCA	PB	PB-3	P3-58	FALSE	3	TRA:CALTRRLGDTDKLIF TRB:CASSKGVLEKLFF	6	CD8-C4

PB-3_CATGGCGGTAAGAGAG	PB	PB-3	P3-1	TRUE	3	TRA:CAVAPISSGSARQLTF TRB:CASSQGMSEAFF	83	CD8-C4
PB-3_CCCAATCGTGATGCCC	PB	PB-3	P3-875	FALSE	1	TRA:CAIGGYQKVTF TRB:CASSDKYNSPLHF	1	CD8-C5
PB-3_CCGTACTGTGAGGGTT	PB	PB-3	P3-1	TRUE	3	TRA:CAVAPISSGSARQLTF TRB:CASSQGMSEAFF	83	CD8-C5
PB-3_CCGTTCATCCTGTACC	PB	PB-3	P3-1	TRUE	3	TRA:CAVAPISSGSARQLTF TRB:CASSQGMSEAFF	83	CD8-C5
PB-3_CCTTACGCACAGATTC	PB	PB-3	P3-909	FALSE	1	TRA:CAVTLAGNMLTF TRB:CASSPGLGGGELFF	1	CD8-C5
PB-3_CCTTCGACAGAAGCAC	PB	PB-3	P3-86	TRUE	3	TRA:CALTNRDDKIIF TRB:CATSRDMGGSYEQYF	5	CD8-C4
PB-3_CGAGCACAGCGGCTTC	PB	PB-3	P3-10	TRUE	3	TRA:CAGHQFSGGYNKLIF TRB:CASRASGTANEQFF	21	CD8-C5
PB-3_CGAGCCATCACTATTC	PB	PB-3	P3-1	TRUE	3	TRA:CAVAPISSGSARQLTF TRB:CASSQGMSEAFF	83	CD8-C5
PB-3_CGATGGCCATCTGGTA	PB	PB-3	P3-939	FALSE	1	TRA:CAVGDQAGTALIF TRB:CASTGGGAQETQYF	1	CD8-C5
PB-3_CGATTGAGTAGAAAGG	PB	PB-3	P3-11	TRUE	3	TRA:CASGGGNKLTF TRB:CASSSDGDEAFF	17	CD8-C5
PB-3_CGCGGTAGTCACCTAA	PB	PB-3	P3-62	TRUE	3	TRA:CAEIPSGAGSYQLTF TRB:CASNGDENTIYF	6	CD8-C5
PB-3_CGCGTTTGTCCAGTAT	PB	PB-3	P3-1	TRUE	3	TRA:CAVAPISSGSARQLTF TRB:CASSQGMSEAFF	83	CD8-C5
PB-3_CGCTTCAAGTTTGCGT	PB	PB-3	P3-271	TRUE	2	TRA:CAQAGGGADGLTF TRB:CATSRGTVSYEQYF	2	CD8-C7
PB-3_CGGACTGTCACCCTCA	PB	PB-3	P3-20	TRUE	3	TRA:CAVSSLQMDSSYKLIF TRB:CASSHRTENSPLHF	12	CD8-C5
PB-3_CGGAGCTAGTGCGATG	PB	PB-3	P3-275	TRUE	2	TRA:CVVIDSGYALNF TRB:CASSQQGFYGYTF	2	CD8-C5
PB-3_CGGCTAGAGCTAGCCC	PB	PB-3	P3-88	TRUE	3	TRA:CAARRGMGGATNKLIF TRB:CASSLTGGTGNTIYF	5	CD8-C5
PB-3_CGGGTCAGTCATGCAT	PB	PB-3	P3-232	FALSE	2	TRA:CALSFSGYSTLTF TRB:CASSLGGRELFF	2	CD8-C4
PB-3_CGTAGGCGTGGTCTCG	PB	PB-3	P3-3	TRUE	3	TRA:CVVPWMDSSYKLIF TRB:CASSQRAGDGNYGYTF	42	CD8-C5
PB-3_CGTAGGCTCCGCTGTT	PB	PB-3	P3-983	FALSE	1	TRA:CIVRVDSGYALNF TRB:CASSQSVGASYNEQFF	1	CD8-C5
PB-3_CGTCACTCAGATCGGA	PB	PB-3	P3-3	TRUE	3	TRA:CVVPWMDSSYKLIF TRB:CASSQRAGDGNYGYTF	42	CD8-C5
PB-3_CGTCCATAGATCACGG	PB	PB-3	P3-989	FALSE	1	TRA:CAGSNSGGSNYKLTF TRB:CASSFIAGGADTQYF	1	CD8-C5
PB-3_CGTCCATAGCCAGTAG	PB	PB-3	P3-990	FALSE	1	TRA:CAESSTGFQKLVF TRB:CASSQRTSNEQFF	1	CD8-C5
PB-3_CGTCCATCAGGTGGAT	PB	PB-3	P3-1	TRUE	3	TRA:CAVAPISSGSARQLTF TRB:CASSQGMSEAFF	83	CD8-C5
PB-3_CGTCTACTCCAAAGTC	PB	PB-3	P3-170	TRUE	3	TRA:CAVDMWLEGGGADGLTF TRB:CASSLRGAIEQFF	3	CD8-C5
PB-3_CGTGAGCTCTTGCATT	PB	PB-3	P3-8	TRUE	3	TRA:CAFDRGSTLGRLYF TRB:CASNPRQGTSTYEQYF	26	CD8-C4
PB-3_CGTGTAAAGTCCGTAT	PB	PB-3	P3-1	TRUE	3	TRA:CAVAPISSGSARQLTF TRB:CASSQGMSEAFF	83	CD8-C5
PB-3_CGTGTAAGTCTTCGTC	PB	PB-3	P3-1	TRUE	3	TRA:CAVAPISSGSARQLTF TRB:CASSQGMSEAFF	83	CD8-C5

PB-3_CGTTGGGGTATCACCA	PB	PB-3	P3-1013	FALSE	1	TRA:CAVSQMYTDKLIF TRB:CASSYNPSLSGNTIYF	1	CD8-C4
PB-3_CGTTGGGTCCGTCATC	PB	PB-3	P3-10	TRUE	3	TRA:CAGHQFSGGYNKLIF TRB:CASRASGTANEQFF	21	CD8-C5
PB-3_CTAAGACCACGGTGTC	PB	PB-3	P3-12	TRUE	3	TRA:CAVGDLGNEKLTF TRB:CASSAEGYYNEQFF	16	CD8-C4
PB-3_CTACCCAAGTGCGTGA	PB	PB-3	P3-1	TRUE	3	TRA:CAVAPISSGSARQLTF TRB:CASSQGMSEAFF	83	CD8-C4
PB-3_CTACGTCGTTACAGAA	PB	PB-3	P3-1028	FALSE	1	TRA:CILRDSDAGNMLTF TRB:CASSLGTGGIGEQYF	1	CD8-C4
PB-3_CTACGTCTCCACTGGG	PB	PB-3	P3-19	FALSE	3	TRA:CALTSGGGADGLTF TRB:CASSQRGGTGLSGANVLTF	12	CD8-C5
PB-3_CTAGCCTAGCAAATCA	PB	PB-3	P3-279	TRUE	2	TRA:CAMRDDQTGANNLFF TRB:CASSFIAGGADTQYF	2	CD8-C5
PB-3_CTCACACGTCACACGC	PB	PB-3	P3-13	TRUE	3	TRA:CAASARGVRGAQKLVF TRB:CASSRTGFQPQHF	15	CD8-C5
PB-3_CTCACACTCTGCAGTA	PB	PB-3	P3-3	TRUE	3	TRA:CVVPWMDSSYKLIF TRB:CASSQRAGDGNYGYTF	42	CD8-C5
PB-3_CTCAGAAGTAGGCATG	PB	PB-3	P3-1043	FALSE	1	TRA:CLGKTSGSRLTF TRB:CASSVGAVNSPLHF	1	CD8-C5
PB-3_CTCAGAAGTGGACGAT	PB	PB-3	P3-171	TRUE	3	TRA:CALSEANARLMF TRB:CASSQVGGGTSDNEQFF	3	CD8-C4
PB-3_CTCGTCAAGTGTACGG	PB	PB-3	P3-230	FALSE	2	TRA:CAASAPNFGNEKLTF TRB:CASSYRGGQGTEAFF	2	CD8-C4
PB-3_CTCTACGTCGGAAATA	PB	PB-3	P3-1080	FALSE	1	TRA:CAGQPGTGANNLFF TRB:CASSETSGGAVQYF	1	CD8-C5
PB-3_CTCTGGTTCAGTACGT	PB	PB-3	P3-16	TRUE	3	TRA:CAASRNNNDMRF TRB:CASTNYQETQYF	14	CD8-C4
PB-3_CTGATAGAGGTTACCT	PB	PB-3	P3-39	TRUE	3	TRA:CAVRDIGGSYIPTF TRB:CASSLGGPEETQYF	8	CD8-C4
PB-3_CTGTGCTGTTCCTCCA	PB	PB-3	P3-31	TRUE	3	TRA:CAVRELKAAGNKLTF TRB:CASRRDSSTGELFF	10	CD8-C2
PB-3_CTGTGCTTCCGCTGTT	PB	PB-3	P3-285	FALSE	2	TRA:CAGKPAGYNKLIF TRB:CASSLGGYTEAFF	2	CD8-C4
PB-3_CTGTGCTTCGCCAGCA	PB	PB-3	P3-39	TRUE	3	TRA:CAVRDIGGSYIPTF TRB:CASSLGGPEETQYF	8	CD8-C4
PB-3_CTGTGCTTCTACGAGT	PB	PB-3	P3-125	TRUE	3	TRA:CVVNIGDSGSARQLTF TRB:CASSFGPRQHF	4	CD8-C5
PB-3_CTGTTTACAATAGCGG	PB	PB-3	P3-42	TRUE	3	TRA:CALGGSTLGRLYF TRB:CASSASTGANSPLHF	8	CD8-C5
PB-3_CTTAACTAGTACGACG	PB	PB-3	P3-39	TRUE	3	TRA:CAVRDIGGSYIPTF TRB:CASSLGGPEETQYF	8	CD8-C4
PB-3_CTTTGCGCAAACAACA	PB	PB-3	P3-1	TRUE	3	TRA:CAVAPISSGSARQLTF TRB:CASSQGMSEAFF	83	CD8-C5
PB-3_CTTTGCGCAGGGTTAG	PB	PB-3	P3-38	FALSE	3	TRA:CALNSGGSNYKLTF TRB:CASSRTGFQPQHF	8	CD8-C5
PB-3_CTTTGCGTCCGTAGTA	PB	PB-3	P3-8	TRUE	3	TRA:CAFDRGSTLGRLYF TRB:CASNPRQGTSTYEQYF	26	CD8-C4
PB-3_GAAATGATCGTCCAGG	PB	PB-3	P3-9	TRUE	3	TRA:CAVRDMDTGRRALTF TRB:CASSWDTRQAEAFF	24	CD8-C5
PB-3_GAACATCCAGGCTGAA	PB	PB-3	P3-1119	FALSE	1	TRA:CALSDRYGNEKLTF TRB:CASSETSGGYLDTQYF	1	CD8-C5
PB-3_GAACATCTCAAACAAG	PB	PB-3	P3-20	TRUE	3	TRA:CAVSSLQMDSSYKLIF TRB:CASSHRTENSPLHF	12	CD8-C5

PB-3_GAAGCAGCAGCTCGAC	PB	PB-3	P3-1	TRUE	3	TRA:CAVAPISSGSARQLTF TRB:CASSQGMSEAFF	83	CD8-C5
PB-3_GAATGAATCACGACTA	PB	PB-3	P3-42	TRUE	3	TRA:CALGGSTLGRLYF TRB:CASSASTGANSPLHF	8	CD8-C5
PB-3_GACCAATCAAATACAG	PB	PB-3	P3-5	TRUE	3	TRA:CALRNSNYQLIW TRB:CASSLGDRAYNEQFF	38	CD8-C5
PB-3_GACCAATGTAGTACCT	PB	PB-3	P3-13	TRUE	3	TRA:CAASARGVRGAQKLVF TRB:CASSRTGFQPQHF	15	CD8-C5
PB-3_GACCAATGTGCACTTA	PB	PB-3	P3-38	FALSE	3	TRA:CALNSGGSNYKLTF TRB:CASSRTGFQPQHF	8	CD8-C5
PB-3_GACCTGGGTTGCGTTA	PB	PB-3	P3-1144	FALSE	1	TRA:CALRGNMLTF TRB:CASSEVGISGFSPLHF	1	CD8-C5
PB-3_GACGCGTTCAGGCAAG	PB	PB-3	P3-38	FALSE	3	TRA:CALNSGGSNYKLTF TRB:CASSRTGFQPQHF	8	CD8-C8
PB-3_GACGCGTTCTTCCTTC	PB	PB-3	P3-292	TRUE	2	TRA:CAFGTYKYIF TRB:CASSLIQGAGNEQFF	2	CD8-C5
PB-3_GACGTGCGTTATCCGA	PB	PB-3	P3-5	TRUE	3	TRA:CALRNSNYQLIW TRB:CASSLGDRAYNEQFF	38	CD8-C5
PB-3_GAGGTGACACGGACAA	PB	PB-3	P3-58	FALSE	3	TRA:CALTRRLGDTDKLIF TRB:CASSKGVLEKLFF	6	CD8-C4
PB-3_GAGTCCGCAGGGCATA	PB	PB-3	P3-1168	FALSE	1	TRA:CAVSGGDGNEKLTF TRB:CATSIGHGYTF	1	CD8-C5
PB-3_GATCAGTTCTGCTGTC	PB	PB-3	P3-8	TRUE	3	TRA:CAFDRGSTLGRLYF TRB:CASNPRQGTSTYEQYF	26	CD8-C4
PB-3_GATCGATAGCGATGAC	PB	PB-3	P3-30	TRUE	3	TRA:CVVSEASGTYKYIF TRB:CASSLNLRKNTEAFF	10	CD8-C5
PB-3_GATTCAGTCACCGGGT	PB	PB-3	P3-1	TRUE	3	TRA:CAVAPISSGSARQLTF TRB:CASSQGMSEAFF	83	CD8-C5
PB-3_GCAATCAGTGCAGGTA	PB	PB-3	P3-153	TRUE	3	TRA:CAENSDFGNVLHC TRB:CASSVGMGNQPQHF	3	CD8-C2
PB-3_GCAATCAGTGTGACGA	PB	PB-3	P3-30	TRUE	3	TRA:CVVSEASGTYKYIF TRB:CASSLNLRKNTEAFF	10	CD8-C5
PB-3_GCGCAGTAGATCCTGT	PB	PB-3	P3-88	TRUE	3	TRA:CAARRGMGGATNKLIF TRB:CASSLTGGTGNTIYF	5	CD8-C5
PB-3_GCTCTGTCAGCTGGCT	PB	PB-3	P3-300	TRUE	2	TRA:CAFMKPIGGNNDMRF TRB:CASSGRQGNTEAFF	2	CD8-C5
PB-3_GCTGCGAAGTGCCAGA	PB	PB-3	P3-1232	FALSE	1	TRA:CAATADFNAGGTSYGKLTF TRB:CASSSPRQGWNEQFF	1	CD8-C4
PB-3_GCTTCCAGTCTACCTC	PB	PB-3	P3-31	TRUE	3	TRA:CAVRELKAAGNKLTF TRB:CASRRDSSTGELFF	10	CD8-C2
PB-3_GGAAAGCCAAACCTAC	PB	PB-3	P3-82	TRUE	3	TRA:CAVYLDRGSTLGRLYF TRB:CASSFGSGPNYGYTF	5	CD8-C4
PB-3_GGAACTTAGTACACCT	PB	PB-3	P3-6	TRUE	3	TRA:CAVRGANNLFF TRB:CASSPGTGYETQYF	29	CD8-C4
PB-3_GGAACTTCAAGTCATC	PB	PB-3	P3-125	TRUE	3	TRA:CVVNIGDSGSARQLTF TRB:CASSFGPRQHF	4	CD8-C5
PB-3_GGAGCAATCAATACCG	PB	PB-3	P3-301	TRUE	2	TRA:CALSVLPMDSNYQLIW TRB:CASSTRTENTEAFF	2	CD8-C5
PB-3_GGAGCAATCCGCATCT	PB	PB-3	P3-1	TRUE	3	TRA:CAVAPISSGSARQLTF TRB:CASSQGMSEAFF	83	CD8-C5
PB-3_GGAGCAATCCGCGGTA	PB	PB-3	P3-5	TRUE	3	TRA:CALRNSNYQLIW TRB:CASSLGDRAYNEQFF	38	CD8-C5
PB-3_GGATGTTGTTGTTTGG	PB	PB-3	P3-32	TRUE	3	TRA:CAASRGGQKLLF TRB:CASSSRTSLVHNEQFF	10	CD8-C4

PB-3_GGATTACGTGAAGGCT	PB	PB-3	P3-3	TRUE	3	TRA:CVVPWMDSSYKLIF TRB:CASSQRAGDGNYGYTF	42	CD8-C5
PB-3_GGGAATGGTCTCACCT	PB	PB-3	P3-1281	FALSE	1	TRA:CGGGADGLTF TRB:CASSETSGGYLDTQYF	1	CD8-C5
PB-3_GGGAATGTCGGCGCTA	PB	PB-3	P3-10	TRUE	3	TRA:CAGHQFSGGYNKLIF TRB:CASRASGTANEQFF	21	CD8-C5
PB-3_GGGCACTAGAGGACGG	PB	PB-3	P3-86	TRUE	3	TRA:CALTNRDDKIIF TRB:CATSRDMGGSYEQYF	5	CD8-C5
PB-3_GGGCATCTCCTGCTTG	PB	PB-3	P3-16	TRUE	3	TRA:CAASRNNNDMRF TRB:CASTNYQETQYF	14	CD8-C4
PB-3_GGGTTGCTCTTCATGT	PB	PB-3	P3-302	FALSE	2	TRA:CAATSEVTGNQFYF TRB:CASSLSPKRPYEQYF	2	CD8-C4
PB-3_GGTATTGAGCGATGAC	PB	PB-3	P3-12	TRUE	3	TRA:CAVGDLGNEKLTF TRB:CASSAEGYYNEQFF	16	CD8-C4
PB-3_GTAACGTCATCTATGG	PB	PB-3	P3-19	FALSE	3	TRA:CALTSGGGADGLTF TRB:CASSQRGGTGLSGANVLTF	12	CD8-C5
PB-3_GTACTTTGTGCGATAG	PB	PB-3	P3-184	TRUE	3	TRA:CACSRYGNNRLAF TRB:CAWSAPTGGGLGSGANVLTF	3	CD8-C5
PB-3_GTAGTCAAGACTCGGA	PB	PB-3	P3-6	TRUE	3	TRA:CAVRGANNLFF TRB:CASSPGTGYETQYF	29	CD8-C4
PB-3_GTATCTTGTTCACCTC	PB	PB-3	P3-61	TRUE	3	TRA:CALIRVDSSYKLIF TRB:CASRLGGGYTF	6	CD8-C4
PB-3_GTATTCTAGCCACCTG	PB	PB-3	P3-86	TRUE	3	TRA:CALTNRDDKIIF TRB:CATSRDMGGSYEQYF	5	CD8-C5
PB-3_GTATTCTTCCGCTGTT	PB	PB-3	P3-1331	FALSE	1	TRA:CLVGDMGQAGTALIF TRB:CASSLAKGSEQYF	1	CD8-C5
PB-3_GTCAAGTTCTCGGACG	PB	PB-3	P3-307	TRUE	2	TRA:CATCRNSGGGADGLTF TRB:CASSSGETSGDADTQYF	2	CD8-C5
PB-3_GTCACAAAGGTTCCTA	PB	PB-3	P3-308	TRUE	2	TRA:CAVEGGSSASKIIF TRB:CASRRTGDGYEQYF	2	CD8-C4
PB-3_GTCGTAAGTCGGATCC	PB	PB-3	P3-1	TRUE	3	TRA:CAVAPISSGSARQLTF TRB:CASSQGMSEAFF	83	CD8-C5
PB-3_GTCGTAATCCGGGTGT	PB	PB-3	P3-103	TRUE	3	TRA:CARDNTGKLIF TRB:CASSPGQGFTEAFF	4	CD8-C5
PB-3_GTCTTCGTCTTCGGTC	PB	PB-3	P3-10	TRUE	3	TRA:CAGHQFSGGYNKLIF TRB:CASRASGTANEQFF	21	CD8-C5
PB-3_GTGCTTCGTAAGCACG	PB	PB-3	P3-13	TRUE	3	TRA:CAASARGVRGAQKLVF TRB:CASSRTGFQPQHF	15	CD8-C5
PB-3_GTGGGTCGTCGTCTTC	PB	PB-3	P3-3	TRUE	3	TRA:CVVPWMDSSYKLIF TRB:CASSQRAGDGNYGYTF	42	CD8-C5
PB-3_GTTCATTCATCGATGT	PB	PB-3	P3-38	FALSE	3	TRA:CALNSGGSNYKLTF TRB:CASSRTGFQPQHF	8	CD8-C5
PB-3_TAAGAGACATGACATC	PB	PB-3	P3-1	TRUE	3	TRA:CAVAPISSGSARQLTF TRB:CASSQGMSEAFF	83	CD8-C5
PB-3_TACCTATGTCTCTCGT	PB	PB-3	P3-313	TRUE	2	TRA:CAVSDWKSRLMF TRB:CASSLSWVEGGGAKGDTQYF	2	CD8-C5
PB-3_TACTCGCTCCTAGAAC	PB	PB-3	P3-3	TRUE	3	TRA:CVVPWMDSSYKLIF TRB:CASSQRAGDGNYGYTF	42	CD8-C5
PB-3_TACTCGCTCGTACCGG	PB	PB-3	P3-6	TRUE	3	TRA:CAVRGANNLFF TRB:CASSPGTGYETQYF	29	CD8-C4
PB-3_TAGACCACACAGACTT	PB	PB-3	P3-113	TRUE	3	TRA:CAVSDYAGGTSYGKLTF TRB:CASSVGGGVREQYF	4	CD8-C4
PB-3_TAGCCGGCATGATCCA	PB	PB-3	P3-1450	FALSE	1	TRA:CAVRPRVNTGFQKLVF TRB:CASSRRAPNGSYNSPLHF	1	CD8-C4

PB-3_TAGGCATCATTGGTAC	PB	PB-3	P3-1	TRUE	3	TRA:CAVAPISSGSARQLTF TRB:CASSQGMSEAFF	83	CD8-C5
PB-3_TAGTGGTAGTCTCAAC	PB	PB-3	P3-14	TRUE	3	TRA:CALPFSGGYNKLIF TRB:CSARKTEPRSYEQYF	15	CD8-C5
PB-3_TATCTCACAGTCAGAG	PB	PB-3	P3-12	TRUE	3	TRA:CAVGDLGNEKLTF TRB:CASSAEGYYNEQFF	16	CD8-C4
PB-3_TCAACGACAGACGTAG	PB	PB-3	P3-13	TRUE	3	TRA:CAASARGVRGAQKLVF TRB:CASSRTGFQPQHF	15	CD8-C5
PB-3_TCAACGATCGAGCCCA	PB	PB-3	P3-1466	FALSE	1	TRA:CAVNDYKLSF TRB:CASSIYSAEYEKLFF	1	CD8-C5
PB-3_TCACGAAGTGGTGTAG	PB	PB-3	P3-47	TRUE	3	TRA:CAVRDSVRVGSARQLTF TRB:CASSASTGANSPLHF	7	CD8-C4
PB-3_TCAGCTCAGTGGCACA	PB	PB-3	P3-302	FALSE	2	TRA:CAATSEVTGNQFYF TRB:CASSLSPKRPYEQYF	2	CD8-C6
PB-3_TCAGGTACAGTTAACC	PB	PB-3	P3-1	TRUE	3	TRA:CAVAPISSGSARQLTF TRB:CASSQGMSEAFF	83	CD8-C5
PB-3_TCAGGTATCAGTTCGA	PB	PB-3	P3-14	TRUE	3	TRA:CALPFSGGYNKLIF TRB:CSARKTEPRSYEQYF	15	CD8-C5
PB-3_TCATTACTCAAGAAGT	PB	PB-3	P3-1501	FALSE	1	TRA:CAASEGHNARLMF TRB:CASSPKRGYPGPEQFF	1	CD8-C4
PB-3_TCCACACGTACCGTTA	PB	PB-3	P3-315	FALSE	2	TRA:CVVSPSYNTDKLIF TRB:CASSRGDMGEYNEQFF	2	CD8-C5
PB-3_TCGAGGCGTTGTCTTT	PB	PB-3	P3-111	TRUE	3	TRA:CALSEARTGNQFYF TRB:CASSYGPGHATQYF	4	CD8-C5
PB-3_TCGCGAGTCAAGGTAA	PB	PB-3	P3-30	TRUE	3	TRA:CVVSEASGTYKYIF TRB:CASSLNLRKNTEAFF	10	CD8-C5
PB-3_TCGTAGACACCACGTG	PB	PB-3	P3-1524	FALSE	1	TRA:CAVRALISGNTPLVF TRB:CASSLGDRAYNEQFF	1	CD8-C5
PB-3_TCTCTAACAATAGCGG	PB	PB-3	P3-58	FALSE	3	TRA:CALTRRLGDTDKLIF TRB:CASSKGVLEKLFF	6	CD8-C5
PB-3_TCTGAGAAGTTATCGC	PB	PB-3	P3-1531	FALSE	1	TRA:CAVSGVTGNQFYF TRB:CASTRLGQGALTGNTIYF	1	CD8-C5
PB-3_TCTGGAAAGCTAACAA	PB	PB-3	P3-12	TRUE	3	TRA:CAVGDLGNEKLTF TRB:CASSAEGYYNEQFF	16	CD8-C4
PB-3_TCTTCGGTCACTCCTG	PB	PB-3	P3-127	TRUE	3	TRA:CVVNPGGYNKLIF TRB:CASSPPGYFYEQYF	4	CD8-C5
PB-3_TCTTTCCAGGGCTTCC	PB	PB-3	P3-1	TRUE	3	TRA:CAVAPISSGSARQLTF TRB:CASSQGMSEAFF	83	CD8-C5
PB-3_TGAGGGAGTAGCCTAT	PB	PB-3	P3-6	TRUE	3	TRA:CAVRGANNLFF TRB:CASSPGTGYETQYF	29	CD8-C4
PB-3_TGCCCTACAATAACGA	PB	PB-3	P3-322	TRUE	2	TRA:CALGEYIF TRB:CASSPPWTDYGQYF	2	CD8-C5
PB-3_TGCTGCTAGCTAAGAT	PB	PB-3	P3-1581	FALSE	1	TRA:CAAAPPGRRALTF TRB:CASRTGTGSYEQYF	1	CD8-C4
PB-3_TGTCCCAAGAGGACGG	PB	PB-3	P3-1	TRUE	3	TRA:CAVAPISSGSARQLTF TRB:CASSQGMSEAFF	83	CD8-C5
PB-3_TGTGTTTAGCGATAGC	PB	PB-3	P3-315	FALSE	2	TRA:CVVSPSYNTDKLIF TRB:CASSRGDMGEYNEQFF	2	CD8-C4
PB-3_TGTTCCGTCTCGTTTA	PB	PB-3	P3-1	TRUE	3	TRA:CAVAPISSGSARQLTF TRB:CASSQGMSEAFF	83	CD8-C5
PB-3_TTAACTCGTGACTACT	PB	PB-3	P3-6	TRUE	3	TRA:CAVRGANNLFF TRB:CASSPGTGYETQYF	29	CD8-C5
PB-3_TTAGGCAAGTGCCATT	PB	PB-3	P3-90	TRUE	3	TRA:CAGRKGDNYGQNFVF TRB:CASSRGTNYGYTF	5	CD8-C5

PB-3_TTAGGCAGTGAAAGAG	PB	PB-3	P3-1	TRUE	3	TRA:CAVAPISSGSARQLTF TRB:CASSQGMSEAFF	83	CD8-C5
PB-3_TTAGTTCAGATCGATA	PB	PB-3	P3-190	TRUE	3	TRA:CAVTEGADGLTF TRB:CASSPGQTYGYTF	3	CD8-C4
PB-3_TTCCCAGGTGCAGTAG	PB	PB-3	P3-325	TRUE	2	TRA:CAMSQETSGSRLTF TRB:CATRVGRGRGSYNSPLHF	2	CD8-C4
PB-3_TTGGCAATCTTAGAGC	PB	PB-3	P3-5	TRUE	3	TRA:CALRNSNYQLIW TRB:CASSLGDRAYNEQFF	38	CD8-C5
PB-3_TTGTAGGAGTTGTCGT	PB	PB-3	P3-1	TRUE	3	TRA:CAVAPISSGSARQLTF TRB:CASSQGMSEAFF	83	CD8-C5
PB-3_TTTGCGCAGGCAGGTT	PB	PB-3	P3-1	TRUE	3	TRA:CAVAPISSGSARQLTF TRB:CASSQGMSEAFF	83	CD8-C5
PB-3_TTTGCGCCATCCAACA	PB	PB-3	P3-10	TRUE	3	TRA:CAGHQFSGGYNKLIF TRB:CASRASGTANEQFF	21	CD8-C5
PB-3_ACATACGCACCGGAAA	PB	PB-3	P3-18	TRUE	3	TRA:CLVGVGNNDMRF TRB:CASSPGTGKYEQYF	12	CD8-C5
PB-3_AAGTCTGGTGTGCCTG	PB	PB-3	P3-146	TRUE	3	TRA:CVVNLYSSASKIIF TRB:CASSLEAGTEAFF	3	CD4-C3
PB-3_AAGTCTGTCGATAGAA	PB	PB-3	P3-537	FALSE	1	TRA:CAASKGSNYQLIW TRB:CASSLEAGQGTYEQYF	1	CD4-C3
PB-3_ACTGAACAGGACATTA	PB	PB-3	P3-605	FALSE	1	TRA:CVVSGRNNARLMF TRB:CASSHIDMVSYNEQFF	1	CD4-C2
PB-3_CAGAGAGGTACAGTGG	PB	PB-3	P3-800	FALSE	1	TRA:CALTEGSNTGKLIF TRB:CATSRDSRRETQYF	1	CD4-C3
PB-3_CCGTGGAAGAAACCGC	PB	PB-3	P3-888	FALSE	1	TRA:CATLSWKETSGSRLTF TRB:CASKRGDYSYEQYF	1	CD4-C3
PB-3_CGCGGTAGTATATGGA	PB	PB-3	P3-948	FALSE	1	TRA:CALSLSSGGSNYKLTF TRB:CASSRRLSGNTIYF	1	CD8-C8
PB-3_CTCATTACAAACGCGA	PB	PB-3	P3-1046	FALSE	1	TRA:CAASGGNQFYF TRB:CASSSMRGGSEQYF	1	CD4-C3
PB-3_CTCGTACAGAGGTTGC	PB	PB-3	P3-1067	FALSE	1	TRA:CAYARNTGANSKLTF TRB:CASSLATGAWETQYF	1	CD4-C3
PB-3_GCACATAGTTCTCATT	PB	PB-3	P3-1191	FALSE	1	TRA:CLVEGFRFQKLVF TRB:CSAAGSNTEAFF	1	CD4-C3
PB-3_GCATGCGAGGTAGCTG	PB	PB-3	P3-297	FALSE	2	TRA:CAATGSNYQLIW TRB:CASSPSSYEQYF	2	CD4-C2
PB-3_TAGGCATCAGCTCGAC	PB	PB-3	P3-182	TRUE	3	TRA:CAVRDGGKSTF TRB:CASSFRDTDTQYF	3	CD4-C3
PB-3_TATCAGGAGTGCGATG	PB	PB-3	P3-281	FALSE	2	TRA:CAASKGGSNYKLTF TRB:CASSPGGSEKLFF	2	CD4-C3
PB-3_TGCTACCCATTAACCG	PB	PB-3	P3-1580	FALSE	1	TRA:CALPSGNTPLVF TRB:CASSMEGREYEKLFF	1	CD4-C3
PB-3_TTGACTTCAAGTACCT	PB	PB-3	P3-1647	FALSE	1	TRA:CAASVYGNKLVF TRB:CASSPQPGSYEQYF	1	CD4-C3
PB-3_AAACCTGCAGCCTTTC	PB	PB-3	P3-482	FALSE	1	TRA:CAFLSGAGSYQLTF TRB:CASSKGQGAIGELFF	1	CD4-C7
PB-3_AAACCTGGTAAGAGAG	PB	PB-3	P3-483	FALSE	1	TRA:CAVRQGFQKLVF TRB:CASSIQGAETQYF	1	CD4-C2
PB-3_AAACGGGTCTGCTGTC	PB	PB-3	P3-485	FALSE	1	TRA:CAVTRSGGSYIPTF TRB:CASSVGQSYGYTF	1	CD4-C2
PB-3_AAAGATGCACTATCTT	PB	PB-3	P3-487	FALSE	1	TRA:CGTPEGGSNYKLTF TRB:CSARAGDRGLYGYTF	1	CD4-C2
PB-3_AAAGATGTCAGAGACG	PB	PB-3	P3-145	FALSE	3	TRA:CALAYNFNKFYF TRB:CASSLSVRDQTQYF	3	CD4-C2

PB-3_AAAGTAGGTATAGTAG	PB	PB-3	P3-489	FALSE	1	TRA:CAMREVQYNFNKFYF TRB:CSARVIPSGGGTDTQYF	1	CD4-C2
PB-3_AACCATGAGGACTGGT	PB	PB-3	P3-495	FALSE	1	TRA:CALGRGSSNTGKLIF TRB:CASRNTGEANTEAFF	1	CD4-C3
PB-3_AACCATGTCCTACAGA	PB	PB-3	P3-231	TRUE	2	TRA:CAVPNAGNNRKLIW TRB:CASSVGAANEQFF	2	CD4-C2
PB-3_AACTCAGTCCGAATGT	PB	PB-3	P3-500	FALSE	1	TRA:CAVSRSGYSTLTF TRB:CASSDTGTGNTEAFF	1	CD4-C1
PB-3_AACTCCCAGTTATCGC	PB	PB-3	P3-501	FALSE	1	TRA:CALWYGQNFVF TRB:CASSQALAGGNDRYNEQFF	1	CD4-C2
PB-3_AACTCTTTCGAATGGG	PB	PB-3	P3-507	FALSE	1	TRA:CAVRADNYGQNFVF TRB:CASREGVQGGSPLHF	1	CD4-C1
PB-3_AACTCTTTCGGCCGAT	PB	PB-3	P3-508	FALSE	1	TRA:CAVSEGTASKLTF TRB:CASSTTGSSAAEAFF	1	CD4-C2
PB-3_AACTGGTAGCGATATA	PB	PB-3	P3-509	FALSE	1	TRA:CARSGGSTGNQFYF TRB:CASSDSRLRGLSNEQFF	1	CD4-C2
PB-3_AACTGGTCAAAGCGGT	PB	PB-3	P3-510	FALSE	1	TRA:CAVEASYSSASKIIF TRB:CASSPRQGVAFF	1	CD4-C2
PB-3_AACTGGTCATCTGGTA	PB	PB-3	P3-511	FALSE	1	TRA:CAVRDGGNNARLMF TRB:CASSLAAGRVRTEAFF	1	CD4-C2
PB-3_AACTGGTGTCATATGC	PB	PB-3	P3-513	FALSE	1	TRA:CAESTGGFKTIF TRB:CASRTPGGERSTQYF	1	CD4-C3
PB-3_AACTGGTGTTACGGAG	PB	PB-3	P3-514	FALSE	1	TRA:CAEIPTGTYKYIF TRB:CASSQGLRSNYGYTF	1	CD4-C2
PB-3_AACTGGTTCTTACCGC	PB	PB-3	P3-517	FALSE	1	TRA:CAVRDQGGGFKTIF TRB:CASSLGAGPTDTQYF	1	CD4-C1
PB-3_AAGACCTAGGTGATAT	PB	PB-3	P3-519	FALSE	1	TRA:CAATPAGGADGLTF TRB:CASSAPGFTTMNEKLFF	1	CD4-C1
PB-3_AAGGAGCAGGGTATCG	PB	PB-3	P3-525	FALSE	1	TRA:CAVLTNARLMF TRB:CASSEALYEQYF	1	CD4-C2
PB-3_AAGGCAGCATTGCGGC	PB	PB-3	P3-530	FALSE	1	TRA:CAASARGSNYKLTF TRB:CASSPSSLTPTQAFF	1	CD4-C2
PB-3_AAGGTTCAGGGCATGT	PB	PB-3	P3-532	FALSE	1	TRA:CAMRGDYKLSF TRB:CASSLTGTVRYEQYF	1	CD4-C2
PB-3_AAGTCTGCAAAGTGCG	PB	PB-3	P3-535	FALSE	1	TRA:CAYRTLTSGTYKYIF TRB:CASSDGYTIYF	1	CD4-C2
PB-3_AAGTCTGTCCGCAAGC	PB	PB-3	P3-536	FALSE	1	TRA:CAATDPGTYKYIF TRB:CASSYSAGRPEQYF	1	CD4-C2
PB-3_AATCGGTAGCTGATAA	PB	PB-3	P3-540	FALSE	1	TRA:CAFKTQGGSEKLVF TRB:CASSSWDRGPAAFF	1	CD4-C2
PB-3_AATCGGTCAAAGGCGT	PB	PB-3	P3-541	FALSE	1	TRA:CAESTGNQFYF TRB:CASSLSQNYEQYF	1	CD8-C4
PB-3_ACACCAAAGGTGGGTT	PB	PB-3	P3-545	FALSE	1	TRA:CIVRAFISSGSARQLTF TRB:CASSSQGQSYTF	1	CD4-C2
PB-3_ACACCAACACAGACTT	PB	PB-3	P3-547	FALSE	1	TRA:CTTASRAIRDDKIIF TRB:CASSRGGGTEAFF	1	CD4-C2
PB-3_ACACCAACAGGGATTG	PB	PB-3	P3-548	FALSE	1	TRA:CAVEETSGSRLTF TRB:CASEGLREPTDTQYF	1	CD4-C2
PB-3_ACACCAATCCGTTGCT	PB	PB-3	P3-549	FALSE	1	TRA:CAVSRSTGTASKLTF TRB:CASSGGTLPYEQYF	1	CD4-C2
PB-3_ACACCCTGTCTTGATG	PB	PB-3	P3-552	FALSE	1	TRA:CAGRTNQGGKLIF TRB:CASSMGQVRSGNQPQHF	1	CD4-C2
PB-3_ACACCGGAGGTAGCTG	PB	PB-3	P3-553	FALSE	1	TRA:CAETGQKLLF TRB:CSAQTGTGSQQYF	1	CD4-C2

PB-3_ACACCGGAGTATCGAA	PB	PB-3	P3-234	FALSE	2	TRA:CAVTPVYGKLTF TRB:CASSLGETQYF	2	CD4-C2
PB-3_ACACCGGGTATTAGCC	PB	PB-3	P3-554	FALSE	1	TRA:CATDPNTGNQFYF TRB:CASSLMIRVNEQFF	1	CD4-C2
PB-3_ACACCGGGTCCAAGTT	PB	PB-3	P3-555	FALSE	1	TRA:CALLSLYNNNDMRF TRB:CASSLNPAANNEQFF	1	CD4-C2
PB-3_ACACCGGGTTGACGTT	PB	PB-3	P3-556	FALSE	1	TRA:CAVSGSKLTF TRB:CASSPSSGKKETQYF	1	CD4-C2
PB-3_ACACCGGTCCATGCTC	PB	PB-3	P3-557	FALSE	1	TRA:CAGPHGSSNTGKLIF TRB:CSARTGGVSGQFF	1	CD4-C2
PB-3_ACACTGAAGCACCGTC	PB	PB-3	P3-558	FALSE	1	TRA:CAGPHSSNTGKLIF TRB:CASSPNPDTQYF	1	CD4-C2
PB-3_ACACTGACAGGTCGTC	PB	PB-3	P3-559	FALSE	1	TRA:CAVGHGTDKLIF TRB:CASSELGARGNTIYF	1	CD4-C2
PB-3_ACAGCCGAGAGGTTAT	PB	PB-3	P3-562	FALSE	1	TRA:CALVIMDSNYQLIW TRB:CATSRGAGRGNQPQHF	1	CD4-C2
PB-3_ACAGCTATCGAACTGT	PB	PB-3	P3-565	FALSE	1	TRA:CVVNIRVADKLIF TRB:CSARLAGTDTQYF	1	CD4-C2
PB-3_ACATCAGGTCGTGGCT	PB	PB-3	P3-568	FALSE	1	TRA:CATEALPQGAQKLVF TRB:CASTKTGRDTQYF	1	CD4-C7
PB-3_ACATCAGTCTTCGAGA	PB	PB-3	P3-570	FALSE	1	TRA:CAVSSLGFGNVLHC TRB:CASSGRLAGNYNEQFF	1	CD4-C1
PB-3_ACATGGTAGATAGTCA	PB	PB-3	P3-571	FALSE	1	TRA:CAVSDLANNNDMRF TRB:CASSQERTRGSYEQYF	1	CD4-C2
PB-3_ACATGGTAGGCAGTCA	PB	PB-3	P3-572	FALSE	1	TRA:CAVPRGNNNDMRF TRB:CASSSNRLAGVQETQYF	1	CD4-C3
PB-3_ACCAGTATCAACTCTT	PB	PB-3	P3-576	FALSE	1	TRA:CAGLAGAQKLVF TRB:CASRRDGTGVDDGYTF	1	CD4-C2
PB-3_ACCAGTATCATGCATG	PB	PB-3	P3-577	FALSE	1	TRA:CAVENRAGGGADGLTF TRB:CASSPRRGNTEAFF	1	CD4-C2
PB-3_ACCAGTATCGGAGCAA	PB	PB-3	P3-578	FALSE	1	TRA:CVVFSGGSYIPTF TRB:CAWSADLFSGNTIYF	1	CD4-C2
PB-3_ACCCACTAGCTCAACT	PB	PB-3	P3-579	FALSE	1	TRA:CAVTLLSGGYNKLIF TRB:CASSRGQLQETQYF	1	CD4-C2
PB-3_ACCTTTATCGGCGCTA	PB	PB-3	P3-582	FALSE	1	TRA:CAFMNPQAGTALIF TRB:CASSEGATQYF	1	CD4-C2
PB-3_ACGAGCCCACCTGGTG	PB	PB-3	P3-583	FALSE	1	TRA:CAPAWNNNARLMF TRB:CASSDHSGGVWTQYF	1	CD4-C2
PB-3_ACGATACCACATCCAA	PB	PB-3	P3-586	FALSE	1	TRA:CALSDRASYDKVIF TRB:CASSVLSGYEQYF	1	CD4-C2
PB-3_ACGCCAGCATACGCCG	PB	PB-3	P3-590	FALSE	1	TRA:CAGRASNYQLIW TRB:CASSSRLGGTDTQYF	1	CD4-C2
PB-3_ACGCCAGTCTAACTGG	PB	PB-3	P3-591	FALSE	1	TRA:CAVSEGGTSGSRLTF TRB:CASVLREGNSPLHF	1	CD4-C2
PB-3_ACGCCGACAGGTCGTC	PB	PB-3	P3-592	FALSE	1	TRA:CAVEGNNAGNMLTF TRB:CASSQVGVGYSPLHF	1	CD4-C1
PB-3_ACGGAGAAGGTCATCT	PB	PB-3	P3-594	FALSE	1	TRA:CVVSGEYNQGGKLIF TRB:CASSLTSRLAGDTQYF	1	CD4-C2
PB-3_ACGGAGAGTGACAAAT	PB	PB-3	P3-596	FALSE	1	TRA:CALSEAGAGSYQLTF TRB:CASSQESSGGVYNEQFF	1	CD4-C2
PB-3_ACGGGTCCATGCAATC	PB	PB-3	P3-601	FALSE	1	TRA:CAVSGGGTGTASKLTF TRB:CASSPRNSYEQYF	1	CD8-C9
PB-3_ACTATCTAGCCAACAG	PB	PB-3	P3-603	FALSE	1	TRA:CAESPEGGATNKLIF TRB:CASSHTGTLTTYEQYF	1	CD4-C2

PB-3_ACTATCTGTCATATCG	PB	PB-3	P3-604	FALSE	1	TRA:CAGENTGNQFYF TRB:CASSRGQGAVEAFF	1	CD4-C2
PB-3_ACTGAGTCACTTAACG	PB	PB-3	P3-608	FALSE	1	TRA:CAVGAGNTDKLIF TRB:CASSRTGSTEAFF	1	CD4-C2
PB-3_ACTGCTCAGTTGCAGG	PB	PB-3	P3-611	FALSE	1	TRA:CAESEYSSASKIIF TRB:CASSIPGGSRGYTF	1	CD4-C2
PB-3_ACTGCTCGTACCCAAT	PB	PB-3	P3-612	FALSE	1	TRA:CAVLNLITPTSSSL TRB:CASTLRGSYNEQFF	1	CD4-C2
PB-3_ACTGTCCAGTACTTGC	PB	PB-3	P3-614	FALSE	1	TRA:CALSVNSGATNKLIF TRB:CASSLGSSTDTQYF	1	CD4-C2
PB-3_ACTTGTTAGCGTAGTG	PB	PB-3	P3-620	FALSE	1	TRA:CAVSGNTPLVF TRB:CASSTGRATNEKLFF	1	CD4-C3
PB-3_AGAATAGAGTGCAAGC	PB	PB-3	P3-623	FALSE	1	TRA:CAVKGNGNKLVF TRB:CATSAGGAPHRNTEAFF	1	CD4-C2
PB-3_AGAGTGGGTACACCGC	PB	PB-3	P3-631	FALSE	1	TRA:CAVQGSTNAGKSTF TRB:CASSQGAPVETQYF	1	CD4-C2
PB-3_AGATCTGAGTGGAGAA	PB	PB-3	P3-632	FALSE	1	TRA:CASNFGNEKLTF TRB:CAWKLILGGVNTEAFF	1	CD4-C2
PB-3_AGATTGCAGGCAATTA	PB	PB-3	P3-633	FALSE	1	TRA:CAAFSGGGADGLTF TRB:CASSHGTAGELFF	1	CD4-C2
PB-3_AGATTGCAGTACGTTC	PB	PB-3	P3-634	FALSE	1	TRA:CAASRRDSNYQLIW TRB:CASSSLRSNTEAFF	1	CD4-C2
PB-3_AGATTGCAGTGGGTTG	PB	PB-3	P3-635	FALSE	1	TRA:CAVRPNSGYALNF TRB:CASSQGGANEYF	1	CD4-C2
PB-3_AGATTGCCATTGGCGC	PB	PB-3	P3-637	FALSE	1	TRA:CAASNTDKLIF TRB:CASSLRAERDEQYF	1	CD4-C2
PB-3_AGCAGCCTCGTGGGAA	PB	PB-3	P3-641	FALSE	1	TRA:CIVVAGGYQKVTF TRB:CASSDRAGGTDTQYF	1	CD4-C3
PB-3_AGCATACGTTCGGCAC	PB	PB-3	P3-645	FALSE	1	TRA:CAEKEDFNKFYF TRB:CASTSGRGSYEQYF	1	CD4-C2
PB-3_AGCATACTCTAGAGTC	PB	PB-3	P3-646	FALSE	1	TRA:CAVPPYNQGGKLIF TRB:CSGGGVTYEQYF	1	CD4-C2
PB-3_AGCCTAAAGCGAAGGG	PB	PB-3	P3-648	FALSE	1	TRA:CVVNNLGYGGSQGNLIF TRB:CASGAGQLTGELFF	1	CD4-C2
PB-3_AGCCTAAGTATAAACG	PB	PB-3	P3-246	TRUE	2	TRA:CVVDTYAGNNRKLIW TRB:CASTVQRVGLGAEAFF	2	CD4-C2
PB-3_AGCGGTCAGATGCCAG	PB	PB-3	P3-154	TRUE	3	TRA:CAVETYNNNDMRF TRB:CASSVRSGTDTQYF	3	CD4-C2
PB-3_AGCGGTCCAATGCCAT	PB	PB-3	P3-650	FALSE	1	TRA:CVVIPAMDSSYKLIF TRB:CASSLGQSINEQFF	1	CD4-C2
PB-3_AGCGGTCCATGCAACT	PB	PB-3	P3-651	FALSE	1	TRA:CAVRDRGDSSYKLIF TRB:CSARDRGSSRMVEQFF	1	CD4-C2
PB-3_AGCGGTCTCATTCACT	PB	PB-3	P3-652	FALSE	1	TRA:CAPSYNFNKFYF TRB:CSVAPASGNTIYF	1	CD4-C2
PB-3_AGCGTATAGGCAAAGA	PB	PB-3	P3-653	FALSE	1	TRA:CAVSDTGNAGKSTF TRB:CASSPQRTSGNTDTQYF	1	CD4-C2
PB-3_AGCGTATTCCACGTTC	PB	PB-3	P3-656	FALSE	1	TRA:CALFYTGTASKLTF TRB:CASSVRQLPDGYTF	1	CD4-C2
PB-3_AGCGTCGCATATACGC	PB	PB-3	P3-657	FALSE	1	TRA:CAVGASGGSYIPTF TRB:CAAGIPQGGNTIYF	1	CD4-C2
PB-3_AGCTCCTAGCCAACAG	PB	PB-3	P3-658	FALSE	1	TRA:CALSRESGGSNYKLTF TRB:CASSSMGYEQYF	1	CD4-C2
PB-3_AGCTCCTTCTTATCTG	PB	PB-3	P3-662	FALSE	1	TRA:CAVRDWVENTPLVF TRB:CASRVVNTGELFF	1	CD4-C2

PB-3_AGCTCTCAGGATGCGT	PB	PB-3	P3-663	FALSE	1	TRA:CAERATGTYKYIF TRB:CASFAGGRAFF	1	CD4-C2
PB-3_AGCTCTCAGTCATCCA	PB	PB-3	P3-664	FALSE	1	TRA:CAVLDAGNNRKLIW TRB:CASSLGLTLSEKLFF	1	CD4-C2
PB-3_AGCTTGATCCGGCACA	PB	PB-3	P3-667	FALSE	1	TRA:CAVVVGNQFYF TRB:CASSYEGYGYTF	1	CD4-C2
PB-3_AGGCCGTGTTAAGGGC	PB	PB-3	P3-670	FALSE	1	TRA:CAMRDASGYALNF TRB:CASSYTSGLPGTQYF	1	CD4-C2
PB-3_AGGCCGTTCTTTAGGG	PB	PB-3	P3-671	FALSE	1	TRA:CAARNYGGSQGNLIF TRB:CASRETVNSPLHF	1	CD4-C2
PB-3_AGGGAGTTCGCTTGTC	PB	PB-3	P3-673	FALSE	1	TRA:CAPSGRDSNYQLIW TRB:CASSVAAGATGELFF	1	CD4-C2
PB-3_AGGGATGAGTGCCATT	PB	PB-3	P3-674	FALSE	1	TRA:CAMSSLTDSWGKFQF TRB:CSARGGQPQHF	1	CD4-C2
PB-3_AGGGATGCATGGGAAC	PB	PB-3	P3-675	FALSE	1	TRA:CAVTTQGGSEKLVF TRB:CSALTSTETQYF	1	CD4-C2
PB-3_AGGGATGGTGGTTTCA	PB	PB-3	P3-676	FALSE	1	TRA:CAASKMEYGNKLVF TRB:CASSGKPGRWPTEAFF	1	CD4-C2
PB-3_AGGGATGTCGGTCTAA	PB	PB-3	P3-677	FALSE	1	TRA:CAPYLGEDTGRRALTF TRB:CASSLTPGQQETQYF	1	CD4-C2
PB-3_AGGGTGAAGAAACCAT	PB	PB-3	P3-679	FALSE	1	TRA:CAVQANTGFQKLVF TRB:CASRSIKREGNSPLHF	1	CD4-C2
PB-3_AGGGTGAGTTTGCATG	PB	PB-3	P3-681	FALSE	1	TRA:CAASIGGNNRKLIW TRB:CASSLTGGRQETQYF	1	CD4-C2
PB-3_AGGGTGATCACCTTAT	PB	PB-3	P3-682	FALSE	1	TRA:CAAISKGGGNKLTF TRB:CASSPGQGTDTQYF	1	CD4-C3
PB-3_AGTAGTCCAGGATCGA	PB	PB-3	P3-687	FALSE	1	TRA:CVVSLFYGGSQGNLIF TRB:CASSLIAGRSYNEQFF	1	CD4-C2
PB-3_AGTCTTTAGTCAATAG	PB	PB-3	P3-688	FALSE	1	TRA:CASPWTSGSRLTF TRB:CASSAGQGGQETQYF	1	CD4-C2
PB-3_AGTGTCACAGTCAGAG	PB	PB-3	P3-694	FALSE	1	TRA:CAVILYNTDKLIF TRB:CASSLKPGAGELFF	1	CD4-C7
PB-3_AGTTGGTGTTCCACAA	PB	PB-3	P3-696	FALSE	1	TRA:CASGGSYIPTF TRB:CSGTGSNTGELFF	1	CD4-C2
PB-3_ATAACGCTCACAGTAC	PB	PB-3	P3-697	FALSE	1	TRA:CAVNEGAGSYQLTF TRB:CASSPNLDGNYGYTF	1	CD4-C2
PB-3_ATAACGCTCACTTCAT	PB	PB-3	P3-698	FALSE	1	TRA:CQGGYNKLIF TRB:CASTNTGGWNQPQHF	1	CD4-C2
PB-3_ATAACGCTCTCAACTT	PB	PB-3	P3-699	FALSE	1	TRA:CAENVGGGGADGLTF TRB:CASSSHGNYGYTF	1	CD4-C2
PB-3_ATAAGAGAGCACACAG	PB	PB-3	P3-700	FALSE	1	TRA:CALSEQGGSEKLVF TRB:CASSEVRTFNNYGYTF	1	CD4-C3
PB-3_ATCACGAGTCTAGTCA	PB	PB-3	P3-249	FALSE	2	TRA:CILRSGAGSYQLTF TRB:CASSLFASPKTDTQYF	2	CD4-C2
PB-3_ATCATCTCAATCCAAC	PB	PB-3	P3-707	FALSE	1	TRA:CAASAATSGTYKYIF TRB:CASRNTYTGRNRLVYF	1	CD4-C2
PB-3_ATCATCTCACCAGCAC	PB	PB-3	P3-708	FALSE	1	TRA:CKGGDTDKLIF TRB:CASSLVQHNTEAFF	1	CD4-C2
PB-3_ATCATCTGTAAGAGAG	PB	PB-3	P3-156	FALSE	3	TRA:CAASSGGGADGLTF TRB:CSASSGVSYTQYF	3	CD4-C2
PB-3_ATCATCTTCGGTGTCG	PB	PB-3	P3-709	FALSE	1	TRA:CAVRGTTSGTYKYIF TRB:CASSPMAVTRSGELFF	1	CD4-C2
PB-3_ATCGAGTAGGGTGTTG	PB	PB-3	P3-717	FALSE	1	TRA:CAVKDRGSTLGRLYF TRB:CASSLGPASPLHF	1	CD4-C2

PB-3_ATCGAGTAGGTGCTTT	PB	PB-3	P3-718	FALSE	1	TRA:CAVSGRTGGSQGNLIF TRB:CASSLGRSFPTDTQYF	1	CD4-C2
PB-3_ATCTGCCTCGGTCCGA	PB	PB-3	P3-157	FALSE	3	TRA:CAASPTIIF TRB:CASSLGYADTQYF	3	CD4-C2
PB-3_ATGGGAGTCCGTTGTC	PB	PB-3	P3-731	FALSE	1	TRA:CIVSGAGSYQLTF TRB:CSATRGQGGQETQYF	1	CD4-C2
PB-3_ATGTGTGGTATAATGG	PB	PB-3	P3-732	FALSE	1	TRA:CAVSMDTGRRALTF TRB:CASSDGGLSADEQYF	1	CD4-C2
PB-3_ATTACTCCATAGTAAG	PB	PB-3	P3-738	FALSE	1	TRA:CAASRGSARQLTF TRB:CASSPDGGAGANVLTF	1	CD4-C1
PB-3_ATTATCCAGTAAGTAC	PB	PB-3	P3-252	FALSE	2	TRA:CAVLGGDYKLSF TRB:CASSPHTQGDEQFF	2	CD4-C2
PB-3_ATTATCCTCTCTGCTG	PB	PB-3	P3-743	FALSE	1	TRA:CAFRNDYKLSF TRB:CASSSARVPSRYTF	1	CD4-C2
PB-3_ATTCTACCATGGGACA	PB	PB-3	P3-744	FALSE	1	TRA:CAGGVGNQGGKLIF TRB:CASKRGNEQFF	1	CD4-C1
PB-3_ATTGGACAGGGTGTGT	PB	PB-3	P3-254	FALSE	2	TRA:CAVRTGGGNKLTF TRB:CASRADSTYEQYF	2	CD4-C2
PB-3_ATTTCTGTCCGCATAA	PB	PB-3	P3-747	FALSE	1	TRA:CAVERETDKLIF TRB:CASSLHRGGDEQFF	1	CD4-C2
PB-3_CAACCTCTCTTTACGT	PB	PB-3	P3-752	FALSE	1	TRA:CAMRESEGAGSYQLTF TRB:CASSPTTVADTQYF	1	CD4-C3
PB-3_CAACTAGGTGTTTGTG	PB	PB-3	P3-157	FALSE	3	TRA:CAASPTIIF TRB:CASSLGYADTQYF	3	CD4-C2
PB-3_CAAGGCCCAATCAGAA	PB	PB-3	P3-115	TRUE	3	TRA:CAVGTNRDDKIIF TRB:CASSGTRLTGELFF	4	CD4-C3
PB-3_CAAGGCCCAGACTCGC	PB	PB-3	P3-759	FALSE	1	TRA:CVGKASGTYKYIF TRB:CSARINQGAGSNQPQHF	1	CD4-C2
PB-3_CAAGTTGAGCCAGTTT	PB	PB-3	P3-760	FALSE	1	TRA:CAYRIYSGGSNYKLTF TRB:CASSVGASGGSYNEQFF	1	CD4-C2
PB-3_CAAGTTGCATTGTGCA	PB	PB-3	P3-761	FALSE	1	TRA:CATDNARLMF TRB:CASSSRESYEKLFF	1	CD4-C2
PB-3_CACAAACAGTACATGA	PB	PB-3	P3-766	FALSE	1	TRA:CAVRDVYGQNFVF TRB:CASSLEGQGYGYTF	1	CD4-C2
PB-3_CACAAACCACGAAGCA	PB	PB-3	P3-768	FALSE	1	TRA:CAYRSPTTSGTYKYIF TRB:CASSIAGTSYEQYF	1	CD4-C2
PB-3_CACACAACATACTACG	PB	PB-3	P3-771	FALSE	1	TRA:CALSDHSGNQFYF TRB:CASSSLLDRGQETQYF	1	CD4-C2
PB-3_CACACAATCGGGAGTA	PB	PB-3	P3-773	FALSE	1	TRA:CLVGASGGYNKLIF TRB:CASTPGVATYEQYF	1	CD4-C2
PB-3_CACACCTTCTTCAACT	PB	PB-3	P3-775	FALSE	1	TRA:CVVSRPQGGKLIF TRB:CASSQEESGTGKLFF	1	CD4-C2
PB-3_CACACTCTCTCGTATT	PB	PB-3	P3-777	FALSE	1	TRA:CAASKKAGNNRKLIW TRB:CASILTVNTEAFF	1	CD4-C2
PB-3_CACAGTACACGAAGCA	PB	PB-3	P3-780	FALSE	1	TRA:CAMSTNTGNQFYF TRB:CASSLEEGAKWNQPQHF	1	CD4-C2
PB-3_CACAGTACATGCTGGC	PB	PB-3	P3-781	FALSE	1	TRA:CAVFGGGTSYGKLTF TRB:CASSQDLSSSYNEQFF	1	CD4-C2
PB-3_CACATAGAGACGCACA	PB	PB-3	P3-782	FALSE	1	TRA:CVVRWNNDMRF TRB:CASSVGTGSTDTQYF	1	CD4-C2
PB-3_CACATAGAGTAGCCGA	PB	PB-3	P3-783	FALSE	1	TRA:CIVDSGNTPLVF TRB:CTISPGGGTRGEQFF	1	CD4-C7
PB-3_CACCACTGTGCCTTGG	PB	PB-3	P3-786	FALSE	1	TRA:CAAYPGAQKLVF TRB:CASSLGLAGFQETQYF	1	CD4-C2

PB-3_CACCACTTCTGGAGCC	PB	PB-3	P3-788	FALSE	1	TRA:CAMREGTNGGSQGNLIF TRB:CASSRQGLYNEQFF	1	CD4-C1
PB-3_CACTCCATCGCACTCT	PB	PB-3	P3-157	FALSE	3	TRA:CAASPTIIF TRB:CASSLGYADTQYF	3	CD4-C2
PB-3_CAGAATCCATACGCTA	PB	PB-3	P3-794	FALSE	1	TRA:CLLGGNNNDMRF TRB:CASSGRVVGTDTQYF	1	CD4-C2
PB-3_CAGAATCGTAAGGATT	PB	PB-3	P3-795	FALSE	1	TRA:CAVNMIEDDKIIF TRB:CASSSLAGEYNEQFF	1	CD4-C2
PB-3_CAGTAACTCATGCAAC	PB	PB-3	P3-817	FALSE	1	TRA:CIVRVDGSSNTGKLIF TRB:CASSRSFRVITGNTIYF	1	CD4-C2
PB-3_CAGTCCTTCGCCTGTT	PB	PB-3	P3-820	FALSE	1	TRA:CAESIRGGGNKLTF TRB:CASSLVGEGYGYTF	1	CD4-C2
PB-3_CATATTCGTCGGCATC	PB	PB-3	P3-823	FALSE	1	TRA:CAGSSGSRLTF TRB:CASSLERAGANVLTF	1	CD4-C2
PB-3_CATCAAGGTGACGGTA	PB	PB-3	P3-824	FALSE	1	TRA:CALSLQNTGNQFYF TRB:CSASGQMNTGELFF	1	CD4-C1
PB-3_CATCAAGTCTTCGGTC	PB	PB-3	P3-825	FALSE	1	TRA:CAAPRYSGGGADGLTF TRB:CASNIGTGYYTF	1	CD4-C2
PB-3_CATCAGAGTCTAGTCA	PB	PB-3	P3-828	FALSE	1	TRA:CAVVPGYALNF TRB:CASSSSGGVTDTQYF	1	CD4-C2
PB-3_CATCCACCACGGCGTT	PB	PB-3	P3-829	FALSE	1	TRA:CAGQLRGTYKYIF TRB:CASSFGGLSGNTIYF	1	CD4-C2
PB-3_CATCCACCATGGTCAT	PB	PB-3	P3-831	FALSE	1	TRA:CAVNRAKGGGADGLTF TRB:CASSLDTNQPQHF	1	CD4-C3
PB-3_CATCCACGTTGCCTCT	PB	PB-3	P3-832	FALSE	1	TRA:CAMREFLTQGGSEKLVF TRB:CASEQAYEQYF	1	CD4-C2
PB-3_CATCGAAAGCGCTTAT	PB	PB-3	P3-834	FALSE	1	TRA:CAVRGLGERF TRB:CASSIATGNEKLFF	1	CD4-C2
PB-3_CATCGAATCACTATTC	PB	PB-3	P3-838	FALSE	1	TRA:CAVEGGGYQKVTF TRB:CASPDREEGYTF	1	CD4-C2
PB-3_CATCGGGAGCTCTCGG	PB	PB-3	P3-839	FALSE	1	TRA:CALPRGGGNKLVF TRB:CASSLNWAGEVDTQYF	1	CD4-C2
PB-3_CATCGGGTCACCGGGT	PB	PB-3	P3-842	FALSE	1	TRA:CAVEDRSRARLMF TRB:CASSPQQPTNEKLFF	1	CD4-C2
PB-3_CATCGGGTCTTCCTTC	PB	PB-3	P3-843	FALSE	1	TRA:CAVGDQTGANNLFF TRB:CASRGLADYEQYF	1	CD4-C2
PB-3_CATGACAAGTAAGTAC	PB	PB-3	P3-844	FALSE	1	TRA:CILRDDAGNNRKLIW TRB:CASSLNSQETQYF	1	CD4-C3
PB-3_CATGACATCATCATTC	PB	PB-3	P3-846	FALSE	1	TRA:CVVSDQDTNAGKSTF TRB:CAIAGTPYEQYF	1	CD4-C2
PB-3_CATGCCTTCTAGCACA	PB	PB-3	P3-847	FALSE	1	TRA:CAMSPNFGNEKLTF TRB:CASVVRGRDTEAFF	1	CD4-C2
PB-3_CATGGCGAGAGTGACC	PB	PB-3	P3-848	FALSE	1	TRA:CALKRPPRLETSGSRLTF TRB:CASNRWTGTNYGYTF	1	CD4-C2
PB-3_CATGGCGCATCCCACT	PB	PB-3	P3-850	FALSE	1	TRA:CAAITKAAGNKLTF TRB:CASTPGGGGTEAFF	1	CD4-C2
PB-3_CATTCGCAGTCTCCTC	PB	PB-3	P3-855	FALSE	1	TRA:CACLSNTGNQFYF TRB:CASSQDNTDTQYF	1	CD4-C2
PB-3_CCAATCCAGTGTACGG	PB	PB-3	P3-858	FALSE	1	TRA:CAVGPNYGQNFVF TRB:CASRPGTFSNEKLFF	1	CD4-C2
PB-3_CCAATCCTCGAACTGT	PB	PB-3	P3-861	FALSE	1	TRA:CALSAPSGGYQKVTF TRB:CSARDRKGVNPDTQYF	1	CD4-C2
PB-3_CCACCTAAGAGTGACC	PB	PB-3	P3-862	FALSE	1	TRA:CIVSLGSARQLTF TRB:CSASLAREDTIYF	1	CD4-C3

PB-3_CCACCTAAGTGTTAGA	PB	PB-3	P3-863	FALSE	1	TRA:CVVNGGRGSTLGRLYF TRB:CASSVEGQDYNEQFF	1	CD4-C2
PB-3_CCACCTACAGCGATCC	PB	PB-3	P3-864	FALSE	1	TRA:CAVQSGGSYKLIF TRB:CSANPAAEAFF	1	CD4-C2
PB-3_CCACCTAGTCTGATTG	PB	PB-3	P3-865	FALSE	1	TRA:CAMSEKNNARLMF TRB:CSVGADGEQYF	1	CD4-C2
PB-3_CCAGCGAAGCGGCTTC	PB	PB-3	P3-869	FALSE	1	TRA:CAAVTGKLIF TRB:CASSFPLSYNEQFF	1	CD4-C3
PB-3_CCATGTCTCGCCAAAT	PB	PB-3	P3-871	FALSE	1	TRA:CAEGAGSYQLTF TRB:CASSLGFSGELFF	1	CD4-C2
PB-3_CCATGTCTCTCCTATA	PB	PB-3	P3-872	FALSE	1	TRA:CAETGGFKTIF TRB:CASSRPGGDAFAEKLFF	1	CD4-C2
PB-3_CCATTCGCACATTCGA	PB	PB-3	P3-873	FALSE	1	TRA:CAMRGVQNFGNEKLTF TRB:CASRAKTDTQYF	1	CD4-C2
PB-3_CCCAGTTAGCTCAACT	PB	PB-3	P3-876	FALSE	1	TRA:CAVVPTLSSQNFVF TRB:CASSPSLNTEAFF	1	CD4-C2
PB-3_CCGGGATGTAGCTCCG	PB	PB-3	P3-881	FALSE	1	TRA:CVVNMESGGYNKLIF TRB:CASTQGRLTEAFF	1	CD4-C2
PB-3_CCGGTAGAGAGCTGCA	PB	PB-3	P3-884	FALSE	1	TRA:CAVLGGSNYKLTF TRB:CASSEFQGSTDTQYF	1	CD4-C2
PB-3_CCGGTAGGTTAAGATG	PB	PB-3	P3-885	FALSE	1	TRA:CALSGDDYKLSF TRB:CASETGYGYTF	1	CD4-C1
PB-3_CCGTACTGTCTGCGGT	PB	PB-3	P3-887	FALSE	1	TRA:CVVKGGAGARLMF TRB:CASSPDRAGANVLTF	1	CD4-C2
PB-3_CCGTGGATCGCCTGAG	PB	PB-3	P3-890	FALSE	1	TRA:CVVYQGSGTYKYIF TRB:CASSSDLWLTQYF	1	CD4-C1
PB-3_CCTACACCAGGCGATA	PB	PB-3	P3-892	FALSE	1	TRA:CAVSDGSGGGADGLTF TRB:CASGSPGLIYEQYF	1	CD4-C2
PB-3_CCTACACTCAACACAC	PB	PB-3	P3-894	FALSE	1	TRA:CALKSQGAQKLVF TRB:CASSLRRTEAFF	1	CD4-C2
PB-3_CCTACCAGTAGAGGAA	PB	PB-3	P3-896	FALSE	1	TRA:CVVNGGYSTLTF TRB:CAISESLGGQETQYF	1	CD4-C2
PB-3_CCTACCAGTCTACCTC	PB	PB-3	P3-897	FALSE	1	TRA:CAVEPTI TRB:CASSAGDQPQHF	1	CD4-C1
PB-3_CCTAGCTGTCGGGTCT	PB	PB-3	P3-156	FALSE	3	TRA:CAASSGGGADGLTF TRB:CSASSGVSYTQYF	3	CD4-C2
PB-3_CCTATTATCTTAGCCC	PB	PB-3	P3-903	FALSE	1	TRA:CAVRDRRNTDKLIF TRB:CASSWGGGGQTITGELFF	1	CD4-C1
PB-3_CCTCAGTCATTCTTAC	PB	PB-3	P3-905	FALSE	1	TRA:CAVRGDSNYQLIW TRB:CASTAGGIDNEQFF	1	CD4-C2
PB-3_CCTCTGACAGGAACGT	PB	PB-3	P3-907	FALSE	1	TRA:CAESIYGNKLVF TRB:CATSREGQDRGDTQYF	1	CD4-C3
PB-3_CCTCTGACAGTCGTGC	PB	PB-3	P3-908	FALSE	1	TRA:CAPERLGVTGKLIF TRB:CASREGWAPLHF	1	CD4-C2
PB-3_CCTTACGTCCAAACTG	PB	PB-3	P3-912	FALSE	1	TRA:CAGPNTGANSKLTF TRB:CASSYPGRDTQYF	1	CD4-C2
PB-3_CCTTCCCTCATACGGT	PB	PB-3	P3-913	FALSE	1	TRA:CAVQGGSLIF TRB:CSAVSTGGAGELFF	1	CD4-C2
PB-3_CCTTTCTCAGTATAAG	PB	PB-3	P3-917	FALSE	1	TRA:CALRSNTGNQFYF TRB:CASSQDTVYNSPLHF	1	CD4-C2
PB-3_CGAACATTCAGAAATG	PB	PB-3	P3-918	FALSE	1	TRA:CVVSRMDSNYQLIW TRB:CASRTDLNTEAFF	1	CD4-C2
PB-3_CGACCTTCAAATACAG	PB	PB-3	P3-923	FALSE	1	TRA:CAVNSDDYKLSF TRB:CASSPLSGTGGSNYGYTF	1	CD4-C2

PB-3_CGACTTCAGAAGGTGA	PB	PB-3	P3-265	FALSE	2	TRA:CAVNNAGNMLTF TRB:CAWRVPGAEAFF	2	CD4-C2
PB-3_CGACTTCCACCCATGG	PB	PB-3	P3-925	FALSE	1	TRA:CATVRSF TRB:CASSLTGELFF	1	CD4-C2
PB-3_CGACTTCGTTCAGACT	PB	PB-3	P3-927	FALSE	1	TRA:CAVSDGGATNKLIF TRB:CASSTRGGRGEQYF	1	CD4-C2
PB-3_CGACTTCTCCTTTCTC	PB	PB-3	P3-928	FALSE	1	TRA:CAVMNSGYSTLTF TRB:CASSQDAGSGAEAFF	1	CD4-C2
PB-3_CGAGCACGTAAGTGGC	PB	PB-3	P3-932	FALSE	1	TRA:CAVRDRNNNARLMF TRB:CASSVGGDDYEQYF	1	CD4-C2
PB-3_CGAGCCAAGGGCATGT	PB	PB-3	P3-935	FALSE	1	TRA:CAENAGTGGFKTIF TRB:CASSQNEKLFF	1	CD4-C2
PB-3_CGAGCCAGTGGCGAAT	PB	PB-3	P3-936	FALSE	1	TRA:CILRVPNNLFF TRB:CASSLGRAYGYTF	1	CD4-C2
PB-3_CGATGTAAGATACACA	PB	PB-3	P3-942	FALSE	1	TRA:CAVRSSGGYQKVTF TRB:CASSPQSYEQYF	1	CD4-C7
PB-3_CGATGTAGTCGCTTTC	PB	PB-3	P3-944	FALSE	1	TRA:CIVRVGRGATNKLIF TRB:CASSLGGVSFSTDTQYF	1	CD4-C2
PB-3_CGCCAAGCAAACCCAT	PB	PB-3	P3-946	FALSE	1	TRA:CATDEGGADGLTF TRB:CASRRDSSDRYEQYF	1	CD4-C7
PB-3_CGCGGTAGTCAACTGT	PB	PB-3	P3-949	FALSE	1	TRA:CAVNTGGFKTIF TRB:CASSWSGTNQPQHF	1	CD4-C2
PB-3_CGCGGTATCAACCATG	PB	PB-3	P3-950	FALSE	1	TRA:CAGPNAGNNRKLIW TRB:CASSWGAGGGELFF	1	CD4-C2
PB-3_CGCTGGATCTCTTGAT	PB	PB-3	P3-955	FALSE	1	TRA:CATYLTGGGNKLTF TRB:CASSLEMDQPQHF	1	CD4-C2
PB-3_CGCTTCAAGTGTGGCA	PB	PB-3	P3-956	FALSE	1	TRA:CAVTGGGNKLTF TRB:CASSSYGTDPGKLFF	1	CD4-C3
PB-3_CGCTTCACAGTTCCCT	PB	PB-3	P3-957	FALSE	1	TRA:CAVNAGNTGRRALTF TRB:CASRSQGAQTDTQYF	1	CD4-C1
PB-3_CGCTTCAGTATGAATG	PB	PB-3	P3-958	FALSE	1	TRA:CAMSGGSNDYKLSF TRB:CASSLNGIETQYF	1	CD4-C2
PB-3_CGGACACCACGCATCG	PB	PB-3	P3-960	FALSE	1	TRA:CAVGNQGGKLIF TRB:CASSLRLAGGVTQYF	1	CD4-C2
PB-3_CGGACGTAGATGGGTC	PB	PB-3	P3-963	FALSE	1	TRA:CAVNAVGAQKLVF TRB:CASSLGFQGETQYF	1	CD4-C2
PB-3_CGGACGTGTCCGCTGA	PB	PB-3	P3-234	FALSE	2	TRA:CAVTPVYGKLTF TRB:CASSLGETQYF	2	CD4-C2
PB-3_CGGAGCTTCTCGCATC	PB	PB-3	P3-969	FALSE	1	TRA:CAASDRYGGSQGNLIF TRB:CASSPTRVQGGNYGYTF	1	CD4-C2
PB-3_CGGCTAGCAGCGTCCA	PB	PB-3	P3-974	FALSE	1	TRA:CAYSAAGKSTF TRB:CASSLGLAGGGTDTQYF	1	CD4-C2
PB-3_CGGGTCACACTTAACG	PB	PB-3	P3-977	FALSE	1	TRA:CILRDTTSGTYKYIF TRB:CASSAVDYGNSPLHF	1	CD4-C2
PB-3_CGGGTCATCATCGATG	PB	PB-3	P3-979	FALSE	1	TRA:CAMRELGSNTGKLIF TRB:CASKGGFGNTIYF	1	CD4-C1
PB-3_CGGTTAAGTACAGTGG	PB	PB-3	P3-980	FALSE	1	TRA:CAMREGQKLLF TRB:CASSEGTTTEAFF	1	CD4-C2
PB-3_CGTCACTTCGTCTGAA	PB	PB-3	P3-985	FALSE	1	TRA:CAVTTGSSNTGKLIF TRB:CASSQDGGLNIQYF	1	CD4-C2
PB-3_CGTCAGGAGCTGTTCA	PB	PB-3	P3-169	FALSE	3	TRA:CAGTKGAQKLVF TRB:CASSPMNTEAFF	3	CD4-C2
PB-3_CGTCAGGAGGTGCAAC	PB	PB-3	P3-986	FALSE	1	TRA:CAENRGNNFNKFYF TRB:CASSPGQPTTQGYTF	1	CD4-C2

PB-3_CGTCCATAGAAGGTGA	PB	PB-3	P3-988	FALSE	1	TRA:CAVNSGGSARQLTF TRB:CASSQVRDSSYEQYF	1	CD4-C2
PB-3_CGTCCATGTAGCGTCC	PB	PB-3	P3-992	FALSE	1	TRA:CAVRSLDYSNYQLIW TRB:CAWSVGPGISYEQYF	1	CD4-C2
PB-3_CGTCCATTCTGAAAGA	PB	PB-3	P3-993	FALSE	1	TRA:CVVSTGNQFYF TRB:CSGGQFSYEQYF	1	CD4-C2
PB-3_CGTCTACTCAACTCTT	PB	PB-3	P3-998	FALSE	1	TRA:CAVSDQGTGNQFYF TRB:CASRAGGDLSLHF	1	CD4-C2
PB-3_CGTGAGCAGACTCGGA	PB	PB-3	P3-999	FALSE	1	TRA:CAVTGYAGKSTF TRB:CALSTSNSNQPQHF	1	CD4-C2
PB-3_CGTGAGCAGATCGATA	PB	PB-3	P3-1000	FALSE	1	TRA:CAVRIYGQNFVF TRB:CASSSGTDNEQFF	1	CD4-C2
PB-3_CGTGAGCTCCGCATCT	PB	PB-3	P3-1001	FALSE	1	TRA:CAVKTAGRRALTF TRB:CASTSDSPNQPQHF	1	CD4-C3
PB-3_CGTGTAAAGAGTGAGA	PB	PB-3	P3-1002	FALSE	1	TRA:CVVSDRGGTYKYIF TRB:CSAGLTGRDTEAFF	1	CD4-C2
PB-3_CGTGTAAGTTATCACG	PB	PB-3	P3-1006	FALSE	1	TRA:CAAGGESGGSNYKLTF TRB:CASSHYNEQFF	1	CD4-C2
PB-3_CGTGTCTCAAAGAATC	PB	PB-3	P3-1007	FALSE	1	TRA:CAVQAGGYQKVTF TRB:CASSIGPRRGGGAQETQYF	1	CD4-C1
PB-3_CGTTAGATCAGGTTCA	PB	PB-3	P3-249	FALSE	2	TRA:CILRSGAGSYQLTF TRB:CASSLFASPKTDTQYF	2	CD4-C2
PB-3_CGTTCTGTCAGTACGT	PB	PB-3	P3-1010	FALSE	1	TRA:CGTERENYGGATNKLIF TRB:CASSLGMTATDTQYF	1	CD4-C2
PB-3_CTAACTTGTCAATACC	PB	PB-3	P3-1016	FALSE	1	TRA:CAASTRAGSYQLTF TRB:CASSPGRIFSGNTIYF	1	CD4-C2
PB-3_CTAACTTGTCCAGTGC	PB	PB-3	P3-1017	FALSE	1	TRA:CSPFGAQKLVF TRB:CAGGAGGFGDTQYF	1	CD4-C2
PB-3_CTACATTAGACCTAGG	PB	PB-3	P3-1023	FALSE	1	TRA:CASGRGQKLLF TRB:CASSETGTAPYEQYF	1	CD4-C2
PB-3_CTACCCAAGTACACCT	PB	PB-3	P3-1025	FALSE	1	TRA:CAARANSGYALNF TRB:CASSPEDGFNSPLHF	1	CD4-C2
PB-3_CTACGTCGTTAGTGGG	PB	PB-3	P3-1029	FALSE	1	TRA:CVRETSGSRLTF TRB:CATSGRLAGDNEQFF	1	CD4-C2
PB-3_CTACGTCTCAAGAAGT	PB	PB-3	P3-1030	FALSE	1	TRA:CIVRVGYSTLTF TRB:CASSLKGASSYEQYF	1	CD4-C2
PB-3_CTAGAGTGTCAGAATA	PB	PB-3	P3-1033	FALSE	1	TRA:CATVIYNQGGKLIF TRB:CASSFLLAGEEHTDTQYF	1	CD4-C2
PB-3_CTAGAGTGTCCGAACC	PB	PB-3	P3-1034	FALSE	1	TRA:CAVSEADFGNVLHC TRB:CASTEYRGWNEQFF	1	CD4-C2
PB-3_CTAGCCTCAACTGGCC	PB	PB-3	P3-1037	FALSE	1	TRA:CAASKKGGANNLFF TRB:CASSLAWNQPQHF	1	CD4-C2
PB-3_CTAGTGACATGGTCAT	PB	PB-3	P3-1040	FALSE	1	TRA:CAVNPPYSSASKIIF TRB:CASSRGGRARTGELFF	1	CD4-C2
PB-3_CTAGTGATCAACGAAA	PB	PB-3	P3-114	TRUE	3	TRA:CVVNGRDDKIIF TRB:CASSLGRGLGNTIYF	4	CD4-C2
PB-3_CTCACACGTTGGTTTG	PB	PB-3	P3-1041	FALSE	1	TRA:CAENIEYGGSQGNLIF TRB:CASSVVDSNTLYNEQFF	1	CD4-C3
PB-3_CTCACACTCTAACTGG	PB	PB-3	P3-1042	FALSE	1	TRA:CVVSGGDYKLSF TRB:CASSPRAILLEQYF	1	CD4-C2
PB-3_CTCATTAAGAGTCTGG	PB	PB-3	P3-1045	FALSE	1	TRA:CAVRRSGGSYIPTF TRB:CASSIGGSTDTQYF	1	CD4-C2
PB-3_CTCCTAGAGACCTTTG	PB	PB-3	P3-1049	FALSE	1	TRA:CAFMKPRGNQFYF TRB:CASSDGTGDYEQYF	1	CD4-C2

PB-3_CTCCTAGCAGTCCTTC	PB	PB-3	P3-1050	FALSE	1	TRA:CIATSGSRLTF TRB:CASSLVTGTEAFF	1	CD4-C2
PB-3_CTCCTAGGTTGCCTCT	PB	PB-3	P3-1052	FALSE	1	TRA:CAALRGARLMF TRB:CASKSGRGSPDTQYF	1	CD4-C1
PB-3_CTCCTAGTCAGAGCTT	PB	PB-3	P3-1053	FALSE	1	TRA:CAVRGTSYGKLTF TRB:CASSRGPSDTDTQYF	1	CD4-C2
PB-3_CTCGAAAGTTCACGGC	PB	PB-3	P3-1054	FALSE	1	TRA:CLVGDSGGTGGFKTIF TRB:CSASYGVEQYF	1	CD4-C2
PB-3_CTCGAAATCGGATGGA	PB	PB-3	P3-1056	FALSE	1	TRA:CAVRLRSGTYKYIF TRB:CASSQDAGSNTEAFF	1	CD4-C2
PB-3_CTCGAGGAGACTTTCG	PB	PB-3	P3-49	TRUE	3	TRA:CAVEDRGNQGGKLIF TRB:CSARALSGGAGEQYF	7	CD4-C2
PB-3_CTCGAGGCAAACTGTC	PB	PB-3	P3-1057	FALSE	1	TRA:CAASEYSSASKIIF TRB:CASSLMAASTDTQYF	1	CD4-C2
PB-3_CTCGGAGAGCGAGAAA	PB	PB-3	P3-1059	FALSE	1	TRA:CAASRYGNKLVF TRB:CAWSGGPNTGELFF	1	CD4-C3
PB-3_CTCGGAGCAGTGAGTG	PB	PB-3	P3-1062	FALSE	1	TRA:CAVRDMGNNDMRF TRB:CATSRDLATGNTIYF	1	CD4-C2
PB-3_CTCGGAGTCCGCAGTG	PB	PB-3	P3-1064	FALSE	1	TRA:CAASRTTGTASKLTF TRB:CSARGRNRGLNSPLHF	1	CD4-C3
PB-3_CTCGGGACAAGGACTG	PB	PB-3	P3-282	TRUE	2	TRA:CAANAGNMLTF TRB:CASSLGGQPNQPQHF	2	CD4-C2
PB-3_CTCTAATCACCAGGTC	PB	PB-3	P3-1074	FALSE	1	TRA:CAVSDPQGAQKLVF TRB:CSARTGHLYGYTF	1	CD4-C1
PB-3_CTCTAATGTATGCTTG	PB	PB-3	P3-1075	FALSE	1	TRA:CAASWKTSYDKVIF TRB:CASSLWGPQETQYF	1	CD4-C2
PB-3_CTCTACGAGGAGTTTA	PB	PB-3	P3-1076	FALSE	1	TRA:CAQNYGGSQGNLIF TRB:CATAPRPGNTGELFF	1	CD4-C1
PB-3_CTCTACGTCGGTGTCG	PB	PB-3	P3-1081	FALSE	1	TRA:CIETGGYQKVTF TRB:CAITPTGEANYGYTF	1	CD4-C2
PB-3_CTGAAGTGTTACCAGT	PB	PB-3	P3-1082	FALSE	1	TRA:CAVRDSNNAGNMLTF TRB:CASSTGGQPQHF	1	CD4-C2
PB-3_CTGAAGTTCCTTCAAT	PB	PB-3	P3-1083	FALSE	1	TRA:CIVRVFNSGAGSYQLTF TRB:CASSLLVTDTQYF	1	CD4-C2
PB-3_CTGATAGCACAGCGTC	PB	PB-3	P3-1085	FALSE	1	TRA:CATDGSGKLTF TRB:CASSVLHVYNEQFF	1	CD4-C2
PB-3_CTGATAGCACGAGGTA	PB	PB-3	P3-1086	FALSE	1	TRA:CAVTGANSKLTF TRB:CSARRGGAGLADTQYF	1	CD4-C2
PB-3_CTGATCCTCTATCCTA	PB	PB-3	P3-1089	FALSE	1	TRA:CAGRGGGSQGNLIF TRB:CASSPSPGPPNTEAFF	1	CD4-C2
PB-3_CTGCCTAAGCCCTAAT	PB	PB-3	P3-1090	FALSE	1	TRA:CATVGGYQKVTF TRB:CASSTSGVQGGFYEQFF	1	CD4-C2
PB-3_CTGCGGAGTGAGTATA	PB	PB-3	P3-1093	FALSE	1	TRA:CAVRDGQKLLF TRB:CASGAHTGGPTDTQYF	1	CD4-C3
PB-3_CTGGTCTAGGGATGGG	PB	PB-3	P3-1095	FALSE	1	TRA:CAVSDRVNGNKLVF TRB:CASSLDPAGEQHF	1	CD4-C2
PB-3_CTGGTCTCACACGCTG	PB	PB-3	P3-1097	FALSE	1	TRA:CLLLGGSNYKLTF TRB:CASSAPGFTTMNEKLFF	1	CD4-C2
PB-3_CTGGTCTCACGACTCG	PB	PB-3	P3-175	TRUE	3	TRA:CIVRSGGSNYKLTF TRB:CASSFLGGGNTIYF	3	CD4-C2
PB-3_CTGTTTATCACCCTCA	PB	PB-3	P3-1102	FALSE	1	TRA:CAYLNYGGSQGNLIF TRB:CASSQDDLNTEAFF	1	CD4-C2
PB-3_CTTCTCTCATGTAGTC	PB	PB-3	P3-287	FALSE	2	TRA:CAVGARDNFNKFYF TRB:CAWRGLGQKFNEQFF	2	CD4-C2

PB-3_CTTGGCTTCTGCCAGG	PB	PB-3	P3-1108	FALSE	1	TRA:CAVPFPSGNTPLVF TRB:CASSSLDGTYEQYF	1	CD4-C2
PB-3_GAAACTCGTGCATCTA	PB	PB-3	P3-1112	FALSE	1	TRA:CAVGGRETSGSRLTF TRB:CATSAPFGGTDTQYF	1	CD4-C2
PB-3_GAAATGATCTCATTCA	PB	PB-3	P3-1116	FALSE	1	TRA:CILPAGGTSYGKLTF TRB:CSARGRNYGYTF	1	CD4-C2
PB-3_GAACATCCAATCCGAT	PB	PB-3	P3-1117	FALSE	1	TRA:CAASNSGYALNF TRB:CSATNGLAGVVETQYF	1	CD4-C2
PB-3_GAACATCCAGCTGGCT	PB	PB-3	P3-1118	FALSE	1	TRA:CAVDRDDKIIF TRB:CASSDPPAGNQPQHF	1	CD4-C2
PB-3_GAACATCTCGAACGGA	PB	PB-3	P3-1120	FALSE	1	TRA:CALSEAWGSARQLTF TRB:CATSSERGRVETQYF	1	CD4-C2
PB-3_GAACCTAAGCACGCCT	PB	PB-3	P3-1121	FALSE	1	TRA:CALSDRRGGADGLTF TRB:CASSPSSSYNSPLHF	1	CD4-C2
PB-3_GAACCTAGTATATGGA	PB	PB-3	P3-1123	FALSE	1	TRA:CAVSETGTASKLTF TRB:CASSLGVGGNIQYF	1	CD4-C2
PB-3_GAACGGATCAAAGTAG	PB	PB-3	P3-1124	FALSE	1	TRA:CILISGAGSYQLTF TRB:CASSQENFPRPGQVKYIQYF	1	CD4-C2
PB-3_GAATAAGAGATCACGG	PB	PB-3	P3-1127	FALSE	1	TRA:CVVNLNDYKLSF TRB:CASSRWSGAEQYF	1	CD4-C2
PB-3_GAATAAGGTCTACCTC	PB	PB-3	P3-1129	FALSE	1	TRA:CAASEDNFNKFYF TRB:CASSIRDYGYTF	1	CD4-C2
PB-3_GAATGAACATTGGGCC	PB	PB-3	P3-265	FALSE	2	TRA:CAVNNAGNMLTF TRB:CAWRVPGAEAFF	2	CD4-C2
PB-3_GACAGAGGTTCCCTTG	PB	PB-3	P3-1135	FALSE	1	TRA:CAYRPGGGADGLTF TRB:CASSLVGGGAETQYF	1	CD4-C3
PB-3_GACAGAGGTTGTTTGG	PB	PB-3	P3-1136	FALSE	1	TRA:CAMMSGGSEKLVF TRB:CSVVRGTHEQYF	1	CD4-C2
PB-3_GACCTGGAGGACAGAA	PB	PB-3	P3-1141	FALSE	1	TRA:CAVDMYNNNDMRF TRB:CASSLRGFTEAFF	1	CD4-C2
PB-3_GACGCGTGTACCGTTA	PB	PB-3	P3-1147	FALSE	1	TRA:CAVSPASYDKVIF TRB:CASSLDPREQYF	1	CD4-C2
PB-3_GACGCGTGTAGATTAG	PB	PB-3	P3-1148	FALSE	1	TRA:CAVSGYLSGTYKYIF TRB:CASSSLSGNTIYF	1	CD4-C3
PB-3_GACGCGTGTCGGCTCA	PB	PB-3	P3-1149	FALSE	1	TRA:CAVSVPSGSARQLTF TRB:CASSQVWGSNEKLFF	1	CD4-C2
PB-3_GACGGCTGTGTAACGG	PB	PB-3	P3-1153	FALSE	1	TRA:CATRFTGGGNKLTF TRB:CAWSVLGYNEQFF	1	CD4-C2
PB-3_GACGTTAGTAAAGGAG	PB	PB-3	P3-1154	FALSE	1	TRA:CAESIAGQKLLF TRB:CASSLGANYEQYF	1	CD4-C2
PB-3_GACTAACGTAGCAAAT	PB	PB-3	P3-1157	FALSE	1	TRA:CAVDSNNAGNMLTF TRB:CASSLIGGATYADTQYF	1	CD4-C7
PB-3_GACTAACTCCACGAAT	PB	PB-3	P3-1158	FALSE	1	TRA:CALSGTNNARLMF TRB:CASREKVFNEKLFF	1	CD4-C2
PB-3_GAGCAGAGTTCGTTGA	PB	PB-3	P3-1163	FALSE	1	TRA:CAPGGKAAGNKLTF TRB:CASSLRGNTEAFF	1	CD4-C2
PB-3_GAGTCCGCAGATCGGA	PB	PB-3	P3-1167	FALSE	1	TRA:CAGDGGSNYKLTF TRB:CASRKSGGYTGELFF	1	CD4-C2
PB-3_GAGTCCGGTCGAAAGC	PB	PB-3	P3-1169	FALSE	1	TRA:CAVRVLRTGGGNKLTF TRB:CASSFRGQGSTYEQYF	1	CD4-C2
PB-3_GATCAGTCACCGCTAG	PB	PB-3	P3-1170	FALSE	1	TRA:CAVNRGRGSTLGRLYF TRB:CSVRGVETQYF	1	CD4-C2
PB-3_GATCGCGGTACCGAGA	PB	PB-3	P3-1174	FALSE	1	TRA:CAVDYGQNFVF TRB:CASSDLYGYTF	1	CD4-C2

PB-3_GCACATACATGGTTGT	PB	PB-3	P3-1190	FALSE	1	TRA:CAAAYSNSGYALNF TRB:CASSFLGVAFF	1	CD4-C1
PB-3_GCACTCTAGCCCAACC	PB	PB-3	P3-1192	FALSE	1	TRA:CARVDNAGKSTF TRB:CASSPQGRVEPLHF	1	CD4-C2
PB-3_GCACTCTGTTAGTGGG	PB	PB-3	P3-296	TRUE	2	TRA:CAVELSSNTGKLIF TRB:CASNSGGITEAFF	2	CD4-C2
PB-3_GCAGTTATCTAACCGA	PB	PB-3	P3-1197	FALSE	1	TRA:CAVGATQGSSNTGKLIF TRB:CAWSVWGGNNSPLHF	1	CD4-C3
PB-3_GCATACAAGGCTATCT	PB	PB-3	P3-1198	FALSE	1	TRA:CGADITTNAGKSTF TRB:CASTPGPLLTGELFF	1	CD4-C2
PB-3_GCATGATCAGGAATCG	PB	PB-3	P3-1201	FALSE	1	TRA:CAASSESGGSYIPTF TRB:CASSWYTGSEAFF	1	CD4-C2
PB-3_GCATGCGCAAGCTGTT	PB	PB-3	P3-248	FALSE	2	TRA:CAVVYTGRRALTF TRB:CAWNQVGADTQYF	2	CD4-C2
PB-3_GCGACCAAGTGTCCCG	PB	PB-3	P3-180	TRUE	3	TRA:CATAVMEYGNKLVF TRB:CASSHVGGVGELFF	3	CD4-C2
PB-3_GCGACCACACATCCGG	PB	PB-3	P3-1210	FALSE	1	TRA:CAYRSAGSNYKLTF TRB:CASSLGGAYNEQFF	1	CD4-C2
PB-3_GCGAGAACAGTGGAGT	PB	PB-3	P3-298	TRUE	2	TRA:CAEEYGNKLVF TRB:CASRNGLAGDTQYF	2	CD4-C3
PB-3_GCGAGAATCTTTACGT	PB	PB-3	P3-1215	FALSE	1	TRA:CNPSSSAGNKLTF TRB:CASTRRAGDTGELFF	1	CD4-C2
PB-3_GCGCAACCACAGACTT	PB	PB-3	P3-1216	FALSE	1	TRA:CVAGLDSNYQLIW TRB:CASSEGFYSGNTIYF	1	CD4-C3
PB-3_GCGCAACTCAAGCCTA	PB	PB-3	P3-1218	FALSE	1	TRA:CAMRDLSYNTDKLIF TRB:CASSRGAYSGNTIYF	1	CD4-C2
PB-3_GCGCCAAAGCAGATCG	PB	PB-3	P3-1220	FALSE	1	TRA:CAVREAVTGGGNKLTF TRB:CASSRLTGGNQPQHF	1	CD4-C2
PB-3_GCGCCAACATGAAGTA	PB	PB-3	P3-291	FALSE	2	TRA:CAVISSGSARQLTF TRB:CASSQGTGVSGGTQYF	2	CD4-C2
PB-3_GCGCCAATCTTTACGT	PB	PB-3	P3-1222	FALSE	1	TRA:CAGANNAGNMLTF TRB:CASSLENLNIQYF	1	CD4-C2
PB-3_GCGCGATAGTGTTTGC	PB	PB-3	P3-1223	FALSE	1	TRA:CLVGDAGGADGLTF TRB:CASSYSGSGVYEQYF	1	CD4-C2
PB-3_GCTCCTATCTGATTCT	PB	PB-3	P3-1226	FALSE	1	TRA:CAVSGDSNYQLIW TRB:CASSLDGWNEQFF	1	CD4-C2
PB-3_GCTCTGTAGCCAGTAG	PB	PB-3	P3-1227	FALSE	1	TRA:CAVGARAAGNKLTF TRB:CASSQEGGGSGTPGNTIYF	1	CD4-C2
PB-3_GCTCTGTTCATCTGCC	PB	PB-3	P3-1228	FALSE	1	TRA:CVVSRSSGGYNKLIF TRB:CASSQDRTGWQETQYF	1	CD4-C2
PB-3_GCTGCAGCAGACAAAT	PB	PB-3	P3-1230	FALSE	1	TRA:CAASGFADKLIF TRB:CSATDGANQPQHF	1	CD4-C2
PB-3_GCTGGGTAGGGTGTGT	PB	PB-3	P3-1237	FALSE	1	TRA:CAAHHNTDKLIF TRB:CASSSNRDGRQYF	1	CD4-C2
PB-3_GGAAAGCTCCTTTCTC	PB	PB-3	P3-156	FALSE	3	TRA:CAASSGGGADGLTF TRB:CSASSGVSYTQYF	3	CD4-C2
PB-3_GGACAAGTCCAGTATG	PB	PB-3	P3-1250	FALSE	1	TRA:CATVLTGRRALTF TRB:CAWSPSGGANVLTF	1	CD4-C2
PB-3_GGACAGACACACCGCA	PB	PB-3	P3-1251	FALSE	1	TRA:CIVRIPNAGNMLTF TRB:CSVETETYEQYF	1	CD4-C2
PB-3_GGACATTTCGCAGGCT	PB	PB-3	P3-1252	FALSE	1	TRA:CAVRDMGYGGATNKLIF TRB:CASSRNRGGIHNEQFF	1	CD4-C2
PB-3_GGACGTCAGCTGCCCA	PB	PB-3	P3-1254	FALSE	1	TRA:CAAYNNNDMRF TRB:CASSQTTNSYEQYF	1	CD4-C2

PB-3_GGAGCAACAGACAAAT	PB	PB-3	P3-1257	FALSE	1	TRA:CVVNAGYSTLTF TRB:CASSLPTGHYGYTF	1	CD4-C2
PB-3_GGAGCAACAGGAACGT	PB	PB-3	P3-1258	FALSE	1	TRA:CATEGPDQGGSEKLVF TRB:CASSHRRQETQYF	1	CD4-C3
PB-3_GGAGCAAGTAGCGTAG	PB	PB-3	P3-1259	FALSE	1	TRA:CALERGNYGQNFVF TRB:CASSLTGLAGGKETQYF	1	CD4-C1
PB-3_GGATTACTCCGTAGGC	PB	PB-3	P3-1265	FALSE	1	TRA:CAENSQGGWGKFQF TRB:CSASLGNQPQHF	1	CD4-C2
PB-3_GGCCGATAGAAACCGC	PB	PB-3	P3-1270	FALSE	1	TRA:CAAKPDGQKLLF TRB:CASSLAAAQETQYF	1	CD4-C2
PB-3_GGCCGATGTCAGAGGT	PB	PB-3	P3-1272	FALSE	1	TRA:CAVTLNQGGKLIF TRB:CSARQLAGASNTGELFF	1	CD4-C1
PB-3_GGCGTGTCATCCCATC	PB	PB-3	P3-1274	FALSE	1	TRA:CAGRRAGGTSYGKLTF TRB:CASSQRGGNNEQFF	1	CD4-C2
PB-3_GGGAGATCAAGTACCT	PB	PB-3	P3-1285	FALSE	1	TRA:CAMREGGNTGGFKTIF TRB:CASSLSPGTGSVNEQFF	1	CD4-C2
PB-3_GGGATGAAGTCCATAC	PB	PB-3	P3-1286	FALSE	1	TRA:CADAYFGNEKLTF TRB:CSTRDRSTTEAFF	1	CD4-C2
PB-3_GGGATGACAGGACGTA	PB	PB-3	P3-1287	FALSE	1	TRA:CIVRPVGGGADGLTF TRB:CASSEIYRNTEAFF	1	CD4-C2
PB-3_GGGCACTAGTGAAGTT	PB	PB-3	P3-1289	FALSE	1	TRA:CAVTGNMLTF TRB:CASSFEGYNSPLHF	1	CD4-C2
PB-3_GGGCACTCAACGATGG	PB	PB-3	P3-1290	FALSE	1	TRA:CAVWAGNNRKLIW TRB:CASSATDRGKLFF	1	CD4-C2
PB-3_GGGTTGCGTGATGTCT	PB	PB-3	P3-1294	FALSE	1	TRA:CVVTPWGLTGGGNKLTF TRB:CASSPGTSGGATGANVLT[illegible]	1	CD4-C1
PB-3_GGTATTGGTCACAAGG	PB	PB-3	P3-1296	FALSE	1	TRA:CAVRQGGSEKLVF TRB:CSASLGRGSEETQYF	1	CD4-C2
PB-3_GGTGCGTTCAAGCCTA	PB	PB-3	P3-1302	FALSE	1	TRA:CVVPQSGGSYIPTF TRB:CAWRKRQGQNSPLHF	1	CD4-C2
PB-3_GGTGTTAGTCGCATCG	PB	PB-3	P3-1304	FALSE	1	TRA:CAVFSGAGSYQLTF TRB:CASSQVFGAGGQETQYF	1	CD4-C2
PB-3_GGTGTTATCAAGAAGT	PB	PB-3	P3-1305	FALSE	1	TRA:CAVSDSSGTASKLTF TRB:CASSRTTYEQYF	1	CD4-C2
PB-3_GGTGTTATCAGTCCCT	PB	PB-3	P3-1306	FALSE	1	TRA:CVVAPGGSQGNLIF TRB:CASSLAPGARYEQYF	1	CD4-C2
PB-3_GTAACGTCAGCTTCGG	PB	PB-3	P3-287	FALSE	2	TRA:CAVGARDNFNKFYF TRB:CAWRGLGQKFNEQFF	2	CD4-C2
PB-3_GTACGTAGTCCCTACT	PB	PB-3	P3-1313	FALSE	1	TRA:CIVRVQRNSGNTPLVF TRB:CATSRDLGKQQPQHF	1	CD4-C2
PB-3_GTACGTATCTCATTCA	PB	PB-3	P3-1314	FALSE	1	TRA:CAMREGNTNAGKSTF TRB:CASSQDMRGNTEAFF	1	CD4-C2
PB-3_GTACTCCGTGCAGTAG	PB	PB-3	P3-1316	FALSE	1	TRA:CAVGADDYKLSF TRB:CASRQGALTEAFF	1	CD4-C2
PB-3_GTACTTTGTCTCGTTC	PB	PB-3	P3-1319	FALSE	1	TRA:CALNLGGGGSNYKLTF TRB:CASSQRRDIQYF	1	CD4-C2
PB-3_GTACTTTGTGATGTGG	PB	PB-3	P3-1320	FALSE	1	TRA:CAGESGDDTDKLIF TRB:CASSMTGEKLFF	1	CD4-C2
PB-3_GTAGGCCGTAAGGGAA	PB	PB-3	P3-1324	FALSE	1	TRA:CVAPLSNFGNEKLTF TRB:CASRAGQAGSGANVLTF	1	CD4-C2
PB-3_GTAGTCATCCTCCTAG	PB	PB-3	P3-304	FALSE	2	TRA:CAGQNTGTASKLTF TRB:CAWSPPRTDTQYF	2	CD4-C1
PB-3_GTATCTTAGACAATAC	PB	PB-3	P3-1326	FALSE	1	TRA:CILRDVWRGGSNYKLTF TRB:CASSQTDTGELFF	1	CD4-C2

PB-3_GTATCTTAGCGTAATA	PB	PB-3	P3-1327	FALSE	1	TRA:CAMRTGAGSYQLTF TRB:CASSQGTGTNYGYTF	1	CD4-C2
PB-3_GTATCTTAGCTGAAAT	PB	PB-3	P3-1328	FALSE	1	TRA:CAASAYSGAGSYQLTF TRB:CASSQSLMGGIDTQYF	1	CD4-C2
PB-3_GTCAAGTTCTTTCCTC	PB	PB-3	P3-1336	FALSE	1	TRA:CAVEAGRTGANNLFF TRB:CASSLGQAGTQYF	1	CD4-C2
PB-3_GTCACGGGTGTTGGGA	PB	PB-3	P3-1338	FALSE	1	TRA:CAGSGGSNYKLTF TRB:CASSYDLRDGYTF	1	CD4-C2
PB-3_GTCATTTAGTGACTCT	PB	PB-3	P3-1341	FALSE	1	TRA:CAVRLNNAGNMLTF TRB:CSARDSNTRITEAFF	1	CD4-C2
PB-3_GTCATTTAGTTGTAGA	PB	PB-3	P3-1342	FALSE	1	TRA:CAVGGGPGGKLIF TRB:CASSQGTGGELFF	1	CD4-C2
PB-3_GTCATTTCAAGGGTCA	PB	PB-3	P3-1343	FALSE	1	TRA:CAIDDYKLSF TRB:CASSEGRASNQPQHF	1	CD4-C2
PB-3_GTCCTCAAGAGGACGG	PB	PB-3	P3-1347	FALSE	1	TRA:CAENMASGGSYIPTF TRB:CASSVDQGRGYTF	1	CD4-C2
PB-3_GTCGGGTGTGGCCCTA	PB	PB-3	P3-1350	FALSE	1	TRA:CAVQANTGKLIF TRB:CAWRGGGNEQFF	1	CD4-C2
PB-3_GTCGTAAAGAATGTTG	PB	PB-3	P3-1352	FALSE	1	TRA:CAVERGSQGNLIF TRB:CASRISGDLQETQYF	1	CD4-C2
PB-3_GTCGTAACAATGTAAG	PB	PB-3	P3-1353	FALSE	1	TRA:CIVRVVSGGGADGLTF TRB:CASSLYVFGTGGSEAFF	1	CD4-C2
PB-3_GTCGTAACATACAGCT	PB	PB-3	P3-1354	FALSE	1	TRA:CAYDVGANNLFF TRB:CASGEGPQYYEQYF	1	CD4-C2
PB-3_GTCGTAAGTAGAGTGC	PB	PB-3	P3-1355	FALSE	1	TRA:CAVQARGAGNMLTF TRB:CASSENHAAEKLFF	1	CD4-C2
PB-3_GTCTCGTGTATCTGCA	PB	PB-3	P3-1357	FALSE	1	TRA:CALSPNQAGTALIF TRB:CSARDWQRGDTDTQYF	1	CD4-C2
PB-3_GTGAAGGAGCGCCTTG	PB	PB-3	P3-1361	FALSE	1	TRA:CAVRAMDSSYKLIF TRB:CASSLSGYEQYF	1	CD4-C2
PB-3_GTGAAGGGTCCCTTGT	PB	PB-3	P3-1363	FALSE	1	TRA:CGEPGSNDYKLSF TRB:CAWRGRGSNYGYTF	1	CD4-C2
PB-3_GTGCAGCCAGGTCCAC	PB	PB-3	P3-1365	FALSE	1	TRA:CAGLPSGYSTLTF TRB:CAWSFVGGRGNEQFF	1	CD4-C3
PB-3_GTGCAGCGTACGAAAT	PB	PB-3	P3-309	TRUE	2	TRA:CATLRSSGYALNF TRB:CASALGGGSKETQYF	2	CD4-C2
PB-3_GTGCATAAGTTCGATC	PB	PB-3	P3-1367	FALSE	1	TRA:CALSDERQGGSEKLVF TRB:CASSLSLLYEQYF	1	CD4-C2
PB-3_GTGCATACACCAGATT	PB	PB-3	P3-1368	FALSE	1	TRA:CAAPPRSASKIIF TRB:CASSLGWTDEQYF	1	CD4-C3
PB-3_GTGCATAGTCCCTACT	PB	PB-3	P3-1369	FALSE	1	TRA:CAVTNNDMRF TRB:CSAQGNRQGGNQPQHF	1	CD4-C2
PB-3_GTGCTTCTCCTGCAGG	PB	PB-3	P3-1375	FALSE	1	TRA:CAVRFSGGYNKLIF TRB:CASSPGQTASYEQYF	1	CD4-C2
PB-3_GTGTGCGCAAGCCATT	PB	PB-3	P3-254	FALSE	2	TRA:CAVRTGGGNKLTF TRB:CASRADSTYEQYF	2	CD4-C2
PB-3_GTGTGCGCAGTTCATG	PB	PB-3	P3-1377	FALSE	1	TRA:CAMKGSYNTDKLIF TRB:CASSYSNVFGDRGEQFF	1	CD4-C3
PB-3_GTGTGCGGTGAGGCTA	PB	PB-3	P3-1379	FALSE	1	TRA:CLVGDPRNQGGKLIF TRB:CASSSPGDPSSYNEQFF	1	CD4-C2
PB-3_GTGTTAGAGAGATGAG	PB	PB-3	P3-1381	FALSE	1	TRA:CAVSPNFGNEKLTF TRB:CASSIDRIQFF	1	CD4-C2
PB-3_GTGTTAGTCGGAGGTA	PB	PB-3	P3-1383	FALSE	1	TRA:CALSGQSGGSYIPTF TRB:CAWELTGFPEAFF	1	CD4-C1

PB-3_GTTAAGCAGTGCCAGA	PB	PB-3	P3-1384	FALSE	1	TRA:CILRELTLSGNTPLVF TRB:CASSLSARGNEKLFF	1	CD4-C2
PB-3_GTTAAGCAGTGGGATC	PB	PB-3	P3-310	FALSE	2	TRA:CAGPLYGGSQGNLIF TRB:CASSPLKRIETQYF	2	CD4-C2
PB-3_GTTACAGTCGTATCAG	PB	PB-3	P3-1386	FALSE	1	TRA:CAASGSARQLTF TRB:CASTGFLEKLFF	1	CD4-C3
PB-3_GTTCATTAGCCACTAT	PB	PB-3	P3-312	TRUE	2	TRA:CAASILTGGGNKLTF TRB:CASGRGITDTQYF	2	CD4-C3
PB-3_GTTCATTTCGCCTGAG	PB	PB-3	P3-1388	FALSE	1	TRA:CAVGAGYGNKLVF TRB:CASSPTGTPEQPQHF	1	CD4-C2
PB-3_GTTCGGGGTTTGGCGC	PB	PB-3	P3-1390	FALSE	1	TRA:CALVAGSYQLTF TRB:CASSLDLRDEQFF	1	CD4-C2
PB-3_GTTCTCGCACGAAGCA	PB	PB-3	P3-1392	FALSE	1	TRA:CAVRVSGANSKLTF TRB:CASSLGGGVEQYF	1	CD4-C2
PB-3_GTTCTCGGTTATCCGA	PB	PB-3	P3-1393	FALSE	1	TRA:CALSSDAGGTSYGKLTF TRB:CASSFGGNTGELFF	1	CD4-C2
PB-3_GTTCTCGGTTATTCTC	PB	PB-3	P3-1394	FALSE	1	TRA:CVVNIGARLMF TRB:CASSSPGGTGPLYGYTF	1	CD4-C2
PB-3_GTTTCTACACCCATTC	PB	PB-3	P3-1395	FALSE	1	TRA:CAVNFNKFYF TRB:CASSDLDTQETQYF	1	CD4-C2
PB-3_GTTTCTATCGCTGATA	PB	PB-3	P3-1396	FALSE	1	TRA:CVVLNNAGNMLTF TRB:CASSQDTPTGTGNTIYF	1	CD4-C2
PB-3_TAAACCGAGAATAGGG	PB	PB-3	P3-1398	FALSE	1	TRA:CAVSEQNTGTASKLTF TRB:CASSLGVMEQETQYF	1	CD4-C2
PB-3_TAAACCGGTCGACTAT	PB	PB-3	P3-1401	FALSE	1	TRA:CAINTGNQFYF TRB:CASSYSAIGPNYNSPLHF	1	CD4-C2
PB-3_TAAGCGTGTAGAGCTG	PB	PB-3	P3-1406	FALSE	1	TRA:CLVQNDYKLSF TRB:CASTKAGGGTDTQYF	1	CD4-C3
PB-3_TAAGCGTTCTGACCTC	PB	PB-3	P3-1407	FALSE	1	TRA:CVASPRSGGYNKLIF TRB:CASGVHTGGANTGELFF	1	CD4-C2
PB-3_TACACGAGTCCTCCAT	PB	PB-3	P3-1409	FALSE	1	TRA:CALDFNQAGTALIF TRB:CASSWQTSTDTQYF	1	CD4-C2
PB-3_TACAGTGAGAGGACGG	PB	PB-3	P3-1411	FALSE	1	TRA:CAVNGRGGTASKLTF TRB:CASSQEIRDSPLHF	1	CD4-C2
PB-3_TACAGTGCAAACAACA	PB	PB-3	P3-1412	FALSE	1	TRA:CAVSVSGANSKLTF TRB:CASSPTGTSGGTDTQYF	1	CD4-C2
PB-3_TACAGTGCAATGGTCT	PB	PB-3	P3-1413	FALSE	1	TRA:CALSERQGGKLIF TRB:CASSQGASGGAGEQFF	1	CD4-C2
PB-3_TACCTTAAGCAGCCTC	PB	PB-3	P3-145	FALSE	3	TRA:CALAYNFNKFYF TRB:CASSLSVRDQTQYF	3	CD4-C2
PB-3_TACCTTACATCCCATC	PB	PB-3	P3-154	TRUE	3	TRA:CAVETYNNNDMRF TRB:CASSVRSGTDTQYF	3	CD4-C2
PB-3_TACCTTAGTAAGAGGA	PB	PB-3	P3-1419	FALSE	1	TRA:CATDAGGGNKLTF TRB:CASSPPRGSYEQYF	1	CD8-C1
PB-3_TACGGGCGTGTATGGG	PB	PB-3	P3-1426	FALSE	1	TRA:CVVSTFSSNDYKLSF TRB:CASSQDGWGSSYNEQFF	1	CD4-C2
PB-3_TACGGTATCTGCTTGC	PB	PB-3	P3-1431	FALSE	1	TRA:CAASDSGGSNYKLTF TRB:CASKEGINAEAFF	1	CD4-C2
PB-3_TACTCATAGTTGTCGT	PB	PB-3	P3-1433	FALSE	1	TRA:CALSGASGTYKYIF TRB:CASRARTGTGDNSPLHF	1	CD4-C1
PB-3_TAGACCAAGACTCGGA	PB	PB-3	P3-1446	FALSE	1	TRA:CAVYPTSGTYKYIF TRB:CASSSTSGGTYEQYF	1	CD4-C2
PB-3_TAGACCATCCGTCATC	PB	PB-3	P3-1447	FALSE	1	TRA:CAGVTGANSKLTF TRB:CASSQPGRQDTEAFF	1	CD4-C2

PB-3_TAGAGCTCATGAAGTA	PB	PB-3	P3-1449	FALSE	1	TRA:CLVGGSGTYKYIF TRB:CASSGGTDFEEQYF	1	CD4-C2
PB-3_TAGGCATTCTTGAGGT	PB	PB-3	P3-1454	FALSE	1	TRA:CALNQAGTALIF TRB:CASRRDSGTGPSEAFF	1	CD4-C2
PB-3_TAGTGGTTCCCATTAT	PB	PB-3	P3-1455	FALSE	1	TRA:CILRALGGGSNYKLTF TRB:CASREGQSTNEKLFF	1	CD4-C2
PB-3_TAGTGGTTCCCGACTT	PB	PB-3	P3-1456	FALSE	1	TRA:CAETSNSGYALNF TRB:CASSLDQGEQYF	1	CD4-C2
PB-3_TAGTTGGCAGGGCATA	PB	PB-3	P3-1457	FALSE	1	TRA:CAMGGWDTGTASKLTF TRB:CASSSGTGGSEAQYF	1	CD4-C2
PB-3_TAGTTGGGTGCATCTA	PB	PB-3	P3-1458	FALSE	1	TRA:CAASRGTGRRALTF TRB:CAILRDLNTEAFF	1	CD4-C2
PB-3_TATCAGGCATCAGTCA	PB	PB-3	P3-1460	FALSE	1	TRA:CAVVGDGATNKLIF TRB:CASSLIAEGSYGYTF	1	CD4-C2
PB-3_TATCTCACACAAGACG	PB	PB-3	P3-1461	FALSE	1	TRA:CATARRDSSYKLIF TRB:CASEGGGLTGELFF	1	CD4-C2
PB-3_TATCTCAGTACAAGTA	PB	PB-3	P3-1463	FALSE	1	TRA:CAMRVETSGSRLTF TRB:CASSLRPRGYTF	1	CD4-C2
PB-3_TCAATCTTCCATGAGT	PB	PB-3	P3-1470	FALSE	1	TRA:CAFMKLGVDAGNMLTF TRB:CASSLSRWQGADTQYF	1	CD4-C2
PB-3_TCACAAGAGATACACA	PB	PB-3	P3-1471	FALSE	1	TRA:CIVRGSGTYKYIF TRB:CASSPGTGAQAFF	1	CD4-C3
PB-3_TCACAAGGTATCAGTC	PB	PB-3	P3-1472	FALSE	1	TRA:CALSDPYGGATNKLIF TRB:CASSLALGQAATEAFF	1	CD4-C1
PB-3_TCACGAACAGGGAGAG	PB	PB-3	P3-1475	FALSE	1	TRA:CAVKAGGSYIPTF TRB:CASTEGVQGTDTQYF	1	CD4-C2
PB-3_TCACGAAGTTATGCGT	PB	PB-3	P3-1477	FALSE	1	TRA:CAMRSNQAGTALIF TRB:CATSMGPNTEAFF	1	CD4-C2
PB-3_TCACGAATCTCATTCA	PB	PB-3	P3-1479	FALSE	1	TRA:CALKAGGGSARQLTF TRB:CASSLGGTQETQYF	1	CD4-C2
PB-3_TCAGATGGTTCCGTCT	PB	PB-3	P3-1482	FALSE	1	TRA:CAMREAYNQGGKLIF TRB:CASSLGQNYGYTF	1	CD4-C2
PB-3_TCAGCAACATCGGAAG	PB	PB-3	P3-1485	FALSE	1	TRA:CAMSATSGNQFYF TRB:CASSQERGQPQHF	1	CD4-C3
PB-3_TCAGCAAGTTGCTCCT	PB	PB-3	P3-1487	FALSE	1	TRA:CAGNFGNEKLTF TRB:CASSALAGVYEQFF	1	CD4-C2
PB-3_TCAGCTCAGTGCAAGC	PB	PB-3	P3-1489	FALSE	1	TRA:CAASGPLGGGADGLTF TRB:CASRQNQGVSQPQHF	1	CD4-C2
PB-3_TCAGCTCAGTTGCAGG	PB	PB-3	P3-1490	FALSE	1	TRA:CLVGDYNQGGKLIF TRB:CSARGDVLDGYTF	1	CD4-C3
PB-3_TCAGCTCCAAGAGTCG	PB	PB-3	P3-1491	FALSE	1	TRA:CLVGGTDSSYKLIF TRB:CASSPHGGQSYEQYF	1	CD4-C2
PB-3_TCAGCTCGTACTCAAC	PB	PB-3	P3-1492	FALSE	1	TRA:CAVILKAAGNKLTF TRB:CASSLADYQETQYF	1	CD4-C2
PB-3_TCAGCTCGTCATGCAT	PB	PB-3	P3-1493	FALSE	1	TRA:CAVRDNAGNMLTF TRB:CASSPRGAAQETQYF	1	CD4-C2
PB-3_TCAGGATCAGATGGGT	PB	PB-3	P3-1495	FALSE	1	TRA:CARTPGGSNYKLTF TRB:CAWSAQTDRNEKLFF	1	CD4-C2
PB-3_TCAGGATTCAGTGCAT	PB	PB-3	P3-1496	FALSE	1	TRA:CAFTFYNNNDMRF TRB:CASTTGLNQETQYF	1	CD4-C2
PB-3_TCATTACTCGTACCGG	PB	PB-3	P3-189	TRUE	3	TRA:CAMTPSGGYQKVTF TRB:CASRPRTGTGVPGELFF	3	CD4-C2
PB-3_TCATTTGAGAAGGGTA	PB	PB-3	P3-256	FALSE	2	TRA:CAAHTGGFKTIF TRB:CAWSRLAGSDTQYF	2	CD4-C2

PB-3_TCATTTGCACCCATGG	PB	PB-3	P3-1502	FALSE	1	TRA:CALGASSYKLIF TRB:CASSLGGGREVSGGYTF	1	CD4-C2
PB-3_TCGAGGCAGAATAGGG	PB	PB-3	P3-1509	FALSE	1	TRA:CAATGSSNTGKLIF TRB:CASSLGFTAGNEQFF	1	CD4-C2
PB-3_TCGAGGCGTAGGGACT	PB	PB-3	P3-1510	FALSE	1	TRA:CAVRRGGANSKLTF TRB:CASSISSGQGFTGELFF	1	CD4-C2
PB-3_TCGCGAGCATTGAGCT	PB	PB-3	P3-1511	FALSE	1	TRA:CAVGLTGGGNKLTF TRB:CASSQVALSSGAYEQYF	1	CD4-C2
PB-3_TCGCGTTAGACCTTTG	PB	PB-3	P3-1513	FALSE	1	TRA:CAGLPSMEYGNKLVF TRB:CASSLVAGRQLAKNIQYF	1	CD4-C5
PB-3_TCGGGACCACATGTGT	PB	PB-3	P3-1515	FALSE	1	TRA:CAVPNLTGNQFYF TRB:CASSFTVKNEQFF	1	CD4-C2
PB-3_TCGGGACGTCTACCTC	PB	PB-3	P3-1516	FALSE	1	TRA:CAVRPGGTSYGKLTF TRB:CASSVSMTKIGENTEAFF	1	CD4-C2
PB-3_TCGGTAAAGCTACCTA	PB	PB-3	P3-1519	FALSE	1	TRA:CATDPTGRRALTF TRB:CASSLGRTVEAFF	1	CD4-C2
PB-3_TCGTACCTCGGCGCTA	PB	PB-3	P3-1523	FALSE	1	TRA:CAASAKGSTLGRLYF TRB:CASSQIKGRPYEQYF	1	CD4-C2
PB-3_TCGTAGATCAGAGGTG	PB	PB-3	P3-1525	FALSE	1	TRA:CLVGEPGFQKLVF TRB:CSARGPRLSSGNTIYF	1	CD4-C2
PB-3_TCTATTGAGACCGGAT	PB	PB-3	P3-1526	FALSE	1	TRA:CAVRSDSNYQLIW TRB:CATSPATSSYNEQFF	1	CD4-C2
PB-3_TCTATTGCACAGATTC	PB	PB-3	P3-1527	FALSE	1	TRA:CAVVPYSSASKIIF TRB:CSARLGDTQYF	1	CD4-C2
PB-3_TCTCATAAGGGTGTTG	PB	PB-3	P3-1528	FALSE	1	TRA:CVVLFSGGYNKLIF TRB:CASSAGQGYIQPQHF	1	CD4-C3
PB-3_TCTCATAGTAAATGTG	PB	PB-3	P3-189	TRUE	3	TRA:CAMTPSGGYQKVTF TRB:CASRPRTGTGVPGELFF	3	CD4-C2
PB-3_TCTCTAAAGGACAGCT	PB	PB-3	P3-169	FALSE	3	TRA:CAGTKGAQKLVF TRB:CASSPMNTEAFF	3	CD4-C3
PB-3_TCTTCGGGTTGTCGCG	PB	PB-3	P3-1534	FALSE	1	TRA:CVVSARTDRGSTLGRLYF TRB:CASSYGLAGVASTDTQYF	1	CD4-C2
PB-3_TCTTCGGTCAGCATGT	PB	PB-3	P3-319	TRUE	2	TRA:CAVLNTGGFKTIF TRB:CASSLTMSSNEKLFF	2	CD4-C3
PB-3_TCTTTCCTCGCAAACT	PB	PB-3	P3-1537	FALSE	1	TRA:CADHSGAGSYQLTF TRB:CASSPPVRDNEQFF	1	CD4-C2
PB-3_TGAAAGATCTGGTTCC	PB	PB-3	P3-1539	FALSE	1	TRA:CAVESSGDKLTF TRB:CASSLGEDNSPLHF	1	CD4-C2
PB-3_TGACAACCAGTCAGAG	PB	PB-3	P3-1540	FALSE	1	TRA:CAVQANNQGGKLIF TRB:CASSWNYRLHF	1	CD4-C2
PB-3_TGACGGCAGTGTGGCA	PB	PB-3	P3-1544	FALSE	1	TRA:CAVGGLTGGGNKLTF TRB:CASSLSRTSDQETQYF	1	CD4-C2
PB-3_TGACGGCCATTCTTAC	PB	PB-3	P3-1545	FALSE	1	TRA:CAATPRDSNYQLIW TRB:CASSPPGSTGRGKLFF	1	CD4-C2
PB-3_TGACGGCTCTGGTGTA	PB	PB-3	P3-252	FALSE	2	TRA:CAVLGGDYKLSF TRB:CASSPHTQGDEQFF	2	CD4-C2
PB-3_TGACTAGAGATGCCAG	PB	PB-3	P3-1546	FALSE	1	TRA:CAVNPRSGGSYIPTF TRB:CATEGGTSDGEQFF	1	CD4-C2
PB-3_TGACTAGTCGCAAACT	PB	PB-3	P3-1547	FALSE	1	TRA:CALSGAVGYNKLIF TRB:CASSPLDTGELFF	1	CD4-C2
PB-3_TGACTTTCAAGTACCT	PB	PB-3	P3-1548	FALSE	1	TRA:CAVRLDYNTDKLIF TRB:CAISESVGFVTEAFF	1	CD4-C2
PB-3_TGACTTTGTATATCCG	PB	PB-3	P3-320	TRUE	2	TRA:CAVPGSSNTGKLIF TRB:CASSQDPGQGEAFF	2	CD4-C2

PB-3_TGAGCATAGAAGGGTA	PB	PB-3	P3-1549	FALSE	1	TRA:CLVGDNAGNMLTF TRB:CASSGTSGGGEETQYF	1	CD4-C2
PB-3_TGAGCCGAGTGCCATT	PB	PB-3	P3-1550	FALSE	1	TRA:CALSEARGNNRLAF TRB:CASSRPFPRGADTEAFF	1	CD4-C7
PB-3_TGAGGGACAATACGCT	PB	PB-3	P3-1551	FALSE	1	TRA:CAVNSWYSSASKIIF TRB:CATGQKRDYGYTF	1	CD4-C2
PB-3_TGATTTCAGCGTAGTG	PB	PB-3	P3-1553	FALSE	1	TRA:CVVSASSGAGSYQLTF TRB:CASSRGLYEQYF	1	CD4-C2
PB-3_TGATTTCAGTCCGGTC	PB	PB-3	P3-1554	FALSE	1	TRA:CAVEESSGTYKYIF TRB:CAISDRDRGTGELFF	1	CD4-C2
PB-3_TGCACCTGTAAATACG	PB	PB-3	P3-1557	FALSE	1	TRA:CALYNQGGKLIF TRB:CASGAPGQGTYEQYF	1	CD4-C2
PB-3_TGCACCTGTAATAGCA	PB	PB-3	P3-1558	FALSE	1	TRA:CALLLSGGYNKLIF TRB:CASSDIPNSPLHF	1	CD4-C2
PB-3_TGCACCTGTAGCGCAA	PB	PB-3	P3-1559	FALSE	1	TRA:CVVNRAGYSTLTF TRB:CASSVRGDNPSTDTQYF	1	CD4-C2
PB-3_TGCCCATCAGCCTGTG	PB	PB-3	P3-1567	FALSE	1	TRA:CAGQKASGGSNYKLTF TRB:CSATGTSSSAKNIQYF	1	CD4-C2
PB-3_TGCCCATGTGGTACAG	PB	PB-3	P3-1569	FALSE	1	TRA:CAVSDSPSGGGADGLTF TRB:CASSLLGRSGAEQFF	1	CD4-C2
PB-3_TGCCCATGTTTAGGAA	PB	PB-3	P3-1570	FALSE	1	TRA:CAAPSNSGYALNF TRB:CASSFGTAEETQYF	1	CD4-C2
PB-3_TGCGCAGTCATCATTC	PB	PB-3	P3-299	FALSE	2	TRA:CAWGIQGAQKLVF TRB:CAISEPRDGTFYTF	2	CD4-C2
PB-3_TGCGCAGTCTTATCTG	PB	PB-3	P3-1574	FALSE	1	TRA:CAVRESYQKVTF TRB:CASSQDRGTGGSQPQHF	1	CD4-C2
PB-3_TGCTACCAGCTAGTTC	PB	PB-3	P3-1576	FALSE	1	TRA:CAASDNARLMF TRB:CASSPRDRGFETQYF	1	CD4-C2
PB-3_TGCTACCCAAGTTAAG	PB	PB-3	P3-1577	FALSE	1	TRA:CAVSFFTNAGKSTF TRB:CASSTGTYEQYF	1	CD4-C2
PB-3_TGCTACCCAATTGCTG	PB	PB-3	P3-1578	FALSE	1	TRA:CAARRAQKLVF TRB:CAISAGSGANEKLFF	1	CD4-C2
PB-3_TGCTACCCACACCGCA	PB	PB-3	P3-1579	FALSE	1	TRA:CAVEAAGNKLTF TRB:CSAVNRVRSNQPQHF	1	CD4-C3
PB-3_TGCTGCTCACGAAACG	PB	PB-3	P3-1582	FALSE	1	TRA:CAVTSMNRDDKIIF TRB:CASTREVGPSYEQYF	1	CD4-C2
PB-3_TGGACGCAGCTAAACA	PB	PB-3	P3-1583	FALSE	1	TRA:CAMRGGGSQGNLIF TRB:CATKREGVEVYTF	1	CD4-C2
PB-3_TGGACGCAGGGCATGT	PB	PB-3	P3-1585	FALSE	1	TRA:CAVSVGSGNTPLVF TRB:CASSPTGGGNTIYF	1	CD4-C1
PB-3_TGGCCAGGTACCGCTG	PB	PB-3	P3-1589	FALSE	1	TRA:CAVRDDMDNAGNMLTF TRB:CASSRSGGMSNQPQHF	1	CD4-C2
PB-3_TGGCGCACACATAACC	PB	PB-3	P3-304	FALSE	2	TRA:CAGQNTGTASKLTF TRB:CAWSPPRTDTQYF	2	CD4-C2
PB-3_TGGGCGTGTTGTGGCC	PB	PB-3	P3-1595	FALSE	1	TRA:CAANNAGNNRKLIW TRB:CASSLGLNQPQHF	1	CD4-C2
PB-3_TGGTTAGGTATGAAAC	PB	PB-3	P3-1597	FALSE	1	TRA:CVVLTSGTYKYIF TRB:CASSDDGGPGGANVLTF	1	CD4-C2
PB-3_TGGTTAGTCCCAAGAT	PB	PB-3	P3-1598	FALSE	1	TRA:CAYRRLTGANNLFF TRB:CATRGGGRSEQFF	1	CD4-C2
PB-3_TGGTTCCAGAGCCCAA	PB	PB-3	P3-1599	FALSE	1	TRA:CAANMIYNQGGKLIF TRB:CAWSAGYEPQHF	1	CD4-C3
PB-3_TGGTTCCTCTGCTGTC	PB	PB-3	P3-1601	FALSE	1	TRA:CALRLRSGGNQFYF TRB:CASSEPLAGGLSSYNEQFF	1	CD4-C2

PB-3_TGTATTCCATGACGGA	PB	PB-3	P3-1603	FALSE	1	TRA:CAVLRAGGYQKVTF TRB:CASSPRTSGTGELFF	1	CD4-C2
PB-3_TGTATTCGTACAGCAG	PB	PB-3	P3-324	TRUE	2	TRA:CATAAFNDYKLSF TRB:CASSVGDYHNEQFF	2	CD4-C2
PB-3_TGTGTTTGTCCCTTGT	PB	PB-3	P3-293	FALSE	2	TRA:CALPISSGSARQLTF TRB:CASSQATGTWGDTQYF	2	CD4-C2
PB-3_TGTGTTTTCTCACATT	PB	PB-3	P3-1615	FALSE	1	TRA:CAVGAGAAGNKLTF TRB:CASSLATGTEAFF	1	CD4-C2
PB-3_TTAACTCGTACGCTGC	PB	PB-3	P3-1618	FALSE	1	TRA:CAVRQNRDDKIIF TRB:CASSQETVGGYQYEQYF	1	CD4-C3
PB-3_TTAGTTCAGCCCAATT	PB	PB-3	P3-1623	FALSE	1	TRA:CAVSRSGGSYIPTF TRB:CASKGGQGNTGELFF	1	CD4-C2
PB-3_TTAGTTCAGCCCGAAA	PB	PB-3	P3-1624	FALSE	1	TRA:CAVPPGGTSYGKLTF TRB:CAWSPGGLKVSYEQYF	1	CD4-C2
PB-3_TTATGCTGTTGCGCAC	PB	PB-3	P3-1625	FALSE	1	TRA:CAALSGTYKYIF TRB:CASSSDSTNEKLFF	1	CD4-C3
PB-3_TTCCCAGCAATGACCT	PB	PB-3	P3-1626	FALSE	1	TRA:CAVDSEKLTF TRB:CASSRTSGGADTQYF	1	CD4-C2
PB-3_TTCCCAGGTCTCACCT	PB	PB-3	P3-1629	FALSE	1	TRA:CAVNNNARLMF TRB:CASIPQSGILRRQYF	1	CD4-C2
PB-3_TTCCCAGGTTAAGTAG	PB	PB-3	P3-1630	FALSE	1	TRA:CAVNIPGPGGGNKLTF TRB:CASSIDRGFYEQYF	1	CD4-C2
PB-3_TTCGAAGAGAAACGAG	PB	PB-3	P3-1632	FALSE	1	TRA:CAVVSGGYQKVTF TRB:CAWMGFGRSEQYF	1	CD4-C2
PB-3_TTCGAAGGTCTAGGTT	PB	PB-3	P3-1633	FALSE	1	TRA:CIVRENQAGTALIF TRB:CSAKQGGQTQYF	1	CD4-C1
PB-3_TTCGGTCGTTAAGATG	PB	PB-3	P3-1635	FALSE	1	TRA:CAVSDYSGGGADGLTF TRB:CAWSTGRHNEQFF	1	CD4-C2
PB-3_TTCTACACAGTAGAGC	PB	PB-3	P3-1637	FALSE	1	TRA:CALSVPSGGSYIPTF TRB:CASSFRDSGNQPQHF	1	CD4-C2
PB-3_TTCTCAAAGAAACGCC	PB	PB-3	P3-1639	FALSE	1	TRA:CAAWAQAGTALIF TRB:CSALRDRDEKLFF	1	CD4-C2
PB-3_TTGAACGCAACGATCT	PB	PB-3	P3-1644	FALSE	1	TRA:CAASTYGNKLVF TRB:CASSFNKDEQYF	1	CD4-C2
PB-3_TTGAACGCATCGATTG	PB	PB-3	P3-1645	FALSE	1	TRA:CAYRSGGGGSNYKLTF TRB:CSAREMGTPGNTIYF	1	CD4-C2
PB-3_TTGAACGGTCTGCGGT	PB	PB-3	P3-1646	FALSE	1	TRA:CARFNNARLMF TRB:CAWAGLDYNEQFF	1	CD4-C2
PB-3_TTGACTTGTCTGGAGA	PB	PB-3	P3-1648	FALSE	1	TRA:CAENSGGSNYKLTF TRB:CAISLHDSYEQYF	1	CD4-C3
PB-3_TTGCCGTCACTCGACG	PB	PB-3	P3-1649	FALSE	1	TRA:CALLANQAGTALIF TRB:CASSEVSSGVSEQFF	1	CD4-C2
PB-3_TTGCCGTCATTTGCTT	PB	PB-3	P3-1650	FALSE	1	TRA:CAYRRTGYALNF TRB:CASSPPDIFNTEAFF	1	CD4-C2
PB-3_TTGCCGTGTAAACACA	PB	PB-3	P3-1651	FALSE	1	TRA:CATGFLLIQGAQKLVF TRB:CSARPDRSNQPQHF	1	CD4-C2
PB-3_TTGCGTCAGTCGATAA	PB	PB-3	P3-1653	FALSE	1	TRA:CVVNTNAGKSTF TRB:CAISESGQTKAFF	1	CD4-C2
PB-3_TTGCGTCTCATGTGGT	PB	PB-3	P3-1656	FALSE	1	TRA:CAGSSGGSNYKLTF TRB:CASSHYRAVTEAFF	1	CD4-C2
PB-3_TTGGCAAAGCGTCAAG	PB	PB-3	P3-326	TRUE	2	TRA:CAVYQAGTALIF TRB:CATGAGPGGANVLTF	2	CD4-C2
PB-3_TTGGCAACAAACGCGA	PB	PB-3	P3-1658	FALSE	1	TRA:CAVLSGAGSYQLTF TRB:CASSLGAGPLYEQYF	1	CD4-C2

PB-3_TTGTAGGAGACTAAGT	PB	PB-3	P3-1659	FALSE	1	TRA:CAASVGGEETSGSRLTF TRB:CASGLESGGSPLHF	1	CD4-C2
PB-3_TTGTAGGAGGCCCGTT	PB	PB-3	P3-1660	FALSE	1	TRA:CAVHTQGGSEKLVF TRB:CASKGFGPVQETQYF	1	CD4-C1
PB-3_TTTACTGAGACGCTTT	PB	PB-3	P3-1666	FALSE	1	TRA:CAASKGYGQNFVF TRB:CASSQYGETQYF	1	CD4-C2
PB-3_TTTACTGCAGCCACCA	PB	PB-3	P3-1667	FALSE	1	TRA:CAAKRGPNNAGNMLTF TRB:CASSVKGRAPYSNQPQHF	1	CD4-C2
PB-3_TTTACTGCAGTACACT	PB	PB-3	P3-1668	FALSE	1	TRA:CAGTNYGGSQGNLIF TRB:CASSAGRMNTGELFF	1	CD4-C2
PB-3_TTTACTGCATACGCCG	PB	PB-3	P3-1669	FALSE	1	TRA:CAAILWGNKLVF TRB:CASSPTGADGNTIYF	1	CD4-C1
PB-3_TTTATGCTCGAATCCA	PB	PB-3	P3-1672	FALSE	1	TRA:CAMREGTVDSSYKLIF TRB:CATTPDRGYTEAFF	1	CD8-C2
PB-3_TTTGGTTAGAAACGCC	PB	PB-3	P3-1675	FALSE	1	TRA:CAGNNAGNMLTF TRB:CASSQDTGGRGAFF	1	CD4-C2
PB-3_TTTGGTTGTAGGACAC	PB	PB-3	P3-327	TRUE	2	TRA:CAGARRETSGSRLTF TRB:CASSQDYGQGFGSYEQYF	2	CD4-C2
PB-3_AACTCCCTCGACAGCC	PB	PB-3	P3-502	FALSE	1	TRA:CAVVDTGGGNKLTF TRB:CASSPGRGYTF	1	CD8-C2
PB-3_AAGCCGCAGGCAGGTT	PB	PB-3	P3-7	TRUE	3	TRA:CALSEARGGSQGNLIF TRB:CASSFVTGGSEAFF	27	CD8-C2
PB-3_ACACCAACAAGAAAGG	PB	PB-3	P3-546	FALSE	1	TRA:CAGSNTGKLIF TRB:CSARGVQASGANVLTF	1	CD8-C2
PB-3_ACACTGAGTACATGTC	PB	PB-3	P3-560	FALSE	1	TRA:CAVADSNYQLIW TRB:CASSNSDRFYGYTF	1	CD8-C2
PB-3_ACGCCAGCAGCCTTTC	PB	PB-3	P3-29	TRUE	3	TRA:CAVSGGNQGAQKLVF TRB:CASSLGDPRGKKLFF	10	CD8-C2
PB-3_ACGCCGACATGTTGAC	PB	PB-3	P3-77	TRUE	3	TRA:CAVNGAGGSYIPTF TRB:CASSLTRKQVDGYTF	5	CD8-C4
PB-3_ACTGCTCAGGCCCGTT	PB	PB-3	P3-610	FALSE	1	TRA:CAMREDTGNQFYF TRB:CASSSKSGWSWETQYF	1	CD4-C3
PB-3_ACTGTCCCAATAAGCA	PB	PB-3	P3-615	FALSE	1	TRA:CAVSDPRNQGGKLIF TRB:CASSRPGTSLSGELFF	1	CD8-C2
PB-3_AGAATAGAGTGTTAGA	PB	PB-3	P3-7	TRUE	3	TRA:CALSEARGGSQGNLIF TRB:CASSFVTGGSEAFF	27	CD8-C2
PB-3_AGAGCGATCAGGCGAA	PB	PB-3	P3-81	TRUE	3	TRA:CAERDSGGYQKVTF TRB:CASSFLGAEQYF	5	CD8-C2
PB-3_AGAGCTTGTATAAACG	PB	PB-3	P3-110	FALSE	3	TRA:CAVRDMAAGNKLTF TRB:CASSFLGQEAFF	4	CD8-C2
PB-3_ATGGGAGAGCGTGAGT	PB	PB-3	P3-29	TRUE	3	TRA:CAVSGGNQGAQKLVF TRB:CASSLGDPRGKKLFF	10	CD8-C2
PB-3_CAAGATCGTTTGGCGC	PB	PB-3	P3-7	TRUE	3	TRA:CALSEARGGSQGNLIF TRB:CASSFVTGGSEAFF	27	CD8-C2
PB-3_CACAGGCCAGCTTAAC	PB	PB-3	P3-117	FALSE	3	TRA:CAENVDTGRRALTF TRB:CASSQDLAQGLSYEQYF	4	CD8-C2
PB-3_CAGAATCGTTAAAGTG	PB	PB-3	P3-29	TRUE	3	TRA:CAVSGGNQGAQKLVF TRB:CASSLGDPRGKKLFF	10	CD8-C2
PB-3_CAGCAGCGTGCAGTAG	PB	PB-3	P3-806	FALSE	1	TRA:CAETLMDTGRRALTF TRB:CASSITGQGYLGF	1	CD8-C2
PB-3_CATCAGAAGGCAAAGA	PB	PB-3	P3-262	FALSE	2	TRA:CAVNEGQTGANNLFF TRB:CASSHPWDSPGEQFF	2	CD4-C6
PB-3_CATTCGCGTGAACCTT	PB	PB-3	P3-241	FALSE	2	TRA:CAAKDYQLIW TRB:CASSEPGQETQYF	2	CD8-C2

PB-3_CATTCGCTCAACTCTT	PB	PB-3	P3-264	TRUE	2	TRA:CAVSDLNTGGFKTIF TRB:CASSQESGPKYEQYF	2	CD8-C2
PB-3_CATTCGCTCTTTCCTC	PB	PB-3	P3-7	TRUE	3	TRA:CALSEARGGSQGNLIF TRB:CASSFVTGGSEAFF	27	CD8-C2
PB-3_CCATGTCAGCTCCTCT	PB	PB-3	P3-29	TRUE	3	TRA:CAVSGGNQGAQKLVF TRB:CASSLGDPRGKKLFF	10	CD8-C2
PB-3_CCATTCGGTCCTCTTG	PB	PB-3	P3-31	TRUE	3	TRA:CAVRELKAAGNKLTF TRB:CASRRDSSTGELFF	10	CD8-C5
PB-3_CCCATACTCTTATCTG	PB	PB-3	P3-878	FALSE	1	TRA:CAVMKNSGGYQKVTF TRB:CASSQGMSEAFF	1	CD8-C2
PB-3_CCGGGATGTTGATTCG	PB	PB-3	P3-882	FALSE	1	TRA:CAVSVTSGGSYIPTF TRB:CASSSRQGLPNQPQHF	1	CD4-C2
PB-3_CCTACACCATACGCCG	PB	PB-3	P3-253	FALSE	2	TRA:CAGAREDNAGNMLTF TRB:CASSRTTGGAGYTF	2	CD8-C2
PB-3_CCTACCATCACATAGC	PB	PB-3	P3-898	FALSE	1	TRA:CAVGETSGSRLTF TRB:CASRGTSGGPYNEQFF	1	CD4-C2
PB-3_CGAGCACTCACTGGGC	PB	PB-3	P3-934	FALSE	1	TRA:CGPSIGDKIIF TRB:CASSLGQTYEQYF	1	CD8-C2
PB-3_CGATGTACAGACTCGC	PB	PB-3	P3-110	FALSE	3	TRA:CAVRDMAAGNKLTF TRB:CASSFLGQEAFF	4	CD8-C2
PB-3_CGCCAAGGTAGGGTAC	PB	PB-3	P3-7	TRUE	3	TRA:CALSEARGGSQGNLIF TRB:CASSFVTGGSEAFF	27	CD8-C2
PB-3_CGCCAAGGTTCCGGCA	PB	PB-3	P3-7	TRUE	3	TRA:CALSEARGGSQGNLIF TRB:CASSFVTGGSEAFF	27	CD8-C2
PB-3_CGGACGTCAAGTACCT	PB	PB-3	P3-273	TRUE	2	TRA:CAEIAAAGNKLTF TRB:CASSWLAGGTNEQFF	2	CD8-C2
PB-3_CGGGTCACATAAAGGT	PB	PB-3	P3-978	FALSE	1	TRA:CLVGDTGDRDDKIIF TRB:CASSYRGSYEQYF	1	CD8-C2
PB-3_CGTCCATGTACCAGTT	PB	PB-3	P3-991	FALSE	1	TRA:CILIVGANSKLTF TRB:CASSVAGEFSGSPLHF	1	CD8-C2
PB-3_CGTGAGCGTATAGGGC	PB	PB-3	P3-110	FALSE	3	TRA:CAVRDMAAGNKLTF TRB:CASSFLGQEAFF	4	CD4-C4
PB-3_CGTTGGGCAGGGATTG	PB	PB-3	P3-1	TRUE	3	TRA:CAVAPISSGSARQLTF TRB:CASSQGMSEAFF	83	CD8-C2
PB-3_CTACACCGTCTAGTCA	PB	PB-3	P3-29	TRUE	3	TRA:CAVSGGNQGAQKLVF TRB:CASSLGDPRGKKLFF	10	CD8-C2
PB-3_CTCAGAAAGAGGACGG	PB	PB-3	P3-276	FALSE	2	TRA:CAEKGGNAGNMLTF TRB:CASSFGLGTEAFF	2	CD8-C2
PB-3_CTCGAGGAGAGACGAA	PB	PB-3	P3-7	TRUE	3	TRA:CALSEARGGSQGNLIF TRB:CASSFVTGGSEAFF	27	CD8-C2
PB-3_CTGATAGAGATATGGT	PB	PB-3	P3-262	FALSE	2	TRA:CAVNEGQTGANNLFF TRB:CASSHPWDSPGEQFF	2	CD4-C4
PB-3_CTGCCTACATCGGGTC	PB	PB-3	P3-17	TRUE	3	TRA:CAVRVRNARLMF TRB:CSVEGPRGDSGNTIYF	14	CD8-C2
PB-3_CTTAGGAGTCCGTTAA	PB	PB-3	P3-7	TRUE	3	TRA:CALSEARGGSQGNLIF TRB:CASSFVTGGSEAFF	27	CD8-C2
PB-3_CTTTGCGAGCGATTCT	PB	PB-3	P3-1110	FALSE	1	TRA:CALSEARGGSQGNLIF TRB:CASSRTGFQPQHF	1	CD8-C2
PB-3_GAACCTAAGTACACCT	PB	PB-3	P3-1122	FALSE	1	TRA:CAVNKGNNARLMF TRB:CASSLLAGGYNEQFF	1	CD8-C2
PB-3_GATCTAGCACGAAAGC	PB	PB-3	P3-29	TRUE	3	TRA:CAVSGGNQGAQKLVF TRB:CASSLGDPRGKKLFF	10	CD8-C2
PB-3_GATCTAGGTCCAGTGC	PB	PB-3	P3-7	TRUE	3	TRA:CALSEARGGSQGNLIF TRB:CASSFVTGGSEAFF	27	CD8-C2

PB-3_GATGAAAAGGGTTTCT	PB	PB-3	P3-1180	FALSE	1	TRA:CALRNSNYQLIW TRB:CASSQNEGETQYF	1	CD8-C2
PB-3_GATGAGGCAGTAACGG	PB	PB-3	P3-117	FALSE	3	TRA:CAENVDTGRRALTF TRB:CASSQDLAQGLSYEQYF	4	CD8-C2
PB-3_GCAAACTGTGACGCCT	PB	PB-3	P3-1187	FALSE	1	TRA:CAVGVRYGGATNKLIF TRB:CSGGEWYEQYF	1	CD8-C2
PB-3_GCACTCTCAATAGCAA	PB	PB-3	P3-1194	FALSE	1	TRA:CVVGGSQGNLIF TRB:CSALHREPGDSPLHF	1	CD8-C2
PB-3_GCGAGAAGTTGCCTCT	PB	PB-3	P3-1214	FALSE	1	TRA:CAVQASGGSNYKLTF TRB:CASSQETTANYGYTF	1	CD8-C2
PB-3_GCGCAACAGCGTCTAT	PB	PB-3	P3-125	TRUE	3	TRA:CVVNIGDSGSARQLTF TRB:CASSFGPRQHF	4	CD8-C2
PB-3_GCGGGTTCACCTATCC	PB	PB-3	P3-1224	FALSE	1	TRA:CAVNRNTGFQKLVF TRB:CAWKAGETSIYF	1	CD8-C2
PB-3_GGAACTTGTGATGTGG	PB	PB-3	P3-84	TRUE	3	TRA:CAVKRNTGFQKLVF TRB:CASSEADTGYTF	5	CD8-C2
PB-3_GTAACTGTCCGTCAAA	PB	PB-3	P3-1312	FALSE	1	TRA:CVLPSRGGYQKVTF TRB:CASSQTGDTDTQYF	1	CD8-C2
PB-3_GTACTCCAGATGTAAC	PB	PB-3	P3-110	FALSE	3	TRA:CAVRDMAAGNKLTF TRB:CASSFLGQEAFF	4	CD8-C2
PB-3_GTACTTTAGGGATACC	PB	PB-3	P3-1317	FALSE	1	TRA:CAAQAPLKTSYDKVIF TRB:CASSLQGQDYEQYF	1	CD8-C2
PB-3_GTCAAGTGTCTTCGTC	PB	PB-3	P3-7	TRUE	3	TRA:CALSEARGGSQGNLIF TRB:CASSFVTGGSEAFF	27	CD8-C2
PB-3_GTGCTTCAGCGTTGCC	PB	PB-3	P3-1372	FALSE	1	TRA:CAVRDSNYQLIW TRB:CASSDGLAADTQYF	1	CD8-C10
PB-3_GTTCATTTCAGCACAT	PB	PB-3	P3-17	TRUE	3	TRA:CAVRVRNARLMF TRB:CSVEGPRGDSGNTIYF	14	CD8-C2
PB-3_TACCTATAGCGTTGCC	PB	PB-3	P3-158	FALSE	3	TRA:CILRDVNTGNQFYF TRB:CASMAPGWANEKLFF	3	CD8-C2
PB-3_TACTTACTCACCTCGT	PB	PB-3	P3-1442	FALSE	1	TRA:CALSEAMGAGSYQLTF TRB:CASSRPGTGDETQYF	1	CD8-C2
PB-3_TACTTGTGTTAAGGGC	PB	PB-3	P3-236	FALSE	2	TRA:CAASMRVGFQKLVF TRB:CASSWADDGELFF	2	CD4-C2
PB-3_TAGACCACAGGGTATG	PB	PB-3	P3-31	TRUE	3	TRA:CAVRELKAAGNKLTF TRB:CASRRDSSTGELFF	10	CD8-C2
PB-3_TAGTTGGTCCGAAGAG	PB	PB-3	P3-7	TRUE	3	TRA:CALSEARGGSQGNLIF TRB:CASSFVTGGSEAFF	27	CD8-C2
PB-3_TCACGAACACGAAATA	PB	PB-3	P3-1474	FALSE	1	TRA:CAENASGGSNYKLTF TRB:CASGISYNSPLHF	1	CD8-C2
PB-3_TCGTACCTCCACGAAT	PB	PB-3	P3-1522	FALSE	1	TRA:CAVAAPIYHGGSQGNLIF TRB:CASRPEVAGINEQFF	1	CD8-C2
PB-3_TGGACGCTCGCCATAA	PB	PB-3	P3-158	FALSE	3	TRA:CILRDVNTGNQFYF TRB:CASMAPGWANEKLFF	3	CD8-C2
PB-3_TGGTTCCGTCAAAGAT	PB	PB-3	P3-7	TRUE	3	TRA:CALSEARGGSQGNLIF TRB:CASSFVTGGSEAFF	27	CD8-C2
PB-3_TTCTCAATCACTGGGC	PB	PB-3	P3-29	TRUE	3	TRA:CAVSGGNQGAQKLVF TRB:CASSLGDPRGKKLFF	10	CD8-C2
PB-3_TTGGAACAGACAGGCT	PB	PB-3	P3-92	TRUE	3	TRA:CAVSDRVSSASKIIF TRB:CASSFSAEQPQHF	5	CD4-C6
PB-3_TTTACTGTCCTAAGTG	PB	PB-3	P3-7	TRUE	3	TRA:CALSEARGGSQGNLIF TRB:CASSFVTGGSEAFF	27	CD8-C2
PB-3_TTTATGCGTCGCCATG	PB	PB-3	P3-117	FALSE	3	TRA:CAENVDTGRRALTF TRB:CASSQDLAQGLSYEQYF	4	CD8-C2

PB-3_TTTGGTTTCTGAAAGA	PB	PB-3	P3-191	TRUE	3	TRA:CAASSNNNDMRF TRB:CASTPTGSGNTEAFF	3	CD8-C2
PB-3_TTTGTCAGTGTGCGTC	PB	PB-3	P3-1682	FALSE	1	TRA:CAVSGSARQLTF TRB:CSALLTTGPEAFF	1	CD8-C2
PB-3_AAACCTGCAATGGAAT	PB	PB-3	P3-2	TRUE	3	TRA:CAASAPNSGGGADGLTF TRB:CSARRGSSNQPQHF	63	CD4-C3
PB-3_AAACCTGGTATCAGTC	PB	PB-3	P3-4	TRUE	3	TRA:CAGHTGANSKLTF TRB:CASSLSTGNYGYTF	39	CD4-C3
PB-3_AAAGATGTCTTAGAGC	PB	PB-3	P3-55	TRUE	3	TRA:CAVSRYNTDKLIF TRB:CASSLGGTGGHNEQFF	6	CD4-C3
PB-3_AAAGCAAAGCTAAACA	PB	PB-3	P3-101	TRUE	3	TRA:CAVRDTSGGSYIPTF TRB:CASSLFGRDTEAFF	4	CD4-C3
PB-3_AAAGCAACATGTCCTC	PB	PB-3	P3-2	TRUE	3	TRA:CAASAPNSGGGADGLTF TRB:CSARRGSSNQPQHF	63	CD4-C3
PB-3_AAAGTAGAGAGTAAGG	PB	PB-3	P3-71	TRUE	3	TRA:CAGDGRSNTGKLIF TRB:CASSLASGQNQPQHF	5	CD4-C3
PB-3_AAAGTAGGTATAATGG	PB	PB-3	P3-488	FALSE	1	TRA:CATAALTGTASKLTF TRB:CASSPDGQLPQYF	1	CD4-C3
PB-3_AAAGTAGTCAAACCAC	PB	PB-3	P3-491	FALSE	1	TRA:CLQGAQKLVF TRB:CASSPTRGGFFF	1	CD4-C3
PB-3_AAATGCCCAGACGCTC	PB	PB-3	P3-492	FALSE	1	TRA:CAVSWGYNNNDMRF TRB:CASSPWDNTEAFF	1	CD4-C3
PB-3_AACCATGGTTCCCGAG	PB	PB-3	P3-56	TRUE	3	TRA:CGAGSYQLTF TRB:CASSEGSGGVYNEQFF	6	CD4-C6
PB-3_AACTGGTGTAACGACG	PB	PB-3	P3-512	FALSE	1	TRA:CAGARTTDSWGKFQF TRB:CASSLAVLEVGTQYF	1	CD4-C3
PB-3_AAGGTTCGTCTAGAGG	PB	PB-3	P3-533	FALSE	1	TRA:CAVTTNTGFQKLVF TRB:CASSFSNPYEQYF	1	CD4-C3
PB-3_AAGTCTGAGAGACTTA	PB	PB-3	P3-2	TRUE	3	TRA:CAASAPNSGGGADGLTF TRB:CSARRGSSNQPQHF	63	CD4-C3
PB-3_AATCCAGCAGCTCGAC	PB	PB-3	P3-538	FALSE	1	TRA:CAVGSSGNTPLVF TRB:CASSLDATSYGYTF	1	CD4-C3
PB-3_AATCGGTGTCAATACC	PB	PB-3	P3-147	FALSE	3	TRA:CALPGSSNTGKLIF TRB:CASRKLTDRGDQPQHF	3	CD4-C3
PB-3_ACACCAACATGCCACG	PB	PB-3	P3-147	FALSE	3	TRA:CALPGSSNTGKLIF TRB:CASRKLTDRGDQPQHF	3	CD4-C3
PB-3_ACACTGAAGGATGGAA	PB	PB-3	P3-4	TRUE	3	TRA:CAGHTGANSKLTF TRB:CASSLSTGNYGYTF	39	CD4-C3
PB-3_ACAGCCGCATTAGCCA	PB	PB-3	P3-2	TRUE	3	TRA:CAASAPNSGGGADGLTF TRB:CSARRGSSNQPQHF	63	CD4-C3
PB-3_ACAGCTATCACGAAGG	PB	PB-3	P3-564	FALSE	1	TRA:CAYGAGGGNKLTF TRB:CSLRGIGVEAFF	1	CD4-C3
PB-3_ACATACGAGCTAACTC	PB	PB-3	P3-2	TRUE	3	TRA:CAASAPNSGGGADGLTF TRB:CSARRGSSNQPQHF	63	CD4-C3
PB-3_ACATACGAGGTGTGGT	PB	PB-3	P3-33	TRUE	3	TRA:CAASAGGANNLFF TRB:CASSLNPGQTDTQYF	9	CD4-C3
PB-3_ACATGGTAGGCCCTTG	PB	PB-3	P3-149	TRUE	3	TRA:CAVRSGAGSYQLTF TRB:CASSEQGVVSRGYTF	3	CD4-C3
PB-3_ACATGGTTCCAACCAA	PB	PB-3	P3-575	FALSE	1	TRA:CALNRVNRDDKIIF TRB:CSVTRMNTEAFF	1	CD4-C3
PB-3_ACGAGCCGTCAGGACA	PB	PB-3	P3-584	FALSE	1	TRA:CAMREAGSSNTGKLIF TRB:CASTPHPGVSNQPQHF	1	CD4-C3
PB-3_ACGATACCAATTGCTG	PB	PB-3	P3-75	TRUE	3	TRA:CAGANTNAGKSTF TRB:CAWSRTPRTDTQYF	5	CD4-C3

PB-3_ACGATGTAGAGGTAGA	PB	PB-3	P3-588	FALSE	1	TRA:CAVEDISGYSTLTF TRB:CASSLGDREEAFF	1	CD4-C3
PB-3_ACGATGTCATGAACCT	PB	PB-3	P3-239	FALSE	2	TRA:CAVLSWGKFQF TRB:CASRPHQAQETQYF	2	CD4-C3
PB-3_ACGCCAGTCCTCGCAT	PB	PB-3	P3-76	TRUE	3	TRA:CAASRIQGAQKLVF TRB:CASSFFGQAHQPQHF	5	CD4-C3
PB-3_ACGGCCAGTAAGTGTA	PB	PB-3	P3-599	FALSE	1	TRA:CALDIGGSGGYNKLIF TRB:CASSLAPQETQYF	1	CD4-C3
PB-3_ACGGGTCGTGATGATA	PB	PB-3	P3-602	FALSE	1	TRA:CVVNNGNNRLAF TRB:CSARDGAGANTGELFF	1	CD4-C3
PB-3_ACTGAACAGTCCGGTC	PB	PB-3	P3-79	TRUE	3	TRA:CVVNPGGSYIPTF TRB:CASSGDYGQIYEQYF	5	CD4-C6
PB-3_ACTGTCCGTAAAGTCA	PB	PB-3	P3-616	FALSE	1	TRA:CATLTGGFKTIF TRB:CASSQDWGANEKLFF	1	CD4-C3
PB-3_ACTTGTTGTCTGATTG	PB	PB-3	P3-109	TRUE	3	TRA:CAGLAGSNYKLTF TRB:CASSSPGGAGGAFF	4	CD4-C3
PB-3_ACTTGTTTCATAACCG	PB	PB-3	P3-621	FALSE	1	TRA:CALSPTVQGAQKLVF TRB:CATSKELRGTTQNSPLHF	1	CD4-C3
PB-3_AGAATAGTCGAATGGG	PB	PB-3	P3-625	FALSE	1	TRA:CAARNTGFQKLVF TRB:CASSGTGNNEQFF	1	CD4-C3
PB-3_AGACGTTTCAATCTCT	PB	PB-3	P3-244	TRUE	2	TRA:CVVSQGGYNKLIF TRB:CASSLSLAGVNEQFF	2	CD4-C3
PB-3_AGAGCTTGTCGACTGC	PB	PB-3	P3-630	FALSE	1	TRA:CAASADRDDKIIF TRB:CASSQGTDNRYF	1	CD4-C2
PB-3_AGATCTGAGAGATGAG	PB	PB-3	P3-4	TRUE	3	TRA:CAGHTGANSKLTF TRB:CASSLSTGNYGYTF	39	CD4-C3
PB-3_AGATTGCAGACACGAC	PB	PB-3	P3-72	FALSE	3	TRA:CAVRRSNYQLIW TRB:CASSPQGETQYF	5	CD4-C3
PB-3_AGCAGCCCATGCGCAC	PB	PB-3	P3-245	FALSE	2	TRA:CALDSGGGADGLTF TRB:CAIGSTGTNEKLFF	2	CD4-C3
PB-3_AGCCTAATCGCGTAGC	PB	PB-3	P3-649	FALSE	1	TRA:CAAQGAAGNKLTF TRB:CASNNPGRNQPQHF	1	CD4-C3
PB-3_AGCCTAATCTGTTTGT	PB	PB-3	P3-247	FALSE	2	TRA:CAVGGGADGLTF TRB:CSARRAITPEQFF	2	CD4-C3
PB-3_AGCGGTCCAATGGACG	PB	PB-3	P3-75	TRUE	3	TRA:CAGANTNAGKSTF TRB:CAWSRTPRTDTQYF	5	CD4-C3
PB-3_AGCGTATGTTCCCGAG	PB	PB-3	P3-655	FALSE	1	TRA:CAVSPNTNAGKSTF TRB:CASRVTGTNPGGFEKLFF	1	CD4-C3
PB-3_AGCTCCTGTGAAGGCT	PB	PB-3	P3-660	FALSE	1	TRA:CATDASDYKLSF TRB:CASYPQGGEKLFF	1	CD4-C3
PB-3_AGGGAGTAGTCTCGGC	PB	PB-3	P3-112	TRUE	3	TRA:CAASINSGNTPLVF TRB:CSARGQGNEQFF	4	CD4-C3
PB-3_AGGGAGTAGTTACGGG	PB	PB-3	P3-672	FALSE	1	TRA:CILRSSNSGYALNF TRB:CASSLDPVRENTEAFF	1	CD4-C3
PB-3_AGGGATGTCTGCTGTC	PB	PB-3	P3-678	FALSE	1	TRA:CVVNPGTGFQKLVF TRB:CASSPGGTLLNEQFF	1	CD4-C3
PB-3_AGGTCATCAATGGAGC	PB	PB-3	P3-685	FALSE	1	TRA:CAVRDRRGYQKVTF TRB:CATKGGTVDQPQHF	1	CD4-C3
PB-3_AGTCTTTTCGGATGTT	PB	PB-3	P3-2	TRUE	3	TRA:CAASAPNSGGGADGLTF TRB:CSARRGSSNQPQHF	63	CD4-C3
PB-3_AGTGAGGCACCGAATT	PB	PB-3	P3-690	FALSE	1	TRA:CAVGAVEYGNKLVF TRB:CASSAFPGMDGYTF	1	CD4-C3
PB-3_AGTTGGTCAATGCCAT	PB	PB-3	P3-695	FALSE	1	TRA:CAVESGTSGSRLTF TRB:CASRTGTGSRRRQPQHF	1	CD4-C3

PB-3_ATAAGAGCATCCCACT	PB	PB-3	P3-702	FALSE	1	TRA:CVVKLTGGGNKLTF TRB:CASSGPFNTGELFF	1	CD4-C3
PB-3_ATAGACCAGGTCATCT	PB	PB-3	P3-704	FALSE	1	TRA:CVVDYNNDMRF TRB:CSARIAGGERGDGYTF	1	CD4-C3
PB-3_ATCACGACAATCGAAA	PB	PB-3	P3-247	FALSE	2	TRA:CAVGGGADGLTF TRB:CSARRAITPEQFF	2	CD4-C3
PB-3_ATCATGGGTAGATTAG	PB	PB-3	P3-711	FALSE	1	TRA:CATPGGYQKVTF TRB:CSVQRGTDTQYF	1	CD4-C3
PB-3_ATCGAGTGTTGATTGC	PB	PB-3	P3-720	FALSE	1	TRA:CAMSHTGGFKTIF TRB:CSAQRTDPGEQYF	1	CD4-C3
PB-3_ATCTACTCAGTGGGAT	PB	PB-3	P3-721	FALSE	1	TRA:CAYRTVTNAGKSTF TRB:CASSALEVNTEAFF	1	CD4-C3
PB-3_ATCTGCCAGAAACGCC	PB	PB-3	P3-722	FALSE	1	TRA:CAVIPSGSARQLTF TRB:CASSFNREKLFF	1	CD4-C3
PB-3_ATGAGGGCACATAACC	PB	PB-3	P3-55	TRUE	3	TRA:CAVSRYNTDKLIF TRB:CASSLGGTGGHNEQFF	6	CD4-C3
PB-3_ATGAGGGGTTTAGGAA	PB	PB-3	P3-725	FALSE	1	TRA:CAPGGSYIPTF TRB:CASSQGELAGSQETQYF	1	CD4-C3
PB-3_ATGAGGGTCATTCACT	PB	PB-3	P3-2	TRUE	3	TRA:CAASAPNSGGGADGLTF TRB:CSARRGSSNQPQHF	63	CD4-C3
PB-3_ATGGGAGGTGCACTTA	PB	PB-3	P3-4	TRUE	3	TRA:CAGHTGANSKLTF TRB:CASSLSTGNYGYTF	39	CD4-C3
PB-3_ATTACTCGTTAGGGTG	PB	PB-3	P3-60	TRUE	3	TRA:CAGARNSGYALNF TRB:CASSQGNGVGKAFF	6	CD4-C3
PB-3_ATTCTACCAATGTAAG	PB	PB-3	P3-4	TRUE	3	TRA:CAGHTGANSKLTF TRB:CASSLSTGNYGYTF	39	CD4-C3
PB-3_CAACCAAAGTGTGAAT	PB	PB-3	P3-748	FALSE	1	TRA:CAVSDVRAGNNRKLIW TRB:CASSLGGTDTQYF	1	CD4-C3
PB-3_CAACCAAGTTGGTAAA	PB	PB-3	P3-749	FALSE	1	TRA:CIVRALI TRB:CASSRSRDYTEAFF	1	CD4-C3
PB-3_CAACCTCTCTGGAGCC	PB	PB-3	P3-751	FALSE	1	TRA:CAVNLGTASKLTF TRB:CASSLMGQRYNEQFF	1	CD4-C3
PB-3_CAAGAAAAGTGGGATC	PB	PB-3	P3-159	TRUE	3	TRA:CAVNVRSWGKFQF TRB:CSVQDNTGELFF	3	CD4-C3
PB-3_CAAGATCCAATAAGCA	PB	PB-3	P3-160	TRUE	3	TRA:CAARGNFNKFYF TRB:CSASTDRVTEAFF	3	CD4-C3
PB-3_CAAGATCCAGTCTTCC	PB	PB-3	P3-756	FALSE	1	TRA:CAVSQDQTGANNLFF TRB:CSARLPGNTEAFF	1	CD4-C3
PB-3_CAAGTTGTCCCAAGTA	PB	PB-3	P3-764	FALSE	1	TRA:CLVPGRGSSNTGKLIF TRB:CASSPDVDRYNEQFF	1	CD4-C3
PB-3_CAAGTTGTCTCGCATC	PB	PB-3	P3-765	FALSE	1	TRA:CAVGAKEYGNKLVF TRB:CASGTGQGAGETQYF	1	CD4-C3
PB-3_CACAAACCAGCTGTAT	PB	PB-3	P3-2	TRUE	3	TRA:CAASAPNSGGGADGLTF TRB:CSARRGSSNQPQHF	63	CD4-C3
PB-3_CACACAAGTATTCTCT	PB	PB-3	P3-772	FALSE	1	TRA:CATDDNTDKLIF TRB:CASSQAGTYSGYTF	1	CD4-C3
PB-3_CACACTCGTTAAAGAC	PB	PB-3	P3-2	TRUE	3	TRA:CAASAPNSGGGADGLTF TRB:CSARRGSSNQPQHF	63	CD4-C3
PB-3_CACAGTAAGACAGACC	PB	PB-3	P3-779	FALSE	1	TRA:CIVRVAGGNQGGKLIF TRB:CSAKVQTGEQYF	1	CD4-C3
PB-3_CACATAGTCGGCTACG	PB	PB-3	P3-785	FALSE	1	TRA:CLLGVNFGNEKLTF TRB:CASSMGPWAFF	1	CD4-C3
PB-3_CACCACTCACTTGGAT	PB	PB-3	P3-118	TRUE	3	TRA:CAESQYGGSQGNLIF TRB:CASSQAYSGGHNEQFF	4	CD4-C3

PB-3_CACCAGGGTCCTCCAT	PB	PB-3	P3-791	FALSE	1	TRA:CAVRRQGGKLIF TRB:CASSRDASGANVLTF	1	CD4-C3
PB-3_CACCTTGTCTTCATGT	PB	PB-3	P3-2	TRUE	3	TRA:CAASAPNSGGGADGLTF TRB:CSARRGSSNQPQHF	63	CD4-C3
PB-3_CAGAATCCACCGCTAG	PB	PB-3	P3-119	TRUE	3	TRA:CAVETAAGNKLTF TRB:CSVPPGQKDRADYGYTF	4	CD4-C3
PB-3_CAGAATCTCGGTGTCG	PB	PB-3	P3-797	FALSE	1	TRA:CAVRDASQTGANNLFF TRB:CASSPTGNSPLHF	1	CD4-C3
PB-3_CAGAGAGAGAATAGGG	PB	PB-3	P3-799	FALSE	1	TRA:CPNFGNEKLTF TRB:CSAGPPPGKNF	1	CD4-C3
PB-3_CAGCAGCAGTCGAGTG	PB	PB-3	P3-162	TRUE	3	TRA:CAARPNQAGTALIF TRB:CSVADRTNTEAFF	3	CD4-C3
PB-3_CAGCCGAGTAAGTTCC	PB	PB-3	P3-4	TRUE	3	TRA:CAGHTGANSKLTF TRB:CASSLSTGNYGYTF	39	CD4-C3
PB-3_CAGCGACCATCCAACA	PB	PB-3	P3-163	TRUE	3	TRA:CAENPTTSGTYKYIF TRB:CASSFRDRFQHF	3	CD4-C3
PB-3_CAGCTGGCAAATACAG	PB	PB-3	P3-811	FALSE	1	TRA:CAVRDIGFGNVLHC TRB:CASSASTGYNEQFF	1	CD4-C3
PB-3_CAGCTGGTCCTCAACC	PB	PB-3	P3-33	TRUE	3	TRA:CAASAGGANNLFF TRB:CASSLNPGQTDTQYF	9	CD4-C3
PB-3_CAGTAACTCATCGCTC	PB	PB-3	P3-816	FALSE	1	TRA:CVVTRNNNARLMF TRB:CASSQRQGTDTQYF	1	CD4-C3
PB-3_CAGTAACTCATCTGTT	PB	PB-3	P3-260	FALSE	2	TRA:CVVNLYSSASKIIF TRB:CASSLEAGTEQYF	2	CD4-C3
PB-3_CATCAAGAGACTAGGC	PB	PB-3	P3-261	TRUE	2	TRA:CAATVYGSSNTGKLIF TRB:CASRRTGGGNQPQHF	2	CD4-C3
PB-3_CATCAGACATGCCCGA	PB	PB-3	P3-827	FALSE	1	TRA:CAVSELDGATNKLIF TRB:CASSQDLGNEQFF	1	CD4-C3
PB-3_CATCCACGTCGAATCT	PB	PB-3	P3-263	TRUE	2	TRA:CALYDTGRRALTF TRB:CASNRGGNTEAFF	2	CD4-C3
PB-3_CATCCACGTTCGTTGA	PB	PB-3	P3-121	TRUE	3	TRA:CAVEEINAGNNRKLIW TRB:CASKGTPGKLFF	4	CD4-C3
PB-3_CATGACACATGGATGG	PB	PB-3	P3-845	FALSE	1	TRA:CAPQTSGSRLTF TRB:CASSQILKQGPGSPLHF	1	CD4-C3
PB-3_CATGGCGAGTCTCGGC	PB	PB-3	P3-2	TRUE	3	TRA:CAASAPNSGGGADGLTF TRB:CSARRGSSNQPQHF	63	CD4-C3
PB-3_CATGGCGTCTCGTATT	PB	PB-3	P3-851	FALSE	1	TRA:CAVGLGSSNTGKLIF TRB:CSARWAANTGELFF	1	CD4-C3
PB-3_CATTATCTCGCCAAAT	PB	PB-3	P3-2	TRUE	3	TRA:CAASAPNSGGGADGLTF TRB:CSARRGSSNQPQHF	63	CD4-C3
PB-3_CATTATCTCGCCTGAG	PB	PB-3	P3-165	TRUE	3	TRA:CAVNGGSQGNLIF TRB:CASSERPRSYNEQFF	3	CD4-C3
PB-3_CATTCGCAGAAGGTGA	PB	PB-3	P3-854	FALSE	1	TRA:CAVNSFQTGANNLFF TRB:CASSSGTSGGADTQYF	1	CD4-C3
PB-3_CCAATCCCACATCCGG	PB	PB-3	P3-859	FALSE	1	TRA:CAASGTSGTYKYIF TRB:CSARDRTGLLGTDTQYF	1	CD4-C3
PB-3_CCAGCGACATAGGATA	PB	PB-3	P3-166	TRUE	3	TRA:CAYRRYNNNDMRF TRB:CASSPTTPRGDTQYF	3	CD4-C3
PB-3_CCATGTCGTTTAGGAA	PB	PB-3	P3-870	FALSE	1	TRA:CAVSDGGFKTIF TRB:CATKTGDQGYTF	1	CD4-C3
PB-3_CCATTCGGTTTCCACC	PB	PB-3	P3-2	TRUE	3	TRA:CAASAPNSGGGADGLTF TRB:CSARRGSSNQPQHF	63	CD4-C3
PB-3_CCCAATCGTTCCAACA	PB	PB-3	P3-2	TRUE	3	TRA:CAASAPNSGGGADGLTF TRB:CSARRGSSNQPQHF	63	CD4-C3

PB-3_CCGGGATCACCAGGTC	PB	PB-3	P3-879	FALSE	1	TRA:CAYSGGGADGLTF TRB:CASSPSQAGSNTEAFF	1	CD4-C3
PB-3_CCGGGATGTTACCAGT	PB	PB-3	P3-146	TRUE	3	TRA:CVVNLYSSASKIIF TRB:CASSLEAGTEAFF	3	CD4-C3
PB-3_CCGTTCACATGTCTCC	PB	PB-3	P3-121	TRUE	3	TRA:CAVEEINAGNNRKLIW TRB:CASKGTPGKLFF	4	CD4-C3
PB-3_CCTACCACAGGCTGAA	PB	PB-3	P3-895	FALSE	1	TRA:CVVSAGSGGYNKLIF TRB:CASSFTDRRDQPQHF	1	CD4-C3
PB-3_CCTACCATCCTCATTA	PB	PB-3	P3-43	TRUE	3	TRA:CAERNSGYSTLTF TRB:CASSAAPSMAGGGGETQYF	8	CD4-C3
PB-3_CCTACCATCCTTTACA	PB	PB-3	P3-900	FALSE	1	TRA:CALYMIYNQGGKLIF TRB:CAGDRGYEQYF	1	CD4-C3
PB-3_CCTATTAGTGACTACT	PB	PB-3	P3-902	FALSE	1	TRA:CAMSVKAAGNKLTF TRB:CASSGRQQYF	1	CD4-C3
PB-3_CCTCAGTAGTATGACA	PB	PB-3	P3-904	FALSE	1	TRA:CAFFSSGSARQLTF TRB:CSAPAGGAPYNEQFF	1	CD4-C3
PB-3_CCTCTGATCGCAGGCT	PB	PB-3	P3-2	TRUE	3	TRA:CAASAPNSGGGADGLTF TRB:CSARRGSSNQPQHF	63	CD4-C3
PB-3_CCTTCGATCCATTCTA	PB	PB-3	P3-914	FALSE	1	TRA:CAMREVYNQGGKLIF TRB:CASSPDRGLPTEAFF	1	CD4-C3
PB-3_CGAACATAGATATGCA	PB	PB-3	P3-122	TRUE	3	TRA:CAGSPPGGGNKLTF TRB:CASSLAAGASTDTQYF	4	CD4-C3
PB-3_CGACTTCTCGTTTATC	PB	PB-3	P3-929	FALSE	1	TRA:CVVNYGGQNFVF TRB:CASSERFAGHIGGYTF	1	CD4-C3
PB-3_CGATCGGGTCCGTGAC	PB	PB-3	P3-937	FALSE	1	TRA:CAVTLGTGRRALTF TRB:CASTVLAGGRTDTQYF	1	CD4-C3
PB-3_CGATGTACACAAGACG	PB	PB-3	P3-266	TRUE	2	TRA:CAVQAGSGGSYIPTF TRB:CASSPRDHHEKLFF	2	CD4-C5
PB-3_CGATGTACAGTCGATT	PB	PB-3	P3-943	FALSE	1	TRA:CAVYKAAGNKLTF TRB:CSARGGGPQETQYF	1	CD4-C3
PB-3_CGATTGACAAATCCGT	PB	PB-3	P3-945	FALSE	1	TRA:CAVRPGVGAQKLVF TRB:CASSSGAGEQYF	1	CD4-C3
PB-3_CGATTGATCTGGAGCC	PB	PB-3	P3-269	TRUE	2	TRA:CAVVHNYGQNFVF TRB:CASSYAIDSANTGELFF	2	CD4-C3
PB-3_CGCGGTAAGTCCAGGA	PB	PB-3	P3-270	TRUE	2	TRA:CAYGKETSGSRLTF TRB:CASSLEWRNEQFF	2	CD4-C3
PB-3_CGCTGGATCTAACTCT	PB	PB-3	P3-954	FALSE	1	TRA:CAMREGGTFGNEKLTF TRB:CASSVFGAGANEQFF	1	CD4-C3
PB-3_CGCTTCAGTCCGAAGA	PB	PB-3	P3-168	TRUE	3	TRA:CAVSKEAAGNKLTF TRB:CSARDLAGFTDTQYF	3	CD4-C3
PB-3_CGCTTCATCGACAGCC	PB	PB-3	P3-79	TRUE	3	TRA:CVVNPGGSYIPTF TRB:CASSGDYGQIYEQYF	5	CD4-C6
PB-3_CGGACACTCACCATAG	PB	PB-3	P3-961	FALSE	1	TRA:CVVNQYSSASKIIF TRB:CASSLEAGTEAFF	1	CD4-C3
PB-3_CGGACGTTCCACGCAG	PB	PB-3	P3-274	TRUE	2	TRA:CAASKVTGGGNKLTF TRB:CASSESAGGGETQYF	2	CD4-C3
PB-3_CGGACTGCAAACCCAT	PB	PB-3	P3-2	TRUE	3	TRA:CAASAPNSGGGADGLTF TRB:CSARRGSSNQPQHF	63	CD4-C3
PB-3_CGGACTGTCCCTAACC	PB	PB-3	P3-87	TRUE	3	TRA:CAAATSGTYKYIF TRB:CASSFRQLRSGANVLTF	5	CD4-C3
PB-3_CGGAGCTAGCAATCTC	PB	PB-3	P3-968	FALSE	1	TRA:CAVSRRRAQKLVF TRB:CASTPSGGGADTQYF	1	CD4-C3
PB-3_CGGAGTCTCCGCGGTA	PB	PB-3	P3-972	FALSE	1	TRA:CAVHGSSNTGKLIF TRB:CASNPRQGALHF	1	CD4-C3

PB-3_CGGGTCACACGAAGCA	PB	PB-3	P3-277	TRUE	2	TRA:CAVSDGGSNYKLTF TRB:CASSNSGAGRTDTQYF	2	CD4-C3
PB-3_CGTCACTGTTTGTTTC	PB	PB-3	P3-72	FALSE	3	TRA:CAVRRSNYQLIW TRB:CASSPQGETQYF	5	CD4-C3
PB-3_CGTCAGGGTCTGCCAG	PB	PB-3	P3-987	FALSE	1	TRA:CALPLAPQGGSEKLVF TRB:CASTKGQYQPQHF	1	CD4-C3
PB-3_CGTCCATTCAGTGTTG	PB	PB-3	P3-2	TRUE	3	TRA:CAASAPNSGGGADGLTF TRB:CSARRGSSNQPQHF	63	CD4-C3
PB-3_CGTGAGCTCTGTCTAT	PB	PB-3	P3-2	TRUE	3	TRA:CAASAPNSGGGADGLTF TRB:CSARRGSSNQPQHF	63	CD4-C3
PB-3_CGTTGGGGTGACCAAG	PB	PB-3	P3-1014	FALSE	1	TRA:CVVSPSYSSASKIIF TRB:CASSQAGRRGPTGELFF	1	CD4-C3
PB-3_CTAACTTAGCGTGTCC	PB	PB-3	P3-1015	FALSE	1	TRA:CATVLMGPLVF TRB:CASRIVDGQNTEAFF	1	CD4-C3
PB-3_CTAACTTAGCTCCTCT	PB	PB-3	P3-4	TRUE	3	TRA:CAGHTGANSKLTF TRB:CASSLSTGNYGYTF	39	CD4-C3
PB-3_CTAAGACGTGAGCGAT	PB	PB-3	P3-1019	FALSE	1	TRA:CAVEQNTNAGKSTF TRB:CASSQEGQRAFF	1	CD4-C3
PB-3_CTAATGGAGCTGAACG	PB	PB-3	P3-1021	FALSE	1	TRA:CAVRRSSGGYQKVTF TRB:CASSAQEDTEAFF	1	CD4-C3
PB-3_CTACCCAGTTGATTGC	PB	PB-3	P3-1026	FALSE	1	TRA:CALSANAGNNRKLIW TRB:CASSESGRGSTANQPQHF	1	CD4-C3
PB-3_CTACGTCCAGGCTGAA	PB	PB-3	P3-4	TRUE	3	TRA:CAGHTGANSKLTF TRB:CASSLSTGNYGYTF	39	CD4-C3
PB-3_CTAGAGTTCCTTTACA	PB	PB-3	P3-4	TRUE	3	TRA:CAGHTGANSKLTF TRB:CASSLSTGNYGYTF	39	CD4-C3
PB-3_CTCACACGTTCCGGCA	PB	PB-3	P3-4	TRUE	3	TRA:CAGHTGANSKLTF TRB:CASSLSTGNYGYTF	39	CD4-C3
PB-3_CTCAGAATCGAACTGT	PB	PB-3	P3-1044	FALSE	1	TRA:CAATTPRGSNFGNEKLTF TRB:CASSYVGGELFF	1	CD4-C3
PB-3_CTCATTAGTCAATACC	PB	PB-3	P3-1047	FALSE	1	TRA:CAVYGANNLFF TRB:CASSPLSRGDTQYF	1	CD4-C3
PB-3_CTCGGAGAGGTGACCA	PB	PB-3	P3-1060	FALSE	1	TRA:CAVVGATGGFKTIF TRB:CSGGDPDTGELFF	1	CD4-C3
PB-3_CTCGGAGGTCGCATAT	PB	PB-3	P3-1063	FALSE	1	TRA:CAVRRSFSGGYNKLIF TRB:CASSMGGEWPQHF	1	CD4-C3
PB-3_CTCGGAGTCAAGGCTT	PB	PB-3	P3-281	FALSE	2	TRA:CAASKGGSNYKLTF TRB:CASSPGGSEKLFF	2	CD4-C3
PB-3_CTCGGGACACATCCGG	PB	PB-3	P3-172	TRUE	3	TRA:CVVSEGGSTLGRLYF TRB:CAWSRQGSSSGNTIYF	3	CD4-C3
PB-3_CTCGGGAGTACATCCA	PB	PB-3	P3-2	TRUE	3	TRA:CAASAPNSGGGADGLTF TRB:CSARRGSSNQPQHF	63	CD4-C4
PB-3_CTCGTACGTGTAATGA	PB	PB-3	P3-76	TRUE	3	TRA:CAASRIQGAQKLVF TRB:CASSFFGQAHQPQHF	5	CD4-C3
PB-3_CTCGTACTCTTTCCTC	PB	PB-3	P3-1071	FALSE	1	TRA:CAVRAVI TRB:CASTSSSGANVLTF	1	CD4-C3
PB-3_CTCGTCAAGAAAGTGG	PB	PB-3	P3-2	TRUE	3	TRA:CAASAPNSGGGADGLTF TRB:CSARRGSSNQPQHF	63	CD4-C3
PB-3_CTCGTCAGTCGATTGT	PB	PB-3	P3-1073	FALSE	1	TRA:CAVSDRGVKAAGNKLTF TRB:CSAPGTYEQYF	1	CD4-C3
PB-3_CTCTACGCAATGAATG	PB	PB-3	P3-1077	FALSE	1	TRA:CALPARYNTNAGKSTF TRB:CASSPSGGITDTQYF	1	CD4-C3
PB-3_CTCTACGCAGGTTTCA	PB	PB-3	P3-173	TRUE	3	TRA:CAVSGPGTYKYIF TRB:CSQTTSGGTYQETQYF	3	CD4-C6

PB-3_CTCTACGGTTTAAGCC	PB	PB-3	P3-1079	FALSE	1	TRA:CIVRAHNNNNDMRF TRB:CASSPDLTGGSGNTIYF	1	CD4-C2
PB-3_CTGATCCAGAACAACT	PB	PB-3	P3-172	TRUE	3	TRA:CVVSEGGSTLGRLYF TRB:CAWSRQGSSSGNTIYF	3	CD4-C3
PB-3_CTGATCCAGGCATGTG	PB	PB-3	P3-284	TRUE	2	TRA:CAVRSNSGGSNYKLTF TRB:CSASGTPITDTQYF	2	CD4-C3
PB-3_CTGATCCGTCCCGACA	PB	PB-3	P3-89	TRUE	3	TRA:CAVEYPDSWGKFQF TRB:CASSPQGETQYF	5	CD4-C3
PB-3_CTGATCCTCCGCGTTT	PB	PB-3	P3-2	TRUE	3	TRA:CAASAPNSGGGADGLTF TRB:CSARRGSSNQPQHF	63	CD4-C6
PB-3_CTGCCTATCTTGACGA	PB	PB-3	P3-1092	FALSE	1	TRA:CAVRRSGTYKYIF TRB:CASSAGTHSPLHF	1	CD4-C3
PB-3_CTGCGGAGTAGTGAAT	PB	PB-3	P3-2	TRUE	3	TRA:CAASAPNSGGGADGLTF TRB:CSARRGSSNQPQHF	63	CD4-C3
PB-3_CTGCTGTCAATACGCT	PB	PB-3	P3-1094	FALSE	1	TRA:CALYSGNTPLVF TRB:CASSSAGGYTGELFF	1	CD4-C3
PB-3_CTGTGCTTCAGAGCTT	PB	PB-3	P3-1099	FALSE	1	TRA:CAASAGFGNVLHC TRB:CASSPRQGAGYEQYF	1	CD4-C3
PB-3_CTTAACTAGCTGCCCA	PB	PB-3	P3-286	TRUE	2	TRA:CAASVSFAYKYIF TRB:CASSPGQMNEKLFF	2	CD4-C3
PB-3_CTTAACTAGGTGCTTT	PB	PB-3	P3-2	TRUE	3	TRA:CAASAPNSGGGADGLTF TRB:CSARRGSSNQPQHF	63	CD4-C3
PB-3_CTTAACTCAAACAACA	PB	PB-3	P3-1103	FALSE	1	TRA:CAVQDSTSGTYKYIF TRB:CAISESPGGYTF	1	CD4-C3
PB-3_CTTAGGACAGCATACT	PB	PB-3	P3-1105	FALSE	1	TRA:CAIQTSYDKVIF TRB:CSARREVYNSPLHF	1	CD4-C3
PB-3_CTTAGGAGTCCGCTGA	PB	PB-3	P3-1106	FALSE	1	TRA:CAVTKGWGATNKLIF TRB:CASSEQAARGETQYF	1	CD4-C3
PB-3_CTTAGGAGTCTAGCCG	PB	PB-3	P3-118	TRUE	3	TRA:CAESQYGGSQGNLIF TRB:CASSQAYSGGHNEQFF	4	CD4-C6
PB-3_CTTAGGATCTTCGAGA	PB	PB-3	P3-2	TRUE	3	TRA:CAASAPNSGGGADGLTF TRB:CSARRGSSNQPQHF	63	CD4-C3
PB-3_CTTTGCGGTCCTAGCG	PB	PB-3	P3-1111	FALSE	1	TRA:CIVLTNNAGNMLTF TRB:CASRPSGGAWELFF	1	CD4-C3
PB-3_GAAACTCAGAGGTTAT	PB	PB-3	P3-289	TRUE	2	TRA:CAVDENTGFQKLVF TRB:CASKGESEQYF	2	CD4-C3
PB-3_GAAATGAAGTCTCCTC	PB	PB-3	P3-1113	FALSE	1	TRA:CIWSQDMRF TRB:CASSKTSRQPQHF	1	CD4-C3
PB-3_GAATAAGCATTCGACA	PB	PB-3	P3-121	TRUE	3	TRA:CAVEEINAGNNRKLIW TRB:CASKGTPGKLFF	4	CD4-C3
PB-3_GAATGAATCAAACCAC	PB	PB-3	P3-1130	FALSE	1	TRA:CIVRVAGGNQGGKLIF TRB:CSATGQHNEQFF	1	CD4-C3
PB-3_GAATGAATCCGTACAA	PB	PB-3	P3-1131	FALSE	1	TRA:CAFAPYTNAGKSTF TRB:CASSLATSPVNEKLFF	1	CD4-C2
PB-3_GACCTGGAGACTGGGT	PB	PB-3	P3-1139	FALSE	1	TRA:CIVRPNTGTASKLTF TRB:CASSEVGTGFYEQYF	1	CD4-C3
PB-3_GACCTGGAGGACATTA	PB	PB-3	P3-1142	FALSE	1	TRA:CAIMDSNYQLIW TRB:CASRTDSRETQYF	1	CD4-C3
PB-3_GACGCGTGTCTCCCTA	PB	PB-3	P3-1150	FALSE	1	TRA:CILSFGGTSYGKLTF TRB:CASSDLWGTPGLPSYEQYF	1	CD4-C3
PB-3_GACGTTACAAGACACG	PB	PB-3	P3-2	TRUE	3	TRA:CAASAPNSGGGADGLTF TRB:CSARRGSSNQPQHF	63	CD4-C3
PB-3_GACTAACCAGTAGAGC	PB	PB-3	P3-1156	FALSE	1	TRA:CAMRAEYGGATNKLIF TRB:CASSQDNLGLSTEAFF	1	CD4-C3

PB-3_GACTAACGTAGCGCTC	PB	PB-3	P3-4	TRUE	3	TRA:CAGHTGANSKLTF TRB:CASSLSTGNYGYTF	39	CD4-C3
PB-3_GACTACAGTGCGGTAA	PB	PB-3	P3-1160	FALSE	1	TRA:CAGGGATNKLIF TRB:CASTRLSSYEQYF	1	CD4-C3
PB-3_GAGTCCGTCCCATTTA	PB	PB-3	P3-105	TRUE	3	TRA:CAVNENSGYSTLTF TRB:CASSFSGGQETQYF	4	CD4-C3
PB-3_GATCAGTCAGTCTTCC	PB	PB-3	P3-1171	FALSE	1	TRA:CAMREENSGGSNYKLTF TRB:CSARDFAESTDTQYF	1	CD4-C3
PB-3_GATCGATCAGGTGCCT	PB	PB-3	P3-119	TRUE	3	TRA:CAVETAAGNKLTF TRB:CSVPPGQKDRADYGYTF	4	CD4-C3
PB-3_GATCGATTCTTGAGGT	PB	PB-3	P3-2	TRUE	3	TRA:CAASAPNSGGGADGLTF TRB:CSARRGSSNQPQHF	63	CD4-C3
PB-3_GATCGCGTCAAACCAC	PB	PB-3	P3-1175	FALSE	1	TRA:CAVSPRRGGSEKLVF TRB:CASSQGPNTEAFF	1	CD4-C3
PB-3_GATCGCGTCTGGTGTA	PB	PB-3	P3-1176	FALSE	1	TRA:CAGGGSTLGRLYF TRB:CASSFGADTQYF	1	CD4-C3
PB-3_GATCTAGGTGTCTGAT	PB	PB-3	P3-4	TRUE	3	TRA:CAGHTGANSKLTF TRB:CASSLSTGNYGYTF	39	CD4-C3
PB-3_GATGAAAGTGACTACT	PB	PB-3	P3-295	TRUE	2	TRA:CAMRERFTGGGNKLTF TRB:CASSQDKGTGTSGANVLTF	2	CD4-C3
PB-3_GATGAAATCCCTGACT	PB	PB-3	P3-1181	FALSE	1	TRA:CAARSPGNKLVF TRB:CASSQDHGGGYGYTF	1	CD4-C3
PB-3_GATGAAATCTAACTTC	PB	PB-3	P3-64	TRUE	3	TRA:CIEQGAQKLVF TRB:CASSRASGTAPYEQYF	6	CD4-C3
PB-3_GATGAGGAGTTGAGAT	PB	PB-3	P3-1182	FALSE	1	TRA:CVVSATTDSWGKFQF TRB:CASMLRGTNQPQHF	1	CD4-C3
PB-3_GATGCTACACAGCGTC	PB	PB-3	P3-7	TRUE	3	TRA:CALSEARGGSQGNLIF TRB:CASSFVTGGSEAFF	27	CD4-C3
PB-3_GATTCAGTCCAAGCCG	PB	PB-3	P3-1186	FALSE	1	TRA:CAVHRADRGSTLGRLYF TRB:CASSESPLRTAGGYTF	1	CD4-C3
PB-3_GCAAACTCAAGTAATG	PB	PB-3	P3-178	TRUE	3	TRA:CAVSANSGNTPLVF TRB:CSASGQVGEQYF	3	CD4-C3
PB-3_GCACTCTAGCCGATTT	PB	PB-3	P3-1193	FALSE	1	TRA:CAVEDGGFKTIF TRB:CASSQETGNSPLHF	1	CD4-C3
PB-3_GCATACACAAATTGCC	PB	PB-3	P3-1199	FALSE	1	TRA:CAVTYGGATNKLIF TRB:CSASGQIGEQFF	1	CD4-C3
PB-3_GCATACACAGCGTCCA	PB	PB-3	P3-2	TRUE	3	TRA:CAASAPNSGGGADGLTF TRB:CSARRGSSNQPQHF	63	CD4-C3
PB-3_GCATACACAGGTCTCG	PB	PB-3	P3-1200	FALSE	1	TRA:CAATALRGGSEKLVF TRB:CASSRDSLHEKLFF	1	CD4-C3
PB-3_GCCAAATCACCATGTA	PB	PB-3	P3-43	TRUE	3	TRA:CAERNSGYSTLTF TRB:CASSAAPSMAGGGGETQYF	8	CD4-C3
PB-3_GCGACCATCAAGATCC	PB	PB-3	P3-1213	FALSE	1	TRA:CATIRTGNQFYF TRB:CASSRPGQGVLNNSPLHF	1	CD4-C3
PB-3_GCGCCAAAGTCCATAC	PB	PB-3	P3-2	TRUE	3	TRA:CAASAPNSGGGADGLTF TRB:CSARRGSSNQPQHF	63	CD4-C3
PB-3_GCGGGTTCATGCTAGT	PB	PB-3	P3-1225	FALSE	1	TRA:CIVRVGGGNQGGKLIF TRB:CSAPDNQETQYF	1	CD4-C3
PB-3_GCTGCTTAGCAAATCA	PB	PB-3	P3-181	TRUE	3	TRA:CAVVPSGSARQLTF TRB:CASRGGTSGGAVDTQYF	3	CD4-C3
PB-3_GCTGGGTCACGACTCG	PB	PB-3	P3-1238	FALSE	1	TRA:CAAPLEGYALNF TRB:CASSYDSGASTDTQYF	1	CD4-C3
PB-3_GCTGGGTCATTGCGGC	PB	PB-3	P3-1239	FALSE	1	TRA:CAVCLYNTDKLIF TRB:CSSAAGLYNEQFF	1	CD4-C3

PB-3_GCTTCCACACGTGAGA	PB	PB-3	P3-1241	FALSE	1	TRA:CALTPRDNNARLMF TRB:CASSGSTGTTGRHGYTF	1	CD4-C3
PB-3_GCTTCCATCAAAGTAG	PB	PB-3	P3-1242	FALSE	1	TRA:CAESFNAGGTSYGKLTF TRB:CSVRRTGYEGYTF	1	CD4-C2
PB-3_GGAACTTTCCTTGCCA	PB	PB-3	P3-1246	FALSE	1	TRA:CIVSVFGGSQGNLIF TRB:CSAPGGGTEAFF	1	CD4-C3
PB-3_GGAATAAGTTCGTTGA	PB	PB-3	P3-2	TRUE	3	TRA:CAASAPNSGGGADGLTF TRB:CSARRGSSNQPQHF	63	CD4-C3
PB-3_GGAATAATCGTAGATC	PB	PB-3	P3-1247	FALSE	1	TRA:CIVRVGNRGSTLGRLYF TRB:CASSPDRNTEAFF	1	CD4-C3
PB-3_GGAATAATCTCGATGA	PB	PB-3	P3-109	TRUE	3	TRA:CAGLAGSNYKLTF TRB:CASSSPGGAGGAFF	4	CD4-C3
PB-3_GGACATTAGAATTGTG	PB	PB-3	P3-163	TRUE	3	TRA:CAENPTTSGTYKYIF TRB:CASSFRDRFQHF	3	CD4-C3
PB-3_GGACATTTCTGGGCCA	PB	PB-3	P3-1253	FALSE	1	TRA:CAEKPDYGGQNFVF TRB:CSVDGQGAYEQYF	1	CD4-C3
PB-3_GGACGTCGTCCAAGTT	PB	PB-3	P3-245	FALSE	2	TRA:CALDSGGGADGLTF TRB:CAIGSTGTNEKLFF	2	CD4-C3
PB-3_GGACGTCGTGGCGAAT	PB	PB-3	P3-1256	FALSE	1	TRA:CAVNPGGYNKLIF TRB:CSARFGTSTDTQYF	1	CD4-C3
PB-3_GGATGTTTCAGCGACC	PB	PB-3	P3-1263	FALSE	1	TRA:CAVSDPGSGGSNYKLTF TRB:CASSGGGNLETQYF	1	CD4-C3
PB-3_GGATTACCATACGCTA	PB	PB-3	P3-2	TRUE	3	TRA:CAASAPNSGGGADGLTF TRB:CSARRGSSNQPQHF	63	CD4-C3
PB-3_GGCAATTCAAGTTAAG	PB	PB-3	P3-1267	FALSE	1	TRA:CAGLKAAGNKLTF TRB:CASSLERLRANTGELFF	1	CD4-C3
PB-3_GGCAATTTCACGACTA	PB	PB-3	P3-1268	FALSE	1	TRA:CAGNQAGTALIF TRB:CASSYGSNNSPLHF	1	CD4-C3
PB-3_GGCAATTTCACTGGGC	PB	PB-3	P3-4	TRUE	3	TRA:CAGHTGANSKLTF TRB:CASSLSTGNYGYTF	39	CD4-C3
PB-3_GGGAGATCACGAGGTA	PB	PB-3	P3-72	FALSE	3	TRA:CAVRRSNYQLIW TRB:CASSPQGETQYF	5	CD4-C3
PB-3_GGGCATCGTACATCCA	PB	PB-3	P3-183	TRUE	3	TRA:CVVNMYSSASKIIF TRB:CASSLEAGTEAFF	3	CD4-C3
PB-3_GGTATTGAGCCACTAT	PB	PB-3	P3-1295	FALSE	1	TRA:CAVQPNQAGTALIF TRB:CASSLSDTLSGNTIYF	1	CD4-C3
PB-3_GGTATTGGTCTAAAGA	PB	PB-3	P3-2	TRUE	3	TRA:CAASAPNSGGGADGLTF TRB:CSARRGSSNQPQHF	63	CD4-C3
PB-3_GGTATTGGTTCAGTAC	PB	PB-3	P3-1297	FALSE	1	TRA:CALRSQSGGSNYKLTF TRB:CASSAGTGPFQETQYF	1	CD4-C2
PB-3_GTAACGTAGGGATCTG	PB	PB-3	P3-303	TRUE	2	TRA:CAVGAAYGGSQGNLIF TRB:CASSPLGGGLTTDTQYF	2	CD4-C3
PB-3_GTAACTGTCATTGCGA	PB	PB-3	P3-4	TRUE	3	TRA:CAGHTGANSKLTF TRB:CASSLSTGNYGYTF	39	CD4-C3
PB-3_GTAACTGTCCCAAGAT	PB	PB-3	P3-1311	FALSE	1	TRA:CAVDTSGSRLTF TRB:CSARQNFVNQYF	1	CD4-C3
PB-3_GTACTCCAGGCCCTTG	PB	PB-3	P3-1315	FALSE	1	TRA:CAVSRRGRRALTF TRB:CSARERGGSGDTQYF	1	CD4-C3
PB-3_GTACTCCTCTTGTTTG	PB	PB-3	P3-2	TRUE	3	TRA:CAASAPNSGGGADGLTF TRB:CSARRGSSNQPQHF	63	CD4-C3
PB-3_GTAGGCCCAGATGGCA	PB	PB-3	P3-1322	FALSE	1	TRA:CAVGANSNYQLIW TRB:CASSRARLAGVGETQYF	1	CD4-C3
PB-3_GTAGGCCCATGCTGGC	PB	PB-3	P3-1323	FALSE	1	TRA:CAVSGNTPLVF TRB:CAGDRGGTGELFF	1	CD4-C3

PB-3_GTATCTTAGCCAGGAT	PB	PB-3	P3-2	TRUE	3	TRA:CAASAPNSGGGADGLTF TRB:CSARRGSSNQPQHF	63	CD4-C3
PB-3_GTATCTTTCAAGCCTA	PB	PB-3	P3-306	TRUE	2	TRA:CALSEAWGQNFVF TRB:CSARDTEGLGTEAFF	2	CD4-C3
PB-3_GTATTCTGTGACTACT	PB	PB-3	P3-1330	FALSE	1	TRA:CAVMLTGTASKLTF TRB:CSYPGVGGYTQYF	1	CD4-C3
PB-3_GTCAAGTCATCTACGA	PB	PB-3	P3-288	FALSE	2	TRA:CAVGIQGAQKLVF TRB:CASSQDIGGEQFF	2	CD4-C3
PB-3_GTCATTTAGTACGATA	PB	PB-3	P3-1340	FALSE	1	TRA:CALNNQGGKLIF TRB:CASRPGGPDNQPQHF	1	CD4-C3
PB-3_GTCCTCATCAACTCTT	PB	PB-3	P3-149	TRUE	3	TRA:CAVRSGAGSYQLTF TRB:CASSEQGVVSRGYTF	3	CD4-C3
PB-3_GTCGTAAAGAAGGTGA	PB	PB-3	P3-1351	FALSE	1	TRA:CAVSSRGDSNYQLIW TRB:CASSPGQGLTDTQYF	1	CD4-C3
PB-3_GTCGTAAAGATATGCA	PB	PB-3	P3-2	TRUE	3	TRA:CAASAPNSGGGADGLTF TRB:CSARRGSSNQPQHF	63	CD4-C3
PB-3_GTCTCGTTCCATGCTC	PB	PB-3	P3-4	TRUE	3	TRA:CAGHTGANSKLTF TRB:CASSLSTGNYGYTF	39	CD4-C3
PB-3_GTGCATAAGCAGATCG	PB	PB-3	P3-4	TRUE	3	TRA:CAGHTGANSKLTF TRB:CASSLSTGNYGYTF	39	CD4-C3
PB-3_GTGCATAGTTTGCATG	PB	PB-3	P3-2	TRUE	3	TRA:CAASAPNSGGGADGLTF TRB:CSARRGSSNQPQHF	63	CD4-C3
PB-3_GTGCGGTGTCAATGTC	PB	PB-3	P3-1370	FALSE	1	TRA:CIVRVGGGNQGGKLIF TRB:CSARDQKSEAFF	1	CD4-C3
PB-3_GTGCTTCTCATGGTCA	PB	PB-3	P3-1374	FALSE	1	TRA:CGIGFQKLVF TRB:CASSLEFLQGSSGANVLTF	1	CD4-C3
PB-3_GTGTGCGGTTCGGGCT	PB	PB-3	P3-1380	FALSE	1	TRA:CAVNMYGGSQGNLIF TRB:CASSLLAGTYNEQFF	1	CD4-C3
PB-3_TAAACCGCACCAGCAC	PB	PB-3	P3-1399	FALSE	1	TRA:CAGGRSWGKFQF TRB:CSASLAGRQETQYF	1	CD4-C3
PB-3_TAAGTGCTCTCTAAGG	PB	PB-3	P3-1408	FALSE	1	TRA:CALDESSGTYKYIF TRB:CASSPRDRITAYEQYF	1	CD4-C3
PB-3_TACACGAGTTAAGATG	PB	PB-3	P3-1410	FALSE	1	TRA:CAYRRQETSGSRLTF TRB:CSARRQLNTEAFF	1	CD4-C3
PB-3_TACAGTGTCAAGAAGT	PB	PB-3	P3-1416	FALSE	1	TRA:CAMRDPFGNEKLTF TRB:CSPRRGSGRTDTQYF	1	CD4-C3
PB-3_TACCTTACAAGCCGCT	PB	PB-3	P3-71	TRUE	3	TRA:CAGDGRSNTGKLIF TRB:CASSLASGQNQPQHF	5	CD4-C3
PB-3_TACGGATAGCTGGAAC	PB	PB-3	P3-1420	FALSE	1	TRA:CAMVAFNDYKLSF TRB:CASNRVDYSNQPQHF	1	CD4-C3
PB-3_TACGGATTCACTTATC	PB	PB-3	P3-177	TRUE	3	TRA:CAVRSNFGNEKLTF TRB:CSVPQGHTEAFF	3	CD4-C3
PB-3_TACGGGCAGGAGTTTA	PB	PB-3	P3-1423	FALSE	1	TRA:CIVRVAAGNQGGKLIF TRB:CSARGNTGELFF	1	CD4-C3
PB-3_TACGGGCGTGCTTCTC	PB	PB-3	P3-1425	FALSE	1	TRA:CLVGGGNAGKSTF TRB:CASSSGTGALALIYEQYF	1	CD4-C3
PB-3_TACTCATTCGCACTCT	PB	PB-3	P3-1435	FALSE	1	TRA:CAFRGSGNKLVF TRB:CASSISGNTDTQYF	1	CD4-C3
PB-3_TACTCATTCTGCGGCA	PB	PB-3	P3-1437	FALSE	1	TRA:CAMRERDGGFKTIF TRB:CASSQVDGLAGSSYNEQFF	1	CD4-C3
PB-3_TACTCGCGTAACGACG	PB	PB-3	P3-1440	FALSE	1	TRA:CAVESREYGNKLVF TRB:CASSVQVNQPQHF	1	CD4-C3
PB-3_TACTTGTAGATCTGAA	PB	PB-3	P3-4	TRUE	3	TRA:CAGHTGANSKLTF TRB:CASSLSTGNYGYTF	39	CD4-C3

PB-3_TAGAGCTCATTAGGCT	PB	PB-3	P3-178	TRUE	3	TRA:CAVSANSGNTPLVF TRB:CSASGQVGEQYF	3	CD4-C3
PB-3_TAGGCATAGAGGTACC	PB	PB-3	P3-1451	FALSE	1	TRA:CVVTWGDDMRF TRB:CASNGPGQESYEQYF	1	CD4-C3
PB-3_TAGGCATCACATAACC	PB	PB-3	P3-1452	FALSE	1	TRA:CVVIHARLMF TRB:CASSLSGQETQYF	1	CD4-C3
PB-3_TAGGCATGTACATGTC	PB	PB-3	P3-2	TRUE	3	TRA:CAASAPNSGGGADGLTF TRB:CSARRGSSNQPQHF	63	CD4-C3
PB-3_TATGCCCCATTCCTGC	PB	PB-3	P3-186	TRUE	3	TRA:CAVALSGGSYIPTF TRB:CAISEREAGSTEAFF	3	CD4-C3
PB-3_TATTACCAGGCAGTCA	PB	PB-3	P3-187	TRUE	3	TRA:CAASAGRARLMF TRB:CSARSPGTGGGTDTQYF	3	CD4-C3
PB-3_TCACAAGGTGAGTATA	PB	PB-3	P3-1473	FALSE	1	TRA:CVVNMYSSASKIIF TRB:CASSLEAGTEQYF	1	CD4-C3
PB-3_TCACGAATCAGGTTCA	PB	PB-3	P3-1478	FALSE	1	TRA:CAVGAFNTGNQFYF TRB:CSVGEPDTGELFF	1	CD4-C3
PB-3_TCAGATGCAAATACAG	PB	PB-3	P3-2	TRUE	3	TRA:CAASAPNSGGGADGLTF TRB:CSARRGSSNQPQHF	63	CD4-C3
PB-3_TCAGCAAAGTTACCCA	PB	PB-3	P3-4	TRUE	3	TRA:CAGHTGANSKLTF TRB:CASSLSTGNYGYTF	39	CD4-C3
PB-3_TCAGCAACATCGACGC	PB	PB-3	P3-1484	FALSE	1	TRA:CAVQKGINTGTASKLTF TRB:CASSWAIDRVKNSPLHF	1	CD4-C3
PB-3_TCAGCAAGTCTGATTG	PB	PB-3	P3-2	TRUE	3	TRA:CAASAPNSGGGADGLTF TRB:CSARRGSSNQPQHF	63	CD4-C3
PB-3_TCAGCTCCATCGGTTA	PB	PB-3	P3-4	TRUE	3	TRA:CAGHTGANSKLTF TRB:CASSLSTGNYGYTF	39	CD4-C3
PB-3_TCAGGTACATTGAGCT	PB	PB-3	P3-2	TRUE	3	TRA:CAASAPNSGGGADGLTF TRB:CSARRGSSNQPQHF	63	CD4-C3
PB-3_TCAGGTAGTGTTCGAT	PB	PB-3	P3-188	TRUE	3	TRA:CGTDNAGNMLTF TRB:CASSPTGTNTEAFF	3	CD4-C3
PB-3_TCATTACAGTGGCACA	PB	PB-3	P3-1499	FALSE	1	TRA:CALRLSGGGADGLTF TRB:CASSPQQGNTEAFF	1	CD4-C3
PB-3_TCATTACCATCGACGC	PB	PB-3	P3-2	TRUE	3	TRA:CAASAPNSGGGADGLTF TRB:CSARRGSSNQPQHF	63	CD4-C3
PB-3_TCATTACGTCAAAGCG	PB	PB-3	P3-260	FALSE	2	TRA:CVVNLYSSASKIIF TRB:CASSLEAGTEQYF	2	CD4-C3
PB-3_TCATTTGTCCAAGCCG	PB	PB-3	P3-1504	FALSE	1	TRA:CAVRHTGANSKLTF TRB:CASSLDNQETQYF	1	CD4-C3
PB-3_TCGAGGCAGGTGGGTT	PB	PB-3	P3-126	TRUE	3	TRA:CAVTMIYNQGGKLIF TRB:CASSGSYEQYF	4	CD4-C3
PB-3_TCGGTAATCCACTCCA	PB	PB-3	P3-1520	FALSE	1	TRA:CIVRTSEKTSGSRLTF TRB:CASSLSQGNEAFF	1	CD4-C3
PB-3_TCGTAGAGTCAGATAA	PB	PB-3	P3-4	TRUE	3	TRA:CAGHTGANSKLTF TRB:CASSLSTGNYGYTF	39	CD4-C3
PB-3_TCTCTAACACTGTTAG	PB	PB-3	P3-1529	FALSE	1	TRA:CALSEGYGGATNKLIF TRB:CASSLGSRDEQYF	1	CD4-C3
PB-3_TCTGAGAGTCTTCTCG	PB	PB-3	P3-1532	FALSE	1	TRA:CAMREISRGQGAQKLVF TRB:CASSEAEATGVDGYTF	1	CD4-C3
PB-3_TCTGAGATCGAGAGCA	PB	PB-3	P3-7	TRUE	3	TRA:CALSEARGGSQGNLIF TRB:CASSFVTGGSEAFF	27	CD8-C2
PB-3_TCTGGAACATTGGTAC	PB	PB-3	P3-21	TRUE	3	TRA:CIVRVAGGNQGGKLIF TRB:CSAQGSKAEQYF	12	CD4-C3
PB-3_TCTTCGGTCCAATGGT	PB	PB-3	P3-1535	FALSE	1	TRA:CAVSGGAGNMLTF TRB:CAGGQGLNTEAFF	1	CD4-C3

PB-3_TCTTTCCGTATTCTCT	PB	PB-3	P3-2	TRUE	3	TRA:CAASAPNSGGGADGLTF TRB:CSARRGSSNQPQHF	63	CD4-C3
PB-3_TCTTTCCTCCTGCTTG	PB	PB-3	P3-160	TRUE	3	TRA:CAARGNFNKFYF TRB:CSASTDRVTEAFF	3	CD4-C6
PB-3_TGAAAGAAGTGGTAGC	PB	PB-3	P3-1538	FALSE	1	TRA:CIVRVAAGNQGGKLIF TRB:CSARDLGNEQYF	1	CD4-C3
PB-3_TGAGGGACACGGCGTT	PB	PB-3	P3-1552	FALSE	1	TRA:CAVDIGGTSGTYKYIF TRB:CASSLRGRSNYGYTF	1	CD4-C3
PB-3_TGCACCTTCCGGCACA	PB	PB-3	P3-1560	FALSE	1	TRA:CAVSDRDSSASKIIF TRB:CSEREGGGEAFF	1	CD4-C3
PB-3_TGCCAAACACTGAAGG	PB	PB-3	P3-1562	FALSE	1	TRA:CAASINSGNTPLVF TRB:CSARAQGNEQYF	1	CD4-C3
PB-3_TGCCAAAGTTAGTGGG	PB	PB-3	P3-1563	FALSE	1	TRA:CAASWGSNYQLIW TRB:CASSIGRGGYNEQFF	1	CD4-C3
PB-3_TGCCCATAGTACACCT	PB	PB-3	P3-1565	FALSE	1	TRA:CAASRGSGGGADGLTF TRB:CSANGQTGELFF	1	CD4-C3
PB-3_TGCCCATAGTCTTGCA	PB	PB-3	P3-1566	FALSE	1	TRA:CAVSRRGSNYKLTF TRB:CASSLVRDVNQPQHF	1	CD4-C3
PB-3_TGCCCATGTATCTGCA	PB	PB-3	P3-1568	FALSE	1	TRA:CAVGTNTGNQFYF TRB:CSGLPGTGRGKLFF	1	CD4-C3
PB-3_TGCGCAGTCACATAGC	PB	PB-3	P3-1573	FALSE	1	TRA:CAMREANSGGYQKVTF TRB:CASSLRLQETQYF	1	CD4-C3
PB-3_TGCTACCTCAACACTG	PB	PB-3	P3-4	TRUE	3	TRA:CAGHTGANSKLTF TRB:CASSLSTGNYGYTF	39	CD4-C3
PB-3_TGGACGCGTTACTGAC	PB	PB-3	P3-1586	FALSE	1	TRA:CAGPGGATNKLIF TRB:CASSPSYHRATNEKLFF	1	CD4-C3
PB-3_TGGCCAGGTGCGGTAA	PB	PB-3	P3-60	TRUE	3	TRA:CAGARNSGYALNF TRB:CASSQGNGVGKAFF	6	CD4-C3
PB-3_TGGCGCAAGACGACGT	PB	PB-3	P3-2	TRUE	3	TRA:CAASAPNSGGGADGLTF TRB:CSARRGSSNQPQHF	63	CD4-C3
PB-3_TGGCTGGGTCGTCTTC	PB	PB-3	P3-1591	FALSE	1	TRA:CATDLTGNQFYF TRB:CASSVFSGDTQYF	1	CD4-C3
PB-3_TGGCTGGTCAATCACG	PB	PB-3	P3-1592	FALSE	1	TRA:CAVLRPGFGNEKLTF TRB:CAISEGTSEGTQYF	1	CD4-C3
PB-3_TGGGAAGGTCGACTAT	PB	PB-3	P3-323	TRUE	2	TRA:CAVSTGGGNKLTF TRB:CASSPRQSTDTQYF	2	CD4-C3
PB-3_TGGGCGTTCTGCAGTA	PB	PB-3	P3-239	FALSE	2	TRA:CAVLSWGKFQF TRB:CASRPHQAQETQYF	2	CD4-C2
PB-3_TGGTTCCGTTGCTCCT	PB	PB-3	P3-2	TRUE	3	TRA:CAASAPNSGGGADGLTF TRB:CSARRGSSNQPQHF	63	CD4-C3
PB-3_TGTGGTAAGCTCCTTC	PB	PB-3	P3-1605	FALSE	1	TRA:CALRPYNFNKFYF TRB:CASSLYGDSNQPQHF	1	CD4-C3
PB-3_TGTGGTACACAGCGTC	PB	PB-3	P3-1608	FALSE	1	TRA:CAEISYNNARLMF TRB:CASSPGTGRPEAFF	1	CD4-C3
PB-3_TGTGGTAGTGTAATGA	PB	PB-3	P3-176	TRUE	3	TRA:CAVTRRGATNKLIF TRB:CASSTRDASGGYEQYF	3	CD4-C3
PB-3_TGTGTTTGTTTGGGCC	PB	PB-3	P3-1614	FALSE	1	TRA:CAAEAGTALIF TRB:CASSGTGDNSPLHF	1	CD4-C3
PB-3_TTAGGCAGTCCAGTAT	PB	PB-3	P3-1622	FALSE	1	TRA:CAVRSPRSGNTPLVF TRB:CSARKHRDKASTDTQYF	1	CD4-C3
PB-3_TTCCCAGCACATAACC	PB	PB-3	P3-91	TRUE	3	TRA:CASYRGGADGLTF TRB:CSASGQVGEAFF	5	CD4-C3
PB-3_TTCCCAGCACGAAACG	PB	PB-3	P3-1627	FALSE	1	TRA:CAETLDSNYQLIW TRB:CAWSGQGIVSGNTIYF	1	CD4-C3

PB-3_TTCCCAGGTAGCTAAA	PB	PB-3	P3-1628	FALSE	1	TRA:CADPNTGRRALTF TRB:CSASGTGGEYGYTF	1	CD4-C3
PB-3_TTCTTAGAGAGTCGGT	PB	PB-3	P3-1641	FALSE	1	TRA:CAVRSNFGNEKLTF TRB:CSANMGWNEQFF	1	CD4-C3
PB-3_TTGTAGGCAGGACGTA	PB	PB-3	P3-44	TRUE	3	TRA:CAGSDEKLTF TRB:CASSSGVRNSPLHF	8	CD4-C3
PB-3_TTTATGCAGAGCTGGT	PB	PB-3	P3-1670	FALSE	1	TRA:CALARDGGGGGNKLTF TRB:CASSFSAGGQETQYF	1	CD4-C3
PB-3_TTTGTCAAGTGGGTTG	PB	PB-3	P3-1678	FALSE	1	TRA:CASLMEYGNKLVF TRB:CSARTHYEQYF	1	CD4-C3
PB-3_TTTGTCAGTGTGAATA	PB	PB-3	P3-1681	FALSE	1	TRA:CAVREDTNAGKSTF TRB:CASSGGTGVIIYNEQFF	1	CD4-C3
PB-3_AAAGATGCATGCTGGC	PB	PB-3	P3-7	TRUE	3	TRA:CALSEARGGSQGNLIF TRB:CASSFVTGGSEAFF	27	CD8-C2
PB-3_AACTCAGCAGTATCTG	PB	PB-3	P3-57	TRUE	3	TRA:CAGMDSSYKLIF TRB:CASSEAGGTSGDEQFF	6	CD8-C10
PB-3_AACTGGTTCCTAGTGA	PB	PB-3	P3-102	TRUE	3	TRA:CAVMDSNYQLIW TRB:CASSDRDNQPQHF	4	CD8-C10
PB-3_AACTTTCAGCGTAGTG	PB	PB-3	P3-73	TRUE	3	TRA:CAVRDSNYQLIW TRB:CASSDRDTGELFF	5	CD8-C10
PB-3_AAGCCGCTCGGAGGTA	PB	PB-3	P3-522	FALSE	1	TRA:CGADANQAGTALIF TRB:CASRLDRGQDTQYF	1	CD4-C3
PB-3_ACACCAACACGTGAGA	PB	PB-3	P3-233	TRUE	2	TRA:CAVTDSNYQLIW TRB:CASSQGEGSYNEQFF	2	CD8-C10
PB-3_ACACTGAAGGCTACGA	PB	PB-3	P3-104	TRUE	3	TRA:CAVLEGDYQLIW TRB:CASAWTASGANVLTF	4	CD8-C10
PB-3_ACGATGTAGACTAGGC	PB	PB-3	P3-151	FALSE	3	TRA:CAVRDSNYQLIW TRB:CASSYRGDTDTQYF	3	CD8-C10
PB-3_ACGCCAGAGCCCGAAA	PB	PB-3	P3-107	TRUE	3	TRA:CAALDSNYQLIW TRB:CSAGTEPYEQYF	4	CD8-C10
PB-3_ACGGAGACAAGCTGTT	PB	PB-3	P3-78	TRUE	3	TRA:CATSDSNYQLIW TRB:CASSGAGGQDTQYF	5	CD8-C2
PB-3_ACTGAACAGTTGCAGG	PB	PB-3	P3-29	TRUE	3	TRA:CAVSGGNQGAQKLVF TRB:CASSLGDPRGKKLFF	10	CD8-C2
PB-3_ACTTGTTTCAAGGCTT	PB	PB-3	P3-80	TRUE	3	TRA:CAVKDSNYQLIW TRB:CASSFLQGDYNEQFF	5	CD8-C10
PB-3_AGACGTTAGACAGGCT	PB	PB-3	P3-626	FALSE	1	TRA:CAVNRGQKVTF TRB:CASSSTSGGRPYEQYF	1	CD8-C10
PB-3_AGCAGCCGTAGATTAG	PB	PB-3	P3-152	FALSE	3	TRA:CARLTF TRB:CASSTTRNTEAFF	3	CD8-C10
PB-3_AGCAGCCGTTCAGACT	PB	PB-3	P3-640	FALSE	1	TRA:CAVMDSNYQLIW TRB:CASSEAPGTNEKLFF	1	CD8-C10
PB-3_AGGGAGTAGTGTTTGC	PB	PB-3	P3-78	TRUE	3	TRA:CATSDSNYQLIW TRB:CASSGAGGQDTQYF	5	CD8-C10
PB-3_AGGGAGTCACTCAGGC	PB	PB-3	P3-40	TRUE	3	TRA:CVVSDRGSTLGRLYF TRB:CASMLQGNTGELFF	8	CD8-C10
PB-3_AGTGAGGGTGGTGTAG	PB	PB-3	P3-57	TRUE	3	TRA:CAGMDSSYKLIF TRB:CASSEAGGTSGDEQFF	6	CD8-C10
PB-3_ATAGACCCAACCGCCA	PB	PB-3	P3-2	TRUE	3	TRA:CAASAPNSGGGADGLTF TRB:CSARRGSSNQPQHF	63	CD4-C3
PB-3_ATCATCTAGACTTGAA	PB	PB-3	P3-152	FALSE	3	TRA:CARLTF TRB:CASSTTRNTEAFF	3	CD8-C10
PB-3_ATCCGAACAGACAAAT	PB	PB-3	P3-716	FALSE	1	TRA:CAVMDSNYQLIW TRB:CASSGTGGTDTQYF	1	CD8-C10

PB-3_ATCTGCCTCTTGTCAT	PB	PB-3	P3-41	TRUE	3	TRA:CAVRDSNYQLIW TRB:CASSLGVENTIYF	8	CD8-C10
PB-3_ATGAGGGGTTATCGGT	PB	PB-3	P3-151	FALSE	3	TRA:CAVRDSNYQLIW TRB:CASSYRGDTDTQYF	3	CD8-C10
PB-3_ATGCGATCAGGGTTAG	PB	PB-3	P3-35	TRUE	3	TRA:CAVRDRDYQLIW TRB:CASSEGAASGANVLTF	9	CD8-C10
PB-3_ATGTGTGCAACTTGAC	PB	PB-3	P3-41	TRUE	3	TRA:CAVRDSNYQLIW TRB:CASSLGVENTIYF	8	CD8-C10
PB-3_ATTATCCGTCTTCAAG	PB	PB-3	P3-253	FALSE	2	TRA:CAGAREDNAGNMLTF TRB:CASSRTTGGAGYTF	2	CD4-C3
PB-3_ATTGGTGTCAGTGTTG	PB	PB-3	P3-158	FALSE	3	TRA:CILRDVNTGNQFYF TRB:CASMAPGWANEKLFF	3	CD8-C10
PB-3_ATTTCTGAGTAAGTAC	PB	PB-3	P3-255	FALSE	2	TRA:CAVRDSNYQLIW TRB:CASSKNEQETQYF	2	CD8-C10
PB-3_CAACCAAAGCCGCCTA	PB	PB-3	P3-35	TRUE	3	TRA:CAVRDRDYQLIW TRB:CASSEGAASGANVLTF	9	CD8-C10
PB-3_CAAGTTGAGCGATGAC	PB	PB-3	P3-107	TRUE	3	TRA:CAALDSNYQLIW TRB:CSAGTEPYEQYF	4	CD8-C10
PB-3_CACAAACTCTTGTATC	PB	PB-3	P3-116	TRUE	3	TRA:CVVSDRGSTLGRLYF TRB:CASNPGTGVYNEQFF	4	CD8-C10
PB-3_CACCAGGCATTGGGCC	PB	PB-3	P3-7	TRUE	3	TRA:CALSEARGGSQGNLIF TRB:CASSFVTGGSEAFF	27	CD8-C2
PB-3_CAGATCAGTAGCTGCC	PB	PB-3	P3-41	TRUE	3	TRA:CAVRDSNYQLIW TRB:CASSLGVENTIYF	8	CD8-C10
PB-3_CAGCTGGGTAAGTAGT	PB	PB-3	P3-7	TRUE	3	TRA:CALSEARGGSQGNLIF TRB:CASSFVTGGSEAFF	27	CD8-C2
PB-3_CAGTAACCACGTCTCT	PB	PB-3	P3-57	TRUE	3	TRA:CAGMDSSYKLIF TRB:CASSEAGGTSGDEQFF	6	CD8-C10
PB-3_CATTCGCCACCAGTTA	PB	PB-3	P3-117	FALSE	3	TRA:CAENVDTGRRALTF TRB:CASSQDLAQGLSYEQYF	4	CD4-C3
PB-3_CCACCTATCAATCTCT	PB	PB-3	P3-78	TRUE	3	TRA:CATSDSNYQLIW TRB:CASSGAGGQDTQYF	5	CD8-C10
PB-3_CCTATTAGTTACGCGC	PB	PB-3	P3-84	TRUE	3	TRA:CAVKRNTGFQKLVF TRB:CASSEADTGYTF	5	CD8-C2
PB-3_CCTTACGTCCAACCAA	PB	PB-3	P3-40	TRUE	3	TRA:CVVSDRGSTLGRLYF TRB:CASMLQGNTGELFF	8	CD8-C10
PB-3_CGATGGCTCGTGGACC	PB	PB-3	P3-940	FALSE	1	TRA:CAVLDSNYQLIW TRB:CASSERSGTGALEQYF	1	CD8-C10
PB-3_CGATGTATCGGAGCAA	PB	PB-3	P3-268	FALSE	2	TRA:CAVQDSNYQLIW TRB:CSARVPGEMTEAFF	2	CD8-C10
PB-3_CGGACACTCTGTTTGT	PB	PB-3	P3-104	TRUE	3	TRA:CAVLEGDYQLIW TRB:CASAWTASGANVLTF	4	CD8-C10
PB-3_CGGACTGTCTTGGGTA	PB	PB-3	P3-967	FALSE	1	TRA:CAVWDSNYQLIW TRB:CASSQEVPGGVSEQYF	1	CD8-C10
PB-3_CGTCTACAGAGTTGGC	PB	PB-3	P3-63	TRUE	3	TRA:CAVVDSNYQLIW TRB:CSARAGGRDYNEQFF	6	CD8-C10
PB-3_CTAGTGAGTGAAGGCT	PB	PB-3	P3-2	TRUE	3	TRA:CAASAPNSGGGADGLTF TRB:CSARRGSSNQPQHF	63	CD4-C3
PB-3_CTCGAAACACCACGTG	PB	PB-3	P3-7	TRUE	3	TRA:CALSEARGGSQGNLIF TRB:CASSFVTGGSEAFF	27	CD8-C2
PB-3_CTGCCTAAGACTAAGT	PB	PB-3	P3-78	TRUE	3	TRA:CATSDSNYQLIW TRB:CASSGAGGQDTQYF	5	CD8-C10
PB-3_GAAATGATCAGCACAT	PB	PB-3	P3-1115	FALSE	1	TRA:CAVLDSNYQLIW TRB:CASSESNEGTGELFF	1	CD8-C10

PB-3_GAACATCTCAGGCGAA	PB	PB-3	P3-290	TRUE	2	TRA:CAVNDKNYGGSQGNLIF TRB:CASSFLRTDTDTQYF	2	CD8-C10
PB-3_GACTAACAGCCTTGAT	PB	PB-3	P3-41	TRUE	3	TRA:CAVRDSNYQLIW TRB:CASSLGVENTIYF	8	CD8-C10
PB-3_GATCGATAGGCACATG	PB	PB-3	P3-120	TRUE	3	TRA:CAVMDSSYKLIF TRB:CASSDGGDTGELFF	4	CD8-C10
PB-3_GCATGATAGGAGTTGC	PB	PB-3	P3-151	FALSE	3	TRA:CAVRDSNYQLIW TRB:CASSYRGDTDTQYF	3	CD8-C10
PB-3_GCATGATCATGGTAGG	PB	PB-3	P3-1202	FALSE	1	TRA:CAVKEDSNYQLIW TRB:CASSPTGNNEKLFF	1	CD8-C2
PB-3_GCTTCCATCGACAGCC	PB	PB-3	P3-255	FALSE	2	TRA:CAVRDSNYQLIW TRB:CASSKNEQETQYF	2	CD8-C10
PB-3_GGAATAAGTCGACTAT	PB	PB-3	P3-80	TRUE	3	TRA:CAVKDSNYQLIW TRB:CASSFLQGDYNEQFF	5	CD8-C10
PB-3_GGGAGATAGCTGCGAA	PB	PB-3	P3-1283	FALSE	1	TRA:CAVRNSNYQLIW TRB:CASSTTLNTEAFF	1	CD8-C10
PB-3_GTATTCTTCCTAGGGC	PB	PB-3	P3-63	TRUE	3	TRA:CAVVDSNYQLIW TRB:CSARAGGRDYNEQFF	6	CD8-C10
PB-3_GTTACAGTCCCTAACC	PB	PB-3	P3-35	TRUE	3	TRA:CAVRDRDYQLIW TRB:CASSEGAASGANVLTF	9	CD8-C10
PB-3_GTTCGGGCAGTGAGTG	PB	PB-3	P3-120	TRUE	3	TRA:CAVMDSSYKLIF TRB:CASSDGGDTGELFF	4	CD8-C10
PB-3_TAAACCGGTCAGGACA	PB	PB-3	P3-104	TRUE	3	TRA:CAVLEGDYQLIW TRB:CASAWTASGANVLTF	4	CD8-C10
PB-3_TACACGACAAGCGTAG	PB	PB-3	P3-2	TRUE	3	TRA:CAASAPNSGGGADGLTF TRB:CSARRGSSNQPQHF	63	CD4-C3
PB-3_TACTTACGTATAATGG	PB	PB-3	P3-268	FALSE	2	TRA:CAVQDSNYQLIW TRB:CSARVPGEMTEAFF	2	CD8-C10
PB-3_TACTTGTAGCCATCGC	PB	PB-3	P3-1443	FALSE	1	TRA:CAVTTSGTYKYIF TRB:CASRRRDRTQETQYF	1	CD4-C3
PB-3_TATGCCCTCAAGGCTT	PB	PB-3	P3-116	TRUE	3	TRA:CVVSDRGSTLGRLYF TRB:CASNPGTGVYNEQFF	4	CD8-C10
PB-3_TATTACCGTAAAGTCA	PB	PB-3	P3-1465	FALSE	1	TRA:CAVRDSNYQLIW TRB:CATSSTGESAEQYF	1	CD8-C10
PB-3_TCACGAAGTGCCTGCA	PB	PB-3	P3-102	TRUE	3	TRA:CAVMDSNYQLIW TRB:CASSDRDNQPQHF	4	CD8-C10
PB-3_TCAGATGAGTTCGATC	PB	PB-3	P3-1480	FALSE	1	TRA:CAVMDSNYQLIW TRB:CSARLAGGQETQYF	1	CD8-C10
PB-3_TCAGATGGTACCGTTA	PB	PB-3	P3-41	TRUE	3	TRA:CAVRDSNYQLIW TRB:CASSLGVENTIYF	8	CD8-C10
PB-3_TCAGCTCTCTATCGCC	PB	PB-3	P3-116	TRUE	3	TRA:CVVSDRGSTLGRLYF TRB:CASNPGTGVYNEQFF	4	CD8-C10
PB-3_TCGCGTTGTACCGAGA	PB	PB-3	P3-1514	FALSE	1	TRA:CVVSDRGSTLGRLYF TRB:CASSVGTSGPSRETQYF	1	CD8-C10
PB-3_TCGGTAATCAACGAAA	PB	PB-3	P3-316	TRUE	2	TRA:CAEGDGANNLFF TRB:CASSITGTDTEAFF	2	CD8-C2
PB-3_TGCCCATCATGTTCCC	PB	PB-3	P3-63	TRUE	3	TRA:CAVVDSNYQLIW TRB:CSARAGGRDYNEQFF	6	CD8-C10
PB-3_TGTCCCACACGTGAGA	PB	PB-3	P3-1604	FALSE	1	TRA:CAVSDSNYQLIW TRB:CASAGDYNSPLHF	1	CD8-C10
PB-3_TTAGGACGTGAGGCTA	PB	PB-3	P3-1619	FALSE	1	TRA:CAVARGGSNYKLTF TRB:CASSLQNGGPSNEQFF	1	CD8-C2
PB-3_TTAGTTCAGAATCTCC	PB	PB-3	P3-152	FALSE	3	TRA:CARLTF TRB:CASSTTRNTEAFF	3	CD8-C10

PB-3_TTGAACGAGTGTTTGC	PB	PB-3	P3-1643	FALSE	1	TRA:CVVSDRGSTLGRLYF TRB:CASSESPGKTGELFF	1	CD4-C3
PB-3_TTGCCGTGTGCTTCTC	PB	PB-3	P3-80	TRUE	3	TRA:CAVKDSNYQLIW TRB:CASSFLQGDYNEQFF	5	CD8-C10
PB-3_AAGGCAGTCGACCAGC	PB	PB-3	P3-531	FALSE	1	TRA:CAVRDARGYKLSF TRB:CSAMPGQGYEQYF	1	CD4-C2
PB-3_ACGGGTCAGCCACGTC	PB	PB-3	P3-600	FALSE	1	TRA:CAMRERDSSYKLIF TRB:CASSLSSGGTTDTQYF	1	CD4-C7
PB-3_AGATTGCAGAGTAAGG	PB	PB-3	P3-79	TRUE	3	TRA:CVVNPGGSYIPTF TRB:CASSGDYGQIYEQYF	5	CD4-C3
PB-3_CACACAATCTGCCAGG	PB	PB-3	P3-774	FALSE	1	TRA:CAYRGPRGNKLTF TRB:CASSLLGTGDSPLHF	1	CD4-C5
PB-3_CAGCTAAAGAAACCTA	PB	PB-3	P3-810	FALSE	1	TRA:CATIQYTGGFKTIF TRB:CASSEGNQPQHF	1	CD4-C6
PB-3_CCGTACTGTAGAAGGA	PB	PB-3	P3-886	FALSE	1	TRA:CAGQLYTNAGKSTF TRB:CASSRGRTEAFF	1	CD4-C2
PB-3_CGAACATTCCCTAACC	PB	PB-3	P3-919	FALSE	1	TRA:CALSERGAQKLVF TRB:CASNVDGNIYEQYF	1	CD4-C5
PB-3_CGAACATTCTTAGCCC	PB	PB-3	P3-920	FALSE	1	TRA:CAVDTGRRALTF TRB:CASSLTGDITEAFF	1	CD4-C7
PB-3_CGTTCTGAGCAGGCTA	PB	PB-3	P3-114	TRUE	3	TRA:CVVNGRDDKIIF TRB:CASSLGRGLGNTIYF	4	CD4-C2
PB-3_CTACATTAGTACGTTC	PB	PB-3	P3-1024	FALSE	1	TRA:CAENIGLNSGYALNF TRB:CASYRDRGSEAFF	1	CD8-C2
PB-3_CTACCCATCGCGTAGC	PB	PB-3	P3-1027	FALSE	1	TRA:CAATVTGTASKLTF TRB:CASSVNSGYLKSDTQYF	1	CD4-C5
PB-3_CTCATTAGTTGGGACA	PB	PB-3	P3-1048	FALSE	1	TRA:CAGGGGADGLTF TRB:CASSRTTSNTEAFF	1	CD4-C5
PB-3_CTGGTCTAGTGACTCT	PB	PB-3	P3-1096	FALSE	1	TRA:CAVRDYAGKSTF TRB:CASRGADEKLFF	1	CD4-C2
PB-3_GACGCGTAGTGGGATC	PB	PB-3	P3-1146	FALSE	1	TRA:CAVKKREYGNKLVF TRB:CASSEVAGASRNQPQHF	1	CD4-C3
PB-3_GCTGCTTAGAAGGACA	PB	PB-3	P3-1234	FALSE	1	TRA:CVRGGSNYKLTF TRB:CASSFLAAGGYEQYF	1	CD4-C5
PB-3_GTACTTTCAGACGTAG	PB	PB-3	P3-1318	FALSE	1	TRA:CAPDTGRRALTF TRB:CASSSSRVTQETQYF	1	CD4-C3
PB-3_TGATTTCAGAAGATTC	PB	PB-3	P3-321	TRUE	2	TRA:CAYRSAYGSSNTGKLIF TRB:CASSRDRGFEKLFF	2	CD4-C7
PB-3_TGCCCTACAATTCCTT	PB	PB-3	P3-1571	FALSE	1	TRA:CAVSDLNTGNQFYF TRB:CASSLLGGVVTDTQYF	1	CD4-C2
PB-3_TGGACGCAGCTAGCCC	PB	PB-3	P3-1584	FALSE	1	TRA:CVVNSRNTGTASKLTF TRB:CASSSTVHEQYF	1	CD4-C2
PB-3_AAGGAGCAGGCAGGTT	PB	PB-3	P3-524	FALSE	1	TRA:CAVKESGGYQKVTF TRB:CASSTTRGETQYF	1	CD4-C7
PB-3_ACATCAGTCTGTGCAA	PB	PB-3	P3-569	FALSE	1	TRA:CVVRGSSGDKLTF TRB:CAWGTGISSGYTF	1	CD4-C7
PB-3_ACGAGCCTCCACTCCA	PB	PB-3	P3-34	TRUE	3	TRA:CAVEGTGGFKTIF TRB:CSVDSQGSNEQFF	9	CD4-C7
PB-3_AGCTCCTGTCTAGCCG	PB	PB-3	P3-659	FALSE	1	TRA:CAVQAYNQFYF TRB:CASSFSELREQYF	1	CD4-C7
PB-3_AGCTCCTTCAGGCAAG	PB	PB-3	P3-661	FALSE	1	TRA:CAAYTNFGNEKLTF TRB:CSARKSNNYGYTF	1	CD4-C7
PB-3_AGGTCATCAAGCGTAG	PB	PB-3	P3-684	FALSE	1	TRA:CAVSGPSSNTGKLIF TRB:CASSEGLFSGNTIYF	1	CD4-C7

PB-3_ATCCGAAAGACCTTTG	PB	PB-3	P3-715	FALSE	1	TRA:CAAFRPPSNTGKLIF TRB:CSVAAQGADTQYF	1	CD4-C7
PB-3_ATCTGCCCACCCATTC	PB	PB-3	P3-724	FALSE	1	TRA:CIVRGREGSSNTGKLIF TRB:CSAMGQLSSPLHF	1	CD4-C7
PB-3_ATTATCCTCGTACCGG	PB	PB-3	P3-114	TRUE	3	TRA:CVVNGRDDKIIF TRB:CASSLGRGLGNTIYF	4	CD4-C2
PB-3_CAAGTTGGTATGCTTG	PB	PB-3	P3-762	FALSE	1	TRA:CAVSYSGGGADGLTF TRB:CASKKSDRKNTEAFF	1	CD4-C7
PB-3_CACAAACCAATAGCGG	PB	PB-3	P3-767	FALSE	1	TRA:CIVRVSGGYNKLIF TRB:CSVGKGSYGADMNTEAFF	1	CD4-C7
PB-3_CACAGGCTCCAGTAGT	PB	PB-3	P3-778	FALSE	1	TRA:CAASSGGSNYKLTF TRB:CATSDSGGQGTYEQYF	1	CD4-C7
PB-3_CAGAATCAGGTGTGGT	PB	PB-3	P3-793	FALSE	1	TRA:CAAKSTNAGKSTF TRB:CSASSPYEQYF	1	CD4-C7
PB-3_CAGATCAGTAAGAGAG	PB	PB-3	P3-805	FALSE	1	TRA:CAVRDSSARQLTF TRB:CASGGGKETQYF	1	CD4-C7
PB-3_CAGTCCTGTCACTTCC	PB	PB-3	P3-818	FALSE	1	TRA:CAVKSSNTGKLIF TRB:CASSLLRLNSPLHF	1	CD4-C7
PB-3_CATATGGAGTGTTTGC	PB	PB-3	P3-822	FALSE	1	TRA:CAVGETGANNLFF TRB:CASKFKRLAKNIQYF	1	CD4-C7
PB-3_CATCCACCAGACGCCT	PB	PB-3	P3-830	FALSE	1	TRA:CAPDRNTGNQFYF TRB:CASSPNRGTGATEAFF	1	CD4-C7
PB-3_CATCGAAAGAAGCCCA	PB	PB-3	P3-833	FALSE	1	TRA:CAVQGWNSGNTPLVF TRB:CASSETPSPSNNEQFF	1	CD4-C7
PB-3_CATTATCCAAGTCATC	PB	PB-3	P3-853	FALSE	1	TRA:CARLGQGAQKLVF TRB:CASSPPGPSSYNSPLHF	1	CD4-C7
PB-3_CCGTGGAGTGGCGAAT	PB	PB-3	P3-889	FALSE	1	TRA:CALRTGNQFYF TRB:CASRERVHTQYF	1	CD4-C7
PB-3_CGACTTCAGGGTTTCT	PB	PB-3	P3-924	FALSE	1	TRA:CLVVTMDSNYQLIW TRB:CASSTLAGANEQFF	1	CD4-C7
PB-3_CGAGAAGTCGCACTCT	PB	PB-3	P3-930	FALSE	1	TRA:CAYRRNSGYALNF TRB:CSVGAGQGHYEQYF	1	CD4-C7
PB-3_CGCTATCAGAGGTACC	PB	PB-3	P3-952	FALSE	1	TRA:CAASKASGANSKLTF TRB:CASSTPAGGTQYF	1	CD4-C7
PB-3_CGGACGTCAGGACCCT	PB	PB-3	P3-964	FALSE	1	TRA:CAYRSPNQYTGTASKLTF TRB:CSARRGSSNQPQHF	1	CD4-C7
PB-3_CGGGTCAAGGCAATTA	PB	PB-3	P3-975	FALSE	1	TRA:CALPYSSASKIIF TRB:CASSEGDYGYTF	1	CD4-C7
PB-3_CGTAGGCGTGGCTCCA	PB	PB-3	P3-982	FALSE	1	TRA:CAVGESGGYNKLIF TRB:CASSRTGDSSYNEQFF	1	CD4-C7
PB-3_CTCCTAGCATTCCTCG	PB	PB-3	P3-1051	FALSE	1	TRA:CAVARSSGSARQLTF TRB:CASSSSGGAWELFF	1	CD4-C7
PB-3_CTCGAAATCGGAATCT	PB	PB-3	P3-1055	FALSE	1	TRA:CAGDGTGGFKTIF TRB:CASSRTGELFF	1	CD4-C2
PB-3_CTCGGAGTCGGAGCAA	PB	PB-3	P3-1065	FALSE	1	TRA:CAVNQGGSEKLVF TRB:CASQKARPQRSNQPQHF	1	CD4-C7
PB-3_CTCGTACCATACTCTT	PB	PB-3	P3-1068	FALSE	1	TRA:CAAHNNARLMF TRB:CASSPGTSADNEQFF	1	CD4-C7
PB-3_CTCGTACCATCGGACC	PB	PB-3	P3-1069	FALSE	1	TRA:CVVSDQWNFNKFYF TRB:CASSGLGLAGDRETQYF	1	CD4-C7
PB-3_CTCGTACTCGGAAACG	PB	PB-3	P3-1070	FALSE	1	TRA:CATDVIGAQKLVF TRB:CSARDPGSPLHF	1	CD4-C7
PB-3_CTCGTCAAGGTCATCT	PB	PB-3	P3-1072	FALSE	1	TRA:CAVGARGFKTIF TRB:CAISPSEGQRNTGELFF	1	CD4-C7

PB-3_CTTGGCTGTTCCGTCT	PB	PB-3	P3-1107	FALSE	1	TRA:CATDAVSGYSTLTF TRB:CASKKTSGGVNTQYF	1	CD4-C7
PB-3_GAAACTCCAGTTAACC	PB	PB-3	P3-145	FALSE	3	TRA:CALAYNFNKFYF TRB:CASSLSVRDQTQYF	3	CD4-C7
PB-3_GAAATGAAGTGTACCT	PB	PB-3	P3-1114	FALSE	1	TRA:CAVTGGGNKLTF TRB:CSARYFQSGNTIYF	1	CD4-C7
PB-3_GAAGCAGGTGACTCAT	PB	PB-3	P3-1126	FALSE	1	TRA:CAPQTGANNLFF TRB:CASTAHRFYEQYF	1	CD4-C2
PB-3_GAATAAGCAAGGGTCA	PB	PB-3	P3-1128	FALSE	1	TRA:CAVTFTGGGNKLTF TRB:CASSVAFDVSGTQYF	1	CD4-C2
PB-3_GACACGCAGAAGGACA	PB	PB-3	P3-1133	FALSE	1	TRA:CAMSQRQGNLIF TRB:CASSEAQPGAGYEQYF	1	CD4-C2
PB-3_GACACGCGTCCATGAT	PB	PB-3	P3-1134	FALSE	1	TRA:CAASRGGATNKLIF TRB:CSASSGRGGQRNIQYF	1	CD4-C2
PB-3_GATCGTACATCAGTAC	PB	PB-3	P3-1177	FALSE	1	TRA:CARGEGSSNTGKLIF TRB:CASSFSRTGYTDTQYF	1	CD4-C7
PB-3_GATGAGGTCGGTTCGG	PB	PB-3	P3-1183	FALSE	1	TRA:CAMREGPNDMRF TRB:CASSFIDRATYEQYF	1	CD4-C7
PB-3_GATTCAGCAATCTACG	PB	PB-3	P3-1185	FALSE	1	TRA:CAVRDSNAGNMLTF TRB:CASGLVERWTSGPQYNEQFF	1	CD4-C7
PB-3_GCACTCTGTACAGCAG	PB	PB-3	P3-1195	FALSE	1	TRA:CAVRHGSSNTGKLIF TRB:CSARWGGRAETQYF	1	CD4-C2
PB-3_GCATGTACACGTTGGC	PB	PB-3	P3-1205	FALSE	1	TRA:CAENSKAAGNKLTF TRB:CASRALGATDTQYF	1	CD4-C7
PB-3_GCGACCAGTCTTGATG	PB	PB-3	P3-1211	FALSE	1	TRA:CASIEDAGNMLTF TRB:CASSVDGTGGSGEQFF	1	CD4-C2
PB-3_GCGCAACCAATCTACG	PB	PB-3	P3-299	FALSE	2	TRA:CAWGIQGAQKLVF TRB:CAISEPRDGTFYTF	2	CD4-C7
PB-3_GCGCAGTGTTCAGTAC	PB	PB-3	P3-1219	FALSE	1	TRA:CAVPSGGSYIPTF TRB:CASSRRQGRGYTF	1	CD4-C7
PB-3_GCTGCTTAGAATAGGG	PB	PB-3	P3-1235	FALSE	1	TRA:CAVYQAGTALIF TRB:CASSQLASGNTIYF	1	CD4-C7
PB-3_GGAATAATCTATCCCG	PB	PB-3	P3-1248	FALSE	1	TRA:CAESNNARLMF TRB:CAWSSDLNGEAFF	1	CD4-C7
PB-3_GGCAATTTCGGCATCG	PB	PB-3	P3-1269	FALSE	1	TRA:CAMSLNNFNKFYF TRB:CASSLVTTDTQYF	1	CD4-C7
PB-3_GGCGTGTCAGTAAGCG	PB	PB-3	P3-1273	FALSE	1	TRA:CAVNNQAGTALIF TRB:CASTPEVNQPQHF	1	CD4-C7
PB-3_GGCTGGTGTCGTTGTA	PB	PB-3	P3-1278	FALSE	1	TRA:CAVVSTGGGNKLTF TRB:CASSLGQANEKLFF	1	CD4-C7
PB-3_GGGAATGAGCTAGTTC	PB	PB-3	P3-1280	FALSE	1	TRA:CAEIPYNQGGKLIF TRB:CSAESRSEQFF	1	CD4-C7
PB-3_GGGCACTTCGGATGTT	PB	PB-3	P3-1293	FALSE	1	TRA:CALSAASGYSTLTF TRB:CSVQTSGPETQYF	1	CD4-C7
PB-3_GGTGAAGTCTTAGAGC	PB	PB-3	P3-1298	FALSE	1	TRA:CAVRPSLNSGNTPLVF TRB:CSARTDYNSPLHF	1	CD4-C7
PB-3_GTAACGTCAACTTGAC	PB	PB-3	P3-1308	FALSE	1	TRA:CALSNQGAQKLVF TRB:CASSPRTGDTGELFF	1	CD4-C2
PB-3_GTCAAGTCAGCTTCGG	PB	PB-3	P3-1332	FALSE	1	TRA:CAFKRGAQKLVF TRB:CASSTAGTGRYEQYF	1	CD4-C2
PB-3_GTCAAGTTCTTGTATC	PB	PB-3	P3-1335	FALSE	1	TRA:CAVLDDKIIF TRB:CASSSGQGTDTQYF	1	CD4-C2
PB-3_GTCATTTGTCGTCTTC	PB	PB-3	P3-1345	FALSE	1	TRA:CALNQAGTALIF TRB:CSAHDSNQETQYF	1	CD4-C2

PB-3_GTCGTAATCTTAACCT	PB	PB-3	P3-1356	FALSE	1	TRA:CATHRTGSSNTGKLIF TRB:CSVAFWGNEQFF	1	CD4-C7
PB-3_GTGTGCGGTCATGCAT	PB	PB-3	P3-1378	FALSE	1	TRA:CATEGTGGTSYGKLTF TRB:CASSGSAYEQYF	1	CD4-C7
PB-3_GTTACAGGTAGAGCTG	PB	PB-3	P3-311	TRUE	2	TRA:CVVSEPPTSGTYKYIF TRB:CASSFRNSNQPQHF	2	CD4-C7
PB-3_GTTCGGGGTGCCTGGT	PB	PB-3	P3-1389	FALSE	1	TRA:CADRGGGADGLTF TRB:CASSLGRSQETQYF	1	CD4-C2
PB-3_TAAACCGGTCAAACTC	PB	PB-3	P3-1400	FALSE	1	TRA:CAVRGVNAGNMLTF TRB:CASSSQQGSGANVLTF	1	CD4-C7
PB-3_TAAGAGACAACGCACC	PB	PB-3	P3-1402	FALSE	1	TRA:CAVRADLRSQGNLIF TRB:CASSLAVSSYNEQFF	1	CD4-C7
PB-3_TACAGTGCAGGCTCAC	PB	PB-3	P3-1414	FALSE	1	TRA:CGVKGDTGNQFYF TRB:CASSLWGTQPQHF	1	CD4-C7
PB-3_TACAGTGGTTGATTGC	PB	PB-3	P3-1415	FALSE	1	TRA:CAVSKGNNRLAF TRB:CASSPQSASYEQYF	1	CD4-C2
PB-3_TACGGATGTAGTAGTA	PB	PB-3	P3-1422	FALSE	1	TRA:CAGAAGGTSYGKLTF TRB:CASSGGHTGELFF	1	CD4-C7
PB-3_TACGGGCCAGCTTCGG	PB	PB-3	P3-1424	FALSE	1	TRA:CAVRDGTNAGKSTF TRB:CASSSLGQGKYEQYF	1	CD4-C2
PB-3_TACGGGCTCCTATTCA	PB	PB-3	P3-1427	FALSE	1	TRA:CAAGGDDKIIF TRB:CASSVTARAGYGYTF	1	CD4-C7
PB-3_TACTCATAGTCCTCCT	PB	PB-3	P3-1432	FALSE	1	TRA:CAVRDIDAGNMLTF TRB:CASSPDRVEQYF	1	CD4-C7
PB-3_TCACGAAGTACCAGTT	PB	PB-3	P3-1476	FALSE	1	TRA:CAVRSGTYKYIF TRB:CASSLGRYHNEQFF	1	CD4-C7
PB-3_TCAGGATTCTGGCGAC	PB	PB-3	P3-1497	FALSE	1	TRA:CAFMKRVGGGATNKLIF TRB:CAGRRSGNTIYF	1	CD4-C7
PB-3_TCAGGTATCGGCTTGG	PB	PB-3	P3-1498	FALSE	1	TRA:CAMSAGGYQKVTF TRB:CASRPRGRQLFF	1	CD4-C7
PB-3_TCATTACCATTACGAC	PB	PB-3	P3-1500	FALSE	1	TRA:CAAPSGSRLTF TRB:CASSLWASGDTQYF	1	CD4-C7
PB-3_TCTGAGAAGAGCTATA	PB	PB-3	P3-1530	FALSE	1	TRA:CILLNAGNMLTF TRB:CSARDAANGQFTEAFF	1	CD4-C7
PB-3_TGACAACGTTACGTCA	PB	PB-3	P3-1541	FALSE	1	TRA:CVVGGSGGSYIPTF TRB:CASSLGSVQETQYF	1	CD4-C7
PB-3_TGACAACTCACGCGGT	PB	PB-3	P3-1542	FALSE	1	TRA:CATDAGRYSTLTF TRB:CSASPHGQGLGQPQHF	1	CD4-C7
PB-3_TGCCCTACATATGGTC	PB	PB-3	P3-1572	FALSE	1	TRA:CAYRSAVYSGGGADGLTF TRB:CATSDPGDRGSDTQYF	1	CD4-C7
PB-3_TGGCCAGAGGACGAAA	PB	PB-3	P3-1587	FALSE	1	TRA:CAVDSSGSRLTF TRB:CASSLDRVQDTQYF	1	CD4-C7
PB-3_TGGCCAGCACACATGT	PB	PB-3	P3-1588	FALSE	1	TRA:CVLNTGGFKTIF TRB:CASSLQRDGEAFF	1	CD4-C7
PB-3_TGTGGTAAGGTCATCT	PB	PB-3	P3-1607	FALSE	1	TRA:CASTIQGAQKLVF TRB:CASSPVGSGDTDTQYF	1	CD4-C7
PB-3_TGTGTTTGTCTAGAGG	PB	PB-3	P3-1612	FALSE	1	TRA:CAAILLGSQGNLIF TRB:CASSYRLAAEQFF	1	CD4-C7
PB-3_TGTTCCGGTTAAGGGC	PB	PB-3	P3-1616	FALSE	1	TRA:CAVERNFNKFYF TRB:CAWSVVGDNYGYTF	1	CD4-C7
PB-3_TTAACTCCAGGTGGAT	PB	PB-3	P3-1617	FALSE	1	TRA:CAMSEI TRB:CASSLGQGAGDTQYF	1	CD4-C7
PB-3_TTAGGACTCGAATCCA	PB	PB-3	P3-1620	FALSE	1	TRA:CADHNAGNMLTF TRB:CASSFTTSTDTQYF	1	CD4-C7

PB-3_TTAGGCAAGATGTCGG	PB	PB-3	P3-1621	FALSE	1	TRA:CAVGYQKVTF TRB:CSARAGRGSYNEQFF	1	CD4-C7
PB-3_TTCGGTCAGATGGCGT	PB	PB-3	P3-1634	FALSE	1	TRA:CALRRGGGFKTIF TRB:CASSLVTGEETQYF	1	CD4-C7
PB-3_TTGTAGGGTTAGAACA	PB	PB-3	P3-1664	FALSE	1	TRA:CAGQTGANNLFF TRB:CASSEAYRNRGKAFF	1	CD4-C7
PB-3_TTGTAGGTCCAGAGGA	PB	PB-3	P3-1665	FALSE	1	TRA:CAGNSGSRLTF TRB:CSARPGASLYNEQFF	1	CD4-C7
PB-3_TTTGGTTAGAACAACT	PB	PB-3	P3-1676	FALSE	1	TRA:CVVRVVDKLIF TRB:CASSKGQGMSGYTF	1	CD4-C2
PB-3_TTTGTCAAGATAGTCA	PB	PB-3	P3-1677	FALSE	1	TRA:CVVSDRTEGGKLIF TRB:CASSFNRFPGDEQYF	1	CD4-C7
PB-3_AAAGTAGGTCTTGCGG	PB	PB-3	P3-490	FALSE	1	TRA:CAQGGSNYKLTF TRB:CASSSRTGNYNEQFF	1	CD4-C1
PB-3_AAATGCCGTCTACCTC	PB	PB-3	P3-493	FALSE	1	TRA:CIVRVGNNNARLMF TRB:CSASLGAGTGEQYF	1	CD4-C1
PB-3_AACCGCGGTACCATCA	PB	PB-3	P3-497	FALSE	1	TRA:CILPDGQKLLF TRB:CASSTSGGAFETQYF	1	CD4-C1
PB-3_AACGTTGCACCATGTA	PB	PB-3	P3-498	FALSE	1	TRA:CAVQAVEGDYKLSF TRB:CASSYTQGAGQPQHF	1	CD4-C2
PB-3_AACTCTTAGATGTGTA	PB	PB-3	P3-504	FALSE	1	TRA:CARNTGGGNKLTF TRB:CASSLDSTRAYEQYF	1	CD4-C1
PB-3_AACTCTTTCAACTCTT	PB	PB-3	P3-506	FALSE	1	TRA:CLVGETTDKLIF TRB:CSVEVPGWGELFF	1	CD4-C1
PB-3_AACTGGTTCAGAGACG	PB	PB-3	P3-515	FALSE	1	TRA:CAVEPGGSYIPTF TRB:CAWKNNEQFF	1	CD4-C1
PB-3_AACTGGTTCGAGCCCA	PB	PB-3	P3-516	FALSE	1	TRA:CAVRNQGGKLIF TRB:CASSLVGGDNEQFF	1	CD4-C1
PB-3_AAGCCGCGTGCCTTGG	PB	PB-3	P3-521	FALSE	1	TRA:CAGDEDQAGTALIF TRB:CASKRGGATGELFF	1	CD4-C1
PB-3_AAGGAGCGTTAAGATG	PB	PB-3	P3-527	FALSE	1	TRA:CAVRSGYGGSQGNLIF TRB:CASSSLPVGYGYTF	1	CD4-C1
PB-3_AAGGAGCTCATAGCAC	PB	PB-3	P3-528	FALSE	1	TRA:CALSSI TRB:CASSFRAGLTEAFF	1	CD4-C1
PB-3_AAGGTTCTCCGTACAA	PB	PB-3	P3-534	FALSE	1	TRA:CAPPSGYALNF TRB:CASSDATQNTEAFF	1	CD4-C1
PB-3_AATCGGTGTCATATCG	PB	PB-3	P3-543	FALSE	1	TRA:CAASMNNDMRF TRB:CASSISGQKNSPLHF	1	CD4-C1
PB-3_ACACCCTAGAAACGCC	PB	PB-3	P3-550	FALSE	1	TRA:CAVEESGGTASKLTF TRB:CASSSGGDYEQYF	1	CD4-C1
PB-3_ACACCCTCACATTAGC	PB	PB-3	P3-551	FALSE	1	TRA:CAASQGGSEKLVF TRB:CASTEGVHPVWGYTF	1	CD4-C1
PB-3_ACAGCTAAGTCTTGCA	PB	PB-3	P3-563	FALSE	1	TRA:CAMRDWTRNAGKSTF TRB:CASSLSSQSYEQYF	1	CD4-C1
PB-3_ACAGCTATCTCGTTTA	PB	PB-3	P3-566	FALSE	1	TRA:CAVEVAGTYKYIF TRB:CASSLRSGYTF	1	CD4-C2
PB-3_ACAGCTATCTTATCTG	PB	PB-3	P3-567	FALSE	1	TRA:CAVNRQGGSEKLVF TRB:CASSYTDRGVGNQPQHF	1	CD4-C1
PB-3_ACATGGTGTCAGGACA	PB	PB-3	P3-573	FALSE	1	TRA:CAVRGNTPLVF TRB:CASSLDRTSGGAYTQYF	1	CD4-C1
PB-3_ACGATACTCCTGCAGG	PB	PB-3	P3-587	FALSE	1	TRA:CAGRSGGSNYKLTF TRB:CASSPEDRFGWAFF	1	CD4-C1
PB-3_ACGGAGACAAGTACCT	PB	PB-3	P3-595	FALSE	1	TRA:CAVTDTGNQFYF TRB:CAISGRYEQYF	1	CD4-C2

PB-3_ACGGCCAAGACTGGGT	PB	PB-3	P3-598	FALSE	1	TRA:CAVWLGSQGNLIF TRB:CASSAGPNHNSPLHF	1	CD4-C1
PB-3_ACTGAGTCAATTGCTG	PB	PB-3	P3-607	FALSE	1	TRA:CAMREGRVAESSGDKLTF TRB:CASRLRTAPTEQYF	1	CD4-C1
PB-3_ACTGTCCTCACAATGC	PB	PB-3	P3-617	FALSE	1	TRA:CAASIRTGFQKLVF TRB:CASSQERSGTVNTEAFF	1	CD4-C1
PB-3_ACTTTCATCGTACGGC	PB	PB-3	P3-622	FALSE	1	TRA:CAENNYGQNFVF TRB:CASSPGTVGEKLFF	1	CD4-C1
PB-3_AGACGTTGTAAGAGGA	PB	PB-3	P3-627	FALSE	1	TRA:CAASVSGNTPLVF TRB:CASSSPRSPEGTGELFF	1	CD4-C1
PB-3_AGATTGCGTGGCCCTA	PB	PB-3	P3-638	FALSE	1	TRA:CAMRGDTGRRALTF TRB:CATSRKSGTGSSGNTIYF	1	CD4-C1
PB-3_AGATTGCTCAAGGTAA	PB	PB-3	P3-639	FALSE	1	TRA:CAVSERSNDYKLSF TRB:CAWSITGLSGNTIYF	1	CD4-C1
PB-3_AGCATACAGGCAGGTT	PB	PB-3	P3-643	FALSE	1	TRA:CILFYGNNRLAF TRB:CASGGRQFETQYF	1	CD4-C1
PB-3_AGCTCTCTCCAAAGTC	PB	PB-3	P3-665	FALSE	1	TRA:CAVKVNFGNEKLTF TRB:CASSHGQDTGELFF	1	CD4-C1
PB-3_AGCTCTCTCTAACCGA	PB	PB-3	P3-666	FALSE	1	TRA:CAASNDYKLSF TRB:CASRGHGGSYEQYF	1	CD4-C1
PB-3_AGGCCACGTGACTACT	PB	PB-3	P3-668	FALSE	1	TRA:CIVRAHSGAGSYQLTF TRB:CASSQGQGVGGYTF	1	CD4-C1
PB-3_AGGCCGTCAATCAGAA	PB	PB-3	P3-669	FALSE	1	TRA:CAASDAGGFKTIF TRB:CASSLGPTGTENEQFF	1	CD4-C1
PB-3_AGTGAGGTCATCTGTT	PB	PB-3	P3-691	FALSE	1	TRA:CVVTENDYKLSF TRB:CASSYLGTAQETQYF	1	CD4-C1
PB-3_ATAAGAGGTACCGAGA	PB	PB-3	P3-703	FALSE	1	TRA:CAYRNTGNQFYF TRB:CASSYGRGVTRLRETQYF	1	CD4-C1
PB-3_ATAGACCCAGTGGAGT	PB	PB-3	P3-705	FALSE	1	TRA:CAVRDRMGGYNKLIF TRB:CASSVGTVTNNSPLHF	1	CD4-C1
PB-3_ATCATGGCAACGCACC	PB	PB-3	P3-710	FALSE	1	TRA:CALSKNTGFQKLVF TRB:CASSLWRTGAGMNTEAFF	1	CD4-C1
PB-3_ATCCACCGTCGACTAT	PB	PB-3	P3-713	FALSE	1	TRA:CAVNRPGGSYIPTF TRB:CASSYDTDTQYF	1	CD4-C1
PB-3_ATCGAGTGTATAGTAG	PB	PB-3	P3-719	FALSE	1	TRA:CALSPSSNTGKLIF TRB:CASSLGLAGLTDTQYF	1	CD4-C1
PB-3_ATCTGCCAGGCAGTCA	PB	PB-3	P3-723	FALSE	1	TRA:CAVEDMEDTGNQFYF TRB:CASSYTPAGDYGYTF	1	CD4-C1
PB-3_ATGGGAGAGGTAGCCA	PB	PB-3	P3-728	FALSE	1	TRA:CAARSLYNNNDMRF TRB:CAWSMNTEAFF	1	CD4-C1
PB-3_ATGTGTGGTCTGATCA	PB	PB-3	P3-734	FALSE	1	TRA:CALSDRDSSASKIIF TRB:CASQSGQNNSPLHF	1	CD4-C1
PB-3_ATGTGTGGTGACTACT	PB	PB-3	P3-735	FALSE	1	TRA:CAVSDRKSTLTF TRB:CASSRGTSVADTQYF	1	CD4-C1
PB-3_ATGTGTGTCCTTCAAT	PB	PB-3	P3-736	FALSE	1	TRA:CAAFSGGYNKLIF TRB:CSAPPEGARTGELFF	1	CD4-C1
PB-3_ATTACTCAGTGTCCAT	PB	PB-3	P3-737	FALSE	1	TRA:CAVEDSNYQLIW TRB:CASRLPRGDNSPLHF	1	CD4-C1
PB-3_ATTACTCGTAGCGTGA	PB	PB-3	P3-739	FALSE	1	TRA:CAVSDYGNKLVF TRB:CASSGDSHGYTF	1	CD4-C1
PB-3_ATTATCCAGGACACCA	PB	PB-3	P3-741	FALSE	1	TRA:CAVQARAAGNKLTF TRB:CASSQNNYGYTF	1	CD4-C1
PB-3_ATTATCCTCAGCGATT	PB	PB-3	P3-742	FALSE	1	TRA:CAVSDRGRGGGNKLTF TRB:CASSLGATDEAFF	1	CD4-C1

PB-3_CAACTAGTCTACTCAT	PB	PB-3	P3-754	FALSE	1	TRA:CAASIGGNTPLVF TRB:CASSMGQEYYEQYF	1	CD4-C1
PB-3_CAAGATCGTAAACACA	PB	PB-3	P3-757	FALSE	1	TRA:CAVRSSARQLTF TRB:CASSPGQVSVIAKNIQYF	1	CD4-C1
PB-3_CACACAACAATGGAAT	PB	PB-3	P3-770	FALSE	1	TRA:CAVNARSNDYKLSF TRB:CASTSTGTEAFF	1	CD4-C1
PB-3_CACACTCTCACCTCGT	PB	PB-3	P3-776	FALSE	1	TRA:CAVGGSSNTGKLIF TRB:CASSAAGGSNQPQHF	1	CD4-C1
PB-3_CACCACTTCGTTTATC	PB	PB-3	P3-787	FALSE	1	TRA:CALSEETDSWGKFQF TRB:CASNLGDSGANVLTF	1	CD4-C1
PB-3_CACTCCAGTAACGTTC	PB	PB-3	P3-792	FALSE	1	TRA:CAVIITHLGGNNRKLIW TRB:CASSRLVAKNIQYF	1	CD4-C1
PB-3_CAGAGAGGTCTTCTCG	PB	PB-3	P3-801	FALSE	1	TRA:CAEPTSSNTGKLIF TRB:CASSPTSGGYEQYF	1	CD4-C1
PB-3_CAGAGAGGTGCAGACA	PB	PB-3	P3-802	FALSE	1	TRA:CAVGGSSNTGKLIF TRB:CATSDTGRGSNQPQHF	1	CD4-C1
PB-3_CAGCCGAAGCAATCTC	PB	PB-3	P3-808	FALSE	1	TRA:CAVRDEGNTPLVF TRB:CSAPPGPLSYEQYF	1	CD4-C1
PB-3_CAGGTGCTCATTGCCC	PB	PB-3	P3-814	FALSE	1	TRA:CALSEGGNEKLTF TRB:CASSSSGILSGNTIYF	1	CD4-C1
PB-3_CATATGGAGTCCGGTC	PB	PB-3	P3-821	FALSE	1	TRA:CAVGIMEYGNKLVF TRB:CASSVAPTGIDSYEQYF	1	CD4-C1
PB-3_CATCGAACAACTTGAC	PB	PB-3	P3-835	FALSE	1	TRA:CAVRSNDYKLSF TRB:CASSLSPGSNQPQHF	1	CD4-C2
PB-3_CATCGAACAAGCGATG	PB	PB-3	P3-836	FALSE	1	TRA:CLVGDILRAGNNRKLIW TRB:CASSQDSTEQFF	1	CD4-C1
PB-3_CATCGAACACCACCAG	PB	PB-3	P3-837	FALSE	1	TRA:CAVYNNNDMRF TRB:CASSSRAGYGYTF	1	CD4-C2
PB-3_CATTCGCGTGCAACTT	PB	PB-3	P3-857	FALSE	1	TRA:CAMREGSGANSKLTF TRB:CASGGWTGKYF	1	CD4-C1
PB-3_CCACCTATCGAACTGT	PB	PB-3	P3-866	FALSE	1	TRA:CAVGAGNNRKLIW TRB:CASNPGAQETQYF	1	CD4-C1
PB-3_CCCAATCCAATGGATA	PB	PB-3	P3-874	FALSE	1	TRA:CAVDTGANSKLTF TRB:CASSLLGNQPQHF	1	CD4-C1
PB-3_CCGGGATCAGGTGCCT	PB	PB-3	P3-880	FALSE	1	TRA:CATEPTTSGTYKYIF TRB:CASSVGHQETQYF	1	CD4-C1
PB-3_CCGTTCAGTACTTAGC	PB	PB-3	P3-891	FALSE	1	TRA:CARRTARKNTGGFKTIF TRB:CASSQTSNSNQPQHF	1	CD4-C1
PB-3_CCTACACGTATAGGTA	PB	PB-3	P3-893	FALSE	1	TRA:CAFQRNTGGFKTIF TRB:CAWSEAGGYEQYF	1	CD4-C1
PB-3_CCTAGCTAGGGATGGG	PB	PB-3	P3-901	FALSE	1	TRA:CILRDGVSSNTGKLIF TRB:CASARSGGYTF	1	CD4-C1
PB-3_CCTCTGAAGCAGACTG	PB	PB-3	P3-906	FALSE	1	TRA:CAASSLTGGGNKLTF TRB:CASSQAPSWVREAFF	1	CD4-C1
PB-3_CCTTCGATCTGTTGAG	PB	PB-3	P3-915	FALSE	1	TRA:CAVRFI TRB:CASSEGLNEQFF	1	CD4-C1
PB-3_CCTTTCTCAGACGCCT	PB	PB-3	P3-916	FALSE	1	TRA:CACSYGQNFVF TRB:CASSFLGTAKEETQYF	1	CD4-C2
PB-3_CGAATGTCAGATAATG	PB	PB-3	P3-922	FALSE	1	TRA:CAVILNTNAGKSTF TRB:CASSYSRADTQYF	1	CD4-C1
PB-3_CGACTTCCACTACAGT	PB	PB-3	P3-926	FALSE	1	TRA:CAVSPMKTSYDKVIF TRB:CASSEDGGGHYNEQFF	1	CD4-C1
PB-3_CGATGTAAGACTCGGA	PB	PB-3	P3-941	FALSE	1	TRA:CAEIAGANSKLTF TRB:CATSDRASPGELFF	1	CD4-C1

PB-3_CGGACACAGCCCAATT	PB	PB-3	P3-959	FALSE	1	TRA:CILRDVKETSGSRLTF TRB:CASSLWGSYGYTF	1	CD4-C1
PB-3_CGGAGTCGTGCTCTTC	PB	PB-3	P3-970	FALSE	1	TRA:CAVSQAGGYSTLTF TRB:CASRGSLGTQETQYF	1	CD4-C1
PB-3_CGGAGTCGTTAAGTAG	PB	PB-3	P3-971	FALSE	1	TRA:CAMSSNSGGSNYKLTF TRB:CASSRTSGGALDTQYF	1	CD4-C1
PB-3_CGTCACTCATCGGAAG	PB	PB-3	P3-984	FALSE	1	TRA:CARDNTNAGKSTF TRB:CASSSGTGGFGELFF	1	CD4-C1
PB-3_CGTCTACAGTCGATAA	PB	PB-3	P3-995	FALSE	1	TRA:CAVLYGQNFVF TRB:CASSLEGTVPYNEQFF	1	CD4-C1
PB-3_CGTCTACAGTTGCAGG	PB	PB-3	P3-996	FALSE	1	TRA:CAGPNSGYALNF TRB:CASSPGQFTQYF	1	CD4-C1
PB-3_CGTCTACCATATACCG	PB	PB-3	P3-997	FALSE	1	TRA:CALKGEYGNKLVF TRB:CASSPSGRNTEAFF	1	CD4-C1
PB-3_CGTGAGCAGGCATGGT	PB	PB-3	P3-278	FALSE	2	TRA:CAVTSGGTSYGKLTF TRB:CASSMSNYGYTF	2	CD4-C1
PB-3_CGTGTAACAACCGCCA	PB	PB-3	P3-1003	FALSE	1	TRA:CAASGGAGNMLTF TRB:CASSPGLYSGNTIYF	1	CD4-C1
PB-3_CGTGTAAGTGGTAACG	PB	PB-3	P3-1005	FALSE	1	TRA:CIVRSMNYGGATNKLIF TRB:CASMPFKTGTAYGYTF	1	CD4-C2
PB-3_CGTTGGGCATGCCTTC	PB	PB-3	P3-1012	FALSE	1	TRA:CAVNMYLDNNARLMF TRB:CASSRDTGPRNTEAFF	1	CD4-C1
PB-3_CTAAGACAGACACGAC	PB	PB-3	P3-1018	FALSE	1	TRA:CAVDRDDKIIF TRB:CASSFYPNSNQPQHF	1	CD4-C1
PB-3_CTAAGACTCCCTCTTT	PB	PB-3	P3-1020	FALSE	1	TRA:CAEATSGSRLTF TRB:CSARDARDFTEAFF	1	CD4-C1
PB-3_CTAATGGTCATAAAGG	PB	PB-3	P3-1022	FALSE	1	TRA:CALTTDGQKLLF TRB:CASRGQGSSYNEQFF	1	CD4-C1
PB-3_CTAGCCTAGATAGTCA	PB	PB-3	P3-1036	FALSE	1	TRA:CAGSGGSNYKLTF TRB:CASSLGTGRSTDTQYF	1	CD4-C2
PB-3_CTAGCCTGTGTGACGA	PB	PB-3	P3-1038	FALSE	1	TRA:CATDAFNDYKLSF TRB:CASRGDKTYEQYF	1	CD4-C1
PB-3_CTCGGGACAATTCCTT	PB	PB-3	P3-1066	FALSE	1	TRA:CAWNSGGYQKVTF TRB:CASSEAGTAFYNEQFF	1	CD4-C1
PB-3_CTGTGCTGTAAACCTC	PB	PB-3	P3-1098	FALSE	1	TRA:CAVEEGSNYQLIW TRB:CASKSGHVNSPLHF	1	CD4-C2
PB-3_CTGTTTAAGCTACCTA	PB	PB-3	P3-1100	FALSE	1	TRA:CVAPGAGSYQLTF TRB:CASSLSTQNTEAFF	1	CD4-C1
PB-3_CTTAACTTCATGCATG	PB	PB-3	P3-1104	FALSE	1	TRA:CALGDMEYGNKLVF TRB:CASSYSIEDRSSKETQYF	1	CD4-C1
PB-3_GACCAATAGGAATGGA	PB	PB-3	P3-1138	FALSE	1	TRA:CAMREDNNAGNMLTF TRB:CASSSRTSGGDEQFF	1	CD4-C1
PB-3_GACCTGGAGCTGTCTA	PB	PB-3	P3-1140	FALSE	1	TRA:CAAKTGGSYIPTF TRB:CAASGAGREKLFF	1	CD4-C1
PB-3_GACTGCGTCCAGAAGG	PB	PB-3	P3-1161	FALSE	1	TRA:CLVGDSAGNNRKLIW TRB:CASTERQGNPSGANVLTF	1	CD4-C1
PB-3_GAGGTGACATCGGGTC	PB	PB-3	P3-1166	FALSE	1	TRA:CAVGIYGNKLVF TRB:CASSLSQGGSYEQYF	1	CD4-C1
PB-3_GATCAGTTCACCATAG	PB	PB-3	P3-293	FALSE	2	TRA:CALPISSGSARQLTF TRB:CASSQATGTWGDTQYF	2	CD4-C1
PB-3_GATCAGTTCTCTTATG	PB	PB-3	P3-1172	FALSE	1	TRA:CAEEGGNEKLTF TRB:CASRPLTGGDIQYF	1	CD4-C1
PB-3_GATCGATTCGGAATCT	PB	PB-3	P3-1173	FALSE	1	TRA:CATDEKARLMF TRB:CASSFTSGRNEQFF	1	CD4-C1

PB-3_GCATGCGTCCTTCAAT	PB	PB-3	P3-1203	FALSE	1	TRA:CAVSRSGGTSYGKLTF TRB:CASSLGTGGPQHF	1	CD4-C1
PB-3_GCCTCTATCAGCTCGG	PB	PB-3	P3-1208	FALSE	1	TRA:CAEIPRSAAGNKLTF TRB:CASSRGVTNEKLFF	1	CD4-C1
PB-3_GCCTCTATCTACGAGT	PB	PB-3	P3-1209	FALSE	1	TRA:CAVTNTGGFKTIF TRB:CSAIEGQATKNIQYF	1	CD4-C1
PB-3_GCGCAACGTCCAAGTT	PB	PB-3	P3-1217	FALSE	1	TRA:CAVKSETGGSYIPTF TRB:CASSPGDRNYGYTF	1	CD4-C1
PB-3_GGAAAGCCATGAGCGA	PB	PB-3	P3-1243	FALSE	1	TRA:CAMRESSGGGKLIF TRB:CSARSLAGGEETQYF	1	CD4-C1
PB-3_GGAAAGCTCTTAGAGC	PB	PB-3	P3-1244	FALSE	1	TRA:CAAAISGNTPLVF TRB:CSAREGRGPSYEQYF	1	CD4-C1
PB-3_GGAAAGCTCTTAGCCC	PB	PB-3	P3-1245	FALSE	1	TRA:CAASGEDSNYQLIW TRB:CASSFKGVPYEQYF	1	CD4-C1
PB-3_GGATGTTCAGATGGGT	PB	PB-3	P3-1260	FALSE	1	TRA:CAVSDRNGGFKTIF TRB:CASSLGGANYEQYF	1	CD4-C1
PB-3_GGATGTTGTATATCCG	PB	PB-3	P3-1261	FALSE	1	TRA:CAASARGSQGNLIF TRB:CASSLLGQDTQYF	1	CD4-C1
PB-3_GGATGTTGTCAACATC	PB	PB-3	P3-1262	FALSE	1	TRA:CAARPADGQKLLF TRB:CSASREKLFF	1	CD4-C1
PB-3_GGCAATTCAAGCTGTT	PB	PB-3	P3-1266	FALSE	1	TRA:CAVVPSGGGADGLTF TRB:CASSAQTGMNTEAFF	1	CD4-C1
PB-3_GGCCGATCAAACGCGA	PB	PB-3	P3-1271	FALSE	1	TRA:CVVALYSGGGADGLTF TRB:CASSPLGTTDTQYF	1	CD4-C1
PB-3_GGCTCGAAGCTGATAA	PB	PB-3	P3-1276	FALSE	1	TRA:CIRRGSQGNLIF TRB:CASSPGLAGGPETQYF	1	CD4-C1
PB-3_GGCTCGATCTGCGACG	PB	PB-3	P3-1277	FALSE	1	TRA:CAGRPLQPGSARQLTF TRB:CASRYAGSSYNSPLHF	1	CD4-C1
PB-3_GGGACCTCAGATGGGT	PB	PB-3	P3-1282	FALSE	1	TRA:CAVQISNFGNEKLTF TRB:CASSPGQQGARNEQFF	1	CD4-C1
PB-3_GGGCACTGTCCGCTGA	PB	PB-3	P3-1291	FALSE	1	TRA:CATNPSGSARQLTF TRB:CASSLEPLANTEAFF	1	CD4-C1
PB-3_GGTGCGTCAGCGAACA	PB	PB-3	P3-1300	FALSE	1	TRA:CAENTGGYNKLIF TRB:CASSLAGRETQYF	1	CD4-C1
PB-3_GGTGTTATCGCCGTGA	PB	PB-3	P3-1307	FALSE	1	TRA:CAENSGNTPLVF TRB:CASRKDGTNEKLFF	1	CD4-C1
PB-3_GTAGTCACACTAAGTC	PB	PB-3	P3-1325	FALSE	1	TRA:CAGDSGNTPLVF TRB:CASSTGVGNTIYF	1	CD4-C1
PB-3_GTCACAAAGCCACGTC	PB	PB-3	P3-1337	FALSE	1	TRA:CAARHETSGSRLTF TRB:CSARVVGRGNSPLHF	1	CD4-C1
PB-3_GTCATTTAGCTAGTCT	PB	PB-3	P3-1339	FALSE	1	TRA:CAVRVIAGFKTIF TRB:CASSLARGTVTNYGYTF	1	CD4-C1
PB-3_GTCATTTCAGATGAGC	PB	PB-3	P3-1344	FALSE	1	TRA:CAVDGGSQGNLIF TRB:CASSFFDRDRDTQYF	1	CD4-C1
PB-3_GTCCTCACAGCCACCA	PB	PB-3	P3-1348	FALSE	1	TRA:CVVRPNAGNMLTF TRB:CASSIHRAGETQYF	1	CD4-C1
PB-3_GTCTCGTTCGGAGGTA	PB	PB-3	P3-1358	FALSE	1	TRA:CLVVMNSGYSTLTF TRB:CATTGGDNSPLHF	1	CD4-C1
PB-3_GTCTTCGAGAGTAAGG	PB	PB-3	P3-1359	FALSE	1	TRA:CALSRSTNAGKSTF TRB:CASRPTSDTEAFF	1	CD4-C1
PB-3_GTCTTCGCAAGCCGCT	PB	PB-3	P3-1360	FALSE	1	TRA:CAFRRNSGSRLTF TRB:CASSIMGQGFGYTF	1	CD4-C1
PB-3_GTGAAGGAGGCCCGTT	PB	PB-3	P3-1362	FALSE	1	TRA:CAARISGGYNKLIF TRB:CASSRESSTDTQYF	1	CD4-C2

PB-3_GTGCAGCAGATGCGAC	PB	PB-3	P3-1364	FALSE	1	TRA:CAGRDNNAGNMLTF TRB:CASSLDGYQSYNSPLHF	1	CD4-C2
PB-3_GTGCATAAGACACTAA	PB	PB-3	P3-1366	FALSE	1	TRA:CAFKGDDKIIF TRB:CSARLAGGVVEQFF	1	CD4-C1
PB-3_GTTAAGCAGTGTTGAA	PB	PB-3	P3-1385	FALSE	1	TRA:CIVEGGSNYKLTF TRB:CSARGSNQPQHF	1	CD4-C1
PB-3_GTTCATTAGAGAGCTC	PB	PB-3	P3-1387	FALSE	1	TRA:CRRKTGRRALTF TRB:CSAHRRYRDRAKNEQFF	1	CD4-C1
PB-3_TAAGAGAGTTCGGCAC	PB	PB-3	P3-1404	FALSE	1	TRA:CAESESYNTDKLIF TRB:CASSPSAASSYEQYF	1	CD4-C1
PB-3_TACCTATAGCCGATTT	PB	PB-3	P3-1417	FALSE	1	TRA:CAASSSFQKLVF TRB:CSAPLPGQGSGYTF	1	CD4-C1
PB-3_TACTCATGTGGAAAGA	PB	PB-3	P3-1434	FALSE	1	TRA:CARTLAGGSEKLVF TRB:CASSQEGASGGRNIQYF	1	CD4-C1
PB-3_TACTCATTCTGATTCT	PB	PB-3	P3-1436	FALSE	1	TRA:CAASYSSASKIIF TRB:CASSTRDTDTQYF	1	CD4-C1
PB-3_TACTCGCCAACGCACC	PB	PB-3	P3-1438	FALSE	1	TRA:CALSEAQNQGGKLIF TRB:CASSRPTVAKNIQYF	1	CD4-C1
PB-3_TACTCGCTCGGAAATA	PB	PB-3	P3-1441	FALSE	1	TRA:CAALYRGSTLGRLYF TRB:CASSLVSDTQYF	1	CD4-C1
PB-3_TACTTGTTCCGTCAAA	PB	PB-3	P3-1444	FALSE	1	TRA:CLIQGAQKLVF TRB:CASGRVGQPYGYTF	1	CD4-C2
PB-3_TACTTGTTCTGAGTGT	PB	PB-3	P3-1445	FALSE	1	TRA:CAASPPGANNLFF TRB:CASSSGQGVSEAFF	1	CD4-C1
PB-3_TATCTCAGTGTTCTTT	PB	PB-3	P3-1464	FALSE	1	TRA:CAMSATGNQFYF TRB:CASTAGSSYEQYF	1	CD4-C2
PB-3_TCAATCTGTGCTCTTC	PB	PB-3	P3-1469	FALSE	1	TRA:CAVRATGGSYIPTF TRB:CASSPPGGQNTEAFF	1	CD4-C1
PB-3_TCACAAGAGACCGGAT	PB	PB-3	P3-278	FALSE	2	TRA:CAVTSGGTSYGKLTF TRB:CASSMSNYGYTF	2	CD4-C1
PB-3_TCAGATGCAGTCAGCC	PB	PB-3	P3-1481	FALSE	1	TRA:CAVVRGGAQKLVF TRB:CASSEGRGEAFF	1	CD4-C1
PB-3_TCAGATGTCACAGGCC	PB	PB-3	P3-1483	FALSE	1	TRA:CIVRVAGGTASKLTF TRB:CASSLELSVRNTQYF	1	CD4-C1
PB-3_TCAGCAAGTTCCATGA	PB	PB-3	P3-1486	FALSE	1	TRA:CAVNYNRYGNKLVF TRB:CATRGGGEYSYEQYF	1	CD4-C1
PB-3_TCATTTGCACGTTGGC	PB	PB-3	P3-1503	FALSE	1	TRA:CAVKMSRGYNKLIF TRB:CASSLRAGDLADTQYF	1	CD4-C1
PB-3_TCCCGATAGACGCTTT	PB	PB-3	P3-1505	FALSE	1	TRA:CVVNSDTGRRALTF TRB:CASSPLTGDLEQFF	1	CD4-C1
PB-3_TCCCGATAGGCACATG	PB	PB-3	P3-1506	FALSE	1	TRA:CVVPFSGGYNKLIF TRB:CASYQGNFANTGELFF	1	CD4-C1
PB-3_TCGTACCCATGCTAGT	PB	PB-3	P3-1521	FALSE	1	TRA:CAAPTSGTYKYIF TRB:CASSRSGGDNEQFF	1	CD4-C1
PB-3_TGATTTCCAGCATGAG	PB	PB-3	P3-1555	FALSE	1	TRA:CATGDSWGKFQF TRB:CASSGTRDETQYF	1	CD4-C1
PB-3_TGCACCTCAGCCTTTC	PB	PB-3	P3-1556	FALSE	1	TRA:CALDRGNTPLVF TRB:CASSYPAGGSNTGELFF	1	CD4-C1
PB-3_TGCGGGTTCCATGAAC	PB	PB-3	P3-1575	FALSE	1	TRA:CALSTFGAGNNRKLIW TRB:CASSLTGTSTDTQYF	1	CD4-C1
PB-3_TGGCTGGCACGCTTTC	PB	PB-3	P3-1590	FALSE	1	TRA:CAPPRAGGYNKLIF TRB:CASSSGRGGKSTDTQYF	1	CD4-C1
PB-3_TGGGAAGCATACGCTA	PB	PB-3	P3-1593	FALSE	1	TRA:CAENINNAGNMLTF TRB:CSAQKQAPQETQYF	1	CD4-C1

PB-3_TGGTTAGCATATGGTC	PB	PB-3	P3-1596	FALSE	1	TRA:CVVNIGTNAGKSTF TRB:CASSLSGGELFF	1	CD4-C1
PB-3_TGGTTCCGTAGGACAC	PB	PB-3	P3-1600	FALSE	1	TRA:CAMRIGTGNTPLVF TRB:CASSLGLSGANVLTF	1	CD4-C1
PB-3_TGTGTTTAGGCTACGA	PB	PB-3	P3-1609	FALSE	1	TRA:CALNGYGQNFVF TRB:CASSEYGTPETQYF	1	CD4-C1
PB-3_TGTGTTTGTCAGAGGT	PB	PB-3	P3-1611	FALSE	1	TRA:CAMREGRGSSNTGKLIF TRB:CSAAGYSNQPQHF	1	CD4-C1
PB-3_TGTGTTTGTTAAAGAC	PB	PB-3	P3-1613	FALSE	1	TRA:CAMTAGSSNTGKLIF TRB:CASMGGEGETQYF	1	CD4-C1
PB-3_TTCCCAGTCAGCTCGG	PB	PB-3	P3-1631	FALSE	1	TRA:CAASKGNGNQFYF TRB:CASTRAFSGNTIYF	1	CD4-C1
PB-3_TTCTTAGTCCAATGGT	PB	PB-3	P3-1642	FALSE	1	TRA:CALSGRGGADGLTF TRB:CASSQDRSNTGELFF	1	CD4-C1
PB-3_TTGCGTCCAGCATGAG	PB	PB-3	P3-1654	FALSE	1	TRA:CAGPRTSYDKVIF TRB:CASSLNGQGYNSPLHF	1	CD4-C1
PB-3_TTGCGTCGTTCCACAA	PB	PB-3	P3-1655	FALSE	1	TRA:CAVPPTSGTYKYIF TRB:CASSYGGNNYGYTF	1	CD4-C1
PB-3_TTGGCAAAGCTCAACT	PB	PB-3	P3-1657	FALSE	1	TRA:CAAPRQAGTALIF TRB:CSALGAGTAYNEQFF	1	CD4-C2
PB-3_TTGTAGGCATGCTGGC	PB	PB-3	P3-1661	FALSE	1	TRA:CVVSAQGAQKLVF TRB:CASSYPNRGNTIYF	1	CD4-C1
PB-3_TTGTAGGGTGAGGGTT	PB	PB-3	P3-1663	FALSE	1	TRA:CAASEKRALTF TRB:CASSQSSNQETQYF	1	CD4-C1
PB-3_TTTATGCGTTCGTTGA	PB	PB-3	P3-1671	FALSE	1	TRA:CAGGGQGAQKLVF TRB:CASSLAGGETQYF	1	CD4-C1
PB-3_TTTGTCACAGTAAGAT	PB	PB-3	P3-1679	FALSE	1	TRA:CAVARPGTGNQFYF TRB:CAISSGDRGAGELFF	1	CD4-C1
PB-3_AACACGTTCGTACCGG	PB	PB-3	P3-494	FALSE	1	TRA:CALSQTDSWGKFQF TRB:CSVETGGYTF	1	CD8-C6
PB-3_AACCATGGTGTGCCTG	PB	PB-3	P3-72	FALSE	3	TRA:CAVRRSNYQLIW TRB:CASSPQGETQYF	5	CD4-C6
PB-3_AACCGCGAGGTGCTAG	PB	PB-3	P3-496	FALSE	1	TRA:CAGRNQAGTALIF TRB:CSASGGGGQPQHF	1	CD8-C6
PB-3_AAGGAGCCACCACCAG	PB	PB-3	P3-526	FALSE	1	TRA:CAVKGSGTYKYIF TRB:CASSLSGRGGRETQYF	1	CD8-C6
PB-3_AATCCAGGTCTTGCGG	PB	PB-3	P3-539	FALSE	1	TRA:CAMSASVGGYNKLIF TRB:CSARLGGRTEAFF	1	CD8-C5
PB-3_AATCGGTTCTATCCCG	PB	PB-3	P3-544	FALSE	1	TRA:CAVDISNFGNEKLTF TRB:CASSLGTTYEQYF	1	CD8-C2
PB-3_ACGCAGCTCAAACGGG	PB	PB-3	P3-589	FALSE	1	TRA:CAVRGSSNTGKLIF TRB:CASSGGWSYNSPLHF	1	CD8-C7
PB-3_AGAATAGTCAAACCGT	PB	PB-3	P3-624	FALSE	1	TRA:CLVGDMDTGRRALTF TRB:CASSSNRARETPLHF	1	CD8-C6
PB-3_AGAGCGACAGGGCATA	PB	PB-3	P3-2	TRUE	3	TRA:CAASAPNSGGGADGLTF TRB:CSARRGSSNQPQHF	63	CD4-C6
PB-3_AGAGCGATCGACGGAA	PB	PB-3	P3-628	FALSE	1	TRA:CAVTLVDFGNEKLTF TRB:CASSQGQGAPNTEAFF	1	CD8-C6
PB-3_AGAGCTTCAGCTGCTG	PB	PB-3	P3-629	FALSE	1	TRA:CATAPNTGGFKTIF TRB:CASSPPGTGNGNTIYF	1	CD8-C6
PB-3_AGGTCATCAGCCTTGG	PB	PB-3	P3-4	TRUE	3	TRA:CAGHTGANSKLTF TRB:CASSLSTGNYGYTF	39	CD8-C10
PB-3_ATGAGGGCAGTAAGCG	PB	PB-3	P3-251	FALSE	2	TRA:CAMSPDNYGQNFVF TRB:CAISESRRQGATTGELFF	2	CD4-C6

PB-3_ATTGGACTCTTGTATC	PB	PB-3	P3-746	FALSE	1	TRA:CAARGSYIPTF TRB:CATSRDLSVGEAFF	1	CD8-C6
PB-3_CAAGATCAGTGCAAGC	PB	PB-3	P3-755	FALSE	1	TRA:CIAGATDSWGKFQF TRB:CASSLEQGGYGYTF	1	CD4-C6
PB-3_CAAGTTGGTTACGTCA	PB	PB-3	P3-763	FALSE	1	TRA:CALRRKGSQGNLIF TRB:CASSLFSQENYGYTF	1	CD8-C6
PB-3_CACAGTAAGGCAATTA	PB	PB-3	P3-2	TRUE	3	TRA:CAASAPNSGGGADGLTF TRB:CSARRGSSNQPQHF	63	CD4-C3
PB-3_CAGAATCTCGCAAACT	PB	PB-3	P3-796	FALSE	1	TRA:CAVGDSNFGNEKLTF TRB:CASSLDSGHEQFF	1	CD8-C6
PB-3_CAGAGAGTCTGCGTAA	PB	PB-3	P3-804	FALSE	1	TRA:CAEIASNTGNQFYF TRB:CASSLGGGRWDYGYTF	1	CD8-C2
PB-3_CAGGTGCCACAGATTC	PB	PB-3	P3-813	FALSE	1	TRA:CAVGKGGSQGNLIF TRB:CASRAGQGEQFF	1	CD8-C2
PB-3_CATCAGAAGCAGCGTA	PB	PB-3	P3-826	FALSE	1	TRA:CAENEGSGNTPLVF TRB:CASRDRDTNEKLFF	1	CD8-C6
PB-3_CATGACAAGCGACGTA	PB	PB-3	P3-2	TRUE	3	TRA:CAASAPNSGGGADGLTF TRB:CSARRGSSNQPQHF	63	CD4-C6
PB-3_CCAATCCTCCCACTTG	PB	PB-3	P3-84	TRUE	3	TRA:CAVKRNTGFQKLVF TRB:CASSEADTGYTF	5	CD4-C3
PB-3_CGAACATCACCAACCG	PB	PB-3	P3-251	FALSE	2	TRA:CAMSPDNYGQNFVF TRB:CAISESRRQGATTGELFF	2	CD4-C6
PB-3_CGAGAAGGTCCCTTGT	PB	PB-3	P3-4	TRUE	3	TRA:CAGHTGANSKLTF TRB:CASSLSTGNYGYTF	39	CD8-C6
PB-3_CGGACTGGTCCCGACA	PB	PB-3	P3-966	FALSE	1	TRA:CAVSPFFGNEKLTF TRB:CASSAGRGSSYNEQFF	1	CD8-C6
PB-3_CGGTTAAGTTCCACTC	PB	PB-3	P3-981	FALSE	1	TRA:CALSEALYNTDKLIF TRB:CSATGEETGYYEQYF	1	CD8-C7
PB-3_CTAGAGTAGCTGCCCA	PB	PB-3	P3-1031	FALSE	1	TRA:CVVNRRFGSGTYKYIF TRB:CATSRDQTVQFF	1	CD8-C6
PB-3_CTAGCCTGTTCAGCGC	PB	PB-3	P3-1039	FALSE	1	TRA:CALSPRGGSEKLVF TRB:CSVPDRSNQPQHF	1	CD8-C6
PB-3_CTCTACGTCCATGCTC	PB	PB-3	P3-174	TRUE	3	TRA:CAMRAGAGGTSYGKLTF TRB:CASSEDYRDTEAFF	3	CD4-C6
PB-3_CTGATCCGTGACTACT	PB	PB-3	P3-1088	FALSE	1	TRA:CAAMGGGNKLTF TRB:CASSRTTQDNNSPLHF	1	CD8-C6
PB-3_CTGCCTAGTAGAGCTG	PB	PB-3	P3-4	TRUE	3	TRA:CAGHTGANSKLTF TRB:CASSLSTGNYGYTF	39	CD4-C6
PB-3_CTTAGGACATTCCTCG	PB	PB-3	P3-2	TRUE	3	TRA:CAASAPNSGGGADGLTF TRB:CSARRGSSNQPQHF	63	CD4-C6
PB-3_CTTGGCTGTGGTCCGT	PB	PB-3	P3-176	TRUE	3	TRA:CAVTRRGATNKLIF TRB:CASSTRDASGGYEQYF	3	CD4-C6
PB-3_CTTTGCGAGATGTCGG	PB	PB-3	P3-1109	FALSE	1	TRA:CAVRPRGSTGKLIF TRB:CASSYEGYGYTF	1	CD8-C6
PB-3_GACGCGTTCCAAGTAC	PB	PB-3	P3-291	FALSE	2	TRA:CAVISSGSARQLTF TRB:CASSQGTGVSGGTQYF	2	CD8-C6
PB-3_GACGGCTGTGAGTATA	PB	PB-3	P3-1152	FALSE	1	TRA:CAASKDQNNNARLMF TRB:CSAADSNQPQHF	1	CD4-C6
PB-3_GATCGCGCACTTCGAA	PB	PB-3	P3-294	TRUE	2	TRA:CAVSDLDGATNKLIF TRB:CASSLEGIETQYF	2	CD8-C6
PB-3_GATCTAGTCTCCTATA	PB	PB-3	P3-1179	FALSE	1	TRA:CAVGGPSGTYKYIF TRB:CASSLSRQETQYF	1	CD8-C6
PB-3_GCATGCGGTGGTGTAG	PB	PB-3	P3-179	TRUE	3	TRA:CAVNSGSSNTGKLIF TRB:CSARDSASTGELFF	3	CD8-C6

PB-3_GCGCCAATCCGGGTGT	PB	PB-3	P3-1221	FALSE	1	TRA:CAASNPRNAGNMLTF TRB:CASSLMQGLKKLFF	1	CD8-C6
PB-3_GCTGCGAGTATGGTTC	PB	PB-3	P3-2	TRUE	3	TRA:CAASAPNSGGGADGLTF TRB:CSARRGSSNQPQHF	63	CD4-C6
PB-3_GGACGTCCATTATCTC	PB	PB-3	P3-1255	FALSE	1	TRA:CALSGYGSARQLTF TRB:CASSVTGLTEAFF	1	CD8-C6
PB-3_GGCAATTCAGGGCATA	PB	PB-3	P3-182	TRUE	3	TRA:CAVRDGGKSTF TRB:CASSFRDTDTQYF	3	CD4-C6
PB-3_GGGATGACATTAACCG	PB	PB-3	P3-1288	FALSE	1	TRA:CAALLSGGSYIPTF TRB:CASSPAEGYTF	1	CD8-C2
PB-3_GGTGCGTGTTGTGGAG	PB	PB-3	P3-1301	FALSE	1	TRA:CALKSGNTPLVF TRB:CASSPMLNTDTQYF	1	CD8-C6
PB-3_GTCATTTTCGGAGGTA	PB	PB-3	P3-1346	FALSE	1	TRA:CAVQAESGNTPLVF TRB:CASSQAWGFGELFF	1	CD4-C2
PB-3_GTCCTCATCAGAGACG	PB	PB-3	P3-1349	FALSE	1	TRA:CAGQSDNQGGKLIF TRB:CASSFGGEAPLHF	1	CD8-C6
PB-3_GTTTCTAAGTCTTGCA	PB	PB-3	P3-72	FALSE	3	TRA:CAVRRSNYQLIW TRB:CASSPQGETQYF	5	CD4-C3
PB-3_TACGGTATCCTAGGGC	PB	PB-3	P3-1430	FALSE	1	TRA:CAVMNYGGSQGNLIF TRB:CASSSALRDIYGYTF	1	CD8-C6
PB-3_TAGAGCTAGTTCGCAT	PB	PB-3	P3-1448	FALSE	1	TRA:CALSSNDYKLSF TRB:CASRDRAGGSYNEQFF	1	CD4-C6
PB-3_TCAGCTCAGTATCTCG	PB	PB-3	P3-1488	FALSE	1	TRA:CAAEITGGGNKLTF TRB:CASSLEEGYGYTF	1	CD8-C2
PB-3_TCGGGACTCGGAAATA	PB	PB-3	P3-1518	FALSE	1	TRA:CAVVSGGSYIPTF TRB:CASSPGGEGWNQPQHF	1	CD8-C6
PB-3_TCTATTGCATGGGACA	PB	PB-3	P3-317	FALSE	2	TRA:CARPGAGGYQKVTF TRB:CASSSRPNTEAFF	2	CD8-C6
PB-3_TCTTCGGGTGTATGGG	PB	PB-3	P3-1533	FALSE	1	TRA:CAMSAETGTASKLTF TRB:CSGRTPGEEEEIYNEQFF	1	CD8-C6
PB-3_TGTATTCCATCTCGCT	PB	PB-3	P3-1602	FALSE	1	TRA:CAVRSPNFGNEKLTF TRB:CASSGSVGGAYEQYF	1	CD8-C6
PB-3_TTCTACAAGAGGGCTT	PB	PB-3	P3-1636	FALSE	1	TRA:CIVRVADNQGGKLIF TRB:CASSRNGRTSNQPQHF	1	CD8-C6
PB-3_TTCTCAAAGCAGATCG	PB	PB-3	P3-2	TRUE	3	TRA:CAASAPNSGGGADGLTF TRB:CSARRGSSNQPQHF	63	CD8-C6
PB-3_TTTGCGCCAAATCCGT	PB	PB-3	P3-1674	FALSE	1	TRA:CAARGGRGSTLGRLYF TRB:CASSLDQGGEKLFF	1	CD8-C2
PB-3_TTTGTCAGTCATACTG	PB	PB-3	P3-1680	FALSE	1	TRA:CAVTMNRDDKIIF TRB:CASSFESTIEETQYF	1	CD8-C6
PB-3_AAAGATGAGCGATGAC	PB	PB-3	P3-486	FALSE	1	TRA:CAVGTGANSKLTF TRB:CASSLGGGGGELFF	1	CD8-C1
PB-3_AACTCAGGTGATGATA	PB	PB-3	P3-499	FALSE	1	TRA:CVVSAAYNTDKLIF TRB:CAWGLESYEQYF	1	CD8-C1
PB-3_AAGGAGCAGCTAAGAT	PB	PB-3	P3-523	FALSE	1	TRA:CAVSPVFMTTDSWGKFQF TRB:CASSVEGTANTEAFF	1	CD8-C1
PB-3_ACACTGAGTGCCTGTG	PB	PB-3	P3-561	FALSE	1	TRA:CAMREGYNTDKLIF TRB:CASSQETLQSYNEQFF	1	CD8-C1
PB-3_ACTATCTGTGATAAGT	PB	PB-3	P3-242	TRUE	2	TRA:CAVLYNQGGKLIF TRB:CSARSFETAIPEAFF	2	CD8-C1
PB-3_AGCCTAAAGATAGCAT	PB	PB-3	P3-153	TRUE	3	TRA:CAENSDFGNVLHC TRB:CASSVGMGNQPQHF	3	CD8-C2
PB-3_AGTGAGGAGAGAACAG	PB	PB-3	P3-689	FALSE	1	TRA:CAVNDNNDMRF TRB:CASIGQGALTEAFF	1	CD8-C1

PB-3_AGTGTCACAGGCGATA	PB	PB-3	P3-693	FALSE	1	TRA:CAVRRILDNYGQNFVF TRB:CASSDIRVHNEQFF	1	CD8-C1
PB-3_ATGTGTGGTCAGTGGA	PB	PB-3	P3-733	FALSE	1	TRA:CIVRGHAGNNRKLIW TRB:CAWTTGTGFEKLFF	1	CD4-C2
PB-3_ATTATCCAGAGTACAT	PB	PB-3	P3-740	FALSE	1	TRA:CAVRDRGATNKLIF TRB:CASSEIRQDYEQYF	1	CD8-C1
PB-3_CACAAACGTATTAGCC	PB	PB-3	P3-769	FALSE	1	TRA:CALSGFASGGSYIPTF TRB:CASSVGQGVDEQYF	1	CD8-C1
PB-3_CAGAGAGTCCTTTCGG	PB	PB-3	P3-803	FALSE	1	TRA:CAASDPYDKVIF TRB:CASSFLGLRTDTQYF	1	CD8-C1
PB-3_CAGTAACTCAGCGATT	PB	PB-3	P3-815	FALSE	1	TRA:CALSGSAGQLTF TRB:CASSFGTGGMNTEAFF	1	CD8-C1
PB-3_CATCGGGGTCCAGTAT	PB	PB-3	P3-840	FALSE	1	TRA:CAVATNAGKSTF TRB:CASSEMRTGGQPQHF	1	CD8-C1
PB-3_CATTATCAGGCTCTTA	PB	PB-3	P3-852	FALSE	1	TRA:CALRGDSNYQLIW TRB:CATTTRQYGYTF	1	CD8-C1
PB-3_CCAATCCGTTCGTGAT	PB	PB-3	P3-860	FALSE	1	TRA:CATVLLMDSSYKLIF TRB:CASSLVVGKYGYTF	1	CD8-C1
PB-3_CCACTACCACATCTTT	PB	PB-3	P3-868	FALSE	1	TRA:CAVIRPDSGNTPLVF TRB:CSVDPTGAPQTQYF	1	CD8-C1
PB-3_CGAGCACAGTCCCACG	PB	PB-3	P3-931	FALSE	1	TRA:CAYDNAGNMLTF TRB:CASSTGTGEREQYF	1	CD4-C2
PB-3_CGAGCACGTATATGGA	PB	PB-3	P3-933	FALSE	1	TRA:CVVNRHNYGQNFVF TRB:CASSQRTGNTEAFF	1	CD8-C1
PB-3_CGCGGTAAGGTAAACT	PB	PB-3	P3-947	FALSE	1	TRA:CALSEGSSASKIIF TRB:CASSYSGTFRNSPLHF	1	CD8-C1
PB-3_CGCTGGAGTAAGTAGT	PB	PB-3	P3-953	FALSE	1	TRA:CAVDRGGSTLGRLYF TRB:CASSLSPFNINEQFF	1	CD8-C1
PB-3_CGGACGTAGATGAGAG	PB	PB-3	P3-962	FALSE	1	TRA:CAVRSRGSGAGSYQLTF TRB:CASSQTGNSNQPQHF	1	CD8-C1
PB-3_CGGGTCAAGTCCTCCT	PB	PB-3	P3-976	FALSE	1	TRA:CAESSGAGSYQLTF TRB:CASSLAGSNNEQFF	1	CD8-C1
PB-3_CTCGAGGGTTCAGGCC	PB	PB-3	P3-1058	FALSE	1	TRA:CAVGNTNAGKSTF TRB:CSVEIPGVSYEQYF	1	CD8-C1
PB-3_CTGCCTAGTGGTCCGT	PB	PB-3	P3-1091	FALSE	1	TRA:CAFSNFGNEKLTF TRB:CAWSEVNEQYF	1	CD8-C1
PB-3_CTGTTTAGTCAGAGGT	PB	PB-3	P3-1101	FALSE	1	TRA:CALGNAGGTSYGKLTF TRB:CAISARGQGGQPQHF	1	CD8-C1
PB-3_GAAGCAGAGCGGCTTC	PB	PB-3	P3-1125	FALSE	1	TRA:CAADNYKLSF TRB:CSAQTRTGNNEQFF	1	CD8-C2
PB-3_GACACGCAGAAAGTGG	PB	PB-3	P3-1132	FALSE	1	TRA:CVVSSGGYQKVTF TRB:CASSTTDWNTEAFF	1	CD8-C1
PB-3_GACTAACCACAGCCCA	PB	PB-3	P3-1155	FALSE	1	TRA:CASGACLGFGNVLHC TRB:CSARDLSDGGWPQHF	1	CD8-C1
PB-3_GAGCAGATCAGGCGAA	PB	PB-3	P3-1164	FALSE	1	TRA:CAWGNSGGSNYKLTF TRB:CASSQDASGHSYEQYF	1	CD8-C1
PB-3_GCAGTTACACAGCGTC	PB	PB-3	P3-1196	FALSE	1	TRA:CALSPWGSSNTGKLIF TRB:CASSLRGQGARGEAFF	1	CD8-C1
PB-3_GCTGCAGTCGCCGTGA	PB	PB-3	P3-1231	FALSE	1	TRA:CAARDSGGGADGLTF TRB:CASSLQGQGETQYF	1	CD8-C2
PB-3_GCTGGGTTCGTTGACA	PB	PB-3	P3-1240	FALSE	1	TRA:CAYYGRGTSGSRLTF TRB:CASSQGPYSYEQYF	1	CD8-C1
PB-3_GGATGTTTCTATCCTA	PB	PB-3	P3-1264	FALSE	1	TRA:CALSEENTDKLIF TRB:CASSDDRFTEAFF	1	CD8-C1

PB-3_GGGAGATCAAGAGTCG	PB	PB-3	P3-1284	FALSE	1	TRA:CAVSLNQAGTALIF TRB:CASSNSGGHQETQYF	1	CD8-C1
PB-3_GTCAAGTTCAAGGTAA	PB	PB-3	P3-1333	FALSE	1	TRA:CAVNAPLDSNYQLIW TRB:CASSLEDYQQETQYF	1	CD4-C2
PB-3_GTGGGTCGTTATCCGA	PB	PB-3	P3-1376	FALSE	1	TRA:CILRDANSGYALNF TRB:CASSKGQGNSPLHF	1	CD4-C1
PB-3_GTTCGGGTCAGTCCCT	PB	PB-3	P3-1391	FALSE	1	TRA:CVVFSFSSGTYKYIF TRB:CASSLGQGPYEQYF	1	CD8-C1
PB-3_TACGGTACATTCACTT	PB	PB-3	P3-1428	FALSE	1	TRA:CVVNSGAGSYQLTF TRB:CAGTRTGSYNEQFF	1	CD8-C1
PB-3_TAGGCATCAGTACACT	PB	PB-3	P3-1453	FALSE	1	TRA:CVVPLSDRGSTLGRLYF TRB:CASSFVGGNYGYTF	1	CD8-C1
PB-3_TCAATCTCATGGATGG	PB	PB-3	P3-1468	FALSE	1	TRA:CAVVGSKLTF TRB:CASNDRGYNEQFF	1	CD8-C1
PB-3_TCCCGATTCTAACTGG	PB	PB-3	P3-1508	FALSE	1	TRA:CAAPRGGGADGLTF TRB:CASSVGGSTDTQYF	1	CD8-C1
PB-3_TCGGGACTCAAACAAG	PB	PB-3	P3-1517	FALSE	1	TRA:CAVSDREGYQKVTF TRB:CASSSLTGGNQETQYF	1	CD8-C1
PB-3_TGCCAAAAGAATCTCC	PB	PB-3	P3-1561	FALSE	1	TRA:CAVIPTYQAGTALIF TRB:CASSDLTENTGELFF	1	CD4-C1
PB-3_TGCCCATAGGTAAACT	PB	PB-3	P3-1564	FALSE	1	TRA:CIVRVPIKAAGNKLTF TRB:CASSPATPSGIQYF	1	CD8-C1
PB-3_TGGGCGTGTAATCACC	PB	PB-3	P3-1594	FALSE	1	TRA:CAATSSYNTDKLIF TRB:CAWRTTGYNSPLHF	1	CD8-C1
PB-3_TTCTCCTGTTGCTCCT	PB	PB-3	P3-1640	FALSE	1	TRA:CAMRELPIYNQGGKLIF TRB:CASSLGSGAYSPLHF	1	CD8-C1
Pla-1_AAACCTGCAAGAGGCT	Pla	Pla-1	P1-16	TRUE	3	TRA:CALSGTPMNRDDKIIF TRB:CASSQSWDLAVLNEQFF	79	CD8-C7
Pla-1_AAAGATGAGCGTAATA	Pla	Pla-1	P1-5	TRUE	3	TRA:CALSGTPMNRDDKIIF TRB:CSARGRGLAGGPAYEQYF	223	CD8-C7
Pla-1_AAAGCAAAGTCCTCCT	Pla	Pla-1	P1-1	TRUE	3	TRA:CAMSDRMTGGFKTIF TRB:CASSQVGRGGEETQYF	1047	CD8-C7
Pla-1_AAATGCCTCAAACCAC	Pla	Pla-1	P1-22	TRUE	3	TRA:CASNSGYSTLTF TRB:CASSQVTGGARDEQYF	65	CD8-C7
Pla-1_AACACGTAGATGAGAG	Pla	Pla-1	P1-26	TRUE	3	TRA:CAPRSGGSYIPTF TRB:CASSQDLGLDYGYTF	56	CD8-C7
Pla-1_AACGTTGTCCGCAGTG	Pla	Pla-1	P1-4	TRUE	3	TRA:CAMKGRDDKIIF TRB:CASTKGVSREAFF	226	CD8-C7
Pla-1_AACTCAGAGAGGTTAT	Pla	Pla-1	P1-17	TRUE	3	TRA:CGTGARGSSGSRLTF TRB:CASSRQGMGAFF	78	CD4-C6
Pla-1_AACTGGTCAATTGCTG	Pla	Pla-1	P1-4	TRUE	3	TRA:CAMKGRDDKIIF TRB:CASTKGVSREAFF	226	CD8-C6
Pla-1_AAGACCTGTGTCAATC	Pla	Pla-1	P1-1	TRUE	3	TRA:CAMSDRMTGGFKTIF TRB:CASSQVGRGGEETQYF	1047	CD8-C7
Pla-1_AAGACCTTCCGCTGTT	Pla	Pla-1	P1-1	TRUE	3	TRA:CAMSDRMTGGFKTIF TRB:CASSQVGRGGEETQYF	1047	CD8-C7
Pla-1_AAGGAGCCATGCAATC	Pla	Pla-1	P1-31	TRUE	3	TRA:CAVEEGADRLMF TRB:CASSLLGGGELFF	46	CD8-C7
Pla-1_AAGGTTCAGCACCGCT	Pla	Pla-1	P1-9	TRUE	3	TRA:CAEAEGGGADGLTF TRB:CATSGGDNEQFF	174	CD8-C7
Pla-1_AAGGTTCAGGAATTAC	Pla	Pla-1	P1-1	TRUE	3	TRA:CAMSDRMTGGFKTIF TRB:CASSQVGRGGEETQYF	1047	CD8-C7
Pla-1_AATCGGTAGATAGCAT	Pla	Pla-1	P1-17	TRUE	3	TRA:CGTGARGSSGSRLTF TRB:CASSRQGMGAFF	78	CD8-C7

Pla-1_ACACCAAGTAGAAAGG	Pla	Pla-1	P1-1141	FALSE	1	TRA:CAVRPPGRNSGGYQKVTF TRB:CASRWTGSGDTQYF	1	CD8-C7
Pla-1_ACACCGGAGTCATGCT	Pla	Pla-1	P1-1	TRUE	3	TRA:CAMSDRMTGGFKTIF TRB:CASSQVGRGGEETQYF	1047	CD8-C7
Pla-1_ACATACGGTCTGATCA	Pla	Pla-1	P1-25	TRUE	3	TRA:CIVGRDSSYKLIF TRB:CASNPQRGAHRDTQYF	58	CD4-C6
Pla-1_ACCAGTAAGAGTGAGA	Pla	Pla-1	P1-2	TRUE	3	TRA:CGQNFVF TRB:CASNQRGGPSLYNEQFF	323	CD8-C7
Pla-1_ACGAGGAGTATGCTTG	Pla	Pla-1	P1-1	TRUE	3	TRA:CAMSDRMTGGFKTIF TRB:CASSQVGRGGEETQYF	1047	CD8-C7
Pla-1_ACGATGTCACGTCTCT	Pla	Pla-1	P1-5	TRUE	3	TRA:CALSGTPMNRDDKIIF TRB:CSARGRGLAGGPAYEQYF	223	CD8-C7
Pla-1_ACGATGTTCGAACTGT	Pla	Pla-1	P1-320	FALSE	2	TRA:CAASGANAGNMLTF TRB:CASSQMAGASTDTQYF	2	CD4-C6
Pla-1_ACGCCAGCATGGTTGT	Pla	Pla-1	P1-31	TRUE	3	TRA:CAVEEGADRLMF TRB:CASSLLGGGELFF	46	CD8-C7
Pla-1_ACGCCGAAGATGCGAC	Pla	Pla-1	P1-1194	FALSE	1	TRA:CAFKISDGQKLLF TRB:CASSRRGVTDTQYF	1	CD8-C7
Pla-1_ACGCCGATCCAAGTAC	Pla	Pla-1	P1-108	TRUE	3	TRA:CAASNGDTGNQFYF TRB:CSASKQGRNQPQHF	5	CD4-C6
Pla-1_ACGGAGACACCATGTA	Pla	Pla-1	P1-36	TRUE	3	TRA:CILRDNAGNNRKLIW TRB:CASSSRQGFPGGANVLTF	40	CD4-C6
Pla-1_ACGGAGATCATCGGAT	Pla	Pla-1	P1-4	TRUE	3	TRA:CAMKGRDDKIIF TRB:CASTKGVSREAFF	226	CD8-C7
Pla-1_ACGGCCAAGATGGGTC	Pla	Pla-1	P1-6	TRUE	3	TRA:CAMREGRERDDKIIF TRB:CASSLPNTEAFF	216	CD8-C5
Pla-1_ACGGCCAGTACTCGCG	Pla	Pla-1	P1-152	TRUE	3	TRA:CALSEAGKPGRKAYLRT TRB:CASSPGTGSYNEQFF	4	CD8-C7
Pla-1_ACGGGCTTCACGCGGT	Pla	Pla-1	P1-1	TRUE	3	TRA:CAMSDRMTGGFKTIF TRB:CASSQVGRGGEETQYF	1047	CD8-C7
Pla-1_ACTATCTCAGTTCATG	Pla	Pla-1	P1-5	TRUE	3	TRA:CALSGTPMNRDDKIIF TRB:CSARGRGLAGGPAYEQYF	223	CD8-C7
Pla-1_ACTGAGTAGGACAGCT	Pla	Pla-1	P1-1	TRUE	3	TRA:CAMSDRMTGGFKTIF TRB:CASSQVGRGGEETQYF	1047	CD8-C7
Pla-1_ACTGAGTCAATCAGAA	Pla	Pla-1	P1-3	TRUE	3	TRA:CAFLSGTYKYIF TRB:CASSQSPGQRNYGYTF	308	CD8-C7
Pla-1_ACTGAGTCACAACGCC	Pla	Pla-1	P1-5	TRUE	3	TRA:CALSGTPMNRDDKIIF TRB:CSARGRGLAGGPAYEQYF	223	CD8-C5
Pla-1_ACTGAGTCATGGTTGT	Pla	Pla-1	P1-264	TRUE	2	TRA:CAVQAGNNDMRF TRB:CASSPGTGSYNEQFF	2	CD8-C7
Pla-1_AGAGCGAGTCTGATCA	Pla	Pla-1	P1-1	TRUE	3	TRA:CAMSDRMTGGFKTIF TRB:CASSQVGRGGEETQYF	1047	CD8-C7
Pla-1_AGAGCTTGTACCGTAT	Pla	Pla-1	P1-19	TRUE	3	TRA:CALGAFGSARQLTF TRB:CASSKGQGPRETQYF	70	CD8-C7
Pla-1_AGAGCTTTCCAGAAGG	Pla	Pla-1	P1-16	TRUE	3	TRA:CALSGTPMNRDDKIIF TRB:CASSQSWDLAVLNEQFF	79	CD8-C7
Pla-1_AGAGTGGCACTTCGAA	Pla	Pla-1	P1-8	TRUE	3	TRA:CAVRPPGRNSGGYQKVTF TRB:CASSRRGVTDTQYF	204	CD8-C7
Pla-1_AGATCTGTCTCGGACG	Pla	Pla-1	P1-8	TRUE	3	TRA:CAVRPPGRNSGGYQKVTF TRB:CASSRRGVTDTQYF	204	CD8-C7
Pla-1_AGATTGCAGCTAACAA	Pla	Pla-1	P1-11	TRUE	3	TRA:CAVEPLYGSGNTGKLIF TRB:CASSSHGQLNQPQHF	147	CD8-C7
Pla-1_AGATTGCTCCTTTCGG	Pla	Pla-1	P1-3	TRUE	3	TRA:CAFLSGTYKYIF TRB:CASSQSPGQRNYGYTF	308	CD8-C6

Pla-1_AGCAGCCCAGATCGGA	Pla	Pla-1	P1-1	TRUE	3	TRA:CAMSDRMTGGFKTIF TRB:CASSQVGRGGEETQYF	1047	CD8-C7
Pla-1_AGCAGCCCAGTGGGAT	Pla	Pla-1	P1-1	TRUE	3	TRA:CAMSDRMTGGFKTIF TRB:CASSQVGRGGEETQYF	1047	CD8-C7
Pla-1_AGCATACGTCGAATCT	Pla	Pla-1	P1-22	TRUE	3	TRA:CASNSGYSTLTF TRB:CASSQVTGGARDEQYF	65	CD8-C7
Pla-1_AGCGTATGTTACCAGT	Pla	Pla-1	P1-1	TRUE	3	TRA:CAMSDRMTGGFKTIF TRB:CASSQVGRGGEETQYF	1047	CD8-C7
Pla-1_AGCTTGACAATCCAAC	Pla	Pla-1	P1-91	TRUE	3	TRA:CVVSDQYNTDKLIF TRB:CASGEGQGASSYEQYF	6	CD8-C5
Pla-1_AGCTTGAGTACTTCTT	Pla	Pla-1	P1-39	TRUE	3	TRA:CAVRDLGGTSYDKVIF TRB:CSARVREVSYEQYF	31	CD8-C7
Pla-1_AGGCCACCAAGGACAC	Pla	Pla-1	P1-5	TRUE	3	TRA:CALSGTPMNRDDKIIF TRB:CSARGRGLAGGPAYEQYF	223	CD8-C7
Pla-1_AGGGATGCAATGAATG	Pla	Pla-1	P1-6	TRUE	3	TRA:CAMREGRERDDKIIF TRB:CASSLPNTEAFF	216	CD8-C5
Pla-1_AGGGTGATCATTGCGA	Pla	Pla-1	P1-17	TRUE	3	TRA:CGTGARGSSGSRLTF TRB:CASSRQGMGAFF	78	CD4-C6
Pla-1_AGTCTTTCACATCCAA	Pla	Pla-1	P1-11	TRUE	3	TRA:CAVEPLYGSGNTGKLIF TRB:CASSSHGQLNQPQHF	147	CD8-C7
Pla-1_AGTCTTTTCATCTGTT	Pla	Pla-1	P1-1	TRUE	3	TRA:CAMSDRMTGGFKTIF TRB:CASSQVGRGGEETQYF	1047	CD8-C7
Pla-1_AGTGAGGAGTGACATA	Pla	Pla-1	P1-7	TRUE	3	TRA:CAVQAGNNDMRF TRB:CASSTTGDFNYGYTF	215	CD8-C7
Pla-1_AGTTGGTAGGTAGCTG	Pla	Pla-1	P1-1304	FALSE	1	TRA:CAMSDRMTGGFKTIF TRB:CAISLTGDGKETQYF	1	CD8-C7
Pla-1_AGTTGGTGTAAACCTC	Pla	Pla-1	P1-2	TRUE	3	TRA:CGQNFVF TRB:CASNQRGGPSLYNEQFF	323	CD8-C7
Pla-1_ATAGACCCAAGGTGTG	Pla	Pla-1	P1-1	TRUE	3	TRA:CAMSDRMTGGFKTIF TRB:CASSQVGRGGEETQYF	1047	CD8-C7
Pla-1_ATAGACCGTAACGTTC	Pla	Pla-1	P1-63	TRUE	3	TRA:CAEPLNTGNQFYF TRB:CASSVGNEQFF	10	CD8-C7
Pla-1_ATCATCTAGGTGGGTT	Pla	Pla-1	P1-4	TRUE	3	TRA:CAMKGRDDKIIF TRB:CASTKGVSREAFF	226	CD8-C7
Pla-1_ATCATGGGTACCGGCT	Pla	Pla-1	P1-1	TRUE	3	TRA:CAMSDRMTGGFKTIF TRB:CASSQVGRGGEETQYF	1047	CD8-C7
Pla-1_ATCCGAAGTAGCCTCG	Pla	Pla-1	P1-1	TRUE	3	TRA:CAMSDRMTGGFKTIF TRB:CASSQVGRGGEETQYF	1047	CD8-C7
Pla-1_ATGAGGGTCTGTCCGT	Pla	Pla-1	P1-1	TRUE	3	TRA:CAMSDRMTGGFKTIF TRB:CASSQVGRGGEETQYF	1047	CD8-C7
Pla-1_ATGGGAGTCTACCTGC	Pla	Pla-1	P1-1	TRUE	3	TRA:CAMSDRMTGGFKTIF TRB:CASSQVGRGGEETQYF	1047	CD8-C7
Pla-1_ATTACTCCATTCGACA	Pla	Pla-1	P1-12	TRUE	3	TRA:CALSDERGDYKLSF TRB:CASSPGTGSYNEQFF	141	CD8-C5
Pla-1_ATTCTACGTCAGGACA	Pla	Pla-1	P1-1	TRUE	3	TRA:CAMSDRMTGGFKTIF TRB:CASSQVGRGGEETQYF	1047	CD8-C7
Pla-1_ATTTCTGCATGACGGA	Pla	Pla-1	P1-10	TRUE	3	TRA:CISNSPMDSSYKLIF TRB:CASSSWGDTDEAFF	162	CD8-C7
Pla-1_ATTTCTGGTCTAGCCG	Pla	Pla-1	P1-14	TRUE	3	TRA:CAASVVNDMRF TRB:CASSHRGGAKNIQYF	109	CD8-C7
Pla-1_CAACCAAGTGTGACGA	Pla	Pla-1	P1-22	TRUE	3	TRA:CASNSGYSTLTF TRB:CASSQVTGGARDEQYF	65	CD8-C7
Pla-1_CAACCTCAGCTGTTCA	Pla	Pla-1	P1-1	TRUE	3	TRA:CAMSDRMTGGFKTIF TRB:CASSQVGRGGEETQYF	1047	CD8-C7

Pla-1_CAACCTCGTGCACTTA	Pla	Pla-1	P1-25	TRUE	3	TRA:CIVGRDSSYKLIF TRB:CASNPQRGAHRDTQYF	58	CD8-C7
Pla-1_CAACTAGTCAGTCCCT	Pla	Pla-1	P1-1	TRUE	3	TRA:CAMSDRMTGGFKTIF TRB:CASSQVGRGGEETQYF	1047	CD8-C7
Pla-1_CAAGAAATCGTACCGG	Pla	Pla-1	P1-1	TRUE	3	TRA:CAMSDRMTGGFKTIF TRB:CASSQVGRGGEETQYF	1047	CD8-C7
Pla-1_CAAGGCCGTATAGTAG	Pla	Pla-1	P1-26	TRUE	3	TRA:CAPRSGGSYIPTF TRB:CASSQDLGLDYGYTF	56	CD8-C7
Pla-1_CACACCTCAGCCTTGG	Pla	Pla-1	P1-21	TRUE	3	TRA:CAASRGTGANNLFF TRB:CATQREGREQYF	67	CD4-C6
Pla-1_CACACTCCACATAACC	Pla	Pla-1	P1-1	TRUE	3	TRA:CAMSDRMTGGFKTIF TRB:CASSQVGRGGEETQYF	1047	CD8-C6
Pla-1_CACAGGCAGACCTAGG	Pla	Pla-1	P1-1	TRUE	3	TRA:CAMSDRMTGGFKTIF TRB:CASSQVGRGGEETQYF	1047	CD8-C7
Pla-1_CACAGGCTCGGAAACG	Pla	Pla-1	P1-1	TRUE	3	TRA:CAMSDRMTGGFKTIF TRB:CASSQVGRGGEETQYF	1047	CD8-C7
Pla-1_CACATAGAGTCAAGCG	Pla	Pla-1	P1-127	FALSE	3	TRA:CALSADNDMRF TRB:CASTRGDWIYNEQFF	5	CD4-C6
Pla-1_CACATAGTCATACGGT	Pla	Pla-1	P1-3	TRUE	3	TRA:CAFLSGTYKYIF TRB:CASSQSPGQRNYGYTF	308	CD8-C6
Pla-1_CACATAGTCTGAGGGA	Pla	Pla-1	P1-1	TRUE	3	TRA:CAMSDRMTGGFKTIF TRB:CASSQVGRGGEETQYF	1047	CD8-C7
Pla-1_CACATTTCAGAGTGTG	Pla	Pla-1	P1-6	TRUE	3	TRA:CAMREGRERDDKIIF TRB:CASSLPNTEAFF	216	CD8-C5
Pla-1_CACATTTTCCTATTCA	Pla	Pla-1	P1-3	TRUE	3	TRA:CAFLSGTYKYIF TRB:CASSQSPGQRNYGYTF	308	CD8-C7
Pla-1_CACCACTTCAAGGTAA	Pla	Pla-1	P1-4	TRUE	3	TRA:CAMKGRDDKIIF TRB:CASTKGVSREAFF	226	CD8-C7
Pla-1_CAGAATCCACATGACT	Pla	Pla-1	P1-1	TRUE	3	TRA:CAMSDRMTGGFKTIF TRB:CASSQVGRGGEETQYF	1047	CD8-C7
Pla-1_CAGAGAGAGGGTTCCC	Pla	Pla-1	P1-5	TRUE	3	TRA:CALSGTPMNRDDKIIF TRB:CSARGRGLAGGPAYEQYF	223	CD8-C7
Pla-1_CAGAGAGCATGCTAGT	Pla	Pla-1	P1-1	TRUE	3	TRA:CAMSDRMTGGFKTIF TRB:CASSQVGRGGEETQYF	1047	CD8-C7
Pla-1_CAGAGAGGTCGCCATG	Pla	Pla-1	P1-1	TRUE	3	TRA:CAMSDRMTGGFKTIF TRB:CASSQVGRGGEETQYF	1047	CD8-C7
Pla-1_CAGCATACACCAGATT	Pla	Pla-1	P1-1421	FALSE	1	TRA:CAMKGRDDKIIF TRB:CSARGRGLAGGPAYEQYF	1	CD8-C6
Pla-1_CAGCGACGTGTGGTTT	Pla	Pla-1	P1-9	TRUE	3	TRA:CAEAEGGGADGLTF TRB:CATSGGDNEQFF	174	CD8-C7
Pla-1_CAGCTAACACAACTGT	Pla	Pla-1	P1-5	TRUE	3	TRA:CALSGTPMNRDDKIIF TRB:CSARGRGLAGGPAYEQYF	223	CD8-C7
Pla-1_CAGGTGCGTGTCGCTG	Pla	Pla-1	P1-2	TRUE	3	TRA:CGQNFVF TRB:CASNQRGGPSLYNEQFF	323	CD8-C7
Pla-1_CAGTAACGTAGAGTGC	Pla	Pla-1	P1-24	TRUE	3	TRA:CAVKGNTDKLIF TRB:CASSLRTGAYNEQFF	62	CD8-C6
Pla-1_CATATGGCAATCCGAT	Pla	Pla-1	P1-1	TRUE	3	TRA:CAMSDRMTGGFKTIF TRB:CASSQVGRGGEETQYF	1047	CD8-C7
Pla-1_CATATGGTCCTCCTAG	Pla	Pla-1	P1-2	TRUE	3	TRA:CGQNFVF TRB:CASNQRGGPSLYNEQFF	323	CD8-C7
Pla-1_CATATTCCAGGAATGC	Pla	Pla-1	P1-8	TRUE	3	TRA:CAVRPPGRNSGGYQKVTF TRB:CASSRRGVTDTQYF	204	CD8-C7
Pla-1_CATCAAGTCGGAATCT	Pla	Pla-1	P1-1438	FALSE	1	TRA:CAVLPETSGSRLTF TRB:CASSQVGRGGEETQYF	1	CD8-C7

Pla-1_CATCAGAAGAGACTTA	Pla	Pla-1	P1-1	TRUE	3	TRA:CAMSDRMTGGFKTIF TRB:CASSQVGRGGEETQYF	1047	CD8-C7
Pla-1_CATCCACCATTTCAGG	Pla	Pla-1	P1-29	TRUE	3	TRA:CATVSFSGGYNKLIF TRB:CASSLDRRGLSYEQYF	50	CD8-C7
Pla-1_CATCCACTCGCCTGAG	Pla	Pla-1	P1-5	TRUE	3	TRA:CALSGTPMNRDDKIIF TRB:CSARGRGLAGGPAYEQYF	223	CD8-C7
Pla-1_CATGACAAGTTTAGGA	Pla	Pla-1	P1-4	TRUE	3	TRA:CAMKGRDDKIIF TRB:CASTKGVSREAFF	226	CD8-C7
Pla-1_CATGACACAACCGCCA	Pla	Pla-1	P1-5	TRUE	3	TRA:CALSGTPMNRDDKIIF TRB:CSARGRGLAGGPAYEQYF	223	CD8-C7
Pla-1_CATGGCGGTACCTACA	Pla	Pla-1	P1-1462	FALSE	1	TRA:CAMSDRMTGGFKTIF TRB:CASSSSEATNEKLFF	1	CD8-C7
Pla-1_CATGGCGTCACAGGCC	Pla	Pla-1	P1-36	TRUE	3	TRA:CILRDNAGNNRKLIW TRB:CASSSRQGFPGGANVLTF	40	CD8-C7
Pla-1_CATTATCAGCCTTGAT	Pla	Pla-1	P1-13	TRUE	3	TRA:CVVMNYGGSQGNLIF TRB:CASSSWGDTDEAFF	112	CD8-C7
Pla-1_CATTCGCCACAGAGGT	Pla	Pla-1	P1-5	TRUE	3	TRA:CALSGTPMNRDDKIIF TRB:CSARGRGLAGGPAYEQYF	223	CD8-C7
Pla-1_CATTCGCGTTAAGGGC	Pla	Pla-1	P1-22	TRUE	3	TRA:CASNSGYSTLTF TRB:CASSQVTGGARDEQYF	65	CD8-C7
Pla-1_CCACCTATCCCTAACC	Pla	Pla-1	P1-1	TRUE	3	TRA:CAMSDRMTGGFKTIF TRB:CASSQVGRGGEETQYF	1047	CD8-C7
Pla-1_CCACTACTCGGTTAAC	Pla	Pla-1	P1-1	TRUE	3	TRA:CAMSDRMTGGFKTIF TRB:CASSQVGRGGEETQYF	1047	CD8-C7
Pla-1_CCCAATCGTCCTAGCG	Pla	Pla-1	P1-5	TRUE	3	TRA:CALSGTPMNRDDKIIF TRB:CSARGRGLAGGPAYEQYF	223	CD8-C7
Pla-1_CCCTCCTCACTTCGAA	Pla	Pla-1	P1-1	TRUE	3	TRA:CAMSDRMTGGFKTIF TRB:CASSQVGRGGEETQYF	1047	CD8-C7
Pla-1_CCCTCCTGTAAGTGTA	Pla	Pla-1	P1-1	TRUE	3	TRA:CAMSDRMTGGFKTIF TRB:CASSQVGRGGEETQYF	1047	CD8-C7
Pla-1_CCGTACTTCTGTCTAT	Pla	Pla-1	P1-13	TRUE	3	TRA:CVVMNYGGSQGNLIF TRB:CASSSWGDTDEAFF	112	CD8-C7
Pla-1_CCGTGGATCTGATACG	Pla	Pla-1	P1-15	TRUE	3	TRA:CAVNSSPLGANSKLTF TRB:CASSDSGNFYNEQFF	92	CD8-C2
Pla-1_CCGTTCACAGATTGCT	Pla	Pla-1	P1-10	TRUE	3	TRA:CISNSPMDSSYKLIF TRB:CASSSWGDTDEAFF	162	CD8-C7
Pla-1_CCGTTCAGTAATCGTC	Pla	Pla-1	P1-1	TRUE	3	TRA:CAMSDRMTGGFKTIF TRB:CASSQVGRGGEETQYF	1047	CD8-C7
Pla-1_CCTAAAGTCCGTCAAA	Pla	Pla-1	P1-1	TRUE	3	TRA:CAMSDRMTGGFKTIF TRB:CASSQVGRGGEETQYF	1047	CD8-C7
Pla-1_CCTAGCTAGCACGCCT	Pla	Pla-1	P1-2	TRUE	3	TRA:CGQNFVF TRB:CASNQRGGPSLYNEQFF	323	CD8-C7
Pla-1_CCTATTAGTCCAGTTA	Pla	Pla-1	P1-2	TRUE	3	TRA:CGQNFVF TRB:CASNQRGGPSLYNEQFF	323	CD8-C7
Pla-1_CCTATTATCACCCTCA	Pla	Pla-1	P1-12	TRUE	3	TRA:CALSDERGDYKLSF TRB:CASSPGTGSYNEQFF	141	CD8-C5
Pla-1_CCTCAGTTCATTATCC	Pla	Pla-1	P1-1525	FALSE	1	TRA:CAVASSNDYKLSF TRB:CASNQRGGPSLYNEQFF	1	CD4-C6
Pla-1_CCTCTGAAGGCAGGTT	Pla	Pla-1	P1-1	TRUE	3	TRA:CAMSDRMTGGFKTIF TRB:CASSQVGRGGEETQYF	1047	CD8-C7
Pla-1_CGAACATCAGTTCATG	Pla	Pla-1	P1-7	TRUE	3	TRA:CAVQAGNNDMRF TRB:CASSTTGDFNYGYTF	215	CD8-C5
Pla-1_CGAACATGTACTCAAC	Pla	Pla-1	P1-8	TRUE	3	TRA:CAVRPPGRNSGGYQKVTF TRB:CASSRRGVTDTQYF	204	CD8-C7

Pla-1_CGAATGTAGATGGGTC	Pla	Pla-1	P1-1	TRUE	3	TRA:CAMSDRMTGGFKTIF TRB:CASSQVGRGGEETQYF	1047	CD8-C7
Pla-1_CGACTTCAGAGCAATT	Pla	Pla-1	P1-6	TRUE	3	TRA:CAMREGRERDDKIIF TRB:CASSLPNTEAFF	216	CD8-C7
Pla-1_CGAGAAGTCCACGTGG	Pla	Pla-1	P1-1	TRUE	3	TRA:CAMSDRMTGGFKTIF TRB:CASSQVGRGGEETQYF	1047	CD8-C5
Pla-1_CGAGCACAGCGGCTTC	Pla	Pla-1	P1-61	TRUE	3	TRA:CAARGGSQGNLIF TRB:CASRPRHTDTQYF	10	CD8-C7
Pla-1_CGAGCCAAGCGCTTAT	Pla	Pla-1	P1-29	TRUE	3	TRA:CATVSFSGGYNKLIF TRB:CASSLDRRGLSYEQYF	50	CD8-C7
Pla-1_CGAGCCAAGGTGCAAC	Pla	Pla-1	P1-2	TRUE	3	TRA:CGQNFVF TRB:CASNQRGGPSLYNEQFF	323	CD8-C7
Pla-1_CGATCGGAGCACCGTC	Pla	Pla-1	P1-8	TRUE	3	TRA:CAVRPPGRNSGGYQKVTF TRB:CASSRRGVTDTQYF	204	CD8-C7
Pla-1_CGATGGCTCACCACCT	Pla	Pla-1	P1-2	TRUE	3	TRA:CGQNFVF TRB:CASNQRGGPSLYNEQFF	323	CD8-C7
Pla-1_CGATGTAAGCGATTCT	Pla	Pla-1	P1-17	TRUE	3	TRA:CGTGARGSSGSRLTF TRB:CASSRQGMGAFF	78	CD8-C7
Pla-1_CGCGGTATCTTGCCGT	Pla	Pla-1	P1-1	TRUE	3	TRA:CAMSDRMTGGFKTIF TRB:CASSQVGRGGEETQYF	1047	CD8-C7
Pla-1_CGCTGGACACCGAAAG	Pla	Pla-1	P1-1	TRUE	3	TRA:CAMSDRMTGGFKTIF TRB:CASSQVGRGGEETQYF	1047	CD8-C7
Pla-1_CGCTTCACAGTAACGG	Pla	Pla-1	P1-1	TRUE	3	TRA:CAMSDRMTGGFKTIF TRB:CASSQVGRGGEETQYF	1047	CD8-C7
Pla-1_CGCTTCACATCAGTCA	Pla	Pla-1	P1-1	TRUE	3	TRA:CAMSDRMTGGFKTIF TRB:CASSQVGRGGEETQYF	1047	CD8-C7
Pla-1_CGGACACAGTGGTCCC	Pla	Pla-1	P1-273	TRUE	2	TRA:CAVPLNTGNQFYF TRB:CAISVGNEQFF	2	CD8-C7
Pla-1_CGGACTGCAAGCCTAT	Pla	Pla-1	P1-5	TRUE	3	TRA:CALSGTPMNRDDKIIF TRB:CSARGRGLAGGPAYEQYF	223	CD8-C7
Pla-1_CGGACTGTCGTTTGCC	Pla	Pla-1	P1-1590	FALSE	1	TRA:CATVSFSGGYNKLIF TRB:CASIEGQGPYNEQFF	1	CD8-C2
Pla-1_CGGAGCTGTAGAGGAA	Pla	Pla-1	P1-8	TRUE	3	TRA:CAVRPPGRNSGGYQKVTF TRB:CASSRRGVTDTQYF	204	CD4-C6
Pla-1_CGGAGCTGTGGCAAAC	Pla	Pla-1	P1-3	TRUE	3	TRA:CAFLSGTYKYIF TRB:CASSQSPGQRNYGYTF	308	CD8-C11
Pla-1_CGGGTCACACGAAGCA	Pla	Pla-1	P1-16	TRUE	3	TRA:CALSGTPMNRDDKIIF TRB:CASSQSWDLAVLNEQFF	79	CD8-C7
Pla-1_CGTAGCGTCCATTCTA	Pla	Pla-1	P1-5	TRUE	3	TRA:CALSGTPMNRDDKIIF TRB:CSARGRGLAGGPAYEQYF	223	CD8-C7
Pla-1_CGTAGGCCAGCCTTTC	Pla	Pla-1	P1-1	TRUE	3	TRA:CAMSDRMTGGFKTIF TRB:CASSQVGRGGEETQYF	1047	CD8-C7
Pla-1_CGTCACTAGGGCACTA	Pla	Pla-1	P1-1609	FALSE	1	TRA:CAVKGNTDKLIF TRB:CSARGRGLAGGPAYEQYF	1	CD8-C7
Pla-1_CGTCAGGCACGACTCG	Pla	Pla-1	P1-1613	FALSE	1	TRA:CAGYTGTASKLTF TRB:CASSPHPRDTDTQYF	1	CD8-C7
Pla-1_CGTCCATTCTTGTATC	Pla	Pla-1	P1-42	TRUE	3	TRA:CASFSGGSQGNLIF TRB:CSAGQGSNQPQHF	24	CD8-C7
Pla-1_CGTGTAAAGTCCAGGA	Pla	Pla-1	P1-1	TRUE	3	TRA:CAMSDRMTGGFKTIF TRB:CASSQVGRGGEETQYF	1047	CD8-C7
Pla-1_CGTGTAATCCTCATTA	Pla	Pla-1	P1-8	TRUE	3	TRA:CAVRPPGRNSGGYQKVTF TRB:CASSRRGVTDTQYF	204	CD8-C7
Pla-1_CGTGTCTTCACATAGC	Pla	Pla-1	P1-1	TRUE	3	TRA:CAMSDRMTGGFKTIF TRB:CASSQVGRGGEETQYF	1047	CD8-C7

Pla-1_CGTTCTGGTAAGTGGC	Pla	Pla-1	P1-9	TRUE	3	TRA:CAEAEGGGADGLTF TRB:CATSGGDNEQFF	174	CD8-C7
Pla-1_CTAACTTCAGCGTCCA	Pla	Pla-1	P1-21	TRUE	3	TRA:CAASRGTGANNLFF TRB:CATQREGREQYF	67	CD8-C7
Pla-1_CTAATGGCAGGGTATG	Pla	Pla-1	P1-9	TRUE	3	TRA:CAEAEGGGADGLTF TRB:CATSGGDNEQFF	174	CD8-C5
Pla-1_CTACACCAGGAGTTTA	Pla	Pla-1	P1-17	TRUE	3	TRA:CGTGARGSSGSRLTF TRB:CASSRQGMGAFF	78	CD8-C7
Pla-1_CTACACCGTGCATCTA	Pla	Pla-1	P1-24	TRUE	3	TRA:CAVKGNTDKLIF TRB:CASSLRTGAYNEQFF	62	CD8-C7
Pla-1_CTACCCACATCACCCT	Pla	Pla-1	P1-5	TRUE	3	TRA:CALSGTPMNRDDKIIF TRB:CSARGRGLAGGPAYEQYF	223	CD8-C7
Pla-1_CTCGAAAGTTGTTTGG	Pla	Pla-1	P1-1681	FALSE	1	TRA:CAVKDGSGGYNKLIF TRB:CSARGRGLAGGPAYEQYF	1	CD8-C7
Pla-1_CTCGAAATCAAGCCTA	Pla	Pla-1	P1-4	TRUE	3	TRA:CAMKGRDDKIIF TRB:CASTKGVSREAFF	226	CD8-C7
Pla-1_CTCGGAGTCGGAAACG	Pla	Pla-1	P1-1689	FALSE	1	TRA:CLVGTGGFKTIF TRB:CSARGRGLAGGPAYEQYF	1	CD8-C7
Pla-1_CTCGGGATCTTAGCCC	Pla	Pla-1	P1-22	TRUE	3	TRA:CASNSGYSTLTF TRB:CASSQVTGGARDEQYF	65	CD8-C7
Pla-1_CTCGTCAAGGTGTTAA	Pla	Pla-1	P1-14	TRUE	3	TRA:CAASVVNDMRF TRB:CASSHRGGAKNIQYF	109	CD8-C7
Pla-1_CTCTACGTCTGTCTAT	Pla	Pla-1	P1-9	TRUE	3	TRA:CAEAEGGGADGLTF TRB:CATSGGDNEQFF	174	CD8-C7
Pla-1_CTGAAGTCACGTCAGC	Pla	Pla-1	P1-134	FALSE	3	TRA:CAGGGGSQGNLIF TRB:CASSQGLIYGYTF	5	CD8-C7
Pla-1_CTGATAGGTTCCTCCA	Pla	Pla-1	P1-50	TRUE	3	TRA:CARWLGSQGNLIF TRB:CASSLTDRVPQFELVYEQYF	15	CD8-C7
Pla-1_CTGCCTATCCACGCAG	Pla	Pla-1	P1-1	TRUE	3	TRA:CAMSDRMTGGFKTIF TRB:CASSQVGRGGEETQYF	1047	CD8-C7
Pla-1_CTGCCTATCTTCCTTC	Pla	Pla-1	P1-2	TRUE	3	TRA:CGQNFVF TRB:CASNQRGGPSLYNEQFF	323	CD8-C7
Pla-1_CTGCGGAAGCGATTCT	Pla	Pla-1	P1-1727	FALSE	1	TRA:CIVRPEGGTYKYIF TRB:CASSRRGVTDTQYF	1	CD8-C7
Pla-1_CTGCGGACAGGCAGTA	Pla	Pla-1	P1-21	TRUE	3	TRA:CAASRGTGANNLFF TRB:CATQREGREQYF	67	CD8-C7
Pla-1_CTGCTGTTCGTACGGC	Pla	Pla-1	P1-12	TRUE	3	TRA:CALSDERGDYKLSF TRB:CASSPGTGSYNEQFF	141	CD8-C5
Pla-1_CTGGTCTTCAATCTCT	Pla	Pla-1	P1-25	TRUE	3	TRA:CIVGRDSSYKLIF TRB:CASNPQRGAHRDTQYF	58	CD8-C7
Pla-1_CTGGTCTTCACCCGAG	Pla	Pla-1	P1-19	TRUE	3	TRA:CALGAFGSARQLTF TRB:CASSKGQGPRETQYF	70	CD8-C7
Pla-1_CTTAGGACAAATACAG	Pla	Pla-1	P1-2	TRUE	3	TRA:CGQNFVF TRB:CASNQRGGPSLYNEQFF	323	CD8-C7
Pla-1_CTTAGGACATTAGCCA	Pla	Pla-1	P1-5	TRUE	3	TRA:CALSGTPMNRDDKIIF TRB:CSARGRGLAGGPAYEQYF	223	CD8-C7
Pla-1_CTTCTCTAGATGCCAG	Pla	Pla-1	P1-7	TRUE	3	TRA:CAVQAGNNDMRF TRB:CASSTTGDFNYGYTF	215	CD8-C5
Pla-1_CTTCTCTCACTGCCAG	Pla	Pla-1	P1-14	TRUE	3	TRA:CAASVVNDMRF TRB:CASSHRGGAKNIQYF	109	CD8-C7
Pla-1_CTTTGCGGTACTCTCC	Pla	Pla-1	P1-63	TRUE	3	TRA:CAEPLNTGNQFYF TRB:CASSVGNEQFF	10	CD8-C7
Pla-1_GAAACTCTCTTATCTG	Pla	Pla-1	P1-4	TRUE	3	TRA:CAMKGRDDKIIF TRB:CASTKGVSREAFF	226	CD8-C7

Pla-1_GAAATGAAGGCTACGA	Pla	Pla-1	P1-1761	FALSE	1	TRA:CAVNSSPLGANSKLTF TRB:CSARGRGLAGGPAYEQYF	1	CD8-C7
Pla-1_GAACATCCAAGAGGCT	Pla	Pla-1	P1-3	TRUE	3	TRA:CAFLSGTYKYIF TRB:CASSQSPGQRNYGYTF	308	CD8-C6
Pla-1_GAACATCGTACCATCA	Pla	Pla-1	P1-12	TRUE	3	TRA:CALSDERGDYKLSF TRB:CASSPGTGSYNEQFF	141	CD8-C5
Pla-1_GAACATCTCGGCGCTA	Pla	Pla-1	P1-11	TRUE	3	TRA:CAVEPLYGSGNTGKLIF TRB:CASSSHGQLNQPQHF	147	CD8-C7
Pla-1_GAATAAGTCACAGGCC	Pla	Pla-1	P1-7	TRUE	3	TRA:CAVQAGNNDMRF TRB:CASSTTGDFNYGYTF	215	CD8-C7
Pla-1_GAATGAAGTTAAAGAC	Pla	Pla-1	P1-1	TRUE	3	TRA:CAMSDRMTGGFKTIF TRB:CASSQVGRGGEETQYF	1047	CD8-C7
Pla-1_GAATGAATCCCATTAT	Pla	Pla-1	P1-7	TRUE	3	TRA:CAVQAGNNDMRF TRB:CASSTTGDFNYGYTF	215	CD8-C5
Pla-1_GACGCGTCAAGTCTAC	Pla	Pla-1	P1-1	TRUE	3	TRA:CAMSDRMTGGFKTIF TRB:CASSQVGRGGEETQYF	1047	CD8-C7
Pla-1_GACGTTACAGCGTTCG	Pla	Pla-1	P1-1	TRUE	3	TRA:CAMSDRMTGGFKTIF TRB:CASSQVGRGGEETQYF	1047	CD8-C7
Pla-1_GACTAACTCCCTCAGT	Pla	Pla-1	P1-1	TRUE	3	TRA:CAMSDRMTGGFKTIF TRB:CASSQVGRGGEETQYF	1047	CD8-C5
Pla-1_GACTGCGTCGGTCTAA	Pla	Pla-1	P1-58	TRUE	3	TRA:CAVRPNRDDKIIF TRB:CASSSRLAGGTDTQYF	12	CD8-C7
Pla-1_GAGTCCGAGATACACA	Pla	Pla-1	P1-3	TRUE	3	TRA:CAFLSGTYKYIF TRB:CASSQSPGQRNYGYTF	308	CD8-C7
Pla-1_GAGTCCGGTCCAGTGC	Pla	Pla-1	P1-63	TRUE	3	TRA:CAEPLNTGNQFYF TRB:CASSVGNEQFF	10	CD8-C7
Pla-1_GATCGATAGGTGTTAA	Pla	Pla-1	P1-1	TRUE	3	TRA:CAMSDRMTGGFKTIF TRB:CASSQVGRGGEETQYF	1047	CD8-C7
Pla-1_GATCGTACACCCATTC	Pla	Pla-1	P1-9	TRUE	3	TRA:CAEAEGGGADGLTF TRB:CATSGGDNEQFF	174	CD8-C7
Pla-1_GATCTAGTCCCAACGG	Pla	Pla-1	P1-1834	FALSE	1	TRA:CAMSDRMTGGFKTIF TRB:CASRPQGSLDTEAFF	1	CD8-C5
Pla-1_GATGAAAAGAGGTTAT	Pla	Pla-1	P1-1	TRUE	3	TRA:CAMSDRMTGGFKTIF TRB:CASSQVGRGGEETQYF	1047	CD8-C7
Pla-1_GATGCTACATTTCACT	Pla	Pla-1	P1-1	TRUE	3	TRA:CAMSDRMTGGFKTIF TRB:CASSQVGRGGEETQYF	1047	CD8-C7
Pla-1_GATGCTATCATTCACT	Pla	Pla-1	P1-24	TRUE	3	TRA:CAVKGNTDKLIF TRB:CASSLRTGAYNEQFF	62	CD8-C7
Pla-1_GATTCAGCAATCAGAA	Pla	Pla-1	P1-7	TRUE	3	TRA:CAVQAGNNDMRF TRB:CASSTTGDFNYGYTF	215	CD8-C5
Pla-1_GCAAACTGTTCCACAA	Pla	Pla-1	P1-1	TRUE	3	TRA:CAMSDRMTGGFKTIF TRB:CASSQVGRGGEETQYF	1047	CD8-C7
Pla-1_GCAAACTTCACGATGT	Pla	Pla-1	P1-2	TRUE	3	TRA:CGQNFVF TRB:CASNQRGGPSLYNEQFF	323	CD8-C7
Pla-1_GCAATCAAGGGATCTG	Pla	Pla-1	P1-25	TRUE	3	TRA:CIVGRDSSYKLIF TRB:CASNPQRGAHRDTQYF	58	CD8-C7
Pla-1_GCAATCACATGCCTAA	Pla	Pla-1	P1-2	TRUE	3	TRA:CGQNFVF TRB:CASNQRGGPSLYNEQFF	323	CD8-C7
Pla-1_GCAGTTAAGGGAAACA	Pla	Pla-1	P1-1	TRUE	3	TRA:CAMSDRMTGGFKTIF TRB:CASSQVGRGGEETQYF	1047	CD8-C7
Pla-1_GCATACAGTGCTAGCC	Pla	Pla-1	P1-1	TRUE	3	TRA:CAMSDRMTGGFKTIF TRB:CASSQVGRGGEETQYF	1047	CD8-C7
Pla-1_GCATGCGCAGTAAGCG	Pla	Pla-1	P1-6	TRUE	3	TRA:CAMREGRERDDKIIF TRB:CASSLPNTEAFF	216	CD8-C5

Pla-1_GCGAGAAAGCGTGAGT	Pla	Pla-1	P1-1	TRUE	3	TRA:CAMSDRMTGGFKTIF TRB:CASSQVGRGGEETQYF	1047	CD8-C7
Pla-1_GCGCAACAGCTAGCCC	Pla	Pla-1	P1-1	TRUE	3	TRA:CAMSDRMTGGFKTIF TRB:CASSQVGRGGEETQYF	1047	CD8-C7
Pla-1_GCGCAGTAGTTCGCAT	Pla	Pla-1	P1-5	TRUE	3	TRA:CALSGTPMNRDDKIIF TRB:CSARGRGLAGGPAYEQYF	223	CD8-C7
Pla-1_GCGCCAAAGTCAAGGC	Pla	Pla-1	P1-30	TRUE	3	TRA:CALPPGGNNRKLIW TRB:CASSFGDRGGTEAFF	47	CD4-C6
Pla-1_GCGCGATAGCTGAACG	Pla	Pla-1	P1-8	TRUE	3	TRA:CAVRPPGRNSGGYQKVTF TRB:CASSRRGVTDTQYF	204	CD8-C7
Pla-1_GCGCGATCAGGTCTCG	Pla	Pla-1	P1-1	TRUE	3	TRA:CAMSDRMTGGFKTIF TRB:CASSQVGRGGEETQYF	1047	CD8-C7
Pla-1_GCGGGTTAGCGAGAAA	Pla	Pla-1	P1-1	TRUE	3	TRA:CAMSDRMTGGFKTIF TRB:CASSQVGRGGEETQYF	1047	CD8-C7
Pla-1_GCGGGTTGTGTGCGTC	Pla	Pla-1	P1-10	TRUE	3	TRA:CISNSPMDSSYKLIF TRB:CASSSWGDTDEAFF	162	CD8-C7
Pla-1_GCTCCTAAGCTGAACG	Pla	Pla-1	P1-8	TRUE	3	TRA:CAVRPPGRNSGGYQKVTF TRB:CASSRRGVTDTQYF	204	CD8-C7
Pla-1_GCTGGGTTCTTTACGT	Pla	Pla-1	P1-8	TRUE	3	TRA:CAVRPPGRNSGGYQKVTF TRB:CASSRRGVTDTQYF	204	CD8-C7
Pla-1_GCTTCCAGTCCAACTA	Pla	Pla-1	P1-7	TRUE	3	TRA:CAVQAGNNDMRF TRB:CASSTTGDFNYGYTF	215	CD8-C5
Pla-1_GGAACTTAGACTAGGC	Pla	Pla-1	P1-1	TRUE	3	TRA:CAMSDRMTGGFKTIF TRB:CASSQVGRGGEETQYF	1047	CD8-C7
Pla-1_GGAACTTAGTGTCCCG	Pla	Pla-1	P1-1	TRUE	3	TRA:CAMSDRMTGGFKTIF TRB:CASSQVGRGGEETQYF	1047	CD8-C7
Pla-1_GGAACTTCAACACCCG	Pla	Pla-1	P1-26	TRUE	3	TRA:CAPRSGGSYIPTF TRB:CASSQDLGLDYGYTF	56	CD8-C7
Pla-1_GGAATAAAGGGTTCCC	Pla	Pla-1	P1-1	TRUE	3	TRA:CAMSDRMTGGFKTIF TRB:CASSQVGRGGEETQYF	1047	CD8-C7
Pla-1_GGAATAACAAAGGAAG	Pla	Pla-1	P1-1	TRUE	3	TRA:CAMSDRMTGGFKTIF TRB:CASSQVGRGGEETQYF	1047	CD8-C7
Pla-1_GGACAGAAGGGATCTG	Pla	Pla-1	P1-1	TRUE	3	TRA:CAMSDRMTGGFKTIF TRB:CASSQVGRGGEETQYF	1047	CD8-C7
Pla-1_GGACAGATCTCGCTTG	Pla	Pla-1	P1-14	TRUE	3	TRA:CAASVVNDMRF TRB:CASSHRGGAKNIQYF	109	CD8-C7
Pla-1_GGACGTCCACCATGTA	Pla	Pla-1	P1-1	TRUE	3	TRA:CAMSDRMTGGFKTIF TRB:CASSQVGRGGEETQYF	1047	CD8-C7
Pla-1_GGACGTCCATCGATTG	Pla	Pla-1	P1-8	TRUE	3	TRA:CAVRPPGRNSGGYQKVTF TRB:CASSRRGVTDTQYF	204	CD8-C7
Pla-1_GGAGCAACAGCTGCTG	Pla	Pla-1	P1-1	TRUE	3	TRA:CAMSDRMTGGFKTIF TRB:CASSQVGRGGEETQYF	1047	CD8-C7
Pla-1_GGATGTTCATCCTTGC	Pla	Pla-1	P1-4	TRUE	3	TRA:CAMKGRDDKIIF TRB:CASTKGVSREAFF	226	CD8-C5
Pla-1_GGATGTTGTGCCTTGG	Pla	Pla-1	P1-283	TRUE	2	TRA:CIVRVPNTGNQFYF TRB:CAIRTADMVIGTDTQYF	2	CD8-C7
Pla-1_GGATTACAGAGTGAGA	Pla	Pla-1	P1-6	TRUE	3	TRA:CAMREGRERDDKIIF TRB:CASSLPNTEAFF	216	CD8-C7
Pla-1_GGATTACGTTGTACAC	Pla	Pla-1	P1-17	TRUE	3	TRA:CGTGARGSSGSRLTF TRB:CASSRQGMGAFF	78	CD4-C6
Pla-1_GGCTGGTCATACCATG	Pla	Pla-1	P1-1	TRUE	3	TRA:CAMSDRMTGGFKTIF TRB:CASSQVGRGGEETQYF	1047	CD8-C7
Pla-1_GGCTGGTGTTATTCTC	Pla	Pla-1	P1-28	TRUE	3	TRA:CAVKKGTGRRALTF TRB:CASETGQGTDTQYF	53	CD8-C7

Pla-1_GGGAATGAGATCGATA	Pla	Pla-1	P1-5	TRUE	3	TRA:CALSGTPMNRDDKIIF TRB:CSARGRGLAGGPAYEQYF	223	CD8-C7
Pla-1_GGGAATGAGGCAAAGA	Pla	Pla-1	P1-9	TRUE	3	TRA:CAEAEGGGADGLTF TRB:CATSGGDNEQFF	174	CD8-C7
Pla-1_GGGAATGCAAGACACG	Pla	Pla-1	P1-29	TRUE	3	TRA:CATVSFSGGYNKLIF TRB:CASSLDRRGLSYEQYF	50	CD8-C7
Pla-1_GGGATGACACTAAGTC	Pla	Pla-1	P1-1	TRUE	3	TRA:CAMSDRMTGGFKTIF TRB:CASSQVGRGGEETQYF	1047	CD8-C7
Pla-1_GGGATGACAGGGAGAG	Pla	Pla-1	P1-1	TRUE	3	TRA:CAMSDRMTGGFKTIF TRB:CASSQVGRGGEETQYF	1047	CD8-C7
Pla-1_GGGCACTAGCGTGAAC	Pla	Pla-1	P1-11	TRUE	3	TRA:CAVEPLYGSGNTGKLIF TRB:CASSSHGQLNQPQHF	147	CD8-C7
Pla-1_GGGCACTTCAACGGGA	Pla	Pla-1	P1-26	TRUE	3	TRA:CAPRSGGSYIPTF TRB:CASSQDLGLDYGYTF	56	CD8-C7
Pla-1_GGGCATCTCCACTGGG	Pla	Pla-1	P1-8	TRUE	3	TRA:CAVRPPGRNSGGYQKVTF TRB:CASSRRGVTDTQYF	204	CD8-C7
Pla-1_GGGCATCTCCTTCAAT	Pla	Pla-1	P1-10	TRUE	3	TRA:CISNSPMDSSYKLIF TRB:CASSSWGDTDEAFF	162	CD8-C7
Pla-1_GGGTTGCTCCCAAGAT	Pla	Pla-1	P1-1	TRUE	3	TRA:CAMSDRMTGGFKTIF TRB:CASSQVGRGGEETQYF	1047	CD8-C7
Pla-1_GGTGTTACATGGAATA	Pla	Pla-1	P1-114	TRUE	3	TRA:CAEPENTGNQFYF TRB:CAISLGNEQFF	5	CD8-C7
Pla-1_GTAACGTAGTGGGATC	Pla	Pla-1	P1-13	TRUE	3	TRA:CVVMNYGGSQGNLIF TRB:CASSSWGDTDEAFF	112	CD8-C7
Pla-1_GTACGTAGTAGGAGTC	Pla	Pla-1	P1-42	TRUE	3	TRA:CASFSGGSQGNLIF TRB:CSAGQGSNQPQHF	24	CD4-C6
Pla-1_GTACTCCAGTTTCCTT	Pla	Pla-1	P1-3	TRUE	3	TRA:CAFLSGTYKYIF TRB:CASSQSPGQRNYGYTF	308	CD8-C5
Pla-1_GTAGGCCGTAGGACAC	Pla	Pla-1	P1-5	TRUE	3	TRA:CALSGTPMNRDDKIIF TRB:CSARGRGLAGGPAYEQYF	223	CD8-C7
Pla-1_GTAGGCCTCCATGAAC	Pla	Pla-1	P1-7	TRUE	3	TRA:CAVQAGNNDMRF TRB:CASSTTGDFNYGYTF	215	CD8-C7
Pla-1_GTCACAATCTCCTATA	Pla	Pla-1	P1-1	TRUE	3	TRA:CAMSDRMTGGFKTIF TRB:CASSQVGRGGEETQYF	1047	CD8-C7
Pla-1_GTCACGGCACAGGCCT	Pla	Pla-1	P1-4	TRUE	3	TRA:CAMKGRDDKIIF TRB:CASTKGVSREAFF	226	CD8-C7
Pla-1_GTCATTTGTATATCCG	Pla	Pla-1	P1-1	TRUE	3	TRA:CAMSDRMTGGFKTIF TRB:CASSQVGRGGEETQYF	1047	CD8-C7
Pla-1_GTCGGGTCATGGTCTA	Pla	Pla-1	P1-5	TRUE	3	TRA:CALSGTPMNRDDKIIF TRB:CSARGRGLAGGPAYEQYF	223	CD8-C7
Pla-1_GTGCATAGTGTGCGTC	Pla	Pla-1	P1-9	TRUE	3	TRA:CAEAEGGGADGLTF TRB:CATSGGDNEQFF	174	CD8-C7
Pla-1_GTGCATATCCCTCAGT	Pla	Pla-1	P1-1	TRUE	3	TRA:CAMSDRMTGGFKTIF TRB:CASSQVGRGGEETQYF	1047	CD8-C7
Pla-1_GTGCTTCAGGCGACAT	Pla	Pla-1	P1-1	TRUE	3	TRA:CAMSDRMTGGFKTIF TRB:CASSQVGRGGEETQYF	1047	CD8-C7
Pla-1_GTGCTTCTCAACGAAA	Pla	Pla-1	P1-1	TRUE	3	TRA:CAMSDRMTGGFKTIF TRB:CASSQVGRGGEETQYF	1047	CD8-C7
Pla-1_GTGCTTCTCCTGCAGG	Pla	Pla-1	P1-8	TRUE	3	TRA:CAVRPPGRNSGGYQKVTF TRB:CASSRRGVTDTQYF	204	CD8-C7
Pla-1_GTGTGCGGTGCCTGGT	Pla	Pla-1	P1-14	TRUE	3	TRA:CAASVVNDMRF TRB:CASSHRGGAKNIQYF	109	CD8-C7
Pla-1_GTTACAGGTCTGCAAT	Pla	Pla-1	P1-1	TRUE	3	TRA:CAMSDRMTGGFKTIF TRB:CASSQVGRGGEETQYF	1047	CD8-C7

Pla-1_GTTACAGTCTACTTAC	Pla	Pla-1	P1-24	TRUE	3	TRA:CAVKGNTDKLIF TRB:CASSLRTGAYNEQFF	62	CD8-C6
Pla-1_GTTCATTTCACGCGGT	Pla	Pla-1	P1-2	TRUE	3	TRA:CGQNFVF TRB:CASNQRGGPSLYNEQFF	323	CD8-C7
Pla-1_GTTCGGGAGAGGTTGC	Pla	Pla-1	P1-10	TRUE	3	TRA:CISNSPMDSSYKLIF TRB:CASSSWGDTDEAFF	162	CD8-C7
Pla-1_GTTCTCGTCTCATTCA	Pla	Pla-1	P1-24	TRUE	3	TRA:CAVKGNTDKLIF TRB:CASSLRTGAYNEQFF	62	CD4-C6
Pla-1_TAAACCGCAGCCAGAA	Pla	Pla-1	P1-73	TRUE	3	TRA:CAGI TRB:CASSRTTGGSSYEQYF	8	CD8-C7
Pla-1_TAAGAGAGTACATCCA	Pla	Pla-1	P1-4	TRUE	3	TRA:CAMKGRDDKIIF TRB:CASTKGVSREAFF	226	CD8-C7
Pla-1_TAAGAGATCATACGGT	Pla	Pla-1	P1-22	TRUE	3	TRA:CASNSGYSTLTF TRB:CASSQVTGGARDEQYF	65	CD8-C7
Pla-1_TAAGCGTAGCCAGTAG	Pla	Pla-1	P1-1	TRUE	3	TRA:CAMSDRMTGGFKTIF TRB:CASSQVGRGGEETQYF	1047	CD8-C7
Pla-1_TAAGCGTCAATCTACG	Pla	Pla-1	P1-1	TRUE	3	TRA:CAMSDRMTGGFKTIF TRB:CASSQVGRGGEETQYF	1047	CD8-C7
Pla-1_TAAGTGCAGCACCGTC	Pla	Pla-1	P1-1	TRUE	3	TRA:CAMSDRMTGGFKTIF TRB:CASSQVGRGGEETQYF	1047	CD8-C6
Pla-1_TAAGTGCCAGCAGTTT	Pla	Pla-1	P1-293	TRUE	2	TRA:CAGWYSGGGADGLTF TRB:CATSAGDNEQFF	2	CD8-C7
Pla-1_TACAGTGTCAGGTTCA	Pla	Pla-1	P1-1	TRUE	3	TRA:CAMSDRMTGGFKTIF TRB:CASSQVGRGGEETQYF	1047	CD8-C7
Pla-1_TACCTATTCTATCCCG	Pla	Pla-1	P1-3	TRUE	3	TRA:CAFLSGTYKYIF TRB:CASSQSPGQRNYGYTF	308	CD8-C7
Pla-1_TACGGATCAATTCCTT	Pla	Pla-1	P1-6	TRUE	3	TRA:CAMREGRERDDKIIF TRB:CASSLPNTEAFF	216	CD8-C7
Pla-1_TACTCGCCAGGCTGAA	Pla	Pla-1	P1-26	TRUE	3	TRA:CAPRSGGSYIPTF TRB:CASSQDLGLDYGYTF	56	CD8-C7
Pla-1_TACTTACCATTATCTC	Pla	Pla-1	P1-3	TRUE	3	TRA:CAFLSGTYKYIF TRB:CASSQSPGQRNYGYTF	308	CD8-C7
Pla-1_TACTTACTCTCGAGTA	Pla	Pla-1	P1-2	TRUE	3	TRA:CGQNFVF TRB:CASNQRGGPSLYNEQFF	323	CD8-C7
Pla-1_TAGACCAAGGGATGGG	Pla	Pla-1	P1-6	TRUE	3	TRA:CAMREGRERDDKIIF TRB:CASSLPNTEAFF	216	CD8-C5
Pla-1_TAGCCGGCAATGTAAG	Pla	Pla-1	P1-35	TRUE	3	TRA:CAVSDRGYFGNEKLTF TRB:CSALDDVRPVIAVYNEQFF	40	CD8-C6
Pla-1_TAGCCGGGTCTGCGGT	Pla	Pla-1	P1-6	TRUE	3	TRA:CAMREGRERDDKIIF TRB:CASSLPNTEAFF	216	CD8-C7
Pla-1_TAGGCATTCACTGGGC	Pla	Pla-1	P1-2113	FALSE	1	TRA:CGTGARGSSGSRLTF TRB:CASSQSPGQRNYGYTF	1	CD8-C7
Pla-1_TAGGCATTCCCAGGTG	Pla	Pla-1	P1-22	TRUE	3	TRA:CASNSGYSTLTF TRB:CASSQVTGGARDEQYF	65	CD8-C7
Pla-1_TAGGCATTCTGATTCT	Pla	Pla-1	P1-1	TRUE	3	TRA:CAMSDRMTGGFKTIF TRB:CASSQVGRGGEETQYF	1047	CD8-C7
Pla-1_TATCAGGAGCAGCCTC	Pla	Pla-1	P1-40	TRUE	3	TRA:CAVRDVGNTGFQKLVF TRB:CASSLGQGSMKTQYF	27	CD8-C7
Pla-1_TATGCCCTCCAATGGT	Pla	Pla-1	P1-1	TRUE	3	TRA:CAMSDRMTGGFKTIF TRB:CASSQVGRGGEETQYF	1047	CD8-C7
Pla-1_TATTACCCAGTCTTCC	Pla	Pla-1	P1-16	TRUE	3	TRA:CALSGTPMNRDDKIIF TRB:CASSQSWDLAVLNEQFF	79	CD8-C7
Pla-1_TATTACCGTAAGGGAA	Pla	Pla-1	P1-1	TRUE	3	TRA:CAMSDRMTGGFKTIF TRB:CASSQVGRGGEETQYF	1047	CD8-C7

Pla-1_TCACAAGAGTTCGATC	Pla	Pla-1	P1-8	TRUE	3	TRA:CAVRPPGRNSGGYQKVTF TRB:CASSRRGVTDTQYF	204	CD8-C7
Pla-1_TCAGATGAGCTTATCG	Pla	Pla-1	P1-22	TRUE	3	TRA:CASNSGYSTLTF TRB:CASSQVTGGARDEQYF	65	CD8-C7
Pla-1_TCAGATGTCCGTAGTA	Pla	Pla-1	P1-88	TRUE	3	TRA:CAVRDRGSGNTPLVF TRB:CASSGDRVYEQYF	6	CD8-C7
Pla-1_TCAGCTCAGAAGGCCT	Pla	Pla-1	P1-1	TRUE	3	TRA:CAMSDRMTGGFKTIF TRB:CASSQVGRGGEETQYF	1047	CD8-C7
Pla-1_TCAGGTAAGCACACAG	Pla	Pla-1	P1-1	TRUE	3	TRA:CAMSDRMTGGFKTIF TRB:CASSQVGRGGEETQYF	1047	CD8-C7
Pla-1_TCCCGATCAGACAAGC	Pla	Pla-1	P1-180	TRUE	3	TRA:CAMSDRMTGGFKTIF TRB:CASSQSPGQRNYGYTF	3	CD8-C7
Pla-1_TCGCGTTAGAGCTGCA	Pla	Pla-1	P1-2156	FALSE	1	TRA:CALSEAGYGGSQGNLIF TRB:CASSQSWDLAVLNEQFF	1	CD8-C7
Pla-1_TCGTACCCAAACGTGG	Pla	Pla-1	P1-5	TRUE	3	TRA:CALSGTPMNRDDKIIF TRB:CSARGRGLAGGPAYEQYF	223	CD8-C7
Pla-1_TCTCATAAGACTGTAA	Pla	Pla-1	P1-3	TRUE	3	TRA:CAFLSGTYKYIF TRB:CASSQSPGQRNYGYTF	308	CD8-C7
Pla-1_TCTGGAATCAATCTCT	Pla	Pla-1	P1-43	TRUE	3	TRA:CAASIIQGAQKLVF TRB:CASSFPGQGILEQYF	23	CD8-C2
Pla-1_TCTTCGGCATACTCTT	Pla	Pla-1	P1-8	TRUE	3	TRA:CAVRPPGRNSGGYQKVTF TRB:CASSRRGVTDTQYF	204	CD8-C7
Pla-1_TGAAAGAGTCTAAAGA	Pla	Pla-1	P1-1	TRUE	3	TRA:CAMSDRMTGGFKTIF TRB:CASSQVGRGGEETQYF	1047	CD8-C7
Pla-1_TGACAACAGCCACGCT	Pla	Pla-1	P1-5	TRUE	3	TRA:CALSGTPMNRDDKIIF TRB:CSARGRGLAGGPAYEQYF	223	CD8-C7
Pla-1_TGACGGCCACCAGATT	Pla	Pla-1	P1-1	TRUE	3	TRA:CAMSDRMTGGFKTIF TRB:CASSQVGRGGEETQYF	1047	CD8-C7
Pla-1_TGACTAGGTCCGTGAC	Pla	Pla-1	P1-1	TRUE	3	TRA:CAMSDRMTGGFKTIF TRB:CASSQVGRGGEETQYF	1047	CD8-C7
Pla-1_TGACTTTCAAGCCCAC	Pla	Pla-1	P1-10	TRUE	3	TRA:CISNSPMDSSYKLIF TRB:CASSSWGDTDEAFF	162	CD8-C7
Pla-1_TGACTTTGTTTGGCGC	Pla	Pla-1	P1-14	TRUE	3	TRA:CAASVVNDMRF TRB:CASSHRGGAKNIQYF	109	CD8-C7
Pla-1_TGAGCATCATTTCAGG	Pla	Pla-1	P1-1	TRUE	3	TRA:CAMSDRMTGGFKTIF TRB:CASSQVGRGGEETQYF	1047	CD8-C7
Pla-1_TGAGGGAAGCCAGGAT	Pla	Pla-1	P1-11	TRUE	3	TRA:CAVEPLYGSGNTGKLIF TRB:CASSSHGQLNQPQHF	147	CD8-C7
Pla-1_TGAGGGACAAGTCATC	Pla	Pla-1	P1-1	TRUE	3	TRA:CAMSDRMTGGFKTIF TRB:CASSQVGRGGEETQYF	1047	CD8-C7
Pla-1_TGAGGGACATGGATGG	Pla	Pla-1	P1-1	TRUE	3	TRA:CAMSDRMTGGFKTIF TRB:CASSQVGRGGEETQYF	1047	CD8-C7
Pla-1_TGAGGGATCGTTGCCT	Pla	Pla-1	P1-2	TRUE	3	TRA:CGQNFVF TRB:CASNQRGGPSLYNEQFF	323	CD8-C7
Pla-1_TGATTTCAGTCGATAA	Pla	Pla-1	P1-152	TRUE	3	TRA:CALSEAGKPGRKAYLRT TRB:CASSPGTGSYNEQFF	4	CD8-C5
Pla-1_TGCCAAACAGTCAGAG	Pla	Pla-1	P1-1	TRUE	3	TRA:CAMSDRMTGGFKTIF TRB:CASSQVGRGGEETQYF	1047	CD8-C5
Pla-1_TGCCCATAGCACCGTC	Pla	Pla-1	P1-5	TRUE	3	TRA:CALSGTPMNRDDKIIF TRB:CSARGRGLAGGPAYEQYF	223	CD8-C7
Pla-1_TGCCCATCATCAGTCA	Pla	Pla-1	P1-5	TRUE	3	TRA:CALSGTPMNRDDKIIF TRB:CSARGRGLAGGPAYEQYF	223	CD8-C7
Pla-1_TGCCCTACAGGAATGC	Pla	Pla-1	P1-1	TRUE	3	TRA:CAMSDRMTGGFKTIF TRB:CASSQVGRGGEETQYF	1047	CD8-C7

Pla-1_TGCCCTATCATTTGGG	Pla	Pla-1	P1-35	TRUE	3	TRA:CAVSDRGYFGNEKLTF TRB:CSALDDVRPVIAVYNEQFF	40	CD8-C6
Pla-1_TGCGCAGGTTCCGTCT	Pla	Pla-1	P1-2225	FALSE	1	TRA:CAVQAGNNDMRF TRB:CASSFGDRGGTEAFF	1	CD8-C7
Pla-1_TGCGGGTAGCAGCCTC	Pla	Pla-1	P1-26	TRUE	3	TRA:CAPRSGGSYIPTF TRB:CASSQDLGLDYGYTF	56	CD8-C7
Pla-1_TGCGGGTCAAACTGCT	Pla	Pla-1	P1-5	TRUE	3	TRA:CALSGTPMNRDDKIIF TRB:CSARGRGLAGGPAYEQYF	223	CD8-C7
Pla-1_TGCGGGTCATGGTAGG	Pla	Pla-1	P1-36	TRUE	3	TRA:CILRDNAGNNRKLIW TRB:CASSSRQGFPGGANVLTF	40	CD4-C6
Pla-1_TGCGGGTGTTCCACTC	Pla	Pla-1	P1-5	TRUE	3	TRA:CALSGTPMNRDDKIIF TRB:CSARGRGLAGGPAYEQYF	223	CD8-C6
Pla-1_TGCGTGGAGTGAAGTT	Pla	Pla-1	P1-53	TRUE	3	TRA:CALSERDTDKLIF TRB:CASSILQGVITEAFF	13	CD8-C7
Pla-1_TGCTGCTCAAACCTAC	Pla	Pla-1	P1-17	TRUE	3	TRA:CGTGARGSSGSRLTF TRB:CASSRQGMGAFF	78	CD8-C7
Pla-1_TGCTGCTCAAAGCAAT	Pla	Pla-1	P1-1	TRUE	3	TRA:CAMSDRMTGGFKTIF TRB:CASSQVGRGGEETQYF	1047	CD8-C7
Pla-1_TGGACGCAGGAATGGA	Pla	Pla-1	P1-30	TRUE	3	TRA:CALPPGGNNRKLIW TRB:CASSFGDRGGTEAFF	47	CD8-C7
Pla-1_TGGCTGGAGCTGCCCA	Pla	Pla-1	P1-1	TRUE	3	TRA:CAMSDRMTGGFKTIF TRB:CASSQVGRGGEETQYF	1047	CD8-C7
Pla-1_TGGCTGGGTACGCTGC	Pla	Pla-1	P1-9	TRUE	3	TRA:CAEAEGGGADGLTF TRB:CATSGGDNEQFF	174	CD8-C7
Pla-1_TGGGAAGGTGTGTGCC	Pla	Pla-1	P1-3	TRUE	3	TRA:CAFLSGTYKYIF TRB:CASSQSPGQRNYGYTF	308	CD8-C7
Pla-1_TGGGCGTTCCTAGAAC	Pla	Pla-1	P1-8	TRUE	3	TRA:CAVRPPGRNSGGYQKVTF TRB:CASSRRGVTDTQYF	204	CD8-C7
Pla-1_TGGTTCCAGCGATATA	Pla	Pla-1	P1-3	TRUE	3	TRA:CAFLSGTYKYIF TRB:CASSQSPGQRNYGYTF	308	CD8-C6
Pla-1_TGTATTCAGGGTCGAT	Pla	Pla-1	P1-13	TRUE	3	TRA:CVVMNYGGSQGNLIF TRB:CASSSWGDTDEAFF	112	CD8-C7
Pla-1_TGTATTCGTCCAGTGC	Pla	Pla-1	P1-1	TRUE	3	TRA:CAMSDRMTGGFKTIF TRB:CASSQVGRGGEETQYF	1047	CD8-C7
Pla-1_TGTCCCAAGCTCCTCT	Pla	Pla-1	P1-3	TRUE	3	TRA:CAFLSGTYKYIF TRB:CASSQSPGQRNYGYTF	308	CD8-C6
Pla-1_TGTGGTAGTAGCTCCG	Pla	Pla-1	P1-47	TRUE	3	TRA:CIVGGNFNKFYF TRB:CASSPEWDTDTQYF	18	CD8-C5
Pla-1_TTAGGACAGACTGTAA	Pla	Pla-1	P1-1	TRUE	3	TRA:CAMSDRMTGGFKTIF TRB:CASSQVGRGGEETQYF	1047	CD8-C7
Pla-1_TTCGGTCTCATGCAAC	Pla	Pla-1	P1-1	TRUE	3	TRA:CAMSDRMTGGFKTIF TRB:CASSQVGRGGEETQYF	1047	CD8-C7
Pla-1_TTCTCAACATGGTCTA	Pla	Pla-1	P1-73	TRUE	3	TRA:CAGI TRB:CASSRTTGGSSYEQYF	8	CD8-C7
Pla-1_TTCTCCTAGGATGCGT	Pla	Pla-1	P1-10	TRUE	3	TRA:CISNSPMDSSYKLIF TRB:CASSSWGDTDEAFF	162	CD8-C7
Pla-1_TTGTAGGCAGGCAGTA	Pla	Pla-1	P1-10	TRUE	3	TRA:CISNSPMDSSYKLIF TRB:CASSSWGDTDEAFF	162	CD8-C7
Pla-1_TTGTAGGGTCAACTGT	Pla	Pla-1	P1-1	TRUE	3	TRA:CAMSDRMTGGFKTIF TRB:CASSQVGRGGEETQYF	1047	CD8-C7
Pla-1_TTTACTGCAGTAACGG	Pla	Pla-1	P1-1	TRUE	3	TRA:CAMSDRMTGGFKTIF TRB:CASSQVGRGGEETQYF	1047	CD8-C7
Pla-1_AAACCTGAGACGCACA	Pla	Pla-1	P1-7	TRUE	3	TRA:CAVQAGNNDMRF TRB:CASSTTGDFNYGYTF	215	CD8-C5

Pla-1_AAACCTGCAGGGCATA	Pla	Pla-1	P1-120	FALSE	3	TRA:CAYRSSYSGYALNF TRB:CAWSPYSEKLFF	5	CD8-C2
Pla-1_AAACCTGTCACCTCGT	Pla	Pla-1	P1-97	FALSE	3	TRA:CAVRTAGNKLTF TRB:CSAQGRSSYEQYF	6	CD8-C4
Pla-1_AAACGGGGTCCTCTTG	Pla	Pla-1	P1-143	TRUE	3	TRA:CAEGNQAGTALIF TRB:CASSTIGQGIPGANVLTF	4	CD8-C5
Pla-1_AAAGCAATCACAACGT	Pla	Pla-1	P1-20	TRUE	3	TRA:CVVVGGRALTF TRB:CASSLATNPHETQYF	69	CD8-C4
Pla-1_AAATGCCGTTTGTTGG	Pla	Pla-1	P1-305	FALSE	2	TRA:CALRVVTQGGSEKLVF TRB:CASSLFPRATGELFF	2	CD8-C2
Pla-1_AACACGTAGTAAGTAC	Pla	Pla-1	P1-6	TRUE	3	TRA:CAMREGRERDDKIIF TRB:CASSLPNTEAFF	216	CD8-C3
Pla-1_AACACGTGTAGCTTGT	Pla	Pla-1	P1-1093	FALSE	1	TRA:CVVMNYGGSQGNLIF TRB:CSARVPGQMKETQYF	1	CD8-C7
Pla-1_AACTCCCCACCACGTG	Pla	Pla-1	P1-37	TRUE	3	TRA:CAVNIVGNQFYF TRB:CASSPLGTEAFF	40	CD8-C5
Pla-1_AACTGGTGTCCCGACA	Pla	Pla-1	P1-310	FALSE	2	TRA:CALIRSAGNNRKLIW TRB:CASRGQGNQPQHF	2	CD8-C3
Pla-1_AACTGGTTCACCTTAT	Pla	Pla-1	P1-1108	FALSE	1	TRA:CAVTPREDYKLSF TRB:CSVVPGLAKNIQYF	1	CD8-C5
Pla-1_AACTTTCTCCGTCATC	Pla	Pla-1	P1-206	FALSE	3	TRA:CALTLPRAAGNKLTF TRB:CASRGQGNQPQHF	3	CD8-C4
Pla-1_AAGGTTCGTGACGCCT	Pla	Pla-1	P1-1124	FALSE	1	TRA:CAAPARGFGNVLHC TRB:CASSFRDTGELFF	1	CD8-C4
Pla-1_AAGGTTCGTTAAAGTG	Pla	Pla-1	P1-37	TRUE	3	TRA:CAVNIVGNQFYF TRB:CASSPLGTEAFF	40	CD8-C4
Pla-1_AAGTCTGAGTGAATTG	Pla	Pla-1	P1-56	TRUE	3	TRA:CVVNPWGSGTYKYIF TRB:CASSRDITDTQYF	13	CD8-C4
Pla-1_AAGTCTGAGTGGTAAT	Pla	Pla-1	P1-70	FALSE	3	TRA:CAMRETPGEAGTALIF TRB:CSARITYLNTEAFF	9	CD8-C5
Pla-1_AATCCAGTCAAAGTAG	Pla	Pla-1	P1-6	TRUE	3	TRA:CAMREGRERDDKIIF TRB:CASSLPNTEAFF	216	CD8-C4
Pla-1_AATCGGTAGTGGTAGC	Pla	Pla-1	P1-154	TRUE	3	TRA:CAGEKSSGDKLTF TRB:CSARGTGLNYGYTF	4	CD8-C4
Pla-1_AATCGGTTCGAACTGT	Pla	Pla-1	P1-20	TRUE	3	TRA:CVVVGGRALTF TRB:CASSLATNPHETQYF	69	CD8-C5
Pla-1_ACACCAAAGGTGTTAA	Pla	Pla-1	P1-36	TRUE	3	TRA:CILRDNAGNNRKLIW TRB:CASSSRQGFPGGANVLTF	40	CD8-C3
Pla-1_ACACCCTCACCGAAAG	Pla	Pla-1	P1-210	FALSE	3	TRA:CAYRRLGNTPLVF TRB:CASSPLDGHSNQPQHF	3	CD8-C5
Pla-1_ACACCGGAGAGGTAGA	Pla	Pla-1	P1-7	TRUE	3	TRA:CAVQAGNNDMRF TRB:CASSTTGDFNYGYTF	215	CD8-C5
Pla-1_ACAGCCGTCATGTGGT	Pla	Pla-1	P1-33	TRUE	3	TRA:CLVGTGGFKTIF TRB:CSARVPGQMKETQYF	41	CD8-C5
Pla-1_ACATACGCATGTAGTC	Pla	Pla-1	P1-1158	FALSE	1	TRA:CAAIPIRDMRF TRB:CASSLGTGGFEQYF	1	CD8-C4
Pla-1_ACATACGTCCGAATGT	Pla	Pla-1	P1-1160	FALSE	1	TRA:CAFNTGTASKLTF TRB:CASSAHPGSQRGNTIYF	1	CD8-C4
Pla-1_ACATCAGAGACTTGAA	Pla	Pla-1	P1-44	TRUE	3	TRA:CAYRGLGAQKLVF TRB:CASSSLDGQDNHYGYTF	22	CD8-C5
Pla-1_ACATCAGTCGTTACGA	Pla	Pla-1	P1-13	TRUE	3	TRA:CVVMNYGGSQGNLIF TRB:CASSSWGDTDEAFF	112	CD8-C5
Pla-1_ACATGGTCAGATGAGC	Pla	Pla-1	P1-60	FALSE	3	TRA:CAYLSSTGTASKLTF TRB:CASSSLDGQDNHYGYTF	11	CD8-C5

Pla-1_ACCAGTACATTGGTAC	Pla	Pla-1	P1-1164	FALSE	1	TRA:CAVLMDSNYQLIW TRB:CASSEDGMNTEAFF	1	CD8-C5
Pla-1_ACCGTAACATCAGTCA	Pla	Pla-1	P1-45	TRUE	3	TRA:CAYRPLGAQKLVF TRB:CASSTLDGRESSYEQYF	20	CD8-C5
Pla-1_ACCTTTATCATAAAGG	Pla	Pla-1	P1-32	FALSE	3	TRA:CAVMNTGFQKLVF TRB:CSVSGLVLGGNEQFF	46	CD8-C4
Pla-1_ACGATACCATGTAGTC	Pla	Pla-1	P1-1185	FALSE	1	TRA:CAEPTNTGNQFYF TRB:CAISVGNEQFF	1	CD8-C5
Pla-1_ACGATGTTCCTATGTT	Pla	Pla-1	P1-1189	FALSE	1	TRA:CVVNIPWNNNARLMF TRB:CSVRLTVSYEQYF	1	CD8-C4
Pla-1_ACGCCAGCACTGCCAG	Pla	Pla-1	P1-1193	FALSE	1	TRA:CAFMKHVASGGSNYKLTF TRB:CAWKGSGNTIYF	1	CD8-C5
Pla-1_ACGCCGACATCCCACT	Pla	Pla-1	P1-99	FALSE	3	TRA:CIVRLLSTASKLTF TRB:CASSELQGKNTEAFF	6	CD4-C5
Pla-1_ACGGAGAGTATAGTAG	Pla	Pla-1	P1-7	TRUE	3	TRA:CAVQAGNNDMRF TRB:CASSTTGDFNYGYTF	215	CD8-C5
Pla-1_ACGGCCAAGCTCCTCT	Pla	Pla-1	P1-81	FALSE	3	TRA:CAVTFAGNQFYF TRB:CSAGTGTSGLSEQFF	7	CD8-C4
Pla-1_ACGGGCTAGAATAGGG	Pla	Pla-1	P1-1203	FALSE	1	TRA:CAVNIRRLTGGGNKLTF TRB:CASSPPQGSNTEAFF	1	CD8-C5
Pla-1_ACGGGCTCAATTGCTG	Pla	Pla-1	P1-45	TRUE	3	TRA:CAYRPLGAQKLVF TRB:CASSTLDGRESSYEQYF	20	CD8-C5
Pla-1_ACGGGCTTCCGTTGCT	Pla	Pla-1	P1-45	TRUE	3	TRA:CAYRPLGAQKLVF TRB:CASSTLDGRESSYEQYF	20	CD8-C3
Pla-1_ACGGGTCAGTAGCGGT	Pla	Pla-1	P1-126	FALSE	3	TRA:CAVGSRPGGGYNKLIF TRB:CASGQGSYGELFF	5	CD8-C4
Pla-1_ACGTCAACACTATCTT	Pla	Pla-1	P1-323	FALSE	2	TRA:CAVWHTDKLIF TRB:CASSLVGGVGTDTQYF	2	CD8-C4
Pla-1_ACTGAGTCATGTTCCC	Pla	Pla-1	P1-23	TRUE	3	TRA:CALRAITQGGSEKLVF TRB:CASSLTGAVYGYTF	65	CD8-C3
Pla-1_ACTGCTCCAGTATGCT	Pla	Pla-1	P1-1223	FALSE	1	TRA:CVVILMDSNYQLIW TRB:CSARGTGSYNEQFF	1	CD8-C5
Pla-1_ACTGCTCTCACCAGGC	Pla	Pla-1	P1-1225	FALSE	1	TRA:CALSSDSNYQLIW TRB:CASRREEPNTGELFF	1	CD8-C4
Pla-1_ACTGTCCCAGACGCAA	Pla	Pla-1	P1-1229	FALSE	1	TRA:CAVSWAAGNKLTF TRB:CASSPGQGNTEAFF	1	CD8-C4
Pla-1_ACTGTCCCATGTCCTC	Pla	Pla-1	P1-9	TRUE	3	TRA:CAEAEGGGADGLTF TRB:CATSGGDNEQFF	174	CD8-C5
Pla-1_ACTGTCCCATTACGAC	Pla	Pla-1	P1-1232	FALSE	1	TRA:CAVSRDDKIIF TRB:CASSSRHPETQYF	1	CD8-C4
Pla-1_ACTTACTAGGAACTGC	Pla	Pla-1	P1-1233	FALSE	1	TRA:CAMSDAGKSTF TRB:CASSDTTGANTGELFF	1	CD8-C2
Pla-1_ACTTACTAGGTAGCTG	Pla	Pla-1	P1-7	TRUE	3	TRA:CAVQAGNNDMRF TRB:CASSTTGDFNYGYTF	215	CD8-C5
Pla-1_ACTTTCACAGTCCTTC	Pla	Pla-1	P1-12	TRUE	3	TRA:CALSDERGDYKLSF TRB:CASSPGTGSYNEQFF	141	CD8-C5
Pla-1_AGACGTTCATCGTCGG	Pla	Pla-1	P1-81	FALSE	3	TRA:CAVTFAGNQFYF TRB:CSAGTGTSGLSEQFF	7	CD8-C4
Pla-1_AGAGCTTAGCTGAAAT	Pla	Pla-1	P1-1246	FALSE	1	TRA:CAYRSPVNRDDKIIF TRB:CASSLYSGRNEQFF	1	CD8-C3
Pla-1_AGAGCTTGTGACGGTA	Pla	Pla-1	P1-23	TRUE	3	TRA:CALRAITQGGSEKLVF TRB:CASSLTGAVYGYTF	65	CD8-C5
Pla-1_AGAGCTTGTTCGAATC	Pla	Pla-1	P1-330	FALSE	2	TRA:CAVNTWRSDKLSF TRB:CAWTPTGTGDHGYTF	2	CD8-C4

Pla-1_AGAGTGGAGTGACATA	Pla	Pla-1	P1-1249	FALSE	1	TRA:CAMRRDSNYQLIW TRB:CASSGRDSNYNEQFF	1	CD8-C3
Pla-1_AGAGTGGGTTCGTTGA	Pla	Pla-1	P1-45	TRUE	3	TRA:CAYRPLGAQKLVF TRB:CASSTLDGRESSYEQYF	20	CD8-C5
Pla-1_AGATCTGCACGTCTCT	Pla	Pla-1	P1-20	TRUE	3	TRA:CVVVGGRALTF TRB:CASSLATNPHETQYF	69	CD8-C4
Pla-1_AGATTGCGTCCAAGTT	Pla	Pla-1	P1-20	TRUE	3	TRA:CVVVGGRALTF TRB:CASSLATNPHETQYF	69	CD8-C4
Pla-1_AGCAGCCCATCACGTA	Pla	Pla-1	P1-12	TRUE	3	TRA:CALSDERGDYKLSF TRB:CASSPGTGSYNEQFF	141	CD8-C5
Pla-1_AGCATACAGAGTAATC	Pla	Pla-1	P1-1265	FALSE	1	TRA:CAETAMDSSYKLIF TRB:CASSPTYGTVATNEKLFF	1	CD8-C4
Pla-1_AGCCTAAAGTGATCGG	Pla	Pla-1	P1-7	TRUE	3	TRA:CAVQAGNNDMRF TRB:CASSTTGDFNYGYTF	215	CD8-C5
Pla-1_AGCGGTCGTCATTAGC	Pla	Pla-1	P1-1268	FALSE	1	TRA:CAVRDTNSGYSTLTF TRB:CASSTPLSGANVLTF	1	CD8-C4
Pla-1_AGCGTATAGTAGTGCG	Pla	Pla-1	P1-1271	FALSE	1	TRA:CAMRGSQVELGGSNYKLTF TRB:CASSPPRASTDTQYF	1	CD8-C5
Pla-1_AGGGAGTGTTAAGAAC	Pla	Pla-1	P1-1284	FALSE	1	TRA:CAYPGANSKLTF TRB:CASSLANFGGVAGANVLTF	1	CD8-C4
Pla-1_AGGTCATAGTAGCGGT	Pla	Pla-1	P1-206	FALSE	3	TRA:CALTLPRAAGNKLTF TRB:CASRGQGNQPQHF	3	CD8-C4
Pla-1_AGGTCATCAACTGGCC	Pla	Pla-1	P1-44	TRUE	3	TRA:CAYRGLGAQKLVF TRB:CASSSLDGQDNHYGYTF	22	CD8-C4
Pla-1_AGGTCATCAGTTCATG	Pla	Pla-1	P1-23	TRUE	3	TRA:CALRAITQGGSEKLVF TRB:CASSLTGAVYGYTF	65	CD8-C4
Pla-1_AGGTCATGTTGGTAAA	Pla	Pla-1	P1-68	TRUE	3	TRA:CAVIPDILTGGGNKLTF TRB:CASSLQGFGNTIYF	9	CD8-C4
Pla-1_AGTAGTCGTCATGCCG	Pla	Pla-1	P1-340	FALSE	2	TRA:CAMSVESGANNLFF TRB:CASSLVLQGSGNTIYF	2	CD8-C5
Pla-1_AGTCTTTGTCCAACTA	Pla	Pla-1	P1-37	TRUE	3	TRA:CAVNIVGNQFYF TRB:CASSPLGTEAFF	40	CD8-C4
Pla-1_AGTCTTTTCTACTTAC	Pla	Pla-1	P1-23	TRUE	3	TRA:CALRAITQGGSEKLVF TRB:CASSLTGAVYGYTF	65	CD8-C4
Pla-1_AGTGGGACACGGCGTT	Pla	Pla-1	P1-223	FALSE	3	TRA:CGTNQAGTALIF TRB:CASSLSRNYNSPLHF	3	CD8-C3
Pla-1_AGTTGGTAGAAACCTA	Pla	Pla-1	P1-12	TRUE	3	TRA:CALSDERGDYKLSF TRB:CASSPGTGSYNEQFF	141	CD8-C4
Pla-1_AGTTGGTTCATCATTC	Pla	Pla-1	P1-52	TRUE	3	TRA:CAETPDYKLSF TRB:CASRDLYEQYF	13	CD8-C4
Pla-1_ATAACGCTCCTCGCAT	Pla	Pla-1	P1-6	TRUE	3	TRA:CAMREGRERDDKIIF TRB:CASSLPNTEAFF	216	CD8-C5
Pla-1_ATAAGAGCATTCGACA	Pla	Pla-1	P1-343	FALSE	2	TRA:CAVPPGNSGYALNF TRB:CASSEGGSSTDTQYF	2	CD8-C4
Pla-1_ATAAGAGTCTTCGAGA	Pla	Pla-1	P1-1315	FALSE	1	TRA:CIAQSTMDSNYQLIW TRB:CASSQEPGSNQPQHF	1	CD8-C5
Pla-1_ATAGACCGTTGTCGCG	Pla	Pla-1	P1-55	TRUE	3	TRA:CALSPQDSNYQLIW TRB:CASSFWTSANEQFF	13	CD8-C5
Pla-1_ATAGACCTCAACACAC	Pla	Pla-1	P1-12	TRUE	3	TRA:CALSDERGDYKLSF TRB:CASSPGTGSYNEQFF	141	CD8-C5
Pla-1_ATCATGGAGCCAACAG	Pla	Pla-1	P1-139	TRUE	3	TRA:CGTERDQFYF TRB:CATSRELFGEKLFF	4	CD8-C2
Pla-1_ATCATGGGTACCAGTT	Pla	Pla-1	P1-20	TRUE	3	TRA:CVVVGGRALTF TRB:CASSLATNPHETQYF	69	CD8-C5

Pla-1_ATCATGGGTCGACTAT	Pla	Pla-1	P1-20	TRUE	3	TRA:CVVVGGRALTF TRB:CASSLATNPHETQYF	69	CD8-C4
Pla-1_ATCATGGTCAAGGTAA	Pla	Pla-1	P1-92	TRUE	3	TRA:CIVQLTGTASKLTF TRB:CASSFKSGRTNEQFF	6	CD4-C5
Pla-1_ATCCACCAGCTGAACG	Pla	Pla-1	P1-12	TRUE	3	TRA:CALSDERGDYKLSF TRB:CASSPGTGSYNEQFF	141	CD8-C5
Pla-1_ATCGAGTTCTAACTGG	Pla	Pla-1	P1-46	FALSE	3	TRA:CAVRGTGTASKLTF TRB:CSVRQGGNEQFF	20	CD8-C4
Pla-1_ATCTGCCAGACTAGAT	Pla	Pla-1	P1-1338	FALSE	1	TRA:CALSDRNNARLMF TRB:CASSIRTDGNTIYF	1	CD8-C4
Pla-1_ATGCGATCACCATCCT	Pla	Pla-1	P1-23	TRUE	3	TRA:CALRAITQGGSEKLVF TRB:CASSLTGAVYGYTF	65	CD8-C5
Pla-1_ATGGGAGAGACAAAGG	Pla	Pla-1	P1-86	TRUE	3	TRA:CAVGGRSNTGRRALTF TRB:CAISLTGDGKETQYF	6	CD8-C5
Pla-1_ATGGGAGAGCCCAACC	Pla	Pla-1	P1-1347	FALSE	1	TRA:CAVRSSSNDYKLSF TRB:CASKRAGYNEQFF	1	CD8-C4
Pla-1_ATGGGAGGTAGCTAAA	Pla	Pla-1	P1-1348	FALSE	1	TRA:CAASRRRTSYDKVIF TRB:CSAIPGTGARNQPQHF	1	CD8-C4
Pla-1_ATGTGTGGTACGACCC	Pla	Pla-1	P1-7	TRUE	3	TRA:CAVQAGNNDMRF TRB:CASSTTGDFNYGYTF	215	CD8-C5
Pla-1_ATGTGTGTCAACACTG	Pla	Pla-1	P1-82	FALSE	3	TRA:CAASEDSNYQLIW TRB:CASSFGDSSSETQYF	7	CD8-C4
Pla-1_ATTACTCAGCAAATCA	Pla	Pla-1	P1-166	FALSE	3	TRA:CAVDGYNKLIF TRB:CASRPDLDRVQETQYF	4	CD8-C4
Pla-1_ATTATCCTCGGTCTAA	Pla	Pla-1	P1-37	TRUE	3	TRA:CAVNIVGNQFYF TRB:CASSPLGTEAFF	40	CD8-C4
Pla-1_ATTGGTGCAAGCGAGT	Pla	Pla-1	P1-1358	FALSE	1	TRA:CATAPTYGNNRLAF TRB:CASSSDRGYNEQFF	1	CD8-C4
Pla-1_ATTTCTGTCCTTTACA	Pla	Pla-1	P1-45	TRUE	3	TRA:CAYRPLGAQKLVF TRB:CASSTLDGRESSYEQYF	20	CD8-C4
Pla-1_CAACCAAAGTACCGGA	Pla	Pla-1	P1-110	TRUE	3	TRA:CAVAPGTGRRALTF TRB:CSARDRTAQETQYF	5	CD8-C5
Pla-1_CAACCTCAGATATACG	Pla	Pla-1	P1-9	TRUE	3	TRA:CAEAEGGGADGLTF TRB:CATSGGDNEQFF	174	CD8-C5
Pla-1_CAACCTCAGATGAGAG	Pla	Pla-1	P1-23	TRUE	3	TRA:CALRAITQGGSEKLVF TRB:CASSLTGAVYGYTF	65	CD8-C5
Pla-1_CAACCTCAGCTACCGC	Pla	Pla-1	P1-75	FALSE	3	TRA:CAMRGGNFNKFYF TRB:CSAQGAYEQYF	8	CD8-C4
Pla-1_CAACCTCTCTTGAGAC	Pla	Pla-1	P1-55	TRUE	3	TRA:CALSPQDSNYQLIW TRB:CASSFWTSANEQFF	13	CD8-C5
Pla-1_CAACTAGCACCGGAAA	Pla	Pla-1	P1-353	FALSE	2	TRA:CAAFTKAAGNKLTF TRB:CASNPGGATYNEQFF	2	CD8-C5
Pla-1_CAAGAAAGTCCTCTTG	Pla	Pla-1	P1-57	FALSE	3	TRA:CAMREGGNTGNQFYF TRB:CASKKDRGTTQAKNIQYF	13	CD8-C5
Pla-1_CAAGAAAGTGTAAGTA	Pla	Pla-1	P1-206	FALSE	3	TRA:CALTLPRAAGNKLTF TRB:CASRGQGNQPQHF	3	CD8-C4
Pla-1_CAAGATCCAATGGAGC	Pla	Pla-1	P1-1373	FALSE	1	TRA:CAVETLRGYNNARLMF TRB:CASSPGTGSYNEQFF	1	CD8-C6
Pla-1_CAAGATCTCGCACTCT	Pla	Pla-1	P1-53	TRUE	3	TRA:CALSERDTDKLIF TRB:CASSILQGVITEAFF	13	CD8-C5
Pla-1_CAAGGCCTCCACGTGG	Pla	Pla-1	P1-1378	FALSE	1	TRA:CAGPPRPGSARQLTF TRB:CASSPEGHRTYEQYF	1	CD4-C5
Pla-1_CAAGTTGAGCTAACAA	Pla	Pla-1	P1-23	TRUE	3	TRA:CALRAITQGGSEKLVF TRB:CASSLTGAVYGYTF	65	CD8-C4

Pla-1_CACAAACTCACATAGC	Pla	Pla-1	P1-356	FALSE	2	TRA:CAVSPTGTASKLTF TRB:CASSEGTAHYQPQHF	2	CD8-C4
Pla-1_CACACTCAGCAGACTG	Pla	Pla-1	P1-60	FALSE	3	TRA:CAYLSSTGTASKLTF TRB:CASSSLDGQDNHYGYTF	11	CD8-C5
Pla-1_CACAGGCAGTGACTCT	Pla	Pla-1	P1-83	FALSE	3	TRA:CAVRDIENTGFQKLVF TRB:CASTSGGFNQPQHF	7	CD8-C5
Pla-1_CACAGTACAGCAGTTT	Pla	Pla-1	P1-227	FALSE	3	TRA:CAVVNAGKSTF TRB:CASSLTGLVSNSPLHF	3	CD8-C3
Pla-1_CACAGTAGTGAAGGCT	Pla	Pla-1	P1-282	TRUE	2	TRA:CAEPRNTGNQFYF TRB:CAISVGNEQFF	2	CD8-C5
Pla-1_CACATAGGTAAAGGAG	Pla	Pla-1	P1-7	TRUE	3	TRA:CAVQAGNNDMRF TRB:CASSTTGDFNYGYTF	215	CD8-C5
Pla-1_CACATAGGTCCGAATT	Pla	Pla-1	P1-1398	FALSE	1	TRA:CAVSDLNNNDMRF TRB:CASSLALPGQGYNEQFF	1	CD8-C5
Pla-1_CACATTTCAAGGCTCC	Pla	Pla-1	P1-37	TRUE	3	TRA:CAVNIVGNQFYF TRB:CASSPLGTEAFF	40	CD8-C4
Pla-1_CACCAGGGTATAGTAG	Pla	Pla-1	P1-4	TRUE	3	TRA:CAMKGRDDKIIF TRB:CASTKGVSREAFF	226	CD8-C2
Pla-1_CAGAATCGTAGCGCTC	Pla	Pla-1	P1-363	FALSE	2	TRA:CGADDGNQFYF TRB:CSAYLRQGGDTQYF	2	CD8-C5
Pla-1_CAGCATACACGCTTTC	Pla	Pla-1	P1-5	TRUE	3	TRA:CALSGTPMNRDDKIIF TRB:CSARGRGLAGGPAYEQYF	223	CD8-C5
Pla-1_CAGCCGACAAAGGCGT	Pla	Pla-1	P1-37	TRUE	3	TRA:CAVNIVGNQFYF TRB:CASSPLGTEAFF	40	CD8-C4
Pla-1_CAGCCGATCAGCAACT	Pla	Pla-1	P1-154	TRUE	3	TRA:CAGEKSSGDKLTF TRB:CSARGTGLNYGYTF	4	CD8-C4
Pla-1_CAGCGACTCCCACTTG	Pla	Pla-1	P1-171	TRUE	3	TRA:CAGQRRANSKLTF TRB:CASSQTTGREAKNIQYF	3	CD8-C5
Pla-1_CAGTCCTGTAGGGTAC	Pla	Pla-1	P1-40	TRUE	3	TRA:CAVRDVGNTGFQKLVF TRB:CASSLGQGSMKTQYF	27	CD8-C2
Pla-1_CATATGGCAAGTACCT	Pla	Pla-1	P1-225	FALSE	3	TRA:CAARYGGSQGNLIF TRB:CASSGLRFSGANVLTF	3	CD8-C4
Pla-1_CATATGGGTGTCGCTG	Pla	Pla-1	P1-1437	FALSE	1	TRA:CAEDSSYKLIF TRB:CSARGTGSYNEQFF	1	CD8-C5
Pla-1_CATCAAGTCGAACTGT	Pla	Pla-1	P1-167	FALSE	3	TRA:CGTGINYGQNFVF TRB:CASSPRDGGGTEAFF	4	CD8-C4
Pla-1_CATCAGACAATAGCAA	Pla	Pla-1	P1-60	FALSE	3	TRA:CAYLSSTGTASKLTF TRB:CASSSLDGQDNHYGYTF	11	CD8-C4
Pla-1_CATCAGAGTCTACCTC	Pla	Pla-1	P1-1440	FALSE	1	TRA:CATRMDSSYKLIF TRB:CASSQDGGRVLGEKLFF	1	CD8-C5
Pla-1_CATCGAACAGTCTTCC	Pla	Pla-1	P1-55	TRUE	3	TRA:CALSPQDSNYQLIW TRB:CASSFWTSANEQFF	13	CD8-C5
Pla-1_CATCGAATCCGAAGAG	Pla	Pla-1	P1-32	FALSE	3	TRA:CAVMNTGFQKLVF TRB:CSVSGLVLGGNEQFF	46	CD8-C4
Pla-1_CATCGGGGTTGTTTGG	Pla	Pla-1	P1-1449	FALSE	1	TRA:CAAKGSQGNLIF TRB:CASSLDGYGKSSYEQYF	1	CD8-C4
Pla-1_CATGACAAGAGCCTAG	Pla	Pla-1	P1-1454	FALSE	1	TRA:CADFLFSNDYKLSF TRB:CASSQSSNSPLHF	1	CD8-C4
Pla-1_CATGACATCTTATCTG	Pla	Pla-1	P1-1457	FALSE	1	TRA:CAPNNNARLMF TRB:CASSSGTAHYNEQFF	1	CD8-C5
Pla-1_CATGCCTCAGATCGGA	Pla	Pla-1	P1-1458	FALSE	1	TRA:CILSGEDGGSQGNLIF TRB:CASSEVGFGDTQYF	1	CD8-C5
Pla-1_CATGCCTGTTCCTCCA	Pla	Pla-1	P1-1459	FALSE	1	TRA:CVGGGADGLTF TRB:CASSLTSGRGNIQYF	1	CD8-C4

Pla-1_CATGGCGCACATGACT	Pla	Pla-1	P1-110	TRUE	3	TRA:CAVAPGTGRRALTF TRB:CSARDRTAQETQYF	5	CD8-C5
Pla-1_CATTATCCAAGCGAGT	Pla	Pla-1	P1-5	TRUE	3	TRA:CALSGTPMNRDDKIIF TRB:CSARGRGLAGGPAYEQYF	223	CD8-C5
Pla-1_CATTATCCACGGTGTC	Pla	Pla-1	P1-235	FALSE	3	TRA:CAATSTGTYKYIF TRB:CASSFGGRNEQYF	3	CD8-C3
Pla-1_CATTATCGTGTGGTTT	Pla	Pla-1	P1-7	TRUE	3	TRA:CAVQAGNNDMRF TRB:CASSTTGDFNYGYTF	215	CD8-C5
Pla-1_CCACCTAAGACTGTAA	Pla	Pla-1	P1-1473	FALSE	1	TRA:CAVQAFGALIF TRB:CASSVGGSNQPQHF	1	CD8-C5
Pla-1_CCACCTAGTCTCATCC	Pla	Pla-1	P1-3	TRUE	3	TRA:CAFLSGTYKYIF TRB:CASSQSPGQRNYGYTF	308	CD8-C2
Pla-1_CCAGCGAAGTTAAGTG	Pla	Pla-1	P1-225	FALSE	3	TRA:CAARYGGSQGNLIF TRB:CASSGLRFSGANVLTF	3	CD8-C4
Pla-1_CCATGTCCACCGAAAG	Pla	Pla-1	P1-7	TRUE	3	TRA:CAVQAGNNDMRF TRB:CASSTTGDFNYGYTF	215	CD8-C3
Pla-1_CCATTCGAGCTGAACG	Pla	Pla-1	P1-6	TRUE	3	TRA:CAMREGRERDDKIIF TRB:CASSLPNTEAFF	216	CD8-C5
Pla-1_CCCAATCGTTTGTTGG	Pla	Pla-1	P1-9	TRUE	3	TRA:CAEAEGGGADGLTF TRB:CATSGGDNEQFF	174	CD8-C7
Pla-1_CCCAGTTCAATAGCGG	Pla	Pla-1	P1-6	TRUE	3	TRA:CAMREGRERDDKIIF TRB:CASSLPNTEAFF	216	CD8-C5
Pla-1_CCCAGTTCACGCGAAA	Pla	Pla-1	P1-20	TRUE	3	TRA:CVVVGGRALTF TRB:CASSLATNPHETQYF	69	CD8-C5
Pla-1_CCGGTAGCATACTCTT	Pla	Pla-1	P1-32	FALSE	3	TRA:CAVMNTGFQKLVF TRB:CSVSGLVLGGNEQFF	46	CD8-C5
Pla-1_CCGGTAGTCTCGCTTG	Pla	Pla-1	P1-132	FALSE	3	TRA:CAVKGSQGNLIF TRB:CSARITYLNTEAFF	5	CD8-C5
Pla-1_CCGTACTGTAAGTAGT	Pla	Pla-1	P1-134	FALSE	3	TRA:CAGGGGSQGNLIF TRB:CASSQGLIYGYTF	5	CD8-C5
Pla-1_CCGTACTGTGTTTGGT	Pla	Pla-1	P1-80	FALSE	3	TRA:CAASAEGSQGNLIF TRB:CASKKDRGTTQAKNIQYF	7	CD8-C5
Pla-1_CCTACACCAGGCTCAC	Pla	Pla-1	P1-1511	FALSE	1	TRA:CAASINNQGGKLIF TRB:CASRPVGTGSEQYF	1	CD8-C4
Pla-1_CCTATTAAGGAGTTGC	Pla	Pla-1	P1-32	FALSE	3	TRA:CAVMNTGFQKLVF TRB:CSVSGLVLGGNEQFF	46	CD8-C4
Pla-1_CCTATTACAATAAGCA	Pla	Pla-1	P1-37	TRUE	3	TRA:CAVNIVGNQFYF TRB:CASSPLGTEAFF	40	CD8-C3
Pla-1_CCTATTATCAAGAAGT	Pla	Pla-1	P1-7	TRUE	3	TRA:CAVQAGNNDMRF TRB:CASSTTGDFNYGYTF	215	CD8-C5
Pla-1_CCTCAGTTCAACCAAC	Pla	Pla-1	P1-1524	FALSE	1	TRA:CAVSDLGNARLMF TRB:CSARDGDTTLYNEQFF	1	CD8-C5
Pla-1_CCTCAGTTCGCATGGC	Pla	Pla-1	P1-363	FALSE	2	TRA:CGADDGNQFYF TRB:CSAYLRQGGDTQYF	2	CD8-C4
Pla-1_CCTTTCTGTTCCACTC	Pla	Pla-1	P1-73	TRUE	3	TRA:CAGI TRB:CASSRTTGGSSYEQYF	8	CD8-C3
Pla-1_CGAATGTCAGGTCGTC	Pla	Pla-1	P1-1540	FALSE	1	TRA:CVVVGGRALTF TRB:CASSGQSYEQYF	1	CD8-C3
Pla-1_CGAATGTGTATTAGCC	Pla	Pla-1	P1-46	FALSE	3	TRA:CAVRGTGTASKLTF TRB:CSVRQGGNEQFF	20	CD8-C4
Pla-1_CGAGAAGCATTTCACT	Pla	Pla-1	P1-45	TRUE	3	TRA:CAYRPLGAQKLVF TRB:CASSTLDGRESSYEQYF	20	CD8-C5
Pla-1_CGAGCACTCGCATGGC	Pla	Pla-1	P1-1559	FALSE	1	TRA:CAVNIGYGGSQGNLIF TRB:CASSLSTSGGANEQFF	1	CD8-C5

Pla-1_CGAGCACTCTGCCCTA	Pla	Pla-1	P1-132	FALSE	3	TRA:CAVKGSQGNLIF TRB:CSARITYLNTEAFF	5	CD8-C5
Pla-1_CGATCGGCAGTAAGAT	Pla	Pla-1	P1-240	FALSE	3	TRA:CAAGDSNYQLIW TRB:CASTPFVGSRDTEAFF	3	CD8-C4
Pla-1_CGATGGCAGTCGATAA	Pla	Pla-1	P1-1563	FALSE	1	TRA:CAASFGGTSYGKLTF TRB:CASSIGGGLAKNIQYF	1	CD8-C5
Pla-1_CGCCAAGCACCAACCG	Pla	Pla-1	P1-99	FALSE	3	TRA:CIVRLLSTASKLTF TRB:CASSELQGKNTEAFF	6	CD8-C3
Pla-1_CGCGGTAGTCTCTCGT	Pla	Pla-1	P1-7	TRUE	3	TRA:CAVQAGNNDMRF TRB:CASSTTGDFNYGYTF	215	CD8-C5
Pla-1_CGCTATCGTCTTCGTC	Pla	Pla-1	P1-1572	FALSE	1	TRA:CAMSHYNTDKLIF TRB:CASKIWGILICGILYNEQFF	1	CD8-C5
Pla-1_CGCTATCTCGGAAATA	Pla	Pla-1	P1-9	TRUE	3	TRA:CAEAEGGGADGLTF TRB:CATSGGDNEQFF	174	CD8-C5
Pla-1_CGCTGGAAGACAAGCC	Pla	Pla-1	P1-44	TRUE	3	TRA:CAYRGLGAQKLVF TRB:CASSSLDGQDNHYGYTF	22	CD8-C5
Pla-1_CGGACACCAACACCTA	Pla	Pla-1	P1-6	TRUE	3	TRA:CAMREGRERDDKIIF TRB:CASSLPNTEAFF	216	CD8-C5
Pla-1_CGGACACGTAGCTTGT	Pla	Pla-1	P1-126	FALSE	3	TRA:CAVGSRPGGGYNKLIF TRB:CASGQGSYGELFF	5	CD8-C4
Pla-1_CGGACACGTGAACCTT	Pla	Pla-1	P1-1581	FALSE	1	TRA:CAVSETKTSGSRLTF TRB:CASSLDGQVNQPQHF	1	CD8-C4
Pla-1_CGGACGTTCGCCAAAT	Pla	Pla-1	P1-21	TRUE	3	TRA:CAASRGTGANNLFF TRB:CATQREGREQYF	67	CD4-C6
Pla-1_CGGACTGCACTTCGAA	Pla	Pla-1	P1-33	TRUE	3	TRA:CLVGTGGFKTIF TRB:CSARVPGQMKETQYF	41	CD4-C5
Pla-1_CGGACTGTCACGCGGT	Pla	Pla-1	P1-7	TRUE	3	TRA:CAVQAGNNDMRF TRB:CASSTTGDFNYGYTF	215	CD8-C5
Pla-1_CGGAGTCCATCCGTGG	Pla	Pla-1	P1-1593	FALSE	1	TRA:CALRAITQGGSEKLVF TRB:CSARGRGLAGGPAYEQYF	1	CD8-C7
Pla-1_CGTAGCGCAGTTTACG	Pla	Pla-1	P1-10	TRUE	3	TRA:CISNSPMDSSYKLIF TRB:CASSSWGDTDEAFF	162	CD8-C5
Pla-1_CGTCACTAGGACATTA	Pla	Pla-1	P1-46	FALSE	3	TRA:CAVRGTGTASKLTF TRB:CSVRQGGNEQFF	20	CD8-C4
Pla-1_CGTCCATAGATGTCGG	Pla	Pla-1	P1-347	FALSE	2	TRA:CALSDRGGNNRLAF TRB:CASSFVGVEVGLPVDTQYF	2	CD8-C4
Pla-1_CGTCCATGTGCAGTAG	Pla	Pla-1	P1-9	TRUE	3	TRA:CAEAEGGGADGLTF TRB:CATSGGDNEQFF	174	CD8-C5
Pla-1_CGTCCATTCTCTTGAT	Pla	Pla-1	P1-32	FALSE	3	TRA:CAVMNTGFQKLVF TRB:CSVSGLVLGGNEQFF	46	CD8-C5
Pla-1_CGTCTACAGCTGCAAG	Pla	Pla-1	P1-67	TRUE	3	TRA:CALNTNQAGTALIF TRB:CASSLGTRYAYEQYF	9	CD8-C3
Pla-1_CGTGAGCCACCGATAT	Pla	Pla-1	P1-32	FALSE	3	TRA:CAVMNTGFQKLVF TRB:CSVSGLVLGGNEQFF	46	CD8-C4
Pla-1_CGTGAGCCAGTATCTG	Pla	Pla-1	P1-1620	FALSE	1	TRA:CAKFSYSGGGADGLTF TRB:CASSVQGISLDTGELFF	1	CD8-C4
Pla-1_CGTTAGATCCCATTTA	Pla	Pla-1	P1-7	TRUE	3	TRA:CAVQAGNNDMRF TRB:CASSTTGDFNYGYTF	215	CD8-C5
Pla-1_CGTTCTGAGGATGTAT	Pla	Pla-1	P1-1631	FALSE	1	TRA:CATDARVGSNYKLTF TRB:CASSNRLKTAEAFF	1	CD8-C5
Pla-1_CGTTCTGTCTTCGGTC	Pla	Pla-1	P1-1632	FALSE	1	TRA:CAVRVGDMRF TRB:CSVETGGYGYTF	1	CD8-C4
Pla-1_CTAACTTCAGATGAGC	Pla	Pla-1	P1-9	TRUE	3	TRA:CAEAEGGGADGLTF TRB:CATSGGDNEQFF	174	CD8-C5

Pla-1_CTAATGGAGTGGTAAT	Pla	Pla-1	P1-388	FALSE	2	TRA:CAYRRNYGGATNKLIF TRB:CASSLSFTSGNTIYF	2	CD8-C4
Pla-1_CTACACCCAGATAATG	Pla	Pla-1	P1-130	FALSE	3	TRA:CAMREPGRALNF TRB:CASIAPGGVNYGYTF	5	CD8-C5
Pla-1_CTACATTCAATGGACG	Pla	Pla-1	P1-37	TRUE	3	TRA:CAVNIVGNQFYF TRB:CASSPLGTEAFF	40	CD8-C4
Pla-1_CTACATTCAGCTGGCT	Pla	Pla-1	P1-39	TRUE	3	TRA:CAVRDLGGTSYDKVIF TRB:CSARVREVSYEQYF	31	CD8-C5
Pla-1_CTACCCAAGCAGATCG	Pla	Pla-1	P1-33	TRUE	3	TRA:CLVGTGGFKTIF TRB:CSARVPGQMKETQYF	41	CD8-C3
Pla-1_CTACGTCGTAAGAGAG	Pla	Pla-1	P1-21	TRUE	3	TRA:CAASRGTGANNLFF TRB:CATQREGREQYF	67	CD4-C6
Pla-1_CTAGAGTAGATACACA	Pla	Pla-1	P1-1653	FALSE	1	TRA:CAVSAVWETSGSRLTF TRB:CASSVRDRSLWNTGELFF	1	CD8-C5
Pla-1_CTAGAGTAGGAGCGAG	Pla	Pla-1	P1-44	TRUE	3	TRA:CAYRGLGAQKLVF TRB:CASSSLDGQDNHYGYTF	22	CD8-C5
Pla-1_CTAGTGACAGTAAGAT	Pla	Pla-1	P1-62	TRUE	3	TRA:CVVNLMEYGNKLVF TRB:CASSGARQGQYEQYF	10	CD8-C4
Pla-1_CTCACACGTCTCAACA	Pla	Pla-1	P1-1662	FALSE	1	TRA:CAEPSQLYNQGGKLIF TRB:CASSSDRKNQPQHF	1	CD8-C5
Pla-1_CTCATTACACGACGAA	Pla	Pla-1	P1-1668	FALSE	1	TRA:CAVRDEDSSASKIIF TRB:CASRPAGGGLEQYF	1	CD8-C5
Pla-1_CTCGAAAAGATCGGGT	Pla	Pla-1	P1-1680	FALSE	1	TRA:CAVEEMDSSYKLIF TRB:CASSLDFMTSGSNEQFF	1	CD8-C3
Pla-1_CTCGAAAAGCTAACTC	Pla	Pla-1	P1-179	TRUE	3	TRA:CALAPTNTGNQFYF TRB:CASQRVGLSRYEQYF	3	CD8-C5
Pla-1_CTCGGAGTCACAGGCC	Pla	Pla-1	P1-20	TRUE	3	TRA:CVVVGGRALTF TRB:CASSLATNPHETQYF	69	CD8-C8
Pla-1_CTCGGGATCTTGACGA	Pla	Pla-1	P1-1692	FALSE	1	TRA:CAWGDSSYKLIF TRB:CATSPGLAGGNTGELFF	1	CD8-C4
Pla-1_CTCGTCAGTGCATCTA	Pla	Pla-1	P1-1697	FALSE	1	TRA:CATPFFTGNQFYF TRB:CASSSGQGRDIQYF	1	CD8-C3
Pla-1_CTCTAATAGAAGGACA	Pla	Pla-1	P1-1699	FALSE	1	TRA:CATALILTGGGNKLTF TRB:CASSEDGYLYEQYF	1	CD8-C4
Pla-1_CTCTACGCAGGAATGC	Pla	Pla-1	P1-45	TRUE	3	TRA:CAYRPLGAQKLVF TRB:CASSTLDGRESSYEQYF	20	CD8-C5
Pla-1_CTCTGGTTCACTTCAT	Pla	Pla-1	P1-392	FALSE	2	TRA:CAVRPLSSGSARQLTF TRB:CSVPAGGPSTDTQYF	2	CD8-C4
Pla-1_CTGATAGCAAGTCTGT	Pla	Pla-1	P1-1716	FALSE	1	TRA:CAARRAGSYQLTF TRB:CASSLVGWTGGSQPQHF	1	CD8-C4
Pla-1_CTGATAGGTTCCAACA	Pla	Pla-1	P1-45	TRUE	3	TRA:CAYRPLGAQKLVF TRB:CASSTLDGRESSYEQYF	20	CD8-C5
Pla-1_CTGATCCTCTTTACAC	Pla	Pla-1	P1-7	TRUE	3	TRA:CAVQAGNNDMRF TRB:CASSTTGDFNYGYTF	215	CD8-C5
Pla-1_CTGGTCTCAGGGCATA	Pla	Pla-1	P1-1733	FALSE	1	TRA:CAMREGSERGSQGNLIF TRB:CASSYSRVYSNQPQHF	1	CD8-C4
Pla-1_CTGGTCTTCACGATGT	Pla	Pla-1	P1-37	TRUE	3	TRA:CAVNIVGNQFYF TRB:CASSPLGTEAFF	40	CD8-C5
Pla-1_CTGTTTACAAGTCTGT	Pla	Pla-1	P1-6	TRUE	3	TRA:CAMREGRERDDKIIF TRB:CASSLPNTEAFF	216	CD8-C4
Pla-1_CTGTTTATCGAATCCA	Pla	Pla-1	P1-134	FALSE	3	TRA:CAGGGGSQGNLIF TRB:CASSQGLIYGYTF	5	CD8-C4
Pla-1_CTTACCGAGATGCCTT	Pla	Pla-1	P1-1	TRUE	3	TRA:CAMSDRMTGGFKTIF TRB:CASSQVGRGGEETQYF	1047	CD8-C5

Pla-1_CTTAGGAAGGTCATCT	Pla	Pla-1	P1-23	TRUE	3	TRA:CALRAITQGGSEKLVF TRB:CASSLTGAVYGYTF	65	CD8-C5
Pla-1_GAAACTCTCGGATGGA	Pla	Pla-1	P1-1760	FALSE	1	TRA:CALSEEGFQKLVF TRB:CASSPHGQPYEQYF	1	CD8-C5
Pla-1_GAAGCAGCAGATCGGA	Pla	Pla-1	P1-75	FALSE	3	TRA:CAMRGGNFNKFYF TRB:CSAQGAYEQYF	8	CD8-C4
Pla-1_GAAGCAGGTACAGCAG	Pla	Pla-1	P1-36	TRUE	3	TRA:CILRDNAGNNRKLIW TRB:CASSSRQGFPGGANVLTF	40	CD8-C4
Pla-1_GAATAAGGTTGAACTC	Pla	Pla-1	P1-37	TRUE	3	TRA:CAVNIVGNQFYF TRB:CASSPLGTEAFF	40	CD8-C4
Pla-1_GAATGAACACCAGGCT	Pla	Pla-1	P1-366	FALSE	2	TRA:CAGMDTGRRALTF TRB:CASSIYRQGSQPQHF	2	CD8-C5
Pla-1_GACAGAGAGATCACGG	Pla	Pla-1	P1-82	FALSE	3	TRA:CAASEDSNYQLIW TRB:CASSFGDSSSETQYF	7	CD8-C4
Pla-1_GACAGAGCATACTACG	Pla	Pla-1	P1-158	FALSE	3	TRA:CALRVITQGGSEKLVF TRB:CASSEGSSYNEQFF	4	CD8-C5
Pla-1_GACCTGGCAAGTACCT	Pla	Pla-1	P1-97	FALSE	3	TRA:CAVRTAGNKLTF TRB:CSAQGRSSYEQYF	6	CD8-C4
Pla-1_GACGCGTAGAGGTAGA	Pla	Pla-1	P1-1791	FALSE	1	TRA:CAMKGGKLIF TRB:CSVHTPGNTEAFF	1	CD8-C4
Pla-1_GACGGCTGTATTCGTG	Pla	Pla-1	P1-343	FALSE	2	TRA:CAVPPGNSGYALNF TRB:CASSEGGSSTDTQYF	2	CD8-C4
Pla-1_GACGTGCTCTCCGGTT	Pla	Pla-1	P1-23	TRUE	3	TRA:CALRAITQGGSEKLVF TRB:CASSLTGAVYGYTF	65	CD8-C4
Pla-1_GACGTTAGTCTCTTTA	Pla	Pla-1	P1-1804	FALSE	1	TRA:CALSEAENSGYSTLTF TRB:CSATGSRNTYEQYF	1	CD8-C4
Pla-1_GACGTTATCTACCAGA	Pla	Pla-1	P1-23	TRUE	3	TRA:CALRAITQGGSEKLVF TRB:CASSLTGAVYGYTF	65	CD8-C5
Pla-1_GACTAACCACCATGTA	Pla	Pla-1	P1-37	TRUE	3	TRA:CAVNIVGNQFYF TRB:CASSPLGTEAFF	40	CD8-C4
Pla-1_GACTACAAGGACACCA	Pla	Pla-1	P1-217	FALSE	3	TRA:CAGGWGGNKLVF TRB:CSVSGLVLGGNEQFF	3	CD8-C4
Pla-1_GACTACATCATTTGGG	Pla	Pla-1	P1-33	TRUE	3	TRA:CLVGTGGFKTIF TRB:CSARVPGQMKETQYF	41	CD8-C3
Pla-1_GACTGCGAGGCCGAAT	Pla	Pla-1	P1-87	TRUE	3	TRA:CGTVYNYGQNFVF TRB:CASSRGRGAQPQHF	6	CD8-C5
Pla-1_GACTGCGAGTCGTACT	Pla	Pla-1	P1-244	FALSE	3	TRA:CAARGYQLTF TRB:CASSPSRKGAPYEQYF	3	CD8-C4
Pla-1_GACTGCGTCCGATATG	Pla	Pla-1	P1-86	TRUE	3	TRA:CAVGGRSNTGRRALTF TRB:CAISLTGDGKETQYF	6	CD8-C4
Pla-1_GAGGTGAAGAGAGCTC	Pla	Pla-1	P1-33	TRUE	3	TRA:CLVGTGGFKTIF TRB:CSARVPGQMKETQYF	41	CD8-C3
Pla-1_GAGGTGATCCGATATG	Pla	Pla-1	P1-33	TRUE	3	TRA:CLVGTGGFKTIF TRB:CSARVPGQMKETQYF	41	CD8-C5
Pla-1_GAGTCCGAGGGTGTTG	Pla	Pla-1	P1-6	TRUE	3	TRA:CAMREGRERDDKIIF TRB:CASSLPNTEAFF	216	CD8-C5
Pla-1_GAGTCCGGTCGGATCC	Pla	Pla-1	P1-128	FALSE	3	TRA:CAVVNTGTASKLTF TRB:CASSEQGYEQYF	5	CD8-C5
Pla-1_GATCAGTGTCAACTGT	Pla	Pla-1	P1-152	TRUE	3	TRA:CALSEAGKPGRKAYLRT TRB:CASSPGTGSYNEQFF	4	CD8-C5
Pla-1_GATCAGTTCGTGACAT	Pla	Pla-1	P1-32	FALSE	3	TRA:CAVMNTGFQKLVF TRB:CSVSGLVLGGNEQFF	46	CD8-C5
Pla-1_GATCGCGCACCGAAAG	Pla	Pla-1	P1-37	TRUE	3	TRA:CAVNIVGNQFYF TRB:CASSPLGTEAFF	40	CD8-C5

Pla-1_GATCGCGCAGGTGCCT	Pla	Pla-1	P1-81	FALSE	3	TRA:CAVTFAGNQFYF TRB:CSAGTGTSGLSEQFF	7	CD8-C3
Pla-1_GATCGTATCCCGGATG	Pla	Pla-1	P1-33	TRUE	3	TRA:CLVGTGGFKTIF TRB:CSARVPGQMKETQYF	41	CD8-C3
Pla-1_GATGCTAAGGGAGTAA	Pla	Pla-1	P1-154	TRUE	3	TRA:CAGEKSSGDKLTF TRB:CSARGTGLNYGYTF	4	CD8-C5
Pla-1_GATGCTATCAATCACG	Pla	Pla-1	P1-23	TRUE	3	TRA:CALRAITQGGSEKLVF TRB:CASSLTGAVYGYTF	65	CD8-C4
Pla-1_GCAATCATCAAGGTAA	Pla	Pla-1	P1-1845	FALSE	1	TRA:CAVRDTGGFKTIF TRB:CASSPGTGFYEQYF	1	CD8-C4
Pla-1_GCACATACAGACGTAG	Pla	Pla-1	P1-20	TRUE	3	TRA:CVVVGGRALTF TRB:CASSLATNPHETQYF	69	CD8-C4
Pla-1_GCACATACATCTATGG	Pla	Pla-1	P1-126	FALSE	3	TRA:CAVGSRPGGGYNKLIF TRB:CASGQGSYGELFF	5	CD8-C5
Pla-1_GCACTCTGTCGATTGT	Pla	Pla-1	P1-155	TRUE	3	TRA:CAVMDTGRRALTF TRB:CAGGWTDEQYF	4	CD8-C4
Pla-1_GCACTCTTCCGATATG	Pla	Pla-1	P1-33	TRUE	3	TRA:CLVGTGGFKTIF TRB:CSARVPGQMKETQYF	41	CD8-C2
Pla-1_GCAGTTACAATGGTCT	Pla	Pla-1	P1-4	TRUE	3	TRA:CAMKGRDDKIIF TRB:CASTKGVSREAFF	226	CD8-C4
Pla-1_GCAGTTACAGATAATG	Pla	Pla-1	P1-9	TRUE	3	TRA:CAEAEGGGADGLTF TRB:CATSGGDNEQFF	174	CD8-C5
Pla-1_GCATGTAAGCGATGAC	Pla	Pla-1	P1-60	FALSE	3	TRA:CAYLSSTGTASKLTF TRB:CASSSLDGQDNHYGYTF	11	CD8-C5
Pla-1_GCCAAATAGTAAGTAC	Pla	Pla-1	P1-41	TRUE	3	TRA:CVVSEPPGGSYIPTF TRB:CASSSRPEDTQYF	27	CD8-C5
Pla-1_GCCAAATCAATCACAC	Pla	Pla-1	P1-1	TRUE	3	TRA:CAMSDRMTGGFKTIF TRB:CASSQVGRGGEETQYF	1047	CD8-C5
Pla-1_GCGAGAACAGACACTT	Pla	Pla-1	P1-222	FALSE	3	TRA:CAVRLPYNTDKLIF TRB:CASSFRHEQYF	3	CD8-C4
Pla-1_GCGGGTTTCCCTTGTG	Pla	Pla-1	P1-23	TRUE	3	TRA:CALRAITQGGSEKLVF TRB:CASSLTGAVYGYTF	65	CD8-C5
Pla-1_GCTCTGTAGTGGCACA	Pla	Pla-1	P1-405	FALSE	2	TRA:CALKGERGGSEKLVF TRB:CASSPAGETGTETQYF	2	CD8-C5
Pla-1_GCTCTGTTCCCTTGCA	Pla	Pla-1	P1-97	FALSE	3	TRA:CAVRTAGNKLTF TRB:CSAQGRSSYEQYF	6	CD8-C4
Pla-1_GCTGCAGCAACTGCGC	Pla	Pla-1	P1-6	TRUE	3	TRA:CAMREGRERDDKIIF TRB:CASSLPNTEAFF	216	CD8-C5
Pla-1_GCTGCGAGTTCAACCA	Pla	Pla-1	P1-93	TRUE	3	TRA:CAFTGGGGADGLTF TRB:CASSVAWGTGGWNTEAFF	6	CD8-C3
Pla-1_GCTGGGTCATACGCTA	Pla	Pla-1	P1-23	TRUE	3	TRA:CALRAITQGGSEKLVF TRB:CASSLTGAVYGYTF	65	CD8-C5
Pla-1_GCTTGAACATTACCTT	Pla	Pla-1	P1-6	TRUE	3	TRA:CAMREGRERDDKIIF TRB:CASSLPNTEAFF	216	CD8-C5
Pla-1_GGAAAGCAGAACTGTA	Pla	Pla-1	P1-1900	FALSE	1	TRA:CADGGSQGNLIF TRB:CASSQAGGTYEQYF	1	CD8-C5
Pla-1_GGAATAAAGACTAGGC	Pla	Pla-1	P1-20	TRUE	3	TRA:CVVVGGRALTF TRB:CASSLATNPHETQYF	69	CD8-C3
Pla-1_GGACAAGAGCCAGAAC	Pla	Pla-1	P1-7	TRUE	3	TRA:CAVQAGNNDMRF TRB:CASSTTGDFNYGYTF	215	CD8-C3
Pla-1_GGACAAGAGTTAGGTA	Pla	Pla-1	P1-269	TRUE	2	TRA:CAFMLTYGGSQGNLIF TRB:CASSLTVLDEQFF	2	CD8-C7
Pla-1_GGACAAGCACCGATAT	Pla	Pla-1	P1-87	TRUE	3	TRA:CGTVYNYGGQNFVF TRB:CASSRGRGAQPQHF	6	CD8-C5

Pla-1_GGACAGAGTCAAGCGA	Pla	Pla-1	P1-183	TRUE	3	TRA:CASYYGGSQGNLIF TRB:CSAGQGSNQPQHF	3	CD8-C10
Pla-1_GGACGTCGTTAAGATG	Pla	Pla-1	P1-3	TRUE	3	TRA:CAFLSGTYKYIF TRB:CASSQSPGQRNYGYTF	308	CD8-C5
Pla-1_GGATGTTAGTCGTTTG	Pla	Pla-1	P1-405	FALSE	2	TRA:CALKGERGGSEKLVF TRB:CASSPAGETGTETQYF	2	CD8-C5
Pla-1_GGATTACCATCCCATC	Pla	Pla-1	P1-12	TRUE	3	TRA:CALSDERGDYKLSF TRB:CASSPGTGSYNEQFF	141	CD8-C5
Pla-1_GGCAATTGTCGCGGTT	Pla	Pla-1	P1-135	FALSE	3	TRA:CAVRASGANSKLTF TRB:CSAQIGTGPEKLFF	5	CD8-C4
Pla-1_GGCGACTAGCCAGAAC	Pla	Pla-1	P1-37	TRUE	3	TRA:CAVNIVGNQFYF TRB:CASSPLGTEAFF	40	CD8-C4
Pla-1_GGCGTGTCACGAGGTA	Pla	Pla-1	P1-130	FALSE	3	TRA:CAMREPGRALNF TRB:CASIAPGGVNYGYTF	5	CD8-C4
Pla-1_GGCTCGAGTTGCCTCT	Pla	Pla-1	P1-20	TRUE	3	TRA:CVVVGGRALTF TRB:CASSLATNPHETQYF	69	CD8-C3
Pla-1_GGCTGGTAGGGAACGG	Pla	Pla-1	P1-1938	FALSE	1	TRA:CVVNLMEYGNKLVF TRB:CASSPWTGVNEQFF	1	CD8-C3
Pla-1_GGGACCTCACATAACC	Pla	Pla-1	P1-60	FALSE	3	TRA:CAYLSSTGTASKLTF TRB:CASSSLDGQDNHYGYTF	11	CD8-C8
Pla-1_GGGAGATCATGGTTGT	Pla	Pla-1	P1-1944	FALSE	1	TRA:CAVRDRGNSSASKIIF TRB:CASSYSGRGDTQYF	1	CD8-C3
Pla-1_GGGATGACAACTGGCC	Pla	Pla-1	P1-23	TRUE	3	TRA:CALRAITQGGSEKLVF TRB:CASSLTGAVYGYTF	65	CD8-C4
Pla-1_GGGATGAGTCAGAGGT	Pla	Pla-1	P1-128	FALSE	3	TRA:CAVVNTGTASKLTF TRB:CASSEQGYEQYF	5	CD8-C4
Pla-1_GGGCACTGTAGCACGA	Pla	Pla-1	P1-73	TRUE	3	TRA:CAGI TRB:CASSRTTGGSSYEQYF	8	CD8-C6
Pla-1_GGTATTGGTGTTAAGA	Pla	Pla-1	P1-15	TRUE	3	TRA:CAVNSSPLGANSKLTF TRB:CASSDSGNFYNEQFF	92	CD8-C5
Pla-1_GGTGAAGCAGATGAGC	Pla	Pla-1	P1-1962	FALSE	1	TRA:CALDGGYQKVTF TRB:CASSPTGDDYLDTQYF	1	CD8-C4
Pla-1_GGTGAAGTCTTACCTA	Pla	Pla-1	P1-7	TRUE	3	TRA:CAVQAGNNDMRF TRB:CASSTTGDFNYGYTF	215	CD8-C5
Pla-1_GGTGCGTAGAGGACGG	Pla	Pla-1	P1-32	FALSE	3	TRA:CAVMNTGFQKLVF TRB:CSVSGLVLGGNEQFF	46	CD8-C5
Pla-1_GGTGCGTAGGCGACAT	Pla	Pla-1	P1-110	TRUE	3	TRA:CAVAPGTGRRALTF TRB:CSARDRTAQETQYF	5	CD8-C5
Pla-1_GGTGCGTCATGGAATA	Pla	Pla-1	P1-407	FALSE	2	TRA:CAEDNNARLMF TRB:CSARVGVGNTIYF	2	CD8-C5
Pla-1_GTAACGTTCGCCATAA	Pla	Pla-1	P1-23	TRUE	3	TRA:CALRAITQGGSEKLVF TRB:CASSLTGAVYGYTF	65	CD8-C4
Pla-1_GTAACTGAGATGCCTT	Pla	Pla-1	P1-44	TRUE	3	TRA:CAYRGLGAQKLVF TRB:CASSSLDGQDNHYGYTF	22	CD8-C4
Pla-1_GTAACTGCATATGGTC	Pla	Pla-1	P1-4	TRUE	3	TRA:CAMKGRDDKIIF TRB:CASTKGVSREAFF	226	CD8-C6
Pla-1_GTAACTGGTAAAGGAG	Pla	Pla-1	P1-412	FALSE	2	TRA:CAVEYGSNYQLIW TRB:CASSLWVDNEQFF	2	CD8-C4
Pla-1_GTAACTGTCATGCTCC	Pla	Pla-1	P1-204	TRUE	3	TRA:CAVRDMMDSNYQLIW TRB:CASSLDGAGGETQYF	3	CD8-C5
Pla-1_GTAACTGTCCTGCAGG	Pla	Pla-1	P1-1977	FALSE	1	TRA:CAVNSSPLGANSKLTF TRB:CASSQTTGREAKNIQYF	1	CD8-C5
Pla-1_GTACTCCGTCGATTGT	Pla	Pla-1	P1-79	TRUE	3	TRA:CAFMKLGGATNKLIF TRB:CASSLDRYSVDEQFF	7	CD4-C6

Pla-1_GTACTTTTCGCGGATC	Pla	Pla-1	P1-1985	FALSE	1	TRA:CAVRETSGSRLTF TRB:CASSPPGWIQYF	1	CD8-C8
Pla-1_GTATTCTAGTGGGATC	Pla	Pla-1	P1-46	FALSE	3	TRA:CAVRGTGTASKLTF TRB:CSVRQGGNEQFF	20	CD8-C5
Pla-1_GTATTCTCAAGCGTAG	Pla	Pla-1	P1-65	FALSE	3	TRA:CAVQAHSGGSNYKLTF TRB:CASAETSYTGELFF	10	CD4-C6
Pla-1_GTCAAGTCACCGGAAA	Pla	Pla-1	P1-1994	FALSE	1	TRA:CALSGTSSSNTGKLIF TRB:CSVVDRDDYGYTF	1	CD8-C4
Pla-1_GTCACAAAGAGTAAGG	Pla	Pla-1	P1-46	FALSE	3	TRA:CAVRGTGTASKLTF TRB:CSVRQGGNEQFF	20	CD8-C4
Pla-1_GTCACAAAGGGAAACA	Pla	Pla-1	P1-33	TRUE	3	TRA:CLVGTGGFKTIF TRB:CSARVPGQMKETQYF	41	CD8-C5
Pla-1_GTCGTAACACAGACTT	Pla	Pla-1	P1-23	TRUE	3	TRA:CALRAITQGGSEKLVF TRB:CASSLTGAVYGYTF	65	CD8-C8
Pla-1_GTGCAGCGTCCAAGTT	Pla	Pla-1	P1-2018	FALSE	1	TRA:CALSEARSNDYKLSF TRB:CASSPVRWGAVVKNIQYF	1	CD8-C5
Pla-1_GTGCATACACAACGCC	Pla	Pla-1	P1-32	FALSE	3	TRA:CAVMNTGFQKLVF TRB:CSVSGLVLGGNEQFF	46	CD8-C3
Pla-1_GTGCATACATATGCTG	Pla	Pla-1	P1-23	TRUE	3	TRA:CALRAITQGGSEKLVF TRB:CASSLTGAVYGYTF	65	CD8-C4
Pla-1_GTGCATATCCAAACAC	Pla	Pla-1	P1-2022	FALSE	1	TRA:CAAIRTGGGNKLTF TRB:CASSKRLAGGSKFGTDTQYF	1	CD8-C4
Pla-1_GTGCTTCAGTGTTAGA	Pla	Pla-1	P1-9	TRUE	3	TRA:CAEAEGGGADGLTF TRB:CATSGGDNEQFF	174	CD8-C5
Pla-1_GTGCTTCCATGCAACT	Pla	Pla-1	P1-323	FALSE	2	TRA:CAVWHTDKLIF TRB:CASSLVGGVGTDTQYF	2	CD8-C5
Pla-1_GTGCTTCTCTTCCTTC	Pla	Pla-1	P1-349	FALSE	2	TRA:CAASKGFGNVLHC TRB:CASSPQGGGRGSYNEQFF	2	CD8-C4
Pla-1_GTGGGTCCAGCTGCTG	Pla	Pla-1	P1-20	TRUE	3	TRA:CVVVGGRALTF TRB:CASSLATNPHETQYF	69	CD8-C4
Pla-1_GTGGGTCGTCGAGATG	Pla	Pla-1	P1-33	TRUE	3	TRA:CLVGTGGFKTIF TRB:CSARVPGQMKETQYF	41	CD8-C3
Pla-1_GTGGGTCTCGAATGCT	Pla	Pla-1	P1-2031	FALSE	1	TRA:CAMRESPNTGNQFYF TRB:CASSLAWTGGSYEQYF	1	CD8-C5
Pla-1_GTGTGCGGTGAGGGTT	Pla	Pla-1	P1-2036	FALSE	1	TRA:CATDVRMDSSYKLIF TRB:CASSQVGGLEKLFF	1	CD8-C4
Pla-1_GTGTTAGAGCAATCTC	Pla	Pla-1	P1-20	TRUE	3	TRA:CVVVGGRALTF TRB:CASSLATNPHETQYF	69	CD8-C4
Pla-1_GTTAAGCAGACTAGAT	Pla	Pla-1	P1-134	FALSE	3	TRA:CAGGGGSQGNLIF TRB:CASSQGLIYGYTF	5	CD8-C4
Pla-1_GTTAAGCTCCCTCTTT	Pla	Pla-1	P1-20	TRUE	3	TRA:CVVVGGRALTF TRB:CASSLATNPHETQYF	69	CD8-C5
Pla-1_GTTACAGTCGAGAACG	Pla	Pla-1	P1-82	FALSE	3	TRA:CAASEDSNYQLIW TRB:CASSFGDSSSETQYF	7	CD8-C4
Pla-1_GTTCATTAGGCCGAAT	Pla	Pla-1	P1-2049	FALSE	1	TRA:CALSEANAGGTSYGKLTF TRB:CASSVVDRTQETQYF	1	CD8-C4
Pla-1_GTTCTCGAGGATGGTC	Pla	Pla-1	P1-2057	FALSE	1	TRA:CAASIESRDDKIIF TRB:CASSQGPGNTIYF	1	CD8-C4
Pla-1_GTTTCTAGTCTCGTTC	Pla	Pla-1	P1-20	TRUE	3	TRA:CVVVGGRALTF TRB:CASSLATNPHETQYF	69	CD8-C9
Pla-1_TAAGAGACAATAGCAA	Pla	Pla-1	P1-23	TRUE	3	TRA:CALRAITQGGSEKLVF TRB:CASSLTGAVYGYTF	65	CD8-C4
Pla-1_TAAGAGACATTCCTGC	Pla	Pla-1	P1-36	TRUE	3	TRA:CILRDNAGNNRKLIW TRB:CASSSRQGFPGGANVLTF	40	CD4-C6

Pla-1_TAAGTGCCAGATCCAT	Pla	Pla-1	P1-12	TRUE	3	TRA:CALSDERGDYKLSF TRB:CASSPGTGSYNEQFF	141	CD8-C4
Pla-1_TACAGTGGTTCGTGAT	Pla	Pla-1	P1-7	TRUE	3	TRA:CAVQAGNNDMRF TRB:CASSTTGDFNYGYTF	215	CD8-C5
Pla-1_TACCTTAAGTTTCCTT	Pla	Pla-1	P1-37	TRUE	3	TRA:CAVNIVGNQFYF TRB:CASSPLGTEAFF	40	CD8-C4
Pla-1_TACGGATCACGGTAAG	Pla	Pla-1	P1-2081	FALSE	1	TRA:CAADTGKLIF TRB:CSARPNRGLGYEQFF	1	CD8-C3
Pla-1_TACGGTAGTCCTGCTT	Pla	Pla-1	P1-2086	FALSE	1	TRA:CALHNFNKFYF TRB:CASSLAQGRNTEAFF	1	CD8-C5
Pla-1_TACGGTATCCTCGCAT	Pla	Pla-1	P1-45	TRUE	3	TRA:CAYRPLGAQKLVF TRB:CASSTLDGRESSYEQYF	20	CD8-C5
Pla-1_TACTCATCATGTAAGA	Pla	Pla-1	P1-247	FALSE	3	TRA:CAARGGSQGNLIF TRB:CASSLGGDTEAFF	3	CD8-C5
Pla-1_TACTCATGTGCAGTAG	Pla	Pla-1	P1-386	FALSE	2	TRA:CVVSSGGYNKLIF TRB:CASRPTGEGNEQFF	2	CD8-C10
Pla-1_TAGGCATAGAGGTACC	Pla	Pla-1	P1-300	TRUE	2	TRA:CAASFNDYKLSF TRB:CASKSVRTPQFF	2	CD8-C5
Pla-1_TAGGCATCAGTAGAGC	Pla	Pla-1	P1-20	TRUE	3	TRA:CVVVGGRALTF TRB:CASSLATNPHETQYF	69	CD8-C5
Pla-1_TAGGCATCAGTCAGCC	Pla	Pla-1	P1-2111	FALSE	1	TRA:CALRRRGQNFVF TRB:CASGAGNTIYF	1	CD4-C6
Pla-1_TAGTGGTGTATGGTTC	Pla	Pla-1	P1-38	TRUE	3	TRA:CAFTAGGATNKLIF TRB:CASIEGQGPYNEQFF	38	CD8-C2
Pla-1_TAGTTGGCACAGACTT	Pla	Pla-1	P1-37	TRUE	3	TRA:CAVNIVGNQFYF TRB:CASSPLGTEAFF	40	CD8-C5
Pla-1_TATCAGGAGTGACATA	Pla	Pla-1	P1-6	TRUE	3	TRA:CAMREGRERDDKIIF TRB:CASSLPNTEAFF	216	CD8-C5
Pla-1_TATCTCACAATCTGCA	Pla	Pla-1	P1-7	TRUE	3	TRA:CAVQAGNNDMRF TRB:CASSTTGDFNYGYTF	215	CD8-C5
Pla-1_TATGCCCAGTGTACGG	Pla	Pla-1	P1-2	TRUE	3	TRA:CGQNFVF TRB:CASNQRGGPSLYNEQFF	323	CD8-C5
Pla-1_TATTACCCATTCACTT	Pla	Pla-1	P1-14	TRUE	3	TRA:CAASVVNDMRF TRB:CASSHRGGAKNIQYF	109	CD8-C7
Pla-1_TCAACGAAGTATCTCG	Pla	Pla-1	P1-2126	FALSE	1	TRA:CAARPNNAGNMLTF TRB:CASSTNRGKANVLTF	1	CD8-C3
Pla-1_TCAATCTAGGAATTAC	Pla	Pla-1	P1-223	FALSE	3	TRA:CGTNQAGTALIF TRB:CASSLSRNYNSPLHF	3	CD8-C5
Pla-1_TCAATCTCATCTACGA	Pla	Pla-1	P1-353	FALSE	2	TRA:CAAFTKAAGNKLTF TRB:CASNPGGATYNEQFF	2	CD8-C5
Pla-1_TCAATCTCATTTCACT	Pla	Pla-1	P1-197	TRUE	3	TRA:CAENFQGAQKLVF TRB:CSVDPSGNTGELFF	3	CD8-C5
Pla-1_TCAATCTGTCGTGGCT	Pla	Pla-1	P1-388	FALSE	2	TRA:CAYRRNYGGATNKLIF TRB:CASSLSFTSGNTIYF	2	CD8-C5
Pla-1_TCATTACCAAGTACCT	Pla	Pla-1	P1-217	FALSE	3	TRA:CAGGWGGNKLVF TRB:CSVSGLVLGGNEQFF	3	CD8-C5
Pla-1_TCATTACCATTCCTGC	Pla	Pla-1	P1-2148	FALSE	1	TRA:CAASGNTPLVF TRB:CASRSSGGLQNEQFF	1	CD8-C4
Pla-1_TCATTACGTCGACTGC	Pla	Pla-1	P1-167	FALSE	3	TRA:CGTGINYGQNFVF TRB:CASSPRDGGGTEAFF	4	CD8-C5
Pla-1_TCCCGATAGACCGGAT	Pla	Pla-1	P1-20	TRUE	3	TRA:CVVVGGRALTF TRB:CASSLATNPHETQYF	69	CD8-C5
Pla-1_TCGAGGCCATTGGCGC	Pla	Pla-1	P1-186	TRUE	3	TRA:CAVPPYMDSNYQLIW TRB:CASSLFGASPLHF	3	CD8-C3

Pla-1_TCGCGAGCAAAGCAAT	Pla	Pla-1	P1-23	TRUE	3	TRA:CALRAITQGGSEKLVF TRB:CASSLTGAVYGYTF	65	CD8-C3
Pla-1_TCGCGTTGTACTCTCC	Pla	Pla-1	P1-2157	FALSE	1	TRA:CAERSEADSWGKLQF TRB:CSARVPGQMKETQYF	1	CD8-C3
Pla-1_TCGGGACAGACTCGGA	Pla	Pla-1	P1-97	FALSE	3	TRA:CAVRTAGNKLTF TRB:CSAQGRSSYEQYF	6	CD8-C5
Pla-1_TCGGGACGTAAATACG	Pla	Pla-1	P1-68	TRUE	3	TRA:CAVIPDILTGGGNKLTF TRB:CASSLQGFGNTIYF	9	CD8-C5
Pla-1_TCGGTAAAGGCTCATT	Pla	Pla-1	P1-211	FALSE	3	TRA:CAVNVQKSNTGNQFYF TRB:CASSYSVSYEQYF	3	CD8-C2
Pla-1_TCTATTGGTCGAATCT	Pla	Pla-1	P1-412	FALSE	2	TRA:CAVEYGSNYQLIW TRB:CASSLWVDNEQFF	2	CD8-C5
Pla-1_TCTCTAAAGGCGTACA	Pla	Pla-1	P1-61	TRUE	3	TRA:CAARGGSQGNLIF TRB:CASRPRHTDTQYF	10	CD8-C7
Pla-1_TCTCTAAAGTTACCCA	Pla	Pla-1	P1-2181	FALSE	1	TRA:CATVSFSGGYNKLIF TRB:CASSSHGQLNQPQHF	1	CD8-C6
Pla-1_TCTCTAATCAGTGCAT	Pla	Pla-1	P1-137	FALSE	3	TRA:CAVSMNRDDKIIF TRB:CASSSGRLSGANVLTF	5	CD8-C3
Pla-1_TGAAAGACAGATCCAT	Pla	Pla-1	P1-7	TRUE	3	TRA:CAVQAGNNDMRF TRB:CASSTTGDFNYGYTF	215	CD8-C5
Pla-1_TGACGGCAGATGTGTA	Pla	Pla-1	P1-2195	FALSE	1	TRA:CAVRHSGGYQKVTF TRB:CASSLGGTGEKIRNEQFF	1	CD8-C5
Pla-1_TGACGGCGTTAAGACA	Pla	Pla-1	P1-118	TRUE	3	TRA:CAVVKAGGTSYGKLTF TRB:CASKSLAGKSSYEQYF	5	CD8-C4
Pla-1_TGACTTTTCTAACGGT	Pla	Pla-1	P1-139	TRUE	3	TRA:CGTERDQFYF TRB:CATSRELFGEKLFF	4	CD8-C3
Pla-1_TGAGCATTCCAAGTAC	Pla	Pla-1	P1-295	TRUE	2	TRA:CALPFISGNTPLVF TRB:CASSLGPLENIQYF	2	CD8-C4
Pla-1_TGAGCATTCGTCTGCT	Pla	Pla-1	P1-62	TRUE	3	TRA:CVVNLMEYGNKLVF TRB:CASSGARQGQYEQYF	10	CD8-C4
Pla-1_TGAGCCGGTGAGCGAT	Pla	Pla-1	P1-56	TRUE	3	TRA:CVVNPWGSGTYKYIF TRB:CASSRDITDTQYF	13	CD8-C5
Pla-1_TGAGCCGTCAACCAAC	Pla	Pla-1	P1-2206	FALSE	1	TRA:CAVNRGNTGFQKLVF TRB:CATSPWGGTDTQYF	1	CD8-C4
Pla-1_TGAGCCGTCTGTACGA	Pla	Pla-1	P1-230	FALSE	3	TRA:CAVDPWGSQGNLIF TRB:CASSPGREQFF	3	CD8-C5
Pla-1_TGAGGGATCCCTAACC	Pla	Pla-1	P1-3	TRUE	3	TRA:CAFLSGTYKYIF TRB:CASSQSPGQRNYGYTF	308	CD8-C7
Pla-1_TGATTTCCAGATTGCT	Pla	Pla-1	P1-6	TRUE	3	TRA:CAMREGRERDDKIIF TRB:CASSLPNTEAFF	216	CD8-C5
Pla-1_TGCCAAAAGATAGGAG	Pla	Pla-1	P1-2212	FALSE	1	TRA:CAVFRLIYNQGGKLIF TRB:CSVEDLPASGRVGETQYF	1	CD8-C3
Pla-1_TGCCCTATCAGATAAG	Pla	Pla-1	P1-9	TRUE	3	TRA:CAEAEGGGADGLTF TRB:CATSGGDNEQFF	174	CD8-C5
Pla-1_TGCTGCTTCGGTGTTA	Pla	Pla-1	P1-99	FALSE	3	TRA:CIVRLLSTASKLTF TRB:CASSELQGKNTEAFF	6	CD8-C4
Pla-1_TGGACGCTCGGCATCG	Pla	Pla-1	P1-125	FALSE	3	TRA:CILKSRTSYDKVIF TRB:CASSFLSGTDTQYF	5	CD8-C5
Pla-1_TGGCCAGAGGAATTAC	Pla	Pla-1	P1-62	TRUE	3	TRA:CVVNLMEYGNKLVF TRB:CASSGARQGQYEQYF	10	CD8-C3
Pla-1_TGGCCAGCAATAAGCA	Pla	Pla-1	P1-7	TRUE	3	TRA:CAVQAGNNDMRF TRB:CASSTTGDFNYGYTF	215	CD8-C5
Pla-1_TGGCGCACAGTAAGAT	Pla	Pla-1	P1-414	FALSE	2	TRA:CAMRANHNAGNNRKLIW TRB:CAWSVPGTGGEKLFF	2	CD8-C4

Pla-1_TGGCGCAGTAAGCACG	Pla	Pla-1	P1-236	FALSE	3	TRA:CAMREGRF TRB:CASSLRVPGSDTQYF	3	CD8-C3
Pla-1_TGGCTGGAGGCATTGG	Pla	Pla-1	P1-424	FALSE	2	TRA:CVAITGGATNKLIF TRB:CAISESRQGEYNEQFF	2	CD8-C3
Pla-1_TGGTTAGCAAGTCTGT	Pla	Pla-1	P1-32	FALSE	3	TRA:CAVMNTGFQKLVF TRB:CSVSGLVLGGNEQFF	46	CD8-C4
Pla-1_TGTATTCGTTCCGGCA	Pla	Pla-1	P1-9	TRUE	3	TRA:CAEAEGGGADGLTF TRB:CATSGGDNEQFF	174	CD8-C5
Pla-1_TGTCCCAGTATTACCG	Pla	Pla-1	P1-2266	FALSE	1	TRA:CAYRRALGGALPGGSEKLVF TRB:CASSYVRGATGELFF	1	CD8-C4
Pla-1_TTAACTCCAAAGGCGT	Pla	Pla-1	P1-2275	FALSE	1	TRA:CAVGGPNDYKLSF TRB:CASRDGAGGYTF	1	CD8-C4
Pla-1_TTAACTCCACGTCAGC	Pla	Pla-1	P1-100	FALSE	3	TRA:CAVRDRITGNQFYF TRB:CASSQDWGSNQPQHF	6	CD8-C4
Pla-1_TTAACTCTCACAACGT	Pla	Pla-1	P1-2277	FALSE	1	TRA:CAVRGTGTASKLTF TRB:CSVSGLVLGGNEQFF	1	CD8-C5
Pla-1_TTAGGACCATGGGAAC	Pla	Pla-1	P1-20	TRUE	3	TRA:CVVVGGRALTF TRB:CASSLATNPHETQYF	69	CD8-C4
Pla-1_TTAGGCACAGTCGTGC	Pla	Pla-1	P1-32	FALSE	3	TRA:CAVMNTGFQKLVF TRB:CSVSGLVLGGNEQFF	46	CD8-C5
Pla-1_TTAGGCAGTCAAAGAT	Pla	Pla-1	P1-7	TRUE	3	TRA:CAVQAGNNDMRF TRB:CASSTTGDFNYGYTF	215	CD8-C5
Pla-1_TTAGTTCCAACGCACC	Pla	Pla-1	P1-316	FALSE	2	TRA:CVVNQGKTSYDKVIF TRB:CATEDREGYEQYF	2	CD8-C2
Pla-1_TTAGTTCCACACCGAC	Pla	Pla-1	P1-37	TRUE	3	TRA:CAVNIVGNQFYF TRB:CASSPLGTEAFF	40	CD8-C4
Pla-1_TTAGTTCTCACGATGT	Pla	Pla-1	P1-56	TRUE	3	TRA:CVVNPWGSGTYKYIF TRB:CASSRDITDTQYF	13	CD8-C4
Pla-1_TTCCCAGCACGGATAG	Pla	Pla-1	P1-2292	FALSE	1	TRA:CIVRALYNQGGKLIF TRB:CASSQDGGQYQPQHF	1	CD8-C5
Pla-1_TTCCCAGCAGGCTCAC	Pla	Pla-1	P1-7	TRUE	3	TRA:CAVQAGNNDMRF TRB:CASSTTGDFNYGYTF	215	CD8-C5
Pla-1_TTCCCAGGTTTAGCTG	Pla	Pla-1	P1-23	TRUE	3	TRA:CALRAITQGGSEKLVF TRB:CASSLTGAVYGYTF	65	CD8-C5
Pla-1_TTCCCAGTCTGCTGTC	Pla	Pla-1	P1-32	FALSE	3	TRA:CAVMNTGFQKLVF TRB:CSVSGLVLGGNEQFF	46	CD8-C4
Pla-1_TTCGGTCAGCGTAATA	Pla	Pla-1	P1-91	TRUE	3	TRA:CVVSDQYNTDKLIF TRB:CASGEGQGASSYEQYF	6	CD8-C5
Pla-1_TTCTACAGTGTTGGGA	Pla	Pla-1	P1-32	FALSE	3	TRA:CAVMNTGFQKLVF TRB:CSVSGLVLGGNEQFF	46	CD8-C5
Pla-1_TTCTCAAGTACCAGTT	Pla	Pla-1	P1-23	TRUE	3	TRA:CALRAITQGGSEKLVF TRB:CASSLTGAVYGYTF	65	CD8-C4
Pla-1_TTCTCCTCAGTCCTTC	Pla	Pla-1	P1-2306	FALSE	1	TRA:CAYRSAWGGGADGLTF TRB:CASSRTDGLGEETQYF	1	CD8-C4
Pla-1_TTGAACGAGGTAGCCA	Pla	Pla-1	P1-2313	FALSE	1	TRA:CAVQAHSGGSNYKLTF TRB:CASSPLDGRSTDTQYF	1	CD8-C5
Pla-1_TTGCGTCAGGCGATAC	Pla	Pla-1	P1-2318	FALSE	1	TRA:CAVEEPAGNKLTF TRB:CASSQDLASGVSYNEQFF	1	CD8-C4
Pla-1_TTGCGTCCATCGGAAG	Pla	Pla-1	P1-75	FALSE	3	TRA:CAMRGGNFNKFYF TRB:CSAQGAYEQYF	8	CD8-C2
Pla-1_TTGGAACCATTTGCCC	Pla	Pla-1	P1-2320	FALSE	1	TRA:CVVNPWGSGTYKYIF TRB:CASSQSWDLAVLNEQFF	1	CD8-C5
Pla-1_TTGTAGGTCTTAGCCC	Pla	Pla-1	P1-23	TRUE	3	TRA:CALRAITQGGSEKLVF TRB:CASSLTGAVYGYTF	65	CD8-C5

Pla-1_TTTACTGCATTGGGCC	Pla	Pla-1	P1-80	FALSE	3	TRA:CAASAEGSQGNLIF TRB:CASKKDRGTTQAKNIQYF	7	CD8-C5
Pla-1_TTTGCGCTCACCAGGC	Pla	Pla-1	P1-6	TRUE	3	TRA:CAMREGRERDDKIIF TRB:CASSLPNTEAFF	216	CD8-C5
Pla-1_AAAGATGTCACCGGGT	Pla	Pla-1	P1-301	FALSE	2	TRA:CVVNRETGANNLFF TRB:CASSYKGPTYEQYF	2	CD8-C5
Pla-1_AAATGCCTCACAAACC	Pla	Pla-1	P1-1091	FALSE	1	TRA:CGTELGGNQFYF TRB:CASSYSRGPSSYNEQFF	1	CD4-C4
Pla-1_AACACGTTCAATAAGG	Pla	Pla-1	P1-1094	FALSE	1	TRA:CVVTRNYNQGGKLIF TRB:CASSTGQGANEKLFF	1	CD8-C3
Pla-1_AACGTTGGTCGGCATC	Pla	Pla-1	P1-121	FALSE	3	TRA:CAASEGGATNKLIF TRB:CSAGTSGNIQYF	5	CD8-C3
Pla-1_AACTCCCGTAGCCTAT	Pla	Pla-1	P1-23	TRUE	3	TRA:CALRAITQGGSEKLVF TRB:CASSLTGAVYGYTF	65	CD8-C3
Pla-1_AACTCCCGTGCAACGA	Pla	Pla-1	P1-10	TRUE	3	TRA:CISNSPMDSSYKLIF TRB:CASSSWGDTDEAFF	162	CD8-C3
Pla-1_AACTCCCTCTCCGGTT	Pla	Pla-1	P1-307	FALSE	2	TRA:CAIYSNYQLIW TRB:CASSTQGLDEQFF	2	CD8-C3
Pla-1_AACTCTTTCAGGATCT	Pla	Pla-1	P1-1105	FALSE	1	TRA:CAASTSNFGNEKLTF TRB:CASRANIQYF	1	CD8-C3
Pla-1_AACTCTTTCTGCCCTA	Pla	Pla-1	P1-56	TRUE	3	TRA:CVVNPWGSGTYKYIF TRB:CASSRDITDTQYF	13	CD8-C3
Pla-1_AACTGGTCATGCCTTC	Pla	Pla-1	P1-23	TRUE	3	TRA:CALRAITQGGSEKLVF TRB:CASSLTGAVYGYTF	65	CD8-C3
Pla-1_AACTGGTGTCTCAACA	Pla	Pla-1	P1-311	FALSE	2	TRA:CAVNSPTNSGYSTLTF TRB:CASRSPSENEQFF	2	CD8-C3
Pla-1_AAGGAGCCAAGAGGCT	Pla	Pla-1	P1-46	FALSE	3	TRA:CAVRGTGTASKLTF TRB:CSVRQGGNEQFF	20	CD8-C3
Pla-1_AAGGAGCCAGCTCCGA	Pla	Pla-1	P1-1118	FALSE	1	TRA:CAVGRFRGGTALIF TRB:CASSILRGRGHTEAFF	1	CD8-C3
Pla-1_AAGGAGCGTGCCTGTG	Pla	Pla-1	P1-117	TRUE	3	TRA:CIVGETSGSRLTF TRB:CASSLVGGVGTDTQYF	5	CD8-C3
Pla-1_AAGGCAGCAAGTCTGT	Pla	Pla-1	P1-158	FALSE	3	TRA:CALRVITQGGSEKLVF TRB:CASSEGSSYNEQFF	4	CD8-C3
Pla-1_AAGGTTCCAGGTGGAT	Pla	Pla-1	P1-20	TRUE	3	TRA:CVVVGGRALTF TRB:CASSLATNPHETQYF	69	CD8-C3
Pla-1_AAGTCTGGTAGGGACT	Pla	Pla-1	P1-1129	FALSE	1	TRA:CVVKSPSGGGADGLTF TRB:CASSLFFRGGHEQYF	1	CD8-C3
Pla-1_AAGTCTGTCTGTCAAG	Pla	Pla-1	P1-314	FALSE	2	TRA:CAEPSNTGNQFYF TRB:CAISLGNEQFF	2	CD8-C3
Pla-1_AATCCAGTCTTTACAC	Pla	Pla-1	P1-32	FALSE	3	TRA:CAVMNTGFQKLVF TRB:CSVSGLVLGGNEQFF	46	CD8-C3
Pla-1_AATCGGTAGGTTACCT	Pla	Pla-1	P1-1136	FALSE	1	TRA:CAASALNNARLMF TRB:CASSQEEAGPAKNIQYF	1	CD8-C3
Pla-1_AATCGGTAGTCGTTTG	Pla	Pla-1	P1-7	TRUE	3	TRA:CAVQAGNNDMRF TRB:CASSTTGDFNYGYTF	215	CD8-C3
Pla-1_ACACCCTCAGCATACT	Pla	Pla-1	P1-1144	FALSE	1	TRA:CAFSVGGGSNYKLTF TRB:CASSGVAGGLYNEQFF	1	CD8-C3
Pla-1_ACACCGGCAGCTCGAC	Pla	Pla-1	P1-20	TRUE	3	TRA:CVVVGGRALTF TRB:CASSLATNPHETQYF	69	CD8-C8
Pla-1_ACACTGAAGTGCCATT	Pla	Pla-1	P1-80	FALSE	3	TRA:CAASAEGSQGNLIF TRB:CASKKDRGTTQAKNIQYF	7	CD8-C3
Pla-1_ACACTGATCCTTGGTC	Pla	Pla-1	P1-1148	FALSE	1	TRA:CAENASGGGNKLTF TRB:CASSRDITDTQYF	1	CD4-C5

Pla-1_ACAGCCGCACTTGGAT	Pla	Pla-1	P1-99	FALSE	3	TRA:CIVRLLSTASKLTF TRB:CASSELQGKNTEAFF	6	CD8-C3
Pla-1_ACAGCCGGTGATAAAC	Pla	Pla-1	P1-100	FALSE	3	TRA:CAVRDRITGNQFYF TRB:CASSQDWGSNQPQHF	6	CD8-C3
Pla-1_ACAGCTAAGCGAAGGG	Pla	Pla-1	P1-44	TRUE	3	TRA:CAYRGLGAQKLVF TRB:CASSSLDGQDNHYGYTF	22	CD8-C3
Pla-1_ACAGCTATCGGCGCTA	Pla	Pla-1	P1-56	TRUE	3	TRA:CVVNPWGSGTYKYIF TRB:CASSRDITDTQYF	13	CD8-C3
Pla-1_ACAGCTATCTGCAGTA	Pla	Pla-1	P1-99	FALSE	3	TRA:CIVRLLSTASKLTF TRB:CASSELQGKNTEAFF	6	CD8-C3
Pla-1_ACATACGAGACTTGAA	Pla	Pla-1	P1-12	TRUE	3	TRA:CALSDERGDYKLSF TRB:CASSPGTGSYNEQFF	141	CD8-C3
Pla-1_ACATACGTCGTCCAGG	Pla	Pla-1	P1-1161	FALSE	1	TRA:CAFPTGTASKLTF TRB:CASSTREKLFF	1	CD8-C8
Pla-1_ACATCAGTCAAAGACA	Pla	Pla-1	P1-316	FALSE	2	TRA:CVVNQGKTSYDKVIF TRB:CATEDREGYEQYF	2	CD8-C3
Pla-1_ACCAGTAAGGTGATAT	Pla	Pla-1	P1-310	FALSE	2	TRA:CALIRSAGNNRKLIW TRB:CASRGQGNQPQHF	2	CD8-C3
Pla-1_ACCCACTAGTTACCCA	Pla	Pla-1	P1-70	FALSE	3	TRA:CAMRETPGEAGTALIF TRB:CSARITYLNTEAFF	9	CD8-C2
Pla-1_ACCGTAAAGATGCGAC	Pla	Pla-1	P1-1172	FALSE	1	TRA:CAESIGASKIIF TRB:CASSRTSTDTQYF	1	CD8-C3
Pla-1_ACCGTAATCTGCGGCA	Pla	Pla-1	P1-74	TRUE	3	TRA:CAVGGGSTLGRLYF TRB:CASSIVAGADTEAFF	8	CD8-C3
Pla-1_ACCTTTAAGAAGGGTA	Pla	Pla-1	P1-56	TRUE	3	TRA:CVVNPWGSGTYKYIF TRB:CASSRDITDTQYF	13	CD8-C3
Pla-1_ACCTTTACAGATCTGT	Pla	Pla-1	P1-200	TRUE	3	TRA:CAVGGNNNDMRF TRB:CASSRQDLHEQYF	3	CD8-C3
Pla-1_ACCTTTATCCTTTCGG	Pla	Pla-1	P1-1177	FALSE	1	TRA:CAVHVYGGSQGNLIF TRB:CASREKDRADTEAFF	1	CD8-C3
Pla-1_ACGAGCCCAGCGTAAG	Pla	Pla-1	P1-20	TRUE	3	TRA:CVVVGGRALTF TRB:CASSLATNPHETQYF	69	CD8-C3
Pla-1_ACGAGCCGTAATCGTC	Pla	Pla-1	P1-125	FALSE	3	TRA:CILKSRTSYDKVIF TRB:CASSFLSGTDTQYF	5	CD8-C2
Pla-1_ACGAGGAAGGCGTACA	Pla	Pla-1	P1-318	FALSE	2	TRA:CAMRRDDKIIF TRB:CASSQDFRQGSLSLHF	2	CD8-C3
Pla-1_ACGAGGAAGTATCGAA	Pla	Pla-1	P1-57	FALSE	3	TRA:CAMREGGNTGNQFYF TRB:CASKKDRGTTQAKNIQYF	13	CD8-C3
Pla-1_ACGAGGAAGTGATCGG	Pla	Pla-1	P1-1178	FALSE	1	TRA:CALHNNAGNMLTF TRB:CAWSRGARVSSEAFF	1	CD8-C3
Pla-1_ACGATGTAGAAGGACA	Pla	Pla-1	P1-1187	FALSE	1	TRA:CIVGYINNDMRF TRB:CASSLLDGKNTEAFF	1	CD8-C3
Pla-1_ACGATGTGTAAACGCG	Pla	Pla-1	P1-215	FALSE	3	TRA:CAVSSNRDDKIIF TRB:CASSPGRLAGENIQYF	3	CD8-C3
Pla-1_ACGCAGCGTGACCAAG	Pla	Pla-1	P1-33	TRUE	3	TRA:CLVGTGGFKTIF TRB:CSARVPGQMKETQYF	41	CD8-C3
Pla-1_ACGGAGATCAGTGCAT	Pla	Pla-1	P1-23	TRUE	3	TRA:CALRAITQGGSEKLVF TRB:CASSLTGAVYGYTF	65	CD8-C3
Pla-1_ACGGGCTCACACTGCG	Pla	Pla-1	P1-55	TRUE	3	TRA:CALSPQDSNYQLIW TRB:CASSFWTSANEQFF	13	CD8-C3
Pla-1_ACGGGTCCAGGATTGG	Pla	Pla-1	P1-1207	FALSE	1	TRA:CAYRYFYGGSQGNLIF TRB:CASKKDRGTTQAKNIQYF	1	CD8-C3
Pla-1_ACTGAACTCACCTCGT	Pla	Pla-1	P1-325	FALSE	2	TRA:CAVRASYNTDKLIF TRB:CASSYPSGKYNEQFF	2	CD8-C3

Pla-1_ACTGATGGTAGCTGCC	Pla	Pla-1	P1-20	TRUE	3	TRA:CVVVGGRALTF TRB:CASSLATNPHETQYF	69	CD8-C3
Pla-1_ACTGCTCAGGGATCTG	Pla	Pla-1	P1-32	FALSE	3	TRA:CAVMNTGFQKLVF TRB:CSVSGLVLGGNEQFF	46	CD8-C3
Pla-1_ACTGCTCAGTGGTAGC	Pla	Pla-1	P1-57	FALSE	3	TRA:CAMREGGNTGNQFYF TRB:CASKKDRGTTQAKNIQYF	13	CD4-C4
Pla-1_ACTGCTCGTCTCGTTC	Pla	Pla-1	P1-1224	FALSE	1	TRA:CAMRESRGNQGGKLIF TRB:CASTLGAGYLSEAFF	1	CD8-C3
Pla-1_ACTGTCCAGGAGCGAG	Pla	Pla-1	P1-1227	FALSE	1	TRA:CAVWVSSGAGSYQLTF TRB:CATSRDQGIEKLFF	1	CD8-C3
Pla-1_ACTGTCCCAAGAGGCT	Pla	Pla-1	P1-3	TRUE	3	TRA:CAFLSGTYKYIF TRB:CASSQSPGQRNYGYTF	308	CD8-C3
Pla-1_ACTTACTAGGACATTA	Pla	Pla-1	P1-33	TRUE	3	TRA:CLVGTGGFKTIF TRB:CSARVPGQMKETQYF	41	CD8-C8
Pla-1_ACTTGTTAGCCAGGAT	Pla	Pla-1	P1-128	FALSE	3	TRA:CAVVNTGTASKLTF TRB:CASSEQGYEQYF	5	CD8-C3
Pla-1_ACTTGTTGTTATCACG	Pla	Pla-1	P1-217	FALSE	3	TRA:CAGGWGGNKLVF TRB:CSVSGLVLGGNEQFF	3	CD8-C3
Pla-1_ACTTTCATCACGAAGG	Pla	Pla-1	P1-52	TRUE	3	TRA:CAETPDYKLSF TRB:CASRDLYEQYF	13	CD8-C3
Pla-1_AGAATAGCAGGCTCAC	Pla	Pla-1	P1-20	TRUE	3	TRA:CVVVGGRALTF TRB:CASSLATNPHETQYF	69	CD8-C3
Pla-1_AGACGTTAGAAACGAG	Pla	Pla-1	P1-210	FALSE	3	TRA:CAYRRLGNTPLVF TRB:CASSPLDGHSNQPQHF	3	CD8-C3
Pla-1_AGAGCTTCATTAGGCT	Pla	Pla-1	P1-218	FALSE	3	TRA:CAYRRLGNTPLVF TRB:CASSLVDGGSSYEQYF	3	CD8-C3
Pla-1_AGAGCTTTCAATCACG	Pla	Pla-1	P1-20	TRUE	3	TRA:CVVVGGRALTF TRB:CASSLATNPHETQYF	69	CD8-C5
Pla-1_AGATCTGTCTAGAGTC	Pla	Pla-1	P1-32	FALSE	3	TRA:CAVMNTGFQKLVF TRB:CSVSGLVLGGNEQFF	46	CD8-C3
Pla-1_AGATTGCAGCGATGAC	Pla	Pla-1	P1-20	TRUE	3	TRA:CVVVGGRALTF TRB:CASSLATNPHETQYF	69	CD8-C3
Pla-1_AGATTGCGTGCGAAAC	Pla	Pla-1	P1-49	FALSE	3	TRA:CAGGGSEKLVF TRB:CASSLFYPAGQQYF	16	CD8-C8
Pla-1_AGCATACGTACAAGTA	Pla	Pla-1	P1-210	FALSE	3	TRA:CAYRRLGNTPLVF TRB:CASSPLDGHSNQPQHF	3	CD8-C3
Pla-1_AGCCTAAAGCAATATG	Pla	Pla-1	P1-9	TRUE	3	TRA:CAEAEGGGADGLTF TRB:CATSGGDNEQFF	174	CD8-C3
Pla-1_AGCGTATGTTCGGCAC	Pla	Pla-1	P1-44	TRUE	3	TRA:CAYRGLGAQKLVF TRB:CASSSLDGQDNHYGYTF	22	CD8-C5
Pla-1_AGCGTATTCCATGAGT	Pla	Pla-1	P1-44	TRUE	3	TRA:CAYRGLGAQKLVF TRB:CASSSLDGQDNHYGYTF	22	CD8-C3
Pla-1_AGCGTCGAGCACACAG	Pla	Pla-1	P1-44	TRUE	3	TRA:CAYRGLGAQKLVF TRB:CASSSLDGQDNHYGYTF	22	CD8-C3
Pla-1_AGCGTCGTCTGCCCTA	Pla	Pla-1	P1-334	FALSE	2	TRA:CIVRVTTDSWGKLQF TRB:CSARVLDTQYF	2	CD8-C3
Pla-1_AGCTCCTGTGGTAACG	Pla	Pla-1	P1-52	TRUE	3	TRA:CAETPDYKLSF TRB:CASRDLYEQYF	13	CD8-C3
Pla-1_AGGCCACAGCAGCGTA	Pla	Pla-1	P1-220	FALSE	3	TRA:CAAGQKNYGGSQGNLIF TRB:CASSIRGESSTEAFF	3	CD8-C3
Pla-1_AGGCCACGTGGTTTCA	Pla	Pla-1	P1-40	TRUE	3	TRA:CAVRDVGNTGFQKLVF TRB:CASSLGQGSMKTQYF	27	CD8-C8
Pla-1_AGGGAGTGTAGAGGAA	Pla	Pla-1	P1-1283	FALSE	1	TRA:CAVNIRGLTGGGNKLTF TRB:CASSPPTGSNTEAFF	1	CD8-C3

Pla-1_AGGGATGAGTAGGCCA	Pla	Pla-1	P1-1285	FALSE	1	TRA:CAVRGRGYSTLTF TRB:CASSQEGSNQPQHF	1	CD8-C3
Pla-1_AGGGATGTCAATAAGG	Pla	Pla-1	P1-1286	FALSE	1	TRA:CAMRGFGNVLHC TRB:CASSTGTQKNYGYTF	1	CD8-C2
Pla-1_AGGGATGTCCGCATCT	Pla	Pla-1	P1-32	FALSE	3	TRA:CAVMNTGFQKLVF TRB:CSVSGLVLGGNEQFF	46	CD8-C3
Pla-1_AGGTCATGTTGGTGGA	Pla	Pla-1	P1-221	FALSE	3	TRA:CAVIENNARLMF TRB:CASSSGTYEQYF	3	CD8-C2
Pla-1_AGGTCATTCAACGGGA	Pla	Pla-1	P1-1289	FALSE	1	TRA:CAGYPSGGYNKLIF TRB:CASSWLAGDNPHEQYF	1	CD8-C3
Pla-1_AGGTCATTCGATGAGG	Pla	Pla-1	P1-32	FALSE	3	TRA:CAVMNTGFQKLVF TRB:CSVSGLVLGGNEQFF	46	CD8-C3
Pla-1_AGGTCCGGTAAGCACG	Pla	Pla-1	P1-120	FALSE	3	TRA:CAYRSSYSGYALNF TRB:CAWSPYSEKLFF	5	CD4-C4
Pla-1_AGGTCCGGTGCCTGTG	Pla	Pla-1	P1-40	TRUE	3	TRA:CAVRDVGNTGFQKLVF TRB:CASSLGQGSMKTQYF	27	CD8-C3
Pla-1_AGTGAGGTCCTATTCA	Pla	Pla-1	P1-222	FALSE	3	TRA:CAVRLPYNTDKLIF TRB:CASSFRHEQYF	3	CD8-C2
Pla-1_ATAACGCGTTAGAACA	Pla	Pla-1	P1-1308	FALSE	1	TRA:CAETPTGKLIF TRB:CASSLEGSSYEQYF	1	CD8-C3
Pla-1_ATAAGAGAGCTGAAAT	Pla	Pla-1	P1-32	FALSE	3	TRA:CAVMNTGFQKLVF TRB:CSVSGLVLGGNEQFF	46	CD8-C3
Pla-1_ATAAGAGAGTCATCCA	Pla	Pla-1	P1-62	TRUE	3	TRA:CVVNLMEYGNKLVF TRB:CASSGARQGQYEQYF	10	CD8-C3
Pla-1_ATAAGAGCAATGTAAG	Pla	Pla-1	P1-37	TRUE	3	TRA:CAVNIVGNQFYF TRB:CASSPLGTEAFF	40	CD8-C3
Pla-1_ATCACGACAGGACGTA	Pla	Pla-1	P1-32	FALSE	3	TRA:CAVMNTGFQKLVF TRB:CSVSGLVLGGNEQFF	46	CD8-C8
Pla-1_ATCATCTGTAGCGCAA	Pla	Pla-1	P1-44	TRUE	3	TRA:CAYRGLGAQKLVF TRB:CASSSLDGQDNHYGYTF	22	CD8-C3
Pla-1_ATCCACCAGTGAAGAG	Pla	Pla-1	P1-1327	FALSE	1	TRA:CAVPPSARQLTF TRB:CASSESGLELDNSYNEQFF	1	CD8-C3
Pla-1_ATCCACCGTACCTACA	Pla	Pla-1	P1-57	FALSE	3	TRA:CAMREGGNTGNQFYF TRB:CASKKDRGTTQAKNIQYF	13	CD8-C3
Pla-1_ATCCGAAAGCTCCCAG	Pla	Pla-1	P1-128	FALSE	3	TRA:CAVVNTGTASKLTF TRB:CASSEQGYEQYF	5	CD8-C3
Pla-1_ATCCGAAGTGTCAATC	Pla	Pla-1	P1-9	TRUE	3	TRA:CAEAEGGGADGLTF TRB:CATSGGDNEQFF	174	CD8-C3
Pla-1_ATCGAGTTCTTGACGA	Pla	Pla-1	P1-347	FALSE	2	TRA:CALSDRGGNNRLAF TRB:CASSFVGVEVGLPVDTQYF	2	CD8-C3
Pla-1_ATCTGCCAGAGCCTAG	Pla	Pla-1	P1-23	TRUE	3	TRA:CALRAITQGGSEKLVF TRB:CASSLTGAVYGYTF	65	CD8-C3
Pla-1_ATGAGGGTCTGGTATG	Pla	Pla-1	P1-40	TRUE	3	TRA:CAVRDVGNTGFQKLVF TRB:CASSLGQGSMKTQYF	27	CD8-C3
Pla-1_ATGCGATTCAGCAACT	Pla	Pla-1	P1-349	FALSE	2	TRA:CAASKGFGNVLHC TRB:CASSPQGGGRGSYNEQFF	2	CD8-C3
Pla-1_ATGGGAGGTTCGTTGA	Pla	Pla-1	P1-75	FALSE	3	TRA:CAMRGGNFNKFYF TRB:CSAQGAYEQYF	8	CD8-C3
Pla-1_ATTACTCAGTGTTAGA	Pla	Pla-1	P1-1352	FALSE	1	TRA:CAVNKDSGTYKYIF TRB:CASEGEGYGYTF	1	CD8-C3
Pla-1_ATTACTCCAAGCTGGA	Pla	Pla-1	P1-125	FALSE	3	TRA:CILKSRTSYDKVIF TRB:CASSFLSGTDTQYF	5	CD8-C8
Pla-1_ATTACTCCAGGCAGTA	Pla	Pla-1	P1-225	FALSE	3	TRA:CAARYGGSQGNLIF TRB:CASSGLRFSGANVLTF	3	CD8-C3

Pla-1_ATTACTCGTCGATTGT	Pla	Pla-1	P1-33	TRUE	3	TRA:CLVGTGGFKTIF TRB:CSARVPGQMKETQYF	41	CD8-C3
Pla-1_ATTACTCTCGCCCTTA	Pla	Pla-1	P1-1353	FALSE	1	TRA:CAASRSDYKLSF TRB:CASSVGTGTTQYF	1	CD8-C8
Pla-1_ATTATCCCAGGACGTA	Pla	Pla-1	P1-40	TRUE	3	TRA:CAVRDVGNTGFQKLVF TRB:CASSLGQGSMKTQYF	27	CD8-C3
Pla-1_ATTATCCGTACAGTGG	Pla	Pla-1	P1-44	TRUE	3	TRA:CAYRGLGAQKLVF TRB:CASSSLDGQDNHYGYTF	22	CD8-C5
Pla-1_ATTGGACCAGTAAGAT	Pla	Pla-1	P1-92	TRUE	3	TRA:CIVQLTGTASKLTF TRB:CASSFKSGRTNEQFF	6	CD4-C4
Pla-1_ATTGGTGCAGGACGTA	Pla	Pla-1	P1-45	TRUE	3	TRA:CAYRPLGAQKLVF TRB:CASSTLDGRESSYEQYF	20	CD8-C3
Pla-1_ATTGGTGGTAGCTTGT	Pla	Pla-1	P1-23	TRUE	3	TRA:CALRAITQGGSEKLVF TRB:CASSLTGAVYGYTF	65	CD8-C3
Pla-1_ATTGGTGTCGAGAGCA	Pla	Pla-1	P1-223	FALSE	3	TRA:CGTNQAGTALIF TRB:CASSLSRNYNSPLHF	3	CD8-C3
Pla-1_ATTTCTGGTAGATTAG	Pla	Pla-1	P1-23	TRUE	3	TRA:CALRAITQGGSEKLVF TRB:CASSLTGAVYGYTF	65	CD8-C3
Pla-1_ATTTCTGTCGCTTAGA	Pla	Pla-1	P1-23	TRUE	3	TRA:CALRAITQGGSEKLVF TRB:CASSLTGAVYGYTF	65	CD8-C5
Pla-1_CAACCTCGTCTTGTCC	Pla	Pla-1	P1-68	TRUE	3	TRA:CAVIPDILTGGGNKLTF TRB:CASSLQGFGNTIYF	9	CD8-C3
Pla-1_CAAGAAAAGCTGAACG	Pla	Pla-1	P1-55	TRUE	3	TRA:CALSPQDSNYQLIW TRB:CASSFWTSANEQFF	13	CD8-C3
Pla-1_CAAGAAAAGTGCGATG	Pla	Pla-1	P1-1371	FALSE	1	TRA:CAVQAWNTGNQFYF TRB:CASSQEILGSVSTDTQYF	1	CD8-C5
Pla-1_CAAGAAACATCGATTG	Pla	Pla-1	P1-133	FALSE	3	TRA:CAFMNRGSTLGRLYF TRB:CASSFLSGTDTQYF	5	CD8-C8
Pla-1_CAAGATCCATGAAGTA	Pla	Pla-1	P1-1374	FALSE	1	TRA:CALTGGDNYGQNFVF TRB:CASSLGLAGTGELFF	1	CD4-C4
Pla-1_CAAGATCGTTGAGTTC	Pla	Pla-1	P1-186	TRUE	3	TRA:CAVPPYMDSNYQLIW TRB:CASSLFGASPLHF	3	CD8-C3
Pla-1_CAAGGCCCAATCAGAA	Pla	Pla-1	P1-20	TRUE	3	TRA:CVVVGGRALTF TRB:CASSLATNPHETQYF	69	CD8-C3
Pla-1_CAAGGCCTCGCTTAGA	Pla	Pla-1	P1-20	TRUE	3	TRA:CVVVGGRALTF TRB:CASSLATNPHETQYF	69	CD8-C3
Pla-1_CAAGTTGAGATATGGT	Pla	Pla-1	P1-1380	FALSE	1	TRA:CIVGGLEYGNKLVF TRB:CASSWDFYNEQFF	1	CD8-C3
Pla-1_CACACAAGTACTTGAC	Pla	Pla-1	P1-23	TRUE	3	TRA:CALRAITQGGSEKLVF TRB:CASSLTGAVYGYTF	65	CD8-C5
Pla-1_CACACTCGTGCCTGCA	Pla	Pla-1	P1-123	FALSE	3	TRA:CAVKRVTGGFKTIF TRB:CATSGDTGTTNYNEQFF	5	CD8-C2
Pla-1_CACAGGCGTACGAAAT	Pla	Pla-1	P1-327	FALSE	2	TRA:CAYLWRGGGADGLTF TRB:CASSQGSGLYNEQFF	2	CD8-C3
Pla-1_CACAGTACAAATACAG	Pla	Pla-1	P1-57	FALSE	3	TRA:CAMREGGNTGNQFYF TRB:CASKKDRGTTQAKNIQYF	13	CD8-C3
Pla-1_CACAGTATCAGTTGAC	Pla	Pla-1	P1-1395	FALSE	1	TRA:CAVNIVGNQFYF TRB:CASSLTGAVYGYTF	1	CD8-C7
Pla-1_CACATAGAGCGTGAAC	Pla	Pla-1	P1-23	TRUE	3	TRA:CALRAITQGGSEKLVF TRB:CASSLTGAVYGYTF	65	CD8-C3
Pla-1_CACCACTGTCTAGTCA	Pla	Pla-1	P1-32	FALSE	3	TRA:CAVMNTGFQKLVF TRB:CSVSGLVLGGNEQFF	46	CD8-C3
Pla-1_CACTCCAAGTTGTCGT	Pla	Pla-1	P1-123	FALSE	3	TRA:CAVKRVTGGFKTIF TRB:CATSGDTGTTNYNEQFF	5	CD8-C2

Pla-1_CACTCCACAAGTAGTA	Pla	Pla-1	P1-75	FALSE	3	TRA:CAMRGGNFNKFYF TRB:CSAQGAYEQYF	8	CD8-C3
Pla-1_CACTCCAGTCGTGGCT	Pla	Pla-1	P1-1414	FALSE	1	TRA:CAGLYSGNTGKLIF TRB:CASSLFYPAGQQYF	1	CD8-C3
Pla-1_CACTCCAGTGAACCTT	Pla	Pla-1	P1-362	FALSE	2	TRA:CAISSSNTGKLIF TRB:CSVSLQGARRSGEKLFF	2	CD8-C2
Pla-1_CAGAATCGTCGCTTTC	Pla	Pla-1	P1-20	TRUE	3	TRA:CVVVGGRALTF TRB:CASSLATNPHETQYF	69	CD8-C4
Pla-1_CAGAGAGAGTCCATAC	Pla	Pla-1	P1-10	TRUE	3	TRA:CISNSPMDSSYKLIF TRB:CASSSWGDTDEAFF	162	CD8-C3
Pla-1_CAGAGAGCATCATCCC	Pla	Pla-1	P1-45	TRUE	3	TRA:CAYRPLGAQKLVF TRB:CASSTLDGRESSYEQYF	20	CD8-C3
Pla-1_CAGATCACACTACAGT	Pla	Pla-1	P1-103	FALSE	3	TRA:CATENDYKLSF TRB:CSVGGTFANTGELFF	6	CD8-C3
Pla-1_CAGCAGCAGACCTAGG	Pla	Pla-1	P1-65	FALSE	3	TRA:CAVQAHSGGSNYKLTF TRB:CASAETSYTGELFF	10	CD4-C4
Pla-1_CAGCCGAGTCTAAAGA	Pla	Pla-1	P1-134	FALSE	3	TRA:CAGGGGSQGNLIF TRB:CASSQGLIYGYTF	5	CD8-C3
Pla-1_CAGCGACGTGTTAAGA	Pla	Pla-1	P1-20	TRUE	3	TRA:CVVVGGRALTF TRB:CASSLATNPHETQYF	69	CD8-C4
Pla-1_CAGCTAAGTCCGTCAG	Pla	Pla-1	P1-366	FALSE	2	TRA:CAGMDTGRRALTF TRB:CASSIYRQGSQPQHF	2	CD8-C3
Pla-1_CAGCTAAGTCTCTTAT	Pla	Pla-1	P1-230	FALSE	3	TRA:CAVDPWGSQGNLIF TRB:CASSPGREQFF	3	CD8-C2
Pla-1_CAGCTAATCTCCAGGG	Pla	Pla-1	P1-20	TRUE	3	TRA:CVVVGGRALTF TRB:CASSLATNPHETQYF	69	CD8-C3
Pla-1_CAGCTGGGTCCGAACC	Pla	Pla-1	P1-1428	FALSE	1	TRA:CALRDISNFGNEKLTF TRB:CASSATGKLYGYTF	1	CD8-C3
Pla-1_CAGCTGGTCGTAGATC	Pla	Pla-1	P1-231	FALSE	3	TRA:CAVGPSDAGNNRKLIW TRB:CASSYSGGGEETQYF	3	CD8-C3
Pla-1_CAGTAACCAAGCTGAG	Pla	Pla-1	P1-1432	FALSE	1	TRA:CATDHTGTASKLTF TRB:CSATEGALSRQPQHF	1	CD8-C3
Pla-1_CAGTCCTGTCGGCATC	Pla	Pla-1	P1-368	FALSE	2	TRA:CAVSDAFYGSSNTGKLIF TRB:CASSLGTRSGREPNQPQH	2	CD8-C3
Pla-1_CAGTCCTGTTCGCTAA	Pla	Pla-1	P1-104	FALSE	3	TRA:CALRGSARQLTF TRB:CASSQEEAGPAKNIQYF	6	CD8-C3
Pla-1_CATATTCAGGGTATCG	Pla	Pla-1	P1-40	TRUE	3	TRA:CAVRDVGNTGFQKLVF TRB:CASSLGQGSMKTQYF	27	CD8-C3
Pla-1_CATCAGACAATGTAAG	Pla	Pla-1	P1-33	TRUE	3	TRA:CLVGTGGFKTIF TRB:CSARVPGQMKETQYF	41	CD8-C3
Pla-1_CATCAGAGTGGAAAGA	Pla	Pla-1	P1-75	FALSE	3	TRA:CAMRGGNFNKFYF TRB:CSAQGAYEQYF	8	CD8-C3
Pla-1_CATCAGATCTACCTGC	Pla	Pla-1	P1-1444	FALSE	1	TRA:CAFSGGYQKVTF TRB:CASSLGTGEPYGYTF	1	CD8-C3
Pla-1_CATCGAAAGAAGGTTT	Pla	Pla-1	P1-49	FALSE	3	TRA:CAGGGSEKLVF TRB:CASSLFYPAGQQYF	16	CD8-C3
Pla-1_CATCGAATCTCGTTTA	Pla	Pla-1	P1-20	TRUE	3	TRA:CVVVGGRALTF TRB:CASSLATNPHETQYF	69	CD8-C3
Pla-1_CATCGGGGTTCGTCTC	Pla	Pla-1	P1-1448	FALSE	1	TRA:CAEVPQFYF TRB:CASSLGQGRRAEAFF	1	CD8-C3
Pla-1_CATGACACAGCATGAG	Pla	Pla-1	P1-45	TRUE	3	TRA:CAYRPLGAQKLVF TRB:CASSTLDGRESSYEQYF	20	CD8-C4
Pla-1_CATGACACATTTCACT	Pla	Pla-1	P1-7	TRUE	3	TRA:CAVQAGNNDMRF TRB:CASSTTGDFNYGYTF	215	CD8-C5

Pla-1_CATGGCGAGAGTCTGG	Pla	Pla-1	P1-1461	FALSE	1	TRA:CAAYREGGAQKLVF TRB:CASSYSMVGTEAFF	1	CD8-C3
Pla-1_CATGGCGAGTGTTGAA	Pla	Pla-1	P1-135	FALSE	3	TRA:CAVRASGANSKLTF TRB:CSAQIGTGPEKLFF	5	CD8-C3
Pla-1_CATTATCCACGGTAAG	Pla	Pla-1	P1-1464	FALSE	1	TRA:CAVEDSGGYQKVTF TRB:CASSPGRADTQYF	1	CD8-C3
Pla-1_CATTCGCCACACTGCG	Pla	Pla-1	P1-158	FALSE	3	TRA:CALRVITQGGSEKLVF TRB:CASSEGSSYNEQFF	4	CD8-C3
Pla-1_CATTCGCGTACCGTAT	Pla	Pla-1	P1-1468	FALSE	1	TRA:CAENQGPGGSYIPTF TRB:CASSEFSGADNQPQHF	1	CD8-C3
Pla-1_CCACGGAGTGTAATGA	Pla	Pla-1	P1-20	TRUE	3	TRA:CVVVGGRALTF TRB:CASSLATNPHETQYF	69	CD8-C3
Pla-1_CCACTACAGAAGATTC	Pla	Pla-1	P1-1478	FALSE	1	TRA:CAATSGSARQLTF TRB:CASRRQAVHTEAFF	1	CD8-C2
Pla-1_CCACTACCAGGTCTCG	Pla	Pla-1	P1-1480	FALSE	1	TRA:CAPLFGGYNKLIF TRB:CSAVPLEDYGYTF	1	CD8-C3
Pla-1_CCAGCGACAATGGACG	Pla	Pla-1	P1-1484	FALSE	1	TRA:CAYYSGAGSYQLTF TRB:CASSLAEKLVYEQYF	1	CD8-C2
Pla-1_CCAGCGACACATTAGC	Pla	Pla-1	P1-80	FALSE	3	TRA:CAASAEGSQGNLIF TRB:CASKKDRGTTQAKNIQYF	7	CD8-C3
Pla-1_CCATGTCGTCTGCAAT	Pla	Pla-1	P1-1485	FALSE	1	TRA:CAVRDIGRNRGSTLGRLYF TRB:CASSLRGPTQRTDTQYF	1	CD8-C3
Pla-1_CCATTCGGTCATCGGC	Pla	Pla-1	P1-120	FALSE	3	TRA:CAYRSSYSGYALNF TRB:CAWSPYSEKLFF	5	CD8-C3
Pla-1_CCCTCCTTCGCCAGCA	Pla	Pla-1	P1-236	FALSE	3	TRA:CAMREGRF TRB:CASSLRVPGSDTQYF	3	CD8-C3
Pla-1_CCGGTAGAGCACAGGT	Pla	Pla-1	P1-1499	FALSE	1	TRA:CAVRVRSNDYKLSF TRB:CASSWGGPYTEAFF	1	CD8-C3
Pla-1_CCGGTAGCATGCTAGT	Pla	Pla-1	P1-33	TRUE	3	TRA:CLVGTGGFKTIF TRB:CSARVPGQMKETQYF	41	CD8-C3
Pla-1_CCGGTAGGTCTAAAGA	Pla	Pla-1	P1-23	TRUE	3	TRA:CALRAITQGGSEKLVF TRB:CASSLTGAVYGYTF	65	CD8-C3
Pla-1_CCGGTAGTCACTTCAT	Pla	Pla-1	P1-32	FALSE	3	TRA:CAVMNTGFQKLVF TRB:CSVSGLVLGGNEQFF	46	CD8-C8
Pla-1_CCGTACTCAAGCCGCT	Pla	Pla-1	P1-1502	FALSE	1	TRA:CAVGSGYSTLTF TRB:CSARDRKGTSGAYEQFF	1	CD8-C3
Pla-1_CCGTACTTCTTACCTA	Pla	Pla-1	P1-132	FALSE	3	TRA:CAVKGSQGNLIF TRB:CSARITYLNTEAFF	5	CD8-C2
Pla-1_CCGTGGAAGTACGTTC	Pla	Pla-1	P1-45	TRUE	3	TRA:CAYRPLGAQKLVF TRB:CASSTLDGRESSYEQYF	20	CD8-C3
Pla-1_CCGTGGACATTTCAGG	Pla	Pla-1	P1-1503	FALSE	1	TRA:CAVGAWEYGNKLVF TRB:CASSQDDSGGGQETQYF	1	CD8-C3
Pla-1_CCGTTCATCGCCTGTT	Pla	Pla-1	P1-7	TRUE	3	TRA:CAVQAGNNDMRF TRB:CASSTTGDFNYGYTF	215	CD8-C3
Pla-1_CCTACACAGAAGCCCA	Pla	Pla-1	P1-23	TRUE	3	TRA:CALRAITQGGSEKLVF TRB:CASSLTGAVYGYTF	65	CD8-C3
Pla-1_CCTACACCAATACGCT	Pla	Pla-1	P1-1509	FALSE	1	TRA:CAARGYQLTF TRB:CASSKGQGPRETQYF	1	CD8-C2
Pla-1_CCTACACTCGTGTAGT	Pla	Pla-1	P1-20	TRUE	3	TRA:CVVVGGRALTF TRB:CASSLATNPHETQYF	69	CD8-C3
Pla-1_CCTATTATCCACGCAG	Pla	Pla-1	P1-376	FALSE	2	TRA:CAYRSAGEGLTF TRB:CASSLPSSGSFTDTQYF	2	CD8-C3
Pla-1_CCTCAGTAGGGTCTCC	Pla	Pla-1	P1-57	FALSE	3	TRA:CAMREGGNTGNQFYF TRB:CASKKDRGTTQAKNIQYF	13	CD8-C3

Pla-1_CCTCAGTCACCGAAAG	Pla	Pla-1	P1-1522	FALSE	1	TRA:CAVRDNNFNKFYF TRB:CSVEDLEGRRAQYF	1	CD8-C8
Pla-1_CCTCAGTCATAAGACA	Pla	Pla-1	P1-7	TRUE	3	TRA:CAVQAGNNDMRF TRB:CASSTTGDFNYGYTF	215	CD8-C3
Pla-1_CCTCAGTTCCAATGGT	Pla	Pla-1	P1-80	FALSE	3	TRA:CAASAEGSQGNLIF TRB:CASKKDRGTTQAKNIQYF	7	CD8-C3
Pla-1_CCTTACGCATGACATC	Pla	Pla-1	P1-37	TRUE	3	TRA:CAVNIVGNQFYF TRB:CASSPLGTEAFF	40	CD8-C3
Pla-1_CCTTCCCGTCCTCCAT	Pla	Pla-1	P1-1532	FALSE	1	TRA:CATDAREGSNYKLTF TRB:CASSNRLKTAEAFF	1	CD8-C3
Pla-1_CCTTCCCTCCTATGTT	Pla	Pla-1	P1-1533	FALSE	1	TRA:CAVQYNTDKLIF TRB:CSAREGWATQYF	1	CD8-C3
Pla-1_CGAACATTCAGGTAAA	Pla	Pla-1	P1-49	FALSE	3	TRA:CAGGGSEKLVF TRB:CASSLFYPAGQQYF	16	CD8-C2
Pla-1_CGAATGTCACAGGCCT	Pla	Pla-1	P1-1539	FALSE	1	TRA:CAFFSQGGSEKLVF TRB:CSARDRLAGSTGELFF	1	CD8-C3
Pla-1_CGAATGTGTGCAGTAG	Pla	Pla-1	P1-23	TRUE	3	TRA:CALRAITQGGSEKLVF TRB:CASSLTGAVYGYTF	65	CD8-C3
Pla-1_CGAGAAGCAGCTCCGA	Pla	Pla-1	P1-1552	FALSE	1	TRA:CAVGATGGSYIPTF TRB:CASSSPNEKLFF	1	CD8-C3
Pla-1_CGAGCACCAAAGAATC	Pla	Pla-1	P1-1555	FALSE	1	TRA:CAAEMDSSYKLIF TRB:CASSLYAGESYNEQFF	1	CD8-C8
Pla-1_CGATCGGAGACCACGA	Pla	Pla-1	P1-1561	FALSE	1	TRA:CAMSDASGGYNKLIF TRB:CASSSRRGVGEAFF	1	CD8-C3
Pla-1_CGATCGGCATGCCTTC	Pla	Pla-1	P1-137	FALSE	3	TRA:CAVSMNRDDKIIF TRB:CASSSGRLSGANVLTF	5	CD8-C3
Pla-1_CGATCGGTCGTTTAGG	Pla	Pla-1	P1-1562	FALSE	1	TRA:CGTGLSNTGNQFYF TRB:CASSLAYPSYNEQFF	1	CD8-C3
Pla-1_CGATGGCTCAGTCAGT	Pla	Pla-1	P1-239	FALSE	3	TRA:CAASLGDTDKLIF TRB:CASSPWTGVNEQFF	3	CD8-C3
Pla-1_CGATGGCTCATGTCTT	Pla	Pla-1	P1-380	FALSE	2	TRA:CADNAGNNRKLIW TRB:CASDSGVSYEQYF	2	CD8-C3
Pla-1_CGATGTAGTGTCCTCT	Pla	Pla-1	P1-135	FALSE	3	TRA:CAVRASGANSKLTF TRB:CSAQIGTGPEKLFF	5	CD8-C3
Pla-1_CGATTGAAGGCTCATT	Pla	Pla-1	P1-83	FALSE	3	TRA:CAVRDIENTGFQKLVF TRB:CASTSGGFNQPQHF	7	CD8-C2
Pla-1_CGATTGAGTCCTCTTG	Pla	Pla-1	P1-236	FALSE	3	TRA:CAMREGRF TRB:CASSLRVPGSDTQYF	3	CD8-C3
Pla-1_CGCCAAGTCAACACGT	Pla	Pla-1	P1-83	FALSE	3	TRA:CAVRDIENTGFQKLVF TRB:CASTSGGFNQPQHF	7	CD8-C8
Pla-1_CGCGGTAAGTACTTGC	Pla	Pla-1	P1-6	TRUE	3	TRA:CAMREGRERDDKIIF TRB:CASSLPNTEAFF	216	CD8-C3
Pla-1_CGCGTTTAGAACAATC	Pla	Pla-1	P1-23	TRUE	3	TRA:CALRAITQGGSEKLVF TRB:CASSLTGAVYGYTF	65	CD8-C3
Pla-1_CGCTATCCACCAGATT	Pla	Pla-1	P1-23	TRUE	3	TRA:CALRAITQGGSEKLVF TRB:CASSLTGAVYGYTF	65	CD8-C3
Pla-1_CGCTATCGTTTGTGTG	Pla	Pla-1	P1-167	FALSE	3	TRA:CGTGINYGGQNFVF TRB:CASSPRDGGGTEAFF	4	CD8-C3
Pla-1_CGCTGGAAGGAGTTTA	Pla	Pla-1	P1-44	TRUE	3	TRA:CAYRGLGAQKLVF TRB:CASSSLDGQDNHYGYTF	22	CD8-C3
Pla-1_CGCTGGACAGAGCCAA	Pla	Pla-1	P1-1575	FALSE	1	TRA:CAYRNPAPNAGNMLTF TRB:CASSPPLHSGRWREQYF	1	CD8-C3
Pla-1_CGCTGGAGTCGCATCG	Pla	Pla-1	P1-49	FALSE	3	TRA:CAGGGSEKLVF TRB:CASSLFYPAGQQYF	16	CD8-C3

Pla-1_CGCTGGATCTGCTTGC	Pla	Pla-1	P1-70	FALSE	3	TRA:CAMRETPGEAGTALIF TRB:CSARITYLNTEAFF	9	CD8-C3
Pla-1_CGCTTCAAGTGAATTG	Pla	Pla-1	P1-197	TRUE	3	TRA:CAENFQGAQKLVF TRB:CSVDPSGNTGELFF	3	CD8-C5
Pla-1_CGGACGTGTAACGACG	Pla	Pla-1	P1-32	FALSE	3	TRA:CAVMNTGFQKLVF TRB:CSVSGLVLGGNEQFF	46	CD8-C4
Pla-1_CGGACGTGTATAATGG	Pla	Pla-1	P1-1584	FALSE	1	TRA:CAVRLNYGGSQGNLIF TRB:CAISLTGDGKETQYF	1	CD8-C3
Pla-1_CGGACGTGTTGATTGC	Pla	Pla-1	P1-1586	FALSE	1	TRA:CAYRYFYGGSQGNLIF TRB:CASSFTPYEQYF	1	CD8-C3
Pla-1_CGGACTGAGTGCGATG	Pla	Pla-1	P1-1588	FALSE	1	TRA:CATDEAEYGNKLVF TRB:CASSGVRSPLHF	1	CD8-C8
Pla-1_CGGACTGGTGCGATAG	Pla	Pla-1	P1-1589	FALSE	1	TRA:CVVEEGKLIF TRB:CASSKGTRTLEAFF	1	CD8-C3
Pla-1_CGGACTGTCCAAACAC	Pla	Pla-1	P1-191	TRUE	3	TRA:CIVSGAYSGAGSYQLTF TRB:CASSLVRGGETQYF	3	CD8-C3
Pla-1_CGGAGCTGTAAGTAGT	Pla	Pla-1	P1-1591	FALSE	1	TRA:CAVHGSGNTGKLIF TRB:CASYASPSGNTIYF	1	CD8-C3
Pla-1_CGGAGCTTCAACGAAA	Pla	Pla-1	P1-8	TRUE	3	TRA:CAVRPPGRNSGGYQKVTF TRB:CASSRRGVTDTQYF	204	CD8-C5
Pla-1_CGGAGTCCATCCGGGT	Pla	Pla-1	P1-74	TRUE	3	TRA:CAVGGGSTLGRLYF TRB:CASSIVAGADTEAFF	8	CD8-C3
Pla-1_CGGCTAGCAGACAAAT	Pla	Pla-1	P1-20	TRUE	3	TRA:CVVVGGRALTF TRB:CASSLATNPHETQYF	69	CD8-C3
Pla-1_CGGCTAGCAGTGAGTG	Pla	Pla-1	P1-1595	FALSE	1	TRA:CVVSPHDMRF TRB:CASSFSGGTEAFF	1	CD8-C3
Pla-1_CGGGTCACAATACGCT	Pla	Pla-1	P1-37	TRUE	3	TRA:CAVNIVGNQFYF TRB:CASSPLGTEAFF	40	CD8-C3
Pla-1_CGGGTCACACCTCGTT	Pla	Pla-1	P1-55	TRUE	3	TRA:CALSPQDSNYQLIW TRB:CASSFWTSANEQFF	13	CD8-C3
Pla-1_CGGTTAAAGACCTAGG	Pla	Pla-1	P1-188	TRUE	3	TRA:CPRNNNARLMF TRB:CASTSGGFNQPQHF	3	CD8-C2
Pla-1_CGGTTAACAGGAATCG	Pla	Pla-1	P1-32	FALSE	3	TRA:CAVMNTGFQKLVF TRB:CSVSGLVLGGNEQFF	46	CD8-C3
Pla-1_CGTAGCGCATTCCTGC	Pla	Pla-1	P1-57	FALSE	3	TRA:CAMREGGNTGNQFYF TRB:CASKKDRGTTQAKNIQYF	13	CD8-C2
Pla-1_CGTAGGCGTAGCCTAT	Pla	Pla-1	P1-100	FALSE	3	TRA:CAVRDRITGNQFYF TRB:CASSQDWGSNQPQHF	6	CD8-C3
Pla-1_CGTAGGCGTGTCCTCT	Pla	Pla-1	P1-1606	FALSE	1	TRA:CAVSGGGNQFYF TRB:CASSSSRGAADTQYF	1	CD8-C3
Pla-1_CGTAGGCGTGTGCCTG	Pla	Pla-1	P1-1607	FALSE	1	TRA:CVVVGGRALTF TRB:CASSQTTGREAKNIQYF	1	CD8-C3
Pla-1_CGTAGGCTCGACAGCC	Pla	Pla-1	P1-49	FALSE	3	TRA:CAGGGSEKLVF TRB:CASSLFYPAGQQYF	16	CD4-C4
Pla-1_CGTCAGGAGTGAACGC	Pla	Pla-1	P1-20	TRUE	3	TRA:CVVVGGRALTF TRB:CASSLATNPHETQYF	69	CD8-C3
Pla-1_CGTCCATAGGTAGCTG	Pla	Pla-1	P1-20	TRUE	3	TRA:CVVVGGRALTF TRB:CASSLATNPHETQYF	69	CD8-C3
Pla-1_CGTCCATAGTAGGCCA	Pla	Pla-1	P1-137	FALSE	3	TRA:CAVSMNRDDKIIF TRB:CASSSGRLSGANVLTF	5	CD8-C5
Pla-1_CGTCCATGTACTTAGC	Pla	Pla-1	P1-386	FALSE	2	TRA:CVVSSGGYNKLIF TRB:CASRPTGEGNEQFF	2	CD8-C5
Pla-1_CGTCCATTCTTTAGTC	Pla	Pla-1	P1-33	TRUE	3	TRA:CLVGTGGFKTIF TRB:CSARVPGQMKETQYF	41	CD8-C3

Pla-1_CGTCTACCACAACTGT	Pla	Pla-1	P1-1614	FALSE	1	TRA:CAMSGGGADGLTF TRB:CAWTPTGTGDHGYTF	1	CD8-C8
Pla-1_CGTCTACGTTCACGGC	Pla	Pla-1	P1-81	FALSE	3	TRA:CAVTFAGNQFYF TRB:CSAGTGTSGLSEQFF	7	CD8-C3
Pla-1_CGTGAGCCACCGAATT	Pla	Pla-1	P1-62	TRUE	3	TRA:CVVNLMEYGNKLVF TRB:CASSGARQGQYEQYF	10	CD8-C3
Pla-1_CGTGTAACACCTCGTT	Pla	Pla-1	P1-1622	FALSE	1	TRA:CATDNSNQAGTALIF TRB:CASSFGQTLDEQYF	1	CD8-C3
Pla-1_CGTGTCTAGTCTTGCA	Pla	Pla-1	P1-1626	FALSE	1	TRA:CAVDQGGKLIF TRB:CSVPASGSVNEQFF	1	CD8-C3
Pla-1_CGTGTCTCAGCTCCGA	Pla	Pla-1	P1-46	FALSE	3	TRA:CAVRGTGTASKLTF TRB:CSVRQGGNEQFF	20	CD8-C3
Pla-1_CGTGTCTGTAAACACA	Pla	Pla-1	P1-60	FALSE	3	TRA:CAYLSSTGTASKLTF TRB:CASSSLDGQDNHYGYTF	11	CD8-C3
Pla-1_CGTTGGGAGTACGACG	Pla	Pla-1	P1-227	FALSE	3	TRA:CAVVNAGKSTF TRB:CASSLTGLVSNSPLHF	3	CD8-C3
Pla-1_CTAACTTCAGGGATTG	Pla	Pla-1	P1-100	FALSE	3	TRA:CAVRDRITGNQFYF TRB:CASSQDWGSNQPQHF	6	CD8-C3
Pla-1_CTAACTTCATAAAGGT	Pla	Pla-1	P1-1633	FALSE	1	TRA:CAVRVLGDSNYQLIW TRB:CASSPPKAAQETQYF	1	CD8-C3
Pla-1_CTAACTTGTGTGCGTC	Pla	Pla-1	P1-20	TRUE	3	TRA:CVVVGGRALTF TRB:CASSLATNPHETQYF	69	CD8-C5
Pla-1_CTAAGACGTAGCAAAT	Pla	Pla-1	P1-37	TRUE	3	TRA:CAVNIVGNQFYF TRB:CASSPLGTEAFF	40	CD8-C3
Pla-1_CTAAGACTCCGAACGC	Pla	Pla-1	P1-86	TRUE	3	TRA:CAVGGRSNTGRRALTF TRB:CAISLTGDGKETQYF	6	CD8-C3
Pla-1_CTAATGGCAAGAAAGG	Pla	Pla-1	P1-104	FALSE	3	TRA:CALRGSARQLTF TRB:CASSQEEAGPAKNIQYF	6	CD4-C4
Pla-1_CTACACCCACTCTGTC	Pla	Pla-1	P1-1639	FALSE	1	TRA:CITFQTGANNLFF TRB:CASSIGTATEAFF	1	CD8-C3
Pla-1_CTACACCTCCCTCTTT	Pla	Pla-1	P1-67	TRUE	3	TRA:CALNTNQAGTALIF TRB:CASSLGTRYAYEQYF	9	CD8-C3
Pla-1_CTACCCACACATGGGA	Pla	Pla-1	P1-125	FALSE	3	TRA:CILKSRTSYDKVIF TRB:CASSFLSGTDTQYF	5	CD8-C8
Pla-1_CTACCCAGTGTGAAAT	Pla	Pla-1	P1-1648	FALSE	1	TRA:CAVRAARGAGGYNKLIF TRB:CASSTIRRTSGGEQYF	1	CD8-C8
Pla-1_CTAGAGTCAGACAGGT	Pla	Pla-1	P1-44	TRUE	3	TRA:CAYRGLGAQKLVF TRB:CASSSLDGQDNHYGYTF	22	CD8-C3
Pla-1_CTAGAGTCAGGAATGC	Pla	Pla-1	P1-133	FALSE	3	TRA:CAFMNRGSTLGRLYF TRB:CASSFLSGTDTQYF	5	CD8-C5
Pla-1_CTAGTGATCAGCTGGC	Pla	Pla-1	P1-60	FALSE	3	TRA:CAYLSSTGTASKLTF TRB:CASSSLDGQDNHYGYTF	11	CD8-C3
Pla-1_CTCATTAGTACTCTCC	Pla	Pla-1	P1-20	TRUE	3	TRA:CVVVGGRALTF TRB:CASSLATNPHETQYF	69	CD8-C4
Pla-1_CTCCTAGCATGCCTAA	Pla	Pla-1	P1-1674	FALSE	1	TRA:CAPQRGSNYKLTF TRB:CASSLVPDQETQYF	1	CD8-C3
Pla-1_CTCGAAAAGGAGTACC	Pla	Pla-1	P1-33	TRUE	3	TRA:CLVGTGGFKTIF TRB:CSARVPGQMKETQYF	41	CD8-C3
Pla-1_CTCGAAACATATGGTC	Pla	Pla-1	P1-166	FALSE	3	TRA:CAVDGYNKLIF TRB:CASRPDLDRVQETQYF	4	CD8-C8
Pla-1_CTCGAGGCATTGGGCC	Pla	Pla-1	P1-340	FALSE	2	TRA:CAMSVESGANNLFF TRB:CASSLVLQGSGNTIYF	2	CD8-C3
Pla-1_CTCGAGGGTGATGCCC	Pla	Pla-1	P1-75	FALSE	3	TRA:CAMRGGNFNKFYF TRB:CSAQGAYEQYF	8	CD8-C3

Pla-1_CTCGGAGAGCTTATCG	Pla	Pla-1	P1-1685	FALSE	1	TRA:CAVGDYGNNRLAF TRB:CASSPGTKPLNTEAFF	1	CD8-C3
Pla-1_CTCGGAGGTTGTTTGG	Pla	Pla-1	P1-1688	FALSE	1	TRA:CAFMTLQLNDYKLSF TRB:CASSGRTDSNQPQHF	1	CD8-C5
Pla-1_CTCGGGAGTATCTGCA	Pla	Pla-1	P1-1691	FALSE	1	TRA:CAVSAMNYGGSQGNLIF TRB:CASSPLLGYGYTF	1	CD4-C4
Pla-1_CTCTAATAGTTGAGAT	Pla	Pla-1	P1-100	FALSE	3	TRA:CAVRDRITGNQFYF TRB:CASSQDWGSNQPQHF	6	CD8-C3
Pla-1_CTGAAGTGTCGGCTCA	Pla	Pla-1	P1-32	FALSE	3	TRA:CAVMNTGFQKLVF TRB:CSVSGLVLGGNEQFF	46	CD8-C3
Pla-1_CTGAAGTGTTCGCTAA	Pla	Pla-1	P1-393	FALSE	2	TRA:CAVNAPGGSYIPTF TRB:CASLEGPYSNQPQHF	2	CD8-C3
Pla-1_CTGATCCTCTCCAACC	Pla	Pla-1	P1-41	TRUE	3	TRA:CVVSEPPGGSYIPTF TRB:CASSSRPEDTQYF	27	CD4-C4
Pla-1_CTGCCTACAACAACCT	Pla	Pla-1	P1-1726	FALSE	1	TRA:CIVITSGTYKYIF TRB:CSAQKPGRYYGYTF	1	CD8-C2
Pla-1_CTGCGGAGTCCTGCTT	Pla	Pla-1	P1-23	TRUE	3	TRA:CALRAITQGGSEKLVF TRB:CASSLTGAVYGYTF	65	CD8-C3
Pla-1_CTGCTGTGTGCGATAG	Pla	Pla-1	P1-44	TRUE	3	TRA:CAYRGLGAQKLVF TRB:CASSSLDGQDNHYGYTF	22	CD8-C3
Pla-1_CTGGTCTAGATACACA	Pla	Pla-1	P1-231	FALSE	3	TRA:CAVGPSDAGNNRKLIW TRB:CASSYSGGGEETQYF	3	CD8-C3
Pla-1_CTGTGCTCACAACGTT	Pla	Pla-1	P1-20	TRUE	3	TRA:CVVVGGRALTF TRB:CASSLATNPHETQYF	69	CD8-C3
Pla-1_CTTAACTTCCCAGGTG	Pla	Pla-1	P1-244	FALSE	3	TRA:CAARGYQLTF TRB:CASSPSRKGAPYEQYF	3	CD8-C3
Pla-1_CTTACCGAGATGGCGT	Pla	Pla-1	P1-311	FALSE	2	TRA:CAVNSPTNSGYSTLTF TRB:CASRSPSENEQFF	2	CD8-C8
Pla-1_CTTACCGTCGCCTGTT	Pla	Pla-1	P1-318	FALSE	2	TRA:CAMRRDDKIIF TRB:CASSQDFRQGSLSLHF	2	CD8-C3
Pla-1_CTTGGCTGTCAAAGAT	Pla	Pla-1	P1-167	FALSE	3	TRA:CGTGINYGQNFVF TRB:CASSPRDGGGTEAFF	4	CD8-C3
Pla-1_GAAACTCGTACTTGAC	Pla	Pla-1	P1-32	FALSE	3	TRA:CAVMNTGFQKLVF TRB:CSVSGLVLGGNEQFF	46	CD8-C3
Pla-1_GAAATGAAGATCCCGC	Pla	Pla-1	P1-23	TRUE	3	TRA:CALRAITQGGSEKLVF TRB:CASSLTGAVYGYTF	65	CD8-C3
Pla-1_GAAATGAGTTCAGGCC	Pla	Pla-1	P1-1763	FALSE	1	TRA:CAFAGGSEKLVF TRB:CASSPPGQGNEKLFF	1	CD8-C2
Pla-1_GAACATCAGTGTCCCG	Pla	Pla-1	P1-23	TRUE	3	TRA:CALRAITQGGSEKLVF TRB:CASSLTGAVYGYTF	65	CD8-C3
Pla-1_GAACATCGTCATACTG	Pla	Pla-1	P1-245	FALSE	3	TRA:CAVASSNDYKLSF TRB:CASSSPNEKLFF	3	CD8-C3
Pla-1_GAACATCGTTACTGAC	Pla	Pla-1	P1-1766	FALSE	1	TRA:CAMKQGAQKLVF TRB:CASSLTLAGVNNEQFF	1	CD8-C3
Pla-1_GAACATCTCTTTAGGG	Pla	Pla-1	P1-1768	FALSE	1	TRA:CALRFNDYKLSF TRB:CSVAEGGASEAFF	1	CD8-C3
Pla-1_GAACGGAAGGCATTGG	Pla	Pla-1	P1-1770	FALSE	1	TRA:CAMREGTGNQFYF TRB:CASSWLAGDNPHEQYF	1	CD8-C3
Pla-1_GAACGGAGTCGAAAGC	Pla	Pla-1	P1-36	TRUE	3	TRA:CILRDNAGNNRKLIW TRB:CASSSRQGFPGGANVLTF	40	CD8-C3
Pla-1_GAAGCAGGTTGAGGTG	Pla	Pla-1	P1-74	TRUE	3	TRA:CAVGGGSTLGRLYF TRB:CASSIVAGADTEAFF	8	CD8-C3
Pla-1_GAATAAGGTATAAACG	Pla	Pla-1	P1-1783	FALSE	1	TRA:CAAPLAASGGSYIPTF TRB:CASSQEFRSSYEQYF	1	CD8-C8

Pla-1_GAATGAATCAACCATG	Pla	Pla-1	P1-49	FALSE	3	TRA:CAGGGSEKLVF TRB:CASSLFYPAGQQYF	16	CD8-C8
Pla-1_GACACGCGTAGAAGGA	Pla	Pla-1	P1-231	FALSE	3	TRA:CAVGPSDAGNNRKLIW TRB:CASSYSGGGEETQYF	3	CD8-C3
Pla-1_GACAGAGCAAACTGTC	Pla	Pla-1	P1-60	FALSE	3	TRA:CAYLSSTGTASKLTF TRB:CASSSLDGQDNHYGYTF	11	CD8-C3
Pla-1_GACCAATTCACCCTCA	Pla	Pla-1	P1-1787	FALSE	1	TRA:CALRAITQGGSEKLVF TRB:CASSPEGHRTYEQYF	1	CD8-C3
Pla-1_GACGCGTAGCCGTCGT	Pla	Pla-1	P1-46	FALSE	3	TRA:CAVRGTGTASKLTF TRB:CSVRQGGNEQFF	20	CD8-C3
Pla-1_GACGTGCAGTACCGGA	Pla	Pla-1	P1-32	FALSE	3	TRA:CAVMNTGFQKLVF TRB:CSVSGLVLGGNEQFF	46	CD8-C3
Pla-1_GACGTTAGTCAGAATA	Pla	Pla-1	P1-1803	FALSE	1	TRA:CAVILNQAGTALIF TRB:CASTQHHEGRTEAFF	1	CD8-C3
Pla-1_GACGTTAGTCGATTGT	Pla	Pla-1	P1-126	FALSE	3	TRA:CAVGSRPGGGYNKLIF TRB:CASGQGSYGELFF	5	CD8-C3
Pla-1_GACTAACCAGGGTTAG	Pla	Pla-1	P1-56	TRUE	3	TRA:CVVNPWGSGTYKYIF TRB:CASSRDITDTQYF	13	CD8-C5
Pla-1_GACTAACCATGCCCGA	Pla	Pla-1	P1-89	TRUE	3	TRA:CAVDVNYGQNFVF TRB:CASSWGDTQYF	6	CD8-C3
Pla-1_GACTACACAGGAACGT	Pla	Pla-1	P1-1810	FALSE	1	TRA:CAVRRYSGGYQKVTF TRB:CASRDLYEQYF	1	CD8-C3
Pla-1_GAGCAGAAGCAGATCG	Pla	Pla-1	P1-135	FALSE	3	TRA:CAVRASGANSKLTF TRB:CSAQIGTGPEKLFF	5	CD8-C3
Pla-1_GAGCAGATCCATTCTA	Pla	Pla-1	P1-92	TRUE	3	TRA:CIVQLTGTASKLTF TRB:CASSFKSGRTNEQFF	6	CD8-C3
Pla-1_GAGTCCGCACACTGCG	Pla	Pla-1	P1-218	FALSE	3	TRA:CAYRRLGNTPLVF TRB:CASSLVDGGSSYEQYF	3	CD8-C5
Pla-1_GATCAGTGTAGGGTAC	Pla	Pla-1	P1-1819	FALSE	1	TRA:CAATPNAGNNRKLIW TRB:CASSELQGKNTEAFF	1	CD8-C3
Pla-1_GATCGATTCGAGAACG	Pla	Pla-1	P1-1827	FALSE	1	TRA:CAERGGSSNTGKLIF TRB:CASTAKHTGAAYEQYF	1	CD8-C3
Pla-1_GATCGATTCTCTTATG	Pla	Pla-1	P1-113	TRUE	3	TRA:CAVNGGSQGNLIF TRB:CASSLAVNYGYTF	5	CD8-C3
Pla-1_GATCGCGAGCTCCTTC	Pla	Pla-1	P1-198	TRUE	3	TRA:CAVRDMDDKIIF TRB:CASRPQGAGGYEQYF	3	CD8-C3
Pla-1_GATCGCGAGCTGCCCA	Pla	Pla-1	P1-1829	FALSE	1	TRA:CGGGSNYKLTF TRB:CSARTSGMETQYF	1	CD8-C3
Pla-1_GATCGCGTCAGGCCCA	Pla	Pla-1	P1-1831	FALSE	1	TRA:CAEGGGGADGLTF TRB:CASSLRSREYEQYF	1	CD8-C3
Pla-1_GATCGTAGTTCAGCGC	Pla	Pla-1	P1-20	TRUE	3	TRA:CVVVGGRALTF TRB:CASSLATNPHETQYF	69	CD8-C3
Pla-1_GATGAAACAAGGTTCT	Pla	Pla-1	P1-44	TRUE	3	TRA:CAYRGLGAQKLVF TRB:CASSSLDGQDNHYGYTF	22	CD8-C3
Pla-1_GATGAAATCAACACCA	Pla	Pla-1	P1-1838	FALSE	1	TRA:CAFTFGGFKTIF TRB:CASSLAGARFRTDTQYF	1	CD8-C3
Pla-1_GCAAACTAGTTATCGC	Pla	Pla-1	P1-45	TRUE	3	TRA:CAYRPLGAQKLVF TRB:CASSTLDGRESSYEQYF	20	CD8-C3
Pla-1_GCAAACTCATACCATG	Pla	Pla-1	P1-97	FALSE	3	TRA:CAVRTAGNKLTF TRB:CSAQGRSSYEQYF	6	CD8-C3
Pla-1_GCAATCACAGCCTATA	Pla	Pla-1	P1-15	TRUE	3	TRA:CAVNSSPLGANSKLTF TRB:CASSDSGNFYNEQFF	92	CD8-C3
Pla-1_GCAATCATCCTTCAAT	Pla	Pla-1	P1-359	FALSE	2	TRA:CAVSRSGGYNKLIF TRB:CASRPTSVRDTQYF	2	CD8-C3

Pla-1_GCACATAAGGCTCAGA	Pla	Pla-1	P1-356	FALSE	2	TRA:CAVSPTGTASKLTF TRB:CASSEGTAHYQPQHF	2	CD8-C3
Pla-1_GCAGCCAAGGGTCTCC	Pla	Pla-1	P1-20	TRUE	3	TRA:CVVVGGRALTF TRB:CASSLATNPHETQYF	69	CD8-C3
Pla-1_GCAGCCACATAAGACA	Pla	Pla-1	P1-1849	FALSE	1	TRA:CAVKRVTGGFKTIF TRB:CATSRDQGAGNTEAFF	1	CD8-C3
Pla-1_GCAGCCACATGCAACT	Pla	Pla-1	P1-137	FALSE	3	TRA:CAVSMNRDDKIIF TRB:CASSSGRLSGANVLTF	5	CD8-C5
Pla-1_GCAGTTACAATAACGA	Pla	Pla-1	P1-45	TRUE	3	TRA:CAYRPLGAQKLVF TRB:CASSTLDGRESSYEQYF	20	CD8-C3
Pla-1_GCAGTTACACTTCGAA	Pla	Pla-1	P1-23	TRUE	3	TRA:CALRAITQGGSEKLVF TRB:CASSLTGAVYGYTF	65	CD8-C3
Pla-1_GCATACACATTGTGCA	Pla	Pla-1	P1-32	FALSE	3	TRA:CAVMNTGFQKLVF TRB:CSVSGLVLGGNEQFF	46	CD8-C3
Pla-1_GCATGTACAACACCCG	Pla	Pla-1	P1-1861	FALSE	1	TRA:CAVEVMSRDDKIIF TRB:CASSLARQVGTDTQYF	1	CD8-C3
Pla-1_GCGACCAGTCTGGAGA	Pla	Pla-1	P1-1868	FALSE	1	TRA:CAILIYNQGGKLIF TRB:CASSGLDPGSGNTIYF	1	CD8-C3
Pla-1_GCGAGAAGTATTACCG	Pla	Pla-1	P1-1869	FALSE	1	TRA:CAVYLERGGSYIPTF TRB:CASRGRTAGNTIYF	1	CD8-C3
Pla-1_GCGAGAATCACGCGGT	Pla	Pla-1	P1-164	FALSE	3	TRA:CAAGENTGTASKLTF TRB:CASSSTYRVQETQYF	4	CD8-C3
Pla-1_GCGCAACAGCAATATG	Pla	Pla-1	P1-20	TRUE	3	TRA:CVVVGGRALTF TRB:CASSLATNPHETQYF	69	CD8-C3
Pla-1_GCGCAACCATGCAATC	Pla	Pla-1	P1-17	TRUE	3	TRA:CGTGARGSSGSRLTF TRB:CASSRQGMGAFF	78	CD8-C3
Pla-1_GCGCAACTCAACACGT	Pla	Pla-1	P1-130	FALSE	3	TRA:CAMREPGRALNF TRB:CASIAPGGVNYGYTF	5	CD8-C3
Pla-1_GCGCCAATCCACGTTC	Pla	Pla-1	P1-60	FALSE	3	TRA:CAYLSSTGTASKLTF TRB:CASSSLDGQDNHYGYTF	11	CD8-C5
Pla-1_GCTCCTATCATACGGT	Pla	Pla-1	P1-32	FALSE	3	TRA:CAVMNTGFQKLVF TRB:CSVSGLVLGGNEQFF	46	CD8-C3
Pla-1_GCTCTGTAGAGTGACC	Pla	Pla-1	P1-33	TRUE	3	TRA:CLVGTGGFKTIF TRB:CSARVPGQMKETQYF	41	CD8-C3
Pla-1_GCTGCAGCAATGTTGC	Pla	Pla-1	P1-86	TRUE	3	TRA:CAVGGRSNTGRRALTF TRB:CAISLTGDGKETQYF	6	CD8-C3
Pla-1_GCTGGGTCAGTCGTGC	Pla	Pla-1	P1-1893	FALSE	1	TRA:CIVRVYNTNAGKSTF TRB:CASSQDLRPQTSAKNIQYF	1	CD8-C2
Pla-1_GGAATAAAGTGATCGG	Pla	Pla-1	P1-46	FALSE	3	TRA:CAVRGTGTASKLTF TRB:CSVRQGGNEQFF	20	CD8-C3
Pla-1_GGACAGACACGGCCAT	Pla	Pla-1	P1-20	TRUE	3	TRA:CVVVGGRALTF TRB:CASSLATNPHETQYF	69	CD8-C3
Pla-1_GGACAGACAGCCAGAA	Pla	Pla-1	P1-33	TRUE	3	TRA:CLVGTGGFKTIF TRB:CSARVPGQMKETQYF	41	CD8-C5
Pla-1_GGACATTAGACGCACA	Pla	Pla-1	P1-235	FALSE	3	TRA:CAATSTGTYKYIF TRB:CASSFGGRNEQYF	3	CD8-C3
Pla-1_GGACATTCACTTAAGC	Pla	Pla-1	P1-1909	FALSE	1	TRA:CALRAITQGGSEKLVF TRB:CASSFAGTYEQYF	1	CD8-C8
Pla-1_GGACATTCATATGGTC	Pla	Pla-1	P1-55	TRUE	3	TRA:CALSPQDSNYQLIW TRB:CASSFWTSANEQFF	13	CD8-C3
Pla-1_GGACATTGTGACGGTA	Pla	Pla-1	P1-407	FALSE	2	TRA:CAEDNNARLMF TRB:CSARVGVGNTIYF	2	CD8-C3
Pla-1_GGACGTCCAATCTACG	Pla	Pla-1	P1-1912	FALSE	1	TRA:CAVRDWAGDMRF TRB:CASSADRDNEQFF	1	CD8-C3

Pla-1_GGACGTCGTACAGTTC	Pla	Pla-1	P1-113	TRUE	3	TRA:CAVNGGSQGNLIF TRB:CASSLAVNYGYTF	5	CD8-C3
Pla-1_GGACGTCGTCAGATAA	Pla	Pla-1	P1-1913	FALSE	1	TRA:CAGVLDSNYQLIW TRB:CASSQDLGVGTQYF	1	CD8-C3
Pla-1_GGATGTTCAGGACCCT	Pla	Pla-1	P1-56	TRUE	3	TRA:CVVNPWGSGTYKYIF TRB:CASSRDITDTQYF	13	CD8-C3
Pla-1_GGATGTTTCACAAACC	Pla	Pla-1	P1-99	FALSE	3	TRA:CIVRLLSTASKLTF TRB:CASSELQGKNTEAFF	6	CD8-C8
Pla-1_GGCAATTCAATGGAAT	Pla	Pla-1	P1-33	TRUE	3	TRA:CLVGTGGFKTIF TRB:CSARVPGQMKETQYF	41	CD8-C3
Pla-1_GGCCGATTCAGCATGT	Pla	Pla-1	P1-20	TRUE	3	TRA:CVVVGGRALTF TRB:CASSLATNPHETQYF	69	CD8-C5
Pla-1_GGCGACTAGCGATAGC	Pla	Pla-1	P1-137	FALSE	3	TRA:CAVSMNRDDKIIF TRB:CASSSGRLSGANVLTF	5	CD8-C3
Pla-1_GGCGACTCACGAGAGT	Pla	Pla-1	P1-34	FALSE	3	TRA:CAMSGGGADGLTF TRB:CASNVGVHEQFF	41	CD8-C2
Pla-1_GGCGACTTCTACTATC	Pla	Pla-1	P1-1930	FALSE	1	TRA:CAAVTGGGNKLTF TRB:CASSLNSYEQYF	1	CD8-C3
Pla-1_GGCGTGTCAGGCGATA	Pla	Pla-1	P1-1932	FALSE	1	TRA:CAVGGPNDYKLSF TRB:CASSVGTGTTQYF	1	CD8-C3
Pla-1_GGCGTGTGTCGCTTTC	Pla	Pla-1	P1-117	TRUE	3	TRA:CIVGETSGSRLTF TRB:CASSLVGGVGTDTQYF	5	CD8-C3
Pla-1_GGCTCGAGTGTTAAGA	Pla	Pla-1	P1-393	FALSE	2	TRA:CAVNAPGGSYIPTF TRB:CASLEGPYSNQPQHF	2	CD8-C3
Pla-1_GGCTGGTAGCTAGTCT	Pla	Pla-1	P1-60	FALSE	3	TRA:CAYLSSTGTASKLTF TRB:CASSSLDGQDNHYGYTF	11	CD8-C5
Pla-1_GGCTGGTTCGTAGGAG	Pla	Pla-1	P1-1940	FALSE	1	TRA:CAAILTGGGNKLTF TRB:CASKGAGGPGETQYF	1	CD8-C8
Pla-1_GGGACCTCATGCAATC	Pla	Pla-1	P1-1942	FALSE	1	TRA:CAVSIIQGAQKLVF TRB:CASSLGGQGLYTDTQYF	1	CD8-C3
Pla-1_GGGAGATCATTTGCCC	Pla	Pla-1	P1-20	TRUE	3	TRA:CVVVGGRALTF TRB:CASSLATNPHETQYF	69	CD8-C3
Pla-1_GGGAGATGTTTGACTG	Pla	Pla-1	P1-68	TRUE	3	TRA:CAVIPDILTGGGNKLTF TRB:CASSLQGFGNTIYF	9	CD8-C3
Pla-1_GGGATGAAGCAGCCTC	Pla	Pla-1	P1-7	TRUE	3	TRA:CAVQAGNNDMRF TRB:CASSTTGDFNYGYTF	215	CD8-C8
Pla-1_GGGCACTCAATCGGTT	Pla	Pla-1	P1-20	TRUE	3	TRA:CVVVGGRALTF TRB:CASSLATNPHETQYF	69	CD8-C3
Pla-1_GGGCACTCACTAGTAC	Pla	Pla-1	P1-49	FALSE	3	TRA:CAGGGSEKLVF TRB:CASSLFYPAGQQYF	16	CD8-C3
Pla-1_GGGCACTCATCGGGTC	Pla	Pla-1	P1-56	TRUE	3	TRA:CVVNPWGSGTYKYIF TRB:CASSRDITDTQYF	13	CD8-C3
Pla-1_GGGCACTTCGCTTGTC	Pla	Pla-1	P1-23	TRUE	3	TRA:CALRAITQGGSEKLVF TRB:CASSLTGAVYGYTF	65	CD8-C3
Pla-1_GGGCATCAGAGCTGCA	Pla	Pla-1	P1-1951	FALSE	1	TRA:CAGREGFGNVLHC TRB:CASSNLHRGRGGPLTEAFF	1	CD8-C3
Pla-1_GGGCATCTCAGTTCGA	Pla	Pla-1	P1-33	TRUE	3	TRA:CLVGTGGFKTIF TRB:CSARVPGQMKETQYF	41	CD8-C3
Pla-1_GGGTCTGGTACCTACA	Pla	Pla-1	P1-80	FALSE	3	TRA:CAASAEGSQGNLIF TRB:CASKKDRGTTQAKNIQYF	7	CD8-C2
Pla-1_GGGTCTGTCCATTCTA	Pla	Pla-1	P1-49	FALSE	3	TRA:CAGGGSEKLVF TRB:CASSLFYPAGQQYF	16	CD8-C3
Pla-1_GGGTCTGTCCGTCAAA	Pla	Pla-1	P1-37	TRUE	3	TRA:CAVNIVGNQFYF TRB:CASSPLGTEAFF	40	CD8-C3

Pla-1_GGTATTGGTGCAACGA	Pla	Pla-1	P1-1959	FALSE	1	TRA:CAENGGYIPTF TRB:CAWSGGTSGRAHVNEQFF	1	CD8-C3
Pla-1_GGTGCGTCAATAACGA	Pla	Pla-1	P1-411	FALSE	2	TRA:CAGARSLISGGSNYKLTF TRB:CASGWTSGSRSPIYNEQFI	2	CD8-C3
Pla-1_GGTGTTATCTCTGTCG	Pla	Pla-1	P1-12	TRUE	3	TRA:CALSDERGDYKLSF TRB:CASSPGTGSYNEQFF	141	CD8-C3
Pla-1_GTAACGTCAATGTAAG	Pla	Pla-1	P1-201	TRUE	3	TRA:CAYVGGNTPLVF TRB:CASSFPPSGGARETQYF	3	CD8-C3
Pla-1_GTAACTGTCGGTCTAA	Pla	Pla-1	P1-222	FALSE	3	TRA:CAVRLPYNTDKLIF TRB:CASSFRHEQYF	3	CD8-C3
Pla-1_GTACGTACATAAAGGT	Pla	Pla-1	P1-128	FALSE	3	TRA:CAVVNTGTASKLTF TRB:CASSEQGYEQYF	5	CD8-C3
Pla-1_GTACGTAGTCCAACTA	Pla	Pla-1	P1-33	TRUE	3	TRA:CLVGTGGFKTIF TRB:CSARVPGQMKETQYF	41	CD8-C3
Pla-1_GTAGGCCGTCTGATTG	Pla	Pla-1	P1-413	FALSE	2	TRA:CALTWANNLFF TRB:CAWSSDRGYNEQFF	2	CD8-C2
Pla-1_GTAGTCAAGCACGCCT	Pla	Pla-1	P1-118	TRUE	3	TRA:CAVVKAGGTSYGKLTF TRB:CASKSLAGKSSYEQYF	5	CD8-C3
Pla-1_GTAGTCAAGCTGCGAA	Pla	Pla-1	P1-42	TRUE	3	TRA:CASFSGGSQGNLIF TRB:CSAGQGSNQPQHF	24	CD4-C4
Pla-1_GTAGTCAGTGTTGGGA	Pla	Pla-1	P1-1989	FALSE	1	TRA:CLVGGPGNTGKLIF TRB:CASSLQANQETQYF	1	CD8-C3
Pla-1_GTATCTTCAATGGAGC	Pla	Pla-1	P1-23	TRUE	3	TRA:CALRAITQGGSEKLVF TRB:CASSLTGAVYGYTF	65	CD8-C3
Pla-1_GTATTCTTCCCAAGAT	Pla	Pla-1	P1-82	FALSE	3	TRA:CAASEDSNYQLIW TRB:CASSFGDSSSETQYF	7	CD8-C3
Pla-1_GTCAAGTCAATAACGA	Pla	Pla-1	P1-46	FALSE	3	TRA:CAVRGTGTASKLTF TRB:CSVRQGGNEQFF	20	CD8-C3
Pla-1_GTCACAAAGACAGAGA	Pla	Pla-1	P1-131	FALSE	3	TRA:CAVRDSGTYKYIF TRB:CASSIDLGGANVLTF	5	CD8-C3
Pla-1_GTCACAACAGAGTGTG	Pla	Pla-1	P1-1997	FALSE	1	TRA:CALSETYNFNKFYF TRB:CASSEDNSNYGYTF	1	CD8-C3
Pla-1_GTCACGGAGGGATACC	Pla	Pla-1	P1-2000	FALSE	1	TRA:CAVVNSGGGADGLTF TRB:CSVRTGFGTEAFF	1	CD8-C3
Pla-1_GTCACGGAGGTGCAAC	Pla	Pla-1	P1-2001	FALSE	1	TRA:CAVKSPLSNYQLIW TRB:CASSLREAPYEQYF	1	CD8-C3
Pla-1_GTCACGGCATTAACCG	Pla	Pla-1	P1-136	FALSE	3	TRA:CAVGGGADGLTF TRB:CSTRGAAYEQFF	5	CD8-C8
Pla-1_GTCATTTAGGACAGAA	Pla	Pla-1	P1-20	TRUE	3	TRA:CVVVGGRALTF TRB:CASSLATNPHETQYF	69	CD8-C3
Pla-1_GTCCTCACAGACGCTC	Pla	Pla-1	P1-57	FALSE	3	TRA:CAMREGGNTGNQFYF TRB:CASKKDRGTTQAKNIQYF	13	CD8-C3
Pla-1_GTCTTCGAGGCTATCT	Pla	Pla-1	P1-32	FALSE	3	TRA:CAVMNTGFQKLVF TRB:CSVSGLVLGGNEQFF	46	CD8-C3
Pla-1_GTCTTCGAGTGTTGAA	Pla	Pla-1	P1-45	TRUE	3	TRA:CAYRPLGAQKLVF TRB:CASSTLDGRESSYEQYF	20	CD8-C3
Pla-1_GTGAAGGCAAGCGAGT	Pla	Pla-1	P1-32	FALSE	3	TRA:CAVMNTGFQKLVF TRB:CSVSGLVLGGNEQFF	46	CD8-C3
Pla-1_GTGAAGGCAATGACCT	Pla	Pla-1	P1-95	TRUE	3	TRA:CIVRVARGNEKLTF TRB:CASSLYGEGHQPQHF	6	CD8-C3
Pla-1_GTGCAGCAGCGTGAGT	Pla	Pla-1	P1-2017	FALSE	1	TRA:CAVAPRGNNRLAF TRB:CASGLDPGQAFAGAFF	1	CD8-C3
Pla-1_GTGCATAGTACAAGTA	Pla	Pla-1	P1-81	FALSE	3	TRA:CAVTFAGNQFYF TRB:CSAGTGTSGLSEQFF	7	CD8-C3

Pla-1_GTGCATAGTTATCACG	Pla	Pla-1	P1-23	TRUE	3	TRA:CALRAITQGGSEKLVF TRB:CASSLTGAVYGYTF	65	CD8-C3
Pla-1_GTGCATATCAGCTGGC	Pla	Pla-1	P1-20	TRUE	3	TRA:CVVVGGRALTF TRB:CASSLATNPHETQYF	69	CD8-C3
Pla-1_GTGCATATCTGTCTCG	Pla	Pla-1	P1-358	FALSE	2	TRA:CAVRVNSGYSTLTF TRB:CSVDGTGVTYEQYF	2	CD8-C3
Pla-1_GTGCGGTGTTCCACGG	Pla	Pla-1	P1-70	FALSE	3	TRA:CAMRETPGEAGTALIF TRB:CSARITYLNTEAFF	9	CD8-C2
Pla-1_GTGCGGTTCATTGCGA	Pla	Pla-1	P1-7	TRUE	3	TRA:CAVQAGNNDMRF TRB:CASSTTGDFNYGYTF	215	CD8-C3
Pla-1_GTGCTTCGTCGTTGTA	Pla	Pla-1	P1-76	FALSE	3	TRA:CAVRVMEYGNKLVF TRB:CASSFGTGFEQYF	8	CD8-C3
Pla-1_GTGCTTCTCAGATAAG	Pla	Pla-1	P1-10	TRUE	3	TRA:CISNSPMDSSYKLIF TRB:CASSSWGDTDEAFF	162	CD8-C3
Pla-1_GTGGGTCCAAGCTGTT	Pla	Pla-1	P1-23	TRUE	3	TRA:CALRAITQGGSEKLVF TRB:CASSLTGAVYGYTF	65	CD8-C4
Pla-1_GTGTGCGTCTGTTTGT	Pla	Pla-1	P1-20	TRUE	3	TRA:CVVVGGRALTF TRB:CASSLATNPHETQYF	69	CD8-C3
Pla-1_GTGTTAGTCGTTGACA	Pla	Pla-1	P1-55	TRUE	3	TRA:CALSPQDSNYQLIW TRB:CASSFWTSANEQFF	13	CD8-C3
Pla-1_GTGTTAGTCTTGGGTA	Pla	Pla-1	P1-2038	FALSE	1	TRA:CAMREDGGYNKLIF TRB:CASGGNTIYF	1	CD8-C3
Pla-1_GTTAAGCCAAGCTGGA	Pla	Pla-1	P1-118	TRUE	3	TRA:CAVVKAGGTSYGKLTF TRB:CASKSLAGKSSYEQYF	5	CD8-C3
Pla-1_GTTAAGCCACAGCCCA	Pla	Pla-1	P1-33	TRUE	3	TRA:CLVGTGGFKTIF TRB:CSARVPGQMKETQYF	41	CD8-C3
Pla-1_GTTACAGAGTACGCCC	Pla	Pla-1	P1-130	FALSE	3	TRA:CAMREPGRALNF TRB:CASIAPGGVNYGYTF	5	CD8-C3
Pla-1_GTTACAGCAGGAATCG	Pla	Pla-1	P1-2047	FALSE	1	TRA:CAASRGTGANNLFF TRB:CASSFLSGTDTQYF	1	CD8-C2
Pla-1_GTTCATTGTAGATTAG	Pla	Pla-1	P1-33	TRUE	3	TRA:CLVGTGGFKTIF TRB:CSARVPGQMKETQYF	41	CD8-C3
Pla-1_GTTCTCGGTGGCTCCA	Pla	Pla-1	P1-2058	FALSE	1	TRA:CADPLTGANNLFF TRB:CASSYPRTGGSYEQYF	1	CD8-C3
Pla-1_GTTTCTATCAGTCCCT	Pla	Pla-1	P1-2060	FALSE	1	TRA:CAVDDNYGQNFVF TRB:CASSPGTIYNEQFF	1	CD8-C3
Pla-1_TAAACCGGTACAGCAG	Pla	Pla-1	P1-20	TRUE	3	TRA:CVVVGGRALTF TRB:CASSLATNPHETQYF	69	CD8-C3
Pla-1_TAAGAGATCATGTCTT	Pla	Pla-1	P1-40	TRUE	3	TRA:CAVRDVGNTGFQKLVF TRB:CASSLGQGSMKTQYF	27	CD8-C2
Pla-1_TAAGCGTCAGGCAGTA	Pla	Pla-1	P1-2066	FALSE	1	TRA:CAGDHTYGNKLVF TRB:CASSESGSQNTEAFF	1	CD8-C3
Pla-1_TAAGCGTTCCGCGGTA	Pla	Pla-1	P1-42	TRUE	3	TRA:CASFSGGSQGNLIF TRB:CSAGQGSNQPQHF	24	CD8-C5
Pla-1_TACACGAGTCACTGGC	Pla	Pla-1	P1-32	FALSE	3	TRA:CAVMNTGFQKLVF TRB:CSVSGLVLGGNEQFF	46	CD8-C3
Pla-1_TACCTATAGATGCCAG	Pla	Pla-1	P1-70	FALSE	3	TRA:CAMRETPGEAGTALIF TRB:CSARITYLNTEAFF	9	CD8-C3
Pla-1_TACCTTAAGGCGCTCT	Pla	Pla-1	P1-294	TRUE	2	TRA:CAMNYGQNFVF TRB:CSARDNGAATDTQYF	2	CD8-C3
Pla-1_TACGGATGTAAGAGGA	Pla	Pla-1	P1-133	FALSE	3	TRA:CAFMNRGSTLGRLYF TRB:CASSFLSGTDTQYF	5	CD8-C3
Pla-1_TACGGGCGTCTCTTTA	Pla	Pla-1	P1-32	FALSE	3	TRA:CAVMNTGFQKLVF TRB:CSVSGLVLGGNEQFF	46	CD8-C3

Pla-1_TACGGGCGTTCGGCAC	Pla	Pla-1	P1-23	TRUE	3	TRA:CALRAITQGGSEKLVF TRB:CASSLTGAVYGYTF	65	CD8-C3
Pla-1_TACGGTAGTAAAGTCA	Pla	Pla-1	P1-2085	FALSE	1	TRA:CALSDFSGGYNKLIF TRB:CASSNCTGHNTGELFF	1	CD8-C3
Pla-1_TACGGTATCTGCAAGT	Pla	Pla-1	P1-2088	FALSE	1	TRA:CAMREVVYNQGGKLIF TRB:CASSLGQRGLQYF	1	CD8-C2
Pla-1_TACTCATGTCCAACTA	Pla	Pla-1	P1-46	FALSE	3	TRA:CAVRGTGTASKLTF TRB:CSVRQGGNEQFF	20	CD8-C3
Pla-1_TACTTGTCAATAGCGG	Pla	Pla-1	P1-2098	FALSE	1	TRA:CIVRVPAYF TRB:CASSDTHHGYEQYF	1	CD8-C3
Pla-1_TAGACCACACATCCAA	Pla	Pla-1	P1-2103	FALSE	1	TRA:CAVNGLNYGQNFVF TRB:CASSSRRGVGEAFF	1	CD8-C3
Pla-1_TAGAGCTAGAGGACGG	Pla	Pla-1	P1-120	FALSE	3	TRA:CAYRSSYSGYALNF TRB:CAWSPYSEKLFF	5	CD8-C5
Pla-1_TAGAGCTCATGGTCAT	Pla	Pla-1	P1-110	TRUE	3	TRA:CAVAPGTGRRALTF TRB:CSARDRTAQETQYF	5	CD8-C3
Pla-1_TAGAGCTTCACCCGAG	Pla	Pla-1	P1-2108	FALSE	1	TRA:CAGRDISSGSARQLTF TRB:CSAGDRSSYEQYF	1	CD8-C3
Pla-1_TAGTTGGCACATTAGC	Pla	Pla-1	P1-74	TRUE	3	TRA:CAVGGGSTLGRLYF TRB:CASSIVAGADTEAFF	8	CD8-C3
Pla-1_TAGTTGGGTCGGCACT	Pla	Pla-1	P1-2114	FALSE	1	TRA:CAFPLRGSQGNLIF TRB:CASSLTEDGTDTQYF	1	CD8-C3
Pla-1_TATCAGGCAGCTGCTG	Pla	Pla-1	P1-296	TRUE	2	TRA:CALSGWDTDKLIF TRB:CASTADREGQETQYF	2	CD8-C3
Pla-1_TATCAGGTCCTCTAGC	Pla	Pla-1	P1-204	TRUE	3	TRA:CAVRDMMDSNYQLIW TRB:CASSLDGAGGETQYF	3	CD8-C3
Pla-1_TATGCCCGTGCTCTTC	Pla	Pla-1	P1-2123	FALSE	1	TRA:CAVPLNKLIF TRB:CASSLEMGLNTEAFF	1	CD8-C3
Pla-1_TATTACCCACGAGAGT	Pla	Pla-1	P1-46	FALSE	3	TRA:CAVRGTGTASKLTF TRB:CSVRQGGNEQFF	20	CD8-C3
Pla-1_TCAACGATCGGCATCG	Pla	Pla-1	P1-307	FALSE	2	TRA:CAIYSNYQLIW TRB:CASSTQGLDEQFF	2	CD8-C3
Pla-1_TCAACGATCTCAACTT	Pla	Pla-1	P1-2130	FALSE	1	TRA:CVVNPWGSGTYKYIF TRB:CASSLATNPHETQYF	1	CD8-C3
Pla-1_TCAATCTTCGAACGGA	Pla	Pla-1	P1-57	FALSE	3	TRA:CAMREGGNTGNQFYF TRB:CASKKDRGTTQAKNIQYF	13	CD8-C2
Pla-1_TCAGATGAGAAACGCC	Pla	Pla-1	P1-218	FALSE	3	TRA:CAYRRLGNTPLVF TRB:CASSLVDGGSSYEQYF	3	CD8-C3
Pla-1_TCAGATGCAGGGTACA	Pla	Pla-1	P1-40	TRUE	3	TRA:CAVRDVGNTGFQKLVF TRB:CASSLGQGSMKTQYF	27	CD8-C8
Pla-1_TCAGCAAAGATGCCAG	Pla	Pla-1	P1-37	TRUE	3	TRA:CAVNIVGNQFYF TRB:CASSPLGTEAFF	40	CD8-C8
Pla-1_TCAGCAAGTCGGGTCT	Pla	Pla-1	P1-171	TRUE	3	TRA:CAGQRRANSKLTF TRB:CASSQTTGREAKNIQYF	3	CD8-C3
Pla-1_TCAGGATAGTACGACG	Pla	Pla-1	P1-2144	FALSE	1	TRA:CALYTGTASKLTF TRB:CAWSTDSGYNEQFF	1	CD8-C2
Pla-1_TCAGGTAGTACCGTTA	Pla	Pla-1	P1-74	TRUE	3	TRA:CAVGGGSTLGRLYF TRB:CASSIVAGADTEAFF	8	CD8-C5
Pla-1_TCATTACTCACTTACT	Pla	Pla-1	P1-44	TRUE	3	TRA:CAYRGLGAQKLVF TRB:CASSSLDGQDNHYGYTF	22	CD8-C8
Pla-1_TCATTACTCAGCGACC	Pla	Pla-1	P1-33	TRUE	3	TRA:CLVGTGGFKTIF TRB:CSARVPGQMKETQYF	41	CD8-C3
Pla-1_TCATTTGTCTGTTGAG	Pla	Pla-1	P1-132	FALSE	3	TRA:CAVKGSQGNLIF TRB:CSARITYLNTEAFF	5	CD8-C3

Pla-1_TCGAGGCAGGCGATAC	Pla	Pla-1	P1-158	FALSE	3	TRA:CALRVITQGGSEKLVF TRB:CASSEGSSYNEQFF	4	CD8-C3
Pla-1_TCGCGAGCAGTCACTA	Pla	Pla-1	P1-32	FALSE	3	TRA:CAVMNTGFQKLVF TRB:CSVSGLVLGGNEQFF	46	CD8-C3
Pla-1_TCGGGACAGACTGTAA	Pla	Pla-1	P1-82	FALSE	3	TRA:CAASEDSNYQLIW TRB:CASSFGDSSSETQYF	7	CD8-C3
Pla-1_TCGGGACGTAGAGCTG	Pla	Pla-1	P1-131	FALSE	3	TRA:CAVRDSGTYKYIF TRB:CASSIDLGGANVLTF	5	CD8-C2
Pla-1_TCGGTAAGTTATCGGT	Pla	Pla-1	P1-2165	FALSE	1	TRA:CLGWGNQAGTALIF TRB:CASSSDINYGYTF	1	CD8-C3
Pla-1_TCGTACCTCACAAACC	Pla	Pla-1	P1-301	FALSE	2	TRA:CVVNRETGANNLFF TRB:CASSYKGPTYEQYF	2	CD8-C3
Pla-1_TCGTAGATCCTTGACC	Pla	Pla-1	P1-330	FALSE	2	TRA:CAVNTWRSDKLSF TRB:CAWTPTGTGDHGYTF	2	CD8-C3
Pla-1_TCTATTGGTGACAAAT	Pla	Pla-1	P1-2175	FALSE	1	TRA:CAVNANAGKSTF TRB:CASSQSPQETQYF	1	CD8-C3
Pla-1_TCTCATACACTTGGAT	Pla	Pla-1	P1-244	FALSE	3	TRA:CAARGYQLTF TRB:CASSPSRKGAPYEQYF	3	CD8-C3
Pla-1_TCTCTAACACGTTGGC	Pla	Pla-1	P1-2182	FALSE	1	TRA:CVVNVSNDYKLSF TRB:CASSLGTGTGTVETQYF	1	CD4-C6
Pla-1_TCTCTAAGTTACTGAC	Pla	Pla-1	P1-2183	FALSE	1	TRA:CAVRDKTGTALIF TRB:CASSLSADTQYF	1	CD8-C3
Pla-1_TCTGAGAAGGGCACTA	Pla	Pla-1	P1-20	TRUE	3	TRA:CVVVGGRALTF TRB:CASSLATNPHETQYF	69	CD8-C3
Pla-1_TCTGAGAAGTAAGTAC	Pla	Pla-1	P1-33	TRUE	3	TRA:CLVGTGGFKTIF TRB:CSARVPGQMKETQYF	41	CD8-C3
Pla-1_TCTGGAAAGACTAGAT	Pla	Pla-1	P1-57	FALSE	3	TRA:CAMREGGNTGNQFYF TRB:CASKKDRGTTQAKNIQYF	13	CD8-C3
Pla-1_TCTGGAACACTGAAGG	Pla	Pla-1	P1-74	TRUE	3	TRA:CAVGGGSTLGRLYF TRB:CASSIVAGADTEAFF	8	CD8-C3
Pla-1_TCTTCGGAGTGGACGT	Pla	Pla-1	P1-2186	FALSE	1	TRA:CALSPTTSGTYKYIF TRB:CASSFPDTDTQYF	1	CD8-C3
Pla-1_TGAAAGATCCGTCATC	Pla	Pla-1	P1-23	TRUE	3	TRA:CALRAITQGGSEKLVF TRB:CASSLTGAVYGYTF	65	CD8-C3
Pla-1_TGACAACCAAGAAGAG	Pla	Pla-1	P1-32	FALSE	3	TRA:CAVMNTGFQKLVF TRB:CSVSGLVLGGNEQFF	46	CD8-C3
Pla-1_TGACAACCACATCCAA	Pla	Pla-1	P1-2192	FALSE	1	TRA:CGADGRGFKTIF TRB:CASSIRTSAEDDYEQYF	1	CD8-C3
Pla-1_TGACGGCTCATGTCTT	Pla	Pla-1	P1-2197	FALSE	1	TRA:CAEKSGGSNYKLTF TRB:CASSGTRDEQYF	1	CD8-C3
Pla-1_TGACTTTTCAACTCTT	Pla	Pla-1	P1-49	FALSE	3	TRA:CAGGGSEKLVF TRB:CASSLFYPAGQQYF	16	CD8-C2
Pla-1_TGAGAGGAGCCAGTTT	Pla	Pla-1	P1-198	TRUE	3	TRA:CAVRDMDDKIIF TRB:CASRPQGAGGYEQYF	3	CD8-C5
Pla-1_TGAGAGGTCTGGGCCA	Pla	Pla-1	P1-20	TRUE	3	TRA:CVVVGGRALTF TRB:CASSLATNPHETQYF	69	CD8-C3
Pla-1_TGAGCATTCGATCCCT	Pla	Pla-1	P1-37	TRUE	3	TRA:CAVNIVGNQFYF TRB:CASSPLGTEAFF	40	CD8-C3
Pla-1_TGAGCATTCGCCTGAG	Pla	Pla-1	P1-49	FALSE	3	TRA:CAGGGSEKLVF TRB:CASSLFYPAGQQYF	16	CD8-C4
Pla-1_TGAGCCGCACCGTTGG	Pla	Pla-1	P1-74	TRUE	3	TRA:CAVGGGSTLGRLYF TRB:CASSIVAGADTEAFF	8	CD8-C3
Pla-1_TGAGCCGCATCCGGGT	Pla	Pla-1	P1-54	TRUE	3	TRA:CAGWYSGGGADGLTF TRB:CATGAGDNEQFF	13	CD8-C2

Pla-1_TGCACCTAGAGGTTGC	Pla	Pla-1	P1-45	TRUE	3	TRA:CAYRPLGAQKLVF TRB:CASSTLDGRESSYEQYF	20	CD8-C3
Pla-1_TGCCCATAGACATAAC	Pla	Pla-1	P1-46	FALSE	3	TRA:CAVRGTGTASKLTF TRB:CSVRQGGNEQFF	20	CD8-C3
Pla-1_TGCCCATAGACTAGGC	Pla	Pla-1	P1-6	TRUE	3	TRA:CAMREGRERDDKIIF TRB:CASSLPNTEAFF	216	CD8-C3
Pla-1_TGCCCATAGCCCGAAA	Pla	Pla-1	P1-2216	FALSE	1	TRA:CAVPPYMDSNYQLIW TRB:CASDSGVSYEQYF	1	CD8-C3
Pla-1_TGCCCATCAAACGTGG	Pla	Pla-1	P1-20	TRUE	3	TRA:CVVVGGRALTF TRB:CASSLATNPHETQYF	69	CD8-C3
Pla-1_TGCGCAGTCTGCCAGG	Pla	Pla-1	P1-2228	FALSE	1	TRA:CAYRWVSRANSKLTF TRB:CASSLATNPHETQYF	1	CD8-C2
Pla-1_TGCGTGGTCGGTGTTA	Pla	Pla-1	P1-400	FALSE	2	TRA:CAVRNNARLMF TRB:CASSWTRGDNEQFF	2	CD8-C8
Pla-1_TGCTGCTGTAAGTGGC	Pla	Pla-1	P1-240	FALSE	3	TRA:CAAGDSNYQLIW TRB:CASTPFVGSRDTEAFF	3	CD8-C3
Pla-1_TGGACGCCAGGCTGAA	Pla	Pla-1	P1-2246	FALSE	1	TRA:CALSAYNFNKFYF TRB:CASISGTGFFEQYF	1	CD8-C3
Pla-1_TGGCGCAAGATTACCC	Pla	Pla-1	P1-2249	FALSE	1	TRA:CAMSGSGYSTLTF TRB:CSGVGTASYEQYF	1	CD8-C3
Pla-1_TGGCTGGCAATGCCAT	Pla	Pla-1	P1-200	TRUE	3	TRA:CAVGGNNNDMRF TRB:CASSRQDLHEQYF	3	CD8-C3
Pla-1_TGGCTGGTCGCATGAT	Pla	Pla-1	P1-8	TRUE	3	TRA:CAVRPPGRNSGGYQKVTF TRB:CASSRRGVTDTQYF	204	CD8-C3
Pla-1_TGTATTCAGAACAATC	Pla	Pla-1	P1-123	FALSE	3	TRA:CAVKRVTGGFKTIF TRB:CATSGDTGTTNYNEQFF	5	CD8-C2
Pla-1_TGTATTCTCATACGGT	Pla	Pla-1	P1-193	TRUE	3	TRA:CAVRFSGGYNKLIF TRB:CASLEGPYSNQPQHF	3	CD8-C3
Pla-1_TGTCCCAAGGCCGAAT	Pla	Pla-1	P1-424	FALSE	2	TRA:CVAITGGATNKLIF TRB:CAISESRQGEYNEQFF	2	CD8-C8
Pla-1_TGTCCCACAGGACCCT	Pla	Pla-1	P1-2265	FALSE	1	TRA:CALRTGGFKTIF TRB:CASSYSGGGEETQYF	1	CD8-C3
Pla-1_TGTGGTAAGGCAATTA	Pla	Pla-1	P1-97	FALSE	3	TRA:CAVRTAGNKLTF TRB:CSAQGRSSYEQYF	6	CD8-C3
Pla-1_TGTGGTATCAACTCTT	Pla	Pla-1	P1-46	FALSE	3	TRA:CAVRGTGTASKLTF TRB:CSVRQGGNEQFF	20	CD8-C3
Pla-1_TGTGTTTAGTTCCACA	Pla	Pla-1	P1-81	FALSE	3	TRA:CAVTFAGNQFYF TRB:CSAGTGTSGLSEQFF	7	CD8-C3
Pla-1_TGTGTTTGTTCGTGAT	Pla	Pla-1	P1-392	FALSE	2	TRA:CAVRPLSSGSARQLTF TRB:CSVPAGGPSTDTQYF	2	CD8-C8
Pla-1_TGTGTTTTCTAGCACA	Pla	Pla-1	P1-33	TRUE	3	TRA:CLVGTGGFKTIF TRB:CSARVPGQMKETQYF	41	CD8-C3
Pla-1_TGTTCCGTCCTCAATT	Pla	Pla-1	P1-20	TRUE	3	TRA:CVVVGGRALTF TRB:CASSLATNPHETQYF	69	CD8-C3
Pla-1_TTAACTCCACACGCTG	Pla	Pla-1	P1-44	TRUE	3	TRA:CAYRGLGAQKLVF TRB:CASSSLDGQDNHYGYTF	22	CD8-C8
Pla-1_TTAACTCTCGTGACAT	Pla	Pla-1	P1-2279	FALSE	1	TRA:CATDGSGGSNYKLTF TRB:CASTLGPGGYTF	1	CD8-C3
Pla-1_TTAACTCTCTTTAGTC	Pla	Pla-1	P1-2281	FALSE	1	TRA:CAASLGDTDKLIF TRB:CASSIDLGGANVLTF	1	CD8-C3
Pla-1_TTAGGCACATCGGACC	Pla	Pla-1	P1-2285	FALSE	1	TRA:CAVKPLDTGRRALTF TRB:CATSAGDNEQFF	1	CD8-C2
Pla-1_TTAGTTCCAAGCCATT	Pla	Pla-1	P1-240	FALSE	3	TRA:CAAGDSNYQLIW TRB:CASTPFVGSRDTEAFF	3	CD8-C3

Pla-1_TTAGTTCGTCGATTGT	Pla	Pla-1	P1-82	FALSE	3	TRA:CAASEDSNYQLIW TRB:CASSFGDSSSETQYF	7	CD8-C3
Pla-1_TTAGTTCTCCCTGACT	Pla	Pla-1	P1-2287	FALSE	1	TRA:CAVRDDKIIF TRB:CASSLTPGATEAFF	1	CD8-C3
Pla-1_TTAGTTCTCTGCAGTA	Pla	Pla-1	P1-33	TRUE	3	TRA:CLVGTGGFKTIF TRB:CSARVPGQMKETQYF	41	CD8-C3
Pla-1_TTCCCAGAGCCAGAAC	Pla	Pla-1	P1-2290	FALSE	1	TRA:CAVRPRSSNTGKLIF TRB:CAWRVQASSYNSPLHF	1	CD8-C3
Pla-1_TTCGAAGTCGAATCCA	Pla	Pla-1	P1-220	FALSE	3	TRA:CAAGQKNYGGSQGNLIF TRB:CASSIRGESSTEAFF	3	CD8-C5
Pla-1_TTCTACACAAGAAAGG	Pla	Pla-1	P1-2302	FALSE	1	TRA:CVVYGYSTLTF TRB:CASSQAWGSYTGELFF	1	CD8-C3
Pla-1_TTCTCAAAGAAAGTGG	Pla	Pla-1	P1-33	TRUE	3	TRA:CLVGTGGFKTIF TRB:CSARVPGQMKETQYF	41	CD8-C8
Pla-1_TTCTCAAAGTGGGATC	Pla	Pla-1	P1-2303	FALSE	1	TRA:CAASDRGAQKLVF TRB:CASSPHVFGTEAFF	1	CD8-C3
Pla-1_TTGAACGAGGTGCAAC	Pla	Pla-1	P1-118	TRUE	3	TRA:CAVVKAGGTSYGKLTF TRB:CASKSLAGKSSYEQYF	5	CD8-C3
Pla-1_TTGACTTGTCTGGAGA	Pla	Pla-1	P1-32	FALSE	3	TRA:CAVMNTGFQKLVF TRB:CSVSGLVLGGNEQFF	46	CD8-C3
Pla-1_TTGCCGTTCGGTTAAC	Pla	Pla-1	P1-33	TRUE	3	TRA:CLVGTGGFKTIF TRB:CSARVPGQMKETQYF	41	CD8-C3
Pla-1_TTGGAACCAGTAAGCG	Pla	Pla-1	P1-33	TRUE	3	TRA:CLVGTGGFKTIF TRB:CSARVPGQMKETQYF	41	CD8-C3
Pla-1_TTGGAACTCGCCCTTA	Pla	Pla-1	P1-55	TRUE	3	TRA:CALSPQDSNYQLIW TRB:CASSFWTSANEQFF	13	CD8-C3
Pla-1_TTGGCAAAGATTACCC	Pla	Pla-1	P1-2321	FALSE	1	TRA:CAASSTGTASKLTF TRB:CASSTERGGPRDEQFF	1	CD8-C2
Pla-1_TTGGCAAAGTCGATAA	Pla	Pla-1	P1-44	TRUE	3	TRA:CAYRGLGAQKLVF TRB:CASSSLDGQDNHYGYTF	22	CD8-C3
Pla-1_TTTACTGAGGTGATTA	Pla	Pla-1	P1-33	TRUE	3	TRA:CLVGTGGFKTIF TRB:CSARVPGQMKETQYF	41	CD8-C3
Pla-1_TTTATGCAGCGATTCT	Pla	Pla-1	P1-411	FALSE	2	TRA:CAGARSLISGGSNYKLTF TRB:CASGWTSGSRSPIYNEQF	2	CD8-C3
Pla-1_TTTATGCAGTCCAGGA	Pla	Pla-1	P1-23	TRUE	3	TRA:CALRAITQGGSEKLVF TRB:CASSLTGAVYGYTF	65	CD8-C3
Pla-1_TTTATGCAGTGGACGT	Pla	Pla-1	P1-2330	FALSE	1	TRA:CILRDPRGLTSGSRLTF TRB:CASSQAPGLADNEQFF	1	CD8-C3
Pla-1_TTTGGTTAGCTCCTCT	Pla	Pla-1	P1-46	FALSE	3	TRA:CAVRGTGTASKLTF TRB:CSVRQGGNEQFF	20	CD8-C3
Pla-1_TTTGGTTCATGGTCTA	Pla	Pla-1	P1-2340	FALSE	1	TRA:CAVDTGANNLFF TRB:CASSADRGSYEQYF	1	CD4-C5
Pla-1_TTTGTCAAGTCATGCT	Pla	Pla-1	P1-191	TRUE	3	TRA:CIVSGAYSGAGSYQLTF TRB:CASSLVRGGETQYF	3	CD8-C3
Pla-1_AAACGGGGTGTGCCTG	Pla	Pla-1	P1-51	FALSE	3	TRA:CAYRSLNNAGNMLTF TRB:CASSRGNNSPLHF	14	CD4-C4
Pla-1_AAAGATGCAGGTCTCG	Pla	Pla-1	P1-1084	FALSE	1	TRA:CGPRVVQGAQKLVF TRB:CASSLDGVGRPGETQYF	1	CD8-C8
Pla-1_AAAGCAACACAACTGT	Pla	Pla-1	P1-1085	FALSE	1	TRA:CAVRVSNNAGNMLTF TRB:CASSEIKTRRDEQFF	1	CD4-C4
Pla-1_AAAGTAGAGAGCAATT	Pla	Pla-1	P1-302	FALSE	2	TRA:CVVILLSTGRRALTF TRB:CASSLGLAGDNEQFF	2	CD4-C4
Pla-1_AAATGCCAGTTTGCGT	Pla	Pla-1	P1-1090	FALSE	1	TRA:CAVKNRPGGTSYGKLTF TRB:CASSPRQGGNNQPQHF	1	CD4-C4

Pla-1_AACCGCGAGTGTGGCA	Pla	Pla-1	P1-1099	FALSE	1	TRA:CAVDDGGTGNQFYF TRB:CASSLWGASDTQYF	1	CD4-C4
Pla-1_AACCGCGCATGCTAGT	Pla	Pla-1	P1-281	TRUE	2	TRA:CVVNMRFTGGGNKLTF TRB:CASSPQGGTSTGELFF	2	CD4-C4
Pla-1_AACGTTGGTAAGTTCC	Pla	Pla-1	P1-1100	FALSE	1	TRA:CAESKAAGNKLTF TRB:CASSPGQGGDTQYF	1	CD4-C4
Pla-1_AACTCAGGTCGACTGC	Pla	Pla-1	P1-306	FALSE	2	TRA:CAFPSPGRGSTLGRLYF TRB:CASSLGLADSTDTQYF	2	CD4-C4
Pla-1_AACTCAGGTGTAAGTA	Pla	Pla-1	P1-122	FALSE	3	TRA:CALTFSGGYQKVTF TRB:CASSPLGTSPYEQYF	5	CD4-C4
Pla-1_AACTCCCAGTAACCCT	Pla	Pla-1	P1-205	FALSE	3	TRA:CAVNTLGNQFYF TRB:CASSPGHGVVGEQYF	3	CD4-C4
Pla-1_AACTCTTGTTTAGGAA	Pla	Pla-1	P1-308	FALSE	2	TRA:CAAGNNNDMRF TRB:CSASFRGGITGELFF	2	CD4-C4
Pla-1_AACTCTTTCTCAACTT	Pla	Pla-1	P1-1106	FALSE	1	TRA:CAPFTGGGNKLTF TRB:CASSGGGQGWETQYF	1	CD4-C4
Pla-1_AACTTTCCACAGACAG	Pla	Pla-1	P1-1110	FALSE	1	TRA:CATDARDSNYQLIW TRB:CASSGEGEGFGYTF	1	CD4-C4
Pla-1_AACTTTCTCTTCGGTC	Pla	Pla-1	P1-313	FALSE	2	TRA:CAVGRFTGGGNKLTF TRB:CASSDRAGNTIYF	2	CD4-C4
Pla-1_AAGACCTGTAAACACA	Pla	Pla-1	P1-96	FALSE	3	TRA:CAAQDTGTASKLTF TRB:CASNPNRDRPSEQFF	6	CD4-C4
Pla-1_AAGACCTTCGAATGCT	Pla	Pla-1	P1-1115	FALSE	1	TRA:CAMTYGSSNTGKLIF TRB:CASSWGGTSGETQYF	1	CD4-C4
Pla-1_AAGGCAGCAAAGTGCG	Pla	Pla-1	P1-1121	FALSE	1	TRA:CAAYNTNAGKSTF TRB:CSAIDRGPGAKNIQYF	1	CD4-C4
Pla-1_AAGGTTCTCCACGACG	Pla	Pla-1	P1-159	FALSE	3	TRA:CAVQGPYKAAGNKLTF TRB:CASSRDLGYEQYF	4	CD4-C4
Pla-1_AAGTCTGAGTCCGGTC	Pla	Pla-1	P1-1127	FALSE	1	TRA:CALRMNTGFQKLVF TRB:CASSGTAQSLTGEQYF	1	CD8-C8
Pla-1_AAGTCTGTCGATAGAA	Pla	Pla-1	P1-1130	FALSE	1	TRA:CVVNQQNSGNTPLVF TRB:CAISESAGRQPQHF	1	CD4-C4
Pla-1_AATCCAGCATGGGAAC	Pla	Pla-1	P1-51	FALSE	3	TRA:CAYRSLNNAGNMLTF TRB:CASSRGNNSPLHF	14	CD4-C4
Pla-1_AATCCAGGTCGCATAT	Pla	Pla-1	P1-95	TRUE	3	TRA:CIVRVARGNEKLTF TRB:CASSLYGEGHQPQHF	6	CD4-C4
Pla-1_AATCCAGTCATAAAGG	Pla	Pla-1	P1-1134	FALSE	1	TRA:CAGQYRGAGSYQLTF TRB:CAWSKGPEQYF	1	CD4-C4
Pla-1_AATCGGTAGGTAGCTG	Pla	Pla-1	P1-1135	FALSE	1	TRA:CALLPVSGNTPLVF TRB:CASRASNSPLHF	1	CD4-C4
Pla-1_AATCGGTCATGCCTAA	Pla	Pla-1	P1-90	TRUE	3	TRA:CAVNNGRGSTLGRLYF TRB:CASSSGPGSGANVLTF	6	CD4-C4
Pla-1_AATCGGTGTTCGCGAC	Pla	Pla-1	P1-1137	FALSE	1	TRA:CVIRMDSSYKLIF TRB:CSATGLAGRSTETQYF	1	CD4-C4
Pla-1_ACACCAAAGCACGCCT	Pla	Pla-1	P1-1138	FALSE	1	TRA:CVVDGSSNTGKLIF TRB:CASRATVVGVKNIQYF	1	CD8-C8
Pla-1_ACACCAAAGCTGCAAG	Pla	Pla-1	P1-1139	FALSE	1	TRA:CAVRRSNAGNMLTF TRB:CASSFGYRTSNNEQFF	1	CD4-C4
Pla-1_ACAGCCGGTACCATCA	Pla	Pla-1	P1-1150	FALSE	1	TRA:CAARSQNAGGTSYGKLTF TRB:CASSNRDRVTDTQYF	1	CD4-C4
Pla-1_ACCAGTAGTCAAAGAT	Pla	Pla-1	P1-1165	FALSE	1	TRA:CAVRDDAAGNKLTF TRB:CASSLLSSGTGHYGYTF	1	CD4-C4
Pla-1_ACCAGTAGTTCACCTC	Pla	Pla-1	P1-1166	FALSE	1	TRA:CAVSIEGGFKTIF TRB:CASSLIYEQYF	1	CD8-C8

Pla-1_ACCAGTATCTGCAAGT	Pla	Pla-1	P1-1169	FALSE	1	TRA:CAAPPTGSGNTGKLIF TRB:CASSLGGRAPGELFF	1	CD4-C4
Pla-1_ACCCACTAGCTTCGCG	Pla	Pla-1	P1-1170	FALSE	1	TRA:CLLGLSYNTDKLIF TRB:CASSRYQENYGYTF	1	CD4-C3
Pla-1_ACCGTAAAGAAGGCCT	Pla	Pla-1	P1-1171	FALSE	1	TRA:CAVSSNYQLIW TRB:CASSHSPYEQYF	1	CD4-C4
Pla-1_ACCTTTACAAAGGTGC	Pla	Pla-1	P1-1175	FALSE	1	TRA:CAMGPGQNFVF TRB:CASSLTSGQYEQYF	1	CD4-C4
Pla-1_ACGAGCCGTAGCTAAA	Pla	Pla-1	P1-19	TRUE	3	TRA:CALGAFGSARQLTF TRB:CASSKGQGPRETQYF	70	CD4-C4
Pla-1_ACGAGGAAGTGTGAAT	Pla	Pla-1	P1-1179	FALSE	1	TRA:CAATGGGNKLTF TRB:CSAIATGVRAIQYF	1	CD8-C8
Pla-1_ACGAGGATCACGACTA	Pla	Pla-1	P1-1182	FALSE	1	TRA:CAGQLRGSNYKLTF TRB:CSVATGEETQYF	1	CD4-C4
Pla-1_ACGATACTCAAACCAC	Pla	Pla-1	P1-160	FALSE	3	TRA:CAASADGGSQGNLIF TRB:CASSPTYGYTF	4	CD4-C4
Pla-1_ACGATGTTCTACCTGC	Pla	Pla-1	P1-1191	FALSE	1	TRA:CVVNPYNNNDMRF TRB:CASSVGGGGTEAFF	1	CD4-C4
Pla-1_ACGCCAGAGGTGCACA	Pla	Pla-1	P1-1192	FALSE	1	TRA:CAENIEGAQKLVF TRB:CASSVARRSYGYTF	1	CD4-C4
Pla-1_ACGCCGAGTGATAAGT	Pla	Pla-1	P1-1196	FALSE	1	TRA:CVVREGAGFQKLVF TRB:CASSEIGTGGYGYTF	1	CD8-C8
Pla-1_ACGGAGAGTAGAGTGC	Pla	Pla-1	P1-1200	FALSE	1	TRA:CAVTRGFKTIF TRB:CASSKVQNGGLNNEQFF	1	CD4-C4
Pla-1_ACGGCCATCCTTGCCA	Pla	Pla-1	P1-1202	FALSE	1	TRA:CIVRVAGSQKVTF TRB:CASSLEGEKLFF	1	CD8-C8
Pla-1_ACGTCAACAGGTCTCG	Pla	Pla-1	P1-41	TRUE	3	TRA:CVVSEPPGGSYIPTF TRB:CASSSRPEDTQYF	27	CD4-C4
Pla-1_ACTGAGTTCGGAAACG	Pla	Pla-1	P1-327	FALSE	2	TRA:CAYLWRGGGADGLTF TRB:CASSQGSGLYNEQFF	2	CD4-C4
Pla-1_ACTGATGGTAAGAGGA	Pla	Pla-1	P1-216	FALSE	3	TRA:CALLFGNVLHC TRB:CASSLAVRANSMNTEAFF	3	CD4-C4
Pla-1_ACTGCTCCACCCAGTG	Pla	Pla-1	P1-1222	FALSE	1	TRA:CAVNLWTAGNKLTF TRB:CASSMGQGGTEAFF	1	CD4-C3
Pla-1_ACTGCTCCATGCTAGT	Pla	Pla-1	P1-162	FALSE	3	TRA:CVVSGGLRGAAGNKLTF TRB:CASSPLTVNTEAFF	4	CD4-C4
Pla-1_ACTGCTCTCGCCTGAG	Pla	Pla-1	P1-1226	FALSE	1	TRA:CAVTSSGGSNYKLTF TRB:CASSLTGDTEAFF	1	CD8-C8
Pla-1_ACTGTCCAGTGTCCCG	Pla	Pla-1	P1-1228	FALSE	1	TRA:CAARGTGYSTLTF TRB:CASSQDMAGGYTF	1	CD4-C4
Pla-1_ACTTGTTTCGGGAGTA	Pla	Pla-1	P1-1236	FALSE	1	TRA:CAASGGSSNTGKLIF TRB:CASSEFGKAQYF	1	CD4-C4
Pla-1_ACTTTCATCACATAGC	Pla	Pla-1	P1-1240	FALSE	1	TRA:CASHSGNTPLVF TRB:CSARVPGQMKETQYF	1	CD4-C4
Pla-1_ACTTTCATCCACTGGG	Pla	Pla-1	P1-129	FALSE	3	TRA:CAFLFYGGSQGNLIF TRB:CASSQGRGRYGNTIYF	5	CD4-C4
Pla-1_AGACGTTAGCCTTGAT	Pla	Pla-1	P1-1242	FALSE	1	TRA:CALDPLGGSQGNLIF TRB:CASAGGTGDEKLFF	1	CD4-C4
Pla-1_AGACGTTCAGTTCCCT	Pla	Pla-1	P1-51	FALSE	3	TRA:CAYRSLNNAGNMLTF TRB:CASSRGNNSPLHF	14	CD4-C4
Pla-1_AGACGTTGTACAGCAG	Pla	Pla-1	P1-65	FALSE	3	TRA:CAVQAHSGGSNYKLTF TRB:CASAETSYTGELFF	10	CD4-C4
Pla-1_AGAGCGAGTCCTGCTT	Pla	Pla-1	P1-329	FALSE	2	TRA:CIVRVAGGGADGLTF TRB:CASSTGTGGPTRETQYF	2	CD4-C4

Pla-1_AGAGCGAGTTGTCTTT	Pla	Pla-1	P1-1245	FALSE	1	TRA:CIVLAGSARQLTF TRB:CASSPATGLNTEAFF	1	CD4-C4
Pla-1_AGAGCTTGTGAGTATA	Pla	Pla-1	P1-1248	FALSE	1	TRA:CAESRGNFNKFYF TRB:CASSSGTSPGTDTQYF	1	CD4-C4
Pla-1_AGAGCTTTCATGGTCA	Pla	Pla-1	P1-331	FALSE	2	TRA:CAVLIAGGYQKVTF TRB:CSASPGAQETQYF	2	CD4-C4
Pla-1_AGAGTGGGTCCGTCAG	Pla	Pla-1	P1-332	FALSE	2	TRA:CALNFSGGYNKLIF TRB:CAISESEDSYEQYF	2	CD4-C4
Pla-1_AGAGTGGTCACGATGT	Pla	Pla-1	P1-219	FALSE	3	TRA:CAVRGADSWGKLQF TRB:CASSDRTEGRAFF	3	CD4-C4
Pla-1_AGATTGCGTAGCCTAT	Pla	Pla-1	P1-1260	FALSE	1	TRA:CVVNIGEEEVTGGGNKLTF TRB:CASSIGARKWESGNTIYF	1	CD4-C4
Pla-1_AGCAGCCAGAAAGTGG	Pla	Pla-1	P1-42	TRUE	3	TRA:CASFSGGSQGNLIF TRB:CSAGQGSNQPQHF	24	CD4-C4
Pla-1_AGCTCTCAGACTACAA	Pla	Pla-1	P1-1273	FALSE	1	TRA:CAGYGGSQGNLIF TRB:CASSLMSYEQYF	1	CD4-C4
Pla-1_AGCTCTCCAAGAGGCT	Pla	Pla-1	P1-1274	FALSE	1	TRA:CAVSRANSGYALNF TRB:CASSWAASQETQYF	1	CD4-C4
Pla-1_AGCTCTCTCTTAGAGC	Pla	Pla-1	P1-1275	FALSE	1	TRA:CAVSVSSGGSYIPTF TRB:CASSWDRGTYGYTF	1	CD4-C4
Pla-1_AGCTTGACATCATCCC	Pla	Pla-1	P1-1278	FALSE	1	TRA:CAVHLGETSGSRLTF TRB:CASSRQGMGNQPQHF	1	CD8-C8
Pla-1_AGGCCACGTTCGTGAT	Pla	Pla-1	P1-163	FALSE	3	TRA:CALRFNDYKLSF TRB:CASTSFETEAFF	4	CD4-C4
Pla-1_AGGGTGACATGCTAGT	Pla	Pla-1	P1-1287	FALSE	1	TRA:CAERPNGGATNKLIF TRB:CASRLTVNRVHEQYF	1	CD4-C4
Pla-1_AGGTCATTCTCTAGGA	Pla	Pla-1	P1-164	FALSE	3	TRA:CAAGENTGTASKLTF TRB:CASSSTYRVQETQYF	4	CD4-C4
Pla-1_AGGTCCGCAGTGGGAT	Pla	Pla-1	P1-1291	FALSE	1	TRA:CAETSSGYALNF TRB:CASSLVATYGEQFF	1	CD8-C8
Pla-1_AGTGAGGCAGGTCCAC	Pla	Pla-1	P1-342	FALSE	2	TRA:CAVPHRF TRB:CSAAGQGVHEQYF	2	CD4-C4
Pla-1_AGTGAGGTCTATGTGG	Pla	Pla-1	P1-1297	FALSE	1	TRA:CAVTLNAGNNRKLIW TRB:CASSRPGQGFSPLHF	1	CD4-C4
Pla-1_AGTGGGAAGTGAATTG	Pla	Pla-1	P1-1298	FALSE	1	TRA:CAVRPRETSGSRLTF TRB:CASSYQRDQPQHF	1	CD4-C4
Pla-1_AGTGTCATCCACGAAT	Pla	Pla-1	P1-1303	FALSE	1	TRA:CAVSHTGGGNKLTF TRB:CSARGRGQETQYF	1	CD4-C4
Pla-1_ATAACGCTCACCACCT	Pla	Pla-1	P1-1309	FALSE	1	TRA:CAVGVRTSYDKVIF TRB:CASSAVAEQFF	1	CD4-C4
Pla-1_ATAAGAGCATTGGTAC	Pla	Pla-1	P1-1314	FALSE	1	TRA:CVVSGLSGGGADGLTF TRB:CASSLQARGGGTEAFF	1	CD4-C3
Pla-1_ATCACGAGTGTTCGAT	Pla	Pla-1	P1-1319	FALSE	1	TRA:CAMKAPMNRDDKIIF TRB:CASSGPTGNNEKLFF	1	CD4-C4
Pla-1_ATCATCTCAATGCCAT	Pla	Pla-1	P1-1321	FALSE	1	TRA:CAVSFSGNTGKLIF TRB:CASRLTGDSNQPQHF	1	CD4-C3
Pla-1_ATCATGGTCGGCGCAT	Pla	Pla-1	P1-1326	FALSE	1	TRA:CAENQGNNARLMF TRB:CASSRKTGEWLFF	1	CD4-C4
Pla-1_ATCCGAATCATGCTCC	Pla	Pla-1	P1-1330	FALSE	1	TRA:CGTGSHGNEKLTF TRB:CASSRQWETQYF	1	CD4-C4
Pla-1_ATCGAGTGTCATATCG	Pla	Pla-1	P1-1332	FALSE	1	TRA:CAESRRGGADGLTF TRB:CASSLALAGTGELFF	1	CD4-C4
Pla-1_ATCTACTAGCTAGTGG	Pla	Pla-1	P1-42	TRUE	3	TRA:CASFSGGSQGNLIF TRB:CSAGQGSNQPQHF	24	CD4-C4

Pla-1_ATCTACTAGGCTACGA	Pla	Pla-1	P1-1334	FALSE	1	TRA:CAFIYTGGFKTIF TRB:CASSLALGQASYEQYF	1	CD8-C8
Pla-1_ATCTACTAGTGTACTC	Pla	Pla-1	P1-1336	FALSE	1	TRA:CALTGAQKLVF TRB:CACRQGGIGEKLFF	1	CD4-C4
Pla-1_ATGCGATTCTGCGGCA	Pla	Pla-1	P1-96	FALSE	3	TRA:CAAQDTGTASKLTF TRB:CASNPNRDRPSEQFF	6	CD8-C2
Pla-1_ATGGGAGAGACTTTCG	Pla	Pla-1	P1-1346	FALSE	1	TRA:CAYRRFGSSNTGKLIF TRB:CASSYGRVYEQYF	1	CD4-C4
Pla-1_ATGGGAGTCGCCGTGA	Pla	Pla-1	P1-165	FALSE	3	TRA:CAMDYNQGGKLIF TRB:CASSSATGNTEAFF	4	CD4-C4
Pla-1_ATTCTACAGTGTGGCA	Pla	Pla-1	P1-1355	FALSE	1	TRA:CALRADTGRRALTF TRB:CASSLGTGTGTVETQYF	1	CD4-C4
Pla-1_ATTCTACGTGTGCGTC	Pla	Pla-1	P1-308	FALSE	2	TRA:CAAGNNNDMRF TRB:CSASFRGGITGELFF	2	CD4-C3
Pla-1_ATTGGACTCCAAGTAC	Pla	Pla-1	P1-1357	FALSE	1	TRA:CAVSPSGGSYIPTF TRB:CASSRSGTENTEAFF	1	CD4-C4
Pla-1_ATTGGTGAGTCAAGGC	Pla	Pla-1	P1-81	FALSE	3	TRA:CAVTFAGNQFYF TRB:CSAGTGTSGLSEQFF	7	CD8-C3
Pla-1_ATTTCTGAGAATAGGG	Pla	Pla-1	P1-351	FALSE	2	TRA:CAESILTGGGNKLTF TRB:CASSLYSYDSPLHF	2	CD4-C4
Pla-1_ATTTCTGAGCGCCTTG	Pla	Pla-1	P1-1360	FALSE	1	TRA:CAVIPEGQNFVF TRB:CAYQPGARESYEQYF	1	CD8-C2
Pla-1_ATTTCTGAGTACGTTC	Pla	Pla-1	P1-1361	FALSE	1	TRA:CAYRNVKTSYDKVIF TRB:CASSARSTNYGYTF	1	CD4-C2
Pla-1_CAACCAAAGTGGCACA	Pla	Pla-1	P1-1363	FALSE	1	TRA:CVVNKGGTSYGKLTF TRB:CASSGTGGRNEKLFF	1	CD4-C4
Pla-1_CAACCTCGTGGACGAT	Pla	Pla-1	P1-228	FALSE	3	TRA:CAVRDPYNQGGKLIF TRB:CASSLGMGSSYEQYF	3	CD4-C4
Pla-1_CAAGGCCGTAAGTAGT	Pla	Pla-1	P1-1377	FALSE	1	TRA:CATDRIQGAQKLVF TRB:CASSLGSTSGNTIYF	1	CD4-C2
Pla-1_CACAAACGTCGAAAGC	Pla	Pla-1	P1-1385	FALSE	1	TRA:CATERGQKLLF TRB:CASSLGRDRDSPLHF	1	CD4-C4
Pla-1_CACAGGCAGCCATCGC	Pla	Pla-1	P1-172	TRUE	3	TRA:CAVRRDDKIIF TRB:CASSPIAGGSFDEQYF	3	CD4-C4
Pla-1_CACAGGCCACAGAGGT	Pla	Pla-1	P1-1393	FALSE	1	TRA:CAAFIYNQGGKLIF TRB:CASSLDGQGARGYTF	1	CD4-C4
Pla-1_CACAGTACAGCTGTAT	Pla	Pla-1	P1-51	FALSE	3	TRA:CAYRSLNNAGNMLTF TRB:CASSRGNNSPLHF	14	CD4-C4
Pla-1_CACAGTAGTCGACTAT	Pla	Pla-1	P1-48	TRUE	3	TRA:CAEVSGYSTLTF TRB:CASSFPTGGRTGRTEAFF	16	CD4-C4
Pla-1_CACATAGAGCTGCCCA	Pla	Pla-1	P1-1397	FALSE	1	TRA:CAGQGGKLIF TRB:CASSRQGAETQYF	1	CD8-C8
Pla-1_CACATAGTCGGAGGTA	Pla	Pla-1	P1-1400	FALSE	1	TRA:CILKYGNEKLTF TRB:CASSRGQGAGEQYF	1	CD4-C4
Pla-1_CACCTTGAGTCCGGTC	Pla	Pla-1	P1-360	FALSE	2	TRA:CAGINYGGQNFVF TRB:CSVAGTLYTGELFF	2	CD4-C4
Pla-1_CACCTTGGTCCTGCTT	Pla	Pla-1	P1-361	FALSE	2	TRA:CAENRNAGNMLTF TRB:CASSYDRVGEQYF	2	CD4-C4
Pla-1_CACTCCAAGGATTCGG	Pla	Pla-1	P1-1411	FALSE	1	TRA:CAGEKGNTGKLIF TRB:CAIRPGLAGGATDTQYF	1	CD4-C4
Pla-1_CAGCAGCCAAGTTCTG	Pla	Pla-1	P1-1419	FALSE	1	TRA:CAVRDSGTYKYIF TRB:CASSTPTPEQYF	1	CD4-C4
Pla-1_CAGCAGCTCCAAGTAC	Pla	Pla-1	P1-1420	FALSE	1	TRA:CASSNSGGYQKVTF TRB:CASSDRGTSGAETQYF	1	CD4-C4

Pla-1_CAGCATATCAAACCGT	Pla	Pla-1	P1-346	FALSE	2	TRA:CGTELGGNQFYF TRB:CASSVGDGTEAFF	2	CD4-C4
Pla-1_CAGCCGACAGTCAGAG	Pla	Pla-1	P1-151	TRUE	3	TRA:CAGTNSGYSTLTF TRB:CASSPKESGQTFQETQYF	4	CD4-C4
Pla-1_CAGCTAAGTCGAGTTT	Pla	Pla-1	P1-51	FALSE	3	TRA:CAYRSLNNAGNMLTF TRB:CASSRGNNSPLHF	14	CD4-C4
Pla-1_CAGCTGGAGTCCTCCT	Pla	Pla-1	P1-163	FALSE	3	TRA:CALRFNDYKLSF TRB:CASTSFETEAFF	4	CD4-C3
Pla-1_CAGGTGCAGATGCCAG	Pla	Pla-1	P1-41	TRUE	3	TRA:CVVSEPPGGSYIPTF TRB:CASSSRPEDTQYF	27	CD4-C6
Pla-1_CAGGTGCCAATGGAGC	Pla	Pla-1	P1-232	FALSE	3	TRA:CAVGASNQGGKLIF TRB:CASSLGMGSSYEQYF	3	CD4-C4
Pla-1_CATATGGTCTGAGGGA	Pla	Pla-1	P1-105	FALSE	3	TRA:CIVRVVSNQGGKLIF TRB:CASSPRTGGVGTQYF	6	CD4-C5
Pla-1_CATCGAAAGTTTAGGA	Pla	Pla-1	P1-214	FALSE	3	TRA:CAMREGRSRNDMRF TRB:CASSLYGPTSGRPLDQETQY	3	CD4-C4
Pla-1_CATCGAATCCTCAACC	Pla	Pla-1	P1-1445	FALSE	1	TRA:CALSDRGNARLMF TRB:CASSPPSSGRPGDTQYF	1	CD4-C4
Pla-1_CATCGGGTCTGCGACG	Pla	Pla-1	P1-51	FALSE	3	TRA:CAYRSLNNAGNMLTF TRB:CASSRGNNSPLHF	14	CD4-C4
Pla-1_CATGACATCGATCCCT	Pla	Pla-1	P1-147	TRUE	3	TRA:CAGQLSGGSNYKLTF TRB:CASSERQGKAYGYTF	4	CD4-C4
Pla-1_CATGGCGTCTGAGGGA	Pla	Pla-1	P1-341	FALSE	2	TRA:CAFKQSGNTPLVF TRB:CASSPWGAGLYNEQFF	2	CD4-C4
Pla-1_CATTATCCACGACGAA	Pla	Pla-1	P1-369	FALSE	2	TRA:CAVNALNFGNEKLTF TRB:CASSLASGSNNEQFF	2	CD4-C4
Pla-1_CATTATCCACGCGAAA	Pla	Pla-1	P1-1463	FALSE	1	TRA:CAATAPSGSRLTF TRB:CASSQDSWGGMGYGYTF	1	CD4-C4
Pla-1_CATTCGCGTAGGCTGA	Pla	Pla-1	P1-1469	FALSE	1	TRA:CAAVSSSGYSTLTF TRB:CASTETAAGALGEQYF	1	CD4-C4
Pla-1_CCACCTACAGGATCGA	Pla	Pla-1	P1-17	TRUE	3	TRA:CGTGARGSSGSRLTF TRB:CASSRQGMGAFF	78	CD4-C4
Pla-1_CCACCTACAGTTTACG	Pla	Pla-1	P1-1474	FALSE	1	TRA:CVVSGAGYSTLTF TRB:CASSLGEETQYF	1	CD4-C4
Pla-1_CCACGGAAGCACCGTC	Pla	Pla-1	P1-1477	FALSE	1	TRA:CAFFDDYKLSF TRB:CASSLSYEQYF	1	CD8-C2
Pla-1_CCACTACAGATCCCGC	Pla	Pla-1	P1-372	FALSE	2	TRA:CAASARYNAGNMLTF TRB:CASSFGKRGGFGFETQYF	2	CD4-C3
Pla-1_CCACTACCACTGTTAG	Pla	Pla-1	P1-1479	FALSE	1	TRA:CAVRPLLNTGFQKLVF TRB:CASSSGTYEQYF	1	CD4-C4
Pla-1_CCCAATCTCATACGGT	Pla	Pla-1	P1-1491	FALSE	1	TRA:CAVEDRKGGSYIPTF TRB:CASSQGLARSSYEQYF	1	CD4-C4
Pla-1_CCCTCCTAGCAATATG	Pla	Pla-1	P1-1493	FALSE	1	TRA:CAYRSAWGSSNTGKLIF TRB:CASSRGTEAYNEQFF	1	CD4-C4
Pla-1_CCGGGATAGAGTCTGG	Pla	Pla-1	P1-1496	FALSE	1	TRA:CVVTRSGGSYIPTF TRB:CSARKDRGSANSPLHF	1	CD4-C4
Pla-1_CCGGTAGGTCCGTCAG	Pla	Pla-1	P1-96	FALSE	3	TRA:CAAQDTGTASKLTF TRB:CASNPNRDRPSEQFF	6	CD4-C4
Pla-1_CCGTACTCACCCATGG	Pla	Pla-1	P1-51	FALSE	3	TRA:CAYRSLNNAGNMLTF TRB:CASSRGNNSPLHF	14	CD4-C4
Pla-1_CCGTGGATCTGGTGTA	Pla	Pla-1	P1-41	TRUE	3	TRA:CVVSEPPGGSYIPTF TRB:CASSSRPEDTQYF	27	CD4-C4
Pla-1_CCTACCAAGACAATAC	Pla	Pla-1	P1-1513	FALSE	1	TRA:CAVLNAGNNRKLIW TRB:CSVVGGLGYTF	1	CD4-C4

Pla-1_CCTCTGAGTCCGTGAC	Pla	Pla-1	P1-1527	FALSE	1	TRA:CALMNSGGYQKVTF TRB:CASSDRRDQNIQYF	1	CD4-C4
Pla-1_CCTCTGAGTCGCTTTC	Pla	Pla-1	P1-159	FALSE	3	TRA:CAVQGPYKAAGNKLTF TRB:CASSRDLGYEQYF	4	CD4-C4
Pla-1_CCTTACGAGTAGATGT	Pla	Pla-1	P1-1529	FALSE	1	TRA:CAASRGWGKLQF TRB:CASSLPNPQGKTEAFF	1	CD4-C4
Pla-1_CCTTTCTGTCAGCTAT	Pla	Pla-1	P1-1535	FALSE	1	TRA:CAVSERYSTLTF TRB:CASSYLGSTEAFF	1	CD8-C8
Pla-1_CGAATGTCATCCGTGG	Pla	Pla-1	P1-104	FALSE	3	TRA:CALRGSARQLTF TRB:CASSQEEAGPAKNIQYF	6	CD4-C4
Pla-1_CGACCTTGTTGATTGC	Pla	Pla-1	P1-238	FALSE	3	TRA:CAVGPWGAQKLVF TRB:CATSPSRGNNEQFF	3	CD4-C4
Pla-1_CGACTTCAGTTACGGG	Pla	Pla-1	P1-1546	FALSE	1	TRA:CAMREGKDGGTSYGKLTF TRB:CASSPSRGSAPTGNTIYF	1	CD8-C8
Pla-1_CGACTTCCAATCACAC	Pla	Pla-1	P1-1547	FALSE	1	TRA:CAGQNQGGKLIF TRB:CAISERANTDTQYF	1	CD4-C4
Pla-1_CGAGAAGCATTTGCTT	Pla	Pla-1	P1-1553	FALSE	1	TRA:CALSSNSGYALNF TRB:CATSRDQGAGNTEAFF	1	CD4-C4
Pla-1_CGAGAAGTCATAACCG	Pla	Pla-1	P1-96	FALSE	3	TRA:CAAQDTGTASKLTF TRB:CASNPNRDRPSEQFF	6	CD4-C4
Pla-1_CGAGCACCAAGTTCTG	Pla	Pla-1	P1-1557	FALSE	1	TRA:CAASSTGGAGNMLTF TRB:CASSMDGQSRERQYF	1	CD4-C4
Pla-1_CGAGCACTCACCATAG	Pla	Pla-1	P1-129	FALSE	3	TRA:CAFLFYGGSQGNLIF TRB:CASSQGRGRYGNTIYF	5	CD4-C4
Pla-1_CGAGCCAGTAAAGTCA	Pla	Pla-1	P1-1560	FALSE	1	TRA:CAETSTMDTGRRALTF TRB:CASSPPGQGSYEQYF	1	CD4-C4
Pla-1_CGATGTATCGAACGGA	Pla	Pla-1	P1-1566	FALSE	1	TRA:CAMSDASGGYNKLIF TRB:CASGAYTRQYF	1	CD4-C4
Pla-1_CGCGTTTAGACAGACC	Pla	Pla-1	P1-381	FALSE	2	TRA:CAEKSKSNTGKLIF TRB:CASLVYRLQAYYNEQFF	2	CD4-C4
Pla-1_CGCGTTTAGAGGTACC	Pla	Pla-1	P1-1571	FALSE	1	TRA:CAASRSGSARQLTF TRB:CASSLIGGGYEQYF	1	CD4-C4
Pla-1_CGCTGGAAGAGTAAGG	Pla	Pla-1	P1-1574	FALSE	1	TRA:CALSGQGDYKLSF TRB:CASSPGSGERDGYTF	1	CD4-C4
Pla-1_CGGACACAGCTTCGCG	Pla	Pla-1	P1-1580	FALSE	1	TRA:CAAGLLSNARLMF TRB:CSAEDRGPNEQFF	1	CD4-C4
Pla-1_CGGACACGTAGCGTAG	Pla	Pla-1	P1-83	FALSE	3	TRA:CAVRDIENTGFQKLVF TRB:CASTSGGFNQPQHF	7	CD4-C4
Pla-1_CGGACGTCAGATGGCA	Pla	Pla-1	P1-1582	FALSE	1	TRA:CAVTSNYQLIW TRB:CASSLGTGDYGYTF	1	CD4-C4
Pla-1_CGGACGTCATATGCTG	Pla	Pla-1	P1-1583	FALSE	1	TRA:CAVGPRGGNKLVF TRB:CASSSGTVRSSYNEQFF	1	CD8-C8
Pla-1_CGGAGTCTCAGCTCGG	Pla	Pla-1	P1-1594	FALSE	1	TRA:CAESRAGGQKLLF TRB:CASSFDGDQPQHF	1	CD4-C4
Pla-1_CGGCTAGCATCCTTGC	Pla	Pla-1	P1-1596	FALSE	1	TRA:CAPEGAARQLTF TRB:CASNTGQDEKLFF	1	CD4-C4
Pla-1_CGGGTCACATGTAGTC	Pla	Pla-1	P1-384	FALSE	2	TRA:CAGDFGNEKLTF TRB:CASSTGTSLFSYNEQFF	2	CD4-C4
Pla-1_CGGTTAACAGTGACAG	Pla	Pla-1	P1-1600	FALSE	1	TRA:CAVPNSGGSNYKLTF TRB:CASRGRDRGSQPQHF	1	CD4-C4
Pla-1_CGTAGCGGTATAAACG	Pla	Pla-1	P1-1602	FALSE	1	TRA:CAFMKVPNNNDMRF TRB:CASSMAASPLHF	1	CD4-C4
Pla-1_CGTAGGCCATCGACGC	Pla	Pla-1	P1-1604	FALSE	1	TRA:CVVSDQRQNFVF TRB:CASSLFRETQYF	1	CD4-C4

Pla-1_CGTAGGCGTCCAGTAT	Pla	Pla-1	P1-385	FALSE	2	TRA:CATGSAGGTSYGKLTF TRB:CASSFRDTQYF	2	CD4-C4
Pla-1_CGTCACTGTCGCTTTC	Pla	Pla-1	P1-21	TRUE	3	TRA:CAASRGTGANNLFF TRB:CATQREGREQYF	67	CD4-C4
Pla-1_CGTCACTTCAGGATCT	Pla	Pla-1	P1-1611	FALSE	1	TRA:CAVSSGNTGKLIF TRB:CASSVGEGQGSYEQYF	1	CD4-C4
Pla-1_CGTGAGCAGCTATGCT	Pla	Pla-1	P1-1619	FALSE	1	TRA:CAASMYGSGANNLFF TRB:CASSYRGNTEAFF	1	CD4-C4
Pla-1_CGTGTAATCCTCTAGC	Pla	Pla-1	P1-196	TRUE	3	TRA:CAVESPGRGGATNKLIF TRB:CASSARGVTYNEQFF	3	CD4-C3
Pla-1_CGTGTAATCGGATGGA	Pla	Pla-1	P1-1624	FALSE	1	TRA:CALSAFSGSARQLTF TRB:CASSEARQRRGAGELFF	1	CD4-C5
Pla-1_CGTTAGATCTTAACCT	Pla	Pla-1	P1-242	FALSE	3	TRA:CAFFHSNTGKLIF TRB:CASSNPGWNNEQFF	3	CD4-C4
Pla-1_CTAACTTTCCTACAGA	Pla	Pla-1	P1-372	FALSE	2	TRA:CAASARYNAGNMLTF TRB:CASSFGKRGGFGFETQYF	2	CD4-C4
Pla-1_CTAATGGAGCAACGGT	Pla	Pla-1	P1-1637	FALSE	1	TRA:CAPWGSGGYNKLIF TRB:CASSQDQGVGATSTDTQYF	1	CD4-C4
Pla-1_CTACACCCAGATCTGT	Pla	Pla-1	P1-1640	FALSE	1	TRA:CVVNKGGATNKLIF TRB:CASSLDGGDSYEQFF	1	CD4-C4
Pla-1_CTACACCGTGCCTTGG	Pla	Pla-1	P1-226	FALSE	3	TRA:CAAVSAFSGGYNKLIF TRB:CASSLAGRAATNEKLFF	3	CD4-C5
Pla-1_CTACACCTCATCTGCC	Pla	Pla-1	P1-1641	FALSE	1	TRA:CAATDSGSSNTGKLIF TRB:CASSATSGGAPNEQFF	1	CD8-C8
Pla-1_CTACATTAGAACAATC	Pla	Pla-1	P1-1642	FALSE	1	TRA:CVVNPGYALNF TRB:CASSFVSGGVGQPQHF	1	CD4-C4
Pla-1_CTACATTTCAGCAACT	Pla	Pla-1	P1-1644	FALSE	1	TRA:CAMSWGAQKLVF TRB:CSARETVGSDTQYF	1	CD4-C4
Pla-1_CTACCCAGTAAGGATT	Pla	Pla-1	P1-1645	FALSE	1	TRA:CAVIEGDYGGSQGNLIF TRB:CSAIRVGDTEAFF	1	CD8-C8
Pla-1_CTACCCAGTAGAGGAA	Pla	Pla-1	P1-1646	FALSE	1	TRA:CAASLNNNDMRF TRB:CASSDPSTDTQYF	1	CD4-C4
Pla-1_CTACGTCCACTCAGGC	Pla	Pla-1	P1-1649	FALSE	1	TRA:CAVGLMDSNYQLIW TRB:CSASEGNYGYTF	1	CD4-C4
Pla-1_CTACGTCGTAATCACC	Pla	Pla-1	P1-1650	FALSE	1	TRA:CATF TRB:CASSLGLGSGNTIYF	1	CD4-C4
Pla-1_CTAGCCTAGCGAAGGG	Pla	Pla-1	P1-351	FALSE	2	TRA:CAESILTGGGNKLTF TRB:CASSLYSYDSPLHF	2	CD4-C4
Pla-1_CTCAGAAGTTATTCTC	Pla	Pla-1	P1-1665	FALSE	1	TRA:CVVPRGYSSASKIIF TRB:CASSQLSGDEQFF	1	CD4-C4
Pla-1_CTCATTAGTCGTTGTA	Pla	Pla-1	P1-169	FALSE	3	TRA:CAYRSANNDMRF TRB:CASRGQLYGNQPQHF	4	CD4-C4
Pla-1_CTCCTAGAGTTTAGGA	Pla	Pla-1	P1-1672	FALSE	1	TRA:CVVIRLLVMDTGRRALTF TRB:CALRGETYEQYF	1	CD4-C5
Pla-1_CTCCTAGGTAGCTGCC	Pla	Pla-1	P1-1676	FALSE	1	TRA:CALPTVYNQGGKLIF TRB:CSASPPWTGYNEQFF	1	CD4-C4
Pla-1_CTCCTAGTCTAACCGA	Pla	Pla-1	P1-1679	FALSE	1	TRA:CAVNKYGNKLVF TRB:CASSVVEGSSTDTQYF	1	CD4-C2
Pla-1_CTCGAAATCGGGAGTA	Pla	Pla-1	P1-1682	FALSE	1	TRA:CAENIQGNQFYF TRB:CASSALGRRQPQHF	1	CD4-C4
Pla-1_CTCGGAGAGGTTACCT	Pla	Pla-1	P1-1686	FALSE	1	TRA:CVVNPNFGNEKLTF TRB:CASSRGGQGAYEQYF	1	CD4-C4
Pla-1_CTCGGGAGTATGAAAC	Pla	Pla-1	P1-104	FALSE	3	TRA:CALRGSARQLTF TRB:CASSQEEAGPAKNIQYF	6	CD4-C4

Pla-1_CTCGTACCAAGTTAAG	Pla	Pla-1	P1-219	FALSE	3	TRA:CAVRGADSWGKLQF TRB:CASSDRTEGRAFF	3	CD4-C4
Pla-1_CTCGTACTCCTGCAGG	Pla	Pla-1	P1-243	FALSE	3	TRA:CALSETSGGSNYKLTF TRB:CASSTRLAGGRISYNEQFF	3	CD4-C4
Pla-1_CTCTAATAGAAGGTGA	Pla	Pla-1	P1-1700	FALSE	1	TRA:CGTGARGSSGSRLTF TRB:CASRRDRDSNQPQHF	1	CD4-C4
Pla-1_CTCTACGGTATCACCA	Pla	Pla-1	P1-1705	FALSE	1	TRA:CASGGIQGAQKLVF TRB:CASSPLGGRGRETQYF	1	CD4-C4
Pla-1_CTCTGGTAGAGAGCTC	Pla	Pla-1	P1-321	FALSE	2	TRA:CAYRSLNNARLMF TRB:CASSLRTSGRARVEQFF	2	CD4-C4
Pla-1_CTCTGGTAGCTGAACG	Pla	Pla-1	P1-1706	FALSE	1	TRA:CAGRLAGNMLTF TRB:CASTLDRGLGGYTF	1	CD4-C4
Pla-1_CTCTGGTTCGCCAAAT	Pla	Pla-1	P1-1709	FALSE	1	TRA:CAEREYGNKLVF TRB:CASNPDRGGTNNSPLHF	1	CD4-C4
Pla-1_CTCTGGTTCGTTGCCT	Pla	Pla-1	P1-1710	FALSE	1	TRA:CAPGGYNKLIF TRB:CASSRRRDRENSPLHF	1	CD4-C4
Pla-1_CTGATAGTCGTAGATC	Pla	Pla-1	P1-394	FALSE	2	TRA:CAYRSAIGPGGTSYGKLTF TRB:CASSPRRKNQPQHF	2	CD4-C4
Pla-1_CTGATCCGTTATTCTC	Pla	Pla-1	P1-51	FALSE	3	TRA:CAYRSLNNAGNMLTF TRB:CASSRGNNSPLHF	14	CD4-C4
Pla-1_CTGCGGAAGTGGGTTG	Pla	Pla-1	P1-1728	FALSE	1	TRA:CATDAGGNKLVF TRB:CASSLTGLGTEAFF	1	CD4-C4
Pla-1_CTGCTGTAGTTTAGGA	Pla	Pla-1	P1-1729	FALSE	1	TRA:CAAGTYGQNFVF TRB:CASSSQGNTGELFF	1	CD4-C4
Pla-1_CTGCTGTCAATCACAC	Pla	Pla-1	P1-122	FALSE	3	TRA:CALTFSGGYQKVTF TRB:CASSPLGTSPYEQYF	5	CD4-C4
Pla-1_CTGGTCTGTGTTCTTT	Pla	Pla-1	P1-1734	FALSE	1	TRA:CVVSPMDSSYKLIF TRB:CASSPSPQTSGRWDTQYF	1	CD4-C4
Pla-1_CTGTGCTCAGTAACGG	Pla	Pla-1	P1-1739	FALSE	1	TRA:CALDTGTASKLTF TRB:CASSQAHRGTEAFF	1	CD4-C4
Pla-1_CTGTTTACAGGAATCG	Pla	Pla-1	P1-1740	FALSE	1	TRA:CAGPVSAGGTSYGKLTF TRB:CASSSAGAILHF	1	CD4-C4
Pla-1_CTTAACTAGGCCGAAT	Pla	Pla-1	P1-1742	FALSE	1	TRA:CGTGGQTNNARLMF TRB:CASSRDRADYNEQFF	1	CD4-C4
Pla-1_CTTCTCTGTATAAACG	Pla	Pla-1	P1-352	FALSE	2	TRA:CAVNNRRGSQGNLIF TRB:CSAKGAVYEQYF	2	CD4-C4
Pla-1_CTTGGCTAGAACAATC	Pla	Pla-1	P1-1755	FALSE	1	TRA:CALGDGGSQGNLIF TRB:CASSYSAYTEAFF	1	CD4-C4
Pla-1_CTTGGCTCAGACAAAT	Pla	Pla-1	P1-1756	FALSE	1	TRA:CVVSDRGSTLGRLYF TRB:CASSINRDTTGPYSTDTQYF	1	CD4-C4
Pla-1_CTTGGCTGTGTTTGTG	Pla	Pla-1	P1-344	FALSE	2	TRA:CAERIKAAGNKLTF TRB:CASSYSSGVRELFF	2	CD4-C4
Pla-1_GAAATGACATCACCCT	Pla	Pla-1	P1-205	FALSE	3	TRA:CAVNTLGNQFYF TRB:CASSPGHGVVGEQYF	3	CD4-C4
Pla-1_GAACGGATCACCGGGT	Pla	Pla-1	P1-1772	FALSE	1	TRA:CAASRRRGSTLGRLYF TRB:CASSLVSPGGTSGHEQYF	1	CD4-C4
Pla-1_GAACGGATCCTACAGA	Pla	Pla-1	P1-122	FALSE	3	TRA:CALTFSGGYQKVTF TRB:CASSPLGTSPYEQYF	5	CD4-C4
Pla-1_GAAGCAGAGTGTACCT	Pla	Pla-1	P1-1775	FALSE	1	TRA:CAMSAGGQKLLF TRB:CSARDSRAGGTDTQYF	1	CD4-C4
Pla-1_GAAGCAGGTGTAAGTA	Pla	Pla-1	P1-1777	FALSE	1	TRA:CLVESSSNTGKLIF TRB:CSAREKTGGNSPLHF	1	CD4-C4
Pla-1_GAAGCAGTCCCAAGAT	Pla	Pla-1	P1-1779	FALSE	1	TRA:CAMSPFNNDMRF TRB:CASSYSGGVYNEQFF	1	CD4-C4

Pla-1_GAATAAGAGCTGTTCA	Pla	Pla-1	P1-1781	FALSE	1	TRA:CAGRSQGAQKLVF TRB:CASSRQGASSSYEQYF	1	CD4-C4
Pla-1_GAATAAGCAGACGCTC	Pla	Pla-1	P1-156	FALSE	3	TRA:CATDRYNNNDMRF TRB:CASSLTSGERGNTGELFF	4	CD4-C3
Pla-1_GACACGCTCCACGCAG	Pla	Pla-1	P1-399	FALSE	2	TRA:CAARGRHGSSNTGKLIF TRB:CASSFHTGGAKRGEQYF	2	CD4-C4
Pla-1_GACCAATTCTGGTTCC	Pla	Pla-1	P1-208	FALSE	3	TRA:CAVRDTGGFKTIF TRB:CAWSVGGGGTNEQFF	3	CD4-C4
Pla-1_GACGGCTCACCAACCG	Pla	Pla-1	P1-51	FALSE	3	TRA:CAYRSLNNAGNMLTF TRB:CASSRGNNSPLHF	14	CD4-C4
Pla-1_GACGTGCAGAGCTGGT	Pla	Pla-1	P1-1796	FALSE	1	TRA:CATVISGYSTLTF TRB:CSAARSLAGVYSEQFF	1	CD4-C4
Pla-1_GACGTGCCATGTCCTC	Pla	Pla-1	P1-1798	FALSE	1	TRA:CAVKEANQAGTALIF TRB:CASNRGTGNGEAFF	1	CD4-C4
Pla-1_GACGTGCTCCCAGGTG	Pla	Pla-1	P1-246	FALSE	3	TRA:CAVVPTPDSWGKLQF TRB:CASSVGRTGQETQYF	3	CD8-C8
Pla-1_GACGTGCTCCGGCACA	Pla	Pla-1	P1-95	TRUE	3	TRA:CIVRVARGNEKLTF TRB:CASSLYGEGHQPQHF	6	CD4-C4
Pla-1_GACGTTAGTACGAAAT	Pla	Pla-1	P1-1802	FALSE	1	TRA:CAFIIHTDKLIF TRB:CASSVAGAGTDTQYF	1	CD4-C4
Pla-1_GACGTTATCACCATAG	Pla	Pla-1	P1-246	FALSE	3	TRA:CAVVPTPDSWGKLQF TRB:CASSVGRTGQETQYF	3	CD4-C4
Pla-1_GAGGTGAAGTTAACGA	Pla	Pla-1	P1-368	FALSE	2	TRA:CAVSDAFYGSSNTGKLIF TRB:CASSLGTRSGREPNQPQH	2	CD4-C4
Pla-1_GAGGTGAGTTATCCGA	Pla	Pla-1	P1-1816	FALSE	1	TRA:CVVFTNTGNQFYF TRB:CAISEWGSGRSSYNEQFF	1	CD4-C4
Pla-1_GAGGTGATCCTTGACC	Pla	Pla-1	P1-1817	FALSE	1	TRA:CAVGGTYKYIF TRB:CASSITSYGYTF	1	CD4-C4
Pla-1_GATCGATAGATGGGTC	Pla	Pla-1	P1-1823	FALSE	1	TRA:CIVRVASGTYKYIF TRB:CASSGGKANSPLHF	1	CD4-C4
Pla-1_GATCGATTCATTGCCC	Pla	Pla-1	P1-151	TRUE	3	TRA:CAGTNSGYSTLTF TRB:CASSPKESGQTFQETQYF	4	CD8-C8
Pla-1_GATCGATTCTGCTGCT	Pla	Pla-1	P1-94	TRUE	3	TRA:CAASIQDFGNEKLTF TRB:CASSLQVPPNTEAFF	6	CD4-C4
Pla-1_GATCTAGGTCTAGTCA	Pla	Pla-1	P1-1833	FALSE	1	TRA:CIVPETNYGQNFVF TRB:CAWSVLQGKPQHF	1	CD4-C4
Pla-1_GATGAAACACTATCTT	Pla	Pla-1	P1-1837	FALSE	1	TRA:CAGPVRSTNAGKSTF TRB:CASSPGTVRGTQYF	1	CD4-C4
Pla-1_GATGAAACAGACGCCT	Pla	Pla-1	P1-51	FALSE	3	TRA:CAYRSLNNAGNMLTF TRB:CASSRGNNSPLHF	14	CD4-C4
Pla-1_GATGAGGAGGAGTACC	Pla	Pla-1	P1-1839	FALSE	1	TRA:CALEGGGADGLTF TRB:CASSPEAPRHSGNTIYF	1	CD4-C4
Pla-1_GATGAGGGTCTAAACC	Pla	Pla-1	P1-157	FALSE	3	TRA:CAGIRAGNQFYF TRB:CASRRDRDSNQPQHF	4	CD4-C4
Pla-1_GCAAACTGTTCTCATT	Pla	Pla-1	P1-320	FALSE	2	TRA:CAASGANAGNMLTF TRB:CASSQMAGASTDTQYF	2	CD8-C2
Pla-1_GCAATCACATGTCCTC	Pla	Pla-1	P1-1844	FALSE	1	TRA:CAVTVNSGYSTLTF TRB:CASSLAAVGGLSGANVLTF	1	CD4-C4
Pla-1_GCAATCATCTACTCAT	Pla	Pla-1	P1-1846	FALSE	1	TRA:CAVDNAGNMLTF TRB:CASSLGRRDEKLFF	1	CD4-C4
Pla-1_GCACATATCCGAGCCA	Pla	Pla-1	P1-1847	FALSE	1	TRA:CIPANDYKLSF TRB:CASSSRYLPNIQYF	1	CD4-C4
Pla-1_GCACTCTGTCCGTCAG	Pla	Pla-1	P1-122	FALSE	3	TRA:CALTFSGGYQKVTF TRB:CASSPLGTSPYEQYF	5	CD4-C4

Pla-1_GCACTCTTCGCGGATC	Pla	Pla-1	P1-348	FALSE	2	TRA:CAMKRGSGGSNYKLTF TRB:CASILRDIYGYTF	2	CD4-C4
Pla-1_GCAGCCACATCTCCCA	Pla	Pla-1	P1-1850	FALSE	1	TRA:CAVWSGNQFYF TRB:CASSSAGANTGELFF	1	CD4-C4
Pla-1_GCAGCCAGTGACCAAG	Pla	Pla-1	P1-28	TRUE	3	TRA:CAVKKGTGRRALTF TRB:CASETGQGTDTQYF	53	CD4-C3
Pla-1_GCATACATCCCATTAT	Pla	Pla-1	P1-403	FALSE	2	TRA:CAMRSGGGADGLTF TRB:CASSQRASYYGYTF	2	CD4-C4
Pla-1_GCATGCGTCTTTAGTC	Pla	Pla-1	P1-404	FALSE	2	TRA:CVVSENTDKLIF TRB:CASRLTGTGLPLHF	2	CD4-C4
Pla-1_GCATGTACATCACGAT	Pla	Pla-1	P1-1862	FALSE	1	TRA:CAVRRQIGFGNEKLTF TRB:CASSYNLGGRGTEAFF	1	CD4-C6
Pla-1_GCATGTAGTACCATCA	Pla	Pla-1	P1-306	FALSE	2	TRA:CAFPSPGRGSTLGRLYF TRB:CASSLGLADSTDTQYF	2	CD4-C4
Pla-1_GCCAAATTCGCCTGAG	Pla	Pla-1	P1-106	TRUE	3	TRA:CAVIEGNQFYF TRB:CASSLTPGLNIQYF	5	CD4-C4
Pla-1_GCCTCTACAAAGGAAG	Pla	Pla-1	P1-1865	FALSE	1	TRA:CAYTGNARLMF TRB:CSASYGSSGNTIYF	1	CD4-C4
Pla-1_GCGCAACGTACAGTTC	Pla	Pla-1	P1-165	FALSE	3	TRA:CAMDYNQGGKLIF TRB:CASSSATGNTEAFF	4	CD4-C4
Pla-1_GCGCAGTAGAATTGTG	Pla	Pla-1	P1-1871	FALSE	1	TRA:CALSDLGSGANFGNEKLTF TRB:CASSFLPGGTEAFF	1	CD4-C4
Pla-1_GCGCAGTAGCATCATC	Pla	Pla-1	P1-1872	FALSE	1	TRA:CAGGRGSNYKLTF TRB:CASSPFGTAYGYTF	1	CD4-C4
Pla-1_GCGCCAAGTGATGATA	Pla	Pla-1	P1-159	FALSE	3	TRA:CAVQGPYKAAGNKLTF TRB:CASSRDLGYEQYF	4	CD8-C8
Pla-1_GCGCGATCAAACGTGG	Pla	Pla-1	P1-78	TRUE	3	TRA:CAAAQGGSEKLVF TRB:CASTRGDWIYNEQFF	7	CD4-C4
Pla-1_GCGGGTTCAGCGAACA	Pla	Pla-1	P1-1879	FALSE	1	TRA:CAATNSGGSNYKLTF TRB:CASSLLDRPLVYGYTF	1	CD4-C4
Pla-1_GCTCCTACACGTTGGC	Pla	Pla-1	P1-1883	FALSE	1	TRA:CAASSFYGGSQGNLIF TRB:CASSLLVGQGSHEQYF	1	CD4-C4
Pla-1_GCTCCTATCGGTTCGG	Pla	Pla-1	P1-1884	FALSE	1	TRA:CAGASSNTGKLIF TRB:CASRDTSGSYREQYF	1	CD4-C4
Pla-1_GCTCTGTTCCTTGCCA	Pla	Pla-1	P1-1885	FALSE	1	TRA:CAFFHSNTGKLIF TRB:CASSFHTGGAKRGEQYF	1	CD4-C4
Pla-1_GCTGCAGCAAACGTGG	Pla	Pla-1	P1-1886	FALSE	1	TRA:CAVRARGEAAGNKLTF TRB:CASSFQDRFYGYTF	1	CD4-C4
Pla-1_GCTGCGACAATCTACG	Pla	Pla-1	P1-1888	FALSE	1	TRA:CADKSGGYQKVTF TRB:CASRGASPVTEAFF	1	CD4-C4
Pla-1_GCTGCGACATCAGTCA	Pla	Pla-1	P1-406	FALSE	2	TRA:CVVNIPFGNEKLTF TRB:CASSRGGQGSYEQYF	2	CD4-C4
Pla-1_GCTGCTTCAAGGGTCA	Pla	Pla-1	P1-1890	FALSE	1	TRA:CAGRNFGNEKLTF TRB:CASSQGTSPGPQYF	1	CD4-C4
Pla-1_GCTTCCATCCAAGTAC	Pla	Pla-1	P1-237	FALSE	3	TRA:CAVNMGGGSYIPTF TRB:CSVEETRSMYF	3	CD4-C4
Pla-1_GGAATAAAGCTGGAAC	Pla	Pla-1	P1-1902	FALSE	1	TRA:CAVRVDPDGGSQGNLIF TRB:CASSLSGGAGQPQHF	1	CD4-C4
Pla-1_GGACAAGCAGGATCGA	Pla	Pla-1	P1-403	FALSE	2	TRA:CAMRSGGGADGLTF TRB:CASSQRASYYGYTF	2	CD4-C4
Pla-1_GGACAGAAGCATGGCA	Pla	Pla-1	P1-1906	FALSE	1	TRA:CAVRDSPRGSASKIIF TRB:CASSLLSGQGLYEQYF	1	CD4-C4
Pla-1_GGACAGACAGGCTGAA	Pla	Pla-1	P1-1907	FALSE	1	TRA:CVVSDEGNYQLIW TRB:CASSFTSEQYF	1	CD4-C4

Pla-1_GGACGTCTCCAAGTAC	Pla	Pla-1	P1-104	FALSE	3	TRA:CALRGSARQLTF TRB:CASSQEEAGPAKNIQYF	6	CD4-C4
Pla-1_GGAGCAACAGCGTTCG	Pla	Pla-1	P1-369	FALSE	2	TRA:CAVNALNFGNEKLTF TRB:CASSLASGSNNEQFF	2	CD4-C4
Pla-1_GGATTACAGGCCGAAT	Pla	Pla-1	P1-1917	FALSE	1	TRA:CARMNTGFQKLVF TRB:CASSLIGQHTDTQYF	1	CD8-C10
Pla-1_GGCCGATAGTTAACGA	Pla	Pla-1	P1-1923	FALSE	1	TRA:CAVRSGGATNKLIF TRB:CASSQLSSTGELFF	1	CD4-C4
Pla-1_GGCCGATCAGCTGCAC	Pla	Pla-1	P1-1924	FALSE	1	TRA:CAVDRLFMKTSYDKVIF TRB:CAISDFGGPGEQYF	1	CD4-C4
Pla-1_GGCGACTCATTCTCAT	Pla	Pla-1	P1-1926	FALSE	1	TRA:CAENGMGYSTLTF TRB:CSANFPGPLYNEQFF	1	CD8-C8
Pla-1_GGCGACTGTTACCGAT	Pla	Pla-1	P1-1928	FALSE	1	TRA:CVQGAQKLVF TRB:CASSPTSEAGYEQYF	1	CD4-C4
Pla-1_GGCTCGAGTCTGATCA	Pla	Pla-1	P1-1934	FALSE	1	TRA:CAATARRAGKSTF TRB:CASSLNGLAGTGAAETQYF	1	CD8-C8
Pla-1_GGCTCGATCTATCCTA	Pla	Pla-1	P1-1937	FALSE	1	TRA:CAMREEGFGNEKLTF TRB:CASSISRPPLNTEAFF	1	CD4-C4
Pla-1_GGGACCTTCAATCACG	Pla	Pla-1	P1-389	FALSE	2	TRA:CAASSRGSARQLTF TRB:CASSLSQGRETQYF	2	CD4-C4
Pla-1_GGGAGATTCAGTACGT	Pla	Pla-1	P1-1945	FALSE	1	TRA:CAVGLSYNFNKFYF TRB:CASSPTPGTGGDYEQYF	1	CD4-C4
Pla-1_GGGATGATCACAAACC	Pla	Pla-1	P1-1946	FALSE	1	TRA:CAASLDNYGQNFVF TRB:CAISEPERAQYGYTF	1	CD8-C8
Pla-1_GGGCATCAGACAAGCC	Pla	Pla-1	P1-1950	FALSE	1	TRA:CAVRNNNARLMF TRB:CASSYQVTGGSPLHF	1	CD4-C4
Pla-1_GGGTCTGTCCTTGCCA	Pla	Pla-1	P1-1956	FALSE	1	TRA:CAVNSGYALNF TRB:CASSHTDMNTEAFF	1	CD8-C8
Pla-1_GGGTTGCCAGCGAACA	Pla	Pla-1	P1-242	FALSE	3	TRA:CAFFHSNTGKLIF TRB:CASSNPGWNNEQFF	3	CD4-C4
Pla-1_GGTATTGAGTGTGGCA	Pla	Pla-1	P1-1958	FALSE	1	TRA:CAVRQAGGGADGLTF TRB:CSASIPRGAGSYEQYF	1	CD4-C4
Pla-1_GGTGAAGCACGGTTTA	Pla	Pla-1	P1-1961	FALSE	1	TRA:CAASPNAGNNRKLIW TRB:CASSTGPSYNSPLHF	1	CD4-C4
Pla-1_GGTGCGTCAAGAAAGG	Pla	Pla-1	P1-1964	FALSE	1	TRA:CALPFQGAQKLVF TRB:CASSSQTPPREQYF	1	CD8-C8
Pla-1_GGTGCGTGTGCAACGA	Pla	Pla-1	P1-1965	FALSE	1	TRA:CAASNGYNKLIF TRB:CASSQAGGGDEQFF	1	CD4-C4
Pla-1_GGTGCGTGTTGTACAC	Pla	Pla-1	P1-103	FALSE	3	TRA:CATENDYKLSF TRB:CSVGGTFANTGELFF	6	CD4-C4
Pla-1_GGTGTTACAGCCACCA	Pla	Pla-1	P1-1968	FALSE	1	TRA:CADDSGGSNYKLTF TRB:CASSLAGNYGYTF	1	CD8-C8
Pla-1_GGTGTTAGTAATTGGA	Pla	Pla-1	P1-1969	FALSE	1	TRA:CALDQNNRLAF TRB:CASGTGRPTNTGELFF	1	CD4-C4
Pla-1_GTAACGTAGAGTACAT	Pla	Pla-1	P1-1972	FALSE	1	TRA:CAVQPLKAAGNKLTF TRB:CASRKTGVSNQPQHF	1	CD8-C8
Pla-1_GTAACGTCAGTACACT	Pla	Pla-1	P1-1973	FALSE	1	TRA:CASNTGNQFYF TRB:CASSLAPQGPSTVYEQYF	1	CD4-C4
Pla-1_GTAACGTGTCGTTGTA	Pla	Pla-1	P1-1974	FALSE	1	TRA:CAVSGSGGYQKVTF TRB:CASSQYGVSENTGELFF	1	CD4-C4
Pla-1_GTAACTGGTTAAAGAC	Pla	Pla-1	P1-1976	FALSE	1	TRA:CAAIGRGATNKLIF TRB:CASSSTGGDQPQHF	1	CD4-C4
Pla-1_GTAACTGTCTATCGCC	Pla	Pla-1	P1-1978	FALSE	1	TRA:CAGNGGFKTIF TRB:CASTRAIGAGTDYGYTF	1	CD4-C4

Pla-1_GTACTCCCACCCATTC	Pla	Pla-1	P1-1983	FALSE	1	TRA:CAILYGGSQGNLIF TRB:CASSQDGAGSPLISDTQYF	1	CD4-C4
Pla-1_GTAGGCCAGCTGAAAT	Pla	Pla-1	P1-367	FALSE	2	TRA:CAENVGNRDDKIIF TRB:CASSREANSGNTIYF	2	CD4-C4
Pla-1_GTAGGCCGTCGTTGTA	Pla	Pla-1	P1-1988	FALSE	1	TRA:CVVRGIKAAGNKLTF TRB:CASRKTEREGQFYEQYF	1	CD4-C5
Pla-1_GTAGTCAGTAGCGTGA	Pla	Pla-1	P1-122	FALSE	3	TRA:CALTFSGGYQKVTF TRB:CASSPLGTSPYEQYF	5	CD4-C4
Pla-1_GTATCTTTCACGATGT	Pla	Pla-1	P1-408	FALSE	2	TRA:CAILSGGYNKLIF TRB:CASSQDPGGGYGYTF	2	CD4-C4
Pla-1_GTATCTTTCATGGTCA	Pla	Pla-1	P1-1992	FALSE	1	TRA:CAVSPNDYKLSF TRB:CASSFGGEIYGYTF	1	CD4-C4
Pla-1_GTATTCTCACAACGCC	Pla	Pla-1	P1-109	TRUE	3	TRA:CAAQQGSGGSNYKLTF TRB:CASSLGDKQYF	5	CD4-C4
Pla-1_GTCAAGTCACGGACAA	Pla	Pla-1	P1-228	FALSE	3	TRA:CAVRDPYNQGGKLIF TRB:CASSLGMGSSYEQYF	3	CD4-C4
Pla-1_GTCAAGTTCATAGCAC	Pla	Pla-1	P1-415	FALSE	2	TRA:CAYRSAYNNNDMRF TRB:CASSRGLAGVVEQFF	2	CD4-C4
Pla-1_GTCAAGTTCCAAATGC	Pla	Pla-1	P1-1995	FALSE	1	TRA:CAMREEGSARQLTF TRB:CASSYSPGGALNEQFF	1	CD4-C4
Pla-1_GTCACAATCGCAGGCT	Pla	Pla-1	P1-1999	FALSE	1	TRA:CALKTDSWGKLQF TRB:CSARATGRGYEQYF	1	CD4-C3
Pla-1_GTCATTTTCCAGAAGG	Pla	Pla-1	P1-105	FALSE	3	TRA:CIVRVVSNQGGKLIF TRB:CASSPRTGGVGTQYF	6	CD4-C4
Pla-1_GTCGGGTGTCCCGACA	Pla	Pla-1	P1-2005	FALSE	1	TRA:CAEIRMNNNAGNMLTF TRB:CASSVAGASTDTQYF	1	CD4-C4
Pla-1_GTCGGGTTCAGGCCCA	Pla	Pla-1	P1-2006	FALSE	1	TRA:CAYRSAWKGGSEKLVF TRB:CASSHLRASGSYNEQFF	1	CD4-C4
Pla-1_GTCTCGTCAGCATGAG	Pla	Pla-1	P1-2011	FALSE	1	TRA:CALTGGFKTIF TRB:CASSPDRTPTDTQYF	1	CD4-C5
Pla-1_GTCTCGTGTGCCTGTG	Pla	Pla-1	P1-2012	FALSE	1	TRA:CVVSDRGSTLGRLYF TRB:CASSTVKRDEQFF	1	CD4-C4
Pla-1_GTCTTCGGTTGATTCG	Pla	Pla-1	P1-416	FALSE	2	TRA:CGITGNQFYF TRB:CASRRLRDRGATNSNQPQHF	2	CD4-C4
Pla-1_GTGAAGGAGATGTGGC	Pla	Pla-1	P1-2014	FALSE	1	TRA:CAGLVGSTLGRLYF TRB:CASSGSAAQPQHF	1	CD4-C4
Pla-1_GTGCAGCAGACCACGA	Pla	Pla-1	P1-2016	FALSE	1	TRA:CAAPPLYNQGGKLIF TRB:CASSFGGRGPAEQYF	1	CD4-C4
Pla-1_GTGCAGCGTTTGACAC	Pla	Pla-1	P1-2019	FALSE	1	TRA:CALYSNTGNQFYF TRB:CASSTRRELDTQYF	1	CD4-C4
Pla-1_GTGCGGTAGCGAAGGG	Pla	Pla-1	P1-2023	FALSE	1	TRA:CAYGRGTSGTYKYIF TRB:CASSPDVNSPLHF	1	CD4-C4
Pla-1_GTGCGGTTCTCGAGTA	Pla	Pla-1	P1-216	FALSE	3	TRA:CALLFGNVLHC TRB:CASSLAVRANSMNTEAFF	3	CD4-C5
Pla-1_GTGCTTCCATCGGTTA	Pla	Pla-1	P1-2027	FALSE	1	TRA:CAENRGGGADGLTF TRB:CASRISRNEQFF	1	CD8-C8
Pla-1_GTGGGTCGTAAGTGTA	Pla	Pla-1	P1-105	FALSE	3	TRA:CIVRVVSNQGGKLIF TRB:CASSPRTGGVGTQYF	6	CD4-C4
Pla-1_GTGTGCGAGGCAGGTT	Pla	Pla-1	P1-2034	FALSE	1	TRA:CATGDYGQNFVF TRB:CASSQQGRNTEAFF	1	CD4-C4
Pla-1_GTTAAGCCACTGTGTA	Pla	Pla-1	P1-2040	FALSE	1	TRA:CGTEHYGGYNKLIF TRB:CASSVGDGNTIYF	1	CD4-C4
Pla-1_GTTAAGCGTTGGGACA	Pla	Pla-1	P1-2041	FALSE	1	TRA:CAASARTSGSRLTF TRB:CASSPLGSSGRALPDTQYF	1	CD4-C4

Pla-1_GTTACAGCAACCGCCA	Pla	Pla-1	P1-2046	FALSE	1	TRA:CAASKSRETSGSRLTF TRB:CASRRPGSLDEQYF	1	CD4-C4
Pla-1_GTTCATTGTAGGAGTC	Pla	Pla-1	P1-2052	FALSE	1	TRA:CAVTRMDSSYKLIF TRB:CASSFSSGISTYNEQFF	1	CD4-C4
Pla-1_GTTCATTGTCATCCCT	Pla	Pla-1	P1-329	FALSE	2	TRA:CIVRVAGGGADGLTF TRB:CASSTGTGGPTRETQYF	2	CD4-C4
Pla-1_GTTCGGGAGATTACCC	Pla	Pla-1	P1-364	FALSE	2	TRA:CAYRSGGINFGNEKLTF TRB:CASSAGLRPEQYF	2	CD4-C3
Pla-1_TAAGAGATCTCGATGA	Pla	Pla-1	P1-127	FALSE	3	TRA:CALSADNDMRF TRB:CASTRGDWIYNEQFF	5	CD4-C4
Pla-1_TACACGACAGAGTGTG	Pla	Pla-1	P1-2070	FALSE	1	TRA:CAVLTGGFKTIF TRB:CASSLATAGYNEQFF	1	CD4-C3
Pla-1_TACAGTGAGCTCCTTC	Pla	Pla-1	P1-2072	FALSE	1	TRA:CAVRDKAAGNKLTF TRB:CASSPLQEGEKLFF	1	CD4-C4
Pla-1_TACAGTGCACAGCCCA	Pla	Pla-1	P1-2073	FALSE	1	TRA:CAMSAVTNRNDMRF TRB:CASSAATSGANVLTF	1	CD4-C4
Pla-1_TACCTATTCTGTCTAT	Pla	Pla-1	P1-2076	FALSE	1	TRA:CATVVLSGGYNKLIF TRB:CASSPRKGWDWYEQFF	1	CD8-C8
Pla-1_TACCTTACAAACGCGA	Pla	Pla-1	P1-42	TRUE	3	TRA:CASFSGGSQGNLIF TRB:CSAGQGSNQPQHF	24	CD4-C4
Pla-1_TACCTTAGTATGCTTG	Pla	Pla-1	P1-109	TRUE	3	TRA:CAAQQGSGGSNYKLTF TRB:CASSLGDKQYF	5	CD4-C4
Pla-1_TACCTTAGTCGTCTTC	Pla	Pla-1	P1-2077	FALSE	1	TRA:CALSEARGADGLTF TRB:CASSHTDMNTEAFF	1	CD4-C4
Pla-1_TACCTTAGTGCGAAAC	Pla	Pla-1	P1-2078	FALSE	1	TRA:CAASMENFNKFYF TRB:CASSWRASYGYTF	1	CD4-C4
Pla-1_TACGGATGTAGGAGTC	Pla	Pla-1	P1-2082	FALSE	1	TRA:CAANAWEFGNEKLTF TRB:CASSPSQGGSPLHF	1	CD4-C4
Pla-1_TACGGTATCACGGTTA	Pla	Pla-1	P1-2087	FALSE	1	TRA:CAENRQNQKLVF TRB:CSVWKGLAGGQETQYF	1	CD4-C4
Pla-1_TACTCATTCACATGCA	Pla	Pla-1	P1-2089	FALSE	1	TRA:CASLRGETSGSRLTF TRB:CASSPRTGTGREKLFF	1	CD4-C4
Pla-1_TACTCATTCTGCGACG	Pla	Pla-1	P1-2090	FALSE	1	TRA:CIVRVSPRQLTF TRB:CASSPNGGDTQYF	1	CD4-C3
Pla-1_TACTCGCGTTCCGGCA	Pla	Pla-1	P1-2092	FALSE	1	TRA:CAAVYNNAGNMLTF TRB:CASSLRTTGSRTDTQYF	1	CD4-C4
Pla-1_TACTTACAGCTGCAAG	Pla	Pla-1	P1-2094	FALSE	1	TRA:CILRDNSNSGYALNF TRB:CASSPNGGRYAEAFF	1	CD4-C4
Pla-1_TACTTACTCGCTTGTC	Pla	Pla-1	P1-418	FALSE	2	TRA:CAASAGGYNKLIF TRB:CASSNRGQGEKLFF	2	CD4-C4
Pla-1_TACTTGTTCTCTGAGA	Pla	Pla-1	P1-2100	FALSE	1	TRA:CAVRDTGFQKLVF TRB:CASRDGTTRSYEQYF	1	CD4-C4
Pla-1_TAGACCACAATGCCAT	Pla	Pla-1	P1-2102	FALSE	1	TRA:CVVLRGDSSYKLIF TRB:CASSQTRDSYYGYTF	1	CD4-C4
Pla-1_TAGAGCTGTTGTTTGG	Pla	Pla-1	P1-2107	FALSE	1	TRA:CALTTRGYSTLTF TRB:CASSISLEGNYGYTF	1	CD4-C4
Pla-1_TAGCCGGCAGTCTTCC	Pla	Pla-1	P1-228	FALSE	3	TRA:CAVRDPYNQGGKLIF TRB:CASSLGMGSSYEQYF	3	CD4-C4
Pla-1_TAGCCGGGTTCCACGG	Pla	Pla-1	P1-381	FALSE	2	TRA:CAEKSKSNTGKLIF TRB:CASLVYRLQAYYNEQFF	2	CD4-C4
Pla-1_TAGGCATGTTCATGGT	Pla	Pla-1	P1-2112	FALSE	1	TRA:CAAVDAGGTSYGKLTF TRB:CASSWTSGKSSYNEQFF	1	CD4-C4
Pla-1_TAGTGGTTCCACTCCA	Pla	Pla-1	P1-41	TRUE	3	TRA:CVVSEPPGGSYIPTF TRB:CASSSRPEDTQYF	27	CD4-C4

Pla-1_TAGTTGGTCCGTCAAA	Pla	Pla-1	P1-2115	FALSE	1	TRA:CIASSGSARQLTF TRB:CASSMGRVKQETQYF	1	CD4-C4
Pla-1_TATCTCACATCTCGCT	Pla	Pla-1	P1-232	FALSE	3	TRA:CAVGASNQGGKLIF TRB:CASSLGMGSSYEQYF	3	CD4-C4
Pla-1_TATTACCAGCTAGTGG	Pla	Pla-1	P1-2125	FALSE	1	TRA:CAVKGAGRRALTF TRB:CASSTRSGDTNEKLFF	1	CD4-C3
Pla-1_TATTACCCATGCTGGC	Pla	Pla-1	P1-404	FALSE	2	TRA:CVVSENTDKLIF TRB:CASRLTGTGLPLHF	2	CD4-C4
Pla-1_TCAATCTGTCGGCACT	Pla	Pla-1	P1-2133	FALSE	1	TRA:CAVDGTSGSRLTF TRB:CASSVNRGLYGYTF	1	CD4-C4
Pla-1_TCACAAGTCCATGAAC	Pla	Pla-1	P1-2136	FALSE	1	TRA:CAVRRGNYGQNFVF TRB:CATSSPGLGYNEQFF	1	CD4-C4
Pla-1_TCACGAACATTATCTC	Pla	Pla-1	P1-421	FALSE	2	TRA:CAVALLATDKLIF TRB:CASSLEARSTDTQYF	2	CD4-C4
Pla-1_TCAGATGCATCACAAC	Pla	Pla-1	P1-2140	FALSE	1	TRA:CAVRDSPGNTPLVF TRB:CAWSAAGRRADTQYF	1	CD4-C4
Pla-1_TCAGATGTCCTTTACA	Pla	Pla-1	P1-415	FALSE	2	TRA:CAYRSAYNNNDMRF TRB:CASSRGLAGVVEQFF	2	CD4-C4
Pla-1_TCAGCAACAAGTAATG	Pla	Pla-1	P1-48	TRUE	3	TRA:CAEVSGYSTLTF TRB:CASSFPTGGRTGRTEAFF	16	CD4-C4
Pla-1_TCAGGATCAGAGCCAA	Pla	Pla-1	P1-406	FALSE	2	TRA:CVVNIPFGNEKLTF TRB:CASSRGGQGSYEQYF	2	CD4-C4
Pla-1_TCAGGTACAGACGCAA	Pla	Pla-1	P1-2145	FALSE	1	TRA:CGADPDGSQGNLIF TRB:CASSYSSWSGQFF	1	CD4-C4
Pla-1_TCAGGTATCGCCCTTA	Pla	Pla-1	P1-2146	FALSE	1	TRA:CAASGPNFGNEKLTF TRB:CASSIVPGYGYTF	1	CD4-C4
Pla-1_TCATTACCATCCTTGC	Pla	Pla-1	P1-2147	FALSE	1	TRA:CAVSEGAAGNKLTF TRB:CASSLGLTGELFF	1	CD4-C4
Pla-1_TCATTTGCACGTCAGC	Pla	Pla-1	P1-2149	FALSE	1	TRA:CAPGGGTYKYIF TRB:CSVSLQGARRSGEKLFF	1	CD4-C4
Pla-1_TCATTTGGTGTGGTTT	Pla	Pla-1	P1-2152	FALSE	1	TRA:CALRSNYGGSQGNLIF TRB:CSKLAGGGETQYF	1	CD8-C8
Pla-1_TCATTTGTCCAGGGCT	Pla	Pla-1	P1-2153	FALSE	1	TRA:CAVGTSNTGKLIF TRB:CASTFGGSSYEQYF	1	CD4-C4
Pla-1_TCGGTAAGTAGCTTGT	Pla	Pla-1	P1-178	TRUE	3	TRA:CAVFLWEARLMF TRB:CASSFGGQGGTEAFF	3	CD4-C6
Pla-1_TCGTACCTCTCGTTTA	Pla	Pla-1	P1-2168	FALSE	1	TRA:CAMNSGTSYGKLTF TRB:CSARDARGQPQHF	1	CD4-C4
Pla-1_TCGTAGACATGTTGAC	Pla	Pla-1	P1-2171	FALSE	1	TRA:CADDSGGSNYKLTF TRB:CASRIFTGDGGYTF	1	CD8-C8
Pla-1_TCTCATACAGATAATG	Pla	Pla-1	P1-212	FALSE	3	TRA:CAYRSARNNNNDMRF TRB:CSAARTGGNLAFF	3	CD4-C5
Pla-1_TCTCATATCTCCCTGA	Pla	Pla-1	P1-422	FALSE	2	TRA:CASFNTGNQFYF TRB:CASSVVGGRATGELFF	2	CD4-C4
Pla-1_TCTCTAACAGAGTGTG	Pla	Pla-1	P1-188	TRUE	3	TRA:CPRNNNARLMF TRB:CASTSGGFNQPQHF	3	CD8-C8
Pla-1_TCTTCGGGTAAACGCG	Pla	Pla-1	P1-2189	FALSE	1	TRA:CAASDSWGKLQF TRB:CASSESGRTYNEQFF	1	CD4-C4
Pla-1_TGACGGCAGATAGTCA	Pla	Pla-1	P1-2194	FALSE	1	TRA:CAVRESGGSNYKLTF TRB:CASSLTGGGGDTQYF	1	CD4-C4
Pla-1_TGACTAGCAGTGGAGT	Pla	Pla-1	P1-160	FALSE	3	TRA:CAASADGGSQGNLIF TRB:CASSPTYGYTF	4	CD4-C4
Pla-1_TGACTAGTCTAACTCT	Pla	Pla-1	P1-2201	FALSE	1	TRA:CAVRDRLISNFGNEKLTF TRB:CASSPSRGNTEAFF	1	CD4-C4

Pla-1_TGACTTTAGATGTGTA	Pla	Pla-1	P1-419	FALSE	2	TRA:CAENRRDDKIIF TRB:CASSETLRNTEAFF	2	CD4-C4
Pla-1_TGACTTTGTCACTGGC	Pla	Pla-1	P1-2203	FALSE	1	TRA:CIVRVGNQGGKLIF TRB:CASETGGANYGYTF	1	CD4-C4
Pla-1_TGAGCATAGGCATGTG	Pla	Pla-1	P1-2205	FALSE	1	TRA:CAETLYQKVTF TRB:CASSSTGVEAFF	1	CD4-C4
Pla-1_TGAGCATTCACTTATC	Pla	Pla-1	P1-41	TRUE	3	TRA:CVVSEPPGGSYIPTF TRB:CASSSRPEDTQYF	27	CD4-C4
Pla-1_TGATTTCTCTTTAGTC	Pla	Pla-1	P1-242	FALSE	3	TRA:CAFFHSNTGKLIF TRB:CASSNPGWNNEQFF	3	CD4-C4
Pla-1_TGCCAAAGTCTCTTAT	Pla	Pla-1	P1-2214	FALSE	1	TRA:CTAGAQKLVF TRB:CASSEPGQYTEAFF	1	CD4-C4
Pla-1_TGCCCATAGTCAATAG	Pla	Pla-1	P1-2217	FALSE	1	TRA:CAFRPNDYKLSF TRB:CATQADNSPLHF	1	CD4-C4
Pla-1_TGCCCATTCTAACTGG	Pla	Pla-1	P1-41	TRUE	3	TRA:CVVSEPPGGSYIPTF TRB:CASSSRPEDTQYF	27	CD4-C4
Pla-1_TGCCCTAAGCGAAGGG	Pla	Pla-1	P1-163	FALSE	3	TRA:CALRFNDYKLSF TRB:CASTSFETEAFF	4	CD4-C4
Pla-1_TGCGCAGAGATGCCAG	Pla	Pla-1	P1-2223	FALSE	1	TRA:CAVKSYNTDKLIF TRB:CASSSRLGGDRDGYTF	1	CD4-C3
Pla-1_TGCGGGTCAAGGTTCT	Pla	Pla-1	P1-2233	FALSE	1	TRA:CAEVSGYSTLTF TRB:CATSRDTGPVVSEQYF	1	CD4-C4
Pla-1_TGCTACCAGAATTCCC	Pla	Pla-1	P1-2238	FALSE	1	TRA:CAVRPSSGYSTLTF TRB:CASSITIGIQVEFWPQHF	1	CD4-C4
Pla-1_TGCTACCCATGGTCAT	Pla	Pla-1	P1-2241	FALSE	1	TRA:CAETPTGGGNKLTF TRB:CATSDYRGGAFPDTQYF	1	CD4-C4
Pla-1_TGCTGCTAGCGTCAAG	Pla	Pla-1	P1-2242	FALSE	1	TRA:CAVTPNRDDKIIF TRB:CASSPQGGYGYTF	1	CD4-C4
Pla-1_TGCTGCTGTGTTGAGG	Pla	Pla-1	P1-331	FALSE	2	TRA:CAVLIAGGYQKVTF TRB:CSASPGAQETQYF	2	CD4-C4
Pla-1_TGCTGCTGTTCCACGG	Pla	Pla-1	P1-94	TRUE	3	TRA:CAASIQDFGNEKLTF TRB:CASSLQVPPNTEAFF	6	CD4-C6
Pla-1_TGGACGCAGGCCGAAT	Pla	Pla-1	P1-2244	FALSE	1	TRA:CPSGGKLIF TRB:CATSQQTGNNQPQHF	1	CD8-C8
Pla-1_TGGCCAGTCCTCCTAG	Pla	Pla-1	P1-2248	FALSE	1	TRA:CGTELGGNQFYF TRB:CASSLAVNYGYTF	1	CD4-C4
Pla-1_TGGCTGGTCTTGTCAT	Pla	Pla-1	P1-127	FALSE	3	TRA:CALSADNDMRF TRB:CASTRGDWIYNEQFF	5	CD4-C3
Pla-1_TGGTTAGGTAAGAGAG	Pla	Pla-1	P1-2260	FALSE	1	TRA:CLVGEGDTGRRALTF TRB:CASRWTGTVNTEAFF	1	CD4-C4
Pla-1_TGTATTCCATCCGTGG	Pla	Pla-1	P1-90	TRUE	3	TRA:CAVNNGRGSTLGRLYF TRB:CASSSGPGSGANVLTF	6	CD4-C4
Pla-1_TGTCCCAAGGCACATG	Pla	Pla-1	P1-2264	FALSE	1	TRA:CALSAPWDGGGSEKLVF TRB:CASRQGQVLTDTQYF	1	CD4-C4
Pla-1_TGTGTTTCAGGTCTCG	Pla	Pla-1	P1-2269	FALSE	1	TRA:CALYNFNKFYF TRB:CASSRWQKNTEAFF	1	CD4-C4
Pla-1_TGTGTTTGTCCGACGT	Pla	Pla-1	P1-2270	FALSE	1	TRA:CALPGGIKAAGNKLTF TRB:CASSLEARVTNNEQFF	1	CD4-C4
Pla-1_TGTTCCGAGGACTGGT	Pla	Pla-1	P1-224	FALSE	3	TRA:CAVGEGYSTLTF TRB:CASMKGQGGSPLHF	3	CD4-C3
Pla-1_TGTTCCGCACCACGTG	Pla	Pla-1	P1-2273	FALSE	1	TRA:CGAAPTQGGSEKLVF TRB:CASSVVGGLSIHEQYF	1	CD4-C4
Pla-1_TTAACTCCAGCATACT	Pla	Pla-1	P1-2276	FALSE	1	TRA:CIVIGSGTYKYIF TRB:CSARREGGETQYF	1	CD4-C4

Pla-1_TTAGGACAGCTCCTTC	Pla	Pla-1	P1-292	TRUE	2	TRA:CAAPSNTNAGKSTF TRB:CASSLDVYQDNEQFF	2	CD4-C4
Pla-1_TTAGGACAGGATTCGG	Pla	Pla-1	P1-2282	FALSE	1	TRA:CVVLFLSGTYKYIF TRB:CASSPGHGDSNQPQHF	1	CD4-C4
Pla-1_TTAGGACAGTAGGCCA	Pla	Pla-1	P1-2283	FALSE	1	TRA:CAGATRTSYDKVIF TRB:CASHPFSTDTQYF	1	CD4-C4
Pla-1_TTAGTTCGTCAGAATA	Pla	Pla-1	P1-120	FALSE	3	TRA:CAYRSSYSGYALNF TRB:CAWSPYSEKLFF	5	CD8-C8
Pla-1_TTCGAAGTCTCGCTTG	Pla	Pla-1	P1-2296	FALSE	1	TRA:CAMRENGNTDKLIF TRB:CSARQGSNVNTEAFF	1	CD4-C4
Pla-1_TTCGGTCCACTTAAGC	Pla	Pla-1	P1-28	TRUE	3	TRA:CAVKKGTGRRALTF TRB:CASETGQGTDTQYF	53	CD4-C4
Pla-1_TTCTACAAGCCATCGC	Pla	Pla-1	P1-2301	FALSE	1	TRA:CALKNTGNQFYF TRB:CASRDRRTGSNTGELFF	1	CD4-C4
Pla-1_TTCTCAAAGTCCGTAT	Pla	Pla-1	P1-361	FALSE	2	TRA:CAENRNAGNMLTF TRB:CASSYDRVGEQYF	2	CD4-C4
Pla-1_TTCTTAGAGATGTAAC	Pla	Pla-1	P1-2310	FALSE	1	TRA:CAVRPSNQAGTALIF TRB:CSADGTGVYNSPLHF	1	CD4-C4
Pla-1_TTCTTAGCAGGCAGTA	Pla	Pla-1	P1-421	FALSE	2	TRA:CAVALLATDKLIF TRB:CASSLEARSTDTQYF	2	CD4-C5
Pla-1_TTGAACGAGCTCCCAG	Pla	Pla-1	P1-2312	FALSE	1	TRA:CAVIDGGSQGNLIF TRB:CASSFTGGNNQPQHF	1	CD4-C4
Pla-1_TTGACTTAGGCAAAGA	Pla	Pla-1	P1-2316	FALSE	1	TRA:CAGARTGNQFYF TRB:CASSLQGGLSGNTEAFF	1	CD4-C4
Pla-1_TTGACTTTCACCTTAT	Pla	Pla-1	P1-399	FALSE	2	TRA:CAARGRHGSSNTGKLIF TRB:CASSFHTGGAKRGEQYF	2	CD4-C4
Pla-1_TTGCCGTAGGCCCTTG	Pla	Pla-1	P1-384	FALSE	2	TRA:CAGDFGNEKLTF TRB:CASSTGTSLFSYNEQFF	2	CD4-C4
Pla-1_TTGCCGTCAATTCCTT	Pla	Pla-1	P1-21	TRUE	3	TRA:CAASRGTGANNLFF TRB:CATQREGREQYF	67	CD4-C4
Pla-1_TTGCCGTCATTATCTC	Pla	Pla-1	P1-335	FALSE	2	TRA:CAASAGGGADGLTF TRB:CASSDRGRGYTF	2	CD4-C4
Pla-1_TTGCCGTGTTCAACCA	Pla	Pla-1	P1-2317	FALSE	1	TRA:CAASFEAAGNKLTF TRB:CASGRTGSKTQYF	1	CD4-C4
Pla-1_TTGGAACAGGACAGAA	Pla	Pla-1	P1-2319	FALSE	1	TRA:CATQFNSNYQLIW TRB:CASTSMGQVNEQFF	1	CD4-C4
Pla-1_TTGGAACCAAGCTGTT	Pla	Pla-1	P1-324	FALSE	2	TRA:CAVGAPLSDGQKLLF TRB:CASSEGTSRNEQFF	2	CD4-C4
Pla-1_TTGGCAACAAGCCGCT	Pla	Pla-1	P1-332	FALSE	2	TRA:CALNFSGGYNKLIF TRB:CAISESEDSYEQYF	2	CD4-C6
Pla-1_TTGGCAACAGGGTACA	Pla	Pla-1	P1-19	TRUE	3	TRA:CALGAFGSARQLTF TRB:CASSKGQGPRETQYF	70	CD4-C4
Pla-1_TTGGCAATCCATGCTC	Pla	Pla-1	P1-2323	FALSE	1	TRA:CAVETQGAQKLVF TRB:CASSSSIRQGKVQPQHF	1	CD4-C4
Pla-1_TTGTAGGGTCTAGGTT	Pla	Pla-1	P1-2324	FALSE	1	TRA:CAASNTDKLVF TRB:CASSFYRDSNQPQHF	1	CD4-C4
Pla-1_TTTACTGAGTCAATAG	Pla	Pla-1	P1-385	FALSE	2	TRA:CATGSAGGTSYGKLTF TRB:CASSFRDTQYF	2	CD4-C4
Pla-1_TTTACTGCACTTCGAA	Pla	Pla-1	P1-2327	FALSE	1	TRA:CAENIYNQGGKLIF TRB:CASSLGPLQEQFF	1	CD4-C4
Pla-1_TTTACTGGTCGACTGC	Pla	Pla-1	P1-2328	FALSE	1	TRA:CAARTQGGSEKLVF TRB:CASSRRDRGFSGVGEQYF	1	CD4-C4
Pla-1_TTTATGCAGGACAGCT	Pla	Pla-1	P1-165	FALSE	3	TRA:CAMDYNQGGKLIF TRB:CASSSATGNTEAFF	4	CD4-C4

Pla-1_TTTCCTCAGGTGATAT	Pla	Pla-1	P1-203	TRUE	3	TRA:CAGVLAGNMLTF TRB:CASRRDRDPNEQFF	3	CD4-C4
Pla-1_TTTCCTCCATCCGTGG	Pla	Pla-1	P1-2333	FALSE	1	TRA:CAVTPLTGGGNKLTF TRB:CASSQFRGGRGTEAFF	1	CD4-C4
Pla-1_TTTGCGCCACCTTGTC	Pla	Pla-1	P1-2334	FALSE	1	TRA:CAFIKAAGNKLTF TRB:CASSSRTSGLYNEQFF	1	CD8-C8
Pla-1_TTTGCGCGTTTCCACC	Pla	Pla-1	P1-2335	FALSE	1	TRA:CAASALQAGTALIF TRB:CASMIGGVMLYGANVLTF	1	CD4-C4
Pla-1_TTTGCGCGTTTGGGCC	Pla	Pla-1	P1-208	FALSE	3	TRA:CAVRDTGGFKTIF TRB:CAWSVGGGGTNEQFF	3	CD4-C4
Pla-1_TTTGGTTAGTCAAGCG	Pla	Pla-1	P1-2339	FALSE	1	TRA:CAVRSNTGFQKLVF TRB:CASSLVRTADEQYF	1	CD4-C4
Pla-1_TTTGGTTGTAACGCGA	Pla	Pla-1	P1-2341	FALSE	1	TRA:CAIWRSRNNNARLMF TRB:CASSVLQRNTEAFF	1	CD4-C4
Pla-1_TTTGTCAAGACTACAA	Pla	Pla-1	P1-238	FALSE	3	TRA:CAVGPWGAQKLVF TRB:CATSPSRGNNEQFF	3	CD4-C4
Pla-1_TTTGTCAAGGTGGGTT	Pla	Pla-1	P1-232	FALSE	3	TRA:CAVGASNQGGKLIF TRB:CASSLGMGSSYEQYF	3	CD4-C4
Pla-1_AAATGCCAGATCGGGT	Pla	Pla-1	P1-1089	FALSE	1	TRA:CAMSPFNTGRRALTF TRB:CSAMTLSGGKDTQYF	1	CD4-C2
Pla-1_AACCATGTCTTGTCAT	Pla	Pla-1	P1-1098	FALSE	1	TRA:CVVSDHPKNAGNMLTF TRB:CASSPTPEGQSYNSPLHF	1	CD4-C2
Pla-1_AACTCCCGTACGACCC	Pla	Pla-1	P1-1102	FALSE	1	TRA:CAVEAFSGYSTLTF TRB:CASSGTGGGLTEAFF	1	CD4-C2
Pla-1_AAGGTTCGTCAATGTC	Pla	Pla-1	P1-1123	FALSE	1	TRA:CPPKEGHFGNEKLTF TRB:CSARDNAGRREKLFF	1	CD4-C5
Pla-1_AAGTCTGCATCATCCC	Pla	Pla-1	P1-1128	FALSE	1	TRA:CAMREDINDYKLSF TRB:CASSLEGQGTYEQYF	1	CD4-C2
Pla-1_ACACCAACACAGCCCA	Pla	Pla-1	P1-1140	FALSE	1	TRA:CAVGTGRRALTF TRB:CASSETGYTYEQYF	1	CD4-C2
Pla-1_ACACTGATCTGCTGTC	Pla	Pla-1	P1-1149	FALSE	1	TRA:CALSGKNTDKLIF TRB:CASSQGQWGEETQYF	1	CD4-C2
Pla-1_ACAGCTACAAAGTGCG	Pla	Pla-1	P1-1153	FALSE	1	TRA:CGTESSQGGSEKLVF TRB:CASSQGTGGFGYTF	1	CD4-C2
Pla-1_ACAGCTACAATAACGA	Pla	Pla-1	P1-1154	FALSE	1	TRA:CALSGAYSGAGSYQLTF TRB:CASSHILAGDTGELFF	1	CD4-C2
Pla-1_ACATACGCACCGAAAG	Pla	Pla-1	P1-1157	FALSE	1	TRA:CAVRDLFSGGYNKLIF TRB:CSALTSGGRGNTGELFF	1	CD4-C2
Pla-1_ACATCAGCAAGGTGTG	Pla	Pla-1	P1-1162	FALSE	1	TRA:CALDSGVYGNNRLAF TRB:CASSFYASTDTQYF	1	CD4-C2
Pla-1_ACGCAGCAGTTCGCGC	Pla	Pla-1	P1-321	FALSE	2	TRA:CAYRSLNNARLMF TRB:CASSLRTSGRARVEQFF	2	CD4-C2
Pla-1_ACGCCGAAGCAGGCTA	Pla	Pla-1	P1-1195	FALSE	1	TRA:CAAAPSGNTPLVF TRB:CASSFGAFSSYEQYF	1	CD4-C2
Pla-1_ACGGGCTAGCCACTAT	Pla	Pla-1	P1-1204	FALSE	1	TRA:CAVPDSGSARQLTF TRB:CASSLGAPNYGYTF	1	CD4-C4
Pla-1_ACGTCAATCTGATTCT	Pla	Pla-1	P1-1212	FALSE	1	TRA:CAMSPGAGGTSYGKLTF TRB:CASSGGLAGADTQYF	1	CD4-C2
Pla-1_ACGTCAATCTTGCCGT	Pla	Pla-1	P1-1213	FALSE	1	TRA:CAFMKSNTDKLIF TRB:CATSDLKVSEAFF	1	CD4-C2
Pla-1_ACTGATGAGTACGCGA	Pla	Pla-1	P1-1220	FALSE	1	TRA:CAENHRDQAGTALIF TRB:CASVRGPDTEAFF	1	CD4-C1
Pla-1_ACTGATGTCATAGCAC	Pla	Pla-1	P1-1221	FALSE	1	TRA:CAGARTGNQFYF TRB:CASSKPLGGRQGRGTQYF	1	CD4-C2

Pla-1_ACTGTCCCAGTATGCT	Pla	Pla-1	P1-1230	FALSE	1	TRA:CAVSPLDQTGANNLFF TRB:CASSPQTGNSPLHF	1	CD4-C2
Pla-1_ACTTACTCAAGGACAC	Pla	Pla-1	P1-1234	FALSE	1	TRA:CALSDQGGGFKTIF TRB:CASRGGQGLQPQHF	1	CD4-C2
Pla-1_ACTTTCAAGGCTACGA	Pla	Pla-1	P1-1238	FALSE	1	TRA:CAMRGAKRDDKIIF TRB:CASSQDPGGTDTQYF	1	CD4-C2
Pla-1_AGGCCGTCACAACGCC	Pla	Pla-1	P1-1280	FALSE	1	TRA:CAEYPGGYNKLIF TRB:CASNKQGVNRQPQHF	1	CD4-C2
Pla-1_AGGGTGACACAGGAGT	Pla	Pla-1	P1-337	FALSE	2	TRA:CVVVRNTGGFKTIF TRB:CASSDRWSSYEQYF	2	CD4-C7
Pla-1_AGTAGTCTCTCCTATA	Pla	Pla-1	P1-1293	FALSE	1	TRA:CIVRLTTDSWGKLQF TRB:CASRPSGSTPTGELFF	1	CD4-C2
Pla-1_AGTGTCAAGCCTTGAT	Pla	Pla-1	P1-1299	FALSE	1	TRA:CATAAGLNEKLTF TRB:CSATFLTEAFF	1	CD4-C2
Pla-1_ATCATCTGTAGAGTGC	Pla	Pla-1	P1-1322	FALSE	1	TRA:CAENGGNNRLAF TRB:CSAIDPGGAGYEQYF	1	CD4-C2
Pla-1_ATCATGGCATCCGTGG	Pla	Pla-1	P1-1323	FALSE	1	TRA:CAANFNKFYF TRB:CASSLRGLAGDTQYF	1	CD4-C2
Pla-1_ATCTACTAGTGTTGAA	Pla	Pla-1	P1-1337	FALSE	1	TRA:CAYRRLQGGSEKLVF TRB:CSVEGTSGRALTYNEQFF	1	CD4-C2
Pla-1_ATCTGCCTCACTTCAT	Pla	Pla-1	P1-1340	FALSE	1	TRA:CAVRETGANNLFF TRB:CASSMVRGHSGNTIYF	1	CD4-C2
Pla-1_ATGTGTGCACCTATCC	Pla	Pla-1	P1-313	FALSE	2	TRA:CAVGRFTGGGNKLTF TRB:CASSDRAGNTIYF	2	CD4-C2
Pla-1_CAAGATCGTGTGGTTT	Pla	Pla-1	P1-1376	FALSE	1	TRA:CAAPLQGAQKLVF TRB:CASSRYPGGNSPLHF	1	CD4-C2
Pla-1_CAAGTTGTCACCCGAG	Pla	Pla-1	P1-1383	FALSE	1	TRA:CAVATVWAGNNRKLIW TRB:CASSLASSSYNEQFF	1	CD4-C2
Pla-1_CACAGGCCAAAGAATC	Pla	Pla-1	P1-1392	FALSE	1	TRA:CAVRVLLPAGNKLTF TRB:CASSNRDSGNYRAKNIQYF	1	CD4-C2
Pla-1_CACCAGGTCATCTGCC	Pla	Pla-1	P1-1408	FALSE	1	TRA:CAVGARGGANNLFF TRB:CASRLDLLNTEAFF	1	CD4-C2
Pla-1_CACCTTGCACACCGAC	Pla	Pla-1	P1-1409	FALSE	1	TRA:CAVKLGIGNTGKLIF TRB:CAISDPQRKQETQYF	1	CD4-C2
Pla-1_CAGAATCGTTACGGAG	Pla	Pla-1	P1-1416	FALSE	1	TRA:CAGLRSGNTGKLIF TRB:CSAEGHHENTGELFF	1	CD4-C2
Pla-1_CATGACATCTTACCGC	Pla	Pla-1	P1-1456	FALSE	1	TRA:CADGYNNNDMRF TRB:CATSDRGWEQFF	1	CD4-C2
Pla-1_CCAATCCCAGTATCTG	Pla	Pla-1	P1-1471	FALSE	1	TRA:CAALNTGNQFYF TRB:CASSPQGGRVDTQYF	1	CD8-C1
Pla-1_CCACCTATCGTTTAGG	Pla	Pla-1	P1-1476	FALSE	1	TRA:CAVRRGSNFGNEKLTF TRB:CASSPTRDRASEQYF	1	CD4-C2
Pla-1_CCCAATCAGTTAAGTG	Pla	Pla-1	P1-1489	FALSE	1	TRA:CAVRWSNYQLIW TRB:CASNKGTDNSPLHF	1	CD4-C2
Pla-1_CCGGGATAGTGGCACA	Pla	Pla-1	P1-1497	FALSE	1	TRA:CAVDRYSSASKIIF TRB:CSARQGLADWTQYF	1	CD4-C2
Pla-1_CCTAGCTCAAGCCATT	Pla	Pla-1	P1-1515	FALSE	1	TRA:CLLAINNDMRF TRB:CASSQTLRGAYEQYF	1	CD4-C4
Pla-1_CCTATTACACCAGTTA	Pla	Pla-1	P1-1518	FALSE	1	TRA:CIVGRSNYKLTF TRB:CASSLNRETQYF	1	CD4-C2
Pla-1_CGATGTATCCTCCTAG	Pla	Pla-1	P1-1565	FALSE	1	TRA:CAASDGGGADGLTF TRB:CASSGALAADTQYF	1	CD4-C2
Pla-1_CGTCACTGTCTTTCAT	Pla	Pla-1	P1-1610	FALSE	1	TRA:CAMREGADTDKLIF TRB:CASSPGGGLHTDTQYF	1	CD4-C2

Pla-1_CTAAGACCATGCCCGA	Pla	Pla-1	P1-1634	FALSE	1	TRA:CAVSPGTASKLTF TRB:CASSLGGRVHQPQHF	1	CD4-C2
Pla-1_CTAGAGTCAACACCTA	Pla	Pla-1	P1-1655	FALSE	1	TRA:CADSGGSYIPTF TRB:CASSSTGRNTEAFF	1	CD4-C2
Pla-1_CTAGCCTTCCTTAATC	Pla	Pla-1	P1-1658	FALSE	1	TRA:CAMREGGQGAQKLVF TRB:CASSSGHRRGRYEQYF	1	CD4-C1
Pla-1_CTCATTAAGAAACCAT	Pla	Pla-1	P1-360	FALSE	2	TRA:CAGINYGGQNFVF TRB:CSVAGTLYTGELFF	2	CD4-C2
Pla-1_CTCATTACATCTACGA	Pla	Pla-1	P1-49	FALSE	3	TRA:CAGGGSEKLVF TRB:CASSLFYPAGQQYF	16	CD4-C2
Pla-1_CTCATTAGTGACCAAG	Pla	Pla-1	P1-1669	FALSE	1	TRA:CAYKRLRGGTYKYIF TRB:CASSREGWETQYF	1	CD4-C2
Pla-1_CTCCTAGCAATAACGA	Pla	Pla-1	P1-1673	FALSE	1	TRA:CAASGANAGGTSYGKLTF TRB:CASGRQGAIQPQHF	1	CD4-C2
Pla-1_CTCCTAGTCCGCTGTT	Pla	Pla-1	P1-1678	FALSE	1	TRA:CAMRPHQGGSEKLVF TRB:CASSLLGGSIGETQYF	1	CD4-C2
Pla-1_CTCGAGGTCTCATTCA	Pla	Pla-1	P1-1684	FALSE	1	TRA:CALSNNAGNNRKLIW TRB:CASSYFRTGGNYGYTF	1	CD4-C2
Pla-1_CTCTACGCAGTAACGG	Pla	Pla-1	P1-1704	FALSE	1	TRA:CLVAIYQTGANNLFF TRB:CASSFVGATNEKLFF	1	CD4-C2
Pla-1_CTGATAGAGTATGACA	Pla	Pla-1	P1-1715	FALSE	1	TRA:CAVQAAGSWGKLQF TRB:CASSLGARMGTEAFF	1	CD4-C3
Pla-1_CTGATAGCACTTGGAT	Pla	Pla-1	P1-1717	FALSE	1	TRA:CAASAHGSGNTGKLIF TRB:CASSLWRGDGQPQHF	1	CD4-C2
Pla-1_CTGATAGCAGGAATGC	Pla	Pla-1	P1-1718	FALSE	1	TRA:CAVGARNRDDKIIF TRB:CASSPAGIGAGGRDTQYF	1	CD4-C2
Pla-1_CTGATCCCATCCTAGA	Pla	Pla-1	P1-1723	FALSE	1	TRA:CAVSGANNLFF TRB:CSAPAGGPRAGSYEQYF	1	CD4-C2
Pla-1_CTGCTGTTCGTAGGAG	Pla	Pla-1	P1-1731	FALSE	1	TRA:CAMRVEYGNKLVF TRB:CASSKTGYRNTGELFF	1	CD4-C2
Pla-1_CTGTTTATCGTCCGTT	Pla	Pla-1	P1-1741	FALSE	1	TRA:CALLIPGNQFYF TRB:CASSQVGTDTQYF	1	CD4-C2
Pla-1_CTTAACTCAGCATACT	Pla	Pla-1	P1-1743	FALSE	1	TRA:CAMSAMDSNYQLIW TRB:CASRKGGGGSTDTQYF	1	CD4-C2
Pla-1_CTTACCGGTGTGAAAT	Pla	Pla-1	P1-1750	FALSE	1	TRA:CAVPPSGSARQLTF TRB:CASSPGQGAPRTEAFF	1	CD4-C2
Pla-1_CTTACCGTCCTTTACA	Pla	Pla-1	P1-1751	FALSE	1	TRA:CAASIGGNTGKLIF TRB:CASRGTGGTAKNIQYF	1	CD4-C2
Pla-1_CTTCTCTCAACGATGG	Pla	Pla-1	P1-1754	FALSE	1	TRA:CAVHLSGSNYKLTF TRB:CASSHRPGDNQPQHF	1	CD4-C1
Pla-1_GAAGCAGGTACAGTTC	Pla	Pla-1	P1-1776	FALSE	1	TRA:CAVESPPGNTGKLIF TRB:CASSAGRETQYF	1	CD4-C3
Pla-1_GAAGCAGTCACTGGGC	Pla	Pla-1	P1-1778	FALSE	1	TRA:CAVQTGANNLFF TRB:CASSLQGGGGETQYF	1	CD4-C2
Pla-1_GAATAAGTCACAGTAC	Pla	Pla-1	P1-1784	FALSE	1	TRA:CALSDPGGGGADGLTF TRB:CASSGGSDTQYF	1	CD4-C2
Pla-1_GACCTGGGTAGCAAAT	Pla	Pla-1	P1-46	FALSE	3	TRA:CAVRGTGTASKLTF TRB:CSVRQGGNEQFF	20	CD4-C2
Pla-1_GACCTGGGTTCGCTAA	Pla	Pla-1	P1-1789	FALSE	1	TRA:CAMREGNFNKFYF TRB:CASSQRQTSGATKNIQYF	1	CD4-C2
Pla-1_GACGGCTTCGATAGAA	Pla	Pla-1	P1-400	FALSE	2	TRA:CAVRNNARLMF TRB:CASSWTRGDNEQFF	2	CD4-C5
Pla-1_GACGTTACATCTCCCA	Pla	Pla-1	P1-1801	FALSE	1	TRA:CATSLPMDSNYQLIW TRB:CASSPNRYDRTGGARTFF	1	CD4-C2

Pla-1_GACTACACATTGAGCT	Pla	Pla-1	P1-1811	FALSE	1	TRA:CAVIPGGGQGGKLIF TRB:CASSLSYEQYF	1	CD4-C3
Pla-1_GATTCAGAGTAGGTGC	Pla	Pla-1	P1-1840	FALSE	1	TRA:CAASSYGTGTASKLTF TRB:CASSPTGTGGRANEKLFF	1	CD4-C2
Pla-1_GCGGGTTTCGAATGCT	Pla	Pla-1	P1-1880	FALSE	1	TRA:CALGAYNNNDMRF TRB:CASGTGTGGFNEQFF	1	CD4-C3
Pla-1_GCTCCTAAGCATCATC	Pla	Pla-1	P1-1881	FALSE	1	TRA:CAETASNTGKLIF TRB:CSARDAVAGYTDTQYF	1	CD4-C5
Pla-1_GCTGGGTTCTTATCTG	Pla	Pla-1	P1-1894	FALSE	1	TRA:CAVGDNYGQNFVF TRB:CATTKGRTGGGSGANVLTF	1	CD4-C2
Pla-1_GGAACTTGTGAGTGAC	Pla	Pla-1	P1-219	FALSE	3	TRA:CAVRGADSWGKLQF TRB:CASSDRTEGRAFF	3	CD4-C3
Pla-1_GGCAATTCACGTAAGG	Pla	Pla-1	P1-1921	FALSE	1	TRA:CATVSFSGGYNKLIF TRB:CASSHEPRTGELFF	1	CD4-C2
Pla-1_GGCTCGATCTATCCCG	Pla	Pla-1	P1-1936	FALSE	1	TRA:CGADTSGAGSYQLTF TRB:CASTIEAYGYTF	1	CD4-C2
Pla-1_GGGAATGGTAGCTAAA	Pla	Pla-1	P1-1941	FALSE	1	TRA:CIVRAGGFKAAGNKLTF TRB:CASSGTGTKTYNEQFF	1	CD4-C2
Pla-1_GGGCACTCATATACCG	Pla	Pla-1	P1-1947	FALSE	1	TRA:CAARSYRGYSTLTF TRB:CATSRGQQETQYF	1	CD4-C5
Pla-1_GGTGCGTTCGTTACAG	Pla	Pla-1	P1-1967	FALSE	1	TRA:CVARDTGRRALTF TRB:CASRLGILEPQHF	1	CD4-C2
Pla-1_GGTGTTAGTACTTAGC	Pla	Pla-1	P1-1970	FALSE	1	TRA:CAVGVQAAGNKLTF TRB:CASSSDGSRGEQYF	1	CD4-C2
Pla-1_GTACGTAAGCTAGTTC	Pla	Pla-1	P1-1979	FALSE	1	TRA:CAVYNFNKFYF TRB:CASSLYSGGFTDTQYF	1	CD4-C3
Pla-1_GTGAAGGAGTACGCGA	Pla	Pla-1	P1-2015	FALSE	1	TRA:CAARPYSGNTPLVF TRB:CASSSPRDRDTEAFF	1	CD4-C2
Pla-1_GTGCTTCGTGTGACCC	Pla	Pla-1	P1-2028	FALSE	1	TRA:CATVGGKLIF TRB:CAWSGGYEETQYF	1	CD4-C2
Pla-1_GTTAAGCTCTCCTATA	Pla	Pla-1	P1-2044	FALSE	1	TRA:CAGPSGGSNYKLTF TRB:CASSQDFPGRGNYGYTF	1	CD4-C2
Pla-1_TAAGAGAAGGACAGAA	Pla	Pla-1	P1-2063	FALSE	1	TRA:CALSDPSGGSYIPTF TRB:CASSIDRNTGELFF	1	CD4-C2
Pla-1_TAGTTGGTCTGTCCGT	Pla	Pla-1	P1-418	FALSE	2	TRA:CAASAGGYNKLIF TRB:CASSNRGQGEKLFF	2	CD4-C5
Pla-1_TATCAGGCACGTCTCT	Pla	Pla-1	P1-2117	FALSE	1	TRA:CAASKGGGGSYIPTF TRB:CASSLGTSAYEQYF	1	CD4-C2
Pla-1_TATCTCAGTTCGTGAT	Pla	Pla-1	P1-2121	FALSE	1	TRA:CLVGDKMDSSYKLIF TRB:CASRIAGGTSNTQYF	1	CD4-C2
Pla-1_TCAACGAGTCCGTGAC	Pla	Pla-1	P1-2127	FALSE	1	TRA:CAVGSSGTYKYIF TRB:CASSTIAGGKYNEQFF	1	CD4-C7
Pla-1_TCAACGAGTCGTCTTC	Pla	Pla-1	P1-2128	FALSE	1	TRA:CAGESGTYKYIF TRB:CSATGQESYQPQHF	1	CD4-C3
Pla-1_TCACAAGCAGACGCCT	Pla	Pla-1	P1-2134	FALSE	1	TRA:CAVMDTGGFKTIF TRB:CASSPSDRVEQPQHF	1	CD4-C2
Pla-1_TCACGAAGTCGTTGTA	Pla	Pla-1	P1-2139	FALSE	1	TRA:CAVIPITGGGNKLTF TRB:CASSDRGQVNTEAFF	1	CD4-C5
Pla-1_TCAGCAAGTACTTCTT	Pla	Pla-1	P1-2142	FALSE	1	TRA:CAFMLYNNNDMRF TRB:CASSLSQTYEQYF	1	CD4-C2
Pla-1_TCCACACTCCACGAAT	Pla	Pla-1	P1-2155	FALSE	1	TRA:CAVEDHRNNDMRF TRB:CASSEGKVNSPLHF	1	CD4-C2
Pla-1_TCGAGGCTCCTATTCA	Pla	Pla-1	P1-417	FALSE	2	TRA:CAVINAGNNRKLIW TRB:CAWSAGSGVFGAFF	2	CD4-C5

Pla-1_TGAGGGAAGGAATCGC	Pla	Pla-1	P1-2207	FALSE	1	TRA:CAVKGYNQGGKLIF TRB:CSARSSRHSNQPQHF	1	CD4-C2
Pla-1_TGCCCTAAGTAGTGCG	Pla	Pla-1	P1-337	FALSE	2	TRA:CVVVRNTGGFKTIF TRB:CASSDRWSSYEQYF	2	CD4-C2
Pla-1_TGCCCTACAGCGTAAG	Pla	Pla-1	P1-2222	FALSE	1	TRA:CVVHGSSNTGKLIF TRB:CASSFPGQGTEAFF	1	CD4-C2
Pla-1_TGCGCAGTCAAAGACA	Pla	Pla-1	P1-2226	FALSE	1	TRA:CAPLVAGNKLTF TRB:CATLVGYGTDTQYF	1	CD4-C2
Pla-1_TGGCTGGGTGCTGTAT	Pla	Pla-1	P1-2253	FALSE	1	TRA:CIRGGQYEKLTF TRB:CASRDTGVAGELFF	1	CD4-C3
Pla-1_TGTGTTTGTTACGCGC	Pla	Pla-1	P1-2271	FALSE	1	TRA:CVVNERGPYNDMRF TRB:CASSATGNGGYTF	1	CD4-C2
Pla-1_TTCCCAGCAAATTGCC	Pla	Pla-1	P1-2291	FALSE	1	TRA:CIAGAGGSNYKLTF TRB:CSAPPRGFGQPQHF	1	CD4-C7
Pla-1_TTCGGTCAGCCCGAAA	Pla	Pla-1	P1-2297	FALSE	1	TRA:CAVRYSSASKIIF TRB:CSASKGEETQYF	1	CD4-C2
Pla-1_TTCTCAACACCTATCC	Pla	Pla-1	P1-2304	FALSE	1	TRA:CIADTGGGNKLTF TRB:CSAKQGQETQYF	1	CD4-C2
Pla-1_TTCTCAAGTTGTTTGG	Pla	Pla-1	P1-2305	FALSE	1	TRA:CAVTSRGGATNKLIF TRB:CASKVGEVDIQYF	1	CD4-C2
Pla-1_TTCTCCTCATAGAAAC	Pla	Pla-1	P1-2307	FALSE	1	TRA:CAVESGTYKYIF TRB:CASSPGTGDLTEQFF	1	CD4-C3
Pla-1_TTGAACGTCTGCCCTA	Pla	Pla-1	P1-2315	FALSE	1	TRA:CVVNMGTGNQFYF TRB:CASSLSDSGNYEQYF	1	CD4-C2
Pla-1_TTGTAGGTCCCAGGTG	Pla	Pla-1	P1-2325	FALSE	1	TRA:CAVQAVRAGGGADGLTF TRB:CASSKGINQPQHF	1	CD4-C3
Pla-1_TTTGCGCTCACTTACT	Pla	Pla-1	P1-2336	FALSE	1	TRA:CAVNPGADGQKLLF TRB:CSARLANEQFF	1	CD4-C3
Pla-1_TTTGGTTTCAGCGATT	Pla	Pla-1	P1-2342	FALSE	1	TRA:CAVIPKAAGNKLTF TRB:CSARDSLGGSPLHF	1	CD4-C2
Pla-1_TTTGGTTTCTTCAACT	Pla	Pla-1	P1-2343	FALSE	1	TRA:CAGVTTDSWGKLQF TRB:CASSLLSLDTQYF	1	CD4-C2
Pla-1_AAAGATGGTATTCTCT	Pla	Pla-1	P1-290	TRUE	2	TRA:CATNTDKLIF TRB:CASSLEGDWNEQYF	2	CD8-C2
Pla-1_AAAGCAACATATACCG	Pla	Pla-1	P1-1086	FALSE	1	TRA:CAGHTGGGNKLTF TRB:CASSPLGAGSNYHNEQFF	1	CD8-C2
Pla-1_AAAGCAAGTCTGCGGT	Pla	Pla-1	P1-62	TRUE	3	TRA:CVVNLMEYGNKLVF TRB:CASSGARQGQYEQYF	10	CD8-C2
Pla-1_AAAGTAGCATTACGAC	Pla	Pla-1	P1-303	FALSE	2	TRA:CAYPDAPTESQGNLIF TRB:CASSQGSGLYNEQFF	2	CD4-C4
Pla-1_AAAGTAGGTTAGATGA	Pla	Pla-1	P1-34	FALSE	3	TRA:CAMSGGGADGLTF TRB:CASNVGVHEQFF	41	CD8-C2
Pla-1_AACCATGTCACAGGCC	Pla	Pla-1	P1-1095	FALSE	1	TRA:CAMSGGGADGLTF TRB:CASSQPWSSWEQYF	1	CD8-C2
Pla-1_AACCATGTCGTGGGAA	Pla	Pla-1	P1-1096	FALSE	1	TRA:CAVEPSGSRLTF TRB:CASSVNWGFEQYF	1	CD8-C2
Pla-1_AACTCCCTCCTGTACC	Pla	Pla-1	P1-1104	FALSE	1	TRA:CARTDSWGKLQF TRB:CASSLRGRTGELFF	1	CD8-C2
Pla-1_AACTGGTAGCGTTCCG	Pla	Pla-1	P1-98	FALSE	3	TRA:CAVTAAGNKLTF TRB:CASSQVGGSEQFF	6	CD8-C2
Pla-1_AACTTTCCACCAGGCT	Pla	Pla-1	P1-34	FALSE	3	TRA:CAMSGGGADGLTF TRB:CASNVGVHEQFF	41	CD8-C2
Pla-1_AAGGAGCTCTCGATGA	Pla	Pla-1	P1-1119	FALSE	1	TRA:CLVGDEVGTTGNQFYF TRB:CASSKGQGPRETQYF	1	CD8-C2

Pla-1_AAGGCAGAGTCTCCTC	Pla	Pla-1	P1-123	FALSE	3	TRA:CAVKRVTGGFKTIF TRB:CATSGDTGTTNYNEQFF	5	CD8-C2
Pla-1_AAGTCTGAGGACCACA	Pla	Pla-1	P1-1126	FALSE	1	TRA:CATDAPLYNQGGKLIF TRB:CASSYRPPRGHFGELFF	1	CD8-C2
Pla-1_AAGTCTGTCCTAAGTG	Pla	Pla-1	P1-124	FALSE	3	TRA:CAYRWVSRANSKLTF TRB:CASIEGQGPYNEQFF	5	CD8-C2
Pla-1_AATCCAGGTTGGTTTG	Pla	Pla-1	P1-1132	FALSE	1	TRA:CAFTAGGATNKLIF TRB:CASSLGQGSMKTQYF	1	CD8-C2
Pla-1_ACACCAAGTATTACCG	Pla	Pla-1	P1-1142	FALSE	1	TRA:CAMRETPGEAGTALIF TRB:CASSIDLGGANVLTF	1	CD8-C2
Pla-1_ACACCCTGTTCGGCAC	Pla	Pla-1	P1-38	TRUE	3	TRA:CAFTAGGATNKLIF TRB:CASIEGQGPYNEQFF	38	CD8-C2
Pla-1_ACACCGGGTTCAGGCC	Pla	Pla-1	P1-1145	FALSE	1	TRA:CVVSPNTGFQKLVF TRB:CASSQANRGPGGYTF	1	CD8-C2
Pla-1_ACACTGACAGACGCCT	Pla	Pla-1	P1-1146	FALSE	1	TRA:CAVTSNTGNQFYF TRB:CASSYTAFQETQYF	1	CD8-C2
Pla-1_ACAGCTATCCTTGACC	Pla	Pla-1	P1-52	TRUE	3	TRA:CAETPDYKLSF TRB:CASRDLYEQYF	13	CD8-C2
Pla-1_ACCGTAAGTAAGTGTA	Pla	Pla-1	P1-317	FALSE	2	TRA:CAVKGYSTLTF TRB:CASSPPKAAQETQYF	2	CD8-C3
Pla-1_ACCTTTACAGCGTTCG	Pla	Pla-1	P1-15	TRUE	3	TRA:CAVNSSPLGANSKLTF TRB:CASSDSGNFYNEQFF	92	CD8-C2
Pla-1_ACCTTTAGTTACTGAC	Pla	Pla-1	P1-214	FALSE	3	TRA:CAMREGRSRNDMRF TRB:CASSLYGPTSGRPLDQETQY	3	CD8-C2
Pla-1_ACGAGCCTCCTGTACC	Pla	Pla-1	P1-34	FALSE	3	TRA:CAMSGGGADGLTF TRB:CASNVGVHEQFF	41	CD8-C2
Pla-1_ACGAGGAGTCTGATTG	Pla	Pla-1	P1-1181	FALSE	1	TRA:CVVSDRGSTLGRLYF TRB:CASSEYHRGLDTEAFF	1	CD8-C2
Pla-1_ACGATACAGACACTAA	Pla	Pla-1	P1-1184	FALSE	1	TRA:CAMREGEDSNYQLIW TRB:CASSPDSYNQPQHF	1	CD8-C3
Pla-1_ACGATACAGCGCCTTG	Pla	Pla-1	P1-38	TRUE	3	TRA:CAFTAGGATNKLIF TRB:CASIEGQGPYNEQFF	38	CD8-C2
Pla-1_ACGATACAGGACATTA	Pla	Pla-1	P1-34	FALSE	3	TRA:CAMSGGGADGLTF TRB:CASNVGVHEQFF	41	CD8-C2
Pla-1_ACGATGTTCGAATGCT	Pla	Pla-1	P1-1190	FALSE	1	TRA:CAVRELADSWGKLQF TRB:CATRRWREGRDEQFF	1	CD8-C2
Pla-1_ACGCAGCCAAGGGTCA	Pla	Pla-1	P1-38	TRUE	3	TRA:CAFTAGGATNKLIF TRB:CASIEGQGPYNEQFF	38	CD8-C2
Pla-1_ACGCAGCTCAACCAAC	Pla	Pla-1	P1-101	FALSE	3	TRA:CAVIRPAGGATNKLIF TRB:CASSLGTGGDTQYF	6	CD8-C2
Pla-1_ACGGAGAAGCTAGGCA	Pla	Pla-1	P1-161	FALSE	3	TRA:CVVTTSQFSGNTPLVF TRB:CASSLYSGALMNTEAFF	4	CD8-C2
Pla-1_ACGGAGATCTGAGTGT	Pla	Pla-1	P1-15	TRUE	3	TRA:CAVNSSPLGANSKLTF TRB:CASSDSGNFYNEQFF	92	CD8-C2
Pla-1_ACGGCCACACCATCCT	Pla	Pla-1	P1-1201	FALSE	1	TRA:CAESIGGNTPLVF TRB:CASSLVGTGYSNQPQHF	1	CD8-C2
Pla-1_ACGGGCTGTTGGTTTG	Pla	Pla-1	P1-38	TRUE	3	TRA:CAFTAGGATNKLIF TRB:CASIEGQGPYNEQFF	38	CD8-C2
Pla-1_ACGTCAATCATAGCAC	Pla	Pla-1	P1-303	FALSE	2	TRA:CAYPDAPTESQGNLIF TRB:CASSQGSGLYNEQFF	2	CD8-C2
Pla-1_ACGTCAATCGCGGATC	Pla	Pla-1	P1-1211	FALSE	1	TRA:CAVRPKAWTSGTYKYIF TRB:CASSTGLEDYNEQFF	1	CD8-C2
Pla-1_ACTGAGTGTGCCTGCA	Pla	Pla-1	P1-326	FALSE	2	TRA:CAVRPYSSNTGKLIF TRB:CASSFGTGYEQYF	2	CD8-C2

Pla-1_ACTGAGTTCCATGAGT	Pla	Pla-1	P1-1219	FALSE	1	TRA:CAERISSGSARQLTF TRB:CASSLTGETQYF	1	CD8-C2
Pla-1_ACTGATGTCTGATACG	Pla	Pla-1	P1-38	TRUE	3	TRA:CAFTAGGATNKLIF TRB:CASIEGQGPYNEQFF	38	CD8-C2
Pla-1_ACTGTCCCATGCGCAC	Pla	Pla-1	P1-1231	FALSE	1	TRA:CALINAGGTSYGKLTF TRB:CASSPPPGRSNYGYTF	1	CD8-C2
Pla-1_ACTGTCCTCCGGGTGT	Pla	Pla-1	P1-34	FALSE	3	TRA:CAMSGGGADGLTF TRB:CASNVGVHEQFF	41	CD8-C2
Pla-1_ACTTGTTCAATAAGCA	Pla	Pla-1	P1-1235	FALSE	1	TRA:CASLNYGGSQGNLIF TRB:CASSPVEGAVNEQFF	1	CD8-C2
Pla-1_AGAATAGCAAGTAGTA	Pla	Pla-1	P1-1241	FALSE	1	TRA:CAEPSYNFNKFYF TRB:CASSAGNEQFF	1	CD8-C2
Pla-1_AGAATAGCATCTGGTA	Pla	Pla-1	P1-116	TRUE	3	TRA:CALFAYSGAGSYQLTF TRB:CASRDDARTDTQYF	5	CD8-C2
Pla-1_AGACGTTTCACAAACC	Pla	Pla-1	P1-1244	FALSE	1	TRA:CALSSNQGGKLIF TRB:CASRKETGTGDYEQYF	1	CD8-C2
Pla-1_AGAGCTTCACTTAAGC	Pla	Pla-1	P1-34	FALSE	3	TRA:CAMSGGGADGLTF TRB:CASNVGVHEQFF	41	CD8-C2
Pla-1_AGAGCTTCATTCCTCG	Pla	Pla-1	P1-1247	FALSE	1	TRA:CAETPPGTYKYIF TRB:CASSYGNTIYF	1	CD8-C2
Pla-1_AGAGTGGCATACGCCG	Pla	Pla-1	P1-1250	FALSE	1	TRA:CAVRGVLMDSSYKLIF TRB:CASSIGHHEQYF	1	CD8-C4
Pla-1_AGATCTGAGGTGATTA	Pla	Pla-1	P1-1255	FALSE	1	TRA:CAETATDSWGKLQF TRB:CASSLLGLGDTQYF	1	CD8-C2
Pla-1_AGATCTGGTTAGTGGG	Pla	Pla-1	P1-1256	FALSE	1	TRA:CAVTHNTDKLIF TRB:CASSARIGAGASGELFF	1	CD8-C3
Pla-1_AGATCTGTCTACTCAT	Pla	Pla-1	P1-49	FALSE	3	TRA:CAGGGSEKLVF TRB:CASSLFYPAGQQYF	16	CD8-C2
Pla-1_AGATTGCCATCCCACT	Pla	Pla-1	P1-1258	FALSE	1	TRA:CAASFTGKLIF TRB:CASNSGTGGKETQYF	1	CD8-C2
Pla-1_AGATTGCTCTATCCTA	Pla	Pla-1	P1-161	FALSE	3	TRA:CVVTTSQFSGNTPLVF TRB:CASSLYSGALMNTEAFF	4	CD8-C2
Pla-1_AGCAGCCCATCCGTGG	Pla	Pla-1	P1-101	FALSE	3	TRA:CAVIRPAGGATNKLIF TRB:CASSLGTGGDTQYF	6	CD8-C2
Pla-1_AGCCTAAGTGACGCCT	Pla	Pla-1	P1-34	FALSE	3	TRA:CAMSGGGADGLTF TRB:CASNVGVHEQFF	41	CD8-C2
Pla-1_AGCGGTCAGAAGGGTA	Pla	Pla-1	P1-43	TRUE	3	TRA:CAASIIQGAQKLVF TRB:CASSFPGQGILEQYF	23	CD8-C2
Pla-1_AGGCCGTAGTGGACGT	Pla	Pla-1	P1-34	FALSE	3	TRA:CAMSGGGADGLTF TRB:CASNVGVHEQFF	41	CD8-C2
Pla-1_AGGGATGAGACATAAC	Pla	Pla-1	P1-38	TRUE	3	TRA:CAFTAGGATNKLIF TRB:CASIEGQGPYNEQFF	38	CD8-C2
Pla-1_AGGGTGAAGAAACCGC	Pla	Pla-1	P1-34	FALSE	3	TRA:CAMSGGGADGLTF TRB:CASNVGVHEQFF	41	CD8-C2
Pla-1_AGGGTGAGTAAGTTCC	Pla	Pla-1	P1-33	TRUE	3	TRA:CLVGTGGFKTIF TRB:CSARVPGQMKETQYF	41	CD8-C2
Pla-1_AGGTCCGGTCGACTAT	Pla	Pla-1	P1-1292	FALSE	1	TRA:CAGPSSGGYNKLIF TRB:CASSQSWDLAVLNEQFF	1	CD8-C2
Pla-1_AGGTCCGGTTCTGTTT	Pla	Pla-1	P1-125	FALSE	3	TRA:CILKSRTSYDKVIF TRB:CASSFLSGTDTQYF	5	CD8-C2
Pla-1_AGGTCCGTCGGAGGTA	Pla	Pla-1	P1-339	FALSE	2	TRA:CAVIPNTGGFKTIF TRB:CASSLRDGAFF	2	CD8-C2
Pla-1_AGTAGTCAGTACGTAA	Pla	Pla-1	P1-38	TRUE	3	TRA:CAFTAGGATNKLIF TRB:CASIEGQGPYNEQFF	38	CD4-C6

Pla-1_AGTCTTTCACATCCGG	Pla	Pla-1	P1-34	FALSE	3	TRA:CAMSGGGADGLTF TRB:CASNVGVHEQFF	41	CD8-C2
Pla-1_AGTCTTTTCCTGCTTG	Pla	Pla-1	P1-1294	FALSE	1	TRA:CAVRFAGQFYF TRB:CASGYTGLRQGAGNSPLHF	1	CD8-C2
Pla-1_AGTCTTTTCTCACATT	Pla	Pla-1	P1-1295	FALSE	1	TRA:CAYRRSPGNMLTF TRB:CASSLQVPPNTEAFF	1	CD8-C2
Pla-1_AGTGGGACACGGTGTC	Pla	Pla-1	P1-140	TRUE	3	TRA:CAYRSGKGGKLIF TRB:CASSDTSSHINEQFF	4	CD8-C2
Pla-1_AGTTGGTCATCGGTTA	Pla	Pla-1	P1-34	FALSE	3	TRA:CAMSGGGADGLTF TRB:CASNVGVHEQFF	41	CD8-C2
Pla-1_ATAACGCCATCATCCC	Pla	Pla-1	P1-1307	FALSE	1	TRA:CVVRKGNKLVF TRB:CASSPGSSGRRVADTQYF	1	CD8-C2
Pla-1_ATAAGAGCACACGCTG	Pla	Pla-1	P1-1313	FALSE	1	TRA:CGADPDGSQGNLIF TRB:CSARDREHYNEQFF	1	CD4-C5
Pla-1_ATAAGAGGTCTAAAGA	Pla	Pla-1	P1-70	FALSE	3	TRA:CAMRETPGEAGTALIF TRB:CSARITYLNTEAFF	9	CD8-C2
Pla-1_ATCATCTAGTGCGATG	Pla	Pla-1	P1-1320	FALSE	1	TRA:CAESINARLMF TRB:CSVEAANTGNTIYF	1	CD8-C2
Pla-1_ATCATCTCAGAGTGTG	Pla	Pla-1	P1-15	TRUE	3	TRA:CAVNSSPLGANSKLTF TRB:CASSDSGNFYNEQFF	92	CD8-C2
Pla-1_ATCCGAAAGGGAACGG	Pla	Pla-1	P1-1328	FALSE	1	TRA:CAVALF TRB:CASRGEEGQGLEAFF	1	CD8-C2
Pla-1_ATCCGAACATTTGCCC	Pla	Pla-1	P1-34	FALSE	3	TRA:CAMSGGGADGLTF TRB:CASNVGVHEQFF	41	CD8-C2
Pla-1_ATCCGAAGTGCTTCTC	Pla	Pla-1	P1-15	TRUE	3	TRA:CAVNSSPLGANSKLTF TRB:CASSDSGNFYNEQFF	92	CD8-C2
Pla-1_ATCGAGTCATATGCTG	Pla	Pla-1	P1-38	TRUE	3	TRA:CAFTAGGATNKLIF TRB:CASIEGQGPYNEQFF	38	CD8-C2
Pla-1_ATCTGCCAGAGCTGGT	Pla	Pla-1	P1-1339	FALSE	1	TRA:CAASKETGGSYIPTF TRB:CASSDLGLAGAGNEQFF	1	CD4-C4
Pla-1_ATCTGCCCAAGCGATG	Pla	Pla-1	P1-348	FALSE	2	TRA:CAMKRGSGGSNYKLTF TRB:CASILRDIYGYTF	2	CD8-C2
Pla-1_ATGAGGGAGTAATCCC	Pla	Pla-1	P1-1342	FALSE	1	TRA:CAVKGSQGNLIF TRB:CSGETDYGYTF	1	CD8-C2
Pla-1_ATGAGGGTCTTAGCCC	Pla	Pla-1	P1-38	TRUE	3	TRA:CAFTAGGATNKLIF TRB:CASIEGQGPYNEQFF	38	CD8-C2
Pla-1_ATGGGAGGTCGAGATG	Pla	Pla-1	P1-15	TRUE	3	TRA:CAVNSSPLGANSKLTF TRB:CASSDSGNFYNEQFF	92	CD8-C2
Pla-1_ATGGGAGTCGCAGGCT	Pla	Pla-1	P1-1349	FALSE	1	TRA:CAVPTRTTSGTYKYIF TRB:CASSDSGNFYNEQFF	1	CD8-C2
Pla-1_ATGTGTGAGATATACG	Pla	Pla-1	P1-1350	FALSE	1	TRA:CAVRDIENTGFQKLVF TRB:CASSQVGRGGEETQYF	1	CD8-C2
Pla-1_ATGTGTGGTCATACTG	Pla	Pla-1	P1-38	TRUE	3	TRA:CAFTAGGATNKLIF TRB:CASIEGQGPYNEQFF	38	CD8-C2
Pla-1_ATGTGTGTCAATCACG	Pla	Pla-1	P1-38	TRUE	3	TRA:CAFTAGGATNKLIF TRB:CASIEGQGPYNEQFF	38	CD8-C2
Pla-1_ATTCTACAGTCCATAC	Pla	Pla-1	P1-15	TRUE	3	TRA:CAVNSSPLGANSKLTF TRB:CASSDSGNFYNEQFF	92	CD8-C2
Pla-1_ATTGGTGAGTGAATTG	Pla	Pla-1	P1-15	TRUE	3	TRA:CAVNSSPLGANSKLTF TRB:CASSDSGNFYNEQFF	92	CD8-C2
Pla-1_ATTTCTGTCCGTTGTC	Pla	Pla-1	P1-38	TRUE	3	TRA:CAFTAGGATNKLIF TRB:CASIEGQGPYNEQFF	38	CD8-C2
Pla-1_CAACCTCTCACAAACC	Pla	Pla-1	P1-132	FALSE	3	TRA:CAVKGSQGNLIF TRB:CSARITYLNTEAFF	5	CD8-C3

Pla-1_CAACTAGGTCTCCACT	Pla	Pla-1	P1-38	TRUE	3	TRA:CAFTAGGATNKLIF TRB:CASIEGQGPYNEQFF	38	CD8-C2
Pla-1_CAAGATCCAACTGCGC	Pla	Pla-1	P1-34	FALSE	3	TRA:CAMSGGGADGLTF TRB:CASNVGVHEQFF	41	CD8-C2
Pla-1_CAAGTTGCAAGTCTGT	Pla	Pla-1	P1-38	TRUE	3	TRA:CAFTAGGATNKLIF TRB:CASIEGQGPYNEQFF	38	CD8-C2
Pla-1_CAAGTTGCATTACGAC	Pla	Pla-1	P1-229	FALSE	3	TRA:CAWGPNNDMRF TRB:CASSVVSGGELFF	3	CD8-C2
Pla-1_CAAGTTGTCATAAAGG	Pla	Pla-1	P1-1384	FALSE	1	TRA:CVVSDHRVDSSYKLIF TRB:CASSDPQTLYNSPLHF	1	CD8-C2
Pla-1_CAAGTTGTCCTTTCGG	Pla	Pla-1	P1-355	FALSE	2	TRA:CALDGGQKLLF TRB:CASSEPDRGRSYEQYF	2	CD8-C2
Pla-1_CAAGTTGTCTCTAAGG	Pla	Pla-1	P1-34	FALSE	3	TRA:CAMSGGGADGLTF TRB:CASNVGVHEQFF	41	CD8-C2
Pla-1_CACAAACGTAAAGTCA	Pla	Pla-1	P1-15	TRUE	3	TRA:CAVNSSPLGANSKLTF TRB:CASSDSGNFYNEQFF	92	CD8-C2
Pla-1_CACACCTAGGATCGCA	Pla	Pla-1	P1-65	FALSE	3	TRA:CAVQAHSGGSNYKLTF TRB:CASAETSYTGELFF	10	CD8-C2
Pla-1_CACACCTCATGTAAGA	Pla	Pla-1	P1-1387	FALSE	1	TRA:CAVPADRGSTLGRLYF TRB:CASSPRQATNQPQHF	1	CD8-C2
Pla-1_CACACTCAGGAGTAGA	Pla	Pla-1	P1-1388	FALSE	1	TRA:CAVTLRRASGGSYIPTF TRB:CASSLGGGPFGEQFF	1	CD4-C4
Pla-1_CACAGGCCAGATCTGT	Pla	Pla-1	P1-1394	FALSE	1	TRA:CAYPPQGAQKLVF TRB:CASSPWDRGYTF	1	CD8-C2
Pla-1_CACATAGCACCAGGCT	Pla	Pla-1	P1-358	FALSE	2	TRA:CAVRVNSGYSTLTF TRB:CSVDGTGVTYEQYF	2	CD8-C2
Pla-1_CACATTTTCAACACTG	Pla	Pla-1	P1-4	TRUE	3	TRA:CAMKGRDDKIIF TRB:CASTKGVSREAFF	226	CD8-C2
Pla-1_CACCACTAGACTTGAA	Pla	Pla-1	P1-1402	FALSE	1	TRA:CAAYSGYSTLTF TRB:CASSGGRDNTEAFF	1	CD8-C2
Pla-1_CACCACTAGCGTCTAT	Pla	Pla-1	P1-1403	FALSE	1	TRA:CLVGDEVGTTGNQFYF TRB:CASSQSPGQRNYGYTF	1	CD8-C2
Pla-1_CACCACTGTAATAGCA	Pla	Pla-1	P1-1404	FALSE	1	TRA:CAMSDTGGGADGLTF TRB:CSARDRVNYNEQFF	1	CD8-C2
Pla-1_CACCACTTCTAACCGA	Pla	Pla-1	P1-76	FALSE	3	TRA:CAVRVMEYGNKLVF TRB:CASSFGTGFEQYF	8	CD8-C2
Pla-1_CACCAGGTCGCATGGC	Pla	Pla-1	P1-359	FALSE	2	TRA:CAVSRSGGYNKLIF TRB:CASRPTSVRDTQYF	2	CD8-C3
Pla-1_CAGAATCGTTGGACCC	Pla	Pla-1	P1-78	TRUE	3	TRA:CAAAQGGSEKLVF TRB:CASTRGDWIYNEQFF	7	CD8-C2
Pla-1_CAGCATATCCTCAACC	Pla	Pla-1	P1-1423	FALSE	1	TRA:CAVRSWDSGGSNYKLTF TRB:CASSLIAGAVGGEQFF	1	CD8-C2
Pla-1_CAGCATATCTGCAGTA	Pla	Pla-1	P1-1424	FALSE	1	TRA:CAMSADDYKLSF TRB:CASRTGYSPLHF	1	CD8-C2
Pla-1_CAGCCGAAGACGCAAC	Pla	Pla-1	P1-15	TRUE	3	TRA:CAVNSSPLGANSKLTF TRB:CASSDSGNFYNEQFF	92	CD8-C2
Pla-1_CAGCCGACAAGCCTAT	Pla	Pla-1	P1-121	FALSE	3	TRA:CAASEGGATNKLIF TRB:CSAGTSGNIQYF	5	CD8-C2
Pla-1_CAGCTAAAGTAATCCC	Pla	Pla-1	P1-15	TRUE	3	TRA:CAVNSSPLGANSKLTF TRB:CASSDSGNFYNEQFF	92	CD8-C2
Pla-1_CAGCTAATCACGCATA	Pla	Pla-1	P1-15	TRUE	3	TRA:CAVNSSPLGANSKLTF TRB:CASSDSGNFYNEQFF	92	CD8-C2
Pla-1_CAGTAACAGGCGTACA	Pla	Pla-1	P1-121	FALSE	3	TRA:CAASEGGATNKLIF TRB:CSAGTSGNIQYF	5	CD8-C2

Pla-1_CAGTAACAGTGTGGCA	Pla	Pla-1	P1-1431	FALSE	1	TRA:CAYRSVRTDNNARLMF TRB:CASSYTAFQETQYF	1	CD8-C2
Pla-1_CAGTAACGTCTCTCTG	Pla	Pla-1	P1-1434	FALSE	1	TRA:CAVDTGTASKLTF TRB:CASSEATYYEQFF	1	CD8-C9
Pla-1_CAGTCCTGTCTAGGTT	Pla	Pla-1	P1-34	FALSE	3	TRA:CAMSGGGADGLTF TRB:CASNVGVHEQFF	41	CD8-C2
Pla-1_CATATGGTCATGGTCA	Pla	Pla-1	P1-15	TRUE	3	TRA:CAVNSSPLGANSKLTF TRB:CASSDSGNFYNEQFF	92	CD8-C2
Pla-1_CATATGGTCGGTGTTA	Pla	Pla-1	P1-70	FALSE	3	TRA:CAMRETPGEAGTALIF TRB:CSARITYLNTEAFF	9	CD8-C2
Pla-1_CATCAGAGTGAGCGAT	Pla	Pla-1	P1-49	FALSE	3	TRA:CAGGGSEKLVF TRB:CASSLFYPAGQQYF	16	CD8-C2
Pla-1_CATCAGATCCTAGAAC	Pla	Pla-1	P1-1443	FALSE	1	TRA:CASNGGYGGSQGNLIF TRB:CASLPGHGVTDTQYF	1	CD8-C2
Pla-1_CATCCACGTGCCTGTG	Pla	Pla-1	P1-3	TRUE	3	TRA:CAFLSGTYKYIF TRB:CASSQSPGQRNYGYTF	308	CD8-C2
Pla-1_CATCGGGGTACTCGCG	Pla	Pla-1	P1-65	FALSE	3	TRA:CAVQAHSGGSNYKLTF TRB:CASAETSYTGELFF	10	CD8-C2
Pla-1_CATCGGGGTTAGAACA	Pla	Pla-1	P1-229	FALSE	3	TRA:CAWGPNNDMRF TRB:CASSVVSGGELFF	3	CD8-C2
Pla-1_CATCGGGTCCGCATAA	Pla	Pla-1	P1-168	FALSE	3	TRA:CLVGDEVGTTGNQFYF TRB:CASSLEDVGGYSNQPQHF	4	CD8-C2
Pla-1_CATCGGGTCGTGGACC	Pla	Pla-1	P1-1453	FALSE	1	TRA:CAMSTGGGNKLTF TRB:CASSLLQGSYEQYF	1	CD8-C2
Pla-1_CATGGCGCAATGTAAG	Pla	Pla-1	P1-34	FALSE	3	TRA:CAMSGGGADGLTF TRB:CASNVGVHEQFF	41	CD8-C2
Pla-1_CATTATCGTAAACCTC	Pla	Pla-1	P1-1465	FALSE	1	TRA:CAMRPSETGNQFYF TRB:CASSLTATEKNYGYTF	1	CD8-C2
Pla-1_CATTCGCCAGCTTAAC	Pla	Pla-1	P1-76	FALSE	3	TRA:CAVRVMEYGNKLVF TRB:CASSFGTGFEQYF	8	CD8-C2
Pla-1_CATTCGCTCGAGCCCA	Pla	Pla-1	P1-52	TRUE	3	TRA:CAETPDYKLSF TRB:CASRDLYEQYF	13	CD8-C2
Pla-1_CCAATCCAGACTACAA	Pla	Pla-1	P1-34	FALSE	3	TRA:CAMSGGGADGLTF TRB:CASNVGVHEQFF	41	CD8-C2
Pla-1_CCAATCCGTGCAACGA	Pla	Pla-1	P1-1472	FALSE	1	TRA:CAMNAFGFLNTGRRALTF TRB:CASSFLAGTDTQYF	1	CD8-C2
Pla-1_CCACCTATCACAACGT	Pla	Pla-1	P1-15	TRUE	3	TRA:CAVNSSPLGANSKLTF TRB:CASSDSGNFYNEQFF	92	CD8-C2
Pla-1_CCACCTATCAGTTCGA	Pla	Pla-1	P1-1475	FALSE	1	TRA:CALRAHFQGGSEKLVF TRB:CASSLAEGREETQYF	1	CD8-C2
Pla-1_CCACGGAAGAACAATC	Pla	Pla-1	P1-38	TRUE	3	TRA:CAFTAGGATNKLIF TRB:CASIEGQGPYNEQFF	38	CD8-C2
Pla-1_CCACGGAGTGGAAAGA	Pla	Pla-1	P1-289	TRUE	2	TRA:CAVNDGSSNTGKLIF TRB:CSVGGDPQETQYF	2	CD8-C2
Pla-1_CCACTACTCAAAGACA	Pla	Pla-1	P1-155	TRUE	3	TRA:CAVMDTGRRALTF TRB:CAGGWTDEQYF	4	CD8-C2
Pla-1_CCCAATCTCGGTTAAC	Pla	Pla-1	P1-98	FALSE	3	TRA:CAVTAAGNKLTF TRB:CASSQVGGSEQFF	6	CD8-C2
Pla-1_CCGGGATAGACTACAA	Pla	Pla-1	P1-373	FALSE	2	TRA:CAVIPNTGGFKTIF TRB:CASSQLAGGLETQYF	2	CD8-C2
Pla-1_CCGTACTGTATATGGA	Pla	Pla-1	P1-15	TRUE	3	TRA:CAVNSSPLGANSKLTF TRB:CASSDSGNFYNEQFF	92	CD8-C2
Pla-1_CCGTGGATCTGGGCCA	Pla	Pla-1	P1-1505	FALSE	1	TRA:CAFIGSNYQLIW TRB:CAWSAQTTWQFF	1	CD8-C2

Pla-1_CCTAAAGTCCCGACTT	Pla	Pla-1	P1-202	TRUE	3	TRA:CAANGRDDKIIF TRB:CASSPSAYLGGTEAFF	3	CD8-C6
Pla-1_CCTACACCAGAAGCAC	Pla	Pla-1	P1-1510	FALSE	1	TRA:CAVTVSNFGNEKLTF TRB:CSAREELAGGPGELFF	1	CD8-C2
Pla-1_CCTACCATCTTCATGT	Pla	Pla-1	P1-15	TRUE	3	TRA:CAVNSSPLGANSKLTF TRB:CASSDSGNFYNEQFF	92	CD8-C2
Pla-1_CCTCAGTCAAGCGAGT	Pla	Pla-1	P1-1520	FALSE	1	TRA:CAMRTYSGAGSYQLTF TRB:CASSRAGNGWTSSPLHF	1	CD8-C2
Pla-1_CCTCAGTCATAACCTG	Pla	Pla-1	P1-1523	FALSE	1	TRA:CLPAGGSARQLTF TRB:CASGAGISYNEQFF	1	CD8-C2
Pla-1_CCTCTGAGTCTCGTTC	Pla	Pla-1	P1-1528	FALSE	1	TRA:CAVKWGQTGANNLFF TRB:CASSLGYGTEAFF	1	CD8-C2
Pla-1_CCTTACGGTGTCCTCT	Pla	Pla-1	P1-1530	FALSE	1	TRA:CAFMKPADDKIIF TRB:CASSSYSGVGNEQFF	1	CD8-C2
Pla-1_CCTTCGAGTCGACTAT	Pla	Pla-1	P1-136	FALSE	3	TRA:CAVGGGADGLTF TRB:CSTRGAAYEQFF	5	CD8-C3
Pla-1_CCTTTCTTCGGCGGTT	Pla	Pla-1	P1-38	TRUE	3	TRA:CAFTAGGATNKLIF TRB:CASIEGQGPYNEQFF	38	CD8-C2
Pla-1_CGAACATGTCTTGATG	Pla	Pla-1	P1-1536	FALSE	1	TRA:CALNTNQAGTALIF TRB:CASSLDGGSSYEQYF	1	CD4-C3
Pla-1_CGAACATGTTACCAGT	Pla	Pla-1	P1-1538	FALSE	1	TRA:CAVYGGSNYKLTF TRB:CASSPLGGLGNQPQHF	1	CD8-C2
Pla-1_CGAATGTGTCTAGCCG	Pla	Pla-1	P1-1542	FALSE	1	TRA:CAMKGRDDKIIF TRB:CASSIGTATEAFF	1	CD8-C6
Pla-1_CGACTTCGTTGGAGGT	Pla	Pla-1	P1-103	FALSE	3	TRA:CATENDYKLSF TRB:CSVGGTFANTGELFF	6	CD8-C2
Pla-1_CGAGCACCAAGCTGTT	Pla	Pla-1	P1-1556	FALSE	1	TRA:CAMREVVYNQGGKLIF TRB:CATSDLNSGGNYGYTF	1	CD8-C2
Pla-1_CGAGCACTCAACACTG	Pla	Pla-1	P1-136	FALSE	3	TRA:CAVGGGADGLTF TRB:CSTRGAAYEQFF	5	CD8-C2
Pla-1_CGAGCACTCTCTTGAT	Pla	Pla-1	P1-103	FALSE	3	TRA:CATENDYKLSF TRB:CSVGGTFANTGELFF	6	CD8-C2
Pla-1_CGATCGGCAAGAGGCT	Pla	Pla-1	P1-15	TRUE	3	TRA:CAVNSSPLGANSKLTF TRB:CASSDSGNFYNEQFF	92	CD8-C2
Pla-1_CGATTGAAGCGAAGGG	Pla	Pla-1	P1-57	FALSE	3	TRA:CAMREGGNTGNQFYF TRB:CASKKDRGTTQAKNIQYF	13	CD8-C2
Pla-1_CGCGGTAGTTATCGGT	Pla	Pla-1	P1-1570	FALSE	1	TRA:CAVTPRSYKLIF TRB:CASSDPQGGTEKETQYF	1	CD8-C2
Pla-1_CGCTATCGTAAATGTG	Pla	Pla-1	P1-38	TRUE	3	TRA:CAFTAGGATNKLIF TRB:CASIEGQGPYNEQFF	38	CD8-C2
Pla-1_CGCTATCTCTTGTACT	Pla	Pla-1	P1-1573	FALSE	1	TRA:CAVLTNRDDKIIF TRB:CATSREAGTGPQHF	1	CD8-C2
Pla-1_CGCTGGAGTGACCAAG	Pla	Pla-1	P1-34	FALSE	3	TRA:CAMSGGGADGLTF TRB:CASNVGVHEQFF	41	CD8-C2
Pla-1_CGGGTCAGTGTTTGTG	Pla	Pla-1	P1-1599	FALSE	1	TRA:CAGARGSSNTGKLIF TRB:CASSIRSAYEQYF	1	CD8-C2
Pla-1_CGGTTAAAGACACTAA	Pla	Pla-1	P1-15	TRUE	3	TRA:CAVNSSPLGANSKLTF TRB:CASSDSGNFYNEQFF	92	CD8-C2
Pla-1_CGGTTAACACGACGAA	Pla	Pla-1	P1-38	TRUE	3	TRA:CAFTAGGATNKLIF TRB:CASIEGQGPYNEQFF	38	CD8-C2
Pla-1_CGTAGCGTCAAGATCC	Pla	Pla-1	P1-1603	FALSE	1	TRA:CAMREGMENNRLAF TRB:CASSLTVGQGKLGYTF	1	CD8-C2
Pla-1_CGTAGGCGTCCCTACT	Pla	Pla-1	P1-1605	FALSE	1	TRA:CLVGITGGFKTIF TRB:CASQGLGGGTEAFF	1	CD8-C2

Pla-1_CGTAGGCTCCGCTGTT	Pla	Pla-1	P1-1608	FALSE	1	TRA:CAMREGPFFNKFYF TRB:CAWSVRRQGGETQYF	1	CD8-C2
Pla-1_CGTCACTTCTGGGCCA	Pla	Pla-1	P1-1612	FALSE	1	TRA:CAATGGSYIPTF TRB:CASSERSQGTSPLHF	1	CD8-C10
Pla-1_CGTCCATCAATCTACG	Pla	Pla-1	P1-34	FALSE	3	TRA:CAMSGGGADGLTF TRB:CASNVGVHEQFF	41	CD8-C2
Pla-1_CGTCTACCAGATCTGT	Pla	Pla-1	P1-326	FALSE	2	TRA:CAVRPYSSNTGKLIF TRB:CASSFGTGYEQYF	2	CD8-C2
Pla-1_CGTCTACCAGGATTGG	Pla	Pla-1	P1-168	FALSE	3	TRA:CLVGDEVGTTGNQFYF TRB:CASSLEDVGGYSNQPQHF	4	CD8-C2
Pla-1_CGTCTACCATCCGCGA	Pla	Pla-1	P1-1615	FALSE	1	TRA:CALRTITQGGSEKLVF TRB:CASSHDGSSYNEQFF	1	CD4-C4
Pla-1_CGTCTACGTGCCTTGG	Pla	Pla-1	P1-1616	FALSE	1	TRA:CAERSEADSWGKLQF TRB:CASSEIPGGTDTQYF	1	CD8-C2
Pla-1_CTAACTTTCCGTAGGC	Pla	Pla-1	P1-387	FALSE	2	TRA:CALSEVTSGSRLTF TRB:CASSSGRGTGAYEQYF	2	CD8-C2
Pla-1_CTACACCTCGGCGCTA	Pla	Pla-1	P1-390	FALSE	2	TRA:CALTLATDSWGKLQF TRB:CASSVLGRGSTDTQYF	2	CD8-C2
Pla-1_CTACGTCCAGTAACGG	Pla	Pla-1	P1-103	FALSE	3	TRA:CATENDYKLSF TRB:CSVGGTFANTGELFF	6	CD8-C2
Pla-1_CTACGTCTCAAGGCTT	Pla	Pla-1	P1-1651	FALSE	1	TRA:CAANSGGGADGLTF TRB:CASRHGLAGTDTQYF	1	CD8-C2
Pla-1_CTAGAGTAGTGAAGTT	Pla	Pla-1	P1-3	TRUE	3	TRA:CAFLSGTYKYIF TRB:CASSQSPGQRNYGYTF	308	CD8-C2
Pla-1_CTAGAGTCAATCAGAA	Pla	Pla-1	P1-101	FALSE	3	TRA:CAVIRPAGGATNKLIF TRB:CASSLGTGGDTQYF	6	CD8-C2
Pla-1_CTAGAGTTCACGAAGG	Pla	Pla-1	P1-1656	FALSE	1	TRA:CAGPGNAGGTSYGKLTF TRB:CASSQSPGQRNYGYTF	1	CD8-C2
Pla-1_CTAGTGAGTTAAGACA	Pla	Pla-1	P1-38	TRUE	3	TRA:CAFTAGGATNKLIF TRB:CASIEGQGPYNEQFF	38	CD8-C2
Pla-1_CTCACACAGTGGTAAT	Pla	Pla-1	P1-138	FALSE	3	TRA:CALGASKIIF TRB:CASGAGISYNEQFF	5	CD8-C2
Pla-1_CTCCTAGAGCGTGAAC	Pla	Pla-1	P1-1671	FALSE	1	TRA:CAVNVNSGNTPLVF TRB:CASSPDGPLDTQYF	1	CD8-C2
Pla-1_CTCCTAGGTTTGCATG	Pla	Pla-1	P1-1677	FALSE	1	TRA:CAIGTGGFKTIF TRB:CASAETSYTGELFF	1	CD8-C2
Pla-1_CTCCTAGTCATCGCTC	Pla	Pla-1	P1-38	TRUE	3	TRA:CAFTAGGATNKLIF TRB:CASIEGQGPYNEQFF	38	CD8-C2
Pla-1_CTCGGAGCATGCTGGC	Pla	Pla-1	P1-15	TRUE	3	TRA:CAVNSSPLGANSKLTF TRB:CASSDSGNFYNEQFF	92	CD8-C2
Pla-1_CTCGTACAGCTAGGCA	Pla	Pla-1	P1-15	TRUE	3	TRA:CAVNSSPLGANSKLTF TRB:CASSDSGNFYNEQFF	92	CD8-C2
Pla-1_CTCGTACCAGACAAAT	Pla	Pla-1	P1-1693	FALSE	1	TRA:CAENEKLTF TRB:CASSPQDGGNRDGAFF	1	CD8-C2
Pla-1_CTCGTACGTAAACACA	Pla	Pla-1	P1-298	TRUE	2	TRA:CVVNDNYGQNFVF TRB:CRPGGGFSYNEQFF	2	CD8-C2
Pla-1_CTCGTCAGTCATCGGC	Pla	Pla-1	P1-1696	FALSE	1	TRA:CAVDVNYGQNFVF TRB:CASRPAGTGDYGYTF	1	CD8-C2
Pla-1_CTGAAGTTCCCGACTT	Pla	Pla-1	P1-15	TRUE	3	TRA:CAVNSSPLGANSKLTF TRB:CASSDSGNFYNEQFF	92	CD8-C2
Pla-1_CTGAAGTTCGCCAGCA	Pla	Pla-1	P1-390	FALSE	2	TRA:CALTLATDSWGKLQF TRB:CASSVLGRGSTDTQYF	2	CD8-C2
Pla-1_CTGATAGAGAGTACAT	Pla	Pla-1	P1-213	FALSE	3	TRA:CAMSSNQAGTALIF TRB:CASSLGQGRRAEAFF	3	CD8-C2

Pla-1_CTGATCCGTTCCACAA	Pla	Pla-1	P1-1724	FALSE	1	TRA:CAVRDSTNAGKSTF TRB:CASSFPRTPSSPGSGNEQFF	1	CD8-C2
Pla-1_CTGCCTAAGTAGGCCA	Pla	Pla-1	P1-314	FALSE	2	TRA:CAEPSNTGNQFYF TRB:CAISLGNEQFF	2	CD8-C2
Pla-1_CTGCCTAGTAATCACC	Pla	Pla-1	P1-38	TRUE	3	TRA:CAFTAGGATNKLIF TRB:CASIEGQGPYNEQFF	38	CD8-C2
Pla-1_CTGTGCTGTCCAGTTA	Pla	Pla-1	P1-136	FALSE	3	TRA:CAVGGGADGLTF TRB:CSTRGAAYEQFF	5	CD8-C2
Pla-1_CTTAACTGTTCCACTC	Pla	Pla-1	P1-1745	FALSE	1	TRA:CAVRGGRNNARLMF TRB:CASSVGDRGGSPLHF	1	CD8-C2
Pla-1_CTTACCGAGCGATGAC	Pla	Pla-1	P1-3	TRUE	3	TRA:CAFLSGTYKYIF TRB:CASSQSPGQRNYGYTF	308	CD8-C7
Pla-1_CTTGGCTGTCATTAGC	Pla	Pla-1	P1-89	TRUE	3	TRA:CAVDVNYGQNFVF TRB:CASSWGDTQYF	6	CD8-C2
Pla-1_CTTGGCTGTTCCATGA	Pla	Pla-1	P1-1757	FALSE	1	TRA:CAVAAGLTGGGNKLTF TRB:CASSLAGGLVVGETQYF	1	CD8-C2
Pla-1_GAAACTCAGAGCTGCA	Pla	Pla-1	P1-34	FALSE	3	TRA:CAMSGGGADGLTF TRB:CASNVGVHEQFF	41	CD8-C2
Pla-1_GAAACTCGTAGCGCAA	Pla	Pla-1	P1-3	TRUE	3	TRA:CAFLSGTYKYIF TRB:CASSQSPGQRNYGYTF	308	CD8-C2
Pla-1_GAAATGACACGCGAAA	Pla	Pla-1	P1-76	FALSE	3	TRA:CAVRVMEYGNKLVF TRB:CASSFGTGFEQYF	8	CD8-C2
Pla-1_GAAATGAGTCCAACTA	Pla	Pla-1	P1-38	TRUE	3	TRA:CAFTAGGATNKLIF TRB:CASIEGQGPYNEQFF	38	CD8-C2
Pla-1_GAACATCCAATCACAC	Pla	Pla-1	P1-38	TRUE	3	TRA:CAFTAGGATNKLIF TRB:CASIEGQGPYNEQFF	38	CD8-C2
Pla-1_GAACATCCACGGCGTT	Pla	Pla-1	P1-1764	FALSE	1	TRA:CAAPPWGSSYKLIF TRB:CASSLTPNTGELFF	1	CD8-C2
Pla-1_GAACATCTCATGCTCC	Pla	Pla-1	P1-305	FALSE	2	TRA:CALRVVTQGGSEKLVF TRB:CASSLFPRATGELFF	2	CD8-C2
Pla-1_GAACATCTCTACCTGC	Pla	Pla-1	P1-1767	FALSE	1	TRA:CAVFDSWGKLQF TRB:CSAREERNEQFF	1	CD8-C2
Pla-1_GAAGCAGCAATCGAAA	Pla	Pla-1	P1-34	FALSE	3	TRA:CAMSGGGADGLTF TRB:CASNVGVHEQFF	41	CD8-C2
Pla-1_GAATAAGAGCGTGAGT	Pla	Pla-1	P1-38	TRUE	3	TRA:CAFTAGGATNKLIF TRB:CASIEGQGPYNEQFF	38	CD8-C2
Pla-1_GAATAAGAGCTAACAA	Pla	Pla-1	P1-1780	FALSE	1	TRA:CAVIPTSGTYKYIF TRB:CASSRDITDTQYF	1	CD8-C2
Pla-1_GACCAATGTCCAAGTT	Pla	Pla-1	P1-76	FALSE	3	TRA:CAVRVMEYGNKLVF TRB:CASSFGTGFEQYF	8	CD8-C2
Pla-1_GACCTGGGTCATATCG	Pla	Pla-1	P1-164	FALSE	3	TRA:CAAGENTGTASKLTF TRB:CASSSTYRVQETQYF	4	CD8-C2
Pla-1_GACGCGTTCTACCTGC	Pla	Pla-1	P1-15	TRUE	3	TRA:CAVNSSPLGANSKLTF TRB:CASSDSGNFYNEQFF	92	CD8-C8
Pla-1_GACGGCTGTAAAGTCA	Pla	Pla-1	P1-1793	FALSE	1	TRA:CALLMNRDDKIIF TRB:CSVCLTAGDTQYF	1	CD8-C2
Pla-1_GACGTGCCACATCCAA	Pla	Pla-1	P1-43	TRUE	3	TRA:CAASIIQGAQKLVF TRB:CASSFPGQGILEQYF	23	CD8-C2
Pla-1_GACGTGCCATAAGACA	Pla	Pla-1	P1-15	TRUE	3	TRA:CAVNSSPLGANSKLTF TRB:CASSDSGNFYNEQFF	92	CD8-C2
Pla-1_GACGTTAAGTCGAGTG	Pla	Pla-1	P1-1800	FALSE	1	TRA:CAVNIRGNDMRF TRB:CASSLNPQTQYF	1	CD8-C2
Pla-1_GACTAACAGACAATAC	Pla	Pla-1	P1-168	FALSE	3	TRA:CLVGDEVGTTGNQFYF TRB:CASSLEDVGGYSNQPQHF	4	CD4-C4

Pla-1_GACTAACGTGGTTTCA	Pla	Pla-1	P1-1807	FALSE	1	TRA:CAAWNTGKLIF TRB:CSADGTSGAEQFF	1	CD8-C2
Pla-1_GACTAACTCCGTCAAA	Pla	Pla-1	P1-211	FALSE	3	TRA:CAVNVQKSNTGNQFYF TRB:CASSYSVSYEQYF	3	CD8-C2
Pla-1_GACTACACATCCTTGC	Pla	Pla-1	P1-28	TRUE	3	TRA:CAVKKGTGRRALTF TRB:CASETGQGTDTQYF	53	CD8-C10
Pla-1_GAGGTGAGTCGAGTTT	Pla	Pla-1	P1-139	TRUE	3	TRA:CGTERDQFYF TRB:CATSRELFGEKLFF	4	CD4-C4
Pla-1_GATCTAGAGAGACTAT	Pla	Pla-1	P1-133	FALSE	3	TRA:CAFMNRGSTLGRLYF TRB:CASSFLSGTDTQYF	5	CD8-C2
Pla-1_GATCTAGTCAATCACG	Pla	Pla-1	P1-34	FALSE	3	TRA:CAMSGGGADGLTF TRB:CASNVGVHEQFF	41	CD8-C2
Pla-1_GATGAAAAGAACTGTA	Pla	Pla-1	P1-1835	FALSE	1	TRA:CAVIPDILTGGGNKLTF TRB:CASSQTGDSTDTQYF	1	CD8-C3
Pla-1_GATTCAGTCCGTTGTC	Pla	Pla-1	P1-15	TRUE	3	TRA:CAVNSSPLGANSKLTF TRB:CASSDSGNFYNEQFF	92	CD8-C2
Pla-1_GCAATCAAGATCGGGT	Pla	Pla-1	P1-28	TRUE	3	TRA:CAVKKGTGRRALTF TRB:CASETGQGTDTQYF	53	CD4-C3
Pla-1_GCACATAGTACAGCAG	Pla	Pla-1	P1-138	FALSE	3	TRA:CALGASKIIF TRB:CASGAGISYNEQFF	5	CD8-C2
Pla-1_GCAGCCAAGATGTGGC	Pla	Pla-1	P1-1848	FALSE	1	TRA:CAESIGNDMRF TRB:CASSPEGLAVGELFF	1	CD8-C2
Pla-1_GCAGCCATCTTTCCTC	Pla	Pla-1	P1-1851	FALSE	1	TRA:CAVQAGNNDMRF TRB:CASSLSLRDIGGEQYF	1	CD8-C2
Pla-1_GCAGTTAGTTTGTTTC	Pla	Pla-1	P1-1854	FALSE	1	TRA:CAVRDSGTDKLIF TRB:CASSLGGTAYGYTF	1	CD8-C2
Pla-1_GCAGTTATCAACCAAC	Pla	Pla-1	P1-80	FALSE	3	TRA:CAASAEGSQGNLIF TRB:CASKKDRGTTQAKNIQYF	7	CD8-C2
Pla-1_GCATGATCAAACGCGA	Pla	Pla-1	P1-1857	FALSE	1	TRA:CALSDPETSGSRLTF TRB:CSAGTSGRGREQYF	1	CD8-C3
Pla-1_GCATGTACATTCTCAT	Pla	Pla-1	P1-395	FALSE	2	TRA:CVDPSKYIF TRB:CASSIRVSKGTDTQYF	2	CD8-C2
Pla-1_GCCTCTAGTAAGAGAG	Pla	Pla-1	P1-1866	FALSE	1	TRA:CAYRGYQGGSEKLVF TRB:CASSLASGGETQYF	1	CD8-C2
Pla-1_GCGAGAAGTAGGGACT	Pla	Pla-1	P1-334	FALSE	2	TRA:CIVRVTTDSWGKLQF TRB:CSARVLDTQYF	2	CD8-C2
Pla-1_GCGAGAAGTCGGCATC	Pla	Pla-1	P1-1870	FALSE	1	TRA:CAVKRVTGGFKTIF TRB:CASIEGQGPYNEQFF	1	CD8-C2
Pla-1_GCGCAACCACACCGCA	Pla	Pla-1	P1-339	FALSE	2	TRA:CAVIPNTGGFKTIF TRB:CASSLRDGAFF	2	CD8-C2
Pla-1_GCGCGATAGGCTATCT	Pla	Pla-1	P1-192	TRUE	3	TRA:CAASGNNAGNMLTF TRB:CASSLMAGGEREQYF	3	CD8-C2
Pla-1_GCGCGATTCCTTTCGG	Pla	Pla-1	P1-101	FALSE	3	TRA:CAVIRPAGGATNKLIF TRB:CASSLGTGGDTQYF	6	CD8-C2
Pla-1_GCGGGTTGTCCAAGTT	Pla	Pla-1	P1-116	TRUE	3	TRA:CALFAYSGAGSYQLTF TRB:CASRDDARTDTQYF	5	CD8-C2
Pla-1_GCTGCTTCAGGTGGAT	Pla	Pla-1	P1-1891	FALSE	1	TRA:CAVYNFNKFYF TRB:CASSSFDTGQGAYEQYF	1	CD8-C2
Pla-1_GCTTCCAAGGAGTTGC	Pla	Pla-1	P1-3	TRUE	3	TRA:CAFLSGTYKYIF TRB:CASSQSPGQRNYGYTF	308	CD8-C2
Pla-1_GCTTCCACAATCGGTT	Pla	Pla-1	P1-1895	FALSE	1	TRA:CAASGANAGNMLTF TRB:CASSDLKPTGSYNSPLHF	1	CD8-C2
Pla-1_GCTTGAACAGTGAGTG	Pla	Pla-1	P1-116	TRUE	3	TRA:CALFAYSGAGSYQLTF TRB:CASRDDARTDTQYF	5	CD8-C2

Pla-1_GGACAAGCAGCATGAG	Pla	Pla-1	P1-15	TRUE	3	TRA:CAVNSSPLGANSKLTF TRB:CASSDSGNFYNEQFF	92	CD4-C4
Pla-1_GGACAGACAAGAAAGG	Pla	Pla-1	P1-15	TRUE	3	TRA:CAVNSSPLGANSKLTF TRB:CASSDSGNFYNEQFF	92	CD8-C2
Pla-1_GGACGTCCAGCCTTGG	Pla	Pla-1	P1-138	FALSE	3	TRA:CALGASKIIF TRB:CASGAGISYNEQFF	5	CD8-C2
Pla-1_GGACGTCTCAGCTGGC	Pla	Pla-1	P1-1914	FALSE	1	TRA:CAVGAPRGYQKVTF TRB:CASSDRGDRAYEQYF	1	CD8-C2
Pla-1_GGATGTTTCCTCCTAG	Pla	Pla-1	P1-34	FALSE	3	TRA:CAMSGGGADGLTF TRB:CASNVGVHEQFF	41	CD8-C2
Pla-1_GGATTACGTGCAGGTA	Pla	Pla-1	P1-1919	FALSE	1	TRA:CAVHENTGGFKTIF TRB:CSAHSDTSTDTQYF	1	CD8-C2
Pla-1_GGCAATTGTTAAAGTG	Pla	Pla-1	P1-123	FALSE	3	TRA:CAVKRVTGGFKTIF TRB:CATSGDTGTTNYNEQFF	5	CD8-C2
Pla-1_GGCCGATCATGCTGGC	Pla	Pla-1	P1-15	TRUE	3	TRA:CAVNSSPLGANSKLTF TRB:CASSDSGNFYNEQFF	92	CD8-C2
Pla-1_GGCGTGTTCAAACAAG	Pla	Pla-1	P1-370	FALSE	2	TRA:CAVNGRTDKLIF TRB:CSATTTTTGLPDTQYF	2	CD8-C3
Pla-1_GGCTCGATCTGGCGAC	Pla	Pla-1	P1-221	FALSE	3	TRA:CAVIENNARLMF TRB:CASSSGTYEQYF	3	CD8-C2
Pla-1_GGGAATGGTGTAATGA	Pla	Pla-1	P1-38	TRUE	3	TRA:CAFTAGGATNKLIF TRB:CASIEGQGPYNEQFF	38	CD8-C2
Pla-1_GGGAGATAGAGACGAA	Pla	Pla-1	P1-1943	FALSE	1	TRA:CVVSDRMGTYKYIF TRB:CASSLSPGLDYNEQFF	1	CD8-C2
Pla-1_GGGCACTTCAACACAC	Pla	Pla-1	P1-1948	FALSE	1	TRA:CAVTPTDKLIF TRB:CSARDPYGQGSDTQYF	1	CD8-C2
Pla-1_GGGCATCAGCTCCCAG	Pla	Pla-1	P1-136	FALSE	3	TRA:CAVGGGADGLTF TRB:CSTRGAAYEQFF	5	CD8-C2
Pla-1_GGGCATCCAGCGTCCA	Pla	Pla-1	P1-43	TRUE	3	TRA:CAASIIQGAQKLVF TRB:CASSFPGQGILEQYF	23	CD8-C2
Pla-1_GGTGCGTAGCTTATCG	Pla	Pla-1	P1-36	TRUE	3	TRA:CILRDNAGNNRKLIW TRB:CASSSRQGFPGGANVLTF	40	CD4-C6
Pla-1_GGTGCGTTCCATGAGT	Pla	Pla-1	P1-1966	FALSE	1	TRA:CAGGRGAQKLVF TRB:CASSLGGGNYYEQYF	1	CD8-C2
Pla-1_GTAACGTGTCTACCTC	Pla	Pla-1	P1-221	FALSE	3	TRA:CAVIENNARLMF TRB:CASSSGTYEQYF	3	CD8-C2
Pla-1_GTAACTGAGGCCCTTG	Pla	Pla-1	P1-76	FALSE	3	TRA:CAVRVMEYGNKLVF TRB:CASSFGTGFEQYF	8	CD8-C2
Pla-1_GTAACTGAGTCGCCGT	Pla	Pla-1	P1-1975	FALSE	1	TRA:CVSTGNTPLVF TRB:CSAIRGDRQETQYF	1	CD8-C2
Pla-1_GTACGTAAGACAAAGG	Pla	Pla-1	P1-43	TRUE	3	TRA:CAASIIQGAQKLVF TRB:CASSFPGQGILEQYF	23	CD8-C5
Pla-1_GTACTCCAGTCCAGGA	Pla	Pla-1	P1-248	FALSE	3	TRA:CGADRGNYGGSQGNLIF TRB:CASSLWAPSAKNIQYF	3	CD8-C2
Pla-1_GTACTCCGTACAGTGG	Pla	Pla-1	P1-3	TRUE	3	TRA:CAFLSGTYKYIF TRB:CASSQSPGQRNYGYTF	308	CD8-C2
Pla-1_GTAGGCCCAAGCGAGT	Pla	Pla-1	P1-121	FALSE	3	TRA:CAASEGGATNKLIF TRB:CSAGTSGNIQYF	5	CD8-C3
Pla-1_GTAGGCCCACTGTGTA	Pla	Pla-1	P1-1987	FALSE	1	TRA:CAERTNAGKSTF TRB:CASSLRWASGIEDTQYF	1	CD8-C2
Pla-1_GTAGGCCGTCTCACCT	Pla	Pla-1	P1-249	FALSE	3	TRA:CALSEERSAGGTSYGKLTF TRB:CATSPPKGGDWRTQYF	3	CD8-C2
Pla-1_GTAGTCATCAATCTCT	Pla	Pla-1	P1-43	TRUE	3	TRA:CAASIIQGAQKLVF TRB:CASSFPGQGILEQYF	23	CD8-C2

Pla-1_GTCAAGTGTAATTGGA	Pla	Pla-1	P1-249	FALSE	3	TRA:CALSEERSAGGTSYGKLTF TRB:CATSPPKGGDWRTQYF	3	CD8-C2
Pla-1_GTCACAACAACTGCTA	Pla	Pla-1	P1-15	TRUE	3	TRA:CAVNSSPLGANSKLTF TRB:CASSDSGNFYNEQFF	92	CD8-C2
Pla-1_GTCACAAGTACGAAAT	Pla	Pla-1	P1-230	FALSE	3	TRA:CAVDPWGSQGNLIF TRB:CASSPGREQFF	3	CD8-C2
Pla-1_GTCACGGGTAAGTGGC	Pla	Pla-1	P1-38	TRUE	3	TRA:CAFTAGGATNKLIF TRB:CASIEGQGPYNEQFF	38	CD8-C2
Pla-1_GTCATTTGTCAGAAGC	Pla	Pla-1	P1-2002	FALSE	1	TRA:CAQRDSNSGYALNF TRB:CASSEAGKRNQPQHF	1	CD8-C2
Pla-1_GTCGGGTCAATGCCAT	Pla	Pla-1	P1-2003	FALSE	1	TRA:CALSEAGYGGSQGNLIF TRB:CASSLQGEYEQYF	1	CD8-C2
Pla-1_GTCTTCGGTGTATGGG	Pla	Pla-1	P1-249	FALSE	3	TRA:CALSEERSAGGTSYGKLTF TRB:CATSPPKGGDWRTQYF	3	CD8-C2
Pla-1_GTGCTTCAGCTAGCCC	Pla	Pla-1	P1-2025	FALSE	1	TRA:CALSPIRGYSGGGADGLTF TRB:CASSLGDTVPNTGELFF	1	CD8-C2
Pla-1_GTGCTTCTCTAACGGT	Pla	Pla-1	P1-34	FALSE	3	TRA:CAMSGGGADGLTF TRB:CASNVGVHEQFF	41	CD8-C2
Pla-1_GTGGGTCAGGATGCGT	Pla	Pla-1	P1-164	FALSE	3	TRA:CAAGENTGTASKLTF TRB:CASSSTYRVQETQYF	4	CD8-C2
Pla-1_GTGTGCGGTTCCATGA	Pla	Pla-1	P1-98	FALSE	3	TRA:CAVTAAGNKLTF TRB:CASSQVGGSEQFF	6	CD8-C2
Pla-1_GTGTTAGGTCCGACGT	Pla	Pla-1	P1-2037	FALSE	1	TRA:CAVRERGGSNYKLTF TRB:CASSRTGDTEAFF	1	CD4-C4
Pla-1_GTTAAGCTCGTTGCCT	Pla	Pla-1	P1-34	FALSE	3	TRA:CAMSGGGADGLTF TRB:CASNVGVHEQFF	41	CD8-C2
Pla-1_GTTCATTCAGTAAGCG	Pla	Pla-1	P1-34	FALSE	3	TRA:CAMSGGGADGLTF TRB:CASNVGVHEQFF	41	CD8-C2
Pla-1_GTTCATTGTAGCGTAG	Pla	Pla-1	P1-2051	FALSE	1	TRA:CAFFFGNEKLTF TRB:CASSDRGGRAFF	1	CD8-C2
Pla-1_GTTCATTGTAGCTCCG	Pla	Pla-1	P1-38	TRUE	3	TRA:CAFTAGGATNKLIF TRB:CASIEGQGPYNEQFF	38	CD8-C2
Pla-1_GTTCATTGTGGCCCTA	Pla	Pla-1	P1-34	FALSE	3	TRA:CAMSGGGADGLTF TRB:CASNVGVHEQFF	41	CD8-C2
Pla-1_GTTCATTGTTAAAGAC	Pla	Pla-1	P1-2053	FALSE	1	TRA:CAGTQTYGQNFVF TRB:CAIKGQGAQGYTF	1	CD8-C2
Pla-1_GTTCATTGTTATCGGT	Pla	Pla-1	P1-98	FALSE	3	TRA:CAVTAAGNKLTF TRB:CASSQVGGSEQFF	6	CD8-C2
Pla-1_GTTCGGGAGGTCGGAT	Pla	Pla-1	P1-65	FALSE	3	TRA:CAVQAHSGGSNYKLTF TRB:CASAETSYTGELFF	10	CD8-C2
Pla-1_GTTCGGGCACTTCTGC	Pla	Pla-1	P1-2054	FALSE	1	TRA:CAMRENPGNQFYF TRB:CASSSGLAANEQFF	1	CD8-C2
Pla-1_TAAACCGCAAGCTGGA	Pla	Pla-1	P1-34	FALSE	3	TRA:CAMSGGGADGLTF TRB:CASNVGVHEQFF	41	CD8-C2
Pla-1_TAAACCGTCACCAGGC	Pla	Pla-1	P1-214	FALSE	3	TRA:CAMREGRSRNDMRF TRB:CASSLYGPTSGRPLDQETQY	3	CD8-C2
Pla-1_TAAGTGCGTAAGTTCC	Pla	Pla-1	P1-387	FALSE	2	TRA:CALSEVTSGSRLTF TRB:CASSSGRGTGAYEQYF	2	CD8-C2
Pla-1_TACCTATAGAGGTACC	Pla	Pla-1	P1-34	FALSE	3	TRA:CAMSGGGADGLTF TRB:CASNVGVHEQFF	41	CD8-C2
Pla-1_TACGGATCAATGGACG	Pla	Pla-1	P1-251	FALSE	3	TRA:CIVRAEGYGQNFVF TRB:CASSIGSLAYEQYF	3	CD8-C2
Pla-1_TACGGATTCCTCAATT	Pla	Pla-1	P1-248	FALSE	3	TRA:CGADRGNYGGSQGNLIF TRB:CASSLWAPSAKNIQYF	3	CD8-C2

Pla-1_TACGGGCGTTGGACCC	Pla	Pla-1	P1-251	FALSE	3	TRA:CIVRAEGYGQNFVF TRB:CASSIGSLAYEQYF	3	CD8-C2
Pla-1_TACTCGCCAGCCTTGG	Pla	Pla-1	P1-34	FALSE	3	TRA:CAMSGGGADGLTF TRB:CASNVGVHEQFF	41	CD8-C2
Pla-1_TACTCGCGTATATCCG	Pla	Pla-1	P1-2091	FALSE	1	TRA:CGAAPELDGKLSF TRB:CASRNPRASGRFNEQFF	1	CD8-C2
Pla-1_TACTCGCGTGAGTGAC	Pla	Pla-1	P1-195	TRUE	3	TRA:CAANLYPHSGAGSYQLTF TRB:CASSSGTGGETQYF	3	CD8-C2
Pla-1_TACTCGCGTTGGACCC	Pla	Pla-1	P1-124	FALSE	3	TRA:CAYRWVSRANSKLTF TRB:CASIEGQGPYNEQFF	5	CD8-C2
Pla-1_TACTTACGTATGGTTC	Pla	Pla-1	P1-2095	FALSE	1	TRA:CALQGAQKLVF TRB:CASSQERRQGGASYYEQYF	1	CD8-C2
Pla-1_TAGAGCTAGCTTATCG	Pla	Pla-1	P1-98	FALSE	3	TRA:CAVTAAGNKLTF TRB:CASSQVGGSEQFF	6	CD8-C2
Pla-1_TAGAGCTGTACAGTGG	Pla	Pla-1	P1-2105	FALSE	1	TRA:CVVVGGRALTF TRB:CASSATGGAGSEAFF	1	CD4-C4
Pla-1_TAGAGCTTCCAAACTG	Pla	Pla-1	P1-2109	FALSE	1	TRA:CIVRVARTSGSRLTF TRB:CASSIGGLSYEQYF	1	CD8-C2
Pla-1_TAGGCATCACGGTAAG	Pla	Pla-1	P1-38	TRUE	3	TRA:CAFTAGGATNKLIF TRB:CASIEGQGPYNEQFF	38	CD8-C2
Pla-1_TAGGCATTCGGATGGA	Pla	Pla-1	P1-38	TRUE	3	TRA:CAFTAGGATNKLIF TRB:CASIEGQGPYNEQFF	38	CD8-C2
Pla-1_TAGTGGTAGTTCGCAT	Pla	Pla-1	P1-13	TRUE	3	TRA:CVVMNYGGSQGNLIF TRB:CASSSWGDTDEAFF	112	CD8-C2
Pla-1_TAGTGGTTCTGTCAAG	Pla	Pla-1	P1-15	TRUE	3	TRA:CAVNSSPLGANSKLTF TRB:CASSDSGNFYNEQFF	92	CD8-C2
Pla-1_TAGTTGGGTCCAAGTT	Pla	Pla-1	P1-34	FALSE	3	TRA:CAMSGGGADGLTF TRB:CASNVGVHEQFF	41	CD8-C2
Pla-1_TAGTTGGTCATCGCTC	Pla	Pla-1	P1-43	TRUE	3	TRA:CAASIIQGAQKLVF TRB:CASSFPGQGILEQYF	23	CD8-C2
Pla-1_TATCTCAAGGTGTTAA	Pla	Pla-1	P1-133	FALSE	3	TRA:CAFMNRGSTLGRLYF TRB:CASSFLSGTDTQYF	5	CD8-C2
Pla-1_TATCTCATCTACCTGC	Pla	Pla-1	P1-317	FALSE	2	TRA:CAVKGYSTLTF TRB:CASSPPKAAQETQYF	2	CD8-C2
Pla-1_TATGCCCTCACATAGC	Pla	Pla-1	P1-2124	FALSE	1	TRA:CAAGDSNYQLIW TRB:CASSLNGGRDTEAFF	1	CD8-C4
Pla-1_TCAGATGGTCTCTTAT	Pla	Pla-1	P1-141	TRUE	3	TRA:CALRILRGSYIPTF TRB:CASSLPGRYSGANVLTF	4	CD8-C2
Pla-1_TCAGATGTCAGGCAAG	Pla	Pla-1	P1-251	FALSE	3	TRA:CIVRAEGYGQNFVF TRB:CASSIGSLAYEQYF	3	CD8-C2
Pla-1_TCAGCAAAGATCCCGC	Pla	Pla-1	P1-2141	FALSE	1	TRA:CLVGDGPSGTYKYIF TRB:CASWSHVGTGDGQETQYF	1	CD8-C2
Pla-1_TCCACACTCGGAGGTA	Pla	Pla-1	P1-124	FALSE	3	TRA:CAYRWVSRANSKLTF TRB:CASIEGQGPYNEQFF	5	CD8-C2
Pla-1_TCGAGGCAGGTGATAT	Pla	Pla-1	P1-38	TRUE	3	TRA:CAFTAGGATNKLIF TRB:CASIEGQGPYNEQFF	38	CD8-C2
Pla-1_TCGAGGCTCACATGCA	Pla	Pla-1	P1-43	TRUE	3	TRA:CAASIIQGAQKLVF TRB:CASSFPGQGILEQYF	23	CD8-C2
Pla-1_TCGCGAGCACATGACT	Pla	Pla-1	P1-67	TRUE	3	TRA:CALNTNQAGTALIF TRB:CASSLGTRYAYEQYF	9	CD8-C8
Pla-1_TCGCGTTCAGGATCGA	Pla	Pla-1	P1-15	TRUE	3	TRA:CAVNSSPLGANSKLTF TRB:CASSDSGNFYNEQFF	92	CD8-C2
Pla-1_TCGGGACGTATATCCG	Pla	Pla-1	P1-2163	FALSE	1	TRA:CAFMKLDEKLTF TRB:CASSSRGSEANYGYTF	1	CD8-C2

Pla-1_TCGTACCAGCTGCCCA	Pla	Pla-1	P1-168	FALSE	3	TRA:CLVGDEVGTTGNQFYF TRB:CASSLEDVGGYSNQPQHF	4	CD8-C2
Pla-1_TCGTACCCACCAGGTC	Pla	Pla-1	P1-76	FALSE	3	TRA:CAVRVMEYGNKLVF TRB:CASSFGTGFEQYF	8	CD8-C2
Pla-1_TCGTACCTCGGAAACG	Pla	Pla-1	P1-2167	FALSE	1	TRA:CAFMMGAGGTSYGKLTF TRB:CASSAGSFGYTF	1	CD8-C2
Pla-1_TCTATTGCAAGTTCTG	Pla	Pla-1	P1-92	TRUE	3	TRA:CIVQLTGTASKLTF TRB:CASSFKSGRTNEQFF	6	CD4-C6
Pla-1_TCTCATACAGCTTAAC	Pla	Pla-1	P1-2177	FALSE	1	TRA:CAVPDRGSTLGRLYF TRB:CASSELIENTGELFF	1	CD8-C2
Pla-1_TCTCATAGTAAGTGTA	Pla	Pla-1	P1-3	TRUE	3	TRA:CAFLSGTYKYIF TRB:CASSQSPGQRNYGYTF	308	CD8-C2
Pla-1_TCTCATATCACGCATA	Pla	Pla-1	P1-2180	FALSE	1	TRA:CALSEPPI TRB:CASWSGHEPYEQYF	1	CD8-C2
Pla-1_TCTGAGAAGCTAGTTC	Pla	Pla-1	P1-138	FALSE	3	TRA:CALGASKIIF TRB:CASGAGISYNEQFF	5	CD8-C2
Pla-1_TGACAACCATCCGCGA	Pla	Pla-1	P1-38	TRUE	3	TRA:CAFTAGGATNKLIF TRB:CASIEGQGPYNEQFF	38	CD8-C9
Pla-1_TGACTAGCAATGGACG	Pla	Pla-1	P1-83	FALSE	3	TRA:CAVRDIENTGFQKLVF TRB:CASTSGGFNQPQHF	7	CD4-C5
Pla-1_TGAGAGGGTATTCGTG	Pla	Pla-1	P1-155	TRUE	3	TRA:CAVMDTGRRALTF TRB:CAGGWTDEQYF	4	CD8-C2
Pla-1_TGAGGGAAGTGCAAGC	Pla	Pla-1	P1-2208	FALSE	1	TRA:CAVSMDSNYQLIW TRB:CASSPRGALYNEQFF	1	CD8-C2
Pla-1_TGATTTCTCACAATGC	Pla	Pla-1	P1-2209	FALSE	1	TRA:CAVRTFSGGYNKLIF TRB:CASSSLAGVLGELFF	1	CD8-C2
Pla-1_TGCACCTCAAGAAGAG	Pla	Pla-1	P1-413	FALSE	2	TRA:CALTWANNLFF TRB:CAWSSDRGYNEQFF	2	CD4-C4
Pla-1_TGCACCTCAGGATCGA	Pla	Pla-1	P1-2211	FALSE	1	TRA:CAGFPSGGSYIPTF TRB:CASSVAGSGGTDTQYF	1	CD8-C2
Pla-1_TGCCAAACACATTTCT	Pla	Pla-1	P1-2213	FALSE	1	TRA:CAEFSGGYNKLIF TRB:CATSREGVSYEQYF	1	CD8-C2
Pla-1_TGCCCTACAAGGCTCC	Pla	Pla-1	P1-2221	FALSE	1	TRA:CAVHQSGYSTLTF TRB:CASSYISGGHYEQYF	1	CD8-C2
Pla-1_TGCTACCGTTTCCACC	Pla	Pla-1	P1-65	FALSE	3	TRA:CAVQAHSGGSNYKLTF TRB:CASAETSYTGELFF	10	CD8-C2
Pla-1_TGCTGCTTCATTTGGG	Pla	Pla-1	P1-2243	FALSE	1	TRA:CAVTSAGNMLTF TRB:CASSVVGGAEAFF	1	CD8-C2
Pla-1_TGGCCAGAGCTATGCT	Pla	Pla-1	P1-83	FALSE	3	TRA:CAVRDIENTGFQKLVF TRB:CASTSGGFNQPQHF	7	CD8-C2
Pla-1_TGGCGCACATACGCCG	Pla	Pla-1	P1-2250	FALSE	1	TRA:CLVGDRFKAAGNKLTF TRB:CASSPSGGQGPSNAGELFF	1	CD8-C2
Pla-1_TGGCTGGTCTGGTTCC	Pla	Pla-1	P1-138	FALSE	3	TRA:CALGASKIIF TRB:CASGAGISYNEQFF	5	CD8-C2
Pla-1_TGGGAAGGTCTCAACA	Pla	Pla-1	P1-43	TRUE	3	TRA:CAASIIQGAQKLVF TRB:CASSFPGQGILEQYF	23	CD8-C2
Pla-1_TGGGAAGTCCTTTCGG	Pla	Pla-1	P1-98	FALSE	3	TRA:CAVTAAGNKLTF TRB:CASSQVGGSEQFF	6	CD8-C2
Pla-1_TGGGCGTCAGACGCTC	Pla	Pla-1	P1-2257	FALSE	1	TRA:CAVSDQSTGKLIF TRB:CASSSRQGFPGGANVLTF	1	CD8-C2
Pla-1_TGGGCGTGTTGGTAAA	Pla	Pla-1	P1-2259	FALSE	1	TRA:CAVLNEKLTF TRB:CASRTLAGDGYEQFF	1	CD8-C2
Pla-1_TGGTTAGCACTTAACG	Pla	Pla-1	P1-161	FALSE	3	TRA:CVVTTSQFSGNTPLVF TRB:CASSLYSGALMNTEAFF	4	CD8-C2

Pla-1_TGTATTCGTACTCAAC	Pla	Pla-1	P1-34	FALSE	3	TRA:CAMSGGGADGLTF TRB:CASNVGVHEQFF	41	CD8-C2
Pla-1_TGTGGTACACCGGAAA	Pla	Pla-1	P1-76	FALSE	3	TRA:CAVRVMEYGNKLVF TRB:CASSFGTGFEQYF	8	CD8-C2
Pla-1_TGTGGTATCAGCGATT	Pla	Pla-1	P1-34	FALSE	3	TRA:CAMSGGGADGLTF TRB:CASNVGVHEQFF	41	CD8-C2
Pla-1_TGTGTTTAGCGTTTAC	Pla	Pla-1	P1-3	TRUE	3	TRA:CAFLSGTYKYIF TRB:CASSQSPGQRNYGYTF	308	CD8-C2
Pla-1_TGTGTTTTCCTTGGTC	Pla	Pla-1	P1-2272	FALSE	1	TRA:CAASVGRDIYDMRF TRB:CASTAGGNTGELFF	1	CD8-C2
Pla-1_TTAACTCTCGGAAACG	Pla	Pla-1	P1-38	TRUE	3	TRA:CAFTAGGATNKLIF TRB:CASIEGQGPYNEQFF	38	CD8-C2
Pla-1_TTAGGACCAGTCCTTC	Pla	Pla-1	P1-15	TRUE	3	TRA:CAVNSSPLGANSKLTF TRB:CASSDSGNFYNEQFF	92	CD8-C2
Pla-1_TTAGGACGTGTTCTTT	Pla	Pla-1	P1-43	TRUE	3	TRA:CAASIIQGAQKLVF TRB:CASSFPGQGILEQYF	23	CD8-C2
Pla-1_TTAGGCAAGTTAACGA	Pla	Pla-1	P1-34	FALSE	3	TRA:CAMSGGGADGLTF TRB:CASNVGVHEQFF	41	CD8-C2
Pla-1_TTCGAAGTCTGTTTGT	Pla	Pla-1	P1-213	FALSE	3	TRA:CAMSSNQAGTALIF TRB:CASSLGQGRRAEAFF	3	CD8-C2
Pla-1_TTCTACACAAACTGTC	Pla	Pla-1	P1-15	TRUE	3	TRA:CAVNSSPLGANSKLTF TRB:CASSDSGNFYNEQFF	92	CD8-C2
Pla-1_TTCTCCTGTCAGAGGT	Pla	Pla-1	P1-88	TRUE	3	TRA:CAVRDRGSGNTPLVF TRB:CASSGDRVYEQYF	6	CD8-C2
Pla-1_TTCTCCTTCCGCGCAA	Pla	Pla-1	P1-103	FALSE	3	TRA:CATENDYKLSF TRB:CSVGGTFANTGELFF	6	CD8-C2
Pla-1_TTGCCGTAGTAGATGT	Pla	Pla-1	P1-166	FALSE	3	TRA:CAVDGYNKLIF TRB:CASRPDLDRVQETQYF	4	CD8-C3
Pla-1_TTGCGTCAGGCTCAGA	Pla	Pla-1	P1-15	TRUE	3	TRA:CAVNSSPLGANSKLTF TRB:CASSDSGNFYNEQFF	92	CD8-C2
Pla-1_TTTATGCCACCAGCAC	Pla	Pla-1	P1-15	TRUE	3	TRA:CAVNSSPLGANSKLTF TRB:CASSDSGNFYNEQFF	92	CD8-C2
Pla-1_TTTGCGCGTAGTGAAT	Pla	Pla-1	P1-101	FALSE	3	TRA:CAVIRPAGGATNKLIF TRB:CASSLGTGGDTQYF	6	CD8-C2
Pla-1_TTTGGTTAGCTGAACG	Pla	Pla-1	P1-95	TRUE	3	TRA:CIVRVARGNEKLTF TRB:CASSLYGEGHQPQHF	6	CD8-C2
Pla-1_TTTGTCATCTTATCTG	Pla	Pla-1	P1-38	TRUE	3	TRA:CAFTAGGATNKLIF TRB:CASIEGQGPYNEQFF	38	CD8-C2
Pla-1_AACACGTCAGATTGCT	Pla	Pla-1	P1-28	TRUE	3	TRA:CAVKKGTGRRALTF TRB:CASETGQGTDTQYF	53	CD4-C3
Pla-1_AACCATGTCTCGCTTG	Pla	Pla-1	P1-1097	FALSE	1	TRA:CAFMTLITGANSKLTF TRB:CASSFGDTYNEQFF	1	CD4-C3
Pla-1_AACCGCGTCCACGACG	Pla	Pla-1	P1-156	FALSE	3	TRA:CATDRYNNNDMRF TRB:CASSLTSGERGNTGELFF	4	CD4-C3
Pla-1_AACTCAGGTGATGTGG	Pla	Pla-1	P1-1101	FALSE	1	TRA:CAVPRGGTSYGKLTF TRB:CASSLAGRRYTDTQYF	1	CD4-C3
Pla-1_AACTGGTAGAGACTAT	Pla	Pla-1	P1-1107	FALSE	1	TRA:CAVRTNTGRRALTF TRB:CATATGTSNTGELFF	1	CD4-C3
Pla-1_AACTTTCCAGGGATTG	Pla	Pla-1	P1-1111	FALSE	1	TRA:CAVTRGGGGNKLTF TRB:CASSLNSGEIIETQYF	1	CD4-C3
Pla-1_AAGACCTAGAGTCTGG	Pla	Pla-1	P1-1112	FALSE	1	TRA:CAENQGGSEKLVF TRB:CASSLRGPGQGAVAFF	1	CD4-C3
Pla-1_ACACTGAGTGCTGTAT	Pla	Pla-1	P1-1147	FALSE	1	TRA:CAVNVYNFNKFYF TRB:CASSLGGTGELFF	1	CD4-C3

Pla-1_ACAGCTATCATGCAAC	Pla	Pla-1	P1-1155	FALSE	1	TRA:CAGVNKFYF TRB:CASSLDLAGMEETQYF	1	CD4-C4
Pla-1_ACCGTAAGTAGCGTAG	Pla	Pla-1	P1-95	TRUE	3	TRA:CIVRVARGNEKLTF TRB:CASSLYGEGHQPQHF	6	CD4-C5
Pla-1_ACGAGGAGTCTACCTC	Pla	Pla-1	P1-1180	FALSE	1	TRA:CGTELSNAGNMLTF TRB:CASSRQTGANVLTF	1	CD4-C3
Pla-1_ACGGGCTCAGCGTTCG	Pla	Pla-1	P1-1205	FALSE	1	TRA:CAVTRNFGNEKLTF TRB:CASSRPDGGSYEQYF	1	CD4-C3
Pla-1_ACGTCAATCCTAGAAC	Pla	Pla-1	P1-1210	FALSE	1	TRA:CAETLQDMRF TRB:CASSVEPSGAEAFF	1	CD4-C3
Pla-1_AGAATAGCACACTGCG	Pla	Pla-1	P1-328	FALSE	2	TRA:CALSPQGGSEKLVF TRB:CSGSEGTDTQYF	2	CD4-C3
Pla-1_AGACGTTAGGCAGGTT	Pla	Pla-1	P1-1243	FALSE	1	TRA:CAVNLAGSYNNNDMRF TRB:CASSLGGGAYEQYF	1	CD4-C3
Pla-1_AGAGCTTAGCCCAACC	Pla	Pla-1	P1-160	FALSE	3	TRA:CAASADGGSQGNLIF TRB:CASSPTYGYTF	4	CD4-C3
Pla-1_AGAGTGGGTAGGCATG	Pla	Pla-1	P1-1252	FALSE	1	TRA:CALYGQNFVF TRB:CASSRLAGAVDTQYF	1	CD4-C3
Pla-1_AGCAGCCAGCGACGTA	Pla	Pla-1	P1-1262	FALSE	1	TRA:CAASLETSGSRLTF TRB:CASRAQLNSPLHF	1	CD4-C4
Pla-1_AGCAGCCGTAACGTTC	Pla	Pla-1	P1-1263	FALSE	1	TRA:CAYRSAYSGGSNYKLTF TRB:CASSLDRGGYNEQFF	1	CD4-C3
Pla-1_AGCATACAGCGACGTA	Pla	Pla-1	P1-333	FALSE	2	TRA:CAMRAGGGNKLTF TRB:CAAGVGNTEAFF	2	CD4-C3
Pla-1_AGCTTGAAGAGAGCTC	Pla	Pla-1	P1-322	FALSE	2	TRA:CAVSEVYAGGYNKLIF TRB:CASSIARWTSGGASEQFF	2	CD4-C3
Pla-1_AGGGTGACACGCGAAA	Pla	Pla-1	P1-28	TRUE	3	TRA:CAVKKGTGRRALTF TRB:CASETGQGTDTQYF	53	CD4-C3
Pla-1_AGTGAGGAGATATGCA	Pla	Pla-1	P1-153	TRUE	3	TRA:CAVKPLDTGRRALTF TRB:CATSLRVTDTQYF	4	CD4-C3
Pla-1_AGTGTCACATGGAATA	Pla	Pla-1	P1-1302	FALSE	1	TRA:CVVSGGSQGNLIF TRB:CSAVAGGSYEQYF	1	CD4-C5
Pla-1_AGTGTCACATTAACCG	Pla	Pla-1	P1-78	TRUE	3	TRA:CAAAQGGSEKLVF TRB:CASTRGDWIYNEQFF	7	CD4-C3
Pla-1_AGTTGGTCAAGGACTG	Pla	Pla-1	P1-1306	FALSE	1	TRA:CAMREVSTGFQKLVF TRB:CASSLDPTGTGELFF	1	CD4-C4
Pla-1_ATCACGAAGGTGTGGT	Pla	Pla-1	P1-1318	FALSE	1	TRA:CAVIYGGATNKLIF TRB:CASSPSLNTEAFF	1	CD4-C4
Pla-1_ATCGAGTCATCGGAAG	Pla	Pla-1	P1-1331	FALSE	1	TRA:CAVNNPDGQKLLF TRB:CASSVGRSAKNIQYF	1	CD4-C3
Pla-1_ATGAGGGAGCCTATGT	Pla	Pla-1	P1-1341	FALSE	1	TRA:CAVGAPGDDKIIF TRB:CSATGTTAGEQFF	1	CD4-C3
Pla-1_ATTGGTGGTCTCCACT	Pla	Pla-1	P1-226	FALSE	3	TRA:CAAVSAFSGGYNKLIF TRB:CASSLAGRAATNEKLFF	3	CD4-C3
Pla-1_CAAGAAAAGCTTTGGT	Pla	Pla-1	P1-1370	FALSE	1	TRA:CAAMNDYKLSF TRB:CASSQASGRGALNYGYTF	1	CD4-C3
Pla-1_CAAGAAACAGGGATTG	Pla	Pla-1	P1-354	FALSE	2	TRA:CAVRQIRSSNTGKLIF TRB:CASSSGLENNEQFF	2	CD4-C3
Pla-1_CAAGATCGTGTGCGTC	Pla	Pla-1	P1-1375	FALSE	1	TRA:CIVDARLMF TRB:CASSSSGGGRTNNEQFF	1	CD4-C6
Pla-1_CACAGTAGTCATCCCT	Pla	Pla-1	P1-357	FALSE	2	TRA:CASPRENYGGATNKLIF TRB:CSMGQSSIETQYF	2	CD4-C3
Pla-1_CACCTTGTCAGTGTTG	Pla	Pla-1	P1-1410	FALSE	1	TRA:CVVSLAGGADGLTF TRB:CSARDGGVSAYGYTF	1	CD4-C3

Pla-1_CAGCTAAGTAACGTTC	Pla	Pla-1	P1-1426	FALSE	1	TRA:CAGHGIGGGYQKVTF TRB:CSMGQSSIETQYF	1	CD4-C3
Pla-1_CATCAGAAGGTGATTA	Pla	Pla-1	P1-1439	FALSE	1	TRA:CAELKLNSGYSTLTF TRB:CASSLAGNYGYTF	1	CD4-C3
Pla-1_CATCGGGTCAACACCA	Pla	Pla-1	P1-1451	FALSE	1	TRA:CATWRDFGNVLHC TRB:CASSLESVQPQHF	1	CD4-C3
Pla-1_CATCGGGTCCGCGGTA	Pla	Pla-1	P1-1452	FALSE	1	TRA:CAVIRSNYQLIW TRB:CASSLLGGSNQPQHF	1	CD4-C3
Pla-1_CATTCGCGTATAATGG	Pla	Pla-1	P1-1470	FALSE	1	TRA:CAVRLKNAGGTSYGKLTF TRB:CASSPHPGRDSYEQYF	1	CD4-C3
Pla-1_CCAATCCCAACACGCC	Pla	Pla-1	P1-371	FALSE	2	TRA:CAVSDNAGNNRKLIW TRB:CASSLRASSTDTQYF	2	CD4-C3
Pla-1_CCACTACAGACACGAC	Pla	Pla-1	P1-169	FALSE	3	TRA:CAYRSANNDMRF TRB:CASRGQLYGNQPQHF	4	CD4-C5
Pla-1_CCCAGTTAGTGCCATT	Pla	Pla-1	P1-1492	FALSE	1	TRA:CAASAIVGAGSYQLTF TRB:CASTRGQYNSPLHF	1	CD8-C9
Pla-1_CCGGGATAGAAACCTA	Pla	Pla-1	P1-1495	FALSE	1	TRA:CAAYNYNQGGKLIF TRB:CASSPGTRPSGYTF	1	CD4-C3
Pla-1_CCTAAAGGTTCGTGAT	Pla	Pla-1	P1-1508	FALSE	1	TRA:CAGSDKLIF TRB:CSARVRDRDYNSPLHF	1	CD4-C3
Pla-1_CCTACACCATAAGACA	Pla	Pla-1	P1-375	FALSE	2	TRA:CALSVRTARQLTF TRB:CASSQTGDSNQPQHF	2	CD4-C3
Pla-1_CCTACCATCCGAGCCA	Pla	Pla-1	P1-189	TRUE	3	TRA:CAPGLLF TRB:CASSLSHQGSPLHF	3	CD4-C5
Pla-1_CCTCAGTCACATAACC	Pla	Pla-1	P1-1521	FALSE	1	TRA:CAASTNSGNTPLVF TRB:CASSPNGGDTQYF	1	CD4-C3
Pla-1_CCTCTGACAGCCAGAA	Pla	Pla-1	P1-237	FALSE	3	TRA:CAVNMGGGSYIPTF TRB:CSVEETRSMYF	3	CD4-C3
Pla-1_CGACCTTAGAGGGCTT	Pla	Pla-1	P1-1544	FALSE	1	TRA:CVVSAPNAGKSTF TRB:CASSSRDSGPQHF	1	CD4-C6
Pla-1_CGACTTCCATGGAATA	Pla	Pla-1	P1-1548	FALSE	1	TRA:CVVSDTGRRALTF TRB:CASSPTSGRAIDTQYF	1	CD4-C5
Pla-1_CGAGAAGAGCATCATC	Pla	Pla-1	P1-1550	FALSE	1	TRA:CAVYNTNAGKSTF TRB:CASRKGDLRDTGYTF	1	CD4-C3
Pla-1_CGAGAAGGTGAACCTT	Pla	Pla-1	P1-1554	FALSE	1	TRA:CVVKRGTSYDKVIF TRB:CAISETGREQYF	1	CD4-C5
Pla-1_CGAGCACGTACTTGAC	Pla	Pla-1	P1-378	FALSE	2	TRA:CAVRPRSNDYKLSF TRB:CASSPDRQGKTEAFF	2	CD4-C3
Pla-1_CGCCAAGGTAGTACCT	Pla	Pla-1	P1-28	TRUE	3	TRA:CAVKKGTGRRALTF TRB:CASETGQGTDTQYF	53	CD4-C3
Pla-1_CGCTTCAAGACAGAGA	Pla	Pla-1	P1-1577	FALSE	1	TRA:CAVRDAGFGNVLHC TRB:CASSRVGGQSTDTQYF	1	CD4-C3
Pla-1_CGCTTCAGTACTCGCG	Pla	Pla-1	P1-1578	FALSE	1	TRA:CAVQVVSDGQKLLF TRB:CASSLGRLAGNTGELFF	1	CD4-C3
Pla-1_CGGAGCTTCATGTAGC	Pla	Pla-1	P1-1592	FALSE	1	TRA:CAVEGDYKLSF TRB:CASSENGHDTGELFF	1	CD4-C3
Pla-1_CGGCTAGTCAAGCCTA	Pla	Pla-1	P1-241	FALSE	3	TRA:CAYRRNTGNQFYF TRB:CASSLVPRGGNIYEQYF	3	CD4-C3
Pla-1_CGTAGGCTCACTGGGC	Pla	Pla-1	P1-355	FALSE	2	TRA:CALDGGQKLLF TRB:CASSEPDRGRSYEQYF	2	CD4-C3
Pla-1_CGTCAGGAGACATAAC	Pla	Pla-1	P1-181	TRUE	3	TRA:CAVTRVMGTYKYIF TRB:CASGTQNSPLHF	3	CD4-C3
Pla-1_CTACACCCATCAGTCA	Pla	Pla-1	P1-389	FALSE	2	TRA:CAASSRGSARQLTF TRB:CASSLSQGRETQYF	2	CD4-C5

Pla-1_CTAGTGAGTCACAAGG	Pla	Pla-1	P1-1659	FALSE	1	TRA:CALNGNTDKLIF TRB:CASRDRAGGPYNEQFF	1	CD4-C3
Pla-1_CTCGAGGAGGGATACC	Pla	Pla-1	P1-1683	FALSE	1	TRA:CALRAAYNQGGKLIF TRB:CASSLGGTATGELFF	1	CD4-C3
Pla-1_CTCGTACGTAGATTAG	Pla	Pla-1	P1-1695	FALSE	1	TRA:CAVEKAAGNKLTF TRB:CASRTGNAEAFF	1	CD4-C3
Pla-1_CTGATAGTCGGAAATA	Pla	Pla-1	P1-1721	FALSE	1	TRA:CVVSGSVSAGNMLTF TRB:CAISVGQGPSGNTIYF	1	CD4-C3
Pla-1_CTGATCCAGCTAGCCC	Pla	Pla-1	P1-395	FALSE	2	TRA:CVDPSKYIF TRB:CASSIRVSKGTDTQYF	2	CD8-C9
Pla-1_CTGGTCTTCAACTCTT	Pla	Pla-1	P1-1735	FALSE	1	TRA:CALRSLGNEKLTF TRB:CASSFDRAPSPLHF	1	CD4-C3
Pla-1_CTGGTCTTCACATACG	Pla	Pla-1	P1-1736	FALSE	1	TRA:CATPPPFIIQGAQKLVF TRB:CSAEIVLANYGYTF	1	CD4-C3
Pla-1_CTGGTCTTCCTAGAAC	Pla	Pla-1	P1-1737	FALSE	1	TRA:CAVQLTGNQFYF TRB:CASSPIPLSGGQAMGDTQYF	1	CD4-C3
Pla-1_CTTACCGCACGAGAGT	Pla	Pla-1	P1-1748	FALSE	1	TRA:CAVADNAGNMLTF TRB:CASSLVLAGYNEQFF	1	CD4-C3
Pla-1_GAAATGACAAGGTTTC	Pla	Pla-1	P1-1762	FALSE	1	TRA:CALARDDKIIF TRB:CSYGRDRNYGYTF	1	CD4-C4
Pla-1_GAATAAGAGTAGGCCA	Pla	Pla-1	P1-245	FALSE	3	TRA:CAVASSNDYKLSF TRB:CASSSPNEKLFF	3	CD4-C3
Pla-1_GACGGCTGTTAAGACA	Pla	Pla-1	P1-1794	FALSE	1	TRA:CAARRLGTSYGKLTF TRB:CASSPDSNYGYTF	1	CD4-C3
Pla-1_GACTACACAAGACGTG	Pla	Pla-1	P1-1809	FALSE	1	TRA:CIVKVPGNTGKLIF TRB:CRVGVPLSSESPLHF	1	CD4-C3
Pla-1_GATCGATAGATTACCC	Pla	Pla-1	P1-1824	FALSE	1	TRA:CAVGLTGGGNKLTF TRB:CASSLQLGQPEAFF	1	CD4-C4
Pla-1_GATCGCGAGAGAACAG	Pla	Pla-1	P1-1828	FALSE	1	TRA:CASTARTYKYIF TRB:CASSQPPMNRDRGLRTDTQYF	1	CD4-C3
Pla-1_GATGAAACAGCCTGTG	Pla	Pla-1	P1-391	FALSE	2	TRA:CGTENMGNQFYF TRB:CASSVGDGVEAFF	2	CD4-C6
Pla-1_GATGAAACATGGAATA	Pla	Pla-1	P1-153	TRUE	3	TRA:CAVKPLDTGRRALTF TRB:CATSLRVTDTQYF	4	CD4-C3
Pla-1_GCAGCCAAGCGTAATA	Pla	Pla-1	P1-153	TRUE	3	TRA:CAVKPLDTGRRALTF TRB:CATSLRVTDTQYF	4	CD4-C4
Pla-1_GCATACACATGTAAGA	Pla	Pla-1	P1-1856	FALSE	1	TRA:CAMFLSNFGNEKLTF TRB:CASSRQGAPDTQYF	1	CD4-C3
Pla-1_GCATGCGCAGTCGATT	Pla	Pla-1	P1-1860	FALSE	1	TRA:CAVRQIRSSNTGKLIF TRB:CSAGTSGNIQYF	1	CD4-C3
Pla-1_GCCAAATTCGTCGTTC	Pla	Pla-1	P1-241	FALSE	3	TRA:CAYRRNTGNQFYF TRB:CASSLVPRGGNIYEQYF	3	CD4-C3
Pla-1_GCGCAGTCACACCGAC	Pla	Pla-1	P1-1873	FALSE	1	TRA:CAVANNAGNMLTF TRB:CASTEGKEKLFF	1	CD4-C3
Pla-1_GCGGGTTTCCTGTAGA	Pla	Pla-1	P1-299	TRUE	2	TRA:CAFMKATGTASKLTF TRB:CASSSPGLAGVSYNEQFF	2	CD4-C2
Pla-1_GCTCCTATCGGGAGTA	Pla	Pla-1	P1-241	FALSE	3	TRA:CAYRRNTGNQFYF TRB:CASSLVPRGGNIYEQYF	3	CD4-C3
Pla-1_GCTGCAGTCAACGCTA	Pla	Pla-1	P1-378	FALSE	2	TRA:CAVRPRSNDYKLSF TRB:CASSPDRQGKTEAFF	2	CD4-C3
Pla-1_GCTGGGTCAATACGCT	Pla	Pla-1	P1-357	FALSE	2	TRA:CASPRENYGGATNKLIF TRB:CSMGQSSIETQYF	2	CD4-C3
Pla-1_GCTGGGTCAATGTAAG	Pla	Pla-1	P1-1892	FALSE	1	TRA:CAVRDIGGATNKLIF TRB:CATSRADLAGGGYNEQFF	1	CD4-C3

Pla-1_GCTTCCAGTGCCTTGG	Pla	Pla-1	P1-1897	FALSE	1	TRA:CAVSDKSGGGADGLTF TRB:CASSRLARTDTQYF	1	CD4-C3
Pla-1_GCTTGAACAGTTAACC	Pla	Pla-1	P1-1899	FALSE	1	TRA:CAVSKGNTPLVF TRB:CSASPEASGAGGYTF	1	CD4-C4
Pla-1_GGAACTTGTAGGCATG	Pla	Pla-1	P1-169	FALSE	3	TRA:CAYRSANNDMRF TRB:CASRGQLYGNQPQHF	4	CD4-C6
Pla-1_GGACAAGCACAGACAG	Pla	Pla-1	P1-338	FALSE	2	TRA:CAEKELTF TRB:CASTSFETEAFF	2	CD4-C5
Pla-1_GGAGCAATCGCGTTTC	Pla	Pla-1	P1-34	FALSE	3	TRA:CAMSGGGADGLTF TRB:CASNVGVHEQFF	41	CD8-C10
Pla-1_GGCGACTTCTCCCTGA	Pla	Pla-1	P1-1931	FALSE	1	TRA:CAVGAGNQGGKLIF TRB:CANSRQTMNTEAFF	1	CD4-C3
Pla-1_GGCTCGATCGATAGAA	Pla	Pla-1	P1-1935	FALSE	1	TRA:CAASRRDSSYKLIF TRB:CASSADRGRNTEAFF	1	CD4-C4
Pla-1_GGGTTGCCATTACCTT	Pla	Pla-1	P1-1957	FALSE	1	TRA:CAPLPQGGSEKLVF TRB:CASSPAGTVNSPLHF	1	CD4-C3
Pla-1_GGTGAAGAGGCGATAC	Pla	Pla-1	P1-1960	FALSE	1	TRA:CIVRVVSSYDKVIF TRB:CASSFGVSTEAFF	1	CD4-C5
Pla-1_GGTGAAGTCCGCGCAA	Pla	Pla-1	P1-1963	FALSE	1	TRA:CAVSPRKDTGRRALTF TRB:CASSSNRGLNEKLFF	1	CD4-C4
Pla-1_GTACTCCAGCGCCTTG	Pla	Pla-1	P1-1981	FALSE	1	TRA:CAVNYLDSSYKLIF TRB:CSQIKASGPSEAFF	1	CD8-C8
Pla-1_GTACTTTAGTTGAGAT	Pla	Pla-1	P1-1984	FALSE	1	TRA:CAMSGGGADGLTF TRB:CASSQGFSYGYTF	1	CD4-C4
Pla-1_GTAGTCAGTTGATTCG	Pla	Pla-1	P1-263	TRUE	2	TRA:CAASKHSWGKLQF TRB:CSAGISSGGRYEQYF	2	CD4-C3
Pla-1_GTCACAAGTACGACCC	Pla	Pla-1	P1-1998	FALSE	1	TRA:CAVTSNQGGKLIF TRB:CASTPRDSTDTQYF	1	CD4-C5
Pla-1_GTCCTCACAGCTCCGA	Pla	Pla-1	P1-156	FALSE	3	TRA:CATDRYNNNDMRF TRB:CASSLTSGERGNTGELFF	4	CD4-C3
Pla-1_GTCCTCATCATTTGGG	Pla	Pla-1	P1-21	TRUE	3	TRA:CAASRGTGANNLFF TRB:CATQREGREQYF	67	CD4-C3
Pla-1_GTCTCGTAGACCTAGG	Pla	Pla-1	P1-246	FALSE	3	TRA:CAVVPTPDSWGKLQF TRB:CASSVGRTGQETQYF	3	CD4-C5
Pla-1_GTCTTCGAGCCAGTAG	Pla	Pla-1	P1-156	FALSE	3	TRA:CATDRYNNNDMRF TRB:CASSLTSGERGNTGELFF	4	CD4-C3
Pla-1_GTCTTCGCACGAGGTA	Pla	Pla-1	P1-2013	FALSE	1	TRA:CAMSYSGNTPLVF TRB:CATSESLAGSSGELFF	1	CD4-C3
Pla-1_GTGCATAAGTAACCCT	Pla	Pla-1	P1-2020	FALSE	1	TRA:CAVSPRGSSYKLIF TRB:CASSLGPVSQETQYF	1	CD4-C4
Pla-1_GTGCTTCTCCGCGGTA	Pla	Pla-1	P1-2029	FALSE	1	TRA:CAVTSSGGSNYKLTF TRB:CASSGTGGTMETQYF	1	CD4-C3
Pla-1_GTTAAGCCAGCTCGCA	Pla	Pla-1	P1-28	TRUE	3	TRA:CAVKKGTGRRALTF TRB:CASETGQGTDTQYF	53	CD4-C4
Pla-1_GTTAAGCTCATGCAAC	Pla	Pla-1	P1-2043	FALSE	1	TRA:CATDEEQGAQKLVF TRB:CASSPLWSTYNSPLHF	1	CD4-C4
Pla-1_GTTCGGGCAGGGTATG	Pla	Pla-1	P1-2055	FALSE	1	TRA:CAARF TRB:CASSATSGSYNEQFF	1	CD4-C3
Pla-1_GTTCTCGTCAACGGCC	Pla	Pla-1	P1-224	FALSE	3	TRA:CAVGEGYSTLTF TRB:CASMKGQGGSPLHF	3	CD4-C3
Pla-1_TAAGAGACAGCCTTTC	Pla	Pla-1	P1-2065	FALSE	1	TRA:CAASARGGSEKLVF TRB:CASSQPGQRMNTEAFF	1	CD4-C3
Pla-1_TAAGCGTCATTTCACT	Pla	Pla-1	P1-2067	FALSE	1	TRA:CAVSRPRGFQKLVF TRB:CSARVVGPGETQYF	1	CD4-C3

Pla-1_TACGGATAGCCTTGAT	Pla	Pla-1	P1-106	TRUE	3	TRA:CAVIEGNQFYF TRB:CASSLTPGLNIQYF	5	CD4-C3
Pla-1_TACGGATTCGTATCAG	Pla	Pla-1	P1-398	FALSE	2	TRA:CAENKGGNMLTF TRB:CATPRGHRSGANVLTF	2	CD4-C4
Pla-1_TACTTACGTTCGGCAC	Pla	Pla-1	P1-2096	FALSE	1	TRA:CAVSDESDYKLSF TRB:CATSERGHGYTF	1	CD4-C4
Pla-1_TAGACCAAGTACCGGA	Pla	Pla-1	P1-2101	FALSE	1	TRA:CAVPPGNTGKLIF TRB:CASSIDGGSTDTQYF	1	CD4-C3
Pla-1_TAGTTGGTCTGGTGTA	Pla	Pla-1	P1-371	FALSE	2	TRA:CAVSDNAGNNRKLIW TRB:CASSLRASSTDTQYF	2	CD4-C3
Pla-1_TATCAGGGTTCCACAA	Pla	Pla-1	P1-2118	FALSE	1	TRA:CAASASGNTPLVF TRB:CASRARGRGEKLFF	1	CD4-C3
Pla-1_TATGCCCCATCGGACC	Pla	Pla-1	P1-169	FALSE	3	TRA:CAYRSANNDMRF TRB:CASRGQLYGNQPQHF	4	CD4-C3
Pla-1_TCAACGAAGCCCTAAT	Pla	Pla-1	P1-238	FALSE	3	TRA:CAVGPWGAQKLVF TRB:CATSPSRGNNEQFF	3	CD4-C3
Pla-1_TCAACGACAGGGATTG	Pla	Pla-1	P1-375	FALSE	2	TRA:CALSVRTARQLTF TRB:CASSQTGDSNQPQHF	2	CD4-C3
Pla-1_TCAACGAGTTGTGGAG	Pla	Pla-1	P1-2129	FALSE	1	TRA:CARISGNTPLVF TRB:CASSEVGHPNTEAFF	1	CD4-C3
Pla-1_TCAATCTAGACTAAGT	Pla	Pla-1	P1-2131	FALSE	1	TRA:CVVPNGRGRRGSTLGRLYF TRB:CSAPTKNTEAFF	1	CD4-C3
Pla-1_TCACGAACAAGCCCAC	Pla	Pla-1	P1-2138	FALSE	1	TRA:CAASLRGSARQLTF TRB:CSAGTSGGTGELFF	1	CD4-C3
Pla-1_TCGGTAAAGACGCTTT	Pla	Pla-1	P1-2164	FALSE	1	TRA:CAMSHGGSQGNLIF TRB:CASSSGNGADSPLHF	1	CD4-C3
Pla-1_TCGTAGAAGATGGGTC	Pla	Pla-1	P1-2169	FALSE	1	TRA:CAVGEKLTF TRB:CASRWRGEPYNEQFF	1	CD4-C3
Pla-1_TCTATTGAGCAGGCTA	Pla	Pla-1	P1-30	TRUE	3	TRA:CALPPGGNNRKLIW TRB:CASSFGDRGGTEAFF	47	CD4-C6
Pla-1_TCTCATAGTTCCTCCA	Pla	Pla-1	P1-2179	FALSE	1	TRA:CAMNSGAAGNKLTF TRB:CSVDGTYEQYF	1	CD4-C3
Pla-1_TCTTTCCCACGAGAGT	Pla	Pla-1	P1-2190	FALSE	1	TRA:CAEKDSRGSGNTGKLIF TRB:CASRPTGGGGYTF	1	CD4-C3
Pla-1_TGCGGGTTCAGAAATG	Pla	Pla-1	P1-2234	FALSE	1	TRA:CIVRVRGNYQLIW TRB:CATSRDRGGRSQETQYF	1	CD4-C5
Pla-1_TGCGGGTTCTGCGACG	Pla	Pla-1	P1-2235	FALSE	1	TRA:CALRPISTDKLIF TRB:CASSLGSAGANVLTF	1	CD4-C3
Pla-1_TGCGTGGTCCAAGCCG	Pla	Pla-1	P1-2237	FALSE	1	TRA:CAVRDGTGFQKLVF TRB:CSARVVGTTDTQYF	1	CD4-C3
Pla-1_TGGACGCCACCGTTGG	Pla	Pla-1	P1-423	FALSE	2	TRA:CAENRNSGYSTLTF TRB:CASSGGGQGWETQYF	2	CD4-C3
Pla-1_TGGACGCGTGTTTGTG	Pla	Pla-1	P1-243	FALSE	3	TRA:CALSETSGGSNYKLTF TRB:CASSTRLAGGRISYNEQFF	3	CD4-C3
Pla-1_TGGCTGGTCAACTCTT	Pla	Pla-1	P1-2254	FALSE	1	TRA:CAGLHNNARLMF TRB:CASSPPIQLLYNEQFF	1	CD4-C3
Pla-1_TGTGGTATCACCGTAA	Pla	Pla-1	P1-423	FALSE	2	TRA:CAENRNSGYSTLTF TRB:CASSGGGQGWETQYF	2	CD4-C3
Pla-1_TTATGCTAGTAGGCCA	Pla	Pla-1	P1-237	FALSE	3	TRA:CAVNMGGGSYIPTF TRB:CSVEETRSMYF	3	CD4-C3
Pla-1_TTCCCAGTCTCAACTT	Pla	Pla-1	P1-2295	FALSE	1	TRA:CAVDTGGFKTIF TRB:CASSPSLAGGGETQYF	1	CD4-C3
Pla-1_TTGACTTGTGTGCCTG	Pla	Pla-1	P1-28	TRUE	3	TRA:CAVKKGTGRRALTF TRB:CASETGQGTDTQYF	53	CD8-C10

Pla-1_TTGCCGTTCGCGTTTC	Pla	Pla-1	P1-28	TRUE	3	TRA:CAVKKGTGRRALTF TRB:CASETGQGTDTQYF	53	CD4-C3
Pla-1_TTTGCGCTCTGCTGTC	Pla	Pla-1	P1-2338	FALSE	1	TRA:CALDSGSSNTGKLIF TRB:CSAHPPGNTIYF	1	CD4-C3
Pla-1_TTTGGTTCAGGGCATA	Pla	Pla-1	P1-129	FALSE	3	TRA:CAFLFYGGSQGNLIF TRB:CASSQGRGRYGNTIYF	5	CD4-C3
Pla-1_TTTGTCACAGGAATCG	Pla	Pla-1	P1-159	FALSE	3	TRA:CAVQGPYKAAGNKLTF TRB:CASSRDLGYEQYF	4	CD4-C4
Pla-1_AAATGCCAGTTATCGC	Pla	Pla-1	P1-304	FALSE	2	TRA:CAVRDGDYKLSF TRB:CASSYTSGGAETQYF	2	CD8-C10
Pla-1_AAGGCAGAGGCCGAAT	Pla	Pla-1	P1-1120	FALSE	1	TRA:CAIMDSNYQLIW TRB:CASSDGDRASQPQHF	1	CD8-C10
Pla-1_AATCCAGGTTTGTGTG	Pla	Pla-1	P1-209	FALSE	3	TRA:CAVGDNAGNMLTF TRB:CASSQTSDTGELFF	3	CD8-C10
Pla-1_AATCCAGTCAGCCTAA	Pla	Pla-1	P1-1133	FALSE	1	TRA:CAALDSNYQLIW TRB:CASSYGQGATDTQYF	1	CD8-C10
Pla-1_ACAGCTAAGGGTGTGT	Pla	Pla-1	P1-70	FALSE	3	TRA:CAMRETPGEAGTALIF TRB:CSARITYLNTEAFF	9	CD4-C4
Pla-1_ACATCAGCAATGGACG	Pla	Pla-1	P1-1163	FALSE	1	TRA:CAVMDSNYQLIW TRB:CASSQDPSGGYEQYF	1	CD8-C10
Pla-1_ACATGGTCATAACCTG	Pla	Pla-1	P1-28	TRUE	3	TRA:CAVKKGTGRRALTF TRB:CASETGQGTDTQYF	53	CD4-C4
Pla-1_ACGCCGATCAACGGGA	Pla	Pla-1	P1-1197	FALSE	1	TRA:CAPSRDSNYQLIW TRB:CASSDNARDTGELFF	1	CD8-C10
Pla-1_ACGGGCTAGACCTAGG	Pla	Pla-1	P1-28	TRUE	3	TRA:CAVKKGTGRRALTF TRB:CASETGQGTDTQYF	53	CD4-C3
Pla-1_ACTGAGTCAAGCGATG	Pla	Pla-1	P1-28	TRUE	3	TRA:CAVKKGTGRRALTF TRB:CASETGQGTDTQYF	53	CD8-C2
Pla-1_AGCGGTCTCGAACGGA	Pla	Pla-1	P1-1269	FALSE	1	TRA:CALMDSSYKLIF TRB:CASSELAGGQDTQYF	1	CD8-C10
Pla-1_AGGGATGTCAACACTG	Pla	Pla-1	P1-336	FALSE	2	TRA:CAVSDNDYKLSF TRB:CASSEFSGGANEQFF	2	CD8-C10
Pla-1_ATGTGTGCATTCGACA	Pla	Pla-1	P1-350	FALSE	2	TRA:CAPLDSNYQLIW TRB:CASSLDREATEAFF	2	CD8-C10
Pla-1_ATTACTCAGGTGGGTT	Pla	Pla-1	P1-28	TRUE	3	TRA:CAVKKGTGRRALTF TRB:CASETGQGTDTQYF	53	CD8-C10
Pla-1_CAACTAGAGGATGGAA	Pla	Pla-1	P1-1366	FALSE	1	TRA:CAVMDSNYQLIW TRB:CASRPQGDGANVLTF	1	CD8-C10
Pla-1_CAGCTGGTCAGCTCTC	Pla	Pla-1	P1-1429	FALSE	1	TRA:CAVRDSNYQLIW TRB:CSARWDRDYEQYF	1	CD8-C10
Pla-1_CAGTAACGTCGAGATG	Pla	Pla-1	P1-28	TRUE	3	TRA:CAVKKGTGRRALTF TRB:CASETGQGTDTQYF	53	CD4-C3
Pla-1_CATCAGAGTCTGATCA	Pla	Pla-1	P1-1441	FALSE	1	TRA:CLYLLYAYSGAGSYQLTF TRB:CASSQSPGQRNYGYTF	1	CD8-C10
Pla-1_CATCGGGTCAAAGTAG	Pla	Pla-1	P1-1450	FALSE	1	TRA:CALSDSNYQLIW TRB:CSARELLAGDTGELFF	1	CD8-C10
Pla-1_CATGACAGTACCGCTG	Pla	Pla-1	P1-28	TRUE	3	TRA:CAVKKGTGRRALTF TRB:CASETGQGTDTQYF	53	CD8-C10
Pla-1_CATTATCTCATGGTCA	Pla	Pla-1	P1-28	TRUE	3	TRA:CAVKKGTGRRALTF TRB:CASETGQGTDTQYF	53	CD4-C4
Pla-1_CCAGCGAAGTTCGCGC	Pla	Pla-1	P1-1483	FALSE	1	TRA:CAVMDSNYQLIW TRB:CASSEQGDTGELFF	1	CD8-C10
Pla-1_CCTTCCCAGACTACAA	Pla	Pla-1	P1-1531	FALSE	1	TRA:CAQVSGGYNKLIF TRB:CASSYSEFPEQYF	1	CD8-C10

Pla-1_CGATCGGCACTTCTGC	Pla	Pla-1	P1-379	FALSE	2	TRA:CAVMDSSYKLIF TRB:CASSERENTEAFF	2	CD8-C10
Pla-1_CGGAGCTGTCGAATCT	Pla	Pla-1	P1-383	FALSE	2	TRA:CAVRDRDYKLSF TRB:CASSELAGVQETQYF	2	CD8-C10
Pla-1_CGGTTAAGTCGTTGTA	Pla	Pla-1	P1-1601	FALSE	1	TRA:CAVRDSNYQLIW TRB:CASSDTSDETDTQYF	1	CD8-C10
Pla-1_CGTAGCGCAGGTCCAC	Pla	Pla-1	P1-383	FALSE	2	TRA:CAVRDRDYKLSF TRB:CASSELAGVQETQYF	2	CD8-C10
Pla-1_CGTCCATGTAGAGTGC	Pla	Pla-1	P1-28	TRUE	3	TRA:CAVKKGTGRRALTF TRB:CASETGQGTDTQYF	53	CD4-C4
Pla-1_CTCATTAAGCTGCGAA	Pla	Pla-1	P1-209	FALSE	3	TRA:CAVGDNAGNMLTF TRB:CASSQTSDTGELFF	3	CD8-C10
Pla-1_CTCGGGACATCACCCT	Pla	Pla-1	P1-28	TRUE	3	TRA:CAVKKGTGRRALTF TRB:CASETGQGTDTQYF	53	CD8-C10
Pla-1_CTGATAGTCCGCTGTT	Pla	Pla-1	P1-336	FALSE	2	TRA:CAVSDNDYKLSF TRB:CASSEFSGGANEQFF	2	CD8-C10
Pla-1_GACGTGCTCGGCCGAT	Pla	Pla-1	P1-401	FALSE	2	TRA:CAVRDSNYQLIW TRB:CASSQTSDTGELFF	2	CD8-C10
Pla-1_GAGCAGATCACCGTAA	Pla	Pla-1	P1-1814	FALSE	1	TRA:CAVRDNNSGYSTLTF TRB:CASSPGTSGGANEQFF	1	CD8-C10
Pla-1_GATCGATGTCAATACC	Pla	Pla-1	P1-1826	FALSE	1	TRA:CAVMDSNYQLIW TRB:CASKTGTGDSQPQHF	1	CD8-C10
Pla-1_GATCGCGAGAGAGCTC	Pla	Pla-1	P1-28	TRUE	3	TRA:CAVKKGTGRRALTF TRB:CASETGQGTDTQYF	53	CD4-C3
Pla-1_GCATGATGTACCCAAT	Pla	Pla-1	P1-1858	FALSE	1	TRA:CAVRDRDYKLSF TRB:CASSEQAGSYNEQFF	1	CD8-C10
Pla-1_GCCTCTAAGCCAGGAT	Pla	Pla-1	P1-1864	FALSE	1	TRA:CAVDTGGFKTIF TRB:CASSPRGTYSNQPQHF	1	CD4-C4
Pla-1_GCGCGATGTAGGGACT	Pla	Pla-1	P1-1877	FALSE	1	TRA:CAVRTSGTYKYIF TRB:CAWSALFSEQYF	1	CD8-C10
Pla-1_GCGGGTTCACTCGACG	Pla	Pla-1	P1-28	TRUE	3	TRA:CAVKKGTGRRALTF TRB:CASETGQGTDTQYF	53	CD4-C3
Pla-1_GGCAATTTCAACACGT	Pla	Pla-1	P1-304	FALSE	2	TRA:CAVRDGDYKLSF TRB:CASSYTSGGAETQYF	2	CD8-C10
Pla-1_GGTGTTACAACTGCTA	Pla	Pla-1	P1-28	TRUE	3	TRA:CAVKKGTGRRALTF TRB:CASETGQGTDTQYF	53	CD8-C10
Pla-1_GTACGTAGTTTGGCGC	Pla	Pla-1	P1-401	FALSE	2	TRA:CAVRDSNYQLIW TRB:CASSQTSDTGELFF	2	CD8-C10
Pla-1_GTGAAGGTCGTACGGC	Pla	Pla-1	P1-28	TRUE	3	TRA:CAVKKGTGRRALTF TRB:CASETGQGTDTQYF	53	CD4-C3
Pla-1_GTGCATAGTCGTTGTA	Pla	Pla-1	P1-2021	FALSE	1	TRA:CAVRDSNYQLIW TRB:CASSDSQDTDTQYF	1	CD8-C10
Pla-1_GTGGGTCTCTCCGGTT	Pla	Pla-1	P1-2033	FALSE	1	TRA:CAVMDSSYKLIF TRB:CASSEDTGGVSPLHF	1	CD8-C10
Pla-1_TACTCGCTCTGGTGTA	Pla	Pla-1	P1-2093	FALSE	1	TRA:CAVPDSNYQLIW TRB:CASSDSGEAEKLFF	1	CD8-C10
Pla-1_TACTTACCAGCATACT	Pla	Pla-1	P1-1	TRUE	3	TRA:CAMSDRMTGGFKTIF TRB:CASSQVGRGGEETQYF	1047	CD8-C10
Pla-1_TAGAGCTGTACCATCA	Pla	Pla-1	P1-28	TRUE	3	TRA:CAVKKGTGRRALTF TRB:CASETGQGTDTQYF	53	CD4-C3
Pla-1_TAGAGCTGTGATAAAC	Pla	Pla-1	P1-2106	FALSE	1	TRA:CGRQMLLFTTVLWRMNRDDKIIF TRB:CASSDRDRDTGE	1	CD8-C10
Pla-1_TCAGATGCATTTCAGG	Pla	Pla-1	P1-409	FALSE	2	TRA:CVVSPILYGGATNKLIF TRB:CASRVGGSGQLSGANVLTF	2	CD8-C10

Pla-1_TCTATTGGTAAGTGGC	Pla	Pla-1	P1-209	FALSE	3	TRA:CAVGDNAGNMLTF TRB:CASSQTSDTGELFF	3	CD8-C10
Pla-1_TGGCGCAGTCGAGTTT	Pla	Pla-1	P1-28	TRUE	3	TRA:CAVKKGTGRRALTF TRB:CASETGQGTDTQYF	53	CD8-C10
Pla-1_TTCCCAGTCAGCGATT	Pla	Pla-1	P1-2294	FALSE	1	TRA:CAVMDSSYKLIF TRB:CASSPGTSGGANEQFF	1	CD8-C10
Pla-1_TTCTTAGGTAGGACAC	Pla	Pla-1	P1-2311	FALSE	1	TRA:CVVSDRGSTLGRLYF TRB:CASSESGPGGELFF	1	CD8-C10
Pla-1_TTTACTGGTTATGCGT	Pla	Pla-1	P1-379	FALSE	2	TRA:CAVMDSSYKLIF TRB:CASSERENTEAFF	2	CD8-C10
Pla-1_TTTATGCGTTGCCTCT	Pla	Pla-1	P1-350	FALSE	2	TRA:CAPLDSNYQLIW TRB:CASSLDREATEAFF	2	CD8-C10
Pla-1_AAACCTGCAATGGACG	Pla	Pla-1	P1-96	FALSE	3	TRA:CAAQDTGTASKLTF TRB:CASNPNRDRPSEQFF	6	CD4-C5
Pla-1_AAACGGGAGAAGATTC	Pla	Pla-1	P1-1082	FALSE	1	TRA:CILVSGFGNEKLTF TRB:CASTKTQSGAYTGELFF	1	CD4-C5
Pla-1_AAAGTAGAGGGTCTCC	Pla	Pla-1	P1-277	TRUE	2	TRA:CGTEWSGANNDMRF TRB:CATAEGDQPQHF	2	CD4-C5
Pla-1_AACTCCCCAAGCGCTC	Pla	Pla-1	P1-157	FALSE	3	TRA:CAGIRAGNQFYF TRB:CASRRDRDSNQPQHF	4	CD4-C5
Pla-1_AAGCCGCTCCGTCAAA	Pla	Pla-1	P1-1116	FALSE	1	TRA:CAVGLGGNTPLVF TRB:CASSAGWQTQYF	1	CD4-C4
Pla-1_AAGGAGCCAAATACAG	Pla	Pla-1	P1-1117	FALSE	1	TRA:CAYRRRETSGSRLTF TRB:CASSLIGVSRNSPLHF	1	CD4-C5
Pla-1_AAGGTTCTCTTGTACT	Pla	Pla-1	P1-208	FALSE	3	TRA:CAVRDTGGFKTIF TRB:CAWSVGGGGTNEQFF	3	CD4-C4
Pla-1_AATCCAGAGGCTCAGA	Pla	Pla-1	P1-1131	FALSE	1	TRA:CAVYHTGGFKTIF TRB:CSAGATSGSLETQYF	1	CD4-C5
Pla-1_ACCAGTATCGGCGCTA	Pla	Pla-1	P1-157	FALSE	3	TRA:CAGIRAGNQFYF TRB:CASRRDRDSNQPQHF	4	CD4-C5
Pla-1_ACCCACTAGATGTGGC	Pla	Pla-1	P1-212	FALSE	3	TRA:CAYRSARNNNNDMRF TRB:CSAARTGGNLAFF	3	CD4-C5
Pla-1_ACCCACTAGCCGATTT	Pla	Pla-1	P1-21	TRUE	3	TRA:CAASRGTGANNLFF TRB:CATQREGREQYF	67	CD4-C6
Pla-1_ACCTTTACAATCAGAA	Pla	Pla-1	P1-213	FALSE	3	TRA:CAMSSNQAGTALIF TRB:CASSLGQGRRAEAFF	3	CD4-C5
Pla-1_ACGAGGATCCGGGTGT	Pla	Pla-1	P1-1183	FALSE	1	TRA:CALTVTGFQKLVF TRB:CASSLEGTGAYGYTF	1	CD4-C4
Pla-1_ACGCAGCCAAGCGATG	Pla	Pla-1	P1-322	FALSE	2	TRA:CAVSEVYAGGYNKLIF TRB:CASSIARWTSGGASEQFF	2	CD4-C5
Pla-1_ACGGAGATCCACTGGG	Pla	Pla-1	P1-102	FALSE	3	TRA:CAENNDAGNMLTF TRB:CASSETWGVTEAFF	6	CD4-C5
Pla-1_ACGGCCATCTGTTTGT	Pla	Pla-1	P1-19	TRUE	3	TRA:CALGAFGSARQLTF TRB:CASSKGQGPRETQYF	70	CD4-C6
Pla-1_ACGGGTCTCGCTAGCG	Pla	Pla-1	P1-1208	FALSE	1	TRA:CAVDPILTGGGNKLTF TRB:CASSLSRQTYNEQFF	1	CD4-C5
Pla-1_ACTGAGTTCAATAAGG	Pla	Pla-1	P1-127	FALSE	3	TRA:CALSADNDMRF TRB:CASTRGDWIYNEQFF	5	CD4-C5
Pla-1_ACTGAGTTCAGTTGAC	Pla	Pla-1	P1-1218	FALSE	1	TRA:CIVRVSGNEKLTF TRB:CASSSTARTDTQYF	1	CD4-C5
Pla-1_AGAGCGAGTCCAAGTT	Pla	Pla-1	P1-309	FALSE	2	TRA:CAVNSPNQAGTALIF TRB:CASSLLPSGTSTDTQYF	2	CD4-C5
Pla-1_AGAGTGGCATTCCTGC	Pla	Pla-1	P1-1251	FALSE	1	TRA:CALSDPSGSRLTF TRB:CASSLEGVYNEQFF	1	CD4-C4

Pla-1_AGCATACCAATGAATG	Pla	Pla-1	P1-196	TRUE	3	TRA:CAVESPGRGGATNKLIF TRB:CASSARGVTYNEQFF	3	CD4-C5
Pla-1_AGCCTAAAGCCCAGCT	Pla	Pla-1	P1-21	TRUE	3	TRA:CAASRGTGANNLFF TRB:CATQREGREQYF	67	CD4-C5
Pla-1_AGCCTAATCTGAGGGA	Pla	Pla-1	P1-1266	FALSE	1	TRA:CAVNGGGFGNVLHC TRB:CASSPRTSGANTGELFF	1	CD4-C4
Pla-1_AGCTTGAAGTACGTAA	Pla	Pla-1	P1-335	FALSE	2	TRA:CAASAGGGADGLTF TRB:CASSDRGRGYTF	2	CD4-C5
Pla-1_AGCTTGACAAGGACTG	Pla	Pla-1	P1-1276	FALSE	1	TRA:CAMSAGSARQLTF TRB:CASSLDGYGTGQDYGYTF	1	CD4-C5
Pla-1_AGGCCACGTTGGTGGA	Pla	Pla-1	P1-178	TRUE	3	TRA:CAVFLWEARLMF TRB:CASSFGGQGGTEAFF	3	CD4-C5
Pla-1_AGGTCATGTGCACGAA	Pla	Pla-1	P1-338	FALSE	2	TRA:CAEKELTF TRB:CASTSFETEAFF	2	CD4-C5
Pla-1_AGGTCCGTCCTGCCAT	Pla	Pla-1	P1-42	TRUE	3	TRA:CASFSGGSQGNLIF TRB:CSAGQGSNQPQHF	24	CD4-C5
Pla-1_AGTAGTCGTTCGGGCT	Pla	Pla-1	P1-341	FALSE	2	TRA:CAFKQSGNTPLVF TRB:CASSPWGAGLYNEQFF	2	CD4-C5
Pla-1_AGTGAGGTCATGCTCC	Pla	Pla-1	P1-1296	FALSE	1	TRA:CAASTNNQGGKLIF TRB:CASSVRGLRDEQFF	1	CD4-C2
Pla-1_AGTGTCACAGGACGTA	Pla	Pla-1	P1-1301	FALSE	1	TRA:CALDMEDGGNEKLTF TRB:CASSFSGHSTDTQYF	1	CD4-C5
Pla-1_ATAAGAGAGGTGATTA	Pla	Pla-1	P1-1311	FALSE	1	TRA:CAGDQGAQKLVF TRB:CASSTRGGGWKTQYF	1	CD4-C5
Pla-1_ATAGACCAGTGGGTTG	Pla	Pla-1	P1-224	FALSE	3	TRA:CAVGEGYSTLTF TRB:CASMKGQGGSPLHF	3	CD4-C4
Pla-1_ATCACGAAGACTTGAA	Pla	Pla-1	P1-1317	FALSE	1	TRA:CAVAGQGAQKLVF TRB:CASSLDRGHHEQYF	1	CD4-C5
Pla-1_ATCATCTCAGCTGGCT	Pla	Pla-1	P1-252	TRUE	2	TRA:CAASINGRGSTLGRLYF TRB:CASLPGHGVTDTQYF	2	CD4-C5
Pla-1_ATCATCTGTCGCTTCT	Pla	Pla-1	P1-345	FALSE	2	TRA:CAESISWTGGGADGLTF TRB:CASSVLGEKLFF	2	CD4-C5
Pla-1_ATCCACCCATGCCTAA	Pla	Pla-1	P1-346	FALSE	2	TRA:CGTELGGNQFYF TRB:CASSVGDGTEAFF	2	CD4-C5
Pla-1_ATGCGATTCGCAAGCC	Pla	Pla-1	P1-1345	FALSE	1	TRA:CAVGSGGSNYKLTF TRB:CASSPGGGANVLTF	1	CD4-C5
Pla-1_ATTATCCAGTTATCGC	Pla	Pla-1	P1-1354	FALSE	1	TRA:CILVRAQKLVF TRB:CASSPPPDRGKETQYF	1	CD4-C5
Pla-1_ATTGGTGGTTGGGACA	Pla	Pla-1	P1-1359	FALSE	1	TRA:CAATHSGYNKLIF TRB:CASSYSRQGEKLFF	1	CD4-C3
Pla-1_ATTTCTGAGGAGCGTT	Pla	Pla-1	P1-162	FALSE	3	TRA:CVVSGGLRGAAGNKLTF TRB:CASSPLTVNTEAFF	4	CD4-C6
Pla-1_ATTTCTGTCAGTTTGG	Pla	Pla-1	P1-268	TRUE	2	TRA:CAASGLDFYNQGGKLIF TRB:CASSSRGGASSPLHF	2	CD4-C5
Pla-1_CAAGAAAGTCCGAGTC	Pla	Pla-1	P1-1372	FALSE	1	TRA:CAVQAGNNDMRF TRB:CASSPRTSGANTGELFF	1	CD4-C5
Pla-1_CAAGGCCTCGAATCCA	Pla	Pla-1	P1-1379	FALSE	1	TRA:CAGQSKNTGNQFYF TRB:CASSDGGNEQFF	1	CD4-C5
Pla-1_CAAGTTGGTAGCTTGT	Pla	Pla-1	P1-1382	FALSE	1	TRA:CAFMKPPYPHSGGGADGLTF TRB:CASSRTGVANTEAFF	1	CD4-C5
Pla-1_CACACAATCTATCCCG	Pla	Pla-1	P1-1386	FALSE	1	TRA:CAVTHAGKSTF TRB:CSAPSGTTTYGYTF	1	CD4-C5
Pla-1_CACACTCTCTGCAGTA	Pla	Pla-1	P1-1389	FALSE	1	TRA:CAGDLSGGFKTIF TRB:CASKKETASNQPQHF	1	CD4-C5

Pla-1_CACAGGCAGGCTCAGA	Pla	Pla-1	P1-1391	FALSE	1	TRA:CAVTVKAAGNKLTF TRB:CASSLGLRYNEQFF	1	CD4-C5
Pla-1_CACATAGAGACTTTCG	Pla	Pla-1	P1-1396	FALSE	1	TRA:CAVGGSPTSGTYKYIF TRB:CASSPVGRYEQYF	1	CD4-C5
Pla-1_CACATAGTCCTAAGTG	Pla	Pla-1	P1-1399	FALSE	1	TRA:CVVSDRGSTLGRLYF TRB:CASTGQGGNSPLHF	1	CD4-C5
Pla-1_CACATAGTCTGGCGAC	Pla	Pla-1	P1-119	TRUE	3	TRA:CVVSQAGSGNTGKLIF TRB:CSAIPWRGYGYTF	5	CD4-C5
Pla-1_CACATTTCACATTCGA	Pla	Pla-1	P1-1401	FALSE	1	TRA:CAFMKHPAVSGTASKLTF TRB:CASSDPRGLNTEAFF	1	CD4-C5
Pla-1_CACCACTGTACTTGAC	Pla	Pla-1	P1-51	FALSE	3	TRA:CAYRSLNNAGNMLTF TRB:CASSRGNNSPLHF	14	CD4-C5
Pla-1_CACCACTGTCGTGGCT	Pla	Pla-1	P1-1405	FALSE	1	TRA:CALPPGGNNRKLIW TRB:CSARERQGHPYEQYF	1	CD4-C6
Pla-1_CACCAGGGTGCCTGGT	Pla	Pla-1	P1-121	FALSE	3	TRA:CAASEGGATNKLIF TRB:CSAGTSGNIQYF	5	CD4-C5
Pla-1_CACTCCAAGTGGCACA	Pla	Pla-1	P1-1413	FALSE	1	TRA:CAGPLNAGNNRKLIW TRB:CASSLGTTSSYEQYF	1	CD4-C5
Pla-1_CAGAATCAGCAGGCTA	Pla	Pla-1	P1-1415	FALSE	1	TRA:CAVSDPQGGSEKLVF TRB:CASSSGVRQGWGYTF	1	CD4-C5
Pla-1_CAGATCAGTCTGGAGA	Pla	Pla-1	P1-364	FALSE	2	TRA:CAYRSGGINFGNEKLTF TRB:CASSAGLRPEQYF	2	CD4-C3
Pla-1_CAGATCATCCCGACTT	Pla	Pla-1	P1-102	FALSE	3	TRA:CAENNDAGNMLTF TRB:CASSETWGVTEAFF	6	CD4-C6
Pla-1_CAGCATAGTCAGTGGA	Pla	Pla-1	P1-365	FALSE	2	TRA:CAALDSYNTDKLIF TRB:CASSSTVNTEAFF	2	CD4-C5
Pla-1_CAGCTAATCAATACCG	Pla	Pla-1	P1-1427	FALSE	1	TRA:CAPPGRGFGNVLHC TRB:CASSSSGASQTQYF	1	CD4-C4
Pla-1_CAGTAACGTCGCATAT	Pla	Pla-1	P1-1433	FALSE	1	TRA:CAGPMGGATNKLIF TRB:CAWGLAGYNEQFF	1	CD4-C5
Pla-1_CAGTCCTCACGTAAGG	Pla	Pla-1	P1-77	FALSE	3	TRA:CALSEAGYGGSQGNLIF TRB:CASSHEPRTGELFF	8	CD4-C2
Pla-1_CCACCTAAGCCATCGC	Pla	Pla-1	P1-40	TRUE	3	TRA:CAVRDVGNTGFQKLVF TRB:CASSLGQGSMKTQYF	27	CD4-C5
Pla-1_CCACTACTCAACGCTA	Pla	Pla-1	P1-1481	FALSE	1	TRA:CAVDSNYGGQNFVF TRB:CASSRGETQYF	1	CD4-C6
Pla-1_CCAGCGAAGGTGATAT	Pla	Pla-1	P1-1482	FALSE	1	TRA:CAVRDDSSASKIIF TRB:CASSRGSQPQHF	1	CD8-C11
Pla-1_CCATGTCAGCACCGTC	Pla	Pla-1	P1-1	TRUE	3	TRA:CAMSDRMTGGFKTIF TRB:CASSQVGRGGEETQYF	1047	CD4-C5
Pla-1_CCCAATCAGGCATGTG	Pla	Pla-1	P1-1488	FALSE	1	TRA:CAVEEGYGGATNKLIF TRB:CASSLARRQETQYF	1	CD4-C5
Pla-1_CCGGGATTCAACGAAA	Pla	Pla-1	P1-1498	FALSE	1	TRA:CAVAPNQAGTALIF TRB:CSARAPLSSYEQYF	1	CD4-C5
Pla-1_CCGGTAGCACTAGTAC	Pla	Pla-1	P1-1501	FALSE	1	TRA:CAESRAAGNKLTF TRB:CASSPDPERNEQFF	1	CD4-C5
Pla-1_CCGTACTAGGGCTTGA	Pla	Pla-1	P1-48	TRUE	3	TRA:CAEVSGYSTLTF TRB:CASSFPTGGRTGRTEAFF	16	CD4-C5
Pla-1_CCGTACTCATGGGAAC	Pla	Pla-1	P1-90	TRUE	3	TRA:CAVNNGRGSTLGRLYF TRB:CASSSGPGSGANVLTF	6	CD4-C5
Pla-1_CCGTTCATCACCTTAT	Pla	Pla-1	P1-1506	FALSE	1	TRA:CAVSEYNQGGKLIF TRB:CASRSAGSFSYNEQFF	1	CD4-C5
Pla-1_CCTAAAGTCCTATGTT	Pla	Pla-1	P1-21	TRUE	3	TRA:CAASRGTGANNLFF TRB:CATQREGREQYF	67	CD4-C4

Pla-1_CCTAGCTGTTCAGACT	Pla	Pla-1	P1-1516	FALSE	1	TRA:CAVSDILTGGGNKLTF TRB:CSARGRGLAGGPAYEQYF	1	CD4-C5
Pla-1_CCTATTAAGTCGTACT	Pla	Pla-1	P1-1517	FALSE	1	TRA:CVVSRSGGSYIPTF TRB:CASSLWGMNTGELFF	1	CD4-C5
Pla-1_CCTTCGATCTTATCTG	Pla	Pla-1	P1-1534	FALSE	1	TRA:CAVNAKDTNAGKSTF TRB:CASSFRDSYNEQFF	1	CD4-C5
Pla-1_CGACCTTAGCTTCGCG	Pla	Pla-1	P1-1545	FALSE	1	TRA:CAVSDASGGYNKLIF TRB:CASRGEYSYNEQFF	1	CD4-C5
Pla-1_CGACCTTAGGTAGCCA	Pla	Pla-1	P1-377	FALSE	2	TRA:CAESFSGYALNF TRB:CASSLPSIRADTQYF	2	CD4-C5
Pla-1_CGACTTCTCTCGGACG	Pla	Pla-1	P1-1549	FALSE	1	TRA:CAMSDASGGYNKLIF TRB:CAASEREVRNEQFF	1	CD4-C5
Pla-1_CGAGCACCAGCTCCGA	Pla	Pla-1	P1-1558	FALSE	1	TRA:CVVTRPSRAGNMLTF TRB:CSATKDRGGSNQPQHF	1	CD4-C5
Pla-1_CGATGGCAGCTAAACA	Pla	Pla-1	P1-107	TRUE	3	TRA:CALSDKEKLTF TRB:CAISEGGGVGYTF	5	CD4-C5
Pla-1_CGATGGCGTCTCAACA	Pla	Pla-1	P1-92	TRUE	3	TRA:CIVQLTGTASKLTF TRB:CASSFKSGRTNEQFF	6	CD4-C6
Pla-1_CGCGGTAGTGACAAAT	Pla	Pla-1	P1-48	TRUE	3	TRA:CAEVSGYSTLTF TRB:CASSFPTGGRTGRTEAFF	16	CD4-C6
Pla-1_CGCTGGATCGCAGGCT	Pla	Pla-1	P1-1576	FALSE	1	TRA:CAASRAGNQFYF TRB:CASSVSTDTQYF	1	CD4-C4
Pla-1_CGGACACGTGCAGACA	Pla	Pla-1	P1-102	FALSE	3	TRA:CAENNDAGNMLTF TRB:CASSETWGVTEAFF	6	CD4-C5
Pla-1_CGGACGTGTCTAGTCA	Pla	Pla-1	P1-1585	FALSE	1	TRA:CVVTLPGGYQKVTF TRB:CSANSGNTIYF	1	CD4-C5
Pla-1_CGGGTCACAAGCGTAG	Pla	Pla-1	P1-1598	FALSE	1	TRA:CAMRETPGEAGTALIF TRB:CASSLAVRANSMNTEAFF	1	CD4-C5
Pla-1_CGTAGGCTCACAGTAC	Pla	Pla-1	P1-51	FALSE	3	TRA:CAYRSLNNAGNMLTF TRB:CASSRGNNSPLHF	14	CD4-C5
Pla-1_CGTCACTCAAGGCTCC	Pla	Pla-1	P1-27	TRUE	3	TRA:CALVDFSGYSTLTF TRB:CASSGRGTNEKLFF	55	CD8-C9
Pla-1_CGTCAGGAGCTTTGGT	Pla	Pla-1	P1-131	FALSE	3	TRA:CAVRDSGTYKYIF TRB:CASSIDLGGANVLTF	5	CD4-C6
Pla-1_CGTCTACTCAGCTGGC	Pla	Pla-1	P1-1618	FALSE	1	TRA:CALSSTGNQFYF TRB:CSAFPGRGETQYF	1	CD4-C5
Pla-1_CGTCTACTCCGAAGAG	Pla	Pla-1	P1-20	TRUE	3	TRA:CVVVGGRALTF TRB:CASSLATNPHETQYF	69	CD4-C5
Pla-1_CGTGAGCAGTGTACTC	Pla	Pla-1	P1-70	FALSE	3	TRA:CAMRETPGEAGTALIF TRB:CSARITYLNTEAFF	9	CD8-C4
Pla-1_CGTGTAAGTGCAACTT	Pla	Pla-1	P1-1623	FALSE	1	TRA:CAASTNQAGTALIF TRB:CSAWQGYEKLFF	1	CD4-C3
Pla-1_CGTGTCTAGACAAAGG	Pla	Pla-1	P1-1625	FALSE	1	TRA:CAVRDKGDSNYQLIW TRB:CQQVSLTETQYF	1	CD4-C5
Pla-1_CGTGTCTCATCAGTAC	Pla	Pla-1	P1-1627	FALSE	1	TRA:CAVSDGSGGYNKLIF TRB:CASRDIYSNQPQHF	1	CD4-C5
Pla-1_CGTTAGAGTCCAAGTT	Pla	Pla-1	P1-1629	FALSE	1	TRA:CAYRSALDGGATNKLIF TRB:CATSDPGTSRSDTQYF	1	CD4-C5
Pla-1_CGTTAGAGTCTGATCA	Pla	Pla-1	P1-1630	FALSE	1	TRA:CAVRPPGRNSGGYQKVTF TRB:CASSYSRGGATNTEAFF	1	CD4-C5
Pla-1_CTACGTCAGGGCACTA	Pla	Pla-1	P1-226	FALSE	3	TRA:CAAVSAFSGGYNKLIF TRB:CASSLAGRAATNEKLFF	3	CD4-C5
Pla-1_CTAGCCTGTGTGGCTC	Pla	Pla-1	P1-1657	FALSE	1	TRA:CAVRDQVGRGSYIPTF TRB:CASSWVADGAYEQYF	1	CD4-C4

Pla-1_CTAGTGAGTACTTGAC	Pla	Pla-1	P1-90	TRUE	3	TRA:CAVNNGRGSTLGRLYF TRB:CASSSGPGSGANVLTF	6	CD4-C6
Pla-1_CTAGTGAGTTGTGGCC	Pla	Pla-1	P1-104	FALSE	3	TRA:CALRGSARQLTF TRB:CASSQEEAGPAKNIQYF	6	CD4-C5
Pla-1_CTAGTGATCAATACCG	Pla	Pla-1	P1-1660	FALSE	1	TRA:CAVGSLKAAGNKLTF TRB:CATSDAGDLRTQYF	1	CD4-C5
Pla-1_CTAGTGATCTACTTAC	Pla	Pla-1	P1-391	FALSE	2	TRA:CGTENMGNQFYF TRB:CASSVGDGVEAFF	2	CD4-C5
Pla-1_CTCAGAATCCGCGTTT	Pla	Pla-1	P1-1666	FALSE	1	TRA:CAVNAYRDDKIIF TRB:CASRRDRVSYEQYF	1	CD8-C3
Pla-1_CTCGGAGGTGTTGAGG	Pla	Pla-1	P1-1687	FALSE	1	TRA:CAYRSGTPNTGTASKLTF TRB:CASRLAGVVYEQYF	1	CD4-C5
Pla-1_CTCGGGATCAGGATCT	Pla	Pla-1	P1-119	TRUE	3	TRA:CVVSQAGSGNTGKLIF TRB:CSAIPWRGYGYTF	5	CD4-C5
Pla-1_CTCTACGAGCTACCGC	Pla	Pla-1	P1-1703	FALSE	1	TRA:CAASFLTGTASKLTF TRB:CASSATGSYEQYF	1	CD4-C5
Pla-1_CTCTGGTTCCTGTAGA	Pla	Pla-1	P1-1708	FALSE	1	TRA:CAVAPLINDMRF TRB:CASSLEARVTNNEQFF	1	CD4-C5
Pla-1_CTGAAACCAGCTGTAT	Pla	Pla-1	P1-1711	FALSE	1	TRA:CAAKGAGFGNVLHC TRB:CASSFPSGTGSYEQYF	1	CD4-C5
Pla-1_CTGAAACGTAACGTTC	Pla	Pla-1	P1-1712	FALSE	1	TRA:CAVQYGGQKLLF TRB:CASKATENQPQHF	1	CD4-C5
Pla-1_CTGAAGTAGTTAGGTA	Pla	Pla-1	P1-1713	FALSE	1	TRA:CAYGTGNQFYF TRB:CASSLPTNTGELFF	1	CD4-C5
Pla-1_CTGATAGGTAAGGATT	Pla	Pla-1	P1-1719	FALSE	1	TRA:CAVRPRDDKIIF TRB:CASSLTHDTGELFF	1	CD4-C5
Pla-1_CTGCTGTCAATGGATA	Pla	Pla-1	P1-1730	FALSE	1	TRA:CALSPGYTGANSKLTF TRB:CASSVGRDEKLFF	1	CD4-C4
Pla-1_CTGTGCTAGAATAGGG	Pla	Pla-1	P1-1738	FALSE	1	TRA:CAESSTGGGNKLTF TRB:CASSPRTGASGANVLTF	1	CD4-C5
Pla-1_CTTAACTGTGCACTTA	Pla	Pla-1	P1-1744	FALSE	1	TRA:CAENRDGQKLLF TRB:CASSPSSVRANVLTF	1	CD4-C5
Pla-1_CTTAGGATCAACACAC	Pla	Pla-1	P1-1753	FALSE	1	TRA:CAPRLGNVLHC TRB:CASSFWTSANEQFF	1	CD4-C4
Pla-1_CTTGGCTGTGCACGAA	Pla	Pla-1	P1-12	TRUE	3	TRA:CALSDERGDYKLSF TRB:CASSPGTGSYNEQFF	141	CD4-C4
Pla-1_CTTGGCTTCATCGATG	Pla	Pla-1	P1-1758	FALSE	1	TRA:CAASADGGSQGNLIF TRB:CASSRGPNSFTDTQYF	1	CD4-C5
Pla-1_GAACGGATCCGTAGGC	Pla	Pla-1	P1-397	FALSE	2	TRA:CAASAYAGGTSYGKLTF TRB:CASSLKGRGRITGGTDTQY	2	CD8-C2
Pla-1_GAACGGATCTCTTGAT	Pla	Pla-1	P1-1773	FALSE	1	TRA:CAMSVTLNSGYSTLTF TRB:CASRPTRQGQGTPDTQYF	1	CD4-C4
Pla-1_GAATAAGCACGGCTAC	Pla	Pla-1	P1-1782	FALSE	1	TRA:CAVYNQGGKLIF TRB:CASRDDARTDTQYF	1	CD4-C5
Pla-1_GACAGAGGTAGCCTCG	Pla	Pla-1	P1-1786	FALSE	1	TRA:CALSDGVTGGGNKLTF TRB:CASSGGQTTNEKLFF	1	CD4-C5
Pla-1_GACAGAGGTCGAACAG	Pla	Pla-1	P1-102	FALSE	3	TRA:CAENNDAGNMLTF TRB:CASSETWGVTEAFF	6	CD4-C5
Pla-1_GACCTGGCATGCTAGT	Pla	Pla-1	P1-1788	FALSE	1	TRA:CGTELAAGNKLTF TRB:CASSPRQANTGELFF	1	CD4-C3
Pla-1_GACGTTAGTGTGCCTG	Pla	Pla-1	P1-1805	FALSE	1	TRA:CADQGGSEKLVF TRB:CASSYRGLAGRDTQYF	1	CD4-C5
Pla-1_GACTAACGTTTCGCTC	Pla	Pla-1	P1-175	TRUE	3	TRA:CAASVVDFGNEKLTF TRB:CASSSQGLSYNEQFF	3	CD4-C4

Pla-1_GACTAACGTTTGACTG	Pla	Pla-1	P1-1808	FALSE	1	TRA:CALSDRGNARLMF TRB:CATQGAGLETQYF	1	CD4-C5
Pla-1_GACTGCGGTCATATGC	Pla	Pla-1	P1-1812	FALSE	1	TRA:CAVLNAGNNRKLIW TRB:CASKSRVDQPQHF	1	CD4-C5
Pla-1_GAGTCCGAGATGAGAG	Pla	Pla-1	P1-212	FALSE	3	TRA:CAYRSARNNNNDMRF TRB:CSAARTGGNLAFF	3	CD4-C5
Pla-1_GATCAGTGTCATGCCG	Pla	Pla-1	P1-1820	FALSE	1	TRA:CAVSAKAAGNKLTF TRB:CASILGGAGTYEQYF	1	CD4-C5
Pla-1_GATCAGTGTCCCGACA	Pla	Pla-1	P1-1821	FALSE	1	TRA:CAYTNRKYGGSQGNLIF TRB:CASSLERGTDTQYF	1	CD4-C6
Pla-1_GATCAGTGTTGTGGAG	Pla	Pla-1	P1-119	TRUE	3	TRA:CVVSQAGSGNTGKLIF TRB:CSAIPWRGYGYTF	5	CD4-C6
Pla-1_GATTCAGTCAGTGTTG	Pla	Pla-1	P1-89	TRUE	3	TRA:CAVDVNYGGQNFVF TRB:CASSWGDTQYF	6	CD4-C5
Pla-1_GCAAACTTCATCATTC	Pla	Pla-1	P1-402	FALSE	2	TRA:CVVNRENGQNFVF TRB:CAWGGLAGFSDTQYF	2	CD4-C5
Pla-1_GCATACAAGGTGCTAG	Pla	Pla-1	P1-1855	FALSE	1	TRA:CAARNNAGNMLTF TRB:CASSSPFFSGAYYNEQFF	1	CD8-C3
Pla-1_GCCTCTAGTAGGGTAC	Pla	Pla-1	P1-1867	FALSE	1	TRA:CAGQVSGTGRRALTF TRB:CASSRDITDTQYF	1	CD4-C5
Pla-1_GCGCAGTTCCGTAGGC	Pla	Pla-1	P1-1875	FALSE	1	TRA:CAVEGDDKIIF TRB:CASSRGTGGLEQFF	1	CD4-C4
Pla-1_GCGGGTTTCCTATTCA	Pla	Pla-1	P1-129	FALSE	3	TRA:CAFLFYGGSQGNLIF TRB:CASSQGRGRYGNTIYF	5	CD4-C5
Pla-1_GCTGCAGTCGCCAGCA	Pla	Pla-1	P1-90	TRUE	3	TRA:CAVNNGRGSTLGRLYF TRB:CASSSGPGSGANVLTF	6	CD4-C6
Pla-1_GCTGCGAAGGATGGTC	Pla	Pla-1	P1-79	TRUE	3	TRA:CAFMKLGGATNKLIF TRB:CASSLDRYSVDEQFF	7	CD8-C3
Pla-1_GCTTCCACACCATCCT	Pla	Pla-1	P1-94	TRUE	3	TRA:CAASIQDFGNEKLTF TRB:CASSLQVPPNTEAFF	6	CD4-C5
Pla-1_GGAAAGCTCGTCCGTT	Pla	Pla-1	P1-1901	FALSE	1	TRA:CAPGVRSQGNLIF TRB:CASSERQAGEQFF	1	CD4-C6
Pla-1_GGACAAGCATACTACG	Pla	Pla-1	P1-129	FALSE	3	TRA:CAFLFYGGSQGNLIF TRB:CASSQGRGRYGNTIYF	5	CD4-C5
Pla-1_GGACATTGTCGATTGT	Pla	Pla-1	P1-1910	FALSE	1	TRA:CASPLGASKIIF TRB:CASREGQAVEKLFF	1	CD4-C5
Pla-1_GGACGTCAGTCCGTAT	Pla	Pla-1	P1-1911	FALSE	1	TRA:CAVEPGSARQLTF TRB:CASSNQGPGANVLTF	1	CD8-C2
Pla-1_GGACGTCGTATCAGTC	Pla	Pla-1	P1-21	TRUE	3	TRA:CAASRGTGANNLFF TRB:CATQREGREQYF	67	CD4-C6
Pla-1_GGAGCAAAGATCGATA	Pla	Pla-1	P1-1915	FALSE	1	TRA:CAVNSLAGRRALTF TRB:CAWALRDRPTEAFF	1	CD4-C2
Pla-1_GGATGTTCAATTCCTT	Pla	Pla-1	P1-397	FALSE	2	TRA:CAASAYAGGTSYGKLTF TRB:CASSLKGRGRITGGTDTQY	2	CD4-C5
Pla-1_GGATGTTCACGACGAA	Pla	Pla-1	P1-215	FALSE	3	TRA:CAVSSNRDDKIIF TRB:CASSPGRLAGENIQYF	3	CD4-C3
Pla-1_GGATTACGTGCACTTA	Pla	Pla-1	P1-1918	FALSE	1	TRA:CAAHTGGFKTIF TRB:CASSLGTVNQPQHF	1	CD4-C5
Pla-1_GGCCGATCACTTAACG	Pla	Pla-1	P1-408	FALSE	2	TRA:CAILSGGYNKLIF TRB:CASSQDPGGGYGYTF	2	CD4-C5
Pla-1_GGCTCGACACACCGCA	Pla	Pla-1	P1-78	TRUE	3	TRA:CAAAQGGSEKLVF TRB:CASTRGDWIYNEQFF	7	CD4-C6
Pla-1_GGCTCGACACAGTCGC	Pla	Pla-1	P1-1933	FALSE	1	TRA:CAVVGSQGNLIF TRB:CASSLRPTDTQYF	1	CD4-C5

Pla-1_GGGACCTTCTTCATGT	Pla	Pla-1	P1-157	FALSE	3	TRA:CAGIRAGNQFYF TRB:CASRRDRDSNQPQHF	4	CD4-C5
Pla-1_GGGCACTGTGAGTGAC	Pla	Pla-1	P1-48	TRUE	3	TRA:CAEVSGYSTLTF TRB:CASSFPTGGRTGRTEAFF	16	CD4-C5
Pla-1_GGGTTGCGTCTCACCT	Pla	Pla-1	P1-394	FALSE	2	TRA:CAYRSAIGPGGTSYGKLTF TRB:CASSPRRKNQPQHF	2	CD4-C4
Pla-1_GGTATTGCATCGATTG	Pla	Pla-1	P1-170	FALSE	3	TRA:CALSGKNTGNQFYF TRB:CASSLDQGGNYGYTF	4	CD4-C5
Pla-1_GTACTCCAGGGAAACA	Pla	Pla-1	P1-1982	FALSE	1	TRA:CAVRSNTGNQFYF TRB:CASRKQGAGETQYF	1	CD4-C5
Pla-1_GTACTTTTCCGTCATC	Pla	Pla-1	P1-362	FALSE	2	TRA:CAISSSNTGKLIF TRB:CSVSLQGARRSGEKLFF	2	CD4-C5
Pla-1_GTATCTTAGAAAGTGG	Pla	Pla-1	P1-1990	FALSE	1	TRA:CASRGTGGFKTIF TRB:CSAGKKTSGVRNTGELFF	1	CD4-C5
Pla-1_GTATCTTAGGGCTTCC	Pla	Pla-1	P1-250	FALSE	3	TRA:CAHYDRGSTLGRLYF TRB:CASSLRIDQPQHF	3	CD4-C5
Pla-1_GTCAAGTAGTACGCCC	Pla	Pla-1	P1-1993	FALSE	1	TRA:CAVQAGNNDMRF TRB:CASSARGVTYNEQFF	1	CD4-C6
Pla-1_GTCGGGTCACGTTGGC	Pla	Pla-1	P1-2004	FALSE	1	TRA:CAMSSSNYKLTF TRB:CAISEDRSSNQPQHF	1	CD4-C5
Pla-1_GTCGGGTTCGTGACAT	Pla	Pla-1	P1-89	TRUE	3	TRA:CAVDVNYGGQNFVF TRB:CASSWGDTQYF	6	CD4-C5
Pla-1_GTCTCGTAGGGATCTG	Pla	Pla-1	P1-328	FALSE	2	TRA:CALSPQGGSEKLVF TRB:CSGSEGTDTQYF	2	CD4-C5
Pla-1_GTGCATATCACATACG	Pla	Pla-1	P1-272	TRUE	2	TRA:CAFMSNNNDMRF TRB:CASKPRDTEAFF	2	CD4-C5
Pla-1_GTGTTAGGTCATATCG	Pla	Pla-1	P1-417	FALSE	2	TRA:CAVINAGNNRKLIW TRB:CAWSAGSGVFGAFF	2	CD4-C5
Pla-1_GTTCATTCATAACCTG	Pla	Pla-1	P1-2050	FALSE	1	TRA:CAVRDGGNEKLTF TRB:CSAKTGGAGTGELFF	1	CD4-C5
Pla-1_GTTCATTTCCCGACTT	Pla	Pla-1	P1-216	FALSE	3	TRA:CALLFGNVLHC TRB:CASSLAVRANSMNTEAFF	3	CD4-C5
Pla-1_GTTCATTTCTACGAGT	Pla	Pla-1	P1-416	FALSE	2	TRA:CGITGNQFYF TRB:CASRRLRDRGATNSNQPQHF	2	CD4-C3
Pla-1_TAAGAGACACAGACAG	Pla	Pla-1	P1-2064	FALSE	1	TRA:CAVSMNRDDKIIF TRB:CASSARGVTYNEQFF	1	CD4-C5
Pla-1_TAAGTGCCACACCGCA	Pla	Pla-1	P1-2068	FALSE	1	TRA:CAGSYGNNRLAF TRB:CASSLTQGAFLRELFF	1	CD4-C5
Pla-1_TACCTTATCATCACCC	Pla	Pla-1	P1-2079	FALSE	1	TRA:CAKNTGNQFYF TRB:CASSPQPTGELFF	1	CD4-C3
Pla-1_TACGGTAGTCAGAGGT	Pla	Pla-1	P1-48	TRUE	3	TRA:CAEVSGYSTLTF TRB:CASSFPTGGRTGRTEAFF	16	CD4-C6
Pla-1_TACTTACTCTCTTATG	Pla	Pla-1	P1-2097	FALSE	1	TRA:CAENRGSGYALNF TRB:CASTPTLRVPEAFF	1	CD4-C5
Pla-1_TAGACCAGTGCGATAG	Pla	Pla-1	P1-127	FALSE	3	TRA:CALSADNDMRF TRB:CASTRGDWIYNEQFF	5	CD4-C5
Pla-1_TAGACCATCCTGCTTG	Pla	Pla-1	P1-190	TRUE	3	TRA:CAEGGVDGQKLLF TRB:CASSEDAGELFF	3	CD4-C6
Pla-1_TAGCCGGGTCAAAGAT	Pla	Pla-1	P1-48	TRUE	3	TRA:CAEVSGYSTLTF TRB:CASSFPTGGRTGRTEAFF	16	CD4-C6
Pla-1_TAGGCATCAAGTTGTC	Pla	Pla-1	P1-2110	FALSE	1	TRA:CAVRGYGGQNFVF TRB:CASSLDHTEAFF	1	CD4-C3
Pla-1_TATCAGGAGGGCATGT	Pla	Pla-1	P1-419	FALSE	2	TRA:CAENRRDDKIIF TRB:CASSETLRNTEAFF	2	CD4-C5

Pla-1_TATCAGGAGTTTCCTT	Pla	Pla-1	P1-2116	FALSE	1	TRA:CAVKNAGNMLTF TRB:CSARDPRGRPDTGELFF	1	CD4-C5
Pla-1_TATCAGGCATCCGTGG	Pla	Pla-1	P1-175	TRUE	3	TRA:CAASVVDFGNEKLTF TRB:CASSSQGLSYNEQFF	3	CD4-C6
Pla-1_TATCTCAAGGTTACCT	Pla	Pla-1	P1-2120	FALSE	1	TRA:CIVTQGGSQGNLIF TRB:CASSSFGRGSTGELFF	1	CD4-C6
Pla-1_TATGCCCAGGCCCTCA	Pla	Pla-1	P1-402	FALSE	2	TRA:CVVNRENGQNFVF TRB:CAWGGLAGFSDTQYF	2	CD4-C5
Pla-1_TCACAAGGTGTGTGCC	Pla	Pla-1	P1-2135	FALSE	1	TRA:CAVEVMDSSYKLIF TRB:CASSLRQGITDTQYF	1	CD4-C4
Pla-1_TCAGCAACAGTGAGTG	Pla	Pla-1	P1-36	TRUE	3	TRA:CILRDNAGNNRKLIW TRB:CASSSRQGFPGGANVLTF	40	CD4-C6
Pla-1_TCAGCAAGTGTAAGTA	Pla	Pla-1	P1-2143	FALSE	1	TRA:CAVGLDTGRRALTF TRB:CASSGERDTDTQYF	1	CD4-C5
Pla-1_TCAGCTCCATACGCTA	Pla	Pla-1	P1-165	FALSE	3	TRA:CAMDYNQGGKLIF TRB:CASSSATGNTEAFF	4	CD4-C5
Pla-1_TCATTTGGTAAATACG	Pla	Pla-1	P1-2150	FALSE	1	TRA:CAASLNDYKLSF TRB:CASSQRASNNEQFF	1	CD4-C5
Pla-1_TCCCGATAGGCATGTG	Pla	Pla-1	P1-105	FALSE	3	TRA:CIVRVVSNQGGKLIF TRB:CASSPRTGGVGTQYF	6	CD4-C5
Pla-1_TCCCGATGTCCAGTAT	Pla	Pla-1	P1-377	FALSE	2	TRA:CAESFSGYALNF TRB:CASSLPSIRADTQYF	2	CD4-C5
Pla-1_TCCCGATTCAGGCAAG	Pla	Pla-1	P1-422	FALSE	2	TRA:CASFNTGNQFYF TRB:CASSVVGGRATGELFF	2	CD4-C5
Pla-1_TCGCGTTGTAAGTGGC	Pla	Pla-1	P1-162	FALSE	3	TRA:CVVSGGLRGAAGNKLTF TRB:CASSPLTVNTEAFF	4	CD4-C5
Pla-1_TCGCGTTGTTGGGACA	Pla	Pla-1	P1-2159	FALSE	1	TRA:CAVKFGNEKLTF TRB:CASSRRQGPSGANVLTF	1	CD4-C6
Pla-1_TCGCGTTTCCGATATG	Pla	Pla-1	P1-2160	FALSE	1	TRA:CALDLDRGSTLGRLYF TRB:CASSPLVGKGYSNQPQHF	1	CD4-C5
Pla-1_TCGGGACCATGCAATC	Pla	Pla-1	P1-2162	FALSE	1	TRA:CAVRASNTGFQKLVF TRB:CASSPPNLHNEQFF	1	CD4-C5
Pla-1_TCGTAGACACGACTCG	Pla	Pla-1	P1-2170	FALSE	1	TRA:CATPVGGYQKVTF TRB:CSGSGTARSNEQFF	1	CD4-C4
Pla-1_TCTATTGTCGTACGGC	Pla	Pla-1	P1-2176	FALSE	1	TRA:CAVGAGNTPLVF TRB:CASSLAMAGGVYEQYF	1	CD4-C5
Pla-1_TCTCTAAAGCGTAGTG	Pla	Pla-1	P1-105	FALSE	3	TRA:CIVRVVSNQGGKLIF TRB:CASSPRTGGVGTQYF	6	CD4-C5
Pla-1_TCTCTAACAGTAAGAT	Pla	Pla-1	P1-163	FALSE	3	TRA:CALRFNDYKLSF TRB:CASTSFETEAFF	4	CD4-C5
Pla-1_TCTCTAATCACGATGT	Pla	Pla-1	P1-2184	FALSE	1	TRA:CADPKTSYDKVIF TRB:CSAFPGRGETQYF	1	CD4-C5
Pla-1_TGACGGCGTTAAGAAC	Pla	Pla-1	P1-2196	FALSE	1	TRA:CALNPAGGYNKLIF TRB:CSAEKTRGQAYEQYF	1	CD4-C5
Pla-1_TGACTAGGTGCAACGA	Pla	Pla-1	P1-2200	FALSE	1	TRA:CAVVRNTPLVF TRB:CASSSGRGTSYEQYF	1	CD4-C5
Pla-1_TGACTTTAGCGTTTAC	Pla	Pla-1	P1-2202	FALSE	1	TRA:CAARSGGSYIPTF TRB:CASTASENTGELFF	1	CD4-C5
Pla-1_TGCCCATGTAGTAGTA	Pla	Pla-1	P1-170	FALSE	3	TRA:CALSGKNTGNQFYF TRB:CASSLDQGGNYGYTF	4	CD4-C6
Pla-1_TGCCCTAAGCGTTTAC	Pla	Pla-1	P1-250	FALSE	3	TRA:CAHYDRGSTLGRLYF TRB:CASSLRIDQPQHF	3	CD4-C6
Pla-1_TGCGCAGTCGCCAGCA	Pla	Pla-1	P1-319	FALSE	2	TRA:CVVSESPASKIIF TRB:CASSLRQGITDTQYF	2	CD4-C5

Pla-1_TGCGGGTAGAAGAAGC	Pla	Pla-1	P1-2230	FALSE	1	TRA:CATGRGSNYKLTF TRB:CASTTEFGELFF	1	CD4-C5
Pla-1_TGCGGGTAGCAGGTCA	Pla	Pla-1	P1-2231	FALSE	1	TRA:CAGAAPAGGTSYGKLTF TRB:CASSRGQTSNQPQHF	1	CD4-C4
Pla-1_TGCGGGTAGTGAAGTT	Pla	Pla-1	P1-2232	FALSE	1	TRA:CAVSPTGGFKTIF TRB:CASSFRQGGEKLFF	1	CD4-C5
Pla-1_TGCGTGGGTCCGTTAA	Pla	Pla-1	P1-2236	FALSE	1	TRA:CAVRDGGSQGNLIF TRB:CASSLTTDTEAFF	1	CD4-C5
Pla-1_TGGCCAGGTACTCAAC	Pla	Pla-1	P1-94	TRUE	3	TRA:CAASIQDFGNEKLTF TRB:CASSLQVPPNTEAFF	6	CD4-C4
Pla-1_TGGCTGGAGCGATTCT	Pla	Pla-1	P1-2251	FALSE	1	TRA:CAVRDGGSGNTGKLIF TRB:CAWFRGTVYTGELFF	1	CD4-C5
Pla-1_TGGCTGGCAGGATTGG	Pla	Pla-1	P1-2252	FALSE	1	TRA:CAENMNSGYSTLTF TRB:CASSIQGAVTDTQYF	1	CD4-C5
Pla-1_TGGGAAGAGAGCCCAA	Pla	Pla-1	P1-2255	FALSE	1	TRA:CVVEVDNTGKLIF TRB:CASSPGVYEQYF	1	CD4-C5
Pla-1_TGGGAAGTCAGTTAGC	Pla	Pla-1	P1-83	FALSE	3	TRA:CAVRDIENTGFQKLVF TRB:CASTSGGFNQPQHF	7	CD4-C5
Pla-1_TGTATTCCACTAGTAC	Pla	Pla-1	P1-2262	FALSE	1	TRA:CAVSEPRNRDDKIIF TRB:CASSPQRRNSYEQYF	1	CD4-C4
Pla-1_TGTCCCAGTACAGTGG	Pla	Pla-1	P1-89	TRUE	3	TRA:CAVDVNYGGQNFVF TRB:CASSWGDTQYF	6	CD4-C5
Pla-1_TTAACTCTCAGATAAG	Pla	Pla-1	P1-2278	FALSE	1	TRA:CAVMDGGSNYKLTF TRB:CASSLARGGTAFF	1	CD4-C5
Pla-1_TTAACTCTCGTTTATC	Pla	Pla-1	P1-2280	FALSE	1	TRA:CIGLAGNNRKLIW TRB:CASSLEGGAGANVLTF	1	CD4-C5
Pla-1_TTAGGACTCGCAAGCC	Pla	Pla-1	P1-2284	FALSE	1	TRA:CAVRAGRRALTF TRB:CASSQDFGATYEQYF	1	CD4-C5
Pla-1_TTATGCTGTTGTGGCC	Pla	Pla-1	P1-51	FALSE	3	TRA:CAYRSLNNAGNMLTF TRB:CASSRGNNSPLHF	14	CD4-C5
Pla-1_TTCGAAGTCCTCAACC	Pla	Pla-1	P1-345	FALSE	2	TRA:CAESISWTGGGADGLTF TRB:CASSVLGEKLFF	2	CD4-C5
Pla-1_TTCGGTCAGTGTTTGC	Pla	Pla-1	P1-2298	FALSE	1	TRA:CAVGLTGTASKLTF TRB:CASSYSRGPSLRAEAFF	1	CD4-C5
Pla-1_TTCTCCTGTAACGCGA	Pla	Pla-1	P1-2309	FALSE	1	TRA:CAVSGGSNYKLTF TRB:CASSSGEGRLHEQYF	1	CD4-C5
Pla-1_TTGAACGGTGGGTATG	Pla	Pla-1	P1-2314	FALSE	1	TRA:CALSDFTGNQFYF TRB:CSARRTENYGYTF	1	CD4-C5
Pla-1_TTGACTTTCGCGATCG	Pla	Pla-1	P1-48	TRUE	3	TRA:CAEVSGYSTLTF TRB:CASSFPTGGRTGRTEAFF	16	CD4-C5
Pla-1_TTTATGCTCGCCAGCA	Pla	Pla-1	P1-2332	FALSE	1	TRA:CAYGGSQGNLIF TRB:CSAMGRDQETQYF	1	CD4-C5
Pla-1_TTTCCTCCATGTTGAC	Pla	Pla-1	P1-65	FALSE	3	TRA:CAVQAHSGGSNYKLTF TRB:CASAETSYTGELFF	10	CD4-C3
Pla-1_TTTGTCAGTCGAAAGC	Pla	Pla-1	P1-2344	FALSE	1	TRA:CAMSAMVQGAQKLVF TRB:CASSPRGTGADTQYF	1	CD4-C5
Pla-1_AAACGGGAGTCTTGCA	Pla	Pla-1	P1-1083	FALSE	1	TRA:CAYRSRDNYGQNFVF TRB:CASSLGQGLISTDTQYF	1	CD4-C7
Pla-1_AAAGCAAAGGGAACGG	Pla	Pla-1	P1-69	FALSE	3	TRA:CAVGVYNTDKLIF TRB:CASSQEEGASSYEQYF	9	CD4-C7
Pla-1_AAATGCCTCTGCCCTA	Pla	Pla-1	P1-1092	FALSE	1	TRA:CAANPNTGTASKLTF TRB:CSAIPGGMNTEAFF	1	CD4-C7
Pla-1_AACTTTCTCTTCATGT	Pla	Pla-1	P1-312	FALSE	2	TRA:CALTHNQAGTALIF TRB:CASSEGTGLGYGYTF	2	CD4-C7

Pla-1_AAGACCTGTAGAGTGC	Pla	Pla-1	P1-1113	FALSE	1	TRA:CALSDRSDSWGKLQF TRB:CASSQGYEQYF	1	CD4-C7
Pla-1_AAGGAGCTCAGTCAGT	Pla	Pla-1	P1-207	FALSE	3	TRA:CAAAGTASKLTF TRB:CASSFDDRGGEQYF	3	CD4-C7
Pla-1_AATCGGTCAAAGGCGT	Pla	Pla-1	P1-69	FALSE	3	TRA:CAVGVYNTDKLIF TRB:CASSQEEGASSYEQYF	9	CD4-C7
Pla-1_ACACCAAGTTACCAGT	Pla	Pla-1	P1-1143	FALSE	1	TRA:CALPYGNNRLAF TRB:CSARAGGNSYEQYF	1	CD4-C7
Pla-1_ACACTGAAGTCGAGTG	Pla	Pla-1	P1-64	FALSE	3	TRA:CALSGTSSSNTGKLIF TRB:CASSFERTSGYTDTQYF	10	CD4-C7
Pla-1_ACAGCCGAGAGCCTAG	Pla	Pla-1	P1-315	FALSE	2	TRA:CALVVGGGYSTLTF TRB:CASSPGQGFNEQFF	2	CD4-C7
Pla-1_ACGATGTAGGACATTA	Pla	Pla-1	P1-1188	FALSE	1	TRA:CAVRGGSYNTDKLIF TRB:CASSQVGRGGEETQYF	1	CD4-C7
Pla-1_ACGGGTCTCCACTCCA	Pla	Pla-1	P1-59	FALSE	3	TRA:CALSGANDYKLSF TRB:CSASGHSSGNTIYF	12	CD4-C7
Pla-1_ACGTCAACAAGGACAC	Pla	Pla-1	P1-1209	FALSE	1	TRA:CATVPGGSYIPTF TRB:CASRITPSTDTQYF	1	CD4-C7
Pla-1_ACTATCTGTGAGTATA	Pla	Pla-1	P1-1217	FALSE	1	TRA:CAASSVNRDDKIIF TRB:CASSHGTGTYEQYF	1	CD4-C2
Pla-1_AGAGCGAAGGGATCTG	Pla	Pla-1	P1-59	FALSE	3	TRA:CALSGANDYKLSF TRB:CSASGHSSGNTIYF	12	CD4-C7
Pla-1_AGATTGCGTCTAGGTT	Pla	Pla-1	P1-1261	FALSE	1	TRA:CAASGRGGTSYGKLTF TRB:CASYGLYYGYTF	1	CD4-C7
Pla-1_AGCGGTCAGAATTGTG	Pla	Pla-1	P1-1267	FALSE	1	TRA:CALSGANDYKLSF TRB:CASSLTGAVYGYTF	1	CD4-C7
Pla-1_AGCTTGACACACATGT	Pla	Pla-1	P1-1277	FALSE	1	TRA:CAVRDTSNTGKLIF TRB:CASSPPGRVGGQPQHF	1	CD4-C7
Pla-1_AGGCCGTAGTGTGGCA	Pla	Pla-1	P1-1279	FALSE	1	TRA:CALSITSGSRLTF TRB:CASSPDRGEQYF	1	CD4-C7
Pla-1_AGGGAGTAGGACCACA	Pla	Pla-1	P1-1281	FALSE	1	TRA:CAVRDVLAAGNKLTF TRB:CASSYRTSGGYNEQFF	1	CD4-C7
Pla-1_AGTGTCACAAGTAATG	Pla	Pla-1	P1-1300	FALSE	1	TRA:CLVGGGAGNMLTF TRB:CSARAGGIYEQYF	1	CD4-C7
Pla-1_ATCACGAAGACAATAC	Pla	Pla-1	P1-1316	FALSE	1	TRA:CILRDDFPGNQFYF TRB:CASRYRNNEQFF	1	CD4-C7
Pla-1_ATCTACTGTAAGGATT	Pla	Pla-1	P1-69	FALSE	3	TRA:CAVGVYNTDKLIF TRB:CASSQEEGASSYEQYF	9	CD4-C7
Pla-1_ATGCGATGTAGAGGAA	Pla	Pla-1	P1-64	FALSE	3	TRA:CALSGTSSSNTGKLIF TRB:CASSFERTSGYTDTQYF	10	CD4-C7
Pla-1_ATGGGAGCAAGTCTAC	Pla	Pla-1	P1-59	FALSE	3	TRA:CALSGANDYKLSF TRB:CSASGHSSGNTIYF	12	CD4-C7
Pla-1_CAACCAAGTAGCTCCG	Pla	Pla-1	P1-1364	FALSE	1	TRA:CAVWGTGFQKLVF TRB:CASRYPARSGTGELFF	1	CD4-C7
Pla-1_CAAGAAAAGGCAATTA	Pla	Pla-1	P1-285	TRUE	2	TRA:CAMSDASGGYNKLIF TRB:CASGTHNRNTGELFF	2	CD4-C7
Pla-1_CACACCTCAAAGTGCG	Pla	Pla-1	P1-59	FALSE	3	TRA:CALSGANDYKLSF TRB:CSASGHSSGNTIYF	12	CD4-C7
Pla-1_CACCACTGTGACGGTA	Pla	Pla-1	P1-1406	FALSE	1	TRA:CAMRGSYNAGNNRKLIW TRB:CASTPQSSGHQETQYF	1	CD4-C7
Pla-1_CAGAATCAGTACTTGC	Pla	Pla-1	P1-69	FALSE	3	TRA:CAVGVYNTDKLIF TRB:CASSQEEGASSYEQYF	9	CD4-C7
Pla-1_CAGAGAGGTCATATCG	Pla	Pla-1	P1-1417	FALSE	1	TRA:CAMLNTNAGKSTF TRB:CASSLPDRGYYEQYF	1	CD4-C7

Pla-1_CAGATCACATTTGCTT	Pla	Pla-1	P1-69	FALSE	3	TRA:CAVGVYNTDKLIF TRB:CASSQEEGASSYEQYF	9	CD4-C7
Pla-1_CAGCATACACTTCTGC	Pla	Pla-1	P1-1422	FALSE	1	TRA:CALSGTSSSNTGKLIF TRB:CASIEGQGPYNEQFF	1	CD4-C7
Pla-1_CAGCCGAGTTATCGGT	Pla	Pla-1	P1-1425	FALSE	1	TRA:CAESNHGQNFVF TRB:CSASVGRGEQFF	1	CD4-C7
Pla-1_CAGCTGGCACAAGTAA	Pla	Pla-1	P1-367	FALSE	2	TRA:CAENVGNRDDKIIF TRB:CASSREANSGNTIYF	2	CD4-C7
Pla-1_CAGCTGGTCAGTGCAT	Pla	Pla-1	P1-1430	FALSE	1	TRA:CAFMKHGQGGSEKLVF TRB:CASSHGFSTDTQYF	1	CD4-C7
Pla-1_CAGTAACTCTGTCAAG	Pla	Pla-1	P1-1436	FALSE	1	TRA:CAASKRSFGNVLHC TRB:CASSQDLGLDYGYTF	1	CD4-C7
Pla-1_CATCAGAGTGATGCCC	Pla	Pla-1	P1-1442	FALSE	1	TRA:CAASMGSGYSTLTF TRB:CASSRNWDREIHEQFF	1	CD4-C7
Pla-1_CATCGGGCAAGGTTCT	Pla	Pla-1	P1-1446	FALSE	1	TRA:CAVGVYNTDKLIF TRB:CASSSGNSPYEQYF	1	CD4-C7
Pla-1_CATGCCTGTCTTCGTC	Pla	Pla-1	P1-234	FALSE	3	TRA:CATWMDSSYKLIF TRB:CASSPLGRQGARTGELFF	3	CD4-C7
Pla-1_CATTATCGTGCTTCTC	Pla	Pla-1	P1-1466	FALSE	1	TRA:CAVRDNAGNMLTF TRB:CASSPDGLGGKKLFF	1	CD4-C7
Pla-1_CCACCTATCATCACCC	Pla	Pla-1	P1-312	FALSE	2	TRA:CALTHNQAGTALIF TRB:CASSEGTGLGYGYTF	2	CD4-C7
Pla-1_CCACGGAGTGTGGTTT	Pla	Pla-1	P1-233	FALSE	3	TRA:CAAILSSGTYKYIF TRB:CASSVGGGSYNEQFF	3	CD4-C7
Pla-1_CCGGTAGCAAATCCGT	Pla	Pla-1	P1-1500	FALSE	1	TRA:CAATYGQNFVF TRB:CASSLQITGGSGANVLTF	1	CD4-C7
Pla-1_CCGGTAGGTGGTGTAG	Pla	Pla-1	P1-374	FALSE	2	TRA:CIVRVNSGNTPLVF TRB:CSASGHSSGNTIYF	2	CD4-C7
Pla-1_CCTAAAGCATATGGTC	Pla	Pla-1	P1-1507	FALSE	1	TRA:CAVKTYGNNRLAF TRB:CASSSGAAGAYQETQYF	1	CD4-C7
Pla-1_CCTACCAGTTGTGGCC	Pla	Pla-1	P1-1514	FALSE	1	TRA:CALSGYSGNTPLVF TRB:CASSRSLSTTNEKLFF	1	CD4-C7
Pla-1_CCTCAGTAGCCTCGTG	Pla	Pla-1	P1-1519	FALSE	1	TRA:CAYRSGLIIQGAQKLVF TRB:CASSSPPGRGDYNEQFF	1	CD4-C7
Pla-1_CGAACATGTGTGCGTC	Pla	Pla-1	P1-1537	FALSE	1	TRA:CAVQARSRTGGSYIPTF TRB:CASSLGQGLADWTQYF	1	CD4-C7
Pla-1_CGAATGTGTGCACGAA	Pla	Pla-1	P1-65	FALSE	3	TRA:CAVQAHSGGSNYKLTF TRB:CASAETSYTGELFF	10	CD4-C7
Pla-1_CGAATGTTCCTCATTA	Pla	Pla-1	P1-77	FALSE	3	TRA:CALSEAGYGGSQGNLIF TRB:CASSHEPRTGELFF	8	CD4-C7
Pla-1_CGACCTTGTCTGCAAT	Pla	Pla-1	P1-233	FALSE	3	TRA:CAAILSSGTYKYIF TRB:CASSVGGGSYNEQFF	3	CD4-C7
Pla-1_CGAGAAGAGCTATGCT	Pla	Pla-1	P1-1551	FALSE	1	TRA:CAMRGTNSGGSNYKLTF TRB:CSVSPWTFLTDTQYF	1	CD4-C7
Pla-1_CGATGGCCACGAAATA	Pla	Pla-1	P1-59	FALSE	3	TRA:CALSGANDYKLSF TRB:CSASGHSSGNTIYF	12	CD4-C7
Pla-1_CGATGTACACTGTGTA	Pla	Pla-1	P1-1564	FALSE	1	TRA:CAVERKNDYKLSF TRB:CASSLDRAFWPQHF	1	CD4-C2
Pla-1_CGCGTTTTCAGAGACG	Pla	Pla-1	P1-234	FALSE	3	TRA:CATWMDSSYKLIF TRB:CASSPLGRQGARTGELFF	3	CD4-C7
Pla-1_CGCTTCAGTTACCAGT	Pla	Pla-1	P1-1579	FALSE	1	TRA:CAVDGSGGYNKLIF TRB:CASSQGQGAGGYTF	1	CD4-C7
Pla-1_CGGACTGTCGTCTGCT	Pla	Pla-1	P1-382	FALSE	2	TRA:CAASKRSFGNVLHC TRB:CASSEAGQGRKTQYF	2	CD4-C7

Pla-1_CGTAGCGTCGATAGAA	Pla	Pla-1	P1-77	FALSE	3	TRA:CALSEAGYGGSQGNLIF TRB:CASSHEPRTGELFF	8	CD8-C8
Pla-1_CGTCAGGAGTCAAGGC	Pla	Pla-1	P1-374	FALSE	2	TRA:CIVRVNSGNTPLVF TRB:CSASGHSSGNTIYF	2	CD4-C7
Pla-1_CGTGTAAAGAGTGAGA	Pla	Pla-1	P1-59	FALSE	3	TRA:CALSGANDYKLSF TRB:CSASGHSSGNTIYF	12	CD4-C7
Pla-1_CTAAGACGTACATGTC	Pla	Pla-1	P1-1635	FALSE	1	TRA:CVVSDQSGTYKYIF TRB:CASRPYLTDITEAFF	1	CD4-C7
Pla-1_CTAATGGAGATATGGT	Pla	Pla-1	P1-1636	FALSE	1	TRA:CAVSRSGGYQKVTF TRB:CASRTTSESTDTQYF	1	CD4-C7
Pla-1_CTACCCAGTACTCTCC	Pla	Pla-1	P1-59	FALSE	3	TRA:CALSGANDYKLSF TRB:CSASGHSSGNTIYF	12	CD4-C7
Pla-1_CTACCCAGTGCACGAA	Pla	Pla-1	P1-1647	FALSE	1	TRA:CALSEETGTASKLTF TRB:CASSIPGQGWGYTF	1	CD4-C7
Pla-1_CTCACACGTAGAGGAA	Pla	Pla-1	P1-1661	FALSE	1	TRA:CAVLHSNYQLIW TRB:CASRWTSGFNTGELFF	1	CD4-C7
Pla-1_CTCACACTCACCTCGT	Pla	Pla-1	P1-1663	FALSE	1	TRA:CIVRVPSYNQGGKLIF TRB:CASRAYNEQFF	1	CD4-C4
Pla-1_CTCAGAAAGCAATCTC	Pla	Pla-1	P1-1664	FALSE	1	TRA:CAVTTGGGNKLTF TRB:CSAAGTSGPRGEQYF	1	CD4-C7
Pla-1_CTCAGAACAAATTGCC	Pla	Pla-1	P1-64	FALSE	3	TRA:CALSGTSSSNTGKLIF TRB:CASSFERTSGYTDTQYF	10	CD4-C7
Pla-1_CTCGAGGGTGATAAAC	Pla	Pla-1	P1-170	FALSE	3	TRA:CALSGKNTGNQFYF TRB:CASSLDQGGNYGYTF	4	CD4-C7
Pla-1_CTCGTCATCGAGGTAG	Pla	Pla-1	P1-1698	FALSE	1	TRA:CAVGAGGSYIPTF TRB:CASSLEWTGYGNEKLFF	1	CD4-C7
Pla-1_CTCTAATAGCAGGTCA	Pla	Pla-1	P1-1701	FALSE	1	TRA:CALGGGSYIPTF TRB:CASIGHASIGSLEQYF	1	CD4-C7
Pla-1_CTCTGGTTCCTAGAAC	Pla	Pla-1	P1-1707	FALSE	1	TRA:CAMSAYTGGFKTIF TRB:CASSLEVRGRNTEAFF	1	CD4-C7
Pla-1_CTGATAGGTCCTAGCG	Pla	Pla-1	P1-1720	FALSE	1	TRA:CVVSSGYSTLTF TRB:CASSDTSGGPTQYF	1	CD4-C7
Pla-1_CTGATCCGTGTTTGGT	Pla	Pla-1	P1-396	FALSE	2	TRA:CIVRPLYNFNKFYF TRB:CASTYTGTGGSEQYF	2	CD4-C7
Pla-1_CTGATCCTCATAAAGG	Pla	Pla-1	P1-1725	FALSE	1	TRA:CAVRNYGGSQGNLIF TRB:CASRPDPADTQYF	1	CD4-C7
Pla-1_CTGGTCTAGCTGCAAG	Pla	Pla-1	P1-1732	FALSE	1	TRA:CVVNMNRDDKIIF TRB:CASSRVDEQYF	1	CD4-C7
Pla-1_CTTAACTTCACTATTC	Pla	Pla-1	P1-1747	FALSE	1	TRA:CAVERWDTGRRALTF TRB:CSAIPGGMNTEAFF	1	CD4-C7
Pla-1_CTTCTCTAGATATGCA	Pla	Pla-1	P1-64	FALSE	3	TRA:CALSGTSSSNTGKLIF TRB:CASSFERTSGYTDTQYF	10	CD4-C7
Pla-1_GAACGGAAGCGGCTTC	Pla	Pla-1	P1-1769	FALSE	1	TRA:CAVPGGTGSSNTGKLIF TRB:CASSRRFGSQETQYF	1	CD4-C7
Pla-1_GAAGCAGAGAGCTGGT	Pla	Pla-1	P1-1774	FALSE	1	TRA:CIVAPSGGSYIPTF TRB:CAWSVGGAKNIQYF	1	CD4-C7
Pla-1_GACGGCTCAAGCCTAT	Pla	Pla-1	P1-1792	FALSE	1	TRA:CAAILSSGTYKYIF TRB:CASNLGGGADNEQFF	1	CD4-C7
Pla-1_GACTGCGTCAATCACG	Pla	Pla-1	P1-1813	FALSE	1	TRA:CIVRVDPSLYGGSQGNLIF TRB:CASSLEGGELFF	1	CD4-C7
Pla-1_GAGGTGAGTACAGTTC	Pla	Pla-1	P1-1815	FALSE	1	TRA:CVVSANNNARLMF TRB:CASSPTASAVSSYEQYF	1	CD4-C7
Pla-1_GATCGATAGACCTTTG	Pla	Pla-1	P1-1822	FALSE	1	TRA:CAVVFLYSSASKIIF TRB:CASRAYNEQFF	1	CD4-C2

Pla-1_GATCGATAGGGTGTGT	Pla	Pla-1	P1-1825	FALSE	1	TRA:CALSEVFSGGYNKLIF TRB:CASRVGRGYQPQHF	1	CD4-C7
Pla-1_GATCGCGGTAGTAGTA	Pla	Pla-1	P1-1830	FALSE	1	TRA:CISNTGNQFYF TRB:CASSPRRTNTEAFF	1	CD4-C7
Pla-1_GATCGTATCTATGTGG	Pla	Pla-1	P1-1832	FALSE	1	TRA:CAVLSGYSTLTF TRB:CASNPTGGIYEQYF	1	CD4-C7
Pla-1_GATGAAACAAGAAGAG	Pla	Pla-1	P1-1836	FALSE	1	TRA:CAVDTTGANNLFF TRB:CSVLPGQETQYF	1	CD4-C7
Pla-1_GATTCAGCAATAGAGT	Pla	Pla-1	P1-1841	FALSE	1	TRA:CAVSGAAGGTSYGKLTF TRB:CASSYGLKVVF	1	CD4-C7
Pla-1_GCAATCAAGCATCATC	Pla	Pla-1	P1-1843	FALSE	1	TRA:CAVEARRRDDKIIF TRB:CASRRDTGEKLFF	1	CD4-C7
Pla-1_GCACTCTCACTCTGTC	Pla	Pla-1	P1-182	TRUE	3	TRA:CAASRGGGADGLTF TRB:CASSLEVGGFGNTIYF	3	CD4-C7
Pla-1_GCGCAGTTCCGCGGTA	Pla	Pla-1	P1-1874	FALSE	1	TRA:CVVRETGANNLFF TRB:CSARLPGLAGGYEQYF	1	CD4-C7
Pla-1_GCTGCGAAGCAAATCA	Pla	Pla-1	P1-1887	FALSE	1	TRA:CAASVGGTSYGKLTF TRB:CASMKVGGNTIYF	1	CD4-C4
Pla-1_GCTGCGATCACAGTAC	Pla	Pla-1	P1-1889	FALSE	1	TRA:CVVSAYEVTGGGNKLTF TRB:CASSLAVRGRVETQYF	1	CD4-C7
Pla-1_GCTTCCACATAAAGGT	Pla	Pla-1	P1-1896	FALSE	1	TRA:CAAPYGGSQGNLIF TRB:CASSYGQPSTDTQYF	1	CD4-C7
Pla-1_GGAATAAGTAGATTAG	Pla	Pla-1	P1-1903	FALSE	1	TRA:CAVRDRITGGGNKLTF TRB:CASSYSSGRAYEQYF	1	CD4-C7
Pla-1_GGAATAAGTTCCGGCA	Pla	Pla-1	P1-1904	FALSE	1	TRA:CLVGSYNFNKFYF TRB:CASSFGGFLPYEQYF	1	CD4-C7
Pla-1_GGACAAGCAATGAAAC	Pla	Pla-1	P1-1905	FALSE	1	TRA:CVVRDGGFKTIF TRB:CASSLGGAYEQYF	1	CD4-C7
Pla-1_GGCCGATGTTATCCGA	Pla	Pla-1	P1-1925	FALSE	1	TRA:CAGLNTGTASKLTF TRB:CASEINRNTEAFF	1	CD4-C7
Pla-1_GGGCATCCATGGAATA	Pla	Pla-1	P1-1952	FALSE	1	TRA:CLVGDSGGFKTIF TRB:CASSVGVSGYEQYF	1	CD4-C7
Pla-1_GGGCATCGTGCGGTAA	Pla	Pla-1	P1-1953	FALSE	1	TRA:CAMREGGYQKVTF TRB:CASSRDSTSQPQHF	1	CD4-C7
Pla-1_GGTATTGCAAGTCATC	Pla	Pla-1	P1-59	FALSE	3	TRA:CALSGANDYKLSF TRB:CSASGHSSGNTIYF	12	CD4-C7
Pla-1_GGTGAAGAGTGATCGG	Pla	Pla-1	P1-410	FALSE	2	TRA:CALDGAGGFKTIF TRB:CASSLQGSTDTQYF	2	CD4-C7
Pla-1_GTAACGTTCTGCTTGC	Pla	Pla-1	P1-170	FALSE	3	TRA:CALSGKNTGNQFYF TRB:CASSLDQGGNYGYTF	4	CD4-C2
Pla-1_GTACTTTGTCATATGC	Pla	Pla-1	P1-315	FALSE	2	TRA:CALVVGGGYSTLTF TRB:CASSPGQGFNEQFF	2	CD4-C7
Pla-1_GTATCTTGTGGAAAGA	Pla	Pla-1	P1-1991	FALSE	1	TRA:CIVRAGAYNQGGKLIF TRB:CASRSQGSHGYTF	1	CD4-C7
Pla-1_GTCGGGTTCGAGAGCA	Pla	Pla-1	P1-2007	FALSE	1	TRA:CALGLLDDKIIF TRB:CSARLAMRMSSYNEQFF	1	CD4-C7
Pla-1_GTCTCGTCACTGTGTA	Pla	Pla-1	P1-2010	FALSE	1	TRA:CAASMGGGADGLTF TRB:CASSRTGEGEQYF	1	CD4-C7
Pla-1_GTCTTCGCAAGGACAC	Pla	Pla-1	P1-77	FALSE	3	TRA:CALSEAGYGGSQGNLIF TRB:CASSHEPRTGELFF	8	CD4-C7
Pla-1_GTGCTTCAGGGCACTA	Pla	Pla-1	P1-2026	FALSE	1	TRA:CAVSNTGGFKTIF TRB:CASKGQVVPYEQYF	1	CD4-C7
Pla-1_GTGTGCGAGTGGCACA	Pla	Pla-1	P1-2035	FALSE	1	TRA:CAVGANDYKLSF TRB:CASSLQTGGDQPQHF	1	CD4-C7

Pla-1_GTTACAGTCGCTGATA	Pla	Pla-1	P1-2048	FALSE	1	TRA:CVVTAASDTNAGKSTF TRB:CSASWGFSYNEQFF	1	CD4-C7
Pla-1_GTTCATTGTCGCGTGT	Pla	Pla-1	P1-69	FALSE	3	TRA:CAVGVYNTDKLIF TRB:CASSQEEGASSYEQYF	9	CD4-C7
Pla-1_GTTTCTAAGCTGGAAC	Pla	Pla-1	P1-2059	FALSE	1	TRA:CASKNAGNMLTF TRB:CASSLGQGGRYEQYF	1	CD4-C7
Pla-1_TAAACCGAGATAGCAT	Pla	Pla-1	P1-2061	FALSE	1	TRA:CAVGVYNTDKLIF TRB:CASSPETGATDTQYF	1	CD4-C7
Pla-1_TAAACCGGTACCCAAT	Pla	Pla-1	P1-2062	FALSE	1	TRA:CIVRVGGGADGLTF TRB:CASSLLFGGPREDTQYF	1	CD4-C7
Pla-1_TAAACCGGTATCGCAT	Pla	Pla-1	P1-207	FALSE	3	TRA:CAAAGTASKLTF TRB:CASSFDDRGGEQYF	3	CD4-C7
Pla-1_TACCTATAGACCACGA	Pla	Pla-1	P1-2074	FALSE	1	TRA:CAVRDNPK TRB:CASSFGTGTNRDTQYF	1	CD4-C7
Pla-1_TACGGATCACAACGCC	Pla	Pla-1	P1-2080	FALSE	1	TRA:CAVNIWDLPKGRANAGGTSYGKLTF TRB:CASSQQGGET[illegible]	1	CD4-C7
Pla-1_TACGGGCGTGCCTGTG	Pla	Pla-1	P1-2084	FALSE	1	TRA:CAVFPLGGTSYGKLTF TRB:CASSLWGAGAGGTYNEQFF	1	CD4-C7
Pla-1_TAGACCATCAGGTAAA	Pla	Pla-1	P1-2104	FALSE	1	TRA:CALARDSNYQLIW TRB:CSVQQGKNTEAFF	1	CD4-C7
Pla-1_TATCTCAAGCGATGAC	Pla	Pla-1	P1-2119	FALSE	1	TRA:CAYGTDKLIF TRB:CASRDRGRSGNIQYF	1	CD4-C7
Pla-1_TATCTCAGTACGAAAT	Pla	Pla-1	P1-420	FALSE	2	TRA:CAVRDLDSNDYKLSF TRB:CASSLDEGSNIQYF	2	CD4-C7
Pla-1_TATGCCCAGCAGATCG	Pla	Pla-1	P1-2122	FALSE	1	TRA:CIVRVEGAAGNKLTF TRB:CSVARSSYNEQFF	1	CD4-C7
Pla-1_TCAATCTGTACGCTGC	Pla	Pla-1	P1-2132	FALSE	1	TRA:CAANTGGFKTIF TRB:CASSLKEYQYF	1	CD4-C7
Pla-1_TCAGCTCCATTGGTAC	Pla	Pla-1	P1-69	FALSE	3	TRA:CAVGVYNTDKLIF TRB:CASSQEEGASSYEQYF	9	CD4-C7
Pla-1_TCATTTGGTCCAGTTA	Pla	Pla-1	P1-2151	FALSE	1	TRA:CAVDSFRPRNKLVF TRB:CASSLLLAGGEQYF	1	CD4-C7
Pla-1_TCCACACCAGGTCTCG	Pla	Pla-1	P1-2154	FALSE	1	TRA:CAASPNNARLMF TRB:CATSYRVQEKLFF	1	CD4-C7
Pla-1_TCGAGGCAGGGCACTA	Pla	Pla-1	P1-59	FALSE	3	TRA:CALSGANDYKLSF TRB:CSASGHSSGNTIYF	12	CD4-C7
Pla-1_TCGAGGCGTAGAGTGC	Pla	Pla-1	P1-396	FALSE	2	TRA:CIVRPLYNFNKFYF TRB:CASTYTGTGGSEQYF	2	CD4-C7
Pla-1_TCGCGTTGTCAAACTC	Pla	Pla-1	P1-2158	FALSE	1	TRA:CIVRPPFGGTYKYIF TRB:CASSYSGTGGREQFF	1	CD4-C7
Pla-1_TCGGGACAGCTGCAAG	Pla	Pla-1	P1-2161	FALSE	1	TRA:CAAETSGSRLTF TRB:CASSERGIGYF	1	CD4-C7
Pla-1_TCGTACCAGGAATTAC	Pla	Pla-1	P1-2166	FALSE	1	TRA:CIVRAHYGGSQGNLIF TRB:CASSLGTGSAETQYF	1	CD4-C7
Pla-1_TCGTAGAGTGTAATGA	Pla	Pla-1	P1-2172	FALSE	1	TRA:CADINMGGADGLTF TRB:CASSEQGRELGSYEQYF	1	CD4-C2
Pla-1_TCGTAGAGTTCTGAAC	Pla	Pla-1	P1-69	FALSE	3	TRA:CAVGVYNTDKLIF TRB:CASSQEEGASSYEQYF	9	CD4-C7
Pla-1_TCTCATAGTGCGGTAA	Pla	Pla-1	P1-2178	FALSE	1	TRA:CALLPKTSYDKVIF TRB:CASRQGNTGELFF	1	CD4-C7
Pla-1_TCTGAGAAGTCGATAA	Pla	Pla-1	P1-69	FALSE	3	TRA:CAVGVYNTDKLIF TRB:CASSQEEGASSYEQYF	9	CD4-C7
Pla-1_TGAGAGGCATAGTAAG	Pla	Pla-1	P1-2204	FALSE	1	TRA:CAASVHFNKFYF TRB:CASSLPRENYGYTF	1	CD4-C7

Pla-1_TGCACCTAGCTGTTCA	Pla	Pla-1	P1-2210	FALSE	1	TRA:CATADRGSTLGRLYF TRB:CASSHGASGGQGAYEQYF	1	CD4-C7
Pla-1_TGCCCATCACTTAACG	Pla	Pla-1	P1-2218	FALSE	1	TRA:CIVTSGTYKYIF TRB:CSARGVGRSADTQYF	1	CD4-C7
Pla-1_TGCGCAGTCGCGATCG	Pla	Pla-1	P1-2227	FALSE	1	TRA:CAGGNQGGKLIF TRB:CASRHDYYGYTF	1	CD4-C7
Pla-1_TGCGCAGTCTTGTACT	Pla	Pla-1	P1-2229	FALSE	1	TRA:CAVSSGGGADGLTF TRB:CAWSNRVGYTF	1	CD4-C7
Pla-1_TGCTACCAGCTGAAAT	Pla	Pla-1	P1-2239	FALSE	1	TRA:CAYRTSGGATNKLIF TRB:CASRQTSGDLYEQYF	1	CD4-C7
Pla-1_TGGGAAGCAAGTCATC	Pla	Pla-1	P1-2256	FALSE	1	TRA:CIPIRSGGGADGLTF TRB:CASSERPWTGDTGELFF	1	CD4-C7
Pla-1_TGGGCGTGTTGCGCAC	Pla	Pla-1	P1-2258	FALSE	1	TRA:CAGPLTGSARQLTF TRB:CAWNRGDNEQFF	1	CD4-C7
Pla-1_TGTCCCAAGTACGACG	Pla	Pla-1	P1-59	FALSE	3	TRA:CALSGANDYKLSF TRB:CSASGHSSGNTIYF	12	CD4-C7
Pla-1_TGTGGTAGTCAAACTC	Pla	Pla-1	P1-410	FALSE	2	TRA:CALDGAGGFKTIF TRB:CASSLQGSTDTQYF	2	CD4-C7
Pla-1_TTAGTTCAGATGCCTT	Pla	Pla-1	P1-2286	FALSE	1	TRA:CAMREGQGGGFKTIF TRB:CASSRNPSSSGANVLTF	1	CD4-C7
Pla-1_TTATGCTGTGATAAAC	Pla	Pla-1	P1-2289	FALSE	1	TRA:CAENFNAGNNRKLIW TRB:CASGHGSNYGYTF	1	CD4-C7
Pla-1_TTCCCAGCAGTAAGCG	Pla	Pla-1	P1-2293	FALSE	1	TRA:CAVRGTGTASKLTF TRB:CSATGVETQYF	1	CD4-C7
Pla-1_TTCTCAATCGTAGGAG	Pla	Pla-1	P1-234	FALSE	3	TRA:CATWMDSSYKLIF TRB:CASSPLGRQGARTGELFF	3	CD4-C7
Pla-1_TTGCCGTAGCTGAACG	Pla	Pla-1	P1-420	FALSE	2	TRA:CAVRDLDSNDYKLSF TRB:CASSLDEGSNIQYF	2	CD4-C7
Pla-1_TTGGCAAGTGGCTCCA	Pla	Pla-1	P1-2322	FALSE	1	TRA:CAGDGSGNTGKLIF TRB:CASSPLGTEAFF	1	CD4-C7
Pla-1_TTGTAGGTCGATGAGG	Pla	Pla-1	P1-2326	FALSE	1	TRA:CAVRVSGNTPLVF TRB:CASSLWQARETQYF	1	CD4-C2
Pla-1_TTTACTGTCCTGCAGG	Pla	Pla-1	P1-2329	FALSE	1	TRA:CAVERGATNKLIF TRB:CSADDRDRGSGYTF	1	CD4-C7
Pla-1_TTTGCGCTCCAGAAGG	Pla	Pla-1	P1-2337	FALSE	1	TRA:CAVRGGSQGNLIF TRB:CASSQVAQARGLIRGDTQYF	1	CD4-C7
Pla-1_AAAGTAGAGGTTACCT	Pla	Pla-1	P1-1087	FALSE	1	TRA:CALSASNKEDTGRRALTF TRB:CSATTTTTGLPDTQYF	1	CD8-C8
Pla-1_AAAGTAGCACCACGTG	Pla	Pla-1	P1-1088	FALSE	1	TRA:CAVSRRISNFGNEKLTF TRB:CASGLTTTNEKLFF	1	CD8-C9
Pla-1_ACACTGAAGATCTGCT	Pla	Pla-1	P1-44	TRUE	3	TRA:CAYRGLGAQKLVF TRB:CASSSLDGQDNHYGYTF	22	CD8-C8
Pla-1_ACAGCCGCAAGGACTG	Pla	Pla-1	P1-211	FALSE	3	TRA:CAVNVQKSNTGNQFYF TRB:CASSYSVSYEQYF	3	CD8-C8
Pla-1_ACATCAGCAAGCCTAT	Pla	Pla-1	P1-193	TRUE	3	TRA:CAVRFSGGYNKLIF TRB:CASLEGPYSNQPQHF	3	CD8-C8
Pla-1_ACCTTTAAGTTCGATC	Pla	Pla-1	P1-1174	FALSE	1	TRA:CAYRSPLTGNQFYF TRB:CASSPPRGKQYF	1	CD8-C5
Pla-1_ACGAGCCTCCTTTCGG	Pla	Pla-1	P1-32	FALSE	3	TRA:CAVMNTGFQKLVF TRB:CSVSGLVLGGNEQFF	46	CD8-C9
Pla-1_ACGATGTCATCGTCGG	Pla	Pla-1	P1-319	FALSE	2	TRA:CVVSESPASKIIF TRB:CASSLRQGITDTQYF	2	CD4-C6
Pla-1_ACTATCTCAGGTCCAC	Pla	Pla-1	P1-1216	FALSE	1	TRA:CALSEGGQKLLF TRB:CASSYTSSYEQYF	1	CD8-C8

Pla-1_ACTTTCAGTCAGATAA	Pla	Pla-1	P1-1239	FALSE	1	TRA:CAVNEGSNYKLTF TRB:CASKPGSGSTEAFF	1	CD8-C9
Pla-1_AGACGTTTCAACGCTA	Pla	Pla-1	P1-116	TRUE	3	TRA:CALFAYSGAGSYQLTF TRB:CASRDDARTDTQYF	5	CD8-C8
Pla-1_AGAGTGGTCGAGAGCA	Pla	Pla-1	P1-1254	FALSE	1	TRA:CAEIPASKLTF TRB:CASSNTGTSGRADVTQYF	1	CD8-C8
Pla-1_AGCAGCCAGTACGTTC	Pla	Pla-1	P1-201	TRUE	3	TRA:CAYVGGNTPLVF TRB:CASSFPPSGGARETQYF	3	CD8-C8
Pla-1_AGCGTATAGGACTGGT	Pla	Pla-1	P1-1270	FALSE	1	TRA:CAASRATAGNKLTF TRB:CSARDRDSSYEQYF	1	CD8-C8
Pla-1_AGCGTCGAGCTTCGCG	Pla	Pla-1	P1-20	TRUE	3	TRA:CVVVGGRALTF TRB:CASSLATNPHETQYF	69	CD8-C9
Pla-1_AGTTGGTAGTCCTCCT	Pla	Pla-1	P1-1305	FALSE	1	TRA:CVVNPWAGANNLFF TRB:CASSLKGQGFTEAFF	1	CD8-C8
Pla-1_ATCATCTAGAGCTATA	Pla	Pla-1	P1-344	FALSE	2	TRA:CAERIKAAGNKLTF TRB:CASSYSSGVRELFF	2	CD8-C9
Pla-1_ATCATCTCATCGGACC	Pla	Pla-1	P1-9	TRUE	3	TRA:CAEAEGGGADGLTF TRB:CATSGGDNEQFF	174	CD8-C9
Pla-1_ATCATGGGTCCGAATT	Pla	Pla-1	P1-1324	FALSE	1	TRA:CAVAPGTGRRALTF TRB:CASSVVAAAMNTEAFF	1	CD8-C5
Pla-1_ATCCGAATCAGGCAAG	Pla	Pla-1	P1-1329	FALSE	1	TRA:CATASGSARQLTF TRB:CASSLSGSDYNEQFF	1	CD8-C9
Pla-1_ATCGAGTGTGTGACGA	Pla	Pla-1	P1-1333	FALSE	1	TRA:CAASPYSNASKIIF TRB:CAWSDRLGGGRSEAFF	1	CD8-C8
Pla-1_ATCTGCCTCAGTCAGT	Pla	Pla-1	P1-78	TRUE	3	TRA:CAAAQGGSEKLVF TRB:CASTRGDWIYNEQFF	7	CD8-C8
Pla-1_ATGAGGGGTAAATGAC	Pla	Pla-1	P1-1343	FALSE	1	TRA:CAVASSGSARQLTF TRB:CASSQRWQGAGFAEAFF	1	CD8-C8
Pla-1_ATTACTCGTTGAACTC	Pla	Pla-1	P1-51	FALSE	3	TRA:CAYRSLNNAGNMLTF TRB:CASSRGNNSPLHF	14	CD8-C9
Pla-1_ATTCTACTCACCTCGT	Pla	Pla-1	P1-1356	FALSE	1	TRA:CAAPLYYQLIW TRB:CSARLGRYGYTF	1	CD8-C8
Pla-1_CAACCAAAGTTGCAGG	Pla	Pla-1	P1-184	TRUE	3	TRA:CILRDGLMGDYKLSF TRB:CASSRDSRNSPLHF	3	CD8-C9
Pla-1_CAACCAATCTCTAGGA	Pla	Pla-1	P1-352	FALSE	2	TRA:CAVNNRRGSQGNLIF TRB:CSAKGAVYEQYF	2	CD8-C8
Pla-1_CAACCAATCTTCGAGA	Pla	Pla-1	P1-227	FALSE	3	TRA:CAVVNAGKSTF TRB:CASSLTGLVSNSPLHF	3	CD8-C8
Pla-1_CAACCTCAGAAACGCC	Pla	Pla-1	P1-1365	FALSE	1	TRA:CALRVITQGGFEKLVF TRB:CASSEGSSYNEQFF	1	CD8-C8
Pla-1_CACACAATCGGTCCGA	Pla	Pla-1	P1-56	TRUE	3	TRA:CVVNPWGSGTYKYIF TRB:CASSRDITDTQYF	13	CD8-C9
Pla-1_CACACTCGTACCTACA	Pla	Pla-1	P1-270	TRUE	2	TRA:CAAKSNTGRRALTF TRB:CASRMGVDQPQHF	2	CD8-C8
Pla-1_CACATTTCAATGTAAG	Pla	Pla-1	P1-55	TRUE	3	TRA:CALSPQDSNYQLIW TRB:CASSFWTSANEQFF	13	CD8-C8
Pla-1_CACATTTTCCGTACAA	Pla	Pla-1	P1-253	TRUE	2	TRA:CAMTRTGGGNKLTF TRB:CASSLGTGKGGPGSYEQYF	2	CD8-C8
Pla-1_CACCAGGGTACCAGTT	Pla	Pla-1	P1-1407	FALSE	1	TRA:CAESIGGSQGNLIF TRB:CAIRGHPSTDTQYF	1	CD8-C8
Pla-1_CAGCATAGTGCATCTA	Pla	Pla-1	P1-5	TRUE	3	TRA:CALSGTPMNRDDKIIF TRB:CSARGRGLAGGPAYEQYF	223	CD8-C8
Pla-1_CAGTAACTCTACTCAT	Pla	Pla-1	P1-1435	FALSE	1	TRA:CAAPGTYKYIF TRB:CASSFAGTYEQYF	1	CD8-C8

Pla-1_CATATTCAGAGACGAA	Pla	Pla-1	P1-160	FALSE	3	TRA:CAASADGGSQGNLIF TRB:CASSPTYGYTF	4	CD8-C9
Pla-1_CATATTCAGCACCGCT	Pla	Pla-1	P1-176	TRUE	3	TRA:CATVRTGANNLFF TRB:CASSQGLYEQYF	3	CD8-C9
Pla-1_CATGCCTAGCAGCGTA	Pla	Pla-1	P1-24	TRUE	3	TRA:CAVKGNTDKLIF TRB:CASSLRTGAYNEQFF	62	CD4-C6
Pla-1_CATGCCTGTTCGTCTC	Pla	Pla-1	P1-30	TRUE	3	TRA:CALPPGGNNRKLIW TRB:CASSFGDRGGTEAFF	47	CD8-C8
Pla-1_CATTCGCTCAGCGACC	Pla	Pla-1	P1-370	FALSE	2	TRA:CAVNGRTDKLIF TRB:CSATTTTTGLPDTQYF	2	CD4-C3
Pla-1_CCCTCCTCACATGGGA	Pla	Pla-1	P1-1494	FALSE	1	TRA:CAVRDNTGKLIF TRB:CAISDRGVNTQYF	1	CD8-C9
Pla-1_CCGTGGAGTCTCGTTC	Pla	Pla-1	P1-1504	FALSE	1	TRA:CAMSGGGADGLTF TRB:CASSFQRGEGTDTQYF	1	CD8-C8
Pla-1_CCTCTGAAGGGCTTCC	Pla	Pla-1	P1-1526	FALSE	1	TRA:CATGTGANSKLTF TRB:CASSLAVGTGGEKLFF	1	CD8-C8
Pla-1_CGAATGTGTCAGATAA	Pla	Pla-1	P1-1541	FALSE	1	TRA:CAVGADTGRRALTF TRB:CASSGERDTDTQYF	1	CD8-C8
Pla-1_CGAGCACAGAATTCCC	Pla	Pla-1	P1-239	FALSE	3	TRA:CAASLGDTDKLIF TRB:CASSPWTGVNEQFF	3	CD8-C9
Pla-1_CGATGTAAGAATTCCC	Pla	Pla-1	P1-101	FALSE	3	TRA:CAVIRPAGGATNKLIF TRB:CASSLGTGGDTQYF	6	CD8-C8
Pla-1_CGCGGTAGTGTAAGTA	Pla	Pla-1	P1-33	TRUE	3	TRA:CLVGTGGFKTIF TRB:CSARVPGQMKETQYF	41	CD8-C9
Pla-1_CGGCTAGCAGTCAGCC	Pla	Pla-1	P1-184	TRUE	3	TRA:CILRDGLMGDYKLSF TRB:CASSRDSRNSPLHF	3	CD8-C9
Pla-1_CGTCACTGTATAGTAG	Pla	Pla-1	P1-33	TRUE	3	TRA:CLVGTGGFKTIF TRB:CSARVPGQMKETQYF	41	CD8-C8
Pla-1_CGTCACTTCGGTTAAC	Pla	Pla-1	P1-15	TRUE	3	TRA:CAVNSSPLGANSKLTF TRB:CASSDSGNFYNEQFF	92	CD8-C9
Pla-1_CGTCTACTCACATACG	Pla	Pla-1	P1-1617	FALSE	1	TRA:CAMTTVLNSGYSTLTF TRB:CASRSLTSGRVRETQYF	1	CD8-C9
Pla-1_CGTGTAAAGTGAAGAG	Pla	Pla-1	P1-1621	FALSE	1	TRA:CAASDLGTGNQFYF TRB:CASSLLGPSNQDTQYF	1	CD8-C8
Pla-1_CGTTAGACAAACCTAC	Pla	Pla-1	P1-1628	FALSE	1	TRA:CALSNFGNEKLTF TRB:CASSFPPSGGARETQYF	1	CD8-C9
Pla-1_CTACACCAGAATAGGG	Pla	Pla-1	P1-1638	FALSE	1	TRA:CAERGGSYIPTF TRB:CSVARGPEDQPQHF	1	CD8-C9
Pla-1_CTAGAGTAGGGTCTCC	Pla	Pla-1	P1-1654	FALSE	1	TRA:CAASGNARLMF TRB:CASSRRSGVIYNEQFF	1	CD8-C9
Pla-1_CTCATTACAACGATGG	Pla	Pla-1	P1-1667	FALSE	1	TRA:CAVVNAGKSTF TRB:CASSPLGTEAFF	1	CD8-C4
Pla-1_CTCCTAGGTACCGTTA	Pla	Pla-1	P1-1675	FALSE	1	TRA:CAVRLGGGSSNTGKLIF TRB:CASSLGGLGSYGYTF	1	CD8-C8
Pla-1_CTCGGAGAGAGAACAG	Pla	Pla-1	P1-49	FALSE	3	TRA:CAGGGSEKLVF TRB:CASSLFYPAGQQYF	16	CD8-C8
Pla-1_CTCGGGAAGTGGCACA	Pla	Pla-1	P1-1690	FALSE	1	TRA:CIVSYAGNMLTF TRB:CSARGTSNYGYTF	1	CD8-C8
Pla-1_CTCGTACGTAATTGGA	Pla	Pla-1	P1-1694	FALSE	1	TRA:CAMSARRGSNYKLTF TRB:CASSLERATEAFF	1	CD8-C8
Pla-1_CTCGTACTCTCTTGAT	Pla	Pla-1	P1-126	FALSE	3	TRA:CAVGSRPGGGYNKLIF TRB:CASGQGSYGELFF	5	CD8-C9
Pla-1_CTCTGGTCATTGAGCT	Pla	Pla-1	P1-8	TRUE	3	TRA:CAVRPPGRNSGGYQKVTF TRB:CASSRRGVTDTQYF	204	CD8-C9

Pla-1_CTGATAGAGCGAGAAA	Pla	Pla-1	P1-1714	FALSE	1	TRA:CAFTAGGTSYGKLTF TRB:CASSTTSGGGTDTQYF	1	CD8-C9
Pla-1_CTTAACTTCACCTTAT	Pla	Pla-1	P1-1746	FALSE	1	TRA:CAVRWYTDKLIF TRB:CASSWRSRDRDQSEQYF	1	CD8-C9
Pla-1_GAAATGACAAGTAGTA	Pla	Pla-1	P1-161	FALSE	3	TRA:CVVTTSQFSGNTPLVF TRB:CASSLYSGALMNTEAFF	4	CD8-C8
Pla-1_GAACGGATCGTATCAG	Pla	Pla-1	P1-32	FALSE	3	TRA:CAVMNTGFQKLVF TRB:CSVSGLVLGGNEQFF	46	CD8-C9
Pla-1_GAAGCAGGTGTTTGTG	Pla	Pla-1	P1-15	TRUE	3	TRA:CAVNSSPLGANSKLTF TRB:CASSDSGNFYNEQFF	92	CD8-C9
Pla-1_GAATAAGAGACCGGAT	Pla	Pla-1	P1-15	TRUE	3	TRA:CAVNSSPLGANSKLTF TRB:CASSDSGNFYNEQFF	92	CD8-C8
Pla-1_GACACGCTCAATCACG	Pla	Pla-1	P1-398	FALSE	2	TRA:CAENKGGNMLTF TRB:CATPRGHRSGANVLTF	2	CD8-C9
Pla-1_GACAGAGCACAAGTAA	Pla	Pla-1	P1-46	FALSE	3	TRA:CAVRGTGTASKLTF TRB:CSVRQGGNEQFF	20	CD8-C4
Pla-1_GACTAACTCACATAGC	Pla	Pla-1	P1-44	TRUE	3	TRA:CAYRGLGAQKLVF TRB:CASSSLDGQDNHYGYTF	22	CD8-C8
Pla-1_GATCAGTAGGTGCTAG	Pla	Pla-1	P1-215	FALSE	3	TRA:CAVSSNRDDKIIF TRB:CASSPGRLAGENIQYF	3	CD8-C8
Pla-1_GCATGTATCTGGCGTG	Pla	Pla-1	P1-23	TRUE	3	TRA:CALRAITQGGSEKLVF TRB:CASSLTGAVYGYTF	65	CD8-C8
Pla-1_GCCAAATCAAGGGTCA	Pla	Pla-1	P1-1863	FALSE	1	TRA:CAMREPMGGGADGLTF TRB:CASSYSRTEAFF	1	CD8-C8
Pla-1_GGAAAGCCACAAGCCC	Pla	Pla-1	P1-44	TRUE	3	TRA:CAYRGLGAQKLVF TRB:CASSSLDGQDNHYGYTF	22	CD8-C8
Pla-1_GGACAGAAGCTCCTCT	Pla	Pla-1	P1-33	TRUE	3	TRA:CLVGTGGFKTIF TRB:CSARVPGQMKETQYF	41	CD8-C9
Pla-1_GGATGTTAGATGTTAG	Pla	Pla-1	P1-235	FALSE	3	TRA:CAATSTGTYKYIF TRB:CASSFGGRNEQYF	3	CD8-C8
Pla-1_GGATTACAGTAGGCCA	Pla	Pla-1	P1-34	FALSE	3	TRA:CAMSGGGADGLTF TRB:CASNVGVHEQFF	41	CD8-C8
Pla-1_GGCAATTAGCCGTCGT	Pla	Pla-1	P1-1920	FALSE	1	TRA:CIVRVAGSSNTGKLIF TRB:CASSRGRQETQYF	1	CD8-C8
Pla-1_GGGCACTTCTGCTGTC	Pla	Pla-1	P1-1949	FALSE	1	TRA:CAVIATGGGNKLTF TRB:CASSKLRDSQYNEQFF	1	CD8-C8
Pla-1_GGGTTGCGTAAATGTG	Pla	Pla-1	P1-239	FALSE	3	TRA:CAASLGDTDKLIF TRB:CASSPWTGVNEQFF	3	CD8-C9
Pla-1_GTATTCTTCTCAAACG	Pla	Pla-1	P1-414	FALSE	2	TRA:CAMRANHNAGNNRKLIW TRB:CAWSVPGTGGEKLFF	2	CD8-C8
Pla-1_GTCACAATCCGTCAAA	Pla	Pla-1	P1-34	FALSE	3	TRA:CAMSGGGADGLTF TRB:CASNVGVHEQFF	41	CD8-C8
Pla-1_GTGCATATCTCTTGAT	Pla	Pla-1	P1-37	TRUE	3	TRA:CAVNIVGNQFYF TRB:CASSPLGTEAFF	40	CD8-C9
Pla-1_GTTACAGAGCAATCTC	Pla	Pla-1	P1-278	TRUE	2	TRA:CAVQAKDMRF TRB:CASSLAELGGNTIYF	2	CD8-C9
Pla-1_TACACGAAGGCAGTCA	Pla	Pla-1	P1-119	TRUE	3	TRA:CVVSQAGSGNTGKLIF TRB:CSAIPWRGYGYTF	5	CD8-C8
Pla-1_TACCTATTCTCTGTCG	Pla	Pla-1	P1-32	FALSE	3	TRA:CAVMNTGFQKLVF TRB:CSVSGLVLGGNEQFF	46	CD8-C8
Pla-1_TACGGGCCAGTATAAG	Pla	Pla-1	P1-2083	FALSE	1	TRA:CATDPPGRRALTF TRB:CASIEGQGPYNEQFF	1	CD8-C8
Pla-1_TACGGTAGTATTACCG	Pla	Pla-1	P1-32	FALSE	3	TRA:CAVMNTGFQKLVF TRB:CSVSGLVLGGNEQFF	46	CD8-C9

Pla-1_TCACGAAAGTACACCT	Pla	Pla-1	P1-2137	FALSE	1	TRA:CILGGTSYGKLTF TRB:CSAPESGGELFF	1	CD8-C8
Pla-1_TCAGGATGTTGATTGC	Pla	Pla-1	P1-49	FALSE	3	TRA:CAGGGSEKLVF TRB:CASSLFYPAGQQYF	16	CD8-C8
Pla-1_TCGCGAGGTCCGTGAC	Pla	Pla-1	P1-28	TRUE	3	TRA:CAVKKGTGRRALTF TRB:CASETGQGTDTQYF	53	CD8-C8
Pla-1_TCTATTGAGTGAATTG	Pla	Pla-1	P1-2174	FALSE	1	TRA:CALSDRGGFGNVLHC TRB:CSVGGTGNFWTEAFF	1	CD8-C9
Pla-1_TGACTAGCAGTCACTA	Pla	Pla-1	P1-2198	FALSE	1	TRA:CAVSRDPQKLVF TRB:CASKRGDYLISEQYF	1	CD8-C9
Pla-1_TGACTAGCATTATCTC	Pla	Pla-1	P1-2199	FALSE	1	TRA:CAVRGTFMDSNYQLIW TRB:CASVGHNEQFF	1	CD8-C5
Pla-1_TGAGGGACAACACCTA	Pla	Pla-1	P1-7	TRUE	3	TRA:CAVQAGNNDMRF TRB:CASSTTGDFNYGYTF	215	CD8-C8
Pla-1_TGCACCTGTTTAGGAA	Pla	Pla-1	P1-185	TRUE	3	TRA:CATDEDKFYF TRB:CASSESWTGGTYEQYF	3	CD8-C9
Pla-1_TGCGCAGAGACTTTCG	Pla	Pla-1	P1-56	TRUE	3	TRA:CVVNPWGSGTYKYIF TRB:CASSRDITDTQYF	13	CD8-C9
Pla-1_TGCTGCTAGGGCTCTC	Pla	Pla-1	P1-46	FALSE	3	TRA:CAVRGTGTASKLTF TRB:CSVRQGGNEQFF	20	CD8-C8
Pla-1_TGGTTAGTCTGTCTCG	Pla	Pla-1	P1-250	FALSE	3	TRA:CAHYDRGSTLGRLYF TRB:CASSLRIDQPQHF	3	CD8-C9
Pla-1_TGGTTCCTCTGCGTAA	Pla	Pla-1	P1-108	TRUE	3	TRA:CAASNGDTGNQFYF TRB:CSASKQGRNQPQHF	5	CD8-C8
Pla-1_TGTTCCGAGTCAATAG	Pla	Pla-1	P1-82	FALSE	3	TRA:CAASEDSNYQLIW TRB:CASSFGDSSSETQYF	7	CD8-C9
Pla-1_TTAGGCAAGGCTATCT	Pla	Pla-1	P1-131	FALSE	3	TRA:CAVRDSGTYKYIF TRB:CASSIDLGGANVLTF	5	CD8-C8
Pla-1_TTATGCTAGTGAATTG	Pla	Pla-1	P1-2288	FALSE	1	TRA:CALRRGSTLGRLYF TRB:CASSQDDVSGYTF	1	CD8-C8
Pla-1_TTCCCAGCATTGTGCA	Pla	Pla-1	P1-117	TRUE	3	TRA:CIVGETSGSRLTF TRB:CASSLVGGVGTDTQYF	5	CD8-C9
Pla-1_TTCGGTCCACTTGGAT	Pla	Pla-1	P1-2299	FALSE	1	TRA:CLVGGENTGFQKLVF TRB:CASEGTYEQYF	1	CD8-C8
Pla-1_TTGGCAACAAGCTGAG	Pla	Pla-1	P1-354	FALSE	2	TRA:CAVRQIRSSNTGKLIF TRB:CASSSGLENNEQFF	2	CD8-C9
Pla-1_TTGTAGGCACTCTGTC	Pla	Pla-1	P1-102	FALSE	3	TRA:CAENNDAGNMLTF TRB:CASSETWGVTEAFF	6	CD8-C9
Pla-1_AACTCCCGTGCCTGGT	Pla	Pla-1	P1-1103	FALSE	1	TRA:CAYKVARVYAGNNRKLIW TRB:CASSYIPGGRFETQYF	1	CD4-C1
Pla-1_AAGTCTGAGAGTGAGA	Pla	Pla-1	P1-1125	FALSE	1	TRA:CAFYLVSSNTGKLIF TRB:CASSLGRTSIHEQYF	1	CD4-C1
Pla-1_ACCAGTATCAGCCTAA	Pla	Pla-1	P1-1167	FALSE	1	TRA:CAARAITGNQFYF TRB:CASSSQGNNQPQHF	1	CD4-C1
Pla-1_ACCAGTATCCTTGCCA	Pla	Pla-1	P1-1168	FALSE	1	TRA:CAAPRSDGGQKLLF TRB:CASSPMGTGGLRETQYF	1	CD4-C1
Pla-1_ACTATCTCAAGCGAGT	Pla	Pla-1	P1-1214	FALSE	1	TRA:CAASYNNAGNMLTF TRB:CASSSIYEQYF	1	CD4-C1
Pla-1_AGATTGCCATTACGAC	Pla	Pla-1	P1-1259	FALSE	1	TRA:CAVGPRGDDKIIF TRB:CASSAFPQGQGHYNEQFF	1	CD4-C1
Pla-1_AGCAGCCTCTTTCCTC	Pla	Pla-1	P1-1264	FALSE	1	TRA:CAMREVFGNEKLTF TRB:CASSLPLGTSGGKDTQYF	1	CD4-C1
Pla-1_AGCTCCTTCTGCAGTA	Pla	Pla-1	P1-1272	FALSE	1	TRA:CVVNTGGGNKLTF TRB:CASSLAGGDYEQYF	1	CD4-C1

Pla-1_AGGGAGTCATTGAGCT	Pla	Pla-1	P1-1282	FALSE	1	TRA:CALTSSSNTGKLIF TRB:CASRLDRLSTEAFF	1	CD4-C1
Pla-1_AGGTCATTCTCGGACG	Pla	Pla-1	P1-1290	FALSE	1	TRA:CAASKKTSYDKVIF TRB:CAWDRNTEAFF	1	CD4-C1
Pla-1_ATAAGAGAGATGCCTT	Pla	Pla-1	P1-1310	FALSE	1	TRA:CAVGASKAAGNKLTF TRB:CASSPARDTEAFF	1	CD4-C1
Pla-1_ATAAGAGAGTAACCCT	Pla	Pla-1	P1-1312	FALSE	1	TRA:CAVGARGSQGNLIF TRB:CASSVGSRISYNEQFF	1	CD4-C1
Pla-1_ATCTACTAGGGTCTCC	Pla	Pla-1	P1-1335	FALSE	1	TRA:CAASGSAGNNRKLIW TRB:CASSPGASGPKETQYF	1	CD4-C1
Pla-1_CAACTAGCAAAGCAAT	Pla	Pla-1	P1-1367	FALSE	1	TRA:CAALNQAGTALIF TRB:CASSWSGQGHYGYTF	1	CD4-C1
Pla-1_CAAGTTGCACAGGAGT	Pla	Pla-1	P1-1381	FALSE	1	TRA:CAGLRSGNTGKLIF TRB:CASSPGQGYGYTF	1	CD4-C1
Pla-1_CCATGTCTCCGCGCAA	Pla	Pla-1	P1-1487	FALSE	1	TRA:CAVRGPNDYKLSF TRB:CASSPPGQGNTGELFF	1	CD4-C1
Pla-1_CCCAATCGTGTGAATA	Pla	Pla-1	P1-1490	FALSE	1	TRA:CAVGSNYGGATNKLIF TRB:CASSPLGGAYNSPLHF	1	CD4-C1
Pla-1_CCTACACGTTCACCTC	Pla	Pla-1	P1-1512	FALSE	1	TRA:CAMRVYSGSARQLTF TRB:CASSEGGAGANVLTF	1	CD4-C1
Pla-1_CGACCTTAGAAACGAG	Pla	Pla-1	P1-1543	FALSE	1	TRA:CAVIRSSGSARQLTF TRB:CASSQLGGTEAFF	1	CD4-C1
Pla-1_CTCATTATCGCATGAT	Pla	Pla-1	P1-1670	FALSE	1	TRA:CALKSGGYQKVTF TRB:CASSLVSESDNEQFF	1	CD4-C1
Pla-1_CTCTAATTCGGTCCGA	Pla	Pla-1	P1-1702	FALSE	1	TRA:CAATDGQKLLF TRB:CASSLGERNEQFF	1	CD4-C1
Pla-1_CTGATCCCAAACTGTC	Pla	Pla-1	P1-1722	FALSE	1	TRA:CIVQGGSEKLVF TRB:CASSTPRDRWGTEAFF	1	CD4-C1
Pla-1_GAACATCGTCTTGATG	Pla	Pla-1	P1-1765	FALSE	1	TRA:CAGHRDGQKLLF TRB:CASSPTASGNTIYF	1	CD4-C1
Pla-1_GACACGCGTTGCTCCT	Pla	Pla-1	P1-1785	FALSE	1	TRA:CAVNANSWGKLQF TRB:CASSDPLLKGGYTF	1	CD4-C1
Pla-1_GACGCGTAGACACTAA	Pla	Pla-1	P1-1790	FALSE	1	TRA:CAVQALPRQFYF TRB:CASSFGTGSGQETQYF	1	CD4-C2
Pla-1_GACGGCTGTTCCGGCA	Pla	Pla-1	P1-1795	FALSE	1	TRA:CATDHNSNSGYALNF TRB:CASSVVMGGSFYGYTF	1	CD4-C1
Pla-1_GACGTGCAGAGTGAGA	Pla	Pla-1	P1-1797	FALSE	1	TRA:CAASKGTGRRALTF TRB:CASSWVSQETQYF	1	CD4-C1
Pla-1_GACGTTAGTTTCCACC	Pla	Pla-1	P1-1806	FALSE	1	TRA:CAASTYFGNEKLTF TRB:CASSLLYGYTF	1	CD4-C1
Pla-1_GCATGATTCGGTTAAC	Pla	Pla-1	P1-1859	FALSE	1	TRA:CLVGDKMGNTPLVF TRB:CSARDAGTGQGDSPLHF	1	CD4-C1
Pla-1_GCTCCTACAATGAAAC	Pla	Pla-1	P1-1882	FALSE	1	TRA:CALRATGFQKLVF TRB:CASSMVSGAKNIQYF	1	CD4-C1
Pla-1_GGATGTTTCAGAGACG	Pla	Pla-1	P1-1916	FALSE	1	TRA:CALCIQGAQKLVF TRB:CASSPALEGRFETQYF	1	CD4-C1
Pla-1_GGCGACTGTATATGGA	Pla	Pla-1	P1-1927	FALSE	1	TRA:CAASPASGGSYIPTF TRB:CASSAPGAGKLFF	1	CD4-C1
Pla-1_GGCGACTTCCTGTAGA	Pla	Pla-1	P1-1929	FALSE	1	TRA:CAENNAGNMLTF TRB:CASSQGSGEYNEQFF	1	CD4-C2
Pla-1_GGGTCTGGTTGCGTTA	Pla	Pla-1	P1-1955	FALSE	1	TRA:CAFDYGQNFVF TRB:CASSQGTTLRNTEAFF	1	CD4-C1
Pla-1_GTCACAAAGTGGGATC	Pla	Pla-1	P1-1996	FALSE	1	TRA:CALSEWTSGSRLTF TRB:CASSLQGPGNTIYF	1	CD4-C1

Pla-1_GTGCGGTGTTACGACT	Pla	Pla-1	P1-2024	FALSE	1	TRA:CAMKTSYDKVIF TRB:CSVEVPHPEINMRDPAPTDTQYF	1	CD4-C1
Pla-1_GTGGGTCTCGCTTGTC	Pla	Pla-1	P1-2032	FALSE	1	TRA:CLVRPSGTYKYIF TRB:CASVPLGGINEQFF	1	CD8-C1
Pla-1_GTTAAGCTCATCTGCC	Pla	Pla-1	P1-2042	FALSE	1	TRA:CVVRVNTGGFKTIF TRB:CASRKTGDQPQHF	1	CD4-C1
Pla-1_TCTGGAATCTGTTTGT	Pla	Pla-1	P1-2185	FALSE	1	TRA:CAVLMNRDDKIIF TRB:CASSFQRPRETQYF	1	CD4-C1
Pla-1_TGCCCATAGCAGGTCA	Pla	Pla-1	P1-2215	FALSE	1	TRA:CALLGRTGNTPLVF TRB:CASSIRTAYNSPLHF	1	CD4-C1
Pla-1_TGCCCTAAGGCTCAGA	Pla	Pla-1	P1-2220	FALSE	1	TRA:CAVILTGTASKLTF TRB:CASSFSSLGYTF	1	CD4-C1
Pla-1_TGCGCAGGTAAACCTC	Pla	Pla-1	P1-2224	FALSE	1	TRA:CAVSGSSNTGKLIF TRB:CAISRDLYEQYF	1	CD4-C1
Pla-1_TGCTACCAGTCCATAC	Pla	Pla-1	P1-2240	FALSE	1	TRA:CLVGEVRGNKLVF TRB:CASTGGAAGYEQYF	1	CD4-C1
Pla-1_TGGTTCCGTGGCGAAT	Pla	Pla-1	P1-2261	FALSE	1	TRA:CAVSPNSGGSNYKLTF TRB:CASPPPYTEAFF	1	CD4-C1
Pla-1_TGTATTCTCACTATTC	Pla	Pla-1	P1-2263	FALSE	1	TRA:CAVAISNFGNEKLTF TRB:CASSENSNSPLHF	1	CD4-C1
Pla-1_TGTGTTTCAAATACAG	Pla	Pla-1	P1-2268	FALSE	1	TRA:CAVRGQAGTALIF TRB:CSARDPTAGAYNEQFF	1	CD4-C1
Pla-1_TGTTCCGTCACTGGGC	Pla	Pla-1	P1-2274	FALSE	1	TRA:CAVQPGNYGGATNKLIF TRB:CASSFNPALPETQYF	1	CD4-C1
Pla-1_TTTATGCCACATCCGG	Pla	Pla-1	P1-2331	FALSE	1	TRA:CAVNARRNTPLVF TRB:CASSLSLSGGGTDTQYF	1	CD4-C1
Pla-1_AACCATGTCACCATAG	Pla	Pla-1	P1-33	TRUE	3	TRA:CLVGTGGFKTIF TRB:CSARVPGQMKETQYF	41	CD8-C8
Pla-1_ACGCCGAAGACTAAGT	Pla	Pla-1	P1-36	TRUE	3	TRA:CILRDNAGNNRKLIW TRB:CASSSRQGFPGGANVLTF	40	CD4-C6
Pla-1_ACTATCTCACGGCCAT	Pla	Pla-1	P1-1215	FALSE	1	TRA:CAFTAGGATNKLIF TRB:CASSSRPEDTQYF	1	CD8-C7
Pla-1_AGAATAGCAGGCTGAA	Pla	Pla-1	P1-162	FALSE	3	TRA:CVVSGGLRGAAGNKLTF TRB:CASSPLTVNTEAFF	4	CD4-C6
Pla-1_AGATTGCCACTTAACG	Pla	Pla-1	P1-21	TRUE	3	TRA:CAASRGTGANNLFF TRB:CATQREGREQYF	67	CD4-C6
Pla-1_AGGCCACAGAAGATTC	Pla	Pla-1	P1-1	TRUE	3	TRA:CAMSDRMTGGFKTIF TRB:CASSQVGRGGEETQYF	1047	CD4-C6
Pla-1_AGTGGGAGTACGAAAT	Pla	Pla-1	P1-21	TRUE	3	TRA:CAASRGTGANNLFF TRB:CATQREGREQYF	67	CD4-C6
Pla-1_ATAACGCCACGTCAGC	Pla	Pla-1	P1-14	TRUE	3	TRA:CAASVVNDMRF TRB:CASSHRGGAKNIQYF	109	CD4-C7
Pla-1_ATGTGTGTCCAAGCCG	Pla	Pla-1	P1-25	TRUE	3	TRA:CIVGRDSSYKLIF TRB:CASNPQRGAHRDTQYF	58	CD4-C3
Pla-1_ATTACTCCAACTGCGC	Pla	Pla-1	P1-205	FALSE	3	TRA:CAVNTLGNQFYF TRB:CASSPGHGVVGEQYF	3	CD4-C6
Pla-1_CAAGATCAGTTGTAGA	Pla	Pla-1	P1-203	TRUE	3	TRA:CAGVLAGNMLTF TRB:CASRRDRDPNEQFF	3	CD4-C6
Pla-1_CATCGAAGTAACGACG	Pla	Pla-1	P1-62	TRUE	3	TRA:CVVNLMEYGNKLVF TRB:CASSGARQGQYEQYF	10	CD8-C5
Pla-1_CCACGGAAGACTTGAA	Pla	Pla-1	P1-36	TRUE	3	TRA:CILRDNAGNNRKLIW TRB:CASSSRQGFPGGANVLTF	40	CD4-C6
Pla-1_CCGTACTGTTGTACAC	Pla	Pla-1	P1-25	TRUE	3	TRA:CIVGRDSSYKLIF TRB:CASNPQRGAHRDTQYF	58	CD4-C6

Pla-1_CCTACACTCCCTTGTG	Pla	Pla-1	P1-21	TRUE	3	TRA:CAASRGTGANNLFF TRB:CATQREGREQYF	67	CD4-C6
Pla-1_CGCCAAGCAAGAAGAG	Pla	Pla-1	P1-1568	FALSE	1	TRA:CAETYSGGYQKVTF TRB:CASSLVAQGEAFF	1	CD8-C2
Pla-1_CGCGGTAGTTGAGGTG	Pla	Pla-1	P1-41	TRUE	3	TRA:CVVSEPPGGSYIPTF TRB:CASSSRPEDTQYF	27	CD4-C6
Pla-1_CGCTGGATCATGTCCC	Pla	Pla-1	P1-17	TRUE	3	TRA:CGTGARGSSGSRLTF TRB:CASSRQGMGAFF	78	CD8-C7
Pla-1_CGGACTGGTTCCCGAG	Pla	Pla-1	P1-111	TRUE	3	TRA:CALSPYGGSQGNLIF TRB:CASSWNLGQAYEQYF	5	CD4-C6
Pla-1_CTCGTCACACATCCAA	Pla	Pla-1	P1-24	TRUE	3	TRA:CAVKGNTDKLIF TRB:CASSLRTGAYNEQFF	62	CD4-C3
Pla-1_CTCTACGTCGATCCCT	Pla	Pla-1	P1-27	TRUE	3	TRA:CALVDFSGYSTLTF TRB:CASSGRGTNEKLFF	55	CD4-C6
Pla-1_CTGCGGACAATAAGCA	Pla	Pla-1	P1-36	TRUE	3	TRA:CILRDNAGNNRKLIW TRB:CASSSRQGFPGGANVLTF	40	CD4-C6
Pla-1_CTGCGGATCGCCCTTA	Pla	Pla-1	P1-41	TRUE	3	TRA:CVVSEPPGGSYIPTF TRB:CASSSRPEDTQYF	27	CD4-C3
Pla-1_CTGGTCTAGTAGCCGA	Pla	Pla-1	P1-17	TRUE	3	TRA:CGTGARGSSGSRLTF TRB:CASSRQGMGAFF	78	CD4-C6
Pla-1_CTGGTCTGTCCGACGT	Pla	Pla-1	P1-342	FALSE	2	TRA:CAVPHRF TRB:CSAAGQGVHEQYF	2	CD4-C4
Pla-1_CTTCTCTAGGGTATCG	Pla	Pla-1	P1-41	TRUE	3	TRA:CVVSEPPGGSYIPTF TRB:CASSSRPEDTQYF	27	CD4-C3
Pla-1_GAAACTCTCTGGTATG	Pla	Pla-1	P1-21	TRUE	3	TRA:CAASRGTGANNLFF TRB:CATQREGREQYF	67	CD4-C3
Pla-1_GAACCTAGTGACTCAT	Pla	Pla-1	P1-102	FALSE	3	TRA:CAENNDAGNMLTF TRB:CASSETWGVTEAFF	6	CD4-C6
Pla-1_GACCTGGGTAGGACAC	Pla	Pla-1	P1-36	TRUE	3	TRA:CILRDNAGNNRKLIW TRB:CASSSRQGFPGGANVLTF	40	CD8-C10
Pla-1_GAGCAGATCCGCGTTT	Pla	Pla-1	P1-245	FALSE	3	TRA:CAVASSNDYKLSF TRB:CASSSPNEKLFF	3	CD4-C6
Pla-1_GATCGCGTCTTGACGA	Pla	Pla-1	P1-17	TRUE	3	TRA:CGTGARGSSGSRLTF TRB:CASSRQGMGAFF	78	CD4-C2
Pla-1_GATCTAGCAATGCCAT	Pla	Pla-1	P1-21	TRUE	3	TRA:CAASRGTGANNLFF TRB:CATQREGREQYF	67	CD4-C6
Pla-1_GATTCAGGTCGAGATG	Pla	Pla-1	P1-24	TRUE	3	TRA:CAVKGNTDKLIF TRB:CASSLRTGAYNEQFF	62	CD4-C6
Pla-1_GCAATCAGTACAGCAG	Pla	Pla-1	P1-36	TRUE	3	TRA:CILRDNAGNNRKLIW TRB:CASSSRQGFPGGANVLTF	40	CD4-C6
Pla-1_GCAGTTAAGGCAATTA	Pla	Pla-1	P1-1852	FALSE	1	TRA:CAMREGLTMGTTDSLL TRB:CASYSMGSYNEQFF	1	CD4-C3
Pla-1_GCTGCGAAGATCGGGT	Pla	Pla-1	P1-17	TRUE	3	TRA:CGTGARGSSGSRLTF TRB:CASSRQGMGAFF	78	CD4-C6
Pla-1_GGGTTGCCAGATCCAT	Pla	Pla-1	P1-17	TRUE	3	TRA:CGTGARGSSGSRLTF TRB:CASSRQGMGAFF	78	CD4-C6
Pla-1_GGTGTTAGTATCTGCA	Pla	Pla-1	P1-19	TRUE	3	TRA:CALGAFGSARQLTF TRB:CASSKGQGPRETQYF	70	CD4-C6
Pla-1_GTAGGCCCACTCAGGC	Pla	Pla-1	P1-1986	FALSE	1	TRA:CAERYTGNQFYF TRB:CASSQVGRGGEETQYF	1	CD4-C3
Pla-1_GTCATTTTCGTCCGTT	Pla	Pla-1	P1-79	TRUE	3	TRA:CAFMKLGGATNKLIF TRB:CASSLDRYSVDEQFF	7	CD4-C6
Pla-1_GTGCTTCAGCGATAGC	Pla	Pla-1	P1-19	TRUE	3	TRA:CALGAFGSARQLTF TRB:CASSKGQGPRETQYF	70	CD4-C6

Pla-1_GTGTTAGAGGATGGAA	Pla	Pla-1	P1-17	TRUE	3	TRA:CGTGARGSSGSRLTF TRB:CASSRQGMGAFF	78	CD4-C6
Pla-1_GTTAAGCAGGGATGGG	Pla	Pla-1	P1-2039	FALSE	1	TRA:CAVGEYGNKLVF TRB:CASSVLGPGITEAFF	1	CD8-C6
Pla-1_TACGGTAAGAGACTAT	Pla	Pla-1	P1-365	FALSE	2	TRA:CAALDSYNTDKLIF TRB:CASSSTVNTEAFF	2	CD4-C6
Pla-1_TACGGTACACTATCTT	Pla	Pla-1	P1-36	TRUE	3	TRA:CILRDNAGNNRKLIW TRB:CASSSRQGFPGGANVLTF	40	CD4-C4
Pla-1_TATTACCTCACAGGCC	Pla	Pla-1	P1-36	TRUE	3	TRA:CILRDNAGNNRKLIW TRB:CASSSRQGFPGGANVLTF	40	CD4-C6
Pla-1_TCAATCTTCCGCGGTA	Pla	Pla-1	P1-79	TRUE	3	TRA:CAFMKLGGATNKLIF TRB:CASSLDRYSVDEQFF	7	CD4-C6
Pla-1_TCTCTAACAACTGCGC	Pla	Pla-1	P1-19	TRUE	3	TRA:CALGAFGSARQLTF TRB:CASSKGQGPRETQYF	70	CD4-C6
Pla-1_TGCCCATGTCGGCTCA	Pla	Pla-1	P1-2219	FALSE	1	TRA:CVVKSGGKLIF TRB:CAIRGLAETTGELFF	1	CD4-C6
Pla-1_TGGACGCAGTACATGA	Pla	Pla-1	P1-2245	FALSE	1	TRA:CIVRSYSNSGYALNF TRB:CASSYQGARGYGYTF	1	CD4-C6
Pla-1_TGGACGCCATGACATC	Pla	Pla-1	P1-24	TRUE	3	TRA:CAVKGNTDKLIF TRB:CASSLRTGAYNEQFF	62	CD4-C6
Pla-1_TGGCCAGGTGACGCCT	Pla	Pla-1	P1-107	TRUE	3	TRA:CALSDKEKLTF TRB:CAISEGGGVGYTF	5	CD4-C6
Pla-1_TGGGAAGAGCTAGCCC	Pla	Pla-1	P1-42	TRUE	3	TRA:CASFSGGSQGNLIF TRB:CSAGQGSNQPQHF	24	CD4-C6
Pla-1_TTGACTTTCCACGCAG	Pla	Pla-1	P1-36	TRUE	3	TRA:CILRDNAGNNRKLIW TRB:CASSSRQGFPGGANVLTF	40	CD4-C6
Pla-1_ACGGGCTTCGAATGGG	Pla	Pla-1	P1-1206	FALSE	1	TRA:CAVQAEGYSTLTF TRB:CASSVAGNEQFF	1	CD4-C1
Pla-1_CTACATTAGGAGCGAG	Pla	Pla-1	P1-1643	FALSE	1	TRA:CAGVVRGSTLGRLYF TRB:CASSVDGGGGSTGELFF	1	CD8-C1
Pla-1_GCTTCCATCGCTTGTC	Pla	Pla-1	P1-1898	FALSE	1	TRA:CAVNTSGSARQLTF TRB:CASSSTSSGGGRSYNEQFF	1	CD8-C1
Pla-1_GGACATTAGCCACTAT	Pla	Pla-1	P1-1908	FALSE	1	TRA:CGTEMRRADSWGKLQF TRB:CASSHPPGSYAEAFF	1	CD8-C1
Pla-1_TACTTGTTCAGAGGTG	Pla	Pla-1	P1-2099	FALSE	1	TRA:CAVGATVNYQLIW TRB:CASSPGTGAEQYF	1	CD4-C1
Pla-1_TGAAAGACAAGCGTAG	Pla	Pla-1	P1-2191	FALSE	1	TRA:CGPPPGTYKYIF TRB:CASSPIPGQRNTEAFF	1	CD8-C1
Pla-1_AACTTTCAGCCACGTC	Pla	Pla-1	P1-20	TRUE	3	TRA:CVVVGGRALTF TRB:CASSLATNPHETQYF	69	CD8-C11
Pla-1_AAGACCTGTCTCGTTC	Pla	Pla-1	P1-1114	FALSE	1	TRA:CAVNNAGNMLTF TRB:CASSAGQGYFDEQYF	1	CD8-C11
Pla-1_ACGATACGTAATCACC	Pla	Pla-1	P1-1186	FALSE	1	TRA:CAENPSGKLIF TRB:CASSPPAIRADTQYF	1	CD8-C11
Pla-1_AGGGTGAGTCAATGTC	Pla	Pla-1	P1-1288	FALSE	1	TRA:CAAWGETSGSRLTF TRB:CASSRFWGPENTGELFF	1	CD8-C11
Pla-1_AGTTGGTTCGCCAAAT	Pla	Pla-1	P1-1	TRUE	3	TRA:CAMSDRMTGGFKTIF TRB:CASSQVGRGGEETQYF	1047	CD8-C11
Pla-1_CAAGATCGTTATCCGA	Pla	Pla-1	P1-8	TRUE	3	TRA:CAVRPPGRNSGGYQKVTF TRB:CASSRRGVTDTQYF	204	CD8-C11
Pla-1_CAGAATCTCCCTTGCA	Pla	Pla-1	P1-220	FALSE	3	TRA:CAAGQKNYGGSQGNLIF TRB:CASSIRGESSTEAFF	3	CD8-C11
Pla-1_CCTATTAAGCAGATCG	Pla	Pla-1	P1-376	FALSE	2	TRA:CAYRSAGEGLTF TRB:CASSLPSSGSFTDTQYF	2	CD8-C11

Pla-1_CCTCAGTGTCAGTGGA	Pla	Pla-1	P1-8	TRUE	3	TRA:CAVRPPGRNSGGYQKVTF TRB:CASSRRGVTDTQYF	204	CD8-C11
Pla-1_CTACGTCTCGCGATCG	Pla	Pla-1	P1-1652	FALSE	1	TRA:CAVSPYGNNRLAF TRB:CASSLQGQGGTEAFF	1	CD8-C11
Pla-1_CTCACACCAATTGCTG	Pla	Pla-1	P1-6	TRUE	3	TRA:CAMREGRERDDKIIF TRB:CASSLPNTEAFF	216	CD8-C11
Pla-1_CTCTAATTCGCCTGTT	Pla	Pla-1	P1-13	TRUE	3	TRA:CVVMNYGGSQGNLIF TRB:CASSSWGDTDEAFF	112	CD8-C11
Pla-1_GATGCTATCGCATGAT	Pla	Pla-1	P1-243	FALSE	3	TRA:CALSETSGGSNYKLTF TRB:CASSTRLAGGRISYNEQFF	3	CD8-C11
Pla-1_GCATGATAGAAACCTA	Pla	Pla-1	P1-46	FALSE	3	TRA:CAVRGTGTASKLTF TRB:CSVRQGGNEQFF	20	CD8-C11
Pla-1_GCCAAATTCTTAACCT	Pla	Pla-1	P1-20	TRUE	3	TRA:CVVVGGRALTF TRB:CASSLATNPHETQYF	69	CD8-C11
Pla-1_GCCTCTAGTCGAAAGC	Pla	Pla-1	P1-135	FALSE	3	TRA:CAVRASGANSKLTF TRB:CSAQIGTGPEKLFF	5	CD8-C11
Pla-1_GCGCCAACAGTCAGAG	Pla	Pla-1	P1-2	TRUE	3	TRA:CGQNFVF TRB:CASNQRGGPSLYNEQFF	323	CD8-C11
Pla-1_GGAATAAGTATGAATG	Pla	Pla-1	P1-20	TRUE	3	TRA:CVVVGGRALTF TRB:CASSLATNPHETQYF	69	CD8-C11
Pla-1_GGTGCGTAGCAACGGT	Pla	Pla-1	P1-23	TRUE	3	TRA:CALRAITQGGSEKLVF TRB:CASSLTGAVYGYTF	65	CD8-C11
Pla-1_GTCTCGTAGGAGCGTT	Pla	Pla-1	P1-9	TRUE	3	TRA:CAEAEGGGADGLTF TRB:CATSGGDNEQFF	174	CD8-C11
Pla-1_GTTCGGGTCAGAGCTT	Pla	Pla-1	P1-2056	FALSE	1	TRA:CLVGFKAAGNKLTF TRB:CASSPASGGADWDTQYF	1	CD8-C11
Pla-1_TAAGCGTTCCGCAAGC	Pla	Pla-1	P1-23	TRUE	3	TRA:CALRAITQGGSEKLVF TRB:CASSLTGAVYGYTF	65	CD8-C11
Pla-1_TATCAGGTCGGAGCAA	Pla	Pla-1	P1-55	TRUE	3	TRA:CALSPQDSNYQLIW TRB:CASSFWTSANEQFF	13	CD8-C11
Pla-1_TGACTAGAGCCAACAG	Pla	Pla-1	P1-247	FALSE	3	TRA:CAARGGSQGNLIF TRB:CASSLGGDTEAFF	3	CD8-C11
Pla-1_TGACTAGGTTGGTTTG	Pla	Pla-1	P1-1	TRUE	3	TRA:CAMSDRMTGGFKTIF TRB:CASSQVGRGGEETQYF	1047	CD8-C11
Pla-1_TTAACTCAGTTTCCTT	Pla	Pla-1	P1-23	TRUE	3	TRA:CALRAITQGGSEKLVF TRB:CASSLTGAVYGYTF	65	CD8-C11
Pla-1_TTTACTGAGGTTCCTA	Pla	Pla-1	P1-26	TRUE	3	TRA:CAPRSGGSYIPTF TRB:CASSQDLGLDYGYTF	56	CD8-C11
Pla-2_AAAGCAAGTGCACCAC	Pla	Pla-2	P2-4	TRUE	3	TRA:CAIRYQSFNFGNEKLTF TRB:CASYRRVTEQYF	148	CD8-C7
Pla-2_AACGTTGCAAGCCATT	Pla	Pla-2	P2-1	TRUE	3	TRA:CVVNIIGYQKVTF TRB:CASTRQGKQPQHF	270	CD8-C7
Pla-2_ACACCGGAGGGCATGT	Pla	Pla-2	P2-76	TRUE	3	TRA:CAGQEQGGSEKLVF TRB:CASSQMGLYGYTF	7	CD4-C6
Pla-2_ACAGCTAAGGACCACA	Pla	Pla-2	P2-32	TRUE	3	TRA:CAMREANVGADGLTF TRB:CASSTRIAGGPFPETQYF	17	CD4-C6
Pla-2_ACCCACTCATCCGGGT	Pla	Pla-2	P2-11	TRUE	3	TRA:CAVHNQGGKLIF TRB:CASSYLGAGYQETQYF	43	CD8-C7
Pla-2_ACGGGCTAGAACAATC	Pla	Pla-2	P2-3569	FALSE	1	TRA:CAMTQNAGKSTF TRB:CASSPGTANTGELFF	1	CD8-C7
Pla-2_ACGGGTCTCAATACCG	Pla	Pla-2	P2-5	TRUE	3	TRA:CASSFSGGYNKLIF TRB:CASSYLGAGYQETQYF	127	CD8-C7
Pla-2_AGTGTCACAAGCCGCT	Pla	Pla-2	P2-3	TRUE	3	TRA:CAVNPLESSYKLIF TRB:CASSPGTANTGELFF	177	CD8-C7

Pla-2_AGTGTCATCTTGCAAG	Pla	Pla-2	P2-1	TRUE	3	TRA:CVVNIIGYQKVTF TRB:CASTRQGKQPQHF	270	CD8-C7
Pla-2_ATCACGAAGACTTTCG	Pla	Pla-2	P2-5	TRUE	3	TRA:CASSFSGGYNKLIF TRB:CASSYLGAGYQETQYF	127	CD8-C7
Pla-2_ATCCACCGTTCCATGA	Pla	Pla-2	P2-1	TRUE	3	TRA:CVVNIIGYQKVTF TRB:CASTRQGKQPQHF	270	CD8-C7
Pla-2_ATCCACCTCCGCGTTT	Pla	Pla-2	P2-4	TRUE	3	TRA:CAIRYQSFNFGNEKLTF TRB:CASYRRVTEQYF	148	CD8-C7
Pla-2_CAACCAATCCGTCAAA	Pla	Pla-2	P2-3	TRUE	3	TRA:CAVNPLESSYKLIF TRB:CASSPGTANTGELFF	177	CD8-C7
Pla-2_CACCTTGGTCCAGTGC	Pla	Pla-2	P2-1	TRUE	3	TRA:CVVNIIGYQKVTF TRB:CASTRQGKQPQHF	270	CD8-C7
Pla-2_CAGTAACTCTAAGCCA	Pla	Pla-2	P2-3	TRUE	3	TRA:CAVNPLESSYKLIF TRB:CASSPGTANTGELFF	177	CD8-C7
Pla-2_CATATTCAGAGCTTCT	Pla	Pla-2	P2-129	TRUE	3	TRA:CARGGTGNQFYF TRB:CAWSPMPNEQFF	4	CD8-C7
Pla-2_CCAATCCGTCTAGCGC	Pla	Pla-2	P2-1	TRUE	3	TRA:CVVNIIGYQKVTF TRB:CASTRQGKQPQHF	270	CD8-C7
Pla-2_CCAGCGAAGATGGGTC	Pla	Pla-2	P2-3	TRUE	3	TRA:CAVNPLESSYKLIF TRB:CASSPGTANTGELFF	177	CD8-C7
Pla-2_CCGGTAGAGCTCTCGG	Pla	Pla-2	P2-3	TRUE	3	TRA:CAVNPLESSYKLIF TRB:CASSPGTANTGELFF	177	CD8-C7
Pla-2_CCTATTAGTGCAGGTA	Pla	Pla-2	P2-3	TRUE	3	TRA:CAVNPLESSYKLIF TRB:CASSPGTANTGELFF	177	CD8-C5
Pla-2_CGATCGGGTCAGTGGA	Pla	Pla-2	P2-202	TRUE	3	TRA:CALTTAGGTSYGKLTF TRB:CASKVSGTSGRNEQFF	3	CD8-C7
Pla-2_CGGAGCTGTATTCGTG	Pla	Pla-2	P2-5	TRUE	3	TRA:CASSFSGGYNKLIF TRB:CASSYLGAGYQETQYF	127	CD8-C7
Pla-2_CGGCTAGTCTCCAGGG	Pla	Pla-2	P2-1	TRUE	3	TRA:CVVNIIGYQKVTF TRB:CASTRQGKQPQHF	270	CD8-C7
Pla-2_CTACACCTCAACTCTT	Pla	Pla-2	P2-5	TRUE	3	TRA:CASSFSGGYNKLIF TRB:CASSYLGAGYQETQYF	127	CD8-C7
Pla-2_CTCAGAAGTGGCCCTA	Pla	Pla-2	P2-293	TRUE	2	TRA:CAVNMGTYKYIF TRB:CASSRTYEQYF	2	CD8-C3
Pla-2_CTCCTAGGTGTTCTTT	Pla	Pla-2	P2-11	TRUE	3	TRA:CAVHNQGGKLIF TRB:CASSYLGAGYQETQYF	43	CD8-C7
Pla-2_CTCGAAAAGACTAAGT	Pla	Pla-2	P2-12	TRUE	3	TRA:CGADAEYGNKLVF TRB:CASSEGQGAIMQPQHF	42	CD8-C7
Pla-2_GAAGCAGGTCCGTCAG	Pla	Pla-2	P2-17	TRUE	3	TRA:CAVQASNAGNMLTF TRB:CASSVEQGNVETQYF	30	CD8-C5
Pla-2_GACGTGCTCGAGAACG	Pla	Pla-2	P2-5	TRUE	3	TRA:CASSFSGGYNKLIF TRB:CASSYLGAGYQETQYF	127	CD8-C7
Pla-2_GATGAAATCTGTGCAA	Pla	Pla-2	P2-7	TRUE	3	TRA:CAESDSGNTPLVF TRB:CASSYSTPRGEAFF	84	CD8-C7
Pla-2_GCGGGTTGTTTGTGTG	Pla	Pla-2	P2-1	TRUE	3	TRA:CVVNIIGYQKVTF TRB:CASTRQGKQPQHF	270	CD8-C7
Pla-2_GGATTACCAGGAACGT	Pla	Pla-2	P2-5	TRUE	3	TRA:CASSFSGGYNKLIF TRB:CASSYLGAGYQETQYF	127	CD8-C7
Pla-2_GGCGACTTCAGTGCAT	Pla	Pla-2	P2-3	TRUE	3	TRA:CAVNPLESSYKLIF TRB:CASSPGTANTGELFF	177	CD8-C5
Pla-2_GTGCTTCGTTCCCGAG	Pla	Pla-2	P2-1	TRUE	3	TRA:CVVNIIGYQKVTF TRB:CASTRQGKQPQHF	270	CD8-C7
Pla-2_TACCTATCATTAGCCA	Pla	Pla-2	P2-5	TRUE	3	TRA:CASSFSGGYNKLIF TRB:CASSYLGAGYQETQYF	127	CD8-C5

Pla-2_TACCTTATCCACGAAT	Pla	Pla-2	P2-7	TRUE	3	TRA:CAESDSGNTPLVF TRB:CASSYSTPRGEAFF	84	CD8-C7
Pla-2_TACGGATAGACGCTTT	Pla	Pla-2	P2-1	TRUE	3	TRA:CVVNIIGYQKVTF TRB:CASTRQGKQPQHF	270	CD8-C7
Pla-2_TCAGGATCACACCGAC	Pla	Pla-2	P2-122	TRUE	3	TRA:CAYAPSGSRLTF TRB:CASSQGGWGRRDEQFF	4	CD8-C7
Pla-2_TCGTAGACAGGTCTCG	Pla	Pla-2	P2-12	TRUE	3	TRA:CGADAEYGNKLVF TRB:CASSEGQGAIMQPQHF	42	CD8-C7
Pla-2_TCTTCGGCATTGGTAC	Pla	Pla-2	P2-11	TRUE	3	TRA:CAVHNQGGKLIF TRB:CASSYLGAGYQETQYF	43	CD8-C7
Pla-2_TGACTTTTCTGTCCGT	Pla	Pla-2	P2-4855	FALSE	1	TRA:CALSEADGYNKLIF TRB:CASSPGQGRDTQYF	1	CD8-C6
Pla-2_TGCGTGGCACTTCGAA	Pla	Pla-2	P2-3	TRUE	3	TRA:CAVNPLESSYKLIF TRB:CASSPGTANTGELFF	177	CD8-C9
Pla-2_TGTATTCGTCTTCAAG	Pla	Pla-2	P2-4943	FALSE	1	TRA:CALSQGGKLIF TRB:CASSLGTGRDDTQYF	1	CD8-C7
Pla-2_TGTATTCTCCTCCTAG	Pla	Pla-2	P2-11	TRUE	3	TRA:CAVHNQGGKLIF TRB:CASSYLGAGYQETQYF	43	CD8-C7
Pla-2_TTCCCAGAGACTTTCG	Pla	Pla-2	P2-1	TRUE	3	TRA:CVVNIIGYQKVTF TRB:CASTRQGKQPQHF	270	CD8-C7
Pla-2_TTCCCAGAGAGAACAG	Pla	Pla-2	P2-246	FALSE	3	TRA:CAIGGAGNMLTF TRB:CASSARDRAYEQYF	3	CD8-C7
Pla-2_TTGCGTCCAGCTGTGC	Pla	Pla-2	P2-3	TRUE	3	TRA:CAVNPLESSYKLIF TRB:CASSPGTANTGELFF	177	CD8-C7
Pla-2_TTTGGTTGTGATGCCC	Pla	Pla-2	P2-389	FALSE	2	TRA:CAATSGTYKYIF TRB:CSATPGLALSYNEQFF	2	CD8-C6
Pla-2_AAACCTGTCGCCTGAG	Pla	Pla-2	P2-13	TRUE	3	TRA:CAYDLLTGNQFYF TRB:CASVRQLNTEAFF	38	CD8-C4
Pla-2_AAACGGGCAGCGAACA	Pla	Pla-2	P2-2	TRUE	3	TRA:CVVPNTNAGKSTF TRB:CASSLQGSTYNEQFF	220	CD8-C5
Pla-2_AAAGCAAAGCAGCGTA	Pla	Pla-2	P2-2	TRUE	3	TRA:CVVPNTNAGKSTF TRB:CASSLQGSTYNEQFF	220	CD8-C5
Pla-2_AAAGTAGCACTTGGAT	Pla	Pla-2	P2-16	FALSE	3	TRA:CAVEMDSSYKLIF TRB:CASSQGRVTQYF	32	CD8-C4
Pla-2_AAATGCCAGTAGGTGC	Pla	Pla-2	P2-3423	FALSE	1	TRA:CAVSGSGGYNKLIF TRB:CASSFEVNTGELFF	1	CD8-C8
Pla-2_AACACGTGTCCAAGTT	Pla	Pla-2	P2-3426	FALSE	1	TRA:CATAGNSGNTPLVF TRB:CASSGAGAGELFF	1	CD8-C5
Pla-2_AACCGCGAGTGTTGAA	Pla	Pla-2	P2-99	TRUE	3	TRA:CLVGDYAGGYQKVTF TRB:CASSEQGPTEAFF	5	CD8-C5
Pla-2_AACGTTGCAGTACACT	Pla	Pla-2	P2-3439	FALSE	1	TRA:CALFGSGNTGKLIF TRB:CASNQQGRSEKLFF	1	CD8-C4
Pla-2_AACTCCCTCTGGGCCA	Pla	Pla-2	P2-209	FALSE	3	TRA:CATGTHQAGTALIF TRB:CSAEEETQYF	3	CD8-C4
Pla-2_AACTTTCGTACACCGC	Pla	Pla-2	P2-35	FALSE	3	TRA:CGTETYTGFQKLVF TRB:CSVGTGSSYNEQFF	15	CD8-C5
Pla-2_AAGACCTGTTACAGAA	Pla	Pla-2	P2-16	FALSE	3	TRA:CAVEMDSSYKLIF TRB:CASSQGRVTQYF	32	CD8-C5
Pla-2_AAGGAGCAGCTGAAAT	Pla	Pla-2	P2-12	TRUE	3	TRA:CGADAEYGNKLVF TRB:CASSEGQGAIMQPQHF	42	CD8-C4
Pla-2_AAGGAGCTCTTGTCAT	Pla	Pla-2	P2-8	TRUE	3	TRA:CAALEGDNYGQNFVF TRB:CASSPTWGGVRDTQYF	53	CD8-C5
Pla-2_AAGGCAGAGCCAGTAG	Pla	Pla-2	P2-3	TRUE	3	TRA:CAVNPLESSYKLIF TRB:CASSPGTANTGELFF	177	CD8-C4

Pla-2_AAGGTTCCAGTGGGAT	Pla	Pla-2	P2-3	TRUE	3	TRA:CAVNPLESSYKLIF TRB:CASSPGTANTGELFF	177	CD8-C5
Pla-2_AAGTCTGAGTCCCACG	Pla	Pla-2	P2-73	FALSE	3	TRA:CAVPGSGNTGKLIF TRB:CASSQVYQGAMNTEAFF	8	CD8-C4
Pla-2_AAGTCTGCATGTCGAT	Pla	Pla-2	P2-318	TRUE	2	TRA:CAVEEGVRLMF TRB:CATSAGQGAFTDTQYF	2	CD8-C4
Pla-2_AAGTCTGTCTGTACGA	Pla	Pla-2	P2-3477	FALSE	1	TRA:CAVRDMENNRLAF TRB:CASSRTGEGRQPQHF	1	CD8-C4
Pla-2_AATCCAGGTAGCACGA	Pla	Pla-2	P2-177	TRUE	3	TRA:CVVSAVSGGSYIPTF TRB:CSARVRSYEQYF	3	CD8-C4
Pla-2_AATCGGTAGAGAACAG	Pla	Pla-2	P2-57	TRUE	3	TRA:CAVRDMSSGAGSYQLTF TRB:CASSELEDQETQYF	9	CD8-C5
Pla-2_AATCGGTGTGTGTGCC	Pla	Pla-2	P2-56	FALSE	3	TRA:CATDLDGQKLLF TRB:CASRERGGNTGELFF	10	CD8-C3
Pla-2_AATCGGTTCATAAAGG	Pla	Pla-2	P2-6	TRUE	3	TRA:CVVSVAAGNKLTF TRB:CASSPGTDLYEQYF	85	CD8-C4
Pla-2_ACACCAACAAGGACTG	Pla	Pla-2	P2-285	TRUE	2	TRA:CAFVQGGSEKLVF TRB:CASSEWIEGADTQYF	2	CD8-C4
Pla-2_ACACCGGTCCAATGGT	Pla	Pla-2	P2-2	TRUE	3	TRA:CVVPNTNAGKSTF TRB:CASSLQGSTYNEQFF	220	CD8-C4
Pla-2_ACAGCCGAGTTTAGGA	Pla	Pla-2	P2-3	TRUE	3	TRA:CAVNPLESSYKLIF TRB:CASSPGTANTGELFF	177	CD8-C7
Pla-2_ACAGCCGTCATTCACT	Pla	Pla-2	P2-127	TRUE	3	TRA:CAARRSGYSTLTF TRB:CASSLFPTGVAYGYTF	4	CD8-C6
Pla-2_ACAGCCGTCTAACTCT	Pla	Pla-2	P2-3	TRUE	3	TRA:CAVNPLESSYKLIF TRB:CASSPGTANTGELFF	177	CD8-C8
Pla-2_ACATCAGGTGTAATGA	Pla	Pla-2	P2-3509	FALSE	1	TRA:CAVNTRISGSARQLTF TRB:CASIGTGLYEQYF	1	CD8-C4
Pla-2_ACATGGTAGTGGAGTC	Pla	Pla-2	P2-3	TRUE	3	TRA:CAVNPLESSYKLIF TRB:CASSPGTANTGELFF	177	CD8-C5
Pla-2_ACATGGTCACATTTCT	Pla	Pla-2	P2-2	TRUE	3	TRA:CVVPNTNAGKSTF TRB:CASSLQGSTYNEQFF	220	CD8-C4
Pla-2_ACATGGTGTGACGCCT	Pla	Pla-2	P2-73	FALSE	3	TRA:CAVPGSGNTGKLIF TRB:CASSQVYQGAMNTEAFF	8	CD8-C4
Pla-2_ACGAGCCCAGACAGGT	Pla	Pla-2	P2-2	TRUE	3	TRA:CVVPNTNAGKSTF TRB:CASSLQGSTYNEQFF	220	CD8-C3
Pla-2_ACGAGGATCAAAGTAG	Pla	Pla-2	P2-20	TRUE	3	TRA:CAYDSNYQLIW TRB:CASSLSIDRADTGELFF	25	CD8-C4
Pla-2_ACGATACAGCTAGCCC	Pla	Pla-2	P2-2	TRUE	3	TRA:CVVPNTNAGKSTF TRB:CASSLQGSTYNEQFF	220	CD8-C5
Pla-2_ACGATACAGTGGTAGC	Pla	Pla-2	P2-55	TRUE	3	TRA:CIVRGIQGAQKLVF TRB:CASSLQGGGSGGLTQYF	10	CD8-C4
Pla-2_ACGATACTCCGCTGTT	Pla	Pla-2	P2-127	TRUE	3	TRA:CAARRSGYSTLTF TRB:CASSLFPTGVAYGYTF	4	CD8-C6
Pla-2_ACGATGTTCAATCACG	Pla	Pla-2	P2-3550	FALSE	1	TRA:CVVPRPGSARQLTF TRB:CAISPDSGTGELFF	1	CD8-C4
Pla-2_ACGATGTTCACCACCT	Pla	Pla-2	P2-81	FALSE	3	TRA:CAVRGNTPLVF TRB:CASSSNRDSYEQYF	7	CD8-C4
Pla-2_ACGCAGCAGGCGATAC	Pla	Pla-2	P2-3553	FALSE	1	TRA:CGTEGNDMRF TRB:CASSYIAGGSNEQFF	1	CD8-C4
Pla-2_ACGCCGAGTGGGTATG	Pla	Pla-2	P2-8	TRUE	3	TRA:CAALEGDNYGQNFVF TRB:CASSPTWGGVRDTQYF	53	CD8-C5
Pla-2_ACGGCCATCCGGCACA	Pla	Pla-2	P2-4	TRUE	3	TRA:CAIRYQSFNFGNEKLTF TRB:CASYRRVTEQYF	148	CD8-C4

Pla-2_ACGGGCTGTAAATACG	Pla	Pla-2	P2-3573	FALSE	1	TRA:CAQGTSGGSYIPTF TRB:CASRAPDRDLNTEAFF	1	CD8-C4
Pla-2_ACGGGCTTCAACCATG	Pla	Pla-2	P2-56	FALSE	3	TRA:CATDLDGQKLLF TRB:CASRERGGNTGELFF	10	CD8-C5
Pla-2_ACGGGTCCATTATCTC	Pla	Pla-2	P2-143	FALSE	3	TRA:CAYMEAAGNKLTF TRB:CASSPGLSTGELFF	4	CD8-C5
Pla-2_ACGGGTCTCCGTCATC	Pla	Pla-2	P2-2	TRUE	3	TRA:CVVPNTNAGKSTF TRB:CASSLQGSTYNEQFF	220	CD8-C3
Pla-2_ACGTCAATCGAACGGA	Pla	Pla-2	P2-275	TRUE	2	TRA:CATGGGADGLTF TRB:CASSYFGENTEAFF	2	CD8-C4
Pla-2_ACGTCAATCTACTCAT	Pla	Pla-2	P2-2	TRUE	3	TRA:CVVPNTNAGKSTF TRB:CASSLQGSTYNEQFF	220	CD8-C4
Pla-2_ACTATCTAGTCAATAG	Pla	Pla-2	P2-22	TRUE	3	TRA:CAMRERSWVRQKLVF TRB:CASSRGTSGTDTQYF	24	CD8-C4
Pla-2_ACTGAACAGACGCAAC	Pla	Pla-2	P2-16	FALSE	3	TRA:CAVEMDSSYKLIF TRB:CASSQGRVTQYF	32	CD8-C5
Pla-2_ACTGAGTAGGTGTGGT	Pla	Pla-2	P2-3	TRUE	3	TRA:CAVNPLESSYKLIF TRB:CASSPGTANTGELFF	177	CD8-C5
Pla-2_ACTGATGCATGATCCA	Pla	Pla-2	P2-1	TRUE	3	TRA:CVVNIIGYQKVTF TRB:CASTRQGKQPQHF	270	CD8-C5
Pla-2_ACTGATGGTCCGACGT	Pla	Pla-2	P2-20	TRUE	3	TRA:CAYDSNYQLIW TRB:CASSLSIDRADTGELFF	25	CD8-C4
Pla-2_ACTGCTCCAAGCCTAT	Pla	Pla-2	P2-3595	FALSE	1	TRA:CAVRDSNDYKLSF TRB:CASSHNPNTGELFF	1	CD8-C5
Pla-2_ACTTGTTTCAATCTCT	Pla	Pla-2	P2-66	TRUE	3	TRA:CAVCRNRDDKIIF TRB:CASSTPQGHYGYTF	8	CD8-C4
Pla-2_ACTTGTTTCACCATAG	Pla	Pla-2	P2-3603	FALSE	1	TRA:CAVGGISDMRF TRB:CASSIQSRDGNNEQFF	1	CD4-C6
Pla-2_ACTTTCAAGGATGCGT	Pla	Pla-2	P2-3	TRUE	3	TRA:CAVNPLESSYKLIF TRB:CASSPGTANTGELFF	177	CD8-C5
Pla-2_ACTTTCAAGTGTTTGC	Pla	Pla-2	P2-46	TRUE	3	TRA:CAFSTWGNQGGKLIF TRB:CASSQDRLGGGTYEQYF	11	CD8-C5
Pla-2_ACTTTCAGTGGTGTAG	Pla	Pla-2	P2-73	FALSE	3	TRA:CAVPGSGNTGKLIF TRB:CASSQVYQGAMNTEAFF	8	CD8-C4
Pla-2_ACTTTCATCTGAAAGA	Pla	Pla-2	P2-3611	FALSE	1	TRA:CEKTGTASKLTF TRB:CASSLGGNGNTIYF	1	CD8-C5
Pla-2_AGACGTTTCGATCCCT	Pla	Pla-2	P2-2	TRUE	3	TRA:CVVPNTNAGKSTF TRB:CASSLQGSTYNEQFF	220	CD8-C5
Pla-2_AGATCTGGTGTTCGAT	Pla	Pla-2	P2-228	FALSE	3	TRA:CAVPPLTGGYQKVTF TRB:CASSGGGGGLETQYF	3	CD8-C4
Pla-2_AGCATACAGTGTCCCG	Pla	Pla-2	P2-3	TRUE	3	TRA:CAVNPLESSYKLIF TRB:CASSPGTANTGELFF	177	CD8-C5
Pla-2_AGCCTAAGTTCCCGAG	Pla	Pla-2	P2-109	TRUE	3	TRA:CAPPFGGSEKLVF TRB:CASSKTGSSYEQYF	5	CD8-C5
Pla-2_AGCGTATAGCGGCTTC	Pla	Pla-2	P2-2	TRUE	3	TRA:CVVPNTNAGKSTF TRB:CASSLQGSTYNEQFF	220	CD8-C4
Pla-2_AGCGTCGAGGATATAC	Pla	Pla-2	P2-3658	FALSE	1	TRA:CAIRNNNARLMF TRB:CASSQGRVTQYF	1	CD8-C7
Pla-2_AGCTCCTAGGTCATCT	Pla	Pla-2	P2-2	TRUE	3	TRA:CVVPNTNAGKSTF TRB:CASSLQGSTYNEQFF	220	CD8-C4
Pla-2_AGCTCCTCAGCGTAAG	Pla	Pla-2	P2-83	FALSE	3	TRA:CAVDTGTASKLTF TRB:CASRGSTDTQYF	7	CD8-C5
Pla-2_AGCTCCTTCCGCAGTG	Pla	Pla-2	P2-13	TRUE	3	TRA:CAYDLLTGNQFYF TRB:CASVRQLNTEAFF	38	CD8-C4

Pla-2_AGGGAGTAGGGTCGAT	Pla	Pla-2	P2-232	FALSE	3	TRA:CAASGGGNKLTF TRB:CASSGNTDTQYF	3	CD8-C5
Pla-2_AGGGAGTGTGTATGGG	Pla	Pla-2	P2-3	TRUE	3	TRA:CAVNPLESSYKLIF TRB:CASSPGTANTGELFF	177	CD8-C5
Pla-2_AGGGTGACAATTGCTG	Pla	Pla-2	P2-233	FALSE	3	TRA:CAASIWNYGQNFVF TRB:CSAIERGGRETQYF	3	CD8-C4
Pla-2_AGGTCCGAGCTGAAAT	Pla	Pla-2	P2-6	TRUE	3	TRA:CVVSVAAGNKLTF TRB:CASSPGTDLYEQYF	85	CD8-C4
Pla-2_AGTCTTTAGAGCAATT	Pla	Pla-2	P2-20	TRUE	3	TRA:CAYDSNYQLIW TRB:CASSLSIDRADTGELFF	25	CD8-C6
Pla-2_AGTTGGTAGCTGGAAC	Pla	Pla-2	P2-2	TRUE	3	TRA:CVVPNTNAGKSTF TRB:CASSLQGSTYNEQFF	220	CD8-C4
Pla-2_AGTTGGTGTTATCCGA	Pla	Pla-2	P2-3	TRUE	3	TRA:CAVNPLESSYKLIF TRB:CASSPGTANTGELFF	177	CD8-C5
Pla-2_AGTTGGTTCGCGCCAA	Pla	Pla-2	P2-3	TRUE	3	TRA:CAVNPLESSYKLIF TRB:CASSPGTANTGELFF	177	CD8-C5
Pla-2_ATAACGCTCTATCCCG	Pla	Pla-2	P2-15	TRUE	3	TRA:CAVGDNFNKFYF TRB:CASSRTSAAGELFF	35	CD8-C4
Pla-2_ATAAGAGAGGACAGAA	Pla	Pla-2	P2-153	FALSE	3	TRA:CAYNNNDMRF TRB:CASSDRGGRQETQYF	4	CD8-C2
Pla-2_ATCGAGTAGATACACA	Pla	Pla-2	P2-2	TRUE	3	TRA:CVVPNTNAGKSTF TRB:CASSLQGSTYNEQFF	220	CD8-C5
Pla-2_ATGCGATCATAGAAAC	Pla	Pla-2	P2-3746	FALSE	1	TRA:CAVARFDDMRF TRB:CASRELAGDYEQYF	1	CD8-C4
Pla-2_ATGTGTGCATTATCTC	Pla	Pla-2	P2-3	TRUE	3	TRA:CAVNPLESSYKLIF TRB:CASSPGTANTGELFF	177	CD8-C7
Pla-2_ATTACTCTCACCACCT	Pla	Pla-2	P2-10	TRUE	3	TRA:CAPLTPNDMRF TRB:CASLNGAGGSYEQYF	45	CD8-C4
Pla-2_ATTACTCTCCATGCTC	Pla	Pla-2	P2-3	TRUE	3	TRA:CAVNPLESSYKLIF TRB:CASSPGTANTGELFF	177	CD8-C5
Pla-2_ATTATCCAGTTCGATC	Pla	Pla-2	P2-55	TRUE	3	TRA:CIVRGIQGAQKLVF TRB:CASSLQGGGSGGLTQYF	10	CD8-C4
Pla-2_ATTATCCAGTTGTCGT	Pla	Pla-2	P2-3758	FALSE	1	TRA:CASTQLDGQKLLF TRB:CASSPTTSGVSYEQYF	1	CD8-C4
Pla-2_ATTGGACAGTGAAGTT	Pla	Pla-2	P2-2	TRUE	3	TRA:CVVPNTNAGKSTF TRB:CASSLQGSTYNEQFF	220	CD8-C4
Pla-2_ATTGGACTCACGAAGG	Pla	Pla-2	P2-2	TRUE	3	TRA:CVVPNTNAGKSTF TRB:CASSLQGSTYNEQFF	220	CD8-C4
Pla-2_ATTGGTGTCCTATGTT	Pla	Pla-2	P2-10	TRUE	3	TRA:CAPLTPNDMRF TRB:CASLNGAGGSYEQYF	45	CD8-C4
Pla-2_ATTTCTGAGTCCAGGA	Pla	Pla-2	P2-8	TRUE	3	TRA:CAALEGDNYGQNFVF TRB:CASSPTWGGVRDTQYF	53	CD8-C5
Pla-2_ATTTCTGCACGTCTCT	Pla	Pla-2	P2-3776	FALSE	1	TRA:CAASGGAGGTSYGKLTF TRB:CASSAGTSGTYEQYF	1	CD8-C4
Pla-2_CAACTAGAGATAGGAG	Pla	Pla-2	P2-156	FALSE	3	TRA:CAMRHHGSGNTGKLIF TRB:CASSLAGASGPNNEQFF	4	CD8-C6
Pla-2_CAAGTTGAGCTCCTTC	Pla	Pla-2	P2-456	FALSE	2	TRA:CAVEGSGLNFNKFYF TRB:CSVVGTEAFF	2	CD8-C5
Pla-2_CACAAACCATAAGACA	Pla	Pla-2	P2-3	TRUE	3	TRA:CAVNPLESSYKLIF TRB:CASSPGTANTGELFF	177	CD8-C5
Pla-2_CACACTCAGCGATTCT	Pla	Pla-2	P2-52	FALSE	3	TRA:CARTTNAGKSTF TRB:CASSETSGRIYEQFF	11	CD8-C4
Pla-2_CACACTCAGGTGTTAA	Pla	Pla-2	P2-69	TRUE	3	TRA:CAVRAGSARQLTF TRB:CATTGTSLDEQYF	8	CD8-C3

Pla-2_CACACTCCAAAGAATC	Pla	Pla-2	P2-60	TRUE	3	TRA:CALSEGSGANSKLTF TRB:CASSSHSGLAGGFF	9	CD4-C5
Pla-2_CACACTCTCAGGCCCA	Pla	Pla-2	P2-3824	FALSE	1	TRA:CVVSVAAGNKLTF TRB:CSARSDRLGYQPQHF	1	CD8-C5
Pla-2_CACAGGCTCGTGGACC	Pla	Pla-2	P2-6	TRUE	3	TRA:CVVSVAAGNKLTF TRB:CASSPGTDLYEQYF	85	CD8-C4
Pla-2_CACAGTATCGGATGGA	Pla	Pla-2	P2-460	FALSE	2	TRA:CAVRDIPRSGGYNKLIF TRB:CASSLSRGGGTEAFF	2	CD8-C5
Pla-2_CACCACTTCGAGGTAG	Pla	Pla-2	P2-29	TRUE	3	TRA:CAVGGDFNKFYF TRB:CSARSDRLGYQPQHF	21	CD8-C5
Pla-2_CACCAGGGTAGCCTAT	Pla	Pla-2	P2-3841	FALSE	1	TRA:CAASKLSSNYKLTF TRB:CASSQDSTSVYNEQFF	1	CD8-C7
Pla-2_CACTCCAAGCGGCTTC	Pla	Pla-2	P2-2	TRUE	3	TRA:CVVPNTNAGKSTF TRB:CASSLQGSTYNEQFF	220	CD8-C4
Pla-2_CACTCCATCGTAGGTT	Pla	Pla-2	P2-2	TRUE	3	TRA:CVVPNTNAGKSTF TRB:CASSLQGSTYNEQFF	220	CD8-C7
Pla-2_CAGAATCGTTTACTCT	Pla	Pla-2	P2-88	TRUE	3	TRA:CAVPGAGNKLTF TRB:CASSRSLAGVDTGELFF	6	CD8-C4
Pla-2_CAGAGAGAGACATAAC	Pla	Pla-2	P2-3851	FALSE	1	TRA:CAVYNAGNMLTF TRB:CASSFGKNTEAFF	1	CD8-C4
Pla-2_CAGAGAGCAAAGAATC	Pla	Pla-2	P2-20	TRUE	3	TRA:CAYDSNYQLIW TRB:CASSLSIDRADTGELFF	25	CD8-C3
Pla-2_CAGAGAGCACCACGTG	Pla	Pla-2	P2-29	TRUE	3	TRA:CAVGGDFNKFYF TRB:CSARSDRLGYQPQHF	21	CD8-C6
Pla-2_CAGAGAGCACCGTTGG	Pla	Pla-2	P2-8	TRUE	3	TRA:CAALEGDNYGQNFVF TRB:CASSPTWGGVRDTQYF	53	CD8-C4
Pla-2_CAGAGAGGTACTCTCC	Pla	Pla-2	P2-13	TRUE	3	TRA:CAYDLLTGNQFYF TRB:CASVRQLNTEAFF	38	CD8-C4
Pla-2_CAGAGAGTCAAACGGG	Pla	Pla-2	P2-3855	FALSE	1	TRA:CAMRERNTNAGKSTF TRB:CASKRDGTQETQYF	1	CD8-C4
Pla-2_CAGAGAGTCACCAGGC	Pla	Pla-2	P2-158	FALSE	3	TRA:CAVGAVRETSGSRLTF TRB:CASSFTDLPYEQYF	4	CD8-C4
Pla-2_CAGCAGCGTAGCGCAA	Pla	Pla-2	P2-161	FALSE	3	TRA:CALSTNAGKSTF TRB:CASSSNRDSYEQYF	4	CD8-C4
Pla-2_CAGCATACAGACGCTC	Pla	Pla-2	P2-3	TRUE	3	TRA:CAVNPLESSYKLIF TRB:CASSPGTANTGELFF	177	CD8-C5
Pla-2_CAGCCGAAGATCGATA	Pla	Pla-2	P2-21	TRUE	3	TRA:CALSSYNNNDMRF TRB:CASSLDLAPSTEAFF	25	CD8-C5
Pla-2_CAGCGACTCATCATTC	Pla	Pla-2	P2-72	FALSE	3	TRA:CATDEETAGNKLTF TRB:CASSEAGWMGALHTEAFF	8	CD8-C4
Pla-2_CAGTAACAGAGTACAT	Pla	Pla-2	P2-4	TRUE	3	TRA:CAIRYQSFNFGNEKLTF TRB:CASYRRVTEQYF	148	CD8-C5
Pla-2_CAGTCCTCATCGACGC	Pla	Pla-2	P2-3888	FALSE	1	TRA:CAASRNFGNEKLTF TRB:CASSYGSSNEQFF	1	CD8-C4
Pla-2_CATATGGTCCTGTACC	Pla	Pla-2	P2-99	TRUE	3	TRA:CLVGDYAGGYQKVTF TRB:CASSEQGPTEAFF	5	CD8-C4
Pla-2_CATATGGTCTCCAGGG	Pla	Pla-2	P2-6	TRUE	3	TRA:CVVSVAAGNKLTF TRB:CASSPGTDLYEQYF	85	CD8-C5
Pla-2_CATATTCAGTAGCGGT	Pla	Pla-2	P2-19	TRUE	3	TRA:CAVRDMENNRLAF TRB:CASSLGGGLTYEQYF	27	CD8-C4
Pla-2_CATCAAGAGACTAAGT	Pla	Pla-2	P2-6	TRUE	3	TRA:CVVSVAAGNKLTF TRB:CASSPGTDLYEQYF	85	CD8-C4
Pla-2_CATCAGAAGTGCAAGC	Pla	Pla-2	P2-2	TRUE	3	TRA:CVVPNTNAGKSTF TRB:CASSLQGSTYNEQFF	220	CD8-C4

Pla-2_CATCGGGGTAGCTGCC	Pla	Pla-2	P2-3	TRUE	3	TRA:CAVNPLESSYKLIF TRB:CASSPGTANTGELFF	177	CD8-C7
Pla-2_CATGACACACATTAGC	Pla	Pla-2	P2-3	TRUE	3	TRA:CAVNPLESSYKLIF TRB:CASSPGTANTGELFF	177	CD8-C5
Pla-2_CATGGCGAGTGTTAGA	Pla	Pla-2	P2-6	TRUE	3	TRA:CVVSVAAGNKLTF TRB:CASSPGTDLYEQYF	85	CD8-C4
Pla-2_CATGGCGCAGAGTGTG	Pla	Pla-2	P2-6	TRUE	3	TRA:CVVSVAAGNKLTF TRB:CASSPGTDLYEQYF	85	CD8-C5
Pla-2_CATTATCAGGAGCGAG	Pla	Pla-2	P2-3927	FALSE	1	TRA:CVVSEEDSNYQLIW TRB:CATSRGGPGETQYF	1	CD8-C10
Pla-2_CCAATCCAGGAGTTGC	Pla	Pla-2	P2-3	TRUE	3	TRA:CAVNPLESSYKLIF TRB:CASSPGTANTGELFF	177	CD8-C5
Pla-2_CCAATCCCACCATGTA	Pla	Pla-2	P2-15	TRUE	3	TRA:CAVGDNFNKFYF TRB:CASSRTSAAGELFF	35	CD8-C4
Pla-2_CCACCTAGTCCGTGAC	Pla	Pla-2	P2-19	TRUE	3	TRA:CAVRDMENNRLAF TRB:CASSLGGGLTYEQYF	27	CD8-C4
Pla-2_CCACGGATCTGCGACG	Pla	Pla-2	P2-6	TRUE	3	TRA:CVVSVAAGNKLTF TRB:CASSPGTDLYEQYF	85	CD8-C4
Pla-2_CCCAATCAGGGTGTGT	Pla	Pla-2	P2-3958	FALSE	1	TRA:CAVRDPLVGYGGSQGNLIF TRB:CASSEDRAGTGELFF	1	CD8-C4
Pla-2_CCGGGATCAGTAGAGC	Pla	Pla-2	P2-156	FALSE	3	TRA:CAMRHHGSGNTGKLIF TRB:CASSLAGASGPNNEQFF	4	CD8-C6
Pla-2_CCGTACTAGACTTTCG	Pla	Pla-2	P2-3975	FALSE	1	TRA:CVVNGRLI TRB:CASSEGGTGELFF	1	CD8-C2
Pla-2_CCGTACTTCTGAGTGT	Pla	Pla-2	P2-10	TRUE	3	TRA:CAPLTPNDMRF TRB:CASLNGAGGSYEQYF	45	CD8-C4
Pla-2_CCGTGGAAGCAGCGTA	Pla	Pla-2	P2-3978	FALSE	1	TRA:CAVRDIHGAGNNRKLIW TRB:CASILGGNEQFF	1	CD8-C4
Pla-2_CCGTGGACAATCTACG	Pla	Pla-2	P2-21	TRUE	3	TRA:CALSSYNNNDMRF TRB:CASSLDLAPSTEAFF	25	CD8-C5
Pla-2_CCGTGGAGTCTCTCTG	Pla	Pla-2	P2-2	TRUE	3	TRA:CVVPNTNAGKSTF TRB:CASSLQGSTYNEQFF	220	CD8-C4
Pla-2_CCGTTCACAATGCCAT	Pla	Pla-2	P2-16	FALSE	3	TRA:CAVEMDSSYKLIF TRB:CASSQGRVTQYF	32	CD8-C4
Pla-2_CCTAAAGTCGGAGGTA	Pla	Pla-2	P2-68	TRUE	3	TRA:CAVYSGYSTLTF TRB:CSVEDVGGKSSYNEQFF	8	CD8-C5
Pla-2_CCTACCATCTCAACTT	Pla	Pla-2	P2-2	TRUE	3	TRA:CVVPNTNAGKSTF TRB:CASSLQGSTYNEQFF	220	CD8-C8
Pla-2_CCTCTGATCCTAGGGC	Pla	Pla-2	P2-6	TRUE	3	TRA:CVVSVAAGNKLTF TRB:CASSPGTDLYEQYF	85	CD8-C4
Pla-2_CCTTACGCAGTATCTG	Pla	Pla-2	P2-3	TRUE	3	TRA:CAVNPLESSYKLIF TRB:CASSPGTANTGELFF	177	CD8-C5
Pla-2_CCTTCCCCACGAAATA	Pla	Pla-2	P2-17	TRUE	3	TRA:CAVQASNAGNMLTF TRB:CASSVEQGNVETQYF	30	CD8-C5
Pla-2_CGAACATCATGACGGA	Pla	Pla-2	P2-158	FALSE	3	TRA:CAVGAVRETSGSRLTF TRB:CASSFTDLPYEQYF	4	CD8-C4
Pla-2_CGAACATGTATTACCG	Pla	Pla-2	P2-4026	FALSE	1	TRA:CVVPNTNAGKSTF TRB:CASSLSIDRADTGELFF	1	CD8-C5
Pla-2_CGAATGTGTGTGGTTT	Pla	Pla-2	P2-29	TRUE	3	TRA:CAVGGDFNKFYF TRB:CSARSDRLGYQPQHF	21	CD8-C5
Pla-2_CGACCTTAGTCCTCCT	Pla	Pla-2	P2-4034	FALSE	1	TRA:CAVIFTGGGNKLTF TRB:CASSFDAGLQETQYF	1	CD8-C3
Pla-2_CGACTTCAGTCCATAC	Pla	Pla-2	P2-495	FALSE	2	TRA:CAVTRYDYKLSF TRB:CASSESDRDSFYQETQYF	2	CD8-C5

Pla-2_CGACTTCCAGTAAGCG	Pla	Pla-2	P2-4037	FALSE	1	TRA:CAMRANNDYKLSF TRB:CASTRQGGSDTQYF	1	CD8-C5
Pla-2_CGACTTCGTTCAGCGC	Pla	Pla-2	P2-15	TRUE	3	TRA:CAVGDNFNKFYF TRB:CASSRTSAAGELFF	35	CD8-C4
Pla-2_CGAGAAGTCAAACCGT	Pla	Pla-2	P2-2	TRUE	3	TRA:CVVPNTNAGKSTF TRB:CASSLQGSTYNEQFF	220	CD8-C5
Pla-2_CGAGCCATCGGAGGTA	Pla	Pla-2	P2-4052	FALSE	1	TRA:CAESYNNNDMRF TRB:CASSLAQSEQYF	1	CD8-C4
Pla-2_CGATGGCAGGTGACCA	Pla	Pla-2	P2-47	TRUE	3	TRA:CAASAGGNKLTF TRB:CASSTEDEQYF	11	CD8-C4
Pla-2_CGATGTAGTGATGCCC	Pla	Pla-2	P2-8	TRUE	3	TRA:CAALEGDNYGQNFVF TRB:CASSPTWGGVRDTQYF	53	CD8-C4
Pla-2_CGATGTAGTGCCTGCA	Pla	Pla-2	P2-16	FALSE	3	TRA:CAVEMDSSYKLIF TRB:CASSQGRVTQYF	32	CD8-C4
Pla-2_CGCGGTAGTATGAAAC	Pla	Pla-2	P2-156	FALSE	3	TRA:CAMRHHGSGNTGKLIF TRB:CASSLAGASGPNNEQFF	4	CD8-C6
Pla-2_CGCGGTATCAACCATG	Pla	Pla-2	P2-3	TRUE	3	TRA:CAVNPLESSYKLIF TRB:CASSPGTANTGELFF	177	CD8-C5
Pla-2_CGCTATCCAACACGCC	Pla	Pla-2	P2-16	FALSE	3	TRA:CAVEMDSSYKLIF TRB:CASSQGRVTQYF	32	CD8-C4
Pla-2_CGGACACCAGATGGGT	Pla	Pla-2	P2-10	TRUE	3	TRA:CAPLTPNDMRF TRB:CASLNGAGGSYEQYF	45	CD8-C4
Pla-2_CGGACACGTACGCACC	Pla	Pla-2	P2-2	TRUE	3	TRA:CVVPNTNAGKSTF TRB:CASSLQGSTYNEQFF	220	CD8-C4
Pla-2_CGGACGTTCCCATTAT	Pla	Pla-2	P2-4101	FALSE	1	TRA:CVVNSRGTYKYIF TRB:CSGSRLADSYEQYF	1	CD8-C4
Pla-2_CGGAGTCCATGTTCCC	Pla	Pla-2	P2-3	TRUE	3	TRA:CAVNPLESSYKLIF TRB:CASSPGTANTGELFF	177	CD8-C5
Pla-2_CGGGTCAAGTAGGTGC	Pla	Pla-2	P2-75	FALSE	3	TRA:CAVNDGRGADGLTF TRB:CASLGGRTDTQYF	8	CD8-C5
Pla-2_CGGGTCACACATTAGC	Pla	Pla-2	P2-15	TRUE	3	TRA:CAVGDNFNKFYF TRB:CASSRTSAAGELFF	35	CD8-C4
Pla-2_CGGGTCAGTCAAACTC	Pla	Pla-2	P2-3	TRUE	3	TRA:CAVNPLESSYKLIF TRB:CASSPGTANTGELFF	177	CD8-C4
Pla-2_CGGGTCATCTATCCTA	Pla	Pla-2	P2-4121	FALSE	1	TRA:CVVSEASGTYKYIF TRB:CASSLNLRKNTEAFF	1	CD8-C4
Pla-2_CGGTTAAGTAAATGTG	Pla	Pla-2	P2-59	TRUE	3	TRA:CAVDEGNTPLVF TRB:CAISESVDNTEAFF	9	CD8-C5
Pla-2_CGTAGCGAGGCAGGTT	Pla	Pla-2	P2-4123	FALSE	1	TRA:CATRVITGNQFYF TRB:CASSPGTGGALGGYTF	1	CD8-C4
Pla-2_CGTAGCGCAGGAATCG	Pla	Pla-2	P2-433	FALSE	2	TRA:CAVSNTDKLIF TRB:CASVGGNTEAFF	2	CD8-C4
Pla-2_CGTAGGCAGGGTGTGT	Pla	Pla-2	P2-6	TRUE	3	TRA:CVVSVAAGNKLTF TRB:CASSPGTDLYEQYF	85	CD8-C4
Pla-2_CGTAGGCCAGCTGGCT	Pla	Pla-2	P2-504	FALSE	2	TRA:CGRQMLLFTTVHRSAGKSTF TRB:CASSPITGDTQYF	2	CD8-C4
Pla-2_CGTAGGCGTAAGAGGA	Pla	Pla-2	P2-2	TRUE	3	TRA:CVVPNTNAGKSTF TRB:CASSLQGSTYNEQFF	220	CD8-C3
Pla-2_CGTAGGCTCATCTGTT	Pla	Pla-2	P2-4131	FALSE	1	TRA:CALSEAVEAGTALIF TRB:CASSPREDQPQHF	1	CD8-C2
Pla-2_CGTAGGCTCATGCTCC	Pla	Pla-2	P2-4132	FALSE	1	TRA:CIVRGSGSARQLTF TRB:CASSSYGGLETQYF	1	CD8-C5
Pla-2_CGTCACTAGGCACATG	Pla	Pla-2	P2-2	TRUE	3	TRA:CVVPNTNAGKSTF TRB:CASSLQGSTYNEQFF	220	CD8-C5

Pla-2_CGTCACTCAGTCTTCC	Pla	Pla-2	P2-15	TRUE	3	TRA:CAVGDNFNKFYF TRB:CASSRTSAAGELFF	35	CD8-C4
Pla-2_CGTCACTTCCTCAATT	Pla	Pla-2	P2-13	TRUE	3	TRA:CAYDLLTGNQFYF TRB:CASVRQLNTEAFF	38	CD8-C5
Pla-2_CGTCAGGGTCAGAAGC	Pla	Pla-2	P2-4	TRUE	3	TRA:CAIRYQSFNFGNEKLTF TRB:CASYRRVTEQYF	148	CD8-C4
Pla-2_CGTCTACTCTTGTTTG	Pla	Pla-2	P2-507	FALSE	2	TRA:CLVGVVNQAGTALIF TRB:CASSLVTGSQETQYF	2	CD8-C4
Pla-2_CGTGAGCCAACTGCTA	Pla	Pla-2	P2-19	TRUE	3	TRA:CAVRDMENNRLAF TRB:CASSLGGGLTYEQYF	27	CD8-C4
Pla-2_CGTGAGCGTAGCACGA	Pla	Pla-2	P2-2	TRUE	3	TRA:CVVPNTNAGKSTF TRB:CASSLQGSTYNEQFF	220	CD8-C4
Pla-2_CGTGTAACAGTGACAG	Pla	Pla-2	P2-4145	FALSE	1	TRA:CAVNSPITSGTYKYIF TRB:CASSDGGGGQETQYF	1	CD8-C5
Pla-2_CGTTAGACACATAACC	Pla	Pla-2	P2-109	TRUE	3	TRA:CAPPFGGSEKLVF TRB:CASSKTGSSYEQYF	5	CD8-C4
Pla-2_CGTTCTGGTGCCTTGG	Pla	Pla-2	P2-16	FALSE	3	TRA:CAVEMDSSYKLIF TRB:CASSQGRVTQYF	32	CD8-C5
Pla-2_CGTTGGGTCAGTACGT	Pla	Pla-2	P2-216	FALSE	3	TRA:CGTEGNDMRF TRB:CASSLTGETQYF	3	CD8-C4
Pla-2_CTAATGGTCGTAGGTT	Pla	Pla-2	P2-21	TRUE	3	TRA:CALSSYNNNDMRF TRB:CASSLDLAPSTEAFF	25	CD8-C4
Pla-2_CTACATTTCATTCACT	Pla	Pla-2	P2-16	FALSE	3	TRA:CAVEMDSSYKLIF TRB:CASSQGRVTQYF	32	CD8-C5
Pla-2_CTACCCATCGGCGCTA	Pla	Pla-2	P2-212	FALSE	3	TRA:CAENIRATNKLIF TRB:CAWSPRGSNQPQHF	3	CD8-C5
Pla-2_CTACGTCGTCGAGTTT	Pla	Pla-2	P2-29	TRUE	3	TRA:CAVGGDFNKFYF TRB:CSARSDRLGYQPQHF	21	CD8-C4
Pla-2_CTAGCCTAGCTCTCGG	Pla	Pla-2	P2-15	TRUE	3	TRA:CAVGDNFNKFYF TRB:CASSRTSAAGELFF	35	CD8-C4
Pla-2_CTAGCCTTCATGGTCA	Pla	Pla-2	P2-4	TRUE	3	TRA:CAIRYQSFNFGNEKLTF TRB:CASYRRVTEQYF	148	CD8-C4
Pla-2_CTAGTGATCCCGGATG	Pla	Pla-2	P2-4193	FALSE	1	TRA:CATEGWGSQGNLIF TRB:CAISESPDYTDTQYF	1	CD8-C4
Pla-2_CTCAGAAGTACTCGCG	Pla	Pla-2	P2-2	TRUE	3	TRA:CVVPNTNAGKSTF TRB:CASSLQGSTYNEQFF	220	CD8-C5
Pla-2_CTCAGAAGTGGAAAGA	Pla	Pla-2	P2-6	TRUE	3	TRA:CVVSVAAGNKLTF TRB:CASSPGTDLYEQYF	85	CD8-C4
Pla-2_CTCATTAAGACATAAC	Pla	Pla-2	P2-6	TRUE	3	TRA:CVVSVAAGNKLTF TRB:CASSPGTDLYEQYF	85	CD8-C4
Pla-2_CTCATTAGTGCTGTAT	Pla	Pla-2	P2-83	FALSE	3	TRA:CAVDTGTASKLTF TRB:CASRGSTDTQYF	7	CD8-C5
Pla-2_CTCCTAGTCCGGCACA	Pla	Pla-2	P2-4206	FALSE	1	TRA:CAVRETSGNTGKLIF TRB:CASSASSGGAGRRELFF	1	CD8-C4
Pla-2_CTCGAGGCACGTGAGA	Pla	Pla-2	P2-63	FALSE	3	TRA:CAASGEAGTALIF TRB:CATSREIQPNEKLFF	9	CD4-C6
Pla-2_CTCGGAGAGGTACTCT	Pla	Pla-2	P2-2	TRUE	3	TRA:CVVPNTNAGKSTF TRB:CASSLQGSTYNEQFF	220	CD8-C4
Pla-2_CTCGGGAGTCTAGCCG	Pla	Pla-2	P2-15	TRUE	3	TRA:CAVGDNFNKFYF TRB:CASSRTSAAGELFF	35	CD8-C4
Pla-2_CTCGTACAGCGAGAAA	Pla	Pla-2	P2-3	TRUE	3	TRA:CAVNPLESSYKLIF TRB:CASSPGTANTGELFF	177	CD8-C5
Pla-2_CTCGTACTCGCTGATA	Pla	Pla-2	P2-3	TRUE	3	TRA:CAVNPLESSYKLIF TRB:CASSPGTANTGELFF	177	CD8-C3

Pla-2_CTCGTACTCTCATTCA	Pla	Pla-2	P2-13	TRUE	3	TRA:CAYDLLTGNQFYF TRB:CASVRQLNTEAFF	38	CD8-C5
Pla-2_CTCTACGGTTAAAGAC	Pla	Pla-2	P2-4225	FALSE	1	TRA:CVVNRDEKLTF TRB:CASRGWGDVSEQFF	1	CD8-C5
Pla-2_CTGAAGTCACCTGGTG	Pla	Pla-2	P2-52	FALSE	3	TRA:CARTTNAGKSTF TRB:CASSETSGRIYEQFF	11	CD8-C4
Pla-2_CTGATAGTCACAGGCC	Pla	Pla-2	P2-4235	FALSE	1	TRA:CVVNKPSNYQLIW TRB:CASSPGTDLYEQYF	1	CD8-C4
Pla-2_CTGCCTAGTTCCACGG	Pla	Pla-2	P2-111	TRUE	3	TRA:CAVYSGGSNYKLTF TRB:CASSLQGGGSGGLTQYF	5	CD8-C4
Pla-2_CTGCGGACAACGCACC	Pla	Pla-2	P2-164	FALSE	3	TRA:CAVGLAGNMLTF TRB:CASSYAPAGERGGRNEQFF	4	CD8-C4
Pla-2_CTGCTGTCATCTACGA	Pla	Pla-2	P2-2	TRUE	3	TRA:CVVPNTNAGKSTF TRB:CASSLQGSTYNEQFF	220	CD8-C5
Pla-2_CTGGTCTGTACCGCTG	Pla	Pla-2	P2-4251	FALSE	1	TRA:CAASGGGNKLTF TRB:CASSQRDEQFF	1	CD8-C4
Pla-2_CTGTTTACACCTCGGA	Pla	Pla-2	P2-2	TRUE	3	TRA:CVVPNTNAGKSTF TRB:CASSLQGSTYNEQFF	220	CD8-C4
Pla-2_CTGTTTATCAATAAGG	Pla	Pla-2	P2-16	FALSE	3	TRA:CAVEMDSSYKLIF TRB:CASSQGRVTQYF	32	CD8-C5
Pla-2_CTTACCGTCAGGCGAA	Pla	Pla-2	P2-4	TRUE	3	TRA:CAIRYQSFNFGNEKLTF TRB:CASYRRVTEQYF	148	CD8-C5
Pla-2_CTTCTCTCACTAGTAC	Pla	Pla-2	P2-10	TRUE	3	TRA:CAPLTPNDMRF TRB:CASLNGAGGSYEQYF	45	CD8-C4
Pla-2_CTTCTCTCATGCAATC	Pla	Pla-2	P2-4274	FALSE	1	TRA:CAYRSALGGNQFYF TRB:CASTPPRGPLHF	1	CD8-C2
Pla-2_CTTGGCTCAAGCGTAG	Pla	Pla-2	P2-3	TRUE	3	TRA:CAVNPLESSYKLIF TRB:CASSPGTANTGELFF	177	CD8-C5
Pla-2_CTTGGCTTCTTCGGTC	Pla	Pla-2	P2-4282	FALSE	1	TRA:CAMRGKNSGYALNF TRB:CASSPAGGNYEQYF	1	CD8-C4
Pla-2_CTTTGCGCACCGATAT	Pla	Pla-2	P2-421	FALSE	2	TRA:CAVQAPDSGGYQKVTF TRB:CASSVTGLGVWGDHQETQY	2	CD8-C4
Pla-2_CTTTGCGCACTTAAGC	Pla	Pla-2	P2-532	FALSE	2	TRA:CAAFSSGGSYIPTF TRB:CASTVGGTGDREQYF	2	CD8-C4
Pla-2_CTTTGCGTCACTTCAT	Pla	Pla-2	P2-533	FALSE	2	TRA:CAASVWGSARQLTF TRB:CASSQESGGLVFEQFF	2	CD8-C5
Pla-2_GAAACTCCAAGCCCAC	Pla	Pla-2	P2-4288	FALSE	1	TRA:CAYDSNYQLIW TRB:CASSLGVGQGFHNEQFF	1	CD8-C5
Pla-2_GAAACTCCAGCTCCGA	Pla	Pla-2	P2-237	FALSE	3	TRA:CALSEALGSGGGADGLTF TRB:CASSTTGDGVVSYEQYF	3	CD8-C5
Pla-2_GAAACTCGTATCAGTC	Pla	Pla-2	P2-81	FALSE	3	TRA:CAVRGNTPLVF TRB:CASSSNRDSYEQYF	7	CD8-C4
Pla-2_GAAATGAAGACTTGAA	Pla	Pla-2	P2-126	TRUE	3	TRA:CATDGDFGNEKLTF TRB:CASSLTGETQYF	4	CD8-C4
Pla-2_GAAATGACAGGGTATG	Pla	Pla-2	P2-2	TRUE	3	TRA:CVVPNTNAGKSTF TRB:CASSLQGSTYNEQFF	220	CD8-C4
Pla-2_GAATAAGGTCAGAATA	Pla	Pla-2	P2-4316	FALSE	1	TRA:CAMRSAGSGNTGKLIF TRB:CASSLGLGTEAFF	1	CD8-C4
Pla-2_GAATGAAAGACTACAA	Pla	Pla-2	P2-4318	FALSE	1	TRA:CAVGGDTGNQFYF TRB:CASSLQGSTYNEQFF	1	CD8-C6
Pla-2_GAATGAAAGTTACCCA	Pla	Pla-2	P2-4	TRUE	3	TRA:CAIRYQSFNFGNEKLTF TRB:CASYRRVTEQYF	148	CD8-C5
Pla-2_GACACGCTCCGTCATC	Pla	Pla-2	P2-6	TRUE	3	TRA:CVVSVAAGNKLTF TRB:CASSPGTDLYEQYF	85	CD8-C5

Pla-2_GACAGAGAGTGTGAAT	Pla	Pla-2	P2-4332	FALSE	1	TRA:CAASNLRGSTLGRLYF TRB:CASSGRTDAYTEAFF	1	CD8-C5
Pla-2_GACAGAGGTGCCTGGT	Pla	Pla-2	P2-73	FALSE	3	TRA:CAVPGSGNTGKLIF TRB:CASSQVYQGAMNTEAFF	8	CD8-C4
Pla-2_GACGCGTTCGTTACAG	Pla	Pla-2	P2-35	FALSE	3	TRA:CGTETYTGFQKLVF TRB:CSVGTGSSYNEQFF	15	CD8-C4
Pla-2_GACGGCTCACAAGCCC	Pla	Pla-2	P2-4340	FALSE	1	TRA:CALNDYKLSF TRB:CASSFGATSGPGNEQFF	1	CD8-C5
Pla-2_GACGGCTCAGGGTATG	Pla	Pla-2	P2-108	TRUE	3	TRA:CAMFPIKAAGNKLTF TRB:CASNTEANYGYTF	5	CD8-C5
Pla-2_GACGGCTCAGGTCGTC	Pla	Pla-2	P2-13	TRUE	3	TRA:CAYDLLTGNQFYF TRB:CASVRQLNTEAFF	38	CD8-C5
Pla-2_GACGTGCAGGTGACCA	Pla	Pla-2	P2-10	TRUE	3	TRA:CAPLTPNDMRF TRB:CASLNGAGGSYEQYF	45	CD8-C4
Pla-2_GACGTGCTCTGCTTGC	Pla	Pla-2	P2-5	TRUE	3	TRA:CASSFSGGYNKLIF TRB:CASSYLGAGYQETQYF	127	CD8-C5
Pla-2_GACGTTACAATGGAAT	Pla	Pla-2	P2-91	FALSE	3	TRA:CAAYPSYNTDKLIF TRB:CASSPGGTVETQYF	6	CD8-C3
Pla-2_GACTACAAGTCCCACG	Pla	Pla-2	P2-539	FALSE	2	TRA:CAMRAGGAEYTDKLIF TRB:CASSLGGTLAKNIQYF	2	CD8-C4
Pla-2_GACTGCGCAATTCCTT	Pla	Pla-2	P2-4362	FALSE	1	TRA:CAMRGAYYGNNRLAF TRB:CASSQHDRNSQETQYF	1	CD8-C5
Pla-2_GAGGTGAAGTTAGCGG	Pla	Pla-2	P2-4368	FALSE	1	TRA:CAMREPLSATNKLIF TRB:CASSLGGANVLTF	1	CD8-C5
Pla-2_GAGGTGACACCTTGTC	Pla	Pla-2	P2-157	FALSE	3	TRA:CAVKSGNTGKLIF TRB:CASSDRNTGELFF	4	CD8-C5
Pla-2_GAGGTGAGTAAAGGAG	Pla	Pla-2	P2-4	TRUE	3	TRA:CAIRYQSFNFGNEKLTF TRB:CASYRRVTEQYF	148	CD8-C4
Pla-2_GAGTCCGCACTGTTAG	Pla	Pla-2	P2-2	TRUE	3	TRA:CVVPNTNAGKSTF TRB:CASSLQGSTYNEQFF	220	CD8-C3
Pla-2_GAGTCCGGTTAGGGTG	Pla	Pla-2	P2-541	FALSE	2	TRA:CAAYKLSF TRB:CASSPGGTVETQYF	2	CD8-C4
Pla-2_GATCGATCACAGGCCT	Pla	Pla-2	P2-161	FALSE	3	TRA:CALSTNAGKSTF TRB:CASSSNRDSYEQYF	4	CD8-C4
Pla-2_GATCGATGTCTTGATG	Pla	Pla-2	P2-75	FALSE	3	TRA:CAVNDGRGADGLTF TRB:CASLGGRTDTQYF	8	CD8-C7
Pla-2_GATCGCGTCCACGACG	Pla	Pla-2	P2-20	TRUE	3	TRA:CAYDSNYQLIW TRB:CASSLSIDRADTGELFF	25	CD8-C3
Pla-2_GATCGTACATCTCCCA	Pla	Pla-2	P2-8	TRUE	3	TRA:CAALEGDNYGQNFVF TRB:CASSPTWGGVRDTQYF	53	CD8-C4
Pla-2_GATCGTATCAACGGGA	Pla	Pla-2	P2-13	TRUE	3	TRA:CAYDLLTGNQFYF TRB:CASVRQLNTEAFF	38	CD8-C5
Pla-2_GATCTAGCAGATGGGT	Pla	Pla-2	P2-228	FALSE	3	TRA:CAVPPLTGGYQKVTF TRB:CASSGGGGGLETQYF	3	CD8-C5
Pla-2_GATCTAGCATATGAGA	Pla	Pla-2	P2-141	FALSE	3	TRA:CAVRDRLENNAGNMLTF TRB:CASSKTGSSYEQYF	4	CD8-C5
Pla-2_GATCTAGTCCGGCACA	Pla	Pla-2	P2-502	FALSE	2	TRA:CLVRHTGFQKLVF TRB:CALVGSSLETQYF	2	CD8-C4
Pla-2_GATGAAAGTAAACGCG	Pla	Pla-2	P2-2	TRUE	3	TRA:CVVPNTNAGKSTF TRB:CASSLQGSTYNEQFF	220	CD8-C5
Pla-2_GATGCTATCCCGACTT	Pla	Pla-2	P2-6	TRUE	3	TRA:CVVSVAAGNKLTF TRB:CASSPGTDLYEQYF	85	CD8-C5
Pla-2_GCACATATCTTGCATT	Pla	Pla-2	P2-4403	FALSE	1	TRA:CAVKNQAGTALIF TRB:CASSHQGRTDTQYF	1	CD8-C4

Pla-2_GCAGCCAAGCTAGTTC	Pla	Pla-2	P2-4410	FALSE	1	TRA:CATWGSQGNLIF TRB:CAISDSRGNNQPQHF	1	CD4-C5
Pla-2_GCAGCCAAGGACATTA	Pla	Pla-2	P2-4411	FALSE	1	TRA:CAVDVYNTDKLIF TRB:CASSPWGGERLRDTQYF	1	CD8-C4
Pla-2_GCAGTTAAGAAACGAG	Pla	Pla-2	P2-6	TRUE	3	TRA:CVVSVAAGNKLTF TRB:CASSPGTDLYEQYF	85	CD8-C4
Pla-2_GCAGTTAAGTACGTTC	Pla	Pla-2	P2-13	TRUE	3	TRA:CAYDLLTGNQFYF TRB:CASVRQLNTEAFF	38	CD8-C5
Pla-2_GCATGTAAGTATTGGA	Pla	Pla-2	P2-75	FALSE	3	TRA:CAVNDGRGADGLTF TRB:CASLGGRTDTQYF	8	CD8-C2
Pla-2_GCATGTAGTCAGGACA	Pla	Pla-2	P2-16	FALSE	3	TRA:CAVEMDSSYKLIF TRB:CASSQGRVTQYF	32	CD8-C5
Pla-2_GCCAAATAGATCTGAA	Pla	Pla-2	P2-73	FALSE	3	TRA:CAVPGSGNTGKLIF TRB:CASSQVYQGAMNTEAFF	8	CD8-C4
Pla-2_GCCAAATTCGTCACGG	Pla	Pla-2	P2-78	TRUE	3	TRA:CAATTDSWGKFQF TRB:CASSSSASDYNEQFF	7	CD8-C4
Pla-2_GCCTCTAAGGTCATCT	Pla	Pla-2	P2-10	TRUE	3	TRA:CAPLTPNDMRF TRB:CASLNGAGGSYEQYF	45	CD8-C4
Pla-2_GCCTCTACATCAGTAC	Pla	Pla-2	P2-4438	FALSE	1	TRA:CVVNPGNQFYF TRB:CASSLATREETQYF	1	CD8-C4
Pla-2_GCGACCAAGAGTCGGT	Pla	Pla-2	P2-4	TRUE	3	TRA:CAIRYQSFNFGNEKLTF TRB:CASYRRVTEQYF	148	CD8-C5
Pla-2_GCGAGAATCAGCTTAG	Pla	Pla-2	P2-29	TRUE	3	TRA:CAVGGDFNKFYF TRB:CSARSDRLGYQPQHF	21	CD8-C5
Pla-2_GCGAGAATCGACGGAA	Pla	Pla-2	P2-2	TRUE	3	TRA:CVVPNTNAGKSTF TRB:CASSLQGSTYNEQFF	220	CD8-C4
Pla-2_GCGCAACGTGGTACAG	Pla	Pla-2	P2-16	FALSE	3	TRA:CAVEMDSSYKLIF TRB:CASSQGRVTQYF	32	CD8-C4
Pla-2_GCGCAGTCATAAAGGT	Pla	Pla-2	P2-16	FALSE	3	TRA:CAVEMDSSYKLIF TRB:CASSQGRVTQYF	32	CD4-C6
Pla-2_GCGCCAAAGTGCGATG	Pla	Pla-2	P2-4453	FALSE	1	TRA:CAGLPAATNFGNEKLTF TRB:CASTLGYSSTDTQYF	1	CD8-C4
Pla-2_GCGCCAATCTCTGCTG	Pla	Pla-2	P2-89	FALSE	3	TRA:CAASAGNTDKLIF TRB:CASSTGLAVSYEQYF	6	CD8-C4
Pla-2_GCGGGTTGTTCTCATT	Pla	Pla-2	P2-3	TRUE	3	TRA:CAVNPLESSYKLIF TRB:CASSPGTANTGELFF	177	CD8-C5
Pla-2_GCTCTGTTCATCGATG	Pla	Pla-2	P2-45	TRUE	3	TRA:CAVSGDSNYQLIW TRB:CASSLGGNGNTIYF	11	CD8-C4
Pla-2_GCTGCAGCAGCCTGTG	Pla	Pla-2	P2-69	TRUE	3	TRA:CAVRAGSARQLTF TRB:CATTGTSLDEQYF	8	CD8-C4
Pla-2_GCTGCGACATCCTAGA	Pla	Pla-2	P2-15	TRUE	3	TRA:CAVGDNFNKFYF TRB:CASSRTSAAGELFF	35	CD8-C4
Pla-2_GCTGGGTCATGTAGTC	Pla	Pla-2	P2-8	TRUE	3	TRA:CAALEGDNYGQNFVF TRB:CASSPTWGGVRDTQYF	53	CD8-C4
Pla-2_GCTTGAAGTTACTGAC	Pla	Pla-2	P2-539	FALSE	2	TRA:CAMRAGGAEYTDKLIF TRB:CASSLGGTLAKNIQYF	2	CD8-C4
Pla-2_GGAACTTAGAGTTGGC	Pla	Pla-2	P2-15	TRUE	3	TRA:CAVGDNFNKFYF TRB:CASSRTSAAGELFF	35	CD8-C5
Pla-2_GGAATAAAGGCCCGTT	Pla	Pla-2	P2-4499	FALSE	1	TRA:CAVGPNYGGSQGNLIF TRB:CASSPGPLYNEQFF	1	CD8-C4
Pla-2_GGACAAGCAACACCCG	Pla	Pla-2	P2-3	TRUE	3	TRA:CAVNPLESSYKLIF TRB:CASSPGTANTGELFF	177	CD8-C5
Pla-2_GGATTACAGAGGTTGC	Pla	Pla-2	P2-68	TRUE	3	TRA:CAVYSGYSTLTF TRB:CSVEDVGGKSSYNEQFF	8	CD8-C5

Pla-2_GGCAATTCAGCCAATT	Pla	Pla-2	P2-4532	FALSE	1	TRA:CAVRDKKGVTGGGNKLTF TRB:CAISPTYAGELFF	1	CD8-C4
Pla-2_GGCGACTAGTGGTCCC	Pla	Pla-2	P2-2	TRUE	3	TRA:CVVPNTNAGKSTF TRB:CASSLQGSTYNEQFF	220	CD8-C4
Pla-2_GGCTCGACAATCTACG	Pla	Pla-2	P2-48	TRUE	3	TRA:CVVGTISGGYNKLIF TRB:CASSPITGDTQYF	11	CD8-C5
Pla-2_GGCTGGTCAGTGGAGT	Pla	Pla-2	P2-47	TRUE	3	TRA:CAASAGGNKLTF TRB:CASSTEDEQYF	11	CD8-C4
Pla-2_GGGAATGAGCGGCTTC	Pla	Pla-2	P2-2	TRUE	3	TRA:CVVPNTNAGKSTF TRB:CASSLQGSTYNEQFF	220	CD8-C5
Pla-2_GGGAATGTCATGGTCA	Pla	Pla-2	P2-10	TRUE	3	TRA:CAPLTPNDMRF TRB:CASLNGAGGSYEQYF	45	CD8-C5
Pla-2_GGGACCTGTAGCGCTC	Pla	Pla-2	P2-30	TRUE	3	TRA:CAVGGDTGNQFYF TRB:CASSLFPTGVAYGYTF	20	CD8-C6
Pla-2_GGGACCTTCATGTGGT	Pla	Pla-2	P2-4553	FALSE	1	TRA:CALSERNTGGFKTIF TRB:CSAREKGGVPLSGANVLTF	1	CD8-C3
Pla-2_GGGACCTTCTGGAGCC	Pla	Pla-2	P2-56	FALSE	3	TRA:CATDLDGQKLLF TRB:CASRERGGNTGELFF	10	CD8-C5
Pla-2_GGGCACTCACCATCCT	Pla	Pla-2	P2-3	TRUE	3	TRA:CAVNPLESSYKLIF TRB:CASSPGTANTGELFF	177	CD8-C5
Pla-2_GGGCATCCATAAAGGT	Pla	Pla-2	P2-3	TRUE	3	TRA:CAVNPLESSYKLIF TRB:CASSPGTANTGELFF	177	CD8-C5
Pla-2_GGGCATCTCCCGACTT	Pla	Pla-2	P2-2	TRUE	3	TRA:CVVPNTNAGKSTF TRB:CASSLQGSTYNEQFF	220	CD8-C4
Pla-2_GGGTTGCCATACGCTA	Pla	Pla-2	P2-107	TRUE	3	TRA:CASGGYQKVTF TRB:CASSQDALAGSYEQYF	5	CD8-C5
Pla-2_GGTATTGGTATTACCG	Pla	Pla-2	P2-2	TRUE	3	TRA:CVVPNTNAGKSTF TRB:CASSLQGSTYNEQFF	220	CD8-C4
Pla-2_GGTGTTACACCGCTAG	Pla	Pla-2	P2-69	TRUE	3	TRA:CAVRAGSARQLTF TRB:CATTGTSLDEQYF	8	CD8-C4
Pla-2_GTACGTAAGAAGGGTA	Pla	Pla-2	P2-2	TRUE	3	TRA:CVVPNTNAGKSTF TRB:CASSLQGSTYNEQFF	220	CD8-C4
Pla-2_GTACGTAAGAGAGCTC	Pla	Pla-2	P2-2	TRUE	3	TRA:CVVPNTNAGKSTF TRB:CASSLQGSTYNEQFF	220	CD8-C5
Pla-2_GTACGTAGTAGCCTAT	Pla	Pla-2	P2-4589	FALSE	1	TRA:CALTEVDQGAQKLVF TRB:CSARDGRGRFGEAFF	1	CD8-C4
Pla-2_GTACTCCGTGATGCCC	Pla	Pla-2	P2-4594	FALSE	1	TRA:CGSGNTGKLIF TRB:CASSVSGTGSYEQYF	1	CD8-C5
Pla-2_GTACTCCTCCTTGCCA	Pla	Pla-2	P2-3	TRUE	3	TRA:CAVNPLESSYKLIF TRB:CASSPGTANTGELFF	177	CD8-C5
Pla-2_GTACTTTCATGTTGAC	Pla	Pla-2	P2-2	TRUE	3	TRA:CVVPNTNAGKSTF TRB:CASSLQGSTYNEQFF	220	CD8-C4
Pla-2_GTCACAATCAAACCGT	Pla	Pla-2	P2-20	TRUE	3	TRA:CAYDSNYQLIW TRB:CASSLSIDRADTGELFF	25	CD8-C4
Pla-2_GTCATTTAGATCACGG	Pla	Pla-2	P2-3	TRUE	3	TRA:CAVNPLESSYKLIF TRB:CASSPGTANTGELFF	177	CD8-C5
Pla-2_GTCGGGTCACTTAAGC	Pla	Pla-2	P2-2	TRUE	3	TRA:CVVPNTNAGKSTF TRB:CASSLQGSTYNEQFF	220	CD8-C4
Pla-2_GTCGTAAAGGCGACAT	Pla	Pla-2	P2-48	TRUE	3	TRA:CVVGTISGGYNKLIF TRB:CASSPITGDTQYF	11	CD8-C5
Pla-2_GTCGTAACAACTGCGC	Pla	Pla-2	P2-15	TRUE	3	TRA:CAVGDNFNKFYF TRB:CASSRTSAAGELFF	35	CD8-C4
Pla-2_GTCGTAACATCCGCGA	Pla	Pla-2	P2-3	TRUE	3	TRA:CAVNPLESSYKLIF TRB:CASSPGTANTGELFF	177	CD8-C5

Pla-2_GTCGTAATCGTGGGAA	Pla	Pla-2	P2-6	TRUE	3	TRA:CVVSVAAGNKLTF TRB:CASSPGTDLYEQYF	85	CD8-C4
Pla-2_GTCTTCGTCACAAACC	Pla	Pla-2	P2-4627	FALSE	1	TRA:CAVEGSDMRF TRB:CASSPGAGGANVLTF	1	CD8-C5
Pla-2_GTGAAGGGTAAGCACG	Pla	Pla-2	P2-4631	FALSE	1	TRA:CAMSSWNRDDKIIF TRB:CASSPTWGGVRDTQYF	1	CD8-C4
Pla-2_GTGCAGCGTACAGTTC	Pla	Pla-2	P2-250	FALSE	3	TRA:CAENKEGSGGSNYKLTF TRB:CASSPPGTPVGRETQYF	3	CD8-C5
Pla-2_GTGCATACACTCAGGC	Pla	Pla-2	P2-460	FALSE	2	TRA:CAVRDIPRSGGYNKLIF TRB:CASSLSRGGGTEAFF	2	CD8-C4
Pla-2_GTGGGTCAGACCTAGG	Pla	Pla-2	P2-6	TRUE	3	TRA:CVVSVAAGNKLTF TRB:CASSPGTDLYEQYF	85	CD8-C4
Pla-2_GTGGGTCTCCAATGGT	Pla	Pla-2	P2-35	FALSE	3	TRA:CGTETYTGFQKLVF TRB:CSVGTGSSYNEQFF	15	CD8-C5
Pla-2_GTTCATTCAACCGCCA	Pla	Pla-2	P2-3	TRUE	3	TRA:CAVNPLESSYKLIF TRB:CASSPGTANTGELFF	177	CD8-C4
Pla-2_GTTCATTGTCAATGTC	Pla	Pla-2	P2-107	TRUE	3	TRA:CASGGYQKVTF TRB:CASSQDALAGSYEQYF	5	CD8-C3
Pla-2_GTTCGGGGTTTGTTGG	Pla	Pla-2	P2-3	TRUE	3	TRA:CAVNPLESSYKLIF TRB:CASSPGTANTGELFF	177	CD8-C5
Pla-2_GTTCGGGTCAATCACG	Pla	Pla-2	P2-12	TRUE	3	TRA:CGADAEYGNKLVF TRB:CASSEGQGAIMQPQHF	42	CD8-C5
Pla-2_GTTCGGGTCCCAAGAT	Pla	Pla-2	P2-2	TRUE	3	TRA:CVVPNTNAGKSTF TRB:CASSLQGSTYNEQFF	220	CD8-C5
Pla-2_TAAACCGAGGATGCGT	Pla	Pla-2	P2-4667	FALSE	1	TRA:CAASVKTDKLIF TRB:CASSTAPDSYTNTGELFF	1	CD8-C5
Pla-2_TAAACCGGTAATCACC	Pla	Pla-2	P2-161	FALSE	3	TRA:CALSTNAGKSTF TRB:CASSSNRDSYEQYF	4	CD8-C5
Pla-2_TAAGAGAAGTGTACTC	Pla	Pla-2	P2-60	TRUE	3	TRA:CALSEGSGANSKLTF TRB:CASSSHSGLAGGFF	9	CD8-C4
Pla-2_TAAGTGCAGCGATGAC	Pla	Pla-2	P2-10	TRUE	3	TRA:CAPLTPNDMRF TRB:CASLNGAGGSYEQYF	45	CD8-C4
Pla-2_TACAGTGGTTCGCTAA	Pla	Pla-2	P2-2	TRUE	3	TRA:CVVPNTNAGKSTF TRB:CASSLQGSTYNEQFF	220	CD8-C5
Pla-2_TACCTATGTTCATGGT	Pla	Pla-2	P2-2	TRUE	3	TRA:CVVPNTNAGKSTF TRB:CASSLQGSTYNEQFF	220	CD8-C4
Pla-2_TACCTTAAGAGCTGGT	Pla	Pla-2	P2-35	FALSE	3	TRA:CGTETYTGFQKLVF TRB:CSVGTGSSYNEQFF	15	CD8-C5
Pla-2_TACCTTAAGAGGACGG	Pla	Pla-2	P2-2	TRUE	3	TRA:CVVPNTNAGKSTF TRB:CASSLQGSTYNEQFF	220	CD8-C8
Pla-2_TACGGTATCTATCCTA	Pla	Pla-2	P2-81	FALSE	3	TRA:CAVRGNTPLVF TRB:CASSSNRDSYEQYF	7	CD8-C5
Pla-2_TACGGTATCTGTGCAA	Pla	Pla-2	P2-2	TRUE	3	TRA:CVVPNTNAGKSTF TRB:CASSLQGSTYNEQFF	220	CD8-C4
Pla-2_TACTCATGTAAACCTC	Pla	Pla-2	P2-2	TRUE	3	TRA:CVVPNTNAGKSTF TRB:CASSLQGSTYNEQFF	220	CD8-C5
Pla-2_TACTCATTCTGTACGA	Pla	Pla-2	P2-6	TRUE	3	TRA:CVVSVAAGNKLTF TRB:CASSPGTDLYEQYF	85	CD8-C5
Pla-2_TACTCGCTCGTAGATC	Pla	Pla-2	P2-129	TRUE	3	TRA:CARGGTGNQFYF TRB:CAWSPMPNEQFF	4	CD8-C4
Pla-2_TACTTGTAGTCCGTAT	Pla	Pla-2	P2-3	TRUE	3	TRA:CAVNPLESSYKLIF TRB:CASSPGTANTGELFF	177	CD8-C4
Pla-2_TAGAGCTGTAATCACC	Pla	Pla-2	P2-1	TRUE	3	TRA:CVVNIIGYQKVTF TRB:CASTRQGKQPQHF	270	CD8-C5

Pla-2_TAGCCGGAGGCTATCT	Pla	Pla-2	P2-2	TRUE	3	TRA:CVVPNTNAGKSTF TRB:CASSLQGSTYNEQFF	220	CD8-C5
Pla-2_TAGCCGGTCTCACATT	Pla	Pla-2	P2-140	FALSE	3	TRA:CAVNSGANNLFF TRB:CSARDEEREGYNEQFF	4	CD8-C4
Pla-2_TAGGCATCAATGAAAC	Pla	Pla-2	P2-20	TRUE	3	TRA:CAYDSNYQLIW TRB:CASSLSIDRADTGELFF	25	CD8-C4
Pla-2_TAGGCATCAGTAGAGC	Pla	Pla-2	P2-4724	FALSE	1	TRA:CAGLSNNDMRF TRB:CASSPGRWYEQYF	1	CD8-C4
Pla-2_TAGTTGGTCGGACAAG	Pla	Pla-2	P2-6	TRUE	3	TRA:CVVSVAAGNKLTF TRB:CASSPGTDLYEQYF	85	CD8-C4
Pla-2_TATCTCAAGGCCCTCA	Pla	Pla-2	P2-20	TRUE	3	TRA:CAYDSNYQLIW TRB:CASSLSIDRADTGELFF	25	CD8-C4
Pla-2_TATCTCAGTTGGTGGA	Pla	Pla-2	P2-29	TRUE	3	TRA:CAVGGDFNKFYF TRB:CSARSDRLGYQPQHF	21	CD8-C5
Pla-2_TATGCCCAGGCATGTG	Pla	Pla-2	P2-47	TRUE	3	TRA:CAASAGGNKLTF TRB:CASSTEDEQYF	11	CD8-C4
Pla-2_TCAACGAGTAGTGAAT	Pla	Pla-2	P2-4745	FALSE	1	TRA:CAMREGRVDSSYKLIF TRB:CASSYSNHPTDTQYF	1	CD8-C3
Pla-2_TCAATCTAGACTGTAA	Pla	Pla-2	P2-6	TRUE	3	TRA:CVVSVAAGNKLTF TRB:CASSPGTDLYEQYF	85	CD8-C4
Pla-2_TCACAAGCATTCCTGC	Pla	Pla-2	P2-4752	FALSE	1	TRA:CAAGLTDKLIF TRB:CASSLYRSDIEQYF	1	CD8-C4
Pla-2_TCACAAGGTTTGGCGC	Pla	Pla-2	P2-69	TRUE	3	TRA:CAVRAGSARQLTF TRB:CATTGTSLDEQYF	8	CD8-C4
Pla-2_TCACAAGTCACATAGC	Pla	Pla-2	P2-3	TRUE	3	TRA:CAVNPLESSYKLIF TRB:CASSPGTANTGELFF	177	CD8-C5
Pla-2_TCACGAATCCTCAACC	Pla	Pla-2	P2-3	TRUE	3	TRA:CAVNPLESSYKLIF TRB:CASSPGTANTGELFF	177	CD8-C5
Pla-2_TCAGATGGTATAAACG	Pla	Pla-2	P2-2	TRUE	3	TRA:CVVPNTNAGKSTF TRB:CASSLQGSTYNEQFF	220	CD8-C5
Pla-2_TCAGCTCTCAACACCA	Pla	Pla-2	P2-3	TRUE	3	TRA:CAVNPLESSYKLIF TRB:CASSPGTANTGELFF	177	CD8-C5
Pla-2_TCAGGATCATGCAATC	Pla	Pla-2	P2-2	TRUE	3	TRA:CVVPNTNAGKSTF TRB:CASSLQGSTYNEQFF	220	CD8-C4
Pla-2_TCAGGTAAGCGTGTCC	Pla	Pla-2	P2-296	TRUE	2	TRA:CALKKSSGDKLTF TRB:CASTSGGGGQETQYF	2	CD8-C4
Pla-2_TCAGGTAGTCTAACGT	Pla	Pla-2	P2-2	TRUE	3	TRA:CVVPNTNAGKSTF TRB:CASSLQGSTYNEQFF	220	CD8-C4
Pla-2_TCATTACCAGATTGCT	Pla	Pla-2	P2-3	TRUE	3	TRA:CAVNPLESSYKLIF TRB:CASSPGTANTGELFF	177	CD8-C7
Pla-2_TCCACACGTGACGCCT	Pla	Pla-2	P2-562	FALSE	2	TRA:CALRAGSARQLTF TRB:CASTGTSLDEQFF	2	CD8-C4
Pla-2_TCCACACTCTGCTGTC	Pla	Pla-2	P2-4	TRUE	3	TRA:CAIRYQSFNFGNEKLTF TRB:CASYRRVTEQYF	148	CD8-C5
Pla-2_TCCCGATCAACTGCGC	Pla	Pla-2	P2-3	TRUE	3	TRA:CAVNPLESSYKLIF TRB:CASSPGTANTGELFF	177	CD8-C5
Pla-2_TCGCGTTAGTGTGAAT	Pla	Pla-2	P2-4798	FALSE	1	TRA:CALSEVDGNNRKLIW TRB:CASSQGSGVTEAFF	1	CD8-C5
Pla-2_TCGTACCTCCATTCTA	Pla	Pla-2	P2-2	TRUE	3	TRA:CVVPNTNAGKSTF TRB:CASSLQGSTYNEQFF	220	CD8-C4
Pla-2_TCGTAGACACTGTCGG	Pla	Pla-2	P2-4811	FALSE	1	TRA:CAASVGIHGEDTGNQFYF TRB:CASSGLAGYSYEQYF	1	CD8-C4
Pla-2_TCTATTGCACATCCGG	Pla	Pla-2	P2-78	TRUE	3	TRA:CAATTDSWGKFQF TRB:CASSSSASDYNEQFF	7	CD8-C4

Pla-2_TCTATTGGTCATATGC	Pla	Pla-2	P2-91	FALSE	3	TRA:CAAYPSYNTDKLIF TRB:CASSPGGTVETQYF	6	CD8-C4
Pla-2_TCTCATATCGAGAGCA	Pla	Pla-2	P2-2	TRUE	3	TRA:CVVPNTNAGKSTF TRB:CASSLQGSTYNEQFF	220	CD8-C4
Pla-2_TCTGGAAAGCCTATGT	Pla	Pla-2	P2-2	TRUE	3	TRA:CVVPNTNAGKSTF TRB:CASSLQGSTYNEQFF	220	CD8-C5
Pla-2_TCTGGAACACCATGTA	Pla	Pla-2	P2-8	TRUE	3	TRA:CAALEGDNYGGQNFVF TRB:CASSPTWGGVRDTQYF	53	CD8-C5
Pla-2_TCTGGAATCAGGCCCA	Pla	Pla-2	P2-216	FALSE	3	TRA:CGTEGNDMRF TRB:CASSLTGETQYF	3	CD8-C5
Pla-2_TCTTCGGCAAAGTCAA	Pla	Pla-2	P2-35	FALSE	3	TRA:CGTETYTGFQKLVF TRB:CSVGTGSSYNEQFF	15	CD8-C5
Pla-2_TCTTCGGTCTTTACAC	Pla	Pla-2	P2-55	TRUE	3	TRA:CIVRGIQGAQKLVF TRB:CASSLQGGGSGGLTQYF	10	CD8-C5
Pla-2_TCTTTCCCATCATCCC	Pla	Pla-2	P2-60	TRUE	3	TRA:CALSEGSGANSKLTF TRB:CASSSHSGLAGGFF	9	CD8-C4
Pla-2_TCTTTCCGTTTAGCTG	Pla	Pla-2	P2-19	TRUE	3	TRA:CAVRDMENNRLAF TRB:CASSLGGGLTYEQYF	27	CD8-C4
Pla-2_TCTTTCCTCACTTATC	Pla	Pla-2	P2-4	TRUE	3	TRA:CAIRYQSFNFGNEKLTF TRB:CASYRRVTEQYF	148	CD8-C5
Pla-2_TGAAAGAAGAAACGAG	Pla	Pla-2	P2-2	TRUE	3	TRA:CVVPNTNAGKSTF TRB:CASSLQGSTYNEQFF	220	CD8-C4
Pla-2_TGACGGCCAAGCCGTC	Pla	Pla-2	P2-6	TRUE	3	TRA:CVVSVAAGNKLTF TRB:CASSPGTDLYEQYF	85	CD8-C4
Pla-2_TGACGGCTCAGTTAGC	Pla	Pla-2	P2-10	TRUE	3	TRA:CAPLTPNDMRF TRB:CASLNGAGGSYEQYF	45	CD8-C4
Pla-2_TGACGGCTCATATCGG	Pla	Pla-2	P2-3	TRUE	3	TRA:CAVNPLESSYKLIF TRB:CASSPGTANTGELFF	177	CD8-C4
Pla-2_TGAGAGGCACAGACTT	Pla	Pla-2	P2-52	FALSE	3	TRA:CARTTNAGKSTF TRB:CASSETSGRIYEQFF	11	CD8-C4
Pla-2_TGAGCCGTCGGCGCTA	Pla	Pla-2	P2-4867	FALSE	1	TRA:CAMREAGGSGAGSYQLTF TRB:CASSLQGSTYNEQFF	1	CD8-C4
Pla-2_TGCACCTTCATCGGAT	Pla	Pla-2	P2-58	TRUE	3	TRA:CAVNERSNDYKLSF TRB:CAISGGDSWGNQPQHF	9	CD8-C5
Pla-2_TGCCAAAAGGGTGTTG	Pla	Pla-2	P2-19	TRUE	3	TRA:CAVRDMENNRLAF TRB:CASSLGGGLTYEQYF	27	CD8-C4
Pla-2_TGCCAAAGTGAGGGAG	Pla	Pla-2	P2-2	TRUE	3	TRA:CVVPNTNAGKSTF TRB:CASSLQGSTYNEQFF	220	CD8-C4
Pla-2_TGCCAAATCACATGCA	Pla	Pla-2	P2-8	TRUE	3	TRA:CAALEGDNYGGQNFVF TRB:CASSPTWGGVRDTQYF	53	CD8-C4
Pla-2_TGCCCATTCCTCAATT	Pla	Pla-2	P2-56	FALSE	3	TRA:CATDLDGQKLLF TRB:CASRERGGNTGELFF	10	CD8-C5
Pla-2_TGCGTGGAGACTCGGA	Pla	Pla-2	P2-6	TRUE	3	TRA:CVVSVAAGNKLTF TRB:CASSPGTDLYEQYF	85	CD8-C4
Pla-2_TGCGTGGTCAAGAAGT	Pla	Pla-2	P2-4895	FALSE	1	TRA:CAVRPLGSGNTGKLIF TRB:CASSSTGGGYNEQFF	1	CD8-C4
Pla-2_TGCTACCAGACTACAA	Pla	Pla-2	P2-52	FALSE	3	TRA:CARTTNAGKSTF TRB:CASSETSGRIYEQFF	11	CD8-C4
Pla-2_TGCTACCAGGGATCTG	Pla	Pla-2	P2-109	TRUE	3	TRA:CAPPFGGSEKLVF TRB:CASSKTGSSYEQYF	5	CD8-C4
Pla-2_TGCTGCTAGTGGGATC	Pla	Pla-2	P2-1	TRUE	3	TRA:CVVNIIGYQKVTF TRB:CASTRQGKQPQHF	270	CD8-C7
Pla-2_TGGCCAGCACGCATCG	Pla	Pla-2	P2-4909	FALSE	1	TRA:CVVPNTNAGKSTF TRB:CASSSSGLAGRETQYF	1	CD4-C5

Pla-2_TGGGAAGCACATTCGA	Pla	Pla-2	P2-3	TRUE	3	TRA:CAVNPLESSYKLIF TRB:CASSPGTANTGELFF	177	CD8-C5
Pla-2_TGGGAAGCAGGTTTCA	Pla	Pla-2	P2-4923	FALSE	1	TRA:CVVMGTYKYIF TRB:CASSINIPGQGKLGRTYNEQFF	1	CD4-C5
Pla-2_TGGGAAGTCTCGGACG	Pla	Pla-2	P2-4927	FALSE	1	TRA:CAVRDIGGSYIPTF TRB:CASSLGGPEETQYF	1	CD8-C5
Pla-2_TGGTTAGAGTTGAGAT	Pla	Pla-2	P2-104	TRUE	3	TRA:CAMREAGGSGAGSYQLTF TRB:CASSLGSGNTIYF	5	CD8-C5
Pla-2_TGTCCCAAGCAGGTCA	Pla	Pla-2	P2-2	TRUE	3	TRA:CVVPNTNAGKSTF TRB:CASSLQGSTYNEQFF	220	CD8-C4
Pla-2_TGTGTTTTCGCGGATC	Pla	Pla-2	P2-68	TRUE	3	TRA:CAVYSGYSTLTF TRB:CSVEDVGGKSSYNEQFF	8	CD8-C5
Pla-2_TGTTCCGTCCAAATGC	Pla	Pla-2	P2-2	TRUE	3	TRA:CVVPNTNAGKSTF TRB:CASSLQGSTYNEQFF	220	CD8-C5
Pla-2_TGTTCCGTCTACTTAC	Pla	Pla-2	P2-4958	FALSE	1	TRA:CAVSPLRNNARLMF TRB:CSAEGQASYEQYF	1	CD8-C4
Pla-2_TTAACTCAGCAGGTCA	Pla	Pla-2	P2-6	TRUE	3	TRA:CVVSVAAGNKLTF TRB:CASSPGTDLYEQYF	85	CD8-C4
Pla-2_TTAGTTCTCTTGTACT	Pla	Pla-2	P2-60	TRUE	3	TRA:CALSEGSGANSKLTF TRB:CASSSHSGLAGGFF	9	CD8-C4
Pla-2_TTCGAAGAGAAGAAGC	Pla	Pla-2	P2-58	TRUE	3	TRA:CAVNERSNDYKLSF TRB:CAISGGDSWGNQPQHF	9	CD8-C2
Pla-2_TTCTCAACACCGCTAG	Pla	Pla-2	P2-72	FALSE	3	TRA:CATDEETAGNKLTF TRB:CASSEAGWMGALHTEAFF	8	CD8-C4
Pla-2_TTGCCGTCATGCCACG	Pla	Pla-2	P2-3	TRUE	3	TRA:CAVNPLESSYKLIF TRB:CASSPGTANTGELFF	177	CD8-C5
Pla-2_TTTACTGAGCGTCAAG	Pla	Pla-2	P2-6	TRUE	3	TRA:CVVSVAAGNKLTF TRB:CASSPGTDLYEQYF	85	CD8-C4
Pla-2_TTTACTGCACGGATAG	Pla	Pla-2	P2-6	TRUE	3	TRA:CVVSVAAGNKLTF TRB:CASSPGTDLYEQYF	85	CD8-C4
Pla-2_TTTACTGCATGGAATA	Pla	Pla-2	P2-161	FALSE	3	TRA:CALSTNAGKSTF TRB:CASSSNRDSYEQYF	4	CD8-C5
Pla-2_TTTATGCTCGCTGATA	Pla	Pla-2	P2-3	TRUE	3	TRA:CAVNPLESSYKLIF TRB:CASSPGTANTGELFF	177	CD8-C5
Pla-2_TTTCCTCGTGCAGACA	Pla	Pla-2	P2-4	TRUE	3	TRA:CAIRYQSFNFGNEKLTF TRB:CASYRRVTEQYF	148	CD8-C4
Pla-2_TTTCCTCTCGCCAGCA	Pla	Pla-2	P2-100	TRUE	3	TRA:CAVGPRLLGNEKLTF TRB:CASSQDSTSVYNEQFF	5	CD8-C4
Pla-2_TTTCCTCTCGCGATCG	Pla	Pla-2	P2-15	TRUE	3	TRA:CAVGDNFNKFYF TRB:CASSRTSAAGELFF	35	CD8-C4
Pla-2_TTTGGTTCAGCTGCTG	Pla	Pla-2	P2-13	TRUE	3	TRA:CAYDLLTGNQFYF TRB:CASVRQLNTEAFF	38	CD8-C5
Pla-2_TTTGTCATCGGATGGA	Pla	Pla-2	P2-2	TRUE	3	TRA:CVVPNTNAGKSTF TRB:CASSLQGSTYNEQFF	220	CD8-C4
Pla-2_AAACCTGTCTGATTCT	Pla	Pla-2	P2-6	TRUE	3	TRA:CVVSVAAGNKLTF TRB:CASSPGTDLYEQYF	85	CD8-C3
Pla-2_AAACGGGAGTGGGTTG	Pla	Pla-2	P2-63	FALSE	3	TRA:CAASGEAGTALIF TRB:CATSREIQPNEKLFF	9	CD4-C5
Pla-2_AAAGATGAGCTGAACG	Pla	Pla-2	P2-13	TRUE	3	TRA:CAYDLLTGNQFYF TRB:CASVRQLNTEAFF	38	CD8-C3
Pla-2_AAAGCAACACCAGGTC	Pla	Pla-2	P2-206	FALSE	3	TRA:CAASGGNSNTGTASKLTF TRB:CASSGLAGYQYEQYF	3	CD8-C3
Pla-2_AAAGCAACATCTCGCT	Pla	Pla-2	P2-10	TRUE	3	TRA:CAPLTPNDMRF TRB:CASLNGAGGSYEQYF	45	CD8-C3

Pla-2_AAAGCAATCCACGACG	Pla	Pla-2	P2-20	TRUE	3	TRA:CAYDSNYQLIW TRB:CASSLSIDRADTGELFF	25	CD8-C3
Pla-2_AAAGCAATCGGCGGTT	Pla	Pla-2	P2-6	TRUE	3	TRA:CVVSVAAGNKLTF TRB:CASSPGTDLYEQYF	85	CD8-C3
Pla-2_AAAGTAGCATCCCACT	Pla	Pla-2	P2-2	TRUE	3	TRA:CVVPNTNAGKSTF TRB:CASSLQGSTYNEQFF	220	CD8-C3
Pla-2_AAAGTAGGTACTCGCG	Pla	Pla-2	P2-2	TRUE	3	TRA:CVVPNTNAGKSTF TRB:CASSLQGSTYNEQFF	220	CD8-C3
Pla-2_AAAGTAGGTGGTTTCA	Pla	Pla-2	P2-6	TRUE	3	TRA:CVVSVAAGNKLTF TRB:CASSPGTDLYEQYF	85	CD8-C3
Pla-2_AAAGTAGTCCTTTCTC	Pla	Pla-2	P2-3	TRUE	3	TRA:CAVNPLESSYKLIF TRB:CASSPGTANTGELFF	177	CD8-C3
Pla-2_AAATGCCAGCGTTCCG	Pla	Pla-2	P2-10	TRUE	3	TRA:CAPLTPNDMRF TRB:CASLNGAGGSYEQYF	45	CD8-C3
Pla-2_AACCATGCAGCTCGAC	Pla	Pla-2	P2-130	TRUE	3	TRA:CAFMTPNGGATNKLIF TRB:CASSQENTAGSYF	4	CD8-C3
Pla-2_AACCATGCATAAGACA	Pla	Pla-2	P2-30	TRUE	3	TRA:CAVGGDTGNQFYF TRB:CASSLFPTGVAYGYTF	20	CD8-C2
Pla-2_AACCATGCATGACATC	Pla	Pla-2	P2-2	TRUE	3	TRA:CVVPNTNAGKSTF TRB:CASSLQGSTYNEQFF	220	CD8-C3
Pla-2_AACCATGGTACTTGAC	Pla	Pla-2	P2-3431	FALSE	1	TRA:CAMSNYGGSQGNLIF TRB:CASSQVLAGVELETQYF	1	CD8-C4
Pla-2_AACCGCGAGAAGGCCT	Pla	Pla-2	P2-3	TRUE	3	TRA:CAVNPLESSYKLIF TRB:CASSPGTANTGELFF	177	CD8-C3
Pla-2_AACTCAGGTTCACCTC	Pla	Pla-2	P2-56	FALSE	3	TRA:CATDLDGQKLLF TRB:CASRERGGNTGELFF	10	CD8-C3
Pla-2_AACTCAGTCGACAGCC	Pla	Pla-2	P2-137	FALSE	3	TRA:CAVRDMVDNFNKFYF TRB:CASSSGTGGFYEQYF	4	CD8-C6
Pla-2_AACTCCCGTAGCGTAG	Pla	Pla-2	P2-385	FALSE	2	TRA:CAVGAETGNQFYF TRB:CASSLYLSYNSPLHF	2	CD8-C2
Pla-2_AACTCTTGTAGAAGGA	Pla	Pla-2	P2-2	TRUE	3	TRA:CVVPNTNAGKSTF TRB:CASSLQGSTYNEQFF	220	CD8-C3
Pla-2_AAGCCGCCATGCCTTC	Pla	Pla-2	P2-212	FALSE	3	TRA:CAENIRATNKLIF TRB:CAWSPRGSNQPQHF	3	CD8-C3
Pla-2_AAGGAGCAGATTACCC	Pla	Pla-2	P2-2	TRUE	3	TRA:CVVPNTNAGKSTF TRB:CASSLQGSTYNEQFF	220	CD8-C2
Pla-2_AAGGCAGAGCTCCCAG	Pla	Pla-2	P2-3461	FALSE	1	TRA:CAMIPRDQAGTALIF TRB:CAWRYGTSPLHF	1	CD8-C3
Pla-2_AAGGCAGCAGCGTTCG	Pla	Pla-2	P2-3463	FALSE	1	TRA:CAVVDSNYQLIW TRB:CASLNGAGGSYEQYF	1	CD8-C3
Pla-2_AAGGTTCAGGAATTAC	Pla	Pla-2	P2-3470	FALSE	1	TRA:CAMRELLKTSYDKVIF TRB:CASSSTGPPHF	1	CD8-C3
Pla-2_AAGGTTCAGTATCGAA	Pla	Pla-2	P2-2	TRUE	3	TRA:CVVPNTNAGKSTF TRB:CASSLQGSTYNEQFF	220	CD8-C3
Pla-2_AAGGTTCTCCAAACTG	Pla	Pla-2	P2-56	FALSE	3	TRA:CATDLDGQKLLF TRB:CASRERGGNTGELFF	10	CD8-C3
Pla-2_AATCCAGAGAAACCGC	Pla	Pla-2	P2-16	FALSE	3	TRA:CAVEMDSSYKLIF TRB:CASSQGRVTQYF	32	CD8-C3
Pla-2_AATCCAGTCTTCCTTC	Pla	Pla-2	P2-47	TRUE	3	TRA:CAASAGGNKLTF TRB:CASSTEDEQYF	11	CD8-C3
Pla-2_AATCGGTAGTACGCCC	Pla	Pla-2	P2-48	TRUE	3	TRA:CVVGTISGGYNKLIF TRB:CASSPITGDTQYF	11	CD8-C3
Pla-2_ACACCAACAGGCGATA	Pla	Pla-2	P2-216	FALSE	3	TRA:CGTEGNDMRF TRB:CASSLTGETQYF	3	CD8-C3

Pla-2_ACACCGGAGTGGACGT	Pla	Pla-2	P2-6	TRUE	3	TRA:CVVSVAAGNKLTF TRB:CASSPGTDLYEQYF	85	CD8-C3
Pla-2_ACACCGGGTCATCCCT	Pla	Pla-2	P2-89	FALSE	3	TRA:CAASAGNTDKLIF TRB:CASSTGLAVSYEQYF	6	CD8-C3
Pla-2_ACACTGAAGCTGCAAG	Pla	Pla-2	P2-2	TRUE	3	TRA:CVVPNTNAGKSTF TRB:CASSLQGSTYNEQFF	220	CD8-C3
Pla-2_ACACTGAGTCCGTGAC	Pla	Pla-2	P2-55	TRUE	3	TRA:CIVRGIQGAQKLVF TRB:CASSLQGGGSGGLTQYF	10	CD8-C4
Pla-2_ACACTGAGTTTACTCT	Pla	Pla-2	P2-48	TRUE	3	TRA:CVVGTISGGYNKLIF TRB:CASSPITGDTQYF	11	CD8-C3
Pla-2_ACACTGATCCCTAATT	Pla	Pla-2	P2-16	FALSE	3	TRA:CAVEMDSSYKLIF TRB:CASSQGRVTQYF	32	CD8-C3
Pla-2_ACAGCTAAGAAGGACA	Pla	Pla-2	P2-52	FALSE	3	TRA:CARTTNAGKSTF TRB:CASSETSGRIYEQFF	11	CD8-C3
Pla-2_ACATCAGAGCTAGGCA	Pla	Pla-2	P2-140	FALSE	3	TRA:CAVNSGANNLFF TRB:CSARDEEREGYNEQFF	4	CD8-C3
Pla-2_ACATCAGAGGAGTAGA	Pla	Pla-2	P2-22	TRUE	3	TRA:CAMRERSWVRQKLVF TRB:CASSRGTSGTDTQYF	24	CD8-C3
Pla-2_ACCAGTATCAACGAAA	Pla	Pla-2	P2-6	TRUE	3	TRA:CVVSVAAGNKLTF TRB:CASSPGTDLYEQYF	85	CD8-C3
Pla-2_ACCGTAATCTGCGGCA	Pla	Pla-2	P2-3	TRUE	3	TRA:CAVNPLESSYKLIF TRB:CASSPGTANTGELFF	177	CD8-C3
Pla-2_ACCTTTAAGATGCCAG	Pla	Pla-2	P2-3528	FALSE	1	TRA:CAVEGSDMRF TRB:CASSVGQARDYTF	1	CD8-C3
Pla-2_ACCTTTACAGAGCCAA	Pla	Pla-2	P2-400	FALSE	2	TRA:CAVRVDSSYKLIF TRB:CAWSRSTGELFF	2	CD8-C2
Pla-2_ACCTTTACAGCGTAAG	Pla	Pla-2	P2-2	TRUE	3	TRA:CVVPNTNAGKSTF TRB:CASSLQGSTYNEQFF	220	CD8-C3
Pla-2_ACGATACCAGTAAGCG	Pla	Pla-2	P2-3544	FALSE	1	TRA:CAVGDSNYQLIW TRB:CASSLQPGTGELFF	1	CD8-C3
Pla-2_ACGATACTCCAAGTAC	Pla	Pla-2	P2-2	TRUE	3	TRA:CVVPNTNAGKSTF TRB:CASSLQGSTYNEQFF	220	CD8-C3
Pla-2_ACGATACTCCCTTGTG	Pla	Pla-2	P2-3546	FALSE	1	TRA:CAVKSGNTGKLIF TRB:CAGSDRNTGELFF	1	CD8-C3
Pla-2_ACGATACTCTGATACG	Pla	Pla-2	P2-3547	FALSE	1	TRA:CAMRELVSQLSGGYNKLIF TRB:CASSLPGQGSQPQHF	1	CD8-C3
Pla-2_ACGATGTCAAAGGTGC	Pla	Pla-2	P2-73	FALSE	3	TRA:CAVPGSGNTGKLIF TRB:CASSQVYQGAMNTEAFF	8	CD8-C3
Pla-2_ACGCCAGCAGCCTGTG	Pla	Pla-2	P2-4	TRUE	3	TRA:CAIRYQSFNFGNEKLTF TRB:CASYRRVTEQYF	148	CD8-C3
Pla-2_ACGCCGAAGTCCATAC	Pla	Pla-2	P2-222	FALSE	3	TRA:CAASEAYSGGGADGLTF TRB:CASSLGGIYNEQFF	3	CD8-C3
Pla-2_ACGGAGATCCGTCAAA	Pla	Pla-2	P2-170	TRUE	3	TRA:CALPAYSGAGSYQLTF TRB:CASSLYENTEAFF	3	CD8-C5
Pla-2_ACGGCCATCCAGGGCT	Pla	Pla-2	P2-30	TRUE	3	TRA:CAVGGDTGNQFYF TRB:CASSLFPTGVAYGYTF	20	CD8-C8
Pla-2_ACTGAACAGCCCGAAA	Pla	Pla-2	P2-2	TRUE	3	TRA:CVVPNTNAGKSTF TRB:CASSLQGSTYNEQFF	220	CD8-C3
Pla-2_ACTGAGTAGAGTCGGT	Pla	Pla-2	P2-68	TRUE	3	TRA:CAVYSGYSTLTF TRB:CSVEDVGGKSSYNEQFF	8	CD8-C3
Pla-2_ACTGATGAGAAACGCC	Pla	Pla-2	P2-4	TRUE	3	TRA:CAIRYQSFNFGNEKLTF TRB:CASYRRVTEQYF	148	CD8-C3
Pla-2_ACTGATGAGAGCTATA	Pla	Pla-2	P2-13	TRUE	3	TRA:CAYDLLTGNQFYF TRB:CASVRQLNTEAFF	38	CD8-C3

Pla-2_ACTGCTCAGACCTTTG	Pla	Pla-2	P2-6	TRUE	3	TRA:CVVSVAAGNKLTF TRB:CASSPGTDLYEQYF	85	CD8-C3
Pla-2_ACTGTCCGTACGAAAT	Pla	Pla-2	P2-6	TRUE	3	TRA:CVVSVAAGNKLTF TRB:CASSPGTDLYEQYF	85	CD8-C3
Pla-2_ACTTACTAGGAACTGC	Pla	Pla-2	P2-60	TRUE	3	TRA:CALSEGSGANSKLTF TRB:CASSSHSGLAGGFF	9	CD8-C3
Pla-2_ACTTACTCATGCCCGA	Pla	Pla-2	P2-209	FALSE	3	TRA:CATGTHQAGTALIF TRB:CSAEEETQYF	3	CD8-C3
Pla-2_ACTTTCAAGCGATGAC	Pla	Pla-2	P2-2	TRUE	3	TRA:CVVPNTNAGKSTF TRB:CASSLQGSTYNEQFF	220	CD8-C3
Pla-2_AGAATAGAGGACACCA	Pla	Pla-2	P2-89	FALSE	3	TRA:CAASAGNTDKLIF TRB:CASSTGLAVSYEQYF	6	CD8-C3
Pla-2_AGAATAGCAGTCACTA	Pla	Pla-2	P2-2	TRUE	3	TRA:CVVPNTNAGKSTF TRB:CASSLQGSTYNEQFF	220	CD8-C3
Pla-2_AGAATAGTCCATGCTC	Pla	Pla-2	P2-42	FALSE	3	TRA:CAVSPRDNNDMRF TRB:CASSARGNSNQPQHF	12	CD4-C4
Pla-2_AGACGTTTCGGATGTT	Pla	Pla-2	P2-10	TRUE	3	TRA:CAPLTPNDMRF TRB:CASLNGAGGSYEQYF	45	CD8-C3
Pla-2_AGACGTTTCGTTACGA	Pla	Pla-2	P2-2	TRUE	3	TRA:CVVPNTNAGKSTF TRB:CASSLQGSTYNEQFF	220	CD8-C5
Pla-2_AGAGCTTCAATGGTCT	Pla	Pla-2	P2-19	TRUE	3	TRA:CAVRDMENNRLAF TRB:CASSLGGGLTYEQYF	27	CD8-C3
Pla-2_AGAGTGGTCCAGTAGT	Pla	Pla-2	P2-6	TRUE	3	TRA:CVVSVAAGNKLTF TRB:CASSPGTDLYEQYF	85	CD8-C3
Pla-2_AGAGTGGTCCCAAGAT	Pla	Pla-2	P2-148	FALSE	3	TRA:CAMRSSGAGSYQLTF TRB:CAILDDVIDTQYF	4	CD8-C3
Pla-2_AGATCTGAGAGTAATC	Pla	Pla-2	P2-59	TRUE	3	TRA:CAVDEGNTPLVF TRB:CAISESVDNTEAFF	9	CD8-C3
Pla-2_AGATCTGCAGATCGGA	Pla	Pla-2	P2-421	FALSE	2	TRA:CAVQAPDSGGYQKVTF TRB:CASSVTGLGVWGDHQETQY	2	CD8-C3
Pla-2_AGATCTGGTAAACGCG	Pla	Pla-2	P2-3	TRUE	3	TRA:CAVNPLESSYKLIF TRB:CASSPGTANTGELFF	177	CD8-C3
Pla-2_AGATCTGGTACACCGC	Pla	Pla-2	P2-73	FALSE	3	TRA:CAVPGSGNTGKLIF TRB:CASSQVYQGAMNTEAFF	8	CD8-C3
Pla-2_AGATTGCAGATAGCAT	Pla	Pla-2	P2-59	TRUE	3	TRA:CAVDEGNTPLVF TRB:CAISESVDNTEAFF	9	CD8-C3
Pla-2_AGCAGCCAGCGCTTAT	Pla	Pla-2	P2-229	FALSE	3	TRA:CAVRDEAAGTYKYIF TRB:CASSQGVSLAGTDTQYF	3	CD8-C3
Pla-2_AGCATACAGCAGATCG	Pla	Pla-2	P2-13	TRUE	3	TRA:CAYDLLTGNQFYF TRB:CASVRQLNTEAFF	38	CD8-C3
Pla-2_AGCCTAAAGACTTTCG	Pla	Pla-2	P2-56	FALSE	3	TRA:CATDLDGQKLLF TRB:CASRERGGNTGELFF	10	CD8-C3
Pla-2_AGCCTAACACCAGTTA	Pla	Pla-2	P2-3641	FALSE	1	TRA:CAANTL TRB:CASLNGAGGSYEQYF	1	CD8-C3
Pla-2_AGCGGTCTCTCGCATC	Pla	Pla-2	P2-22	TRUE	3	TRA:CAMRERSWVRQKLVF TRB:CASSRGTSGTDTQYF	24	CD8-C3
Pla-2_AGCGTATAGATGTTAG	Pla	Pla-2	P2-3655	FALSE	1	TRA:CAVTGAYNNNDMRF TRB:CASLNGAGGSYEQYF	1	CD8-C3
Pla-2_AGCGTATAGCGGATCA	Pla	Pla-2	P2-3656	FALSE	1	TRA:CATDGDFGNEKLTF TRB:CASSLQGSTYNEQFF	1	CD8-C3
Pla-2_AGCGTATAGCTAAACA	Pla	Pla-2	P2-6	TRUE	3	TRA:CVVSVAAGNKLTF TRB:CASSPGTDLYEQYF	85	CD8-C3
Pla-2_AGCGTATGTGTATGGG	Pla	Pla-2	P2-8	TRUE	3	TRA:CAALEGDNYGGQNFVF TRB:CASSPTWGGVRDTQYF	53	CD8-C3

Pla-2_AGCTCCTCATCGTCGG	Pla	Pla-2	P2-87	TRUE	3	TRA:CAFMKPIGTDKLIF TRB:CASRLPGTGSRETQYF	6	CD8-C3
Pla-2_AGCTCCTTCATGTAGC	Pla	Pla-2	P2-3	TRUE	3	TRA:CAVNPLESSYKLIF TRB:CASSPGTANTGELFF	177	CD8-C3
Pla-2_AGCTCTCTCAGCAACT	Pla	Pla-2	P2-6	TRUE	3	TRA:CVVSVAAGNKLTF TRB:CASSPGTDLYEQYF	85	CD8-C3
Pla-2_AGGCCACGTATCGCAT	Pla	Pla-2	P2-3	TRUE	3	TRA:CAVNPLESSYKLIF TRB:CASSPGTANTGELFF	177	CD8-C3
Pla-2_AGGCCGTAGTCGTACT	Pla	Pla-2	P2-3678	FALSE	1	TRA:CALSEQVDSSYKLIF TRB:CASSQDPTTTRSTDTQYF	1	CD8-C3
Pla-2_AGGCCGTTCTGTTGAG	Pla	Pla-2	P2-16	FALSE	3	TRA:CAVEMDSSYKLIF TRB:CASSQGRVTQYF	32	CD8-C3
Pla-2_AGGGAGTAGTCTCCTC	Pla	Pla-2	P2-433	FALSE	2	TRA:CAVSNTDKLIF TRB:CASVGGNTEAFF	2	CD8-C3
Pla-2_AGGGATGAGGCCATAG	Pla	Pla-2	P2-2	TRUE	3	TRA:CVVPNTNAGKSTF TRB:CASSLQGSTYNEQFF	220	CD8-C3
Pla-2_AGGGATGGTTACGGAG	Pla	Pla-2	P2-8	TRUE	3	TRA:CAALEGDNYGQNFVF TRB:CASSPTWGGVRDTQYF	53	CD8-C3
Pla-2_AGGTCATTCGTATCAG	Pla	Pla-2	P2-143	FALSE	3	TRA:CAYMEAAGNKLTF TRB:CASSPGLSTGELFF	4	CD8-C3
Pla-2_AGGTCATTCTTAGCCC	Pla	Pla-2	P2-10	TRUE	3	TRA:CAPLTPNDMRF TRB:CASLNGAGGSYEQYF	45	CD8-C3
Pla-2_AGGTCCGCATGTTGAC	Pla	Pla-2	P2-87	TRUE	3	TRA:CAFMKPIGTDKLIF TRB:CASRLPGTGSRETQYF	6	CD8-C3
Pla-2_AGTAGTCAGATCGGGT	Pla	Pla-2	P2-8	TRUE	3	TRA:CAALEGDNYGQNFVF TRB:CASSPTWGGVRDTQYF	53	CD8-C3
Pla-2_AGTAGTCCAGTACACT	Pla	Pla-2	P2-4	TRUE	3	TRA:CAIRYQSFNFGNEKLTF TRB:CASYRRVTEQYF	148	CD8-C3
Pla-2_AGTGAGGAGGACGAAA	Pla	Pla-2	P2-19	TRUE	3	TRA:CAVRDMENNRLAF TRB:CASSLGGGLTYEQYF	27	CD8-C3
Pla-2_AGTGAGGCAGTTAACC	Pla	Pla-2	P2-3702	FALSE	1	TRA:CIVRVAPRAAGNKLTF TRB:CSVSGRATDTQYF	1	CD8-C3
Pla-2_AGTGAGGGTCCATGAT	Pla	Pla-2	P2-200	TRUE	3	TRA:CAFNSKEVGANNLFF TRB:CATSRGRGREGYTF	3	CD8-C3
Pla-2_AGTGGGACATGCTAGT	Pla	Pla-2	P2-437	FALSE	2	TRA:CALGPNTGNQFYF TRB:CASSLGPDTIFYEQYF	2	CD8-C8
Pla-2_AGTGGGATCCTCCTAG	Pla	Pla-2	P2-3711	FALSE	1	TRA:CVVTYSGAGSYQLTF TRB:CASSETSGRIYEQFF	1	CD8-C2
Pla-2_AGTTGGTAGCCGGTAA	Pla	Pla-2	P2-91	FALSE	3	TRA:CAAYPSYNTDKLIF TRB:CASSPGGTVETQYF	6	CD8-C3
Pla-2_AGTTGGTCATCATCCC	Pla	Pla-2	P2-48	TRUE	3	TRA:CVVGTISGGYNKLIF TRB:CASSPITGDTQYF	11	CD8-C3
Pla-2_ATAACGCAGTTCGATC	Pla	Pla-2	P2-2	TRUE	3	TRA:CVVPNTNAGKSTF TRB:CASSLQGSTYNEQFF	220	CD8-C3
Pla-2_ATAAGAGAGATGTCGG	Pla	Pla-2	P2-3718	FALSE	1	TRA:CAVRDGVSFKTIF TRB:CASSDGQGGEQYF	1	CD8-C3
Pla-2_ATAAGAGTCCGAAGAG	Pla	Pla-2	P2-3722	FALSE	1	TRA:CVVSKAAGNKLTF TRB:CASSPGTDLYEQYF	1	CD8-C3
Pla-2_ATAGACCAGTCATCCA	Pla	Pla-2	P2-3723	FALSE	1	TRA:CAVRPLLGFGNVLHC TRB:CASSSMDSYNEQFF	1	CD8-C3
Pla-2_ATCACGACAAGGTTCT	Pla	Pla-2	P2-65	TRUE	3	TRA:CAPNQAGTALIF TRB:CASKLSGTSGSYEQYF	8	CD8-C3
Pla-2_ATCATCTCAGCCTTTC	Pla	Pla-2	P2-16	FALSE	3	TRA:CAVEMDSSYKLIF TRB:CASSQGRVTQYF	32	CD8-C3

Pla-2_ATCATCTCAGCTTAAC	Pla	Pla-2	P2-229	FALSE	3	TRA:CAVRDEAAGTYKYIF TRB:CASSQGVSLAGTDTQYF	3	CD8-C3
Pla-2_ATCATCTGTCCGAACC	Pla	Pla-2	P2-2	TRUE	3	TRA:CVVPNTNAGKSTF TRB:CASSLQGSTYNEQFF	220	CD8-C3
Pla-2_ATCATCTTCTTCGGTC	Pla	Pla-2	P2-2	TRUE	3	TRA:CVVPNTNAGKSTF TRB:CASSLQGSTYNEQFF	220	CD8-C3
Pla-2_ATCATGGGTCCTCCAT	Pla	Pla-2	P2-3729	FALSE	1	TRA:CAVQAPDSGGYQKVTF TRB:CASSPGTDLYEQYF	1	CD8-C3
Pla-2_ATCCACCCAGCTATTG	Pla	Pla-2	P2-33	TRUE	3	TRA:CAFKLSDGQKLLF TRB:CAWSRAALTEAFF	17	CD8-C8
Pla-2_ATCCGAAAGGCGATAC	Pla	Pla-2	P2-55	TRUE	3	TRA:CIVRGIQGAQKLVF TRB:CASSLQGGGSGGLTQYF	10	CD8-C3
Pla-2_ATCCGAACAGCTTCGG	Pla	Pla-2	P2-6	TRUE	3	TRA:CVVSVAAGNKLTF TRB:CASSPGTDLYEQYF	85	CD8-C3
Pla-2_ATCCGAATCCATGAGT	Pla	Pla-2	P2-6	TRUE	3	TRA:CVVSVAAGNKLTF TRB:CASSPGTDLYEQYF	85	CD8-C3
Pla-2_ATCGAGTTCTACTTAC	Pla	Pla-2	P2-3740	FALSE	1	TRA:CAERTGANNLFF TRB:CASSLGFSARKQETQYF	1	CD8-C3
Pla-2_ATCTACTCATGCTAGT	Pla	Pla-2	P2-42	FALSE	3	TRA:CAVSPRDNNDMRF TRB:CASSARGNSNQPQHF	12	CD4-C4
Pla-2_ATCTGCCCATTAACCG	Pla	Pla-2	P2-15	TRUE	3	TRA:CAVGDNFNKFYF TRB:CASSRTSAAGELFF	35	CD8-C3
Pla-2_ATGAGGGAGCGTAGTG	Pla	Pla-2	P2-8	TRUE	3	TRA:CAALEGDNYGGQNFVF TRB:CASSPTWGGVRDTQYF	53	CD8-C3
Pla-2_ATGAGGGGTGTTCTTT	Pla	Pla-2	P2-10	TRUE	3	TRA:CAPLTPNDMRF TRB:CASLNGAGGSYEQYF	45	CD8-C3
Pla-2_ATGCGATAGTGGTAAT	Pla	Pla-2	P2-126	TRUE	3	TRA:CATDGDFGNEKLTF TRB:CASSLTGETQYF	4	CD8-C3
Pla-2_ATGCGATTCGCCGTGA	Pla	Pla-2	P2-89	FALSE	3	TRA:CAASAGNTDKLIF TRB:CASSTGLAVSYEQYF	6	CD4-C7
Pla-2_ATGGGAGGTTTGACAC	Pla	Pla-2	P2-13	TRUE	3	TRA:CAYDLLTGNQFYF TRB:CASVRQLNTEAFF	38	CD8-C2
Pla-2_ATGTGTGCATTGTGCA	Pla	Pla-2	P2-3	TRUE	3	TRA:CAVNPLESSYKLIF TRB:CASSPGTANTGELFF	177	CD4-C4
Pla-2_ATGTGTGTCAGAAATG	Pla	Pla-2	P2-13	TRUE	3	TRA:CAYDLLTGNQFYF TRB:CASVRQLNTEAFF	38	CD8-C3
Pla-2_ATTACTCGTTGGTGGA	Pla	Pla-2	P2-3	TRUE	3	TRA:CAVNPLESSYKLIF TRB:CASSPGTANTGELFF	177	CD8-C5
Pla-2_ATTATCCGTTCAGACT	Pla	Pla-2	P2-66	TRUE	3	TRA:CAVCRNRDDKIIF TRB:CASSTPQGHYGYTF	8	CD8-C3
Pla-2_ATTCTACAGTCTTGCA	Pla	Pla-2	P2-6	TRUE	3	TRA:CVVSVAAGNKLTF TRB:CASSPGTDLYEQYF	85	CD8-C3
Pla-2_ATTCTACGTGTAATGA	Pla	Pla-2	P2-6	TRUE	3	TRA:CVVSVAAGNKLTF TRB:CASSPGTDLYEQYF	85	CD8-C3
Pla-2_ATTGGACAGAGTCTGG	Pla	Pla-2	P2-72	FALSE	3	TRA:CATDEETAGNKLTF TRB:CASSEAGWMGALHTEAFF	8	CD8-C3
Pla-2_ATTGGACGTTCCGTCT	Pla	Pla-2	P2-3	TRUE	3	TRA:CAVNPLESSYKLIF TRB:CASSPGTANTGELFF	177	CD8-C3
Pla-2_ATTGGTGAGTGTTTGC	Pla	Pla-2	P2-13	TRUE	3	TRA:CAYDLLTGNQFYF TRB:CASVRQLNTEAFF	38	CD8-C3
Pla-2_ATTGGTGCACGCGAAA	Pla	Pla-2	P2-2	TRUE	3	TRA:CVVPNTNAGKSTF TRB:CASSLQGSTYNEQFF	220	CD8-C3
Pla-2_ATTTCTGCACACTGCG	Pla	Pla-2	P2-2	TRUE	3	TRA:CVVPNTNAGKSTF TRB:CASSLQGSTYNEQFF	220	CD8-C3

Pla-2_ATTTCTGGTAAAGTCA	Pla	Pla-2	P2-3778	FALSE	1	TRA:CAVATGGFKTIF TRB:CASRPQGMNTEAFF	1	CD8-C3
Pla-2_CAACCTCAGCGCCTCA	Pla	Pla-2	P2-45	TRUE	3	TRA:CAVSGDSNYQLIW TRB:CASSLGGNGNTIYF	11	CD8-C3
Pla-2_CAACCTCGTGGTGTAG	Pla	Pla-2	P2-3	TRUE	3	TRA:CAVNPLESSYKLIF TRB:CASSPGTANTGELFF	177	CD8-C3
Pla-2_CAACCTCGTTCAGTAC	Pla	Pla-2	P2-20	TRUE	3	TRA:CAYDSNYQLIW TRB:CASSLSIDRADTGELFF	25	CD8-C3
Pla-2_CAACTAGAGTGAAGTT	Pla	Pla-2	P2-3787	FALSE	1	TRA:CAVHTGRRALTF TRB:CASTGTSLETQYF	1	CD8-C3
Pla-2_CAACTAGCAACAACCT	Pla	Pla-2	P2-13	TRUE	3	TRA:CAYDLLTGNQFYF TRB:CASVRQLNTEAFF	38	CD8-C3
Pla-2_CAACTAGTCTACGAGT	Pla	Pla-2	P2-13	TRUE	3	TRA:CAYDLLTGNQFYF TRB:CASVRQLNTEAFF	38	CD8-C3
Pla-2_CAAGATCAGATCACGG	Pla	Pla-2	P2-8	TRUE	3	TRA:CAALEGDNYGQNFVF TRB:CASSPTWGGVRDTQYF	53	CD8-C3
Pla-2_CAAGATCAGCGTGAAC	Pla	Pla-2	P2-13	TRUE	3	TRA:CAYDLLTGNQFYF TRB:CASVRQLNTEAFF	38	CD8-C3
Pla-2_CAAGGCCGTTGTACAC	Pla	Pla-2	P2-3799	FALSE	1	TRA:CAASMGYGGATNKLIF TRB:CASSNSGGRGYNEQFF	1	CD8-C3
Pla-2_CAAGGCCTCGCCCTTA	Pla	Pla-2	P2-19	TRUE	3	TRA:CAVRDMENNRLAF TRB:CASSLGGGLTYEQYF	27	CD8-C5
Pla-2_CAAGGCCTCGGCCGAT	Pla	Pla-2	P2-367	TRUE	2	TRA:CALSDPYNTDKLIF TRB:CASSFYKGELFF	2	CD8-C3
Pla-2_CAAGTTGAGAGTGAGA	Pla	Pla-2	P2-157	FALSE	3	TRA:CAVKSGNTGKLIF TRB:CASSDRNTGELFF	4	CD8-C3
Pla-2_CAAGTTGGTGGTCCGT	Pla	Pla-2	P2-158	FALSE	3	TRA:CAVGAVRETSGSRLTF TRB:CASSFTDLPYEQYF	4	CD8-C3
Pla-2_CAAGTTGTCCGGCACA	Pla	Pla-2	P2-3804	FALSE	1	TRA:CIATPSLYNQGGKLIF TRB:CSARATGGELFF	1	CD8-C3
Pla-2_CACACTCAGATGCGAC	Pla	Pla-2	P2-68	TRUE	3	TRA:CAVYSGYSTLTF TRB:CSVEDVGGKSSYNEQFF	8	CD8-C3
Pla-2_CACAGGCTCACGACTA	Pla	Pla-2	P2-6	TRUE	3	TRA:CVVSVAAGNKLTF TRB:CASSPGTDLYEQYF	85	CD8-C3
Pla-2_CACAGGCTCCTTAATC	Pla	Pla-2	P2-2	TRUE	3	TRA:CVVPNTNAGKSTF TRB:CASSLQGSTYNEQFF	220	CD8-C3
Pla-2_CACAGGCTCGCTTAGA	Pla	Pla-2	P2-237	FALSE	3	TRA:CALSEALGSGGGADGLTF TRB:CASSTTGDGVVSYEQYF	3	CD8-C5
Pla-2_CACAGGCTCTCTGAGA	Pla	Pla-2	P2-8	TRUE	3	TRA:CAALEGDNYGQNFVF TRB:CASSPTWGGVRDTQYF	53	CD8-C3
Pla-2_CACATAGTCGCGTTTC	Pla	Pla-2	P2-10	TRUE	3	TRA:CAPLTPNDMRF TRB:CASLNGAGGSYEQYF	45	CD8-C3
Pla-2_CACATTTAGAGACTTA	Pla	Pla-2	P2-3832	FALSE	1	TRA:CAGTSWGKFQF TRB:CAISGGLVGTDTQYF	1	CD8-C3
Pla-2_CACATTTTCACGCATA	Pla	Pla-2	P2-55	TRUE	3	TRA:CIVRGIQGAQKLVF TRB:CASSLQGGGSGGLTQYF	10	CD8-C3
Pla-2_CACATTTTCGCCTGTT	Pla	Pla-2	P2-153	FALSE	3	TRA:CAYNNNDMRF TRB:CASSDRGGRQETQYF	4	CD8-C3
Pla-2_CACCACTCAGCCAATT	Pla	Pla-2	P2-6	TRUE	3	TRA:CVVSVAAGNKLTF TRB:CASSPGTDLYEQYF	85	CD8-C3
Pla-2_CACCACTGTACGCACC	Pla	Pla-2	P2-45	TRUE	3	TRA:CAVSGDSNYQLIW TRB:CASSLGGNGNTIYF	11	CD8-C4
Pla-2_CACCAGGTCTCTGTCG	Pla	Pla-2	P2-82	FALSE	3	TRA:CVADTGNQFYF TRB:CASSSALTGTYEQYF	7	CD8-C2

Pla-2_CACTCCATCACCGTAA	Pla	Pla-2	P2-3846	FALSE	1	TRA:CAASYTGTASKLTF TRB:CASSISPGLFGEKLFF	1	CD8-C3
Pla-2_CAGAATCCACTGTTAG	Pla	Pla-2	P2-56	FALSE	3	TRA:CATDLDGQKLLF TRB:CASRERGGNTGELFF	10	CD8-C8
Pla-2_CAGAATCGTCGCATAT	Pla	Pla-2	P2-72	FALSE	3	TRA:CATDEETAGNKLTF TRB:CASSEAGWMGALHTEAFF	8	CD8-C3
Pla-2_CAGAGAGAGCATGGCA	Pla	Pla-2	P2-156	FALSE	3	TRA:CAMRHHGSGNTGKLIF TRB:CASSLAGASGPNNEQFF	4	CD8-C3
Pla-2_CAGAGAGGTCATCGGC	Pla	Pla-2	P2-6	TRUE	3	TRA:CVVSVAAGNKLTF TRB:CASSPGTDLYEQYF	85	CD8-C3
Pla-2_CAGATCACAGCTGCAC	Pla	Pla-2	P2-30	TRUE	3	TRA:CAVGGDTGNQFYF TRB:CASSLFPTGVAYGYTF	20	CD8-C3
Pla-2_CAGCATACACGGTTTA	Pla	Pla-2	P2-369	TRUE	2	TRA:CVVSDDANDTDKLIF TRB:CASSVPGLAGGLLVFNEQFF	2	CD8-C3
Pla-2_CAGCATAGTAAGAGGA	Pla	Pla-2	P2-22	TRUE	3	TRA:CAMRERSWVRQKLVF TRB:CASSRGTSGTDTQYF	24	CD8-C3
Pla-2_CAGCCGAAGCGGCTTC	Pla	Pla-2	P2-2	TRUE	3	TRA:CVVPNTNAGKSTF TRB:CASSLQGSTYNEQFF	220	CD8-C3
Pla-2_CAGCCGACATGGTCTA	Pla	Pla-2	P2-468	FALSE	2	TRA:CAASGNGAGSYQLTF TRB:CASSGLAGYAYEQYF	2	CD8-C3
Pla-2_CAGCCGAGTCGGGTCT	Pla	Pla-2	P2-6	TRUE	3	TRA:CVVSVAAGNKLTF TRB:CASSPGTDLYEQYF	85	CD8-C3
Pla-2_CAGCGACAGAAGGACA	Pla	Pla-2	P2-48	TRUE	3	TRA:CVVGTISGGYNKLIF TRB:CASSPITGDTQYF	11	CD8-C3
Pla-2_CAGCGACCAAGTCTAC	Pla	Pla-2	P2-3874	FALSE	1	TRA:CAVSDLYGNARLMF TRB:CASRAGTGSDTQYF	1	CD8-C3
Pla-2_CAGCGACCATTCACTT	Pla	Pla-2	P2-60	TRUE	3	TRA:CALSEGSGANSKLTF TRB:CASSSHSGLAGGFF	9	CD8-C3
Pla-2_CAGGTGCTCAACGGCC	Pla	Pla-2	P2-206	FALSE	3	TRA:CAASGGNSNTGTASKLTF TRB:CASSGLAGYQYEQYF	3	CD8-C3
Pla-2_CATATGGCATTATCTC	Pla	Pla-2	P2-6	TRUE	3	TRA:CVVSVAAGNKLTF TRB:CASSPGTDLYEQYF	85	CD8-C3
Pla-2_CATATGGTCATACGGT	Pla	Pla-2	P2-69	TRUE	3	TRA:CAVRAGSARQLTF TRB:CATTGTSLDEQYF	8	CD8-C3
Pla-2_CATATTCAGGTGTGGT	Pla	Pla-2	P2-29	TRUE	3	TRA:CAVGGDFNKFYF TRB:CSARSDRLGYQPQHF	21	CD8-C2
Pla-2_CATATTCCAGTACACT	Pla	Pla-2	P2-3895	FALSE	1	TRA:CVVPNTNAGKSTF TRB:CASLNGAGGSYEQYF	1	CD8-C3
Pla-2_CATATTCCATTTGCTT	Pla	Pla-2	P2-48	TRUE	3	TRA:CVVGTISGGYNKLIF TRB:CASSPITGDTQYF	11	CD8-C3
Pla-2_CATATTCGTTGGGACA	Pla	Pla-2	P2-6	TRUE	3	TRA:CVVSVAAGNKLTF TRB:CASSPGTDLYEQYF	85	CD8-C3
Pla-2_CATCCACAGTGAAGAG	Pla	Pla-2	P2-6	TRUE	3	TRA:CVVSVAAGNKLTF TRB:CASSPGTDLYEQYF	85	CD8-C3
Pla-2_CATCCACCATCGTCGG	Pla	Pla-2	P2-22	TRUE	3	TRA:CAMRERSWVRQKLVF TRB:CASSRGTSGTDTQYF	24	CD8-C3
Pla-2_CATCCACTCCAATGGT	Pla	Pla-2	P2-22	TRUE	3	TRA:CAMRERSWVRQKLVF TRB:CASSRGTSGTDTQYF	24	CD8-C3
Pla-2_CATGACAAGAAACCAT	Pla	Pla-2	P2-15	TRUE	3	TRA:CAVGDNFNKFYF TRB:CASSRTSAAGELFF	35	CD8-C3
Pla-2_CATGCCTCATCGGACC	Pla	Pla-2	P2-3919	FALSE	1	TRA:CAYRGDNYGQNFVF TRB:CATSDHPRGADTQYF	1	CD8-C3
Pla-2_CATGGCGTCAGGTTCA	Pla	Pla-2	P2-3924	FALSE	1	TRA:CAVGDNFNKFCF TRB:CASSRTSAAGELFF	1	CD8-C3

Pla-2_CATTCGCAGATATACG	Pla	Pla-2	P2-189	TRUE	3	TRA:CAVRDWGRDDKIIF TRB:CASSSKPRSGANVLTF	3	CD8-C3
Pla-2_CCACTACAGGGATCTG	Pla	Pla-2	P2-13	TRUE	3	TRA:CAYDLLTGNQFYF TRB:CASVRQLNTEAFF	38	CD8-C3
Pla-2_CCACTACTCGTGGACC	Pla	Pla-2	P2-13	TRUE	3	TRA:CAYDLLTGNQFYF TRB:CASVRQLNTEAFF	38	CD8-C3
Pla-2_CCAGCGACATATGCTG	Pla	Pla-2	P2-3	TRUE	3	TRA:CAVNPLESSYKLIF TRB:CASSPGTANTGELFF	177	CD8-C3
Pla-2_CCAGCGAGTTGCGTTA	Pla	Pla-2	P2-68	TRUE	3	TRA:CAVYSGYSTLTF TRB:CSVEDVGGKSSYNEQFF	8	CD8-C3
Pla-2_CCATGTCGTCCAGTGC	Pla	Pla-2	P2-2	TRUE	3	TRA:CVVPNTNAGKSTF TRB:CASSLQGSTYNEQFF	220	CD8-C3
Pla-2_CCCAATCCACAGGTTT	Pla	Pla-2	P2-481	FALSE	2	TRA:CALSEYTGGFKTIF TRB:CSAPKPGQGLNTEAFF	2	CD8-C3
Pla-2_CCCAGTTCACGCATCG	Pla	Pla-2	P2-6	TRUE	3	TRA:CVVSVAAGNKLTF TRB:CASSPGTDLYEQYF	85	CD8-C3
Pla-2_CCCAGTTTCCAAGCCG	Pla	Pla-2	P2-6	TRUE	3	TRA:CVVSVAAGNKLTF TRB:CASSPGTDLYEQYF	85	CD8-C3
Pla-2_CCCAGTTTCGTATCAG	Pla	Pla-2	P2-59	TRUE	3	TRA:CAVDEGNTPLVF TRB:CAISESVDNTEAFF	9	CD8-C3
Pla-2_CCCATACCAGCTTAAC	Pla	Pla-2	P2-3967	FALSE	1	TRA:CATPSGTYKYIF TRB:CASSFLSEANEQFF	1	CD8-C3
Pla-2_CCGTGGACACAACGCC	Pla	Pla-2	P2-10	TRUE	3	TRA:CAPLTPNDMRF TRB:CASLNGAGGSYEQYF	45	CD8-C3
Pla-2_CCGTGGACACTTCGAA	Pla	Pla-2	P2-6	TRUE	3	TRA:CVVSVAAGNKLTF TRB:CASSPGTDLYEQYF	85	CD8-C3
Pla-2_CCGTGGAGTATCACCA	Pla	Pla-2	P2-3979	FALSE	1	TRA:CATLSGGSYIPTF TRB:CASSVGTGTGELFF	1	CD8-C3
Pla-2_CCGTGGATCATTCACT	Pla	Pla-2	P2-484	FALSE	2	TRA:CALSGWNNNARLMF TRB:CASTPLRQGYFGGYTF	2	CD8-C3
Pla-2_CCGTTCATCGATAGAA	Pla	Pla-2	P2-16	FALSE	3	TRA:CAVEMDSSYKLIF TRB:CASSQGRVTQYF	32	CD8-C3
Pla-2_CCTAAAGAGGGCTTCC	Pla	Pla-2	P2-3982	FALSE	1	TRA:CAASAGGNKLVF TRB:CASVRQLNTEAFF	1	CD8-C3
Pla-2_CCTACCATCTCCCTGA	Pla	Pla-2	P2-2	TRUE	3	TRA:CVVPNTNAGKSTF TRB:CASSLQGSTYNEQFF	220	CD8-C3
Pla-2_CCTAGCTAGAAACGCC	Pla	Pla-2	P2-3992	FALSE	1	TRA:CAYMGNRDDKIIF TRB:CASSQSGSNEQFF	1	CD8-C3
Pla-2_CCTAGCTCAGCTCGAC	Pla	Pla-2	P2-196	TRUE	3	TRA:CAVYNAGNMLTF TRB:CASSSRQVYF	3	CD8-C3
Pla-2_CCTAGCTGTAAGTGTA	Pla	Pla-2	P2-245	FALSE	3	TRA:CKRDNNDMRF TRB:CASSSTGESYTF	3	CD8-C3
Pla-2_CCTATTAGTGCGAAAC	Pla	Pla-2	P2-29	TRUE	3	TRA:CAVGGDFNKFYF TRB:CSARSDRLGYQPQHF	21	CD8-C3
Pla-2_CCTATTATCTTGAGAC	Pla	Pla-2	P2-30	TRUE	3	TRA:CAVGGDTGNQFYF TRB:CASSLFPTGVAYGYTF	20	CD8-C6
Pla-2_CCTCTGAGTCATCCCT	Pla	Pla-2	P2-10	TRUE	3	TRA:CAPLTPNDMRF TRB:CASLNGAGGSYEQYF	45	CD8-C3
Pla-2_CCTCTGATCCCAAGTA	Pla	Pla-2	P2-16	FALSE	3	TRA:CAVEMDSSYKLIF TRB:CASSQGRVTQYF	32	CD8-C3
Pla-2_CCTTACGCAACTTGAC	Pla	Pla-2	P2-2	TRUE	3	TRA:CVVPNTNAGKSTF TRB:CASSLQGSTYNEQFF	220	CD8-C3
Pla-2_CCTTACGCAAGTCATC	Pla	Pla-2	P2-22	TRUE	3	TRA:CAMRERSWVRQKLVF TRB:CASSRGTSGTDTQYF	24	CD8-C3

Pla-2_CCTTCGAGTGAGTGAC	Pla	Pla-2	P2-4017	FALSE	1	TRA:CAMREDLDKIIF TRB:CASRRDRGGLGEAFF	1	CD8-C3
Pla-2_CCTTCGATCGAGGTAG	Pla	Pla-2	P2-130	TRUE	3	TRA:CAFMTPNGGATNKLIF TRB:CASSQENTAGSYF	4	CD8-C3
Pla-2_CCTTTCTTCAGGATCT	Pla	Pla-2	P2-4024	FALSE	1	TRA:CIVRASYNTDKLIF TRB:CASSKAVSFPDTQYF	1	CD8-C3
Pla-2_CGAATGTAGCTAGTGG	Pla	Pla-2	P2-20	TRUE	3	TRA:CAYDSNYQLIW TRB:CASSLSIDRADTGELFF	25	CD8-C3
Pla-2_CGACCTTAGAAGGACA	Pla	Pla-2	P2-4033	FALSE	1	TRA:CAVSSRRNTGNQFYF TRB:CASLNGAGGSYEQYF	1	CD8-C8
Pla-2_CGACTTCAGAGGTTAT	Pla	Pla-2	P2-6	TRUE	3	TRA:CVVSVAAGNKLTF TRB:CASSPGTDLYEQYF	85	CD8-C3
Pla-2_CGACTTCCACACCGAC	Pla	Pla-2	P2-206	FALSE	3	TRA:CAASGGNSNTGTASKLTF TRB:CASSGLAGYQYEQYF	3	CD8-C3
Pla-2_CGAGAAGCAGCTTCGG	Pla	Pla-2	P2-8	TRUE	3	TRA:CAALEGDNYGQNFVF TRB:CASSPTWGGVRDTQYF	53	CD8-C3
Pla-2_CGAGCACAGTAGATGT	Pla	Pla-2	P2-4041	FALSE	1	TRA:CAYDSNYQLIW TRB:CASSLQQQETQYF	1	CD8-C3
Pla-2_CGAGCCATCTTATCTG	Pla	Pla-2	P2-484	FALSE	2	TRA:CALSGWNNNARLMF TRB:CASTPLRQGYFGGYTF	2	CD8-C3
Pla-2_CGATCGGAGATCACGG	Pla	Pla-2	P2-59	TRUE	3	TRA:CAVDEGNTPLVF TRB:CAISESVDNTEAFF	9	CD8-C3
Pla-2_CGATCGGGTTGCGCAC	Pla	Pla-2	P2-16	FALSE	3	TRA:CAVEMDSSYKLIF TRB:CASSQGRVTQYF	32	CD8-C3
Pla-2_CGATGGCGTCGCTTCT	Pla	Pla-2	P2-2	TRUE	3	TRA:CVVPNTNAGKSTF TRB:CASSLQGSTYNEQFF	220	CD8-C5
Pla-2_CGATGTACAGTTTACG	Pla	Pla-2	P2-45	TRUE	3	TRA:CAVSGDSNYQLIW TRB:CASSLGGNGNTIYF	11	CD8-C3
Pla-2_CGCCAAGGTAAGCACG	Pla	Pla-2	P2-2	TRUE	3	TRA:CVVPNTNAGKSTF TRB:CASSLQGSTYNEQFF	220	CD8-C3
Pla-2_CGCGGTATCCCTAATT	Pla	Pla-2	P2-2	TRUE	3	TRA:CVVPNTNAGKSTF TRB:CASSLQGSTYNEQFF	220	CD8-C3
Pla-2_CGCGTTTAGCTGAAAT	Pla	Pla-2	P2-137	FALSE	3	TRA:CAVRDMVDNFNKFYF TRB:CASSSGTGGFYEQYF	4	CD4-C4
Pla-2_CGCTATCGTGACGGTA	Pla	Pla-2	P2-22	TRUE	3	TRA:CAMRERSWVRQKLVF TRB:CASSRGTSGTDTQYF	24	CD8-C3
Pla-2_CGCTGGAAGCTGCCCA	Pla	Pla-2	P2-72	FALSE	3	TRA:CATDEETAGNKLTF TRB:CASSEAGWMGALHTEAFF	8	CD8-C3
Pla-2_CGCTGGAAGGAATTAC	Pla	Pla-2	P2-2	TRUE	3	TRA:CVVPNTNAGKSTF TRB:CASSLQGSTYNEQFF	220	CD8-C8
Pla-2_CGCTGGAGTCCAGTTA	Pla	Pla-2	P2-4085	FALSE	1	TRA:CAVDNNNDMRF TRB:CAISGTFGTEAFF	1	CD8-C8
Pla-2_CGCTTCAGTTGCGTTA	Pla	Pla-2	P2-4092	FALSE	1	TRA:CAVRDMRYGGATNKLIF TRB:CASSVDLAASYEQYF	1	CD8-C3
Pla-2_CGGACACAGTGGGCTA	Pla	Pla-2	P2-20	TRUE	3	TRA:CAYDSNYQLIW TRB:CASSLSIDRADTGELFF	25	CD8-C3
Pla-2_CGGACACCACGCCAGT	Pla	Pla-2	P2-100	TRUE	3	TRA:CAVGPRLLGNEKLTF TRB:CASSQDSTSVYNEQFF	5	CD8-C3
Pla-2_CGGACGTCACAGATTC	Pla	Pla-2	P2-20	TRUE	3	TRA:CAYDSNYQLIW TRB:CASSLSIDRADTGELFF	25	CD8-C3
Pla-2_CGGACTGAGTGTCCCG	Pla	Pla-2	P2-502	FALSE	2	TRA:CLVRHTGFQKLVF TRB:CALVGSSLETQYF	2	CD8-C3
Pla-2_CGGACTGGTGCGGTAA	Pla	Pla-2	P2-4103	FALSE	1	TRA:CVVPNTNAGKSTF TRB:CASSYGARNEQFF	1	CD8-C3

Pla-2_CGGAGTCAGAGGTTGC	Pla	Pla-2	P2-8	TRUE	3	TRA:CAALEGDNYGQNFVF TRB:CASSPTWGGVRDTQYF	53	CD8-C3
Pla-2_CGGAGTCCATGGTCTA	Pla	Pla-2	P2-4111	FALSE	1	TRA:CAMREAGGSGAGSYQLTF TRB:CAISESVDNTEAFF	1	CD8-C3
Pla-2_CGGAGTCTCCACGAAT	Pla	Pla-2	P2-6	TRUE	3	TRA:CVVSVAAGNKLTF TRB:CASSPGTDLYEQYF	85	CD8-C3
Pla-2_CGGCTAGCATCATCCC	Pla	Pla-2	P2-8	TRUE	3	TRA:CAALEGDNYGQNFVF TRB:CASSPTWGGVRDTQYF	53	CD8-C3
Pla-2_CGGCTAGTCTGGGCCA	Pla	Pla-2	P2-107	TRUE	3	TRA:CASGGYQKVTF TRB:CASSQDALAGSYEQYF	5	CD8-C3
Pla-2_CGGTTAAAGGACAGAA	Pla	Pla-2	P2-15	TRUE	3	TRA:CAVGDNFNKFYF TRB:CASSRTSAAGELFF	35	CD8-C8
Pla-2_CGTAGCGAGGCTCAGA	Pla	Pla-2	P2-4124	FALSE	1	TRA:CAFNYGQNFVF TRB:CASSLWGQSQGTQYF	1	CD8-C3
Pla-2_CGTAGCGTCCAGAGGA	Pla	Pla-2	P2-222	FALSE	3	TRA:CAASEAYSGGGADGLTF TRB:CASSLGGIYNEQFF	3	CD8-C3
Pla-2_CGTAGGCTCCTGTAGA	Pla	Pla-2	P2-83	FALSE	3	TRA:CAVDTGTASKLTF TRB:CASRGSTDTQYF	7	CD8-C3
Pla-2_CGTCACTCACGTGAGA	Pla	Pla-2	P2-16	FALSE	3	TRA:CAVEMDSSYKLIF TRB:CASSQGRVTQYF	32	CD8-C3
Pla-2_CGTCAGGCATCTCCCA	Pla	Pla-2	P2-73	FALSE	3	TRA:CAVPGSGNTGKLIF TRB:CASSQVYQGAMNTEAFF	8	CD8-C3
Pla-2_CGTCAGGTCCTCGCAT	Pla	Pla-2	P2-365	TRUE	2	TRA:CAMREASLSGGYNKLIF TRB:CASRQDRQETQYF	2	CD8-C3
Pla-2_CGTCCATAGTTAAGTG	Pla	Pla-2	P2-10	TRUE	3	TRA:CAPLTPNDMRF TRB:CASLNGAGGSYEQYF	45	CD8-C3
Pla-2_CGTCTACCACTTGGAT	Pla	Pla-2	P2-20	TRUE	3	TRA:CAYDSNYQLIW TRB:CASSLSIDRADTGELFF	25	CD8-C3
Pla-2_CGTCTACGTGATAAGT	Pla	Pla-2	P2-52	FALSE	3	TRA:CARTTNAGKSTF TRB:CASSETSGRIYEQFF	11	CD8-C3
Pla-2_CGTGTAAGTTCACGGC	Pla	Pla-2	P2-29	TRUE	3	TRA:CAVGGDFNKFYF TRB:CSARSDRLGYQPQHF	21	CD8-C5
Pla-2_CGTTCTGTCTTAGCCC	Pla	Pla-2	P2-4160	FALSE	1	TRA:CILSIGAGSYQLTF TRB:CASTVVGEDNEQFF	1	CD8-C3
Pla-2_CGTTGGGCACAGGTTT	Pla	Pla-2	P2-29	TRUE	3	TRA:CAVGGDFNKFYF TRB:CSARSDRLGYQPQHF	21	CD8-C3
Pla-2_CTAACTTGTATCTGCA	Pla	Pla-2	P2-10	TRUE	3	TRA:CAPLTPNDMRF TRB:CASLNGAGGSYEQYF	45	CD8-C3
Pla-2_CTAAGACAGATGTGGC	Pla	Pla-2	P2-6	TRUE	3	TRA:CVVSVAAGNKLTF TRB:CASSPGTDLYEQYF	85	CD8-C3
Pla-2_CTAAGACAGGCAGGTT	Pla	Pla-2	P2-3	TRUE	3	TRA:CAVNPLESSYKLIF TRB:CASSPGTANTGELFF	177	CD8-C3
Pla-2_CTACCCAAGATCTGCT	Pla	Pla-2	P2-19	TRUE	3	TRA:CAVRDMENNRLAF TRB:CASSLGGGLTYEQYF	27	CD8-C3
Pla-2_CTACCCACAAGCCGTC	Pla	Pla-2	P2-6	TRUE	3	TRA:CVVSVAAGNKLTF TRB:CASSPGTDLYEQYF	85	CD8-C3
Pla-2_CTACCCATCCCTTGTG	Pla	Pla-2	P2-4178	FALSE	1	TRA:CAFSKGNNRLAF TRB:CASSPFMSGGELFF	1	CD8-C2
Pla-2_CTACGTCAGCGAAGGG	Pla	Pla-2	P2-22	TRUE	3	TRA:CAMRERSWVRQKLVF TRB:CASSRGTSGTDTQYF	24	CD8-C3
Pla-2_CTACGTCCACAGTCGC	Pla	Pla-2	P2-19	TRUE	3	TRA:CAVRDMENNRLAF TRB:CASSLGGGLTYEQYF	27	CD8-C3
Pla-2_CTACGTCCATTCCTCG	Pla	Pla-2	P2-4180	FALSE	1	TRA:CAVRLYGNNRLAF TRB:CASSLTGNQNTEAFF	1	CD8-C3

Pla-2_CTAGAGTCAAGGCTCC	Pla	Pla-2	P2-15	TRUE	3	TRA:CAVGDNFNKFYF TRB:CASSRTSAAGELFF	35	CD8-C3
Pla-2_CTAGAGTTCACCCTCA	Pla	Pla-2	P2-20	TRUE	3	TRA:CAYDSNYQLIW TRB:CASSLSIDRADTGELFF	25	CD8-C3
Pla-2_CTAGCCTGTGATGTCT	Pla	Pla-2	P2-137	FALSE	3	TRA:CAVRDMVDNFNKFYF TRB:CASSSGTGGFYEQYF	4	CD8-C3
Pla-2_CTAGTGAGTAGGGACT	Pla	Pla-2	P2-245	FALSE	3	TRA:CKRDNNDMRF TRB:CASSSTGESYTF	3	CD8-C3
Pla-2_CTAGTGATCCTTGCCA	Pla	Pla-2	P2-29	TRUE	3	TRA:CAVGGDFNKFYF TRB:CSARSDRLGYQPQHF	21	CD8-C7
Pla-2_CTCACACTCGCCTGTT	Pla	Pla-2	P2-4198	FALSE	1	TRA:CIVGTSYGKLTF TRB:CASSETSGRIYEQFF	1	CD4-C7
Pla-2_CTCATTATCTACGAGT	Pla	Pla-2	P2-133	TRUE	3	TRA:CAVNKPAGNKLTF TRB:CASSTRPGGANVLTF	4	CD4-C4
Pla-2_CTCCTAGAGTCCGGTC	Pla	Pla-2	P2-13	TRUE	3	TRA:CAYDLLTGNQFYF TRB:CASVRQLNTEAFF	38	CD8-C3
Pla-2_CTCCTAGGTGTGACGA	Pla	Pla-2	P2-16	FALSE	3	TRA:CAVEMDSSYKLIF TRB:CASSQGRVTQYF	32	CD8-C8
Pla-2_CTCCTAGTCCTGTACC	Pla	Pla-2	P2-6	TRUE	3	TRA:CVVSVAAGNKLTF TRB:CASSPGTDLYEQYF	85	CD8-C3
Pla-2_CTCGGAGCAACTGGCC	Pla	Pla-2	P2-4211	FALSE	1	TRA:CIVRVLYNFNKFYF TRB:CASSQSALGLVLGQETQYF	1	CD8-C3
Pla-2_CTCGTCAAGAGTCTGG	Pla	Pla-2	P2-390	FALSE	2	TRA:CAVRGRGGSGNTGKLIF TRB:CSARDLWLAGGPNPQETQ	2	CD8-C5
Pla-2_CTCGTCATCTTCCTTC	Pla	Pla-2	P2-2	TRUE	3	TRA:CVVPNTNAGKSTF TRB:CASSLQGSTYNEQFF	220	CD8-C3
Pla-2_CTCTAATGTGCACCAC	Pla	Pla-2	P2-16	FALSE	3	TRA:CAVEMDSSYKLIF TRB:CASSQGRVTQYF	32	CD8-C3
Pla-2_CTCTACGGTTGGTTTG	Pla	Pla-2	P2-6	TRUE	3	TRA:CVVSVAAGNKLTF TRB:CASSPGTDLYEQYF	85	CD8-C3
Pla-2_CTGAAACCAAGCTGTT	Pla	Pla-2	P2-522	FALSE	2	TRA:CALSAAGGTSYGKLTF TRB:CASKLSGTSGSYEQYF	2	CD8-C3
Pla-2_CTGAAACCAGACAGGT	Pla	Pla-2	P2-41	TRUE	3	TRA:CAVRDSDYKLSF TRB:CASSQGESSYEQYF	12	CD8-C5
Pla-2_CTGATCCCACCCATGG	Pla	Pla-2	P2-2	TRUE	3	TRA:CVVPNTNAGKSTF TRB:CASSLQGSTYNEQFF	220	CD8-C3
Pla-2_CTGCCTAAGGTGCACA	Pla	Pla-2	P2-2	TRUE	3	TRA:CVVPNTNAGKSTF TRB:CASSLQGSTYNEQFF	220	CD8-C3
Pla-2_CTGCCTAGTCCGTGAC	Pla	Pla-2	P2-4	TRUE	3	TRA:CAIRYQSFNFGNEKLTF TRB:CASYRRVTEQYF	148	CD8-C3
Pla-2_CTGCCTATCCGCATCT	Pla	Pla-2	P2-33	TRUE	3	TRA:CAFKLSDGQKLLF TRB:CAWSRAALTEAFF	17	CD4-C4
Pla-2_CTGCTGTCAGCCACCA	Pla	Pla-2	P2-8	TRUE	3	TRA:CAALEGDNYGQNFVF TRB:CASSPTWGGVRDTQYF	53	CD8-C3
Pla-2_CTGCTGTCATTCACTT	Pla	Pla-2	P2-2	TRUE	3	TRA:CVVPNTNAGKSTF TRB:CASSLQGSTYNEQFF	220	CD8-C3
Pla-2_CTGGTCTGTGGGTATG	Pla	Pla-2	P2-2	TRUE	3	TRA:CVVPNTNAGKSTF TRB:CASSLQGSTYNEQFF	220	CD8-C3
Pla-2_CTTAACTGTCTGATCA	Pla	Pla-2	P2-6	TRUE	3	TRA:CVVSVAAGNKLTF TRB:CASSPGTDLYEQYF	85	CD8-C3
Pla-2_CTTACCGAGCGTAGTG	Pla	Pla-2	P2-10	TRUE	3	TRA:CAPLTPNDMRF TRB:CASLNGAGGSYEQYF	45	CD8-C3
Pla-2_CTTCTCTGTCCGAAGA	Pla	Pla-2	P2-4275	FALSE	1	TRA:CAIIQGAQKLVF TRB:CASSVGPGSGELFF	1	CD8-C3

Pla-2_CTTCTCTGTCGAGATG	Pla	Pla-2	P2-3	TRUE	3	TRA:CAVNPLESSYKLIF TRB:CASSPGTANTGELFF	177	CD8-C3
Pla-2_CTTTGCGCATGGTAGG	Pla	Pla-2	P2-3	TRUE	3	TRA:CAVNPLESSYKLIF TRB:CASSPGTANTGELFF	177	CD8-C3
Pla-2_GAAACTCCACGACTCG	Pla	Pla-2	P2-4289	FALSE	1	TRA:CAASQAIWNAGNMLTF TRB:CASSFFPAGGRQTKNIQYF	1	CD8-C3
Pla-2_GAAACTCCAGGATCGA	Pla	Pla-2	P2-2	TRUE	3	TRA:CVVPNTNAGKSTF TRB:CASSLQGSTYNEQFF	220	CD8-C3
Pla-2_GAACATCCACTTGGAT	Pla	Pla-2	P2-52	FALSE	3	TRA:CARTTNAGKSTF TRB:CASSETSGRIYEQFF	11	CD8-C3
Pla-2_GAACATCGTGTTAAGA	Pla	Pla-2	P2-4299	FALSE	1	TRA:CAVNSFNKFYF TRB:CAWRRDLQGNTEAFF	1	CD8-C3
Pla-2_GAACCTACAAACCCAT	Pla	Pla-2	P2-6	TRUE	3	TRA:CVVSVAAGNKLTF TRB:CASSPGTDLYEQYF	85	CD8-C3
Pla-2_GAACCTAGTGGCGAAT	Pla	Pla-2	P2-13	TRUE	3	TRA:CAYDLLTGNQFYF TRB:CASVRQLNTEAFF	38	CD8-C3
Pla-2_GAACCTATCAATCTCT	Pla	Pla-2	P2-16	FALSE	3	TRA:CAVEMDSSYKLIF TRB:CASSQGRVTQYF	32	CD8-C3
Pla-2_GAAGCAGGTGTAAGTA	Pla	Pla-2	P2-20	TRUE	3	TRA:CAYDSNYQLIW TRB:CASSLSIDRADTGELFF	25	CD8-C3
Pla-2_GAATAAGAGTCAAGGC	Pla	Pla-2	P2-176	TRUE	3	TRA:CAMREPGANNLFF TRB:CASSLGGDGNTIYF	3	CD8-C3
Pla-2_GAATAAGCAGCGATCC	Pla	Pla-2	P2-354	TRUE	2	TRA:CALRQGARQLTF TRB:CALVGSSLETQYF	2	CD8-C3
Pla-2_GAATAAGCAGCTCGCA	Pla	Pla-2	P2-16	FALSE	3	TRA:CAVEMDSSYKLIF TRB:CASSQGRVTQYF	32	CD8-C3
Pla-2_GAATAAGTCCGGGTGT	Pla	Pla-2	P2-20	TRUE	3	TRA:CAYDSNYQLIW TRB:CASSLSIDRADTGELFF	25	CD8-C3
Pla-2_GACACGCCAAACGTGG	Pla	Pla-2	P2-125	TRUE	3	TRA:CAASEGNFGNEKLTF TRB:CASSQSGGPFREQYF	4	CD8-C3
Pla-2_GACAGAGAGCTGAACG	Pla	Pla-2	P2-201	TRUE	3	TRA:CAFRTDSNYQLIW TRB:CASSLASGNQETQYF	3	CD8-C3
Pla-2_GACCAATCAATAACGA	Pla	Pla-2	P2-87	TRUE	3	TRA:CAFMKPIGTDKLIF TRB:CASRLPGTGSRETQYF	6	CD8-C3
Pla-2_GACCAATTCGCTAGCG	Pla	Pla-2	P2-19	TRUE	3	TRA:CAVRDMENNRLAF TRB:CASSLGGGLTYEQYF	27	CD8-C3
Pla-2_GACCTGGCACGGACAA	Pla	Pla-2	P2-22	TRUE	3	TRA:CAMRERSWVRQKLVF TRB:CASSRGTSGTDTQYF	24	CD8-C3
Pla-2_GACGCGTAGTGTCCAT	Pla	Pla-2	P2-3	TRUE	3	TRA:CAVNPLESSYKLIF TRB:CASSPGTANTGELFF	177	CD8-C3
Pla-2_GACGCGTCATCCGTGG	Pla	Pla-2	P2-2	TRUE	3	TRA:CVVPNTNAGKSTF TRB:CASSLQGSTYNEQFF	220	CD8-C3
Pla-2_GACGGCTCAAGGACAC	Pla	Pla-2	P2-63	FALSE	3	TRA:CAASGEAGTALIF TRB:CATSREIQPNEKLFF	9	CD4-C4
Pla-2_GACGGCTGTCTGCGGT	Pla	Pla-2	P2-13	TRUE	3	TRA:CAYDLLTGNQFYF TRB:CASVRQLNTEAFF	38	CD8-C3
Pla-2_GACGTTAAGCAAATCA	Pla	Pla-2	P2-4348	FALSE	1	TRA:CAEVEPDYKLSF TRB:CASILGGNEQFF	1	CD8-C3
Pla-2_GACTAACGTTTGCATG	Pla	Pla-2	P2-20	TRUE	3	TRA:CAYDSNYQLIW TRB:CASSLSIDRADTGELFF	25	CD8-C3
Pla-2_GACTAACGTTTGGCGC	Pla	Pla-2	P2-2	TRUE	3	TRA:CVVPNTNAGKSTF TRB:CASSLQGSTYNEQFF	220	CD8-C3
Pla-2_GACTACAAGAACAATC	Pla	Pla-2	P2-4351	FALSE	1	TRA:CAVTSLNTGFQKLVF TRB:CSGQLSSFPDTQYF	1	CD8-C3

Pla-2_GACTGCGCAATGAAAC	Pla	Pla-2	P2-2	TRUE	3	TRA:CVVPNTNAGKSTF TRB:CASSLQGSTYNEQFF	220	CD8-C3
Pla-2_GACTGCGCACGAAGCA	Pla	Pla-2	P2-4363	FALSE	1	TRA:CAVLHLDSNYQLIW TRB:CASNRPMVGQGASTDTQYF	1	CD8-C3
Pla-2_GAGCAGATCGAACTGT	Pla	Pla-2	P2-196	TRUE	3	TRA:CAVYNAGNMLTF TRB:CASSSRQVYF	3	CD8-C3
Pla-2_GAGGTGAAGGGTTTCT	Pla	Pla-2	P2-6	TRUE	3	TRA:CVVSVAAGNKLTF TRB:CASSPGTDLYEQYF	85	CD8-C3
Pla-2_GAGTCCGGTTGTGGAG	Pla	Pla-2	P2-228	FALSE	3	TRA:CAVPPLTGGYQKVTF TRB:CASSGGGGGLETQYF	3	CD8-C3
Pla-2_GATCAGTCAAGGTGTG	Pla	Pla-2	P2-29	TRUE	3	TRA:CAVGGDFNKFYF TRB:CSARSDRLGYQPQHF	21	CD8-C3
Pla-2_GATCAGTGTCATTAGC	Pla	Pla-2	P2-2	TRUE	3	TRA:CVVPNTNAGKSTF TRB:CASSLQGSTYNEQFF	220	CD8-C3
Pla-2_GATCGCGCACCCATGG	Pla	Pla-2	P2-8	TRUE	3	TRA:CAALEGDNYGQNFVF TRB:CASSPTWGGVRDTQYF	53	CD8-C3
Pla-2_GATCGCGGTTCAGGCC	Pla	Pla-2	P2-2	TRUE	3	TRA:CVVPNTNAGKSTF TRB:CASSLQGSTYNEQFF	220	CD8-C3
Pla-2_GATCGTATCACGATGT	Pla	Pla-2	P2-4383	FALSE	1	TRA:CAVCHSSYKLIF TRB:CASSLGGTGRDLYEQYF	1	CD8-C3
Pla-2_GATCGTATCACTTCAT	Pla	Pla-2	P2-72	FALSE	3	TRA:CATDEETAGNKLTF TRB:CASSEAGWMGALHTEAFF	8	CD8-C3
Pla-2_GATGAAAAGCCAGTAG	Pla	Pla-2	P2-6	TRUE	3	TRA:CVVSVAAGNKLTF TRB:CASSPGTDLYEQYF	85	CD8-C3
Pla-2_GATGAGGGTCTAAAGA	Pla	Pla-2	P2-72	FALSE	3	TRA:CATDEETAGNKLTF TRB:CASSEAGWMGALHTEAFF	8	CD8-C3
Pla-2_GATGCTAAGTCTCGGC	Pla	Pla-2	P2-250	FALSE	3	TRA:CAENKEGSGGSNYKLTF TRB:CASSPPGTPVGRETQYF	3	CD8-C3
Pla-2_GCAAACTAGTGTCCCG	Pla	Pla-2	P2-10	TRUE	3	TRA:CAPLTPNDMRF TRB:CASLNGAGGSYEQYF	45	CD8-C3
Pla-2_GCAAACTCACGTCTCT	Pla	Pla-2	P2-52	FALSE	3	TRA:CARTTNAGKSTF TRB:CASSETSGRIYEQFF	11	CD8-C3
Pla-2_GCAATCACATCCGGGT	Pla	Pla-2	P2-4399	FALSE	1	TRA:CAMREDSGGYQKVTF TRB:CASSLWGSSTGNTEAFF	1	CD8-C3
Pla-2_GCACATAAGGAGTCTG	Pla	Pla-2	P2-10	TRUE	3	TRA:CAPLTPNDMRF TRB:CASLNGAGGSYEQYF	45	CD8-C5
Pla-2_GCACATACAACCGCCA	Pla	Pla-2	P2-2	TRUE	3	TRA:CVVPNTNAGKSTF TRB:CASSLQGSTYNEQFF	220	CD8-C3
Pla-2_GCACATACAAGACGTG	Pla	Pla-2	P2-4402	FALSE	1	TRA:CLVGDPPRYQLIW TRB:CASSYNLGLGRTYEQYF	1	CD8-C3
Pla-2_GCACATATCCACGACG	Pla	Pla-2	P2-2	TRUE	3	TRA:CVVPNTNAGKSTF TRB:CASSLQGSTYNEQFF	220	CD8-C3
Pla-2_GCACTCTAGTTCGCAT	Pla	Pla-2	P2-4404	FALSE	1	TRA:CAVRGNTPLVF TRB:CASSLQGSTYNEQFF	1	CD8-C3
Pla-2_GCACTCTCAGCTGCAC	Pla	Pla-2	P2-4405	FALSE	1	TRA:CAYRTPYSGGGADGLTF TRB:CASSDQPGAWSYEQYF	1	CD8-C3
Pla-2_GCACTCTCAGTCTTCC	Pla	Pla-2	P2-10	TRUE	3	TRA:CAPLTPNDMRF TRB:CASLNGAGGSYEQYF	45	CD8-C3
Pla-2_GCAGCCATCAAGCCTA	Pla	Pla-2	P2-100	TRUE	3	TRA:CAVGPRLLGNEKLTF TRB:CASSQDSTSVYNEQFF	5	CD8-C3
Pla-2_GCAGTTAAGTATTGGA	Pla	Pla-2	P2-4413	FALSE	1	TRA:CATWGGAQKLVF TRB:CASSLFAGQGDYEQYF	1	CD8-C3
Pla-2_GCAGTTAAGTGTTGAA	Pla	Pla-2	P2-35	FALSE	3	TRA:CGTETYTGFQKLVF TRB:CSVGTGSSYNEQFF	15	CD8-C3

Pla-2_GCATACACAAGGGTCA	Pla	Pla-2	P2-10	TRUE	3	TRA:CAPLTPNDMRF TRB:CASLNGAGGSYEQYF	45	CD8-C3
Pla-2_GCATGATGTCGAGTTT	Pla	Pla-2	P2-2	TRUE	3	TRA:CVVPNTNAGKSTF TRB:CASSLQGSTYNEQFF	220	CD8-C3
Pla-2_GCATGATGTTTAGCTG	Pla	Pla-2	P2-89	FALSE	3	TRA:CAASAGNTDKLIF TRB:CASSTGLAVSYEQYF	6	CD8-C8
Pla-2_GCATGATTCAACGGCC	Pla	Pla-2	P2-4422	FALSE	1	TRA:CAMGTYYGGATNKLIF TRB:CASSLVGGTQDYMNTEAFF	1	CD8-C3
Pla-2_GCATGTAAGACACGAC	Pla	Pla-2	P2-140	FALSE	3	TRA:CAVNSGANNLFF TRB:CSARDEEREGYNEQFF	4	CD8-C2
Pla-2_GCCAAATAGCTATGCT	Pla	Pla-2	P2-75	FALSE	3	TRA:CAVNDGRGADGLTF TRB:CASLGGRTDTQYF	8	CD8-C2
Pla-2_GCCAAATCAGCTGTTA	Pla	Pla-2	P2-91	FALSE	3	TRA:CAAYPSYNTDKLIF TRB:CASSPGGTVETQYF	6	CD8-C8
Pla-2_GCCAAATGTAGAGCTG	Pla	Pla-2	P2-68	TRUE	3	TRA:CAVYSGYSTLTF TRB:CSVEDVGGKSSYNEQFF	8	CD8-C3
Pla-2_GCCAAATTCTCACATT	Pla	Pla-2	P2-468	FALSE	2	TRA:CAASGNGAGSYQLTF TRB:CASSGLAGYAYEQYF	2	CD8-C3
Pla-2_GCCTCTACAAGTTGTC	Pla	Pla-2	P2-60	TRUE	3	TRA:CALSEGSGANSKLTF TRB:CASSSHSGLAGGFF	9	CD8-C8
Pla-2_GCCTCTAGTTTCGCTC	Pla	Pla-2	P2-2	TRUE	3	TRA:CVVPNTNAGKSTF TRB:CASSLQGSTYNEQFF	220	CD8-C3
Pla-2_GCCTCTATCTGTCAAG	Pla	Pla-2	P2-507	FALSE	2	TRA:CLVGVVNQAGTALIF TRB:CASSLVTGSQETQYF	2	CD8-C3
Pla-2_GCGACCACACGTCAGC	Pla	Pla-2	P2-10	TRUE	3	TRA:CAPLTPNDMRF TRB:CASLNGAGGSYEQYF	45	CD8-C3
Pla-2_GCGCAGTTCCAAGTAC	Pla	Pla-2	P2-16	FALSE	3	TRA:CAVEMDSSYKLIF TRB:CASSQGRVTQYF	32	CD8-C3
Pla-2_GCGCGATAGCACACAG	Pla	Pla-2	P2-111	TRUE	3	TRA:CAVYSGGSNYKLTF TRB:CASSLQGGGSGGLTQYF	5	CD8-C3
Pla-2_GCGCGATTCAAGATCC	Pla	Pla-2	P2-385	FALSE	2	TRA:CAVGAETGNQFYF TRB:CASSLYLSYNSPLHF	2	CD8-C3
Pla-2_GCGGGTTGTAGGGTAC	Pla	Pla-2	P2-2	TRUE	3	TRA:CVVPNTNAGKSTF TRB:CASSLQGSTYNEQFF	220	CD8-C3
Pla-2_GCTCTGTGTGCATCTA	Pla	Pla-2	P2-2	TRUE	3	TRA:CVVPNTNAGKSTF TRB:CASSLQGSTYNEQFF	220	CD8-C3
Pla-2_GCTCTGTTCCCTCTTT	Pla	Pla-2	P2-4466	FALSE	1	TRA:CAVEMDSSYKLIF TRB:CASSLGGGLTYEQYF	1	CD8-C3
Pla-2_GCTGCAGGTTGTCTTT	Pla	Pla-2	P2-22	TRUE	3	TRA:CAMRERSWVRQKLVF TRB:CASSRGTSGTDTQYF	24	CD8-C8
Pla-2_GCTGCTTAGACGCAAC	Pla	Pla-2	P2-4471	FALSE	1	TRA:CAMREMEGSYIPTF TRB:CASSLTGVGIYGYTF	1	CD8-C3
Pla-2_GCTGCTTGTACTCTCC	Pla	Pla-2	P2-87	TRUE	3	TRA:CAFMKPIGTDKLIF TRB:CASRLPGTGSRETQYF	6	CD8-C3
Pla-2_GCTTCCATCTGCAGTA	Pla	Pla-2	P2-16	FALSE	3	TRA:CAVEMDSSYKLIF TRB:CASSQGRVTQYF	32	CD8-C3
Pla-2_GGAAAGCAGAAGAAGC	Pla	Pla-2	P2-4489	FALSE	1	TRA:CAYDSNYQLIW TRB:CASSLDGMNTEAFF	1	CD8-C3
Pla-2_GGAAAGCAGTGCAAGC	Pla	Pla-2	P2-4	TRUE	3	TRA:CAIRYQSFNFGNEKLTF TRB:CASYRRVTEQYF	148	CD8-C3
Pla-2_GGAAAGCGTTCCAACA	Pla	Pla-2	P2-4490	FALSE	1	TRA:CAVTGNQFYF TRB:CSAEGQGATQYF	1	CD8-C5
Pla-2_GGAAAGCTCGTCCAGG	Pla	Pla-2	P2-4492	FALSE	1	TRA:CAASGGGTSYGKLTF TRB:CASSRSGAGVSEQYF	1	CD4-C4

Pla-2_GGAACTTAGGAGTAGA	Pla	Pla-2	P2-6	TRUE	3	TRA:CVVSVAAGNKLTF TRB:CASSPGTDLYEQYF	85	CD8-C3
Pla-2_GGAATAATCACGCGGT	Pla	Pla-2	P2-47	TRUE	3	TRA:CAASAGGNKLTF TRB:CASSTEDEQYF	11	CD8-C3
Pla-2_GGACAAGAGAGTACAT	Pla	Pla-2	P2-8	TRUE	3	TRA:CAALEGDNYGGQNFVF TRB:CASSPTWGGVRDTQYF	53	CD8-C3
Pla-2_GGACAAGCATTTCAGG	Pla	Pla-2	P2-2	TRUE	3	TRA:CVVPNTNAGKSTF TRB:CASSLQGSTYNEQFF	220	CD8-C3
Pla-2_GGACAAGGTCGCCATG	Pla	Pla-2	P2-2	TRUE	3	TRA:CVVPNTNAGKSTF TRB:CASSLQGSTYNEQFF	220	CD8-C3
Pla-2_GGACAAGGTGCGCTTG	Pla	Pla-2	P2-203	TRUE	3	TRA:CAFIGGQNFVF TRB:CASSQNRPQGMSNQPQHF	3	CD8-C3
Pla-2_GGACAGACAATCTGCA	Pla	Pla-2	P2-6	TRUE	3	TRA:CVVSVAAGNKLTF TRB:CASSPGTDLYEQYF	85	CD8-C3
Pla-2_GGACATTAGGTGCTAG	Pla	Pla-2	P2-10	TRUE	3	TRA:CAPLTPNDMRF TRB:CASLNGAGGSYEQYF	45	CD8-C3
Pla-2_GGACATTTCACTGGGC	Pla	Pla-2	P2-2	TRUE	3	TRA:CVVPNTNAGKSTF TRB:CASSLQGSTYNEQFF	220	CD8-C3
Pla-2_GGAGCAAGTGCACCAC	Pla	Pla-2	P2-81	FALSE	3	TRA:CAVRGNTPLVF TRB:CASSSNRDSYEQYF	7	CD8-C3
Pla-2_GGATGTTTCTTTAGTC	Pla	Pla-2	P2-4522	FALSE	1	TRA:CAGRLGAGGYQKVTF TRB:CSASWEDRAHEQYF	1	CD8-C3
Pla-2_GGATTACGTAAGAGGA	Pla	Pla-2	P2-2	TRUE	3	TRA:CVVPNTNAGKSTF TRB:CASSLQGSTYNEQFF	220	CD8-C3
Pla-2_GGATTACTCTGTCCGT	Pla	Pla-2	P2-100	TRUE	3	TRA:CAVGPRLLGNEKLTF TRB:CASSQDSTSVYNEQFF	5	CD8-C3
Pla-2_GGCAATTAGTCTTGCA	Pla	Pla-2	P2-4530	FALSE	1	TRA:CAMRERSWVGQKLVF TRB:CASSRGTSGTDTQYF	1	CD8-C3
Pla-2_GGCCGATAGATCCTGT	Pla	Pla-2	P2-42	FALSE	3	TRA:CAVSPRDNNDMRF TRB:CASSARGNSNQPQHF	12	CD8-C3
Pla-2_GGCGACTCATATGAGA	Pla	Pla-2	P2-10	TRUE	3	TRA:CAPLTPNDMRF TRB:CASLNGAGGSYEQYF	45	CD8-C3
Pla-2_GGCGTGTGTGACTCAT	Pla	Pla-2	P2-61	TRUE	3	TRA:CAVSFITQGGSEKLVF TRB:CASSRDRGTYNEQFF	9	CD8-C3
Pla-2_GGCGTGTTCCTAGAAC	Pla	Pla-2	P2-555	FALSE	2	TRA:CAMRAMDSSYKLIF TRB:CASSTTDRPSHYGYTF	2	CD8-C3
Pla-2_GGCTGGTTCGTTACAG	Pla	Pla-2	P2-2	TRUE	3	TRA:CVVPNTNAGKSTF TRB:CASSLQGSTYNEQFF	220	CD8-C5
Pla-2_GGGATGAAGAAGGTGA	Pla	Pla-2	P2-10	TRUE	3	TRA:CAPLTPNDMRF TRB:CASLNGAGGSYEQYF	45	CD8-C3
Pla-2_GGGCATCCATTCGACA	Pla	Pla-2	P2-6	TRUE	3	TRA:CVVSVAAGNKLTF TRB:CASSPGTDLYEQYF	85	CD8-C4
Pla-2_GGGTCTGCATCTACGA	Pla	Pla-2	P2-16	FALSE	3	TRA:CAVEMDSSYKLIF TRB:CASSQGRVTQYF	32	CD8-C3
Pla-2_GGGTCTGTCCGCATCT	Pla	Pla-2	P2-4567	FALSE	1	TRA:CAASLISGGGADGLTF TRB:CASSTSGTRNQPQHF	1	CD8-C3
Pla-2_GGGTTGCCAGCTTAAC	Pla	Pla-2	P2-4569	FALSE	1	TRA:CAASDRNQFYF TRB:CASSLRPSGGLGNEQFF	1	CD8-C5
Pla-2_GGTATTGGTGTGTGCC	Pla	Pla-2	P2-181	TRUE	3	TRA:CAVEWTDSSYKLIF TRB:CASRYGTTYEQYF	3	CD4-C4
Pla-2_GGTGCGTGTACCGAGA	Pla	Pla-2	P2-6	TRUE	3	TRA:CVVSVAAGNKLTF TRB:CASSPGTDLYEQYF	85	CD8-C4
Pla-2_GTAACGTGTCTCTTAT	Pla	Pla-2	P2-6	TRUE	3	TRA:CVVSVAAGNKLTF TRB:CASSPGTDLYEQYF	85	CD8-C3

Pla-2_GTAACTGTCACCTTAT	Pla	Pla-2	P2-4587	FALSE	1	TRA:CAVGDNFNKFYF TRB:CASSLQGSTYNEQFF	1	CD8-C3
Pla-2_GTACGTAGTCAATGTC	Pla	Pla-2	P2-88	TRUE	3	TRA:CAVPGAGNKLTF TRB:CASSRSLAGVDTGELFF	6	CD8-C3
Pla-2_GTATCTTCAGCCTATA	Pla	Pla-2	P2-3	TRUE	3	TRA:CAVNPLESSYKLIF TRB:CASSPGTANTGELFF	177	CD8-C2
Pla-2_GTATTCTCAACTGCTA	Pla	Pla-2	P2-69	TRUE	3	TRA:CAVRAGSARQLTF TRB:CATTGTSLDEQYF	8	CD8-C3
Pla-2_GTATTCTTCACATAGC	Pla	Pla-2	P2-30	TRUE	3	TRA:CAVGGDTGNQFYF TRB:CASSLFPTGVAYGYTF	20	CD8-C2
Pla-2_GTCAAGTGTATAGGGC	Pla	Pla-2	P2-20	TRUE	3	TRA:CAYDSNYQLIW TRB:CASSLSIDRADTGELFF	25	CD8-C3
Pla-2_GTCACAAAGTCTCCTC	Pla	Pla-2	P2-3	TRUE	3	TRA:CAVNPLESSYKLIF TRB:CASSPGTANTGELFF	177	CD8-C3
Pla-2_GTCATTTAGGATGGTC	Pla	Pla-2	P2-22	TRUE	3	TRA:CAMRERSWVRQKLVF TRB:CASSRGTSGTDTQYF	24	CD8-C4
Pla-2_GTCATTTGTGGTACAG	Pla	Pla-2	P2-15	TRUE	3	TRA:CAVGDNFNKFYF TRB:CASSRTSAAGELFF	35	CD8-C3
Pla-2_GTCCTCACAACTGCTA	Pla	Pla-2	P2-75	FALSE	3	TRA:CAVNDGRGADGLTF TRB:CASLGGRTDTQYF	8	CD8-C3
Pla-2_GTCGGGTCAGCGTCCA	Pla	Pla-2	P2-222	FALSE	3	TRA:CAASEAYSGGGADGLTF TRB:CASSLGGIYNEQFF	3	CD8-C3
Pla-2_GTCGGGTGTCTTTCAT	Pla	Pla-2	P2-22	TRUE	3	TRA:CAMRERSWVRQKLVF TRB:CASSRGTSGTDTQYF	24	CD8-C3
Pla-2_GTCGGGTGTGCTAGCC	Pla	Pla-2	P2-19	TRUE	3	TRA:CAVRDMENNRLAF TRB:CASSLGGGLTYEQYF	27	CD8-C3
Pla-2_GTCGGGTGTTCTGGTA	Pla	Pla-2	P2-2	TRUE	3	TRA:CVVPNTNAGKSTF TRB:CASSLQGSTYNEQFF	220	CD8-C3
Pla-2_GTCGTAAAGATATGCA	Pla	Pla-2	P2-6	TRUE	3	TRA:CVVSVAAGNKLTF TRB:CASSPGTDLYEQYF	85	CD8-C3
Pla-2_GTGAAGGCAACACCTA	Pla	Pla-2	P2-4630	FALSE	1	TRA:CAVNAGNARLMF TRB:CASSSRQVYF	1	CD8-C3
Pla-2_GTGCATAGTCGCATAT	Pla	Pla-2	P2-109	TRUE	3	TRA:CAPPFGGSEKLVF TRB:CASSKTGSSYEQYF	5	CD8-C3
Pla-2_GTGCATATCCCAGGTG	Pla	Pla-2	P2-8	TRUE	3	TRA:CAALEGDNYGQNFVF TRB:CASSPTWGGVRDTQYF	53	CD8-C3
Pla-2_GTGCGGTCATGGGAAC	Pla	Pla-2	P2-22	TRUE	3	TRA:CAMRERSWVRQKLVF TRB:CASSRGTSGTDTQYF	24	CD8-C3
Pla-2_GTGGGTCAGCTGTCTA	Pla	Pla-2	P2-19	TRUE	3	TRA:CAVRDMENNRLAF TRB:CASSLGGGLTYEQYF	27	CD8-C3
Pla-2_GTGTGCGGTGTTCTTT	Pla	Pla-2	P2-45	TRUE	3	TRA:CAVSGDSNYQLIW TRB:CASSLGGNGNTIYF	11	CD8-C3
Pla-2_GTTAAGCGTCTACCTC	Pla	Pla-2	P2-532	FALSE	2	TRA:CAAFSSGGSYIPTF TRB:CASTVGGTGDREQYF	2	CD8-C3
Pla-2_GTTACAGAGTCGATAA	Pla	Pla-2	P2-82	FALSE	3	TRA:CVADTGNQFYF TRB:CASSSALTGTYEQYF	7	CD8-C3
Pla-2_GTTACAGGTGCAACGA	Pla	Pla-2	P2-3	TRUE	3	TRA:CAVNPLESSYKLIF TRB:CASSPGTANTGELFF	177	CD8-C5
Pla-2_GTTCATTAGTTGTCGT	Pla	Pla-2	P2-8	TRUE	3	TRA:CAALEGDNYGQNFVF TRB:CASSPTWGGVRDTQYF	53	CD8-C3
Pla-2_GTTCATTGTGAGTGAC	Pla	Pla-2	P2-4663	FALSE	1	TRA:CAVSGGGFKTIF TRB:CASSYSAGGGSNSNQPQHF	1	CD8-C4
Pla-2_GTTCTCGAGAGTGAGA	Pla	Pla-2	P2-22	TRUE	3	TRA:CAMRERSWVRQKLVF TRB:CASSRGTSGTDTQYF	24	CD8-C3

Pla-2_GTTCTCGTCGCGATCG	Pla	Pla-2	P2-3	TRUE	3	TRA:CAVNPLESSYKLIF TRB:CASSPGTANTGELFF	177	CD8-C2
Pla-2_TAAACCGAGTGTTAGA	Pla	Pla-2	P2-2	TRUE	3	TRA:CVVPNTNAGKSTF TRB:CASSLQGSTYNEQFF	220	CD8-C3
Pla-2_TAAACCGCACGGCCAT	Pla	Pla-2	P2-245	FALSE	3	TRA:CKRDNNDMRF TRB:CASSSTGESYTF	3	CD8-C3
Pla-2_TAAACCGGTACTCAAC	Pla	Pla-2	P2-10	TRUE	3	TRA:CAPLTPNDMRF TRB:CASLNGAGGSYEQYF	45	CD8-C3
Pla-2_TAAGCGTAGGCCCTTG	Pla	Pla-2	P2-2	TRUE	3	TRA:CVVPNTNAGKSTF TRB:CASSLQGSTYNEQFF	220	CD8-C3
Pla-2_TAAGCGTCAGGGTTAG	Pla	Pla-2	P2-3	TRUE	3	TRA:CAVNPLESSYKLIF TRB:CASSPGTANTGELFF	177	CD8-C3
Pla-2_TAAGCGTCAGTGGAGT	Pla	Pla-2	P2-4672	FALSE	1	TRA:CAMREAGGSGAGSYQLTF TRB:CATRGTVGRAQETQYF	1	CD8-C3
Pla-2_TAAGCGTTCCGTCAAA	Pla	Pla-2	P2-22	TRUE	3	TRA:CAMRERSWVRQKLVF TRB:CASSRGTSGTDTQYF	24	CD8-C3
Pla-2_TAAGCGTTCTCGGACG	Pla	Pla-2	P2-3	TRUE	3	TRA:CAVNPLESSYKLIF TRB:CASSPGTANTGELFF	177	CD8-C3
Pla-2_TAAGTGCAGGCAAAGA	Pla	Pla-2	P2-2	TRUE	3	TRA:CVVPNTNAGKSTF TRB:CASSLQGSTYNEQFF	220	CD8-C3
Pla-2_TAAGTGCCAGTGGAGT	Pla	Pla-2	P2-91	FALSE	3	TRA:CAAYPSYNTDKLIF TRB:CASSPGGTVETQYF	6	CD8-C3
Pla-2_TAAGTGCGTTGGTAAA	Pla	Pla-2	P2-4678	FALSE	1	TRA:CAAHYYGGSQGNLIF TRB:CASSLSGGGYTDTQYF	1	CD8-C3
Pla-2_TACCTATTCTGCAGTA	Pla	Pla-2	P2-2	TRUE	3	TRA:CVVPNTNAGKSTF TRB:CASSLQGSTYNEQFF	220	CD8-C5
Pla-2_TACCTTAGTAGAAGGA	Pla	Pla-2	P2-58	TRUE	3	TRA:CAVNERSNDYKLSF TRB:CAISGGDSWGNQPQHF	9	CD8-C2
Pla-2_TACCTTAGTAGAGGAA	Pla	Pla-2	P2-55	TRUE	3	TRA:CIVRGIQGAQKLVF TRB:CASSLQGGGSGGLTQYF	10	CD8-C3
Pla-2_TACGGATGTGAGGCTA	Pla	Pla-2	P2-4692	FALSE	1	TRA:CAVEPYNNNDMRF TRB:CASTDDNRYGYTF	1	CD8-C3
Pla-2_TACGGATTCAAAGTAG	Pla	Pla-2	P2-58	TRUE	3	TRA:CAVNERSNDYKLSF TRB:CAISGGDSWGNQPQHF	9	CD8-C3
Pla-2_TACGGGCTCGAATGCT	Pla	Pla-2	P2-2	TRUE	3	TRA:CVVPNTNAGKSTF TRB:CASSLQGSTYNEQFF	220	CD8-C3
Pla-2_TACGGTACATGGAATA	Pla	Pla-2	P2-22	TRUE	3	TRA:CAMRERSWVRQKLVF TRB:CASSRGTSGTDTQYF	24	CD8-C3
Pla-2_TACGGTATCCACGTTC	Pla	Pla-2	P2-8	TRUE	3	TRA:CAALEGDNYGQNFVF TRB:CASSPTWGGVRDTQYF	53	CD8-C3
Pla-2_TACTCATAGGATATAC	Pla	Pla-2	P2-2	TRUE	3	TRA:CVVPNTNAGKSTF TRB:CASSLQGSTYNEQFF	220	CD8-C3
Pla-2_TACTTACTCAGTGTTG	Pla	Pla-2	P2-6	TRUE	3	TRA:CVVSVAAGNKLTF TRB:CASSPGTDLYEQYF	85	CD8-C3
Pla-2_TACTTGTGTAAACGCG	Pla	Pla-2	P2-3	TRUE	3	TRA:CAVNPLESSYKLIF TRB:CASSPGTANTGELFF	177	CD8-C3
Pla-2_TAGACCAAGCTAACAA	Pla	Pla-2	P2-4716	FALSE	1	TRA:CAMREIPFNFGNEKLTF TRB:CAISEGTAITDTQYF	1	CD8-C5
Pla-2_TAGACCAGTAAGGATT	Pla	Pla-2	P2-35	FALSE	3	TRA:CGTETYTGFQKLVF TRB:CSVGTGSSYNEQFF	15	CD8-C2
Pla-2_TAGAGCTCACAGGTTT	Pla	Pla-2	P2-3	TRUE	3	TRA:CAVNPLESSYKLIF TRB:CASSPGTANTGELFF	177	CD8-C3
Pla-2_TAGCCGGTCAGGATCT	Pla	Pla-2	P2-16	FALSE	3	TRA:CAVEMDSSYKLIF TRB:CASSQGRVTQYF	32	CD8-C3

Pla-2_TATCAGGGTAAACGCG	Pla	Pla-2	P2-33	TRUE	3	TRA:CAFKLSDGQKLLF TRB:CAWSRAALTEAFF	17	CD8-C3
Pla-2_TATCTCATCCGCGCAA	Pla	Pla-2	P2-2	TRUE	3	TRA:CVVPNTNAGKSTF TRB:CASSLQGSTYNEQFF	220	CD8-C8
Pla-2_TATTACCCATCACGAT	Pla	Pla-2	P2-86	TRUE	3	TRA:CAVTPLRNTDKLIF TRB:CSASPSGRGRGEQFF	6	CD8-C2
Pla-2_TCAATCTCAATCTGCA	Pla	Pla-2	P2-48	TRUE	3	TRA:CVVGTISGGYNKLIF TRB:CASSPITGDTQYF	11	CD8-C3
Pla-2_TCAATCTCACATTCGA	Pla	Pla-2	P2-52	FALSE	3	TRA:CARTTNAGKSTF TRB:CASSETSGRIYEQFF	11	CD8-C3
Pla-2_TCAATCTCATCCAACA	Pla	Pla-2	P2-19	TRUE	3	TRA:CAVRDMENNRLAF TRB:CASSLGGGLTYEQYF	27	CD8-C3
Pla-2_TCACAAGAGGCTACGA	Pla	Pla-2	P2-2	TRUE	3	TRA:CVVPNTNAGKSTF TRB:CASSLQGSTYNEQFF	220	CD8-C3
Pla-2_TCACAAGGTTGAACTC	Pla	Pla-2	P2-4754	FALSE	1	TRA:CAVPNSNSGYALNF TRB:CSARDPYFPGTGFSWEQYF	1	CD8-C3
Pla-2_TCACGAAAGTGCGATG	Pla	Pla-2	P2-16	FALSE	3	TRA:CAVEMDSSYKLIF TRB:CASSQGRVTQYF	32	CD8-C3
Pla-2_TCACGAACATGCCTTC	Pla	Pla-2	P2-10	TRUE	3	TRA:CAPLTPNDMRF TRB:CASLNGAGGSYEQYF	45	CD8-C8
Pla-2_TCACGAAGTTCCACTC	Pla	Pla-2	P2-52	FALSE	3	TRA:CARTTNAGKSTF TRB:CASSETSGRIYEQFF	11	CD8-C3
Pla-2_TCAGATGCACATCTTT	Pla	Pla-2	P2-32	TRUE	3	TRA:CAMREANVGADGLTF TRB:CASSTRIAGGPFPETQYF	17	CD4-C4
Pla-2_TCAGATGGTCTCACCT	Pla	Pla-2	P2-15	TRUE	3	TRA:CAVGDNFNKFYF TRB:CASSRTSAAGELFF	35	CD8-C3
Pla-2_TCAGCAAAGTCACGCC	Pla	Pla-2	P2-22	TRUE	3	TRA:CAMRERSWVRQKLVF TRB:CASSRGTSGTDTQYF	24	CD8-C3
Pla-2_TCAGCAATCAGCGACC	Pla	Pla-2	P2-6	TRUE	3	TRA:CVVSVAAGNKLTF TRB:CASSPGTDLYEQYF	85	CD8-C3
Pla-2_TCATTACTCAGCGACC	Pla	Pla-2	P2-81	FALSE	3	TRA:CAVRGNTPLVF TRB:CASSSNRDSYEQYF	7	CD8-C3
Pla-2_TCATTTGGTTGCGTTA	Pla	Pla-2	P2-374	TRUE	2	TRA:CALSEAFNTNAGKSTF TRB:CASSLKWTGGAYQPQHF	2	CD8-C3
Pla-2_TCCACACAGGCCGAAT	Pla	Pla-2	P2-3	TRUE	3	TRA:CAVNPLESSYKLIF TRB:CASSPGTANTGELFF	177	CD8-C3
Pla-2_TCCACACAGTGAAGTT	Pla	Pla-2	P2-56	FALSE	3	TRA:CATDLDGQKLLF TRB:CASRERGGNTGELFF	10	CD8-C3
Pla-2_TCCACACGTATTCGTG	Pla	Pla-2	P2-4786	FALSE	1	TRA:CAVYLGGNQFYF TRB:CASTQSLGYTF	1	CD8-C3
Pla-2_TCCACACTCTCTGAGA	Pla	Pla-2	P2-55	TRUE	3	TRA:CIVRGIQGAQKLVF TRB:CASSLQGGGSGGLTQYF	10	CD8-C8
Pla-2_TCCCGATCACACCGAC	Pla	Pla-2	P2-2	TRUE	3	TRA:CVVPNTNAGKSTF TRB:CASSLQGSTYNEQFF	220	CD8-C3
Pla-2_TCCCGATGTTACGCGC	Pla	Pla-2	P2-6	TRUE	3	TRA:CVVSVAAGNKLTF TRB:CASSPGTDLYEQYF	85	CD8-C8
Pla-2_TCGAGGCTCAGGCAAG	Pla	Pla-2	P2-4792	FALSE	1	TRA:CIVRGFSSGSARQLTF TRB:CSASLGQVFVGQFF	1	CD8-C3
Pla-2_TCGCGAGAGCACACAG	Pla	Pla-2	P2-158	FALSE	3	TRA:CAVGAVRETSGSRLTF TRB:CASSFTDLPYEQYF	4	CD8-C3
Pla-2_TCGCGAGAGTAGGTGC	Pla	Pla-2	P2-231	FALSE	3	TRA:CAVWYSGGGADGLTF TRB:CASSQESQREIYEQYF	3	CD4-C4
Pla-2_TCGCGAGCATTGGTAC	Pla	Pla-2	P2-2	TRUE	3	TRA:CVVPNTNAGKSTF TRB:CASSLQGSTYNEQFF	220	CD8-C3

Pla-2_TCGGGACAGGAGTACC	Pla	Pla-2	P2-2	TRUE	3	TRA:CVVPNTNAGKSTF TRB:CASSLQGSTYNEQFF	220	CD8-C3
Pla-2_TCGGTAATCTCGCATC	Pla	Pla-2	P2-3	TRUE	3	TRA:CAVNPLESSYKLIF TRB:CASSPGTANTGELFF	177	CD8-C3
Pla-2_TCGTAGATCAAACGGG	Pla	Pla-2	P2-15	TRUE	3	TRA:CAVGDNFNKFYF TRB:CASSRTSAAGELFF	35	CD8-C3
Pla-2_TCGTAGATCAGCACAT	Pla	Pla-2	P2-107	TRUE	3	TRA:CASGGYQKVTF TRB:CASSQDALAGSYEQYF	5	CD8-C3
Pla-2_TCTCATAGTGGTCTCG	Pla	Pla-2	P2-48	TRUE	3	TRA:CVVGTISGGYNKLIF TRB:CASSPITGDTQYF	11	CD8-C3
Pla-2_TCTGAGAAGCAACGGT	Pla	Pla-2	P2-4	TRUE	3	TRA:CAIRYQSFNFGNEKLTF TRB:CASYRRVTEQYF	148	CD8-C3
Pla-2_TCTGAGAAGCCAGTAG	Pla	Pla-2	P2-8	TRUE	3	TRA:CAALEGDNYGGQNFVF TRB:CASSPTWGGVRDTQYF	53	CD8-C3
Pla-2_TCTGAGAAGGTGCTAG	Pla	Pla-2	P2-78	TRUE	3	TRA:CAATTDSWGKFQF TRB:CASSSSASDYNEQFF	7	CD8-C3
Pla-2_TCTTTCCAGTCTCGGC	Pla	Pla-2	P2-2	TRUE	3	TRA:CVVPNTNAGKSTF TRB:CASSLQGSTYNEQFF	220	CD8-C3
Pla-2_TCTTTCCAGTTGAGTA	Pla	Pla-2	P2-6	TRUE	3	TRA:CVVSVAAGNKLTF TRB:CASSPGTDLYEQYF	85	CD8-C4
Pla-2_TCTTTCCCACGAGGTA	Pla	Pla-2	P2-4845	FALSE	1	TRA:CAVRSGTYKYIF TRB:CASTLTGKAFF	1	CD8-C3
Pla-2_TCTTTCCTCCTCTAGC	Pla	Pla-2	P2-233	FALSE	3	TRA:CAASIWNYGQNFVF TRB:CSAIERGGRETQYF	3	CD8-C3
Pla-2_TGACAACTCAAACAAG	Pla	Pla-2	P2-4848	FALSE	1	TRA:CAVRDRGNNRLAF TRB:CASSFDAGLQETQYF	1	CD8-C3
Pla-2_TGACGGCAGTGGTAAT	Pla	Pla-2	P2-200	TRUE	3	TRA:CAFNSKEVGANNLFF TRB:CATSRGRGREGYTF	3	CD8-C3
Pla-2_TGAGAGGAGAAACCAT	Pla	Pla-2	P2-19	TRUE	3	TRA:CAVRDMENNRLAF TRB:CASSLGGGLTYEQYF	27	CD8-C3
Pla-2_TGAGAGGCACTTGGAT	Pla	Pla-2	P2-106	TRUE	3	TRA:CAAFSGNTPLVF TRB:CASSTSGGMNEQFF	5	CD8-C2
Pla-2_TGAGCATTCTATCGCC	Pla	Pla-2	P2-75	FALSE	3	TRA:CAVNDGRGADGLTF TRB:CASLGGRTDTQYF	8	CD8-C2
Pla-2_TGAGCCGTCTTCATGT	Pla	Pla-2	P2-4868	FALSE	1	TRA:CALNRSGNNRKLIW TRB:CASSGPGLAAPEQFF	1	CD8-C3
Pla-2_TGCACCTCAGCTATTG	Pla	Pla-2	P2-111	TRUE	3	TRA:CAVYSGGSNYKLTF TRB:CASSLQGGGSGGLTQYF	5	CD8-C3
Pla-2_TGCCAAAAGACCGGAT	Pla	Pla-2	P2-2	TRUE	3	TRA:CVVPNTNAGKSTF TRB:CASSLQGSTYNEQFF	220	CD8-C3
Pla-2_TGCCCTAGTCCAGTAT	Pla	Pla-2	P2-81	FALSE	3	TRA:CAVRGNTPLVF TRB:CASSSNRDSYEQYF	7	CD8-C3
Pla-2_TGCGCAGCATCCAACA	Pla	Pla-2	P2-78	TRUE	3	TRA:CAATTDSWGKFQF TRB:CASSSSASDYNEQFF	7	CD8-C3
Pla-2_TGCGGGTTCCTATTCA	Pla	Pla-2	P2-203	TRUE	3	TRA:CAFIGGQNFVF TRB:CASSQNRPQGMSNQPQHF	3	CD8-C3
Pla-2_TGCTACCGTTGTCGCG	Pla	Pla-2	P2-141	FALSE	3	TRA:CAVRDRLENNAGNMLTF TRB:CASSKTGSSYEQYF	4	CD8-C3
Pla-2_TGCTGCTAGCTAAACA	Pla	Pla-2	P2-6	TRUE	3	TRA:CVVSVAAGNKLTF TRB:CASSPGTDLYEQYF	85	CD8-C3
Pla-2_TGCTGCTCAATCGAAA	Pla	Pla-2	P2-10	TRUE	3	TRA:CAPLTPNDMRF TRB:CASLNGAGGSYEQYF	45	CD8-C3
Pla-2_TGCTGCTCAGCTTCGG	Pla	Pla-2	P2-91	FALSE	3	TRA:CAAYPSYNTDKLIF TRB:CASSPGGTVETQYF	6	CD8-C3

Pla-2_TGCTGCTGTCGAGTTT	Pla	Pla-2	P2-6	TRUE	3	TRA:CVVSVAAGNKLTF TRB:CASSPGTDLYEQYF	85	CD8-C3
Pla-2_TGGACGCAGAAACCTA	Pla	Pla-2	P2-4904	FALSE	1	TRA:CAFADPRGSNYKLTF TRB:CASSSRRNTGELFF	1	CD8-C3
Pla-2_TGGACGCCAGATGGGT	Pla	Pla-2	P2-2	TRUE	3	TRA:CVVPNTNAGKSTF TRB:CASSLQGSTYNEQFF	220	CD8-C3
Pla-2_TGGACGCGTCACTGGC	Pla	Pla-2	P2-2	TRUE	3	TRA:CVVPNTNAGKSTF TRB:CASSLQGSTYNEQFF	220	CD8-C3
Pla-2_TGGCGCACAGACAGGT	Pla	Pla-2	P2-3	TRUE	3	TRA:CAVNPLESSYKLIF TRB:CASSPGTANTGELFF	177	CD8-C3
Pla-2_TGGCTGGCACAGTCGC	Pla	Pla-2	P2-102	TRUE	3	TRA:CAVIYGSGNTGKLIF TRB:CASSLVNTIYF	5	CD4-C4
Pla-2_TGGCTGGTCGATAGAA	Pla	Pla-2	P2-55	TRUE	3	TRA:CIVRGIQGAQKLVF TRB:CASSLQGGGSGGLTQYF	10	CD8-C3
Pla-2_TGGCTGGTCGGCCGAT	Pla	Pla-2	P2-15	TRUE	3	TRA:CAVGDNFNKFYF TRB:CASSRTSAAGELFF	35	CD8-C3
Pla-2_TGGGAAGGTCAGATAA	Pla	Pla-2	P2-2	TRUE	3	TRA:CVVPNTNAGKSTF TRB:CASSLQGSTYNEQFF	220	CD8-C3
Pla-2_TGGGAAGTCAACGCTA	Pla	Pla-2	P2-10	TRUE	3	TRA:CAPLTPNDMRF TRB:CASLNGAGGSYEQYF	45	CD8-C3
Pla-2_TGGGCGTGTGCAACTT	Pla	Pla-2	P2-47	TRUE	3	TRA:CAASAGGNKLTF TRB:CASSTEDEQYF	11	CD8-C3
Pla-2_TGGGCGTTCCTATTCA	Pla	Pla-2	P2-16	FALSE	3	TRA:CAVEMDSSYKLIF TRB:CASSQGRVTQYF	32	CD8-C3
Pla-2_TGGTTAGGTAGCTTGT	Pla	Pla-2	P2-522	FALSE	2	TRA:CALSAAGGTSYGKLTF TRB:CASKLSGTSGSYEQYF	2	CD8-C3
Pla-2_TGGTTAGGTATGAAAC	Pla	Pla-2	P2-4934	FALSE	1	TRA:CAVRIQGAQKLVF TRB:CASSLTGNMNTEAFF	1	CD8-C2
Pla-2_TGGTTAGTCCAGTAGT	Pla	Pla-2	P2-4938	FALSE	1	TRA:CAASAGGNKLVF TRB:CASSGGTDTQYF	1	CD8-C3
Pla-2_TGGTTCCAGAATTGTG	Pla	Pla-2	P2-141	FALSE	3	TRA:CAVRDRLENNAGNMLTF TRB:CASSKTGSSYEQYF	4	CD8-C3
Pla-2_TGTATTCAGCCAGGAT	Pla	Pla-2	P2-32	TRUE	3	TRA:CAMREANVGADGLTF TRB:CASSTRIAGGPFPETQYF	17	CD4-C4
Pla-2_TGTATTCGTGCGATAG	Pla	Pla-2	P2-458	FALSE	2	TRA:CAVRDQYTDKLIF TRB:CASSLSPRQGARVEQYF	2	CD8-C3
Pla-2_TGTATTCTCACTATTC	Pla	Pla-2	P2-4944	FALSE	1	TRA:CAAIVWGTGANNLFF TRB:CASSLQGSTYNEQFF	1	CD8-C3
Pla-2_TGTATTCTCGACCAGC	Pla	Pla-2	P2-19	TRUE	3	TRA:CAVRDMENNRLAF TRB:CASSLGGGLTYEQYF	27	CD8-C3
Pla-2_TGTCCCATCTCTTGAT	Pla	Pla-2	P2-400	FALSE	2	TRA:CAVRVDSSYKLIF TRB:CAWSRSTGELFF	2	CD8-C3
Pla-2_TGTGGTATCTCTGAGA	Pla	Pla-2	P2-125	TRUE	3	TRA:CAASEGNFGNEKLTF TRB:CASSQSGGPFREQYF	4	CD8-C3
Pla-2_TGTTCCGCAGCGTTCG	Pla	Pla-2	P2-555	FALSE	2	TRA:CAMRAMDSSYKLIF TRB:CASSTTDRPSHYGYTF	2	CD8-C3
Pla-2_TTAGGACAGAAACCGC	Pla	Pla-2	P2-35	FALSE	3	TRA:CGTETYTGFQKLVF TRB:CSVGTGSSYNEQFF	15	CD8-C3
Pla-2_TTAGGACTCTGCTTGC	Pla	Pla-2	P2-12	TRUE	3	TRA:CGADAEYGNKLVF TRB:CASSEGQGAIMQPQHF	42	CD8-C3
Pla-2_TTAGGCAAGCGTTCCG	Pla	Pla-2	P2-562	FALSE	2	TRA:CALRAGSARQLTF TRB:CASTGTSLDEQFF	2	CD8-C3
Pla-2_TTAGTTCAGGACAGCT	Pla	Pla-2	P2-2	TRUE	3	TRA:CVVPNTNAGKSTF TRB:CASSLQGSTYNEQFF	220	CD8-C3

Pla-2_TTAGTTCCACCTGGTG	Pla	Pla-2	P2-16	FALSE	3	TRA:CAVEMDSSYKLIF TRB:CASSQGRVTQYF	32	CD8-C3
Pla-2_TTAGTTCGTCGTCTTC	Pla	Pla-2	P2-48	TRUE	3	TRA:CVVGTISGGYNKLIF TRB:CASSPITGDTQYF	11	CD8-C3
Pla-2_TTATGCTCACCGGAAA	Pla	Pla-2	P2-83	FALSE	3	TRA:CAVDTGTASKLTF TRB:CASRGSTDTQYF	7	CD8-C2
Pla-2_TTATGCTTCAGCTGGC	Pla	Pla-2	P2-2	TRUE	3	TRA:CVVPNTNAGKSTF TRB:CASSLQGSTYNEQFF	220	CD8-C3
Pla-2_TTCCCAGAGAAGATTC	Pla	Pla-2	P2-2	TRUE	3	TRA:CVVPNTNAGKSTF TRB:CASSLQGSTYNEQFF	220	CD8-C3
Pla-2_TTCCCAGGTAGGAGTC	Pla	Pla-2	P2-111	TRUE	3	TRA:CAVYSGGSNYKLTF TRB:CASSLQGGGSGGLTQYF	5	CD8-C3
Pla-2_TTCGAAGCATACTACG	Pla	Pla-2	P2-209	FALSE	3	TRA:CATGTHQAGTALIF TRB:CSAEEETQYF	3	CD8-C3
Pla-2_TTCGGTCTCGTATCAG	Pla	Pla-2	P2-10	TRUE	3	TRA:CAPLTPNDMRF TRB:CASLNGAGGSYEQYF	45	CD8-C8
Pla-2_TTCTACACAATCTGCA	Pla	Pla-2	P2-533	FALSE	2	TRA:CAASVWGSARQLTF TRB:CASSQESGGLVFEQFF	2	CD8-C3
Pla-2_TTCTCAACAAATCCGT	Pla	Pla-2	P2-15	TRUE	3	TRA:CAVGDNFNKFYF TRB:CASSRTSAAGELFF	35	CD8-C3
Pla-2_TTCTCAATCCTCATTA	Pla	Pla-2	P2-157	FALSE	3	TRA:CAVKSGNTGKLIF TRB:CASSDRNTGELFF	4	CD8-C3
Pla-2_TTCTCCTCAGATGGCA	Pla	Pla-2	P2-4991	FALSE	1	TRA:CAVTRGRGSNYKLTF TRB:CASSVARAYNEQFF	1	CD8-C3
Pla-2_TTCTTAGTCAATCTCT	Pla	Pla-2	P2-78	TRUE	3	TRA:CAATTDSWGKFQF TRB:CASSSSASDYNEQFF	7	CD8-C3
Pla-2_TTGAACGCACAACGTT	Pla	Pla-2	P2-5000	FALSE	1	TRA:CAENGVGAAGNKLTF TRB:CASSGDSSGETQYF	1	CD8-C8
Pla-2_TTGACTTAGTGACATA	Pla	Pla-2	P2-6	TRUE	3	TRA:CVVSVAAGNKLTF TRB:CASSPGTDLYEQYF	85	CD8-C3
Pla-2_TTGCGTCTCCAAATGC	Pla	Pla-2	P2-10	TRUE	3	TRA:CAPLTPNDMRF TRB:CASLNGAGGSYEQYF	45	CD8-C3
Pla-2_TTGGAACCAAGTCATC	Pla	Pla-2	P2-47	TRUE	3	TRA:CAASAGGNKLTF TRB:CASSTEDEQYF	11	CD8-C3
Pla-2_TTGGAACCAGTGAGTG	Pla	Pla-2	P2-153	FALSE	3	TRA:CAYNNNDMRF TRB:CASSDRGGRQETQYF	4	CD8-C2
Pla-2_TTGGAACTCTGGTTCC	Pla	Pla-2	P2-10	TRUE	3	TRA:CAPLTPNDMRF TRB:CASLNGAGGSYEQYF	45	CD8-C3
Pla-2_TTGGCAACACCGTTGG	Pla	Pla-2	P2-6	TRUE	3	TRA:CVVSVAAGNKLTF TRB:CASSPGTDLYEQYF	85	CD8-C3
Pla-2_TTTACTGTCTGTCCGT	Pla	Pla-2	P2-6	TRUE	3	TRA:CVVSVAAGNKLTF TRB:CASSPGTDLYEQYF	85	CD8-C3
Pla-2_TTTATGCTCCTAGGGC	Pla	Pla-2	P2-5029	FALSE	1	TRA:CAFMTVNQAGTALIF TRB:CASSVGTPGTDTQYF	1	CD8-C3
Pla-2_TTTATGCTCCTCGCAT	Pla	Pla-2	P2-157	FALSE	3	TRA:CAVKSGNTGKLIF TRB:CASSDRNTGELFF	4	CD8-C3
Pla-2_TTTCCTCTCATAACCG	Pla	Pla-2	P2-81	FALSE	3	TRA:CAVRGNTPLVF TRB:CASSSNRDSYEQYF	7	CD8-C3
Pla-2_TTTCCTCTCGGAGGTA	Pla	Pla-2	P2-481	FALSE	2	TRA:CALSEYTGGFKTIF TRB:CSAPKPGQGLNTEAFF	2	CD8-C3
Pla-2_TTTGCGCGTTATCCGA	Pla	Pla-2	P2-30	TRUE	3	TRA:CAVGGDTGNQFYF TRB:CASSLFPTGVAYGYTF	20	CD8-C3
Pla-2_TTTGCGCTCGGTTCGG	Pla	Pla-2	P2-2	TRUE	3	TRA:CVVPNTNAGKSTF TRB:CASSLQGSTYNEQFF	220	CD8-C3

Pla-2_TTTGTCACAGGTGCCT	Pla	Pla-2	P2-5038	FALSE	1	TRA:CAVRDMENNRLAF TRB:CASLNGAGGSYEQYF	1	CD8-C3
Pla-2_TTTGTCATCCGTCATC	Pla	Pla-2	P2-565	FALSE	2	TRA:CAVEDRTGANNLFF TRB:CATSDLNSSYNEQFF	2	CD8-C3
Pla-2_AAACCTGGTCTGATTG	Pla	Pla-2	P2-3412	FALSE	1	TRA:CAVNGGNNRLAF TRB:CASSPTVLADTQYF	1	CD4-C4
Pla-2_AAACCTGTCTCTGAGA	Pla	Pla-2	P2-3413	FALSE	1	TRA:CAVKTSGSRLTF TRB:CASSLAAGAYGEQYF	1	CD4-C4
Pla-2_AAAGATGCATTAACCG	Pla	Pla-2	P2-124	TRUE	3	TRA:CAFFNTDKLIF TRB:CASSPRPNYGYTF	4	CD4-C4
Pla-2_AAATGCCCATCGGGTC	Pla	Pla-2	P2-3424	FALSE	1	TRA:CAADLNNNDMRF TRB:CATSRGTGNQPQHF	1	CD4-C4
Pla-2_AACACGTTCATCGCTC	Pla	Pla-2	P2-379	FALSE	2	TRA:CAVRDQTNTDKLIF TRB:CASSSGGGIQFF	2	CD4-C4
Pla-2_AACACGTTCCTTTCGG	Pla	Pla-2	P2-271	TRUE	2	TRA:CALSTPNTDKLIF TRB:CASSRGQGLTDTQYF	2	CD4-C4
Pla-2_AACCATGTCAGCTTAG	Pla	Pla-2	P2-3432	FALSE	1	TRA:CVVNNNARLMF TRB:CASSLMASGGKVLGGGEQYF	1	CD4-C7
Pla-2_AACCATGTCCCAACGG	Pla	Pla-2	P2-3433	FALSE	1	TRA:CAVGTTGNQFYF TRB:CASRRTGPTYEQYF	1	CD4-C4
Pla-2_AACCGCGAGGGCTTGA	Pla	Pla-2	P2-72	FALSE	3	TRA:CATDEETAGNKLTF TRB:CASSEAGWMGALHTEAFF	8	CD4-C4
Pla-2_AACCGCGCATTCTCAT	Pla	Pla-2	P2-3435	FALSE	1	TRA:CALGSGNTGKLIF TRB:CASSLGYDRAGGLAGDPAYEQYF	1	CD4-C4
Pla-2_AACGTTGGTGATGATA	Pla	Pla-2	P2-382	FALSE	2	TRA:CAATRYSGGGADGLTF TRB:CASSPGTGTQVRNTGELFF	2	CD4-C4
Pla-2_AACGTTGGTTGGGACA	Pla	Pla-2	P2-3441	FALSE	1	TRA:CAVRAIQTGANNLFF TRB:CASSFTGDPGANVLTF	1	CD4-C4
Pla-2_AACTCAGAGATGGGTC	Pla	Pla-2	P2-3443	FALSE	1	TRA:CAVEGPGGTDKLIF TRB:CASSLGQDQYEQYF	1	CD4-C4
Pla-2_AACTCAGCACCGAATT	Pla	Pla-2	P2-3444	FALSE	1	TRA:CAFIDSGGYQKVTF TRB:CASGNPRQGHEQYF	1	CD4-C4
Pla-2_AACTCAGGTGTATGGG	Pla	Pla-2	P2-3446	FALSE	1	TRA:CATDARNTGTASKLTF TRB:CASPPRGGRWAEAFF	1	CD4-C4
Pla-2_AACTCCCGTATTCTCT	Pla	Pla-2	P2-3447	FALSE	1	TRA:CACYNAGNMLTF TRB:CASSGTTSEETQYF	1	CD8-C11
Pla-2_AACTCCCTCGTCTGCT	Pla	Pla-2	P2-3448	FALSE	1	TRA:CAVNNARLMF TRB:CSASEATPGANVLTF	1	CD4-C4
Pla-2_AACTCTTGTAGGACAC	Pla	Pla-2	P2-211	FALSE	3	TRA:CAESNSGAGSYQLTF TRB:CASRQQGAREKLFF	3	CD4-C4
Pla-2_AACTTTCGTCCATCCT	Pla	Pla-2	P2-42	FALSE	3	TRA:CAVSPRDNNDMRF TRB:CASSARGNSNQPQHF	12	CD4-C4
Pla-2_AACTTTCGTGTGTGCC	Pla	Pla-2	P2-3454	FALSE	1	TRA:CAVNAGSGNTGKLIF TRB:CASSPRGHGDIQYF	1	CD4-C4
Pla-2_AAGGAGCCAGGGCATA	Pla	Pla-2	P2-387	FALSE	2	TRA:CAVWSDGQKLLF TRB:CASSIMGQSNQPQHF	2	CD4-C4
Pla-2_AAGGCAGAGAGTAAGG	Pla	Pla-2	P2-3460	FALSE	1	TRA:CAVGYNTNAGKSTF TRB:CASSFAGGTEAFF	1	CD4-C4
Pla-2_AAGGCAGCAGATCTGT	Pla	Pla-2	P2-3462	FALSE	1	TRA:CALPGFGNVLHC TRB:CATNPTGAGGELFF	1	CD4-C2
Pla-2_AAGGCAGTCCACGTTC	Pla	Pla-2	P2-3465	FALSE	1	TRA:CAAHWGNTGFQKLVF TRB:CASSLYGDRIFNEQFF	1	CD8-C8
Pla-2_AAGGCAGTCTTGCCGT	Pla	Pla-2	P2-3467	FALSE	1	TRA:CAVEDPGSQGNLIF TRB:CASRVGTGVHEQYF	1	CD4-C4

Pla-2_AAGGTTCGTACCGCTG	Pla	Pla-2	P2-214	FALSE	3	TRA:CAASPMFSGGYNKLIF TRB:CASSLRTGEQFF	3	CD4-C4
Pla-2_AATCCAGAGGACATTA	Pla	Pla-2	P2-3478	FALSE	1	TRA:CILRAGDTDKLIF TRB:CASSYQRGPYNEQFF	1	CD8-C8
Pla-2_AATCCAGTCGCAAACT	Pla	Pla-2	P2-3479	FALSE	1	TRA:CAEGETGANNLFF TRB:CASSQEWTEQETQYF	1	CD4-C4
Pla-2_ACACCAACACCAGATT	Pla	Pla-2	P2-3483	FALSE	1	TRA:CAYRSGEYNTDKLIF TRB:CATRQDGAQSPLHF	1	CD4-C4
Pla-2_ACACCAATCCCTTGCA	Pla	Pla-2	P2-3485	FALSE	1	TRA:CIVKSSQGGKLIF TRB:CASSGPSGGANEQFF	1	CD4-C4
Pla-2_ACACCCTAGTTGAGTA	Pla	Pla-2	P2-3488	FALSE	1	TRA:CAVQSWGNSGYSTLTF TRB:CATSGSDQSVQARETQYF	1	CD4-C4
Pla-2_ACACCCTCATTAGGCT	Pla	Pla-2	P2-3489	FALSE	1	TRA:CAVRDVINAGNNRKLIW TRB:CASSSGRGELFF	1	CD4-C4
Pla-2_ACACCGGCACATCCAA	Pla	Pla-2	P2-3491	FALSE	1	TRA:CAVQESAYNTNAGKSTF TRB:CASSVDSSYEQFF	1	CD4-C5
Pla-2_ACACCGGTCTCTTGAT	Pla	Pla-2	P2-138	FALSE	3	TRA:CAARNTGTASKLTF TRB:CASSLDGMNTEAFF	4	CD8-C8
Pla-2_ACACTGAAGCGTTGCC	Pla	Pla-2	P2-39	TRUE	3	TRA:CAARAVTGGGNKLTF TRB:CASSGRSSAWNEQFF	13	CD4-C4
Pla-2_ACACTGACATCACAAC	Pla	Pla-2	P2-396	FALSE	2	TRA:CAVSGGGADGLTF TRB:CASSFQWTERGGYTF	2	CD4-C4
Pla-2_ACAGCCGCACTGCCAG	Pla	Pla-2	P2-3499	FALSE	1	TRA:CAASKETSGSRLTF TRB:CASSPYVGTEAFF	1	CD4-C4
Pla-2_ACATACGAGCTAGTGG	Pla	Pla-2	P2-3502	FALSE	1	TRA:CAVREGAGSYQLTF TRB:CASSDRTAEQFF	1	CD4-C4
Pla-2_ACATCAGAGAGCTTCT	Pla	Pla-2	P2-42	FALSE	3	TRA:CAVSPRDNNDMRF TRB:CASSARGNSNQPQHF	12	CD4-C4
Pla-2_ACATCAGGTACTCTCC	Pla	Pla-2	P2-217	FALSE	3	TRA:CAIRDNQGGKLIF TRB:CASTGQLTDTQYF	3	CD4-C4
Pla-2_ACATGGTGTAATCACC	Pla	Pla-2	P2-3513	FALSE	1	TRA:CAVRASGGSYIPTF TRB:CASSLAPLAGVGGELFF	1	CD4-C4
Pla-2_ACCAGTAAGCAGCGTA	Pla	Pla-2	P2-3519	FALSE	1	TRA:CAVSPSGGSNYKLTF TRB:CASSPLGERVTDTQYF	1	CD4-C4
Pla-2_ACCAGTAAGTCCGGTC	Pla	Pla-2	P2-398	FALSE	2	TRA:CAITGTASKLTF TRB:CASSLWTGPSSYEQYF	2	CD4-C4
Pla-2_ACCAGTACAGAGCCAA	Pla	Pla-2	P2-396	FALSE	2	TRA:CAVSGGGADGLTF TRB:CASSFQWTERGGYTF	2	CD4-C4
Pla-2_ACCAGTATCTGTCAAG	Pla	Pla-2	P2-3522	FALSE	1	TRA:CAVRGWGGSNYKLTF TRB:CAISEGTSGRSYNEQFF	1	CD4-C4
Pla-2_ACCGTAAAGGGTGTTG	Pla	Pla-2	P2-40	TRUE	3	TRA:CALRETSGSRLTF TRB:CASSPGRYEQYF	12	CD4-C4
Pla-2_ACCTTTAAGTCACGCC	Pla	Pla-2	P2-3529	FALSE	1	TRA:CAMRVRENYGQNFVF TRB:CASSRTSAAGELFF	1	CD4-C4
Pla-2_ACCTTTATCTTGCCGT	Pla	Pla-2	P2-3535	FALSE	1	TRA:CAASKDYGQNFVF TRB:CASTEPGLTSGGTGELFF	1	CD4-C4
Pla-2_ACGAGCCAGGGCTTGA	Pla	Pla-2	P2-386	FALSE	2	TRA:CAVRVEDKIIF TRB:CAWRTPGQGSGKNTIYF	2	CD4-C4
Pla-2_ACGAGCCTCGTAGATC	Pla	Pla-2	P2-401	FALSE	2	TRA:CAVNPSGGGADGLTF TRB:CAISPRQLYSNQPQHF	2	CD4-C4
Pla-2_ACGAGGATCGCTTAGA	Pla	Pla-2	P2-219	FALSE	3	TRA:CAVEVPGSQGNLIF TRB:CASRAGTAVYEQYF	3	CD4-C4
Pla-2_ACGATGTCATCGATTG	Pla	Pla-2	P2-402	FALSE	2	TRA:CALSDILTGGGNKLTF TRB:CATSRGGQANSPLHF	2	CD8-C8

Pla-2_ACGATGTTCAGAGCTT	Pla	Pla-2	P2-3551	FALSE	1	TRA:CAMRGVNFGNEKLTF TRB:CASSTSDTRIYEQYF	1	CD4-C4
Pla-2_ACGCAGCGTTGTACAC	Pla	Pla-2	P2-3557	FALSE	1	TRA:CAVRPGTYKYIF TRB:CASSQSAGGSTDTQYF	1	CD4-C4
Pla-2_ACGCCAGAGTTGCAGG	Pla	Pla-2	P2-405	FALSE	2	TRA:CAYRKTSYDKVIF TRB:CASSILRGELFF	2	CD4-C4
Pla-2_ACGCCAGCATGCTAGT	Pla	Pla-2	P2-220	FALSE	3	TRA:CAMRVPLGGYGQNFVF TRB:CATRDFQETQYF	3	CD4-C4
Pla-2_ACGCCAGCATGGTAGG	Pla	Pla-2	P2-221	FALSE	3	TRA:CASSGNTGKLIF TRB:CASSTRPGGTDTQYF	3	CD4-C4
Pla-2_ACGCCGACATGAACCT	Pla	Pla-2	P2-3564	FALSE	1	TRA:CAMREGLGTGTASKLTF TRB:CSARDRNTPISGNTIYF	1	CD4-C4
Pla-2_ACGCCGAGTGACTCAT	Pla	Pla-2	P2-3567	FALSE	1	TRA:CAVSVGDTGRRALTF TRB:CSASPGLAGKEQFF	1	CD4-C4
Pla-2_ACGGAGAAGGCAGGTT	Pla	Pla-2	P2-64	FALSE	3	TRA:CAATSLISDGQKLLF TRB:CSARDSFNYGYTF	9	CD4-C4
Pla-2_ACGGCCAGTAGTAGTA	Pla	Pla-2	P2-353	TRUE	2	TRA:CAVSTDKLIF TRB:CASSLAYLGGGANVLTF	2	CD8-C3
Pla-2_ACGGGCTTCCACGACG	Pla	Pla-2	P2-1	TRUE	3	TRA:CVVNIIGYQKVTF TRB:CASTRQGKQPQHF	270	CD4-C4
Pla-2_ACGGGTCAGGGTCTCC	Pla	Pla-2	P2-3577	FALSE	1	TRA:CAVQARGGTSYGKLTF TRB:CASSQDPGLQQFF	1	CD4-C4
Pla-2_ACGTCAAAGACTAGGC	Pla	Pla-2	P2-33	TRUE	3	TRA:CAFKLSDGQKLLF TRB:CAWSRAALTEAFF	17	CD4-C4
Pla-2_ACTATCTAGCGTGAGT	Pla	Pla-2	P2-3578	FALSE	1	TRA:CGRGSNYKLTF TRB:CASSLWLAGGLTDTQYF	1	CD4-C4
Pla-2_ACTATCTTCCCATTTA	Pla	Pla-2	P2-28	TRUE	3	TRA:CATDANTGNQFYF TRB:CASSLGQWVEQYF	22	CD4-C4
Pla-2_ACTGAACTCCATGCTC	Pla	Pla-2	P2-3581	FALSE	1	TRA:CAVGSGSARQLTF TRB:CASSHSDRDFYQPQHF	1	CD4-C4
Pla-2_ACTGAGTCAAGGTGTG	Pla	Pla-2	P2-3587	FALSE	1	TRA:CAVVSFGNEKLTF TRB:CASSMTSGSGEQFF	1	CD4-C4
Pla-2_ACTGATGGTACTTGAC	Pla	Pla-2	P2-225	FALSE	3	TRA:CAVRDVGGRRALTF TRB:CASSNSGGTEAFF	3	CD4-C4
Pla-2_ACTGTCCCAGCTGTAT	Pla	Pla-2	P2-371	TRUE	2	TRA:CLVGNQGGKLIF TRB:CASTPGRESDIQYF	2	CD8-C8
Pla-2_ACTGTCCGTGACCAAG	Pla	Pla-2	P2-3598	FALSE	1	TRA:CAVRRIGMIYNQGGKLIF TRB:CASASPSVWDHNEQFF	1	CD4-C4
Pla-2_ACTTTCAAGTGAATTG	Pla	Pla-2	P2-3604	FALSE	1	TRA:CAASYKAAGNKLTF TRB:CASSLWGQGGNEQFF	1	CD4-C3
Pla-2_ACTTTCACACTTGGAT	Pla	Pla-2	P2-3606	FALSE	1	TRA:CPRPLNSNSGYALNF TRB:CASRANRDPAYEQYF	1	CD4-C4
Pla-2_ACTTTCAGTTGTCTTT	Pla	Pla-2	P2-50	FALSE	3	TRA:CAPIRAQKLVF TRB:CASSSPPKSGELFF	11	CD4-C4
Pla-2_ACTTTCATCAATACCG	Pla	Pla-2	P2-3608	FALSE	1	TRA:CILRDREKTGGFKTIF TRB:CASSVGHTHEQYF	1	CD4-C4
Pla-2_AGAATAGCAAATACAG	Pla	Pla-2	P2-36	FALSE	3	TRA:CAAQAGNTPLVF TRB:CSAGVGGTIQYF	15	CD4-C4
Pla-2_AGAGCGAGTAGGAGTC	Pla	Pla-2	P2-3616	FALSE	1	TRA:CAMILPQGGSEKLVF TRB:CASSRGQNQPQHF	1	CD4-C4
Pla-2_AGAGCGATCATGCATG	Pla	Pla-2	P2-3617	FALSE	1	TRA:CALPPNTGTASKLTF TRB:CATTGATYEQYF	1	CD4-C4
Pla-2_AGAGCTTTCCTAGTGA	Pla	Pla-2	P2-419	FALSE	2	TRA:CAVEDGTGGFKTIF TRB:CAISESRPGQGNTEAFF	2	CD4-C4

Pla-2_AGAGTGGAGCTGGAAC	Pla	Pla-2	P2-38	TRUE	3	TRA:CAVRDGSDYKLSF TRB:CASKADLNNQPQHF	14	CD4-C4
Pla-2_AGATCTGCATTTGCTT	Pla	Pla-2	P2-3628	FALSE	1	TRA:CAVLDAGNNRKLIW TRB:CASSYRVTPEKLFF	1	CD4-C4
Pla-2_AGATCTGTCACTCCTG	Pla	Pla-2	P2-211	FALSE	3	TRA:CAESNSGAGSYQLTF TRB:CASRQQGAREKLFF	3	CD4-C4
Pla-2_AGATCTGTCGAACGGA	Pla	Pla-2	P2-149	FALSE	3	TRA:CAVTNRGNNRLAF TRB:CSARVPGGLSDEQFF	4	CD4-C4
Pla-2_AGATTGCAGTTGTAGA	Pla	Pla-2	P2-3633	FALSE	1	TRA:CATVGNDMRF TRB:CASSSIGSNYNEQFF	1	CD8-C8
Pla-2_AGCAGCCCAAGCGAGT	Pla	Pla-2	P2-3635	FALSE	1	TRA:CAAGDQTGANNLFF TRB:CASSSGYGYTF	1	CD4-C4
Pla-2_AGCAGCCCACCTATCC	Pla	Pla-2	P2-33	TRUE	3	TRA:CAFKLSDGQKLLF TRB:CAWSRAALTEAFF	17	CD4-C4
Pla-2_AGCCTAAAGGAGTTGC	Pla	Pla-2	P2-3639	FALSE	1	TRA:CAVGPNTNAGKSTF TRB:CASSPGFTPRATNEKLFF	1	CD4-C4
Pla-2_AGCCTAAAGTAGCCGA	Pla	Pla-2	P2-3640	FALSE	1	TRA:CAASEETSGSRLTF TRB:CASSFFLDTQYF	1	CD4-C4
Pla-2_AGCCTAACAAGAGTCG	Pla	Pla-2	P2-88	TRUE	3	TRA:CAVPGAGNKLTF TRB:CASSRSLAGVDTGELFF	6	CD4-C4
Pla-2_AGCGGTCCAATGGATA	Pla	Pla-2	P2-3646	FALSE	1	TRA:CAVRGAGSYQLTF TRB:CASSRDIDSDYGYTF	1	CD8-C8
Pla-2_AGCGGTCCAGATCCAT	Pla	Pla-2	P2-306	TRUE	2	TRA:CAVVGNTDKLIF TRB:CASSLKGSGANVLTF	2	CD4-C4
Pla-2_AGCGGTCGTAAGAGAG	Pla	Pla-2	P2-3649	FALSE	1	TRA:CAVLMIKAAGNKLTF TRB:CASSFRDRGKYYQETQYF	1	CD4-C4
Pla-2_AGCGGTCGTCGCGGTT	Pla	Pla-2	P2-31	FALSE	3	TRA:CAASDNTDKLIF TRB:CASSQVHEQYF	20	CD4-C4
Pla-2_AGCGGTCGTCTGATTG	Pla	Pla-2	P2-3651	FALSE	1	TRA:CAVSDTGGFKTIF TRB:CASSRDYTDTQYF	1	CD4-C4
Pla-2_AGCGGTCTCTAACTCT	Pla	Pla-2	P2-428	FALSE	2	TRA:CALTANTDKLIF TRB:CASSRLSGTLENEQFF	2	CD4-C4
Pla-2_AGCGTATAGAGCCCAA	Pla	Pla-2	P2-3654	FALSE	1	TRA:CAVRNSGNTPLVF TRB:CAISESGTEQYF	1	CD4-C4
Pla-2_AGCGTCGAGAAGGACA	Pla	Pla-2	P2-3657	FALSE	1	TRA:CAPTTNFGNEKLTF TRB:CSASSSLDGYTF	1	CD4-C4
Pla-2_AGCGTCGAGACTCGGA	Pla	Pla-2	P2-272	TRUE	2	TRA:CAMKGDDKIIF TRB:CSARDASFASYEQYF	2	CD4-C4
Pla-2_AGCTCCTAGGTGGGTT	Pla	Pla-2	P2-3662	FALSE	1	TRA:CAASDNNDMRF TRB:CASSRGQLRIHNEQFF	1	CD4-C4
Pla-2_AGCTTGACATGCCCGA	Pla	Pla-2	P2-197	TRUE	3	TRA:CVVSARAGGFKTIF TRB:CASSEAFGGSYNEQFF	3	CD4-C4
Pla-2_AGCTTGAGTCGCTTTC	Pla	Pla-2	P2-3669	FALSE	1	TRA:CAVSRSDDKIIF TRB:CASRSTVSSYNEQFF	1	CD4-C4
Pla-2_AGGCCACGTACTCAAC	Pla	Pla-2	P2-3673	FALSE	1	TRA:CATDAPNTNAGKSTF TRB:CASSWGDQPQHF	1	CD4-C4
Pla-2_AGGGAGTTCACCATAG	Pla	Pla-2	P2-3683	FALSE	1	TRA:CAVEDVGTYKYIF TRB:CASSFQAGRGTGELFF	1	CD4-C4
Pla-2_AGGGATGCATACAGCT	Pla	Pla-2	P2-3685	FALSE	1	TRA:CALSPGQGGSEKLVF TRB:CASSRQVVGSNEKLFF	1	CD4-C4
Pla-2_AGGGATGTCACGAAGG	Pla	Pla-2	P2-3687	FALSE	1	TRA:CVVSDRGSTLGRLYF TRB:CASRNTRNEQYF	1	CD4-C4
Pla-2_AGGGTGAAGTTCCACA	Pla	Pla-2	P2-3688	FALSE	1	TRA:CAASMRLDSNYQLIW TRB:CSARDEGTQYF	1	CD4-C4

Pla-2_AGGGTGACAAGAGTCG	Pla	Pla-2	P2-3689	FALSE	1	TRA:CAPSEGSGTYKYIF TRB:CAIRAASATGELFF	1	CD4-C4
Pla-2_AGGGTGACAATGACCT	Pla	Pla-2	P2-133	TRUE	3	TRA:CAVNKPAGNKLTF TRB:CASSTRPGGANVLTF	4	CD4-C4
Pla-2_AGGTCATGTCGGGTCT	Pla	Pla-2	P2-39	TRUE	3	TRA:CAARAVTGGGNKLTF TRB:CASSGRSSAWNEQFF	13	CD4-C4
Pla-2_AGTAGTCAGTAAGTAC	Pla	Pla-2	P2-3695	FALSE	1	TRA:CATDETSGSRLTF TRB:CASSWRRGTWETQYF	1	CD8-C2
Pla-2_AGTAGTCAGTGGGATC	Pla	Pla-2	P2-3696	FALSE	1	TRA:CAVRKLVTGGGNKLTF TRB:CASSSPGEKLFF	1	CD4-C4
Pla-2_AGTCTTTAGCAACGGT	Pla	Pla-2	P2-3699	FALSE	1	TRA:CAASIWNARLMF TRB:CASSLTDTQYF	1	CD4-C7
Pla-2_AGTCTTTAGCTGCCCA	Pla	Pla-2	P2-435	FALSE	2	TRA:CAVTGFQKLVF TRB:CSARPPGQGADEQYF	2	CD4-C4
Pla-2_AGTCTTTGTTATGTGC	Pla	Pla-2	P2-152	FALSE	3	TRA:CALKGSSGYSTLTF TRB:CSAIPSGGIGTDTQYF	4	CD4-C4
Pla-2_AGTGAGGTCGCACTCT	Pla	Pla-2	P2-3705	FALSE	1	TRA:CAIAIDSNYQLIW TRB:CAWSSTLAGGRRQYF	1	CD4-C4
Pla-2_AGTTGGTCATCCCACT	Pla	Pla-2	P2-32	TRUE	3	TRA:CAMREANVGADGLTF TRB:CASSTRIAGGPFPETQYF	17	CD4-C4
Pla-2_ATAACGCTCACAGGCC	Pla	Pla-2	P2-3717	FALSE	1	TRA:CAENNKIQGAQKLVF TRB:CASSRLAGGLGEQFF	1	CD8-C8
Pla-2_ATAAGAGTCCCAAGAT	Pla	Pla-2	P2-3721	FALSE	1	TRA:CATDAKYGGQNFVF TRB:CASSPGQGRGETQYF	1	CD4-C2
Pla-2_ATCATCTAGGTTCCTA	Pla	Pla-2	P2-92	FALSE	3	TRA:CAMREGRAANNLFF TRB:CAWSVRGWTRRMSSTDTQY	6	CD4-C4
Pla-2_ATCATCTCACTTAACG	Pla	Pla-2	P2-3726	FALSE	1	TRA:CAVSPKTGGGNKLTF TRB:CASSPRQGERETQYF	1	CD4-C4
Pla-2_ATCATCTGTTCTGTTT	Pla	Pla-2	P2-3728	FALSE	1	TRA:CAVSDPRTGNTGKLIF TRB:CASSLDAGGAYEQYF	1	CD4-C4
Pla-2_ATCATCTTCTGCCAGG	Pla	Pla-2	P2-278	TRUE	2	TRA:CAAGQGAQKLVF TRB:CASSLDGLLNTGELFF	2	CD4-C4
Pla-2_ATCATGGTCTAACCGA	Pla	Pla-2	P2-3730	FALSE	1	TRA:CAVGAKGSGTYKYIF TRB:CASRYTDSQETQYF	1	CD4-C4
Pla-2_ATCGAGTCATCGATGT	Pla	Pla-2	P2-3737	FALSE	1	TRA:CATDEIGSGNTGKLIF TRB:CASSFVSRVNAPLDQPQHF	1	CD4-C4
Pla-2_ATCGAGTGTGGCCCTA	Pla	Pla-2	P2-3739	FALSE	1	TRA:CATDRYSGAGSYQLTF TRB:CASSFGNYGYTF	1	CD4-C4
Pla-2_ATCTGCCAGTGTACGG	Pla	Pla-2	P2-180	TRUE	3	TRA:CAVNGNNFNKFYF TRB:CASSSGQDYEQYF	3	CD4-C4
Pla-2_ATCTGCCGTCGATTGT	Pla	Pla-2	P2-3742	FALSE	1	TRA:CAATAGGTGNQFYF TRB:CASSSGTGPGEQYF	1	CD4-C4
Pla-2_ATGAGGGAGCCACTAT	Pla	Pla-2	P2-3743	FALSE	1	TRA:CATDHGSGNTGKLIF TRB:CASSYHRLAGGELFF	1	CD4-C4
Pla-2_ATGCGATCAAACCTAC	Pla	Pla-2	P2-3745	FALSE	1	TRA:CAVNRMDSSYKLIF TRB:CASSDTLFETQYF	1	CD4-C4
Pla-2_ATGCGATTCTAAGCCA	Pla	Pla-2	P2-3747	FALSE	1	TRA:CAVSLGGTSYGKLTF TRB:CASILYEQYF	1	CD4-C4
Pla-2_ATGGGAGTCAGCAACT	Pla	Pla-2	P2-444	FALSE	2	TRA:CAVIPNAGGTSYGKLTF TRB:CASKSENNSPLHF	2	CD4-C4
Pla-2_ATGTGTGGTTCAGGCC	Pla	Pla-2	P2-3750	FALSE	1	TRA:CIVRRGLGNDMRF TRB:CASSLGKGLAETQYF	1	CD4-C5
Pla-2_ATTACTCAGATAGGAG	Pla	Pla-2	P2-3752	FALSE	1	TRA:CVVNKNGQNFVF TRB:CASSLLAGQETQYF	1	CD4-C4

Pla-2_ATTACTCAGTCGTACT	Pla	Pla-2	P2-3753	FALSE	1	TRA:CATDKGAQKLVF TRB:CASSPDAGNTQYF	1	CD4-C4
Pla-2_ATTACTCTCTTGCAAG	Pla	Pla-2	P2-446	FALSE	2	TRA:CVVRGNQGGKLIF TRB:CATDQGNSITGYTF	2	CD4-C4
Pla-2_ATTATCCAGATCCGAG	Pla	Pla-2	P2-3755	FALSE	1	TRA:CAVTHNTGRRALTF TRB:CASSIGTAGANVLTF	1	CD4-C4
Pla-2_ATTGGACAGCCGATTT	Pla	Pla-2	P2-33	TRUE	3	TRA:CAFKLSDGQKLLF TRB:CAWSRAALTEAFF	17	CD4-C4
Pla-2_ATTGGACCAAACTGTC	Pla	Pla-2	P2-3760	FALSE	1	TRA:CALVVGGANSKLTF TRB:CASSSAGGGNYGYTF	1	CD4-C4
Pla-2_ATTGGACGTGTCTGAT	Pla	Pla-2	P2-139	FALSE	3	TRA:CAVATGFQKLVF TRB:CASTLGGQGNNEQFF	4	CD4-C4
Pla-2_ATTGGTGAGCTTATCG	Pla	Pla-2	P2-3769	FALSE	1	TRA:CAVEEGSSYKLIF TRB:CASSGQGADGYGYTF	1	CD4-C4
Pla-2_ATTGGTGGTTACGACT	Pla	Pla-2	P2-448	FALSE	2	TRA:CATVLMNSGYSTLTF TRB:CASSLIGGGTEAFF	2	CD4-C4
Pla-2_ATTGGTGTCTAGAGTC	Pla	Pla-2	P2-3772	FALSE	1	TRA:CAVRVRGSWGKFQF TRB:CASSSGTGGNGELFF	1	CD4-C4
Pla-2_ATTTCTGGTAATTGGA	Pla	Pla-2	P2-3779	FALSE	1	TRA:CAGSDPGSQGNLIF TRB:CASSSRRIPGDYGYTF	1	CD4-C4
Pla-2_ATTTCTGGTAGCTAAA	Pla	Pla-2	P2-182	TRUE	3	TRA:CAPNFGNEKLTF TRB:CASSFSSAYEQYF	3	CD4-C4
Pla-2_CAACCTCAGTCAAGGC	Pla	Pla-2	P2-155	FALSE	3	TRA:CAGGRSNDYKLSF TRB:CASSGGAGGPGNEQFF	4	CD4-C4
Pla-2_CAACTAGAGATGGGTC	Pla	Pla-2	P2-450	FALSE	2	TRA:CAVKGGNNRLAF TRB:CASNRDKSTDTQYF	2	CD4-C4
Pla-2_CAAGAAACAAGCTGTT	Pla	Pla-2	P2-452	FALSE	2	TRA:CALVPNNAGNMLTF TRB:CASSTGQTYEQYF	2	CD4-C4
Pla-2_CAAGAAACATGAGCGA	Pla	Pla-2	P2-3794	FALSE	1	TRA:CAVNPERGSTLGRLYF TRB:CASSRRETQYF	1	CD4-C4
Pla-2_CAAGATCTCGTCGTTC	Pla	Pla-2	P2-3797	FALSE	1	TRA:CAVRDGRGSQGNLIF TRB:CASSEAYLGGLPSGANVLTF	1	CD4-C4
Pla-2_CAAGGCCAGTGCCATT	Pla	Pla-2	P2-455	FALSE	2	TRA:CAVIRTGANSKLTF TRB:CASSSQGAYGYTF	2	CD4-C4
Pla-2_CAAGTTGAGTTGAGTA	Pla	Pla-2	P2-3800	FALSE	1	TRA:CAAIHGGNQFYF TRB:CASTGGANYGYTF	1	CD4-C4
Pla-2_CACAAACTCTTCATGT	Pla	Pla-2	P2-220	FALSE	3	TRA:CAMRVPLGGYGQNFVF TRB:CATRDFQETQYF	3	CD4-C4
Pla-2_CACACAAAGGCTAGGT	Pla	Pla-2	P2-3812	FALSE	1	TRA:CAVQGYSSASKIIF TRB:CASSSQGGQTYNEQFF	1	CD4-C4
Pla-2_CACACAAAGTAGATGT	Pla	Pla-2	P2-3813	FALSE	1	TRA:CAVIQGGSEKLVF TRB:CASSRGTGASPLHF	1	CD8-C3
Pla-2_CACACCTGTTATCACG	Pla	Pla-2	P2-3818	FALSE	1	TRA:CVVNRAPGDSWGKFQF TRB:CAGMGQGENIQYF	1	CD4-C4
Pla-2_CACACCTTCGCCAGCA	Pla	Pla-2	P2-3821	FALSE	1	TRA:CAATGTGTASKLTF TRB:CASSQDPILTVRNQPQHF	1	CD4-C4
Pla-2_CACACCTTCGTAGGTT	Pla	Pla-2	P2-3822	FALSE	1	TRA:CAVRGPSGNNARLMF TRB:CASSFPPSYEQYF	1	CD4-C4
Pla-2_CACACTCAGTACTTGC	Pla	Pla-2	P2-3823	FALSE	1	TRA:CAASKGSNDYKLSF TRB:CASSQRGRGETQYF	1	CD4-C4
Pla-2_CACAGGCTCTTTAGGG	Pla	Pla-2	P2-459	FALSE	2	TRA:CIVSVDNYGQNFVF TRB:CAWSGLLGETQYF	2	CD4-C4
Pla-2_CACATAGAGGTGCAAC	Pla	Pla-2	P2-160	FALSE	3	TRA:CAAIRISVDGNVLHC TRB:CASSRPGYEQFF	4	CD4-C4

Pla-2_CACATAGCAGGCGATA	Pla	Pla-2	P2-462	FALSE	2	TRA:CAVPNNNDMRF TRB:CASSPGLANTGELFF	2	CD4-C4
Pla-2_CACATTTAGAAGAAGC	Pla	Pla-2	P2-3831	FALSE	1	TRA:CVVNKEGSARQLTF TRB:CAISVGGGVDTQYF	1	CD4-C4
Pla-2_CACATTTCAGCCTATA	Pla	Pla-2	P2-3836	FALSE	1	TRA:CAVNLRGYQKVTF TRB:CASSLVLGEGSPLHF	1	CD4-C4
Pla-2_CACCAGGGTTGATTGC	Pla	Pla-2	P2-3842	FALSE	1	TRA:CAASREGQGAQKLVF TRB:CASRTGTVNTGELFF	1	CD4-C4
Pla-2_CACCAGGTCCAAATGC	Pla	Pla-2	P2-3843	FALSE	1	TRA:CAERPQAGGGNKLTF TRB:CASKTGTVNEQFF	1	CD4-C4
Pla-2_CACCTTGAGGACATTA	Pla	Pla-2	P2-38	TRUE	3	TRA:CAVRDGSDYKLSF TRB:CASKADLNNQPQHF	14	CD4-C4
Pla-2_CACCTTGCATGCAATC	Pla	Pla-2	P2-32	TRUE	3	TRA:CAMREANVGADGLTF TRB:CASSTRIAGGPFPETQYF	17	CD4-C4
Pla-2_CACTCCAAGATATACG	Pla	Pla-2	P2-18	TRUE	3	TRA:CAATSSSNFGNEKLTF TRB:CAWSRAALTEAFF	28	CD4-C4
Pla-2_CACTCCAGTCGGATCC	Pla	Pla-2	P2-18	TRUE	3	TRA:CAATSSSNFGNEKLTF TRB:CAWSRAALTEAFF	28	CD4-C4
Pla-2_CACTCCATCCACTGGG	Pla	Pla-2	P2-3847	FALSE	1	TRA:CASWQANAGKSTF TRB:CASSLQGSTYNEQFF	1	CD4-C4
Pla-2_CAGAATCAGAAGGACA	Pla	Pla-2	P2-461	FALSE	2	TRA:CAVPAGTALIF TRB:CASSLSGAYQETQYF	2	CD4-C4
Pla-2_CAGAGAGAGGCAGGTT	Pla	Pla-2	P2-398	FALSE	2	TRA:CAITGTASKLTF TRB:CASSLWTGPSSYEQYF	2	CD4-C4
Pla-2_CAGAGAGTCAGGATCT	Pla	Pla-2	P2-3856	FALSE	1	TRA:CAYGGATNKLIF TRB:CASSTGQTYEQYF	1	CD4-C4
Pla-2_CAGATCAAGTGAACGC	Pla	Pla-2	P2-3858	FALSE	1	TRA:CAVSDYYNTDKLIF TRB:CASSVSGRSEETQYF	1	CD4-C4
Pla-2_CAGATCACAAATACAG	Pla	Pla-2	P2-3859	FALSE	1	TRA:CALSVGSQNFVF TRB:CSVNERGDFTDTQYF	1	CD4-C5
Pla-2_CAGATCACACGACGAA	Pla	Pla-2	P2-3860	FALSE	1	TRA:CAAPRRKAAGNKLTF TRB:CASSLSTDTQYF	1	CD4-C4
Pla-2_CAGATCACACTCGACG	Pla	Pla-2	P2-38	TRUE	3	TRA:CAVRDGSDYKLSF TRB:CASKADLNNQPQHF	14	CD4-C4
Pla-2_CAGCAGCCAATGACCT	Pla	Pla-2	P2-38	TRUE	3	TRA:CAVRDGSDYKLSF TRB:CASKADLNNQPQHF	14	CD4-C4
Pla-2_CAGCAGCCACCTGGTG	Pla	Pla-2	P2-3864	FALSE	1	TRA:CVVLLNFNKFYF TRB:CASSLVASSSGLSYEQYF	1	CD4-C4
Pla-2_CAGCATAAGTAGTGCG	Pla	Pla-2	P2-3868	FALSE	1	TRA:CATAIFNNNDMRF TRB:CASSLGLGGGTDIQYF	1	CD4-C4
Pla-2_CAGCATAGTGGCAAAC	Pla	Pla-2	P2-18	TRUE	3	TRA:CAATSSSNFGNEKLTF TRB:CAWSRAALTEAFF	28	CD4-C4
Pla-2_CAGCATAGTGTTTGTG	Pla	Pla-2	P2-32	TRUE	3	TRA:CAMREANVGADGLTF TRB:CASSTRIAGGPFPETQYF	17	CD4-C4
Pla-2_CAGCCGACAAACCCAT	Pla	Pla-2	P2-3870	FALSE	1	TRA:CAMSEGAGGTSYGKLTF TRB:CASKQRVLSYEQYF	1	CD4-C4
Pla-2_CAGCGACAGTTTGCGT	Pla	Pla-2	P2-162	FALSE	3	TRA:CIVRAMNSGYSTLTF TRB:CSAPPWQGAYEQYF	4	CD4-C6
Pla-2_CAGCGACTCCTCGCAT	Pla	Pla-2	P2-62	FALSE	3	TRA:CAVITDTGRRALTF TRB:CASSIGTSGYNEQFF	9	CD8-C8
Pla-2_CAGCTAAGTGTCAATC	Pla	Pla-2	P2-471	FALSE	2	TRA:CAVRDGSDYKLSF TRB:CASSDGTGGGYTF	2	CD4-C4
Pla-2_CAGCTGGCACCCATGG	Pla	Pla-2	P2-90	FALSE	3	TRA:CIGNTPLVF TRB:CASSDRDRVLNTEAFF	6	CD4-C4

Pla-2_CAGCTGGTCCGCAGTG	Pla	Pla-2	P2-1	TRUE	3	TRA:CVVNIIGYQKVTF TRB:CASTRQGKQPQHF	270	CD4-C4
Pla-2_CAGCTGGTCTCCAACC	Pla	Pla-2	P2-92	FALSE	3	TRA:CAMREGRAANNLFF TRB:CAWSVRGWTRRMSSTDTQY[illegible]	6	CD4-C4
Pla-2_CAGCTGGTCTTAACCT	Pla	Pla-2	P2-3880	FALSE	1	TRA:CAVLRGSTLGRLYF TRB:CSVAGTPSGNTIYF	1	CD4-C4
Pla-2_CAGTAACTCTTCCTTC	Pla	Pla-2	P2-273	TRUE	2	TRA:CAVFDRGSTLGRLYF TRB:CASSASQSARDSPTQYF	2	CD4-C4
Pla-2_CAGTCCTAGCATCATC	Pla	Pla-2	P2-452	FALSE	2	TRA:CALVPNNAGNMLTF TRB:CASSTGQTYEQYF	2	CD4-C4
Pla-2_CAGTCCTTCCAAGTAC	Pla	Pla-2	P2-3891	FALSE	1	TRA:CAENSGPGRRALTF TRB:CASSPMHRGADQPQHF	1	CD4-C4
Pla-2_CATATGGAGTTTGCGT	Pla	Pla-2	P2-363	TRUE	2	TRA:CAVTSTGGFKTIF TRB:CASSLGASGSLYEQYF	2	CD4-C4
Pla-2_CATATGGTCCTTGCCA	Pla	Pla-2	P2-3894	FALSE	1	TRA:CAYGGGATNKLIF TRB:CASSQGTGGYGYTF	1	CD4-C4
Pla-2_CATCAAGCACCGAATT	Pla	Pla-2	P2-3901	FALSE	1	TRA:CALPPNTGTASKLTF TRB:CASIGPAYEQYF	1	CD4-C4
Pla-2_CATCAGACATAAGACA	Pla	Pla-2	P2-18	TRUE	3	TRA:CAATSSSNFGNEKLTF TRB:CAWSRAALTEAFF	28	CD4-C4
Pla-2_CATCAGAGTCCGTCAG	Pla	Pla-2	P2-3902	FALSE	1	TRA:CAVNAYSGAGSYQLTF TRB:CASSPSGTGRSEQYF	1	CD4-C4
Pla-2_CATCGGGCAGCTTAAC	Pla	Pla-2	P2-3909	FALSE	1	TRA:CALSDLSSKLTF TRB:CASSLELAGTYEQYF	1	CD4-C4
Pla-2_CATGACAAGGATCGCA	Pla	Pla-2	P2-3910	FALSE	1	TRA:CAVLWDKLVF TRB:CASSRDRYNEQFF	1	CD4-C4
Pla-2_CATGACAAGGGTGTTG	Pla	Pla-2	P2-444	FALSE	2	TRA:CAVIPNAGGTSYGKLTF TRB:CASKSENNSPLHF	2	CD4-C4
Pla-2_CATGACACAGAGTGTG	Pla	Pla-2	P2-3911	FALSE	1	TRA:CAVTRYTGNQFYF TRB:CASSLAGVYNEQFF	1	CD4-C4
Pla-2_CATGACACAGGTCGTC	Pla	Pla-2	P2-3912	FALSE	1	TRA:CAVNRAGGTSYGKLTF TRB:CASSLRVGETQYF	1	CD4-C4
Pla-2_CATGACATCTGCAGTA	Pla	Pla-2	P2-3917	FALSE	1	TRA:CAVSLYSAGNMLTF TRB:CASSEHRVQETQYF	1	CD4-C4
Pla-2_CATGACATCTTAGCCC	Pla	Pla-2	P2-3918	FALSE	1	TRA:CAENRGTSRGGYQKVTF TRB:CASGGGPGTDTQYF	1	CD8-C8
Pla-2_CATGGCGGTGGGTATG	Pla	Pla-2	P2-219	FALSE	3	TRA:CAVEVPGSQGNLIF TRB:CASRAGTAVYEQYF	3	CD4-C4
Pla-2_CATGGCGTCATCTGTT	Pla	Pla-2	P2-3925	FALSE	1	TRA:CAVRSSLNTGTASKLTF TRB:CASSLRLAGGSSYNEQFF	1	CD4-C4
Pla-2_CATGGCGTCTGCTTGC	Pla	Pla-2	P2-184	TRUE	3	TRA:CARYQGGKLIF TRB:CASSPRWGLGSYNEQFF	3	CD4-C4
Pla-2_CATTCGCAGAACTCGG	Pla	Pla-2	P2-3931	FALSE	1	TRA:CVVSDRGSTLGRLYF TRB:CASSEYLRGDLYEQYF	1	CD8-C10
Pla-2_CATTCGCAGGGAACGG	Pla	Pla-2	P2-3932	FALSE	1	TRA:CAVRGNNARLMF TRB:CASSQGGSNQPQHF	1	CD4-C4
Pla-2_CATTCGCTCTCTGTCG	Pla	Pla-2	P2-3934	FALSE	1	TRA:CAVESRGAQKLVF TRB:CSARGLAGGSETQYF	1	CD4-C4
Pla-2_CCAATCCAGATCGATA	Pla	Pla-2	P2-479	FALSE	2	TRA:CAVQARDNNARLMF TRB:CAWSVPQGRAFF	2	CD4-C4
Pla-2_CCAATCCAGCCAGGAT	Pla	Pla-2	P2-3935	FALSE	1	TRA:CAVGAGGNTGKLIF TRB:CASSGLAGLQETQYF	1	CD4-C4
Pla-2_CCAATCCCACATTAGC	Pla	Pla-2	P2-3936	FALSE	1	TRA:CAVSGGGFKTIF TRB:CASSREGETQYF	1	CD4-C3

Pla-2_CCAATCCCAGGTGCCT	Pla	Pla-2	P2-38	TRUE	3	TRA:CAVRDGSDYKLSF TRB:CASKADLNNQPQHF	14	CD4-C4
Pla-2_CCAATCCTCATCTGCC	Pla	Pla-2	P2-3939	FALSE	1	TRA:CAVLNGGNQFYF TRB:CASSVRDRGDNQPQHF	1	CD4-C4
Pla-2_CCAATCCTCTTCTGGC	Pla	Pla-2	P2-3941	FALSE	1	TRA:CAETHRDDKIIF TRB:CASSSPQREGGNEQYF	1	CD8-C8
Pla-2_CCACCTAAGCTAGTTC	Pla	Pla-2	P2-61	TRUE	3	TRA:CAVSFITQGGSEKLVF TRB:CASSRDRGTYNEQFF	9	CD4-C5
Pla-2_CCACGGATCCTATTCA	Pla	Pla-2	P2-3946	FALSE	1	TRA:CALSDRNVGNNRKLIW TRB:CASSDGRGGAKNIQYF	1	CD4-C4
Pla-2_CCACTACCAGCGAACA	Pla	Pla-2	P2-3947	FALSE	1	TRA:CAEFETNFGNEKLTF TRB:CASSLGRARSTDTQYF	1	CD4-C4
Pla-2_CCACTACTCCTTAATC	Pla	Pla-2	P2-70	FALSE	3	TRA:CAVPVAGTASKLTF TRB:CASREVSYNEQFF	8	CD4-C4
Pla-2_CCAGCGAAGGGATACC	Pla	Pla-2	P2-3950	FALSE	1	TRA:CAASAAGGGADGLTF TRB:CASSWTGEGYQPQHF	1	CD4-C3
Pla-2_CCAGCGAGTAGCTAAA	Pla	Pla-2	P2-440	FALSE	2	TRA:CALTDGRSNDYKLSF TRB:CASSKRGVKGQAFF	2	CD4-C4
Pla-2_CCAGCGATCCGTACAA	Pla	Pla-2	P2-160	FALSE	3	TRA:CAAIRISVDGNVLHC TRB:CASSRPGYEQFF	4	CD4-C4
Pla-2_CCAGCGATCTACGAGT	Pla	Pla-2	P2-214	FALSE	3	TRA:CAASPMFSGGYNKLIF TRB:CASSLRTGEQFF	3	CD4-C4
Pla-2_CCATTCGGTAGCTTGT	Pla	Pla-2	P2-382	FALSE	2	TRA:CAATRYSGGGADGLTF TRB:CASSPGTGTQVRNTGELFF	2	CD4-C5
Pla-2_CCCAATCAGCGTGTCC	Pla	Pla-2	P2-3957	FALSE	1	TRA:CALSSDTGRRALTF TRB:CASSSDLGKNIQYF	1	CD4-C4
Pla-2_CCCAGTTTCTGCAGTA	Pla	Pla-2	P2-3965	FALSE	1	TRA:CALMPVSGNTGKLIF TRB:CASSAGKRDIGDTQYF	1	CD4-C4
Pla-2_CCCATACCACAGGCCT	Pla	Pla-2	P2-483	FALSE	2	TRA:CAPERVNQGGKLIF TRB:CASSNTYEQYF	2	CD4-C4
Pla-2_CCCATACGTCCTAGCG	Pla	Pla-2	P2-215	FALSE	3	TRA:CAANNNARLMF TRB:CASSLSGARLVTGELFF	3	CD4-C4
Pla-2_CCGGTAGCACAGAGGT	Pla	Pla-2	P2-3972	FALSE	1	TRA:CIVRPYNNNDMRF TRB:CASSLKVAGADTQYF	1	CD4-C4
Pla-2_CCGGTAGCATCTGGTA	Pla	Pla-2	P2-3973	FALSE	1	TRA:CAENKGSFQKLVF TRB:CASSVTSGYEQYF	1	CD4-C4
Pla-2_CCGTTCAGTTACAGAA	Pla	Pla-2	P2-381	FALSE	2	TRA:CAVRDFNNAGNMLTF TRB:CASSLEAFYEQYF	2	CD4-C4
Pla-2_CCTAAAGTCCAATGGT	Pla	Pla-2	P2-3985	FALSE	1	TRA:CAVGPGGATNKLIF TRB:CASSLAGTSGNEQFF	1	CD4-C4
Pla-2_CCTACCACATCCAACA	Pla	Pla-2	P2-3989	FALSE	1	TRA:CAVLGGSQGNLIF TRB:CASSIQGAQTEAFF	1	CD4-C4
Pla-2_CCTAGCTTCAGGCGAA	Pla	Pla-2	P2-488	FALSE	2	TRA:CAVSYQAGTALIF TRB:CSAFRRKIQANYGYTF	2	CD4-C4
Pla-2_CCTCAGTGTTATTCTC	Pla	Pla-2	P2-492	FALSE	2	TRA:CAVPSYNTDKLIF TRB:CAWSVLTGELFF	2	CD4-C4
Pla-2_CCTCTGAAGTACGCGA	Pla	Pla-2	P2-4001	FALSE	1	TRA:CALTPSGAGSYQLTF TRB:CAWSVQAGFRETQYF	1	CD4-C4
Pla-2_CCTCTGAGTAGCTGCC	Pla	Pla-2	P2-4004	FALSE	1	TRA:CALSERGKTGTASKLTF TRB:CASSPYTDTQYF	1	CD4-C4
Pla-2_CCTTACGAGTTCGATC	Pla	Pla-2	P2-4006	FALSE	1	TRA:CLVGPKGGGNKLTF TRB:CASSVDLGQGNTDTQYF	1	CD4-C4
Pla-2_CCTTACGGTGTAATGA	Pla	Pla-2	P2-4009	FALSE	1	TRA:CAAGNNDMRF TRB:CASTRDKAYGYTF	1	CD4-C4

Pla-2_CCTTCCCGTGGCAAAC	Pla	Pla-2	P2-62	FALSE	3	TRA:CAVITDTGRRALTF TRB:CASSIGTSGYNEQFF	9	CD4-C4
Pla-2_CCTTCGATCACAACGT	Pla	Pla-2	P2-4018	FALSE	1	TRA:CALYVAAGNKLTF TRB:CASSPQRTGLNTEAFF	1	CD4-C4
Pla-2_CCTTCGATCGGCGCAT	Pla	Pla-2	P2-4019	FALSE	1	TRA:CAVSSGNQGGKLIF TRB:CASSPPTGGKETQYF	1	CD4-C4
Pla-2_CCTTTCTAGGCAGGTT	Pla	Pla-2	P2-116	FALSE	3	TRA:CAVSPPAGSNYQLIW TRB:CATSRETGARYEQYF	5	CD4-C4
Pla-2_CCTTTCTCAGTACACT	Pla	Pla-2	P2-399	FALSE	2	TRA:CAVTDGFYDKVIF TRB:CASSMDGETQYF	2	CD4-C4
Pla-2_CGAATGTAGCTCTCGG	Pla	Pla-2	P2-4028	FALSE	1	TRA:CAASEWGSGYSTLTF TRB:CSAGEGTGAGELFF	1	CD4-C4
Pla-2_CGAATGTAGGCAATTA	Pla	Pla-2	P2-207	FALSE	3	TRA:CAVKAEYGGQNFVF TRB:CASSAGPYNSPLHF	3	CD4-C4
Pla-2_CGACCTTCACGGCCAT	Pla	Pla-2	P2-335	TRUE	2	TRA:CIVRTGEYGNKLVF TRB:CAWRGQGTSTQYF	2	CD4-C4
Pla-2_CGAGAAGCACGTGAGA	Pla	Pla-2	P2-18	TRUE	3	TRA:CAATSSSNFGNEKLTF TRB:CAWSRAALTEAFF	28	CD4-C4
Pla-2_CGAGCACAGTGACTCT	Pla	Pla-2	P2-4042	FALSE	1	TRA:CAGADYDYKLSF TRB:CASSLGLAGGNTQYF	1	CD4-C4
Pla-2_CGAGCACAGTTGTAGA	Pla	Pla-2	P2-459	FALSE	2	TRA:CIVSVDNYGGQNFVF TRB:CAWSGLLGETQYF	2	CD4-C4
Pla-2_CGAGCACGTTCCCGAG	Pla	Pla-2	P2-4044	FALSE	1	TRA:CAVSDNTGGFKTIF TRB:CASSPRTTGANEQFF	1	CD4-C4
Pla-2_CGAGCACTCACCAGGC	Pla	Pla-2	P2-4045	FALSE	1	TRA:CAPLAPRENAGNMLTF TRB:CASRRARGLAGGTDTQYF	1	CD4-C4
Pla-2_CGATGGCAGCCATCGC	Pla	Pla-2	P2-4058	FALSE	1	TRA:CAVREGGGFKTIF TRB:CATSREGGGGAFF	1	CD4-C4
Pla-2_CGATGTAGTAGAAGGA	Pla	Pla-2	P2-4061	FALSE	1	TRA:CALSGTGNQFYF TRB:CASSYLPGLAAYEQYF	1	CD4-C4
Pla-2_CGATTGAGTTCGGCAC	Pla	Pla-2	P2-497	FALSE	2	TRA:CAYRTPPNTPLVF TRB:CASSRTLGTSTDTQYF	2	CD4-C4
Pla-2_CGATTGATCACTATTC	Pla	Pla-2	P2-4065	FALSE	1	TRA:CATLTTGNQFYF TRB:CASSLQASSYNEQFF	1	CD4-C4
Pla-2_CGCCAAGAGTATCGAA	Pla	Pla-2	P2-4067	FALSE	1	TRA:CAAKDNYGGQNFVF TRB:CASSLEGHANSPLHF	1	CD4-C4
Pla-2_CGCGGTACATGACGGA	Pla	Pla-2	P2-4073	FALSE	1	TRA:CAVLILTGGGNKLTF TRB:CASAQGHANTEAFF	1	CD4-C4
Pla-2_CGCGTTTAGGGTGTGT	Pla	Pla-2	P2-4074	FALSE	1	TRA:CAVEEPNTDKLIF TRB:CASSFGGMAAGELFF	1	CD4-C4
Pla-2_CGCGTTTCACCAGATT	Pla	Pla-2	P2-4076	FALSE	1	TRA:CACNDYKLSF TRB:CASTHGQVNEQFF	1	CD4-C5
Pla-2_CGCTATCAGATGGCGT	Pla	Pla-2	P2-4078	FALSE	1	TRA:CAVNGGGGADGLTF TRB:CASEFTSSYNEQFF	1	CD4-C4
Pla-2_CGCTATCCATGCAATC	Pla	Pla-2	P2-4081	FALSE	1	TRA:CAVNGGFGNVLHC TRB:CAWSFPSGRAYTQYF	1	CD4-C4
Pla-2_CGCTTCAAGGCACATG	Pla	Pla-2	P2-4086	FALSE	1	TRA:CAVKGNTGKLIF TRB:CASSQRGAGENEQFF	1	CD4-C4
Pla-2_CGCTTCACACAGGAGT	Pla	Pla-2	P2-4088	FALSE	1	TRA:CALNNQGGKLIF TRB:CSASPLDRDNSPLHF	1	CD4-C4
Pla-2_CGCTTCACAGCATGAG	Pla	Pla-2	P2-4089	FALSE	1	TRA:CAVEDDSTLTF TRB:CASSGPVRTGELFF	1	CD4-C4
Pla-2_CGCTTCACATCGACGC	Pla	Pla-2	P2-4090	FALSE	1	TRA:CVVSATGGFKTIF TRB:CASSNRYYNEQFF	1	CD4-C4

Pla-2_CGCTTCATCTCGTTTA	Pla	Pla-2	P2-4094	FALSE	1	TRA:CAVKGTGGFKTIF TRB:CASSLASTTNEKLFF	1	CD4-C4
Pla-2_CGGACACAGATATGCA	Pla	Pla-2	P2-4095	FALSE	1	TRA:CAASRTGANNLFF TRB:CASSLGQSWSTEAFF	1	CD4-C4
Pla-2_CGGACACTCCTCTAGC	Pla	Pla-2	P2-4097	FALSE	1	TRA:CASAPFQKLVF TRB:CASSLDVPGTSGAYNEQFF	1	CD4-C4
Pla-2_CGGAGCTCAGTATGCT	Pla	Pla-2	P2-145	FALSE	3	TRA:CAMRRSSGGSNYKLTF TRB:CASSSIGRGSKRETQYF	4	CD4-C4
Pla-2_CGGCTAGAGGAGCGAG	Pla	Pla-2	P2-4113	FALSE	1	TRA:CAGLSGSARQLTF TRB:CASSSGQVLSGELFF	1	CD4-C4
Pla-2_CGGCTAGGTCAGATAA	Pla	Pla-2	P2-4116	FALSE	1	TRA:CAEGGATNKLIF TRB:CASSQQGNSNQPQHF	1	CD4-C4
Pla-2_CGGGTCAAGCCAGGAT	Pla	Pla-2	P2-251	FALSE	3	TRA:CADNNAGNMLTF TRB:CASSPNLPLLADNQETQYF	3	CD4-C4
Pla-2_CGGGTCATCATGTGGT	Pla	Pla-2	P2-4120	FALSE	1	TRA:CALDGDGNEKLTF TRB:CASSLAPGLAGRETQYF	1	CD4-C5
Pla-2_CGGGTCATCTCTGCTG	Pla	Pla-2	P2-32	TRUE	3	TRA:CAMREANVGADGLTF TRB:CASSTRIAGGPFPETQYF	17	CD4-C4
Pla-2_CGGTTAAAGAAGCCCA	Pla	Pla-2	P2-4122	FALSE	1	TRA:CALSLRNQGGKLIF TRB:CSARDPGLAGVTDTQYF	1	CD4-C4
Pla-2_CGGTTAAAGCTAACTC	Pla	Pla-2	P2-105	TRUE	3	TRA:CAYRRREGGSEKLVF TRB:CASSTSGAIANYGYTF	5	CD4-C4
Pla-2_CGGTTAATCAACACTG	Pla	Pla-2	P2-36	FALSE	3	TRA:CAAQAGNTPLVF TRB:CSAGVGGTIQYF	15	CD4-C4
Pla-2_CGTAGGCAGCCACGTC	Pla	Pla-2	P2-184	TRUE	3	TRA:CARYQGGKLIF TRB:CASSPRWGLGSYNEQFF	3	CD4-C4
Pla-2_CGTAGGCGTGAAGGCT	Pla	Pla-2	P2-32	TRUE	3	TRA:CAMREANVGADGLTF TRB:CASSTRIAGGPFPETQYF	17	CD4-C4
Pla-2_CGTAGGCTCATAGCAC	Pla	Pla-2	P2-4130	FALSE	1	TRA:CGTTNSGGSNYKLTF TRB:CASSVVPIQNNEQFF	1	CD4-C4
Pla-2_CGTCACTAGAGCTTCT	Pla	Pla-2	P2-344	TRUE	2	TRA:CAASNRNYGQNFVF TRB:CASSPRDGYGYTF	2	CD4-C4
Pla-2_CGTCACTAGTCACGCC	Pla	Pla-2	P2-4134	FALSE	1	TRA:CALDMGHNAGNMLTF TRB:CASTVNQGGRGYTF	1	CD4-C4
Pla-2_CGTCAGGAGTTTGCGT	Pla	Pla-2	P2-4137	FALSE	1	TRA:CATDRGGADGLTF TRB:CATLRGGSGYTF	1	CD8-C2
Pla-2_CGTCAGGTCTATCCTA	Pla	Pla-2	P2-4139	FALSE	1	TRA:CAFMKHGGYGQNFVF TRB:CASSEATRGYATPKTNYGYT	1	CD4-C4
Pla-2_CGTGTAAGTTGTCGCG	Pla	Pla-2	P2-510	FALSE	2	TRA:CAVRDPGDDKIIF TRB:CASSVASGKSYEQYF	2	CD4-C4
Pla-2_CGTGTCTAGCCATCGC	Pla	Pla-2	P2-416	FALSE	2	TRA:CAVEDRSYQKVTF TRB:CASSAGGDGFEQFF	2	CD4-C4
Pla-2_CGTGTCTGTTCGGGCT	Pla	Pla-2	P2-4151	FALSE	1	TRA:CAVSHGAQKLVF TRB:CASSKGQETQYF	1	CD4-C4
Pla-2_CGTTAGAAGTTAGGTA	Pla	Pla-2	P2-4153	FALSE	1	TRA:CAVPGYSSASKIIF TRB:CASSEGTALERKQFF	1	CD4-C4
Pla-2_CGTTCTGTCAATCTCT	Pla	Pla-2	P2-4159	FALSE	1	TRA:CAARYSGYSTLTF TRB:CASSQEGETQYF	1	CD4-C4
Pla-2_CTAACTTAGCAACGGT	Pla	Pla-2	P2-4163	FALSE	1	TRA:CAVRRTGGFKTIF TRB:CASSQVGTDFYEQYF	1	CD4-C4
Pla-2_CTAACTTTCCCTCTTT	Pla	Pla-2	P2-4164	FALSE	1	TRA:CALSDRGGDDKIIF TRB:CASTDPRTYNEQFF	1	CD4-C4
Pla-2_CTAACTTTCCGTTGTC	Pla	Pla-2	P2-4165	FALSE	1	TRA:CAVGPIQGAQKLVF TRB:CASTDRSYQPQHF	1	CD4-C4

Pla-2_CTAATGGCAGCGTCCA	Pla	Pla-2	P2-253	FALSE	3	TRA:CAMRESGGGADGLTF TRB:CAWRRGNTEAFF	3	CD4-C4
Pla-2_CTACACCCATTAGCCA	Pla	Pla-2	P2-14	FALSE	3	TRA:CALSEAPGGSEKLVF TRB:CASSIGHPNSPLHF	37	CD4-C4
Pla-2_CTACACCGTATAAACG	Pla	Pla-2	P2-4172	FALSE	1	TRA:CALSGAAITQGGSEKLVF TRB:CASSYWFPGQKSSYNSPLI	1	CD4-C4
Pla-2_CTACATTAGTGGAGAA	Pla	Pla-2	P2-188	TRUE	3	TRA:CAVRRQGGNAGNMLTF TRB:CASSEQGSGSPYEQYF	3	CD4-C4
Pla-2_CTACATTTCAGCTCGG	Pla	Pla-2	P2-78	TRUE	3	TRA:CAATTDSWGKFQF TRB:CASSSSASDYNEQFF	7	CD4-C4
Pla-2_CTACCCATCCAGAAGG	Pla	Pla-2	P2-4177	FALSE	1	TRA:CAGQLGGATNKLIF TRB:CASSLEAFYEQYF	1	CD4-C4
Pla-2_CTACCCATCTAGAGTC	Pla	Pla-2	P2-4179	FALSE	1	TRA:CVVLRRGSGNTGKLIF TRB:CATSDRGRSLNQPQHF	1	CD4-C4
Pla-2_CTAGAGTCAGGTGGAT	Pla	Pla-2	P2-4182	FALSE	1	TRA:CAFYTGGFKTIF TRB:CAWGKGQFSGIQYF	1	CD4-C4
Pla-2_CTAGAGTCAGTAAGAT	Pla	Pla-2	P2-4183	FALSE	1	TRA:CAASMNTGGFKTIF TRB:CASSLGPFGNTIYF	1	CD4-C4
Pla-2_CTAGTGACACGTCAGC	Pla	Pla-2	P2-4189	FALSE	1	TRA:CALVRGSTLGRLYF TRB:CASSPRGDSYNEQFF	1	CD4-C4
Pla-2_CTAGTGATCGGATGGA	Pla	Pla-2	P2-4194	FALSE	1	TRA:CAAGGGADGLTF TRB:CSALSGANVLTF	1	CD4-C4
Pla-2_CTCACACAGCCAGAAC	Pla	Pla-2	P2-4196	FALSE	1	TRA:CAVRTGGYNKLIF TRB:CASSLGGSGRSELFF	1	CD4-C4
Pla-2_CTCAGAACAGGTGCCT	Pla	Pla-2	P2-453	FALSE	2	TRA:CAVSSDTGGFKTIF TRB:CASSATGSSRSGELFF	2	CD4-C4
Pla-2_CTCAGAATCCGTAGGC	Pla	Pla-2	P2-4199	FALSE	1	TRA:CAVHNQGGKLIF TRB:CAISDPRVWPNYEQYF	1	CD4-C4
Pla-2_CTCGAGGAGCCTTGAT	Pla	Pla-2	P2-520	FALSE	2	TRA:CAVRARGTDKLIF TRB:CASSPQAAGTGELFF	2	CD4-C4
Pla-2_CTCGGAGTCATCGGAT	Pla	Pla-2	P2-4214	FALSE	1	TRA:CAPRLNSGGYQKVTF TRB:CASRSTGSNQPQHF	1	CD4-C4
Pla-2_CTCGGGAAGGGTCTCC	Pla	Pla-2	P2-4216	FALSE	1	TRA:CIVIPNFGNEKLTF TRB:CASSLSILNTEAFF	1	CD4-C4
Pla-2_CTCGGGAGTCGAATCT	Pla	Pla-2	P2-64	FALSE	3	TRA:CAATSLISDGQKLLF TRB:CSARDSFNYGYTF	9	CD4-C5
Pla-2_CTCGGGATCAGCTGGC	Pla	Pla-2	P2-4217	FALSE	1	TRA:CAVKRDDFNKFYF TRB:CASESGGPPSYEQYF	1	CD4-C4
Pla-2_CTCGTACAGGACATTA	Pla	Pla-2	P2-92	FALSE	3	TRA:CAMREGRAANNLFF TRB:CAWSVRGWTRRMSSTDTQYI	6	CD4-C4
Pla-2_CTCTAATGTAGAAGGA	Pla	Pla-2	P2-4223	FALSE	1	TRA:CASNSGAGSYQLTF TRB:CASSLLGQYYEQYF	1	CD4-C4
Pla-2_CTCTAATTCAGGCCCA	Pla	Pla-2	P2-4224	FALSE	1	TRA:CAASLLSGGYNKLIF TRB:CASSGLPSQVVGSEAFF	1	CD4-C4
Pla-2_CTCTGGTAGTGGCACA	Pla	Pla-2	P2-4227	FALSE	1	TRA:CAVERKRIYNQGGKLIF TRB:CASSRPRQGRSYNEQFF	1	CD8-C2
Pla-2_CTCTGGTCATGAGCGA	Pla	Pla-2	P2-428	FALSE	2	TRA:CALTANTDKLIF TRB:CASSRLSGTLENEQFF	2	CD4-C4
Pla-2_CTGATCCAGTCAAGCG	Pla	Pla-2	P2-4236	FALSE	1	TRA:CAVRGPGGYQKVTF TRB:CASSPRTGIEQYF	1	CD4-C2
Pla-2_CTGATCCGTTCGGCAC	Pla	Pla-2	P2-64	FALSE	3	TRA:CAATSLISDGQKLLF TRB:CSARDSFNYGYTF	9	CD4-C4
Pla-2_CTGCCTAAGTAGCGGT	Pla	Pla-2	P2-36	FALSE	3	TRA:CAAQAGNTPLVF TRB:CSAGVGGTIQYF	15	CD4-C4

Pla-2_CTGCGGAAGGCCGAAT	Pla	Pla-2	P2-169	TRUE	3	TRA:CGARNNARLMF TRB:CASSRLGAANTGELFF	3	CD4-C6
Pla-2_CTGCGGACACGCCAGT	Pla	Pla-2	P2-85	TRUE	3	TRA:CAVTAYTNAGKSTF TRB:CASRGRSSGNTIYF	6	CD4-C4
Pla-2_CTGCGGACAGCTGGCT	Pla	Pla-2	P2-526	FALSE	2	TRA:CAVLSGTYKYIF TRB:CASSFGGYTDTQYF	2	CD4-C4
Pla-2_CTGCGGATCAAGCCTA	Pla	Pla-2	P2-4246	FALSE	1	TRA:CAARVNARLMF TRB:CSAIASGWGGNGYTF	1	CD4-C4
Pla-2_CTGCGGATCCTTAATC	Pla	Pla-2	P2-214	FALSE	3	TRA:CAASPMFSGGYNKLIF TRB:CASSLRTGEQFF	3	CD4-C4
Pla-2_CTGGTCTCACCATCCT	Pla	Pla-2	P2-526	FALSE	2	TRA:CAVLSGTYKYIF TRB:CASSFGGYTDTQYF	2	CD8-C8
Pla-2_CTGGTCTTCGCGTTTC	Pla	Pla-2	P2-339	TRUE	2	TRA:CAESNPSNFGNEKLTF TRB:CASSSAGDGYTF	2	CD4-C4
Pla-2_CTGTTTATCACAATGC	Pla	Pla-2	P2-4259	FALSE	1	TRA:CATVAGQKLLF TRB:CASSPRAYEQYF	1	CD4-C4
Pla-2_CTTAACTCAAAGCGGT	Pla	Pla-2	P2-4262	FALSE	1	TRA:CAYNNNDMRF TRB:CASSGRSSAWNEQFF	1	CD4-C4
Pla-2_CTTACCGCATGTAAGA	Pla	Pla-2	P2-4266	FALSE	1	TRA:CAVRSSDGQKLLF TRB:CASSLPVGEQFF	1	CD4-C4
Pla-2_CTTACCGTCCTAGAAC	Pla	Pla-2	P2-4267	FALSE	1	TRA:CAVKSEAGRRALTF TRB:CASSMAGVETQYF	1	CD4-C4
Pla-2_CTTACCGTCGCTTGTC	Pla	Pla-2	P2-4269	FALSE	1	TRA:CAVVPGNQGGKLIF TRB:CASSSHAGSYNEQFF	1	CD4-C4
Pla-2_CTTCTCTAGCTGTCTA	Pla	Pla-2	P2-4272	FALSE	1	TRA:CVRTVMSYYTDKLIF TRB:CASSLPQETQYF	1	CD8-C8
Pla-2_CTTGGCTCATATGCTG	Pla	Pla-2	P2-62	FALSE	3	TRA:CAVITDTGRRALTF TRB:CASSIGTSGYNEQFF	9	CD4-C4
Pla-2_CTTGGCTGTTTCGCTC	Pla	Pla-2	P2-4279	FALSE	1	TRA:CAVAAGAQKLVF TRB:CASSPAVGQGLAYGYTF	1	CD4-C4
Pla-2_CTTGGCTGTTTGTTTC	Pla	Pla-2	P2-530	FALSE	2	TRA:CAVGGDTNAGKSTF TRB:CASNAGTSGRAFQFF	2	CD4-C4
Pla-2_CTTGGCTTCCAGATCA	Pla	Pla-2	P2-4280	FALSE	1	TRA:CAVRGNAGNMLTF TRB:CASSLATGTDEQFF	1	CD4-C4
Pla-2_CTTTGCGAGATCCCGC	Pla	Pla-2	P2-4283	FALSE	1	TRA:CATSGEETSGSRLTF TRB:CASSQHAQASETQYF	1	CD4-C4
Pla-2_CTTTGCGAGGAGTAGA	Pla	Pla-2	P2-4284	FALSE	1	TRA:CAKRPGGGNKLTF TRB:CSAPPRTGEALETQYF	1	CD4-C4
Pla-2_CTTTGCGAGTTAAGTG	Pla	Pla-2	P2-531	FALSE	2	TRA:CALSDPRSGGSNYKLTF TRB:CAWAGGFTDTQYF	2	CD4-C4
Pla-2_GAAACTCCAAACCCAT	Pla	Pla-2	P2-4287	FALSE	1	TRA:CAERSSGTYKYIF TRB:CASSKEGANTIYF	1	CD4-C4
Pla-2_GAAACTCGTCTTCTCG	Pla	Pla-2	P2-4290	FALSE	1	TRA:CAPQGGYQKVTF TRB:CAWRSGTSGRWETQYF	1	CD4-C4
Pla-2_GAAATGATCTGCGTAA	Pla	Pla-2	P2-4294	FALSE	1	TRA:CAVTTNFGNEKLTF TRB:CATSDSTGDSFYNEQFF	1	CD4-C4
Pla-2_GAACATCCACTAGTAC	Pla	Pla-2	P2-4297	FALSE	1	TRA:CVVNPGNTGKLIF TRB:CASSSGTLYEQYF	1	CD4-C4
Pla-2_GAACATCCAGCTGGCT	Pla	Pla-2	P2-4298	FALSE	1	TRA:CAVGNQGGKLIF TRB:CATAGGPGTDTQYF	1	CD4-C4
Pla-2_GAACCTACACACCGAC	Pla	Pla-2	P2-4301	FALSE	1	TRA:CAVVNQAGTALIF TRB:CSARRLAGANEQFF	1	CD4-C4
Pla-2_GAATAAGAGTTTAGGA	Pla	Pla-2	P2-4312	FALSE	1	TRA:CAEALVF TRB:CASGRGGLSYNEQFF	1	CD4-C4

Pla-2_GAATAAGGTCTAAACC	Pla	Pla-2	P2-4317	FALSE	1	TRA:CAVRASGAGSYQLTF TRB:CSIGTGGSPDTQYF	1	CD4-C4
Pla-2_GAATGAAAGCTATGCT	Pla	Pla-2	P2-101	TRUE	3	TRA:CATDASGNEKLTF TRB:CASSLGLADTQYF	5	CD4-C4
Pla-2_GAATGAACAGATCCAT	Pla	Pla-2	P2-4322	FALSE	1	TRA:CAVAITGGGNKLTF TRB:CSASNRNQETQYF	1	CD4-C4
Pla-2_GAATGAACATCCCACT	Pla	Pla-2	P2-4323	FALSE	1	TRA:CAVAKDGSGNTGKLIF TRB:CASSGLNPYEQYF	1	CD4-C4
Pla-2_GAATGAAGTGCCTGCA	Pla	Pla-2	P2-417	FALSE	2	TRA:CAALGGSNFGNEKLTF TRB:CASSPGTFGYTF	2	CD4-C4
Pla-2_GACACGCAGTGTCTCA	Pla	Pla-2	P2-4324	FALSE	1	TRA:CAVSRNSGGYQKVTF TRB:CASSLTGQSYEQYF	1	CD4-C4
Pla-2_GACACGCCACCGTTGG	Pla	Pla-2	P2-4325	FALSE	1	TRA:CATVNTNAGKSTF TRB:CASSLVGVGNQPQHF	1	CD4-C2
Pla-2_GACACGCGTAAGGATT	Pla	Pla-2	P2-4326	FALSE	1	TRA:CAFMKNKETSGSRLTF TRB:CASSFERGPPNTQYF	1	CD4-C4
Pla-2_GACACGCTCGGTCCGA	Pla	Pla-2	P2-4331	FALSE	1	TRA:CAVGDSNYQLIW TRB:CAIRQGNYGYTF	1	CD4-C4
Pla-2_GACAGAGGTCAGAGGT	Pla	Pla-2	P2-4333	FALSE	1	TRA:CAYRSGDNTDKLIF TRB:CSASKALGTGDTDTQYF	1	CD4-C4
Pla-2_GACGGCTGTGCAACTT	Pla	Pla-2	P2-4343	FALSE	1	TRA:CAVGPTSGTYKYIF TRB:CATSDHRVGSNQPQHF	1	CD4-C4
Pla-2_GACGTTATCCAGAAGG	Pla	Pla-2	P2-536	FALSE	2	TRA:CIVTAGGTSYGKLTF TRB:CASSSGWGGYEQYF	2	CD4-C4
Pla-2_GACGTTATCGCTAGCG	Pla	Pla-2	P2-4350	FALSE	1	TRA:CAASYKAAGNKLTF TRB:CASSSFDRFGPQHF	1	CD4-C4
Pla-2_GACTAACTCTGCGACG	Pla	Pla-2	P2-50	FALSE	3	TRA:CAPIRAQKLVF TRB:CASSSPPKSGELFF	11	CD4-C4
Pla-2_GACTACAAGAACTCGG	Pla	Pla-2	P2-4352	FALSE	1	TRA:CAMTQNAGKSTF TRB:CASSLRGRAGEQYF	1	CD4-C4
Pla-2_GACTACATCCTCATTA	Pla	Pla-2	P2-443	FALSE	2	TRA:CAVGGGTASKLTF TRB:CASSLPDRGAFF	2	CD4-C4
Pla-2_GACTACATCGTCACGG	Pla	Pla-2	P2-4360	FALSE	1	TRA:CAYPLTQGGSEKLVF TRB:CASSYDRISNGYTF	1	CD4-C4
Pla-2_GACTGCGAGCCAGGAT	Pla	Pla-2	P2-38	TRUE	3	TRA:CAVRDGSDYKLSF TRB:CASKADLNNQPQHF	14	CD4-C4
Pla-2_GACTGCGCAGTACACT	Pla	Pla-2	P2-4364	FALSE	1	TRA:CAVNRVRASGTYKYIF TRB:CASSQDLTGYNEQFF	1	CD4-C4
Pla-2_GAGCAGAAGACAGACC	Pla	Pla-2	P2-4365	FALSE	1	TRA:CAESPPGKAAGNKLTF TRB:CSQDSHEDTEAFF	1	CD4-C4
Pla-2_GAGCAGAGTGCAGGTA	Pla	Pla-2	P2-190	TRUE	3	TRA:CAVSHSGGYQKVTF TRB:CASRRAAHEQYF	3	CD4-C4
Pla-2_GAGGTGACACCGTTGG	Pla	Pla-2	P2-258	FALSE	3	TRA:CVVNGAGGYNKLIF TRB:CASSLRSTGNTEAFF	3	CD4-C4
Pla-2_GAGGTGACAGCTTAAC	Pla	Pla-2	P2-4369	FALSE	1	TRA:CAYRSTSGTYKYIF TRB:CASSSDPRDTQYF	1	CD4-C4
Pla-2_GAGTCCGGTACTTCTT	Pla	Pla-2	P2-39	TRUE	3	TRA:CAARAVTGGGNKLTF TRB:CASSGRSSAWNEQFF	13	CD4-C4
Pla-2_GATCGATTCTTTAGTC	Pla	Pla-2	P2-4377	FALSE	1	TRA:CAVRDIGNTGGFKTIF TRB:CASSPTGVNQPQHF	1	CD4-C4
Pla-2_GATCGTAAGATCTGAA	Pla	Pla-2	P2-4381	FALSE	1	TRA:CATILTEGAQKLVF TRB:CASSVALHTDTQYF	1	CD4-C4
Pla-2_GATCTAGCATTGGGCC	Pla	Pla-2	P2-4384	FALSE	1	TRA:CAMSGVDSSYKLIF TRB:CASSQEAGLAGLNEQFF	1	CD4-C4

Pla-2_GATGAAACACTTACGA	Pla	Pla-2	P2-4385	FALSE	1	TRA:CATGTSGSRLTF TRB:CASSLFLAEQETQYF	1	CD4-C4
Pla-2_GATGAAAGTCACTGGC	Pla	Pla-2	P2-332	TRUE	2	TRA:CAVPGDQAGTALIF TRB:CASSLANAGPVEKLFF	2	CD4-C4
Pla-2_GATGAGGGTGTTCGAT	Pla	Pla-2	P2-4389	FALSE	1	TRA:CAVKAEYGQNFVF TRB:CASSLYPSGGGTDTQYF	1	CD4-C4
Pla-2_GATGCTAAGAGACTAT	Pla	Pla-2	P2-4390	FALSE	1	TRA:CAEKRTGRRALTF TRB:CASSEQGPTNEKLFF	1	CD4-C4
Pla-2_GATGCTACACAAGCCC	Pla	Pla-2	P2-4392	FALSE	1	TRA:CAVRGIQGAQKLVF TRB:CASSTQTGNTGELFF	1	CD4-C4
Pla-2_GATTCAGTCTCGAGTA	Pla	Pla-2	P2-4397	FALSE	1	TRA:CAGVHNTNAGKSTF TRB:CAGRTSASDTQYF	1	CD8-C8
Pla-2_GCACATAGTTGTCGCG	Pla	Pla-2	P2-152	FALSE	3	TRA:CALKGSSGYSTLTF TRB:CSAIPSGGIGTDTQYF	4	CD4-C4
Pla-2_GCACTCTGTCGGGTCT	Pla	Pla-2	P2-462	FALSE	2	TRA:CAVPNNNDMRF TRB:CASSPGLANTGELFF	2	CD4-C4
Pla-2_GCACTCTTCAACACCA	Pla	Pla-2	P2-4406	FALSE	1	TRA:CAVVPTGGTYKYIF TRB:CASSAGQNYGYTF	1	CD4-C4
Pla-2_GCAGCCAAGAGTGACC	Pla	Pla-2	P2-4409	FALSE	1	TRA:CAVGGDFNKFYF TRB:CAISAATGYGYTF	1	CD4-C3
Pla-2_GCAGCCAAGGCTCATT	Pla	Pla-2	P2-4412	FALSE	1	TRA:CAVEDDADKLIF TRB:CSARDPGGGTSYEQYF	1	CD4-C3
Pla-2_GCATGATAGTGCTGCC	Pla	Pla-2	P2-4421	FALSE	1	TRA:CAMRGHRDYGQNFVF TRB:CSATPTGSRETQYF	1	CD4-C4
Pla-2_GCATGCGAGAAGGTTT	Pla	Pla-2	P2-4426	FALSE	1	TRA:CAVEMDSSYKLIF TRB:CSAGVGGTIQYF	1	CD4-C4
Pla-2_GCATGCGAGTCCGGTC	Pla	Pla-2	P2-36	FALSE	3	TRA:CAAQAGNTPLVF TRB:CSAGVGGTIQYF	15	CD4-C5
Pla-2_GCATGTATCCTTAATC	Pla	Pla-2	P2-488	FALSE	2	TRA:CAVSYQAGTALIF TRB:CSAFRRKIQANYGYTF	2	CD4-C4
Pla-2_GCCAAATTCAATAAGG	Pla	Pla-2	P2-4436	FALSE	1	TRA:CAARGVTGTASKLTF TRB:CASRTRETPYNEQFF	1	CD4-C4
Pla-2_GCCTCTACACAGGAGT	Pla	Pla-2	P2-4437	FALSE	1	TRA:CGTEVHTGFQKLVF TRB:CASSRRAGSSYEQYF	1	CD4-C2
Pla-2_GCGACCAGTTCAGCGC	Pla	Pla-2	P2-133	TRUE	3	TRA:CAVNKPAGNKLTF TRB:CASSTRPGGANVLTF	4	CD4-C4
Pla-2_GCGAGAATCATGTCCC	Pla	Pla-2	P2-348	TRUE	2	TRA:CAVSDSNYQLIW TRB:CASGEGTGSDTQYF	2	CD4-C4
Pla-2_GCGCAACTCATCGGAT	Pla	Pla-2	P2-4447	FALSE	1	TRA:CAVTPSWGTDKLIF TRB:CSARDASFASYEQYF	1	CD4-C4
Pla-2_GCGCAGTAGCGATGAC	Pla	Pla-2	P2-530	FALSE	2	TRA:CAVGGDTNAGKSTF TRB:CASNAGTSGRAFQFF	2	CD4-C4
Pla-2_GCGCCAAAGGAATGGA	Pla	Pla-2	P2-4452	FALSE	1	TRA:CAVNRVRASGTYKYIF TRB:CASSPRTVRIGYTQYF	1	CD4-C4
Pla-2_GCGCCAATCCCTGACT	Pla	Pla-2	P2-4455	FALSE	1	TRA:CAVGYSGAGSYQLTF TRB:CASSGETGNTIYF	1	CD4-C4
Pla-2_GCGGGTTCAGGCGATA	Pla	Pla-2	P2-276	TRUE	2	TRA:CALSEAYTGANSKLTF TRB:CASSLLGTSYEQYF	2	CD4-C4
Pla-2_GCGGGTTGTCAGATAA	Pla	Pla-2	P2-50	FALSE	3	TRA:CAPIRAQKLVF TRB:CASSSPPKSGELFF	11	CD4-C4
Pla-2_GCTCCTAGTCTCACCT	Pla	Pla-2	P2-551	FALSE	2	TRA:CAVGPTSGTYKYIF TRB:CASSQLYDEQFF	2	CD4-C4
Pla-2_GCTCCTATCAGCCTAA	Pla	Pla-2	P2-63	FALSE	3	TRA:CAASGEAGTALIF TRB:CATSREIQPNEKLFF	9	CD4-C4

Pla-2_GCTCTGTAGAATGTTG	Pla	Pla-2	P2-4462	FALSE	1	TRA:CAVFNYQLIW TRB:CASSLGGFGYTF	1	CD4-C3
Pla-2_GCTCTGTCAAACCTAC	Pla	Pla-2	P2-172	TRUE	3	TRA:CAVSAKTSYDKVIF TRB:CASSQGGGTGTDTQYF	3	CD4-C4
Pla-2_GCTCTGTCAGATAATG	Pla	Pla-2	P2-116	FALSE	3	TRA:CAVSPPAGSNYQLIW TRB:CATSRETGARYEQYF	5	CD4-C4
Pla-2_GCTGCAGGTCAGAGGT	Pla	Pla-2	P2-146	FALSE	3	TRA:CAMTPGANSKLTF TRB:CASSSPPKSGELFF	4	CD8-C8
Pla-2_GCTGCAGGTTGCGCAC	Pla	Pla-2	P2-4468	FALSE	1	TRA:CAATIDTYKYIF TRB:CASSPSPSYEQYF	1	CD4-C4
Pla-2_GCTGCGAGTCACAAGG	Pla	Pla-2	P2-4470	FALSE	1	TRA:CAVTLTTDSWGKFQF TRB:CASSPGTDLYEQYF	1	CD8-C8
Pla-2_GCTGCTTAGGCGTACA	Pla	Pla-2	P2-4472	FALSE	1	TRA:CAVGQTGANNLFF TRB:CASRRGTGGADTQYF	1	CD4-C4
Pla-2_GCTGCTTCAGGACGTA	Pla	Pla-2	P2-4473	FALSE	1	TRA:CAVISYNTDKLIF TRB:CASSPITGDTQYF	1	CD4-C3
Pla-2_GCTGCTTTCACGCGGT	Pla	Pla-2	P2-4474	FALSE	1	TRA:CAVCGNNNARLMF TRB:CASSSLAGGPRELFF	1	CD4-C4
Pla-2_GCTTCCACATTGAGCT	Pla	Pla-2	P2-4482	FALSE	1	TRA:CAVESEANNLFF TRB:CATATADSYEQYF	1	CD4-C4
Pla-2_GCTTCCAGTACAGACG	Pla	Pla-2	P2-128	TRUE	3	TRA:CAYKKTSYDKVIF TRB:CASSPTSQPQHF	4	CD4-C4
Pla-2_GCTTCCATCCCAGGTG	Pla	Pla-2	P2-4485	FALSE	1	TRA:CAVNRPGTYKYIF TRB:CATSRGQGDHGYRGYTF	1	CD4-C4
Pla-2_GCTTGAAAGCGATGAC	Pla	Pla-2	P2-384	FALSE	2	TRA:CAVSRSNDYKLSF TRB:CASRDPGVYTEAFF	2	CD4-C4
Pla-2_GCTTGAACACGAAAGC	Pla	Pla-2	P2-39	TRUE	3	TRA:CAARAVTGGGNKLTF TRB:CASSGRSSAWNEQFF	13	CD8-C8
Pla-2_GGAAAGCTCCACGTGG	Pla	Pla-2	P2-4491	FALSE	1	TRA:CAEEPLGYSTLTF TRB:CASSLGASGSLYEQYF	1	CD4-C4
Pla-2_GGAACTTCAGCTTCGG	Pla	Pla-2	P2-201	TRUE	3	TRA:CAFRTDSNYQLIW TRB:CASSLASGNQETQYF	3	CD4-C4
Pla-2_GGAATAAAGCGACGTA	Pla	Pla-2	P2-38	TRUE	3	TRA:CAVRDGSDYKLSF TRB:CASKADLNNQPQHF	14	CD4-C4
Pla-2_GGAATAATCTGGCGTG	Pla	Pla-2	P2-220	FALSE	3	TRA:CAMRVPLGGYGQNFVF TRB:CATRDFQETQYF	3	CD4-C4
Pla-2_GGACAGAAGCTCAACT	Pla	Pla-2	P2-14	FALSE	3	TRA:CALSEAPGGSEKLVF TRB:CASSIGHPNSPLHF	37	CD4-C7
Pla-2_GGACAGACAGTCGTGC	Pla	Pla-2	P2-4505	FALSE	1	TRA:CAFMNHNNARLMF TRB:CASSTGDLGNTIYF	1	CD4-C4
Pla-2_GGACAGAGTCTAGGTT	Pla	Pla-2	P2-4506	FALSE	1	TRA:CAVIGSGNTGKLIF TRB:CASSYRREVRAEQYF	1	CD8-C8
Pla-2_GGACATTTCGCCCTTA	Pla	Pla-2	P2-260	FALSE	3	TRA:CAAREVNTGKLIF TRB:CASSEETGRYF	3	CD4-C4
Pla-2_GGACATTTCTGCTGCT	Pla	Pla-2	P2-84	FALSE	3	TRA:CAVSDLTGGGNKLTF TRB:CSVQTEMNTEAFF	7	CD4-C7
Pla-2_GGAGCAACAATCACAC	Pla	Pla-2	P2-4516	FALSE	1	TRA:CAVISYNTDKLIF TRB:CAWSRADTQYF	1	CD4-C4
Pla-2_GGAGCAAGTGATGCCC	Pla	Pla-2	P2-4518	FALSE	1	TRA:CAVTNTGNQFYF TRB:CASRRTGSSEKLFF	1	CD4-C4
Pla-2_GGATGTTCAACGCACC	Pla	Pla-2	P2-42	FALSE	3	TRA:CAVSPRDNNDMRF TRB:CASSARGNSNQPQHF	12	CD4-C4
Pla-2_GGATGTTCAATTGCTG	Pla	Pla-2	P2-4520	FALSE	1	TRA:CAASVGTSGTYKYIF TRB:CASSLGRGTMNTEAFF	1	CD4-C4

Pla-2_GGATGTTCACCGAATT	Pla	Pla-2	P2-531	FALSE	2	TRA:CALSDPRSGGSNYKLTF TRB:CAWAGGFTDTQYF	2	CD4-C4
Pla-2_GGCAATTAGAATTGTG	Pla	Pla-2	P2-4527	FALSE	1	TRA:CAYKPTSYDKVIF TRB:CASSIQGRPGELFF	1	CD4-C4
Pla-2_GGCCGATAGTAGGCCA	Pla	Pla-2	P2-4535	FALSE	1	TRA:CAVSGDNYGQNFVF TRB:CASSRKLGTEAFF	1	CD4-C4
Pla-2_GGCCGATGTATGCTTG	Pla	Pla-2	P2-148	FALSE	3	TRA:CAMRSSGAGSYQLTF TRB:CAILDDVIDTQYF	4	CD8-C2
Pla-2_GGCCGATGTGAAATCA	Pla	Pla-2	P2-554	FALSE	2	TRA:CALSDVSNQFYF TRB:CSARVLANIPGELFF	2	CD4-C4
Pla-2_GGCCGATTCCGAAGAG	Pla	Pla-2	P2-535	FALSE	2	TRA:CAVNRGSTLGRLYF TRB:CASRNDLSGANGYTF	2	CD4-C3
Pla-2_GGCGACTAGCTAGTTC	Pla	Pla-2	P2-4538	FALSE	1	TRA:CAYRSAEGGSEKLVF TRB:CASSLARGLKIYNEQFF	1	CD4-C4
Pla-2_GGCGTGTGTCCGAACC	Pla	Pla-2	P2-519	FALSE	2	TRA:CAYNFGNEKLTF TRB:CASSPAGGPYNEQFF	2	CD4-C4
Pla-2_GGCTCGACATCGGTTA	Pla	Pla-2	P2-4547	FALSE	1	TRA:CAVPNAGGTSYGKLTF TRB:CASSFTPLNTGELFF	1	CD4-C4
Pla-2_GGCTCGAGTTGCGTTA	Pla	Pla-2	P2-528	FALSE	2	TRA:CAVRDGRGFGNVLHC TRB:CSAVGNSGGSSVDTQYF	2	CD4-C4
Pla-2_GGCTCGATCAAACCGT	Pla	Pla-2	P2-4548	FALSE	1	TRA:CATSGNQFYF TRB:CASSARGSPGNTIYF	1	CD4-C4
Pla-2_GGCTGGTTCCTCAACC	Pla	Pla-2	P2-173	TRUE	3	TRA:CAENNGHGGSTLGRLYF TRB:CSLRGDSGYTF	3	CD4-C4
Pla-2_GGCTGGTTCTCTAGGA	Pla	Pla-2	P2-4551	FALSE	1	TRA:CAVRDIGFGNVLHC TRB:CAISVGGGVDTQYF	1	CD4-C4
Pla-2_GGGAATGTCTTGGGTA	Pla	Pla-2	P2-4552	FALSE	1	TRA:CAEKGSRNTDKLIF TRB:CASTLGRGWNEQFF	1	CD4-C4
Pla-2_GGGAGATTCGGAGGTA	Pla	Pla-2	P2-185	TRUE	3	TRA:CAPSGNTPLVF TRB:CSARKTTSGNTIYF	3	CD4-C4
Pla-2_GGGATGATCTGTCTCG	Pla	Pla-2	P2-253	FALSE	3	TRA:CAMRESGGGADGLTF TRB:CAWRRGNTEAFF	3	CD4-C4
Pla-2_GGGCATCAGTAGGCCA	Pla	Pla-2	P2-4562	FALSE	1	TRA:CAVGGNEKLTF TRB:CASSQASSRSIDTQYF	1	CD4-C4
Pla-2_GGGTTGCCATTGTGCA	Pla	Pla-2	P2-4570	FALSE	1	TRA:CAVNAYSGAGSYQLTF TRB:CASSRTGEGRQPQHF	1	CD4-C4
Pla-2_GGGTTGCGTATGAATG	Pla	Pla-2	P2-258	FALSE	3	TRA:CVVNGAGGYNKLIF TRB:CASSLRSTGNTEAFF	3	CD4-C4
Pla-2_GGTATTGAGCGATATA	Pla	Pla-2	P2-32	TRUE	3	TRA:CAMREANVGADGLTF TRB:CASSTRIAGGPFPETQYF	17	CD4-C6
Pla-2_GGTATTGCAATCGGTT	Pla	Pla-2	P2-36	FALSE	3	TRA:CAAQAGNTPLVF TRB:CSAGVGGTIQYF	15	CD4-C4
Pla-2_GTAACGTAGACTTGAA	Pla	Pla-2	P2-62	FALSE	3	TRA:CAVITDTGRRALTF TRB:CASSIGTSGYNEQFF	9	CD4-C4
Pla-2_GTAACGTAGAGCTATA	Pla	Pla-2	P2-149	FALSE	3	TRA:CAVTNRGNNRLAF TRB:CSARVPGGLSDEQFF	4	CD4-C4
Pla-2_GTAACTGCAGACGTAG	Pla	Pla-2	P2-4585	FALSE	1	TRA:CAVDMTSGSEKLVF TRB:CASSVQPGLADKETQYF	1	CD8-C8
Pla-2_GTAACTGTCGCGGATC	Pla	Pla-2	P2-4588	FALSE	1	TRA:CVVNDNTDKLIF TRB:CASSQEAGQGLGYTF	1	CD8-C8
Pla-2_GTACTCCAGACCTAGG	Pla	Pla-2	P2-4592	FALSE	1	TRA:CIVRALTDSWGKFQF TRB:CASSIAAGESYEQYF	1	CD4-C4
Pla-2_GTACTCCCAGCTCCGA	Pla	Pla-2	P2-39	TRUE	3	TRA:CAARAVTGGGNKLTF TRB:CASSGRSSAWNEQFF	13	CD4-C4

Pla-2_GTACTCCTCATTGCGA	Pla	Pla-2	P2-4596	FALSE	1	TRA:CAPDKAGNQFYF TRB:CASRQADYNEQFF	1	CD4-C4
Pla-2_GTACTCCTCTGCCCTA	Pla	Pla-2	P2-556	FALSE	2	TRA:CAAIEGGDRGSTLGRLYF TRB:CASSLRNLGLAGKNSYEQ	2	CD4-C4
Pla-2_GTACTTTTCAACGGCC	Pla	Pla-2	P2-85	TRUE	3	TRA:CAVTAYTNAGKSTF TRB:CASRGRSSGNTIYF	6	CD4-C4
Pla-2_GTAGTCAAGGACCACA	Pla	Pla-2	P2-4602	FALSE	1	TRA:CAVINAGGTSYGKLTF TRB:CASSVRVGETQYF	1	CD4-C4
Pla-2_GTAGTCAGTCGGCATC	Pla	Pla-2	P2-82	FALSE	3	TRA:CVADTGNQFYF TRB:CASSSALTGTYEQYF	7	CD4-C4
Pla-2_GTAGTCATCATAAAGG	Pla	Pla-2	P2-4603	FALSE	1	TRA:CAFSDSNYQLIW TRB:CASSALRINEQFF	1	CD8-C8
Pla-2_GTATCTTAGATGAGAG	Pla	Pla-2	P2-4604	FALSE	1	TRA:CAASGRAGSYQLTF TRB:CASMEVNEQFF	1	CD4-C4
Pla-2_GTCACAATCATGCAAC	Pla	Pla-2	P2-90	FALSE	3	TRA:CIGNTPLVF TRB:CASSDRDRVLNTEAFF	6	CD8-C8
Pla-2_GTCACGGAGCAGCCTC	Pla	Pla-2	P2-4613	FALSE	1	TRA:CALHMDSNYQLIW TRB:CASTRTSGSSYEQYF	1	CD4-C4
Pla-2_GTCACGGCACTTCTGC	Pla	Pla-2	P2-4616	FALSE	1	TRA:CAVIRFNKFYF TRB:CASNIQGAGELFF	1	CD4-C4
Pla-2_GTCATTTAGACAAAGG	Pla	Pla-2	P2-4617	FALSE	1	TRA:CAASTGSARQLTF TRB:CASSQPTGTGTGELFF	1	CD4-C4
Pla-2_GTCATTTCATCGGAAG	Pla	Pla-2	P2-124	TRUE	3	TRA:CAFFNTDKLIF TRB:CASSPRPNYGYTF	4	CD4-C4
Pla-2_GTCCTCACATCGACGC	Pla	Pla-2	P2-4620	FALSE	1	TRA:CAVDHNQAGTALIF TRB:CASSIGGNIQYF	1	CD4-C4
Pla-2_GTCCTCAGTCCGAAGA	Pla	Pla-2	P2-558	FALSE	2	TRA:CIVRVGNSGYALNF TRB:CASSQVPAAGELFF	2	CD4-C4
Pla-2_GTCGGGTGTGATGATA	Pla	Pla-2	P2-4621	FALSE	1	TRA:CAMRGLNTGGFKTIF TRB:CASSPVSAQSTDTQYF	1	CD4-C4
Pla-2_GTCGTAATCCCAACGG	Pla	Pla-2	P2-4622	FALSE	1	TRA:CAERSTYGQNFVF TRB:CASSIEDRDRQPQHF	1	CD4-C4
Pla-2_GTCTCGTAGCCACTAT	Pla	Pla-2	P2-327	TRUE	2	TRA:CAVHSNYQLIW TRB:CASSTMGQAQETQYF	2	CD4-C4
Pla-2_GTCTCGTGTGCCTGTG	Pla	Pla-2	P2-407	FALSE	2	TRA:CAMSAGTGRRALTF TRB:CASSHYRATFDQYF	2	CD4-C4
Pla-2_GTGAAGGAGGCTAGGT	Pla	Pla-2	P2-479	FALSE	2	TRA:CAVQARDNNARLMF TRB:CAWSVPQGRAFF	2	CD4-C4
Pla-2_GTGCAGCCAAAGAATC	Pla	Pla-2	P2-4633	FALSE	1	TRA:CVVNLGGGGADGLTF TRB:CASSPAGQNYGYTF	1	CD4-C4
Pla-2_GTGCATACACAGAGGT	Pla	Pla-2	P2-4635	FALSE	1	TRA:CAVSEGNNDMRF TRB:CASSLGQGYNEKLFF	1	CD4-C4
Pla-2_GTGCATACAGATGGGT	Pla	Pla-2	P2-217	FALSE	3	TRA:CAIRDNQGGKLIF TRB:CASTGQLTDTQYF	3	CD4-C4
Pla-2_GTGCATACATGGTAGG	Pla	Pla-2	P2-4638	FALSE	1	TRA:CAMRGMTQKLVF TRB:CASRTSGGAVSTGELFF	1	CD4-C4
Pla-2_GTGCGGTAGAGACTTA	Pla	Pla-2	P2-28	TRUE	3	TRA:CATDANTGNQFYF TRB:CASSLGQWVEQYF	22	CD4-C4
Pla-2_GTGCGGTCACAGCGTC	Pla	Pla-2	P2-4639	FALSE	1	TRA:CAVNIDGNKLVF TRB:CASSFGGGGEQYF	1	CD4-C4
Pla-2_GTGCGGTGTCATGCAT	Pla	Pla-2	P2-4640	FALSE	1	TRA:CAVGFAGGTSYGKLTF TRB:CASSLRTGGDEQYF	1	CD4-C4
Pla-2_GTGGGTCAGTGCCATT	Pla	Pla-2	P2-4644	FALSE	1	TRA:CGTETTSGTSYGKLTF TRB:CASSDHYEQYF	1	CD4-C4

Pla-2_GTGTGCGCAGACGCAA	Pla	Pla-2	P2-551	FALSE	2	TRA:CAVGPTSGTYKYIF TRB:CASSQLYDEQFF	2	CD4-C4
Pla-2_GTGTGCGGTGACTACT	Pla	Pla-2	P2-4650	FALSE	1	TRA:CAVEDLKNNFNKFYF TRB:CASSPGQGARGETQYF	1	CD4-C4
Pla-2_GTGTTAGAGATGGGTC	Pla	Pla-2	P2-165	FALSE	3	TRA:CAAHEGWAYKYIF TRB:CASSLGQTGQFHF	4	CD4-C4
Pla-2_GTGTTAGGTAAGAGGA	Pla	Pla-2	P2-195	TRUE	3	TRA:CAVSKGGSYIPTF TRB:CASRSGSSTDTQYF	3	CD4-C4
Pla-2_GTTAAGCGTTCGCTAA	Pla	Pla-2	P2-4655	FALSE	1	TRA:CAVQRLRDDKIIF TRB:CASSLAQGSEQYF	1	CD4-C4
Pla-2_GTTAAGCTCCGCAAGC	Pla	Pla-2	P2-4656	FALSE	1	TRA:CALIRYNFNKFYF TRB:CSAREDRGFSPLHF	1	CD4-C4
Pla-2_GTTACAGAGTGCCAGA	Pla	Pla-2	P2-4660	FALSE	1	TRA:CAVRDLMNYGGSQGNLIF TRB:CASSLSASGGLGEQFF	1	CD4-C4
Pla-2_GTTCATTAGAGATGAG	Pla	Pla-2	P2-4662	FALSE	1	TRA:CALKETGANNLFF TRB:CASSLEAFYEQYF	1	CD4-C4
Pla-2_GTTCATTTCGCGTAGC	Pla	Pla-2	P2-4664	FALSE	1	TRA:CSAWSDGQKLLF TRB:CSAGSLGGGELFF	1	CD4-C4
Pla-2_GTTCGGGTCAACGAAA	Pla	Pla-2	P2-560	FALSE	2	TRA:CAVNAQGRDDKIIF TRB:CASSSGRTTHSYEQYF	2	CD4-C4
Pla-2_GTTTCTACATTGTGCA	Pla	Pla-2	P2-561	FALSE	2	TRA:CAETFPWGFQKLVF TRB:CAWSVPGTGDVF	2	CD4-C4
Pla-2_GTTTCTAGTCACACGC	Pla	Pla-2	P2-4666	FALSE	1	TRA:CALSEPNDTGRRALTF TRB:CASSGDTFSYEQYF	1	CD4-C4
Pla-2_GTTTCTATCGCTAGCG	Pla	Pla-2	P2-556	FALSE	2	TRA:CAAIEGGDRGSTLGRLYF TRB:CASSLRNLGLAGKNSYEQ[illegible]	2	CD4-C4
Pla-2_TAAACCGCAGCTATTG	Pla	Pla-2	P2-4668	FALSE	1	TRA:CAASAPGTYKYIF TRB:CASSLQGSTYNEQFF	1	CD4-C4
Pla-2_TAAGAGATCATTTGGG	Pla	Pla-2	P2-4670	FALSE	1	TRA:CAMDTGRRALTF TRB:CASSPQERGTLQQYF	1	CD4-C4
Pla-2_TAAGCGTGTACCGAGA	Pla	Pla-2	P2-4673	FALSE	1	TRA:CAVTNNARLMF TRB:CASSRDINQPQHF	1	CD4-C4
Pla-2_TAAGCGTGTGCATCTA	Pla	Pla-2	P2-4675	FALSE	1	TRA:CAATYQGGSEKLVF TRB:CASTLGLAGPRGEQFF	1	CD4-C4
Pla-2_TAAGTGCGTAACGACG	Pla	Pla-2	P2-32	TRUE	3	TRA:CAMREANVGADGLTF TRB:CASSTRIAGGPFPETQYF	17	CD4-C4
Pla-2_TAAGTGCGTACATGTC	Pla	Pla-2	P2-4677	FALSE	1	TRA:CVVSETTGGFKTIF TRB:CASSLEASGGYEQYF	1	CD4-C4
Pla-2_TAAGTGCTCCGTAGTA	Pla	Pla-2	P2-253	FALSE	3	TRA:CAMRESGGGADGLTF TRB:CAWRRGNTEAFF	3	CD4-C4
Pla-2_TACAGTGCAGTCCTTC	Pla	Pla-2	P2-236	FALSE	3	TRA:CALPPPTGTASKLTF TRB:CASTGPSYEQYF	3	CD4-C4
Pla-2_TACAGTGGTAAGGGCT	Pla	Pla-2	P2-511	FALSE	2	TRA:CAASPASGGSYIPTF TRB:CASSSHQGGSGYTF	2	CD8-C8
Pla-2_TACAGTGGTGATGCCC	Pla	Pla-2	P2-18	TRUE	3	TRA:CAATSSSNFGNEKLTF TRB:CAWSRAALTEAFF	28	CD4-C4
Pla-2_TACAGTGTCTTGCCGT	Pla	Pla-2	P2-4682	FALSE	1	TRA:CAVRDFNQGGKLIF TRB:CASSFAGNEKLFF	1	CD4-C4
Pla-2_TACCTATTCTTCTGGC	Pla	Pla-2	P2-124	TRUE	3	TRA:CAFFNTDKLIF TRB:CASSPRPNYGYTF	4	CD8-C8
Pla-2_TACCTTACAGCTGTGC	Pla	Pla-2	P2-185	TRUE	3	TRA:CAPSGNTPLVF TRB:CSARKTTSGNTIYF	3	CD4-C3
Pla-2_TACGGGCTCTTGCCGT	Pla	Pla-2	P2-4694	FALSE	1	TRA:CALSEASHSGYALNF TRB:CASSLMPLAGGTRLTDTQYF	1	CD4-C4

Pla-2_TACGGTAGTGTAACGG	Pla	Pla-2	P2-4696	FALSE	1	TRA:CAVPGAGSYQLTF TRB:CSARDSGGGHYNEQFF	1	CD4-C4
Pla-2_TACTCATAGACGCACA	Pla	Pla-2	P2-4697	FALSE	1	TRA:CATAGNSRGKLIF TRB:CASSPATGGIYEQYF	1	CD4-C4
Pla-2_TACTCGCAGTGAACAT	Pla	Pla-2	P2-217	FALSE	3	TRA:CAIRDNQGGKLIF TRB:CASTGQLTDTQYF	3	CD4-C4
Pla-2_TACTCGCCACCACGTG	Pla	Pla-2	P2-28	TRUE	3	TRA:CATDANTGNQFYF TRB:CASSLGQWVEQYF	22	CD4-C4
Pla-2_TACTCGCCATCTCCCA	Pla	Pla-2	P2-4703	FALSE	1	TRA:CAVKKETSGSRLTF TRB:CASSLAGSGYEQYF	1	CD4-C4
Pla-2_TACTCGCCATTGAGCT	Pla	Pla-2	P2-4705	FALSE	1	TRA:CVFNAGGTSYGKLTF TRB:CASSALAGGYNEQFF	1	CD4-C4
Pla-2_TACTCGCTCATGTGGT	Pla	Pla-2	P2-4706	FALSE	1	TRA:CAVGPMNYGGSQGNLIF TRB:CASRRQGQREQYF	1	CD8-C8
Pla-2_TACTCGCTCCAAGCCG	Pla	Pla-2	P2-4707	FALSE	1	TRA:CAMSVGGGFKTIF TRB:CASSPRDRGETQYF	1	CD4-C4
Pla-2_TACTTACTCAAGGCTT	Pla	Pla-2	P2-38	TRUE	3	TRA:CAVRDGSDYKLSF TRB:CASKADLNNQPQHF	14	CD4-C4
Pla-2_TACTTGTAGCTGGAAC	Pla	Pla-2	P2-4711	FALSE	1	TRA:CAVRTSYDKVIF TRB:CASSGTYGGRSGETQYF	1	CD4-C4
Pla-2_TACTTGTGTGCCTGCA	Pla	Pla-2	P2-4713	FALSE	1	TRA:CATDADTDKLIF TRB:CAWNRGPNTGELFF	1	CD4-C5
Pla-2_TAGACCAAGTCAATAG	Pla	Pla-2	P2-262	FALSE	3	TRA:CAVKKRDNQGGKLIF TRB:CASSTIARDTQYF	3	CD4-C4
Pla-2_TAGACCATCACGGTTA	Pla	Pla-2	P2-18	TRUE	3	TRA:CAATSSSNFGNEKLTF TRB:CAWSRAALTEAFF	28	CD4-C4
Pla-2_TAGAGCTGTTAAGACA	Pla	Pla-2	P2-4718	FALSE	1	TRA:CAVRDIHGAGNNRKLIW TRB:CASRRGTVINSPLHF	1	CD8-C8
Pla-2_TAGAGCTTCCCACTTG	Pla	Pla-2	P2-4719	FALSE	1	TRA:CAVEDPGSQGNLIF TRB:CAGNYRTEKLFF	1	CD8-C8
Pla-2_TAGCCGGCAGATCTGT	Pla	Pla-2	P2-4720	FALSE	1	TRA:CAMSEKTSYDKVIF TRB:CSARLFGRVEQYF	1	CD4-C4
Pla-2_TAGCCGGTCTGTCAAG	Pla	Pla-2	P2-4721	FALSE	1	TRA:CALSPFNYQLIW TRB:CASSRDSTGNYEQYF	1	CD4-C4
Pla-2_TAGGCATAGCGATGAC	Pla	Pla-2	P2-4722	FALSE	1	TRA:CAFMKRDSNYQLIW TRB:CAWSERTGWSNQPQHF	1	CD4-C4
Pla-2_TAGGCATTCTCTAGGA	Pla	Pla-2	P2-32	TRUE	3	TRA:CAMREANVGADGLTF TRB:CASSTRIAGGPFPETQYF	17	CD4-C5
Pla-2_TAGTGGTGTCACTTCC	Pla	Pla-2	P2-50	FALSE	3	TRA:CAPIRAQKLVF TRB:CASSSPPKSGELFF	11	CD4-C4
Pla-2_TAGTGGTGTTCCGTCT	Pla	Pla-2	P2-4727	FALSE	1	TRA:CAVNKPGRRALTF TRB:CASSLASGEQYF	1	CD4-C4
Pla-2_TAGTTGGTCACGAAGG	Pla	Pla-2	P2-4730	FALSE	1	TRA:CAAVSNFGNEKLTF TRB:CASSFRASTDTQYF	1	CD4-C4
Pla-2_TATCAGGGTAAGGATT	Pla	Pla-2	P2-4731	FALSE	1	TRA:CALIKAAGNKLTF TRB:CASSLSQGESQPQHF	1	CD4-C4
Pla-2_TATCTCAAGACCACGA	Pla	Pla-2	P2-4733	FALSE	1	TRA:CILRIIQGAQKLVF TRB:CASSSGGYNEQFF	1	CD4-C4
Pla-2_TATCTCAAGGAGCGTT	Pla	Pla-2	P2-4734	FALSE	1	TRA:CAVNGVNFGNEKLTF TRB:CASSQETGTAVIF	1	CD4-C7
Pla-2_TATCTCAAGTAGATGT	Pla	Pla-2	P2-192	TRUE	3	TRA:CAMKHDYKLSF TRB:CASSQDPNQPQHF	3	CD4-C4
Pla-2_TCAACGAGTACCTACA	Pla	Pla-2	P2-4744	FALSE	1	TRA:CIVRGSGSARQLTF TRB:CASSQGTGRQETQYF	1	CD4-C4

Pla-2_TCAACGAGTCATACTG	Pla	Pla-2	P2-4746	FALSE	1	TRA:CAVYLSSASKIIF TRB:CSASGWTGFDYGYTF	1	CD4-C4
Pla-2_TCAATCTAGGAATTAC	Pla	Pla-2	P2-4748	FALSE	1	TRA:CALSGGGFKTIF TRB:CSAKPPSGDYNEQFF	1	CD4-C4
Pla-2_TCAATCTGTAAGTGTA	Pla	Pla-2	P2-409	FALSE	2	TRA:CAPDTGNQFYF TRB:CAWSSGQGIYNEQFF	2	CD4-C4
Pla-2_TCACAAGAGACCCACC	Pla	Pla-2	P2-4749	FALSE	1	TRA:CAMANSGYALNF TRB:CAWSGGRAGRNEQFF	1	CD8-C3
Pla-2_TCACAAGCACAGCGTC	Pla	Pla-2	P2-4751	FALSE	1	TRA:CAITGNQFYF TRB:CASRKTGAITGELFF	1	CD4-C4
Pla-2_TCACGAAAGTCCGTAT	Pla	Pla-2	P2-4756	FALSE	1	TRA:CAVRYSGAGSYQLTF TRB:CASSPGTGYTF	1	CD4-C4
Pla-2_TCACGAACATTATCTC	Pla	Pla-2	P2-4757	FALSE	1	TRA:CALVANAGNMLTF TRB:CASSLGVEKLFF	1	CD4-C4
Pla-2_TCAGCAAAGTGCGATG	Pla	Pla-2	P2-4763	FALSE	1	TRA:CAVGVGAAGNKLTF TRB:CASSPSLPFETQYF	1	CD4-C4
Pla-2_TCAGCTCGTCGGATCC	Pla	Pla-2	P2-4771	FALSE	1	TRA:CAGGGPETGTASKLTF TRB:CASSETTGGWEKLFF	1	CD4-C4
Pla-2_TCAGGATGTCCGTTAA	Pla	Pla-2	P2-50	FALSE	3	TRA:CAPIRAQKLVF TRB:CASSSPPKSGELFF	11	CD4-C4
Pla-2_TCAGGATGTGCCTGCA	Pla	Pla-2	P2-98	TRUE	3	TRA:CATDAIRGNTPLVF TRB:CASSDPGGPLHF	5	CD4-C4
Pla-2_TCAGGATTCATTGCCC	Pla	Pla-2	P2-4775	FALSE	1	TRA:CALSPGTSYGKLTF TRB:CASRGGASYGYTF	1	CD4-C4
Pla-2_TCAGGTAAGAGCAATT	Pla	Pla-2	P2-260	FALSE	3	TRA:CAAREVNTGKLIF TRB:CASSEETGRYF	3	CD4-C4
Pla-2_TCATTACTCCGTCAAA	Pla	Pla-2	P2-4780	FALSE	1	TRA:CAVTGGNARLMF TRB:CASSREGANSGNTIYF	1	CD8-C8
Pla-2_TCATTTGAGCTGAAAT	Pla	Pla-2	P2-28	TRUE	3	TRA:CATDANTGNQFYF TRB:CASSLGQWVEQYF	22	CD4-C4
Pla-2_TCATTTGTCCGTTGTC	Pla	Pla-2	P2-426	FALSE	2	TRA:CVVRESGAGSYQLTF TRB:CASSRGTGYTF	2	CD4-C4
Pla-2_TCCACACAGAGGTTGC	Pla	Pla-2	P2-4785	FALSE	1	TRA:CALSEGLGRGSTLGRLYF TRB:CASSRPAGVRNEQYF	1	CD4-C4
Pla-2_TCCACACCACCCAGTG	Pla	Pla-2	P2-28	TRUE	3	TRA:CATDANTGNQFYF TRB:CASSLGQWVEQYF	22	CD4-C4
Pla-2_TCGAGGCCAATGGTCT	Pla	Pla-2	P2-186	TRUE	3	TRA:CAVRADTNAGKSTF TRB:CASSKVGTEAFF	3	CD4-C4
Pla-2_TCGCGTTTCTACTTAC	Pla	Pla-2	P2-218	FALSE	3	TRA:CVVSPYTGGGNKLTF TRB:CASWGDSYNSPLHF	3	CD4-C4
Pla-2_TCGCGTTTCTCCAACC	Pla	Pla-2	P2-146	FALSE	3	TRA:CAMTPGANSKLTF TRB:CASSSPPKSGELFF	4	CD4-C4
Pla-2_TCGGGACTCGCAAGCC	Pla	Pla-2	P2-546	FALSE	2	TRA:CAVPGAQKLVF TRB:CASSEALLGSASNEKLFF	2	CD8-C8
Pla-2_TCGGTAACAAGAAGAG	Pla	Pla-2	P2-4801	FALSE	1	TRA:CALSPSVQGAQKLVF TRB:CSASSPGRLNEQYF	1	CD4-C4
Pla-2_TCGGTAACAGCTGGCT	Pla	Pla-2	P2-4802	FALSE	1	TRA:CAPSANKFYF TRB:CSVVRDNEQFF	1	CD8-C8
Pla-2_TCGGTAACATGCCTAA	Pla	Pla-2	P2-162	FALSE	3	TRA:CIVRAMNSGYSTLTF TRB:CSAPPWQGAYEQYF	4	CD4-C4
Pla-2_TCGTACCGTTAAGATG	Pla	Pla-2	P2-4808	FALSE	1	TRA:CAMRDADLDSSYKLIF TRB:CASSLQGSKLYNEQFF	1	CD4-C4
Pla-2_TCGTAGACAAGTAGTA	Pla	Pla-2	P2-4810	FALSE	1	TRA:CAVTPPSGTYKYIF TRB:CASSLDGKPQHF	1	CD4-C4

Pla-2_TCTATTGAGGCAGTCA	Pla	Pla-2	P2-4815	FALSE	1	TRA:CAVEDLNTDKLIF TRB:CAWSPGNQPQHF	1	CD4-C4
Pla-2_TCTCATAGTCTCTTTA	Pla	Pla-2	P2-4817	FALSE	1	TRA:CAVSGSTGANSKLTF TRB:CASSWGTSGGETQYF	1	CD4-C4
Pla-2_TCTCTAACACACATGT	Pla	Pla-2	P2-4819	FALSE	1	TRA:CAVRVNTNAGKSTF TRB:CASSAVGTEAFF	1	CD4-C4
Pla-2_TCTGAGACACAACGTT	Pla	Pla-2	P2-160	FALSE	3	TRA:CAAIRISVDGNVLHC TRB:CASSRPGYEQFF	4	CD4-C4
Pla-2_TCTGAGAGTAAGTTCC	Pla	Pla-2	P2-218	FALSE	3	TRA:CVVSPYTGGGNKLTF TRB:CASWGDSYNSPLHF	3	CD4-C4
Pla-2_TCTTCGGAGGATGGTC	Pla	Pla-2	P2-4835	FALSE	1	TRA:CAGKAWNSGYALNF TRB:CASRVGTGVHEQYF	1	CD4-C4
Pla-2_TCTTTCCGTGGCAAAC	Pla	Pla-2	P2-4846	FALSE	1	TRA:CAATAVTGTASKLTF TRB:CASSAGQGYYNEQFF	1	CD4-C4
Pla-2_TGAAAGATCAGTTGAC	Pla	Pla-2	P2-139	FALSE	3	TRA:CAVATGFQKLVF TRB:CASTLGGQGNNEQFF	4	CD4-C4
Pla-2_TGACGGCAGCGTCAAG	Pla	Pla-2	P2-4849	FALSE	1	TRA:CAASKGRGAGTALIF TRB:CSASRVETQYF	1	CD4-C4
Pla-2_TGACGGCAGCGTGAAC	Pla	Pla-2	P2-4850	FALSE	1	TRA:CAVDGARLMF TRB:CASSLSRGGRETQYF	1	CD8-C8
Pla-2_TGACGGCAGGGCTCTC	Pla	Pla-2	P2-131	TRUE	3	TRA:CATVGQIQGAQKLVF TRB:CASRTGTSSYEQYF	4	CD4-C4
Pla-2_TGACGGCGTAAACACA	Pla	Pla-2	P2-4852	FALSE	1	TRA:CAETPDGGATNKLIF TRB:CASSTGQGNPTQYF	1	CD4-C4
Pla-2_TGACTAGCACGGTTTA	Pla	Pla-2	P2-137	FALSE	3	TRA:CAVRDMVDNFNKFYF TRB:CASSSGTGGFYEQYF	4	CD4-C4
Pla-2_TGAGCATTCAGCTCGG	Pla	Pla-2	P2-4864	FALSE	1	TRA:CAVRRPNAGGTSYGKLTF TRB:CSARSAWYYEQYF	1	CD4-C4
Pla-2_TGAGCCGGTGACGCCT	Pla	Pla-2	P2-262	FALSE	3	TRA:CAVKKRDNQGGKLIF TRB:CASSTIARDTQYF	3	CD4-C4
Pla-2_TGAGGGAGTAAATACG	Pla	Pla-2	P2-146	FALSE	3	TRA:CAMTPGANSKLTF TRB:CASSSPPKSGELFF	4	CD4-C4
Pla-2_TGAGGGAGTACCTACA	Pla	Pla-2	P2-28	TRUE	3	TRA:CATDANTGNQFYF TRB:CASSLGQWVEQYF	22	CD4-C4
Pla-2_TGATTTCGTGTCTGAT	Pla	Pla-2	P2-4873	FALSE	1	TRA:CAFEDNNARLMF TRB:CASSLDRGNEQFF	1	CD4-C4
Pla-2_TGATTTCGTTACAGAA	Pla	Pla-2	P2-4874	FALSE	1	TRA:CAVEVGGATNKLIF TRB:CASTLSGANVLTF	1	CD4-C4
Pla-2_TGCCAAACACCTTGTC	Pla	Pla-2	P2-4881	FALSE	1	TRA:CAGDRSGGSNYKLTF TRB:CASSTLGDRSRETQYF	1	CD4-C4
Pla-2_TGCCCTAAGCCGATTT	Pla	Pla-2	P2-4887	FALSE	1	TRA:CALDDRETSYDKVIF TRB:CASSYLGNNEQFF	1	CD4-C4
Pla-2_TGCCCTAAGCTAAGAT	Pla	Pla-2	P2-483	FALSE	2	TRA:CAPERVNQGGKLIF TRB:CASSNTYEQYF	2	CD4-C4
Pla-2_TGCGCAGTCACCGTAA	Pla	Pla-2	P2-4891	FALSE	1	TRA:CAMREASNFNKFYF TRB:CSASISRWDTQYF	1	CD4-C4
Pla-2_TGCGTGGTCATTGCCC	Pla	Pla-2	P2-4896	FALSE	1	TRA:CAVGPSYNTDKLIF TRB:CASRRPGQGIEQYF	1	CD4-C4
Pla-2_TGCGTGGTCGCGGATC	Pla	Pla-2	P2-36	FALSE	3	TRA:CAAQAGNTPLVF TRB:CSAGVGGTIQYF	15	CD4-C4
Pla-2_TGCTACCAGACAATAC	Pla	Pla-2	P2-236	FALSE	3	TRA:CALPPPTGTASKLTF TRB:CASTGPSYEQYF	3	CD4-C4
Pla-2_TGCTACCCATGTCTCC	Pla	Pla-2	P2-4899	FALSE	1	TRA:CAVNPSGGYNKLIF TRB:CASSPFGNQPQHF	1	CD4-C4

Pla-2_TGGACGCTCTCTGCTG	Pla	Pla-2	P2-267	TRUE	2	TRA:CIVRVNYGNNRLAF TRB:CASSFSGAWNEQFF	2	CD4-C4
Pla-2_TGGCCAGAGTGGAGTC	Pla	Pla-2	P2-552	FALSE	2	TRA:CAGGRSGNTGKLIF TRB:CASGTTGSSYEQYF	2	CD4-C4
Pla-2_TGGCCAGCATGGTCTA	Pla	Pla-2	P2-151	FALSE	3	TRA:CAVRFGGNQFYF TRB:CASSLAGTQNEQFF	4	CD4-C4
Pla-2_TGGCCAGGTCTAACGT	Pla	Pla-2	P2-4910	FALSE	1	TRA:CAPSTSNTDKLIF TRB:CAWSARQGANYGYTF	1	CD4-C4
Pla-2_TGGCGCAAGTCCATAC	Pla	Pla-2	P2-132	TRUE	3	TRA:CAANRAGNNRKLIW TRB:CASSFKKAGGPVGELFF	4	CD4-C4
Pla-2_TGGCGCACACTCGACG	Pla	Pla-2	P2-4914	FALSE	1	TRA:CAVSVYSGAGSYQLTF TRB:CASSPTEGKGRNIQYF	1	CD4-C4
Pla-2_TGGGAAGCATTGTGCA	Pla	Pla-2	P2-4925	FALSE	1	TRA:CAVEDSGNTPLVF TRB:CASTSRGQTTYEQYF	1	CD4-C4
Pla-2_TGGGAAGTCGCAAACT	Pla	Pla-2	P2-4926	FALSE	1	TRA:CAGLRNYGGSQGNLIF TRB:CSARGQTGNTEAFF	1	CD4-C4
Pla-2_TGGGCGTGTCCGACGT	Pla	Pla-2	P2-4929	FALSE	1	TRA:CAVRDLAGGGNKLTF TRB:CASGGTAANTGELFF	1	CD4-C4
Pla-2_TGTCCCACATTTGCCC	Pla	Pla-2	P2-4949	FALSE	1	TRA:CAVRENTGFQKLVF TRB:CASSEGPEKETQYF	1	CD4-C4
Pla-2_TGTCCCAGTCAACATC	Pla	Pla-2	P2-40	TRUE	3	TRA:CALRETSGSRLTF TRB:CASSPGRYEQYF	12	CD4-C4
Pla-2_TGTCCCATCTTGTACT	Pla	Pla-2	P2-4951	FALSE	1	TRA:CAASSVGNEKLTF TRB:CASSPGQEGPPLHF	1	CD4-C4
Pla-2_TGTGTTTGTACTTCTT	Pla	Pla-2	P2-64	FALSE	3	TRA:CAATSLISDGQKLLF TRB:CSARDSFNYGYTF	9	CD4-C4
Pla-2_TGTTCCGAGTGTTAGA	Pla	Pla-2	P2-4956	FALSE	1	TRA:CERSTQAGTALIF TRB:CASSPGGLETQYF	1	CD4-C4
Pla-2_TGTTCCGCAGCCTTTC	Pla	Pla-2	P2-128	TRUE	3	TRA:CAYKKTSYDKVIF TRB:CASSPTSQPQHF	4	CD4-C4
Pla-2_TTAGGCACACTGTCGG	Pla	Pla-2	P2-1	TRUE	3	TRA:CVVNIIGYQKVTF TRB:CASTRQGKQPQHF	270	CD4-C4
Pla-2_TTATGCTTCAACGCTA	Pla	Pla-2	P2-4971	FALSE	1	TRA:CAVTNDYKLSF TRB:CASSLGLRGKPQHF	1	CD4-C4
Pla-2_TTATGCTTCAGTGTTG	Pla	Pla-2	P2-411	FALSE	2	TRA:CAHGNTGGFKTIF TRB:CASSLGNTGELFF	2	CD4-C4
Pla-2_TTCCCAGTCATTGCCC	Pla	Pla-2	P2-4974	FALSE	1	TRA:CAVEDKNTDKLIF TRB:CASRAGNQPQHF	1	CD4-C2
Pla-2_TTCGAAGTCTTTAGGG	Pla	Pla-2	P2-4982	FALSE	1	TRA:CAVRDPTGAGSYQLTF TRB:CASSQGSQGAYGYTF	1	CD4-C4
Pla-2_TTCGGTCAGGTGCTAG	Pla	Pla-2	P2-4983	FALSE	1	TRA:CALLTYNTDKLIF TRB:CASKDGSNTGELFF	1	CD4-C4
Pla-2_TTCGGTCCAGACAAGC	Pla	Pla-2	P2-4984	FALSE	1	TRA:CAARVQNAGNMLTF TRB:CASSHSDRDFYQPQHF	1	CD4-C3
Pla-2_TTCTACAAGCCCGAAA	Pla	Pla-2	P2-4986	FALSE	1	TRA:CAMSTNTNAGKSTF TRB:CASSLTGAVVTEAFF	1	CD4-C4
Pla-2_TTCTACAGTATGCTTG	Pla	Pla-2	P2-513	FALSE	2	TRA:CAPAPDQFYF TRB:CASSFPRRGPMNTEAFF	2	CD4-C4
Pla-2_TTCTACATCACAACGT	Pla	Pla-2	P2-4988	FALSE	1	TRA:CAANSGGGADGLTF TRB:CAIRGLGQGIETQYF	1	CD4-C4
Pla-2_TTCTCCTCAGTTCATG	Pla	Pla-2	P2-4992	FALSE	1	TRA:CAASYQGGSEKLVF TRB:CASSVGLAAHNEQFF	1	CD4-C4
Pla-2_TTCTTAGTCAGTCCCT	Pla	Pla-2	P2-4997	FALSE	1	TRA:CAGPEGGGSNYKLTF TRB:CSVEGAPGQKETQYF	1	CD4-C4

Pla-2_TTGAACGTCAAAGTAG	Pla	Pla-2	P2-5001	FALSE	1	TRA:CATVRAGGTSYGKLTF TRB:CASSPGQSIGTGELFF	1	CD4-C4
Pla-2_TTGCCGTAGATGTAAC	Pla	Pla-2	P2-5008	FALSE	1	TRA:CAGMFSGGYNKLIF TRB:CASSWGQSGYTF	1	CD4-C4
Pla-2_TTGGAACAGCGAAGGG	Pla	Pla-2	P2-445	FALSE	2	TRA:CAVPPKGGNNRLAF TRB:CASTSFSNQPQHF	2	CD4-C4
Pla-2_TTGGCAACAGTCACTA	Pla	Pla-2	P2-5017	FALSE	1	TRA:CAVNGPGGANSKLTF TRB:CASSETGNQPQHF	1	CD4-C4
Pla-2_TTGTAGGCACATTAGC	Pla	Pla-2	P2-5021	FALSE	1	TRA:CAVINGGSQGNLIF TRB:CAISESRASSGNTIYF	1	CD4-C4
Pla-2_TTGTAGGCAGTAAGCG	Pla	Pla-2	P2-101	TRUE	3	TRA:CATDASGNEKLTF TRB:CASSLGLADTQYF	5	CD8-C8
Pla-2_TTTACTGAGTGTACCT	Pla	Pla-2	P2-5022	FALSE	1	TRA:CAPNFSGGYNKLIF TRB:CASSRDTEAFF	1	CD4-C4
Pla-2_TTTCCTCGTATCGCAT	Pla	Pla-2	P2-5030	FALSE	1	TRA:CAVNAQSGGGADGLTF TRB:CASSFGGLGNQPQHF	1	CD4-C4
Pla-2_TTTGCGCGTCTTGCGG	Pla	Pla-2	P2-5032	FALSE	1	TRA:CAASRGSGYSTLTF TRB:CASSQGGLVYEQYF	1	CD4-C4
Pla-2_TTTGCGCTCACCTCGT	Pla	Pla-2	P2-5033	FALSE	1	TRA:CIAFRAGNTGKLIF TRB:CASSFAQGGGEQYF	1	CD4-C4
Pla-2_TTTGGTTCACGGTAGA	Pla	Pla-2	P2-5036	FALSE	1	TRA:CAVRRGGANSKLTF TRB:CASSVWGTSGNVDTQYF	1	CD4-C5
Pla-2_AAACCTGAGGCATGGT	Pla	Pla-2	P2-3411	FALSE	1	TRA:CAVESYNTDKLIF TRB:CASSPRLNYGYTF	1	CD4-C2
Pla-2_AAACGGGAGCACAGGT	Pla	Pla-2	P2-192	TRUE	3	TRA:CAMKHDYKLSF TRB:CASSQDPNQPQHF	3	CD4-C2
Pla-2_AAACGGGAGTAGGCCA	Pla	Pla-2	P2-377	FALSE	2	TRA:CAGGDYNQGGKLIF TRB:CASTLTQGALTGELFF	2	CD4-C2
Pla-2_AACCATGTCGGTCCGA	Pla	Pla-2	P2-381	FALSE	2	TRA:CAVRDFNNAGNMLTF TRB:CASSLEAFYEQYF	2	CD4-C4
Pla-2_AACGTTGCACCGCTAG	Pla	Pla-2	P2-3438	FALSE	1	TRA:CAVRATGGGNKLTF TRB:CSAAGGTLEKLFF	1	CD4-C2
Pla-2_AACTCTTAGCCATCGC	Pla	Pla-2	P2-3449	FALSE	1	TRA:CAVLVGNNRKLIW TRB:CASSFPQGAYEQYF	1	CD4-C2
Pla-2_AAGCCGCCAGCCTTTC	Pla	Pla-2	P2-3458	FALSE	1	TRA:CATDEGGKLSF TRB:CSAWSRGLGETQYF	1	CD4-C1
Pla-2_AAGGTTCAGCCAGTAG	Pla	Pla-2	P2-3469	FALSE	1	TRA:CAAINWGSQGNLIF TRB:CATSETGGYEQYF	1	CD4-C7
Pla-2_ACACCAATCTCCGGTT	Pla	Pla-2	P2-3486	FALSE	1	TRA:CAVIPNSGYSTLTF TRB:CASSPQGRNYGYTF	1	CD4-C2
Pla-2_ACACTGAAGCTGATAA	Pla	Pla-2	P2-3495	FALSE	1	TRA:CAVLGTYKYIF TRB:CSARDYYEQYF	1	CD4-C2
Pla-2_ACCGTAAGTTCAGCGC	Pla	Pla-2	P2-399	FALSE	2	TRA:CAVTDGFYDKVIF TRB:CASSMDGETQYF	2	CD4-C3
Pla-2_ACGAGCCTCACCAGGC	Pla	Pla-2	P2-3539	FALSE	1	TRA:CAPPGGNTGKLIF TRB:CAWSTLAGVHAF	1	CD4-C4
Pla-2_ACGAGGACACATCCGG	Pla	Pla-2	P2-3541	FALSE	1	TRA:CALSEAVGSGNTGKLIF TRB:CASYRRVTEQYF	1	CD4-C6
Pla-2_ACGAGGACACTTAAGC	Pla	Pla-2	P2-3542	FALSE	1	TRA:CATDVGAQKLVF TRB:CASSQQAGGEKLFF	1	CD4-C2
Pla-2_ACGATGTGTCTCGTTC	Pla	Pla-2	P2-3549	FALSE	1	TRA:CAVGVTRGWGGQNFVF TRB:CASSPQFGRGTNEQFF	1	CD4-C2
Pla-2_ACGCCAGCAAGCTGGA	Pla	Pla-2	P2-3560	FALSE	1	TRA:CAILETGANNLFF TRB:CASRPHDRRMNTEAFF	1	CD4-C2

Pla-2_ACGGGCTGTTCACGGC	Pla	Pla-2	P2-3574	FALSE	1	TRA:CILTEGAGNNRKLIW TRB:CASGQGALGETQYF	1	CD4-C2
Pla-2_ACGGGCTGTTCTGTTT	Pla	Pla-2	P2-3575	FALSE	1	TRA:CAASAQSGGSNYKLTF TRB:CASRLAGARHEQYF	1	CD4-C2
Pla-2_ACTGAGTAGAGCCCAA	Pla	Pla-2	P2-3583	FALSE	1	TRA:CAVRLSNTDKLIF TRB:CASRLNDVAFF	1	CD4-C3
Pla-2_ACTGAGTAGATGAGAG	Pla	Pla-2	P2-3585	FALSE	1	TRA:CAVISAGGTSYGKLTF TRB:CASSSQGISNQPQHF	1	CD4-C2
Pla-2_ACTGATGCAATGCCAT	Pla	Pla-2	P2-3592	FALSE	1	TRA:CIVRASGNTGKLIF TRB:CASSPGPGATYNEQFF	1	CD4-C2
Pla-2_ACTTTCAGTCTACCTC	Pla	Pla-2	P2-3607	FALSE	1	TRA:CAVSDQFPGGGADGLTF TRB:CSARDLQRGRNEQFF	1	CD4-C2
Pla-2_AGAGCGACATTTGCCC	Pla	Pla-2	P2-3614	FALSE	1	TRA:CAVFSGGYNKLIF TRB:CASSLEGGLGGANVLTF	1	CD4-C2
Pla-2_AGAGCTTGTTGCGTTA	Pla	Pla-2	P2-3620	FALSE	1	TRA:CAALVSGGSNYKLTF TRB:CASSQYKGSNSPLHF	1	CD4-C5
Pla-2_AGATCTGGTGCATCTA	Pla	Pla-2	P2-3629	FALSE	1	TRA:CAADAGGTSYGKLTF TRB:CASSMTGVGGYTF	1	CD4-C5
Pla-2_AGATTGCAGAATTCCC	Pla	Pla-2	P2-3631	FALSE	1	TRA:CAGYNFNKFYF TRB:CASSIAAGGDTGELFF	1	CD4-C4
Pla-2_AGCCTAAAGCTGCGAA	Pla	Pla-2	P2-3638	FALSE	1	TRA:CGADIGGGSYIPTF TRB:CASSSSWAKNIQYF	1	CD4-C4
Pla-2_AGCGGTCCACCCTATC	Pla	Pla-2	P2-3647	FALSE	1	TRA:CVCRDCYNQGGKLIF TRB:CASSLGSQGHYEQYF	1	CD4-C2
Pla-2_AGCGGTCGTTACCGAT	Pla	Pla-2	P2-427	FALSE	2	TRA:CAMRLGSNYKLTF TRB:CASSSLAGPYEQYF	2	CD4-C2
Pla-2_AGGCCGTCAGACGCCT	Pla	Pla-2	P2-431	FALSE	2	TRA:CAMREGGRSQGNLIF TRB:CASSETTGGWEKLFF	2	CD4-C6
Pla-2_AGGTCCGCATAACCTG	Pla	Pla-2	P2-3693	FALSE	1	TRA:CASVIGGYNKLIF TRB:CSARDQGGGSYEQYF	1	CD4-C2
Pla-2_ATCTGCCTCCTAGTGA	Pla	Pla-2	P2-198	TRUE	3	TRA:CAVRDGDYKLSF TRB:CASSLEGVETQYF	3	CD4-C2
Pla-2_ATTGGACCAATGCCAT	Pla	Pla-2	P2-3761	FALSE	1	TRA:CATGARGAQKLVF TRB:CASSSGGGGETQYF	1	CD4-C2
Pla-2_CAACCAAGTTAAAGTG	Pla	Pla-2	P2-3781	FALSE	1	TRA:CAASMRAVTGGGNKLTF TRB:CASSIAVSRVSNQPQHF	1	CD4-C3
Pla-2_CAACTAGTCACGCATA	Pla	Pla-2	P2-3791	FALSE	1	TRA:CIVRSRNTGTASKLTF TRB:CASSIAGQGAKNEQFF	1	CD4-C3
Pla-2_CACAGTACACAAGTAA	Pla	Pla-2	P2-3827	FALSE	1	TRA:CLVGPPSFYGGATNKLIF TRB:CASSLELRGGRNTDTQYF	1	CD4-C2
Pla-2_CACATAGGTTCGGCAC	Pla	Pla-2	P2-448	FALSE	2	TRA:CATVLMNSGYSTLTF TRB:CASSLIGGGTEAFF	2	CD4-C3
Pla-2_CACCACTAGTGGTCCC	Pla	Pla-2	P2-427	FALSE	2	TRA:CAMRLGSNYKLTF TRB:CASSSLAGPYEQYF	2	CD4-C4
Pla-2_CAGAGAGCAGCTATTG	Pla	Pla-2	P2-3853	FALSE	1	TRA:CAGGAGNNRKLIW TRB:CASSRDRNTGELFF	1	CD4-C3
Pla-2_CAGCAGCCATACTACG	Pla	Pla-2	P2-3865	FALSE	1	TRA:CAGQAGSFNKFYF TRB:CASSLLGRKETQYF	1	CD4-C2
Pla-2_CAGCCGATCACCGTAA	Pla	Pla-2	P2-3871	FALSE	1	TRA:CAENRSGNQFYF TRB:CAIRPGTSGYEQFF	1	CD4-C4
Pla-2_CAGGTGCAGAGTAAGG	Pla	Pla-2	P2-3881	FALSE	1	TRA:CAMDTGRRALTF TRB:CASSLSTDTQYF	1	CD4-C2
Pla-2_CAGGTGCCACCGTTGG	Pla	Pla-2	P2-3882	FALSE	1	TRA:CATDAGGNEKLTF TRB:CASSFTNTGELFF	1	CD4-C2

Pla-2_CAGTAACTCTGTCCGT	Pla	Pla-2	P2-373	TRUE	2	TRA:CAEHNARLMF TRB:CASSYLDGTEAFF	2	CD4-C2
Pla-2_CAGTCCTCATGGTCTA	Pla	Pla-2	P2-3889	FALSE	1	TRA:CVVRDSSYKLIF TRB:CASSLRRGDTGELFF	1	CD4-C2
Pla-2_CATATTCCATGGTAGG	Pla	Pla-2	P2-3896	FALSE	1	TRA:CIVRVEAGGTSYGKLTF TRB:CSARDNFGRPEAFF	1	CD4-C2
Pla-2_CATCCACAGAAGGACA	Pla	Pla-2	P2-3904	FALSE	1	TRA:CIVRVASSGTASKLTF TRB:CASSPPLGGRITDTQYF	1	CD4-C2
Pla-2_CATGGCGTCCCACTTG	Pla	Pla-2	P2-302	TRUE	2	TRA:CADNNAGNMLTF TRB:CATSEFLFEQFF	2	CD4-C4
Pla-2_CCAATCCTCCTCAACC	Pla	Pla-2	P2-3940	FALSE	1	TRA:CAVSHFGNEKLTF TRB:CASNVRSSYEQYF	1	CD4-C2
Pla-2_CCACGGATCCAGTATG	Pla	Pla-2	P2-3945	FALSE	1	TRA:CAVSDRGSGANSKLTF TRB:CAAGRENYEQYF	1	CD4-C3
Pla-2_CCAGCGACACCCATTC	Pla	Pla-2	P2-3951	FALSE	1	TRA:CVVSENAGNMLTF TRB:CASSFSSPTDTQYF	1	CD4-C7
Pla-2_CCGGTAGTCAACACTG	Pla	Pla-2	P2-3974	FALSE	1	TRA:CAEEGRYGGATNKLIF TRB:CASRHDRGLVGELFF	1	CD4-C4
Pla-2_CCGTTCATCAGCACAT	Pla	Pla-2	P2-3981	FALSE	1	TRA:CIVVQWGDNYGQNFVF TRB:CASSPDSPSYNEQFF	1	CD4-C2
Pla-2_CCGTTCATCCAGATCA	Pla	Pla-2	P2-486	FALSE	2	TRA:CAVNPSGGGADGLTF TRB:CAVREDSFGYTF	2	CD4-C2
Pla-2_CCTCAGTAGCTACCTA	Pla	Pla-2	P2-3998	FALSE	1	TRA:CAAPQHTGRRALTF TRB:CASSPGTGDTEAFF	1	CD4-C3
Pla-2_CCTCTGAGTACCCAAT	Pla	Pla-2	P2-4003	FALSE	1	TRA:CVVGRPGTYKYIF TRB:CASSVDRGTGELFF	1	CD4-C2
Pla-2_CCTCTGATCACGCGGT	Pla	Pla-2	P2-4005	FALSE	1	TRA:CAVSYSRGAGSYQLTF TRB:CASYGTGDVYEQYF	1	CD4-C2
Pla-2_CCTTACGCAGTAACGG	Pla	Pla-2	P2-368	TRUE	2	TRA:CAVNAPTGGFKTIF TRB:CASSAWTGIGDEQYF	2	CD4-C5
Pla-2_CCTTACGGTTTGCATG	Pla	Pla-2	P2-4010	FALSE	1	TRA:CAVGPDNFGNEKLTF TRB:CASSLDNQPQHF	1	CD4-C4
Pla-2_CCTTCCCCAGTCTTCC	Pla	Pla-2	P2-4014	FALSE	1	TRA:CAVSETGGFKTIF TRB:CATSDLGYGYTF	1	CD4-C2
Pla-2_CCTTCCCTCTGATACG	Pla	Pla-2	P2-277	TRUE	2	TRA:CATALYNFNKFYF TRB:CASGSTGTSNQPQHF	2	CD4-C2
Pla-2_CCTTCGAAGGGTTTCT	Pla	Pla-2	P2-4016	FALSE	1	TRA:CGTVTNDYKLSF TRB:CASRKQGGAYEQYF	1	CD4-C3
Pla-2_CCTTTCTCATTTGCTT	Pla	Pla-2	P2-4022	FALSE	1	TRA:CAASKGQGAQKLVF TRB:CASYPQAGEQFF	1	CD4-C2
Pla-2_CGAGAAGTCTCGATGA	Pla	Pla-2	P2-4040	FALSE	1	TRA:CAANNYGQNFVF TRB:CATRADRGSTDTQYF	1	CD4-C2
Pla-2_CGAGCCAAGTGGAGAA	Pla	Pla-2	P2-4048	FALSE	1	TRA:CAVNTEADYKLSF TRB:CASTGRGEQFF	1	CD4-C2
Pla-2_CGATGTATCTCCGGTT	Pla	Pla-2	P2-4063	FALSE	1	TRA:CAVEDLGGGADGLTF TRB:CASSTGQGGVQYF	1	CD4-C2
Pla-2_CGCCAAGGTCTCCATC	Pla	Pla-2	P2-4068	FALSE	1	TRA:CAFLRSQGGSEKLVF TRB:CASRGQGDQPQHF	1	CD4-C3
Pla-2_CGCTATCTCTGATACG	Pla	Pla-2	P2-4083	FALSE	1	TRA:CAVKGSGGATNKLIF TRB:CASTPVQGTYEQYF	1	CD4-C2
Pla-2_CGGAGCTAGTCAAGGC	Pla	Pla-2	P2-4106	FALSE	1	TRA:CAMKYSSASKIIF TRB:CASSKSGLVNTGELFF	1	CD4-C4
Pla-2_CGGCTAGCACGCTTTC	Pla	Pla-2	P2-4114	FALSE	1	TRA:CALKTSYQKVTF TRB:CASSSLTYTDTQYF	1	CD4-C3

Pla-2_CGTAGGCTCGATGAGG	Pla	Pla-2	P2-506	FALSE	2	TRA:CAVGEGNAGKSTF TRB:CASSLQLNSYEQYF	2	CD4-C5
Pla-2_CGTGTAATCCTACAGA	Pla	Pla-2	P2-4146	FALSE	1	TRA:CAAFYKAAGNKLTF TRB:CSARRENTGELFF	1	CD4-C7
Pla-2_CGTGTCTTCTGTCAAG	Pla	Pla-2	P2-4152	FALSE	1	TRA:CATPNAGKSTF TRB:CASSLGTSVSTDTQYF	1	CD4-C2
Pla-2_CGTTCTGAGAAACCTA	Pla	Pla-2	P2-4154	FALSE	1	TRA:CALSPDSYGNKLVF TRB:CASSWRYEQFF	1	CD4-C2
Pla-2_CGTTGGGTCTTAACCT	Pla	Pla-2	P2-4162	FALSE	1	TRA:CAGVWGTGNQFYF TRB:CASSRTGGYQPQHF	1	CD4-C1
Pla-2_CTAAGACCACGCGAAA	Pla	Pla-2	P2-4167	FALSE	1	TRA:CVVNVGGSQGNLIF TRB:CASSLLSSGGGRQDTQYF	1	CD4-C2
Pla-2_CTAATGGAGCGGATCA	Pla	Pla-2	P2-4169	FALSE	1	TRA:CIVGSFNAGGTSYGKLTF TRB:CASGFRFARARGLISYEQY	1	CD4-C2
Pla-2_CTAATGGTCCTGTACC	Pla	Pla-2	P2-4170	FALSE	1	TRA:CVVIPNTGNQFYF TRB:CASSPRRGVDQPQHF	1	CD4-C2
Pla-2_CTACACCTCAAACAAG	Pla	Pla-2	P2-4173	FALSE	1	TRA:CAFMDAGTGTASKLTF TRB:CAWSGQGWQPQHF	1	CD4-C2
Pla-2_CTAGCCTTCGGTTAAC	Pla	Pla-2	P2-4186	FALSE	1	TRA:CAVAAYYGQNFVF TRB:CASLNGAGGSYEQYF	1	CD4-C2
Pla-2_CTAGTGATCAAACGGG	Pla	Pla-2	P2-4192	FALSE	1	TRA:CAVSFTGGGNKLTF TRB:CASRDRERPEAFF	1	CD4-C2
Pla-2_CTCAGAACACGCATCG	Pla	Pla-2	P2-518	FALSE	2	TRA:CALEGDSGYSTLTF TRB:CASSAGGQAGELFF	2	CD4-C2
Pla-2_CTCCTAGCAGATCTGT	Pla	Pla-2	P2-4203	FALSE	1	TRA:CAPCGSDGGTSYGKLTF TRB:CASSPGTAYGYTF	1	CD4-C2
Pla-2_CTCGAAATCACTCTTA	Pla	Pla-2	P2-121	TRUE	3	TRA:CAGASRDTDKLIF TRB:CSVESFLRGASLMTEAFF	4	CD4-C4
Pla-2_CTCGAAATCGTGGGAA	Pla	Pla-2	P2-4208	FALSE	1	TRA:CAVGAYGGATNKLIF TRB:CASSLSGAGMGNQPQHF	1	CD4-C7
Pla-2_CTGAAACGTACGACCC	Pla	Pla-2	P2-244	FALSE	3	TRA:CAVRGGSYIPTF TRB:CASSQGTGRQETQYF	3	CD4-C2
Pla-2_CTGATAGAGAGACTTA	Pla	Pla-2	P2-4233	FALSE	1	TRA:CALKDEAGGGNKLTF TRB:CARQGKDTGELFF	1	CD4-C2
Pla-2_CTGCCTAAGAATTGTG	Pla	Pla-2	P2-4242	FALSE	1	TRA:CAVEAIQGGNNRKLIW TRB:CASSARTGDSNQPQHF	1	CD4-C2
Pla-2_CTGCTGTGTTAAGAAC	Pla	Pla-2	P2-4248	FALSE	1	TRA:CALSERINTGNQFYF TRB:CASSLGRQNYGYTF	1	CD4-C5
Pla-2_CTGGTCTGTCCTAGCG	Pla	Pla-2	P2-268	TRUE	2	TRA:CAEKNGANSKLTF TRB:CASSLVGTGLGEQFF	2	CD4-C2
Pla-2_CTGTTTAAGACTTGAA	Pla	Pla-2	P2-4255	FALSE	1	TRA:CAAKWTGANSKLTF TRB:CASSQSWQGAYEQYF	1	CD4-C7
Pla-2_CTGTTTAAGAGACGAA	Pla	Pla-2	P2-4256	FALSE	1	TRA:CALRPGFNNAGNMLTF TRB:CASSPGGSGGASTGELFF	1	CD4-C4
Pla-2_CTTACCGAGTAGCGGT	Pla	Pla-2	P2-4265	FALSE	1	TRA:CAGANQGGKLIF TRB:CSAIRPVGYGYTF	1	CD4-C7
Pla-2_CTTAGGAGTGTGACGA	Pla	Pla-2	P2-4271	FALSE	1	TRA:CAVSDGGNEKLTF TRB:CSAEPGTGKYNEQFF	1	CD4-C5
Pla-2_CTTCTCTTCTGCCAGG	Pla	Pla-2	P2-4276	FALSE	1	TRA:CAVSDITGGGNKLTF TRB:CASSLLAGGGEQYF	1	CD4-C2
Pla-2_CTTGGCTTCTGATTCT	Pla	Pla-2	P2-180	TRUE	3	TRA:CAVNGNNFNKFYF TRB:CASSSGQDYEQYF	3	CD4-C5
Pla-2_GAAACTCGTTTGGGCC	Pla	Pla-2	P2-4291	FALSE	1	TRA:CAGSQGSQGNLIF TRB:CASRSPGRNTEAFF	1	CD4-C4

Pla-2_GAACATCAGACGCACA	Pla	Pla-2	P2-4295	FALSE	1	TRA:CALTASYGNNRLAF TRB:CASSGTGGNQPQHF	1	CD4-C2
Pla-2_GAAGCAGTCTGTCAAG	Pla	Pla-2	P2-4311	FALSE	1	TRA:CAVRPVKHIF TRB:CASSSTEATYGYTF	1	CD4-C2
Pla-2_GAATGAAAGCCGTCGT	Pla	Pla-2	P2-4320	FALSE	1	TRA:CAVYGGATNKLIF TRB:CASSLGPLTPQHF	1	CD4-C2
Pla-2_GACACGCGTAGAAGGA	Pla	Pla-2	P2-4327	FALSE	1	TRA:CAASNSGYSTLTF TRB:CSAPPLGLSEQYF	1	CD4-C2
Pla-2_GACACGCGTCTCGTTC	Pla	Pla-2	P2-4328	FALSE	1	TRA:CAVQTGFQKLVF TRB:CASSMPRGSGELFF	1	CD4-C2
Pla-2_GACAGAGTCCTCAACC	Pla	Pla-2	P2-4334	FALSE	1	TRA:CAVSARLMF TRB:CASSLGQGGYNEQFF	1	CD4-C2
Pla-2_GACCTGGAGAAGGACA	Pla	Pla-2	P2-4337	FALSE	1	TRA:CAVSDPTNAGKSTF TRB:CSVGWGLDSPLHF	1	CD4-C2
Pla-2_GACGTGCCATACTCTT	Pla	Pla-2	P2-4345	FALSE	1	TRA:CAGVNTGFQKLVF TRB:CACGRGEGLDEQFF	1	CD4-C2
Pla-2_GACTACACAATCCAAC	Pla	Pla-2	P2-4355	FALSE	1	TRA:CAASKSQGNLIF TRB:CASSLSGGAASEQYF	1	CD4-C2
Pla-2_GACTACATCAGGTAAA	Pla	Pla-2	P2-4358	FALSE	1	TRA:CAEIHLGRAGIIF TRB:CSARVYGLTPSYNEQFF	1	CD4-C2
Pla-2_GAGGTGAGTCAGAATA	Pla	Pla-2	P2-4371	FALSE	1	TRA:CAVRPYSSASKIIF TRB:CASSHVNPIGRSSYEQYF	1	CD4-C2
Pla-2_GATCAGTCATATGCTG	Pla	Pla-2	P2-219	FALSE	3	TRA:CAVEVPGSQGNLIF TRB:CASRAGTAVYEQYF	3	CD4-C4
Pla-2_GATCAGTTCCGCGGTA	Pla	Pla-2	P2-4374	FALSE	1	TRA:CAFRGSGAGSYQLTF TRB:CASKGYEGNRNTEAFF	1	CD4-C3
Pla-2_GATCGATCAAGGCTCC	Pla	Pla-2	P2-4376	FALSE	1	TRA:CIVKETSGSRLTF TRB:CSAQAEPKETQYF	1	CD4-C2
Pla-2_GATCGCGGTCAGAATA	Pla	Pla-2	P2-4379	FALSE	1	TRA:CATGSDGTSYGKLTF TRB:CASSQDLGTINSPLHF	1	CD4-C2
Pla-2_GATCGCGTCGGATGTT	Pla	Pla-2	P2-4380	FALSE	1	TRA:CIVRVLSWSGSEKLVF TRB:CASSPTAGGGALGRNGYTF	1	CD4-C2
Pla-2_GATCGTAAGAAACCAT	Pla	Pla-2	P2-485	FALSE	2	TRA:CALTPTGGGNKLTF TRB:CASSIRQRPGELFF	2	CD4-C2
Pla-2_GATCGTAGTCTTGCGG	Pla	Pla-2	P2-4382	FALSE	1	TRA:CAASLGSGGTSYGKLTF TRB:CASSPTRQGALAGANVLTF	1	CD4-C2
Pla-2_GATGAAATCCCGACTT	Pla	Pla-2	P2-4386	FALSE	1	TRA:CALVNNNARLMF TRB:CASSLATVKNNEQFF	1	CD4-C2
Pla-2_GATGAGGAGAACTCGG	Pla	Pla-2	P2-4387	FALSE	1	TRA:CAVRDGDYKLSF TRB:CASSANIGNTIYF	1	CD4-C5
Pla-2_GATGCTACAACACCCG	Pla	Pla-2	P2-4391	FALSE	1	TRA:CAVASRETSGSRLTF TRB:CASSLGGPSNQPQHF	1	CD4-C5
Pla-2_GCGACCATCGGGAGTA	Pla	Pla-2	P2-4443	FALSE	1	TRA:CIVIGNQGGKLIF TRB:CASRDYGVSNQPQHF	1	CD4-C5
Pla-2_GCGCAACTCCCAACGG	Pla	Pla-2	P2-131	TRUE	3	TRA:CATVGQIQGAQKLVF TRB:CASRTGTSSYEQYF	4	CD4-C2
Pla-2_GCGGGTTTCAGGCAAG	Pla	Pla-2	P2-4459	FALSE	1	TRA:CAVGYVEDDYKLSF TRB:CASRKQGPVYNSPLHF	1	CD4-C2
Pla-2_GCTCTGTCAGAAGCAC	Pla	Pla-2	P2-4463	FALSE	1	TRA:CAVHPSLKNRDDKIIF TRB:CASSFSGRKNIQYF	1	CD4-C7
Pla-2_GCTCTGTCATTCGACA	Pla	Pla-2	P2-415	FALSE	2	TRA:CAENPNSGGSNYKLTF TRB:CASMDRAPGTSGFLF	2	CD4-C4
Pla-2_GCTCTGTCATTGGCGC	Pla	Pla-2	P2-4464	FALSE	1	TRA:CALETGGFKTIF TRB:CASSLVVGPDNEQFF	1	CD4-C2

Pla-2_GCTGCAGGTCAGCTAT	Pla	Pla-2	P2-549	FALSE	2	TRA:CAMREGRTGFQKLVF TRB:CSAGQVGSNQPQHF	2	CD4-C2
Pla-2_GCTGCAGTCGTCCGTT	Pla	Pla-2	P2-4469	FALSE	1	TRA:CAVLLKTSYDKVIF TRB:CASSYRTGMGDTQYF	1	CD4-C7
Pla-2_GCTGGGTAGGAGTCTG	Pla	Pla-2	P2-4479	FALSE	1	TRA:CAYRRPGTYKYIF TRB:CASSEASGAAGANVLTF	1	CD4-C3
Pla-2_GGAAAGCTCTAGAGTC	Pla	Pla-2	P2-4493	FALSE	1	TRA:CAVRTDTDKLIF TRB:CASSLTGRETQYF	1	CD4-C5
Pla-2_GGAACTTAGTGATCGG	Pla	Pla-2	P2-4494	FALSE	1	TRA:CAVRTNAGGTSYGKLTF TRB:CASSYKTGLDTDTQYF	1	CD4-C2
Pla-2_GGAACTTTCAGTACGT	Pla	Pla-2	P2-4495	FALSE	1	TRA:CAYRRGYGGSQGNLIF TRB:CASSVSGTGNTEAFF	1	CD4-C4
Pla-2_GGACATTTCAAGGTAA	Pla	Pla-2	P2-4510	FALSE	1	TRA:CAVGVNNARLMF TRB:CASSVSTNLGEQYF	1	CD4-C2
Pla-2_GGATGTTTCATCTGTT	Pla	Pla-2	P2-4521	FALSE	1	TRA:CAVRHRRGNTGKLIF TRB:CASRPAGGLIEDTQYF	1	CD4-C4
Pla-2_GGCGTGTAGCACAGGT	Pla	Pla-2	P2-4543	FALSE	1	TRA:CAVRDRDTDKLIF TRB:CASSQVKGSTGELFF	1	CD4-C3
Pla-2_GGCGTGTCAAAGCGGT	Pla	Pla-2	P2-4545	FALSE	1	TRA:CAVERIGFQKLVF TRB:CASSPPSAREQYF	1	CD4-C2
Pla-2_GGCTCGAAGTCCGTAT	Pla	Pla-2	P2-4546	FALSE	1	TRA:CAVGGANSKLTF TRB:CASSLNRWNEQFF	1	CD4-C2
Pla-2_GGGCACTTCAGTCAGT	Pla	Pla-2	P2-553	FALSE	2	TRA:CAVSKGGGGNKLTF TRB:CASSFSGHEQYF	2	CD4-C7
Pla-2_GGTGAAGAGAATCTCC	Pla	Pla-2	P2-4573	FALSE	1	TRA:CAILRHYKLTF TRB:CASTSGGNRRSYNEQFF	1	CD4-C2
Pla-2_GTAACGTTCCCTAACC	Pla	Pla-2	P2-4584	FALSE	1	TRA:CAVSQMQTGANNLFF TRB:CASSLGRQTFF	1	CD4-C2
Pla-2_GTACTCCAGGTTACCT	Pla	Pla-2	P2-4593	FALSE	1	TRA:CAVNKAGTALIF TRB:CSARMGGQGGSGANVLTF	1	CD4-C7
Pla-2_GTACTTTGTCCGTTAA	Pla	Pla-2	P2-4598	FALSE	1	TRA:CGATTTSGTYKYIF TRB:CASSKEFMNTEAFF	1	CD4-C2
Pla-2_GTACTTTGTTGGTGGA	Pla	Pla-2	P2-4599	FALSE	1	TRA:CAVSDPQLQGAQKLVF TRB:CASKVGGFSGANVLTF	1	CD4-C2
Pla-2_GTCATTTTCTGCAAGT	Pla	Pla-2	P2-4618	FALSE	1	TRA:CAVSSPGYSTLTF TRB:CASSRTGGASSYEQYF	1	CD4-C3
Pla-2_GTCTCGTGTGCGAAAC	Pla	Pla-2	P2-4624	FALSE	1	TRA:CALGGQKLLF TRB:CASSSGLAGRSSYEQYF	1	CD4-C2
Pla-2_GTCTCGTTCTGCCAGG	Pla	Pla-2	P2-14	FALSE	3	TRA:CALSEAPGGSEKLVF TRB:CASSIGHPNSPLHF	37	CD4-C7
Pla-2_GTGCTTCTCACCTTAT	Pla	Pla-2	P2-4643	FALSE	1	TRA:CATCPSGNTGKLIF TRB:CSVATSGRALEQYF	1	CD4-C2
Pla-2_GTGTGCGGTTGAGTTC	Pla	Pla-2	P2-4651	FALSE	1	TRA:CAVSRAGGTSYGKLTF TRB:CASSKQETQYF	1	CD4-C4
Pla-2_TAAGCGTCAAACGTGG	Pla	Pla-2	P2-198	TRUE	3	TRA:CAVRDGDYKLSF TRB:CASSLEGVETQYF	3	CD4-C2
Pla-2_TAAGCGTGTGCACTTA	Pla	Pla-2	P2-4674	FALSE	1	TRA:CAVVLNNAGNMLTF TRB:CASYSVGGQYF	1	CD4-C4
Pla-2_TACACGAGTACTTCTT	Pla	Pla-2	P2-4681	FALSE	1	TRA:CARRNQAGTALIF TRB:CASSSSTGPQETQYF	1	CD4-C3
Pla-2_TACGGTAAGTCTCGGC	Pla	Pla-2	P2-4695	FALSE	1	TRA:CAGRTGGFKTIF TRB:CASSQTGGTGPYEQYF	1	CD4-C2
Pla-2_TAGGCATGTATGAAAC	Pla	Pla-2	P2-274	TRUE	2	TRA:CAMREGSLKTGNQFYF TRB:CASSLVPGTGYSPRTDTQY[illegible]	2	CD4-C2

Pla-2_TATCTCACATTGAGCT	Pla	Pla-2	P2-4736	FALSE	1	TRA:CATSGGSNYKLTF TRB:CASSSTGGGQETQYF	1	CD4-C2
Pla-2_TATGCCCAGAGCCTAG	Pla	Pla-2	P2-4738	FALSE	1	TRA:CAASFSNTGKLIF TRB:CASSPGTGSIYF	1	CD4-C2
Pla-2_TATTACCTCCTAGTGA	Pla	Pla-2	P2-4741	FALSE	1	TRA:CAVNPPRGGGGNKLTF TRB:CASSFAGNSGANVLTF	1	CD4-C3
Pla-2_TCACAAGAGCAGGCTA	Pla	Pla-2	P2-4750	FALSE	1	TRA:CVVSERGGKLIF TRB:CASSQGVGYEQYF	1	CD4-C2
Pla-2_TCAGCAACAAAGGTGC	Pla	Pla-2	P2-4764	FALSE	1	TRA:CATEGGGSNYKLTF TRB:CAINSGGRNNEQFF	1	CD4-C4
Pla-2_TCAGCAACAAGCCGCT	Pla	Pla-2	P2-4765	FALSE	1	TRA:CAVPVNNNARLMF TRB:CASSLRDLNTEAFF	1	CD4-C2
Pla-2_TCAGGTAGTGACAAAT	Pla	Pla-2	P2-473	FALSE	2	TRA:CGTDIYNFNKFYF TRB:CASRKNTEAFF	2	CD4-C7
Pla-2_TCAGGTATCGGTCTAA	Pla	Pla-2	P2-4776	FALSE	1	TRA:CGTGGGTSYGKLTF TRB:CAWSIGSGSYNEQFF	1	CD4-C2
Pla-2_TCCCGATCAATAGCAA	Pla	Pla-2	P2-4788	FALSE	1	TRA:CAVQSNSGNTPLVF TRB:CASSPITGDTQYF	1	CD4-C5
Pla-2_TCGCGAGGTGCGGTAA	Pla	Pla-2	P2-4796	FALSE	1	TRA:CALRGSGNTGKLIF TRB:CASSQAGIGSEAFF	1	CD4-C2
Pla-2_TCGTACCCAAGTTCTG	Pla	Pla-2	P2-4804	FALSE	1	TRA:CAVDDKIIF TRB:CASSMAGYEQYF	1	CD4-C2
Pla-2_TCGTACCGTCTAGCGC	Pla	Pla-2	P2-4806	FALSE	1	TRA:CALRETGANSKLTF TRB:CASSLLQGSGYTF	1	CD4-C2
Pla-2_TCGTAGACACAAGTAA	Pla	Pla-2	P2-166	FALSE	3	TRA:CAVSWGTGYSTLTF TRB:CASSPDAGNTQYF	4	CD4-C5
Pla-2_TCGTAGAGTAGAGGAA	Pla	Pla-2	P2-4812	FALSE	1	TRA:CAVKLQGGSEKLVF TRB:CAWTRPGFGEQYF	1	CD4-C2
Pla-2_TCTGAGATCTGTTGAG	Pla	Pla-2	P2-4826	FALSE	1	TRA:CAMREGRRSDGGQKLLF TRB:CASSLRTSTDTQYF	1	CD4-C2
Pla-2_TCTTCGGAGTGTACCT	Pla	Pla-2	P2-4836	FALSE	1	TRA:CAGRYNFNKFYF TRB:CASSWDAWDTDTQYF	1	CD4-C2
Pla-2_TCTTCGGGTCCCTACT	Pla	Pla-2	P2-4839	FALSE	1	TRA:CAALEGDNYGQNFVF TRB:CASSLGGPFTEAFF	1	CD4-C2
Pla-2_TCTTCGGGTTACGACT	Pla	Pla-2	P2-4840	FALSE	1	TRA:CAYRSQGGSEKLVF TRB:CASSLGTALSYGGYTF	1	CD4-C2
Pla-2_TCTTCGGTCCGTAGTA	Pla	Pla-2	P2-4841	FALSE	1	TRA:CAAVSTSGTYKYIF TRB:CASSQSGLGREVDSPLHF	1	CD4-C2
Pla-2_TCTTTCCAGCTAAGAT	Pla	Pla-2	P2-4843	FALSE	1	TRA:CAQIMNRDDKIIF TRB:CASSIEGGTQYF	1	CD4-C2
Pla-2_TGACAACAGGTTCCTA	Pla	Pla-2	P2-4847	FALSE	1	TRA:CAASEGGGNNRKLIW TRB:CASSWGASGDTQYF	1	CD4-C3
Pla-2_TGAGCATCACGTGAGA	Pla	Pla-2	P2-4859	FALSE	1	TRA:CAVNLMDSSYKLIF TRB:CASNRQPAYEQYF	1	CD4-C3
Pla-2_TGAGCATCAGGCTCAC	Pla	Pla-2	P2-4860	FALSE	1	TRA:CALDSNYQLIW TRB:CASISGTDLGRELFF	1	CD4-C2
Pla-2_TGAGGGAAGGGAAACA	Pla	Pla-2	P2-172	TRUE	3	TRA:CAVSAKTSYDKVIF TRB:CASSQGGGTGTDTQYF	3	CD4-C2
Pla-2_TGCCAAAGTTTGACAC	Pla	Pla-2	P2-4883	FALSE	1	TRA:CAASPWAAGNKLTF TRB:CASSSSGGRNEQFF	1	CD4-C2
Pla-2_TGCGCAGGTTTGTTGG	Pla	Pla-2	P2-4890	FALSE	1	TRA:CVVTLNTGGFKTIF TRB:CAWTPKSGGANTGELFF	1	CD4-C2
Pla-2_TGCTACCCACTCTGTC	Pla	Pla-2	P2-4898	FALSE	1	TRA:CAVDTQGGSEKLVF TRB:CSVRPWEGPSYEQYF	1	CD4-C2

Pla-2_TGCTACCGTCTGATTG	Pla	Pla-2	P2-4900	FALSE	1	TRA:CASMGTNAGKSTF TRB:CASSLTGDNTGELFF	1	CD4-C2
Pla-2_TGGACGCAGGTCGGAT	Pla	Pla-2	P2-4905	FALSE	1	TRA:CATDLIREGDFGNEKLTF TRB:CATSGQNTGELFF	1	CD4-C2
Pla-2_TGGACGCAGGTGCTAG	Pla	Pla-2	P2-4906	FALSE	1	TRA:CAVSRTGFQKLVF TRB:CASSMKINEKLFF	1	CD4-C5
Pla-2_TGGACGCCACTGTGTA	Pla	Pla-2	P2-4908	FALSE	1	TRA:CAVKGQAGTALIF TRB:CASSTTQRNWVLDTQYF	1	CD4-C2
Pla-2_TGGCGCACACAGTCGC	Pla	Pla-2	P2-4913	FALSE	1	TRA:CAGEDAGGTSYGKLTF TRB:CASRRAAYNSPLHF	1	CD4-C5
Pla-2_TGGCGCACAGCCTTTC	Pla	Pla-2	P2-4915	FALSE	1	TRA:CAYPEAQGGSEKLVF TRB:CASSRGTSGTDTQYF	1	CD4-C4
Pla-2_TGGCGCAGTCTTGATG	Pla	Pla-2	P2-2	TRUE	3	TRA:CVVPNTNAGKSTF TRB:CASSLQGSTYNEQFF	220	CD4-C2
Pla-2_TGGGAAGCAGCTTCGG	Pla	Pla-2	P2-4922	FALSE	1	TRA:CAVRASGYSTLTF TRB:CSARARSSGLGTDTQYF	1	CD4-C2
Pla-2_TGGGCGTTCTACTCAT	Pla	Pla-2	P2-4931	FALSE	1	TRA:CAARSFGNEKLTF TRB:CASSLAGASSYEQYF	1	CD4-C4
Pla-2_TGTTCCGAGAGAACAG	Pla	Pla-2	P2-4955	FALSE	1	TRA:CAMSAGNQGGKLIF TRB:CAYSAGGITQETQYF	1	CD4-C2
Pla-2_TTAACTCCATCCGTGG	Pla	Pla-2	P2-4959	FALSE	1	TRA:CAYRSPGGSEKLVF TRB:CATSEGYGYTF	1	CD4-C2
Pla-2_TTAGGCAGTAAATGTG	Pla	Pla-2	P2-4966	FALSE	1	TRA:CAVRGSGTYKYIF TRB:CAWQDTGGAYGYTF	1	CD4-C2
Pla-2_TTATGCTTCCAGTAGT	Pla	Pla-2	P2-4972	FALSE	1	TRA:CALSDLENSGGSNYKLTF TRB:CSAKVVETRMGQYF	1	CD4-C2
Pla-2_TTCTACATCATATCGG	Pla	Pla-2	P2-4989	FALSE	1	TRA:CVVSSEKLVF TRB:CASSEDWAGFIFGNQPQHF	1	CD4-C2
Pla-2_TTCTTAGAGCACCGCT	Pla	Pla-2	P2-4995	FALSE	1	TRA:CALSRNYGGATNKLIF TRB:CSVPALPTYEQYF	1	CD4-C7
Pla-2_TTCTTAGTCATAAAGG	Pla	Pla-2	P2-4998	FALSE	1	TRA:CVVSEQGGSEKLVF TRB:CASSPQGADGYTF	1	CD4-C2
Pla-2_TTCTTAGTCGATCCCT	Pla	Pla-2	P2-4999	FALSE	1	TRA:CIVRDRPAGGTSYGKLTF TRB:CASSLTGLAGGNEQFF	1	CD4-C2
Pla-2_TTGGCAATCTTAGCCC	Pla	Pla-2	P2-5020	FALSE	1	TRA:CAVRSYNTDKLIF TRB:CAFRDSTYNEQFF	1	CD4-C5
Pla-2_TTTATGCCACGTAAGG	Pla	Pla-2	P2-5028	FALSE	1	TRA:CIVRVYSGGSNYKLTF TRB:CSVYPRKGVYEQYF	1	CD4-C7
Pla-2_TTTATGCCACGTGAGA	Pla	Pla-2	P2-537	FALSE	2	TRA:CAGGYNNNDMRF TRB:CATSDFVTGEAFF	2	CD4-C3
Pla-2_TTTGGTTTCGGTGTTA	Pla	Pla-2	P2-5037	FALSE	1	TRA:CALSEVRTYKYIF TRB:CAIKGGLPMDEQFF	1	CD4-C3
Pla-2_AAAGATGCAGTCGATT	Pla	Pla-2	P2-49	FALSE	3	TRA:CATDEGAQKLVF TRB:CASSQDWGGAWGYTF	11	CD8-C2
Pla-2_AAAGTAGTCTTACCGC	Pla	Pla-2	P2-79	FALSE	3	TRA:CAGAGRNDMRF TRB:CSATPGLALSYNEQFF	7	CD8-C2
Pla-2_AAATGCCGTAAGTAGT	Pla	Pla-2	P2-70	FALSE	3	TRA:CAVPVAGTASKLTF TRB:CASREVSYNEQFF	8	CD8-C2
Pla-2_AACTCTTAGGCAGGTT	Pla	Pla-2	P2-210	FALSE	3	TRA:CAVRRPNSGYSTLTF TRB:CSARDTERTGNIQYF	3	CD8-C2
Pla-2_AAGACCTTCCCATTTA	Pla	Pla-2	P2-3457	FALSE	1	TRA:CAASEGTSGTYKYIF TRB:CSAEGQASYEQYF	1	CD8-C2
Pla-2_AAGCCGCGTCAGAGGT	Pla	Pla-2	P2-49	FALSE	3	TRA:CATDEGAQKLVF TRB:CASSQDWGGAWGYTF	11	CD8-C2

Pla-2_AAGGCAGCATGCCCGA	Pla	Pla-2	P2-389	FALSE	2	TRA:CAATSGTYKYIF TRB:CSATPGLALSYNEQFF	2	CD8-C5
Pla-2_AAGGCAGGTTAAAGAC	Pla	Pla-2	P2-49	FALSE	3	TRA:CATDEGAQKLVF TRB:CASSQDWGGAWGYTF	11	CD8-C2
Pla-2_AAGGCAGGTTACCGAT	Pla	Pla-2	P2-49	FALSE	3	TRA:CATDEGAQKLVF TRB:CASSQDWGGAWGYTF	11	CD8-C2
Pla-2_AAGGTTCGTAGAAAGG	Pla	Pla-2	P2-58	TRUE	3	TRA:CAVNERSNDYKLSF TRB:CAISGGDSWGNQPQHF	9	CD4-C5
Pla-2_AAGTCTGGTTGCGTTA	Pla	Pla-2	P2-3475	FALSE	1	TRA:CAFLSTDNDMRF TRB:CSASGWTGFDYGYTF	1	CD8-C2
Pla-2_AATCGGTCAGCTGTAT	Pla	Pla-2	P2-79	FALSE	3	TRA:CAGAGRNDMRF TRB:CSATPGLALSYNEQFF	7	CD8-C2
Pla-2_ACATCAGAGCTGTTCA	Pla	Pla-2	P2-3507	FALSE	1	TRA:CAFLYTGANSKLTF TRB:CASSPGAPEAFF	1	CD8-C2
Pla-2_ACATGGTCAGATTGCT	Pla	Pla-2	P2-3512	FALSE	1	TRA:CAFHPPSFGNEKLTF TRB:CASSLDRNTGELFF	1	CD8-C2
Pla-2_ACCCACTAGCTGAACG	Pla	Pla-2	P2-3523	FALSE	1	TRA:CAYDLLTGNQFYF TRB:CASTRQGKQPQHF	1	CD8-C2
Pla-2_ACCTTTACAGTAACGG	Pla	Pla-2	P2-3530	FALSE	1	TRA:CVIPYTGTASKLTF TRB:CASSLSQAYNEQFF	1	CD8-C2
Pla-2_ACCTTTATCCATTCTA	Pla	Pla-2	P2-3534	FALSE	1	TRA:CGNDYKLSF TRB:CASSLEASGRSGELFF	1	CD8-C2
Pla-2_ACGAGCCAGAGTTGGC	Pla	Pla-2	P2-80	FALSE	3	TRA:CAVLGRGGGATNKLIF TRB:CASSQIGSSPDTQYF	7	CD8-C2
Pla-2_ACGAGCCGTTCCACAA	Pla	Pla-2	P2-3538	FALSE	1	TRA:CAAFSGNTPLVF TRB:CASSSALTGTYEQYF	1	CD8-C2
Pla-2_ACGAGGAAGAGGTAGA	Pla	Pla-2	P2-75	FALSE	3	TRA:CAVNDGRGADGLTF TRB:CASLGGRTDTQYF	8	CD8-C2
Pla-2_ACGCCAGAGACCACGA	Pla	Pla-2	P2-404	FALSE	2	TRA:CAARVDDNNARLMF TRB:CASSRDLLIQSQYF	2	CD8-C2
Pla-2_ACGCCGAGTCAGGACA	Pla	Pla-2	P2-3565	FALSE	1	TRA:CAVRDTGGFKTIF TRB:CASSLEFGEQYF	1	CD8-C4
Pla-2_ACGCCGAGTTCGCTAA	Pla	Pla-2	P2-406	FALSE	2	TRA:CAVSLPTGGGNKLTF TRB:CASSYHSGDTGELFF	2	CD8-C2
Pla-2_ACTATCTAGGACAGAA	Pla	Pla-2	P2-3579	FALSE	1	TRA:CLVGDGGFKTIF TRB:CASSPGGTVETQYF	1	CD8-C2
Pla-2_ACTATCTGTCAAGCGA	Pla	Pla-2	P2-47	TRUE	3	TRA:CAASAGGNKLTF TRB:CASSTEDEQYF	11	CD8-C3
Pla-2_ACTGAACGTAGCGTCC	Pla	Pla-2	P2-82	FALSE	3	TRA:CVADTGNQFYF TRB:CASSSALTGTYEQYF	7	CD8-C2
Pla-2_ACTGAGTAGAGTACCG	Pla	Pla-2	P2-3584	FALSE	1	TRA:CAVEVNFGNEKLTF TRB:CASSQDSTSVYNEQFF	1	CD4-C4
Pla-2_ACTTACTCAAGGCTCC	Pla	Pla-2	P2-108	TRUE	3	TRA:CAMFPIKAAGNKLTF TRB:CASNTEANYGYTF	5	CD8-C2
Pla-2_ACTTACTTCTTCTGGC	Pla	Pla-2	P2-413	FALSE	2	TRA:CLVGFRGTYKYIF TRB:CASSYAPAGERGGRNEQFF	2	CD8-C2
Pla-2_ACTTGTTCATGGTCTA	Pla	Pla-2	P2-83	FALSE	3	TRA:CAVDTGTASKLTF TRB:CASRGSTDTQYF	7	CD8-C2
Pla-2_ACTTGTTGTGTTAAGA	Pla	Pla-2	P2-83	FALSE	3	TRA:CAVDTGTASKLTF TRB:CASRGSTDTQYF	7	CD8-C2
Pla-2_ACTTTCACAAGCTGGA	Pla	Pla-2	P2-3605	FALSE	1	TRA:CAASDTGFQKLVF TRB:CASSAGQGLYEQYF	1	CD8-C2
Pla-2_AGACGTTGTAAACACA	Pla	Pla-2	P2-105	TRUE	3	TRA:CAYRRREGGSEKLVF TRB:CASSTSGAIANYGYTF	5	CD8-C2

Pla-2_AGAGCGAAGTCGCCGT	Pla	Pla-2	P2-3613	FALSE	1	TRA:CVVDPGDGGTSYGKLTF TRB:CAISKLASTDTQYF	1	CD8-C2
Pla-2_AGAGTGGGTGAGGCTA	Pla	Pla-2	P2-12	TRUE	3	TRA:CGADAEYGNKLVF TRB:CASSEGQGAIMQPQHF	42	CD8-C2
Pla-2_AGAGTGGTCTAACTGG	Pla	Pla-2	P2-3625	FALSE	1	TRA:CAVRVGSTLTF TRB:CSVEDGSLNTEAFF	1	CD8-C2
Pla-2_AGATCTGAGTTGTAGA	Pla	Pla-2	P2-13	TRUE	3	TRA:CAYDLLTGNQFYF TRB:CASVRQLNTEAFF	38	CD8-C4
Pla-2_AGATTGCAGGAATGGA	Pla	Pla-2	P2-3632	FALSE	1	TRA:CGTVSVTGANSKLTF TRB:CASSFTGGVYGYTF	1	CD8-C2
Pla-2_AGCAGCCTCTCGGACG	Pla	Pla-2	P2-49	FALSE	3	TRA:CATDEGAQKLVF TRB:CASSQDWGGAWGYTF	11	CD8-C2
Pla-2_AGCGTATTCATTCACT	Pla	Pla-2	P2-45	TRUE	3	TRA:CAVSGDSNYQLIW TRB:CASSLGGNGNTIYF	11	CD8-C2
Pla-2_AGCTCCTAGTCATCCA	Pla	Pla-2	P2-230	FALSE	3	TRA:CAVSPLRNNARLMF TRB:CSAEGGHSYEQYF	3	CD8-C2
Pla-2_AGCTTGAGTGCACGAA	Pla	Pla-2	P2-13	TRUE	3	TRA:CAYDLLTGNQFYF TRB:CASVRQLNTEAFF	38	CD8-C2
Pla-2_AGGCCGTAGTGGGCTA	Pla	Pla-2	P2-3679	FALSE	1	TRA:CAVRDQPSGNTPLVF TRB:CSIRTVAKNIQYF	1	CD8-C2
Pla-2_AGGGAGTTCAATAAGG	Pla	Pla-2	P2-80	FALSE	3	TRA:CAVLGRGGGATNKLIF TRB:CASSQIGSSPDTQYF	7	CD8-C2
Pla-2_AGGGATGCAGTCACTA	Pla	Pla-2	P2-3684	FALSE	1	TRA:CAVRDSRDTGFQKLVF TRB:CASRPGGAVGNEQFF	1	CD8-C2
Pla-2_AGGGTGACACAGAGGT	Pla	Pla-2	P2-29	TRUE	3	TRA:CAVGGDFNKFYF TRB:CSARSDRLGYQPQHF	21	CD8-C5
Pla-2_AGTAGTCGTATCGCAT	Pla	Pla-2	P2-49	FALSE	3	TRA:CATDEGAQKLVF TRB:CASSQDWGGAWGYTF	11	CD8-C2
Pla-2_AGTGAGGGTAGATTAG	Pla	Pla-2	P2-108	TRUE	3	TRA:CAMFPIKAAGNKLTF TRB:CASNTEANYGYTF	5	CD8-C10
Pla-2_AGTGGGAAGTTGCAGG	Pla	Pla-2	P2-3708	FALSE	1	TRA:CAMRELVSQLSGGYNKLIF TRB:CASSFSSGGDTQYF	1	CD8-C2
Pla-2_AGTTGGTAGATAGGAG	Pla	Pla-2	P2-3713	FALSE	1	TRA:CAAMDSNYQLIW TRB:CSARGPGSSTEAFF	1	CD8-C2
Pla-2_AGTTGGTGTACTCAAC	Pla	Pla-2	P2-2	TRUE	3	TRA:CVVPNTNAGKSTF TRB:CASSLQGSTYNEQFF	220	CD8-C2
Pla-2_ATAACGCCATCCCACT	Pla	Pla-2	P2-438	FALSE	2	TRA:CAVRAFSNDYKLSF TRB:CASSHVRDSQGRHTEAFF	2	CD8-C2
Pla-2_ATGGGAGTCGTCGTTC	Pla	Pla-2	P2-3749	FALSE	1	TRA:CVVSASGGGADGLTF TRB:CATGDGRYYNEQFF	1	CD8-C2
Pla-2_ATTACTCTCCTTGACC	Pla	Pla-2	P2-3754	FALSE	1	TRA:CAVRRLEADSWGKFQF TRB:CASSLGLDEQYF	1	CD8-C2
Pla-2_ATTGGACAGGAGTAGA	Pla	Pla-2	P2-49	FALSE	3	TRA:CATDEGAQKLVF TRB:CASSQDWGGAWGYTF	11	CD8-C2
Pla-2_ATTGGACTCAGTTTGG	Pla	Pla-2	P2-3766	FALSE	1	TRA:CALSDYGKGGSEKLVF TRB:CASSSRLGSDTQYF	1	CD8-C3
Pla-2_ATTTCTGCACAGACAG	Pla	Pla-2	P2-3775	FALSE	1	TRA:CAYDLLTGNQFYF TRB:CASSRGTSGTDTQYF	1	CD8-C2
Pla-2_ATTTCTGTCTAACTTC	Pla	Pla-2	P2-29	TRUE	3	TRA:CAVGGDFNKFYF TRB:CSARSDRLGYQPQHF	21	CD8-C2
Pla-2_CAACCAAGTGTAACGG	Pla	Pla-2	P2-3780	FALSE	1	TRA:CAVRDRNDKIIF TRB:CASSLPNQPQHF	1	CD8-C2
Pla-2_CAACTAGAGATCACGG	Pla	Pla-2	P2-29	TRUE	3	TRA:CAVGGDFNKFYF TRB:CSARSDRLGYQPQHF	21	CD8-C7

Pla-2_CAAGTTGCACAGACAG	Pla	Pla-2	P2-3801	FALSE	1	TRA:CGTGTGGGADGLTF TRB:CASSYGTGKTDTQYF	1	CD8-C2
Pla-2_CACACCTAGGAGTACC	Pla	Pla-2	P2-3816	FALSE	1	TRA:CALSDSAGGTSYGKLTF TRB:CASSAYIAGGTGELFF	1	CD8-C2
Pla-2_CACACCTCAAACAACA	Pla	Pla-2	P2-458	FALSE	2	TRA:CAVRDQYTDKLIF TRB:CASSLSPRQGARVEQYF	2	CD8-C2
Pla-2_CACACCTGTCTCCACT	Pla	Pla-2	P2-79	FALSE	3	TRA:CAGAGRNDMRF TRB:CSATPGLALSYNEQFF	7	CD8-C2
Pla-2_CACATAGTCTGTCTAT	Pla	Pla-2	P2-35	FALSE	3	TRA:CGTETYTGFQKLVF TRB:CSVGTGSSYNEQFF	15	CD8-C3
Pla-2_CAGAATCTCATTGCGA	Pla	Pla-2	P2-3849	FALSE	1	TRA:CALSEAFNTNAGKSTF TRB:CASSPGTANTGELFF	1	CD8-C7
Pla-2_CAGCATAAGGTGGGTT	Pla	Pla-2	P2-3867	FALSE	1	TRA:CAVRSYAGGYSTLTF TRB:CASSPLGQGNEQFF	1	CD8-C2
Pla-2_CAGCCGATCTGGTATG	Pla	Pla-2	P2-3872	FALSE	1	TRA:CAVSSTDSWGKFQF TRB:CASSKGTGGSSYNEQFF	1	CD8-C2
Pla-2_CAGCTAAGTATGAATG	Pla	Pla-2	P2-3876	FALSE	1	TRA:CAVRMDSNYQLIW TRB:CASSLNSPVGPAGDEQFF	1	CD8-C2
Pla-2_CATCGAATCGAATGGG	Pla	Pla-2	P2-478	FALSE	2	TRA:CAVRRDTGRRALTF TRB:CASSQQETSGNEQFF	2	CD8-C2
Pla-2_CATGGCGCAGTTCATG	Pla	Pla-2	P2-3923	FALSE	1	TRA:CVVPNTNAGKSTF TRB:CASSRADTQYF	1	CD8-C2
Pla-2_CATTATCTCCTCATTA	Pla	Pla-2	P2-3930	FALSE	1	TRA:CALSGGTSGSRLTF TRB:CASLGGRTDTQYF	1	CD8-C2
Pla-2_CCACTACTCGTGGTCG	Pla	Pla-2	P2-2	TRUE	3	TRA:CVVPNTNAGKSTF TRB:CASSLQGSTYNEQFF	220	CD4-C4
Pla-2_CCACTACTCTTGTCAT	Pla	Pla-2	P2-480	FALSE	2	TRA:CAVSTTDSWGKFQF TRB:CATSRGRGGTSYNEQFF	2	CD8-C2
Pla-2_CCAGCGAGTCCATCCT	Pla	Pla-2	P2-3953	FALSE	1	TRA:CAVRPDPVSGNTPLVF TRB:CASSFQSGRLHEQYF	1	CD8-C2
Pla-2_CCAGCGATCCGAGCCA	Pla	Pla-2	P2-3954	FALSE	1	TRA:CLVGALRWNASKIIF TRB:CASSSDRGRTGELFF	1	CD8-C2
Pla-2_CCATGTCCATACCATG	Pla	Pla-2	P2-3	TRUE	3	TRA:CAVNPLESSYKLIF TRB:CASSPGTANTGELFF	177	CD8-C2
Pla-2_CCATTCGGTCGAGTTT	Pla	Pla-2	P2-346	TRUE	2	TRA:CAVSDGYTNAGKSTF TRB:CASSQDSRQGLNTEAFF	2	CD8-C2
Pla-2_CCATTCGGTTGGAGGT	Pla	Pla-2	P2-80	FALSE	3	TRA:CAVLGRGGGATNKLIF TRB:CASSQIGSSPDTQYF	7	CD8-C2
Pla-2_CCCAATCCACGTAAGG	Pla	Pla-2	P2-3960	FALSE	1	TRA:CALSEDGTDKLIF TRB:CASTEPGQRTDTQYF	1	CD8-C2
Pla-2_CCCTCCTCAGCTATTG	Pla	Pla-2	P2-3970	FALSE	1	TRA:CAFSTWGNQGGKLIF TRB:CSASPSGRGRGEQFF	1	CD8-C2
Pla-2_CCGGGATTCTGCGGCA	Pla	Pla-2	P2-49	FALSE	3	TRA:CATDEGAQKLVF TRB:CASSQDWGGAWGYTF	11	CD8-C2
Pla-2_CCGTGGACACCAGTTA	Pla	Pla-2	P2-58	TRUE	3	TRA:CAVNERSNDYKLSF TRB:CAISGGDSWGNQPQHF	9	CD8-C2
Pla-2_CCTACACGTCCGAATT	Pla	Pla-2	P2-487	FALSE	2	TRA:CVVMRRNYGQNFVF TRB:CASSLAGVSSGKGAYEQYF	2	CD8-C2
Pla-2_CCTACCAGTGCAACTT	Pla	Pla-2	P2-3990	FALSE	1	TRA:CAMTVYSSASKIIF TRB:CASSPGLAGESSYNEQFF	1	CD8-C2
Pla-2_CCTAGCTCAATTGCTG	Pla	Pla-2	P2-3993	FALSE	1	TRA:CAVSYGQAGTALIF TRB:CASSYGTELFF	1	CD8-C2
Pla-2_CCTATTACAGCCACCA	Pla	Pla-2	P2-246	FALSE	3	TRA:CAIGGAGNMLTF TRB:CASSARDRAYEQYF	3	CD8-C2

Pla-2_CCTTACGCACTCAGGC	Pla	Pla-2	P2-106	TRUE	3	TRA:CAAFSGNTPLVF TRB:CASSTSGGMNEQFF	5	CD8-C4
Pla-2_CCTTTCTAGACAAAGG	Pla	Pla-2	P2-4021	FALSE	1	TRA:CAANGPADSWGKFQF TRB:CSARDPGGRYEQYF	1	CD8-C2
Pla-2_CGAGCACGTACCGAGA	Pla	Pla-2	P2-383	FALSE	2	TRA:CATGMIKAAGNKLTF TRB:CASSHSGTSGSWYEQYF	2	CD4-C4
Pla-2_CGAGCCAAGTTAGGTA	Pla	Pla-2	P2-4049	FALSE	1	TRA:CIVRPPTDSWGKFQF TRB:CSVEGVSGGPTYEQYF	1	CD8-C2
Pla-2_CGAGCCAAGTTATCGC	Pla	Pla-2	P2-496	FALSE	2	TRA:CNELFGNEKLTF TRB:CASSGSTDTQYF	2	CD8-C2
Pla-2_CGAGCCACATGAACCT	Pla	Pla-2	P2-4051	FALSE	1	TRA:CAVRDNNAGNMLTF TRB:CASSLVRNSYNEQFF	1	CD8-C2
Pla-2_CGATCGGGTGGACGAT	Pla	Pla-2	P2-4056	FALSE	1	TRA:CAVGDNFNKFYF TRB:CASSPRGHGDTQYF	1	CD4-C4
Pla-2_CGATTGACACTTAAGC	Pla	Pla-2	P2-487	FALSE	2	TRA:CVVMRRNYGQNFVF TRB:CASSLAGVSSGKGAYEQYF	2	CD8-C2
Pla-2_CGCTATCAGTCCAGGA	Pla	Pla-2	P2-230	FALSE	3	TRA:CAVSPLRNNARLMF TRB:CSAEGGHSYEQYF	3	CD8-C2
Pla-2_CGCTATCCACCTATCC	Pla	Pla-2	P2-500	FALSE	2	TRA:CAEKRMYSGGGADGLTF TRB:CASSLYLSYNSPLHF	2	CD8-C2
Pla-2_CGCTGGACACAGCCCA	Pla	Pla-2	P2-480	FALSE	2	TRA:CAVSTTDSWGKFQF TRB:CATSRGRGGTSYNEQFF	2	CD8-C2
Pla-2_CGGAGTCCATGCCACG	Pla	Pla-2	P2-4110	FALSE	1	TRA:CAVTSGGYNKLIF TRB:CSADSSGTYNEQFF	1	CD8-C2
Pla-2_CGGCTAGGTATAATGG	Pla	Pla-2	P2-4115	FALSE	1	TRA:CAASATGFNKFYF TRB:CASSSGSDSGNTIYF	1	CD4-C4
Pla-2_CGGGTCACAAAGAATC	Pla	Pla-2	P2-58	TRUE	3	TRA:CAVNERSNDYKLSF TRB:CAISGGDSWGNQPQHF	9	CD8-C3
Pla-2_CGTAGGCCAAATTGCC	Pla	Pla-2	P2-80	FALSE	3	TRA:CAVLGRGGGATNKLIF TRB:CASSQIGSSPDTQYF	7	CD8-C2
Pla-2_CGTAGGCCACCCATGG	Pla	Pla-2	P2-79	FALSE	3	TRA:CAGAGRNDMRF TRB:CSATPGLALSYNEQFF	7	CD8-C2
Pla-2_CGTCAGGCAATAGCAA	Pla	Pla-2	P2-49	FALSE	3	TRA:CATDEGAQKLVF TRB:CASSQDWGGAWGYTF	11	CD8-C2
Pla-2_CGTCAGGCAGACACTT	Pla	Pla-2	P2-4138	FALSE	1	TRA:CAVRDSDNDKVIF TRB:CASSLGFVGGYGYTF	1	CD8-C2
Pla-2_CGTCCATTCGAGCCCA	Pla	Pla-2	P2-4141	FALSE	1	TRA:CAVSGATWSNNDMRF TRB:CASSYSGLFPYNEQFF	1	CD8-C2
Pla-2_CGTGTAAAGTAACCCT	Pla	Pla-2	P2-509	FALSE	2	TRA:CAVATTDSWGKFQF TRB:CATSRGTGGASYNEQFF	2	CD8-C2
Pla-2_CGTTCTGGTGCGAAAC	Pla	Pla-2	P2-4157	FALSE	1	TRA:CATDEGAQKLVF TRB:CASSPGAKNIQYF	1	CD8-C2
Pla-2_CGTTGGGGTGTAACGG	Pla	Pla-2	P2-70	FALSE	3	TRA:CAVPVAGTASKLTF TRB:CASREVSYNEQFF	8	CD8-C2
Pla-2_CTAATGGAGAGCTATA	Pla	Pla-2	P2-3	TRUE	3	TRA:CAVNPLESSYKLIF TRB:CASSPGTANTGELFF	177	CD8-C3
Pla-2_CTACATTAGAAAGTGG	Pla	Pla-2	P2-143	FALSE	3	TRA:CAYMEAAGNKLTF TRB:CASSPGLSTGELFF	4	CD8-C2
Pla-2_CTAGCCTCACCGATAT	Pla	Pla-2	P2-515	FALSE	2	TRA:CAATSLAGNMLTF TRB:CASSADRVGGSSPYEQYF	2	CD8-C2
Pla-2_CTCACACCAAGCGAGT	Pla	Pla-2	P2-164	FALSE	3	TRA:CAVGLAGNMLTF TRB:CASSYAPAGERGGRNEQFF	4	CD8-C2
Pla-2_CTCCTAGCAATGGAGC	Pla	Pla-2	P2-4202	FALSE	1	TRA:CVVRSGTYKYIF TRB:CASSEQGSNEKLFF	1	CD8-C2

Pla-2_CTCCTAGGTAGCCTCG	Pla	Pla-2	P2-4205	FALSE	1	TRA:CAVKKGYQKVTF TRB:CASSLAKTGGNTGELFF	1	CD8-C2
Pla-2_CTCCTAGGTTTGTGTG	Pla	Pla-2	P2-13	TRUE	3	TRA:CAYDLLTGNQFYF TRB:CASVRQLNTEAFF	38	CD8-C2
Pla-2_CTCGGAGGTTGAACTC	Pla	Pla-2	P2-4213	FALSE	1	TRA:CAVNWDKTSYDKVIF TRB:CSAGWNRVGSPLHF	1	CD8-C2
Pla-2_CTCGTACGTTACCAGT	Pla	Pla-2	P2-49	FALSE	3	TRA:CATDEGAQKLVF TRB:CASSQDWGGAWGYTF	11	CD8-C2
Pla-2_CTCGTCATCCTTGGTC	Pla	Pla-2	P2-3	TRUE	3	TRA:CAVNPLESSYKLIF TRB:CASSPGTANTGELFF	177	CD8-C2
Pla-2_CTCTGGTGTGTGGTTT	Pla	Pla-2	P2-4228	FALSE	1	TRA:CAVIPGGSYIPTF TRB:CASTIDLGTDTQYF	1	CD8-C2
Pla-2_CTGAAGTTCCGTAGTA	Pla	Pla-2	P2-4232	FALSE	1	TRA:CAVRSYGGSQGNLIF TRB:CASSLSASREPQHF	1	CD8-C2
Pla-2_CTGATAGAGTCCAGGA	Pla	Pla-2	P2-86	TRUE	3	TRA:CAVTPLRNTDKLIF TRB:CSASPSGRGRGEQFF	6	CD8-C2
Pla-2_CTGATCCCACTCTGTC	Pla	Pla-2	P2-4238	FALSE	1	TRA:CAGVNFNKFYF TRB:CASSRGTGYTF	1	CD8-C2
Pla-2_CTGCGGAGTTGTCTTT	Pla	Pla-2	P2-29	TRUE	3	TRA:CAVGGDFNKFYF TRB:CSARSDRLGYQPQHF	21	CD8-C2
Pla-2_CTGCTGTCAGACGCCT	Pla	Pla-2	P2-4247	FALSE	1	TRA:CLVGDMLSDARLMF TRB:CSARDGATGADTQYF	1	CD8-C2
Pla-2_CTGCTGTGTTCCACGG	Pla	Pla-2	P2-80	FALSE	3	TRA:CAVLGRGGGATNKLIF TRB:CASSQIGSSPDTQYF	7	CD8-C2
Pla-2_CTGTGCTAGAACAACT	Pla	Pla-2	P2-4252	FALSE	1	TRA:CVVNGNYQLIW TRB:CASGTGLAGGGGELFF	1	CD8-C3
Pla-2_CTGTTTACACTTACGA	Pla	Pla-2	P2-4258	FALSE	1	TRA:CAMRERSWVRQKLVF TRB:CASNYDLNSPLHF	1	CD8-C3
Pla-2_CTGTTTATCAGCATGT	Pla	Pla-2	P2-4260	FALSE	1	TRA:CLVGDGGYGQNFVF TRB:CASSLGTSGSSYNEQFF	1	CD8-C2
Pla-2_CTTACCGAGGAACTGC	Pla	Pla-2	P2-4264	FALSE	1	TRA:CVYYGGSQGNLIF TRB:CASGASADYEQYF	1	CD8-C2
Pla-2_CTTAGGAAGACCCACC	Pla	Pla-2	P2-46	TRUE	3	TRA:CAFSTWGNQGGKLIF TRB:CASSQDRLGGGTYEQYF	11	CD8-C3
Pla-2_CTTAGGATCAAGGCTT	Pla	Pla-2	P2-529	FALSE	2	TRA:CGTETGNQFYF TRB:CASSMVGGVTGELFF	2	CD8-C2
Pla-2_CTTAGGATCCTTCAAT	Pla	Pla-2	P2-13	TRUE	3	TRA:CAYDLLTGNQFYF TRB:CASVRQLNTEAFF	38	CD8-C2
Pla-2_CTTCTCTTCGCTGATA	Pla	Pla-2	P2-438	FALSE	2	TRA:CAVRAFSNDYKLSF TRB:CASSHVRDSQGRHTEAFF	2	CD8-C2
Pla-2_CTTGGCTCAACACCCG	Pla	Pla-2	P2-504	FALSE	2	TRA:CGRQMLLFTTVHRSAGKSTF TRB:CASSPITGDTQYF	2	CD8-C2
Pla-2_GAAGCAGAGATCCCGC	Pla	Pla-2	P2-13	TRUE	3	TRA:CAYDLLTGNQFYF TRB:CASVRQLNTEAFF	38	CD8-C2
Pla-2_GAAGCAGTCGTTTGCC	Pla	Pla-2	P2-534	FALSE	2	TRA:CAEIPPNRDDKIIF TRB:CASSQGSSGRAETQYF	2	CD8-C2
Pla-2_GAATAAGCAGAGCCAA	Pla	Pla-2	P2-4315	FALSE	1	TRA:CAVRDAGNNRKLIW TRB:CASSPRGAYETQYF	1	CD8-C2
Pla-2_GAATGAAAGTCTCGGC	Pla	Pla-2	P2-29	TRUE	3	TRA:CAVGGDFNKFYF TRB:CSARSDRLGYQPQHF	21	CD8-C5
Pla-2_GAATGAATCCGTAGGC	Pla	Pla-2	P2-207	FALSE	3	TRA:CAVKAEYGQNFVF TRB:CASSAGPYNSPLHF	3	CD4-C4
Pla-2_GACACGCTCACTTATC	Pla	Pla-2	P2-408	FALSE	2	TRA:CAVEDLGTYKYIF TRB:CASSPNEAPQYF	2	CD8-C2

Pla-2_GACTAACCAGTGGGAT	Pla	Pla-2	P2-79	FALSE	3	TRA:CAGAGRNDMRF TRB:CSATPGLALSYNEQFF	7	CD8-C2
Pla-2_GACTACAAGGCAGGTT	Pla	Pla-2	P2-538	FALSE	2	TRA:CAASEGTSGTYKYIF TRB:CASSLMHPNTGELFF	2	CD8-C2
Pla-2_GAGCAGATCCAGTATG	Pla	Pla-2	P2-4366	FALSE	1	TRA:CAVVDSNYQLIW TRB:CASSKAEPYEQYF	1	CD8-C2
Pla-2_GAGTCCGTCACCGTAA	Pla	Pla-2	P2-35	FALSE	3	TRA:CGTETYTGFQKLVF TRB:CSVGTGSSYNEQFF	15	CD8-C2
Pla-2_GATCAGTCAGGATTGG	Pla	Pla-2	P2-4372	FALSE	1	TRA:CAYDLLTGNQFYF TRB:CASSTSGGMNEQFF	1	CD8-C2
Pla-2_GATGAGGAGCTTATCG	Pla	Pla-2	P2-6	TRUE	3	TRA:CVVSVAAGNKLTF TRB:CASSPGTDLYEQYF	85	CD8-C10
Pla-2_GATGCTACATCAGTCA	Pla	Pla-2	P2-229	FALSE	3	TRA:CAVRDEAAGTYKYIF TRB:CASSQGVSLAGTDTQYF	3	CD8-C2
Pla-2_GATTCAGAGATAGCAT	Pla	Pla-2	P2-4393	FALSE	1	TRA:CAVGVRGGGNKLTF TRB:CASSLPPGLAGGPYNEQFF	1	CD8-C2
Pla-2_GCAGCCACATCGATTG	Pla	Pla-2	P2-90	FALSE	3	TRA:CIGNTPLVF TRB:CASSDRDRVLNTEAFF	6	CD4-C4
Pla-2_GCATGCGAGTTGTCGT	Pla	Pla-2	P2-4427	FALSE	1	TRA:CLVGPRDDKIIF TRB:CAWSPNYSGELFF	1	CD8-C1
Pla-2_GCCAAATAGCGTCAAG	Pla	Pla-2	P2-4433	FALSE	1	TRA:CAVVGGASGTYKYIF TRB:CASSLGHTYNEQFF	1	CD8-C2
Pla-2_GCGACCAGTCCAACTA	Pla	Pla-2	P2-70	FALSE	3	TRA:CAVPVAGTASKLTF TRB:CASREVSYNEQFF	8	CD8-C2
Pla-2_GCGCAACAGTCCGGTC	Pla	Pla-2	P2-499	FALSE	2	TRA:CALSEVDGGGGADGLTF TRB:CASREVSYNEQFF	2	CD8-C2
Pla-2_GCTCCTACAAGAAAGG	Pla	Pla-2	P2-495	FALSE	2	TRA:CAVTRYDYKLSF TRB:CASSESDRDSFYQETQYF	2	CD8-C2
Pla-2_GCTCTGTAGTGTACTC	Pla	Pla-2	P2-82	FALSE	3	TRA:CVADTGNQFYF TRB:CASSSALTGTYEQYF	7	CD8-C2
Pla-2_GCTGGGTTCGACCAGC	Pla	Pla-2	P2-13	TRUE	3	TRA:CAYDLLTGNQFYF TRB:CASVRQLNTEAFF	38	CD8-C2
Pla-2_GCTGGGTTCTCGCATC	Pla	Pla-2	P2-4480	FALSE	1	TRA:CILSPGNTPLVF TRB:CASSLQGSTYNEQFF	1	CD8-C2
Pla-2_GGAACTTTCGGCGCTA	Pla	Pla-2	P2-52	FALSE	3	TRA:CARTTNAGKSTF TRB:CASSETSGRIYEQFF	11	CD8-C2
Pla-2_GGACAAGAGTGGGTTG	Pla	Pla-2	P2-4502	FALSE	1	TRA:CLVGRMNRDDKIIF TRB:CASRGKVVYEQYF	1	CD8-C2
Pla-2_GGACATTTCCTATTCA	Pla	Pla-2	P2-246	FALSE	3	TRA:CAIGGAGNMLTF TRB:CASSARDRAYEQYF	3	CD8-C7
Pla-2_GGATGTTTCTCTGAGA	Pla	Pla-2	P2-500	FALSE	2	TRA:CAEKRMYSGGGADGLTF TRB:CASSLYLSYNSPLHF	2	CD8-C4
Pla-2_GGATTACCAAACCCAT	Pla	Pla-2	P2-79	FALSE	3	TRA:CAGAGRNDMRF TRB:CSATPGLALSYNEQFF	7	CD8-C2
Pla-2_GGATTACCACTGAAGG	Pla	Pla-2	P2-4524	FALSE	1	TRA:CLVGQNTDKLIF TRB:CASSPKVVVAGYEQYF	1	CD8-C2
Pla-2_GGCCGATTCTGGCGAC	Pla	Pla-2	P2-4537	FALSE	1	TRA:CVVSANGAGSYQLTF TRB:CASSYTRQDPYSYTF	1	CD8-C2
Pla-2_GGCGACTCATGCCTAA	Pla	Pla-2	P2-4540	FALSE	1	TRA:CAMRPNTNAGKSTF TRB:CASSIVGGVTGELFF	1	CD8-C2
Pla-2_GGCGTGTAGTTTCCTT	Pla	Pla-2	P2-4544	FALSE	1	TRA:CALDNAGGTSYGKLTF TRB:CASSPGGTVETQYF	1	CD8-C2
Pla-2_GGGATGAGTAATCACC	Pla	Pla-2	P2-70	FALSE	3	TRA:CAVPVAGTASKLTF TRB:CASREVSYNEQFF	8	CD8-C2

Pla-2_GGGCACTTCCTTTCGG	Pla	Pla-2	P2-70	FALSE	3	TRA:CAVPVAGTASKLTF TRB:CASREVSYNEQFF	8	CD8-C2
Pla-2_GGGTCTGCATTTCAGG	Pla	Pla-2	P2-4565	FALSE	1	TRA:CAASESDYGGSQGNLIF TRB:CASSQDRLGGGTYEQYF	1	CD8-C2
Pla-2_GGGTTGCCAGCGATCC	Pla	Pla-2	P2-538	FALSE	2	TRA:CAASEGTSGTYKYIF TRB:CASSLMHPNTGELFF	2	CD8-C2
Pla-2_GGTATTGTCCACGACG	Pla	Pla-2	P2-450	FALSE	2	TRA:CAVKGGNNRLAF TRB:CASNRDKSTDTQYF	2	CD4-C3
Pla-2_GGTGAAGAGCAATATG	Pla	Pla-2	P2-230	FALSE	3	TRA:CAVSPLRNNARLMF TRB:CSAEGGHSYEQYF	3	CD8-C2
Pla-2_GGTGAAGCACGAAAGC	Pla	Pla-2	P2-4575	FALSE	1	TRA:CAPKMDSNYQLIW TRB:CASSQTGADTQYF	1	CD8-C2
Pla-2_GGTGAAGGTAGGCTGA	Pla	Pla-2	P2-4576	FALSE	1	TRA:CVVSEAGGADGLTF TRB:CASHRTGNTEAFF	1	CD8-C2
Pla-2_GTAACTGAGAAGGCCT	Pla	Pla-2	P2-547	FALSE	2	TRA:CAVRPLRNTDKLIF TRB:CASGHLQETQYF	2	CD8-C7
Pla-2_GTACGTAGTTGTCGCG	Pla	Pla-2	P2-4590	FALSE	1	TRA:CADATGNQFYF TRB:CATRAPSGTGELFF	1	CD8-C2
Pla-2_GTAGGCCTCGCAAACT	Pla	Pla-2	P2-210	FALSE	3	TRA:CAVRRPNSGYSTLTF TRB:CSARDTERTGNIQYF	3	CD8-C2
Pla-2_GTAGTCACATTCACTT	Pla	Pla-2	P2-70	FALSE	3	TRA:CAVPVAGTASKLTF TRB:CASREVSYNEQFF	8	CD8-C2
Pla-2_GTATCTTTCACTATTC	Pla	Pla-2	P2-456	FALSE	2	TRA:CAVEGSGLNFNKFYF TRB:CSVVGTEAFF	2	CD8-C8
Pla-2_GTATTCTAGAAGGACA	Pla	Pla-2	P2-80	FALSE	3	TRA:CAVLGRGGGATNKLIF TRB:CASSQIGSSPDTQYF	7	CD8-C2
Pla-2_GTATTCTTCATCGCTC	Pla	Pla-2	P2-49	FALSE	3	TRA:CATDEGAQKLVF TRB:CASSQDWGGAWGYTF	11	CD8-C2
Pla-2_GTCACGGAGTGGTAGC	Pla	Pla-2	P2-140	FALSE	3	TRA:CAVNSGANNLFF TRB:CSARDEEREGYNEQFF	4	CD8-C5
Pla-2_GTCGGGTAGCGTAATA	Pla	Pla-2	P2-232	FALSE	3	TRA:CAASGGGNKLTF TRB:CASSGNTDTQYF	3	CD8-C2
Pla-2_GTCTTCGCATGGTCAT	Pla	Pla-2	P2-4625	FALSE	1	TRA:CAVDTGTASKLTF TRB:CASRGLADTQYF	1	CD8-C2
Pla-2_GTGAAGGAGCTAAGAT	Pla	Pla-2	P2-18	TRUE	3	TRA:CAATSSSNFGNEKLTF TRB:CAWSRAALTEAFF	28	CD8-C2
Pla-2_GTGCATAAGGGAGTAA	Pla	Pla-2	P2-88	TRUE	3	TRA:CAVPGAGNKLTF TRB:CASSRSLAGVDTGELFF	6	CD4-C4
Pla-2_GTGGGTCAGTGTGGCA	Pla	Pla-2	P2-56	FALSE	3	TRA:CATDLDGQKLLF TRB:CASRERGGNTGELFF	10	CD8-C2
Pla-2_GTTAAGCAGACTACAA	Pla	Pla-2	P2-35	FALSE	3	TRA:CGTETYTGFQKLVF TRB:CSVGTGSSYNEQFF	15	CD8-C2
Pla-2_GTTACAGCATGCGCAC	Pla	Pla-2	P2-106	TRUE	3	TRA:CAAFSGNTPLVF TRB:CASSTSGGMNEQFF	5	CD8-C2
Pla-2_GTTCATTAGAGGTAGA	Pla	Pla-2	P2-1	TRUE	3	TRA:CVVNIIGYQKVTF TRB:CASTRQGKQPQHF	270	CD8-C6
Pla-2_TAAACCGAGTATCGAA	Pla	Pla-2	P2-79	FALSE	3	TRA:CAGAGRNDMRF TRB:CSATPGLALSYNEQFF	7	CD8-C2
Pla-2_TAAGAGAAGTCCAGGA	Pla	Pla-2	P2-4669	FALSE	1	TRA:CAVGLYNQGGKLIF TRB:CASSLAGNTEAFF	1	CD4-C4
Pla-2_TAAGTGCCAAGTCATC	Pla	Pla-2	P2-4676	FALSE	1	TRA:CALNTGGFKTIF TRB:CASTRGGTANEKLFF	1	CD8-C2
Pla-2_TACCTATGTTACGGAG	Pla	Pla-2	P2-237	FALSE	3	TRA:CALSEALGSGGGADGLTF TRB:CASSTTGDGVVSYEQYF	3	CD8-C2

Pla-2_TACCTTACAGGTCTCG	Pla	Pla-2	P2-46	TRUE	3	TRA:CAFSTWGNQGGKLIF TRB:CASSQDRLGGGTYEQYF	11	CD8-C2
Pla-2_TACGGATCAGGCGATA	Pla	Pla-2	P2-4690	FALSE	1	TRA:CVVSDPPGGFKTIF TRB:CAWTSGTGWKKETQYF	1	CD8-C2
Pla-2_TACGGGCAGAACTCGG	Pla	Pla-2	P2-413	FALSE	2	TRA:CLVGFRGTYKYIF TRB:CASSYAPAGERGGRNEQFF	2	CD8-C2
Pla-2_TACTCATGTATATGAG	Pla	Pla-2	P2-164	FALSE	3	TRA:CAVGLAGNMLTF TRB:CASSYAPAGERGGRNEQFF	4	CD8-C2
Pla-2_TACTCATGTCAGAAGC	Pla	Pla-2	P2-121	TRUE	3	TRA:CAGASRDTDKLIF TRB:CSVESFLRGASLMTEAFF	4	CD4-C4
Pla-2_TACTCGCCATTATCTC	Pla	Pla-2	P2-4704	FALSE	1	TRA:CVVSARDSSYKLIF TRB:CASRMGLAGHNEQFF	1	CD8-C2
Pla-2_TACTTACGTTCTGGTA	Pla	Pla-2	P2-4709	FALSE	1	TRA:CAVRPLRNTDKLIF TRB:CSARDTWDVTDTQYF	1	CD8-C2
Pla-2_TAGGCATGTGCCTGGT	Pla	Pla-2	P2-35	FALSE	3	TRA:CGTETYTGFQKLVF TRB:CSVGTGSSYNEQFF	15	CD8-C2
Pla-2_TCAACGAAGACGCTTT	Pla	Pla-2	P2-4742	FALSE	1	TRA:CVVSAGGGNKLTF TRB:CASSLERSSGRVGETQYF	1	CD4-C4
Pla-2_TCAACGAAGTGACATA	Pla	Pla-2	P2-4743	FALSE	1	TRA:CAYDLLTGNQFYF TRB:CASSLYPSGGGTDTQYF	1	CD8-C2
Pla-2_TCAATCTTCGTTGCCT	Pla	Pla-2	P2-342	TRUE	2	TRA:CAVSEGTGRRALTF TRB:CASSLGTQRSTDTQYF	2	CD4-C5
Pla-2_TCAGATGGTTCCCTTG	Pla	Pla-2	P2-4760	FALSE	1	TRA:CAPGGTSYGKLTF TRB:CASSLGQGVGYEQYF	1	CD8-C2
Pla-2_TCAGATGTCATCATTC	Pla	Pla-2	P2-4761	FALSE	1	TRA:CAASMVETSGSRLTF TRB:CASSQGRVTQYF	1	CD8-C2
Pla-2_TCAGCTCCAGATTGCT	Pla	Pla-2	P2-4769	FALSE	1	TRA:CAVPVAGTASKLTF TRB:CASSEAEGWNTEAFF	1	CD8-C2
Pla-2_TCAGGATAGAACAACT	Pla	Pla-2	P2-4772	FALSE	1	TRA:CAESISSGYSTLTF TRB:CATSDPVDRATGELFF	1	CD8-C2
Pla-2_TCAGGTACATCCGCGA	Pla	Pla-2	P2-13	TRUE	3	TRA:CAYDLLTGNQFYF TRB:CASVRQLNTEAFF	38	CD8-C2
Pla-2_TCATTTGGTGACTACT	Pla	Pla-2	P2-4784	FALSE	1	TRA:CAASEGGGADGLTF TRB:CASSVDRGWYEQYF	1	CD4-C4
Pla-2_TCCCGATAGCCACTAT	Pla	Pla-2	P2-58	TRUE	3	TRA:CAVNERSNDYKLSF TRB:CAISGGDSWGNQPQHF	9	CD4-C4
Pla-2_TCGAGGCGTTTGACAC	Pla	Pla-2	P2-106	TRUE	3	TRA:CAAFSGNTPLVF TRB:CASSTSGGMNEQFF	5	CD8-C2
Pla-2_TCGTACCGTCCTCCAT	Pla	Pla-2	P2-232	FALSE	3	TRA:CAASGGGNKLTF TRB:CASSGNTDTQYF	3	CD8-C2
Pla-2_TCTGGAATCGTCTGCT	Pla	Pla-2	P2-4834	FALSE	1	TRA:CAVRRLEADSWGKFQF TRB:CASLNGAGGSYEQYF	1	CD8-C2
Pla-2_TCTTTCCAGATGAGAG	Pla	Pla-2	P2-70	FALSE	3	TRA:CAVPVAGTASKLTF TRB:CASREVSYNEQFF	8	CD8-C2
Pla-2_TGACAACAGTACATGA	Pla	Pla-2	P2-35	FALSE	3	TRA:CGTETYTGFQKLVF TRB:CSVGTGSSYNEQFF	15	CD8-C4
Pla-2_TGACAACCAATGGTCT	Pla	Pla-2	P2-496	FALSE	2	TRA:CNELFGNEKLTF TRB:CASSGSTDTQYF	2	CD8-C2
Pla-2_TGACTAGCACATTAGC	Pla	Pla-2	P2-80	FALSE	3	TRA:CAVLGRGGGATNKLIF TRB:CASSQIGSSPDTQYF	7	CD8-C2
Pla-2_TGACTTTCATACGCTA	Pla	Pla-2	P2-564	FALSE	2	TRA:CAVRDPNSGYALNF TRB:CASSLTVSGSNQPQHF	2	CD8-C2
Pla-2_TGATTTCAGGCTCATT	Pla	Pla-2	P2-86	TRUE	3	TRA:CAVTPLRNTDKLIF TRB:CSASPSGRGRGEQFF	6	CD8-C2

Pla-2_TGATTTCCAAGTACCT	Pla	Pla-2	P2-4872	FALSE	1	TRA:CAVNTDKLIF TRB:CSARGLAGAASYNEQFF	1	CD8-C2
Pla-2_TGATTTCTCTAACCGA	Pla	Pla-2	P2-4875	FALSE	1	TRA:CAASRAGSQGNLIF TRB:CSALPRDRVSYEQYF	1	CD8-C3
Pla-2_TGCCAAATCCAAATGC	Pla	Pla-2	P2-86	TRUE	3	TRA:CAVTPLRNTDKLIF TRB:CSASPSGRGRGEQFF	6	CD8-C2
Pla-2_TGCCCATGTAGCCTCG	Pla	Pla-2	P2-187	TRUE	3	TRA:CAVLTGTASKLTF TRB:CASSGSTDTQYF	3	CD8-C2
Pla-2_TGCCCATGTTCGTGAT	Pla	Pla-2	P2-83	FALSE	3	TRA:CAVDTGTASKLTF TRB:CASRGSTDTQYF	7	CD8-C2
Pla-2_TGCGCAGGTACTCGCG	Pla	Pla-2	P2-4889	FALSE	1	TRA:CAVNPLESSYKLIF TRB:CASRSGGTEAFF	1	CD8-C2
Pla-2_TGCGCAGGTATAGTAG	Pla	Pla-2	P2-509	FALSE	2	TRA:CAVATTDSWGKFQF TRB:CATSRGTGGASYNEQFF	2	CD8-C2
Pla-2_TGCGTGGGTCTTTCAT	Pla	Pla-2	P2-4893	FALSE	1	TRA:CLVDGGYNKLIF TRB:CASSYSGGDGTGELFF	1	CD8-C7
Pla-2_TGCTGCTTCATCATTC	Pla	Pla-2	P2-515	FALSE	2	TRA:CAATSLAGNMLTF TRB:CASSADRVGGSSPYEQYF	2	CD8-C2
Pla-2_TGGCGCAAGTCGCCGT	Pla	Pla-2	P2-4911	FALSE	1	TRA:CAVSSGSRLTF TRB:CASSPGGTVETQYF	1	CD8-C2
Pla-2_TGGGCGTCAGCGATCC	Pla	Pla-2	P2-565	FALSE	2	TRA:CAVEDRTGANNLFF TRB:CATSDLNSSYNEQFF	2	CD8-C2
Pla-2_TGTCCCAGTACGAAAT	Pla	Pla-2	P2-478	FALSE	2	TRA:CAVRRDTGRRALTF TRB:CASSQQETSGNEQFF	2	CD8-C2
Pla-2_TTAACTCTCTTATCTG	Pla	Pla-2	P2-187	TRUE	3	TRA:CAVLTGTASKLTF TRB:CASSGSTDTQYF	3	CD8-C2
Pla-2_TTAGGACGTAGGCATG	Pla	Pla-2	P2-4963	FALSE	1	TRA:CATDMRF TRB:CASSMRLDEQYF	1	CD8-C3
Pla-2_TTAGGCAGTTAGATGA	Pla	Pla-2	P2-4967	FALSE	1	TRA:CAVLVDDKIIF TRB:CASSSRDSPSYNEQFF	1	CD8-C2
Pla-2_TTAGTTCCACAGGTTT	Pla	Pla-2	P2-564	FALSE	2	TRA:CAVRDPNSGYALNF TRB:CASSLTVSGSNQPQHF	2	CD8-C2
Pla-2_TTATGCTAGAGTTGGC	Pla	Pla-2	P2-4969	FALSE	1	TRA:CAVDSNAGNMLTF TRB:CASSVEWGAQETQYF	1	CD8-C2
Pla-2_TTATGCTCATACAGCT	Pla	Pla-2	P2-317	TRUE	2	TRA:CAGYQIKAAGNKLTF TRB:CASSWRQKDTQYF	2	CD8-C2
Pla-2_TTCGAAGGTAACGCGA	Pla	Pla-2	P2-4977	FALSE	1	TRA:CAVPRTGRRALTF TRB:CAISESDPVRLNEQFF	1	CD8-C2
Pla-2_TTGACTTTCGTTTGCC	Pla	Pla-2	P2-13	TRUE	3	TRA:CAYDLLTGNQFYF TRB:CASVRQLNTEAFF	38	CD8-C2
Pla-2_TTGACTTTCTTACCGC	Pla	Pla-2	P2-82	FALSE	3	TRA:CVADTGNQFYF TRB:CASSSALTGTYEQYF	7	CD8-C2
Pla-2_TTGCCGTCAATTGCTG	Pla	Pla-2	P2-5009	FALSE	1	TRA:CVVPNTNAGKSTF TRB:CSVPGQGDYEQYF	1	CD8-C10
Pla-2_TTGGAACTCCTGTACC	Pla	Pla-2	P2-529	FALSE	2	TRA:CGTETGNQFYF TRB:CASSMVGGVTGELFF	2	CD8-C2
Pla-2_TTGTAGGAGAAGAAGC	Pla	Pla-2	P2-350	TRUE	2	TRA:CAVKVDSNYQLIW TRB:CASSPEGAGGGTGELFF	2	CD8-C10
Pla-2_TTTGCGCCAATGACCT	Pla	Pla-2	P2-3	TRUE	3	TRA:CAVNPLESSYKLIF TRB:CASSPGTANTGELFF	177	CD8-C10
Pla-2_TTTGGTTCAAGTTGTC	Pla	Pla-2	P2-5035	FALSE	1	TRA:CALSGRRDTGRRALTF TRB:CASSLGGKDYGYTF	1	CD4-C4
Pla-2_AAACGGGAGTACGATA	Pla	Pla-2	P2-62	FALSE	3	TRA:CAVITDTGRRALTF TRB:CASSIGTSGYNEQFF	9	CD4-C6

Pla-2_AAAGTAGCATTACCTT	Pla	Pla-2	P2-207	FALSE	3	TRA:CAVKAEYGQNFVF TRB:CASSAGPYNSPLHF	3	CD4-C6
Pla-2_AAAGTAGGTATTCGTG	Pla	Pla-2	P2-3420	FALSE	1	TRA:CAVRPGNQFYF TRB:CASSTGQGALSGYTF	1	CD4-C3
Pla-2_AAATGCCGTGAAATCA	Pla	Pla-2	P2-128	TRUE	3	TRA:CAYKKTSYDKVIF TRB:CASSPTSQPQHF	4	CD4-C3
Pla-2_AACACGTTCATGCTCC	Pla	Pla-2	P2-3429	FALSE	1	TRA:CAVLSQGSNYKLTF TRB:CASIHSGTGRIRDGNTIYF	1	CD4-C4
Pla-2_AACCGCGGTTTGTTGG	Pla	Pla-2	P2-3436	FALSE	1	TRA:CAVKPTGGSYIPTF TRB:CASRWGTEDTEAFF	1	CD4-C3
Pla-2_AACGTTGAGAATCTCC	Pla	Pla-2	P2-3437	FALSE	1	TRA:CAALMDSSYKLIF TRB:CASNARESYNSPLHF	1	CD4-C3
Pla-2_AACTCAGGTAGGACAC	Pla	Pla-2	P2-3445	FALSE	1	TRA:CATDAASGGSNYKLTF TRB:CASSLALDRSETQYF	1	CD4-C3
Pla-2_AACTCTTGTCGAAAGC	Pla	Pla-2	P2-345	TRUE	2	TRA:CAVSSGSRLTF TRB:CASSPNVGGFTYEQYF	2	CD4-C3
Pla-2_AACTGGTTCCTCAATT	Pla	Pla-2	P2-3453	FALSE	1	TRA:CVVGKGSQGNLIF TRB:CASTDGGDTQYF	1	CD4-C3
Pla-2_AACTTTCAGAGTACAT	Pla	Pla-2	P2-197	TRUE	3	TRA:CVVSARAGGFKTIF TRB:CASSEAFGGSYNEQFF	3	CD4-C3
Pla-2_AAGCCGCAGAACTCGG	Pla	Pla-2	P2-110	TRUE	3	TRA:CAVSTSGTYKYIF TRB:CASSSSGGDNSPLHF	5	CD4-C3
Pla-2_AAGCCGCCATGGAATA	Pla	Pla-2	P2-386	FALSE	2	TRA:CAVRVEDKIIF TRB:CAWRTPGQGSGKNTIYF	2	CD4-C3
Pla-2_AAGTCTGCATCAGTAC	Pla	Pla-2	P2-215	FALSE	3	TRA:CAANNNARLMF TRB:CASSLSGARLVTGELFF	3	CD4-C3
Pla-2_AAGTCTGGTGTTGGGA	Pla	Pla-2	P2-3474	FALSE	1	TRA:CLVARKGDTGFQKLVF TRB:CASSSPGKETQYF	1	CD4-C3
Pla-2_ACACCGGCAATAGCAA	Pla	Pla-2	P2-131	TRUE	3	TRA:CATVGQIQGAQKLVF TRB:CASRTGTSSYEQYF	4	CD8-C2
Pla-2_ACACTGACATGTAGTC	Pla	Pla-2	P2-139	FALSE	3	TRA:CAVATGFQKLVF TRB:CASTLGGQGNNEQFF	4	CD4-C3
Pla-2_ACATACGCAAACTGTC	Pla	Pla-2	P2-3503	FALSE	1	TRA:CATDKAMKTSYDKVIF TRB:CAISESGDPEAFF	1	CD4-C3
Pla-2_ACATACGCAGGATTGG	Pla	Pla-2	P2-3504	FALSE	1	TRA:CAVRDTQGLVF TRB:CASGLRDGHMNTEAFF	1	CD4-C3
Pla-2_ACATACGGTAGGAGTC	Pla	Pla-2	P2-3505	FALSE	1	TRA:CAVKRWTSGTYKYIF TRB:CASSPWDRGSTDTQYF	1	CD4-C3
Pla-2_ACATACGGTGCCTGGT	Pla	Pla-2	P2-3506	FALSE	1	TRA:CAVRERNAGNMLTF TRB:CASSSAAGGYNEQFF	1	CD4-C2
Pla-2_ACCAGTAAGAGCTTCT	Pla	Pla-2	P2-3518	FALSE	1	TRA:CAVFSQNTDKLIF TRB:CAWSVLGNTEAFF	1	CD4-C5
Pla-2_ACCAGTAGTTACTGAC	Pla	Pla-2	P2-3521	FALSE	1	TRA:CATGGLAGGGNKLTF TRB:CASSLEAGNTQYF	1	CD4-C3
Pla-2_ACCCACTAGCACGCCT	Pla	Pla-2	P2-62	FALSE	3	TRA:CAVITDTGRRALTF TRB:CASSIGTSGYNEQFF	9	CD4-C3
Pla-2_ACGAGCCGTACTTCTT	Pla	Pla-2	P2-323	TRUE	2	TRA:CLVGPAYNQGGKLIF TRB:CASSSSTANSGNTIYF	2	CD4-C3
Pla-2_ACGAGGAAGGCGACAT	Pla	Pla-2	P2-218	FALSE	3	TRA:CVVSPYTGGGNKLTF TRB:CASWGDSYNSPLHF	3	CD4-C3
Pla-2_ACGCAGCCAAGCTGAG	Pla	Pla-2	P2-3554	FALSE	1	TRA:CAVGSNSGGSNYKLTF TRB:CASSLEARQGPARAAKNIQY	1	CD4-C3
Pla-2_ACGCAGCCACCTATCC	Pla	Pla-2	P2-90	FALSE	3	TRA:CIGNTPLVF TRB:CASSDRDRVLNTEAFF	6	CD4-C3

Pla-2_ACGCAGCCATGCCTTC	Pla	Pla-2	P2-3555	FALSE	1	TRA:CAVGDSSASKIIF TRB:CSGNRDRGTDTQYF	1	CD4-C3
Pla-2_ACGCAGCGTTACGACT	Pla	Pla-2	P2-3556	FALSE	1	TRA:CAYSTYKYIF TRB:CASSSTSSNTEAFF	1	CD4-C3
Pla-2_ACGCAGCGTTGTCGCG	Pla	Pla-2	P2-3558	FALSE	1	TRA:CAFISIQGAQKLVF TRB:CSAPPAGGPNEQYF	1	CD4-C3
Pla-2_ACGCCAGCATAACCTG	Pla	Pla-2	P2-3561	FALSE	1	TRA:CVVPNTNAGKSTF TRB:CATSIGAAGANVLTF	1	CD4-C3
Pla-2_ACGCCGAGTCTTGATG	Pla	Pla-2	P2-3566	FALSE	1	TRA:CAVDSWGYQKVTF TRB:CASSQTGLATSYNEQFF	1	CD4-C3
Pla-2_ACGGAGATCCACGTTC	Pla	Pla-2	P2-407	FALSE	2	TRA:CAMSAGTGRRALTF TRB:CASSHYRATFDQYF	2	CD4-C4
Pla-2_ACGGCCATCAACGGGA	Pla	Pla-2	P2-194	TRUE	3	TRA:CAGRLQGAQKLVF TRB:CAWSQSQGYYGYTF	3	CD4-C3
Pla-2_ACGGGCTAGATATGGT	Pla	Pla-2	P2-3570	FALSE	1	TRA:CAAIVWGTGANNLFF TRB:CSARTATNEKLFF	1	CD4-C3
Pla-2_ACGGGCTAGCAACGGT	Pla	Pla-2	P2-3571	FALSE	1	TRA:CAFNPGYSSASKIIF TRB:CASSLDGQGGAFF	1	CD4-C3
Pla-2_ACGGGCTCATACCATG	Pla	Pla-2	P2-3572	FALSE	1	TRA:CAVKRSGAGSYQLTF TRB:CASSEQDSNQPQHF	1	CD4-C3
Pla-2_ACGTCAAAGCAGCCTC	Pla	Pla-2	P2-224	FALSE	3	TRA:CAVSGGGFKTIF TRB:CASSQEGETQYF	3	CD4-C4
Pla-2_ACGTCAATCCACGTTC	Pla	Pla-2	P2-90	FALSE	3	TRA:CIGNTPLVF TRB:CASSDRDRVLNTEAFF	6	CD4-C3
Pla-2_ACTGAACCATTACCTT	Pla	Pla-2	P2-31	FALSE	3	TRA:CAASDNTDKLIF TRB:CASSQVHEQYF	20	CD4-C7
Pla-2_ACTGAACTCTTCGAGA	Pla	Pla-2	P2-3582	FALSE	1	TRA:CAVSYFGNEKLTF TRB:CASSPLTGNTEAFF	1	CD4-C3
Pla-2_ACTGCTCAGCGAGAAA	Pla	Pla-2	P2-3593	FALSE	1	TRA:CLVVPGYSSASKIIF TRB:CSARRQGGGNQPQHF	1	CD4-C3
Pla-2_ACTGCTCGTCCGTTAA	Pla	Pla-2	P2-226	FALSE	3	TRA:CAVDNTGAGSYQLTF TRB:CASSQAGGSYEQYF	3	CD4-C3
Pla-2_ACTTACTAGGTGCTAG	Pla	Pla-2	P2-411	FALSE	2	TRA:CAHGNTGGFKTIF TRB:CASSLGNTGELFF	2	CD4-C4
Pla-2_ACTTGTTCATCACGAT	Pla	Pla-2	P2-414	FALSE	2	TRA:CAVGTTSGTYKYIF TRB:CASSQLYTEAFF	2	CD4-C3
Pla-2_ACTTTCACATTCCTGC	Pla	Pla-2	P2-415	FALSE	2	TRA:CAENPNSGGSNYKLTF TRB:CASMDRAPGTSGFLF	2	CD4-C3
Pla-2_ACTTTCATCACCTCGT	Pla	Pla-2	P2-3609	FALSE	1	TRA:CAYRSVTKGSARQLTF TRB:CASSHDRGAYEQYF	1	CD4-C3
Pla-2_ACTTTCATCCACTCCA	Pla	Pla-2	P2-3610	FALSE	1	TRA:CIVRVANKLSF TRB:CAWSTRGRGIYNEQFF	1	CD4-C3
Pla-2_AGAATAGTCCACTGGG	Pla	Pla-2	P2-416	FALSE	2	TRA:CAVEDRSYQKVTF TRB:CASSAGGDGFEQFF	2	CD4-C3
Pla-2_AGAGCGAGTACCCAAT	Pla	Pla-2	P2-3615	FALSE	1	TRA:CAVSEDTDKLIF TRB:CASRTVGYGEKLFF	1	CD4-C4
Pla-2_AGAGCTTAGGGTTCCC	Pla	Pla-2	P2-3619	FALSE	1	TRA:CVVSIAGGSYIPTF TRB:CASSLDRVHNEQFF	1	CD4-C4
Pla-2_AGAGTGGTCCAAGTAC	Pla	Pla-2	P2-3623	FALSE	1	TRA:CAGPGETSGSRLTF TRB:CASRSQHAGGSRGLFF	1	CD4-C3
Pla-2_AGATCTGCATTCTTAC	Pla	Pla-2	P2-3627	FALSE	1	TRA:CAVQALFNTDKLIF TRB:CASRVESYGYTF	1	CD4-C3
Pla-2_AGATCTGGTGCACCAC	Pla	Pla-2	P2-123	TRUE	3	TRA:CAASNLGTYKYIF TRB:CASSPPIGSPLHF	4	CD4-C2

Pla-2_AGATTGCAGGTTCCTA	Pla	Pla-2	P2-423	FALSE	2	TRA:CAVNPPSGYSTLTF TRB:CASSQGGGGAYEQYF	2	CD4-C3
Pla-2_AGCAGCCCATGCTAGT	Pla	Pla-2	P2-3636	FALSE	1	TRA:CAVMANDYKLSF TRB:CASSRMGQLNQPQHF	1	CD4-C3
Pla-2_AGCAGCCGTCTCTCGT	Pla	Pla-2	P2-3637	FALSE	1	TRA:CAEILNDYKLSF TRB:CASSLKETQYF	1	CD4-C3
Pla-2_AGCAGCCTCACAGTAC	Pla	Pla-2	P2-15	TRUE	3	TRA:CAVGDNFNKFYF TRB:CASSRTSAAGELFF	35	CD4-C3
Pla-2_AGCCTAACAGCCACCA	Pla	Pla-2	P2-3642	FALSE	1	TRA:CAGFSSNTGKLIF TRB:CASSTGQQANTGELFF	1	CD4-C3
Pla-2_AGCGGTCGTCCCTTGT	Pla	Pla-2	P2-3650	FALSE	1	TRA:CAVRNFGNEKLTF TRB:CASSEWGQGANEQFF	1	CD4-C2
Pla-2_AGCGGTCGTTGTCGCG	Pla	Pla-2	P2-3652	FALSE	1	TRA:CVVSDTDKLIF TRB:CAIRNGGMGETQYF	1	CD4-C2
Pla-2_AGCGTATCAACACCCG	Pla	Pla-2	P2-401	FALSE	2	TRA:CAVNPSGGGADGLTF TRB:CAISPRQLYSNQPQHF	2	CD4-C4
Pla-2_AGCTCCTAGACGCAAC	Pla	Pla-2	P2-3661	FALSE	1	TRA:CAVKPGGSYIPTF TRB:CSARVFSGSGGTDTQYF	1	CD4-C3
Pla-2_AGCTCTCCACATTTCT	Pla	Pla-2	P2-3665	FALSE	1	TRA:CILRDGAAGNKLTF TRB:CASSPGGLGELFF	1	CD4-C5
Pla-2_AGGCCACTCGGAGCAA	Pla	Pla-2	P2-3675	FALSE	1	TRA:CAVRDKGNSGNTPLVF TRB:CASSARTGGKNTGELFF	1	CD4-C3
Pla-2_AGGCCGTAGGTGATAT	Pla	Pla-2	P2-3677	FALSE	1	TRA:CAVGASGGSYIPTF TRB:CASSQPKTGAHDEQFF	1	CD4-C3
Pla-2_AGGGAGTCACAGACTT	Pla	Pla-2	P2-403	FALSE	2	TRA:CIAFAGNKLTF TRB:CASNLGVSEAFF	2	CD4-C5
Pla-2_AGGGAGTGTTGTCGCG	Pla	Pla-2	P2-3682	FALSE	1	TRA:CAMREEYSSASKIIF TRB:CASSQDSSGGAAIQYF	1	CD4-C3
Pla-2_AGGGATGTCTGAAAGA	Pla	Pla-2	P2-191	TRUE	3	TRA:CAPLTGSSGDKLTF TRB:CASSLTGNYGYTF	3	CD4-C3
Pla-2_AGGTCATTCGTGGTCG	Pla	Pla-2	P2-28	TRUE	3	TRA:CATDANTGNQFYF TRB:CASSLGQWVEQYF	22	CD4-C3
Pla-2_AGTAGTCAGCAATCTC	Pla	Pla-2	P2-28	TRUE	3	TRA:CATDANTGNQFYF TRB:CASSLGQWVEQYF	22	CD4-C3
Pla-2_AGTAGTCTCAGCATGT	Pla	Pla-2	P2-3697	FALSE	1	TRA:CAVGTGGFKTIF TRB:CASSPGNSNYGYTF	1	CD4-C3
Pla-2_AGTCTTTAGTTCGCAT	Pla	Pla-2	P2-3700	FALSE	1	TRA:CAENISRGTYKYIF TRB:CASSSLGQNNEQFF	1	CD4-C3
Pla-2_AGTCTTTGTCTGCCAG	Pla	Pla-2	P2-436	FALSE	2	TRA:CAAATSGTYKYIF TRB:CASLGRLGAGNTIYF	2	CD4-C3
Pla-2_AGTCTTTTCGAGCCCA	Pla	Pla-2	P2-3701	FALSE	1	TRA:CAVSRGNNNDMRF TRB:CSAIKAVSSTDTQYF	1	CD4-C3
Pla-2_AGTGAGGGTCGATTGT	Pla	Pla-2	P2-3704	FALSE	1	TRA:CAVAGNQFYF TRB:CASSYSSREMADTQYF	1	CD4-C3
Pla-2_AGTTGGTGTTCCCTTG	Pla	Pla-2	P2-36	FALSE	3	TRA:CAAQAGNTPLVF TRB:CSAGVGGTIQYF	15	CD4-C3
Pla-2_AGTTGGTGTTCTGGTA	Pla	Pla-2	P2-167	TRUE	3	TRA:CGTRLMRRSSGSARQLTF TRB:CASSRGLALTQGETQYF	3	CD4-C3
Pla-2_ATAAGAGCACCAGGTC	Pla	Pla-2	P2-439	FALSE	2	TRA:CVVRKAGTALIF TRB:CATSDSGAGGSNQPQHF	2	CD4-C3
Pla-2_ATCCGAATCCGTTGCT	Pla	Pla-2	P2-3732	FALSE	1	TRA:CAVEQQGGSEKLVF TRB:CASSSRTGLSETQYF	1	CD4-C3
Pla-2_ATCCGAATCCTATGTT	Pla	Pla-2	P2-442	FALSE	2	TRA:CATDAIRGNTPLVF TRB:CASSGQGGTEAFF	2	CD4-C4

Pla-2_ATCGAGTCAATGTTGC	Pla	Pla-2	P2-3736	FALSE	1	TRA:CARGSSASKIIF TRB:CASSLLQNIQYF	1	CD4-C3
Pla-2_ATTATCCGTGTCTGAT	Pla	Pla-2	P2-62	FALSE	3	TRA:CAVITDTGRRALTF TRB:CASSIGTSGYNEQFF	9	CD4-C3
Pla-2_CAACCTCCATTTGCCC	Pla	Pla-2	P2-333	TRUE	2	TRA:CAVSVSGGSYIPTF TRB:CASSVGAQSPGANVLTF	2	CD4-C3
Pla-2_CAACCTCGTTAAAGTG	Pla	Pla-2	P2-370	TRUE	2	TRA:CAVGAGGGSQGNLIF TRB:CSARTGLGGYTF	2	CD4-C4
Pla-2_CAACTAGCAACTGCTA	Pla	Pla-2	P2-3788	FALSE	1	TRA:CIVRDGGSNYKLTF TRB:CASSFNTDTQYF	1	CD4-C3
Pla-2_CAACTAGGTGTCGCTG	Pla	Pla-2	P2-451	FALSE	2	TRA:CAAGLSGRSYQLTF TRB:CASSSPNTGELFF	2	CD4-C3
Pla-2_CAAGATCAGGAATCGC	Pla	Pla-2	P2-40	TRUE	3	TRA:CALRETSGSRLTF TRB:CASSPGRYEQYF	12	CD4-C3
Pla-2_CACATAGAGGCTAGGT	Pla	Pla-2	P2-190	TRUE	3	TRA:CAVSHSGGYQKVTF TRB:CASRRAAHEQYF	3	CD4-C3
Pla-2_CACATTTAGGGTTTCT	Pla	Pla-2	P2-3834	FALSE	1	TRA:CATGPTSSSL TRB:CASSVGQVYLNSPLHF	1	CD4-C2
Pla-2_CACATTTTCAGTACGT	Pla	Pla-2	P2-138	FALSE	3	TRA:CAARNTGTASKLTF TRB:CASSLDGMNTEAFF	4	CD4-C3
Pla-2_CACATTTTCCATGAAC	Pla	Pla-2	P2-3837	FALSE	1	TRA:CAPGGYQKVTF TRB:CAIKGPSPDTQYF	1	CD4-C3
Pla-2_CAGAGAGAGAGGGATA	Pla	Pla-2	P2-3852	FALSE	1	TRA:CAVRRTGGNQGGKLIF TRB:CATSRGGQANSPLHF	1	CD4-C3
Pla-2_CAGCAGCCATGGAATA	Pla	Pla-2	P2-3866	FALSE	1	TRA:CAKDTSGTYKYIF TRB:CAWTGGLGSPLHF	1	CD4-C3
Pla-2_CAGCATATCAGGTTCA	Pla	Pla-2	P2-3869	FALSE	1	TRA:CATAGGTSYGKLTF TRB:CAWSMGLYEQYF	1	CD4-C3
Pla-2_CAGCGACAGGCAAAGA	Pla	Pla-2	P2-3873	FALSE	1	TRA:CAASKWGGADGLTF TRB:CSAAGDRGRNEQFF	1	CD4-C3
Pla-2_CAGCTAACATGAGCGA	Pla	Pla-2	P2-3875	FALSE	1	TRA:CAFPYSSASKIIF TRB:CASSVEQYQETQYF	1	CD4-C3
Pla-2_CAGCTAAGTTTGGCGC	Pla	Pla-2	P2-3877	FALSE	1	TRA:CAVIDSNYQLIW TRB:CATSDTGTDYRNEQFF	1	CD4-C3
Pla-2_CATCAAGCAAACCCAT	Pla	Pla-2	P2-3900	FALSE	1	TRA:CAGTSTDKLIF TRB:CASSPRNYGYTF	1	CD4-C3
Pla-2_CATCAGAGTCTCGTTC	Pla	Pla-2	P2-85	TRUE	3	TRA:CAVTAYTNAGKSTF TRB:CASRGRSSGNTIYF	6	CD4-C6
Pla-2_CATCCACGTACCAGTT	Pla	Pla-2	P2-476	FALSE	2	TRA:CIVRVGGGNAGNMLTF TRB:CSAPDHTDTQYF	2	CD4-C3
Pla-2_CATCGAAGTAAACGCG	Pla	Pla-2	P2-3908	FALSE	1	TRA:CLVGSMNNNAGNMLTF TRB:CASSYPGSTNEKLFF	1	CD4-C4
Pla-2_CATCGAAGTGACGGTA	Pla	Pla-2	P2-477	FALSE	2	TRA:CAEKRSGSARQLTF TRB:CASSVTQDEQYF	2	CD4-C6
Pla-2_CATGACAGTCCGAACC	Pla	Pla-2	P2-3913	FALSE	1	TRA:CATDKAGGGNKLTF TRB:CASSWSTGFANTGELFF	1	CD4-C3
Pla-2_CATGACAGTCGATTGT	Pla	Pla-2	P2-3914	FALSE	1	TRA:CAMSPSGGYNKLIF TRB:CASSPEGGNTEAFF	1	CD4-C3
Pla-2_CATGCCTTCATACGGT	Pla	Pla-2	P2-3921	FALSE	1	TRA:CAASKDKLSF TRB:CASRSSGGEKPTQYF	1	CD4-C3
Pla-2_CCACTACCAGCGTAAG	Pla	Pla-2	P2-3948	FALSE	1	TRA:CAVRGLNNNARLMF TRB:CASSSINEQYF	1	CD4-C3
Pla-2_CCACTACTCGCTAGCG	Pla	Pla-2	P2-449	FALSE	2	TRA:CAVQISGSARQLTF TRB:CASSASGMTYNEQFF	2	CD4-C5

Pla-2_CCCAATCCATCAGTCA	Pla	Pla-2	P2-3961	FALSE	1	TRA:CAFVNQAGTALIF TRB:CTPTGDSVGGSGYTF	1	CD4-C3
Pla-2_CCGTACTAGTGGGCTA	Pla	Pla-2	P2-3976	FALSE	1	TRA:CAVNRADSSYKLIF TRB:CASSWELGQPQHF	1	CD4-C3
Pla-2_CCTACACTCGAGCCCA	Pla	Pla-2	P2-3987	FALSE	1	TRA:CAVNESNQGGKLIF TRB:CASSLYAGAQTRGYTF	1	CD4-C3
Pla-2_CCTACCATCTCGAGTA	Pla	Pla-2	P2-98	TRUE	3	TRA:CATDAIRGNTPLVF TRB:CASSDPGGPLHF	5	CD4-C4
Pla-2_CCTACCATCTTTCCTC	Pla	Pla-2	P2-85	TRUE	3	TRA:CAVTAYTNAGKSTF TRB:CASRGRSSGNTIYF	6	CD4-C3
Pla-2_CCTAGCTAGCGATAGC	Pla	Pla-2	P2-244	FALSE	3	TRA:CAVRGGSYIPTF TRB:CASSQGTGRQETQYF	3	CD4-C3
Pla-2_CCTATTATCTCCCTGA	Pla	Pla-2	P2-489	FALSE	2	TRA:CAVRDSNYQLIW TRB:CASSEVGGGQETQYF	2	CD4-C3
Pla-2_CCTCAGTCAGGGATTG	Pla	Pla-2	P2-490	FALSE	2	TRA:CAAAATLTQGAQKLVF TRB:CASSRGGGGRSDTQYF	2	CD4-C3
Pla-2_CCTTACGTCACATACG	Pla	Pla-2	P2-4011	FALSE	1	TRA:CAVRDQGQAGTALIF TRB:CATSPGLAAYEQYF	1	CD4-C3
Pla-2_CCTTCCCCAATGACCT	Pla	Pla-2	P2-4012	FALSE	1	TRA:CVVNLRQNFVF TRB:CASRLGTSGFYEQYF	1	CD4-C3
Pla-2_CCTTTCTAGAGTCTGG	Pla	Pla-2	P2-289	TRUE	2	TRA:CAASYRGAGNNRKLIW TRB:CSARTSGGGNNEQFF	2	CD4-C6
Pla-2_CGAACATGTCCGAATT	Pla	Pla-2	P2-4027	FALSE	1	TRA:CASYRGGADGLTF TRB:CSASGQVGEAFF	1	CD4-C3
Pla-2_CGAATGTCATTCGACA	Pla	Pla-2	P2-4030	FALSE	1	TRA:CAVEVKSGINDMRF TRB:CASSFSGANYGYTF	1	CD4-C3
Pla-2_CGAATGTTCATCGATG	Pla	Pla-2	P2-4032	FALSE	1	TRA:CAVNIPYNFNKFYF TRB:CASVGDGYEQYF	1	CD4-C3
Pla-2_CGACCTTAGTGTTGAA	Pla	Pla-2	P2-4035	FALSE	1	TRA:CAVGNQAGTALIF TRB:CASSQGQGNLEKLFF	1	CD4-C3
Pla-2_CGACTTCCAGACAAAT	Pla	Pla-2	P2-4036	FALSE	1	TRA:CLVVNRDDKIIF TRB:CASSYSRSRFTGELFF	1	CD4-C3
Pla-2_CGACTTCTCTTCGAGA	Pla	Pla-2	P2-4038	FALSE	1	TRA:CIAFAGGTSYGKLTF TRB:CASSSGWAGYEQYF	1	CD4-C3
Pla-2_CGATCGGAGAAGGCCT	Pla	Pla-2	P2-4053	FALSE	1	TRA:CAENHNTDKLIF TRB:CASSEGTGQRVTGELFF	1	CD4-C4
Pla-2_CGATCGGCAAAGCGGT	Pla	Pla-2	P2-4055	FALSE	1	TRA:CAVKGWGYNKLIF TRB:CASSLDPGGQNEQFF	1	CD4-C3
Pla-2_CGATGGCCAGCCTATA	Pla	Pla-2	P2-4059	FALSE	1	TRA:CAVRSTYNAGNMLTF TRB:CASRGAGGGYEQYF	1	CD4-C4
Pla-2_CGATGGCTCACTTATC	Pla	Pla-2	P2-165	FALSE	3	TRA:CAAHEGWAYKYIF TRB:CASSLGQTGQFHF	4	CD4-C3
Pla-2_CGATGTATCCGTACAA	Pla	Pla-2	P2-4062	FALSE	1	TRA:CAATLMGAGTALIF TRB:CASSLAEPYEQYF	1	CD4-C4
Pla-2_CGATTGATCTGTCTAT	Pla	Pla-2	P2-4066	FALSE	1	TRA:CALSLSNSGNTPLVF TRB:CASSFTDLPYEQYF	1	CD4-C3
Pla-2_CGCCAAGTCGAGAACG	Pla	Pla-2	P2-4069	FALSE	1	TRA:CAVGDSNYQLIW TRB:CASSETGTGHYNEQFF	1	CD4-C3
Pla-2_CGCGGTACACAAGCCC	Pla	Pla-2	P2-244	FALSE	3	TRA:CAVRGGSYIPTF TRB:CASSQGTGRQETQYF	3	CD4-C3
Pla-2_CGCGGTACACAGAGGT	Pla	Pla-2	P2-4072	FALSE	1	TRA:CAEIPPSGGSNYKLTF TRB:CASNNALNTEAFF	1	CD4-C3
Pla-2_CGCGTTTAGTCGATAA	Pla	Pla-2	P2-4075	FALSE	1	TRA:CAVRRQGGNAGNMLTF TRB:CASGVWQPRDQETQYF	1	CD4-C5

Pla-2_CGCTGGACAATGTAAG	Pla	Pla-2	P2-4084	FALSE	1	TRA:CAVTAYTNAGKSTF TRB:CASRGRSSGNTTYF	1	CD4-C4
Pla-2_CGGACACAGGCTCTTA	Pla	Pla-2	P2-18	TRUE	3	TRA:CAATSSSNFGNEKLTF TRB:CAWSRAALTEAFF	28	CD4-C3
Pla-2_CGGACGTGTATCTGCA	Pla	Pla-2	P2-4099	FALSE	1	TRA:CAVEPSNNYGQNFVF TRB:CASTRQGKQPQHF	1	CD4-C3
Pla-2_CGGACTGAGAATGTTG	Pla	Pla-2	P2-167	TRUE	3	TRA:CGTRLMRRSSGSARQLTF TRB:CASSRGLALTQGETQYF	3	CD4-C3
Pla-2_CGGAGTCAGGTGCACA	Pla	Pla-2	P2-85	TRUE	3	TRA:CAVTAYTNAGKSTF TRB:CASRGRSSGNTIYF	6	CD4-C3
Pla-2_CGGAGTCTCATGGTCA	Pla	Pla-2	P2-4112	FALSE	1	TRA:CAVSENTGRRALTF TRB:CASSLPGPGNTIYF	1	CD4-C6
Pla-2_CGGGTCAGTGTTTGGT	Pla	Pla-2	P2-503	FALSE	2	TRA:CAVNAYSGAGSYQLTF TRB:CASSPTGQGRNEQYF	2	CD4-C3
Pla-2_CGTAGCGCATTCTCAT	Pla	Pla-2	P2-4126	FALSE	1	TRA:CALLTSGNTGKLIF TRB:CASSRGLTNEKLFF	1	CD4-C4
Pla-2_CGTAGCGTCGGCTTGG	Pla	Pla-2	P2-4127	FALSE	1	TRA:CAMSPSGTYKYIF TRB:CSAVLANSPLHF	1	CD4-C2
Pla-2_CGTCAGGGTTTGGGCC	Pla	Pla-2	P2-116	FALSE	3	TRA:CAVSPPAGSNYQLIW TRB:CATSRETGARYEQYF	5	CD4-C4
Pla-2_CGTCCATCATACGCCG	Pla	Pla-2	P2-298	TRUE	2	TRA:CALDAVRSASKIIF TRB:CSARLGTSQETQYF	2	CD4-C3
Pla-2_CGTGAGCAGTCATGCT	Pla	Pla-2	P2-4144	FALSE	1	TRA:CAVRPVVTGNQFYF TRB:CASSMVSQPQETQYF	1	CD4-C3
Pla-2_CGTGTAATCGCAAGCC	Pla	Pla-2	P2-4147	FALSE	1	TRA:CAEITGANNLFF TRB:CSARGLGYEQFF	1	CD4-C3
Pla-2_CGTGTCTAGATCACGG	Pla	Pla-2	P2-511	FALSE	2	TRA:CAASPASGGSYIPTF TRB:CASSSHQGGSGYTF	2	CD4-C3
Pla-2_CGTTCTGAGGTGCACA	Pla	Pla-2	P2-489	FALSE	2	TRA:CAVRDSNYQLIW TRB:CASSEVGGGQETQYF	2	CD4-C3
Pla-2_CTAAGACTCGCAGGCT	Pla	Pla-2	P2-4168	FALSE	1	TRA:CAVERGGSQGNLIF TRB:CASSLVGGYTDTQYF	1	CD4-C2
Pla-2_CTACACCAGAAGAAGC	Pla	Pla-2	P2-4171	FALSE	1	TRA:CAYRRHTGTASKLTF TRB:CASSQQGAKRETQYF	1	CD4-C3
Pla-2_CTAGTGACAAGCGATG	Pla	Pla-2	P2-4188	FALSE	1	TRA:CVVGRPRKLVF TRB:CASSQTQDRVREQYF	1	CD4-C3
Pla-2_CTAGTGATCCTATGTT	Pla	Pla-2	P2-62	FALSE	3	TRA:CAVITDTGRRALTF TRB:CASSIGTSGYNEQFF	9	CD4-C3
Pla-2_CTCACACCAATCACAC	Pla	Pla-2	P2-4197	FALSE	1	TRA:CAVNPLESSYKLIF TRB:CASSQALGQNSGNTIYF	1	CD4-C4
Pla-2_CTCGAGGGTTTAGCTG	Pla	Pla-2	P2-282	TRUE	2	TRA:CAMRELILTGANSKLTF TRB:CASSLGVGQGFHNEQFF	2	CD4-C3
Pla-2_CTCGGAGGTCATGCCG	Pla	Pla-2	P2-39	TRUE	3	TRA:CAARAVTGGGNKLTF TRB:CASSGRSSAWNEQFF	13	CD4-C3
Pla-2_CTCTACGCACTTAAGC	Pla	Pla-2	P2-387	FALSE	2	TRA:CAVWSDGQKLLF TRB:CASSIMGQSNQPQHF	2	CD8-C8
Pla-2_CTGAAACGTCCGACGT	Pla	Pla-2	P2-4231	FALSE	1	TRA:CAVEDQGYSTLTF TRB:CASSDSGGSGGNEQFF	1	CD4-C3
Pla-2_CTGAAGTCATTCACTT	Pla	Pla-2	P2-523	FALSE	2	TRA:CAVSPFSGAGSYQLTF TRB:CASSVADGLGRNEQFF	2	CD4-C3
Pla-2_CTGCGGAGTCTTGATG	Pla	Pla-2	P2-4245	FALSE	1	TRA:CVKAAGNKLTF TRB:CASSRDRSTDTQYF	1	CD4-C4
Pla-2_CTGTGCTGTCGGCACT	Pla	Pla-2	P2-4254	FALSE	1	TRA:CAASISGNTGKLIF TRB:CASSFSSGGDTQYF	1	CD4-C3

Pla-2_CTTACCGAGCGATGAC	Pla	Pla-2	P2-528	FALSE	2	TRA:CAVRDGRGFGNVLHC TRB:CSAVGNSGGSSVDTQYF	2	CD4-C3
Pla-2_CTTGGCTGTCGGCACT	Pla	Pla-2	P2-4278	FALSE	1	TRA:CLVGARDTGRRALTF TRB:CSASPPASGGYNEQFF	1	CD4-C3
Pla-2_GAACGGACAAACAACA	Pla	Pla-2	P2-4303	FALSE	1	TRA:CAVTRFQGGSEKLVF TRB:CASSFRQGANNEQFF	1	CD4-C3
Pla-2_GAACGGACACGACTCG	Pla	Pla-2	P2-4304	FALSE	1	TRA:CIVRVAQTQGAQKLVF TRB:CASSLSAGQPQHF	1	CD4-C3
Pla-2_GAAGCAGAGATATGCA	Pla	Pla-2	P2-4309	FALSE	1	TRA:CALSGLGNQGGKLIF TRB:CASSSTQGPYEQYF	1	CD4-C3
Pla-2_GAAGCAGGTTGGAGGT	Pla	Pla-2	P2-4310	FALSE	1	TRA:CAVTPNTGFQKLVF TRB:CASSLESGGRSAQHF	1	CD4-C3
Pla-2_GAATAAGAGGATCGCA	Pla	Pla-2	P2-535	FALSE	2	TRA:CAVNRGSTLGRLYF TRB:CASRNDLSGANGYTF	2	CD4-C3
Pla-2_GACCAATTCATTGCCC	Pla	Pla-2	P2-4336	FALSE	1	TRA:CAVLYNAGNMLTF TRB:CAISESGLAGYNEQFF	1	CD4-C4
Pla-2_GACGCGTGTAACGACG	Pla	Pla-2	P2-183	TRUE	3	TRA:CAASTGTGGFKTIF TRB:CASKPTSGTYEQYF	3	CD4-C3
Pla-2_GACGGCTCACGAGAGT	Pla	Pla-2	P2-4341	FALSE	1	TRA:CAVNKYGQKLLF TRB:CASSATGGQGEKLFF	1	CD4-C3
Pla-2_GACGTGCCAATTGCTG	Pla	Pla-2	P2-455	FALSE	2	TRA:CAVIRTGANSKLTF TRB:CASSSQGAYGYTF	2	CD4-C3
Pla-2_GACGTTAGTTGTCTTT	Pla	Pla-2	P2-537	FALSE	2	TRA:CAGGYNNNDMRF TRB:CATSDFVTGEAFF	2	CD4-C3
Pla-2_GACTACATCCGCTGTT	Pla	Pla-2	P2-540	FALSE	2	TRA:CAMSSSGGSNYKLTF TRB:CASSLASQGYTGELFF	2	CD4-C3
Pla-2_GAGGTGAGTAGCGTAG	Pla	Pla-2	P2-4370	FALSE	1	TRA:CAVRGANSGYSTLTF TRB:CASSWDRGSTEAFF	1	CD4-C2
Pla-2_GATCAGTTCAACGCTA	Pla	Pla-2	P2-423	FALSE	2	TRA:CAVNPPSGYSTLTF TRB:CASSQGGGGAYEQYF	2	CD4-C3
Pla-2_GATCAGTTCGGAATCT	Pla	Pla-2	P2-544	FALSE	2	TRA:CALSGGSNYKLTF TRB:CASSMTGSGANVLTF	2	CD4-C3
Pla-2_GATCAGTTCTACTATC	Pla	Pla-2	P2-4375	FALSE	1	TRA:CAVSDAGSYQLTF TRB:CASSQGPGRYEQYF	1	CD4-C3
Pla-2_GATTCAGTCGCCCTTA	Pla	Pla-2	P2-4395	FALSE	1	TRA:CVVGGGTSYGKLTF TRB:CAWSSGQGIYNEQFF	1	CD4-C3
Pla-2_GCAAACTCAGTCTTCC	Pla	Pla-2	P2-477	FALSE	2	TRA:CAEKRSGSARQLTF TRB:CASSVTQDEQYF	2	CD4-C5
Pla-2_GCAATCACATGCTAGT	Pla	Pla-2	P2-28	TRUE	3	TRA:CATDANTGNQFYF TRB:CASSLGQWVEQYF	22	CD4-C3
Pla-2_GCACATATCTCGTTTA	Pla	Pla-2	P2-62	FALSE	3	TRA:CAVITDTGRRALTF TRB:CASSIGTSGYNEQFF	9	CD4-C3
Pla-2_GCAGTTACAGGTCGTC	Pla	Pla-2	P2-132	TRUE	3	TRA:CAANRAGNNRKLIW TRB:CASSFKKAGGPVGELFF	4	CD4-C3
Pla-2_GCATACAAGCGCCTCA	Pla	Pla-2	P2-4417	FALSE	1	TRA:CAVSRGYSGGGADGLTF TRB:CASSLDLTGTADTQYF	1	CD4-C3
Pla-2_GCATGATTCCTTGCCA	Pla	Pla-2	P2-4423	FALSE	1	TRA:CAVGAPGGFKTIF TRB:CASSVDQRDTQYF	1	CD4-C3
Pla-2_GCATGATTCTCTGAGA	Pla	Pla-2	P2-4425	FALSE	1	TRA:CAMIQGSNYKLTF TRB:CSARTQGPRSGNTIYF	1	CD4-C3
Pla-2_GCATGCGCAACGATGG	Pla	Pla-2	P2-4428	FALSE	1	TRA:CAVRVFI TRB:CASSPTSTGTGELFF	1	CD4-C3
Pla-2_GCATGCGCATTTCAGG	Pla	Pla-2	P2-4429	FALSE	1	TRA:CALSEAFNTNAGKSTF TRB:CASSEAGLVSEQFF	1	CD8-C2

Pla-2_GCATGTAAGGCCCTCA	Pla	Pla-2	P2-4430	FALSE	1	TRA:CAVDGWTGNQFYF TRB:CASSDPMTGRYEQYF	1	CD4-C3
Pla-2_GCCAAATTCCTCTAGC	Pla	Pla-2	P2-98	TRUE	3	TRA:CATDAIRGNTPLVF TRB:CASSDPGGPLHF	5	CD4-C3
Pla-2_GCGACCACAGTCGATT	Pla	Pla-2	P2-4441	FALSE	1	TRA:CAMRGANTGNQFYF TRB:CASSQGGRTETQYF	1	CD4-C3
Pla-2_GCGACCAGTTCCCTTG	Pla	Pla-2	P2-549	FALSE	2	TRA:CAMREGRTGFQKLVF TRB:CSAGQVGSNQPQHF	2	CD4-C3
Pla-2_GCGCAGTGTATCAGTC	Pla	Pla-2	P2-4449	FALSE	1	TRA:CAVGEDTDKLIF TRB:CASTERVNSNQPQHF	1	CD4-C4
Pla-2_GCGCCAACATATACGC	Pla	Pla-2	P2-540	FALSE	2	TRA:CAMSSSGGSNYKLTF TRB:CASSLASQGYTGELFF	2	CD4-C3
Pla-2_GCGCCAATCGGCCGAT	Pla	Pla-2	P2-4456	FALSE	1	TRA:CLVGGTGGGNKLTF TRB:CASRVGGTDTQYF	1	CD4-C2
Pla-2_GCTGCAGGTAAGGGAA	Pla	Pla-2	P2-4467	FALSE	1	TRA:CALSSLLYNFNKFYF TRB:CASSLAGEQYF	1	CD4-C3
Pla-2_GCTGCGACACTGCCAG	Pla	Pla-2	P2-39	TRUE	3	TRA:CAARAVTGGGNKLTF TRB:CASSGRSSAWNEQFF	13	CD4-C4
Pla-2_GCTGCTTTCGGTTCGG	Pla	Pla-2	P2-4476	FALSE	1	TRA:CALTAPGAGSYQLTF TRB:CASSLQGSTYNEQFF	1	CD4-C5
Pla-2_GCTTCCATCACGACTA	Pla	Pla-2	P2-503	FALSE	2	TRA:CAVNAYSGAGSYQLTF TRB:CASSPTGQGRNEQYF	2	CD4-C3
Pla-2_GCTTCCATCATGGTCA	Pla	Pla-2	P2-4484	FALSE	1	TRA:CATDKAGGTSYGKLTF TRB:CSAGTGVGPLHF	1	CD4-C3
Pla-2_GCTTCCATCCGCGCAA	Pla	Pla-2	P2-4486	FALSE	1	TRA:CAVRGNYGQNFVF TRB:CASTGQGWMAFF	1	CD4-C3
Pla-2_GCTTGAAAGTCGTACT	Pla	Pla-2	P2-4488	FALSE	1	TRA:CAVINTNAGKSTF TRB:CASSLRGGSNEQFF	1	CD4-C3
Pla-2_GCTTGAATCTGTGCAA	Pla	Pla-2	P2-165	FALSE	3	TRA:CAAHEGWAYKYIF TRB:CASSLGQTGQFHF	4	CD4-C3
Pla-2_GGAACTTTCTCTAAGG	Pla	Pla-2	P2-4497	FALSE	1	TRA:CATPPDAGGTSYGKLTF TRB:CASQSSLGEQFF	1	CD4-C3
Pla-2_GGACAGAAGGCATGTG	Pla	Pla-2	P2-4504	FALSE	1	TRA:CAVNGNRNDMRF TRB:CASSLPGNTIYF	1	CD4-C3
Pla-2_GGATTACGTTAGATGA	Pla	Pla-2	P2-4526	FALSE	1	TRA:CAVNLYQAGTALIF TRB:CASRQGDADTQYF	1	CD4-C3
Pla-2_GGCTCGAAGTCATCCA	Pla	Pla-2	P2-39	TRUE	3	TRA:CAARAVTGGGNKLTF TRB:CASSGRSSAWNEQFF	13	CD4-C3
Pla-2_GGCTGGTTCATAAAGG	Pla	Pla-2	P2-4550	FALSE	1	TRA:CALSGASGGYNKLIF TRB:CASSHPGTVAETQYF	1	CD4-C3
Pla-2_GGGAATGCAATCGGTT	Pla	Pla-2	P2-138	FALSE	3	TRA:CAARNTGTASKLTF TRB:CASSLDGMNTEAFF	4	CD4-C4
Pla-2_GGGCACTTCTTCGGTC	Pla	Pla-2	P2-4560	FALSE	1	TRA:CALMGSARQLTF TRB:CASSLLRGDTYNEQFF	1	CD4-C3
Pla-2_GGGCATCCACAGTCGC	Pla	Pla-2	P2-139	FALSE	3	TRA:CAVATGFQKLVF TRB:CASTLGGQGNNEQFF	4	CD4-C3
Pla-2_GGGTCTGGTCCGCTGA	Pla	Pla-2	P2-4566	FALSE	1	TRA:CAVTTVGNFNKFYF TRB:CASSQGRDRTSGANVLTF	1	CD4-C3
Pla-2_GGGTTGCGTATTAGCC	Pla	Pla-2	P2-149	FALSE	3	TRA:CAVTNRGNNRLAF TRB:CSARVPGGLSDEQFF	4	CD4-C5
Pla-2_GGTGAAGGTGCTAGCC	Pla	Pla-2	P2-4578	FALSE	1	TRA:CAVNIGTNAGKSTF TRB:CASSSTSSNTEAFF	1	CD4-C3
Pla-2_GTACTTTCATCCTAGA	Pla	Pla-2	P2-4597	FALSE	1	TRA:CAVNPLESSYKLIF TRB:CSARAGQGSHSGANVLTF	1	CD4-C3

Pla-2_GTACTTTTCAGCACAT	Pla	Pla-2	P2-4600	FALSE	1	TRA:CALVGYSTLTF TRB:CASSERDTGGGGQPQHF	1	CD4-C3
Pla-2_GTCACAAGTCGTCTTC	Pla	Pla-2	P2-4610	FALSE	1	TRA:CAVELIYNTDKLIF TRB:CASRKDRGTEAFF	1	CD4-C4
Pla-2_GTCACAATCCACGAAT	Pla	Pla-2	P2-4611	FALSE	1	TRA:CIEGFNKFYF TRB:CASSLAGAKQFF	1	CD4-C3
Pla-2_GTCACAATCCGTCAAA	Pla	Pla-2	P2-4612	FALSE	1	TRA:CAVRFGNTPLVF TRB:CSASADTDTQYF	1	CD4-C3
Pla-2_GTCACGGCAAAGAATC	Pla	Pla-2	P2-4614	FALSE	1	TRA:CAVTHNTGRRALTF TRB:CASRIDPGVDQPQHF	1	CD4-C3
Pla-2_GTCATTTCAGGTCCAC	Pla	Pla-2	P2-543	FALSE	2	TRA:CAMSARGGAGNMLTF TRB:CASSRGQGLYGYTF	2	CD4-C3
Pla-2_GTCCTCAAGAAAGTGG	Pla	Pla-2	P2-4619	FALSE	1	TRA:CAPLTPNDMRF TRB:CSAPRLAGGREQFF	1	CD4-C3
Pla-2_GTCTTCGTCAGGCAAG	Pla	Pla-2	P2-191	TRUE	3	TRA:CAPLTGSSGDKLTF TRB:CASSLTGNYGYTF	3	CD4-C3
Pla-2_GTGAAGGTCCGTAGTA	Pla	Pla-2	P2-435	FALSE	2	TRA:CAVTGFQKLVF TRB:CSARPPGQGADEQYF	2	CD4-C4
Pla-2_GTGCAGCCAATAACGA	Pla	Pla-2	P2-251	FALSE	3	TRA:CADNNAGNMLTF TRB:CASSPNLPLLADNQETQYF	3	CD4-C4
Pla-2_GTGCATAGTACATGTC	Pla	Pla-2	P2-224	FALSE	3	TRA:CAVSGGGFKTIF TRB:CASSQEGETQYF	3	CD4-C3
Pla-2_GTGGGTCTCTGAGTGT	Pla	Pla-2	P2-4646	FALSE	1	TRA:CAARYTGANSKLTF TRB:CASSLDGMNTEAFF	1	CD4-C3
Pla-2_GTGTTAGCACAGGAGT	Pla	Pla-2	P2-4652	FALSE	1	TRA:CAVSPRGTGGFKTIF TRB:CASSLTGERSETQYF	1	CD4-C5
Pla-2_GTTCATTAGTAGGTGC	Pla	Pla-2	P2-414	FALSE	2	TRA:CAVGTTSGTYKYIF TRB:CASSQLYTEAFF	2	CD4-C3
Pla-2_TAAGAGACAAACGTGG	Pla	Pla-2	P2-39	TRUE	3	TRA:CAARAVTGGGNKLTF TRB:CASSGRSSAWNEQFF	13	CD4-C3
Pla-2_TAAGAGAGTACAGCAG	Pla	Pla-2	P2-558	FALSE	2	TRA:CIVRVGNSGYALNF TRB:CASSQVPAAGELFF	2	CD4-C3
Pla-2_TAAGTGCGTAGCGTCC	Pla	Pla-2	P2-544	FALSE	2	TRA:CALSGGSNYKLTF TRB:CASSMTGSGANVLTF	2	CD4-C3
Pla-2_TACACGAGTGTAAGTA	Pla	Pla-2	P2-442	FALSE	2	TRA:CATDAIRGNTPLVF TRB:CASSGQGGTEAFF	2	CD4-C3
Pla-2_TACCTTACACCTTGTC	Pla	Pla-2	P2-4686	FALSE	1	TRA:CAVNMGTNAGKSTF TRB:CASSSTGTNTEAFF	1	CD4-C3
Pla-2_TACCTTACATGGGACA	Pla	Pla-2	P2-4687	FALSE	1	TRA:CATVEGLVF TRB:CASSSWTGGEQYF	1	CD4-C3
Pla-2_TACGGATCATGACATC	Pla	Pla-2	P2-4691	FALSE	1	TRA:CAVWNTDKLIF TRB:CASSYGNSTTEAFF	1	CD4-C3
Pla-2_TACTCGCCAAAGCGGT	Pla	Pla-2	P2-4701	FALSE	1	TRA:CAMRDWDFGNEKLTF TRB:CASIGGLVGVNEQFF	1	CD4-C4
Pla-2_TACTTACCAAGAAGAG	Pla	Pla-2	P2-39	TRUE	3	TRA:CAARAVTGGGNKLTF TRB:CASSGRSSAWNEQFF	13	CD4-C3
Pla-2_TACTTGTTCTGCCAGG	Pla	Pla-2	P2-260	FALSE	3	TRA:CAAREVNTGKLIF TRB:CASSEETGRYF	3	CD4-C3
Pla-2_TAGCCGGCAGTATGCT	Pla	Pla-2	P2-110	TRUE	3	TRA:CAVSTSGTYKYIF TRB:CASSSSGGDNSPLHF	5	CD4-C3
Pla-2_TATCAGGGTGCGCTTG	Pla	Pla-2	P2-194	TRUE	3	TRA:CAGRLQGAQKLVF TRB:CAWSQSQGYYGYTF	3	CD4-C4
Pla-2_TATCAGGTCGAGAACG	Pla	Pla-2	P2-4732	FALSE	1	TRA:CAASAIRRKNQGGKLIF TRB:CASSLTSYEQYF	1	CD4-C3

Pla-2_TATCTCACAACTGCTA	Pla	Pla-2	P2-4735	FALSE	1	TRA:CALEQTGANNLFF TRB:CASSWGQGKLNTQYF	1	CD4-C3
Pla-2_TATTACCTCGATCCCT	Pla	Pla-2	P2-193	TRUE	3	TRA:CAPGGSGGSNYKLTF TRB:CASSPGTGGNEQFF	3	CD4-C3
Pla-2_TCAACGATCTTACCGC	Pla	Pla-2	P2-4747	FALSE	1	TRA:CAIPNQAGTALIF TRB:CASSLGPSGNTIYF	1	CD4-C3
Pla-2_TCACAAGCACATGTGT	Pla	Pla-2	P2-110	TRUE	3	TRA:CAVSTSGTYKYIF TRB:CASSSSGGDNSPLHF	5	CD4-C3
Pla-2_TCACGAAAGGGCACTA	Pla	Pla-2	P2-183	TRUE	3	TRA:CAASTGTGGFKTIF TRB:CASKPTSGTYEQYF	3	CD4-C3
Pla-2_TCACGAATCCGCTGTT	Pla	Pla-2	P2-4759	FALSE	1	TRA:CAVRDSDYKLSF TRB:CASSLWTGPSSYEQYF	1	CD8-C10
Pla-2_TCAGCAACAAAGCAAT	Pla	Pla-2	P2-90	FALSE	3	TRA:CIGNTPLVF TRB:CASSDRDRVLNTEAFF	6	CD4-C3
Pla-2_TCAGGATGTGACGCCT	Pla	Pla-2	P2-226	FALSE	3	TRA:CAVDNTGAGSYQLTF TRB:CASSQAGGSYEQYF	3	CD4-C3
Pla-2_TCCACACTCGGCGCTA	Pla	Pla-2	P2-4787	FALSE	1	TRA:CAMTTDSWGKFQF TRB:CSATRPRDSGGSQYF	1	CD4-C3
Pla-2_TCGAGGCAGGACAGAA	Pla	Pla-2	P2-563	FALSE	2	TRA:CAVGAPERSQGNLIF TRB:CASSDRDRVLNTEAFF	2	CD4-C3
Pla-2_TCGCGAGCAAAGGCGT	Pla	Pla-2	P2-476	FALSE	2	TRA:CIVRVGGGNAGNMLTF TRB:CSAPDHTDTQYF	2	CD4-C3
Pla-2_TCGGGACTCTGTTGAG	Pla	Pla-2	P2-39	TRUE	3	TRA:CAARAVTGGGNKLTF TRB:CASSGRSSAWNEQFF	13	CD4-C3
Pla-2_TCGGTAAGTCGTCTTC	Pla	Pla-2	P2-226	FALSE	3	TRA:CAVDNTGAGSYQLTF TRB:CASSQAGGSYEQYF	3	CD4-C4
Pla-2_TCTATTGAGATAGTCA	Pla	Pla-2	P2-4814	FALSE	1	TRA:CIVRSSSSL TRB:CASSTRTVGTGELFF	1	CD4-C3
Pla-2_TCTATTGGTTTGTGTG	Pla	Pla-2	P2-188	TRUE	3	TRA:CAVRRQGGNAGNMLTF TRB:CASSEQGSGSPYEQYF	3	CD4-C3
Pla-2_TCTCATAGTGAAATCA	Pla	Pla-2	P2-4818	FALSE	1	TRA:CAEPENYGQNFVF TRB:CASSRGPRSYTF	1	CD4-C4
Pla-2_TCTCTAAAGCGCTCCA	Pla	Pla-2	P2-402	FALSE	2	TRA:CALSDILTGGGNKLTF TRB:CATSRGGQANSPLHF	2	CD4-C2
Pla-2_TCTGGAAAGTCCATAC	Pla	Pla-2	P2-4827	FALSE	1	TRA:CAMREGEGANSKLTF TRB:CASRVDKFEKLFF	1	CD4-C3
Pla-2_TCTTCGGCAGCTGTTA	Pla	Pla-2	P2-4837	FALSE	1	TRA:CAVDDGQKLLF TRB:CSAPLAGGSTDTQYF	1	CD4-C3
Pla-2_TGACTTTCAAACCCAT	Pla	Pla-2	P2-165	FALSE	3	TRA:CAAHEGWAYKYIF TRB:CASSLGQTGQFHF	4	CD4-C3
Pla-2_TGAGGGATCGGATGTT	Pla	Pla-2	P2-4870	FALSE	1	TRA:CATETQGGSEKLVF TRB:CASSPAAGKWTQYF	1	CD4-C3
Pla-2_TGCCAAAGTAGAGTGC	Pla	Pla-2	P2-561	FALSE	2	TRA:CAETFPWGFQKLVF TRB:CAWSVPGTGDVF	2	CD4-C3
Pla-2_TGCCCTAGTCTCAACA	Pla	Pla-2	P2-486	FALSE	2	TRA:CAVNPSGGGADGLTF TRB:CAVREDSFGYTF	2	CD4-C4
Pla-2_TGCGCAGTCGTCTGCT	Pla	Pla-2	P2-292	TRUE	2	TRA:CAAREGASKIIF TRB:CASSLVGQGAFF	2	CD4-C4
Pla-2_TGCTACCTCGGCATCG	Pla	Pla-2	P2-4901	FALSE	1	TRA:CAAGSGAGSYQLTF TRB:CASSPRGQGFRETQYF	1	CD4-C3
Pla-2_TGCTGCTAGATGTAAC	Pla	Pla-2	P2-4902	FALSE	1	TRA:CAARRTGRRALTF TRB:CSADDSREAFF	1	CD4-C3
Pla-2_TGGGAAGAGACAAAGG	Pla	Pla-2	P2-4919	FALSE	1	TRA:CAVGITGANSKLTF TRB:CASSVGGTSFF	1	CD4-C3

Pla-2_TGGGAAGCACGCCAGT	Pla	Pla-2	P2-4921	FALSE	1	TRA:CAVNAGNMLTF TRB:CASSLHTQGNEQFF	1	CD4-C3
Pla-2_TGGTTAGGTAGCGTGA	Pla	Pla-2	P2-4933	FALSE	1	TRA:CAVTLFSGGYNKLIF TRB:CASSQDGIRNRYNEQFF	1	CD4-C3
Pla-2_TGGTTAGGTCACTTCC	Pla	Pla-2	P2-138	FALSE	3	TRA:CAARNTGTASKLTF TRB:CASSLDGMNTEAFF	4	CD4-C3
Pla-2_TGGTTAGGTGGTTTCA	Pla	Pla-2	P2-4936	FALSE	1	TRA:CVVSLAGTALIF TRB:CASSIVGTDYNEQFF	1	CD4-C3
Pla-2_TGTATTCAGTGGCACA	Pla	Pla-2	P2-4940	FALSE	1	TRA:CALSRNNNARLMF TRB:CSVKEGSKNIQYF	1	CD4-C3
Pla-2_TGTATTCCATGGTCAT	Pla	Pla-2	P2-4942	FALSE	1	TRA:CVVNPGNNRKLIW TRB:CASSVGLMETQYF	1	CD4-C3
Pla-2_TGTCCCAAGATGTTAG	Pla	Pla-2	P2-4945	FALSE	1	TRA:CAEKGDNQAGTALIF TRB:CAISELSSGELFF	1	CD4-C3
Pla-2_TGTCCCACAGGCGATA	Pla	Pla-2	P2-4948	FALSE	1	TRA:CATDNYARLMF TRB:CASRPTGGSYEQYF	1	CD4-C3
Pla-2_TTAGGACAGGCGACAT	Pla	Pla-2	P2-4962	FALSE	1	TRA:CAETWSGTYKYIF TRB:CAWSLGTGNTEAFF	1	CD4-C4
Pla-2_TTAGGCAGTACAGTGG	Pla	Pla-2	P2-36	FALSE	3	TRA:CAAQAGNTPLVF TRB:CSAGVGGTIQYF	15	CD4-C5
Pla-2_TTAGTTCCAATGGAGC	Pla	Pla-2	P2-518	FALSE	2	TRA:CALEGDSGYSTLTF TRB:CASSAGGQAGELFF	2	CD4-C3
Pla-2_TTATGCTTCCATGAGT	Pla	Pla-2	P2-4973	FALSE	1	TRA:CAVSGALTGGGNKLTF TRB:CASSEGGRPKQFF	1	CD4-C3
Pla-2_TTCGAAGAGATAGTCA	Pla	Pla-2	P2-4975	FALSE	1	TRA:CVVSVRRKGGKLIF TRB:CASEAFF	1	CD4-C3
Pla-2_TTCGAAGCACATTCGA	Pla	Pla-2	P2-4976	FALSE	1	TRA:CALSETK TRB:CATKEGTGGFSHEQYF	1	CD4-C3
Pla-2_TTCGAAGTCTCACATT	Pla	Pla-2	P2-4981	FALSE	1	TRA:CAVGAYSGGGADGLTF TRB:CASSPRATSYEQYF	1	CD4-C3
Pla-2_TTCTACAAGAAGGGTA	Pla	Pla-2	P2-520	FALSE	2	TRA:CAVRARGTDKLIF TRB:CASSPQAAGTGELFF	2	CD4-C3
Pla-2_TTCTACAGTGAGGGAG	Pla	Pla-2	P2-523	FALSE	2	TRA:CAVSPFSGAGSYQLTF TRB:CASSVADGLGRNEQFF	2	CD4-C3
Pla-2_TTCTCCTTCTGCGACG	Pla	Pla-2	P2-4994	FALSE	1	TRA:CALSEARRADSWGKFQF TRB:CASRTGPGNQYF	1	CD4-C3
Pla-2_TTCTTAGAGTGTGAAT	Pla	Pla-2	P2-4996	FALSE	1	TRA:CAMREGFKLWGWELPTHF TRB:CASSQRQGGLLYGYTF	1	CD4-C3
Pla-2_TTGAACGTCGCACTCT	Pla	Pla-2	P2-5002	FALSE	1	TRA:CAVSGYQKVTF TRB:CASSQVGTSGGFQETQYF	1	CD4-C3
Pla-2_TTGAACGTCTCGATGA	Pla	Pla-2	P2-5003	FALSE	1	TRA:CIVRVRGGSNYKLTF TRB:CSVAALGGNEQFF	1	CD4-C3
Pla-2_TTGCGTCTCCTTCAAT	Pla	Pla-2	P2-5012	FALSE	1	TRA:CATDARRGSGNTGKLIF TRB:CSAGTATYGYTF	1	CD4-C3
Pla-2_TTGCGTCTCTTCCTTC	Pla	Pla-2	P2-5013	FALSE	1	TRA:CAVRGSWGKFQF TRB:CASSPLEGERQFF	1	CD4-C3
Pla-2_TTTACTGTCGGATGGA	Pla	Pla-2	P2-5025	FALSE	1	TRA:CGTENTNAGKSTF TRB:CASGGGRAEETQYF	1	CD4-C3
Pla-2_TTTGCGCCACCGGAAA	Pla	Pla-2	P2-563	FALSE	2	TRA:CAVGAPERSQGNLIF TRB:CASSDRDRVLNTEAFF	2	CD4-C3
Pla-2_TTTGCGCCAGGGTTAG	Pla	Pla-2	P2-5031	FALSE	1	TRA:CAVEDGGATNKLIF TRB:CASSDTAEGLLFGNTIYF	1	CD4-C3
Pla-2_AAACGGGTCAGGCAAG	Pla	Pla-2	P2-3415	FALSE	1	TRA:CAAVDSNYQLIW TRB:CASSVAGGGNEQFF	1	CD8-C10

Pla-2_AACGTTGGTATCAGTC	Pla	Pla-2	P2-3440	FALSE	1	TRA:CAVRDSNYQLIW TRB:CATTYTQSGANVLTF	1	CD8-C10
Pla-2_AACTCAGAGAGACTAT	Pla	Pla-2	P2-3442	FALSE	1	TRA:CAVMDSNYQLIW TRB:CASSSSGGSSYEQYF	1	CD8-C10
Pla-2_AACTCTTTCGGCGGTT	Pla	Pla-2	P2-3452	FALSE	1	TRA:CAVIATNKFYF TRB:CASSEGTGDYNEQFF	1	CD8-C10
Pla-2_AAGACCTAGTGTACTC	Pla	Pla-2	P2-3455	FALSE	1	TRA:CVPDRGNNRLAF TRB:CASSEAVQDSSYEQYF	1	CD8-C10
Pla-2_AAGTCTGTCAACGCTA	Pla	Pla-2	P2-3476	FALSE	1	TRA:CAGLYNFNKFYF TRB:CASSDSTSGGADTQYF	1	CD8-C10
Pla-2_AATCGGTGTACAGTGG	Pla	Pla-2	P2-41	TRUE	3	TRA:CAVRDSDYKLSF TRB:CASSQGESSYEQYF	12	CD8-C10
Pla-2_ACACCCTCAAGCCGTC	Pla	Pla-2	P2-392	FALSE	2	TRA:CAFMDSNYQLIW TRB:CASSHGTGTADEQYF	2	CD8-C10
Pla-2_ACACCCTGTCTACCTC	Pla	Pla-2	P2-395	FALSE	2	TRA:CAVKDSNYQLIW TRB:CASSQDGTSGSGELFF	2	CD8-C10
Pla-2_ACACTGAAGGCCCTCA	Pla	Pla-2	P2-24	FALSE	3	TRA:CAVMDSSYKLIF TRB:CASSELAGSGDTQYF	23	CD8-C10
Pla-2_ACACTGACAATGAATG	Pla	Pla-2	P2-3497	FALSE	1	TRA:CAVEVTDSWGKFQF TRB:CASSGTGESGELFF	1	CD8-C10
Pla-2_ACATGGTGTATTACCG	Pla	Pla-2	P2-3514	FALSE	1	TRA:CAVRDEDYKLSF TRB:CASSRTSGGADTQYF	1	CD8-C10
Pla-2_ACCAGTACATTTCACT	Pla	Pla-2	P2-3520	FALSE	1	TRA:CAMPAGKLIF TRB:CASSPTRGEVGELFF	1	CD8-C10
Pla-2_ACCCACTGTTTCGCTC	Pla	Pla-2	P2-3526	FALSE	1	TRA:CAAMDSNYQLIW TRB:CSARDPQGFHEQYF	1	CD8-C10
Pla-2_ACCGTAAGTCCGAATT	Pla	Pla-2	P2-141	FALSE	3	TRA:CAVRDRLENNAGNMLTF TRB:CASSKTGSSYEQYF	4	CD8-C10
Pla-2_ACGAGGAAGACTAAGT	Pla	Pla-2	P2-3540	FALSE	1	TRA:CAVIDSNYQLIW TRB:CASSDSTSGSTDTQYF	1	CD8-C10
Pla-2_ACGATGTTCAAAGTAG	Pla	Pla-2	P2-41	TRUE	3	TRA:CAVRDSDYKLSF TRB:CASSQGESSYEQYF	12	CD8-C10
Pla-2_ACGCCGATCTGCAAGT	Pla	Pla-2	P2-3568	FALSE	1	TRA:CAVMDSNYQLIW TRB:CSAPSGRASETQYF	1	CD8-C10
Pla-2_ACTGCTCAGTACGTAA	Pla	Pla-2	P2-3594	FALSE	1	TRA:CAASARKAYLRT TRB:CASGQGVDTGELFF	1	CD8-C10
Pla-2_ACTGTCCCAATAGCGG	Pla	Pla-2	P2-3597	FALSE	1	TRA:CAVEYMIGLGRGTSGSRLTF TRB:CASSLNRGPATEAFF	1	CD8-C10
Pla-2_ACTTACTAGGTACTCT	Pla	Pla-2	P2-3600	FALSE	1	TRA:CAVMDSNYQLIW TRB:CASSEAGHSGANVLTF	1	CD8-C10
Pla-2_AGAGCGATCCGTCATC	Pla	Pla-2	P2-24	FALSE	3	TRA:CAVMDSSYKLIF TRB:CASSELAGSGDTQYF	23	CD8-C10
Pla-2_AGATCTGAGAACTCGG	Pla	Pla-2	P2-24	FALSE	3	TRA:CAVMDSSYKLIF TRB:CASSELAGSGDTQYF	23	CD8-C10
Pla-2_AGATCTGCAAATCCGT	Pla	Pla-2	P2-24	FALSE	3	TRA:CAVMDSSYKLIF TRB:CASSELAGSGDTQYF	23	CD8-C10
Pla-2_AGATCTGTCACATGCA	Pla	Pla-2	P2-3630	FALSE	1	TRA:CAVRDSNYQLIW TRB:CASSYSASSSSGANVLTF	1	CD8-C10
Pla-2_AGATTGCAGCGTGTCC	Pla	Pla-2	P2-148	FALSE	3	TRA:CAMRSSGAGSYQLTF TRB:CAILDDVIDTQYF	4	CD8-C10
Pla-2_AGATTGCTCGGCATCG	Pla	Pla-2	P2-3634	FALSE	1	TRA:CAVRDSNYQLIW TRB:CASSETPGQGAETQYF	1	CD8-C10
Pla-2_AGCCTAAGTATTACCG	Pla	Pla-2	P2-3644	FALSE	1	TRA:CAVMDSNYQLIW TRB:CSARTGRGDTDTQYF	1	CD8-C10

Pla-2_AGCGGTCCATAGGATA	Pla	Pla-2	P2-3648	FALSE	1	TRA:CAVMDSNYQLIW TRB:CASSQIGSSPDTQYF	1	CD8-C2
Pla-2_AGGGTGATCAGCTGGC	Pla	Pla-2	P2-341	TRUE	2	TRA:CAGMDSNYQLIW TRB:CSARDLSEPYEQYF	2	CD8-C10
Pla-2_AGTGGGAAGCGATCCC	Pla	Pla-2	P2-375	TRUE	2	TRA:CAVGDSNYQLIW TRB:CASSDSGGATDTQYF	2	CD8-C10
Pla-2_ATGGGAGAGATTACCC	Pla	Pla-2	P2-118	FALSE	3	TRA:CAVMDSNYQLIW TRB:CASSEGSGGVSEQFF	5	CD8-C10
Pla-2_ATGGGAGAGGATGGAA	Pla	Pla-2	P2-3748	FALSE	1	TRA:CAVPNTNAGKSTF TRB:CASTHADRGSSPLHF	1	CD8-C10
Pla-2_ATGTGTGTCGTTTGCC	Pla	Pla-2	P2-3751	FALSE	1	TRA:CALTDSNYQLIW TRB:CASSEVYSGANVLTF	1	CD8-C10
Pla-2_ATTATCCAGTGATCGG	Pla	Pla-2	P2-3757	FALSE	1	TRA:CLKKIIF TRB:CASSRNPGGGTDTQYF	1	CD8-C10
Pla-2_CAACCAATCCGTTGTC	Pla	Pla-2	P2-3783	FALSE	1	TRA:CAVMDSNYQLIW TRB:CASRITSGETGELFF	1	CD8-C10
Pla-2_CAACTAGGTCTGCAAT	Pla	Pla-2	P2-3790	FALSE	1	TRA:CAVLDSNYQLIW TRB:CASSPGGEVETQYF	1	CD8-C10
Pla-2_CAAGAAACATTCTTAC	Pla	Pla-2	P2-94	FALSE	3	TRA:CAAMDSNYQLIW TRB:CASSQDIELAGPIYNEQFF	6	CD8-C10
Pla-2_CAAGTTGCATACTCTT	Pla	Pla-2	P2-3802	FALSE	1	TRA:CAVLDSNYQLIW TRB:CASSPGNREISEQFF	1	CD8-C10
Pla-2_CACAAACCAATCTACG	Pla	Pla-2	P2-3805	FALSE	1	TRA:CAVRDSNYQLIW TRB:CASSEAGEAYNEQFF	1	CD8-C10
Pla-2_CACAAACTCCAATGGT	Pla	Pla-2	P2-3809	FALSE	1	TRA:CAPLMDTGRRALTF TRB:CASARGSSGANVLTF	1	CD8-C10
Pla-2_CACACCTAGCCAGAAC	Pla	Pla-2	P2-3815	FALSE	1	TRA:CAVMDSNYQLIW TRB:CASSETSGGGSETQYF	1	CD8-C10
Pla-2_CACCACTCAAGGTGTG	Pla	Pla-2	P2-463	FALSE	2	TRA:CAVLDSNYQLIW TRB:CSTSMGDRDYNEQFF	2	CD8-C10
Pla-2_CAGAGAGCAGCTGCAC	Pla	Pla-2	P2-3854	FALSE	1	TRA:CAGVDSNYQLIW TRB:CASSITGDNTGELFF	1	CD8-C10
Pla-2_CAGATCAAGCAGGCTA	Pla	Pla-2	P2-3857	FALSE	1	TRA:CVVSVAAGNKLTF TRB:CASSELAGGPDTQYF	1	CD8-C10
Pla-2_CAGCAGCTCCTGTAGA	Pla	Pla-2	P2-467	FALSE	2	TRA:CAVRDSNYQLIW TRB:CASSSRIEGEVDTQYF	2	CD4-C4
Pla-2_CAGCTAAAGATATACG	Pla	Pla-2	P2-24	FALSE	3	TRA:CAVMDSSYKLIF TRB:CASSELAGSGDTQYF	23	CD8-C10
Pla-2_CAGCTGGCACAGGAGT	Pla	Pla-2	P2-152	FALSE	3	TRA:CALKGSSGYSTLTF TRB:CSAIPSGGIGTDTQYF	4	CD4-C4
Pla-2_CAGCTGGCACTAAGTC	Pla	Pla-2	P2-3878	FALSE	1	TRA:CAVMDSNYQLIW TRB:CASSIAETGAGYGYTF	1	CD8-C10
Pla-2_CAGCTGGTCCCTCTTT	Pla	Pla-2	P2-118	FALSE	3	TRA:CAVMDSNYQLIW TRB:CASSEGSGGVSEQFF	5	CD8-C10
Pla-2_CATATTCGTAGCCTCG	Pla	Pla-2	P2-474	FALSE	2	TRA:CAVVDSNYQLIW TRB:CAWSVGGQGYQPQHF	2	CD8-C10
Pla-2_CATATTCGTGCACCAC	Pla	Pla-2	P2-3898	FALSE	1	TRA:CAVEDRGSTLGRLYF TRB:CASSTTSGGADTQYF	1	CD8-C10
Pla-2_CATGACATCTCGTATT	Pla	Pla-2	P2-3916	FALSE	1	TRA:CAVIDSNYQLIW TRB:CSASGEASYNEQFF	1	CD8-C10
Pla-2_CCAGCGAAGCGTTTAC	Pla	Pla-2	P2-3949	FALSE	1	TRA:CAVMDSNYQLIW TRB:CASSIQQGGTSGANVLTF	1	CD8-C10
Pla-2_CCCAATCTCTATCCTA	Pla	Pla-2	P2-3962	FALSE	1	TRA:CAVMDSSYKLIF TRB:CASLNGAGGSYEQYF	1	CD8-C10

Pla-2_CCCTCCTAGGCAAAGA	Pla	Pla-2	P2-24	FALSE	3	TRA:CAVMDSSYKLIF TRB:CASSELAGSGDTQYF	23	CD8-C10
Pla-2_CCGGTAGAGATGTGTA	Pla	Pla-2	P2-34	TRUE	3	TRA:CAVRDSNYQLIW TRB:CASSLYPSGGGTDTQYF	15	CD8-C10
Pla-2_CCGGTAGCATCTCCCA	Pla	Pla-2	P2-34	TRUE	3	TRA:CAVRDSNYQLIW TRB:CASSLYPSGGGTDTQYF	15	CD8-C10
Pla-2_CCGTACTCACCCAGTG	Pla	Pla-2	P2-3977	FALSE	1	TRA:CAVMDSNYQLIW TRB:CASSQVLELTGELFF	1	CD8-C10
Pla-2_CCTAAAGCAGGATTGG	Pla	Pla-2	P2-3983	FALSE	1	TRA:CAVMDSNYQLIW TRB:CASRESDTGELFF	1	CD8-C10
Pla-2_CCTACCAAGGATTCGG	Pla	Pla-2	P2-120	FALSE	3	TRA:CAVRDSNYQLIW TRB:CASSHREIDEQFF	5	CD8-C10
Pla-2_CCTCAGTTCCGTAGGC	Pla	Pla-2	P2-41	TRUE	3	TRA:CAVRDSDYKLSF TRB:CASSQGESSYEQYF	12	CD8-C10
Pla-2_CCTTCCCAGAGGTTAT	Pla	Pla-2	P2-301	TRUE	2	TRA:CAVMDSNYQLIW TRB:CASSRTGGSYNEQFF	2	CD8-C10
Pla-2_CCTTTCTGTAGGGACT	Pla	Pla-2	P2-4023	FALSE	1	TRA:CAVRDSNYQLIW TRB:CSASTGTGSSPLHF	1	CD4-C4
Pla-2_CGAATGTCACACCGCA	Pla	Pla-2	P2-4029	FALSE	1	TRA:CAVRDSDYKLSF TRB:CSARTSGGGNNEQFF	1	CD8-C10
Pla-2_CGACCTTGTGTGCCTG	Pla	Pla-2	P2-439	FALSE	2	TRA:CVVRKAGTALIF TRB:CATSDSGAGGSNQPQHF	2	CD4-C3
Pla-2_CGAGAAGGTCGACTAT	Pla	Pla-2	P2-4039	FALSE	1	TRA:CAAMDSNYQLIW TRB:CASSPSENYGYTF	1	CD8-C10
Pla-2_CGAGAAGTCCGAAGAG	Pla	Pla-2	P2-34	TRUE	3	TRA:CAVRDSNYQLIW TRB:CASSLYPSGGGTDTQYF	15	CD8-C10
Pla-2_CGAGCACTCATGGTCA	Pla	Pla-2	P2-4046	FALSE	1	TRA:CAVMDSNYQLIW TRB:CASSTQGGGTGELFF	1	CD8-C10
Pla-2_CGAGCCAAGATGTAAC	Pla	Pla-2	P2-4047	FALSE	1	TRA:CAVRDGDYKLSF TRB:CASSEWAEETQYF	1	CD8-C10
Pla-2_CGAGCCAGTTGTGGAG	Pla	Pla-2	P2-248	FALSE	3	TRA:CPDRGSTLGRLYF TRB:CASSVLAGEEGEQFF	3	CD8-C10
Pla-2_CGATCGGAGGCAGTCA	Pla	Pla-2	P2-4054	FALSE	1	TRA:CAPMDSNYQLIW TRB:CASSEVEGTGTDTQYF	1	CD8-C10
Pla-2_CGATGGCCACATGGGA	Pla	Pla-2	P2-24	FALSE	3	TRA:CAVMDSSYKLIF TRB:CASSELAGSGDTQYF	23	CD8-C10
Pla-2_CGGACGTTCATGCATG	Pla	Pla-2	P2-24	FALSE	3	TRA:CAVMDSSYKLIF TRB:CASSELAGSGDTQYF	23	CD8-C10
Pla-2_CGGAGCTAGTGCAAGC	Pla	Pla-2	P2-4107	FALSE	1	TRA:CAVGQGGTASKLTF TRB:CASSEGDRDTGELFF	1	CD8-C10
Pla-2_CGGGTCACACATCTTT	Pla	Pla-2	P2-4117	FALSE	1	TRA:CAVNDYKLSF TRB:CASSELAGADTQYF	1	CD8-C10
Pla-2_CGGGTCAGTATTAGCC	Pla	Pla-2	P2-4119	FALSE	1	TRA:CAVLDSNYQLIW TRB:CATHTGGNEQFF	1	CD8-C10
Pla-2_CGTAGGCTCGGACAAG	Pla	Pla-2	P2-4133	FALSE	1	TRA:CAPLDSNYQLIW TRB:CASSSGQGETGELFF	1	CD8-C10
Pla-2_CGTGAGCTCTGGTATG	Pla	Pla-2	P2-392	FALSE	2	TRA:CAFMDSNYQLIW TRB:CASSHGTGTADEQYF	2	CD8-C10
Pla-2_CGTGTCTAGGACCACA	Pla	Pla-2	P2-4148	FALSE	1	TRA:CATSSGGYQKVTF TRB:CSARGLAGNEQFF	1	CD8-C10
Pla-2_CGTGTCTAGTCCTCCT	Pla	Pla-2	P2-4150	FALSE	1	TRA:CAALDSNYQLIW TRB:CASSYYRDPNTDTQYF	1	CD8-C10
Pla-2_CGTTGGGAGAAACGAG	Pla	Pla-2	P2-4161	FALSE	1	TRA:CAAMDSNYQLIW TRB:CSARDRGDTEAFF	1	CD8-C10

Pla-2_CGTTGGGTCCTAAGTG	Pla	Pla-2	P2-174	TRUE	3	TRA:CAVRDGDYKLSF TRB:CSAKDGGQDYEQYF	3	CD8-C10
Pla-2_CTAAGACTCGTTACAG	Pla	Pla-2	P2-514	FALSE	2	TRA:CAVRDSNYQLIW TRB:CASSDSNTGELFF	2	CD8-C10
Pla-2_CTACATTGTGCAACGA	Pla	Pla-2	P2-470	FALSE	2	TRA:CAPMDSSYKLIF TRB:CASSEGEVGELFF	2	CD8-C10
Pla-2_CTACCCAAGATATACG	Pla	Pla-2	P2-364	TRUE	2	TRA:CAAMDSNYQLIW TRB:CASSEEGGSYDEQFF	2	CD8-C10
Pla-2_CTAGAGTGTCAACTGT	Pla	Pla-2	P2-24	FALSE	3	TRA:CAVMDSSYKLIF TRB:CASSELAGSGDTQYF	23	CD8-C10
Pla-2_CTAGCCTGTTTACTCT	Pla	Pla-2	P2-4185	FALSE	1	TRA:CAVRDSNYQLIW TRB:CASSNRENTEAFF	1	CD8-C10
Pla-2_CTCACACTCATGCAAC	Pla	Pla-2	P2-41	TRUE	3	TRA:CAVRDSDYKLSF TRB:CASSQGESSYEQYF	12	CD8-C10
Pla-2_CTCGAAATCTAACTCT	Pla	Pla-2	P2-280	TRUE	2	TRA:CASMDSNYQLIW TRB:CASSVLAGEEGEQFF	2	CD8-C10
Pla-2_CTCGAGGAGCGCTCCA	Pla	Pla-2	P2-120	FALSE	3	TRA:CAVRDSNYQLIW TRB:CASSHREIDEQFF	5	CD8-C10
Pla-2_CTCGGAGAGACTAGGC	Pla	Pla-2	P2-120	FALSE	3	TRA:CAVRDSNYQLIW TRB:CASSHREIDEQFF	5	CD8-C10
Pla-2_CTCGTACCAGCTGCAC	Pla	Pla-2	P2-4218	FALSE	1	TRA:CVIMDSNYQLIW TRB:CASTRDPSGATDTQYF	1	CD8-C10
Pla-2_CTCTGGTTCCAACCAA	Pla	Pla-2	P2-4229	FALSE	1	TRA:CAVPDSNYQLIW TRB:CASNQDGSGANVLTF	1	CD8-C10
Pla-2_CTGATAGAGTACGCCC	Pla	Pla-2	P2-4234	FALSE	1	TRA:CAVRDTNAGKSTF TRB:CASSEGTSDTGELFF	1	CD8-C10
Pla-2_CTGTGCTAGCGTCTAT	Pla	Pla-2	P2-4253	FALSE	1	TRA:CASMDSNYQLIW TRB:CASSYGDTYEQYF	1	CD8-C10
Pla-2_CTTAACTCATAGTAAG	Pla	Pla-2	P2-4263	FALSE	1	TRA:CAVGARYNTDKLIF TRB:CASSHRGDRAQNYGYTF	1	CD8-C10
Pla-2_CTTACCGCAGCTGCTG	Pla	Pla-2	P2-94	FALSE	3	TRA:CAAMDSNYQLIW TRB:CASSQDIELAGPIYNEQFF	6	CD8-C10
Pla-2_CTTTGCGAGACACTAA	Pla	Pla-2	P2-463	FALSE	2	TRA:CAVLDSNYQLIW TRB:CSTSMGDRDYNEQFF	2	CD8-C10
Pla-2_GAACATCTCCGTCAAA	Pla	Pla-2	P2-4300	FALSE	1	TRA:CAWAAGNMLTF TRB:CAWSGHGGNTIYF	1	CD8-C10
Pla-2_GAACGGAAGAAACCTA	Pla	Pla-2	P2-4302	FALSE	1	TRA:CAVMDSNYQLIW TRB:CSARSPERDTEAFF	1	CD8-C10
Pla-2_GAACGGACATTTGCTT	Pla	Pla-2	P2-257	FALSE	3	TRA:CAAKDSNYQLIW TRB:CASSEGDTGELFF	3	CD8-C10
Pla-2_GACAGAGGTGGTTTCA	Pla	Pla-2	P2-536	FALSE	2	TRA:CIVTAGGTSYGKLTF TRB:CASSSGWGGYEQYF	2	CD8-C10
Pla-2_GACGCGTTCATCATTC	Pla	Pla-2	P2-4339	FALSE	1	TRA:CAVMDSNYQLIW TRB:CASSWDYTDTQYF	1	CD8-C10
Pla-2_GACTACACACGAAACG	Pla	Pla-2	P2-4356	FALSE	1	TRA:CAERSAAGGYNKLIF TRB:CASSEDAGGEAGELFF	1	CD8-C10
Pla-2_GACTACATCTCTGCTG	Pla	Pla-2	P2-4361	FALSE	1	TRA:CAVLDSNYQLIW TRB:CASSFGTGDTGELFF	1	CD8-C10
Pla-2_GAGCAGAAGTAAGTAC	Pla	Pla-2	P2-174	TRUE	3	TRA:CAVRDGDYKLSF TRB:CSAKDGGQDYEQYF	3	CD8-C10
Pla-2_GATCAGTGTCTAACGT	Pla	Pla-2	P2-120	FALSE	3	TRA:CAVRDSNYQLIW TRB:CASSHREIDEQFF	5	CD8-C10
Pla-2_GATCGCGTCTTTAGTC	Pla	Pla-2	P2-94	FALSE	3	TRA:CAAMDSNYQLIW TRB:CASSQDIELAGPIYNEQFF	6	CD8-C10

Pla-2_GCAGTTACATTTGCCC	Pla	Pla-2	P2-94	FALSE	3	TRA:CAAMDSNYQLIW TRB:CASSQDIELAGPIYNEQFF	6	CD8-C10
Pla-2_GCAGTTAGTCGCGGTT	Pla	Pla-2	P2-4415	FALSE	1	TRA:CASMDSNYQLIW TRB:CSALMGREPYEQYF	1	CD8-C10
Pla-2_GCAGTTATCTGGTTCC	Pla	Pla-2	P2-4416	FALSE	1	TRA:CAVVDSSYKLIF TRB:CASNALEGGADTQYF	1	CD8-C10
Pla-2_GCATACAGTACCATCA	Pla	Pla-2	P2-4418	FALSE	1	TRA:CAETLEGNTNAGKSTF TRB:CASSEAPADPGNYGYTF	1	CD8-C10
Pla-2_GCATGTAAGGTTCCTA	Pla	Pla-2	P2-248	FALSE	3	TRA:CPDRGSTLGRLYF TRB:CASSVLAGEEGEQFF	3	CD8-C10
Pla-2_GCCAAATGTAACGACG	Pla	Pla-2	P2-41	TRUE	3	TRA:CAVRDSDYKLSF TRB:CASSQGESSYEQYF	12	CD8-C10
Pla-2_GCCTCTAGTCTAAAGA	Pla	Pla-2	P2-24	FALSE	3	TRA:CAVMDSSYKLIF TRB:CASSELAGSGDTQYF	23	CD8-C10
Pla-2_GCCTCTAGTTCCACTC	Pla	Pla-2	P2-4439	FALSE	1	TRA:CAVSDSNYQLIW TRB:CASSEAQGTASYEQYF	1	CD8-C10
Pla-2_GCGACCATCCTTCAAT	Pla	Pla-2	P2-4442	FALSE	1	TRA:CAVLDSNYQLIW TRB:CSASRGDRGDYNEQFF	1	CD8-C10
Pla-2_GCGAGAAAGCTCAACT	Pla	Pla-2	P2-310	TRUE	2	TRA:CAVMDSNYQLIW TRB:CASSAQGTDTGELFF	2	CD8-C10
Pla-2_GCGCAACCACAGATTC	Pla	Pla-2	P2-4445	FALSE	1	TRA:CACMDSNYQLIW TRB:CASSEWEYNEQFF	1	CD8-C10
Pla-2_GCGCAACCATCGACGC	Pla	Pla-2	P2-4446	FALSE	1	TRA:CAVLDSNYQLIW TRB:CASSTGQGGETQYF	1	CD8-C10
Pla-2_GCGCAGTCAGCCTATA	Pla	Pla-2	P2-199	TRUE	3	TRA:CAVMDSSYKLIF TRB:CSVPGQGDYEQYF	3	CD8-C10
Pla-2_GCGCAGTTCACCTTAT	Pla	Pla-2	P2-4450	FALSE	1	TRA:CAGMDSNYQLIW TRB:CASSETAGTHTDTQYF	1	CD8-C10
Pla-2_GCGCGATTCGAGAACG	Pla	Pla-2	P2-4458	FALSE	1	TRA:CAVIDNDYKLSF TRB:CASSELGGSSYEQYF	1	CD8-C10
Pla-2_GCGGGTTTCTACGAGT	Pla	Pla-2	P2-4460	FALSE	1	TRA:CAVMDSNYQLIW TRB:CASVLAGDVDTQYF	1	CD8-C10
Pla-2_GGAAAGCAGTCTCCTC	Pla	Pla-2	P2-24	FALSE	3	TRA:CAVMDSSYKLIF TRB:CASSELAGSGDTQYF	23	CD8-C10
Pla-2_GGAAAGCGTCTAACGT	Pla	Pla-2	P2-24	FALSE	3	TRA:CAVMDSSYKLIF TRB:CASSELAGSGDTQYF	23	CD8-C10
Pla-2_GGAACTTTCCAATGGT	Pla	Pla-2	P2-4496	FALSE	1	TRA:CAVVDSNYQLIW TRB:CASTLTGENNQPQHF	1	CD8-C8
Pla-2_GGAACTTTCTTGACGA	Pla	Pla-2	P2-4498	FALSE	1	TRA:CAVVDSNYQLIW TRB:CASSHSTGDINEQYF	1	CD8-C10
Pla-2_GGAATAACACCCAGTG	Pla	Pla-2	P2-4500	FALSE	1	TRA:CAVMDSNYQLIW TRB:CASSIEDRDRQPQHF	1	CD4-C4
Pla-2_GGACAAGGTAGCTGCC	Pla	Pla-2	P2-4503	FALSE	1	TRA:CVNMDSNYQLIW TRB:CSANQGLASSEEQYF	1	CD8-C10
Pla-2_GGACATTCACCCATTC	Pla	Pla-2	P2-4509	FALSE	1	TRA:CACMDSNYQLIW TRB:CSARDPSEIDEQYF	1	CD8-C10
Pla-2_GGACGTCAGGAGTTTA	Pla	Pla-2	P2-4511	FALSE	1	TRA:CAVEDSSYKLIF TRB:CASSPTENQPQHF	1	CD8-C10
Pla-2_GGACGTCGTGACAAAT	Pla	Pla-2	P2-4514	FALSE	1	TRA:CAVMDSNYQLIW TRB:CSVEDVGGKSSYNEQFF	1	CD8-C10
Pla-2_GGACGTCTCATGTAGC	Pla	Pla-2	P2-94	FALSE	3	TRA:CAAMDSNYQLIW TRB:CASSQDIELAGPIYNEQFF	6	CD8-C10
Pla-2_GGAGCAATCGATAGAA	Pla	Pla-2	P2-41	TRUE	3	TRA:CAVRDSDYKLSF TRB:CASSQGESSYEQYF	12	CD8-C10

Pla-2_GGCAATTCACTGTGTA	Pla	Pla-2	P2-4531	FALSE	1	TRA:CAVRDSNYQLIW TRB:CASSERGSTDTQYF	1	CD8-C10
Pla-2_GGCAATTGTTCGCTAA	Pla	Pla-2	P2-4533	FALSE	1	TRA:CAVMDSNYQLIW TRB:CASTQHRDSYNSPLHF	1	CD8-C10
Pla-2_GGCTGGTTCCAAGCCG	Pla	Pla-2	P2-2	TRUE	3	TRA:CVVPNTNAGKSTF TRB:CASSLQGSTYNEQFF	220	CD8-C10
Pla-2_GGGATGACATGGTCTA	Pla	Pla-2	P2-34	TRUE	3	TRA:CAVRDSNYQLIW TRB:CASSLYPSGGGTDTQYF	15	CD8-C10
Pla-2_GGGCATCCATGGTCTA	Pla	Pla-2	P2-4563	FALSE	1	TRA:CAALDSNYQLIW TRB:CATSRGEFGEQYF	1	CD8-C10
Pla-2_GGGTCTGTCGTACGGC	Pla	Pla-2	P2-474	FALSE	2	TRA:CAVVDSNYQLIW TRB:CAWSVGGQGYQPQHF	2	CD8-C10
Pla-2_GGTGAAGAGATATACG	Pla	Pla-2	P2-24	FALSE	3	TRA:CAVMDSSYKLIF TRB:CASSELAGSGDTQYF	23	CD8-C10
Pla-2_GGTGTTACACGACTCG	Pla	Pla-2	P2-4579	FALSE	1	TRA:CAMRAGRGYQKVTF TRB:CASSSRIEGEVDTQYF	1	CD8-C10
Pla-2_GTAACGTAGGGCTCTC	Pla	Pla-2	P2-118	FALSE	3	TRA:CAVMDSNYQLIW TRB:CASSEGSGGVSEQFF	5	CD8-C10
Pla-2_GTACGTATCGATGAGG	Pla	Pla-2	P2-4591	FALSE	1	TRA:CAVRDSNYQLIW TRB:CASSESEEGQPQHF	1	CD8-C10
Pla-2_GTACTCCGTGCAACTT	Pla	Pla-2	P2-378	FALSE	2	TRA:CAYRSGTSYGKLTF TRB:CSARETGLSYEQYF	2	CD8-C10
Pla-2_GTAGTCAAGCGATTCT	Pla	Pla-2	P2-118	FALSE	3	TRA:CAVMDSNYQLIW TRB:CASSEGSGGVSEQFF	5	CD8-C10
Pla-2_GTATTCTAGACGACGT	Pla	Pla-2	P2-4607	FALSE	1	TRA:CPDRGSTLGRLYF TRB:CASLNGAGGSYEQYF	1	CD8-C10
Pla-2_GTCACAACATCACGAT	Pla	Pla-2	P2-24	FALSE	3	TRA:CAVMDSSYKLIF TRB:CASSELAGSGDTQYF	23	CD8-C10
Pla-2_GTGAAGGGTTCCTCCA	Pla	Pla-2	P2-34	TRUE	3	TRA:CAVRDSNYQLIW TRB:CASSLYPSGGGTDTQYF	15	CD8-C10
Pla-2_GTGCAGCTCGCCAGCA	Pla	Pla-2	P2-4634	FALSE	1	TRA:CAVRDGDYQLIW TRB:CASSASGGAVDTQYF	1	CD8-C10
Pla-2_GTGCATACACGTAAGG	Pla	Pla-2	P2-4636	FALSE	1	TRA:CAVMDSNYQLIW TRB:CSARTSGSFYEQYF	1	CD8-C10
Pla-2_GTGCATATCTTGCATT	Pla	Pla-2	P2-24	FALSE	3	TRA:CAVMDSSYKLIF TRB:CASSELAGSGDTQYF	23	CD8-C10
Pla-2_GTGTGCGGTAAACACA	Pla	Pla-2	P2-4648	FALSE	1	TRA:CAVEMDSSYKLIF TRB:CSARNLEGVAGGLQGETQYF	1	CD8-C10
Pla-2_GTTACAGAGGCTAGGT	Pla	Pla-2	P2-4659	FALSE	1	TRA:CAVRDSNYQLIW TRB:CASSELAGDSSYEQYF	1	CD8-C10
Pla-2_TAAGAGATCCTGTAGA	Pla	Pla-2	P2-34	TRUE	3	TRA:CAVRDSNYQLIW TRB:CASSLYPSGGGTDTQYF	15	CD8-C10
Pla-2_TAAGTGCTCGGCTTGG	Pla	Pla-2	P2-4679	FALSE	1	TRA:CVILDSNYQLIW TRB:CSARDRYSGRDHTGELFF	1	CD8-C10
Pla-2_TACACGAAGCGCTCCA	Pla	Pla-2	P2-4680	FALSE	1	TRA:CAVRDSNYQLIW TRB:CATTPNEREVGEQYF	1	CD8-C10
Pla-2_TACAGTGCATCACCCT	Pla	Pla-2	P2-41	TRUE	3	TRA:CAVRDSDYKLSF TRB:CASSQGESSYEQYF	12	CD8-C10
Pla-2_TACGGTAAGGTTCCTA	Pla	Pla-2	P2-24	FALSE	3	TRA:CAVMDSSYKLIF TRB:CASSELAGSGDTQYF	23	CD8-C10
Pla-2_TACTCGCCATGTCTCC	Pla	Pla-2	P2-199	TRUE	3	TRA:CAVMDSSYKLIF TRB:CSVPGQGDYEQYF	3	CD8-C10
Pla-2_TACTTGTTCAATACCG	Pla	Pla-2	P2-4714	FALSE	1	TRA:CAVRFGGNQFYF TRB:CASTMGGRDTGELFF	1	CD8-C10

Pla-2_TAGGCATCAAGCCTAT	Pla	Pla-2	P2-4723	FALSE	1	TRA:CASSGNTGKLIF TRB:CASSSSGDLPYNEQFF	1	CD8-C10
Pla-2_TAGTGGTAGCTGAACG	Pla	Pla-2	P2-347	TRUE	2	TRA:CAVMDSNYQLIW TRB:CSARDHRELYTDTQYF	2	CD8-C10
Pla-2_TCACAAGGTGTTGGGA	Pla	Pla-2	P2-248	FALSE	3	TRA:CPDRGSTLGRLYF TRB:CASSVLAGEEGEQFF	3	CD8-C10
Pla-2_TCACGAAGTCAACTGT	Pla	Pla-2	P2-4758	FALSE	1	TRA:CAVTDSNYQLIW TRB:CSARDIGGETEPQHF	1	CD8-C10
Pla-2_TCAGCAAAGAGTCTGG	Pla	Pla-2	P2-24	FALSE	3	TRA:CAVMDSSYKLIF TRB:CASSELAGSGDTQYF	23	CD8-C10
Pla-2_TCAGGTATCCGTTGCT	Pla	Pla-2	P2-34	TRUE	3	TRA:CAVRDSNYQLIW TRB:CASSLYPSGGGTDTQYF	15	CD8-C10
Pla-2_TCATTACGTAAACACA	Pla	Pla-2	P2-4778	FALSE	1	TRA:CAVRDSNYQLIW TRB:CASSRGTSGTDTQYF	1	CD8-C10
Pla-2_TCGCGAGAGGGATCTG	Pla	Pla-2	P2-4794	FALSE	1	TRA:CAVKDSNYQLIW TRB:CAWSVQRDYNEQFF	1	CD8-C10
Pla-2_TCGCGTTGTACCGAGA	Pla	Pla-2	P2-554	FALSE	2	TRA:CALSDVSNQFYF TRB:CSARVLANIPGELFF	2	CD8-C10
Pla-2_TCGTACCGTCCTAGCG	Pla	Pla-2	P2-4805	FALSE	1	TRA:CAVSRASGNTGKLIF TRB:CASSFSWRGALQETQYF	1	CD8-C10
Pla-2_TCTCATACAGACACTT	Pla	Pla-2	P2-41	TRUE	3	TRA:CAVRDSDYKLSF TRB:CASSQGESSYEQYF	12	CD8-C10
Pla-2_TCTCTAACACTCGACG	Pla	Pla-2	P2-4821	FALSE	1	TRA:CAGSDSNYQLIW TRB:CSARGDSNMETQYF	1	CD8-C10
Pla-2_TCTGAGATCGCTTGTC	Pla	Pla-2	P2-257	FALSE	3	TRA:CAAKDSNYQLIW TRB:CASSEGDTGELFF	3	CD8-C10
Pla-2_TGAAAGACAACAACCT	Pla	Pla-2	P2-129	TRUE	3	TRA:CARGGTGNQFYF TRB:CAWSPMPNEQFF	4	CD8-C10
Pla-2_TGACAACTCCAGAAGG	Pla	Pla-2	P2-34	TRUE	3	TRA:CAVRDSNYQLIW TRB:CASSLYPSGGGTDTQYF	15	CD8-C10
Pla-2_TGACGGCCATACGCTA	Pla	Pla-2	P2-4851	FALSE	1	TRA:CAALDSNYQLIW TRB:CASSPVLGTGDEKLFF	1	CD8-C10
Pla-2_TGATTTCTCCTATGTT	Pla	Pla-2	P2-467	FALSE	2	TRA:CAVRDSNYQLIW TRB:CASSSRIEGEVDTQYF	2	CD8-C10
Pla-2_TGCCAAAAGGTGCTAG	Pla	Pla-2	P2-4879	FALSE	1	TRA:CAVKDSNYQLIW TRB:CASSESEGGTDTQYF	1	CD8-C10
Pla-2_TGCCAAAAGTCACGCC	Pla	Pla-2	P2-120	FALSE	3	TRA:CAVRDSNYQLIW TRB:CASSHREIDEQFF	5	CD8-C10
Pla-2_TGCTACCCAGCGATCC	Pla	Pla-2	P2-24	FALSE	3	TRA:CAVMDSSYKLIF TRB:CASSELAGSGDTQYF	23	CD8-C10
Pla-2_TGCTGCTCAATGTAAG	Pla	Pla-2	P2-4903	FALSE	1	TRA:CAALDSNYQLIW TRB:CSARVGTGGFEKLFF	1	CD8-C10
Pla-2_TGGCCAGCACACAGAG	Pla	Pla-2	P2-24	FALSE	3	TRA:CAVMDSSYKLIF TRB:CASSELAGSGDTQYF	23	CD8-C10
Pla-2_TGGCGCACAATCGGTT	Pla	Pla-2	P2-4912	FALSE	1	TRA:CAALDSNYQLIW TRB:CASSETGTQNTEAFF	1	CD8-C10
Pla-2_TGGCGCATCACAGGCC	Pla	Pla-2	P2-4917	FALSE	1	TRA:CAVRDGDYKLSF TRB:CASSEGLDWETQYF	1	CD8-C10
Pla-2_TGGGCGTCACATCCGG	Pla	Pla-2	P2-24	FALSE	3	TRA:CAVMDSSYKLIF TRB:CASSELAGSGDTQYF	23	CD8-C10
Pla-2_TGGTTAGGTTGTGGCC	Pla	Pla-2	P2-4937	FALSE	1	TRA:CPDRGSTLGRLYF TRB:CASSRPLGLAVLEQFF	1	CD8-C10
Pla-2_TGGTTCCAGCGTTCCG	Pla	Pla-2	P2-118	FALSE	3	TRA:CAVMDSNYQLIW TRB:CASSEGSGGVSEQFF	5	CD8-C10

Pla-2_TGTGGTACACTGTGTA	Pla	Pla-2	P2-4952	FALSE	1	TRA:CAVMDSNYQLIW TRB:CASSYSMDGAETQYF	1	CD8-C10
Pla-2_TGTGGTAGTCCATCCT	Pla	Pla-2	P2-4953	FALSE	1	TRA:CAVLDSNYQLIW TRB:CSANKAGADYNEQFF	1	CD8-C10
Pla-2_TTAACTCTCTGCAGTA	Pla	Pla-2	P2-4961	FALSE	1	TRA:CAVVDSSYKLIF TRB:CASSEAGGTDTQYF	1	CD8-C10
Pla-2_TTAGGACCACGTTGGC	Pla	Pla-2	P2-257	FALSE	3	TRA:CAAKDSNYQLIW TRB:CASSEGDTGELFF	3	CD8-C10
Pla-2_TTAGTTCTCCTCGCAT	Pla	Pla-2	P2-4968	FALSE	1	TRA:CAETSPGTASKLTF TRB:CASSRTSGTYNEQFF	1	CD8-C10
Pla-2_TTCGAAGGTCCCTTGT	Pla	Pla-2	P2-4978	FALSE	1	TRA:CAVGGYGGATNKLIF TRB:CASSQDGTSGSGELFF	1	CD8-C10
Pla-2_TTCTACAGTCTTCTCG	Pla	Pla-2	P2-4987	FALSE	1	TRA:CAVLDSNYQLIW TRB:CASSEGGPDTQYF	1	CD8-C10
Pla-2_TTCTACATCCGTTGCT	Pla	Pla-2	P2-4990	FALSE	1	TRA:CAVRDSNYQLIW TRB:CASNPGEASGADTQYF	1	CD8-C10
Pla-2_TTCTCAATCGGAAATA	Pla	Pla-2	P2-24	FALSE	3	TRA:CAVMDSSYKLIF TRB:CASSELAGSGDTQYF	23	CD8-C10
Pla-2_TTGACTTTCTATGTGG	Pla	Pla-2	P2-5006	FALSE	1	TRA:CAGMDSNYQLIW TRB:CSASLLGDYNEQFF	1	CD8-C10
Pla-2_TTGCCGTCACCGGAAA	Pla	Pla-2	P2-94	FALSE	3	TRA:CAAMDSNYQLIW TRB:CASSQDIELAGPIYNEQFF	6	CD8-C10
Pla-2_TTGGAACTCTCTAAGG	Pla	Pla-2	P2-5015	FALSE	1	TRA:CAVIDSNYQLIW TRB:CASSPGTSGGKETQYF	1	CD8-C10
Pla-2_TTGGCAAGTATTAGCC	Pla	Pla-2	P2-5018	FALSE	1	TRA:CAVLDSNYQLIW TRB:CASSDSGTEGNEQFF	1	CD8-C10
Pla-2_TTTACTGTCACTTACT	Pla	Pla-2	P2-5024	FALSE	1	TRA:CVNMDSNYQLIW TRB:CAWSRAALTEAFF	1	CD8-C10
Pla-2_TTTATGCCACCGTTGG	Pla	Pla-2	P2-5027	FALSE	1	TRA:CAPLDSNYQLIW TRB:CASSEGEGLANQPQHF	1	CD8-C10
Pla-2_TTTGTCAGTGTAATGA	Pla	Pla-2	P2-5039	FALSE	1	TRA:CAVMDSNYQLIW TRB:CASSTGTSEAGELFF	1	CD8-C10
Pla-2_AAAGATGAGTGAATTG	Pla	Pla-2	P2-3417	FALSE	1	TRA:CAAALDTGRRALTF TRB:CSATGGMNTEAFF	1	CD4-C5
Pla-2_AAAGCAATCAAGCCTA	Pla	Pla-2	P2-3419	FALSE	1	TRA:CAVQRTGFQKLVF TRB:CASSLDGTYDNEQFF	1	CD8-C2
Pla-2_AAATGCCAGCGTAATA	Pla	Pla-2	P2-3422	FALSE	1	TRA:CAEYDPSNFGNEKLTF TRB:CASSSAGTYGYTF	1	CD4-C5
Pla-2_AAATGCCTCTTTACGT	Pla	Pla-2	P2-112	FALSE	3	TRA:CAVRFSGNTGKLIF TRB:CSVGDAGTEAFF	5	CD4-C5
Pla-2_AACCATGAGCCACTAT	Pla	Pla-2	P2-63	FALSE	3	TRA:CAASGEAGTALIF TRB:CATSREIQPNEKLFF	9	CD4-C5
Pla-2_AACCATGTCCAGAGGA	Pla	Pla-2	P2-380	FALSE	2	TRA:CAVSEETDSWGKFQF TRB:CATSRDRGRSYGYTF	2	CD4-C5
Pla-2_AACCGCGCATGCTGGC	Pla	Pla-2	P2-3434	FALSE	1	TRA:CAVEDQSQGNLIF TRB:CASSQQGSREQYF	1	CD4-C5
Pla-2_AACTCAGGTCGGGTCT	Pla	Pla-2	P2-383	FALSE	2	TRA:CATGMIKAAGNKLTF TRB:CASSHSGTSGSWYEQYF	2	CD4-C5
Pla-2_AACTCAGGTGGACGAT	Pla	Pla-2	P2-64	FALSE	3	TRA:CAATSLISDGQKLLF TRB:CSARDSFNYGYTF	9	CD4-C6
Pla-2_AACTCCCAGACTAGAT	Pla	Pla-2	P2-384	FALSE	2	TRA:CAVSRSNDYKLSF TRB:CASRDPGVYTEAFF	2	CD4-C6
Pla-2_AAGGAGCGTTAAGACA	Pla	Pla-2	P2-213	FALSE	3	TRA:CAAAGNRDDKIIF TRB:CASSQSAETQYF	3	CD4-C6

Pla-2_AAGGCAGTCTGGTTCC	Pla	Pla-2	P2-3466	FALSE	1	TRA:CALRGNNFNKFYF TRB:CAWSKTGTGEQFF	1	CD4-C5
Pla-2_AAGGTTCAGCAATATG	Pla	Pla-2	P2-33	TRUE	3	TRA:CAFKLSDGQKLLF TRB:CAWSRAALTEAFF	17	CD4-C4
Pla-2_AATCGGTCAAAGTCAA	Pla	Pla-2	P2-3480	FALSE	1	TRA:CAASKTPGAGSYQLTF TRB:CASSPAVNTEAFF	1	CD4-C5
Pla-2_ACACCAAGTTCCCGAG	Pla	Pla-2	P2-3484	FALSE	1	TRA:CALTPWGSGTYKYIF TRB:CASSPSGGTDTQYF	1	CD4-C4
Pla-2_ACACCAATCTGCGACG	Pla	Pla-2	P2-3487	FALSE	1	TRA:CAVKGWGYGGSQGNLIF TRB:CASSDLGLAVTDTQYF	1	CD4-C5
Pla-2_ACACCCTCACTTACGA	Pla	Pla-2	P2-393	FALSE	2	TRA:CIVRVYRAGNMLTF TRB:CASSERGTSGGAYTGELFF	2	CD4-C5
Pla-2_ACACCCTCATCTCGCT	Pla	Pla-2	P2-394	FALSE	2	TRA:CAAGAPGRAGRRALTF TRB:CASSPEAVYSGNTIYF	2	CD4-C3
Pla-2_ACACCCTTCATGCAAC	Pla	Pla-2	P2-3490	FALSE	1	TRA:CAVRDPADTGRRALTF TRB:CASSEMVAGGYEQYF	1	CD4-C5
Pla-2_ACAGCTAAGGTGACCA	Pla	Pla-2	P2-3501	FALSE	1	TRA:CAVEDYDYKLSF TRB:CASSEFRVSETQYF	1	CD4-C5
Pla-2_ACATCAGCATCGATGT	Pla	Pla-2	P2-3508	FALSE	1	TRA:CAAPQVEGTASKLTF TRB:CASSSGQGYFYEQYF	1	CD4-C3
Pla-2_ACATCAGGTTCCACGG	Pla	Pla-2	P2-3510	FALSE	1	TRA:CGRQMLLFTTVLWRPLGTSGTYKYIF TRB:CASSLGLSYE[illegible]	1	CD8-C4
Pla-2_ACATGGTAGAGTTGGC	Pla	Pla-2	P2-61	TRUE	3	TRA:CAVSFITQGGSEKLVF TRB:CASSRDRGTYNEQFF	9	CD4-C5
Pla-2_ACCAGTAAGACCGGAT	Pla	Pla-2	P2-3516	FALSE	1	TRA:CAENTPGNQFYF TRB:CSAAKITGTGTTF	1	CD4-C5
Pla-2_ACCAGTAAGACTAGGC	Pla	Pla-2	P2-3517	FALSE	1	TRA:CAVVSNTGNQFYF TRB:CASSYSDGYTGELFF	1	CD4-C5
Pla-2_ACCCACTCACCTTGTC	Pla	Pla-2	P2-112	FALSE	3	TRA:CAVRFSGNTGKLIF TRB:CSVGDAGTEAFF	5	CD4-C5
Pla-2_ACCTTTACATCGGACC	Pla	Pla-2	P2-3531	FALSE	1	TRA:CAASNSGNTPLVF TRB:CASSPPALGLAGTDTQYF	1	CD4-C5
Pla-2_ACCTTTAGTCGCTTCT	Pla	Pla-2	P2-3532	FALSE	1	TRA:CALKEPGNTGKLIF TRB:CASNKAGIEEKLFF	1	CD4-C5
Pla-2_ACCTTTAGTGCTAGCC	Pla	Pla-2	P2-3533	FALSE	1	TRA:CAFMKHNNNDMRF TRB:CAWSVLSGRLEAFF	1	CD4-C5
Pla-2_ACGAGCCAGGAGTTTA	Pla	Pla-2	P2-3536	FALSE	1	TRA:CADFSGNTPLVF TRB:CASSLEGRRNTEAFF	1	CD4-C3
Pla-2_ACGAGCCAGGGTCGAT	Pla	Pla-2	P2-3537	FALSE	1	TRA:CAVGPWGGGNKLTF TRB:CASKGEMNTEAFF	1	CD4-C5
Pla-2_ACGAGGATCTGTACGA	Pla	Pla-2	P2-3543	FALSE	1	TRA:CAMSDSGGSNYKLTF TRB:CASRRDMASGEKLFF	1	CD4-C5
Pla-2_ACGATACGTAAACGCG	Pla	Pla-2	P2-18	TRUE	3	TRA:CAATSSSNFGNEKLTF TRB:CAWSRAALTEAFF	28	CD4-C5
Pla-2_ACGATGTAGGAATGGA	Pla	Pla-2	P2-3548	FALSE	1	TRA:CAVGSSNDYKLSF TRB:CASSGTGNYGYTF	1	CD4-C4
Pla-2_ACGATGTTCAGTACGT	Pla	Pla-2	P2-3552	FALSE	1	TRA:CAVHGNTGKLIF TRB:CASSQDRHRGASTDTQYF	1	CD4-C4
Pla-2_ACGCAGCCAGCTGTTA	Pla	Pla-2	P2-403	FALSE	2	TRA:CIAFAGNKLTF TRB:CASNLGVSEAFF	2	CD4-C3
Pla-2_ACGCAGCGTACAGTGG	Pla	Pla-2	P2-97	TRUE	3	TRA:CALLQGGSYIPTF TRB:CASSKTGGTDTQYF	5	CD4-C5
Pla-2_ACGCCAGGTCAGAAGC	Pla	Pla-2	P2-3563	FALSE	1	TRA:CAVRLTQGGSEKLVF TRB:CASSISDRSFQPQHF	1	CD4-C5

Pla-2_ACGGCCAAGAGAACAG	Pla	Pla-2	P2-50	FALSE	3	TRA:CAPIRAQKLVF TRB:CASSSPPKSGELFF	11	CD4-C5
Pla-2_ACGGCCACAGGAATGC	Pla	Pla-2	P2-76	TRUE	3	TRA:CAGQEQGGSEKLVF TRB:CASSQMGLYGYTF	7	CD4-C5
Pla-2_ACGGGTCAGATTACCC	Pla	Pla-2	P2-3576	FALSE	1	TRA:CVVSDMNTGFQKLVF TRB:CASSQDRTQAYGYTF	1	CD4-C4
Pla-2_ACTATCTAGCCCTAAT	Pla	Pla-2	P2-393	FALSE	2	TRA:CIVRVYRAGNMLTF TRB:CASSERGTSGGAYTGELFF	2	CD8-C3
Pla-2_ACTGAACAGTAGGCCA	Pla	Pla-2	P2-146	FALSE	3	TRA:CAMTPGANSKLTF TRB:CASSSPPKSGELFF	4	CD4-C5
Pla-2_ACTGAACTCAGCTGGC	Pla	Pla-2	P2-3580	FALSE	1	TRA:CAMREGDFGNEKLTF TRB:CSATPGTFIYEQYF	1	CD4-C5
Pla-2_ACTGAGTAGGAATTAC	Pla	Pla-2	P2-3586	FALSE	1	TRA:CAVEDPNRDDKIIF TRB:CASSSTETGTQYF	1	CD4-C5
Pla-2_ACTGAGTAGGCTAGGT	Pla	Pla-2	P2-42	FALSE	3	TRA:CAVSPRDNNDMRF TRB:CASSARGNSNQPQHF	12	CD4-C5
Pla-2_ACTGAGTCATCCGTGG	Pla	Pla-2	P2-3588	FALSE	1	TRA:CAVSSNTPLVF TRB:CAISMGQDQETQYF	1	CD4-C5
Pla-2_ACTGAGTTCGATCCCT	Pla	Pla-2	P2-409	FALSE	2	TRA:CAPDTGNQFYF TRB:CAWSSGQGIYNEQFF	2	CD4-C3
Pla-2_ACTGTCCTCGATAGAA	Pla	Pla-2	P2-211	FALSE	3	TRA:CAESNSGAGSYQLTF TRB:CASRQQGAREKLFF	3	CD4-C5
Pla-2_AGAATAGAGTACGACG	Pla	Pla-2	P2-3612	FALSE	1	TRA:CAEDTGRRALTF TRB:CASSVAGEVRGTDTQYF	1	CD4-C5
Pla-2_AGACGTTAGCTGAAAT	Pla	Pla-2	P2-405	FALSE	2	TRA:CAYRKTSYDKVIF TRB:CASSILRGELFF	2	CD4-C5
Pla-2_AGACGTTCACCGAATT	Pla	Pla-2	P2-417	FALSE	2	TRA:CAALGGSNFGNEKLTF TRB:CASSPGTFGYTF	2	CD4-C5
Pla-2_AGAGCTTTCATCATTC	Pla	Pla-2	P2-221	FALSE	3	TRA:CASSGNTGKLIF TRB:CASSTRPGGTDTQYF	3	CD4-C5
Pla-2_AGAGTGGTCATCGCTC	Pla	Pla-2	P2-3622	FALSE	1	TRA:CAVSGTNTGNQFYF TRB:CASSSIGGELYEQYF	1	CD4-C5
Pla-2_AGATCTGCAAACGTGG	Pla	Pla-2	P2-36	FALSE	3	TRA:CAAQAGNTPLVF TRB:CSAGVGGTIQYF	15	CD4-C5
Pla-2_AGCCTAACAGGGATTG	Pla	Pla-2	P2-3643	FALSE	1	TRA:CAVALSSYNTDKLIF TRB:CASSFGTGGGGNQPQHF	1	CD4-C5
Pla-2_AGCCTAAGTCCAAGTT	Pla	Pla-2	P2-426	FALSE	2	TRA:CVVRESGAGSYQLTF TRB:CASSRGTGYTF	2	CD4-C5
Pla-2_AGCGTCGGTCGAGTTT	Pla	Pla-2	P2-3659	FALSE	1	TRA:CAVGTDTGRRALTF TRB:CASSGERDSDTQYF	1	CD4-C5
Pla-2_AGCTCCTCATCACGAT	Pla	Pla-2	P2-3663	FALSE	1	TRA:CAVTRGAGNMLTF TRB:CASSPKWGSRSYNEQFF	1	CD4-C5
Pla-2_AGCTTGAGTAGTACCT	Pla	Pla-2	P2-3668	FALSE	1	TRA:CATHSWGLTGGGNKLTF TRB:CASRSGYNEQFF	1	CD4-C5
Pla-2_AGCTTGAGTGACAAAT	Pla	Pla-2	P2-3670	FALSE	1	TRA:CAVRGASSGGSYIPTF TRB:CASSFLTGAARNEQFF	1	CD4-C5
Pla-2_AGCTTGATCTTGTATC	Pla	Pla-2	P2-3671	FALSE	1	TRA:CAVSLYAGNNRKLIW TRB:CAWSVGGSANYGYTF	1	CD4-C5
Pla-2_AGGCCACGTCTCTCGT	Pla	Pla-2	P2-3674	FALSE	1	TRA:CALLQGGSYIPTF TRB:CASSRTGGTDTQYF	1	CD4-C5
Pla-2_AGGCCGTAGTTAACGA	Pla	Pla-2	P2-3680	FALSE	1	TRA:CAVNGNARLMF TRB:CASSLASGGGNTIYF	1	CD4-C5
Pla-2_AGGCCGTGTTGGGACA	Pla	Pla-2	P2-231	FALSE	3	TRA:CAVWYSGGGADGLTF TRB:CASSQESQREIYEQYF	3	CD4-C5

Pla-2_AGGGAGTAGAGTAAGG	Pla	Pla-2	P2-221	FALSE	3	TRA:CASSGNTGKLIF TRB:CASSTRPGGTDTQYF	3	CD4-C5
Pla-2_AGGGATGCAGCCTTGG	Pla	Pla-2	P2-116	FALSE	3	TRA:CAVSPPAGSNYQLIW TRB:CATSRETGARYEQYF	5	CD4-C5
Pla-2_AGGGTGACACCAGCAC	Pla	Pla-2	P2-3690	FALSE	1	TRA:CAPFMDSNYQLIW TRB:CASSSRTSLRGETQYF	1	CD4-C4
Pla-2_AGGTCATGTAGAAGGA	Pla	Pla-2	P2-18	TRUE	3	TRA:CAATSSSNFGNEKLTF TRB:CAWSRAALTEAFF	28	CD4-C4
Pla-2_AGGTCCGCACGTAAGG	Pla	Pla-2	P2-434	FALSE	2	TRA:CAVEDSGSGNTGKLIF TRB:CASSLGSGGTGELFF	2	CD4-C5
Pla-2_AGTAGTCAGTAGGCCA	Pla	Pla-2	P2-151	FALSE	3	TRA:CAVRFGGNQFYF TRB:CASSLAGTQNEQFF	4	CD4-C6
Pla-2_AGTCTTTCATGATCCA	Pla	Pla-2	P2-38	TRUE	3	TRA:CAVRDGSDYKLSF TRB:CASKADLNNQPQHF	14	CD4-C5
Pla-2_AGTGAGGGTACGCTGC	Pla	Pla-2	P2-3703	FALSE	1	TRA:CALSFRPLIF TRB:CASSTSGGAETQYF	1	CD4-C5
Pla-2_AGTGGGAAGGCGTACA	Pla	Pla-2	P2-3706	FALSE	1	TRA:CAVRDRGEGGKLIF TRB:CASSLSGIYEQYF	1	CD4-C5
Pla-2_AGTGGGACAAGGTTCT	Pla	Pla-2	P2-3709	FALSE	1	TRA:CAMREGINSGAGSYQLTF TRB:CASASGELYGYTF	1	CD4-C5
Pla-2_AGTGTCAGTACAGACG	Pla	Pla-2	P2-3712	FALSE	1	TRA:CALRRGSARQLTF TRB:CASSEVGTDTQYF	1	CD4-C3
Pla-2_ATAACGCCAGCCTGTG	Pla	Pla-2	P2-3716	FALSE	1	TRA:CAVETKTSYDKVIF TRB:CASSWGQSGYTF	1	CD4-C5
Pla-2_ATAACGCTCAACGAAA	Pla	Pla-2	P2-33	TRUE	3	TRA:CAFKLSDGQKLLF TRB:CAWSRAALTEAFF	17	CD4-C5
Pla-2_ATAAGAGCACTGCCAG	Pla	Pla-2	P2-3719	FALSE	1	TRA:CAVSDPNNNARLMF TRB:CASSQEGFTQYF	1	CD4-C5
Pla-2_ATAGACCTCTCCCTGA	Pla	Pla-2	P2-3725	FALSE	1	TRA:CGTEINNDMRF TRB:CASSIGTRRGTDTQYF	1	CD4-C5
Pla-2_ATCACGAGTTACGGAG	Pla	Pla-2	P2-145	FALSE	3	TRA:CAMRRSSGGSNYKLTF TRB:CASSSIGRGSKRETQYF	4	CD4-C6
Pla-2_ATCATCTCAGGGTACA	Pla	Pla-2	P2-3727	FALSE	1	TRA:CAMREDNSGNTPLVF TRB:CASSPGQEAGANVLTF	1	CD4-C5
Pla-2_ATCATGGTCAACCATG	Pla	Pla-2	P2-441	FALSE	2	TRA:CAASETGANSKLTF TRB:CASSFSRPGGMNTEAFF	2	CD4-C5
Pla-2_ATCATGGTCGAACGGA	Pla	Pla-2	P2-20	TRUE	3	TRA:CAYDSNYQLIW TRB:CASSLSIDRADTGELFF	25	CD4-C5
Pla-2_ATCGAGTAGCTCTCGG	Pla	Pla-2	P2-3735	FALSE	1	TRA:CAPPFGGSEKLVF TRB:CASSEGSGQVNIQYF	1	CD4-C5
Pla-2_ATCGAGTGTCTACCTC	Pla	Pla-2	P2-3738	FALSE	1	TRA:CALGKTGRRALTF TRB:CAWSSLGENTEAFF	1	CD4-C5
Pla-2_ATCTACTAGGTAAACT	Pla	Pla-2	P2-151	FALSE	3	TRA:CAVRFGGNQFYF TRB:CASSLAGTQNEQFF	4	CD4-C5
Pla-2_ATGAGGGAGCCAGTAG	Pla	Pla-2	P2-3744	FALSE	1	TRA:CALSELTNSGGYNKLIF TRB:CASSLEGTNYGYTF	1	CD4-C2
Pla-2_ATGTGTGTCTTGCCGT	Pla	Pla-2	P2-445	FALSE	2	TRA:CAVPPKGGNNRLAF TRB:CASTSFSNQPQHF	2	CD4-C5
Pla-2_ATTATCCAGGATCGCA	Pla	Pla-2	P2-3756	FALSE	1	TRA:CAVSGGSQGNLIF TRB:CASSDRANYGYTF	1	CD4-C5
Pla-2_ATTGGACTCACGATGT	Pla	Pla-2	P2-3765	FALSE	1	TRA:CALNPYGGSQGNLIF TRB:CASNTGTTNTEAFF	1	CD4-C5
Pla-2_ATTGGTGGTCAGAGGT	Pla	Pla-2	P2-3771	FALSE	1	TRA:CAYRSAAGNTPLVF TRB:CASSEMLLGEQYF	1	CD4-C6

Pla-2_ATTGGTGTCTTGAGAC	Pla	Pla-2	P2-3773	FALSE	1	TRA:CAVRSRDDKIIF TRB:CASSGGGGLSGYTF	1	CD4-C5
Pla-2_ATTTCTGTCAACGGCC	Pla	Pla-2	P2-33	TRUE	3	TRA:CAFKLSDGQKLLF TRB:CAWSRAALTEAFF	17	CD4-C5
Pla-2_CAACCTCTCTATCGCC	Pla	Pla-2	P2-449	FALSE	2	TRA:CAVQISGSARQLTF TRB:CASSASGMTYNEQFF	2	CD4-C5
Pla-2_CAACTAGAGTCAAGCG	Pla	Pla-2	P2-3786	FALSE	1	TRA:CALSKEGNQFYF TRB:CASSPRSGTGELFF	1	CD4-C5
Pla-2_CAAGAAATCTTGTATC	Pla	Pla-2	P2-155	FALSE	3	TRA:CAGGRSNDYKLSF TRB:CASSGGAGGPGNEQFF	4	CD4-C6
Pla-2_CAAGATCCACCTATCC	Pla	Pla-2	P2-454	FALSE	2	TRA:CAVDSFNTDKLIF TRB:CAWSVLSGRLEAFF	2	CD4-C5
Pla-2_CAAGTTGTCTCTAAGG	Pla	Pla-2	P2-32	TRUE	3	TRA:CAMREANVGADGLTF TRB:CASSTRIAGGPFPETQYF	17	CD4-C5
Pla-2_CACAAACCACAAGTAA	Pla	Pla-2	P2-3806	FALSE	1	TRA:CAVTRGGSARQLTF TRB:CASSLTQDEQYF	1	CD8-C2
Pla-2_CACAAACGTGGTCTCG	Pla	Pla-2	P2-3808	FALSE	1	TRA:CAASIDVADSWGKFQF TRB:CASSRDGYEQYF	1	CD4-C5
Pla-2_CACAAACTCTCGCATC	Pla	Pla-2	P2-3810	FALSE	1	TRA:CAVSFTGGGNKLTF TRB:CASNPRGRELAGELFF	1	CD4-C5
Pla-2_CACACAATCTAGCACA	Pla	Pla-2	P2-3814	FALSE	1	TRA:CAMREANVGADGLTF TRB:CASRTTWTDNQPQHF	1	CD4-C5
Pla-2_CACACCTGTCAGTGGA	Pla	Pla-2	P2-32	TRUE	3	TRA:CAMREANVGADGLTF TRB:CASSTRIAGGPFPETQYF	17	CD4-C5
Pla-2_CACACCTGTCTTGATG	Pla	Pla-2	P2-3817	FALSE	1	TRA:CAVLQAGTALIF TRB:CASSLEGRTIYF	1	CD4-C4
Pla-2_CACAGGCGTTGGTAAA	Pla	Pla-2	P2-236	FALSE	3	TRA:CALPPPTGTASKLTF TRB:CASTGPSYEQYF	3	CD4-C5
Pla-2_CACAGTACAAGTTCTG	Pla	Pla-2	P2-3826	FALSE	1	TRA:CAMRGEAGFQKLVF TRB:CASRGEGSISGNTIYF	1	CD4-C5
Pla-2_CACAGTATCTCGTATT	Pla	Pla-2	P2-349	TRUE	2	TRA:CAMRDRGANSKLTF TRB:CSASSSSGGANYIGELFF	2	CD4-C5
Pla-2_CACATAGAGCCGATTT	Pla	Pla-2	P2-3828	FALSE	1	TRA:CAASKGNTPLVF TRB:CASSLTSAYFEALHF	1	CD4-C5
Pla-2_CACATAGAGCTGCAAG	Pla	Pla-2	P2-461	FALSE	2	TRA:CAVPAGTALIF TRB:CASSLSGAYQETQYF	2	CD4-C5
Pla-2_CACATAGCATATGCTG	Pla	Pla-2	P2-3829	FALSE	1	TRA:CVVRESGAGSYQLTF TRB:CASSQDALAGSYEQYF	1	CD8-C2
Pla-2_CACATAGTCCCAAGTA	Pla	Pla-2	P2-3830	FALSE	1	TRA:CALRGNTGGFKTIF TRB:CAISELSSGELFF	1	CD4-C5
Pla-2_CACATTTAGCGGCTTC	Pla	Pla-2	P2-64	FALSE	3	TRA:CAATSLISDGQKLLF TRB:CSARDSFNYGYTF	9	CD4-C5
Pla-2_CACCACTAGCGTAGTG	Pla	Pla-2	P2-3838	FALSE	1	TRA:CAATLQGAQKLVF TRB:CASSLRLLAGGTNEQFF	1	CD4-C5
Pla-2_CACCACTAGGGAACGG	Pla	Pla-2	P2-451	FALSE	2	TRA:CAAGLSGRSYQLTF TRB:CASSSPNTGELFF	2	CD4-C5
Pla-2_CACCACTCAGCGTAAG	Pla	Pla-2	P2-186	TRUE	3	TRA:CAVRADTNAGKSTF TRB:CASSKVGTEAFF	3	CD4-C5
Pla-2_CACCAGGAGAAGCCCA	Pla	Pla-2	P2-3839	FALSE	1	TRA:CAMRDLLGRARLTF TRB:CASSVDPSQPQHF	1	CD4-C5
Pla-2_CACCTTGAGGCAGTCA	Pla	Pla-2	P2-3844	FALSE	1	TRA:CAVEDFSSGSARQLTF TRB:CASSGTGAVNEKLFF	1	CD4-C5
Pla-2_CACTCCATCTGTGCAA	Pla	Pla-2	P2-32	TRUE	3	TRA:CAMREANVGADGLTF TRB:CASSTRIAGGPFPETQYF	17	CD4-C5

Pla-2_CAGAATCTCTGTCCGT	Pla	Pla-2	P2-3850	FALSE	1	TRA:CAGQPFSGSARQLTF TRB:CASSPPQGGIRKLFF	1	CD4-C5
Pla-2_CAGCGACTCGGCGGTT	Pla	Pla-2	P2-1	TRUE	3	TRA:CVVNIIGYQKVTF TRB:CASTRQGKQPQHF	270	CD4-C5
Pla-2_CAGCTGGTCACTGGGC	Pla	Pla-2	P2-3879	FALSE	1	TRA:CVVPDGFQKLVF TRB:CASSLRSSEAEQFF	1	CD4-C5
Pla-2_CAGGTGCGTTCCCTTG	Pla	Pla-2	P2-3883	FALSE	1	TRA:CAFFGNEKLTF TRB:CAIGRPRTDLTDTQYF	1	CD4-C5
Pla-2_CAGGTGCTCTGCTTGC	Pla	Pla-2	P2-151	FALSE	3	TRA:CAVRFGGNQFYF TRB:CASSLAGTQNEQFF	4	CD4-C5
Pla-2_CAGTAACAGGTAAACT	Pla	Pla-2	P2-3884	FALSE	1	TRA:CAMRASGGWYDMRF TRB:CASSLKGATISGANVLTF	1	CD4-C5
Pla-2_CAGTAACGTCCGAATT	Pla	Pla-2	P2-3885	FALSE	1	TRA:CAVNPGGYQKVTF TRB:CASSPWDRNSPLHF	1	CD4-C5
Pla-2_CAGTCCTCATGCCCGA	Pla	Pla-2	P2-239	FALSE	3	TRA:CAVYNAGNMLTF TRB:CATTGSLHNEQFF	3	CD4-C5
Pla-2_CATATGGCAAGCTGGA	Pla	Pla-2	P2-3892	FALSE	1	TRA:CALSERTDNAGNMLTF TRB:CASSAGKRDIGDTQYF	1	CD4-C5
Pla-2_CATATGGTCCGGCACA	Pla	Pla-2	P2-3893	FALSE	1	TRA:CAMRERNRDDKIIF TRB:CASSQTSGGADNEQFF	1	CD4-C4
Pla-2_CATATTCTCGTGGTCG	Pla	Pla-2	P2-3899	FALSE	1	TRA:CAVLLGYGNKLVF TRB:CASSSTGGAGELFF	1	CD4-C5
Pla-2_CATCAGAAGGAGCGTT	Pla	Pla-2	P2-169	TRUE	3	TRA:CGARNNARLMF TRB:CASSRLGAANTGELFF	3	CD4-C5
Pla-2_CATCCACCACCAACCG	Pla	Pla-2	P2-3905	FALSE	1	TRA:CAFMEGFGNVLHC TRB:CASSLAGGSYNEQFF	1	CD4-C5
Pla-2_CATCGAAAGCTATGCT	Pla	Pla-2	P2-3906	FALSE	1	TRA:CAVQATGANNLFF TRB:CATSRDHGQAPIQYF	1	CD4-C5
Pla-2_CATCGAAGTGTTCGAT	Pla	Pla-2	P2-36	FALSE	3	TRA:CAAQAGNTPLVF TRB:CSAGVGGTIQYF	15	CD4-C5
Pla-2_CATCGGGTCGATGAGG	Pla	Pla-2	P2-18	TRUE	3	TRA:CAATSSSNFGNEKLTF TRB:CAWSRAALTEAFF	28	CD4-C5
Pla-2_CATGCCTGTTCAACCA	Pla	Pla-2	P2-3920	FALSE	1	TRA:CAVERGGSNYKLTF TRB:CASSRLRDTGELFF	1	CD8-C3
Pla-2_CATGGCGAGTCGATAA	Pla	Pla-2	P2-3922	FALSE	1	TRA:CAVQEIGSGNTGKLIF TRB:CASSRDSFYGYTF	1	CD4-C5
Pla-2_CATTATCAGCACAGGT	Pla	Pla-2	P2-3926	FALSE	1	TRA:CALTLYGGQNFVF TRB:CASSDEGQGGYNEQFF	1	CD4-C5
Pla-2_CATTCGCGTCCGAACC	Pla	Pla-2	P2-3933	FALSE	1	TRA:CAVQAGKFYF TRB:CASTGQPSLSGNTIYF	1	CD4-C5
Pla-2_CCACCTACAGACGCCT	Pla	Pla-2	P2-3943	FALSE	1	TRA:CVVSDPTGTASKLTF TRB:CASSQALSPSNQPQHF	1	CD4-C5
Pla-2_CCAGCGACAGGTCTCG	Pla	Pla-2	P2-3952	FALSE	1	TRA:CAASLAGNQFYF TRB:CASRPRAAGAADTQYF	1	CD4-C5
Pla-2_CCATGTCAGCGCCTCA	Pla	Pla-2	P2-33	TRUE	3	TRA:CAFKLSDGQKLLF TRB:CAWSRAALTEAFF	17	CD4-C5
Pla-2_CCATGTCCACATTTCT	Pla	Pla-2	P2-3955	FALSE	1	TRA:CAVGGTSYDKVIF TRB:CASSLNRGTNEKLFF	1	CD4-C5
Pla-2_CCCAATCAGGATATAC	Pla	Pla-2	P2-38	TRUE	3	TRA:CAVRDGSDYKLSF TRB:CASKADLNNQPQHF	14	CD4-C5
Pla-2_CCCAATCTCTTCATGT	Pla	Pla-2	P2-380	FALSE	2	TRA:CAVSEETDSWGKFQF TRB:CATSRDRGRSYGYTF	2	CD4-C5
Pla-2_CCCAGTTCACAAGTAA	Pla	Pla-2	P2-3963	FALSE	1	TRA:CAVGGNNYGQNFVF TRB:CASSLVPERQFF	1	CD4-C4

Pla-2_CCCTCCTAGGCCGAAT	Pla	Pla-2	P2-3969	FALSE	1	TRA:CAVSSIQGAQKLVF TRB:CASSLGKGLAETQYF	1	CD4-C5
Pla-2_CCGTGGATCGTCCGTT	Pla	Pla-2	P2-243	FALSE	3	TRA:CALYSGNTPLVF TRB:CASNTDRVLEQYF	3	CD4-C5
Pla-2_CCGTTCAAGACTACAA	Pla	Pla-2	P2-3980	FALSE	1	TRA:CATVRRSGTYKYIF TRB:CASSYSNTAQETQYF	1	CD4-C5
Pla-2_CCGTTCAGTTACGTCA	Pla	Pla-2	P2-485	FALSE	2	TRA:CALTPTGGGNKLTF TRB:CASSIRQRPGELFF	2	CD8-C2
Pla-2_CCTATTAGTTTACTCT	Pla	Pla-2	P2-3997	FALSE	1	TRA:CALYEYMLTF TRB:CASTTAGGLYNEQFF	1	CD4-C5
Pla-2_CCTCAGTGTCTCAACA	Pla	Pla-2	P2-491	FALSE	2	TRA:CAVGGTGGFKTIF TRB:CASSPEGPGALGGYTF	2	CD4-C6
Pla-2_CCTCAGTGTTAAGGGC	Pla	Pla-2	P2-204	TRUE	3	TRA:CAVGRRGSGGSNYKLTF TRB:CASQQGYSGNTIYF	3	CD4-C5
Pla-2_CCTCAGTTCTCGTATT	Pla	Pla-2	P2-4000	FALSE	1	TRA:CAMREASYGNNRLAF TRB:CASSLSGSYNEQFF	1	CD4-C5
Pla-2_CCTTCCCAGACTGTAA	Pla	Pla-2	P2-92	FALSE	3	TRA:CAMREGRAANNLFF TRB:CAWSVRGWTRRMSSTDTQY	6	CD4-C5
Pla-2_CCTTCGATCTCATTCA	Pla	Pla-2	P2-4020	FALSE	1	TRA:CAARNQGAQKLVF TRB:CASSRELAGAHETQYF	1	CD4-C5
Pla-2_CGAATGTAGACTAAGT	Pla	Pla-2	P2-18	TRUE	3	TRA:CAATSSSNFGNEKLTF TRB:CAWSRAALTEAFF	28	CD4-C4
Pla-2_CGACTTCCAATAAGCA	Pla	Pla-2	P2-50	FALSE	3	TRA:CAPIRAQKLVF TRB:CASSSPPKSGELFF	11	CD4-C5
Pla-2_CGATCGGAGCAATATG	Pla	Pla-2	P2-112	FALSE	3	TRA:CAVRFSGNTGKLIF TRB:CSVGDAGTEAFF	5	CD4-C5
Pla-2_CGATCGGTCAGTTCGA	Pla	Pla-2	P2-4057	FALSE	1	TRA:CASGGGADGLTF TRB:CASSLEEGSYGYTF	1	CD4-C5
Pla-2_CGCCAAGTCTGCAAGT	Pla	Pla-2	P2-4070	FALSE	1	TRA:CAFMKRSGGGADGLTF TRB:CASRTYTGELFF	1	CD4-C5
Pla-2_CGCTATCAGCCCTAAT	Pla	Pla-2	P2-4079	FALSE	1	TRA:CAVEDPGFQKLVF TRB:CSARWLAGAGSYEQYF	1	CD4-C5
Pla-2_CGCTATCGTAGTGAAT	Pla	Pla-2	P2-250	FALSE	3	TRA:CAENKEGSGGSNYKLTF TRB:CASSPPGTPVGRETQYF	3	CD4-C4
Pla-2_CGCTATCTCAGAGCTT	Pla	Pla-2	P2-4082	FALSE	1	TRA:CAGARGATNKLIF TRB:CASSFRTGLNEQFF	1	CD4-C5
Pla-2_CGCTGGAAGGTTCCTA	Pla	Pla-2	P2-501	FALSE	2	TRA:CAMEKGYSTLTF TRB:CSARAGQGSHSGANVLTF	2	CD4-C3
Pla-2_CGCTTCAAGTGTACTC	Pla	Pla-2	P2-4087	FALSE	1	TRA:CAVEMDSSYKLIF TRB:CASSEQGGGNQPQHF	1	CD4-C5
Pla-2_CGCTTCAGTAGAGCTG	Pla	Pla-2	P2-4091	FALSE	1	TRA:CAVRFGGNQFYF TRB:CASSLLPSEGEQFF	1	CD4-C5
Pla-2_CGGACACAGTTAAGTG	Pla	Pla-2	P2-4096	FALSE	1	TRA:CAVTRMEYGNKLVF TRB:CASSFLGGGDEQFF	1	CD4-C5
Pla-2_CGGACGTAGAGCCTAG	Pla	Pla-2	P2-4098	FALSE	1	TRA:CAASLSNSGYALNF TRB:CASSQGRVSLMNTEAFF	1	CD4-C5
Pla-2_CGGACGTGTGGCGAAT	Pla	Pla-2	P2-4100	FALSE	1	TRA:CAYQGNYKYIF TRB:CASSDGRAMNEQFF	1	CD4-C5
Pla-2_CGGACTGAGCGCCTTG	Pla	Pla-2	P2-239	FALSE	3	TRA:CAVYNAGNMLTF TRB:CATTGSLHNEQFF	3	CD4-C6
Pla-2_CGGACTGAGTGACATA	Pla	Pla-2	P2-4102	FALSE	1	TRA:CAEIETNAGGTSYGKLTF TRB:CASSPGLAGGSARF	1	CD8-C2
Pla-2_CGGAGCTCAAACCCAT	Pla	Pla-2	P2-4108	FALSE	1	TRA:CATAGRSSNDYKLSF TRB:CASTHGANQPQHF	1	CD4-C4

Pla-2_CGGAGCTGTACCGCTG	Pla	Pla-2	P2-4109	FALSE	1	TRA:CATDYSGGYQKVTF TRB:CASSQSGTAASKLFF	1	CD4-C5
Pla-2_CGGTTAAAGTTGTCGT	Pla	Pla-2	P2-33	TRUE	3	TRA:CAFKLSDGQKLLF TRB:CAWSRAALTEAFF	17	CD4-C5
Pla-2_CGGTTAAGTACCAGTT	Pla	Pla-2	P2-18	TRUE	3	TRA:CAATSSSNFGNEKLTF TRB:CAWSRAALTEAFF	28	CD4-C5
Pla-2_CGTAGCGAGTCCTCCT	Pla	Pla-2	P2-4125	FALSE	1	TRA:CAVITNAGKSTF TRB:CASSLGRTAFQETQYF	1	CD4-C4
Pla-2_CGTAGGCCATCTATGG	Pla	Pla-2	P2-4129	FALSE	1	TRA:CAVEDNAGNTPLVF TRB:CASSYSGEETQYF	1	CD4-C5
Pla-2_CGTCACTCAGCCAATT	Pla	Pla-2	P2-101	TRUE	3	TRA:CATDASGNEKLTF TRB:CASSLGLADTQYF	5	CD4-C5
Pla-2_CGTCCATAGCACCGTC	Pla	Pla-2	P2-4140	FALSE	1	TRA:CAVQAGAGSYQLTF TRB:CATSDGGPATQYF	1	CD4-C5
Pla-2_CGTCTACTCCGAACGC	Pla	Pla-2	P2-4142	FALSE	1	TRA:CAVNRAGNNRKLIW TRB:CSPSSGGYEQYF	1	CD4-C7
Pla-2_CGTCTACTCGCAGGCT	Pla	Pla-2	P2-4143	FALSE	1	TRA:CAESQAGNMLTF TRB:CASSLGSGTEKLFF	1	CD4-C5
Pla-2_CGTGAGCAGGACAGCT	Pla	Pla-2	P2-213	FALSE	3	TRA:CAAAGNRDDKIIF TRB:CASSQSAETQYF	3	CD4-C5
Pla-2_CGTGTAAAGAGGACGG	Pla	Pla-2	P2-508	FALSE	2	TRA:CAMRDLTGRRALTF TRB:CASSPGLALSSYEQYF	2	CD4-C4
Pla-2_CGTTCTGAGGGTCGAT	Pla	Pla-2	P2-179	TRUE	3	TRA:CAVEDTDKLIF TRB:CASSRQGSDTQYF	3	CD4-C5
Pla-2_CGTTCTGGTCTTCGTC	Pla	Pla-2	P2-4156	FALSE	1	TRA:CATEHLGAGSYQLTF TRB:CASSFVGGGGFDTQYF	1	CD4-C4
Pla-2_CGTTCTGTCAGTACGT	Pla	Pla-2	P2-18	TRUE	3	TRA:CAATSSSNFGNEKLTF TRB:CAWSRAALTEAFF	28	CD4-C5
Pla-2_CTAACTTGTATCAGTC	Pla	Pla-2	P2-225	FALSE	3	TRA:CAVRDVGGRRALTF TRB:CASSNSGGTEAFF	3	CD4-C5
Pla-2_CTAACTTTCGTGGTCG	Pla	Pla-2	P2-4166	FALSE	1	TRA:CALINSYDKVIF TRB:CASSNPGTGSTDTQYF	1	CD4-C5
Pla-2_CTAAGACTCCTAAGTG	Pla	Pla-2	P2-50	FALSE	3	TRA:CAPIRAQKLVF TRB:CASSSPPKSGELFF	11	CD4-C5
Pla-2_CTACCCAAGAGTCTGG	Pla	Pla-2	P2-4174	FALSE	1	TRA:CAASDNTDKLIF TRB:CASSHGSGRGYEQFF	1	CD4-C5
Pla-2_CTACCCACACATGGGA	Pla	Pla-2	P2-4175	FALSE	1	TRA:CAVSEAGANSKLTF TRB:CASSYSIYGNQPQHF	1	CD4-C4
Pla-2_CTACCCAGTTATGCGT	Pla	Pla-2	P2-4176	FALSE	1	TRA:CAENILGGSQGNLIF TRB:CASSQQSGNQPQHF	1	CD4-C4
Pla-2_CTAGAGTCAGACGCTC	Pla	Pla-2	P2-4181	FALSE	1	TRA:CTRQSDGSNYKLTF TRB:CASSLDGLLNTGELFF	1	CD4-C3
Pla-2_CTAGCCTAGCTAACTC	Pla	Pla-2	P2-4184	FALSE	1	TRA:CAVRMDSSYKLIF TRB:CASSINDGSVPQHF	1	CD4-C5
Pla-2_CTAGTGAAGCTAGTGG	Pla	Pla-2	P2-516	FALSE	2	TRA:CASNRDDKIIF TRB:CASTHGQVNEQFF	2	CD4-C5
Pla-2_CTAGTGACAAAGGCGT	Pla	Pla-2	P2-4187	FALSE	1	TRA:CAMRETYSSASKIIF TRB:CASSQDVGQETQYF	1	CD4-C5
Pla-2_CTAGTGACACTAAGTC	Pla	Pla-2	P2-1	TRUE	3	TRA:CVVNIIGYQKVTF TRB:CASTRQGKQPQHF	270	CD4-C5
Pla-2_CTAGTGACAGCCTTTC	Pla	Pla-2	P2-4190	FALSE	1	TRA:CAVNPLESSYKLIF TRB:CASSQVHEQYF	1	CD4-C7
Pla-2_CTCAGAAGTACCGTTA	Pla	Pla-2	P2-64	FALSE	3	TRA:CAATSLISDGQKLLF TRB:CSARDSFNYGYTF	9	CD4-C5

Pla-2_CTCAGAAGTTATGTGC	Pla	Pla-2	P2-505	FALSE	2	TRA:CAVDNQGGKLIF TRB:CASRPGGGSYEQYF	2	CD4-C6
Pla-2_CTCGAGGCAGCTGTGC	Pla	Pla-2	P2-4210	FALSE	1	TRA:CAEIDPHTGANSKLTF TRB:CASSQDLVGTSSYEQYF	1	CD4-C5
Pla-2_CTCGGAGCAGCCACCA	Pla	Pla-2	P2-4212	FALSE	1	TRA:CATVNNAGKSTF TRB:CASSQDKGGYTF	1	CD4-C5
Pla-2_CTCGGGAGTCACCTAA	Pla	Pla-2	P2-521	FALSE	2	TRA:CAVEDRLGTGGFKTIF TRB:CASSESFGELFF	2	CD4-C5
Pla-2_CTCGTACAGTTATCGC	Pla	Pla-2	P2-110	TRUE	3	TRA:CAVSTSGTYKYIF TRB:CASSSSGGDNSPLHF	5	CD4-C5
Pla-2_CTCGTACTCTCAACTT	Pla	Pla-2	P2-4219	FALSE	1	TRA:CATDEVKRGAQKLVF TRB:CSAGRMGTGELFF	1	CD4-C5
Pla-2_CTCGTCAAGCTATGCT	Pla	Pla-2	P2-4220	FALSE	1	TRA:CAGLITASKLTF TRB:CATSRSGTSGEQYF	1	CD4-C5
Pla-2_CTCTGGTCATTATCTC	Pla	Pla-2	P2-377	FALSE	2	TRA:CAGGDYNQGGKLIF TRB:CASTLTQGALTGELFF	2	CD4-C5
Pla-2_CTGAAACAGTCTCCTC	Pla	Pla-2	P2-4230	FALSE	1	TRA:CIATLSSGNTPLVF TRB:CASSLGLGDTQYF	1	CD4-C5
Pla-2_CTGATCCCACATGTGT	Pla	Pla-2	P2-524	FALSE	2	TRA:CAVEGSGGYQKVTF TRB:CASSLQGSKLYNEQFF	2	CD4-C5
Pla-2_CTGCCTACATTCACTT	Pla	Pla-2	P2-4243	FALSE	1	TRA:CAVLDSNYRLIW TRB:CSALEAAGLTRGNQPQHF	1	CD4-C3
Pla-2_CTGCCTATCGGAATCT	Pla	Pla-2	P2-42	FALSE	3	TRA:CAVSPRDNNDMRF TRB:CASSARGNSNQPQHF	12	CD4-C5
Pla-2_CTGCTGTTCAACACGT	Pla	Pla-2	P2-204	TRUE	3	TRA:CAVGRRGSGGSNYKLTF TRB:CASQQGYSGNTIYF	3	CD4-C5
Pla-2_CTGCTGTTCTCTTGAT	Pla	Pla-2	P2-4249	FALSE	1	TRA:CVVSGNTGTASKLTF TRB:CASSPQGGTQYF	1	CD4-C5
Pla-2_CTGTTTAAGTGGTAGC	Pla	Pla-2	P2-4257	FALSE	1	TRA:CAVDASYSNYQLIW TRB:CASSEFTTLQETQYF	1	CD4-C5
Pla-2_CTTAACTAGTTGAGTA	Pla	Pla-2	P2-4261	FALSE	1	TRA:CAMREVAGTASKLTF TRB:CASSSGPGRSYGYTF	1	CD4-C5
Pla-2_CTTCTCTCACTGTCGG	Pla	Pla-2	P2-4273	FALSE	1	TRA:CAVSDPPIRSGGGADGLTF TRB:CASSPMSGEKLFF	1	CD4-C5
Pla-2_CTTGGCTAGAGAGCTC	Pla	Pla-2	P2-162	FALSE	3	TRA:CIVRAMNSGYSTLTF TRB:CSAPPWQGAYEQYF	4	CD4-C3
Pla-2_CTTGGCTCACTAGTAC	Pla	Pla-2	P2-4277	FALSE	1	TRA:CAGDAGGTSYGKLTF TRB:CASSRTGATEAFF	1	CD4-C5
Pla-2_CTTGGCTTCTGACCTC	Pla	Pla-2	P2-4281	FALSE	1	TRA:CAVSSDNAGNMLTF TRB:CASSPLIGELFF	1	CD4-C5
Pla-2_CTTTGCGGTCTAAAGA	Pla	Pla-2	P2-431	FALSE	2	TRA:CAMREGGRSQGNLIF TRB:CASSETTGGWEKLFF	2	CD4-C5
Pla-2_GAAACTCAGTACGACG	Pla	Pla-2	P2-4286	FALSE	1	TRA:CAVNVAGKLIF TRB:CASSDRGGEDTNEKLFF	1	CD4-C5
Pla-2_GAAATGAAGAACTCGG	Pla	Pla-2	P2-4292	FALSE	1	TRA:CASSGEETSGSRLTF TRB:CATSRGGNPFNEQFF	1	CD4-C6
Pla-2_GAAATGAAGGAGTTGC	Pla	Pla-2	P2-4293	FALSE	1	TRA:CAVSDRGAGNNRKLIW TRB:CATSRTGTAAPEAFF	1	CD4-C5
Pla-2_GAACGGAAGACCGGAT	Pla	Pla-2	P2-26	TRUE	3	TRA:CALVNTGGFKTIF TRB:CASSPPSSGGANEQYF	22	CD8-C3
Pla-2_GAACGGACACGGCTAC	Pla	Pla-2	P2-64	FALSE	3	TRA:CAATSLISDGQKLLF TRB:CSARDSFNYGYTF	9	CD4-C4
Pla-2_GAACGGATCAACCAAC	Pla	Pla-2	P2-4305	FALSE	1	TRA:CAVSEENSNSGYALNF TRB:CASSNGGVNYNEQFF	1	CD4-C5

Pla-2_GAACGGATCACATAGC	Pla	Pla-2	P2-4306	FALSE	1	TRA:CAMSEWASGGSYIPTF TRB:CASSQGVSLVPVNEQFF	1	CD4-C5
Pla-2_GAAGCAGAGACGACGT	Pla	Pla-2	P2-4308	FALSE	1	TRA:CAASNKGGGNKLTF TRB:CASSLWGQGGNEQFF	1	CD4-C5
Pla-2_GAATAAGCACGGCGTT	Pla	Pla-2	P2-4314	FALSE	1	TRA:CAVRDWGGAGNNRKLIW TRB:CASRTGLVTGELFF	1	CD4-C4
Pla-2_GAATGAAAGCGTAATA	Pla	Pla-2	P2-4321	FALSE	1	TRA:CAARSSAGGSNYKLTF TRB:CAISEGQPYGYTF	1	CD4-C5
Pla-2_GAATGAAGTTCCTCCA	Pla	Pla-2	P2-18	TRUE	3	TRA:CAATSSSNFGNEKLTF TRB:CAWSRAALTEAFF	28	CD4-C5
Pla-2_GACACGCGTGCGCTTG	Pla	Pla-2	P2-4329	FALSE	1	TRA:CAVSPNTDKLIF TRB:CASSSGQIYGYTF	1	CD4-C5
Pla-2_GACACGCGTTAAGATG	Pla	Pla-2	P2-4330	FALSE	1	TRA:CIVLMNRDDKIIF TRB:CSALEAAGLTRGNQPQHF	1	CD4-C5
Pla-2_GACCAATGTCATGCCG	Pla	Pla-2	P2-4335	FALSE	1	TRA:CATAMEYGNKLVF TRB:CASSQDFVWTSVYEQYF	1	CD4-C5
Pla-2_GACCTGGAGTGAACGC	Pla	Pla-2	P2-155	FALSE	3	TRA:CAGGRSNDYKLSF TRB:CASSGGAGGPGNEQFF	4	CD4-C5
Pla-2_GACCTGGTCGAACTGT	Pla	Pla-2	P2-61	TRUE	3	TRA:CAVSFITQGGSEKLVF TRB:CASSRDRGTYNEQFF	9	CD4-C5
Pla-2_GACGCGTGTCTCCACT	Pla	Pla-2	P2-454	FALSE	2	TRA:CAVDSFNTDKLIF TRB:CAWSVLSGRLEAFF	2	CD4-C5
Pla-2_GACGCGTTCACATGCA	Pla	Pla-2	P2-4338	FALSE	1	TRA:CAASMRSGYALNF TRB:CASAEGGPLDEQYF	1	CD4-C5
Pla-2_GACGTGCGTAGTAGTA	Pla	Pla-2	P2-4346	FALSE	1	TRA:CAMGVGGGTSYGKLTF TRB:CASTGGLSSYEQYF	1	CD4-C5
Pla-2_GACGTGCGTCGCGTGT	Pla	Pla-2	P2-4347	FALSE	1	TRA:CAVTRNTGRRALTF TRB:CASSPDGQGGDTQYF	1	CD4-C2
Pla-2_GACTACAAGCCTATGT	Pla	Pla-2	P2-4353	FALSE	1	TRA:CAMSVGGGFKTIF TRB:CASSSRDRGETQYF	1	CD4-C5
Pla-2_GACTACATCCCACTTG	Pla	Pla-2	P2-4359	FALSE	1	TRA:CAARPDNFNKFYF TRB:CSAAQGRLNEQFF	1	CD4-C5
Pla-2_GATCAGTAGTTACGGG	Pla	Pla-2	P2-543	FALSE	2	TRA:CAMSARGGAGNMLTF TRB:CASSRGQGLYGYTF	2	CD4-C3
Pla-2_GATCAGTGTAGCAAAT	Pla	Pla-2	P2-4373	FALSE	1	TRA:CAVRDYNQGGKLIF TRB:CASSFAGGTEAFF	1	CD4-C4
Pla-2_GATCGCGAGTTGCAGG	Pla	Pla-2	P2-4378	FALSE	1	TRA:CALSEGGGYSTLTF TRB:CASSLRSMYNEQFF	1	CD4-C5
Pla-2_GATCGTAGTCTACCTC	Pla	Pla-2	P2-546	FALSE	2	TRA:CAVPGAQKLVF TRB:CASSEALLGSASNEKLFF	2	CD4-C5
Pla-2_GATCTAGCACGAAGCA	Pla	Pla-2	P2-92	FALSE	3	TRA:CAMREGRAANNLFF TRB:CAWSVRGWTRRMSSTDTQY	6	CD4-C2
Pla-2_GATGAGGGTAGCCTAT	Pla	Pla-2	P2-4388	FALSE	1	TRA:CAVSLSNTDKLIF TRB:CASSSRDPPYGYTF	1	CD4-C5
Pla-2_GATTCAGGTCAACTGT	Pla	Pla-2	P2-4394	FALSE	1	TRA:CAMFRVNDYKLSF TRB:CASSLDLGTRDSGYTF	1	CD4-C5
Pla-2_GATTCAGTCGGTTAAC	Pla	Pla-2	P2-4396	FALSE	1	TRA:CLVGDALTSGSARQLTF TRB:CASSQAGTGVRNIQYF	1	CD8-C2
Pla-2_GCAAACTGTGCCTGCA	Pla	Pla-2	P2-4398	FALSE	1	TRA:CAASRSDNAGNMLTF TRB:CASTRGGGANVLTF	1	CD4-C5
Pla-2_GCAAACTGTTTAAGCC	Pla	Pla-2	P2-76	TRUE	3	TRA:CAGQEQGGSEKLVF TRB:CASSQMGLYGYTF	7	CD4-C6
Pla-2_GCAATCAGTTGAACTC	Pla	Pla-2	P2-4401	FALSE	1	TRA:CAFMKDSGAGSYQLTF TRB:CASRRQGSNEQFF	1	CD4-C5

Pla-2_GCACTCTTCGCAAGCC	Pla	Pla-2	P2-4407	FALSE	1	TRA:CAVEAGNMLTF TRB:CASSQRTGGGAEAFF	1	CD4-C7
Pla-2_GCAGCCAGTACTCTCC	Pla	Pla-2	P2-231	FALSE	3	TRA:CAVWYSGGGADGLTF TRB:CASSQESQREIYEQYF	3	CD4-C5
Pla-2_GCAGCCAGTTCCAACA	Pla	Pla-2	P2-42	FALSE	3	TRA:CAVSPRDNNDMRF TRB:CASSARGNSNQPQHF	12	CD4-C5
Pla-2_GCATACACATTTGCTT	Pla	Pla-2	P2-112	FALSE	3	TRA:CAVRFSGNTGKLIF TRB:CSVGDAGTEAFF	5	CD4-C5
Pla-2_GCATGATTCGGTCCGA	Pla	Pla-2	P2-4424	FALSE	1	TRA:CALGPPYGGATNKLIF TRB:CSANYYEQFF	1	CD4-C5
Pla-2_GCATGTATCAGAAATG	Pla	Pla-2	P2-4431	FALSE	1	TRA:CALKRSGNNNDMRF TRB:CASRKDSPGEAFF	1	CD4-C5
Pla-2_GCCAAATAGAATGTTG	Pla	Pla-2	P2-497	FALSE	2	TRA:CAYRTPPNTPLVF TRB:CASSRTLGTSTDTQYF	2	CD4-C5
Pla-2_GCCAAATAGTCACGCC	Pla	Pla-2	P2-4434	FALSE	1	TRA:CVVPNTNAGKSTF TRB:CASSPPSSGGANEQFF	1	CD4-C5
Pla-2_GCCAAATTCTCTGTCG	Pla	Pla-2	P2-166	FALSE	3	TRA:CAVSWGTGYSTLTF TRB:CASSPDAGNTQYF	4	CD4-C5
Pla-2_GCCTCTATCAACTCTT	Pla	Pla-2	P2-548	FALSE	2	TRA:CAVRAARTGNNRLAF TRB:CSATTSGEYNEQFF	2	CD4-C5
Pla-2_GCCTCTATCACCCTCA	Pla	Pla-2	P2-4440	FALSE	1	TRA:CAYRSTYSGAGSYQLTF TRB:CSARAPGGGYNSPLHF	1	CD4-C5
Pla-2_GCCTCTATCCCATTAT	Pla	Pla-2	P2-534	FALSE	2	TRA:CAEIPPNRDDKIIF TRB:CASSQGSSGRAETQYF	2	CD4-C5
Pla-2_GCGAGAATCTGCTGTC	Pla	Pla-2	P2-437	FALSE	2	TRA:CALGPNTGNQFYF TRB:CASSLGPDTIFYEQYF	2	CD4-C5
Pla-2_GCGCAGTGTCGGATCC	Pla	Pla-2	P2-179	TRUE	3	TRA:CAVEDTDKLIF TRB:CASSRQGSDTQYF	3	CD4-C5
Pla-2_GCGCCAACAGCCAATT	Pla	Pla-2	P2-4454	FALSE	1	TRA:CAAGGYQKVTF TRB:CATSVGGLGDTQYF	1	CD4-C5
Pla-2_GCTCTGTAGGCTAGAC	Pla	Pla-2	P2-42	FALSE	3	TRA:CAVSPRDNNDMRF TRB:CASSARGNSNQPQHF	12	CD4-C6
Pla-2_GCTGCAGTCTTATCTG	Pla	Pla-2	P2-521	FALSE	2	TRA:CAVEDRLGTGGFKTIF TRB:CASSESFGELFF	2	CD4-C5
Pla-2_GCTGCGAGTAGGCTGA	Pla	Pla-2	P2-36	FALSE	3	TRA:CAAQAGNTPLVF TRB:CSAGVGGTIQYF	15	CD4-C5
Pla-2_GCTGCTTCAGGGAGAG	Pla	Pla-2	P2-243	FALSE	3	TRA:CALYSGNTPLVF TRB:CASNTDRVLEQYF	3	CD4-C5
Pla-2_GCTGGGTAGAGGGATA	Pla	Pla-2	P2-4478	FALSE	1	TRA:CAVSRTDGQKLLF TRB:CASSLAGSGDIQYF	1	CD4-C5
Pla-2_GCTGGGTTCGTAGGTT	Pla	Pla-2	P2-213	FALSE	3	TRA:CAAAGNRDDKIIF TRB:CASSQSAETQYF	3	CD4-C5
Pla-2_GCTTCCACAAGTTCTG	Pla	Pla-2	P2-18	TRUE	3	TRA:CAATSSSNFGNEKLTF TRB:CAWSRAALTEAFF	28	CD4-C5
Pla-2_GCTTCCAGTAGCTCCG	Pla	Pla-2	P2-4483	FALSE	1	TRA:CAARAGNNRKLIW TRB:CASSLREVFTEAFF	1	CD4-C5
Pla-2_GCTTCCATCCTGCCAT	Pla	Pla-2	P2-4487	FALSE	1	TRA:CAVASDTGGFKTIF TRB:CASSFSGESSPLHF	1	CD4-C6
Pla-2_GCTTGAAAGTTATCGC	Pla	Pla-2	P2-419	FALSE	2	TRA:CAVEDGTGGFKTIF TRB:CAISESRPGQGNTEAFF	2	CD4-C4
Pla-2_GCTTGAACAATTCCTT	Pla	Pla-2	P2-181	TRUE	3	TRA:CAVEWTDSSYKLIF TRB:CASRYGTTYEQYF	3	CD4-C5
Pla-2_GGAAAGCTCGTCACGG	Pla	Pla-2	P2-552	FALSE	2	TRA:CAGGRSGNTGKLIF TRB:CASGTTGSSYEQYF	2	CD4-C6

Pla-2_GGACAAGAGCCAGGAT	Pla	Pla-2	P2-36	FALSE	3	TRA:CAAQAGNTPLVF TRB:CSAGVGGTIQYF	15	CD4-C5
Pla-2_GGACAAGTCATAGCAC	Pla	Pla-2	P2-436	FALSE	2	TRA:CAAATSGTYKYIF TRB:CASLGRLGAGNTIYF	2	CD4-C3
Pla-2_GGACAGATCTATCCCG	Pla	Pla-2	P2-4507	FALSE	1	TRA:CVVSESTGGFKTIF TRB:CASSLEASGGYEQYF	1	CD4-C5
Pla-2_GGACGTCCATGCTGGC	Pla	Pla-2	P2-92	FALSE	3	TRA:CAMREGRAANNLFF TRB:CAWSVRGWTRRMSSTDTQY	6	CD4-C5
Pla-2_GGACGTCTCGTAGATC	Pla	Pla-2	P2-4515	FALSE	1	TRA:CAYRSSAGTASKLTF TRB:CSVESLGGDTQYF	1	CD4-C5
Pla-2_GGAGCAATCGGATGGA	Pla	Pla-2	P2-4519	FALSE	1	TRA:CAVRDIRGGSYIPTF TRB:CASSQSASGGTYEQYF	1	CD4-C5
Pla-2_GGATTACAGACTAGAT	Pla	Pla-2	P2-4523	FALSE	1	TRA:CAASRGDSSYKLIF TRB:CASSQVAGHTEAFF	1	CD4-C5
Pla-2_GGATTACCATTGAGCT	Pla	Pla-2	P2-516	FALSE	2	TRA:CASNRDDKIIF TRB:CASTHGQVNEQFF	2	CD4-C5
Pla-2_GGATTACGTAAGAGAG	Pla	Pla-2	P2-4525	FALSE	1	TRA:CIVRAIYGNKLVF TRB:CSATPFGGQTYNEQFF	1	CD4-C5
Pla-2_GGCAATTAGCACAGGT	Pla	Pla-2	P2-4528	FALSE	1	TRA:CALGGAGNNRKLIW TRB:CASSHGSGRGYEQFF	1	CD4-C5
Pla-2_GGCGACTTCGCAAGCC	Pla	Pla-2	P2-4541	FALSE	1	TRA:CAFGVGFGNVLHC TRB:CASASGDSSNQPQHF	1	CD4-C5
Pla-2_GGCGTGTTCATCGCTC	Pla	Pla-2	P2-26	TRUE	3	TRA:CALVNTGGFKTIF TRB:CASSPPSSGGANEQYF	22	CD4-C6
Pla-2_GGCTGGTCAACACGCC	Pla	Pla-2	P2-471	FALSE	2	TRA:CAVRDGSDYKLSF TRB:CASSDGTGGGYTF	2	CD4-C5
Pla-2_GGGACCTGTACAGTTC	Pla	Pla-2	P2-404	FALSE	2	TRA:CAARVDDNNARLMF TRB:CASSRDLLIQSQYF	2	CD4-C6
Pla-2_GGGACCTGTCACTTCC	Pla	Pla-2	P2-61	TRUE	3	TRA:CAVSFITQGGSEKLVF TRB:CASSRDRGTYNEQFF	9	CD4-C5
Pla-2_GGGATGAGTGAGGCTA	Pla	Pla-2	P2-4555	FALSE	1	TRA:CGADTPSGTYKYIF TRB:CASSQMGGGELFF	1	CD4-C5
Pla-2_GGGATGAGTTGGTAAA	Pla	Pla-2	P2-4557	FALSE	1	TRA:CAASTMDSSYKLIF TRB:CASSAQTGSYGYTF	1	CD4-C5
Pla-2_GGGCACTAGCGCCTTG	Pla	Pla-2	P2-4558	FALSE	1	TRA:CAVSEGLYNQGGKLIF TRB:CASSPQKGTSGANVLTF	1	CD4-C3
Pla-2_GGGCATCAGCCAGGAT	Pla	Pla-2	P2-4561	FALSE	1	TRA:CATGLVTGANSKLTF TRB:CASKTQNTDTQYF	1	CD4-C5
Pla-2_GGGCATCTCCTAGTGA	Pla	Pla-2	P2-379	FALSE	2	TRA:CAVRDQTNTDKLIF TRB:CASSSGGGIQFF	2	CD4-C5
Pla-2_GGGTTGCTCGGAGGTA	Pla	Pla-2	P2-4572	FALSE	1	TRA:CAVKNAGGTSYGKLTF TRB:CASSVITPGETQYF	1	CD4-C5
Pla-2_GGTGAAGCAAACGTGG	Pla	Pla-2	P2-4574	FALSE	1	TRA:CALKNNAGNMLTF TRB:CASAGSALDTQYF	1	CD4-C5
Pla-2_GGTGAAGGTCCGAGTC	Pla	Pla-2	P2-4577	FALSE	1	TRA:CAVEEDGGFKTIF TRB:CASLNGAGGSYEQYF	1	CD4-C5
Pla-2_GGTGCGTAGAAACCAT	Pla	Pla-2	P2-358	TRUE	2	TRA:CGTEETSGSRLTF TRB:CASSVQRGVEQYF	2	CD4-C5
Pla-2_GGTGTTAGTAACGTTC	Pla	Pla-2	P2-4580	FALSE	1	TRA:CIVRVAHSNDYKLSF TRB:CASLNGAGGSYEQYF	1	CD4-C7
Pla-2_GGTGTTAGTCACACGC	Pla	Pla-2	P2-4581	FALSE	1	TRA:CAVEDPGSQGNLIF TRB:CASRAGTGVHEQFF	1	CD4-C5
Pla-2_GGTGTTATCTGTTTGT	Pla	Pla-2	P2-4582	FALSE	1	TRA:CAASRRWDNQGGKLIF TRB:CASRSRVLGELFF	1	CD4-C5

Pla-2_GTAACGTCACATCCGG	Pla	Pla-2	P2-4583	FALSE	1	TRA:CARAYNNAGNMLTF TRB:CASKPGPNYYEQYF	1	CD4-C5
Pla-2_GTAACTGGTGGTTTCA	Pla	Pla-2	P2-33	TRUE	3	TRA:CAFKLSDGQKLLF TRB:CAWSRAALTEAFF	17	CD4-C5
Pla-2_GTACGTAGTCCAGTGC	Pla	Pla-2	P2-42	FALSE	3	TRA:CAVSPRDNNDMRF TRB:CASSARGNSNQPQHF	12	CD4-C5
Pla-2_GTACTCCGTGCTTCTC	Pla	Pla-2	P2-4595	FALSE	1	TRA:CAENRSSGTYKYIF TRB:CASSLAEGNEQFF	1	CD4-C5
Pla-2_GTATCTTCAATCCGAT	Pla	Pla-2	P2-524	FALSE	2	TRA:CAVEGSGGYQKVTF TRB:CASSLQGSKLYNEQFF	2	CD4-C5
Pla-2_GTATCTTTCTCTTATG	Pla	Pla-2	P2-338	TRUE	2	TRA:CAVAGRGYSTLTF TRB:CAWSVTDRGSYNEQFF	2	CD4-C5
Pla-2_GTCACAAAGGTACTCT	Pla	Pla-2	P2-4609	FALSE	1	TRA:CAVGGKTGGGNKLTF TRB:CSVTSGGLYNEQFF	1	CD4-C5
Pla-2_GTCATTTCACGAGGTA	Pla	Pla-2	P2-145	FALSE	3	TRA:CAMRRSSGGSNYKLTF TRB:CASSSIGRGSKRETQYF	4	CD4-C4
Pla-2_GTCATTTGTTGGTGGA	Pla	Pla-2	P2-33	TRUE	3	TRA:CAFKLSDGQKLLF TRB:CAWSRAALTEAFF	17	CD4-C4
Pla-2_GTCATTTTCCGCGTTT	Pla	Pla-2	P2-441	FALSE	2	TRA:CAASETGANSKLTF TRB:CASSFSRPGGMNTEAFF	2	CD4-C5
Pla-2_GTCGGGTGTACAGCAG	Pla	Pla-2	P2-61	TRUE	3	TRA:CAVSFITQGGSEKLVF TRB:CASSRDRGTYNEQFF	9	CD4-C5
Pla-2_GTCGTAAGTCGAGTTT	Pla	Pla-2	P2-61	TRUE	3	TRA:CAVSFITQGGSEKLVF TRB:CASSRDRGTYNEQFF	9	CD4-C5
Pla-2_GTCTCGTCACCAGGTC	Pla	Pla-2	P2-4623	FALSE	1	TRA:CAAHRTNDYKLSF TRB:CSPRGGATNTEAFF	1	CD4-C5
Pla-2_GTGAAGGAGTGAAGTT	Pla	Pla-2	P2-4629	FALSE	1	TRA:CAVSSLSGYSTLTF TRB:CASSYRGLGQETQYF	1	CD4-C5
Pla-2_GTGCATAAGCTGTCTA	Pla	Pla-2	P2-1	TRUE	3	TRA:CVVNIIGYQKVTF TRB:CASTRQGKQPQHF	270	CD4-C6
Pla-2_GTGCTTCTCCCTTGCA	Pla	Pla-2	P2-50	FALSE	3	TRA:CAPIRAQKLVF TRB:CASSSPPKSGELFF	11	CD4-C5
Pla-2_GTGTGCGCATCTCGCT	Pla	Pla-2	P2-4647	FALSE	1	TRA:CADAHYGGSQGNLIF TRB:CAISPGQERRYEQYF	1	CD4-C5
Pla-2_GTTACAGAGCGTCAAG	Pla	Pla-2	P2-4658	FALSE	1	TRA:CVGRVLTGGGNKLTF TRB:CASSPPSSGGANEQYF	1	CD4-C6
Pla-2_GTTACAGGTAGGCATG	Pla	Pla-2	P2-262	FALSE	3	TRA:CAVKKRDNQGGKLIF TRB:CASSTIARDTQYF	3	CD4-C5
Pla-2_GTTTCTATCCTTGCCA	Pla	Pla-2	P2-491	FALSE	2	TRA:CAVGGTGGFKTIF TRB:CASSPEGPGALGGYTF	2	CD4-C5
Pla-2_TAAGAGATCCCTGACT	Pla	Pla-2	P2-560	FALSE	2	TRA:CAVNAQGRDDKIIF TRB:CASSSGRTTHSYEQYF	2	CD4-C5
Pla-2_TAAGAGATCGATGAGG	Pla	Pla-2	P2-4671	FALSE	1	TRA:CAASKYGGSQGNLIF TRB:CASGPTDTQYF	1	CD4-C5
Pla-2_TACCTATTCACCACCT	Pla	Pla-2	P2-4685	FALSE	1	TRA:CVVSEPPGGTSYGKLTF TRB:CASSDRGNTEAFF	1	CD4-C5
Pla-2_TACCTTAGTAGCTTGT	Pla	Pla-2	P2-102	TRUE	3	TRA:CAVIYGSGNTGKLIF TRB:CASSLVNTIYF	5	CD4-C5
Pla-2_TACGGATGTCCGAGTC	Pla	Pla-2	P2-112	FALSE	3	TRA:CAVRFSGNTGKLIF TRB:CSVGDAGTEAFF	5	CD4-C5
Pla-2_TACTCGCAGTCGATAA	Pla	Pla-2	P2-4698	FALSE	1	TRA:CALVDSSYKLIF TRB:CASRVERERGEQFF	1	CD4-C5
Pla-2_TACTCGCCAATCCGAT	Pla	Pla-2	P2-4702	FALSE	1	TRA:CVVRAFGNEKLTF TRB:CASSQVSGVEGGYTF	1	CD8-C4

Pla-2_TACTTACAGAATTGTG	Pla	Pla-2	P2-36	FALSE	3	TRA:CAAQAGNTPLVF TRB:CSAGVGGTIQYF	15	CD4-C5
Pla-2_TACTTGTAGCTGAACG	Pla	Pla-2	P2-4710	FALSE	1	TRA:CALVQGAQKLVF TRB:CASSAQIGAQETQYF	1	CD4-C5
Pla-2_TAGACCAAGCAGCCTC	Pla	Pla-2	P2-166	FALSE	3	TRA:CAVSWGTGYSTLTF TRB:CASSPDAGNTQYF	4	CD4-C5
Pla-2_TAGCCGGGTAGTAGTA	Pla	Pla-2	P2-101	TRUE	3	TRA:CATDASGNEKLTF TRB:CASSLGLADTQYF	5	CD4-C5
Pla-2_TAGTGGTCATTGTGCA	Pla	Pla-2	P2-4726	FALSE	1	TRA:CTRQSDGSNYKLTF TRB:CASSPGVGSGETQYF	1	CD4-C4
Pla-2_TAGTTGGCAAATACAG	Pla	Pla-2	P2-4728	FALSE	1	TRA:CAVCPRF TRB:CASTTAGGLYNEQFF	1	CD4-C5
Pla-2_TATTACCGTACTTGAC	Pla	Pla-2	P2-160	FALSE	3	TRA:CAAIRISVDGNVLHC TRB:CASSRPGYEQFF	4	CD4-C5
Pla-2_TATTACCGTCAACTGT	Pla	Pla-2	P2-4739	FALSE	1	TRA:CAVATSGGSYIPTF TRB:CASGPLGTSGEQYF	1	CD4-C5
Pla-2_TATTACCGTGTGAATA	Pla	Pla-2	P2-4740	FALSE	1	TRA:CAVRDLDFGNEKLTF TRB:CASSGEQGFGYEQYF	1	CD4-C5
Pla-2_TCAACGATCACTGGGC	Pla	Pla-2	P2-63	FALSE	3	TRA:CAASGEAGTALIF TRB:CATSREIQPNEKLFF	9	CD4-C5
Pla-2_TCACAAGGTTTGTTTC	Pla	Pla-2	P2-4755	FALSE	1	TRA:CAVARNQFYF TRB:CASHPQGGRTDTQYF	1	CD4-C4
Pla-2_TCACAAGTCACTATTC	Pla	Pla-2	P2-105	TRUE	3	TRA:CAYRRREGGSEKLVF TRB:CASSTSGAIANYGYTF	5	CD4-C3
Pla-2_TCACAAGTCCTAGGGC	Pla	Pla-2	P2-1	TRUE	3	TRA:CVVNIIGYQKVTF TRB:CASTRQGKQPQHF	270	CD4-C5
Pla-2_TCAGATGTCTAACTTC	Pla	Pla-2	P2-4762	FALSE	1	TRA:CIVRVWWNNARLMF TRB:CASSEGGGNNEKLFF	1	CD4-C5
Pla-2_TCAGCAATCACTCTTA	Pla	Pla-2	P2-102	TRUE	3	TRA:CAVIYGSGNTGKLIF TRB:CASSLVNTIYF	5	CD4-C5
Pla-2_TCAGCTCCACTGTGTA	Pla	Pla-2	P2-4768	FALSE	1	TRA:CAVRGQTSGTYKYIF TRB:CASSFQAGTGELFF	1	CD4-C5
Pla-2_TCAGGATCAGGCTCAC	Pla	Pla-2	P2-4773	FALSE	1	TRA:CAARMPGSNYKLTF TRB:CASSPNTLSYNEQFF	1	CD4-C5
Pla-2_TCAGGATCATCTCGCT	Pla	Pla-2	P2-492	FALSE	2	TRA:CAVPSYNTDKLIF TRB:CAWSVLTGELFF	2	CD8-C2
Pla-2_TCAGGATGTGCATCTA	Pla	Pla-2	P2-331	TRUE	2	TRA:CAAHWGNTGFQKLVF TRB:CSASAGLINQPQHF	2	CD4-C5
Pla-2_TCAGGATGTGCCTTGG	Pla	Pla-2	P2-4774	FALSE	1	TRA:CAVNPISSGSARQLTF TRB:CASSPSPGGEQYF	1	CD4-C5
Pla-2_TCAGGTATCAGCAACT	Pla	Pla-2	P2-149	FALSE	3	TRA:CAVTNRGNNRLAF TRB:CSARVPGGLSDEQFF	4	CD4-C5
Pla-2_TCAGGTATCTTCCTTC	Pla	Pla-2	P2-4777	FALSE	1	TRA:CAVNSNSGYALNF TRB:CASSLGVQGKYEQFF	1	CD4-C5
Pla-2_TCATTACGTTTGACAC	Pla	Pla-2	P2-4779	FALSE	1	TRA:CAGDSGNTGKLIF TRB:CASSTTNYGYTF	1	CD4-C3
Pla-2_TCATTTGAGGACACCA	Pla	Pla-2	P2-4781	FALSE	1	TRA:CGFGNVLHC TRB:CASSQLYTSGSFSGANVLTF	1	CD4-C5
Pla-2_TCATTTGCACATGACT	Pla	Pla-2	P2-26	TRUE	3	TRA:CALVNTGGFKTIF TRB:CASSPPSSGGANEQYF	22	CD4-C5
Pla-2_TCCACACTCATGGTCA	Pla	Pla-2	P2-63	FALSE	3	TRA:CAASGEAGTALIF TRB:CATSREIQPNEKLFF	9	CD4-C4
Pla-2_TCCCGATGTGTCCTCT	Pla	Pla-2	P2-4789	FALSE	1	TRA:CAVGGPNAGGTSYGKLTF TRB:CASSNTGSPNEKLFF	1	CD4-C5

Pla-2_TCCCGATTCACGACTA	Pla	Pla-2	P2-4790	FALSE	1	TRA:CAVYMNRDDKIIF TRB:CATSWPGQGSTEAFF	1	CD4-C5
Pla-2_TCGAGGCTCTGCCAGG	Pla	Pla-2	P2-4793	FALSE	1	TRA:CAVRDTGTASKLTF TRB:CASSLYKFQEEQYF	1	CD4-C5
Pla-2_TCGCGTTCACATTAGC	Pla	Pla-2	P2-4799	FALSE	1	TRA:CAVSDRNQGGKLIF TRB:CSVPGPDYYEQYF	1	CD4-C5
Pla-2_TCGGGACGTAGCGTGA	Pla	Pla-2	P2-33	TRUE	3	TRA:CAFKLSDGQKLLF TRB:CAWSRAALTEAFF	17	CD4-C5
Pla-2_TCGTACCCACTTCTGC	Pla	Pla-2	P2-47	TRUE	3	TRA:CAASAGGNKLTF TRB:CASSTEDEQYF	11	CD4-C5
Pla-2_TCGTACCGTGCTCTTC	Pla	Pla-2	P2-4807	FALSE	1	TRA:CAVRDIGGQKLLF TRB:CASSSQETQYF	1	CD4-C5
Pla-2_TCGTAGAAGTAGGCCA	Pla	Pla-2	P2-4809	FALSE	1	TRA:CAFLRGSGDKLTF TRB:CSASYQGRTYEQYF	1	CD4-C5
Pla-2_TCTATTGGTGACTACT	Pla	Pla-2	P2-1	TRUE	3	TRA:CVVNIIGYQKVTF TRB:CASTRQGKQPQHF	270	CD8-C7
Pla-2_TCTATTGGTTGTCTTT	Pla	Pla-2	P2-506	FALSE	2	TRA:CAVGEGNAGKSTF TRB:CASSLQLNSYEQYF	2	CD4-C5
Pla-2_TCTCATAAGGCTCTTA	Pla	Pla-2	P2-32	TRUE	3	TRA:CAMREANVGADGLTF TRB:CASSTRIAGGPFPETQYF	17	CD4-C5
Pla-2_TCTCTAAGTGAGTGAC	Pla	Pla-2	P2-4822	FALSE	1	TRA:CAMKNTGKLIF TRB:CASSPPYGTGETQYF	1	CD4-C5
Pla-2_TCTGAGACATTATCTC	Pla	Pla-2	P2-61	TRUE	3	TRA:CAVSFITQGGSEKLVF TRB:CASSRDRGTYNEQFF	9	CD4-C5
Pla-2_TCTGAGAGTACCGTTA	Pla	Pla-2	P2-501	FALSE	2	TRA:CAMEKGYSTLTF TRB:CSARAGQGSHSGANVLTF	2	CD4-C5
Pla-2_TCTGAGAGTATTACCG	Pla	Pla-2	P2-4824	FALSE	1	TRA:CAVGETSGSRLTF TRB:CASSELHGTGGEQYF	1	CD4-C3
Pla-2_TCTGGAAAGTTCGATC	Pla	Pla-2	P2-548	FALSE	2	TRA:CAVRAARTGNNRLAF TRB:CSATTSGEYNEQFF	2	CD4-C5
Pla-2_TCTGGAACACACTGCG	Pla	Pla-2	P2-4828	FALSE	1	TRA:CAYRSISGSRLTF TRB:CSARAGASRPEQYF	1	CD4-C3
Pla-2_TCTGGAACAGGTGCCT	Pla	Pla-2	P2-4829	FALSE	1	TRA:CAVLLSGSEKLVF TRB:CASVKEQTGELFF	1	CD4-C5
Pla-2_TCTGGAACATTCCTGC	Pla	Pla-2	P2-4830	FALSE	1	TRA:CAVGAPPSGTYKYIF TRB:CASSFLAGGSTDTQYF	1	CD4-C5
Pla-2_TCTTTCCCAGTATGCT	Pla	Pla-2	P2-239	FALSE	3	TRA:CAVYNAGNMLTF TRB:CATTGSLHNEQFF	3	CD4-C5
Pla-2_TCTTTCCGTAGCGCAA	Pla	Pla-2	P2-155	FALSE	3	TRA:CAGGRSNDYKLSF TRB:CASSGGAGGPGNEQFF	4	CD4-C5
Pla-2_TGACTTTAGTACATGA	Pla	Pla-2	P2-4853	FALSE	1	TRA:CIVRVGLDSSYKLIF TRB:CSVERQKNTEAFF	1	CD4-C5
Pla-2_TGACTTTCAGCTATTG	Pla	Pla-2	P2-4854	FALSE	1	TRA:CAVRSGYSGAGSYQLTF TRB:CASSTRILLAVNNEQFF	1	CD4-C4
Pla-2_TGAGAGGCATCCGTGG	Pla	Pla-2	P2-4856	FALSE	1	TRA:CAVWGQFYF TRB:CSARDLGTYGYTF	1	CD4-C5
Pla-2_TGAGCATAGCGTTCCG	Pla	Pla-2	P2-4857	FALSE	1	TRA:CAASMYNNNDMRF TRB:CAWSPRGSNQPQHF	1	CD4-C3
Pla-2_TGAGCATGTCCATGAT	Pla	Pla-2	P2-4862	FALSE	1	TRA:CAFNGNQFYF TRB:CSASYQGRTYEQYF	1	CD4-C4
Pla-2_TGAGCATGTGTGCGTC	Pla	Pla-2	P2-4863	FALSE	1	TRA:CLYSGGGADGLTF TRB:CASSKGLVPDTQYF	1	CD4-C5
Pla-2_TGAGGGAGTCGGCACT	Pla	Pla-2	P2-4869	FALSE	1	TRA:CAVSDENSGYALNF TRB:CASSLQQGTYEQYF	1	CD8-C4

Pla-2_TGATTTCGTGGCAAAC	Pla	Pla-2	P2-1	TRUE	3	TRA:CVVNIIGYQKVTF TRB:CASTRQGKQPQHF	270	CD4-C5
Pla-2_TGCACCTTCTGTACGA	Pla	Pla-2	P2-4878	FALSE	1	TRA:CAVRDVSDSNYQLIW TRB:CASSPYREDSPLHF	1	CD4-C3
Pla-2_TGCCAAAAGTTGAGAT	Pla	Pla-2	P2-4880	FALSE	1	TRA:CAVINQAGTALIF TRB:CASSDQGEGLAGELFF	1	CD4-C5
Pla-2_TGCCAAAGTTCTCATT	Pla	Pla-2	P2-4882	FALSE	1	TRA:CVVRYSGAGSYQLTF TRB:CASSPGTGYTF	1	CD4-C4
Pla-2_TGCCCATAGGAATGGA	Pla	Pla-2	P2-64	FALSE	3	TRA:CAATSLISDGQKLLF TRB:CSARDSFNYGYTF	9	CD4-C5
Pla-2_TGCCCTAAGGAGTACC	Pla	Pla-2	P2-18	TRUE	3	TRA:CAATSSSNFGNEKLTF TRB:CAWSRAALTEAFF	28	CD4-C5
Pla-2_TGCTACCGTAGCTAAA	Pla	Pla-2	P2-224	FALSE	3	TRA:CAVSGGGFKTIF TRB:CASSQEGETQYF	3	CD4-C2
Pla-2_TGGACGCAGTCACGCC	Pla	Pla-2	P2-4907	FALSE	1	TRA:CALVLYNQGGKLIF TRB:CASSVDSGGTGNEQFF	1	CD4-C5
Pla-2_TGGACGCCACCAGATT	Pla	Pla-2	P2-243	FALSE	3	TRA:CALYSGNTPLVF TRB:CASNTDRVLEQYF	3	CD4-C5
Pla-2_TGGCCAGAGACGCTTT	Pla	Pla-2	P2-9	FALSE	3	TRA:CAMTQNAGKSTF TRB:CASSPDRDYEQYF	47	CD4-C7
Pla-2_TGGCTGGTCAAACCGT	Pla	Pla-2	P2-4918	FALSE	1	TRA:CGSGRGQFYF TRB:CASSTDGFYEQYF	1	CD4-C5
Pla-2_TGGCTGGTCGCATGGC	Pla	Pla-2	P2-162	FALSE	3	TRA:CIVRAMNSGYSTLTF TRB:CSAPPWQGAYEQYF	4	CD4-C5
Pla-2_TGGGAAGCAGTAAGCG	Pla	Pla-2	P2-4924	FALSE	1	TRA:CVVSARGGSYIPTF TRB:CASSQEQTGETQYF	1	CD4-C5
Pla-2_TGGGCGTTCTAACTCT	Pla	Pla-2	P2-4930	FALSE	1	TRA:CAVNSGGSNYKLTF TRB:CASSPSQTTETQYF	1	CD4-C2
Pla-2_TGGTTAGAGTTGCAGG	Pla	Pla-2	P2-4932	FALSE	1	TRA:CAVRDKSSGGYNKLIF TRB:CASSLTVGGQGGETQYF	1	CD4-C5
Pla-2_TGGTTCCCAAGGACTG	Pla	Pla-2	P2-116	FALSE	3	TRA:CAVSPPAGSNYQLIW TRB:CATSRETGARYEQYF	5	CD4-C5
Pla-2_TGGTTCCGTACCTACA	Pla	Pla-2	P2-4939	FALSE	1	TRA:CALNTGRRALTF TRB:CASSLQQQETQYF	1	CD4-C4
Pla-2_TGGTTCCGTGTCGCTG	Pla	Pla-2	P2-233	FALSE	3	TRA:CAASIWNYGQNFVF TRB:CSAIERGGRETQYF	3	CD4-C4
Pla-2_TGTCCCAAGGCATGTG	Pla	Pla-2	P2-4946	FALSE	1	TRA:CAASEGNGGSQGNLIF TRB:CASTLPRGGELETQYF	1	CD4-C4
Pla-2_TGTTCCGAGTGTTTGC	Pla	Pla-2	P2-212	FALSE	3	TRA:CAENIRATNKLIF TRB:CAWSPRGSNQPQHF	3	CD4-C5
Pla-2_TTAGGACGTCAAGCGA	Pla	Pla-2	P2-4964	FALSE	1	TRA:CALMSVANSGYALNF TRB:CASSLGHQETQYF	1	CD4-C5
Pla-2_TTAGGCACATCGTCGG	Pla	Pla-2	P2-4965	FALSE	1	TRA:CAVYSGGYQKVTF TRB:CASSRDTTYEQYF	1	CD4-C5
Pla-2_TTCGAAGCATCCTAGA	Pla	Pla-2	P2-434	FALSE	2	TRA:CAVEDSGSGNTGKLIF TRB:CASSLGSGGTGELFF	2	CD4-C5
Pla-2_TTCTCCTCATCCGTGG	Pla	Pla-2	P2-4993	FALSE	1	TRA:CAPDNARLMF TRB:CASSYPGQGAPGELFF	1	CD4-C5
Pla-2_TTGAACGGTCTTTCAT	Pla	Pla-2	P2-508	FALSE	2	TRA:CAMRDLTGRRALTF TRB:CASSPGLALSSYEQYF	2	CD4-C6
Pla-2_TTGACTTCAAAGCGGT	Pla	Pla-2	P2-5005	FALSE	1	TRA:CAGNFGNEKLTF TRB:CAWSRRVYDGYTF	1	CD4-C5
Pla-2_TTGACTTTCTTCCTTC	Pla	Pla-2	P2-5007	FALSE	1	TRA:CIVRVRMVNRDDKIIF TRB:CASRAGDTGELFF	1	CD4-C5

Pla-2_TTGGAACAGACGACGT	Pla	Pla-2	P2-5014	FALSE	1	TRA:CAVDNDYKLSF TRB:CASSFGLAGGNEQYF	1	CD4-C5
Pla-2_TTGGCAAGTGGTGTAG	Pla	Pla-2	P2-5019	FALSE	1	TRA:CAASYSNYQLIW TRB:CASSYGQGTSEQFF	1	CD4-C5
Pla-2_TTTACTGGTTCCCGAG	Pla	Pla-2	P2-50	FALSE	3	TRA:CAPIRAQKLVF TRB:CASSSPPKSGELFF	11	CD4-C5
Pla-2_TTTATGCCAATTGCTG	Pla	Pla-2	P2-5026	FALSE	1	TRA:CVVRESGAGSYQLTF TRB:CASSFGTGGGGNQPQHF	1	CD4-C5
Pla-2_TTTATGCCATAAAGGT	Pla	Pla-2	P2-360	TRUE	2	TRA:CAASAPGTYKYIF TRB:CASSLLRPSADTQYF	2	CD4-C5
Pla-2_TTTGCGCTCTCAAACG	Pla	Pla-2	P2-5034	FALSE	1	TRA:CAVNSYNYGQNFVF TRB:CASSAQTGGTEAFF	1	CD4-C5
Pla-2_AAACGGGAGATGTAAC	Pla	Pla-2	P2-376	FALSE	2	TRA:CAISGNTPLVF TRB:CASSLLRARGETQYF	2	CD4-C7
Pla-2_AAACGGGTCTAACTGG	Pla	Pla-2	P2-3416	FALSE	1	TRA:CAAKKGDTGRRALTF TRB:CSADRGETQYF	1	CD4-C7
Pla-2_AAAGATGAGTGCTGCC	Pla	Pla-2	P2-378	FALSE	2	TRA:CAYRSGTSYGKLTF TRB:CSARETGLSYEQYF	2	CD4-C7
Pla-2_AAAGTAGGTGTAATGA	Pla	Pla-2	P2-3421	FALSE	1	TRA:CAASRSRAGSYQLTF TRB:CASSFESVGYNEQFF	1	CD4-C7
Pla-2_AAATGCCGTGTGCCTG	Pla	Pla-2	P2-3425	FALSE	1	TRA:CARIIQGAQKLVF TRB:CASNPGLAGGPGEQYF	1	CD4-C7
Pla-2_AACACGTGTCCTGCTT	Pla	Pla-2	P2-3427	FALSE	1	TRA:CAVGPHTGGFKTIF TRB:CASTAGQGVTYEQYF	1	CD4-C7
Pla-2_AACACGTTCATCACCC	Pla	Pla-2	P2-3428	FALSE	1	TRA:CAVQGYQKVTF TRB:CASSAGTEGYEQYF	1	CD4-C7
Pla-2_AACACGTTCCTATGTT	Pla	Pla-2	P2-113	FALSE	3	TRA:CADVDSNYQLIW TRB:CASSPRTAGGYTF	5	CD4-C7
Pla-2_AACCATGCACAGCCCA	Pla	Pla-2	P2-208	FALSE	3	TRA:CAESRTGGGADGLTF TRB:CASSLGGRALRDTQYF	3	CD4-C7
Pla-2_AACCATGGTAATTGGA	Pla	Pla-2	P2-71	FALSE	3	TRA:CAARNTGGFKTIF TRB:CAWSEGTSGGTDTQYF	8	CD4-C7
Pla-2_AACGTTGGTTGGTTTG	Pla	Pla-2	P2-135	FALSE	3	TRA:CAVATYSGAGSYQLTF TRB:CASSYGPSGGLNEQFF	4	CD4-C7
Pla-2_AACTCAGGTCTCTTTA	Pla	Pla-2	P2-136	FALSE	3	TRA:CAASVRSQGNLIF TRB:CASSLGPNRYRQRNTIYF	4	CD4-C7
Pla-2_AACTCTTAGCGATCCC	Pla	Pla-2	P2-3450	FALSE	1	TRA:CAVSYSGAGSYQLTF TRB:CSARDPVFVEQYF	1	CD4-C7
Pla-2_AACTTTCTCATCTGCC	Pla	Pla-2	P2-9	FALSE	3	TRA:CAMTQNAGKSTF TRB:CASSPDRDYEQYF	47	CD4-C7
Pla-2_AAGACCTCATAGAAAC	Pla	Pla-2	P2-3456	FALSE	1	TRA:CIVRGEKLTF TRB:CATSDPRDRVYEQYF	1	CD4-C7
Pla-2_AAGGAGCAGACGCACA	Pla	Pla-2	P2-3459	FALSE	1	TRA:CALSTSGTYKYIF TRB:CASSSTSGRADTQYF	1	CD4-C7
Pla-2_AAGGAGCTCGCCTGAG	Pla	Pla-2	P2-388	FALSE	2	TRA:CAANSGGSNYKLTF TRB:CASSLWGTGLETQYF	2	CD4-C7
Pla-2_AAGGCAGCATCGATGT	Pla	Pla-2	P2-3464	FALSE	1	TRA:CAVKQAGAGSYQLTF TRB:CASSDPGQGPETQYF	1	CD4-C7
Pla-2_AAGGTTCAGCAGCGTA	Pla	Pla-2	P2-3468	FALSE	1	TRA:CAESLLNAGNMLTF TRB:CASSMGWTSRNEQFF	1	CD4-C7
Pla-2_AAGTCTGAGGAGTTGC	Pla	Pla-2	P2-51	FALSE	3	TRA:CAENNDTGGFKTIF TRB:CATRGAHEKLFF	11	CD4-C7
Pla-2_AAGTCTGGTGATGTGG	Pla	Pla-2	P2-3473	FALSE	1	TRA:CALSDSGAGSYQLTF TRB:CASSPRQISDTQYF	1	CD4-C7

Pla-2_AAGTCTGTCGATCCCT	Pla	Pla-2	P2-31	FALSE	3	TRA:CAASDNTDKLIF TRB:CASSQVHEQYF	20	CD4-C7
Pla-2_AATCCAGAGGTCGGAT	Pla	Pla-2	P2-391	FALSE	2	TRA:CAGALYGNKLVF TRB:CSGQGAMNTEAFF	2	CD4-C7
Pla-2_AATCGGTGTAAGGATT	Pla	Pla-2	P2-3481	FALSE	1	TRA:CVVTYSGAGSYQLTF TRB:CASSLRFGETQYF	1	CD4-C7
Pla-2_ACACCAAAGAGACTTA	Pla	Pla-2	P2-3482	FALSE	1	TRA:CAASSGIYGGATNKLIF TRB:CASSQGTSGTTNTGELFF	1	CD4-C7
Pla-2_ACACCAAAGCTAAGAT	Pla	Pla-2	P2-51	FALSE	3	TRA:CAENNDTGGFKTIF TRB:CATRGAHEKLFF	11	CD4-C7
Pla-2_ACACCCTCAAACTGCT	Pla	Pla-2	P2-74	FALSE	3	TRA:CAIRNNNARLMF TRB:CASSLERHTGLYGYTF	8	CD4-C7
Pla-2_ACACCGGCATAACCTG	Pla	Pla-2	P2-3492	FALSE	1	TRA:CALDMLSYQKVTF TRB:CAISGQGQATGNTIYF	1	CD4-C7
Pla-2_ACACCGGTCACTTATC	Pla	Pla-2	P2-3493	FALSE	1	TRA:CAASSSNSGYALNF TRB:CATDRGFGNSPLHF	1	CD4-C7
Pla-2_ACACTGAAGCGAGAAA	Pla	Pla-2	P2-3494	FALSE	1	TRA:CAPSGGGADGLTF TRB:CASRGAGLVYEQYF	1	CD4-C7
Pla-2_ACACTGAAGGAATCGC	Pla	Pla-2	P2-3496	FALSE	1	TRA:CAEKADTGRRALTF TRB:CASSFDGQPQHF	1	CD4-C7
Pla-2_ACAGCTAAGGCACATG	Pla	Pla-2	P2-31	FALSE	3	TRA:CAASDNTDKLIF TRB:CASSQVHEQYF	20	CD4-C7
Pla-2_ACATACGCACTTCGAA	Pla	Pla-2	P2-53	FALSE	3	TRA:CAVPFNFGNEKLTF TRB:CASSFRGPYNEQFF	11	CD4-C7
Pla-2_ACATACGGTCGAATCT	Pla	Pla-2	P2-9	FALSE	3	TRA:CAMTQNAGKSTF TRB:CASSPDRDYEQYF	47	CD4-C7
Pla-2_ACATGGTCACCGAATT	Pla	Pla-2	P2-23	FALSE	3	TRA:CAVYSGGGADGLTF TRB:CASSLRFGETQYF	24	CD4-C7
Pla-2_ACATGGTGTTCCATGA	Pla	Pla-2	P2-3515	FALSE	1	TRA:CIVRVMGGGSNYKLTF TRB:CASSYSVGYEQYF	1	CD4-C7
Pla-2_ACCAGTAAGCTGCGAA	Pla	Pla-2	P2-397	FALSE	2	TRA:CAASDWGRGGSNYKLTF TRB:CASSYRLAETGEQFF	2	CD4-C7
Pla-2_ACCCACTCACAGTCGC	Pla	Pla-2	P2-3524	FALSE	1	TRA:CAPLLFNYGGSQGNLIF TRB:CASSQDSRPNTEAFF	1	CD4-C7
Pla-2_ACCCACTGTTACGGAG	Pla	Pla-2	P2-3525	FALSE	1	TRA:CALNPLQKLVF TRB:CASSLMDFYTGELFF	1	CD4-C7
Pla-2_ACCGTAAGTAAACACA	Pla	Pla-2	P2-3527	FALSE	1	TRA:CAGQQDSGGSNYKLTF TRB:CSGRQGWDTEAFF	1	CD4-C7
Pla-2_ACGAGCCGTTGGACCC	Pla	Pla-2	P2-71	FALSE	3	TRA:CAARNTGGFKTIF TRB:CAWSEGTSGGTDTQYF	8	CD4-C7
Pla-2_ACGAGGAGTAATCACC	Pla	Pla-2	P2-23	FALSE	3	TRA:CAVYSGGGADGLTF TRB:CASSLRFGETQYF	24	CD4-C7
Pla-2_ACGATACCAGTCGTGC	Pla	Pla-2	P2-3545	FALSE	1	TRA:CAASAVSGNQFYF TRB:CASSQDWATNEKLFF	1	CD4-C7
Pla-2_ACGATGTTCCAGTAGT	Pla	Pla-2	P2-31	FALSE	3	TRA:CAASDNTDKLIF TRB:CASSQVHEQYF	20	CD4-C7
Pla-2_ACGCCAGGTAAATGTG	Pla	Pla-2	P2-3562	FALSE	1	TRA:CAGGSGTYKYIF TRB:CASSVVPQDGSSGNTIYF	1	CD4-C7
Pla-2_ACGCCGACATCGGTTA	Pla	Pla-2	P2-31	FALSE	3	TRA:CAASDNTDKLIF TRB:CASSQVHEQYF	20	CD4-C7
Pla-2_ACGCCGAGTGGCCCTA	Pla	Pla-2	P2-142	FALSE	3	TRA:CAEKNWNGNKLVF TRB:CASSPMEVWGQPQHF	4	CD4-C7
Pla-2_ACGGAGAAGAGCTGCA	Pla	Pla-2	P2-223	FALSE	3	TRA:CAQNTGNQFYF TRB:CASSSIDTQYF	3	CD4-C7

Pla-2_ACGGGTCCAAATACAG	Pla	Pla-2	P2-23	FALSE	3	TRA:CAVYSGGGADGLTF TRB:CASSLRFGETQYF	24	CD4-C7
Pla-2_ACGGGTCTCGGACAAG	Pla	Pla-2	P2-114	FALSE	3	TRA:CAAAYNNNDMRF TRB:CSARPGSSYQPQHF	5	CD4-C7
Pla-2_ACTATCTTCGTCTGAA	Pla	Pla-2	P2-144	FALSE	3	TRA:CAYRGAYSGAGSYQLTF TRB:CASSPPYWQADTQYF	4	CD4-C7
Pla-2_ACTGAGTTCAAGAAGT	Pla	Pla-2	P2-14	FALSE	3	TRA:CALSEAPGGSEKLVF TRB:CASSIGHPNSPLHF	37	CD4-C7
Pla-2_ACTGATGCAACTGGCC	Pla	Pla-2	P2-3591	FALSE	1	TRA:CATDMGGAQKLVF TRB:CSASYQGRTYEQYF	1	CD4-C7
Pla-2_ACTGCTCTCATGTCCC	Pla	Pla-2	P2-410	FALSE	2	TRA:CLVGDRSYNTDKLIF TRB:CASSQGHEKLFF	2	CD4-C7
Pla-2_ACTGTCCTCTACCTGC	Pla	Pla-2	P2-3599	FALSE	1	TRA:CALKGGSEKLVF TRB:CASSFDRDYEQYF	1	CD4-C7
Pla-2_ACTTACTAGTTTCCTT	Pla	Pla-2	P2-3601	FALSE	1	TRA:CAMPAGGTSYGKLTF TRB:CASSSETGANVLTF	1	CD4-C7
Pla-2_ACTTACTTCAACGAAA	Pla	Pla-2	P2-412	FALSE	2	TRA:CAVDGDGGSQGNLIF TRB:CASSERGRIYGYTF	2	CD4-C7
Pla-2_ACTTTCATCTTACCTA	Pla	Pla-2	P2-31	FALSE	3	TRA:CAASDNTDKLIF TRB:CASSQVHEQYF	20	CD4-C7
Pla-2_AGACGTTAGAGTGACC	Pla	Pla-2	P2-115	FALSE	3	TRA:CAVRDEDNQGGKLIF TRB:CASSQDQGLAGGFSFKETQYF	5	CD4-C7
Pla-2_AGACGTTCAGCTCGCA	Pla	Pla-2	P2-14	FALSE	3	TRA:CALSEAPGGSEKLVF TRB:CASSIGHPNSPLHF	37	CD4-C7
Pla-2_AGAGCGAAGCAGATCG	Pla	Pla-2	P2-418	FALSE	2	TRA:CAVKNFGNEKLTF TRB:CASSKDHFVEQYF	2	CD4-C7
Pla-2_AGAGCTTAGCAATATG	Pla	Pla-2	P2-227	FALSE	3	TRA:CAVRSSTGKLIF TRB:CASSYRQATNEKLFF	3	CD4-C7
Pla-2_AGAGCTTAGCGTAATA	Pla	Pla-2	P2-3618	FALSE	1	TRA:CAASAYQGAQKLVF TRB:CASSFQGGISYNEQFF	1	CD4-C7
Pla-2_AGAGTGGCACGAAATA	Pla	Pla-2	P2-147	FALSE	3	TRA:CAGPPGGYNKLIF TRB:CAIRQGDRQPQHF	4	CD4-C7
Pla-2_AGAGTGGCATCATCCC	Pla	Pla-2	P2-420	FALSE	2	TRA:CALSEYLQGAQKLVF TRB:CASSRGGGRTEAFF	2	CD4-C7
Pla-2_AGAGTGGTCGAACTGT	Pla	Pla-2	P2-3624	FALSE	1	TRA:CIVRVLIQGAQKLVF TRB:CASYRRVTEQYF	1	CD4-C7
Pla-2_AGATCTGAGTTTAGGA	Pla	Pla-2	P2-23	FALSE	3	TRA:CAVYSGGGADGLTF TRB:CASSLRFGETQYF	24	CD4-C7
Pla-2_AGATTGCAGAAACCAT	Pla	Pla-2	P2-53	FALSE	3	TRA:CAVPFNFGNEKLTF TRB:CASSFRGPYNEQFF	11	CD4-C7
Pla-2_AGATTGCAGACCTAGG	Pla	Pla-2	P2-14	FALSE	3	TRA:CALSEAPGGSEKLVF TRB:CASSIGHPNSPLHF	37	CD4-C7
Pla-2_AGATTGCAGACTAGAT	Pla	Pla-2	P2-422	FALSE	2	TRA:CAVNREGGGADGLTF TRB:CSAYGSRTDTQYF	2	CD4-C7
Pla-2_AGCAGCCCATGTCGAT	Pla	Pla-2	P2-14	FALSE	3	TRA:CALSEAPGGSEKLVF TRB:CASSIGHPNSPLHF	37	CD4-C7
Pla-2_AGCCTAAAGAGGTAGA	Pla	Pla-2	P2-424	FALSE	2	TRA:CAVLKAAGNKLTF TRB:CAWSGGLTDTQYF	2	CD4-C7
Pla-2_AGCCTAACATAGGATA	Pla	Pla-2	P2-425	FALSE	2	TRA:CAVKNGGSQGNLIF TRB:CASSYGLAGPSYNEQFF	2	CD4-C7
Pla-2_AGCCTAATCGGTGTTA	Pla	Pla-2	P2-3645	FALSE	1	TRA:CAVQNTGNQFYF TRB:CSASRPPPYNEQFF	1	CD4-C7
Pla-2_AGCGGTCTCTCTTGAT	Pla	Pla-2	P2-3653	FALSE	1	TRA:CLVSTNQAGTALIF TRB:CASSAGLGEDNEQFF	1	CD4-C7

Pla-2_AGCGTCGAGATGGCGT	Pla	Pla-2	P2-429	FALSE	2	TRA:CVVSARGATNKLIF TRB:CASSELQHNEQFF	2	CD4-C7
Pla-2_AGCTCCTGTAATCGTC	Pla	Pla-2	P2-430	FALSE	2	TRA:CAVRDGGNNARLMF TRB:CASSQGSAGANVLTF	2	CD4-C7
Pla-2_AGCTCCTTCGCTTAGA	Pla	Pla-2	P2-3664	FALSE	1	TRA:CAFTATGANSKLTF TRB:CASAVPQGQTYNEQFF	1	CD4-C7
Pla-2_AGCTCTCCAGTCCTTC	Pla	Pla-2	P2-3666	FALSE	1	TRA:CAGDRAGNNRKLIW TRB:CASSPPDVAEAFF	1	CD4-C7
Pla-2_AGCTCTCTCCTCAACC	Pla	Pla-2	P2-9	FALSE	3	TRA:CAMTQNAGKSTF TRB:CASSPDRDYEQYF	47	CD4-C7
Pla-2_AGCTTGACAGCGAACA	Pla	Pla-2	P2-3667	FALSE	1	TRA:CAVGANDYKLSF TRB:CAWSGGRGTIYEQYF	1	CD4-C7
Pla-2_AGGCCACCAATGGAGC	Pla	Pla-2	P2-3672	FALSE	1	TRA:CAENGNYKLSF TRB:CASSQEGGPDTQYF	1	CD4-C7
Pla-2_AGGCCGTAGAAGGACA	Pla	Pla-2	P2-3676	FALSE	1	TRA:CAVGQTTGTASKLTF TRB:CASSEGQGYGYTF	1	CD4-C7
Pla-2_AGGCCGTCATACTCTT	Pla	Pla-2	P2-53	FALSE	3	TRA:CAVPFNFGNEKLTF TRB:CASSFRGPYNEQFF	11	CD4-C7
Pla-2_AGGCCGTGTCTAGGTT	Pla	Pla-2	P2-150	FALSE	3	TRA:CVVRYAGNNRKLIW TRB:CASSQGQPPYEQYF	4	CD4-C7
Pla-2_AGGCCGTTCACCGTAA	Pla	Pla-2	P2-432	FALSE	2	TRA:CAATDSWGKFQF TRB:CSARGDSNQPQHF	2	CD4-C7
Pla-2_AGGGTGAGTGCCTTGG	Pla	Pla-2	P2-430	FALSE	2	TRA:CAVRDGGNNARLMF TRB:CASSQGSAGANVLTF	2	CD4-C7
Pla-2_AGGTCATGTACTTCTT	Pla	Pla-2	P2-3692	FALSE	1	TRA:CAAQSRGNTPLVF TRB:CSVEGGNYGYTF	1	CD4-C7
Pla-2_AGGTCCGGTGAGGGAG	Pla	Pla-2	P2-3694	FALSE	1	TRA:CAVGARARLMF TRB:CASSYLADSYEQYF	1	CD4-C7
Pla-2_AGTAGTCTCTGGAGCC	Pla	Pla-2	P2-3698	FALSE	1	TRA:CAAKRTGTASKLTF TRB:CASSDRDGTDTQYF	1	CD4-C7
Pla-2_AGTCTTTTCACAGTAC	Pla	Pla-2	P2-14	FALSE	3	TRA:CALSEAPGGSEKLVF TRB:CASSIGHPNSPLHF	37	CD4-C7
Pla-2_AGTGGGAAGTCGTTTG	Pla	Pla-2	P2-205	TRUE	3	TRA:CALSESTNAGKSTF TRB:CAWSYADTQYF	3	CD4-C7
Pla-2_AGTGTCACATTCTCAT	Pla	Pla-2	P2-376	FALSE	2	TRA:CAISGNTPLVF TRB:CASSLLRARGETQYF	2	CD4-C7
Pla-2_AGTGTCAGTAGCTAAA	Pla	Pla-2	P2-31	FALSE	3	TRA:CAASDNTDKLIF TRB:CASSQVHEQYF	20	CD4-C7
Pla-2_AGTGTCAGTCGCGAAA	Pla	Pla-2	P2-113	FALSE	3	TRA:CADVDSNYQLIW TRB:CASSPRTAGGYTF	5	CD4-C7
Pla-2_AGTGTCAGTCTGGAGA	Pla	Pla-2	P2-9	FALSE	3	TRA:CAMTQNAGKSTF TRB:CASSPDRDYEQYF	47	CD4-C7
Pla-2_AGTGTCATCTAACTGG	Pla	Pla-2	P2-14	FALSE	3	TRA:CALSEAPGGSEKLVF TRB:CASSIGHPNSPLHF	37	CD4-C7
Pla-2_ATAACGCAGACAGACC	Pla	Pla-2	P2-3715	FALSE	1	TRA:CAVRGATNKLIF TRB:CASTLDAAYSGNTIYF	1	CD4-C7
Pla-2_ATAAGAGCATTAGGCT	Pla	Pla-2	P2-74	FALSE	3	TRA:CAIRNNNARLMF TRB:CASSLERHTGLYGYTF	8	CD4-C7
Pla-2_ATAAGAGGTCCGAATT	Pla	Pla-2	P2-9	FALSE	3	TRA:CAMTQNAGKSTF TRB:CASSPDRDYEQYF	47	CD4-C7
Pla-2_ATAGACCGTCCGAATT	Pla	Pla-2	P2-51	FALSE	3	TRA:CAENNDTGGFKTIF TRB:CATRGAHEKLFF	11	CD4-C7
Pla-2_ATCACGAGTTAGAACA	Pla	Pla-2	P2-117	FALSE	3	TRA:CAVDITGGGNKLTF TRB:CSAREDTDTQYF	5	CD4-C7

Pla-2_ATCCACCAGGCTAGCA	Pla	Pla-2	P2-14	FALSE	3	TRA:CALSEAPGGSEKLVF TRB:CASSIGHPNSPLHF	37	CD4-C7
Pla-2_ATCCACCTCCTATGTT	Pla	Pla-2	P2-3731	FALSE	1	TRA:CVVNIIGYQKVTF TRB:CASKGVGQPQHF	1	CD4-C7
Pla-2_ATCCGAATCGCTTAGA	Pla	Pla-2	P2-3734	FALSE	1	TRA:CVVSAITGGGNKLTF TRB:CASSPPGFSGNTGELFF	1	CD4-C7
Pla-2_ATCTACTAGCTAGGCA	Pla	Pla-2	P2-134	FALSE	3	TRA:CAVNPPNARLMF TRB:CASSSLGRNTEAFF	4	CD4-C7
Pla-2_ATCTACTAGGGCATGT	Pla	Pla-2	P2-3741	FALSE	1	TRA:CAVSSGVPGNNRLAF TRB:CSARAIGQFEQYF	1	CD4-C7
Pla-2_ATCTACTCAAAGCAAT	Pla	Pla-2	P2-71	FALSE	3	TRA:CAARNTGGFKTIF TRB:CAWSEGTSGGTDTQYF	8	CD4-C7
Pla-2_ATCTGCCTCAACCATG	Pla	Pla-2	P2-9	FALSE	3	TRA:CAMTQNAGKSTF TRB:CASSPDRDYEQYF	47	CD4-C7
Pla-2_ATCTGCCTCGTCGTTC	Pla	Pla-2	P2-234	FALSE	3	TRA:CAMREVTTDSWGKFQF TRB:CASSPGAKNIQYF	3	CD4-C7
Pla-2_ATGGGAGCAGCTCGAC	Pla	Pla-2	P2-51	FALSE	3	TRA:CAENNDTGGFKTIF TRB:CATRGAHEKLFF	11	CD4-C7
Pla-2_ATGTGTGCAAACTGTC	Pla	Pla-2	P2-14	FALSE	3	TRA:CALSEAPGGSEKLVF TRB:CASSIGHPNSPLHF	37	CD4-C7
Pla-2_ATGTGTGGTCGGCTCA	Pla	Pla-2	P2-154	FALSE	3	TRA:CAMSGGGADGLTF TRB:CASSFLRGGINEQYF	4	CD4-C7
Pla-2_ATGTGTGGTTACGCGC	Pla	Pla-2	P2-74	FALSE	3	TRA:CAIRNNNARLMF TRB:CASSLERHTGLYGYTF	8	CD4-C7
Pla-2_ATTACTCTCGTATCAG	Pla	Pla-2	P2-114	FALSE	3	TRA:CAAAYNNNDMRF TRB:CSARPGSSYQPQHF	5	CD4-C7
Pla-2_ATTATCCGTCGAACAG	Pla	Pla-2	P2-447	FALSE	2	TRA:CIVRPYNTDKLIF TRB:CASGEGSTDTQYF	2	CD4-C7
Pla-2_ATTGGACGTAGGCTGA	Pla	Pla-2	P2-3763	FALSE	1	TRA:CAPRPHNLEYGNKLVF TRB:CASSEKGPRRGYGYTF	1	CD4-C7
Pla-2_ATTGGACGTGATGTGG	Pla	Pla-2	P2-3764	FALSE	1	TRA:CAVRDIRAGSYQLTF TRB:CASLPPGGFNQPQHF	1	CD4-C7
Pla-2_ATTGGACTCGTTTGCC	Pla	Pla-2	P2-235	FALSE	3	TRA:CATENNARLMF TRB:CSRDRGLQTEAFF	3	CD4-C7
Pla-2_ATTGGACTCTGACCTC	Pla	Pla-2	P2-3767	FALSE	1	TRA:CALGGGYQKVTF TRB:CASSRGELSGRAYNEQFF	1	CD4-C7
Pla-2_ATTGGTGAGCAATCTC	Pla	Pla-2	P2-3768	FALSE	1	TRA:CAPPLEYGNKLVF TRB:CASTRTSRGPTYEQYF	1	CD4-C7
Pla-2_ATTGGTGCAATCAGAA	Pla	Pla-2	P2-23	FALSE	3	TRA:CAVYSGGGADGLTF TRB:CASSLRFGETQYF	24	CD4-C7
Pla-2_ATTGGTGCACTTGGAT	Pla	Pla-2	P2-3770	FALSE	1	TRA:CAVGATYNAGNMLTF TRB:CATSRDLRTGNEAADTQYF	1	CD4-C7
Pla-2_ATTTCTGAGAGTTGGC	Pla	Pla-2	P2-3774	FALSE	1	TRA:CAVRDFLSGAGSYQLTF TRB:CASSLAQGLISPGEQFF	1	CD4-C7
Pla-2_ATTTCTGCATTCCTGC	Pla	Pla-2	P2-3777	FALSE	1	TRA:CAGGSNYKLTF TRB:CASSQVGLAGISTDTQYF	1	CD4-C7
Pla-2_CAACCTCAGCAGCCTC	Pla	Pla-2	P2-23	FALSE	3	TRA:CAVYSGGGADGLTF TRB:CASSLRFGETQYF	24	CD4-C7
Pla-2_CAACCTCGTCTGGAGA	Pla	Pla-2	P2-93	FALSE	3	TRA:CLVGDRATGGFKTIF TRB:CSALAGTGFNEQFF	6	CD4-C7
Pla-2_CAACCTCGTGGGTATG	Pla	Pla-2	P2-3784	FALSE	1	TRA:CALSEWEGAQKLVF TRB:CASRRVQAKELFF	1	CD4-C7
Pla-2_CAACTAGCATCAGTCA	Pla	Pla-2	P2-3789	FALSE	1	TRA:CARRSRGNNRLAF TRB:CASSRGVGELFF	1	CD4-C7

Pla-2_CAAGAAAAGACAGAGA	Pla	Pla-2	P2-3792	FALSE	1	TRA:CALDFNKFYF TRB:CASSQDALAGGRGNEQFF	1	CD4-C7
Pla-2_CAAGATCCAATCGAAA	Pla	Pla-2	P2-14	FALSE	3	TRA:CALSEAPGGSEKLVF TRB:CASSIGHPNSPLHF	37	CD4-C7
Pla-2_CAAGATCTCTCTAGGA	Pla	Pla-2	P2-3798	FALSE	1	TRA:CAGEVRARLMF TRB:CASSRGTSGGYNEQFF	1	CD4-C7
Pla-2_CACAAACCACTTCGAA	Pla	Pla-2	P2-457	FALSE	2	TRA:CATDLNFGNEKLTF TRB:CASSSMTGNNEQFF	2	CD4-C7
Pla-2_CACAAACGTCTCAACA	Pla	Pla-2	P2-3807	FALSE	1	TRA:CAESLPFSGGYQKVTF TRB:CSARDLAVGNEQFF	1	CD4-C7
Pla-2_CACACAAAGCGACGTA	Pla	Pla-2	P2-3811	FALSE	1	TRA:CALSNRDDKIIF TRB:CASSLAFGLAYEQFF	1	CD4-C7
Pla-2_CACACAAAGCTACCTA	Pla	Pla-2	P2-159	FALSE	3	TRA:CAASENNNARLMF TRB:CSAREDTDTQYF	4	CD4-C7
Pla-2_CACACCTGTACAGCAG	Pla	Pla-2	P2-14	FALSE	3	TRA:CALSEAPGGSEKLVF TRB:CASSIGHPNSPLHF	37	CD4-C7
Pla-2_CACACCTGTTGTGGCC	Pla	Pla-2	P2-3819	FALSE	1	TRA:CVVNSGAGNMLTF TRB:CASSTATSNQPQHF	1	CD4-C7
Pla-2_CACACCTTCCTTAATC	Pla	Pla-2	P2-3820	FALSE	1	TRA:CILRDVNGGATNKLIF TRB:CASSITGTVNTEAFF	1	CD4-C7
Pla-2_CACACTCTCTTGCAAG	Pla	Pla-2	P2-3825	FALSE	1	TRA:CAAFNARLMF TRB:CASSFQVPSDTQYF	1	CD4-C7
Pla-2_CACATAGAGACAAGCC	Pla	Pla-2	P2-14	FALSE	3	TRA:CALSEAPGGSEKLVF TRB:CASSIGHPNSPLHF	37	CD4-C7
Pla-2_CACATTTAGCATGGCA	Pla	Pla-2	P2-3833	FALSE	1	TRA:CAGHSDGQKLLF TRB:CASSLEWVFTGELFF	1	CD4-C7
Pla-2_CACATTTAGTCGTACT	Pla	Pla-2	P2-3835	FALSE	1	TRA:CALSETKSGFGNVLHC TRB:CASTYQLSGANVLTF	1	CD4-C7
Pla-2_CACCAGGCACGCCAGT	Pla	Pla-2	P2-3840	FALSE	1	TRA:CAVYSGAGSYQLTF TRB:CASSLDTVNTEAFF	1	CD4-C7
Pla-2_CACCAGGTCATTTGGG	Pla	Pla-2	P2-464	FALSE	2	TRA:CAMRYLWDDYKLSF TRB:CASSAQVVTQYF	2	CD4-C7
Pla-2_CACCTTGTCAACCAAC	Pla	Pla-2	P2-3845	FALSE	1	TRA:CAVSAHNAGNNRKLIW TRB:CASSLAASVDTQYF	1	CD4-C7
Pla-2_CACTCCATCTTAGCCC	Pla	Pla-2	P2-3848	FALSE	1	TRA:CAVGEYGNKLVF TRB:CASSLTGADTQYF	1	CD4-C7
Pla-2_CAGAGAGCACCCATGG	Pla	Pla-2	P2-14	FALSE	3	TRA:CALSEAPGGSEKLVF TRB:CASSIGHPNSPLHF	37	CD4-C7
Pla-2_CAGATCACAGCTTCGG	Pla	Pla-2	P2-465	FALSE	2	TRA:CAVSYSGAGSYQLTF TRB:CASSEWWRTTYEQYF	2	CD4-C7
Pla-2_CAGATCAGTCCGCTGA	Pla	Pla-2	P2-466	FALSE	2	TRA:CIVPKFGNEKLTF TRB:CASSPRTAGGYTF	2	CD4-C7
Pla-2_CAGATCAGTCCGTGAC	Pla	Pla-2	P2-3861	FALSE	1	TRA:CAVNLLGSYQLTF TRB:CASFTGTPGNTIYF	1	CD4-C7
Pla-2_CAGATCATCACCGTAA	Pla	Pla-2	P2-53	FALSE	3	TRA:CAVPFNFGNEKLTF TRB:CASSFRGPYNEQFF	11	CD4-C7
Pla-2_CAGCAGCAGACTAAGT	Pla	Pla-2	P2-3862	FALSE	1	TRA:CAVSDGQKLLF TRB:CASSITWTGGTDTQYF	1	CD4-C7
Pla-2_CAGCAGCAGCTAGTGG	Pla	Pla-2	P2-3863	FALSE	1	TRA:CAVNLGSGGYQKVTF TRB:CAIRDRENTEAFF	1	CD4-C7
Pla-2_CAGCATATCACTCCTG	Pla	Pla-2	P2-238	FALSE	3	TRA:CAVGRGGTSYGKLTF TRB:CASSLEWVFTGELFF	3	CD4-C7
Pla-2_CAGCGACCATCCAACA	Pla	Pla-2	P2-469	FALSE	2	TRA:CAVCAGGTSYGKLTF TRB:CASRPTSGYEQFF	2	CD4-C7

Pla-2_CAGCTGGTCGTTTAGG	Pla	Pla-2	P2-163	FALSE	3	TRA:CAVRDSRQAGTALIF TRB:CASRHDRQTLAGWETQYF	4	CD4-C7
Pla-2_CAGGTGCTCGGTCTAA	Pla	Pla-2	P2-472	FALSE	2	TRA:CAARRTGTASKLTF TRB:CSARDPTSGRVEQFF	2	CD4-C7
Pla-2_CAGTAACAGGTGCTAG	Pla	Pla-2	P2-473	FALSE	2	TRA:CGTDIYNFNKFYF TRB:CASRKNTEAFF	2	CD4-C7
Pla-2_CAGTAACAGTGCGTGA	Pla	Pla-2	P2-163	FALSE	3	TRA:CAVRDSRQAGTALIF TRB:CASRHDRQTLAGWETQYF	4	CD4-C7
Pla-2_CAGTAACCAAACCCAT	Pla	Pla-2	P2-9	FALSE	3	TRA:CAMTQNAGKSTF TRB:CASSPDRDYEQYF	47	CD4-C7
Pla-2_CAGTAACTCCGTAGGC	Pla	Pla-2	P2-3886	FALSE	1	TRA:CVVSETSGTYKYIF TRB:CASSRGLQETQYF	1	CD4-C7
Pla-2_CAGTCCTAGAGCCTAG	Pla	Pla-2	P2-23	FALSE	3	TRA:CAVYSGGGADGLTF TRB:CASSLRFGETQYF	24	CD4-C7
Pla-2_CAGTCCTGTAAATGAC	Pla	Pla-2	P2-3890	FALSE	1	TRA:CAVNSQGGKLIF TRB:CSARDSSYNSPLHF	1	CD4-C7
Pla-2_CATATGGGTAAAGTCA	Pla	Pla-2	P2-422	FALSE	2	TRA:CAVNREGGGADGLTF TRB:CSAYGSRTDTQYF	2	CD4-C7
Pla-2_CATATTCAGATATGGT	Pla	Pla-2	P2-144	FALSE	3	TRA:CAYRGAYSGAGSYQLTF TRB:CASSPPYWQADTQYF	4	CD4-C7
Pla-2_CATCAAGTCTCTGAGA	Pla	Pla-2	P2-397	FALSE	2	TRA:CAASDWGRGGSNYKLTF TRB:CASSYRLAETGEQFF	2	CD4-C7
Pla-2_CATCAGATCCACGACG	Pla	Pla-2	P2-3903	FALSE	1	TRA:CIVRVIPLRPGANSKLTF TRB:CSAETGWSEQYF	1	CD4-C7
Pla-2_CATCCACAGTCAAGGC	Pla	Pla-2	P2-475	FALSE	2	TRA:CGTANSGGYQKVTF TRB:CASMGHFNEKLFF	2	CD4-C7
Pla-2_CATCGAACACCGAATT	Pla	Pla-2	P2-3907	FALSE	1	TRA:CAVRDISNFGNEKLTF TRB:CASSLQGSTYNEQFF	1	CD4-C7
Pla-2_CATCGAATCCTCGCAT	Pla	Pla-2	P2-84	FALSE	3	TRA:CAVSDLTGGGNKLTF TRB:CSVQTEMNTEAFF	7	CD4-C7
Pla-2_CATTATCAGCTAGGCA	Pla	Pla-2	P2-424	FALSE	2	TRA:CAVLKAAGNKLTF TRB:CAWSGGLTDTQYF	2	CD4-C7
Pla-2_CATTATCCATTACCTT	Pla	Pla-2	P2-412	FALSE	2	TRA:CAVDGDGGSQGNLIF TRB:CASSERGRIYGYTF	2	CD4-C7
Pla-2_CATTATCGTAAACCTC	Pla	Pla-2	P2-240	FALSE	3	TRA:CALIHQRALTF TRB:CASSPQDRISTDTQYF	3	CD4-C7
Pla-2_CATTATCGTAATAGCA	Pla	Pla-2	P2-3928	FALSE	1	TRA:CAAENSGGYQKVTF TRB:CASSIGDSSYEQYF	1	CD4-C7
Pla-2_CATTATCGTGGGTCAA	Pla	Pla-2	P2-3929	FALSE	1	TRA:CALSEAPGGSEKLVF TRB:CASSLDGMNTEAFF	1	CD4-C7
Pla-2_CATTCGCAGCCGGTAA	Pla	Pla-2	P2-23	FALSE	3	TRA:CAVYSGGGADGLTF TRB:CASSLRFGETQYF	24	CD4-C7
Pla-2_CATTCGCTCTGTCTCG	Pla	Pla-2	P2-241	FALSE	3	TRA:CAAYTSGTYKYIF TRB:CASSAQVVTQYF	3	CD4-C7
Pla-2_CCAATCCCAGCTGGCT	Pla	Pla-2	P2-3938	FALSE	1	TRA:CIVTISGNTGKLIF TRB:CAGGDLLGRAYNEQFF	1	CD4-C7
Pla-2_CCACCTACACGTCTCT	Pla	Pla-2	P2-3942	FALSE	1	TRA:CAVRDIREYGNKLVF TRB:CASSPQGAETQYF	1	CD4-C7
Pla-2_CCACCTAGTAAGTGGC	Pla	Pla-2	P2-9	FALSE	3	TRA:CAMTQNAGKSTF TRB:CASSPDRDYEQYF	47	CD4-C7
Pla-2_CCACCTAGTCACCCAG	Pla	Pla-2	P2-3944	FALSE	1	TRA:CAVSNANVVSGSARQLTF TRB:CASSSTVTEAFF	1	CD4-C7
Pla-2_CCACCTAGTCTGGAGA	Pla	Pla-2	P2-9	FALSE	3	TRA:CAMTQNAGKSTF TRB:CASSPDRDYEQYF	47	CD4-C7

Pla-2_CCACTACAGATCTGCT	Pla	Pla-2	P2-14	FALSE	3	TRA:CALSEAPGGSEKLVF TRB:CASSIGHPNSPLHF	37	CD4-C7
Pla-2_CCACTACCAACTGGCC	Pla	Pla-2	P2-23	FALSE	3	TRA:CAVYSGGGADGLTF TRB:CASSLRFGETQYF	24	CD4-C7
Pla-2_CCACTACCAAGCGCTC	Pla	Pla-2	P2-115	FALSE	3	TRA:CAVRDEDNQGGKLIF TRB:CASSQDQGLAGGFSFKETQYI	5	CD4-C7
Pla-2_CCAGCGACAGAGCCAA	Pla	Pla-2	P2-14	FALSE	3	TRA:CALSEAPGGSEKLVF TRB:CASSIGHPNSPLHF	37	CD4-C7
Pla-2_CCATGTCGTCTACCTC	Pla	Pla-2	P2-14	FALSE	3	TRA:CALSEAPGGSEKLVF TRB:CASSIGHPNSPLHF	37	CD4-C7
Pla-2_CCATGTCGTTAAGAAC	Pla	Pla-2	P2-23	FALSE	3	TRA:CAVYSGGGADGLTF TRB:CASSLRFGETQYF	24	CD4-C7
Pla-2_CCATGTCTCGCGGATC	Pla	Pla-2	P2-3956	FALSE	1	TRA:CAVSAGATNKLIF TRB:CASSQGGGGGLAQYF	1	CD4-C7
Pla-2_CCCAATCAGCAAATCA	Pla	Pla-2	P2-432	FALSE	2	TRA:CAATDSWGKFQF TRB:CSARGDSNQPQHF	2	CD4-C7
Pla-2_CCCAATCAGTGCGTGA	Pla	Pla-2	P2-3959	FALSE	1	TRA:CVVNQGTGGFKTIF TRB:CSANLAGGYEQYF	1	CD4-C7
Pla-2_CCCAGTTTCTGGGCCA	Pla	Pla-2	P2-482	FALSE	2	TRA:CALYNFNKFYF TRB:CASSPRLAGGTDTQYF	2	CD4-C7
Pla-2_CCCAGTTTCTTGTTTG	Pla	Pla-2	P2-3966	FALSE	1	TRA:CATDLIREGDFGNEKLTF TRB:CASSGRSRGVHEQYF	1	CD4-C7
Pla-2_CCGTACTCAGCAGTTT	Pla	Pla-2	P2-242	FALSE	3	TRA:CAVMRGGSEKLVF TRB:CASIVGRSSYNEQFF	3	CD4-C7
Pla-2_CCGTGGAAGAGTACAT	Pla	Pla-2	P2-23	FALSE	3	TRA:CAVYSGGGADGLTF TRB:CASSLRFGETQYF	24	CD4-C7
Pla-2_CCGTGGAGTATTCGTG	Pla	Pla-2	P2-23	FALSE	3	TRA:CAVYSGGGADGLTF TRB:CASSLRFGETQYF	24	CD4-C7
Pla-2_CCGTTCAAGATGTAAC	Pla	Pla-2	P2-136	FALSE	3	TRA:CAASVRSQGNLIF TRB:CASSLGPNRYRQRNTIYF	4	CD4-C7
Pla-2_CCGTTCAGTTAAGAAC	Pla	Pla-2	P2-163	FALSE	3	TRA:CAVRDSRQAGTALIF TRB:CASRHDRQTLAGWETQYF	4	CD4-C7
Pla-2_CCTAAAGGTCTTCAAG	Pla	Pla-2	P2-3984	FALSE	1	TRA:CAVTRGDDKIIF TRB:CAWSPRGRGQYF	1	CD4-C7
Pla-2_CCTAAAGTCCGTAGGC	Pla	Pla-2	P2-93	FALSE	3	TRA:CLVGDRATGGFKTIF TRB:CSALAGTGFNEQFF	6	CD4-C7
Pla-2_CCTACACTCACGCATA	Pla	Pla-2	P2-3986	FALSE	1	TRA:CAEYGDSSASKIIF TRB:CSVCTGEEENTGELFF	1	CD4-C7
Pla-2_CCTACCAAGTGCGTGA	Pla	Pla-2	P2-3988	FALSE	1	TRA:CIVRGRHTDKLIF TRB:CASSLSTRYNEQFF	1	CD4-C7
Pla-2_CCTACCATCACTATTC	Pla	Pla-2	P2-136	FALSE	3	TRA:CAASVRSQGNLIF TRB:CASSLGPNRYRQRNTIYF	4	CD4-C7
Pla-2_CCTAGCTTCAACACCA	Pla	Pla-2	P2-3994	FALSE	1	TRA:CIVRGWNNDMRF TRB:CASSRQGSYEQYF	1	CD4-C7
Pla-2_CCTAGCTTCCATGAAC	Pla	Pla-2	P2-3995	FALSE	1	TRA:CAASRGDRDDKIIF TRB:CAWSWTASYEQYF	1	CD4-C7
Pla-2_CCTCAGTCAGCGTTCG	Pla	Pla-2	P2-3999	FALSE	1	TRA:CATGNTGTASKLTF TRB:CASSFGSAYEQYF	1	CD4-C7
Pla-2_CCTTACGAGCGAAGGG	Pla	Pla-2	P2-493	FALSE	2	TRA:CAVGPTNFGNEKLTF TRB:CASSPQGRDTEAFF	2	CD4-C7
Pla-2_CCTTACGAGTGTACTC	Pla	Pla-2	P2-93	FALSE	3	TRA:CLVGDRATGGFKTIF TRB:CSALAGTGFNEQFF	6	CD4-C7
Pla-2_CCTTACGCAAATACAG	Pla	Pla-2	P2-4007	FALSE	1	TRA:CAPLSGGSNYKLTF TRB:CASSVHSGSYEQYF	1	CD4-C7

Pla-2_CCTTACGGTCCAAGTT	Pla	Pla-2	P2-4008	FALSE	1	TRA:CAGVTLGAQKLVF TRB:CSAIPGQVNTEAFF	1	CD4-C7
Pla-2_CCTTACGGTCGGCACT	Pla	Pla-2	P2-9	FALSE	3	TRA:CAMTQNAGKSTF TRB:CASSPDRDYEQYF	47	CD4-C7
Pla-2_CCTTCCCCAGAAGCAC	Pla	Pla-2	P2-4013	FALSE	1	TRA:CGTIDDSWGKFQF TRB:CASSSRESTNTGELFF	1	CD4-C7
Pla-2_CCTTCGAAGAAGGGTA	Pla	Pla-2	P2-14	FALSE	3	TRA:CALSEAPGGSEKLVF TRB:CASSIGHPNSPLHF	37	CD4-C7
Pla-2_CCTTTCTAGCACACAG	Pla	Pla-2	P2-247	FALSE	3	TRA:CIVRVLIQGAQKLVF TRB:CASSEGGTLYGYTF	3	CD4-C7
Pla-2_CGAACATAGGCCCTCA	Pla	Pla-2	P2-51	FALSE	3	TRA:CAENNDTGGFKTIF TRB:CATRGAHEKLFF	11	CD4-C7
Pla-2_CGAACATAGTGTACGG	Pla	Pla-2	P2-241	FALSE	3	TRA:CAAYTSGTYKYIF TRB:CASSAQVVTQYF	3	CD4-C7
Pla-2_CGAACATCAATTGCTG	Pla	Pla-2	P2-4025	FALSE	1	TRA:CAMRERSWVRQKLVF TRB:CASSQAAGVTDTQYF	1	CD4-C7
Pla-2_CGAATGTGTAAATACG	Pla	Pla-2	P2-4031	FALSE	1	TRA:CALGGMNSGYSTLTF TRB:CASSPGTAVGETQYF	1	CD4-C7
Pla-2_CGAATGTGTCTCACCT	Pla	Pla-2	P2-71	FALSE	3	TRA:CAARNTGGFKTIF TRB:CAWSEGTSGGTDTQYF	8	CD4-C7
Pla-2_CGACCTTCAGTGAGTG	Pla	Pla-2	P2-494	FALSE	2	TRA:CAFAPASGAGGTSYGKLTF TRB:CASSYLAETQYF	2	CD4-C7
Pla-2_CGACCTTGTTAGAACA	Pla	Pla-2	P2-154	FALSE	3	TRA:CAMSGGGADGLTF TRB:CASSFLRGGINEQYF	4	CD4-C7
Pla-2_CGAGCACCATCCAACA	Pla	Pla-2	P2-4043	FALSE	1	TRA:CAVMHIQGAQKLVF TRB:CASSLAGNSPLHF	1	CD4-C7
Pla-2_CGAGCACTCCGTAGGC	Pla	Pla-2	P2-9	FALSE	3	TRA:CAMTQNAGKSTF TRB:CASSPDRDYEQYF	47	CD4-C7
Pla-2_CGAGCCAAGTGTGGCA	Pla	Pla-2	P2-23	FALSE	3	TRA:CAVYSGGGADGLTF TRB:CASSLRFGETQYF	24	CD4-C7
Pla-2_CGATTGAGTAAACACA	Pla	Pla-2	P2-4064	FALSE	1	TRA:CALANQAGTALIF TRB:CASSTDRGFSGYTF	1	CD4-C7
Pla-2_CGCCAAGAGGTGTTAA	Pla	Pla-2	P2-498	FALSE	2	TRA:CAFMKHDRFYF TRB:CASSFYLWGAHPDTEAFF	2	CD4-C7
Pla-2_CGCCAAGTCCAGGGCT	Pla	Pla-2	P2-418	FALSE	2	TRA:CAVKNFGNEKLTF TRB:CASSKDHFVEQYF	2	CD4-C7
Pla-2_CGCGGTAAGCCCAGCT	Pla	Pla-2	P2-223	FALSE	3	TRA:CAQNTGNQFYF TRB:CASSSIDTQYF	3	CD4-C7
Pla-2_CGCGGTAAGTCGATAA	Pla	Pla-2	P2-235	FALSE	3	TRA:CATENNARLMF TRB:CSRDRGLQTEAFF	3	CD4-C7
Pla-2_CGCGGTATCAAGGCTT	Pla	Pla-2	P2-249	FALSE	3	TRA:CAESRDQTGANNLFF TRB:CASSSLGRNTEAFF	3	CD4-C7
Pla-2_CGCGTTTCAATGGATA	Pla	Pla-2	P2-9	FALSE	3	TRA:CAMTQNAGKSTF TRB:CASSPDRDYEQYF	47	CD4-C7
Pla-2_CGCGTTTCACGGTGTC	Pla	Pla-2	P2-4077	FALSE	1	TRA:CATAPSGGYQKVTF TRB:CASSYFPGPGETQYF	1	CD4-C7
Pla-2_CGCTATCGTACACCGC	Pla	Pla-2	P2-494	FALSE	2	TRA:CAFAPASGAGGTSYGKLTF TRB:CASSYLAETQYF	2	CD4-C7
Pla-2_CGGACACCACACATGT	Pla	Pla-2	P2-144	FALSE	3	TRA:CAYRGAYSGAGSYQLTF TRB:CASSPPYWQADTQYF	4	CD4-C7
Pla-2_CGGAGCTAGCCAACAG	Pla	Pla-2	P2-4105	FALSE	1	TRA:CAVRSRPDSWGKFQF TRB:CASRKLAGVDGPNNEQFF	1	CD4-C7
Pla-2_CGGAGCTCACTAGTAC	Pla	Pla-2	P2-95	FALSE	3	TRA:CAENIANYGGSQGNLIF TRB:CASSEAVQQFF	6	CD4-C7

Pla-2_CGGAGCTTCCTTGCCA	Pla	Pla-2	P2-9	FALSE	3	TRA:CAMTQNAGKSTF TRB:CASSPDRDYEQYF	47	CD4-C7
Pla-2_CGGGTCAGTCTGATTG	Pla	Pla-2	P2-53	FALSE	3	TRA:CAVPFNFGNEKLTF TRB:CASSFRGPYNEQFF	11	CD4-C7
Pla-2_CGGGTCATCCGTACAA	Pla	Pla-2	P2-74	FALSE	3	TRA:CAIRNNNARLMF TRB:CASSLERHTGLYGYTF	8	CD4-C7
Pla-2_CGGTTAACACGTCTCT	Pla	Pla-2	P2-14	FALSE	3	TRA:CALSEAPGGSEKLVF TRB:CASSIGHPNSPLHF	37	CD4-C7
Pla-2_CGGTTAAGTCTCTCGT	Pla	Pla-2	P2-31	FALSE	3	TRA:CAASDNTDKLIF TRB:CASSQVHEQYF	20	CD4-C7
Pla-2_CGTAGCGGTGTTCGAT	Pla	Pla-2	P2-9	FALSE	3	TRA:CAMTQNAGKSTF TRB:CASSPDRDYEQYF	47	CD4-C7
Pla-2_CGTCACTAGATAGTCA	Pla	Pla-2	P2-119	FALSE	3	TRA:CAASIMDDMRF TRB:CSAAIGYGYTF	5	CD4-C7
Pla-2_CGTCACTCAAGTCATC	Pla	Pla-2	P2-252	FALSE	3	TRA:CAVTPTSGTYKYIF TRB:CASSYGPSGGLNEQFF	3	CD4-C7
Pla-2_CGTCCATAGTGGGTTG	Pla	Pla-2	P2-14	FALSE	3	TRA:CALSEAPGGSEKLVF TRB:CASSIGHPNSPLHF	37	CD4-C7
Pla-2_CGTCTACCAGTTCCCT	Pla	Pla-2	P2-9	FALSE	3	TRA:CAMTQNAGKSTF TRB:CASSPDRDYEQYF	47	CD4-C7
Pla-2_CGTGAGCTCTCTGTCG	Pla	Pla-2	P2-252	FALSE	3	TRA:CAVTPTSGTYKYIF TRB:CASSYGPSGGLNEQFF	3	CD4-C7
Pla-2_CGTGTCTAGGCTAGCA	Pla	Pla-2	P2-512	FALSE	2	TRA:CAIMNTGGFKTIF TRB:CASSFLRGGINEQYF	2	CD4-C7
Pla-2_CGTGTCTAGTCAAGCG	Pla	Pla-2	P2-4149	FALSE	1	TRA:CGTEIRTGNQFYF TRB:CASSIGMSSYTGELFF	1	CD4-C7
Pla-2_CGTTAGACAAGGCTCC	Pla	Pla-2	P2-14	FALSE	3	TRA:CALSEAPGGSEKLVF TRB:CASSIGHPNSPLHF	37	CD4-C7
Pla-2_CGTTCTGAGAAGAAGC	Pla	Pla-2	P2-4155	FALSE	1	TRA:CAASVAGSARQLTF TRB:CAWSVVHGWSPLHF	1	CD4-C7
Pla-2_CGTTCTGGTAAACCTC	Pla	Pla-2	P2-23	FALSE	3	TRA:CAVYSGGGADGLTF TRB:CASSLRFGETQYF	24	CD4-C7
Pla-2_CGTTCTGGTTACAGAA	Pla	Pla-2	P2-4158	FALSE	1	TRA:CAENMVGNAGNMLTF TRB:CASSLSTGGIYEQYF	1	CD4-C7
Pla-2_CTAAGACAGCTGAAAT	Pla	Pla-2	P2-119	FALSE	3	TRA:CAASIMDDMRF TRB:CSAAIGYGYTF	5	CD4-C7
Pla-2_CTACCCACACTTGGAT	Pla	Pla-2	P2-95	FALSE	3	TRA:CAENIANYGGSQGNLIF TRB:CASSEAVQQQFF	6	CD4-C7
Pla-2_CTACGTCTCCTTCAAT	Pla	Pla-2	P2-117	FALSE	3	TRA:CAVDITGGGNKLTF TRB:CSAREDTDTQYF	5	CD4-C7
Pla-2_CTAGAGTCAGTGGGAT	Pla	Pla-2	P2-14	FALSE	3	TRA:CALSEAPGGSEKLVF TRB:CASSIGHPNSPLHF	37	CD4-C7
Pla-2_CTAGAGTTCCTATTCA	Pla	Pla-2	P2-135	FALSE	3	TRA:CAVATYSGAGSYQLTF TRB:CASSYGPSGGLNEQFF	4	CD4-C7
Pla-2_CTAGCCTGTCATCCCT	Pla	Pla-2	P2-53	FALSE	3	TRA:CAVPFNFGNEKLTF TRB:CASSFRGPYNEQFF	11	CD4-C7
Pla-2_CTAGTGACAGCTGTAT	Pla	Pla-2	P2-4191	FALSE	1	TRA:CAPSLMDSSYKLIF TRB:CSARDPAVSNQPQHF	1	CD4-C7
Pla-2_CTCACACCATCATCCC	Pla	Pla-2	P2-517	FALSE	2	TRA:CAAKAGTALIF TRB:CASSPATYNYEQYF	2	CD4-C7
Pla-2_CTCAGAAAGCTGCAAG	Pla	Pla-2	P2-23	FALSE	3	TRA:CAVYSGGGADGLTF TRB:CASSLRFGETQYF	24	CD4-C7
Pla-2_CTCATTAGTATAAACG	Pla	Pla-2	P2-4200	FALSE	1	TRA:CGADMQGAQKLVF TRB:CSATPEVGKGGAFF	1	CD4-C7

Pla-2_CTCATTAGTCGTCTTC	Pla	Pla-2	P2-4201	FALSE	1	TRA:CAMTQNAGKSTF TRB:CASSQESGGLVFEQFF	1	CD4-C7
Pla-2_CTCCTAGCAGGTGGAT	Pla	Pla-2	P2-4204	FALSE	1	TRA:CAGRHAGNMLTF TRB:CSANQRDTQYF	1	CD4-C7
Pla-2_CTCCTAGTCTACCTGC	Pla	Pla-2	P2-4207	FALSE	1	TRA:CSQKAGGTSYGKLTF TRB:CASSYQTGVNTEAFF	1	CD4-C7
Pla-2_CTCGAGGAGTGAATTG	Pla	Pla-2	P2-429	FALSE	2	TRA:CVVSARGATNKLIF TRB:CASSELQHNEQFF	2	CD4-C7
Pla-2_CTCGAGGGTCGGGTCT	Pla	Pla-2	P2-95	FALSE	3	TRA:CAENIANYGGSQGNLIF TRB:CASSEAVQQQFF	6	CD4-C7
Pla-2_CTCGGAGAGCTAAACA	Pla	Pla-2	P2-9	FALSE	3	TRA:CAMTQNAGKSTF TRB:CASSPDRDYEQYF	47	CD4-C7
Pla-2_CTCGGAGGTGTTCGAT	Pla	Pla-2	P2-14	FALSE	3	TRA:CALSEAPGGSEKLVF TRB:CASSIGHPNSPLHF	37	CD4-C7
Pla-2_CTCGGAGGTTAAAGTG	Pla	Pla-2	P2-227	FALSE	3	TRA:CAVRSSTGKLIF TRB:CASSYRQATNEKLFF	3	CD4-C7
Pla-2_CTCGGGAAGACTCGGA	Pla	Pla-2	P2-4215	FALSE	1	TRA:CAASTNFGNEKLTF TRB:CASSPGQTQYF	1	CD4-C7
Pla-2_CTCGGGAGTACCGAGA	Pla	Pla-2	P2-9	FALSE	3	TRA:CAMTQNAGKSTF TRB:CASSPDRDYEQYF	47	CD4-C7
Pla-2_CTCGGGAGTAGGAGTC	Pla	Pla-2	P2-150	FALSE	3	TRA:CVVRYAGNNRKLIW TRB:CASSQGQPPYEQYF	4	CD4-C7
Pla-2_CTCGTACGTTCCACGG	Pla	Pla-2	P2-9	FALSE	3	TRA:CAMTQNAGKSTF TRB:CASSPDRDYEQYF	47	CD4-C7
Pla-2_CTCGTACTCTATCCCG	Pla	Pla-2	P2-9	FALSE	3	TRA:CAMTQNAGKSTF TRB:CASSPDRDYEQYF	47	CD4-C7
Pla-2_CTCGTCACAATAGAGT	Pla	Pla-2	P2-4221	FALSE	1	TRA:CAARVSDGGQKLLF TRB:CASGVDSNQPQHF	1	CD4-C7
Pla-2_CTCTAATAGAGATGAG	Pla	Pla-2	P2-4222	FALSE	1	TRA:CALLTVQGAQKLVF TRB:CASSRSIRDLRDEQYF	1	CD4-C7
Pla-2_CTGATCCCAACGCACC	Pla	Pla-2	P2-4237	FALSE	1	TRA:CAFRMDSSYKLIF TRB:CSARQGANTGELFF	1	CD4-C7
Pla-2_CTGATCCCAGCCTATA	Pla	Pla-2	P2-9	FALSE	3	TRA:CAMTQNAGKSTF TRB:CASSPDRDYEQYF	47	CD4-C7
Pla-2_CTGATCCGTCTGGAGA	Pla	Pla-2	P2-4240	FALSE	1	TRA:CAAPRGGSGTYKYIF TRB:CASSVAGADQPQHF	1	CD4-C7
Pla-2_CTGATCCTCTCCGGTT	Pla	Pla-2	P2-4241	FALSE	1	TRA:CAASKVYKLSF TRB:CASSPGTGRSNIQYF	1	CD4-C7
Pla-2_CTGCCTACAGATCTGT	Pla	Pla-2	P2-254	FALSE	3	TRA:CAVEASNNDMRF TRB:CASRFSGANVLTF	3	CD4-C7
Pla-2_CTGCGGACAACACCTA	Pla	Pla-2	P2-4244	FALSE	1	TRA:CALRDSGTYKYIF TRB:CASKLGASGGDYEQYF	1	CD4-C7
Pla-2_CTGCGGAGTATGCTTG	Pla	Pla-2	P2-527	FALSE	2	TRA:CAVSPATGRRALTF TRB:CAITRDYSYEQYF	2	CD4-C7
Pla-2_CTGCTGTGTCGGCTCA	Pla	Pla-2	P2-113	FALSE	3	TRA:CADVDSNYQLIW TRB:CASSPRTAGGYTF	5	CD4-C7
Pla-2_CTGGTCTGTAAGGGCT	Pla	Pla-2	P2-4250	FALSE	1	TRA:CAGGTYMRF TRB:CASSSPRDRGRETQYF	1	CD4-C7
Pla-2_CTGGTCTGTCCATGAT	Pla	Pla-2	P2-84	FALSE	3	TRA:CAVSDLTGGGNKLTF TRB:CSVQTEMNTEAFF	7	CD4-C7
Pla-2_CTGGTCTTCTCTGCTG	Pla	Pla-2	P2-208	FALSE	3	TRA:CAESRTGGGADGLTF TRB:CASSLGGRALRDTQYF	3	CD4-C7
Pla-2_CTGTTTACACCCTATC	Pla	Pla-2	P2-223	FALSE	3	TRA:CAQNTGNQFYF TRB:CASSSIDTQYF	3	CD4-C7

Pla-2_CTTAACTTCGCCGTGA	Pla	Pla-2	P2-147	FALSE	3	TRA:CAGPPGGYNKLIF TRB:CAIRQGDRQPQHF	4	CD4-C7
Pla-2_CTTACCGCATAGTAAG	Pla	Pla-2	P2-255	FALSE	3	TRA:CAVRDMNSGGYQKVTF TRB:CASSLGTNTEAFF	3	CD4-C7
Pla-2_CTTACCGTCGCACTCT	Pla	Pla-2	P2-4268	FALSE	1	TRA:CAVEGTNAGKSTF TRB:CASSLTGGTEAFF	1	CD4-C7
Pla-2_CTTAGGACAGATGAGC	Pla	Pla-2	P2-4270	FALSE	1	TRA:CAVGRLYSGAGSYQLTF TRB:CSARDTRLGNEQFF	1	CD4-C7
Pla-2_CTTAGGATCCTCCTAG	Pla	Pla-2	P2-9	FALSE	3	TRA:CAMTQNAGKSTF TRB:CASSPDRDYEQYF	47	CD4-C7
Pla-2_CTTCTCTAGACCCACC	Pla	Pla-2	P2-472	FALSE	2	TRA:CAARRTGTASKLTF TRB:CSARDPTSGRVEQFF	2	CD4-C7
Pla-2_CTTGGCTAGTAAGTAC	Pla	Pla-2	P2-247	FALSE	3	TRA:CIVRVLIQGAQKLVF TRB:CASSEGGTLYGYTF	3	CD4-C7
Pla-2_GAAACTCAGTTACGGG	Pla	Pla-2	P2-425	FALSE	2	TRA:CAVKNGGSQGNLIF TRB:CASSYGLAGPSYNEQFF	2	CD4-C7
Pla-2_GAAACTCGTGATGTGG	Pla	Pla-2	P2-447	FALSE	2	TRA:CIVRPYNTDKLIF TRB:CASGEGSTDTQYF	2	CD4-C7
Pla-2_GAACATCAGGCGACAT	Pla	Pla-2	P2-256	FALSE	3	TRA:CAASMGTGGFKTIF TRB:CSAGGQDTQYF	3	CD4-C7
Pla-2_GAACCTACACTTACGA	Pla	Pla-2	P2-9	FALSE	3	TRA:CAMTQNAGKSTF TRB:CASSPDRDYEQYF	47	CD4-C7
Pla-2_GAACCTAGTCACCTAA	Pla	Pla-2	P2-493	FALSE	2	TRA:CAVGPTNFGNEKLTF TRB:CASSPQGRDTEAFF	2	CD4-C7
Pla-2_GAACGGATCGGAGGTA	Pla	Pla-2	P2-4307	FALSE	1	TRA:CAVKADSSYKLIF TRB:CATSSATRAGESGTQYF	1	CD4-C7
Pla-2_GAATGAAAGATGAGAG	Pla	Pla-2	P2-4319	FALSE	1	TRA:CAESPGGFKTIF TRB:CSVESGGGGGYTF	1	CD4-C7
Pla-2_GAATGAATCAGGCCCA	Pla	Pla-2	P2-14	FALSE	3	TRA:CALSEAPGGSEKLVF TRB:CASSIGHPNSPLHF	37	CD4-C7
Pla-2_GACCAATCACACCGCA	Pla	Pla-2	P2-234	FALSE	3	TRA:CAMREVTTDSWGKFQF TRB:CASSPGAKNIQYF	3	CD4-C7
Pla-2_GACCAATTCGGACAAG	Pla	Pla-2	P2-498	FALSE	2	TRA:CAFMKHDRFYF TRB:CASSFYLWGAHPDTEAFF	2	CD4-C7
Pla-2_GACCTGGCAATCACAC	Pla	Pla-2	P2-117	FALSE	3	TRA:CAVDITGGGNKLTF TRB:CSAREDTDTQYF	5	CD4-C7
Pla-2_GACCTGGGTGCCTGCA	Pla	Pla-2	P2-84	FALSE	3	TRA:CAVSDLTGGGNKLTF TRB:CSVQTEMNTEAFF	7	CD4-C7
Pla-2_GACGCGTCACATTTCT	Pla	Pla-2	P2-31	FALSE	3	TRA:CAASDNTDKLIF TRB:CASSQVHEQYF	20	CD4-C7
Pla-2_GACGTGCCAGTACACT	Pla	Pla-2	P2-4344	FALSE	1	TRA:CAASYQGGSEKLVF TRB:CASLGGRTDTQYF	1	CD4-C7
Pla-2_GAGCAGACAGGCTGAA	Pla	Pla-2	P2-115	FALSE	3	TRA:CAVRDEDNQGGKLIF TRB:CASSQDQGLAGGFSFKETQYF	5	CD4-C7
Pla-2_GAGCAGAGTAGTACCT	Pla	Pla-2	P2-388	FALSE	2	TRA:CAANSGGSNYKLTF TRB:CASSLWGTGLETQYF	2	CD4-C7
Pla-2_GAGGTGAAGCTAGTCT	Pla	Pla-2	P2-4367	FALSE	1	TRA:CAASLSGGYQKVTF TRB:CASSFTGSSYNSPLHF	1	CD4-C7
Pla-2_GAGGTGATCCGTTGCT	Pla	Pla-2	P2-14	FALSE	3	TRA:CALSEAPGGSEKLVF TRB:CASSIGHPNSPLHF	37	CD4-C7
Pla-2_GAGTCCGGTCCAGTTA	Pla	Pla-2	P2-154	FALSE	3	TRA:CAMSGGGADGLTF TRB:CASSFLRGGINEQYF	4	CD4-C7
Pla-2_GAGTCCGTCCCTGACT	Pla	Pla-2	P2-542	FALSE	2	TRA:CAASKVYKLSF TRB:CASSLGLADITDTQYF	2	CD4-C7

Pla-2_GATCGATAGCGATCCC	Pla	Pla-2	P2-545	FALSE	2	TRA:CAVVFQNQGGKLIF TRB:CASSERRNEQFF	2	CD4-C7
Pla-2_GATCGATAGTACGCCC	Pla	Pla-2	P2-113	FALSE	3	TRA:CADVDSNYQLIW TRB:CASSPRTAGGYTF	5	CD4-C7
Pla-2_GATCTAGGTCTCCCTA	Pla	Pla-2	P2-9	FALSE	3	TRA:CAMTQNAGKSTF TRB:CASSPDRDYEQYF	47	CD4-C7
Pla-2_GATGAGGAGTGTACTC	Pla	Pla-2	P2-238	FALSE	3	TRA:CAVGRGGTSYGKLTF TRB:CASSLEWVFTGELFF	3	CD4-C7
Pla-2_GCAAACTAGTGTACCT	Pla	Pla-2	P2-410	FALSE	2	TRA:CLVGDRSYNTDKLIF TRB:CASSQGHEKLFF	2	CD4-C7
Pla-2_GCAATCAGTGACGGTA	Pla	Pla-2	P2-4400	FALSE	1	TRA:CAVGLNDMRF TRB:CASSLSPSYEQYF	1	CD4-C7
Pla-2_GCACATACATGTCGAT	Pla	Pla-2	P2-9	FALSE	3	TRA:CAMTQNAGKSTF TRB:CASSPDRDYEQYF	47	CD4-C7
Pla-2_GCACATATCAAACGGG	Pla	Pla-2	P2-9	FALSE	3	TRA:CAMTQNAGKSTF TRB:CASSPDRDYEQYF	47	CD4-C7
Pla-2_GCACTCTAGGATGCGT	Pla	Pla-2	P2-240	FALSE	3	TRA:CALIHQRALTF TRB:CASSPQDRISTDTQYF	3	CD4-C7
Pla-2_GCACTCTCATGCCACG	Pla	Pla-2	P2-159	FALSE	3	TRA:CAASENNNARLMF TRB:CSAREDTDTQYF	4	CD4-C7
Pla-2_GCACTCTTCTACTTAC	Pla	Pla-2	P2-4408	FALSE	1	TRA:CAGQLGDMRF TRB:CASSGRQVEAFF	1	CD4-C7
Pla-2_GCAGTTACAGGTGCCT	Pla	Pla-2	P2-4414	FALSE	1	TRA:CALDPLNSNSGYALNF TRB:CSVGVGGQVSDTQYF	1	CD4-C7
Pla-2_GCATACACAGCCACCA	Pla	Pla-2	P2-51	FALSE	3	TRA:CAENNDTGGFKTIF TRB:CATRGAHEKLFF	11	CD4-C7
Pla-2_GCATACAGTGACAAAT	Pla	Pla-2	P2-4419	FALSE	1	TRA:CAASPTGANSKLTF TRB:CASSEGRASSQFF	1	CD4-C7
Pla-2_GCATGATAGCTAGTTC	Pla	Pla-2	P2-4420	FALSE	1	TRA:CAVNTGFQKLVF TRB:CASSPTSAQTYNEQFF	1	CD4-C7
Pla-2_GCATGCGGTCCTCTTG	Pla	Pla-2	P2-205	TRUE	3	TRA:CALSESTNAGKSTF TRB:CAWSYADTQYF	3	CD4-C7
Pla-2_GCATGTAAGAGGACGG	Pla	Pla-2	P2-9	FALSE	3	TRA:CAMTQNAGKSTF TRB:CASSPDRDYEQYF	47	CD4-C7
Pla-2_GCATGTACAAGAGGCT	Pla	Pla-2	P2-242	FALSE	3	TRA:CAVMRGGSEKLVF TRB:CASIVGRSSYNEQFF	3	CD4-C7
Pla-2_GCATGTATCGGAAATA	Pla	Pla-2	P2-4432	FALSE	1	TRA:CALSGGPGYSTLTF TRB:CSAMDTYEQYF	1	CD4-C7
Pla-2_GCGAGAAAGAAACCAT	Pla	Pla-2	P2-4444	FALSE	1	TRA:CAVQGVIQGAQKLVF TRB:CASSRVDEQFF	1	CD4-C7
Pla-2_GCGCAGTAGGCAGGTT	Pla	Pla-2	P2-4448	FALSE	1	TRA:CAETSGGSNYKLTF TRB:CAWSPLDRSNQPQHF	1	CD4-C7
Pla-2_GCGCAGTTCCATGCTC	Pla	Pla-2	P2-4451	FALSE	1	TRA:CAVRQTGFQKLVF TRB:CAAGGGRETQYF	1	CD4-C7
Pla-2_GCGCCAATCAAGATCC	Pla	Pla-2	P2-14	FALSE	3	TRA:CALSEAPGGSEKLVF TRB:CASSIGHPNSPLHF	37	CD4-C7
Pla-2_GCGCGATAGATGTTAG	Pla	Pla-2	P2-4457	FALSE	1	TRA:CAVRDPDTGRRALTF TRB:CASSLVAGPDTQYF	1	CD4-C7
Pla-2_GCGGGTTCAGCCTATA	Pla	Pla-2	P2-234	FALSE	3	TRA:CAMREVTTDSWGKFQF TRB:CASSPGAKNIQYF	3	CD4-C7
Pla-2_GCTCCTAAGCCTATGT	Pla	Pla-2	P2-469	FALSE	2	TRA:CAVCAGGTSYGKLTF TRB:CASRPTSGYEQFF	2	CD4-C7
Pla-2_GCTCTGTCAAGCGAGT	Pla	Pla-2	P2-254	FALSE	3	TRA:CAVEASNNDMRF TRB:CASRFSGANVLTF	3	CD4-C7

Pla-2_GCTCTGTGTTGATTCG	Pla	Pla-2	P2-4465	FALSE	1	TRA:CAGAGHARLMF TRB:CASSAVNSNQPQHF	1	CD4-C7
Pla-2_GCTCTGTTCGTAGGTT	Pla	Pla-2	P2-31	FALSE	3	TRA:CAASDNTDKLIF TRB:CASSQVHEQYF	20	CD4-C7
Pla-2_GCTCTGTTCTGCCCTA	Pla	Pla-2	P2-259	FALSE	3	TRA:CALSDPLTGANNLFF TRB:CSVQTEMNTEAFF	3	CD4-C7
Pla-2_GCTGCGACACTACAGT	Pla	Pla-2	P2-53	FALSE	3	TRA:CAVPFNFGNEKLTF TRB:CASSFRGPYNEQFF	11	CD4-C7
Pla-2_GCTGCTTAGAAACCAT	Pla	Pla-2	P2-114	FALSE	3	TRA:CAAAYNNNDMRF TRB:CSARPGSSYQPQHF	5	CD4-C7
Pla-2_GCTGCTTTCGAGAGCA	Pla	Pla-2	P2-4475	FALSE	1	TRA:CAVMGGSYIPTF TRB:CASSPPGQADPEAFF	1	CD4-C7
Pla-2_GCTTCCACAAGCTGTT	Pla	Pla-2	P2-4481	FALSE	1	TRA:CAVDSNRDSNYQLIW TRB:CASSLARDSTFF	1	CD4-C7
Pla-2_GGAATAAGTTCTGGTA	Pla	Pla-2	P2-553	FALSE	2	TRA:CAVSKGGGGNKLTF TRB:CASSFSGHEQYF	2	CD4-C7
Pla-2_GGACAGAGTCCGAGTC	Pla	Pla-2	P2-147	FALSE	3	TRA:CAGPPGGYNKLIF TRB:CAIRQGDRQPQHF	4	CD4-C7
Pla-2_GGACGTCAGTAGCCGA	Pla	Pla-2	P2-4512	FALSE	1	TRA:CAGGSNYKLTF TRB:CASSAGWETQYF	1	CD4-C2
Pla-2_GGACGTCCACCGATAT	Pla	Pla-2	P2-4513	FALSE	1	TRA:CAFMKLGAGSYQLTF TRB:CASSRQGGGETQYF	1	CD4-C7
Pla-2_GGAGCAATCGCCTGAG	Pla	Pla-2	P2-114	FALSE	3	TRA:CAAAYNNNDMRF TRB:CSARPGSSYQPQHF	5	CD4-C7
Pla-2_GGATGTTGTGCAGACA	Pla	Pla-2	P2-527	FALSE	2	TRA:CAVSPATGRRALTF TRB:CAITRDYSYEQYF	2	CD4-C7
Pla-2_GGATTACAGAAGCCCA	Pla	Pla-2	P2-163	FALSE	3	TRA:CAVRDSRQAGTALIF TRB:CASRHDRQTLAGWETQYF	4	CD4-C7
Pla-2_GGCCGATAGCTGCAAG	Pla	Pla-2	P2-4534	FALSE	1	TRA:CAVSNAGNMLTF TRB:CASSPDRDYEQYF	1	CD4-C7
Pla-2_GGCCGATAGCTTCGCG	Pla	Pla-2	P2-247	FALSE	3	TRA:CIVRVLIQGAQKLVF TRB:CASSEGGTLYGYTF	3	CD4-C7
Pla-2_GGCCGATGTCGCTTTC	Pla	Pla-2	P2-4536	FALSE	1	TRA:CAASYQGGSEKLVF TRB:CASDSAGYRYEQYF	1	CD4-C7
Pla-2_GGCGACTAGCCAGTTT	Pla	Pla-2	P2-517	FALSE	2	TRA:CAAKAGTALIF TRB:CASSPATYNYEQYF	2	CD4-C7
Pla-2_GGCGACTCACCTCGTT	Pla	Pla-2	P2-4539	FALSE	1	TRA:CAATSGGSNYKLTF TRB:CASSLPAGNTEAFF	1	CD4-C7
Pla-2_GGCGTGTCAGGGAGAG	Pla	Pla-2	P2-259	FALSE	3	TRA:CALSDPLTGANNLFF TRB:CSVQTEMNTEAFF	3	CD4-C7
Pla-2_GGCTCGATCACTGGGC	Pla	Pla-2	P2-465	FALSE	2	TRA:CAVSYSGAGSYQLTF TRB:CASSEWWRTTYEQYF	2	CD4-C7
Pla-2_GGCTGGTCATACAGCT	Pla	Pla-2	P2-4549	FALSE	1	TRA:CAVGPQTGANNLFF TRB:CASSQGQEGTQYF	1	CD4-C7
Pla-2_GGCTGGTTCCGCAAGC	Pla	Pla-2	P2-9	FALSE	3	TRA:CAMTQNAGKSTF TRB:CASSPDRDYEQYF	47	CD4-C7
Pla-2_GGGAATGAGAGTTGGC	Pla	Pla-2	P2-9	FALSE	3	TRA:CAMTQNAGKSTF TRB:CASSPDRDYEQYF	47	CD4-C7
Pla-2_GGGACCTTCGAGGTAG	Pla	Pla-2	P2-512	FALSE	2	TRA:CAIMNTGGFKTIF TRB:CASSFLRGGINEQYF	2	CD4-C7
Pla-2_GGGAGATGTATCAGTC	Pla	Pla-2	P2-475	FALSE	2	TRA:CGTANSGGYQKVTF TRB:CASMGHFNEKLFF	2	CD4-C7
Pla-2_GGGAGATTCACTATTC	Pla	Pla-2	P2-545	FALSE	2	TRA:CAVVFQNQGGKLIF TRB:CASSERRNEQFF	2	CD4-C7

Pla-2_GGGATGAGTTAGAACA	Pla	Pla-2	P2-4556	FALSE	1	TRA:CAGRAPGSARQLTF TRB:CASSYSIPGVNTEAFF	1	CD4-C7
Pla-2_GGGCACTCAGGGTATG	Pla	Pla-2	P2-144	FALSE	3	TRA:CAYRGAYSGAGSYQLTF TRB:CASSPPYWQADTQYF	4	CD4-C7
Pla-2_GGGCATCTCGAACTGT	Pla	Pla-2	P2-74	FALSE	3	TRA:CAIRNNNARLMF TRB:CASSLERHTGLYGYTF	8	CD4-C7
Pla-2_GGGTCTGTCACCATAG	Pla	Pla-2	P2-53	FALSE	3	TRA:CAVPFNFGNEKLTF TRB:CASSFRGPYNEQFF	11	CD4-C7
Pla-2_GGGTTGCCAACTGCGC	Pla	Pla-2	P2-4568	FALSE	1	TRA:CAASRLHAGNMLTF TRB:CASSPADSQLNQPQHF	1	CD4-C7
Pla-2_GGGTTGCGTTGGACCC	Pla	Pla-2	P2-9	FALSE	3	TRA:CAMTQNAGKSTF TRB:CASSPDRDYEQYF	47	CD4-C7
Pla-2_GGGTTGCTCGCGATCG	Pla	Pla-2	P2-4571	FALSE	1	TRA:CAVTPPGGTSYGKLTF TRB:CSASTQGSNQPQHF	1	CD4-C7
Pla-2_GGTATTGAGATGCCAG	Pla	Pla-2	P2-9	FALSE	3	TRA:CAMTQNAGKSTF TRB:CASSPDRDYEQYF	47	CD4-C7
Pla-2_GGTGTTATCGTTTAGG	Pla	Pla-2	P2-9	FALSE	3	TRA:CAMTQNAGKSTF TRB:CASSPDRDYEQYF	47	CD4-C7
Pla-2_GTAACGTGTCTAGAGG	Pla	Pla-2	P2-31	FALSE	3	TRA:CAASDNTDKLIF TRB:CASSQVHEQYF	20	CD4-C7
Pla-2_GTAACGTGTGCCTGTG	Pla	Pla-2	P2-9	FALSE	3	TRA:CAMTQNAGKSTF TRB:CASSPDRDYEQYF	47	CD4-C7
Pla-2_GTACGTAAGGTGCAAC	Pla	Pla-2	P2-227	FALSE	3	TRA:CAVRSSTGKLIF TRB:CASSYRQATNEKLFF	3	CD4-C7
Pla-2_GTACTTTTCAACCAAC	Pla	Pla-2	P2-14	FALSE	3	TRA:CALSEAPGGSEKLVF TRB:CASSIGHPNSPLHF	37	CD4-C7
Pla-2_GTAGGCCGTTTGACAC	Pla	Pla-2	P2-4601	FALSE	1	TRA:CAMREGSARTGFQKLVF TRB:CSALFRAPDSNNEQFF	1	CD4-C7
Pla-2_GTAGTCATCATTGCCC	Pla	Pla-2	P2-14	FALSE	3	TRA:CALSEAPGGSEKLVF TRB:CASSIGHPNSPLHF	37	CD4-C7
Pla-2_GTATCTTCACAGGCCT	Pla	Pla-2	P2-4605	FALSE	1	TRA:CAENNDTGGFKTIF TRB:CATRESYEQYF	1	CD4-C7
Pla-2_GTATCTTGTTCCCGAG	Pla	Pla-2	P2-4606	FALSE	1	TRA:CAGRNQAGTALIF TRB:CASSLGLPTDTQYF	1	CD4-C7
Pla-2_GTATTCTCAGGTGGAT	Pla	Pla-2	P2-4608	FALSE	1	TRA:CAVRVDSNYQLIW TRB:CSAPKPGQGLNTEAFF	1	CD4-C7
Pla-2_GTCACAAAGACAAGCC	Pla	Pla-2	P2-9	FALSE	3	TRA:CAMTQNAGKSTF TRB:CASSPDRDYEQYF	47	CD4-C7
Pla-2_GTCACAAAGATCTGCT	Pla	Pla-2	P2-114	FALSE	3	TRA:CAAAYNNNDMRF TRB:CSARPGSSYQPQHF	5	CD4-C7
Pla-2_GTCACAACACAGAGGT	Pla	Pla-2	P2-31	FALSE	3	TRA:CAASDNTDKLIF TRB:CASSQVHEQYF	20	CD4-C7
Pla-2_GTCACAACAGCTTAAC	Pla	Pla-2	P2-391	FALSE	2	TRA:CAGALYGNKLVF TRB:CSGQGAMNTEAFF	2	CD4-C7
Pla-2_GTCATTTTCTGTCAAG	Pla	Pla-2	P2-14	FALSE	3	TRA:CALSEAPGGSEKLVF TRB:CASSIGHPNSPLHF	37	CD4-C7
Pla-2_GTCCTCACAAATACAG	Pla	Pla-2	P2-557	FALSE	2	TRA:CAVSPLPNTDKLIF TRB:CASSQDSRPNTEAFF	2	CD4-C7
Pla-2_GTCGGGTAGGCATGTG	Pla	Pla-2	P2-420	FALSE	2	TRA:CALSEYLQGAQKLVF TRB:CASSRGGGRTEAFF	2	CD4-C7
Pla-2_GTCGGGTGTTAGTGGG	Pla	Pla-2	P2-23	FALSE	3	TRA:CAVYSGGGADGLTF TRB:CASSLRFGETQYF	24	CD4-C7
Pla-2_GTCTCGTTCATGCAAC	Pla	Pla-2	P2-71	FALSE	3	TRA:CAARNTGGFKTIF TRB:CAWSEGTSGGTDTQYF	8	CD4-C7

Pla-2_GTCTTCGAGCCGATTT	Pla	Pla-2	P2-9	FALSE	3	TRA:CAMTQNAGKSTF TRB:CASSPDRDYEQYF	47	CD4-C7
Pla-2_GTCTTCGTCGGCGGTT	Pla	Pla-2	P2-559	FALSE	2	TRA:CAPNTGNQFYF TRB:CSAMGGGGYTF	2	CD4-C7
Pla-2_GTGAAGGAGAATGTTG	Pla	Pla-2	P2-95	FALSE	3	TRA:CAENIANYGGSQGNLIF TRB:CASSEAVQQQFF	6	CD4-C7
Pla-2_GTGAAGGAGCGATAGC	Pla	Pla-2	P2-23	FALSE	3	TRA:CAVYSGGGADGLTF TRB:CASSLRFGETQYF	24	CD4-C7
Pla-2_GTGAAGGTCCGGGTGT	Pla	Pla-2	P2-134	FALSE	3	TRA:CAVNPPNARLMF TRB:CASSSLGRNTEAFF	4	CD4-C7
Pla-2_GTGAAGGTCGCCTGAG	Pla	Pla-2	P2-154	FALSE	3	TRA:CAMSGGGADGLTF TRB:CASSFLRGGINEQYF	4	CD4-C7
Pla-2_GTGCATAAGGTGTTAA	Pla	Pla-2	P2-256	FALSE	3	TRA:CAASMGTGGFKTIF TRB:CSAGGQDTQYF	3	CD4-C7
Pla-2_GTGCATACACATGGGA	Pla	Pla-2	P2-252	FALSE	3	TRA:CAVTPTSGTYKYIF TRB:CASSYGPSGGLNEQFF	3	CD4-C7
Pla-2_GTGCATACAGACAAGC	Pla	Pla-2	P2-9	FALSE	3	TRA:CAMTQNAGKSTF TRB:CASSPDRDYEQYF	47	CD4-C7
Pla-2_GTGCATACAGGGAGAG	Pla	Pla-2	P2-4637	FALSE	1	TRA:CAVRTGANNLFF TRB:CASSLGLAGETQYF	1	CD4-C7
Pla-2_GTGCATATCAGTTAGC	Pla	Pla-2	P2-542	FALSE	2	TRA:CAASKVYKLSF TRB:CASSLGLADITDTQYF	2	CD4-C7
Pla-2_GTGCGGTGTCCCGACA	Pla	Pla-2	P2-4641	FALSE	1	TRA:CALMIGSNTDKLIF TRB:CASSLTGASTGELFF	1	CD4-C7
Pla-2_GTGCGGTGTTCCACAA	Pla	Pla-2	P2-249	FALSE	3	TRA:CAESRDQTGANNLFF TRB:CASSSLGRNTEAFF	3	CD4-C7
Pla-2_GTGCTTCGTAGGAGTC	Pla	Pla-2	P2-4642	FALSE	1	TRA:CAMTQNAGKSTF TRB:CASSPNDRGGFGANVLTF	1	CD4-C7
Pla-2_GTGGGTCCACTATCTT	Pla	Pla-2	P2-4645	FALSE	1	TRA:CAVNTGNQFYF TRB:CASSLSWGAYGYTF	1	CD4-C7
Pla-2_GTGTGCGAGTGCTGCC	Pla	Pla-2	P2-31	FALSE	3	TRA:CAASDNTDKLIF TRB:CASSQVHEQYF	20	CD4-C7
Pla-2_GTGTGCGGTCACAAGG	Pla	Pla-2	P2-4649	FALSE	1	TRA:CAYRSAATNKLIF TRB:CASSLGQGSTGELFF	1	CD4-C7
Pla-2_GTGTTAGTCACTGGGC	Pla	Pla-2	P2-255	FALSE	3	TRA:CAVRDMNSGGYQKVTF TRB:CASSLGTNTEAFF	3	CD4-C7
Pla-2_GTGTTAGTCGCCGTGA	Pla	Pla-2	P2-261	FALSE	3	TRA:CAVDPPMHTGNQFYF TRB:CSALTGWETQYF	3	CD4-C7
Pla-2_GTTAAGCCATGTAGTC	Pla	Pla-2	P2-4654	FALSE	1	TRA:CAESWGYQKVTF TRB:CASSEKGRLADNKETQYF	1	CD4-C7
Pla-2_GTTACAGTCCCAAGAT	Pla	Pla-2	P2-4661	FALSE	1	TRA:CVVSYNTDKLIF TRB:CATSDGHSTGELFF	1	CD4-C7
Pla-2_GTTCATTTCCTTTACA	Pla	Pla-2	P2-238	FALSE	3	TRA:CAVGRGGTSYGKLTF TRB:CASSLEWVFTGELFF	3	CD4-C7
Pla-2_TAAACCGAGGCCCGTT	Pla	Pla-2	P2-142	FALSE	3	TRA:CAEKNWNGNKLVF TRB:CASSPMEVWGQPQHF	4	CD4-C7
Pla-2_TAAACCGGTACGACCC	Pla	Pla-2	P2-51	FALSE	3	TRA:CAENNDTGGFKTIF TRB:CATRGAHEKLFF	11	CD4-C7
Pla-2_TAAACCGTCACTTATC	Pla	Pla-2	P2-9	FALSE	3	TRA:CAMTQNAGKSTF TRB:CASSPDRDYEQYF	47	CD4-C7
Pla-2_TACAGTGCATCACAAC	Pla	Pla-2	P2-51	FALSE	3	TRA:CAENNDTGGFKTIF TRB:CATRGAHEKLFF	11	CD4-C7
Pla-2_TACCTATAGGGTTTCT	Pla	Pla-2	P2-4683	FALSE	1	TRA:CAGRSGANSKLTF TRB:CASSQKDRGFEKLFF	1	CD4-C7

Pla-2_TACCTATCAGCTGTAT	Pla	Pla-2	P2-113	FALSE	3	TRA:CADVDSNYQLIW TRB:CASSPRTAGGYTF	5	CD4-C7
Pla-2_TACCTTAGTGTAATGA	Pla	Pla-2	P2-31	FALSE	3	TRA:CAASDNTDKLIF TRB:CASSQVHEQYF	20	CD4-C7
Pla-2_TACCTTATCTCAAGTG	Pla	Pla-2	P2-4688	FALSE	1	TRA:CALNLNNAGNMLTF TRB:CASSHFGTAVAEAFF	1	CD4-C7
Pla-2_TACGGATCACAACTGT	Pla	Pla-2	P2-4689	FALSE	1	TRA:CAANQAGTALIF TRB:CASSLGQGNTEAFF	1	CD4-C7
Pla-2_TACGGGCAGTTCGATC	Pla	Pla-2	P2-4693	FALSE	1	TRA:CAVRDPEVGGFKTIF TRB:CSVKYGQGASGANVLTF	1	CD4-C7
Pla-2_TACGGTATCTGCTGTC	Pla	Pla-2	P2-9	FALSE	3	TRA:CAMTQNAGKSTF TRB:CASSPDRDYEQYF	47	CD4-C7
Pla-2_TACTCGCAGTGCGTGA	Pla	Pla-2	P2-4699	FALSE	1	TRA:CARPKGDNTNAGKSTF TRB:CASSIRDKPEKLFF	1	CD4-C7
Pla-2_TACTCGCAGTTACCCA	Pla	Pla-2	P2-4700	FALSE	1	TRA:CAASRGSFGNVLHC TRB:CSAPAPPGLAGGHGKNIQYF	1	CD4-C7
Pla-2_TACTTGTCACGTGAGA	Pla	Pla-2	P2-74	FALSE	3	TRA:CAIRNNNARLMF TRB:CASSLERHTGLYGYTF	8	CD4-C7
Pla-2_TACTTGTTCACATGCA	Pla	Pla-2	P2-4715	FALSE	1	TRA:CAVSAYSGAGSYQLTF TRB:CASSPTRTGSPLHF	1	CD4-C7
Pla-2_TACTTGTTCCGCGCAA	Pla	Pla-2	P2-466	FALSE	2	TRA:CIVPKFGNEKLTF TRB:CASSPRTAGGYTF	2	CD4-C7
Pla-2_TAGACCACAGCTCGAC	Pla	Pla-2	P2-147	FALSE	3	TRA:CAGPPGGYNKLIF TRB:CAIRQGDRQPQHF	4	CD4-C7
Pla-2_TAGACCAGTAGCACGA	Pla	Pla-2	P2-4717	FALSE	1	TRA:CALSEAPGGSEKLVF TRB:CASSQGESSYEQYF	1	CD4-C7
Pla-2_TAGAGCTAGCGAAGGG	Pla	Pla-2	P2-242	FALSE	3	TRA:CAVMRGGSEKLVF TRB:CASIVGRSSYNEQFF	3	CD4-C7
Pla-2_TATCAGGAGGCATTGG	Pla	Pla-2	P2-74	FALSE	3	TRA:CAIRNNNARLMF TRB:CASSLERHTGLYGYTF	8	CD4-C7
Pla-2_TATCTCAGTCTTTCAT	Pla	Pla-2	P2-4737	FALSE	1	TRA:CAVSDTGRRALTF TRB:CASSIRDRGREQYF	1	CD4-C7
Pla-2_TCAACGATCGCCAAAT	Pla	Pla-2	P2-241	FALSE	3	TRA:CAAYTSGTYKYIF TRB:CASSAQVVTQYF	3	CD4-C7
Pla-2_TCAATCTCAGATCTGT	Pla	Pla-2	P2-31	FALSE	3	TRA:CAASDNTDKLIF TRB:CASSQVHEQYF	20	CD4-C7
Pla-2_TCACAAGGTCAATGTC	Pla	Pla-2	P2-4753	FALSE	1	TRA:CAVEGGGSYIPTF TRB:CASSAGLGEDNEQFF	1	CD4-C7
Pla-2_TCACGAACAGCCTTTC	Pla	Pla-2	P2-9	FALSE	3	TRA:CAMTQNAGKSTF TRB:CASSPDRDYEQYF	47	CD4-C7
Pla-2_TCAGATGTCATCGCTC	Pla	Pla-2	P2-115	FALSE	3	TRA:CAVRDEDNQGGKLIF TRB:CASSQDQGLAGGFSFKETQYF	5	CD4-C7
Pla-2_TCAGCAAAGATCCTGT	Pla	Pla-2	P2-14	FALSE	3	TRA:CALSEAPGGSEKLVF TRB:CASSIGHPNSPLHF	37	CD4-C7
Pla-2_TCAGCAAAGGTGTGGT	Pla	Pla-2	P2-135	FALSE	3	TRA:CAVATYSGAGSYQLTF TRB:CASSYGPSGGLNEQFF	4	CD4-C7
Pla-2_TCAGCAAGTGGTCCGT	Pla	Pla-2	P2-9	FALSE	3	TRA:CAMTQNAGKSTF TRB:CASSPDRDYEQYF	47	CD4-C7
Pla-2_TCAGCTCGTCGCTTTC	Pla	Pla-2	P2-4770	FALSE	1	TRA:CAVGLAGNMLTF TRB:CASFTGTPGNTIYF	1	CD4-C7
Pla-2_TCAGCTCTCACCCTCA	Pla	Pla-2	P2-93	FALSE	3	TRA:CLVGDRATGGFKTIF TRB:CSALAGTGFNEQFF	6	CD4-C7
Pla-2_TCAGGTACAGTGGAGT	Pla	Pla-2	P2-208	FALSE	3	TRA:CAESRTGGGADGLTF TRB:CASSLGGRALRDTQYF	3	CD4-C7

Pla-2_TCATTACGTACGCACC	Pla	Pla-2	P2-71	FALSE	3	TRA:CAARNTGGFKTIF TRB:CAWSEGTSGGTDTQYF	8	CD4-C7
Pla-2_TCATTACTCTGCCCTA	Pla	Pla-2	P2-23	FALSE	3	TRA:CAVYSGGGADGLTF TRB:CASSLRFGETQYF	24	CD4-C7
Pla-2_TCATTTGAGTAGTGCG	Pla	Pla-2	P2-171	TRUE	3	TRA:CAVNILDGNKLVF TRB:CAHASGTSVYEQYF	3	CD4-C7
Pla-2_TCATTTGAGTTTCCTT	Pla	Pla-2	P2-4782	FALSE	1	TRA:CAMTQNAGKSTF TRB:CASSFSSAYEQYF	1	CD4-C7
Pla-2_TCATTTGCAGACGTAG	Pla	Pla-2	P2-71	FALSE	3	TRA:CAARNTGGFKTIF TRB:CAWSEGTSGGTDTQYF	8	CD4-C7
Pla-2_TCATTTGCAGCTTAAC	Pla	Pla-2	P2-4783	FALSE	1	TRA:CAASIGDYKLSF TRB:CASKETGGSPLHF	1	CD4-C7
Pla-2_TCCACACGTGCACTTA	Pla	Pla-2	P2-457	FALSE	2	TRA:CATDLNFGNEKLTF TRB:CASSSMTGNNEQFF	2	CD4-C7
Pla-2_TCCACACTCAGCATGT	Pla	Pla-2	P2-159	FALSE	3	TRA:CAASENNNARLMF TRB:CSAREDTDTQYF	4	CD4-C7
Pla-2_TCCACACTCTGTTTGT	Pla	Pla-2	P2-254	FALSE	3	TRA:CAVEASNNDMRF TRB:CASRFSGANVLTF	3	CD4-C7
Pla-2_TCGAGGCCACGTAAGG	Pla	Pla-2	P2-4791	FALSE	1	TRA:CAVGRHRSGGSYIPTF TRB:CASSISHPSNRETGANVLTF	1	CD4-C7
Pla-2_TCGAGGCGTAGGGACT	Pla	Pla-2	P2-14	FALSE	3	TRA:CALSEAPGGSEKLVF TRB:CASSIGHPNSPLHF	37	CD4-C7
Pla-2_TCGCGAGTCGTACCGG	Pla	Pla-2	P2-256	FALSE	3	TRA:CAASMGTGGFKTIF TRB:CSAGGQDTQYF	3	CD4-C7
Pla-2_TCGCGTTTCTTGACGA	Pla	Pla-2	P2-4800	FALSE	1	TRA:CAASLAGGSQGNLIF TRB:CASSDGAGPQYF	1	CD4-C7
Pla-2_TCGTACCAGGTGCTTT	Pla	Pla-2	P2-4803	FALSE	1	TRA:CAFMNVGSQGNLIF TRB:CASSRTGFGQFF	1	CD4-C7
Pla-2_TCGTACCCAAGCTGGA	Pla	Pla-2	P2-9	FALSE	3	TRA:CAMTQNAGKSTF TRB:CASSPDRDYEQYF	47	CD4-C7
Pla-2_TCGTAGACACATAACC	Pla	Pla-2	P2-31	FALSE	3	TRA:CAASDNTDKLIF TRB:CASSQVHEQYF	20	CD4-C7
Pla-2_TCTATTGTCCGTCAAA	Pla	Pla-2	P2-4816	FALSE	1	TRA:CEGGSYIPTF TRB:CASSLSNLAGDEQYF	1	CD4-C7
Pla-2_TCTATTGTCGGTGTCG	Pla	Pla-2	P2-51	FALSE	3	TRA:CAENNDTGGFKTIF TRB:CATRGAHEKLFF	11	CD4-C7
Pla-2_TCTCTAACACGAGAGT	Pla	Pla-2	P2-4820	FALSE	1	TRA:CAASLHGSGNTGKLIF TRB:CASSQVLAGGIQYF	1	CD4-C7
Pla-2_TCTCTAATCGGAAATA	Pla	Pla-2	P2-171	TRUE	3	TRA:CAVNILDGNKLVF TRB:CAHASGTSVYEQYF	3	CD4-C7
Pla-2_TCTGAGACACTTAACG	Pla	Pla-2	P2-4823	FALSE	1	TRA:CALSDLINSGYALNF TRB:CASSPARLAGLYEQYF	1	CD4-C7
Pla-2_TCTGGAAAGGCGTACA	Pla	Pla-2	P2-482	FALSE	2	TRA:CALYNFNKFYF TRB:CASSPRLAGGTDTQYF	2	CD4-C7
Pla-2_TCTGGAAGTCCAGTGC	Pla	Pla-2	P2-4831	FALSE	1	TRA:CAEHTGGFKTIF TRB:CSAYETDLATNEKLFF	1	CD4-C7
Pla-2_TCTTCGGAGGACACCA	Pla	Pla-2	P2-95	FALSE	3	TRA:CAENIANYGGSQGNLIF TRB:CASSEAVQQQFF	6	CD4-C7
Pla-2_TCTTCGGGTACCGTAT	Pla	Pla-2	P2-4838	FALSE	1	TRA:CAVEDFGGFQKLVF TRB:CASRNYPGQADSNQPQHF	1	CD4-C7
Pla-2_TCTTCGGTCCTCCTAG	Pla	Pla-2	P2-4842	FALSE	1	TRA:CAVRDGSGYNKLIF TRB:CASSLAGGYGYTF	1	CD4-C7
Pla-2_TGAAAGACAAGTCTGT	Pla	Pla-2	P2-142	FALSE	3	TRA:CAEKNWNGNKLVF TRB:CASSPMEVWGQPQHF	4	CD4-C7

Pla-2_TGACGGCAGTAGCGGT	Pla	Pla-2	P2-255	FALSE	3	TRA:CAVRDMNSGGYQKVTF TRB:CASSLGTNTEAFF	3	CD4-C7
Pla-2_TGACGGCCATTCCTCG	Pla	Pla-2	P2-261	FALSE	3	TRA:CAVDPPMHTGNQFYF TRB:CSALTGWETQYF	3	CD4-C7
Pla-2_TGACTAGTCCGCATAA	Pla	Pla-2	P2-53	FALSE	3	TRA:CAVPFNFGNEKLTF TRB:CASSFRGPYNEQFF	11	CD4-C7
Pla-2_TGACTAGTCTGATACG	Pla	Pla-2	P2-464	FALSE	2	TRA:CAMRYLWDDYKLSF TRB:CASSAQVVTQYF	2	CD4-C7
Pla-2_TGAGAGGGTAAGCACG	Pla	Pla-2	P2-84	FALSE	3	TRA:CAVSDLTGGGNKLTF TRB:CSVQTEMNTEAFF	7	CD4-C7
Pla-2_TGAGCATAGGACATTA	Pla	Pla-2	P2-4858	FALSE	1	TRA:CAVDSNYQLIW TRB:CASSDGEGAGANVLTF	1	CD4-C7
Pla-2_TGAGCATCATTGCGGC	Pla	Pla-2	P2-4861	FALSE	1	TRA:CAVSGSNYQLIW TRB:CASSMGTVYSNQPQHF	1	CD4-C7
Pla-2_TGAGCCGAGATGGGTC	Pla	Pla-2	P2-4865	FALSE	1	TRA:CAVRDMNTGFQKLVF TRB:CASSAPGQGGGTDTQYF	1	CD4-C7
Pla-2_TGAGGGATCGTTGCCT	Pla	Pla-2	P2-240	FALSE	3	TRA:CALIHQRALTF TRB:CASSPQDRISTDTQYF	3	CD4-C7
Pla-2_TGATTTCAGTTCGATC	Pla	Pla-2	P2-119	FALSE	3	TRA:CAASIMDDMRF TRB:CSAAIGYGYTF	5	CD4-C7
Pla-2_TGCACCTCATCACAAC	Pla	Pla-2	P2-4876	FALSE	1	TRA:CIATNRDDKIIF TRB:CASSMDGPQETQYF	1	CD4-C7
Pla-2_TGCACCTGTCTAACGT	Pla	Pla-2	P2-136	FALSE	3	TRA:CAASVRSQGNLIF TRB:CASSLGPNRYRQRNTIYF	4	CD4-C7
Pla-2_TGCACCTTCCCAACGG	Pla	Pla-2	P2-4877	FALSE	1	TRA:CARTTNAGKSTF TRB:CASSLLRARGETQYF	1	CD4-C7
Pla-2_TGCACCTTCTAGAGTC	Pla	Pla-2	P2-259	FALSE	3	TRA:CALSDPLTGANNLFF TRB:CSVQTEMNTEAFF	3	CD4-C7
Pla-2_TGCCAAATCATGCTCC	Pla	Pla-2	P2-4884	FALSE	1	TRA:CAVRGYSGGGADGLTF TRB:CASSLVAGEQYF	1	CD4-C7
Pla-2_TGCCCATAGGGAGTAA	Pla	Pla-2	P2-4885	FALSE	1	TRA:CAASVIRGNTPLVF TRB:CASSLVAGPSNTEAFF	1	CD4-C7
Pla-2_TGCCCATTCGGAAACG	Pla	Pla-2	P2-4886	FALSE	1	TRA:CFMYSGGGADGLTF TRB:CASSGGGAMSLETQYF	1	CD4-C7
Pla-2_TGCCCTATCCACGAAT	Pla	Pla-2	P2-93	FALSE	3	TRA:CLVGDRATGGFKTIF TRB:CSALAGTGFNEQFF	6	CD4-C7
Pla-2_TGCGCAGAGATATGGT	Pla	Pla-2	P2-4888	FALSE	1	TRA:CAFRTNARLMF TRB:CASSYGRDIAYEQYF	1	CD4-C7
Pla-2_TGCGCAGGTTAAGTAG	Pla	Pla-2	P2-23	FALSE	3	TRA:CAVYSGGGADGLTF TRB:CASSLRFGETQYF	24	CD4-C7
Pla-2_TGCGTGGGTAAACGCG	Pla	Pla-2	P2-4892	FALSE	1	TRA:CAVSPSGGGFKTIF TRB:CASSFSGNYEQYF	1	CD4-C7
Pla-2_TGCGTGGGTTTAGCTG	Pla	Pla-2	P2-4894	FALSE	1	TRA:CAILAVTNTGNQFYF TRB:CASSRWDSLNQPQHF	1	CD4-C7
Pla-2_TGCTACCAGGATATAC	Pla	Pla-2	P2-9	FALSE	3	TRA:CAMTQNAGKSTF TRB:CASSPDRDYEQYF	47	CD4-C7
Pla-2_TGCTACCCACAGACTT	Pla	Pla-2	P2-4897	FALSE	1	TRA:CAISPYDKVIF TRB:CSARGGQTFSNEKLFF	1	CD4-C7
Pla-2_TGCTGCTTCAATACCG	Pla	Pla-2	P2-135	FALSE	3	TRA:CAVATYSGAGSYQLTF TRB:CASSYGPSGGLNEQFF	4	CD4-C7
Pla-2_TGCTGCTTCACGAAGG	Pla	Pla-2	P2-9	FALSE	3	TRA:CAMTQNAGKSTF TRB:CASSPDRDYEQYF	47	CD4-C7
Pla-2_TGCTGCTTCTCCAACC	Pla	Pla-2	P2-159	FALSE	3	TRA:CAASENNNARLMF TRB:CSAREDTDTQYF	4	CD4-C7

Pla-2_TGGCCAGTCATTCACT	Pla	Pla-2	P2-71	FALSE	3	TRA:CAARNTGGFKTIF TRB:CAWSEGTSGGTDTQYF	8	CD4-C7
Pla-2_TGGCGCAAGACTACAA	Pla	Pla-2	P2-31	FALSE	3	TRA:CAASDNTDKLIF TRB:CASSQVHEQYF	20	CD4-C7
Pla-2_TGGCGCAGTTACCAGT	Pla	Pla-2	P2-4916	FALSE	1	TRA:CIVRVEGYSTLTF TRB:CSARDPLAGGLEQYF	1	CD4-C7
Pla-2_TGGCTGGCATCACCCT	Pla	Pla-2	P2-84	FALSE	3	TRA:CAVSDLTGGGNKLTF TRB:CSVQTEMNTEAFF	7	CD8-C4
Pla-2_TGGGAAGAGACCCACC	Pla	Pla-2	P2-4920	FALSE	1	TRA:CAVRDRGRLAF TRB:CSVWDRGSVEQYF	1	CD4-C7
Pla-2_TGGGAAGGTATGCTTG	Pla	Pla-2	P2-119	FALSE	3	TRA:CAASIMDDMRF TRB:CSAAIGYGYTF	5	CD4-C7
Pla-2_TGGGCGTAGAGCAATT	Pla	Pla-2	P2-51	FALSE	3	TRA:CAENNDTGGFKTIF TRB:CATRGAHEKLFF	11	CD4-C7
Pla-2_TGGTTAGCAATCTACG	Pla	Pla-2	P2-14	FALSE	3	TRA:CALSEAPGGSEKLVF TRB:CASSIGHPNSPLHF	37	CD4-C7
Pla-2_TGGTTAGGTCTTCAAG	Pla	Pla-2	P2-4935	FALSE	1	TRA:CAMREGVENRDDKIIF TRB:CASTFRPTSYEQYF	1	CD4-C7
Pla-2_TGGTTCCGTTAAGGGC	Pla	Pla-2	P2-95	FALSE	3	TRA:CAENIANYGGSQGNLIF TRB:CASSEAVQQQFF	6	CD4-C7
Pla-2_TGTATTCTCACCTCGT	Pla	Pla-2	P2-14	FALSE	3	TRA:CALSEAPGGSEKLVF TRB:CASSIGHPNSPLHF	37	CD4-C7
Pla-2_TGTCCCACACTGCCAG	Pla	Pla-2	P2-4947	FALSE	1	TRA:CAGGRGGSYIPTF TRB:CASAPGHTGEKLFF	1	CD4-C7
Pla-2_TGTCCCAGTTAAGTAG	Pla	Pla-2	P2-134	FALSE	3	TRA:CAVNPPNARLMF TRB:CASSSLGRNTEAFF	4	CD4-C7
Pla-2_TGTCCCATCATGCATG	Pla	Pla-2	P2-4950	FALSE	1	TRA:CAGQNSGGSNYKLTF TRB:CASSYRLGGTDTQYF	1	CD4-C7
Pla-2_TGTGGTATCGCATGAT	Pla	Pla-2	P2-14	FALSE	3	TRA:CALSEAPGGSEKLVF TRB:CASSIGHPNSPLHF	37	CD4-C7
Pla-2_TGTGTTTCACCGGAAA	Pla	Pla-2	P2-4954	FALSE	1	TRA:CAGVRGYGQNFVF TRB:CASRRLAGGSETQYF	1	CD4-C7
Pla-2_TGTTCCGCACAGTCGC	Pla	Pla-2	P2-53	FALSE	3	TRA:CAVPFNFGNEKLTF TRB:CASSFRGPYNEQFF	11	CD4-C7
Pla-2_TGTTCCGCAGTAAGAT	Pla	Pla-2	P2-4957	FALSE	1	TRA:CAVAMNQAGTALIF TRB:CASRRSGASSYEQYF	1	CD4-C7
Pla-2_TTAACTCCAGCCAGAA	Pla	Pla-2	P2-84	FALSE	3	TRA:CAVSDLTGGGNKLTF TRB:CSVQTEMNTEAFF	7	CD4-C7
Pla-2_TTAACTCTCTGACCTC	Pla	Pla-2	P2-4960	FALSE	1	TRA:CAVVSTGGGNKLTF TRB:CASSLGQANEKLFF	1	CD4-C7
Pla-2_TTAGGACAGCCACCTG	Pla	Pla-2	P2-9	FALSE	3	TRA:CAMTQNAGKSTF TRB:CASSPDRDYEQYF	47	CD4-C7
Pla-2_TTAGGACCAGCTTCGG	Pla	Pla-2	P2-9	FALSE	3	TRA:CAMTQNAGKSTF TRB:CASSPDRDYEQYF	47	CD4-C7
Pla-2_TTATGCTAGATACACA	Pla	Pla-2	P2-235	FALSE	3	TRA:CATENNARLMF TRB:CSRDRGLQTEAFF	3	CD4-C7
Pla-2_TTATGCTAGCAGCGTA	Pla	Pla-2	P2-9	FALSE	3	TRA:CAMTQNAGKSTF TRB:CASSPDRDYEQYF	47	CD4-C7
Pla-2_TTATGCTCACGGTAAG	Pla	Pla-2	P2-93	FALSE	3	TRA:CLVGDRATGGFKTIF TRB:CSALAGTGFNEQFF	6	CD4-C7
Pla-2_TTATGCTGTGCGCTTG	Pla	Pla-2	P2-261	FALSE	3	TRA:CAVDPPMHTGNQFYF TRB:CSALTGWETQYF	3	CD4-C7
Pla-2_TTATGCTGTTCCACGG	Pla	Pla-2	P2-4970	FALSE	1	TRA:CAVRDLQGAQKLVF TRB:CASSLTTSHTDTQYF	1	CD4-C7

Pla-2_TTCCCAGAGTGGGATC	Pla	Pla-2	P2-14	FALSE	3	TRA:CALSEAPGGSEKLVF TRB:CASSIGHPNSPLHF	37	CD4-C4
Pla-2_TTCCCAGCAATAACGA	Pla	Pla-2	P2-9	FALSE	3	TRA:CAMTQNAGKSTF TRB:CASSPDRDYEQYF	47	CD4-C7
Pla-2_TTCCCAGTCCTGTACC	Pla	Pla-2	P2-23	FALSE	3	TRA:CAVYSGGGADGLTF TRB:CASSLRFGETQYF	24	CD4-C7
Pla-2_TTCGAAGGTGCACCAC	Pla	Pla-2	P2-4979	FALSE	1	TRA:CAGVKETSGSRLTF TRB:CASSPTGAWGPYNEQFF	1	CD4-C7
Pla-2_TTCGAAGTCATGTAGC	Pla	Pla-2	P2-4980	FALSE	1	TRA:CVVSEGATDKLIF TRB:CASSDRLTEAFF	1	CD4-C7
Pla-2_TTCGGTCCAGCTGCAC	Pla	Pla-2	P2-117	FALSE	3	TRA:CAVDITGGGNKLTF TRB:CSAREDTDTQYF	5	CD4-C7
Pla-2_TTCTACAAGAGGGCTT	Pla	Pla-2	P2-150	FALSE	3	TRA:CVVRYAGNNRKLIW TRB:CASSQGQPPYEQYF	4	CD4-C7
Pla-2_TTCTACAGTCATATGC	Pla	Pla-2	P2-249	FALSE	3	TRA:CAESRDQTGANNLFF TRB:CASSSLGRNTEAFF	3	CD4-C7
Pla-2_TTCTCCTCATCCTTGC	Pla	Pla-2	P2-74	FALSE	3	TRA:CAIRNNNARLMF TRB:CASSLERHTGLYGYTF	8	CD4-C7
Pla-2_TTCTTAGTCTGGTTCC	Pla	Pla-2	P2-53	FALSE	3	TRA:CAVPFNFGNEKLTF TRB:CASSFRGPYNEQFF	11	CD4-C7
Pla-2_TTGACTTAGAGAGCTC	Pla	Pla-2	P2-5004	FALSE	1	TRA:CAENNDTGGFKTIF TRB:CASSLAAGASTDTQYF	1	CD4-C7
Pla-2_TTGCCGTTCGGCGCTA	Pla	Pla-2	P2-557	FALSE	2	TRA:CAVSPLPNTDKLIF TRB:CASSQDSRPNTEAFF	2	CD4-C7
Pla-2_TTGCCGTTCTCGCTTG	Pla	Pla-2	P2-5010	FALSE	1	TRA:CVVSSVSGGYNKLIF TRB:CASSPPNLGSYNEQFF	1	CD4-C7
Pla-2_TTGCGTCAGATCGGGT	Pla	Pla-2	P2-5011	FALSE	1	TRA:CAGAGGTSYGKLTF TRB:CSARADTGTQGGGKQFF	1	CD4-C7
Pla-2_TTGCGTCCACAGAGGT	Pla	Pla-2	P2-23	FALSE	3	TRA:CAVYSGGGADGLTF TRB:CASSLRFGETQYF	24	CD4-C7
Pla-2_TTGCGTCTCAAACCAC	Pla	Pla-2	P2-23	FALSE	3	TRA:CAVYSGGGADGLTF TRB:CASSLRFGETQYF	24	CD4-C7
Pla-2_TTGCGTCTCAAGGTAA	Pla	Pla-2	P2-14	FALSE	3	TRA:CALSEAPGGSEKLVF TRB:CASSIGHPNSPLHF	37	CD4-C7
Pla-2_TTGGCAAAGGCACATG	Pla	Pla-2	P2-5016	FALSE	1	TRA:CAVRDRNTGFQKLVF TRB:CASRLKRDRVSVFYGYTF	1	CD4-C7
Pla-2_TTGGCAAAGTTTGCGT	Pla	Pla-2	P2-559	FALSE	2	TRA:CAPNTGNQFYF TRB:CSAMGGGGYTF	2	CD4-C7
Pla-2_TTTACTGAGATGCCAG	Pla	Pla-2	P2-23	FALSE	3	TRA:CAVYSGGGADGLTF TRB:CASSLRFGETQYF	24	CD4-C7
Pla-2_TTTACTGCAGCGTAAG	Pla	Pla-2	P2-5023	FALSE	1	TRA:CAAREAGTYKYIF TRB:CEQFF	1	CD4-C7
Pla-2_TTTACTGCATCCGGGT	Pla	Pla-2	P2-115	FALSE	3	TRA:CAVRDEDNQGGKLIF TRB:CASSQDQGLAGGFSFKETQYI	5	CD4-C7
Pla-2_TTTATGCGTAAAGGAG	Pla	Pla-2	P2-142	FALSE	3	TRA:CAEKNWNGNKLVF TRB:CASSPMEVWGQPQHF	4	CD4-C7
Pla-2_TTTGCGCTCCTTTACA	Pla	Pla-2	P2-117	FALSE	3	TRA:CAVDITGGGNKLTF TRB:CSAREDTDTQYF	5	CD4-C7
Pla-2_AAACCTGCAAATACAG	Pla	Pla-2	P2-134	FALSE	3	TRA:CAVNPPNARLMF TRB:CASSSLGRNTEAFF	4	CD8-C8
Pla-2_AAACGGGCATTTCAGG	Pla	Pla-2	P2-3414	FALSE	1	TRA:CAMRGRNNNARLMF TRB:CASSLIGENTEAFF	1	CD8-C8
Pla-2_AACACGTTCCAAGCCG	Pla	Pla-2	P2-3430	FALSE	1	TRA:CAYRRAAGNKLTF TRB:CASSFPASGVTDTQYF	1	CD8-C8

Pla-2_AACCGCGCATCTATGG	Pla	Pla-2	P2-2	TRUE	3	TRA:CVVPNTNAGKSTF TRB:CASSLQGSTYNEQFF	220	CD8-C8
Pla-2_AAGCCGCGTGTTTGGT	Pla	Pla-2	P2-22	TRUE	3	TRA:CAMRERSWVRQKLVF TRB:CASSRGTSGTDTQYF	24	CD8-C8
Pla-2_AAGGTTCCAGTCAGAG	Pla	Pla-2	P2-3471	FALSE	1	TRA:CAVRDSNYQLIW TRB:CASSQEARDPFYNEQFF	1	CD8-C8
Pla-2_AATCCAGGTTAAGATG	Pla	Pla-2	P2-284	TRUE	2	TRA:CAASMAGSQGNLIF TRB:CASSDPPTYEQYF	2	CD8-C8
Pla-2_ACACCCTGTGGAAAGA	Pla	Pla-2	P2-13	TRUE	3	TRA:CAYDLLTGNQFYF TRB:CASVRQLNTEAFF	38	CD8-C8
Pla-2_ACACTGACAGCGTCCA	Pla	Pla-2	P2-89	FALSE	3	TRA:CAASAGNTDKLIF TRB:CASSTGLAVSYEQYF	6	CD8-C8
Pla-2_ACACTGAGTCTCTCTG	Pla	Pla-2	P2-3498	FALSE	1	TRA:CAVLFMDTGRRALTF TRB:CASSLGGAQNIQYF	1	CD8-C8
Pla-2_ACATGGTAGGTAGCTG	Pla	Pla-2	P2-319	TRUE	2	TRA:CALKGSSGYSTLTF TRB:CASSYSIVRGQQPQHF	2	CD8-C9
Pla-2_ACGCCAGAGAATAGGG	Pla	Pla-2	P2-2	TRUE	3	TRA:CVVPNTNAGKSTF TRB:CASSLQGSTYNEQFF	220	CD8-C8
Pla-2_ACTATCTGTAAGGGAA	Pla	Pla-2	P2-2	TRUE	3	TRA:CVVPNTNAGKSTF TRB:CASSLQGSTYNEQFF	220	CD8-C8
Pla-2_ACTGAACCAAGCCATT	Pla	Pla-2	P2-408	FALSE	2	TRA:CAVEDLGTYKYIF TRB:CASSPNEAPQYF	2	CD8-C9
Pla-2_ACTGAGTTCAACTCTT	Pla	Pla-2	P2-3589	FALSE	1	TRA:CAASWLLGNTGKLIF TRB:CASSLGSYEQYF	1	CD8-C8
Pla-2_ACTGAGTTCTCGAGTA	Pla	Pla-2	P2-3590	FALSE	1	TRA:CAVVPGGTSYGKLTF TRB:CASSTQGRGITEAFF	1	CD8-C8
Pla-2_ACTTACTCATGGATGG	Pla	Pla-2	P2-3602	FALSE	1	TRA:CAFKTGYSTLTF TRB:CASSLGGVFGTYEQYF	1	CD8-C9
Pla-2_AGAGCTTTCTAACTGG	Pla	Pla-2	P2-3621	FALSE	1	TRA:CALSKHGNDMRF TRB:CASSSGQGGEQYF	1	CD8-C8
Pla-2_AGCGTCGTCTTGTTTG	Pla	Pla-2	P2-3660	FALSE	1	TRA:CAAPHTGNQFYF TRB:CSARSDRLGYQPQHF	1	CD8-C7
Pla-2_AGGTCATGTCAACATC	Pla	Pla-2	P2-395	FALSE	2	TRA:CAVKDSNYQLIW TRB:CASSQDGTSGSGELFF	2	CD8-C9
Pla-2_AGTGGGACATTTGCTT	Pla	Pla-2	P2-3710	FALSE	1	TRA:CASSPGGSEKLVF TRB:CASSLAGGSDTQYF	1	CD8-C8
Pla-2_ATAAGAGCATAAGACA	Pla	Pla-2	P2-3720	FALSE	1	TRA:CAVRDLYNFNKFYF TRB:CASTPPGQGQTQYF	1	CD8-C8
Pla-2_ATAGACCCACAGATTC	Pla	Pla-2	P2-3724	FALSE	1	TRA:CAMILGYSTLTF TRB:CASSFGSTDTQYF	1	CD8-C8
Pla-2_ATCATCTTCAGCATGT	Pla	Pla-2	P2-20	TRUE	3	TRA:CAYDSNYQLIW TRB:CASSLSIDRADTGELFF	25	CD8-C9
Pla-2_CAACCAAGTTCCACTC	Pla	Pla-2	P2-3782	FALSE	1	TRA:CAVMDSNYQLIW TRB:CASSSGGATDTQYF	1	CD8-C8
Pla-2_CAACCTCGTTCACCTC	Pla	Pla-2	P2-3785	FALSE	1	TRA:CAASGGGNKLTF TRB:CASSKDLDGTGCTDTQYF	1	CD8-C9
Pla-2_CAAGAAACACCAGGCT	Pla	Pla-2	P2-453	FALSE	2	TRA:CAVSSDTGGFKTIF TRB:CASSATGSSRSGELFF	2	CD8-C8
Pla-2_CAAGATCGTGTCAATC	Pla	Pla-2	P2-3796	FALSE	1	TRA:CAVRDSNYQLIW TRB:CASSDSATGELFF	1	CD8-C8
Pla-2_CACAAACAGTGTCTCA	Pla	Pla-2	P2-87	TRUE	3	TRA:CAFMKPIGTDKLIF TRB:CASRLPGTGSRETQYF	6	CD8-C9
Pla-2_CAGAATCTCAACACCA	Pla	Pla-2	P2-3	TRUE	3	TRA:CAVNPLESSYKLIF TRB:CASSPGTANTGELFF	177	CD8-C9

Pla-2_CAGCGACCATGCCCGA	Pla	Pla-2	P2-470	FALSE	2	TRA:CAPMDSSYKLIF TRB:CASSEGEVGELFF	2	CD8-C8
Pla-2_CAGCTGGCACTACAGT	Pla	Pla-2	P2-19	TRUE	3	TRA:CAVRDMENNRLAF TRB:CASSLGGGLTYEQYF	27	CD8-C8
Pla-2_CATATTCGTACCGAGA	Pla	Pla-2	P2-3897	FALSE	1	TRA:CAVRQAEDSSYKLIF TRB:CASSTGLEPYQETQYF	1	CD8-C9
Pla-2_CATCGAATCGGCGGTT	Pla	Pla-2	P2-164	FALSE	3	TRA:CAVGLAGNMLTF TRB:CASSYAPAGERGGRNEQFF	4	CD8-C8
Pla-2_CATGACATCAGATAAG	Pla	Pla-2	P2-3915	FALSE	1	TRA:CALRETSGSRLTF TRB:CASNPTGGHNQPQHF	1	CD8-C8
Pla-2_CCAATCCCACGGCGTT	Pla	Pla-2	P2-3937	FALSE	1	TRA:CAAGLGKSTF TRB:CASSAQTGSYGYTF	1	CD8-C9
Pla-2_CCACGGAGTGCACCAC	Pla	Pla-2	P2-10	TRUE	3	TRA:CAPLTPNDMRF TRB:CASLNGAGGSYEQYF	45	CD8-C4
Pla-2_CCCAGTTCACCGAATT	Pla	Pla-2	P2-3964	FALSE	1	TRA:CAVRVYNFNKFYF TRB:CASSQELGFYEQYF	1	CD8-C8
Pla-2_CCTAGCTTCAACCATG	Pla	Pla-2	P2-22	TRUE	3	TRA:CAMRERSWVRQKLVF TRB:CASSRGTSGTDTQYF	24	CD8-C9
Pla-2_CCTATTATCAATCACG	Pla	Pla-2	P2-75	FALSE	3	TRA:CAVNDGRGADGLTF TRB:CASLGGRTDTQYF	8	CD8-C8
Pla-2_CCTTCCCTCCCGGATG	Pla	Pla-2	P2-4015	FALSE	1	TRA:CAVQAADNARLMF TRB:CACNSQVQETQYF	1	CD8-C9
Pla-2_CCTTCGAGTATATGGA	Pla	Pla-2	P2-34	TRUE	3	TRA:CAVRDSNYQLIW TRB:CASSLYPSGGGTDTQYF	15	CD8-C10
Pla-2_CCTTCGATCGCTTAGA	Pla	Pla-2	P2-2	TRUE	3	TRA:CVVPNTNAGKSTF TRB:CASSLQGSTYNEQFF	220	CD8-C9
Pla-2_CGATGTACACGGCTAC	Pla	Pla-2	P2-4060	FALSE	1	TRA:CAFLRNYGQNFVF TRB:CASSLSAGEAGELFF	1	CD8-C8
Pla-2_CGATTGAAGTTGAGAT	Pla	Pla-2	P2-104	TRUE	3	TRA:CAMREAGGSGAGSYQLTF TRB:CASSLGSGNTIYF	5	CD8-C8
Pla-2_CGCGGTACAACCGCCA	Pla	Pla-2	P2-499	FALSE	2	TRA:CALSEVDGGGGADGLTF TRB:CASREVSYNEQFF	2	CD8-C8
Pla-2_CGGACTGCAGTGAGTG	Pla	Pla-2	P2-251	FALSE	3	TRA:CADNNAGNMLTF TRB:CASSPNLPLLADNQETQYF	3	CD8-C8
Pla-2_CGTTCTGGTGTCCTCT	Pla	Pla-2	P2-182	TRUE	3	TRA:CAPNFGNEKLTF TRB:CASSFSSAYEQYF	3	CD8-C8
Pla-2_CGTTGGGCAAACAACA	Pla	Pla-2	P2-36	FALSE	3	TRA:CAAQAGNTPLVF TRB:CSAGVGGTIQYF	15	CD8-C9
Pla-2_CTAACTTGTGGCCCTA	Pla	Pla-2	P2-513	FALSE	2	TRA:CAPAPDQFYF TRB:CASSFPRRGPMNTEAFF	2	CD8-C8
Pla-2_CTACATTAGGATCGCA	Pla	Pla-2	P2-2	TRUE	3	TRA:CVVPNTNAGKSTF TRB:CASSLQGSTYNEQFF	220	CD8-C8
Pla-2_CTAGTGATCTTACCGC	Pla	Pla-2	P2-4195	FALSE	1	TRA:CAVHNNARLMF TRB:CASSQQGLDSPLHF	1	CD8-C8
Pla-2_CTCACACAGCGTGAGT	Pla	Pla-2	P2-126	TRUE	3	TRA:CATDGDFGNEKLTF TRB:CASSLTGETQYF	4	CD8-C8
Pla-2_CTGATAGGTGGAAAGA	Pla	Pla-2	P2-82	FALSE	3	TRA:CVADTGNQFYF TRB:CASSSALTGTYEQYF	7	CD8-C5
Pla-2_CTGCGGAGTCTGGTCG	Pla	Pla-2	P2-2	TRUE	3	TRA:CVVPNTNAGKSTF TRB:CASSLQGSTYNEQFF	220	CD8-C9
Pla-2_CTTACCGAGGAATTAC	Pla	Pla-2	P2-10	TRUE	3	TRA:CAPLTPNDMRF TRB:CASLNGAGGSYEQYF	45	CD8-C8
Pla-2_GAACCTAGTGATGTGG	Pla	Pla-2	P2-153	FALSE	3	TRA:CAYNNNDMRF TRB:CASSDRGGRQETQYF	4	CD8-C8

Pla-2_GAATAAGCAAGTCATC	Pla	Pla-2	P2-4313	FALSE	1	TRA:CIVSAF TRB:CASNRGNEQFF	1	CD8-C8
Pla-2_GACCTGGAGTGAAGAG	Pla	Pla-2	P2-510	FALSE	2	TRA:CAVRDPGDDKIIF TRB:CASSVASGKSYEQYF	2	CD8-C8
Pla-2_GACGTTAGTGTAAGTA	Pla	Pla-2	P2-4349	FALSE	1	TRA:CALSDQGRGNQGGKLIF TRB:CASSLMGGSTDTQYF	1	CD8-C9
Pla-2_GAGCAGACACGTCAGC	Pla	Pla-2	P2-34	TRUE	3	TRA:CAVRDSNYQLIW TRB:CASSLYPSGGGTDTQYF	15	CD8-C8
Pla-2_GAGCAGACAGCTCGCA	Pla	Pla-2	P2-46	TRUE	3	TRA:CAFSTWGNQGGKLIF TRB:CASSQDRLGGGTYEQYF	11	CD8-C8
Pla-2_GAGGTGAGTGCGCTTG	Pla	Pla-2	P2-6	TRUE	3	TRA:CVVSVAAGNKLTF TRB:CASSPGTDLYEQYF	85	CD8-C8
Pla-2_GATGAAACACCGAATT	Pla	Pla-2	P2-150	FALSE	3	TRA:CVVRYAGNNRKLIW TRB:CASSQGQPPYEQYF	4	CD8-C8
Pla-2_GCATGATGTCGAGATG	Pla	Pla-2	P2-547	FALSE	2	TRA:CAVRPLRNTDKLIF TRB:CASGHLQETQYF	2	CD8-C9
Pla-2_GCGACCACAATCCGAT	Pla	Pla-2	P2-45	TRUE	3	TRA:CAVSGDSNYQLIW TRB:CASSLGGNGNTIYF	11	CD8-C8
Pla-2_GCGCAGTAGGTGGGTT	Pla	Pla-2	P2-1	TRUE	3	TRA:CVVNIIGYQKVTF TRB:CASTRQGKQPQHF	270	CD4-C6
Pla-2_GCTGGGTGTCGCGAAA	Pla	Pla-2	P2-30	TRUE	3	TRA:CAVGGDTGNQFYF TRB:CASSLFPTGVAYGYTF	20	CD8-C9
Pla-2_GGAGCAACACGAGAGT	Pla	Pla-2	P2-4517	FALSE	1	TRA:CAIMDSNYQLIW TRB:CASSHLAGGSTGELFF	1	CD8-C8
Pla-2_GGATGTTTCTGTTTGT	Pla	Pla-2	P2-446	FALSE	2	TRA:CVVRGNQGGKLIF TRB:CATDQGNSITGYTF	2	CD8-C9
Pla-2_GGCTCGAAGAGAACAG	Pla	Pla-2	P2-28	TRUE	3	TRA:CATDANTGNQFYF TRB:CASSLGQWVEQYF	22	CD8-C9
Pla-2_GGGAGATTCCAGGGCT	Pla	Pla-2	P2-4554	FALSE	1	TRA:CALTGNQFYF TRB:CSARYPRRGTEAFF	1	CD8-C8
Pla-2_GGGCACTTCCATGAAC	Pla	Pla-2	P2-4559	FALSE	1	TRA:CAAEEGKIIF TRB:CASSVLQGSGNTIYF	1	CD8-C9
Pla-2_GGGCATCCAGTCAGAG	Pla	Pla-2	P2-541	FALSE	2	TRA:CAAYKLSF TRB:CASSPGGTVETQYF	2	CD8-C8
Pla-2_GGGCATCCATGTAGTC	Pla	Pla-2	P2-4564	FALSE	1	TRA:CAMRRSSGNTGKLIF TRB:CSARDYLKTSSGNTIYF	1	CD8-C8
Pla-2_GGTGCGTTCCTATTCA	Pla	Pla-2	P2-2	TRUE	3	TRA:CVVPNTNAGKSTF TRB:CASSLQGSTYNEQFF	220	CD8-C9
Pla-2_GTAACTGGTGCATCTA	Pla	Pla-2	P2-4586	FALSE	1	TRA:CAAKTGGFKTIF TRB:CSVGGGTGVAGELFF	1	CD8-C8
Pla-2_GTCACGGCAAGTTAAG	Pla	Pla-2	P2-4615	FALSE	1	TRA:CLVGDMAYLNTNAGKSTF TRB:CAWSGPEANYGYTF	1	CD8-C8
Pla-2_GTCTTCGAGGAATTAC	Pla	Pla-2	P2-215	FALSE	3	TRA:CAANNNARLMF TRB:CASSLSGARLVTGELFF	3	CD8-C8
Pla-2_GTCTTCGGTTGACGTT	Pla	Pla-2	P2-4626	FALSE	1	TRA:CAPRHGGATNKLIF TRB:CASSPRDSFSSYNEQFF	1	CD8-C8
Pla-2_GTTAAGCTCTTATCTG	Pla	Pla-2	P2-4657	FALSE	1	TRA:CAVKVDNQGGKLIF TRB:CASSQASGGLGEQFF	1	CD8-C9
Pla-2_TACGGATCAGTTAACC	Pla	Pla-2	P2-394	FALSE	2	TRA:CAAGAPGRAGRRALTF TRB:CASSPEAVYSGNTIYF	2	CD8-C8
Pla-2_TACGGATTCCCGACTT	Pla	Pla-2	P2-550	FALSE	2	TRA:CAVRFSNTNAGKSTF TRB:CASSSNTGFGTDTQYF	2	CD8-C9
Pla-2_TACTCGCTCTTGGGTA	Pla	Pla-2	P2-4708	FALSE	1	TRA:CAVPFNFGNEKLTF TRB:CASSQGHDRNLYNEQFF	1	CD8-C8

Pla-2_TACTTGTGTATTACCG	Pla	Pla-2	P2-4712	FALSE	1	TRA:CAASGAGGTSYGKLTF TRB:CASSQHPAGLNQPQHF	1	CD8-C9
Pla-2_TCAGCAACATAGAAAC	Pla	Pla-2	P2-4766	FALSE	1	TRA:CAVTAGNKLTF TRB:CASSGLAGDVGELFF	1	CD8-C9
Pla-2_TCAGCAATCCAGTAGT	Pla	Pla-2	P2-4767	FALSE	1	TRA:CAYRAGAQKLVF TRB:CASSLRWGSSVYNAEAFF	1	CD8-C8
Pla-2_TCATTTGAGCTGAACG	Pla	Pla-2	P2-33	TRUE	3	TRA:CAFKLSDGQKLLF TRB:CAWSRAALTEAFF	17	CD8-C8
Pla-2_TCTGAGAGTCACTGGC	Pla	Pla-2	P2-4825	FALSE	1	TRA:CVVPLGSSASKIIF TRB:CASSRNAGGGTDTQYF	1	CD8-C10
Pla-2_TCTTTCCAGTCTCCTC	Pla	Pla-2	P2-4844	FALSE	1	TRA:CEGGKLIF TRB:CASSQTGGTGPYEQYF	1	CD8-C8
Pla-2_TGAGAGGTCAGGCAAG	Pla	Pla-2	P2-10	TRUE	3	TRA:CAPLTPNDMRF TRB:CASLNGAGGSYEQYF	45	CD8-C8
Pla-2_TGATTTCAGAATAGGG	Pla	Pla-2	P2-69	TRUE	3	TRA:CAVRAGSARQLTF TRB:CATTGTSLDEQYF	8	CD8-C8
Pla-2_TGGCCAGAGACTTTCG	Pla	Pla-2	P2-3	TRUE	3	TRA:CAVNPLESSYKLIF TRB:CASSPGTANTGELFF	177	CD8-C9
Pla-2_TGGCGCAGTGCGAAAC	Pla	Pla-2	P2-15	TRUE	3	TRA:CAVGDNFNKFYF TRB:CASSRTSAAGELFF	35	CD8-C9
Pla-2_TGGGAAGCAAGCCGCT	Pla	Pla-2	P2-8	TRUE	3	TRA:CAALEGDNYGQNFVF TRB:CASSPTWGGVRDTQYF	53	CD8-C9
Pla-2_TGTCCCACATAGAAAC	Pla	Pla-2	P2-132	TRUE	3	TRA:CAANRAGNNRKLIW TRB:CASSFKKAGGPVGELFF	4	CD8-C8
Pla-2_TTCGGTCCATAGAAAC	Pla	Pla-2	P2-4985	FALSE	1	TRA:CAFMKPYNNNDMRF TRB:CASSQDRTGGQYF	1	CD8-C8
Pla-2_TTCTACAGTGCGGTAA	Pla	Pla-2	P2-18	TRUE	3	TRA:CAATSSSNFGNEKLTF TRB:CAWSRAALTEAFF	28	CD8-C8
Pla-2_AAAGCAAAGCCACGTC	Pla	Pla-2	P2-3418	FALSE	1	TRA:CAYRSAAAGNQFYF TRB:CASSKKAIYGGPQFF	1	CD4-C1
Pla-2_ACAGCCGTCCCGACTT	Pla	Pla-2	P2-3500	FALSE	1	TRA:CAVYNNNDMRF TRB:CSGGDGGREQYF	1	CD4-C1
Pla-2_AGATCTGAGGTGCAAC	Pla	Pla-2	P2-3626	FALSE	1	TRA:CAASYKAAGNKLTF TRB:CASSRGLAGNEQFF	1	CD4-C1
Pla-2_AGGGATGTCAATCACG	Pla	Pla-2	P2-3686	FALSE	1	TRA:CAVPGTDNYGQNFVF TRB:CASSRDRVNTEAFF	1	CD4-C2
Pla-2_AGTGGGAAGTCGAGTG	Pla	Pla-2	P2-3707	FALSE	1	TRA:CAVTRRTGTASKLTF TRB:CASRGAGERNTEAFF	1	CD4-C1
Pla-2_CAGTCCTAGCAGCGTA	Pla	Pla-2	P2-3887	FALSE	1	TRA:CAVTGAGGTSYGKLTF TRB:CSAIPLLTAQNQPQHF	1	CD4-C2
Pla-2_CGAGCCACACCGAAAG	Pla	Pla-2	P2-4050	FALSE	1	TRA:CVVRKLKDDKIIF TRB:CASSSGLADYNEQFF	1	CD4-C1
Pla-2_CGCTATCCAGCTGCAC	Pla	Pla-2	P2-4080	FALSE	1	TRA:CAASNKIQGAQKLVF TRB:CASSADSIYGYTF	1	CD4-C1
Pla-2_CGCTTCATCAAGAAGT	Pla	Pla-2	P2-4093	FALSE	1	TRA:CVVASNTGNQFYF TRB:CATSDSRPVGAYNEQFF	1	CD4-C1
Pla-2_CGGGTCACAGTAGAGC	Pla	Pla-2	P2-4118	FALSE	1	TRA:CAVAILTGGGNKLTF TRB:CASSQGGSSSYEQYF	1	CD4-C1
Pla-2_CTGATCCCAGTAAGAT	Pla	Pla-2	P2-4239	FALSE	1	TRA:CAAHSGGSNYKLTF TRB:CAWSGLAGAAGELFF	1	CD4-C1
Pla-2_CTTTGCGCAGTCACTA	Pla	Pla-2	P2-4285	FALSE	1	TRA:CILRRGNNRLAF TRB:CASSQEGTGGETQYF	1	CD4-C1
Pla-2_GACGGCTCAGCTGGCT	Pla	Pla-2	P2-4342	FALSE	1	TRA:CALSEANQAGTALIF TRB:CAISMGDRVAYEQYF	1	CD8-C1

Pla-2_TACCTATCACGCATCG	Pla	Pla-2	P2-4684	FALSE	1	TRA:CAVPGRDSWGKFQF TRB:CASSLNGNSETQYF	1	CD4-C1
Pla-2_TAGTTGGTCAACACTG	Pla	Pla-2	P2-4729	FALSE	1	TRA:CAASKGSSNTGKLIF TRB:CASRPRGSTYEQYF	1	CD4-C1
Pla-2_TCGCGAGCAGATCCAT	Pla	Pla-2	P2-4795	FALSE	1	TRA:CASIYTGANSKLTF TRB:CASSYGRSYNSPLHF	1	CD4-C1
Pla-2_TCGTAGAGTTCCTCCA	Pla	Pla-2	P2-4813	FALSE	1	TRA:CAENLEYGNKLVF TRB:CASSLDSSSYNSPLHF	1	CD4-C1
Pla-2_TGATTTCCAAAGTCAA	Pla	Pla-2	P2-4871	FALSE	1	TRA:CAMRAGANSKLTF TRB:CASSLAAGGYEQYF	1	CD4-C1
Pla-2_TGGGCGTCATCACAAC	Pla	Pla-2	P2-4928	FALSE	1	TRA:CIVKSGLDQFYF TRB:CASSKLQGAGDDEQFF	1	CD4-C7
Pla-2_AAAGATGGTACAGCAG	Pla	Pla-2	P2-26	TRUE	3	TRA:CALVNTGGFKTIF TRB:CASSPPSSGGANEQYF	22	CD4-C6
Pla-2_AACTCAGGTGCTTCTC	Pla	Pla-2	P2-63	FALSE	3	TRA:CAASGEAGTALIF TRB:CATSREIQPNEKLFF	9	CD4-C6
Pla-2_ACTGAACAGCGATCCC	Pla	Pla-2	P2-145	FALSE	3	TRA:CAMRRSSGGSNYKLTF TRB:CASSSIGRGSKRETQYF	4	CD4-C6
Pla-2_ATCCGAATCGAGAACG	Pla	Pla-2	P2-3733	FALSE	1	TRA:CAVTRPGGYNKLIF TRB:CASSPTGLDFSGANVLTF	1	CD8-C6
Pla-2_ATTGGACCAGATCTGT	Pla	Pla-2	P2-3762	FALSE	1	TRA:CALSSDSNYQLIW TRB:CASSFGGSGIYYEQYF	1	CD8-C6
Pla-2_CCTCTGACATATGAGA	Pla	Pla-2	P2-4002	FALSE	1	TRA:CAESKGSSASKIIF TRB:CASSEGTTRGDTQYF	1	CD8-C5
Pla-2_CCTTCCCAGAATGTGT	Pla	Pla-2	P2-18	TRUE	3	TRA:CAATSSSNFGNEKLTF TRB:CAWSRAALTEAFF	28	CD4-C6
Pla-2_CGTAGGCCATCCCACT	Pla	Pla-2	P2-505	FALSE	2	TRA:CAVDNQGGKLIF TRB:CASRPGGGSYEQYF	2	CD4-C6
Pla-2_CGTCAGGAGAAACCGC	Pla	Pla-2	P2-4136	FALSE	1	TRA:CAVLSGAGSYQLTF TRB:CAISSSSGEDTQYF	1	CD8-C6
Pla-2_CTCGAAACAGTGACAG	Pla	Pla-2	P2-519	FALSE	2	TRA:CAYNFGNEKLTF TRB:CASSPAGGPYNEQFF	2	CD8-C6
Pla-2_CTGATCCGTCATATCG	Pla	Pla-2	P2-525	FALSE	2	TRA:CVVNIIGYQKVTF TRB:CAIRQGDRQPQHF	2	CD4-C6
Pla-2_GCAGCCAAGTGACTCT	Pla	Pla-2	P2-32	TRUE	3	TRA:CAMREANVGADGLTF TRB:CASSTRIAGGPFPETQYF	17	CD4-C6
Pla-2_GCAGTTAAGAGGTACC	Pla	Pla-2	P2-525	FALSE	2	TRA:CVVNIIGYQKVTF TRB:CAIRQGDRQPQHF	2	CD4-C6
Pla-2_GGCCGATGTCCCGACA	Pla	Pla-2	P2-1	TRUE	3	TRA:CVVNIIGYQKVTF TRB:CASTRQGKQPQHF	270	CD4-C6
Pla-2_TAAGCGTCACAAGTAA	Pla	Pla-2	P2-76	TRUE	3	TRA:CAGQEQGGSEKLVF TRB:CASSQMGLYGYTF	7	CD4-C6
Pla-2_TCGCGAGTCCGTTGCT	Pla	Pla-2	P2-4797	FALSE	1	TRA:CATDGVQGAQKLVF TRB:CASSLQGSTYNEQFF	1	CD8-C2
Pla-2_TCTGGAAGTGTGACGA	Pla	Pla-2	P2-4832	FALSE	1	TRA:CAVGLYNQGGKLIF TRB:CASSQPDRVHEQYF	1	CD8-C6
Pla-2_TGACGGCCACTGTGTA	Pla	Pla-2	P2-304	TRUE	2	TRA:CVVSLKPGGGNKLTF TRB:CASSLQGAKNTGELFF	2	CD8-C6
Pla-2_TGACTTTGTGCTCTTC	Pla	Pla-2	P2-258	FALSE	3	TRA:CVVNGAGGYNKLIF TRB:CASSLRSTGNTEAFF	3	CD4-C6
Pla-2_TTCTCCTGTGTCGCTG	Pla	Pla-2	P2-1	TRUE	3	TRA:CVVNIIGYQKVTF TRB:CASTRQGKQPQHF	270	CD4-C6
Pla-2_TAGGCATTCCGTCATC	Pla	Pla-2	P2-4725	FALSE	1	TRA:CGTLGVSGSARQLTF TRB:CASSVRTANTGELFF	1	CD8-C1

Pla-2_AACTTTCTCTTTACGT	Pla	Pla-2	P2-1	TRUE	3	TRA:CVVNIIGYQKVTF TRB:CASTRQGKQPQHF	270	CD8-C11
Pla-2_ACATGGTAGCATCATC	Pla	Pla-2	P2-3511	FALSE	1	TRA:CATDLDGQKLLF TRB:CASSQVLTSGGAVRLNEQFF	1	CD8-C11
Pla-2_ACTGCTCTCAGAGCTT	Pla	Pla-2	P2-3596	FALSE	1	TRA:CIVRVEFGNEKLTF TRB:CASSMLNVSGTDHGANVLTF	1	CD8-C11
Pla-2_AGGGTGAAGTCCGTAT	Pla	Pla-2	P2-24	FALSE	3	TRA:CAVMDSSYKLIF TRB:CASSELAGSGDTQYF	23	CD8-C11
Pla-2_AGGTCATAGCACCGTC	Pla	Pla-2	P2-2	TRUE	3	TRA:CVVPNTNAGKSTF TRB:CASSLQGSTYNEQFF	220	CD8-C11
Pla-2_CAGAGAGCACTGCCAG	Pla	Pla-2	P2-119	FALSE	3	TRA:CAASIMDDMRF TRB:CSAAIGYGYTF	5	CD8-C11
Pla-2_CCCAATCCATTGGGCC	Pla	Pla-2	P2-152	FALSE	3	TRA:CALKGSSGYSTLTF TRB:CSAIPSGGIGTDTQYF	4	CD8-C11
Pla-2_CCCTCCTAGACCTTTG	Pla	Pla-2	P2-3968	FALSE	1	TRA:CAMGGSARQLTF TRB:CASSPKFTGTPEAFF	1	CD8-C11
Pla-2_CGCCAAGTCACAGGCC	Pla	Pla-2	P2-1	TRUE	3	TRA:CVVNIIGYQKVTF TRB:CASTRQGKQPQHF	270	CD8-C11
Pla-2_CGCGGTAAGGCTAGAC	Pla	Pla-2	P2-4071	FALSE	1	TRA:CAVNAVIKAAGNKLTF TRB:CAWERSGALFF	1	CD8-C11
Pla-2_CGTAGGCCACCATGTA	Pla	Pla-2	P2-4128	FALSE	1	TRA:CAENGGLTGGGNKLTF TRB:CASSLIGNEQFF	1	CD8-C11
Pla-2_CGTCAGGGTTCGGCAC	Pla	Pla-2	P2-1	TRUE	3	TRA:CVVNIIGYQKVTF TRB:CASTRQGKQPQHF	270	CD8-C11
Pla-2_CTGAAGTGTTGGTTTG	Pla	Pla-2	P2-6	TRUE	3	TRA:CVVSVAAGNKLTF TRB:CASSPGTDLYEQYF	85	CD8-C11
Pla-2_GACGCGTTCAGAAATG	Pla	Pla-2	P2-1	TRUE	3	TRA:CVVNIIGYQKVTF TRB:CASTRQGKQPQHF	270	CD4-C5
Pla-2_GCCAAATCACTAAGTC	Pla	Pla-2	P2-4435	FALSE	1	TRA:CAVSDISGAGSYQLTF TRB:CASSQTSGPYNEQFF	1	CD8-C11
Pla-2_GCGACCACAATAACGA	Pla	Pla-2	P2-36	FALSE	3	TRA:CAAQAGNTPLVF TRB:CSAGVGGTIQYF	15	CD8-C11
Pla-2_GCTGCGACACGTGAGA	Pla	Pla-2	P2-3	TRUE	3	TRA:CAVNPLESSYKLIF TRB:CASSPGTANTGELFF	177	CD8-C11
Pla-2_GGAAAGCGTGTCCTCT	Pla	Pla-2	P2-320	TRUE	2	TRA:CAGEADKLIF TRB:CASSQETAGGDTQYF	2	CD8-C11
Pla-2_GGGACCTAGGGAAACA	Pla	Pla-2	P2-6	TRUE	3	TRA:CVVSVAAGNKLTF TRB:CASSPGTDLYEQYF	85	CD8-C11
Pla-2_GGGTCTGAGTCGAGTG	Pla	Pla-2	P2-6	TRUE	3	TRA:CVVSVAAGNKLTF TRB:CASSPGTDLYEQYF	85	CD8-C11
Pla-2_TGACTTTCAAAGGAAG	Pla	Pla-2	P2-30	TRUE	3	TRA:CAVGGDTGNQFYF TRB:CASSLFPTGVAYGYTF	20	CD8-C11
Pla-2_TGCGTGGCAAAGGCGT	Pla	Pla-2	P2-2	TRUE	3	TRA:CVVPNTNAGKSTF TRB:CASSLQGSTYNEQFF	220	CD8-C11
Pla-2_TGTCCCATCCATGCTC	Pla	Pla-2	P2-1	TRUE	3	TRA:CVVNIIGYQKVTF TRB:CASTRQGKQPQHF	270	CD8-C11
Pla-2_TTTACTGCAATGGAGC	Pla	Pla-2	P2-2	TRUE	3	TRA:CVVPNTNAGKSTF TRB:CASSLQGSTYNEQFF	220	CD8-C11
Pla-3_AGAGCTTCAAGGCTCC	Pla	Pla-3	P3-61	TRUE	3	TRA:CALIRVDSSYKLIF TRB:CASRLGGGYTF	6	CD8-C7
Pla-3_AGCGTATCAGAAGCAC	Pla	Pla-3	P3-129	FALSE	3	TRA:CAVRGDSYSGAGSYQLTF TRB:CASSGSGASTDTQYF	4	CD8-C6
Pla-3_AGCTTGAAGGGCACTA	Pla	Pla-3	P3-74	TRUE	3	TRA:CALSQGGKLIF TRB:CASSLGTGRDDTQYF	5	CD8-C7

Pla-3_AGGCCGTAGAAACCTA	Pla	Pla-3	P3-1907	FALSE	1	TRA:CALTGANNLFF TRB:CASSYGFGFDTEAFF	1	CD8-C6
Pla-3_ATGTGTGCACTATCTT	Pla	Pla-3	P3-1973	FALSE	1	TRA:CAVRAFF TRB:CASSLTSTGEAFF	1	CD8-C6
Pla-3_CAAGTTGCAGCTGCAC	Pla	Pla-3	P3-99	FALSE	3	TRA:CAASDTGAQKLVF TRB:CASSLPADGDNSPLHF	5	CD8-C6
Pla-3_CGTCACTAGCCTTGAT	Pla	Pla-3	P3-46	FALSE	3	TRA:CAVRRGILTGGGNKLTF TRB:CASSLVSSGGNPSYEQYF	8	CD8-C6
Pla-3_CGTCACTAGGTGCTTT	Pla	Pla-3	P3-2227	FALSE	1	TRA:CAAMVSGTYKYIF TRB:CASSLWTSPEETQYF	1	CD8-C7
Pla-3_CTCAGAAAGAAGCCCA	Pla	Pla-3	P3-2281	FALSE	1	TRA:CAEPMNNNAGNMLTF TRB:CASSLDADYNEQFF	1	CD8-C7
Pla-3_GAAGCAGAGGTCGGAT	Pla	Pla-3	P3-45	FALSE	3	TRA:CAVRVLMAGGSYIPTF TRB:CSAKSNTIAGSTDTQYF	8	CD8-C6
Pla-3_GATCGTACACGACTCG	Pla	Pla-3	P3-2442	FALSE	1	TRA:CAAYNTDKLIF TRB:CASSAGITGELFF	1	CD8-C7
Pla-3_GCATGATCAGCGAACA	Pla	Pla-3	P3-79	TRUE	3	TRA:CVVNPGGSYIPTF TRB:CASSGDYGQIYEQYF	5	CD8-C6
Pla-3_GGTGCGTGTAAGTTCC	Pla	Pla-3	P3-2626	FALSE	1	TRA:CAYFGTASKLTF TRB:CASSYWTGPIYEQYF	1	CD8-C6
Pla-3_GTAGGCCGTATATGAG	Pla	Pla-3	P3-448	FALSE	2	TRA:CAVRDVGNQFYF TRB:CASSLIARGTEAFF	2	CD8-C7
Pla-3_TACGGGCAGAGTCTGG	Pla	Pla-3	P3-111	TRUE	3	TRA:CALSEARTGNQFYF TRB:CASSYGPGHATQYF	4	CD8-C7
Pla-3_TACTCATGTCGAGATG	Pla	Pla-3	P3-466	FALSE	2	TRA:CAGAIEAQKLVF TRB:CATRPGSGMNTEAFF	2	CD8-C5
Pla-3_TCAGGATAGACAGACC	Pla	Pla-3	P3-5	TRUE	3	TRA:CALRNSNYQLIW TRB:CASSLGDRAYNEQFF	38	CD8-C7
Pla-3_TCCACACCACAGGAGT	Pla	Pla-3	P3-1	TRUE	3	TRA:CAVAPISSGSARQLTF TRB:CASSQGMSEAFF	83	CD8-C7
Pla-3_TGAGGGATCGCGGATC	Pla	Pla-3	P3-1	TRUE	3	TRA:CAVAPISSGSARQLTF TRB:CASSQGMSEAFF	83	CD8-C7
Pla-3_TGGCCAGTCAAGATCC	Pla	Pla-3	P3-11	TRUE	3	TRA:CASGGGNKLTF TRB:CASSSDGDEAFF	17	CD8-C7
Pla-3_AAACCTGAGTTAAGTG	Pla	Pla-3	P3-1684	FALSE	1	TRA:CAFDRGSTLGRLYF TRB:CASSLQGASTEAFF	1	CD8-C5
Pla-3_AAACGGGAGGATGGAA	Pla	Pla-3	P3-1686	FALSE	1	TRA:CAALRSSASKIIF TRB:CASGGMGGTEAFF	1	CD8-C5
Pla-3_AAACGGGTCGACAGCC	Pla	Pla-3	P3-328	FALSE	2	TRA:CAVEHPKGGKLIF TRB:CASSPGSYGYTF	2	CD8-C4
Pla-3_AAAGCAATCAAAGTAG	Pla	Pla-3	P3-103	TRUE	3	TRA:CARDNTGKLIF TRB:CASSPGQGFTEAFF	4	CD8-C3
Pla-3_AAATGCCAGGCGACAT	Pla	Pla-3	P3-1698	FALSE	1	TRA:CAVEAHGNNDMRF TRB:CASSFWPIDPNYGYTF	1	CD8-C4
Pla-3_AAATGCCCATCCTTGC	Pla	Pla-3	P3-25	FALSE	3	TRA:CIVRVGSNTGKLIF TRB:CASTNYQETQYF	11	CD8-C5
Pla-3_AACACGTGTGATGCCC	Pla	Pla-3	P3-1704	FALSE	1	TRA:CAASRNNNDMRF TRB:CASSVGTGTEAFF	1	CD8-C4
Pla-3_AACGTTGGTGGTACAG	Pla	Pla-3	P3-1714	FALSE	1	TRA:CAVRSDYGGSQGNLIF TRB:CASSRDRRGYGYTF	1	CD8-C4
Pla-3_AACTCAGGTGCTAGCC	Pla	Pla-3	P3-3	TRUE	3	TRA:CVVPWMDSSYKLIF TRB:CASSQRAGDGNYGYTF	42	CD8-C5
Pla-3_AACTCTTAGTTCGCGC	Pla	Pla-3	P3-12	TRUE	3	TRA:CAVGDLGNEKLTF TRB:CASSAEGYYNEQFF	16	CD8-C4

Pla-3_AACTCTTCAGACGCTC	Pla	Pla-3	P3-11	TRUE	3	TRA:CASGGGNKLTF TRB:CASSSDGDEAFF	17	CD8-C4
Pla-3_AACTCTTTCTCCGGTT	Pla	Pla-3	P3-259	TRUE	2	TRA:CAPGGSNYKLTF TRB:CASSLAQQAALNEQFF	2	CD8-C4
Pla-3_AAGACCTTCGTCTGCT	Pla	Pla-3	P3-1733	FALSE	1	TRA:CAVIISNFGNEKLTF TRB:CSAAPRIATNEKLFF	1	CD8-C4
Pla-3_AAGCCGCTCGCCCTTA	Pla	Pla-3	P3-195	FALSE	3	TRA:CAASDTGAQKLVF TRB:CASSPNTGVNSPLHF	3	CD8-C5
Pla-3_AAGGCAGAGGACACCA	Pla	Pla-3	P3-1743	FALSE	1	TRA:CAMREYSGGSNYKLTF TRB:CASSTDSAVGTGELFF	1	CD8-C4
Pla-3_AAGGTTCGTATCAGTC	Pla	Pla-3	P3-150	TRUE	3	TRA:CVVNRGYGGSQGNLIF TRB:CASSLRGNNQPQHF	3	CD8-C5
Pla-3_AAGTCTGGTACCAGTT	Pla	Pla-3	P3-1	TRUE	3	TRA:CAVAPISSGSARQLTF TRB:CASSQGMSEAFF	83	CD8-C5
Pla-3_AATCCAGCAGACGCTC	Pla	Pla-3	P3-1748	FALSE	1	TRA:CADGGGSARQLTF TRB:CASSTDRLSGGNTIYF	1	CD8-C4
Pla-3_AATCGGTAGCAAATCA	Pla	Pla-3	P3-36	TRUE	3	TRA:CARGAGSYQLTF TRB:CASSVGTGTEAFF	9	CD8-C5
Pla-3_ACACCAAAGGACACCA	Pla	Pla-3	P3-1751	FALSE	1	TRA:CGTERGANNLFF TRB:CASSPADRDYGYTF	1	CD8-C4
Pla-3_ACACCGGTCGCACTCT	Pla	Pla-3	P3-52	FALSE	3	TRA:CAYRSPLTGNQFYF TRB:CASSPPRGMLYF	7	CD8-C5
Pla-3_ACACTGAGTAGGACAC	Pla	Pla-3	P3-1768	FALSE	1	TRA:CVVSWINAGNNRKLIW TRB:CATSRDGLYEQYF	1	CD8-C4
Pla-3_ACAGCCGAGAAGCCCA	Pla	Pla-3	P3-46	FALSE	3	TRA:CAVRRGILTGGGNKLTF TRB:CASSLVSSGGNPSYEQYF	8	CD8-C5
Pla-3_ACATACGAGACTGTAA	Pla	Pla-3	P3-32	TRUE	3	TRA:CAASRGGQKLLF TRB:CASSSRTSLVHNEQFF	10	CD8-C5
Pla-3_ACATGGTTCTCGTATT	Pla	Pla-3	P3-353	FALSE	2	TRA:CSQYNNNDMRF TRB:CASSPGSYGYTF	2	CD8-C4
Pla-3_ACCGTAATCTCAACTT	Pla	Pla-3	P3-5	TRUE	3	TRA:CALRNSNYQLIW TRB:CASSLGDRAYNEQFF	38	CD8-C5
Pla-3_ACCTTTACACGTCTCT	Pla	Pla-3	P3-1792	FALSE	1	TRA:CVVVSGGGADGLTF TRB:CASSLGQGGGYTF	1	CD8-C5
Pla-3_ACGAGCCAGATCACGG	Pla	Pla-3	P3-131	FALSE	3	TRA:CVVNRDEKLTF TRB:CASRGWGDVSEQFF	4	CD8-C5
Pla-3_ACGAGCCCAATCTACG	Pla	Pla-3	P3-5	TRUE	3	TRA:CALRNSNYQLIW TRB:CASSLGDRAYNEQFF	38	CD8-C5
Pla-3_ACGAGCCTCCGAATGT	Pla	Pla-3	P3-20	TRUE	3	TRA:CAVSSLQMDSSYKLIF TRB:CASSHRTENSPLHF	12	CD8-C4
Pla-3_ACGAGGATCGTTACGA	Pla	Pla-3	P3-32	TRUE	3	TRA:CAASRGGQKLLF TRB:CASSSRTSLVHNEQFF	10	CD8-C5
Pla-3_ACGATACAGGCGTACA	Pla	Pla-3	P3-20	TRUE	3	TRA:CAVSSLQMDSSYKLIF TRB:CASSHRTENSPLHF	12	CD8-C5
Pla-3_ACGATGTTCTCCTATA	Pla	Pla-3	P3-62	TRUE	3	TRA:CAEIPSGAGSYQLTF TRB:CASNGDENTIYF	6	CD8-C3
Pla-3_ACGCAGCCAAATTGCC	Pla	Pla-3	P3-90	TRUE	3	TRA:CAGRKGDNYGQNFVF TRB:CASSRGTNYGYTF	5	CD8-C3
Pla-3_ACGCAGCCATTTCACT	Pla	Pla-3	P3-358	FALSE	2	TRA:CAMREGTSGTYKYIF TRB:CATSSGGALDTQYF	2	CD8-C4
Pla-3_ACGCCAGCACGGTAGA	Pla	Pla-3	P3-52	FALSE	3	TRA:CAYRSPLTGNQFYF TRB:CASSPPRGMLYF	7	CD8-C4
Pla-3_ACGCCAGTCTCTTATG	Pla	Pla-3	P3-14	TRUE	3	TRA:CALPFSGGYNKLIF TRB:CSARKTEPRSYEQYF	15	CD8-C5

Pla-3_ACGGGCTGTCTGATCA	Pla	Pla-3	P3-32	TRUE	3	TRA:CAASRGGQKLLF TRB:CASSSRTSLVHNEQFF	10	CD8-C4
Pla-3_ACGGGCTGTGCGAAAC	Pla	Pla-3	P3-42	TRUE	3	TRA:CALGGSTLGRLYF TRB:CASSASTGANSPLHF	8	CD8-C4
Pla-3_ACGTCAAAGATGCCTT	Pla	Pla-3	P3-3	TRUE	3	TRA:CVVPWMDSSYKLIF TRB:CASSQRAGDGNYGYTF	42	CD8-C5
Pla-3_ACTGAGTAGTCCATAC	Pla	Pla-3	P3-1829	FALSE	1	TRA:CAVKNNAGNMLTF TRB:CASSMEDGGLYEQFF	1	CD8-C4
Pla-3_ACTGAGTCAGCCTGTG	Pla	Pla-3	P3-363	FALSE	2	TRA:CAYRTYYNTDKLIF TRB:CASSIADRGGNEQFF	2	CD8-C5
Pla-3_ACTGATGAGATGTCGG	Pla	Pla-3	P3-25	FALSE	3	TRA:CIVRVGSNTGKLIF TRB:CASTNYQETQYF	11	CD8-C4
Pla-3_ACTGATGTCTATGTGG	Pla	Pla-3	P3-1	TRUE	3	TRA:CAVAPISSGSARQLTF TRB:CASSQGMSEAFF	83	CD8-C5
Pla-3_ACTGCTCGTCTCTCTG	Pla	Pla-3	P3-1	TRUE	3	TRA:CAVAPISSGSARQLTF TRB:CASSQGMSEAFF	83	CD8-C7
Pla-3_ACTGCTCGTTCGTTGA	Pla	Pla-3	P3-50	FALSE	3	TRA:CAYRRGNQGGKLIF TRB:CASSVGSGGGYNEQFF	7	CD8-C3
Pla-3_ACTGCTCTCTTAGAGC	Pla	Pla-3	P3-8	TRUE	3	TRA:CAFDRGSTLGRLYF TRB:CASNPRQGTSTYEQYF	26	CD8-C4
Pla-3_ACTTGTTGTGCCTGGT	Pla	Pla-3	P3-12	TRUE	3	TRA:CAVGDLGNEKLTF TRB:CASSAEGYYNEQFF	16	CD8-C4
Pla-3_AGAGTGGCATTTGCTT	Pla	Pla-3	P3-1861	FALSE	1	TRA:CATATFSGGYNKLIF TRB:CASSRGTGDQPQHF	1	CD8-C5
Pla-3_AGATTGCTCCATGAAC	Pla	Pla-3	P3-1867	FALSE	1	TRA:CVVSDPGTSYDKVIF TRB:CASSALAGGDQPQHF	1	CD8-C5
Pla-3_AGATTGCTCGACCAGC	Pla	Pla-3	P3-334	FALSE	2	TRA:CALNTDKLIF TRB:CASDSVGVNEQFF	2	CD8-C5
Pla-3_AGCAGCCGTGGCCCTA	Pla	Pla-3	P3-1872	FALSE	1	TRA:CAVSDYAGGTSYGKLTF TRB:CASSYPPGQGGSNPYEQYF	1	CD8-C5
Pla-3_AGCAGCCTCTGGCGTG	Pla	Pla-3	P3-1874	FALSE	1	TRA:CALSDPNNNARLMF TRB:CASSVSGAPGGYTF	1	CD8-C5
Pla-3_AGCATACGTATTCGTG	Pla	Pla-3	P3-9	TRUE	3	TRA:CAVRDMDTGRRALTF TRB:CASSWDTRQAEAFF	24	CD8-C5
Pla-3_AGCCTAAAGGGTGTTG	Pla	Pla-3	P3-18	TRUE	3	TRA:CLVGVGNNDMRF TRB:CASSPGTGKYEQYF	12	CD8-C5
Pla-3_AGCGGTCCACGAGAGT	Pla	Pla-3	P3-3	TRUE	3	TRA:CVVPWMDSSYKLIF TRB:CASSQRAGDGNYGYTF	42	CD8-C5
Pla-3_AGCGGTCTCGCCCTTA	Pla	Pla-3	P3-377	FALSE	2	TRA:CAWEGKLIF TRB:CASSGEYWNEQFF	2	CD8-C4
Pla-3_AGCTCCTGTTAGTGGG	Pla	Pla-3	P3-209	FALSE	3	TRA:CALSDHSVNTGTASKLTF TRB:CASSINRGPTEAFF	3	CD8-C4
Pla-3_AGGCCGTTCCTTGGTC	Pla	Pla-3	P3-1911	FALSE	1	TRA:CAASIDSGAGSYQLTF TRB:CATSDMGGLYNEQFF	1	CD8-C4
Pla-3_AGGTCCGCACCTGGTG	Pla	Pla-3	P3-90	TRUE	3	TRA:CAGRKGDNYGQNFVF TRB:CASSRGTNYGYTF	5	CD8-C4
Pla-3_AGGTCCGGTCACCTAA	Pla	Pla-3	P3-108	TRUE	3	TRA:CILRDPLKDGQKLLF TRB:CASSPSDSGANVLTF	4	CD8-C5
Pla-3_AGGTCCGGTGCGGTAA	Pla	Pla-3	P3-384	FALSE	2	TRA:CAASADSGNTPLVF TRB:CASRDKGNEQFF	2	CD8-C4
Pla-3_AGTAGTCTCGAACTGT	Pla	Pla-3	P3-9	TRUE	3	TRA:CAVRDMDTGRRALTF TRB:CASSWDTRQAEAFF	24	CD8-C4
Pla-3_AGTCTTTAGATGTCGG	Pla	Pla-3	P3-1920	FALSE	1	TRA:CAAQDGQKLLF TRB:CASSQESGGIGELFF	1	CD8-C4

Pla-3_AGTGAGGAGGATGTAT	Pla	Pla-3	P3-9	TRUE	3	TRA:CAVRDMDTGRRALTF TRB:CASSWDTRQAEAFF	24	CD8-C8
Pla-3_AGTGAGGGTTAAGGGC	Pla	Pla-3	P3-1	TRUE	3	TRA:CAVAPISSGSARQLTF TRB:CASSQGMSEAFF	83	CD8-C3
Pla-3_AGTGGGAAGGCCGAAT	Pla	Pla-3	P3-5	TRUE	3	TRA:CALRNSNYQLIW TRB:CASSLGDRAYNEQFF	38	CD8-C5
Pla-3_AGTGGGATCGCAGGCT	Pla	Pla-3	P3-3	TRUE	3	TRA:CVVPWMDSSYKLIF TRB:CASSQRAGDGNYGYTF	42	CD8-C4
Pla-3_AGTGTCAAGGGCATGT	Pla	Pla-3	P3-36	TRUE	3	TRA:CARGAGSYQLTF TRB:CASSVGTGTEAFF	9	CD8-C5
Pla-3_AGTTGGTAGGAGCGTT	Pla	Pla-3	P3-211	FALSE	3	TRA:CVVNPPGYSSASKIIF TRB:CASSLSRGVGTNEKLFF	3	CD8-C4
Pla-3_AGTTGGTCACCAGCAC	Pla	Pla-3	P3-14	TRUE	3	TRA:CALPFSGGYNKLIF TRB:CSARKTEPRSYEQYF	15	CD8-C5
Pla-3_ATAACGCGTGCTAGCC	Pla	Pla-3	P3-1939	FALSE	1	TRA:CYSTWRLSCLLLCRESRGFGNVLHC TRB:CASSGSGGGN	1	CD8-C5
Pla-3_ATAAGAGAGACAGGCT	Pla	Pla-3	P3-32	TRUE	3	TRA:CAASRGGQKLLF TRB:CASSSRTSLVHNEQFF	10	CD8-C3
Pla-3_ATAAGAGGTTTGACAC	Pla	Pla-3	P3-328	FALSE	2	TRA:CAVEHPKGGKLIF TRB:CASSPGSYGYTF	2	CD8-C4
Pla-3_ATCACGACAAACTGTC	Pla	Pla-3	P3-20	TRUE	3	TRA:CAVSSLQMDSSYKLIF TRB:CASSHRTENSPLHF	12	CD8-C5
Pla-3_ATCACGAGTATTACCG	Pla	Pla-3	P3-62	TRUE	3	TRA:CAEIPSGAGSYQLTF TRB:CASNGDENTIYF	6	CD8-C3
Pla-3_ATCACGATCAGGTTCA	Pla	Pla-3	P3-8	TRUE	3	TRA:CAFDRGSTLGRLYF TRB:CASNPRQGTSTYEQYF	26	CD8-C8
Pla-3_ATCATGGAGGCAGTCA	Pla	Pla-3	P3-52	FALSE	3	TRA:CAYRSPLTGNQFYF TRB:CASSPPRGMLYF	7	CD8-C5
Pla-3_ATCATGGGTATCGCAT	Pla	Pla-3	P3-14	TRUE	3	TRA:CALPFSGGYNKLIF TRB:CSARKTEPRSYEQYF	15	CD8-C5
Pla-3_ATCCACCCAGCTGTGC	Pla	Pla-3	P3-3	TRUE	3	TRA:CVVPWMDSSYKLIF TRB:CASSQRAGDGNYGYTF	42	CD8-C4
Pla-3_ATCCGAACACGGTTTA	Pla	Pla-3	P3-82	TRUE	3	TRA:CAVYLDRGSTLGRLYF TRB:CASSFGSGPNYGYTF	5	CD8-C4
Pla-3_ATCCGAATCGGCTACG	Pla	Pla-3	P3-1955	FALSE	1	TRA:CAVGFSGGYQKVTF TRB:CASSLDLDSSNEQFF	1	CD8-C4
Pla-3_ATCGAGTGTACCATCA	Pla	Pla-3	P3-31	TRUE	3	TRA:CAVRELKAAGNKLTF TRB:CASRRDSSTGELFF	10	CD8-C2
Pla-3_ATCTGCCAGGCCGAAT	Pla	Pla-3	P3-353	FALSE	2	TRA:CSQYNNNDMRF TRB:CASSPGSYGYTF	2	CD8-C4
Pla-3_ATTATCCGTAGCAAAT	Pla	Pla-3	P3-6	TRUE	3	TRA:CAVRGANNLFF TRB:CASSPGTGYETQYF	29	CD8-C5
Pla-3_ATTGGACCACAAGCCC	Pla	Pla-3	P3-399	FALSE	2	TRA:CASPSGGYQKVTF TRB:CASSTLVVYGGPRERNEKLFF	2	CD8-C5
Pla-3_ATTGGTGAGAGCTATA	Pla	Pla-3	P3-1980	FALSE	1	TRA:CAMTSYSGTYKYIF TRB:CASSAEGYYNEQFF	1	CD8-C5
Pla-3_ATTGGTGGTAAGAGGA	Pla	Pla-3	P3-1983	FALSE	1	TRA:CVVSSGNQFYF TRB:CASSLRVVTGELFF	1	CD8-C4
Pla-3_ATTGGTGGTTGGTTTG	Pla	Pla-3	P3-25	FALSE	3	TRA:CIVRVGSNTGKLIF TRB:CASTNYQETQYF	11	CD8-C3
Pla-3_ATTTCTGTCACAATGC	Pla	Pla-3	P3-211	FALSE	3	TRA:CVVNPPGYSSASKIIF TRB:CASSLSRGVGTNEKLFF	3	CD8-C5
Pla-3_CAACCTCGTGATAAGT	Pla	Pla-3	P3-14	TRUE	3	TRA:CALPFSGGYNKLIF TRB:CSARKTEPRSYEQYF	15	CD8-C4

Pla-3_CAAGATCTCGGACAAG	Pla	Pla-3	P3-1996	FALSE	1	TRA:CATDALYTGANSKLTF TRB:CASSLVAEREQYF	1	CD8-C4
Pla-3_CAAGGCCAGAGGTTAT	Pla	Pla-3	P3-9	TRUE	3	TRA:CAVRDMDTGRRALTF TRB:CASSWDTRQAEAFF	24	CD8-C4
Pla-3_CAAGTTGAGGGAAACA	Pla	Pla-3	P3-3	TRUE	3	TRA:CVVPWMDSSYKLIF TRB:CASSQRAGDGNYGYTF	42	CD8-C5
Pla-3_CAAGTTGCATGGAATA	Pla	Pla-3	P3-5	TRUE	3	TRA:CALRNSNYQLIW TRB:CASSLGDRAYNEQFF	38	CD8-C3
Pla-3_CACAAACCAATCACAC	Pla	Pla-3	P3-5	TRUE	3	TRA:CALRNSNYQLIW TRB:CASSLGDRAYNEQFF	38	CD8-C5
Pla-3_CACACCTTCTCCGGTT	Pla	Pla-3	P3-2008	FALSE	1	TRA:CVVSSPDGGSQGNLIF TRB:CASSDNVGVVTEAFF	1	CD8-C5
Pla-3_CACACTCCACCAGTTA	Pla	Pla-3	P3-2010	FALSE	1	TRA:CAPLVRDDKIIF TRB:CASRGTDYTF	1	CD8-C5
Pla-3_CACAGTAGTGCTAGCC	Pla	Pla-3	P3-325	TRUE	2	TRA:CAMSQETSGSRLTF TRB:CATRVGRGRGSYNSPLHF	2	CD8-C4
Pla-3_CACAGTATCAACACGT	Pla	Pla-3	P3-11	TRUE	3	TRA:CASGGGNKLTF TRB:CASSSDGDEAFF	17	CD8-C4
Pla-3_CACATTTAGACGCAAC	Pla	Pla-3	P3-405	FALSE	2	TRA:CASDSGGSNYKLTF TRB:CASSYRGGTHQPQHF	2	CD8-C4
Pla-3_CACATTTAGGCCCGTT	Pla	Pla-3	P3-6	TRUE	3	TRA:CAVRGANNLFF TRB:CASSPGTGYETQYF	29	CD8-C4
Pla-3_CACTCCATCACCACCT	Pla	Pla-3	P3-25	FALSE	3	TRA:CIVRVGSNTGKLIF TRB:CASTNYQETQYF	11	CD8-C4
Pla-3_CACTCCATCGAATGGG	Pla	Pla-3	P3-32	TRUE	3	TRA:CAASRGGQKLLF TRB:CASSSRTSLVHNEQFF	10	CD8-C4
Pla-3_CAGAATCCACGTTGGC	Pla	Pla-3	P3-2032	FALSE	1	TRA:CAASRTGGGNKLTF TRB:CASSLVRYGSSGEQETQYF	1	CD4-C5
Pla-3_CAGAATCCATCCCACT	Pla	Pla-3	P3-42	TRUE	3	TRA:CALGGSTLGRLYF TRB:CASSASTGANSPLHF	8	CD8-C5
Pla-3_CAGAATCTCTGGTATG	Pla	Pla-3	P3-2	TRUE	3	TRA:CAASAPNSGGGADGLTF TRB:CSARRGSSNQPQHF	63	CD8-C4
Pla-3_CAGATCATCATCACCC	Pla	Pla-3	P3-6	TRUE	3	TRA:CAVRGANNLFF TRB:CASSPGTGYETQYF	29	CD8-C4
Pla-3_CAGATCATCGATCCCT	Pla	Pla-3	P3-15	FALSE	3	TRA:CAVRDWDNAGNMLTF TRB:CASSEALGGAGETQYF	15	CD8-C5
Pla-3_CAGCATAAGTCGTTTG	Pla	Pla-3	P3-1	TRUE	3	TRA:CAVAPISSGSARQLTF TRB:CASSQGMSEAFF	83	CD8-C7
Pla-3_CAGCGACTCTTGCAAG	Pla	Pla-3	P3-2051	FALSE	1	TRA:CAYSIGTYKYIF TRB:CASSSAGGTEAFF	1	CD8-C5
Pla-3_CAGCTGGAGAACAATC	Pla	Pla-3	P3-14	TRUE	3	TRA:CALPFSGGYNKLIF TRB:CSARKTEPRSYEQYF	15	CD8-C5
Pla-3_CAGCTGGAGAATTCCC	Pla	Pla-3	P3-174	TRUE	3	TRA:CAMRAGAGGTSYGKLTF TRB:CASSEDYRDTEAFF	3	CD8-C7
Pla-3_CAGCTGGCAGCATGAG	Pla	Pla-3	P3-141	FALSE	3	TRA:CAVEGGGTSYGKLTF TRB:CAWSVAGNQPQHF	4	CD8-C5
Pla-3_CAGCTGGCAGTGGGAT	Pla	Pla-3	P3-2058	FALSE	1	TRA:CAVDNNNDMRF TRB:CSARDLRVSTEAFF	1	CD8-C4
Pla-3_CAGTAACGTCACCTAA	Pla	Pla-3	P3-142	FALSE	3	TRA:CAVGAGTGTASKLTF TRB:CASSLELAGGRDTQYF	4	CD8-C4
Pla-3_CATCAAGAGCCTTGAT	Pla	Pla-3	P3-3	TRUE	3	TRA:CVVPWMDSSYKLIF TRB:CASSQRAGDGNYGYTF	42	CD8-C5
Pla-3_CATCAGACACTCGACG	Pla	Pla-3	P3-416	FALSE	2	TRA:CALGSSNTGKLIF TRB:CASSSGQGTYNSPLHF	2	CD8-C4

Pla-3_CATGACACATTTCAGG	Pla	Pla-3	P3-384	FALSE	2	TRA:CAASADSGNTPLVF TRB:CASRDKGNEQFF	2	CD8-C4
Pla-3_CATGACATCCCACTTG	Pla	Pla-3	P3-6	TRUE	3	TRA:CAVRGANNLFF TRB:CASSPGTGYETQYF	29	CD8-C4
Pla-3_CATGGCGAGTGTACTC	Pla	Pla-3	P3-313	TRUE	2	TRA:CAVSDWKSRLMF TRB:CASSLSWVEGGGAKGDTQYF	2	CD8-C5
Pla-3_CATGGCGAGTGTTAGA	Pla	Pla-3	P3-2093	FALSE	1	TRA:CAASLTL TRB:CASSADRGRSPLHF	1	CD8-C4
Pla-3_CATGGCGCATCATCCC	Pla	Pla-3	P3-419	FALSE	2	TRA:CAVIQGGTSYGKLTF TRB:CASSQELDSYNEQFF	2	CD8-C5
Pla-3_CATTATCCATACCATG	Pla	Pla-3	P3-2097	FALSE	1	TRA:CAGTGGYQKVTF TRB:CASRPAGGGRGYEQYF	1	CD8-C4
Pla-3_CATTATCGTACAGTGG	Pla	Pla-3	P3-47	TRUE	3	TRA:CAVRDSVRVGSARQLTF TRB:CASSASTGANSPLHF	7	CD8-C4
Pla-3_CCAATCCAGCTTTGGT	Pla	Pla-3	P3-2101	FALSE	1	TRA:CAVSRAGRRALTF TRB:CASSQDGGVQETQYF	1	CD8-C4
Pla-3_CCAATCCGTCTCATCC	Pla	Pla-3	P3-422	FALSE	2	TRA:CAASRTTSGTYKYIF TRB:CAGQGGSNEKLFF	2	CD8-C5
Pla-3_CCACGGACAGGAACGT	Pla	Pla-3	P3-12	TRUE	3	TRA:CAVGDLGNEKLTF TRB:CASSAEGYYNEQFF	16	CD8-C3
Pla-3_CCACGGATCCGCATCT	Pla	Pla-3	P3-2109	FALSE	1	TRA:CAVNALGGTSYGKLTF TRB:CASRRGSYNEQFF	1	CD8-C4
Pla-3_CCAGCGATCGGCCGAT	Pla	Pla-3	P3-2112	FALSE	1	TRA:CAASHNTNAGKSTF TRB:CASSSQTSWETQYF	1	CD8-C5
Pla-3_CCATGTCAGTCCTCCT	Pla	Pla-3	P3-279	TRUE	2	TRA:CAMRDDQTGANNLFF TRB:CASSFIAGGADTQYF	2	CD8-C5
Pla-3_CCCATACGTACGCACC	Pla	Pla-3	P3-138	FALSE	3	TRA:CAYDTNAGKSTF TRB:CASSTTGTGHNQPQHF	4	CD8-C5
Pla-3_CCGGGATAGTACATGA	Pla	Pla-3	P3-47	TRUE	3	TRA:CAVRDSVRVGSARQLTF TRB:CASSASTGANSPLHF	7	CD8-C4
Pla-3_CCTAAAGAGTGCCATT	Pla	Pla-3	P3-62	TRUE	3	TRA:CAEIPSGAGSYQLTF TRB:CASNGDENTIYF	6	CD8-C4
Pla-3_CCTACACGTCATGCAT	Pla	Pla-3	P3-77	TRUE	3	TRA:CAVNGAGGSYIPTF TRB:CASSLTRKQVDGYTF	5	CD8-C4
Pla-3_CCTATTACATCTCCCA	Pla	Pla-3	P3-2144	FALSE	1	TRA:CAVSDWKSRLMF TRB:CASSDPAEQETQYF	1	CD8-C5
Pla-3_CCTATTAGTGACAAAT	Pla	Pla-3	P3-5	TRUE	3	TRA:CALRNSNYQLIW TRB:CASSLGDRAYNEQFF	38	CD8-C4
Pla-3_CCTCAGTAGACTACAA	Pla	Pla-3	P3-16	TRUE	3	TRA:CAASRNNNDMRF TRB:CASTNYQETQYF	14	CD8-C4
Pla-3_CCTTCCCCATCACAAC	Pla	Pla-3	P3-2149	FALSE	1	TRA:CAAGSSASKIIF TRB:CSARDLSLRNNEQFF	1	CD8-C4
Pla-3_CGACTTCAGCCGGTAA	Pla	Pla-3	P3-5	TRUE	3	TRA:CALRNSNYQLIW TRB:CASSLGDRAYNEQFF	38	CD8-C5
Pla-3_CGAGCCAAGTCATGCT	Pla	Pla-3	P3-1	TRUE	3	TRA:CAVAPISSGSARQLTF TRB:CASSQGMSEAFF	83	CD8-C5
Pla-3_CGAGCCACATTTCACT	Pla	Pla-3	P3-12	TRUE	3	TRA:CAVGDLGNEKLTF TRB:CASSAEGYYNEQFF	16	CD8-C5
Pla-3_CGATGGCTCTTTAGGG	Pla	Pla-3	P3-6	TRUE	3	TRA:CAVRGANNLFF TRB:CASSPGTGYETQYF	29	CD8-C4
Pla-3_CGATGTACATCACCCT	Pla	Pla-3	P3-6	TRUE	3	TRA:CAVRGANNLFF TRB:CASSPGTGYETQYF	29	CD8-C4
Pla-3_CGATGTAGTCTCATCC	Pla	Pla-3	P3-264	TRUE	2	TRA:CAVSDLNTGGFKTIF TRB:CASSQESGPKYEQYF	2	CD8-C5

Pla-3_CGATGTATCATATCGG	Pla	Pla-3	P3-2180	FALSE	1	TRA:CAVSGNSGGSNYKLTF TRB:CASRGSWGSETQYF	1	CD8-C5
Pla-3_CGATTGAAGCCGCCTA	Pla	Pla-3	P3-25	FALSE	3	TRA:CIVRVGSNTGKLIF TRB:CASTNYQETQYF	11	CD8-C4
Pla-3_CGCGGTAAGGCCCTCA	Pla	Pla-3	P3-11	TRUE	3	TRA:CASGGGNKLTF TRB:CASSSDGDEAFF	17	CD8-C3
Pla-3_CGCGGTACACAGGAGT	Pla	Pla-3	P3-227	FALSE	3	TRA:CAMRDQNAGGTSYGKLTF TRB:CASSENRGSEQFF	3	CD8-C5
Pla-3_CGCGTTTGTCATATCG	Pla	Pla-3	P3-164	TRUE	3	TRA:CALILTGGGNKLTF TRB:CASSWDTRQAEAFF	3	CD8-C4
Pla-3_CGGACTGTCGGTCTAA	Pla	Pla-3	P3-6	TRUE	3	TRA:CAVRGANNLFF TRB:CASSPGTGYETQYF	29	CD8-C4
Pla-3_CGGAGCTGTTAAGATG	Pla	Pla-3	P3-2203	FALSE	1	TRA:CAVNGESNTGKLIF TRB:CASSSRTSLVHNEQFF	1	CD8-C5
Pla-3_CGGAGCTTCATCATTC	Pla	Pla-3	P3-431	FALSE	2	TRA:CALSGSNNAGNMLTF TRB:CASSFFAGLSYEQYF	2	CD8-C5
Pla-3_CGGAGCTTCTCGGACG	Pla	Pla-3	P3-6	TRUE	3	TRA:CAVRGANNLFF TRB:CASSPGTGYETQYF	29	CD8-C5
Pla-3_CGGAGTCAGAGAGCTC	Pla	Pla-3	P3-3	TRUE	3	TRA:CVVPWMDSSYKLIF TRB:CASSQRAGDGNYGYTF	42	CD8-C4
Pla-3_CGGAGTCCAGCTGCAC	Pla	Pla-3	P3-36	TRUE	3	TRA:CARGAGSYQLTF TRB:CASSVGTGTEAFF	9	CD8-C4
Pla-3_CGGCTAGAGAGACGAA	Pla	Pla-3	P3-2208	FALSE	1	TRA:CAENEGGGADGLTF TRB:CASSQGRNFEAFF	1	CD8-C4
Pla-3_CGGCTAGAGGGCACTA	Pla	Pla-3	P3-2210	FALSE	1	TRA:CAYRRNDRGSTLGRLYF TRB:CASSFIYSLEYEQYF	1	CD8-C4
Pla-3_CGGGTCATCTGCTGCT	Pla	Pla-3	P3-15	FALSE	3	TRA:CAVRDWDNAGNMLTF TRB:CASSEALGGAGETQYF	15	CD8-C5
Pla-3_CGGTTAAAGATGCCAG	Pla	Pla-3	P3-13	TRUE	3	TRA:CAASARGVRGAQKLVF TRB:CASSRTGFQPQHF	15	CD8-C5
Pla-3_CGTAGCGCAAGAGGCT	Pla	Pla-3	P3-2222	FALSE	1	TRA:CATRVPSGTYKYIF TRB:CASSFGSSVYNEQFF	1	CD8-C5
Pla-3_CGTAGCGGTCCAGTTA	Pla	Pla-3	P3-153	TRUE	3	TRA:CAENSDFGNVLHC TRB:CASSVGMGNQPQHF	3	CD8-C5
Pla-3_CGTAGGCAGCCGATTT	Pla	Pla-3	P3-204	FALSE	3	TRA:CALSETYYGGSQGNLIF TRB:CASSPQAYEQYF	3	CD8-C5
Pla-3_CGTCACTTCTTTAGGG	Pla	Pla-3	P3-2228	FALSE	1	TRA:CAVSDVTDKLIF TRB:CASSHNPRIAKNIQYF	1	CD8-C3
Pla-3_CGTCTACCATGCATGT	Pla	Pla-3	P3-209	FALSE	3	TRA:CALSDHSVNTGTASKLTF TRB:CASSINRGPTEAFF	3	CD8-C4
Pla-3_CGTGAGCTCTAACTGG	Pla	Pla-3	P3-2237	FALSE	1	TRA:CILRDYGQNFVF TRB:CASSDGWGNSPLHF	1	CD8-C4
Pla-3_CGTGTCTTCCGTTGTC	Pla	Pla-3	P3-3	TRUE	3	TRA:CVVPWMDSSYKLIF TRB:CASSQRAGDGNYGYTF	42	CD8-C3
Pla-3_CGTTCTGAGAAACCGC	Pla	Pla-3	P3-210	FALSE	3	TRA:CVVSPTSNTGKLIF TRB:CSGKGGTEAFF	3	CD8-C4
Pla-3_CGTTGGGAGTGACATA	Pla	Pla-3	P3-370	FALSE	2	TRA:CVVNKNQAGTALIF TRB:CASREDSFSSYNEQFF	2	CD8-C5
Pla-3_CGTTGGGAGTGGAGTC	Pla	Pla-3	P3-47	TRUE	3	TRA:CAVRDSVRVGSARQLTF TRB:CASSASTGANSPLHF	7	CD8-C5
Pla-3_CTAACTTAGATCTGCT	Pla	Pla-3	P3-442	FALSE	2	TRA:CAVQGSGGSNYKLTF TRB:CASSSSGANYGYTF	2	CD8-C5
Pla-3_CTAACTTGTGTGACGA	Pla	Pla-3	P3-1	TRUE	3	TRA:CAVAPISSGSARQLTF TRB:CASSQGMSEAFF	83	CD8-C5

Pla-3_CTAAGACAGGTGTTAA	Pla	Pla-3	P3-8	TRUE	3	TRA:CAFDRGSTLGRLYF TRB:CASNPRQGTSTYEQYF	26	CD8-C4
Pla-3_CTACACCAGAGCCCAA	Pla	Pla-3	P3-5	TRUE	3	TRA:CALRNSNYQLIW TRB:CASSLGDRAYNEQFF	38	CD8-C5
Pla-3_CTACACCCAGACGCCT	Pla	Pla-3	P3-208	FALSE	3	TRA:CAERNYGNNRLAF TRB:CASSQGNAEAFF	3	CD8-C5
Pla-3_CTACACCCAGCTTAAC	Pla	Pla-3	P3-1	TRUE	3	TRA:CAVAPISSGSARQLTF TRB:CASSQGMSEAFF	83	CD8-C5
Pla-3_CTACACCCATTGGGCC	Pla	Pla-3	P3-12	TRUE	3	TRA:CAVGDLGNEKLTF TRB:CASSAEGYYNEQFF	16	CD8-C4
Pla-3_CTACCCATCCTAGAAC	Pla	Pla-3	P3-18	TRUE	3	TRA:CLVGVGNNDMRF TRB:CASSPGTGKYEQYF	12	CD8-C5
Pla-3_CTACGTCAGAGGGATA	Pla	Pla-3	P3-2265	FALSE	1	TRA:CAMKSSGGSNYKLTF TRB:CASSPEGGRTEAFF	1	CD8-C4
Pla-3_CTACGTCCAAGAAGAG	Pla	Pla-3	P3-404	FALSE	2	TRA:CAFMIRGSTLGRLYF TRB:CASSFLAGTDTQYF	2	CD8-C2
Pla-3_CTAGAGTCATGCTAGT	Pla	Pla-3	P3-5	TRUE	3	TRA:CALRNSNYQLIW TRB:CASSLGDRAYNEQFF	38	CD8-C4
Pla-3_CTAGAGTTCAGGCCCA	Pla	Pla-3	P3-1	TRUE	3	TRA:CAVAPISSGSARQLTF TRB:CASSQGMSEAFF	83	CD8-C5
Pla-3_CTAGCCTTCTTGAGGT	Pla	Pla-3	P3-46	FALSE	3	TRA:CAVRRGILTGGGNKLTF TRB:CASSLVSSGGNPSYEQYF	8	CD8-C4
Pla-3_CTCACACGTCGGCTCA	Pla	Pla-3	P3-36	TRUE	3	TRA:CARGAGSYQLTF TRB:CASSVGTGTEAFF	9	CD8-C4
Pla-3_CTCACACGTCTAACGT	Pla	Pla-3	P3-9	TRUE	3	TRA:CAVRDMDTGRRALTF TRB:CASSWDTRQAEAFF	24	CD8-C4
Pla-3_CTCAGAACATCGTCGG	Pla	Pla-3	P3-2282	FALSE	1	TRA:CATDAGGSNYKLTF TRB:CASSDDNWYYGYTF	1	CD8-C4
Pla-3_CTCCTAGCAGGAATGC	Pla	Pla-3	P3-15	FALSE	3	TRA:CAVRDWDNAGNMLTF TRB:CASSEALGGAGETQYF	15	CD8-C4
Pla-3_CTCGAGGGTGCGGTAA	Pla	Pla-3	P3-2291	FALSE	1	TRA:CASSGSARQLTF TRB:CASSESFQNLGQGNGYEQYF	1	CD8-C4
Pla-3_CTCGGAGAGCCGTCGT	Pla	Pla-3	P3-2292	FALSE	1	TRA:CAAATYNTDKLIF TRB:CASSASTGANSPLHF	1	CD8-C5
Pla-3_CTCGGGACAGTCGATT	Pla	Pla-3	P3-6	TRUE	3	TRA:CAVRGANNLFF TRB:CASSPGTGYETQYF	29	CD8-C4
Pla-3_CTCGTCAGTGACTCAT	Pla	Pla-3	P3-362	FALSE	2	TRA:CAMREGWTGANNLFF TRB:CASSHREGNRNTEAFF	2	CD8-C4
Pla-3_CTCTAATCATGTCCTC	Pla	Pla-3	P3-2310	FALSE	1	TRA:CASMRTDSWGKFQF TRB:CASSLSPSGGAGQETQYF	1	CD8-C4
Pla-3_CTCTAATGTCTGGAGA	Pla	Pla-3	P3-131	FALSE	3	TRA:CVVNRDEKLTF TRB:CASRGWGDVSEQFF	4	CD8-C4
Pla-3_CTCTGGTTCACCAGGC	Pla	Pla-3	P3-171	TRUE	3	TRA:CALSEANARLMF TRB:CASSQVGGGTSDNEQFF	3	CD8-C5
Pla-3_CTCTGGTTCCCGGATG	Pla	Pla-3	P3-25	FALSE	3	TRA:CIVRVGSNTGKLIF TRB:CASTNYQETQYF	11	CD8-C4
Pla-3_CTGAAACGTTGTCTTT	Pla	Pla-3	P3-25	FALSE	3	TRA:CIVRVGSNTGKLIF TRB:CASTNYQETQYF	11	CD8-C4
Pla-3_CTGAAGTCAACTGCTA	Pla	Pla-3	P3-2324	FALSE	1	TRA:CAALNQAGTALIF TRB:CASSPGQGYNEQFF	1	CD8-C4
Pla-3_CTGATAGAGCTAGTCT	Pla	Pla-3	P3-18	TRUE	3	TRA:CLVGVGNNDMRF TRB:CASSPGTGKYEQYF	12	CD8-C4
Pla-3_CTGATAGCATTTGCCC	Pla	Pla-3	P3-11	TRUE	3	TRA:CASGGGNKLTF TRB:CASSSDGDEAFF	17	CD8-C5

Pla-3_CTGATAGGTCTCATCC	Pla	Pla-3	P3-2327	FALSE	1	TRA:CAATNDMRF TRB:CAWSVLNEQFF	1	CD8-C4
Pla-3_CTGCCTAAGGATGGTC	Pla	Pla-3	P3-5	TRUE	3	TRA:CALRNSNYQLIW TRB:CASSLGDRAYNEQFF	38	CD8-C5
Pla-3_CTGCCTATCTGGTTCC	Pla	Pla-3	P3-2333	FALSE	1	TRA:CAVSNQAGTALIF TRB:CASSYVADEQYF	1	CD8-C5
Pla-3_CTGCTGTAGATAGTCA	Pla	Pla-3	P3-3	TRUE	3	TRA:CVVPWMDSSYKLIF TRB:CASSQRAGDGNYGYTF	42	CD8-C4
Pla-3_CTGGTCTTCGTACCGG	Pla	Pla-3	P3-1	TRUE	3	TRA:CAVAPISSGSARQLTF TRB:CASSQGMSEAFF	83	CD8-C4
Pla-3_CTGTGCTAGACTCGGA	Pla	Pla-3	P3-2344	FALSE	1	TRA:CAVRGSQGNLIF TRB:CASSLDRVSNQPQHF	1	CD8-C3
Pla-3_CTGTGCTAGCTATGCT	Pla	Pla-3	P3-5	TRUE	3	TRA:CALRNSNYQLIW TRB:CASSLGDRAYNEQFF	38	CD8-C4
Pla-3_CTGTTTAAGTTAAGTG	Pla	Pla-3	P3-3	TRUE	3	TRA:CVVPWMDSSYKLIF TRB:CASSQRAGDGNYGYTF	42	CD8-C5
Pla-3_CTGTTTAGTAATCACC	Pla	Pla-3	P3-450	FALSE	2	TRA:CALRNSNYQLIW TRB:CQQQRPGEGNQPQHF	2	CD8-C3
Pla-3_CTTAGGATCCTAGTGA	Pla	Pla-3	P3-2362	FALSE	1	TRA:CVVRLSGNTPLVF TRB:CASSSVRARGTSGYNEQFF	1	CD8-C4
Pla-3_CTTAGGATCTTCAACT	Pla	Pla-3	P3-258	TRUE	2	TRA:CAVSGDSNYQLIW TRB:CASSLATGPYNEQFF	2	CD8-C5
Pla-3_CTTCTCTTCATTCACT	Pla	Pla-3	P3-414	FALSE	2	TRA:CAVTTNNDMRF TRB:CASSIASGGELFF	2	CD8-C8
Pla-3_CTTCTCTTCCACGTTC	Pla	Pla-3	P3-142	FALSE	3	TRA:CAVGAGTGTASKLTF TRB:CASSLELAGGRDTQYF	4	CD4-C5
Pla-3_CTTCTCTTCGCCTGTT	Pla	Pla-3	P3-16	TRUE	3	TRA:CAASRNNNDMRF TRB:CASTNYQETQYF	14	CD8-C5
Pla-3_GAACATCTCATTATCC	Pla	Pla-3	P3-1	TRUE	3	TRA:CAVAPISSGSARQLTF TRB:CASSQGMSEAFF	83	CD8-C7
Pla-3_GAACCTACACCGATAT	Pla	Pla-3	P3-2380	FALSE	1	TRA:CAMREWGGSQGNLIF TRB:CASSSTSSNTEAFF	1	CD8-C4
Pla-3_GAAGCAGTCCGTCATC	Pla	Pla-3	P3-142	FALSE	3	TRA:CAVGAGTGTASKLTF TRB:CASSLELAGGRDTQYF	4	CD8-C5
Pla-3_GAATAAGTCGTAGGAG	Pla	Pla-3	P3-8	TRUE	3	TRA:CAFDRGSTLGRLYF TRB:CASNPRQGTSTYEQYF	26	CD8-C3
Pla-3_GAATGAACAATACGCT	Pla	Pla-3	P3-2388	FALSE	1	TRA:CAVTNSNYQLIW TRB:CASSSGGVPDTQYF	1	CD8-C4
Pla-3_GAATGAATCGTTGCCT	Pla	Pla-3	P3-2390	FALSE	1	TRA:CAVNGRTGGSYIPTF TRB:CASSPPGYFYEQYF	1	CD8-C5
Pla-3_GACACGCTCAGGCGAA	Pla	Pla-3	P3-454	FALSE	2	TRA:CAVPHGSSNTGKLIF TRB:CASSLGDSVTGELFF	2	CD8-C5
Pla-3_GACAGAGTCCCTAATT	Pla	Pla-3	P3-6	TRUE	3	TRA:CAVRGANNLFF TRB:CASSPGTGYETQYF	29	CD8-C4
Pla-3_GACCTGGAGGAATGGA	Pla	Pla-3	P3-2398	FALSE	1	TRA:CVVNPDNFNKFYF TRB:CASSLQEFSYEQYF	1	CD8-C8
Pla-3_GACCTGGGTTCCGTCT	Pla	Pla-3	P3-2401	FALSE	1	TRA:CAYREYGNKLVF TRB:CASSPTTGPGGNTIYF	1	CD8-C4
Pla-3_GACCTGGTCTATCCCG	Pla	Pla-3	P3-2403	FALSE	1	TRA:CAVGASHNTDKLIF TRB:CSARGGGRGRLSYGYTF	1	CD8-C4
Pla-3_GACGGCTCAGATCTGT	Pla	Pla-3	P3-36	TRUE	3	TRA:CARGAGSYQLTF TRB:CASSVGTGTEAFF	9	CD8-C5
Pla-3_GACGTGCAGCCACTAT	Pla	Pla-3	P3-3	TRUE	3	TRA:CVVPWMDSSYKLIF TRB:CASSQRAGDGNYGYTF	42	CD8-C4

Pla-3_GACGTGCGTGCTAGCC	Pla	Pla-3	P3-5	TRUE	3	TRA:CALRNSNYQLIW TRB:CASSLGDRAYNEQFF	38	CD8-C5
Pla-3_GACGTGCTCAGCATGT	Pla	Pla-3	P3-1	TRUE	3	TRA:CAVAPISSGSARQLTF TRB:CASSQGMSEAFF	83	CD8-C5
Pla-3_GACTAACCACGGTGTC	Pla	Pla-3	P3-80	TRUE	3	TRA:CAVKDSNYQLIW TRB:CASSFLQGDYNEQFF	5	CD8-C5
Pla-3_GACTACACAATGGACG	Pla	Pla-3	P3-2417	FALSE	1	TRA:CATDTGRRALTF TRB:CASSQGMSEAFF	1	CD8-C5
Pla-3_GACTACAGTCTTGTCC	Pla	Pla-3	P3-14	TRUE	3	TRA:CALPFSGGYNKLIF TRB:CSARKTEPRSYEQYF	15	CD8-C5
Pla-3_GACTACAGTTTGGGCC	Pla	Pla-3	P3-3	TRUE	3	TRA:CVVPWMDSSYKLIF TRB:CASSQRAGDGNYGYTF	42	CD8-C5
Pla-3_GACTGCGGTTCGCTAA	Pla	Pla-3	P3-2419	FALSE	1	TRA:CAVLCLSGTNAGKSTF TRB:CASSTGTGNEQYF	1	CD8-C4
Pla-3_GACTGCGTCCAAGCCG	Pla	Pla-3	P3-18	TRUE	3	TRA:CLVGVGNNDMRF TRB:CASSPGTGKYEQYF	12	CD8-C5
Pla-3_GAGCAGAAGTGTTGAA	Pla	Pla-3	P3-2421	FALSE	1	TRA:CAVPGAGSYQLTF TRB:CASSLETGELFF	1	CD8-C7
Pla-3_GATCGATAGGACTGGT	Pla	Pla-3	P3-2437	FALSE	1	TRA:CAIGGGYSTLTF TRB:CASSQDRDTSYEQYF	1	CD8-C4
Pla-3_GATCTAGAGCGTTCCG	Pla	Pla-3	P3-39	TRUE	3	TRA:CAVRDIGGSYIPTF TRB:CASSLGGPEETQYF	8	CD8-C4
Pla-3_GATCTAGAGTAGTGCG	Pla	Pla-3	P3-2446	FALSE	1	TRA:CAVSRDNYGQNFVF TRB:CASSYQTSGFNEQFF	1	CD8-C5
Pla-3_GATCTAGCAAGACACG	Pla	Pla-3	P3-6	TRUE	3	TRA:CAVRGANNLFF TRB:CASSPGTGYETQYF	29	CD8-C4
Pla-3_GATCTAGTCTTAACCT	Pla	Pla-3	P3-2448	FALSE	1	TRA:CATDGYTGNQFYF TRB:CASSLGQNNEQFF	1	CD8-C4
Pla-3_GATGAGGGTCATATCG	Pla	Pla-3	P3-2452	FALSE	1	TRA:CAIFPGPGRDDKIIF TRB:CAWMVDSNEQFF	1	CD8-C5
Pla-3_GATTCAGTCAAGCCTA	Pla	Pla-3	P3-12	TRUE	3	TRA:CAVGDLGNEKLTF TRB:CASSAEGYYNEQFF	16	CD8-C4
Pla-3_GCAATCAGTACGAAAT	Pla	Pla-3	P3-11	TRUE	3	TRA:CASGGGNKLTF TRB:CASSSDGDEAFF	17	CD8-C4
Pla-3_GCAATCATCTTCGAGA	Pla	Pla-3	P3-52	FALSE	3	TRA:CAYRSPLTGNQFYF TRB:CASSPPRGMLYF	7	CD8-C5
Pla-3_GCACTCTGTTAAAGTG	Pla	Pla-3	P3-2474	FALSE	1	TRA:CAERGQAGTALIF TRB:CASSPAAGSYEQYF	1	CD8-C4
Pla-3_GCAGCCACAGACAGGT	Pla	Pla-3	P3-8	TRUE	3	TRA:CAFDRGSTLGRLYF TRB:CASNPRQGTSTYEQYF	26	CD8-C4
Pla-3_GCAGCCACATGAACCT	Pla	Pla-3	P3-48	TRUE	3	TRA:CAYDGATNKLIF TRB:CASSLYGHQPQHF	7	CD8-C5
Pla-3_GCAGCCATCCAGATCA	Pla	Pla-3	P3-358	FALSE	2	TRA:CAMREGTSGTYKYIF TRB:CATSSGGALDTQYF	2	CD8-C4
Pla-3_GCATACAGTCAGGACA	Pla	Pla-3	P3-2481	FALSE	1	TRA:CAASGETSGSRLTF TRB:CASSSDGDEAFF	1	CD8-C4
Pla-3_GCATACATCGTACGGC	Pla	Pla-3	P3-16	TRUE	3	TRA:CAASRNNNDMRF TRB:CASTNYQETQYF	14	CD8-C3
Pla-3_GCATGATGTGTAATGA	Pla	Pla-3	P3-2483	FALSE	1	TRA:CATDSDGQKLLF TRB:CASREVSGAFF	1	CD8-C4
Pla-3_GCGCCAACAGATAATG	Pla	Pla-3	P3-11	TRUE	3	TRA:CASGGGNKLTF TRB:CASSSDGDEAFF	17	CD8-C4
Pla-3_GCGCCAAGTTTCCACC	Pla	Pla-3	P3-1	TRUE	3	TRA:CAVAPISSGSARQLTF TRB:CASSQGMSEAFF	83	CD8-C4

Pla-3_GCGCCAATCTCCTATA	Pla	Pla-3	P3-2517	FALSE	1	TRA:CAVRIGNSNSGYALNF TRB:CASSTGGTEAFF	1	CD8-C5
Pla-3_GCGCGATCACCAGTTA	Pla	Pla-3	P3-374	FALSE	2	TRA:CAMREGQKNNNARLMF TRB:CASSSPLAGGEYNEQFF	2	CD8-C5
Pla-3_GCGGGTTGTAGCCTCG	Pla	Pla-3	P3-86	TRUE	3	TRA:CALTNRDDKIIF TRB:CATSRDMGGSYEQYF	5	CD8-C5
Pla-3_GCTCTGTCACGGTTTA	Pla	Pla-3	P3-2525	FALSE	1	TRA:CAVESNAGNMLTF TRB:CASSLAGRTGLVPDTQYF	1	CD8-C5
Pla-3_GCTCTGTTCGTACCGG	Pla	Pla-3	P3-128	FALSE	3	TRA:CAVIQAGTALIF TRB:CASSLTAGRADTQYF	4	CD8-C5
Pla-3_GCTGGGTCACGTGAGA	Pla	Pla-3	P3-2534	FALSE	1	TRA:CAVILPWGGSYIPTF TRB:CAWSSQLGNTIYF	1	CD8-C4
Pla-3_GCTTCCACAATTGCTG	Pla	Pla-3	P3-6	TRUE	3	TRA:CAVRGANNLFF TRB:CASSPGTGYETQYF	29	CD8-C4
Pla-3_GCTTGAATCTTGTTTG	Pla	Pla-3	P3-12	TRUE	3	TRA:CAVGDLGNEKLTF TRB:CASSAEGYYNEQFF	16	CD8-C8
Pla-3_GGACGTCAGCTAACAA	Pla	Pla-3	P3-2559	FALSE	1	TRA:CAGPDTGNQFYF TRB:CASSKGQGIRELFF	1	CD8-C3
Pla-3_GGATGTTAGTATGACA	Pla	Pla-3	P3-2569	FALSE	1	TRA:CAVENRDDKIIF TRB:CASSTYPEGMGLDYGYTF	1	CD8-C4
Pla-3_GGATTACAGGTAAACT	Pla	Pla-3	P3-2574	FALSE	1	TRA:CAVRDIRRGSQGNLIF TRB:CASSEMGTPYEQYF	1	CD8-C4
Pla-3_GGATTACAGTCCGGTC	Pla	Pla-3	P3-39	TRUE	3	TRA:CAVRDIGGSYIPTF TRB:CASSLGGPEETQYF	8	CD8-C4
Pla-3_GGATTACGTCAGAGGT	Pla	Pla-3	P3-18	TRUE	3	TRA:CLVGVGNNDMRF TRB:CASSPGTGKYEQYF	12	CD8-C4
Pla-3_GGCGACTCAATCCGAT	Pla	Pla-3	P3-2582	FALSE	1	TRA:CAVRVYARLMF TRB:CAISEVSADTQYF	1	CD8-C4
Pla-3_GGCGTGTTCAGAGCTT	Pla	Pla-3	P3-2587	FALSE	1	TRA:CAFDRGSTLGRLYF TRB:CASSNGQGLAFF	1	CD8-C4
Pla-3_GGCTGGTCAAGTTAAG	Pla	Pla-3	P3-6	TRUE	3	TRA:CAVRGANNLFF TRB:CASSPGTGYETQYF	29	CD8-C4
Pla-3_GGGAATGAGTACGCGA	Pla	Pla-3	P3-15	FALSE	3	TRA:CAVRDWDNAGNMLTF TRB:CASSEALGGAGETQYF	15	CD8-C5
Pla-3_GGGACCTAGTACACCT	Pla	Pla-3	P3-161	TRUE	3	TRA:CAFMKPGNTGNQFYF TRB:CASSPGPIGTVNYGYTF	3	CD8-C5
Pla-3_GGGACCTCACGAAACG	Pla	Pla-3	P3-15	FALSE	3	TRA:CAVRDWDNAGNMLTF TRB:CASSEALGGAGETQYF	15	CD8-C5
Pla-3_GGGAGATAGCTCTCGG	Pla	Pla-3	P3-2606	FALSE	1	TRA:CAVNKYNNNDMRF TRB:CASSSDSDTNTGELFF	1	CD8-C4
Pla-3_GGGAGATCAATGACCT	Pla	Pla-3	P3-9	TRUE	3	TRA:CAVRDMDTGRRALTF TRB:CASSWDTRQAEAFF	24	CD8-C4
Pla-3_GGGCACTAGATCACGG	Pla	Pla-3	P3-6	TRUE	3	TRA:CAVRGANNLFF TRB:CASSPGTGYETQYF	29	CD8-C4
Pla-3_GGGTCTGCACCATGTA	Pla	Pla-3	P3-2617	FALSE	1	TRA:CGTEQPNDYKLSF TRB:CASSPPGGRVTEAFF	1	CD8-C4
Pla-3_GGTATTGCACAGTCGC	Pla	Pla-3	P3-2623	FALSE	1	TRA:CLYNFNKFYF TRB:CASSIDLDSGRGTYNEQFF	1	CD8-C4
Pla-3_GGTGCGTCACGCATCG	Pla	Pla-3	P3-2625	FALSE	1	TRA:CAVRDSVRVGSARQLTF TRB:CASSPSLAGTGELFF	1	CD8-C5
Pla-3_GGTGCGTCATGCCCGA	Pla	Pla-3	P3-1	TRUE	3	TRA:CAVAPISSGSARQLTF TRB:CASSQGMSEAFF	83	CD8-C5
Pla-3_GTACGTATCACAGTAC	Pla	Pla-3	P3-32	TRUE	3	TRA:CAASRGGQKLLF TRB:CASSSRTSLVHNEQFF	10	CD8-C5

Pla-3_GTATCTTGTTCCAACA	Pla	Pla-3	P3-128	FALSE	3	TRA:CAVIQAGTALIF TRB:CASSLTAGRADTQYF	4	CD8-C4
Pla-3_GTATCTTGTTCCACAA	Pla	Pla-3	P3-32	TRUE	3	TRA:CAASRGGQKLLF TRB:CASSSRTSLVHNEQFF	10	CD8-C4
Pla-3_GTATTCTTCCTGCAGG	Pla	Pla-3	P3-9	TRUE	3	TRA:CAVRDMDTGRRALTF TRB:CASSWDTRQAEAFF	24	CD8-C4
Pla-3_GTCAAGTTCTATCCCG	Pla	Pla-3	P3-2655	FALSE	1	TRA:CAVRDRADARLMF TRB:CASSLANTGELFF	1	CD8-C4
Pla-3_GTCACGGGTTATTCTC	Pla	Pla-3	P3-2661	FALSE	1	TRA:CAVIGFGNVLHC TRB:CSARDLNARSHPAETQYF	1	CD8-C5
Pla-3_GTCTCGTCAGACAAAT	Pla	Pla-3	P3-14	TRUE	3	TRA:CALPFSGGYNKLIF TRB:CSARKTEPRSYEQYF	15	CD8-C5
Pla-3_GTCTTCGGTCCTCCAT	Pla	Pla-3	P3-2683	FALSE	1	TRA:CIVRVGSNTGKLIF TRB:CASSINRGPTEAFF	1	CD8-C5
Pla-3_GTGAAGGAGGTTACCT	Pla	Pla-3	P3-39	TRUE	3	TRA:CAVRDIGGSYIPTF TRB:CASSLGGPEETQYF	8	CD8-C4
Pla-3_GTGAAGGCAATGAAAC	Pla	Pla-3	P3-15	FALSE	3	TRA:CAVRDWDNAGNMLTF TRB:CASSEALGGAGETQYF	15	CD8-C5
Pla-3_GTGAAGGGTGCCTGGT	Pla	Pla-3	P3-2684	FALSE	1	TRA:CAMEGTYKYIF TRB:CASSPISGTSGGAVGETQYF	1	CD8-C5
Pla-3_GTGCGGTCACAGATTC	Pla	Pla-3	P3-106	TRUE	3	TRA:CAVQTWNSGNTPLVF TRB:CASSPRLAGDNTGELFF	4	CD8-C5
Pla-3_GTGGGTCGTAAGAGAG	Pla	Pla-3	P3-25	FALSE	3	TRA:CIVRVGSNTGKLIF TRB:CASTNYQETQYF	11	CD8-C4
Pla-3_GTGTGCGCACTTCTGC	Pla	Pla-3	P3-2698	FALSE	1	TRA:CAAPSYSSASKIIF TRB:CASSQDRRAGYEQYF	1	CD8-C4
Pla-3_GTTAAGCGTTCCACTC	Pla	Pla-3	P3-5	TRUE	3	TRA:CALRNSNYQLIW TRB:CASSLGDRAYNEQFF	38	CD8-C5
Pla-3_GTTACAGAGAATTGTG	Pla	Pla-3	P3-108	TRUE	3	TRA:CILRDPLKDGQKLLF TRB:CASSPSDSGANVLTF	4	CD8-C5
Pla-3_GTTCGGGGTTTAGGAA	Pla	Pla-3	P3-142	FALSE	3	TRA:CAVGAGTGTASKLTF TRB:CASSLELAGGRDTQYF	4	CD8-C5
Pla-3_GTTCTCGAGTGGAGAA	Pla	Pla-3	P3-10	TRUE	3	TRA:CAGHQFSGGYNKLIF TRB:CASRASGTANEQFF	21	CD8-C5
Pla-3_TAAGAGAAGGCACATG	Pla	Pla-3	P3-30	TRUE	3	TRA:CVVSEASGTYKYIF TRB:CASSLNLRKNTEAFF	10	CD8-C3
Pla-3_TAAGCGTAGATATGGT	Pla	Pla-3	P3-3	TRUE	3	TRA:CVVPWMDSSYKLIF TRB:CASSQRAGDGNYGYTF	42	CD8-C5
Pla-3_TACCTTACAGTGGAGT	Pla	Pla-3	P3-6	TRUE	3	TRA:CAVRGANNLFF TRB:CASSPGTGYETQYF	29	CD8-C5
Pla-3_TACCTTATCCGCGCAA	Pla	Pla-3	P3-308	TRUE	2	TRA:CAVEGGSSASKIIF TRB:CASRRTGDGYEQYF	2	CD8-C4
Pla-3_TACGGATAGAACTCGG	Pla	Pla-3	P3-1	TRUE	3	TRA:CAVAPISSGSARQLTF TRB:CASSQGMSEAFF	83	CD8-C4
Pla-3_TACGGATTCCGTACAA	Pla	Pla-3	P3-111	TRUE	3	TRA:CALSEARTGNQFYF TRB:CASSYGPGHATQYF	4	CD8-C5
Pla-3_TACTCGCAGGAATCGC	Pla	Pla-3	P3-5	TRUE	3	TRA:CALRNSNYQLIW TRB:CASSLGDRAYNEQFF	38	CD8-C5
Pla-3_TACTTACAGGTACTCT	Pla	Pla-3	P3-2761	FALSE	1	TRA:CAMREEDSNYQLIW TRB:CSVGPVSSEAFF	1	CD8-C5
Pla-3_TACTTACTCAACACAC	Pla	Pla-3	P3-408	FALSE	2	TRA:CAVRFGQNFVF TRB:CASSIGQETQYF	2	CD8-C5
Pla-3_TACTTACTCCAAATGC	Pla	Pla-3	P3-422	FALSE	2	TRA:CAASRTTSGTYKYIF TRB:CAGQGGSNEKLFF	2	CD4-C5

Pla-3_TAGACCATCACTTCAT	Pla	Pla-3	P3-190	TRUE	3	TRA:CAVTEGADGLTF TRB:CASSPGQTYGYTF	3	CD8-C4
Pla-3_TAGTGGTCAAACCCAT	Pla	Pla-3	P3-454	FALSE	2	TRA:CAVPHGSSNTGKLIF TRB:CASSLGDSVTGELFF	2	CD8-C4
Pla-3_TAGTTGGAGACAAAGG	Pla	Pla-3	P3-2780	FALSE	1	TRA:CVVSTSGTYKYIF TRB:CASSQDHRRETQYF	1	CD8-C4
Pla-3_TATCAGGGTCGACTGC	Pla	Pla-3	P3-127	TRUE	3	TRA:CVVNPGGYNKLIF TRB:CASSPPGYFYEQYF	4	CD8-C5
Pla-3_TATCTCAAGCGTTGCC	Pla	Pla-3	P3-450	FALSE	2	TRA:CALRNSNYQLIW TRB:CQQQRPGEGNQPQHF	2	CD8-C8
Pla-3_TATCTCATCTAACTCT	Pla	Pla-3	P3-1	TRUE	3	TRA:CAVAPISSGSARQLTF TRB:CASSQGMSEAFF	83	CD8-C5
Pla-3_TATGCCCAGCGTTTAC	Pla	Pla-3	P3-1	TRUE	3	TRA:CAVAPISSGSARQLTF TRB:CASSQGMSEAFF	83	CD8-C5
Pla-3_TATTACCCAGATCTGT	Pla	Pla-3	P3-185	TRUE	3	TRA:CVVNMDEGSQGNLIF TRB:CASSWGVPPRGYTF	3	CD8-C4
Pla-3_TATTACCGTCTTGTCC	Pla	Pla-3	P3-15	FALSE	3	TRA:CAVRDWDNAGNMLTF TRB:CASSEALGGAGETQYF	15	CD8-C4
Pla-3_TATTACCGTGCTCTTC	Pla	Pla-3	P3-11	TRUE	3	TRA:CASGGGNKLTF TRB:CASSSDGDEAFF	17	CD8-C4
Pla-3_TCACAAGAGAACAACT	Pla	Pla-3	P3-16	TRUE	3	TRA:CAASRNNNDMRF TRB:CASTNYQETQYF	14	CD8-C4
Pla-3_TCACAAGAGCTAGCCC	Pla	Pla-3	P3-2802	FALSE	1	TRA:CAARANTGNQFYF TRB:CASSLLGPNEQFF	1	CD8-C4
Pla-3_TCACAAGCAAGTAATG	Pla	Pla-3	P3-1	TRUE	3	TRA:CAVAPISSGSARQLTF TRB:CASSQGMSEAFF	83	CD8-C5
Pla-3_TCACGAAAGGAGTACC	Pla	Pla-3	P3-2806	FALSE	1	TRA:CAVRDSRGSTLGRLYF TRB:CASSRTGDSYEQYF	1	CD8-C4
Pla-3_TCAGCTCCACATCCGG	Pla	Pla-3	P3-5	TRUE	3	TRA:CALRNSNYQLIW TRB:CASSLGDRAYNEQFF	38	CD8-C4
Pla-3_TCATTACCACCACGTG	Pla	Pla-3	P3-6	TRUE	3	TRA:CAVRGANNLFF TRB:CASSPGTGYETQYF	29	CD8-C4
Pla-3_TCATTTGAGGTACTCT	Pla	Pla-3	P3-2830	FALSE	1	TRA:CAVEPPLGSARQLTF TRB:CASSKKTSGGSTDTQYF	1	CD8-C2
Pla-3_TCCACACTCAGCGACC	Pla	Pla-3	P3-185	TRUE	3	TRA:CVVNMDEGSQGNLIF TRB:CASSWGVPPRGYTF	3	CD8-C4
Pla-3_TCCACACTCTGTCAAG	Pla	Pla-3	P3-6	TRUE	3	TRA:CAVRGANNLFF TRB:CASSPGTGYETQYF	29	CD8-C7
Pla-3_TCGCGAGGTACATGTC	Pla	Pla-3	P3-184	TRUE	3	TRA:CACSRYGNNRLAF TRB:CAWSAPTGGGLGSGANVLTF	3	CD8-C5
Pla-3_TCGCGAGGTCCAGTGC	Pla	Pla-3	P3-15	FALSE	3	TRA:CAVRDWDNAGNMLTF TRB:CASSEALGGAGETQYF	15	CD8-C5
Pla-3_TCGGGACAGCCCTAAT	Pla	Pla-3	P3-47	TRUE	3	TRA:CAVRDSVRVGSARQLTF TRB:CASSASTGANSPLHF	7	CD8-C5
Pla-3_TCGGGACCACCCATTC	Pla	Pla-3	P3-2846	FALSE	1	TRA:CAASRTTSGTYKYIF TRB:CASSQAGTGSYEQYF	1	CD8-C5
Pla-3_TCGTACCGTGGGTCAA	Pla	Pla-3	P3-2855	FALSE	1	TRA:CAVNMDQGGKLIF TRB:CAIIDGSGNTIYF	1	CD8-C4
Pla-3_TCTATTGAGACTACAA	Pla	Pla-3	P3-32	TRUE	3	TRA:CAASRGGQKLLF TRB:CASSSRTSLVHNEQFF	10	CD8-C5
Pla-3_TCTCTAATCACGGTTA	Pla	Pla-3	P3-9	TRUE	3	TRA:CAVRDMDTGRRALTF TRB:CASSWDTRQAEAFF	24	CD8-C4
Pla-3_TCTGAGAGTGAGTATA	Pla	Pla-3	P3-11	TRUE	3	TRA:CASGGGNKLTF TRB:CASSSDGDEAFF	17	CD8-C5

Pla-3_TCTGAGATCTCGATGA	Pla	Pla-3	P3-62	TRUE	3	TRA:CAEIPSGAGSYQLTF TRB:CASNGDENTIYF	6	CD8-C4
Pla-3_TCTTCGGTCAGAGACG	Pla	Pla-3	P3-9	TRUE	3	TRA:CAVRDMDTGRRALTF TRB:CASSWDTRQAEAFF	24	CD8-C4
Pla-3_TGAAAGATCAAACAAG	Pla	Pla-3	P3-2880	FALSE	1	TRA:CAVSGYGQNFVF TRB:CASSLTVNQPQHF	1	CD8-C4
Pla-3_TGACTAGGTCTCCATC	Pla	Pla-3	P3-301	TRUE	2	TRA:CALSVLPMDSNYQLIW TRB:CASSTRTENTEAFF	2	CD8-C4
Pla-3_TGAGCATCATCGGAAG	Pla	Pla-3	P3-2898	FALSE	1	TRA:CAENSYTGNQFYF TRB:CASSYRGGTHQPQHF	1	CD8-C5
Pla-3_TGCCAAATCATTCACT	Pla	Pla-3	P3-46	FALSE	3	TRA:CAVRRGILTGGGNKLTF TRB:CASSLVSSGGNPSYEQYF	8	CD8-C4
Pla-3_TGCCCATGTACTCTCC	Pla	Pla-3	P3-2909	FALSE	1	TRA:CAVQAGTDKLIF TRB:CASRASGTANEQFF	1	CD8-C4
Pla-3_TGCCCTATCCCTCTTT	Pla	Pla-3	P3-9	TRUE	3	TRA:CAVRDMDTGRRALTF TRB:CASSWDTRQAEAFF	24	CD8-C4
Pla-3_TGCGCAGAGACTAGGC	Pla	Pla-3	P3-2911	FALSE	1	TRA:CAVDDTGTASKLTF TRB:CASSSIVLQGAKTQYF	1	CD8-C5
Pla-3_TGCGCAGCATCTGGTA	Pla	Pla-3	P3-20	TRUE	3	TRA:CAVSSLQMDSSYKLIF TRB:CASSHRTENSPLHF	12	CD8-C4
Pla-3_TGCTACCAGGGTTTCT	Pla	Pla-3	P3-53	FALSE	3	TRA:CAVVISNYQLIW TRB:CASSLSSYYGYTF	7	CD8-C4
Pla-3_TGCTACCCAAAGAATC	Pla	Pla-3	P3-2919	FALSE	1	TRA:CAAFDMRF TRB:CASSLGRITDTQYF	1	CD8-C4
Pla-3_TGCTACCGTCCTCCAT	Pla	Pla-3	P3-8	TRUE	3	TRA:CAFDRGSTLGRLYF TRB:CASNPRQGTSTYEQYF	26	CD8-C3
Pla-3_TGCTGCTAGAGGTACC	Pla	Pla-3	P3-141	FALSE	3	TRA:CAVEGGGTSYGKLTF TRB:CAWSVAGNQPQHF	4	CD8-C4
Pla-3_TGGCCAGCAGAGTGTG	Pla	Pla-3	P3-190	TRUE	3	TRA:CAVTEGADGLTF TRB:CASSPGQTYGYTF	3	CD8-C4
Pla-3_TGGCCAGGTACCGTTA	Pla	Pla-3	P3-2927	FALSE	1	TRA:CAVAPTRGGTASKLTF TRB:CAISESTGVNTEAFF	1	CD8-C4
Pla-3_TGGCCAGTCAACGCTA	Pla	Pla-3	P3-5	TRUE	3	TRA:CALRNSNYQLIW TRB:CASSLGDRAYNEQFF	38	CD8-C5
Pla-3_TGGCCAGTCTCTGTCG	Pla	Pla-3	P3-2929	FALSE	1	TRA:CAVTEAGTALIF TRB:CASSLGGQENSPLHF	1	CD8-C4
Pla-3_TGGGAAGCATCACAAC	Pla	Pla-3	P3-2933	FALSE	1	TRA:CAVANFNKFYF TRB:CASSRPSGGRDQPQHF	1	CD8-C5
Pla-3_TGGGCGTCACCGATAT	Pla	Pla-3	P3-11	TRUE	3	TRA:CASGGGNKLTF TRB:CASSSDGDEAFF	17	CD8-C3
Pla-3_TGTATTCCACGTGAGA	Pla	Pla-3	P3-15	FALSE	3	TRA:CAVRDWDNAGNMLTF TRB:CASSEALGGAGETQYF	15	CD8-C4
Pla-3_TGTGGTACACCATCCT	Pla	Pla-3	P3-9	TRUE	3	TRA:CAVRDMDTGRRALTF TRB:CASSWDTRQAEAFF	24	CD8-C4
Pla-3_TGTGTTTAGTAGGTGC	Pla	Pla-3	P3-48	TRUE	3	TRA:CAYDGATNKLIF TRB:CASSLYGHQPQHF	7	CD8-C4
Pla-3_TGTTCCGAGGGTGTGT	Pla	Pla-3	P3-1	TRUE	3	TRA:CAVAPISSGSARQLTF TRB:CASSQGMSEAFF	83	CD8-C5
Pla-3_TTAGGCAAGAATGTTG	Pla	Pla-3	P3-1	TRUE	3	TRA:CAVAPISSGSARQLTF TRB:CASSQGMSEAFF	83	CD8-C5
Pla-3_TTAGTTCAGTGGGATC	Pla	Pla-3	P3-240	TRUE	2	TRA:CALYYGNNRLAF TRB:CASSVGQGQGYEQYF	2	CD8-C5
Pla-3_TTATGCTCACACATGT	Pla	Pla-3	P3-2964	FALSE	1	TRA:CAMRDDQTGANNLFF TRB:CASSYLAGGLGTQYF	1	CD8-C5

Pla-3_TTCGAAGCAAGCCGCT	Pla	Pla-3	P3-5	TRUE	3	TRA:CALRNSNYQLIW TRB:CASSLGDRAYNEQFF	38	CD8-C4
Pla-3_TTCGGTCAGCGTTTAC	Pla	Pla-3	P3-9	TRUE	3	TRA:CAVRDMDTGRRALTF TRB:CASSWDTRQAEAFF	24	CD8-C5
Pla-3_TTCTCAACAGACACTT	Pla	Pla-3	P3-3	TRUE	3	TRA:CVVPWMDSSYKLIF TRB:CASSQRAGDGNYGYTF	42	CD8-C5
Pla-3_TTCTCAACAGTATCTG	Pla	Pla-3	P3-3	TRUE	3	TRA:CVVPWMDSSYKLIF TRB:CASSQRAGDGNYGYTF	42	CD8-C5
Pla-3_TTCTCAACATGGGAAC	Pla	Pla-3	P3-36	TRUE	3	TRA:CARGAGSYQLTF TRB:CASSVGTGTEAFF	9	CD8-C3
Pla-3_TTCTTAGGTAGCCTCG	Pla	Pla-3	P3-82	TRUE	3	TRA:CAVYLDRGSTLGRLYF TRB:CASSFGSGPNYGYTF	5	CD8-C3
Pla-3_TTGACTTAGTTACGGG	Pla	Pla-3	P3-2982	FALSE	1	TRA:CVVSANNQGGKLIF TRB:CASSQPAFYGYTF	1	CD8-C5
Pla-3_TTGCCGTGTCTAAAGA	Pla	Pla-3	P3-2986	FALSE	1	TRA:CAVRKAAGNKLTF TRB:CAAGAGQGANEQYF	1	CD8-C4
Pla-3_TTGCCGTTCGCATGAT	Pla	Pla-3	P3-10	TRUE	3	TRA:CAGHQFSGGYNKLIF TRB:CASRASGTANEQFF	21	CD8-C4
Pla-3_TTGGAACTCACTTACT	Pla	Pla-3	P3-2992	FALSE	1	TRA:CAGRYRDDKIIF TRB:CASNPSRTGELFF	1	CD8-C4
Pla-3_TTGGCAACACTGTTAG	Pla	Pla-3	P3-352	FALSE	2	TRA:CAVEGVYNQGGKLIF TRB:CASSLDPGEKTQYF	2	CD8-C4
[truncated: 630,165 more chars]
